# Supplementary material for: Developmental phylotranscriptomics in grapevine suggests an ancestral role of somatic embryogenesis
Source: Commun Biol. 2025 Feb 20;8:265. doi: 10.1038/s42003-025-07712-w (PMC11839975; doi:10.1038/s42003-025-07712-w)
Supplement: Supplementary file 2 — Supplementary Information [file 42003_2025_7712_MOESM2_ESM.pdf]

Supplementary Information

Developmental phylotranscriptomics in grapevine suggests an ancestral role of somatic embryogenesis

Sara Koska[1§], Dunja Leljak-Levanić[2§*], Nenad Malenica[2§*], Kian Bigović Villi[1§], Momir Futo[3,1], Nina Čorak[1], Mateja Jagić[2], Ariana Ivanić[2], Anja Tušar[1], Niko Kasalo[1], Mirjana Domazet-Lošo[4], Kristian Vlahoviček[5], Tomislav Domazet-Lošo[1,3*]

[1]Laboratory of Evolutionary Genetics, Division of Molecular Biology, Ruđer Bošković Institute, Bijenička cesta 54, HR-10000 Zagreb, Croatia

[2]Division of Molecular Biology, Department of Biology, Faculty of Science, University of Zagreb, Horvatovac 102a, HR-10000 Zagreb, Croatia

[3]School of Medicine, Catholic University of Croatia, Ilica 242, HR-10000 Zagreb, Croatia

[4]Faculty of Electrical Engineering and Computing, University of Zagreb, Unska 3, HR-10000 Zagreb, Croatia

[5]Bioinformatics Group, Division of Molecular Biology, Department of Biology, Faculty of Science, University of Zagreb, Horvatovac 102a, HR-10000 Zagreb, Croatia

[§]These authors contributed equally to this work.

[*]Corresponding authors: dunja@zg.biol.pmf.hr, malenica@biol.pmf.hr, tdomazet@irb.hr

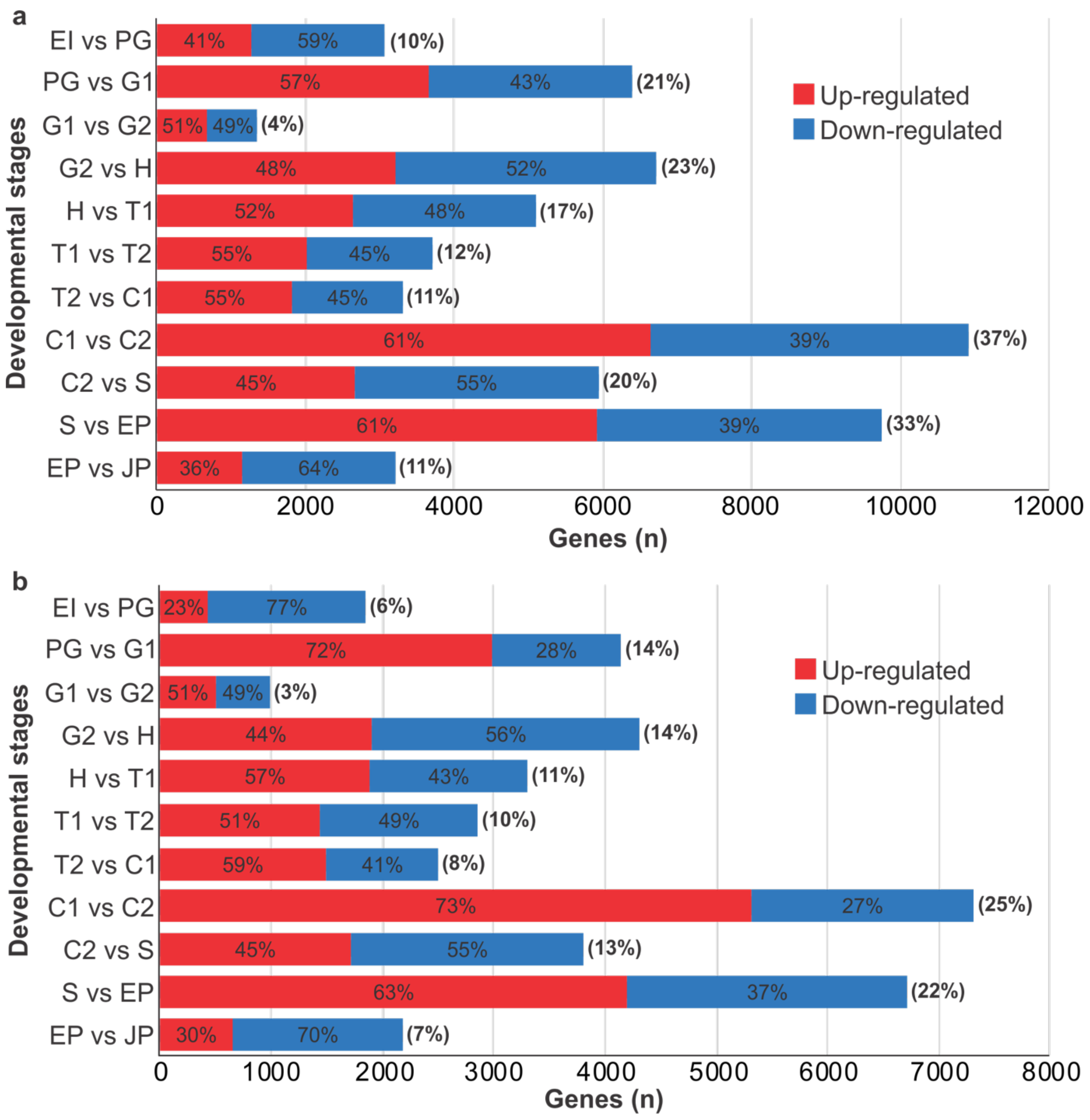

Supplementary Figure 1. Differentially expressed genes between the successive developmental stages of *V. vinifera* somatic embryogenesis. The percentages in parenthesis show the percentage of differentially expressed genes between the two successive developmental stages calculated by comparison to all expressed genes (29,839). Red bars denote the percentage of up-regulated genes, while blue bars mark percentage of down-regulated genes between the two successive stages in reference to all differentially expressed genes during SE. We estimated pairwise differential expression using a pipeline implemented in the *DESeq2* R package (**a,** p-value < 0.05; **b,** p-value < 0.05 and fold change > 2).

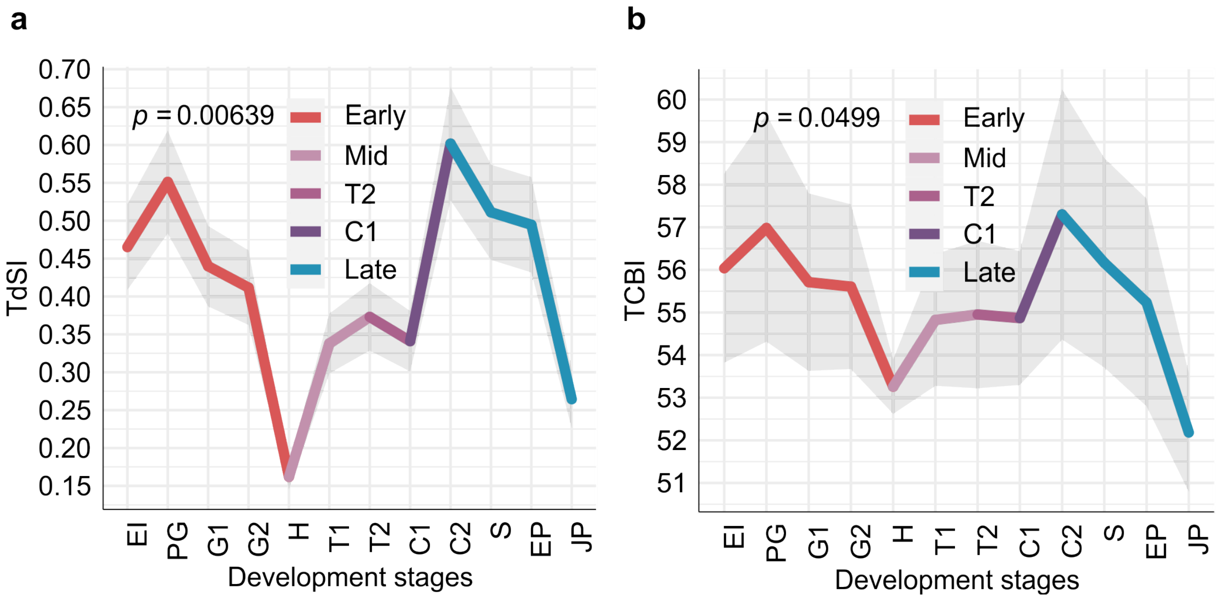

Supplementary Figure 2. The synonymous divergence and codon bias exhibit an hourglass pattern. a, The transcriptome nonsynonymous divergence index (TdSI), and **b,** the transcriptome codon bias index (TCBI) profile exhibit statistically significant hourglass patterns, with genes conserved at synonymous sites and genes expressing strong codon usage bias preferentially expressed during mid-development. Divergence rates for TdSI were estimated by *V. vinifera* – *Vitis arizonica* comparison (see Material and Methods). Codon usage bias was estimated for *V. vinifera* genes using ENC measure (see Material and Methods). The *p* values were calculated using the flat line test while the grey shaded area represents ± one standard deviation estimated using permutation analysis (see Material and Methods). Periods of similar gene expression within the somatic embryogenesis are color-coded: early (red), mid, T2, and C1 (different shades of purple), and late (blue).

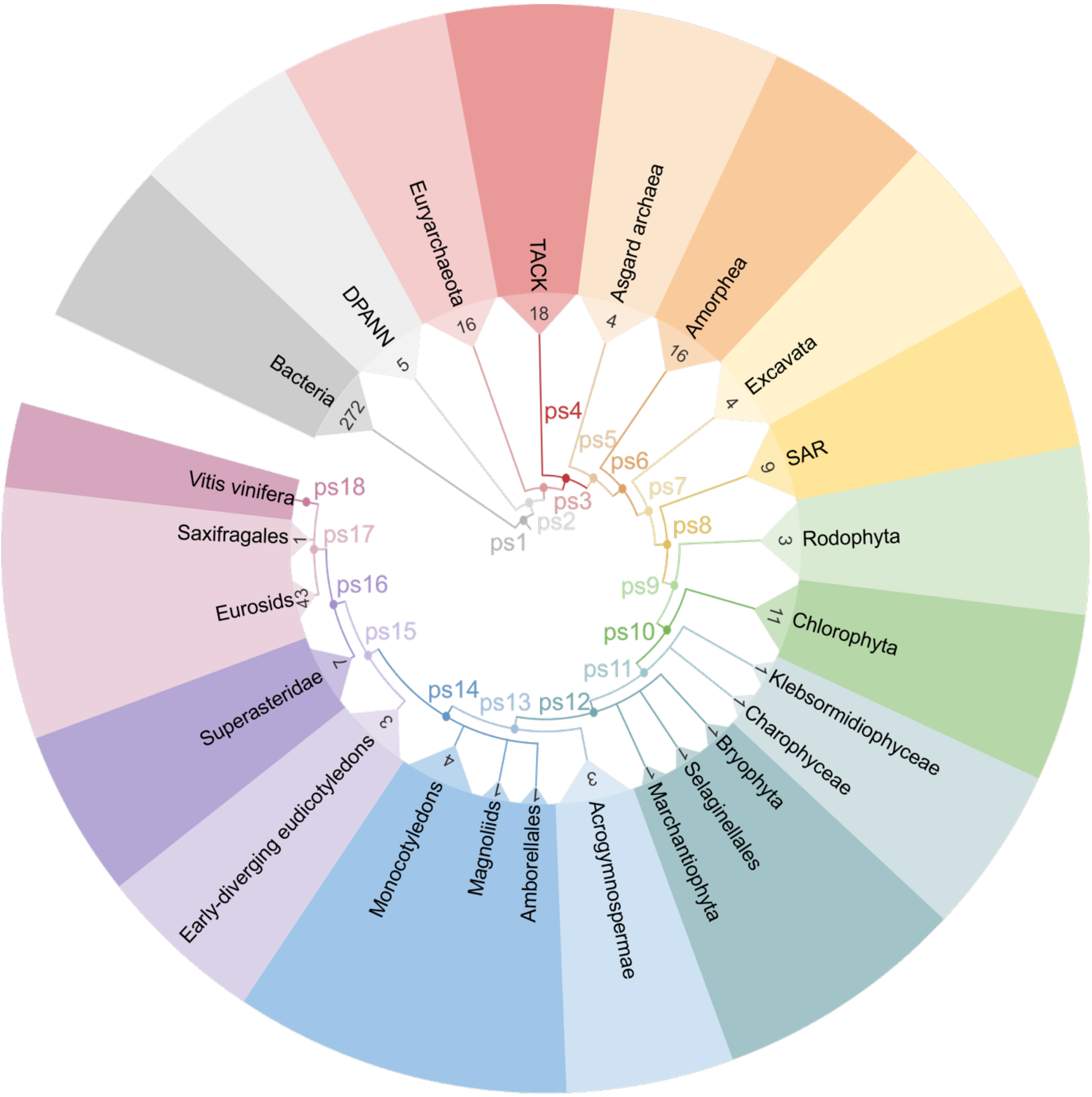

ps1 Cellular organisms
ps2 DPANN/Euryarchaeota/TACK/Asgard archaea/Eukaryota
ps3 Euryarchaeota/TACK/Asgard archaea/Eukaryota
ps4 TACK/Asgard archaea/Eukaryota
ps5 Asgard archaea/Eukaryota
ps6 Eukaryota
ps7 Excavata/Diaphoretickes
ps8 Diaphoretickes
ps9 Archaeplastida
ps10 Viridiplantae
ps11 Streptophyta
ps12 Embryophyta
ps13 Spermatophyta
ps14 Magnoliophyta
ps15 Eudicots
ps16 Core eudicots
ps17 Superrosids
ps18 Vitis vinifera

Supplementary Figure 3. *Vitis vinifera* consensus phylogeny used for the phylostratigraphic analysis. The consensus phylogeny covers divergence from the last common ancestor of cellular organisms to *V. vinifera* as a focal organism. Phylogeny is constructed based on the relevant phylogenetic literature, importance of evolutionary transitions and availability of reference genomes. Eighteen internodes (phylostrata) are marked as ps1 –

ps18. The numbers on the terminal nodes represent the number of species in the matching node and correspond to the genomes used to populate the reference database for sequence similarity searches.

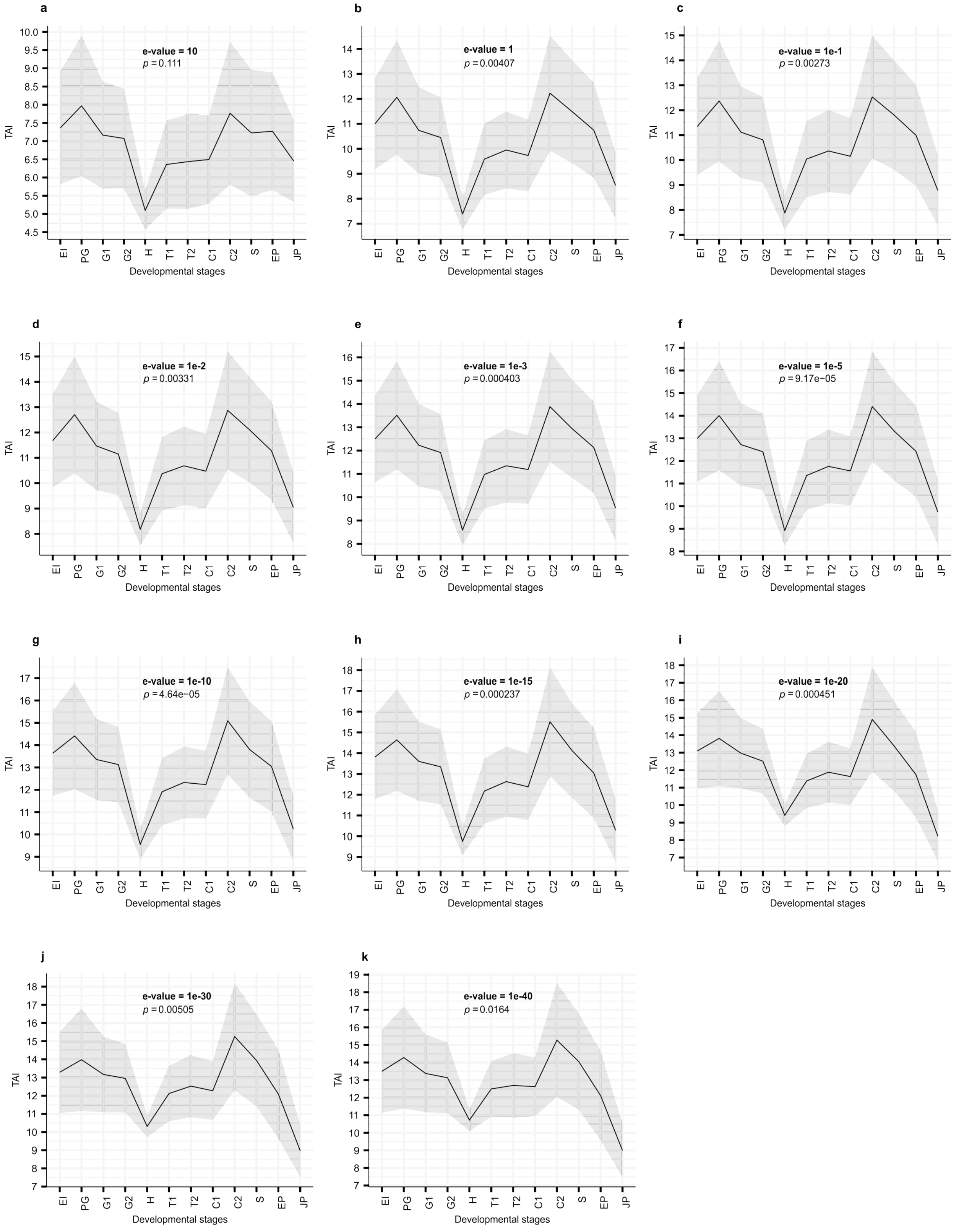

Supplementary Figure 4. Transcriptome age index (TAI) profiles of *Vitis vinifera* somatic embryogenesis calculated from phylostratigraphy maps obtained with different blastp e-value thresholds. **a**, e-value = 10 (n = 29,659); **b**, e-value = 1 (n = 29,625); **c**, e-value = 10e-1 (n = 29,502); **d**, e-value = 10e-2 (n = 29,542); **e**, e-value = 10e-3 (n = 29,496); **f**, e-value = 10e-5 (n = 29,408); **g**, e-value = 10e-10 (n = 29,123); **h**, e-value = 10e-15 (n = 28,771); **i**, e-value = 10e-20 (n = 28,382); **j**, e-value = 10e-30 (n = 27,529); **k**, e-value = 10e-40 (n = 26,550). The *p* values shown were calculated using the flat line test while the grey shaded area represents ± one standard deviation estimated using permutation.

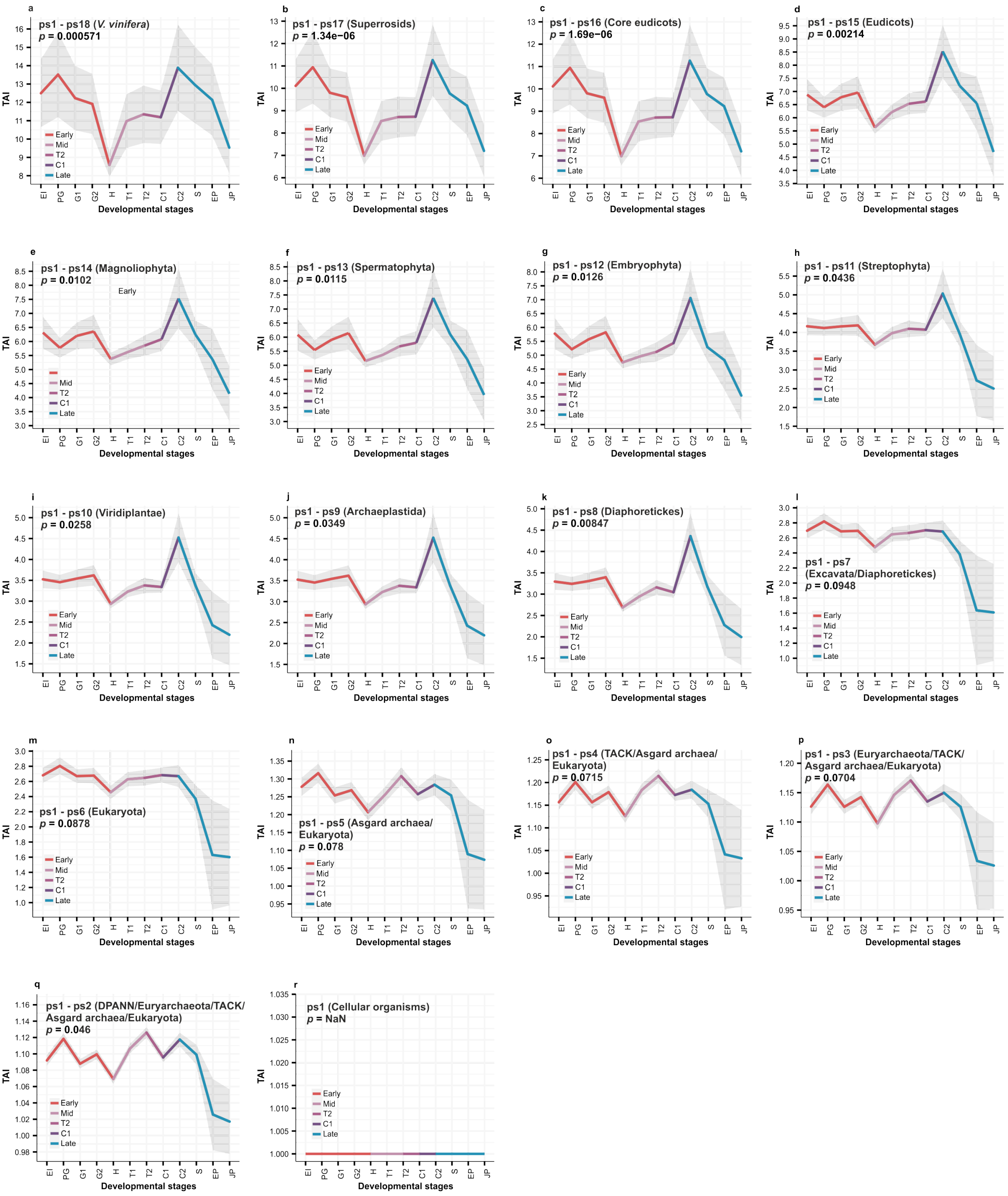

Supplementary Figure 5. The hourglass pattern is significant from the origin of Diaphoretickes at ps8. Transcriptome age indices (TAI) were calculated using reduced datasets obtained by increasingly removing genes from the youngest phylostratum. This process was repeated until only genes from the oldest phylostratum (ps1) remained. **a**, ps1-ps18 (n=29,623); **b**, ps1-ps17 (n = 26,316); **c**, ps1-ps16 (n = 26,081); **d**, ps1-ps15 (n = 25,881); **e**, ps1-ps14 (n = 25,586); **f**, ps1-ps13 (n = 24,813); **g**, ps1-ps12 (n = 23,757); **h**, ps1-ps11 (n = 21,762); **i**, ps1-ps10 (n = 19,982); **j**, ps1-ps9 (n = 19,059); **k**, ps1-ps8 (n = 18,965); **l**, ps1-ps7

(n = 18,573); **m**, ps1-ps6 (n = 18,439); **n**, ps1-ps5 (n = 12,279); **o**, ps1-ps4 (n = 12,161); **p**, ps1-ps3 (n = 12,108); **q**, ps1-ps2 (n = 11,993); **r**, ps1 (n = 11,789). The *p* values were calculated using the flat line test while the grey shaded area represents ± one standard deviation estimated using permutation analysis (see Material and Methods). Expression phases of SE, as depicted in Fig. 1a, are color coded: early (red), mid, T2 and C1 (different shades of purple), and late (blue).

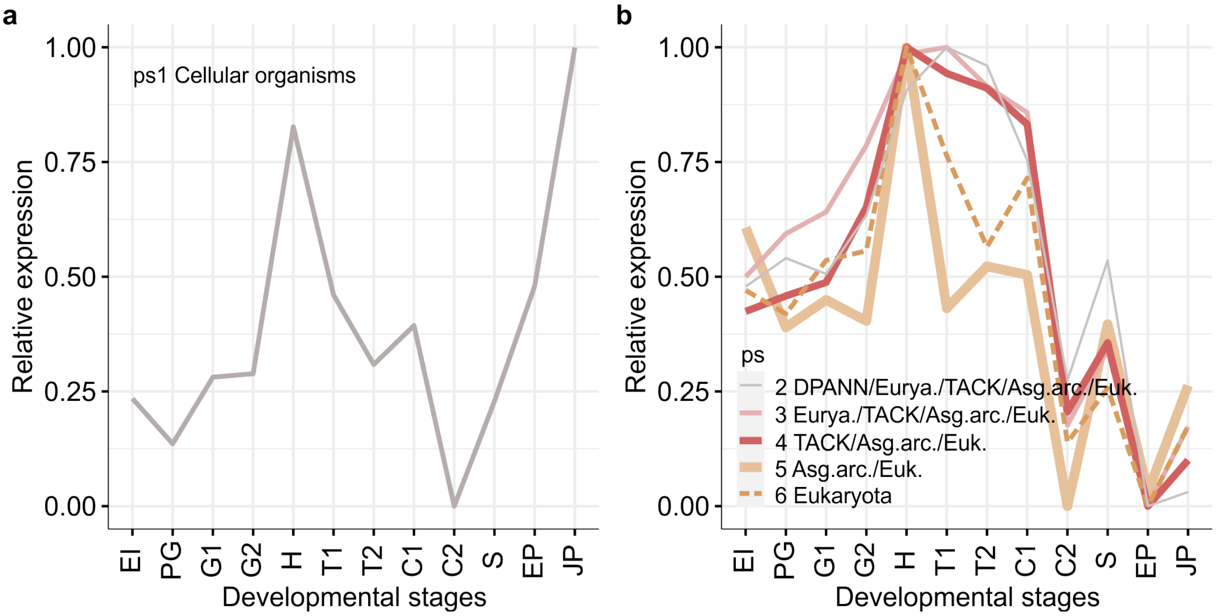

Supplementary Figure 6. Relative expression (RE) profiles along *V. vinifera* somatic embryogenesis. Genes from phylostrata ps1 to ps6 are shown. **a**, RE profiles of the genes from Cellular organisms (ps1). **b**, RE profiles of the genes from DPANN (ps2) to Eukaryota (ps6). These older genes (ps1-ps6) generally peak at the heart stage.

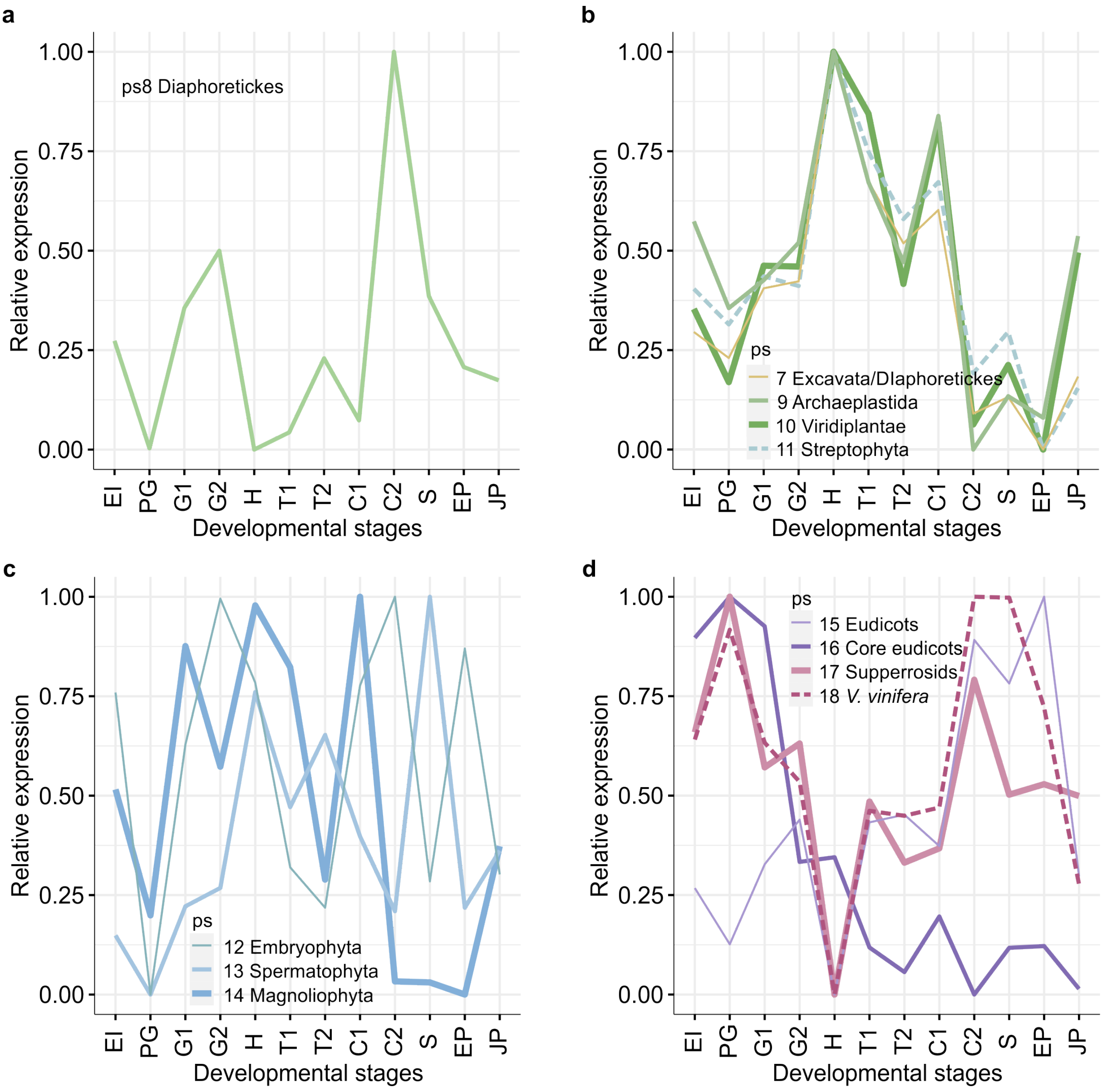

Supplementary Figure 7. Relative expression (RE) profiles along *V. vinifera* somatic embryogenesis. Genes from phylostrata ps7 to ps18 are shown **a**, RE profile of the genes from Diaphoretickes (ps8). **b**, RE profiles of the genes from Excavata (ps7) and Archaeplastida (ps9) to Streptophyta (ps11). **c**, RE profiles of the genes from Embryophyta (ps12) to Magnoliophyta (ps14). **d**, RE profiles of the genes from Eudycots (ps15) to *V. vinifera* (ps18).

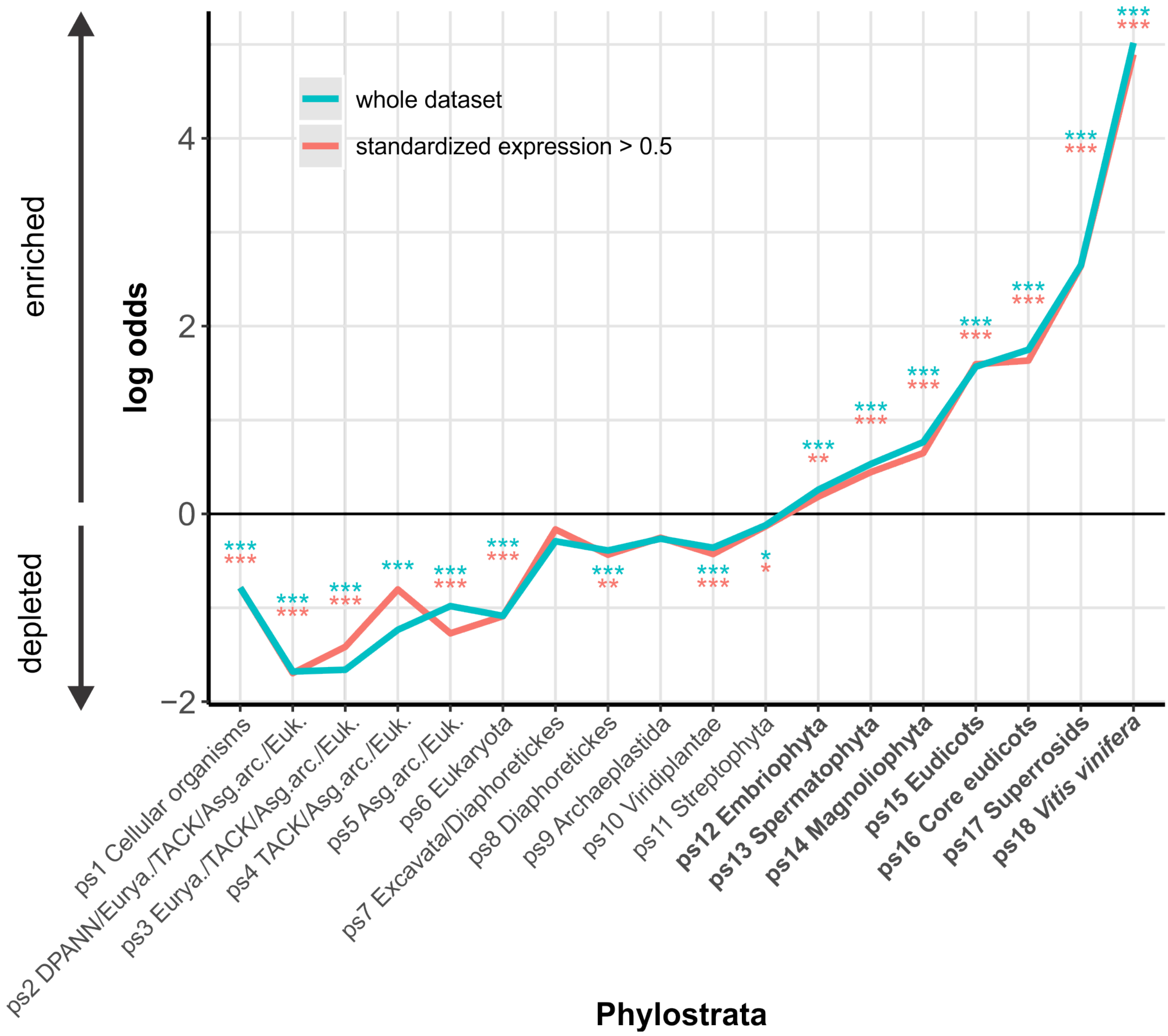

Supplementary Figure 8. *V. vinifera* genes that emerged during the diversification of land plants tend to be functionally unstudied. The enrichment of genes with unknown function across phylostrata (n = 11,224, blue line), and for the subset of genes which standardized expression are at least in one developmental stage 0.5 or more above the median (n = 8,070, red line). We tested the enrichments, i.e., the significance of deviations from the expected values, by two-tailed hypergeometric test and *p* values are corrected for multiple comparisons at 0.05 level (*p < 0.05; **p < 0.01; ***p < 0.001). The enrichments are shown as log-odds (y-axis).
