# Supplementary material for: Developmental phylotranscriptomics in grapevine suggests an ancestral role of somatic embryogenesis
Source: Commun Biol. 2025 Feb 20;8:265. doi: 10.1038/s42003-025-07712-w (PMC11839975; doi:10.1038/s42003-025-07712-w)
Supplement: Supplementary file 3 — Description of Additional Supplementary Files [file 42003_2025_7712_MOESM3_ESM.docx]

Description of Additional Supplementary Files

**File name:** Supplementary Data 1

**Description:** Raw transcriptome counts plus mapping statistics.

**File name:** Supplementary Data 2

**Description:** Differential expression statistics obtained by LRT test (DeSeq2).

**File name:** Supplementary Data 3

**Description:** Standardized expression profiles of 85 recovered gene clusters.

**File name:** Supplementary Data 4

**Description:** Composition of 85 gene clusters and all standardized expression values.

**File name:** Supplementary Data 5

**Description:** Input data used to calculate evolutionary indices.

**File name:** Supplementary Data 6

**Description:** Full consensus phylogeny used in the phylostratigraphic analysis of Vitis vinifera.

**File name:** Supplementary Data 7

**Description:** Phylostratigraphic results and database content.

**File name:** Supplementary Data 8

**Description:** Functional enrichment data and results.

**File name:** Supplementary Data 9

**Description:** Individual standardized expression profiles of all Vitis vinifera genes.

**File name:** Supplementary Data 10

**Description:** Arabidopsis thaliana embryo induction potential (wild type and drm1/drm2 double mutant values).

**File name:** Supplementary Data 11

**Description:** The numerical source data underlying the graphs presented in the paper.
