# Supplementary material for: Developmental phylotranscriptomics in grapevine suggests an ancestral role of somatic embryogenesis
Source: Commun Biol. 2025 Feb 20;8:265. doi: 10.1038/s42003-025-07712-w (PMC11839975; doi:10.1038/s42003-025-07712-w)
Supplement: Supplementary file 6 — Supplementary Data 3 [file 42003_2025_7712_MOESM6_ESM.zip › Supplementary_Data_3_clusters.pdf]

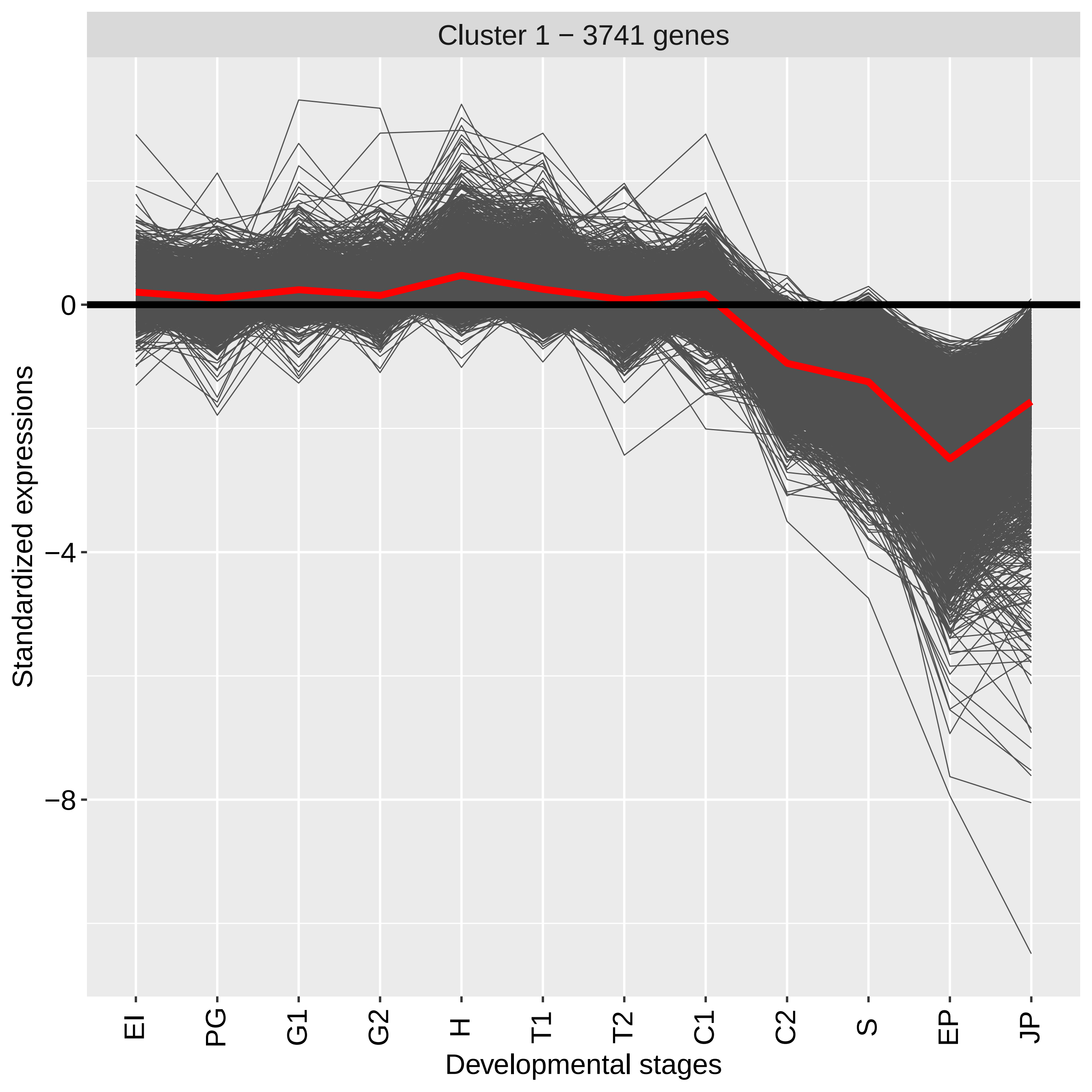

Cluster 1 − 3741 genes
Standardized expressions
0
−4
−8
EI
PG
G1
G2
H
T1
T2
C1
C2
S
EP
JP
Developmental stages

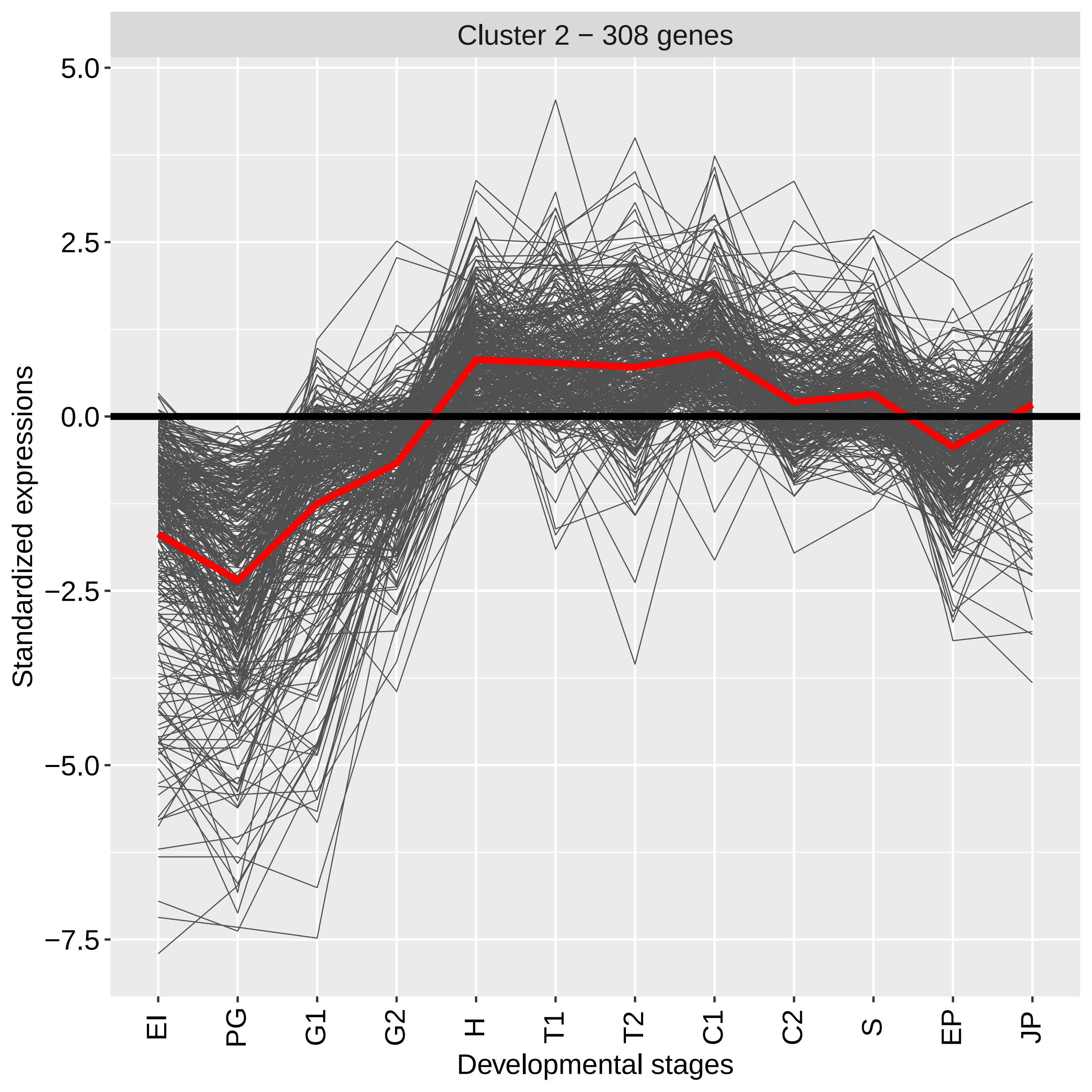

Cluster 2 − 308 genes
5.0
2.5
0.0
−2.5
−5.0
−7.5
Standardized expressions
EI
PG
G1
G2
H
T1
T2
C1
C2
S
EP
JP
Developmental stages

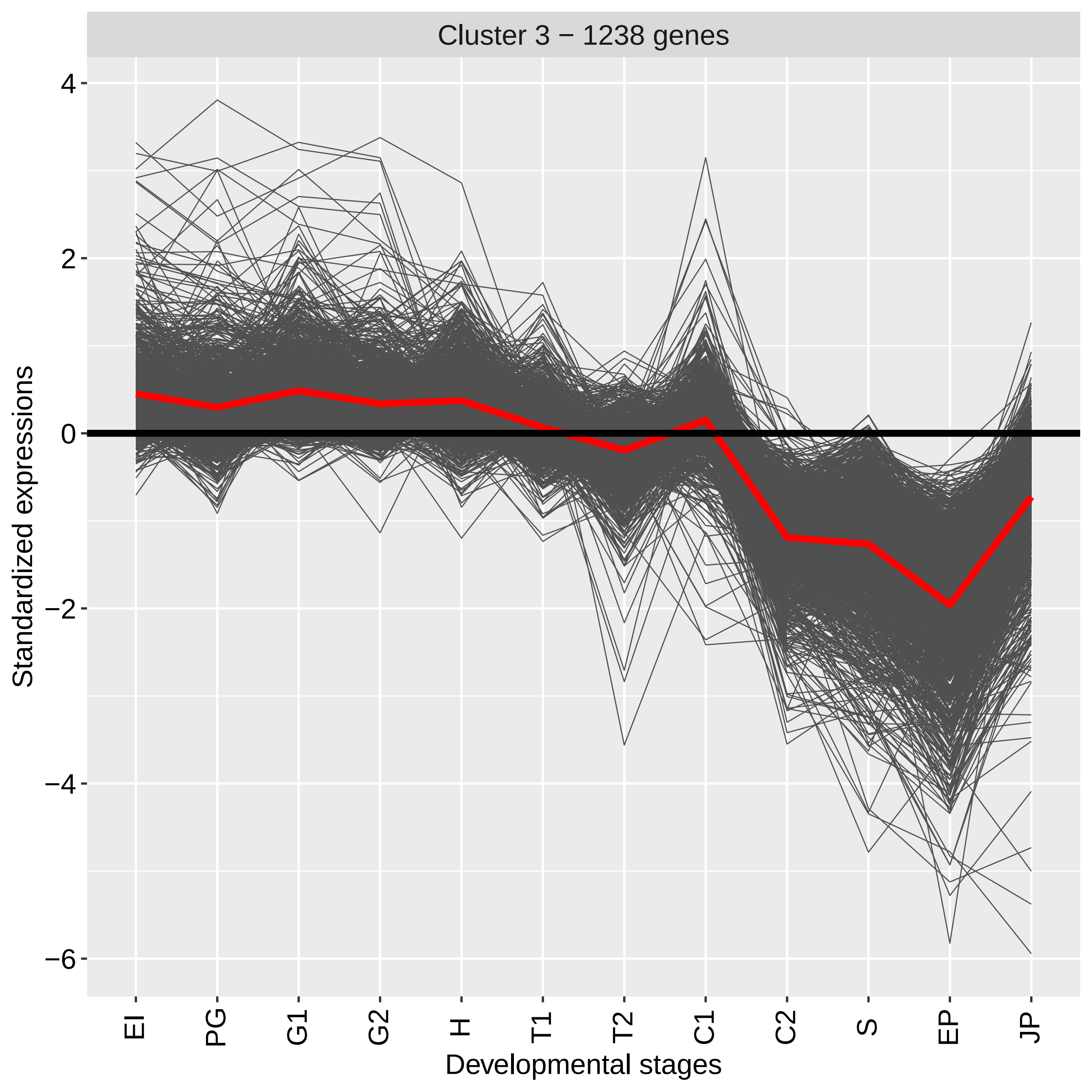
Cluster 3 − 1238 genes
Standardized expressions
4
2
0
−2
−4
−6
EI
PG
G1
G2
H
T1
T2
C1
C2
S
EP
JP
Developmental stages

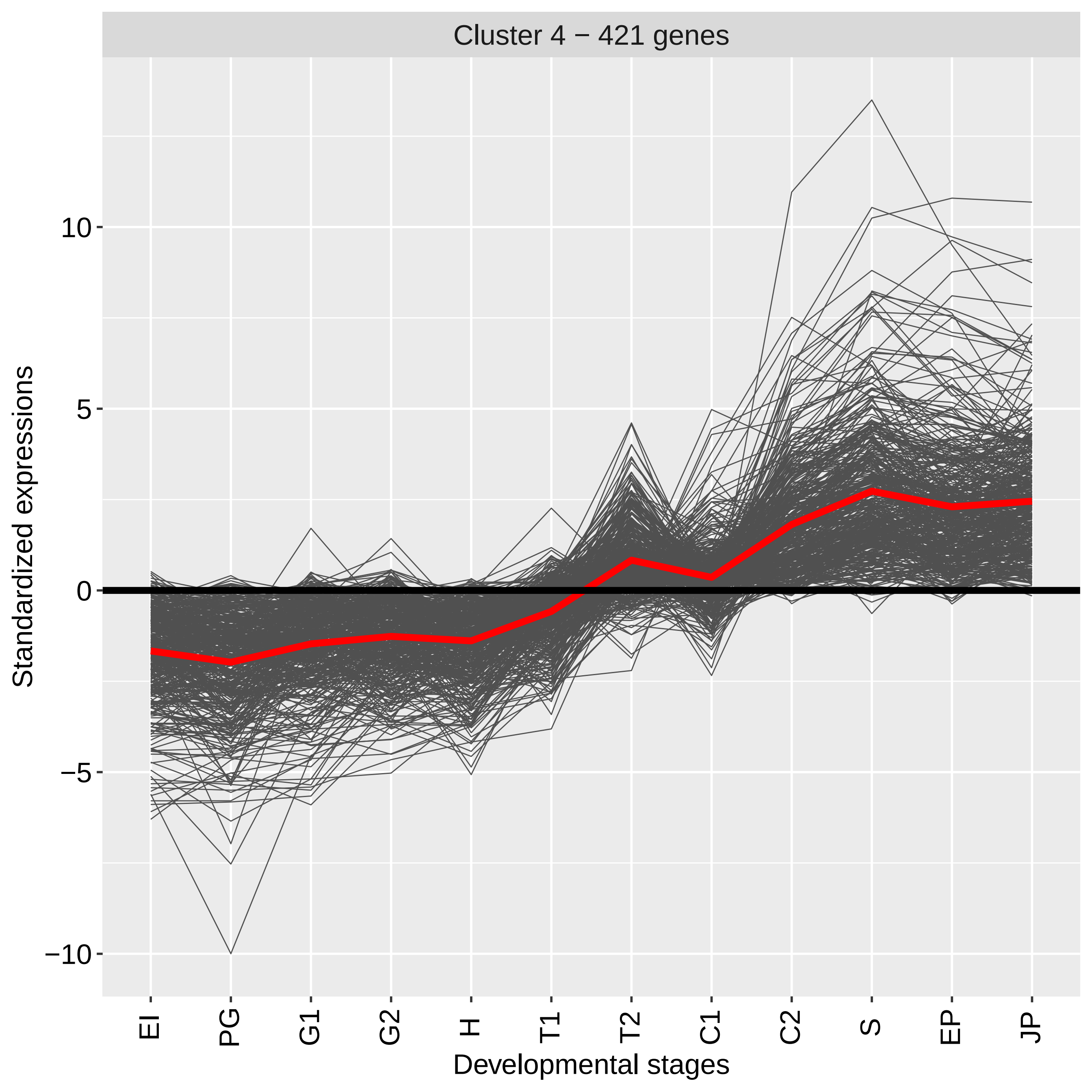
Cluster 4 − 421 genes
Standardized expressions
10
5
0
−5
−10
EI
PG
G1
G2
H
T1
T2
C1
C2
S
EP
JP
Developmental stages

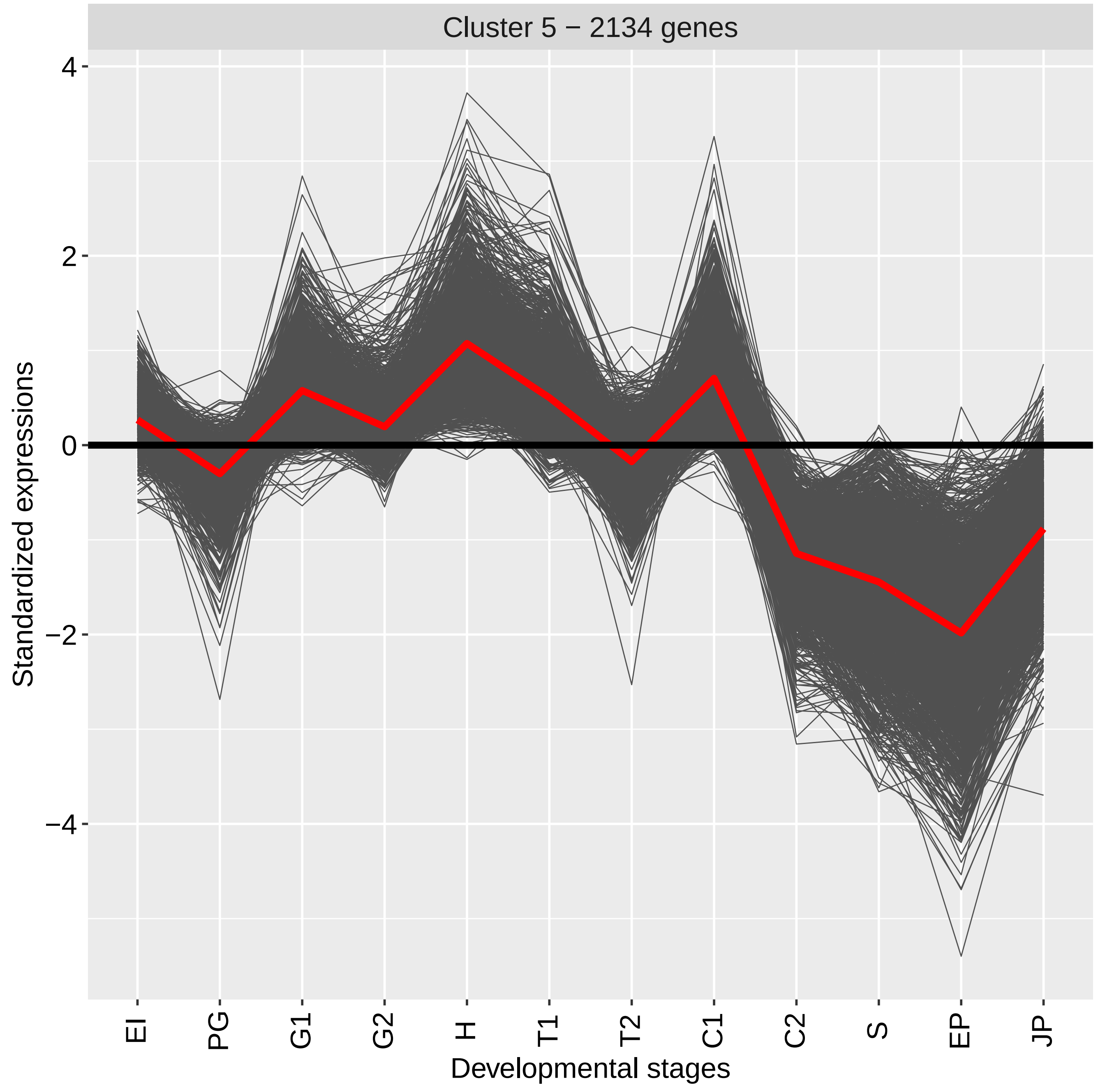

Cluster 5 − 2134 genes
Standardized expressions
4
2
0
−2
−4
EI
PG
G1
G2
H
T1
T2
C1
C2
S
EP
JP
Developmental stages

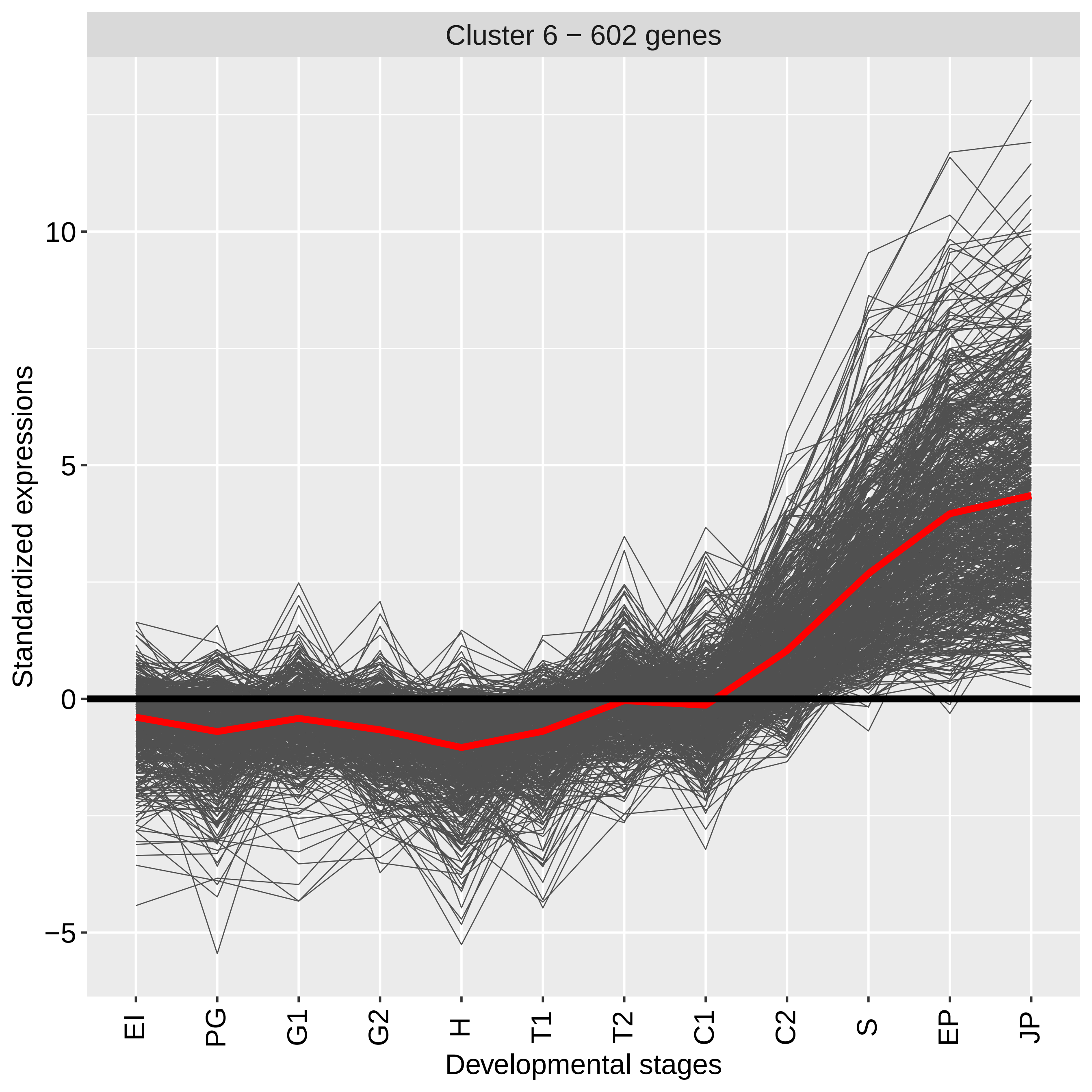
Cluster 6 − 602 genes
Standardized expressions
10
5
0
−5
EI
PG
G1
G2
H
T1
T2
C1
C2
S
EP
JP
Developmental stages

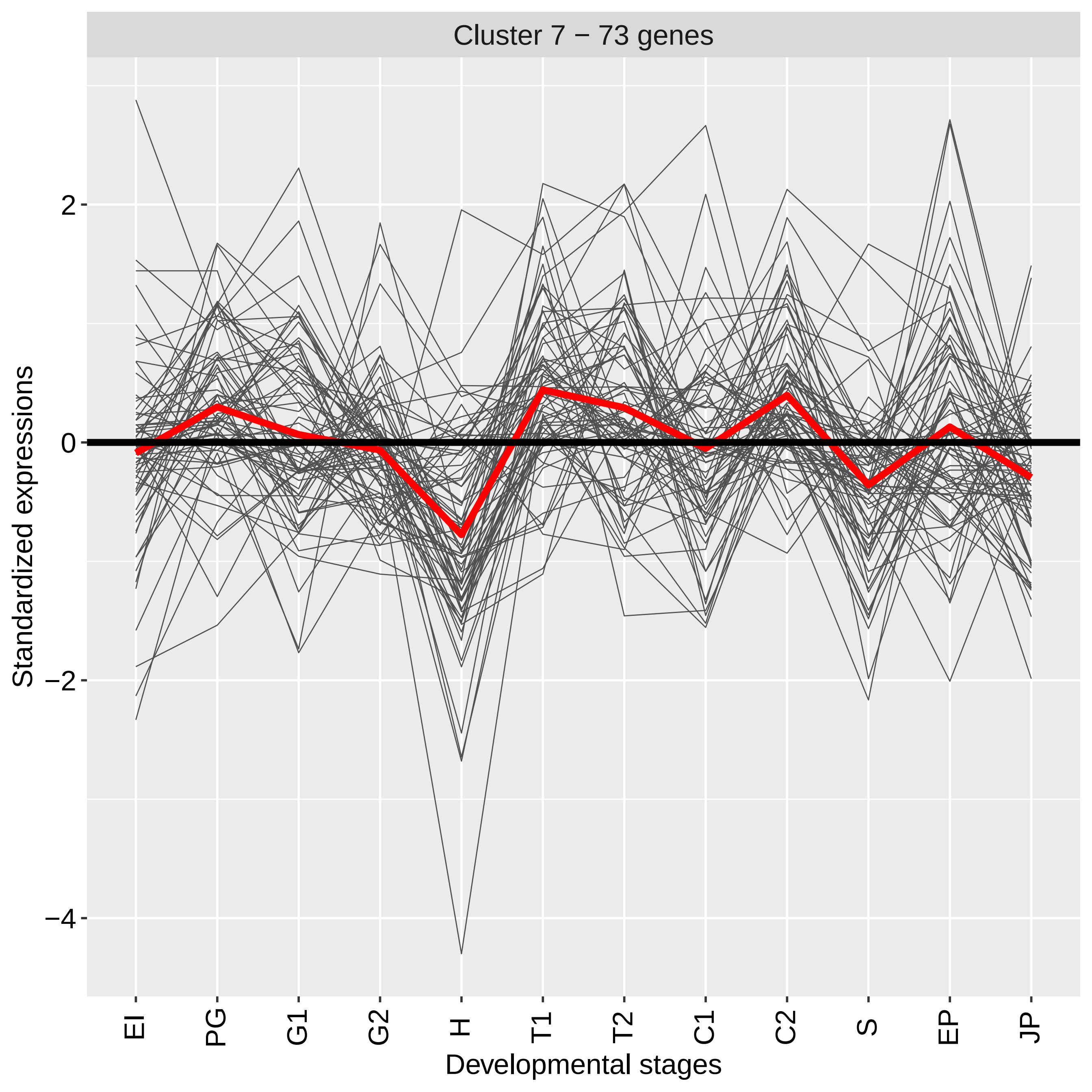
Cluster 7 − 73 genes
Standardized expressions
2
0
−2
−4
EI
PG
G1
G2
H
T1
T2
C1
C2
S
EP
JP
Developmental stages

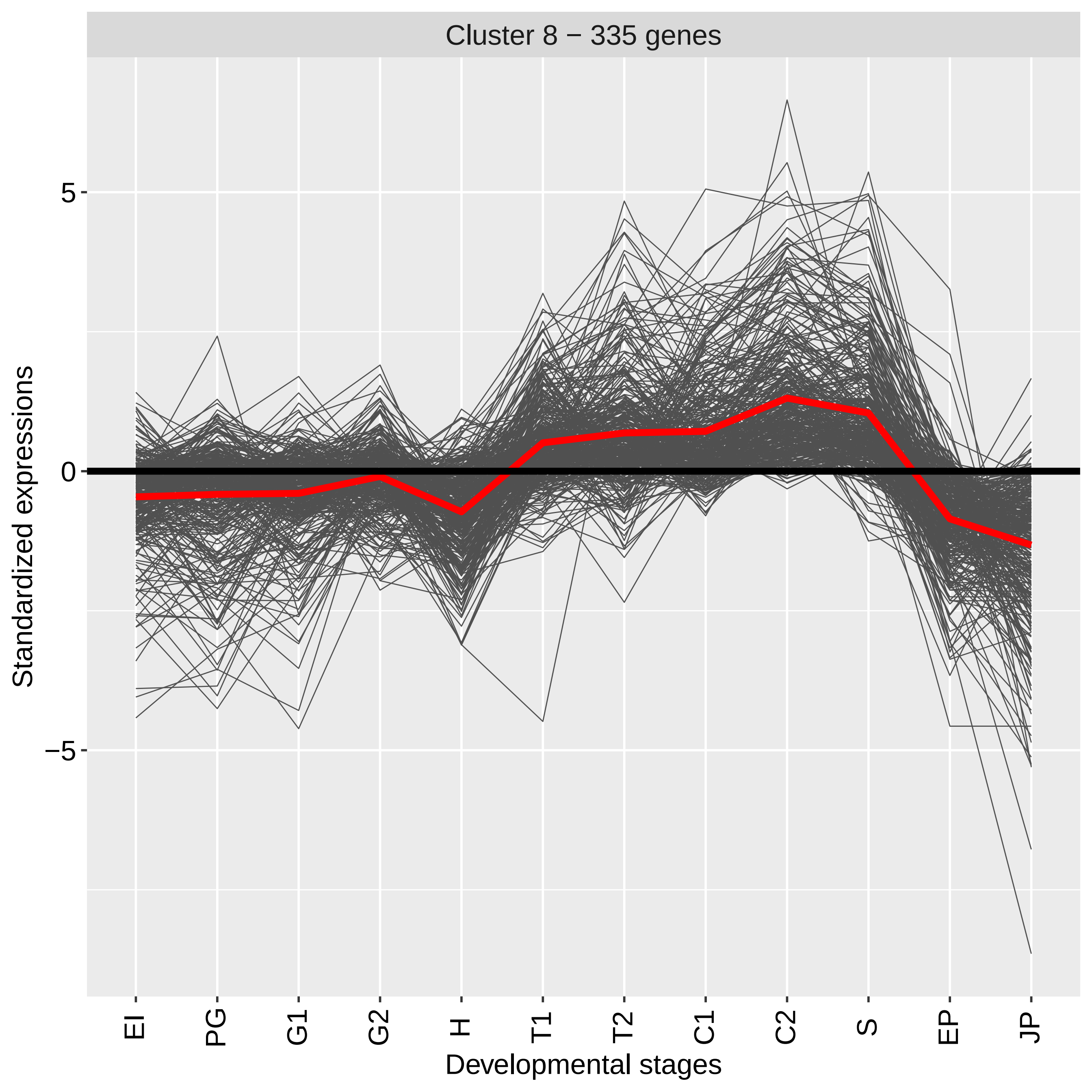

Cluster 8 − 335 genes
Standardized expressions
5
0
−5
EI
PG
G1
G2
H
T1
T2
C1
C2
S
EP
JP
Developmental stages

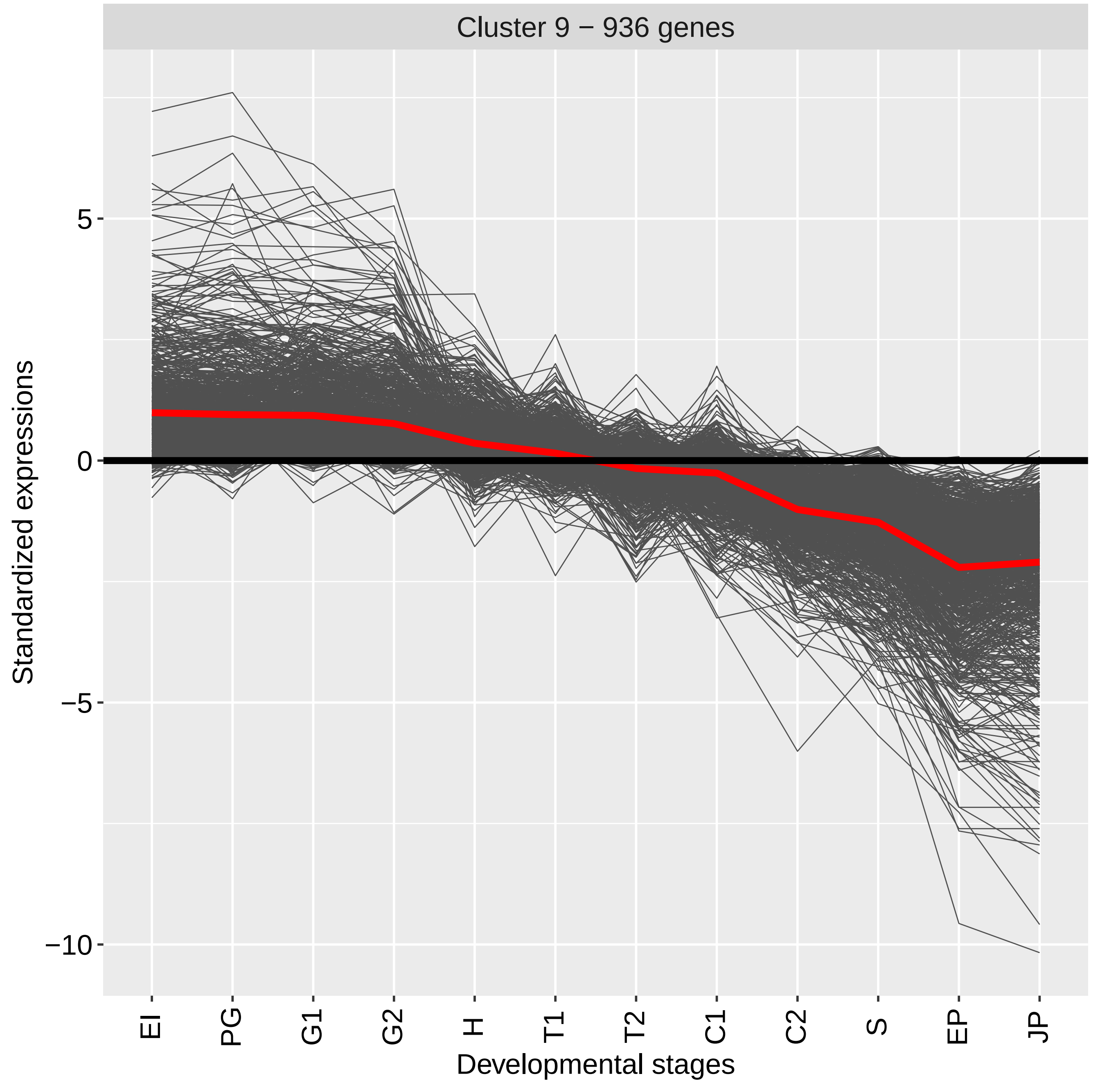

Cluster 9 − 936 genes
Standardized expressions
5
0
−5
−10
EI
PG
G1
G2
H
T1
T2
C1
C2
S
EP
JP
Developmental stages

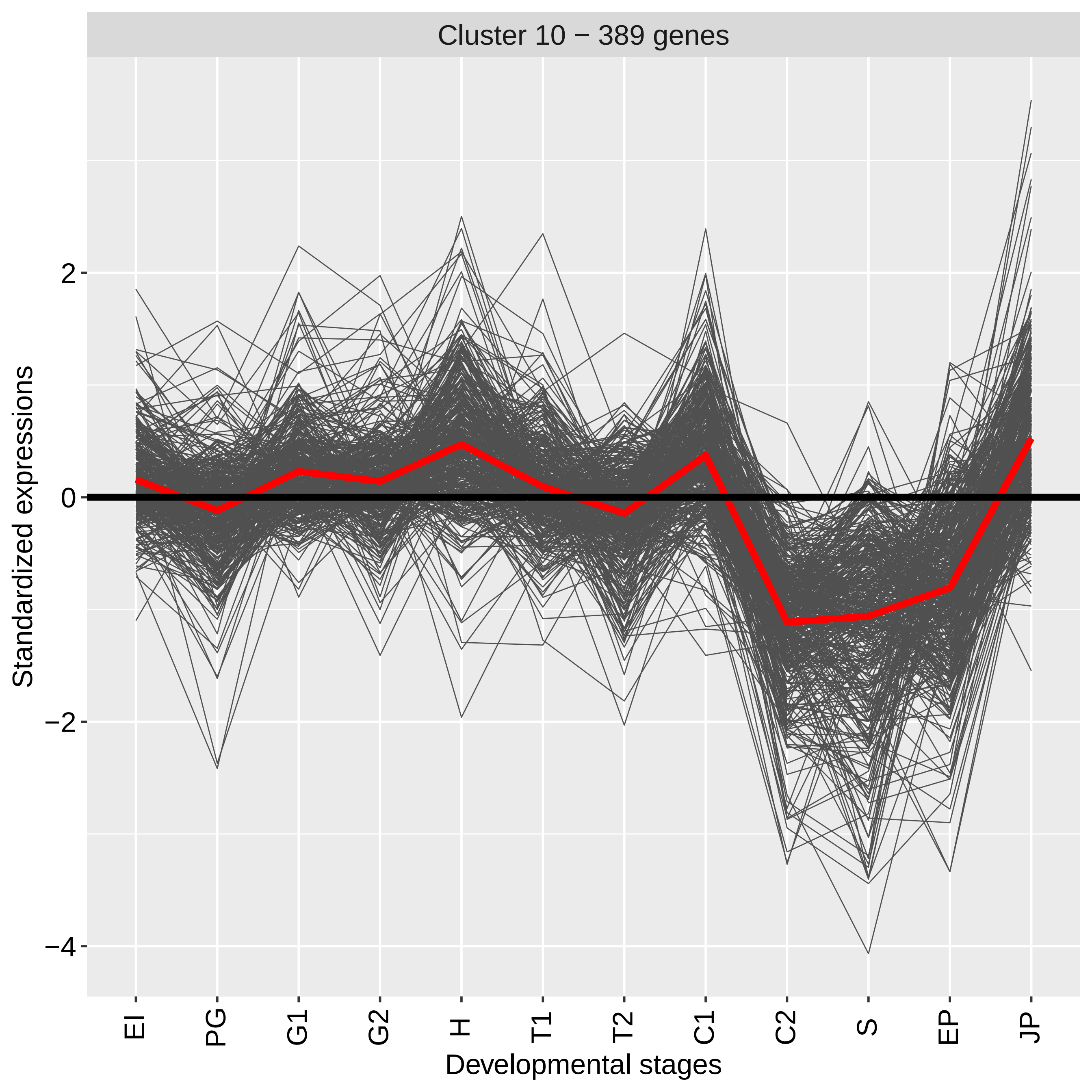
Cluster 10 − 389 genes
Standardized expressions
2
0
−2
−4
EI
PG
G1
G2
H
T1
T2
C1
C2
S
EP
JP
Developmental stages

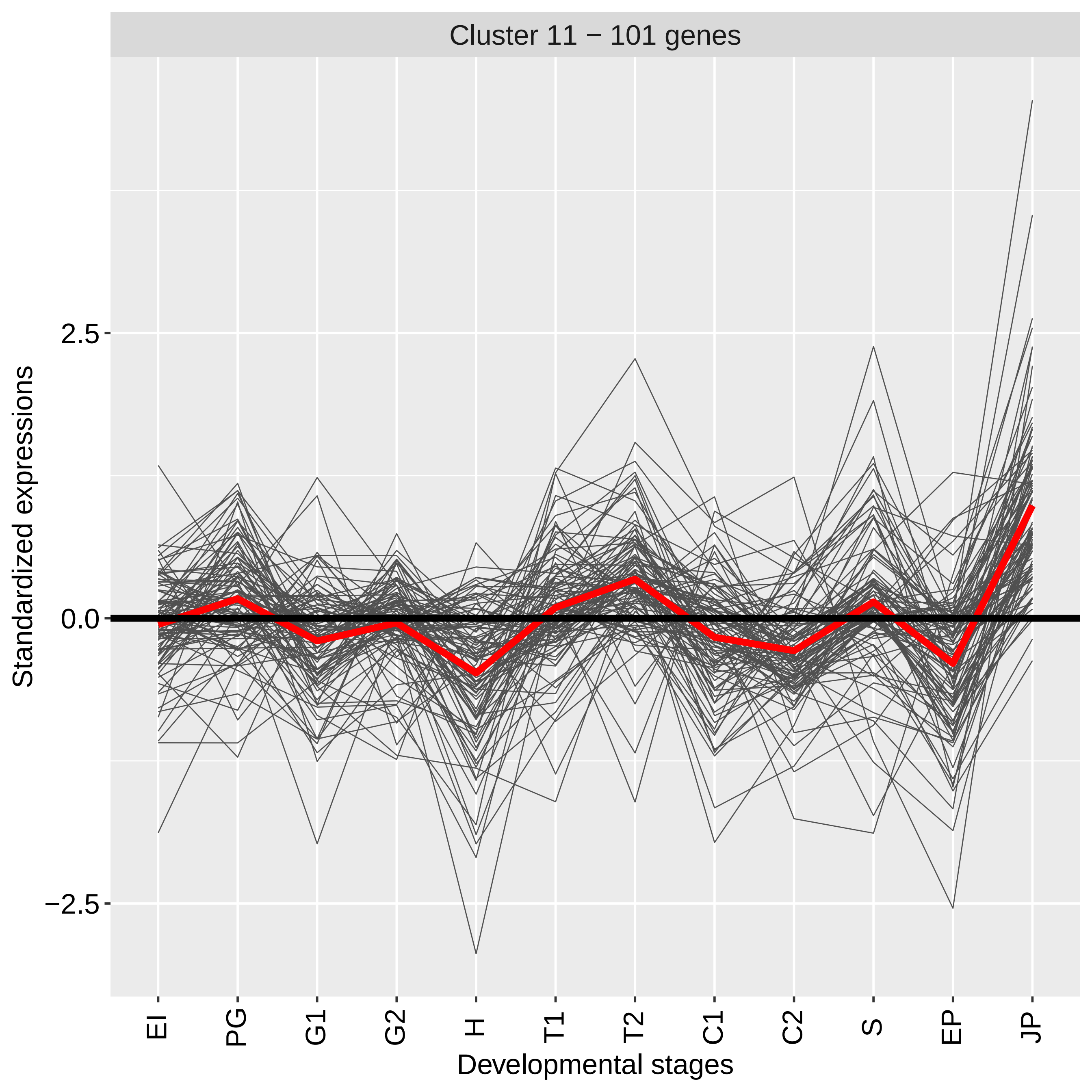
Cluster 11 − 101 genes
Standardized expressions
2.5
0.0
−2.5
EI
PG
G1
G2
H
T1
T2
C1
C2
S
EP
JP
Developmental stages

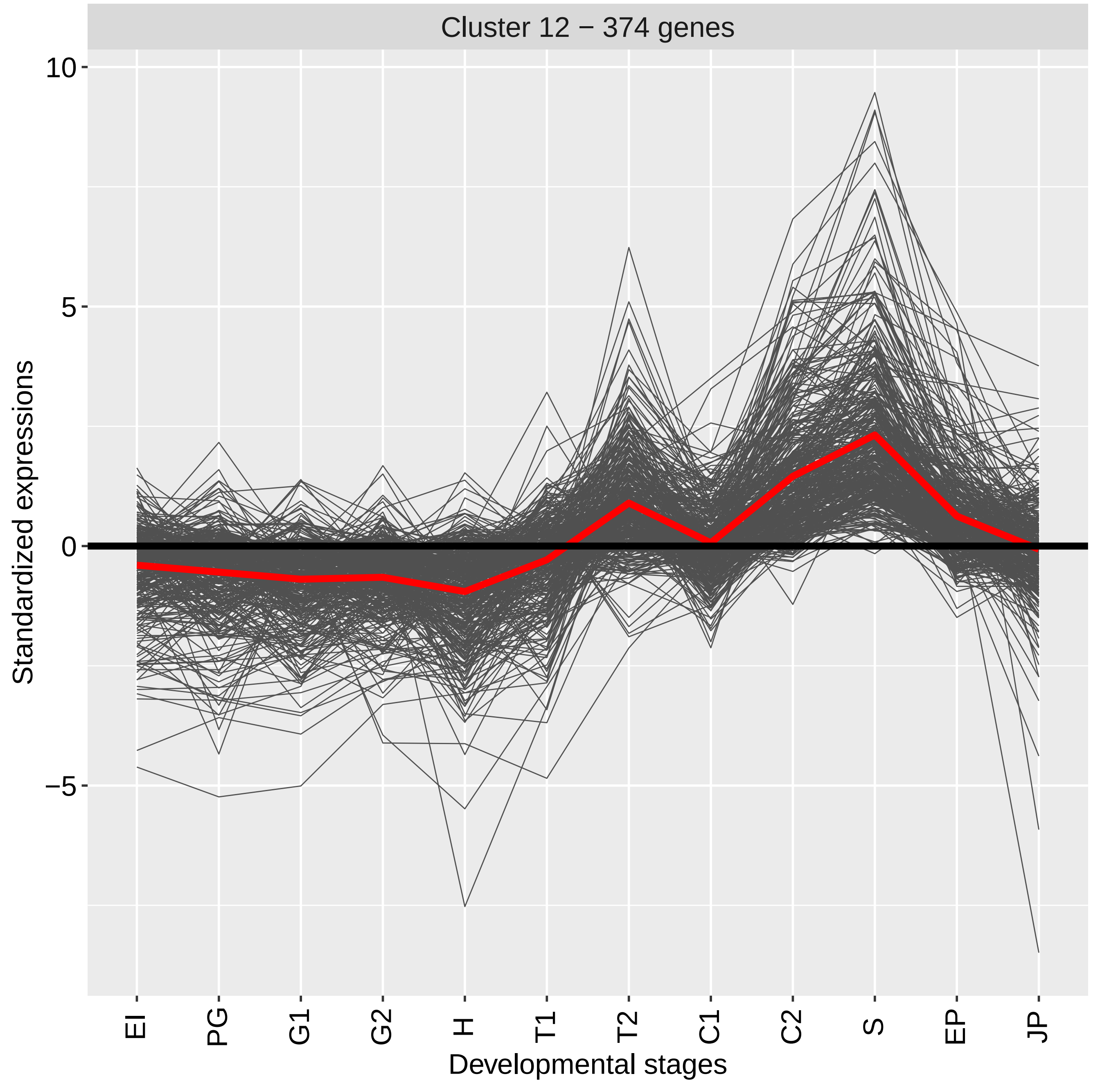

Cluster 12 − 374 genes
10
5
0
−5
Standardized expressions
EI
PG
G1
G2
H
T1
T2
C1
C2
S
EP
JP
Developmental stages

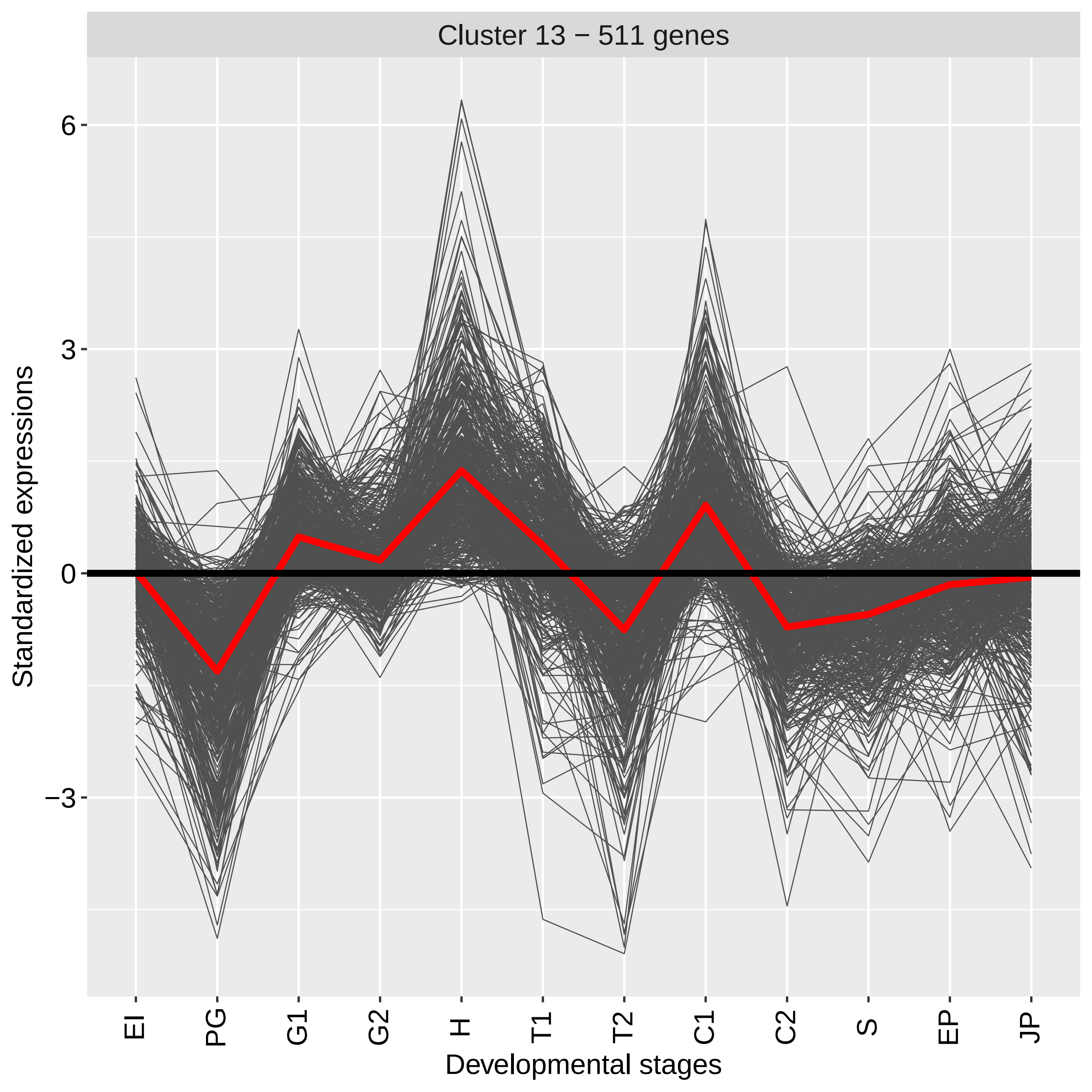

Cluster 13 − 511 genes
Standardized expressions
6
3
0
−3
EI
PG
G1
G2
H
T1
T2
C1
C2
S
EP
JP
Developmental stages

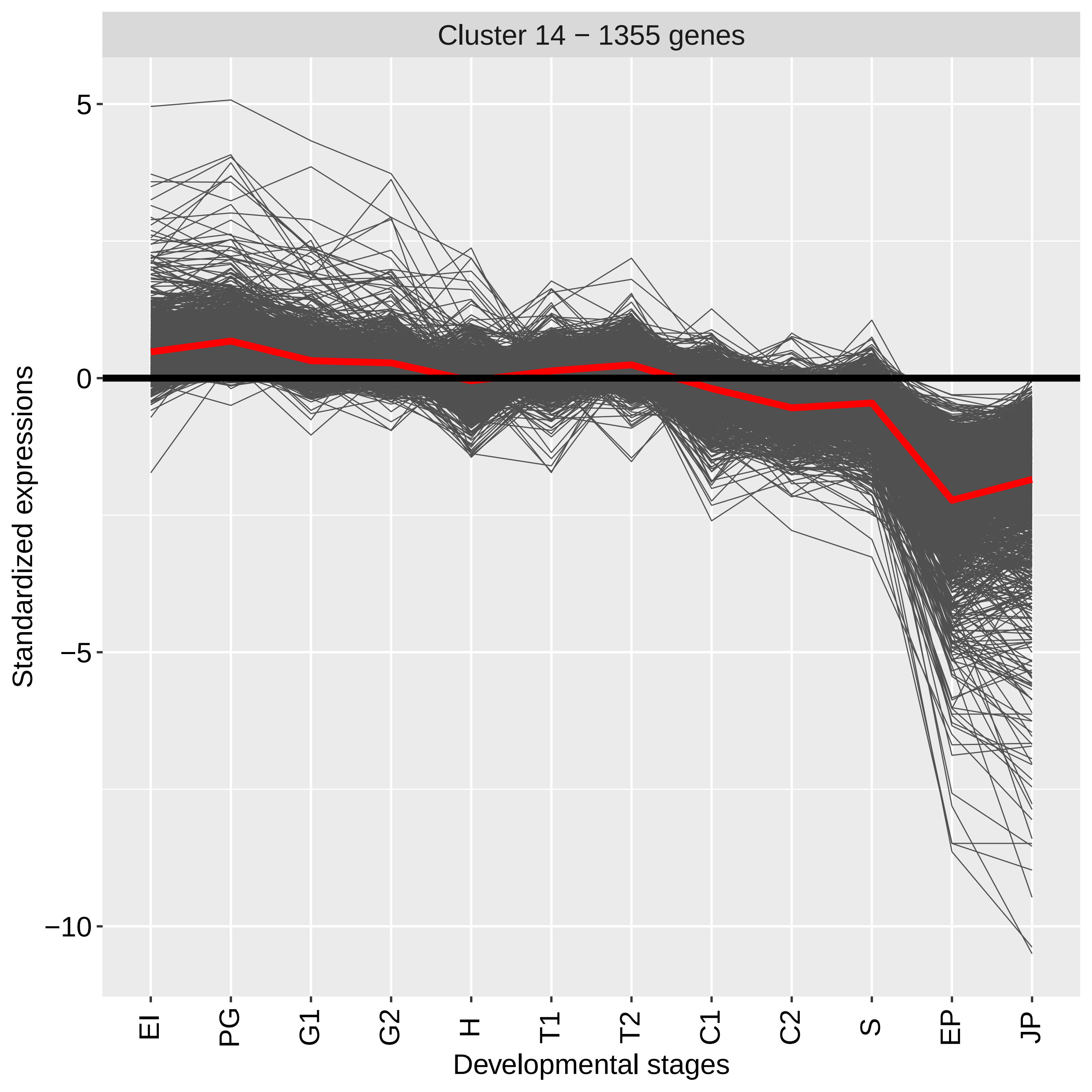

Cluster 14 − 1355 genes
5
0
−5
−10
Standardized expressions
EI
PG
G1
G2
H
T1
T2
C1
C2
S
EP
JP
Developmental stages

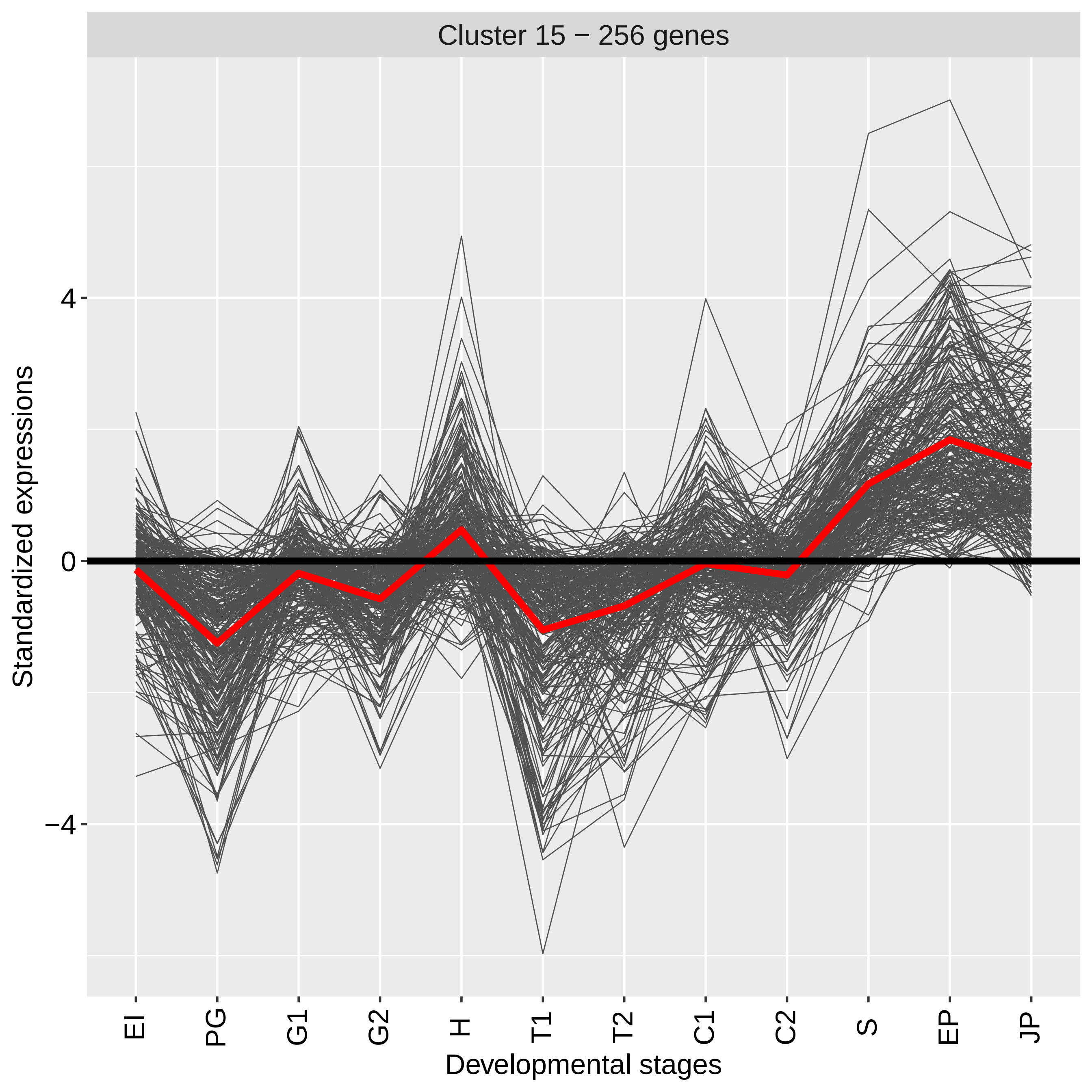
Cluster 15 − 256 genes
Standardized expressions
4
0
−4
EI
PG
G1
G2
H
T1
T2
C1
C2
S
EP
JP
Developmental stages

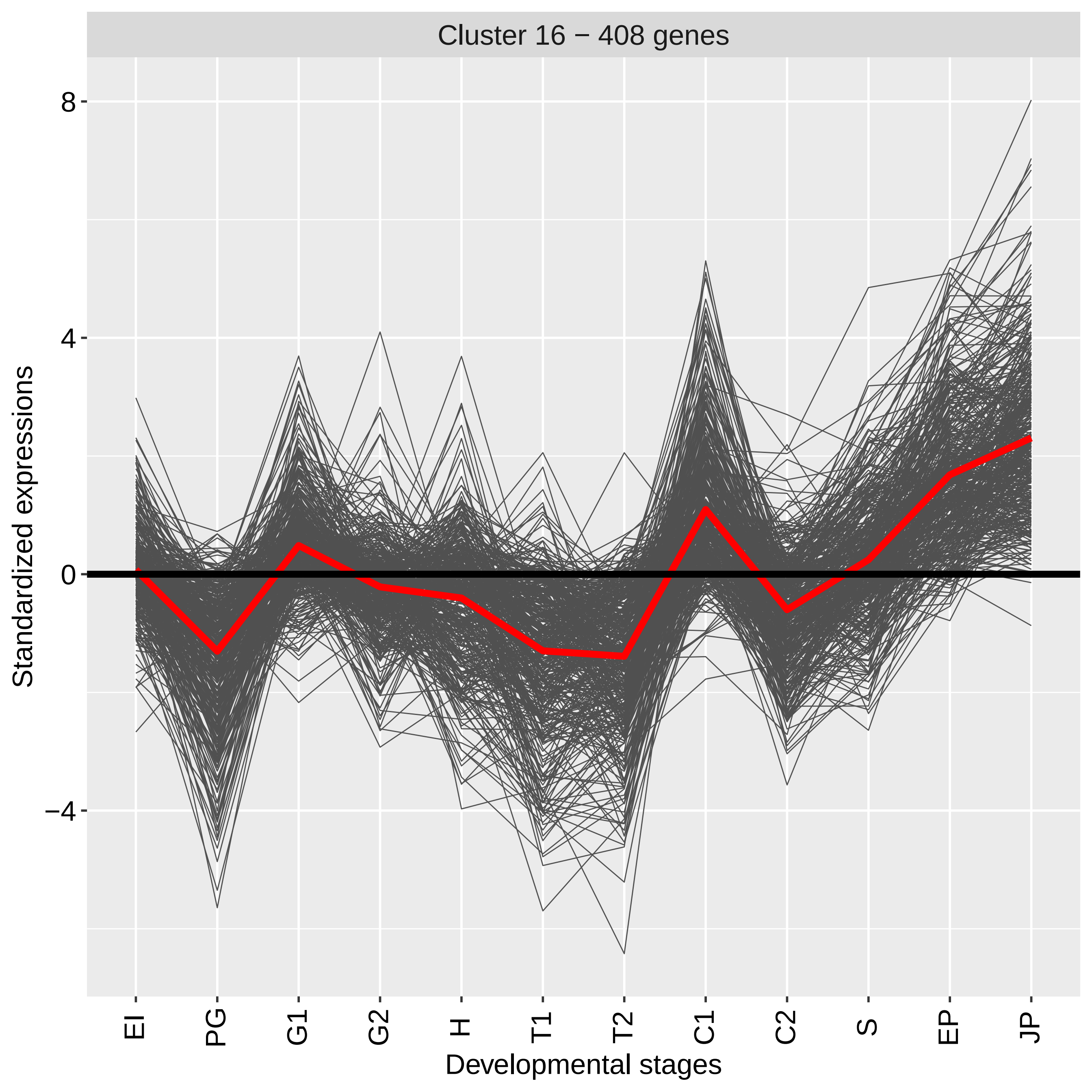
Cluster 16 − 408 genes
8
4
0
−4
Standardized expressions
EI
PG
G1
G2
H
T1
T2
C1
C2
S
EP
JP
Developmental stages

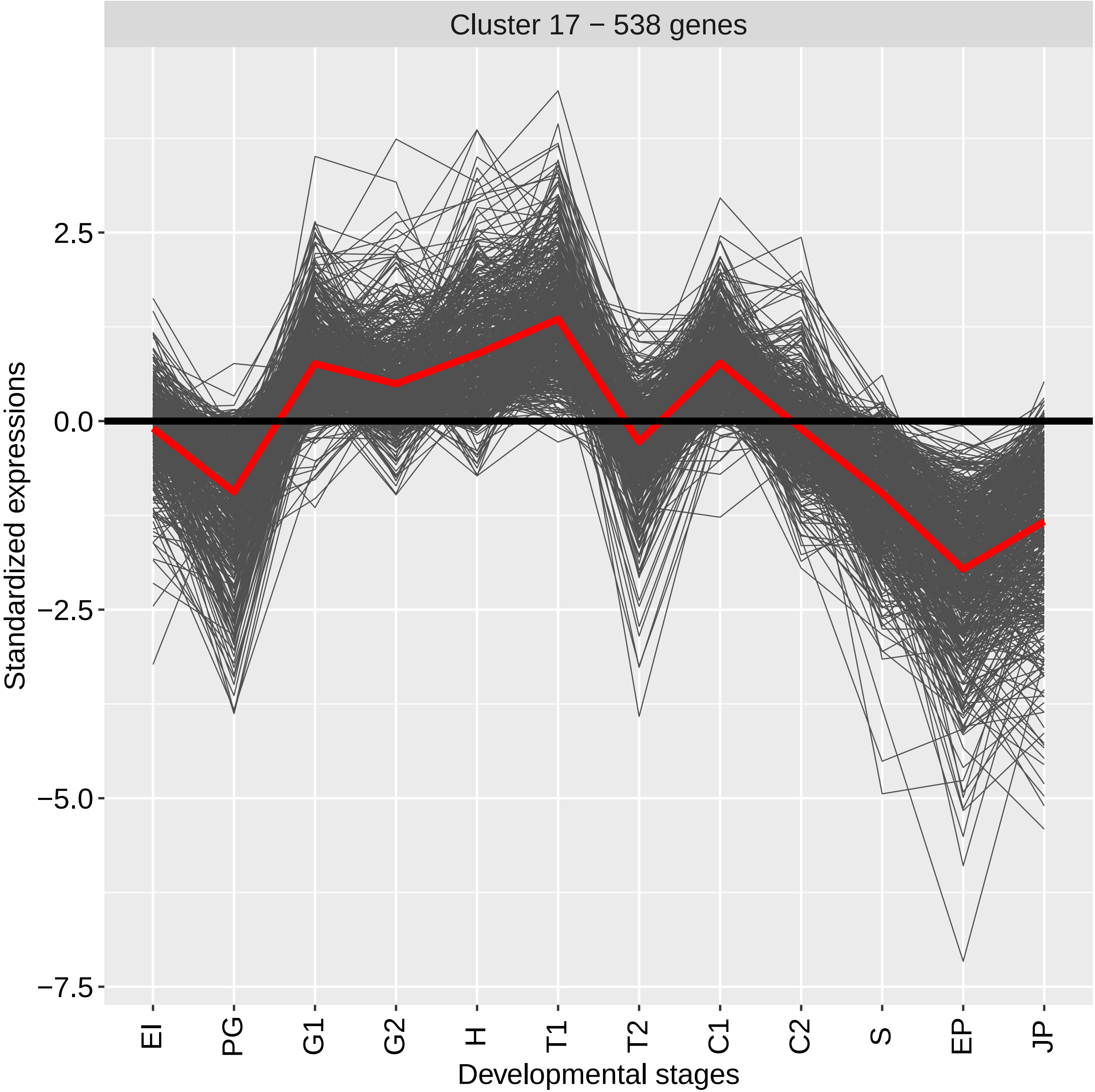
Cluster 17 − 538 genes
Standardized expressions
2.5
0.0
−2.5
−5.0
−7.5
EI
PG
G1
G2
H
T1
T2
C1
C2
S
EP
JP
Developmental stages

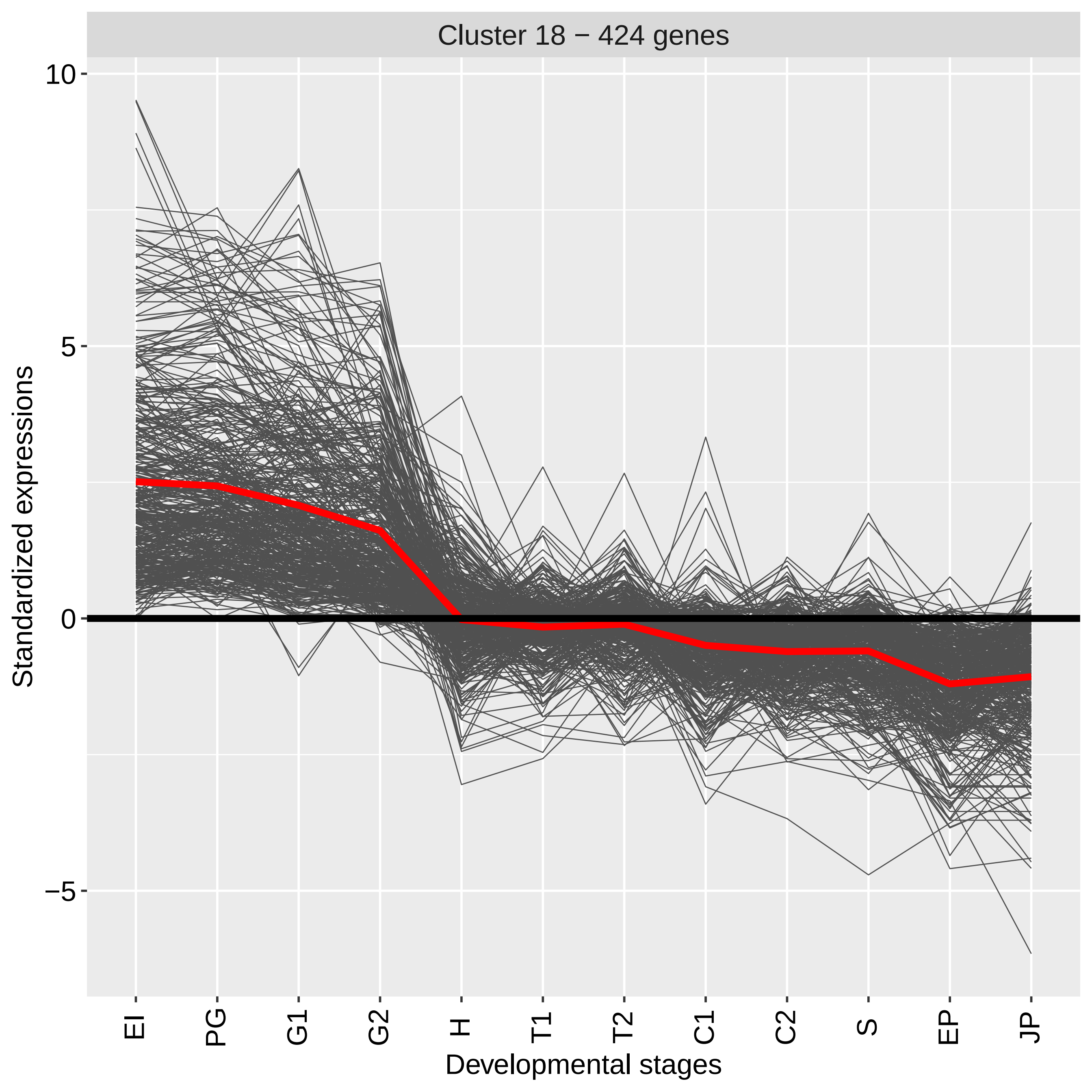
Cluster 18 − 424 genes
Standardized expressions
10
5
0
−5
EI
PG
G1
G2
H
T1
T2
C1
C2
S
EP
JP
Developmental stages

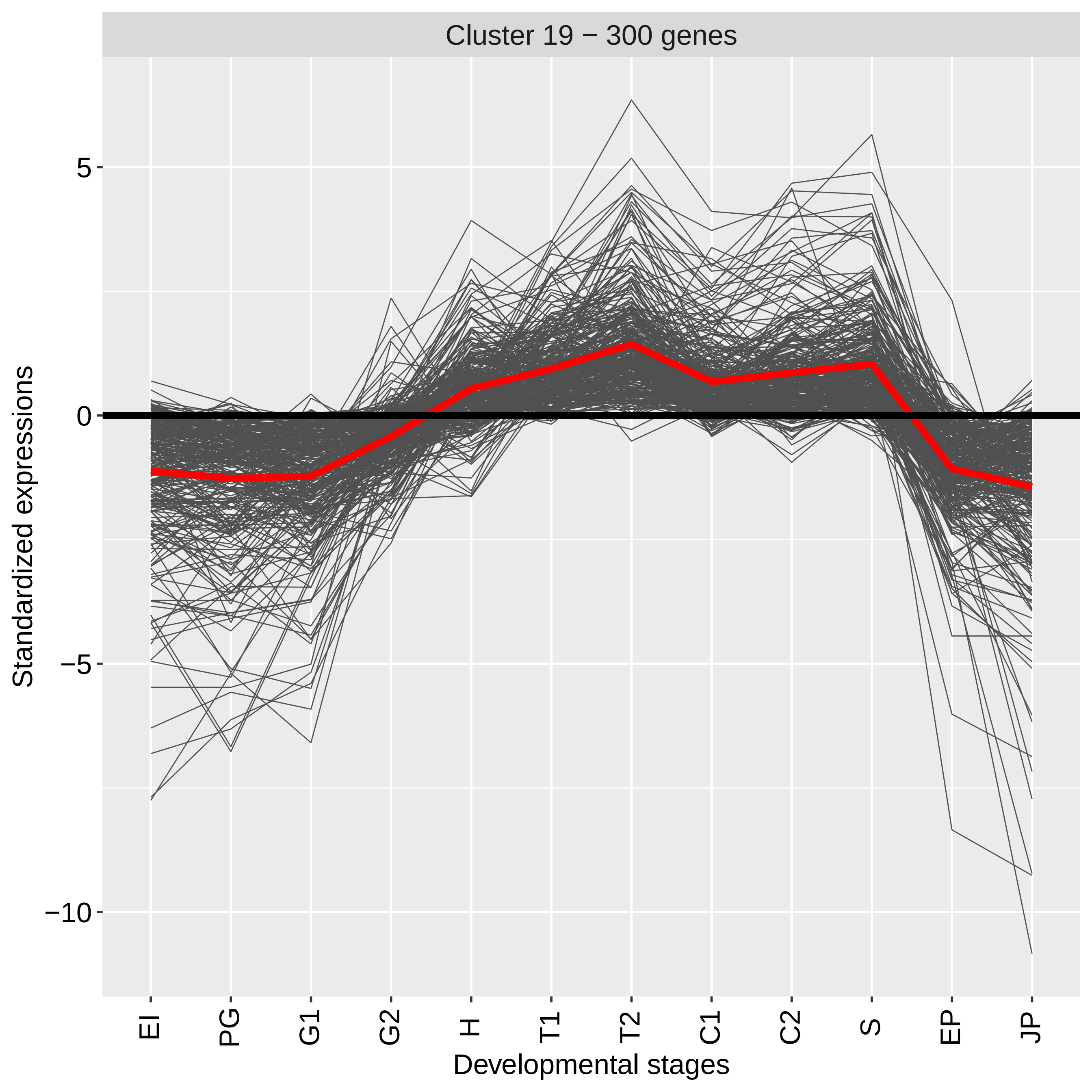

Cluster 19 − 300 genes
5
0
−5
−10
Standardized expressions
EI
PG
G1
G2
H
T1
T2
C1
C2
S
EP
JP
Developmental stages

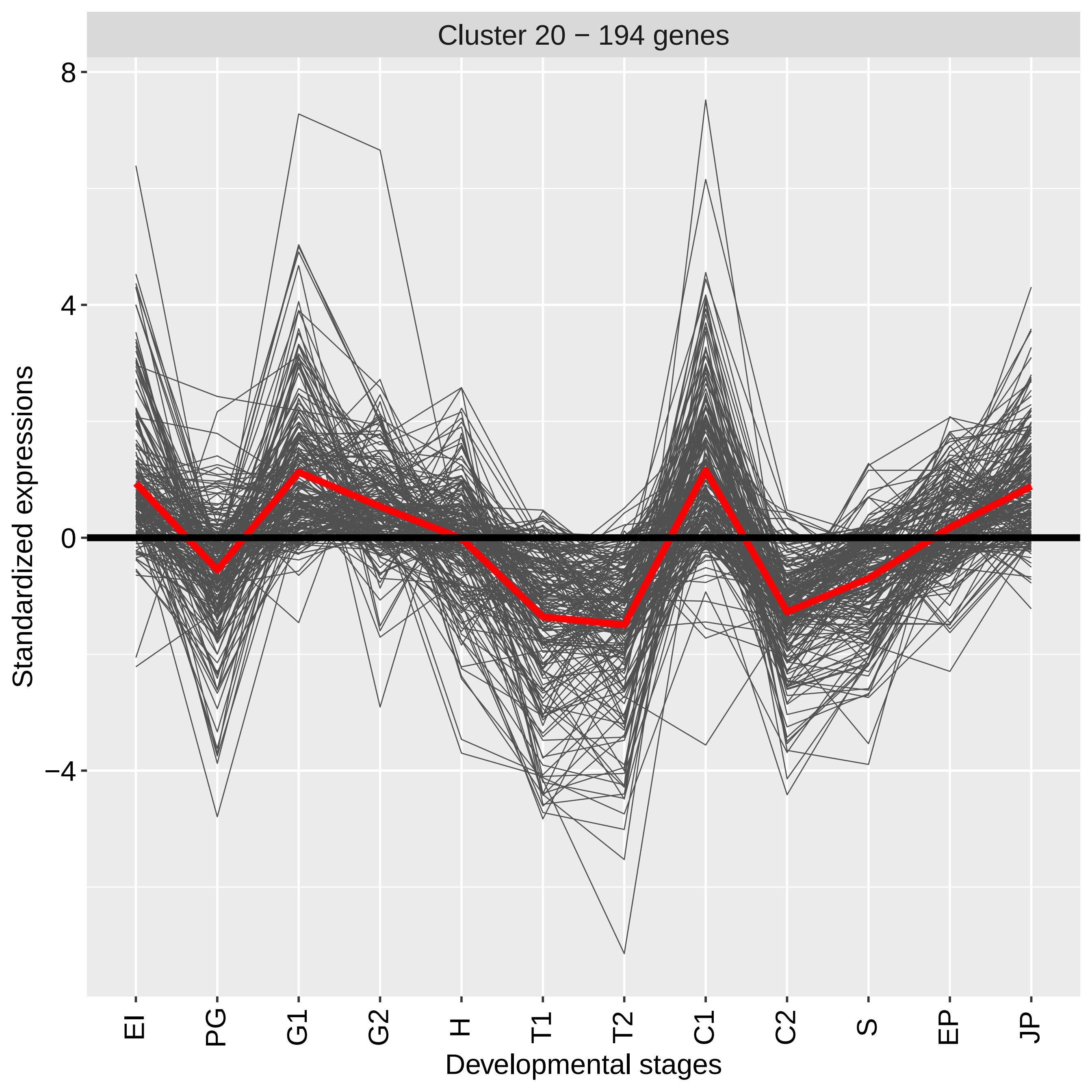

Cluster 20 − 194 genes
Standardized expressions
8
4
0
−4
EI
PG
G1
G2
H
T1
T2
C1
C2
S
EP
JP
Developmental stages

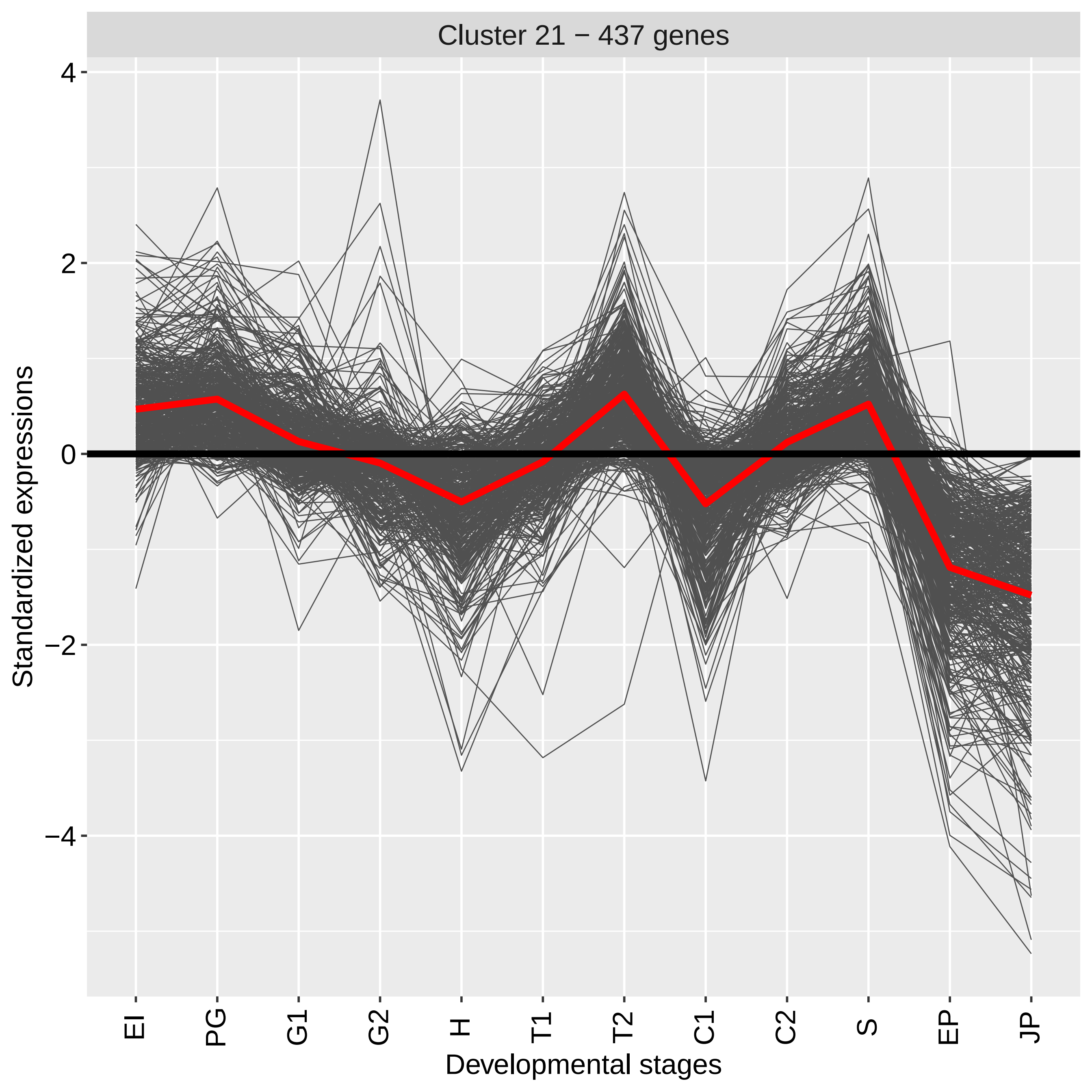

Cluster 21 − 437 genes
Standardized expressions
4
2
0
−2
−4
EI
PG
G1
G2
H
T1
T2
C1
C2
S
EP
JP
Developmental stages

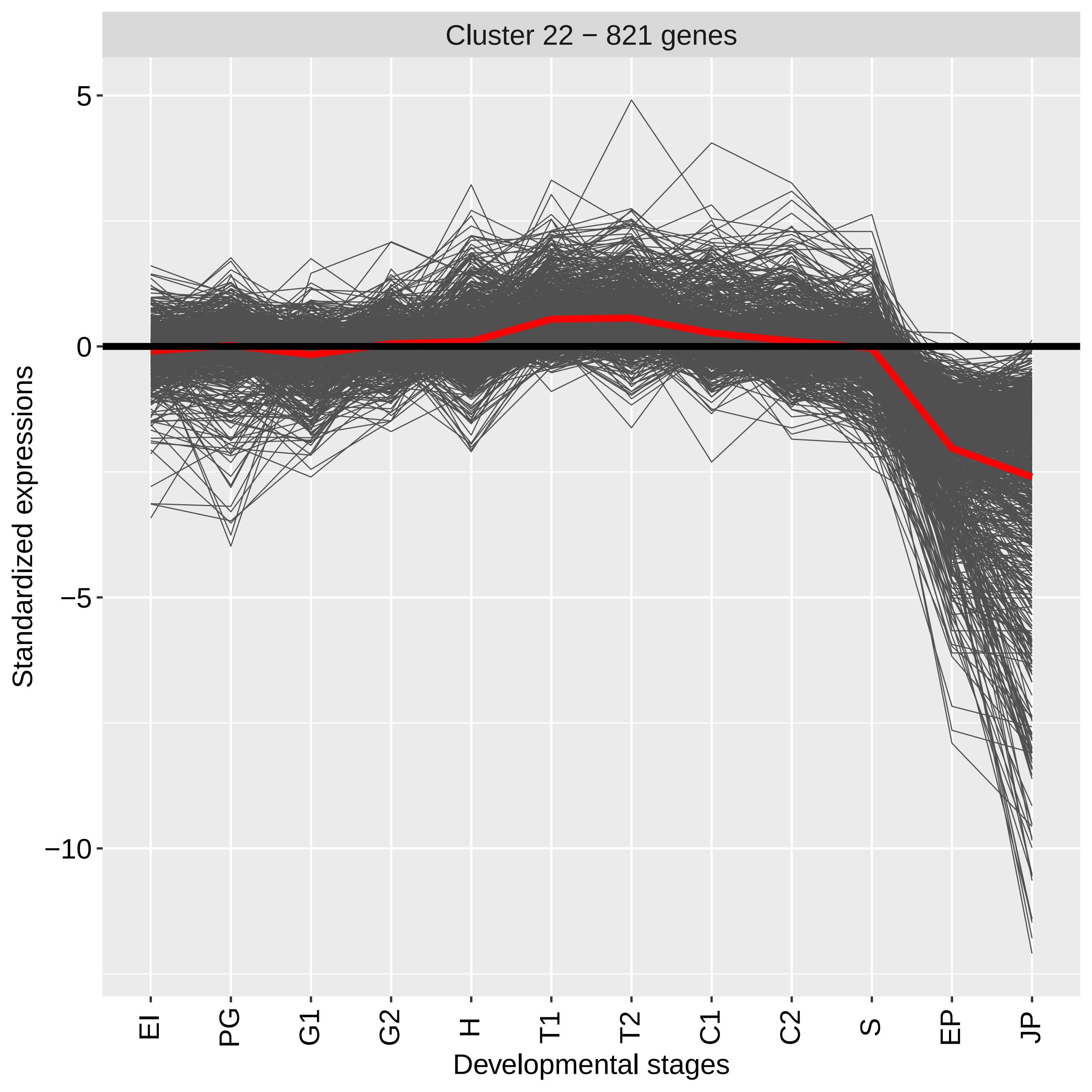

Cluster 22 − 821 genes
Standardized expressions
5
0
−5
−10
EI
PG
G1
G2
H
T1
T2
C1
C2
S
EP
JP
Developmental stages

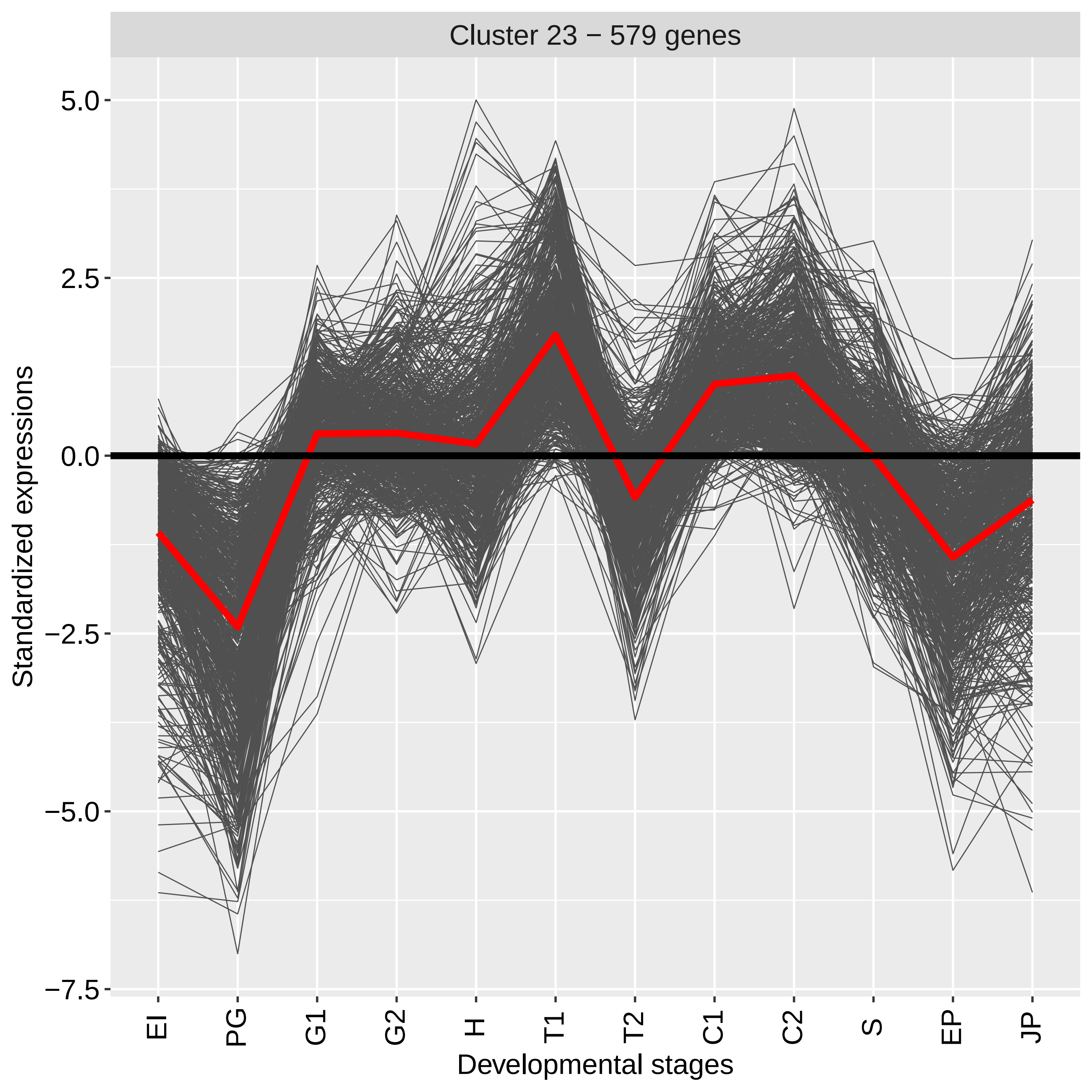

Cluster 23 − 579 genes
5.0
2.5
0.0
−2.5
−5.0
−7.5
Standardized expressions
EI
PG
G1
G2
H
T1
T2
C1
C2
S
EP
JP
Developmental stages

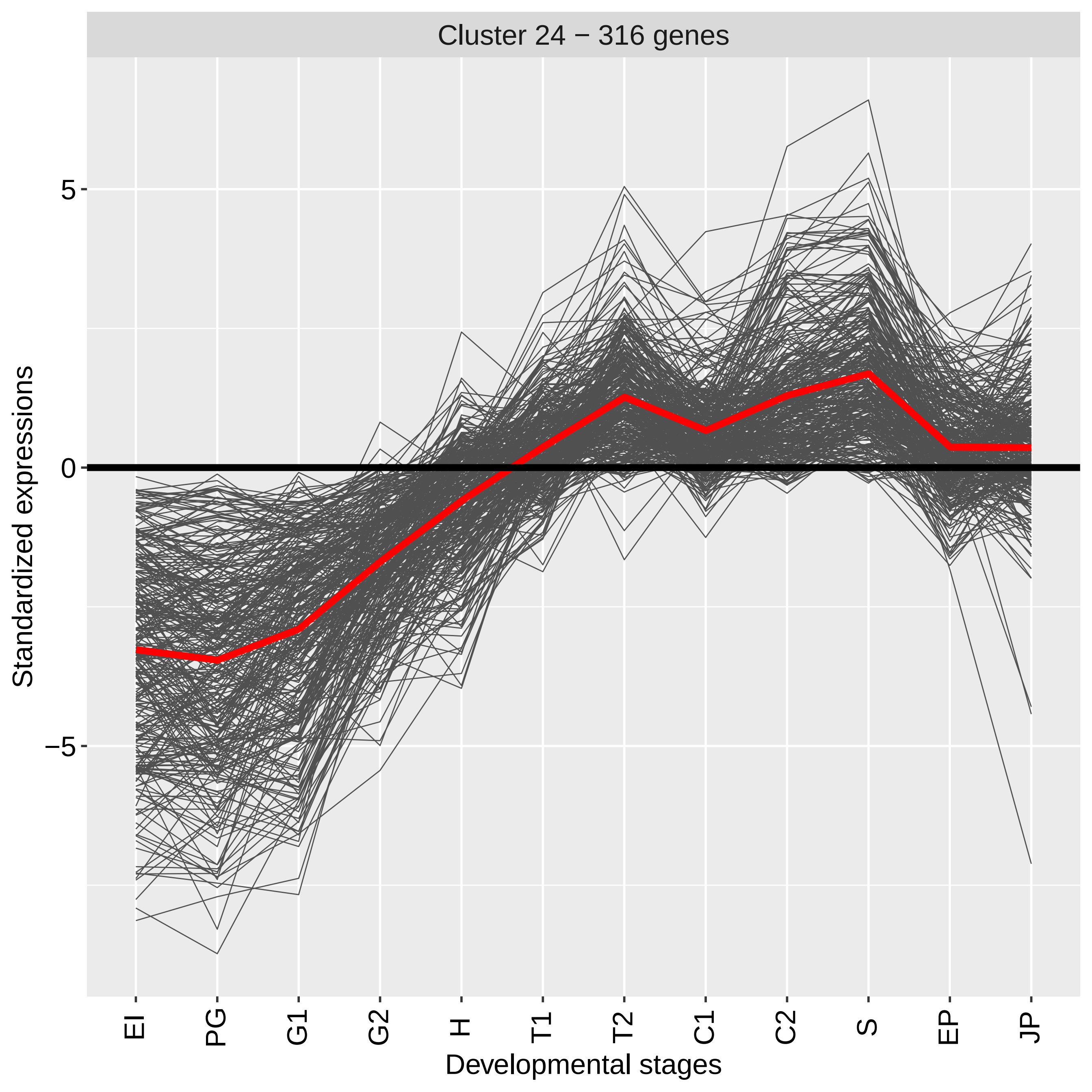
Cluster 24 − 316 genes
Standardized expressions
5
0
−5
EI
PG
G1
G2
H
T1
T2
C1
C2
S
EP
JP
Developmental stages

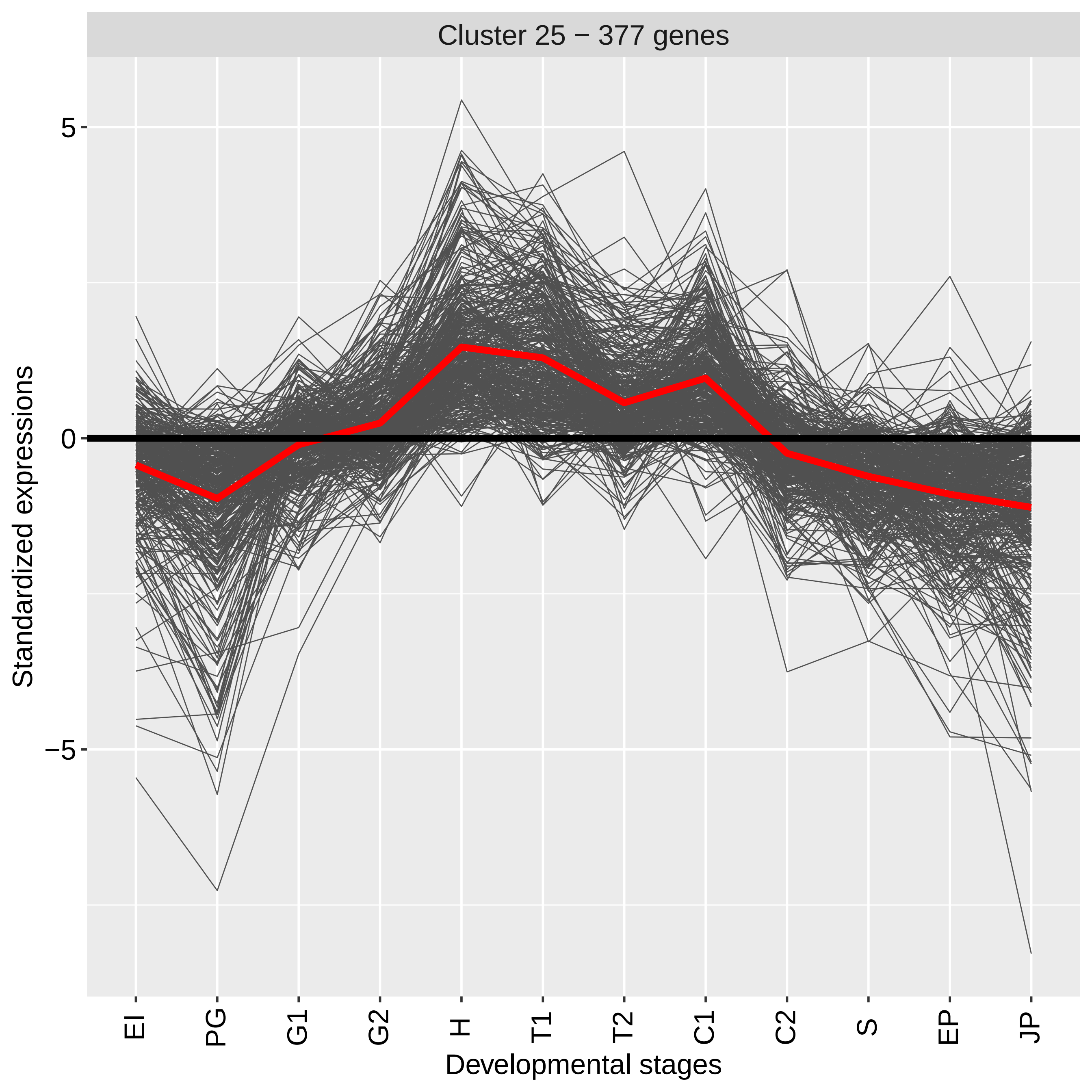
Cluster 25 − 377 genes
Standardized expressions
5
0
−5
EI
PG
G1
G2
H
T1
T2
C1
C2
S
EP
JP
Developmental stages

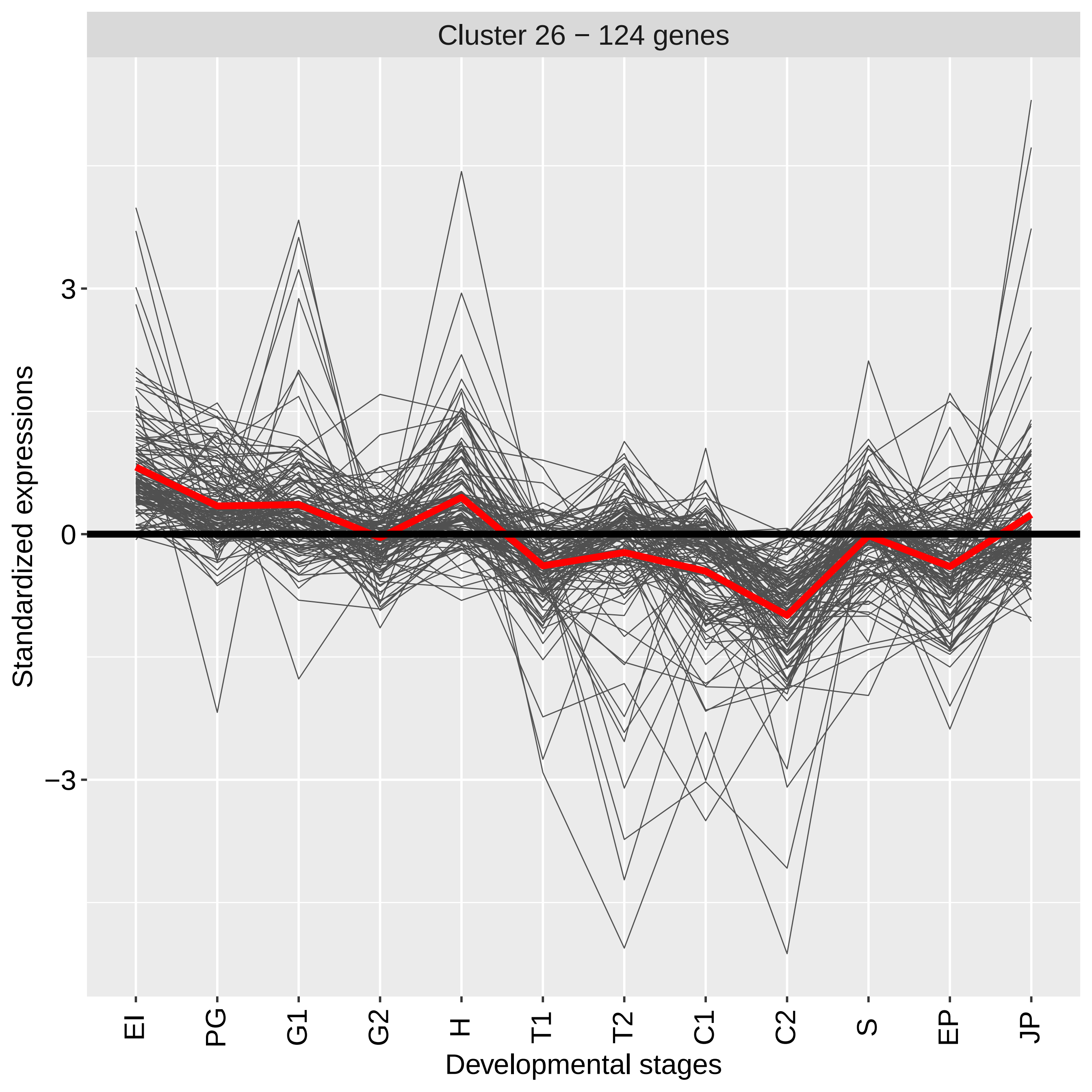
Cluster 26 − 124 genes
Standardized expressions
3
0
−3
EI
PG
G1
G2
H
T1
T2
C1
C2
S
EP
JP
Developmental stages

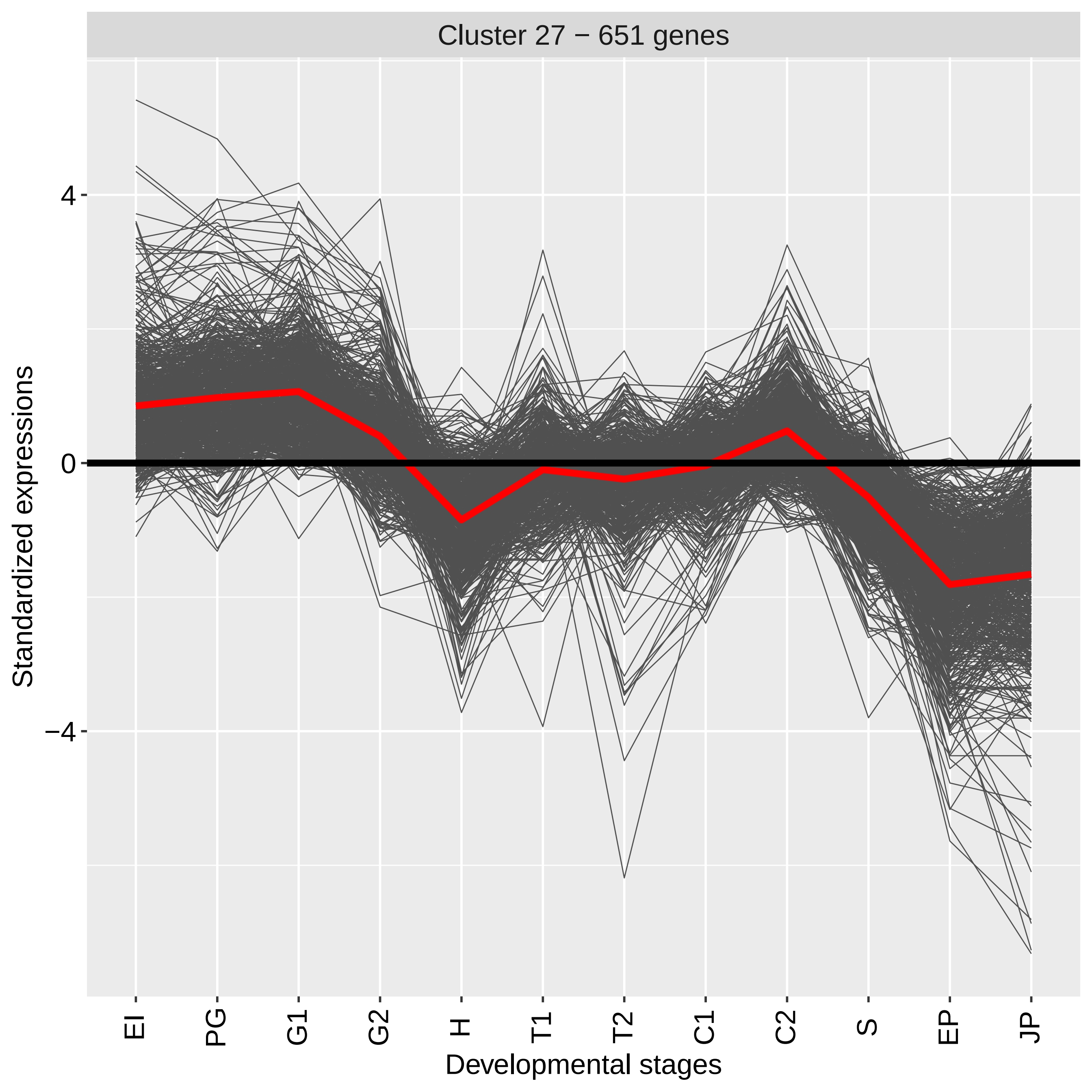

Cluster 27 − 651 genes
Standardized expressions
4
0
−4
EI
PG
G1
G2
H
T1
T2
C1
C2
S
EP
JP
Developmental stages

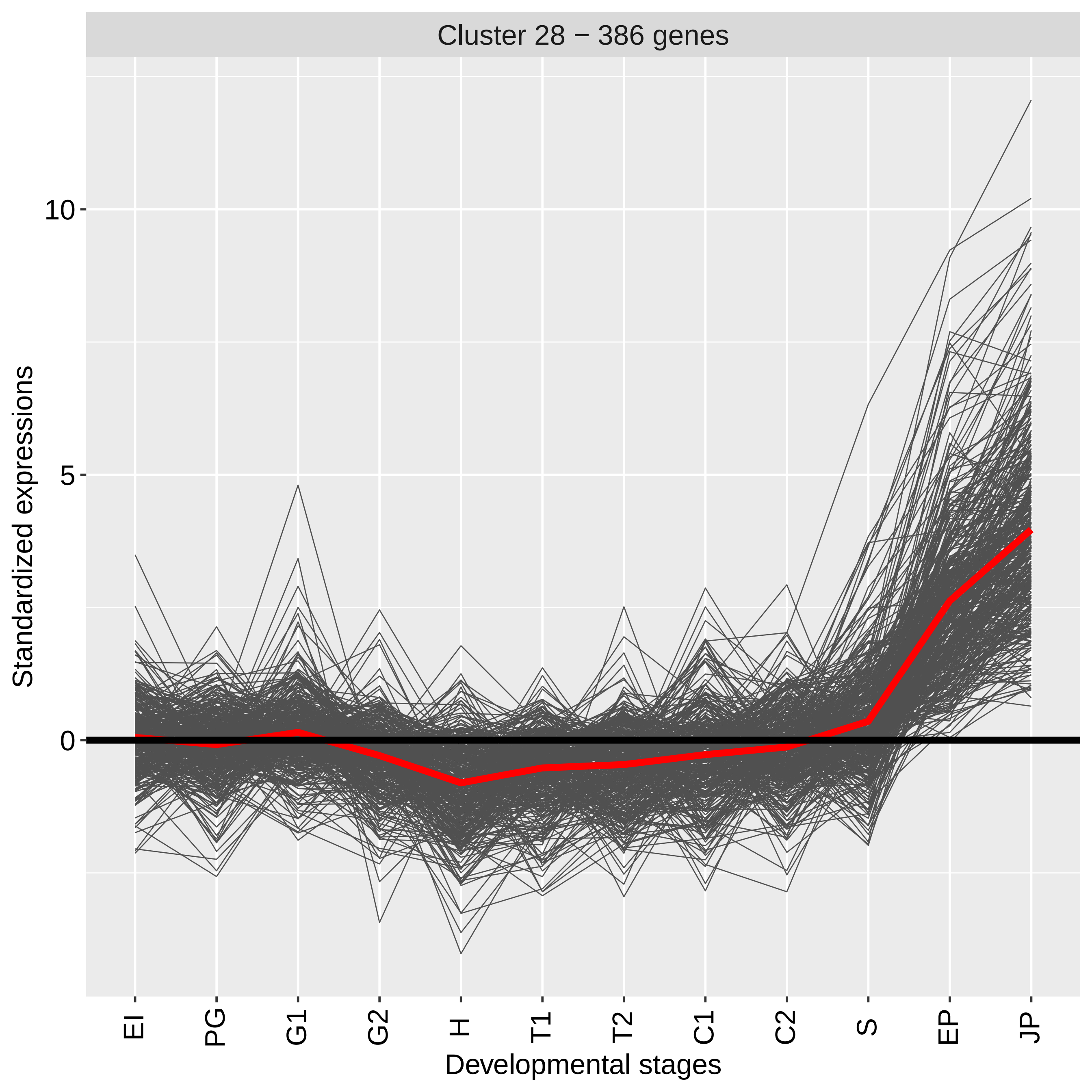

Cluster 28 − 386 genes
Standardized expressions
10
5
0
EI
PG
G1
G2
H
T1
T2
C1
C2
S
EP
JP
Developmental stages

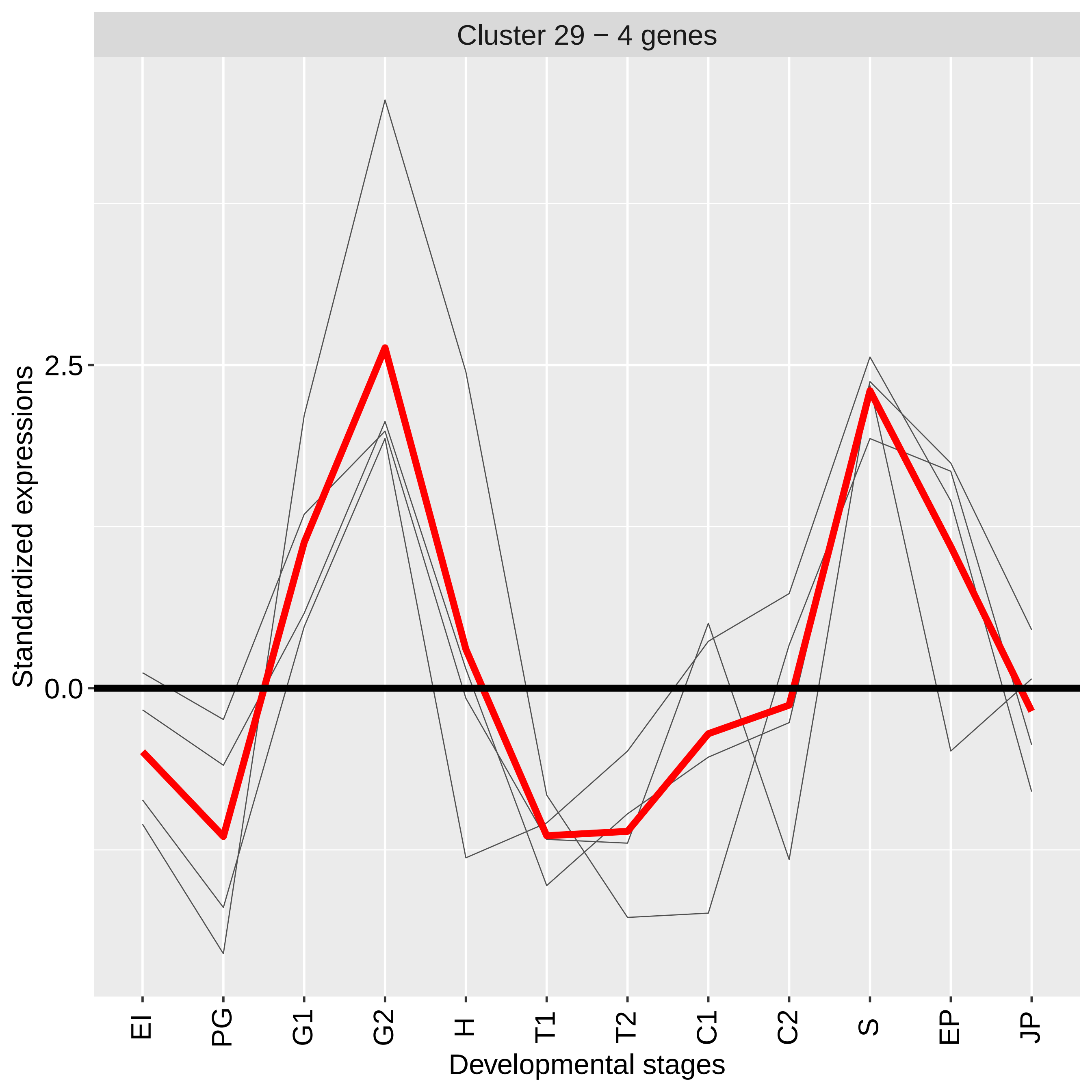

Cluster 29 − 4 genes
Standardized expressions
2.5
0.0
EI
PG
G1
G2
H
T1
T2
C1
C2
S
EP
JP
Developmental stages

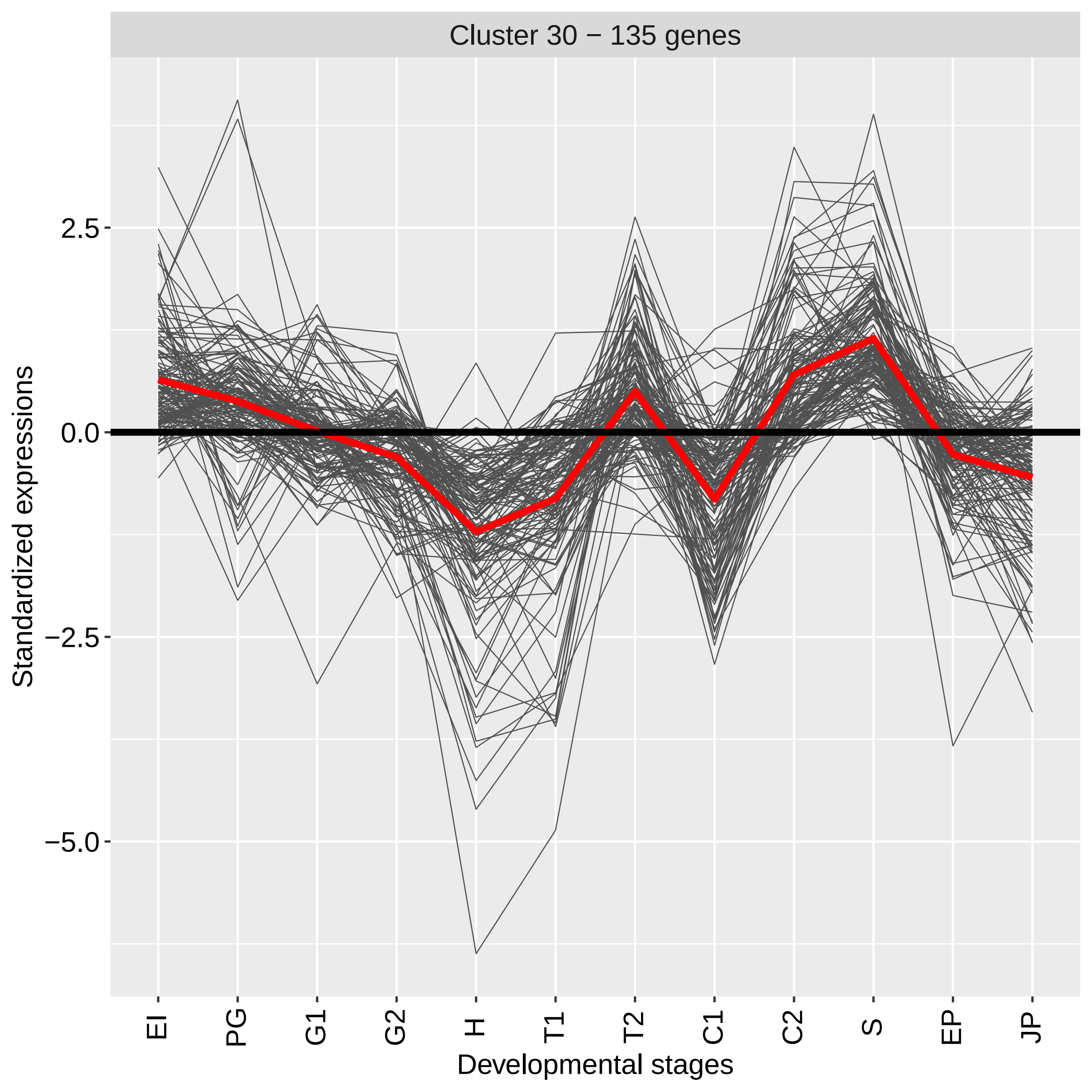

Cluster 30 − 135 genes
Standardized expressions
2.5
0.0
−2.5
−5.0
EI
PG
G1
G2
H
T1
T2
C1
C2
S
EP
JP
Developmental stages

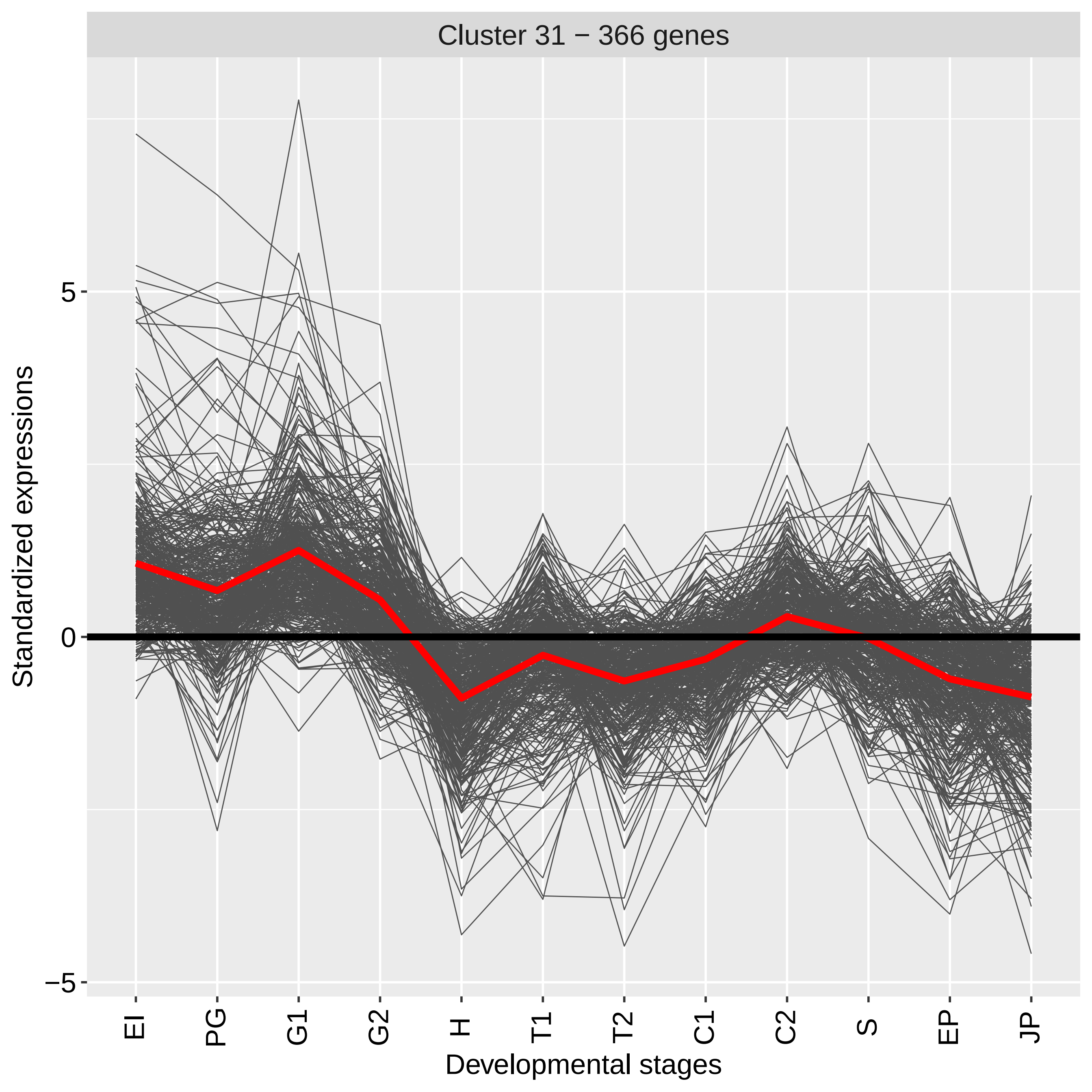

Cluster 31 − 366 genes
Standardized expressions
5
0
−5
EI
PG
G1
G2
H
T1
T2
C1
C2
S
EP
JP
Developmental stages

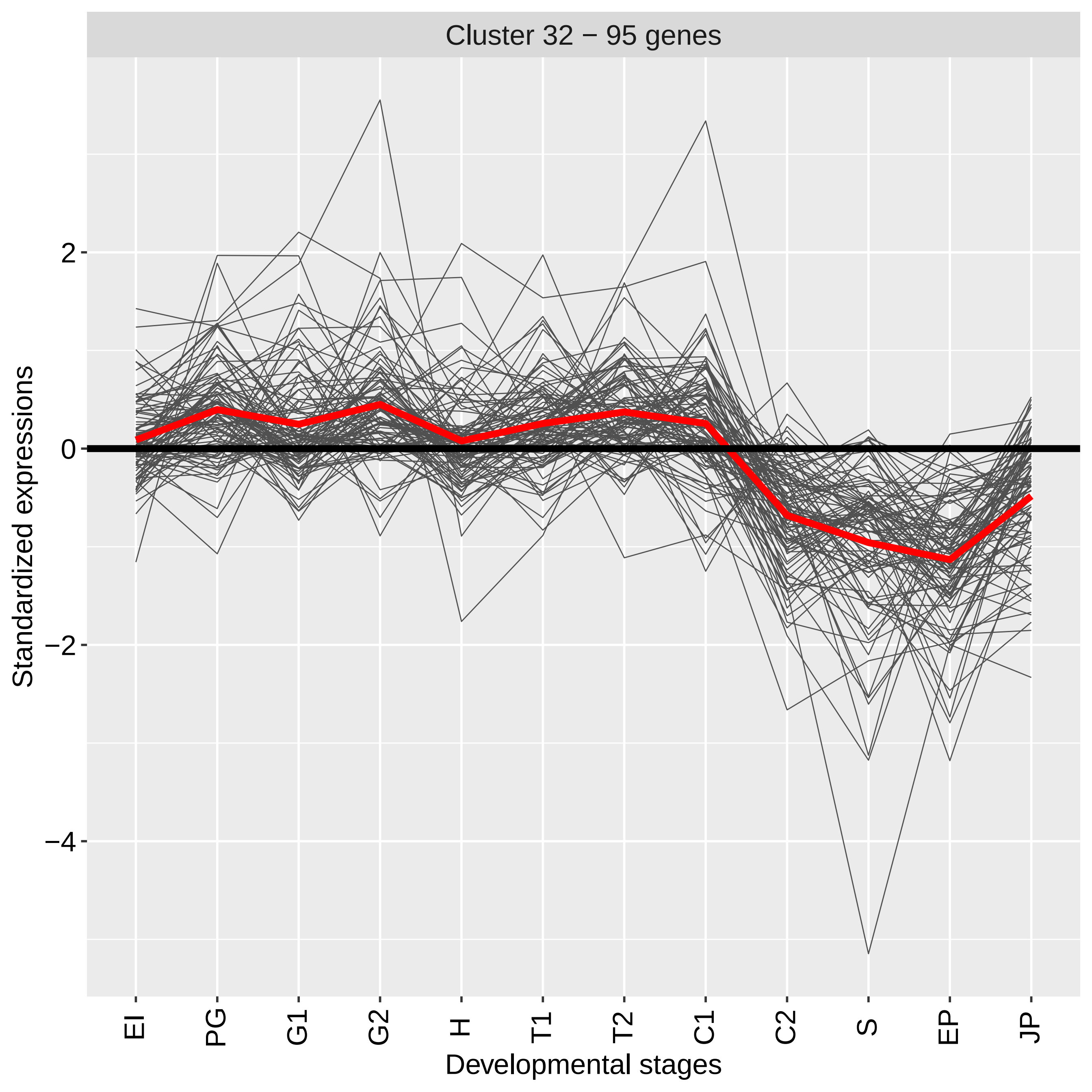
Cluster 32 − 95 genes
Standardized expressions
2
0
−2
−4
EI
PG
G1
G2
H
T1
T2
C1
C2
S
EP
JP
Developmental stages

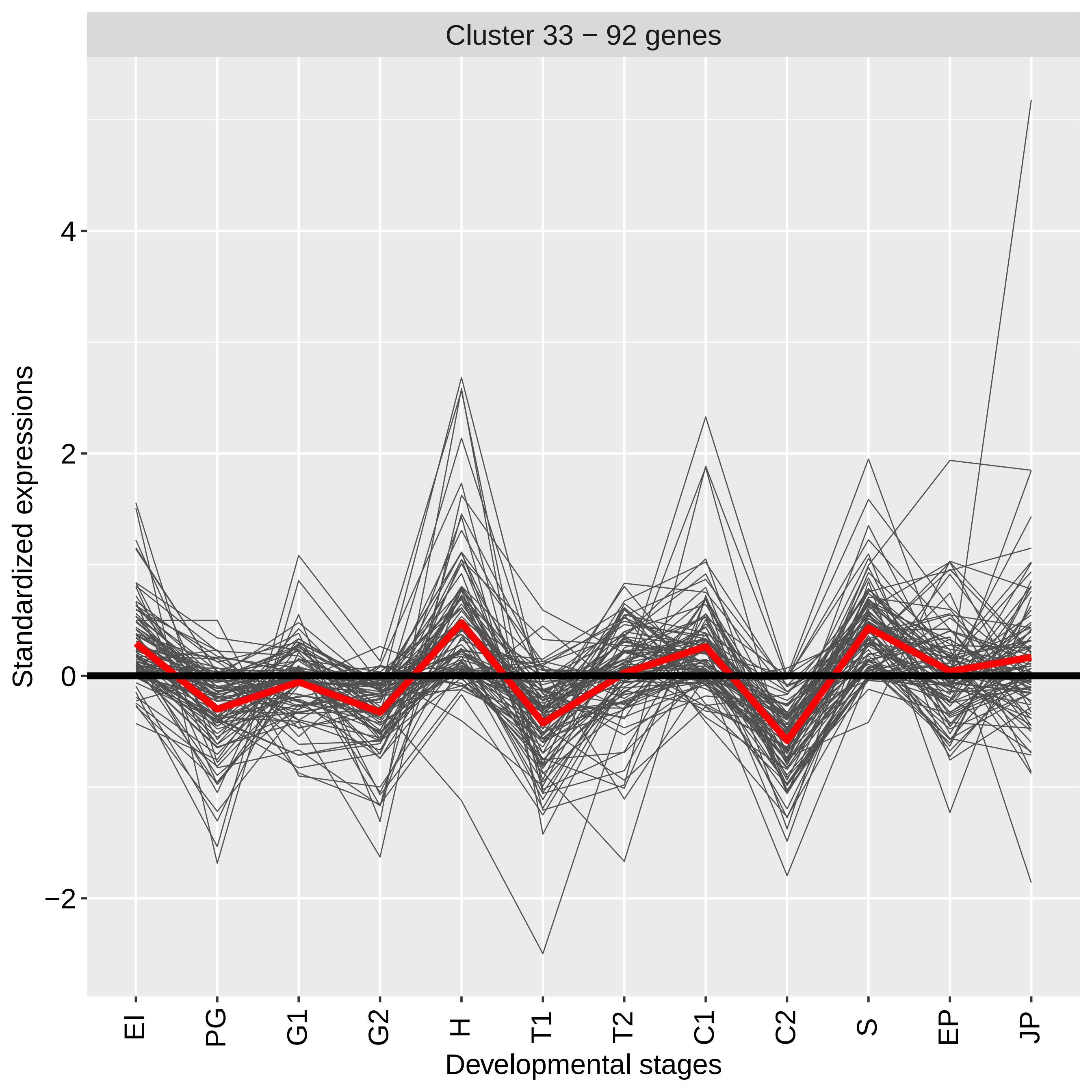
Cluster 33 − 92 genes
Standardized expressions
4
2
0
−2
EI
PG
G1
G2
H
T1
T2
C1
C2
S
EP
JP
Developmental stages

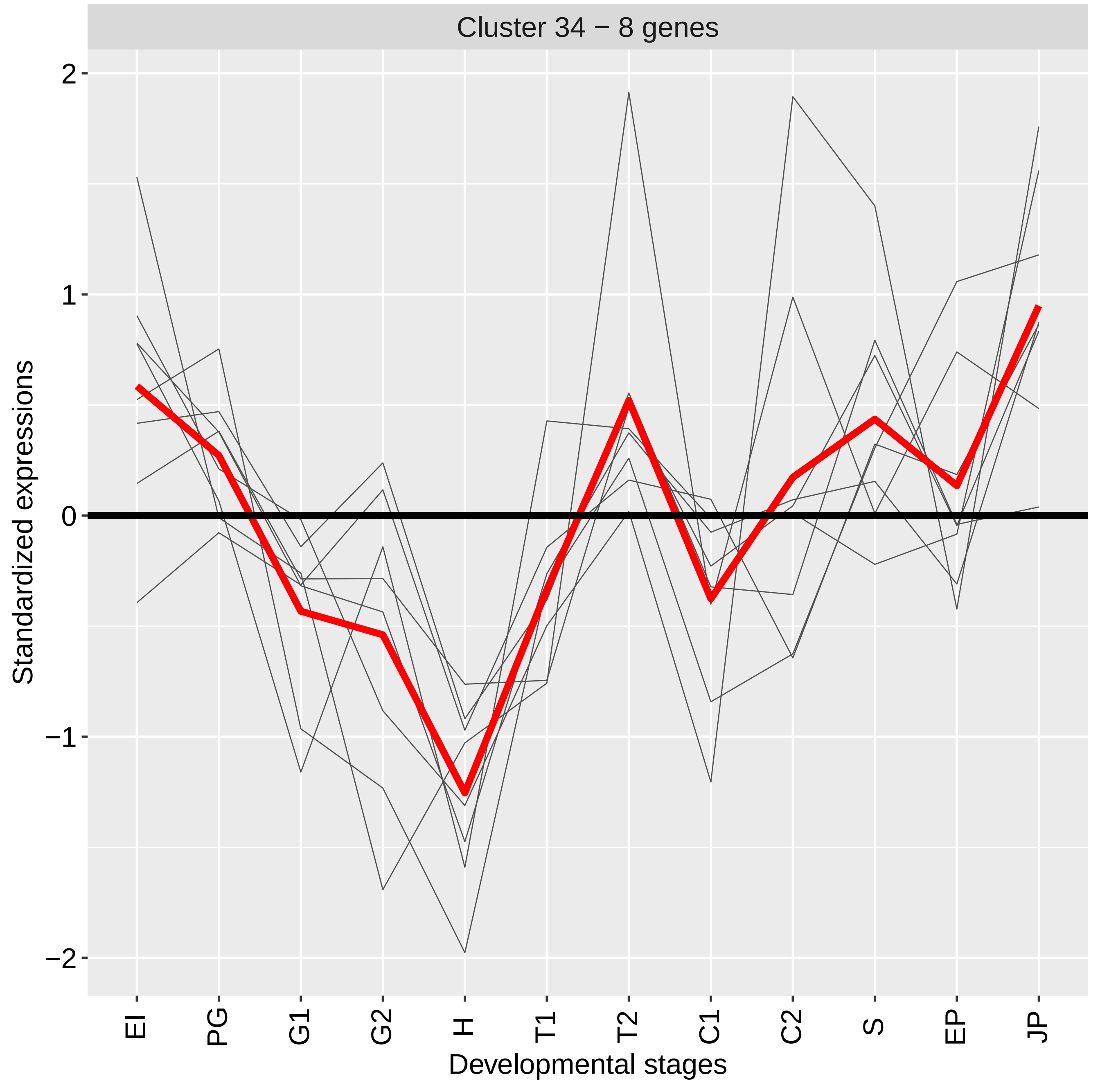
Cluster 34 − 8 genes
Standardized expressions
2
1
0
−1
−2
EI
PG
G1
G2
H
T1
T2
C1
C2
S
EP
JP
Developmental stages

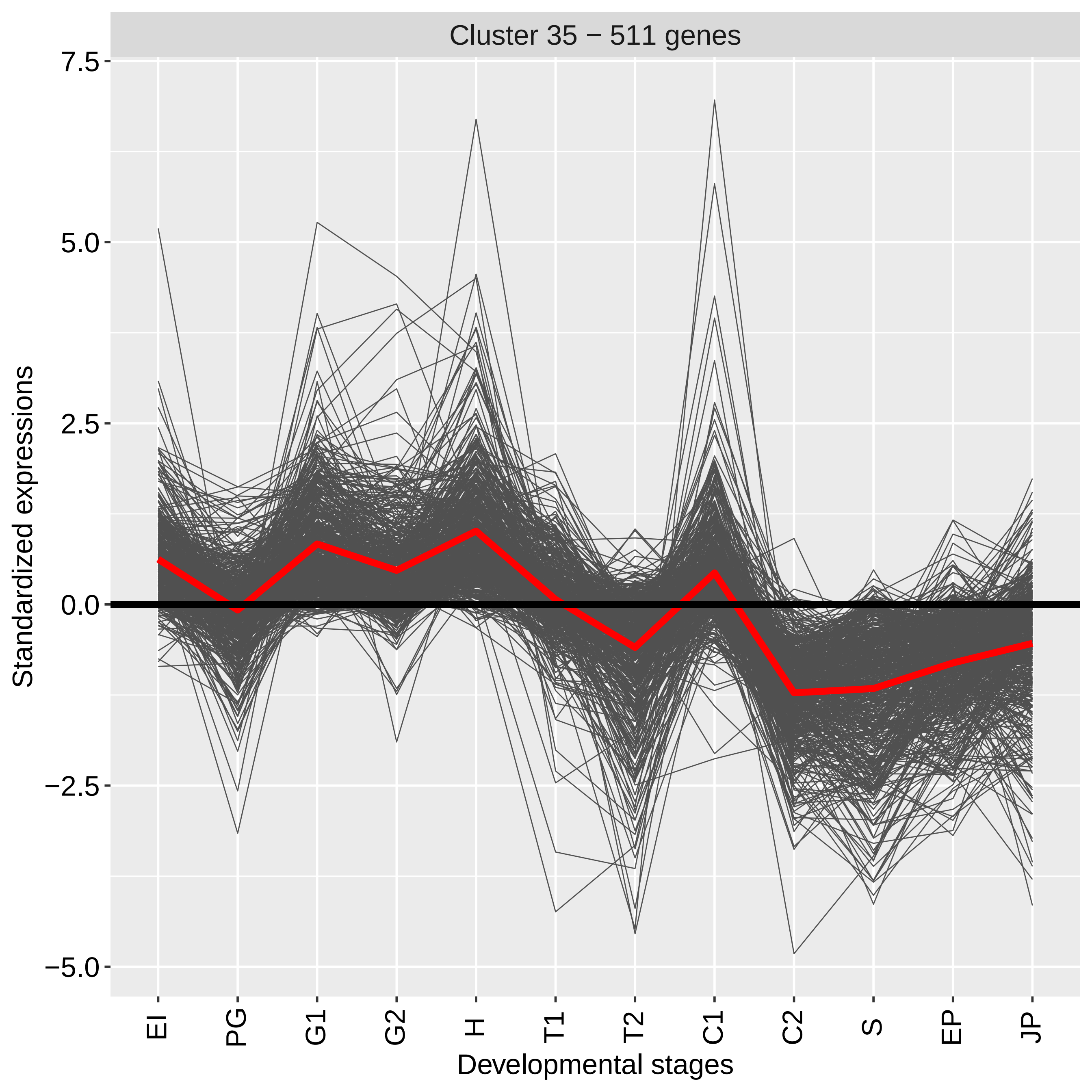

Cluster 35 − 511 genes
7.5
5.0
2.5
0.0
−2.5
−5.0
Standardized expressions
EI
PG
G1
G2
H
T1
T2
C1
C2
S
EP
JP
Developmental stages

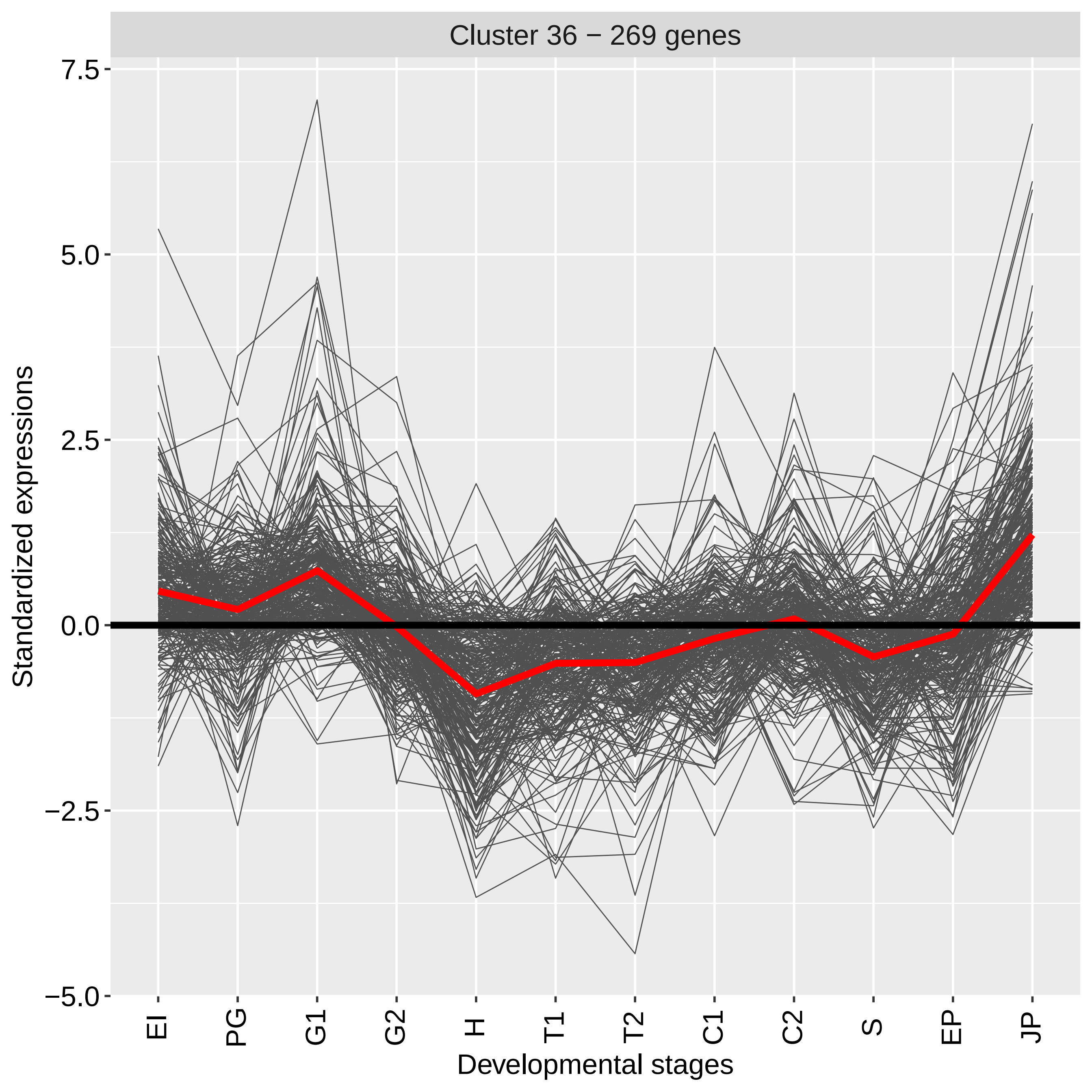

Cluster 36 − 269 genes
Standardized expressions
7.5
5.0
2.5
0.0
−2.5
−5.0
EI
PG
G1
G2
H
T1
T2
C1
C2
S
EP
JP
Developmental stages

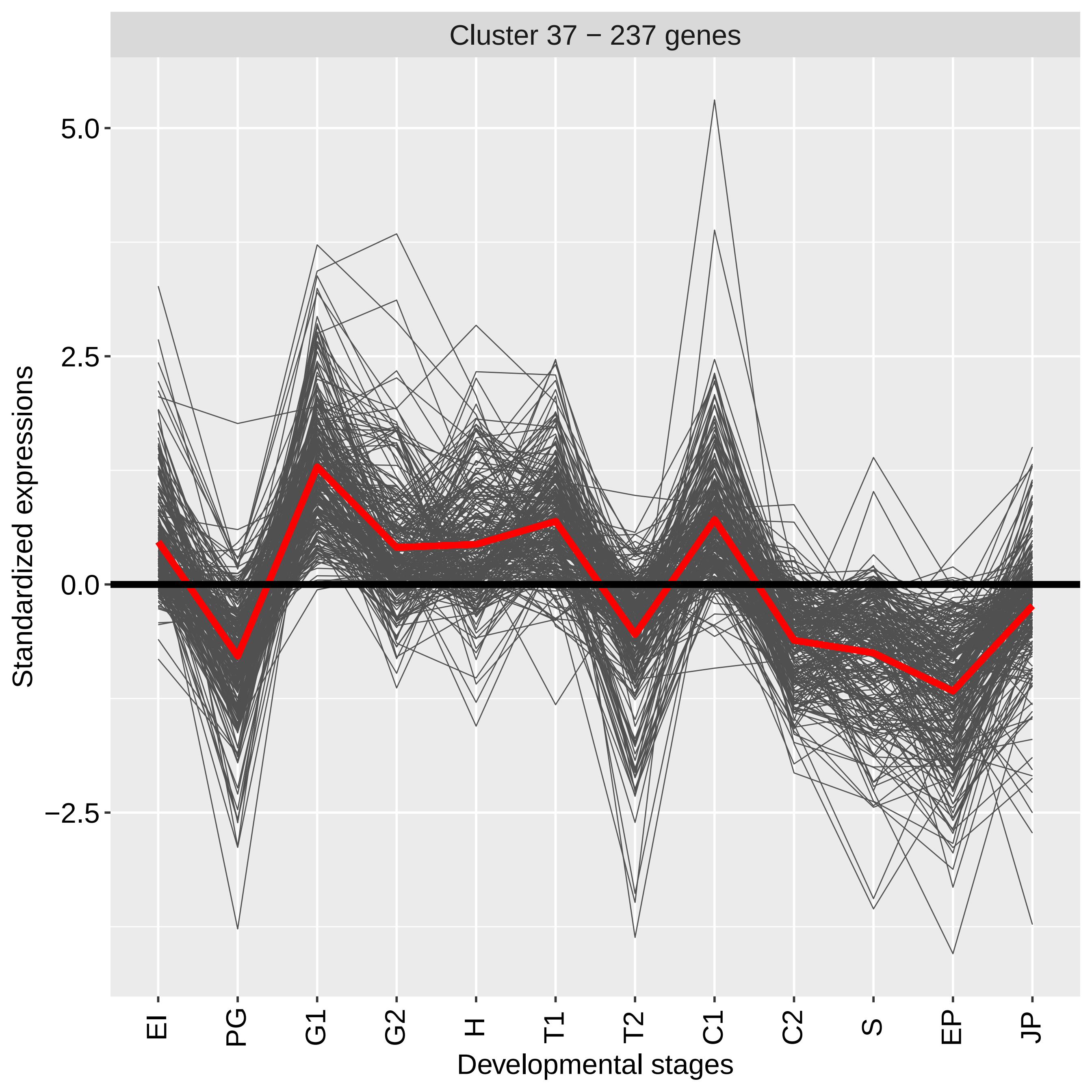
Cluster 37 − 237 genes
Standardized expressions
5.0
2.5
0.0
−2.5
EI
PG
G1
G2
H
T1
T2
C1
C2
S
EP
JP
Developmental stages

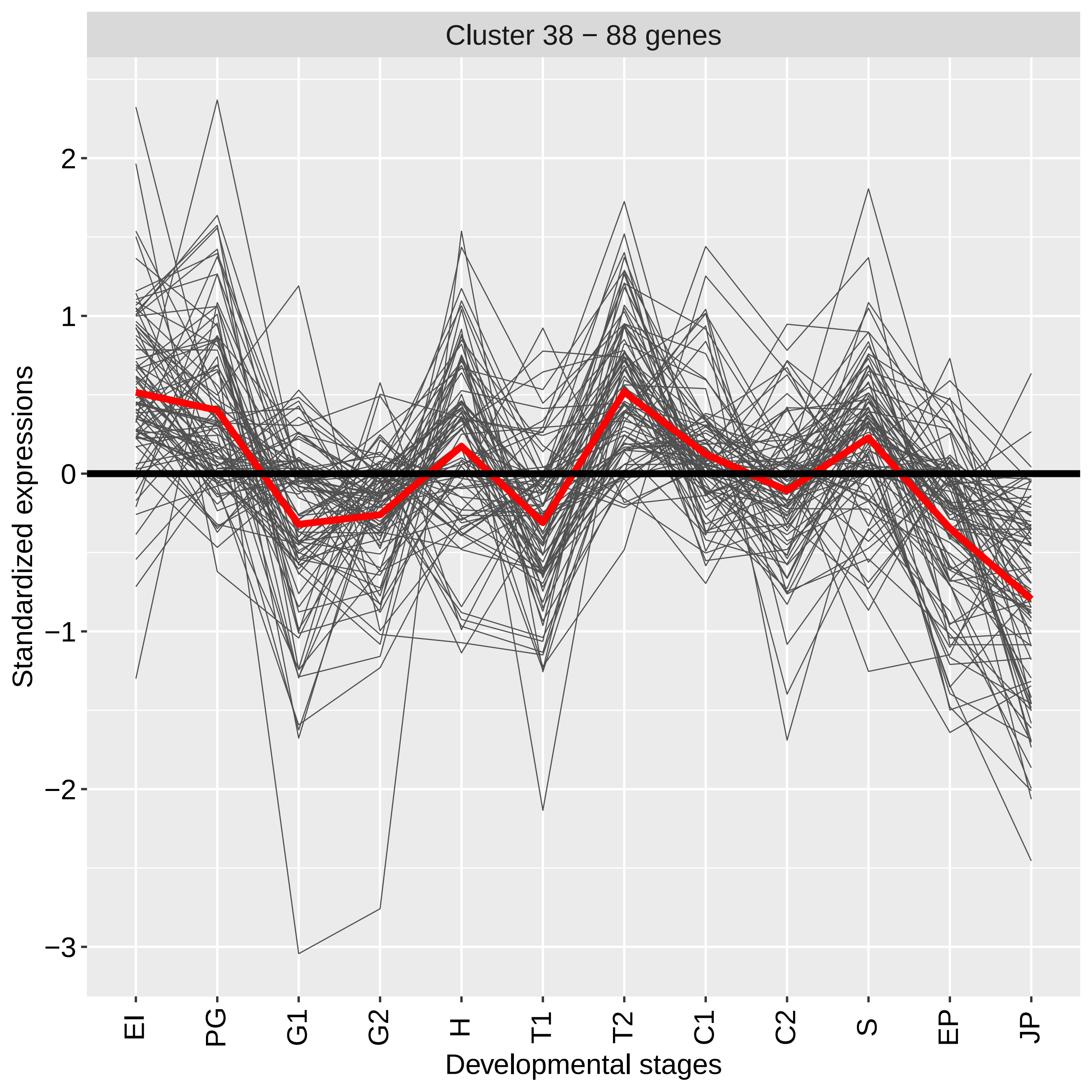
Cluster 38 − 88 genes
Standardized expressions
2
1
0
−1
−2
−3
EI
PG
G1
G2
H
T1
T2
C1
C2
S
EP
JP
Developmental stages

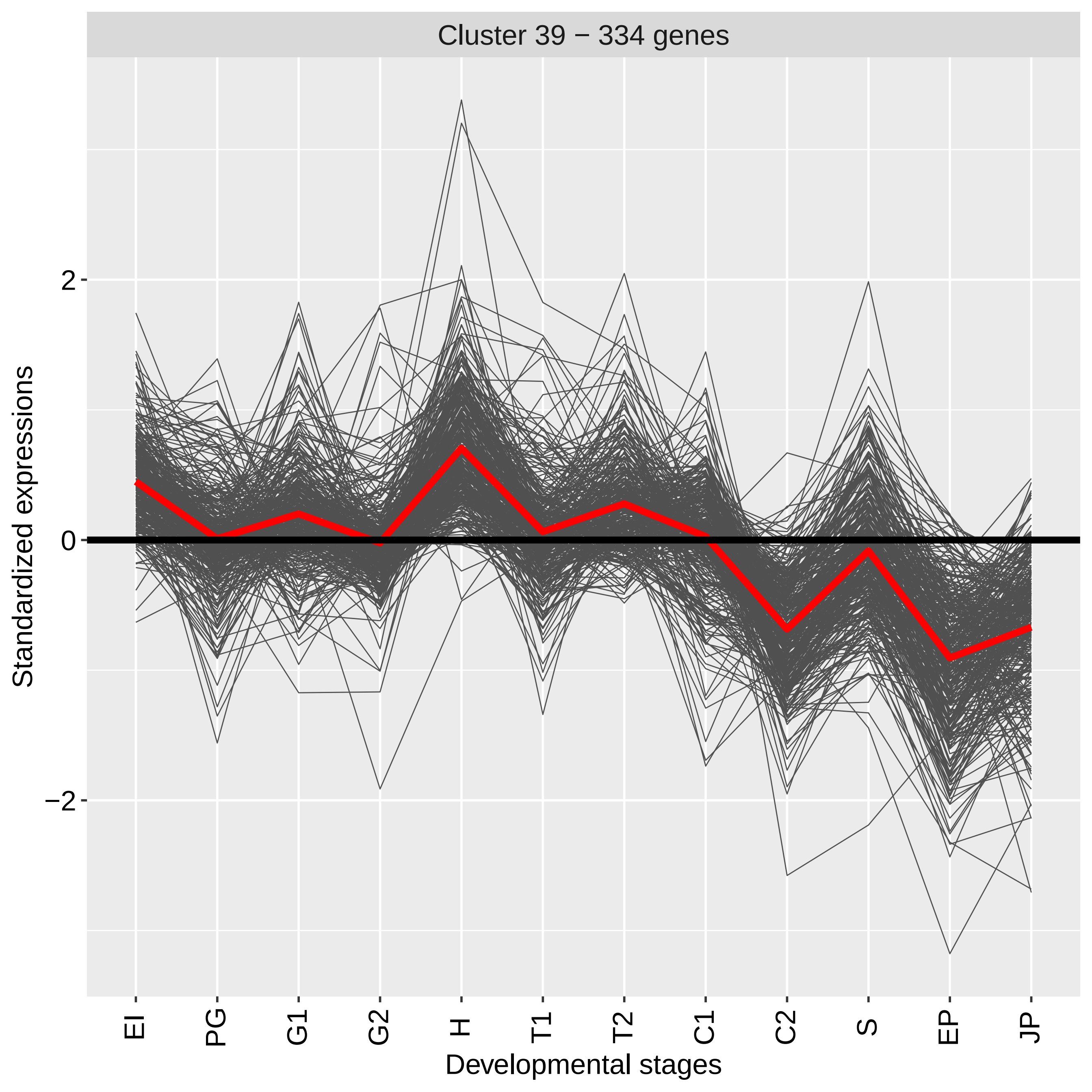

Cluster 39 − 334 genes
Standardized expressions
2
0
−2
EI
PG
G1
G2
H
T1
T2
C1
C2
S
EP
JP
Developmental stages

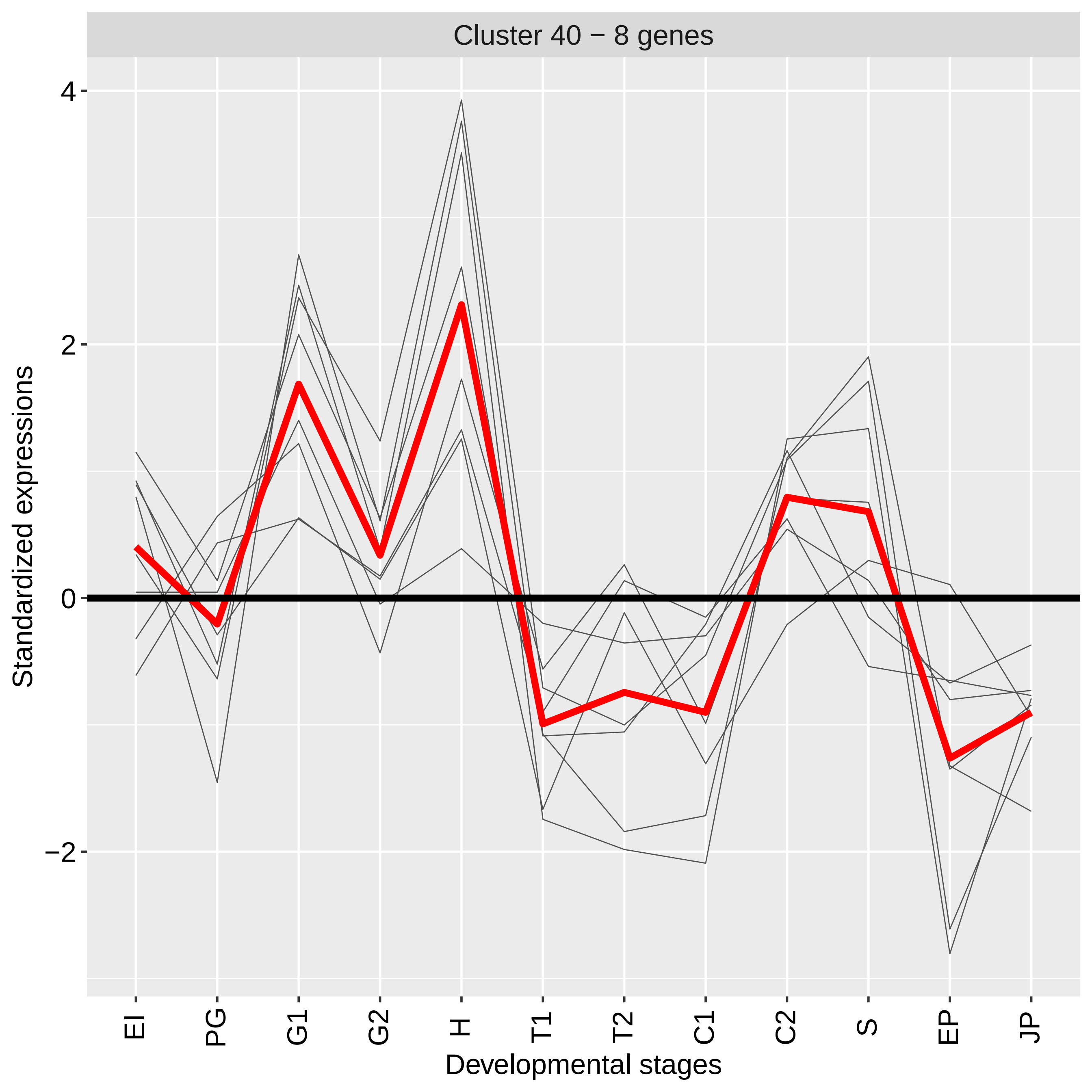

Cluster 40 − 8 genes
Standardized expressions
4
2
0
−2
EI
PG
G1
G2
H
T1
T2
C1
C2
S
EP
JP
Developmental stages

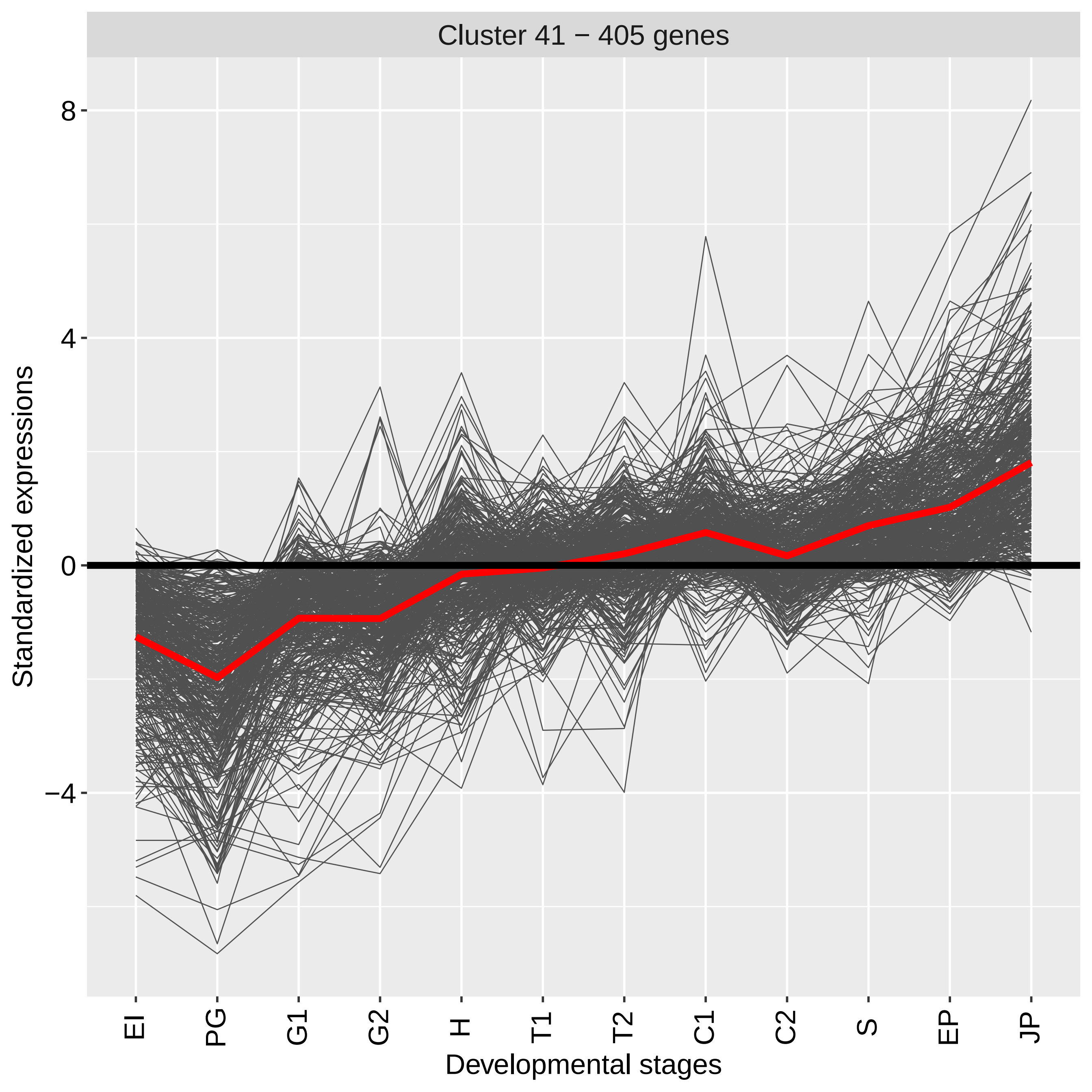
Cluster 41 − 405 genes
8
4
0
−4
Standardized expressions
EI
PG
G1
G2
H
T1
T2
C1
C2
S
EP
JP
Developmental stages

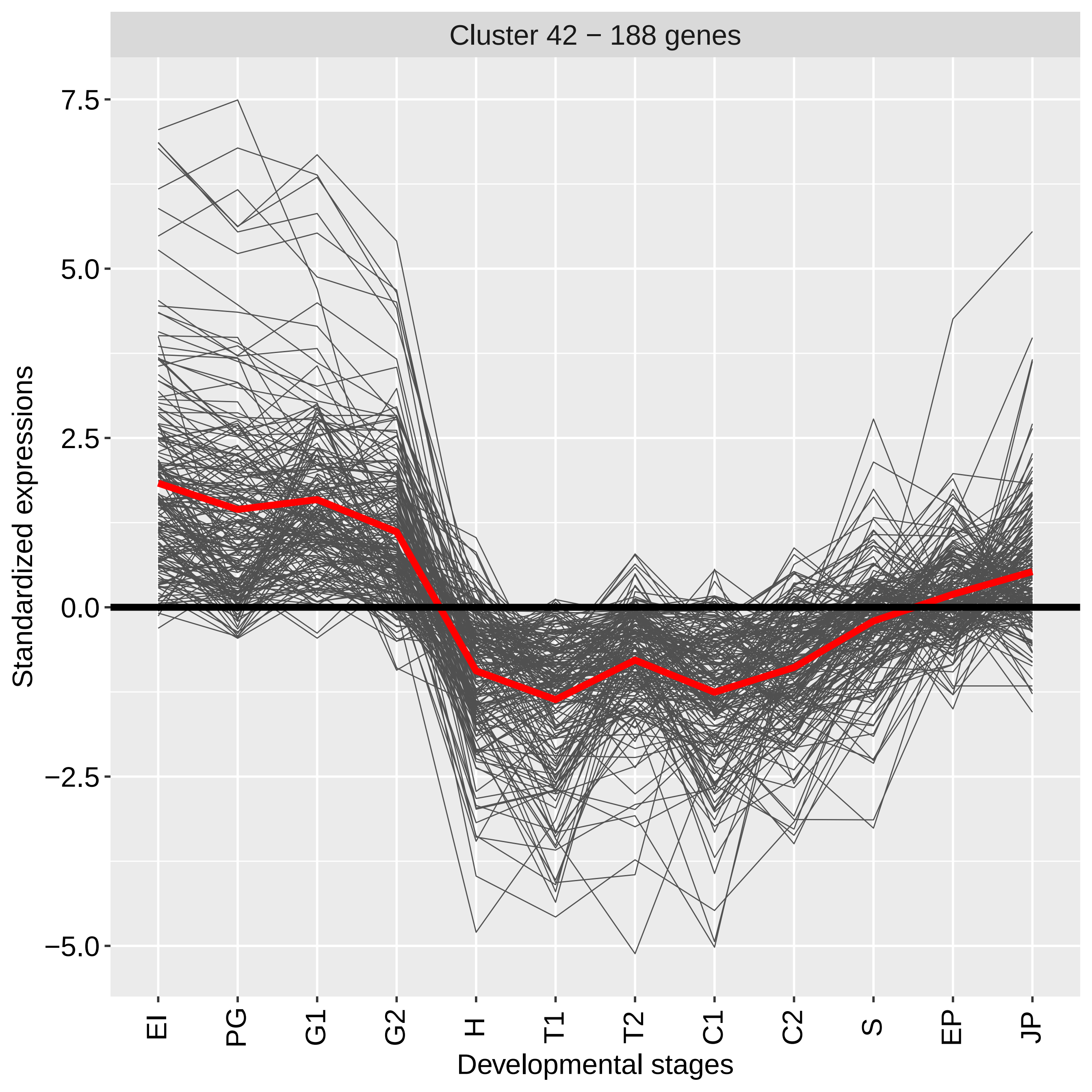
Cluster 42 − 188 genes
7.5
5.0
2.5
0.0
−2.5
−5.0
Standardized expressions
EI
PG
G1
G2
H
T1
T2
C1
C2
S
EP
JP
Developmental stages

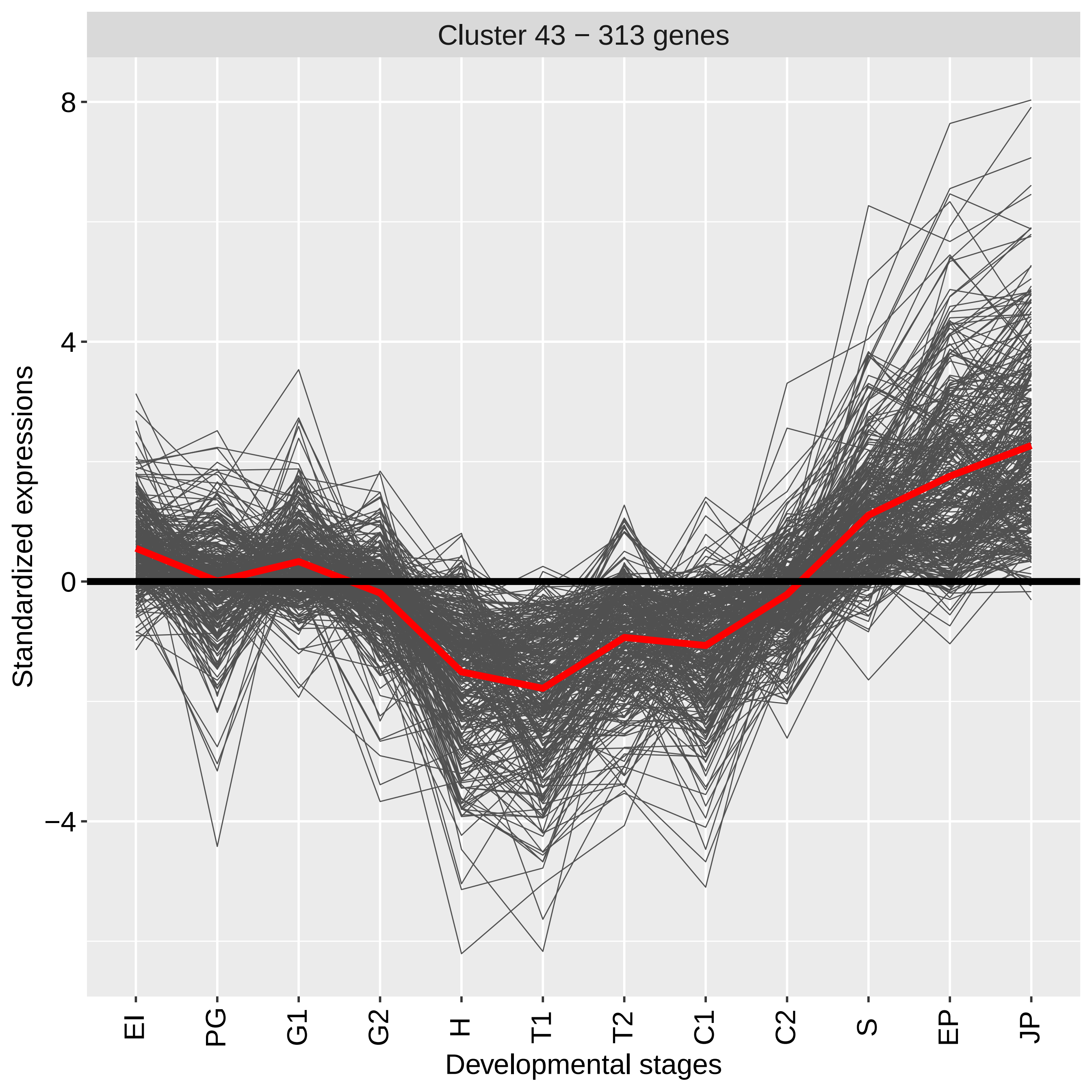
Cluster 43 − 313 genes
Standardized expressions
8
4
0
−4
EI
PG
G1
G2
H
T1
T2
C1
C2
S
EP
JP
Developmental stages

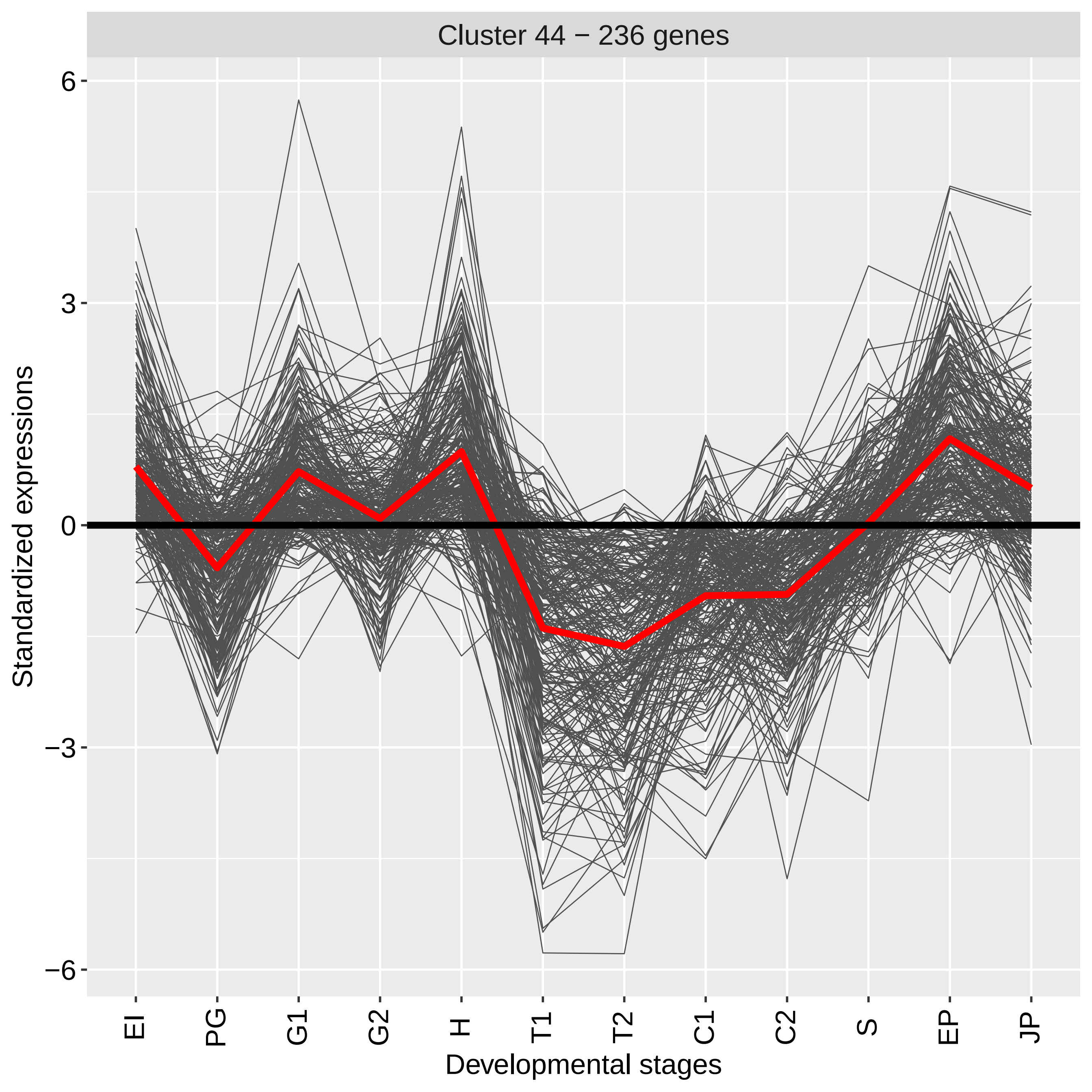
Cluster 44 − 236 genes
6
3
0
−3
−6
Standardized expressions
EI
PG
G1
G2
H
T1
T2
C1
C2
S
EP
JP
Developmental stages

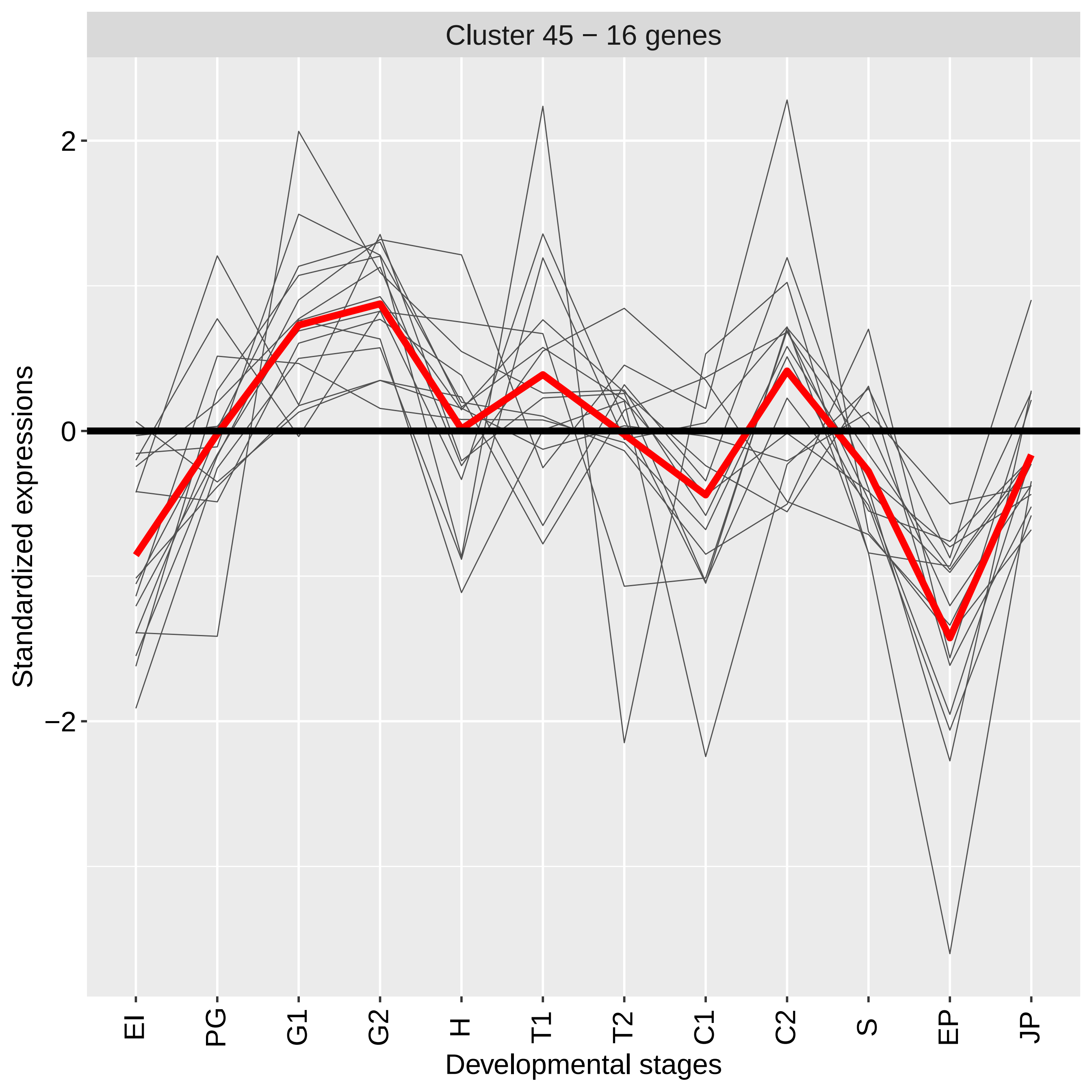

Cluster 45 − 16 genes
Standardized expressions
2
0
−2
EI
PG
G1
G2
H
T1
T2
C1
C2
S
EP
JP
Developmental stages

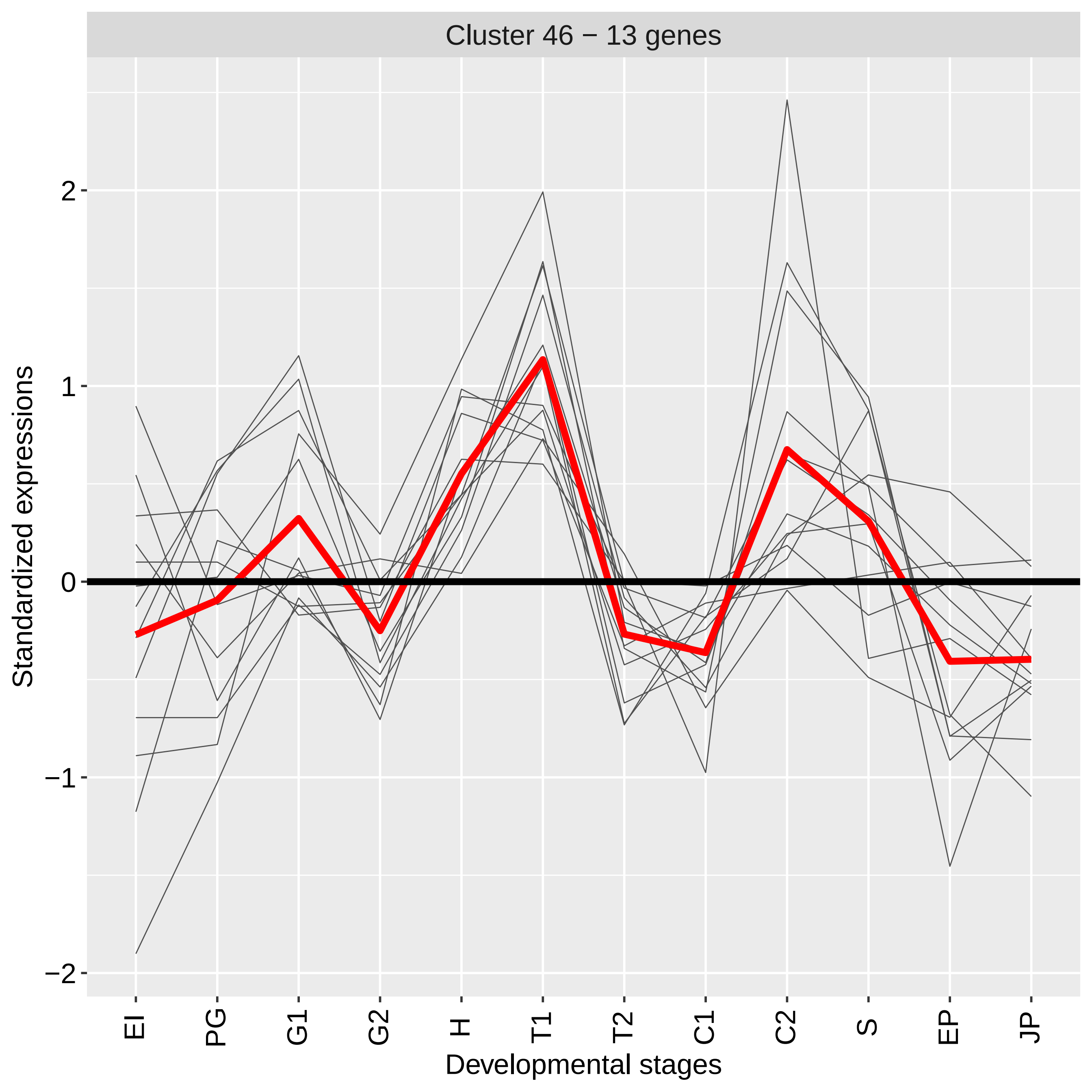

Cluster 46 − 13 genes
Standardized expressions
2
1
0
−1
−2
EI
PG
G1
G2
H
T1
T2
C1
C2
S
EP
JP
Developmental stages

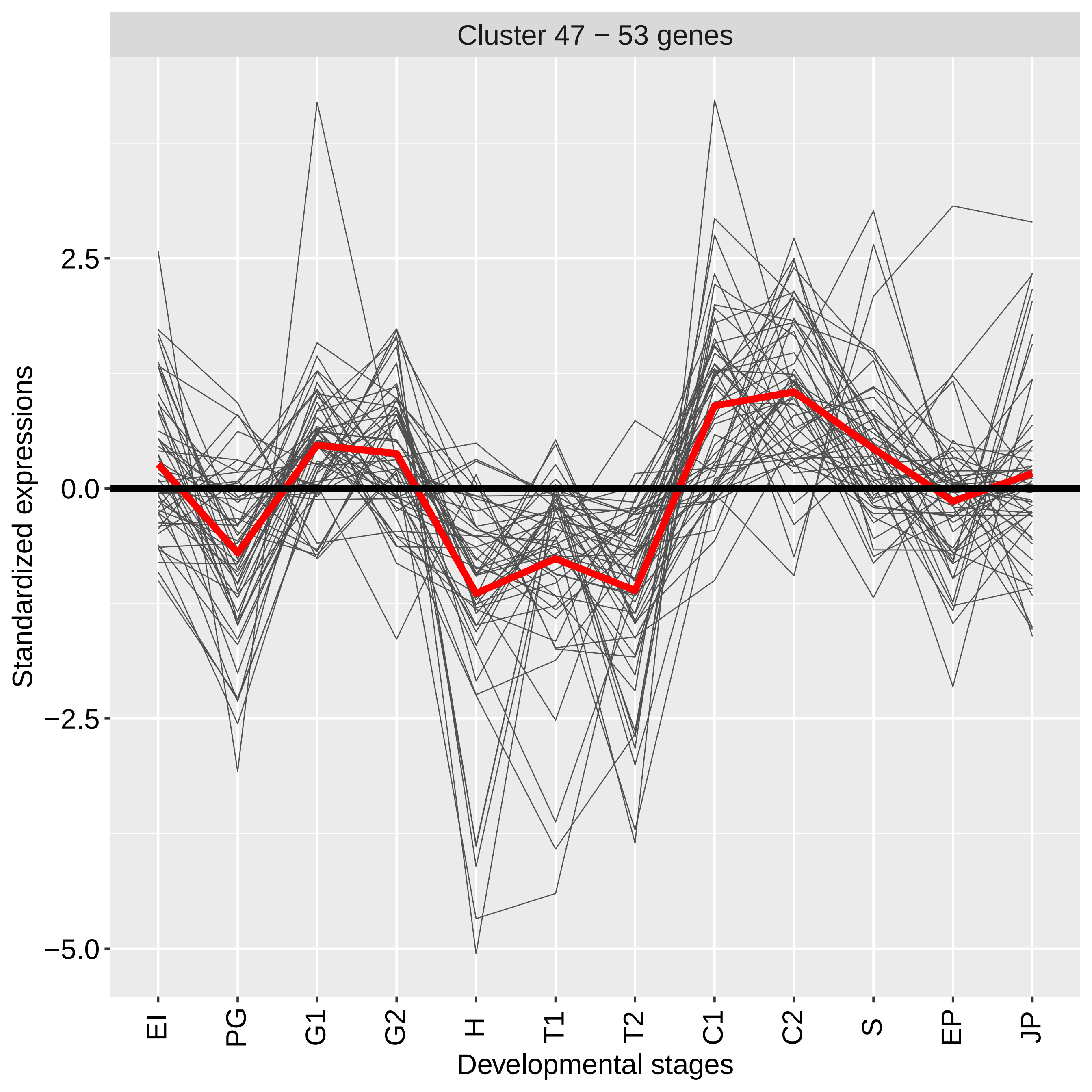

Cluster 47 − 53 genes
Standardized expressions
2.5
0.0
−2.5
−5.0
EI
PG
G1
G2
H
T1
T2
C1
C2
S
EP
JP
Developmental stages

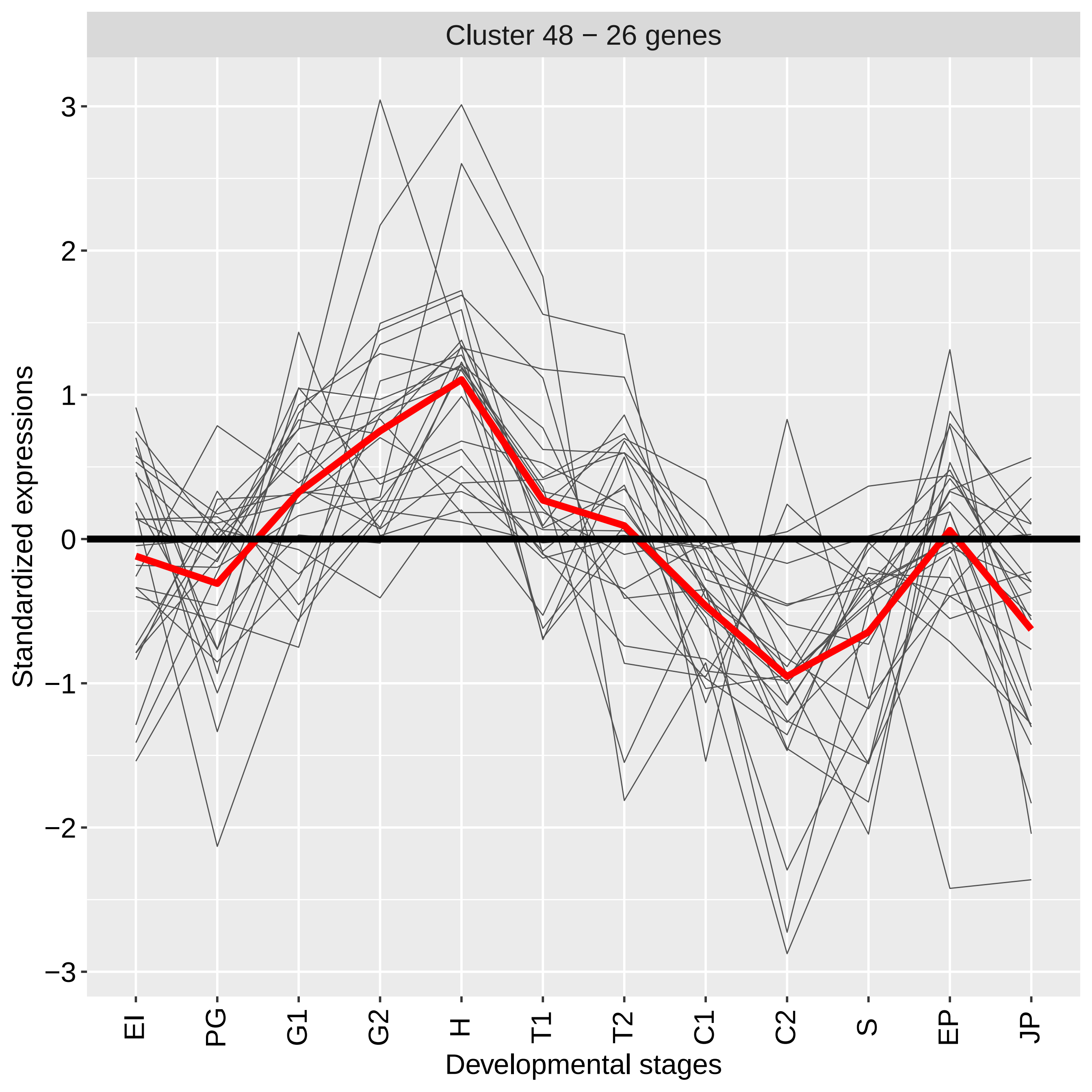

Cluster 48 − 26 genes
Standardized expressions
3
2
1
0
−1
−2
−3
EI
PG
G1
G2
H
T1
T2
C1
C2
S
EP
JP
Developmental stages

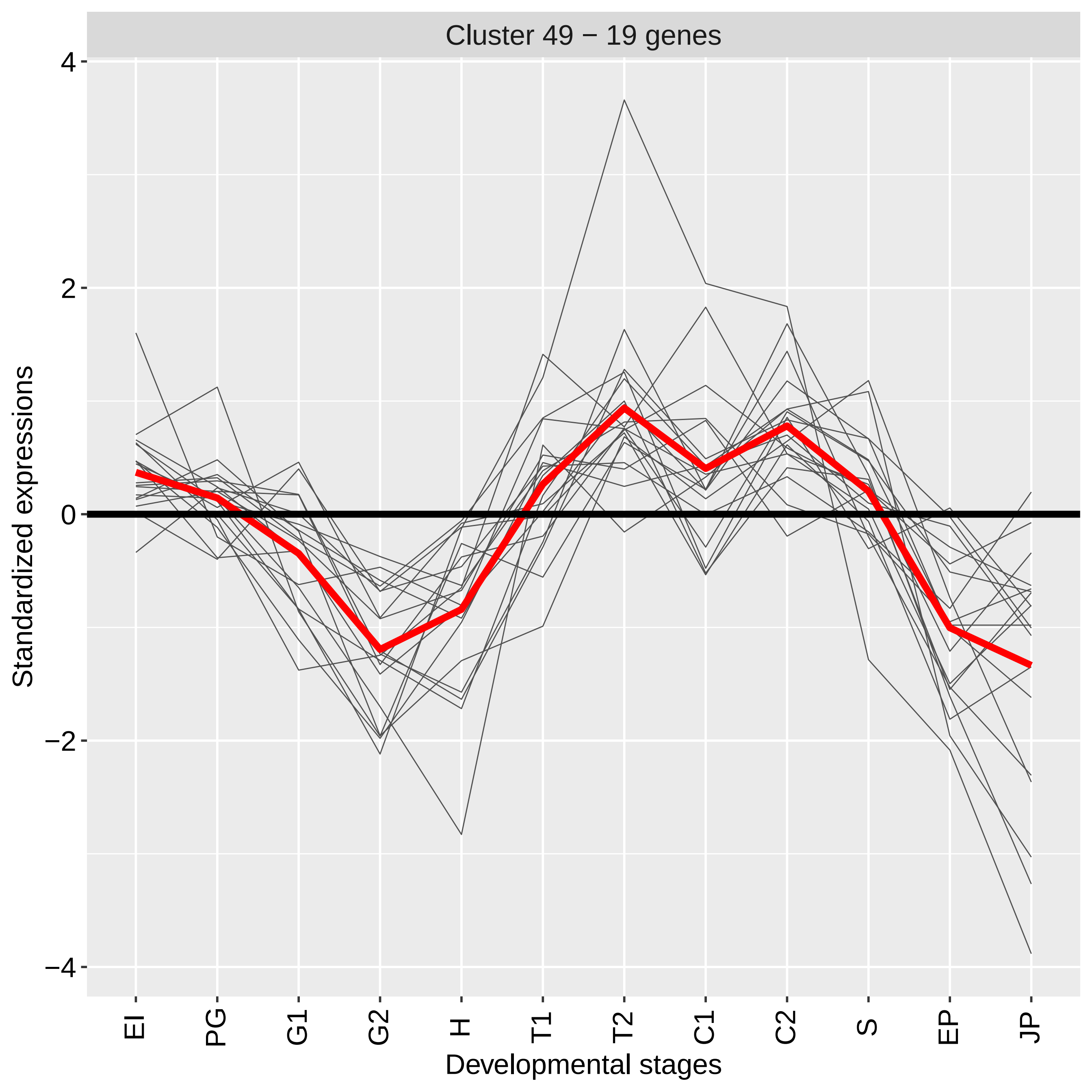
Cluster 49 − 19 genes
Standardized expressions
4
2
0
−2
−4
EI
PG
G1
G2
H
T1
T2
C1
C2
S
EP
JP
Developmental stages

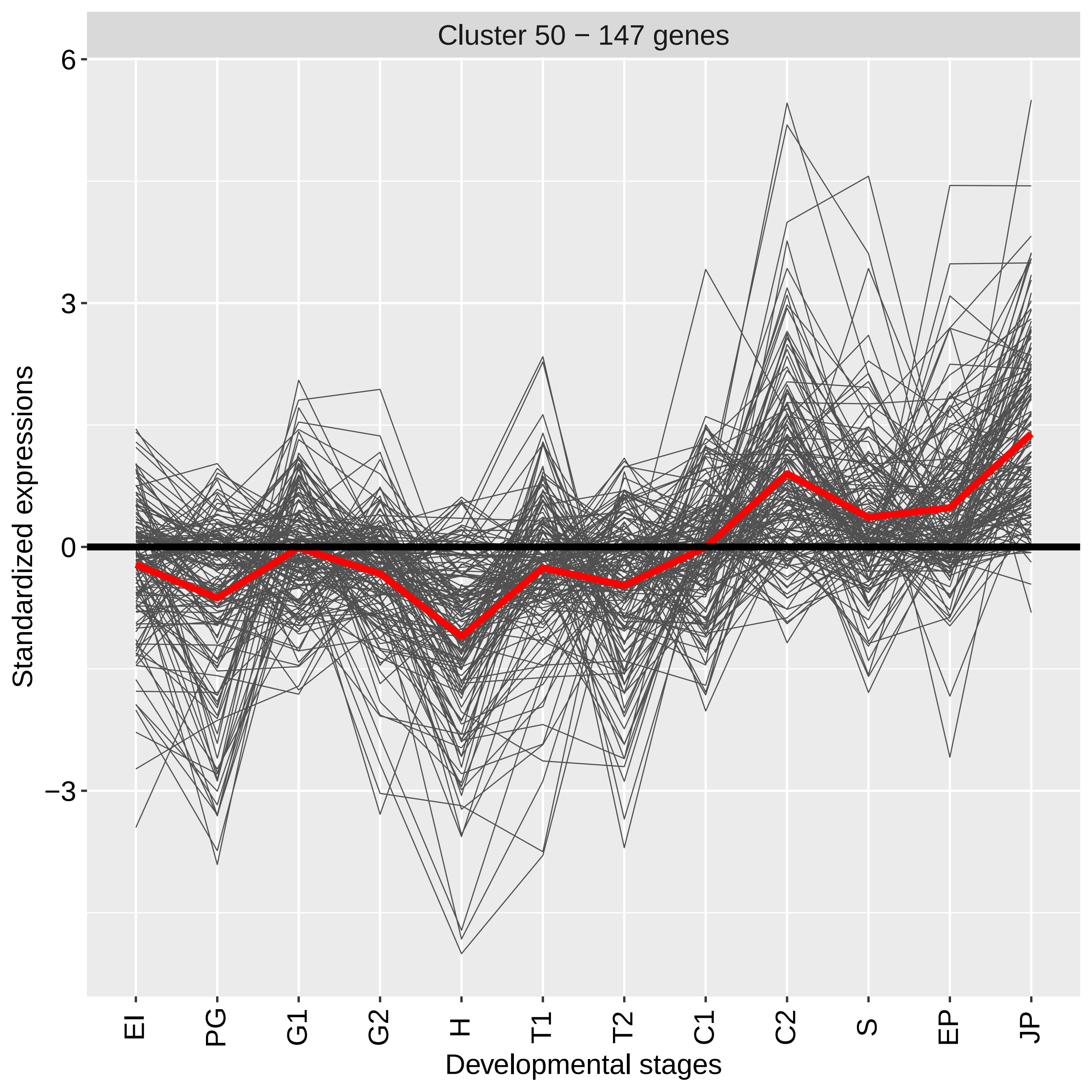
Cluster 50 − 147 genes
Standardized expressions
6
3
0
−3
EI
PG
G1
G2
H
T1
T2
C1
C2
S
EP
JP
Developmental stages

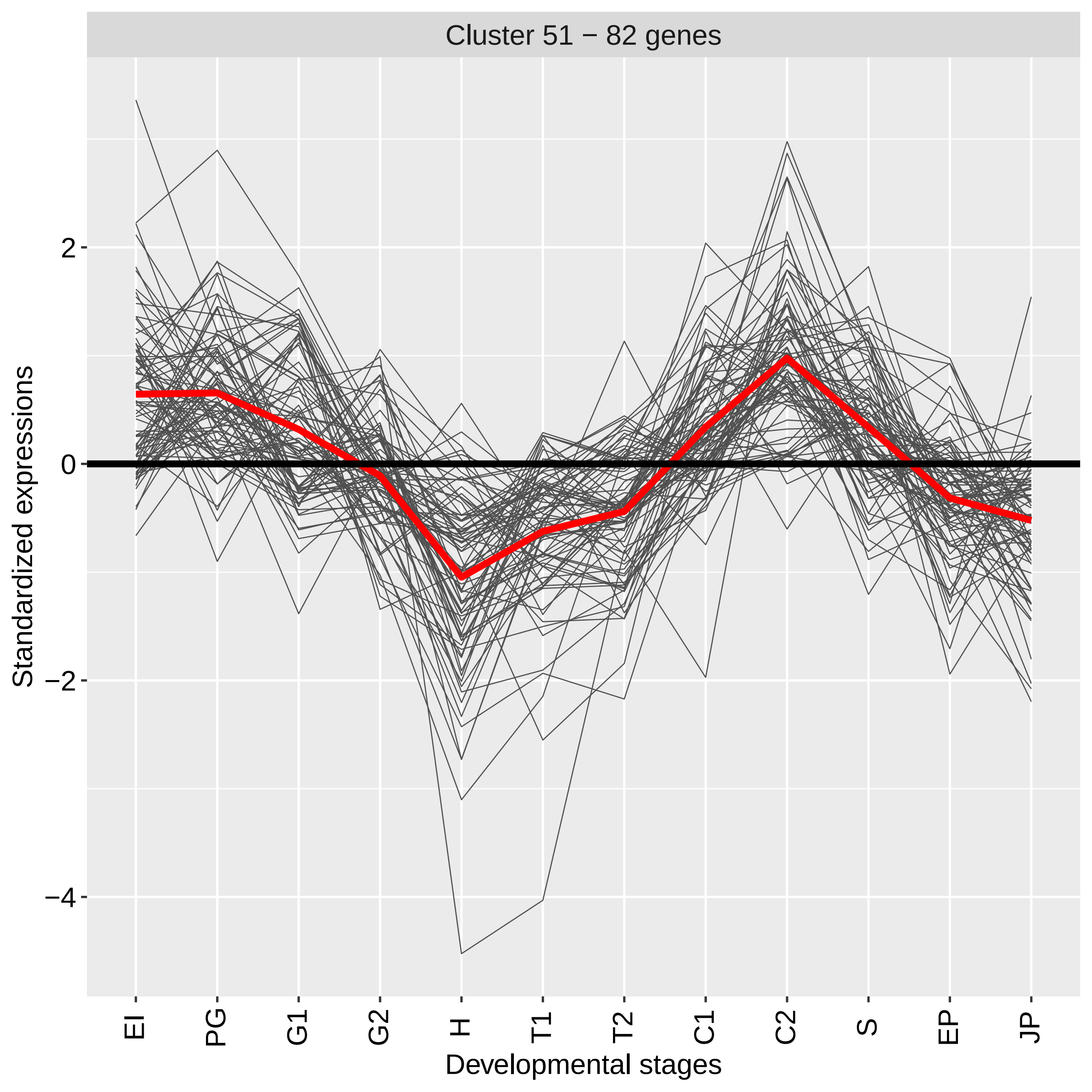

Cluster 51 − 82 genes
Standardized expressions
2
0
−2
−4
EI
PG
G1
G2
H
T1
T2
C1
C2
S
EP
JP
Developmental stages

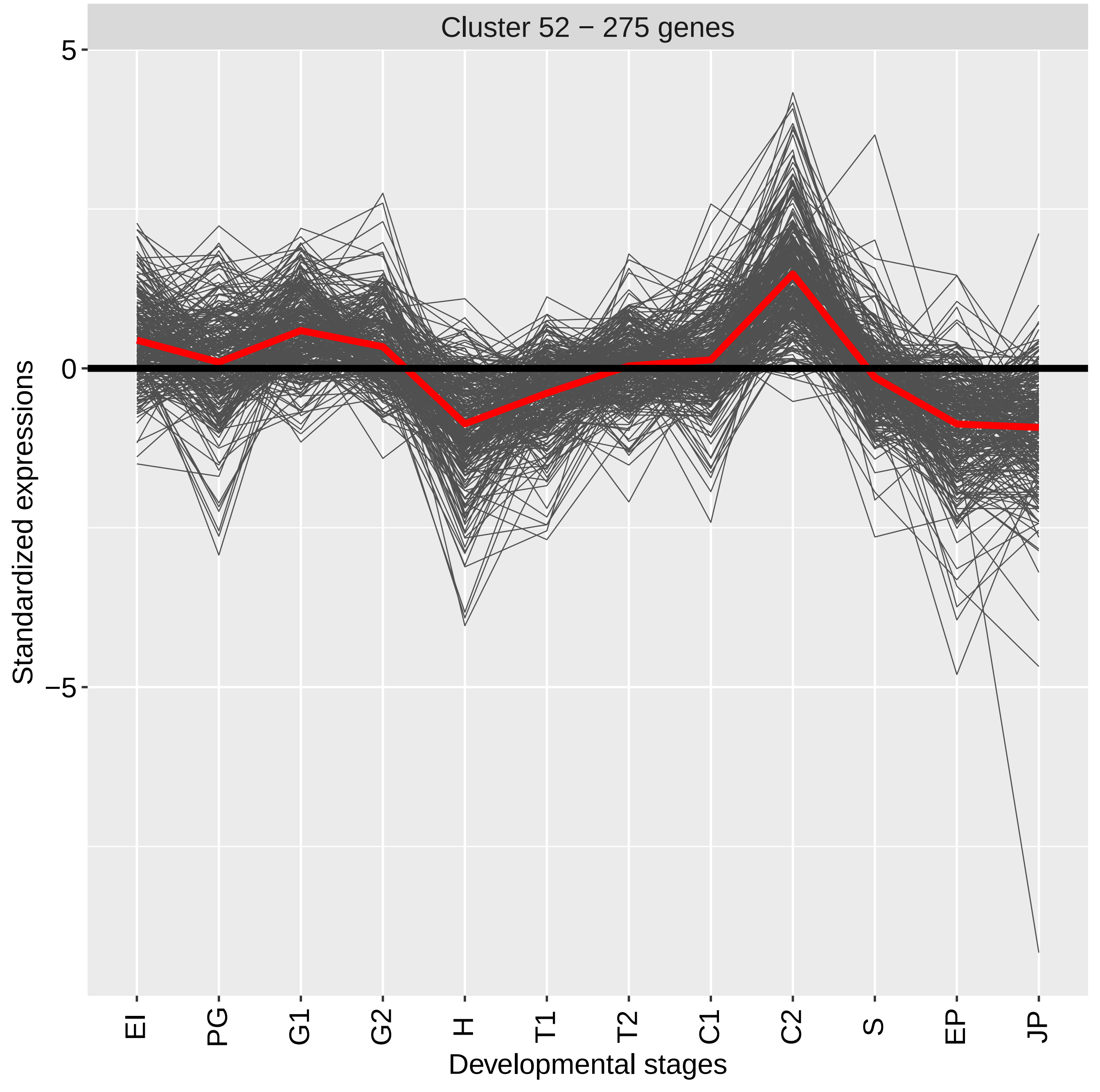
Cluster 52 − 275 genes
5
0
−5
Standardized expressions
EI
PG
G1
G2
H
T1
T2
C1
C2
S
EP
JP
Developmental stages

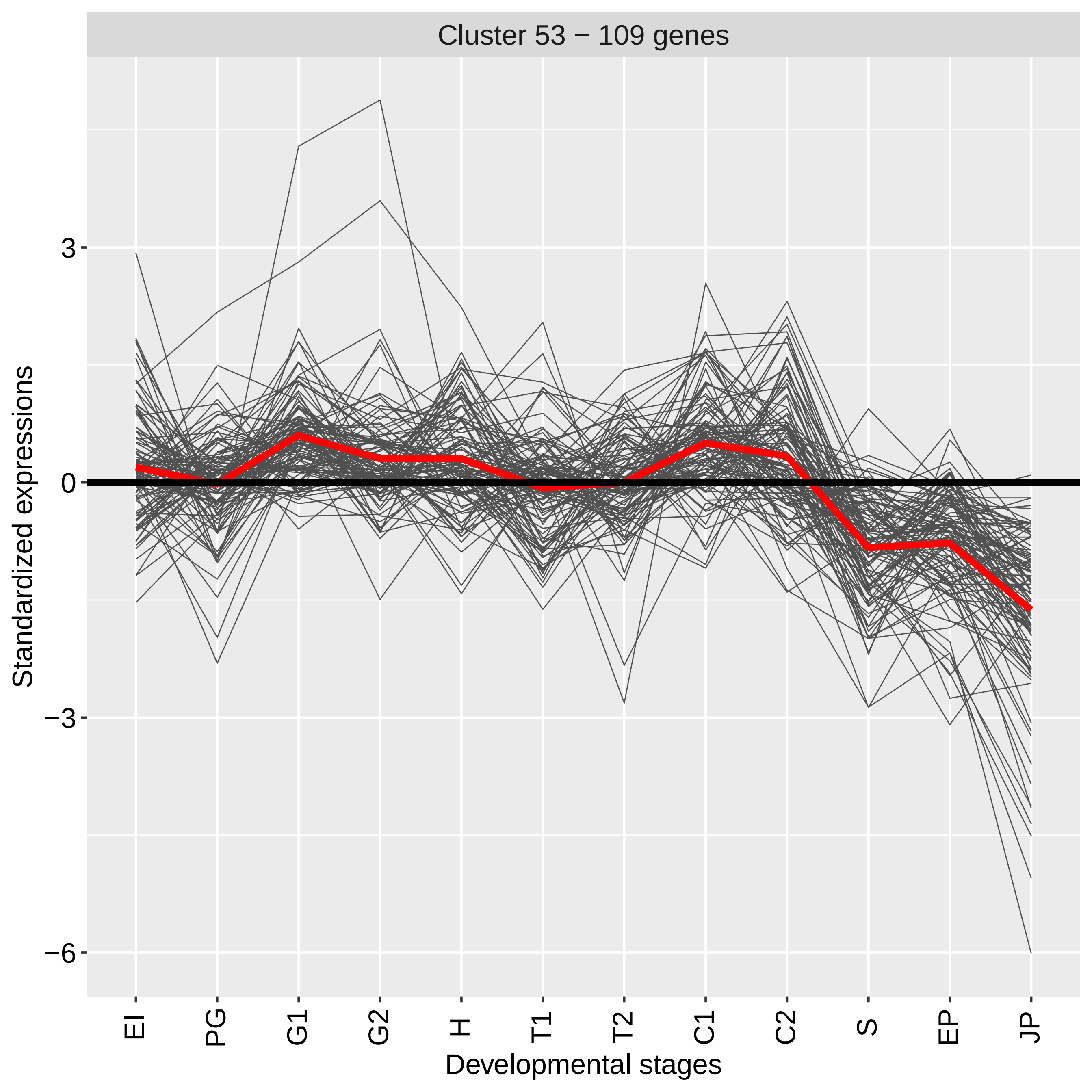
Cluster 53 − 109 genes
Standardized expressions
3
0
−3
−6
EI
PG
G1
G2
H
T1
T2
C1
C2
S
EP
JP
Developmental stages

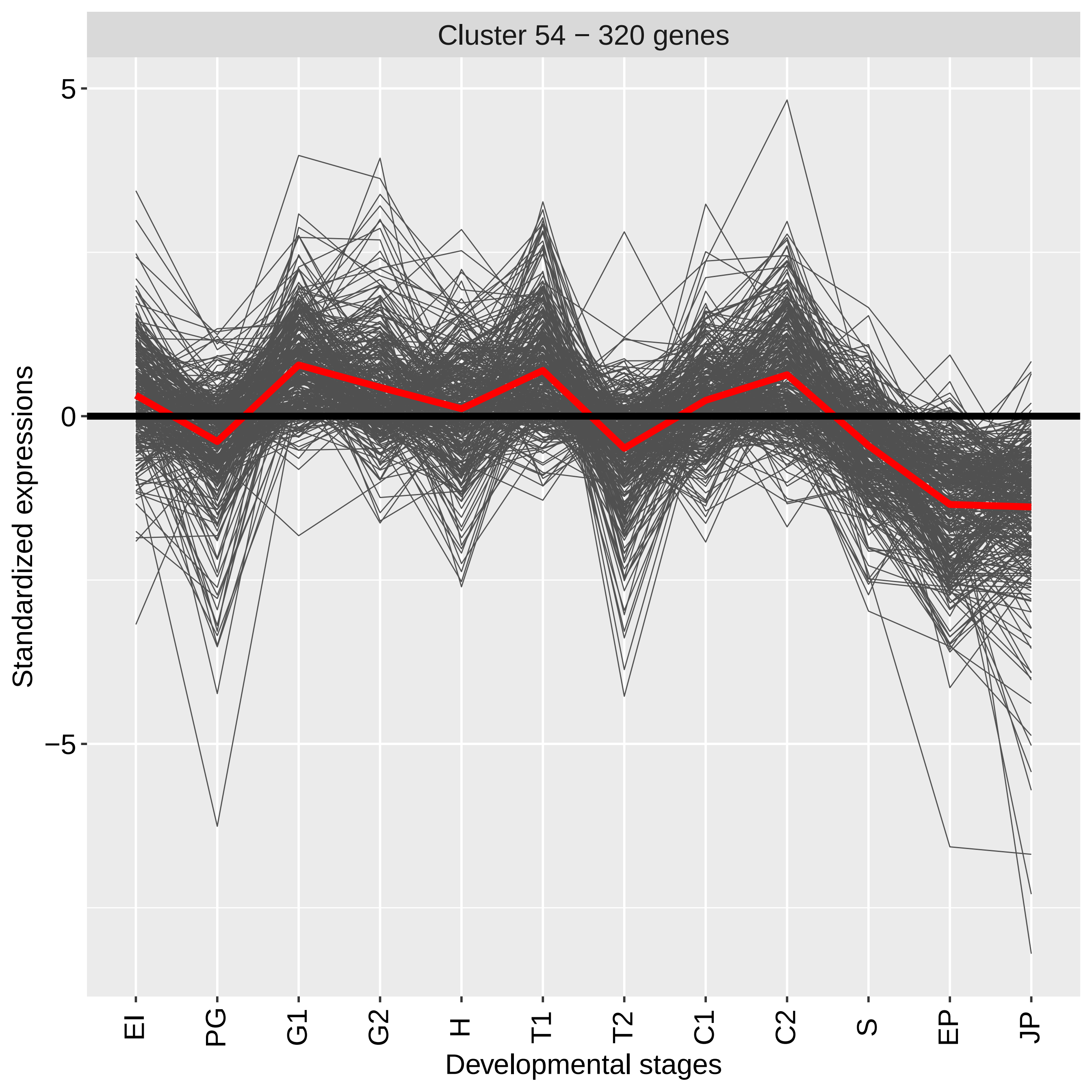

Cluster 54 − 320 genes
5
0
−5
Standardized expressions
EI
PG
G1
G2
H
T1
T2
C1
C2
S
EP
JP
Developmental stages

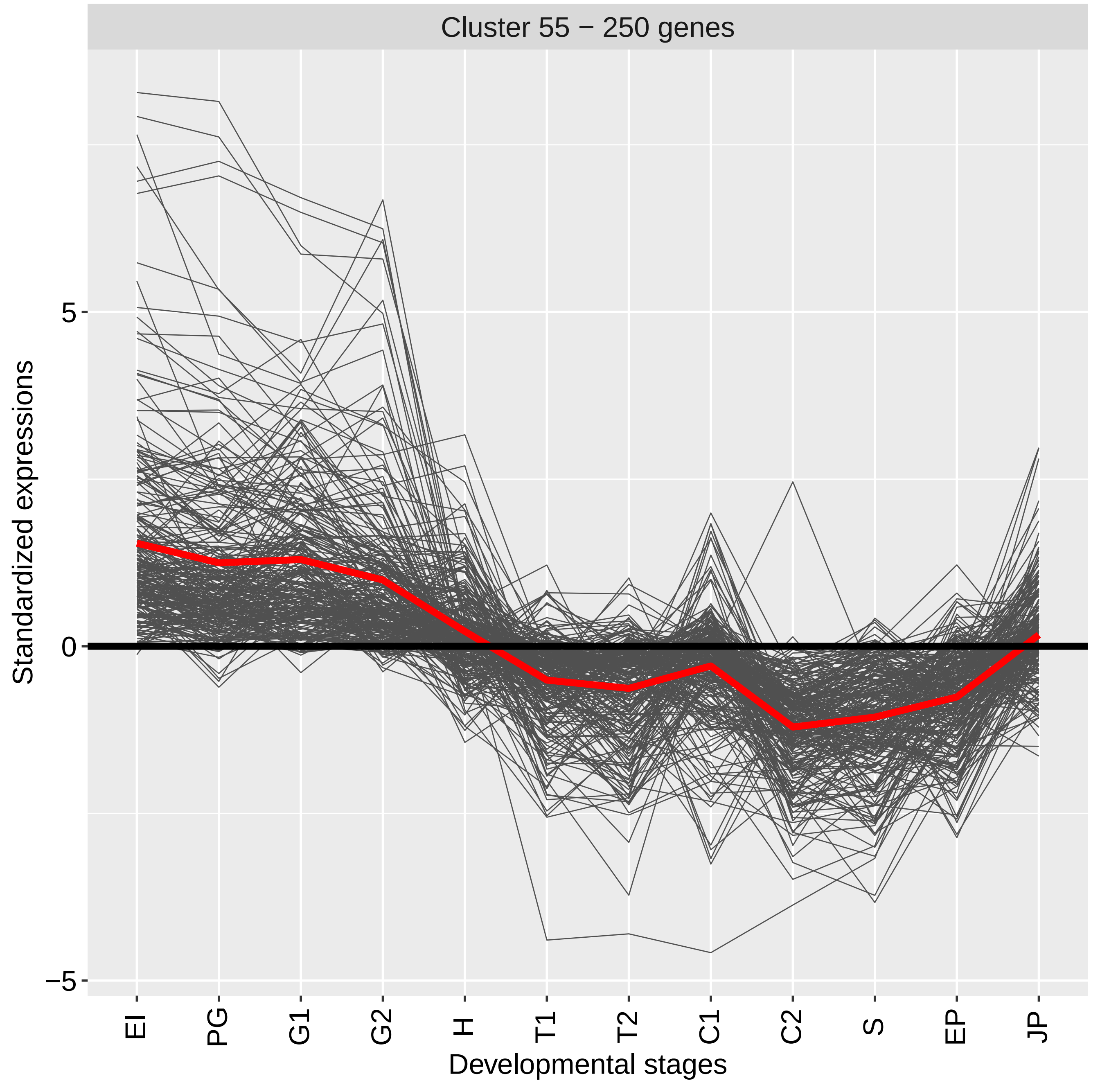
Cluster 55 − 250 genes
Standardized expressions
5
0
−5
EI
PG
G1
G2
H
T1
T2
C1
C2
S
EP
JP
Developmental stages

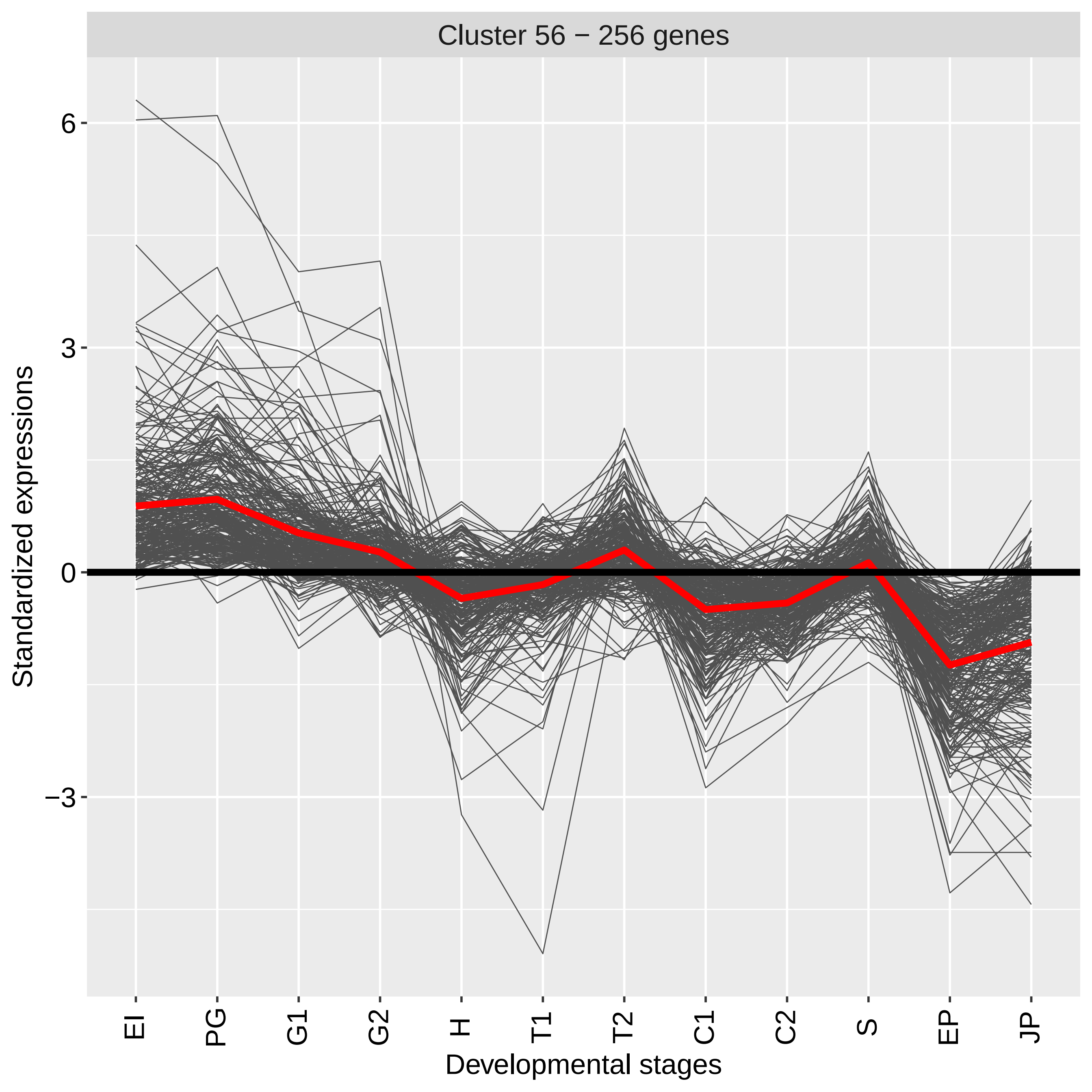
Cluster 56 − 256 genes
Standardized expressions
6
3
0
−3
EI
PG
G1
G2
H
T1
T2
C1
C2
S
EP
JP
Developmental stages

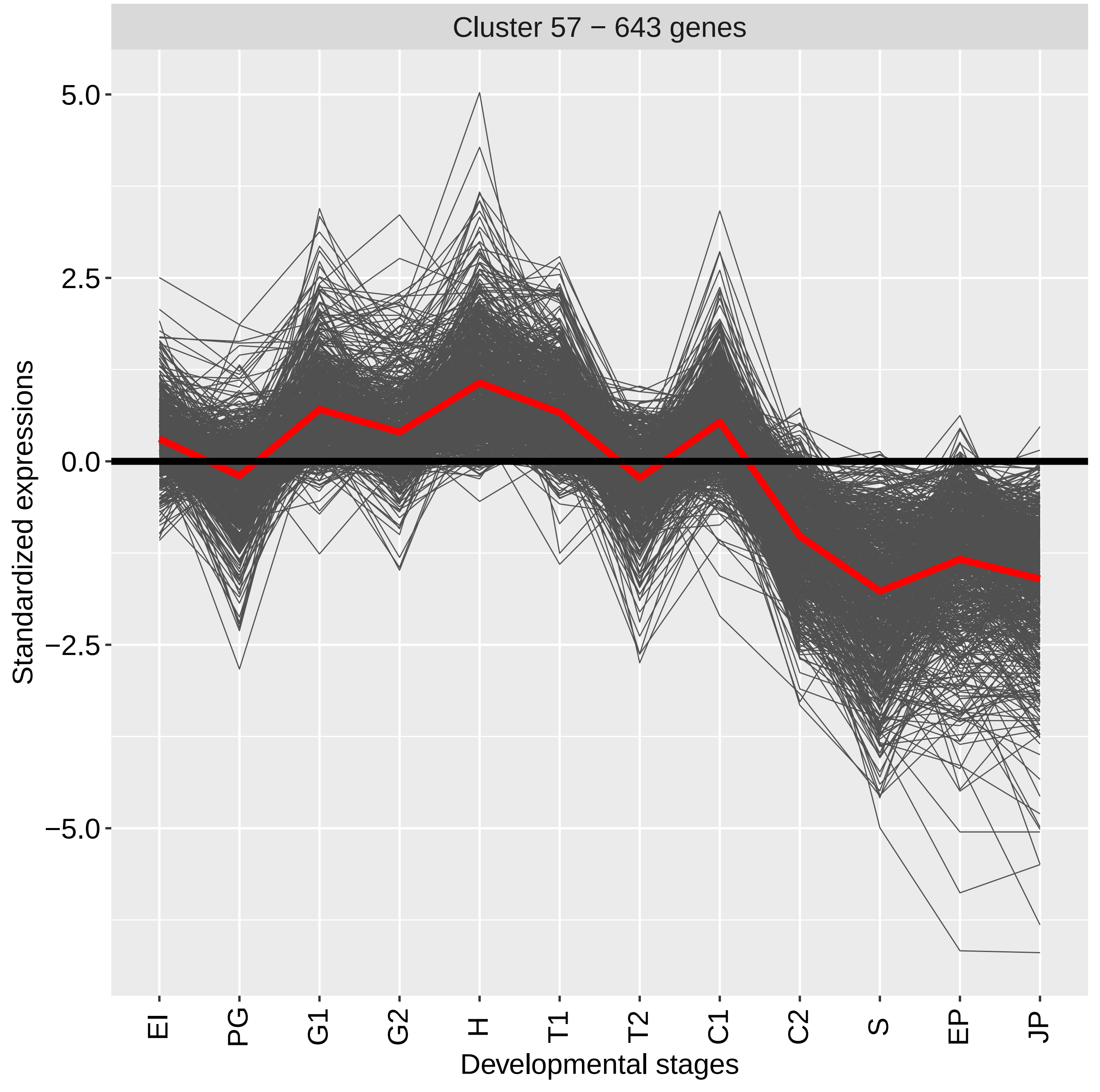

Cluster 57 − 643 genes
Standardized expressions
5.0
2.5
0.0
−2.5
−5.0
EI
PG
G1
G2
H
T1
T2
C1
C2
S
EP
JP
Developmental stages

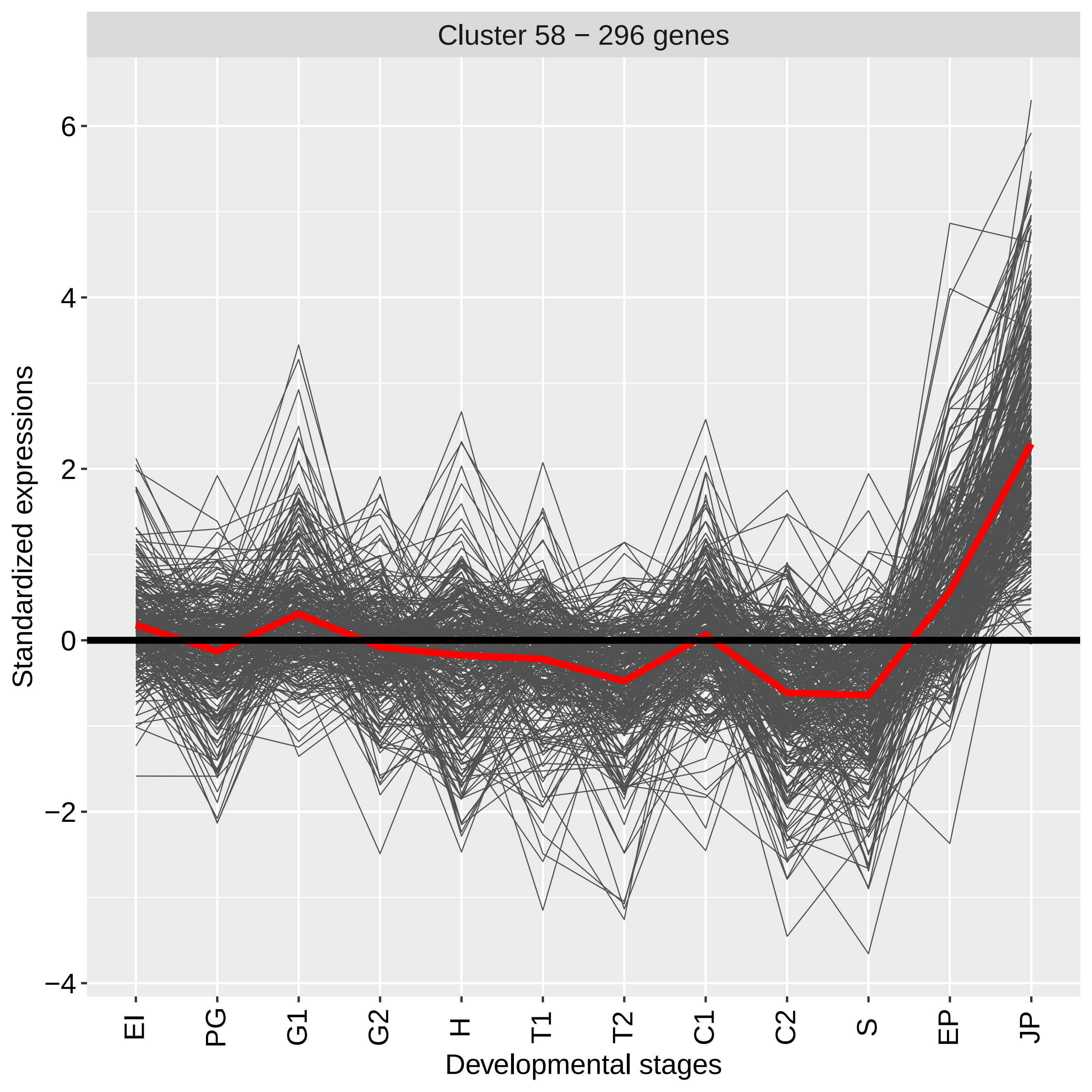

Cluster 58 − 296 genes
Standardized expressions
6
4
2
0
−2
−4
EI
PG
G1
G2
H
T1
T2
C1
C2
S
EP
JP
Developmental stages

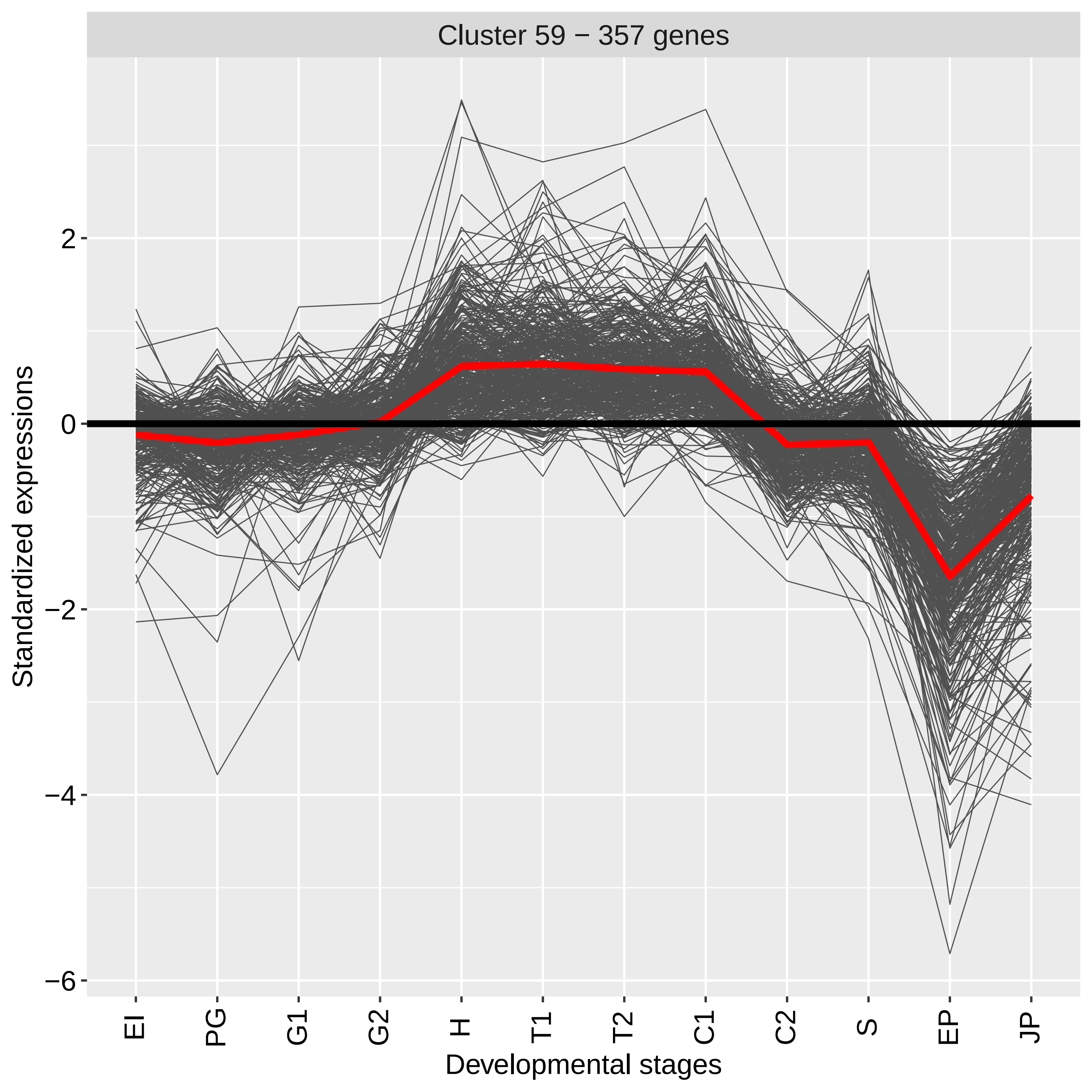

Cluster 59 − 357 genes
Standardized expressions
2
0
−2
−4
−6
EI
PG
G1
G2
H
T1
T2
C1
C2
S
EP
JP
Developmental stages

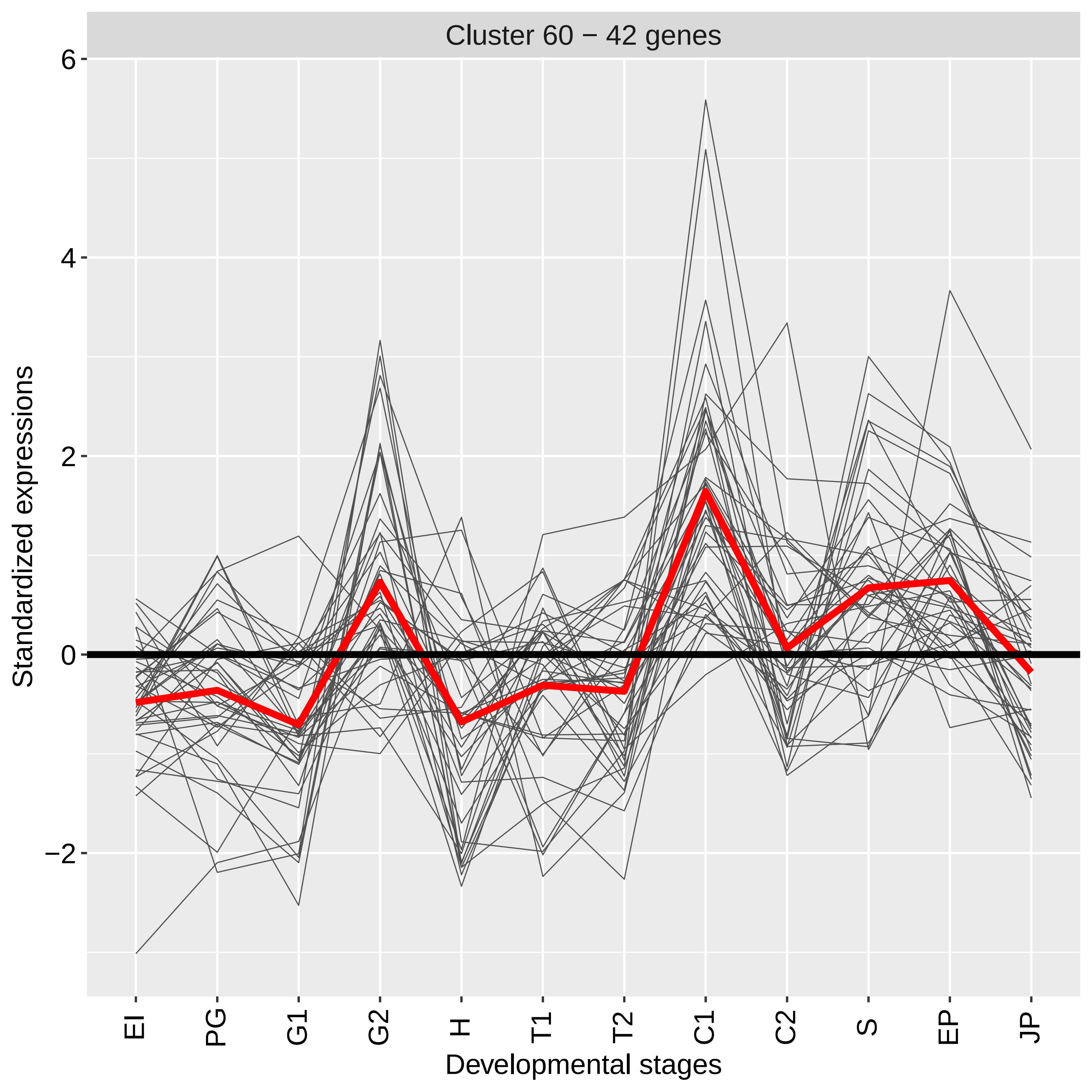

Cluster 60 − 42 genes
Standardized expressions
6
4
2
0
−2
EI
PG
G1
G2
H
T1
T2
C1
C2
S
EP
JP
Developmental stages

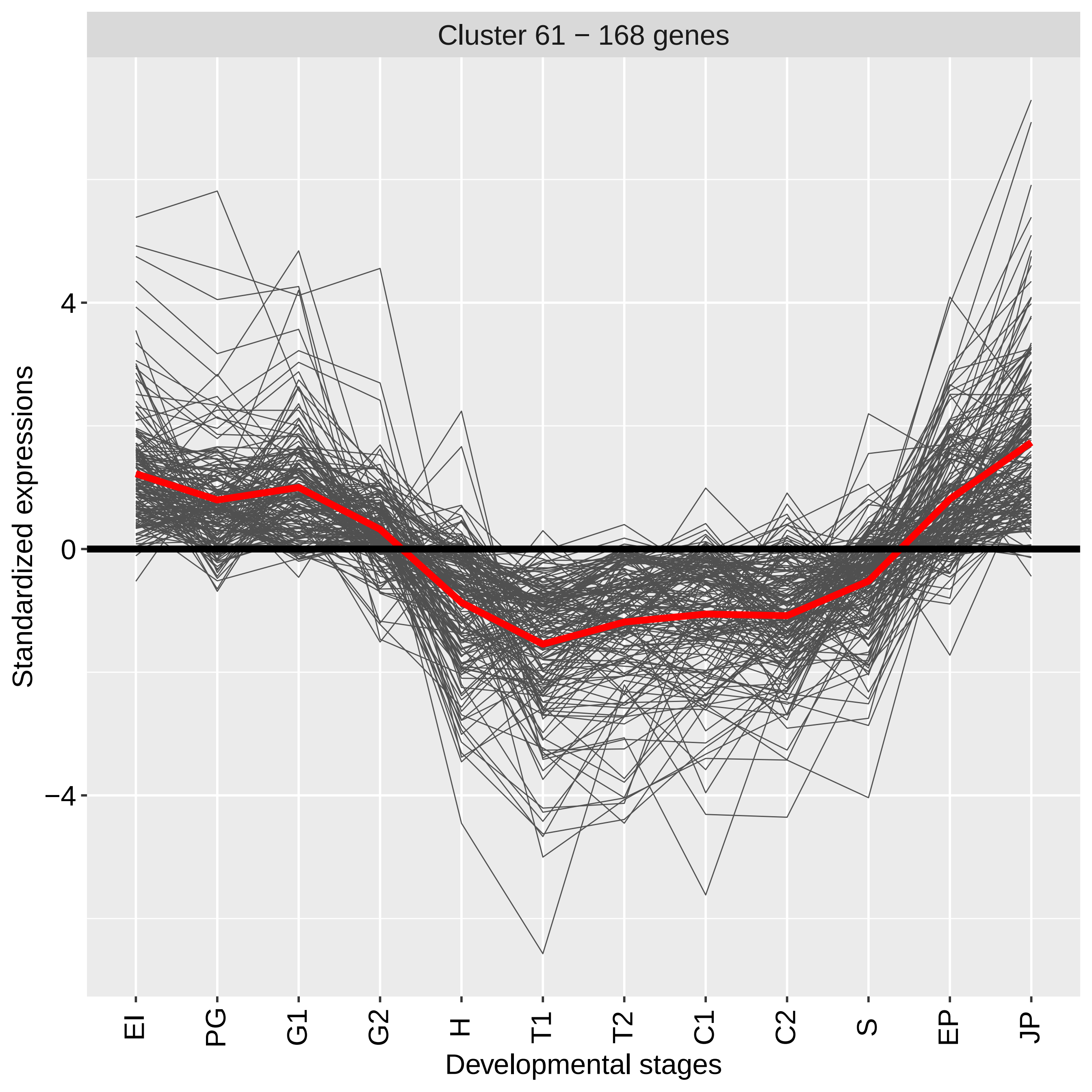

Cluster 61 − 168 genes
Standardized expressions
4
0
−4
EI
PG
G1
G2
H
T1
T2
C1
C2
S
EP
JP
Developmental stages

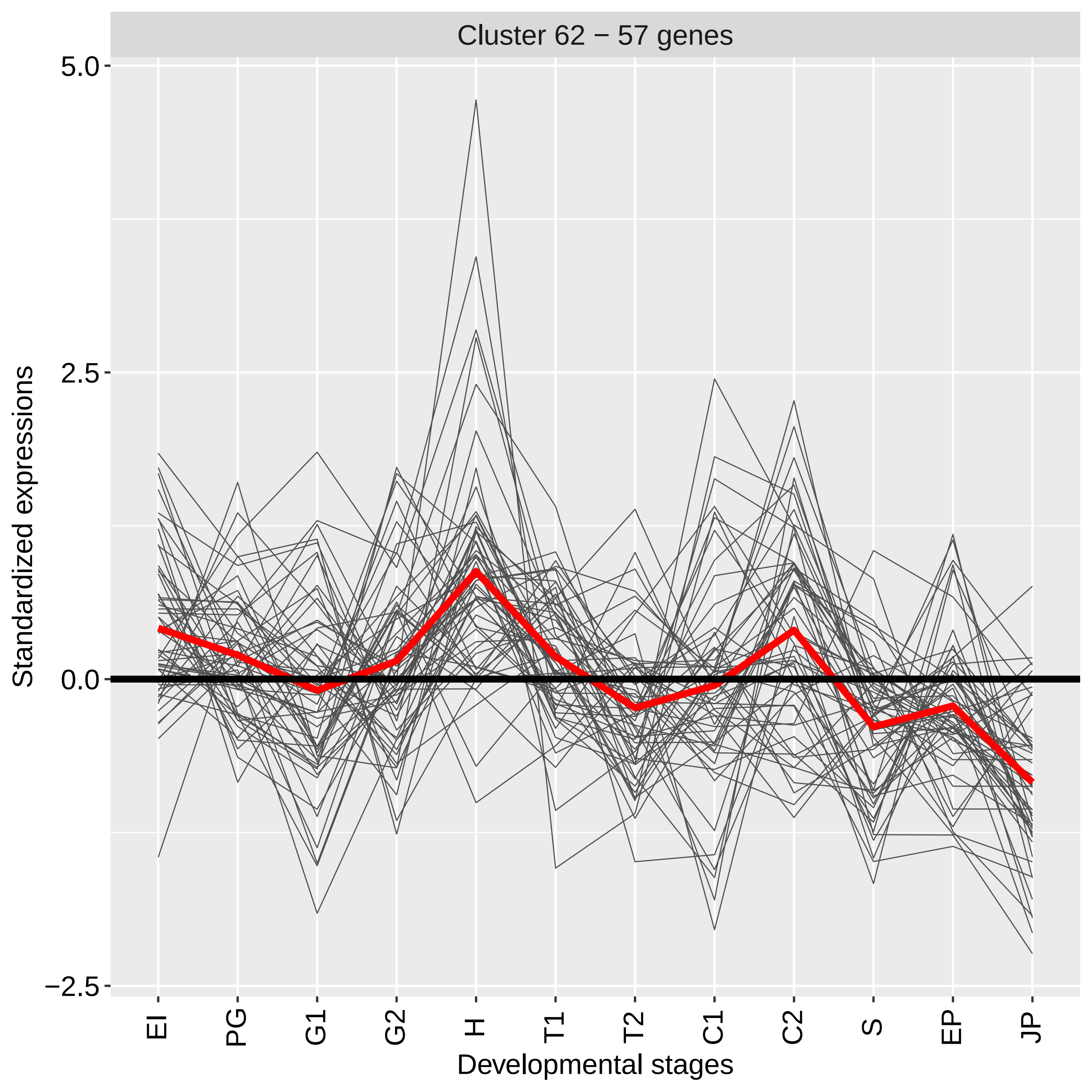

Cluster 62 − 57 genes
5.0
2.5
0.0
−2.5
Standardized expressions
EI
PG
G1
G2
H
T1
T2
C1
C2
S
EP
JP
Developmental stages

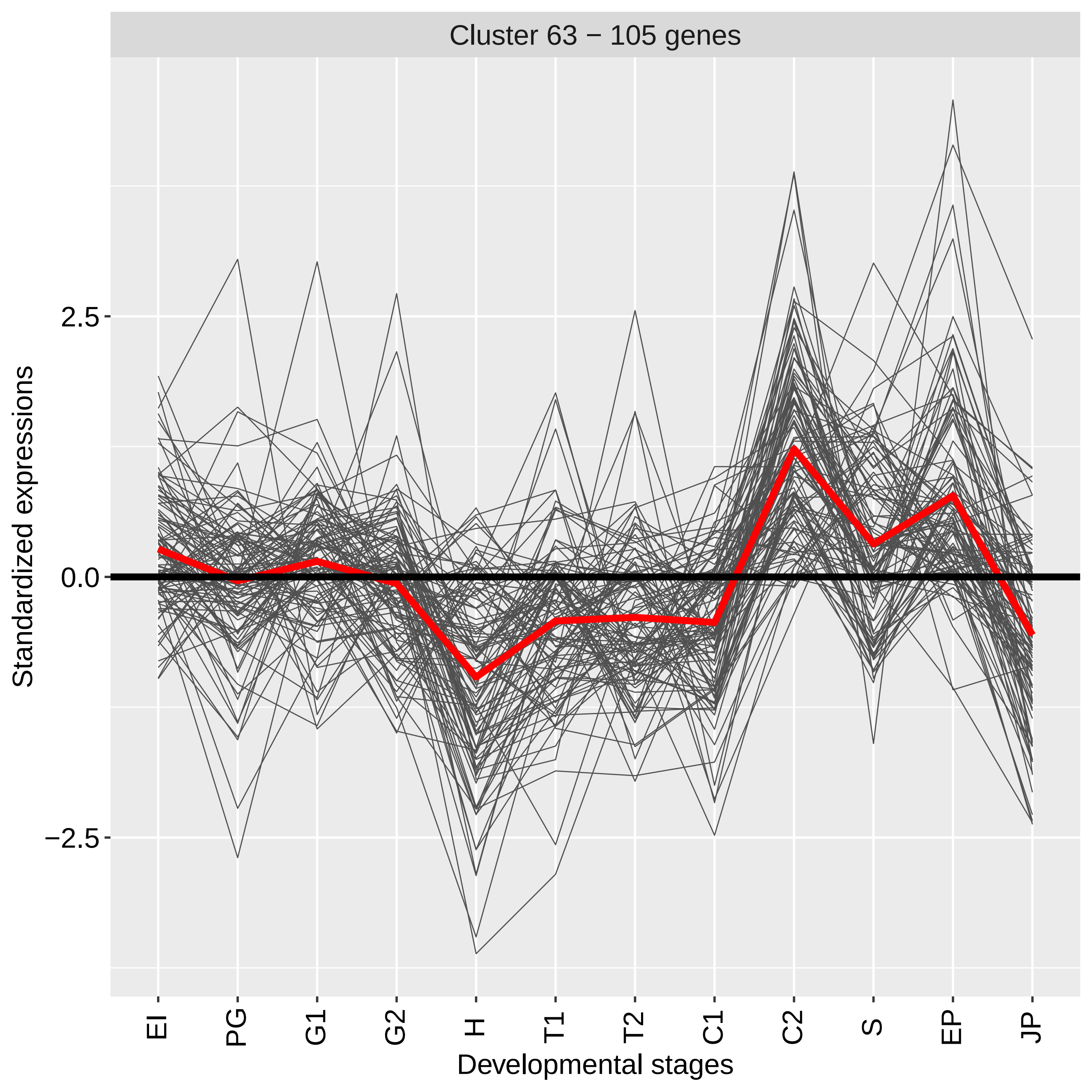
Cluster 63 − 105 genes
Standardized expressions
2.5
0.0
−2.5
EI
PG
G1
G2
H
T1
T2
C1
C2
S
EP
JP
Developmental stages

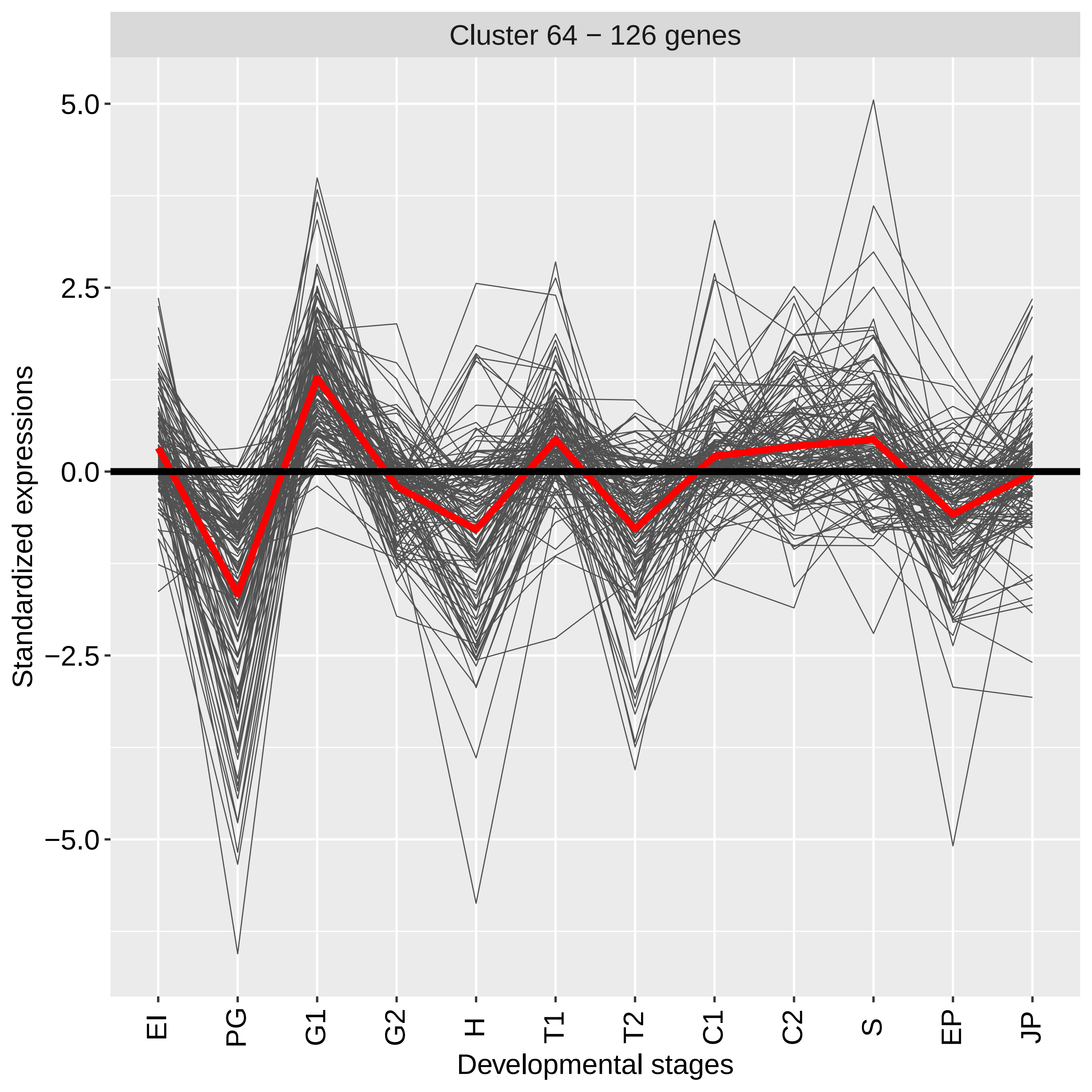
Cluster 64 − 126 genes
Standardized expressions
5.0
2.5
0.0
−2.5
−5.0
EI
PG
G1
G2
H
T1
T2
C1
C2
S
EP
JP
Developmental stages

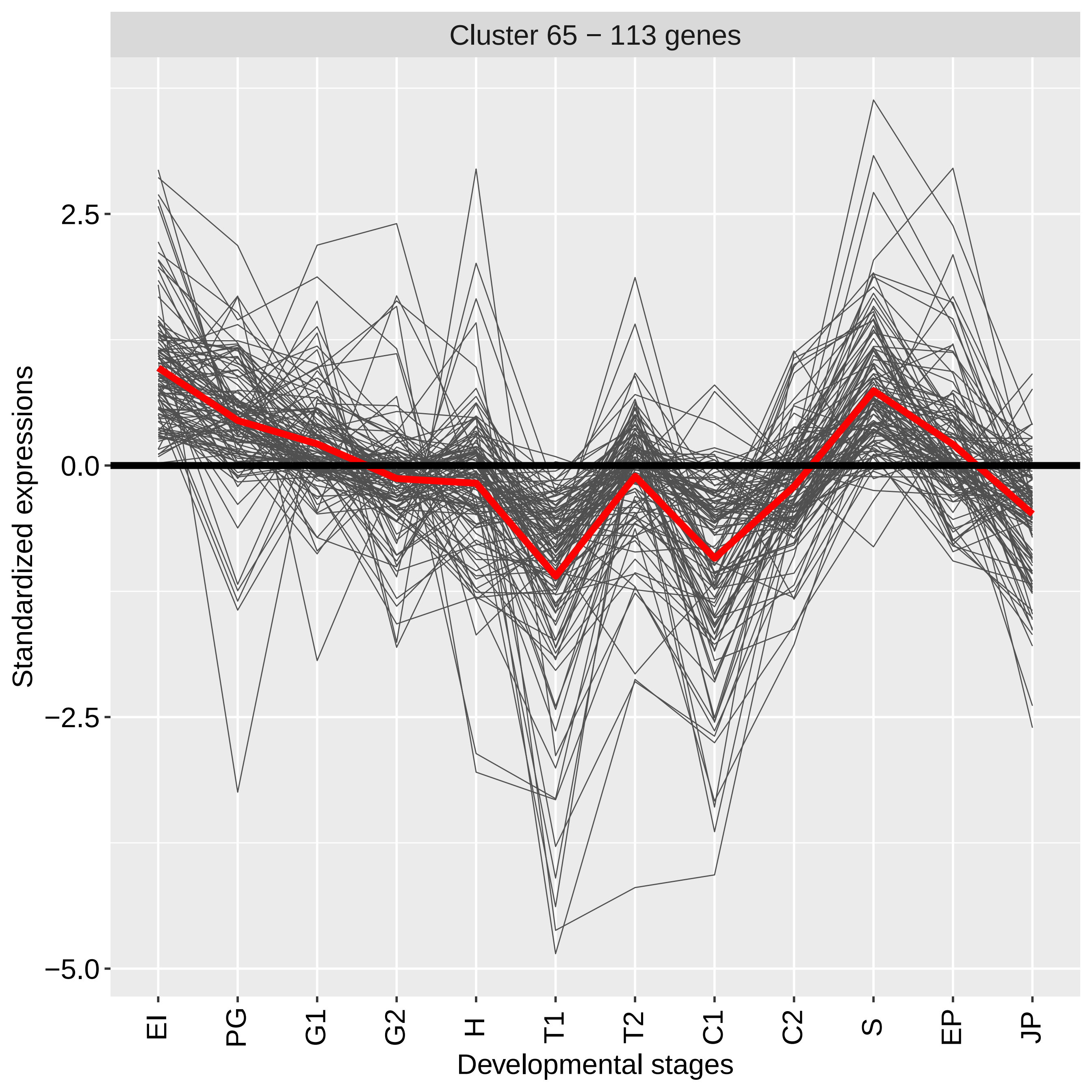

Cluster 65 − 113 genes
Standardized expressions
2.5
0.0
−2.5
−5.0
EI
PG
G1
G2
H
T1
T2
C1
C2
S
EP
JP
Developmental stages

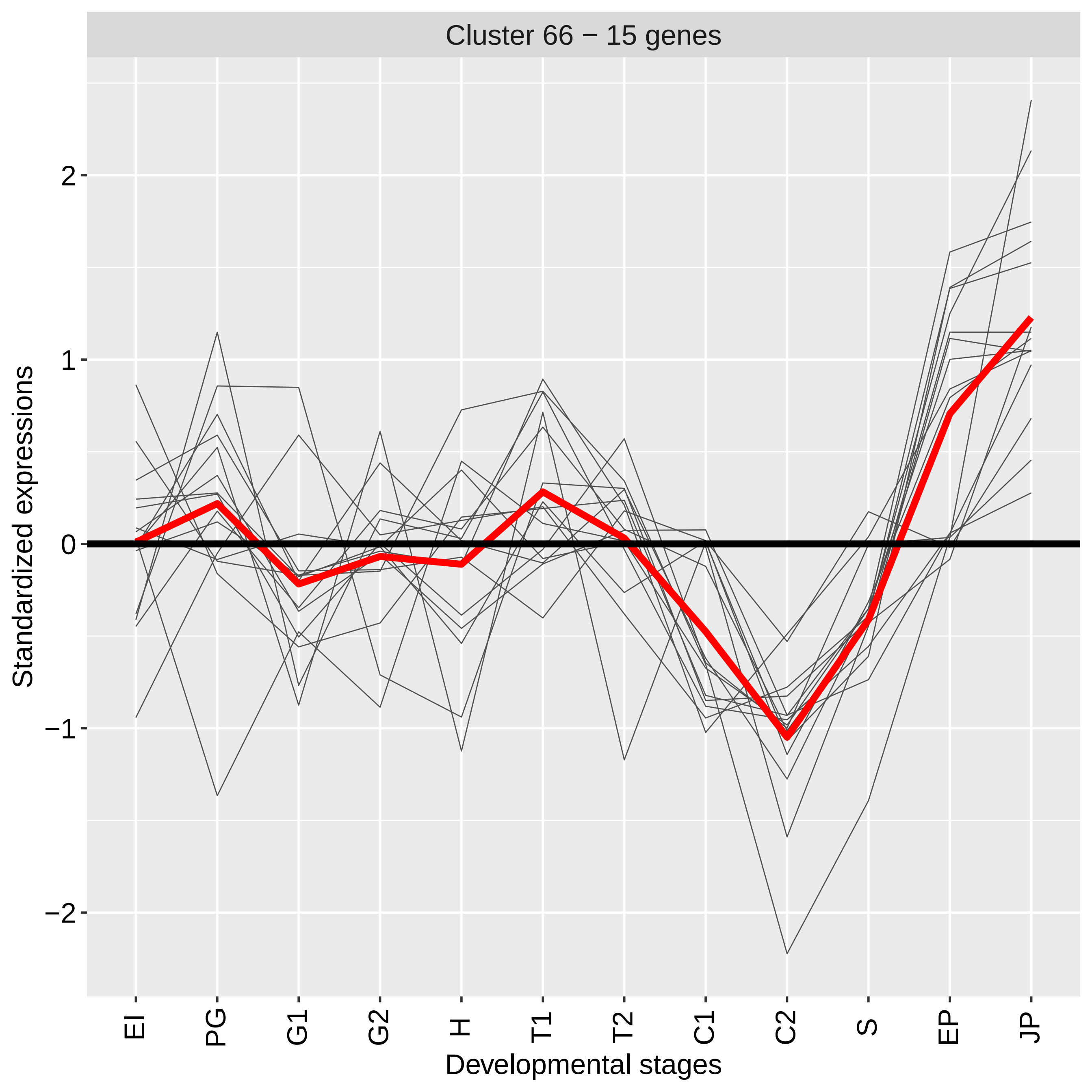
Cluster 66 − 15 genes
Standardized expressions
2
1
0
−1
−2
EI
PG
G1
G2
H
T1
T2
C1
C2
S
EP
JP
Developmental stages

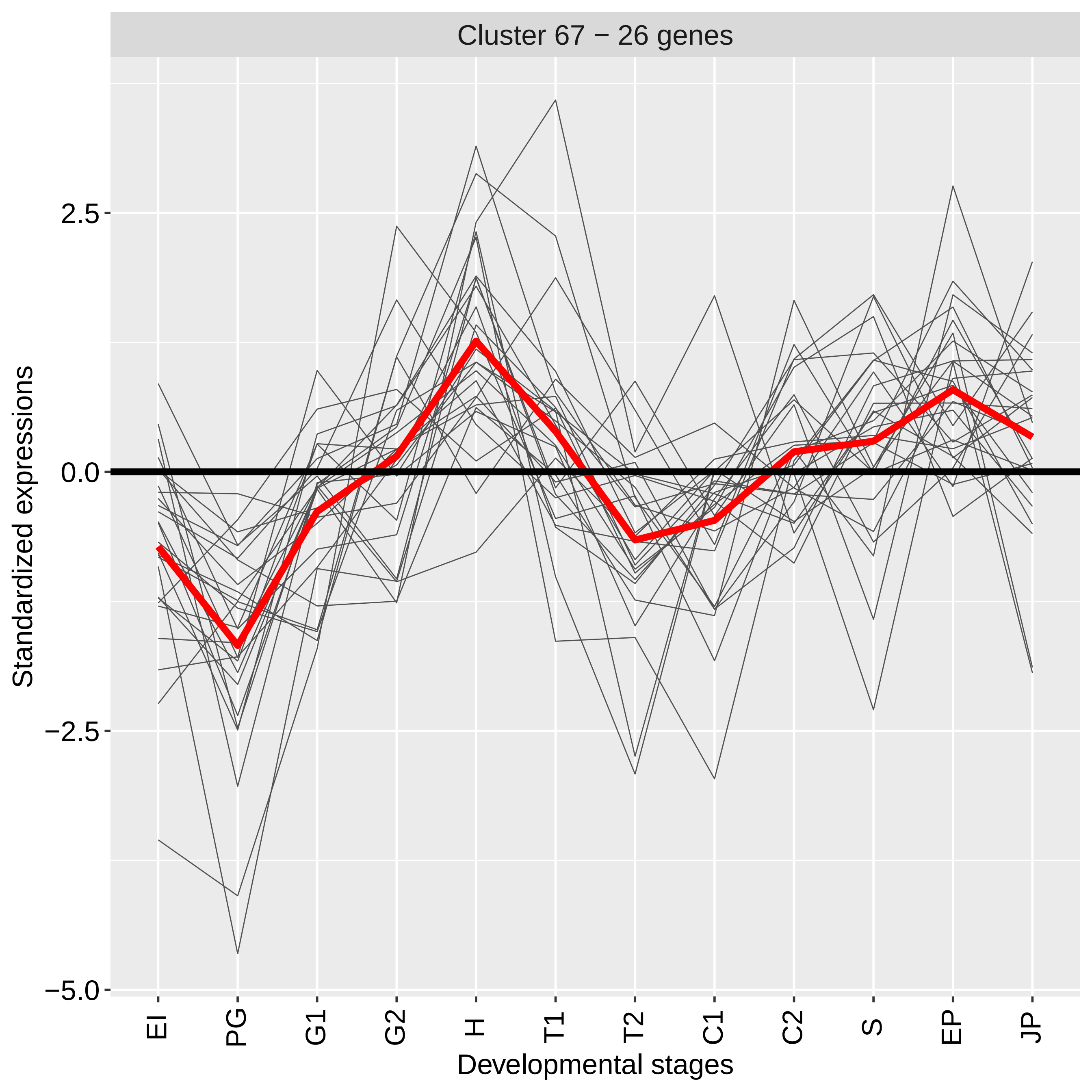
Cluster 67 − 26 genes
Standardized expressions
2.5
0.0
−2.5
−5.0
EI
PG
G1
G2
H
T1
T2
C1
C2
S
EP
JP
Developmental stages

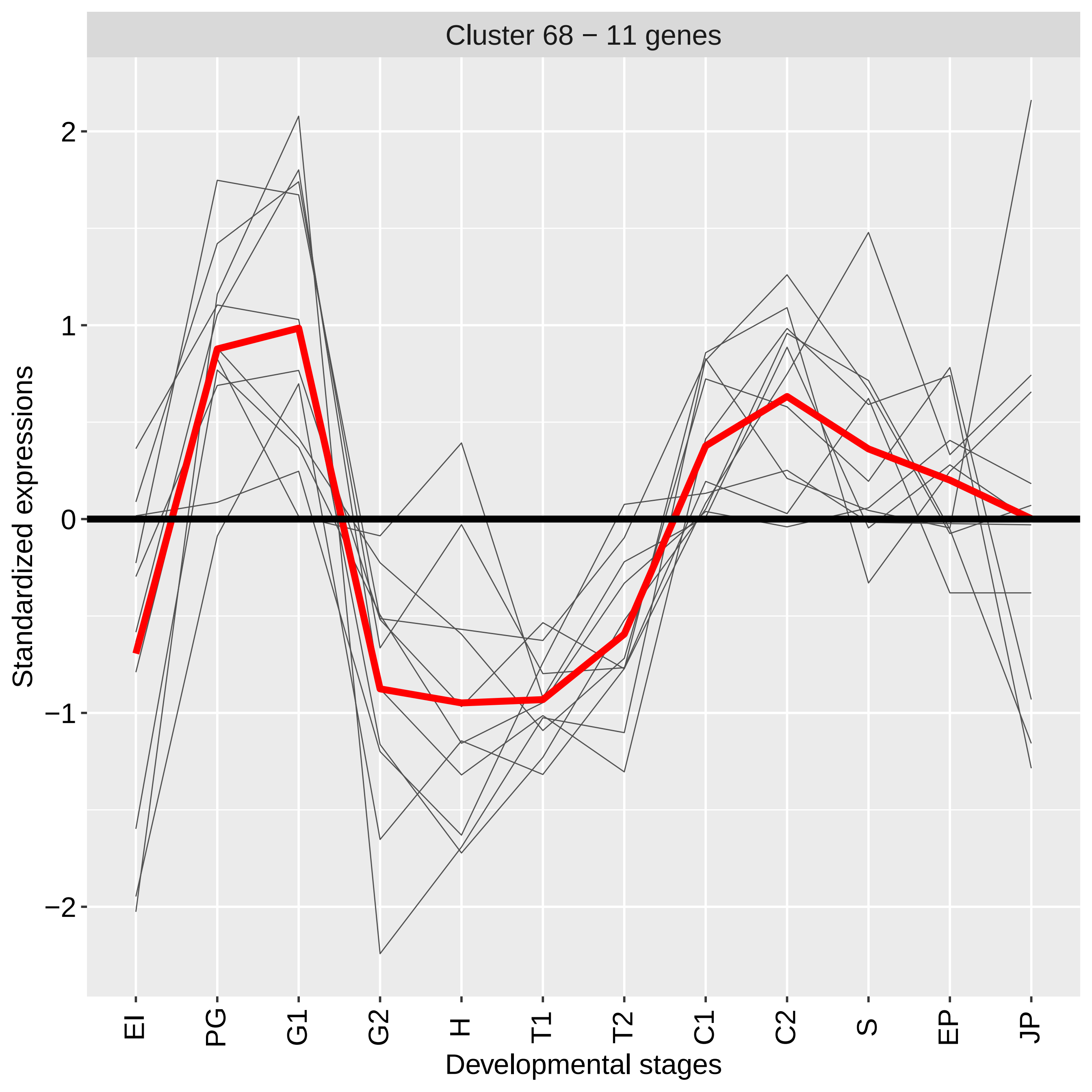

Cluster 68 − 11 genes
Standardized expressions
2
1
0
−1
−2
EI
PG
G1
G2
H
T1
T2
C1
C2
S
EP
JP
Developmental stages

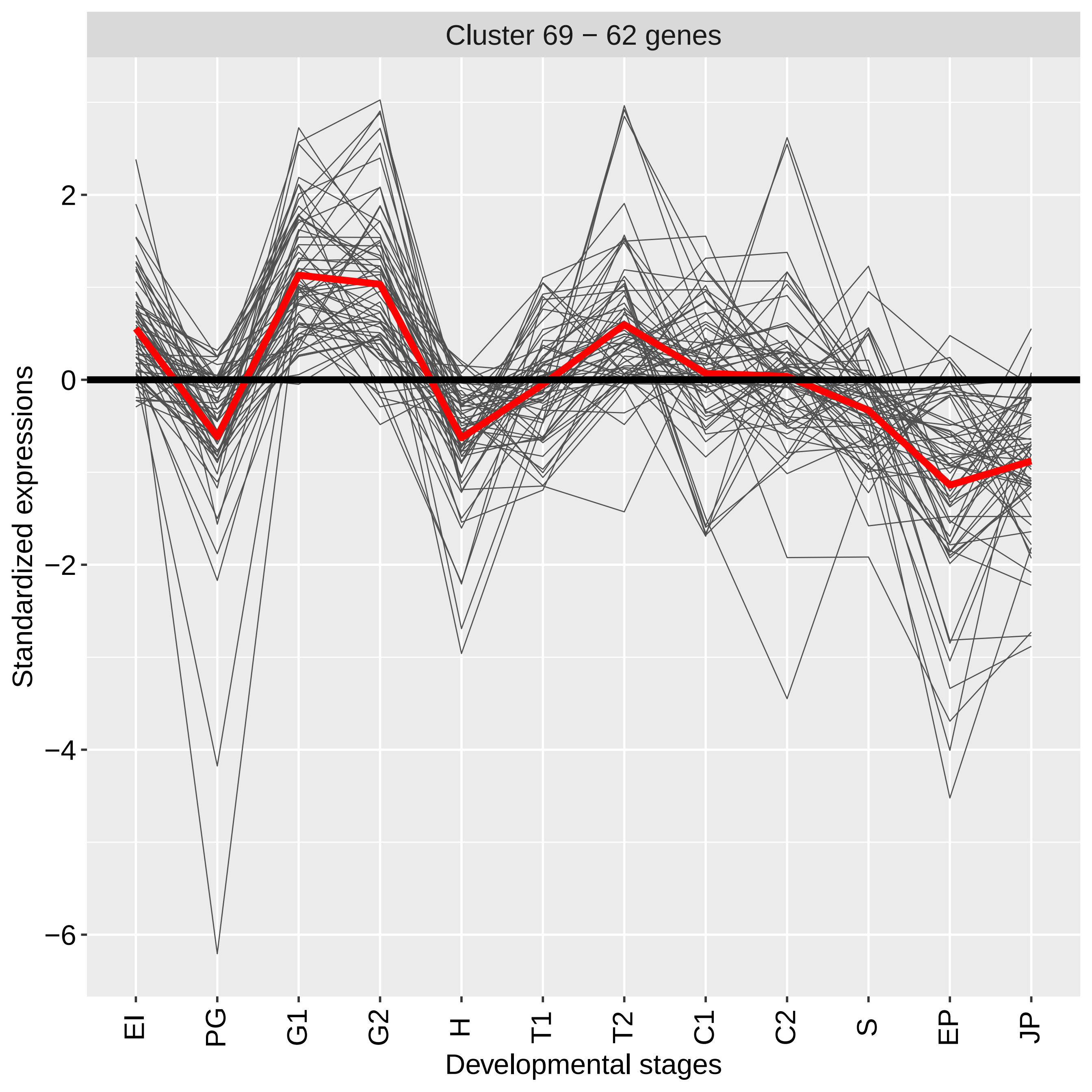

Cluster 69 − 62 genes
Standardized expressions
2
0
−2
−4
−6
EI
PG
G1
G2
H
T1
T2
C1
C2
S
EP
JP
Developmental stages

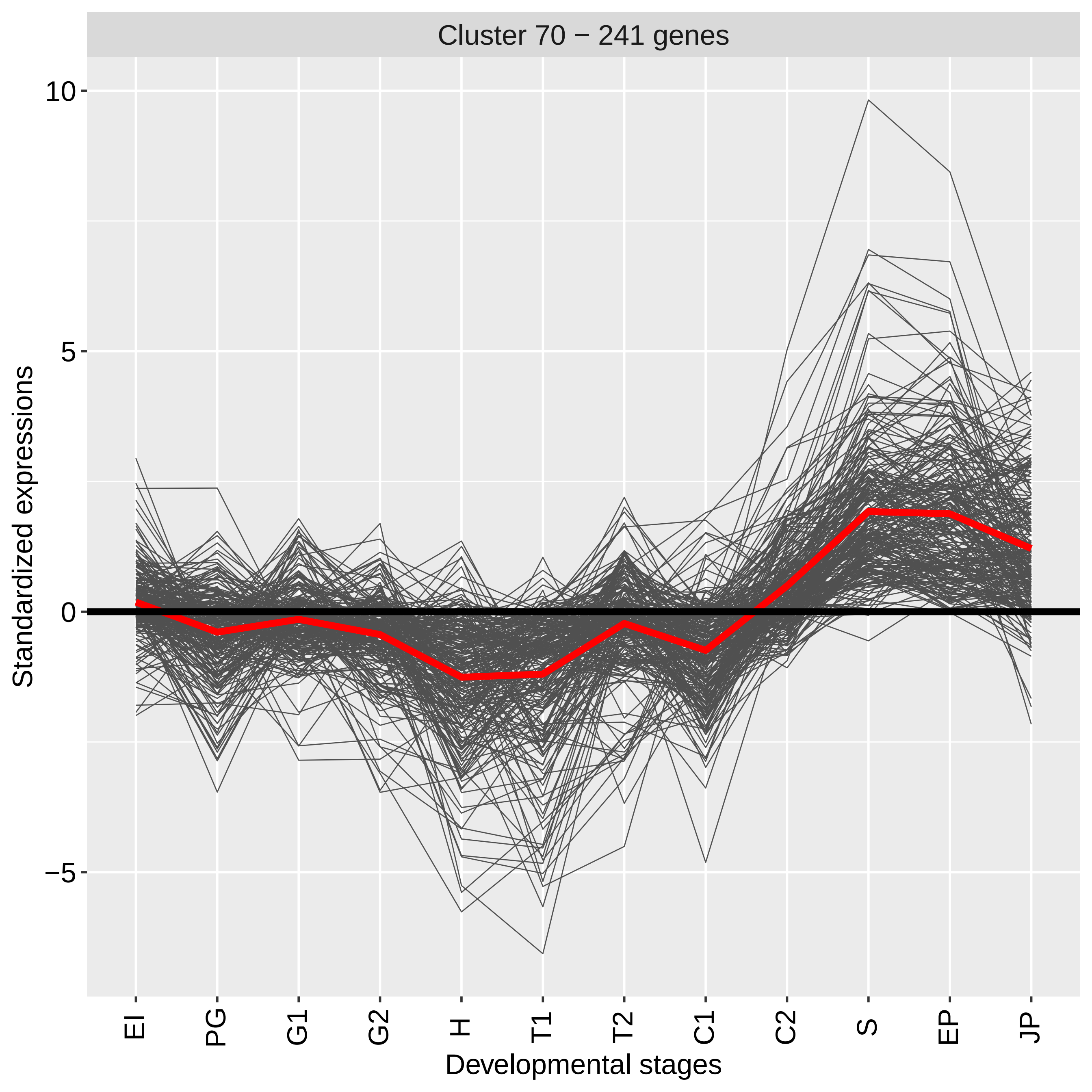

Cluster 70 − 241 genes
10
5
0
−5
Standardized expressions
EI
PG
G1
G2
H
T1
T2
C1
C2
S
EP
JP
Developmental stages

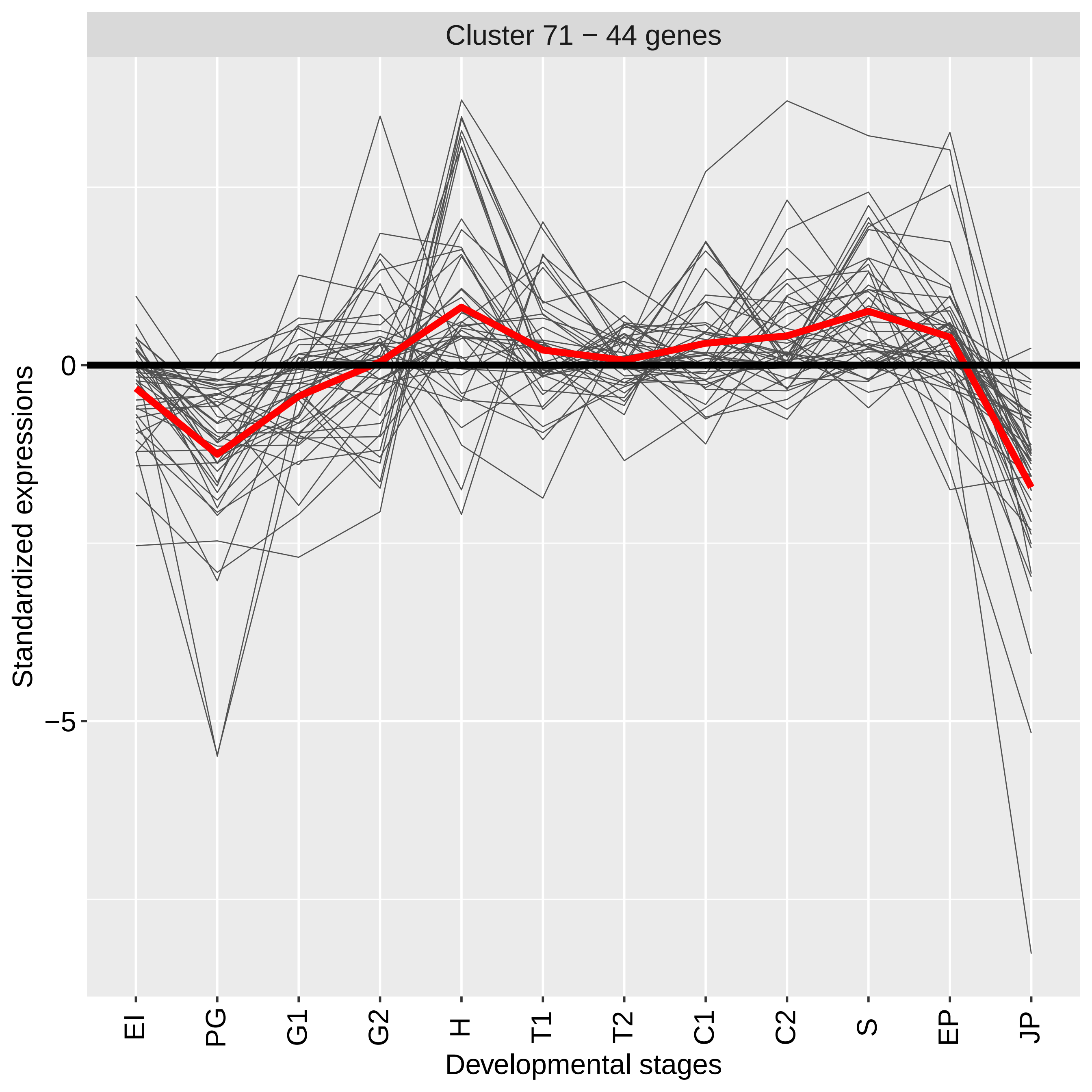
Cluster 71 − 44 genes
Standardized expressions
0
−5
EI
PG
G1
G2
H
T1
T2
C1
C2
S
EP
JP
Developmental stages

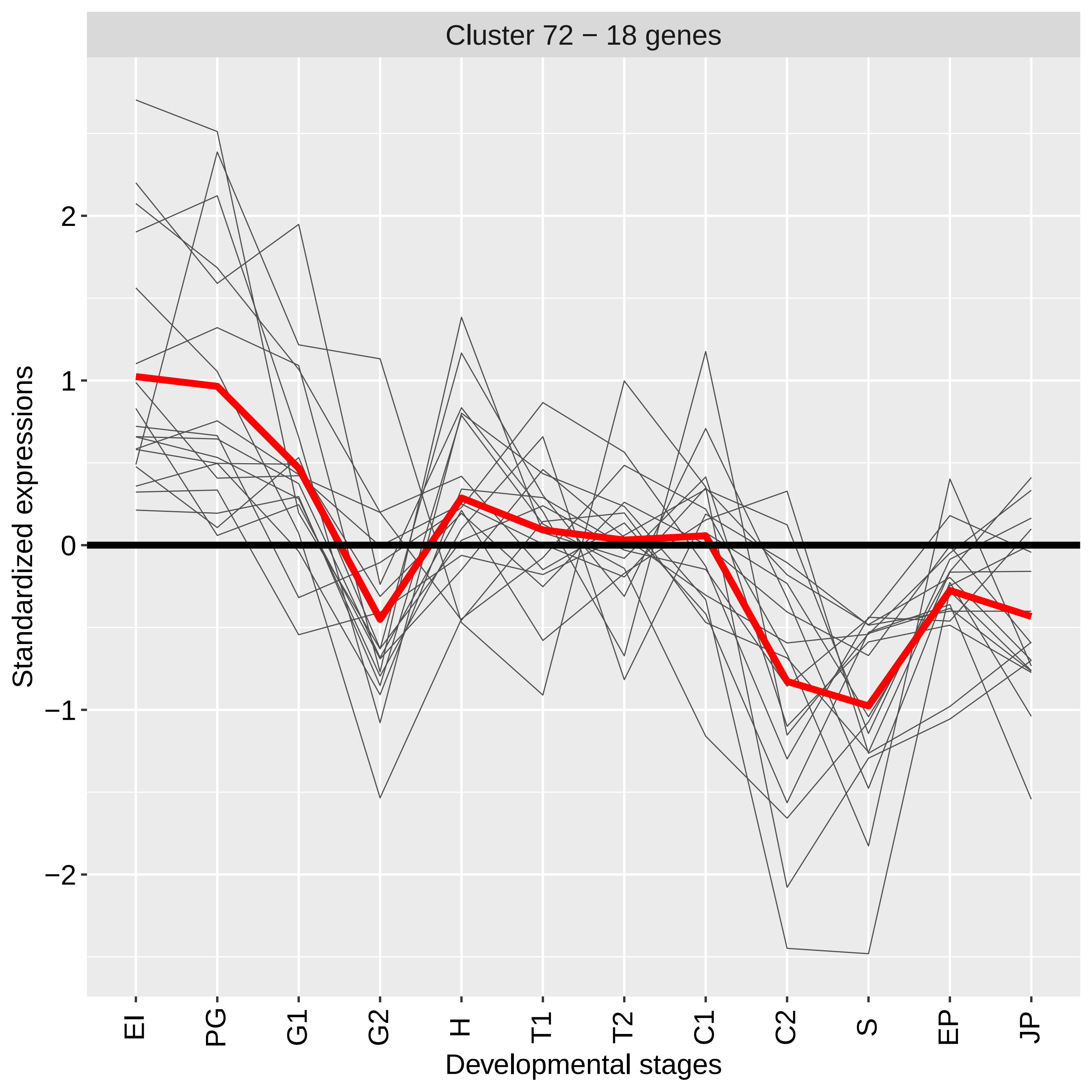

Cluster 72 − 18 genes
Standardized expressions
2
1
0
−1
−2
EI
PG
G1
G2
H
T1
T2
C1
C2
S
EP
JP
Developmental stages

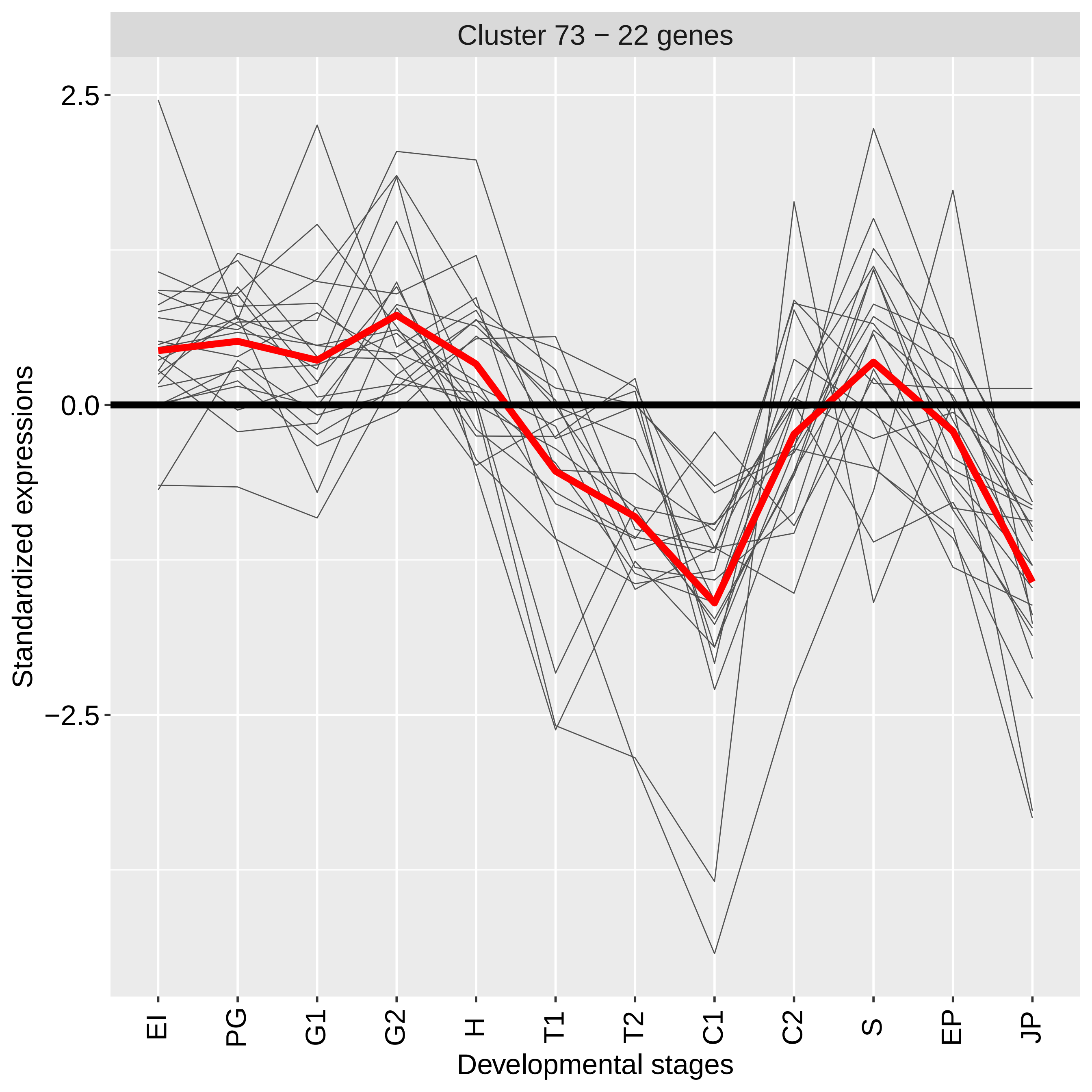

Cluster 73 − 22 genes
Standardized expressions
2.5
0.0
−2.5
EI
PG
G1
G2
H
T1
T2
C1
C2
S
EP
JP
Developmental stages

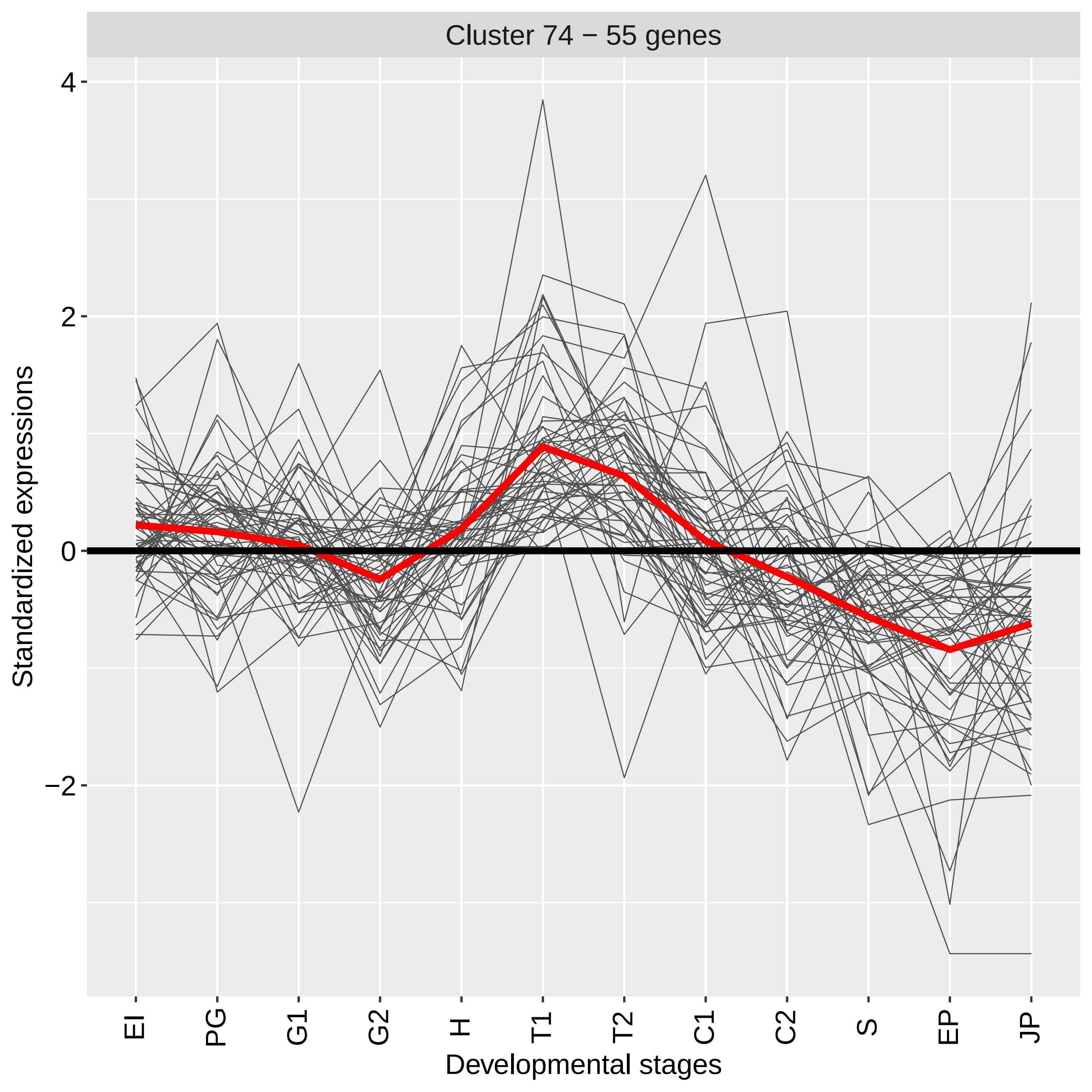
Cluster 74 − 55 genes
Standardized expressions
4
2
0
−2
EI
PG
G1
G2
H
T1
T2
C1
C2
S
EP
JP
Developmental stages

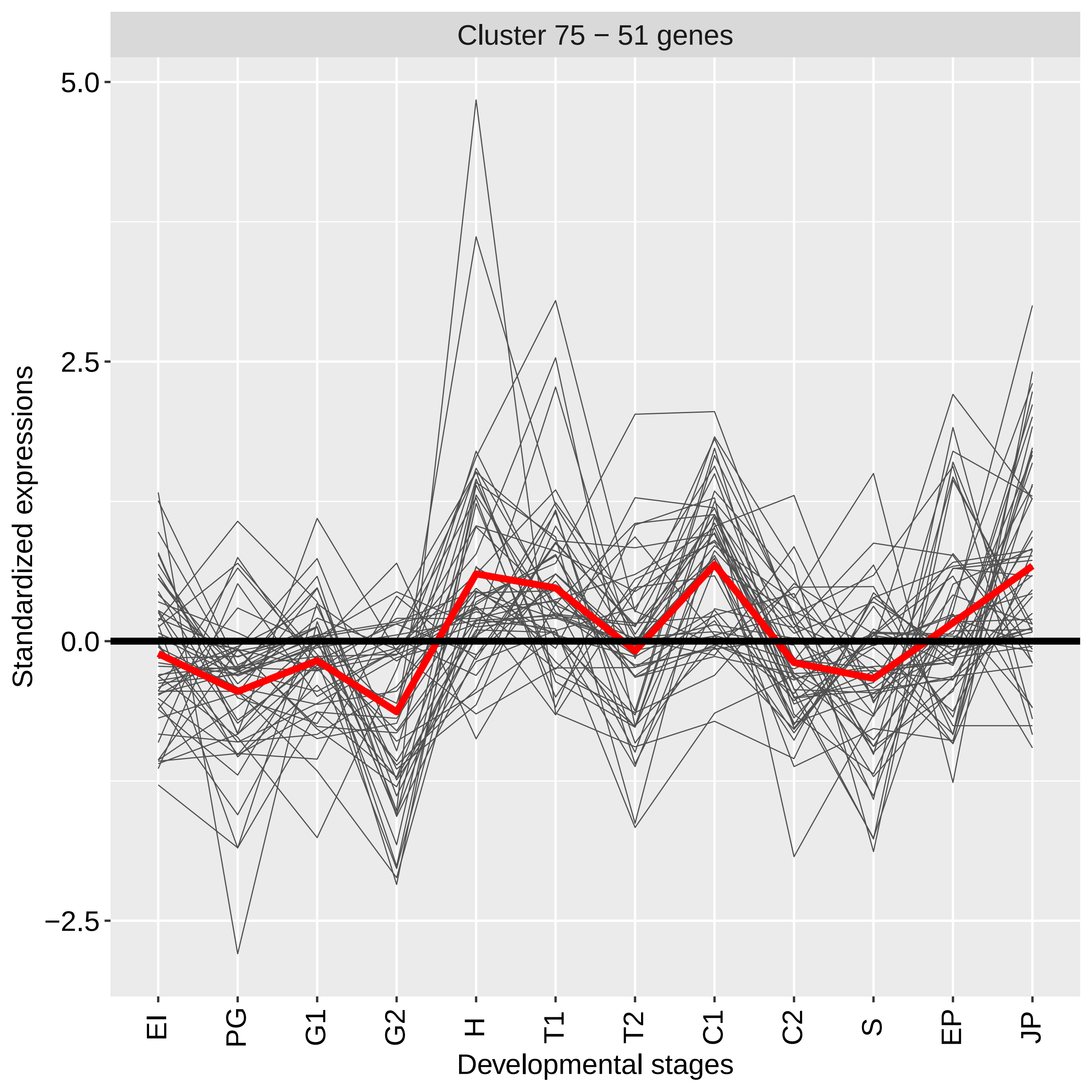
Cluster 75 − 51 genes
Standardized expressions
5.0
2.5
0.0
−2.5
EI
PG
G1
G2
H
T1
T2
C1
C2
S
EP
JP
Developmental stages

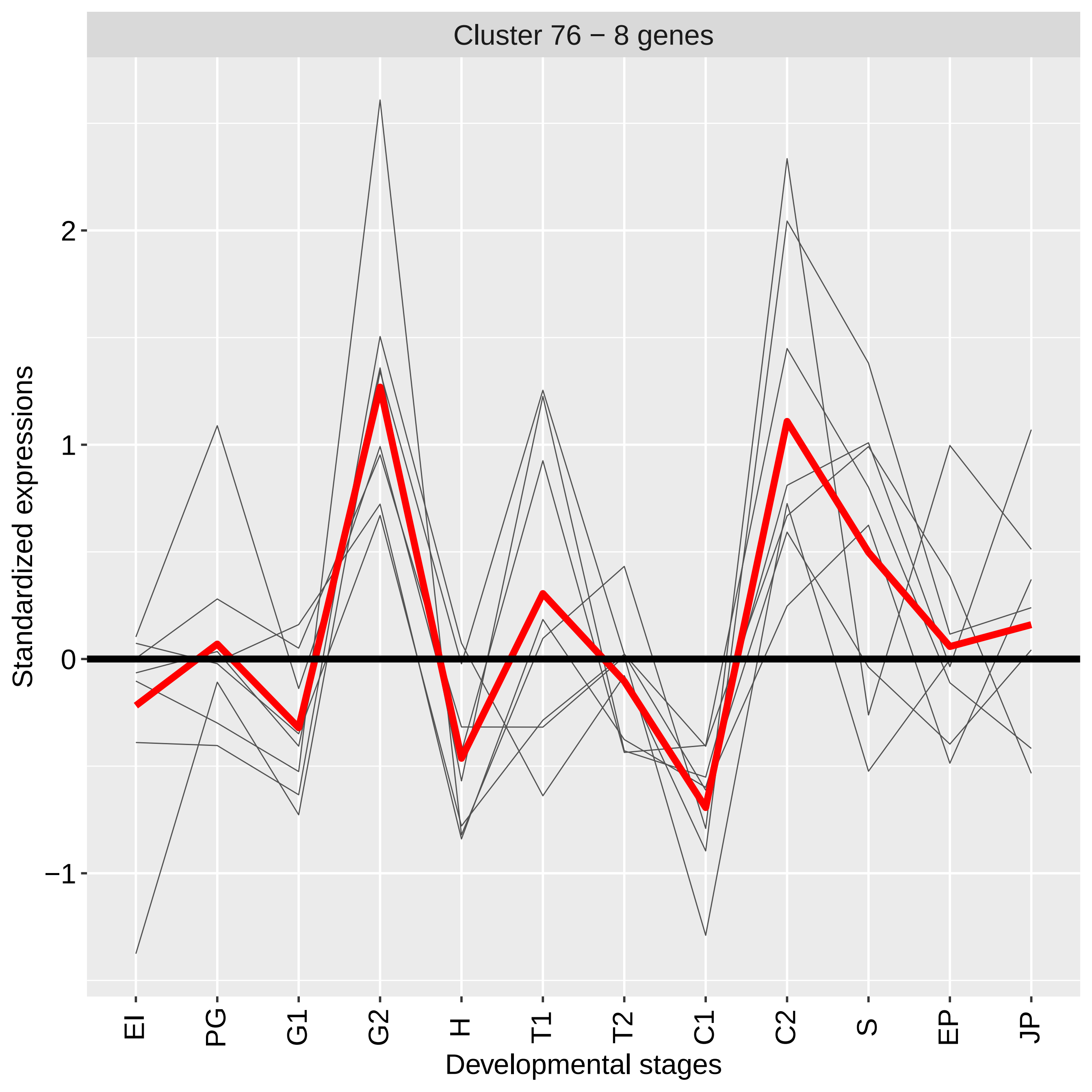

Cluster 76 − 8 genes
Standardized expressions
2
1
0
−1
EI
PG
G1
G2
H
T1
T2
C1
C2
S
EP
JP
Developmental stages

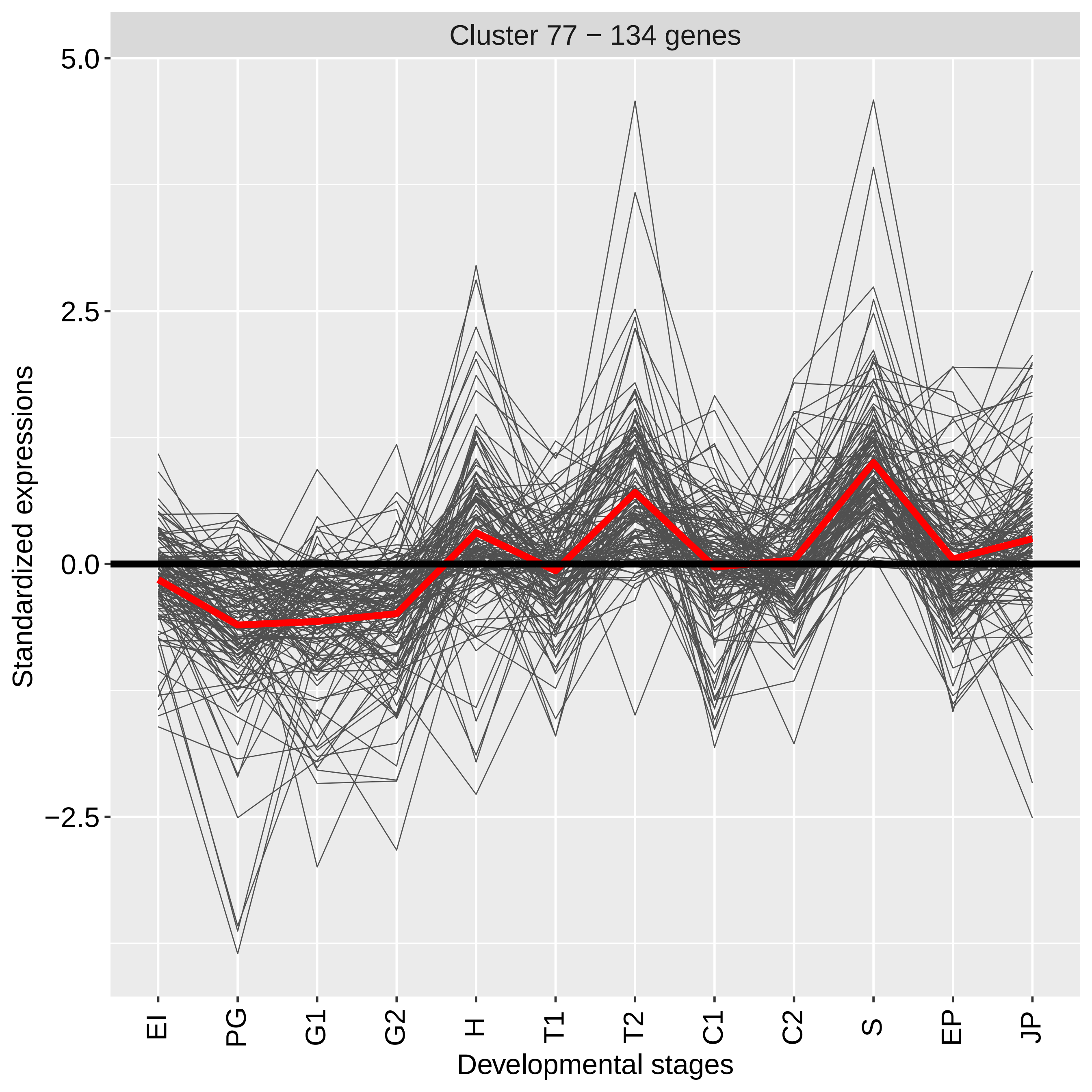
Cluster 77 − 134 genes
5.0
2.5
0.0
−2.5
Standardized expressions
EI
PG
G1
G2
H
T1
T2
C1
C2
S
EP
JP
Developmental stages

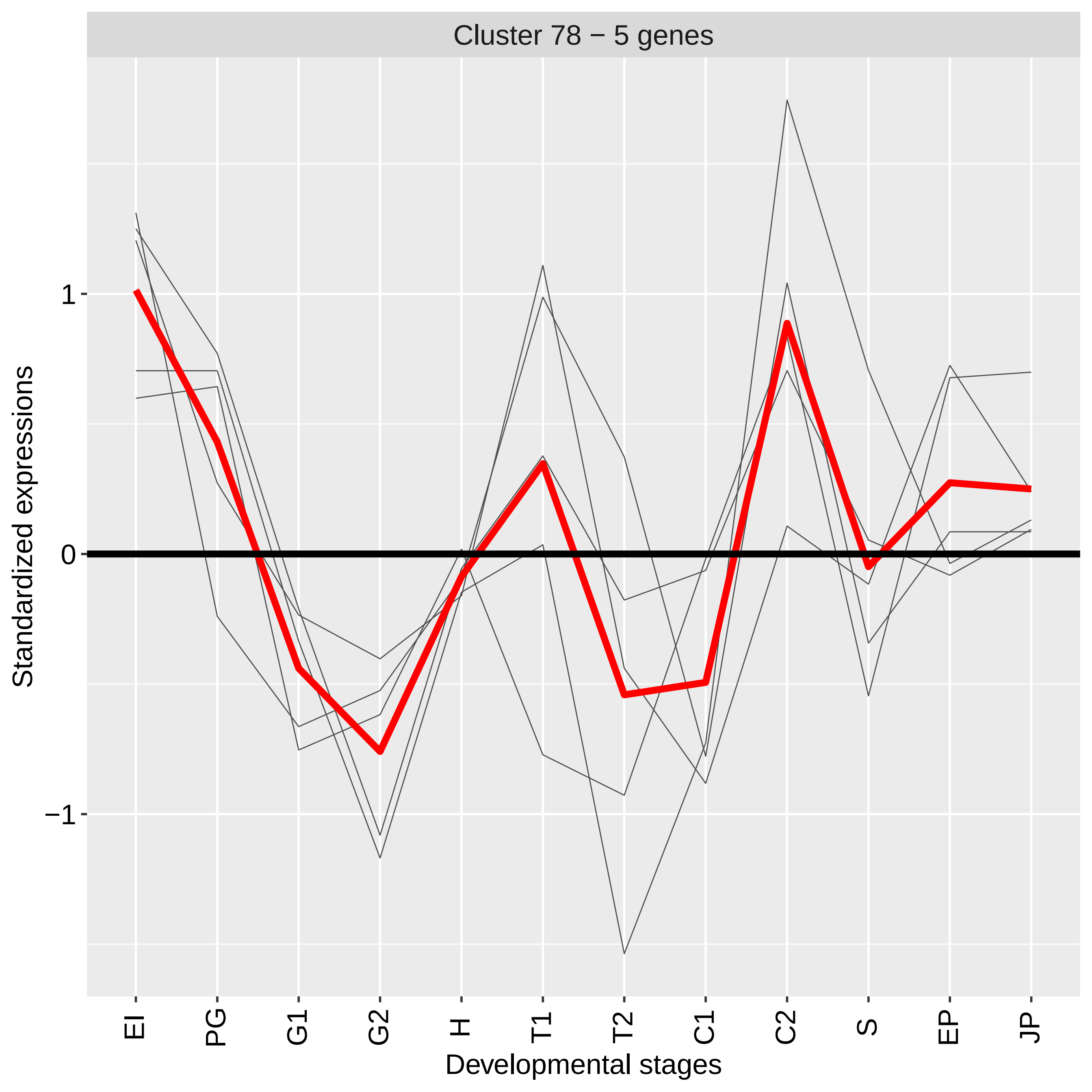

Cluster 78 − 5 genes
Standardized expressions
1
0
−1
EI
PG
G1
G2
H
T1
T2
C1
C2
S
EP
JP
Developmental stages

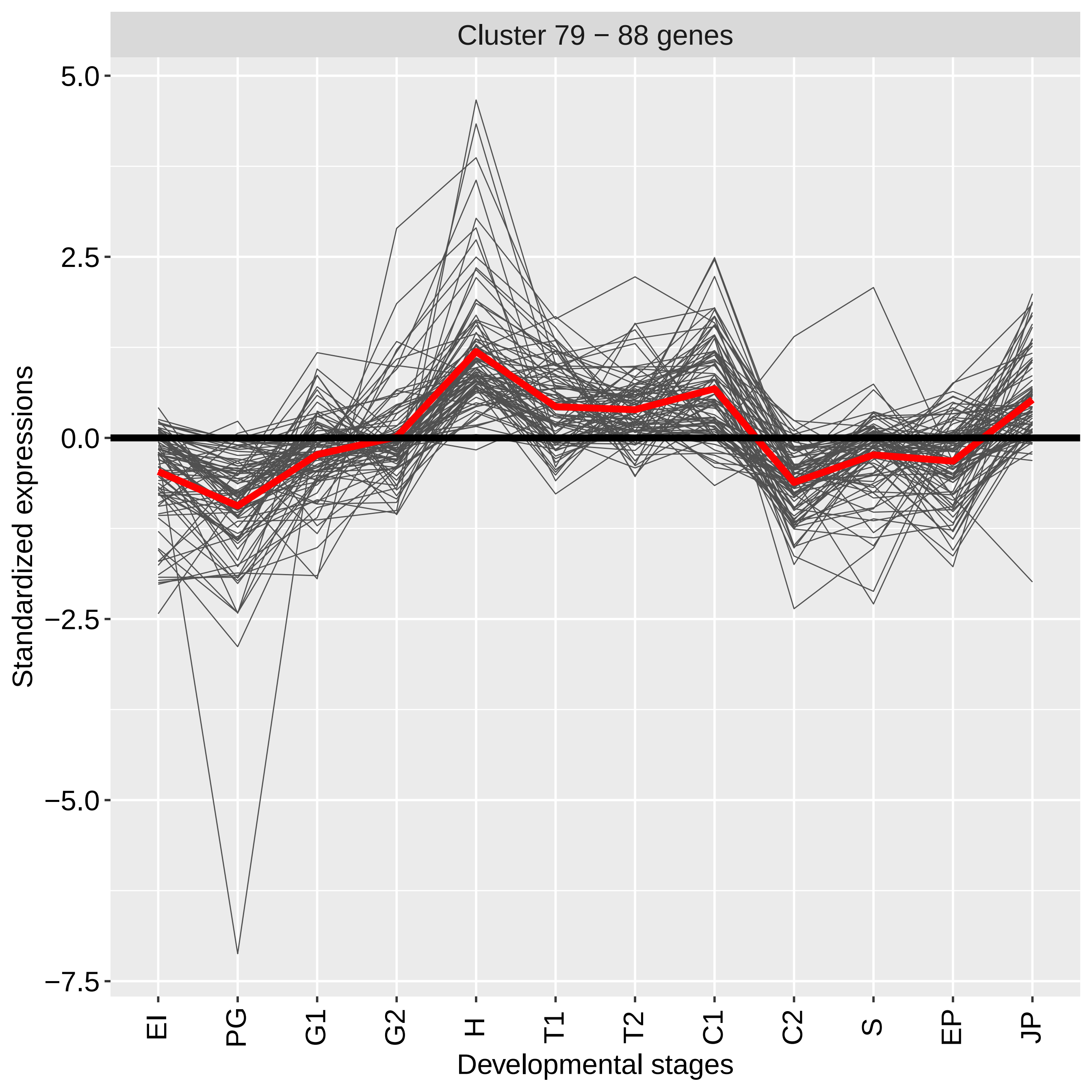
Cluster 79 − 88 genes
5.0
2.5
0.0
−2.5
−5.0
−7.5
Standardized expressions
EI
PG
G1
G2
H
T1
T2
C1
C2
S
EP
JP
Developmental stages

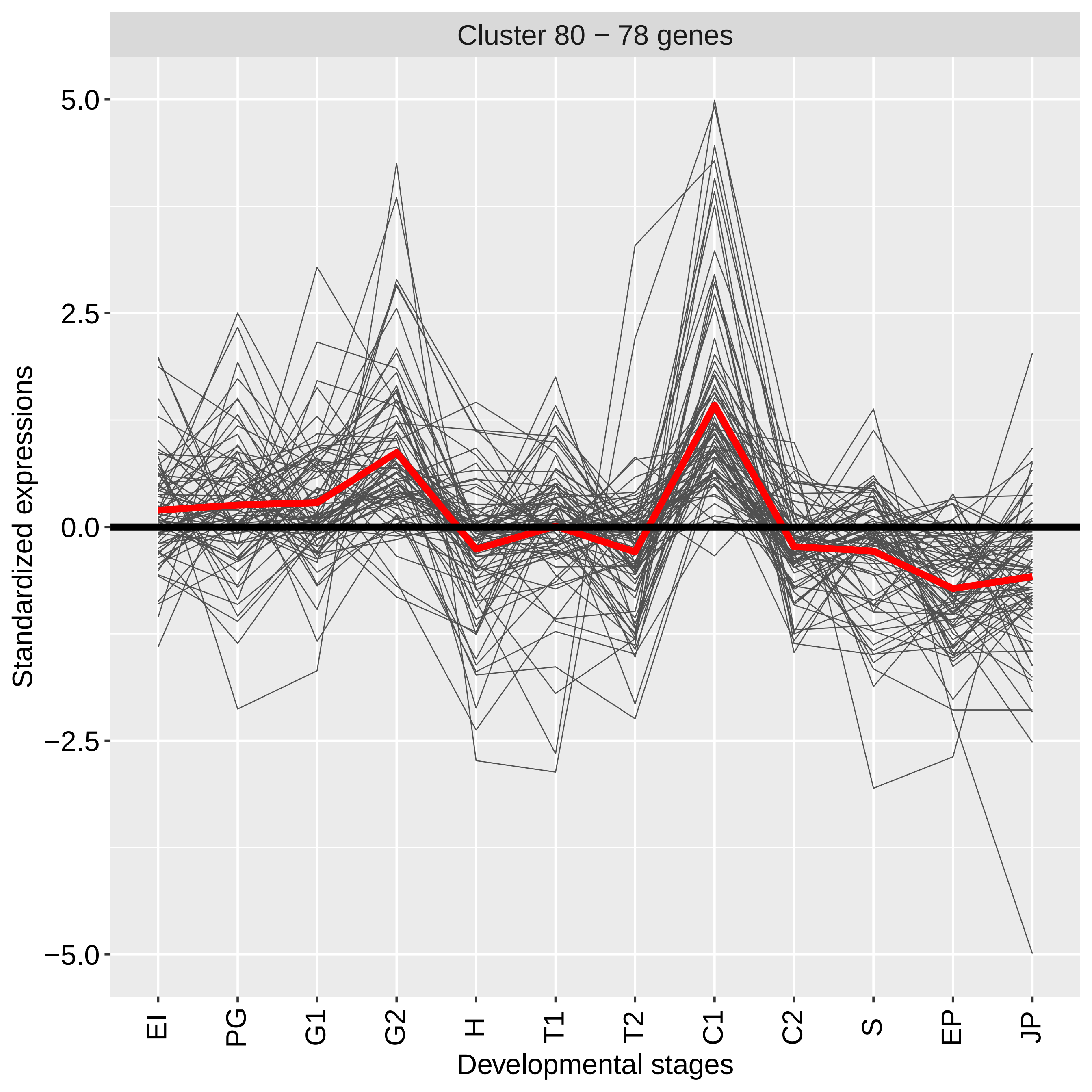
Cluster 80 − 78 genes
5.0
2.5
0.0
−2.5
−5.0
Standardized expressions
EI
PG
G1
G2
H
T1
T2
C1
C2
S
EP
JP
Developmental stages

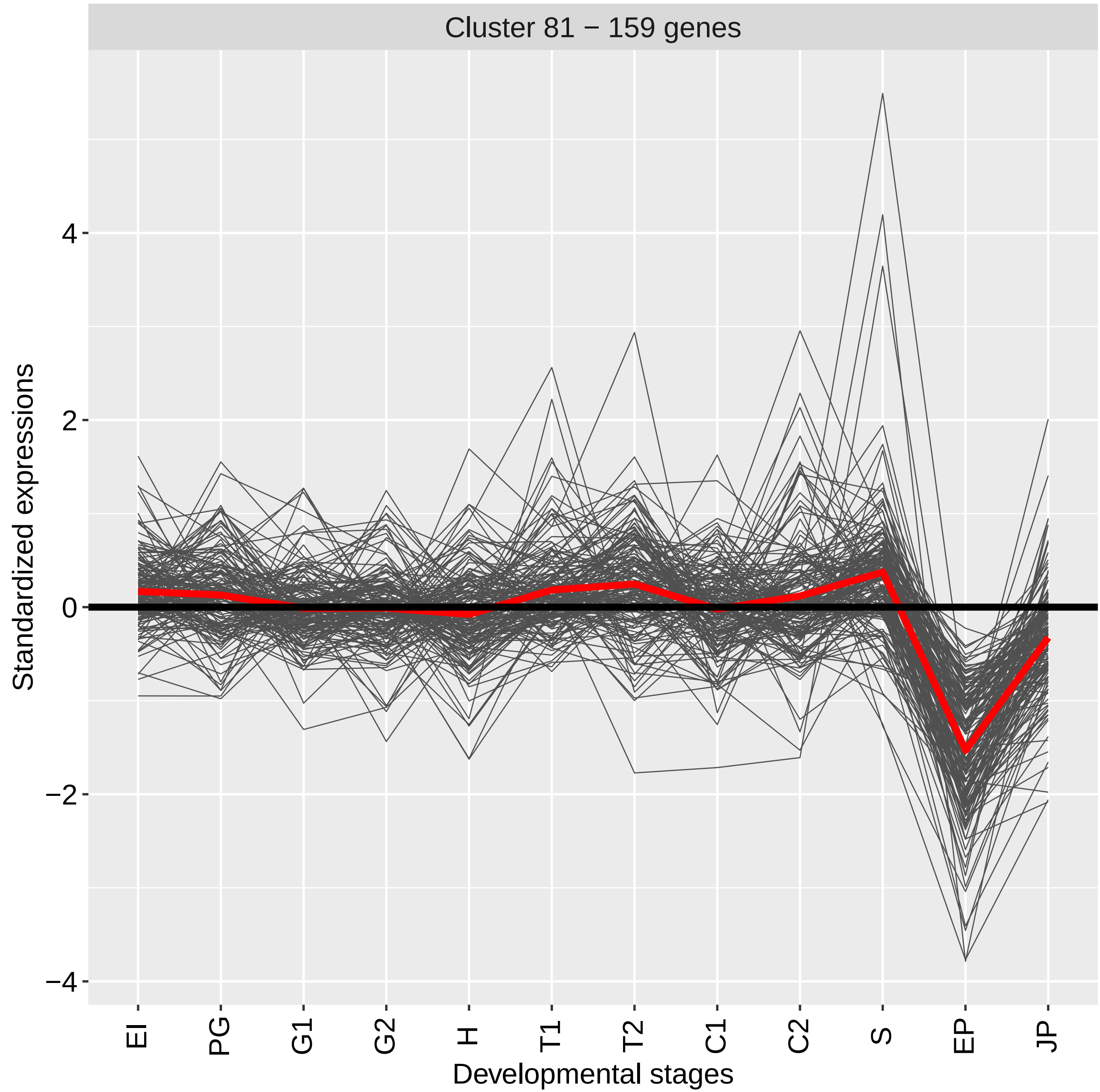
Cluster 81 − 159 genes
Standardized expressions
4
2
0
−2
−4
EI
PG
G1
G2
H
T1
T2
C1
C2
S
EP
JP
Developmental stages

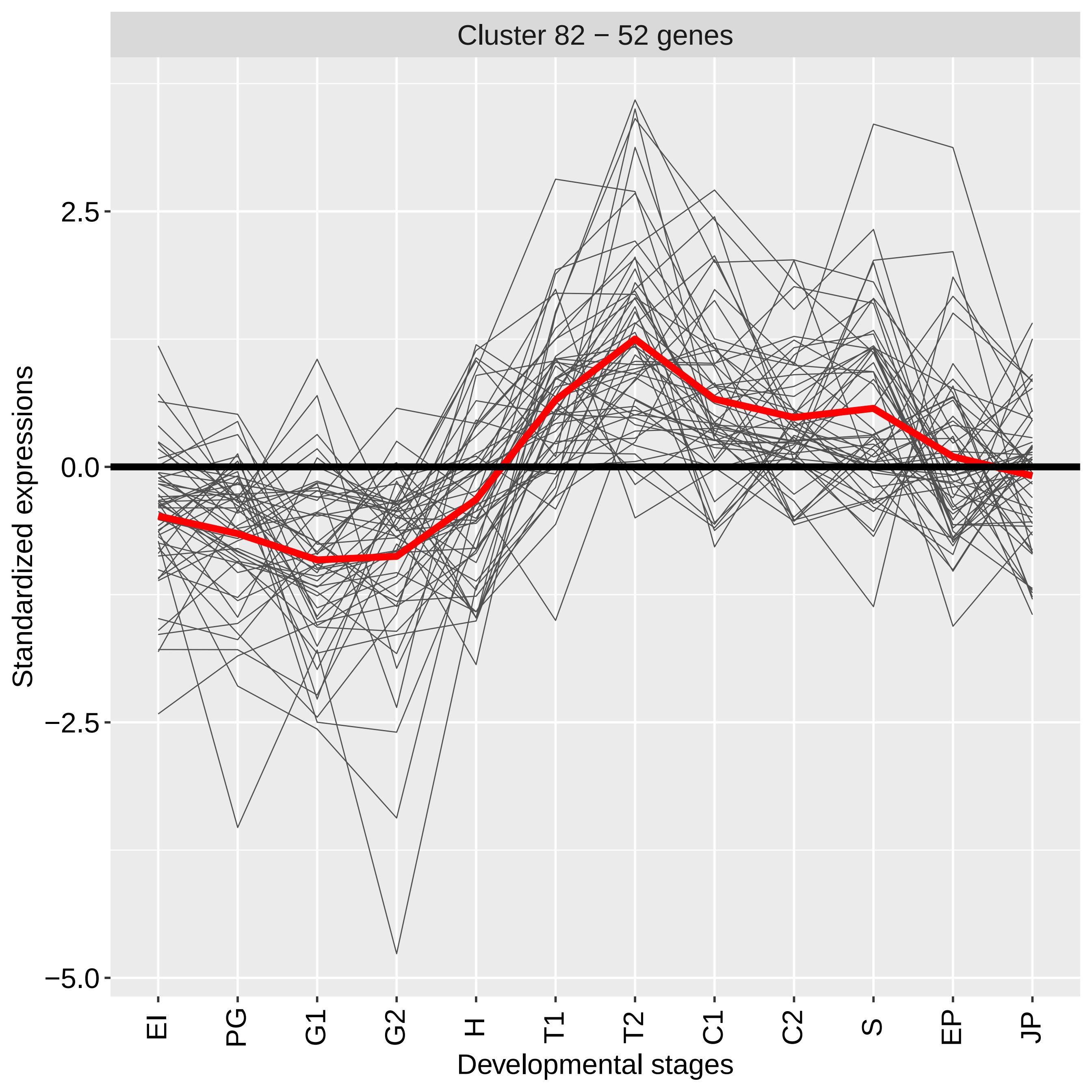
Cluster 82 − 52 genes
Standardized expressions
2.5
0.0
−2.5
−5.0
EI
PG
G1
G2
H
T1
T2
C1
C2
S
EP
JP
Developmental stages

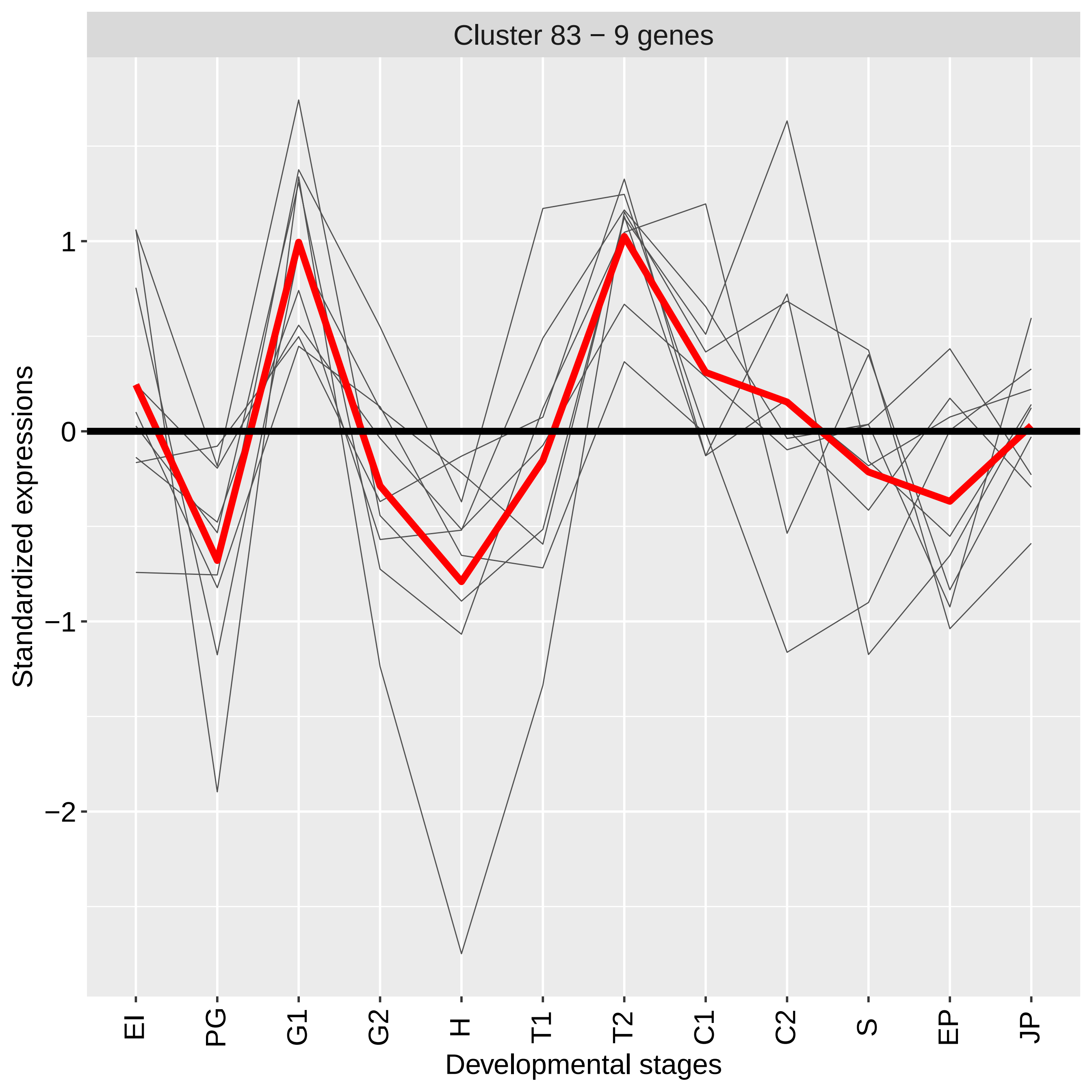

Cluster 83 − 9 genes
Standardized expressions
1
0
−1
−2
EI
PG
G1
G2
H
T1
T2
C1
C2
S
EP
JP
Developmental stages

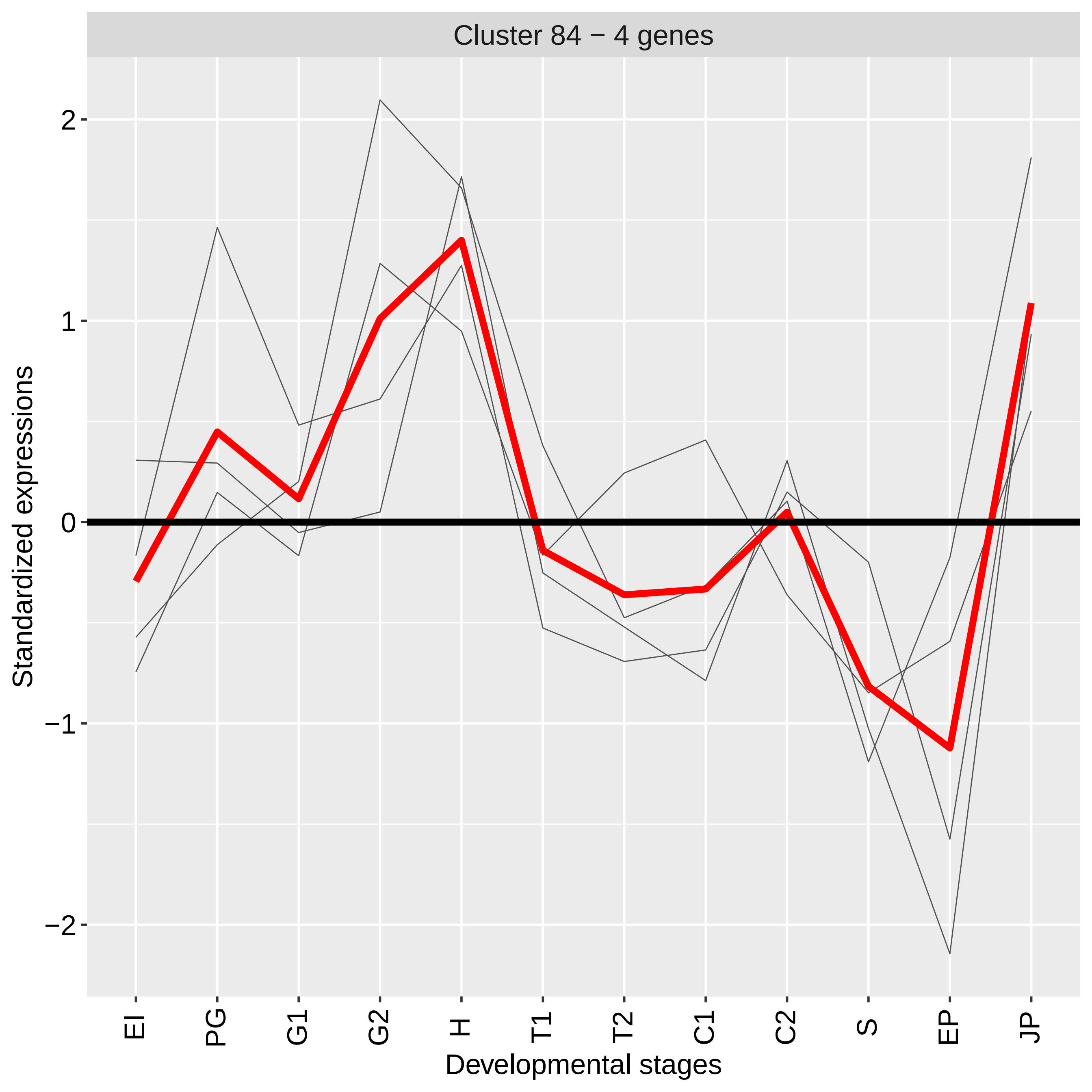

Cluster 84 − 4 genes
Standardized expressions
2
1
0
−1
−2
EI
PG
G1
G2
H
T1
T2
C1
C2
S
EP
JP
Developmental stages

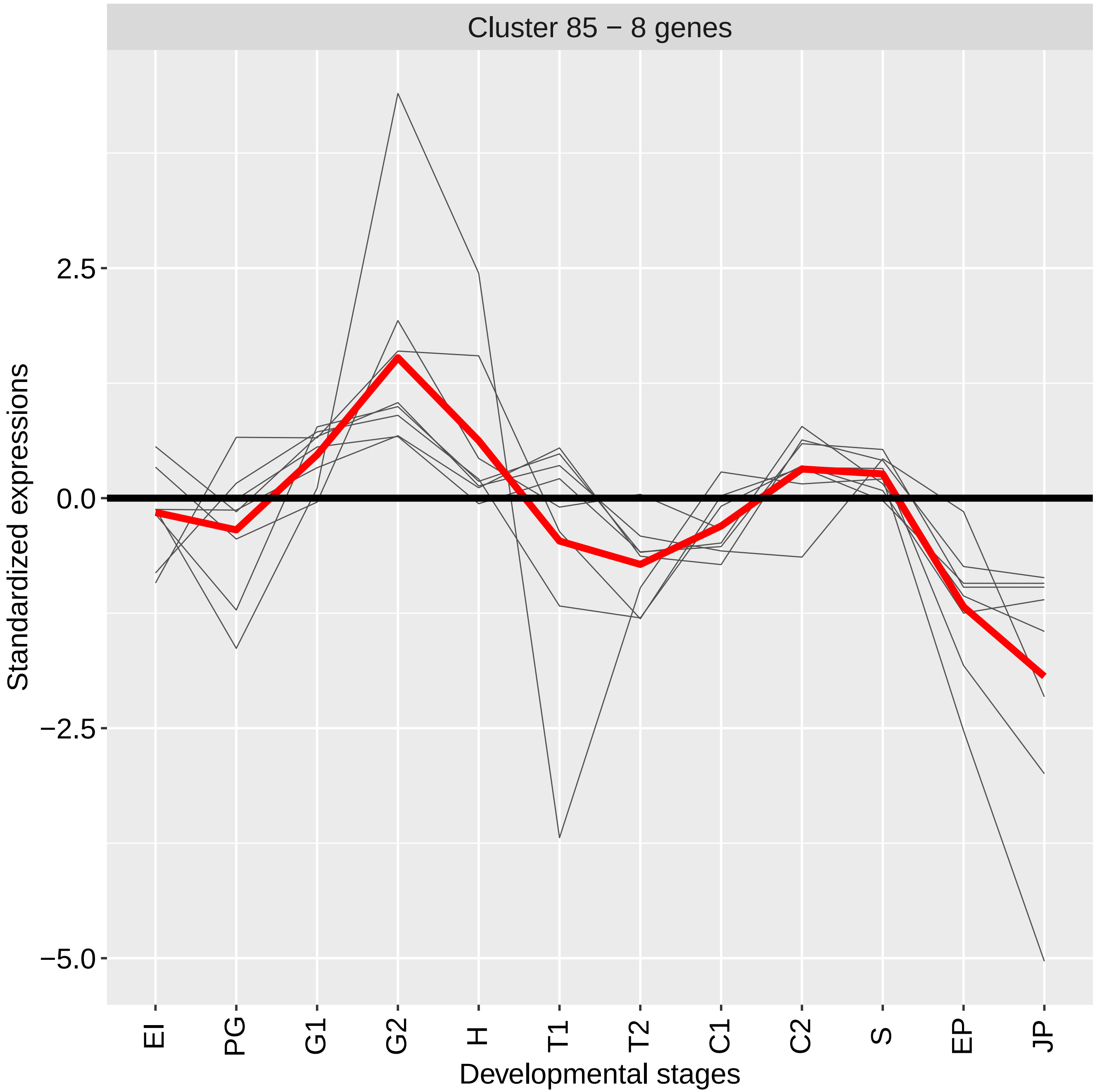

Cluster 85 − 8 genes
Standardized expressions
2.5
0.0
−2.5
−5.0
EI
PG
G1
G2
H
T1
T2
C1
C2
S
EP
JP
Developmental stages
