# Supplementary material for: Developmental phylotranscriptomics in grapevine suggests an ancestral role of somatic embryogenesis
Source: Commun Biol. 2025 Feb 20;8:265. doi: 10.1038/s42003-025-07712-w (PMC11839975; doi:10.1038/s42003-025-07712-w)
Supplement: Supplementary file 9 — Supplementary Data 6 [file 42003_2025_7712_MOESM9_ESM.zip › Supplementary_Data_6_linear_phylogeny.pdf]

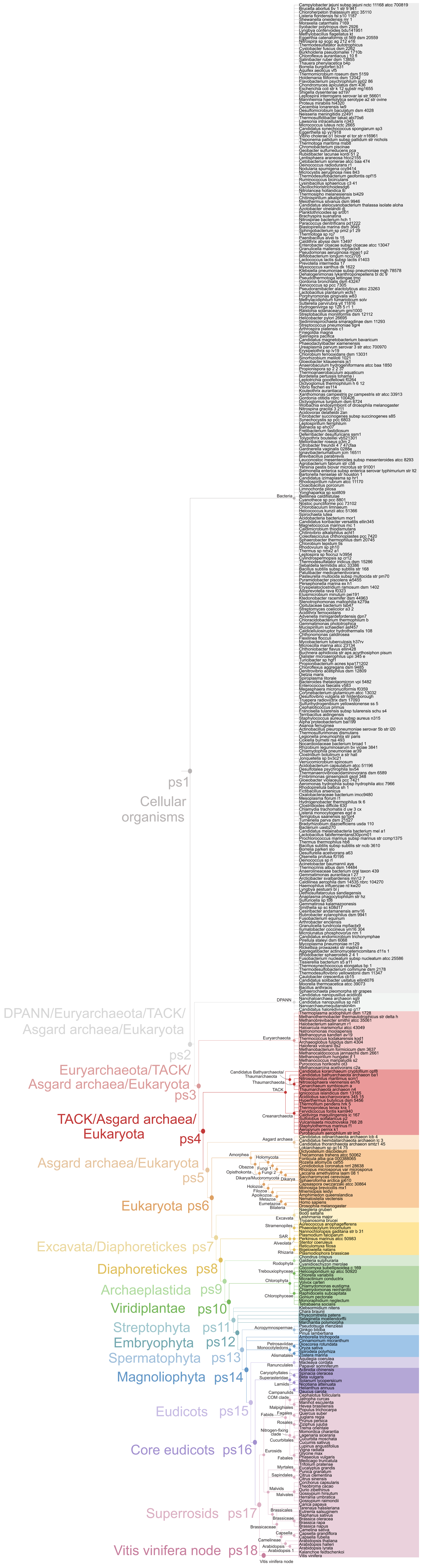

ps1
Cellular organisms
Bacteria
DPANN
DPANN/Euryarchaeota/TACK/ Asgard archaea/Eukaryota
ps2
Euryarchaeota/TACK/ Asgard archaea/Eukaryota
ps3
Euryarchaeota
TACK/Asgard archaea/ Eukaryota
ps4
TACK
Crenarchaeota
Asgard archaea
Asgard archaea/Eukaryota
ps5
Eukaryota ps6
Excavata
Stramenopiles
SAR
Alveolata
Rhizaria
Excavata/Diaphoretickes ps7
Diaphoretickes ps8
Rodophyta
Archaeplastida ps9
Chlorophyta
Viridiplantae ps10
Streptophyta ps11
Embryophyta ps12
Acrogymnospermae
Spermatophyta ps13
Magnoliophyta ps14
Eudicots ps15
Core eudicots ps16
Superrosids ps17
Vitis vinifera node ps18
Vitis vinifera node
