# Supplementary material for: Developmental phylotranscriptomics in grapevine suggests an ancestral role of somatic embryogenesis
Source: Commun Biol. 2025 Feb 20;8:265. doi: 10.1038/s42003-025-07712-w (PMC11839975; doi:10.1038/s42003-025-07712-w)
Supplement: Supplementary file 15 — Reporting Summary [file 42003_2025_7712_MOESM15_ESM.pdf]

nature portfolio

Corresponding author(s): Tomislav Domazet-Lošo

Last updated by author(s): Jan 10, 2025

Reporting Summary

Nature Portfolio wishes to improve the reproducibility of the work that we publish. This form provides structure for consistency and transparency in reporting. For further information on Nature Portfolio policies, see our Editorial Policies and the Editorial Policy Checklist.

Statistics

For all statistical analyses, confirm that the following items are present in the figure legend, table legend, main text, or Methods section.

n/a	Confirmed	
☐	☒	The exact sample size (*n*) for each experimental group/condition, given as a discrete number and unit of measurement
☐	☒	A statement on whether measurements were taken from distinct samples or whether the same sample was measured repeatedly
☐	☒	The statistical test(s) used AND whether they are one- or two-sided<br>*Only common tests should be described solely by name; describe more complex techniques in the Methods section.*
☒	☐	A description of all covariates tested
☐	☒	A description of any assumptions or corrections, such as tests of normality and adjustment for multiple comparisons
☐	☒	A full description of the statistical parameters including central tendency (e.g. means) or other basic estimates (e.g. regression coefficient) AND variation (e.g. standard deviation) or associated estimates of uncertainty (e.g. confidence intervals)
☐	☒	For null hypothesis testing, the test statistic (e.g. *F*, *t*, *r*) with confidence intervals, effect sizes, degrees of freedom and *P* value noted<br>*Give P values as exact values whenever suitable.*
☒	☐	For Bayesian analysis, information on the choice of priors and Markov chain Monte Carlo settings
☒	☐	For hierarchical and complex designs, identification of the appropriate level for tests and full reporting of outcomes
☐	☒	Estimates of effect sizes (e.g. Cohen's *d*, Pearson's *r*), indicating how they were calculated

Our web collection on statistics for biologists contains articles on many of the points above.

Software and code

Policy information about availability of computer code

Data collection	No specific software was used for data collection.
Data analysis	Sequence quality and read coverage were checked using the FastQC V0.11.9. Paired-end sequences were mapped onto the V. vinifera reference genome using BBMap V38.75. Generating, sorting, and indexing of BAM files was done by using SAMtools V1.11. Quantification of mapped reads for each V. vinifera open reading frame was done using the R rsamtools package V2.10.0 and open reading frames were retrieved using the GenomicAlignments R package V1.30.0. We estimated expression similarity between replicates and developmental stages using the PCA analysis implemented in the R package DESeq2 V1.34.0. We clustered the standardized expression profiles of genes using the DP_GP_cluster. We estimated the pairwise differential gene expression between the individual developmental stages, as well as overall differential expression for every gene across all developmental stages using DESeq2 V1.30.1 R package. We used eggNOG-mapper V2.0 (Cantalapiedra et al. 2021) to annotate the V. vinifera genome. We used the blastp algorithm V2.9.0 to compare V. vinifera protein sequences against the referent protein sequence database and construct the phylostratigraphic map. The effect size was calculated using the effectsize V0.8.9 R package. The custom-made code used in this study is available in GitHub at https://github.com/bacillus-biofilms/biofilm-data-analysis and Zenodo at https://doi.org/10.5281/zenodo.14718116.

For manuscripts utilizing custom algorithms or software that are central to the research but not yet described in published literature, software must be made available to editors and reviewers. We strongly encourage code deposition in a community repository (e.g. GitHub). See the Nature Portfolio guidelines for submitting code & software for further information.

Data

Policy information about availability of data

All manuscripts must include a data availability statement. This statement should provide the following information, where applicable:

- Accession codes, unique identifiers, or web links for publicly available datasets
- A description of any restrictions on data availability
- For clinical datasets or third party data, please ensure that the statement adheres to our policy

All transcriptome data have been deposited in NCBI's Gene Expression Omnibus under accession number GSE234231 and are available at the following URL: https://www.ncbi.nlm.nih.gov/geo/query/acc.cgi?acc=GSE234231. Source data underlying the graphs presented in the main figures can be found in Supplementary Data 11. All other data are available in Figshare at https://doi.org/10.6084/m9.figshare.28309805.v1 or from the corresponding authors on reasonable request.

Human research participants

Policy information about studies involving human research participants and Sex and Gender in Research.

Reporting on sex and gender	This study did not involve human participants.
Population characteristics	This study did not involve human participants.
Recruitment	This study did not involve human participants.
Ethics oversight	This study did not involve human participants.

Note that full information on the approval of the study protocol must also be provided in the manuscript.

Field-specific reporting

Please select the one below that is the best fit for your research. If you are not sure, read the appropriate sections before making your selection.

☒ Life sciences ☐ Behavioural & social sciences ☐ Ecological, evolutionary & environmental sciences

For a reference copy of the document with all sections, see nature.com/documents/nr-reporting-summary-flat.pdf

Life sciences study design

All studies must disclose on these points even when the disclosure is negative.

Sample size	To assemble the phylostratigraphic database, we retrieved the full set of protein sequences for 427 terminal taxa, five from NCBI and 422 from Ensembl database. The choice of taxa was dictated by phylogenetic nodes that were present along lineages of focal species and by the availability of reference genomes. To ascertain differences in embryo induction potentials between the wild type and the drm1/drm2 double mutant, we performed three individual experiments of somatic embryogenesis for each line, each conducted on 50 to 100 embryos per line, which allowed us to calculate the percentage of successful embryo induction in each experiment.
Data exclusions	No data were excluded from the analyses.
Replication	The comparison of differences in embryo induction potentials between the wild type and the drm1/drm2 double mutant was done by repeating the somatic embryogenesis experiment three times for each line. The two groups were found to have statistically significant differences in their somatic embryogenesis induction potentials.
Randomization	No randomization was required in the analysis.
Blinding	No blinding was required.

Reporting for specific materials, systems and methods

We require information from authors about some types of materials, experimental systems and methods used in many studies. Here, indicate whether each material, system or method listed is relevant to your study. If you are not sure if a list item applies to your research, read the appropriate section before selecting a response.

Materials & experimental systems

n/a	Involved in the study
☒	☐ Antibodies
☒	☐ Eukaryotic cell lines
☒	☐ Palaeontology and archaeology
☒	☐ Animals and other organisms
☒	☐ Clinical data
☒	☐ Dual use research of concern

Methods

n/a	Involved in the study
☒	☐ ChIP-seq
☒	☐ Flow cytometry
☒	☐ MRI-based neuroimaging
