# Supplementary material for: Developmental phylotranscriptomics in grapevine suggests an ancestral role of somatic embryogenesis
Source: Commun Biol. 2025 Feb 20;8:265. doi: 10.1038/s42003-025-07712-w (PMC11839975; doi:10.1038/s42003-025-07712-w)
Supplement: Supplementary file 12 — Supplementary Data 9 [file Supplementary_Data_9_vitis_profiles_all_genes.pdf › Supplementary_Data_9_vitis_profiles_all_genes.pdf]

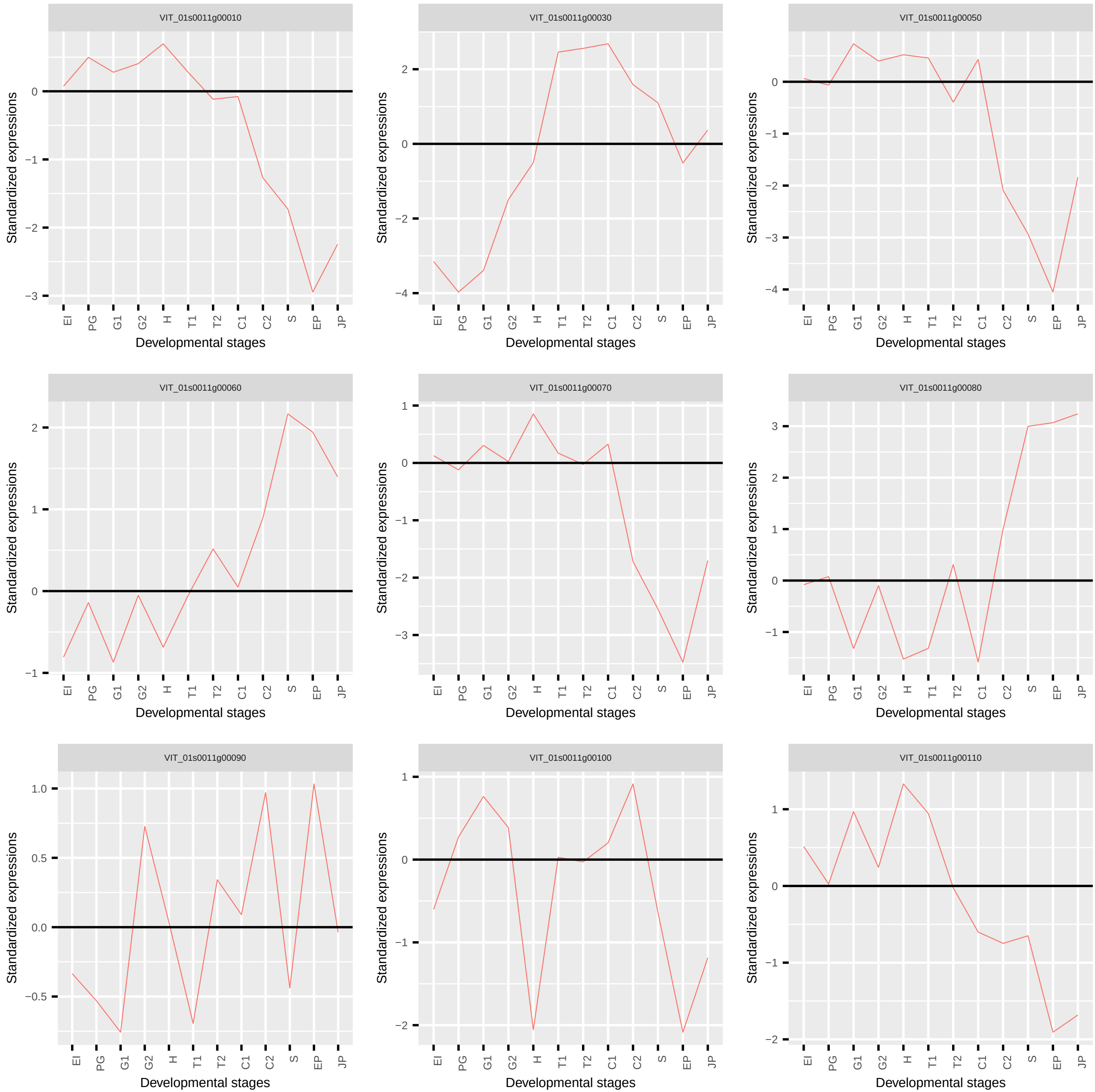
VIT_01s0011g00010
VIT_01s0011g00030
VIT_01s0011g00050
VIT_01s0011g00060
VIT_01s0011g00070
VIT_01s0011g00080
VIT_01s0011g00090
VIT_01s0011g00100
VIT_01s0011g00110
Standardized expressions
Developmental stages
EI
PG
G1
G2
H
T1
T2
C1
C2
S
EP
JP

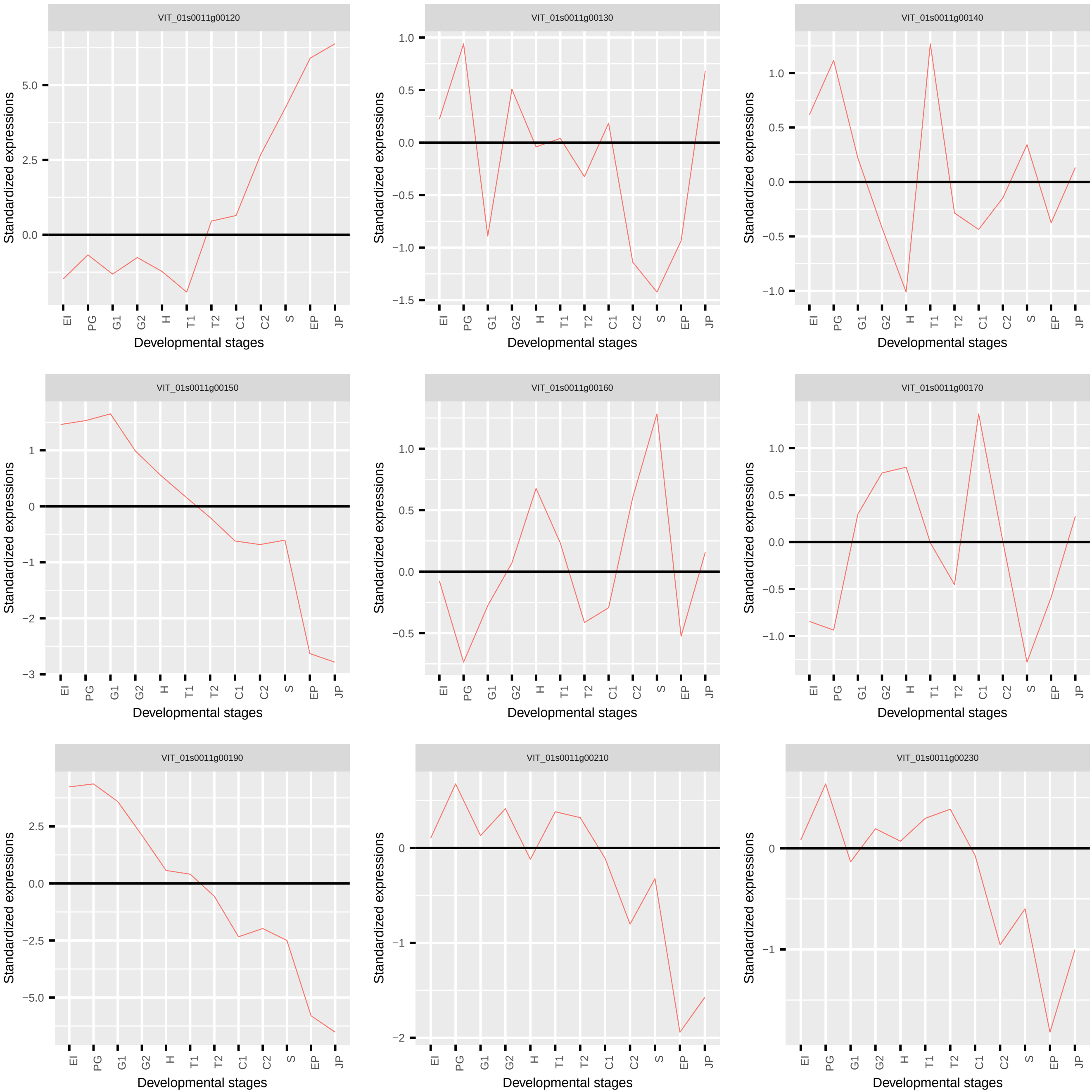
VIT_01s0011g00120
VIT_01s0011g00130
VIT_01s0011g00140
VIT_01s0011g00150
VIT_01s0011g00160
VIT_01s0011g00170
VIT_01s0011g00190
VIT_01s0011g00210
VIT_01s0011g00230
Standardized expressions
Developmental stages
EI PG G1 G2 H T1 T2 C1 C2 S EP JP

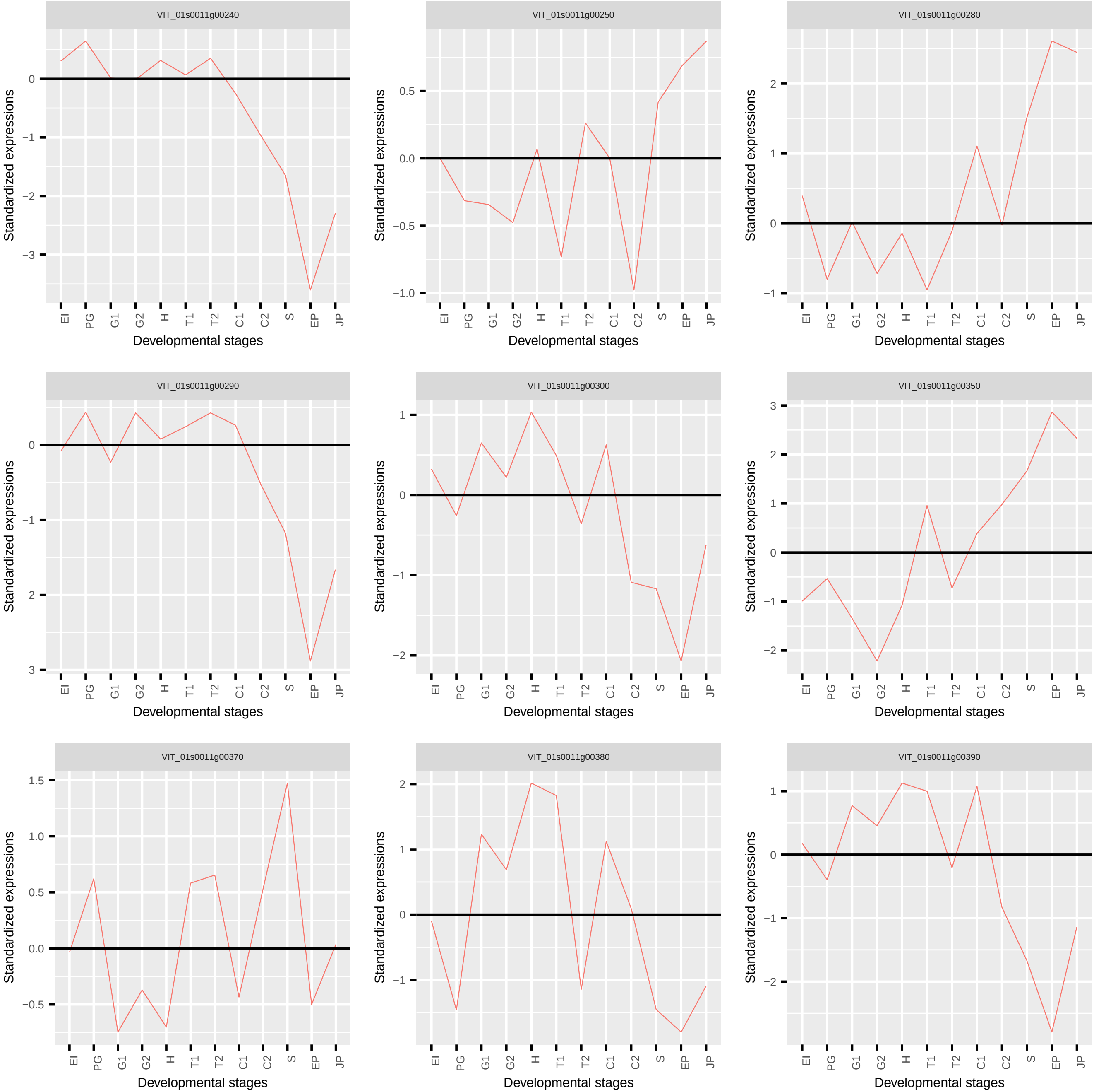
VIT_01s0011g00240
VIT_01s0011g00250
VIT_01s0011g00280
VIT_01s0011g00290
VIT_01s0011g00300
VIT_01s0011g00350
VIT_01s0011g00370
VIT_01s0011g00380
VIT_01s0011g00390
Standardized expressions
Developmental stages
EI PG G1 G2 H T1 T2 C1 C2 S EP JP

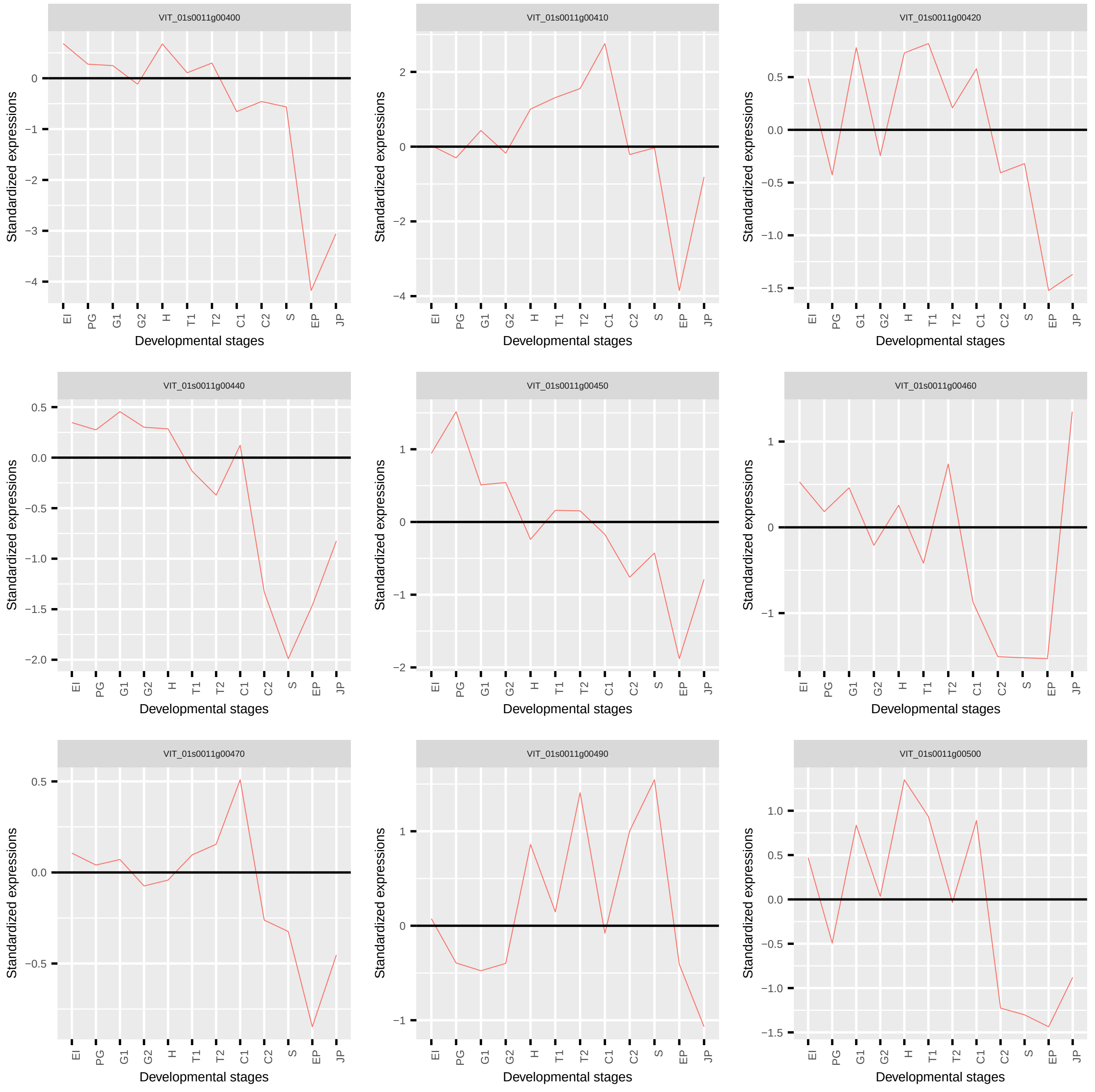
VIT_01s0011g00400
VIT_01s0011g00410
VIT_01s0011g00420
VIT_01s0011g00440
VIT_01s0011g00450
VIT_01s0011g00460
VIT_01s0011g00470
VIT_01s0011g00490
VIT_01s0011g00500
Standardized expressions
Developmental stages
EI PG G1 G2 H T1 T2 C1 C2 S EP JP

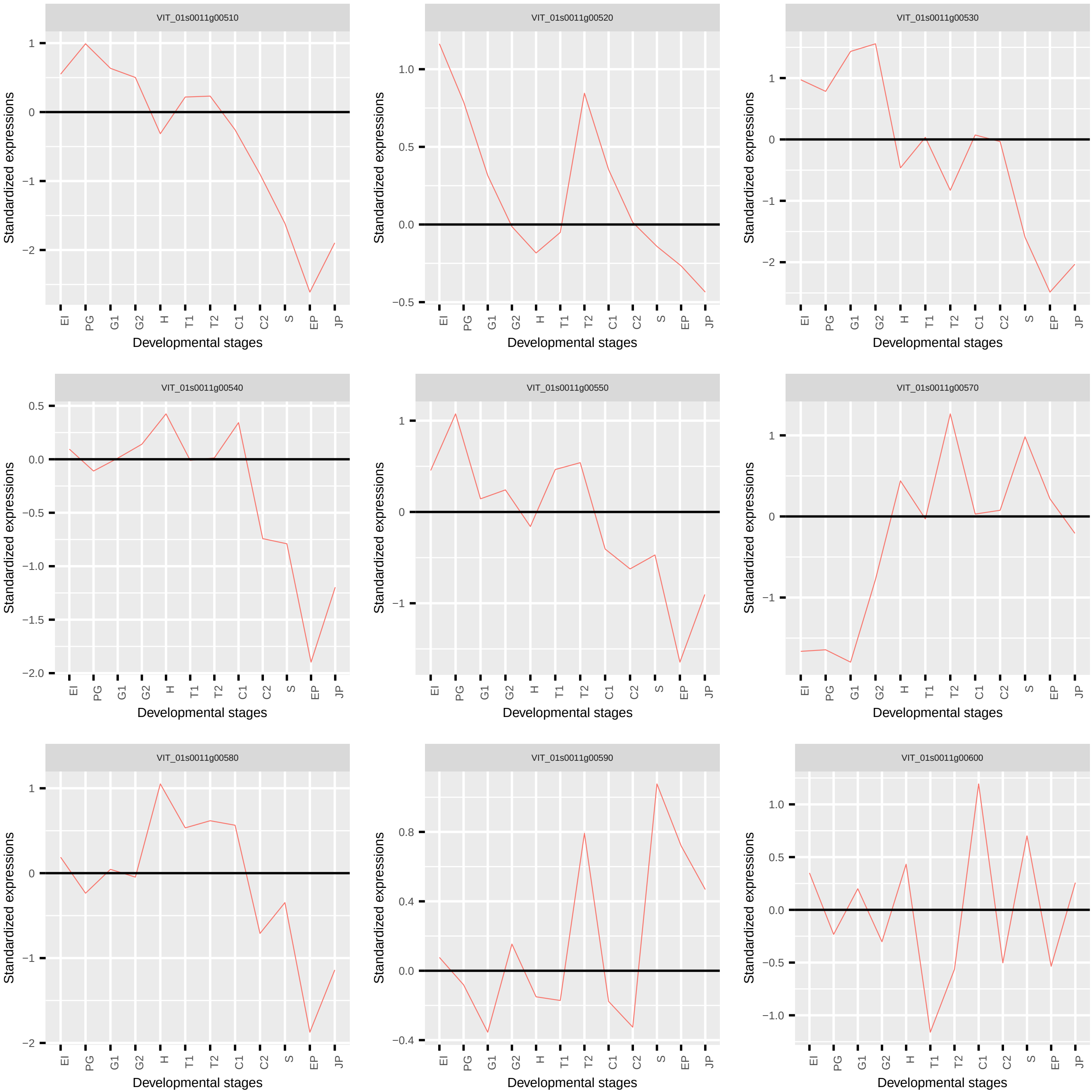

VIT_01s0011g00510
VIT_01s0011g00520
VIT_01s0011g00530
VIT_01s0011g00540
VIT_01s0011g00550
VIT_01s0011g00570
VIT_01s0011g00580
VIT_01s0011g00590
VIT_01s0011g00600
Standardized expressions
Developmental stages
EI
PG
G1
G2
H
T1
T2
C1
C2
S
EP
JP

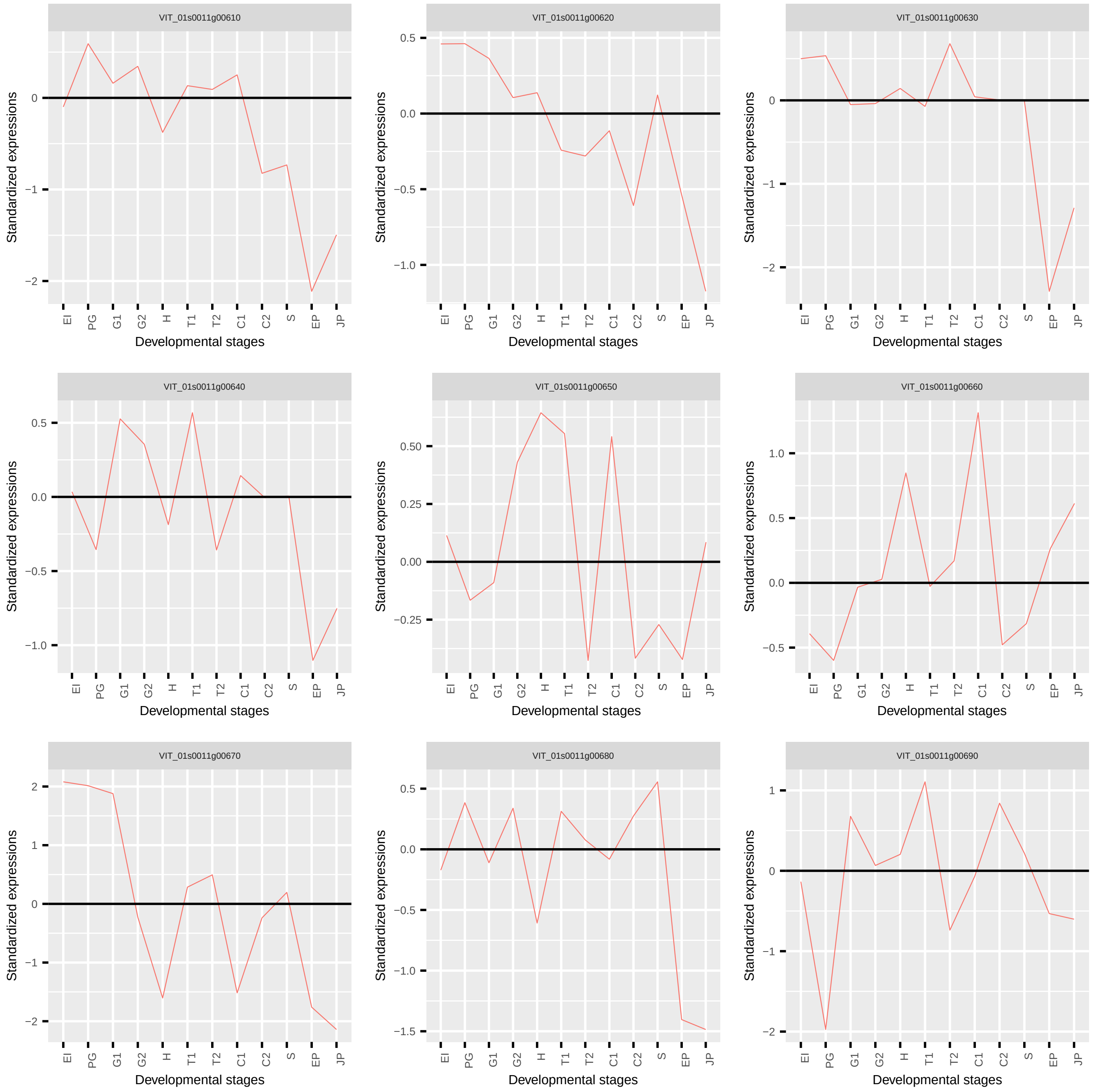
VIT_01s0011g00610
VIT_01s0011g00620
VIT_01s0011g00630
VIT_01s0011g00640
VIT_01s0011g00650
VIT_01s0011g00660
VIT_01s0011g00670
VIT_01s0011g00680
VIT_01s0011g00690
Standardized expressions
Developmental stages
EI PG G1 G2 H T1 T2 C1 C2 S EP JP

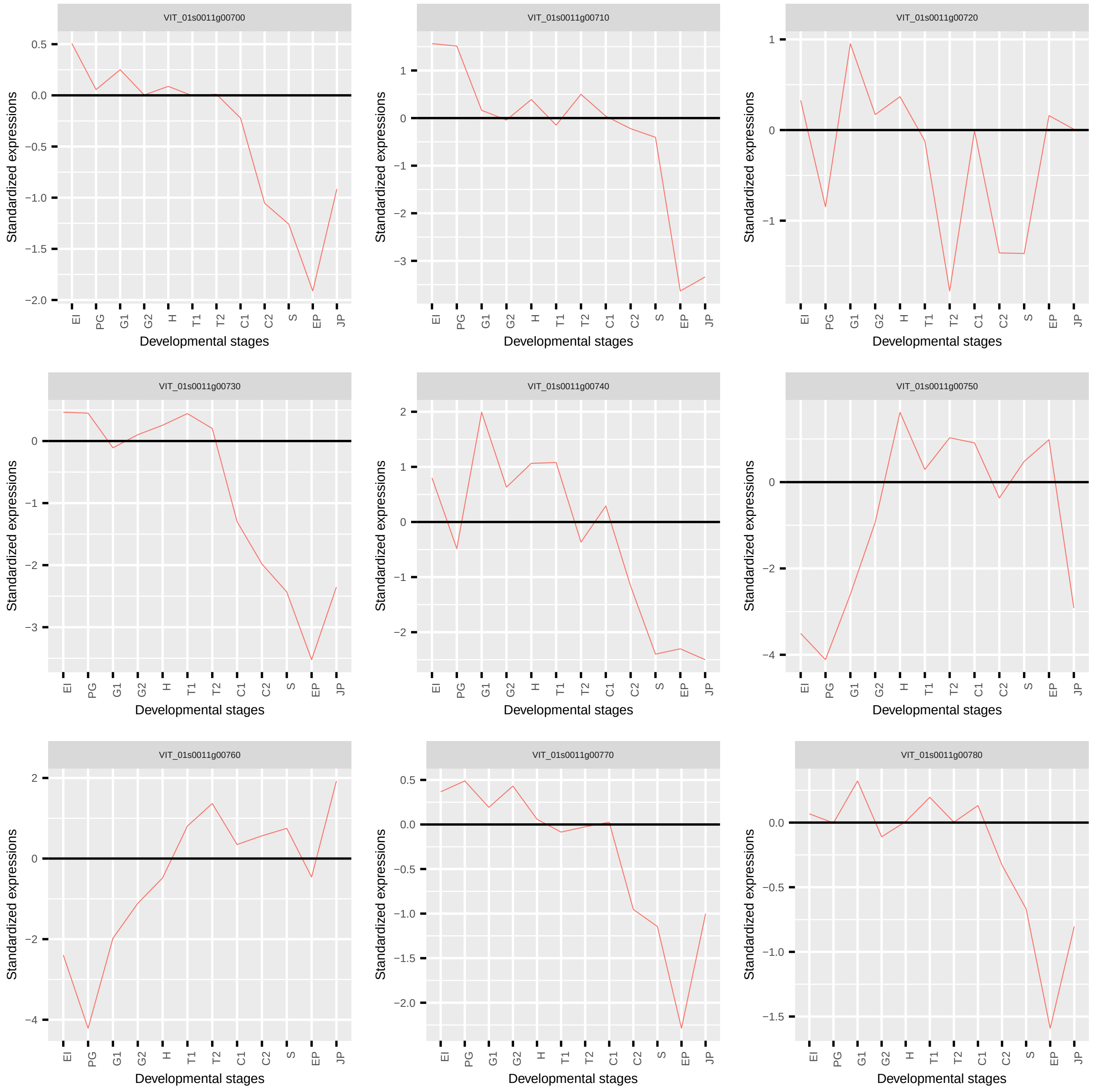
VIT_01s0011g00700
VIT_01s0011g00710
VIT_01s0011g00720
VIT_01s0011g00730
VIT_01s0011g00740
VIT_01s0011g00750
VIT_01s0011g00760
VIT_01s0011g00770
VIT_01s0011g00780
Standardized expressions
Developmental stages
EI PG G1 G2 H T1 T2 C1 C2 S EP JP

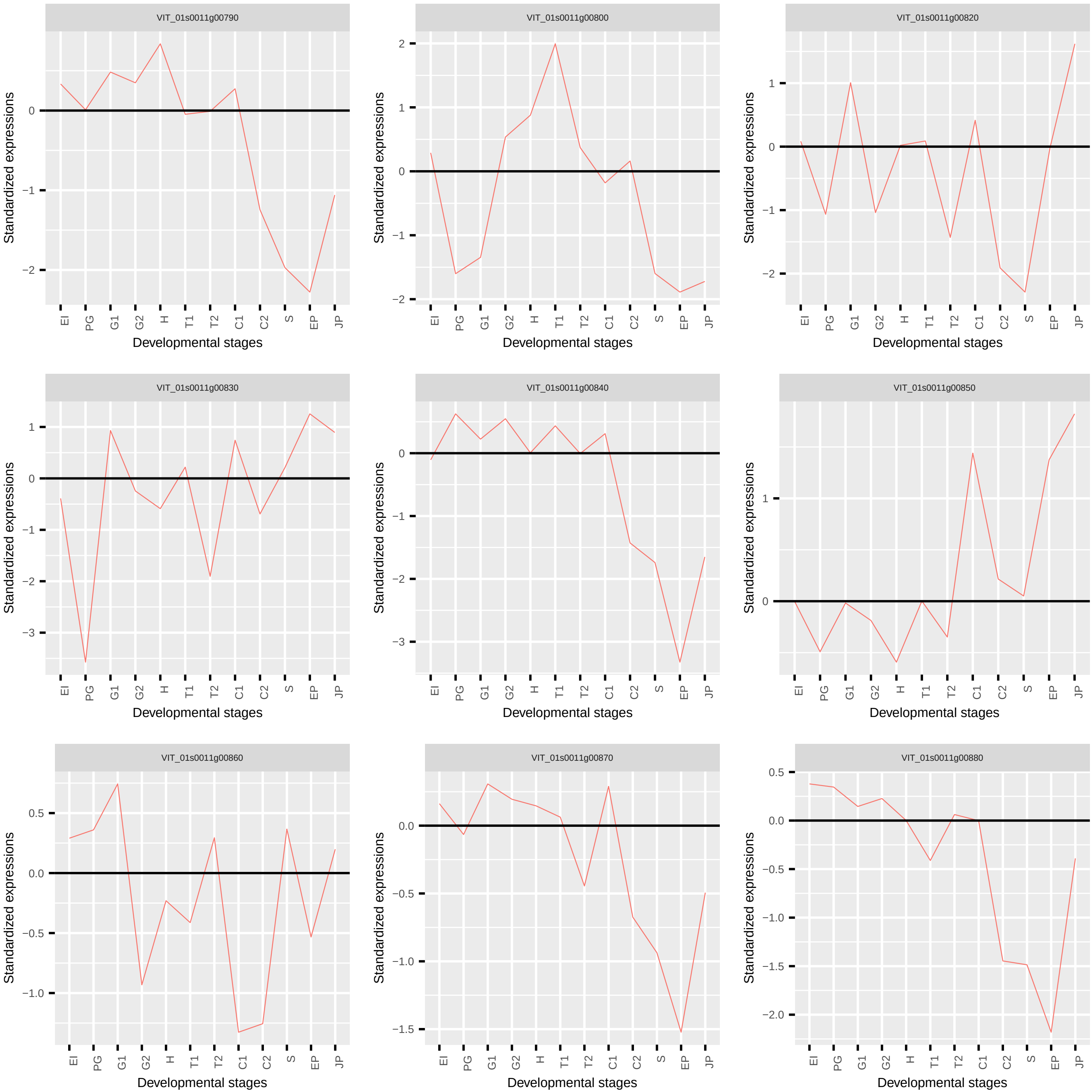
VIT_01s0011g00790
VIT_01s0011g00800
VIT_01s0011g00820
VIT_01s0011g00830
VIT_01s0011g00840
VIT_01s0011g00850
VIT_01s0011g00860
VIT_01s0011g00870
VIT_01s0011g00880
Standardized expressions
Developmental stages
EI PG G1 G2 H T1 T2 C1 C2 S EP JP

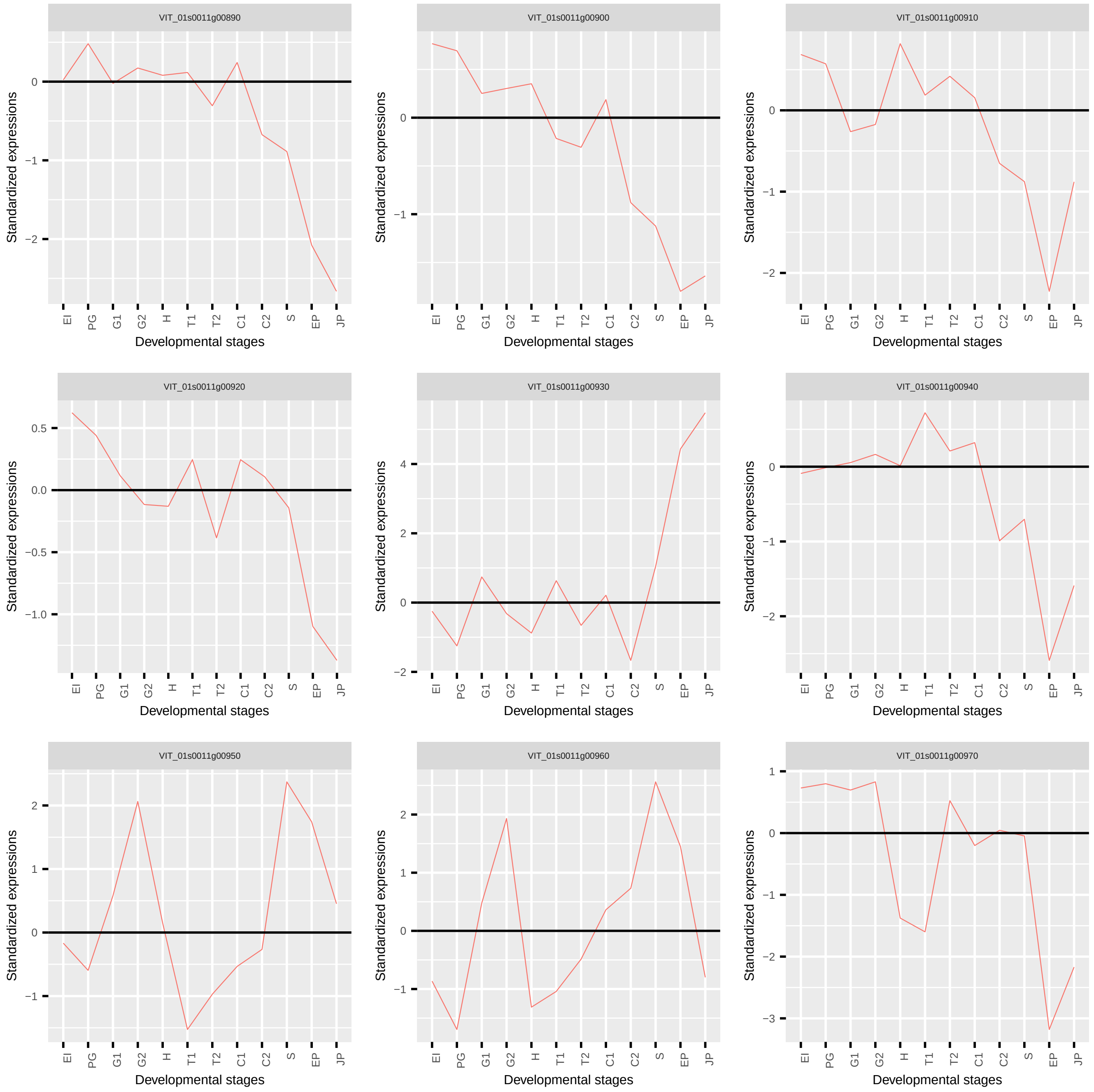
VIT_01s0011g00890
VIT_01s0011g00900
VIT_01s0011g00910
VIT_01s0011g00920
VIT_01s0011g00930
VIT_01s0011g00940
VIT_01s0011g00950
VIT_01s0011g00960
VIT_01s0011g00970
Standardized expressions
Developmental stages
EI
PG
G1
G2
H
T1
T2
C1
C2
S
EP
JP

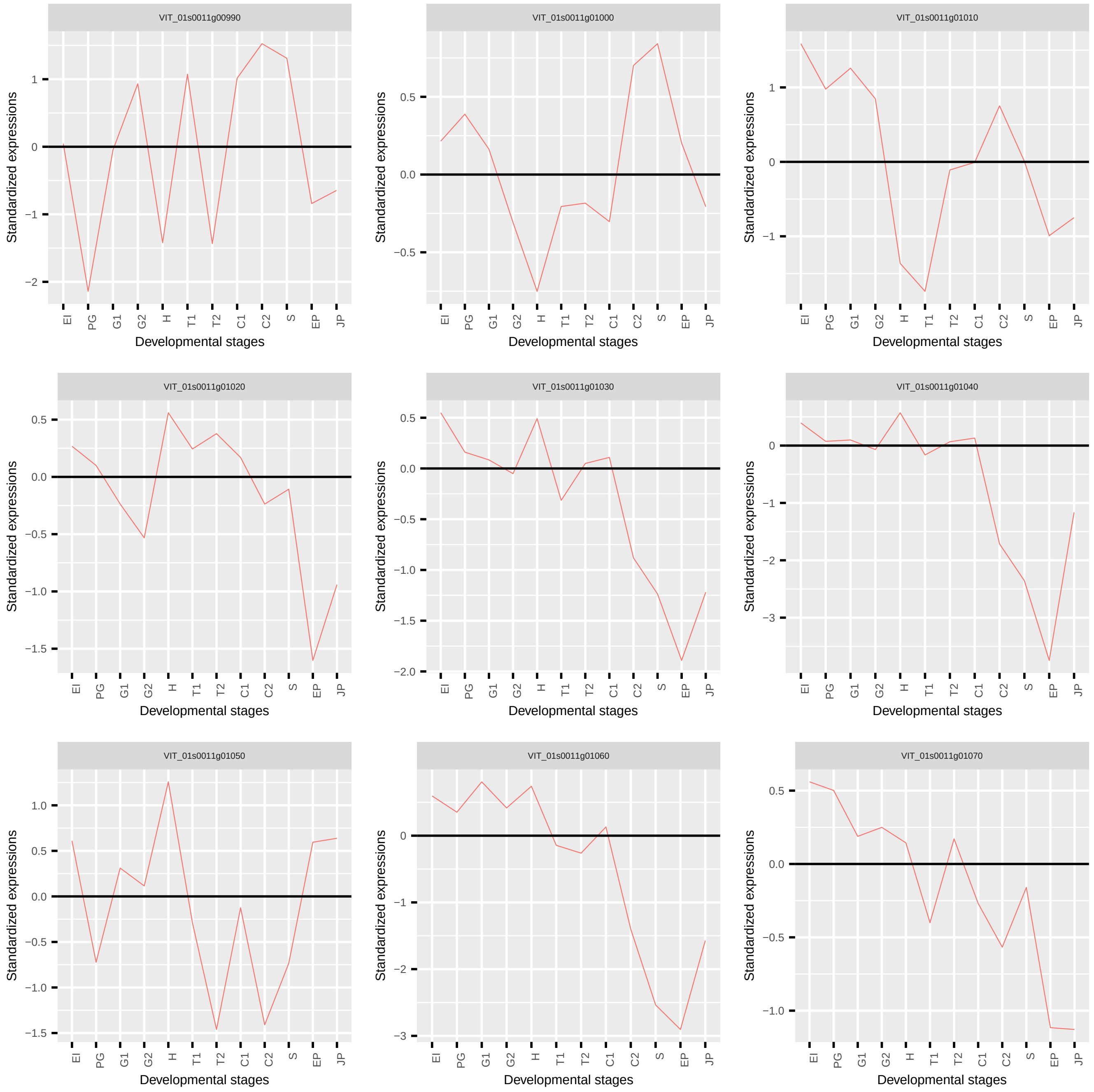
VIT_01s0011g00990
VIT_01s0011g01000
VIT_01s0011g01010
VIT_01s0011g01020
VIT_01s0011g01030
VIT_01s0011g01040
VIT_01s0011g01050
VIT_01s0011g01060
VIT_01s0011g01070
Standardized expressions
Developmental stages
EI PG G1 G2 H T1 T2 C1 C2 S EP JP

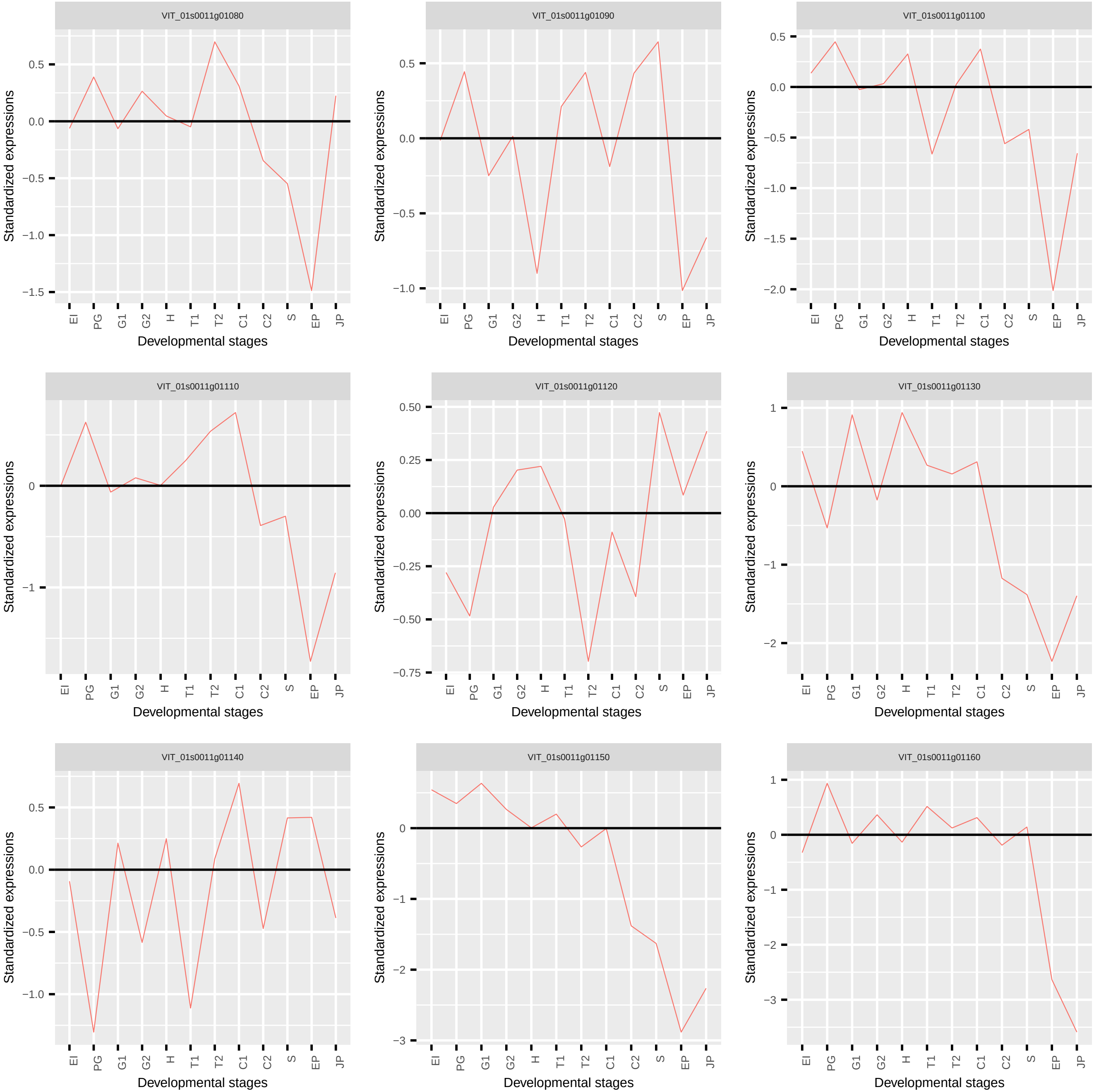
VIT_01s0011g01080
VIT_01s0011g01090
VIT_01s0011g01100
VIT_01s0011g01110
VIT_01s0011g01120
VIT_01s0011g01130
VIT_01s0011g01140
VIT_01s0011g01150
VIT_01s0011g01160
Standardized expressions
Developmental stages
EI
PG
G1
G2
H
T1
T2
C1
C2
S
EP
JP

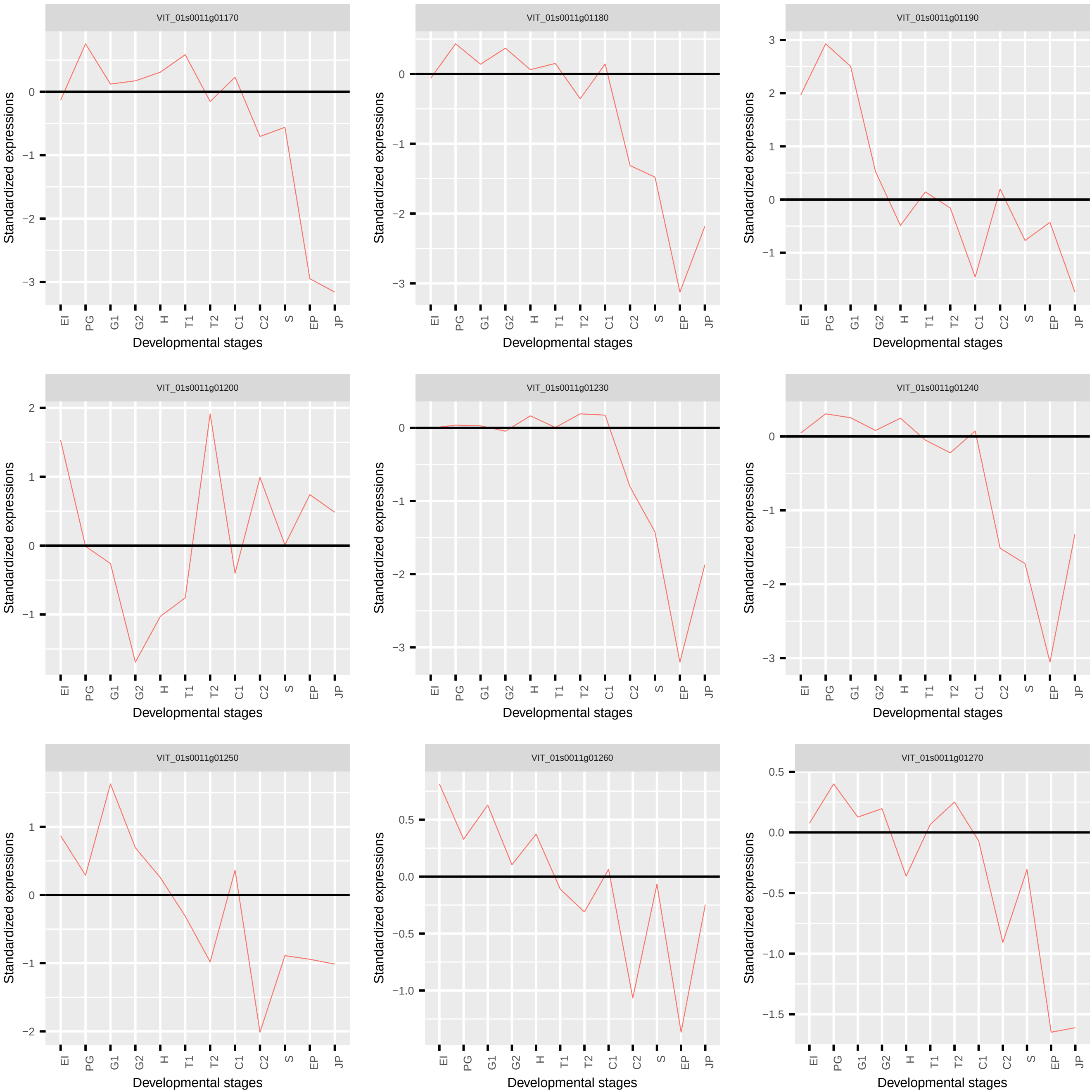
VIT_01s0011g01170
VIT_01s0011g01180
VIT_01s0011g01190
VIT_01s0011g01200
VIT_01s0011g01230
VIT_01s0011g01240
VIT_01s0011g01250
VIT_01s0011g01260
VIT_01s0011g01270
Standardized expressions
Developmental stages
EI PG G1 G2 H T1 T2 C1 C2 S EP JP

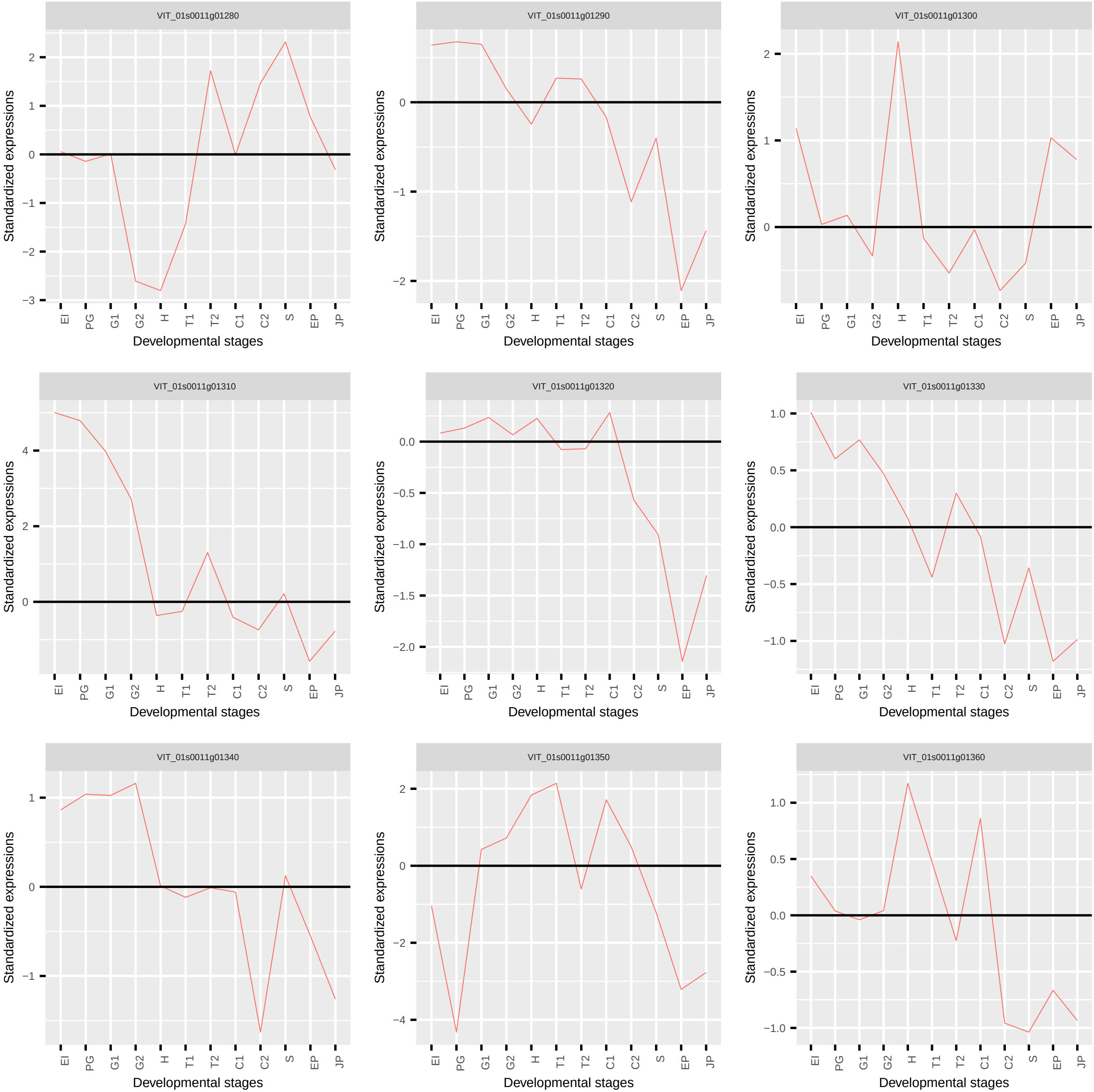
VIT_01s0011g01280
VIT_01s0011g01290
VIT_01s0011g01300
VIT_01s0011g01310
VIT_01s0011g01320
VIT_01s0011g01330
VIT_01s0011g01340
VIT_01s0011g01350
VIT_01s0011g01360
Standardized expressions
Developmental stages
EI PG G1 G2 H T1 T2 C1 C2 S EP JP

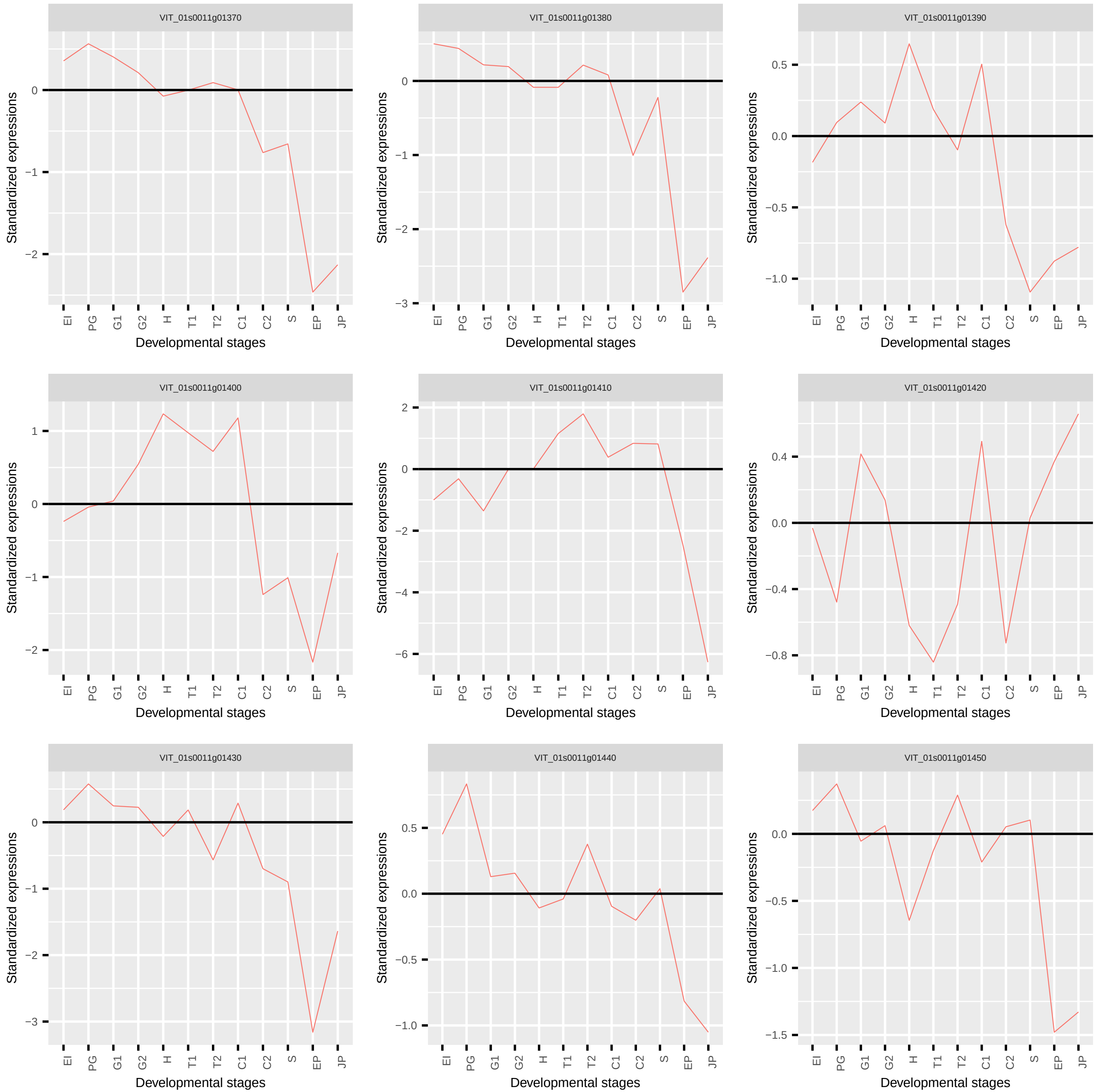

VIT_01s0011g01370
VIT_01s0011g01380
VIT_01s0011g01390
VIT_01s0011g01400
VIT_01s0011g01410
VIT_01s0011g01420
VIT_01s0011g01430
VIT_01s0011g01440
VIT_01s0011g01450
Standardized expressions
Developmental stages
EI PG G1 G2 H T1 T2 C1 C2 S EP JP

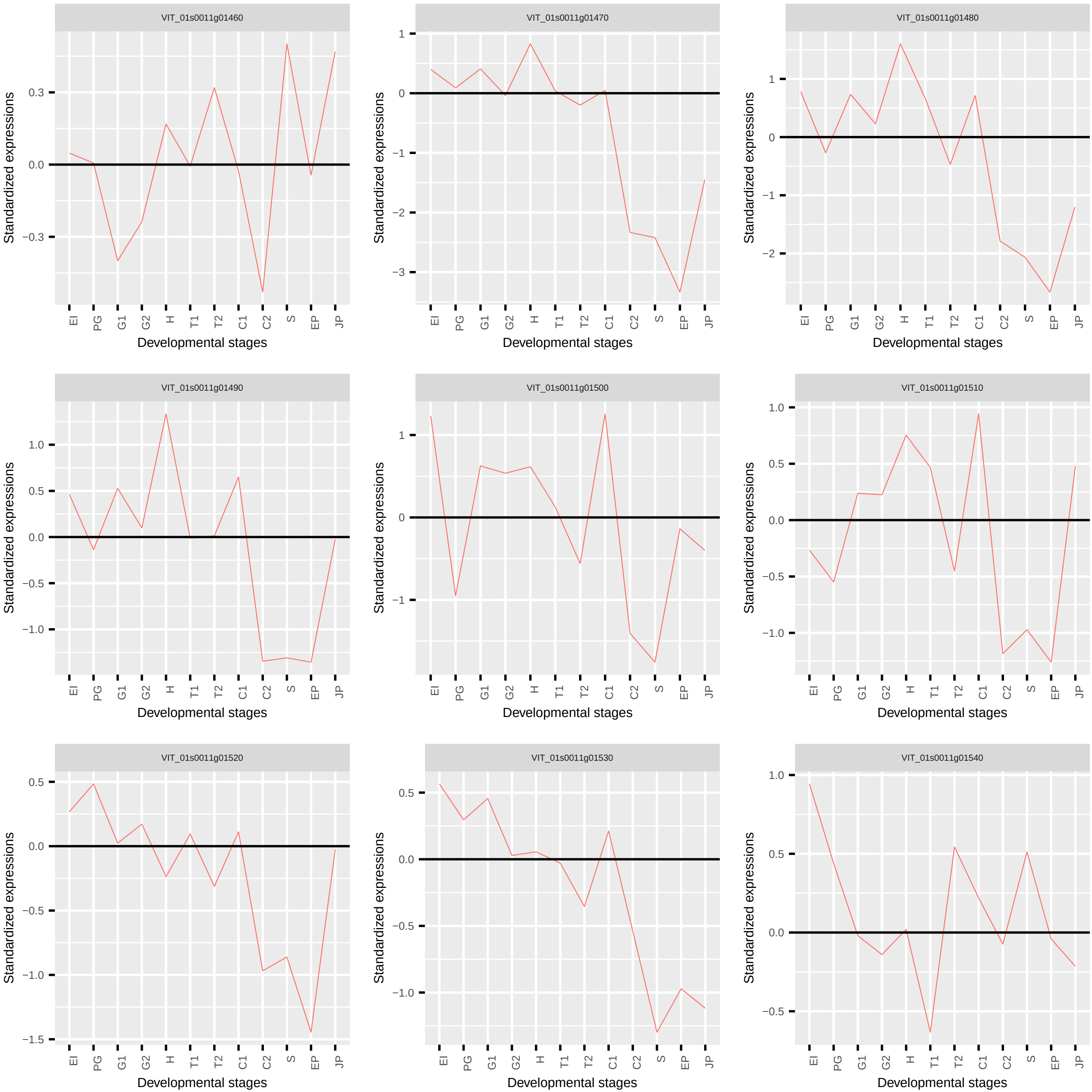
VIT_01s0011g01460
VIT_01s0011g01470
VIT_01s0011g01480
VIT_01s0011g01490
VIT_01s0011g01500
VIT_01s0011g01510
VIT_01s0011g01520
VIT_01s0011g01530
VIT_01s0011g01540
Standardized expressions
Developmental stages
EI
PG
G1
G2
H
T1
T2
C1
C2
S
EP
JP
0.3
0.0
−0.3
1
0
−1
−2
−3
1.0
0.5
−0.5
−1.0
−1.5

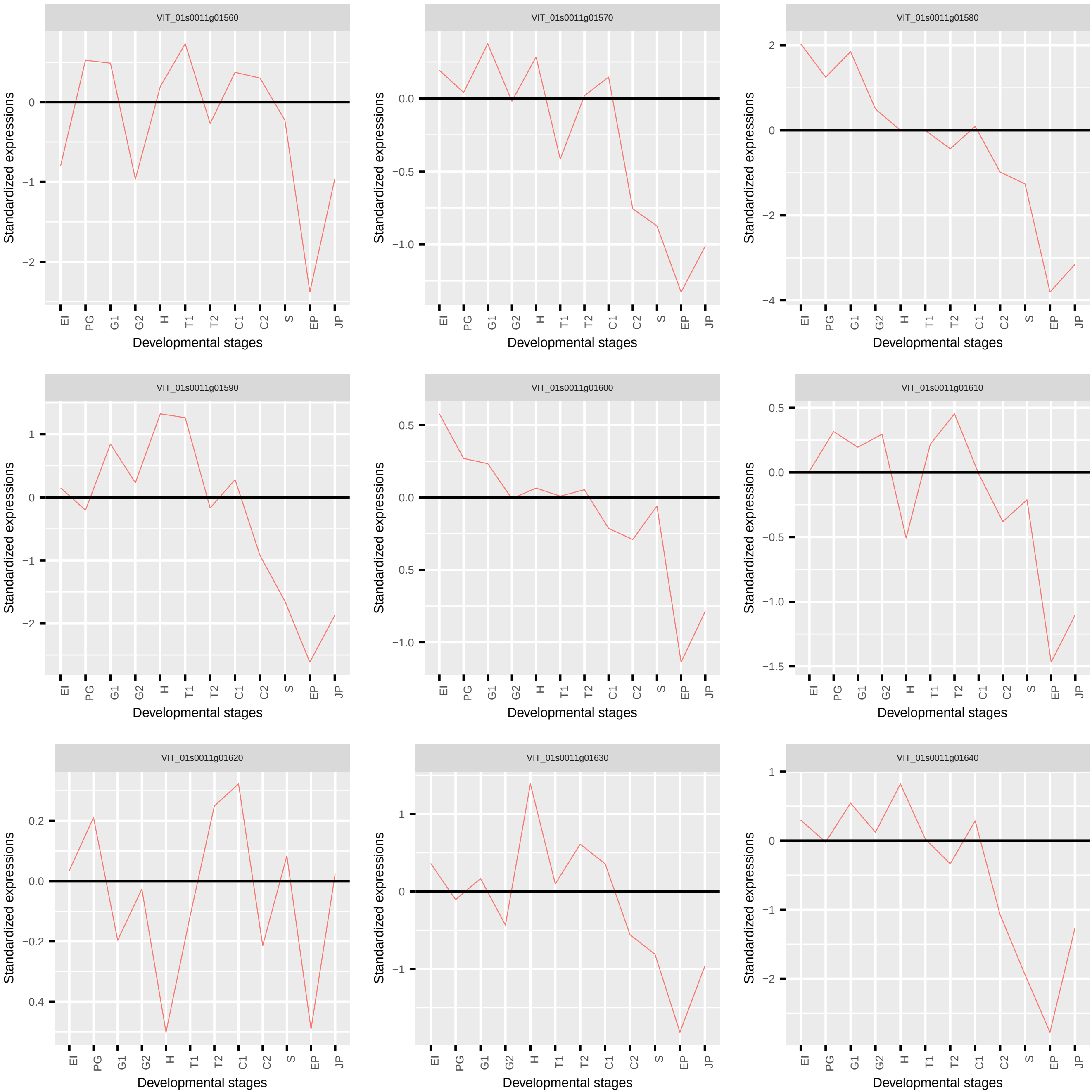
VIT_01s0011g01560
VIT_01s0011g01570
VIT_01s0011g01580
VIT_01s0011g01590
VIT_01s0011g01600
VIT_01s0011g01610
VIT_01s0011g01620
VIT_01s0011g01630
VIT_01s0011g01640
Standardized expressions
Developmental stages
EI PG G1 G2 H T1 T2 C1 C2 S EP JP

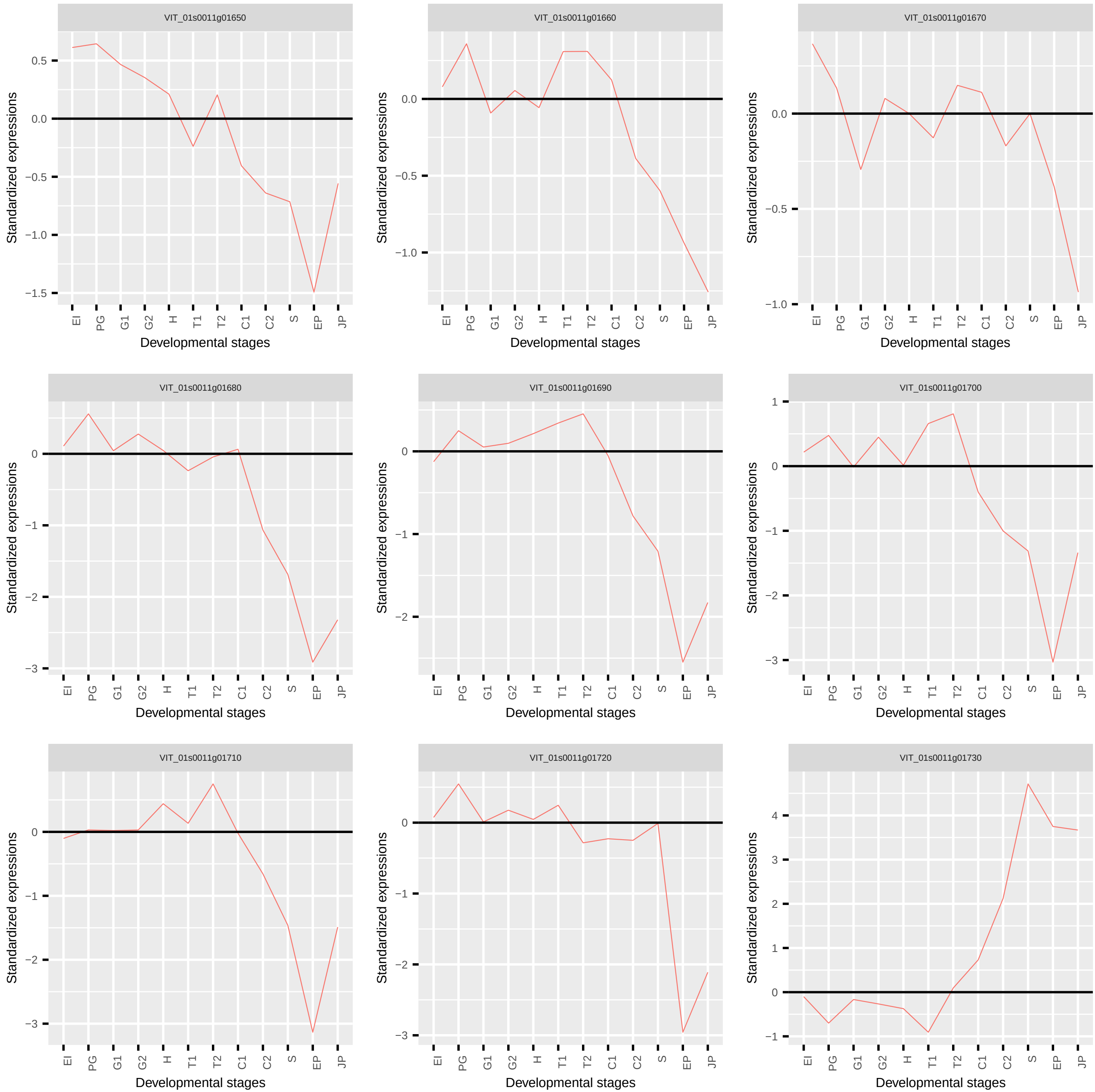
VIT_01s0011g01650
VIT_01s0011g01660
VIT_01s0011g01670
VIT_01s0011g01680
VIT_01s0011g01690
VIT_01s0011g01700
VIT_01s0011g01710
VIT_01s0011g01720
VIT_01s0011g01730
Standardized expressions
Developmental stages
EI
PG
G1
G2
H
T1
T2
C1
C2
S
EP
JP

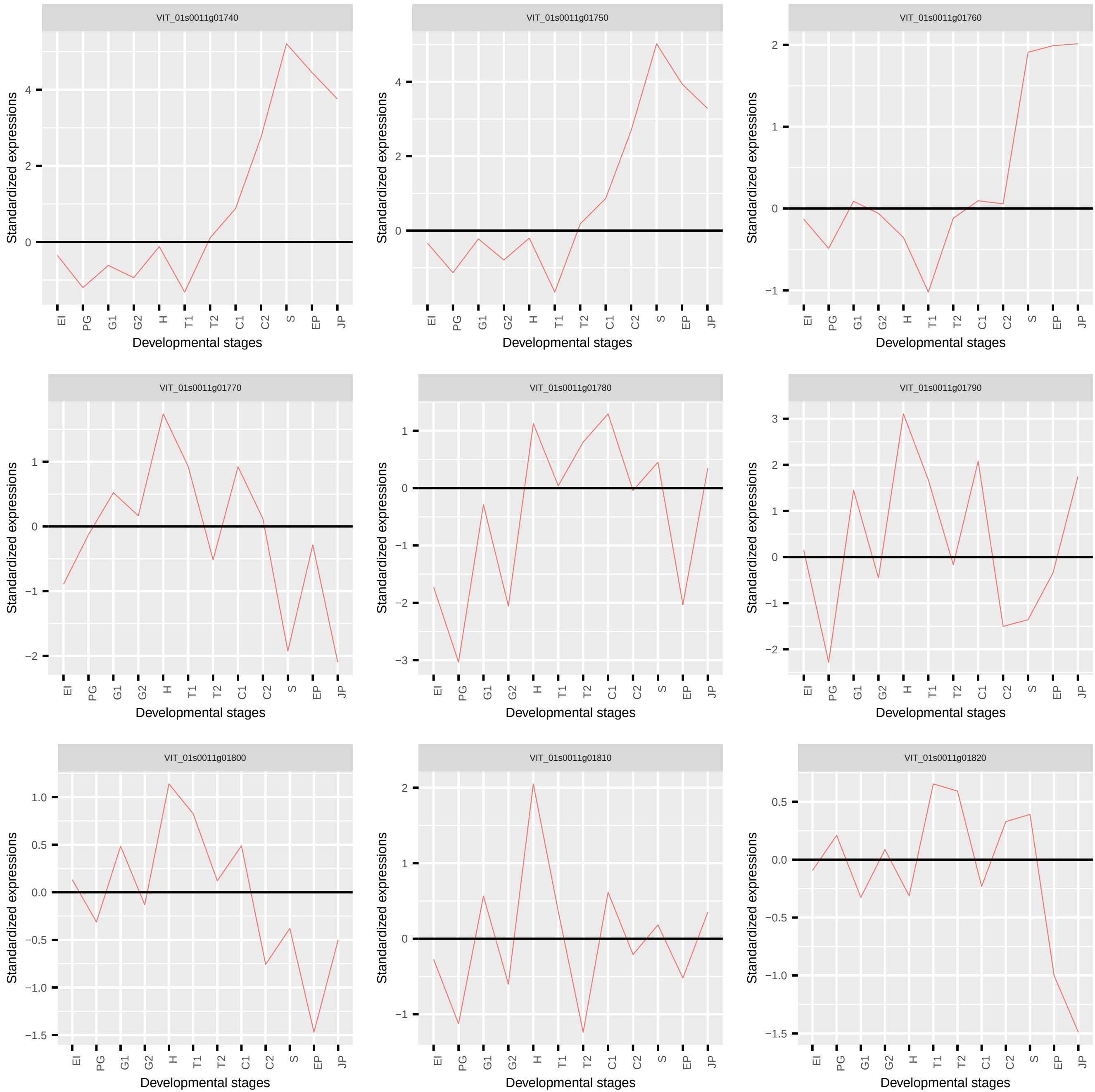
VIT_01s0011g01740
VIT_01s0011g01750
VIT_01s0011g01760
VIT_01s0011g01770
VIT_01s0011g01780
VIT_01s0011g01790
VIT_01s0011g01800
VIT_01s0011g01810
VIT_01s0011g01820
Standardized expressions
Developmental stages
EI PG G1 G2 H T1 T2 C1 C2 S EP JP

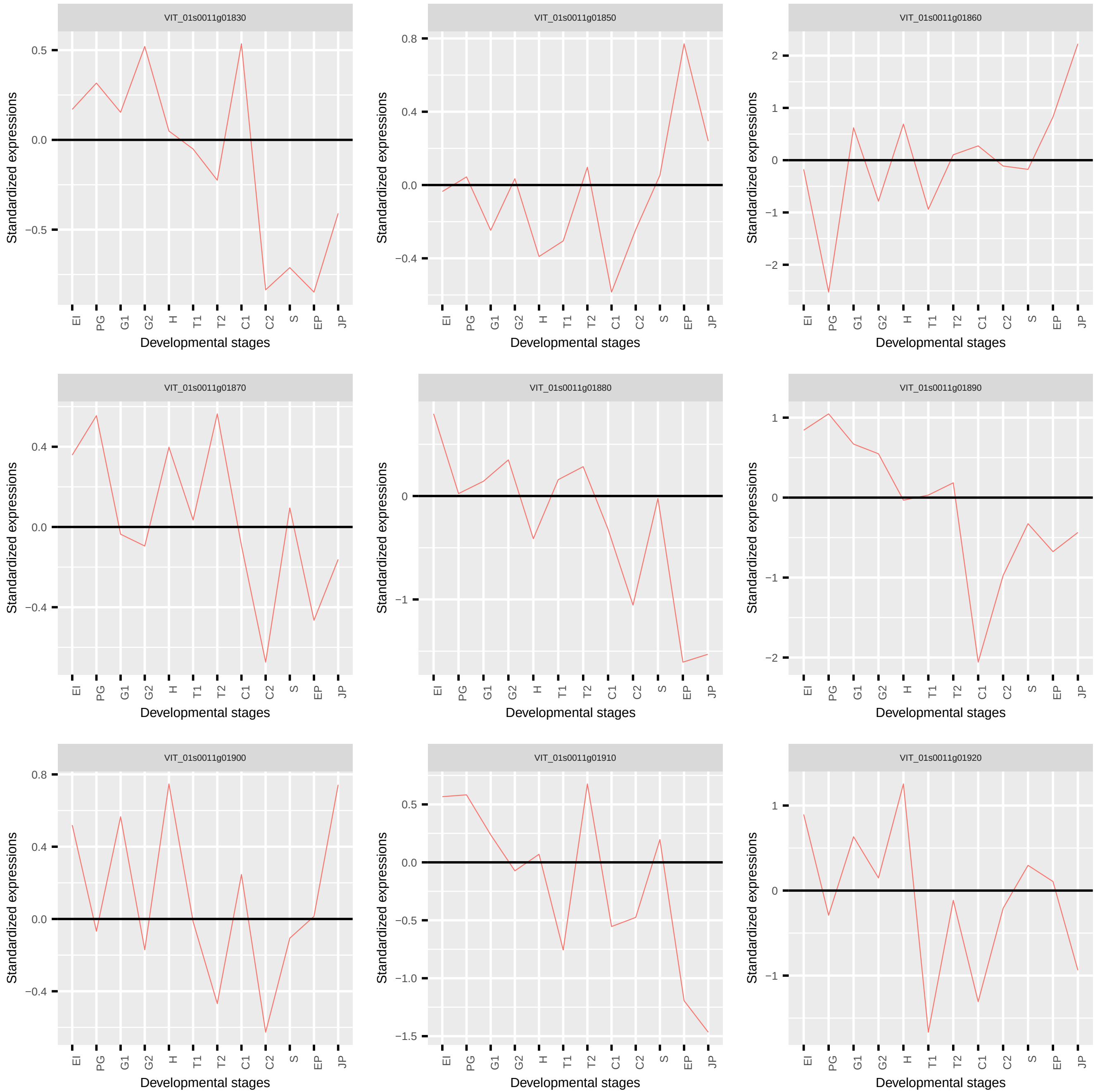

VIT_01s0011g01830
VIT_01s0011g01850
VIT_01s0011g01860
VIT_01s0011g01870
VIT_01s0011g01880
VIT_01s0011g01890
VIT_01s0011g01900
VIT_01s0011g01910
VIT_01s0011g01920
Standardized expressions
Developmental stages
EI
PG
G1
G2
H
T1
T2
C1
C2
S
EP
JP

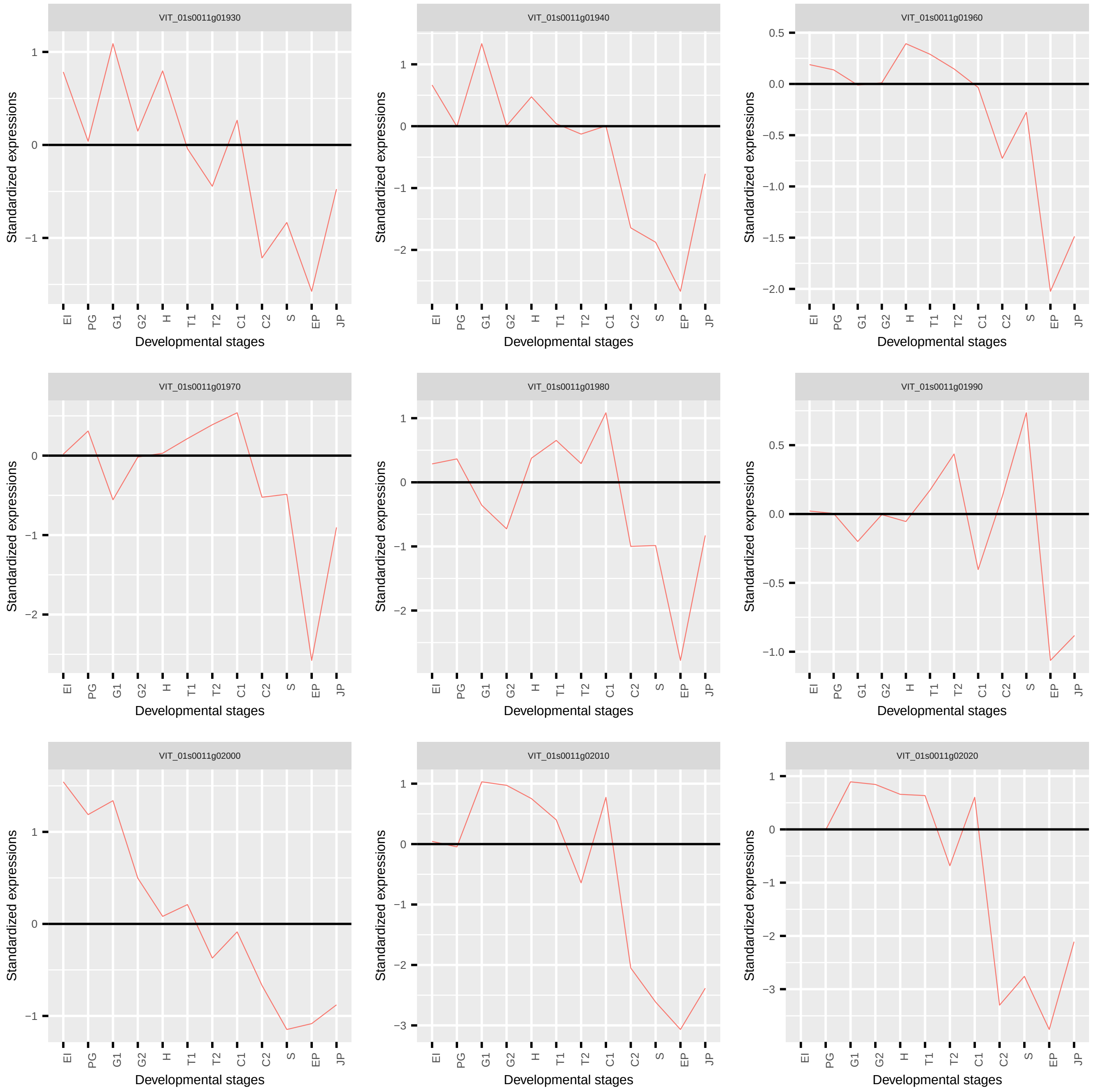

VIT_01s0011g01930
VIT_01s0011g01940
VIT_01s0011g01960
VIT_01s0011g01970
VIT_01s0011g01980
VIT_01s0011g01990
VIT_01s0011g02000
VIT_01s0011g02010
VIT_01s0011g02020
Standardized expressions
Developmental stages
EI PG G1 G2 H T1 T2 C1 C2 S EP JP

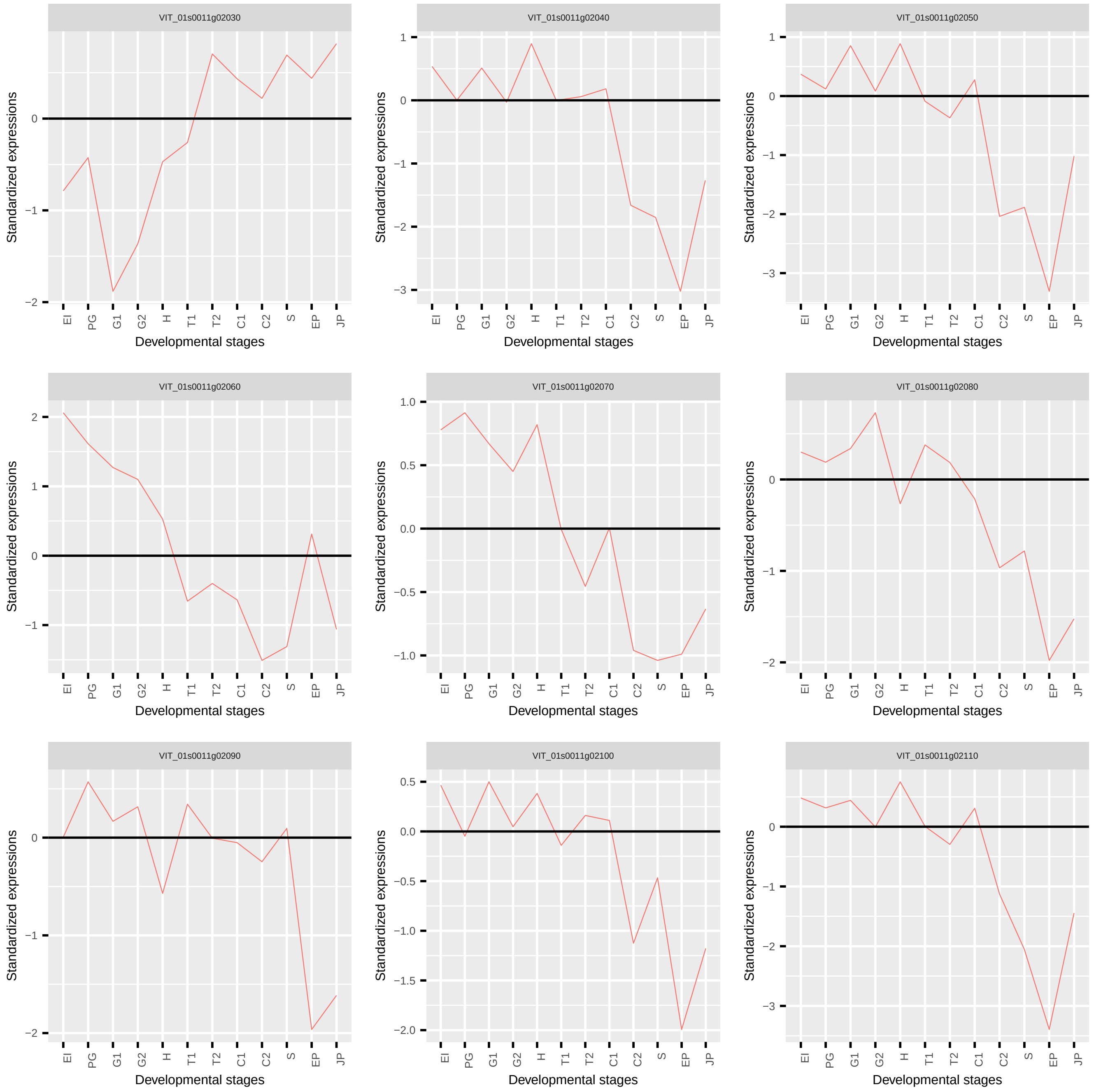
VIT_01s0011g02030
VIT_01s0011g02040
VIT_01s0011g02050
VIT_01s0011g02060
VIT_01s0011g02070
VIT_01s0011g02080
VIT_01s0011g02090
VIT_01s0011g02100
VIT_01s0011g02110
Standardized expressions
Developmental stages
EI
PG
G1
G2
H
T1
T2
C1
C2
S
EP
JP

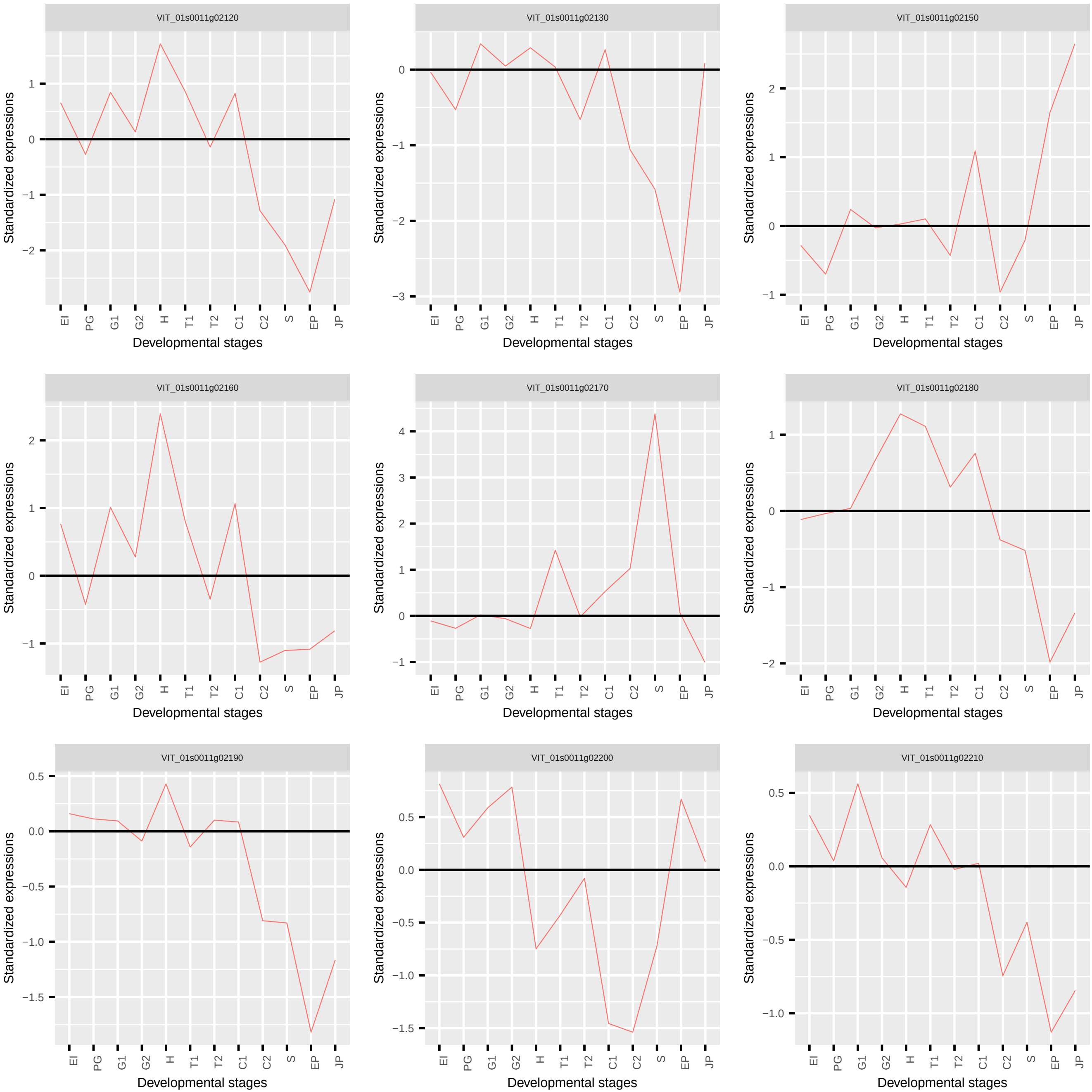
VIT_01s0011g02120
VIT_01s0011g02130
VIT_01s0011g02150
VIT_01s0011g02160
VIT_01s0011g02170
VIT_01s0011g02180
VIT_01s0011g02190
VIT_01s0011g02200
VIT_01s0011g02210
Standardized expressions
Developmental stages
EI PG G1 G2 H T1 T2 C1 C2 S EP JP

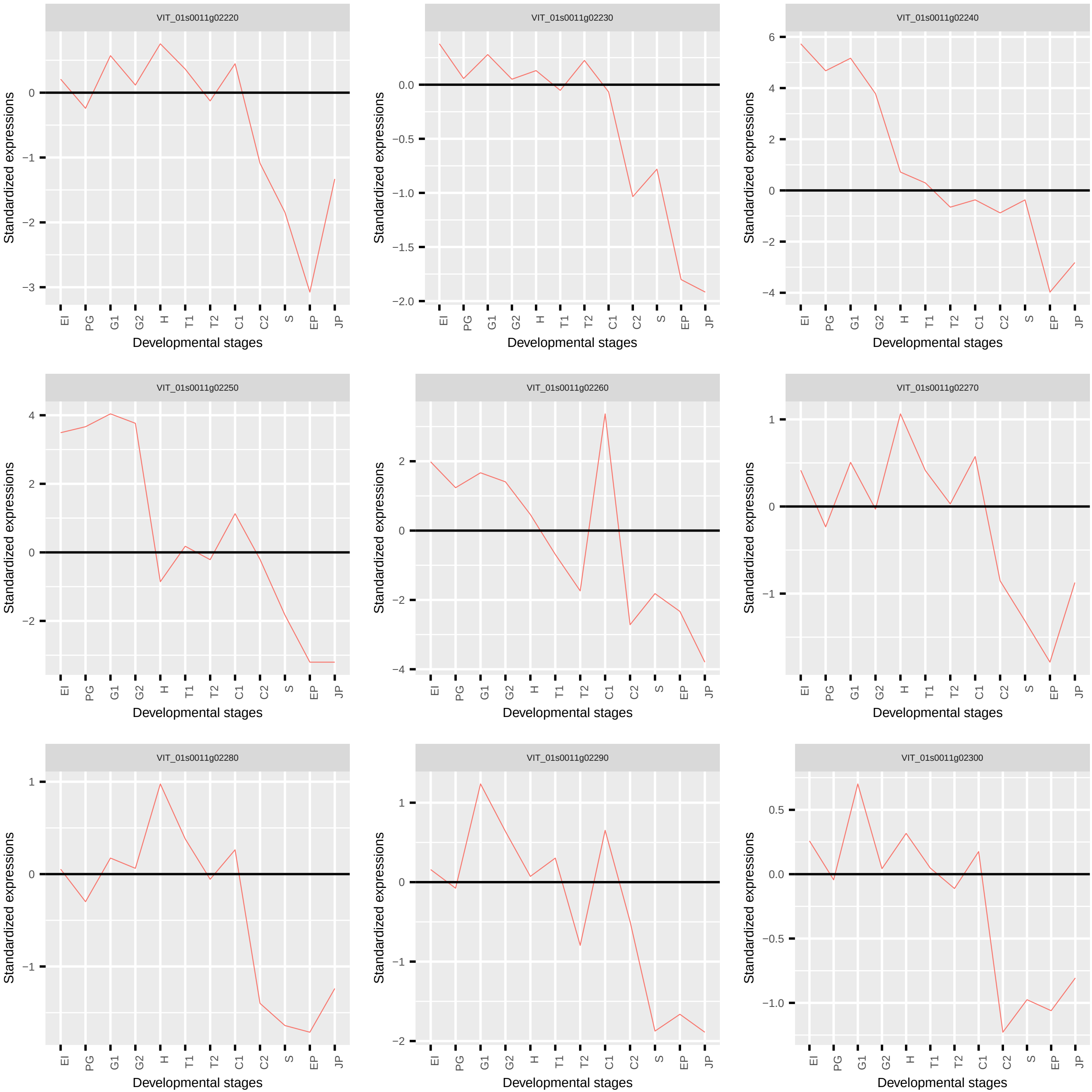
VIT_01s0011g02220
VIT_01s0011g02230
VIT_01s0011g02240
VIT_01s0011g02250
VIT_01s0011g02260
VIT_01s0011g02270
VIT_01s0011g02280
VIT_01s0011g02290
VIT_01s0011g02300
Standardized expressions
Developmental stages
EI PG G1 G2 H T1 T2 C1 C2 S EP JP

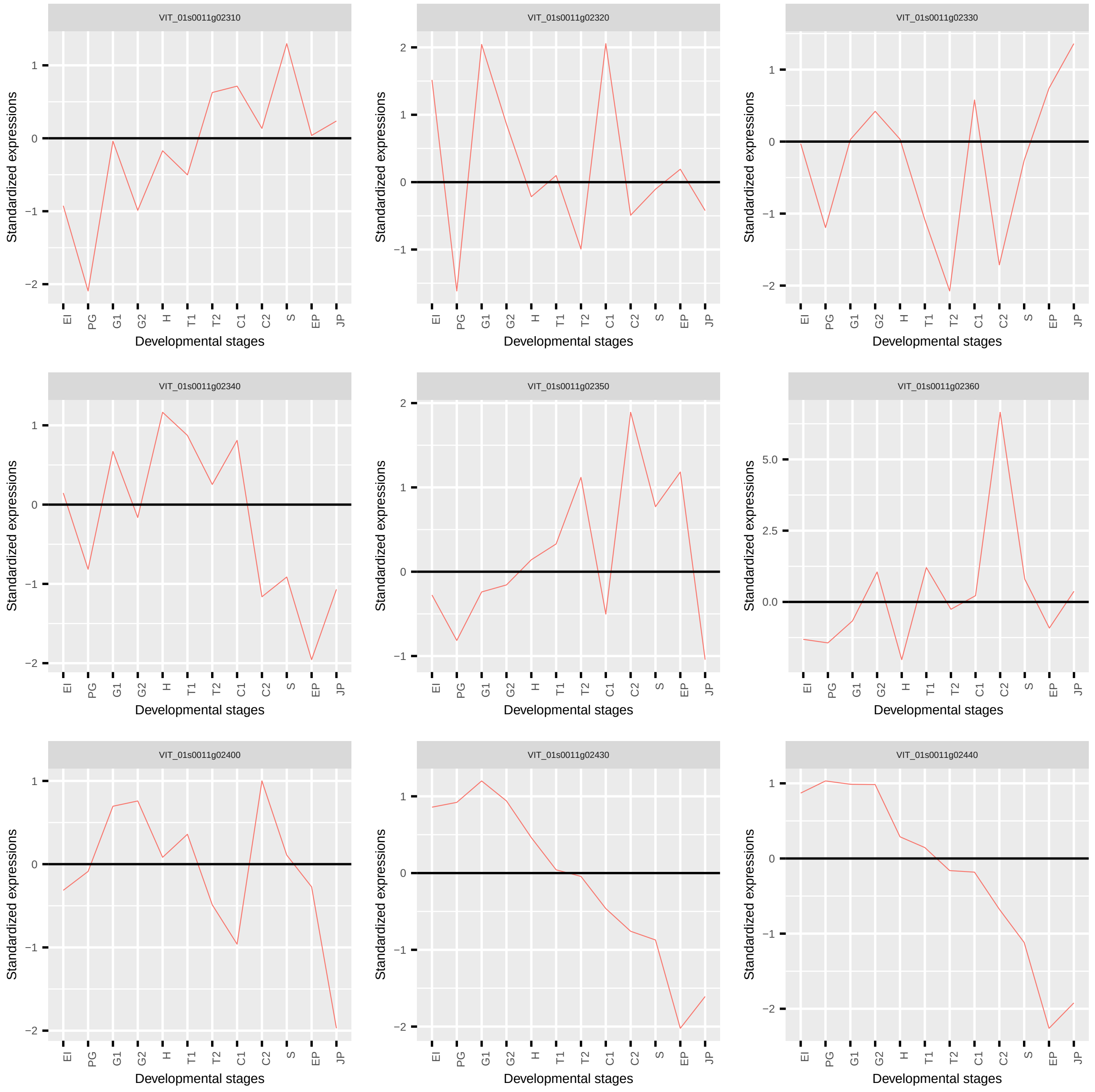
VIT_01s0011g02310
VIT_01s0011g02320
VIT_01s0011g02330
VIT_01s0011g02340
VIT_01s0011g02350
VIT_01s0011g02360
VIT_01s0011g02400
VIT_01s0011g02430
VIT_01s0011g02440
Standardized expressions
Developmental stages
EI
PG
G1
G2
H
T1
T2
C1
C2
S
EP
JP

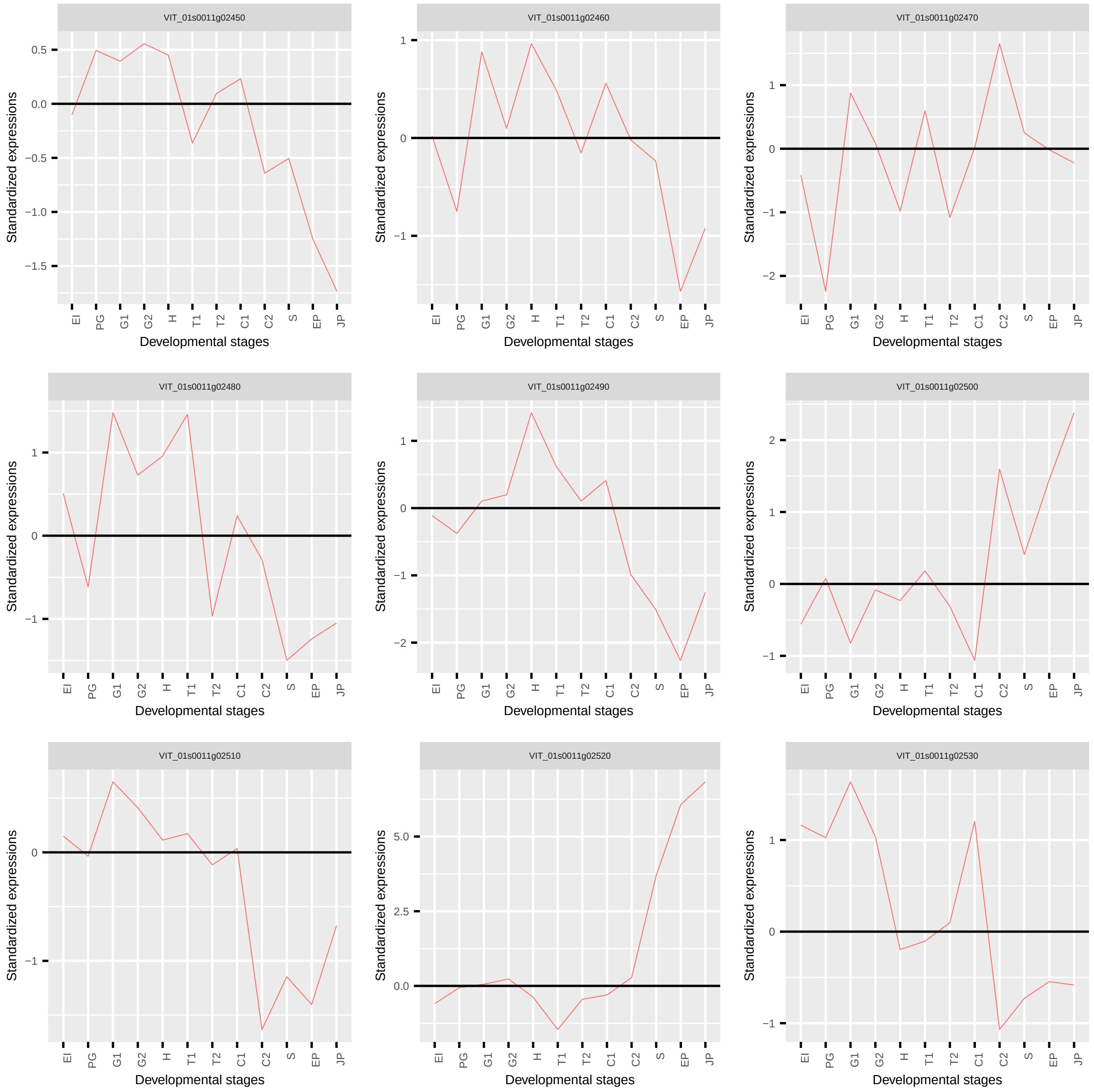

VIT_01s0011g02450
VIT_01s0011g02460
VIT_01s0011g02470
VIT_01s0011g02480
VIT_01s0011g02490
VIT_01s0011g02500
VIT_01s0011g02510
VIT_01s0011g02520
VIT_01s0011g02530
Standardized expressions
Developmental stages
EI
PG
G1
G2
H
T1
T2
C1
C2
S
EP
JP

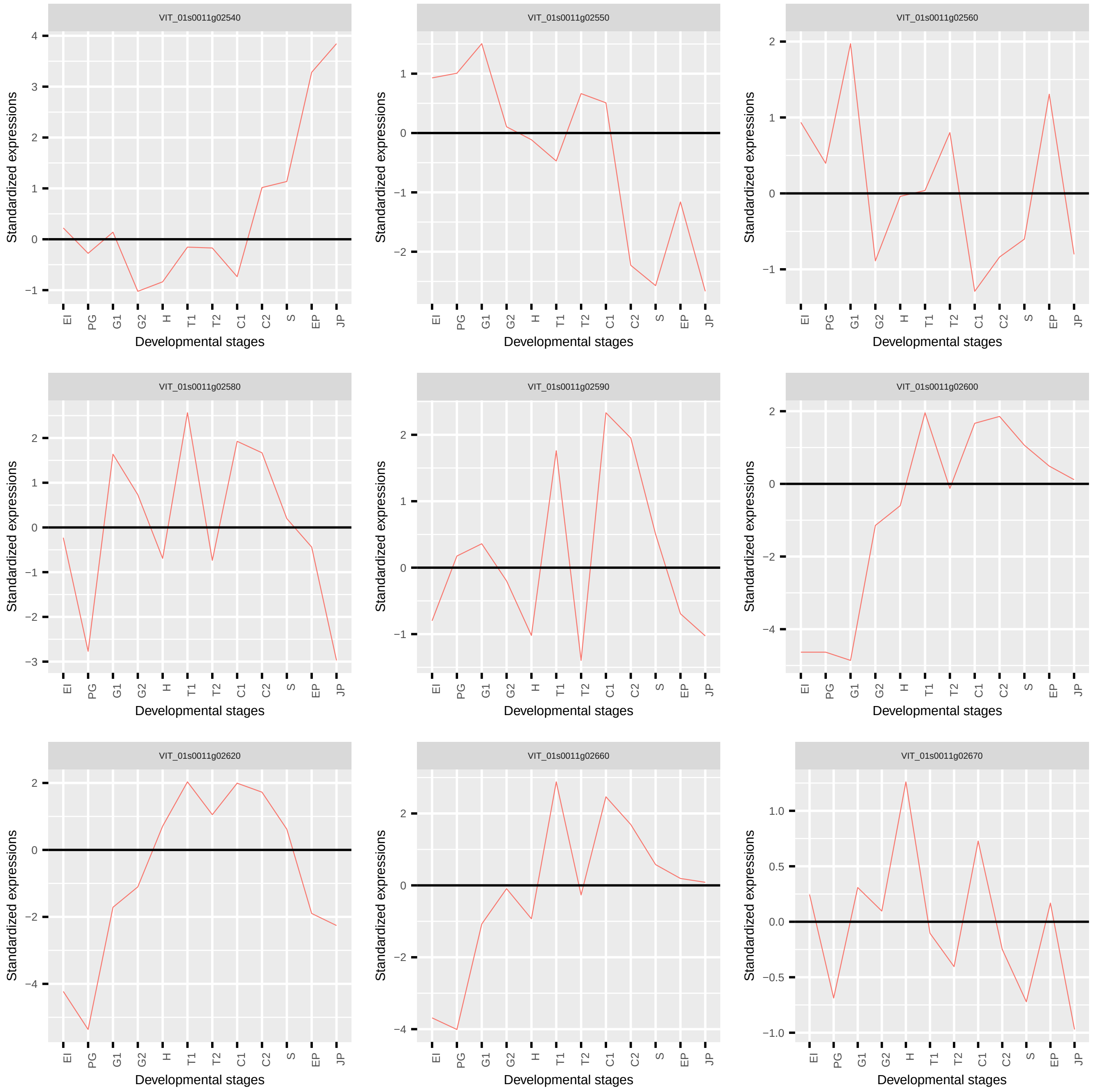
VIT_01s0011g02540
VIT_01s0011g02550
VIT_01s0011g02560
VIT_01s0011g02580
VIT_01s0011g02590
VIT_01s0011g02600
VIT_01s0011g02620
VIT_01s0011g02660
VIT_01s0011g02670
Standardized expressions
Developmental stages
EI PG G1 G2 H T1 T2 C1 C2 S EP JP

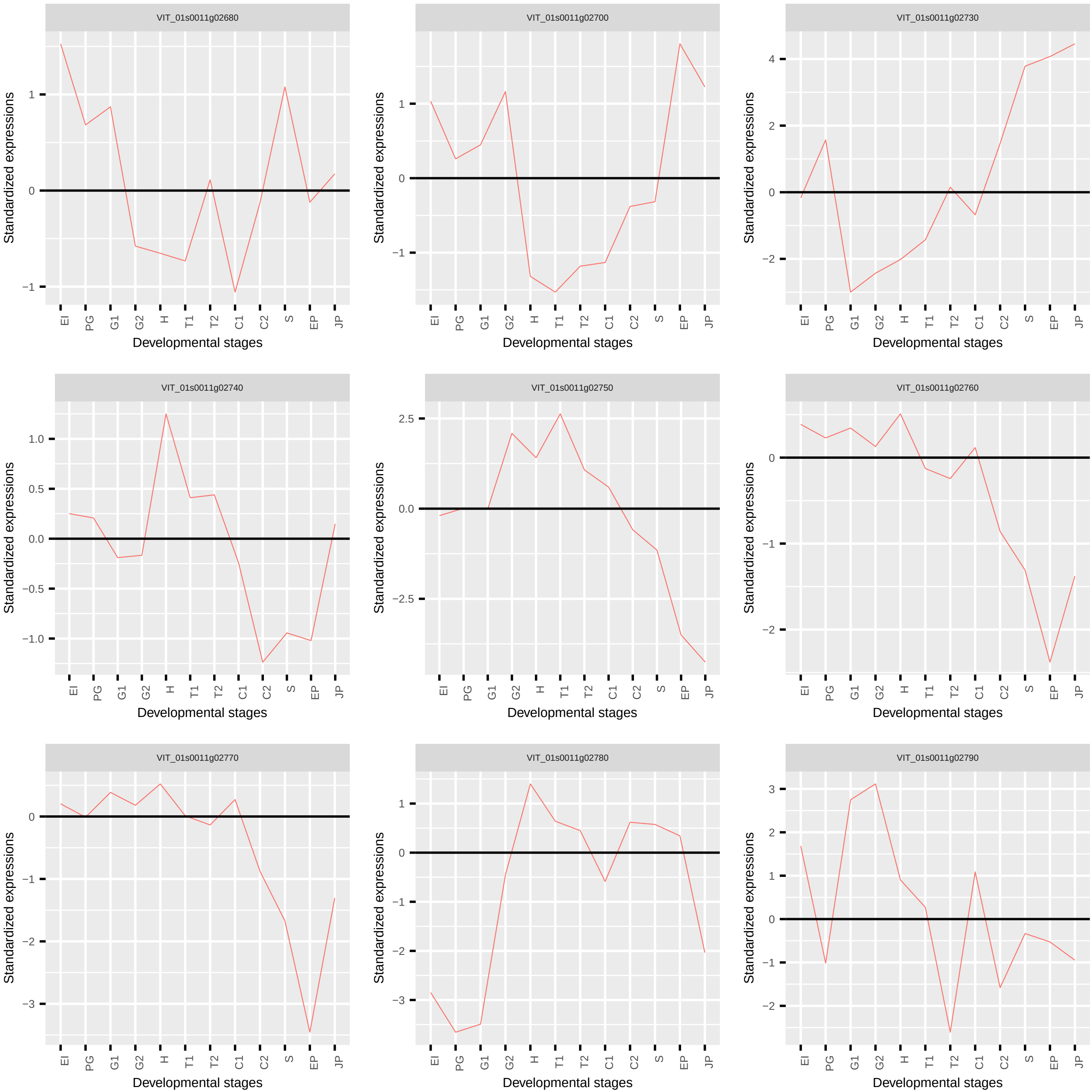
VIT_01s0011g02680
VIT_01s0011g02700
VIT_01s0011g02730
VIT_01s0011g02740
VIT_01s0011g02750
VIT_01s0011g02760
VIT_01s0011g02770
VIT_01s0011g02780
VIT_01s0011g02790
Standardized expressions
Developmental stages
EI PG G1 G2 H T1 T2 C1 C2 S EP JP

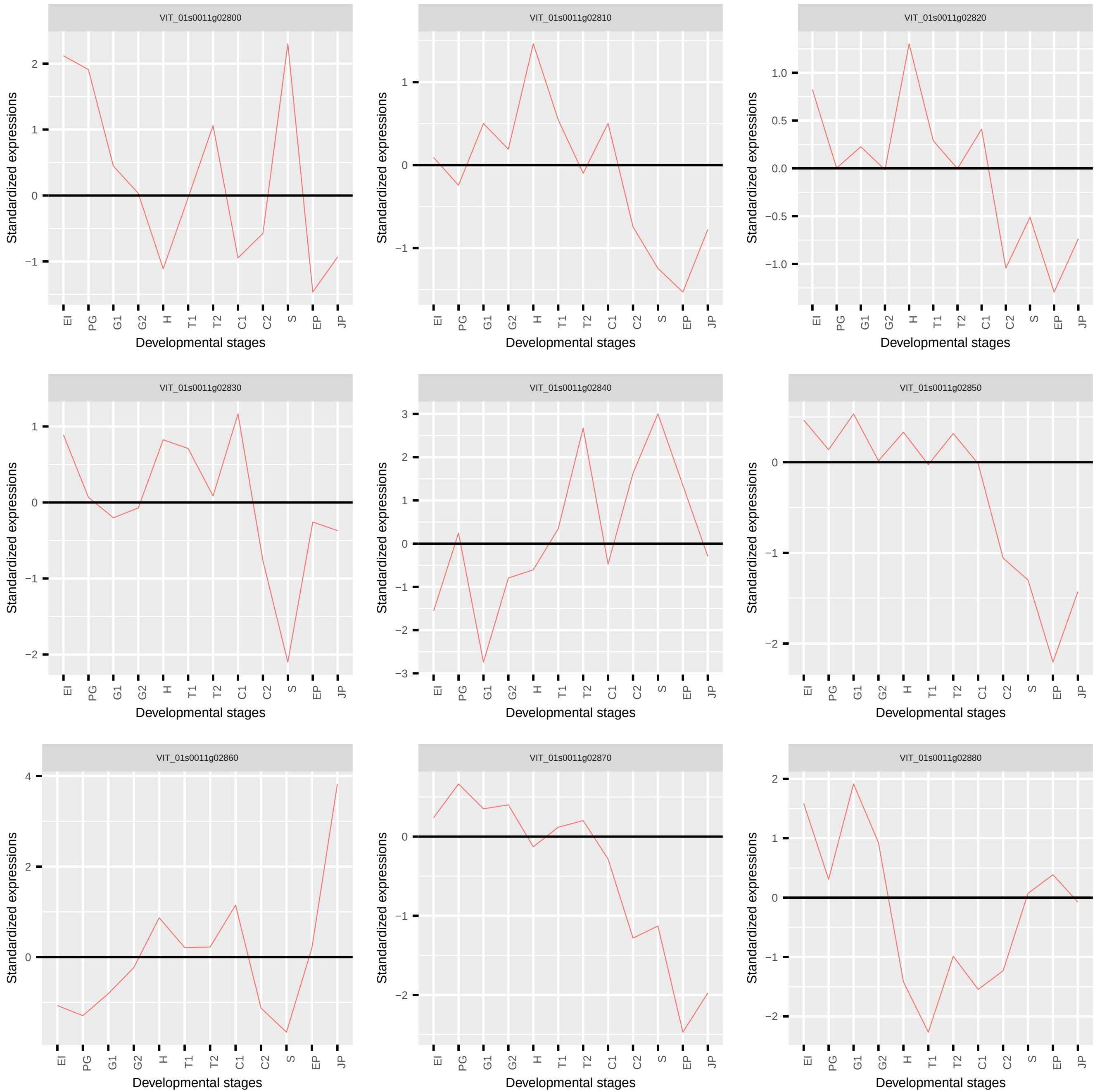

VIT_01s0011g02800
VIT_01s0011g02810
VIT_01s0011g02820
VIT_01s0011g02830
VIT_01s0011g02840
VIT_01s0011g02850
VIT_01s0011g02860
VIT_01s0011g02870
VIT_01s0011g02880
Standardized expressions
Developmental stages
EI
PG
G1
G2
H
T1
T2
C1
C2
S
EP
JP

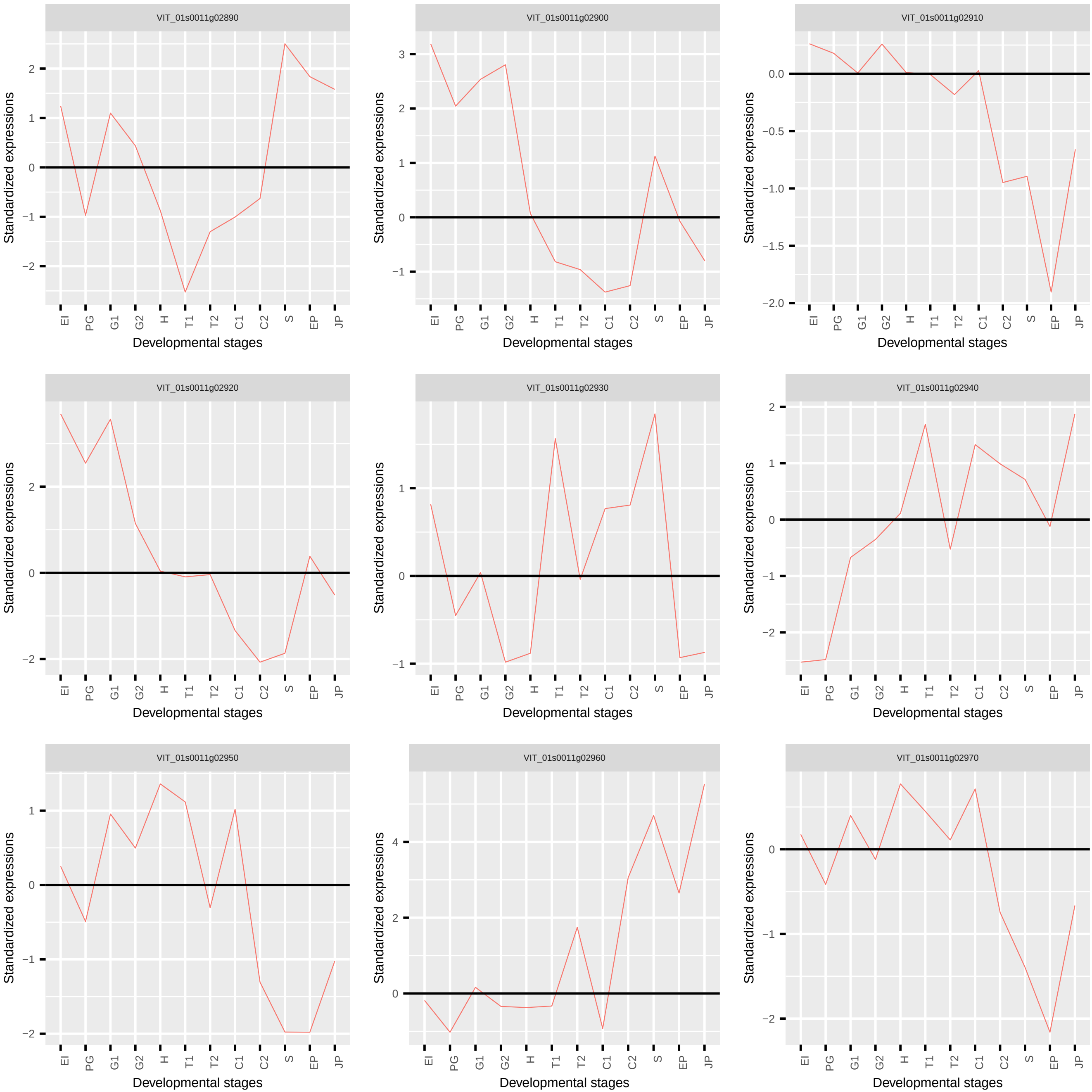
VIT_01s0011g02890
VIT_01s0011g02900
VIT_01s0011g02910
VIT_01s0011g02920
VIT_01s0011g02930
VIT_01s0011g02940
VIT_01s0011g02950
VIT_01s0011g02960
VIT_01s0011g02970
Standardized expressions
Developmental stages
EI
PG
G1
G2
H
T1
T2
C1
C2
S
EP
JP

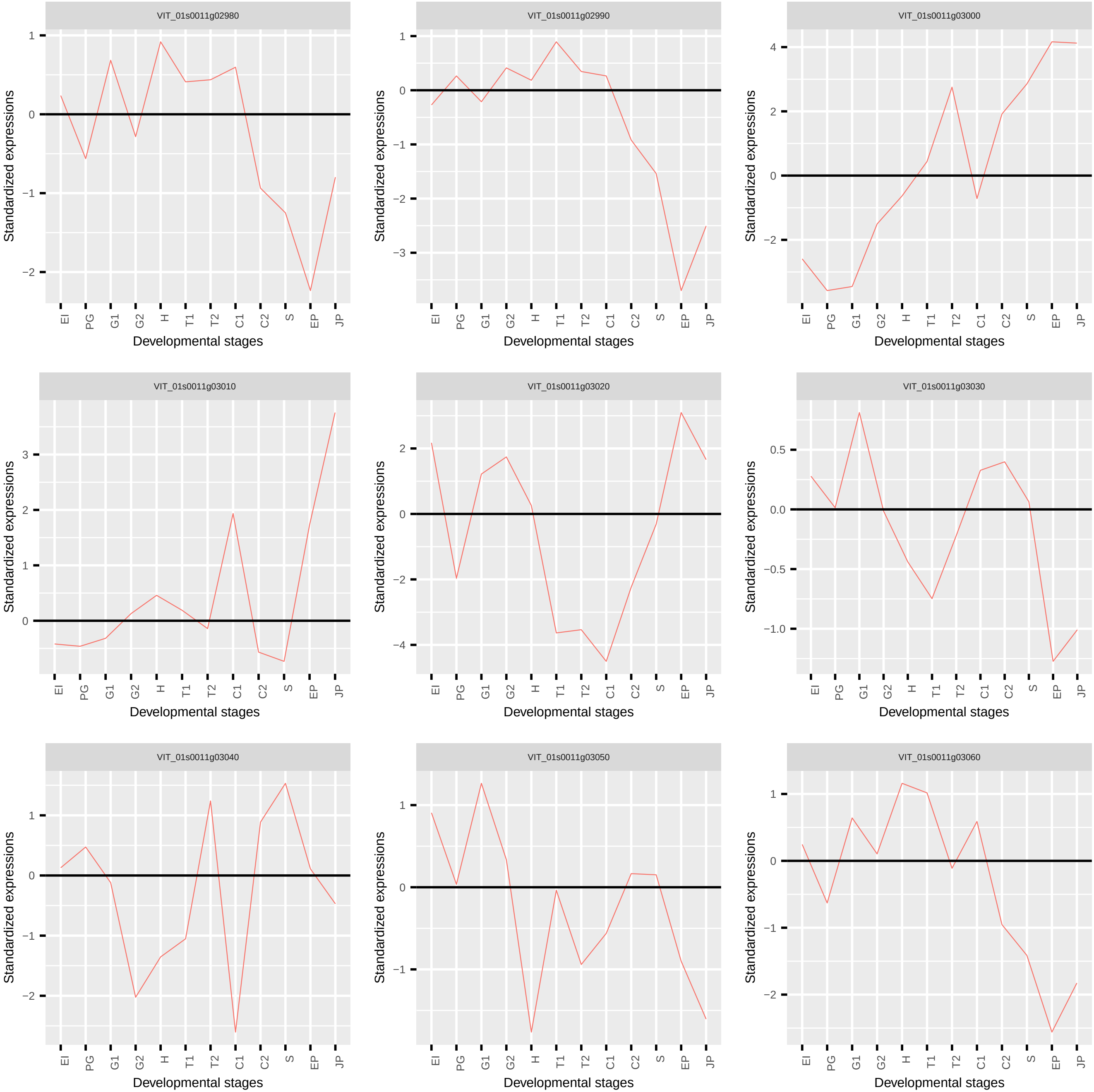
VIT_01s0011g02980
VIT_01s0011g02990
VIT_01s0011g03000
VIT_01s0011g03010
VIT_01s0011g03020
VIT_01s0011g03030
VIT_01s0011g03040
VIT_01s0011g03050
VIT_01s0011g03060
Standardized expressions
Developmental stages
EI PG G1 G2 H T1 T2 C1 C2 S EP JP

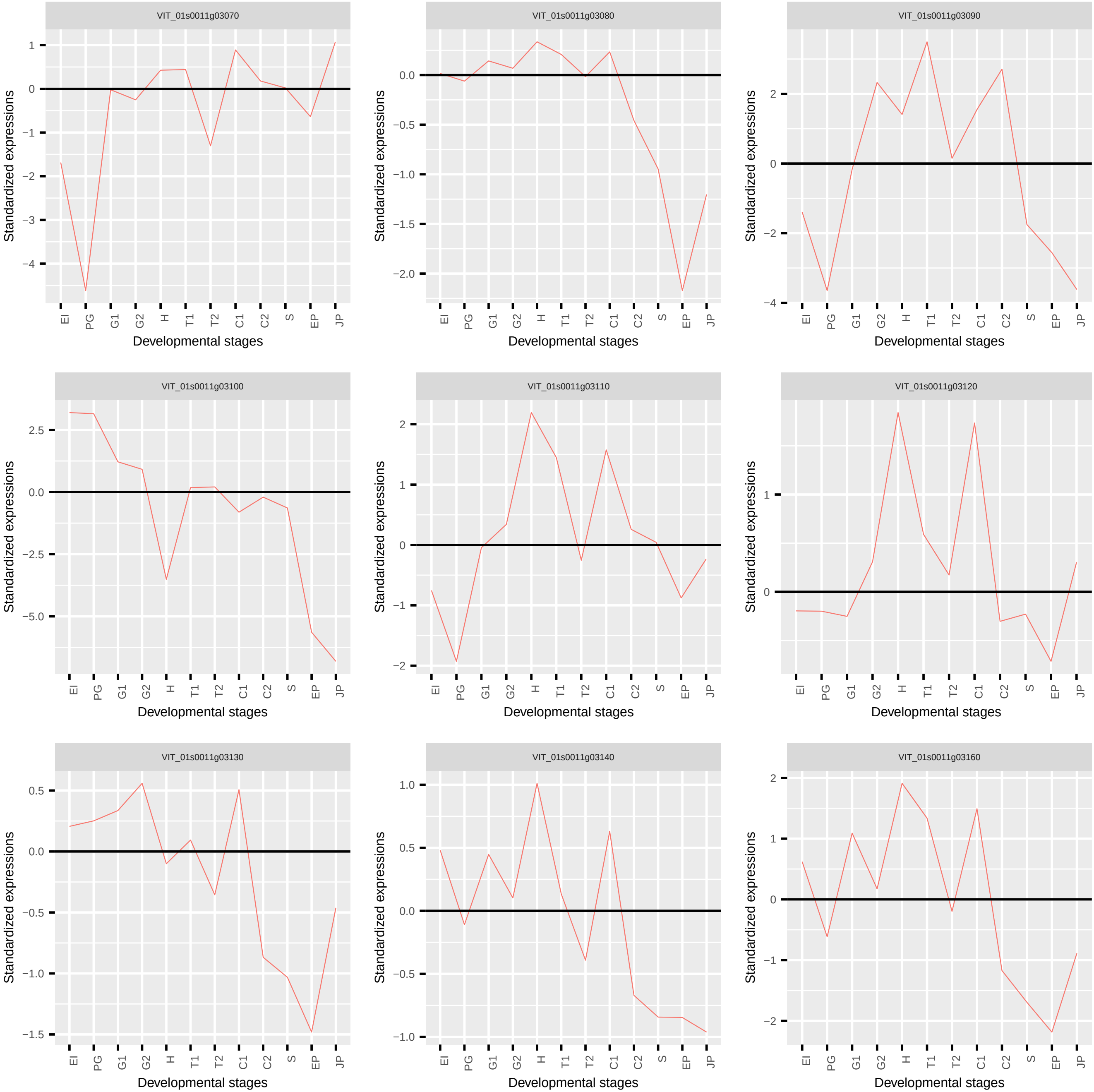
VIT_01s0011g03070
VIT_01s0011g03080
VIT_01s0011g03090
VIT_01s0011g03100
VIT_01s0011g03110
VIT_01s0011g03120
VIT_01s0011g03130
VIT_01s0011g03140
VIT_01s0011g03160
Standardized expressions
Developmental stages
EI
PG
G1
G2
H
T1
T2
C1
C2
S
EP
JP

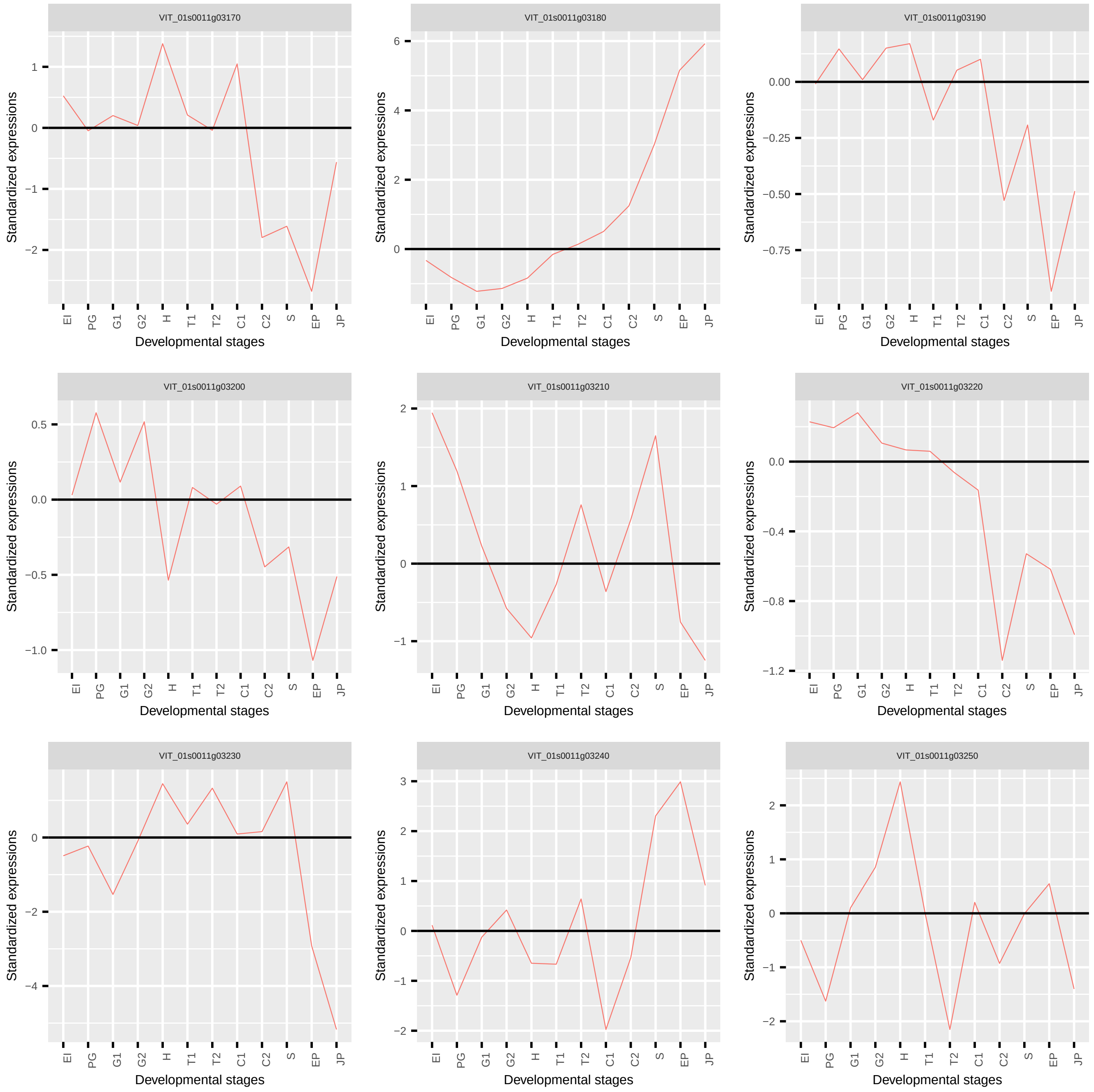

VIT_01s0011g03170
VIT_01s0011g03180
VIT_01s0011g03190
VIT_01s0011g03200
VIT_01s0011g03210
VIT_01s0011g03220
VIT_01s0011g03230
VIT_01s0011g03240
VIT_01s0011g03250
Standardized expressions
Developmental stages
EI PG G1 G2 H T1 T2 C1 C2 S EP JP

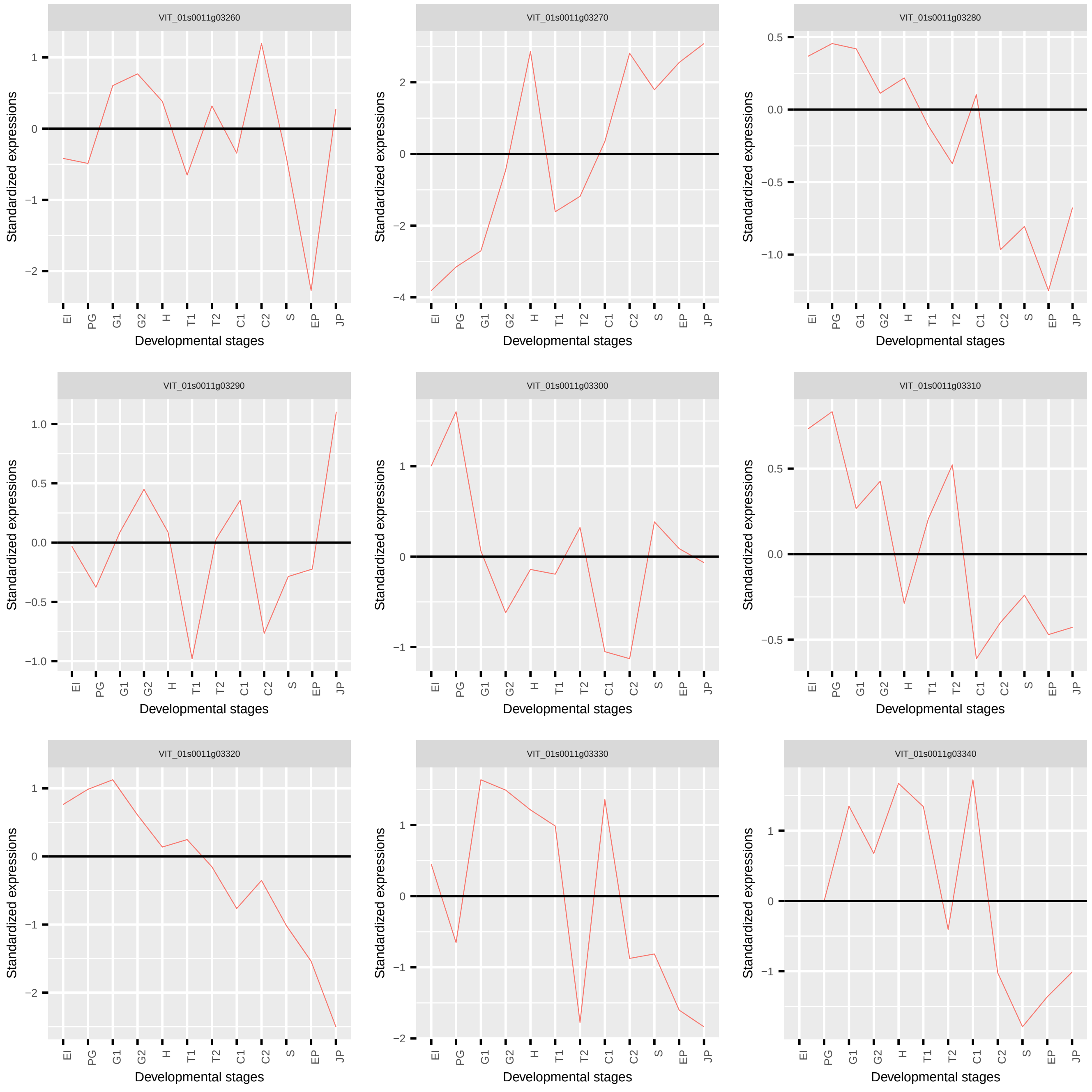
VIT_01s0011g03260
VIT_01s0011g03270
VIT_01s0011g03280
VIT_01s0011g03290
VIT_01s0011g03300
VIT_01s0011g03310
VIT_01s0011g03320
VIT_01s0011g03330
VIT_01s0011g03340
Standardized expressions
Developmental stages
EI PG G1 G2 H T1 T2 C1 C2 S EP JP

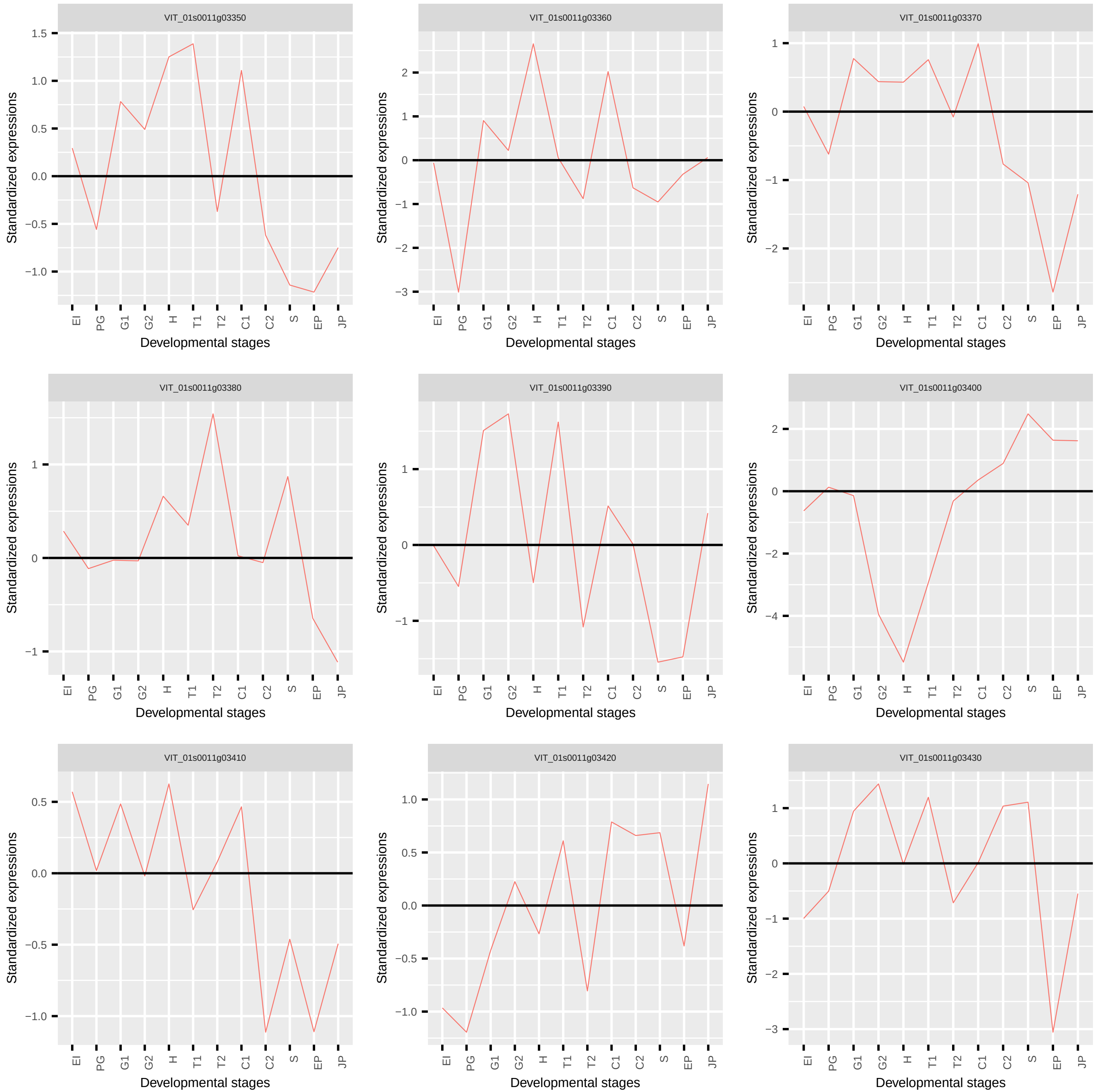
VIT_01s0011g03350
Standardized expressions
1.5
1.0
0.5
0.0
−0.5
−1.0
EI PG G1 G2 H T1 T2 C1 C2 S EP JP
Developmental stages
VIT_01s0011g03360
Standardized expressions
2
1
0
−1
−2
−3
EI PG G1 G2 H T1 T2 C1 C2 S EP JP
Developmental stages
VIT_01s0011g03370
Standardized expressions
1
0
−1
−2
EI PG G1 G2 H T1 T2 C1 C2 S EP JP
Developmental stages
VIT_01s0011g03380
Standardized expressions
1
0
−1
EI PG G1 G2 H T1 T2 C1 C2 S EP JP
Developmental stages
VIT_01s0011g03390
Standardized expressions
1
0
−1
EI PG G1 G2 H T1 T2 C1 C2 S EP JP
Developmental stages
VIT_01s0011g03400
Standardized expressions
2
0
−2
−4
EI PG G1 G2 H T1 T2 C1 C2 S EP JP
Developmental stages
VIT_01s0011g03410
Standardized expressions
0.5
0.0
−0.5
−1.0
EI PG G1 G2 H T1 T2 C1 C2 S EP JP
Developmental stages
VIT_01s0011g03420
Standardized expressions
1.0
0.5
0.0
−0.5
−1.0
EI PG G1 G2 H T1 T2 C1 C2 S EP JP
Developmental stages
VIT_01s0011g03430
Standardized expressions
1
0
−1
−2
−3
EI PG G1 G2 H T1 T2 C1 C2 S EP JP
Developmental stages

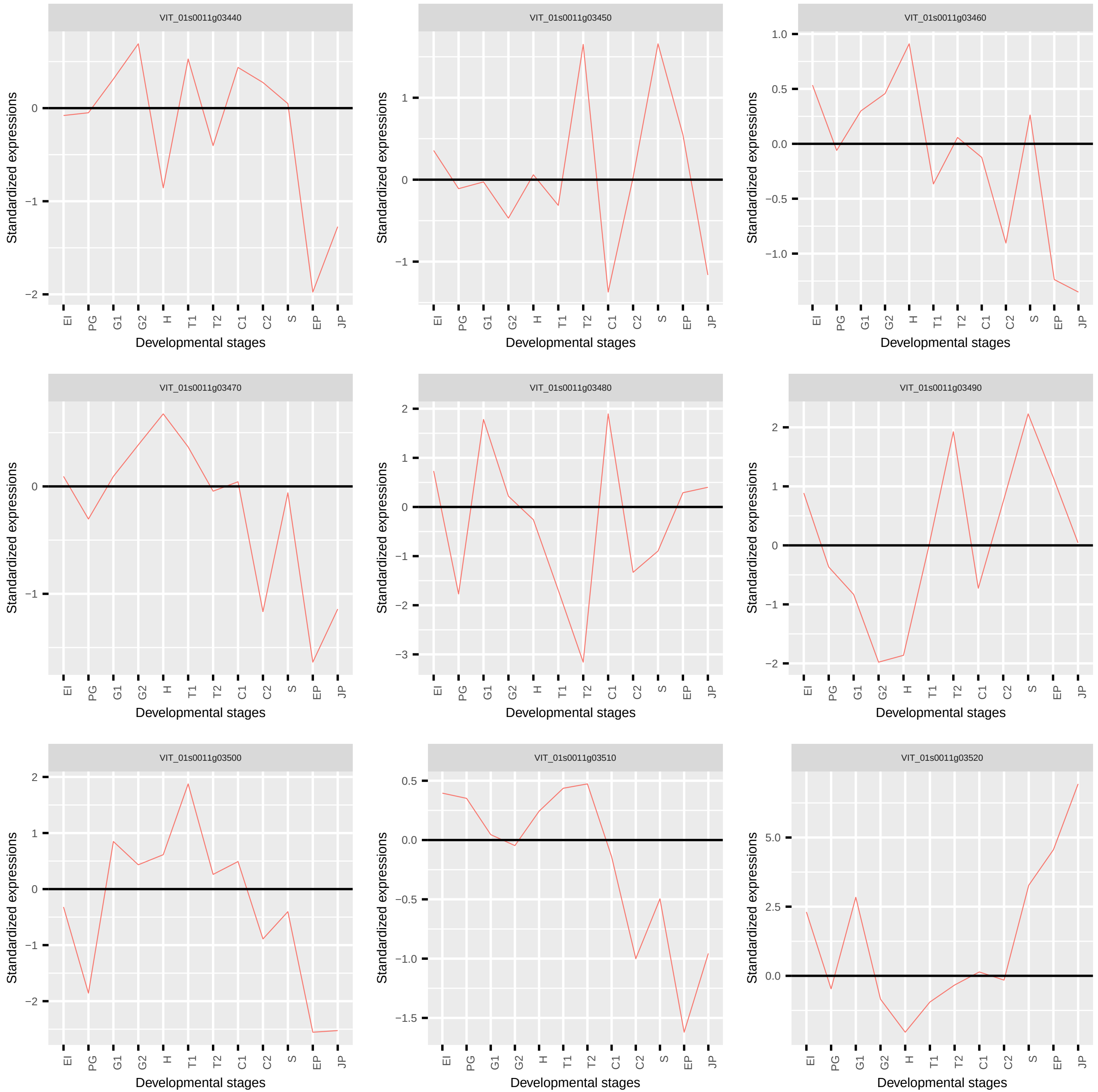
VIT_01s0011g03440
VIT_01s0011g03450
VIT_01s0011g03460
VIT_01s0011g03470
VIT_01s0011g03480
VIT_01s0011g03490
VIT_01s0011g03500
VIT_01s0011g03510
VIT_01s0011g03520
Standardized expressions
Developmental stages
EI PG G1 G2 H T1 T2 C1 C2 S EP JP

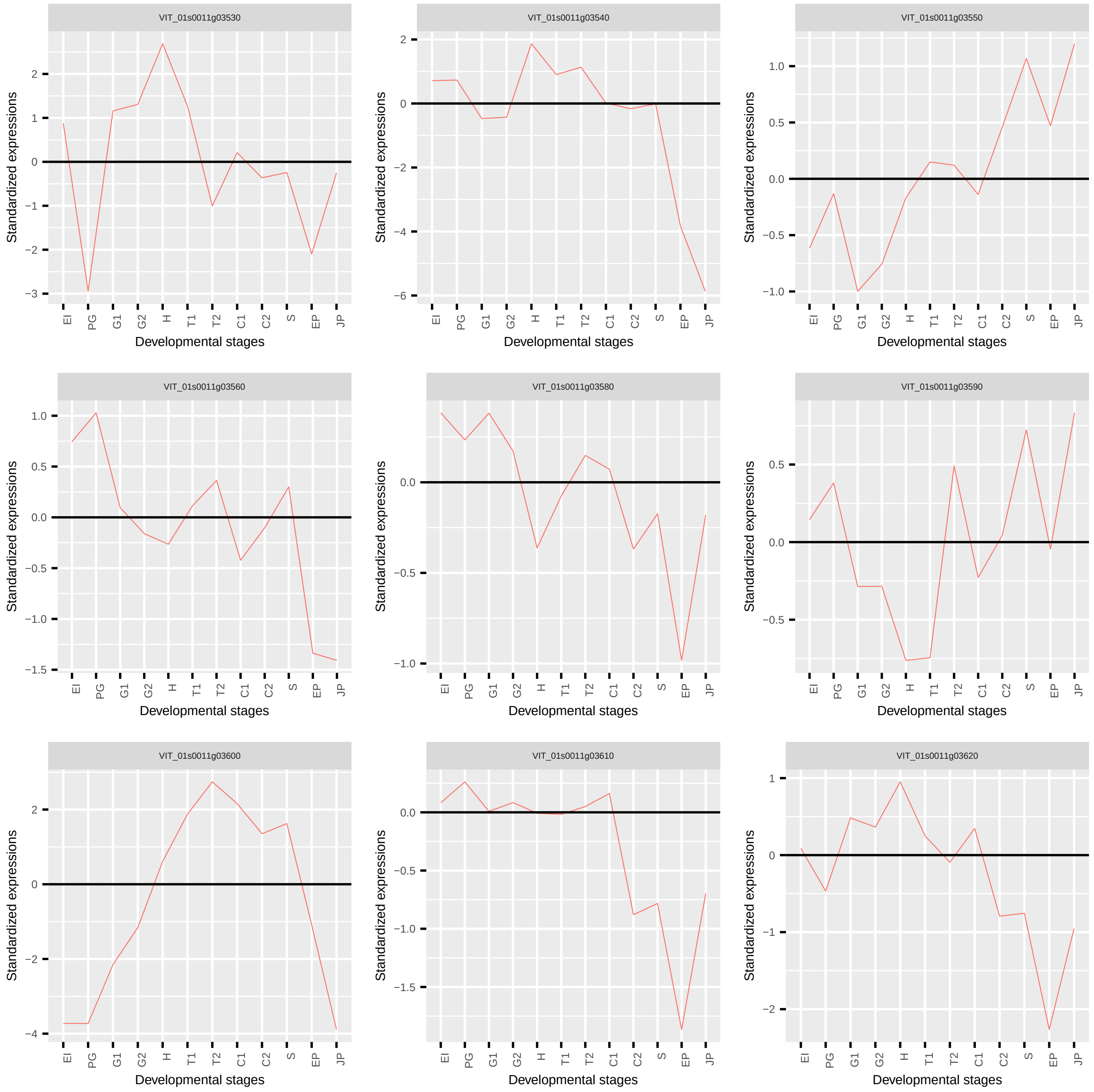
VIT_01s0011g03530
VIT_01s0011g03540
VIT_01s0011g03550
VIT_01s0011g03560
VIT_01s0011g03580
VIT_01s0011g03590
VIT_01s0011g03600
VIT_01s0011g03610
VIT_01s0011g03620
Standardized expressions
EI PG G1 G2 H T1 T2 C1 C2 S EP JP
Developmental stages

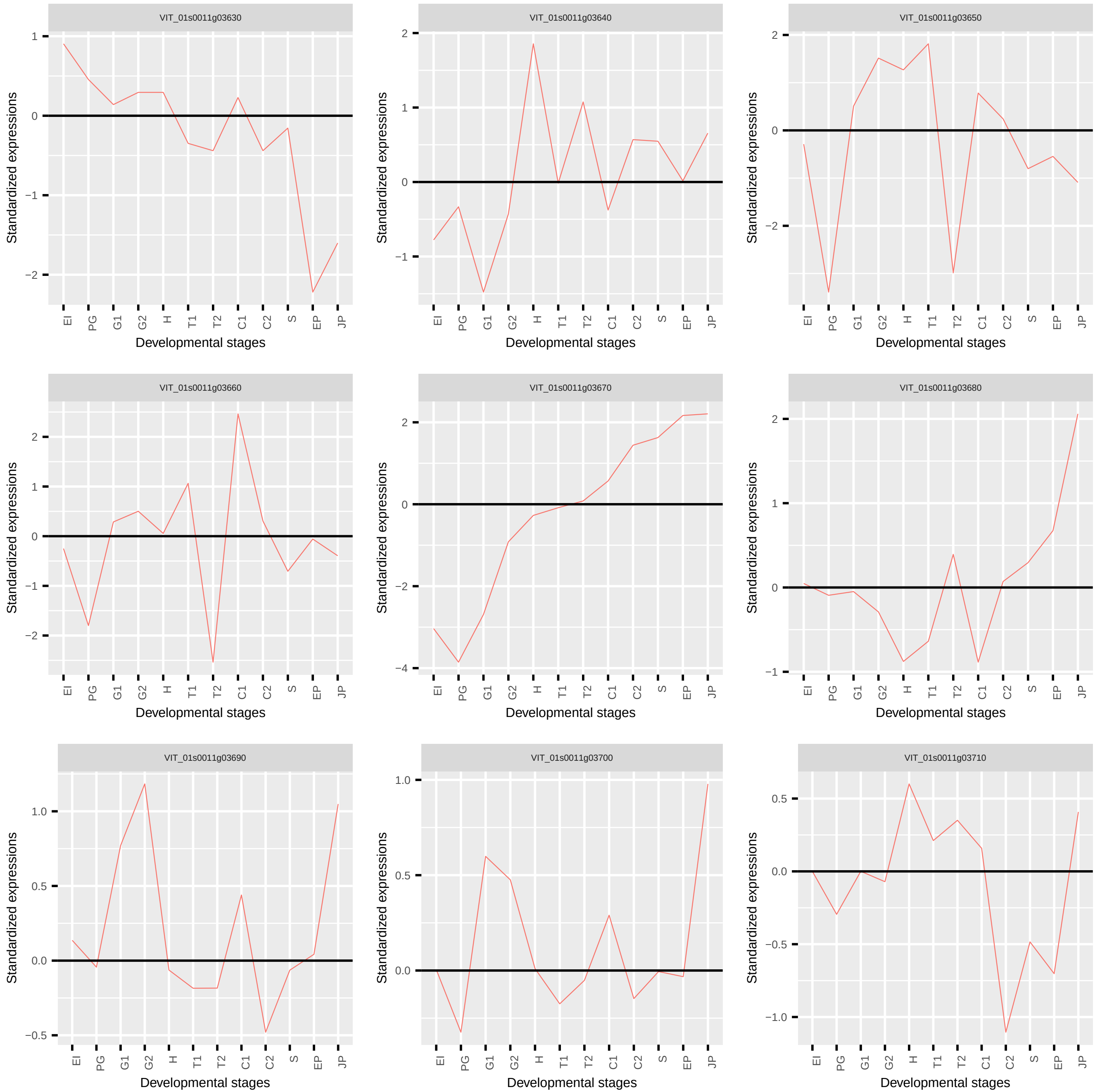
VIT_01s0011g03630
VIT_01s0011g03640
VIT_01s0011g03650
VIT_01s0011g03660
VIT_01s0011g03670
VIT_01s0011g03680
VIT_01s0011g03690
VIT_01s0011g03700
VIT_01s0011g03710
Standardized expressions
Developmental stages
EI
PG
G1
G2
H
T1
T2
C1
C2
S
EP
JP

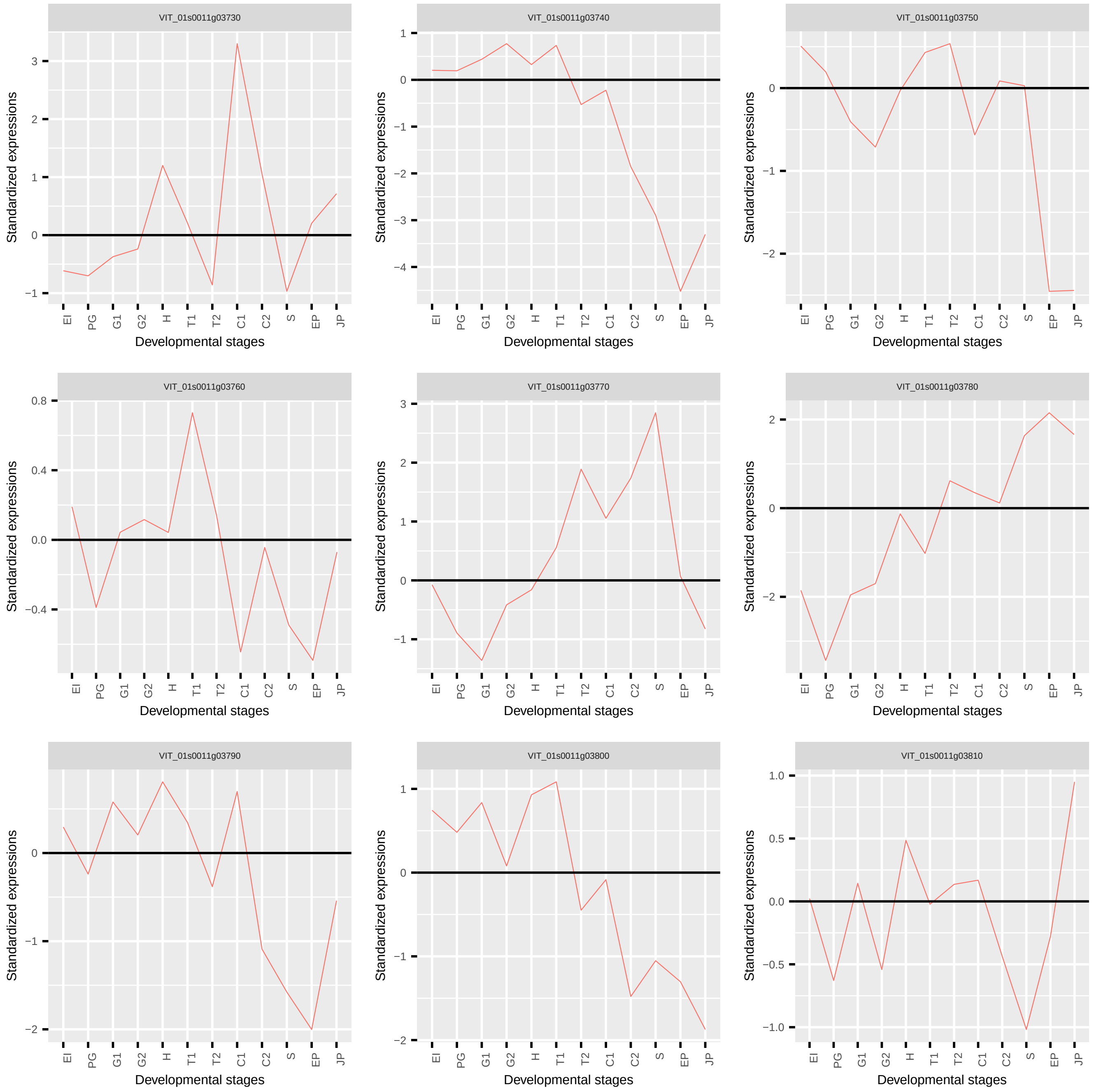

VIT_01s0011g03730
Standardized expressions
3
2
1
0
−1
EI PG G1 G2 H T1 T2 C1 C2 S EP JP
Developmental stages
VIT_01s0011g03740
Standardized expressions
1
0
−1
−2
−3
−4
EI PG G1 G2 H T1 T2 C1 C2 S EP JP
Developmental stages
VIT_01s0011g03750
Standardized expressions
0
−1
−2
EI PG G1 G2 H T1 T2 C1 C2 S EP JP
Developmental stages
VIT_01s0011g03760
Standardized expressions
0.8
0.4
0.0
−0.4
EI PG G1 G2 H T1 T2 C1 C2 S EP JP
Developmental stages
VIT_01s0011g03770
Standardized expressions
3
2
1
0
−1
EI PG G1 G2 H T1 T2 C1 C2 S EP JP
Developmental stages
VIT_01s0011g03780
Standardized expressions
2
0
−2
EI PG G1 G2 H T1 T2 C1 C2 S EP JP
Developmental stages
VIT_01s0011g03790
Standardized expressions
0
−1
−2
EI PG G1 G2 H T1 T2 C1 C2 S EP JP
Developmental stages
VIT_01s0011g03800
Standardized expressions
1
0
−1
−2
EI PG G1 G2 H T1 T2 C1 C2 S EP JP
Developmental stages
VIT_01s0011g03810
Standardized expressions
1.0
0.5
0.0
−0.5
−1.0
EI PG G1 G2 H T1 T2 C1 C2 S EP JP
Developmental stages

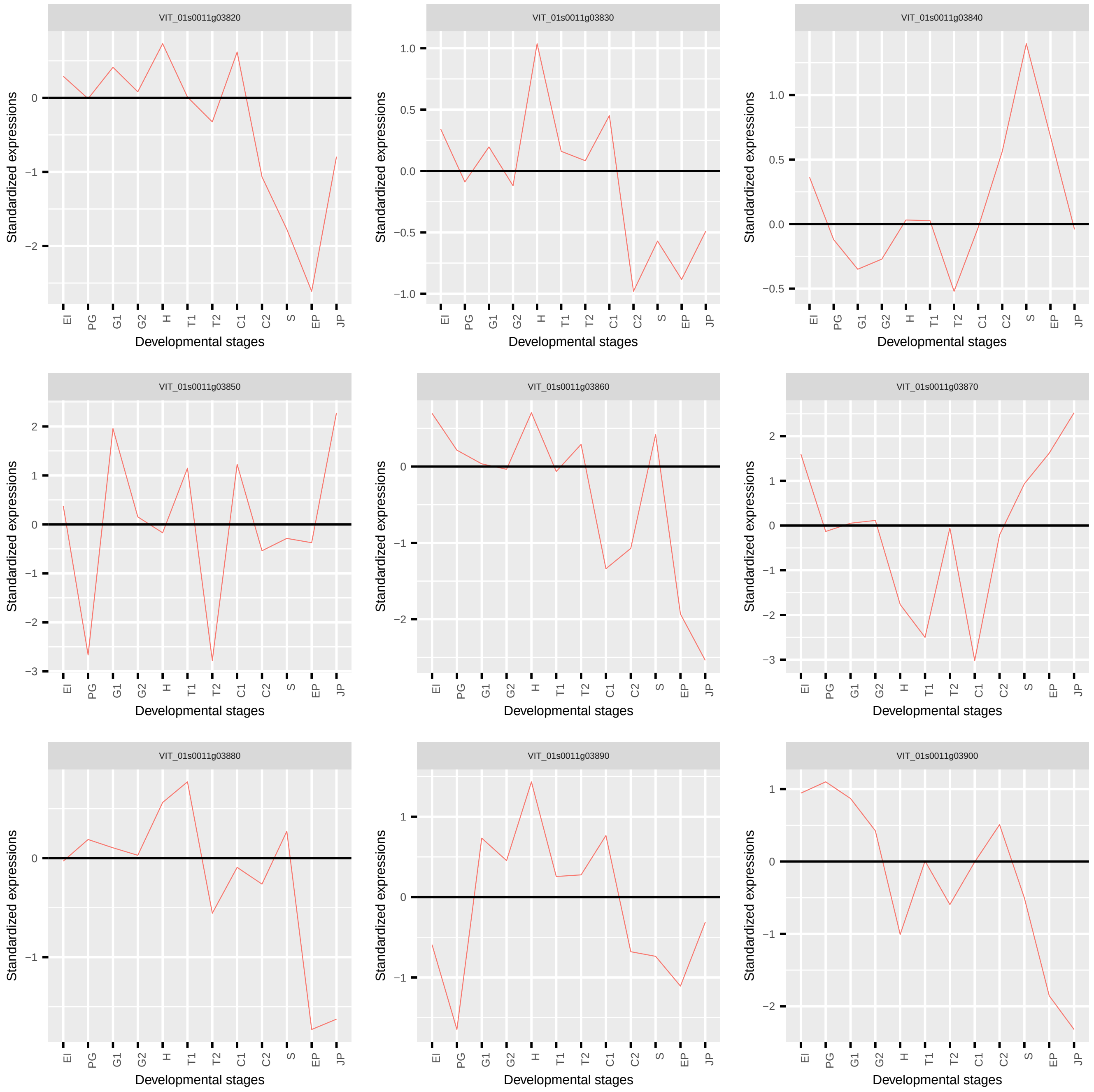
VIT_01s0011g03820
VIT_01s0011g03830
VIT_01s0011g03840
VIT_01s0011g03850
VIT_01s0011g03860
VIT_01s0011g03870
VIT_01s0011g03880
VIT_01s0011g03890
VIT_01s0011g03900
Standardized expressions
Developmental stages
EI PG G1 G2 H T1 T2 C1 C2 S EP JP

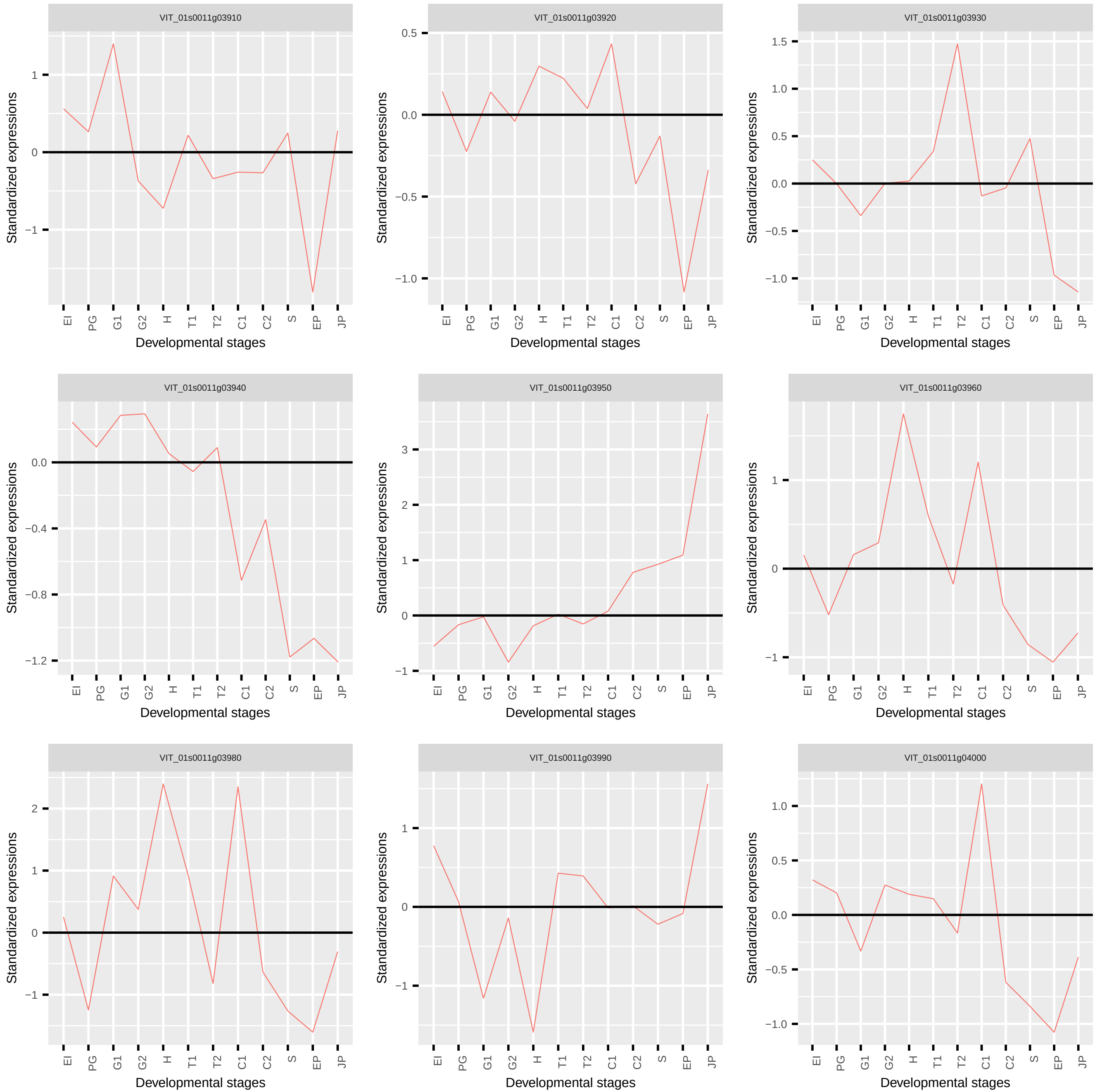
VIT_01s0011g03910
VIT_01s0011g03920
VIT_01s0011g03930
VIT_01s0011g03940
VIT_01s0011g03950
VIT_01s0011g03960
VIT_01s0011g03980
VIT_01s0011g03990
VIT_01s0011g04000
Standardized expressions
Developmental stages
EI PG G1 G2 H T1 T2 C1 C2 S EP JP

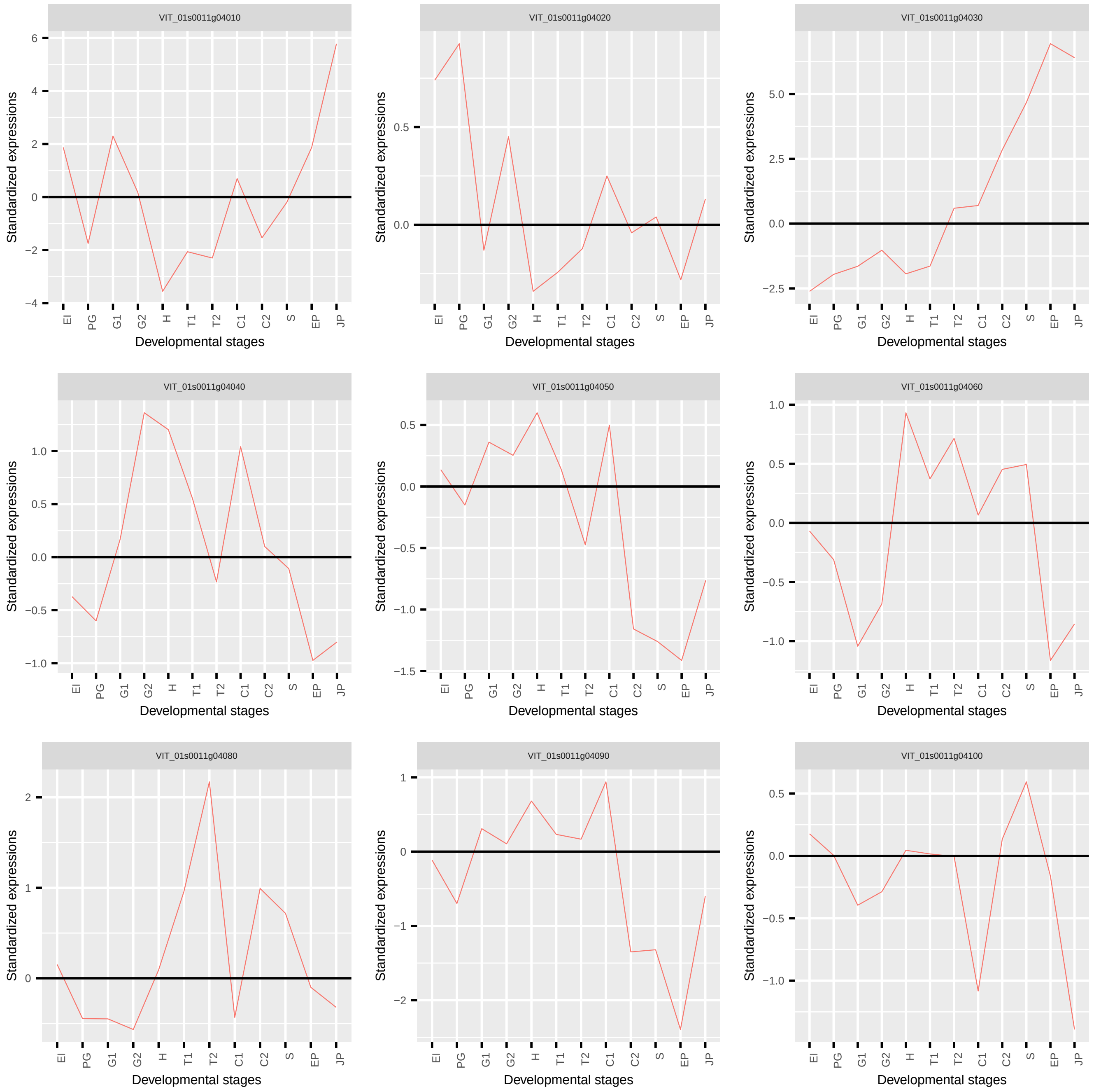
VIT_01s0011g04010
VIT_01s0011g04020
VIT_01s0011g04030
VIT_01s0011g04040
VIT_01s0011g04050
VIT_01s0011g04060
VIT_01s0011g04080
VIT_01s0011g04090
VIT_01s0011g04100
Standardized expressions
Developmental stages
EI PG G1 G2 H T1 T2 C1 C2 S EP JP
6 4 2 0 −2 −4
0.5 0.0
5.0 2.5 0.0 −2.5
1.0 0.5 0.0 −0.5 −1.0
0.5 0.0 −0.5 −1.0 −1.5
1.0 0.5 0.0 −0.5 −1.0
2 1 0
1 0 −1 −2
0.5 0.0 −0.5 −1.0

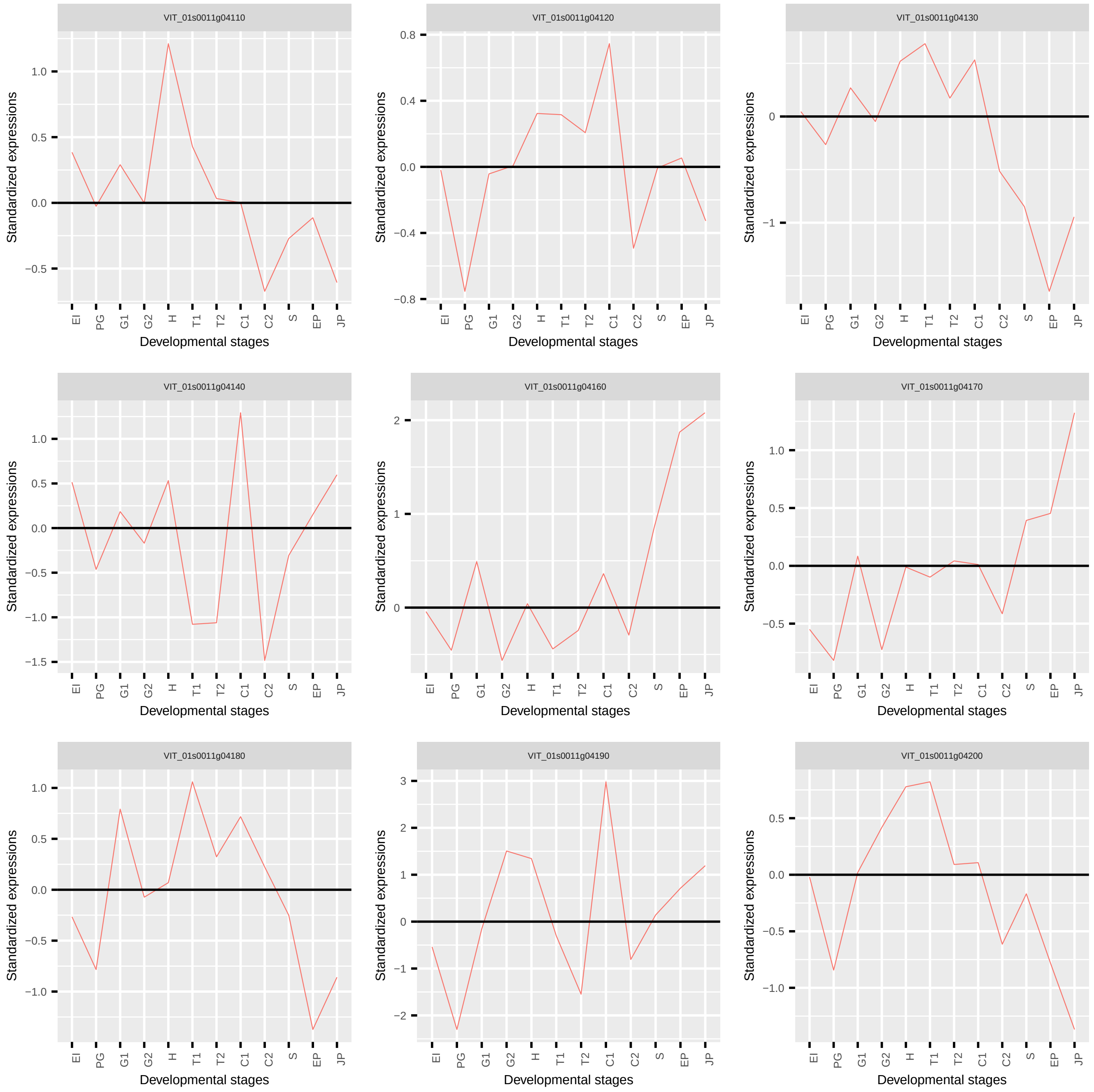
VIT_01s0011g04110
VIT_01s0011g04120
VIT_01s0011g04130
VIT_01s0011g04140
VIT_01s0011g04160
VIT_01s0011g04170
VIT_01s0011g04180
VIT_01s0011g04190
VIT_01s0011g04200
Standardized expressions
Developmental stages
EI PG G1 G2 H T1 T2 C1 C2 S EP JP

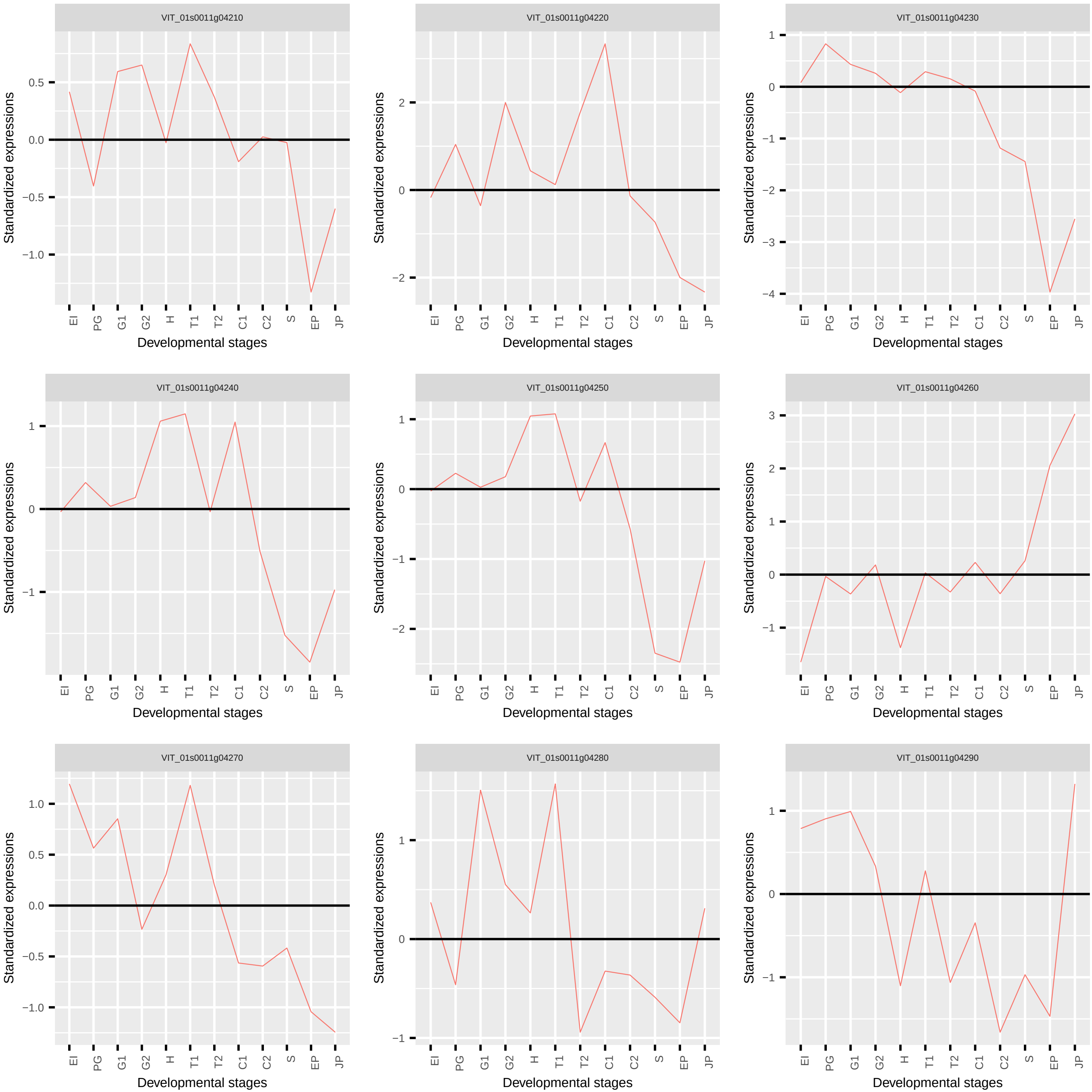

VIT_01s0011g04210
VIT_01s0011g04220
VIT_01s0011g04230
VIT_01s0011g04240
VIT_01s0011g04250
VIT_01s0011g04260
VIT_01s0011g04270
VIT_01s0011g04280
VIT_01s0011g04290
Standardized expressions
Developmental stages
EI
PG
G1
G2
H
T1
T2
C1
C2
S
EP
JP

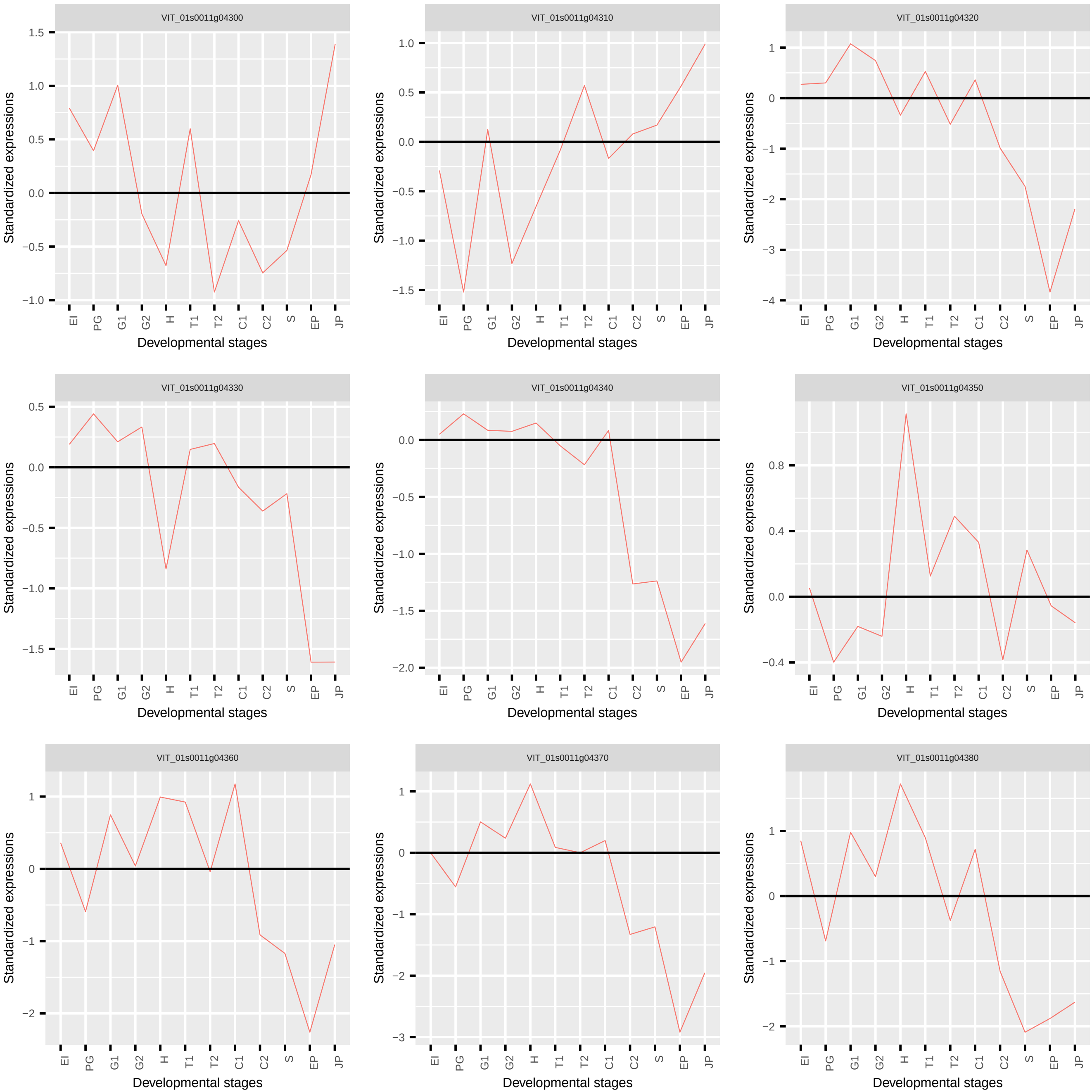
VIT_01s0011g04300
VIT_01s0011g04310
VIT_01s0011g04320
VIT_01s0011g04330
VIT_01s0011g04340
VIT_01s0011g04350
VIT_01s0011g04360
VIT_01s0011g04370
VIT_01s0011g04380
Standardized expressions
Developmental stages
EI
PG
G1
G2
H
T1
T2
C1
C2
S
EP
JP

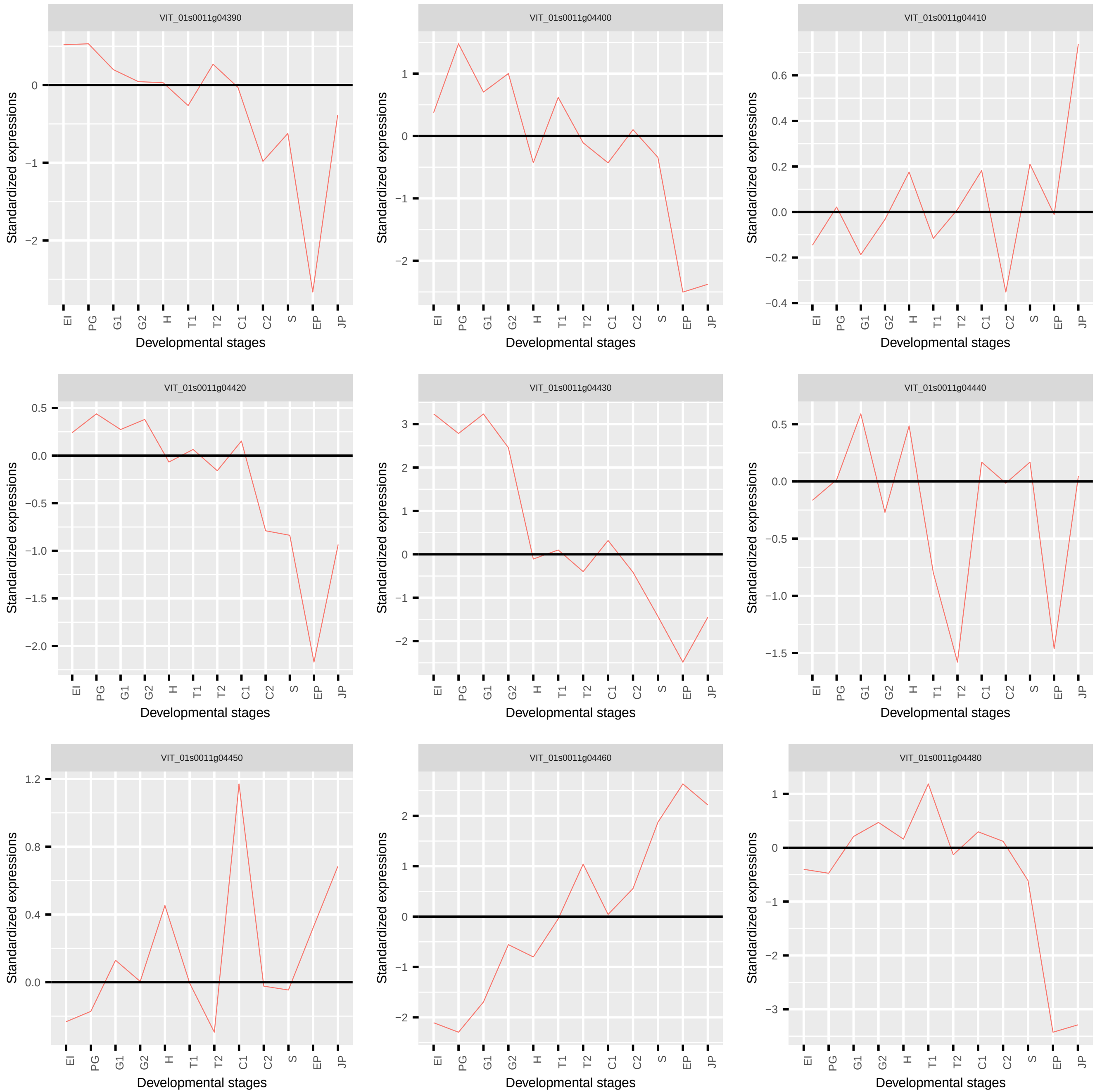
VIT_01s0011g04390
VIT_01s0011g04400
VIT_01s0011g04410
VIT_01s0011g04420
VIT_01s0011g04430
VIT_01s0011g04440
VIT_01s0011g04450
VIT_01s0011g04460
VIT_01s0011g04480
Standardized expressions
Developmental stages
EI
PG
G1
G2
H
T1
T2
C1
C2
S
EP
JP

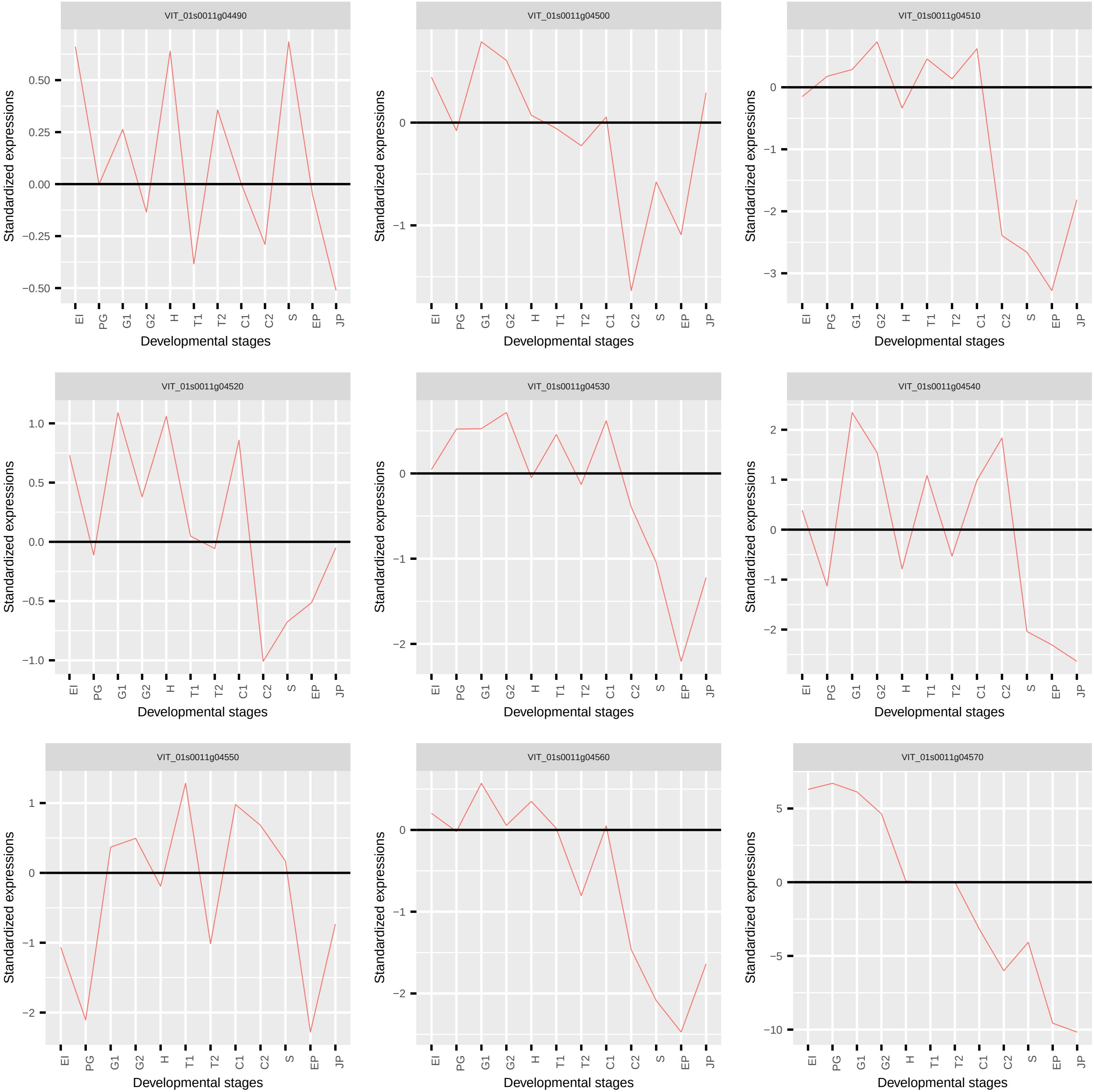

VIT_01s0011g04490
VIT_01s0011g04500
VIT_01s0011g04510
VIT_01s0011g04520
VIT_01s0011g04530
VIT_01s0011g04540
VIT_01s0011g04550
VIT_01s0011g04560
VIT_01s0011g04570
Standardized expressions
Developmental stages
EI PG G1 G2 H T1 T2 C1 C2 S EP JP
0.50 0.25 0.00 −0.25 −0.50
0 −1
0 −1 −2 −3
1.0 0.5 0.0 −0.5 −1.0
0 −1 −2
2 1 0 −1 −2
1 0 −1 −2
0 −1 −2
5 0 −5 −10

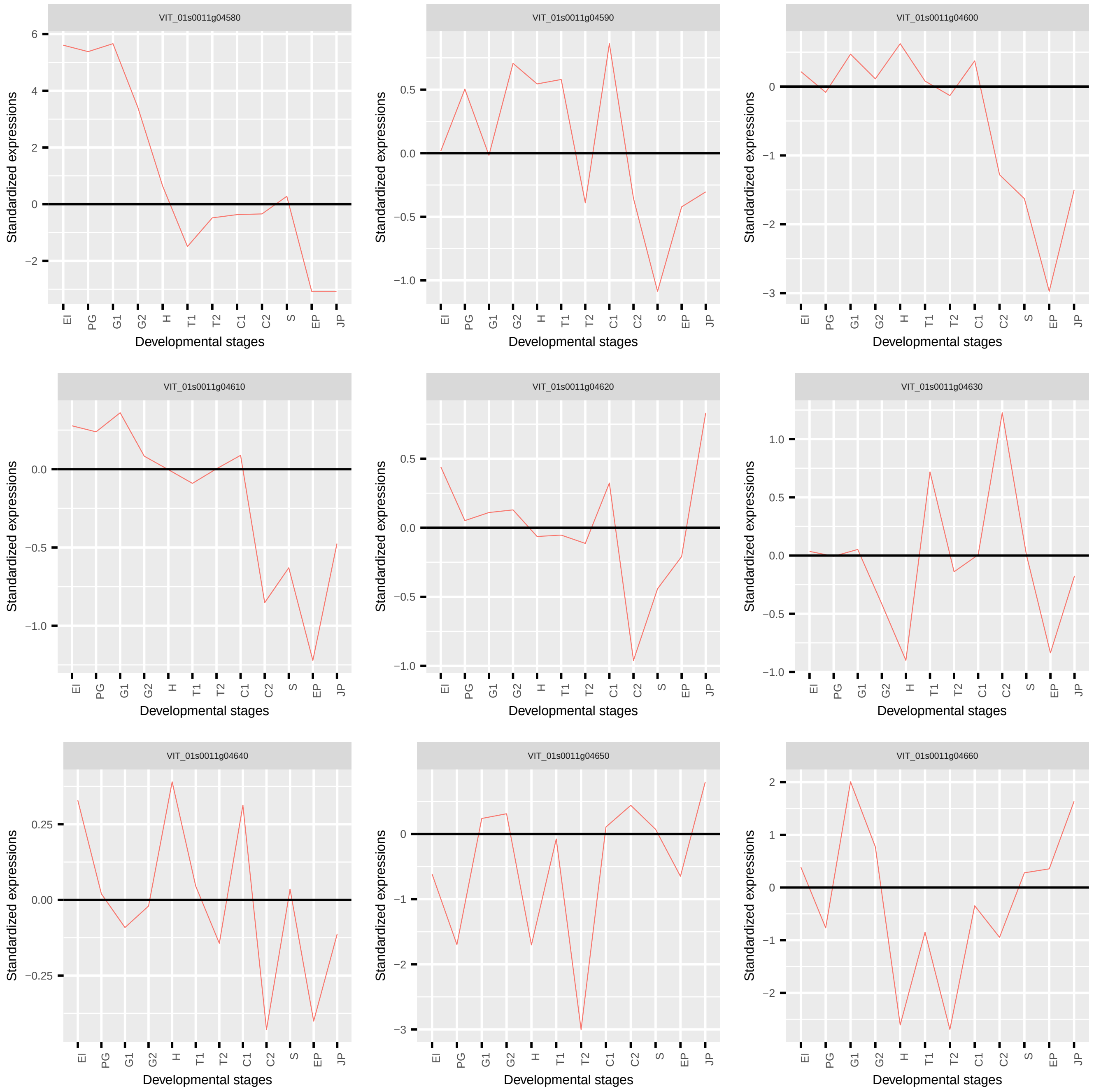
VIT_01s0011g04580
VIT_01s0011g04590
VIT_01s0011g04600
VIT_01s0011g04610
VIT_01s0011g04620
VIT_01s0011g04630
VIT_01s0011g04640
VIT_01s0011g04650
VIT_01s0011g04660
Standardized expressions
Developmental stages
EI
PG
G1
G2
H
T1
T2
C1
C2
S
EP
JP

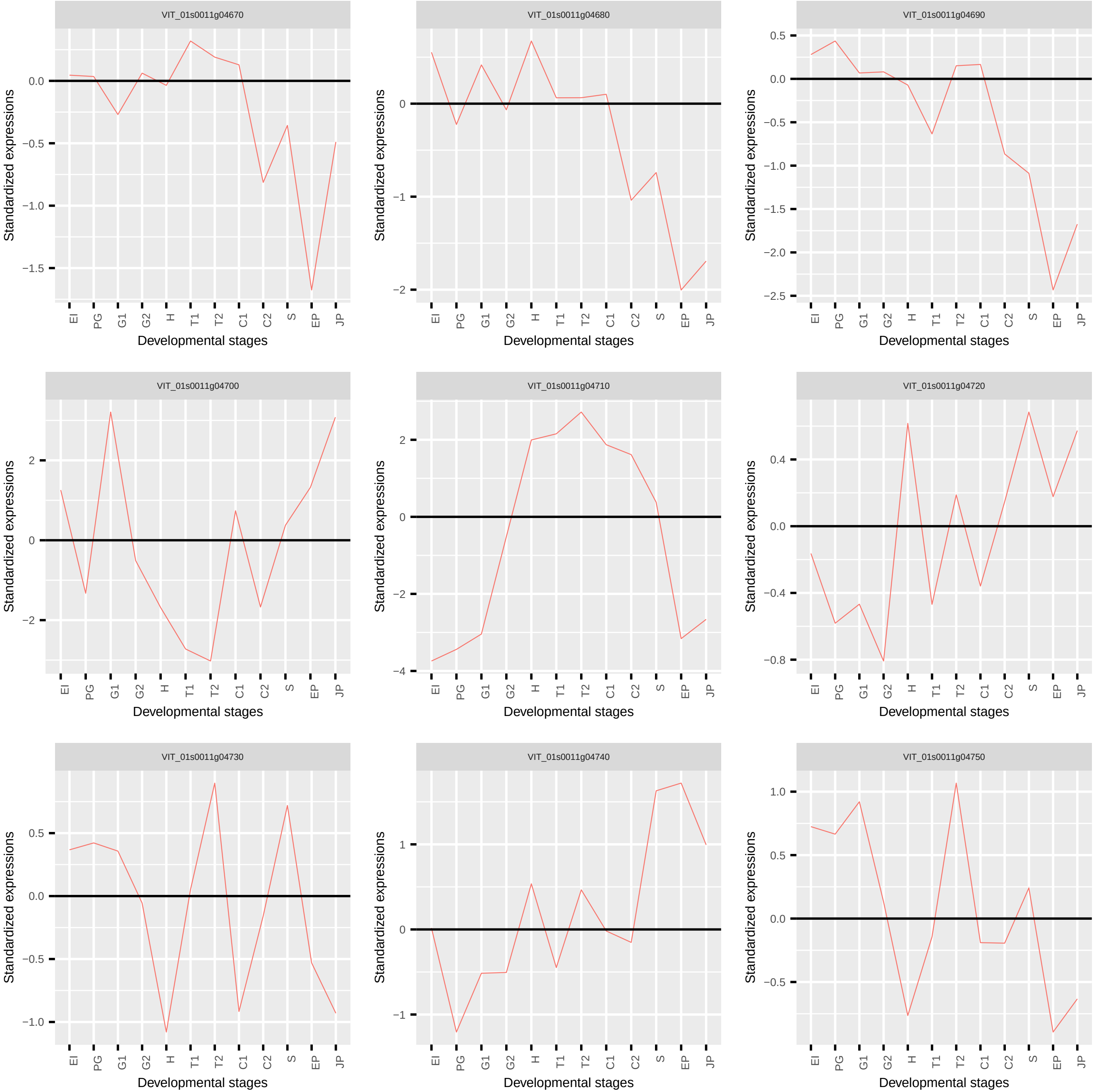

VIT_01s0011g04670
VIT_01s0011g04680
VIT_01s0011g04690
VIT_01s0011g04700
VIT_01s0011g04710
VIT_01s0011g04720
VIT_01s0011g04730
VIT_01s0011g04740
VIT_01s0011g04750
Standardized expressions
Developmental stages
EI
PG
G1
G2
H
T1
T2
C1
C2
S
EP
JP

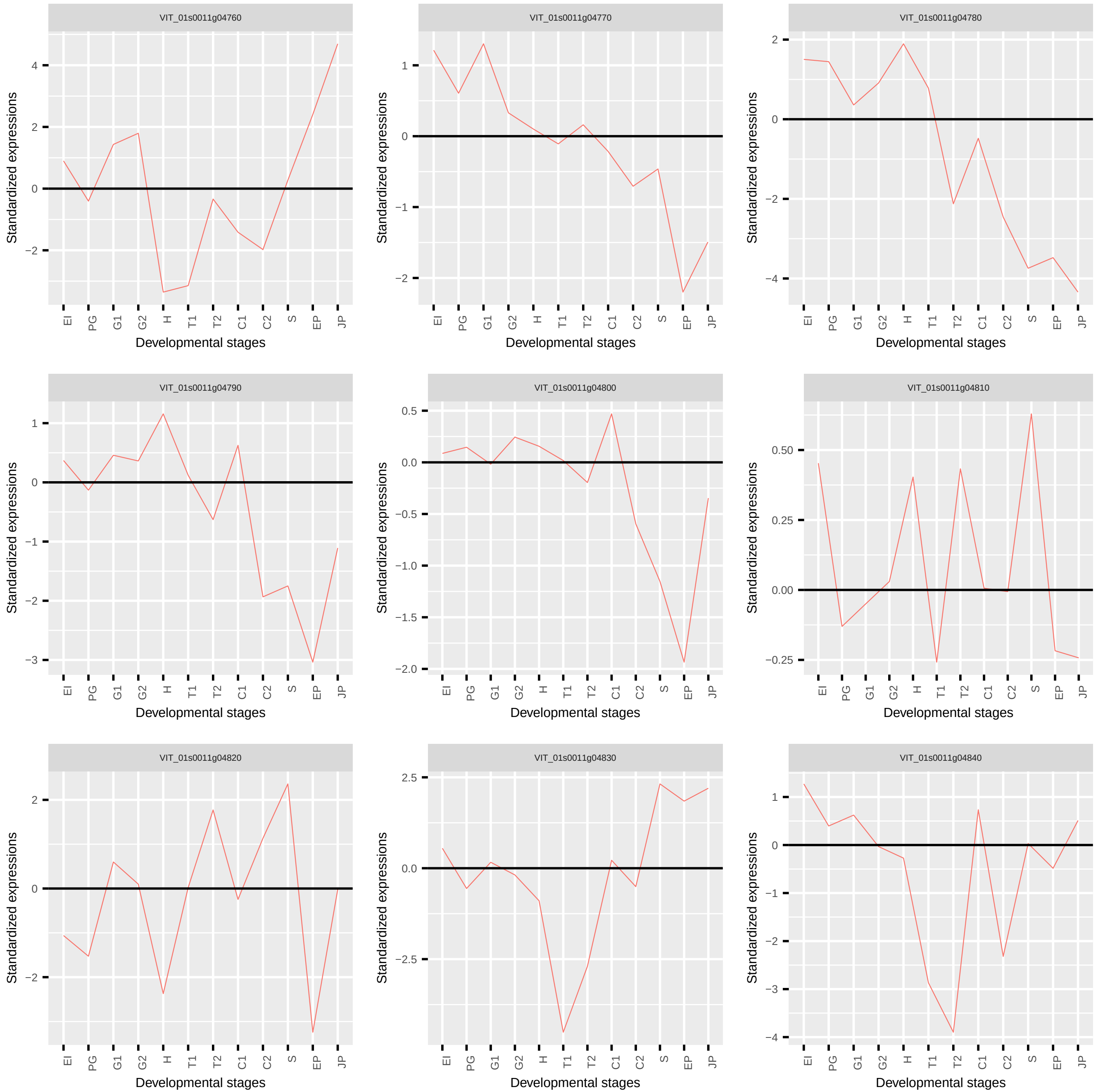
VIT_01s0011g04760
Standardized expressions
4
2
0
−2
EI PG G1 G2 H T1 T2 C1 C2 S EP JP
Developmental stages
VIT_01s0011g04770
Standardized expressions
1
0
−1
−2
EI PG G1 G2 H T1 T2 C1 C2 S EP JP
Developmental stages
VIT_01s0011g04780
Standardized expressions
2
0
−2
−4
EI PG G1 G2 H T1 T2 C1 C2 S EP JP
Developmental stages
VIT_01s0011g04790
Standardized expressions
1
0
−1
−2
−3
EI PG G1 G2 H T1 T2 C1 C2 S EP JP
Developmental stages
VIT_01s0011g04800
Standardized expressions
0.5
0.0
−0.5
−1.0
−1.5
−2.0
EI PG G1 G2 H T1 T2 C1 C2 S EP JP
Developmental stages
VIT_01s0011g04810
Standardized expressions
0.50
0.25
0.00
−0.25
EI PG G1 G2 H T1 T2 C1 C2 S EP JP
Developmental stages
VIT_01s0011g04820
Standardized expressions
2
0
−2
EI PG G1 G2 H T1 T2 C1 C2 S EP JP
Developmental stages
VIT_01s0011g04830
Standardized expressions
2.5
0.0
−2.5
EI PG G1 G2 H T1 T2 C1 C2 S EP JP
Developmental stages
VIT_01s0011g04840
Standardized expressions
1
0
−1
−2
−3
−4
EI PG G1 G2 H T1 T2 C1 C2 S EP JP
Developmental stages

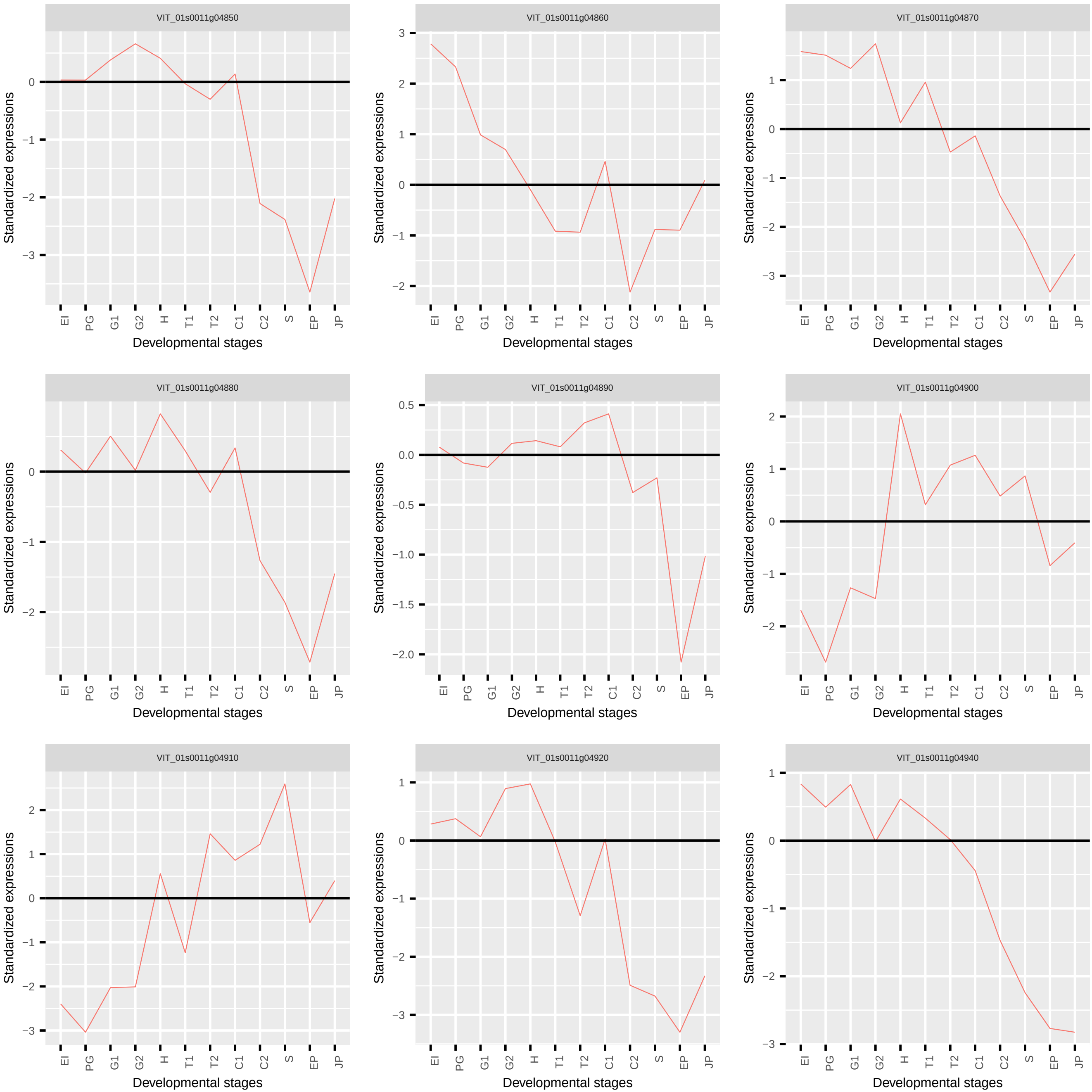

VIT_01s0011g04850
VIT_01s0011g04860
VIT_01s0011g04870
VIT_01s0011g04880
VIT_01s0011g04890
VIT_01s0011g04900
VIT_01s0011g04910
VIT_01s0011g04920
VIT_01s0011g04940
Standardized expressions
Developmental stages
EI
PG
G1
G2
H
T1
T2
C1
C2
S
EP
JP

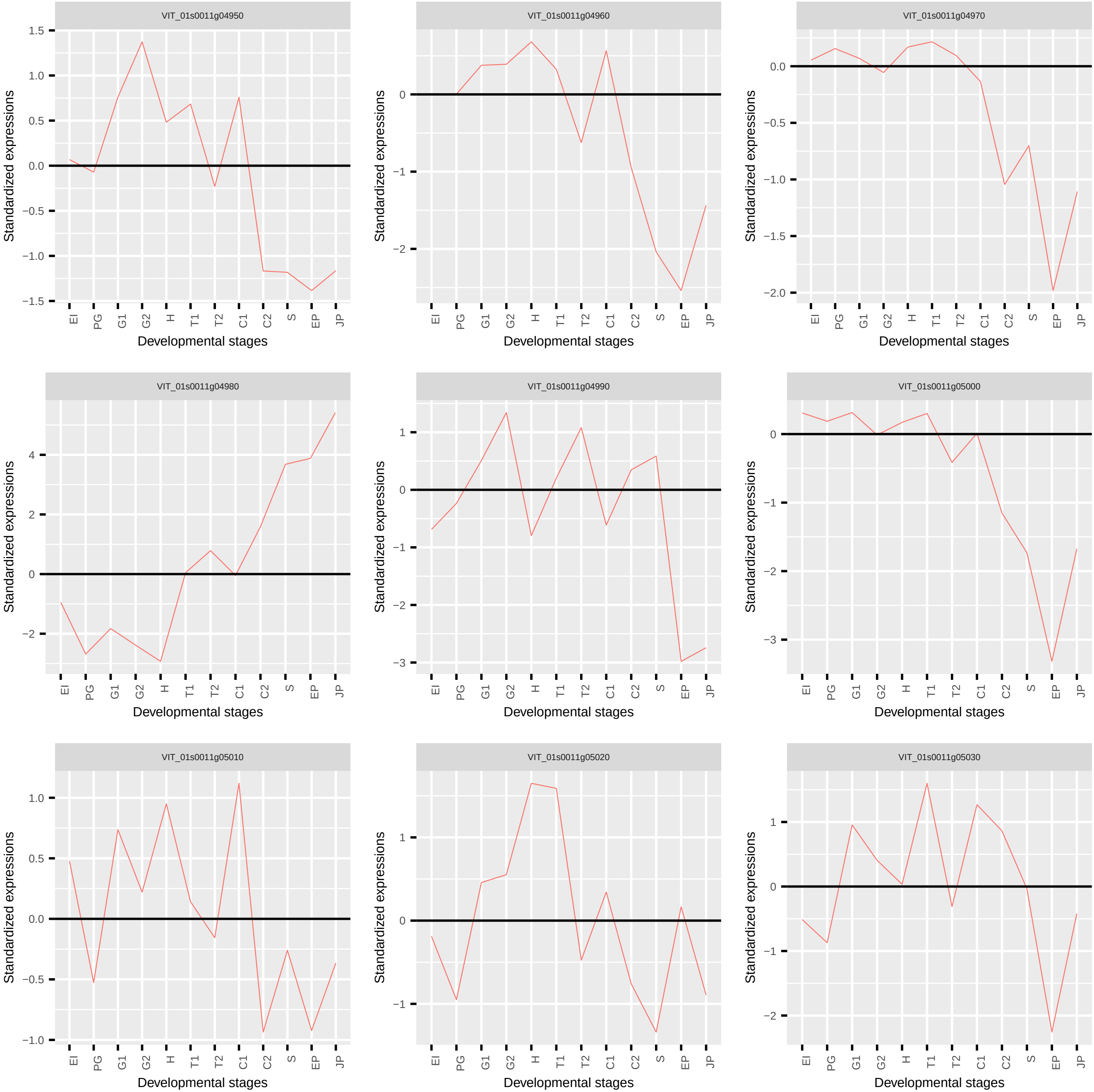
VIT_01s0011g04950
VIT_01s0011g04960
VIT_01s0011g04970
VIT_01s0011g04980
VIT_01s0011g04990
VIT_01s0011g05000
VIT_01s0011g05010
VIT_01s0011g05020
VIT_01s0011g05030
Standardized expressions
Developmental stages
EI PG G1 G2 H T1 T2 C1 C2 S EP JP

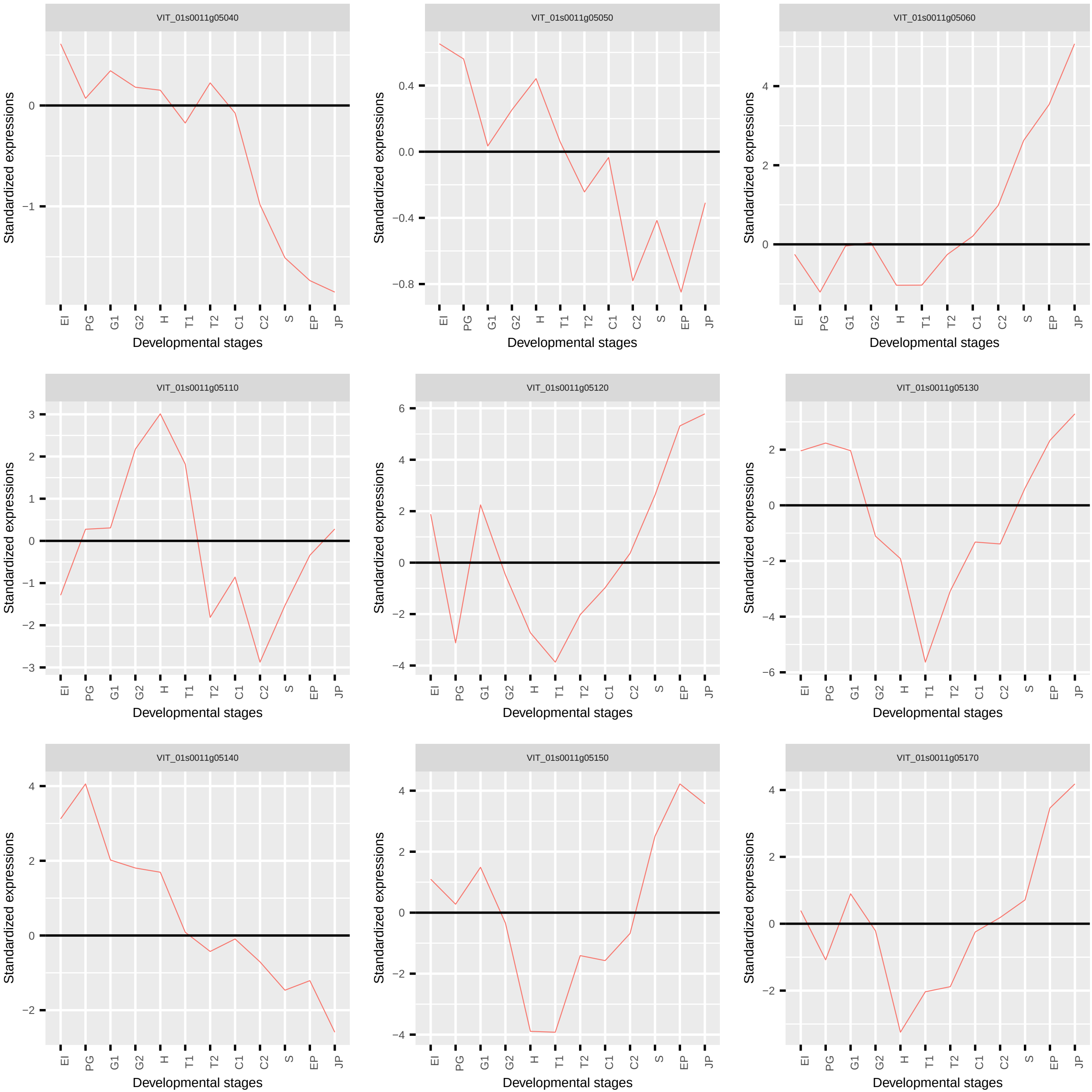
VIT_01s0011g05040
VIT_01s0011g05050
VIT_01s0011g05060
VIT_01s0011g05110
VIT_01s0011g05120
VIT_01s0011g05130
VIT_01s0011g05140
VIT_01s0011g05150
VIT_01s0011g05170
Standardized expressions
Developmental stages
EI
PG
G1
G2
H
T1
T2
C1
C2
S
EP
JP

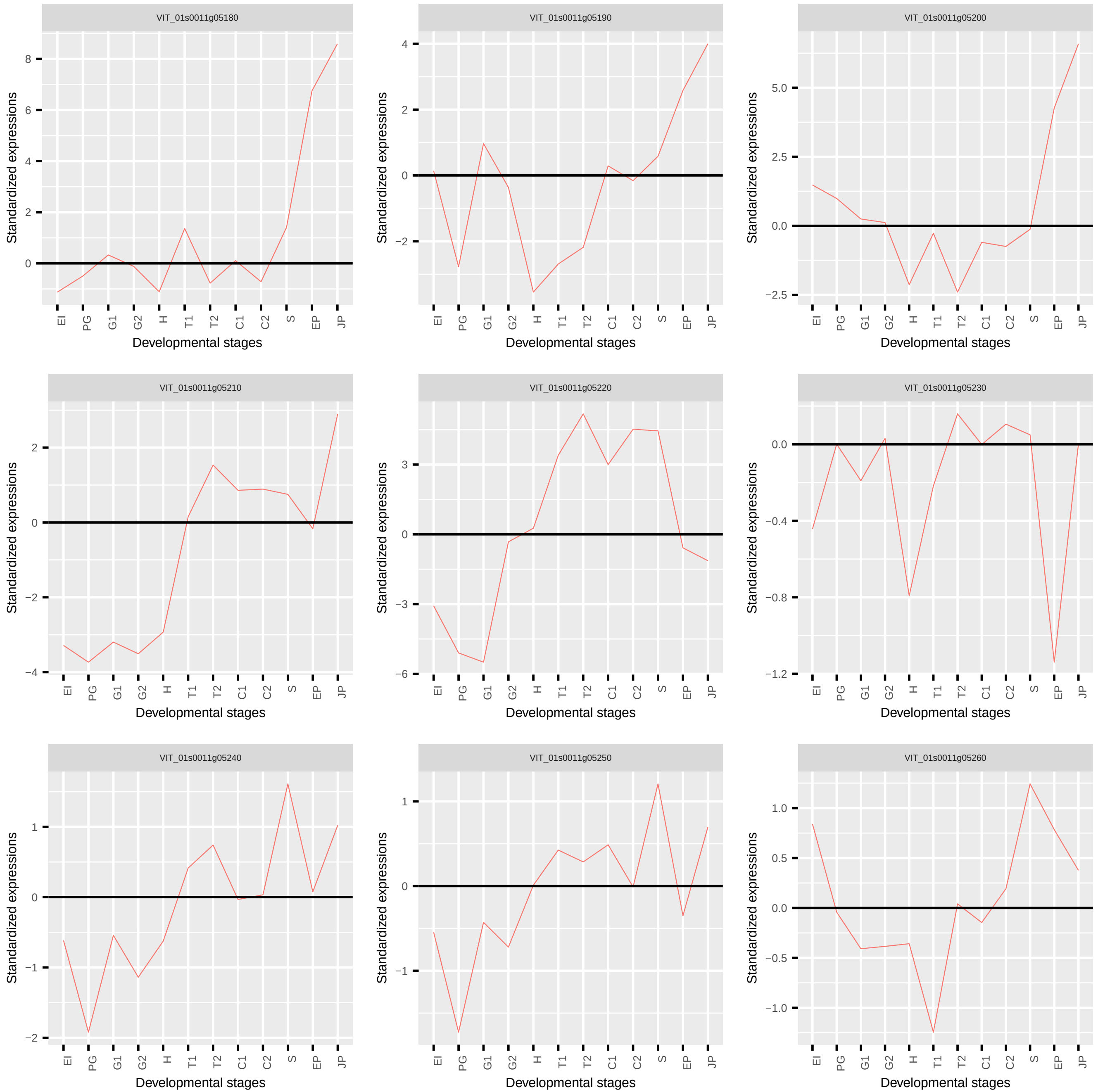
VIT_01s0011g05180
VIT_01s0011g05190
VIT_01s0011g05200
VIT_01s0011g05210
VIT_01s0011g05220
VIT_01s0011g05230
VIT_01s0011g05240
VIT_01s0011g05250
VIT_01s0011g05260
Standardized expressions
Developmental stages
EI PG G1 G2 H T1 T2 C1 C2 S EP JP
8 6 4 2 0
4 2 0 −2
5.0 2.5 0.0 −2.5
2 0 −2 −4
3 0 −3 −6
0.0 −0.4 −0.8 −1.2
1 0 −1 −2
1 0 −1
1.0 0.5 0.0 −0.5 −1.0

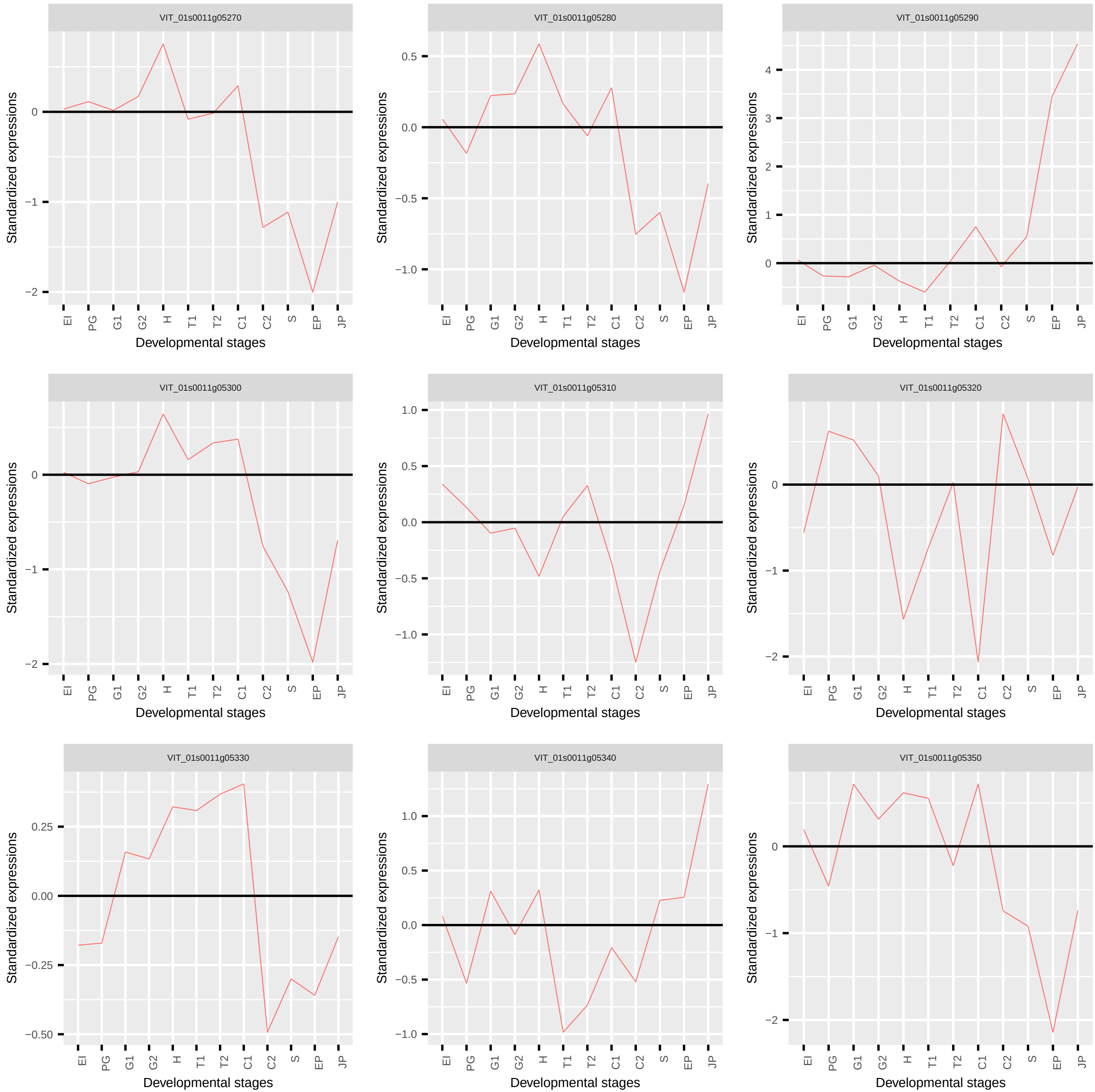

VIT_01s0011g05270
Standardized expressions
0
−1
−2
EI PG G1 G2 H T1 T2 C1 C2 S EP JP
Developmental stages
VIT_01s0011g05280
Standardized expressions
0.5
0.0
−0.5
−1.0
EI PG G1 G2 H T1 T2 C1 C2 S EP JP
Developmental stages
VIT_01s0011g05290
Standardized expressions
4
3
2
1
0
EI PG G1 G2 H T1 T2 C1 C2 S EP JP
Developmental stages
VIT_01s0011g05300
Standardized expressions
0
−1
−2
EI PG G1 G2 H T1 T2 C1 C2 S EP JP
Developmental stages
VIT_01s0011g05310
Standardized expressions
1.0
0.5
0.0
−0.5
−1.0
EI PG G1 G2 H T1 T2 C1 C2 S EP JP
Developmental stages
VIT_01s0011g05320
Standardized expressions
0
−1
−2
EI PG G1 G2 H T1 T2 C1 C2 S EP JP
Developmental stages
VIT_01s0011g05330
Standardized expressions
0.25
0.00
−0.25
−0.50
EI PG G1 G2 H T1 T2 C1 C2 S EP JP
Developmental stages
VIT_01s0011g05340
Standardized expressions
1.0
0.5
0.0
−0.5
−1.0
EI PG G1 G2 H T1 T2 C1 C2 S EP JP
Developmental stages
VIT_01s0011g05350
Standardized expressions
0
−1
−2
EI PG G1 G2 H T1 T2 C1 C2 S EP JP
Developmental stages

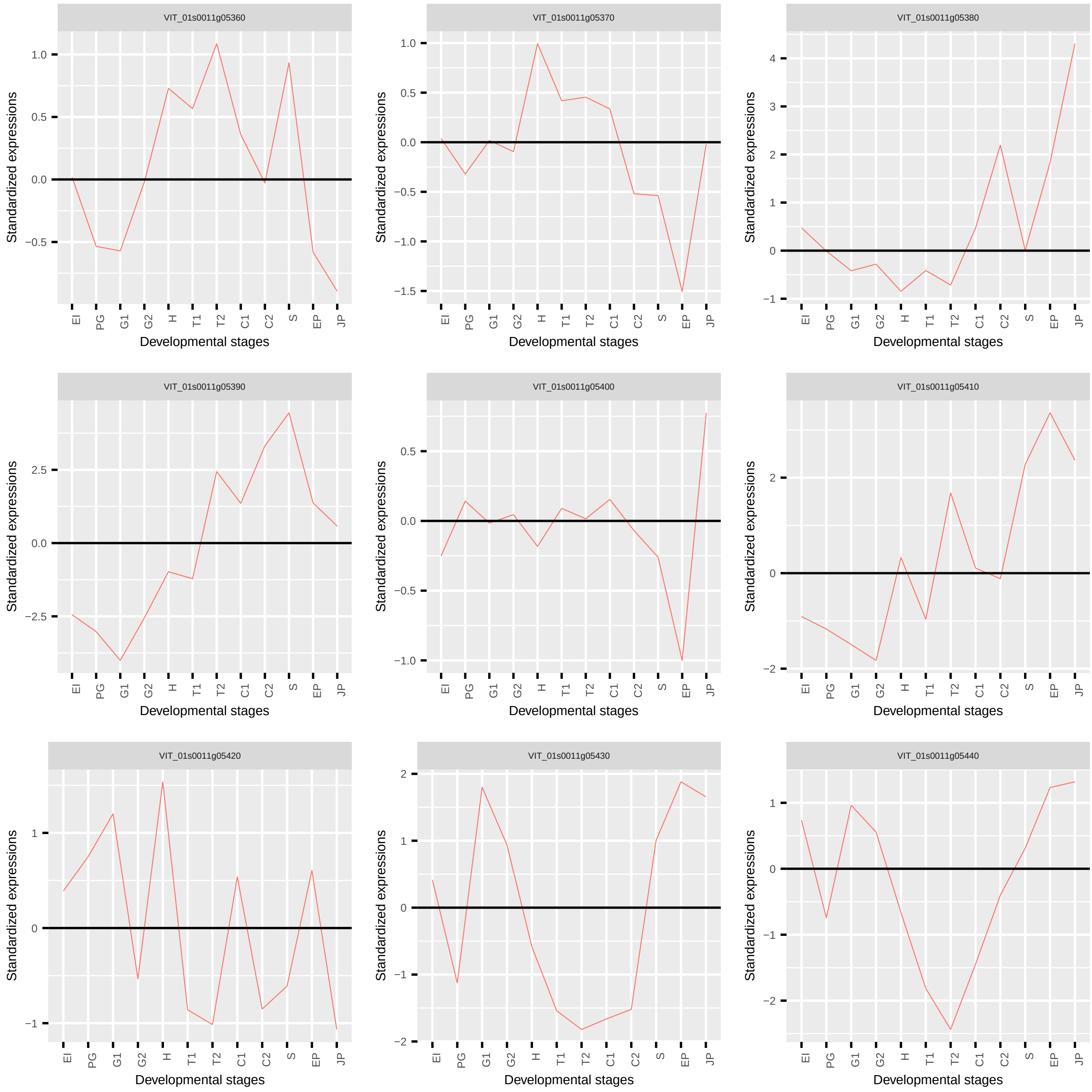
VIT_01s0011g05360
VIT_01s0011g05370
VIT_01s0011g05380
VIT_01s0011g05390
VIT_01s0011g05400
VIT_01s0011g05410
VIT_01s0011g05420
VIT_01s0011g05430
VIT_01s0011g05440
Standardized expressions
Developmental stages
EI PG G1 G2 H T1 T2 C1 C2 S EP JP

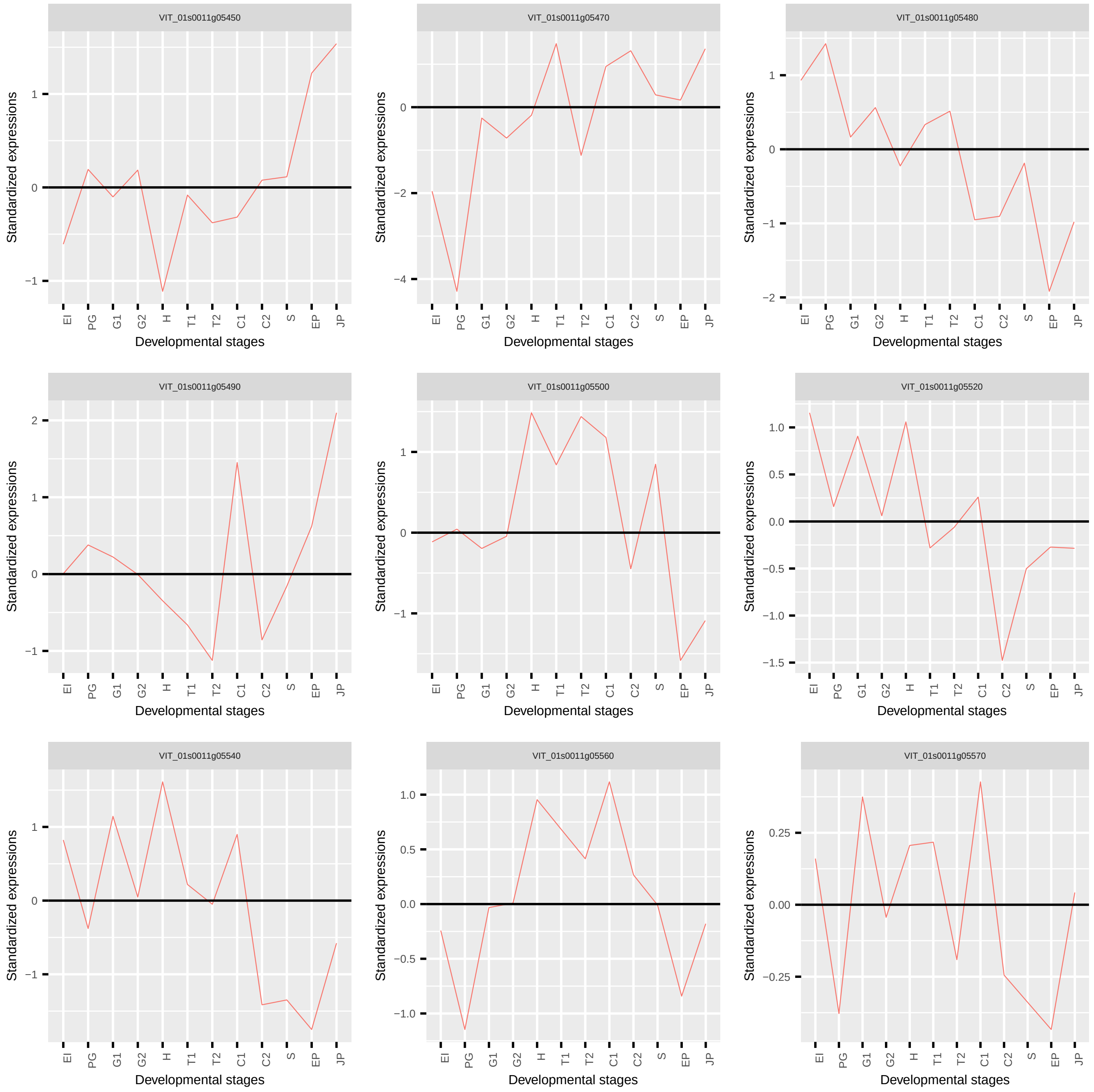
VIT_01s0011g05450
VIT_01s0011g05470
VIT_01s0011g05480
VIT_01s0011g05490
VIT_01s0011g05500
VIT_01s0011g05520
VIT_01s0011g05540
VIT_01s0011g05560
VIT_01s0011g05570
Standardized expressions
Developmental stages
EI PG G1 G2 H T1 T2 C1 C2 S EP JP

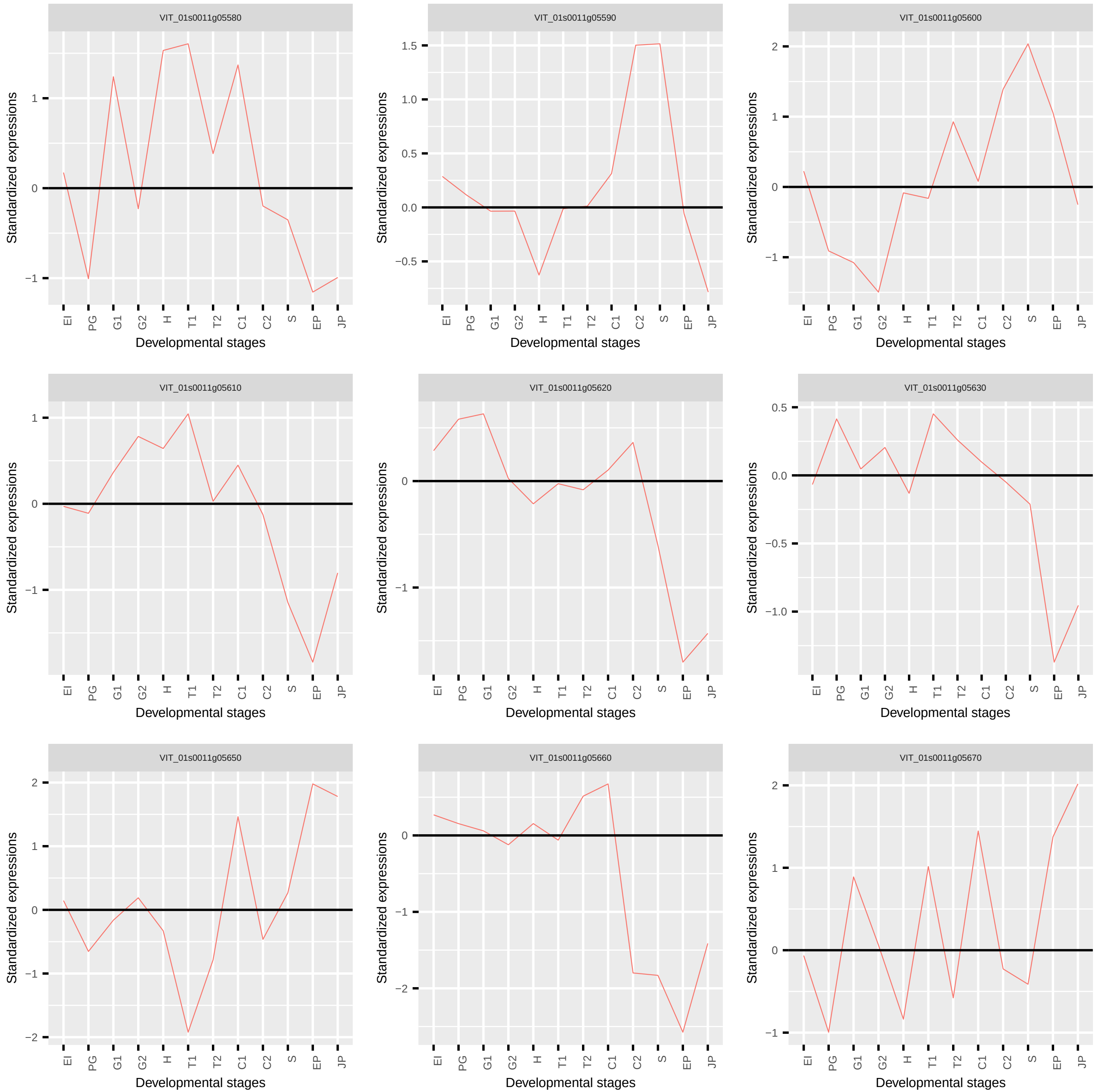
VIT_01s0011g05580
VIT_01s0011g05590
VIT_01s0011g05600
VIT_01s0011g05610
VIT_01s0011g05620
VIT_01s0011g05630
VIT_01s0011g05650
VIT_01s0011g05660
VIT_01s0011g05670
Standardized expressions
Developmental stages
EI PG G1 G2 H T1 T2 C1 C2 S EP JP

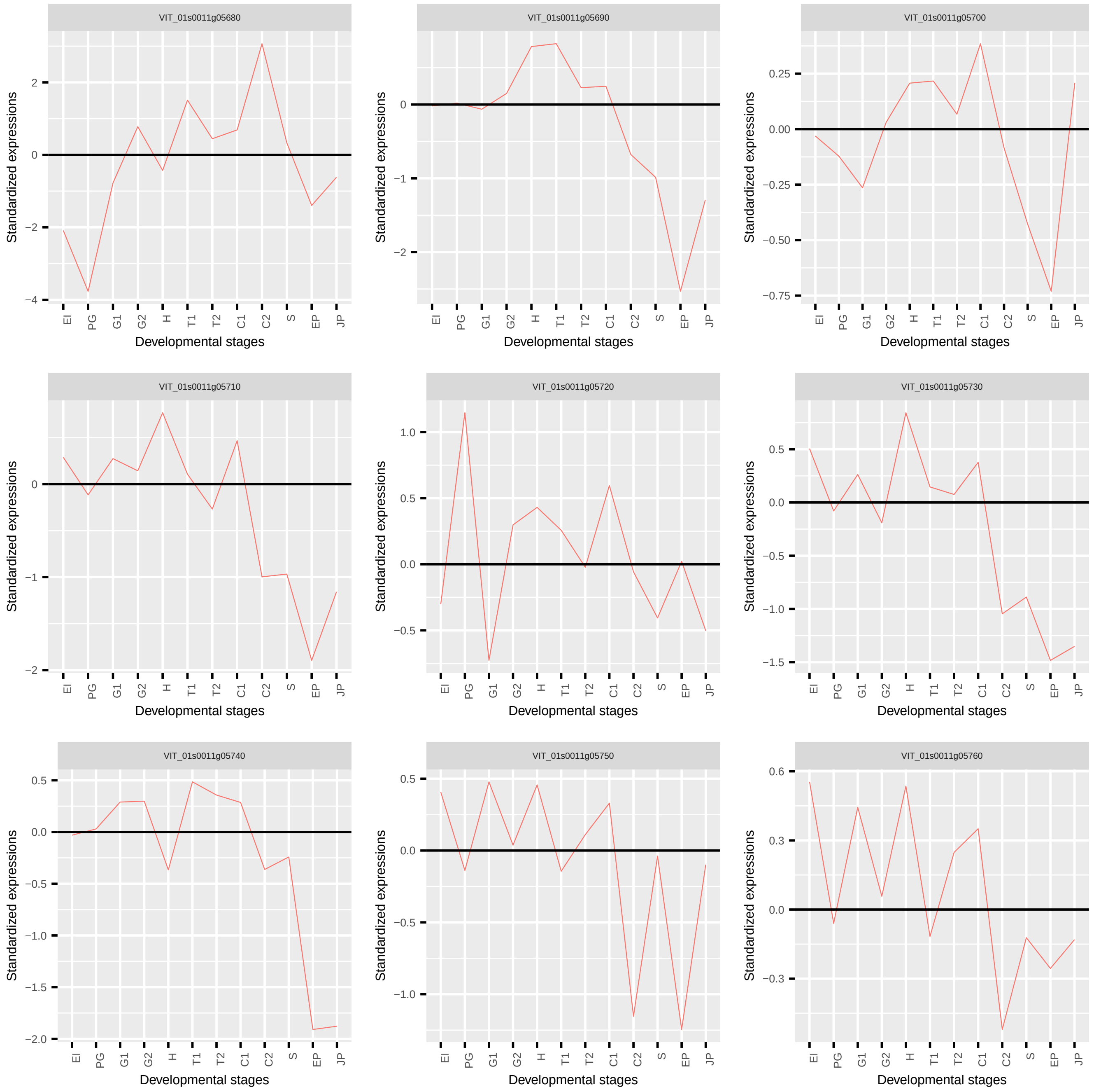
VIT_01s0011g05680
VIT_01s0011g05690
VIT_01s0011g05700
VIT_01s0011g05710
VIT_01s0011g05720
VIT_01s0011g05730
VIT_01s0011g05740
VIT_01s0011g05750
VIT_01s0011g05760
Standardized expressions
Developmental stages
EI PG G1 G2 H T1 T2 C1 C2 S EP JP

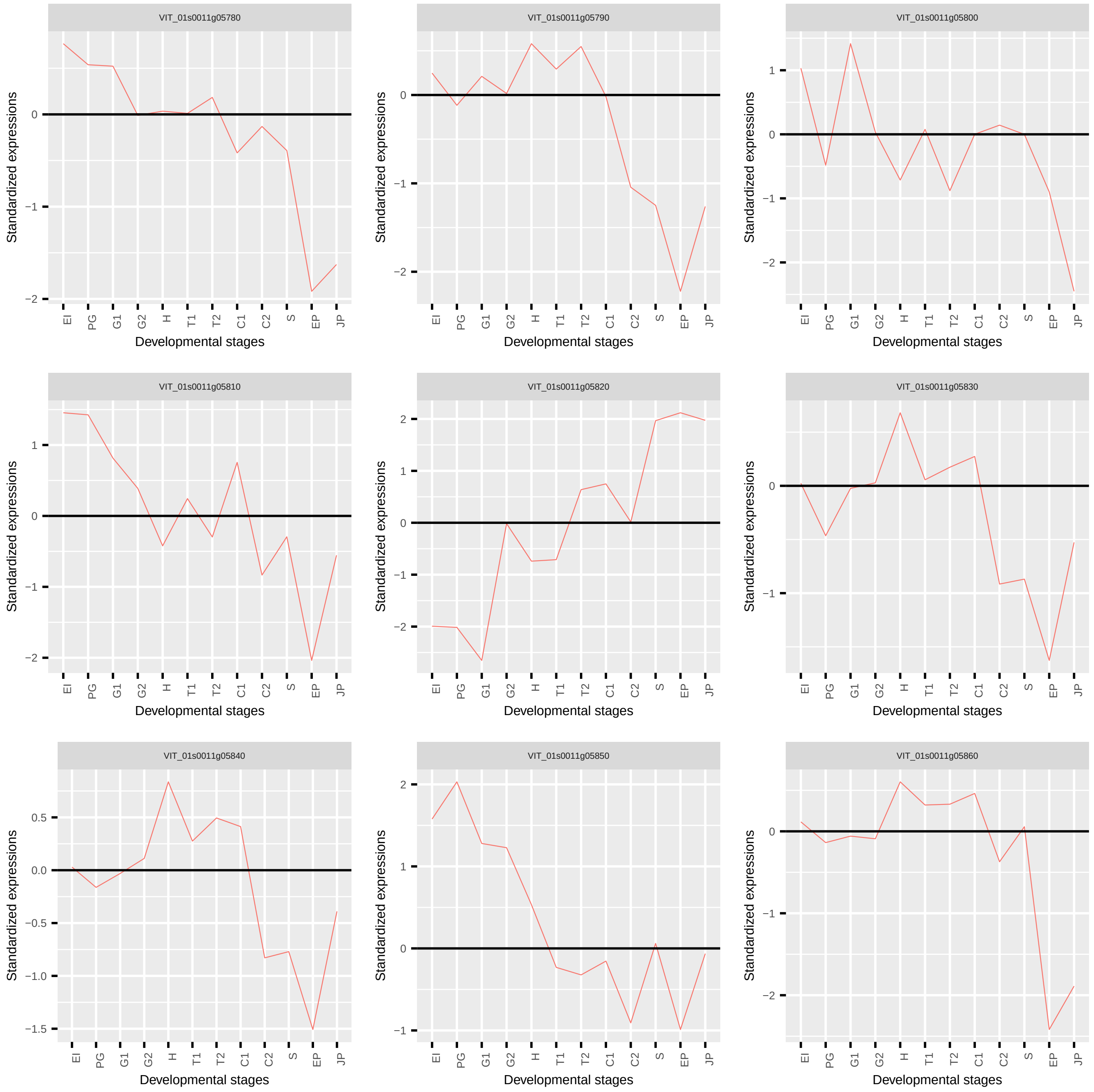
VIT_01s0011g05780
VIT_01s0011g05790
VIT_01s0011g05800
VIT_01s0011g05810
VIT_01s0011g05820
VIT_01s0011g05830
VIT_01s0011g05840
VIT_01s0011g05850
VIT_01s0011g05860
Standardized expressions
Developmental stages
EI PG G1 G2 H T1 T2 C1 C2 S EP JP

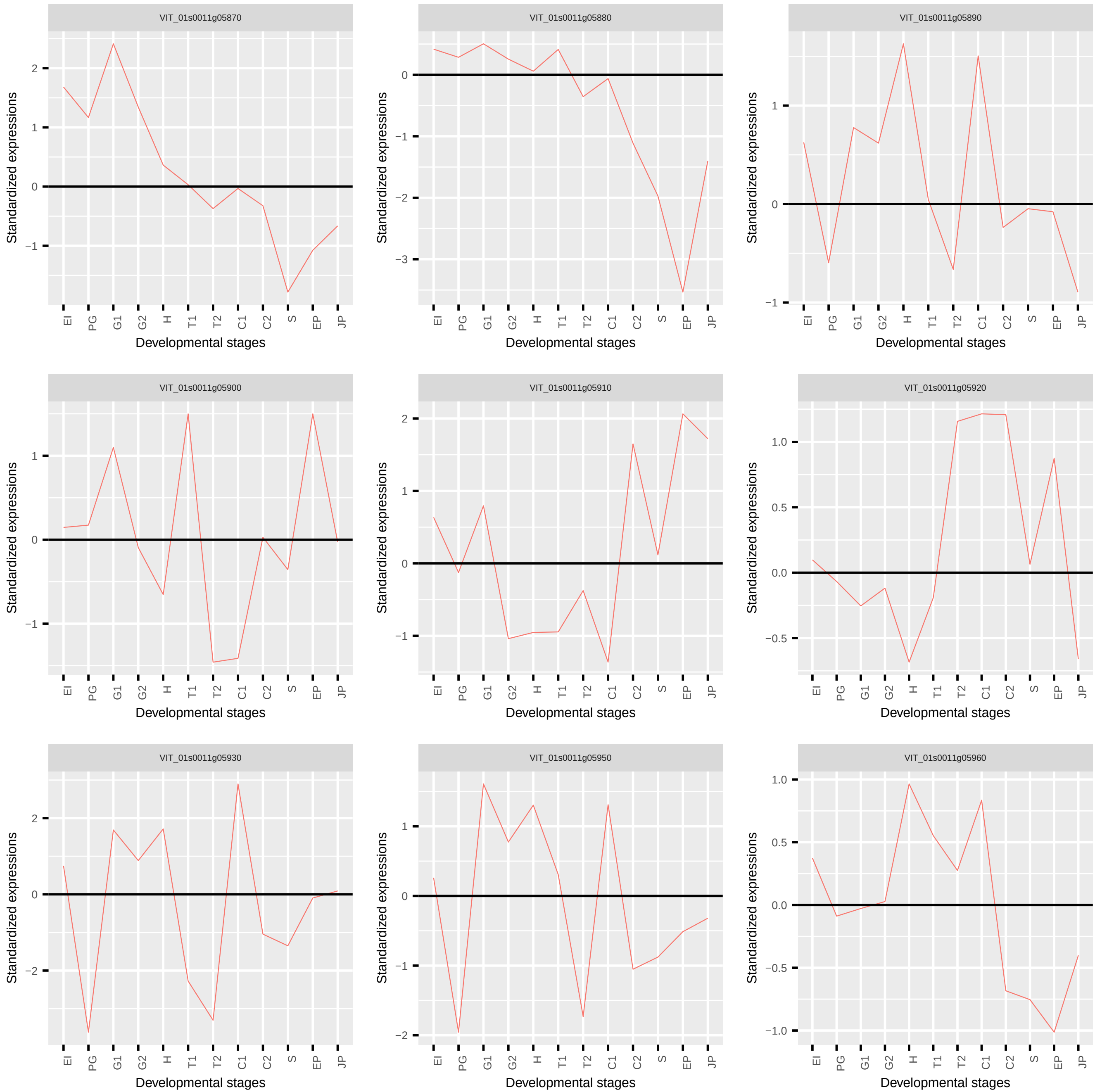
VIT_01s0011g05870
VIT_01s0011g05880
VIT_01s0011g05890
VIT_01s0011g05900
VIT_01s0011g05910
VIT_01s0011g05920
VIT_01s0011g05930
VIT_01s0011g05950
VIT_01s0011g05960
Standardized expressions
Developmental stages
EI PG G1 G2 H T1 T2 C1 C2 S EP JP

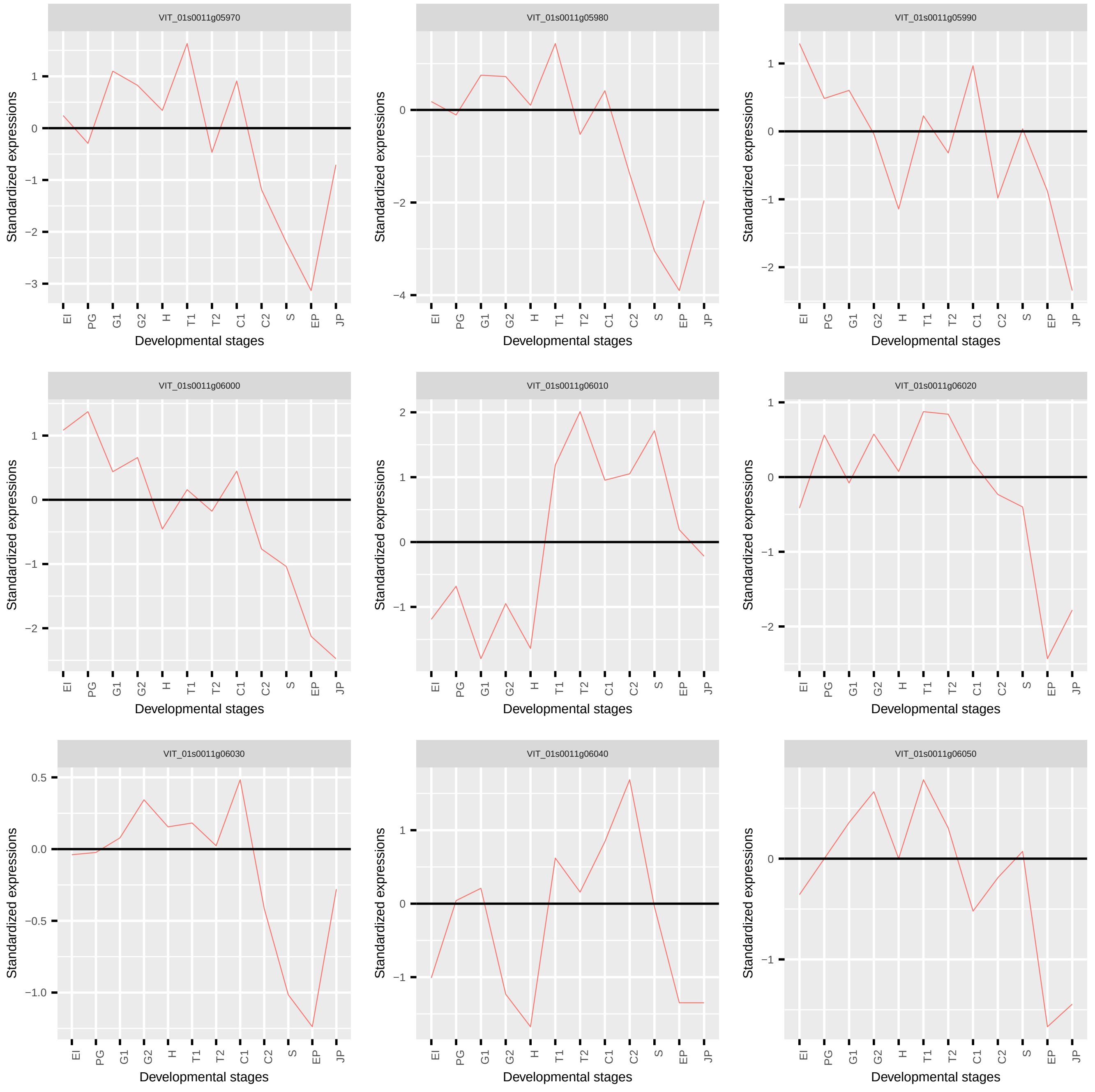
VIT_01s0011g05970
VIT_01s0011g05980
VIT_01s0011g05990
VIT_01s0011g06000
VIT_01s0011g06010
VIT_01s0011g06020
VIT_01s0011g06030
VIT_01s0011g06040
VIT_01s0011g06050
Standardized expressions
Developmental stages
EI
PG
G1
G2
H
T1
T2
C1
C2
S
EP
JP

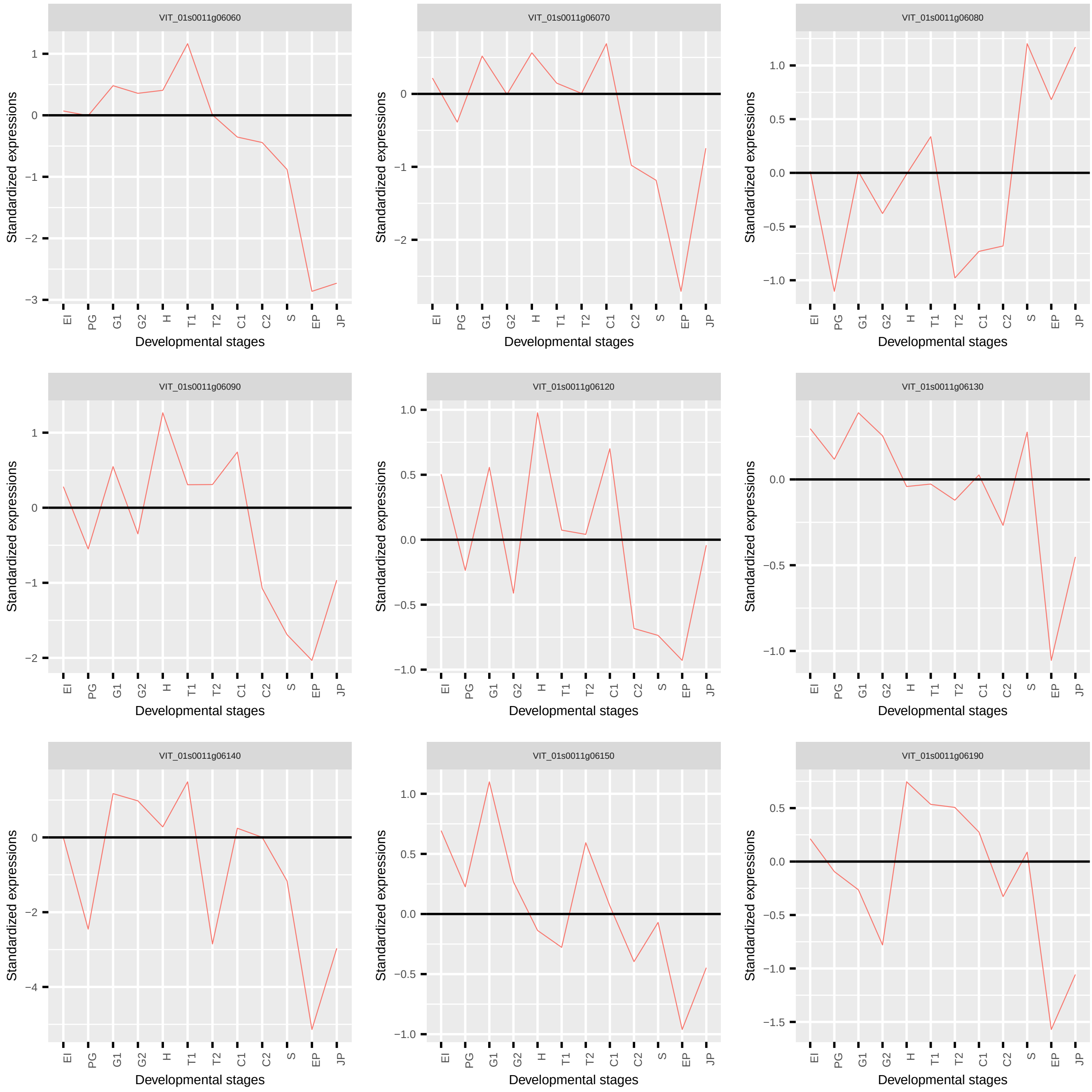
VIT_01s0011g06060
VIT_01s0011g06070
VIT_01s0011g06080
VIT_01s0011g06090
VIT_01s0011g06120
VIT_01s0011g06130
VIT_01s0011g06140
VIT_01s0011g06150
VIT_01s0011g06190
Standardized expressions
Developmental stages
EI PG G1 G2 H T1 T2 C1 C2 S EP JP

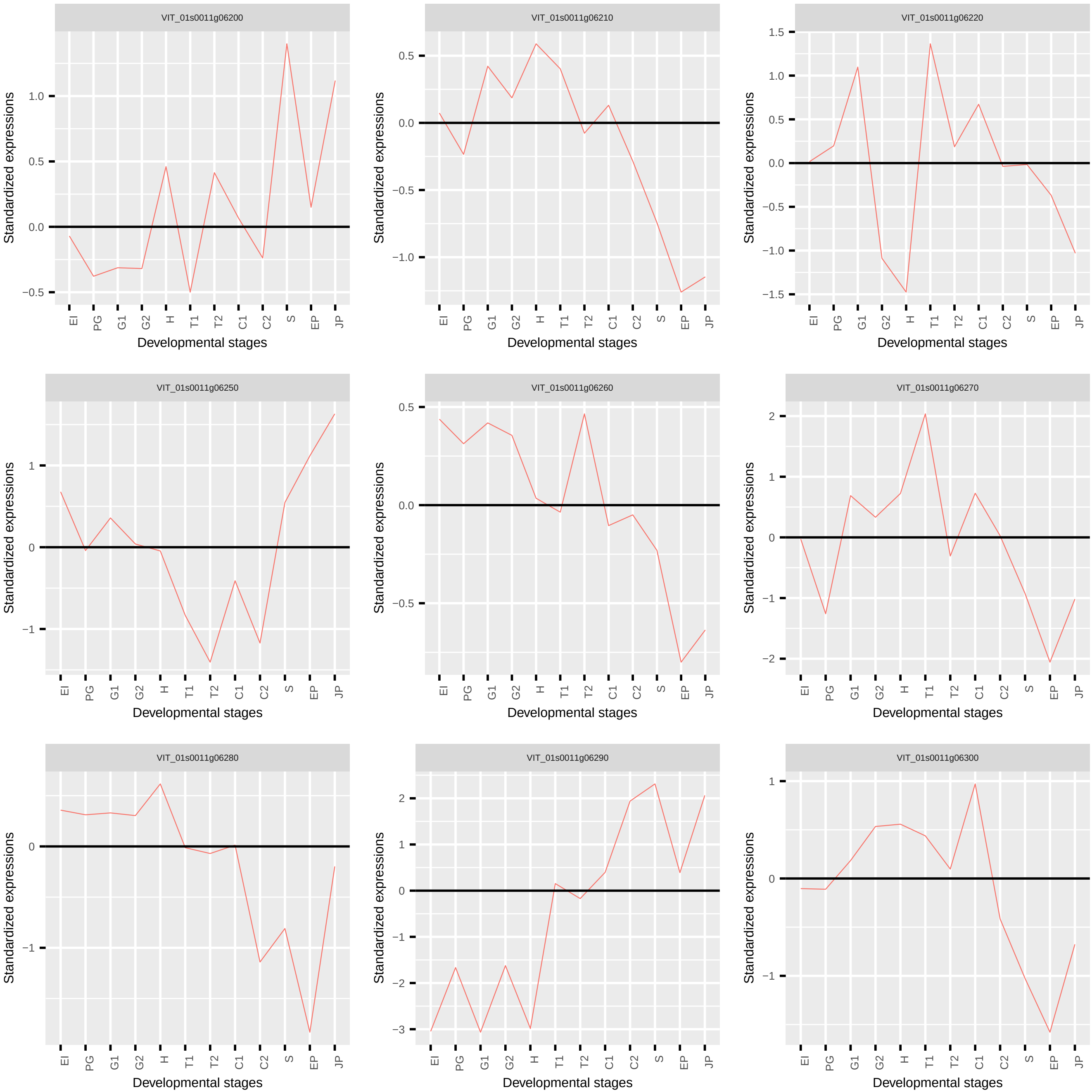
VIT_01s0011g06200
VIT_01s0011g06210
VIT_01s0011g06220
VIT_01s0011g06250
VIT_01s0011g06260
VIT_01s0011g06270
VIT_01s0011g06280
VIT_01s0011g06290
VIT_01s0011g06300
Standardized expressions
Developmental stages
EI PG G1 G2 H T1 T2 C1 C2 S EP JP

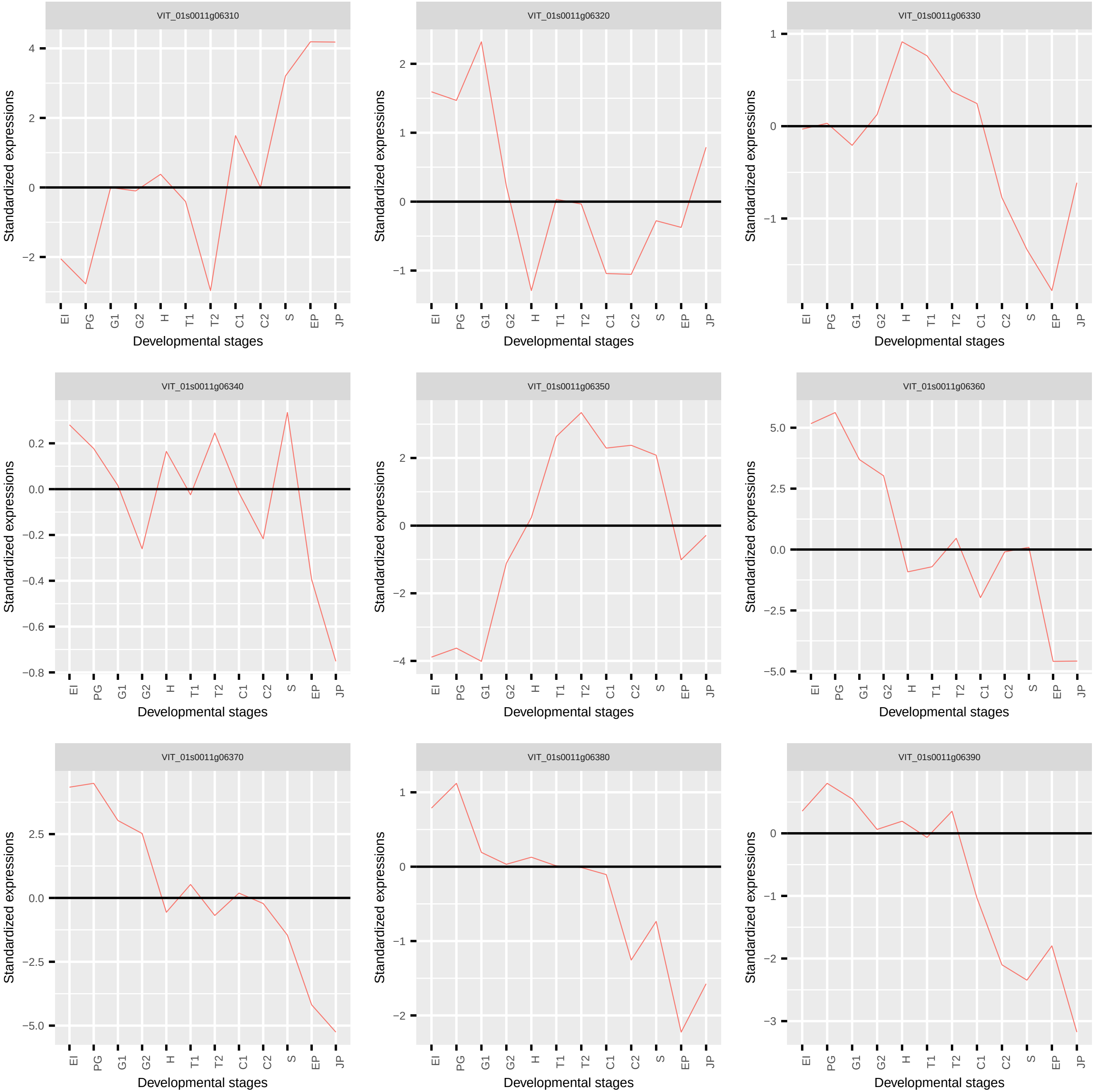
VIT_01s0011g06310
VIT_01s0011g06320
VIT_01s0011g06330
VIT_01s0011g06340
VIT_01s0011g06350
VIT_01s0011g06360
VIT_01s0011g06370
VIT_01s0011g06380
VIT_01s0011g06390
Standardized expressions
Developmental stages
EI PG G1 G2 H T1 T2 C1 C2 S EP JP

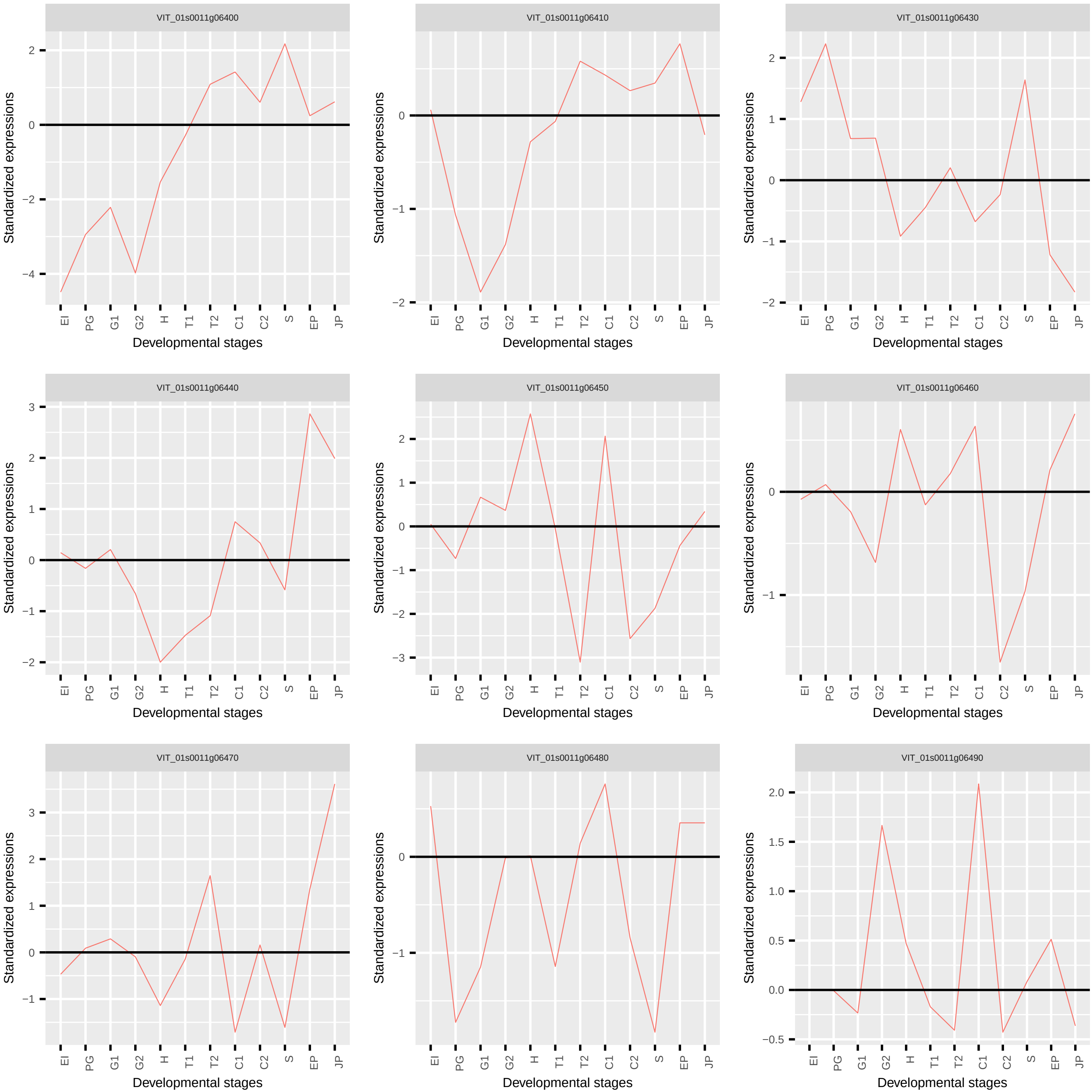
VIT_01s0011g06400
VIT_01s0011g06410
VIT_01s0011g06430
VIT_01s0011g06440
VIT_01s0011g06450
VIT_01s0011g06460
VIT_01s0011g06470
VIT_01s0011g06480
VIT_01s0011g06490
Standardized expressions
Developmental stages
EI PG G1 G2 H T1 T2 C1 C2 S EP JP

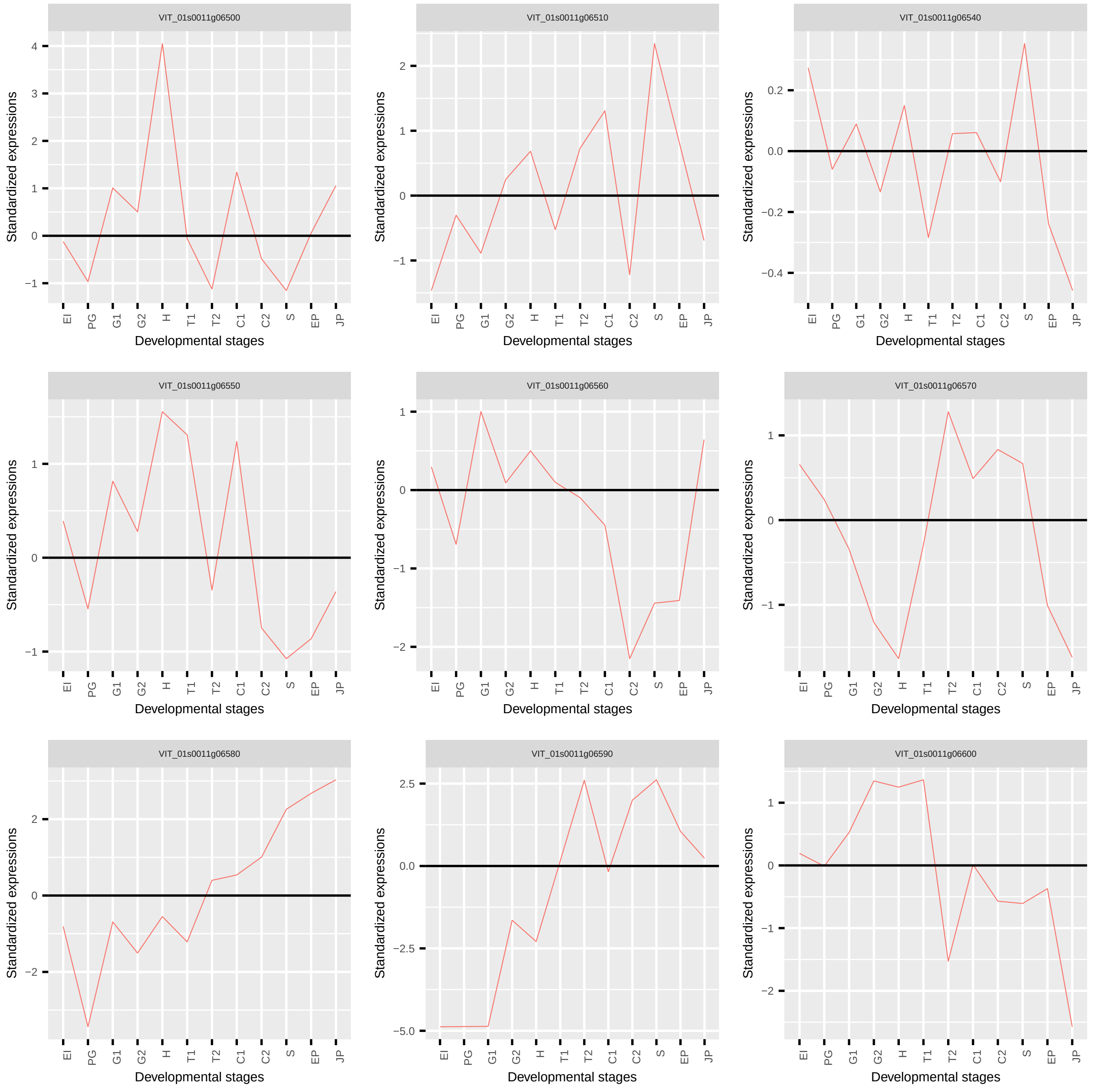
VIT_01s0011g06500
4
3
2
1
0
−1
Standardized expressions
EI
PG
G1
G2
H
T1
T2
C1
C2
S
EP
JP
Developmental stages
VIT_01s0011g06510
2
1
0
−1
Standardized expressions
Developmental stages
VIT_01s0011g06540
0.2
0.0
−0.2
−0.4
Standardized expressions
Developmental stages
VIT_01s0011g06550
1
0
−1
Standardized expressions
Developmental stages
VIT_01s0011g06560
1
0
−1
−2
Standardized expressions
Developmental stages
VIT_01s0011g06570
1
0
−1
Standardized expressions
Developmental stages
VIT_01s0011g06580
2
0
−2
Standardized expressions
Developmental stages
VIT_01s0011g06590
2.5
0.0
−2.5
−5.0
Standardized expressions
Developmental stages
VIT_01s0011g06600
1
0
−1
−2
Standardized expressions
Developmental stages

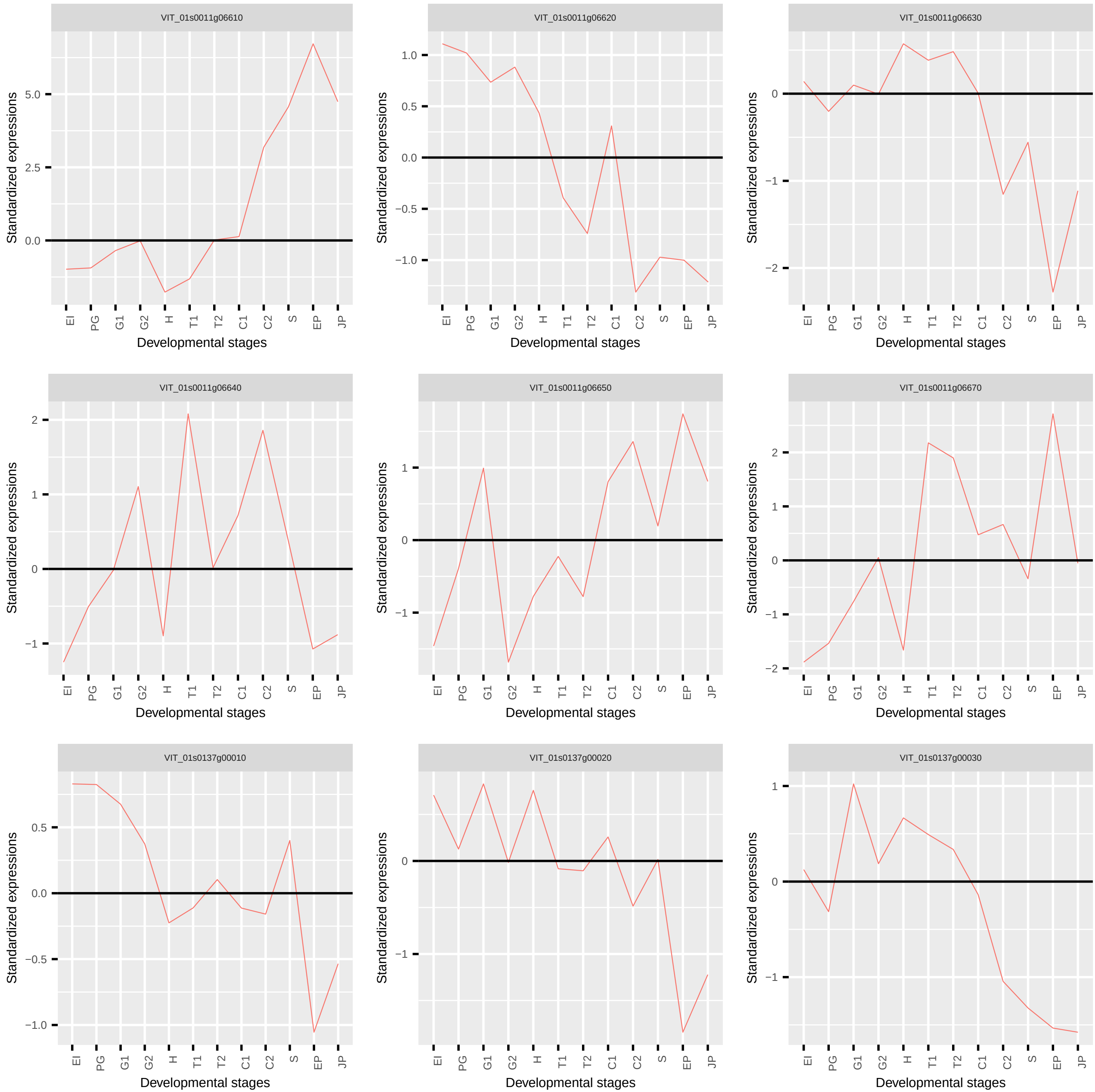
VIT_01s0011g06610
VIT_01s0011g06620
VIT_01s0011g06630
VIT_01s0011g06640
VIT_01s0011g06650
VIT_01s0011g06670
VIT_01s0137g00010
VIT_01s0137g00020
VIT_01s0137g00030
Standardized expressions
Developmental stages
EI
PG
G1
G2
H
T1
T2
C1
C2
S
EP
JP
5.0
2.5
0.0
1.0
0.5
−0.5
−1.0
0
−1
−2
2
1

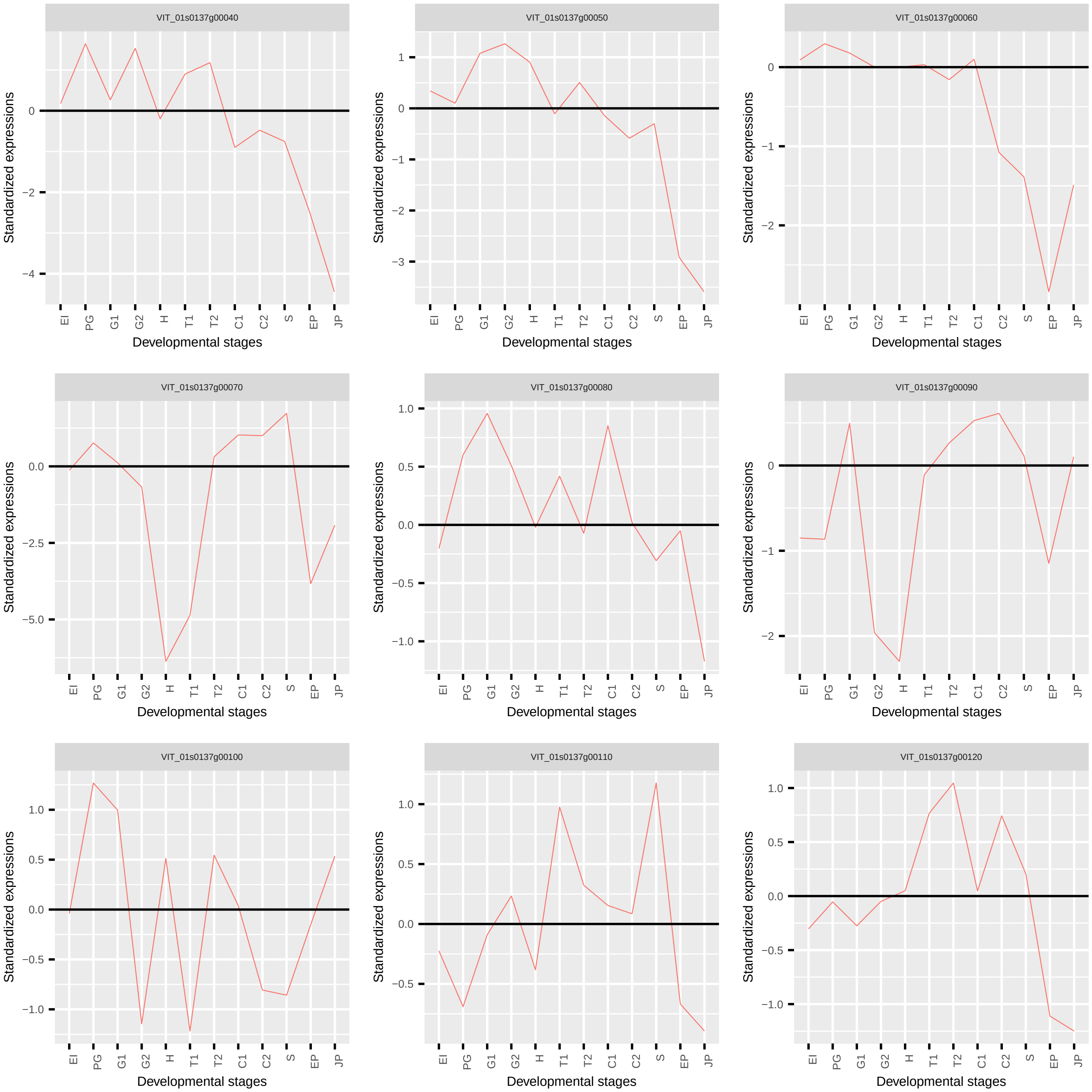

VIT_01s0137g00040
Standardized expressions
0
−2
−4
EI PG G1 G2 H T1 T2 C1 C2 S EP JP
Developmental stages
VIT_01s0137g00050
Standardized expressions
1
0
−1
−2
−3
EI PG G1 G2 H T1 T2 C1 C2 S EP JP
Developmental stages
VIT_01s0137g00060
Standardized expressions
0
−1
−2
EI PG G1 G2 H T1 T2 C1 C2 S EP JP
Developmental stages
VIT_01s0137g00070
Standardized expressions
0.0
−2.5
−5.0
EI PG G1 G2 H T1 T2 C1 C2 S EP JP
Developmental stages
VIT_01s0137g00080
Standardized expressions
1.0
0.5
0.0
−0.5
−1.0
EI PG G1 G2 H T1 T2 C1 C2 S EP JP
Developmental stages
VIT_01s0137g00090
Standardized expressions
0
−1
−2
EI PG G1 G2 H T1 T2 C1 C2 S EP JP
Developmental stages
VIT_01s0137g00100
Standardized expressions
1.0
0.5
0.0
−0.5
−1.0
EI PG G1 G2 H T1 T2 C1 C2 S EP JP
Developmental stages
VIT_01s0137g00110
Standardized expressions
1.0
0.5
0.0
−0.5
EI PG G1 G2 H T1 T2 C1 C2 S EP JP
Developmental stages
VIT_01s0137g00120
Standardized expressions
1.0
0.5
0.0
−0.5
−1.0
EI PG G1 G2 H T1 T2 C1 C2 S EP JP
Developmental stages

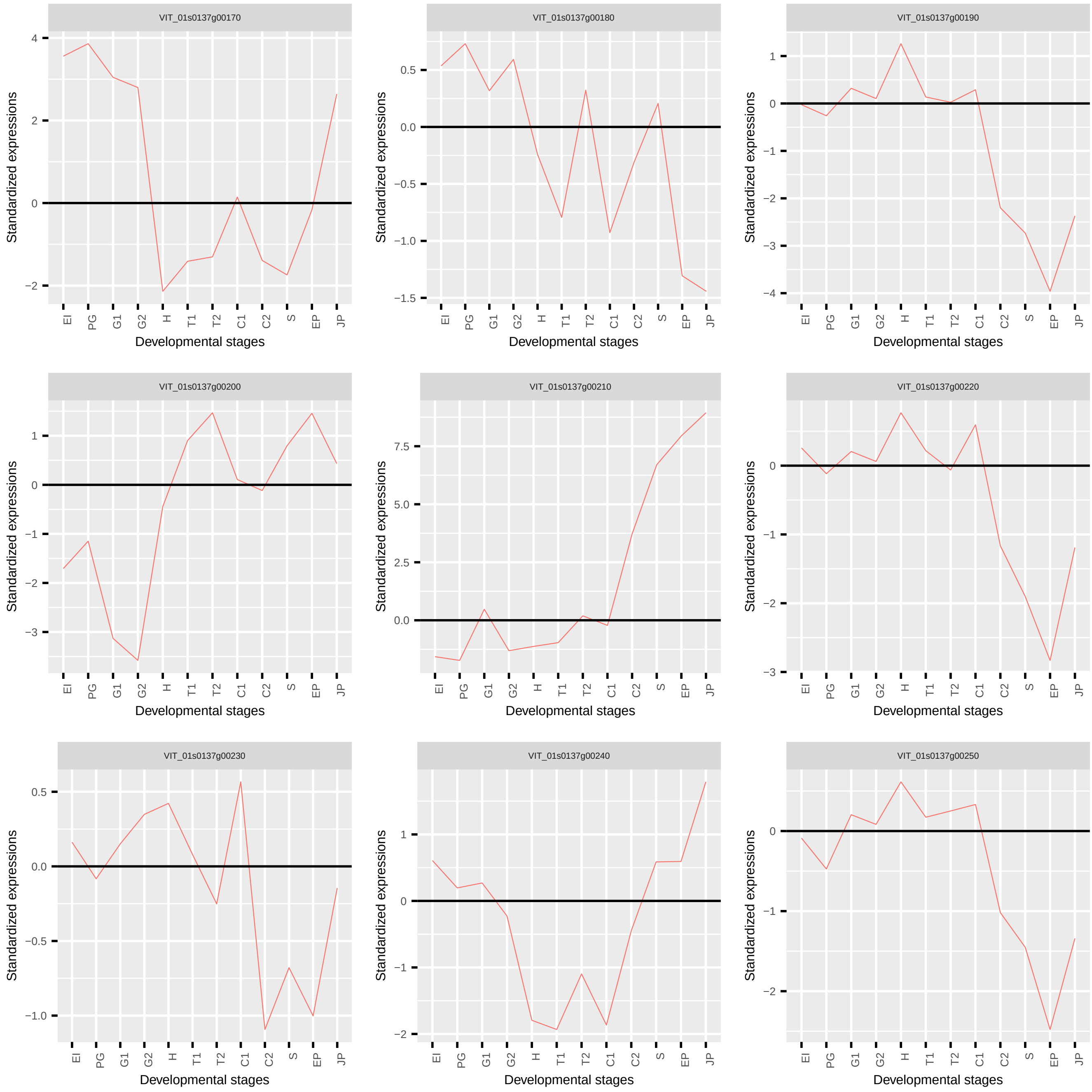
VIT_01s0137g00170
4
2
0
−2
Standardized expressions
EI PG G1 G2 H T1 T2 C1 C2 S EP JP
Developmental stages
VIT_01s0137g00180
0.5
0.0
−0.5
−1.0
−1.5
Standardized expressions
EI PG G1 G2 H T1 T2 C1 C2 S EP JP
Developmental stages
VIT_01s0137g00190
1
0
−1
−2
−3
−4
Standardized expressions
EI PG G1 G2 H T1 T2 C1 C2 S EP JP
Developmental stages
VIT_01s0137g00200
1
0
−1
−2
−3
Standardized expressions
EI PG G1 G2 H T1 T2 C1 C2 S EP JP
Developmental stages
VIT_01s0137g00210
7.5
5.0
2.5
0.0
Standardized expressions
EI PG G1 G2 H T1 T2 C1 C2 S EP JP
Developmental stages
VIT_01s0137g00220
0
−1
−2
−3
Standardized expressions
EI PG G1 G2 H T1 T2 C1 C2 S EP JP
Developmental stages
VIT_01s0137g00230
0.5
0.0
−0.5
−1.0
Standardized expressions
EI PG G1 G2 H T1 T2 C1 C2 S EP JP
Developmental stages
VIT_01s0137g00240
1
0
−1
−2
Standardized expressions
EI PG G1 G2 H T1 T2 C1 C2 S EP JP
Developmental stages
VIT_01s0137g00250
0
−1
−2
Standardized expressions
EI PG G1 G2 H T1 T2 C1 C2 S EP JP
Developmental stages

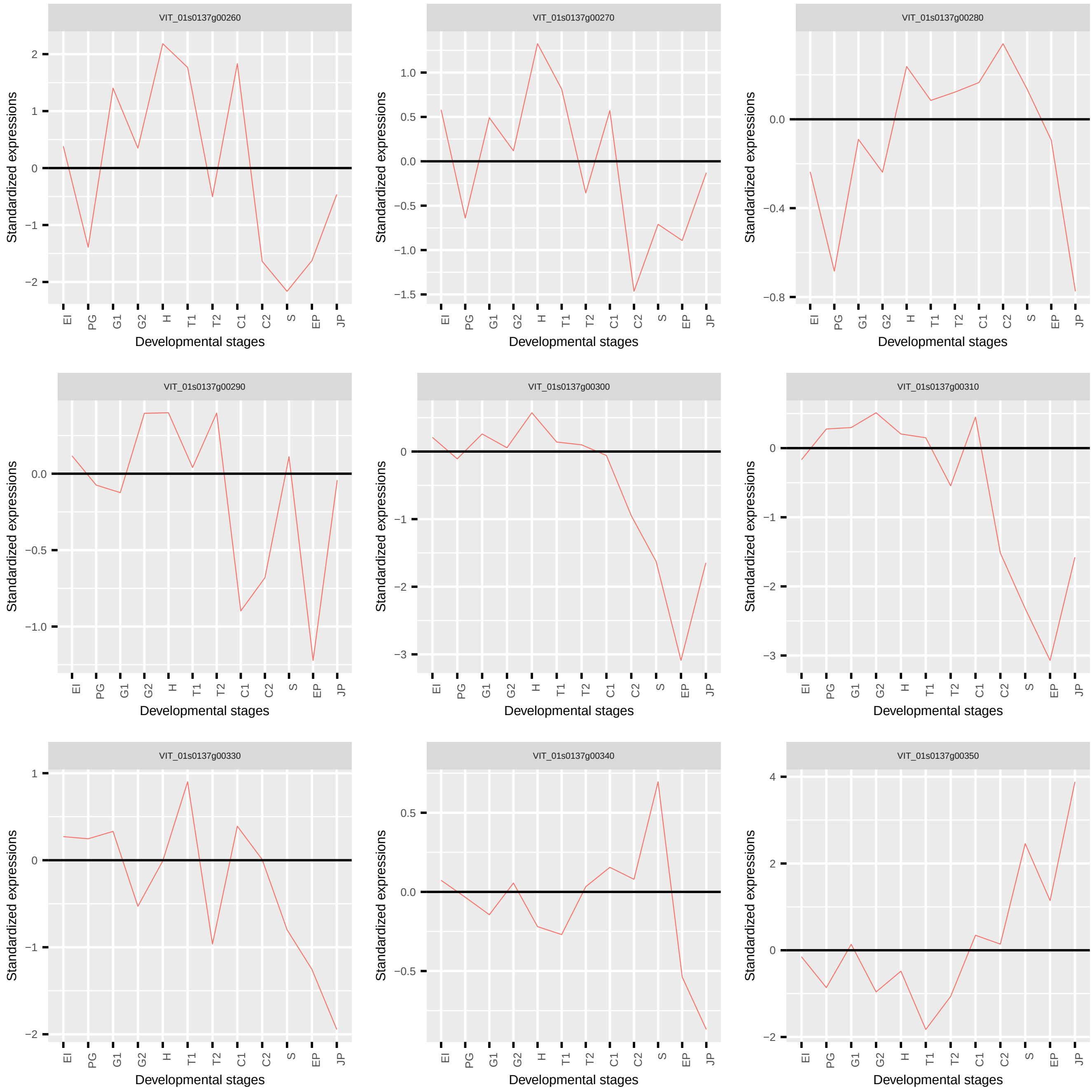
VIT_01s0137g00260
VIT_01s0137g00270
VIT_01s0137g00280
VIT_01s0137g00290
VIT_01s0137g00300
VIT_01s0137g00310
VIT_01s0137g00330
VIT_01s0137g00340
VIT_01s0137g00350
Standardized expressions
Developmental stages
EI
PG
G1
G2
H
T1
T2
C1
C2
S
EP
JP

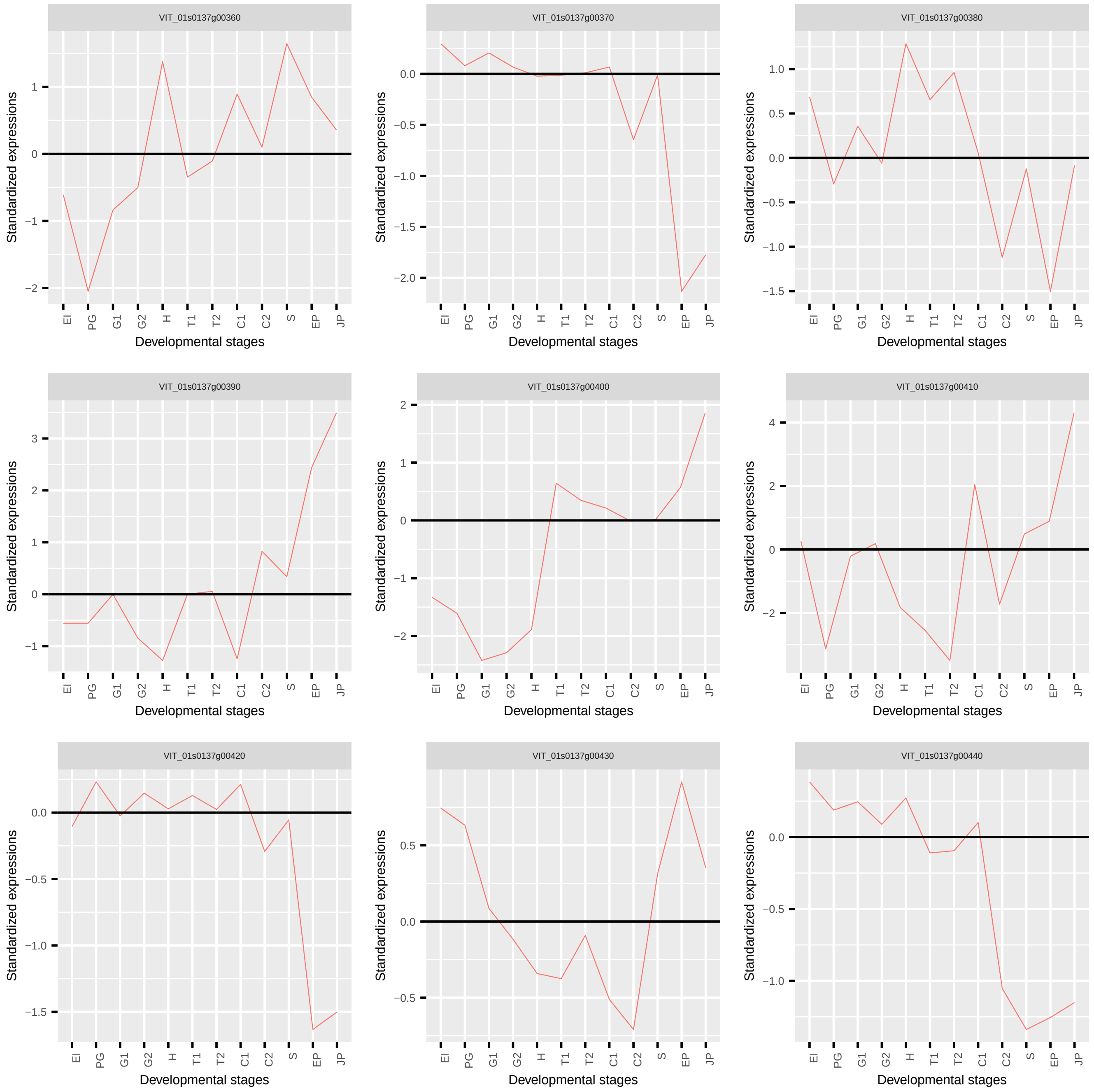
VIT_01s0137g00360
VIT_01s0137g00370
VIT_01s0137g00380
VIT_01s0137g00390
VIT_01s0137g00400
VIT_01s0137g00410
VIT_01s0137g00420
VIT_01s0137g00430
VIT_01s0137g00440
Standardized expressions
Developmental stages
EI PG G1 G2 H T1 T2 C1 C2 S EP JP

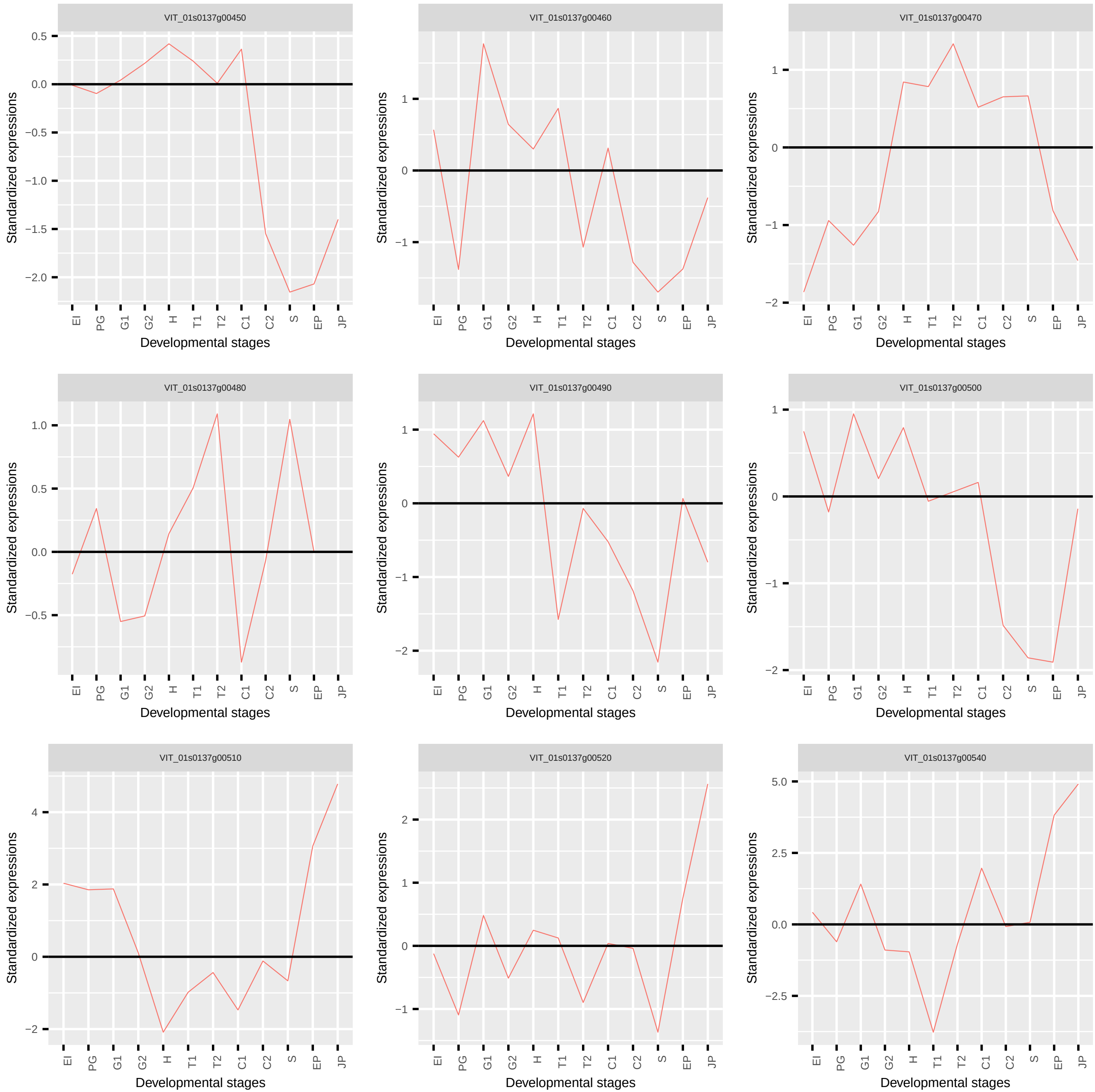
VIT_01s0137g00450
VIT_01s0137g00460
VIT_01s0137g00470
VIT_01s0137g00480
VIT_01s0137g00490
VIT_01s0137g00500
VIT_01s0137g00510
VIT_01s0137g00520
VIT_01s0137g00540
Standardized expressions
Developmental stages
EI
PG
G1
G2
H
T1
T2
C1
C2
S
EP
JP

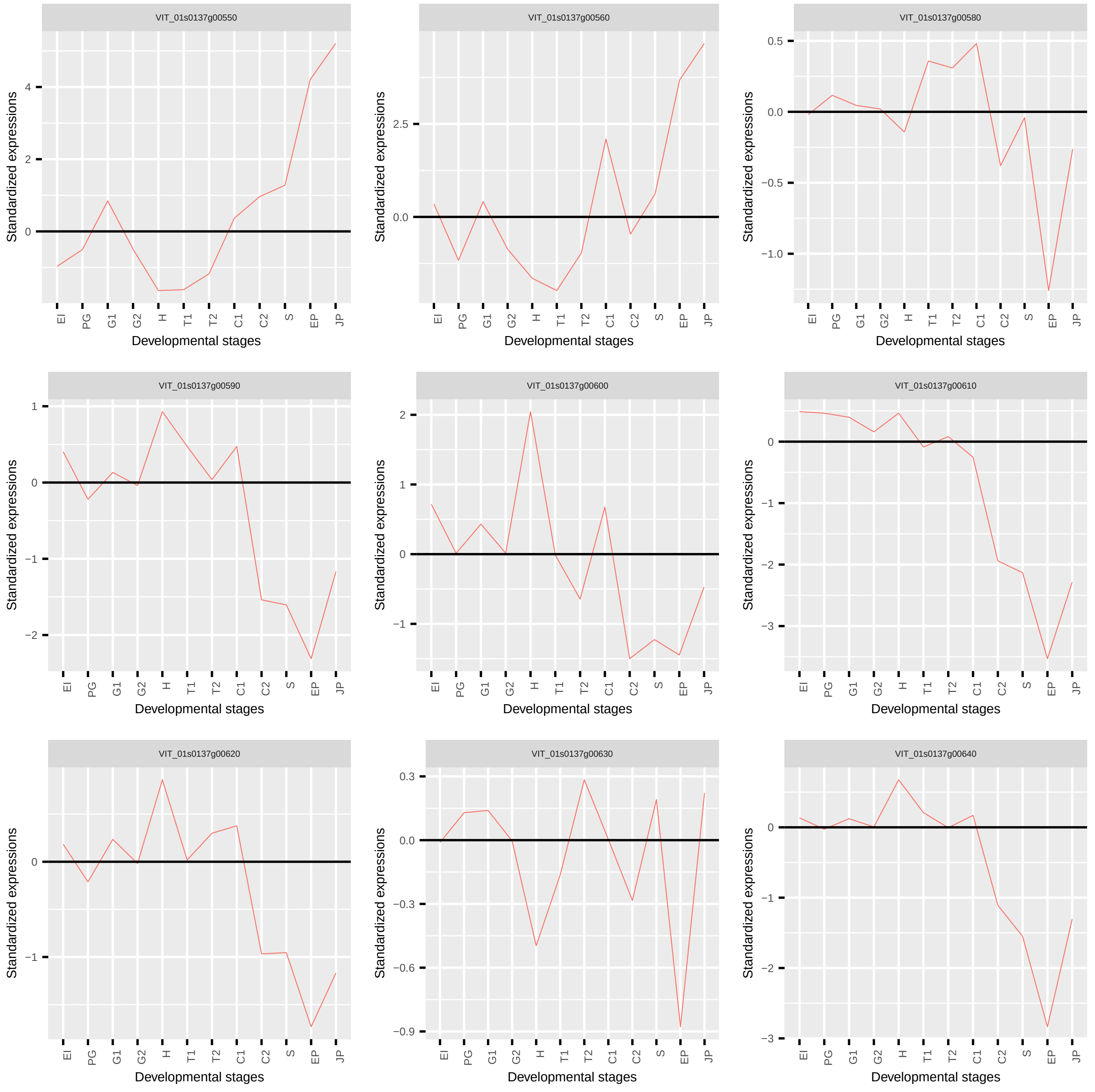

VIT_01s0137g00550
VIT_01s0137g00560
VIT_01s0137g00580
VIT_01s0137g00590
VIT_01s0137g00600
VIT_01s0137g00610
VIT_01s0137g00620
VIT_01s0137g00630
VIT_01s0137g00640
Standardized expressions
Developmental stages
EI PG G1 G2 H T1 T2 C1 C2 S EP JP

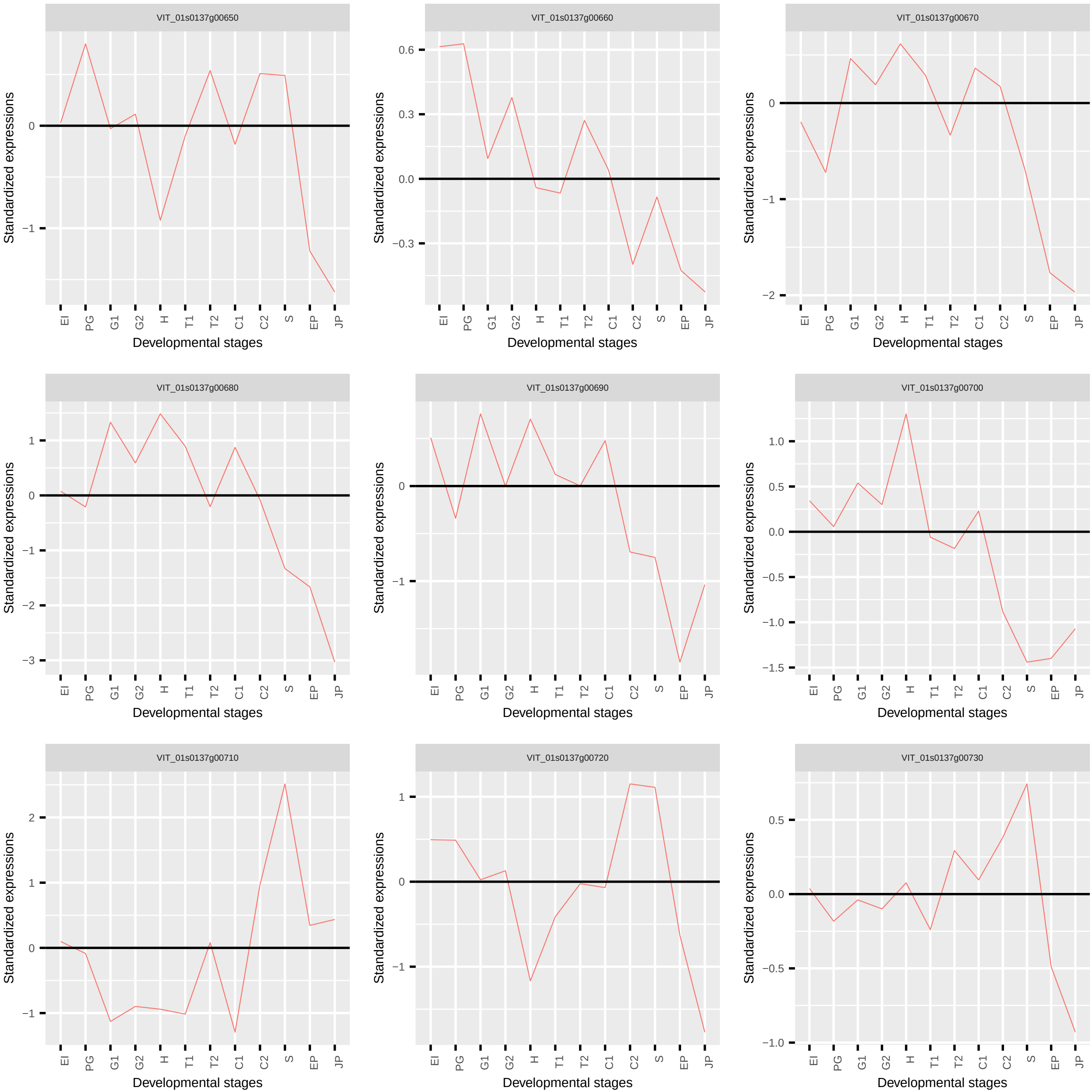
VIT_01s0137g00650
VIT_01s0137g00660
VIT_01s0137g00670
VIT_01s0137g00680
VIT_01s0137g00690
VIT_01s0137g00700
VIT_01s0137g00710
VIT_01s0137g00720
VIT_01s0137g00730
Standardized expressions
Developmental stages
EI
PG
G1
G2
H
T1
T2
C1
C2
S
EP
JP

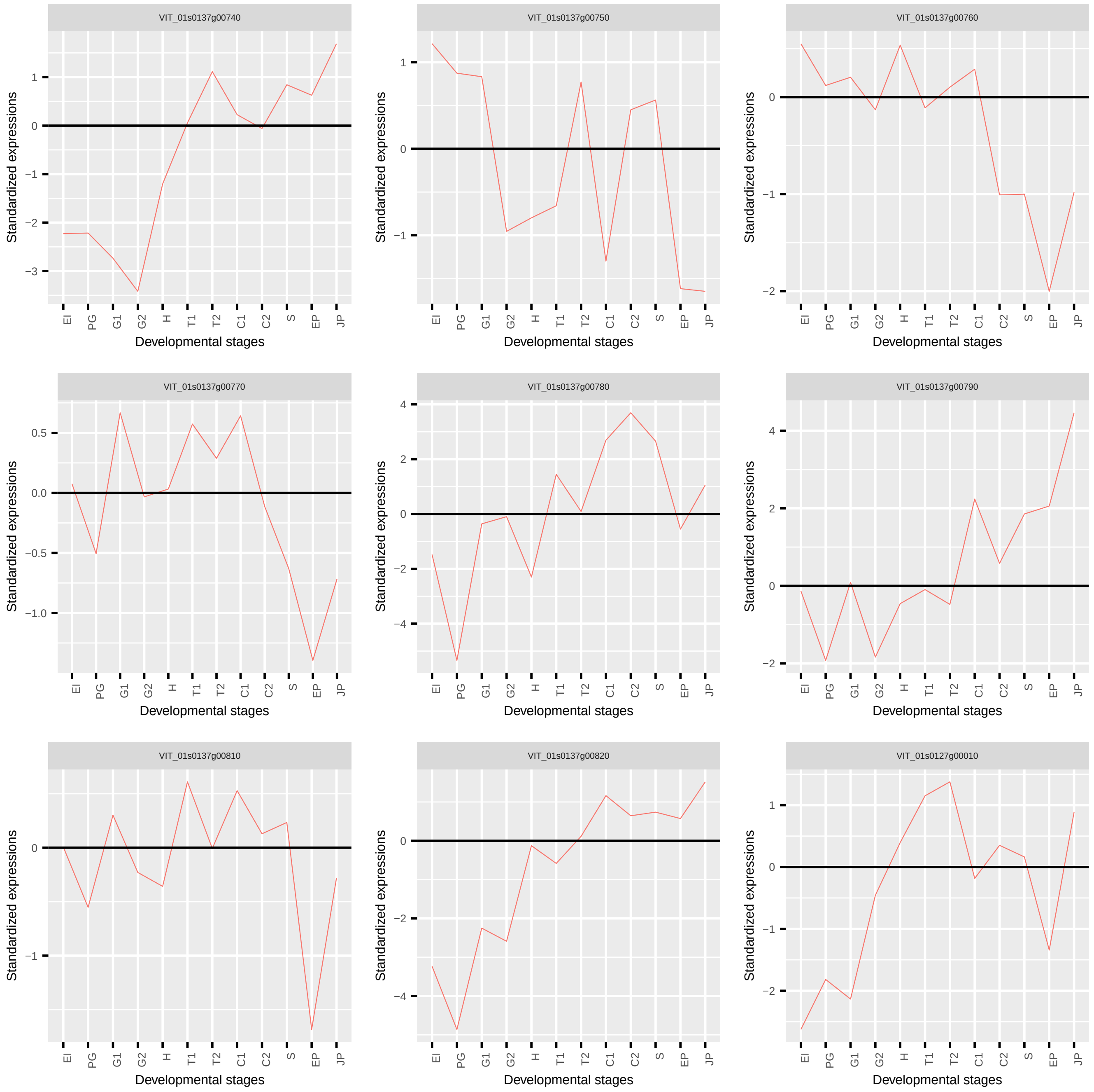

VIT_01s0137g00740
Standardized expressions
1
0
−1
−2
−3
EI PG G1 G2 H T1 T2 C1 C2 S EP JP
Developmental stages
VIT_01s0137g00750
Standardized expressions
1
0
−1
EI PG G1 G2 H T1 T2 C1 C2 S EP JP
Developmental stages
VIT_01s0137g00760
Standardized expressions
0
−1
−2
EI PG G1 G2 H T1 T2 C1 C2 S EP JP
Developmental stages
VIT_01s0137g00770
Standardized expressions
0.5
0.0
−0.5
−1.0
EI PG G1 G2 H T1 T2 C1 C2 S EP JP
Developmental stages
VIT_01s0137g00780
Standardized expressions
4
2
0
−2
−4
EI PG G1 G2 H T1 T2 C1 C2 S EP JP
Developmental stages
VIT_01s0137g00790
Standardized expressions
4
2
0
−2
EI PG G1 G2 H T1 T2 C1 C2 S EP JP
Developmental stages
VIT_01s0137g00810
Standardized expressions
0
−1
EI PG G1 G2 H T1 T2 C1 C2 S EP JP
Developmental stages
VIT_01s0137g00820
Standardized expressions
0
−2
−4
EI PG G1 G2 H T1 T2 C1 C2 S EP JP
Developmental stages
VIT_01s0127g00010
Standardized expressions
1
0
−1
−2
EI PG G1 G2 H T1 T2 C1 C2 S EP JP
Developmental stages

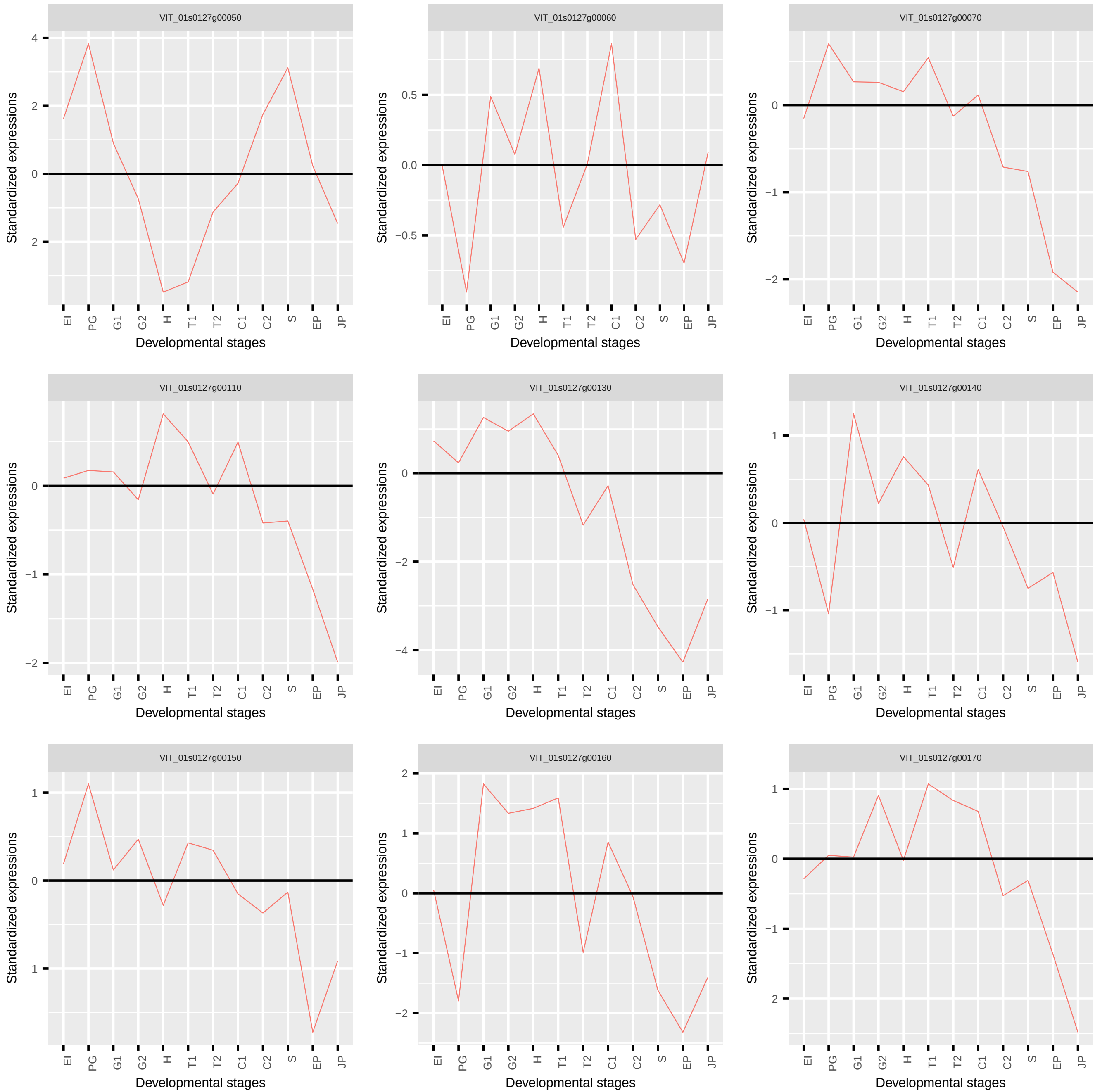
VIT_01s0127g00050
VIT_01s0127g00060
VIT_01s0127g00070
VIT_01s0127g00110
VIT_01s0127g00130
VIT_01s0127g00140
VIT_01s0127g00150
VIT_01s0127g00160
VIT_01s0127g00170
Standardized expressions
Developmental stages
EI PG G1 G2 H T1 T2 C1 C2 S EP JP

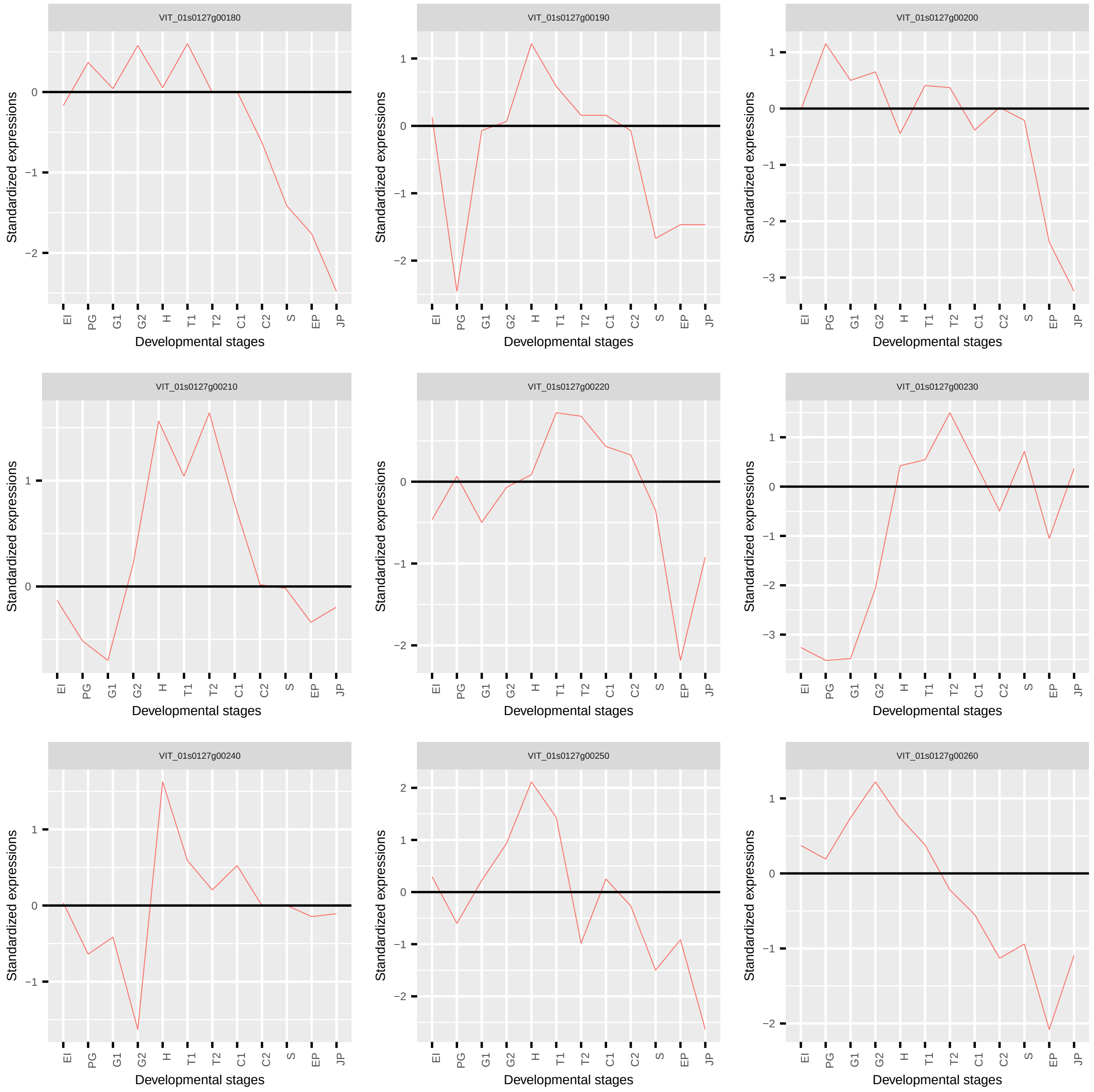
VIT_01s0127g00180
VIT_01s0127g00190
VIT_01s0127g00200
VIT_01s0127g00210
VIT_01s0127g00220
VIT_01s0127g00230
VIT_01s0127g00240
VIT_01s0127g00250
VIT_01s0127g00260
Standardized expressions
Developmental stages
EI PG G1 G2 H T1 T2 C1 C2 S EP JP

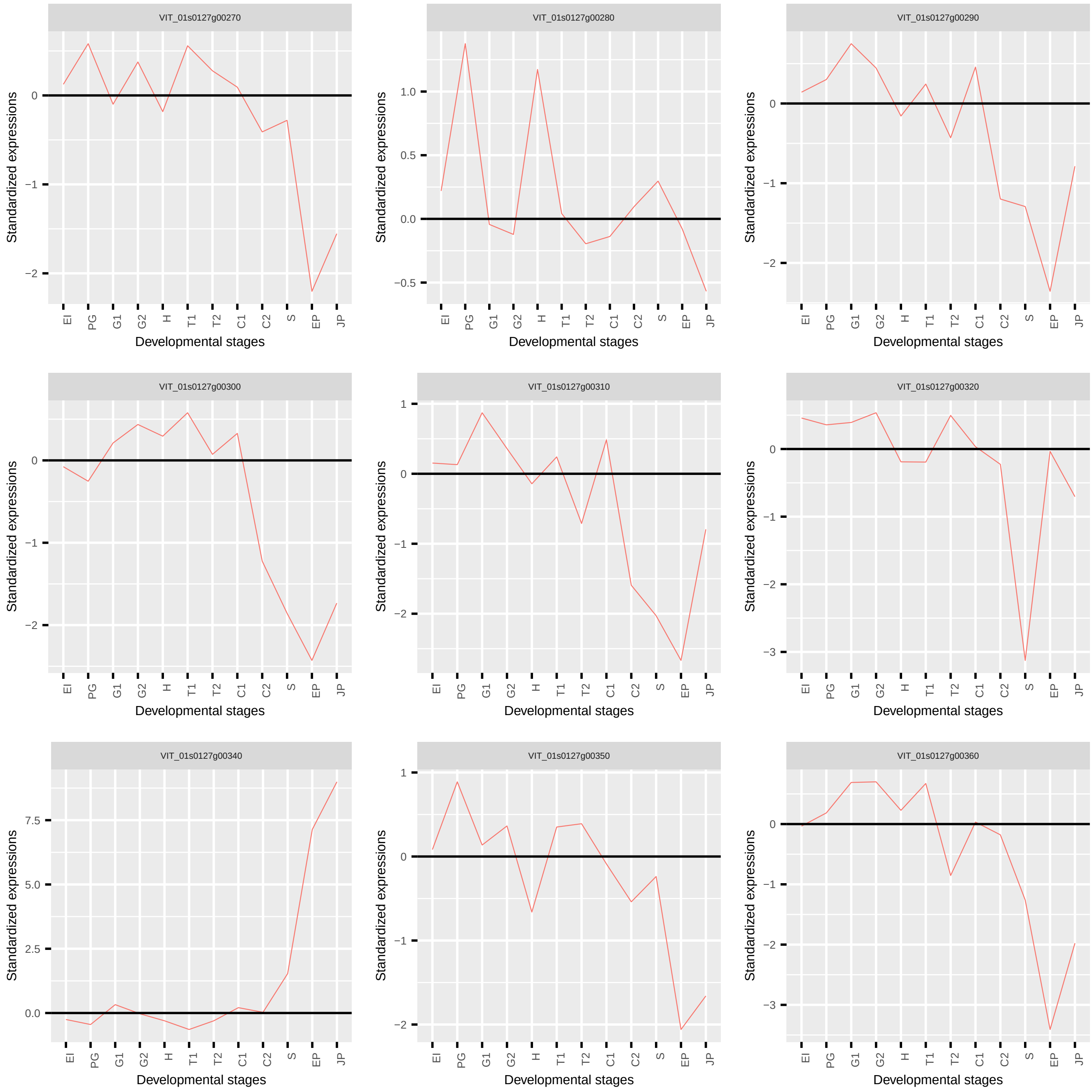

VIT_01s0127g00270
VIT_01s0127g00280
VIT_01s0127g00290
VIT_01s0127g00300
VIT_01s0127g00310
VIT_01s0127g00320
VIT_01s0127g00340
VIT_01s0127g00350
VIT_01s0127g00360
Standardized expressions
Developmental stages
EI PG G1 G2 H T1 T2 C1 C2 S EP JP
0
−1
−2
−3
1
1.0
0.5
0.0
−0.5
7.5
5.0
2.5
0.0

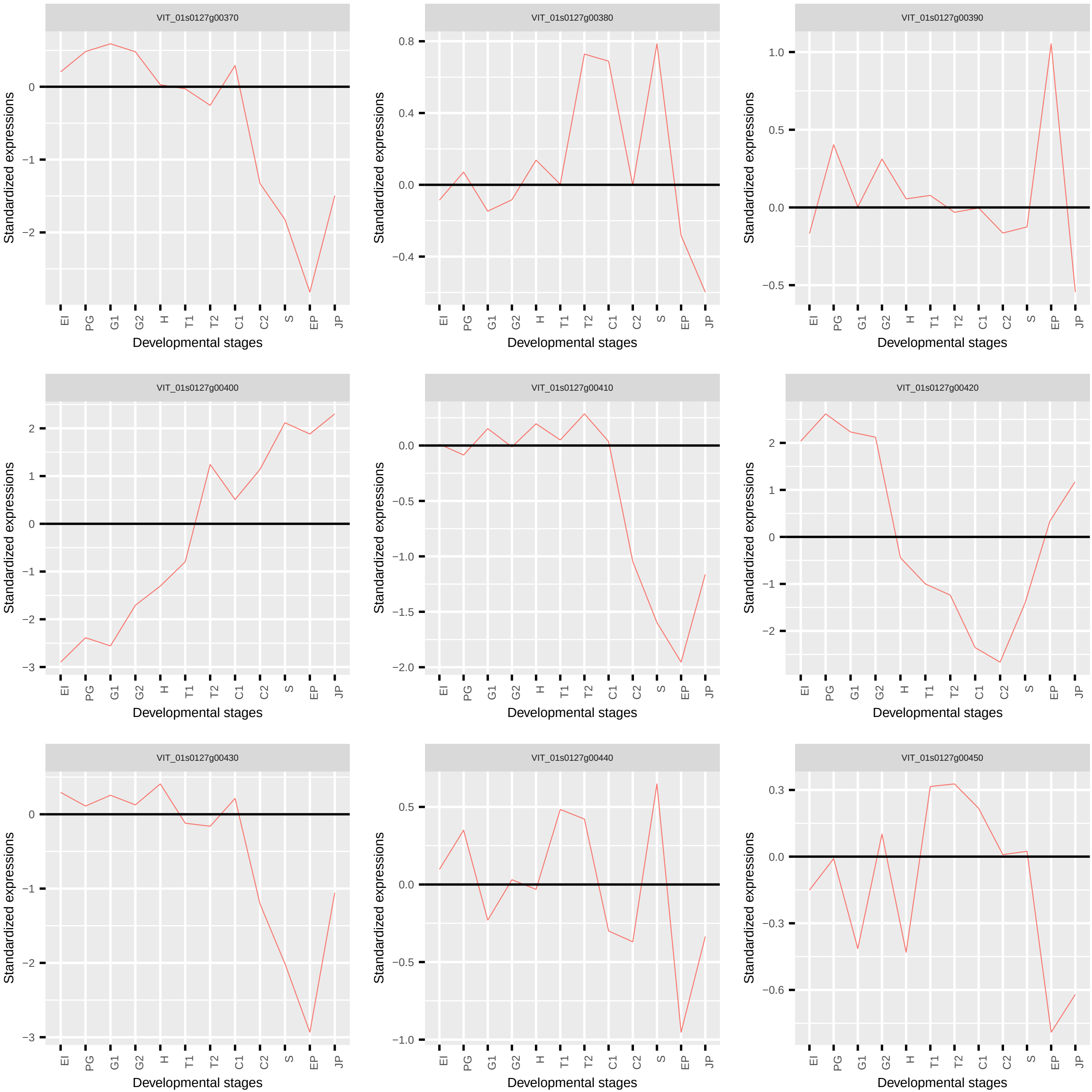
VIT_01s0127g00370
VIT_01s0127g00380
VIT_01s0127g00390
VIT_01s0127g00400
VIT_01s0127g00410
VIT_01s0127g00420
VIT_01s0127g00430
VIT_01s0127g00440
VIT_01s0127g00450
Standardized expressions
Developmental stages
EI
PG
G1
G2
H
T1
T2
C1
C2
S
EP
JP

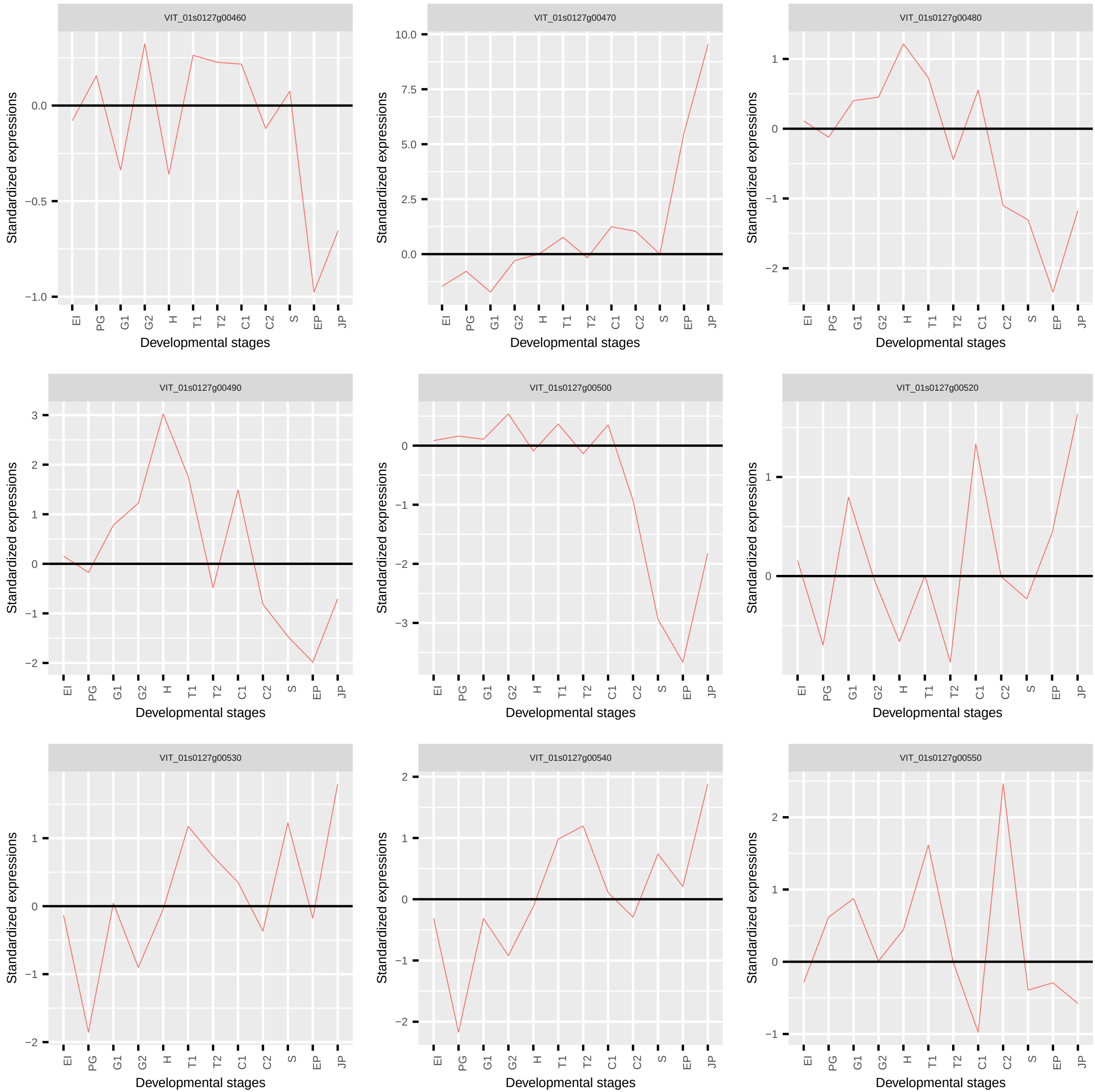

VIT_01s0127g00460
VIT_01s0127g00470
VIT_01s0127g00480
VIT_01s0127g00490
VIT_01s0127g00500
VIT_01s0127g00520
VIT_01s0127g00530
VIT_01s0127g00540
VIT_01s0127g00550
Standardized expressions
Developmental stages
EI
PG
G1
G2
H
T1
T2
C1
C2
S
EP
JP

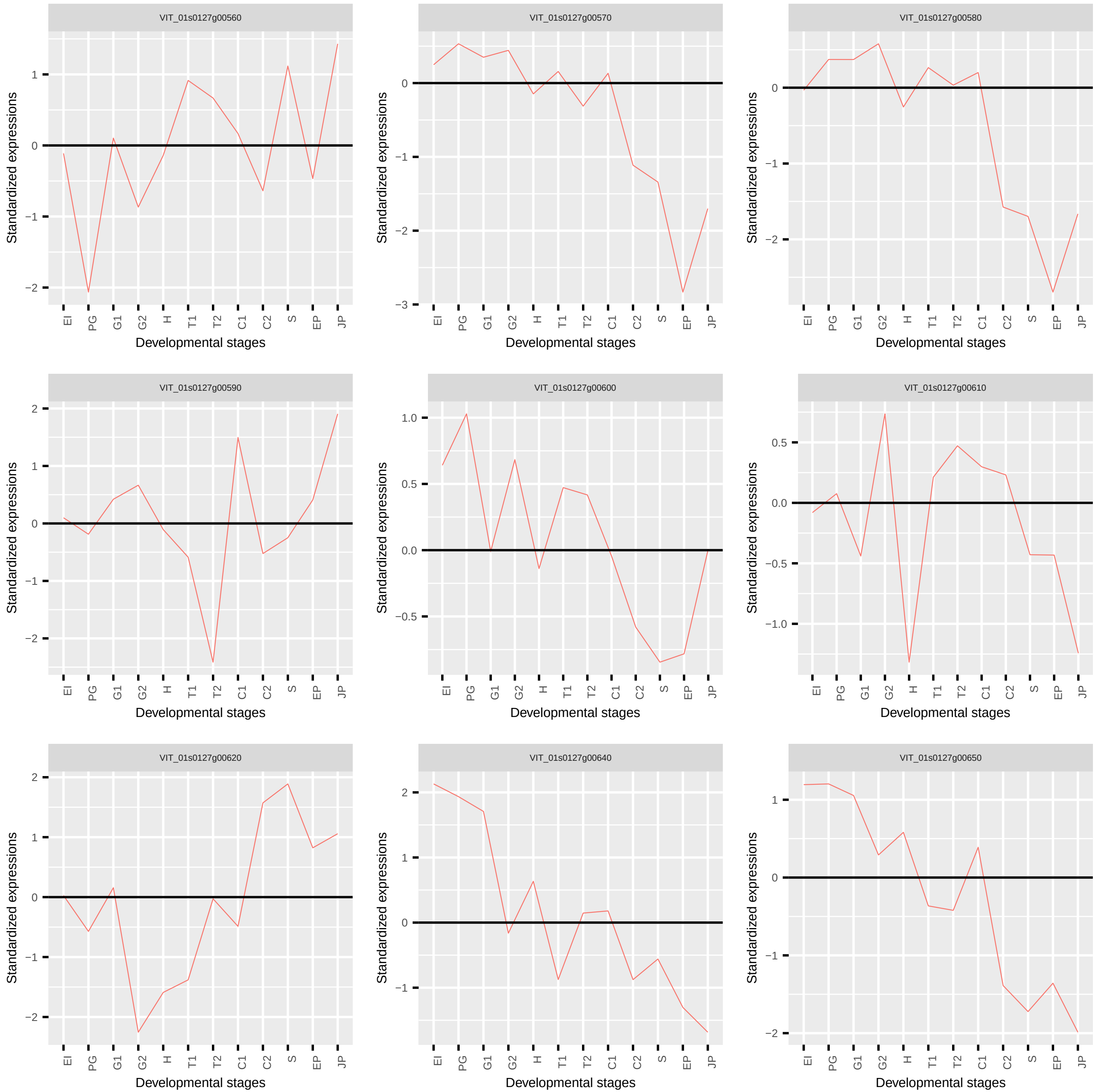
VIT_01s0127g00560
VIT_01s0127g00570
VIT_01s0127g00580
VIT_01s0127g00590
VIT_01s0127g00600
VIT_01s0127g00610
VIT_01s0127g00620
VIT_01s0127g00640
VIT_01s0127g00650
Standardized expressions
Developmental stages
EI PG G1 G2 H T1 T2 C1 C2 S EP JP

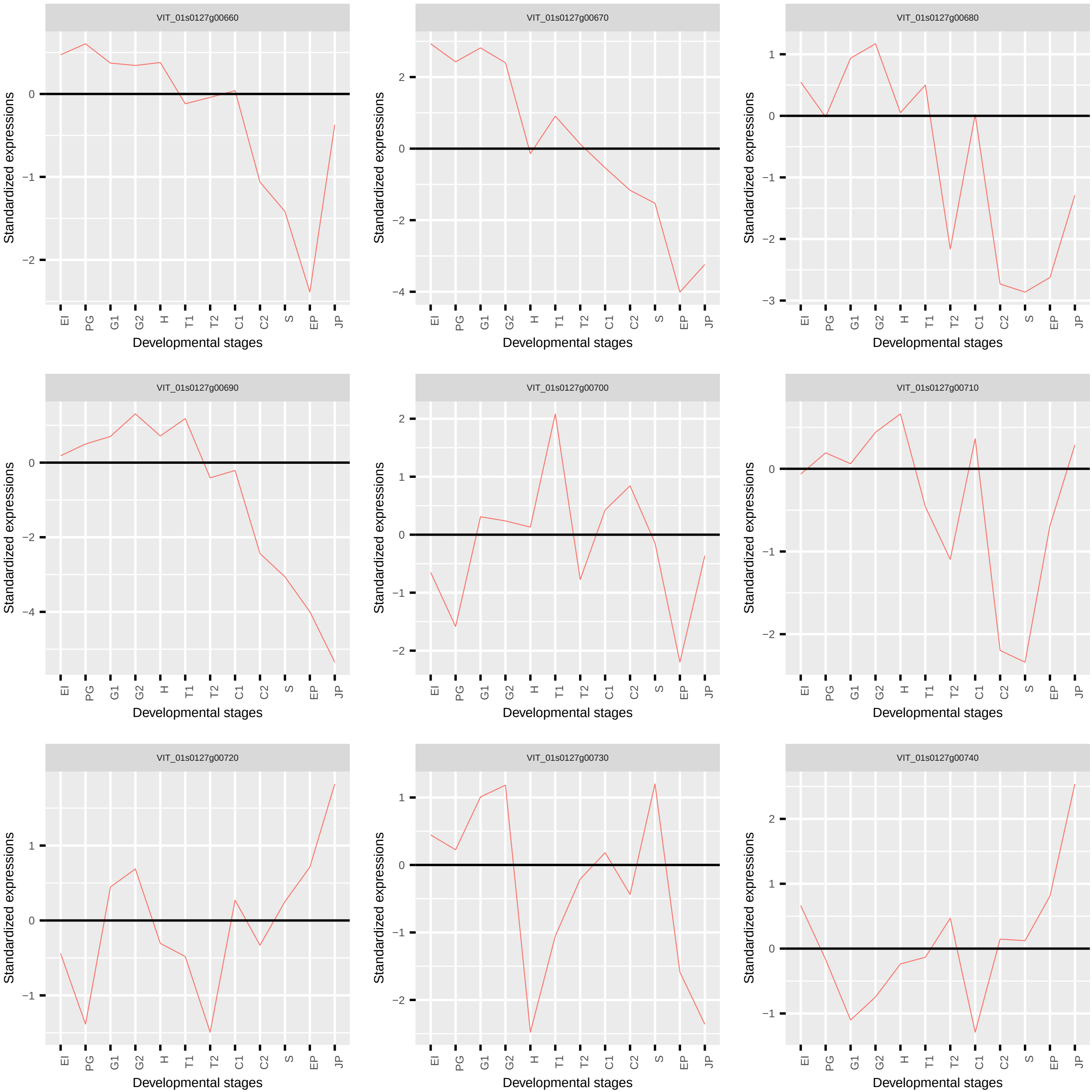
VIT_01s0127g00660
VIT_01s0127g00670
VIT_01s0127g00680
VIT_01s0127g00690
VIT_01s0127g00700
VIT_01s0127g00710
VIT_01s0127g00720
VIT_01s0127g00730
VIT_01s0127g00740
Standardized expressions
Developmental stages
EI PG G1 G2 H T1 T2 C1 C2 S EP JP

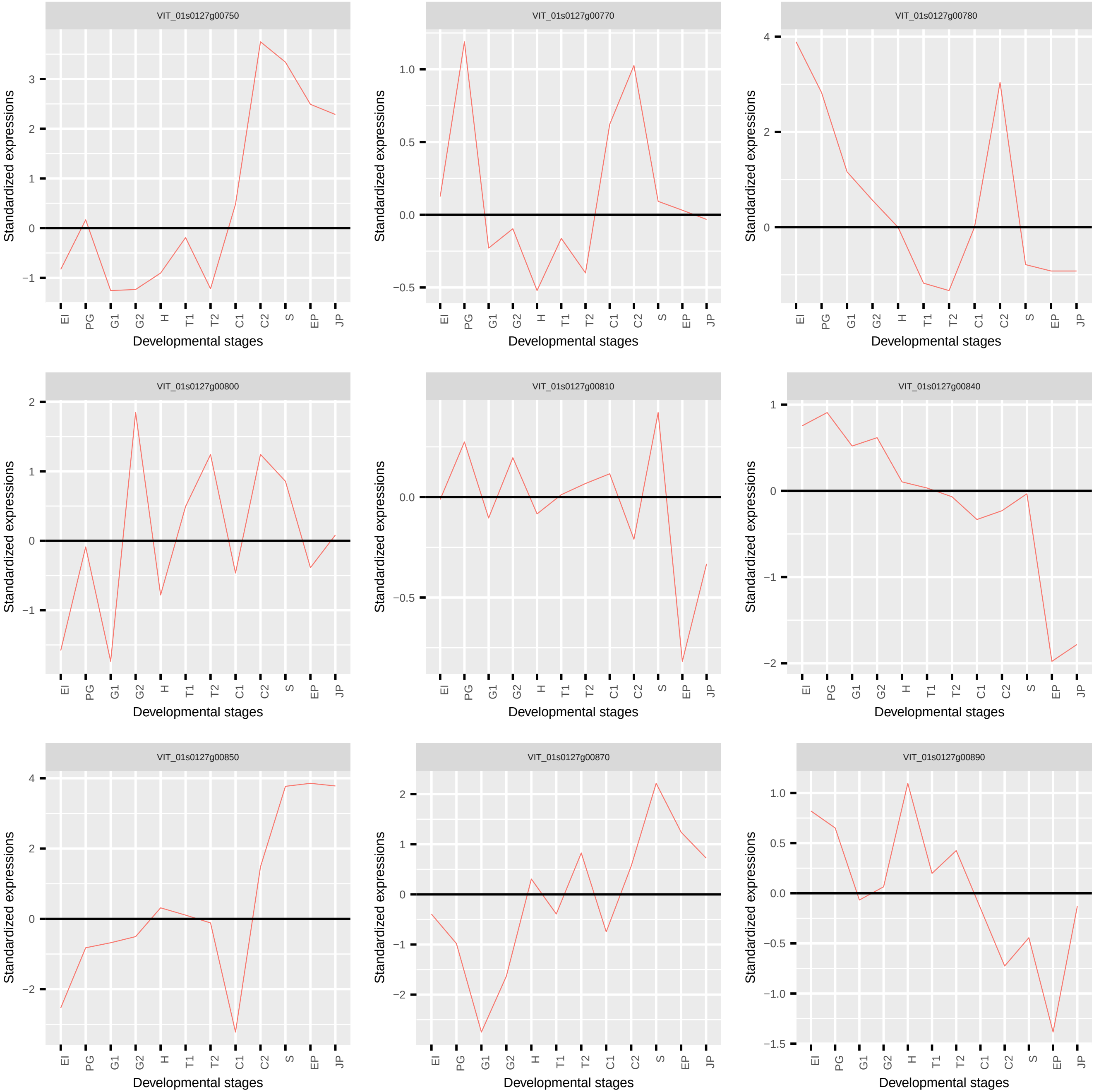
VIT_01s0127g00750
VIT_01s0127g00770
VIT_01s0127g00780
VIT_01s0127g00800
VIT_01s0127g00810
VIT_01s0127g00840
VIT_01s0127g00850
VIT_01s0127g00870
VIT_01s0127g00890
Standardized expressions
Developmental stages
EI PG G1 G2 H T1 T2 C1 C2 S EP JP

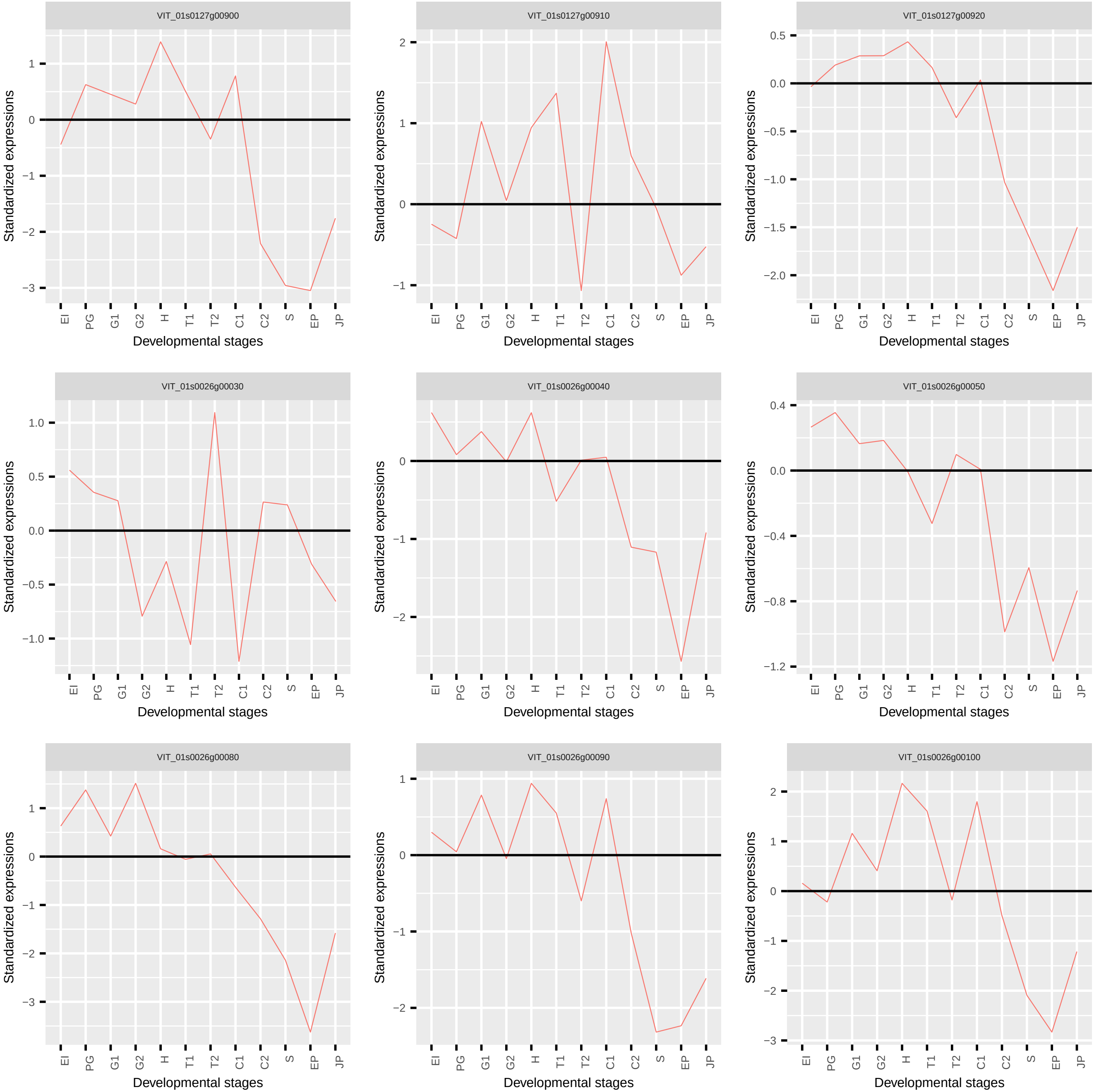

VIT_01s0127g00900
VIT_01s0127g00910
VIT_01s0127g00920
VIT_01s0026g00030
VIT_01s0026g00040
VIT_01s0026g00050
VIT_01s0026g00080
VIT_01s0026g00090
VIT_01s0026g00100
Standardized expressions
Developmental stages
EI
PG
G1
G2
H
T1
T2
C1
C2
S
EP
JP

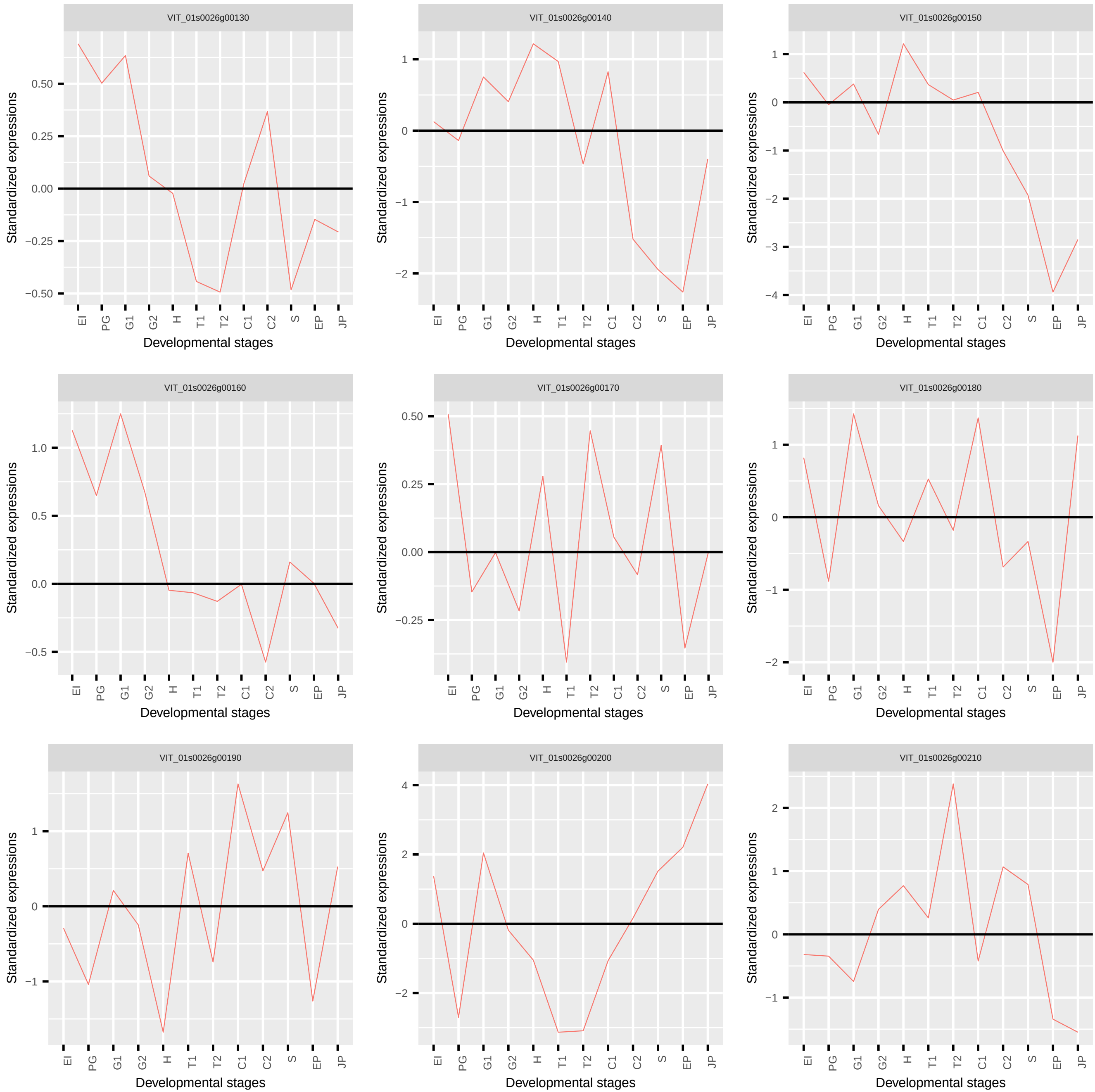
VIT_01s0026g00130
VIT_01s0026g00140
VIT_01s0026g00150
VIT_01s0026g00160
VIT_01s0026g00170
VIT_01s0026g00180
VIT_01s0026g00190
VIT_01s0026g00200
VIT_01s0026g00210
Standardized expressions
Developmental stages
EI PG G1 G2 H T1 T2 C1 C2 S EP JP
0.50 0.25 0.00 −0.25 −0.50
1 0 −1 −2
1 0 −1 −2 −3 −4
1.0 0.5 0.0 −0.5
0.50 0.25 0.00 −0.25
1 0 −1 −2
1 0 −1
4 2 0 −2
2 1 0 −1

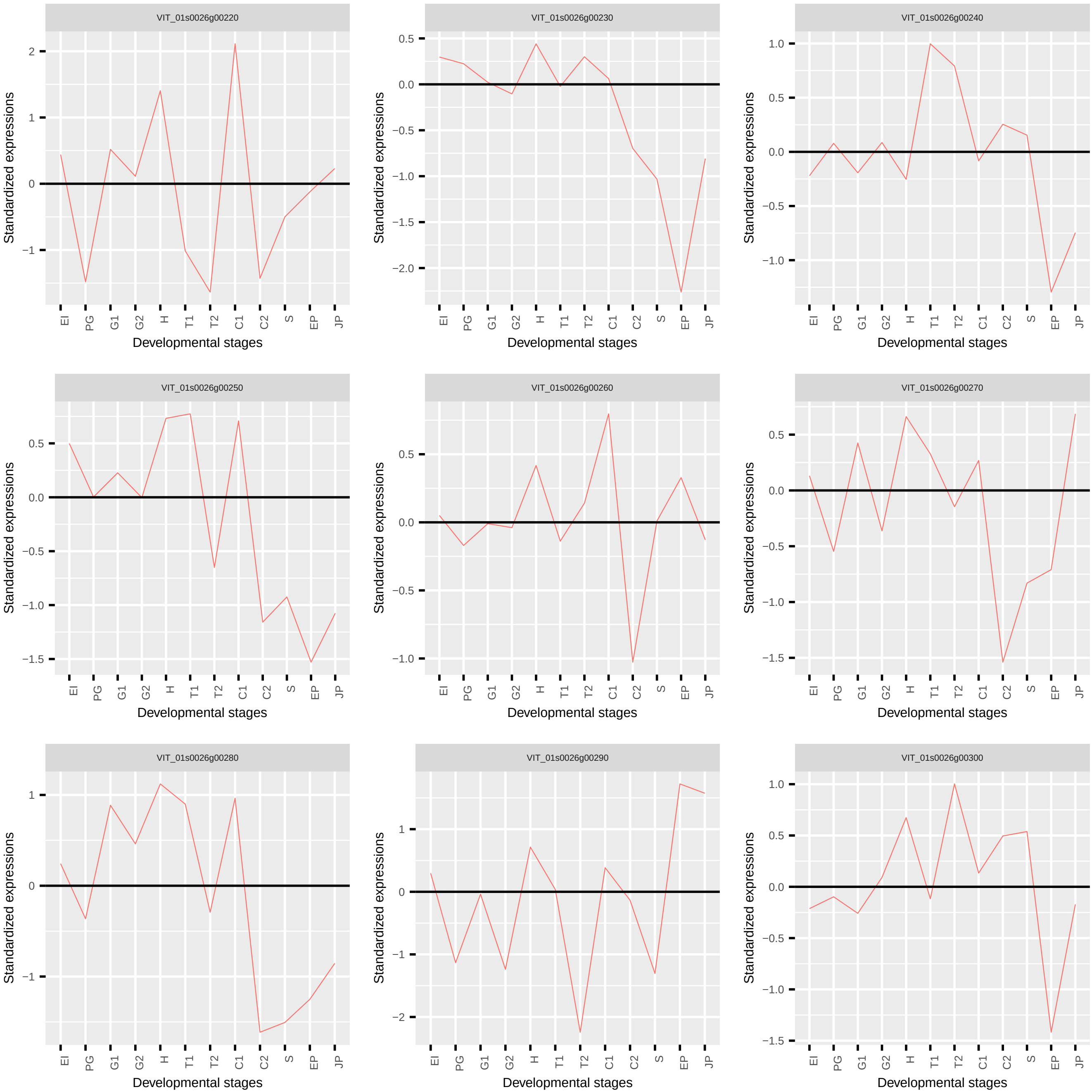

VIT_01s0026g00220
VIT_01s0026g00230
VIT_01s0026g00240
VIT_01s0026g00250
VIT_01s0026g00260
VIT_01s0026g00270
VIT_01s0026g00280
VIT_01s0026g00290
VIT_01s0026g00300
Standardized expressions
Developmental stages
EI PG G1 G2 H T1 T2 C1 C2 S EP JP

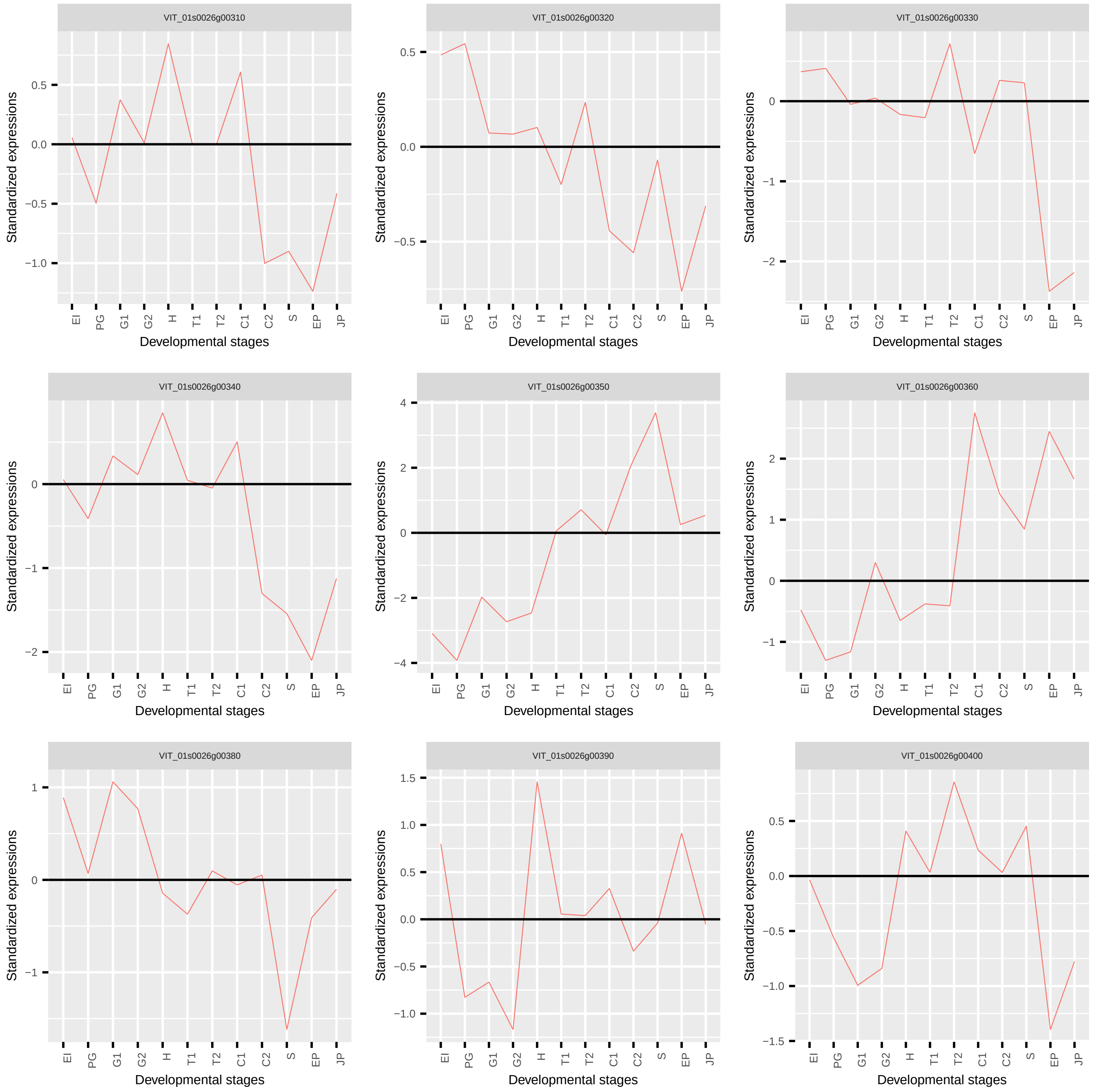
VIT_01s0026g00310
VIT_01s0026g00320
VIT_01s0026g00330
VIT_01s0026g00340
VIT_01s0026g00350
VIT_01s0026g00360
VIT_01s0026g00380
VIT_01s0026g00390
VIT_01s0026g00400
Standardized expressions
Developmental stages
EI PG G1 G2 H T1 T2 C1 C2 S EP JP

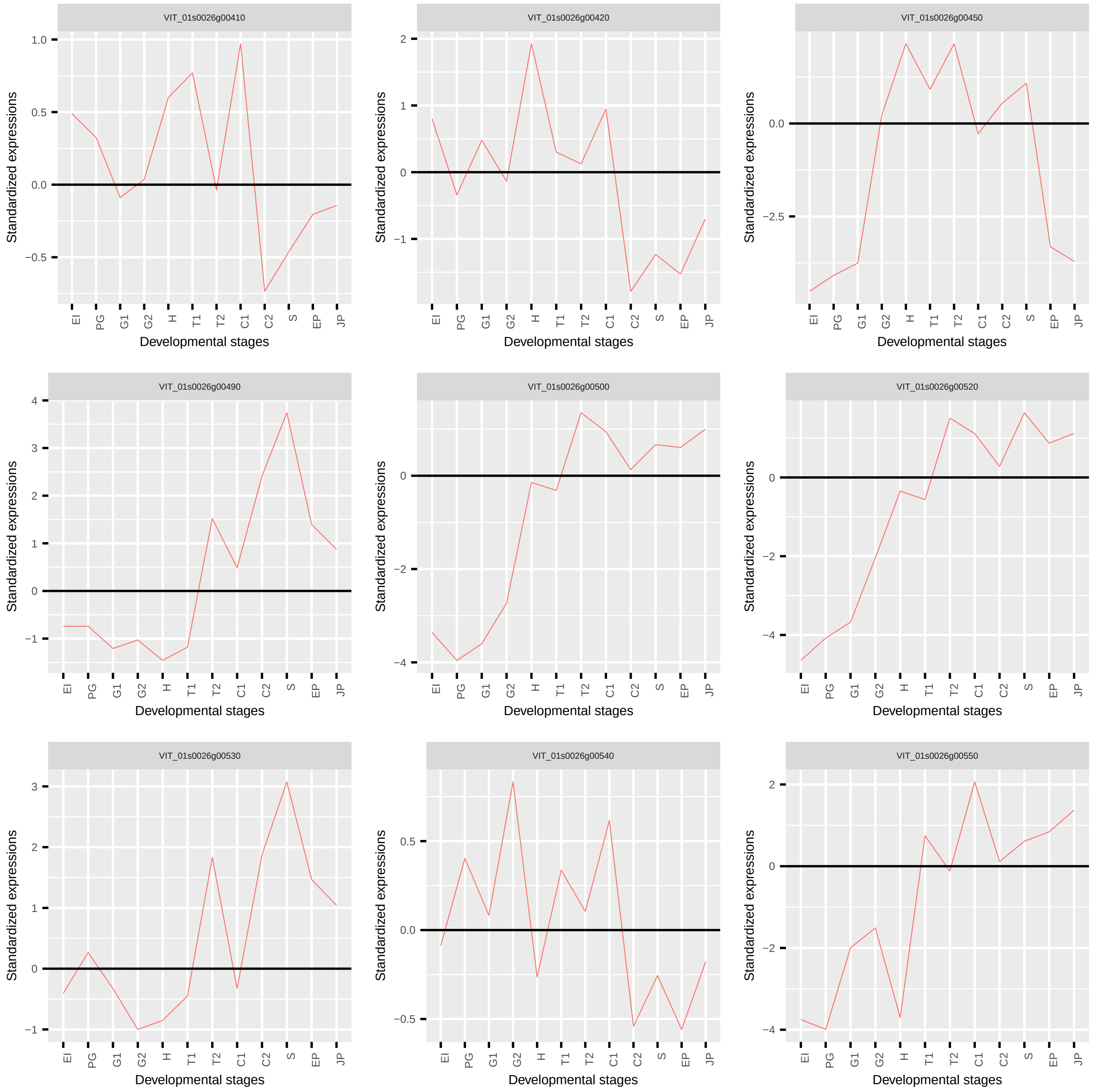
VIT_01s0026g00410
VIT_01s0026g00420
VIT_01s0026g00450
VIT_01s0026g00490
VIT_01s0026g00500
VIT_01s0026g00520
VIT_01s0026g00530
VIT_01s0026g00540
VIT_01s0026g00550
Standardized expressions
Developmental stages
EI PG G1 G2 H T1 T2 C1 C2 S EP JP

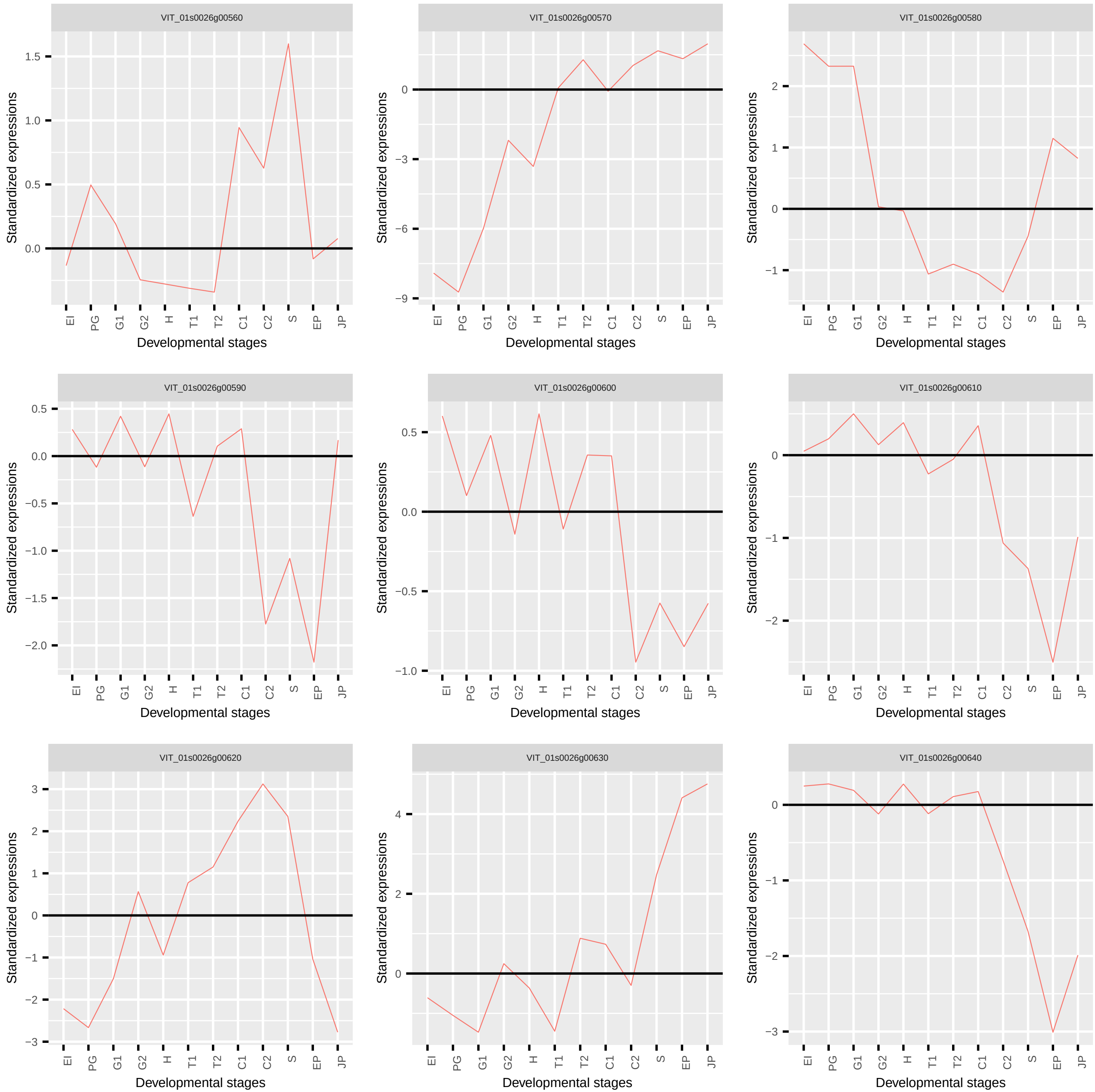
VIT_01s0026g00560
VIT_01s0026g00570
VIT_01s0026g00580
VIT_01s0026g00590
VIT_01s0026g00600
VIT_01s0026g00610
VIT_01s0026g00620
VIT_01s0026g00630
VIT_01s0026g00640
Standardized expressions
Developmental stages
EI PG G1 G2 H T1 T2 C1 C2 S EP JP
0.0 0.5 1.0 1.5
−9 −6 −3 0
−1 0 1 2
−2.0 −1.5 −1.0 −0.5 0.0 0.5
−1.0 −0.5 0.0 0.5
−2 −1 0
−3 −2 −1 0 1 2 3
0 2 4
−3 −2 −1 0

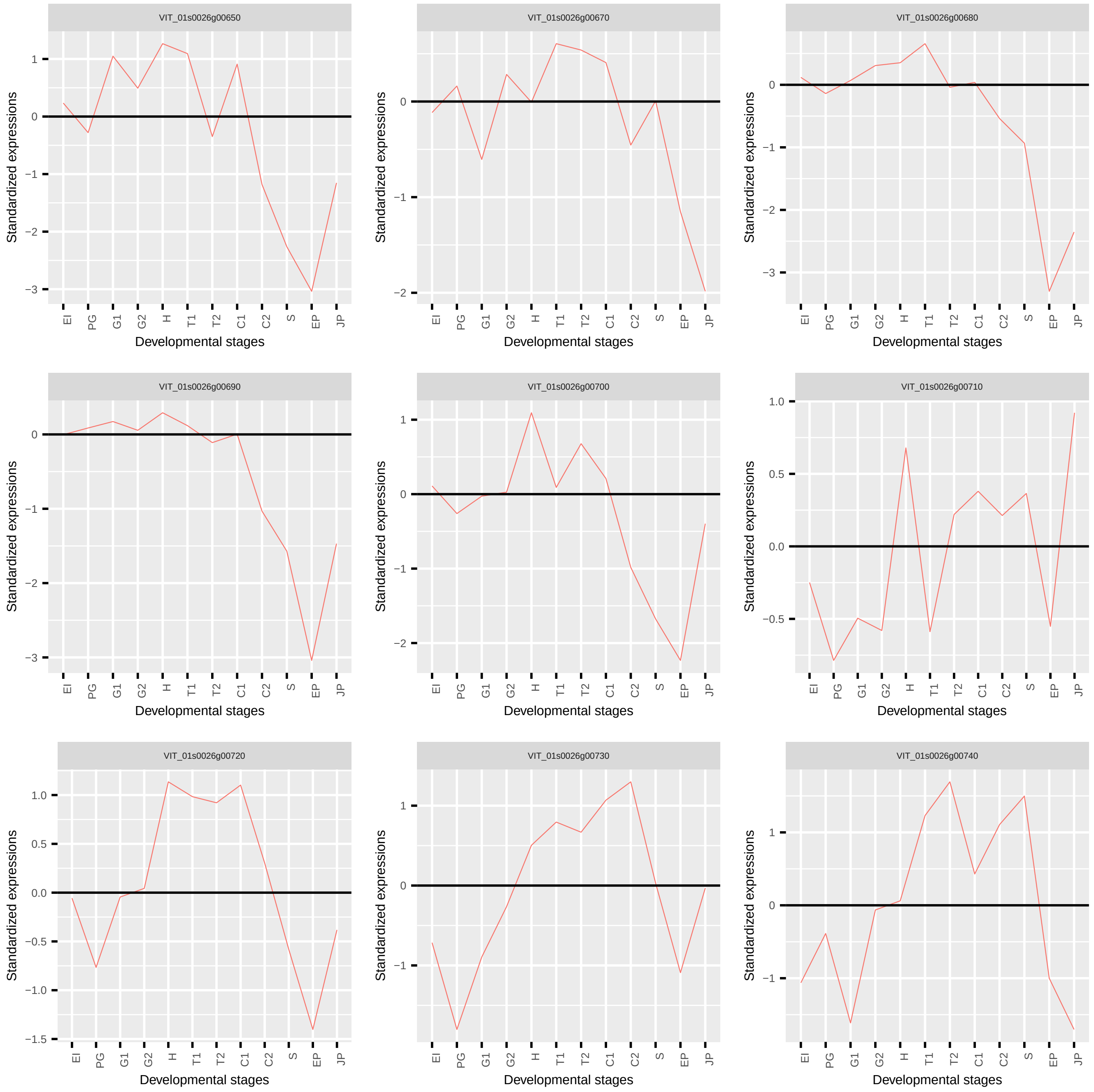
VIT_01s0026g00650
VIT_01s0026g00670
VIT_01s0026g00680
VIT_01s0026g00690
VIT_01s0026g00700
VIT_01s0026g00710
VIT_01s0026g00720
VIT_01s0026g00730
VIT_01s0026g00740
Standardized expressions
Developmental stages
EI PG G1 G2 H T1 T2 C1 C2 S EP JP

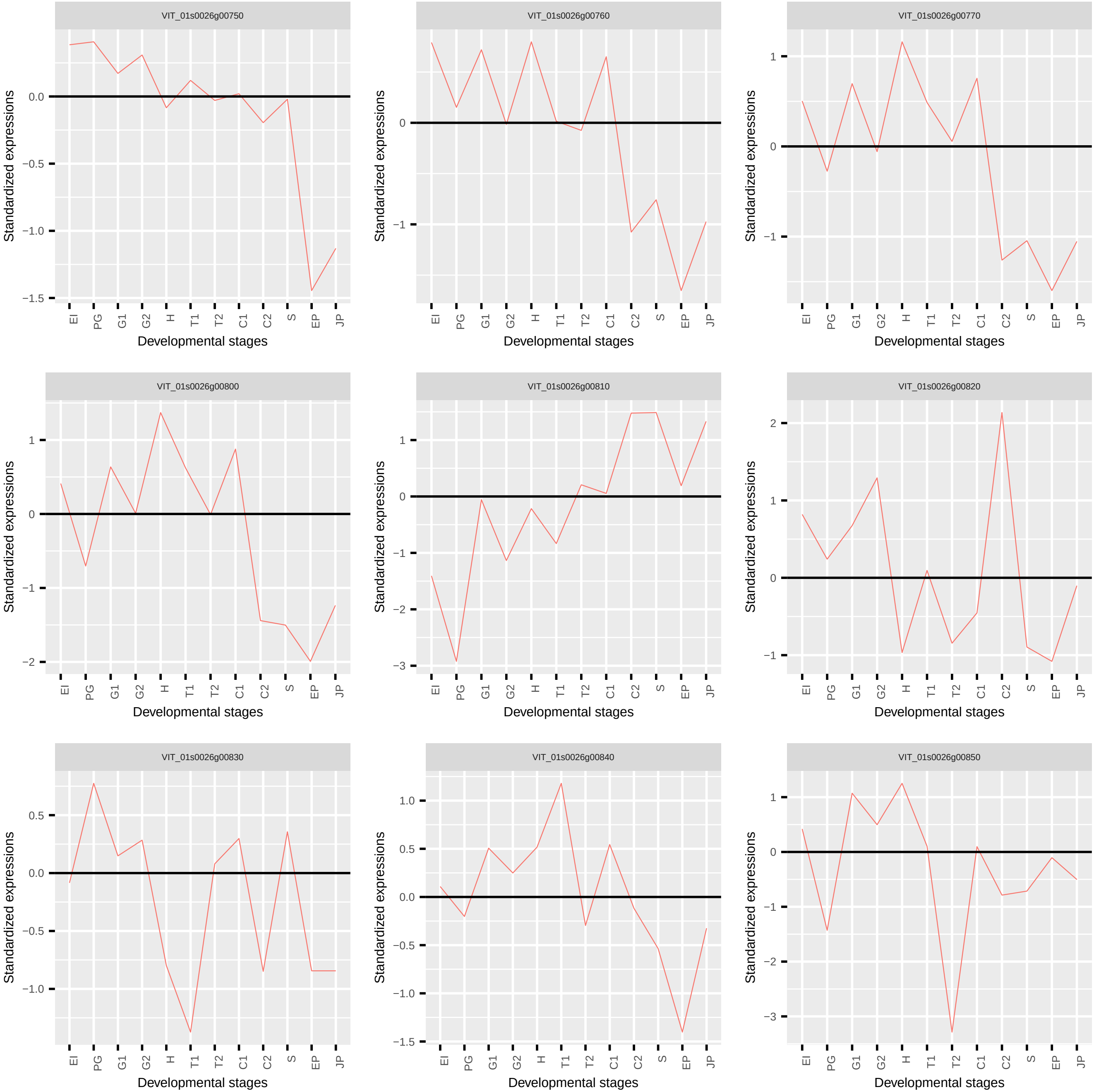
VIT_01s0026g00750
VIT_01s0026g00760
VIT_01s0026g00770
VIT_01s0026g00800
VIT_01s0026g00810
VIT_01s0026g00820
VIT_01s0026g00830
VIT_01s0026g00840
VIT_01s0026g00850
Standardized expressions
Developmental stages
EI PG G1 G2 H T1 T2 C1 C2 S EP JP

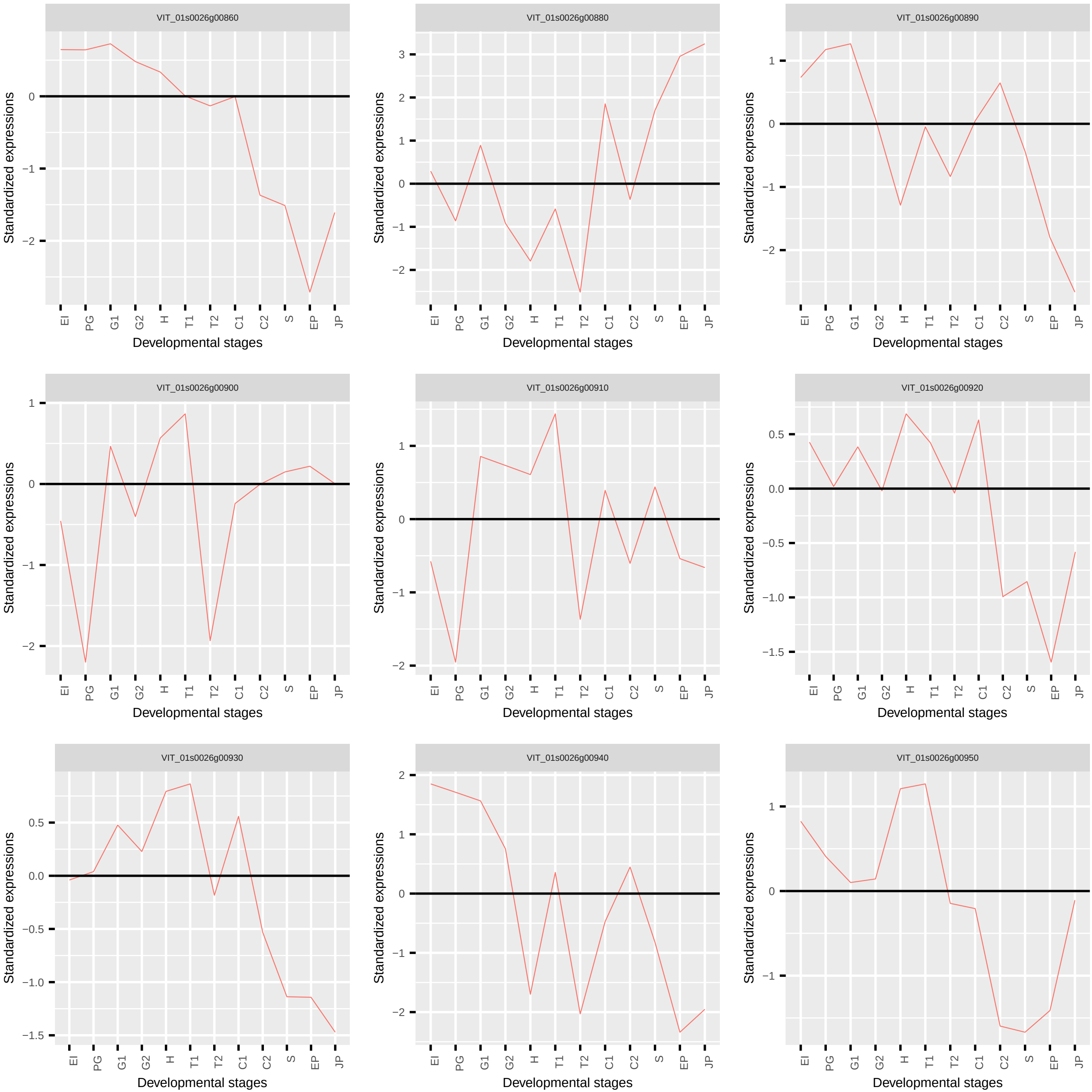
VIT_01s0026g00860
VIT_01s0026g00880
VIT_01s0026g00890
VIT_01s0026g00900
VIT_01s0026g00910
VIT_01s0026g00920
VIT_01s0026g00930
VIT_01s0026g00940
VIT_01s0026g00950
Standardized expressions
Developmental stages
EI PG G1 G2 H T1 T2 C1 C2 S EP JP

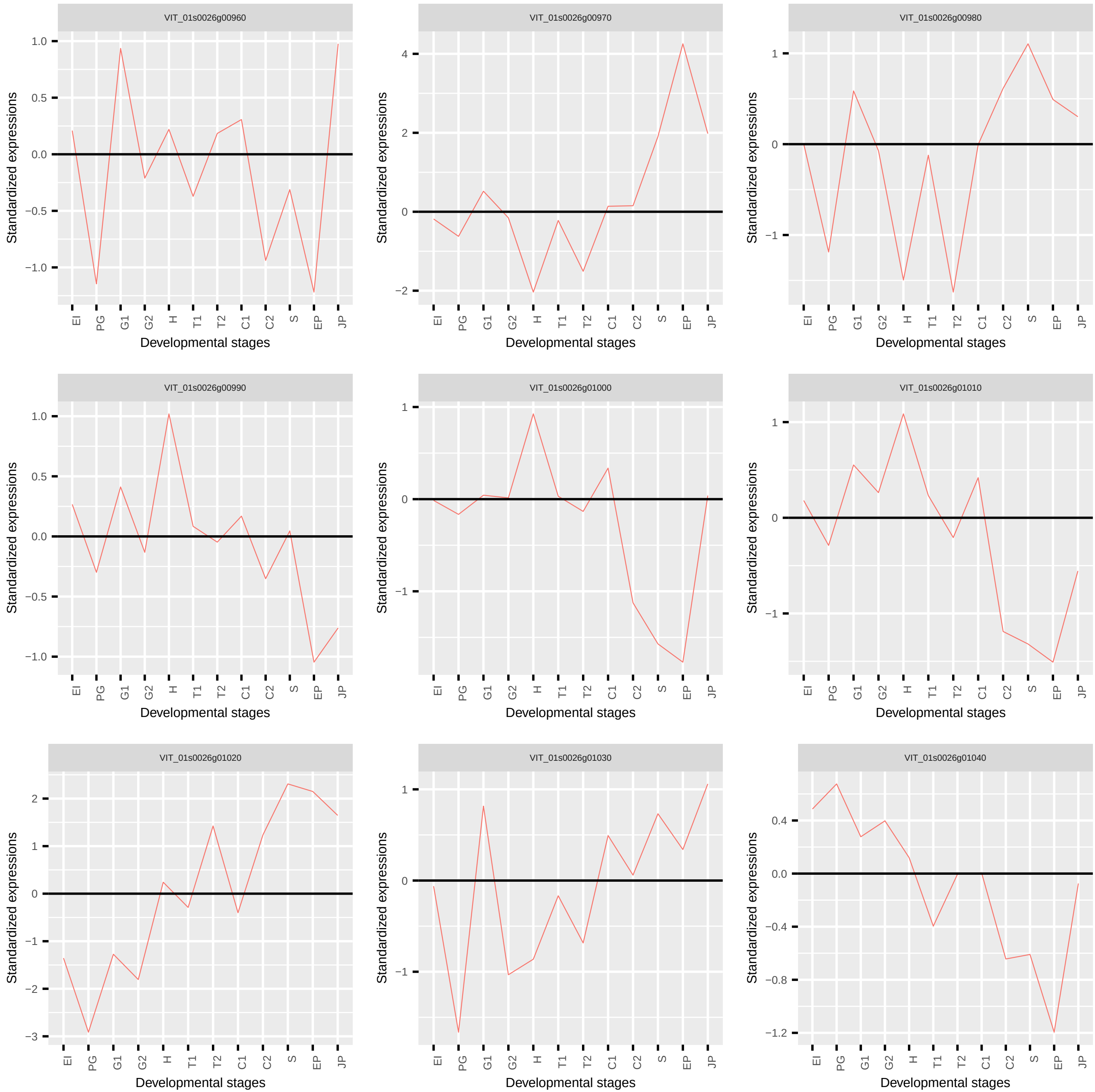
VIT_01s0026g00960
VIT_01s0026g00970
VIT_01s0026g00980
VIT_01s0026g00990
VIT_01s0026g01000
VIT_01s0026g01010
VIT_01s0026g01020
VIT_01s0026g01030
VIT_01s0026g01040
Standardized expressions
Developmental stages
EI
PG
G1
G2
H
T1
T2
C1
C2
S
EP
JP

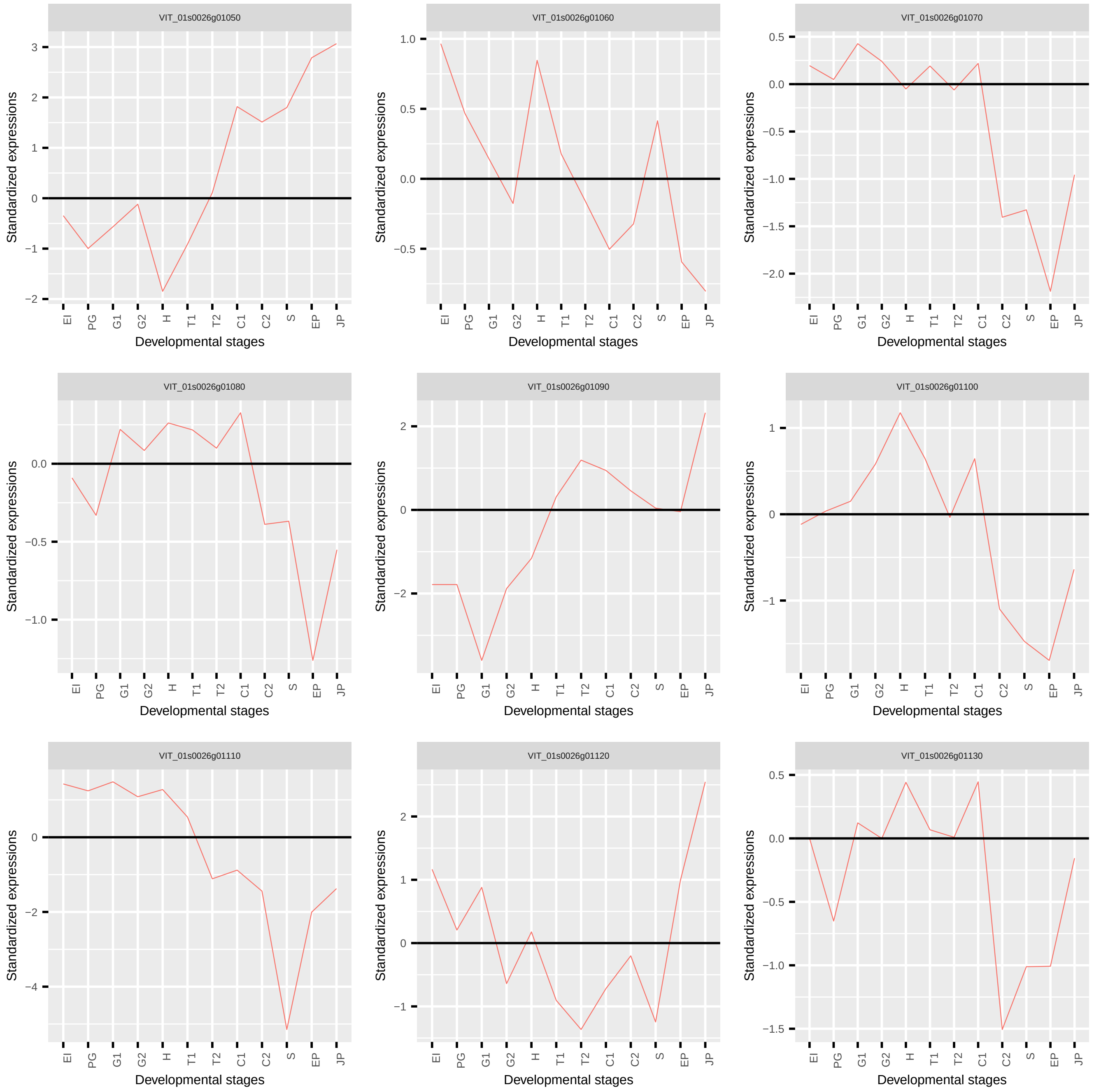
VIT_01s0026g01050
VIT_01s0026g01060
VIT_01s0026g01070
VIT_01s0026g01080
VIT_01s0026g01090
VIT_01s0026g01100
VIT_01s0026g01110
VIT_01s0026g01120
VIT_01s0026g01130
Standardized expressions
Developmental stages
EI PG G1 G2 H T1 T2 C1 C2 S EP JP

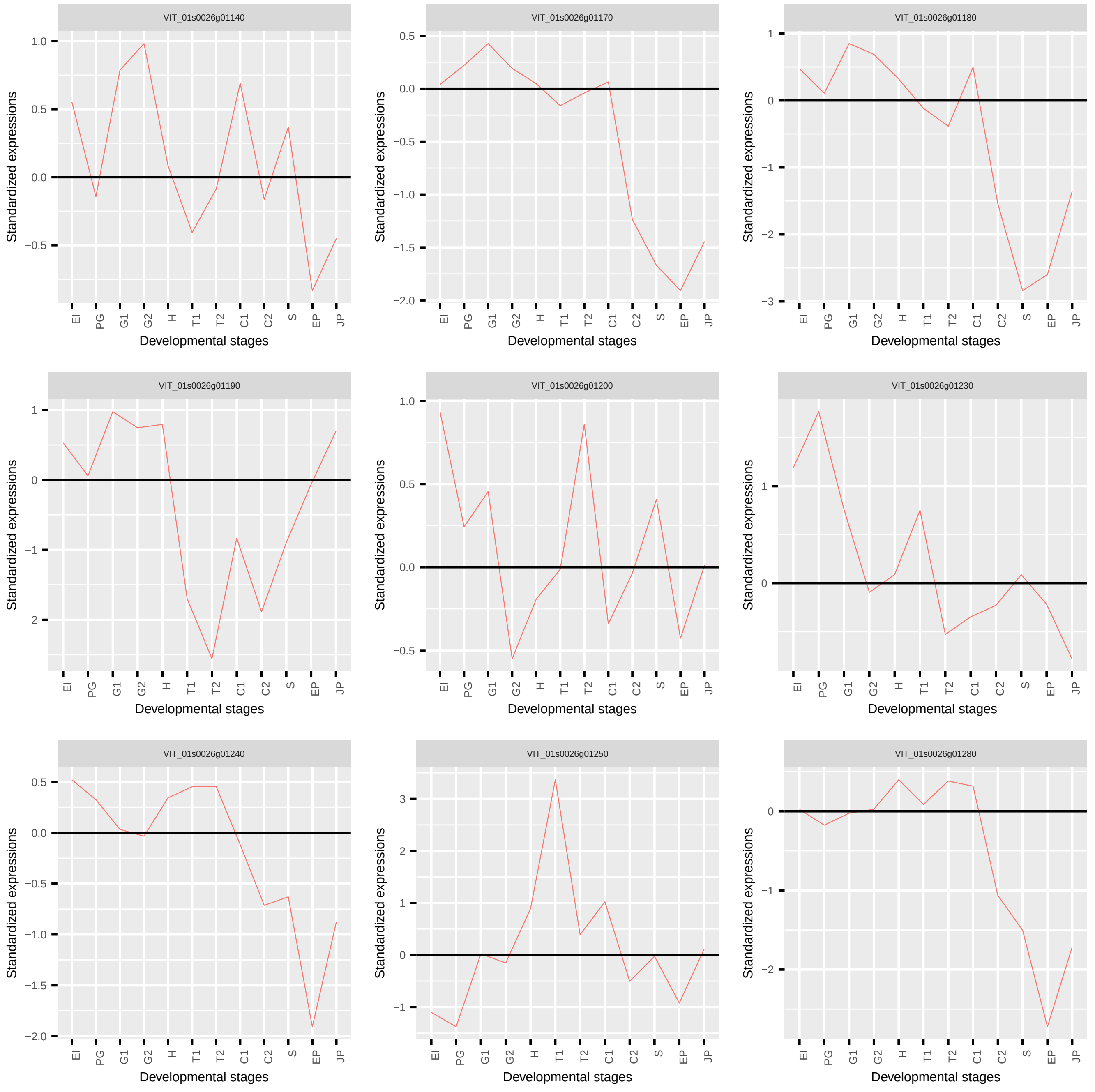

VIT_01s0026g01140
VIT_01s0026g01170
VIT_01s0026g01180
VIT_01s0026g01190
VIT_01s0026g01200
VIT_01s0026g01230
VIT_01s0026g01240
VIT_01s0026g01250
VIT_01s0026g01280
Standardized expressions
Developmental stages
EI PG G1 G2 H T1 T2 C1 C2 S EP JP

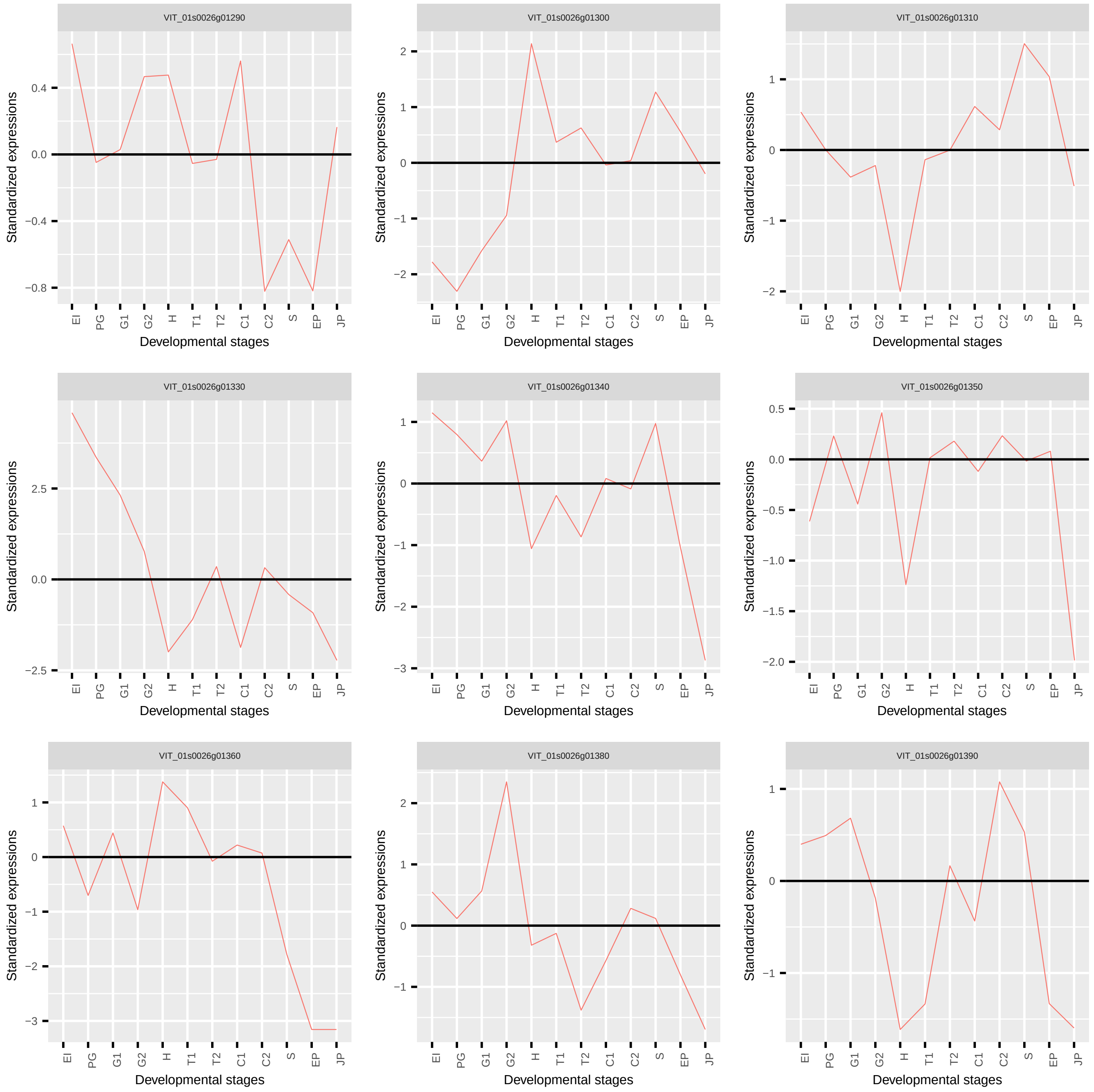
VIT_01s0026g01290
VIT_01s0026g01300
VIT_01s0026g01310
VIT_01s0026g01330
VIT_01s0026g01340
VIT_01s0026g01350
VIT_01s0026g01360
VIT_01s0026g01380
VIT_01s0026g01390
Standardized expressions
Developmental stages
EI PG G1 G2 H T1 T2 C1 C2 S EP JP

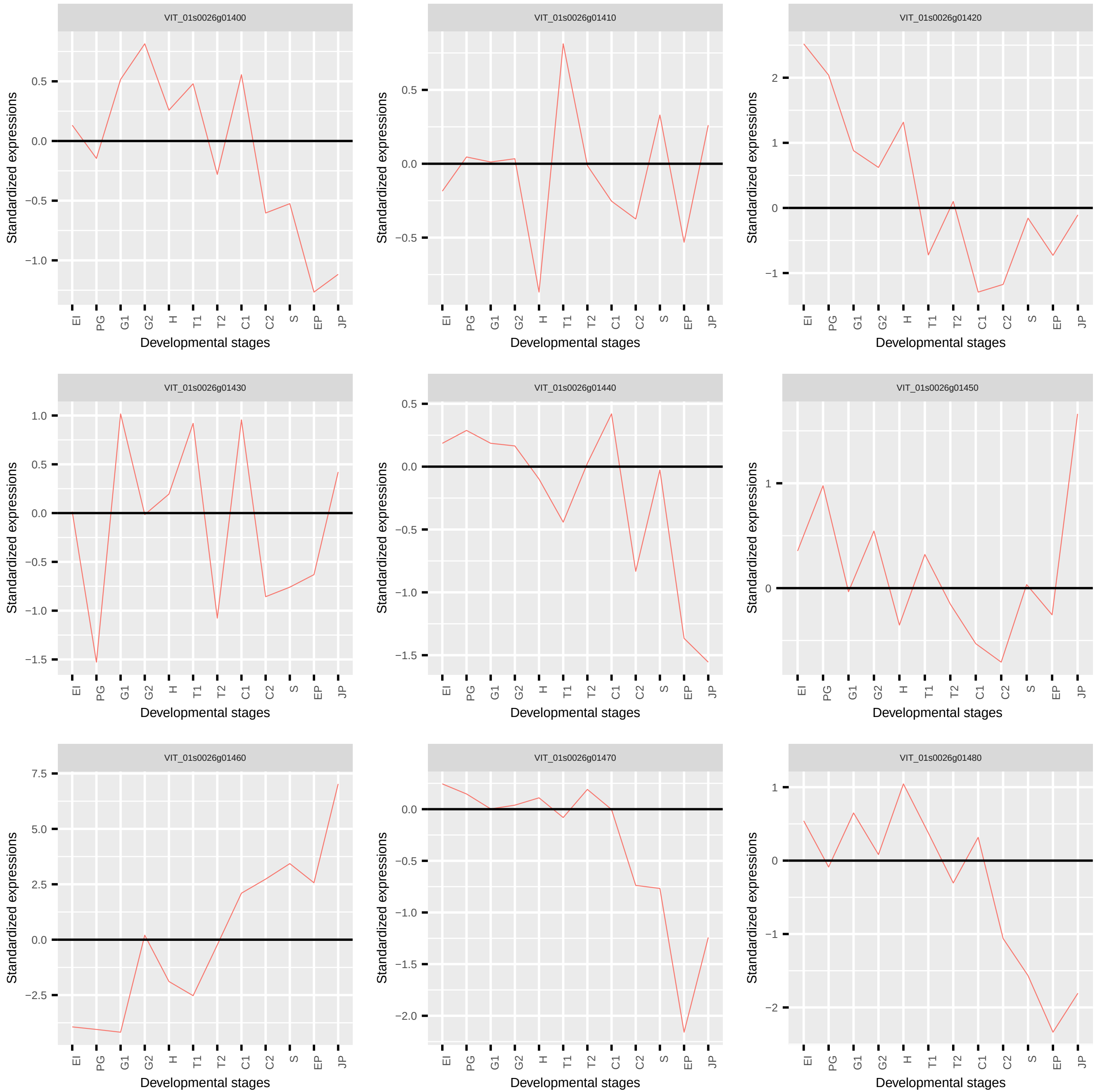
VIT_01s0026g01400
VIT_01s0026g01410
VIT_01s0026g01420
VIT_01s0026g01430
VIT_01s0026g01440
VIT_01s0026g01450
VIT_01s0026g01460
VIT_01s0026g01470
VIT_01s0026g01480
Standardized expressions
Developmental stages
EI PG G1 G2 H T1 T2 C1 C2 S EP JP

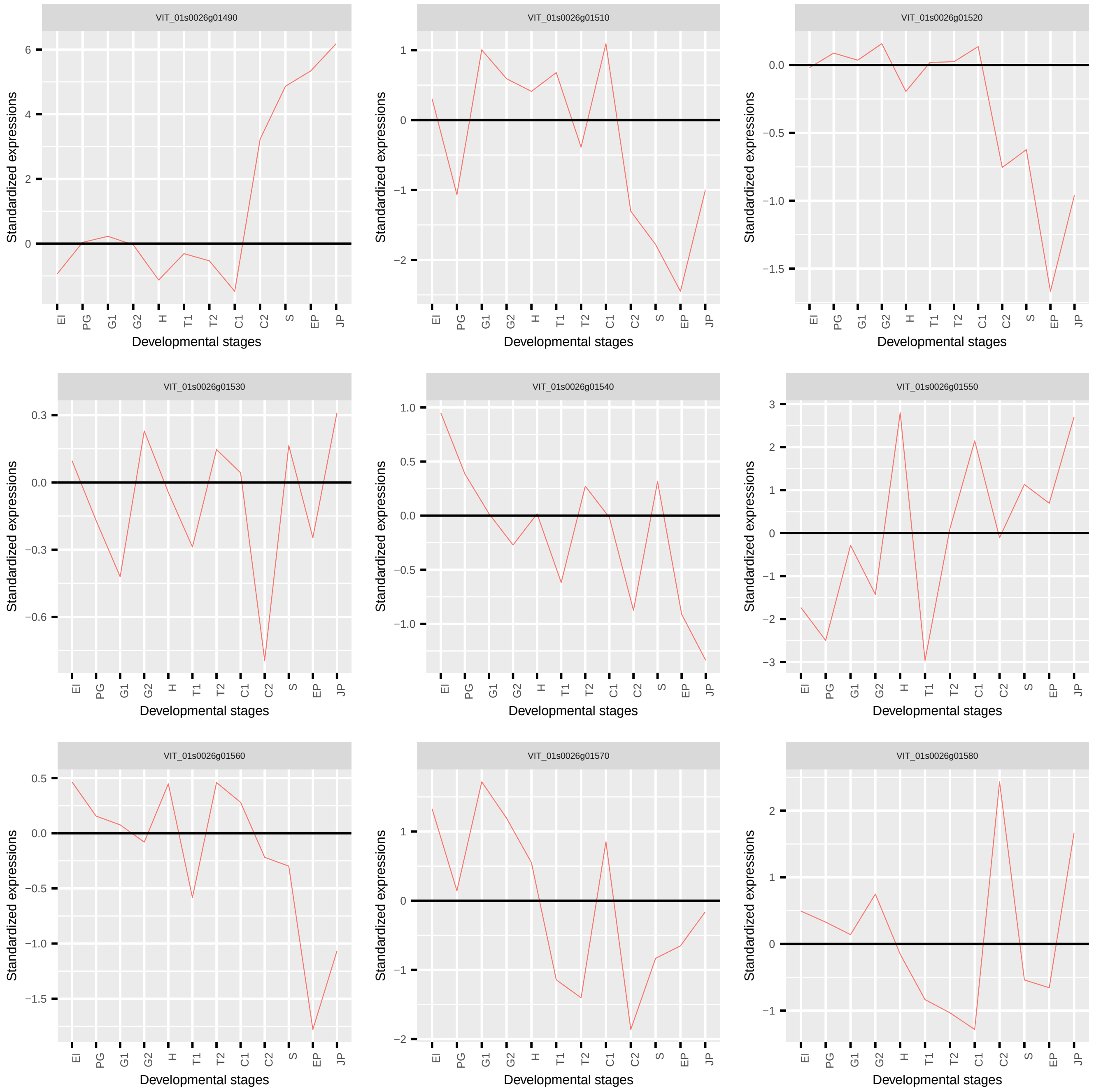
VIT_01s0026g01490
VIT_01s0026g01510
VIT_01s0026g01520
VIT_01s0026g01530
VIT_01s0026g01540
VIT_01s0026g01550
VIT_01s0026g01560
VIT_01s0026g01570
VIT_01s0026g01580
Standardized expressions
Developmental stages
EI
PG
G1
G2
H
T1
T2
C1
C2
S
EP
JP

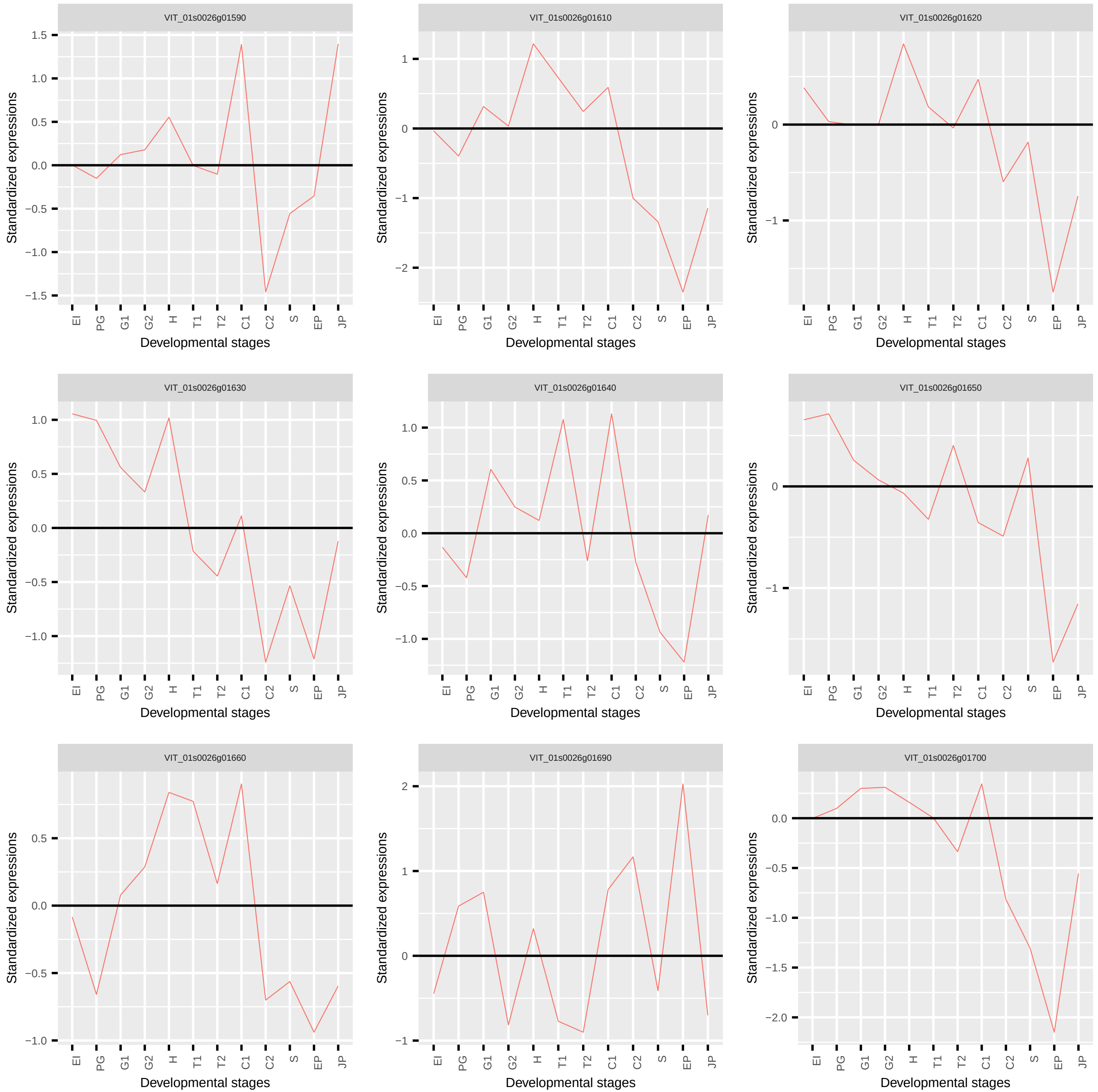

VIT_01s0026g01590
VIT_01s0026g01610
VIT_01s0026g01620
VIT_01s0026g01630
VIT_01s0026g01640
VIT_01s0026g01650
VIT_01s0026g01660
VIT_01s0026g01690
VIT_01s0026g01700
Standardized expressions
Developmental stages
EI
PG
G1
G2
H
T1
T2
C1
C2
S
EP
JP

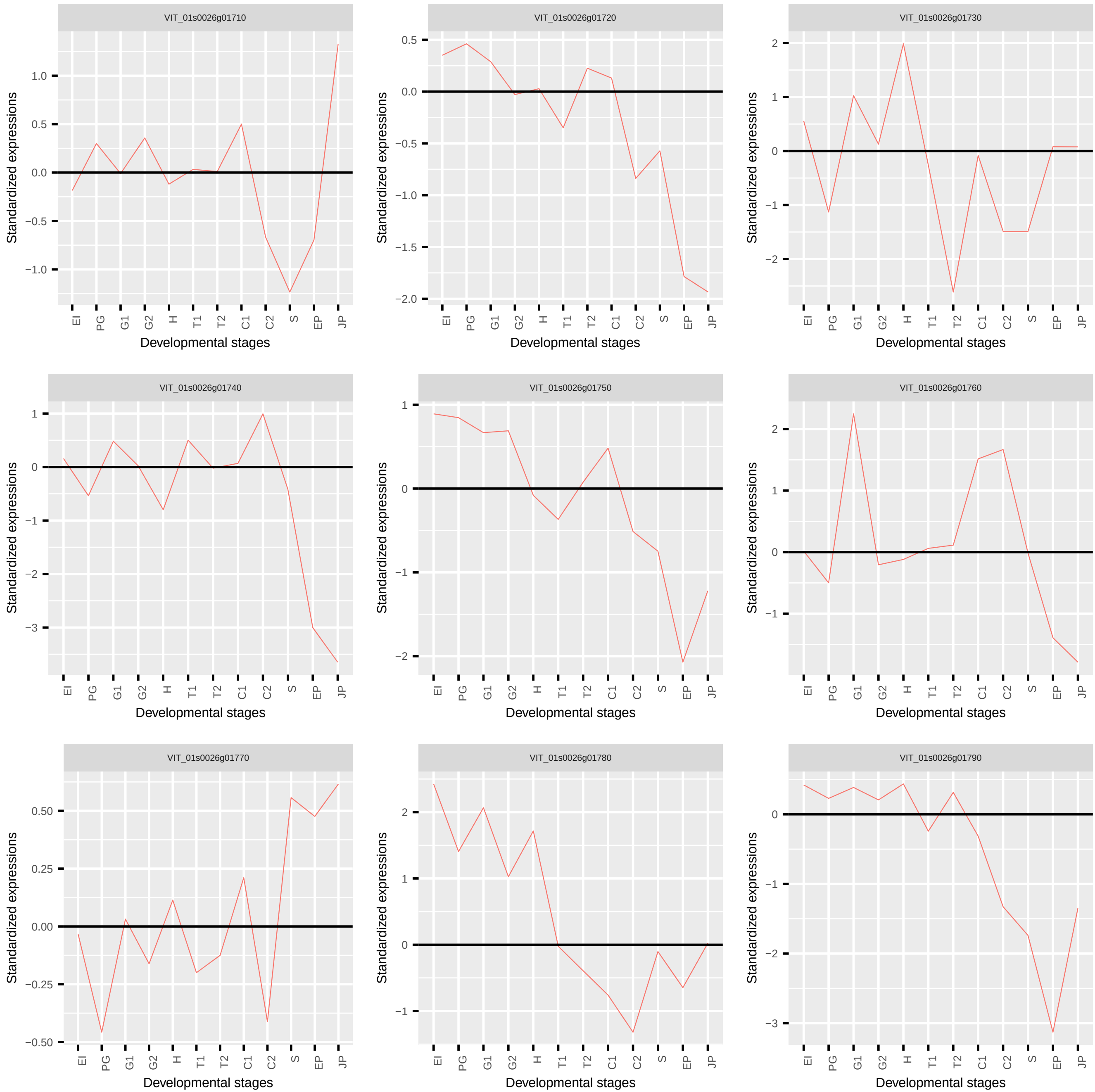
VIT_01s0026g01710
1.0
0.5
0.0
−0.5
−1.0
Standardized expressions
EI PG G1 G2 H T1 T2 C1 C2 S EP JP
Developmental stages
VIT_01s0026g01720
0.5
0.0
−0.5
−1.0
−1.5
−2.0
Standardized expressions
EI PG G1 G2 H T1 T2 C1 C2 S EP JP
Developmental stages
VIT_01s0026g01730
2
1
0
−1
−2
Standardized expressions
EI PG G1 G2 H T1 T2 C1 C2 S EP JP
Developmental stages
VIT_01s0026g01740
1
0
−1
−2
−3
Standardized expressions
EI PG G1 G2 H T1 T2 C1 C2 S EP JP
Developmental stages
VIT_01s0026g01750
1
0
−1
−2
Standardized expressions
EI PG G1 G2 H T1 T2 C1 C2 S EP JP
Developmental stages
VIT_01s0026g01760
2
1
0
−1
Standardized expressions
EI PG G1 G2 H T1 T2 C1 C2 S EP JP
Developmental stages
VIT_01s0026g01770
0.50
0.25
0.00
−0.25
−0.50
Standardized expressions
EI PG G1 G2 H T1 T2 C1 C2 S EP JP
Developmental stages
VIT_01s0026g01780
2
1
0
−1
Standardized expressions
EI PG G1 G2 H T1 T2 C1 C2 S EP JP
Developmental stages
VIT_01s0026g01790
0
−1
−2
−3
Standardized expressions
EI PG G1 G2 H T1 T2 C1 C2 S EP JP
Developmental stages

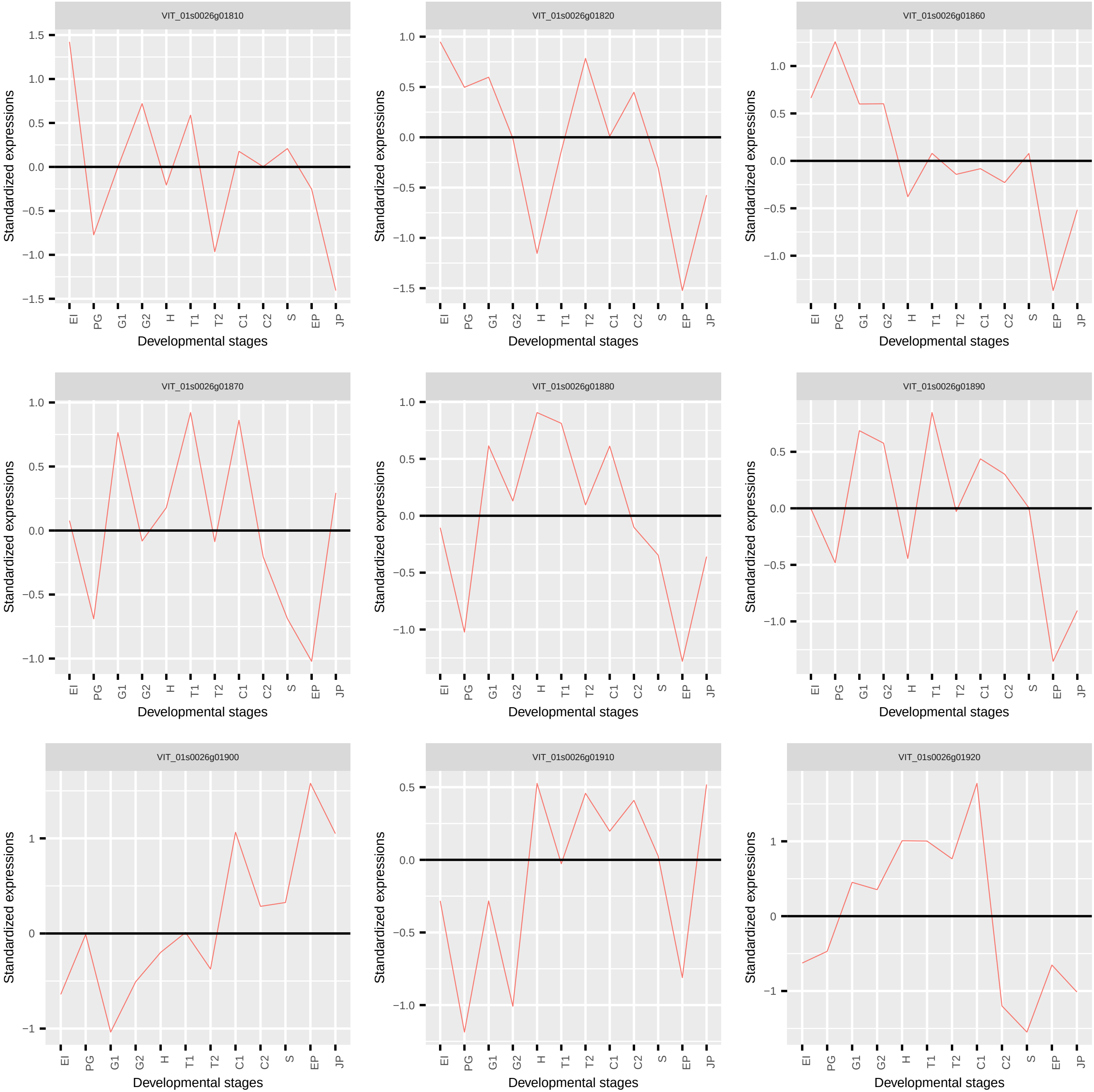
VIT_01s0026g01810
VIT_01s0026g01820
VIT_01s0026g01860
VIT_01s0026g01870
VIT_01s0026g01880
VIT_01s0026g01890
VIT_01s0026g01900
VIT_01s0026g01910
VIT_01s0026g01920
Standardized expressions
Developmental stages
EI PG G1 G2 H T1 T2 C1 C2 S EP JP

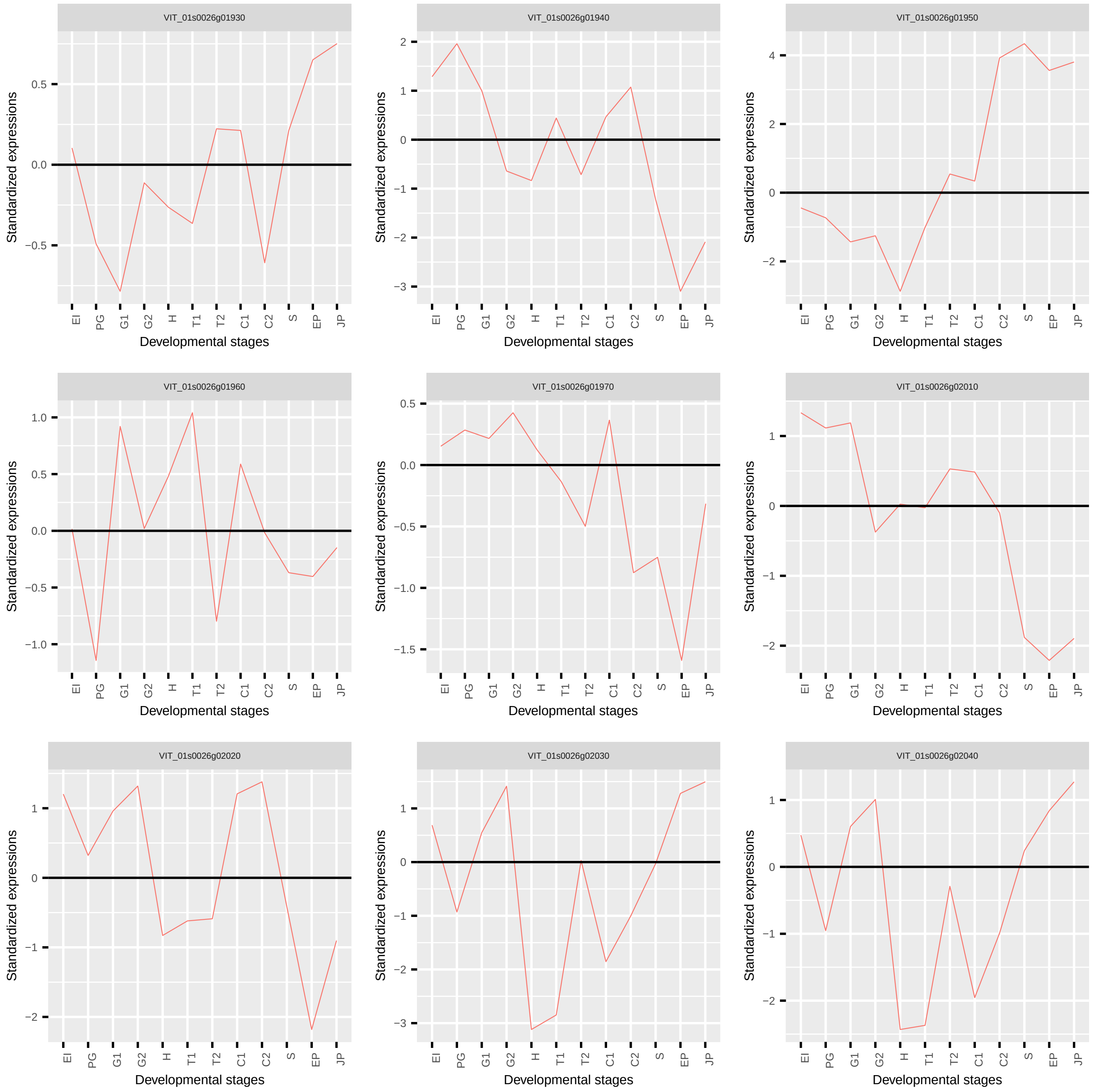
VIT_01s0026g01930
VIT_01s0026g01940
VIT_01s0026g01950
VIT_01s0026g01960
VIT_01s0026g01970
VIT_01s0026g02010
VIT_01s0026g02020
VIT_01s0026g02030
VIT_01s0026g02040
Standardized expressions
Developmental stages
EI PG G1 G2 H T1 T2 C1 C2 S EP JP

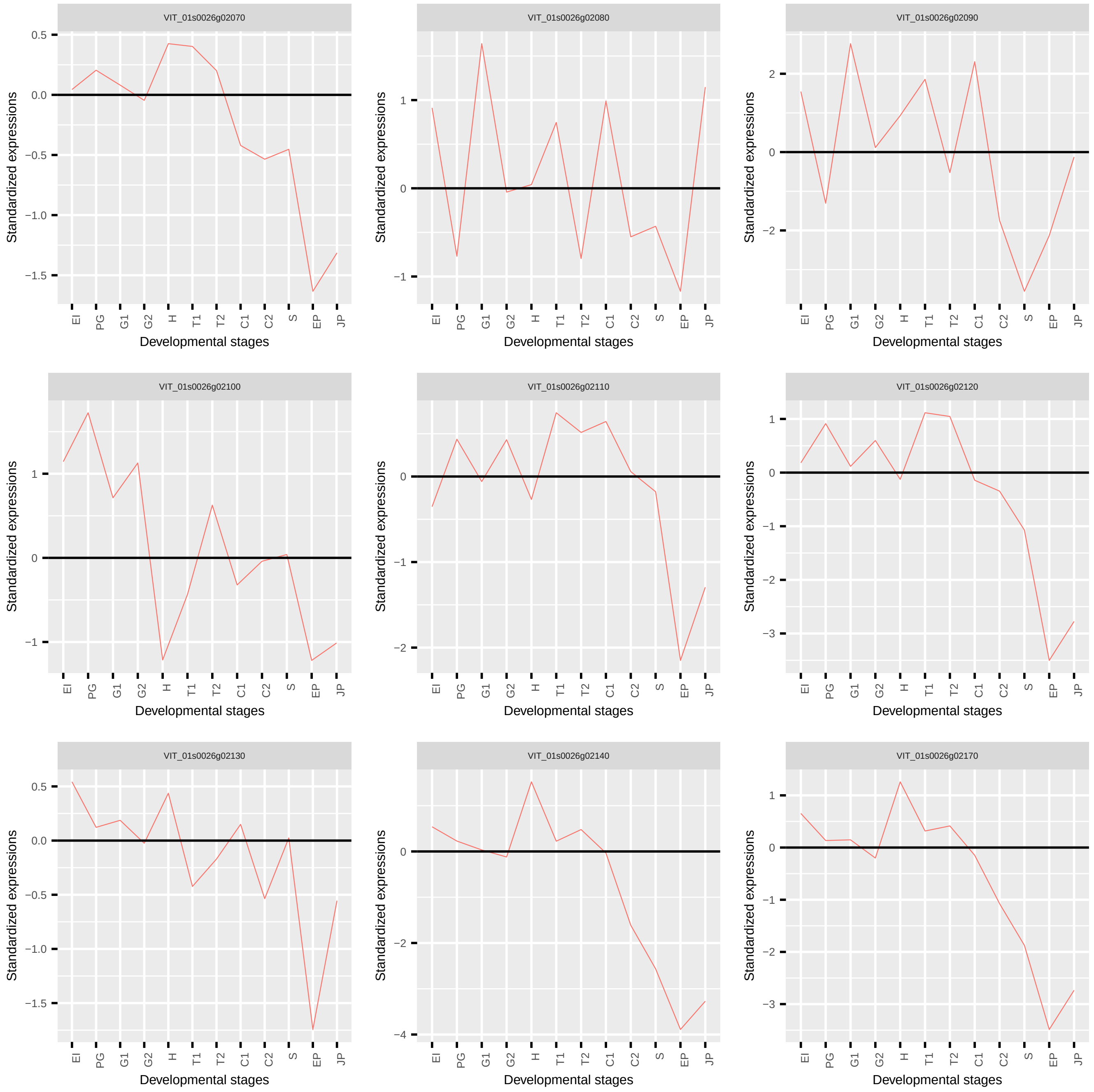
VIT_01s0026g02070
VIT_01s0026g02080
VIT_01s0026g02090
VIT_01s0026g02100
VIT_01s0026g02110
VIT_01s0026g02120
VIT_01s0026g02130
VIT_01s0026g02140
VIT_01s0026g02170
Standardized expressions
Developmental stages
EI PG G1 G2 H T1 T2 C1 C2 S EP JP
0.5 0.0 −0.5 −1.0 −1.5
1 0 −1
2 0 −2
1 0 −1 −2
1 0 −1 −2 −3
0 −2 −4

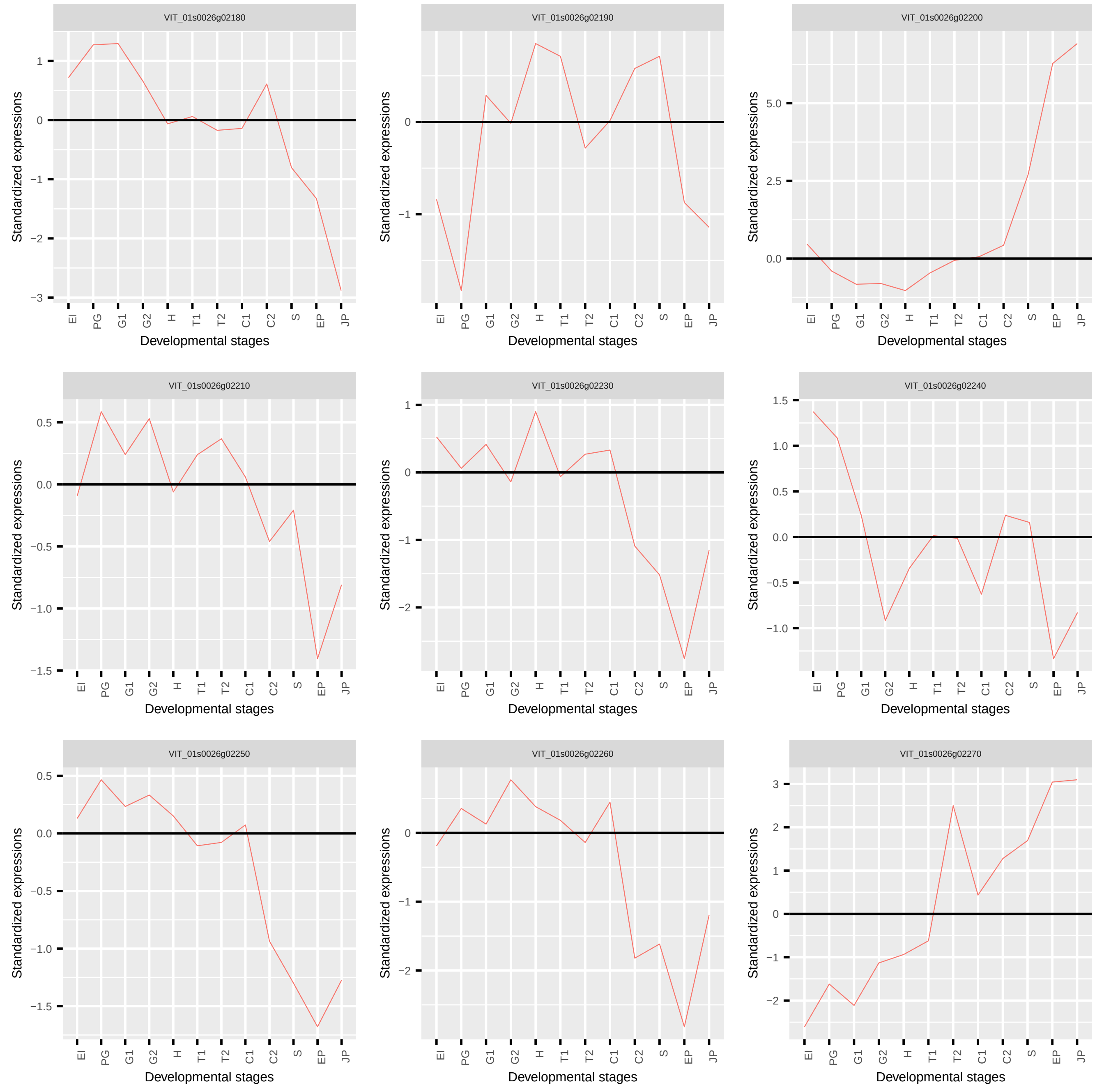
VIT_01s0026g02180
VIT_01s0026g02190
VIT_01s0026g02200
VIT_01s0026g02210
VIT_01s0026g02230
VIT_01s0026g02240
VIT_01s0026g02250
VIT_01s0026g02260
VIT_01s0026g02270
Standardized expressions
Developmental stages
EI
PG
G1
G2
H
T1
T2
C1
C2
S
EP
JP

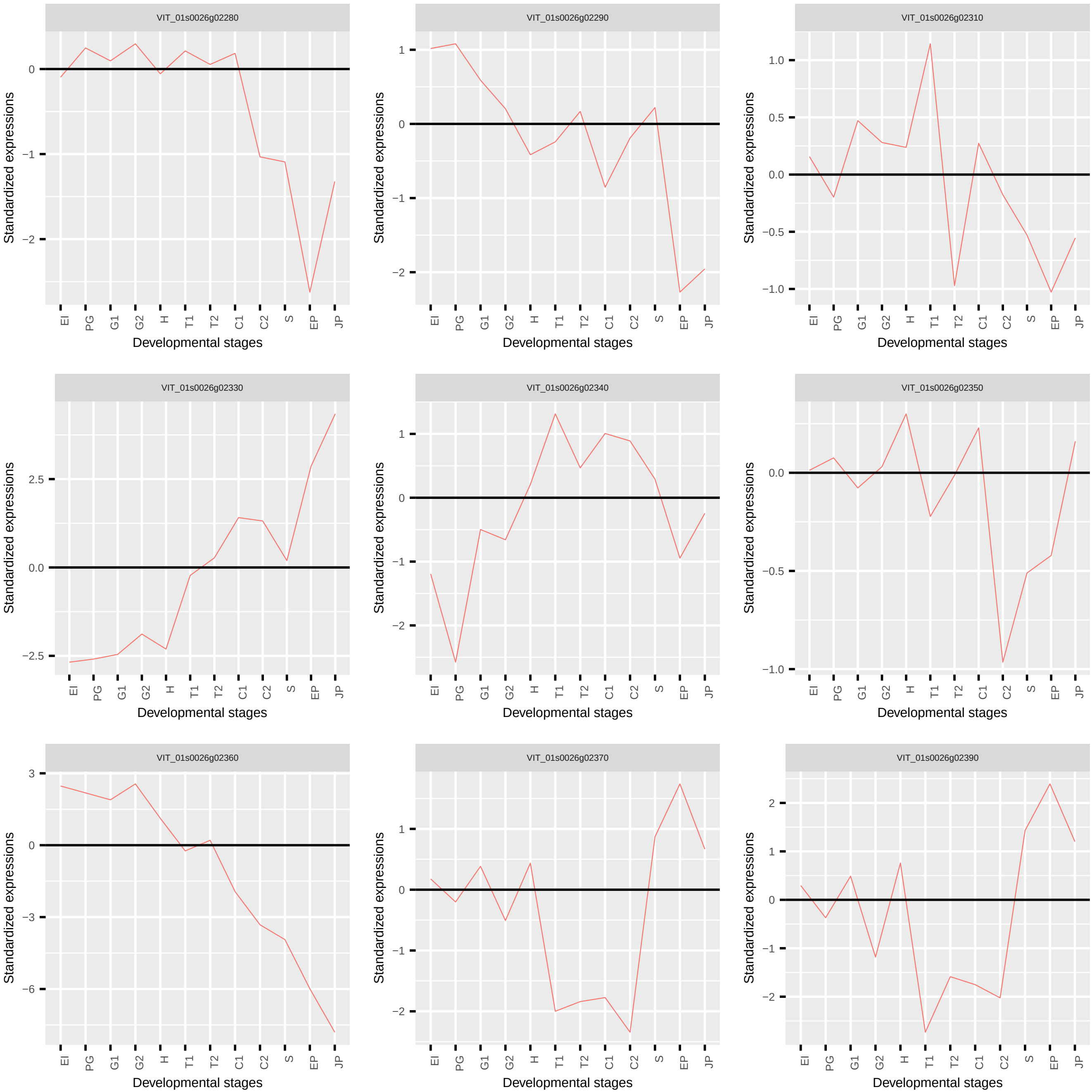
VIT_01s0026g02280
Standardized expressions
0
−1
−2
EI PG G1 G2 H T1 T2 C1 C2 S EP JP
Developmental stages
VIT_01s0026g02290
Standardized expressions
1
0
−1
−2
EI PG G1 G2 H T1 T2 C1 C2 S EP JP
Developmental stages
VIT_01s0026g02310
Standardized expressions
1.0
0.5
0.0
−0.5
−1.0
EI PG G1 G2 H T1 T2 C1 C2 S EP JP
Developmental stages
VIT_01s0026g02330
Standardized expressions
2.5
0.0
−2.5
EI PG G1 G2 H T1 T2 C1 C2 S EP JP
Developmental stages
VIT_01s0026g02340
Standardized expressions
1
0
−1
−2
EI PG G1 G2 H T1 T2 C1 C2 S EP JP
Developmental stages
VIT_01s0026g02350
Standardized expressions
0.0
−0.5
−1.0
EI PG G1 G2 H T1 T2 C1 C2 S EP JP
Developmental stages
VIT_01s0026g02360
Standardized expressions
3
0
−3
−6
EI PG G1 G2 H T1 T2 C1 C2 S EP JP
Developmental stages
VIT_01s0026g02370
Standardized expressions
1
0
−1
−2
EI PG G1 G2 H T1 T2 C1 C2 S EP JP
Developmental stages
VIT_01s0026g02390
Standardized expressions
2
1
0
−1
−2
EI PG G1 G2 H T1 T2 C1 C2 S EP JP
Developmental stages

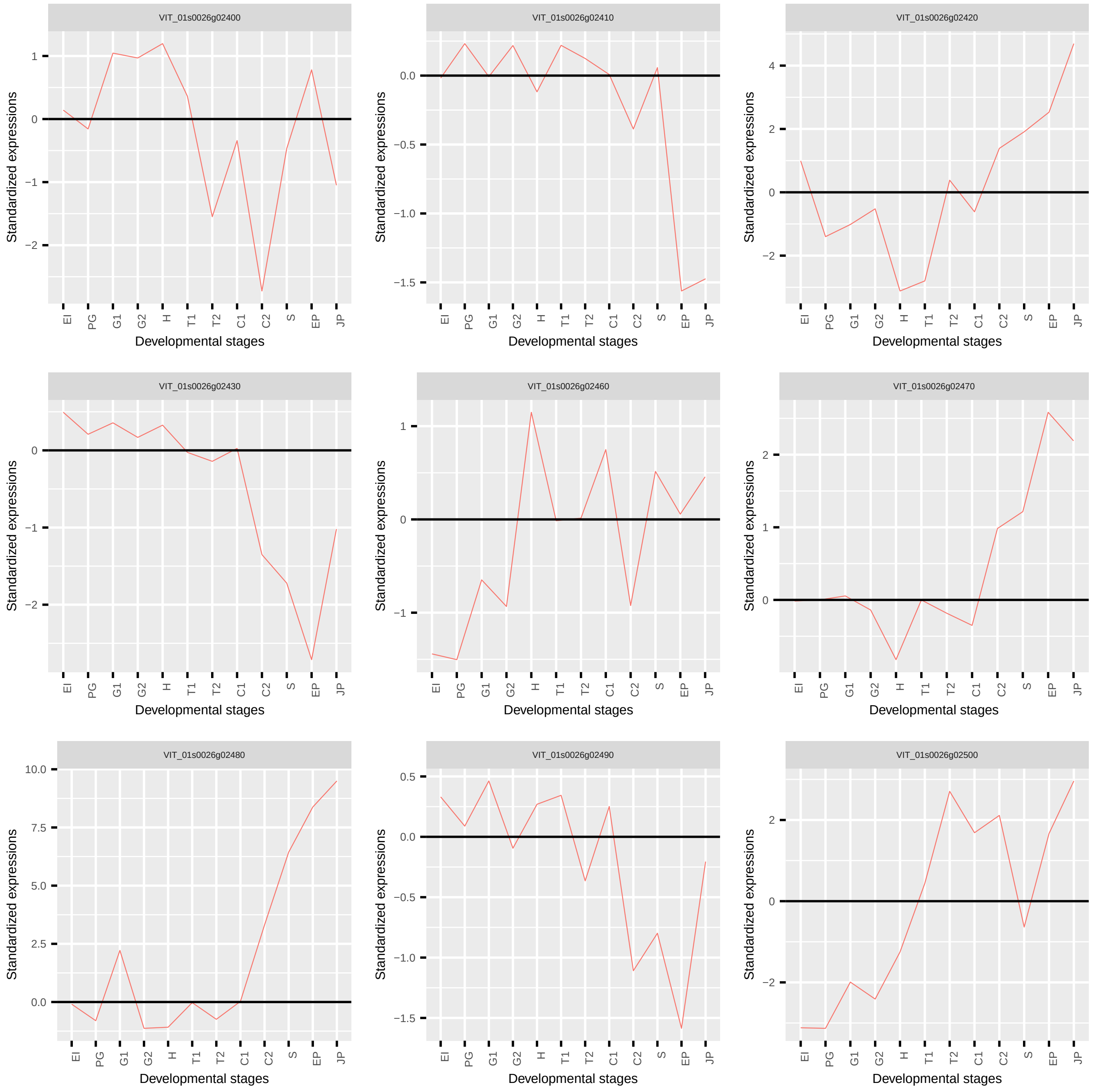
VIT_01s0026g02400
VIT_01s0026g02410
VIT_01s0026g02420
VIT_01s0026g02430
VIT_01s0026g02460
VIT_01s0026g02470
VIT_01s0026g02480
VIT_01s0026g02490
VIT_01s0026g02500
Standardized expressions
Developmental stages
EI PG G1 G2 H T1 T2 C1 C2 S EP JP

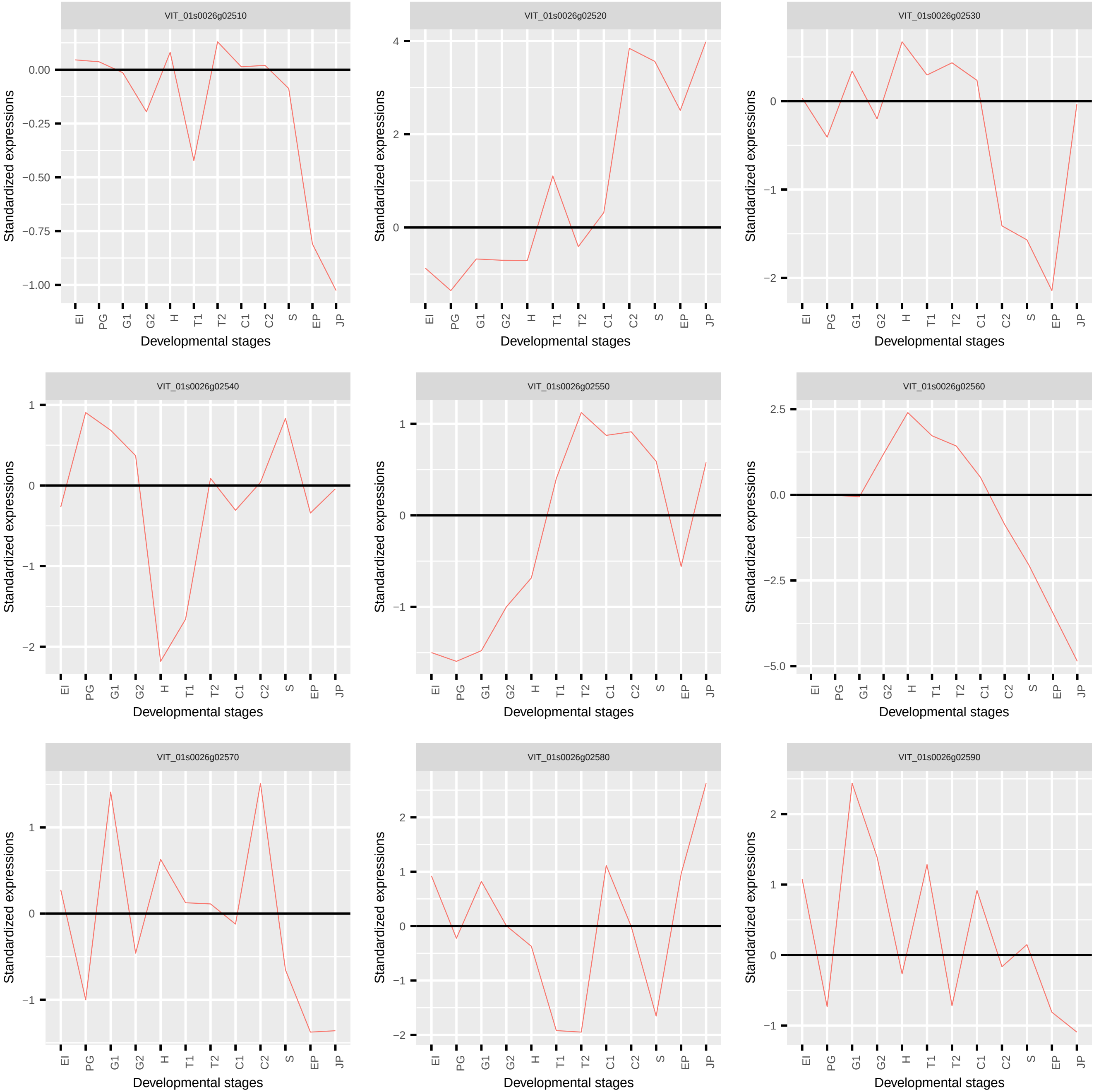
VIT_01s0026g02510
VIT_01s0026g02520
VIT_01s0026g02530
VIT_01s0026g02540
VIT_01s0026g02550
VIT_01s0026g02560
VIT_01s0026g02570
VIT_01s0026g02580
VIT_01s0026g02590
Standardized expressions
Developmental stages
EI PG G1 G2 H T1 T2 C1 C2 S EP JP
0.00 −0.25 −0.50 −0.75 −1.00
4 2 0
0 −1 −2
1 0 −1 −2
1 0 −1
2.5 0.0 −2.5 −5.0
1 0 −1
2 1 0 −1 −2
2 1 0 −1

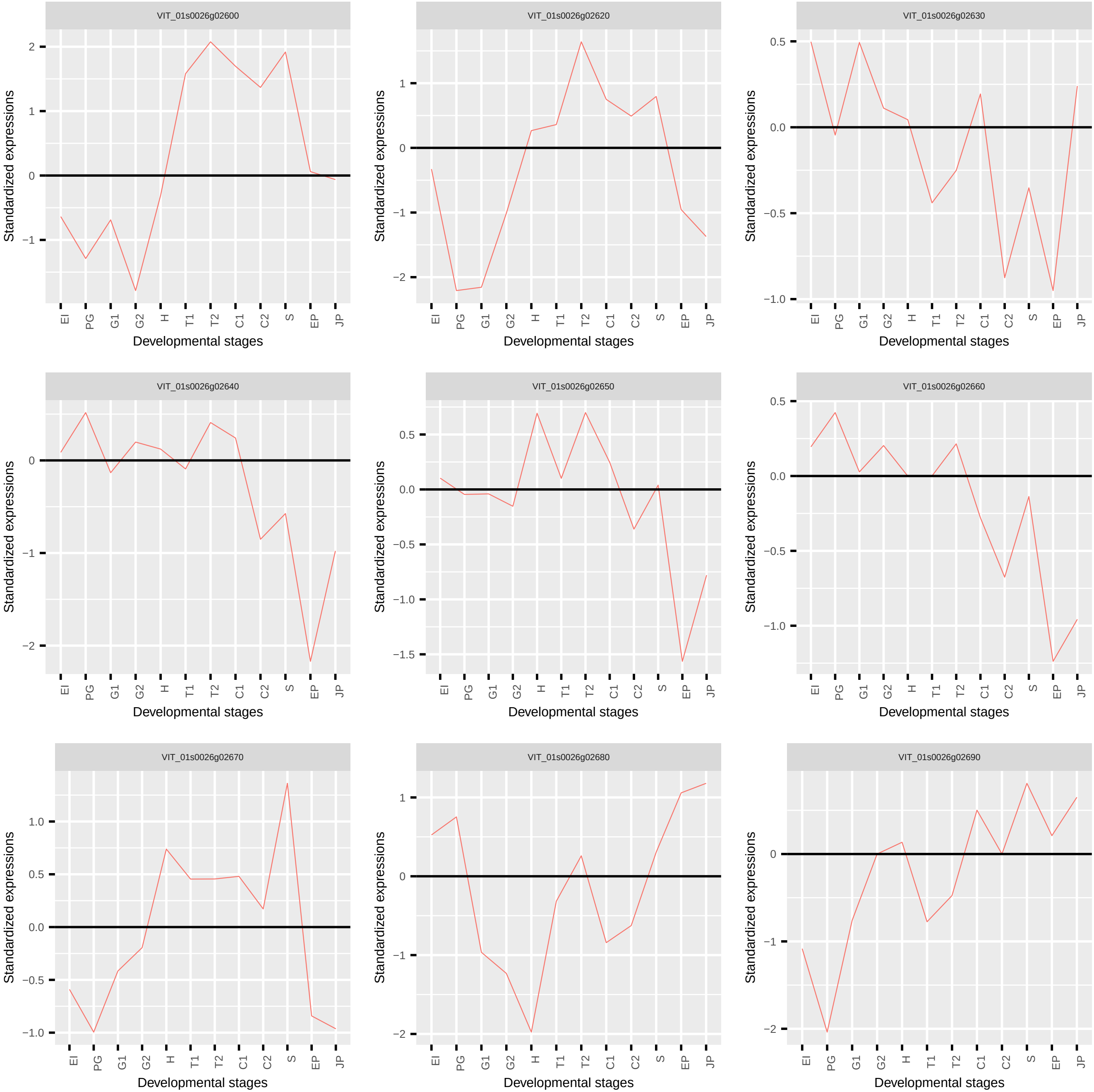
VIT_01s0026g02600
Standardized expressions
2
1
0
−1
EI
PG
G1
G2
H
T1
T2
C1
C2
S
EP
JP
Developmental stages
VIT_01s0026g02620
Standardized expressions
1
0
−1
−2
EI
PG
G1
G2
H
T1
T2
C1
C2
S
EP
JP
Developmental stages
VIT_01s0026g02630
Standardized expressions
0.5
0.0
−0.5
−1.0
EI
PG
G1
G2
H
T1
T2
C1
C2
S
EP
JP
Developmental stages
VIT_01s0026g02640
Standardized expressions
0
−1
−2
EI
PG
G1
G2
H
T1
T2
C1
C2
S
EP
JP
Developmental stages
VIT_01s0026g02650
Standardized expressions
0.5
0.0
−0.5
−1.0
−1.5
EI
PG
G1
G2
H
T1
T2
C1
C2
S
EP
JP
Developmental stages
VIT_01s0026g02660
Standardized expressions
0.5
0.0
−0.5
−1.0
EI
PG
G1
G2
H
T1
T2
C1
C2
S
EP
JP
Developmental stages
VIT_01s0026g02670
Standardized expressions
1.0
0.5
0.0
−0.5
−1.0
EI
PG
G1
G2
H
T1
T2
C1
C2
S
EP
JP
Developmental stages
VIT_01s0026g02680
Standardized expressions
1
0
−1
−2
EI
PG
G1
G2
H
T1
T2
C1
C2
S
EP
JP
Developmental stages
VIT_01s0026g02690
Standardized expressions
0
−1
−2
EI
PG
G1
G2
H
T1
T2
C1
C2
S
EP
JP
Developmental stages

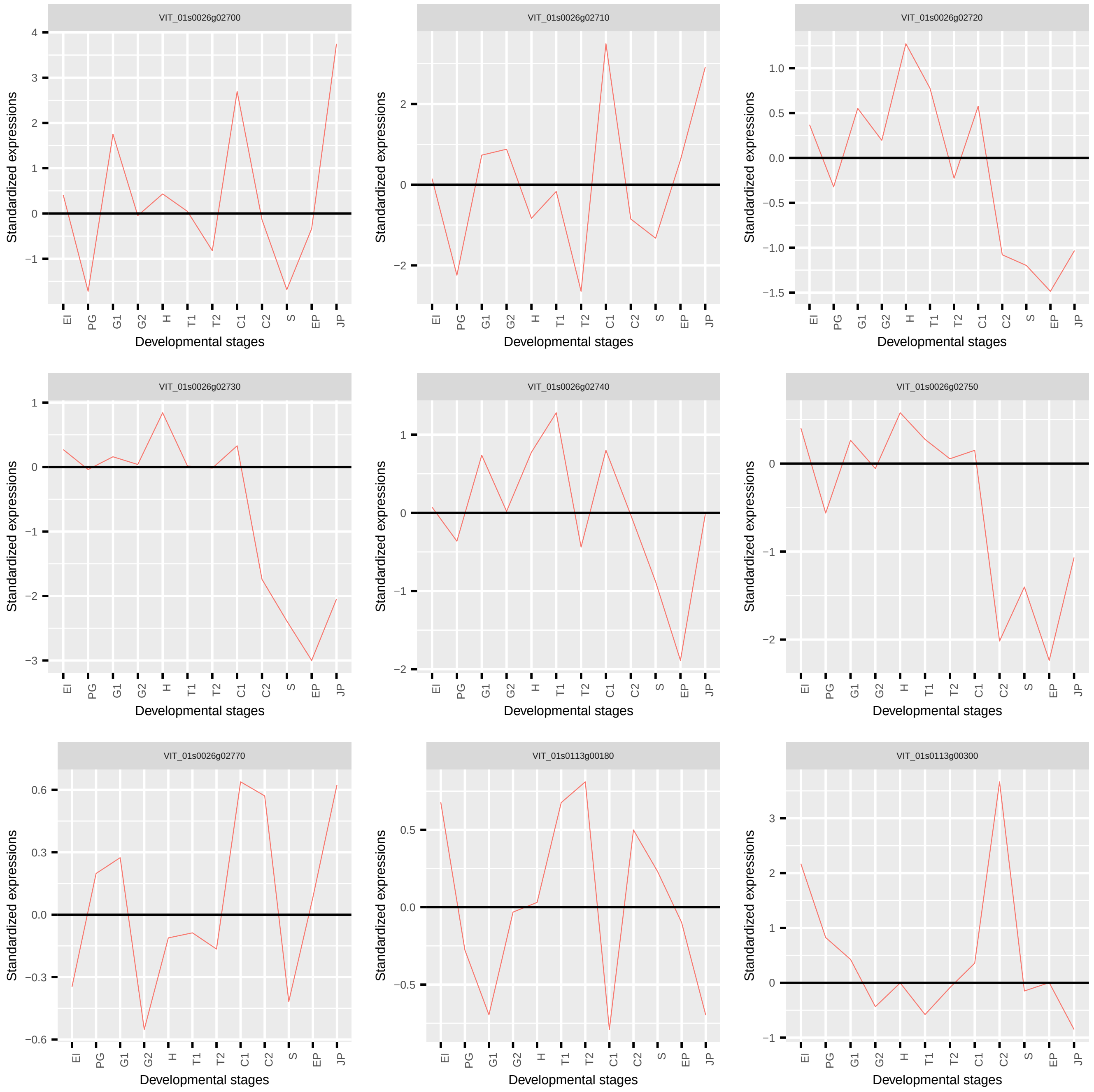
VIT_01s0026g02700
VIT_01s0026g02710
VIT_01s0026g02720
VIT_01s0026g02730
VIT_01s0026g02740
VIT_01s0026g02750
VIT_01s0026g02770
VIT_01s0113g00180
VIT_01s0113g00300
Standardized expressions
Developmental stages
EI PG G1 G2 H T1 T2 C1 C2 S EP JP

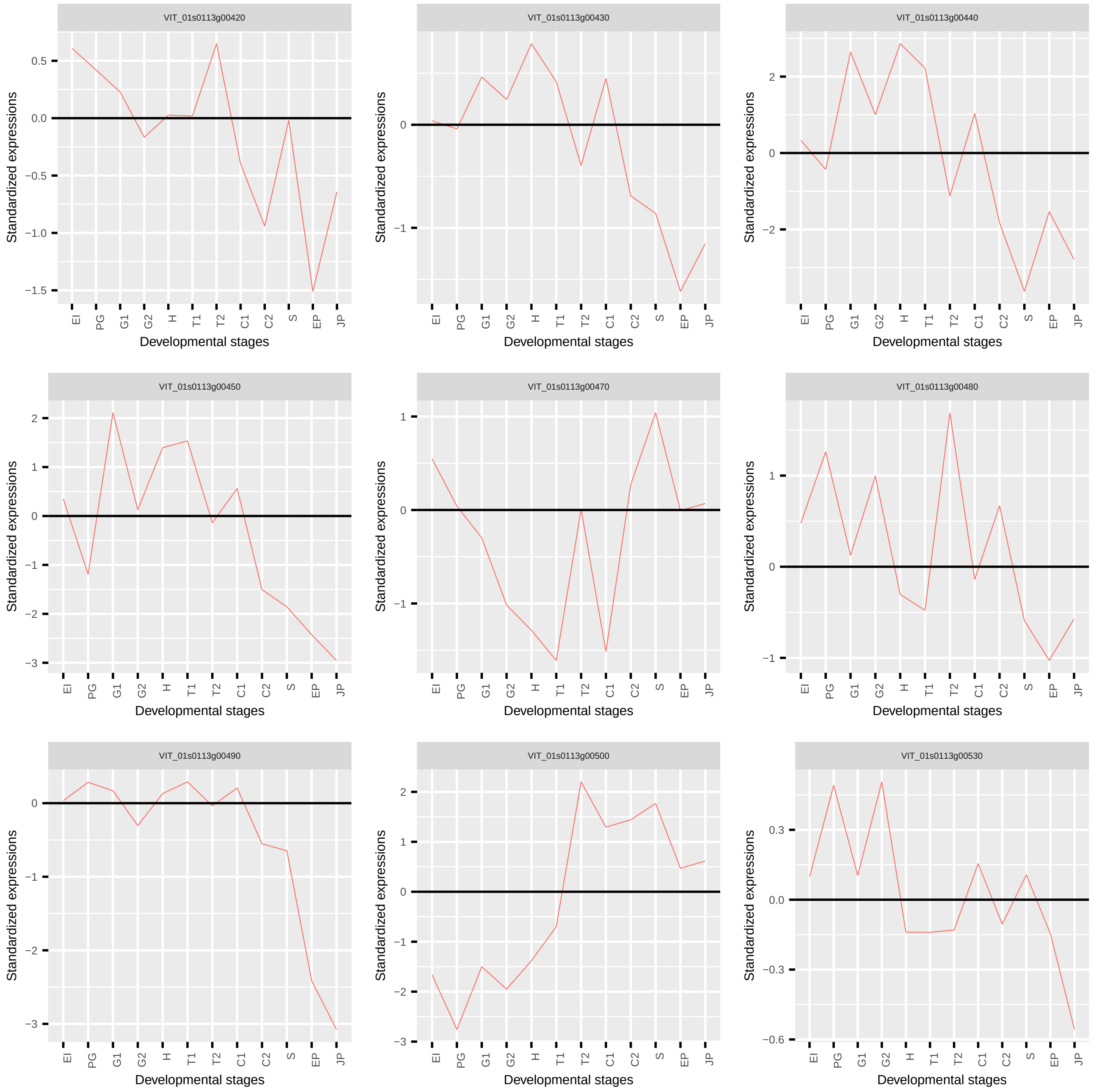
VIT_01s0113g00420
Standardized expressions
0.5
0.0
−0.5
−1.0
−1.5
EI PG G1 G2 H T1 T2 C1 C2 S EP JP
Developmental stages
VIT_01s0113g00430
Standardized expressions
0
−1
EI PG G1 G2 H T1 T2 C1 C2 S EP JP
Developmental stages
VIT_01s0113g00440
Standardized expressions
2
0
−2
EI PG G1 G2 H T1 T2 C1 C2 S EP JP
Developmental stages
VIT_01s0113g00450
Standardized expressions
2
1
0
−1
−2
−3
EI PG G1 G2 H T1 T2 C1 C2 S EP JP
Developmental stages
VIT_01s0113g00470
Standardized expressions
1
0
−1
EI PG G1 G2 H T1 T2 C1 C2 S EP JP
Developmental stages
VIT_01s0113g00480
Standardized expressions
1
0
−1
EI PG G1 G2 H T1 T2 C1 C2 S EP JP
Developmental stages
VIT_01s0113g00490
Standardized expressions
0
−1
−2
−3
EI PG G1 G2 H T1 T2 C1 C2 S EP JP
Developmental stages
VIT_01s0113g00500
Standardized expressions
2
1
0
−1
−2
−3
EI PG G1 G2 H T1 T2 C1 C2 S EP JP
Developmental stages
VIT_01s0113g00530
Standardized expressions
0.3
0.0
−0.3
−0.6
EI PG G1 G2 H T1 T2 C1 C2 S EP JP
Developmental stages

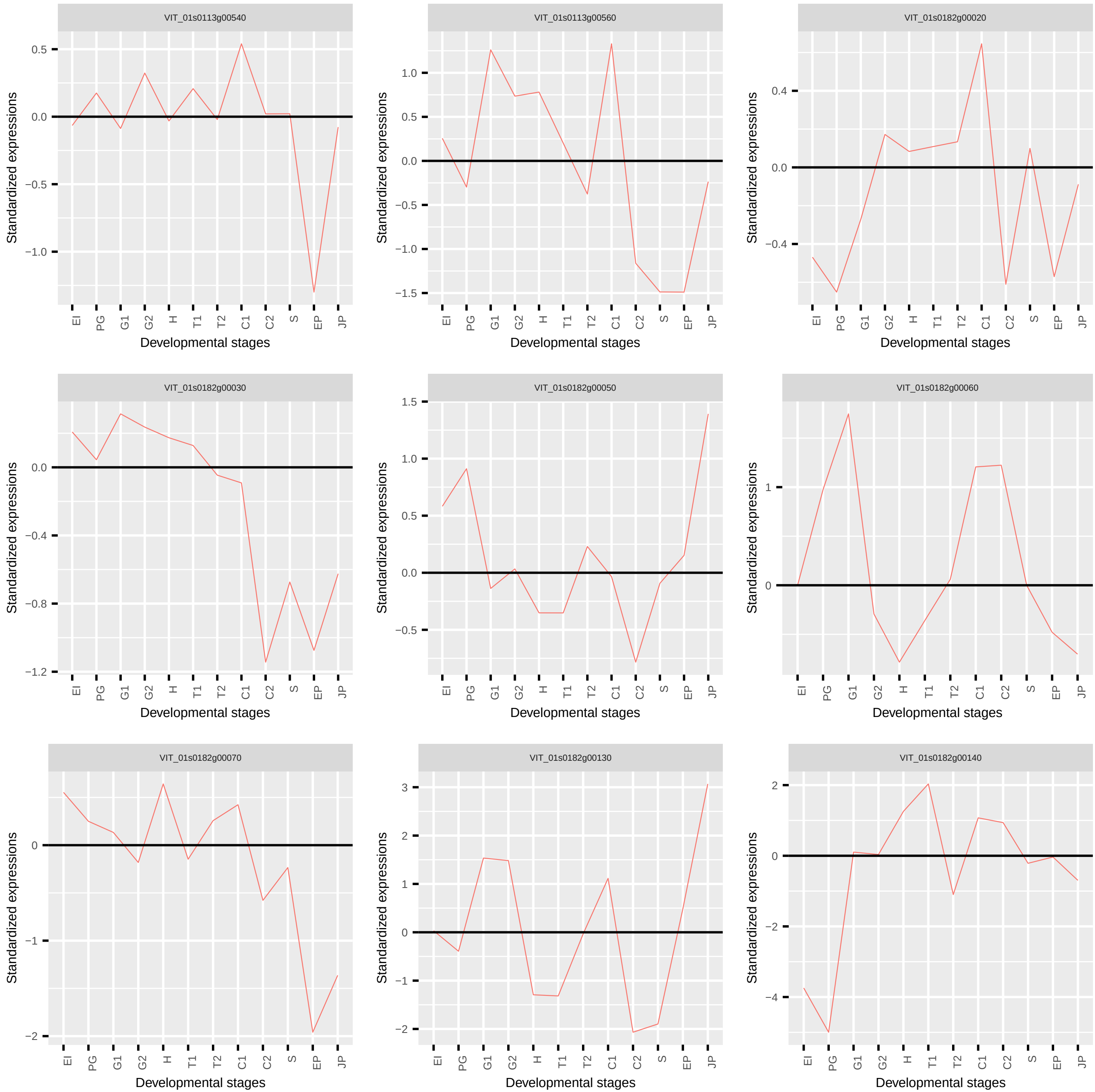

VIT_01s0113g00540
VIT_01s0113g00560
VIT_01s0182g00020
VIT_01s0182g00030
VIT_01s0182g00050
VIT_01s0182g00060
VIT_01s0182g00070
VIT_01s0182g00130
VIT_01s0182g00140
Standardized expressions
Developmental stages
EI
PG
G1
G2
H
T1
T2
C1
C2
S
EP
JP
0.5
0.0
−0.5
−1.0
−1.5
1.0
0.4
−0.4
−0.8
−1.2
1.5
1
0
−1
−2
3
2
−4

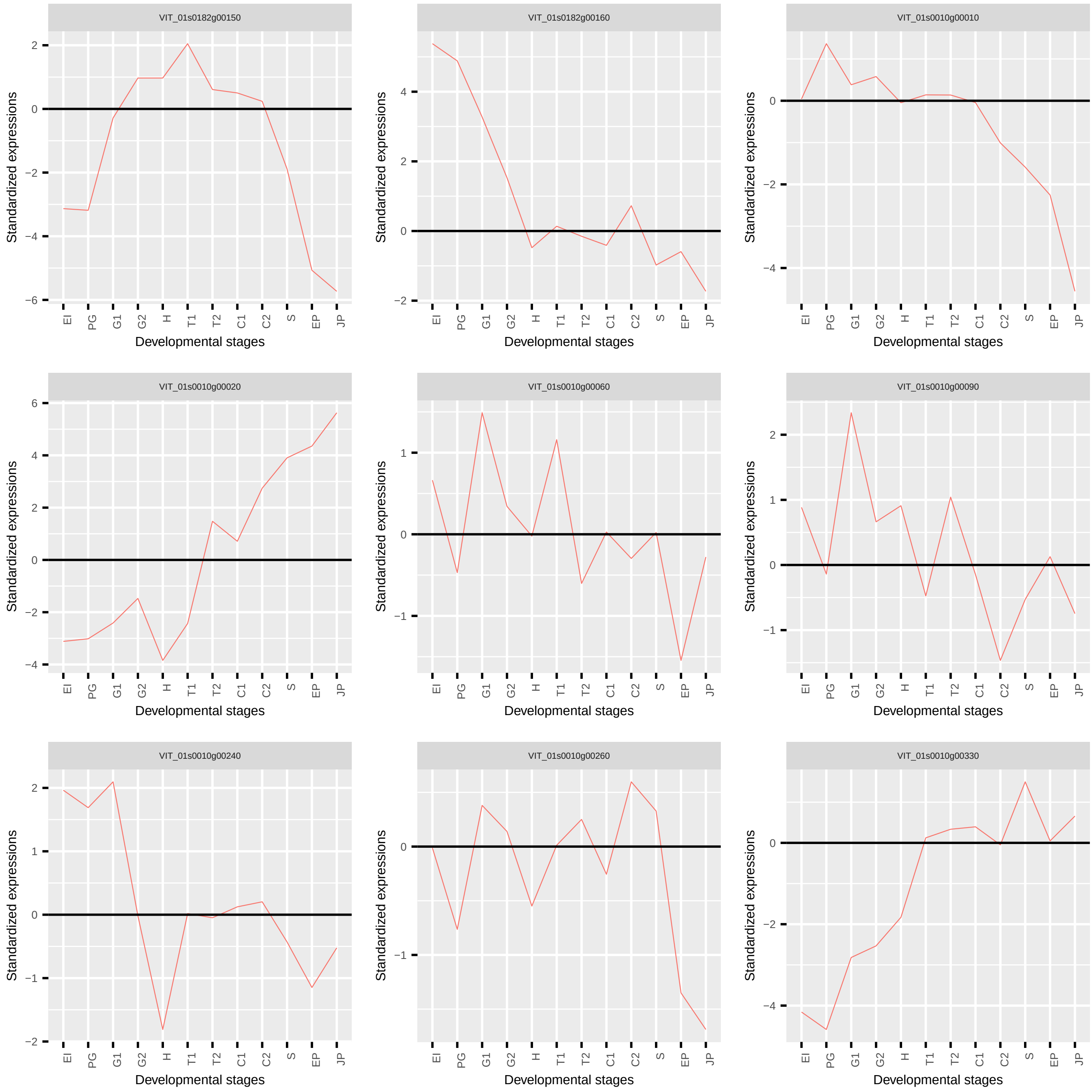
VIT_01s0182g00150
Standardized expressions
2
0
−2
−4
−6
EI PG G1 G2 H T1 T2 C1 C2 S EP JP
Developmental stages
VIT_01s0182g00160
Standardized expressions
4
2
0
−2
EI PG G1 G2 H T1 T2 C1 C2 S EP JP
Developmental stages
VIT_01s0010g00010
Standardized expressions
0
−2
−4
EI PG G1 G2 H T1 T2 C1 C2 S EP JP
Developmental stages
VIT_01s0010g00020
Standardized expressions
6
4
2
0
−2
−4
EI PG G1 G2 H T1 T2 C1 C2 S EP JP
Developmental stages
VIT_01s0010g00060
Standardized expressions
1
0
−1
EI PG G1 G2 H T1 T2 C1 C2 S EP JP
Developmental stages
VIT_01s0010g00090
Standardized expressions
2
1
0
−1
EI PG G1 G2 H T1 T2 C1 C2 S EP JP
Developmental stages
VIT_01s0010g00240
Standardized expressions
2
1
0
−1
−2
EI PG G1 G2 H T1 T2 C1 C2 S EP JP
Developmental stages
VIT_01s0010g00260
Standardized expressions
0
−1
EI PG G1 G2 H T1 T2 C1 C2 S EP JP
Developmental stages
VIT_01s0010g00330
Standardized expressions
0
−2
−4
EI PG G1 G2 H T1 T2 C1 C2 S EP JP
Developmental stages

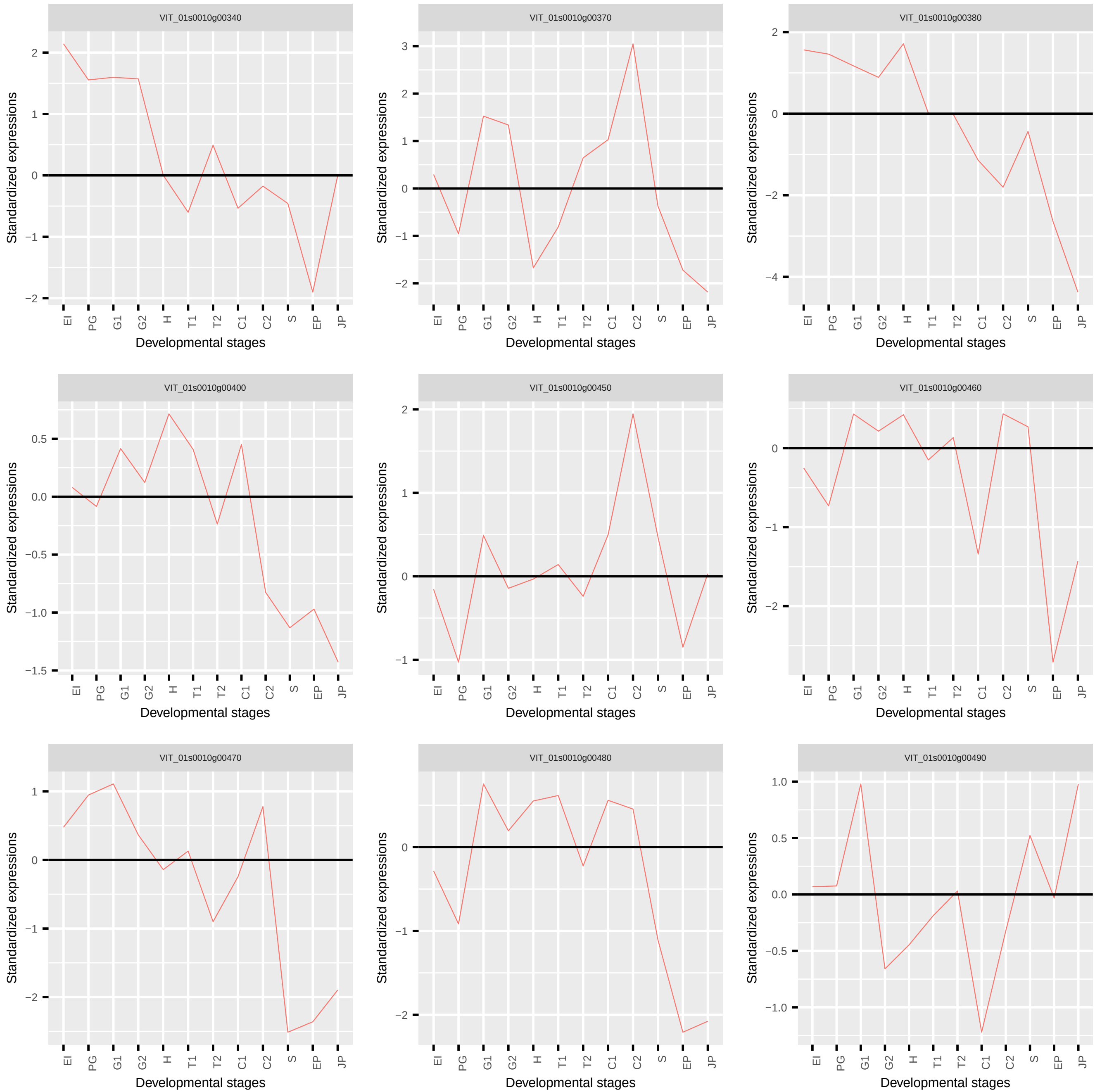
VIT_01s0010g00340
VIT_01s0010g00370
VIT_01s0010g00380
VIT_01s0010g00400
VIT_01s0010g00450
VIT_01s0010g00460
VIT_01s0010g00470
VIT_01s0010g00480
VIT_01s0010g00490
Standardized expressions
Developmental stages
EI
PG
G1
G2
H
T1
T2
C1
C2
S
EP
JP

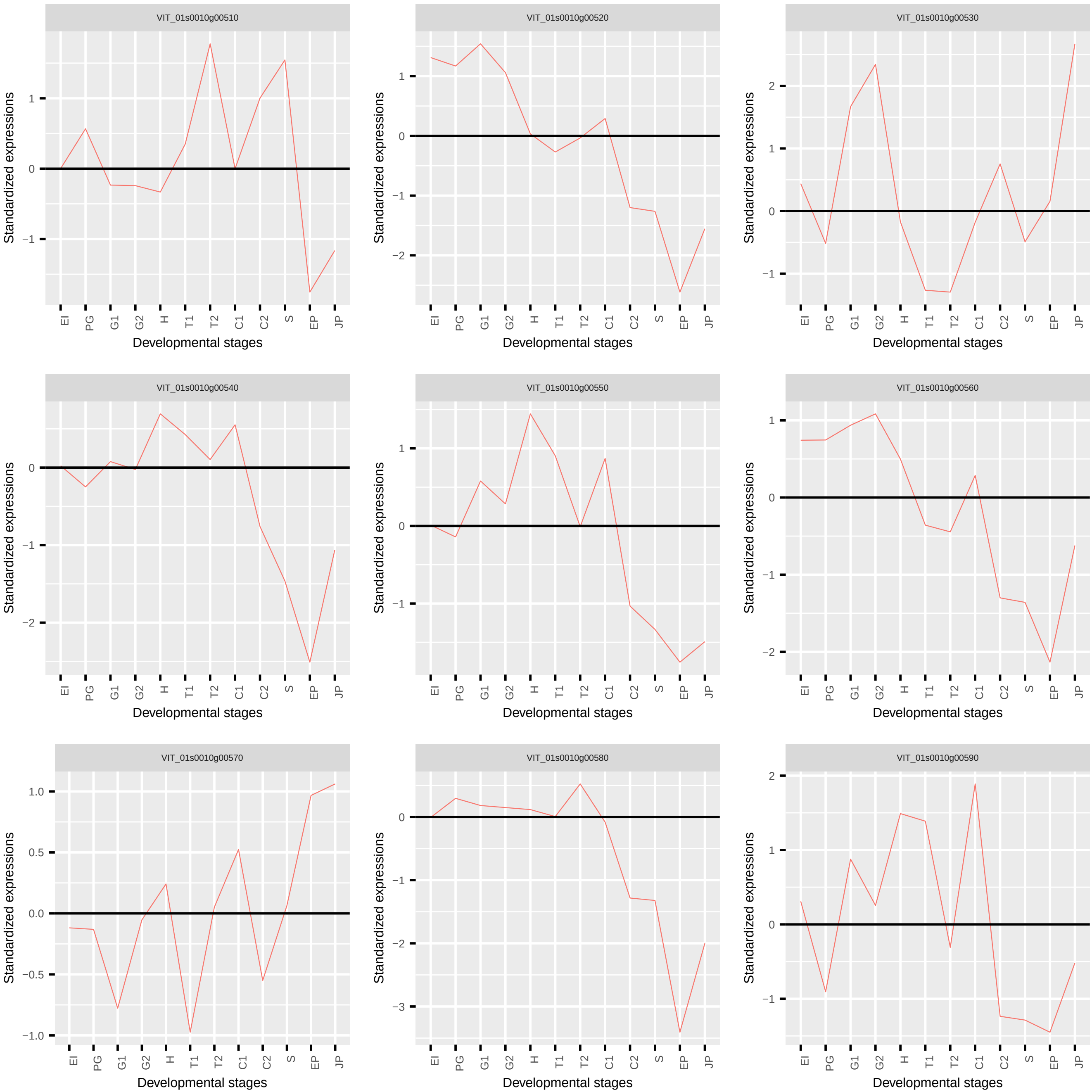
VIT_01s0010g00510
VIT_01s0010g00520
VIT_01s0010g00530
VIT_01s0010g00540
VIT_01s0010g00550
VIT_01s0010g00560
VIT_01s0010g00570
VIT_01s0010g00580
VIT_01s0010g00590
Standardized expressions
Developmental stages
EI PG G1 G2 H T1 T2 C1 C2 S EP JP

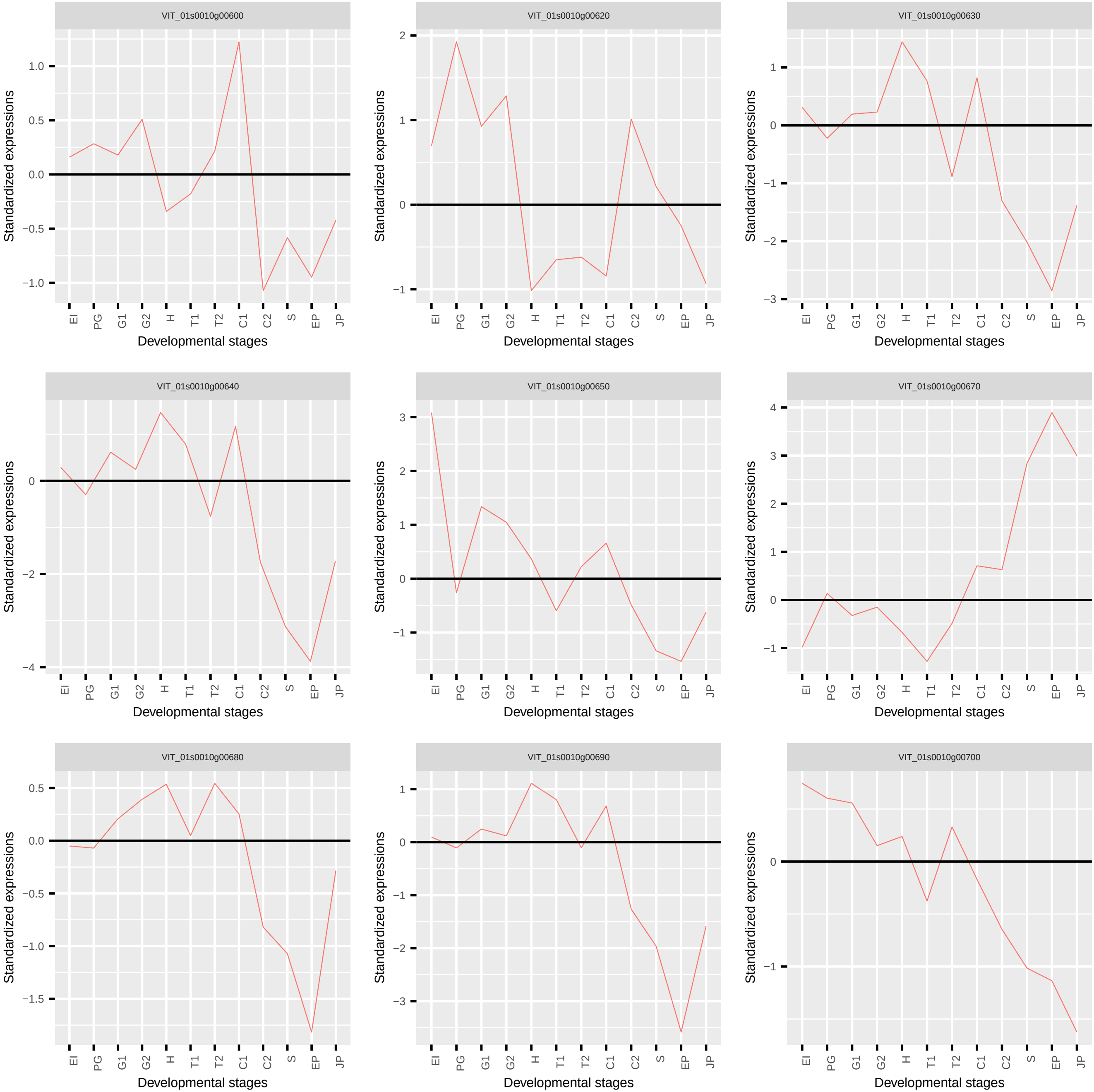
VIT_01s0010g00600
VIT_01s0010g00620
VIT_01s0010g00630
VIT_01s0010g00640
VIT_01s0010g00650
VIT_01s0010g00670
VIT_01s0010g00680
VIT_01s0010g00690
VIT_01s0010g00700
Standardized expressions
Developmental stages
EI PG G1 G2 H T1 T2 C1 C2 S EP JP

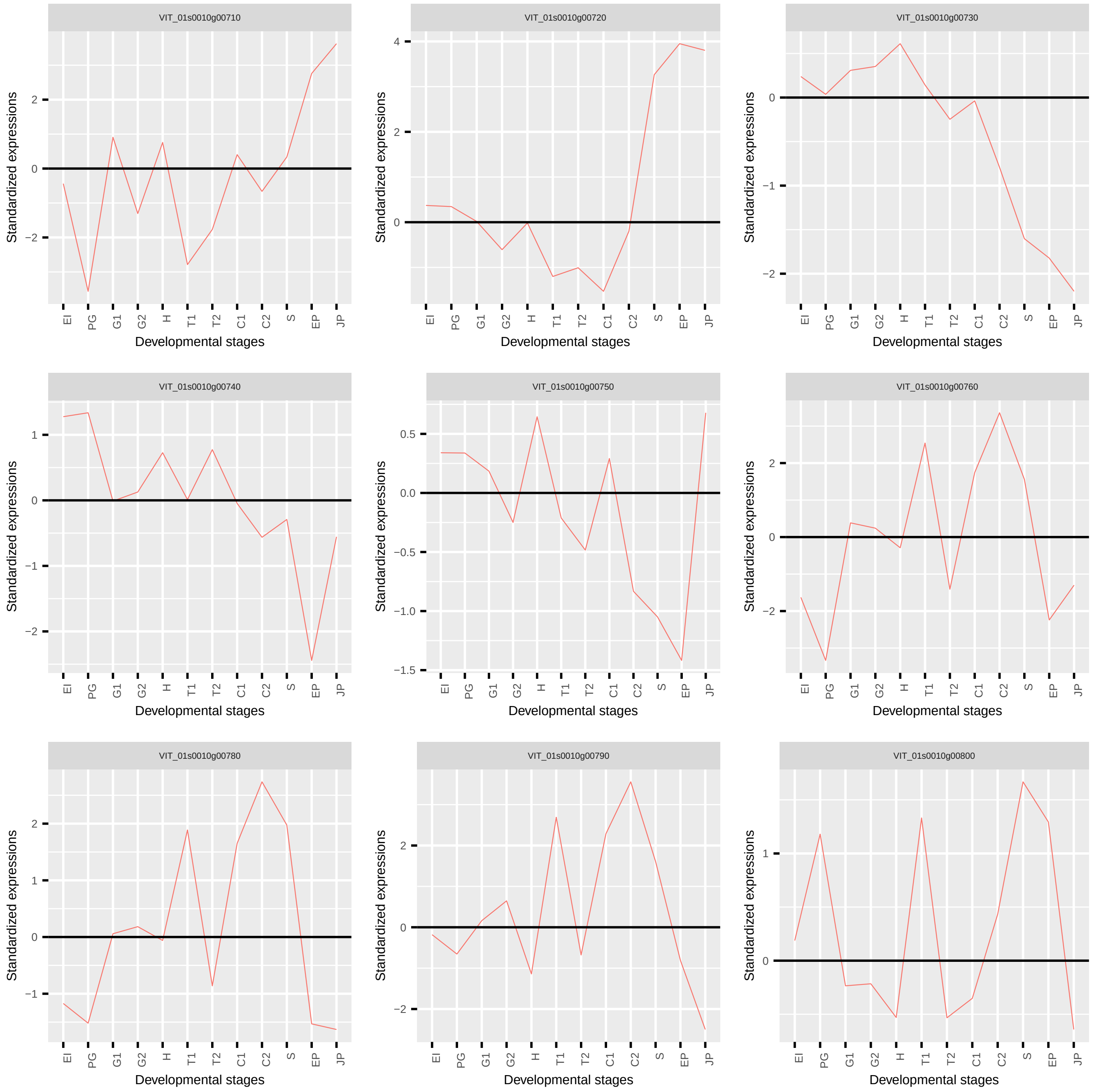

VIT_01s0010g00710
VIT_01s0010g00720
VIT_01s0010g00730
VIT_01s0010g00740
VIT_01s0010g00750
VIT_01s0010g00760
VIT_01s0010g00780
VIT_01s0010g00790
VIT_01s0010g00800
Standardized expressions
Developmental stages
EI
PG
G1
G2
H
T1
T2
C1
C2
S
EP
JP

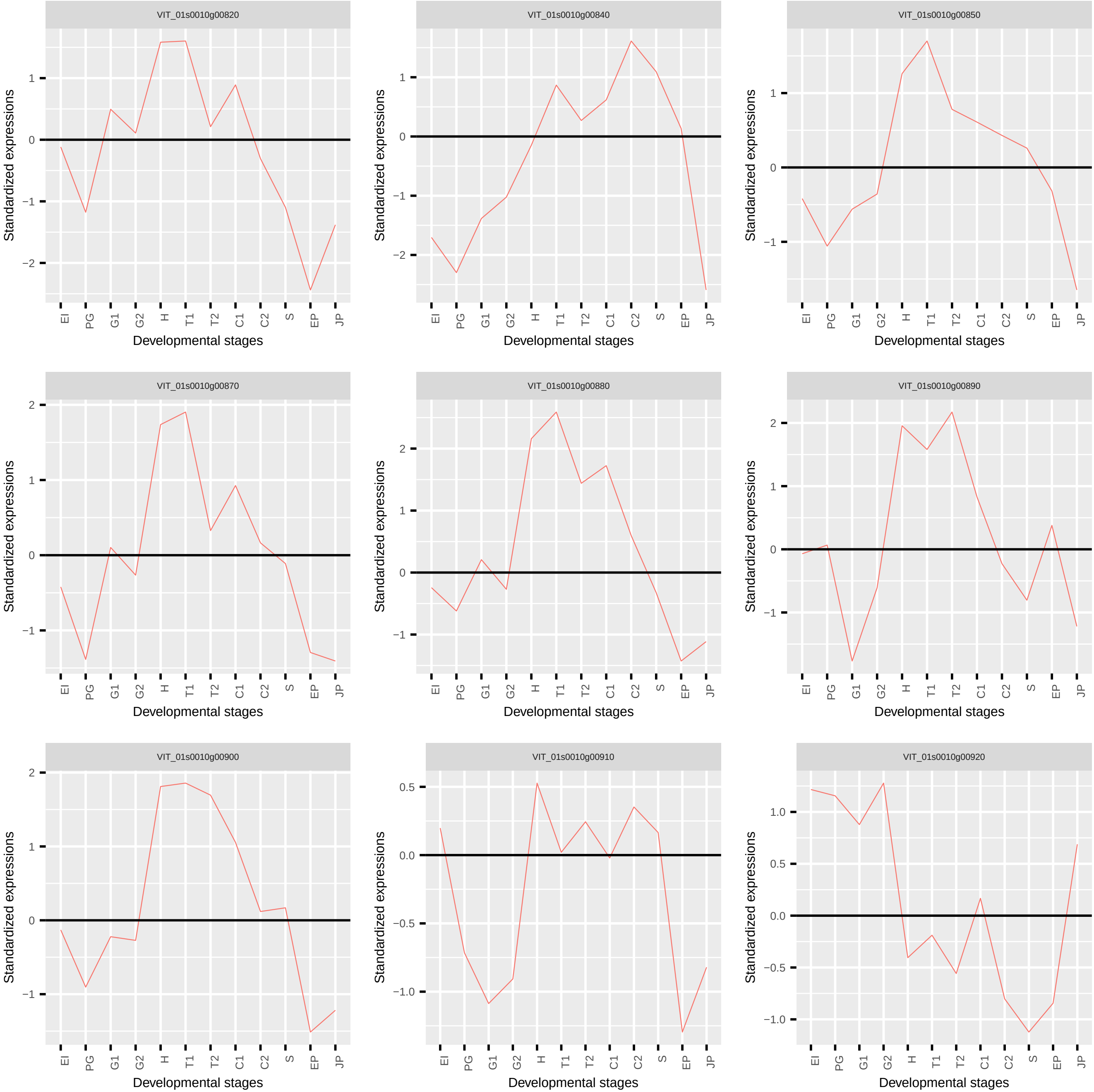

VIT_01s0010g00820
VIT_01s0010g00840
VIT_01s0010g00850
VIT_01s0010g00870
VIT_01s0010g00880
VIT_01s0010g00890
VIT_01s0010g00900
VIT_01s0010g00910
VIT_01s0010g00920
Standardized expressions
Developmental stages
EI PG G1 G2 H T1 T2 C1 C2 S EP JP

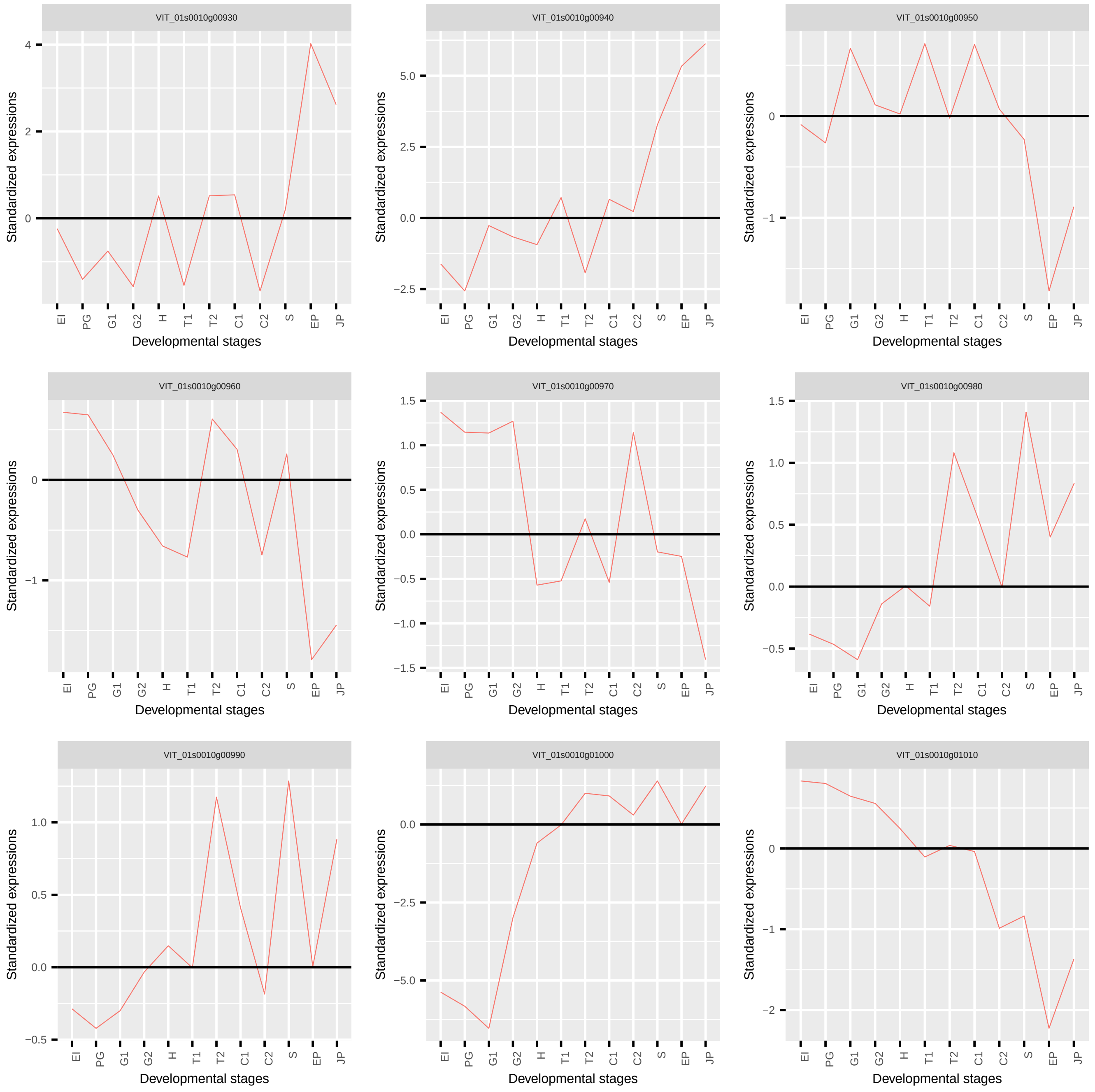

VIT_01s0010g00930
VIT_01s0010g00940
VIT_01s0010g00950
VIT_01s0010g00960
VIT_01s0010g00970
VIT_01s0010g00980
VIT_01s0010g00990
VIT_01s0010g01000
VIT_01s0010g01010
Standardized expressions
Developmental stages
EI PG G1 G2 H T1 T2 C1 C2 S EP JP

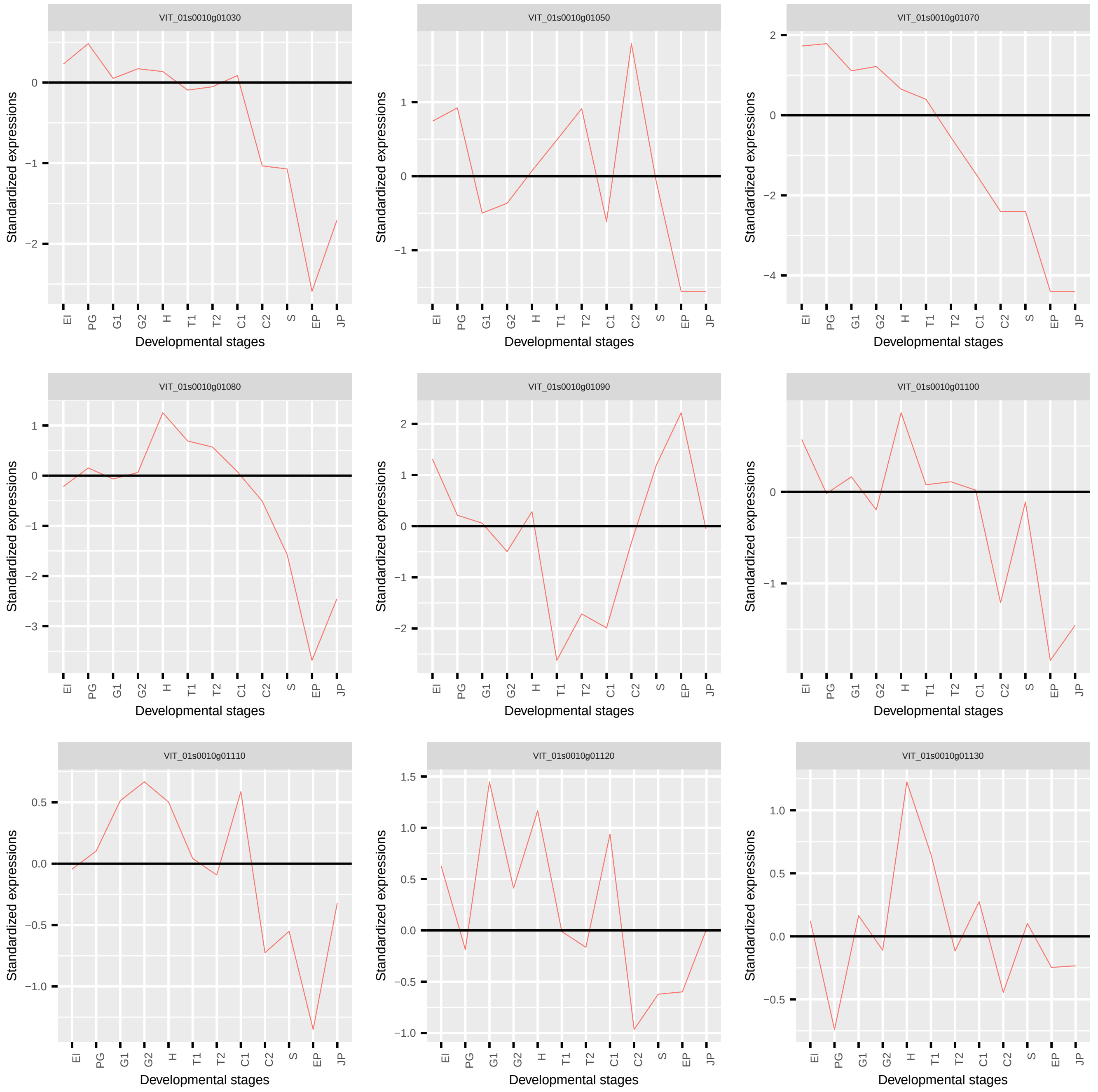
VIT_01s0010g01030
VIT_01s0010g01050
VIT_01s0010g01070
VIT_01s0010g01080
VIT_01s0010g01090
VIT_01s0010g01100
VIT_01s0010g01110
VIT_01s0010g01120
VIT_01s0010g01130
Standardized expressions
Developmental stages
EI PG G1 G2 H T1 T2 C1 C2 S EP JP

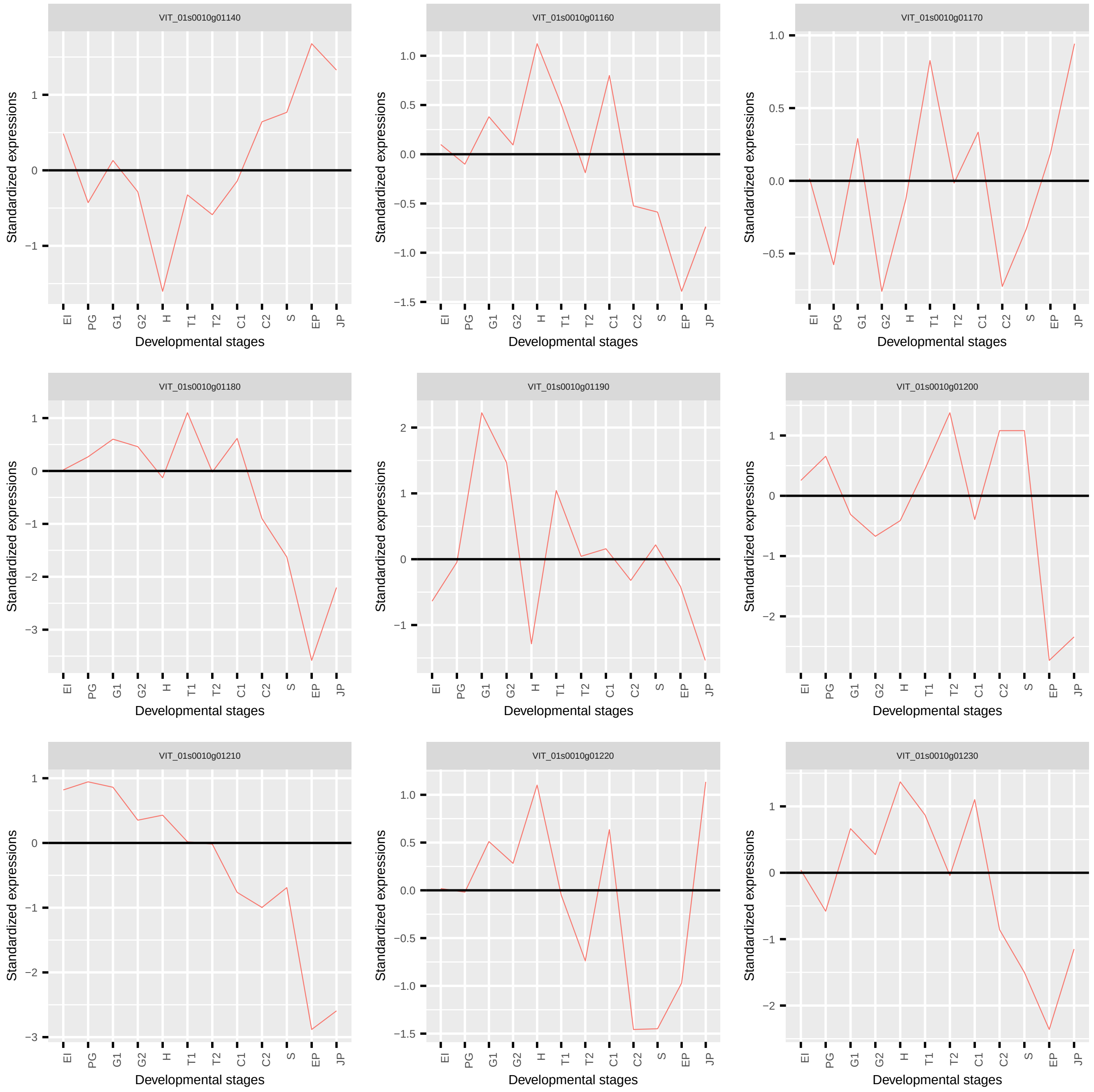
VIT_01s0010g01140
VIT_01s0010g01160
VIT_01s0010g01170
VIT_01s0010g01180
VIT_01s0010g01190
VIT_01s0010g01200
VIT_01s0010g01210
VIT_01s0010g01220
VIT_01s0010g01230
Standardized expressions
Developmental stages
EI
PG
G1
G2
H
T1
T2
C1
C2
S
EP
JP

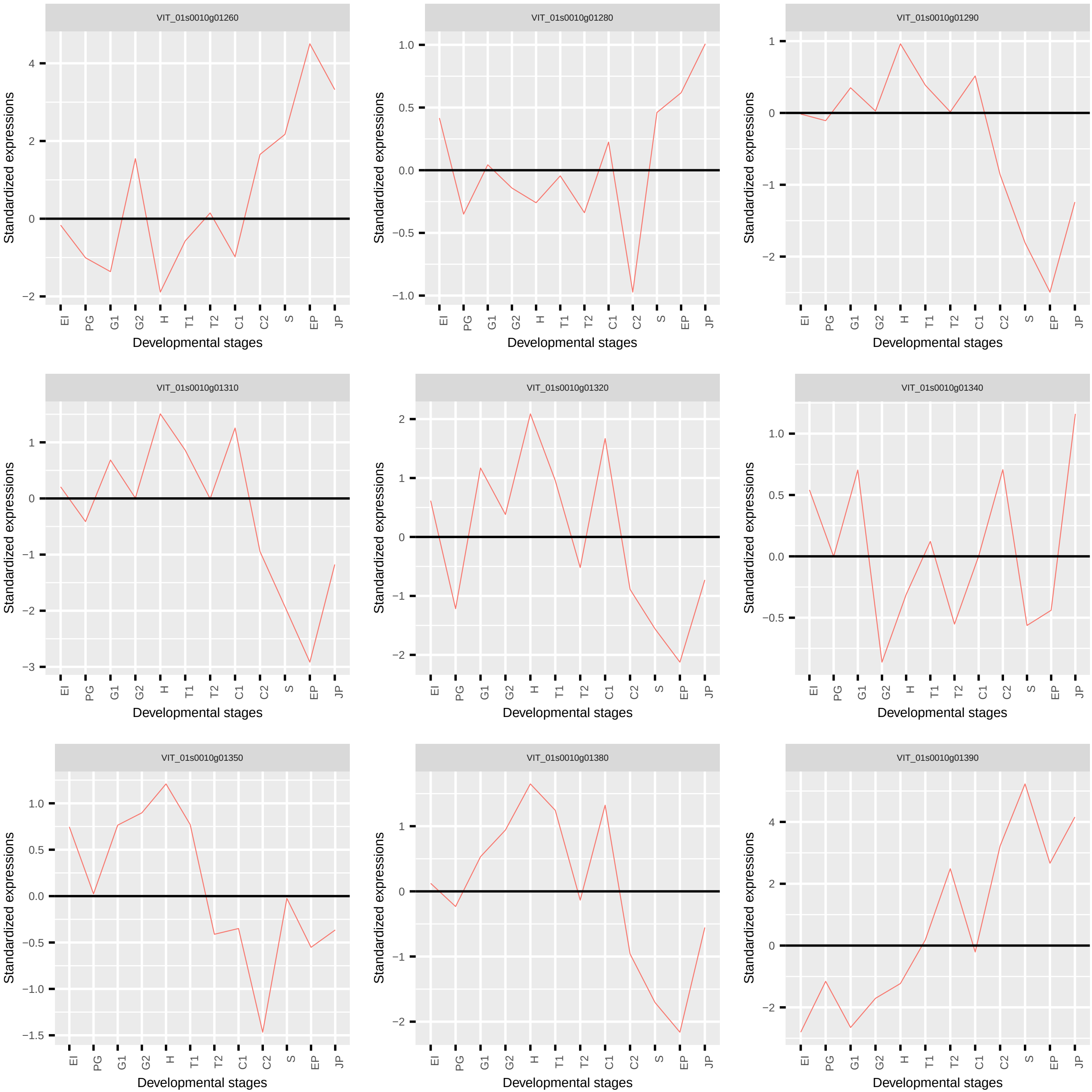
VIT_01s0010g01260
VIT_01s0010g01280
VIT_01s0010g01290
VIT_01s0010g01310
VIT_01s0010g01320
VIT_01s0010g01340
VIT_01s0010g01350
VIT_01s0010g01380
VIT_01s0010g01390
Standardized expressions
Developmental stages
EI
PG
G1
G2
H
T1
T2
C1
C2
S
EP
JP

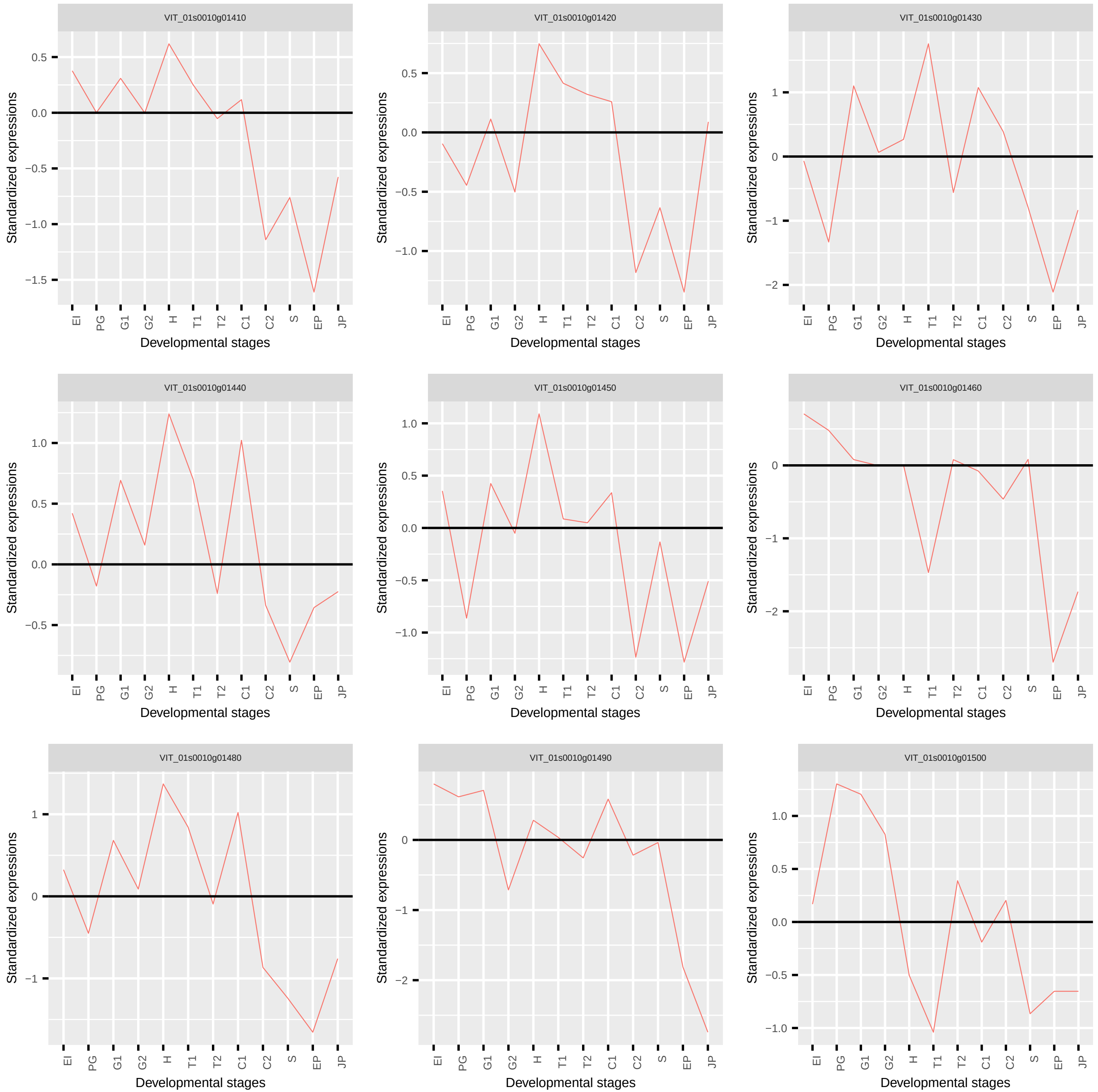
VIT_01s0010g01410
VIT_01s0010g01420
VIT_01s0010g01430
VIT_01s0010g01440
VIT_01s0010g01450
VIT_01s0010g01460
VIT_01s0010g01480
VIT_01s0010g01490
VIT_01s0010g01500
Standardized expressions
Developmental stages
EI PG G1 G2 H T1 T2 C1 C2 S EP JP

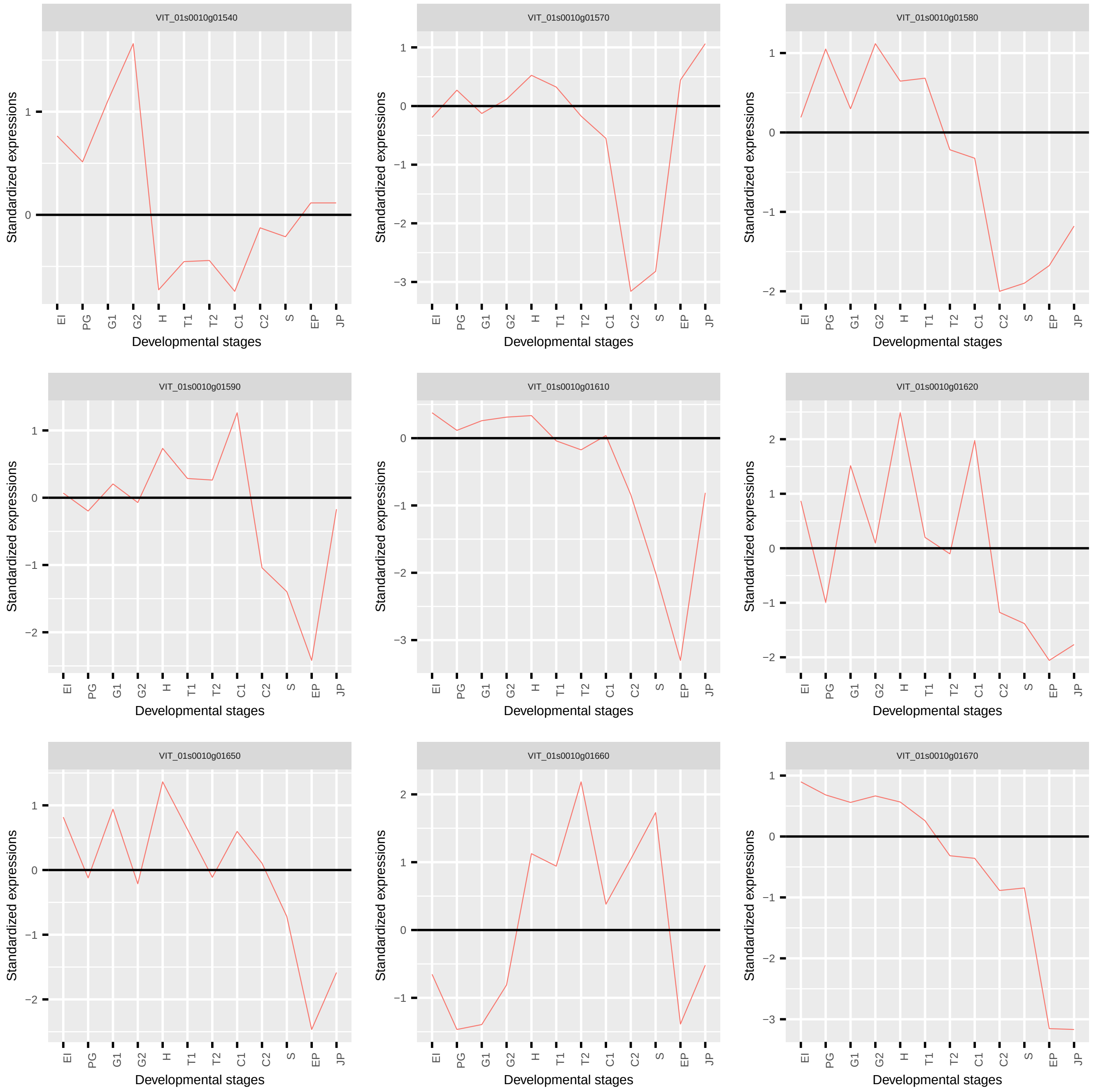
VIT_01s0010g01540
VIT_01s0010g01570
VIT_01s0010g01580
VIT_01s0010g01590
VIT_01s0010g01610
VIT_01s0010g01620
VIT_01s0010g01650
VIT_01s0010g01660
VIT_01s0010g01670
Standardized expressions
Developmental stages
EI PG G1 G2 H T1 T2 C1 C2 S EP JP

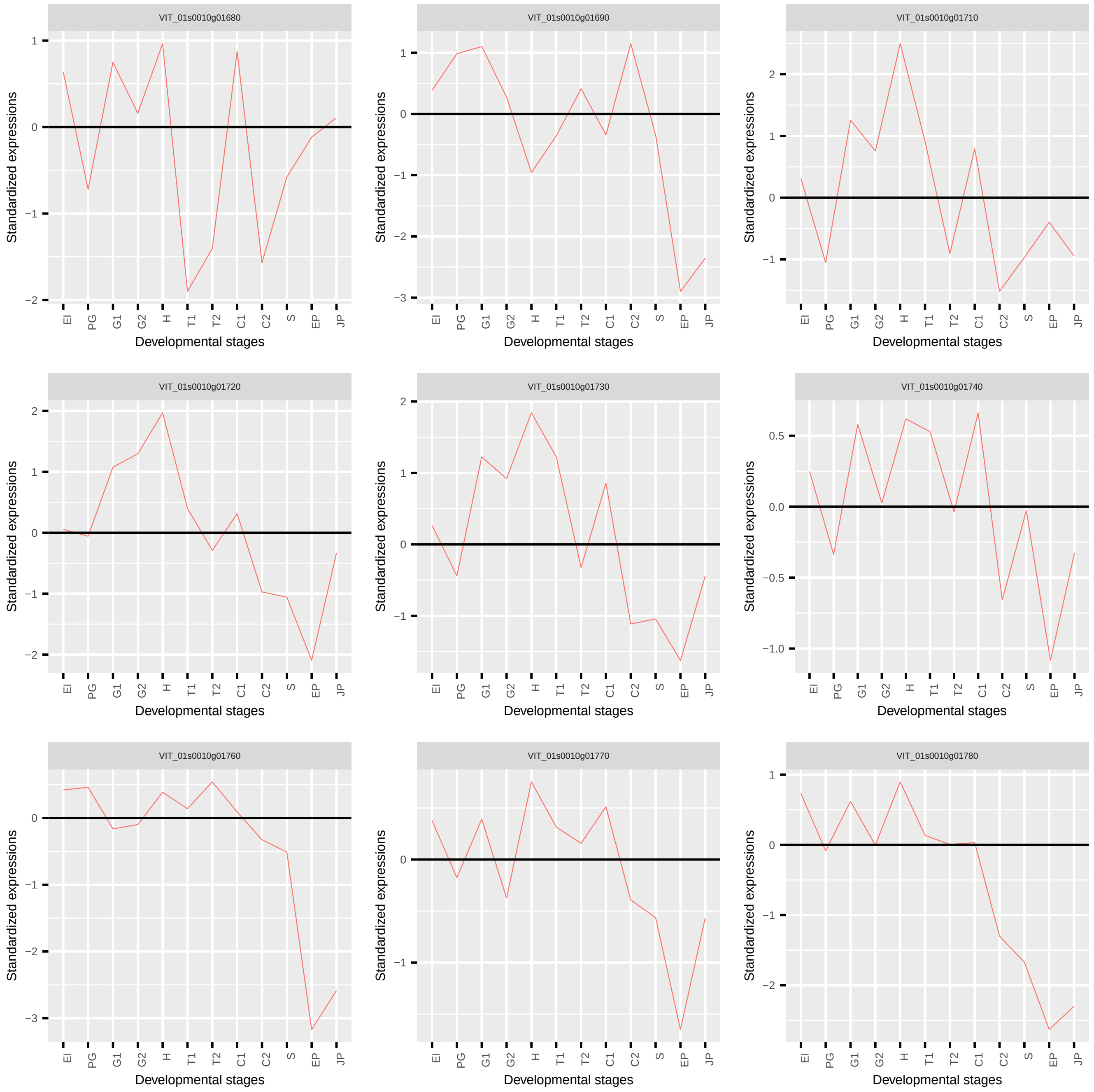

VIT_01s0010g01680
VIT_01s0010g01690
VIT_01s0010g01710
VIT_01s0010g01720
VIT_01s0010g01730
VIT_01s0010g01740
VIT_01s0010g01760
VIT_01s0010g01770
VIT_01s0010g01780
Standardized expressions
Developmental stages
EI PG G1 G2 H T1 T2 C1 C2 S EP JP

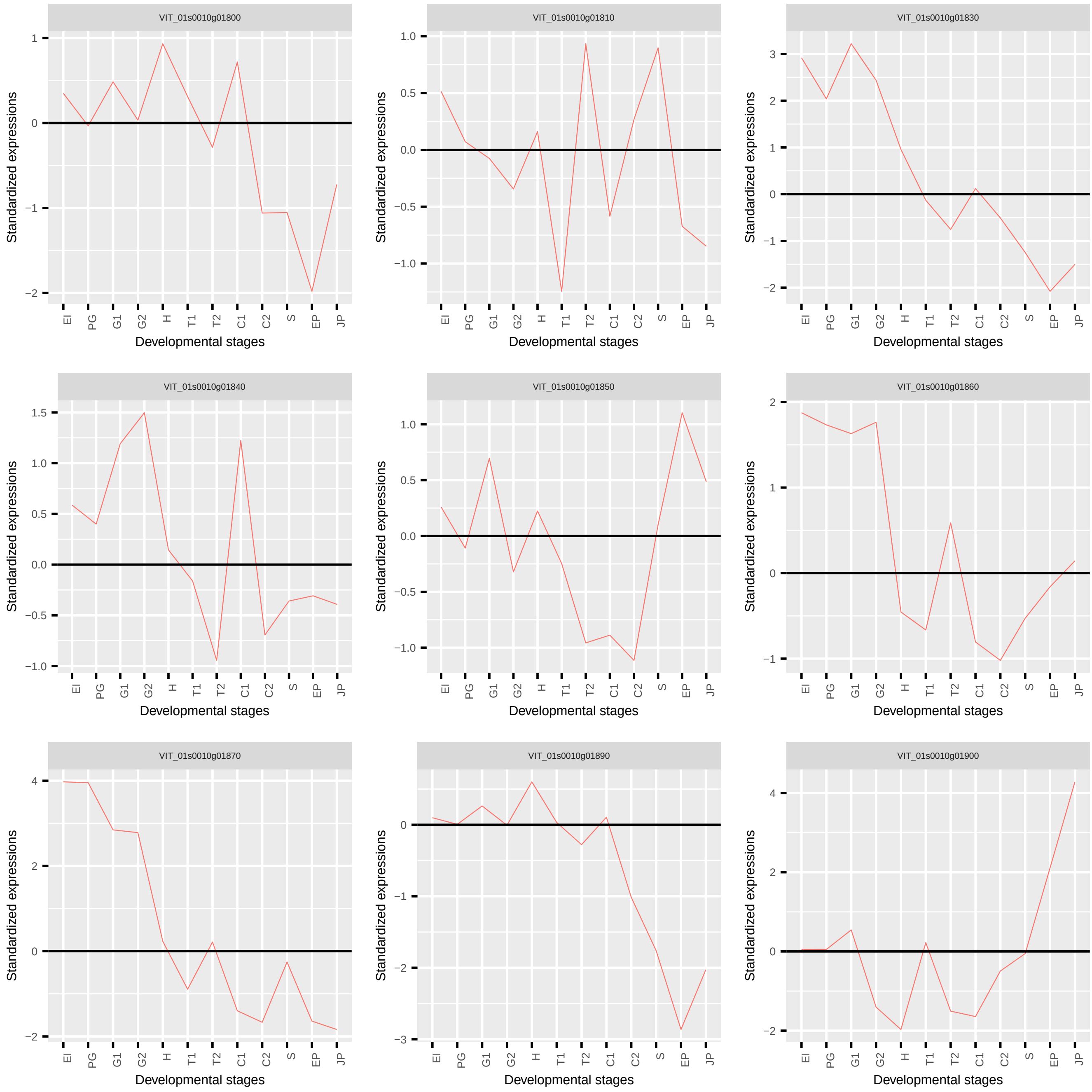
VIT_01s0010g01800
VIT_01s0010g01810
VIT_01s0010g01830
VIT_01s0010g01840
VIT_01s0010g01850
VIT_01s0010g01860
VIT_01s0010g01870
VIT_01s0010g01890
VIT_01s0010g01900
Standardized expressions
Developmental stages
EI PG G1 G2 H T1 T2 C1 C2 S EP JP

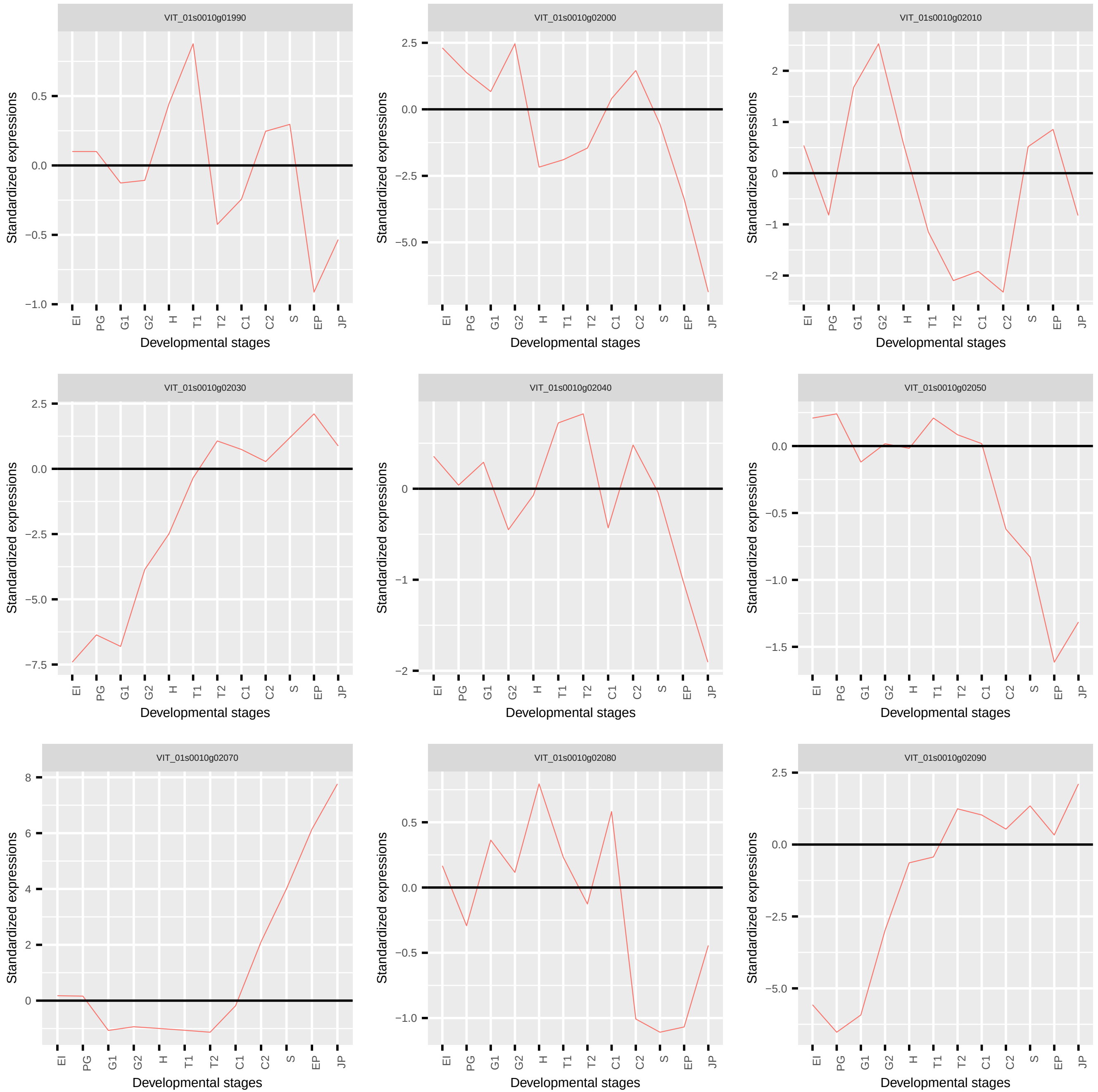
VIT_01s0010g01990
VIT_01s0010g02000
VIT_01s0010g02010
VIT_01s0010g02030
VIT_01s0010g02040
VIT_01s0010g02050
VIT_01s0010g02070
VIT_01s0010g02080
VIT_01s0010g02090
Standardized expressions
Developmental stages
EI PG G1 G2 H T1 T2 C1 C2 S EP JP
0.5
0.0
−0.5
−1.0
2.5
−2.5
−5.0
−7.5
2
1
0
−1
−2
−1.5
8
6
4

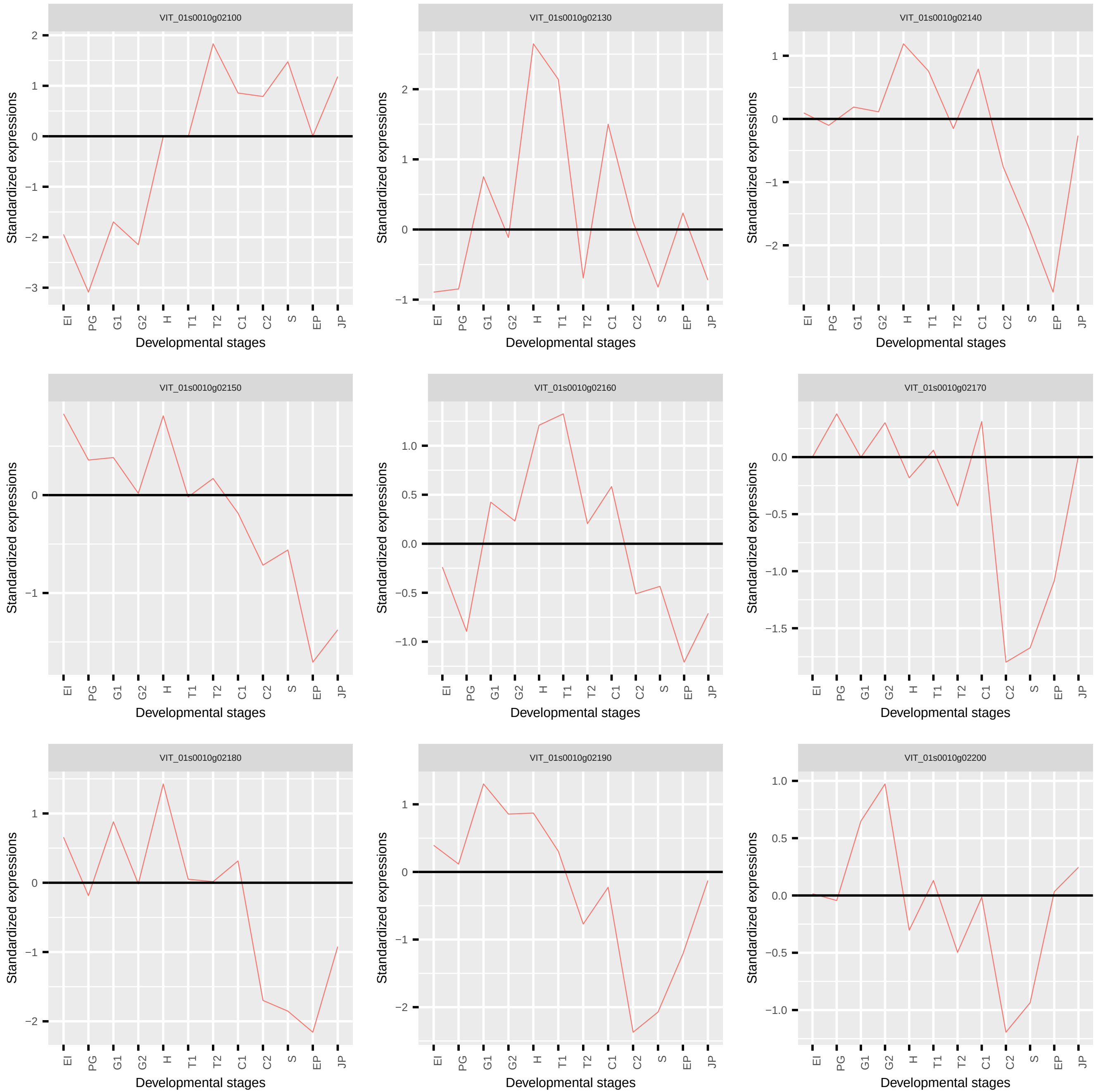
VIT_01s0010g02100
VIT_01s0010g02130
VIT_01s0010g02140
VIT_01s0010g02150
VIT_01s0010g02160
VIT_01s0010g02170
VIT_01s0010g02180
VIT_01s0010g02190
VIT_01s0010g02200
Standardized expressions
Developmental stages
EI PG G1 G2 H T1 T2 C1 C2 S EP JP

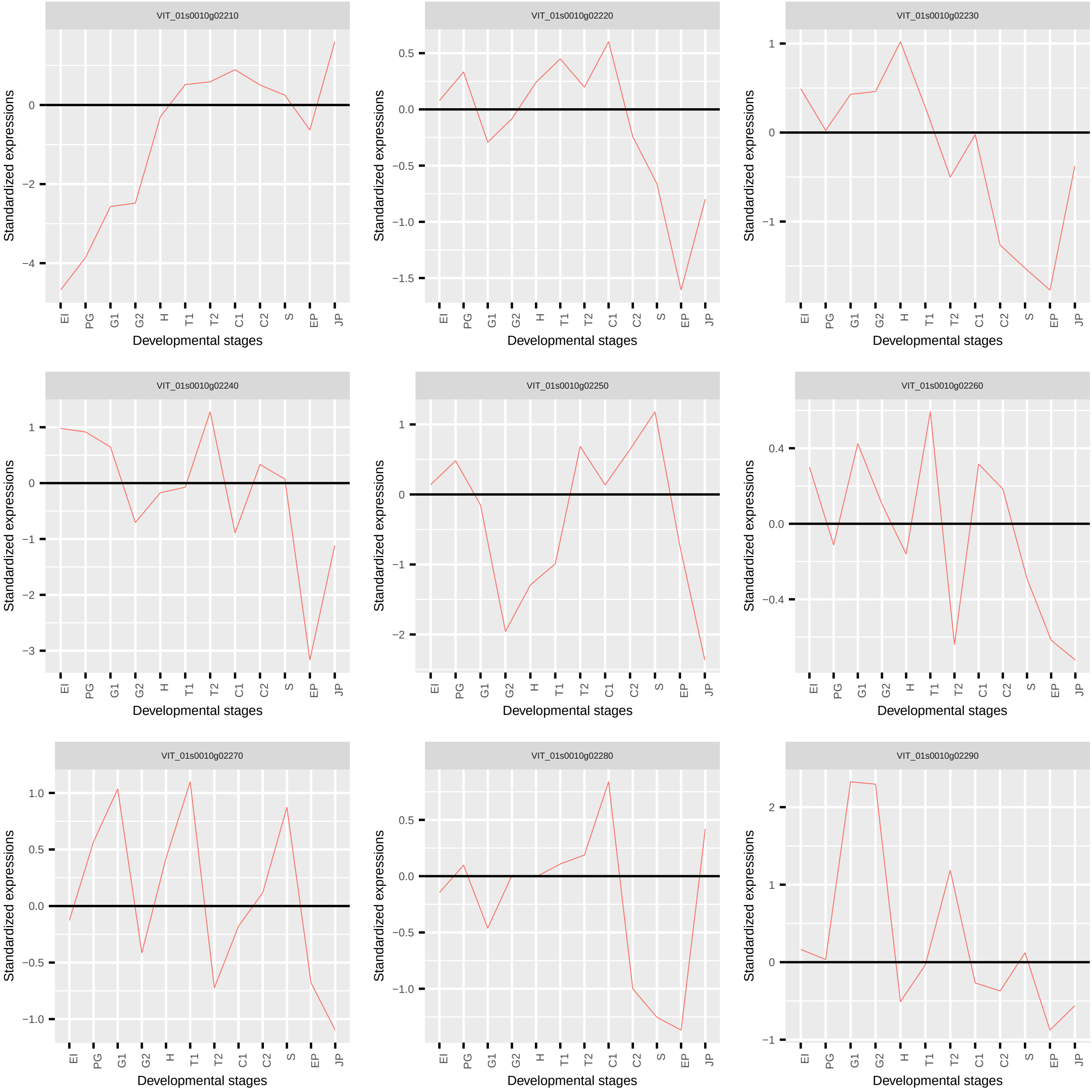
VIT_01s0010g02210
VIT_01s0010g02220
VIT_01s0010g02230
VIT_01s0010g02240
VIT_01s0010g02250
VIT_01s0010g02260
VIT_01s0010g02270
VIT_01s0010g02280
VIT_01s0010g02290
Standardized expressions
Developmental stages
EI PG G1 G2 H T1 T2 C1 C2 S EP JP

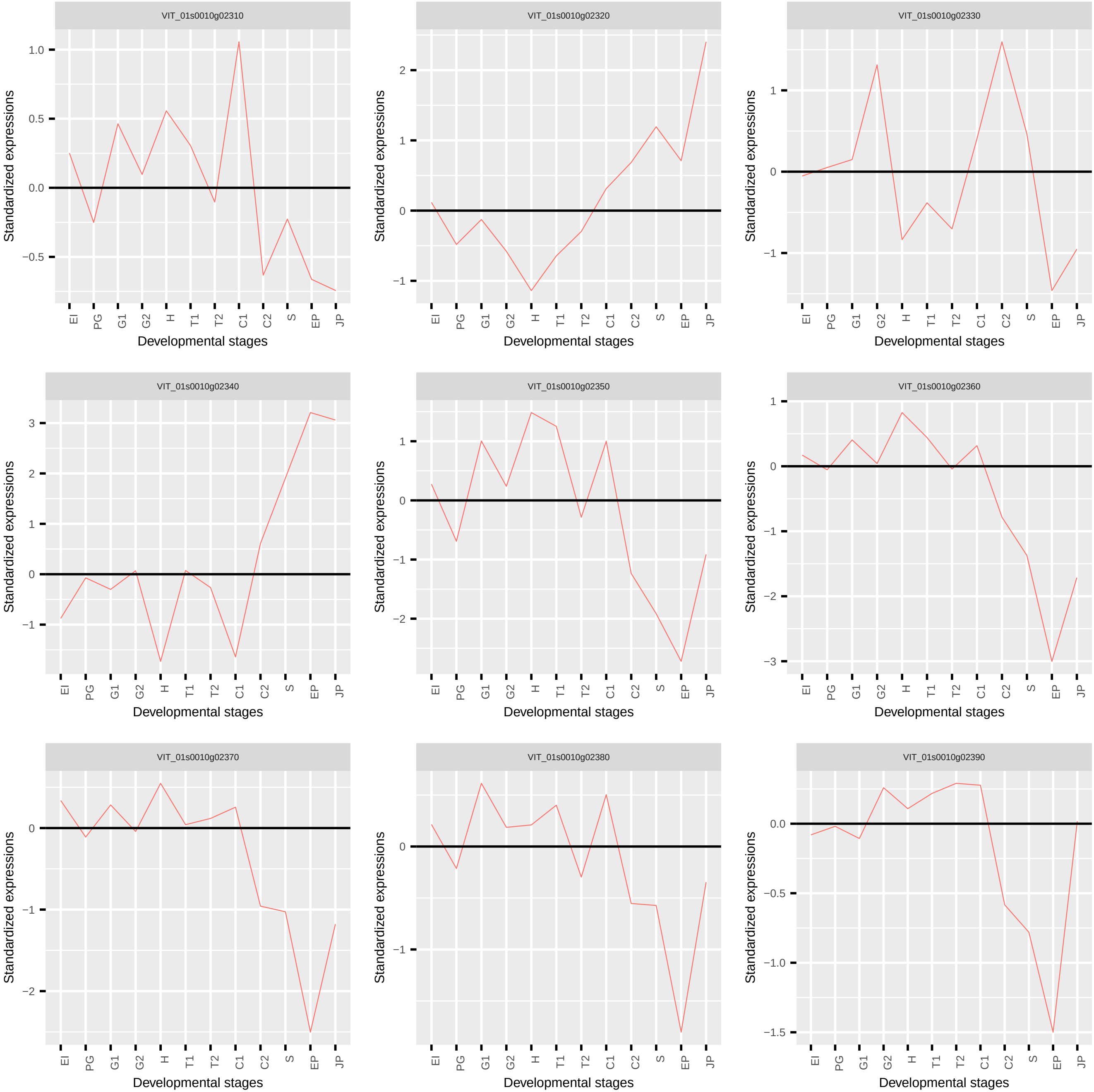
VIT_01s0010g02310
VIT_01s0010g02320
VIT_01s0010g02330
VIT_01s0010g02340
VIT_01s0010g02350
VIT_01s0010g02360
VIT_01s0010g02370
VIT_01s0010g02380
VIT_01s0010g02390
Standardized expressions
Developmental stages
EI PG G1 G2 H T1 T2 C1 C2 S EP JP

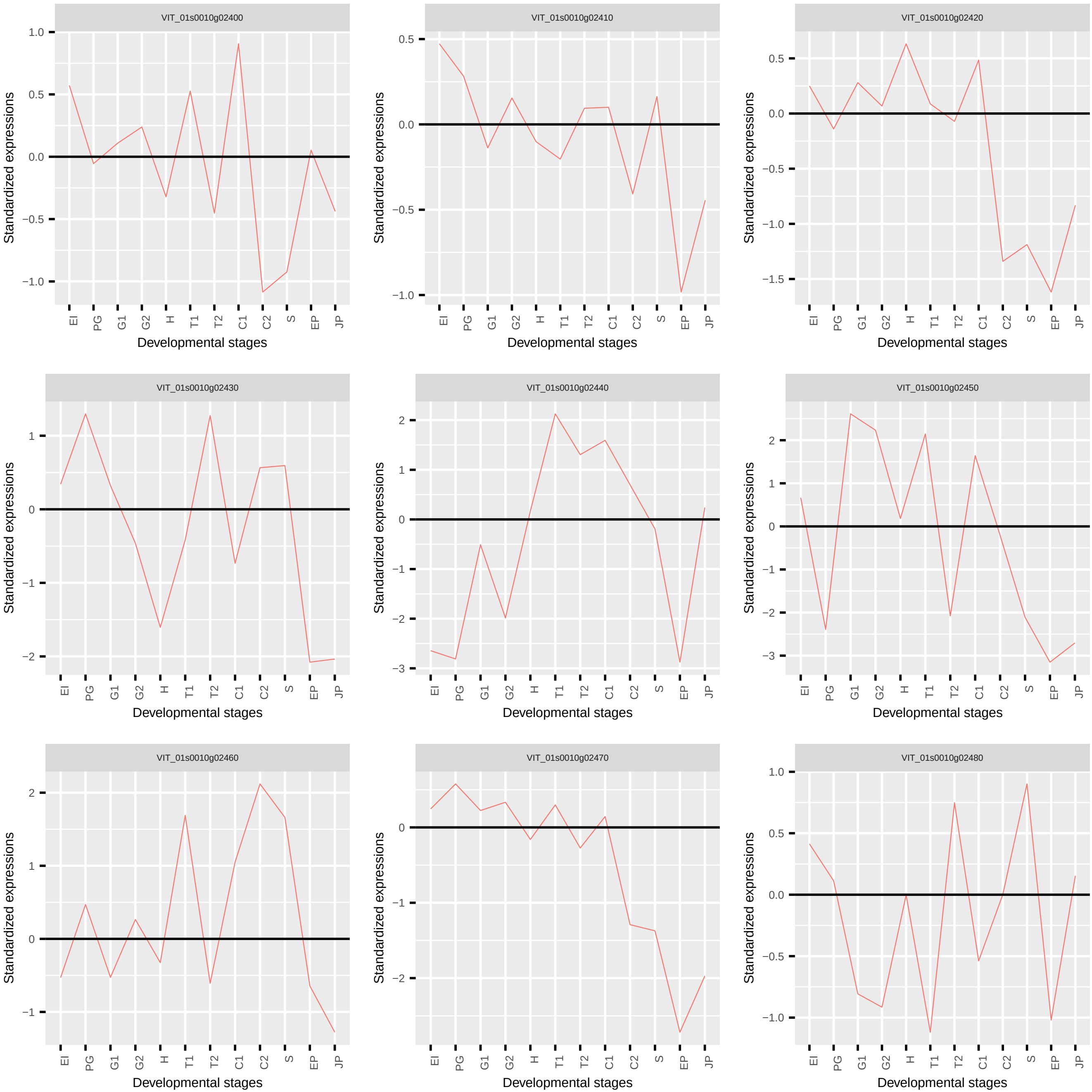
VIT_01s0010g02400
VIT_01s0010g02410
VIT_01s0010g02420
VIT_01s0010g02430
VIT_01s0010g02440
VIT_01s0010g02450
VIT_01s0010g02460
VIT_01s0010g02470
VIT_01s0010g02480
Standardized expressions
Developmental stages
EI PG G1 G2 H T1 T2 C1 C2 S EP JP
1.0
0.5
0.0
−0.5
−1.0
−1.5
2
1
0
−1
−2
−3

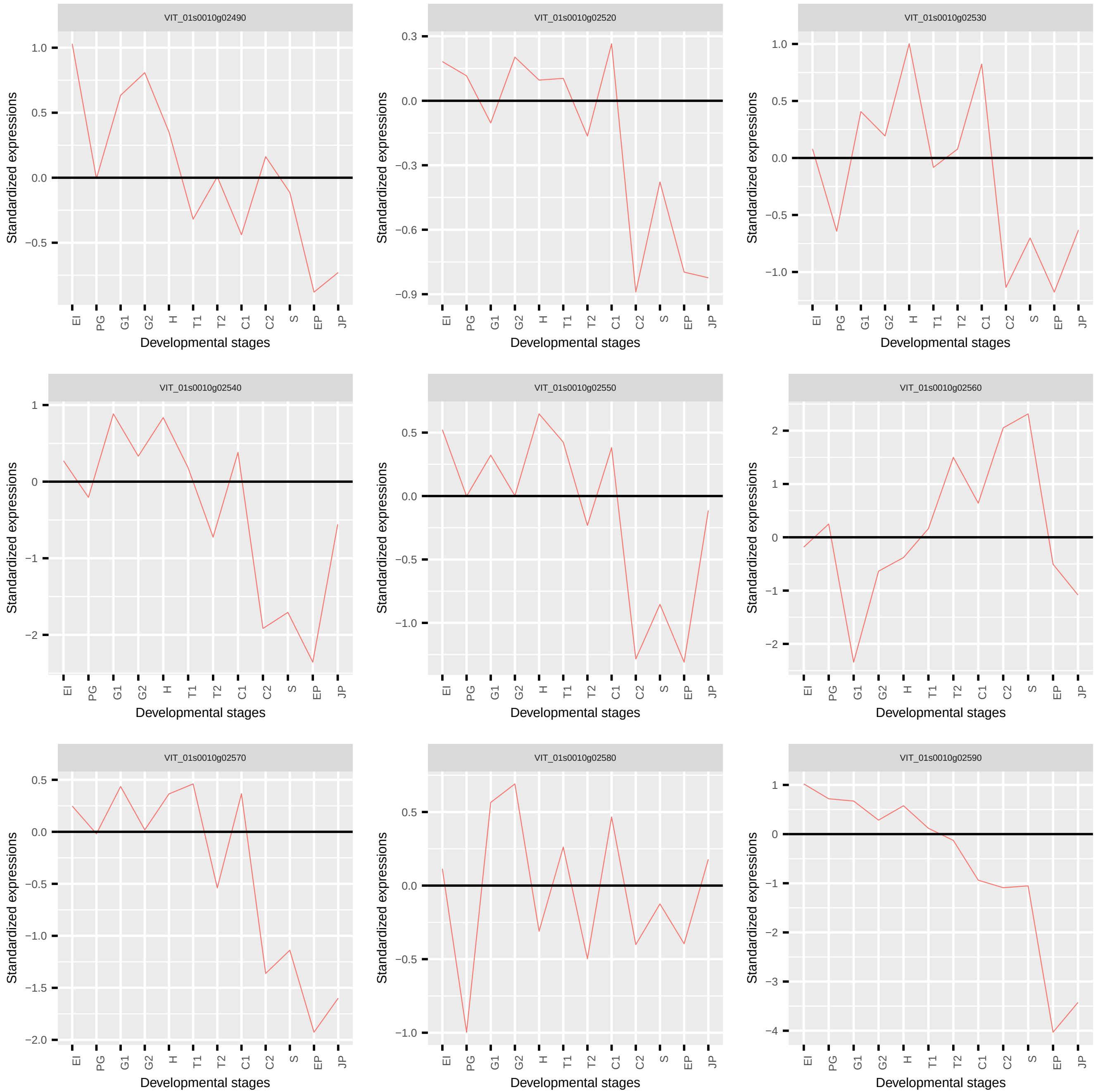
VIT_01s0010g02490
VIT_01s0010g02520
VIT_01s0010g02530
VIT_01s0010g02540
VIT_01s0010g02550
VIT_01s0010g02560
VIT_01s0010g02570
VIT_01s0010g02580
VIT_01s0010g02590
Standardized expressions
Developmental stages
EI PG G1 G2 H T1 T2 C1 C2 S EP JP

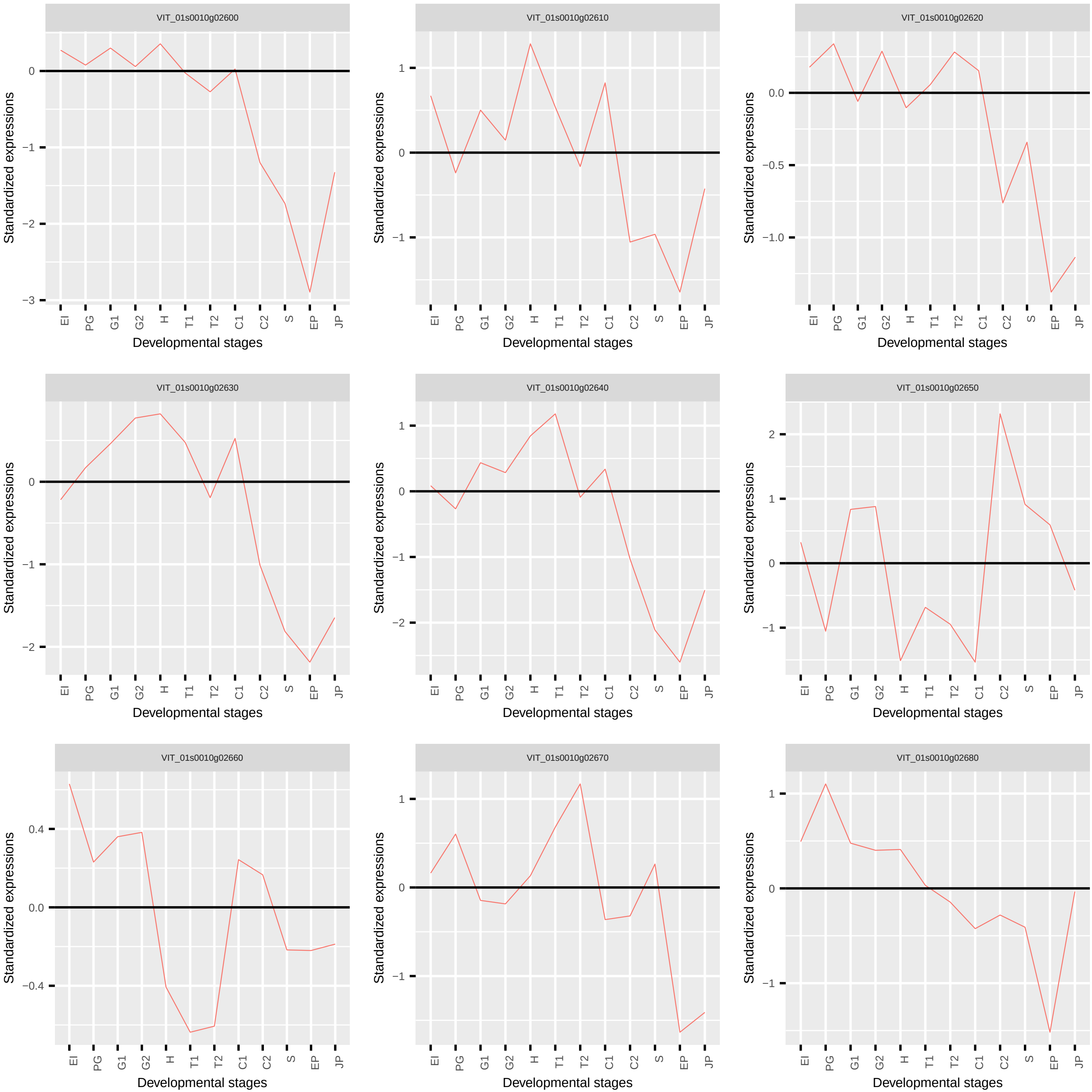
VIT_01s0010g02600
VIT_01s0010g02610
VIT_01s0010g02620
VIT_01s0010g02630
VIT_01s0010g02640
VIT_01s0010g02650
VIT_01s0010g02660
VIT_01s0010g02670
VIT_01s0010g02680
Standardized expressions
Developmental stages
EI PG G1 G2 H T1 T2 C1 C2 S EP JP

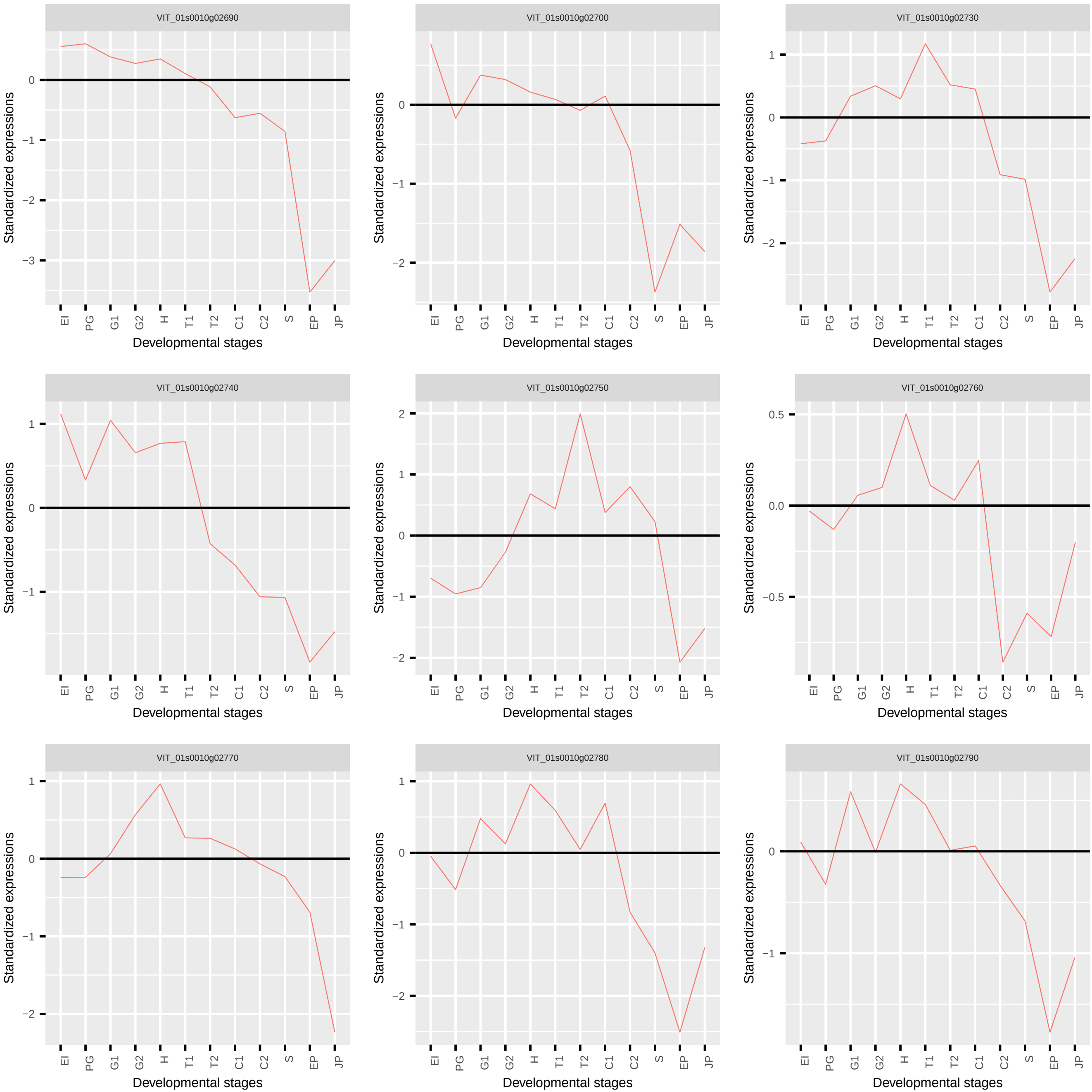

VIT_01s0010g02690
VIT_01s0010g02700
VIT_01s0010g02730
VIT_01s0010g02740
VIT_01s0010g02750
VIT_01s0010g02760
VIT_01s0010g02770
VIT_01s0010g02780
VIT_01s0010g02790
Standardized expressions
Developmental stages
EI
PG
G1
G2
H
T1
T2
C1
C2
S
EP
JP

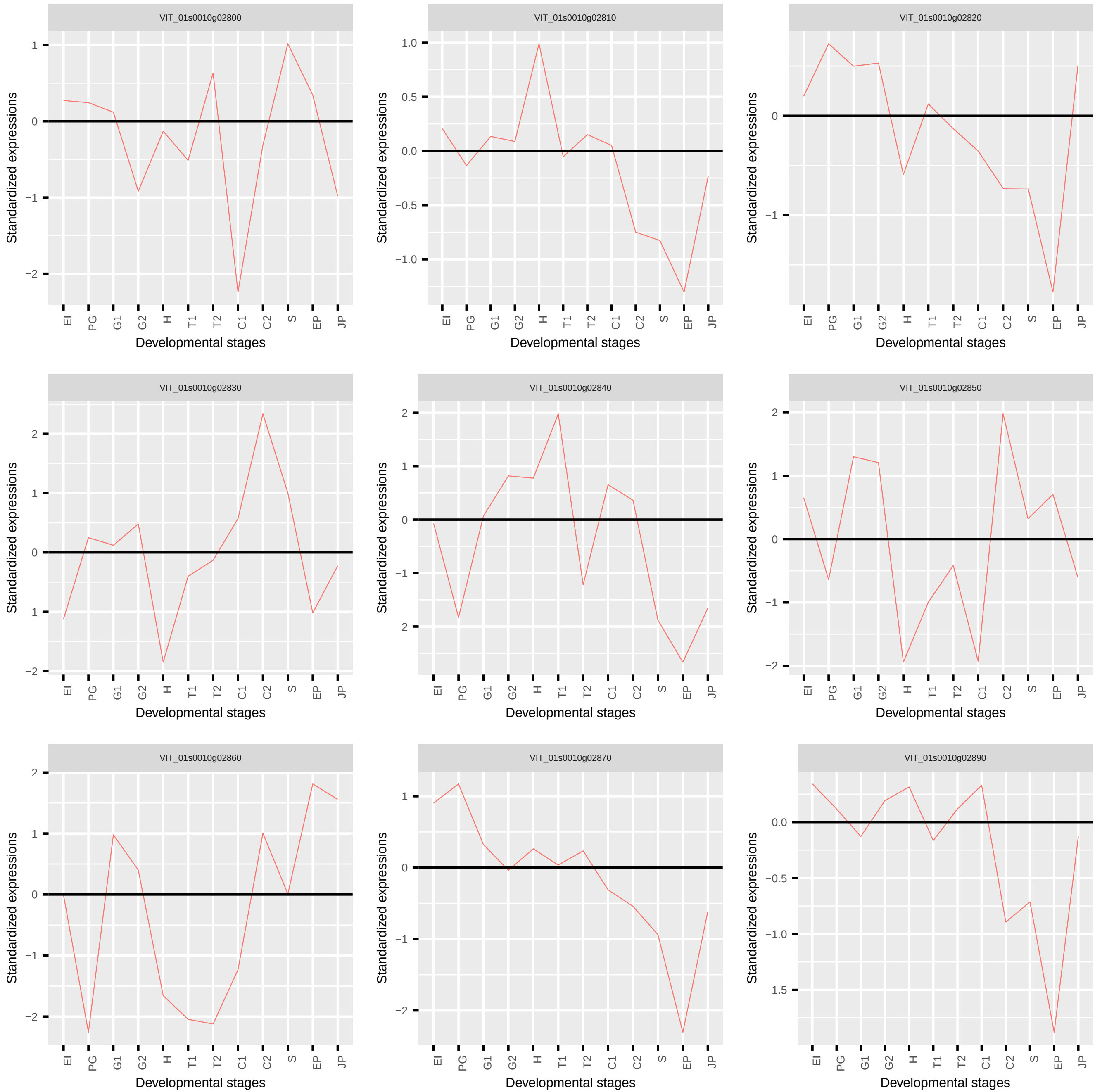

VIT_01s0010g02800
VIT_01s0010g02810
VIT_01s0010g02820
VIT_01s0010g02830
VIT_01s0010g02840
VIT_01s0010g02850
VIT_01s0010g02860
VIT_01s0010g02870
VIT_01s0010g02890
Standardized expressions
Developmental stages
EI PG G1 G2 H T1 T2 C1 C2 S EP JP

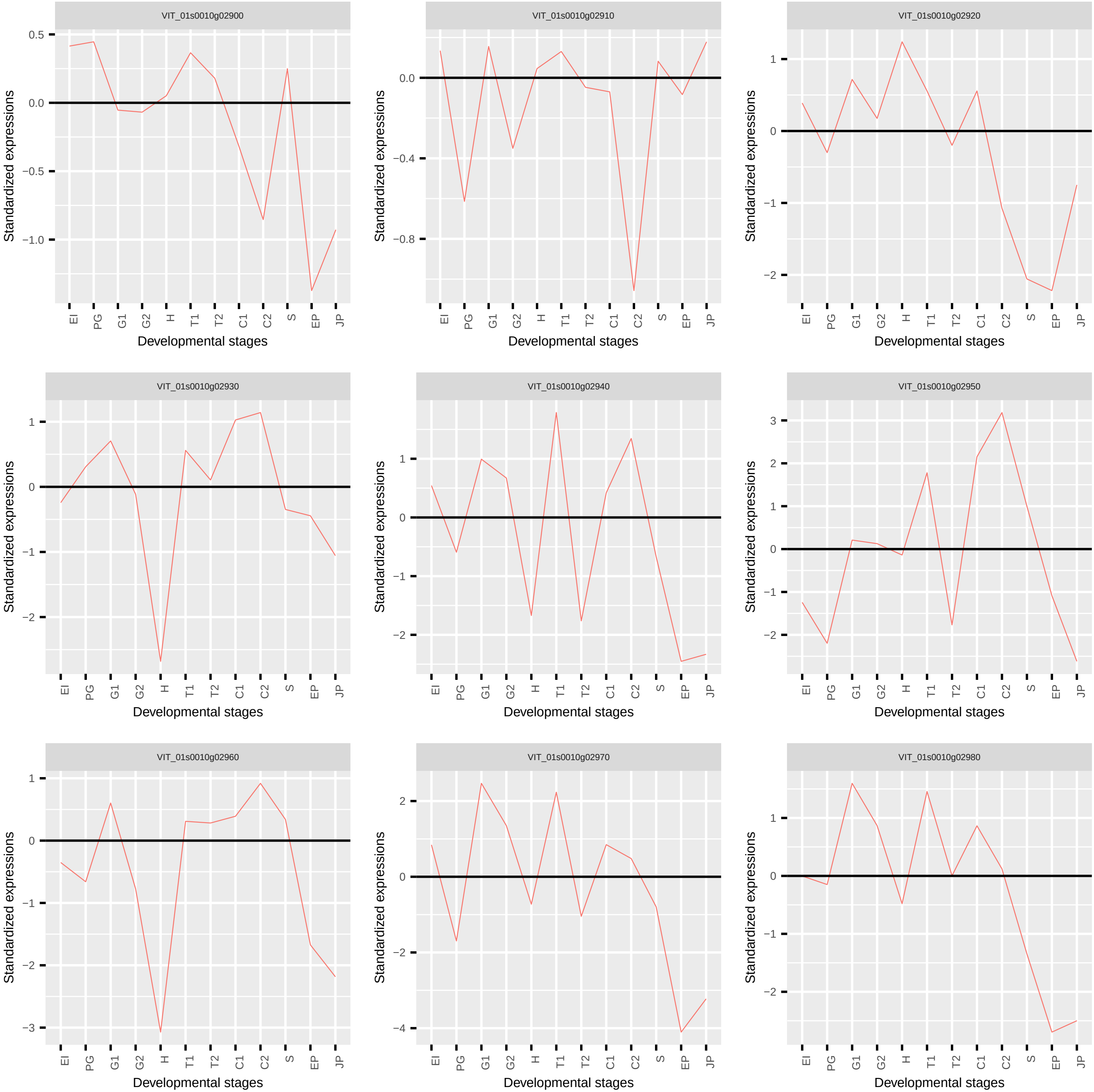
VIT_01s0010g02900
VIT_01s0010g02910
VIT_01s0010g02920
VIT_01s0010g02930
VIT_01s0010g02940
VIT_01s0010g02950
VIT_01s0010g02960
VIT_01s0010g02970
VIT_01s0010g02980
Standardized expressions
Developmental stages
EI
PG
G1
G2
H
T1
T2
C1
C2
S
EP
JP

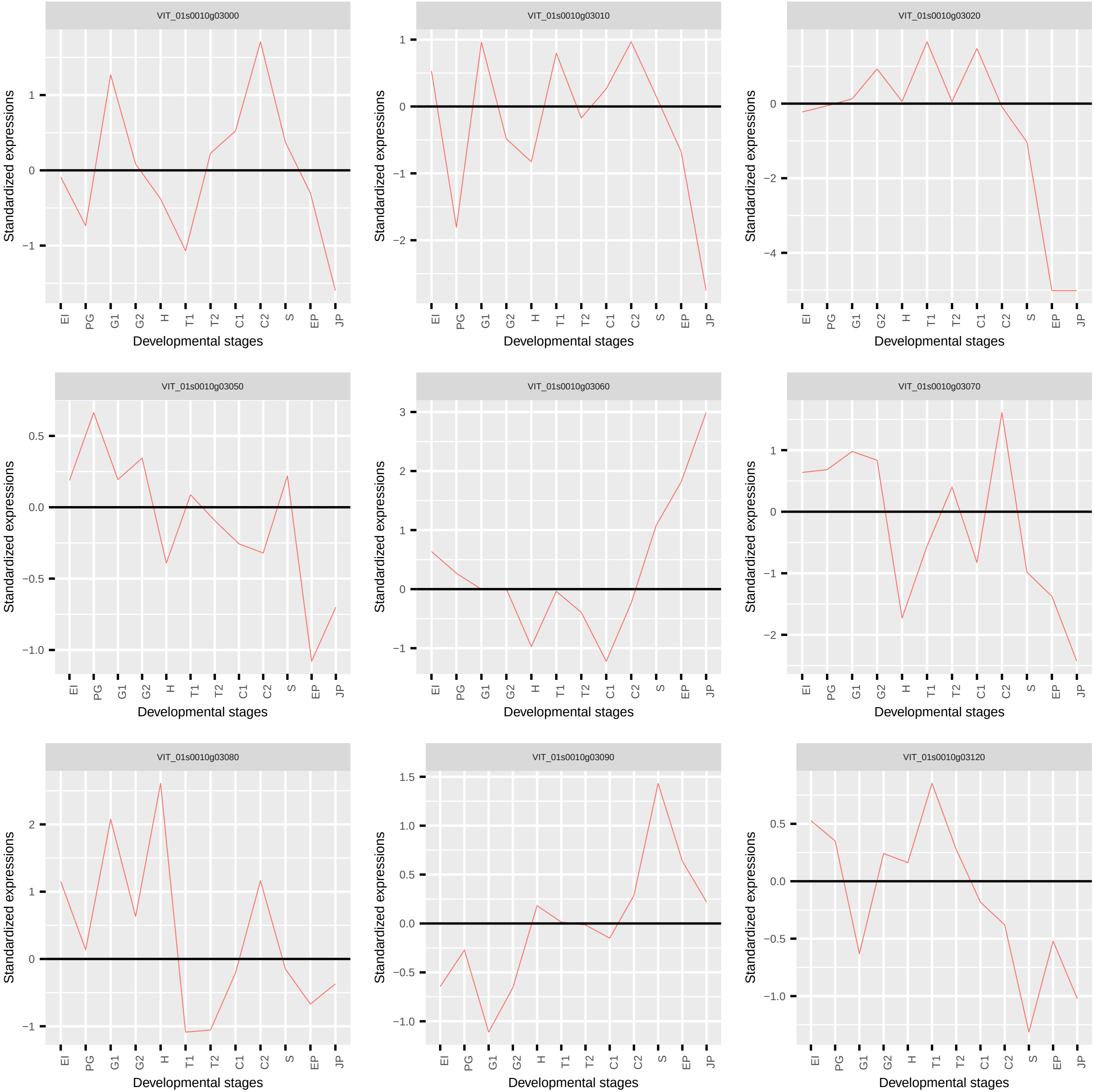

VIT_01s0010g03000
Standardized expressions
1
0
−1
EI PG G1 G2 H T1 T2 C1 C2 S EP JP
Developmental stages
VIT_01s0010g03010
Standardized expressions
1
0
−1
−2
EI PG G1 G2 H T1 T2 C1 C2 S EP JP
Developmental stages
VIT_01s0010g03020
Standardized expressions
0
−2
−4
EI PG G1 G2 H T1 T2 C1 C2 S EP JP
Developmental stages
VIT_01s0010g03050
Standardized expressions
0.5
0.0
−0.5
−1.0
EI PG G1 G2 H T1 T2 C1 C2 S EP JP
Developmental stages
VIT_01s0010g03060
Standardized expressions
3
2
1
0
−1
EI PG G1 G2 H T1 T2 C1 C2 S EP JP
Developmental stages
VIT_01s0010g03070
Standardized expressions
1
0
−1
−2
EI PG G1 G2 H T1 T2 C1 C2 S EP JP
Developmental stages
VIT_01s0010g03080
Standardized expressions
2
1
0
−1
EI PG G1 G2 H T1 T2 C1 C2 S EP JP
Developmental stages
VIT_01s0010g03090
Standardized expressions
1.5
1.0
0.5
0.0
−0.5
−1.0
EI PG G1 G2 H T1 T2 C1 C2 S EP JP
Developmental stages
VIT_01s0010g03120
Standardized expressions
0.5
0.0
−0.5
−1.0
EI PG G1 G2 H T1 T2 C1 C2 S EP JP
Developmental stages

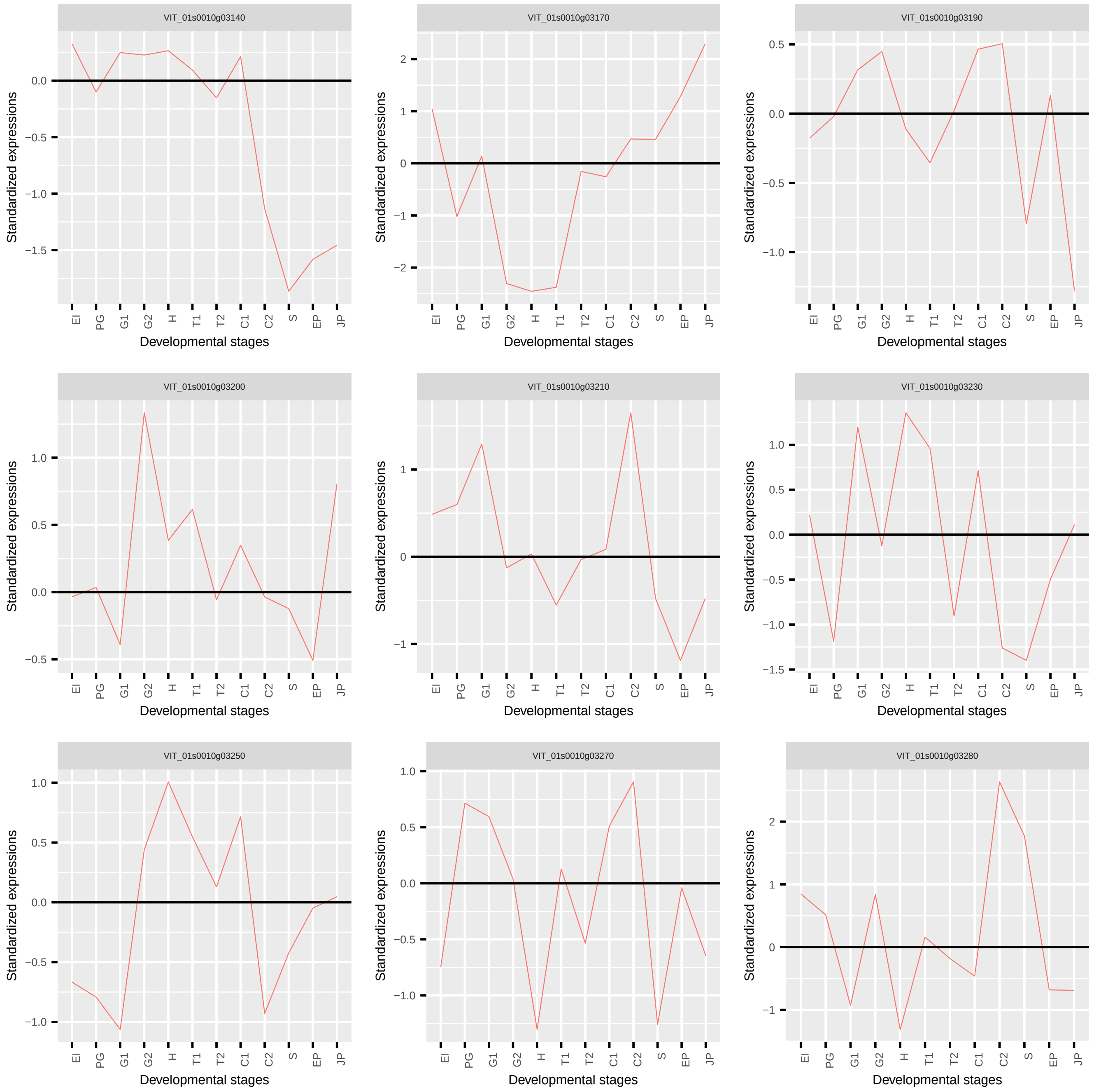
VIT_01s0010g03140
VIT_01s0010g03170
VIT_01s0010g03190
VIT_01s0010g03200
VIT_01s0010g03210
VIT_01s0010g03230
VIT_01s0010g03250
VIT_01s0010g03270
VIT_01s0010g03280
Standardized expressions
Developmental stages
EI PG G1 G2 H T1 T2 C1 C2 S EP JP

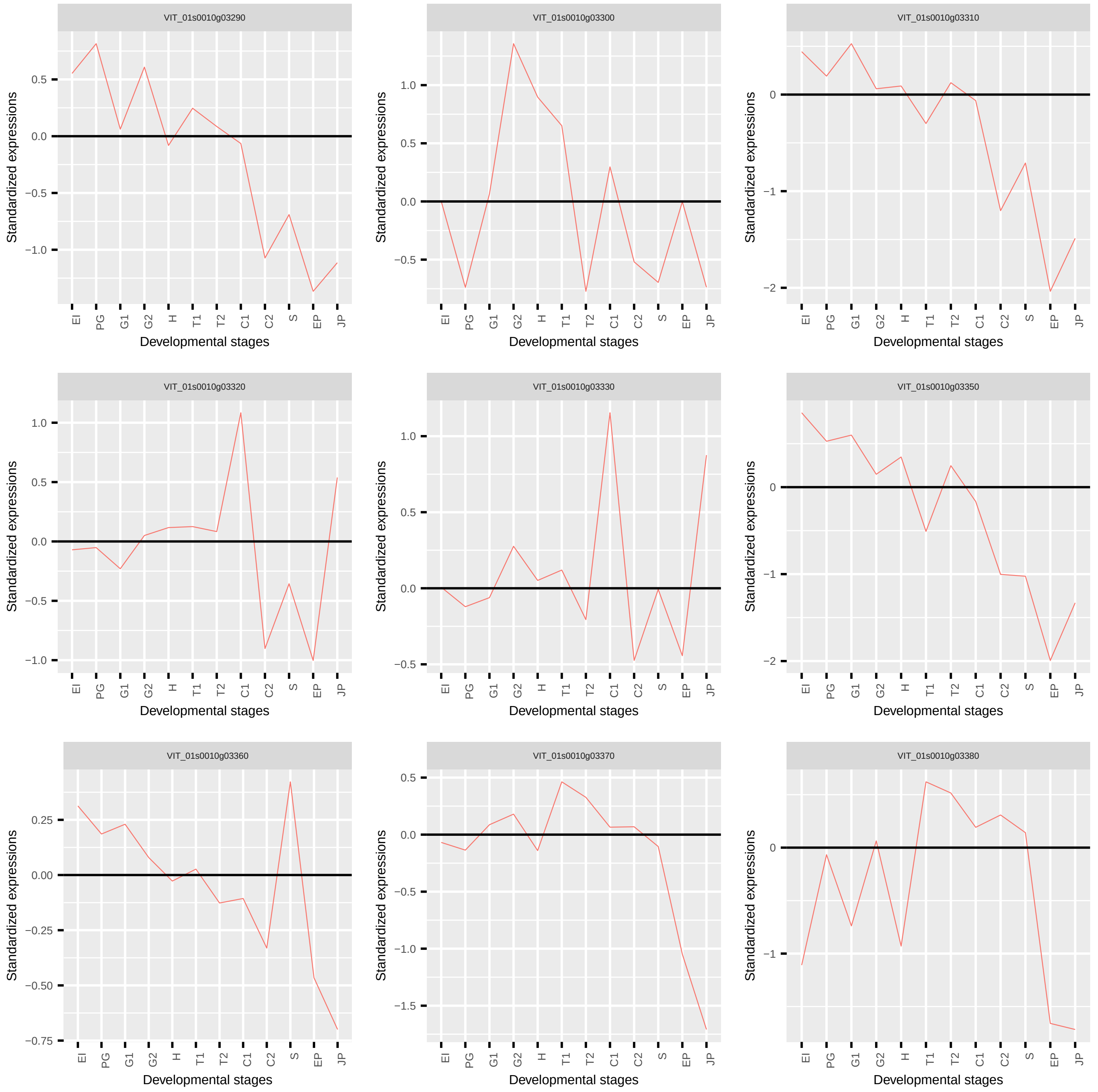
VIT_01s0010g03290
VIT_01s0010g03300
VIT_01s0010g03310
VIT_01s0010g03320
VIT_01s0010g03330
VIT_01s0010g03350
VIT_01s0010g03360
VIT_01s0010g03370
VIT_01s0010g03380
Standardized expressions
Developmental stages
EI
PG
G1
G2
H
T1
T2
C1
C2
S
EP
JP

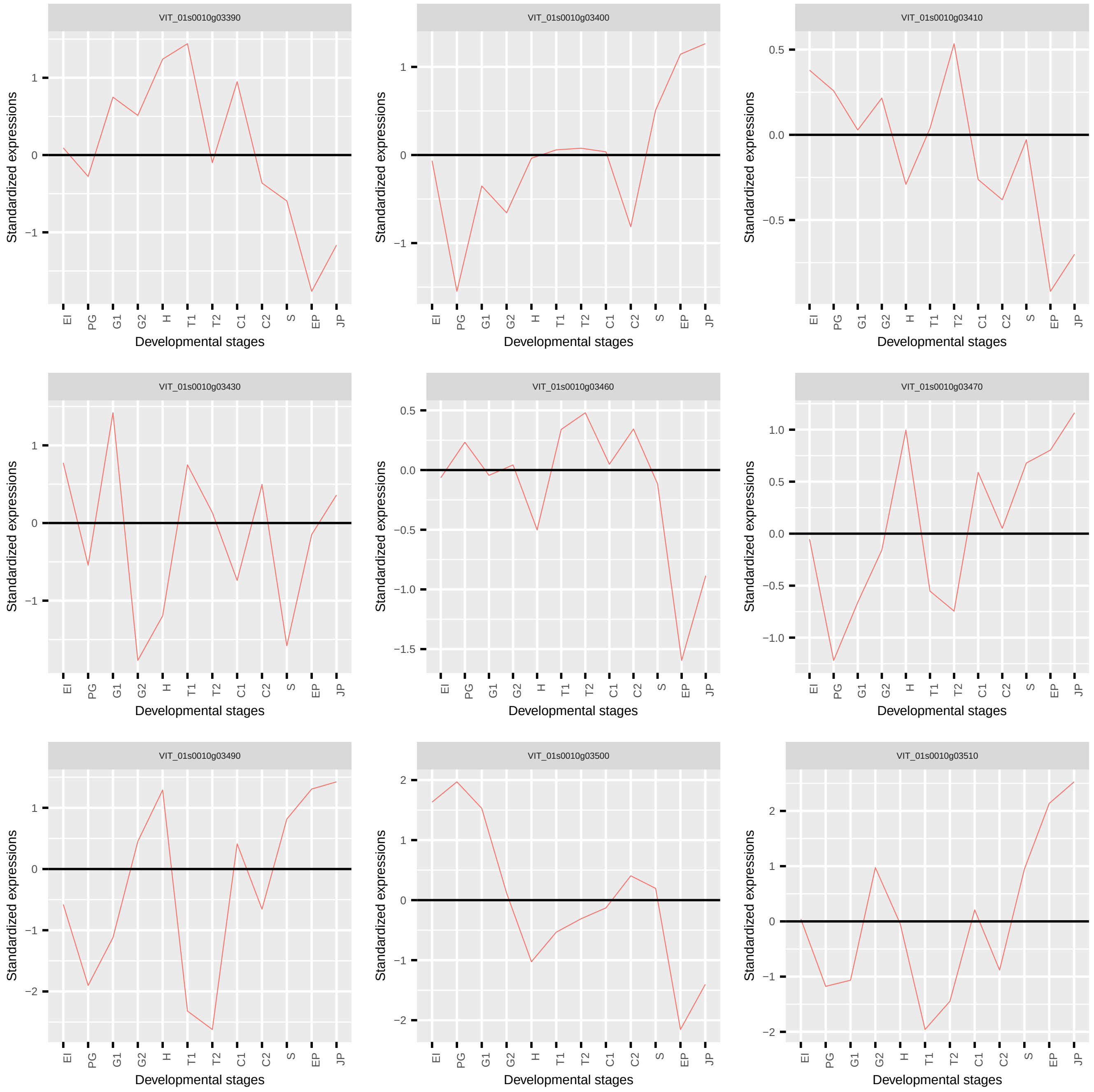

VIT_01s0010g03390
VIT_01s0010g03400
VIT_01s0010g03410
VIT_01s0010g03430
VIT_01s0010g03460
VIT_01s0010g03470
VIT_01s0010g03490
VIT_01s0010g03500
VIT_01s0010g03510
Standardized expressions
Developmental stages
EI
PG
G1
G2
H
T1
T2
C1
C2
S
EP
JP

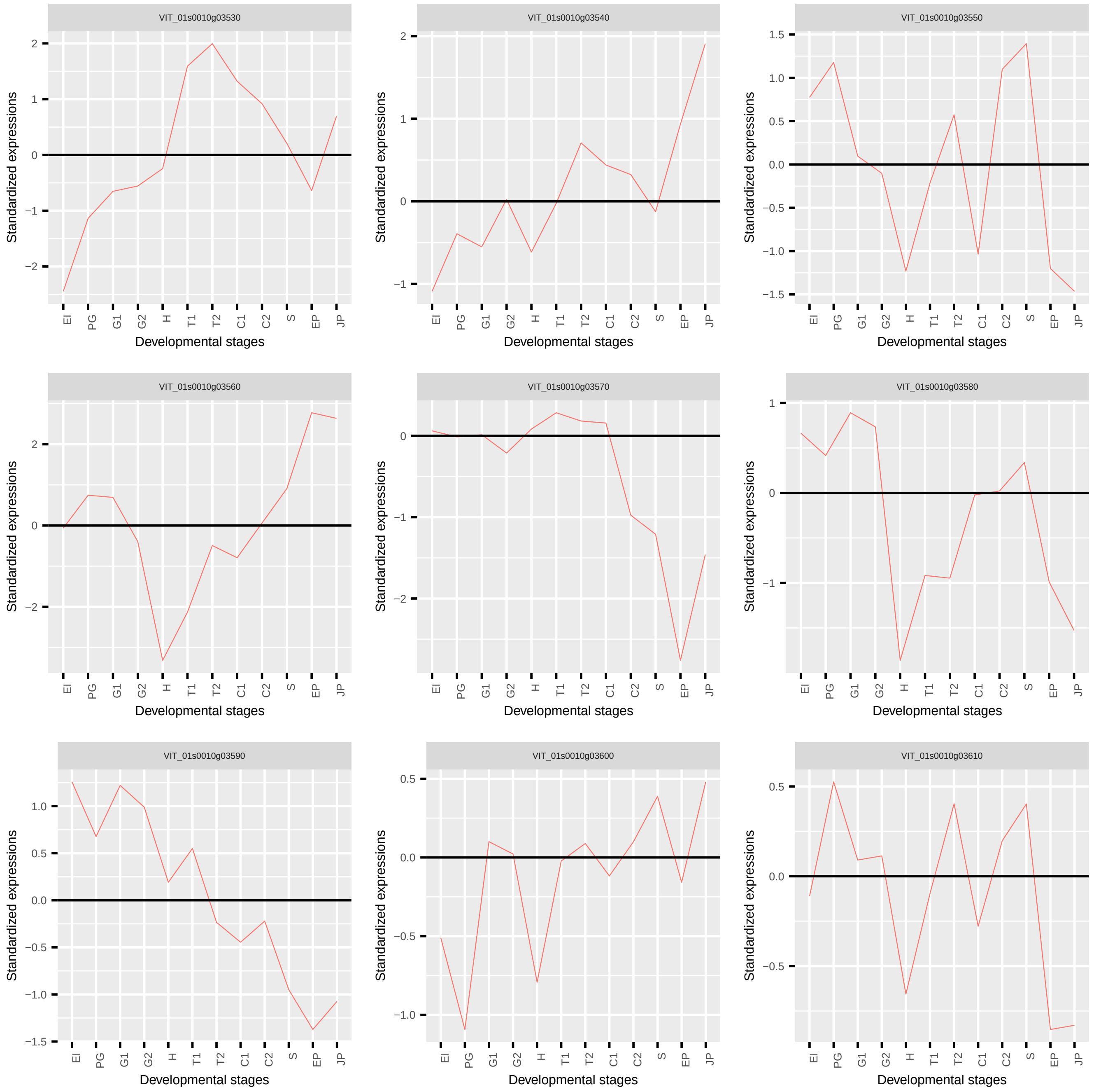
VIT_01s0010g03530
VIT_01s0010g03540
VIT_01s0010g03550
VIT_01s0010g03560
VIT_01s0010g03570
VIT_01s0010g03580
VIT_01s0010g03590
VIT_01s0010g03600
VIT_01s0010g03610
Standardized expressions
Developmental stages
EI PG G1 G2 H T1 T2 C1 C2 S EP JP

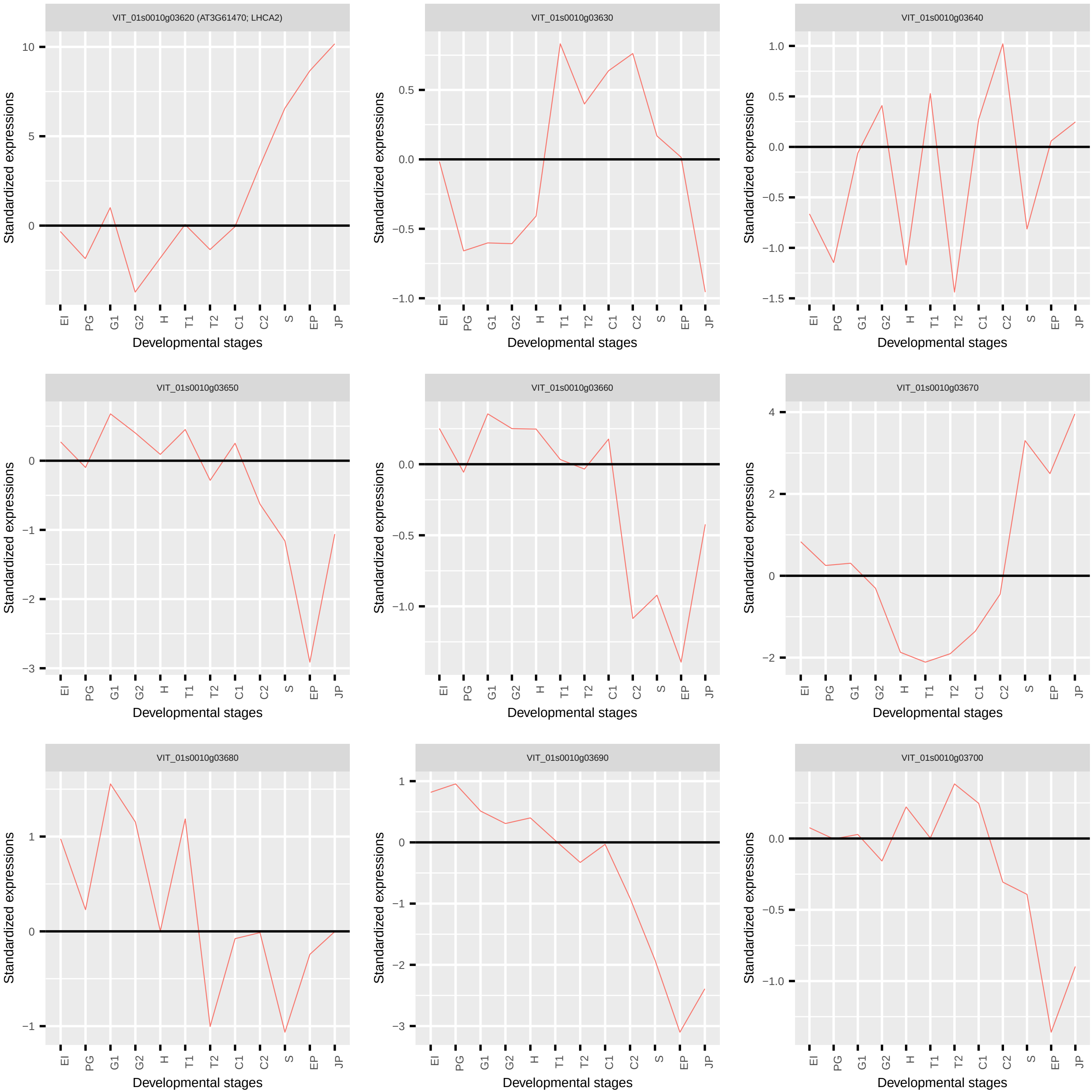
VIT_01s0010g03620 (AT3G61470; LHCA2)
VIT_01s0010g03630
VIT_01s0010g03640
VIT_01s0010g03650
VIT_01s0010g03660
VIT_01s0010g03670
VIT_01s0010g03680
VIT_01s0010g03690
VIT_01s0010g03700
Standardized expressions
Developmental stages
EI
PG
G1
G2
H
T1
T2
C1
C2
S
EP
JP

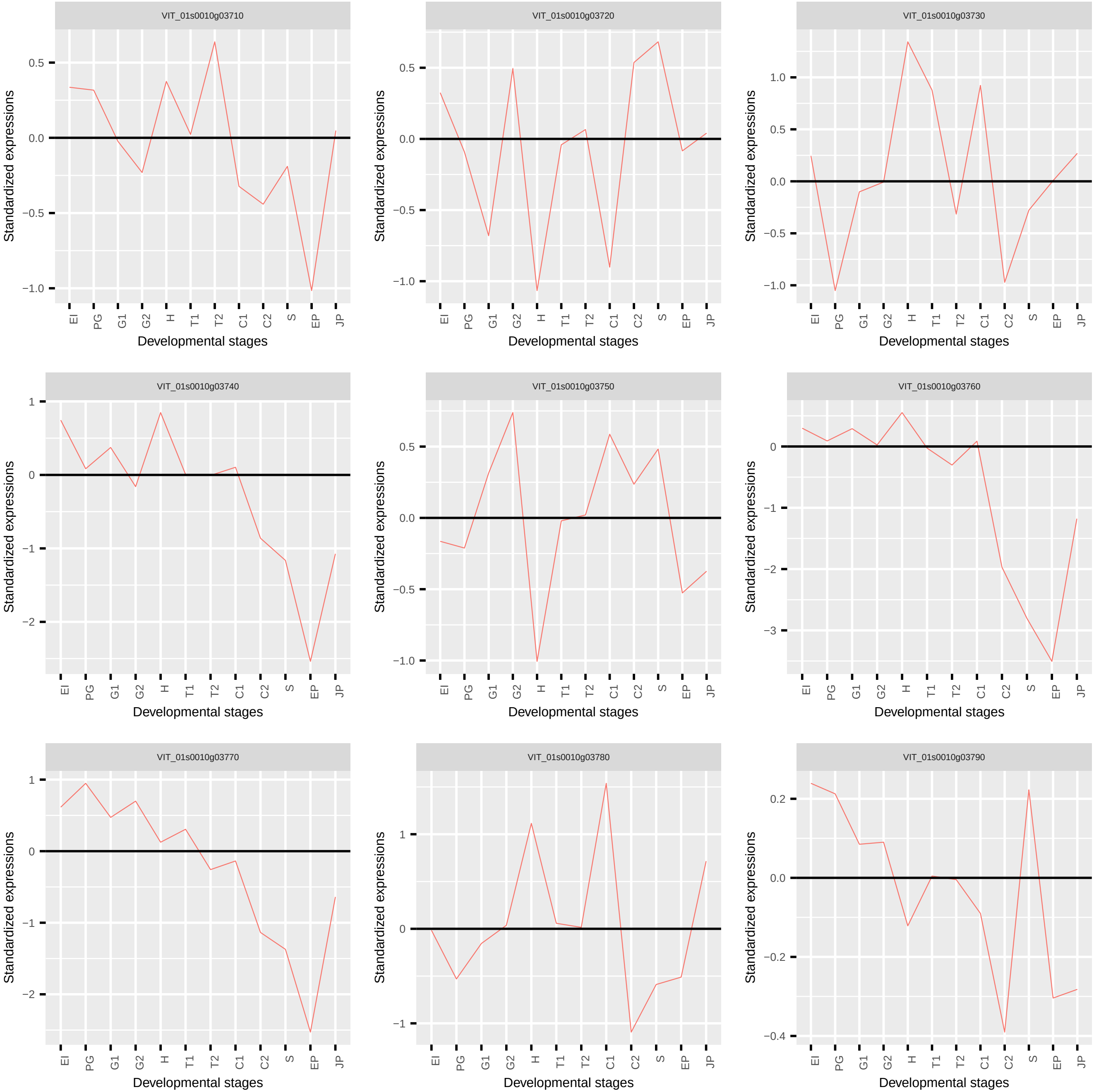
VIT_01s0010g03710
VIT_01s0010g03720
VIT_01s0010g03730
VIT_01s0010g03740
VIT_01s0010g03750
VIT_01s0010g03760
VIT_01s0010g03770
VIT_01s0010g03780
VIT_01s0010g03790
Standardized expressions
Developmental stages
EI PG G1 G2 H T1 T2 C1 C2 S EP JP

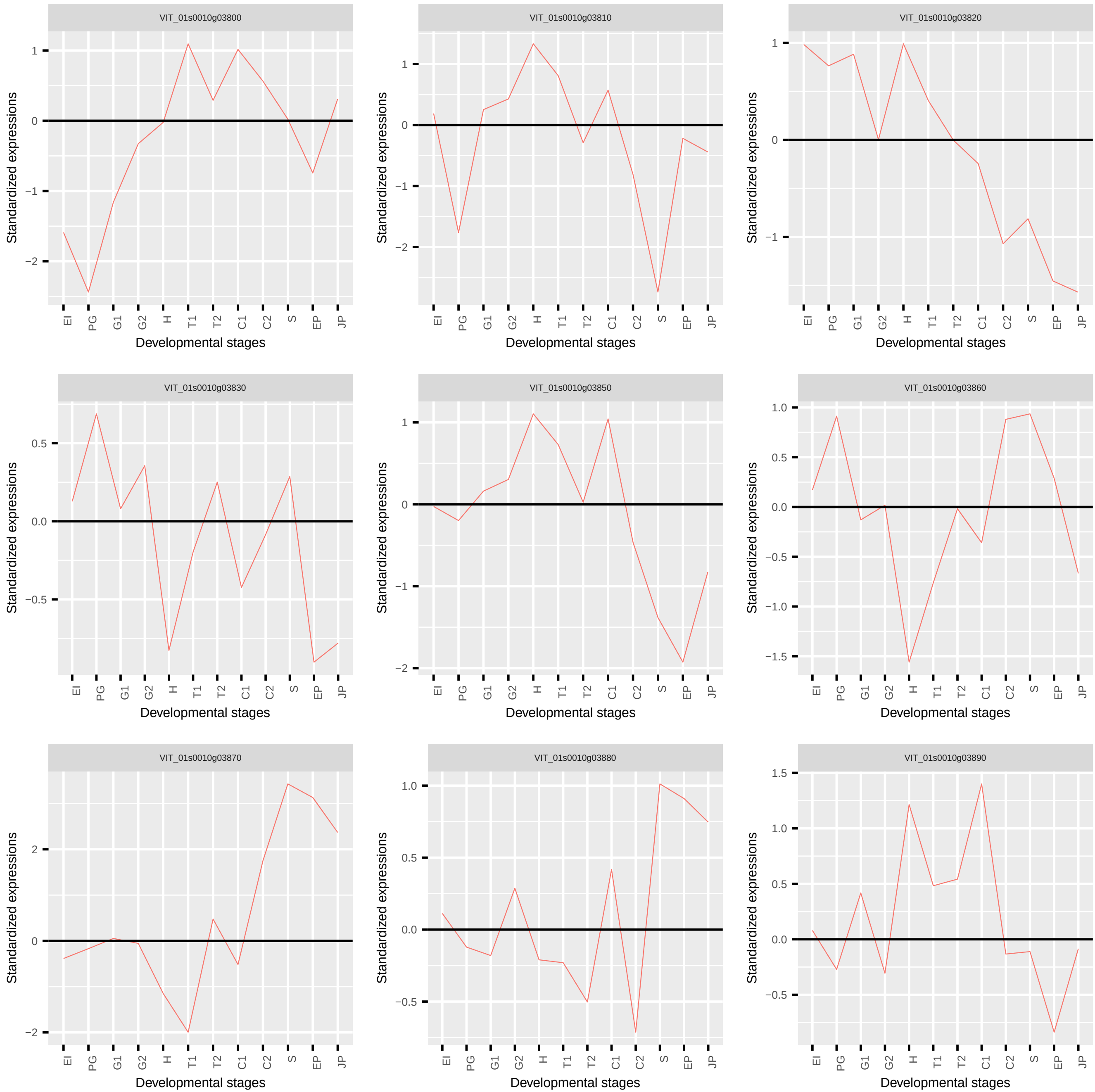
VIT_01s0010g03800
VIT_01s0010g03810
VIT_01s0010g03820
VIT_01s0010g03830
VIT_01s0010g03850
VIT_01s0010g03860
VIT_01s0010g03870
VIT_01s0010g03880
VIT_01s0010g03890
Standardized expressions
Developmental stages
EI
PG
G1
G2
H
T1
T2
C1
C2
S
EP
JP

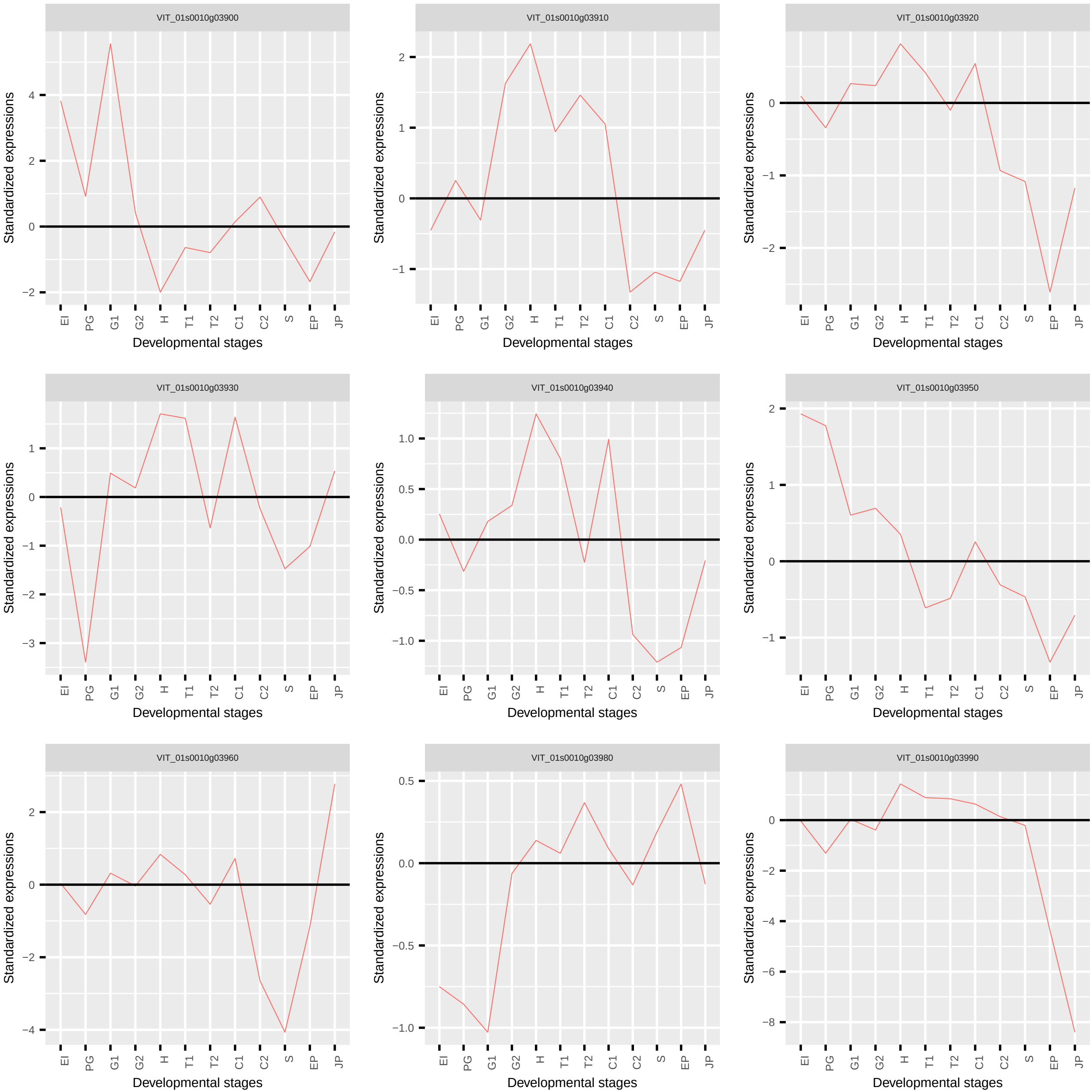
VIT_01s0010g03900
4
2
0
−2
Standardized expressions
EI PG G1 G2 H T1 T2 C1 C2 S EP JP
Developmental stages
VIT_01s0010g03910
2
1
0
−1
Standardized expressions
EI PG G1 G2 H T1 T2 C1 C2 S EP JP
Developmental stages
VIT_01s0010g03920
0
−1
−2
Standardized expressions
EI PG G1 G2 H T1 T2 C1 C2 S EP JP
Developmental stages
VIT_01s0010g03930
1
0
−1
−2
−3
Standardized expressions
EI PG G1 G2 H T1 T2 C1 C2 S EP JP
Developmental stages
VIT_01s0010g03940
1.0
0.5
0.0
−0.5
−1.0
Standardized expressions
EI PG G1 G2 H T1 T2 C1 C2 S EP JP
Developmental stages
VIT_01s0010g03950
2
1
0
−1
Standardized expressions
EI PG G1 G2 H T1 T2 C1 C2 S EP JP
Developmental stages
VIT_01s0010g03960
2
0
−2
−4
Standardized expressions
EI PG G1 G2 H T1 T2 C1 C2 S EP JP
Developmental stages
VIT_01s0010g03980
0.5
0.0
−0.5
−1.0
Standardized expressions
EI PG G1 G2 H T1 T2 C1 C2 S EP JP
Developmental stages
VIT_01s0010g03990
0
−2
−4
−6
−8
Standardized expressions
EI PG G1 G2 H T1 T2 C1 C2 S EP JP
Developmental stages

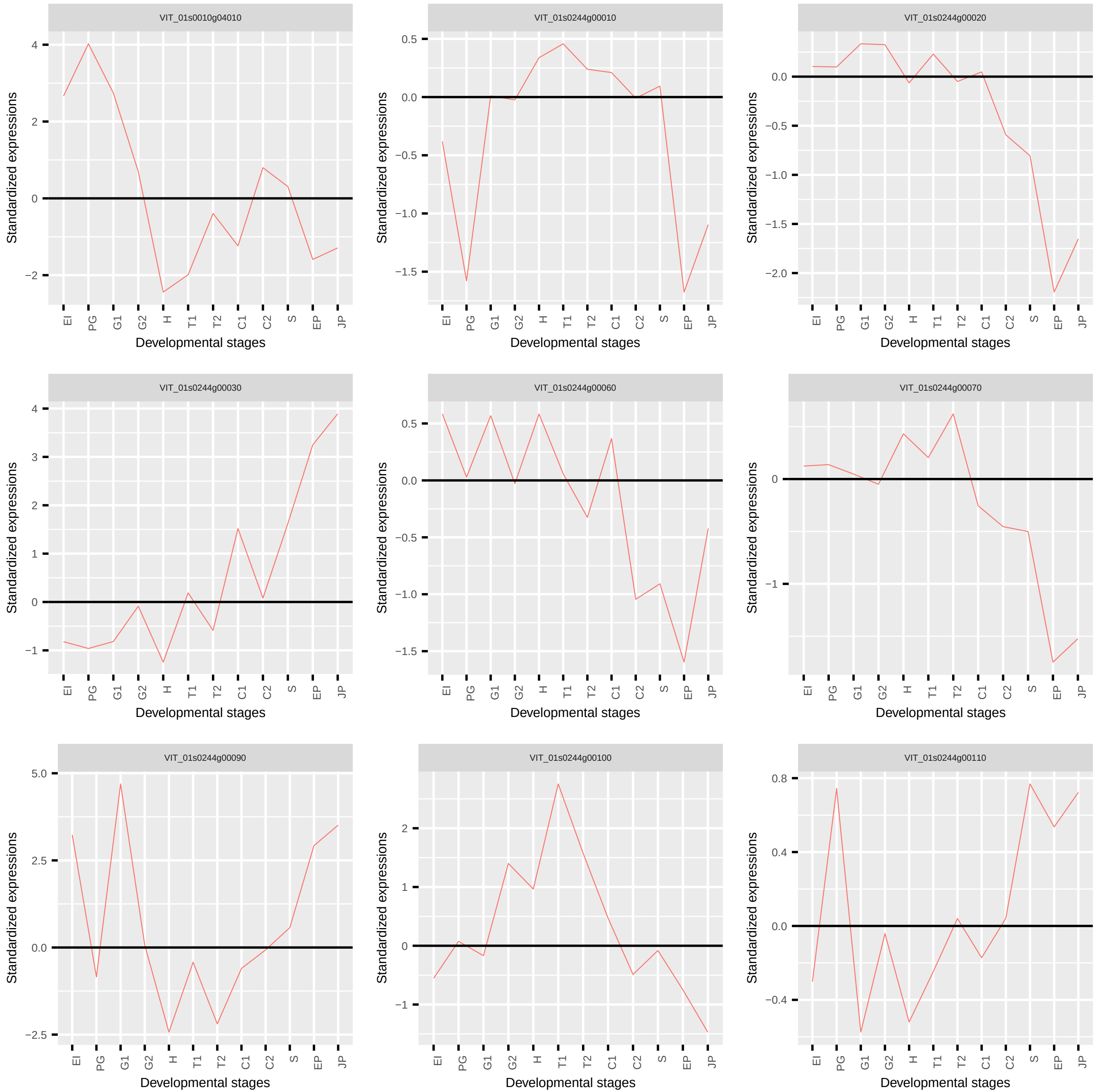
VIT_01s0010g04010
VIT_01s0244g00010
VIT_01s0244g00020
VIT_01s0244g00030
VIT_01s0244g00060
VIT_01s0244g00070
VIT_01s0244g00090
VIT_01s0244g00100
VIT_01s0244g00110
Standardized expressions
Developmental stages
EI PG G1 G2 H T1 T2 C1 C2 S EP JP

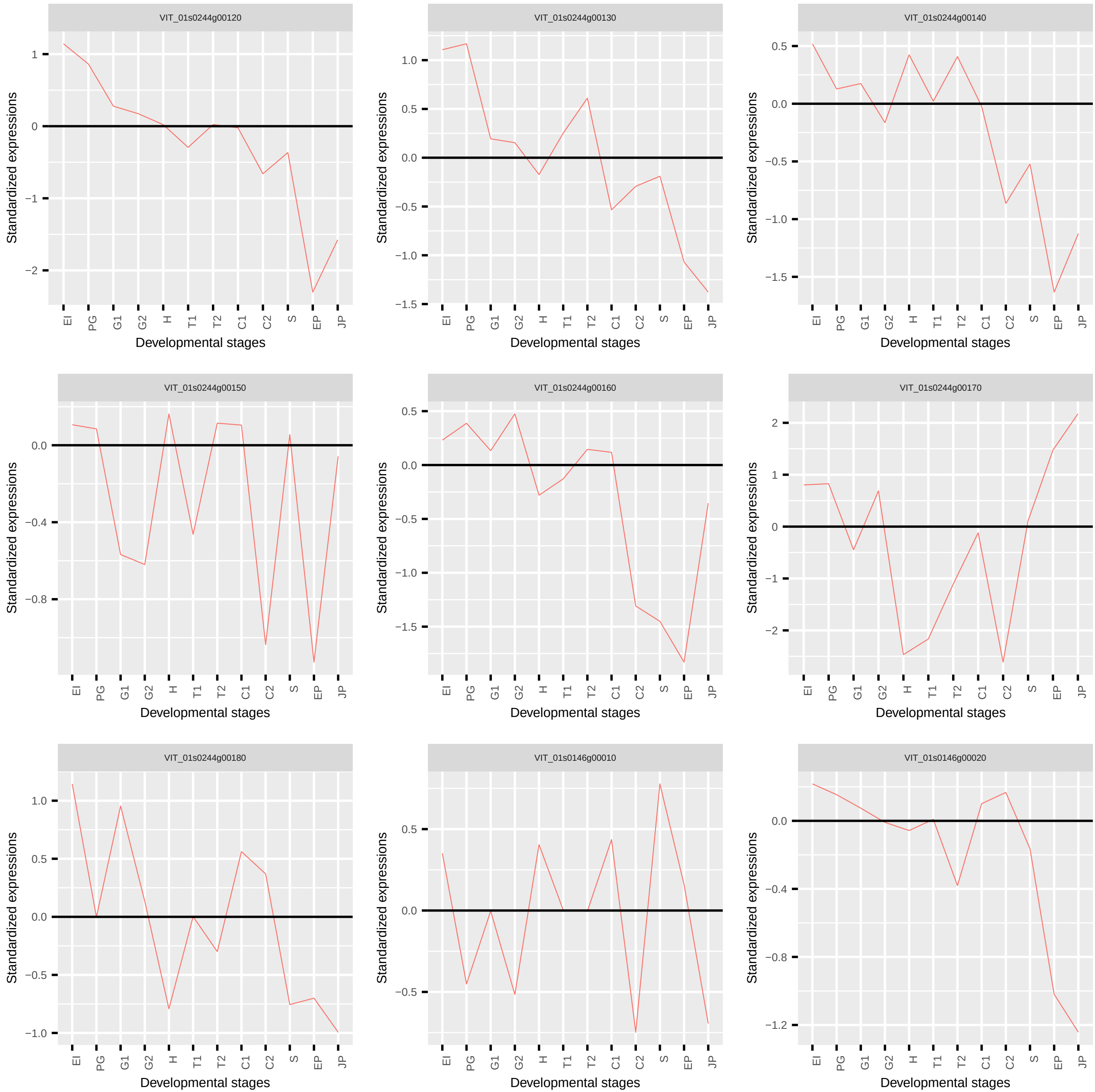
VIT_01s0244g00120
VIT_01s0244g00130
VIT_01s0244g00140
VIT_01s0244g00150
VIT_01s0244g00160
VIT_01s0244g00170
VIT_01s0244g00180
VIT_01s0146g00010
VIT_01s0146g00020
Standardized expressions
Developmental stages
EI PG G1 G2 H T1 T2 C1 C2 S EP JP

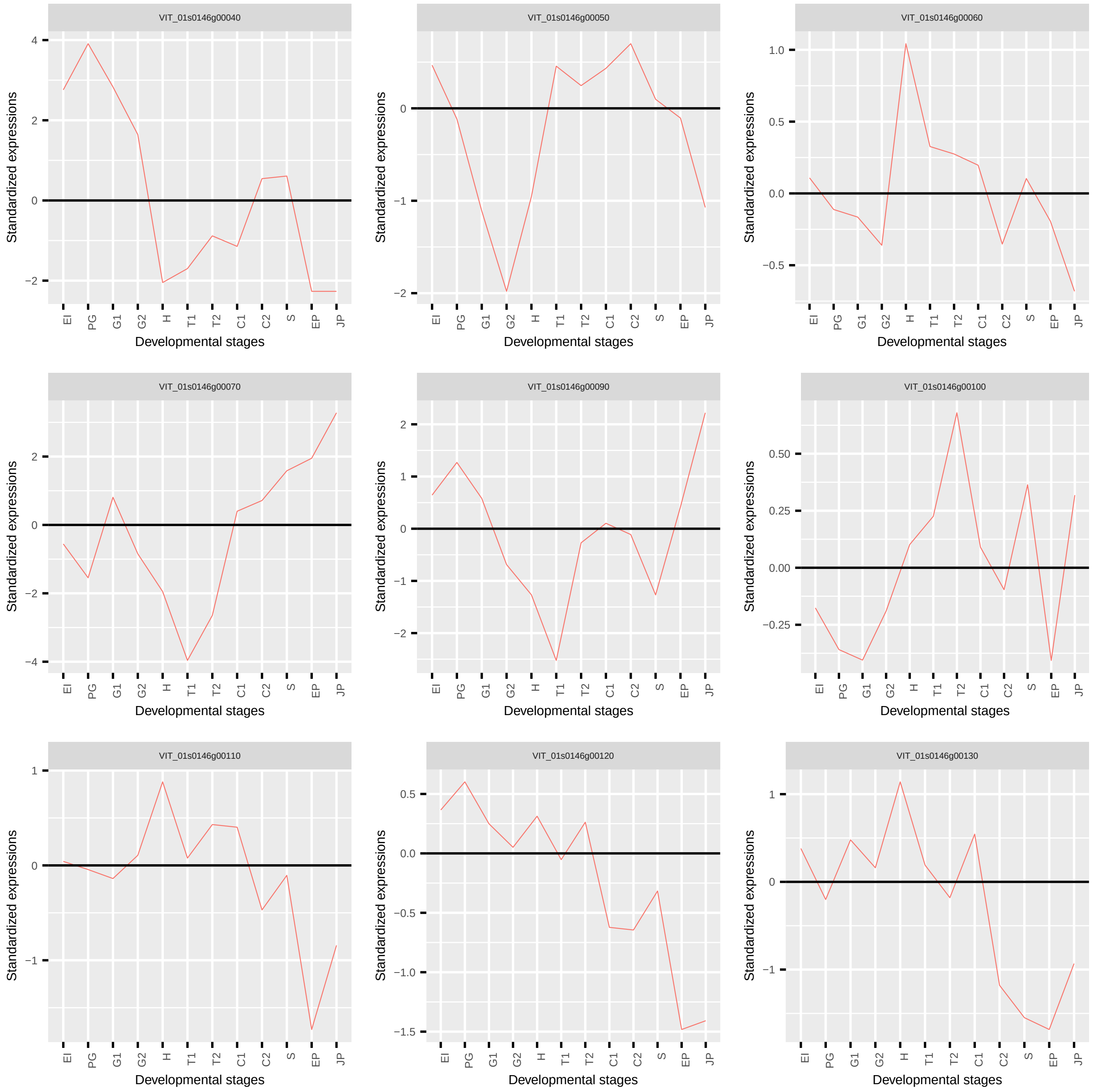
VIT_01s0146g00040
VIT_01s0146g00050
VIT_01s0146g00060
VIT_01s0146g00070
VIT_01s0146g00090
VIT_01s0146g00100
VIT_01s0146g00110
VIT_01s0146g00120
VIT_01s0146g00130
Standardized expressions
Developmental stages
EI PG G1 G2 H T1 T2 C1 C2 S EP JP

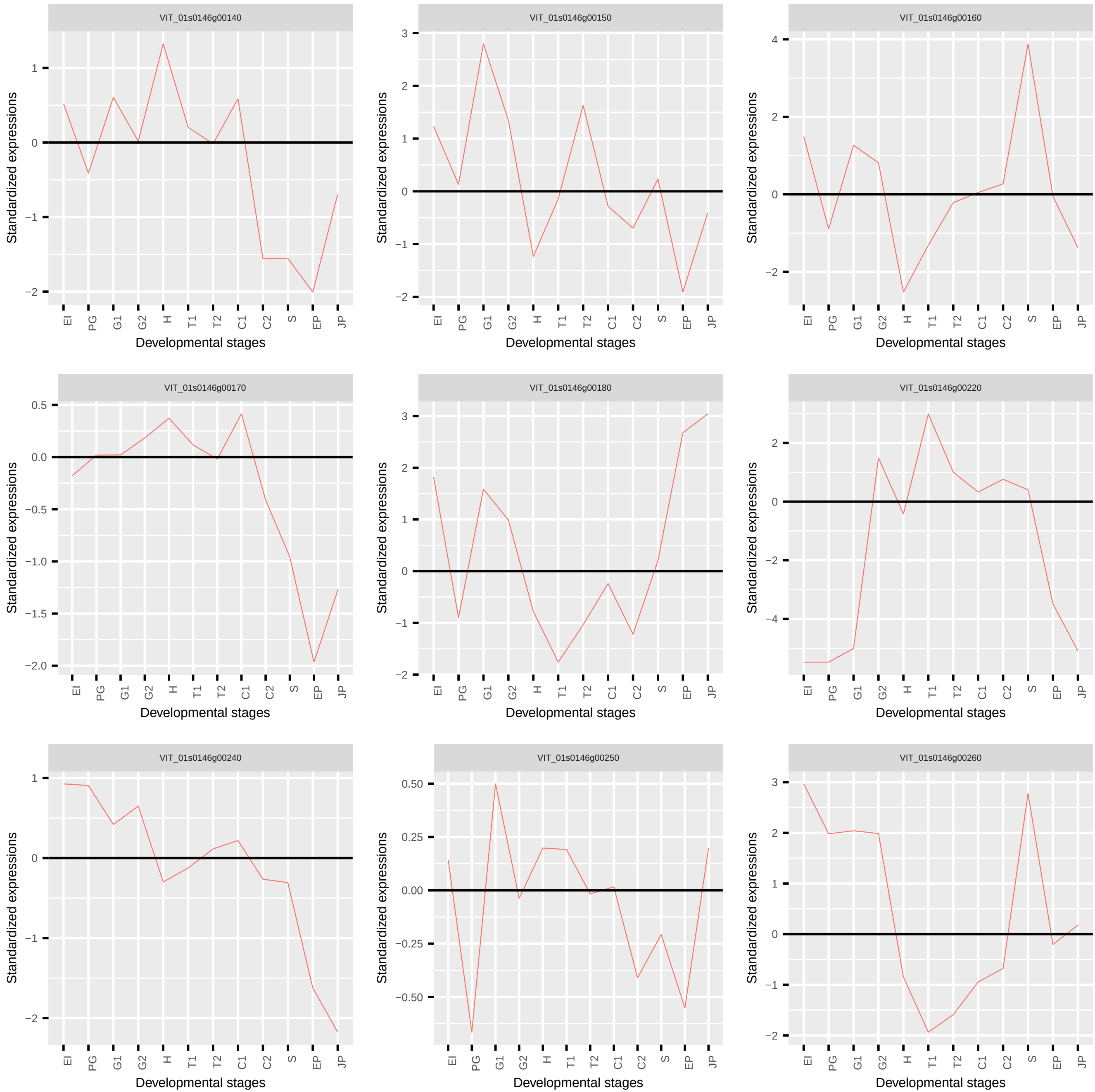
VIT_01s0146g00140
VIT_01s0146g00150
VIT_01s0146g00160
VIT_01s0146g00170
VIT_01s0146g00180
VIT_01s0146g00220
VIT_01s0146g00240
VIT_01s0146g00250
VIT_01s0146g00260
Standardized expressions
Developmental stages
EI PG G1 G2 H T1 T2 C1 C2 S EP JP
1 0 −1 −2
3 2 1 0 −1 −2
4 2 0 −2
0.5 0.0 −0.5 −1.0 −1.5 −2.0
3 2 1 0 −1 −2
2 0 −2 −4
1 0 −1 −2
0.50 0.25 0.00 −0.25 −0.50
3 2 1 0 −1 −2

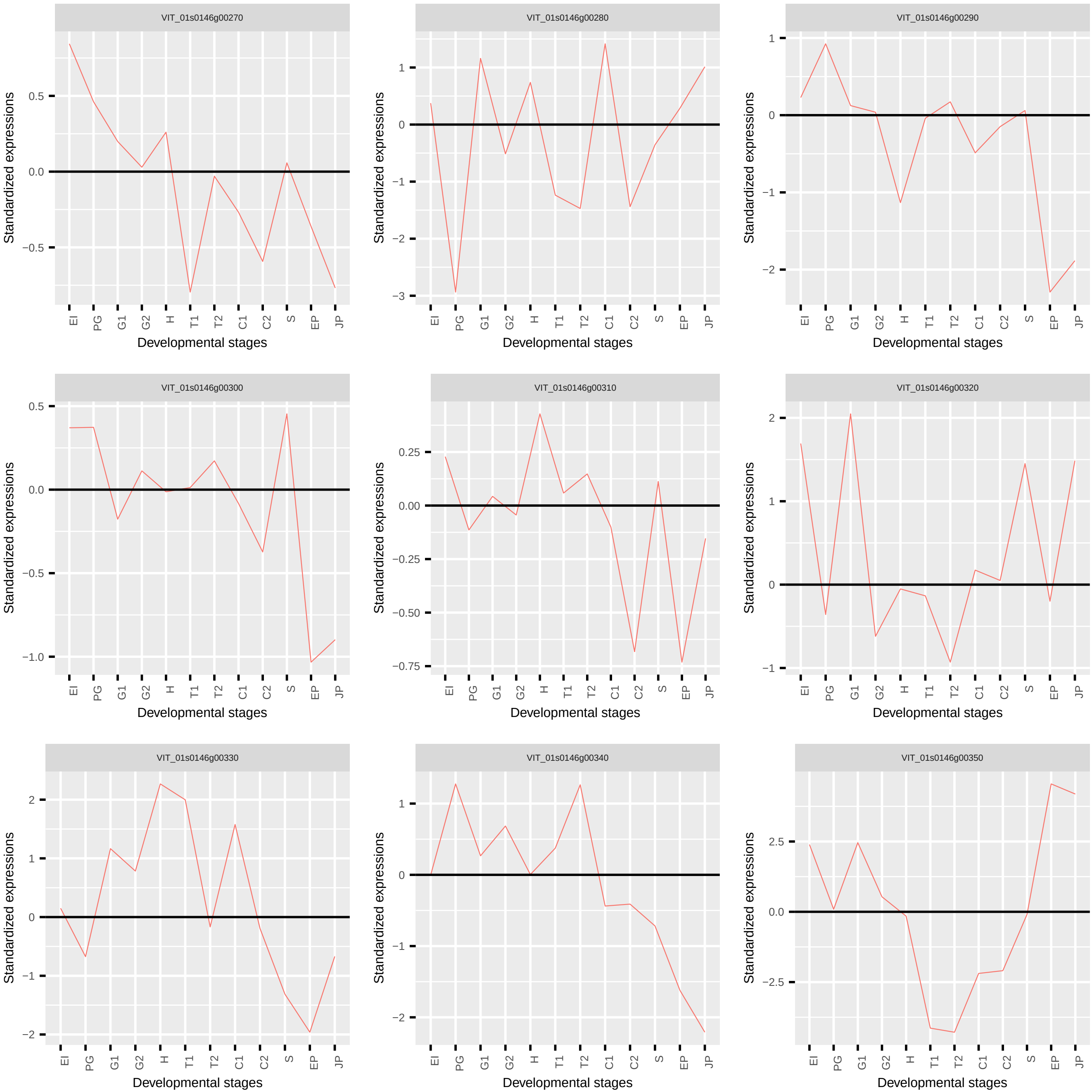
VIT_01s0146g00270
VIT_01s0146g00280
VIT_01s0146g00290
VIT_01s0146g00300
VIT_01s0146g00310
VIT_01s0146g00320
VIT_01s0146g00330
VIT_01s0146g00340
VIT_01s0146g00350
Standardized expressions
Developmental stages
EI PG G1 G2 H T1 T2 C1 C2 S EP JP

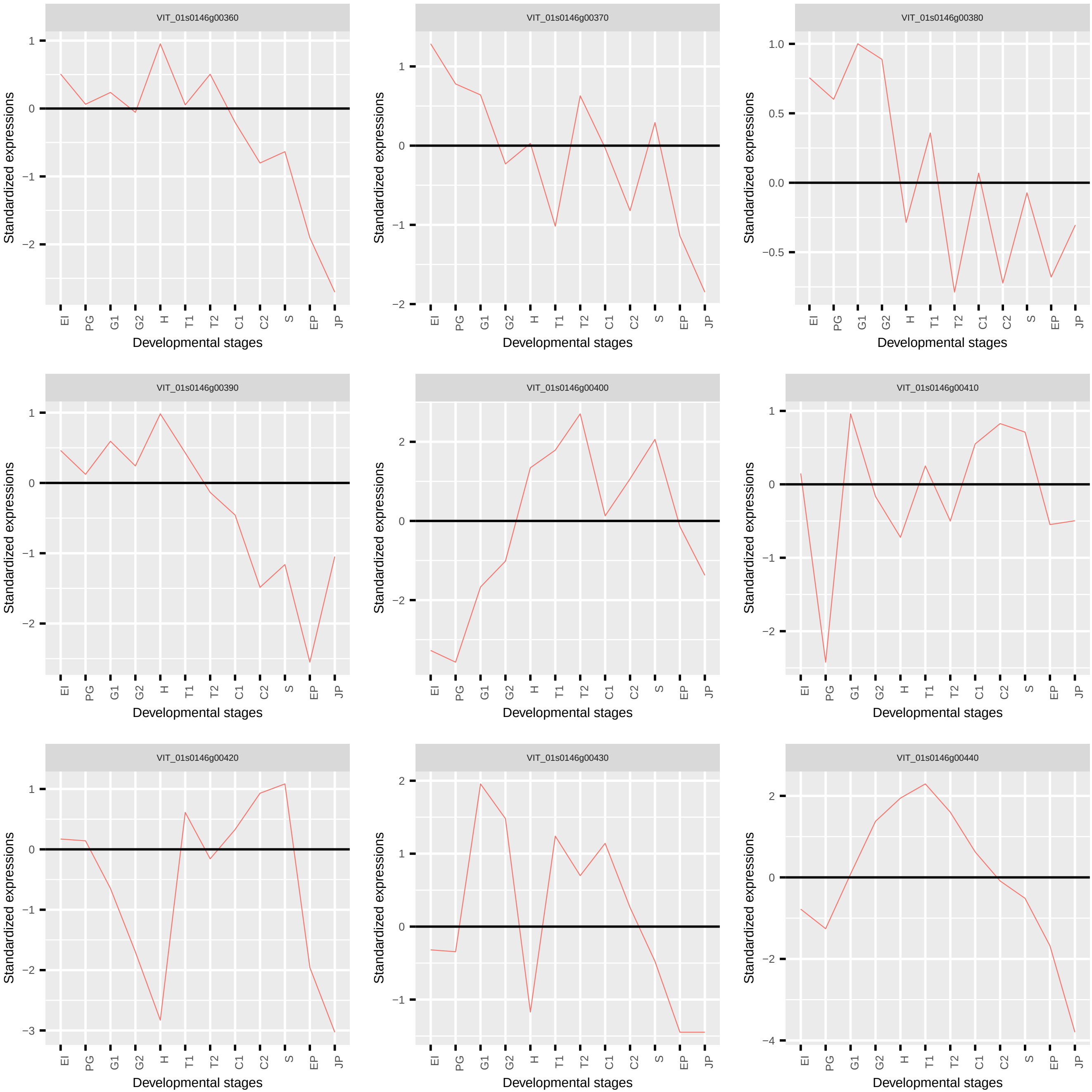
VIT_01s0146g00360
VIT_01s0146g00370
VIT_01s0146g00380
VIT_01s0146g00390
VIT_01s0146g00400
VIT_01s0146g00410
VIT_01s0146g00420
VIT_01s0146g00430
VIT_01s0146g00440
Standardized expressions
Developmental stages
EI PG G1 G2 H T1 T2 C1 C2 S EP JP

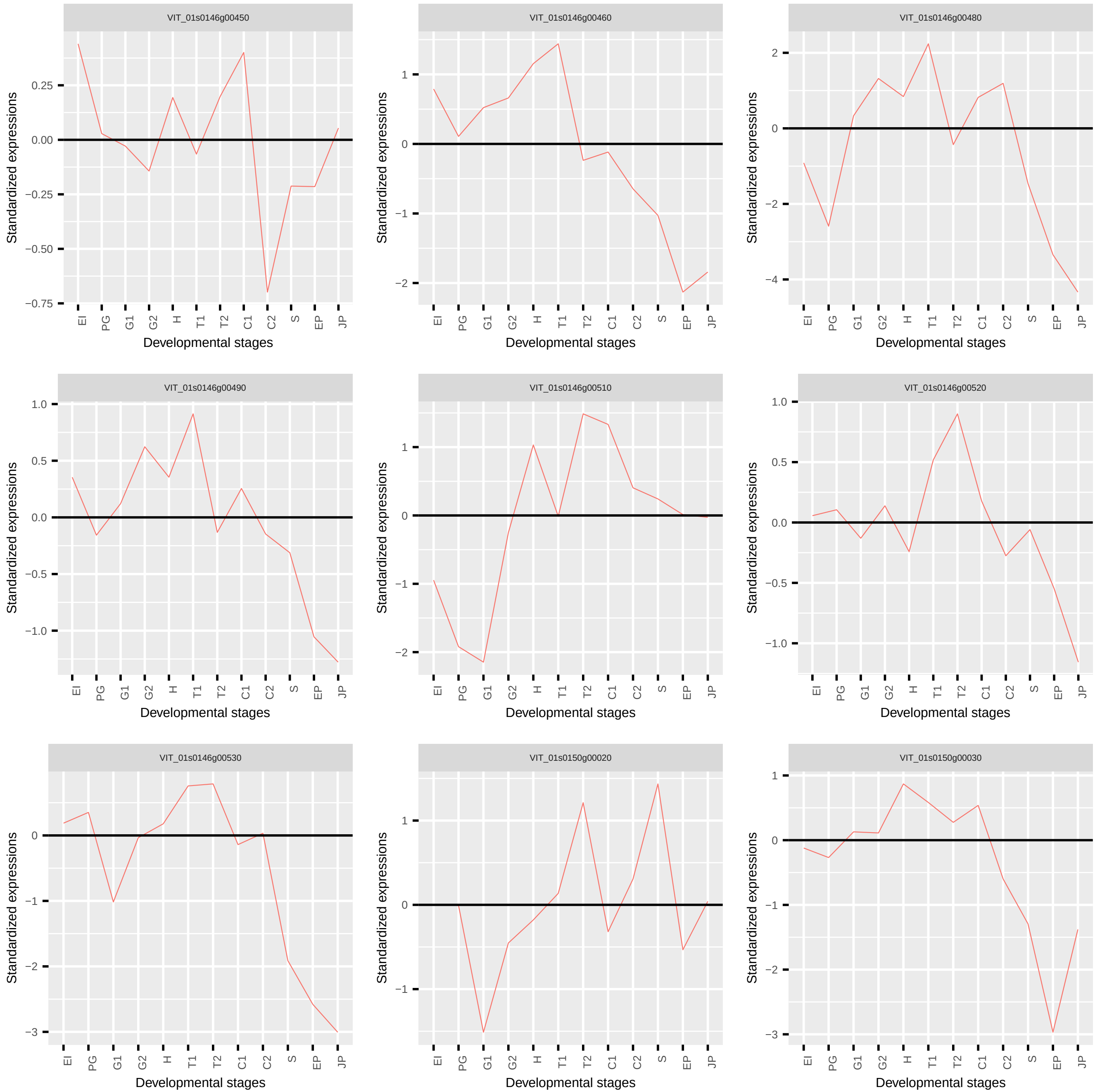
VIT_01s0146g00450
VIT_01s0146g00460
VIT_01s0146g00480
VIT_01s0146g00490
VIT_01s0146g00510
VIT_01s0146g00520
VIT_01s0146g00530
VIT_01s0150g00020
VIT_01s0150g00030
Standardized expressions
Developmental stages
EI PG G1 G2 H T1 T2 C1 C2 S EP JP

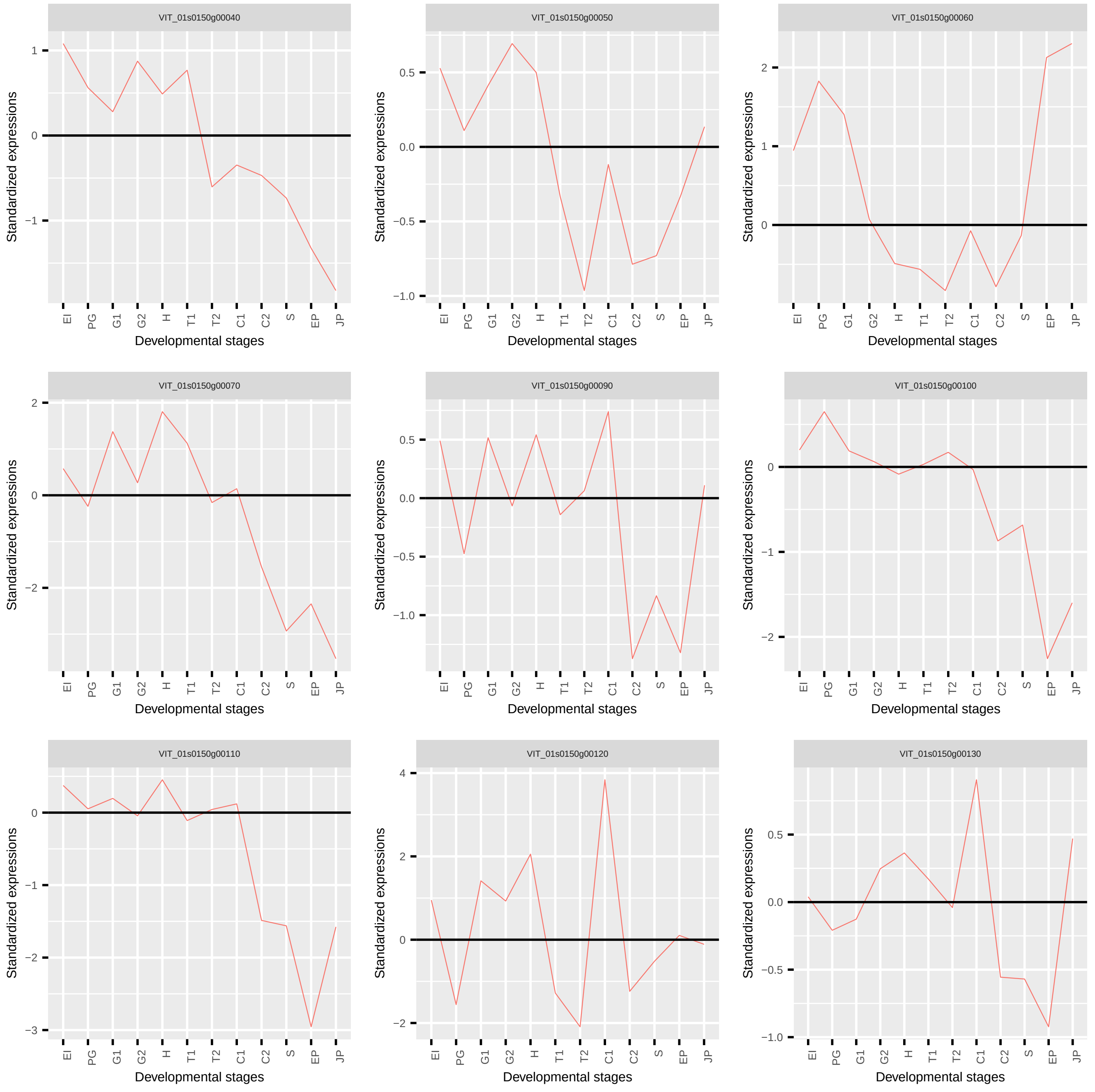
VIT_01s0150g00040
VIT_01s0150g00050
VIT_01s0150g00060
VIT_01s0150g00070
VIT_01s0150g00090
VIT_01s0150g00100
VIT_01s0150g00110
VIT_01s0150g00120
VIT_01s0150g00130
Standardized expressions
Developmental stages
EI PG G1 G2 H T1 T2 C1 C2 S EP JP

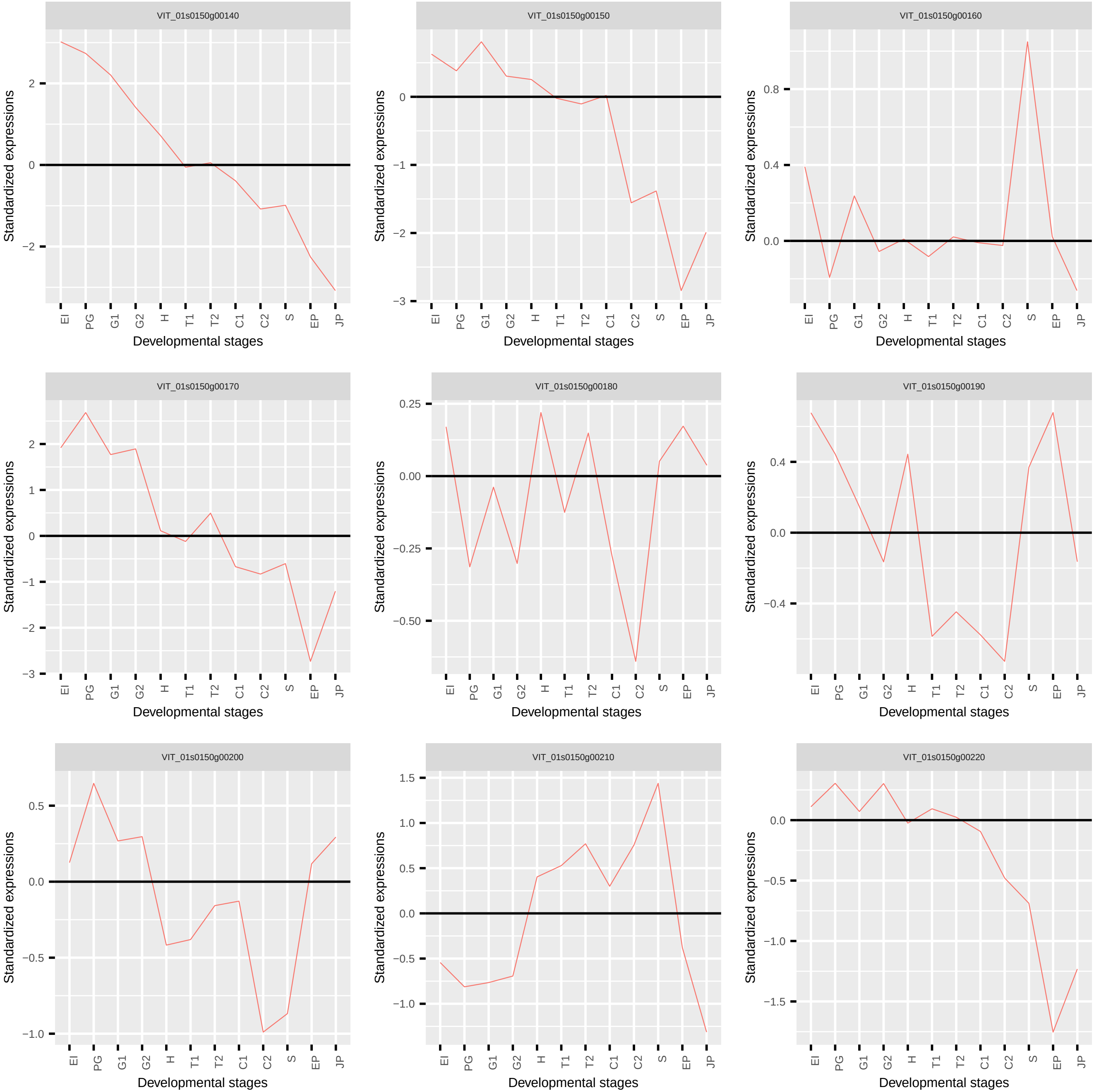
VIT_01s0150g00140
VIT_01s0150g00150
VIT_01s0150g00160
VIT_01s0150g00170
VIT_01s0150g00180
VIT_01s0150g00190
VIT_01s0150g00200
VIT_01s0150g00210
VIT_01s0150g00220
Standardized expressions
Developmental stages
EI PG G1 G2 H T1 T2 C1 C2 S EP JP

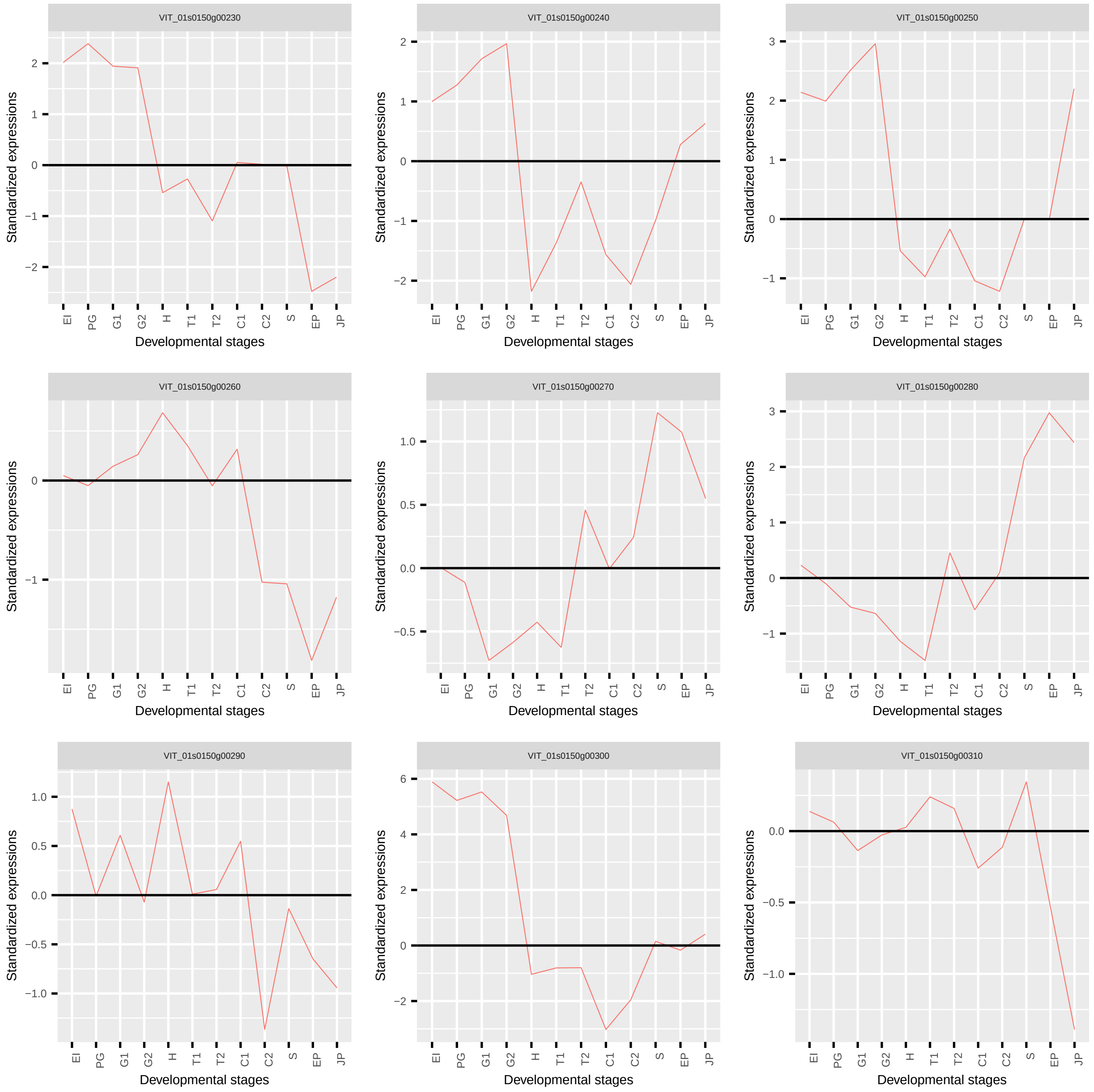
VIT_01s0150g00230
VIT_01s0150g00240
VIT_01s0150g00250
VIT_01s0150g00260
VIT_01s0150g00270
VIT_01s0150g00280
VIT_01s0150g00290
VIT_01s0150g00300
VIT_01s0150g00310
Standardized expressions
Developmental stages
EI
PG
G1
G2
H
T1
T2
C1
C2
S
EP
JP

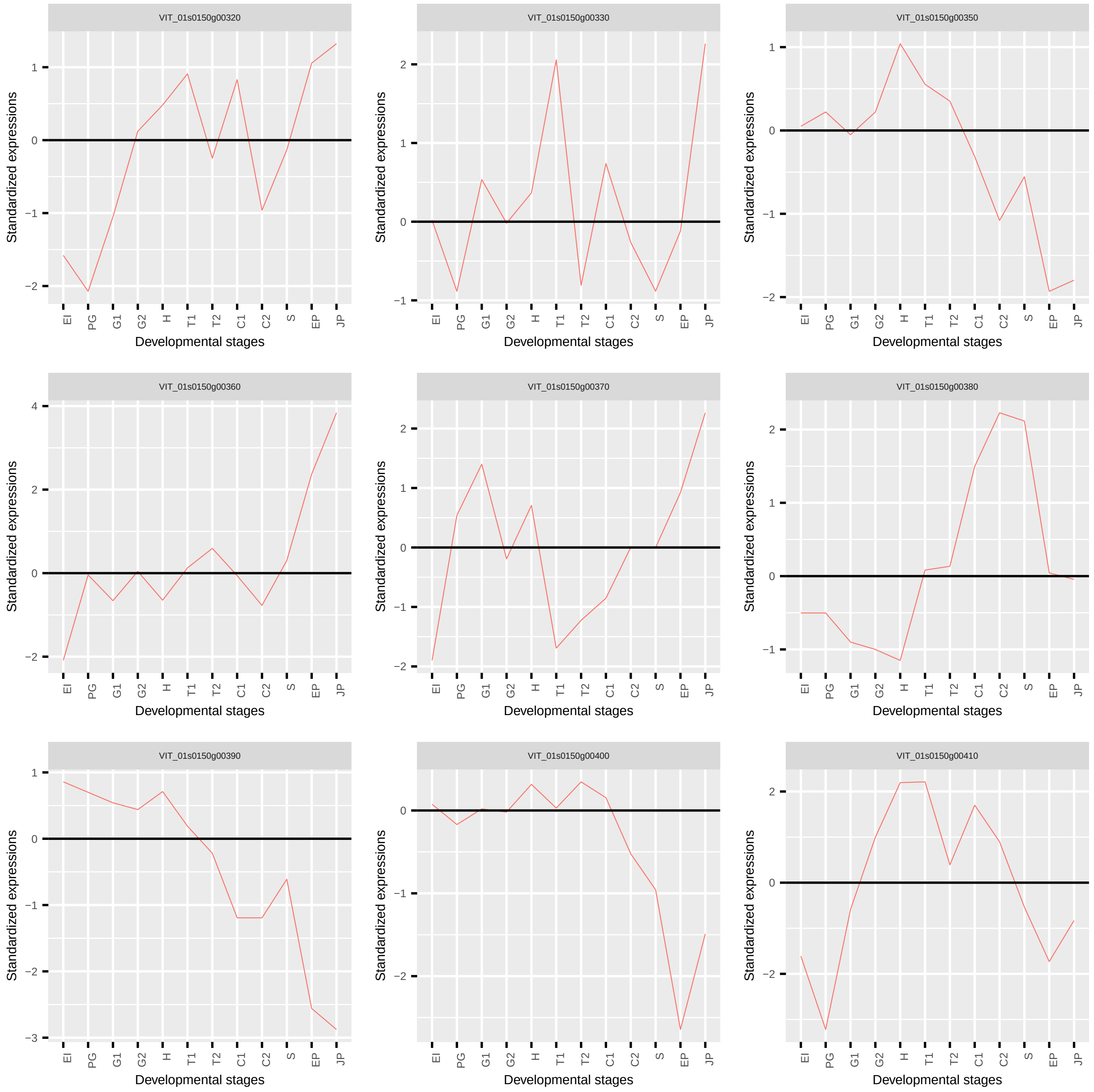
VIT_01s0150g00320
VIT_01s0150g00330
VIT_01s0150g00350
VIT_01s0150g00360
VIT_01s0150g00370
VIT_01s0150g00380
VIT_01s0150g00390
VIT_01s0150g00400
VIT_01s0150g00410
Standardized expressions
Developmental stages
EI PG G1 G2 H T1 T2 C1 C2 S EP JP

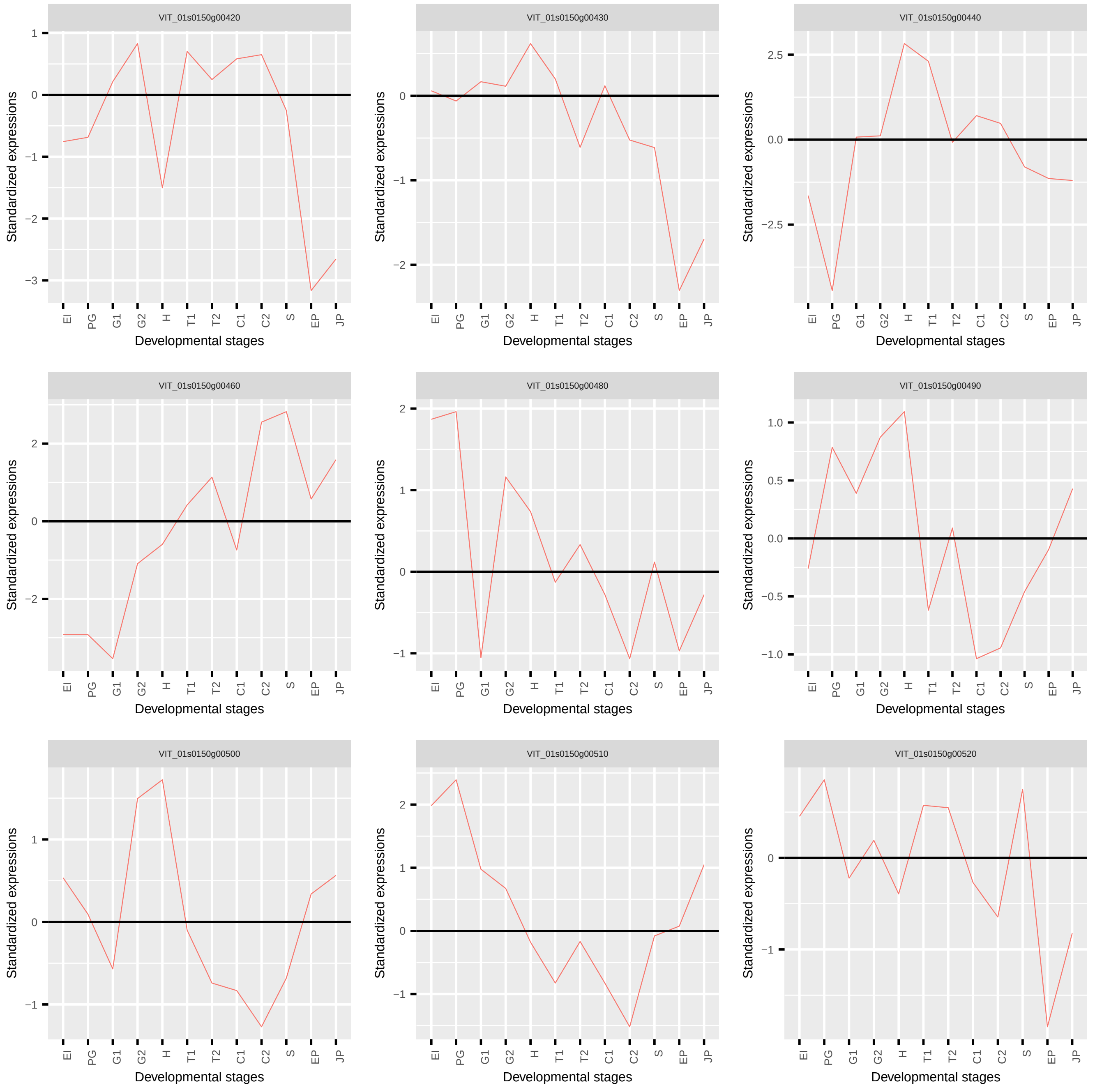
VIT_01s0150g00420
VIT_01s0150g00430
VIT_01s0150g00440
VIT_01s0150g00460
VIT_01s0150g00480
VIT_01s0150g00490
VIT_01s0150g00500
VIT_01s0150g00510
VIT_01s0150g00520
Standardized expressions
Developmental stages
EI PG G1 G2 H T1 T2 C1 C2 S EP JP

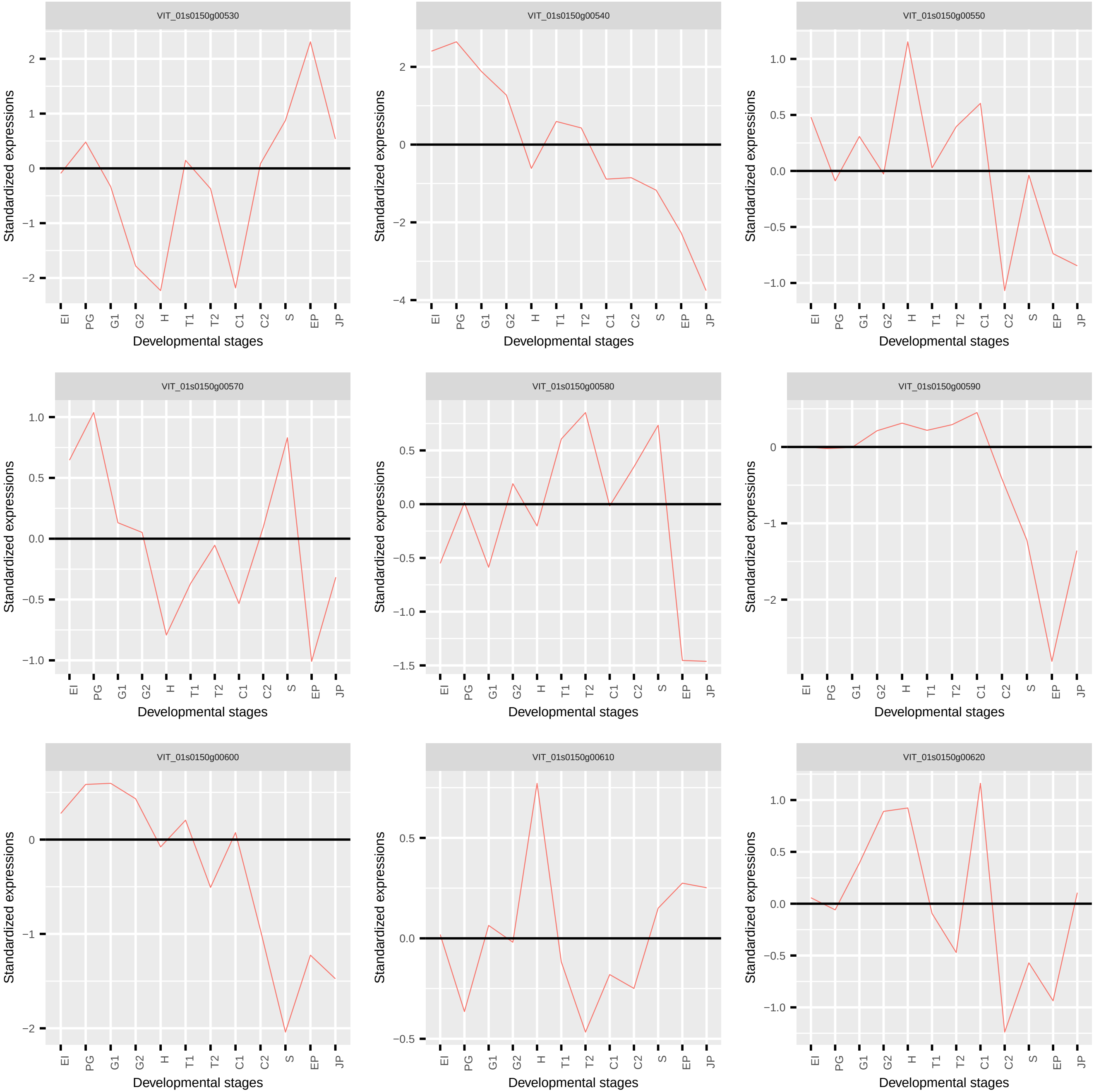
VIT_01s0150g00530
VIT_01s0150g00540
VIT_01s0150g00550
VIT_01s0150g00570
VIT_01s0150g00580
VIT_01s0150g00590
VIT_01s0150g00600
VIT_01s0150g00610
VIT_01s0150g00620
Standardized expressions
Developmental stages
EI PG G1 G2 H T1 T2 C1 C2 S EP JP

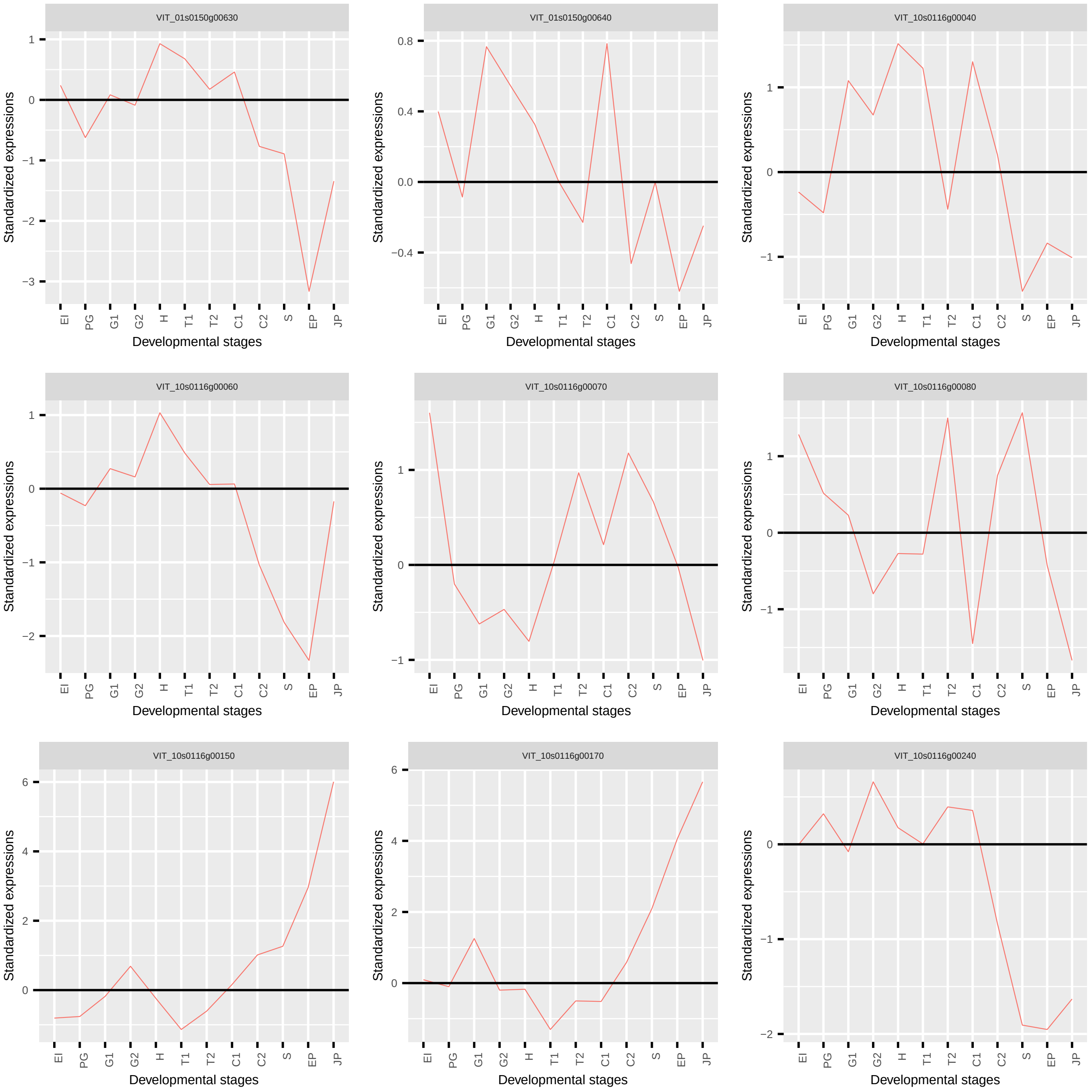
VIT_01s0150g00630
VIT_01s0150g00640
VIT_10s0116g00040
VIT_10s0116g00060
VIT_10s0116g00070
VIT_10s0116g00080
VIT_10s0116g00150
VIT_10s0116g00170
VIT_10s0116g00240
Standardized expressions
Developmental stages
EI PG G1 G2 H T1 T2 C1 C2 S EP JP

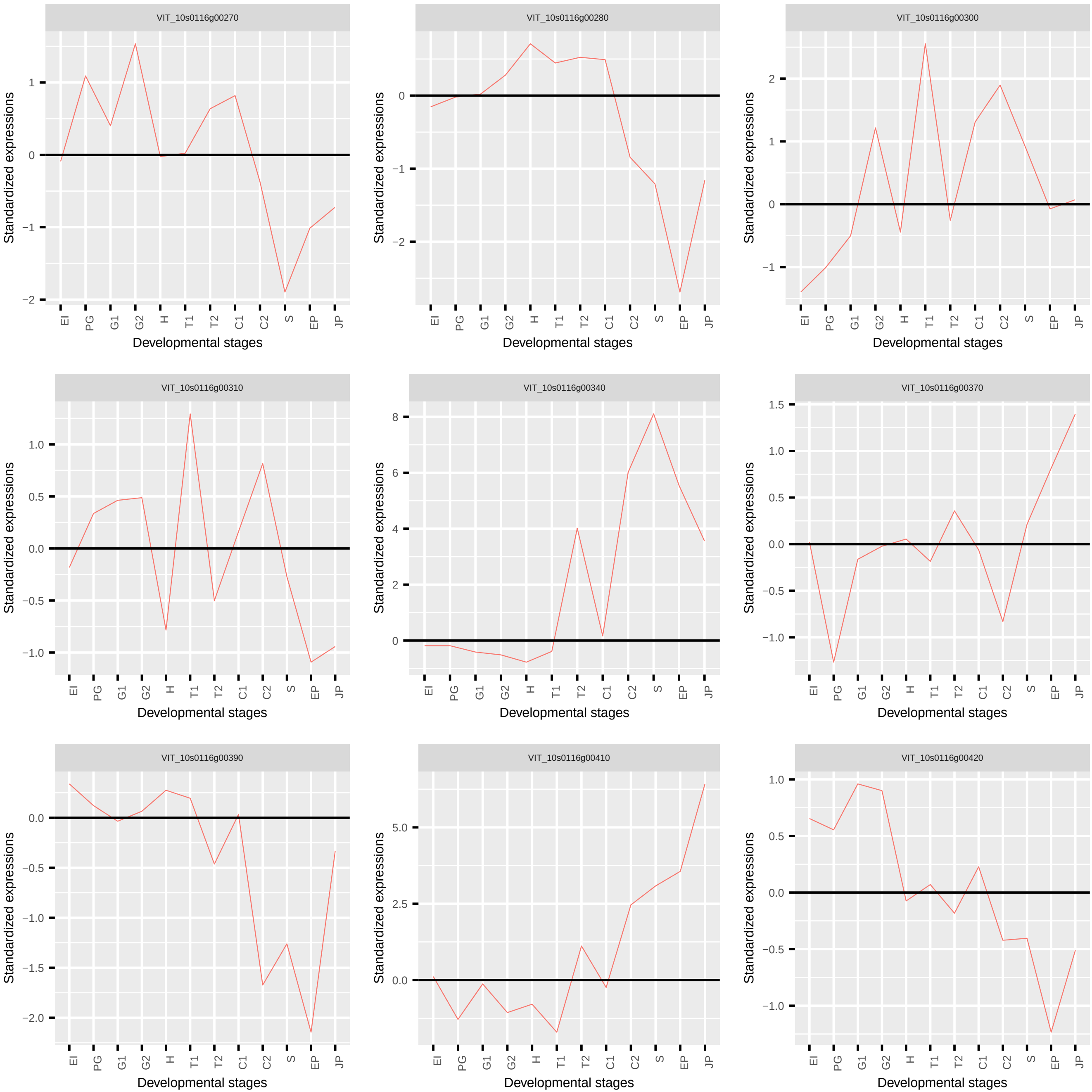
VIT_10s0116g00270
VIT_10s0116g00280
VIT_10s0116g00300
VIT_10s0116g00310
VIT_10s0116g00340
VIT_10s0116g00370
VIT_10s0116g00390
VIT_10s0116g00410
VIT_10s0116g00420
Standardized expressions
Developmental stages
EI PG G1 G2 H T1 T2 C1 C2 S EP JP

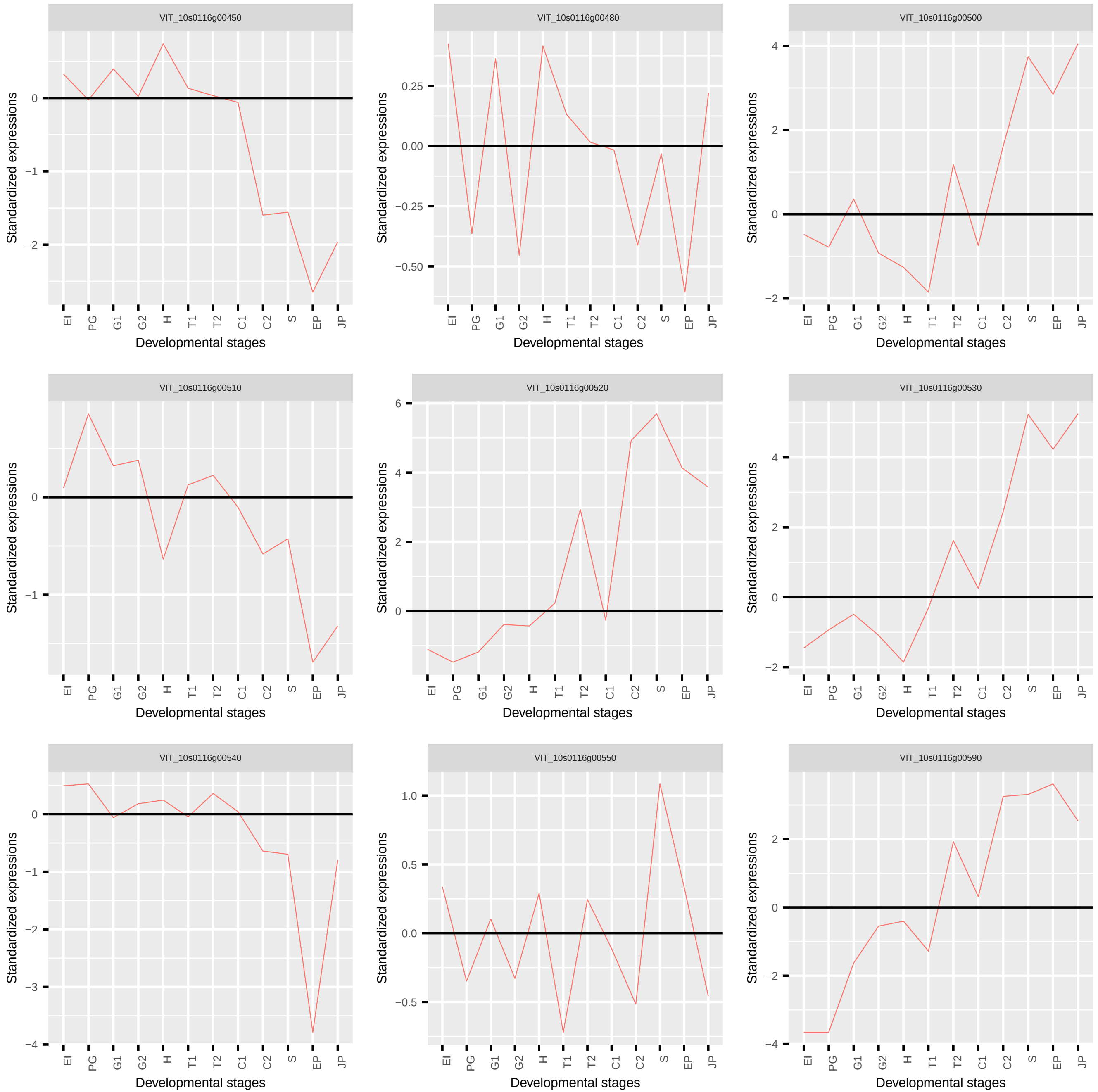
VIT_10s0116g00450
VIT_10s0116g00480
VIT_10s0116g00500
VIT_10s0116g00510
VIT_10s0116g00520
VIT_10s0116g00530
VIT_10s0116g00540
VIT_10s0116g00550
VIT_10s0116g00590
Standardized expressions
Developmental stages
EI PG G1 G2 H T1 T2 C1 C2 S EP JP

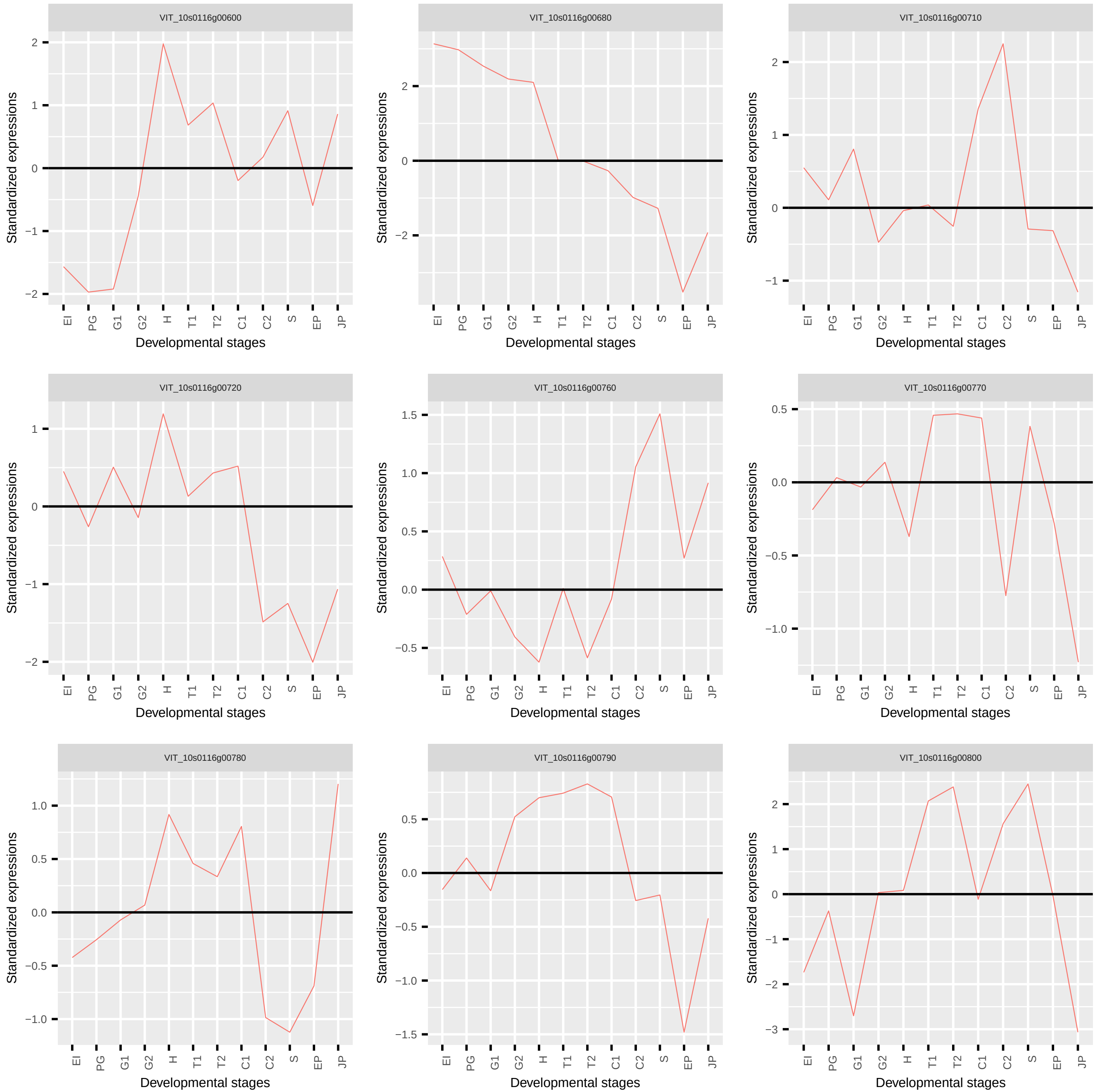
VIT_10s0116g00600
VIT_10s0116g00680
VIT_10s0116g00710
VIT_10s0116g00720
VIT_10s0116g00760
VIT_10s0116g00770
VIT_10s0116g00780
VIT_10s0116g00790
VIT_10s0116g00800
Standardized expressions
EI PG G1 G2 H T1 T2 C1 C2 S EP JP
Developmental stages

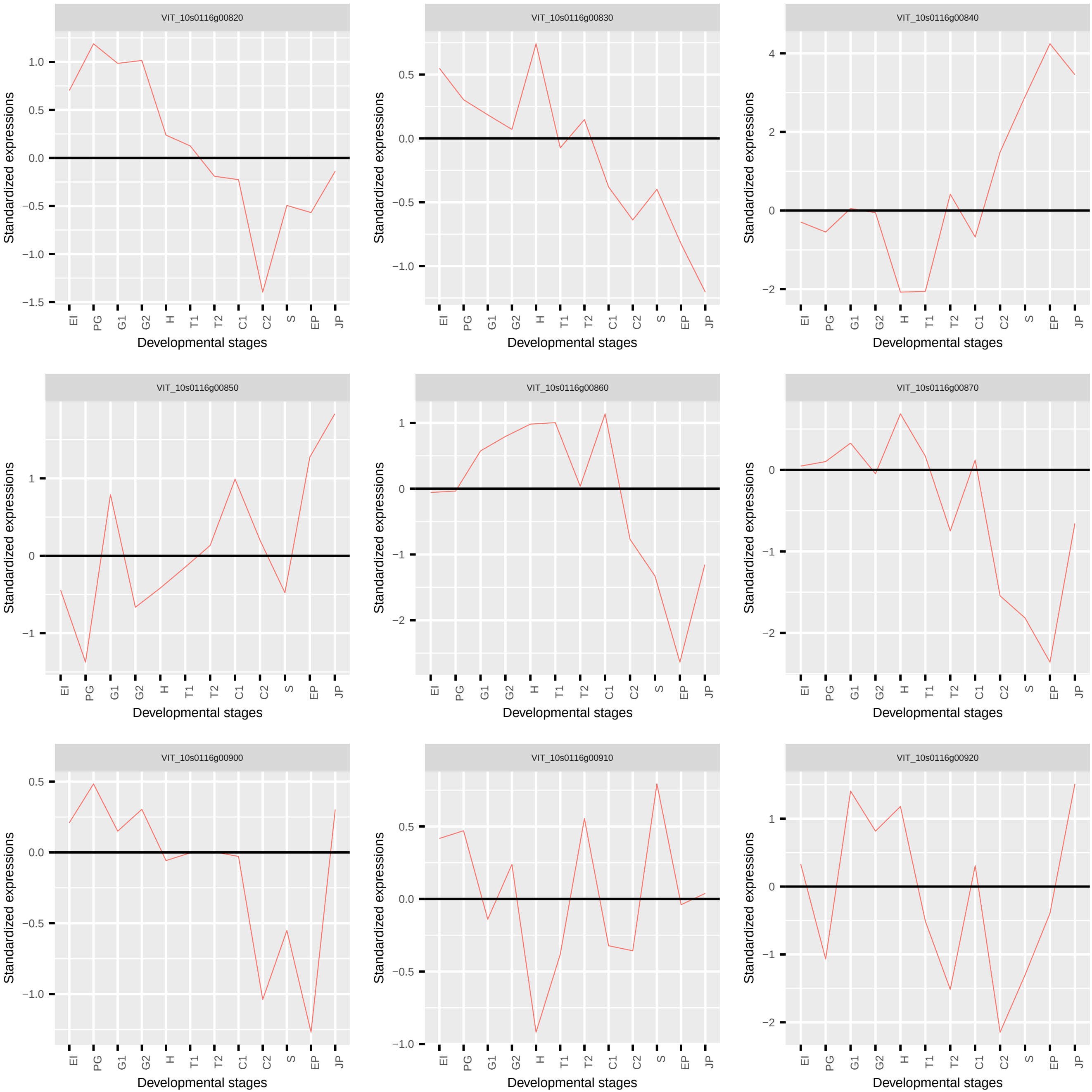

VIT_10s0116g00820
1.0
0.5
0.0
−0.5
−1.0
−1.5
VIT_10s0116g00830
0.5
0.0
−0.5
−1.0
VIT_10s0116g00840
4
2
0
−2
VIT_10s0116g00850
1
0
−1
VIT_10s0116g00860
1
0
−1
−2
VIT_10s0116g00870
0
−1
−2
VIT_10s0116g00900
0.5
0.0
−0.5
−1.0
VIT_10s0116g00910
0.5
0.0
−0.5
−1.0
VIT_10s0116g00920
1
0
−1
−2
Standardized expressions
EI PG G1 G2 H T1 T2 C1 C2 S EP JP
Developmental stages

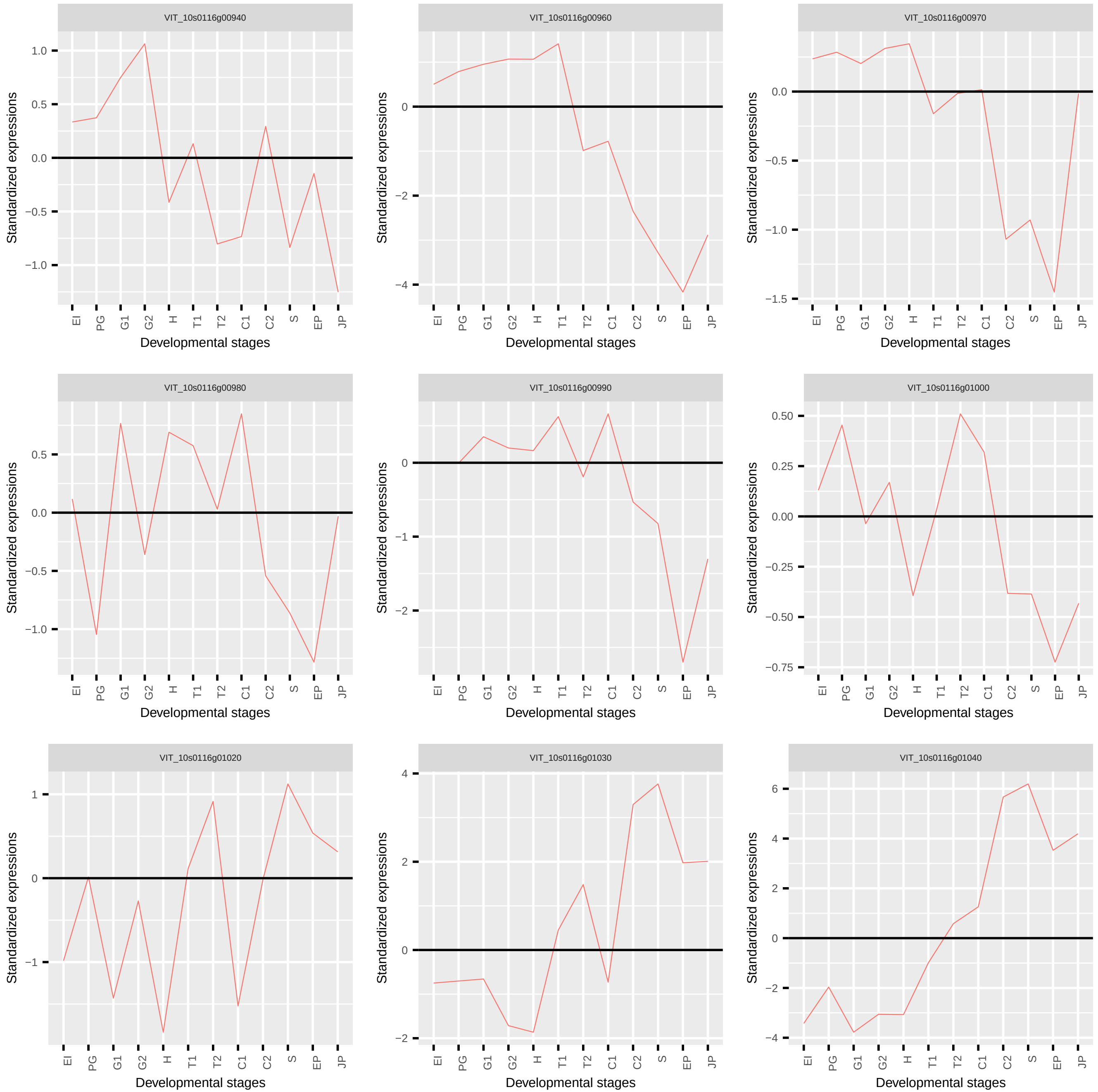
VIT_10s0116g00940
VIT_10s0116g00960
VIT_10s0116g00970
VIT_10s0116g00980
VIT_10s0116g00990
VIT_10s0116g01000
VIT_10s0116g01020
VIT_10s0116g01030
VIT_10s0116g01040
Standardized expressions
Developmental stages
EI PG G1 G2 H T1 T2 C1 C2 S EP JP

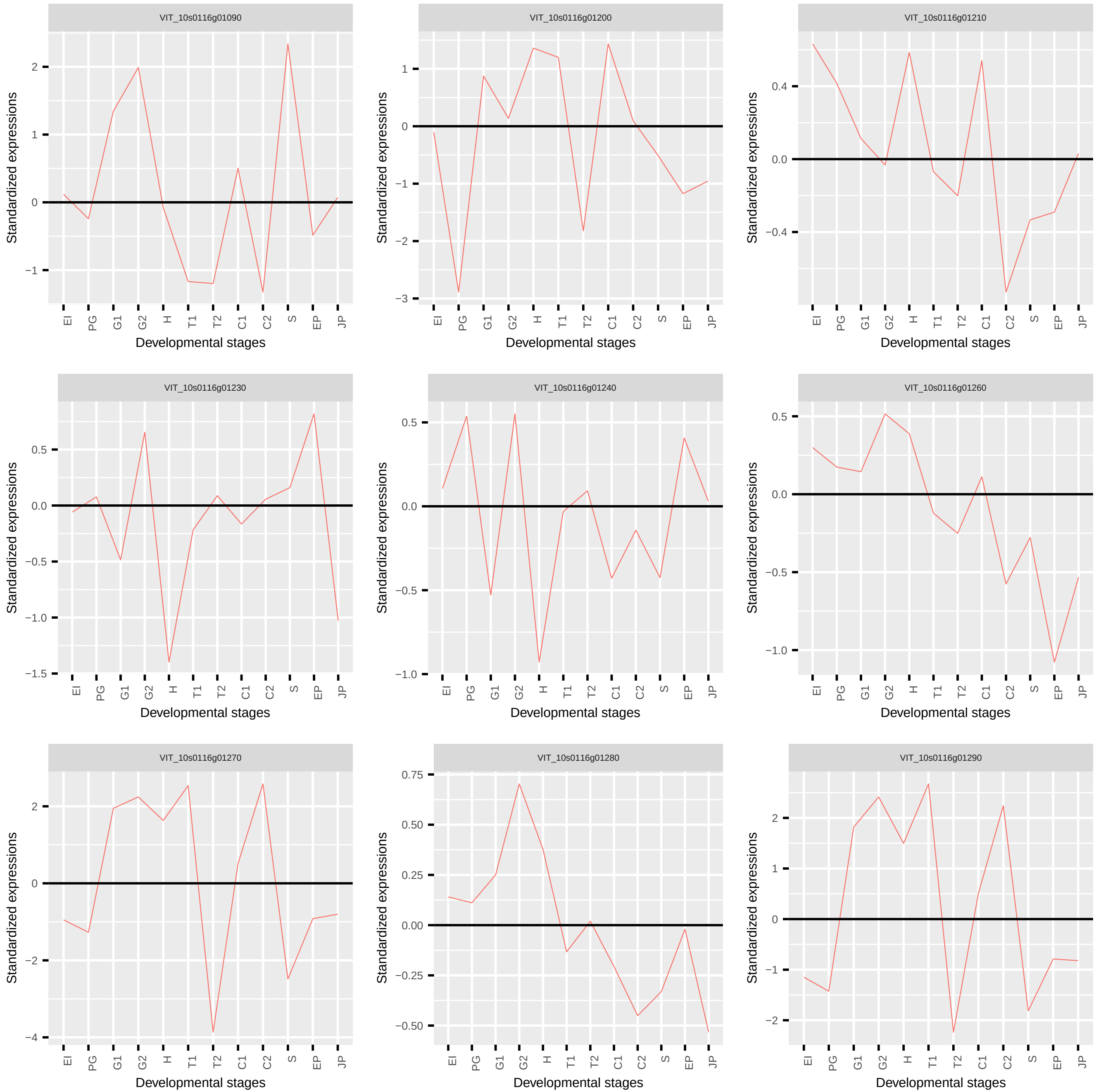
VIT_10s0116g01090
VIT_10s0116g01200
VIT_10s0116g01210
VIT_10s0116g01230
VIT_10s0116g01240
VIT_10s0116g01260
VIT_10s0116g01270
VIT_10s0116g01280
VIT_10s0116g01290
Standardized expressions
Developmental stages
EI PG G1 G2 H T1 T2 C1 C2 S EP JP

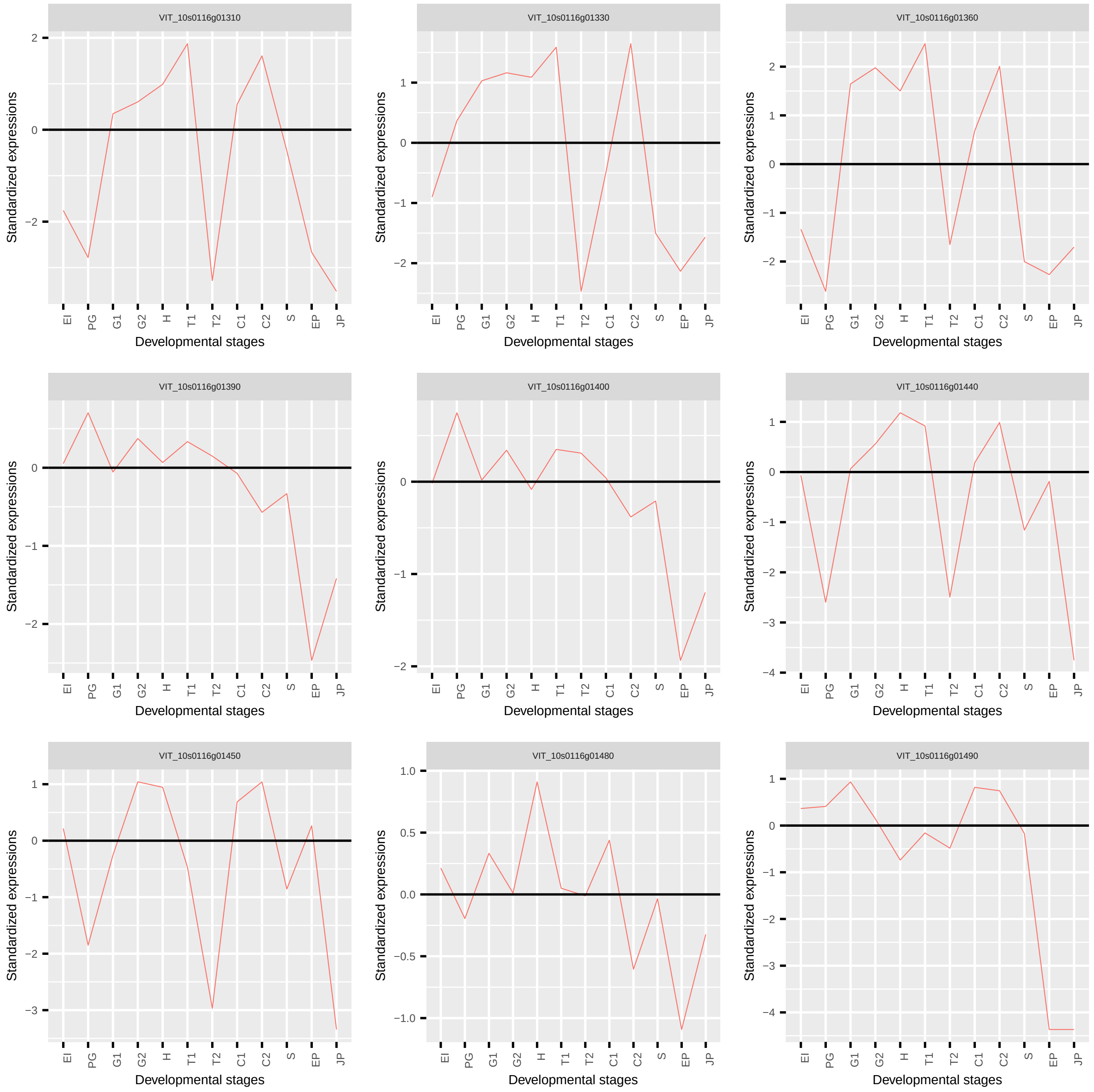
VIT_10s0116g01310
VIT_10s0116g01330
VIT_10s0116g01360
VIT_10s0116g01390
VIT_10s0116g01400
VIT_10s0116g01440
VIT_10s0116g01450
VIT_10s0116g01480
VIT_10s0116g01490
Standardized expressions
Developmental stages
EI
PG
G1
G2
H
T1
T2
C1
C2
S
EP
JP

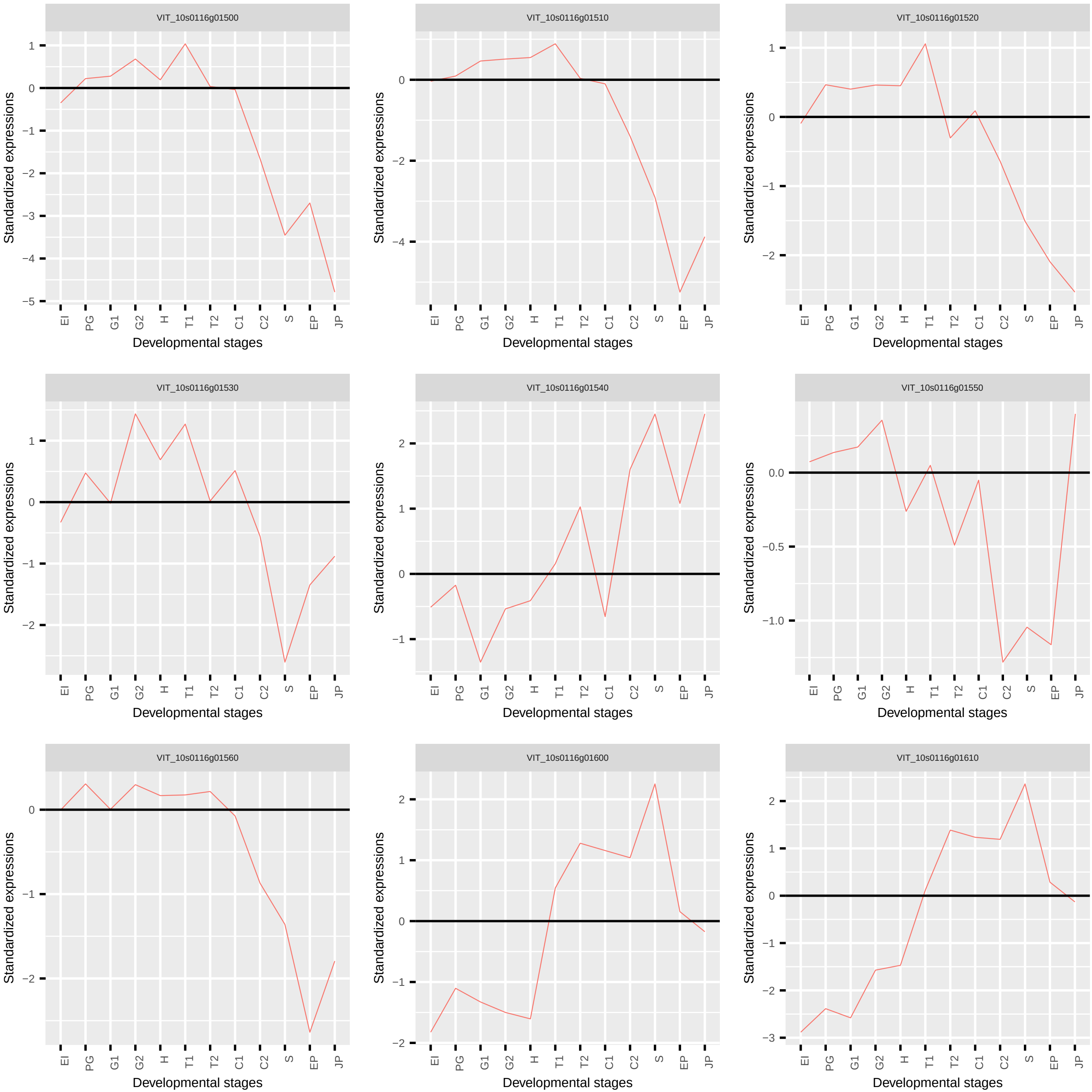
VIT_10s0116g01500
VIT_10s0116g01510
VIT_10s0116g01520
VIT_10s0116g01530
VIT_10s0116g01540
VIT_10s0116g01550
VIT_10s0116g01560
VIT_10s0116g01600
VIT_10s0116g01610
Standardized expressions
Developmental stages
EI
PG
G1
G2
H
T1
T2
C1
C2
S
EP
JP

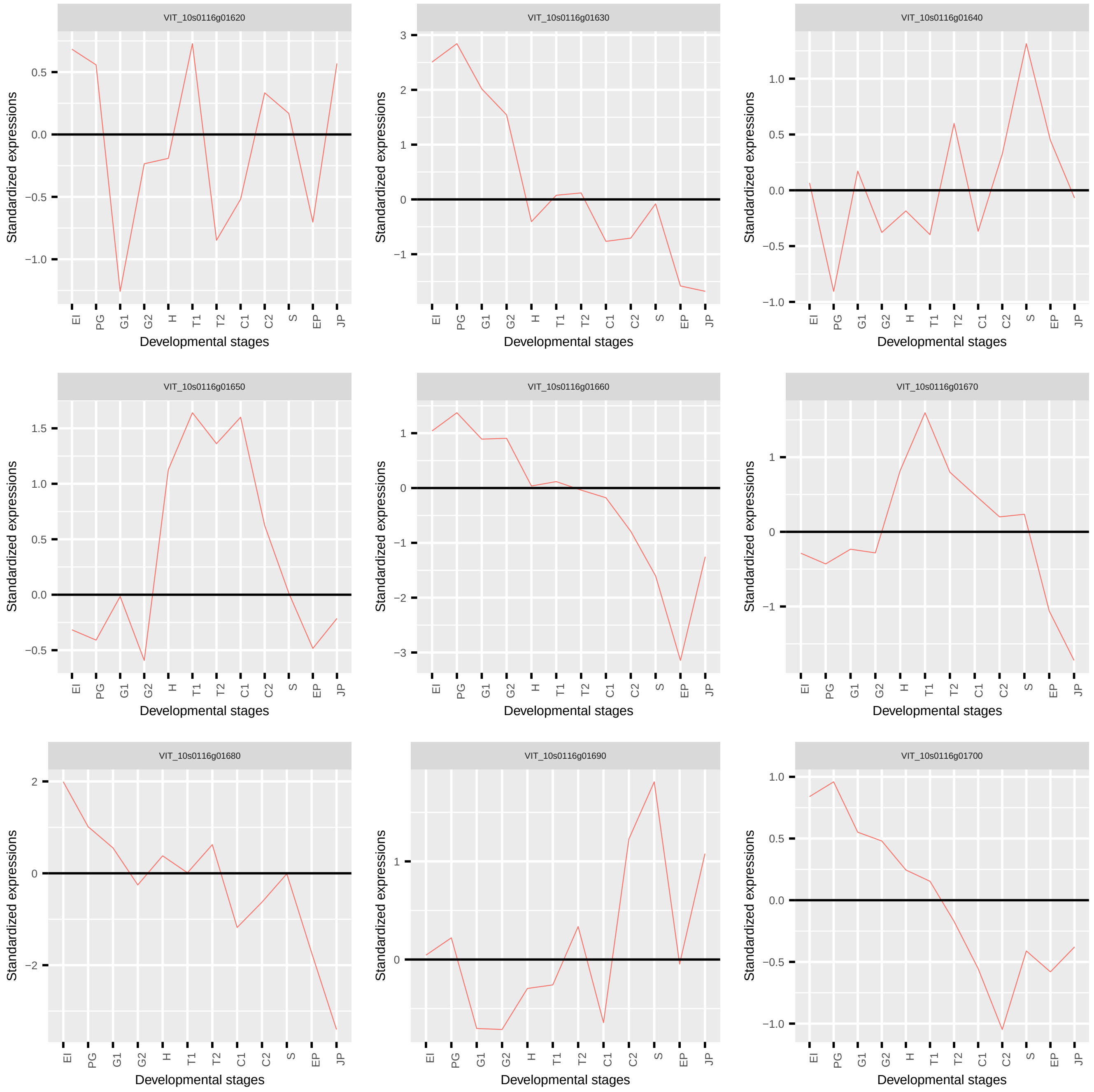

VIT_10s0116g01620
VIT_10s0116g01630
VIT_10s0116g01640
VIT_10s0116g01650
VIT_10s0116g01660
VIT_10s0116g01670
VIT_10s0116g01680
VIT_10s0116g01690
VIT_10s0116g01700
Standardized expressions
Developmental stages
EI PG G1 G2 H T1 T2 C1 C2 S EP JP

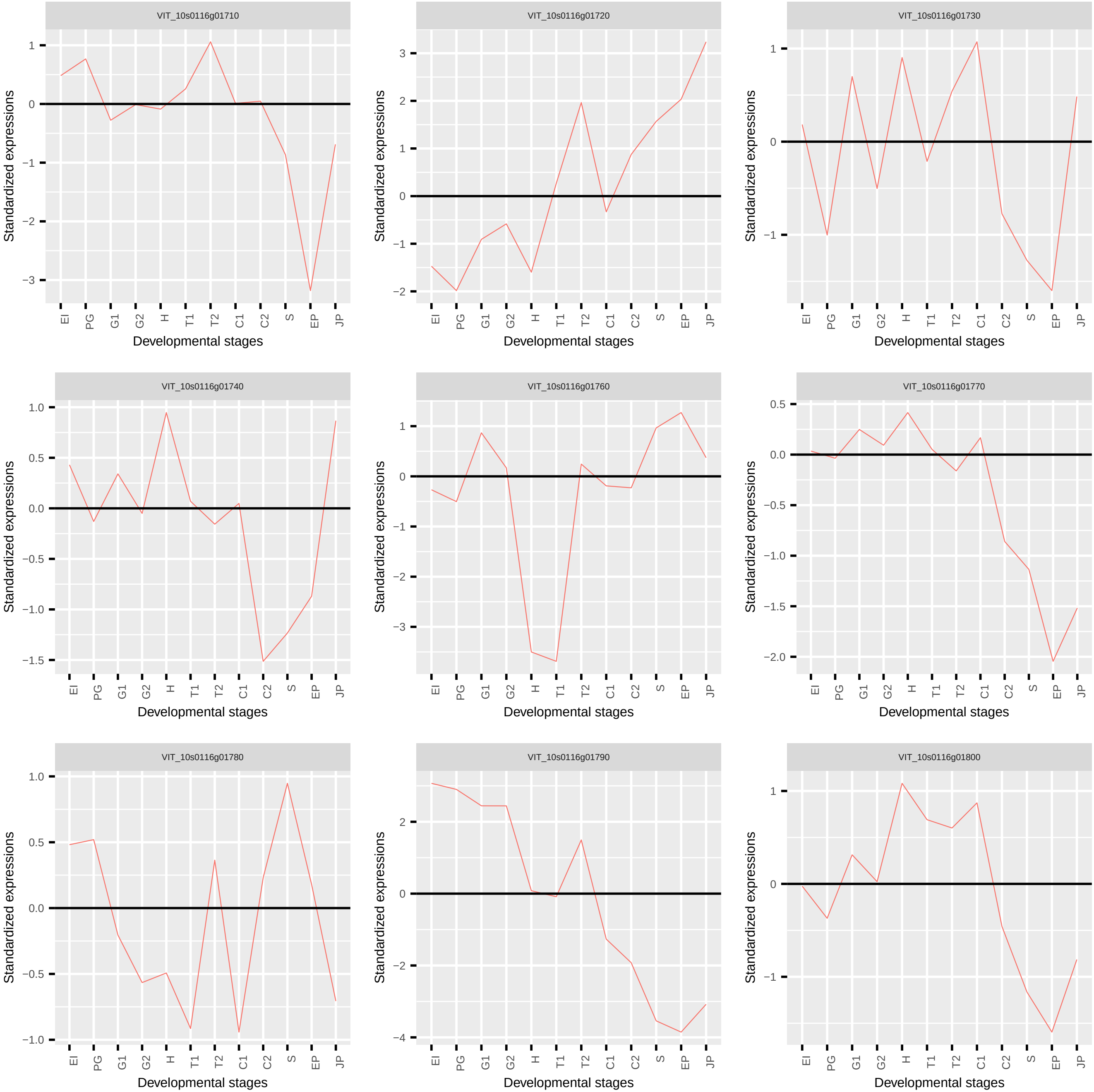
VIT_10s0116g01710
VIT_10s0116g01720
VIT_10s0116g01730
VIT_10s0116g01740
VIT_10s0116g01760
VIT_10s0116g01770
VIT_10s0116g01780
VIT_10s0116g01790
VIT_10s0116g01800
Standardized expressions
Developmental stages
EI
PG
G1
G2
H
T1
T2
C1
C2
S
EP
JP

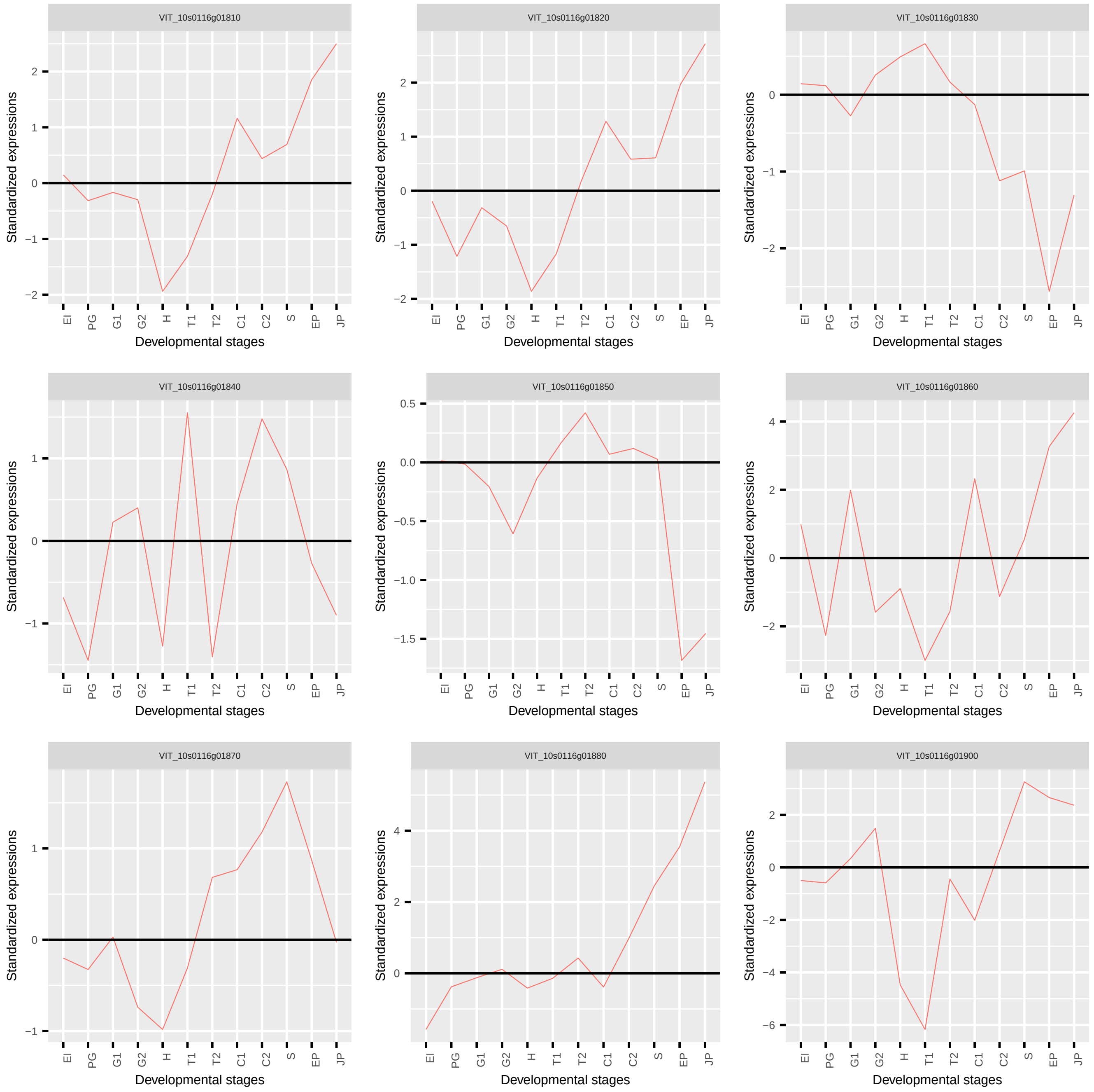
VIT_10s0116g01810
VIT_10s0116g01820
VIT_10s0116g01830
VIT_10s0116g01840
VIT_10s0116g01850
VIT_10s0116g01860
VIT_10s0116g01870
VIT_10s0116g01880
VIT_10s0116g01900
Standardized expressions
Developmental stages
EI PG G1 G2 H T1 T2 C1 C2 S EP JP

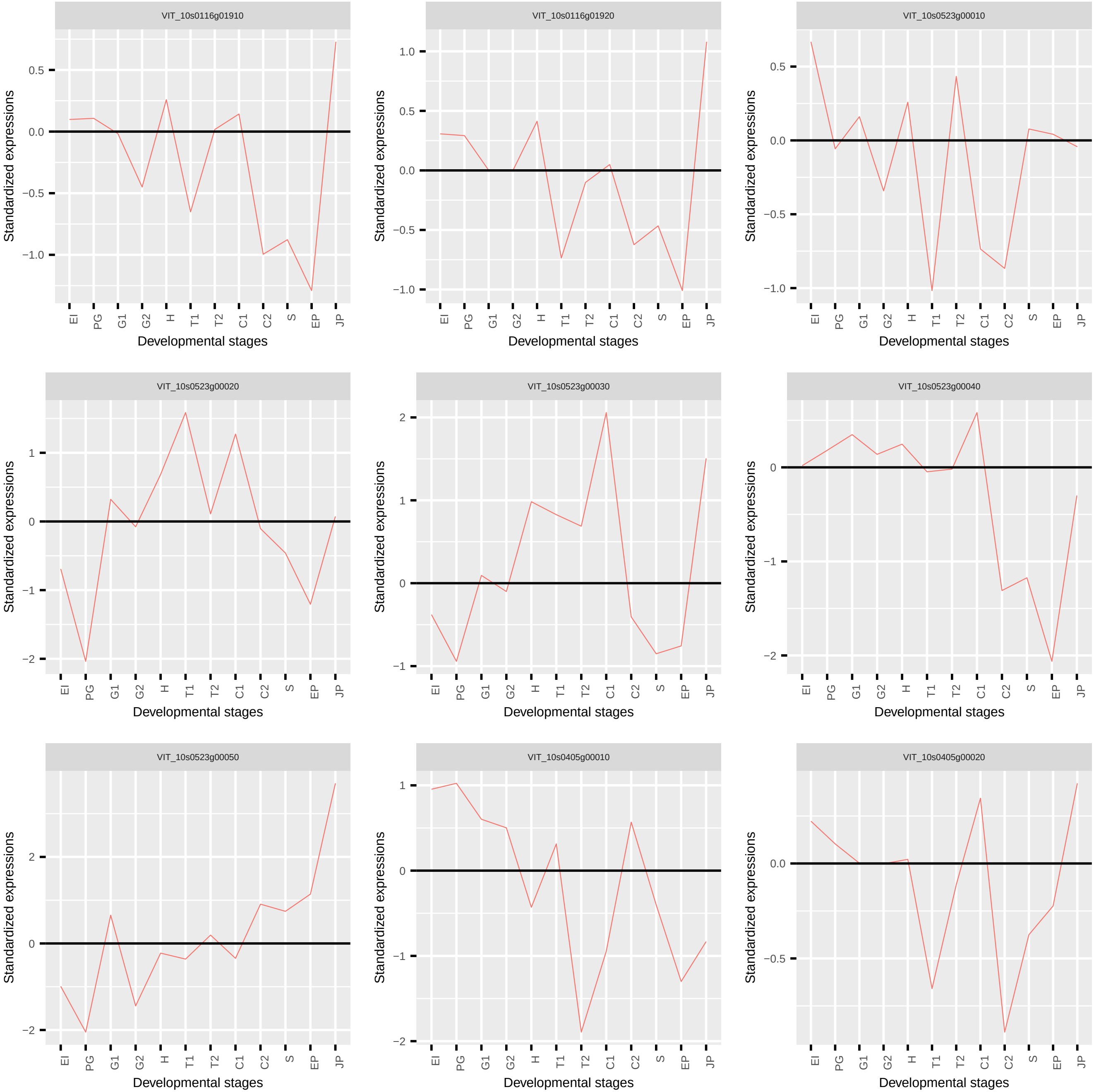
VIT_10s0116g01910
VIT_10s0116g01920
VIT_10s0523g00010
VIT_10s0523g00020
VIT_10s0523g00030
VIT_10s0523g00040
VIT_10s0523g00050
VIT_10s0405g00010
VIT_10s0405g00020
Standardized expressions
Developmental stages
EI PG G1 G2 H T1 T2 C1 C2 S EP JP

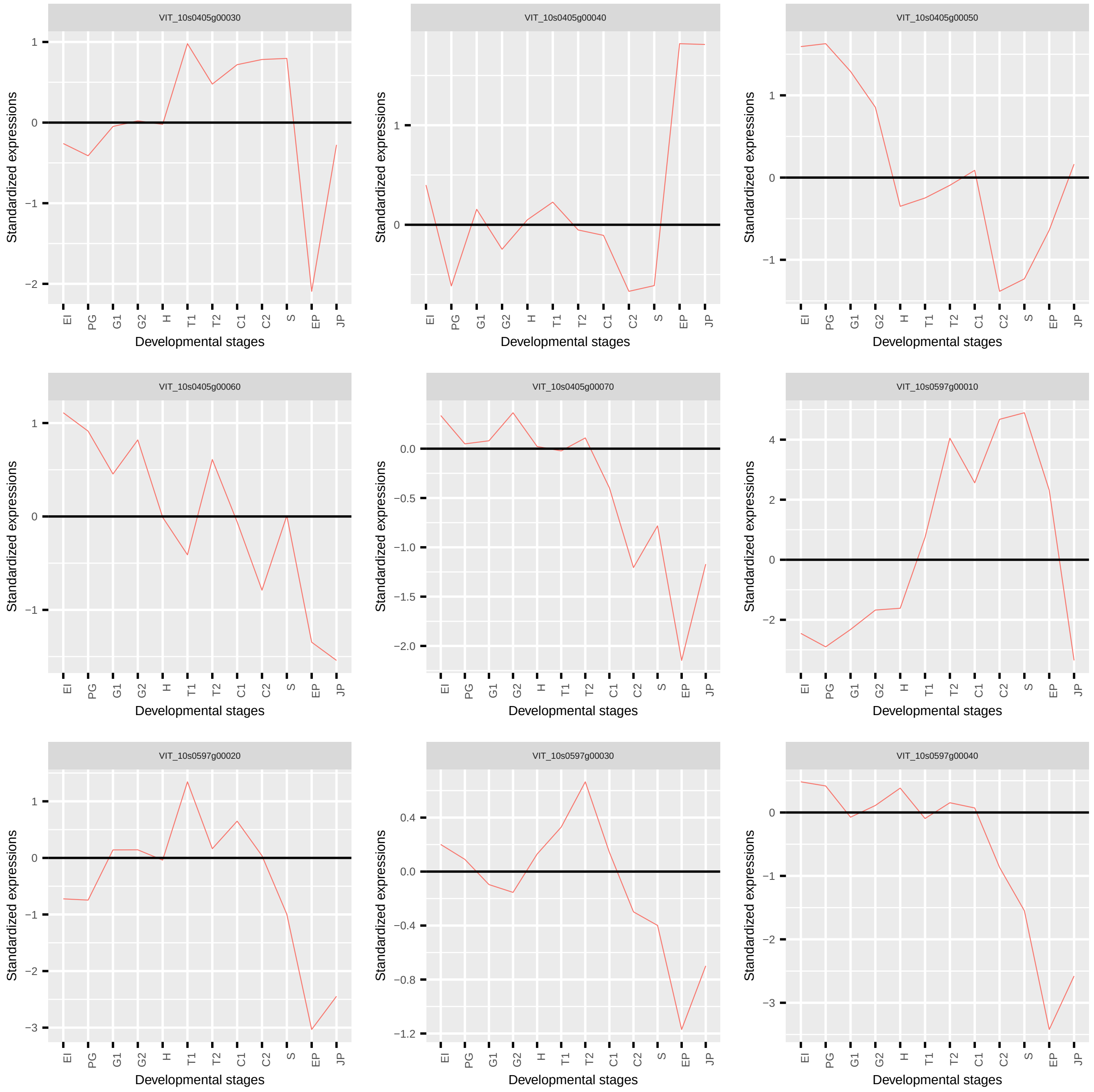

VIT_10s0405g00030
VIT_10s0405g00040
VIT_10s0405g00050
VIT_10s0405g00060
VIT_10s0405g00070
VIT_10s0597g00010
VIT_10s0597g00020
VIT_10s0597g00030
VIT_10s0597g00040
Standardized expressions
Developmental stages
EI
PG
G1
G2
H
T1
T2
C1
C2
S
EP
JP

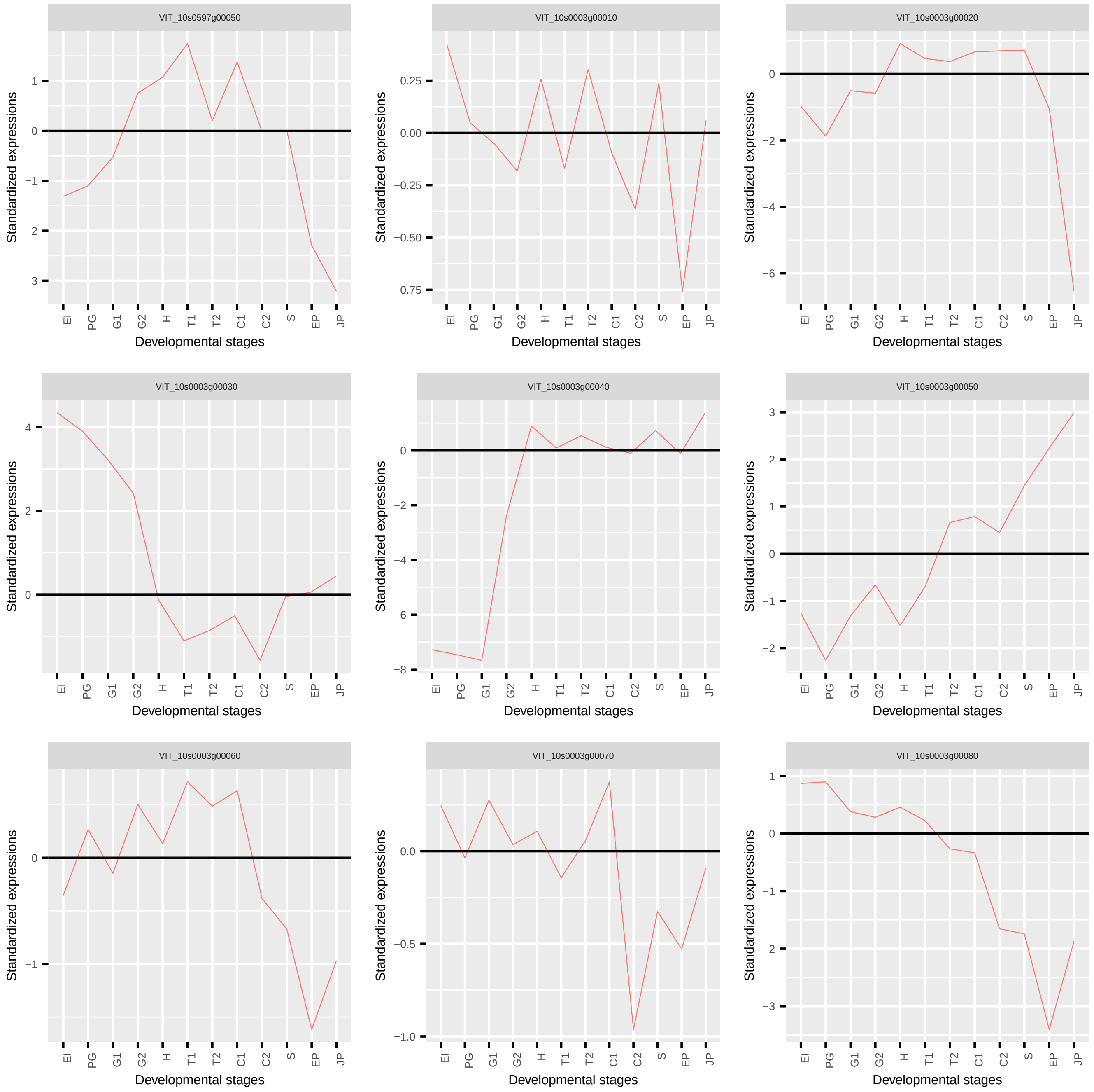
VIT_10s0597g00050
VIT_10s0003g00010
VIT_10s0003g00020
VIT_10s0003g00030
VIT_10s0003g00040
VIT_10s0003g00050
VIT_10s0003g00060
VIT_10s0003g00070
VIT_10s0003g00080
Standardized expressions
Developmental stages
EI PG G1 G2 H T1 T2 C1 C2 S EP JP

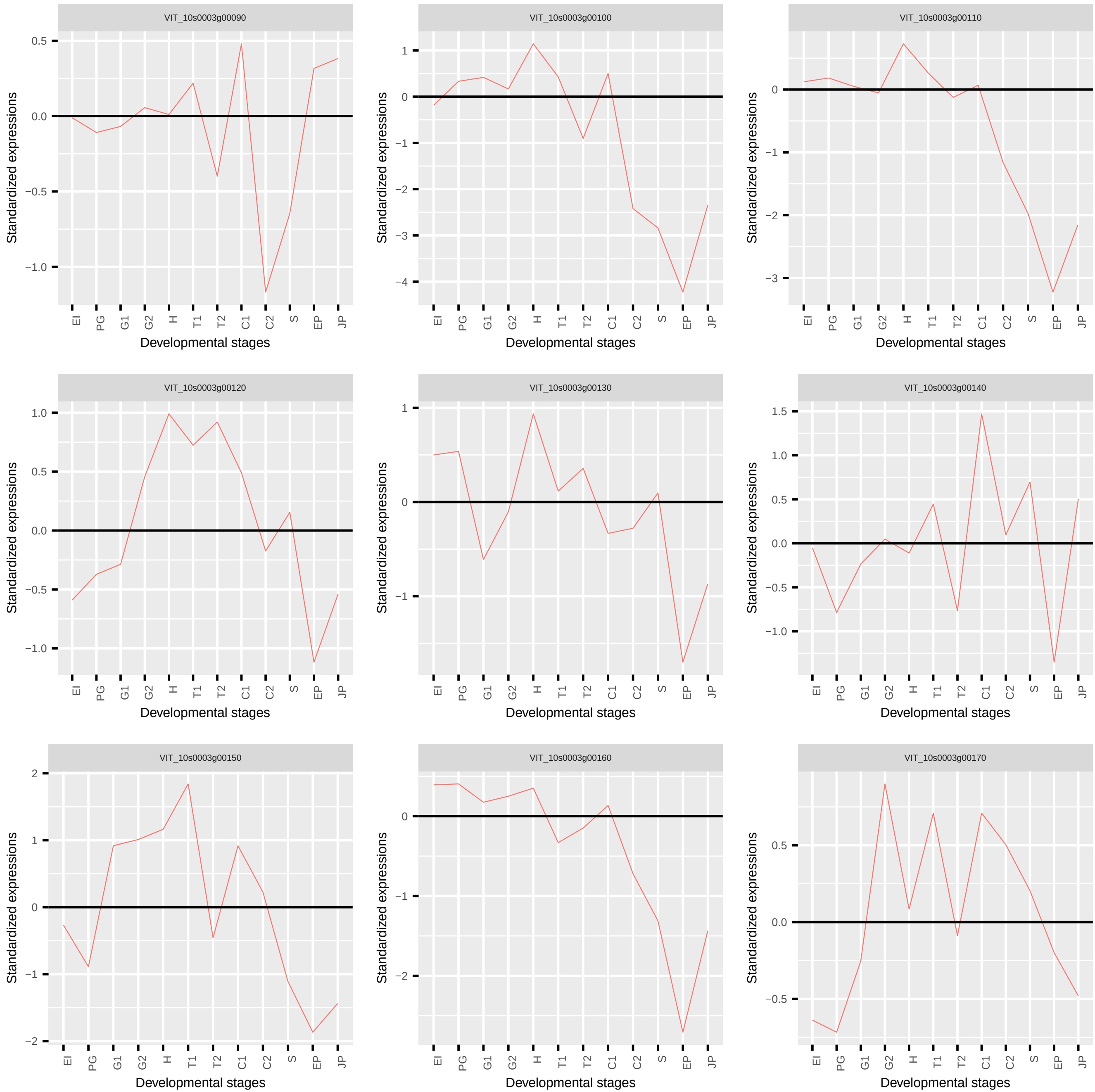
VIT_10s0003g00090
VIT_10s0003g00100
VIT_10s0003g00110
VIT_10s0003g00120
VIT_10s0003g00130
VIT_10s0003g00140
VIT_10s0003g00150
VIT_10s0003g00160
VIT_10s0003g00170
Standardized expressions
Developmental stages
EI
PG
G1
G2
H
T1
T2
C1
C2
S
EP
JP

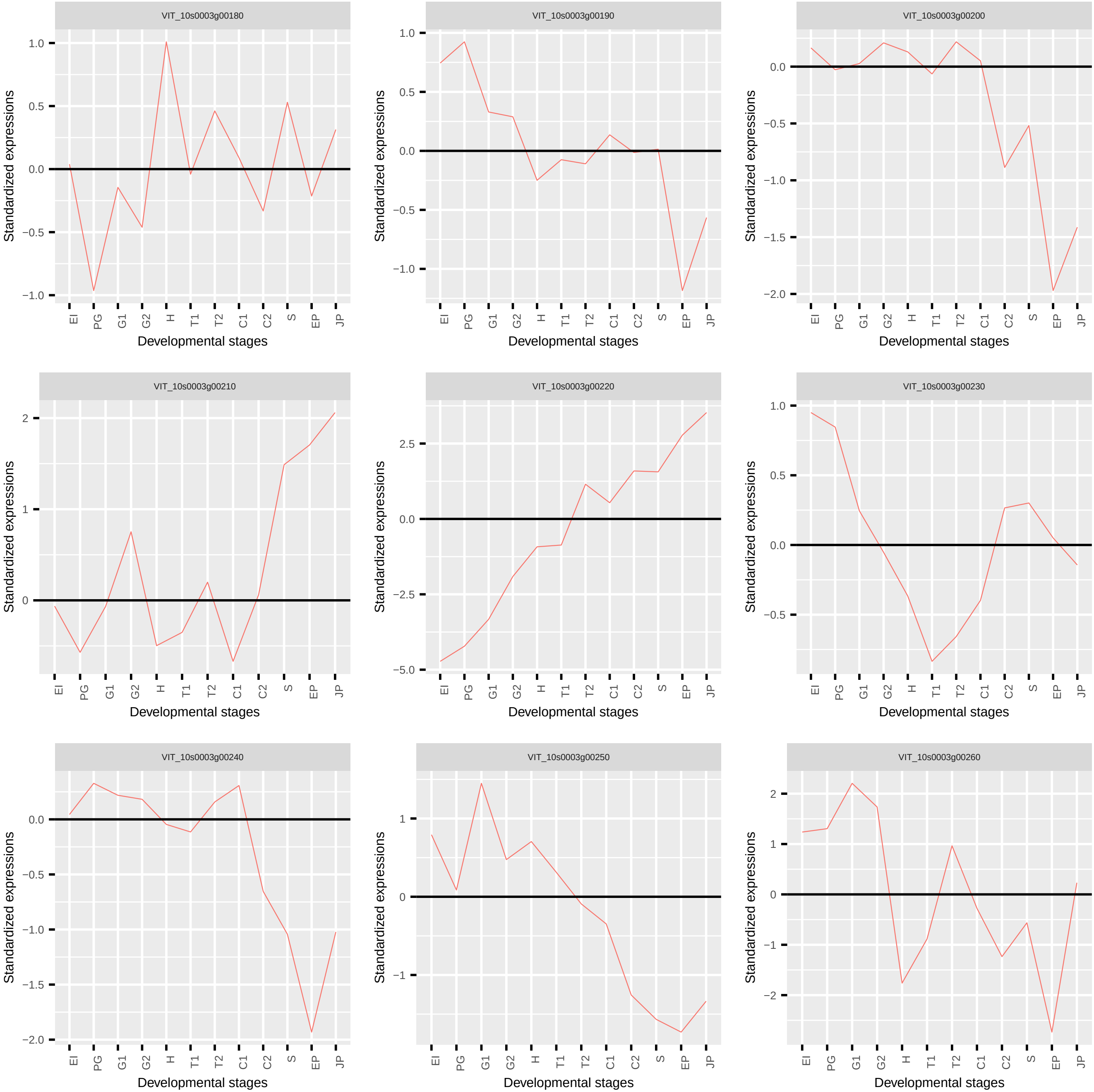

VIT_10s0003g00180
VIT_10s0003g00190
VIT_10s0003g00200
VIT_10s0003g00210
VIT_10s0003g00220
VIT_10s0003g00230
VIT_10s0003g00240
VIT_10s0003g00250
VIT_10s0003g00260
Standardized expressions
Developmental stages
EI
PG
G1
G2
H
T1
T2
C1
C2
S
EP
JP
1.0
0.5
0.0
−0.5
−1.0
−1.5
−2.0
2.5
−2.5
−5.0
2
1
0
−1
−2

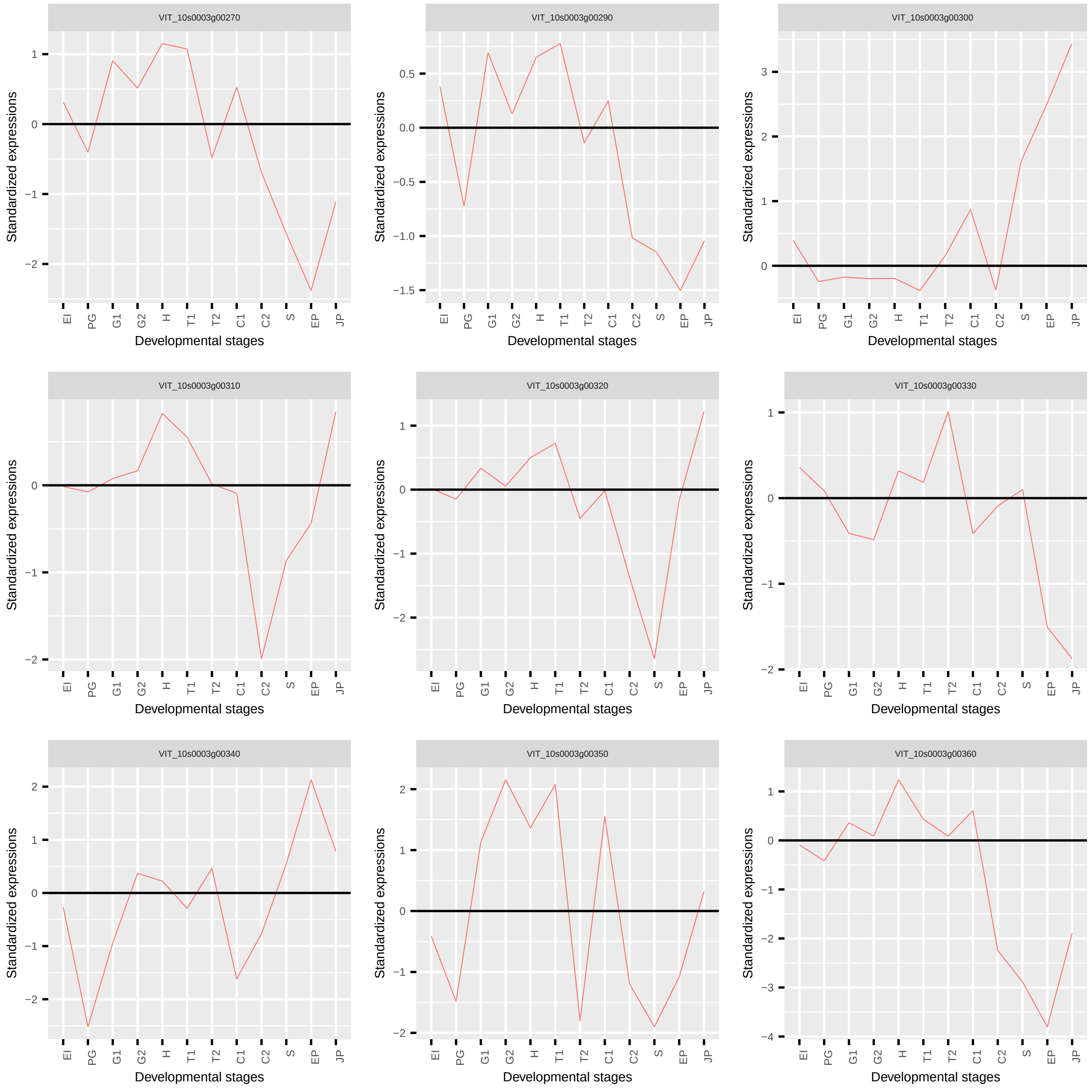
VIT_10s0003g00270
VIT_10s0003g00290
VIT_10s0003g00300
VIT_10s0003g00310
VIT_10s0003g00320
VIT_10s0003g00330
VIT_10s0003g00340
VIT_10s0003g00350
VIT_10s0003g00360
Standardized expressions
Developmental stages
EI PG G1 G2 H T1 T2 C1 C2 S EP JP

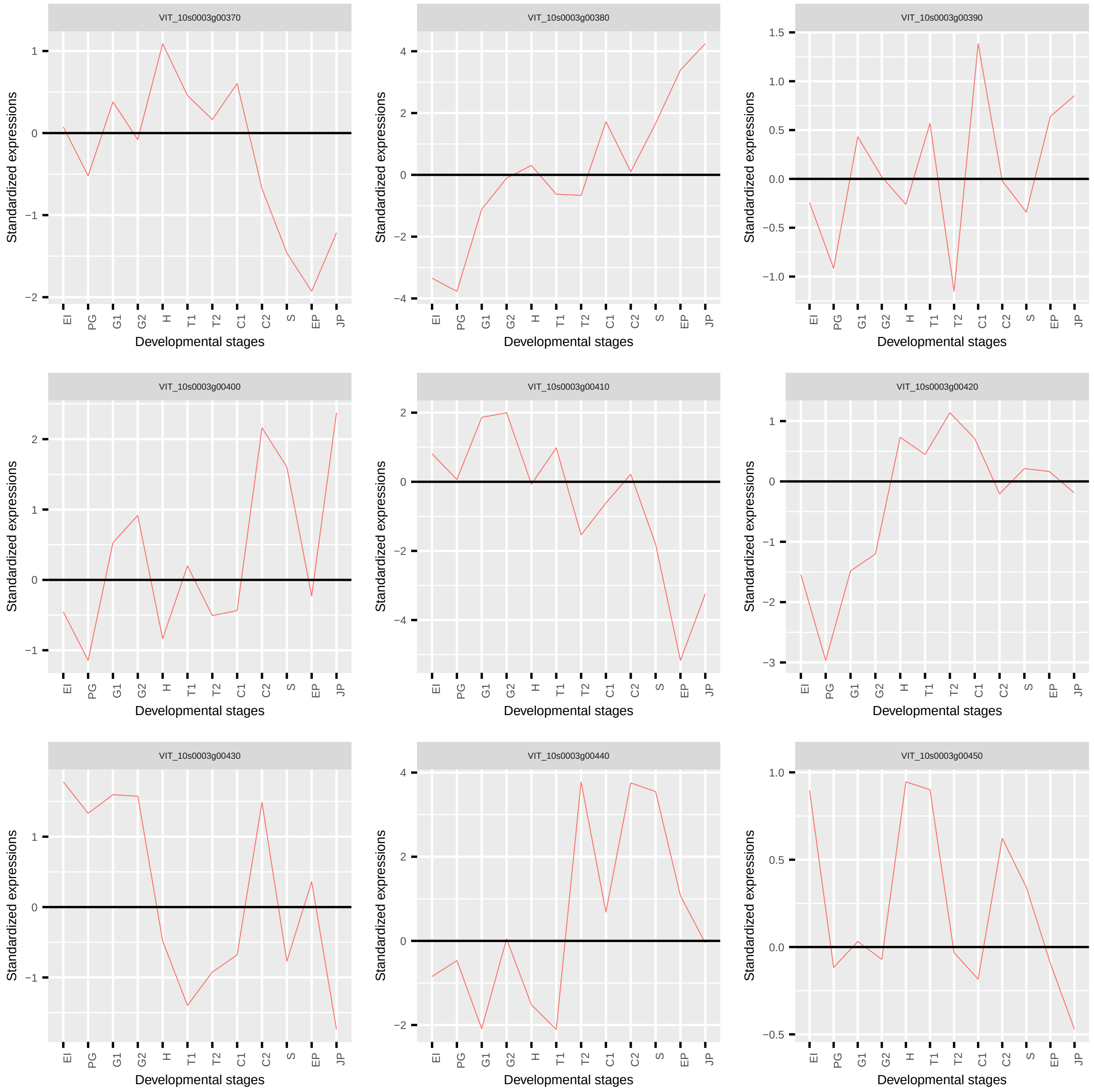
VIT_10s0003g00370
VIT_10s0003g00380
VIT_10s0003g00390
VIT_10s0003g00400
VIT_10s0003g00410
VIT_10s0003g00420
VIT_10s0003g00430
VIT_10s0003g00440
VIT_10s0003g00450
Standardized expressions
Developmental stages
EI PG G1 G2 H T1 T2 C1 C2 S EP JP

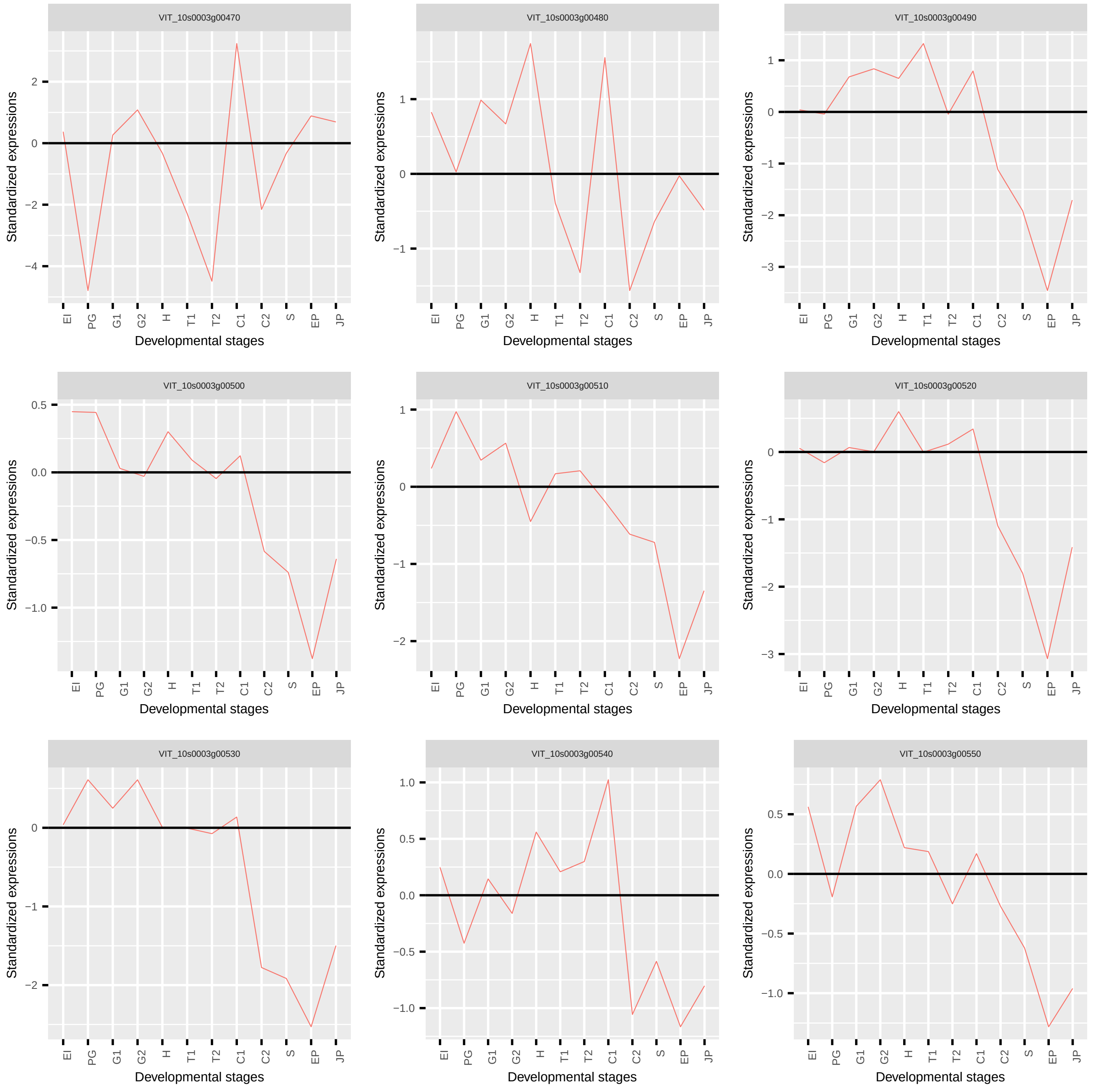
VIT_10s0003g00470
VIT_10s0003g00480
VIT_10s0003g00490
VIT_10s0003g00500
VIT_10s0003g00510
VIT_10s0003g00520
VIT_10s0003g00530
VIT_10s0003g00540
VIT_10s0003g00550
Standardized expressions
Developmental stages
EI PG G1 G2 H T1 T2 C1 C2 S EP JP

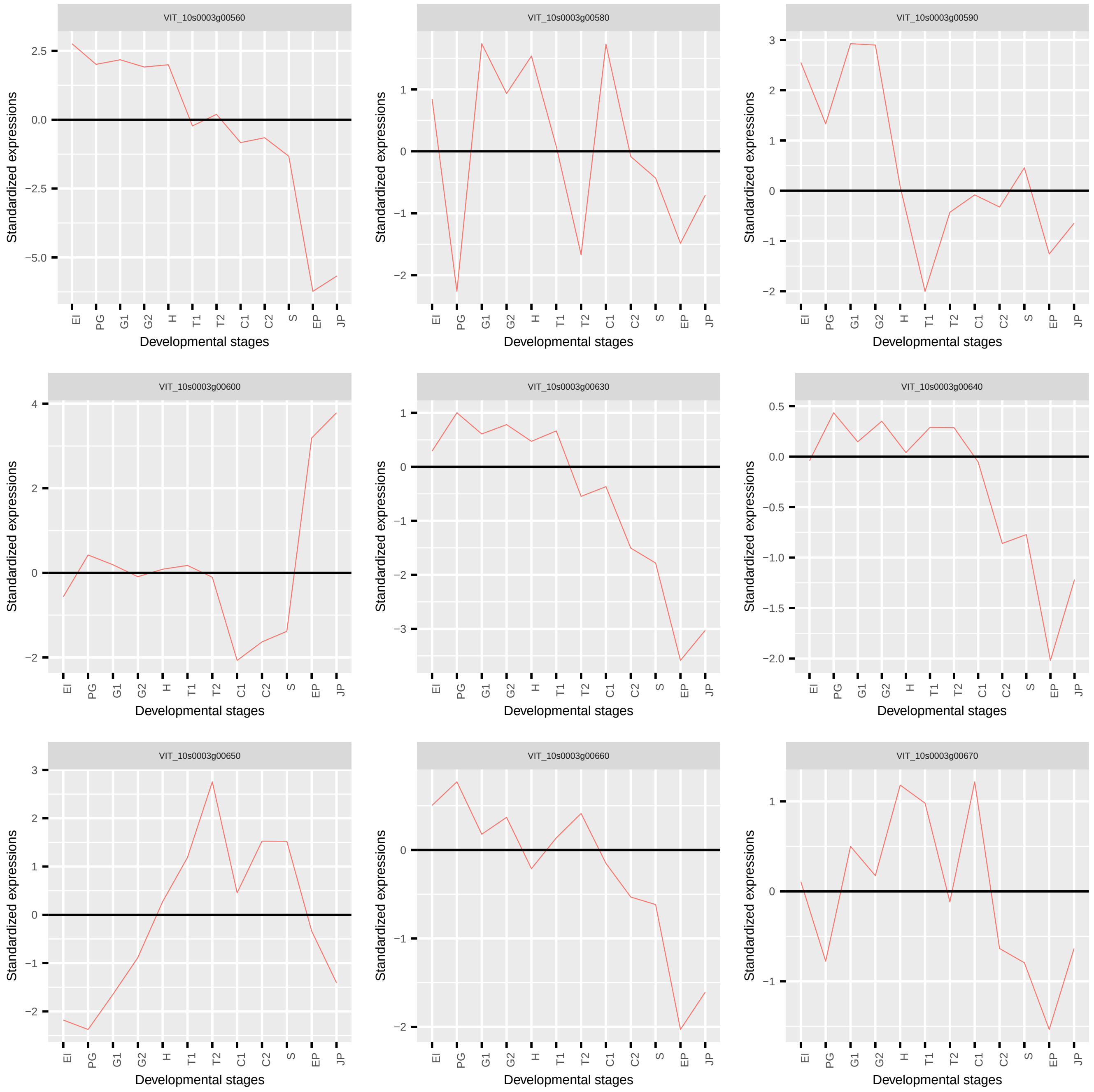
VIT_10s0003g00560
2.5
0.0
−2.5
−5.0
Standardized expressions
EI PG G1 G2 H T1 T2 C1 C2 S EP JP
Developmental stages
VIT_10s0003g00580
1
0
−1
−2
Standardized expressions
EI PG G1 G2 H T1 T2 C1 C2 S EP JP
Developmental stages
VIT_10s0003g00590
3
2
1
0
−1
−2
Standardized expressions
EI PG G1 G2 H T1 T2 C1 C2 S EP JP
Developmental stages
VIT_10s0003g00600
4
2
0
−2
Standardized expressions
EI PG G1 G2 H T1 T2 C1 C2 S EP JP
Developmental stages
VIT_10s0003g00630
1
0
−1
−2
−3
Standardized expressions
EI PG G1 G2 H T1 T2 C1 C2 S EP JP
Developmental stages
VIT_10s0003g00640
0.5
0.0
−0.5
−1.0
−1.5
−2.0
Standardized expressions
EI PG G1 G2 H T1 T2 C1 C2 S EP JP
Developmental stages
VIT_10s0003g00650
3
2
1
0
−1
−2
Standardized expressions
EI PG G1 G2 H T1 T2 C1 C2 S EP JP
Developmental stages
VIT_10s0003g00660
0
−1
−2
Standardized expressions
EI PG G1 G2 H T1 T2 C1 C2 S EP JP
Developmental stages
VIT_10s0003g00670
1
0
−1
Standardized expressions
EI PG G1 G2 H T1 T2 C1 C2 S EP JP
Developmental stages

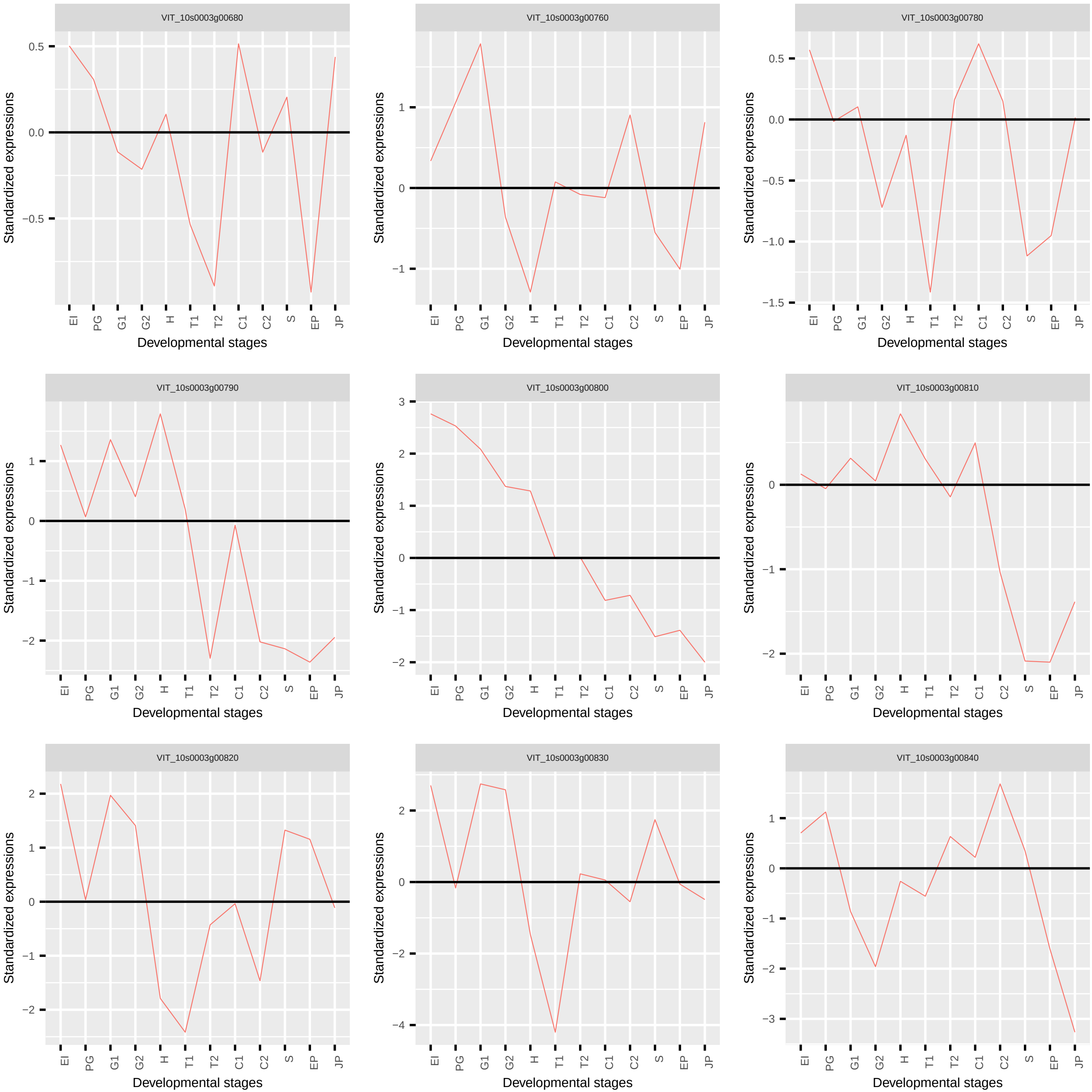
VIT_10s0003g00680
VIT_10s0003g00760
VIT_10s0003g00780
VIT_10s0003g00790
VIT_10s0003g00800
VIT_10s0003g00810
VIT_10s0003g00820
VIT_10s0003g00830
VIT_10s0003g00840
Standardized expressions
Developmental stages
EI
PG
G1
G2
H
T1
T2
C1
C2
S
EP
JP
0.5
0.0
−0.5
1
0
−1
0.5
0.0
−0.5
−1.0
−1.5
1
0
−1
−2
3
2
1
0
−1
−2
0
−1
−2
2
1
0
−1
−2
2
0
−2
−4
1
0
−1
−2
−3

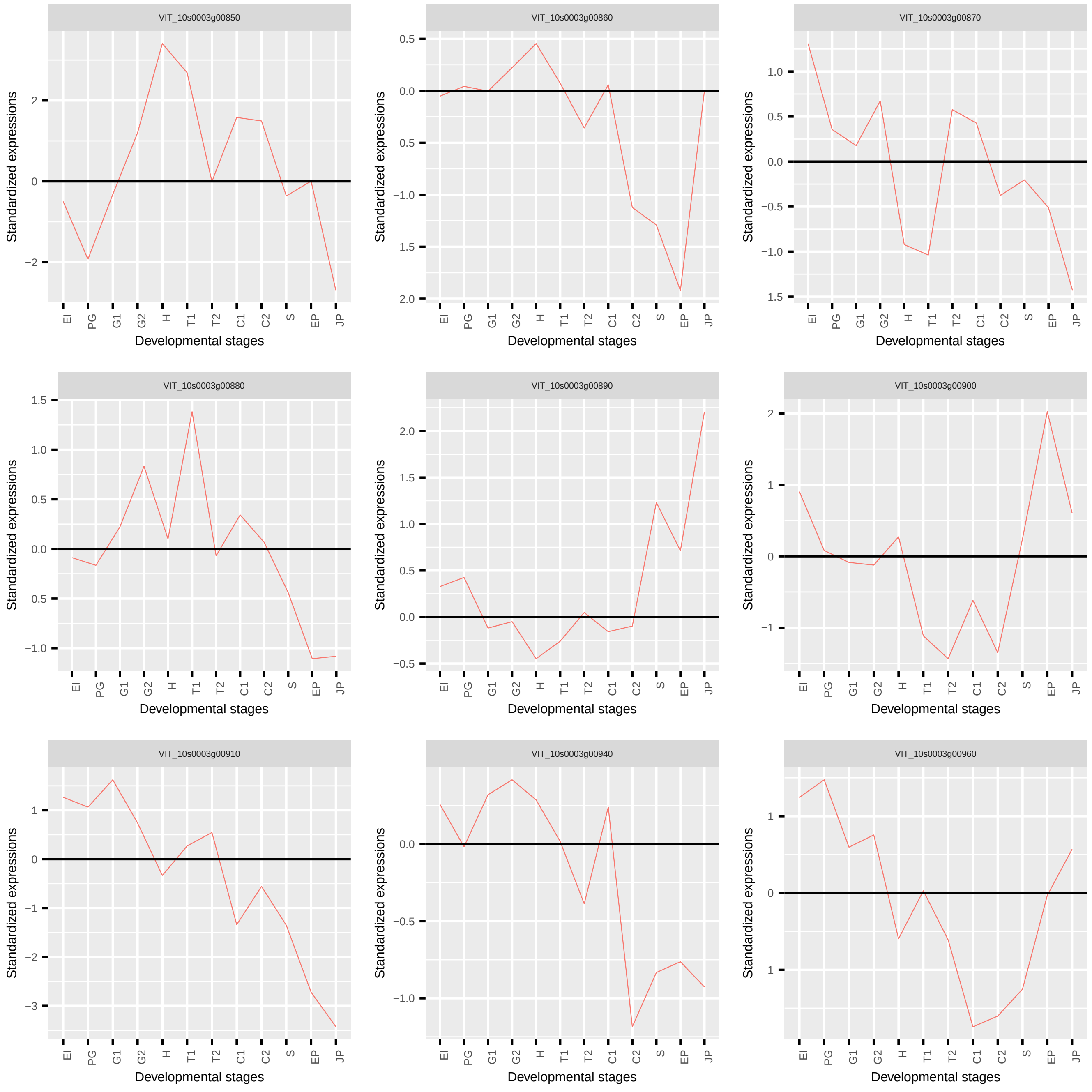
VIT_10s0003g00850
VIT_10s0003g00860
VIT_10s0003g00870
VIT_10s0003g00880
VIT_10s0003g00890
VIT_10s0003g00900
VIT_10s0003g00910
VIT_10s0003g00940
VIT_10s0003g00960
Standardized expressions
Developmental stages
EI PG G1 G2 H T1 T2 C1 C2 S EP JP

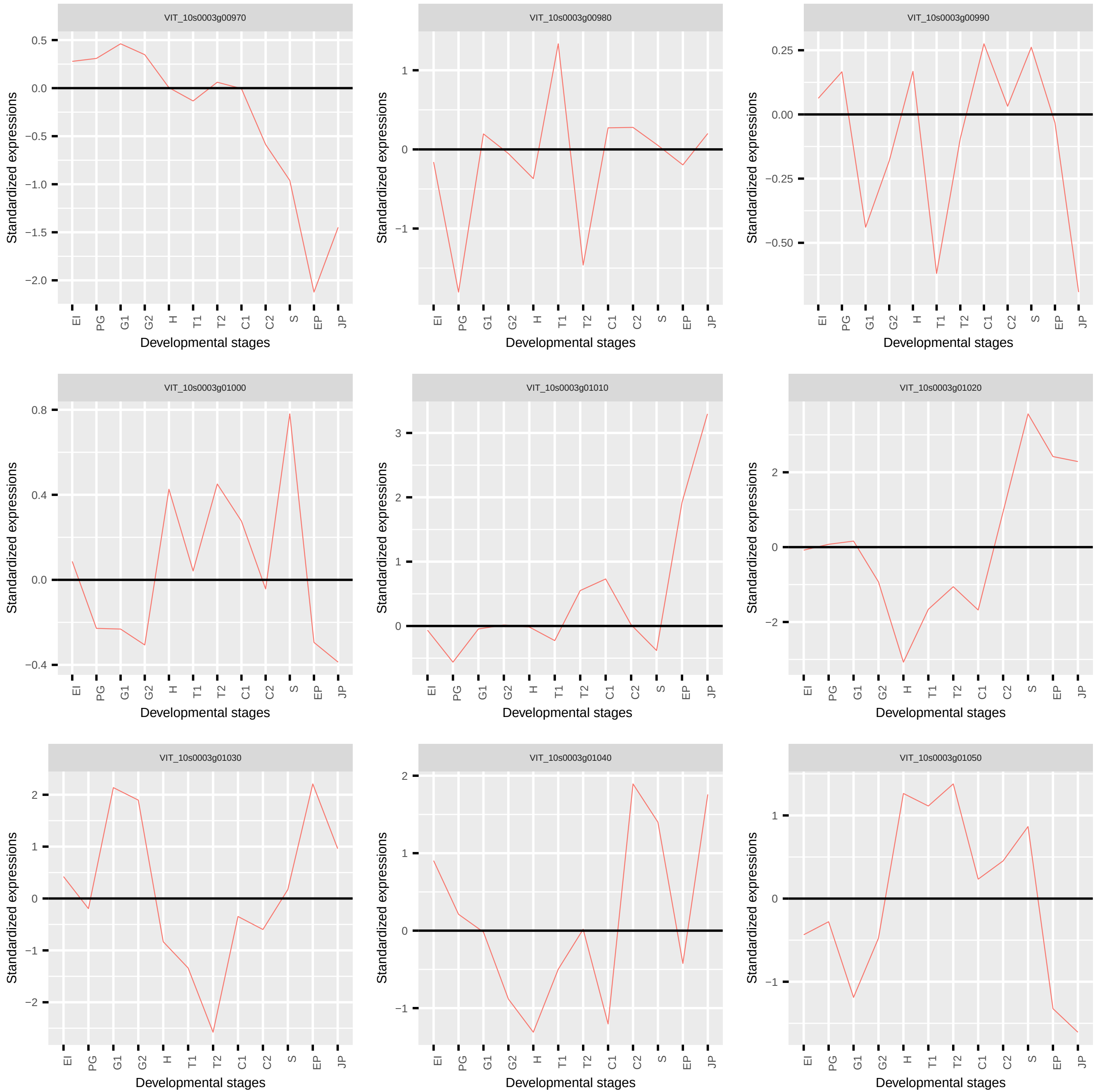
VIT_10s0003g00970
0.5
0.0
−0.5
−1.0
−1.5
−2.0
Standardized expressions
EI PG G1 G2 H T1 T2 C1 C2 S EP JP
Developmental stages
VIT_10s0003g00980
1
0
−1
Standardized expressions
EI PG G1 G2 H T1 T2 C1 C2 S EP JP
Developmental stages
VIT_10s0003g00990
0.25
0.00
−0.25
−0.50
Standardized expressions
EI PG G1 G2 H T1 T2 C1 C2 S EP JP
Developmental stages
VIT_10s0003g01000
0.8
0.4
0.0
−0.4
Standardized expressions
EI PG G1 G2 H T1 T2 C1 C2 S EP JP
Developmental stages
VIT_10s0003g01010
3
2
1
0
Standardized expressions
EI PG G1 G2 H T1 T2 C1 C2 S EP JP
Developmental stages
VIT_10s0003g01020
2
0
−2
Standardized expressions
EI PG G1 G2 H T1 T2 C1 C2 S EP JP
Developmental stages
VIT_10s0003g01030
2
1
0
−1
−2
Standardized expressions
EI PG G1 G2 H T1 T2 C1 C2 S EP JP
Developmental stages
VIT_10s0003g01040
2
1
0
−1
Standardized expressions
EI PG G1 G2 H T1 T2 C1 C2 S EP JP
Developmental stages
VIT_10s0003g01050
1
0
−1
Standardized expressions
EI PG G1 G2 H T1 T2 C1 C2 S EP JP
Developmental stages

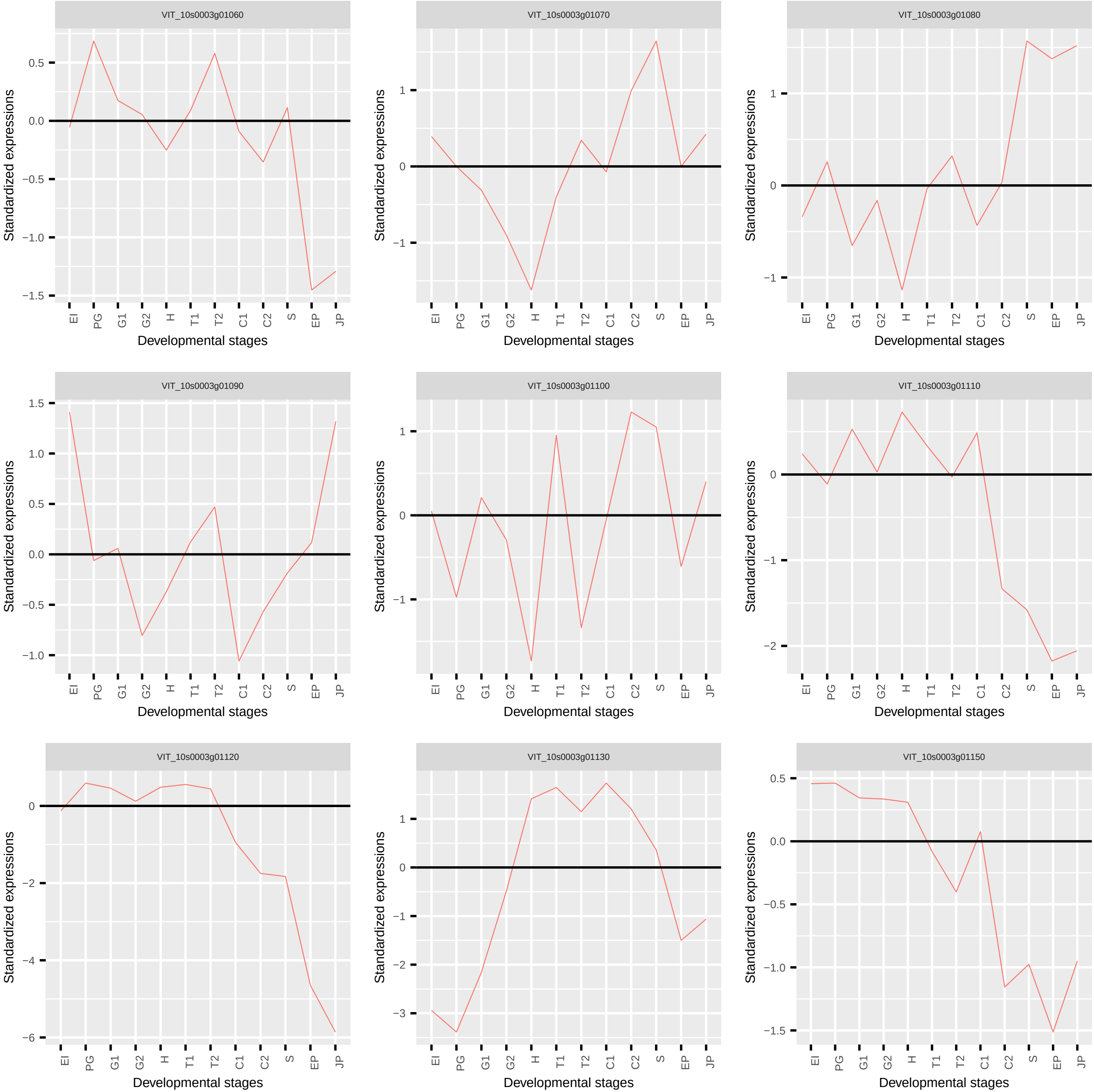
VIT_10s0003g01060
VIT_10s0003g01070
VIT_10s0003g01080
VIT_10s0003g01090
VIT_10s0003g01100
VIT_10s0003g01110
VIT_10s0003g01120
VIT_10s0003g01130
VIT_10s0003g01150
Standardized expressions
Developmental stages
EI PG G1 G2 H T1 T2 C1 C2 S EP JP

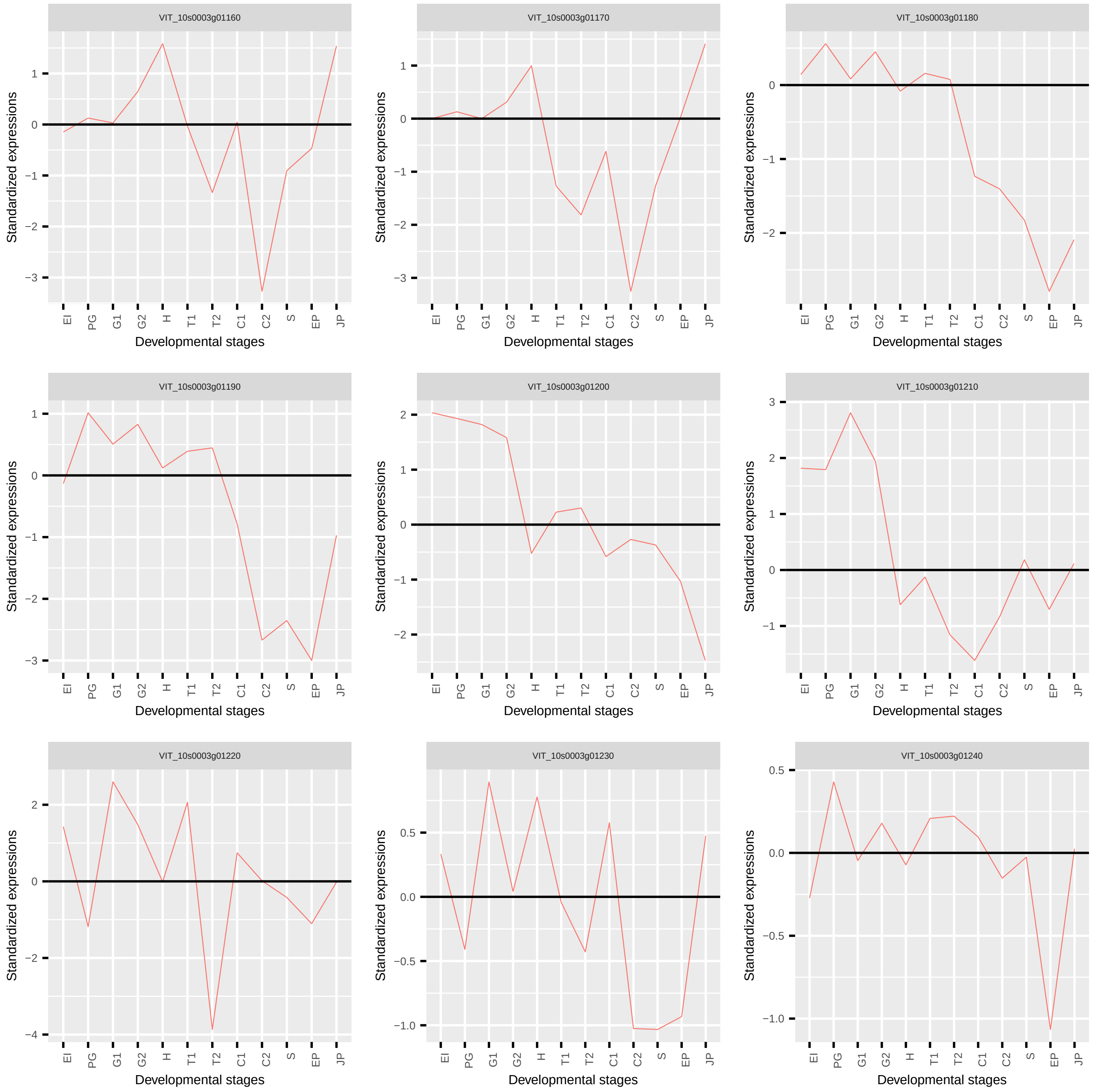
VIT_10s0003g01160
VIT_10s0003g01170
VIT_10s0003g01180
VIT_10s0003g01190
VIT_10s0003g01200
VIT_10s0003g01210
VIT_10s0003g01220
VIT_10s0003g01230
VIT_10s0003g01240
Standardized expressions
Developmental stages
EI
PG
G1
G2
H
T1
T2
C1
C2
S
EP
JP

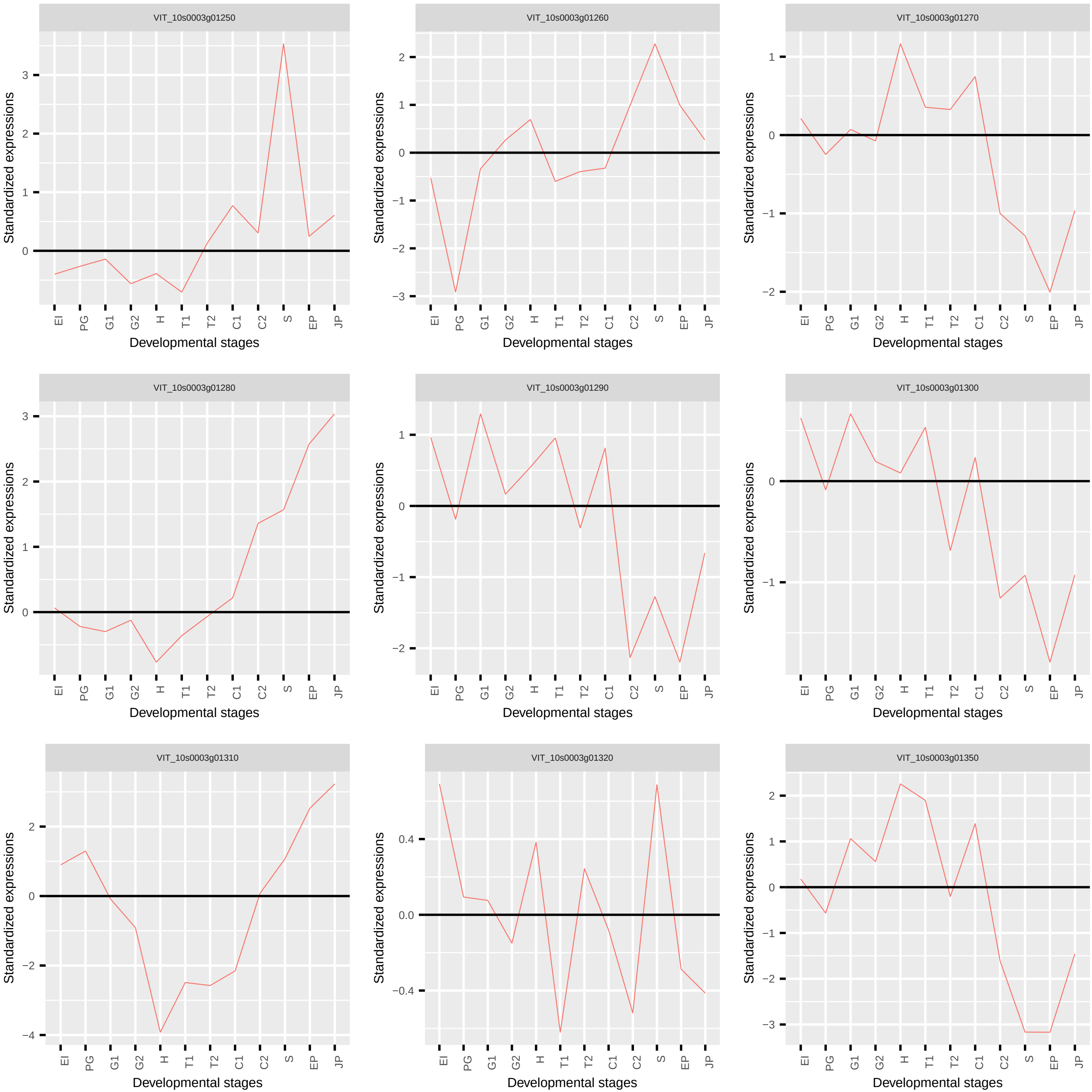
VIT_10s0003g01250
VIT_10s0003g01260
VIT_10s0003g01270
VIT_10s0003g01280
VIT_10s0003g01290
VIT_10s0003g01300
VIT_10s0003g01310
VIT_10s0003g01320
VIT_10s0003g01350
Standardized expressions
Developmental stages
EI PG G1 G2 H T1 T2 C1 C2 S EP JP

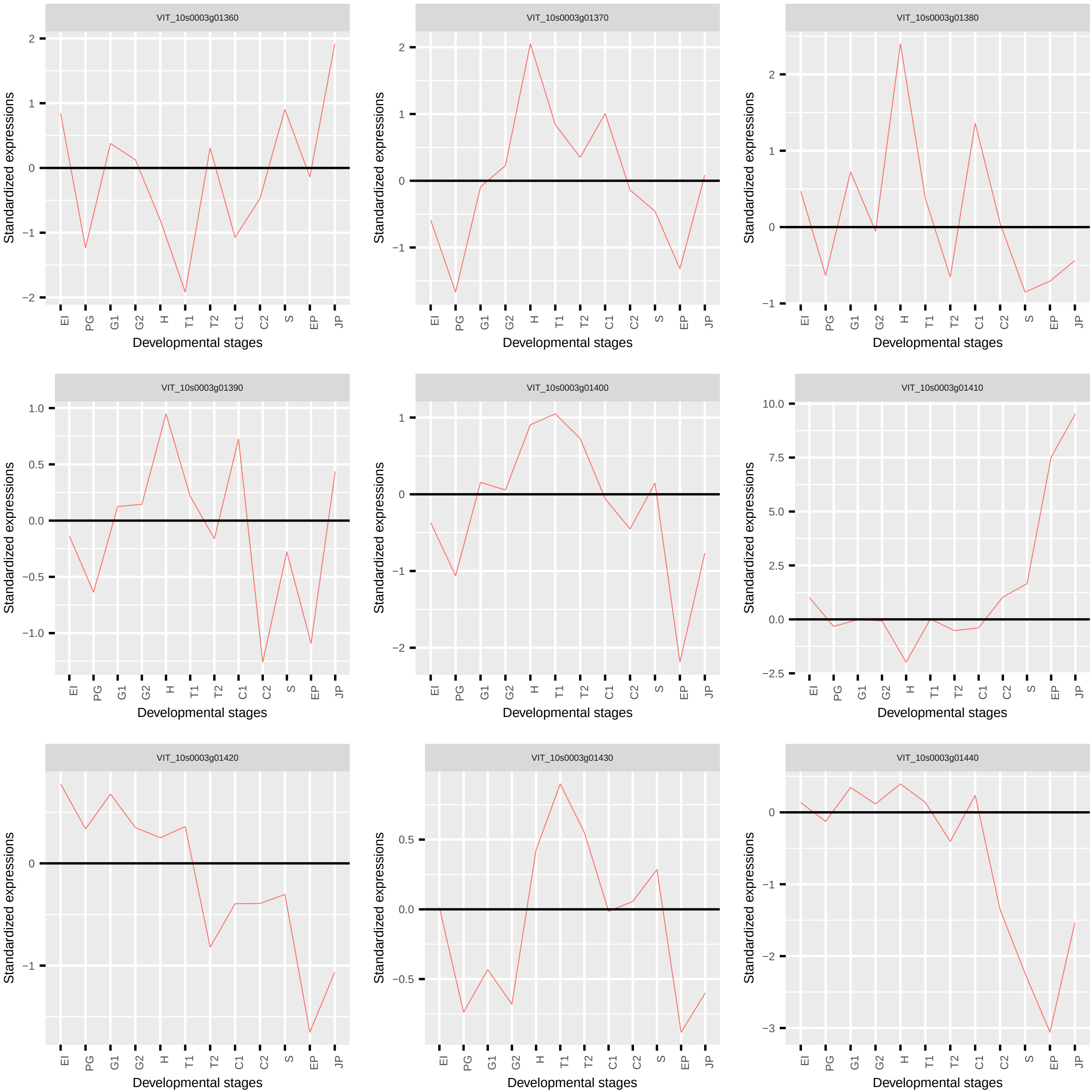
VIT_10s0003g01360
Standardized expressions
2
1
0
−1
−2
EI PG G1 G2 H T1 T2 C1 C2 S EP JP
Developmental stages
VIT_10s0003g01370
Standardized expressions
2
1
0
−1
EI PG G1 G2 H T1 T2 C1 C2 S EP JP
Developmental stages
VIT_10s0003g01380
Standardized expressions
2
1
0
−1
EI PG G1 G2 H T1 T2 C1 C2 S EP JP
Developmental stages
VIT_10s0003g01390
Standardized expressions
1.0
0.5
0.0
−0.5
−1.0
EI PG G1 G2 H T1 T2 C1 C2 S EP JP
Developmental stages
VIT_10s0003g01400
Standardized expressions
1
0
−1
−2
EI PG G1 G2 H T1 T2 C1 C2 S EP JP
Developmental stages
VIT_10s0003g01410
Standardized expressions
10.0
7.5
5.0
2.5
0.0
−2.5
EI PG G1 G2 H T1 T2 C1 C2 S EP JP
Developmental stages
VIT_10s0003g01420
Standardized expressions
0
−1
EI PG G1 G2 H T1 T2 C1 C2 S EP JP
Developmental stages
VIT_10s0003g01430
Standardized expressions
0.5
0.0
−0.5
EI PG G1 G2 H T1 T2 C1 C2 S EP JP
Developmental stages
VIT_10s0003g01440
Standardized expressions
0
−1
−2
−3
EI PG G1 G2 H T1 T2 C1 C2 S EP JP
Developmental stages

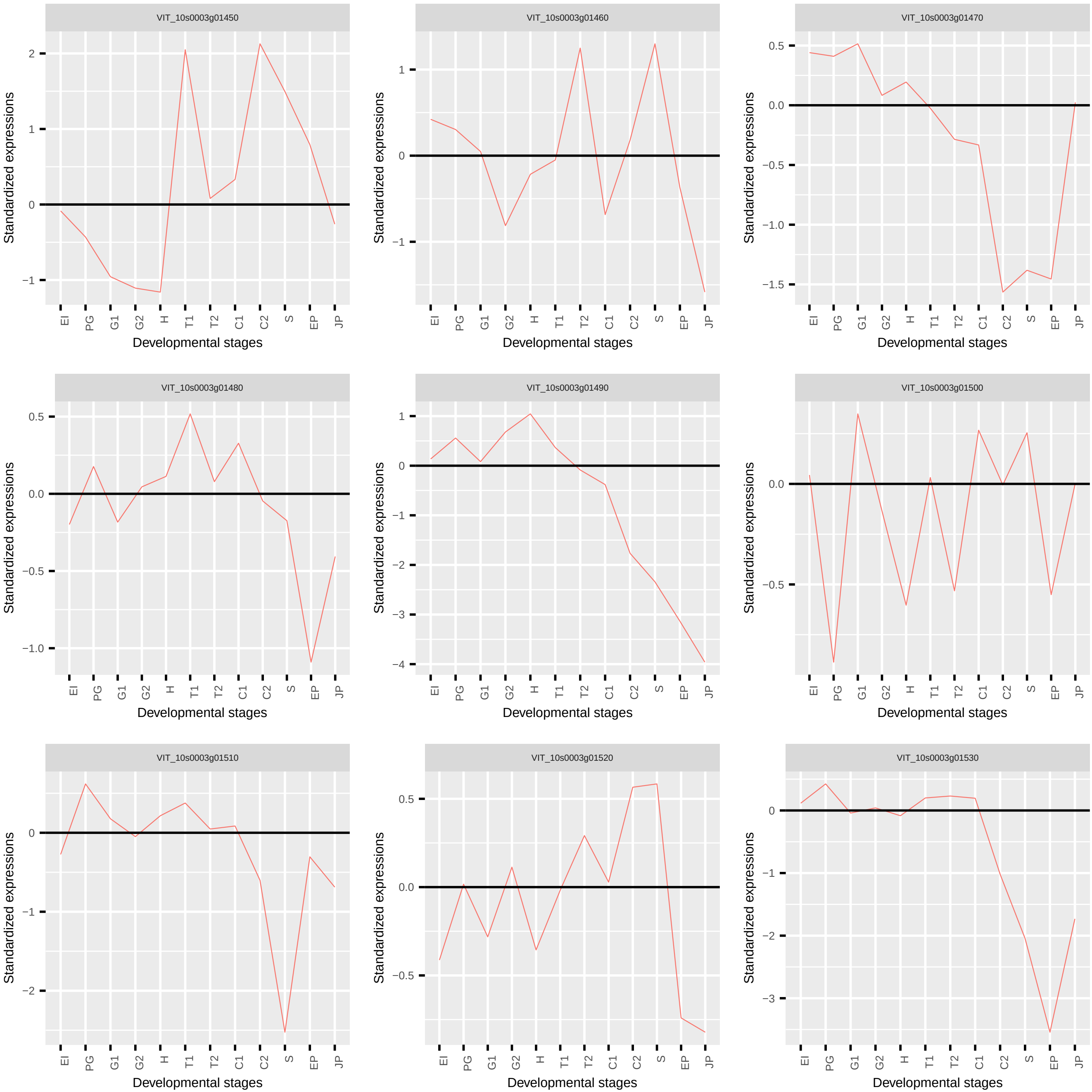
VIT_10s0003g01450
VIT_10s0003g01460
VIT_10s0003g01470
VIT_10s0003g01480
VIT_10s0003g01490
VIT_10s0003g01500
VIT_10s0003g01510
VIT_10s0003g01520
VIT_10s0003g01530
Standardized expressions
Developmental stages
EI PG G1 G2 H T1 T2 C1 C2 S EP JP

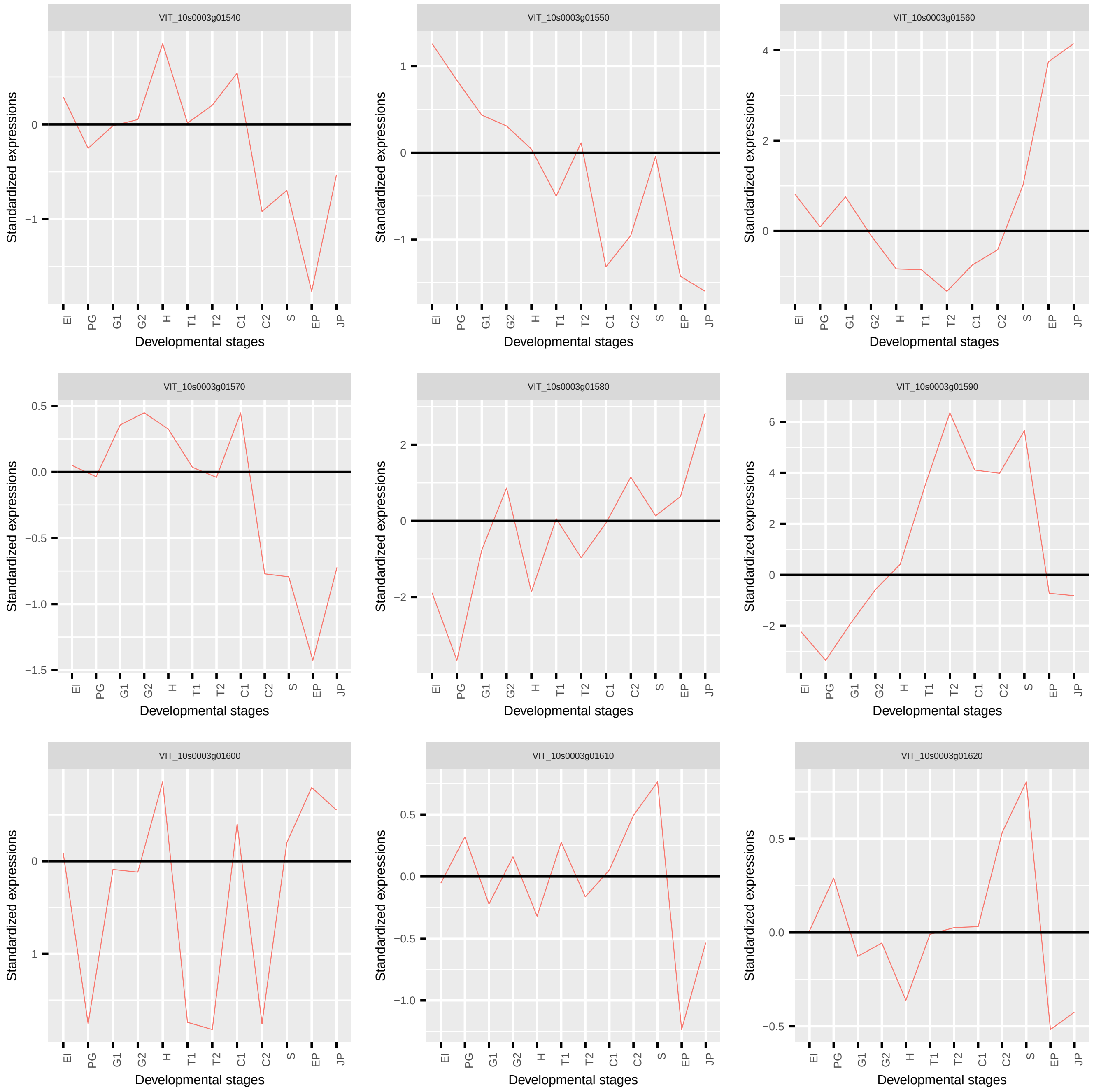

VIT_10s0003g01540
VIT_10s0003g01550
VIT_10s0003g01560
VIT_10s0003g01570
VIT_10s0003g01580
VIT_10s0003g01590
VIT_10s0003g01600
VIT_10s0003g01610
VIT_10s0003g01620
Standardized expressions
Developmental stages
EI PG G1 G2 H T1 T2 C1 C2 S EP JP

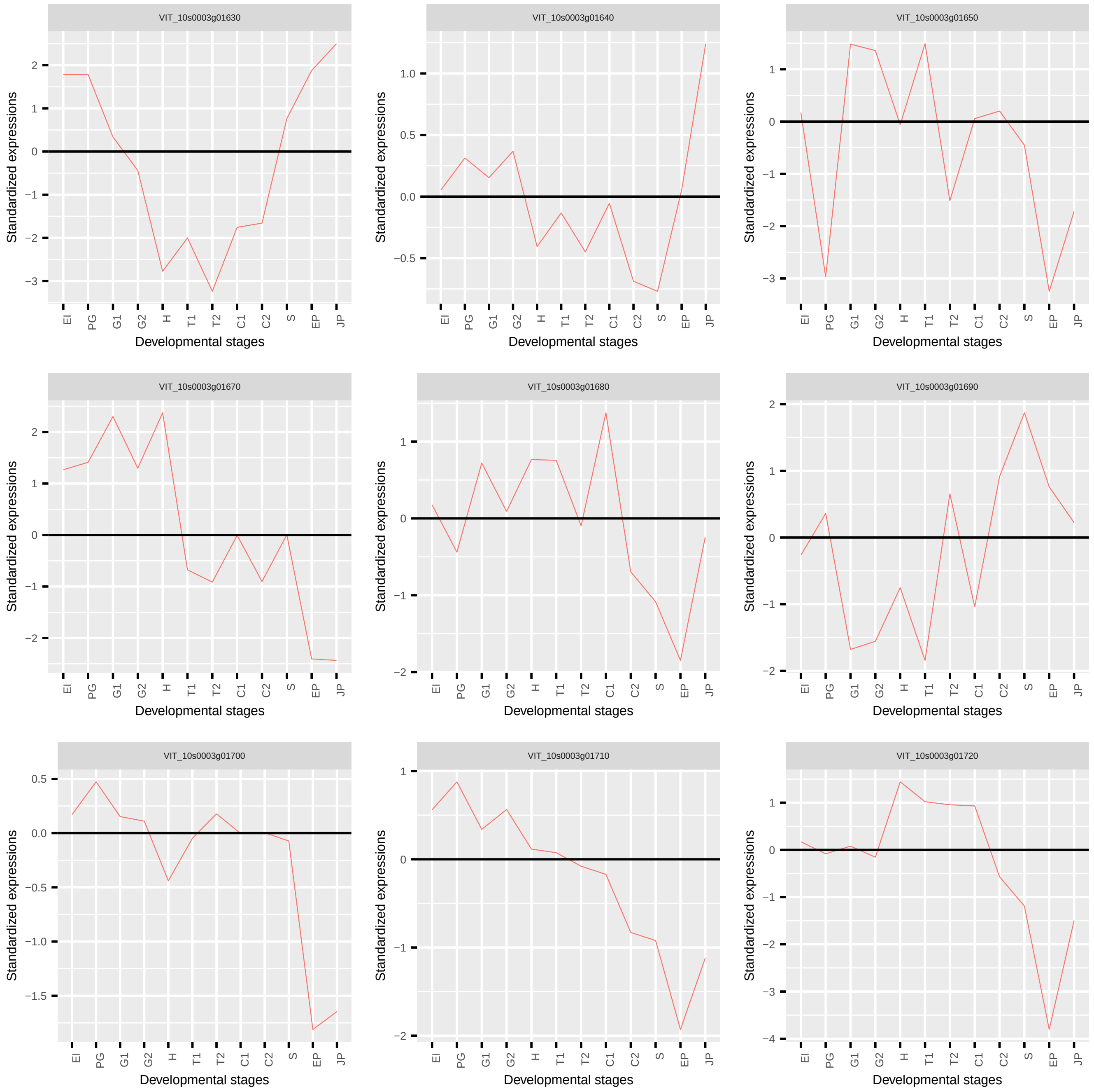
VIT_10s0003g01630
VIT_10s0003g01640
VIT_10s0003g01650
VIT_10s0003g01670
VIT_10s0003g01680
VIT_10s0003g01690
VIT_10s0003g01700
VIT_10s0003g01710
VIT_10s0003g01720
Standardized expressions
Developmental stages
EI PG G1 G2 H T1 T2 C1 C2 S EP JP

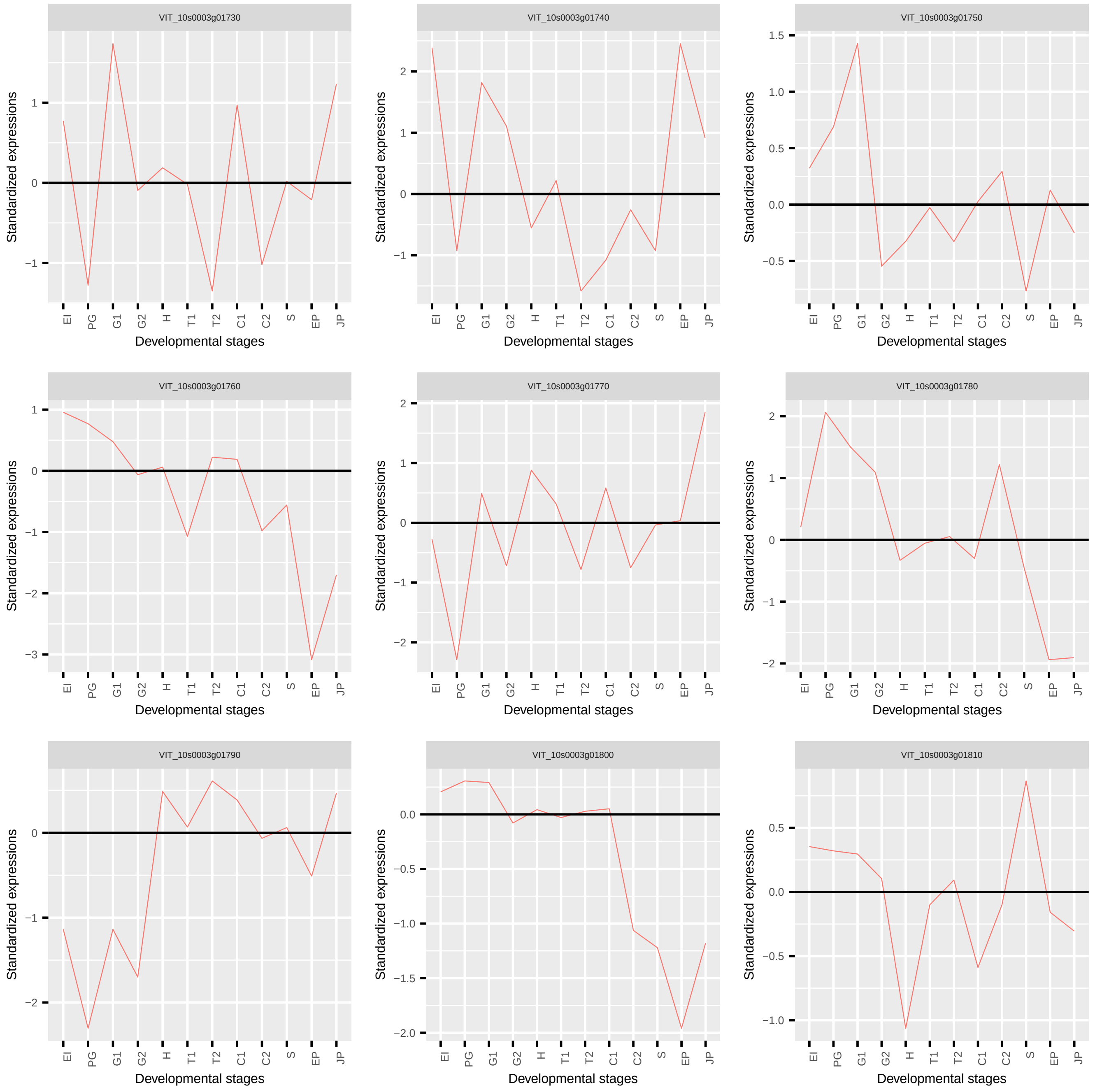

VIT_10s0003g01730
VIT_10s0003g01740
VIT_10s0003g01750
VIT_10s0003g01760
VIT_10s0003g01770
VIT_10s0003g01780
VIT_10s0003g01790
VIT_10s0003g01800
VIT_10s0003g01810
Standardized expressions
Developmental stages
EI
PG
G1
G2
H
T1
T2
C1
C2
S
EP
JP

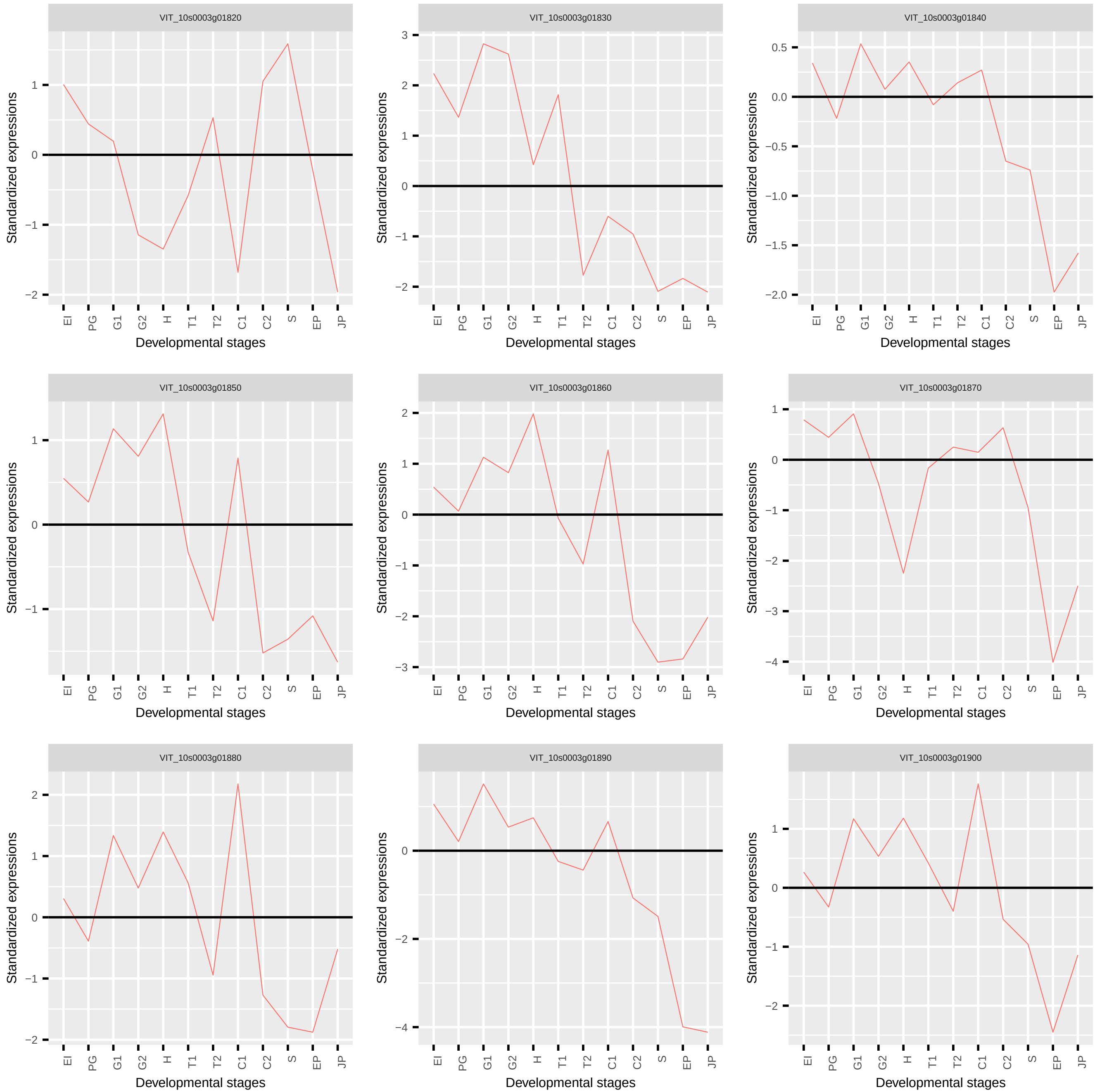
VIT_10s0003g01820
VIT_10s0003g01830
VIT_10s0003g01840
VIT_10s0003g01850
VIT_10s0003g01860
VIT_10s0003g01870
VIT_10s0003g01880
VIT_10s0003g01890
VIT_10s0003g01900
Standardized expressions
Developmental stages
EI PG G1 G2 H T1 T2 C1 C2 S EP JP

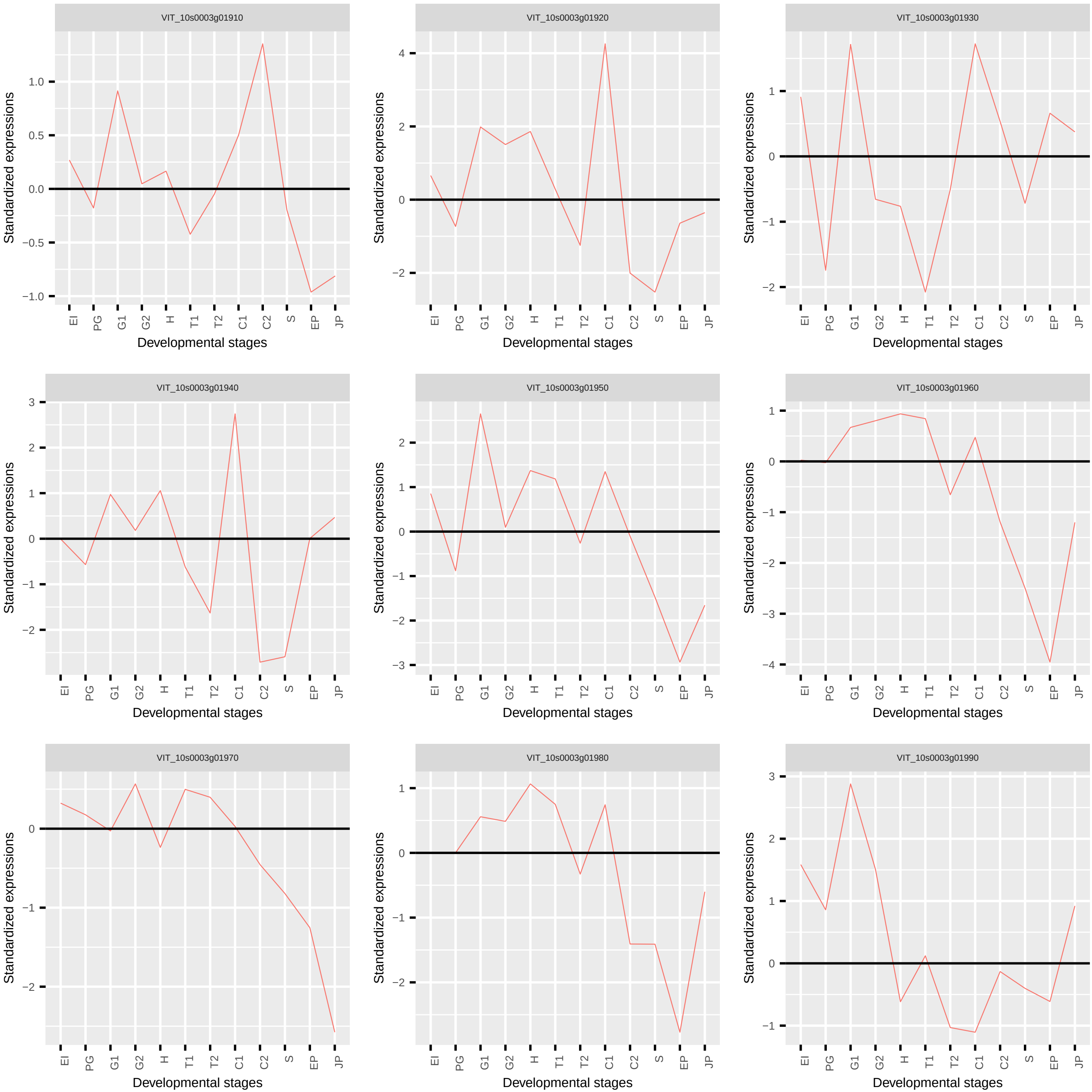
VIT_10s0003g01910
Standardized expressions
1.0
0.5
0.0
−0.5
−1.0
EI PG G1 G2 H T1 T2 C1 C2 S EP JP
Developmental stages
VIT_10s0003g01920
Standardized expressions
4
2
0
−2
EI PG G1 G2 H T1 T2 C1 C2 S EP JP
Developmental stages
VIT_10s0003g01930
Standardized expressions
1
0
−1
−2
EI PG G1 G2 H T1 T2 C1 C2 S EP JP
Developmental stages
VIT_10s0003g01940
Standardized expressions
3
2
1
0
−1
−2
EI PG G1 G2 H T1 T2 C1 C2 S EP JP
Developmental stages
VIT_10s0003g01950
Standardized expressions
2
1
0
−1
−2
−3
EI PG G1 G2 H T1 T2 C1 C2 S EP JP
Developmental stages
VIT_10s0003g01960
Standardized expressions
1
0
−1
−2
−3
−4
EI PG G1 G2 H T1 T2 C1 C2 S EP JP
Developmental stages
VIT_10s0003g01970
Standardized expressions
0
−1
−2
EI PG G1 G2 H T1 T2 C1 C2 S EP JP
Developmental stages
VIT_10s0003g01980
Standardized expressions
1
0
−1
−2
EI PG G1 G2 H T1 T2 C1 C2 S EP JP
Developmental stages
VIT_10s0003g01990
Standardized expressions
3
2
1
0
−1
EI PG G1 G2 H T1 T2 C1 C2 S EP JP
Developmental stages

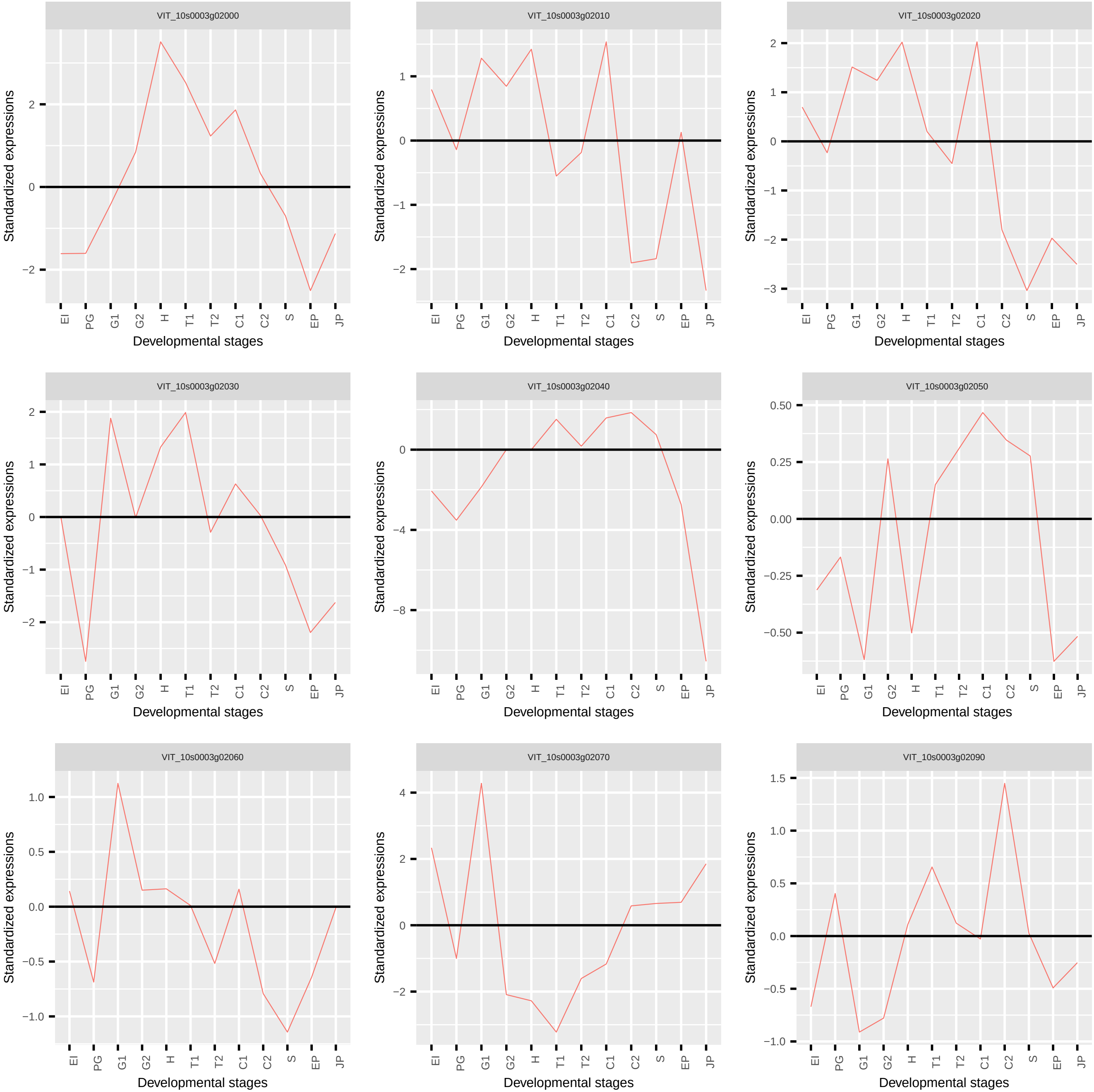
VIT_10s0003g02000
VIT_10s0003g02010
VIT_10s0003g02020
VIT_10s0003g02030
VIT_10s0003g02040
VIT_10s0003g02050
VIT_10s0003g02060
VIT_10s0003g02070
VIT_10s0003g02090
Standardized expressions
Developmental stages
EI PG G1 G2 H T1 T2 C1 C2 S EP JP
2 0 −2
1 0 −1 −2
2 1 0 −1 −2 −3
2 1 0 −1 −2
0 −4 −8
0.50 0.25 0.00 −0.25 −0.50
1.0 0.5 0.0 −0.5 −1.0
4 2 0 −2
1.5 1.0 0.5 0.0 −0.5 −1.0

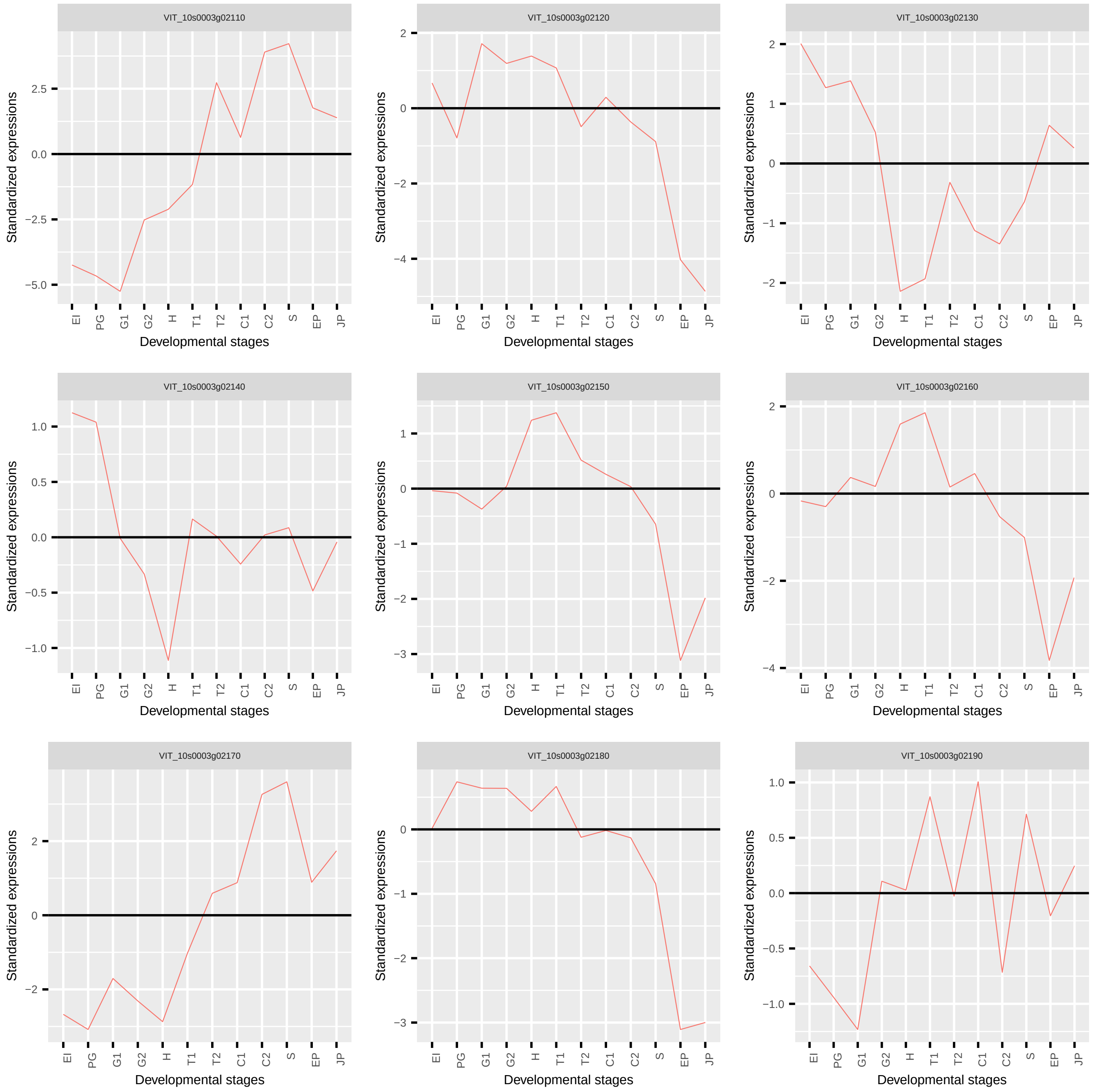
VIT_10s0003g02110
2.5
0.0
−2.5
−5.0
Standardized expressions
EI PG G1 G2 H T1 T2 C1 C2 S EP JP
Developmental stages
VIT_10s0003g02120
2
0
−2
−4
Standardized expressions
EI PG G1 G2 H T1 T2 C1 C2 S EP JP
Developmental stages
VIT_10s0003g02130
2
1
0
−1
−2
Standardized expressions
EI PG G1 G2 H T1 T2 C1 C2 S EP JP
Developmental stages
VIT_10s0003g02140
1.0
0.5
0.0
−0.5
−1.0
Standardized expressions
EI PG G1 G2 H T1 T2 C1 C2 S EP JP
Developmental stages
VIT_10s0003g02150
1
0
−1
−2
−3
Standardized expressions
EI PG G1 G2 H T1 T2 C1 C2 S EP JP
Developmental stages
VIT_10s0003g02160
2
0
−2
−4
Standardized expressions
EI PG G1 G2 H T1 T2 C1 C2 S EP JP
Developmental stages
VIT_10s0003g02170
2
0
−2
Standardized expressions
EI PG G1 G2 H T1 T2 C1 C2 S EP JP
Developmental stages
VIT_10s0003g02180
0
−1
−2
−3
Standardized expressions
EI PG G1 G2 H T1 T2 C1 C2 S EP JP
Developmental stages
VIT_10s0003g02190
1.0
0.5
0.0
−0.5
−1.0
Standardized expressions
EI PG G1 G2 H T1 T2 C1 C2 S EP JP
Developmental stages

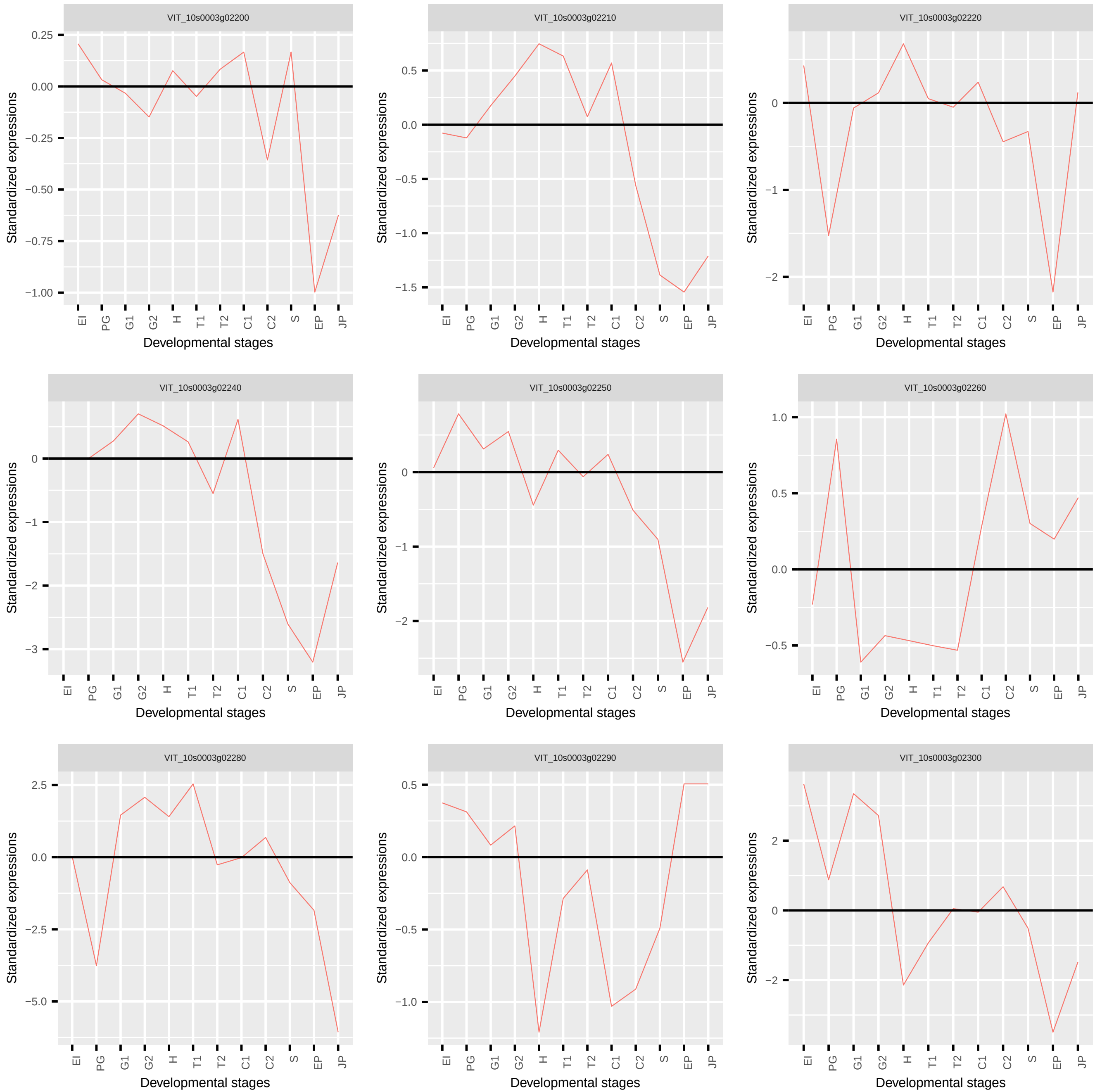
VIT_10s0003g02200
VIT_10s0003g02210
VIT_10s0003g02220
VIT_10s0003g02240
VIT_10s0003g02250
VIT_10s0003g02260
VIT_10s0003g02280
VIT_10s0003g02290
VIT_10s0003g02300
Standardized expressions
Developmental stages
EI PG G1 G2 H T1 T2 C1 C2 S EP JP

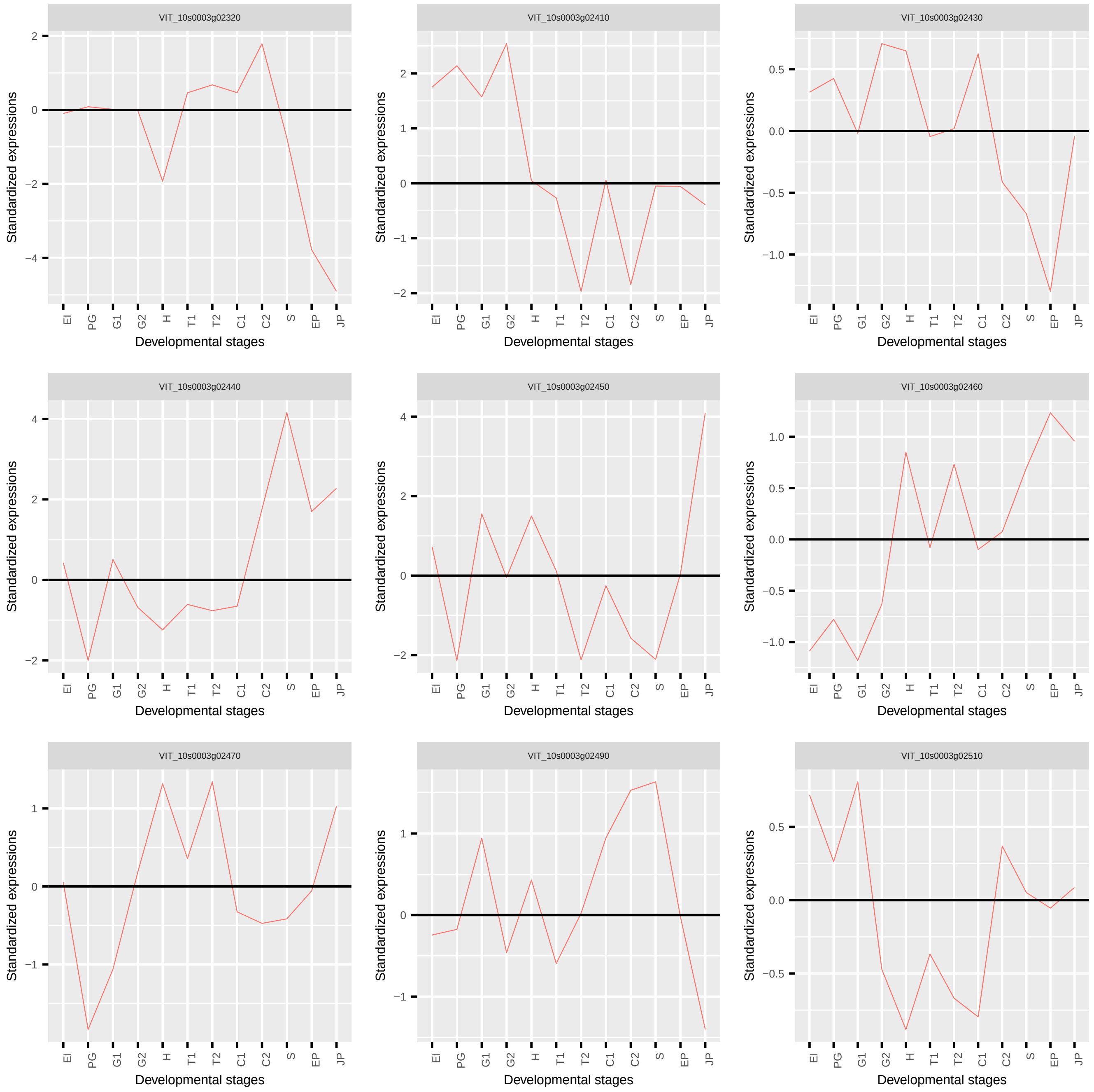
VIT_10s0003g02320
VIT_10s0003g02410
VIT_10s0003g02430
VIT_10s0003g02440
VIT_10s0003g02450
VIT_10s0003g02460
VIT_10s0003g02470
VIT_10s0003g02490
VIT_10s0003g02510
Standardized expressions
Developmental stages
EI PG G1 G2 H T1 T2 C1 C2 S EP JP

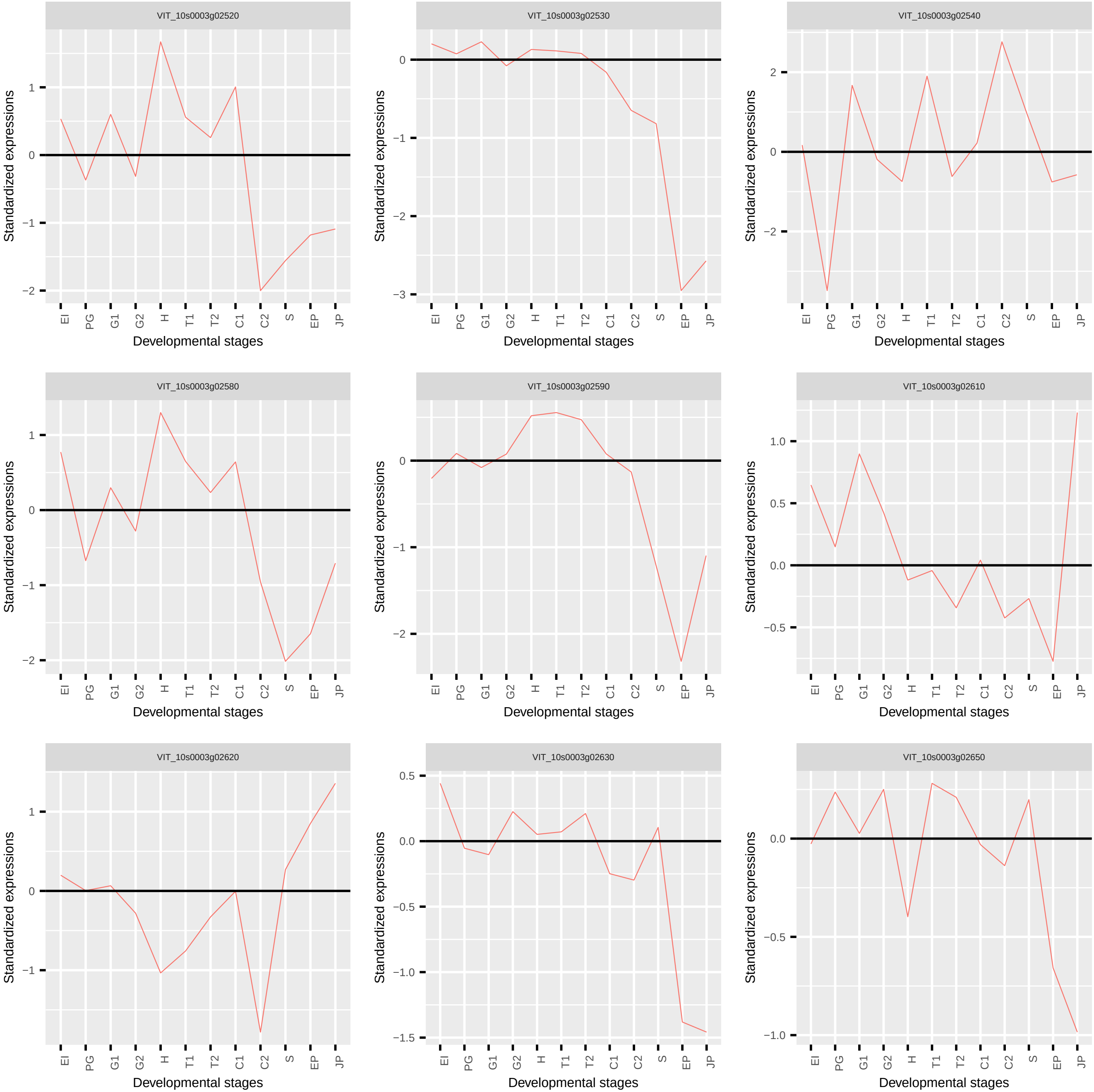
VIT_10s0003g02520
VIT_10s0003g02530
VIT_10s0003g02540
VIT_10s0003g02580
VIT_10s0003g02590
VIT_10s0003g02610
VIT_10s0003g02620
VIT_10s0003g02630
VIT_10s0003g02650
Standardized expressions
Developmental stages
EI
PG
G1
G2
H
T1
T2
C1
C2
S
EP
JP

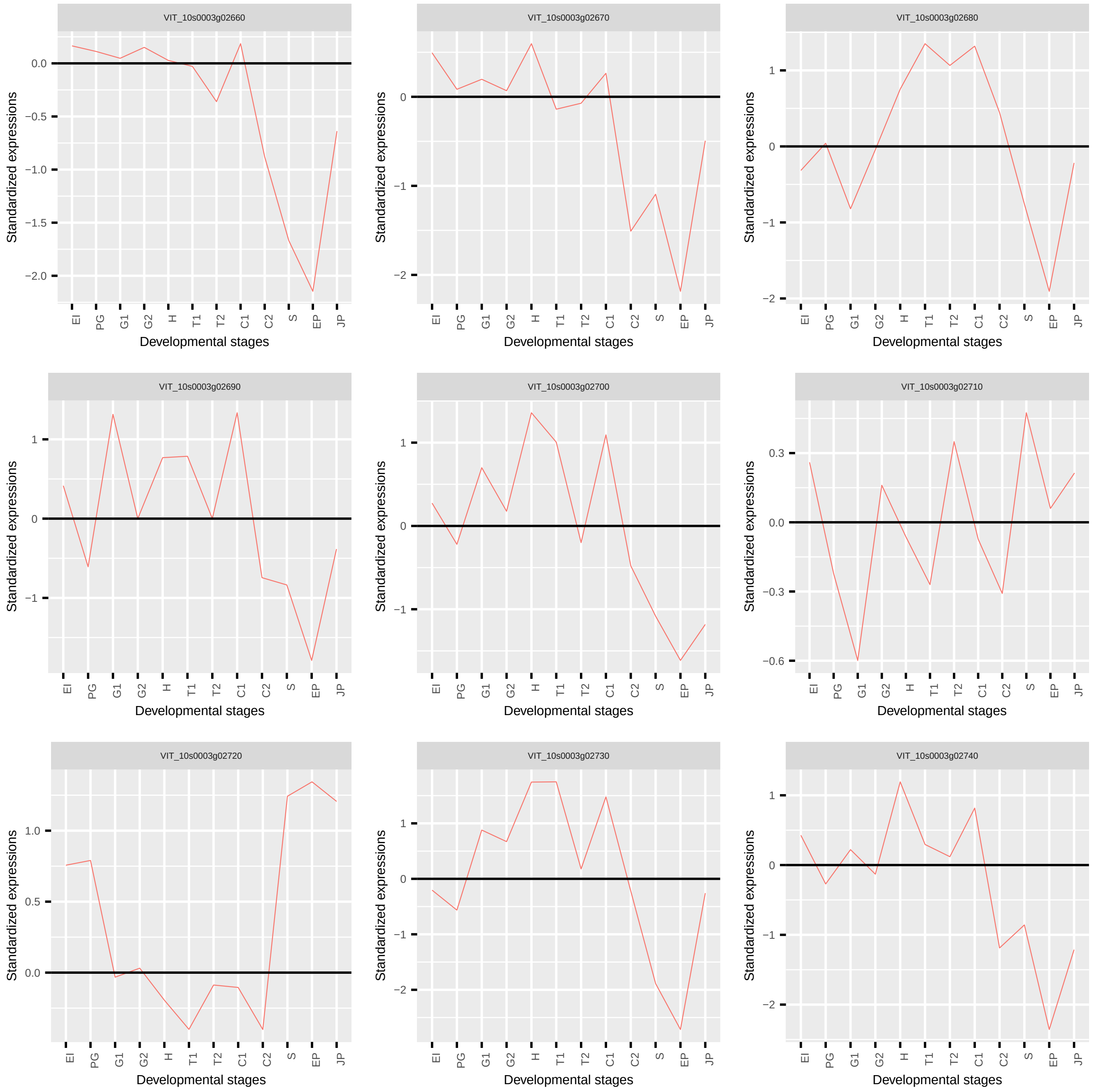
VIT_10s0003g02660
VIT_10s0003g02670
VIT_10s0003g02680
VIT_10s0003g02690
VIT_10s0003g02700
VIT_10s0003g02710
VIT_10s0003g02720
VIT_10s0003g02730
VIT_10s0003g02740
Standardized expressions
Developmental stages
EI
PG
G1
G2
H
T1
T2
C1
C2
S
EP
JP
0.0
−0.5
−1.0
−1.5
−2.0
1
0
−1
−2
0.3
−0.3
−0.6
1.0
0.5

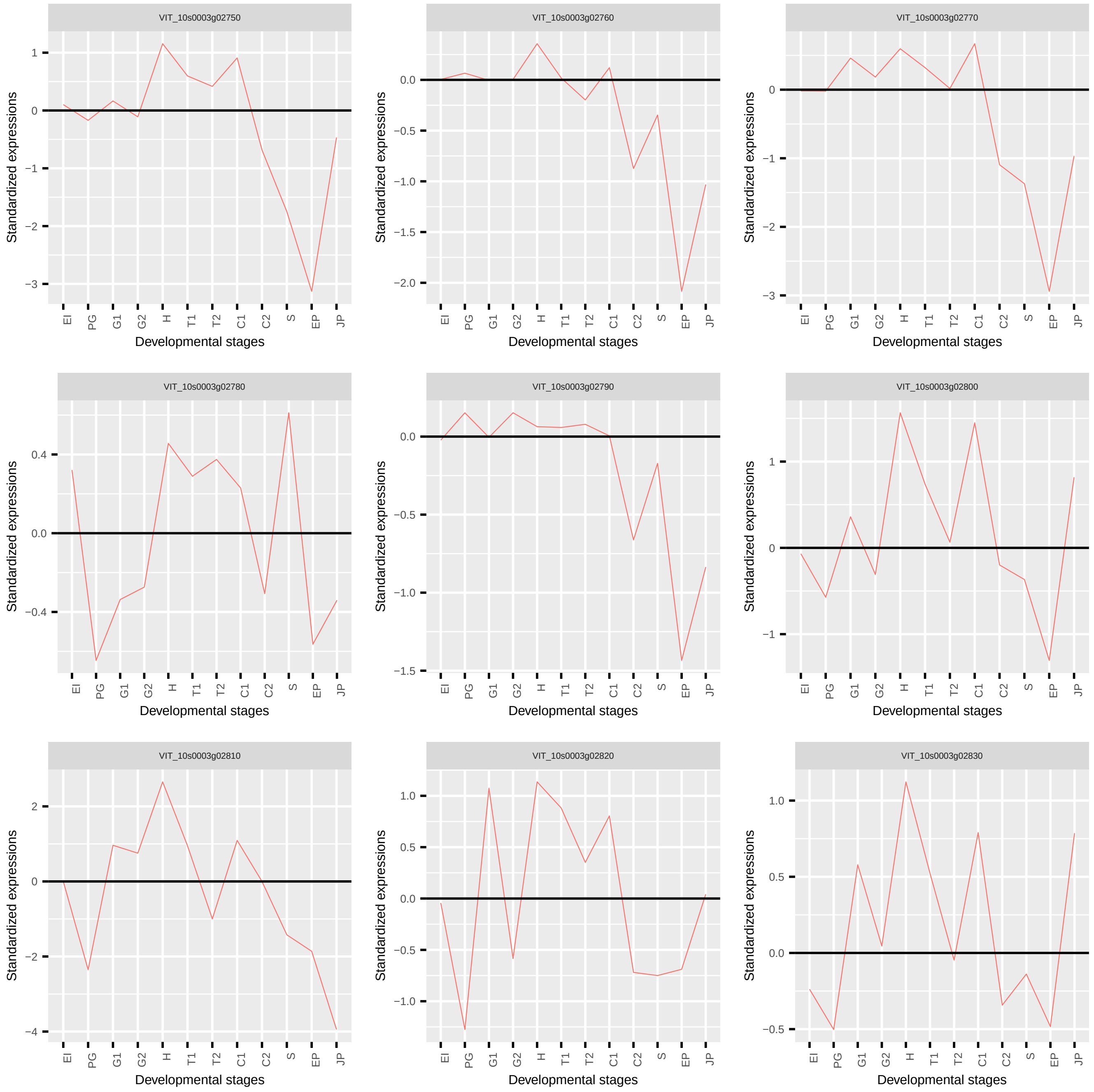
VIT_10s0003g02750
VIT_10s0003g02760
VIT_10s0003g02770
VIT_10s0003g02780
VIT_10s0003g02790
VIT_10s0003g02800
VIT_10s0003g02810
VIT_10s0003g02820
VIT_10s0003g02830
Standardized expressions
Developmental stages
EI PG G1 G2 H T1 T2 C1 C2 S EP JP

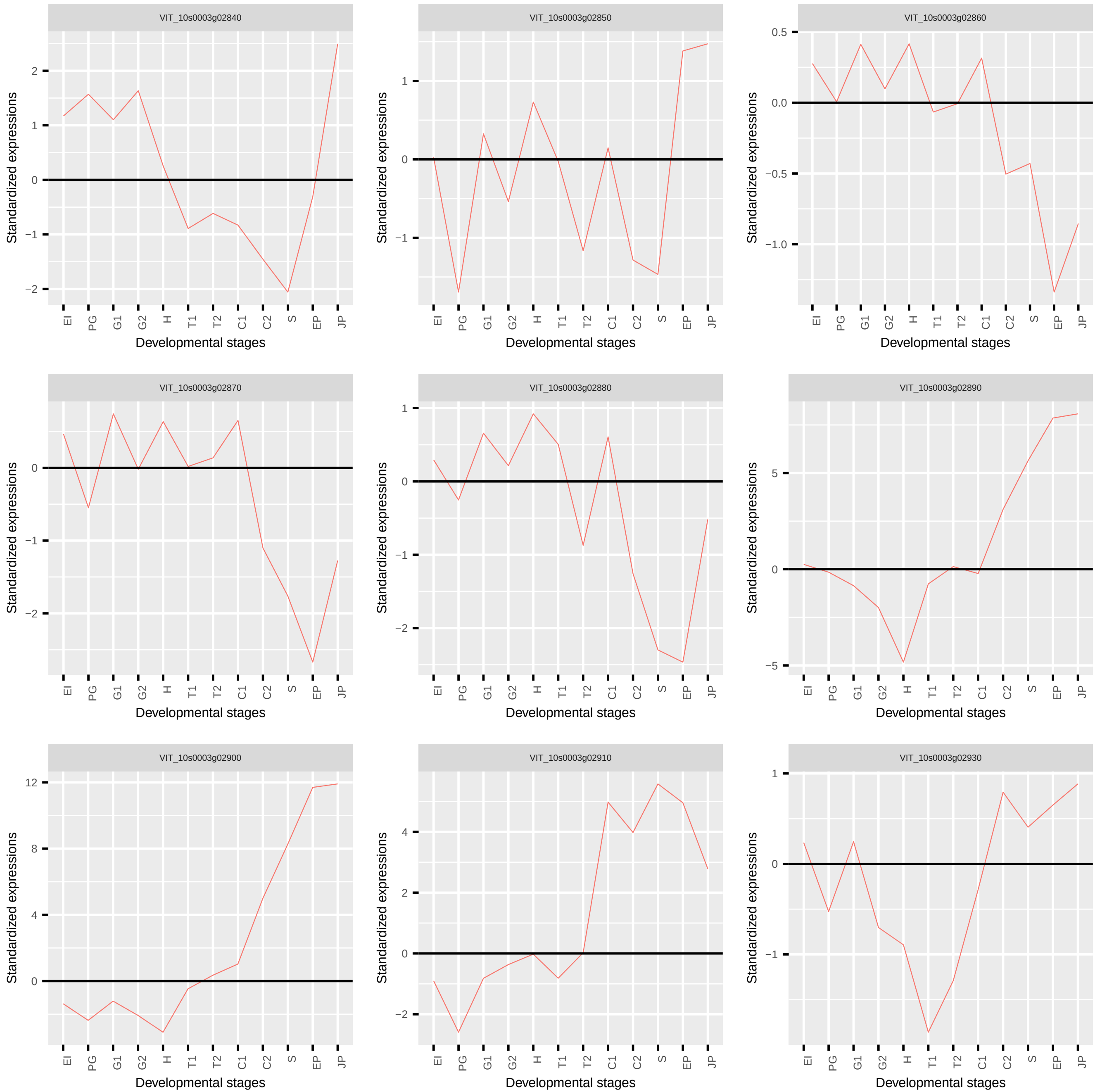

VIT_10s0003g02840
VIT_10s0003g02850
VIT_10s0003g02860
VIT_10s0003g02870
VIT_10s0003g02880
VIT_10s0003g02890
VIT_10s0003g02900
VIT_10s0003g02910
VIT_10s0003g02930
Standardized expressions
Developmental stages
EI
PG
G1
G2
H
T1
T2
C1
C2
S
EP
JP

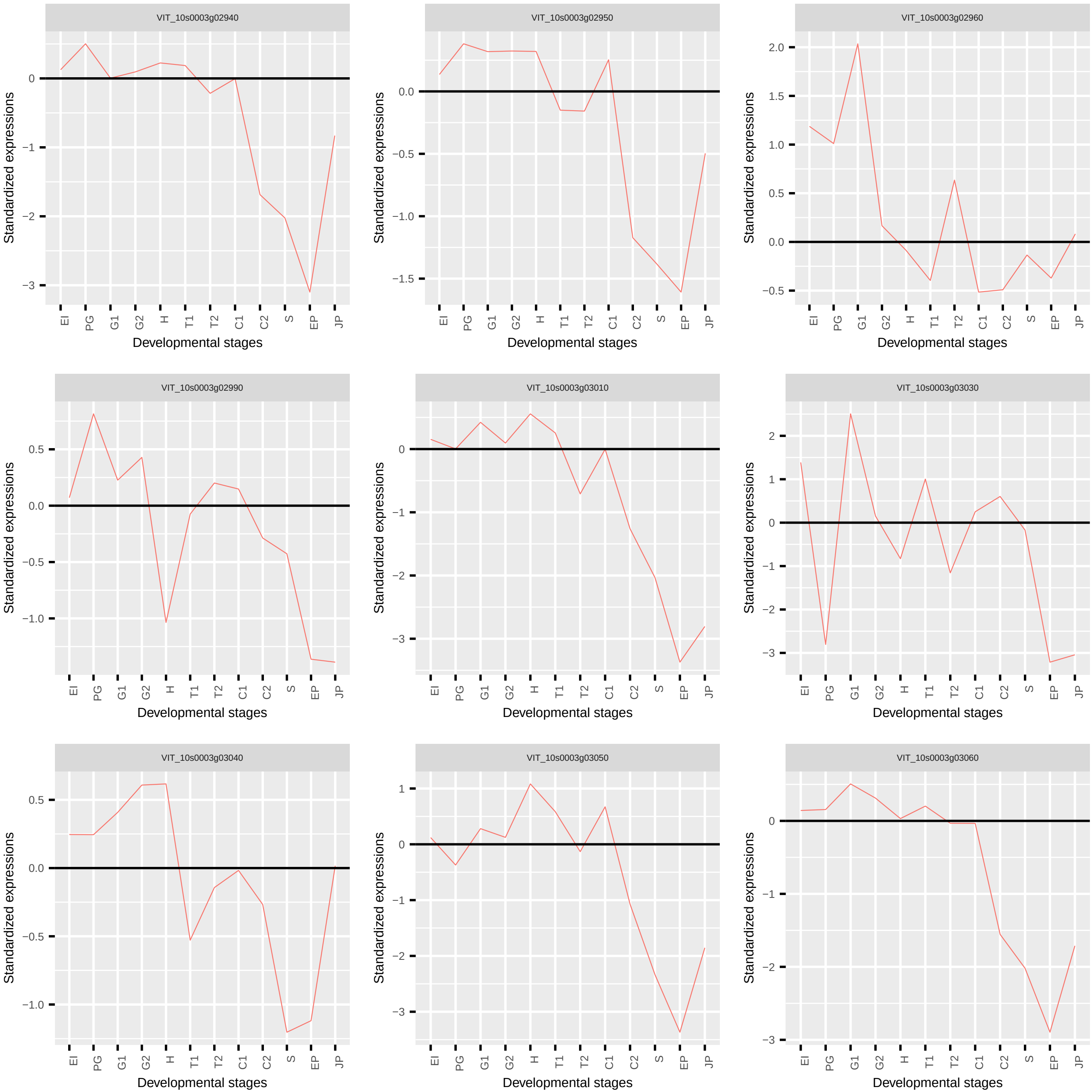
VIT_10s0003g02940
VIT_10s0003g02950
VIT_10s0003g02960
VIT_10s0003g02990
VIT_10s0003g03010
VIT_10s0003g03030
VIT_10s0003g03040
VIT_10s0003g03050
VIT_10s0003g03060
Standardized expressions
Developmental stages
EI PG G1 G2 H T1 T2 C1 C2 S EP JP

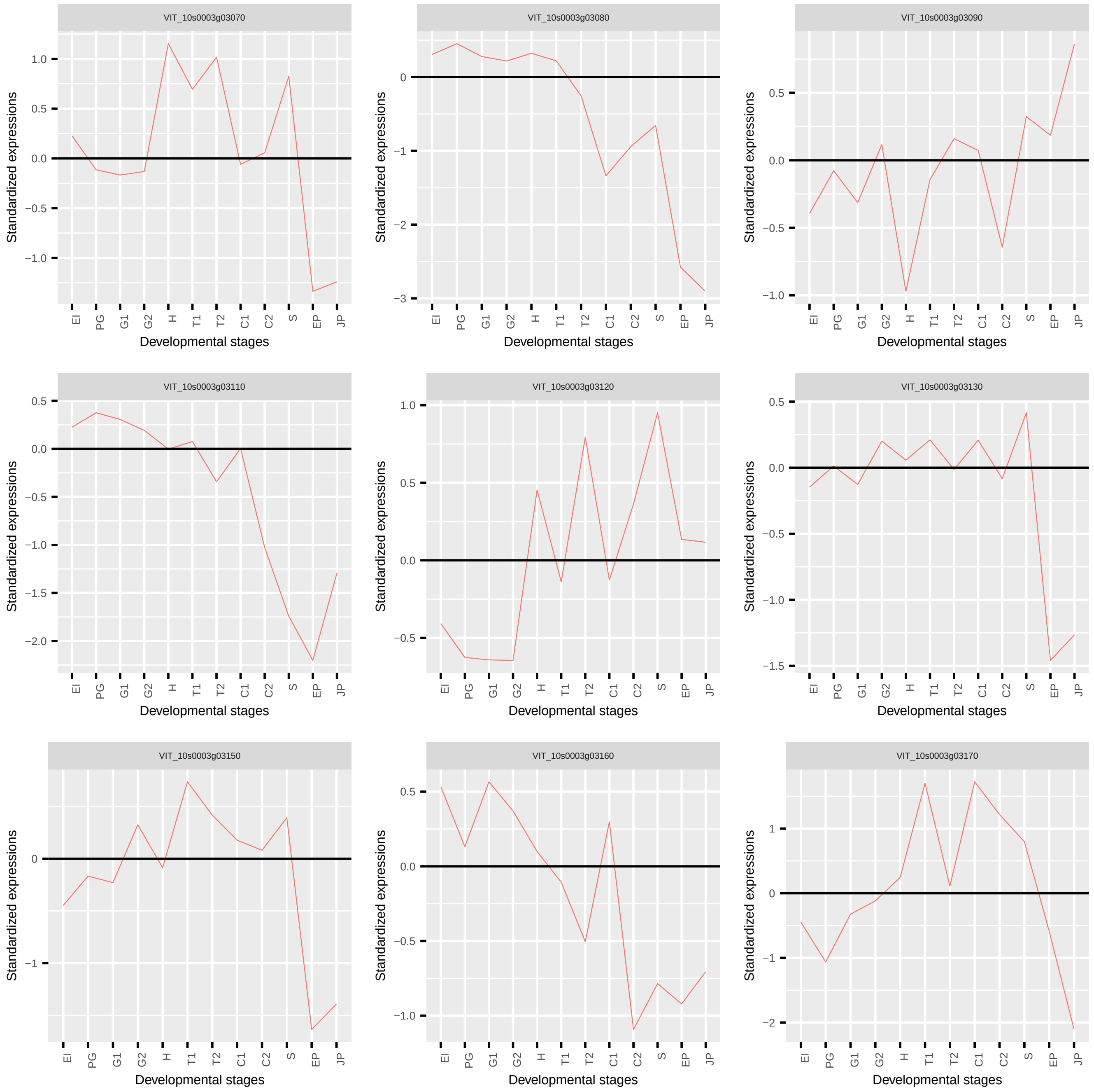

VIT_10s0003g03070
VIT_10s0003g03080
VIT_10s0003g03090
VIT_10s0003g03110
VIT_10s0003g03120
VIT_10s0003g03130
VIT_10s0003g03150
VIT_10s0003g03160
VIT_10s0003g03170
Standardized expressions
Developmental stages
EI PG G1 G2 H T1 T2 C1 C2 S EP JP

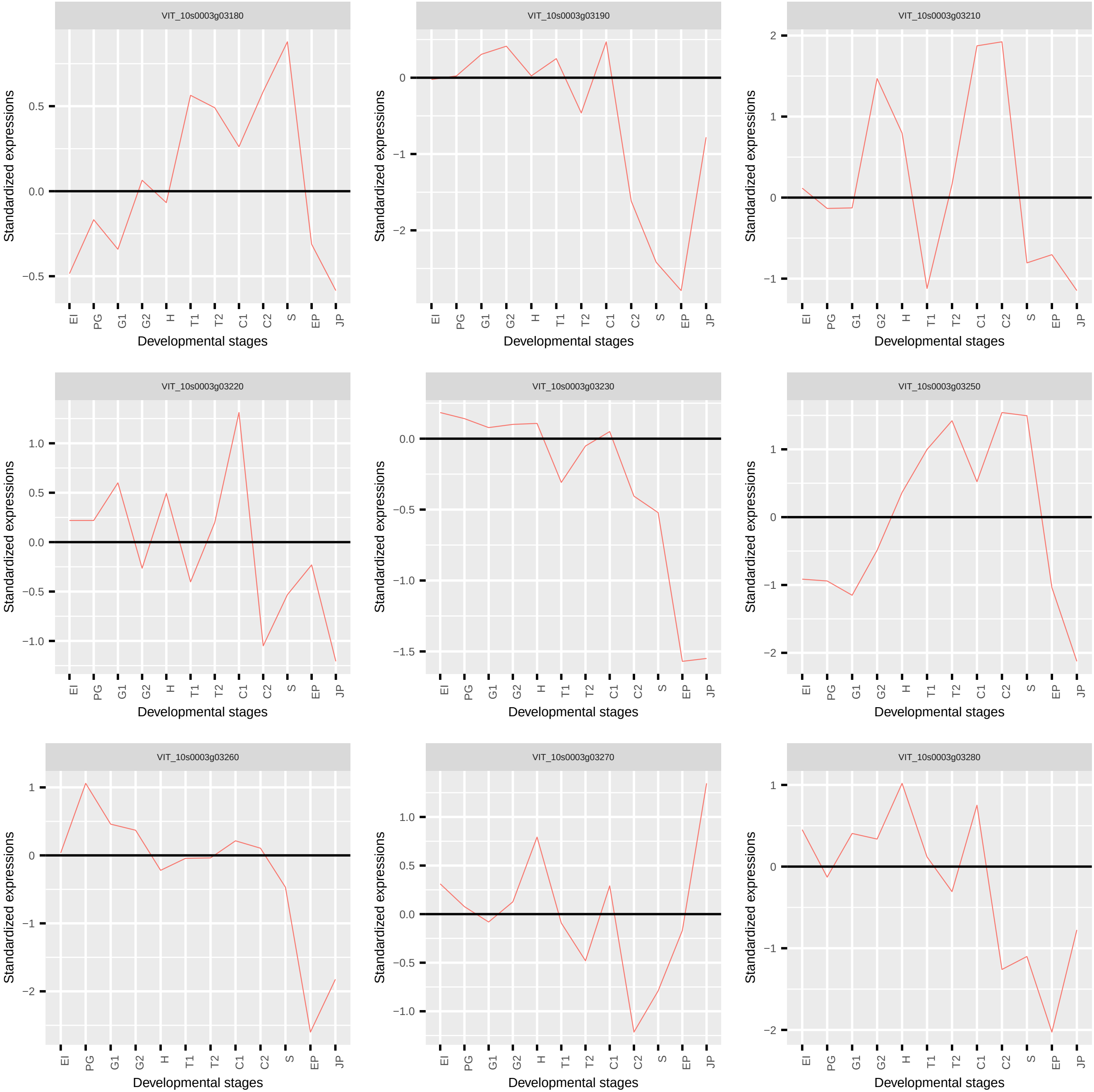
VIT_10s0003g03180
VIT_10s0003g03190
VIT_10s0003g03210
VIT_10s0003g03220
VIT_10s0003g03230
VIT_10s0003g03250
VIT_10s0003g03260
VIT_10s0003g03270
VIT_10s0003g03280
Standardized expressions
Developmental stages
EI PG G1 G2 H T1 T2 C1 C2 S EP JP

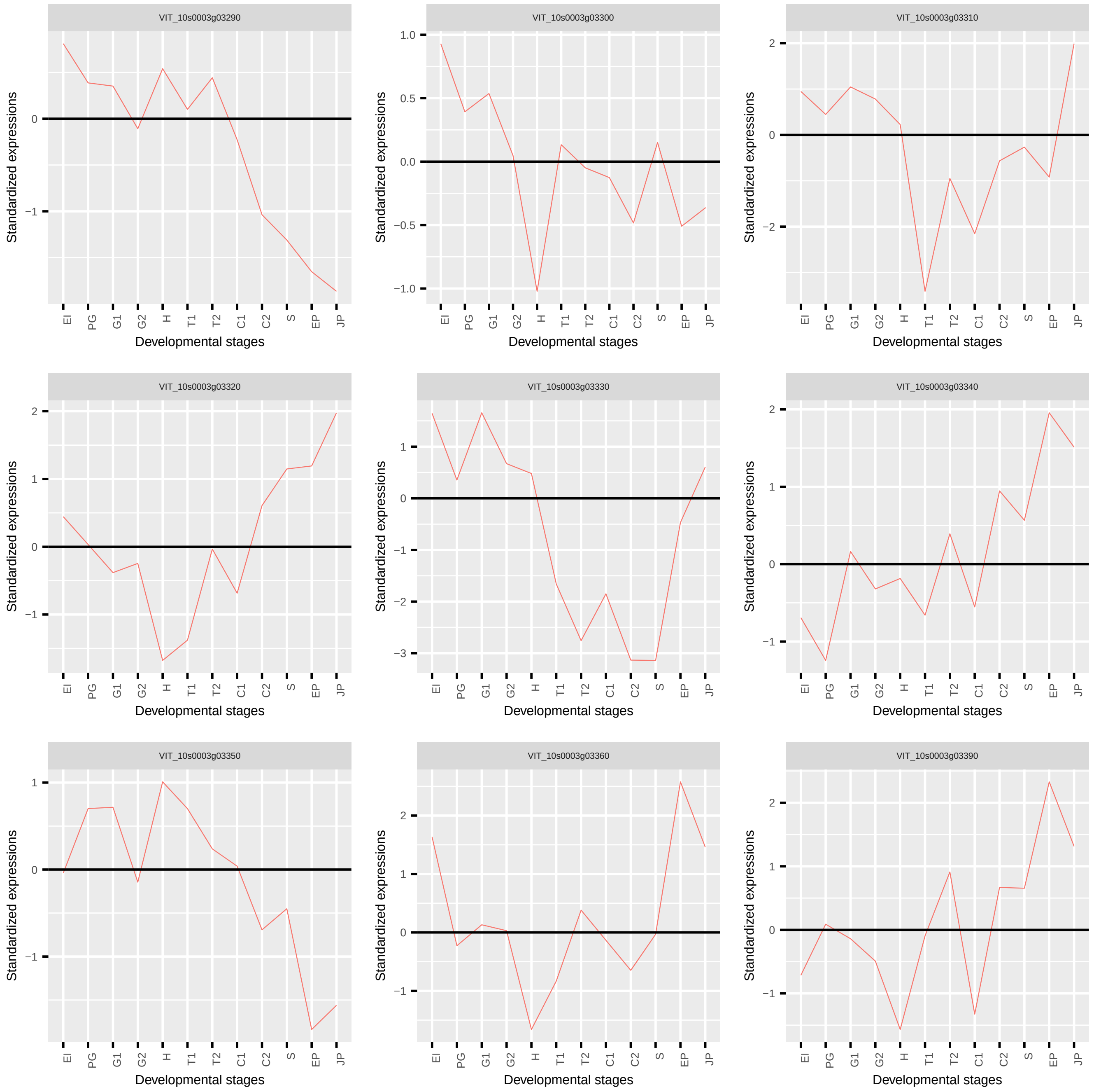
VIT_10s0003g03290
VIT_10s0003g03300
VIT_10s0003g03310
VIT_10s0003g03320
VIT_10s0003g03330
VIT_10s0003g03340
VIT_10s0003g03350
VIT_10s0003g03360
VIT_10s0003g03390
Standardized expressions
Developmental stages
EI PG G1 G2 H T1 T2 C1 C2 S EP JP

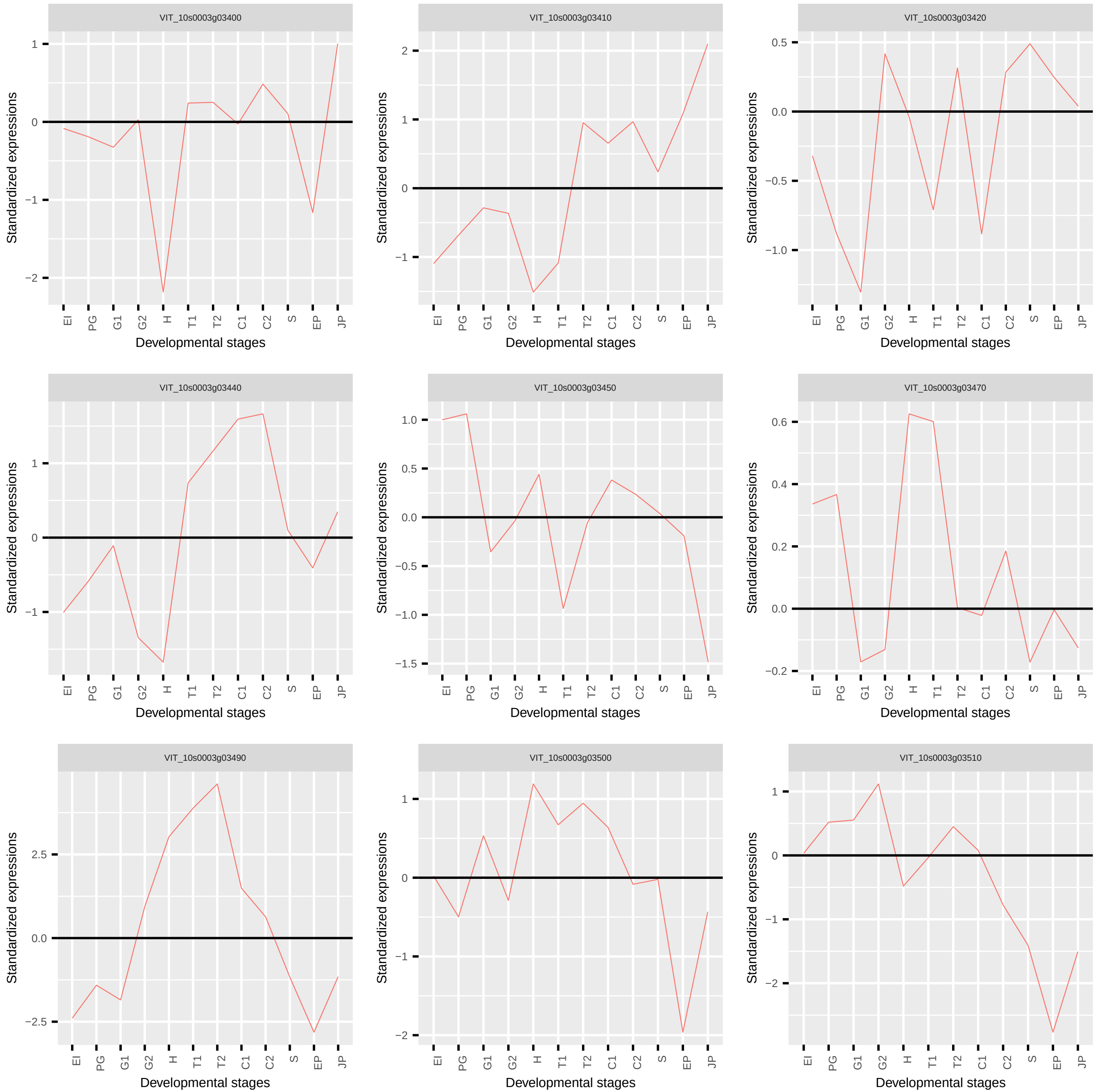
VIT_10s0003g03400
VIT_10s0003g03410
VIT_10s0003g03420
VIT_10s0003g03440
VIT_10s0003g03450
VIT_10s0003g03470
VIT_10s0003g03490
VIT_10s0003g03500
VIT_10s0003g03510
Standardized expressions
Developmental stages
EI
PG
G1
G2
H
T1
T2
C1
C2
S
EP
JP

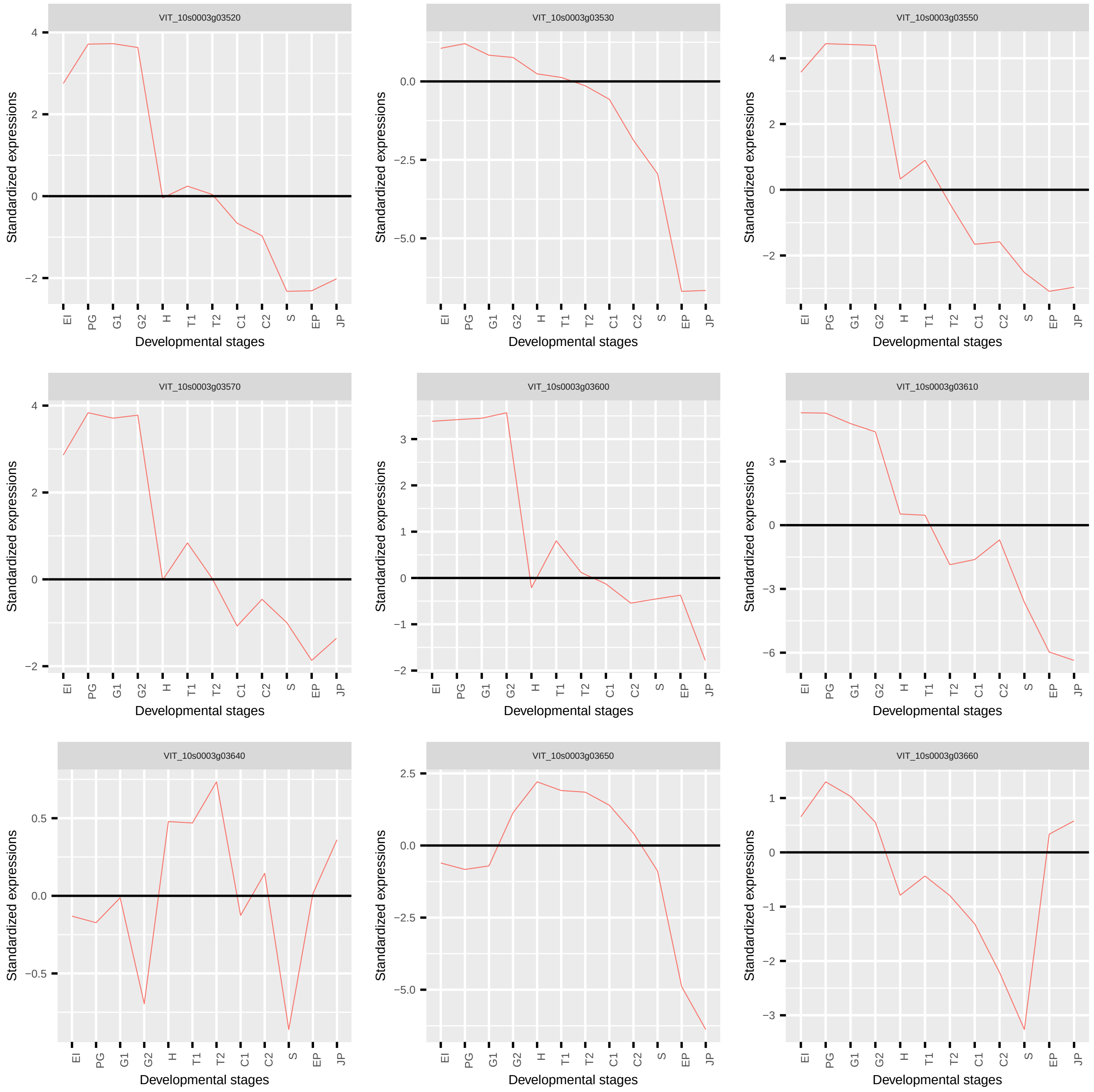
VIT_10s0003g03520
VIT_10s0003g03530
VIT_10s0003g03550
VIT_10s0003g03570
VIT_10s0003g03600
VIT_10s0003g03610
VIT_10s0003g03640
VIT_10s0003g03650
VIT_10s0003g03660
Standardized expressions
Developmental stages
EI PG G1 G2 H T1 T2 C1 C2 S EP JP

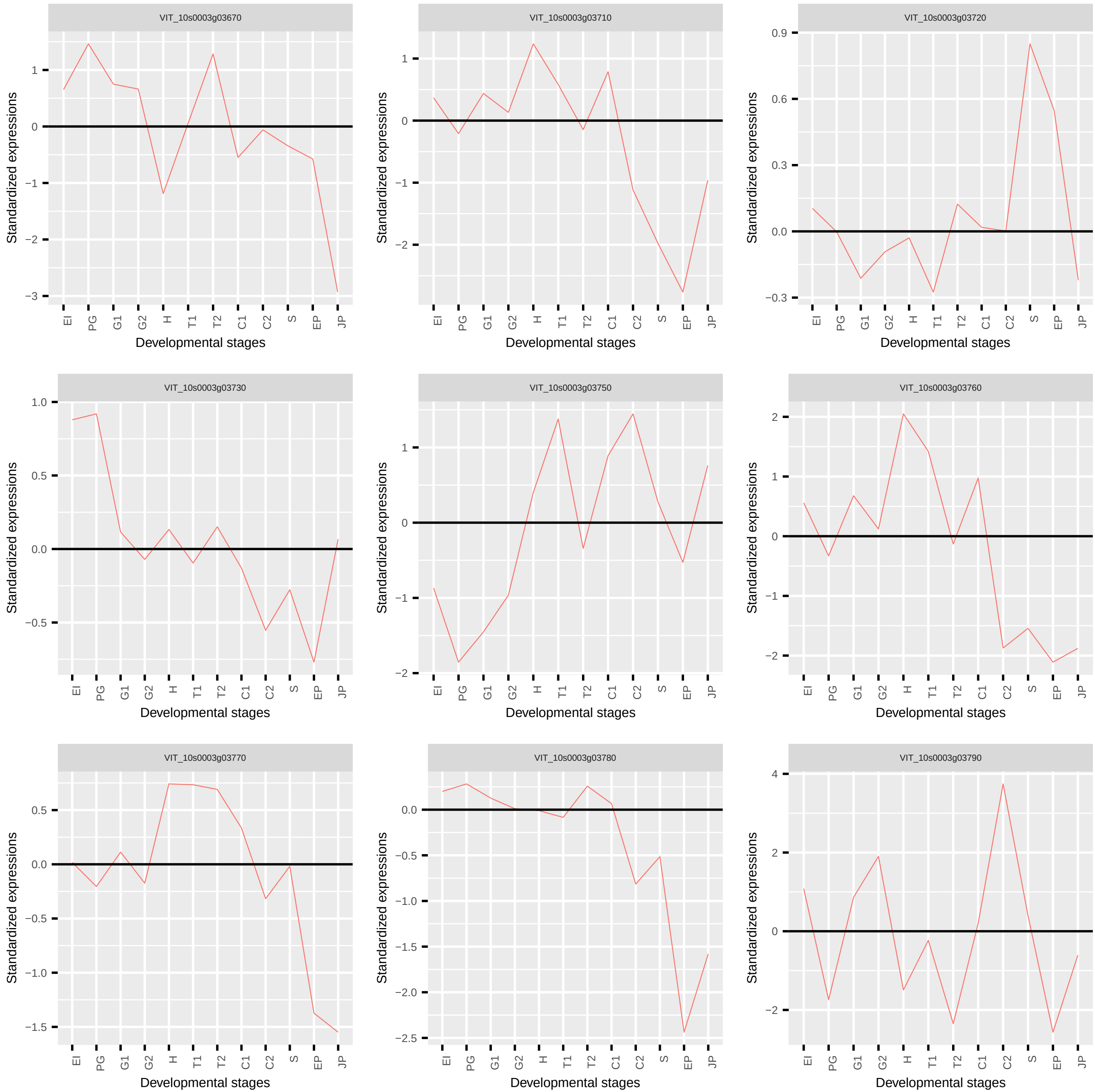
VIT_10s0003g03670
VIT_10s0003g03710
VIT_10s0003g03720
VIT_10s0003g03730
VIT_10s0003g03750
VIT_10s0003g03760
VIT_10s0003g03770
VIT_10s0003g03780
VIT_10s0003g03790
Standardized expressions
Developmental stages
EI PG G1 G2 H T1 T2 C1 C2 S EP JP

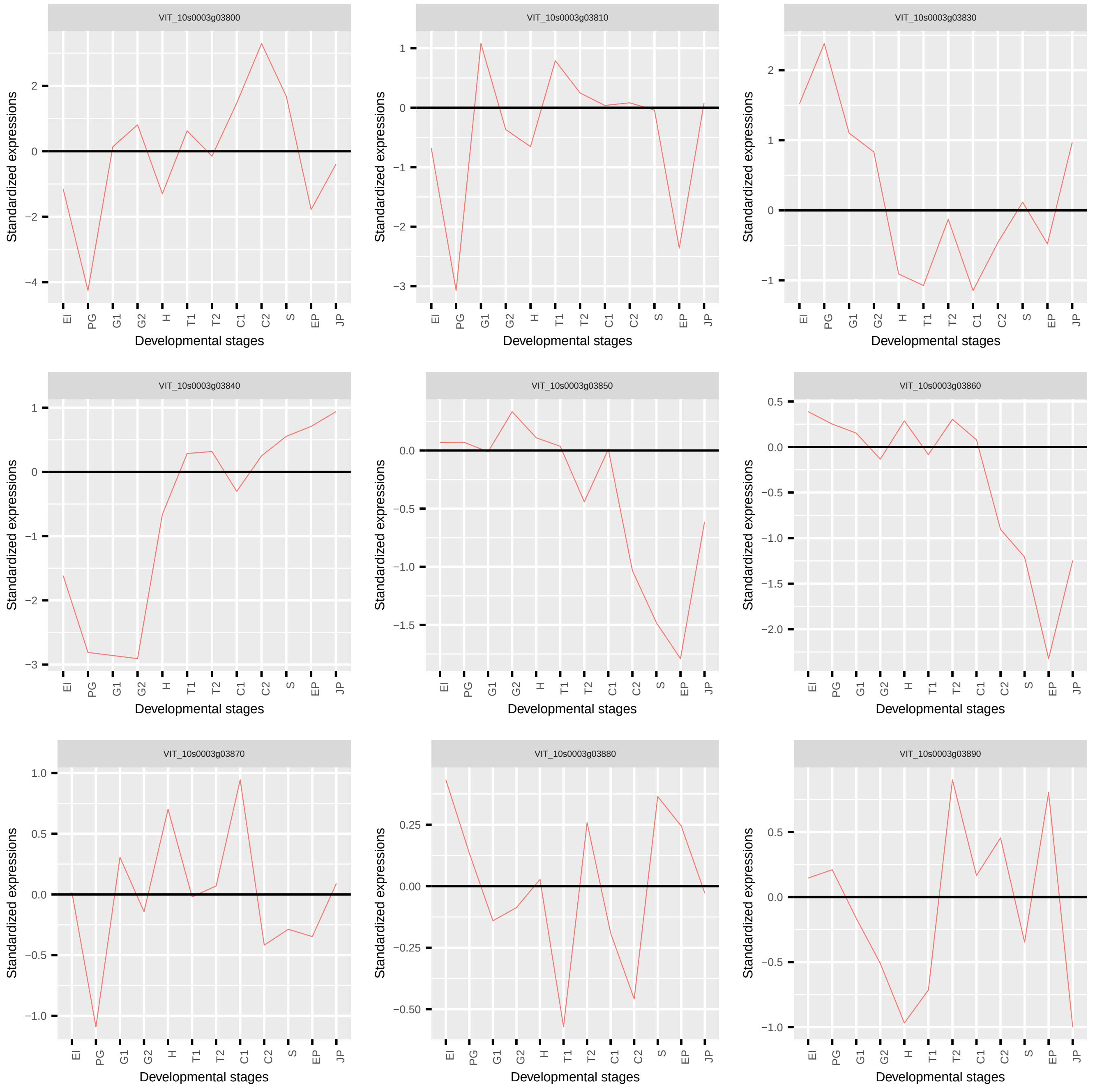
VIT_10s0003g03800
VIT_10s0003g03810
VIT_10s0003g03830
VIT_10s0003g03840
VIT_10s0003g03850
VIT_10s0003g03860
VIT_10s0003g03870
VIT_10s0003g03880
VIT_10s0003g03890
Standardized expressions
Developmental stages
EI PG G1 G2 H T1 T2 C1 C2 S EP JP

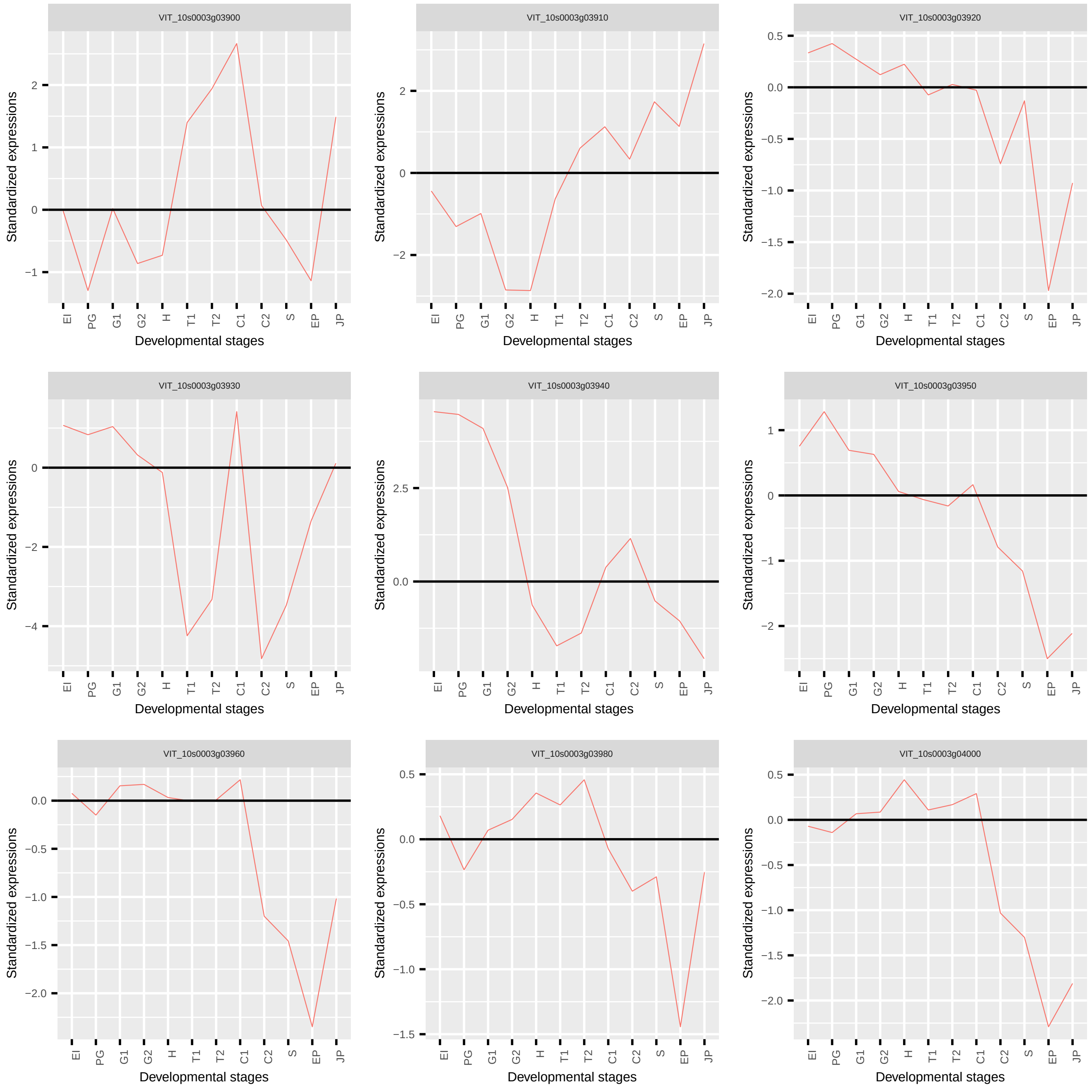
VIT_10s0003g03900
VIT_10s0003g03910
VIT_10s0003g03920
VIT_10s0003g03930
VIT_10s0003g03940
VIT_10s0003g03950
VIT_10s0003g03960
VIT_10s0003g03980
VIT_10s0003g04000
Standardized expressions
Developmental stages
EI
PG
G1
G2
H
T1
T2
C1
C2
S
EP
JP

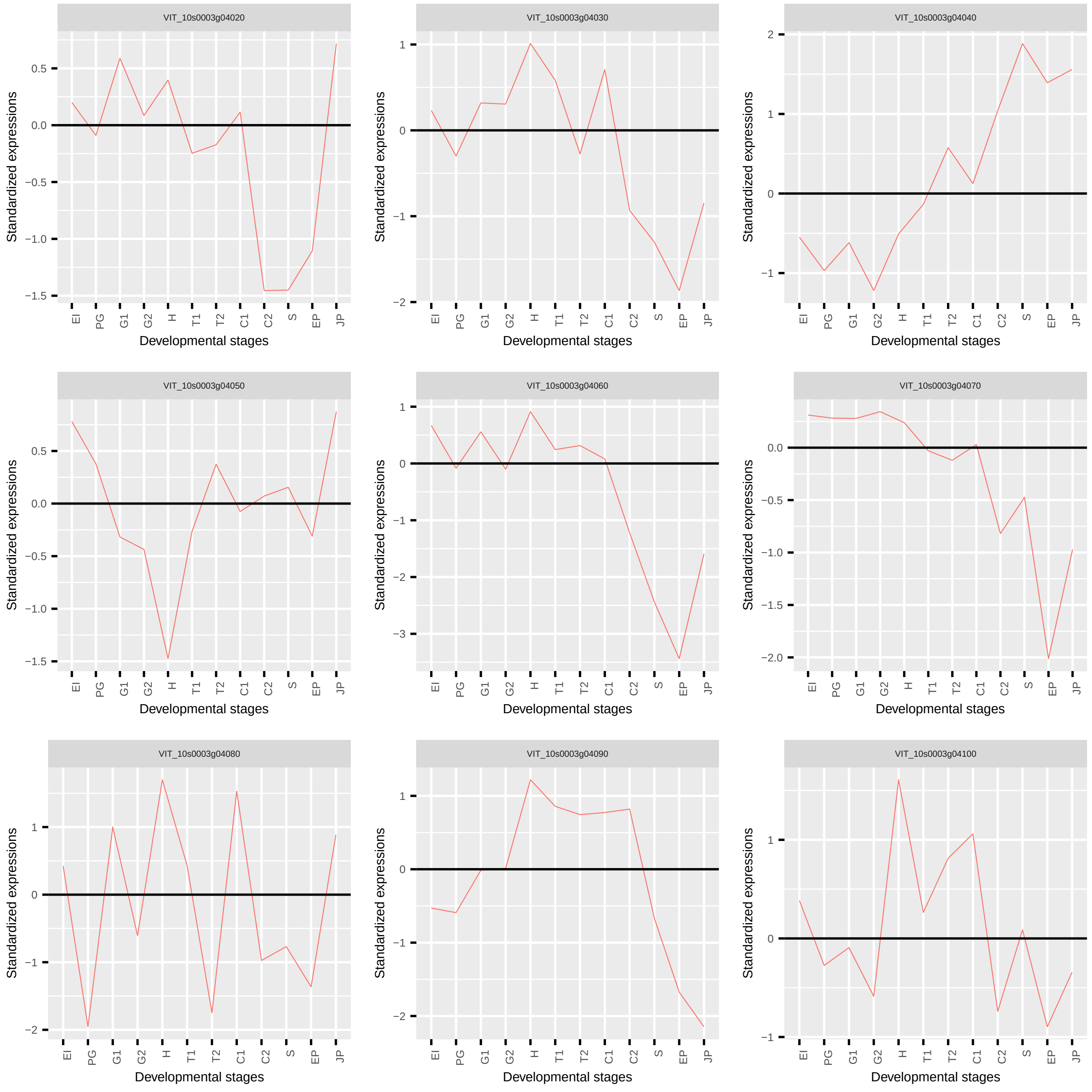
VIT_10s0003g04020
VIT_10s0003g04030
VIT_10s0003g04040
VIT_10s0003g04050
VIT_10s0003g04060
VIT_10s0003g04070
VIT_10s0003g04080
VIT_10s0003g04090
VIT_10s0003g04100
Standardized expressions
Developmental stages
EI PG G1 G2 H T1 T2 C1 C2 S EP JP

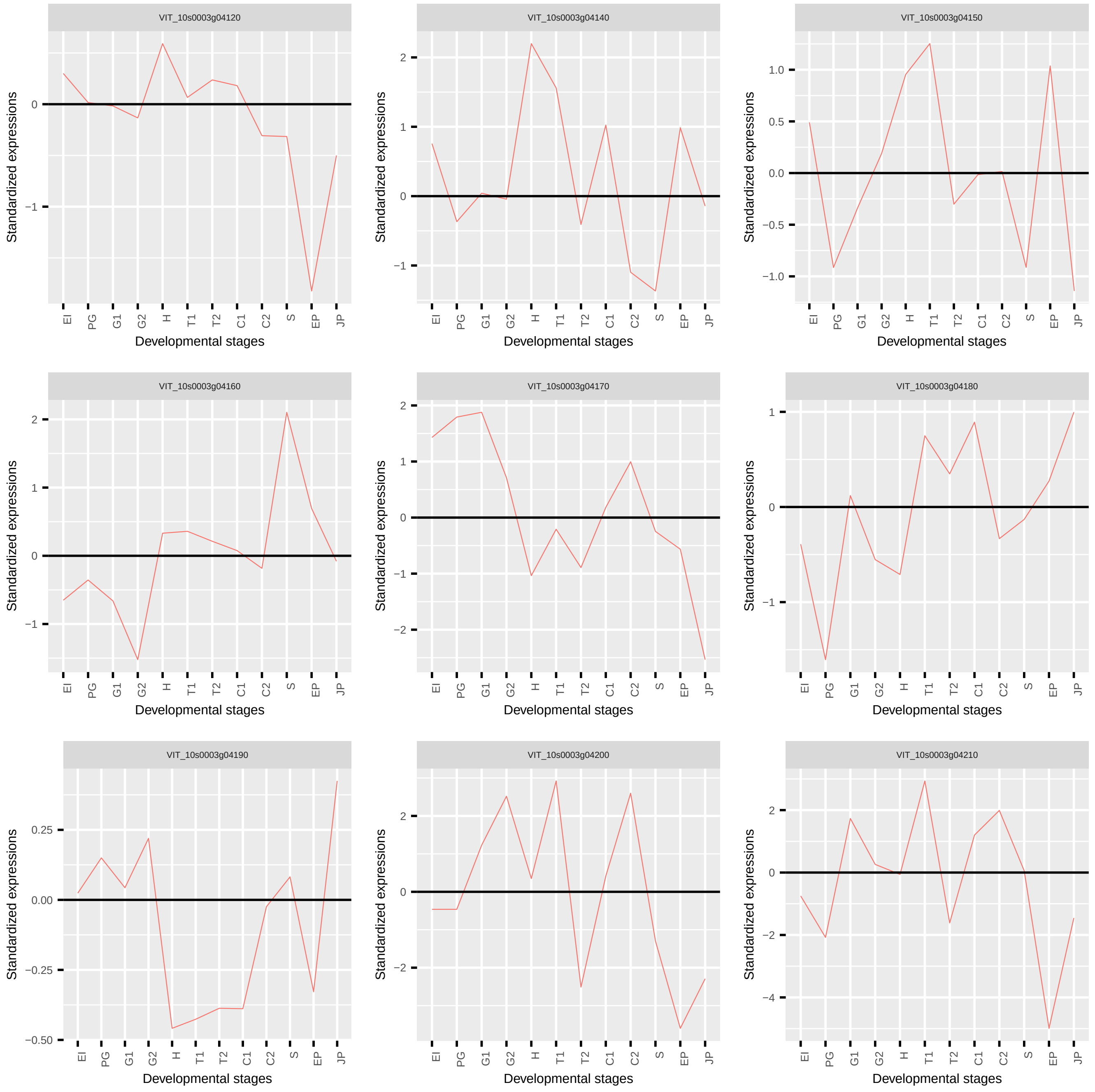
VIT_10s0003g04120
VIT_10s0003g04140
VIT_10s0003g04150
VIT_10s0003g04160
VIT_10s0003g04170
VIT_10s0003g04180
VIT_10s0003g04190
VIT_10s0003g04200
VIT_10s0003g04210
Standardized expressions
Developmental stages
EI
PG
G1
G2
H
T1
T2
C1
C2
S
EP
JP

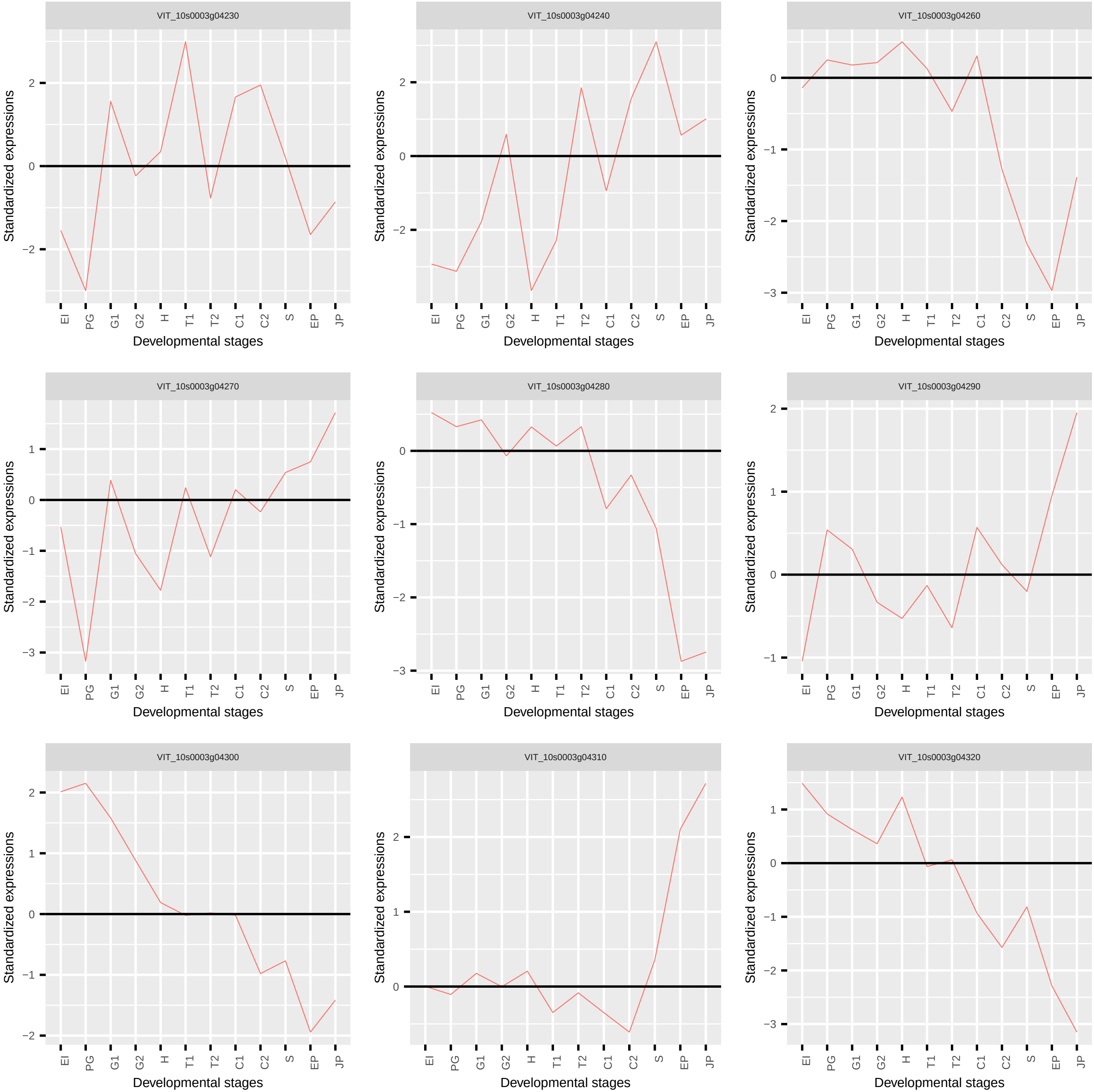
VIT_10s0003g04230
VIT_10s0003g04240
VIT_10s0003g04260
VIT_10s0003g04270
VIT_10s0003g04280
VIT_10s0003g04290
VIT_10s0003g04300
VIT_10s0003g04310
VIT_10s0003g04320
Standardized expressions
Developmental stages
EI PG G1 G2 H T1 T2 C1 C2 S EP JP
2
1
0
−1
−2
−3

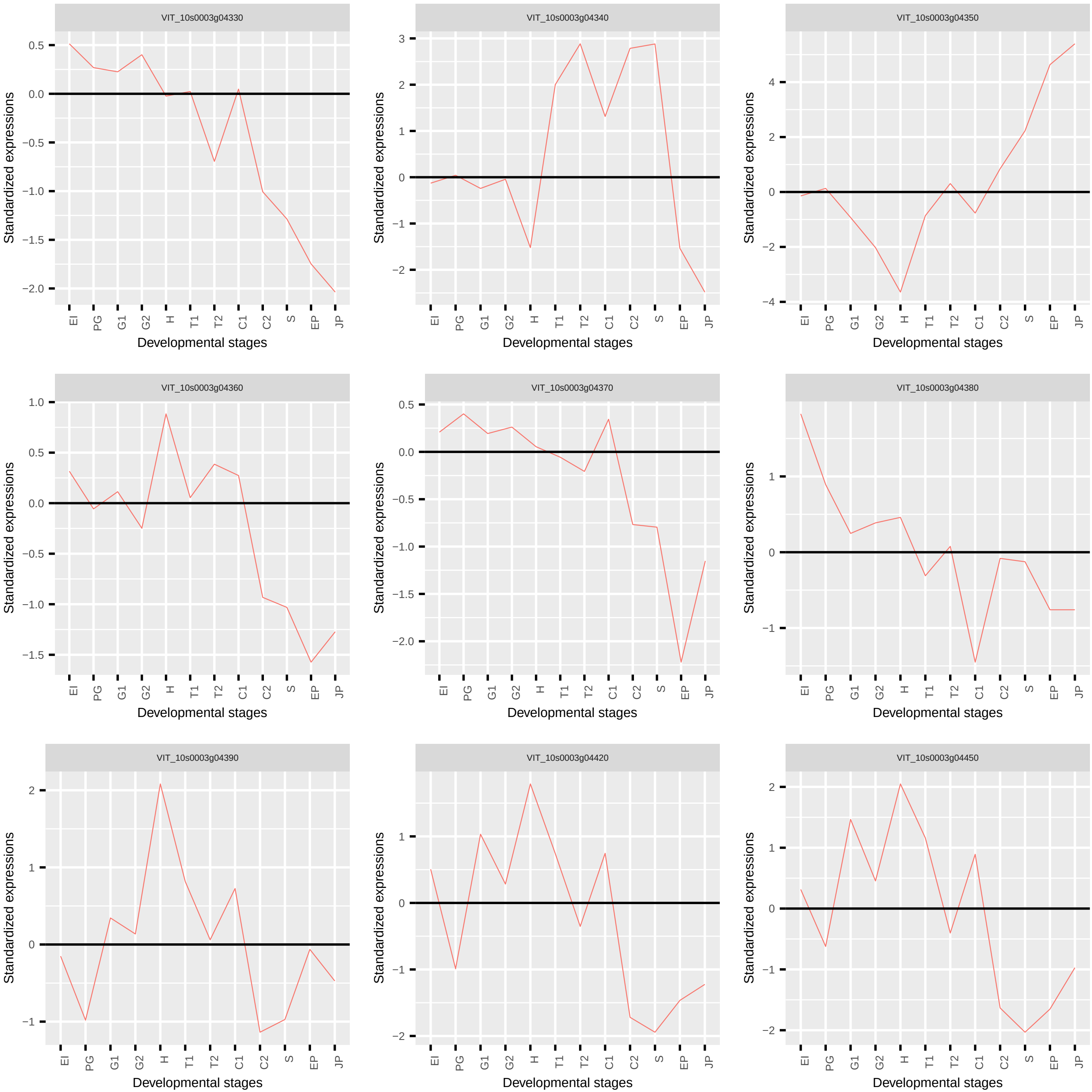
VIT_10s0003g04330
VIT_10s0003g04340
VIT_10s0003g04350
VIT_10s0003g04360
VIT_10s0003g04370
VIT_10s0003g04380
VIT_10s0003g04390
VIT_10s0003g04420
VIT_10s0003g04450
Standardized expressions
Developmental stages
EI
PG
G1
G2
H
T1
T2
C1
C2
S
EP
JP

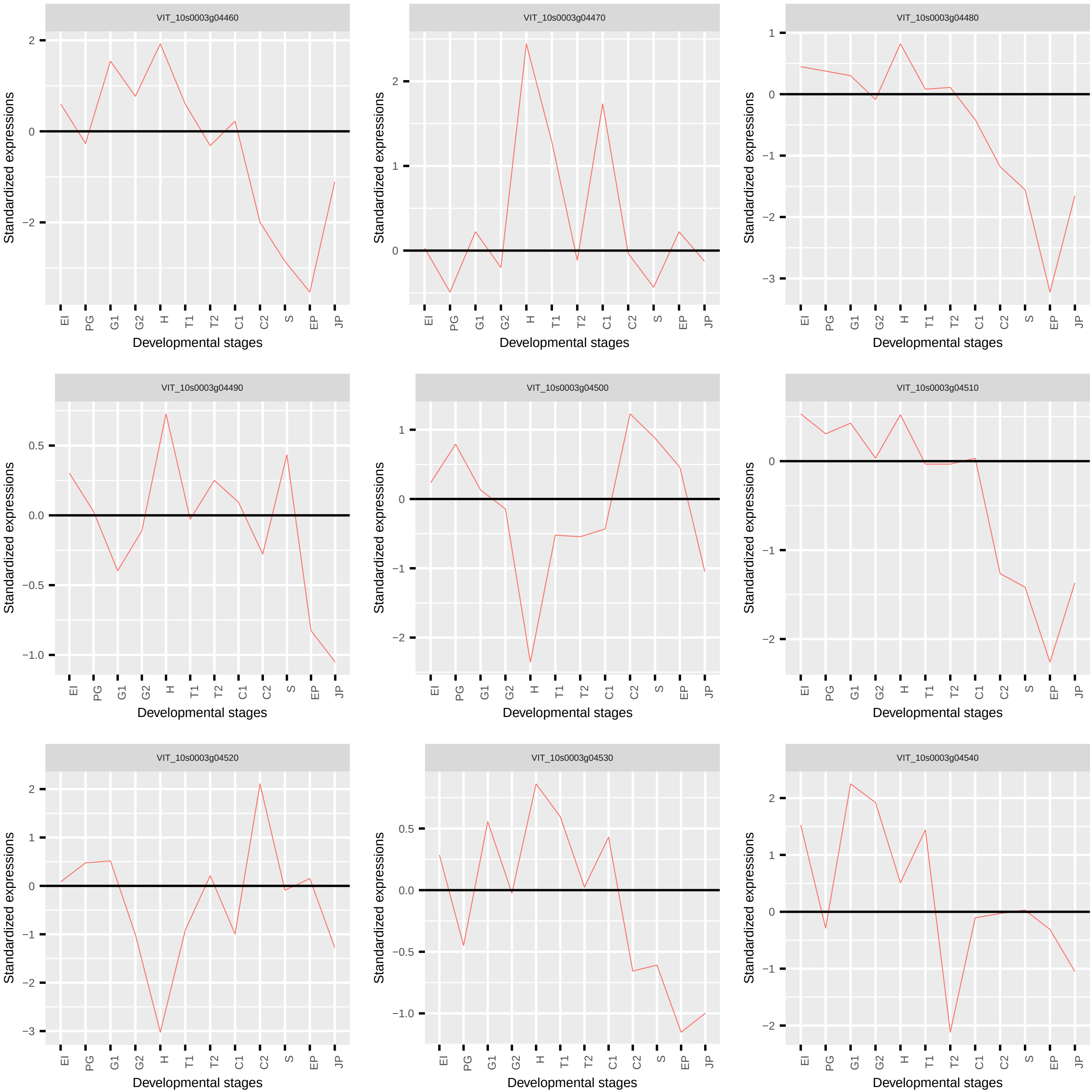
VIT_10s0003g04460
VIT_10s0003g04470
VIT_10s0003g04480
VIT_10s0003g04490
VIT_10s0003g04500
VIT_10s0003g04510
VIT_10s0003g04520
VIT_10s0003g04530
VIT_10s0003g04540
Standardized expressions
Developmental stages
EI PG G1 G2 H T1 T2 C1 C2 S EP JP

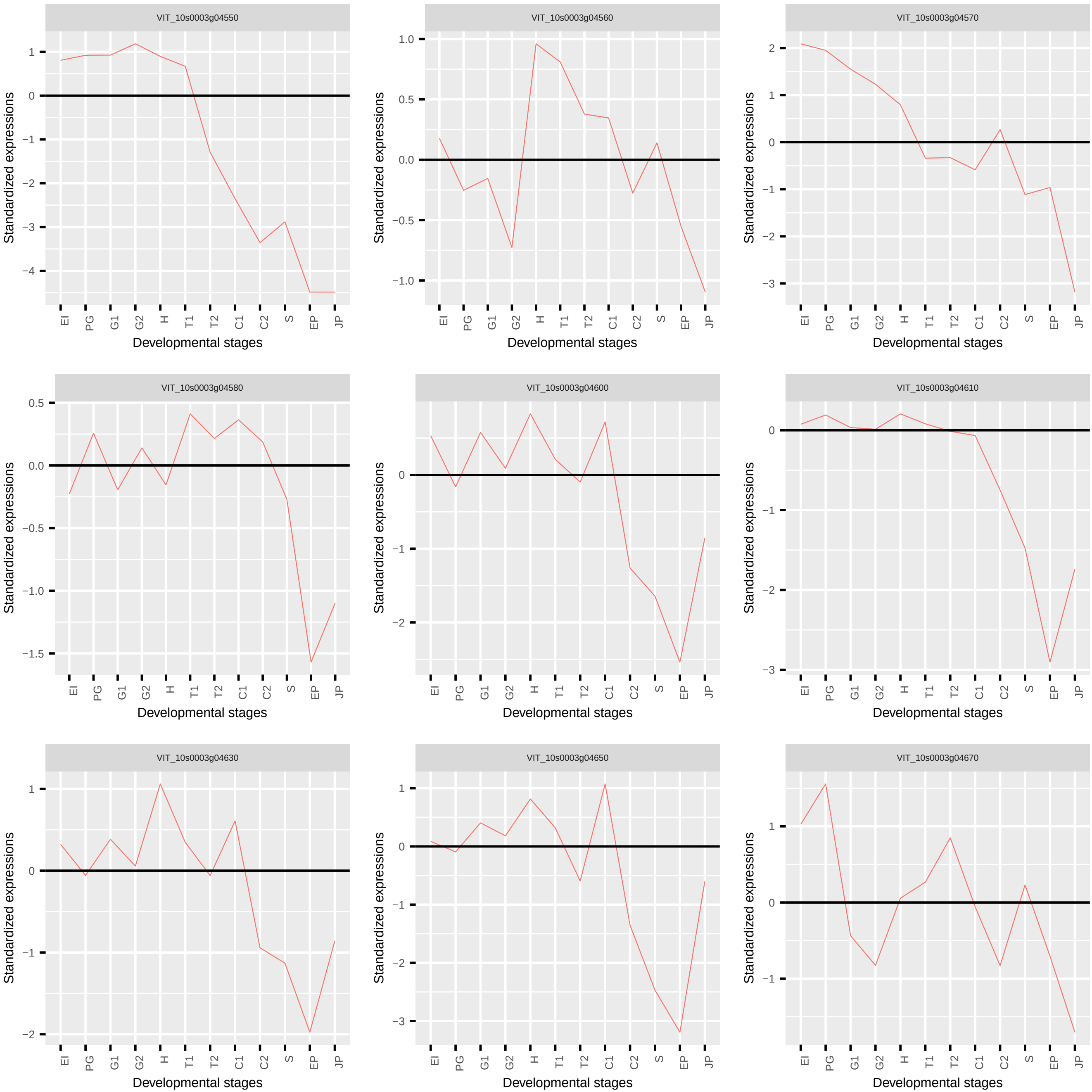
VIT_10s0003g04550
VIT_10s0003g04560
VIT_10s0003g04570
VIT_10s0003g04580
VIT_10s0003g04600
VIT_10s0003g04610
VIT_10s0003g04630
VIT_10s0003g04650
VIT_10s0003g04670
Standardized expressions
Developmental stages
EI PG G1 G2 H T1 T2 C1 C2 S EP JP

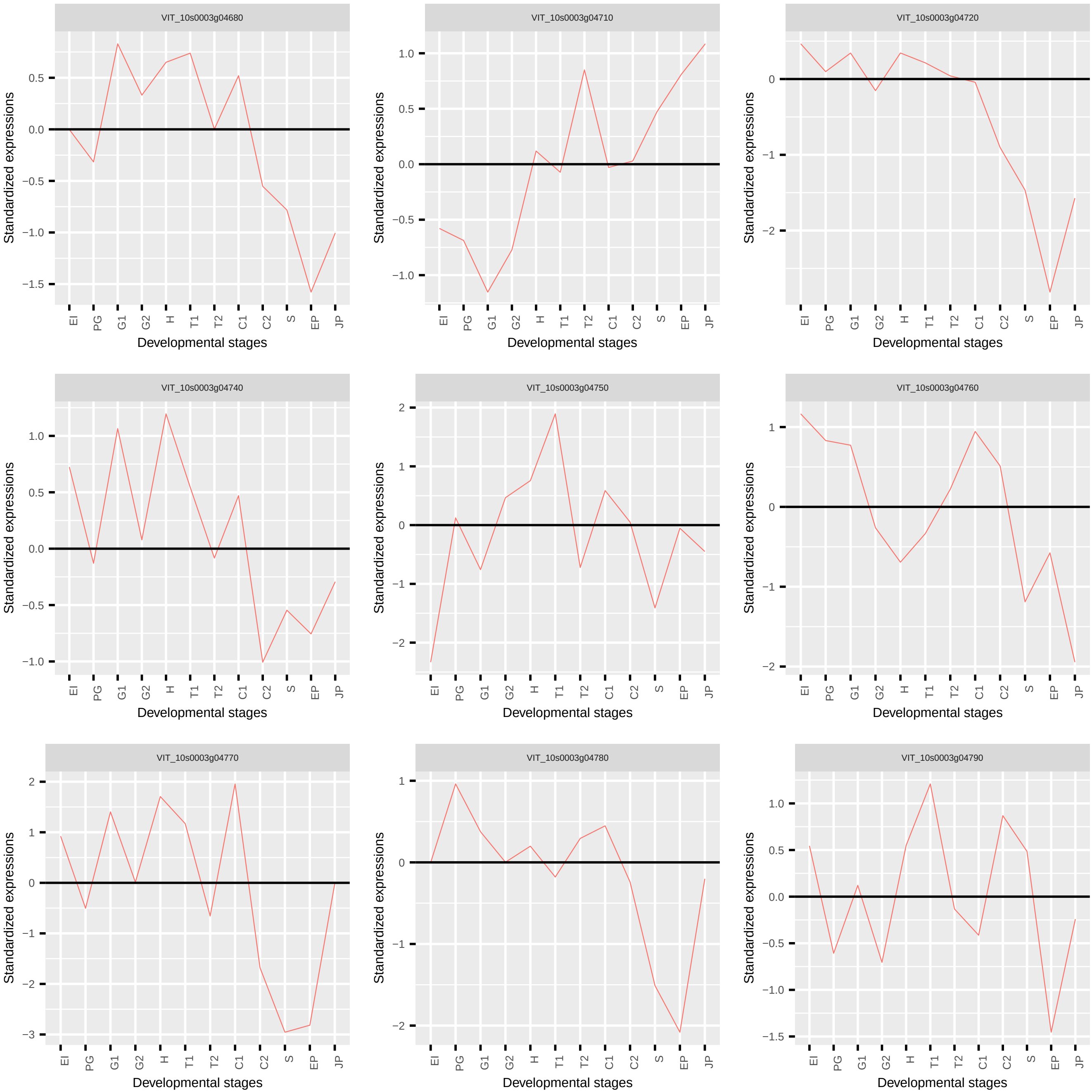

VIT_10s0003g04680
VIT_10s0003g04710
VIT_10s0003g04720
VIT_10s0003g04740
VIT_10s0003g04750
VIT_10s0003g04760
VIT_10s0003g04770
VIT_10s0003g04780
VIT_10s0003g04790
Standardized expressions
Developmental stages
EI PG G1 G2 H T1 T2 C1 C2 S EP JP

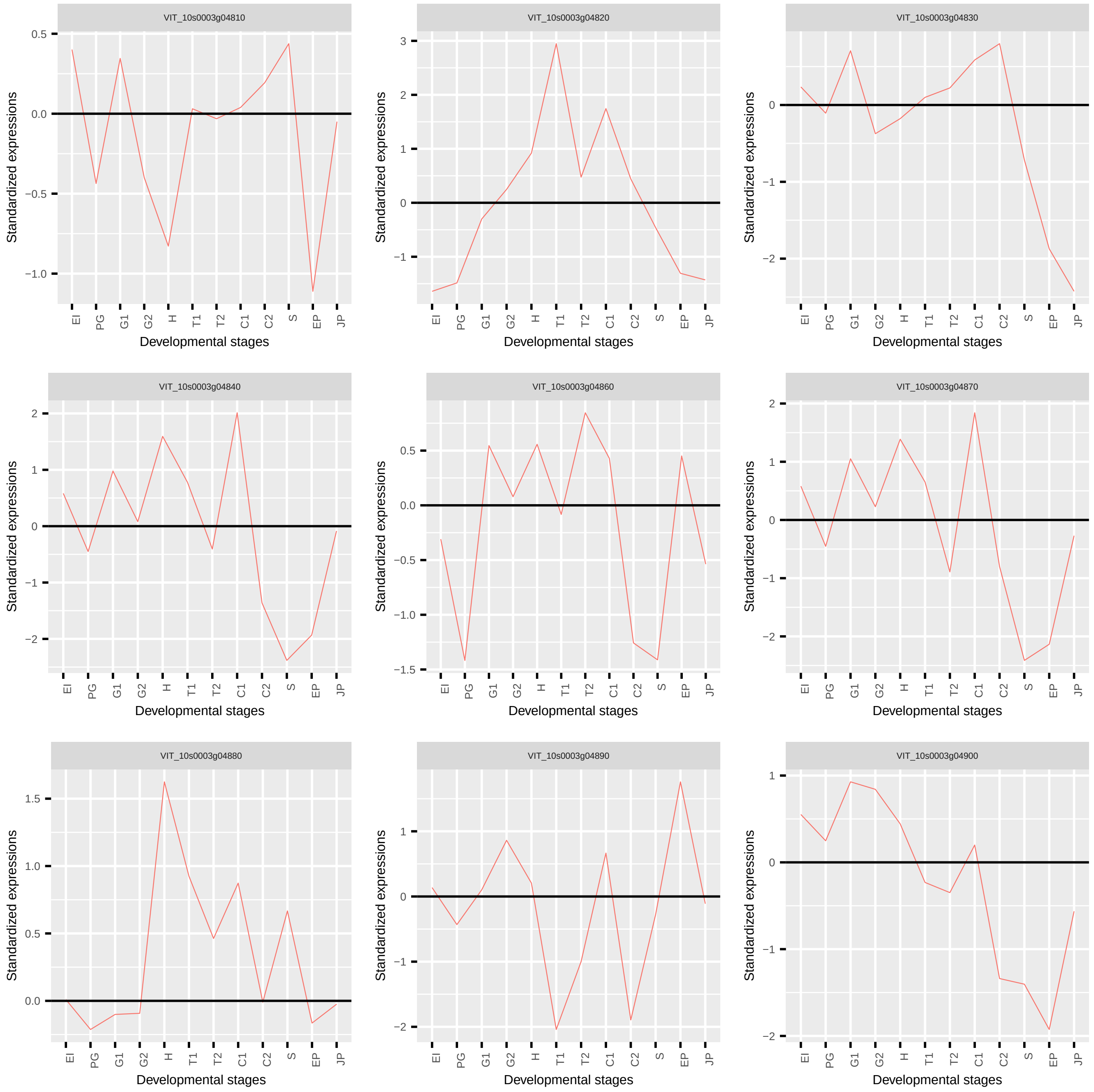
VIT_10s0003g04810
0.5
0.0
−0.5
−1.0
Standardized expressions
EI PG G1 G2 H T1 T2 C1 C2 S EP JP
Developmental stages
VIT_10s0003g04820
3
2
1
0
−1
Standardized expressions
EI PG G1 G2 H T1 T2 C1 C2 S EP JP
Developmental stages
VIT_10s0003g04830
0
−1
−2
Standardized expressions
EI PG G1 G2 H T1 T2 C1 C2 S EP JP
Developmental stages
VIT_10s0003g04840
2
1
0
−1
−2
Standardized expressions
EI PG G1 G2 H T1 T2 C1 C2 S EP JP
Developmental stages
VIT_10s0003g04860
0.5
0.0
−0.5
−1.0
−1.5
Standardized expressions
EI PG G1 G2 H T1 T2 C1 C2 S EP JP
Developmental stages
VIT_10s0003g04870
2
1
0
−1
−2
Standardized expressions
EI PG G1 G2 H T1 T2 C1 C2 S EP JP
Developmental stages
VIT_10s0003g04880
1.5
1.0
0.5
0.0
Standardized expressions
EI PG G1 G2 H T1 T2 C1 C2 S EP JP
Developmental stages
VIT_10s0003g04890
1
0
−1
−2
Standardized expressions
EI PG G1 G2 H T1 T2 C1 C2 S EP JP
Developmental stages
VIT_10s0003g04900
1
0
−1
−2
Standardized expressions
EI PG G1 G2 H T1 T2 C1 C2 S EP JP
Developmental stages

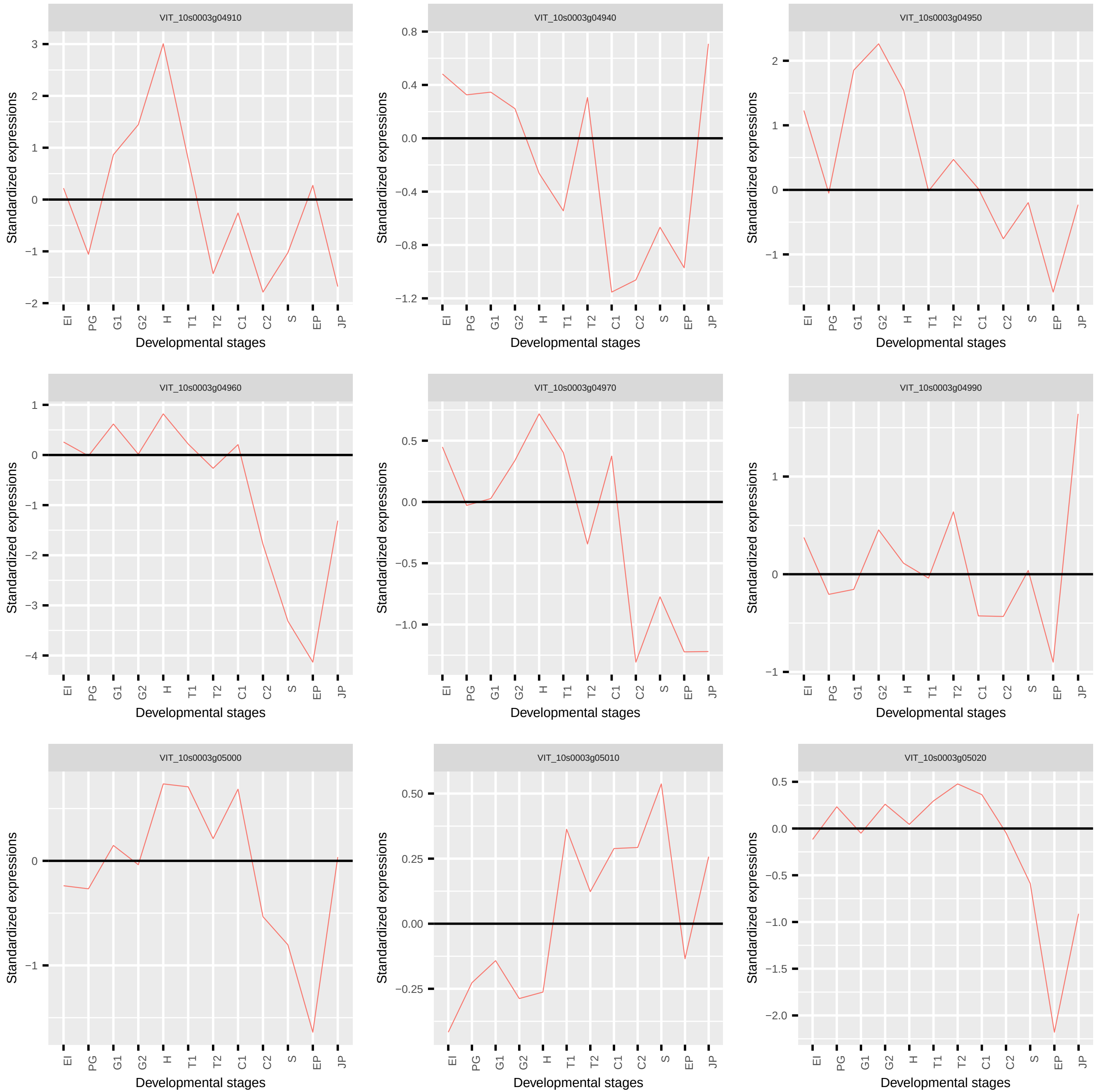
VIT_10s0003g04910
VIT_10s0003g04940
VIT_10s0003g04950
VIT_10s0003g04960
VIT_10s0003g04970
VIT_10s0003g04990
VIT_10s0003g05000
VIT_10s0003g05010
VIT_10s0003g05020
Standardized expressions
Developmental stages
EI PG G1 G2 H T1 T2 C1 C2 S EP JP

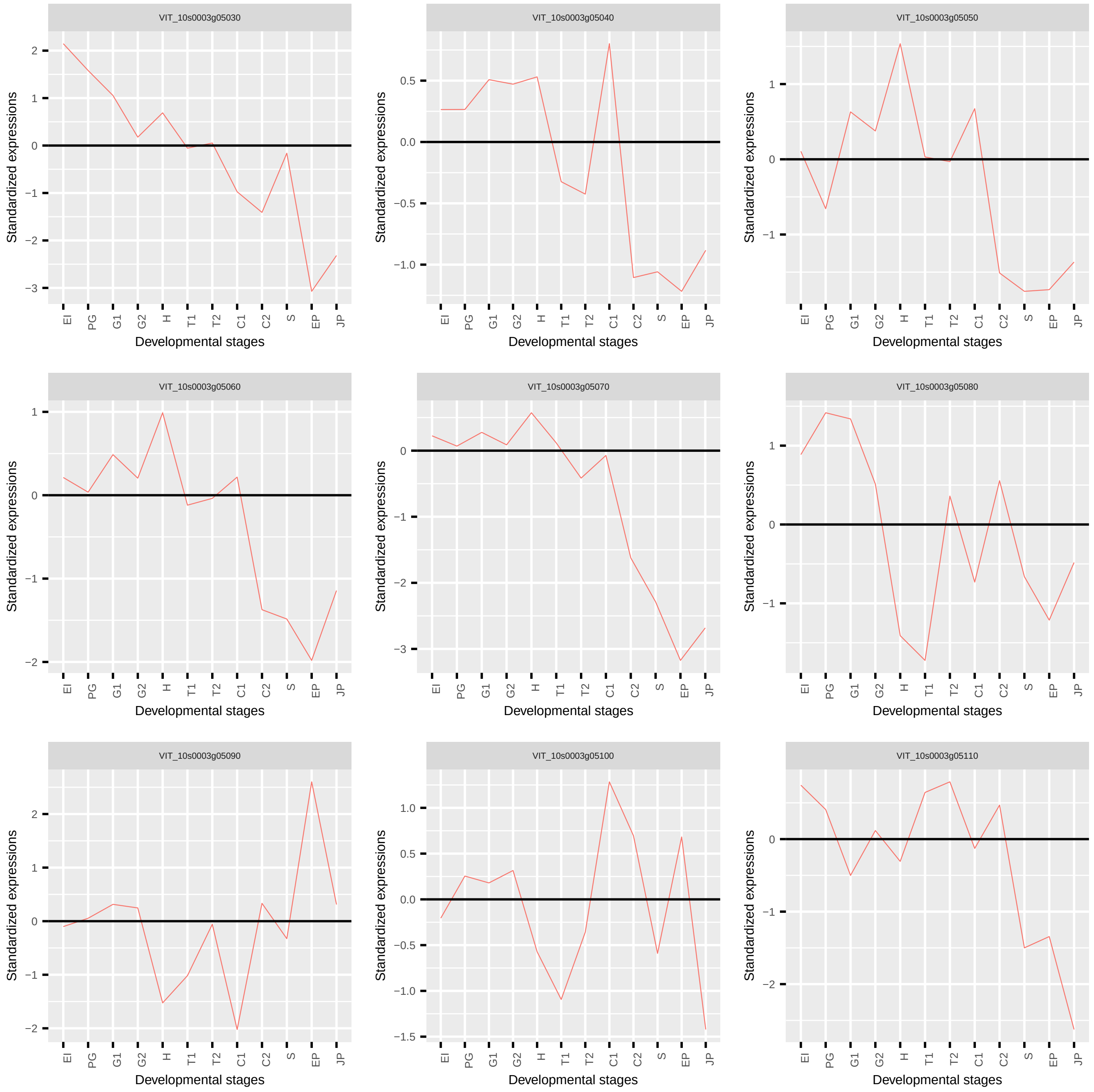
VIT_10s0003g05030
VIT_10s0003g05040
VIT_10s0003g05050
VIT_10s0003g05060
VIT_10s0003g05070
VIT_10s0003g05080
VIT_10s0003g05090
VIT_10s0003g05100
VIT_10s0003g05110
Standardized expressions
Developmental stages
EI PG G1 G2 H T1 T2 C1 C2 S EP JP

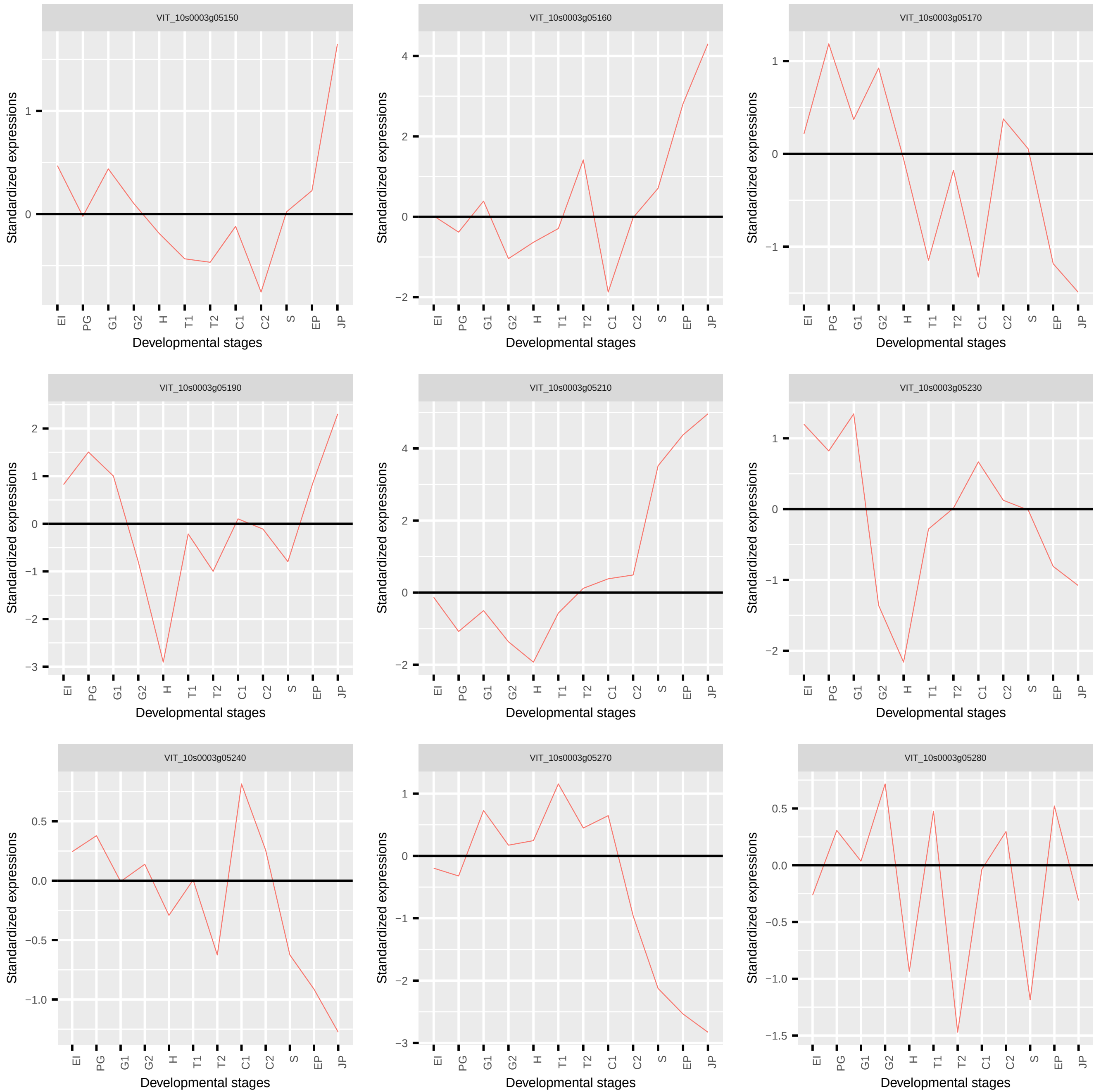

VIT_10s0003g05150
VIT_10s0003g05160
VIT_10s0003g05170
VIT_10s0003g05190
VIT_10s0003g05210
VIT_10s0003g05230
VIT_10s0003g05240
VIT_10s0003g05270
VIT_10s0003g05280
Standardized expressions
Developmental stages
EI PG G1 G2 H T1 T2 C1 C2 S EP JP

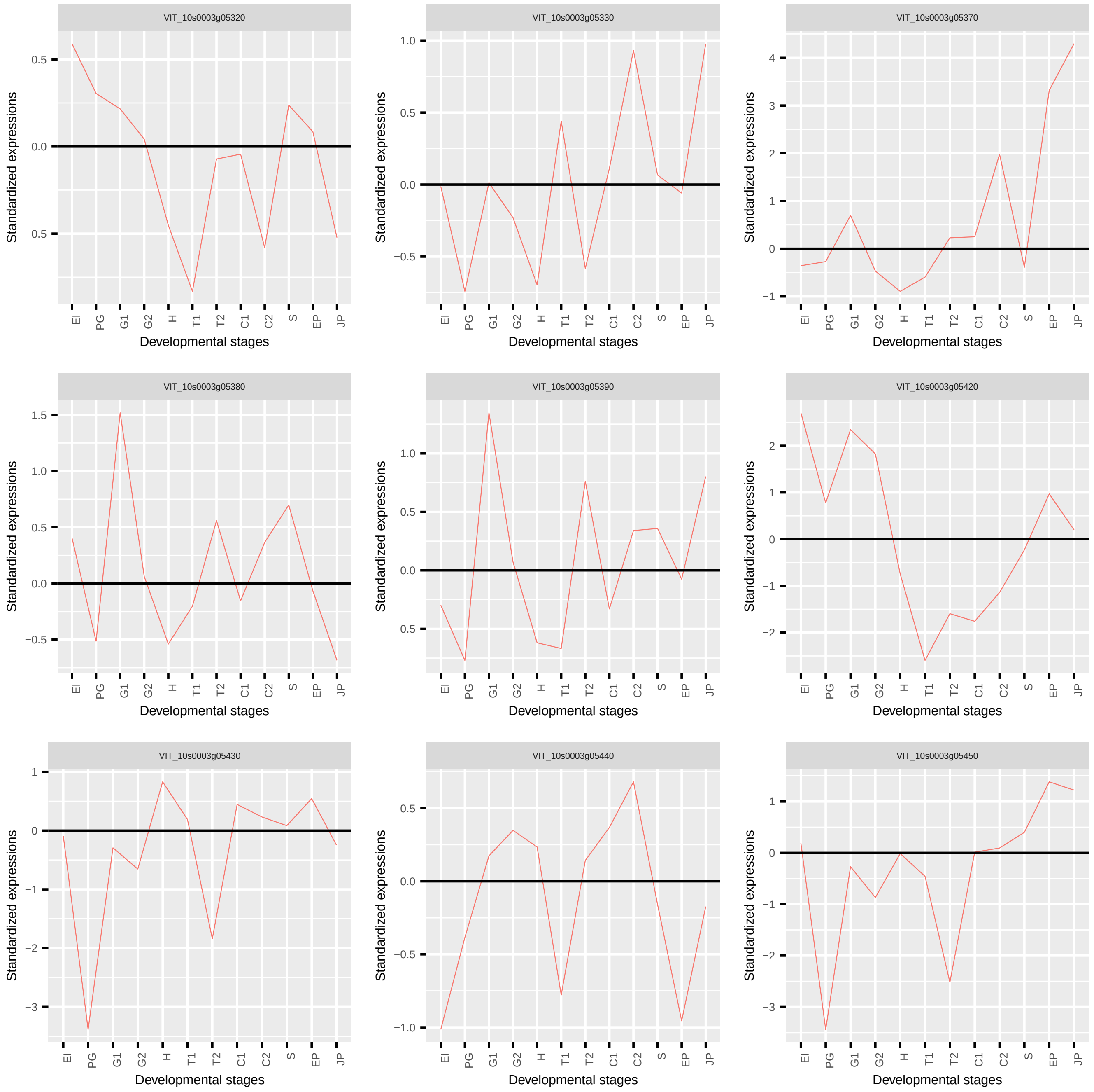
VIT_10s0003g05320
VIT_10s0003g05330
VIT_10s0003g05370
VIT_10s0003g05380
VIT_10s0003g05390
VIT_10s0003g05420
VIT_10s0003g05430
VIT_10s0003g05440
VIT_10s0003g05450
Standardized expressions
Developmental stages
EI PG G1 G2 H T1 T2 C1 C2 S EP JP

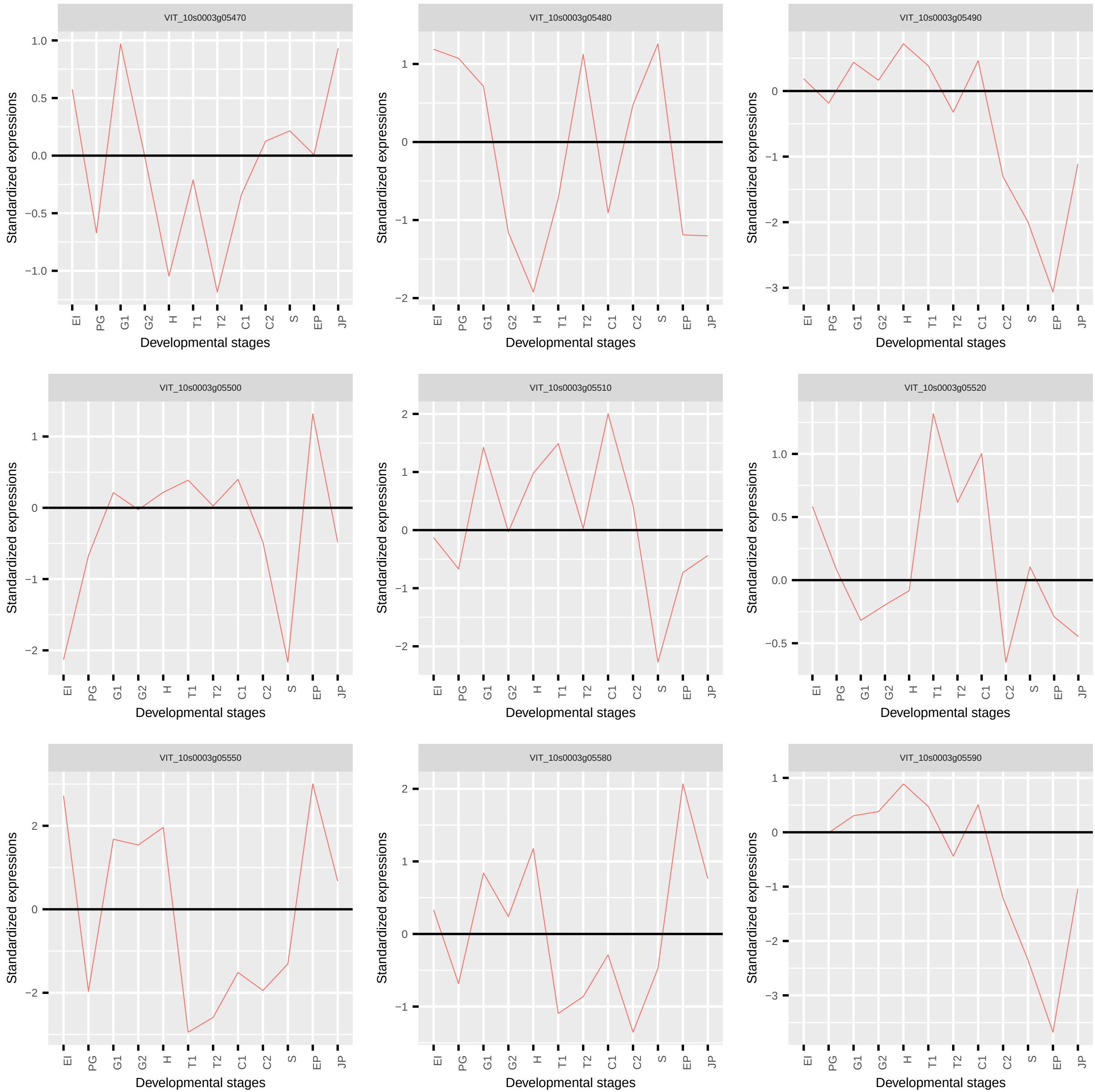
VIT_10s0003g05470
1.0
0.5
0.0
−0.5
−1.0
VIT_10s0003g05480
1
0
−1
−2
VIT_10s0003g05490
0
−1
−2
−3
VIT_10s0003g05500
1
0
−1
−2
VIT_10s0003g05510
2
1
0
−1
−2
VIT_10s0003g05520
1.0
0.5
0.0
−0.5
VIT_10s0003g05550
2
0
−2
VIT_10s0003g05580
2
1
0
−1
VIT_10s0003g05590
1
0
−1
−2
−3
Standardized expressions
EI
PG
G1
G2
H
T1
T2
C1
C2
S
EP
JP
Developmental stages

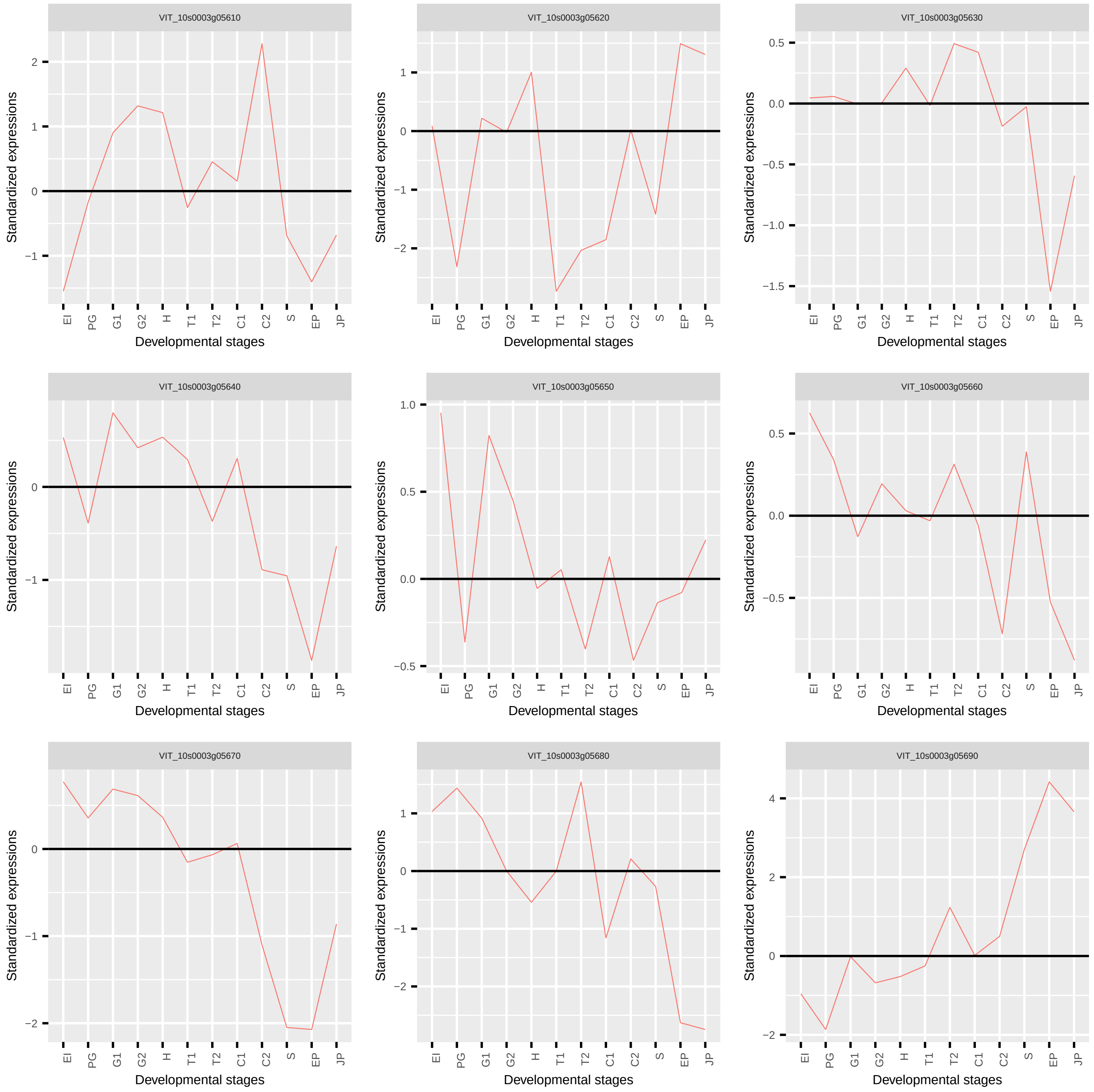

VIT_10s0003g05610
VIT_10s0003g05620
VIT_10s0003g05630
VIT_10s0003g05640
VIT_10s0003g05650
VIT_10s0003g05660
VIT_10s0003g05670
VIT_10s0003g05680
VIT_10s0003g05690
Standardized expressions
Developmental stages
EI PG G1 G2 H T1 T2 C1 C2 S EP JP

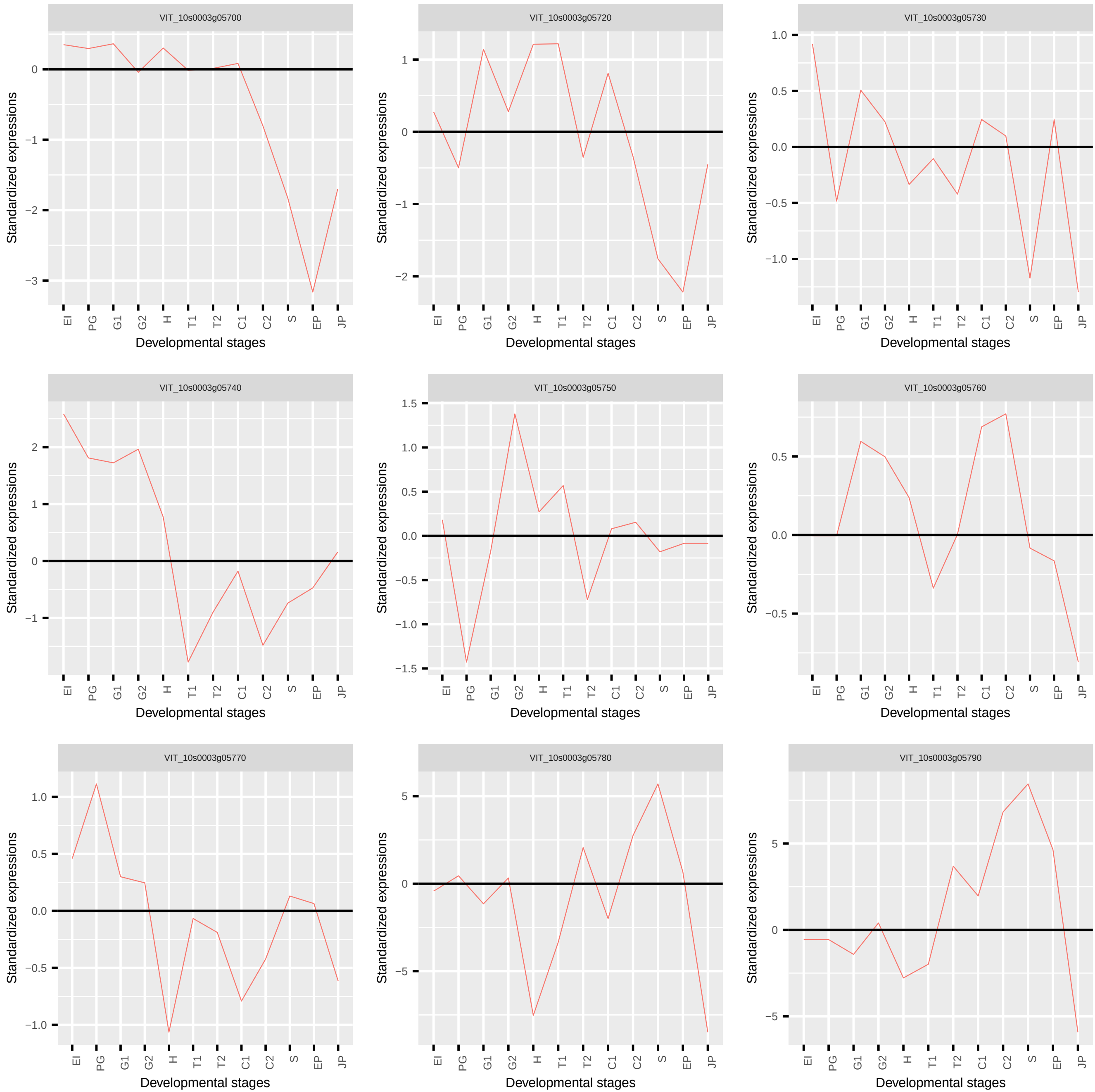
VIT_10s0003g05700
VIT_10s0003g05720
VIT_10s0003g05730
VIT_10s0003g05740
VIT_10s0003g05750
VIT_10s0003g05760
VIT_10s0003g05770
VIT_10s0003g05780
VIT_10s0003g05790
Standardized expressions
Developmental stages
EI PG G1 G2 H T1 T2 C1 C2 S EP JP

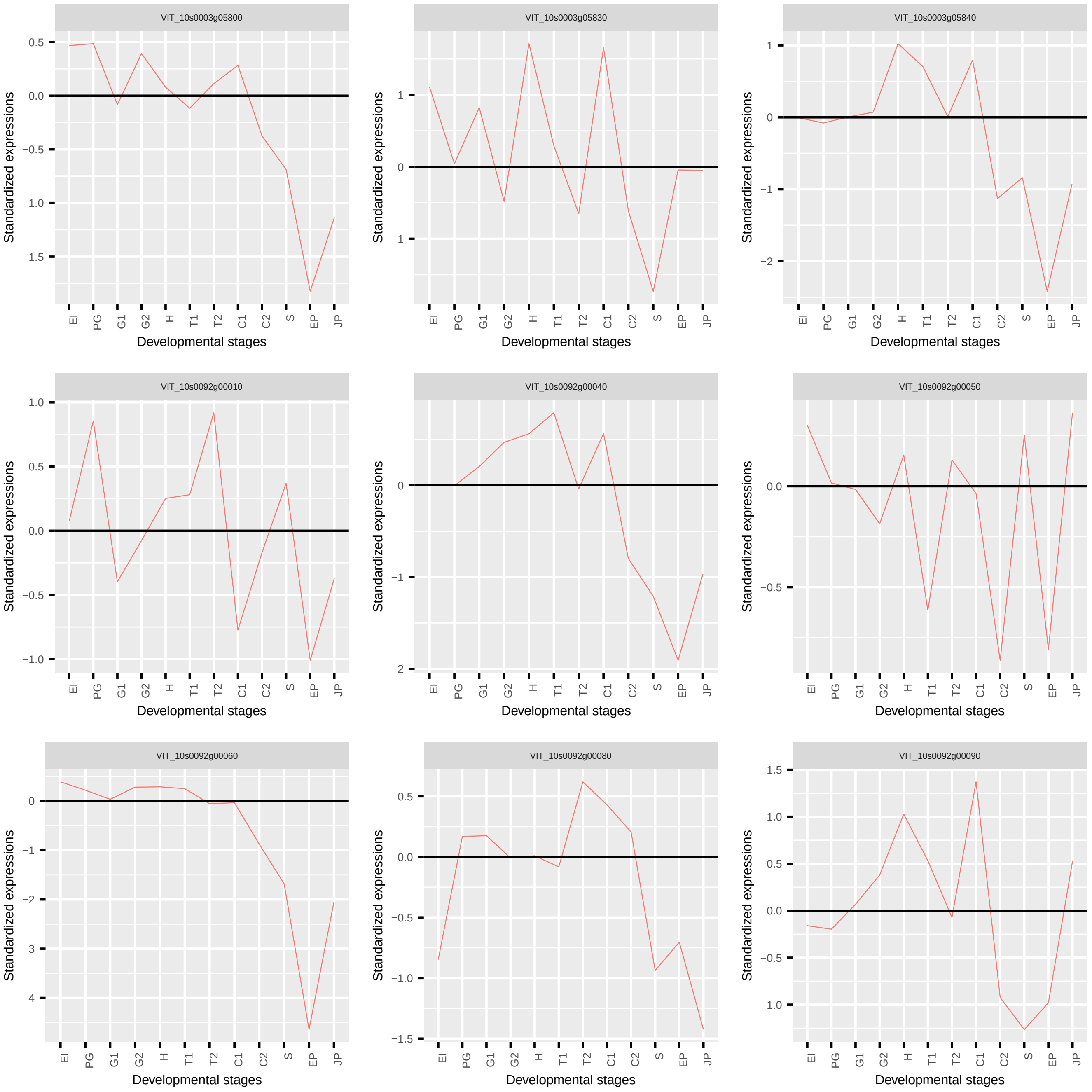
VIT_10s0003g05800
VIT_10s0003g05830
VIT_10s0003g05840
VIT_10s0092g00010
VIT_10s0092g00040
VIT_10s0092g00050
VIT_10s0092g00060
VIT_10s0092g00080
VIT_10s0092g00090
Standardized expressions
Developmental stages
EI PG G1 G2 H T1 T2 C1 C2 S EP JP
0.5 0.0 −0.5 −1.0 −1.5
1 0 −1 −2
1.0 0.5 0.0 −0.5 −1.0
0 −1 −2 −3 −4
1.5 1.0 0.5 0.0 −0.5 −1.0 −1.5

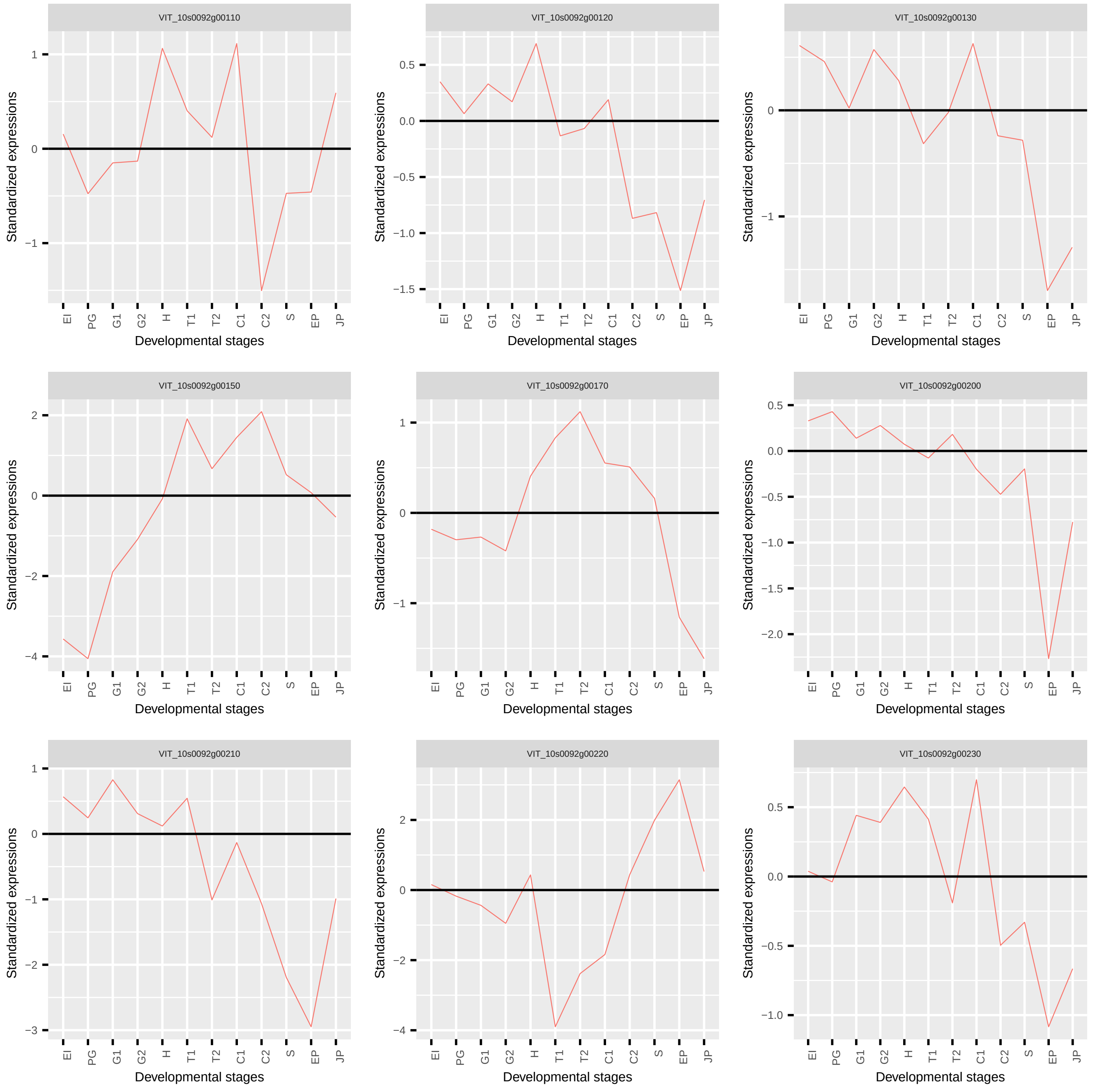
VIT_10s0092g00110
VIT_10s0092g00120
VIT_10s0092g00130
VIT_10s0092g00150
VIT_10s0092g00170
VIT_10s0092g00200
VIT_10s0092g00210
VIT_10s0092g00220
VIT_10s0092g00230
Standardized expressions
Developmental stages
EI PG G1 G2 H T1 T2 C1 C2 S EP JP

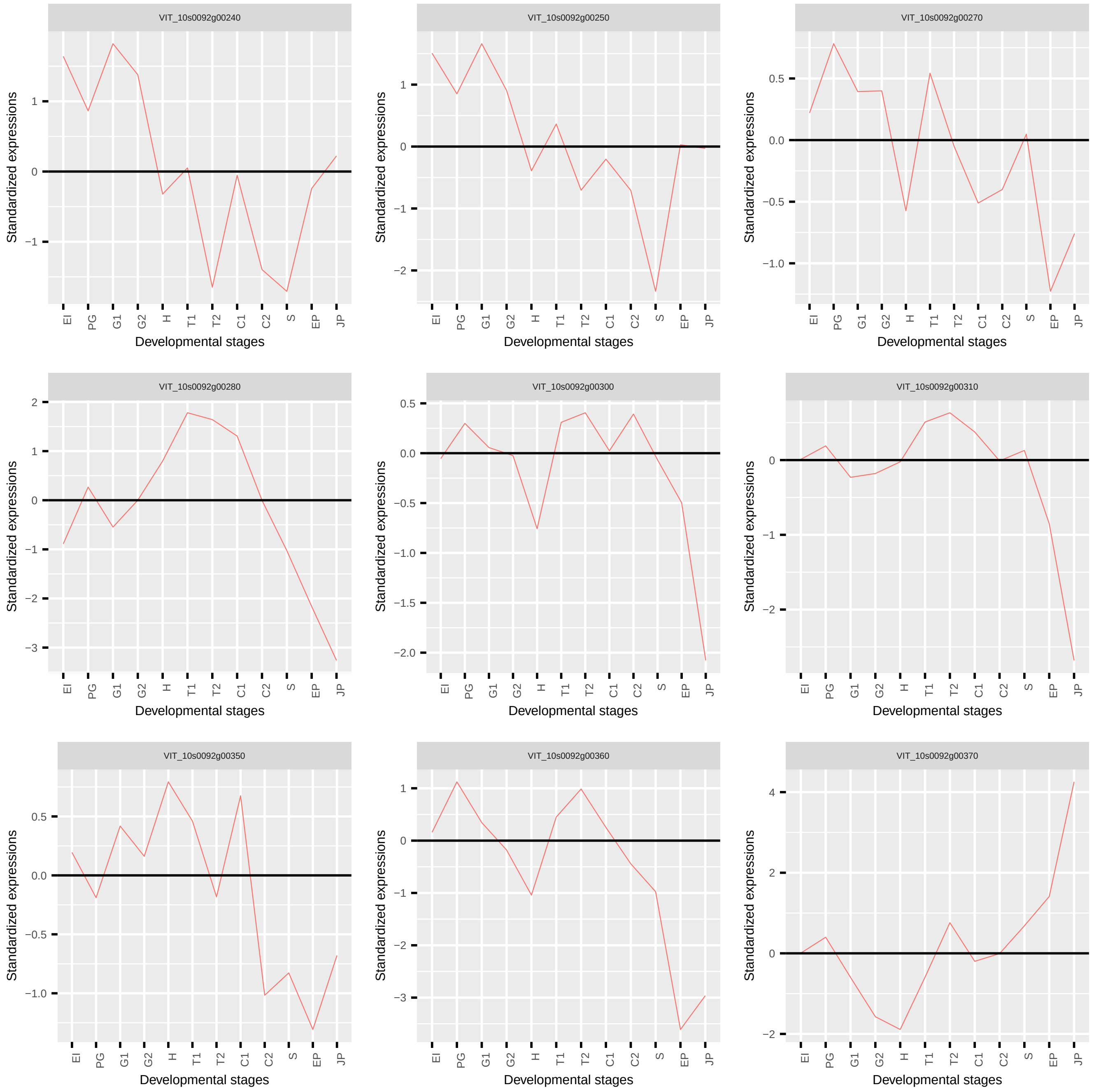

VIT_10s0092g00240
VIT_10s0092g00250
VIT_10s0092g00270
VIT_10s0092g00280
VIT_10s0092g00300
VIT_10s0092g00310
VIT_10s0092g00350
VIT_10s0092g00360
VIT_10s0092g00370
Standardized expressions
Developmental stages
EI PG G1 G2 H T1 T2 C1 C2 S EP JP

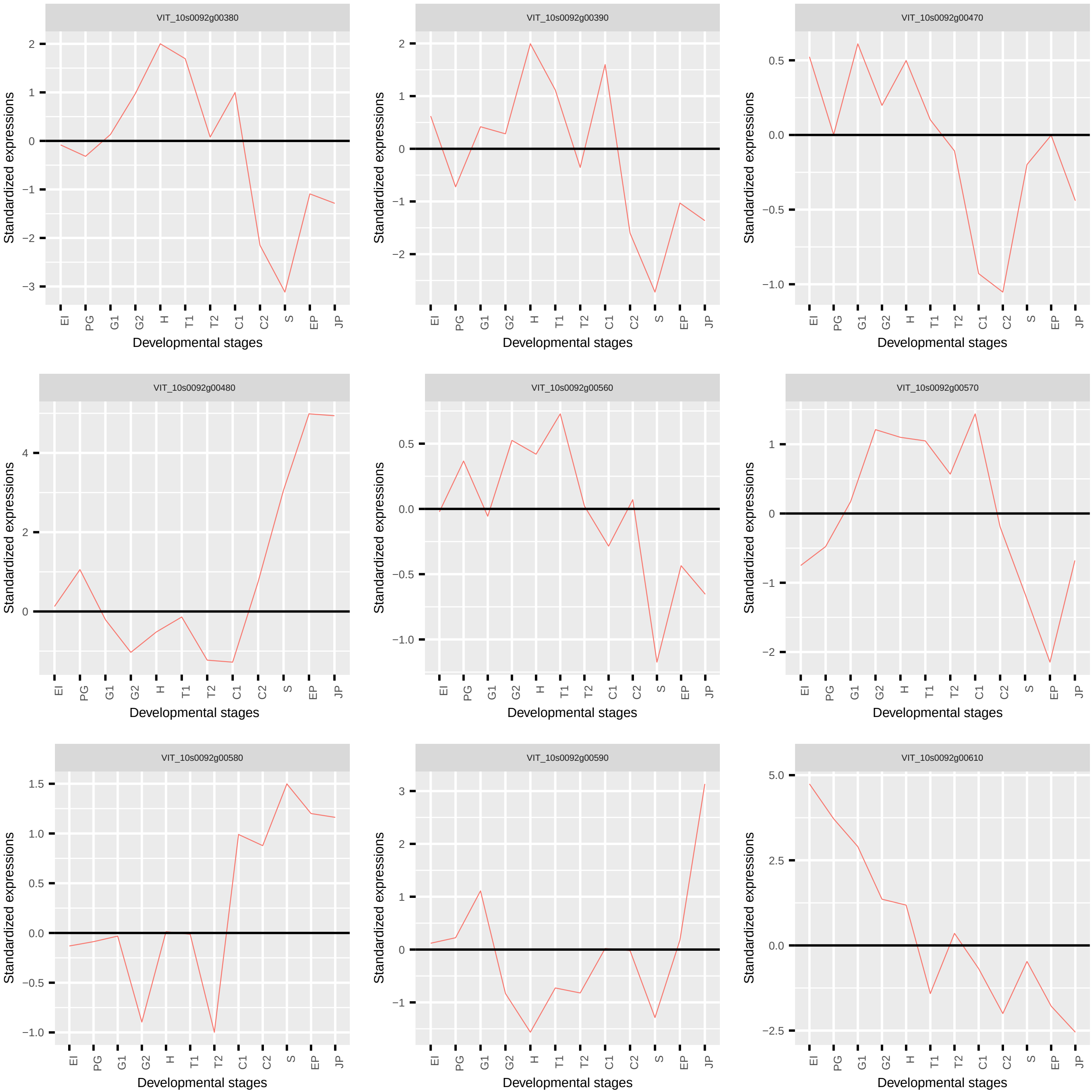
VIT_10s0092g00380
VIT_10s0092g00390
VIT_10s0092g00470
VIT_10s0092g00480
VIT_10s0092g00560
VIT_10s0092g00570
VIT_10s0092g00580
VIT_10s0092g00590
VIT_10s0092g00610
Standardized expressions
Developmental stages
EI PG G1 G2 H T1 T2 C1 C2 S EP JP

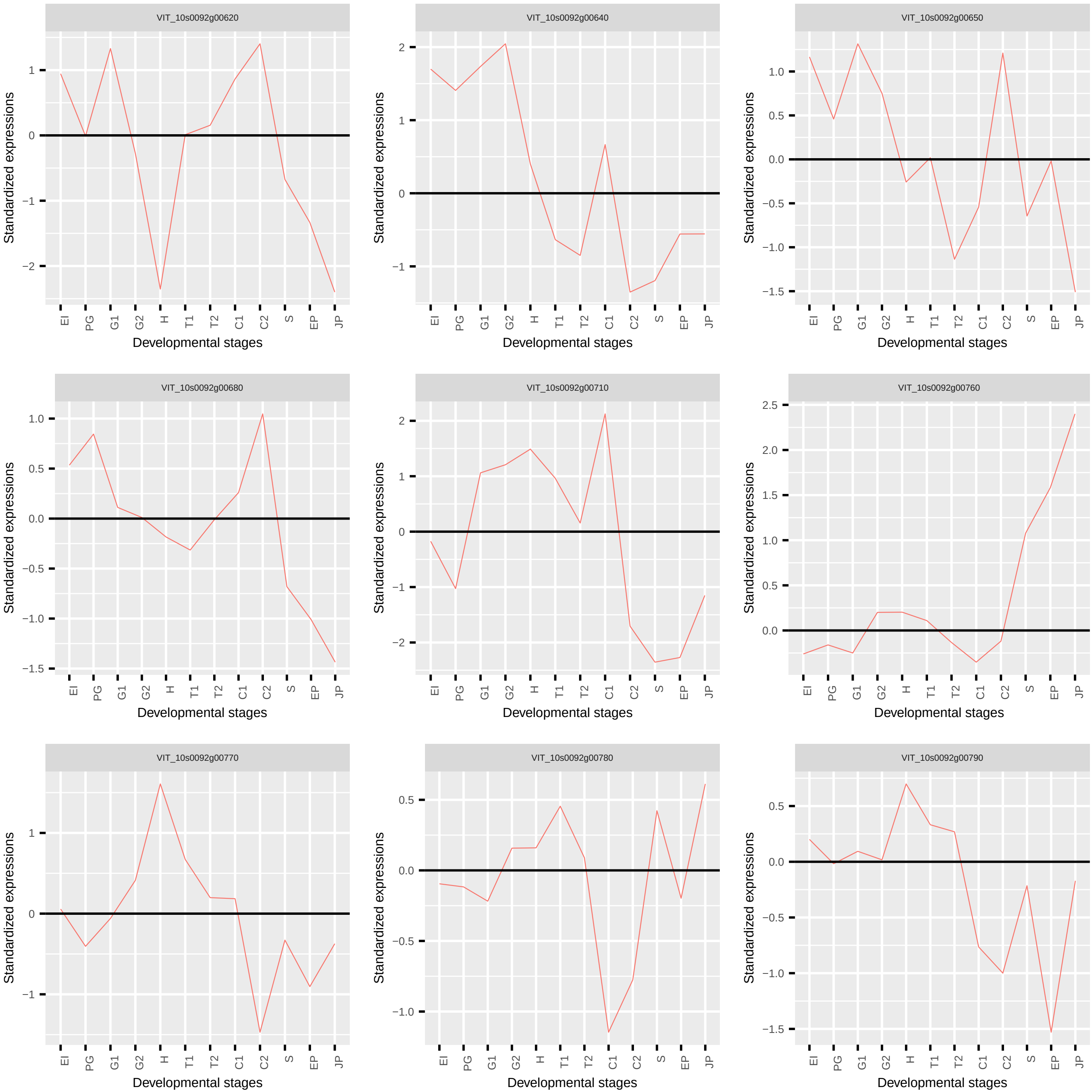
VIT_10s0092g00620
VIT_10s0092g00640
VIT_10s0092g00650
VIT_10s0092g00680
VIT_10s0092g00710
VIT_10s0092g00760
VIT_10s0092g00770
VIT_10s0092g00780
VIT_10s0092g00790
Standardized expressions
Developmental stages
EI PG G1 G2 H T1 T2 C1 C2 S EP JP

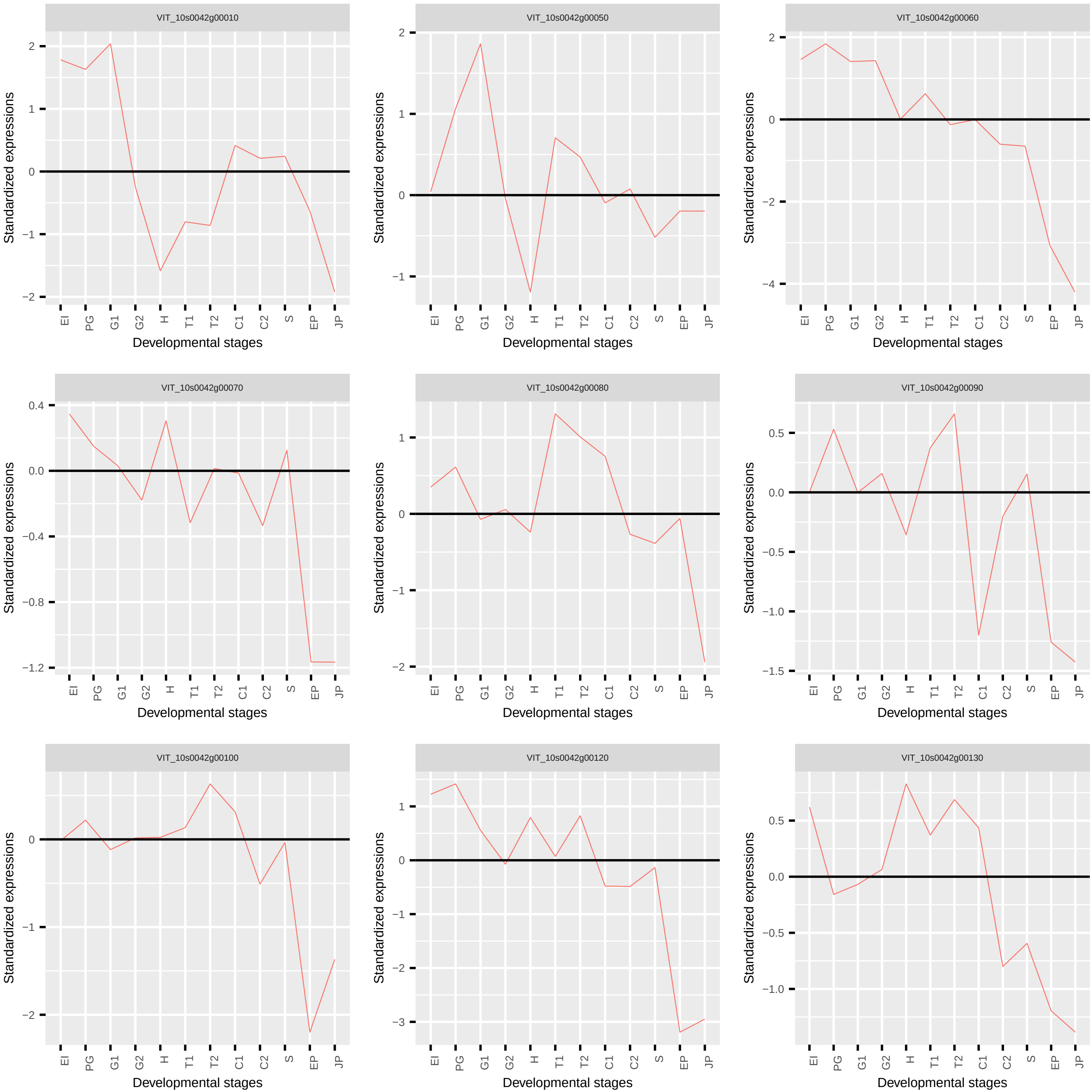
VIT_10s0042g00010
VIT_10s0042g00050
VIT_10s0042g00060
VIT_10s0042g00070
VIT_10s0042g00080
VIT_10s0042g00090
VIT_10s0042g00100
VIT_10s0042g00120
VIT_10s0042g00130
Standardized expressions
Developmental stages
EI PG G1 G2 H T1 T2 C1 C2 S EP JP

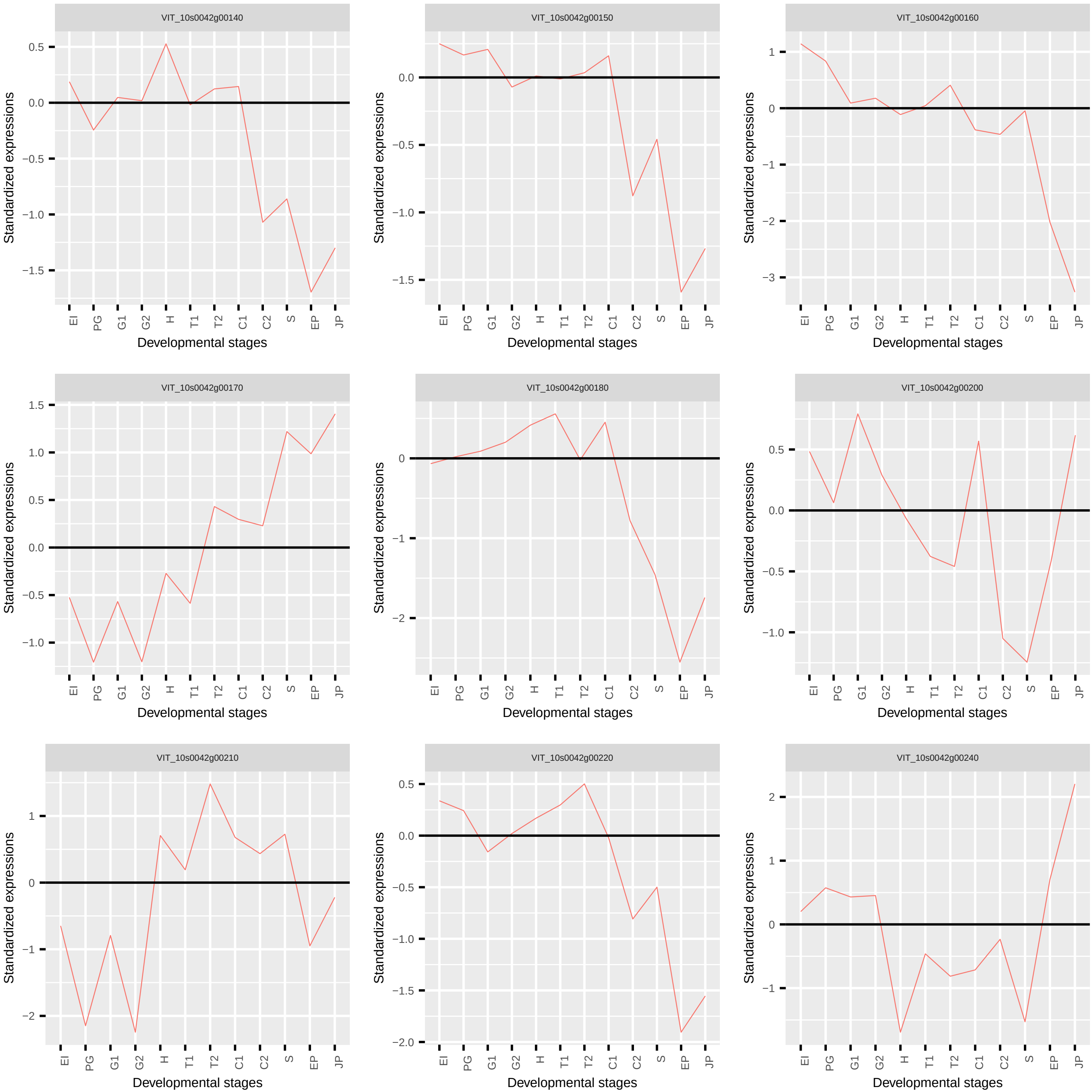

VIT_10s0042g00140
VIT_10s0042g00150
VIT_10s0042g00160
VIT_10s0042g00170
VIT_10s0042g00180
VIT_10s0042g00200
VIT_10s0042g00210
VIT_10s0042g00220
VIT_10s0042g00240
Standardized expressions
Developmental stages
EI PG G1 G2 H T1 T2 C1 C2 S EP JP

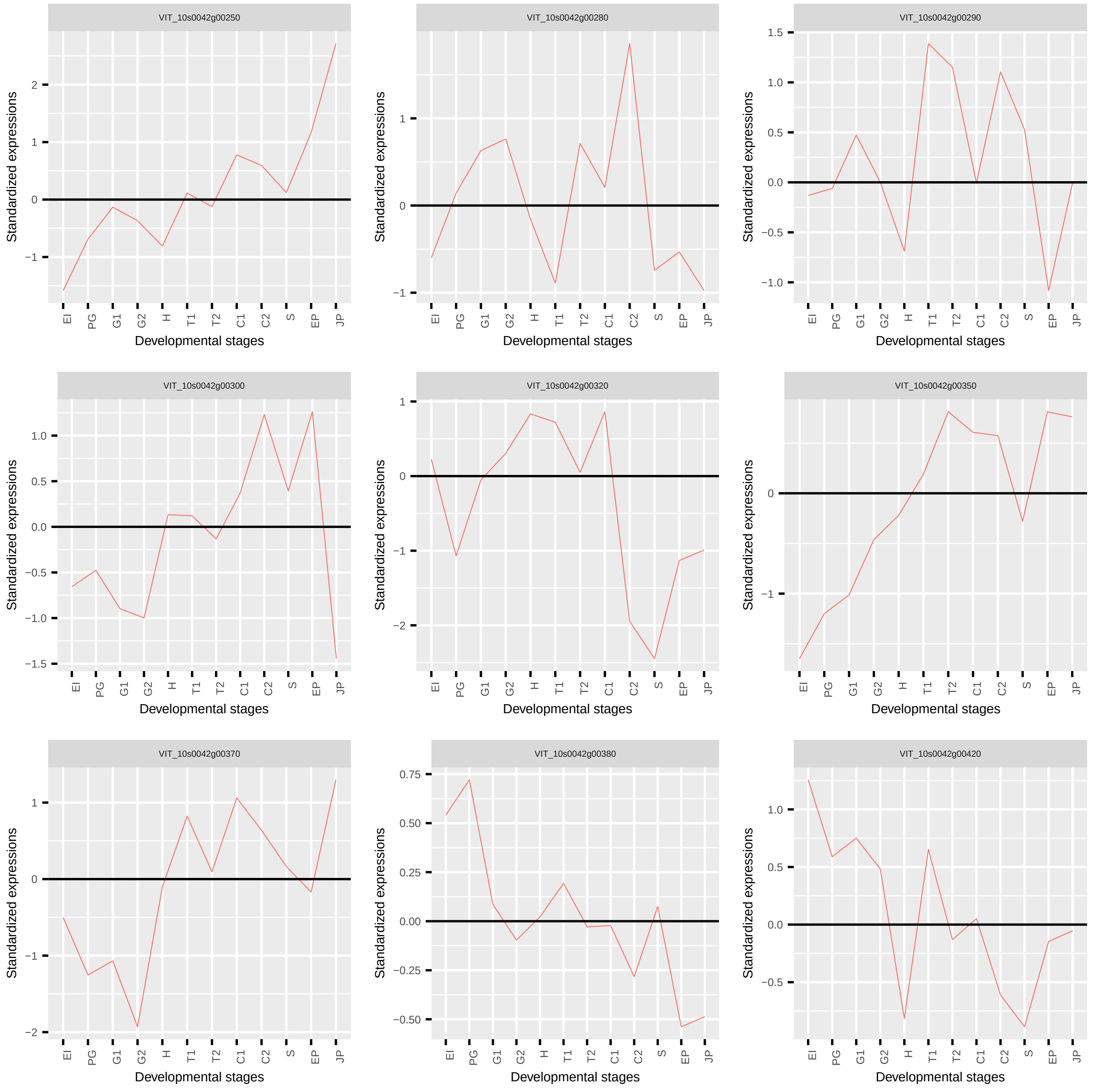
VIT_10s0042g00250
Standardized expressions
−1 0 1 2
EI PG G1 G2 H T1 T2 C1 C2 S EP JP
Developmental stages
VIT_10s0042g00280
Standardized expressions
−1 0 1
EI PG G1 G2 H T1 T2 C1 C2 S EP JP
Developmental stages
VIT_10s0042g00290
Standardized expressions
−1.0 −0.5 0.0 0.5 1.0 1.5
EI PG G1 G2 H T1 T2 C1 C2 S EP JP
Developmental stages
VIT_10s0042g00300
Standardized expressions
−1.5 −1.0 −0.5 0.0 0.5 1.0
EI PG G1 G2 H T1 T2 C1 C2 S EP JP
Developmental stages
VIT_10s0042g00320
Standardized expressions
−2 −1 0 1
EI PG G1 G2 H T1 T2 C1 C2 S EP JP
Developmental stages
VIT_10s0042g00350
Standardized expressions
−1 0
EI PG G1 G2 H T1 T2 C1 C2 S EP JP
Developmental stages
VIT_10s0042g00370
Standardized expressions
−2 −1 0 1
EI PG G1 G2 H T1 T2 C1 C2 S EP JP
Developmental stages
VIT_10s0042g00380
Standardized expressions
−0.50 −0.25 0.00 0.25 0.50 0.75
EI PG G1 G2 H T1 T2 C1 C2 S EP JP
Developmental stages
VIT_10s0042g00420
Standardized expressions
−0.5 0.0 0.5 1.0
EI PG G1 G2 H T1 T2 C1 C2 S EP JP
Developmental stages

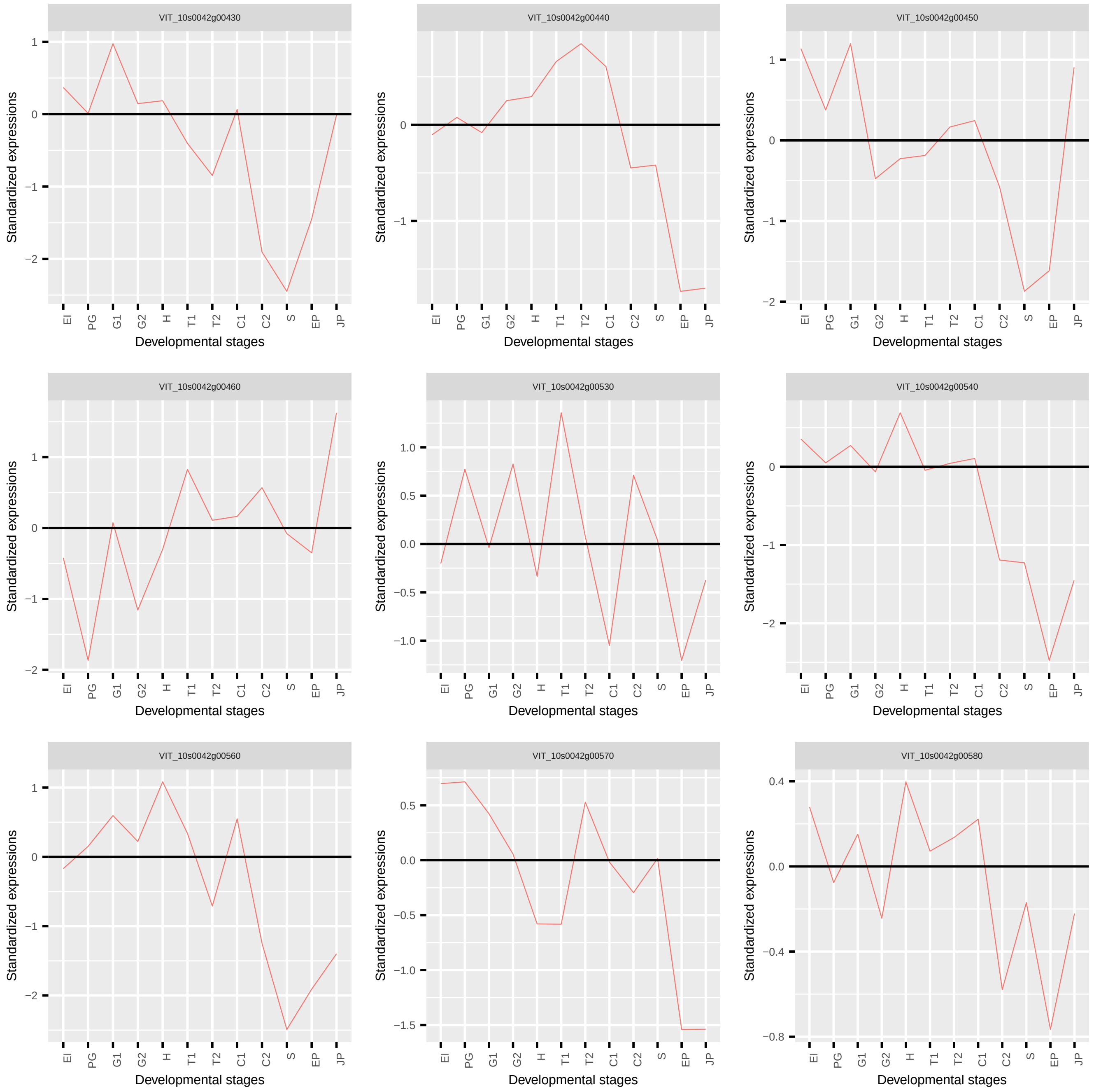

VIT_10s0042g00430
VIT_10s0042g00440
VIT_10s0042g00450
VIT_10s0042g00460
VIT_10s0042g00530
VIT_10s0042g00540
VIT_10s0042g00560
VIT_10s0042g00570
VIT_10s0042g00580
Standardized expressions
Developmental stages
EI PG G1 G2 H T1 T2 C1 C2 S EP JP

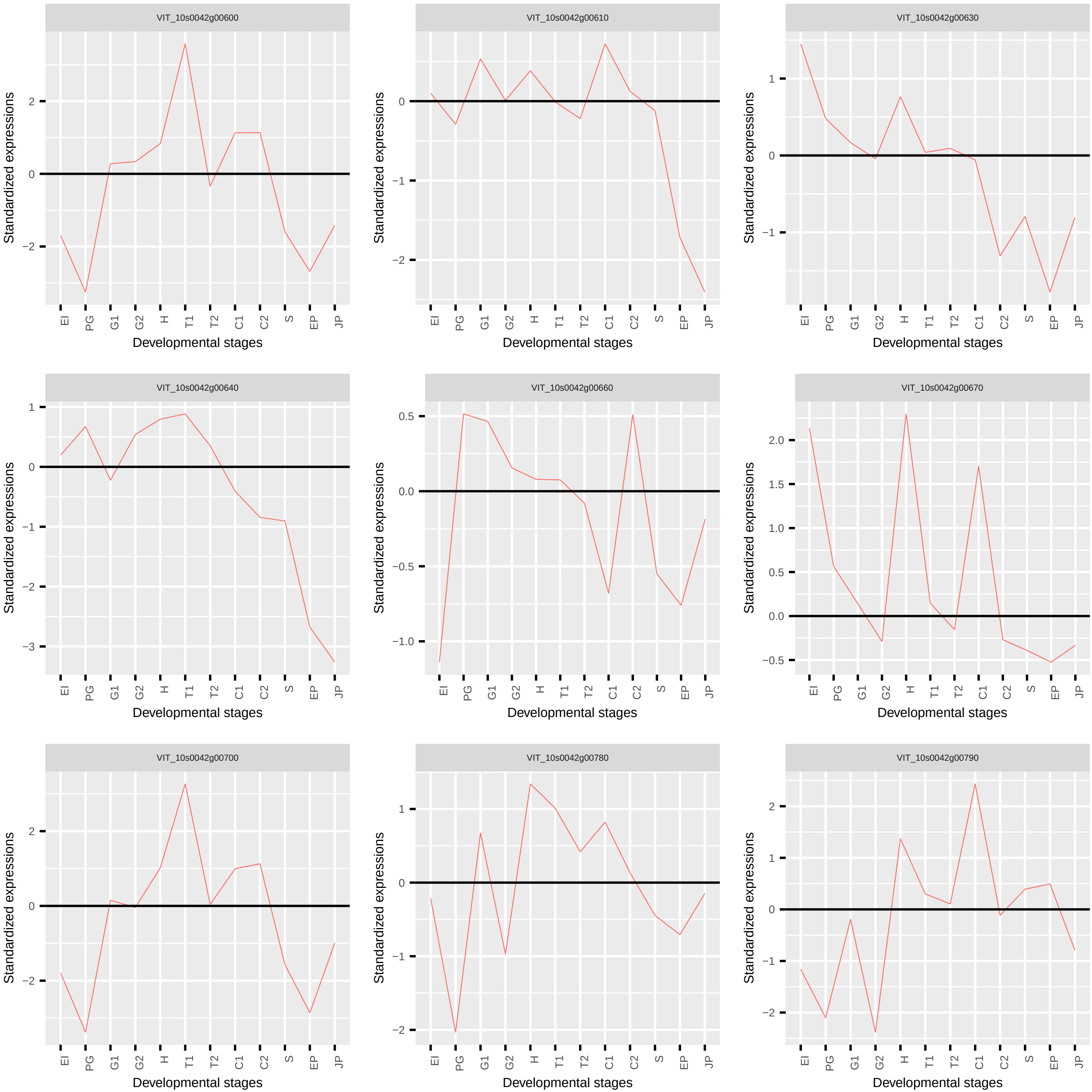
VIT_10s0042g00600
VIT_10s0042g00610
VIT_10s0042g00630
VIT_10s0042g00640
VIT_10s0042g00660
VIT_10s0042g00670
VIT_10s0042g00700
VIT_10s0042g00780
VIT_10s0042g00790
Standardized expressions
Developmental stages
EI PG G1 G2 H T1 T2 C1 C2 S EP JP

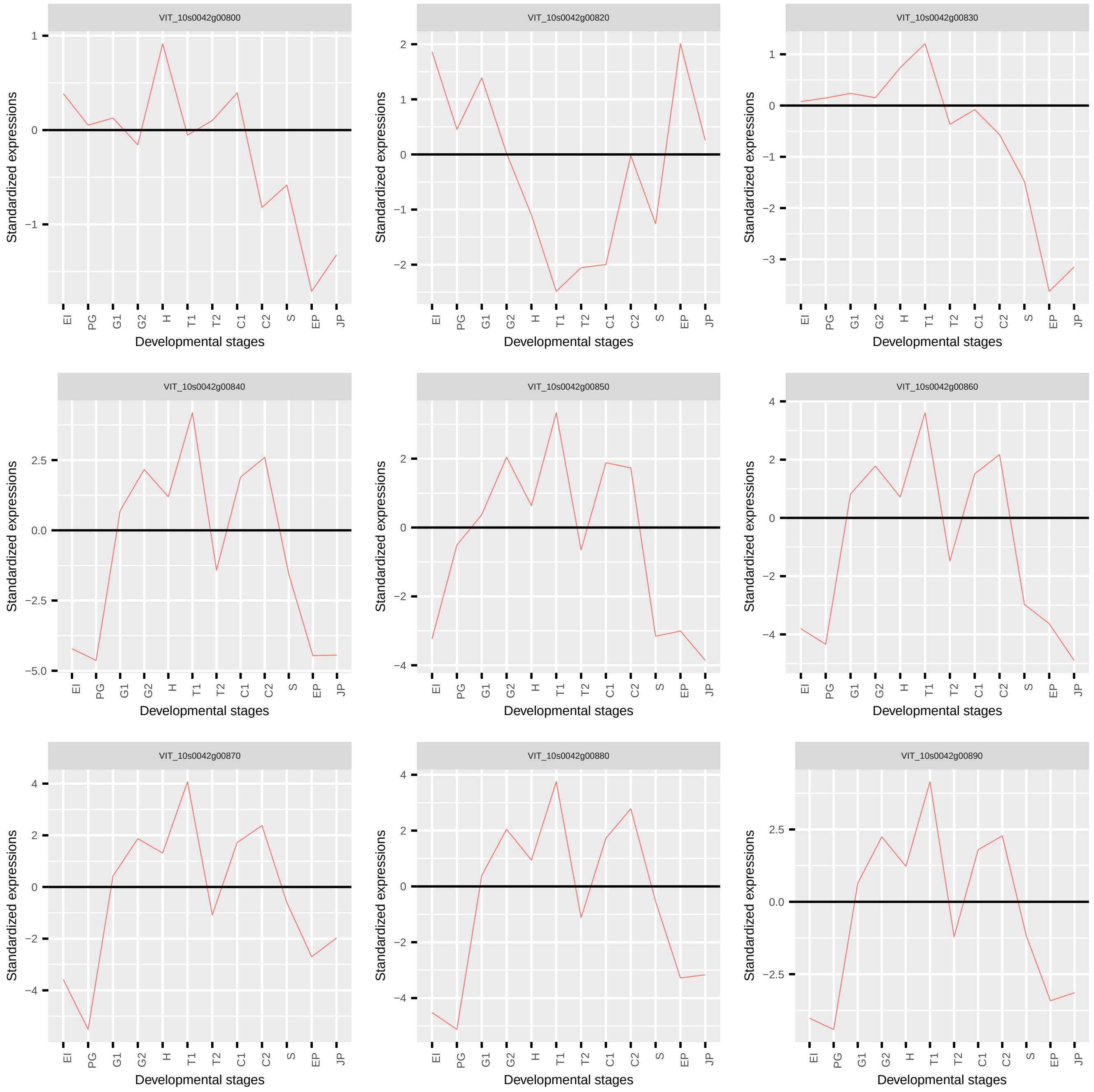
VIT_10s0042g00800
VIT_10s0042g00820
VIT_10s0042g00830
VIT_10s0042g00840
VIT_10s0042g00850
VIT_10s0042g00860
VIT_10s0042g00870
VIT_10s0042g00880
VIT_10s0042g00890
Standardized expressions
Developmental stages
EI
PG
G1
G2
H
T1
T2
C1
C2
S
EP
JP

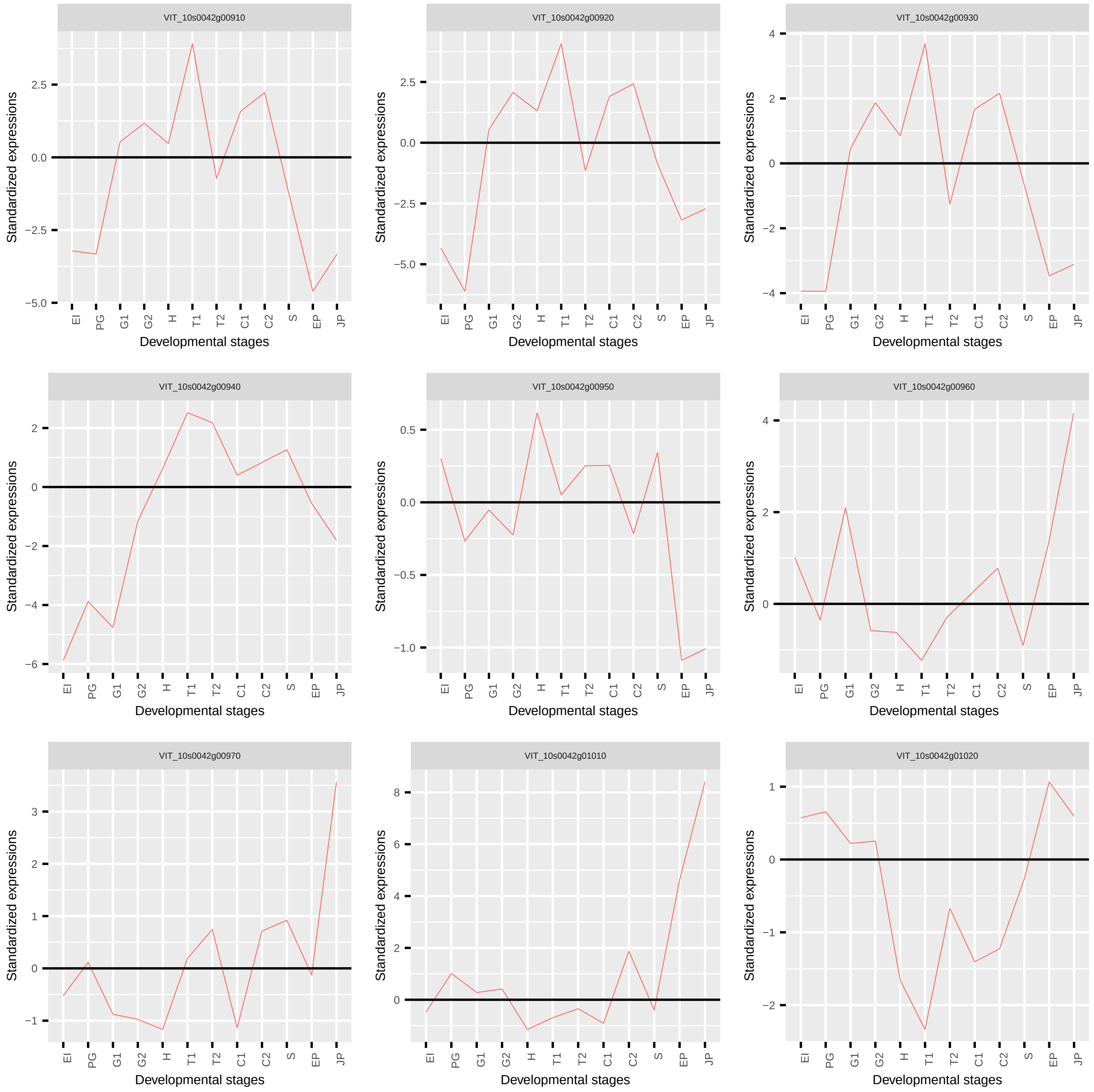

VIT_10s0042g00910
VIT_10s0042g00920
VIT_10s0042g00930
VIT_10s0042g00940
VIT_10s0042g00950
VIT_10s0042g00960
VIT_10s0042g00970
VIT_10s0042g01010
VIT_10s0042g01020
Standardized expressions
Developmental stages
EI PG G1 G2 H T1 T2 C1 C2 S EP JP

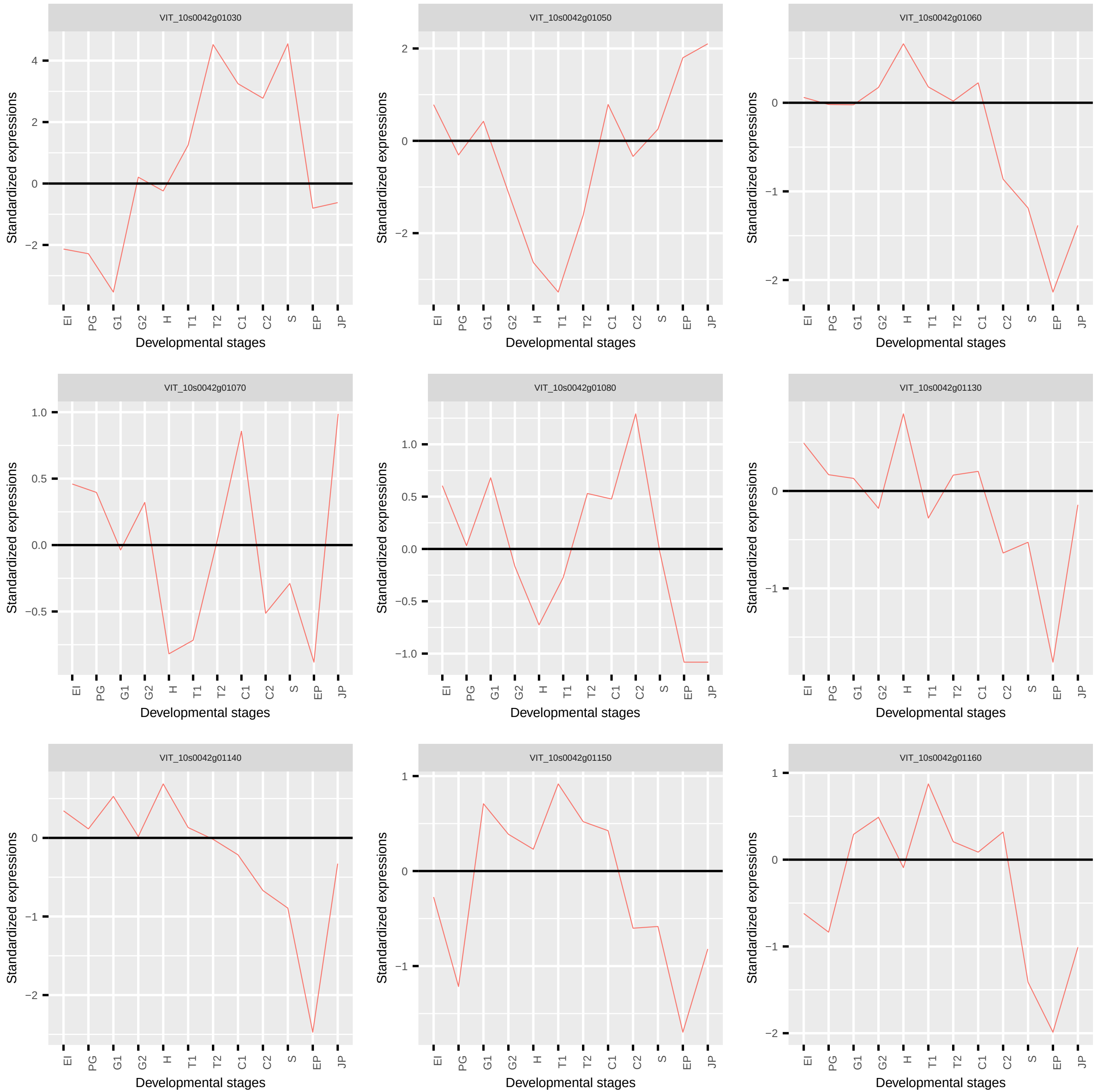

VIT_10s0042g01030
VIT_10s0042g01050
VIT_10s0042g01060
VIT_10s0042g01070
VIT_10s0042g01080
VIT_10s0042g01130
VIT_10s0042g01140
VIT_10s0042g01150
VIT_10s0042g01160
Standardized expressions
Developmental stages
EI
PG
G1
G2
H
T1
T2
C1
C2
S
EP
JP

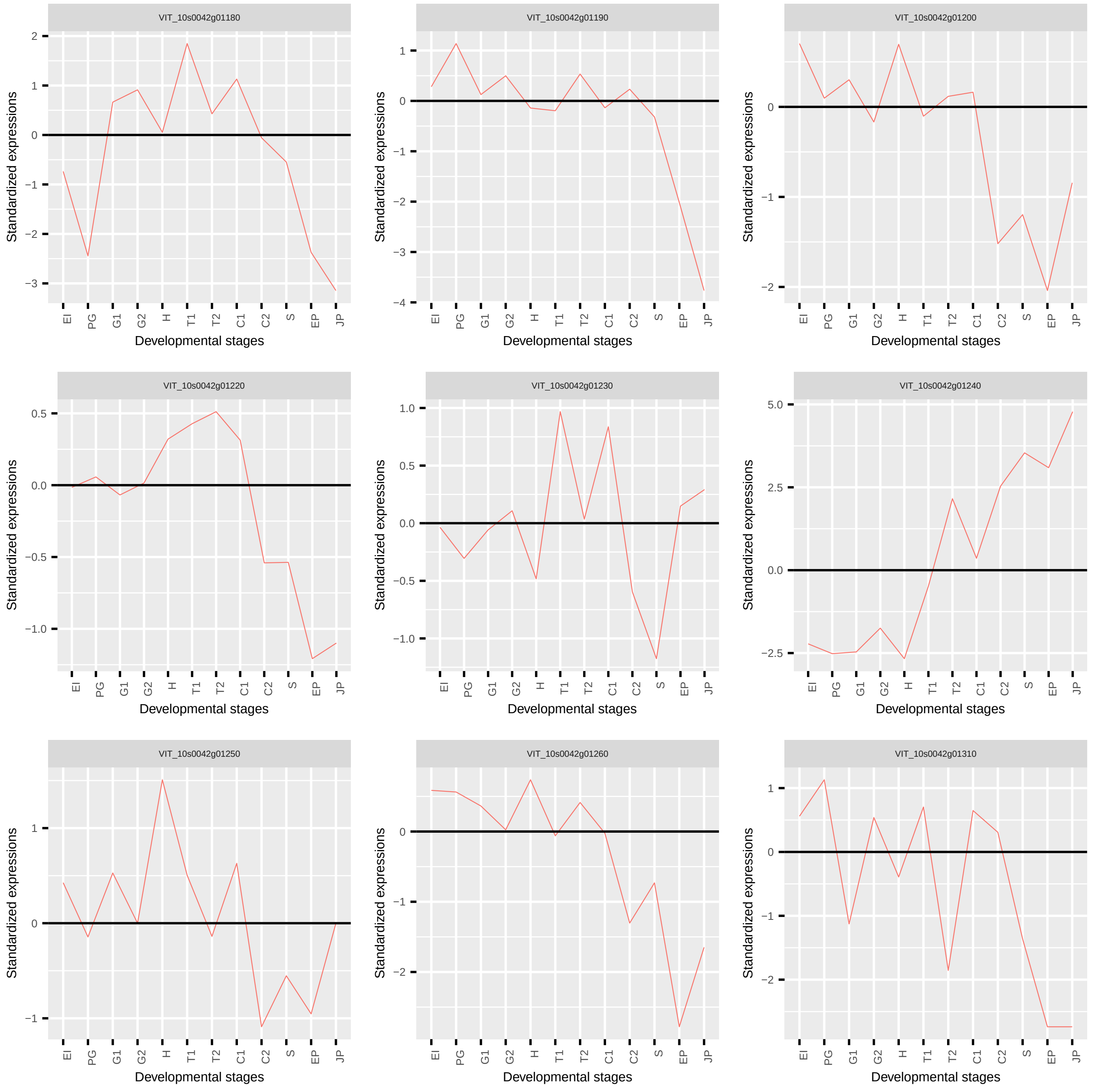
VIT_10s0042g01180
VIT_10s0042g01190
VIT_10s0042g01200
VIT_10s0042g01220
VIT_10s0042g01230
VIT_10s0042g01240
VIT_10s0042g01250
VIT_10s0042g01260
VIT_10s0042g01310
Standardized expressions
Developmental stages
EI PG G1 G2 H T1 T2 C1 C2 S EP JP

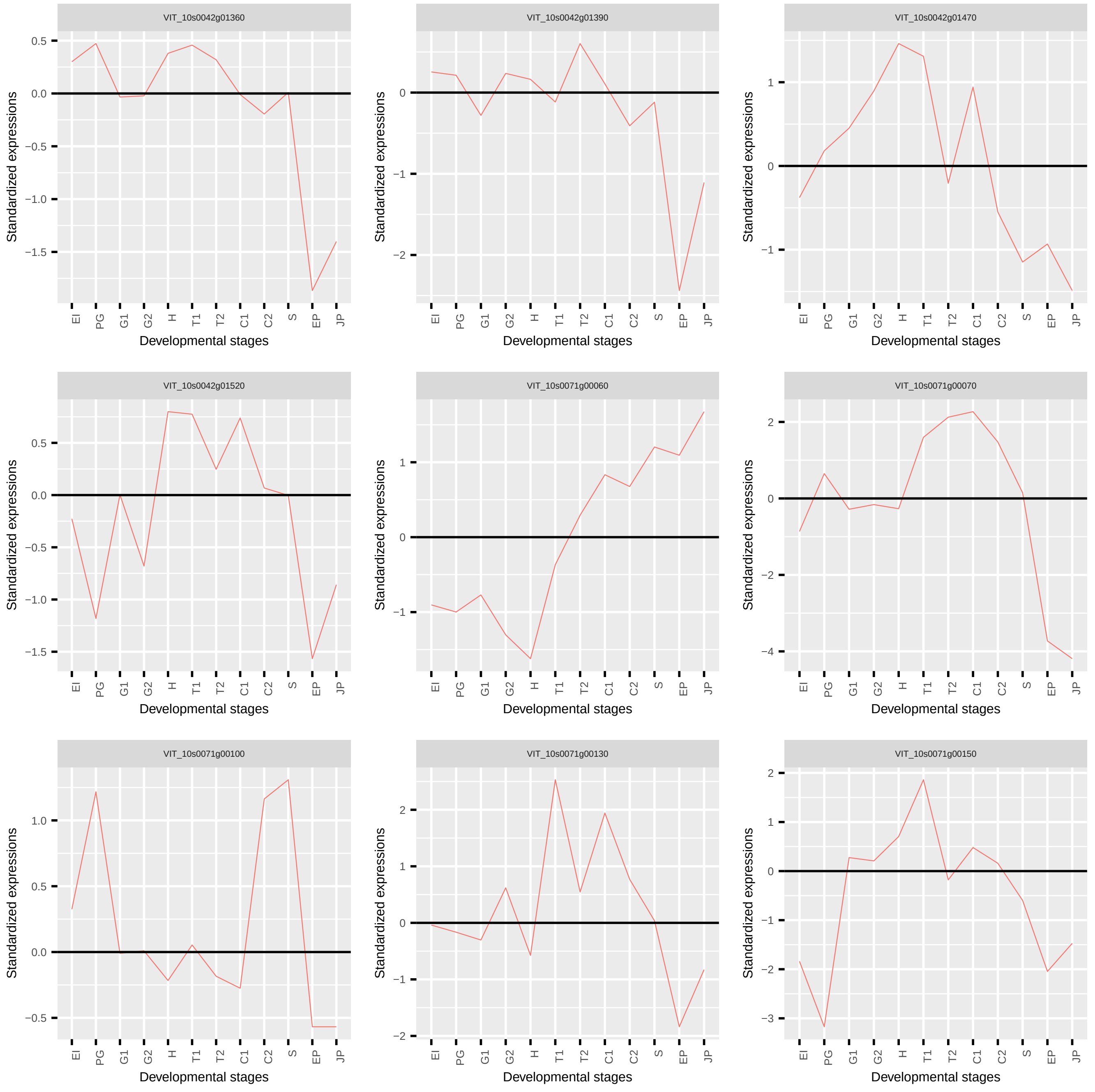

VIT_10s0042g01360
VIT_10s0042g01390
VIT_10s0042g01470
VIT_10s0042g01520
VIT_10s0071g00060
VIT_10s0071g00070
VIT_10s0071g00100
VIT_10s0071g00130
VIT_10s0071g00150
Standardized expressions
Developmental stages
EI PG G1 G2 H T1 T2 C1 C2 S EP JP

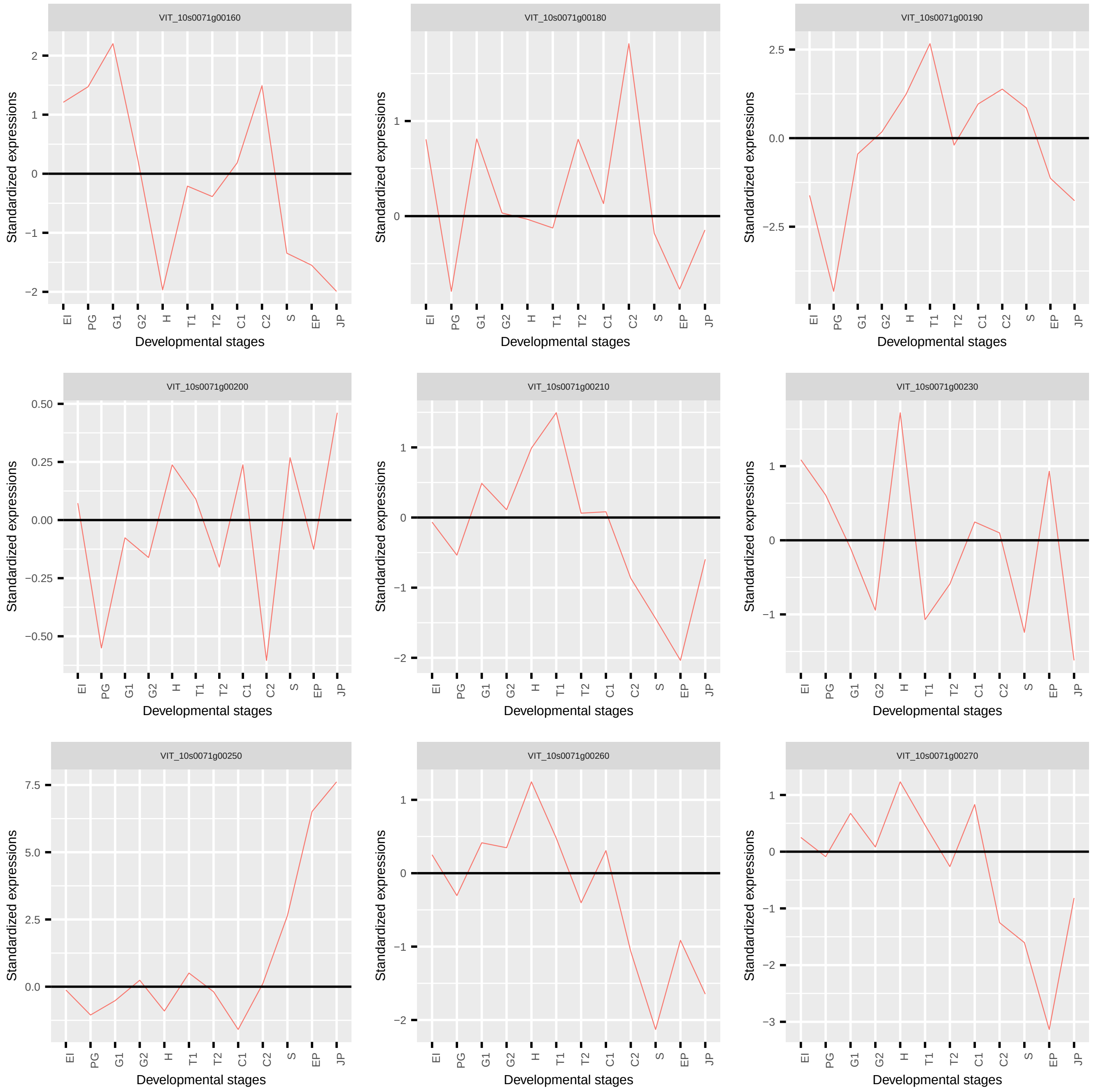
VIT_10s0071g00160
VIT_10s0071g00180
VIT_10s0071g00190
VIT_10s0071g00200
VIT_10s0071g00210
VIT_10s0071g00230
VIT_10s0071g00250
VIT_10s0071g00260
VIT_10s0071g00270
Standardized expressions
Developmental stages
EI PG G1 G2 H T1 T2 C1 C2 S EP JP

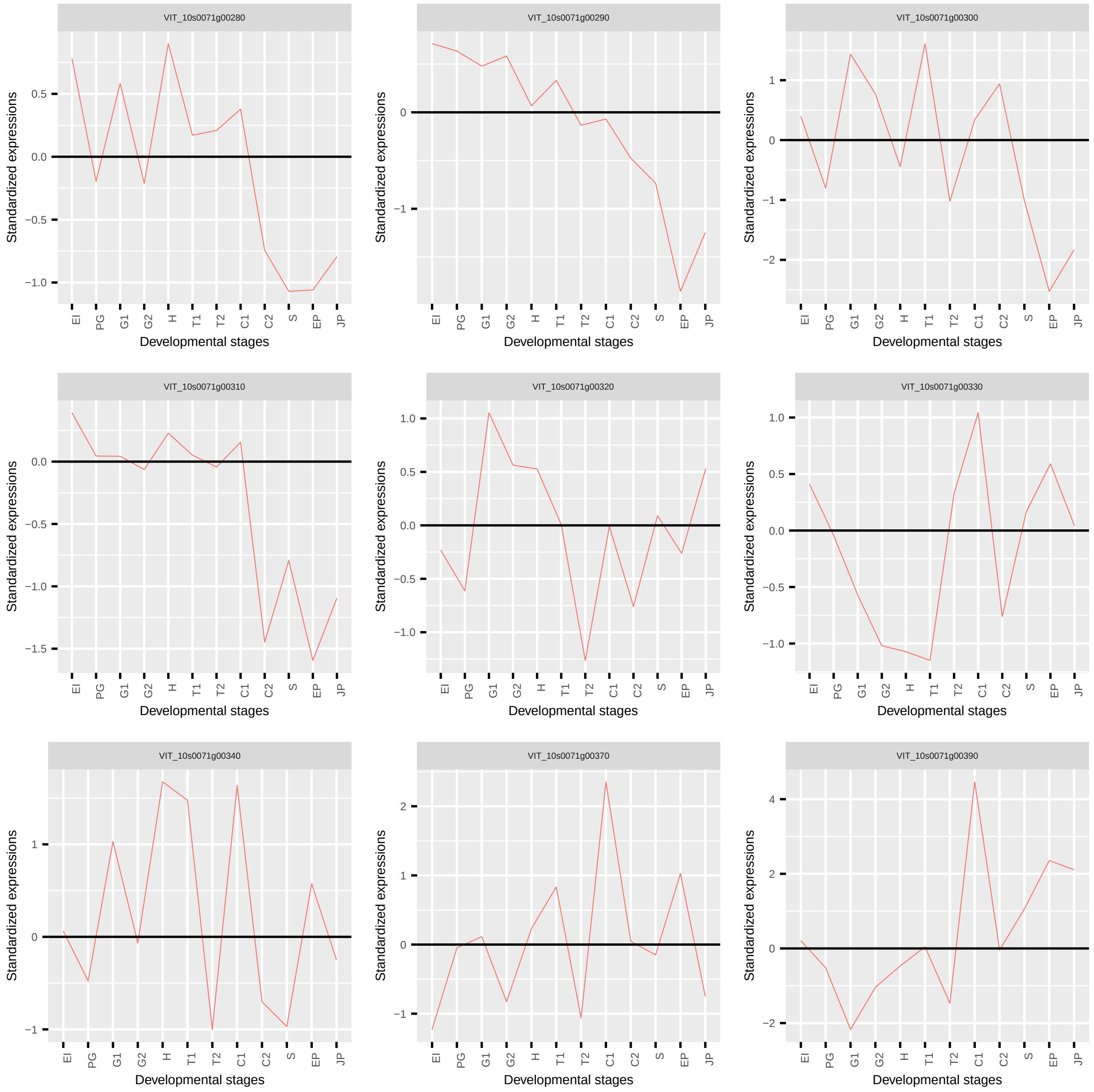
VIT_10s0071g00280
VIT_10s0071g00290
VIT_10s0071g00300
VIT_10s0071g00310
VIT_10s0071g00320
VIT_10s0071g00330
VIT_10s0071g00340
VIT_10s0071g00370
VIT_10s0071g00390
Standardized expressions
Developmental stages
EI PG G1 G2 H T1 T2 C1 C2 S EP JP

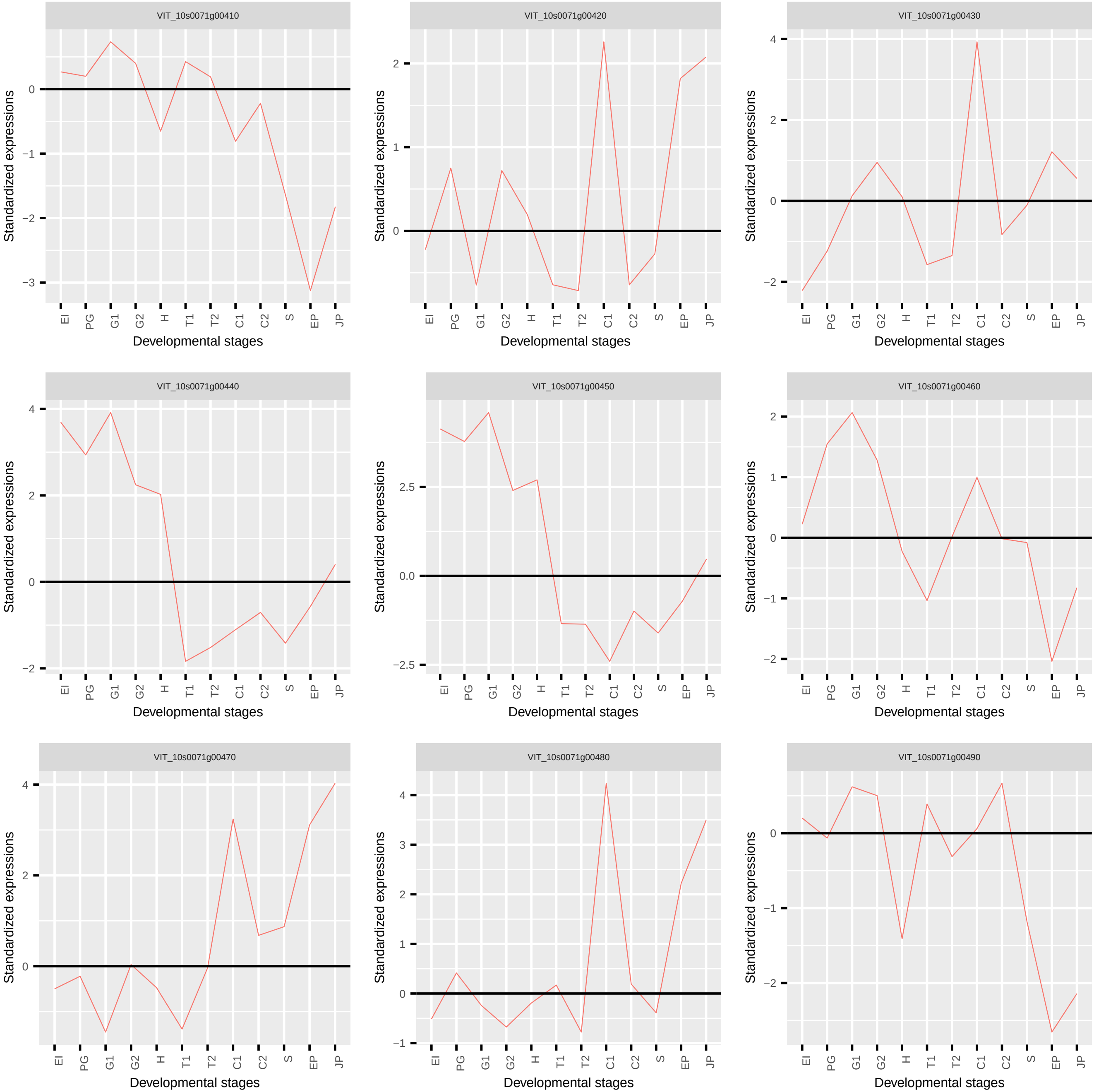
VIT_10s0071g00410
VIT_10s0071g00420
VIT_10s0071g00430
VIT_10s0071g00440
VIT_10s0071g00450
VIT_10s0071g00460
VIT_10s0071g00470
VIT_10s0071g00480
VIT_10s0071g00490
Standardized expressions
Developmental stages
EI PG G1 G2 H T1 T2 C1 C2 S EP JP
0
−1
−2
−3
1
2
4
2.5
0.0
−2.5
3

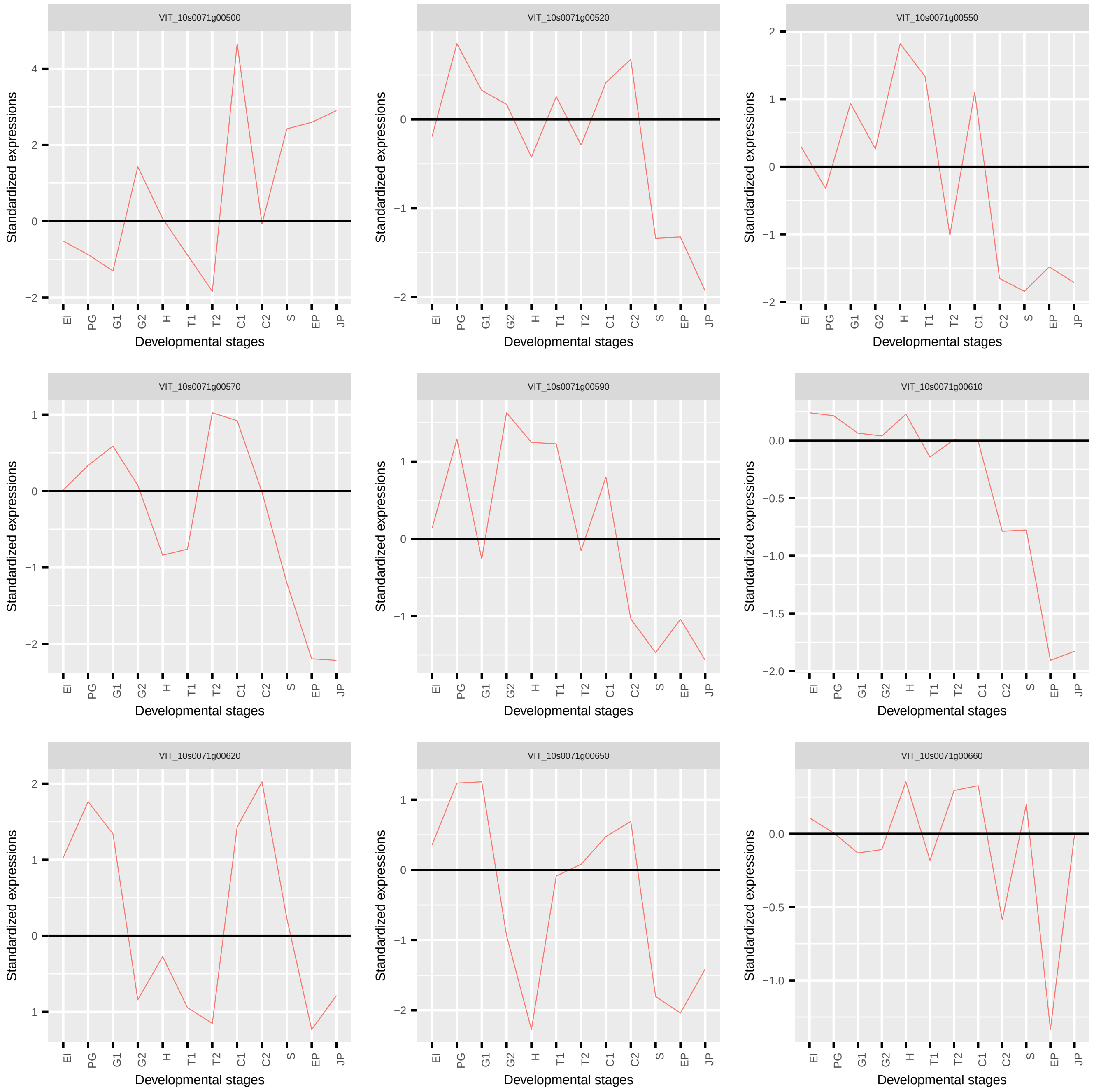
VIT_10s0071g00500
VIT_10s0071g00520
VIT_10s0071g00550
VIT_10s0071g00570
VIT_10s0071g00590
VIT_10s0071g00610
VIT_10s0071g00620
VIT_10s0071g00650
VIT_10s0071g00660
Standardized expressions
Developmental stages
EI
PG
G1
G2
H
T1
T2
C1
C2
S
EP
JP

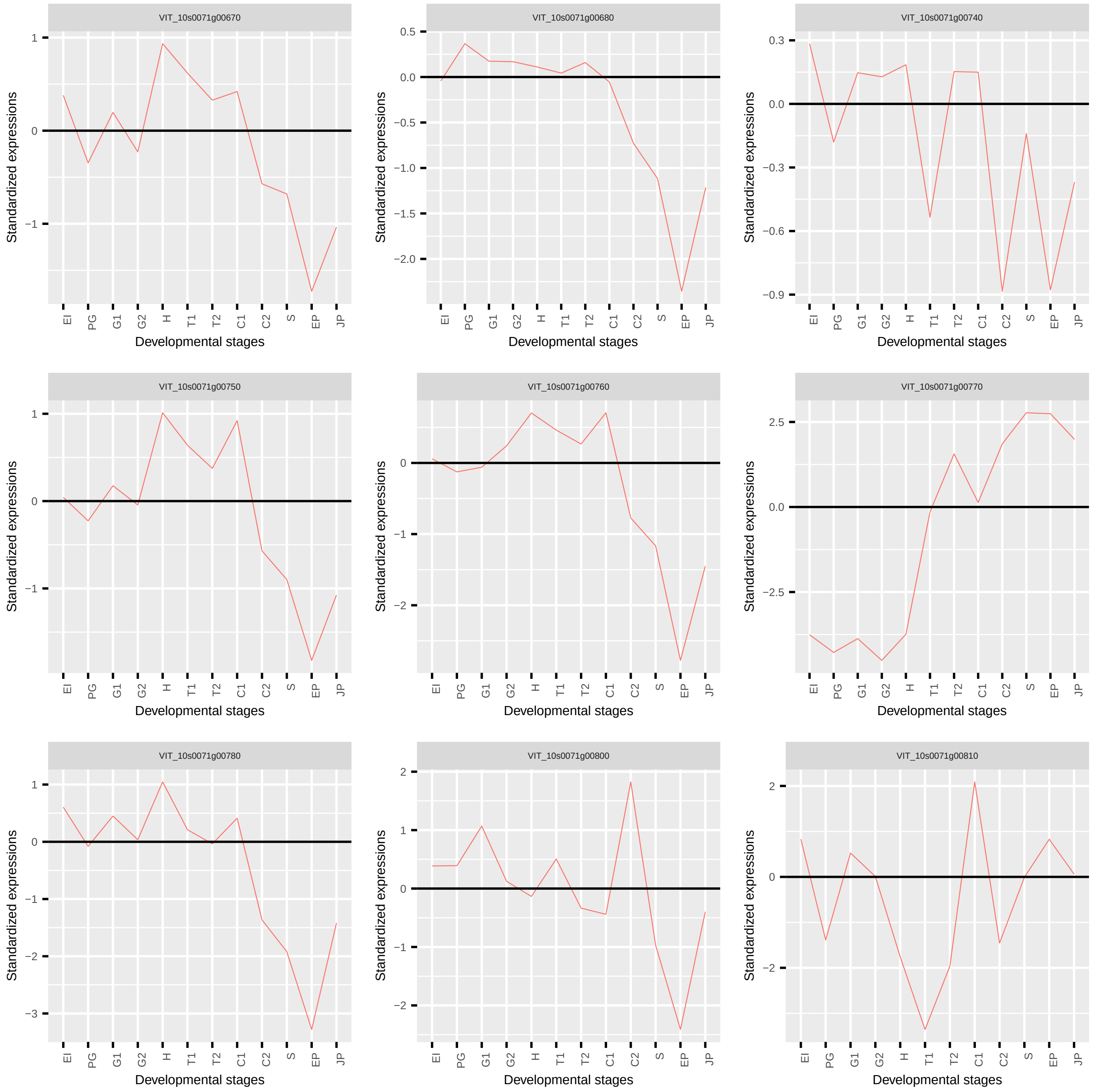
VIT_10s0071g00670
VIT_10s0071g00680
VIT_10s0071g00740
VIT_10s0071g00750
VIT_10s0071g00760
VIT_10s0071g00770
VIT_10s0071g00780
VIT_10s0071g00800
VIT_10s0071g00810
Standardized expressions
Developmental stages
EI PG G1 G2 H T1 T2 C1 C2 S EP JP

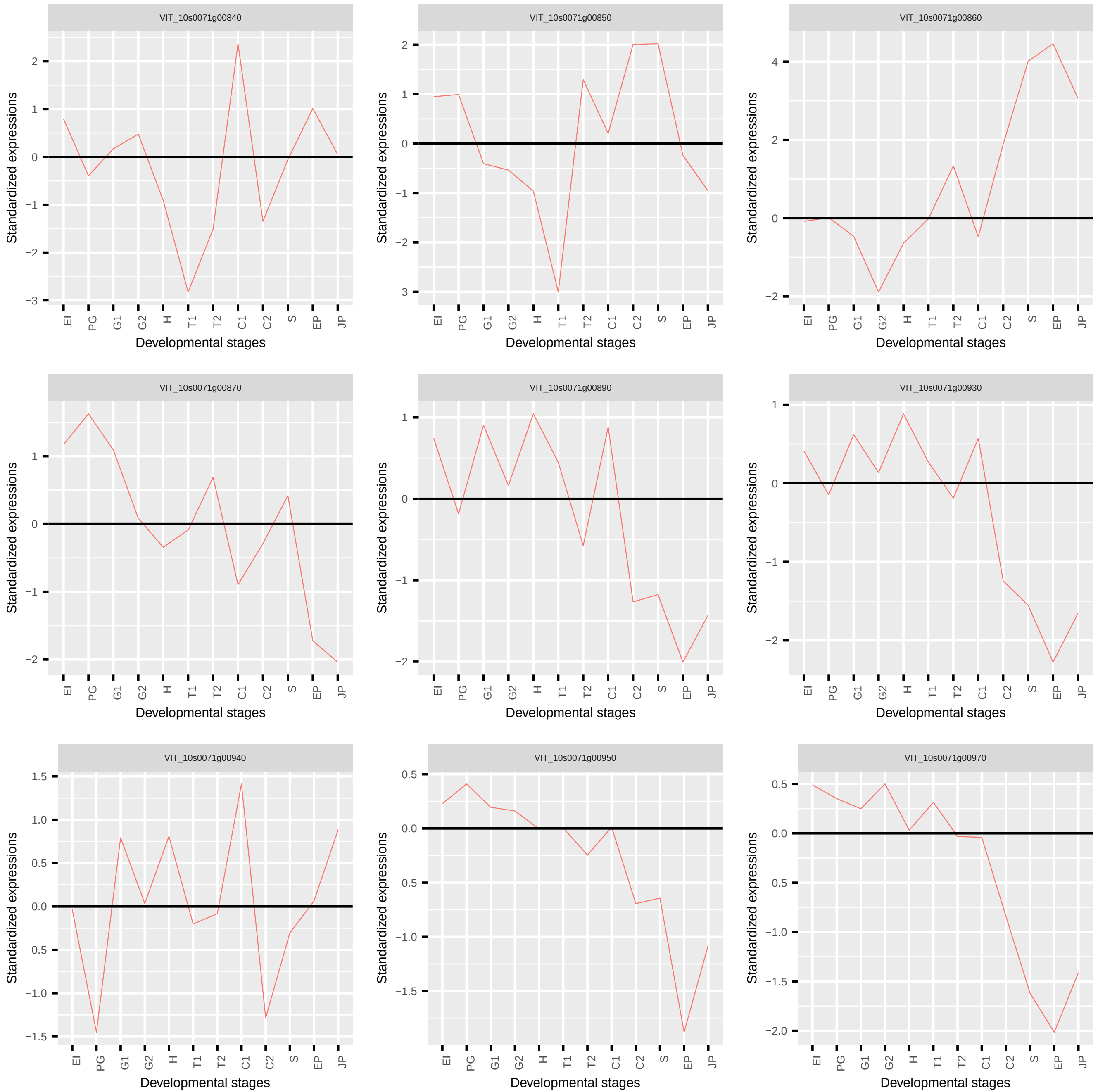

VIT_10s0071g00840
VIT_10s0071g00850
VIT_10s0071g00860
VIT_10s0071g00870
VIT_10s0071g00890
VIT_10s0071g00930
VIT_10s0071g00940
VIT_10s0071g00950
VIT_10s0071g00970
Standardized expressions
Developmental stages
EI
PG
G1
G2
H
T1
T2
C1
C2
S
EP
JP

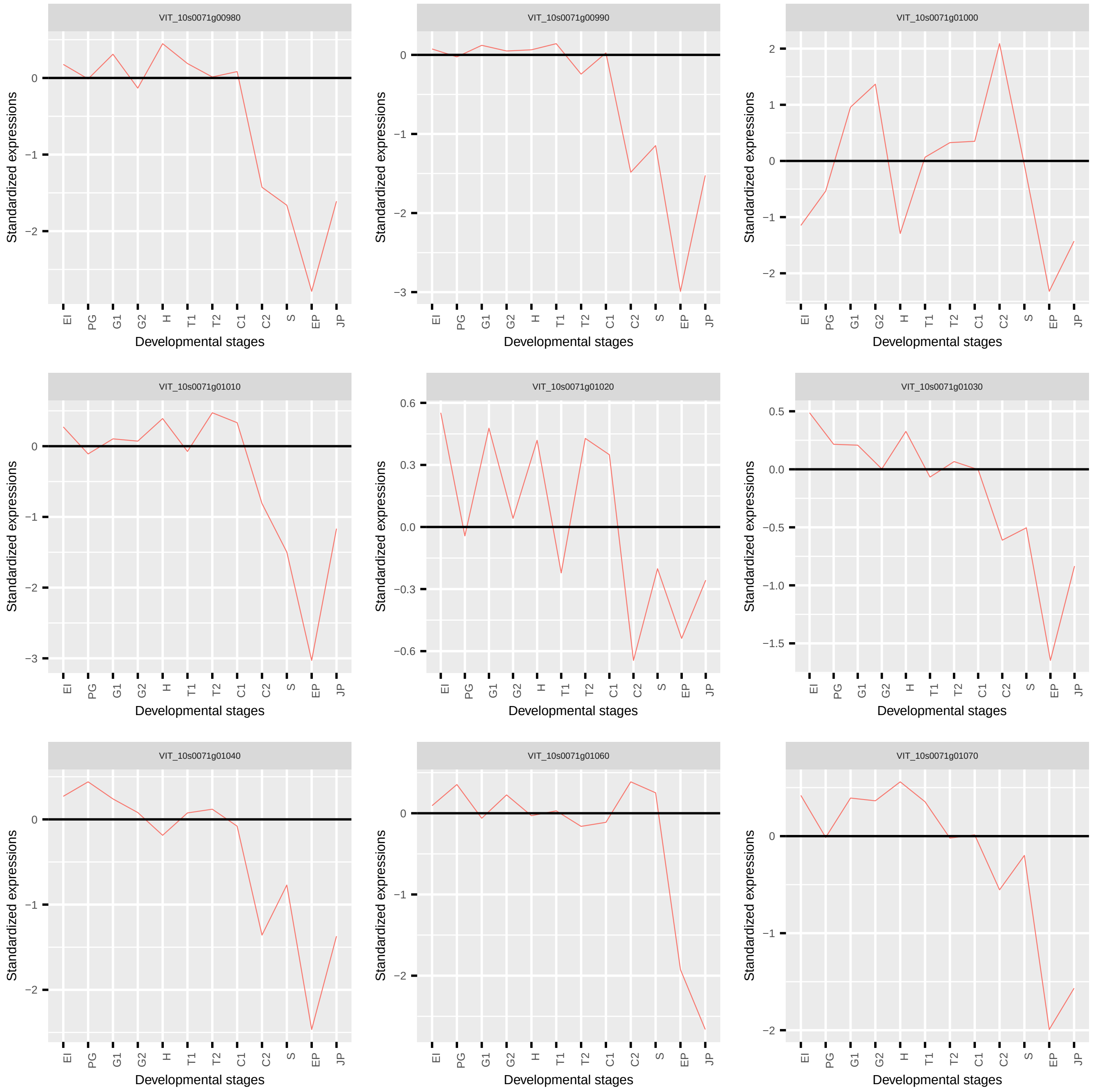
VIT_10s0071g00980
VIT_10s0071g00990
VIT_10s0071g01000
VIT_10s0071g01010
VIT_10s0071g01020
VIT_10s0071g01030
VIT_10s0071g01040
VIT_10s0071g01060
VIT_10s0071g01070
Standardized expressions
Developmental stages
EI PG G1 G2 H T1 T2 C1 C2 S EP JP

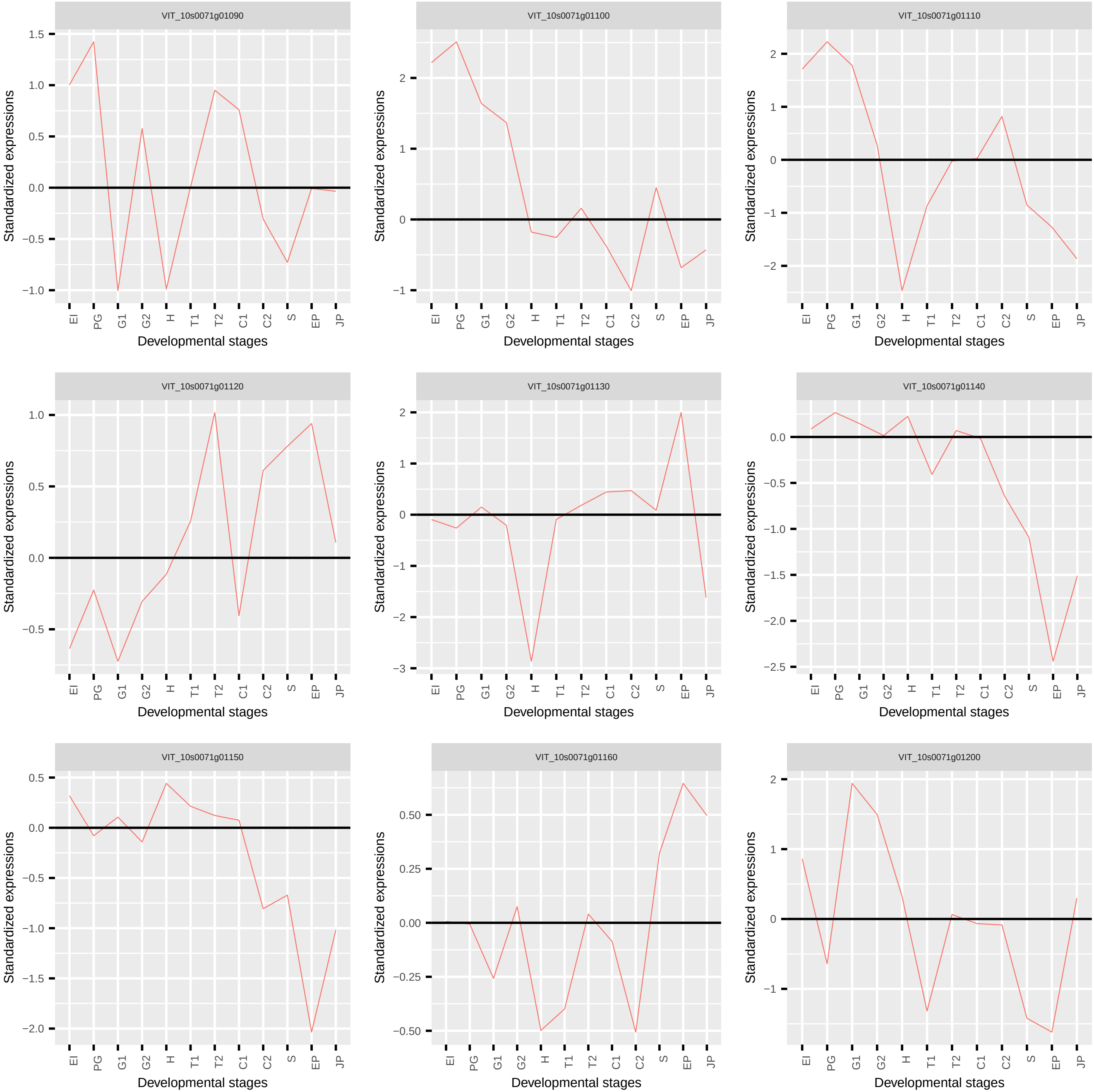
VIT_10s0071g01090
VIT_10s0071g01100
VIT_10s0071g01110
VIT_10s0071g01120
VIT_10s0071g01130
VIT_10s0071g01140
VIT_10s0071g01150
VIT_10s0071g01160
VIT_10s0071g01200
Standardized expressions
Developmental stages
EI PG G1 G2 H T1 T2 C1 C2 S EP JP

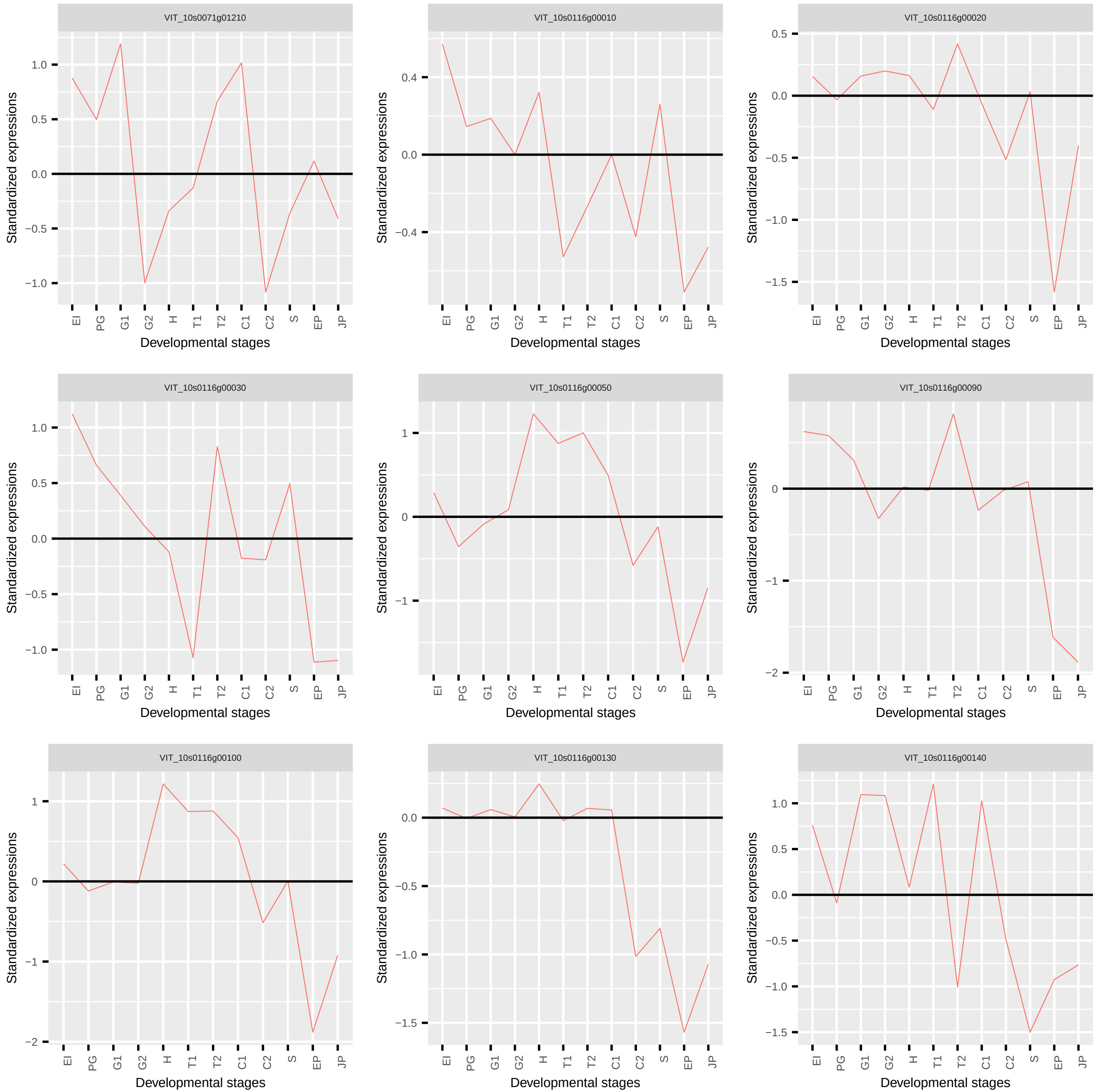
VIT_10s0071g01210
VIT_10s0116g00010
VIT_10s0116g00020
VIT_10s0116g00030
VIT_10s0116g00050
VIT_10s0116g00090
VIT_10s0116g00100
VIT_10s0116g00130
VIT_10s0116g00140
Standardized expressions
Developmental stages
EI PG G1 G2 H T1 T2 C1 C2 S EP JP
1.0 0.5 0.0 −0.5 −1.0
0.4 0.0 −0.4
0.5 0.0 −0.5 −1.0 −1.5
1 0 −1 −2

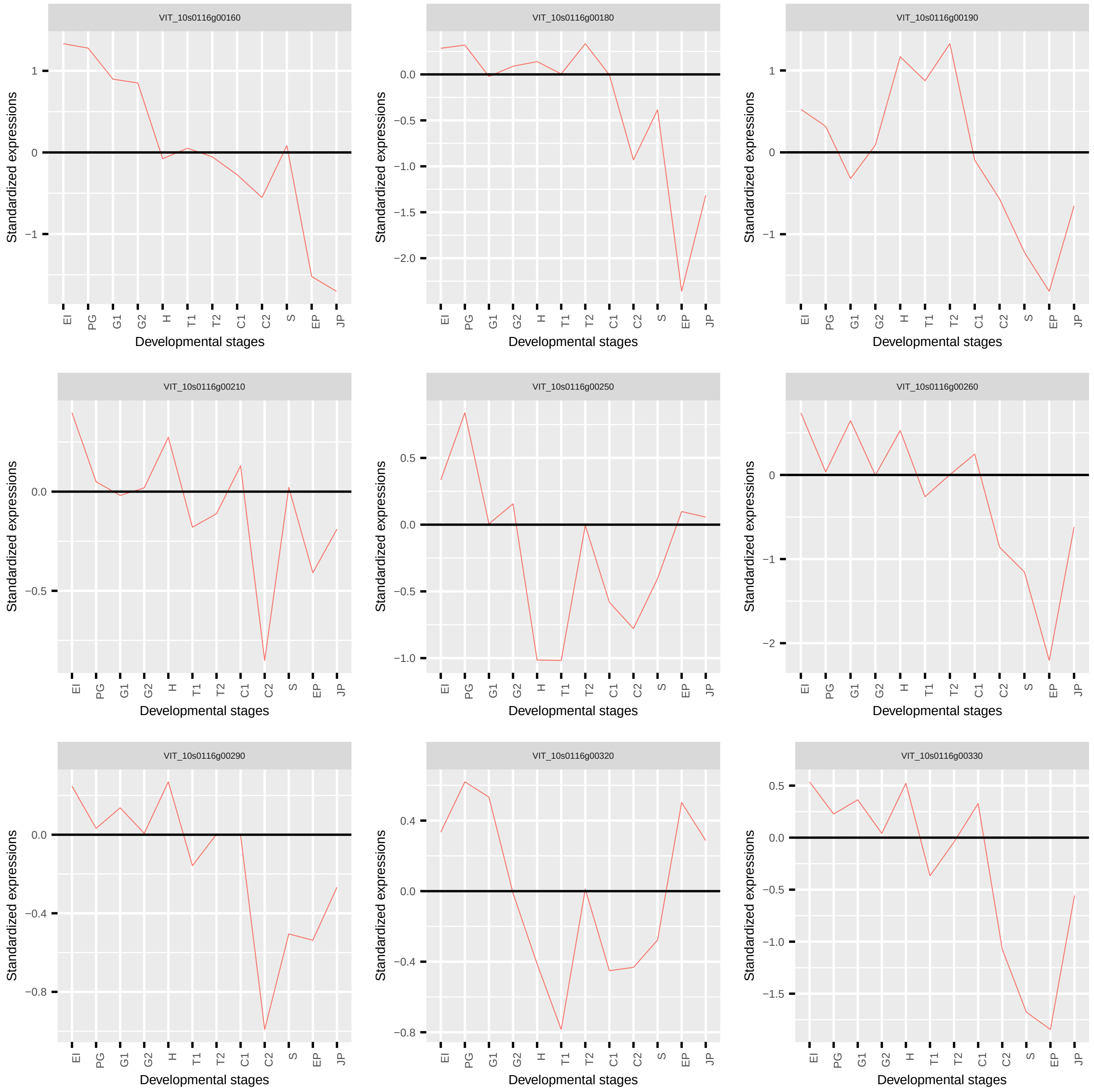
VIT_10s0116g00160
Standardized expressions
1
0
−1
EI PG G1 G2 H T1 T2 C1 C2 S EP JP
Developmental stages
VIT_10s0116g00180
Standardized expressions
0.0
−0.5
−1.0
−1.5
−2.0
EI PG G1 G2 H T1 T2 C1 C2 S EP JP
Developmental stages
VIT_10s0116g00190
Standardized expressions
1
0
−1
EI PG G1 G2 H T1 T2 C1 C2 S EP JP
Developmental stages
VIT_10s0116g00210
Standardized expressions
0.0
−0.5
EI PG G1 G2 H T1 T2 C1 C2 S EP JP
Developmental stages
VIT_10s0116g00250
Standardized expressions
0.5
0.0
−0.5
−1.0
EI PG G1 G2 H T1 T2 C1 C2 S EP JP
Developmental stages
VIT_10s0116g00260
Standardized expressions
0
−1
−2
EI PG G1 G2 H T1 T2 C1 C2 S EP JP
Developmental stages
VIT_10s0116g00290
Standardized expressions
0.0
−0.4
−0.8
EI PG G1 G2 H T1 T2 C1 C2 S EP JP
Developmental stages
VIT_10s0116g00320
Standardized expressions
0.4
0.0
−0.4
−0.8
EI PG G1 G2 H T1 T2 C1 C2 S EP JP
Developmental stages
VIT_10s0116g00330
Standardized expressions
0.5
0.0
−0.5
−1.0
−1.5
EI PG G1 G2 H T1 T2 C1 C2 S EP JP
Developmental stages

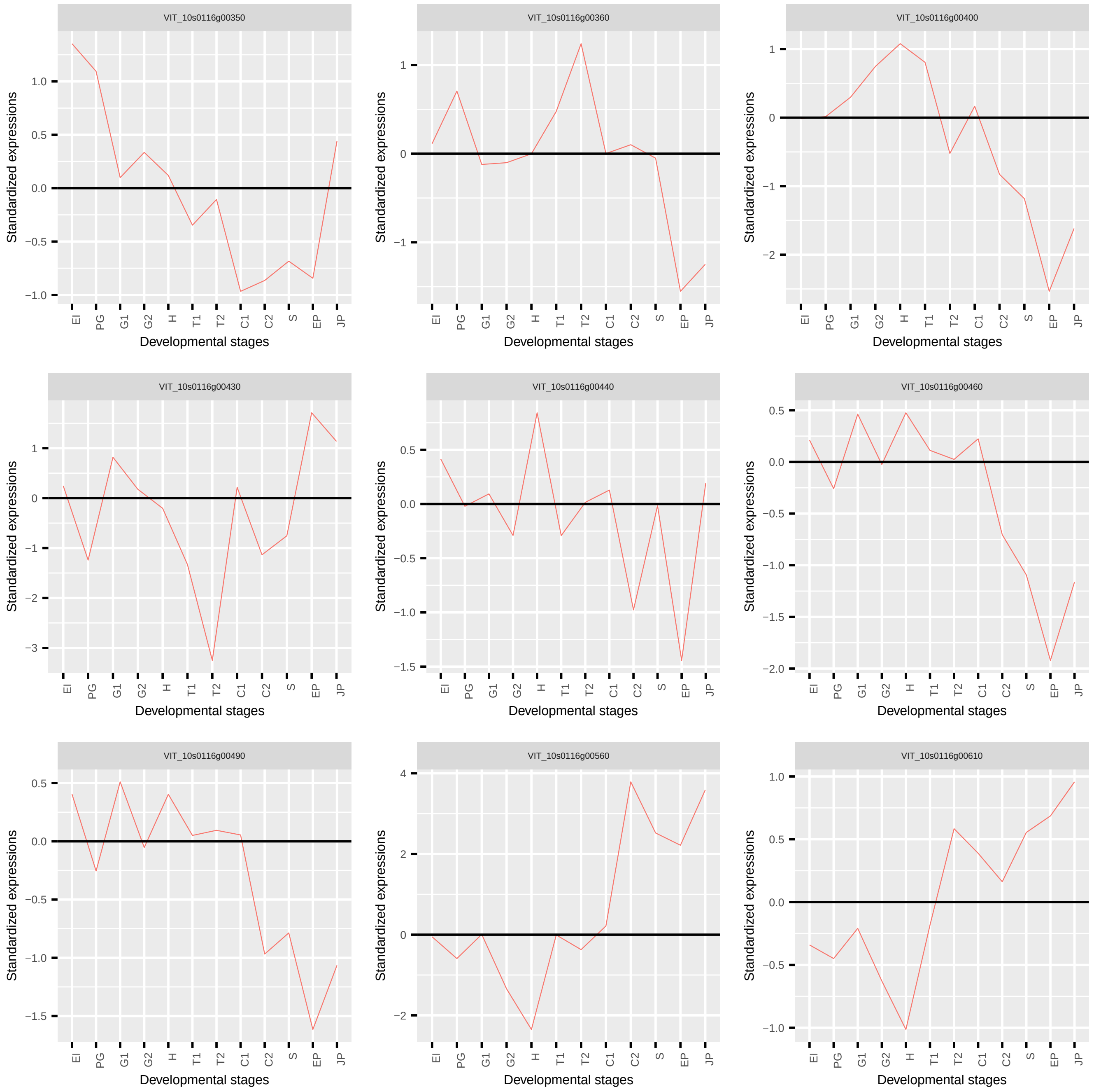
VIT_10s0116g00350
VIT_10s0116g00360
VIT_10s0116g00400
VIT_10s0116g00430
VIT_10s0116g00440
VIT_10s0116g00460
VIT_10s0116g00490
VIT_10s0116g00560
VIT_10s0116g00610
Standardized expressions
Developmental stages
EI
PG
G1
G2
H
T1
T2
C1
C2
S
EP
JP

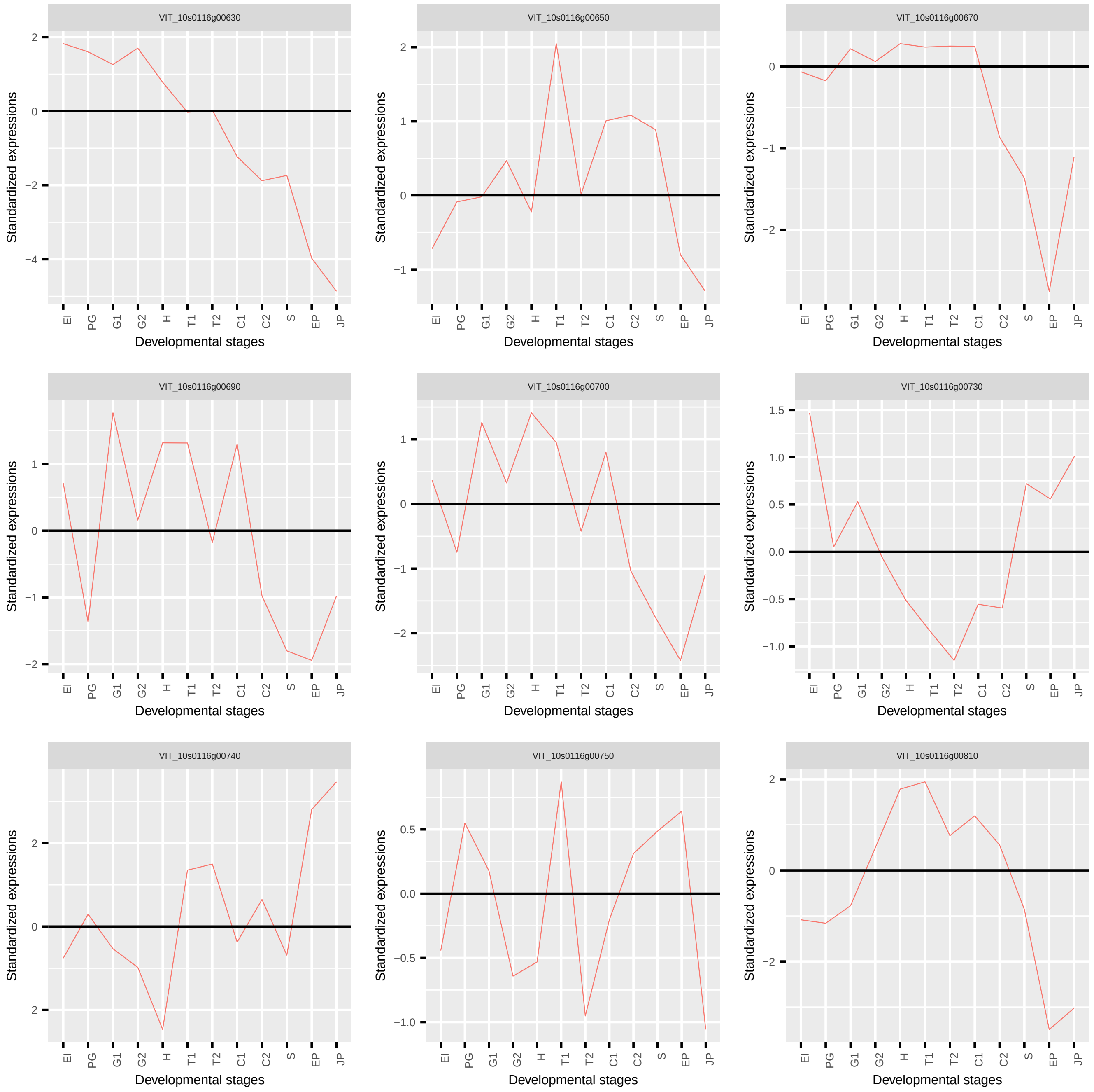
VIT_10s0116g00630
VIT_10s0116g00650
VIT_10s0116g00670
VIT_10s0116g00690
VIT_10s0116g00700
VIT_10s0116g00730
VIT_10s0116g00740
VIT_10s0116g00750
VIT_10s0116g00810
Standardized expressions
Developmental stages
EI PG G1 G2 H T1 T2 C1 C2 S EP JP

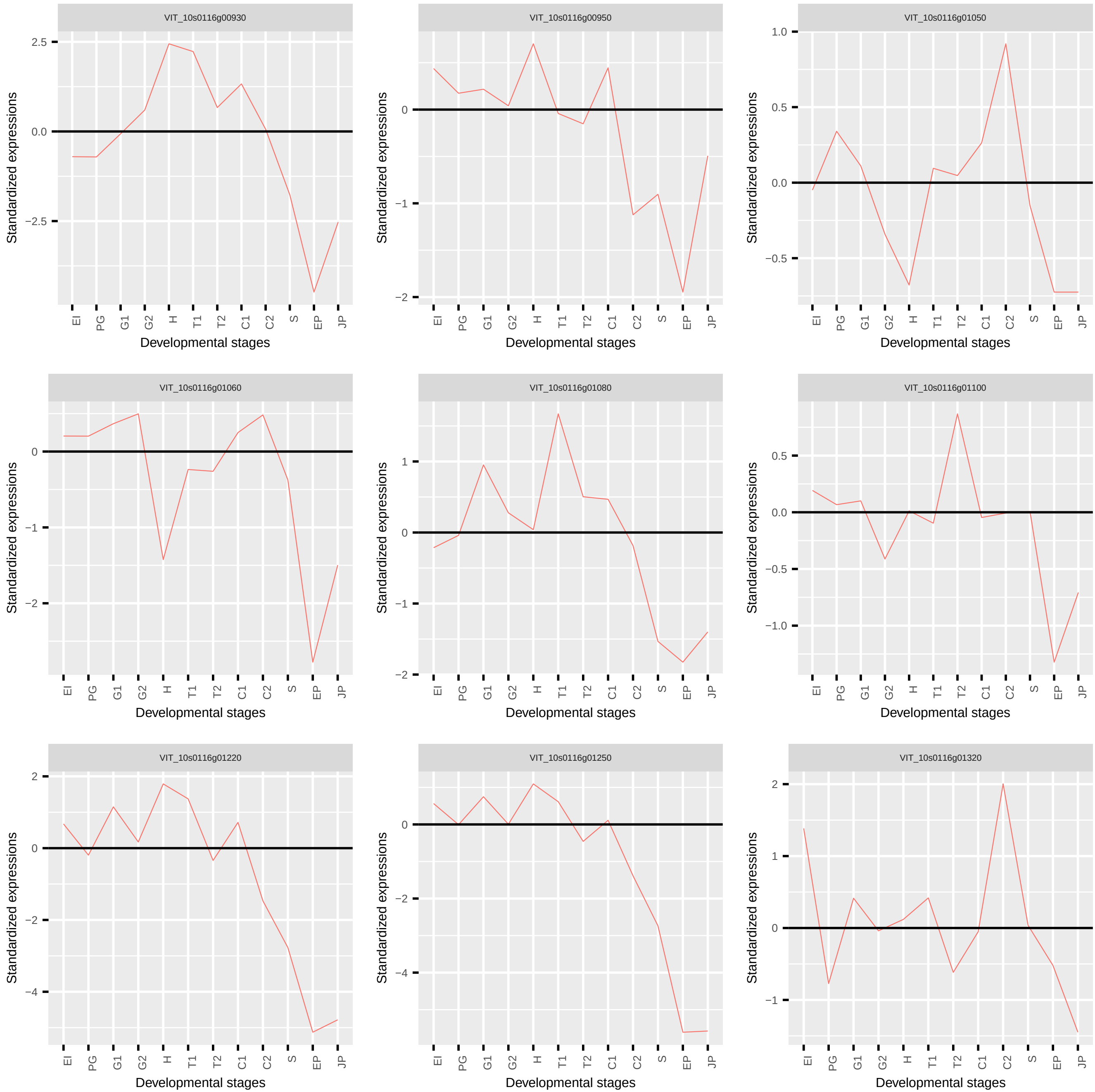
VIT_10s0116g00930
2.5
0.0
−2.5
VIT_10s0116g00950
0
−1
−2
VIT_10s0116g01050
1.0
0.5
0.0
−0.5
VIT_10s0116g01060
0
−1
−2
VIT_10s0116g01080
1
0
−1
−2
VIT_10s0116g01100
0.5
0.0
−0.5
−1.0
VIT_10s0116g01220
2
0
−2
−4
VIT_10s0116g01250
0
−2
−4
VIT_10s0116g01320
2
1
0
−1
Standardized expressions
EI PG G1 G2 H T1 T2 C1 C2 S EP JP
Developmental stages

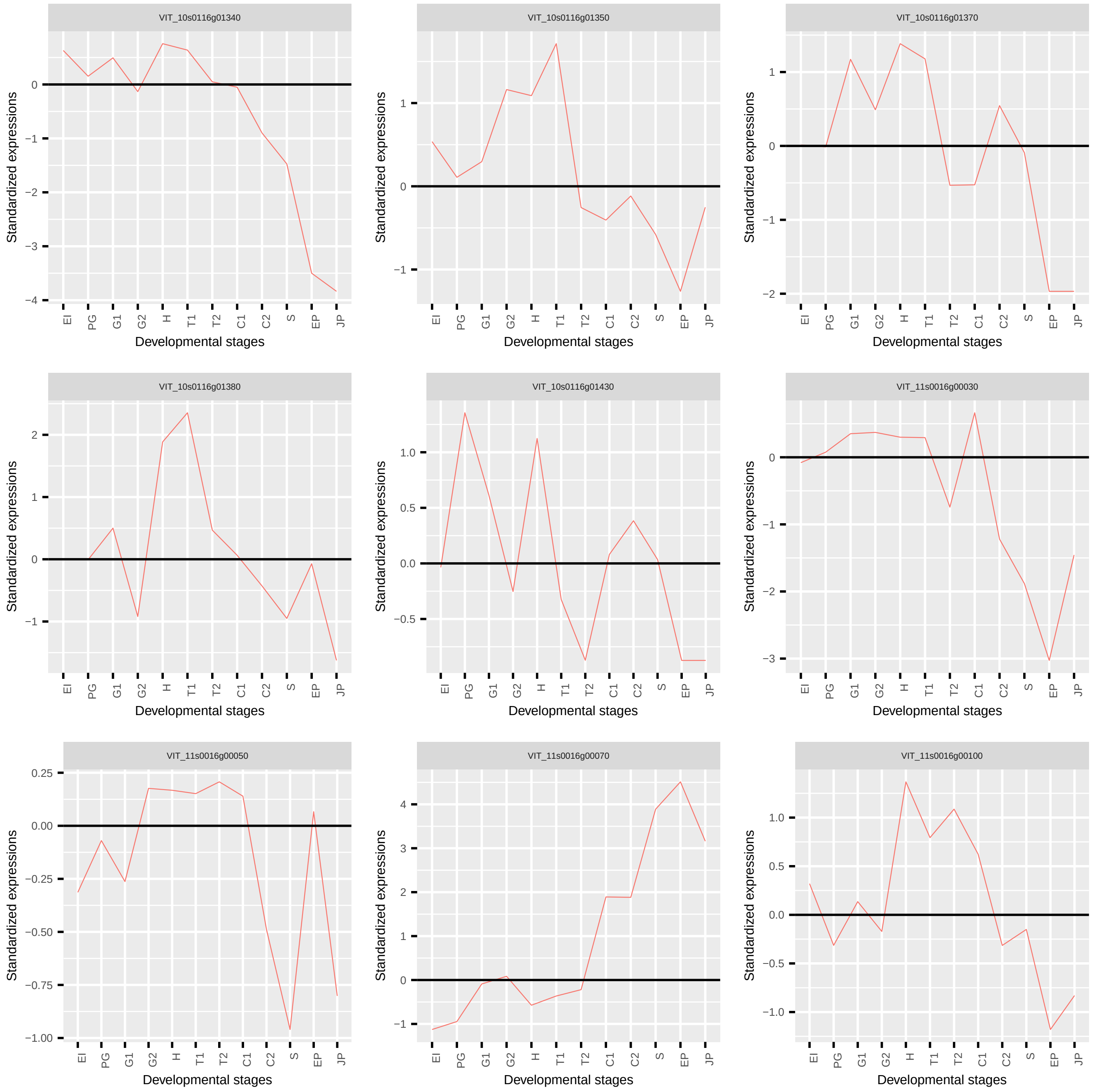
VIT_10s0116g01340
VIT_10s0116g01350
VIT_10s0116g01370
VIT_10s0116g01380
VIT_10s0116g01430
VIT_11s0016g00030
VIT_11s0016g00050
VIT_11s0016g00070
VIT_11s0016g00100
Standardized expressions
Developmental stages
EI PG G1 G2 H T1 T2 C1 C2 S EP JP

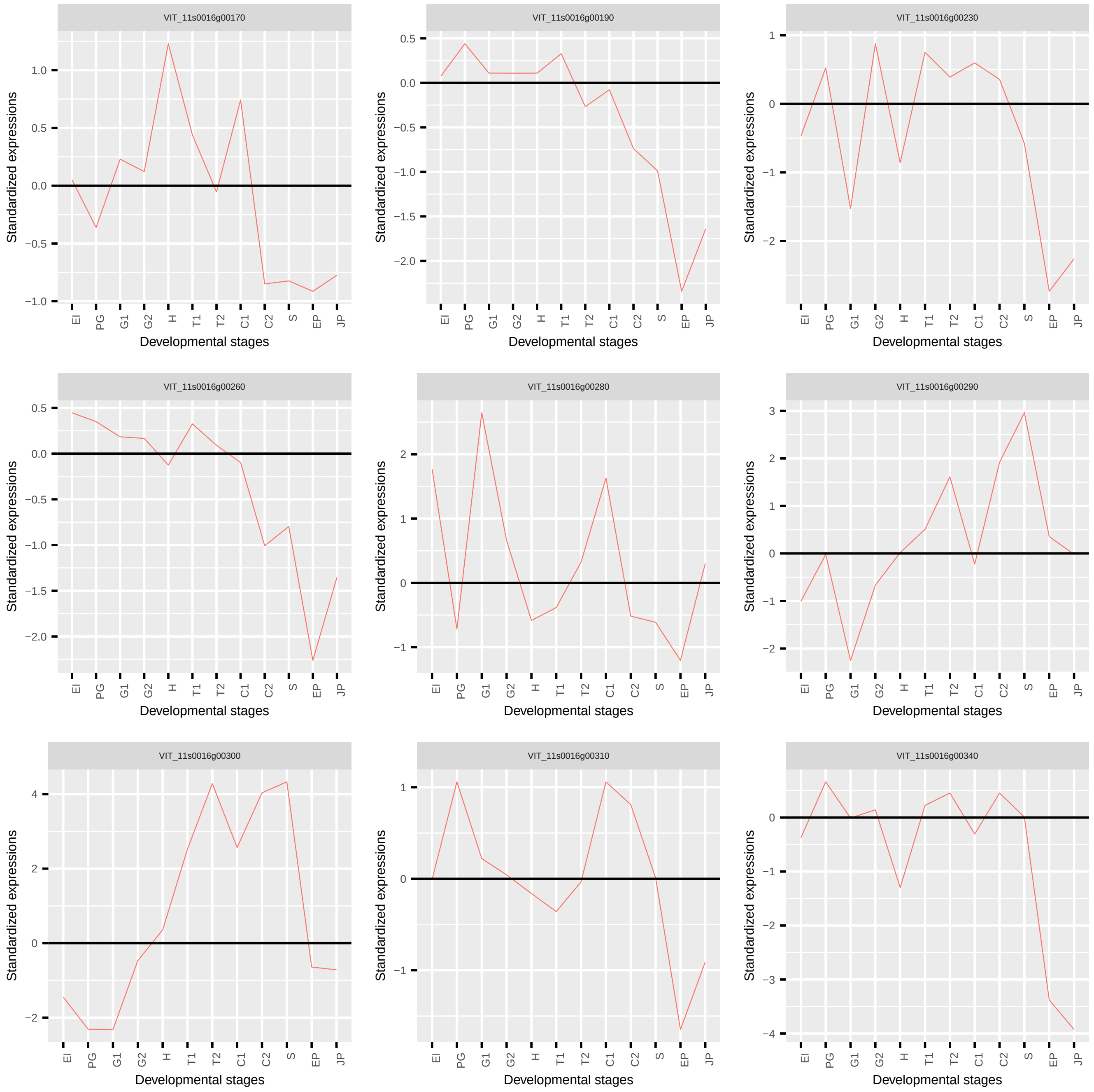
VIT_11s0016g00170
VIT_11s0016g00190
VIT_11s0016g00230
VIT_11s0016g00260
VIT_11s0016g00280
VIT_11s0016g00290
VIT_11s0016g00300
VIT_11s0016g00310
VIT_11s0016g00340
Standardized expressions
Developmental stages
EI PG G1 G2 H T1 T2 C1 C2 S EP JP

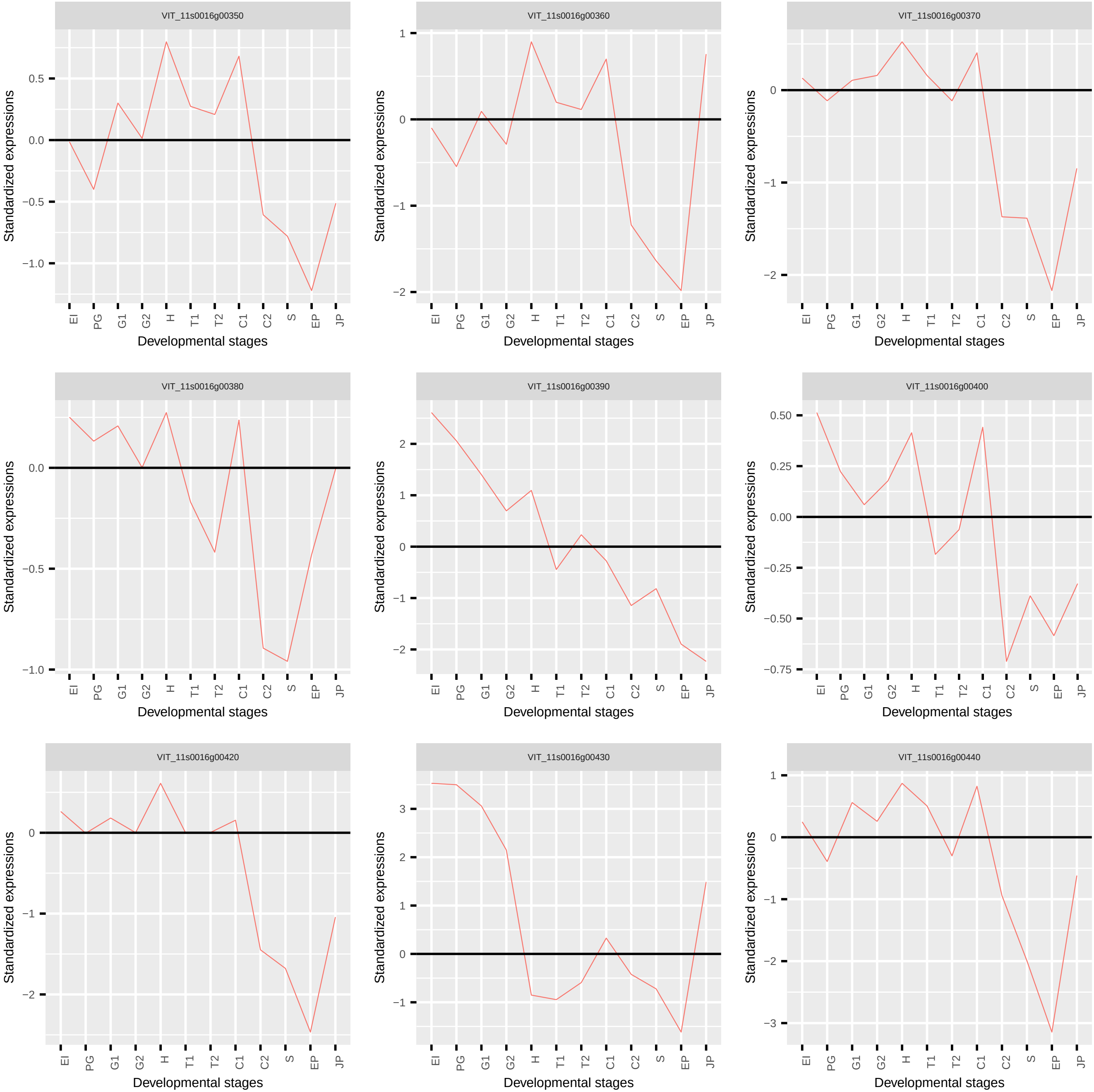

VIT_11s0016g00350
VIT_11s0016g00360
VIT_11s0016g00370
VIT_11s0016g00380
VIT_11s0016g00390
VIT_11s0016g00400
VIT_11s0016g00420
VIT_11s0016g00430
VIT_11s0016g00440
Standardized expressions
Developmental stages
EI PG G1 G2 H T1 T2 C1 C2 S EP JP

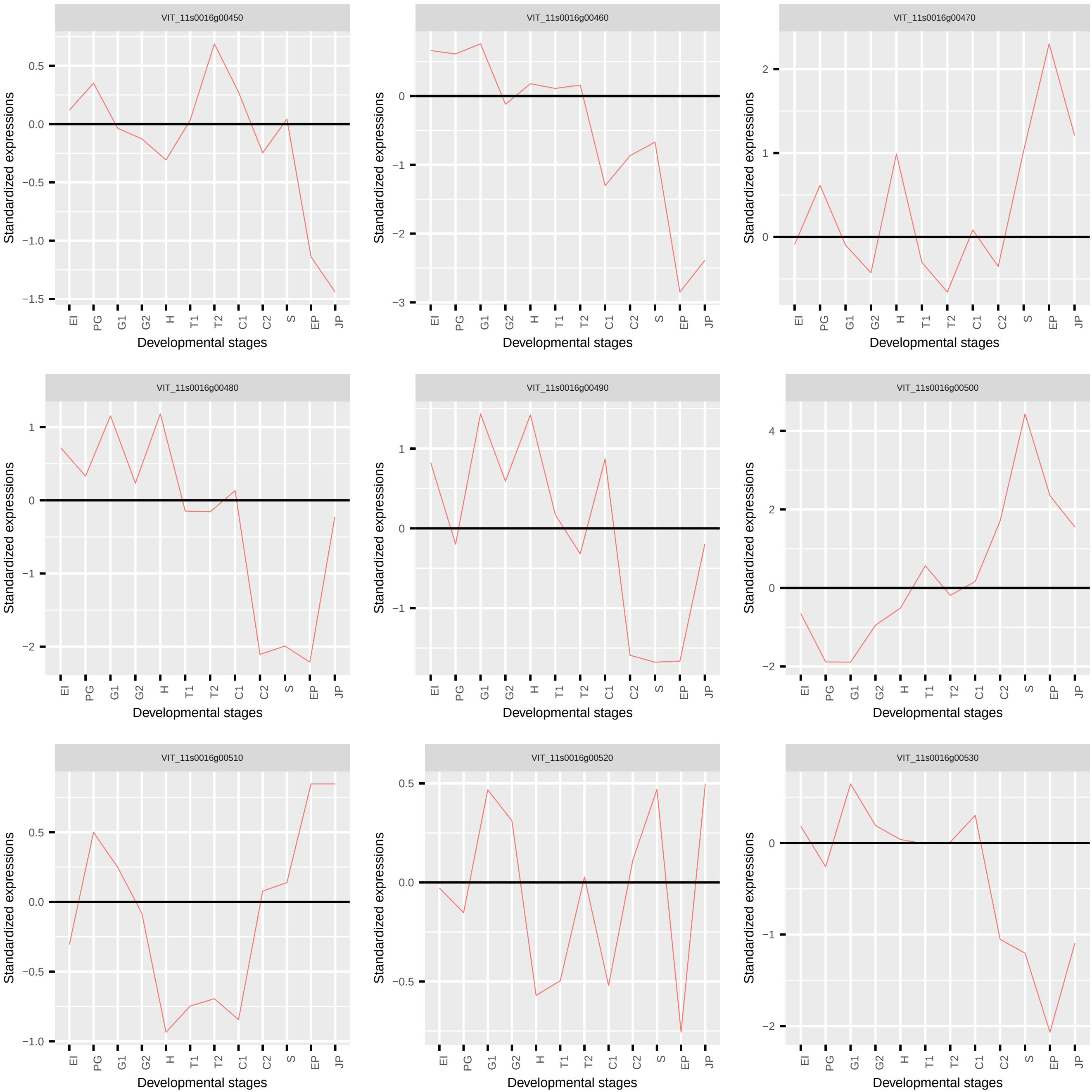
VIT_11s0016g00450
VIT_11s0016g00460
VIT_11s0016g00470
VIT_11s0016g00480
VIT_11s0016g00490
VIT_11s0016g00500
VIT_11s0016g00510
VIT_11s0016g00520
VIT_11s0016g00530
Standardized expressions
Developmental stages
EI
PG
G1
G2
H
T1
T2
C1
C2
S
EP
JP
0.5
0.0
−0.5
−1.0
−1.5
0
−1
−2
−3
2
1
4

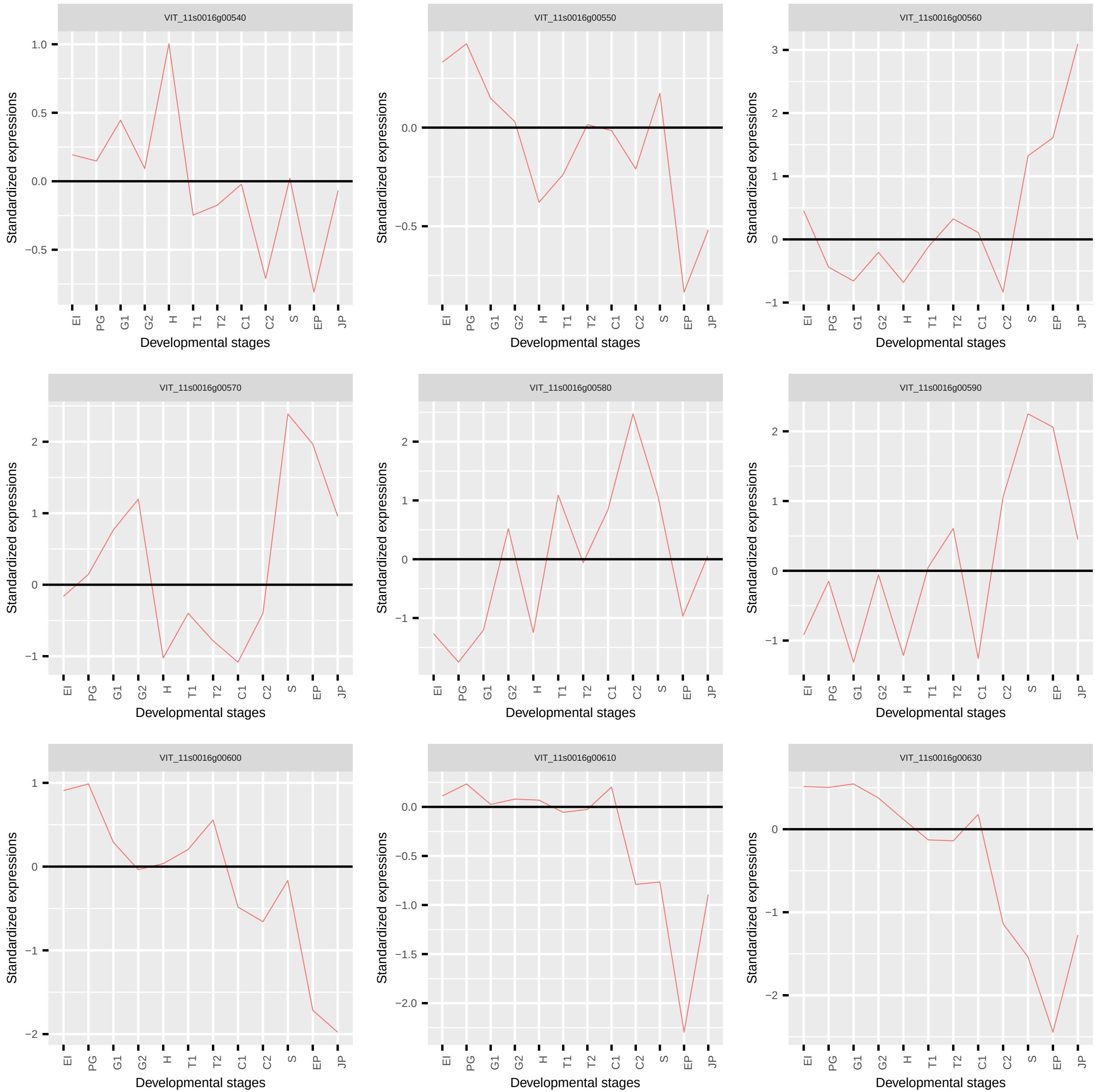

VIT_11s0016g00540
VIT_11s0016g00550
VIT_11s0016g00560
VIT_11s0016g00570
VIT_11s0016g00580
VIT_11s0016g00590
VIT_11s0016g00600
VIT_11s0016g00610
VIT_11s0016g00630
Standardized expressions
Developmental stages
EI PG G1 G2 H T1 T2 C1 C2 S EP JP

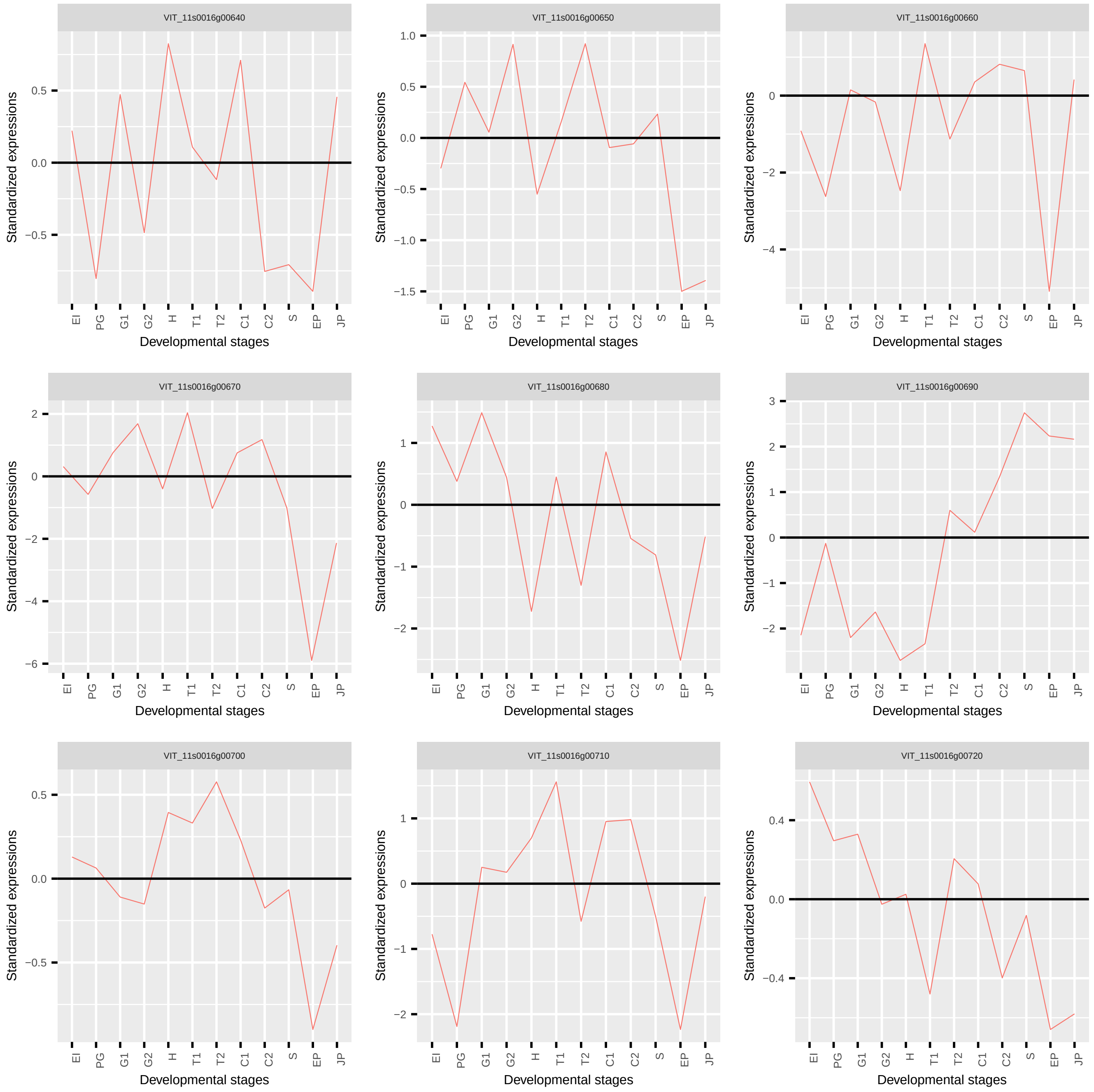
VIT_11s0016g00640
VIT_11s0016g00650
VIT_11s0016g00660
VIT_11s0016g00670
VIT_11s0016g00680
VIT_11s0016g00690
VIT_11s0016g00700
VIT_11s0016g00710
VIT_11s0016g00720
Standardized expressions
Developmental stages
EI PG G1 G2 H T1 T2 C1 C2 S EP JP

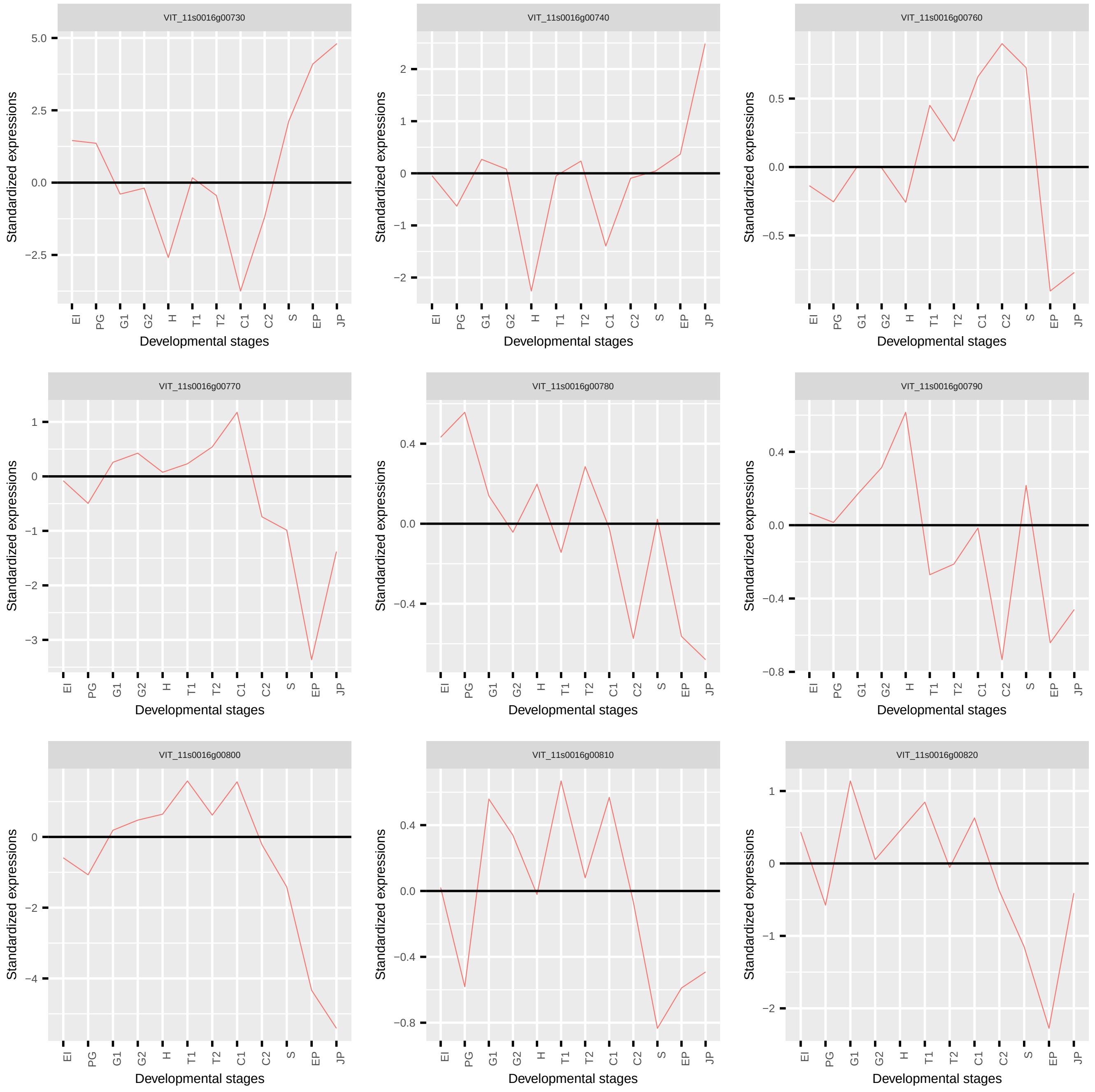
VIT_11s0016g00730
VIT_11s0016g00740
VIT_11s0016g00760
VIT_11s0016g00770
VIT_11s0016g00780
VIT_11s0016g00790
VIT_11s0016g00800
VIT_11s0016g00810
VIT_11s0016g00820
Standardized expressions
Developmental stages
EI
PG
G1
G2
H
T1
T2
C1
C2
S
EP
JP
5.0
2.5
0.0
−2.5
2
1
0
−1
−2
0.5
−0.5
−3
0.4
−0.4
−0.8
−4

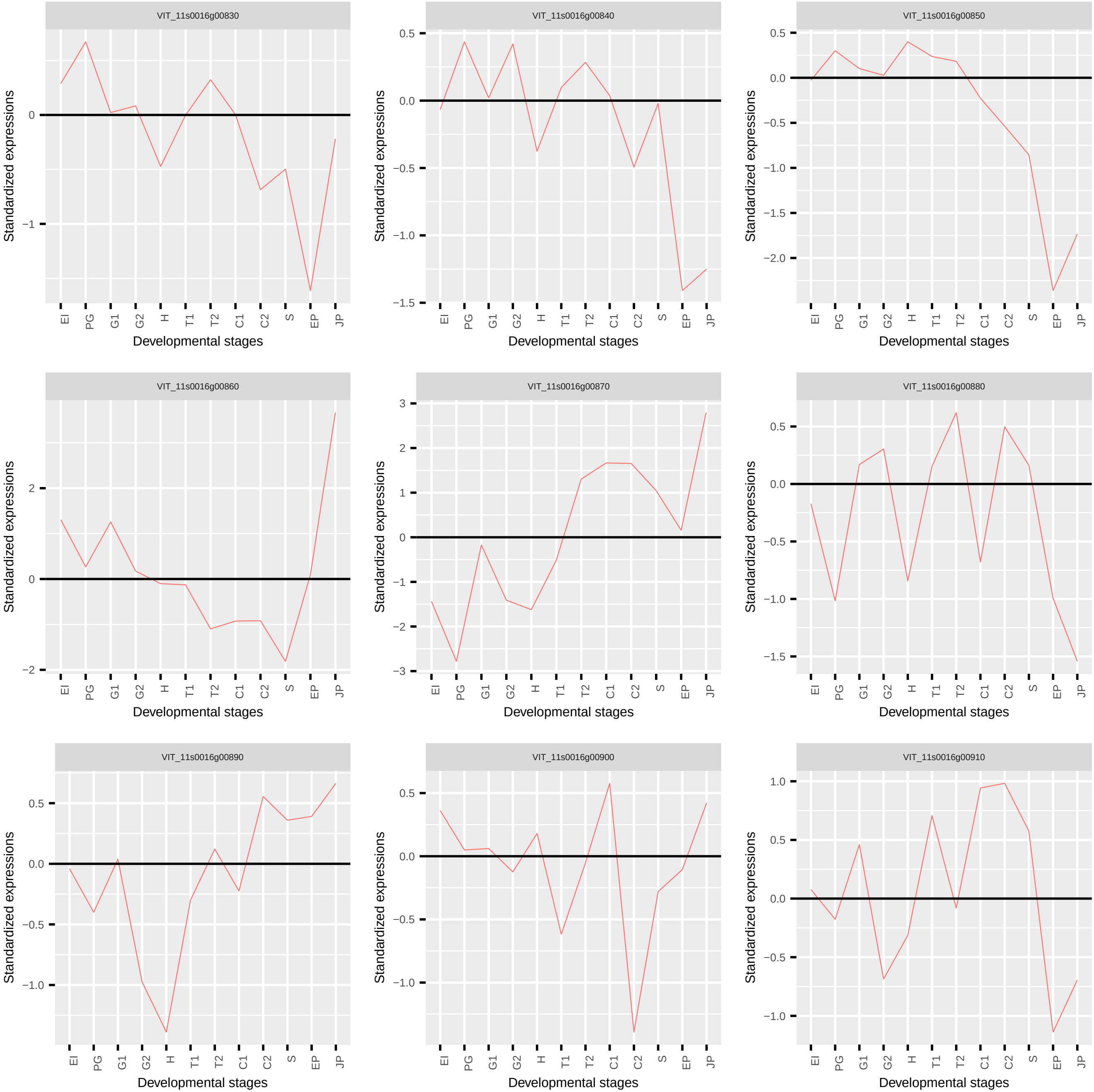
VIT_11s0016g00830
VIT_11s0016g00840
VIT_11s0016g00850
VIT_11s0016g00860
VIT_11s0016g00870
VIT_11s0016g00880
VIT_11s0016g00890
VIT_11s0016g00900
VIT_11s0016g00910
Standardized expressions
Developmental stages
EI PG G1 G2 H T1 T2 C1 C2 S EP JP

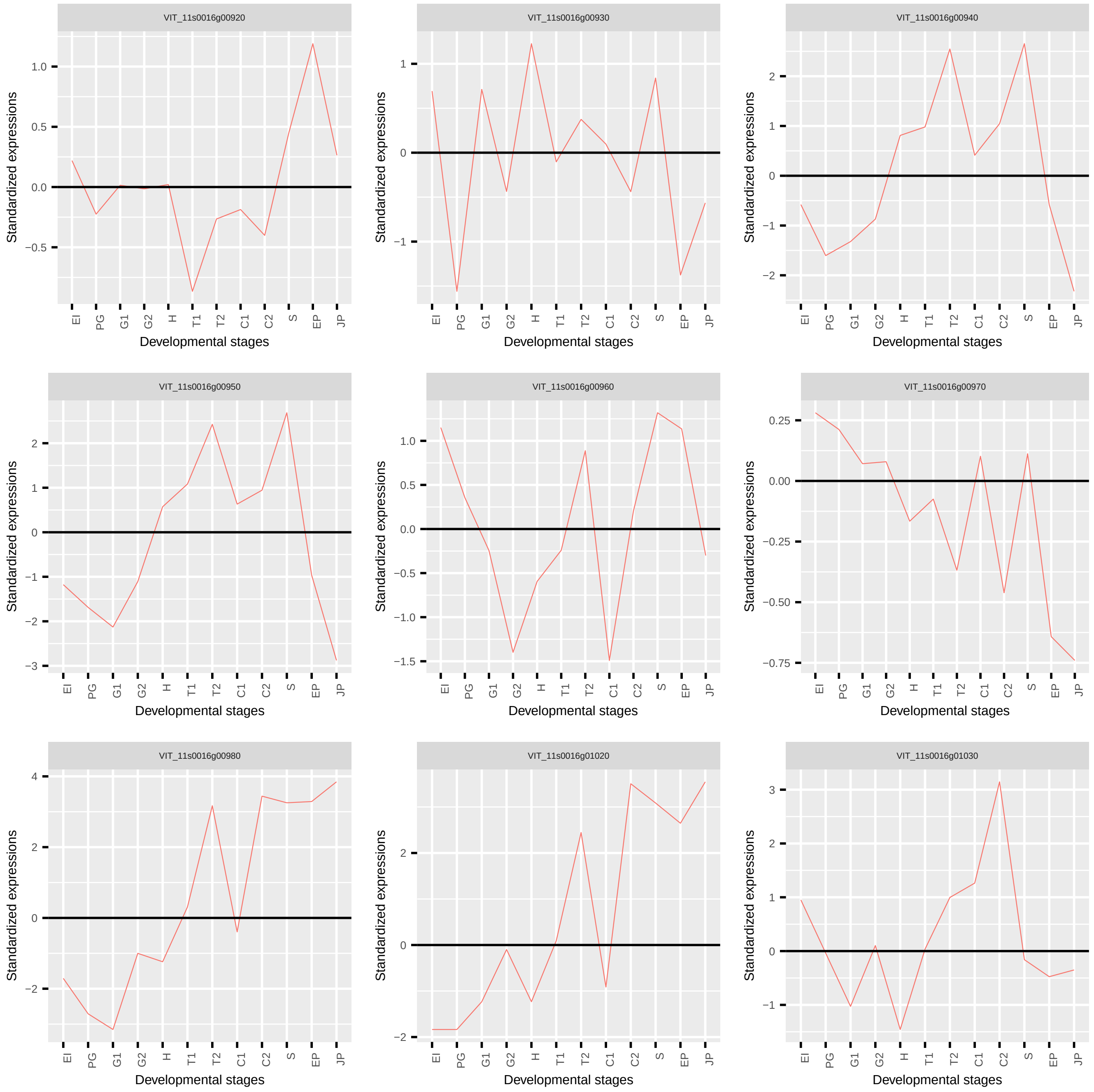
VIT_11s0016g00920
VIT_11s0016g00930
VIT_11s0016g00940
VIT_11s0016g00950
VIT_11s0016g00960
VIT_11s0016g00970
VIT_11s0016g00980
VIT_11s0016g01020
VIT_11s0016g01030
Standardized expressions
Developmental stages
EI
PG
G1
G2
H
T1
T2
C1
C2
S
EP
JP

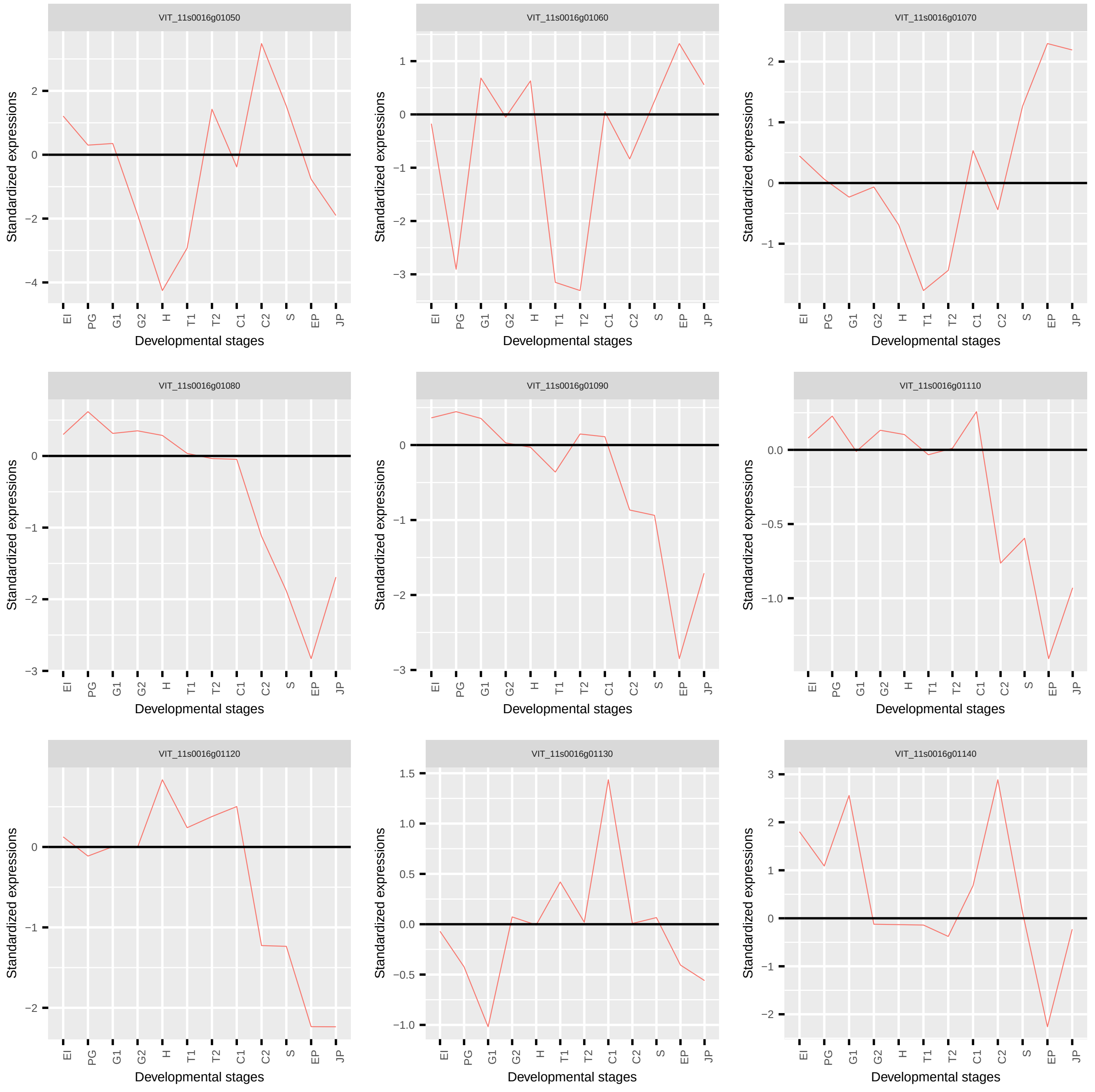
VIT_11s0016g01050
VIT_11s0016g01060
VIT_11s0016g01070
VIT_11s0016g01080
VIT_11s0016g01090
VIT_11s0016g01110
VIT_11s0016g01120
VIT_11s0016g01130
VIT_11s0016g01140
Standardized expressions
Developmental stages
EI PG G1 G2 H T1 T2 C1 C2 S EP JP

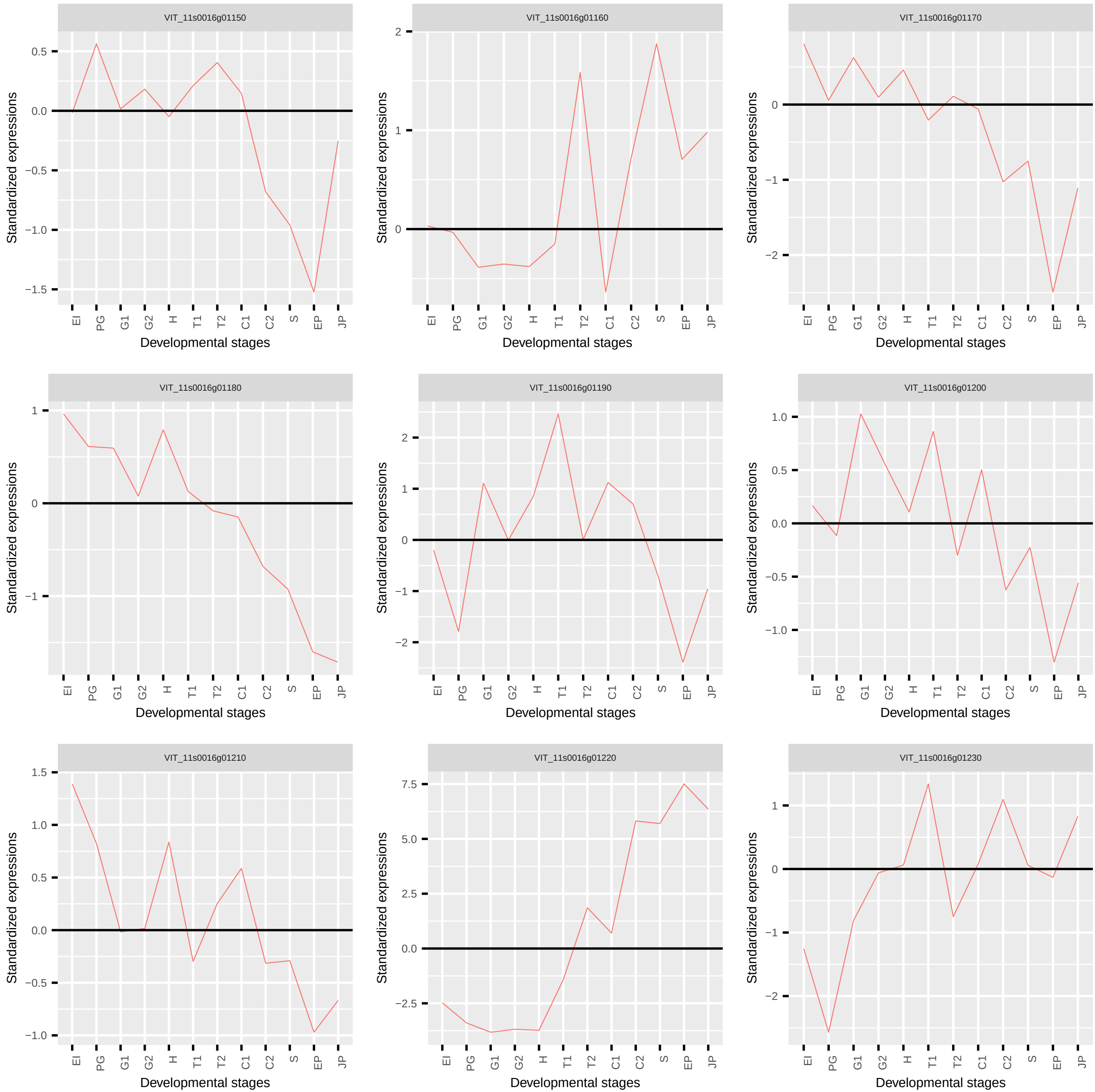
VIT_11s0016g01150
VIT_11s0016g01160
VIT_11s0016g01170
VIT_11s0016g01180
VIT_11s0016g01190
VIT_11s0016g01200
VIT_11s0016g01210
VIT_11s0016g01220
VIT_11s0016g01230
Standardized expressions
Developmental stages
EI PG G1 G2 H T1 T2 C1 C2 S EP JP

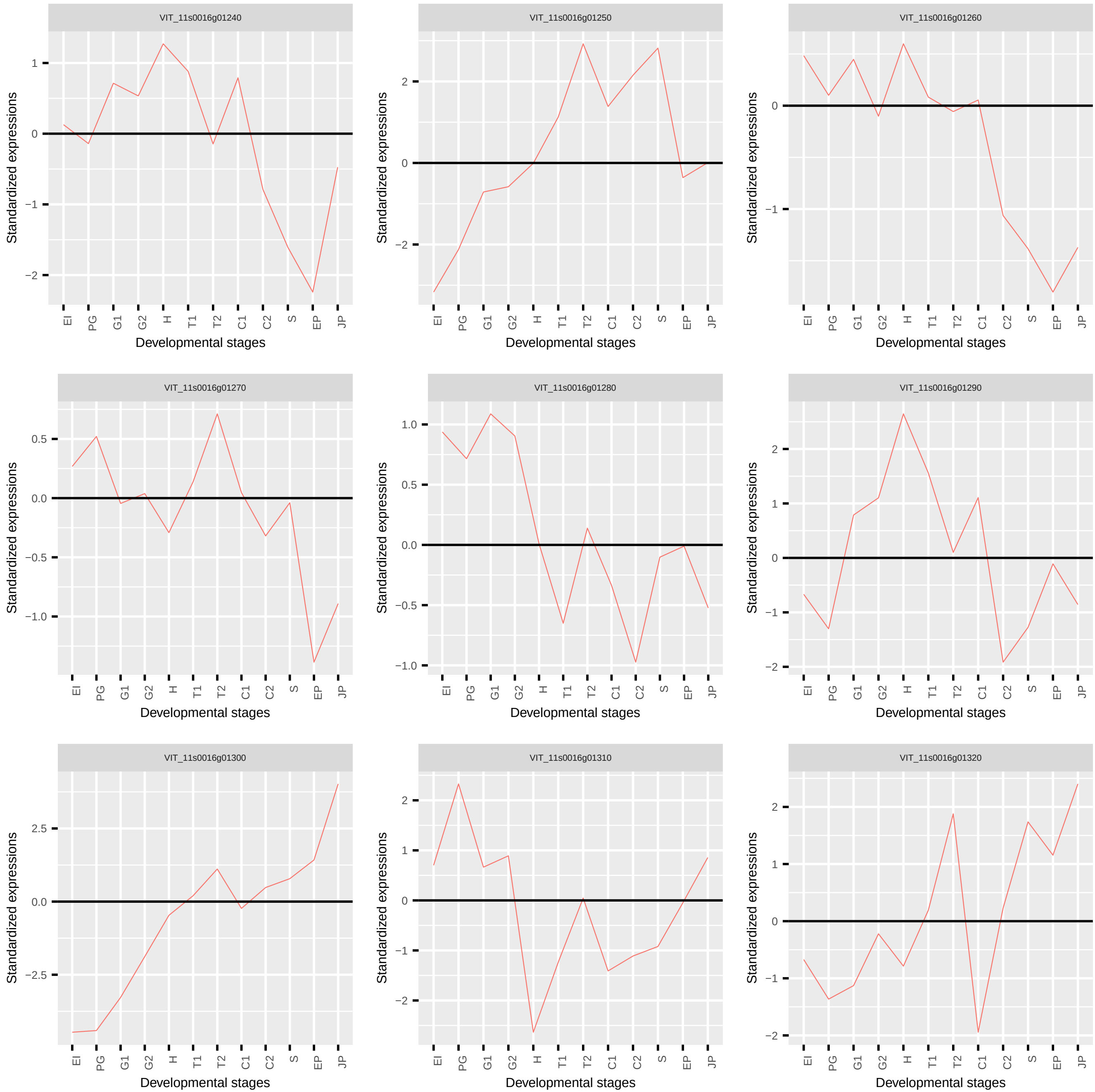
VIT_11s0016g01240
VIT_11s0016g01250
VIT_11s0016g01260
VIT_11s0016g01270
VIT_11s0016g01280
VIT_11s0016g01290
VIT_11s0016g01300
VIT_11s0016g01310
VIT_11s0016g01320
Standardized expressions
Developmental stages
EI PG G1 G2 H T1 T2 C1 C2 S EP JP

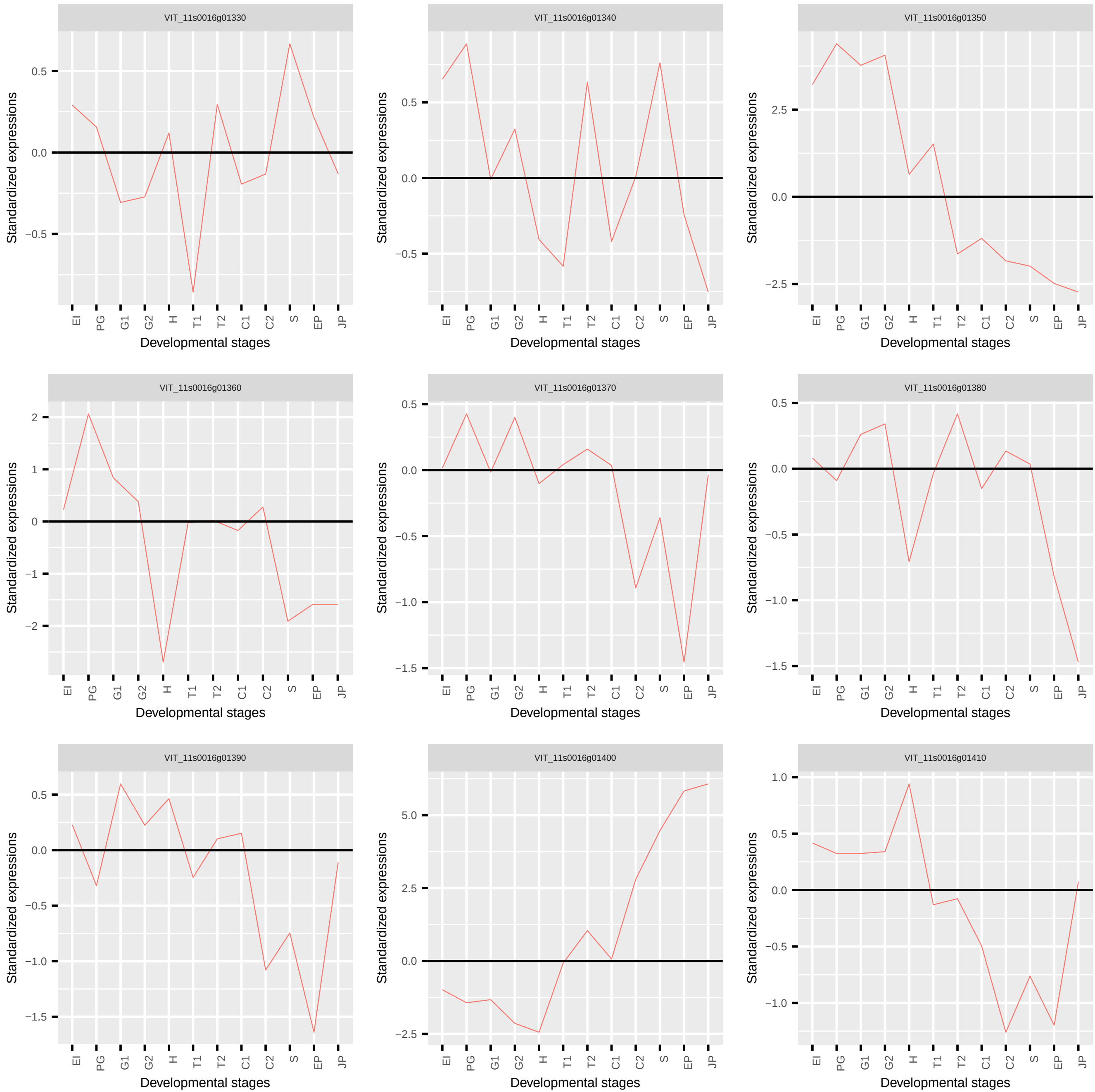
VIT_11s0016g01330
VIT_11s0016g01340
VIT_11s0016g01350
VIT_11s0016g01360
VIT_11s0016g01370
VIT_11s0016g01380
VIT_11s0016g01390
VIT_11s0016g01400
VIT_11s0016g01410
Standardized expressions
Developmental stages
EI PG G1 G2 H T1 T2 C1 C2 S EP JP

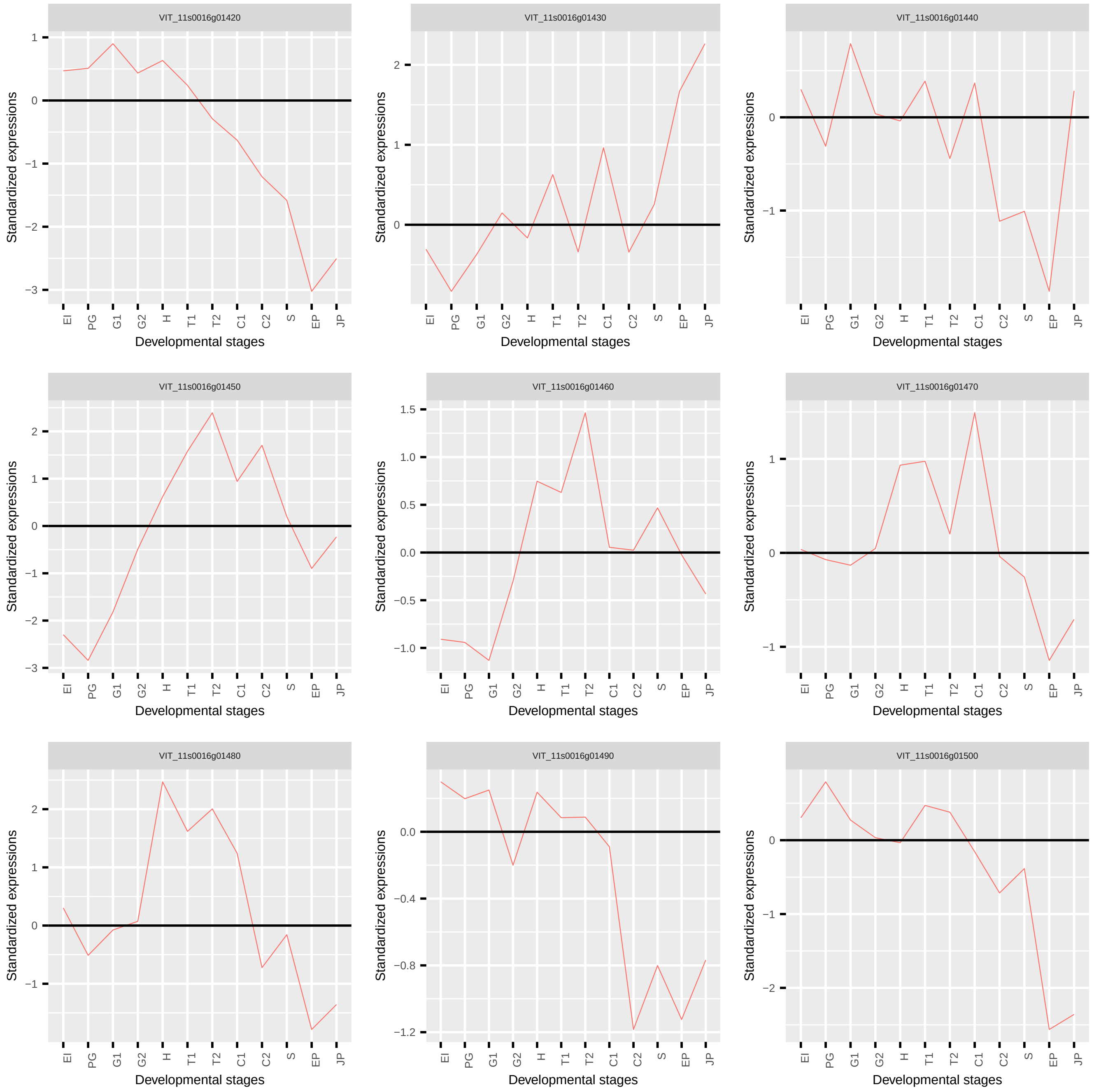
VIT_11s0016g01420
VIT_11s0016g01430
VIT_11s0016g01440
VIT_11s0016g01450
VIT_11s0016g01460
VIT_11s0016g01470
VIT_11s0016g01480
VIT_11s0016g01490
VIT_11s0016g01500
Standardized expressions
Developmental stages
EI PG G1 G2 H T1 T2 C1 C2 S EP JP

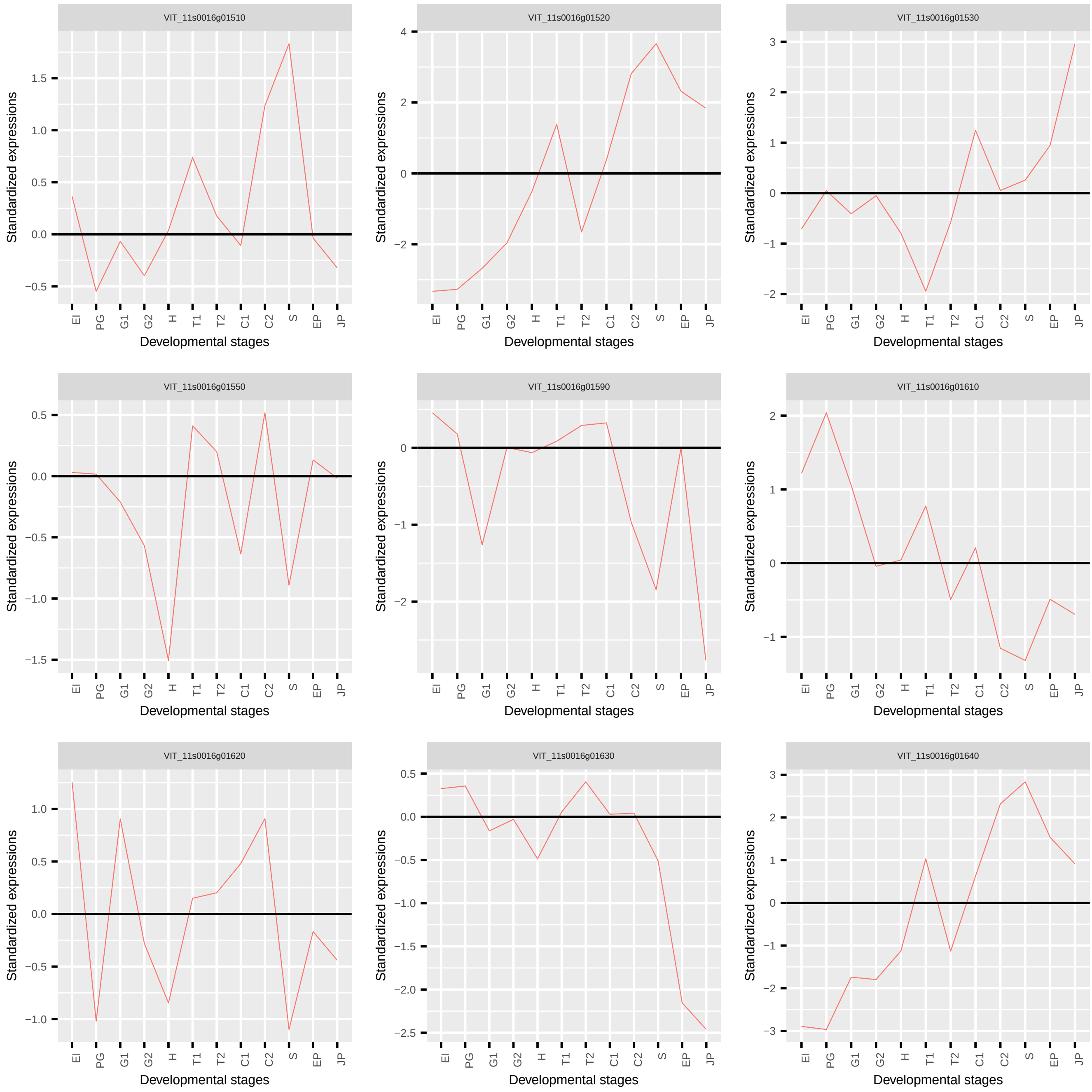
VIT_11s0016g01510
1.5
1.0
0.5
0.0
−0.5
Standardized expressions
EI PG G1 G2 H T1 T2 C1 C2 S EP JP
Developmental stages
VIT_11s0016g01520
4
2
0
−2
Standardized expressions
EI PG G1 G2 H T1 T2 C1 C2 S EP JP
Developmental stages
VIT_11s0016g01530
3
2
1
0
−1
−2
Standardized expressions
EI PG G1 G2 H T1 T2 C1 C2 S EP JP
Developmental stages
VIT_11s0016g01550
0.5
0.0
−0.5
−1.0
−1.5
Standardized expressions
EI PG G1 G2 H T1 T2 C1 C2 S EP JP
Developmental stages
VIT_11s0016g01590
0
−1
−2
Standardized expressions
EI PG G1 G2 H T1 T2 C1 C2 S EP JP
Developmental stages
VIT_11s0016g01610
2
1
0
−1
Standardized expressions
EI PG G1 G2 H T1 T2 C1 C2 S EP JP
Developmental stages
VIT_11s0016g01620
1.0
0.5
0.0
−0.5
−1.0
Standardized expressions
EI PG G1 G2 H T1 T2 C1 C2 S EP JP
Developmental stages
VIT_11s0016g01630
0.5
0.0
−0.5
−1.0
−1.5
−2.0
−2.5
Standardized expressions
EI PG G1 G2 H T1 T2 C1 C2 S EP JP
Developmental stages
VIT_11s0016g01640
3
2
1
0
−1
−2
−3
Standardized expressions
EI PG G1 G2 H T1 T2 C1 C2 S EP JP
Developmental stages

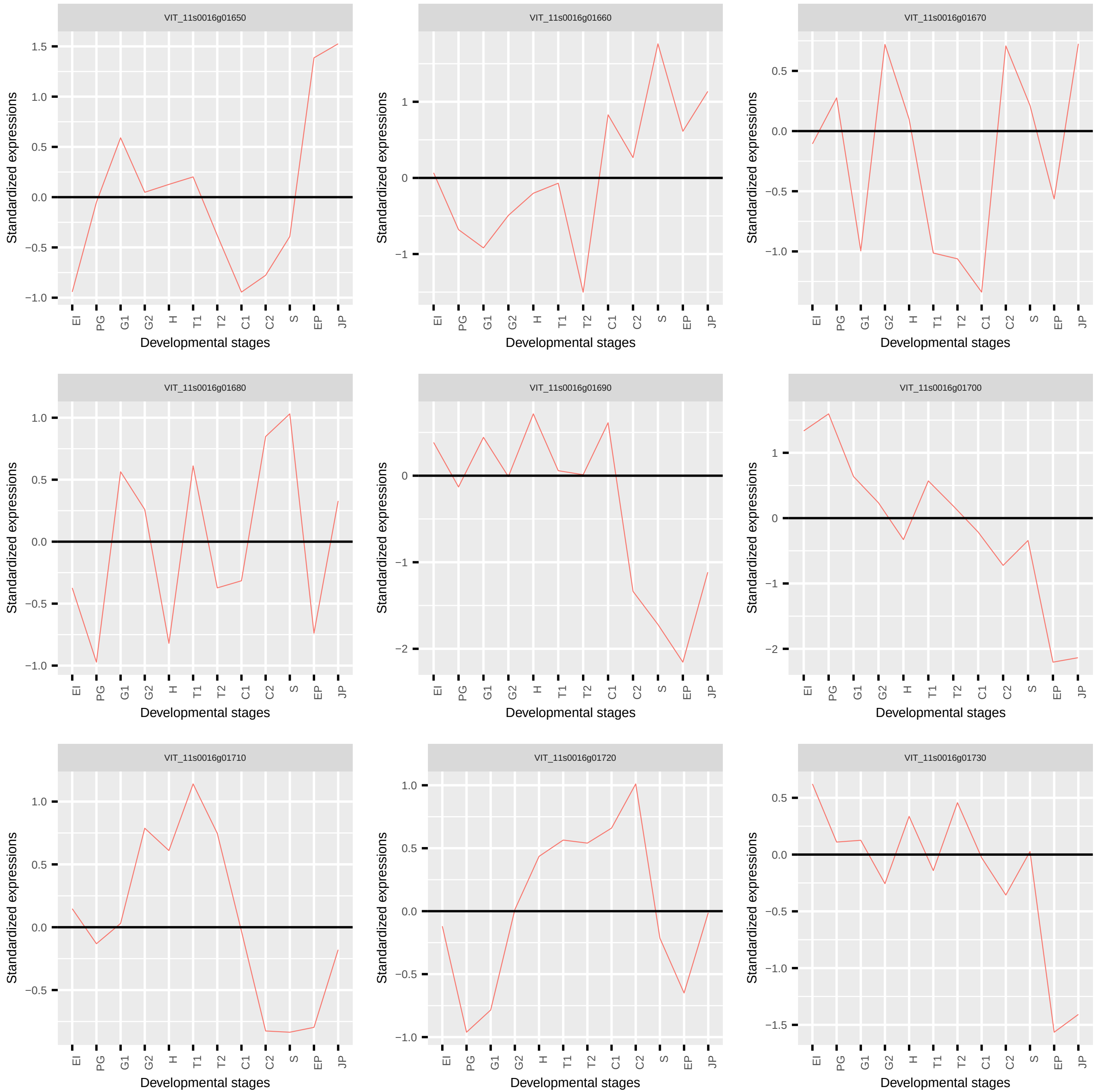
VIT_11s0016g01650
VIT_11s0016g01660
VIT_11s0016g01670
VIT_11s0016g01680
VIT_11s0016g01690
VIT_11s0016g01700
VIT_11s0016g01710
VIT_11s0016g01720
VIT_11s0016g01730
Standardized expressions
Developmental stages
EI PG G1 G2 H T1 T2 C1 C2 S EP JP

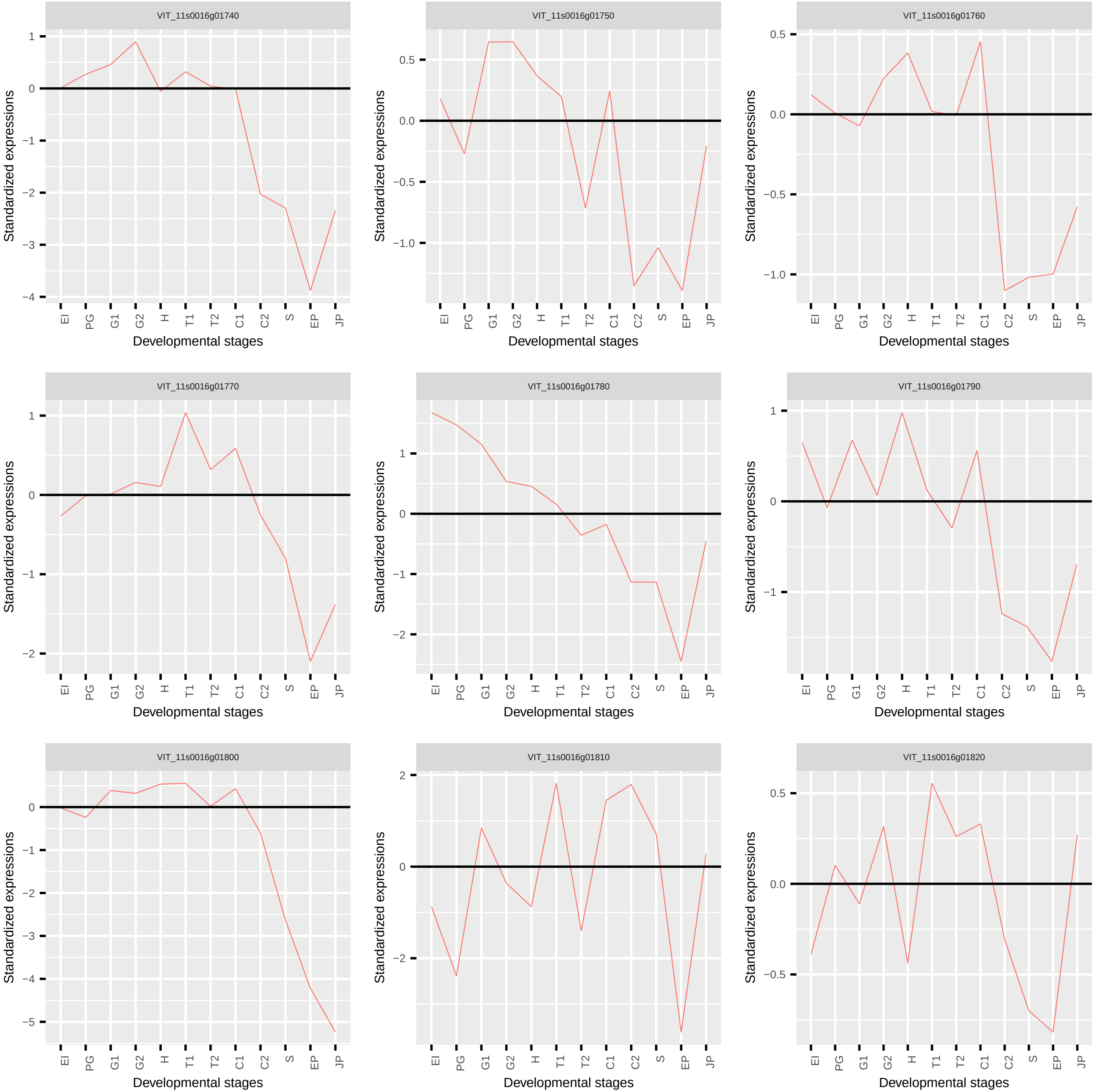

VIT_11s0016g01740
VIT_11s0016g01750
VIT_11s0016g01760
VIT_11s0016g01770
VIT_11s0016g01780
VIT_11s0016g01790
VIT_11s0016g01800
VIT_11s0016g01810
VIT_11s0016g01820
Standardized expressions
Developmental stages
EI PG G1 G2 H T1 T2 C1 C2 S EP JP

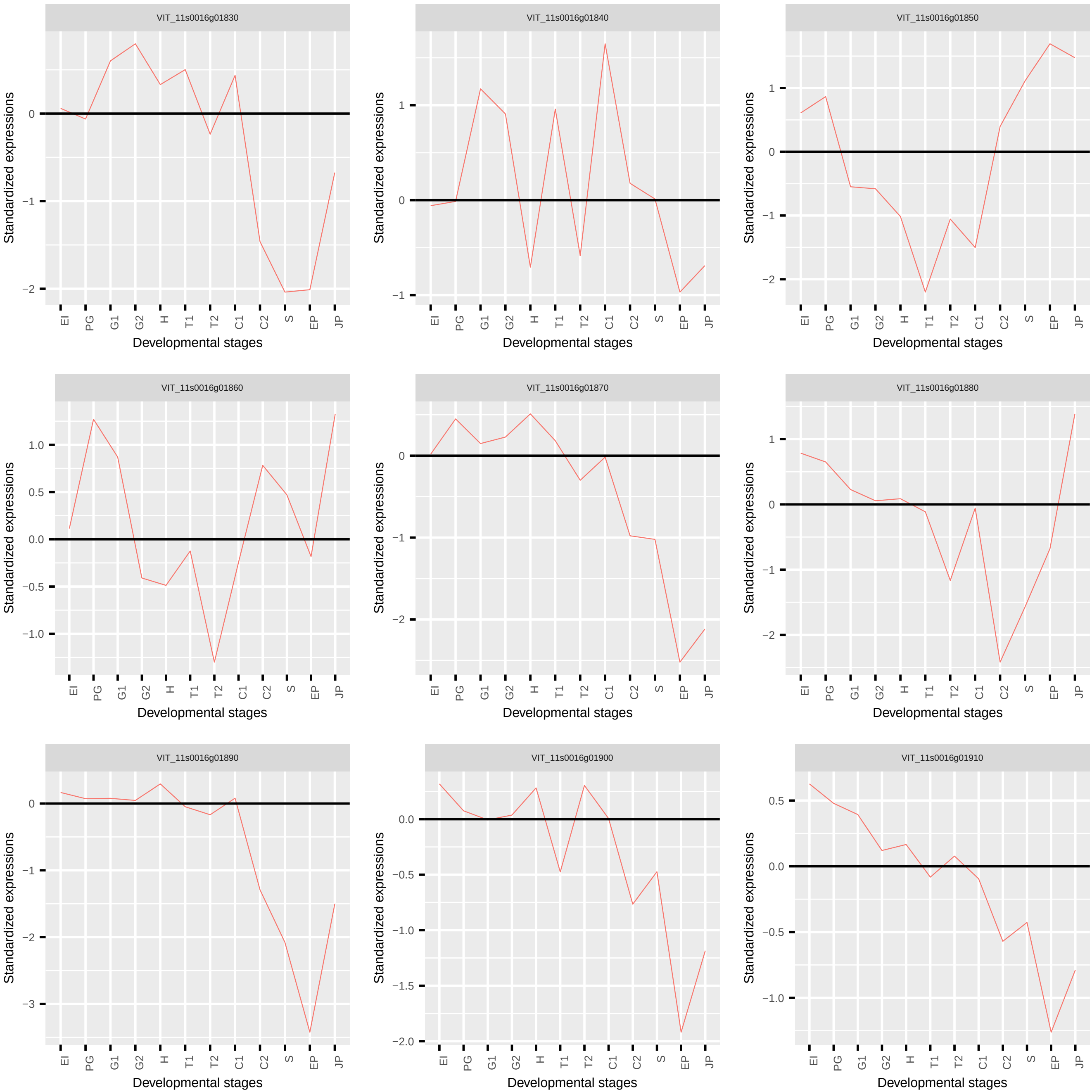
VIT_11s0016g01830
VIT_11s0016g01840
VIT_11s0016g01850
VIT_11s0016g01860
VIT_11s0016g01870
VIT_11s0016g01880
VIT_11s0016g01890
VIT_11s0016g01900
VIT_11s0016g01910
Standardized expressions
Developmental stages
EI
PG
G1
G2
H
T1
T2
C1
C2
S
EP
JP

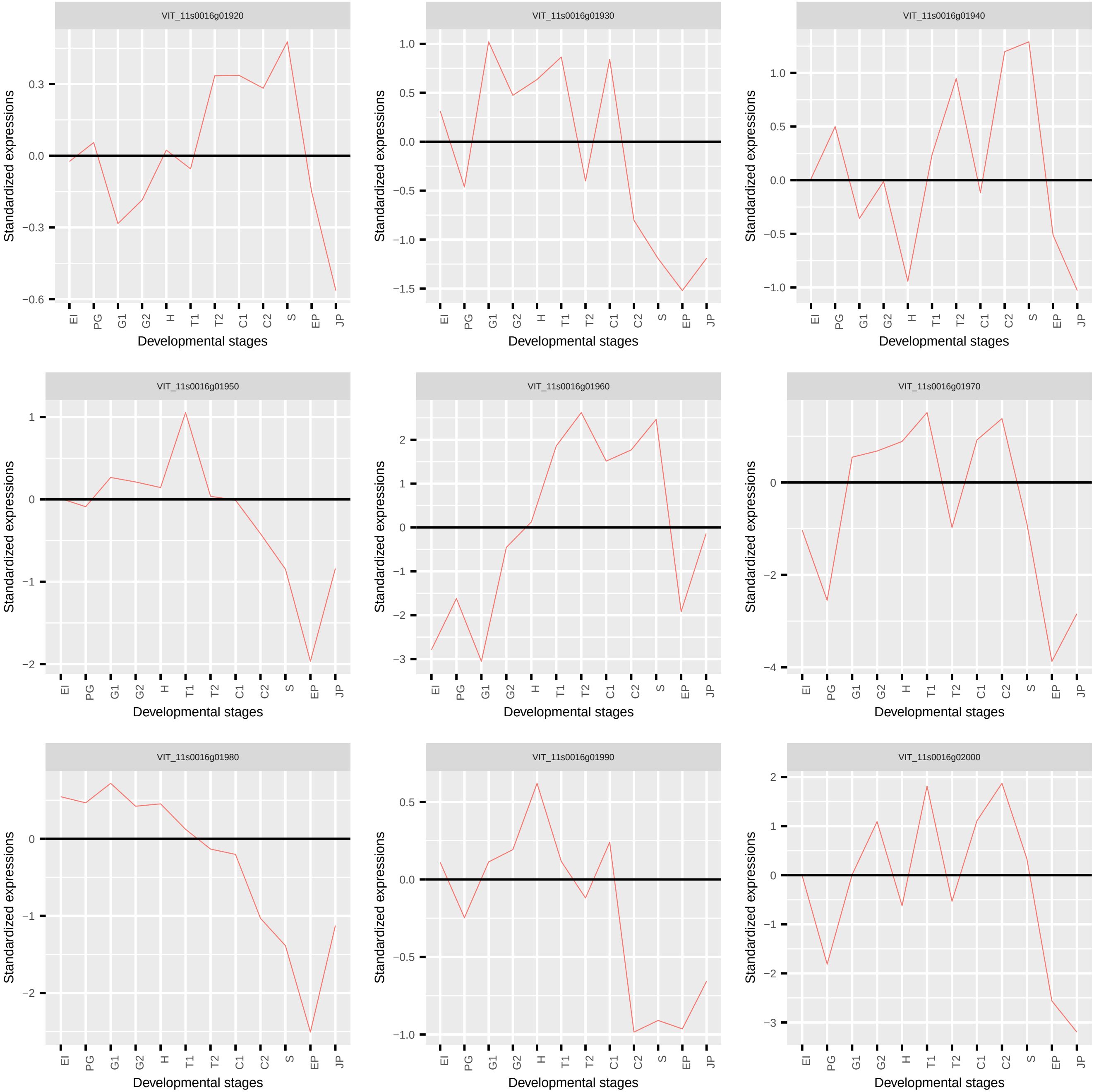

VIT_11s0016g01920
VIT_11s0016g01930
VIT_11s0016g01940
VIT_11s0016g01950
VIT_11s0016g01960
VIT_11s0016g01970
VIT_11s0016g01980
VIT_11s0016g01990
VIT_11s0016g02000
Standardized expressions
Developmental stages
EI PG G1 G2 H T1 T2 C1 C2 S EP JP
0.3 0.0 −0.3 −0.6
1.0 0.5 0.0 −0.5 −1.0 −1.5
1.0 0.5 0.0 −0.5 −1.0
1 0 −1 −2
2 1 0 −1 −2 −3
0 −2 −4
0 −1 −2
0.5 0.0 −0.5 −1.0
2 1 0 −1 −2 −3

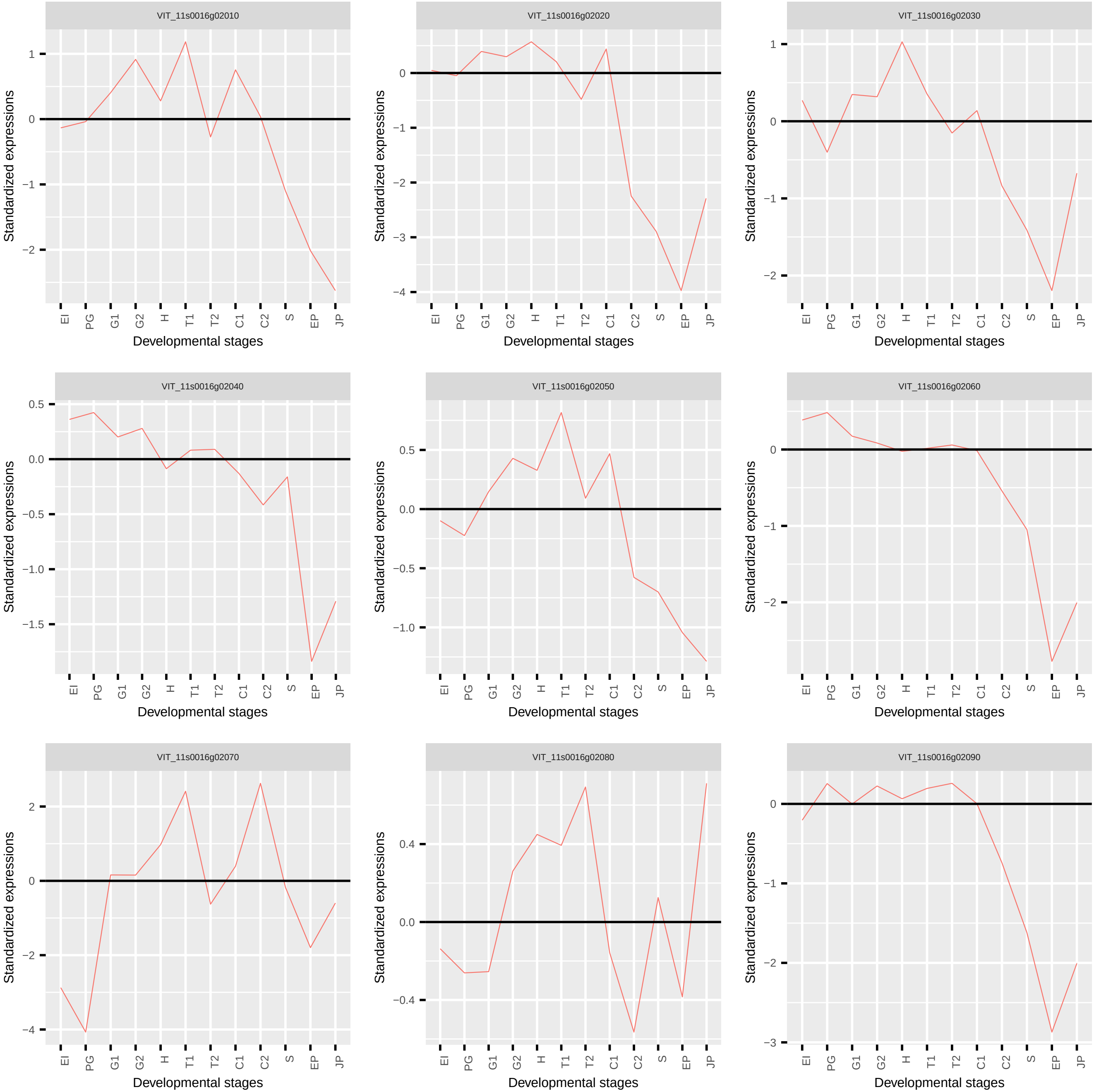

VIT_11s0016g02010
VIT_11s0016g02020
VIT_11s0016g02030
VIT_11s0016g02040
VIT_11s0016g02050
VIT_11s0016g02060
VIT_11s0016g02070
VIT_11s0016g02080
VIT_11s0016g02090
Standardized expressions
Developmental stages
EI PG G1 G2 H T1 T2 C1 C2 S EP JP

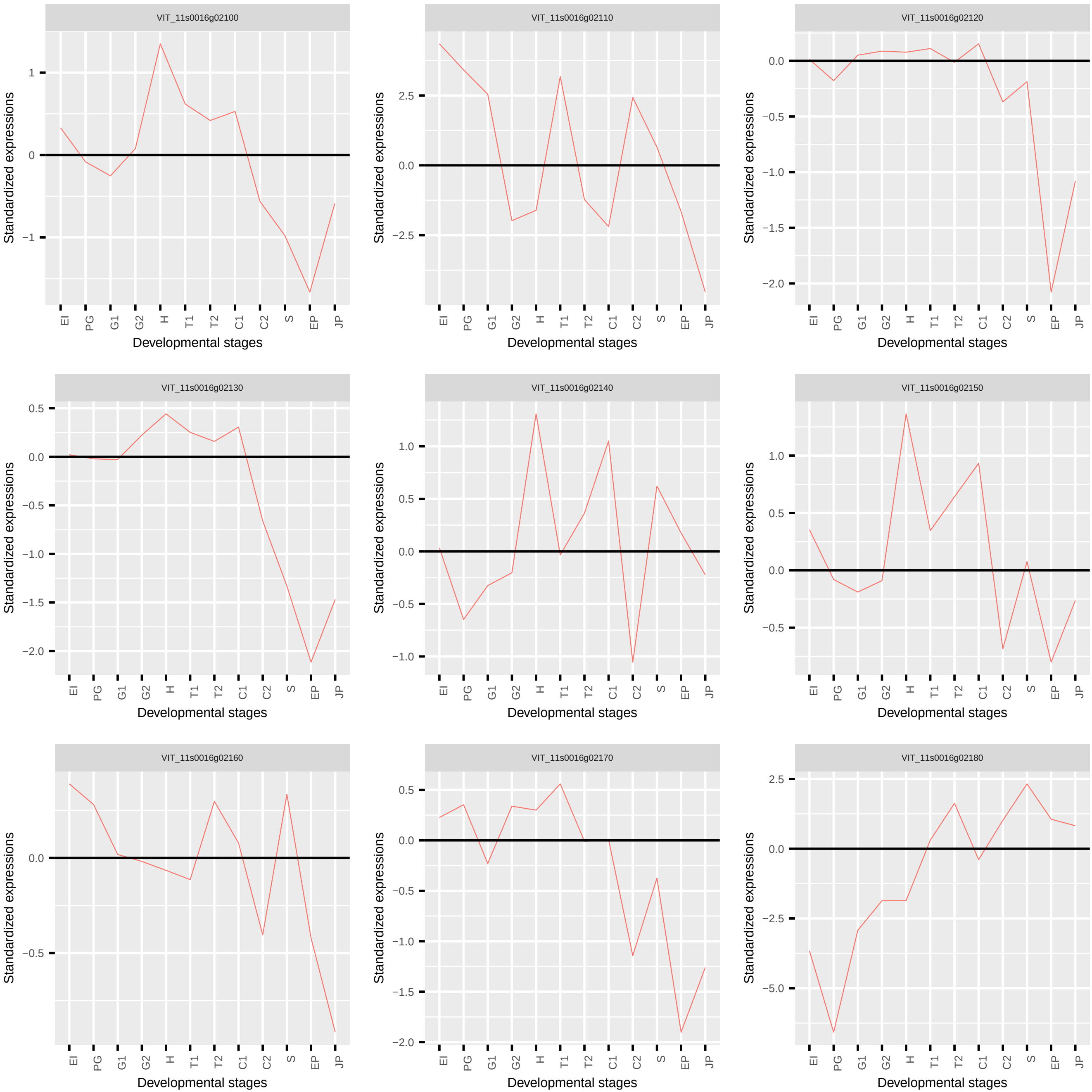

VIT_11s0016g02100
VIT_11s0016g02110
VIT_11s0016g02120
VIT_11s0016g02130
VIT_11s0016g02140
VIT_11s0016g02150
VIT_11s0016g02160
VIT_11s0016g02170
VIT_11s0016g02180
Standardized expressions
Developmental stages
EI PG G1 G2 H T1 T2 C1 C2 S EP JP
1
0
−1
2.5
0.0
−2.5
0.0
−0.5
−1.0
−1.5
−2.0
0.5
0.0
−0.5
−1.0
−1.5
−2.0
1.0
0.5
0.0
−0.5
−1.0
1.0
0.5
0.0
−0.5
0.0
−0.5
0.5
0.0
−0.5
−1.0
−1.5
−2.0
2.5
0.0
−2.5
−5.0

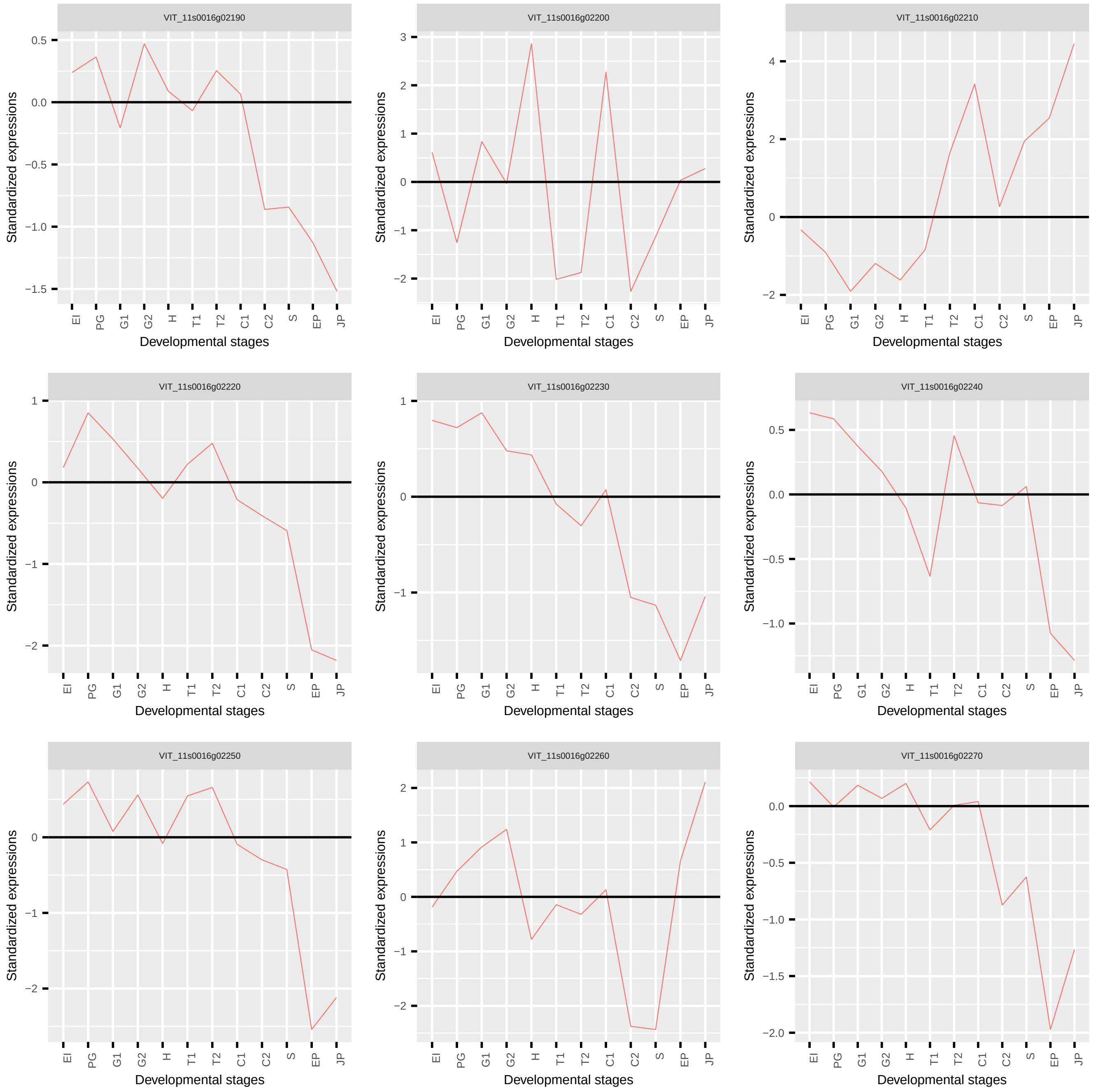

VIT_11s0016g02190
VIT_11s0016g02200
VIT_11s0016g02210
VIT_11s0016g02220
VIT_11s0016g02230
VIT_11s0016g02240
VIT_11s0016g02250
VIT_11s0016g02260
VIT_11s0016g02270
Standardized expressions
Developmental stages
EI
PG
G1
G2
H
T1
T2
C1
C2
S
EP
JP

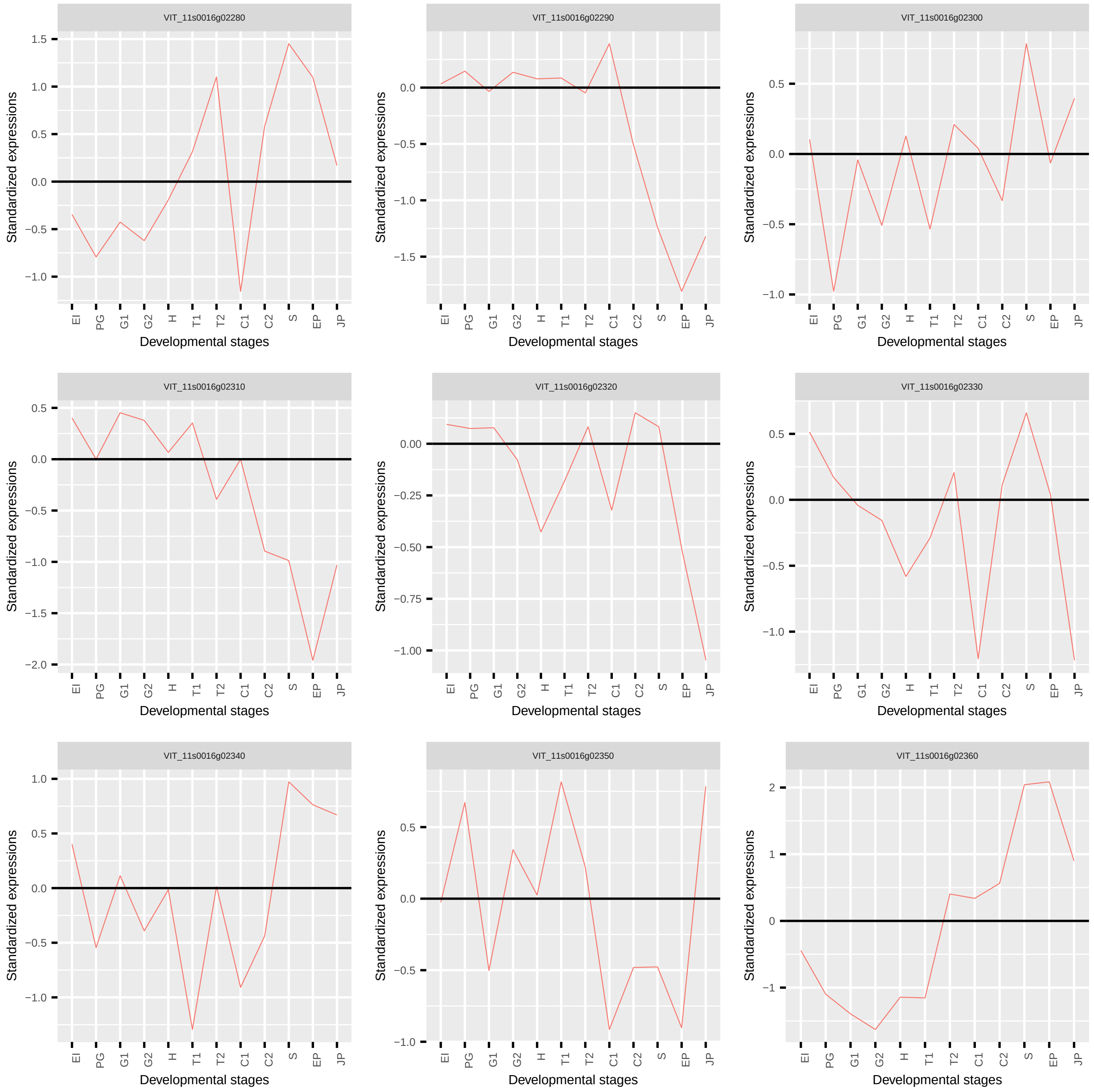
VIT_11s0016g02280
VIT_11s0016g02290
VIT_11s0016g02300
VIT_11s0016g02310
VIT_11s0016g02320
VIT_11s0016g02330
VIT_11s0016g02340
VIT_11s0016g02350
VIT_11s0016g02360
Standardized expressions
Developmental stages
EI PG G1 G2 H T1 T2 C1 C2 S EP JP
1.5
1.0
0.5
0.0
−0.5
−1.0
−1.5
−2.0
0.00
−0.25
−0.50
−0.75
−1.00
2
1
0
−1

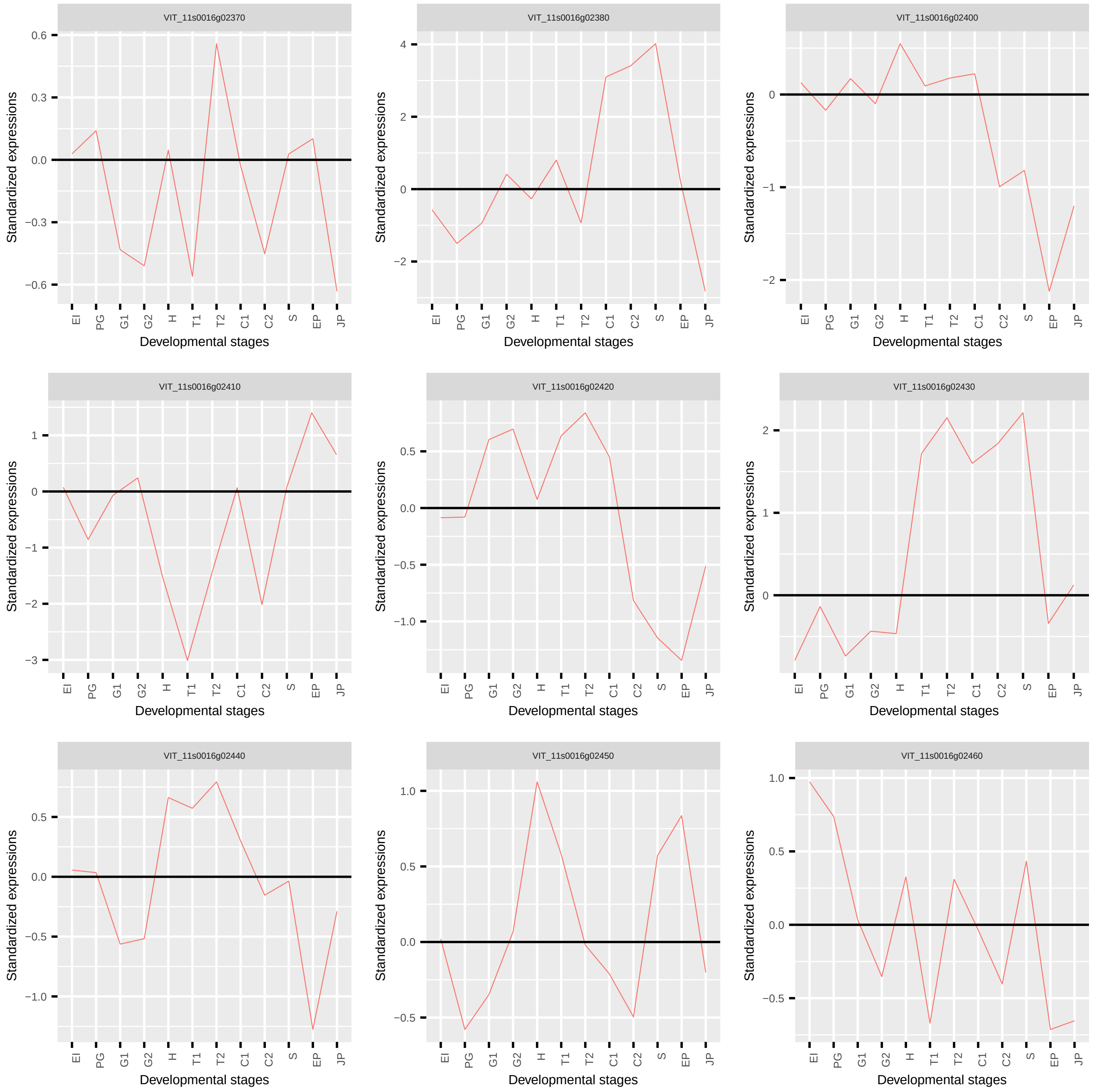
VIT_11s0016g02370
VIT_11s0016g02380
VIT_11s0016g02400
VIT_11s0016g02410
VIT_11s0016g02420
VIT_11s0016g02430
VIT_11s0016g02440
VIT_11s0016g02450
VIT_11s0016g02460
Standardized expressions
Developmental stages
EI PG G1 G2 H T1 T2 C1 C2 S EP JP

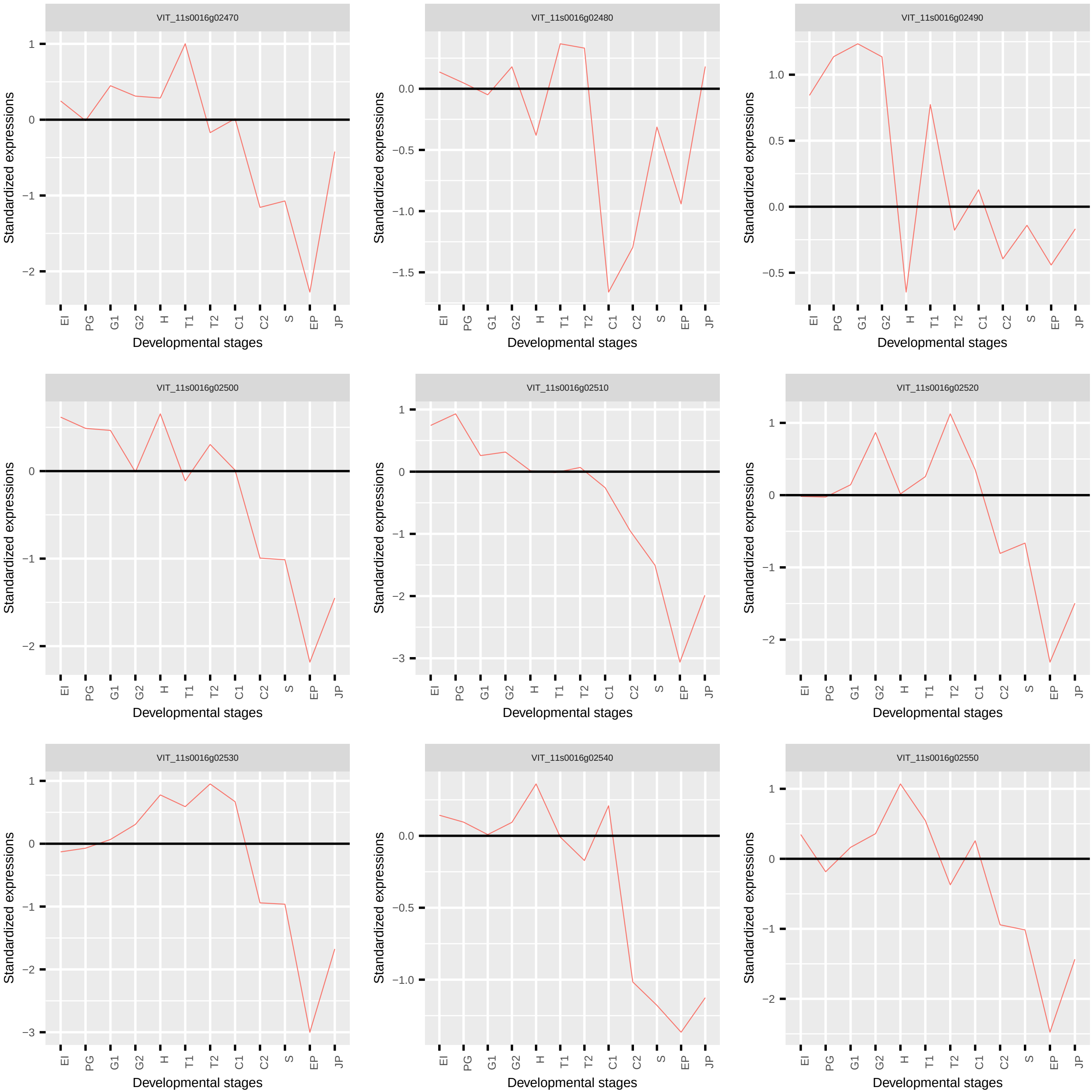

VIT_11s0016g02470
VIT_11s0016g02480
VIT_11s0016g02490
VIT_11s0016g02500
VIT_11s0016g02510
VIT_11s0016g02520
VIT_11s0016g02530
VIT_11s0016g02540
VIT_11s0016g02550
Standardized expressions
Developmental stages
EI PG G1 G2 H T1 T2 C1 C2 S EP JP

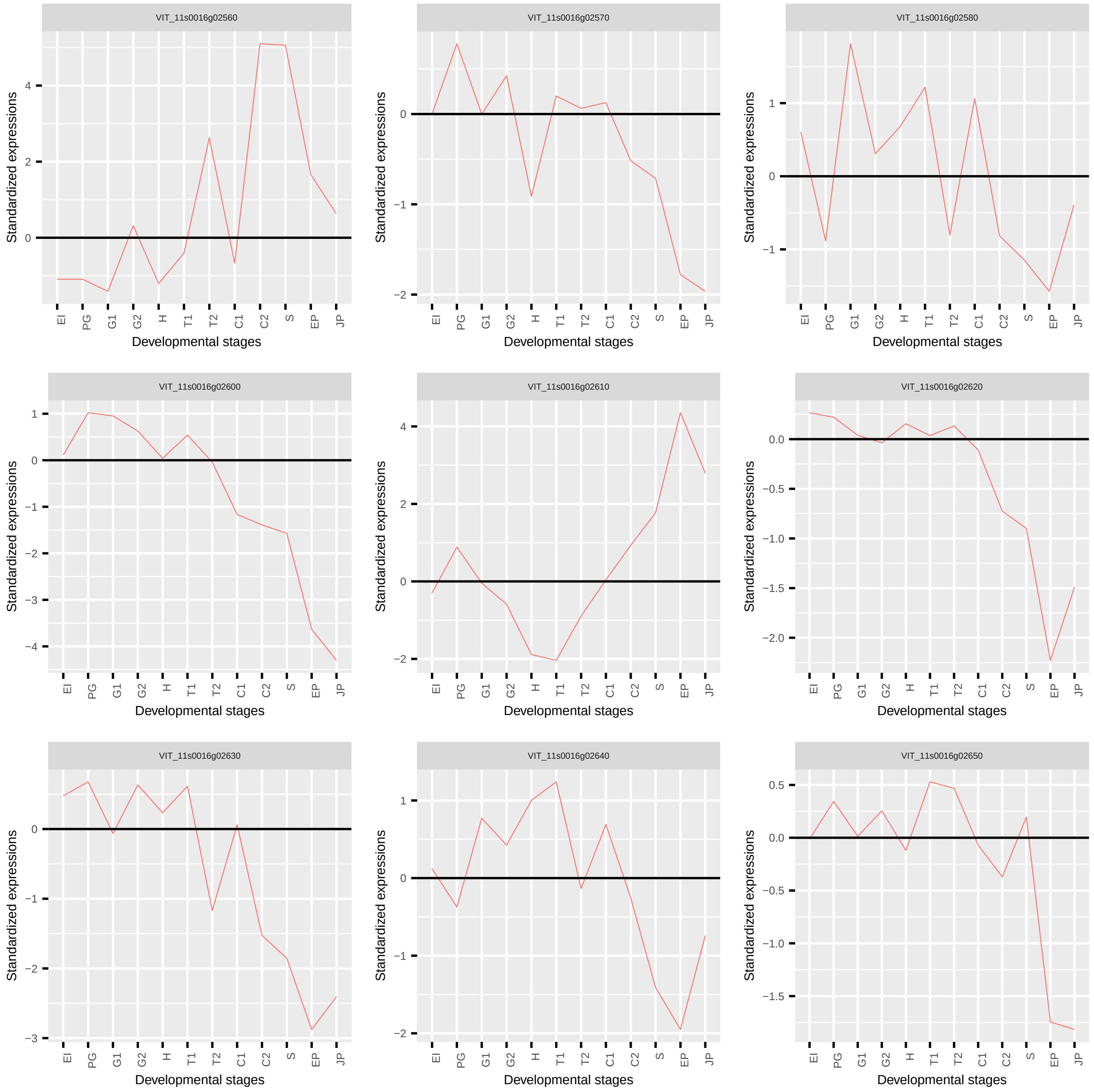

VIT_11s0016g02560
VIT_11s0016g02570
VIT_11s0016g02580
VIT_11s0016g02600
VIT_11s0016g02610
VIT_11s0016g02620
VIT_11s0016g02630
VIT_11s0016g02640
VIT_11s0016g02650
Standardized expressions
Developmental stages
EI PG G1 G2 H T1 T2 C1 C2 S EP JP

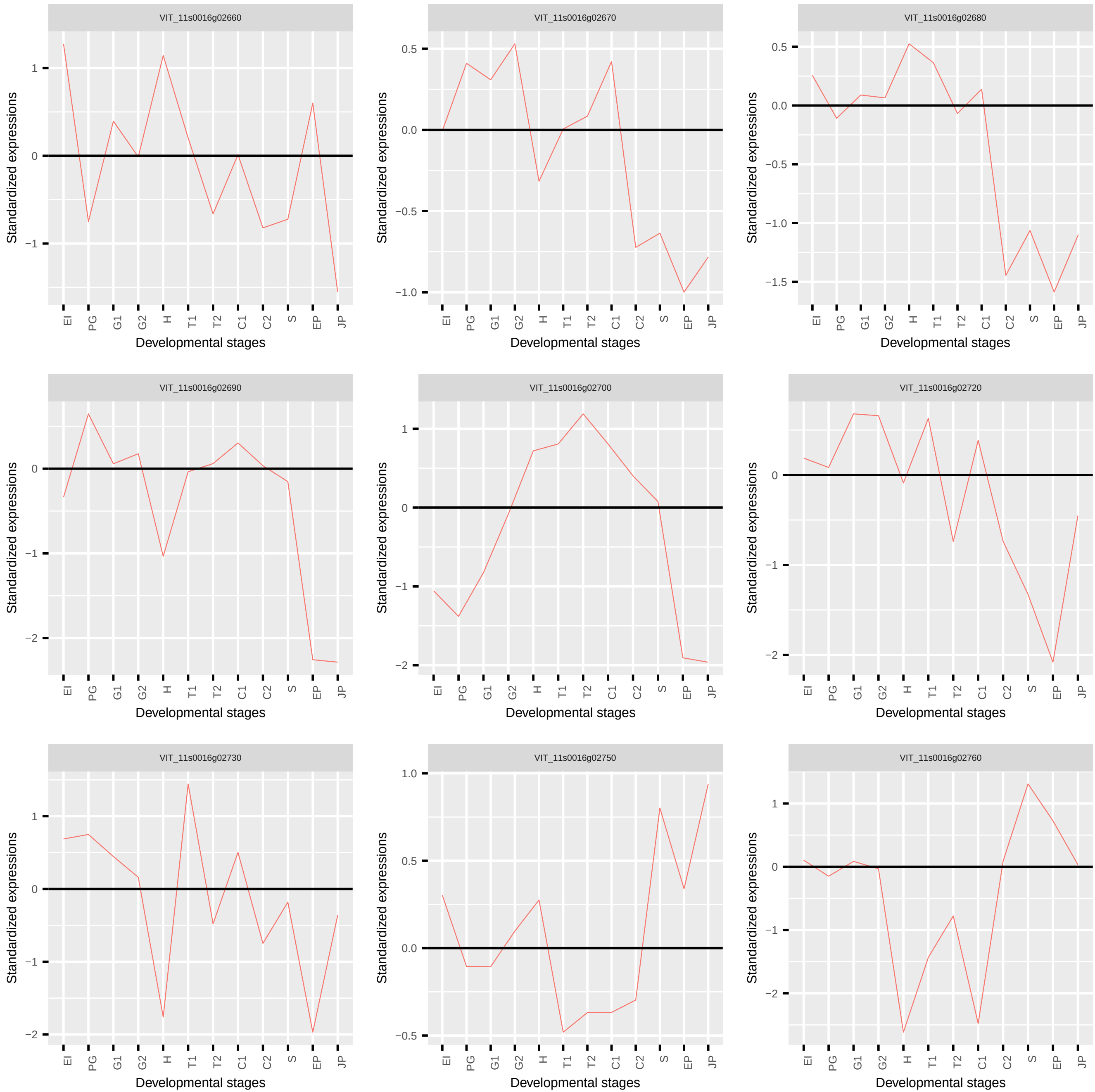
VIT_11s0016g02660
VIT_11s0016g02670
VIT_11s0016g02680
VIT_11s0016g02690
VIT_11s0016g02700
VIT_11s0016g02720
VIT_11s0016g02730
VIT_11s0016g02750
VIT_11s0016g02760
Standardized expressions
Developmental stages
EI PG G1 G2 H T1 T2 C1 C2 S EP JP

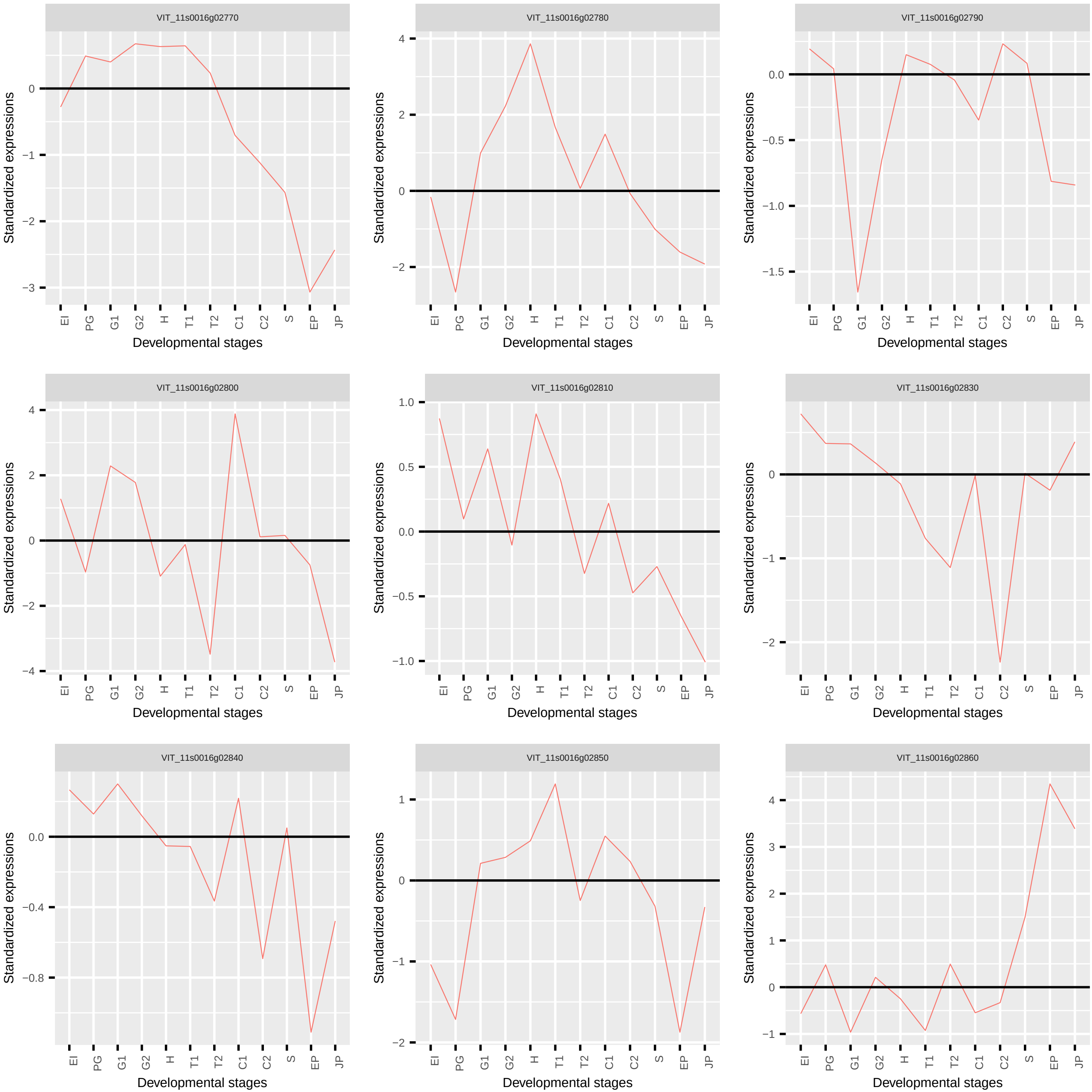
VIT_11s0016g02770
VIT_11s0016g02780
VIT_11s0016g02790
VIT_11s0016g02800
VIT_11s0016g02810
VIT_11s0016g02830
VIT_11s0016g02840
VIT_11s0016g02850
VIT_11s0016g02860
Standardized expressions
Developmental stages
EI PG G1 G2 H T1 T2 C1 C2 S EP JP

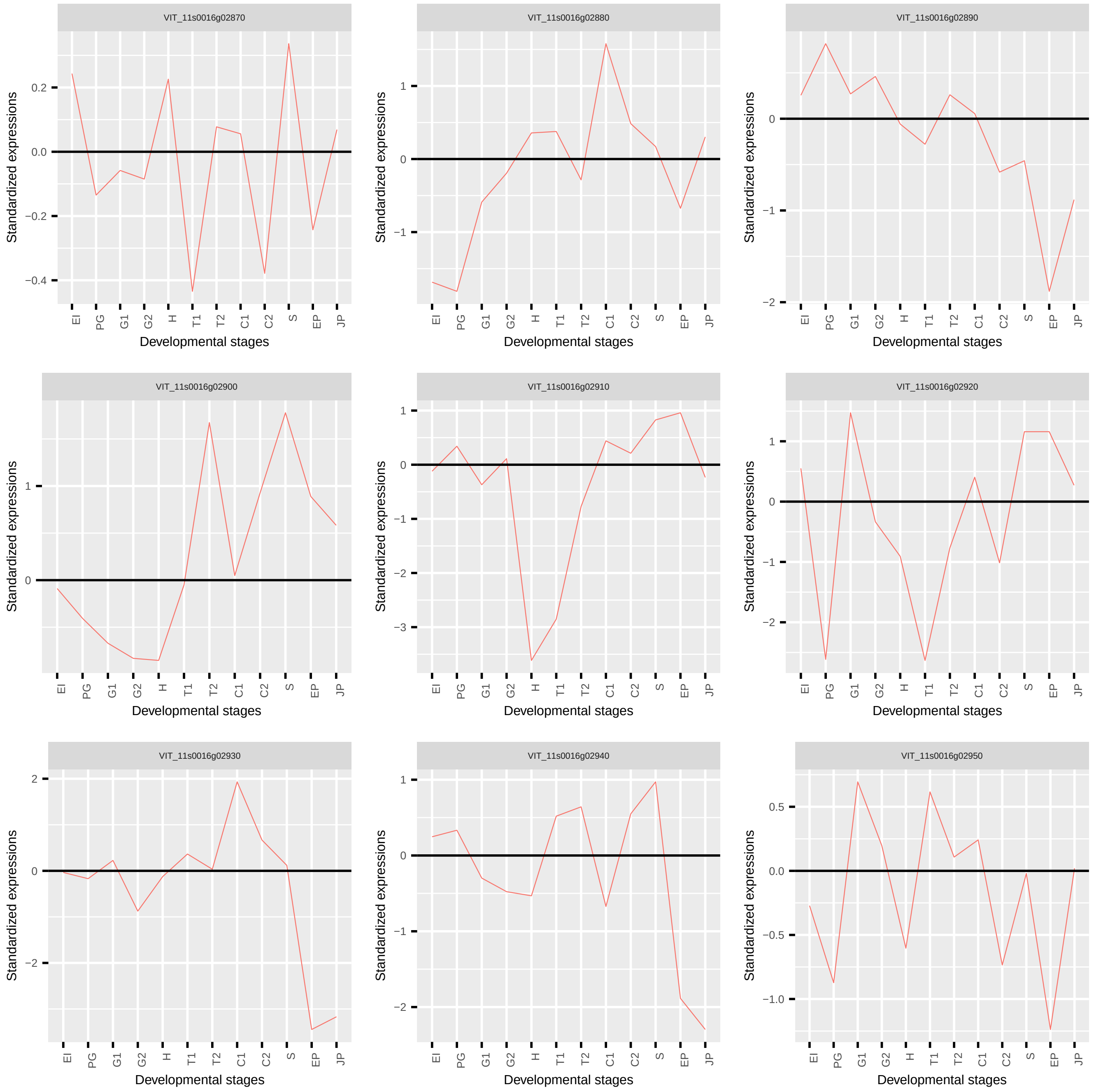
VIT_11s0016g02870
VIT_11s0016g02880
VIT_11s0016g02890
VIT_11s0016g02900
VIT_11s0016g02910
VIT_11s0016g02920
VIT_11s0016g02930
VIT_11s0016g02940
VIT_11s0016g02950
Standardized expressions
Developmental stages
EI PG G1 G2 H T1 T2 C1 C2 S EP JP

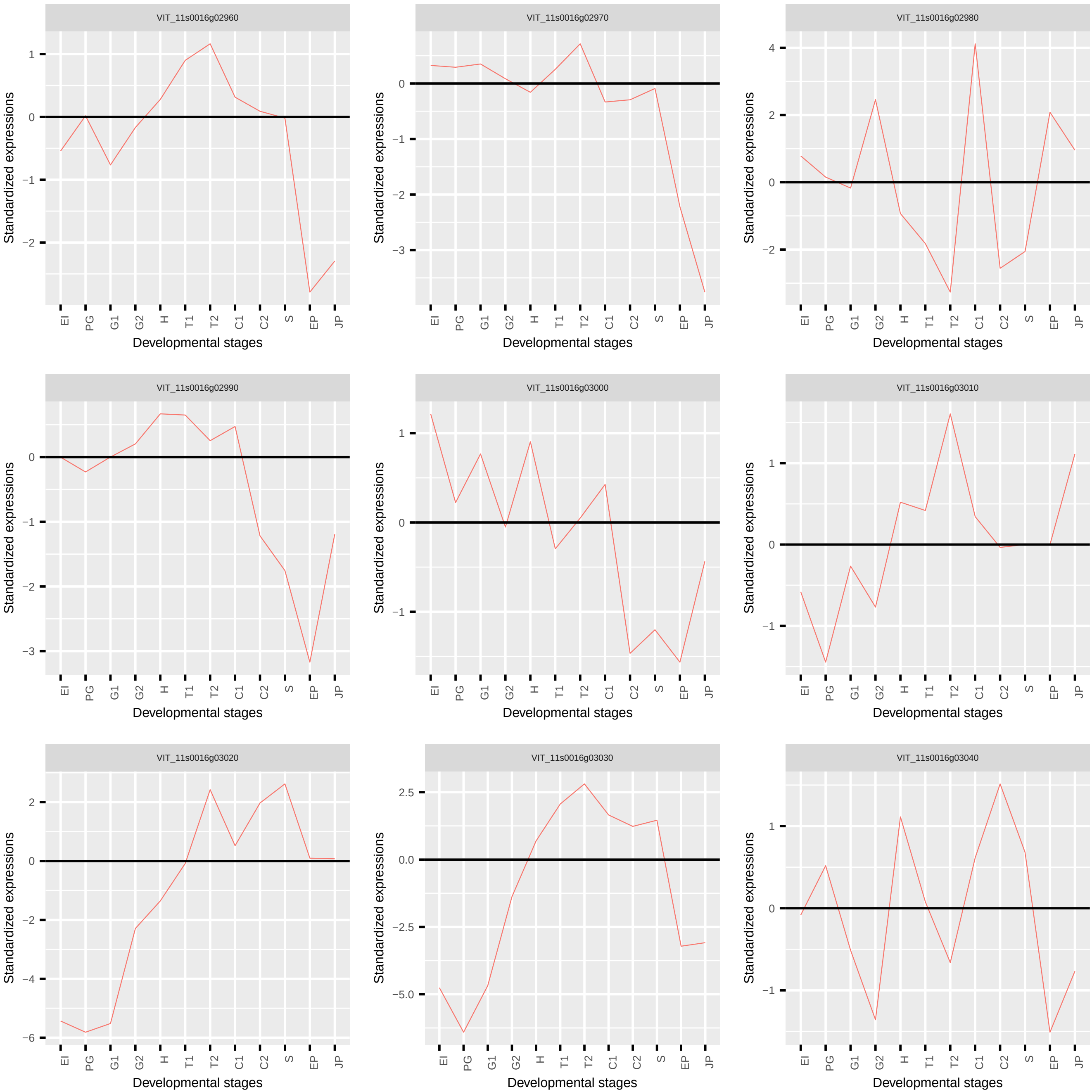
VIT_11s0016g02960
VIT_11s0016g02970
VIT_11s0016g02980
VIT_11s0016g02990
VIT_11s0016g03000
VIT_11s0016g03010
VIT_11s0016g03020
VIT_11s0016g03030
VIT_11s0016g03040
Standardized expressions
Developmental stages
EI PG G1 G2 H T1 T2 C1 C2 S EP JP

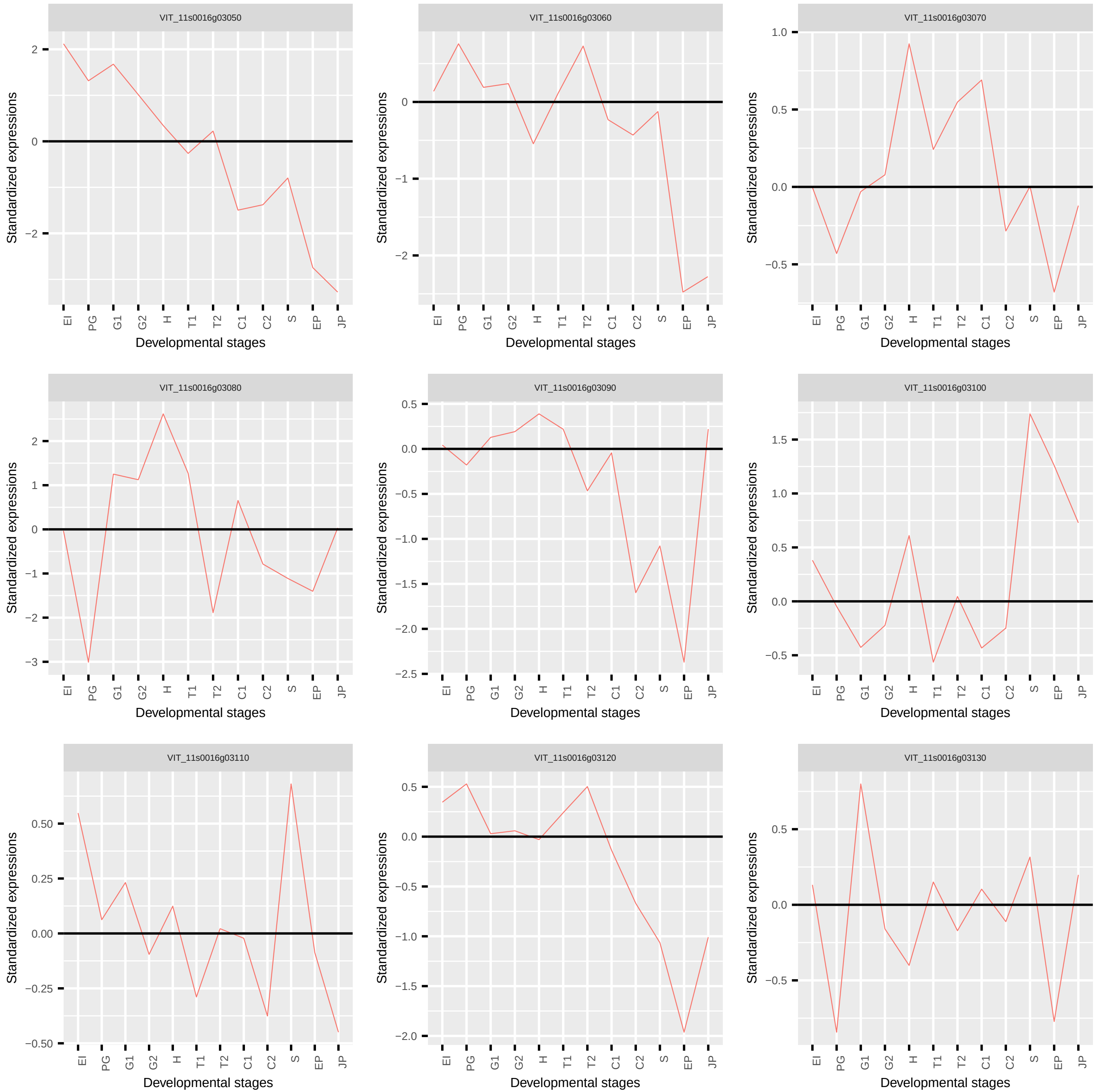

VIT_11s0016g03050
VIT_11s0016g03060
VIT_11s0016g03070
VIT_11s0016g03080
VIT_11s0016g03090
VIT_11s0016g03100
VIT_11s0016g03110
VIT_11s0016g03120
VIT_11s0016g03130
Standardized expressions
Developmental stages
EI
PG
G1
G2
H
T1
T2
C1
C2
S
EP
JP

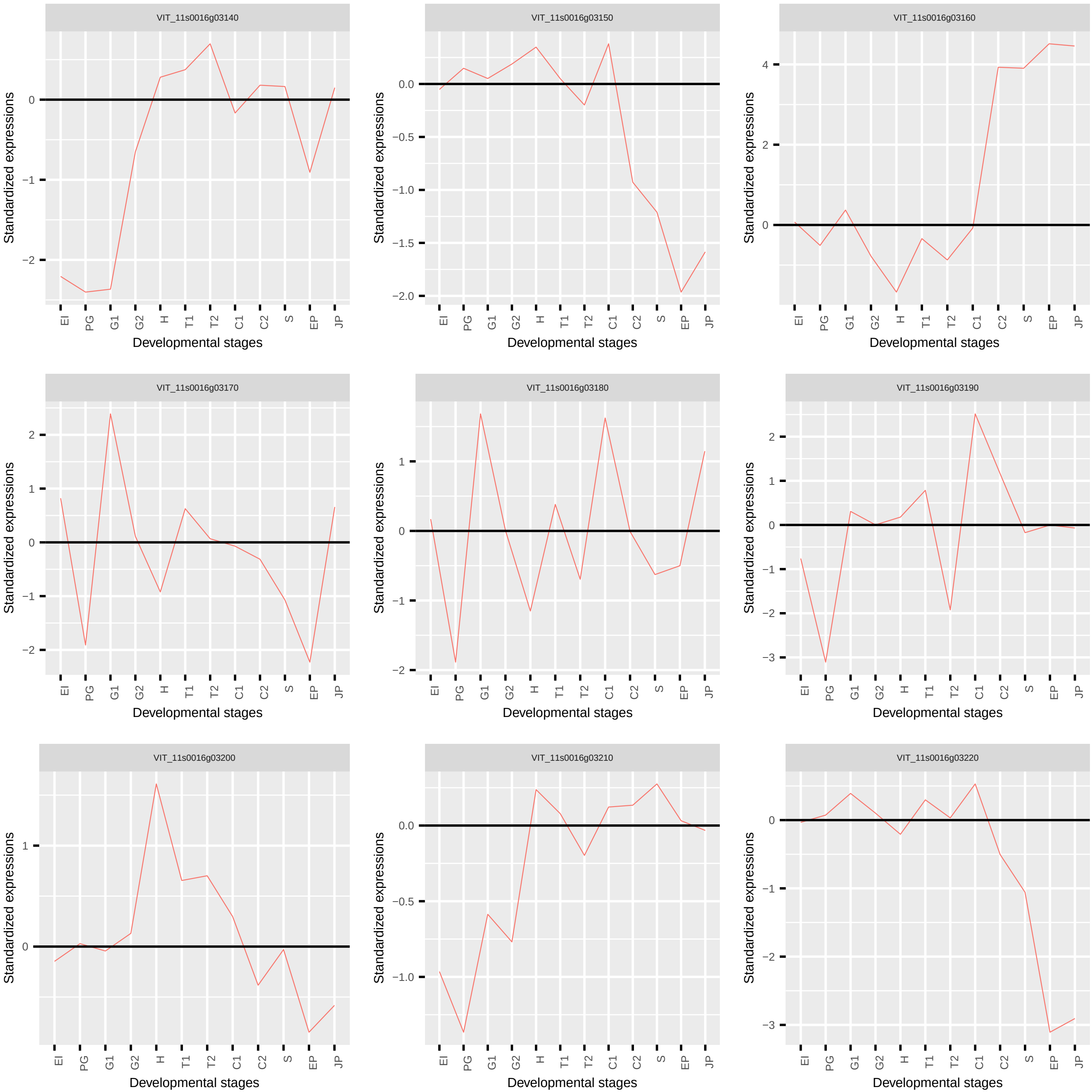
VIT_11s0016g03140
VIT_11s0016g03150
VIT_11s0016g03160
VIT_11s0016g03170
VIT_11s0016g03180
VIT_11s0016g03190
VIT_11s0016g03200
VIT_11s0016g03210
VIT_11s0016g03220
Standardized expressions
Developmental stages
EI
PG
G1
G2
H
T1
T2
C1
C2
S
EP
JP

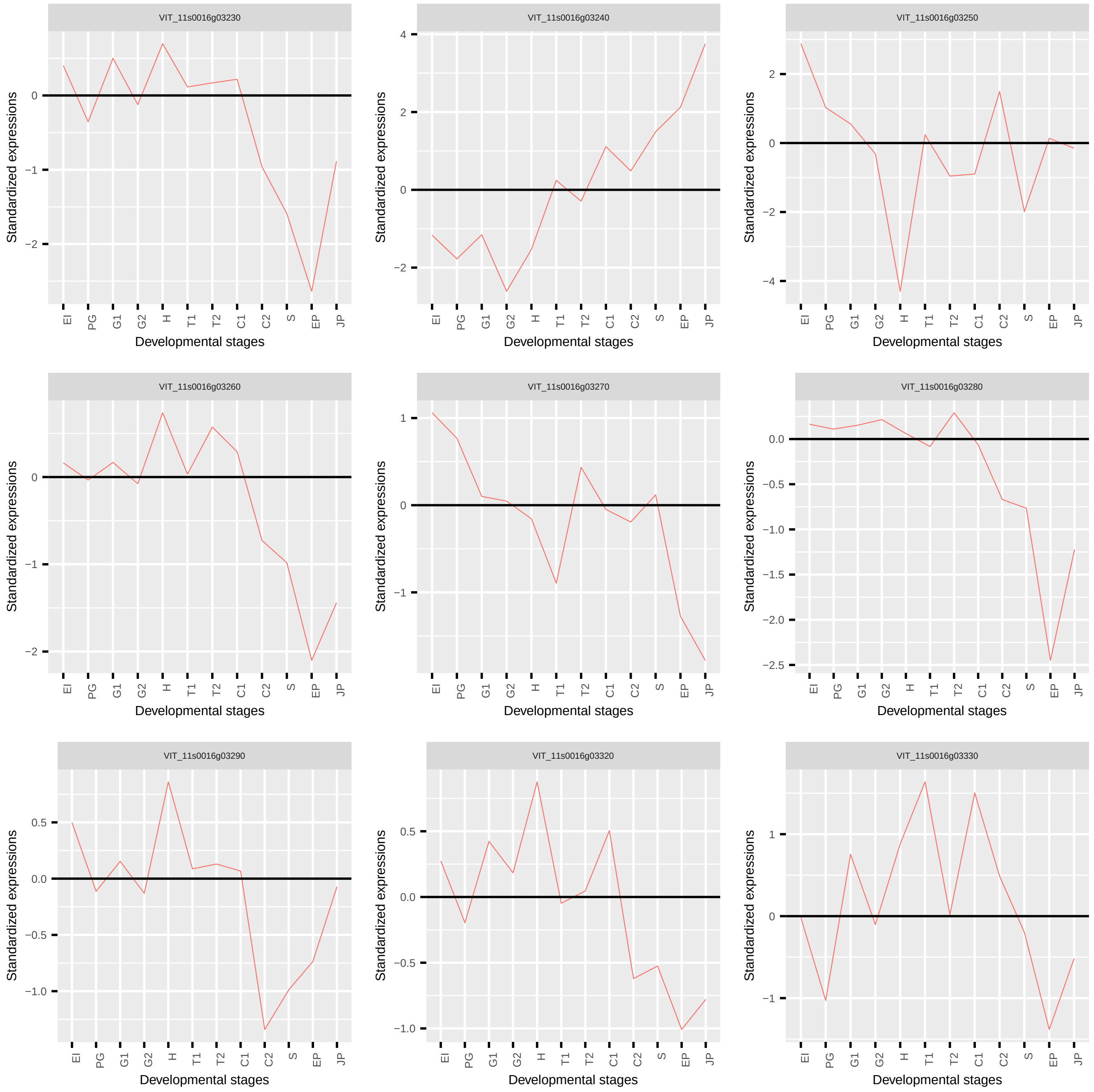
VIT_11s0016g03230
VIT_11s0016g03240
VIT_11s0016g03250
VIT_11s0016g03260
VIT_11s0016g03270
VIT_11s0016g03280
VIT_11s0016g03290
VIT_11s0016g03320
VIT_11s0016g03330
Standardized expressions
Developmental stages
EI
PG
G1
G2
H
T1
T2
C1
C2
S
EP
JP

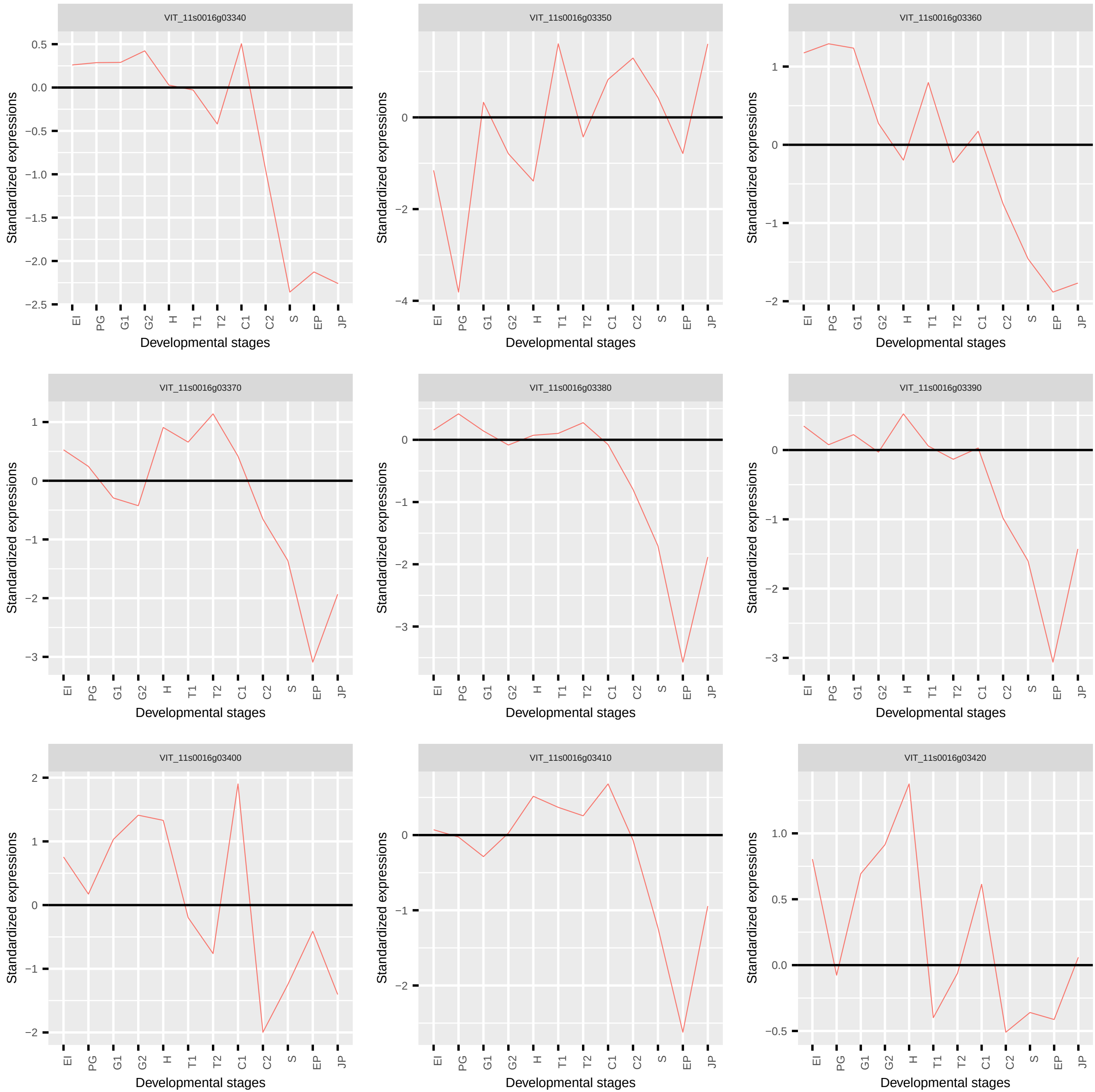

VIT_11s0016g03340
0.5
0.0
−0.5
−1.0
−1.5
−2.0
−2.5
Standardized expressions
EI PG G1 G2 H T1 T2 C1 C2 S EP JP
Developmental stages
VIT_11s0016g03350
0
−2
−4
Standardized expressions
EI PG G1 G2 H T1 T2 C1 C2 S EP JP
Developmental stages
VIT_11s0016g03360
1
0
−1
−2
Standardized expressions
EI PG G1 G2 H T1 T2 C1 C2 S EP JP
Developmental stages
VIT_11s0016g03370
1
0
−1
−2
−3
Standardized expressions
EI PG G1 G2 H T1 T2 C1 C2 S EP JP
Developmental stages
VIT_11s0016g03380
0
−1
−2
−3
Standardized expressions
EI PG G1 G2 H T1 T2 C1 C2 S EP JP
Developmental stages
VIT_11s0016g03390
0
−1
−2
−3
Standardized expressions
EI PG G1 G2 H T1 T2 C1 C2 S EP JP
Developmental stages
VIT_11s0016g03400
2
1
0
−1
−2
Standardized expressions
EI PG G1 G2 H T1 T2 C1 C2 S EP JP
Developmental stages
VIT_11s0016g03410
0
−1
−2
Standardized expressions
EI PG G1 G2 H T1 T2 C1 C2 S EP JP
Developmental stages
VIT_11s0016g03420
1.0
0.5
0.0
−0.5
Standardized expressions
EI PG G1 G2 H T1 T2 C1 C2 S EP JP
Developmental stages

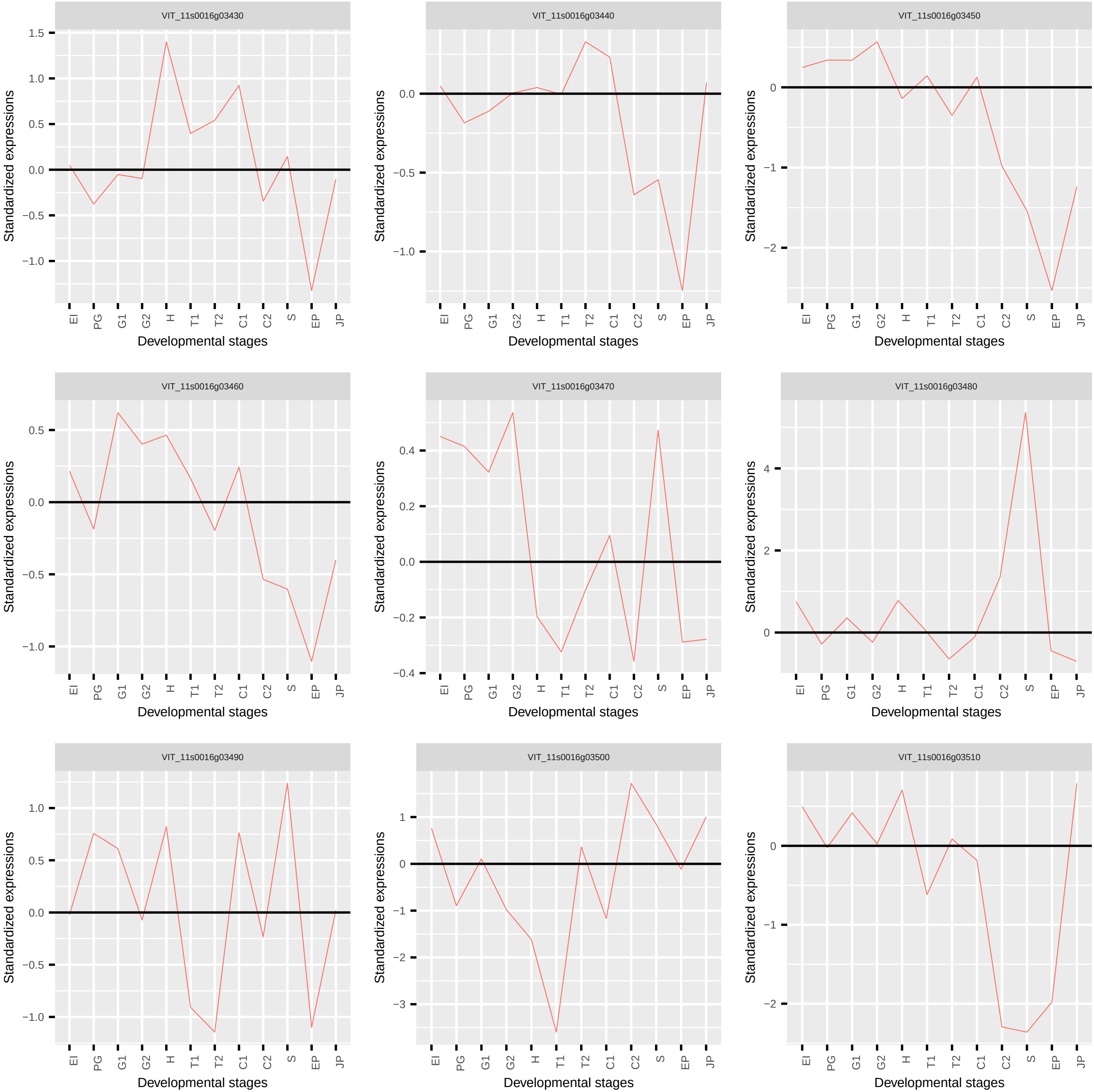
VIT_11s0016g03430
VIT_11s0016g03440
VIT_11s0016g03450
VIT_11s0016g03460
VIT_11s0016g03470
VIT_11s0016g03480
VIT_11s0016g03490
VIT_11s0016g03500
VIT_11s0016g03510
Standardized expressions
Developmental stages
EI PG G1 G2 H T1 T2 C1 C2 S EP JP

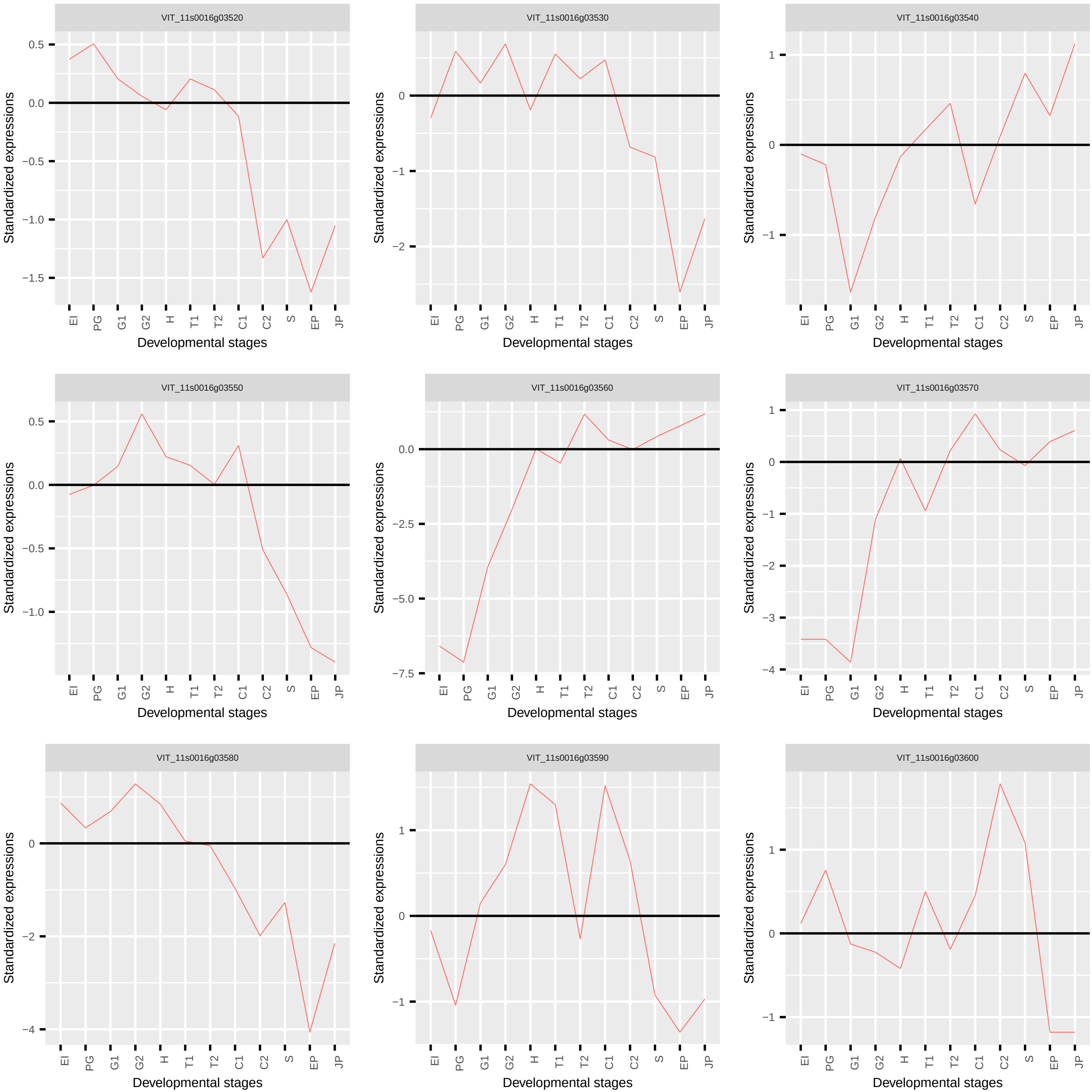

VIT_11s0016g03520
VIT_11s0016g03530
VIT_11s0016g03540
VIT_11s0016g03550
VIT_11s0016g03560
VIT_11s0016g03570
VIT_11s0016g03580
VIT_11s0016g03590
VIT_11s0016g03600
Standardized expressions
Developmental stages
EI PG G1 G2 H T1 T2 C1 C2 S EP JP

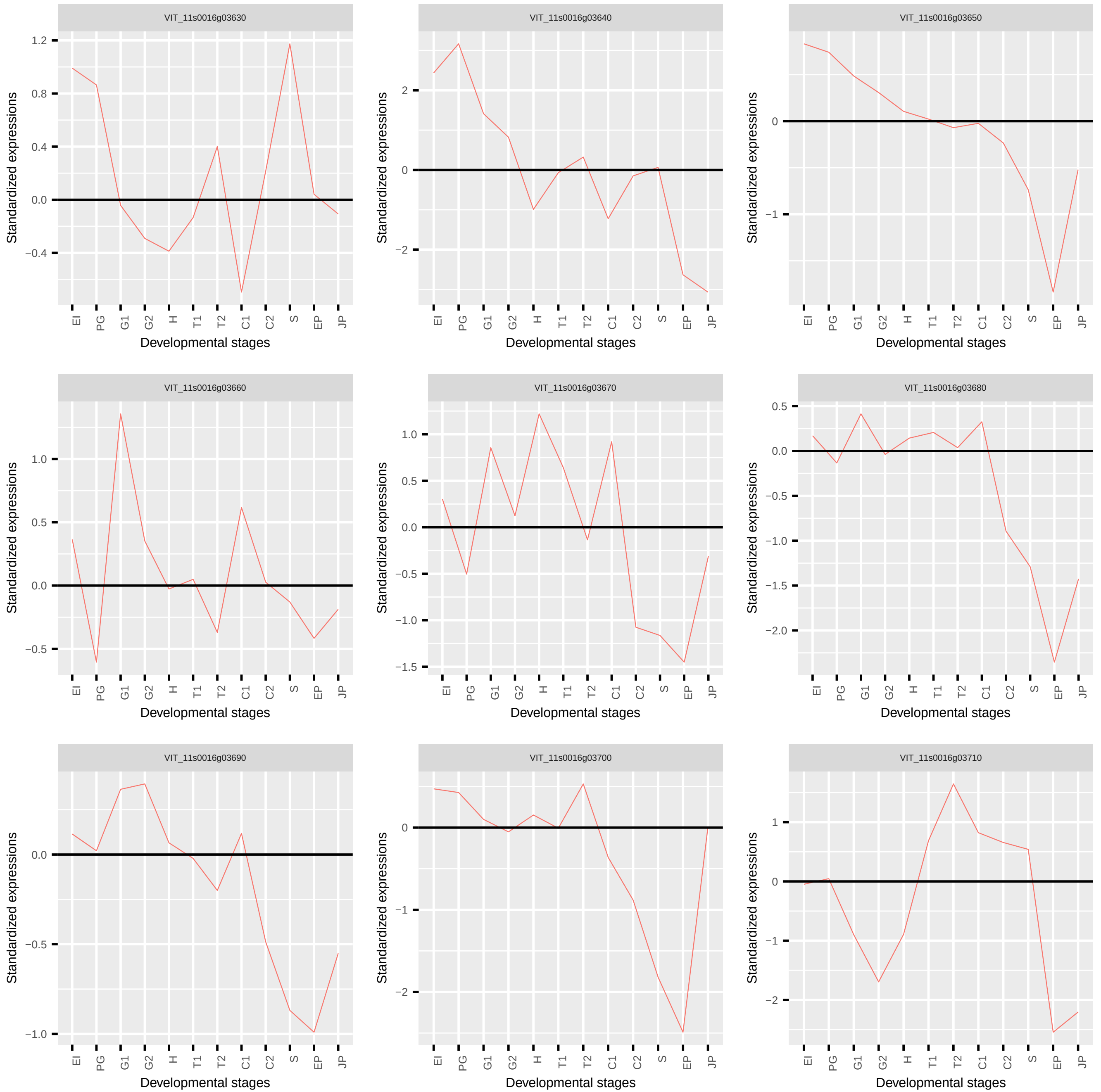
VIT_11s0016g03630
VIT_11s0016g03640
VIT_11s0016g03650
VIT_11s0016g03660
VIT_11s0016g03670
VIT_11s0016g03680
VIT_11s0016g03690
VIT_11s0016g03700
VIT_11s0016g03710
Standardized expressions
Developmental stages
EI PG G1 G2 H T1 T2 C1 C2 S EP JP

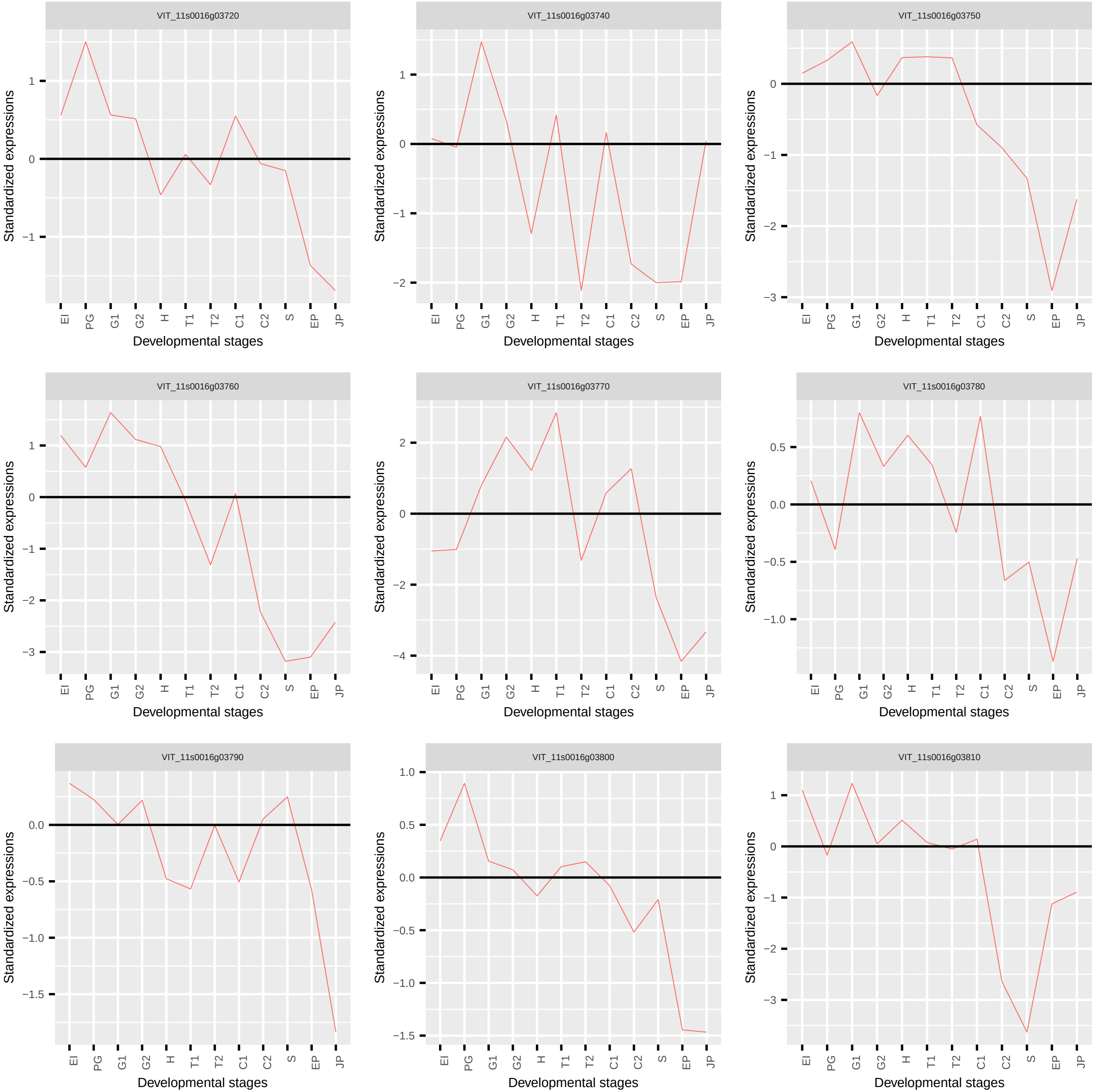

VIT_11s0016g03720
Standardized expressions
1
0
−1
EI PG G1 G2 H T1 T2 C1 C2 S EP JP
Developmental stages
VIT_11s0016g03740
Standardized expressions
1
0
−1
−2
EI PG G1 G2 H T1 T2 C1 C2 S EP JP
Developmental stages
VIT_11s0016g03750
Standardized expressions
0
−1
−2
−3
EI PG G1 G2 H T1 T2 C1 C2 S EP JP
Developmental stages
VIT_11s0016g03760
Standardized expressions
1
0
−1
−2
−3
EI PG G1 G2 H T1 T2 C1 C2 S EP JP
Developmental stages
VIT_11s0016g03770
Standardized expressions
2
0
−2
−4
EI PG G1 G2 H T1 T2 C1 C2 S EP JP
Developmental stages
VIT_11s0016g03780
Standardized expressions
0.5
0.0
−0.5
−1.0
EI PG G1 G2 H T1 T2 C1 C2 S EP JP
Developmental stages
VIT_11s0016g03790
Standardized expressions
0.0
−0.5
−1.0
−1.5
EI PG G1 G2 H T1 T2 C1 C2 S EP JP
Developmental stages
VIT_11s0016g03800
Standardized expressions
1.0
0.5
0.0
−0.5
−1.0
−1.5
EI PG G1 G2 H T1 T2 C1 C2 S EP JP
Developmental stages
VIT_11s0016g03810
Standardized expressions
1
0
−1
−2
−3
EI PG G1 G2 H T1 T2 C1 C2 S EP JP
Developmental stages

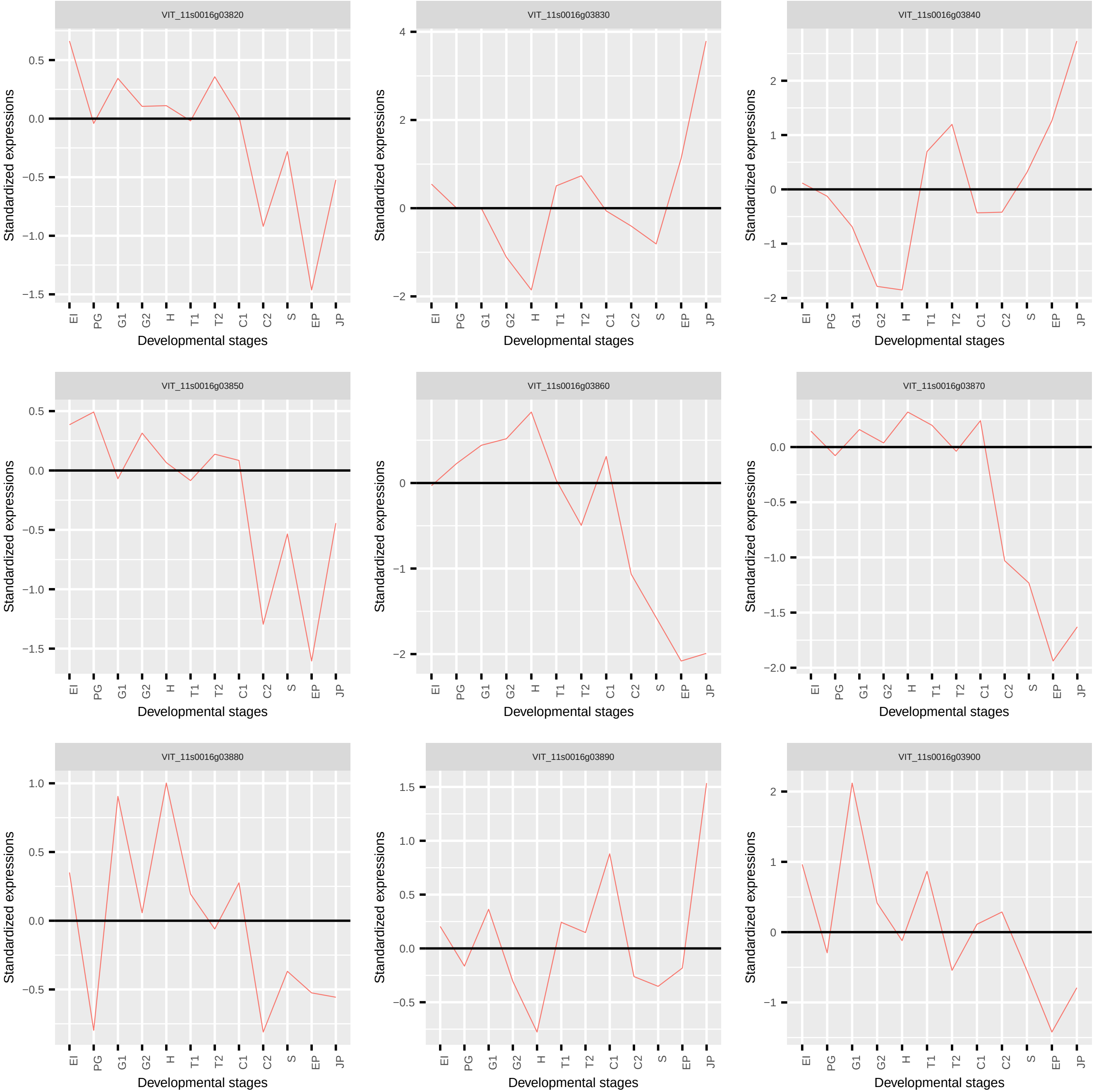
VIT_11s0016g03820
VIT_11s0016g03830
VIT_11s0016g03840
VIT_11s0016g03850
VIT_11s0016g03860
VIT_11s0016g03870
VIT_11s0016g03880
VIT_11s0016g03890
VIT_11s0016g03900
Standardized expressions
Developmental stages
EI
PG
G1
G2
H
T1
T2
C1
C2
S
EP
JP

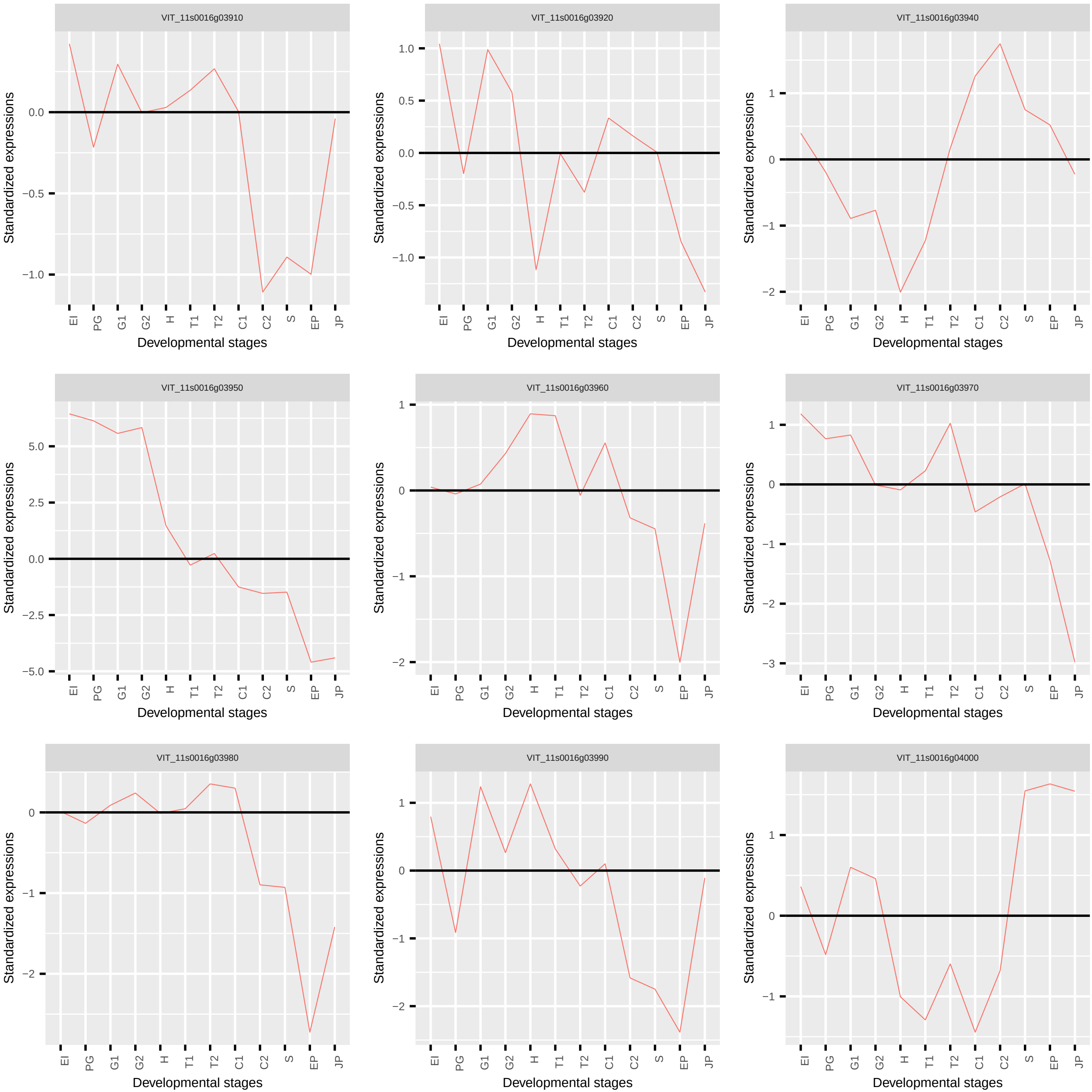
VIT_11s0016g03910
VIT_11s0016g03920
VIT_11s0016g03940
VIT_11s0016g03950
VIT_11s0016g03960
VIT_11s0016g03970
VIT_11s0016g03980
VIT_11s0016g03990
VIT_11s0016g04000
Standardized expressions
Developmental stages
EI PG G1 G2 H T1 T2 C1 C2 S EP JP
0.0 −0.5 −1.0
1.0 0.5 0.0 −0.5 −1.0
1 0 −1 −2
5.0 2.5 0.0 −2.5 −5.0
1 0 −1 −2
1 0 −1 −2 −3
0 −1 −2
1 0 −1 −2
1 0 −1

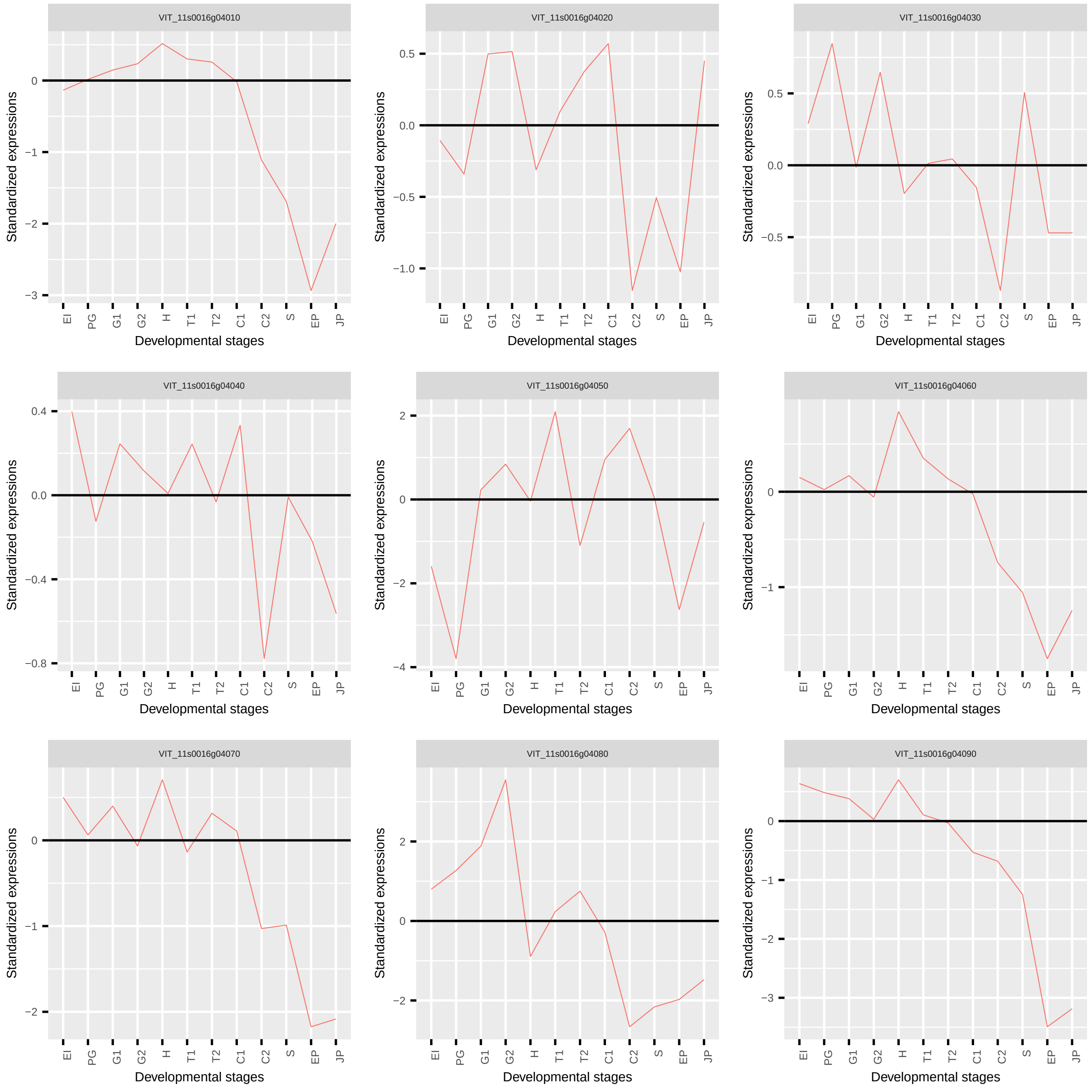
VIT_11s0016g04010
VIT_11s0016g04020
VIT_11s0016g04030
VIT_11s0016g04040
VIT_11s0016g04050
VIT_11s0016g04060
VIT_11s0016g04070
VIT_11s0016g04080
VIT_11s0016g04090
Standardized expressions
Developmental stages
EI PG G1 G2 H T1 T2 C1 C2 S EP JP

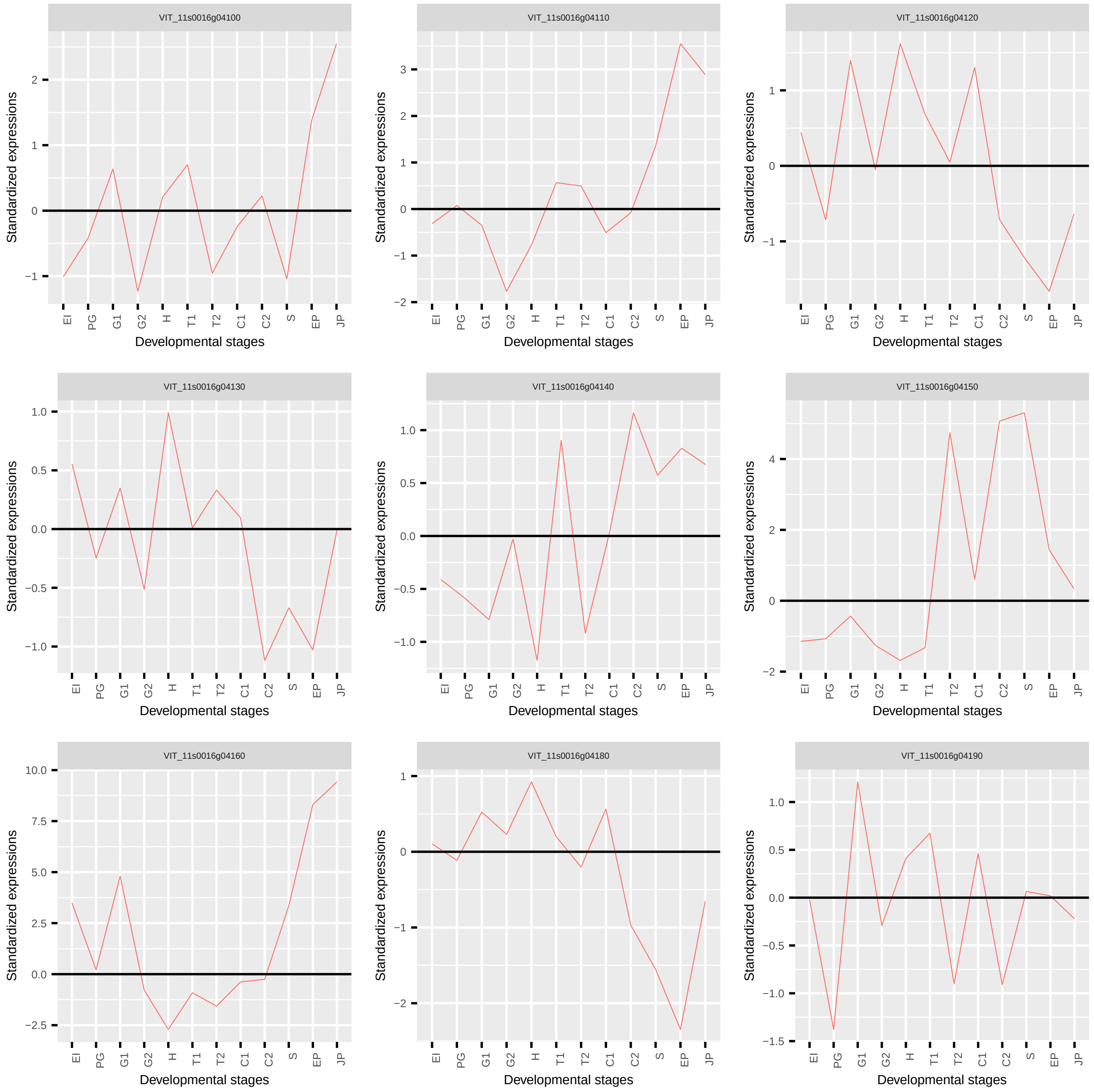

VIT_11s0016g04100
VIT_11s0016g04110
VIT_11s0016g04120
VIT_11s0016g04130
VIT_11s0016g04140
VIT_11s0016g04150
VIT_11s0016g04160
VIT_11s0016g04180
VIT_11s0016g04190
Standardized expressions
Developmental stages
EI PG G1 G2 H T1 T2 C1 C2 S EP JP

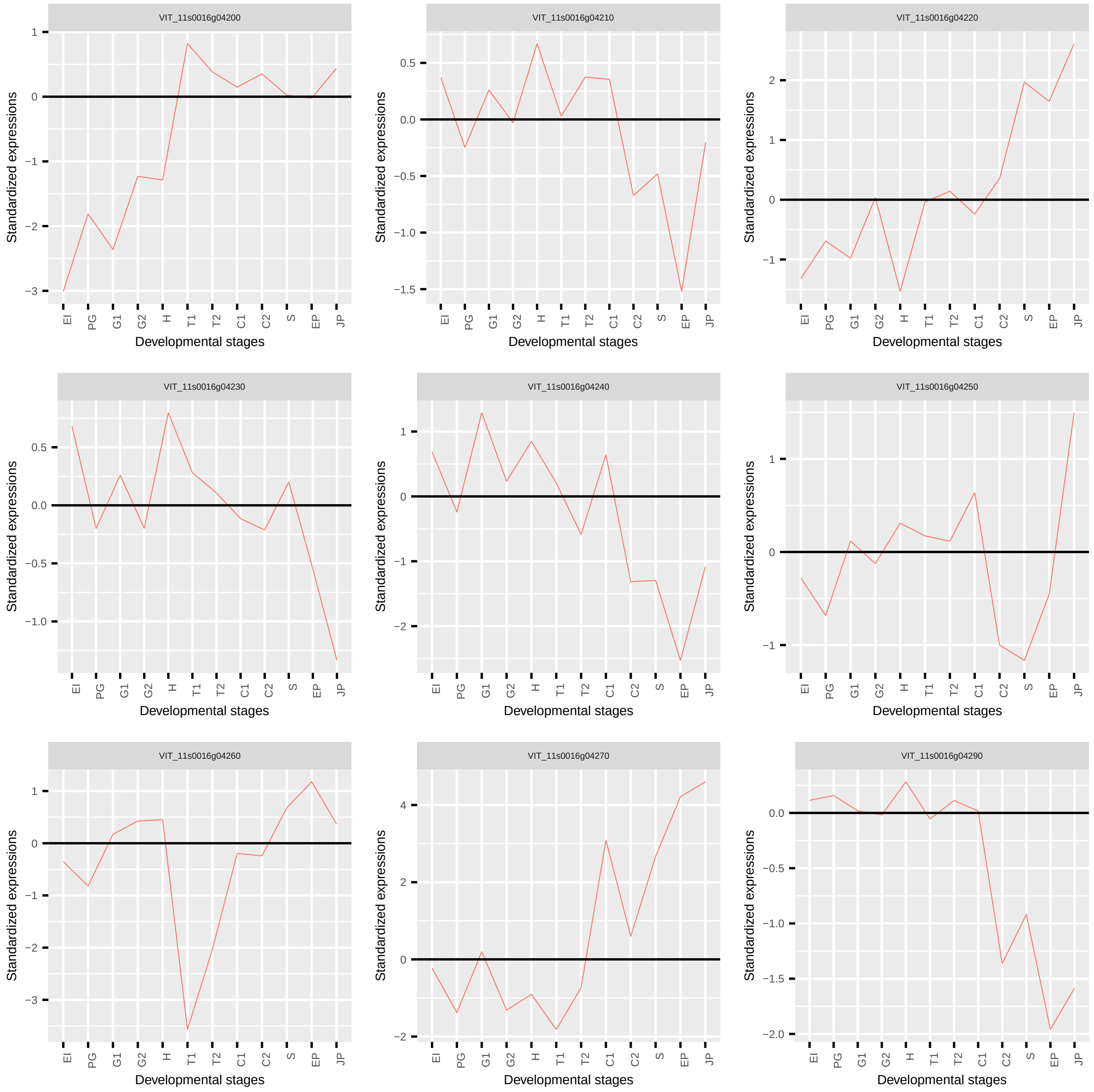

VIT_11s0016g04200
VIT_11s0016g04210
VIT_11s0016g04220
VIT_11s0016g04230
VIT_11s0016g04240
VIT_11s0016g04250
VIT_11s0016g04260
VIT_11s0016g04270
VIT_11s0016g04290
Standardized expressions
Developmental stages
EI PG G1 G2 H T1 T2 C1 C2 S EP JP

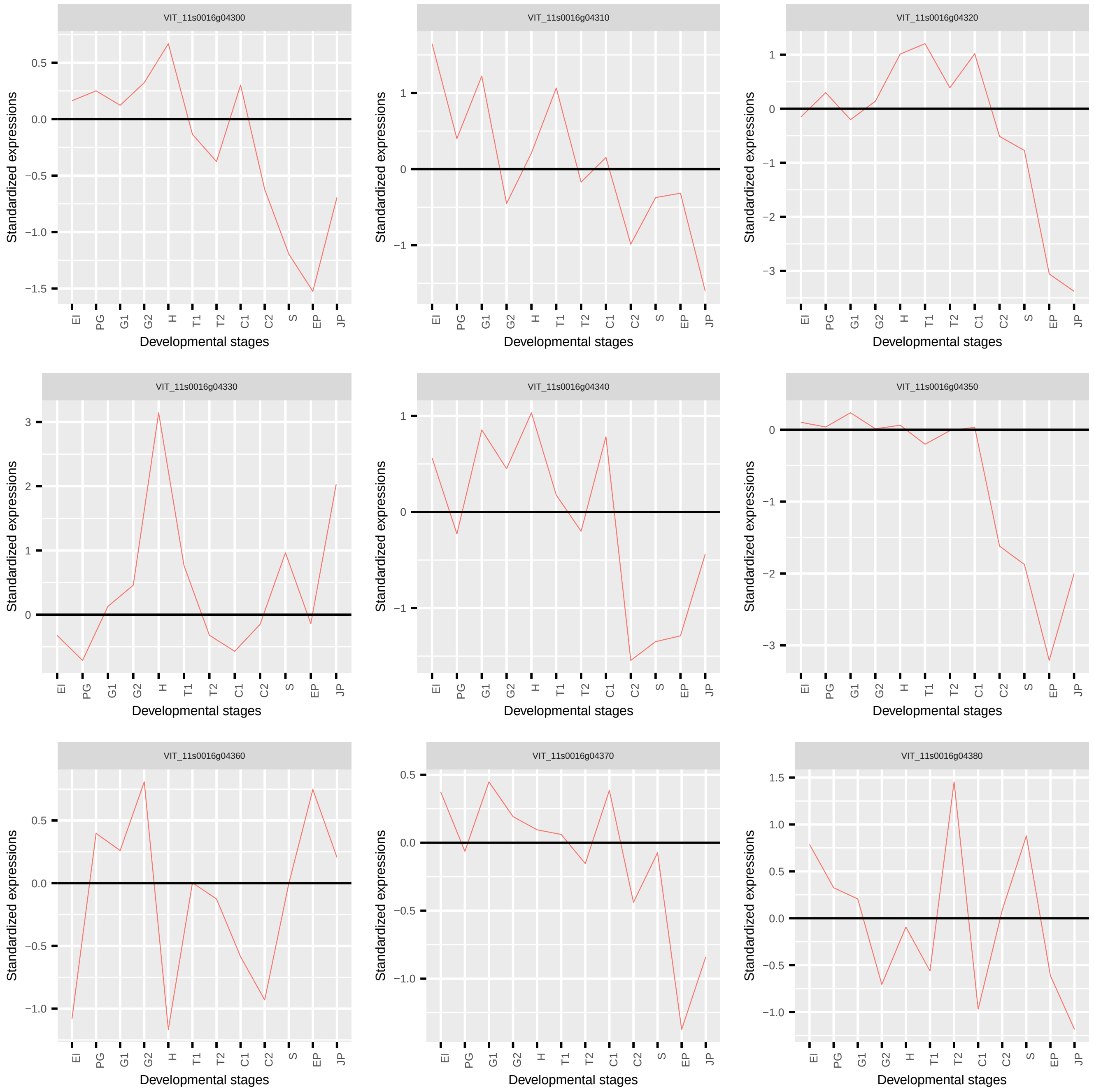

VIT_11s0016g04300
VIT_11s0016g04310
VIT_11s0016g04320
VIT_11s0016g04330
VIT_11s0016g04340
VIT_11s0016g04350
VIT_11s0016g04360
VIT_11s0016g04370
VIT_11s0016g04380
Standardized expressions
Developmental stages
EI PG G1 G2 H T1 T2 C1 C2 S EP JP

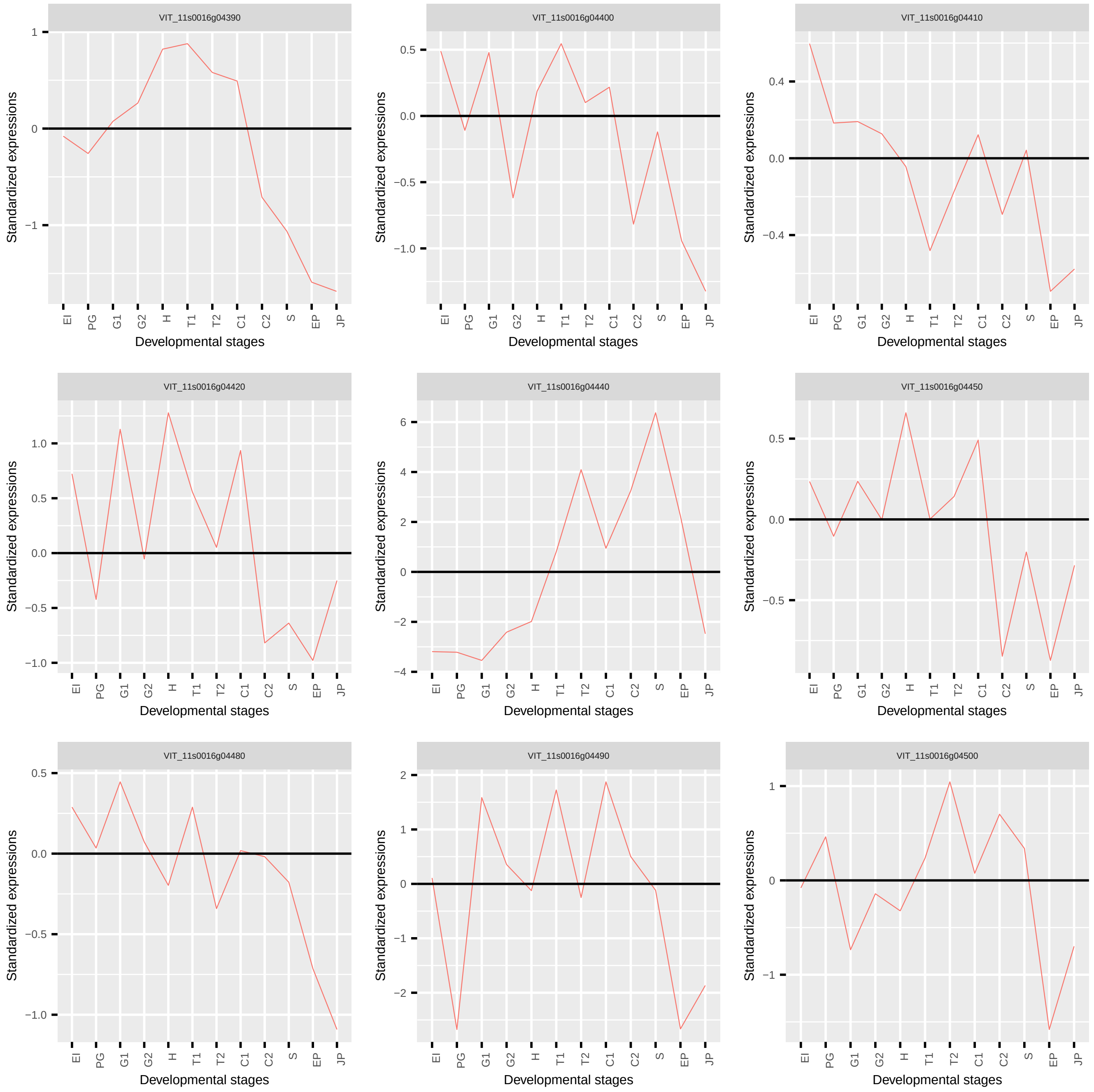

VIT_11s0016g04390
VIT_11s0016g04400
VIT_11s0016g04410
VIT_11s0016g04420
VIT_11s0016g04440
VIT_11s0016g04450
VIT_11s0016g04480
VIT_11s0016g04490
VIT_11s0016g04500
Standardized expressions
Developmental stages
EI PG G1 G2 H T1 T2 C1 C2 S EP JP

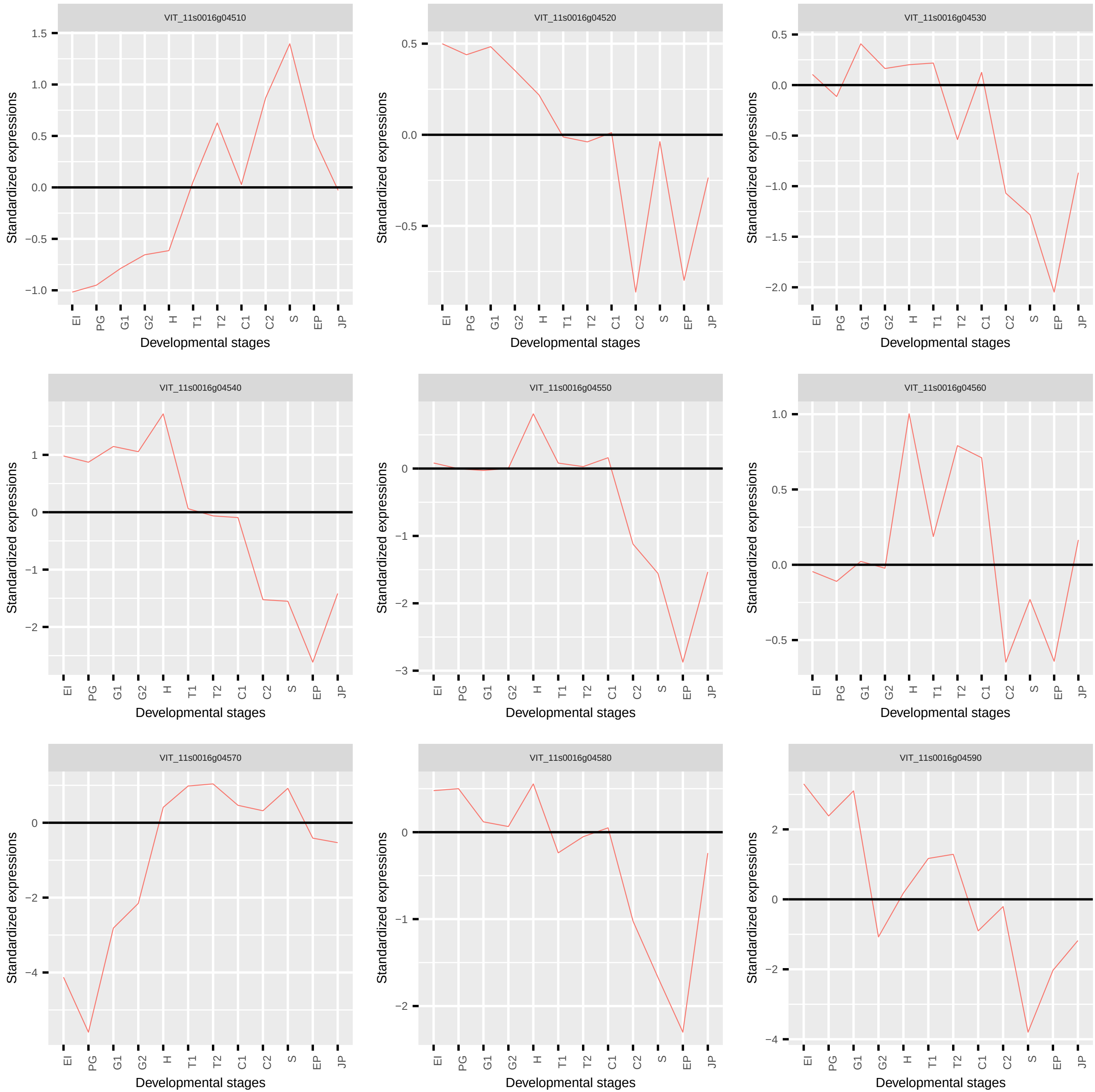
VIT_11s0016g04510
VIT_11s0016g04520
VIT_11s0016g04530
VIT_11s0016g04540
VIT_11s0016g04550
VIT_11s0016g04560
VIT_11s0016g04570
VIT_11s0016g04580
VIT_11s0016g04590
Standardized expressions
Developmental stages
EI PG G1 G2 H T1 T2 C1 C2 S EP JP

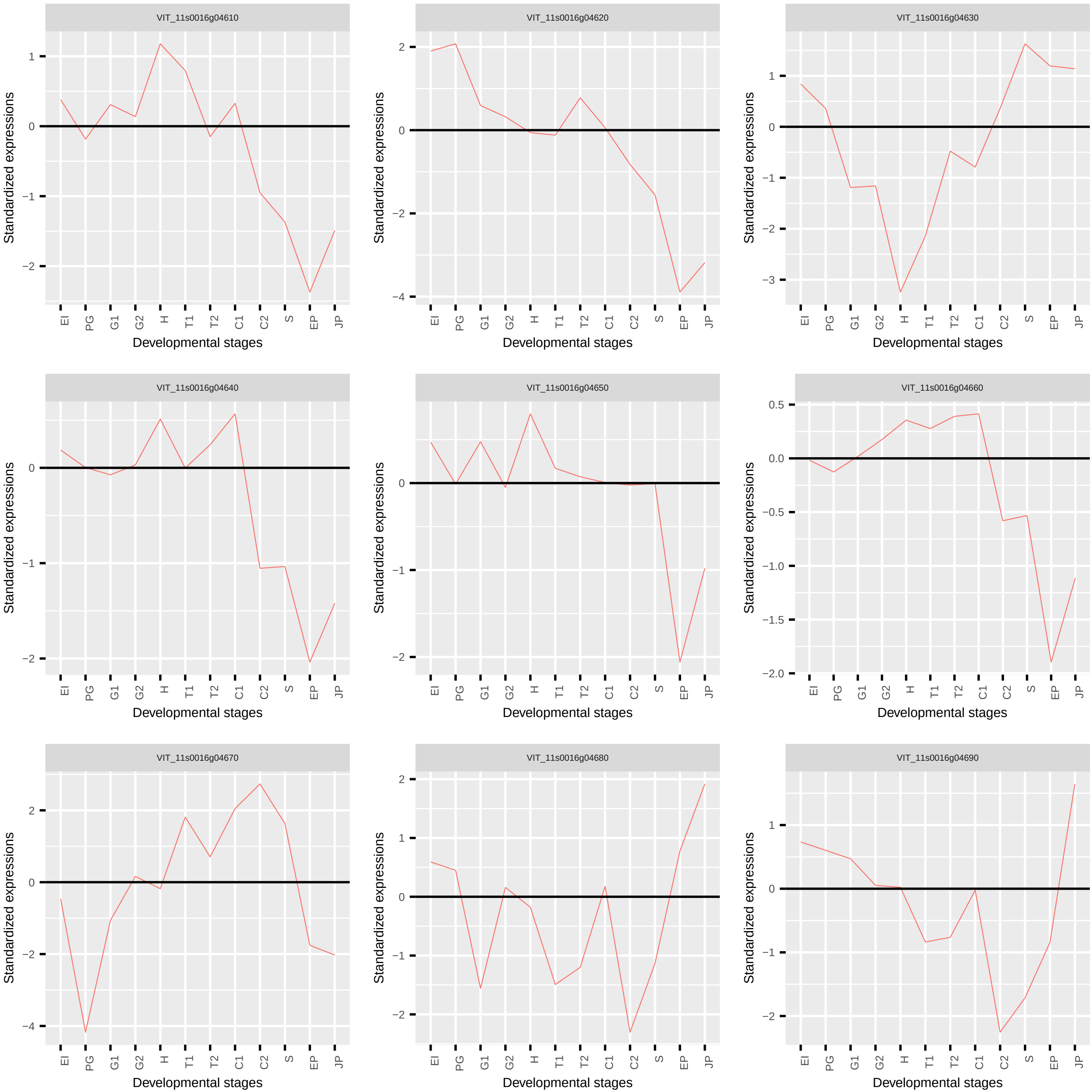
VIT_11s0016g04610
VIT_11s0016g04620
VIT_11s0016g04630
VIT_11s0016g04640
VIT_11s0016g04650
VIT_11s0016g04660
VIT_11s0016g04670
VIT_11s0016g04680
VIT_11s0016g04690
Standardized expressions
Developmental stages
EI
PG
G1
G2
H
T1
T2
C1
C2
S
EP
JP

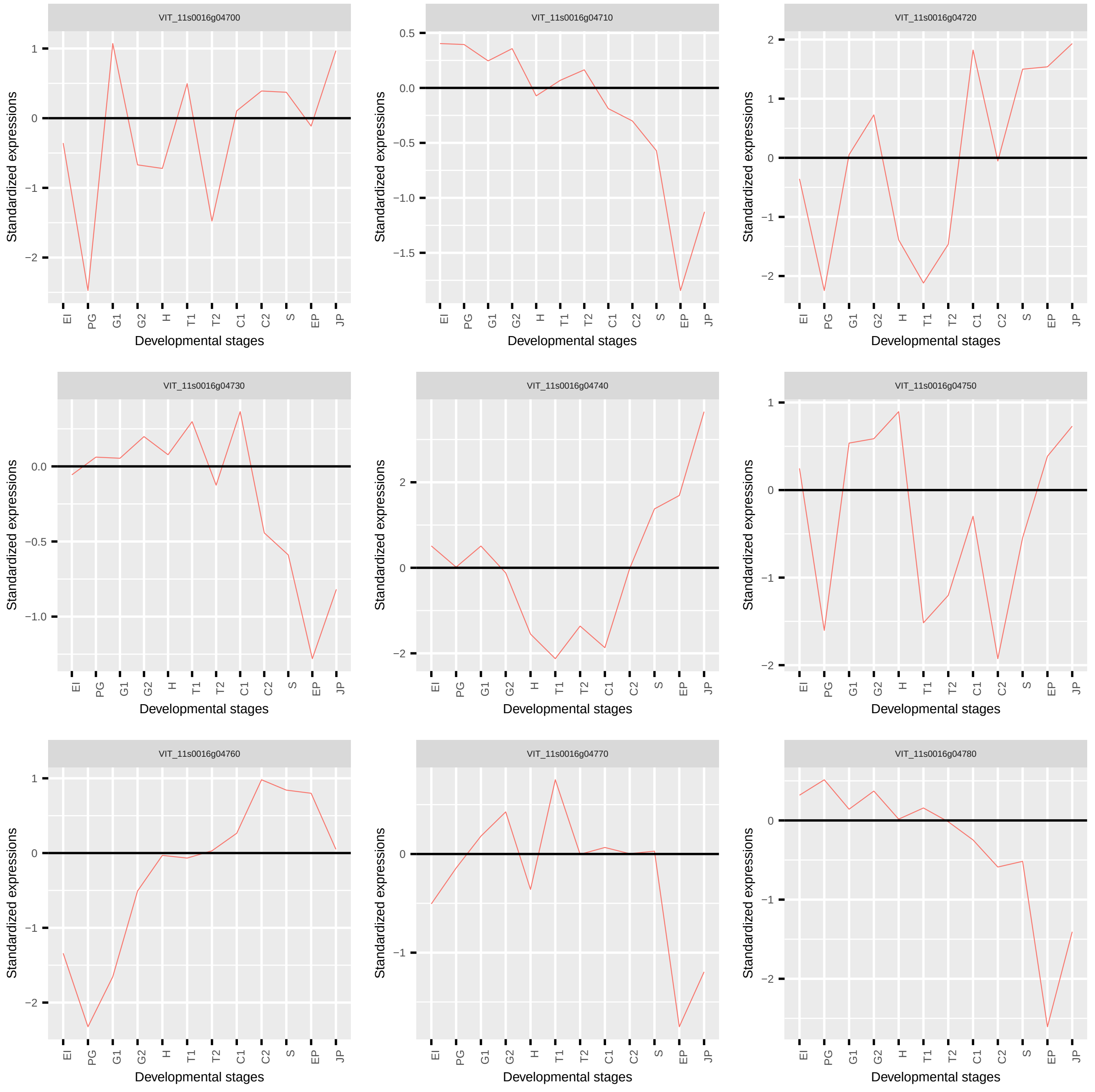
VIT_11s0016g04700
VIT_11s0016g04710
VIT_11s0016g04720
VIT_11s0016g04730
VIT_11s0016g04740
VIT_11s0016g04750
VIT_11s0016g04760
VIT_11s0016g04770
VIT_11s0016g04780
Standardized expressions
Developmental stages
EI
PG
G1
G2
H
T1
T2
C1
C2
S
EP
JP

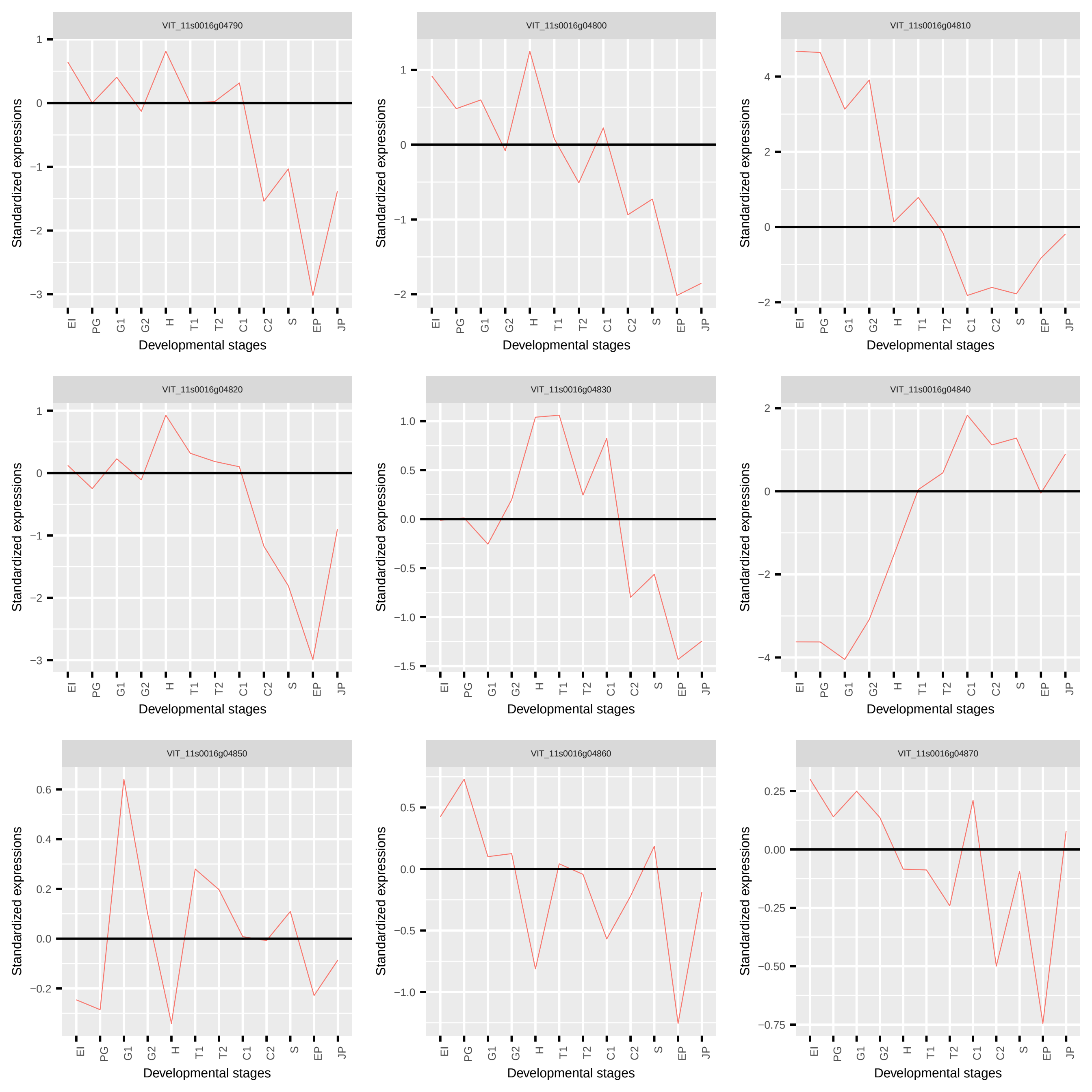
VIT_11s0016g04790
VIT_11s0016g04800
VIT_11s0016g04810
VIT_11s0016g04820
VIT_11s0016g04830
VIT_11s0016g04840
VIT_11s0016g04850
VIT_11s0016g04860
VIT_11s0016g04870
Standardized expressions
Developmental stages
EI PG G1 G2 H T1 T2 C1 C2 S EP JP

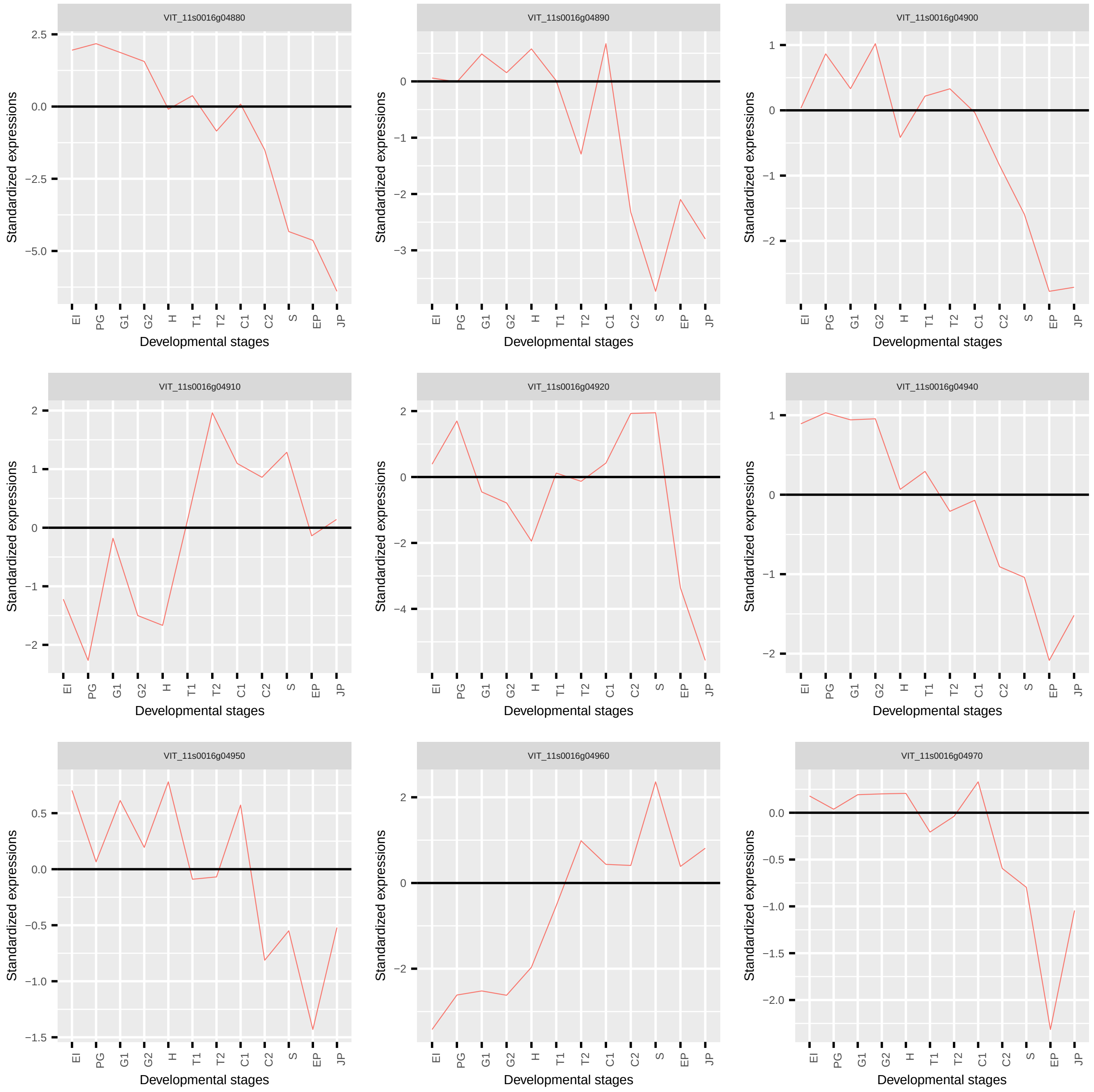

VIT_11s0016g04880
VIT_11s0016g04890
VIT_11s0016g04900
VIT_11s0016g04910
VIT_11s0016g04920
VIT_11s0016g04940
VIT_11s0016g04950
VIT_11s0016g04960
VIT_11s0016g04970
Standardized expressions
Developmental stages
EI
PG
G1
G2
H
T1
T2
C1
C2
S
EP
JP

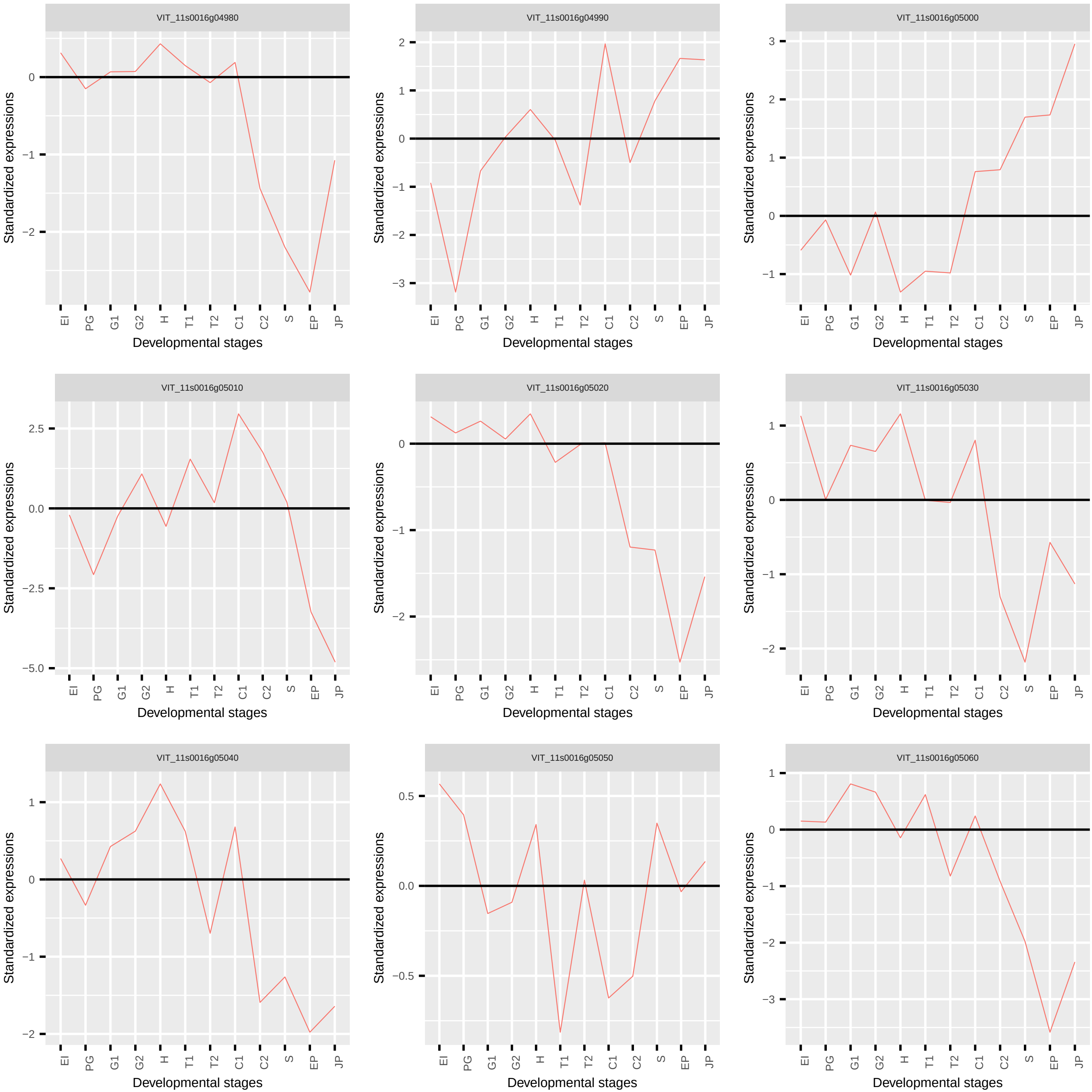
VIT_11s0016g04980
VIT_11s0016g04990
VIT_11s0016g05000
VIT_11s0016g05010
VIT_11s0016g05020
VIT_11s0016g05030
VIT_11s0016g05040
VIT_11s0016g05050
VIT_11s0016g05060
Standardized expressions
Developmental stages
EI PG G1 G2 H T1 T2 C1 C2 S EP JP

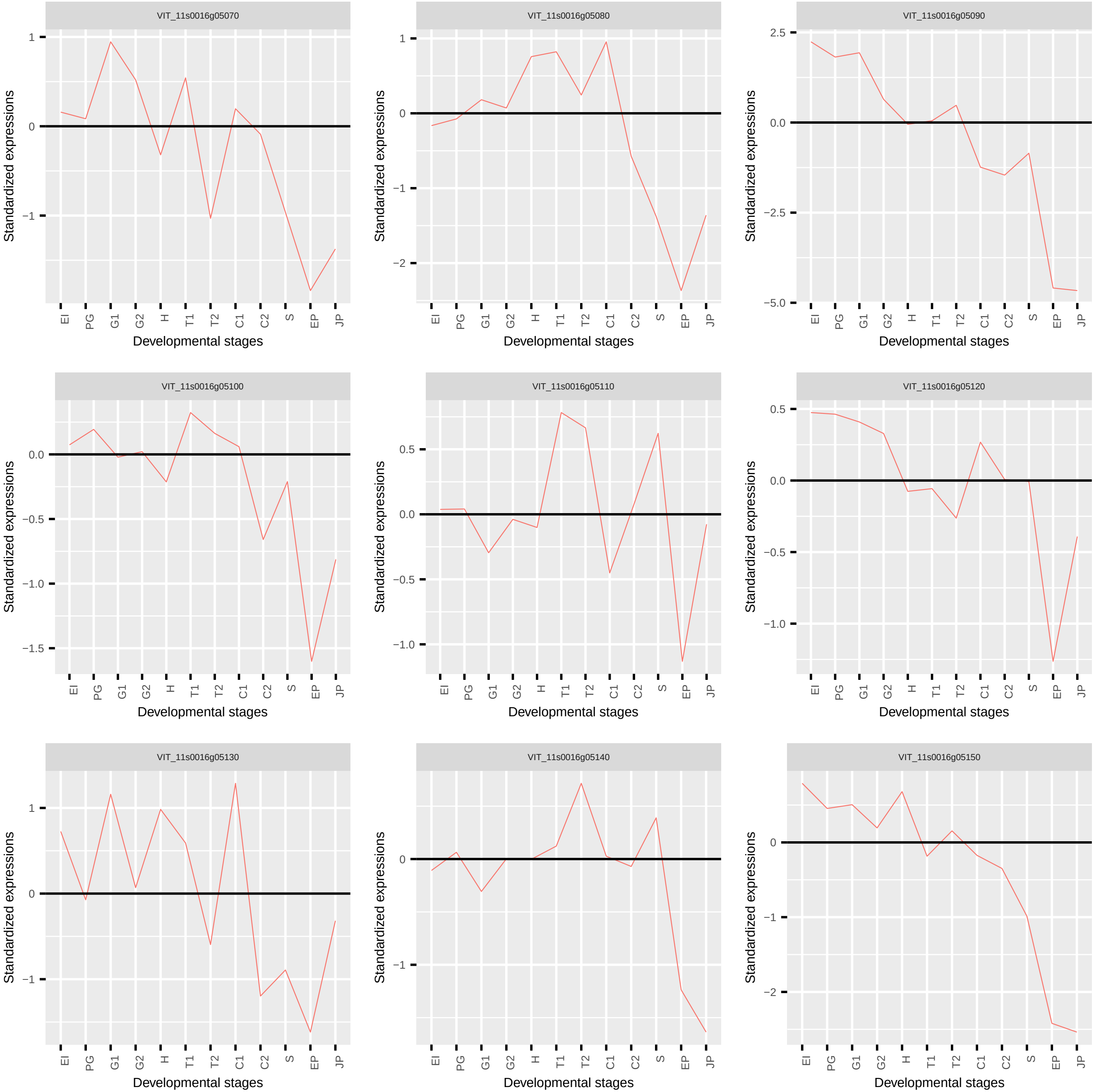
VIT_11s0016g05070
VIT_11s0016g05080
VIT_11s0016g05090
VIT_11s0016g05100
VIT_11s0016g05110
VIT_11s0016g05120
VIT_11s0016g05130
VIT_11s0016g05140
VIT_11s0016g05150
Standardized expressions
Developmental stages
EI
PG
G1
G2
H
T1
T2
C1
C2
S
EP
JP
1
0
−1
−2
2.5
0.0
−2.5
−5.0
0.5
−0.5
−1.0
−1.5

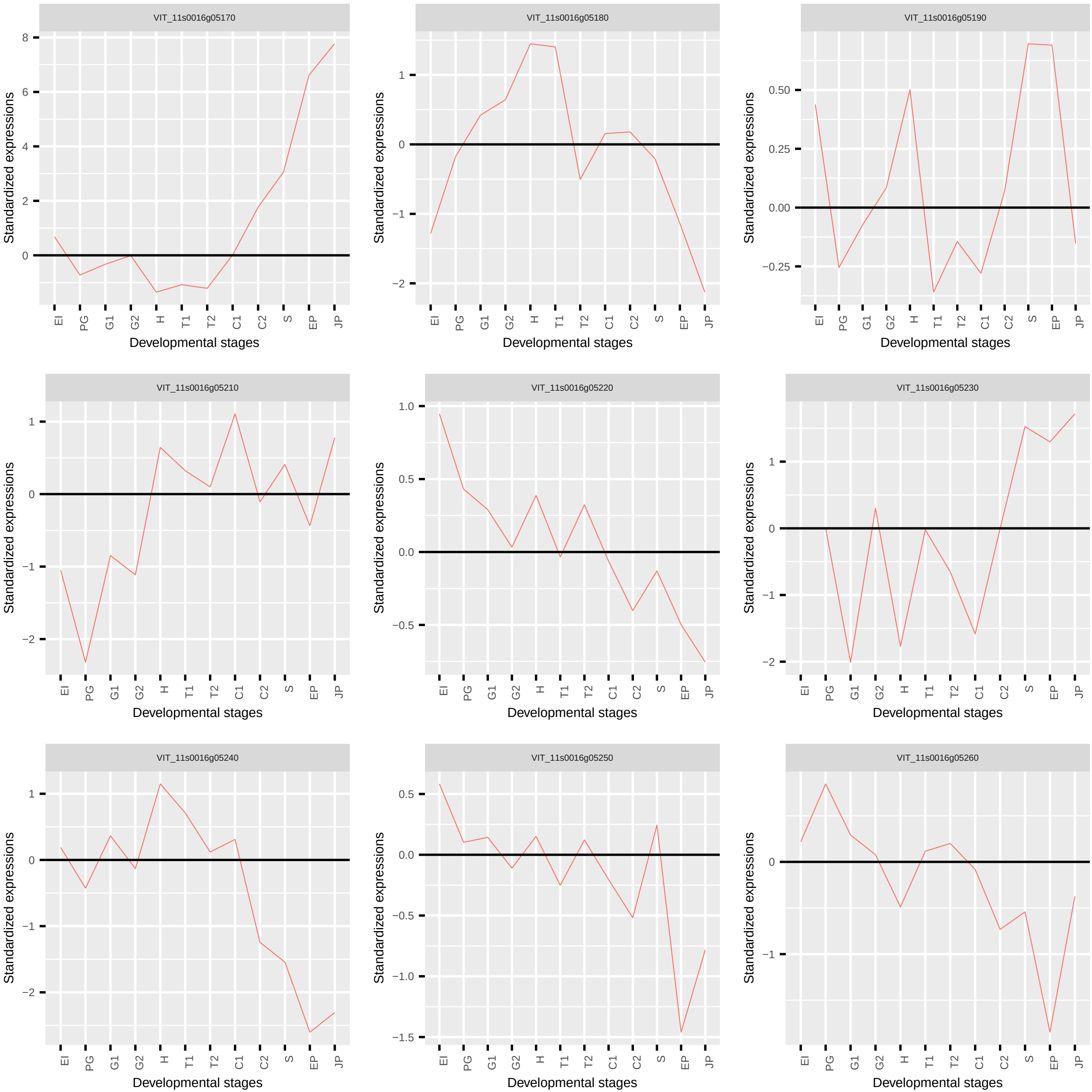
VIT_11s0016g05170
VIT_11s0016g05180
VIT_11s0016g05190
VIT_11s0016g05210
VIT_11s0016g05220
VIT_11s0016g05230
VIT_11s0016g05240
VIT_11s0016g05250
VIT_11s0016g05260
Standardized expressions
Developmental stages
EI PG G1 G2 H T1 T2 C1 C2 S EP JP

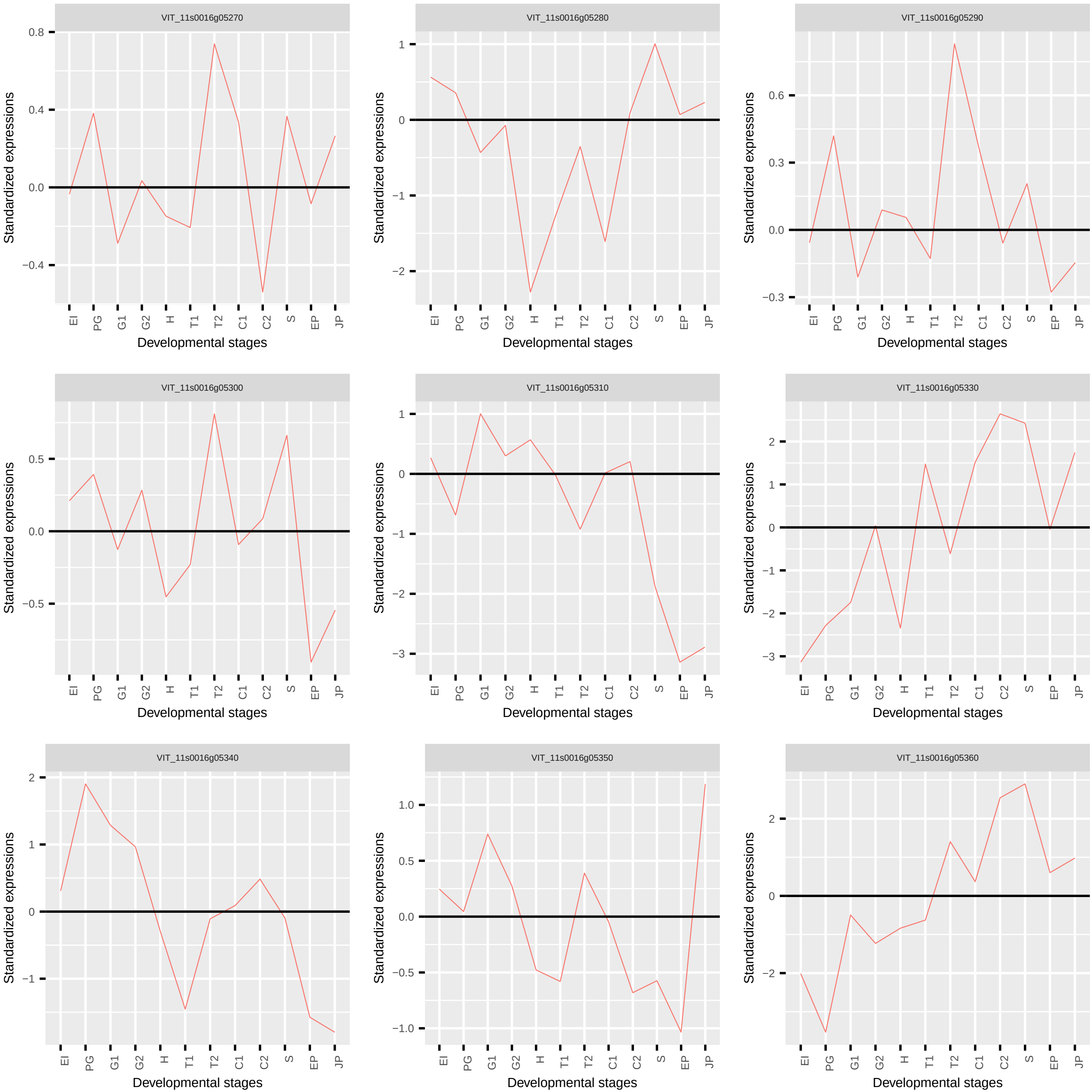
VIT_11s0016g05270
VIT_11s0016g05280
VIT_11s0016g05290
VIT_11s0016g05300
VIT_11s0016g05310
VIT_11s0016g05330
VIT_11s0016g05340
VIT_11s0016g05350
VIT_11s0016g05360
Standardized expressions
Developmental stages
EI
PG
G1
G2
H
T1
T2
C1
C2
S
EP
JP

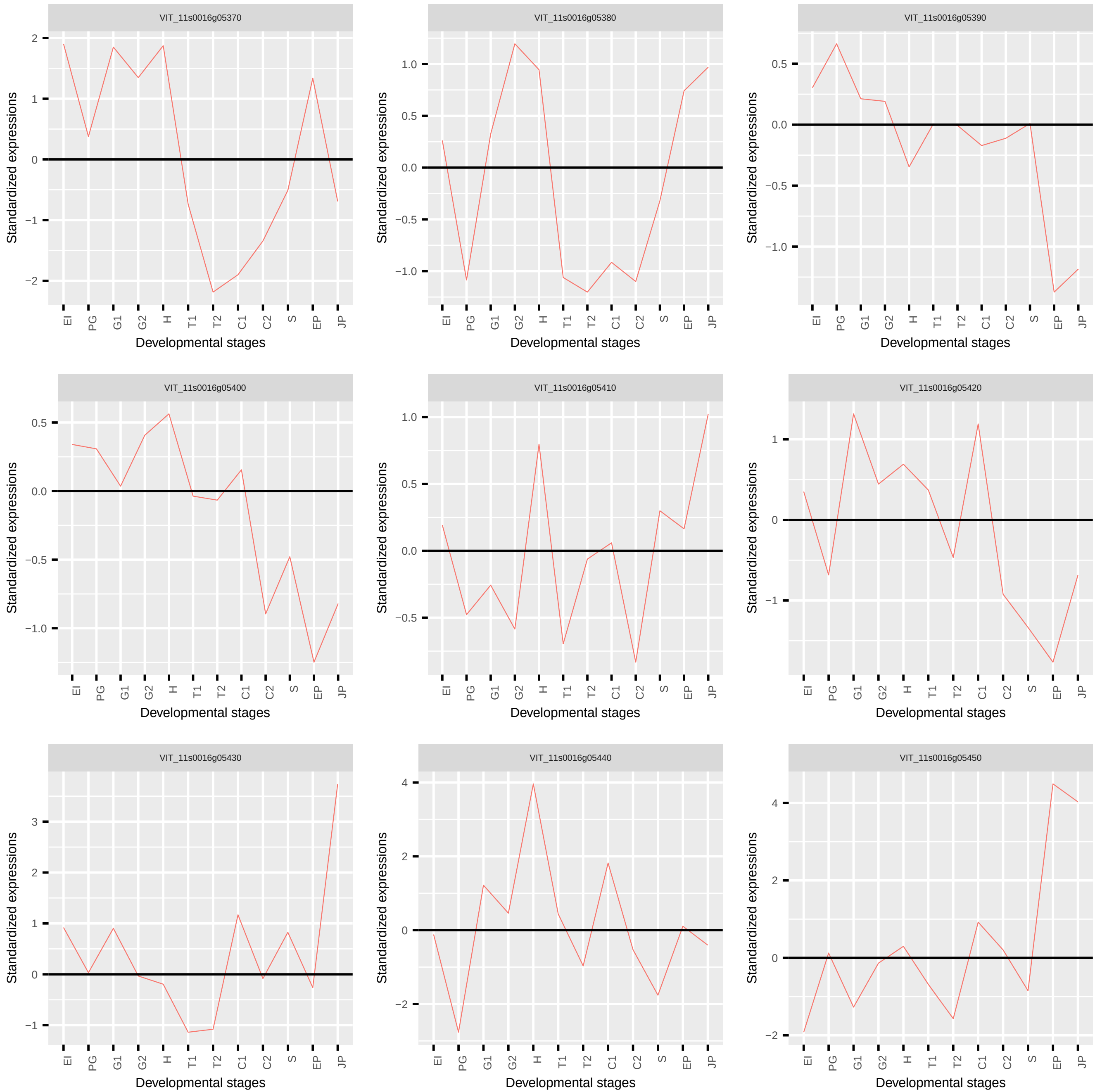

VIT_11s0016g05370
VIT_11s0016g05380
VIT_11s0016g05390
VIT_11s0016g05400
VIT_11s0016g05410
VIT_11s0016g05420
VIT_11s0016g05430
VIT_11s0016g05440
VIT_11s0016g05450
Standardized expressions
Developmental stages
EI
PG
G1
G2
H
T1
T2
C1
C2
S
EP
JP

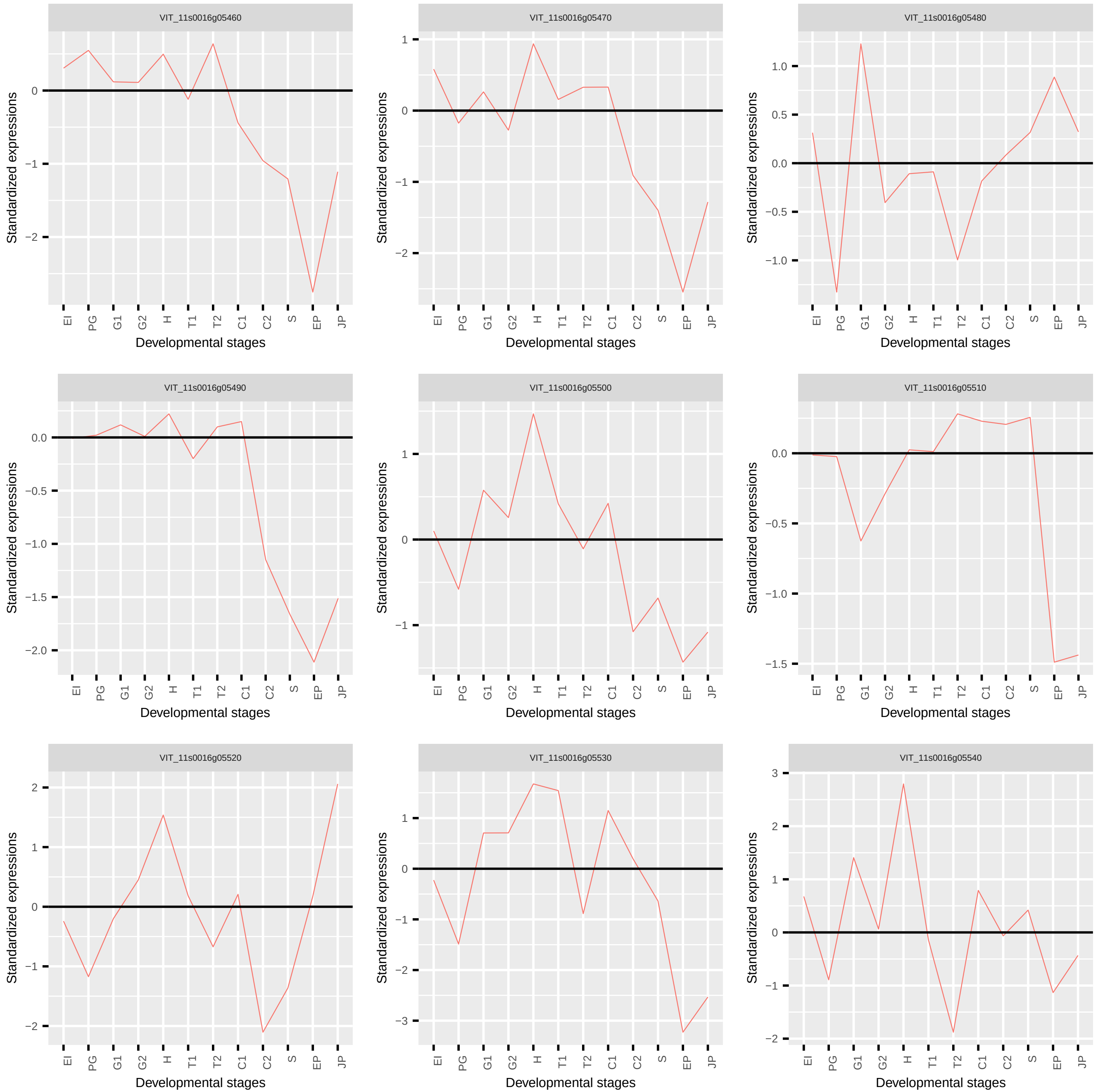
VIT_11s0016g05460
VIT_11s0016g05470
VIT_11s0016g05480
VIT_11s0016g05490
VIT_11s0016g05500
VIT_11s0016g05510
VIT_11s0016g05520
VIT_11s0016g05530
VIT_11s0016g05540
Standardized expressions
Developmental stages
EI
PG
G1
G2
H
T1
T2
C1
C2
S
EP
JP

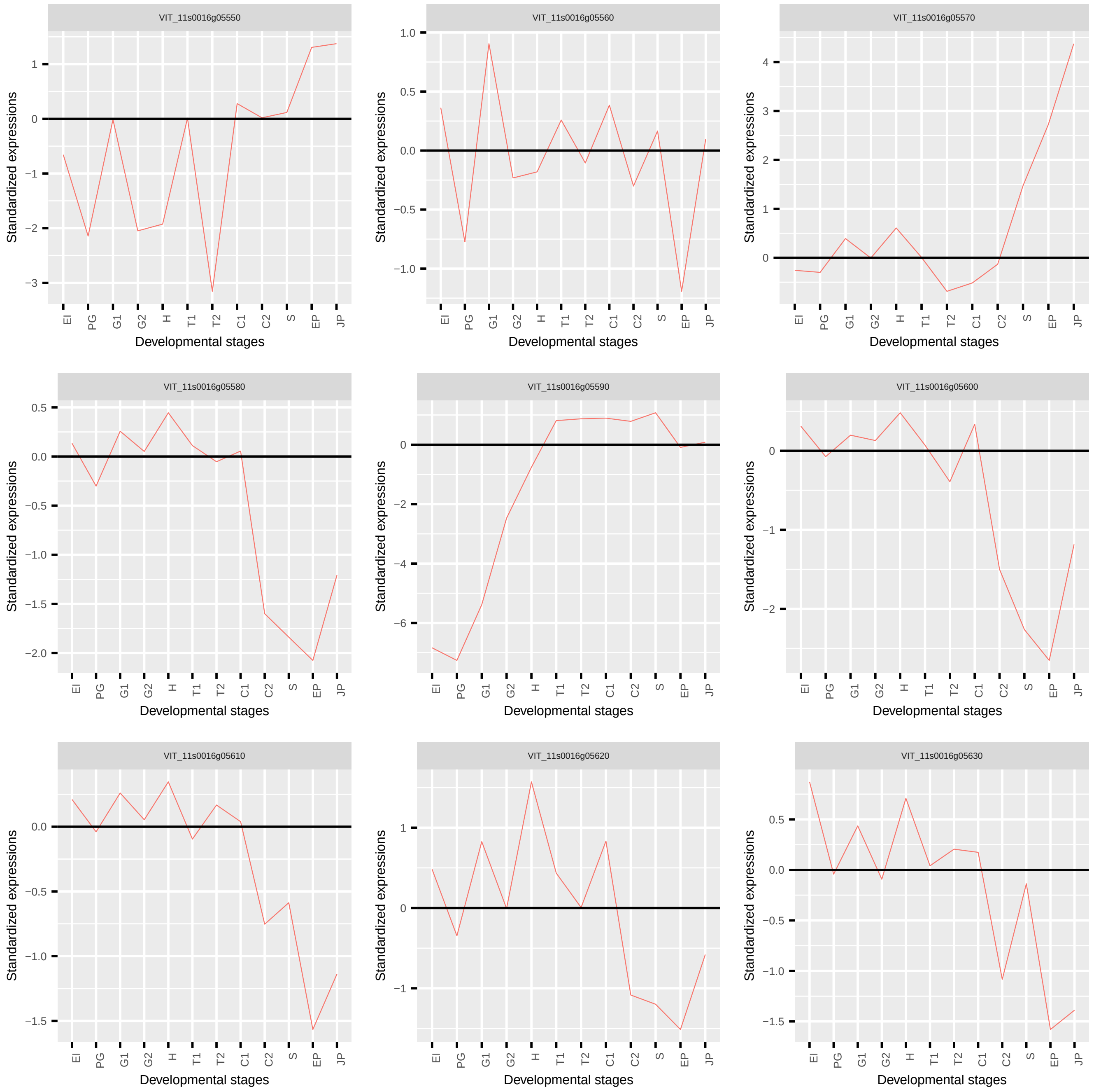

VIT_11s0016g05550
VIT_11s0016g05560
VIT_11s0016g05570
VIT_11s0016g05580
VIT_11s0016g05590
VIT_11s0016g05600
VIT_11s0016g05610
VIT_11s0016g05620
VIT_11s0016g05630
Standardized expressions
Developmental stages
EI PG G1 G2 H T1 T2 C1 C2 S EP JP

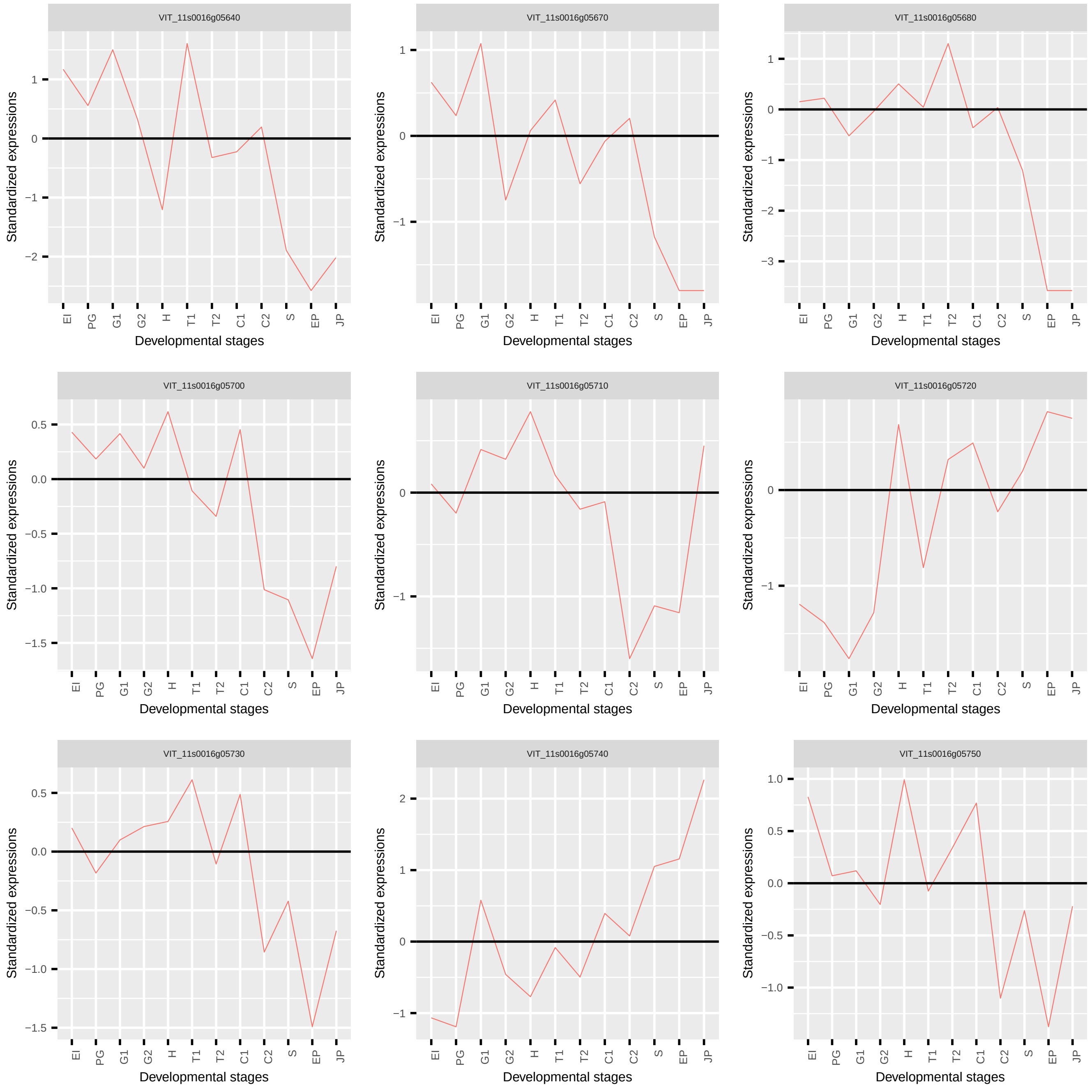
VIT_11s0016g05640
VIT_11s0016g05670
VIT_11s0016g05680
VIT_11s0016g05700
VIT_11s0016g05710
VIT_11s0016g05720
VIT_11s0016g05730
VIT_11s0016g05740
VIT_11s0016g05750
Standardized expressions
Developmental stages
EI PG G1 G2 H T1 T2 C1 C2 S EP JP

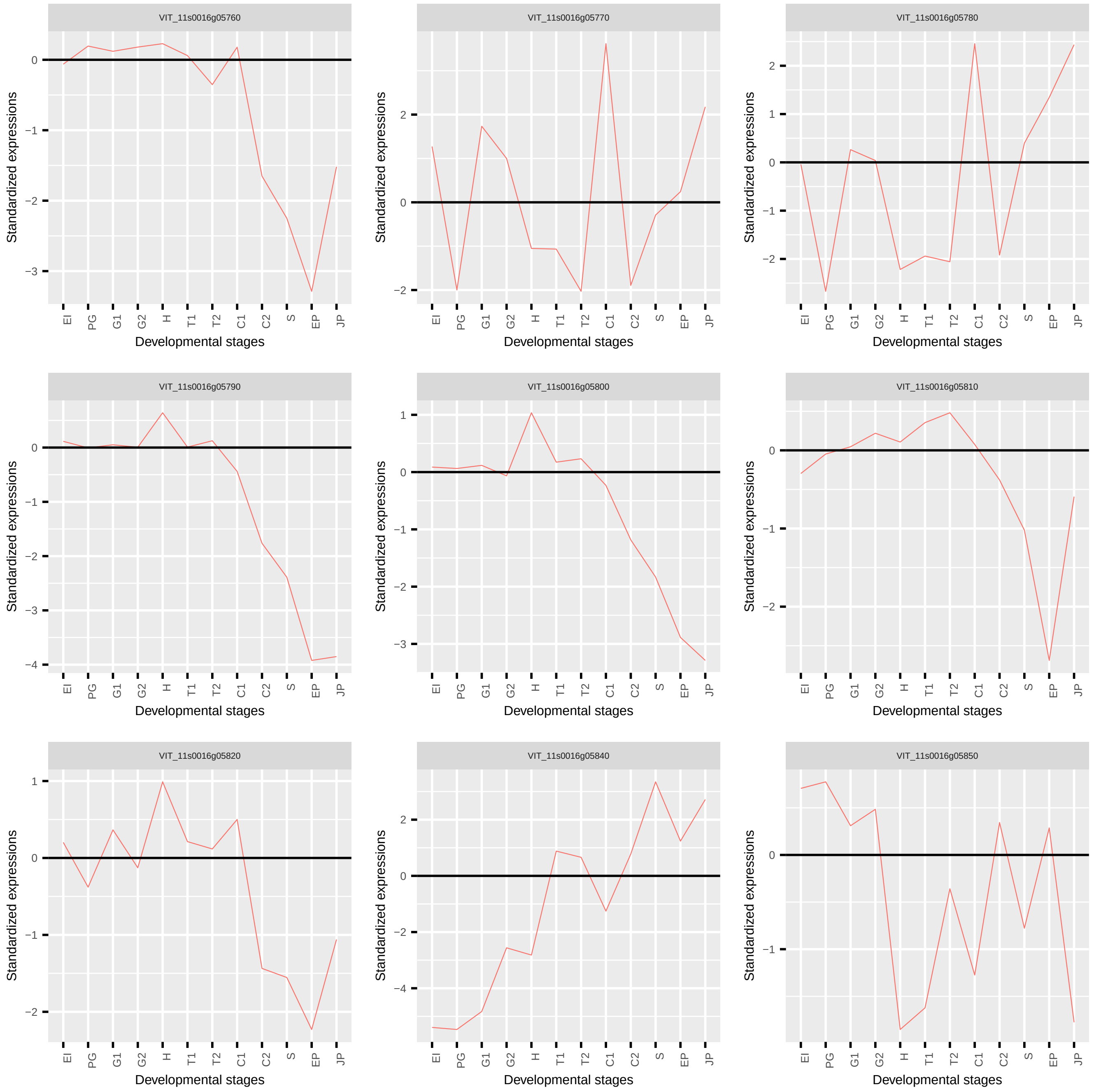
VIT_11s0016g05760
VIT_11s0016g05770
VIT_11s0016g05780
VIT_11s0016g05790
VIT_11s0016g05800
VIT_11s0016g05810
VIT_11s0016g05820
VIT_11s0016g05840
VIT_11s0016g05850
Standardized expressions
Developmental stages
EI PG G1 G2 H T1 T2 C1 C2 S EP JP

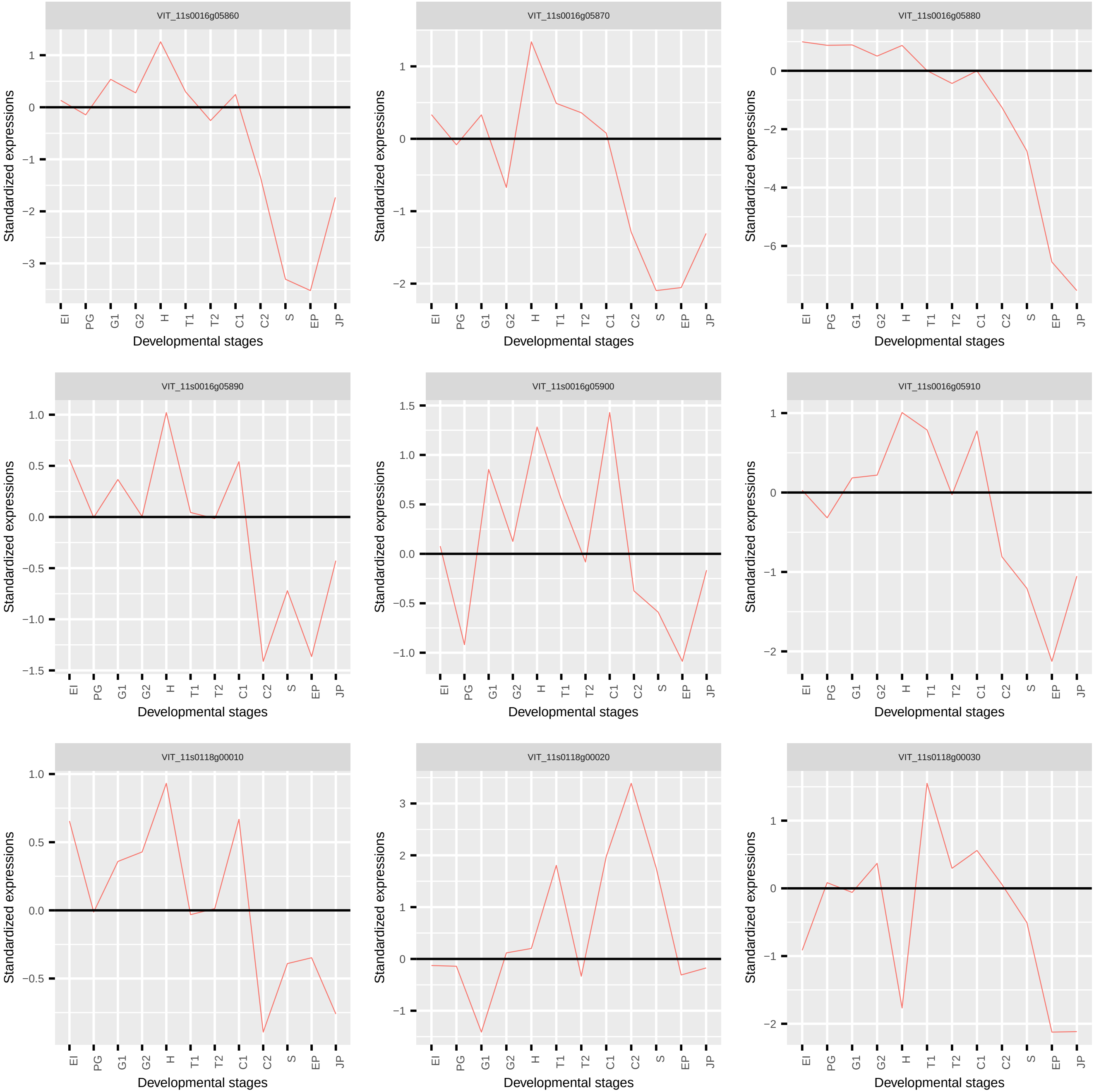
VIT_11s0016g05860
VIT_11s0016g05870
VIT_11s0016g05880
VIT_11s0016g05890
VIT_11s0016g05900
VIT_11s0016g05910
VIT_11s0118g00010
VIT_11s0118g00020
VIT_11s0118g00030
Standardized expressions
Developmental stages
EI
PG
G1
G2
H
T1
T2
C1
C2
S
EP
JP

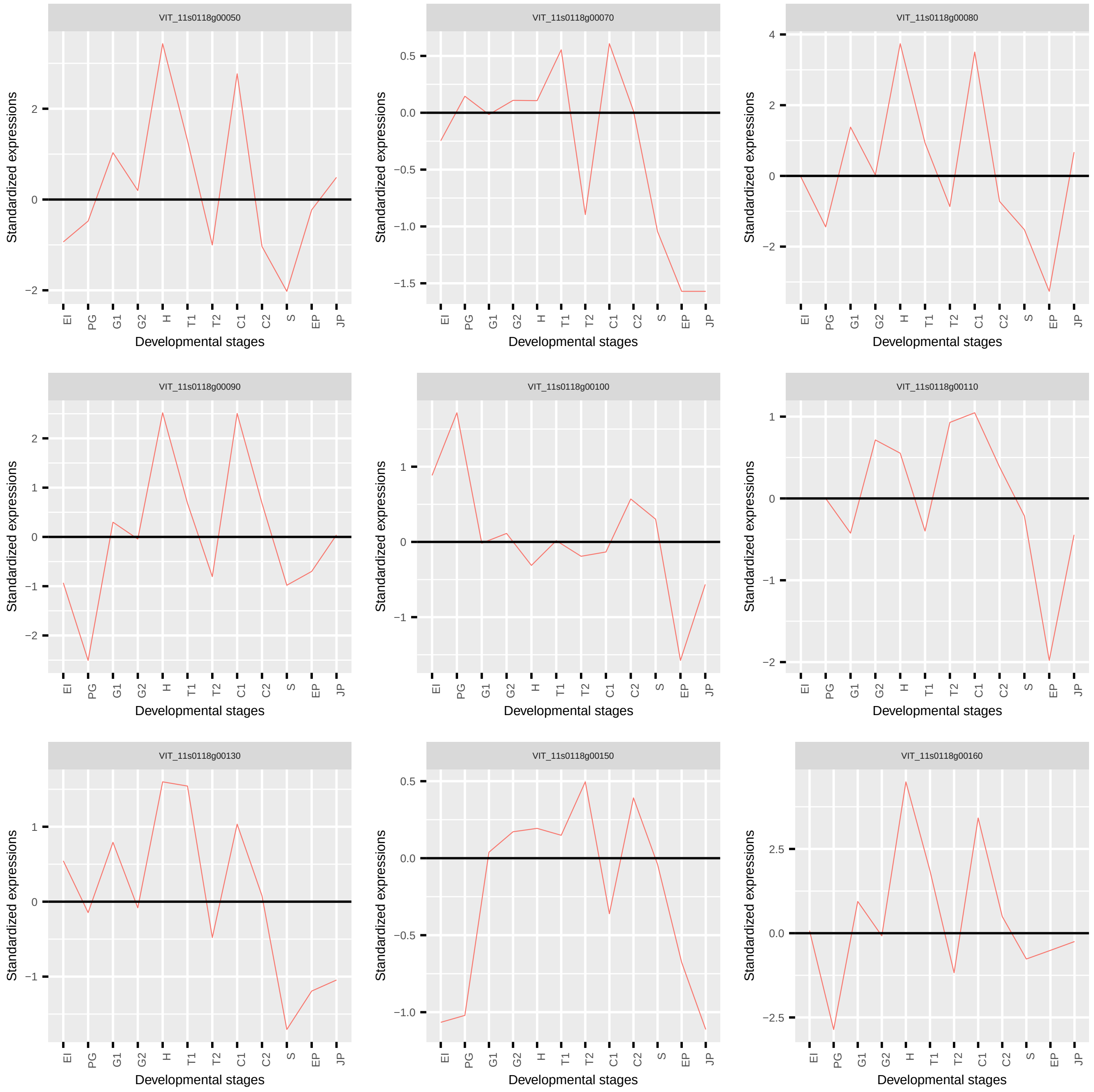

VIT_11s0118g00050
VIT_11s0118g00070
VIT_11s0118g00080
VIT_11s0118g00090
VIT_11s0118g00100
VIT_11s0118g00110
VIT_11s0118g00130
VIT_11s0118g00150
VIT_11s0118g00160
Standardized expressions
Developmental stages
EI PG G1 G2 H T1 T2 C1 C2 S EP JP

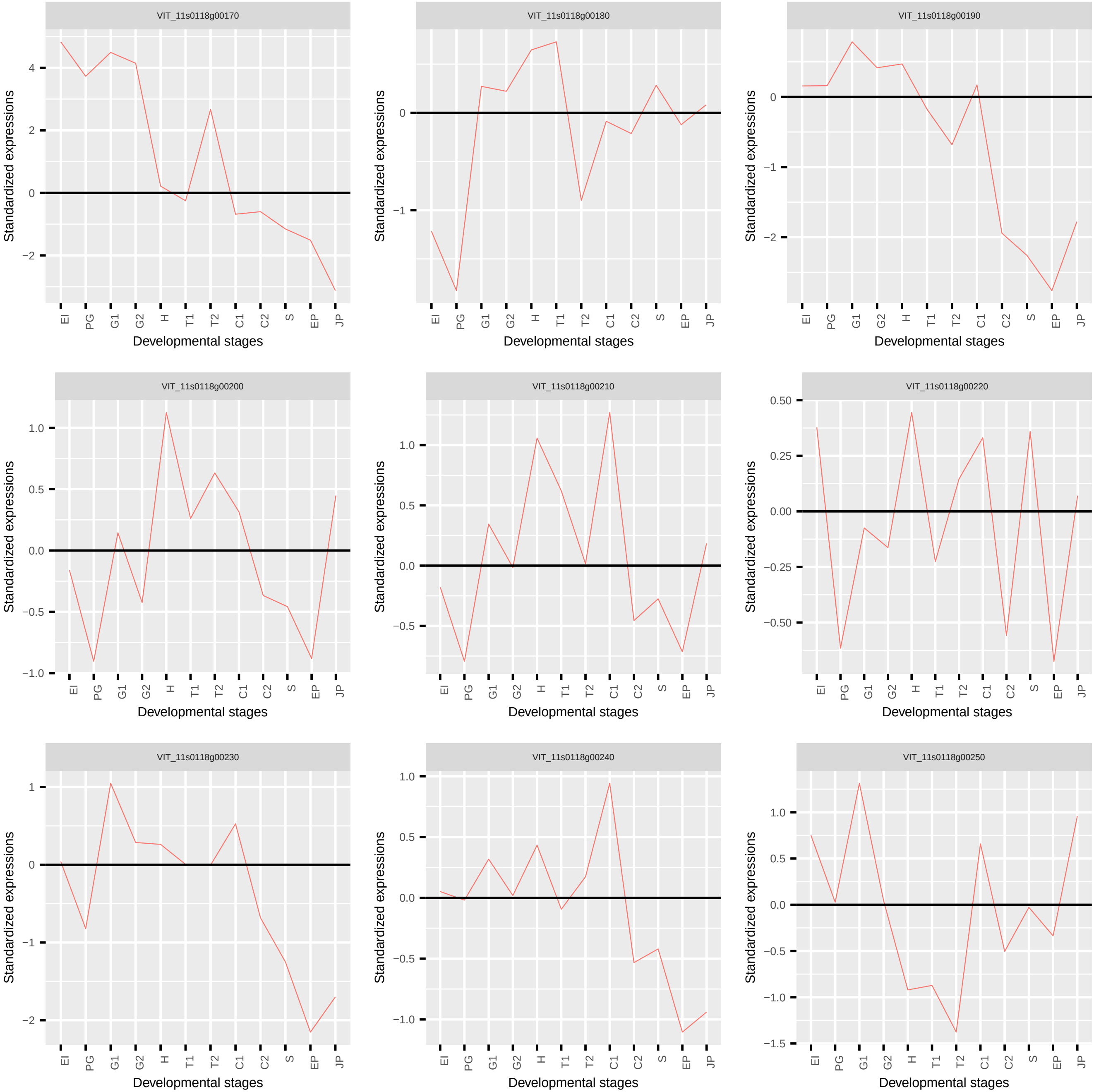
VIT_11s0118g00170
VIT_11s0118g00180
VIT_11s0118g00190
VIT_11s0118g00200
VIT_11s0118g00210
VIT_11s0118g00220
VIT_11s0118g00230
VIT_11s0118g00240
VIT_11s0118g00250
Standardized expressions
Developmental stages
EI PG G1 G2 H T1 T2 C1 C2 S EP JP

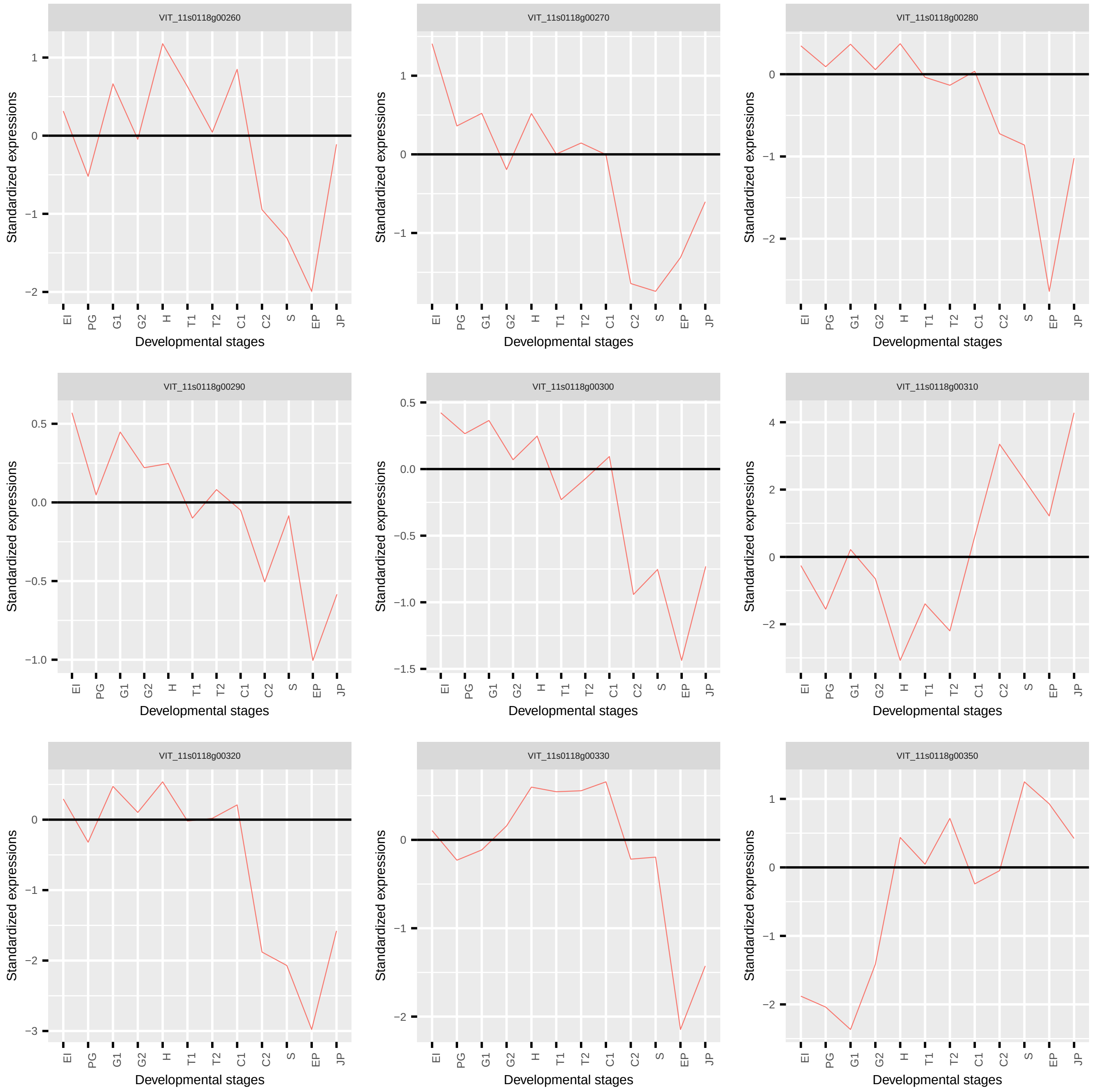
VIT_11s0118g00260
VIT_11s0118g00270
VIT_11s0118g00280
VIT_11s0118g00290
VIT_11s0118g00300
VIT_11s0118g00310
VIT_11s0118g00320
VIT_11s0118g00330
VIT_11s0118g00350
Standardized expressions
Developmental stages
EI PG G1 G2 H T1 T2 C1 C2 S EP JP

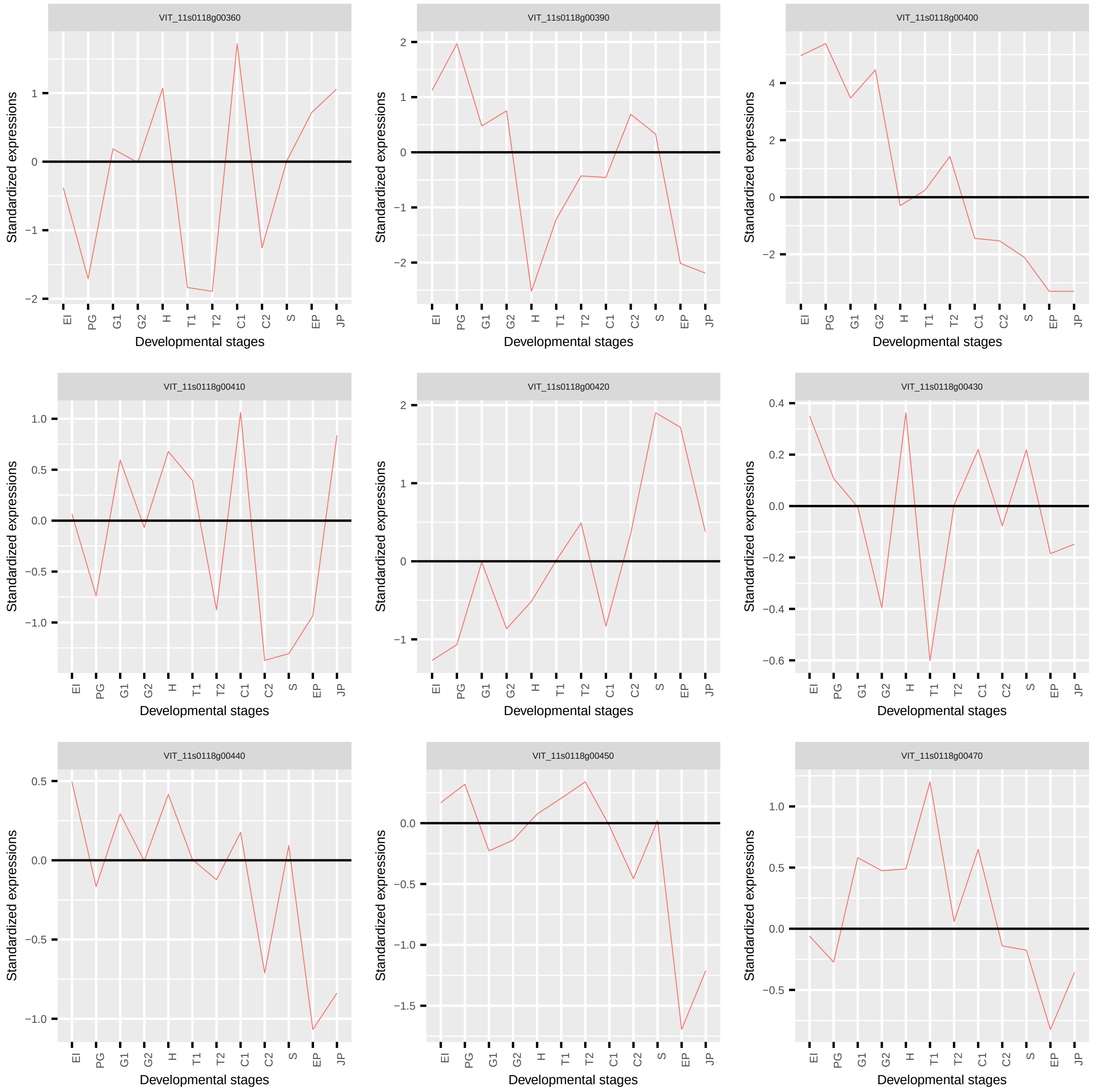
VIT_11s0118g00360
Standardized expressions
Developmental stages
VIT_11s0118g00390
Standardized expressions
Developmental stages
VIT_11s0118g00400
Standardized expressions
Developmental stages
VIT_11s0118g00410
Standardized expressions
Developmental stages
VIT_11s0118g00420
Standardized expressions
Developmental stages
VIT_11s0118g00430
Standardized expressions
Developmental stages
VIT_11s0118g00440
Standardized expressions
Developmental stages
VIT_11s0118g00450
Standardized expressions
Developmental stages
VIT_11s0118g00470
Standardized expressions
Developmental stages
EI PG G1 G2 H T1 T2 C1 C2 S EP JP

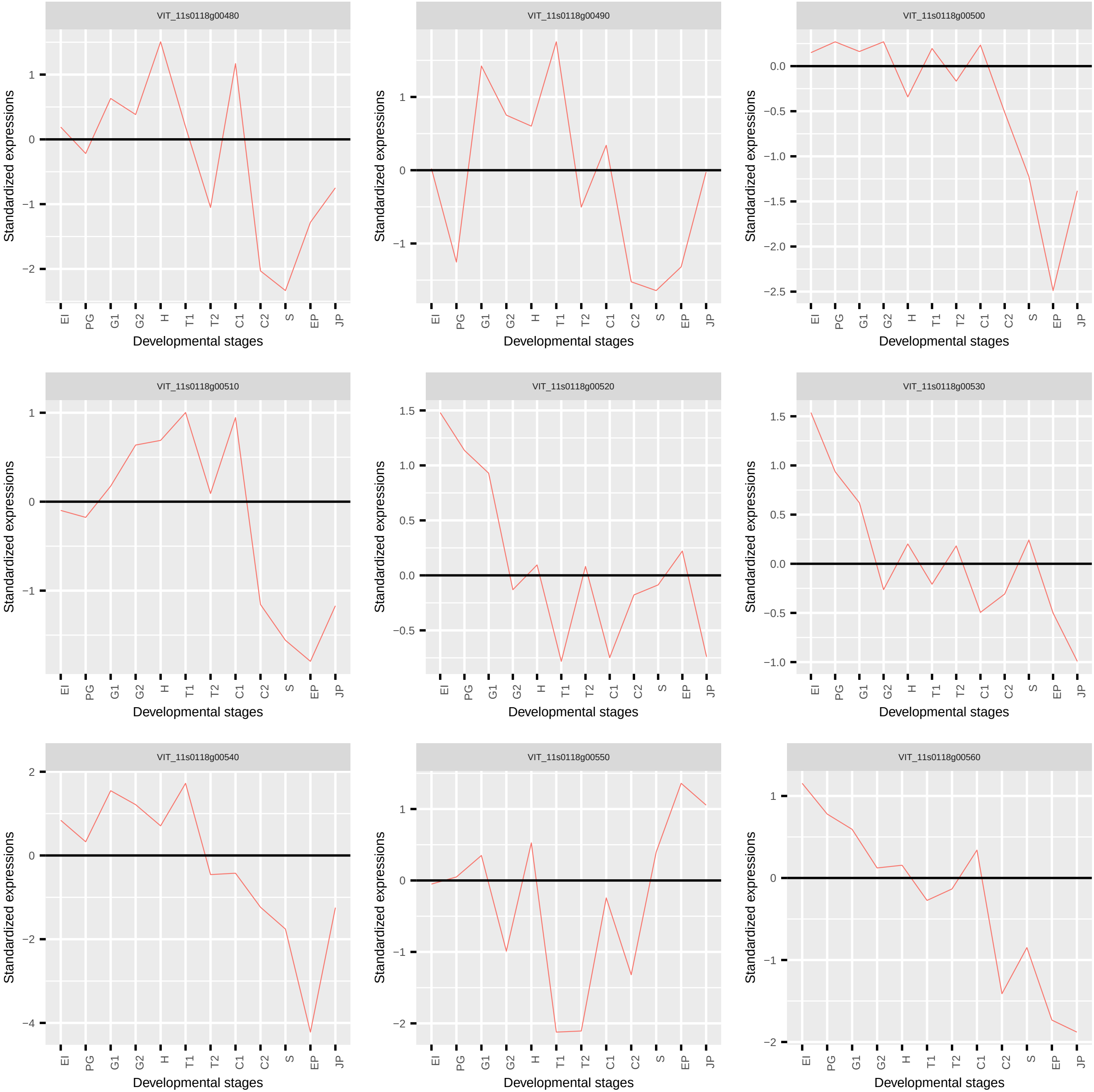
VIT_11s0118g00480
VIT_11s0118g00490
VIT_11s0118g00500
VIT_11s0118g00510
VIT_11s0118g00520
VIT_11s0118g00530
VIT_11s0118g00540
VIT_11s0118g00550
VIT_11s0118g00560
Standardized expressions
Developmental stages
EI PG G1 G2 H T1 T2 C1 C2 S EP JP

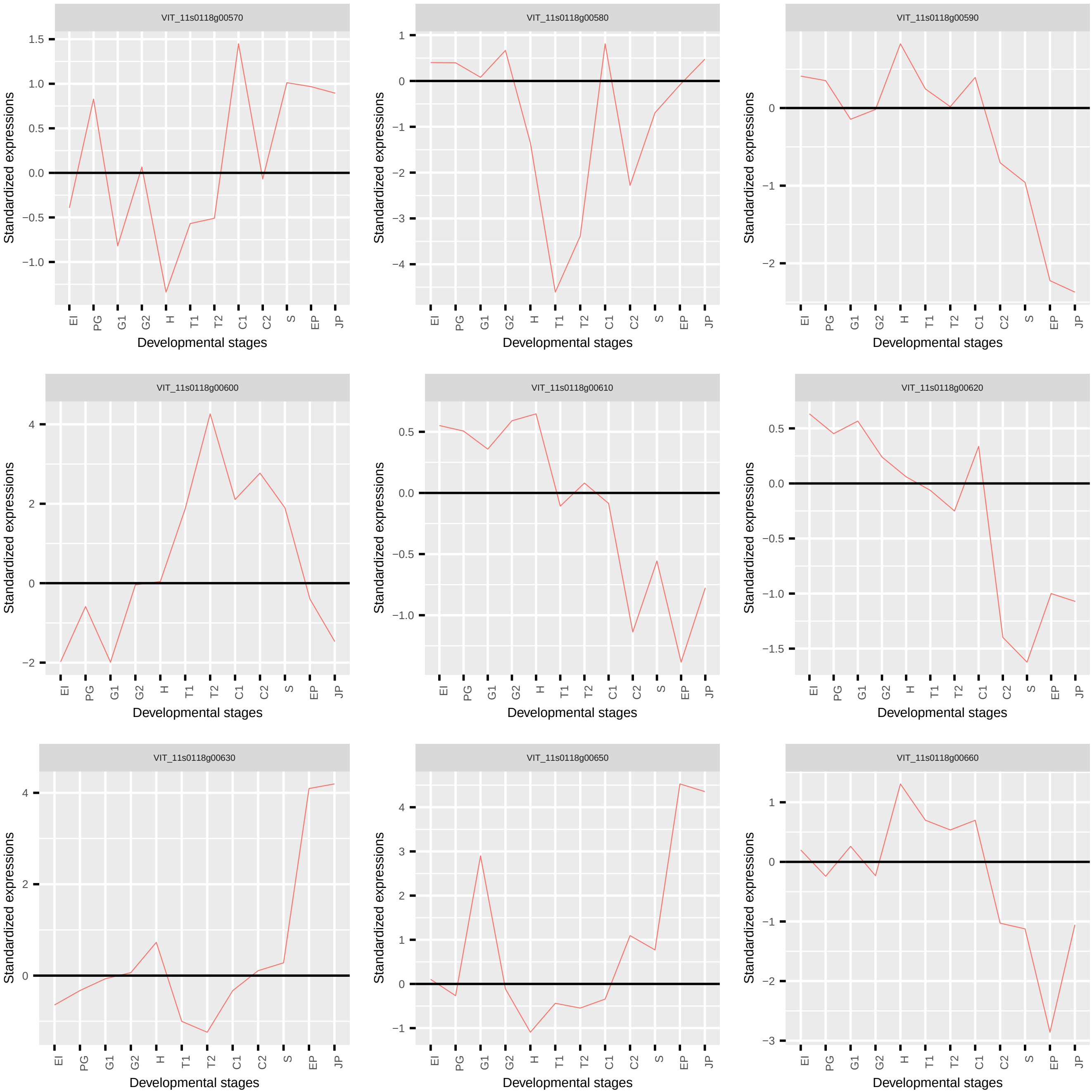

VIT_11s0118g00570
VIT_11s0118g00580
VIT_11s0118g00590
VIT_11s0118g00600
VIT_11s0118g00610
VIT_11s0118g00620
VIT_11s0118g00630
VIT_11s0118g00650
VIT_11s0118g00660
Standardized expressions
Developmental stages
EI PG G1 G2 H T1 T2 C1 C2 S EP JP

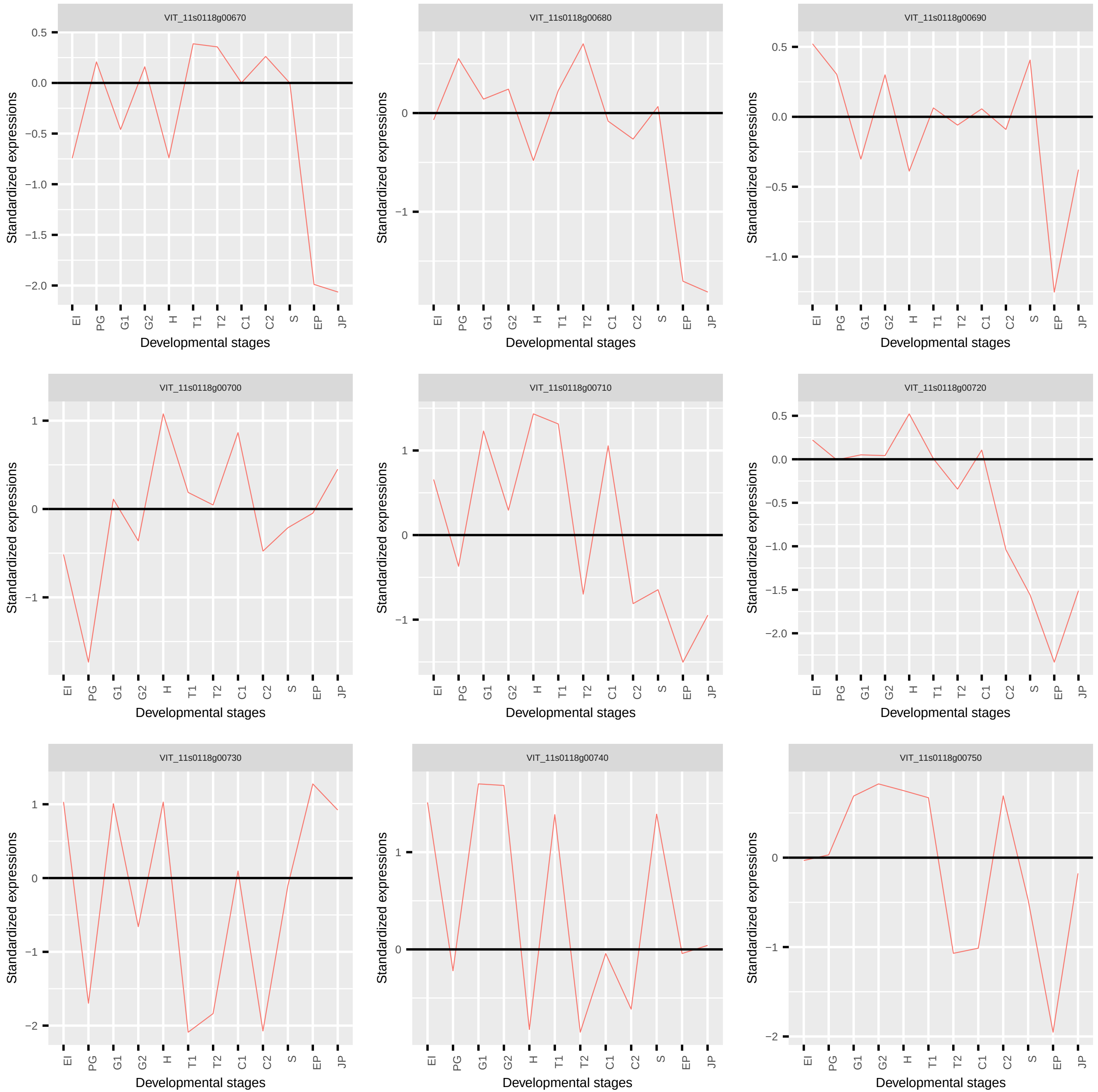
VIT_11s0118g00670
VIT_11s0118g00680
VIT_11s0118g00690
VIT_11s0118g00700
VIT_11s0118g00710
VIT_11s0118g00720
VIT_11s0118g00730
VIT_11s0118g00740
VIT_11s0118g00750
Standardized expressions
Developmental stages
EI
PG
G1
G2
H
T1
T2
C1
C2
S
EP
JP

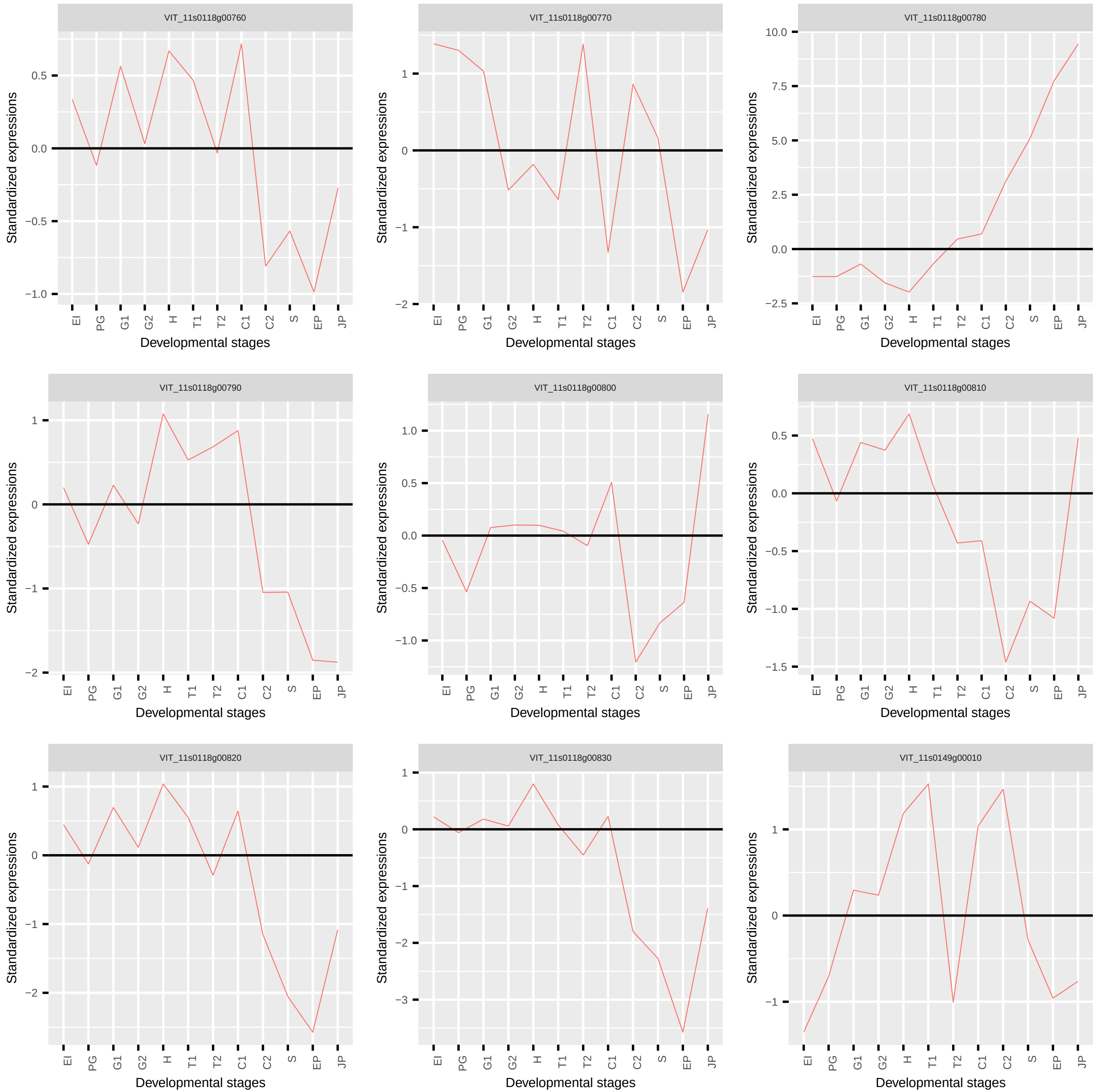
VIT_11s0118g00760
VIT_11s0118g00770
VIT_11s0118g00780
VIT_11s0118g00790
VIT_11s0118g00800
VIT_11s0118g00810
VIT_11s0118g00820
VIT_11s0118g00830
VIT_11s0149g00010
Standardized expressions
Developmental stages
EI
PG
G1
G2
H
T1
T2
C1
C2
S
EP
JP

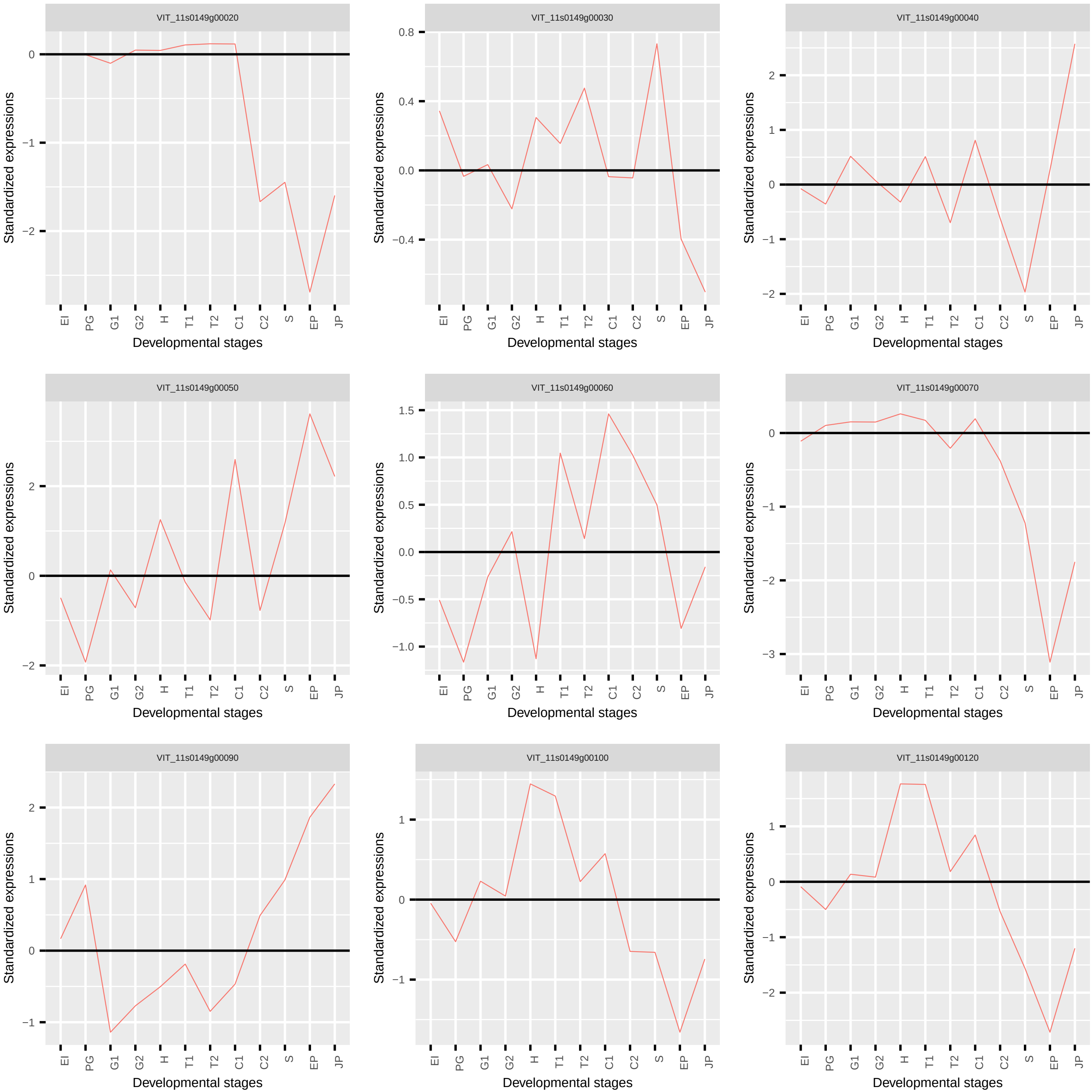
VIT_11s0149g00020
VIT_11s0149g00030
VIT_11s0149g00040
VIT_11s0149g00050
VIT_11s0149g00060
VIT_11s0149g00070
VIT_11s0149g00090
VIT_11s0149g00100
VIT_11s0149g00120
Standardized expressions
Developmental stages
EI
PG
G1
G2
H
T1
T2
C1
C2
S
EP
JP

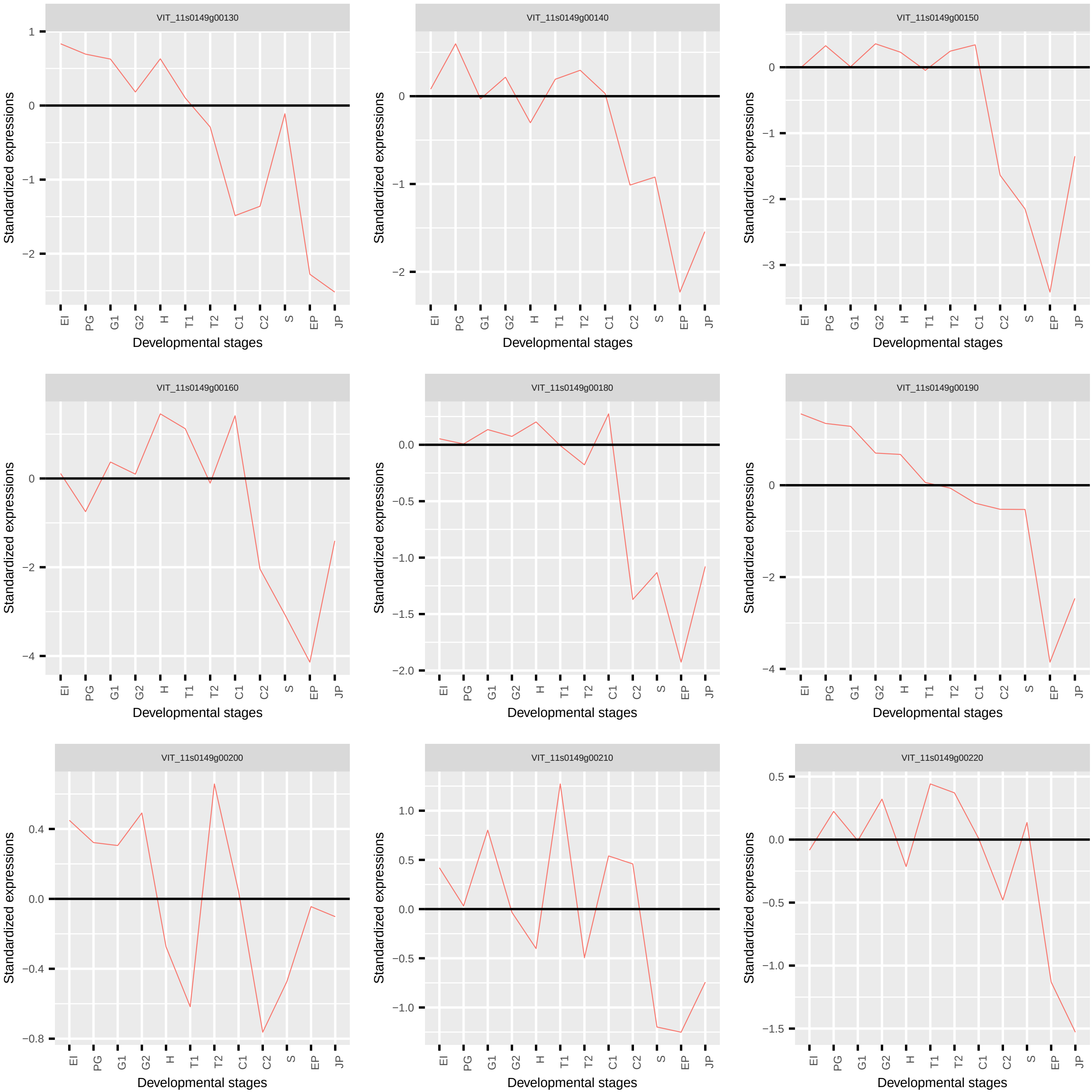

VIT_11s0149g00130
VIT_11s0149g00140
VIT_11s0149g00150
VIT_11s0149g00160
VIT_11s0149g00180
VIT_11s0149g00190
VIT_11s0149g00200
VIT_11s0149g00210
VIT_11s0149g00220
Standardized expressions
Developmental stages
EI PG G1 G2 H T1 T2 C1 C2 S EP JP

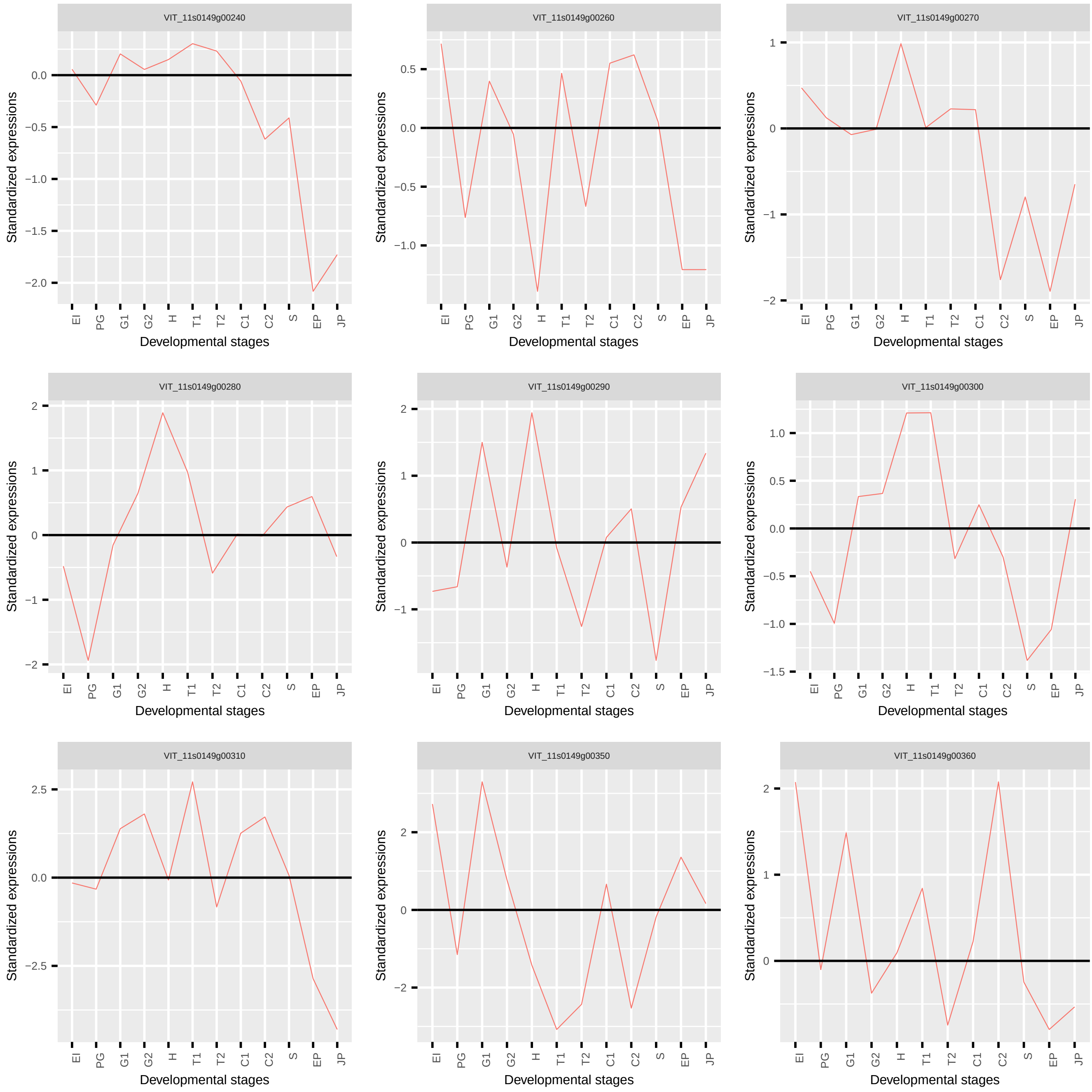
VIT_11s0149g00240
Standardized expressions
0.0
−0.5
−1.0
−1.5
−2.0
EI
PG
G1
G2
H
T1
T2
C1
C2
S
EP
JP
Developmental stages
VIT_11s0149g00260
0.5
0.0
−0.5
−1.0
VIT_11s0149g00270
1
0
−1
−2
VIT_11s0149g00280
2
1
0
−1
−2
VIT_11s0149g00290
VIT_11s0149g00300
1.0
0.5
0.0
−0.5
−1.0
−1.5
VIT_11s0149g00310
2.5
0.0
−2.5
VIT_11s0149g00350
2
0
−2
VIT_11s0149g00360
2
1
0

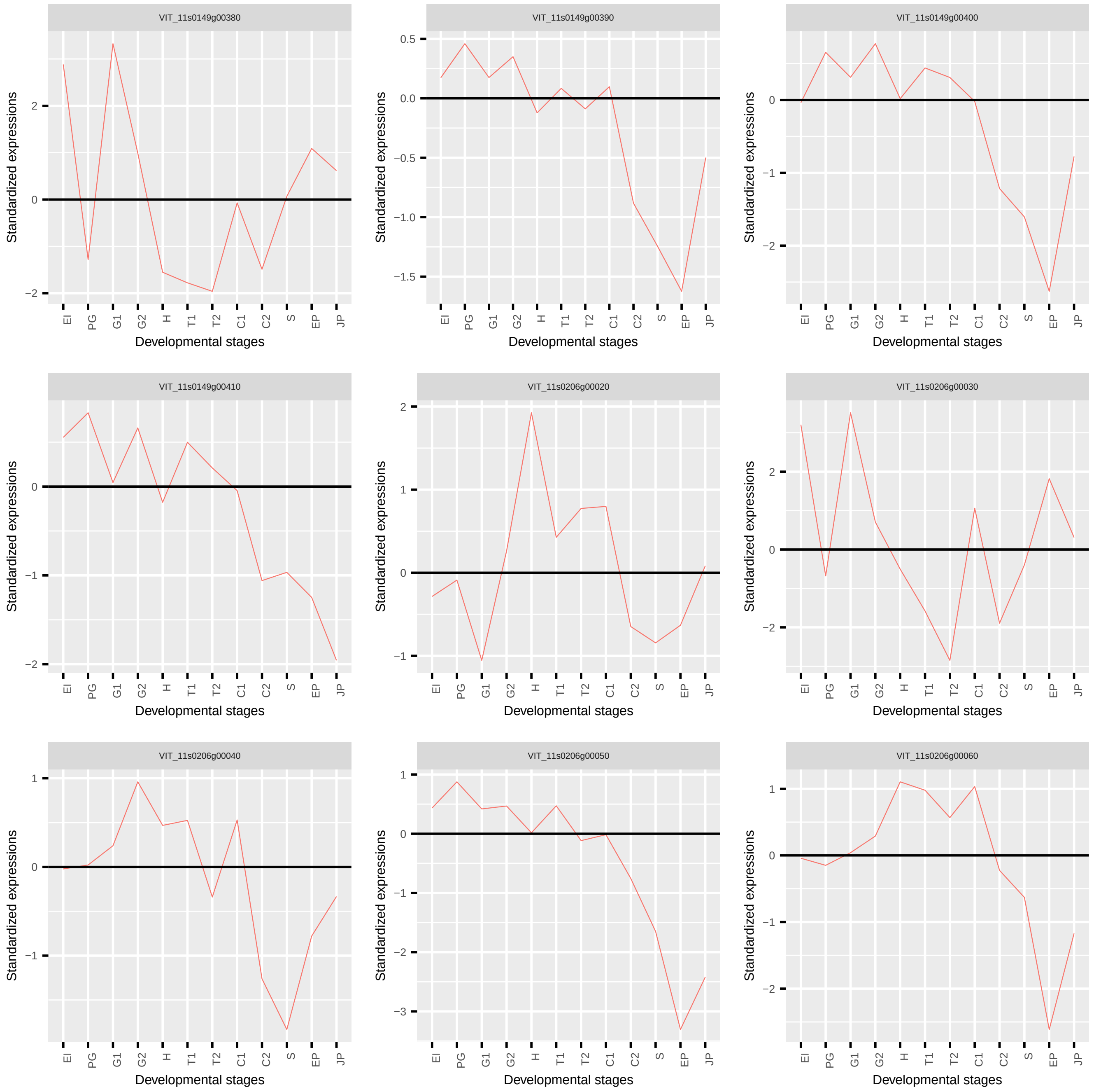
VIT_11s0149g00380
VIT_11s0149g00390
VIT_11s0149g00400
VIT_11s0149g00410
VIT_11s0206g00020
VIT_11s0206g00030
VIT_11s0206g00040
VIT_11s0206g00050
VIT_11s0206g00060
Standardized expressions
Developmental stages
EI PG G1 G2 H T1 T2 C1 C2 S EP JP
2 0 −2
0.5 0.0 −0.5 −1.0 −1.5
0 −1 −2
0 −1 −2
2 1 0 −1
2 0 −2
1 0 −1
1 0 −1 −2 −3
1 0 −1 −2

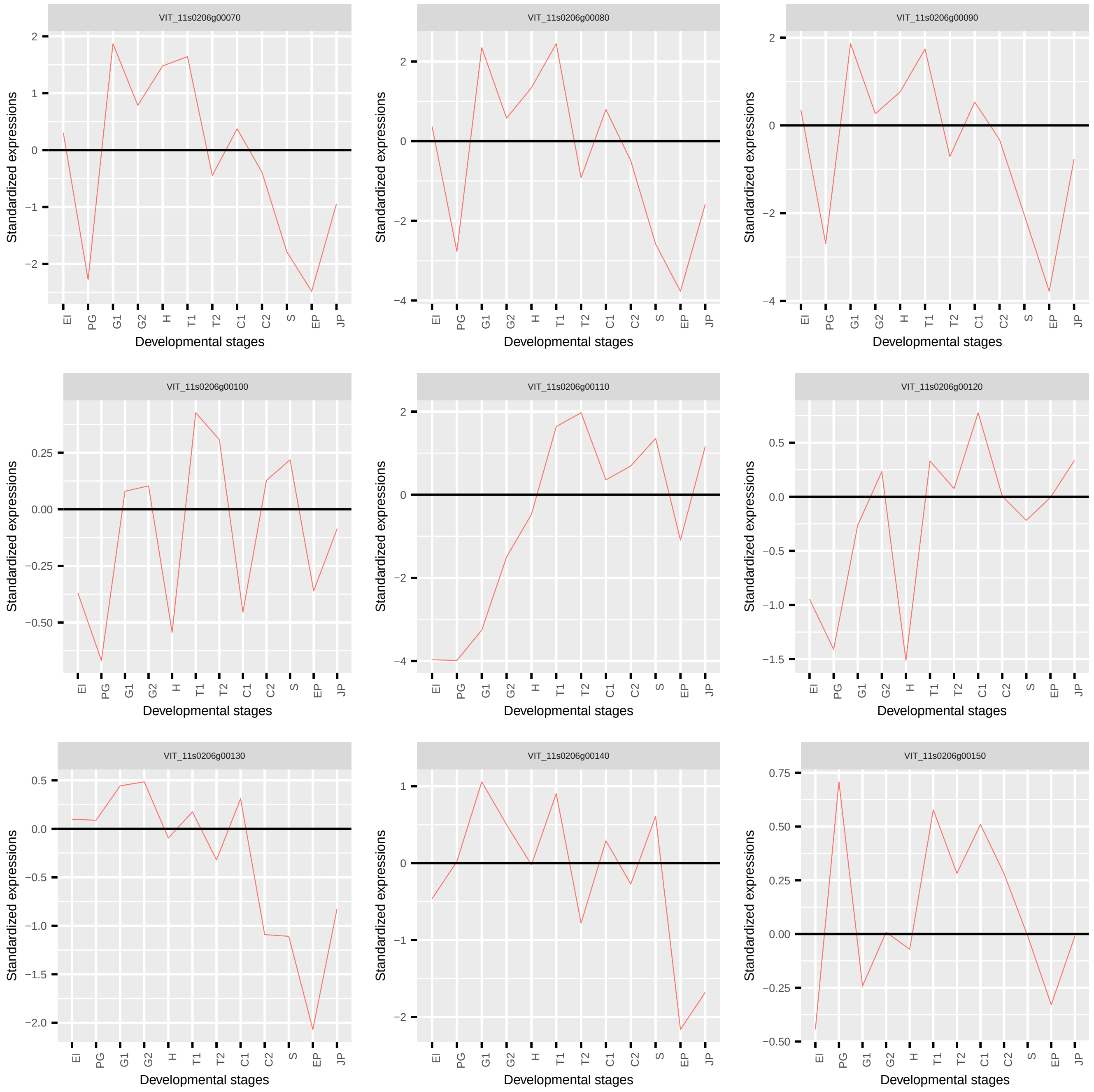

VIT_11s0206g00070
VIT_11s0206g00080
VIT_11s0206g00090
VIT_11s0206g00100
VIT_11s0206g00110
VIT_11s0206g00120
VIT_11s0206g00130
VIT_11s0206g00140
VIT_11s0206g00150
Standardized expressions
Developmental stages
EI PG G1 G2 H T1 T2 C1 C2 S EP JP

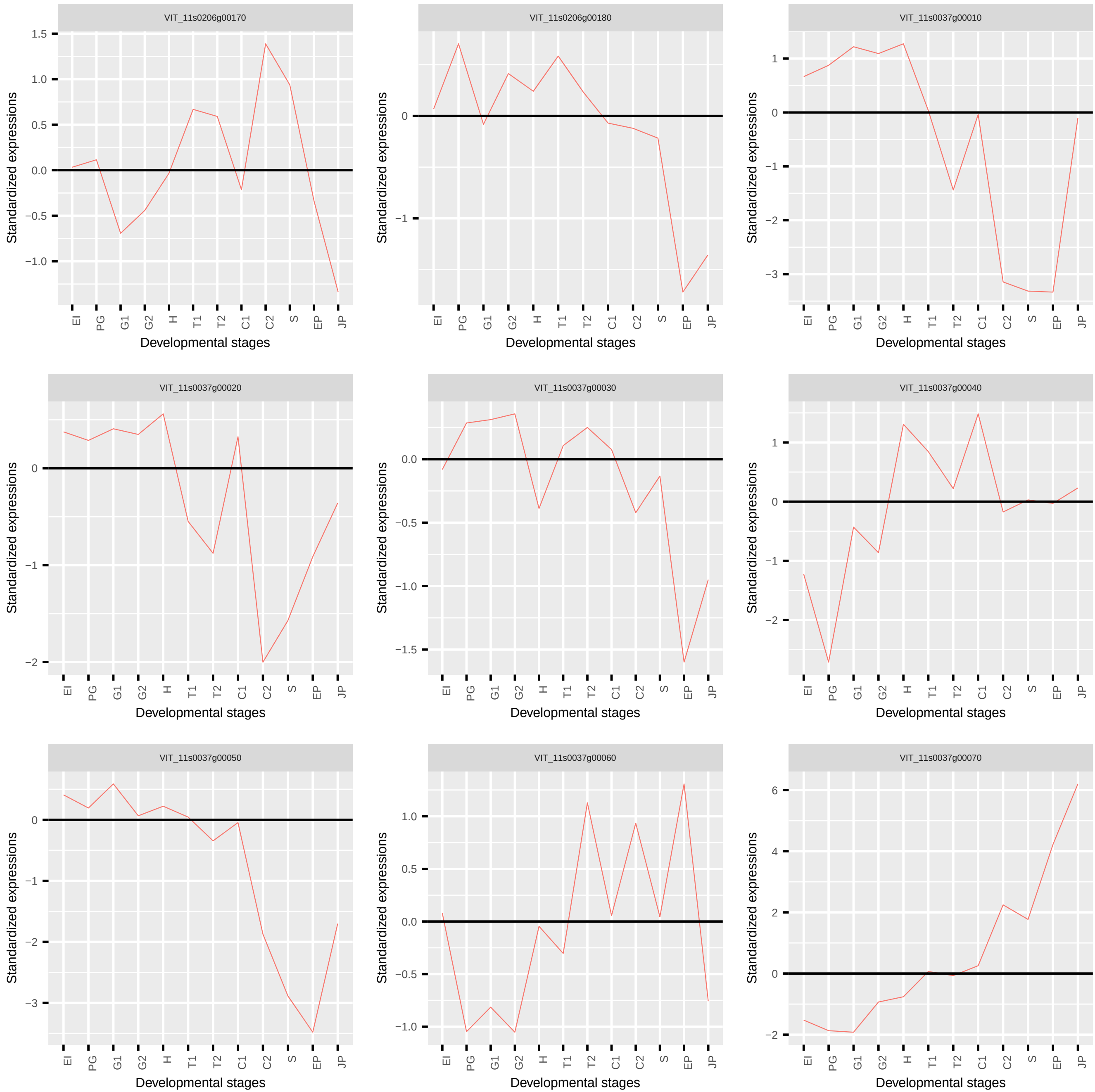
VIT_11s0206g00170
VIT_11s0206g00180
VIT_11s0037g00010
VIT_11s0037g00020
VIT_11s0037g00030
VIT_11s0037g00040
VIT_11s0037g00050
VIT_11s0037g00060
VIT_11s0037g00070
Standardized expressions
Developmental stages
EI
PG
G1
G2
H
T1
T2
C1
C2
S
EP
JP
1.5
1.0
0.5
0.0
−0.5
−1.0
−1.5
0
−1
−2
−3
1
2
4
6

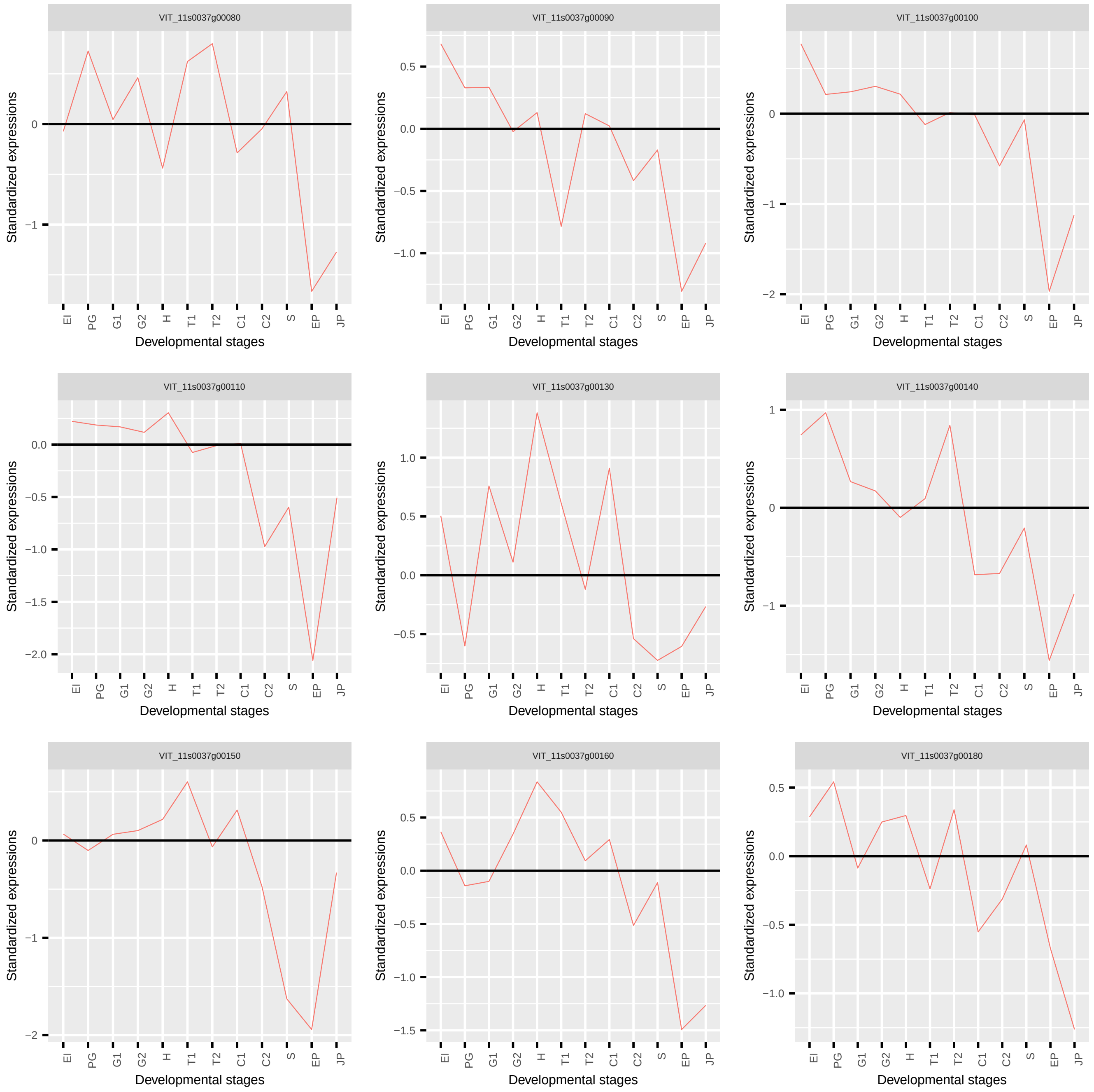
VIT_11s0037g00080
VIT_11s0037g00090
VIT_11s0037g00100
VIT_11s0037g00110
VIT_11s0037g00130
VIT_11s0037g00140
VIT_11s0037g00150
VIT_11s0037g00160
VIT_11s0037g00180
Standardized expressions
Developmental stages
EI PG G1 G2 H T1 T2 C1 C2 S EP JP

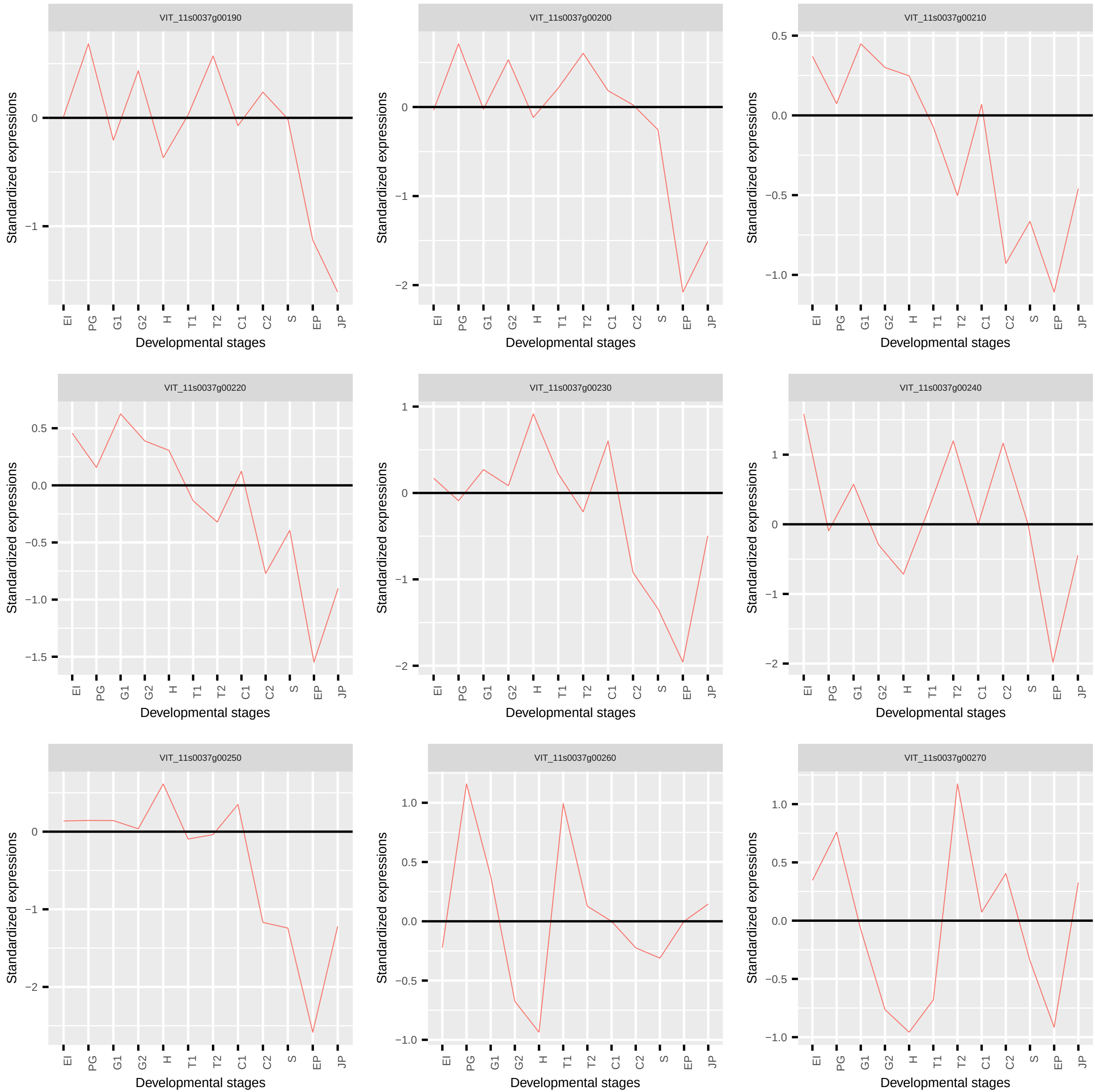
VIT_11s0037g00190
VIT_11s0037g00200
VIT_11s0037g00210
VIT_11s0037g00220
VIT_11s0037g00230
VIT_11s0037g00240
VIT_11s0037g00250
VIT_11s0037g00260
VIT_11s0037g00270
Standardized expressions
Developmental stages
EI PG G1 G2 H T1 T2 C1 C2 S EP JP

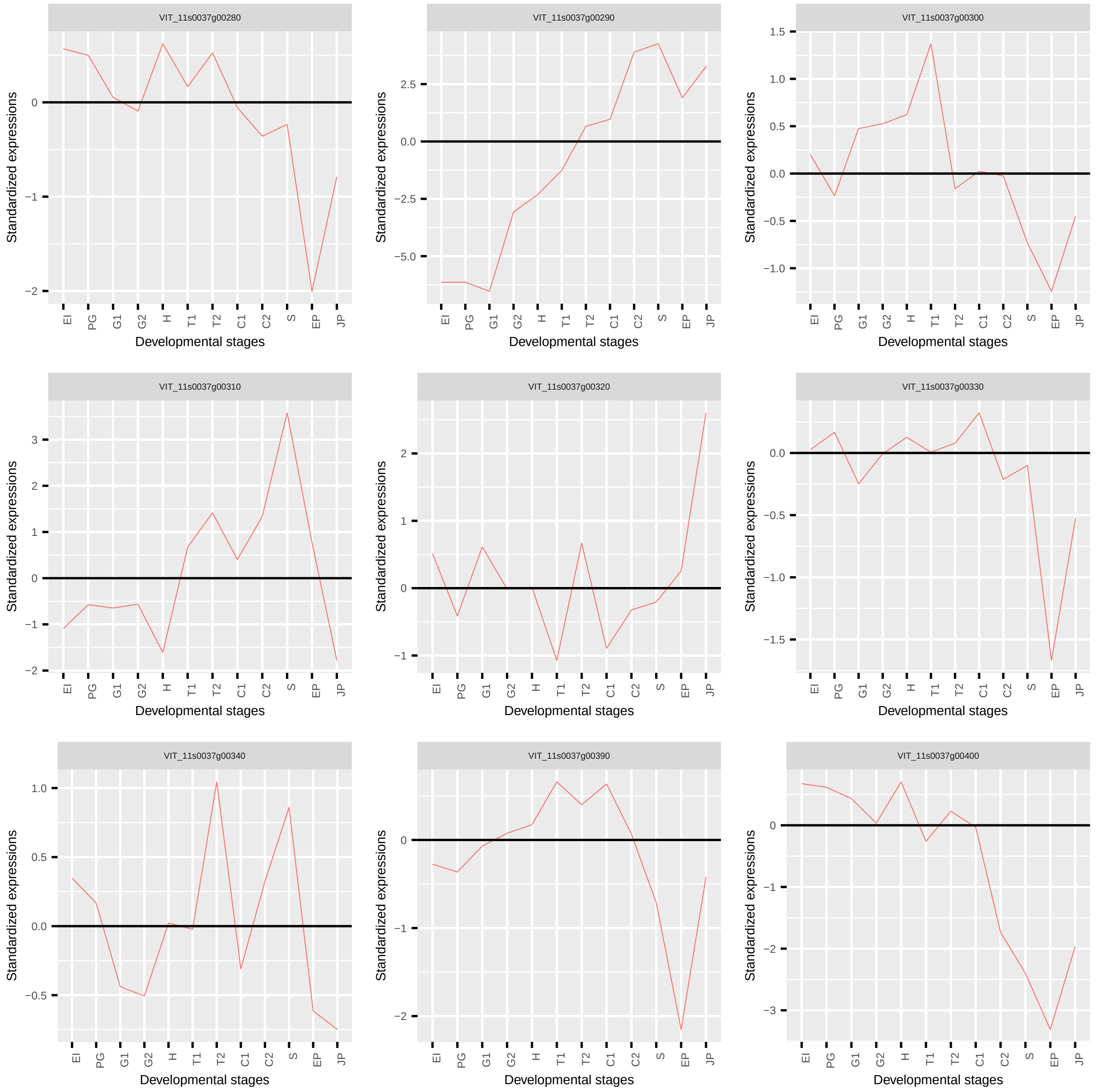
VIT_11s0037g00280
VIT_11s0037g00290
VIT_11s0037g00300
VIT_11s0037g00310
VIT_11s0037g00320
VIT_11s0037g00330
VIT_11s0037g00340
VIT_11s0037g00390
VIT_11s0037g00400
Standardized expressions
Developmental stages
EI PG G1 G2 H T1 T2 C1 C2 S EP JP

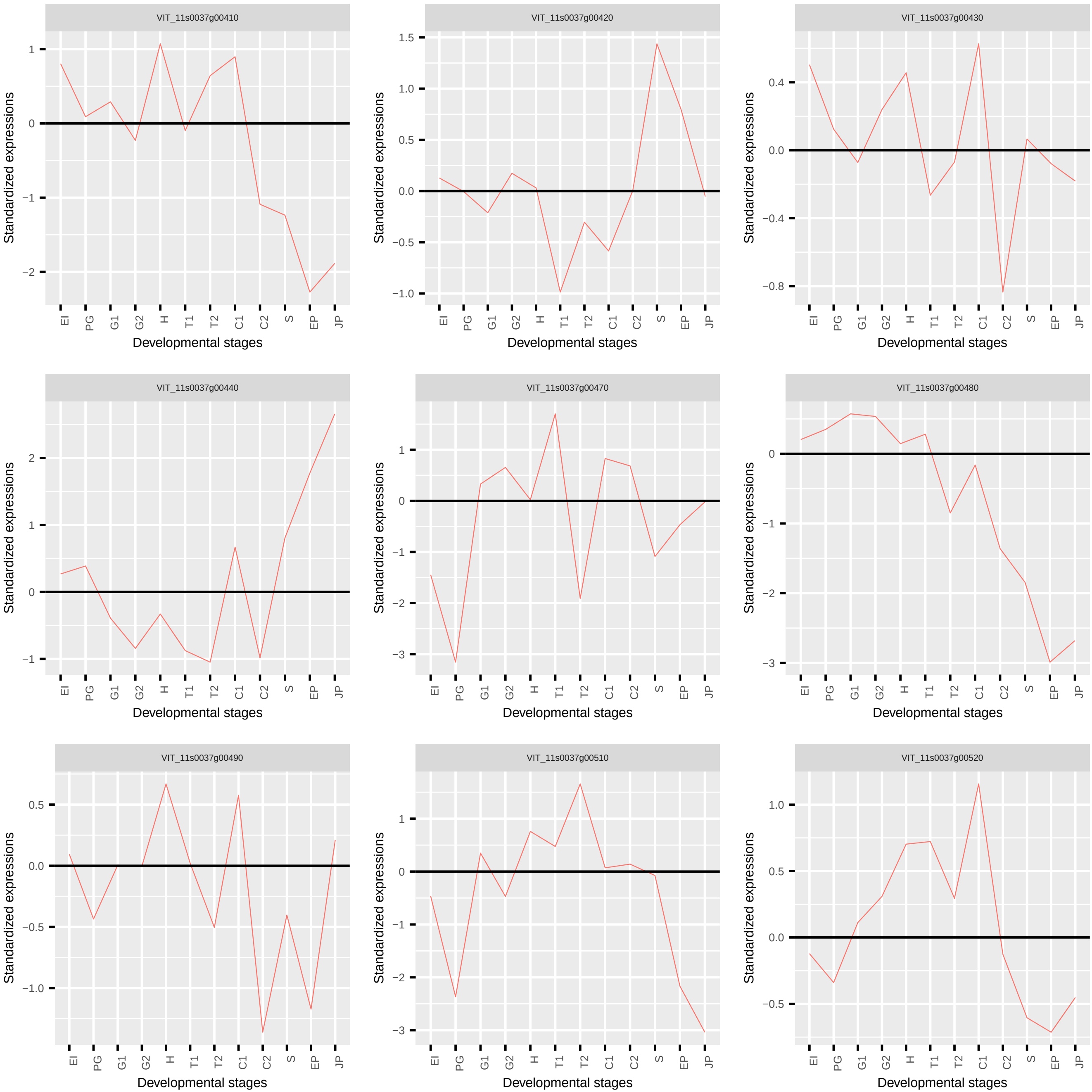

VIT_11s0037g00410
VIT_11s0037g00420
VIT_11s0037g00430
VIT_11s0037g00440
VIT_11s0037g00470
VIT_11s0037g00480
VIT_11s0037g00490
VIT_11s0037g00510
VIT_11s0037g00520
Standardized expressions
Developmental stages
EI PG G1 G2 H T1 T2 C1 C2 S EP JP

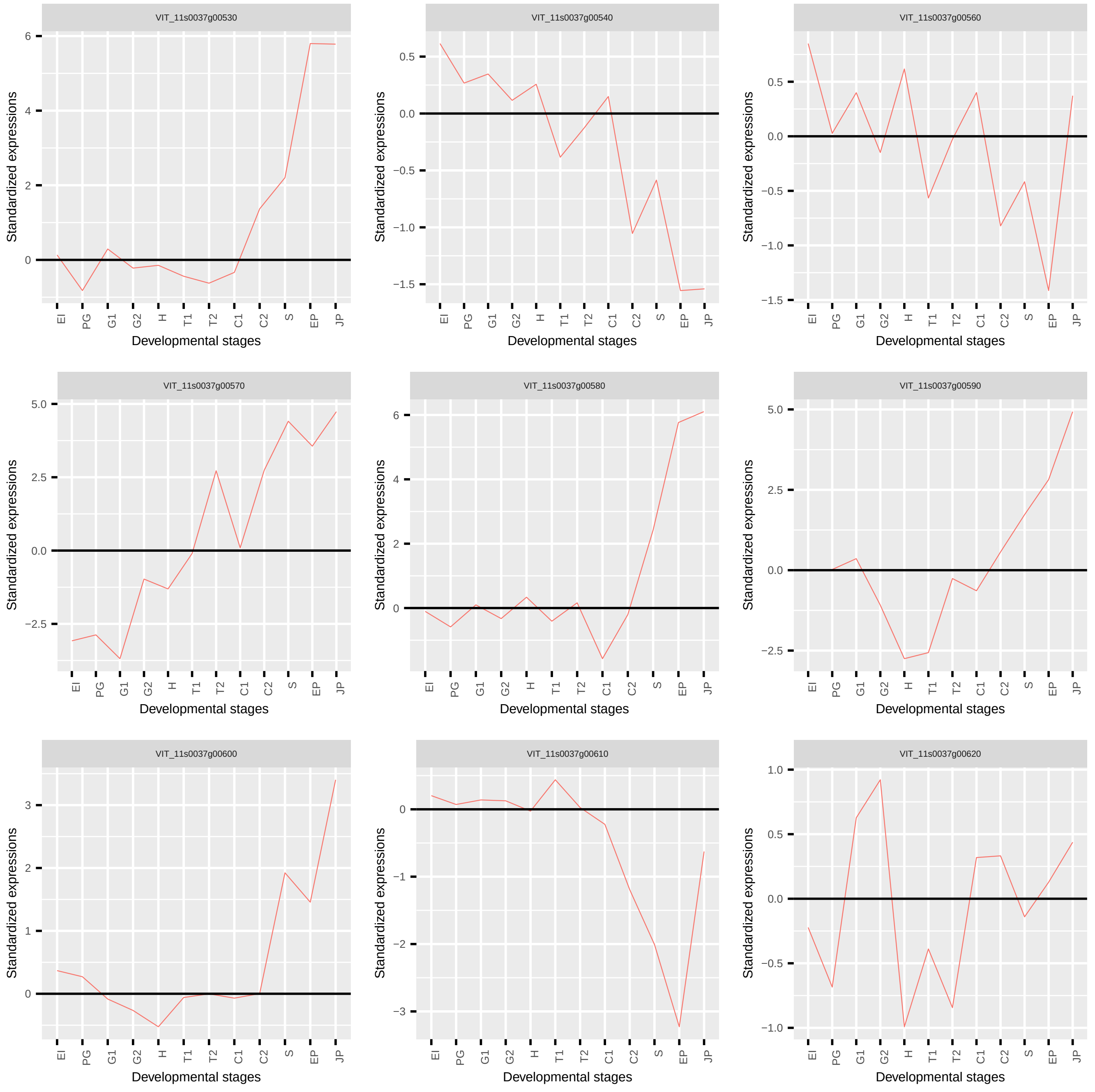
VIT_11s0037g00530
VIT_11s0037g00540
VIT_11s0037g00560
VIT_11s0037g00570
VIT_11s0037g00580
VIT_11s0037g00590
VIT_11s0037g00600
VIT_11s0037g00610
VIT_11s0037g00620
Standardized expressions
Developmental stages
EI
PG
G1
G2
H
T1
T2
C1
C2
S
EP
JP

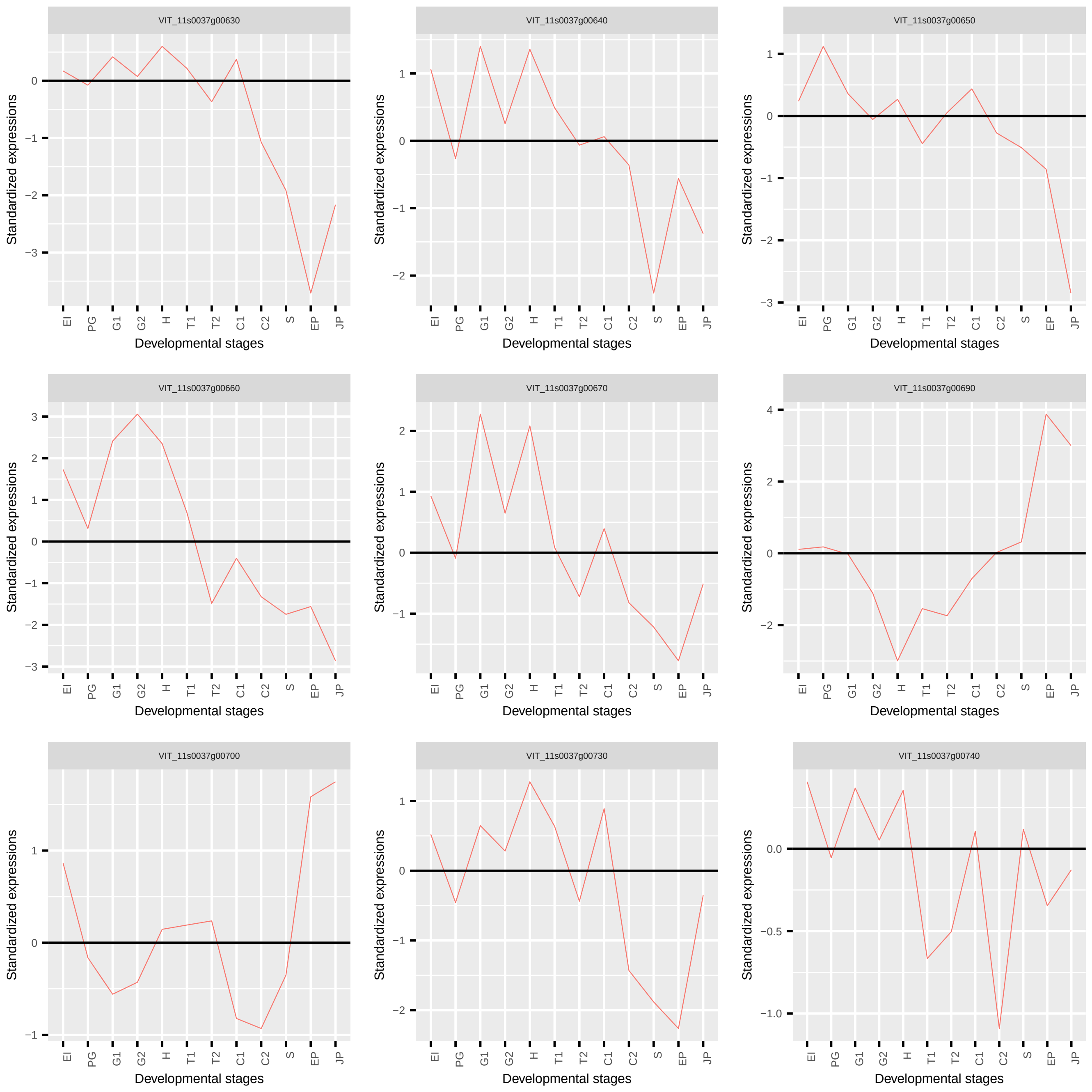
VIT_11s0037g00630
VIT_11s0037g00640
VIT_11s0037g00650
VIT_11s0037g00660
VIT_11s0037g00670
VIT_11s0037g00690
VIT_11s0037g00700
VIT_11s0037g00730
VIT_11s0037g00740
Standardized expressions
Developmental stages
EI PG G1 G2 H T1 T2 C1 C2 S EP JP

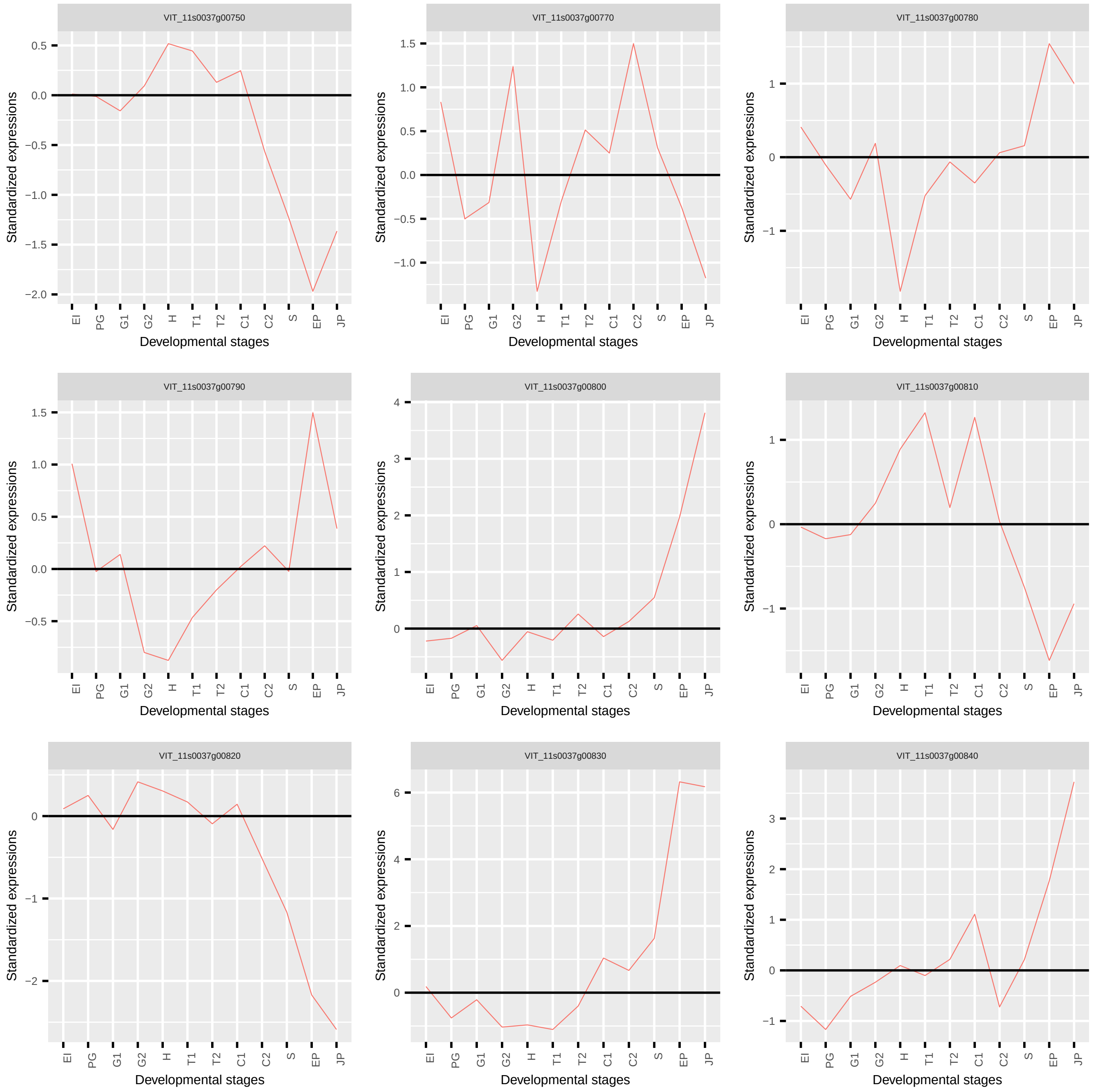

VIT_11s0037g00750
VIT_11s0037g00770
VIT_11s0037g00780
VIT_11s0037g00790
VIT_11s0037g00800
VIT_11s0037g00810
VIT_11s0037g00820
VIT_11s0037g00830
VIT_11s0037g00840
Standardized expressions
Developmental stages
EI
PG
G1
G2
H
T1
T2
C1
C2
S
EP
JP

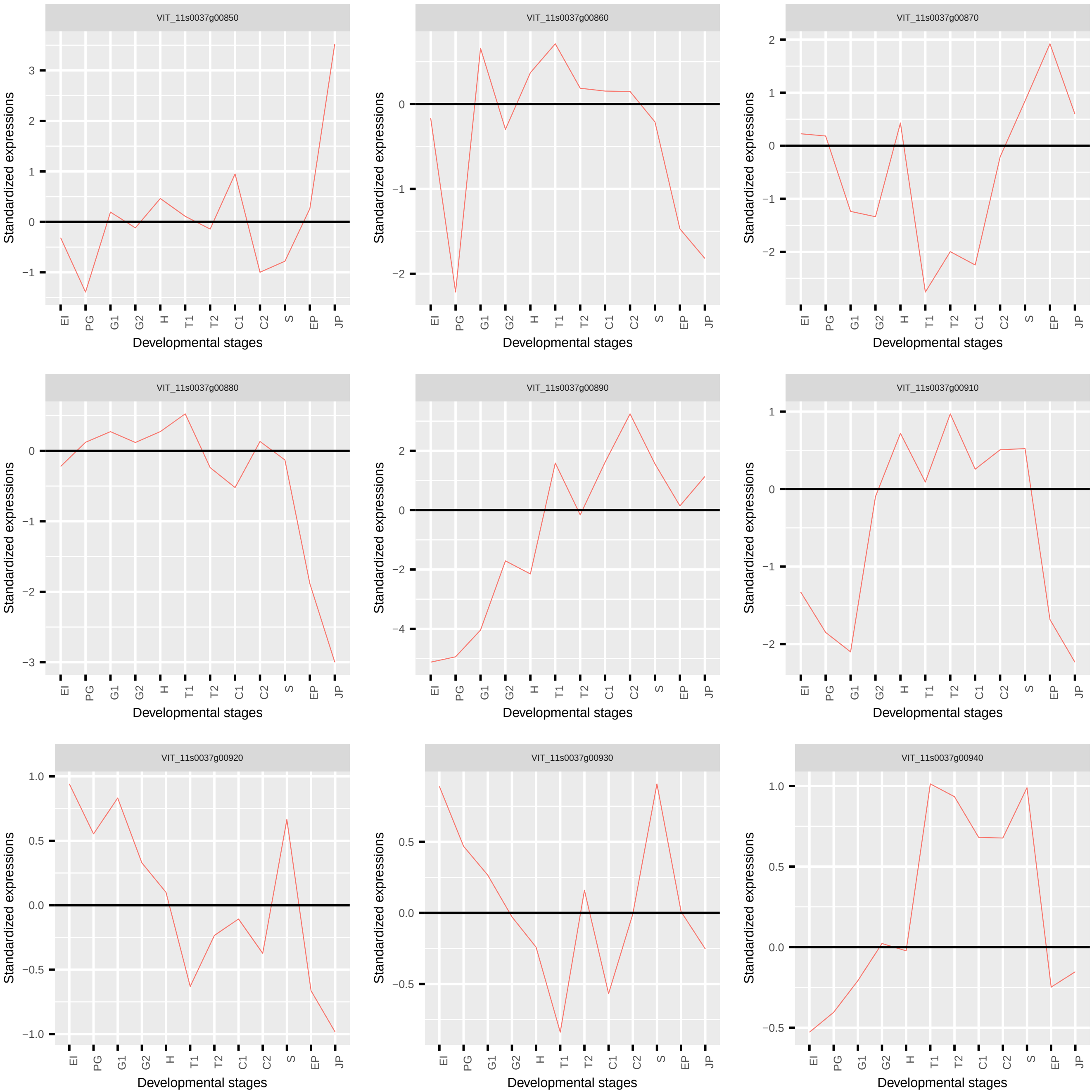

VIT_11s0037g00850
VIT_11s0037g00860
VIT_11s0037g00870
VIT_11s0037g00880
VIT_11s0037g00890
VIT_11s0037g00910
VIT_11s0037g00920
VIT_11s0037g00930
VIT_11s0037g00940
Standardized expressions
Developmental stages
EI PG G1 G2 H T1 T2 C1 C2 S EP JP

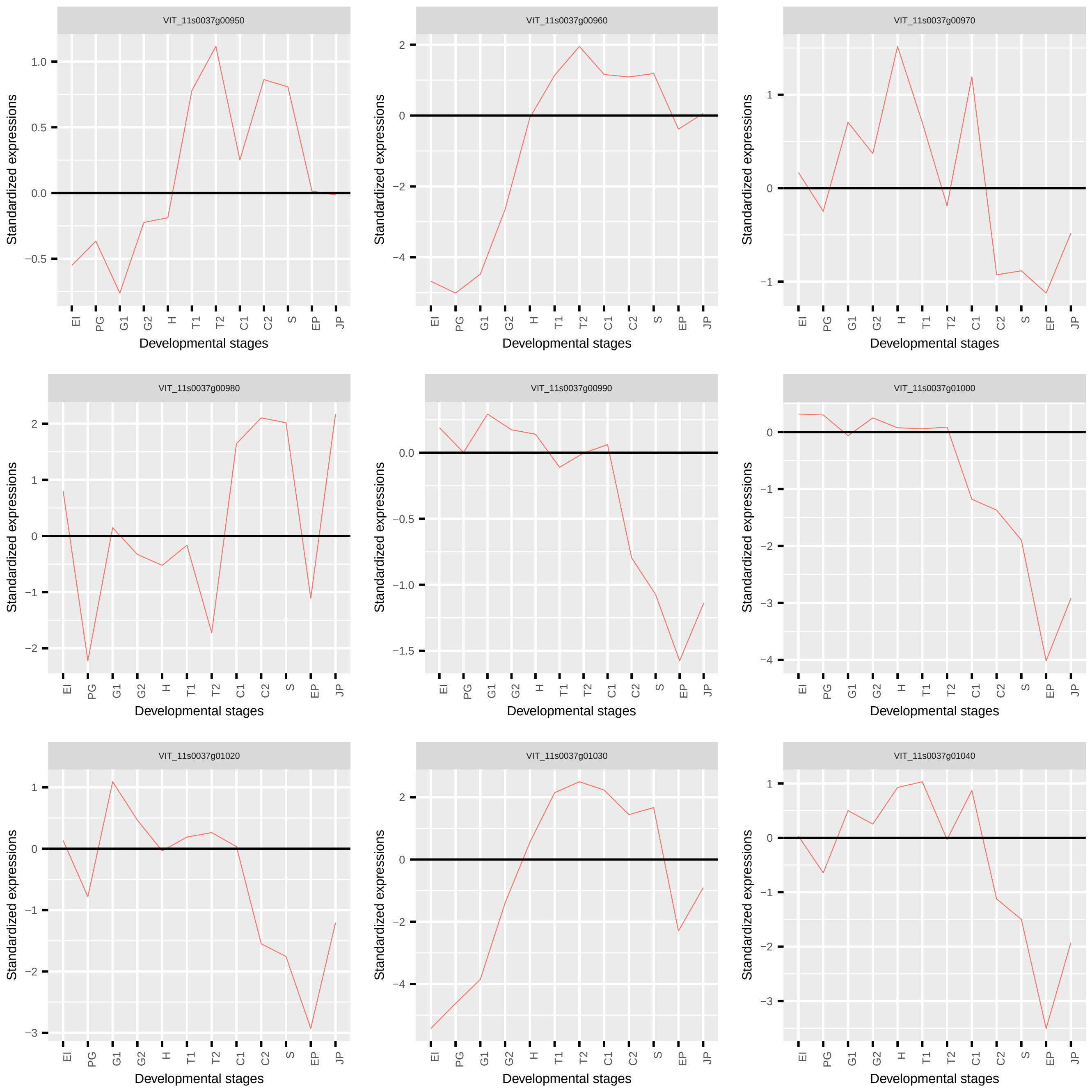
VIT_11s0037g00950
VIT_11s0037g00960
VIT_11s0037g00970
VIT_11s0037g00980
VIT_11s0037g00990
VIT_11s0037g01000
VIT_11s0037g01020
VIT_11s0037g01030
VIT_11s0037g01040
Standardized expressions
Developmental stages
EI
PG
G1
G2
H
T1
T2
C1
C2
S
EP
JP

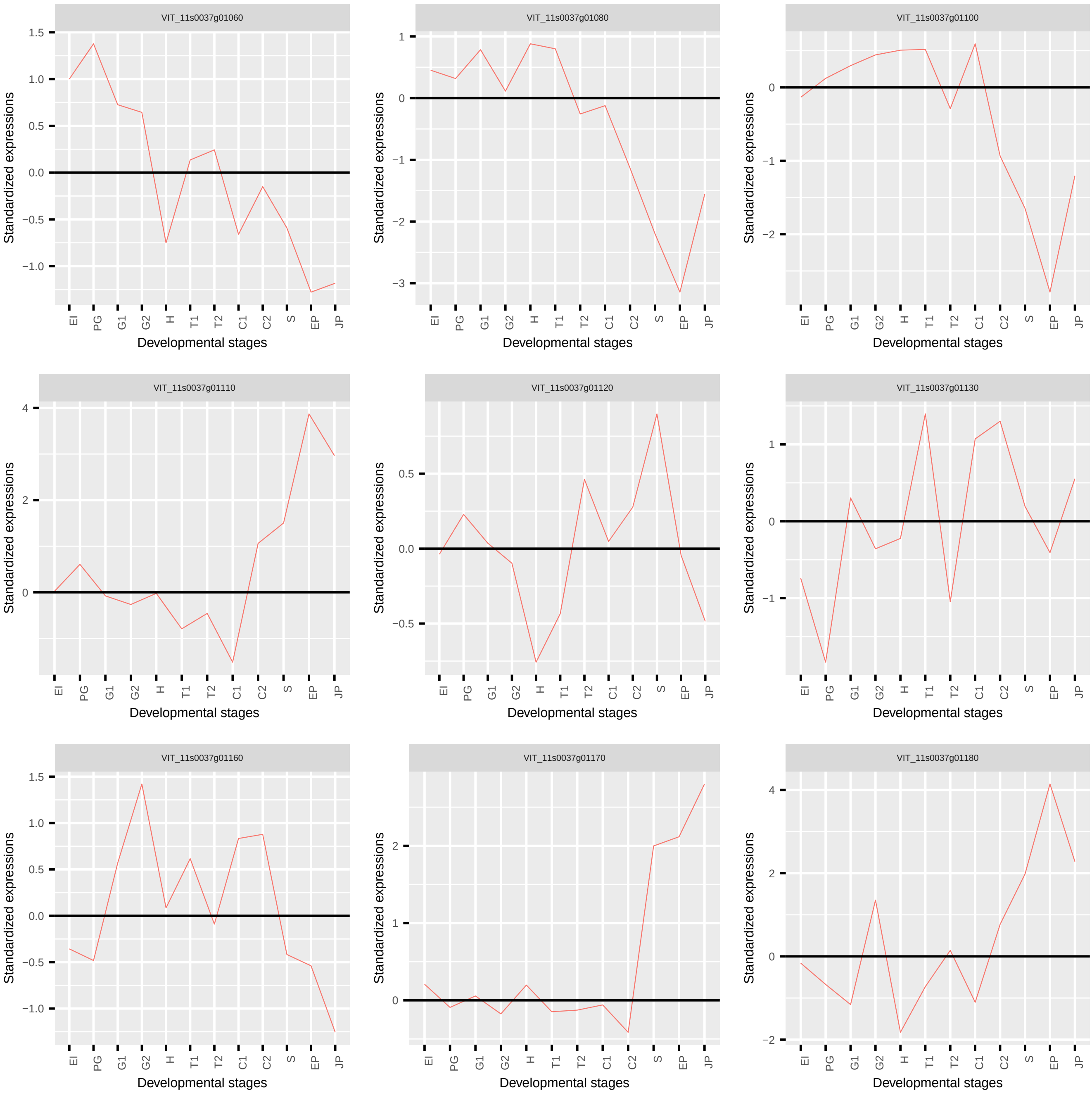

VIT_11s0037g01060
VIT_11s0037g01080
VIT_11s0037g01100
VIT_11s0037g01110
VIT_11s0037g01120
VIT_11s0037g01130
VIT_11s0037g01160
VIT_11s0037g01170
VIT_11s0037g01180
Standardized expressions
Developmental stages
EI PG G1 G2 H T1 T2 C1 C2 S EP JP

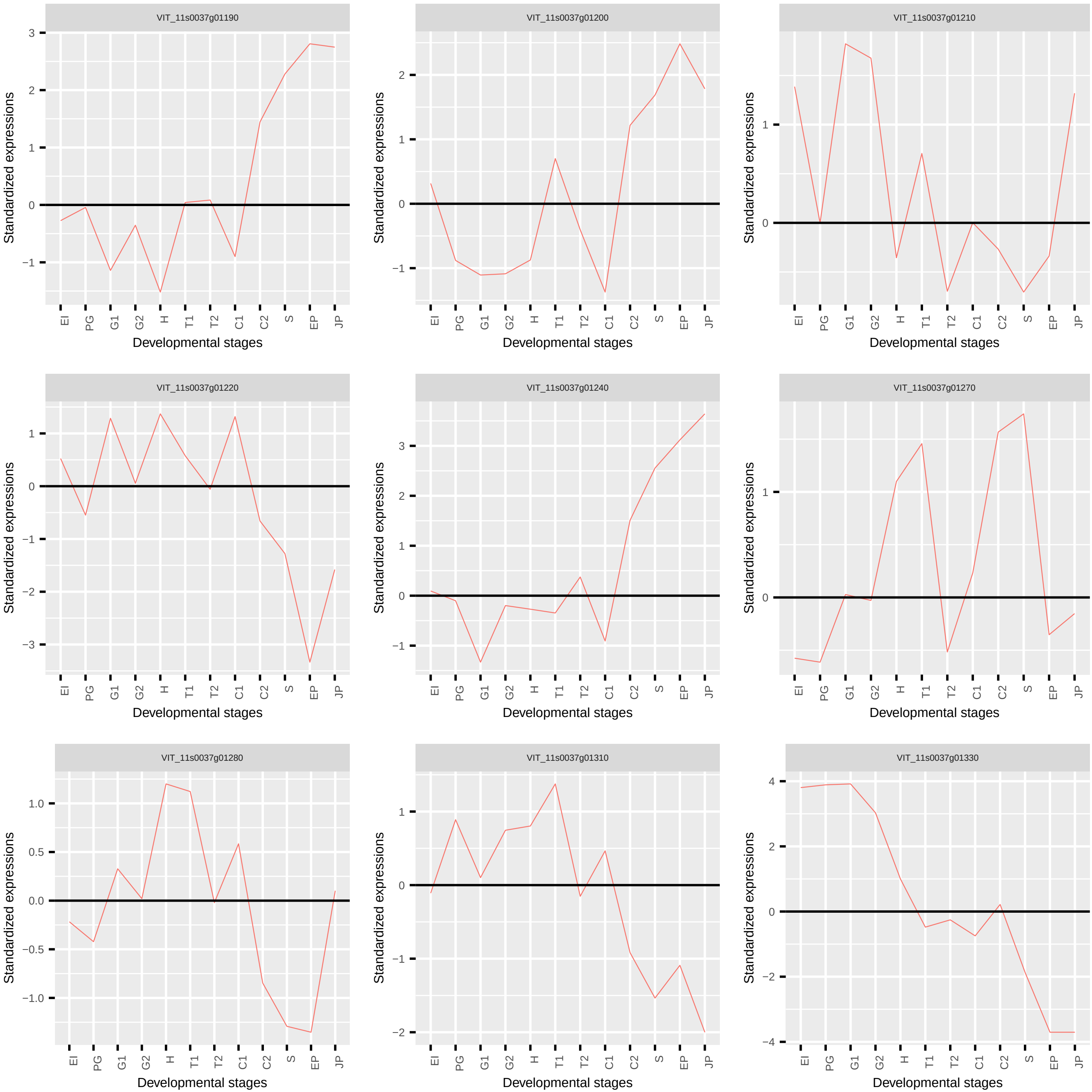
VIT_11s0037g01190
VIT_11s0037g01200
VIT_11s0037g01210
VIT_11s0037g01220
VIT_11s0037g01240
VIT_11s0037g01270
VIT_11s0037g01280
VIT_11s0037g01310
VIT_11s0037g01330
Standardized expressions
Developmental stages
EI PG G1 G2 H T1 T2 C1 C2 S EP JP

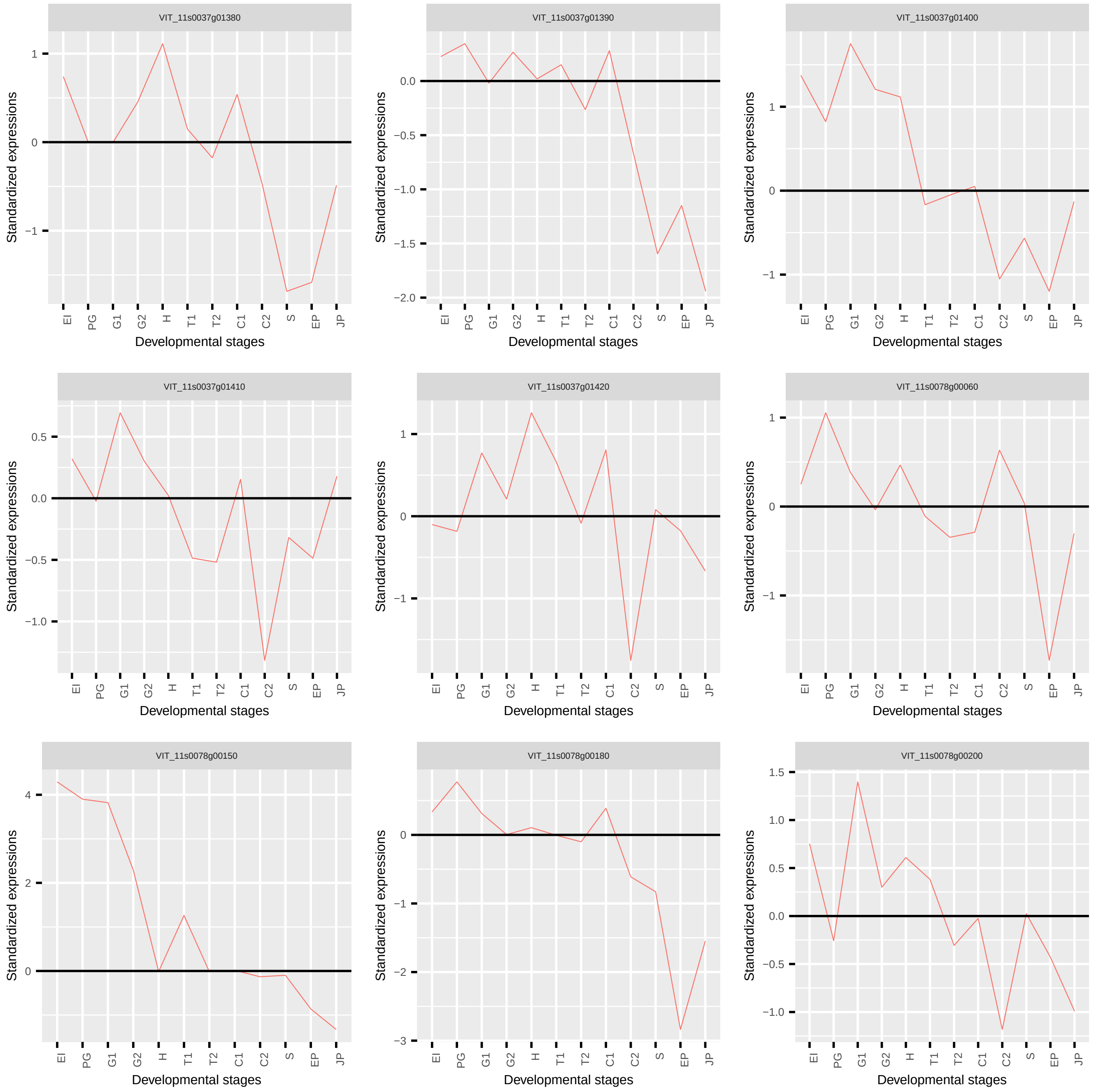
VIT_11s0037g01380
VIT_11s0037g01390
VIT_11s0037g01400
VIT_11s0037g01410
VIT_11s0037g01420
VIT_11s0078g00060
VIT_11s0078g00150
VIT_11s0078g00180
VIT_11s0078g00200
Standardized expressions
Developmental stages
EI PG G1 G2 H T1 T2 C1 C2 S EP JP

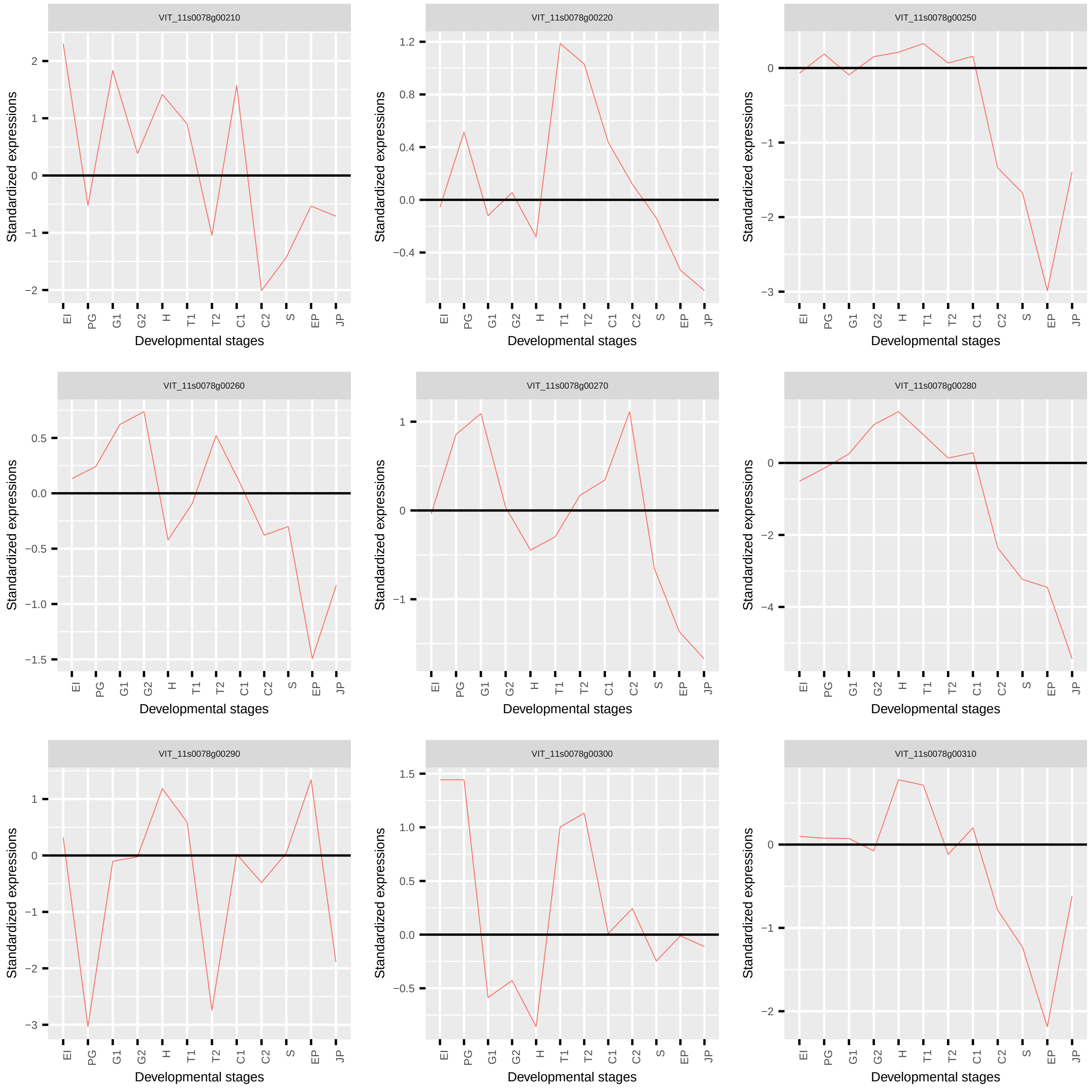
VIT_11s0078g00210
VIT_11s0078g00220
VIT_11s0078g00250
VIT_11s0078g00260
VIT_11s0078g00270
VIT_11s0078g00280
VIT_11s0078g00290
VIT_11s0078g00300
VIT_11s0078g00310
Standardized expressions
Developmental stages
EI PG G1 G2 H T1 T2 C1 C2 S EP JP

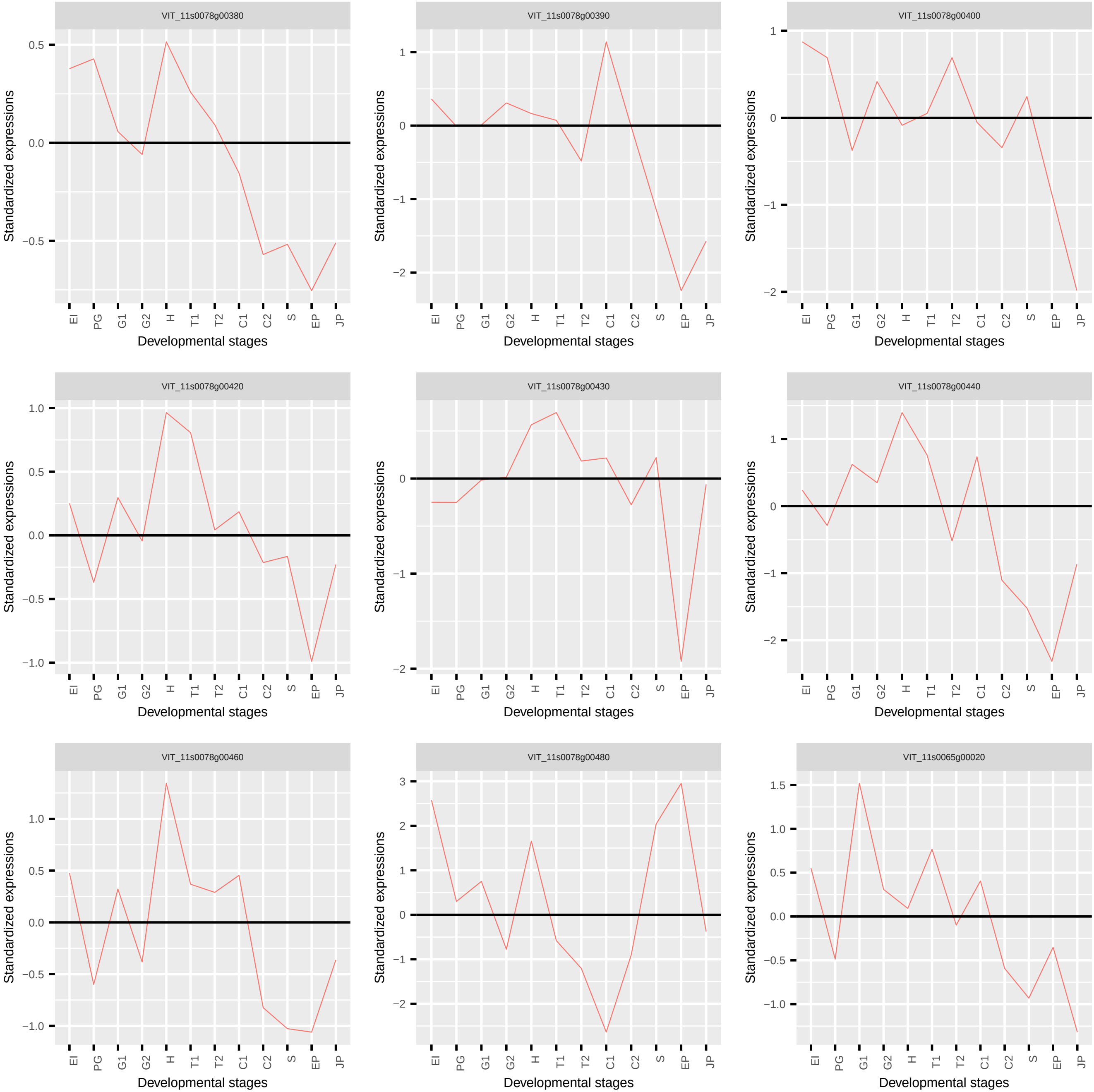

VIT_11s0078g00380
VIT_11s0078g00390
VIT_11s0078g00400
VIT_11s0078g00420
VIT_11s0078g00430
VIT_11s0078g00440
VIT_11s0078g00460
VIT_11s0078g00480
VIT_11s0065g00020
Standardized expressions
Developmental stages
EI
PG
G1
G2
H
T1
T2
C1
C2
S
EP
JP

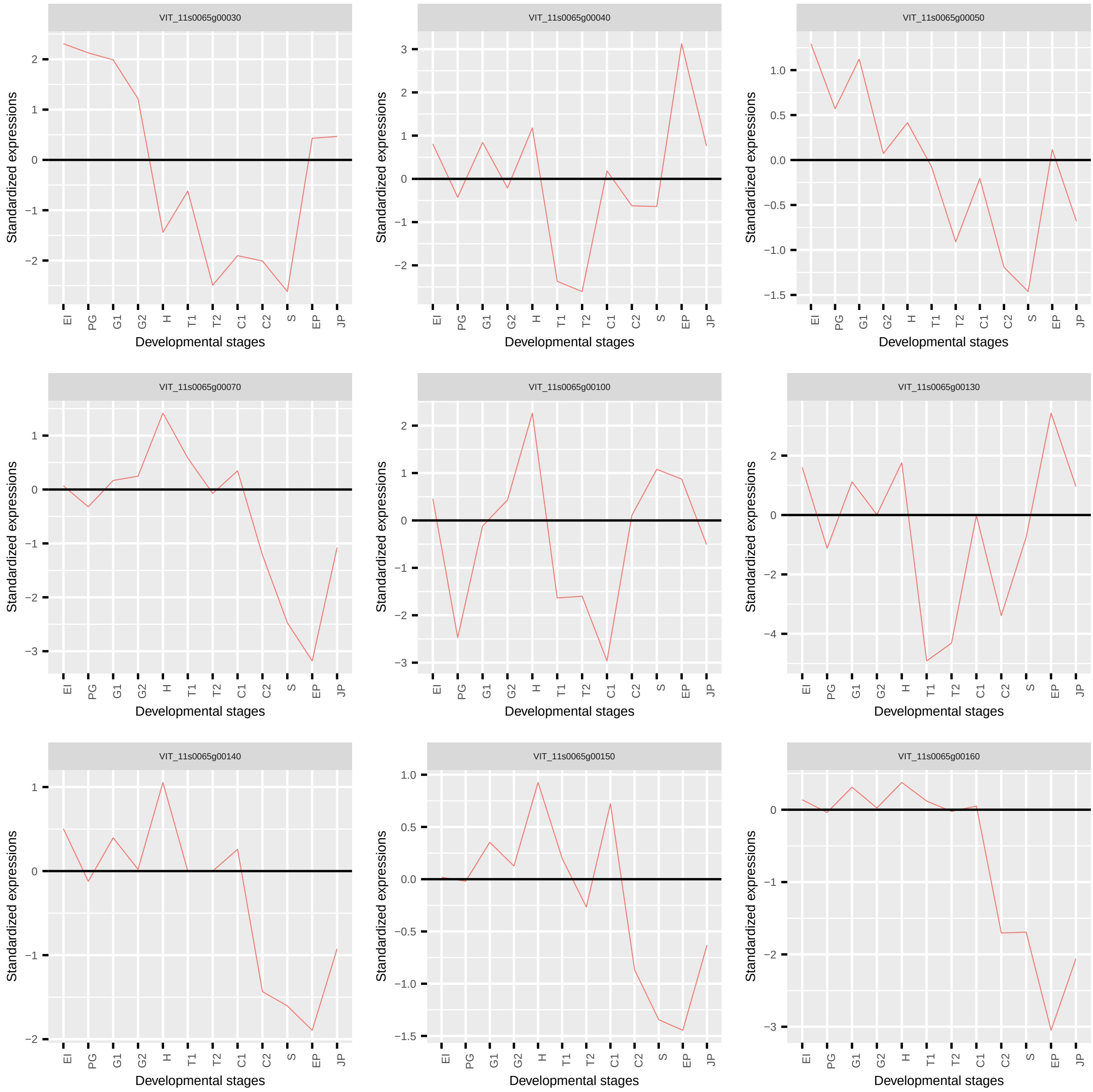
VIT_11s0065g00030
VIT_11s0065g00040
VIT_11s0065g00050
VIT_11s0065g00070
VIT_11s0065g00100
VIT_11s0065g00130
VIT_11s0065g00140
VIT_11s0065g00150
VIT_11s0065g00160
Standardized expressions
Developmental stages
EI PG G1 G2 H T1 T2 C1 C2 S EP JP

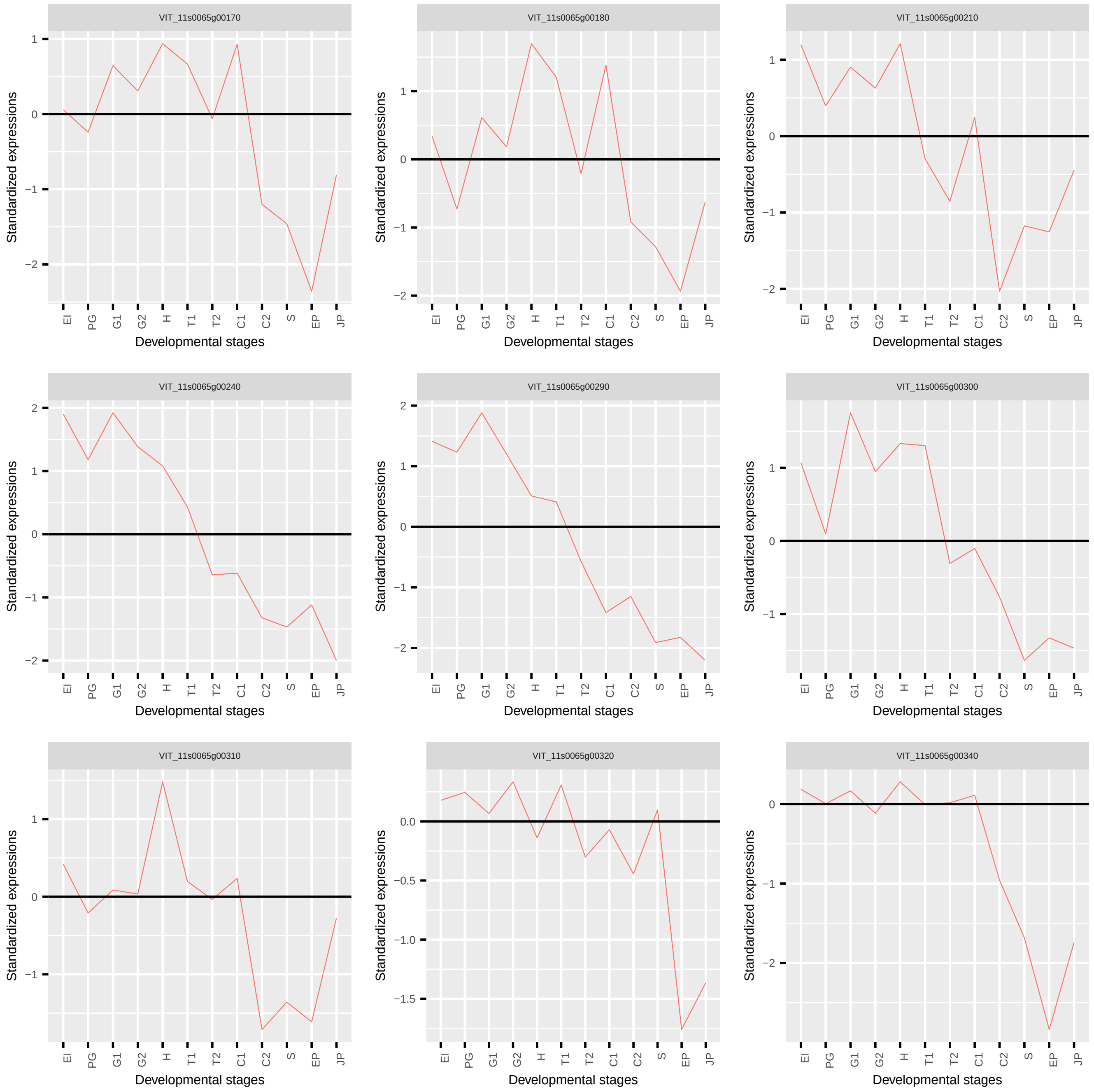
VIT_11s0065g00170
VIT_11s0065g00180
VIT_11s0065g00210
VIT_11s0065g00240
VIT_11s0065g00290
VIT_11s0065g00300
VIT_11s0065g00310
VIT_11s0065g00320
VIT_11s0065g00340
Standardized expressions
Developmental stages
EI
PG
G1
G2
H
T1
T2
C1
C2
S
EP
JP

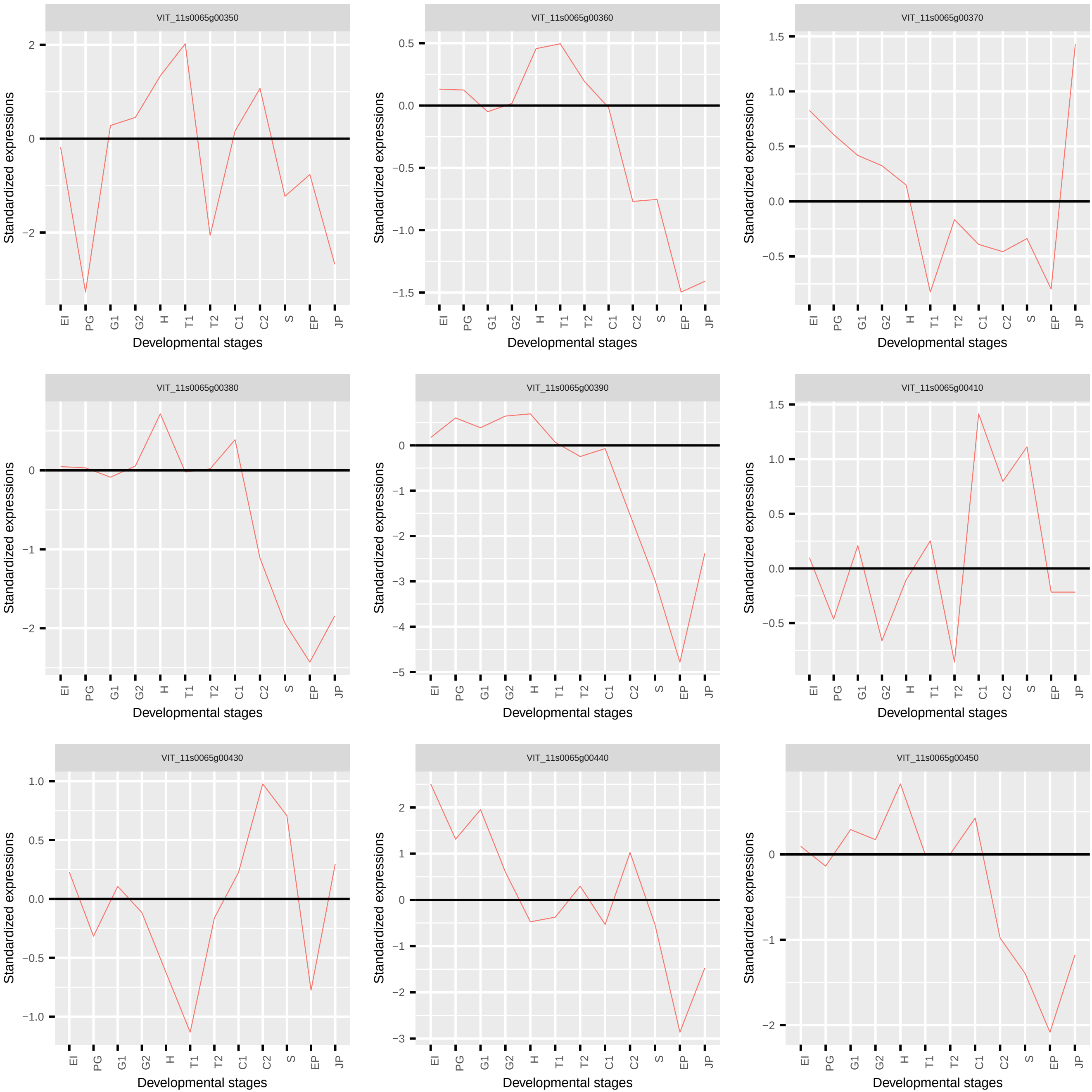
VIT_11s0065g00350
VIT_11s0065g00360
VIT_11s0065g00370
VIT_11s0065g00380
VIT_11s0065g00390
VIT_11s0065g00410
VIT_11s0065g00430
VIT_11s0065g00440
VIT_11s0065g00450
Standardized expressions
Developmental stages
EI
PG
G1
G2
H
T1
T2
C1
C2
S
EP
JP

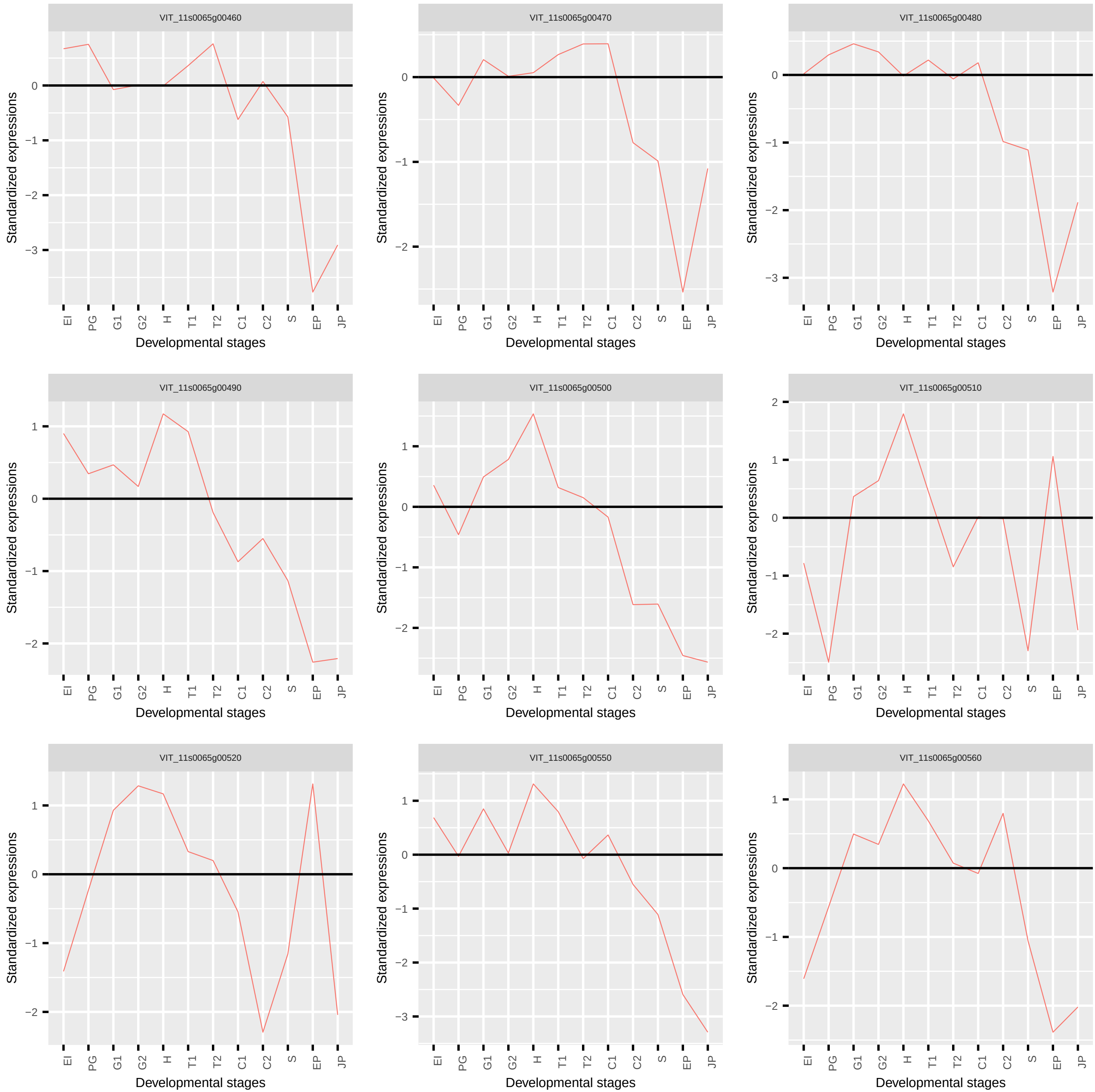
VIT_11s0065g00460
VIT_11s0065g00470
VIT_11s0065g00480
VIT_11s0065g00490
VIT_11s0065g00500
VIT_11s0065g00510
VIT_11s0065g00520
VIT_11s0065g00550
VIT_11s0065g00560
Standardized expressions
Developmental stages
EI PG G1 G2 H T1 T2 C1 C2 S EP JP

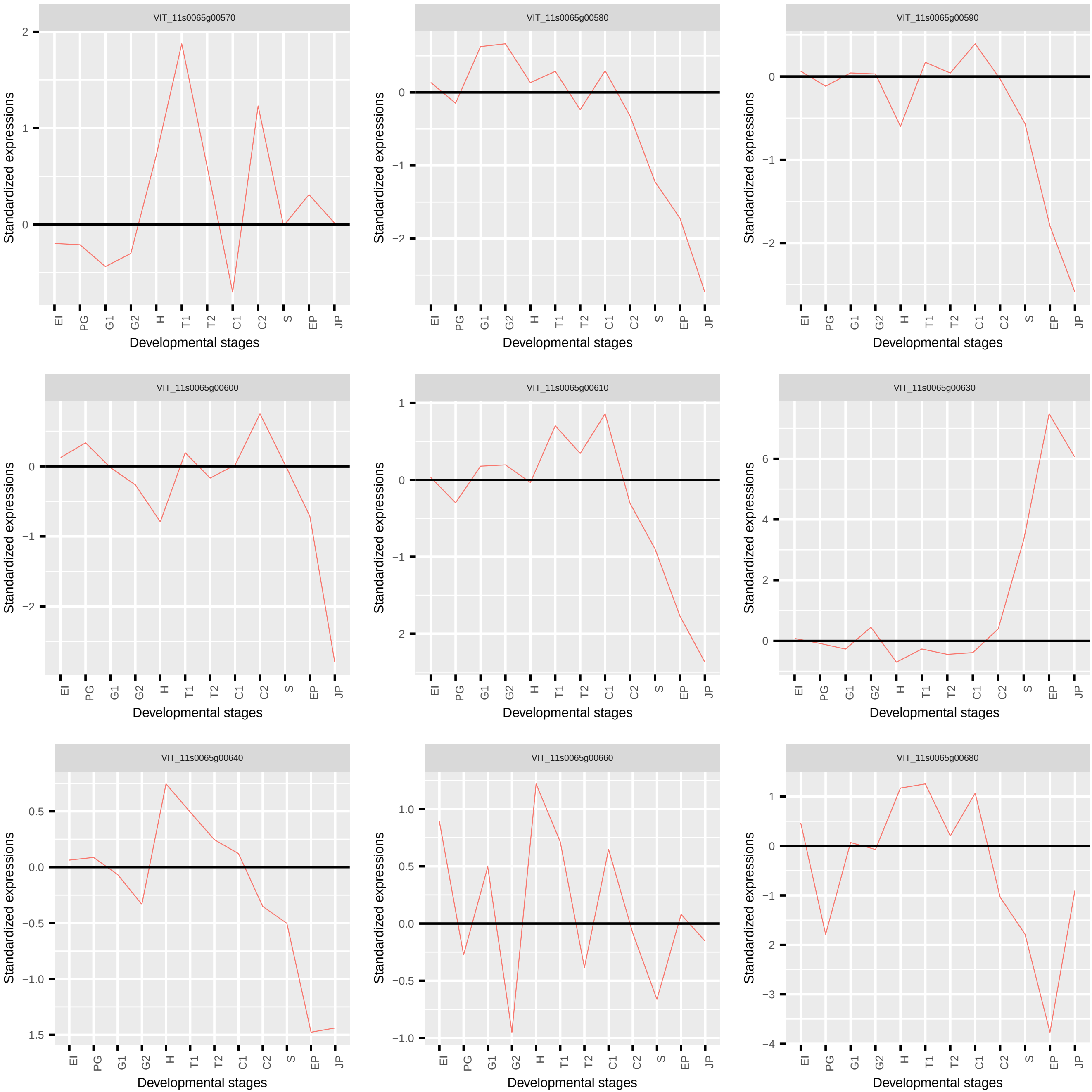

VIT_11s0065g00570
VIT_11s0065g00580
VIT_11s0065g00590
VIT_11s0065g00600
VIT_11s0065g00610
VIT_11s0065g00630
VIT_11s0065g00640
VIT_11s0065g00660
VIT_11s0065g00680
Standardized expressions
Developmental stages
EI PG G1 G2 H T1 T2 C1 C2 S EP JP

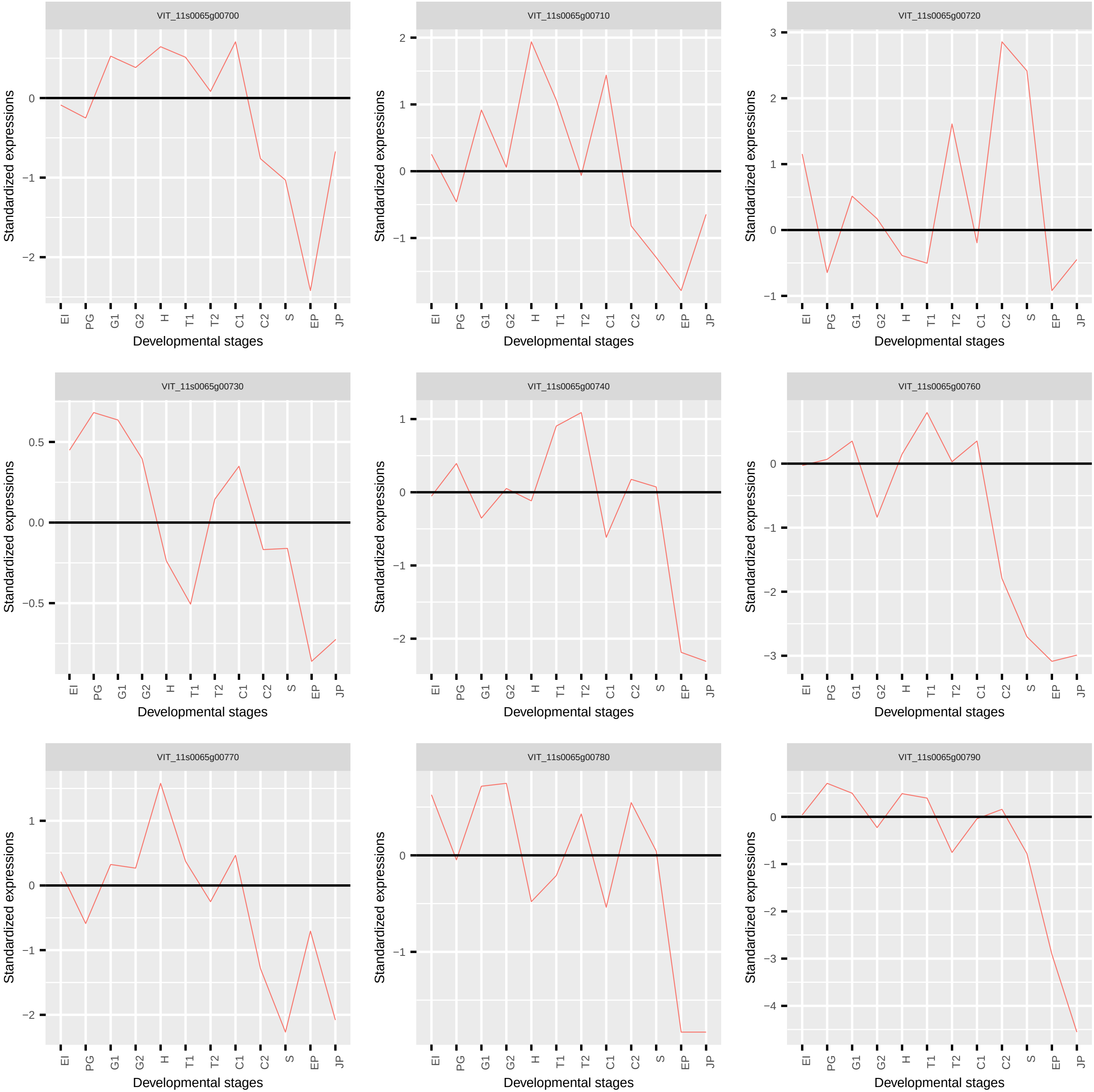

VIT_11s0065g00700
VIT_11s0065g00710
VIT_11s0065g00720
VIT_11s0065g00730
VIT_11s0065g00740
VIT_11s0065g00760
VIT_11s0065g00770
VIT_11s0065g00780
VIT_11s0065g00790
Standardized expressions
Developmental stages
EI PG G1 G2 H T1 T2 C1 C2 S EP JP

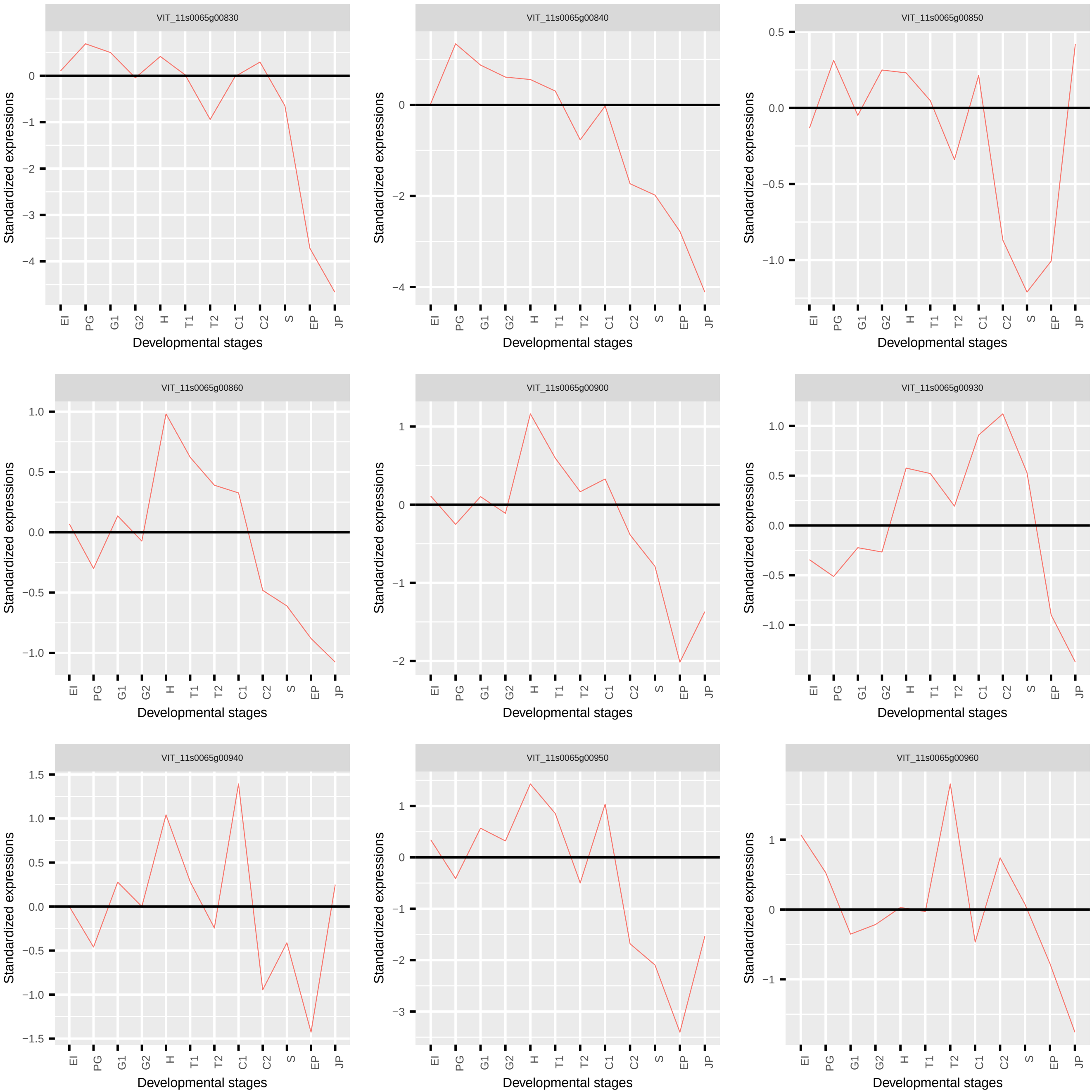
VIT_11s0065g00830
VIT_11s0065g00840
VIT_11s0065g00850
VIT_11s0065g00860
VIT_11s0065g00900
VIT_11s0065g00930
VIT_11s0065g00940
VIT_11s0065g00950
VIT_11s0065g00960
Standardized expressions
Developmental stages
EI PG G1 G2 H T1 T2 C1 C2 S EP JP

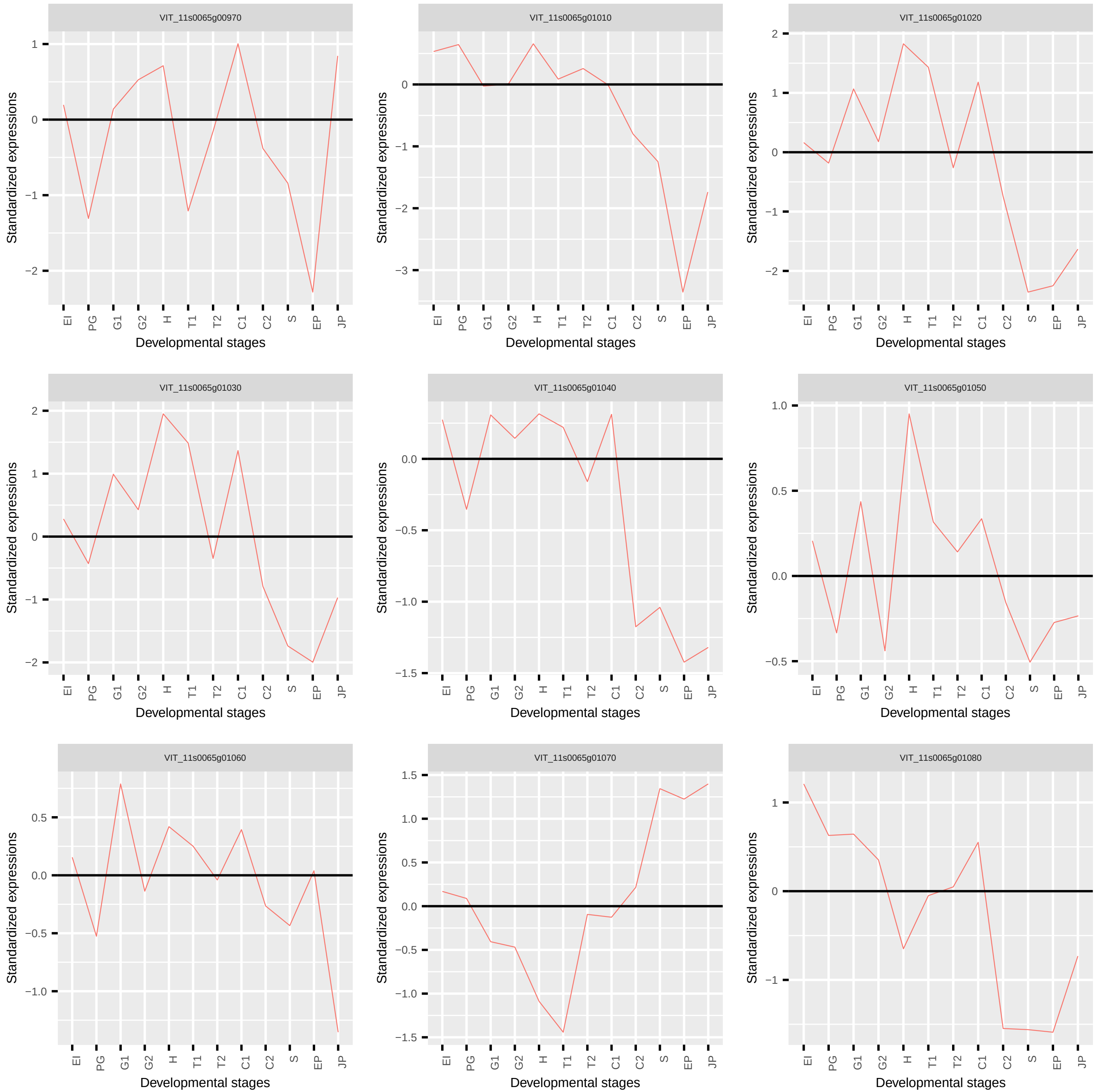

VIT_11s0065g00970
VIT_11s0065g01010
VIT_11s0065g01020
VIT_11s0065g01030
VIT_11s0065g01040
VIT_11s0065g01050
VIT_11s0065g01060
VIT_11s0065g01070
VIT_11s0065g01080
Standardized expressions
Developmental stages
EI
PG
G1
G2
H
T1
T2
C1
C2
S
EP
JP

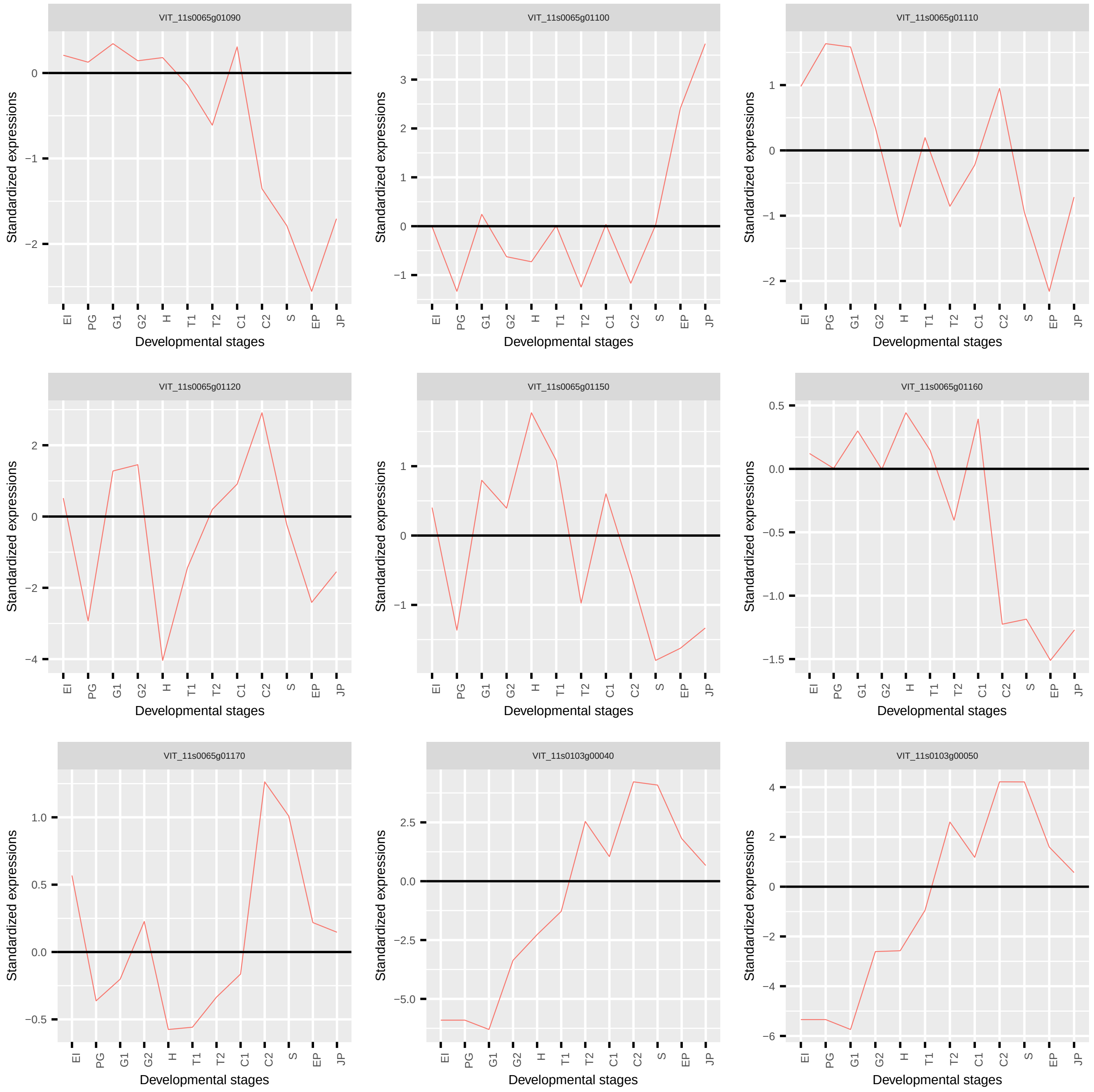
VIT_11s0065g01090
VIT_11s0065g01100
VIT_11s0065g01110
VIT_11s0065g01120
VIT_11s0065g01150
VIT_11s0065g01160
VIT_11s0065g01170
VIT_11s0103g00040
VIT_11s0103g00050
Standardized expressions
Developmental stages
EI PG G1 G2 H T1 T2 C1 C2 S EP JP

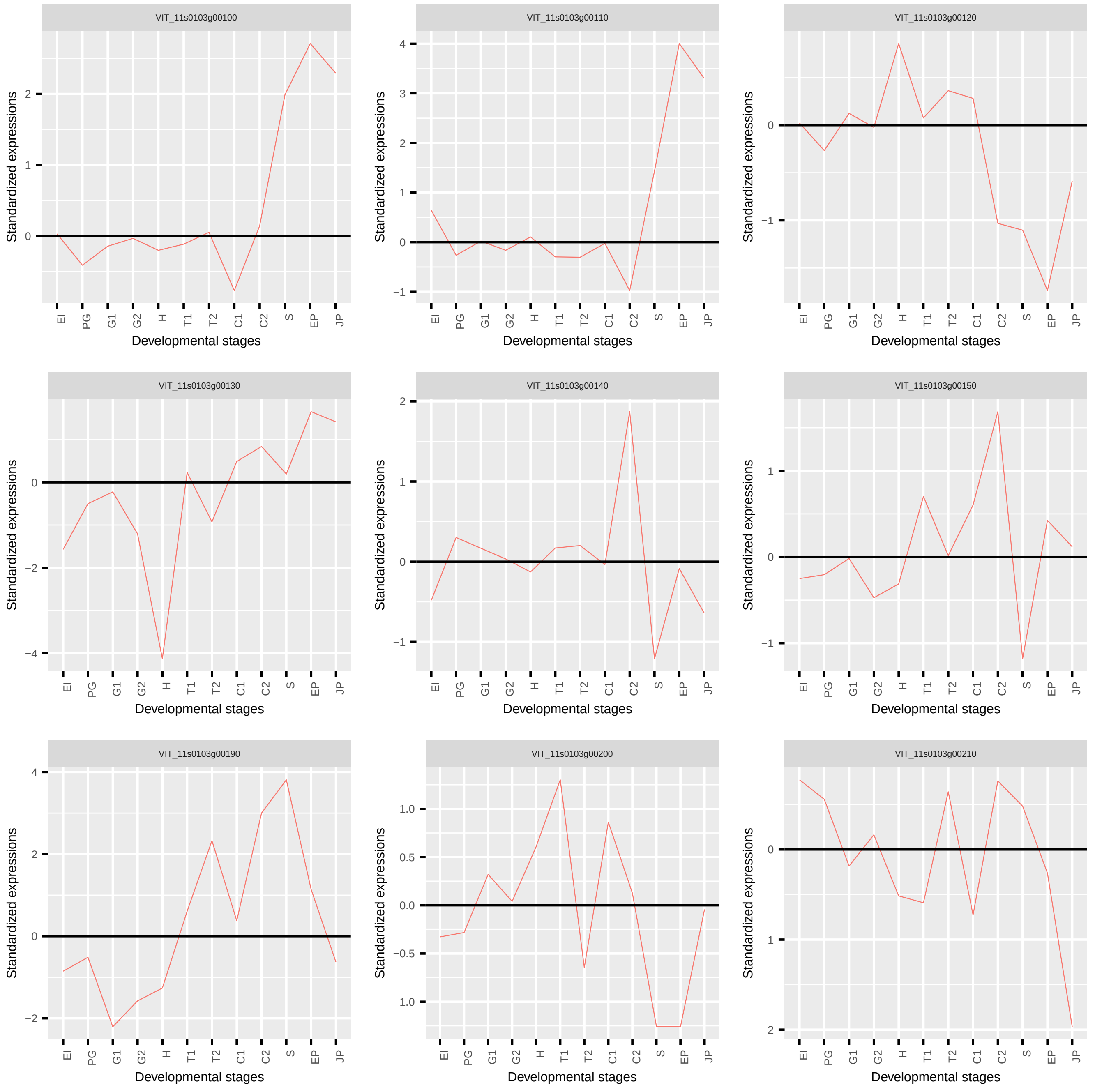
VIT_11s0103g00100
VIT_11s0103g00110
VIT_11s0103g00120
VIT_11s0103g00130
VIT_11s0103g00140
VIT_11s0103g00150
VIT_11s0103g00190
VIT_11s0103g00200
VIT_11s0103g00210
Standardized expressions
Developmental stages
EI PG G1 G2 H T1 T2 C1 C2 S EP JP
−4 −2 −1 0 1 2 3 4
−1.0 −0.5 0.0 0.5 1.0

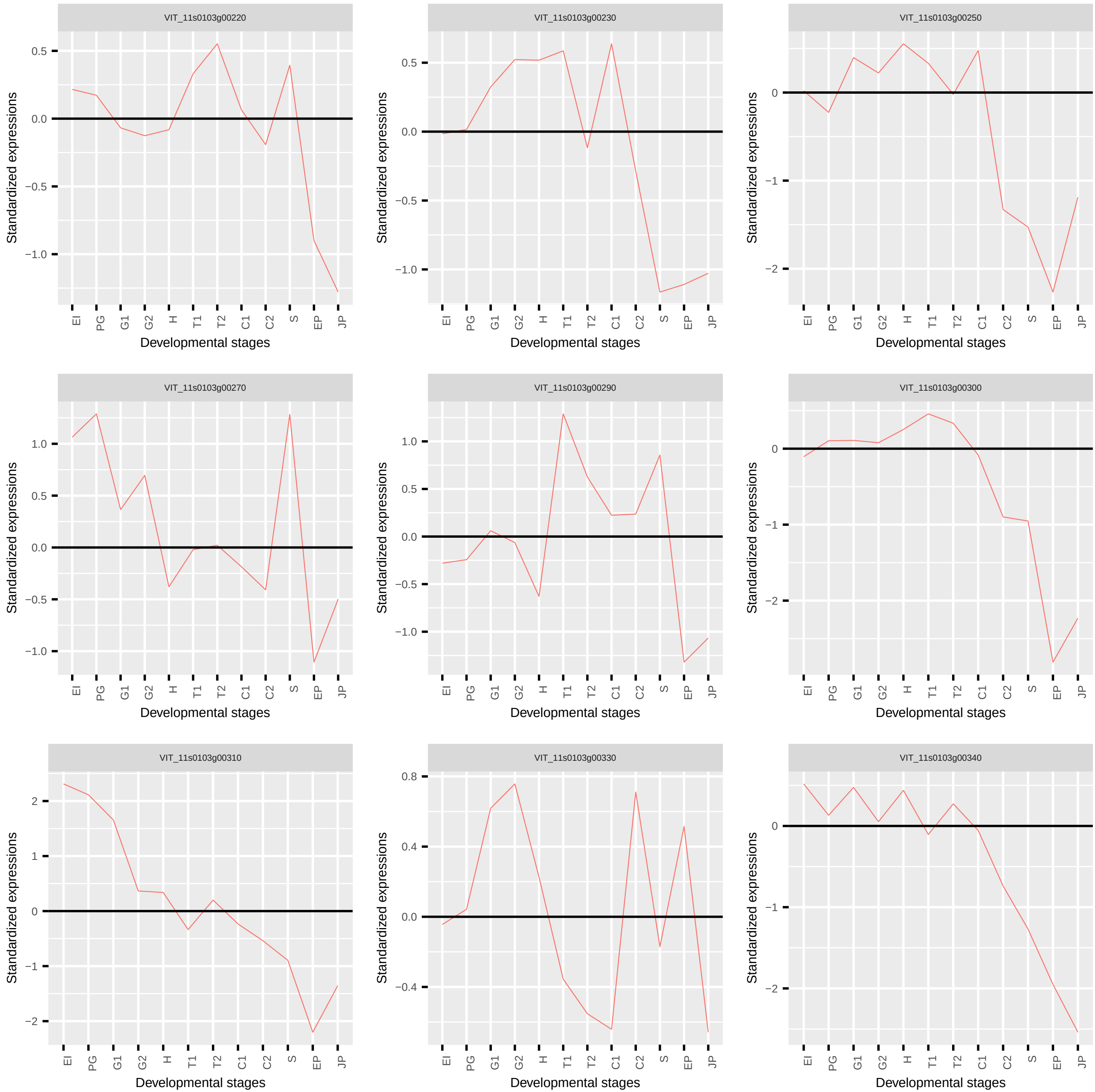
VIT_11s0103g00220
VIT_11s0103g00230
VIT_11s0103g00250
VIT_11s0103g00270
VIT_11s0103g00290
VIT_11s0103g00300
VIT_11s0103g00310
VIT_11s0103g00330
VIT_11s0103g00340
Standardized expressions
Developmental stages
EI PG G1 G2 H T1 T2 C1 C2 S EP JP

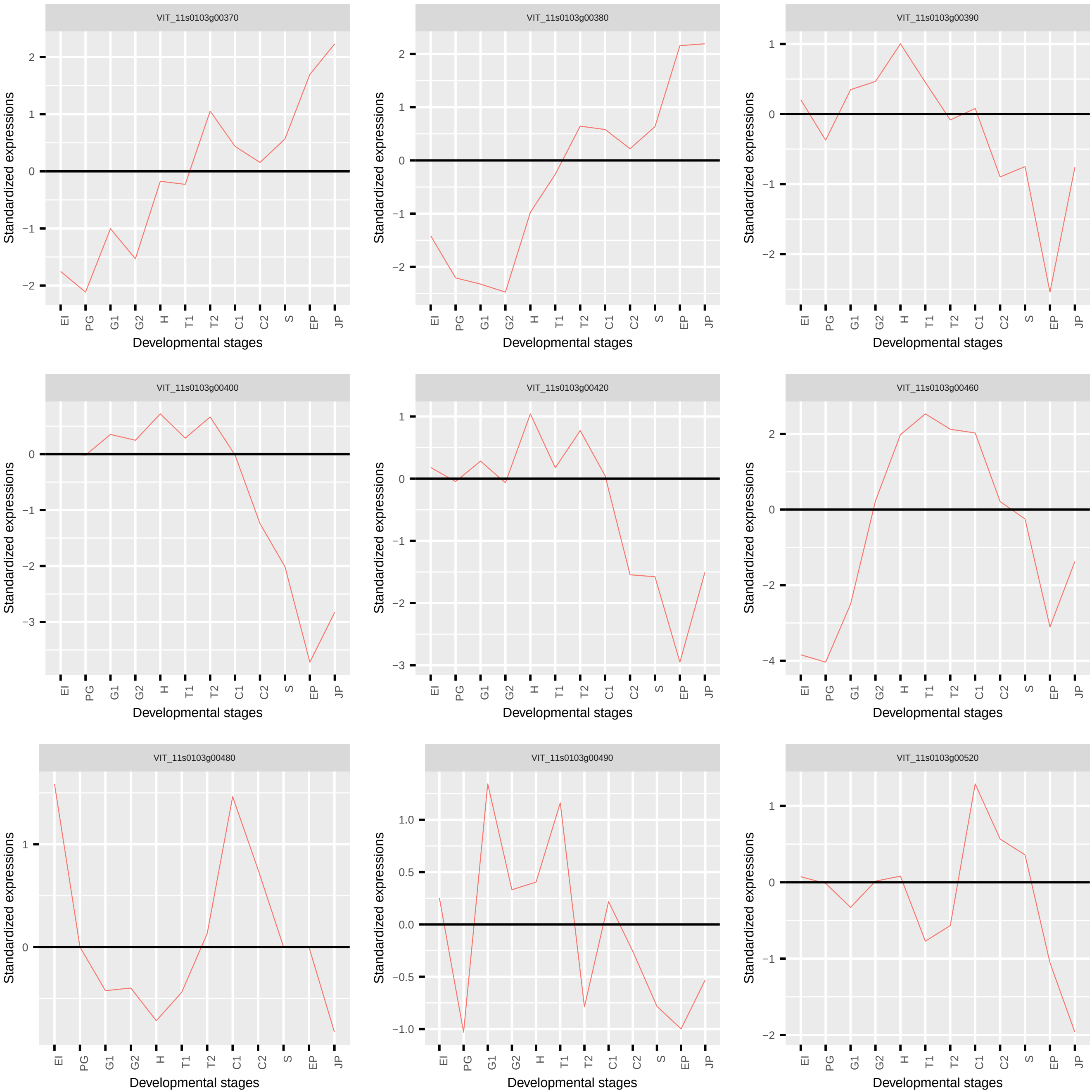
VIT_11s0103g00370
2
1
0
−1
−2
Standardized expressions
EI PG G1 G2 H T1 T2 C1 C2 S EP JP
Developmental stages
VIT_11s0103g00380
2
1
0
−1
−2
Standardized expressions
EI PG G1 G2 H T1 T2 C1 C2 S EP JP
Developmental stages
VIT_11s0103g00390
1
0
−1
−2
Standardized expressions
EI PG G1 G2 H T1 T2 C1 C2 S EP JP
Developmental stages
VIT_11s0103g00400
0
−1
−2
−3
Standardized expressions
EI PG G1 G2 H T1 T2 C1 C2 S EP JP
Developmental stages
VIT_11s0103g00420
1
0
−1
−2
−3
Standardized expressions
EI PG G1 G2 H T1 T2 C1 C2 S EP JP
Developmental stages
VIT_11s0103g00460
2
0
−2
−4
Standardized expressions
EI PG G1 G2 H T1 T2 C1 C2 S EP JP
Developmental stages
VIT_11s0103g00480
1
0
Standardized expressions
EI PG G1 G2 H T1 T2 C1 C2 S EP JP
Developmental stages
VIT_11s0103g00490
1.0
0.5
0.0
−0.5
−1.0
Standardized expressions
EI PG G1 G2 H T1 T2 C1 C2 S EP JP
Developmental stages
VIT_11s0103g00520
1
0
−1
−2
Standardized expressions
EI PG G1 G2 H T1 T2 C1 C2 S EP JP
Developmental stages

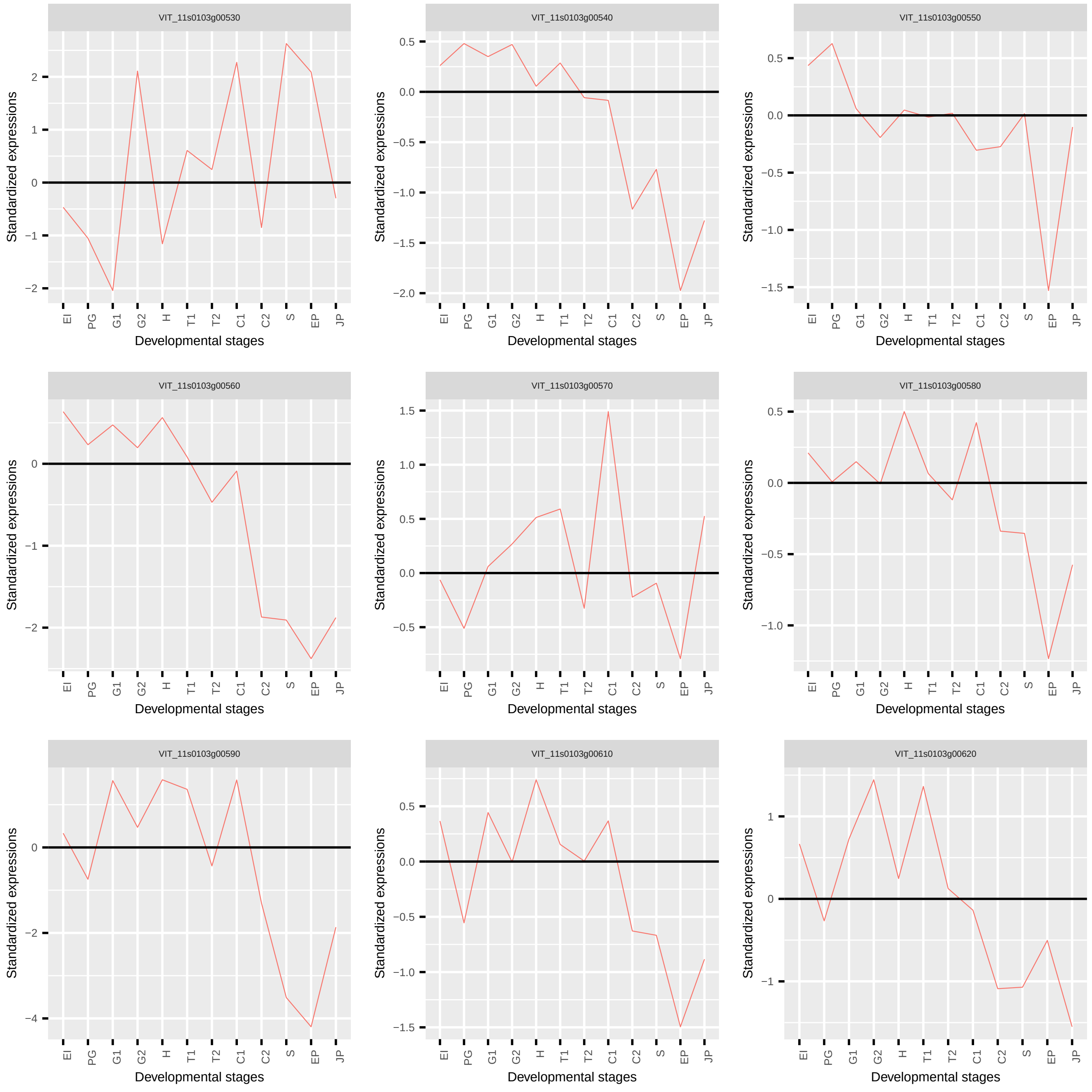
VIT_11s0103g00530
VIT_11s0103g00540
VIT_11s0103g00550
VIT_11s0103g00560
VIT_11s0103g00570
VIT_11s0103g00580
VIT_11s0103g00590
VIT_11s0103g00610
VIT_11s0103g00620
Standardized expressions
Developmental stages
EI PG G1 G2 H T1 T2 C1 C2 S EP JP

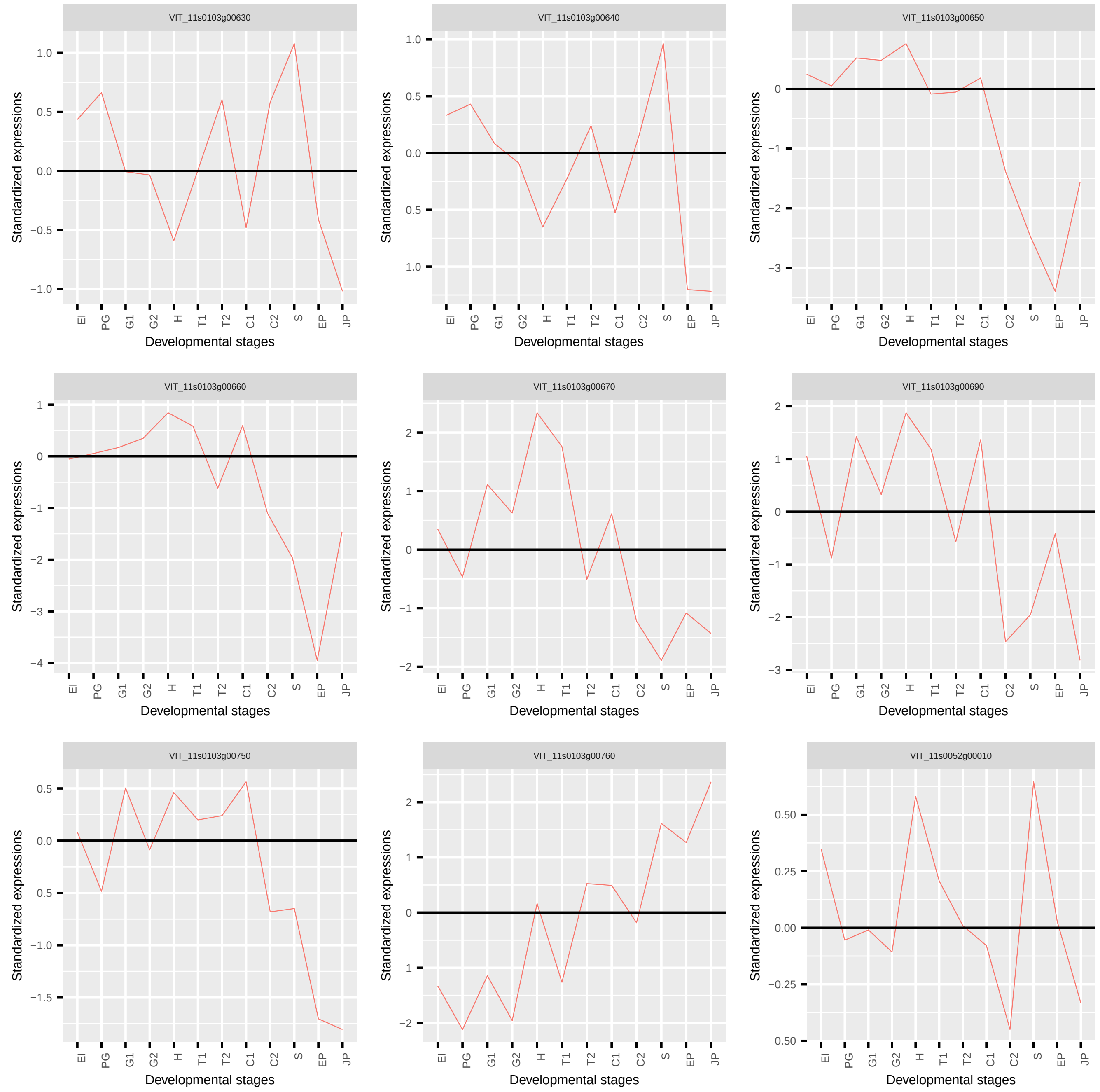
VIT_11s0103g00630
VIT_11s0103g00640
VIT_11s0103g00650
VIT_11s0103g00660
VIT_11s0103g00670
VIT_11s0103g00690
VIT_11s0103g00750
VIT_11s0103g00760
VIT_11s0052g00010
Standardized expressions
Developmental stages
EI PG G1 G2 H T1 T2 C1 C2 S EP JP

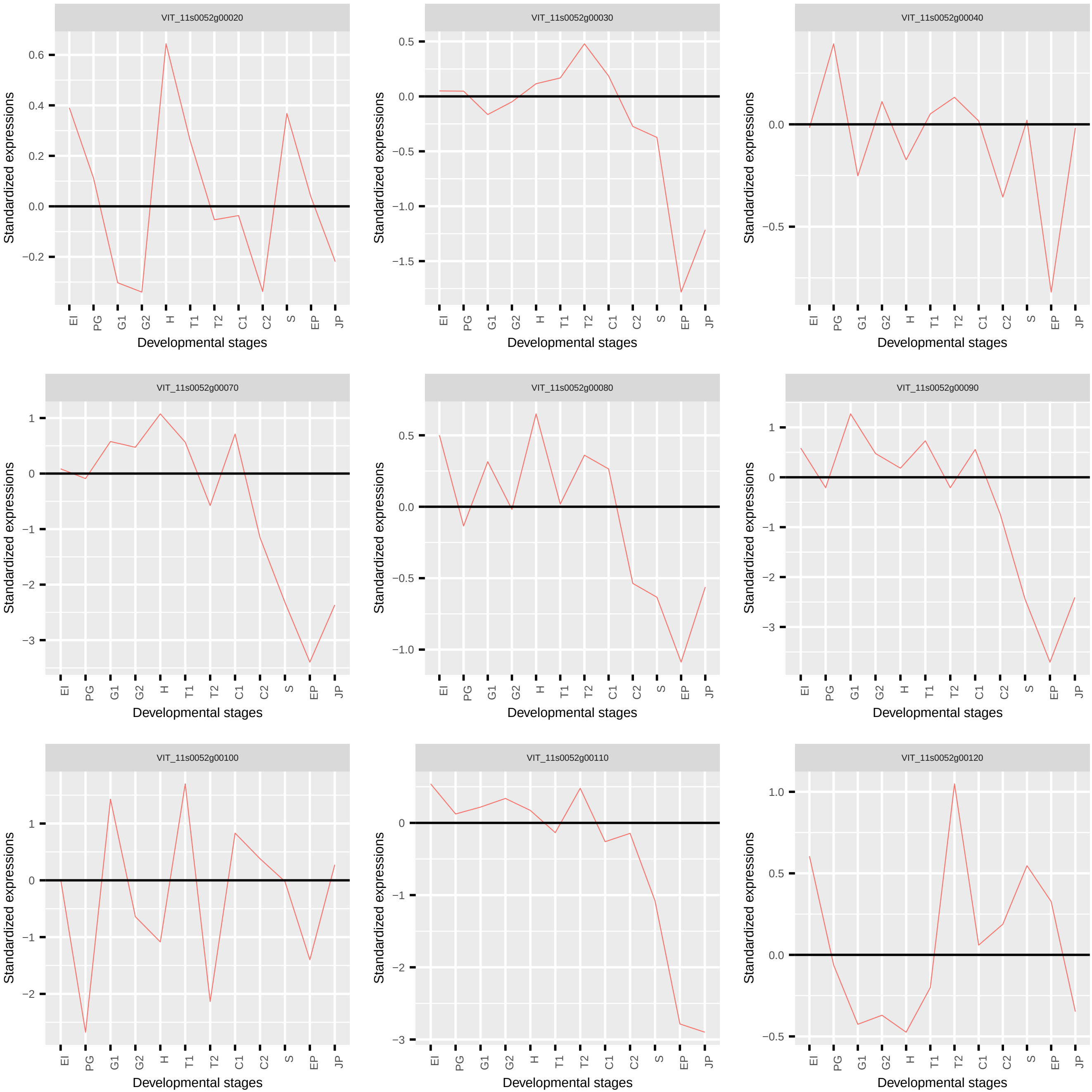

VIT_11s0052g00020
VIT_11s0052g00030
VIT_11s0052g00040
VIT_11s0052g00070
VIT_11s0052g00080
VIT_11s0052g00090
VIT_11s0052g00100
VIT_11s0052g00110
VIT_11s0052g00120
Standardized expressions
Developmental stages
EI PG G1 G2 H T1 T2 C1 C2 S EP JP

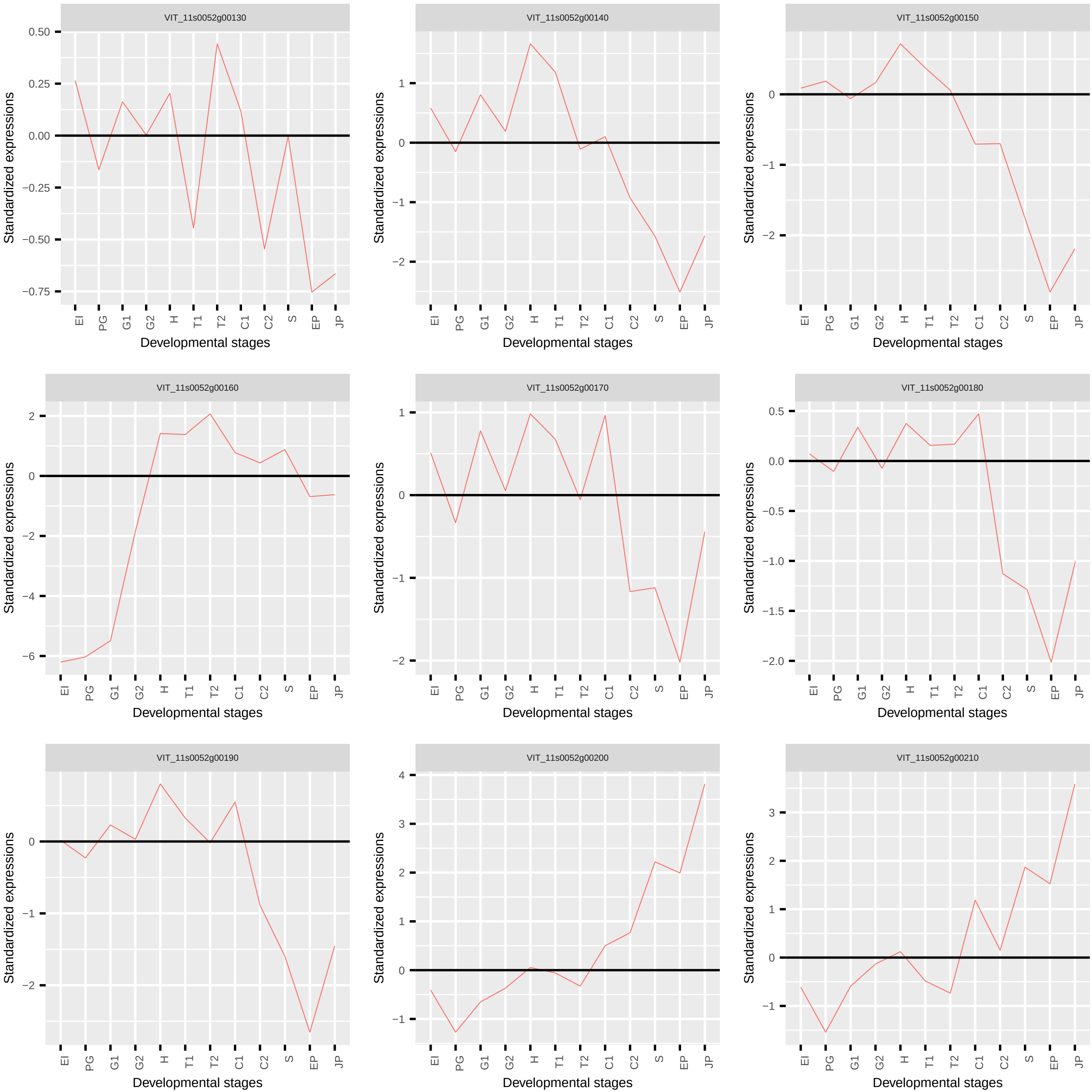
VIT_11s0052g00130
VIT_11s0052g00140
VIT_11s0052g00150
VIT_11s0052g00160
VIT_11s0052g00170
VIT_11s0052g00180
VIT_11s0052g00190
VIT_11s0052g00200
VIT_11s0052g00210
Standardized expressions
Developmental stages
EI PG G1 G2 H T1 T2 C1 C2 S EP JP

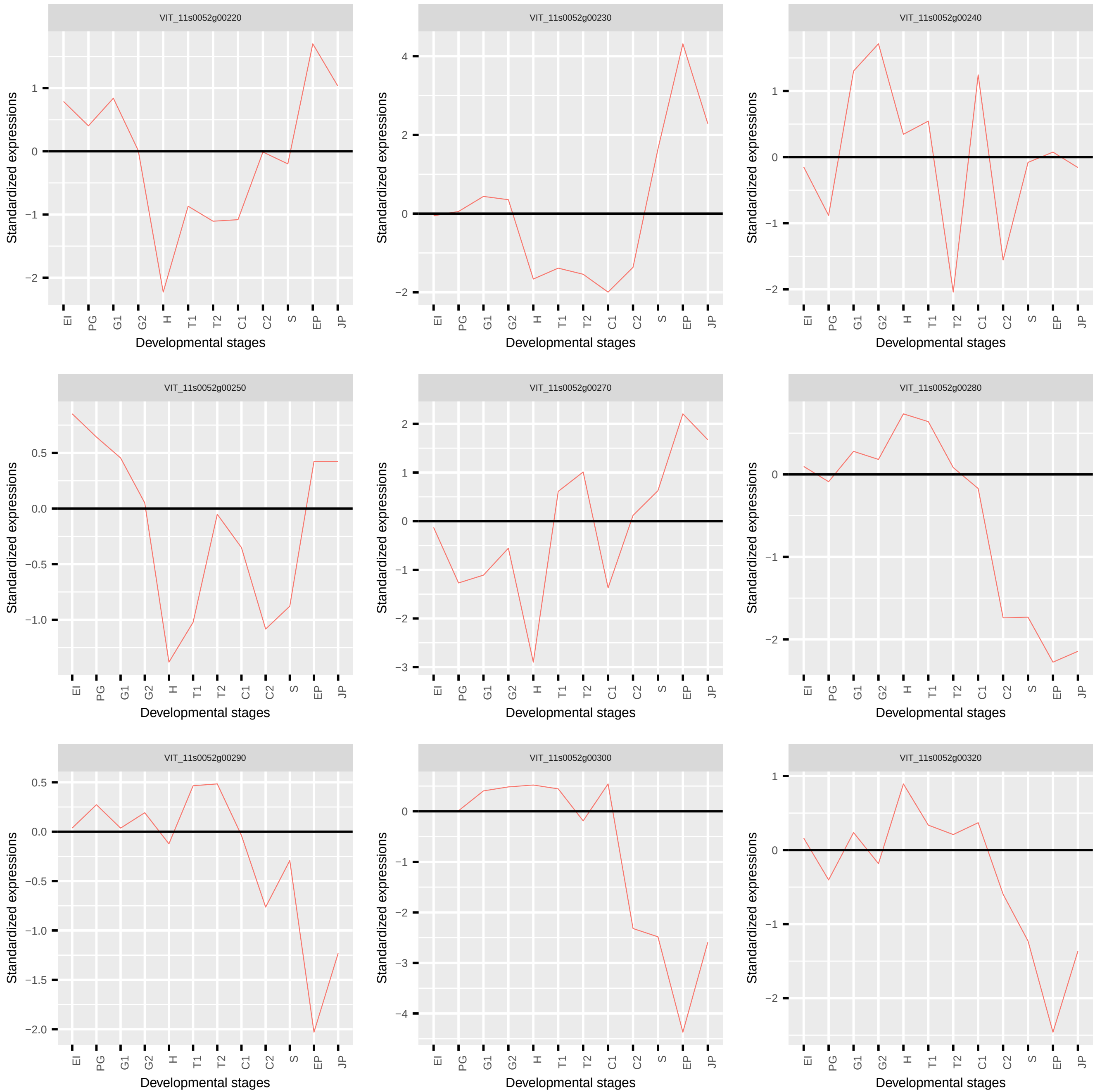
VIT_11s0052g00220
VIT_11s0052g00230
VIT_11s0052g00240
VIT_11s0052g00250
VIT_11s0052g00270
VIT_11s0052g00280
VIT_11s0052g00290
VIT_11s0052g00300
VIT_11s0052g00320
Standardized expressions
Developmental stages
EI PG G1 G2 H T1 T2 C1 C2 S EP JP

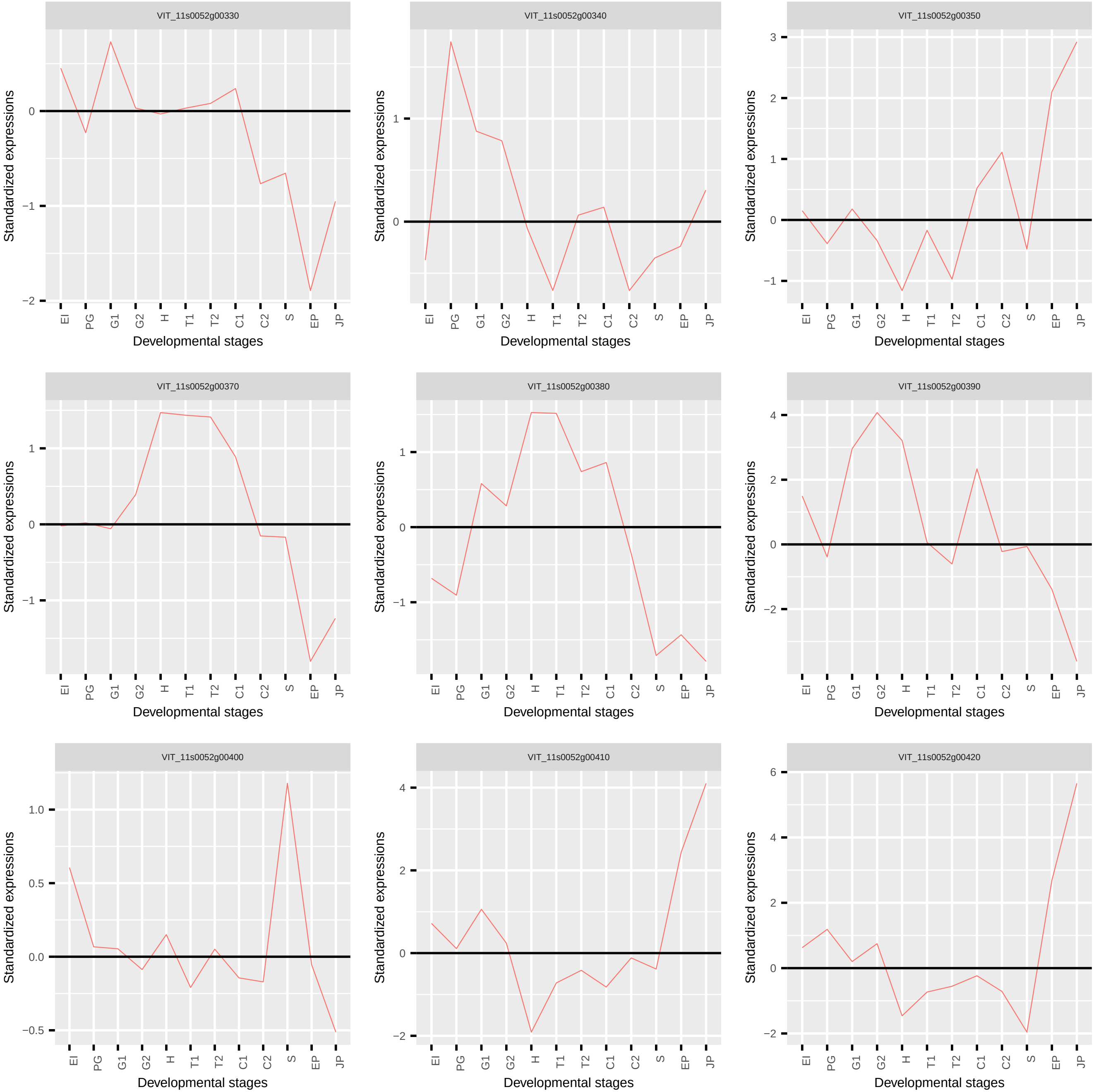
VIT_11s0052g00330
VIT_11s0052g00340
VIT_11s0052g00350
VIT_11s0052g00370
VIT_11s0052g00380
VIT_11s0052g00390
VIT_11s0052g00400
VIT_11s0052g00410
VIT_11s0052g00420
Standardized expressions
Developmental stages
EI PG G1 G2 H T1 T2 C1 C2 S EP JP

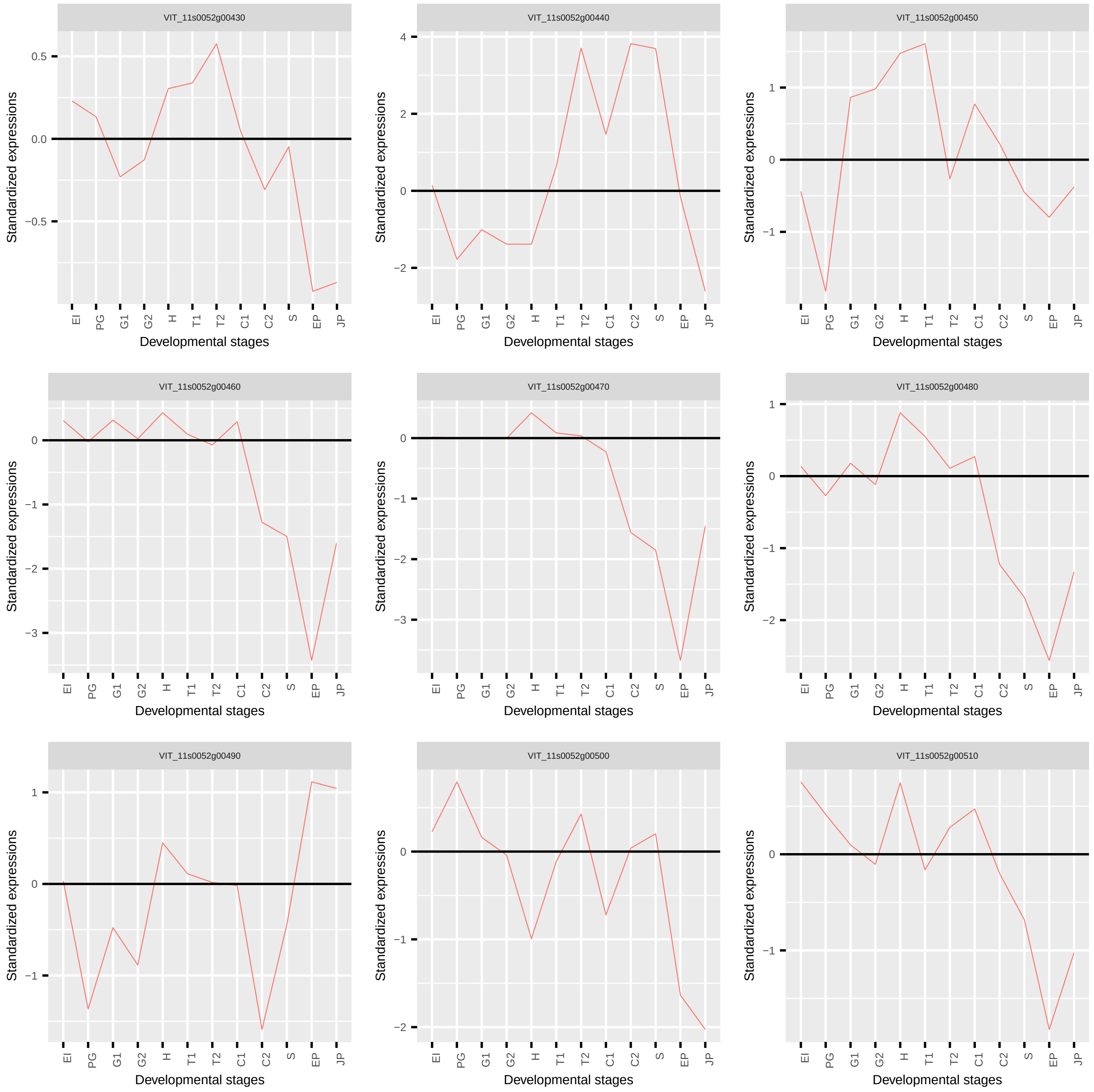

VIT_11s0052g00430
VIT_11s0052g00440
VIT_11s0052g00450
VIT_11s0052g00460
VIT_11s0052g00470
VIT_11s0052g00480
VIT_11s0052g00490
VIT_11s0052g00500
VIT_11s0052g00510
Standardized expressions
Developmental stages
EI PG G1 G2 H T1 T2 C1 C2 S EP JP

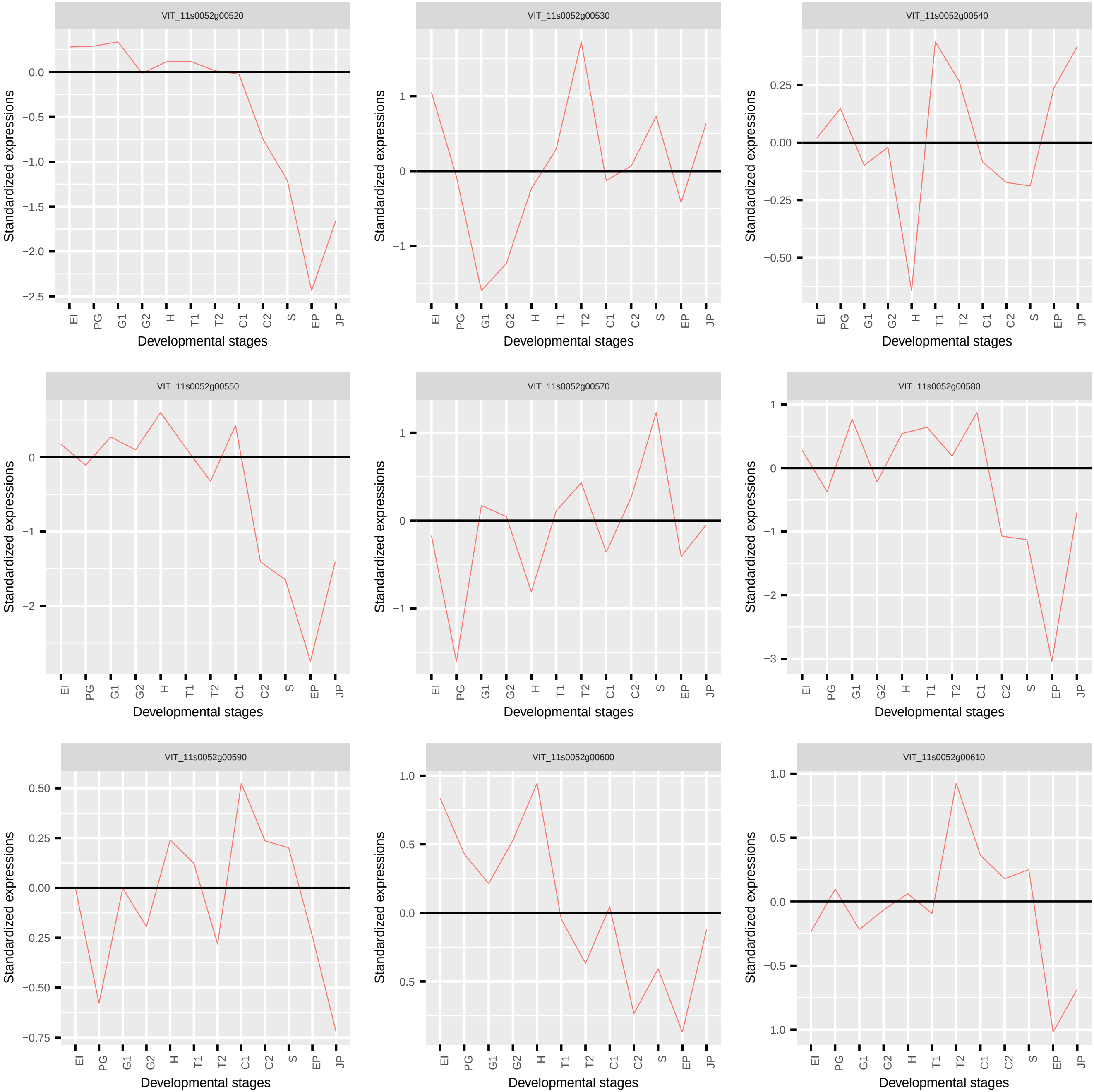

VIT_11s0052g00520
VIT_11s0052g00530
VIT_11s0052g00540
VIT_11s0052g00550
VIT_11s0052g00570
VIT_11s0052g00580
VIT_11s0052g00590
VIT_11s0052g00600
VIT_11s0052g00610
Standardized expressions
Developmental stages
EI
PG
G1
G2
H
T1
T2
C1
C2
S
EP
JP

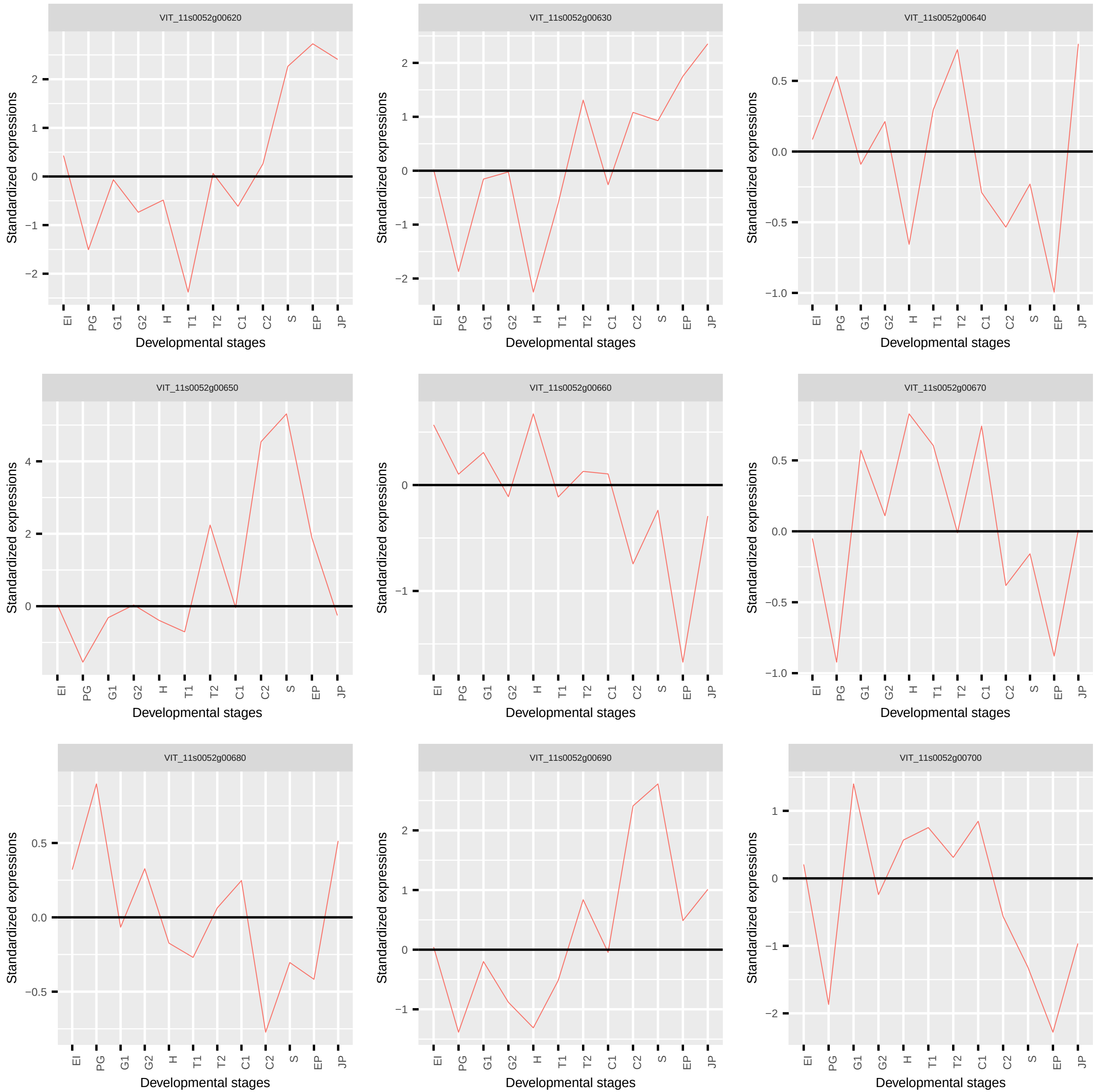
VIT_11s0052g00620
VIT_11s0052g00630
VIT_11s0052g00640
VIT_11s0052g00650
VIT_11s0052g00660
VIT_11s0052g00670
VIT_11s0052g00680
VIT_11s0052g00690
VIT_11s0052g00700
Standardized expressions
Developmental stages
EI
PG
G1
G2
H
T1
T2
C1
C2
S
EP
JP

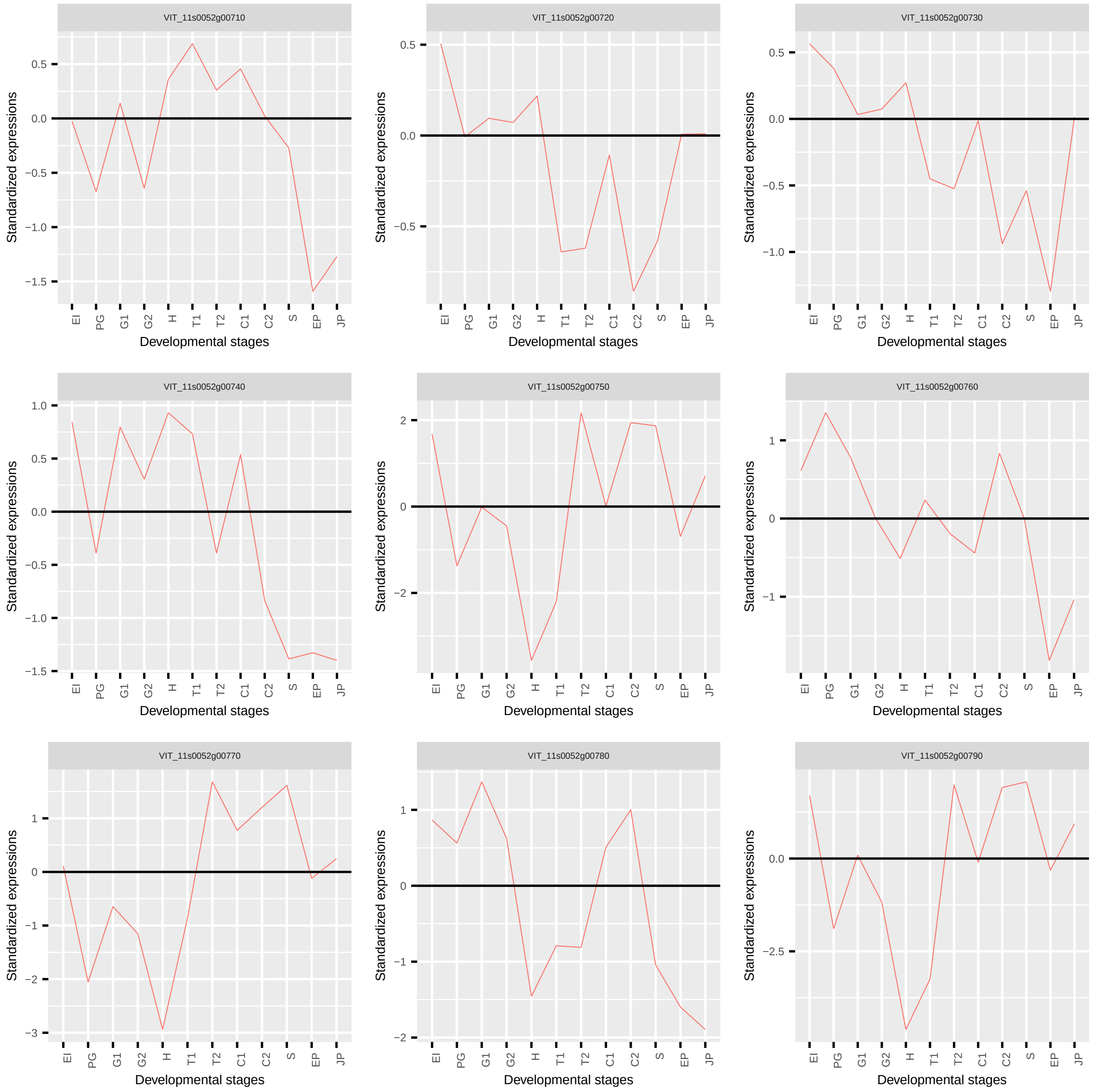
VIT_11s0052g00710
VIT_11s0052g00720
VIT_11s0052g00730
VIT_11s0052g00740
VIT_11s0052g00750
VIT_11s0052g00760
VIT_11s0052g00770
VIT_11s0052g00780
VIT_11s0052g00790
Standardized expressions
Developmental stages
EI PG G1 G2 H T1 T2 C1 C2 S EP JP
0.5 0.0 −0.5 −1.0 −1.5
2 0 −2
1 0 −1 −2 −3
0.0 −2.5

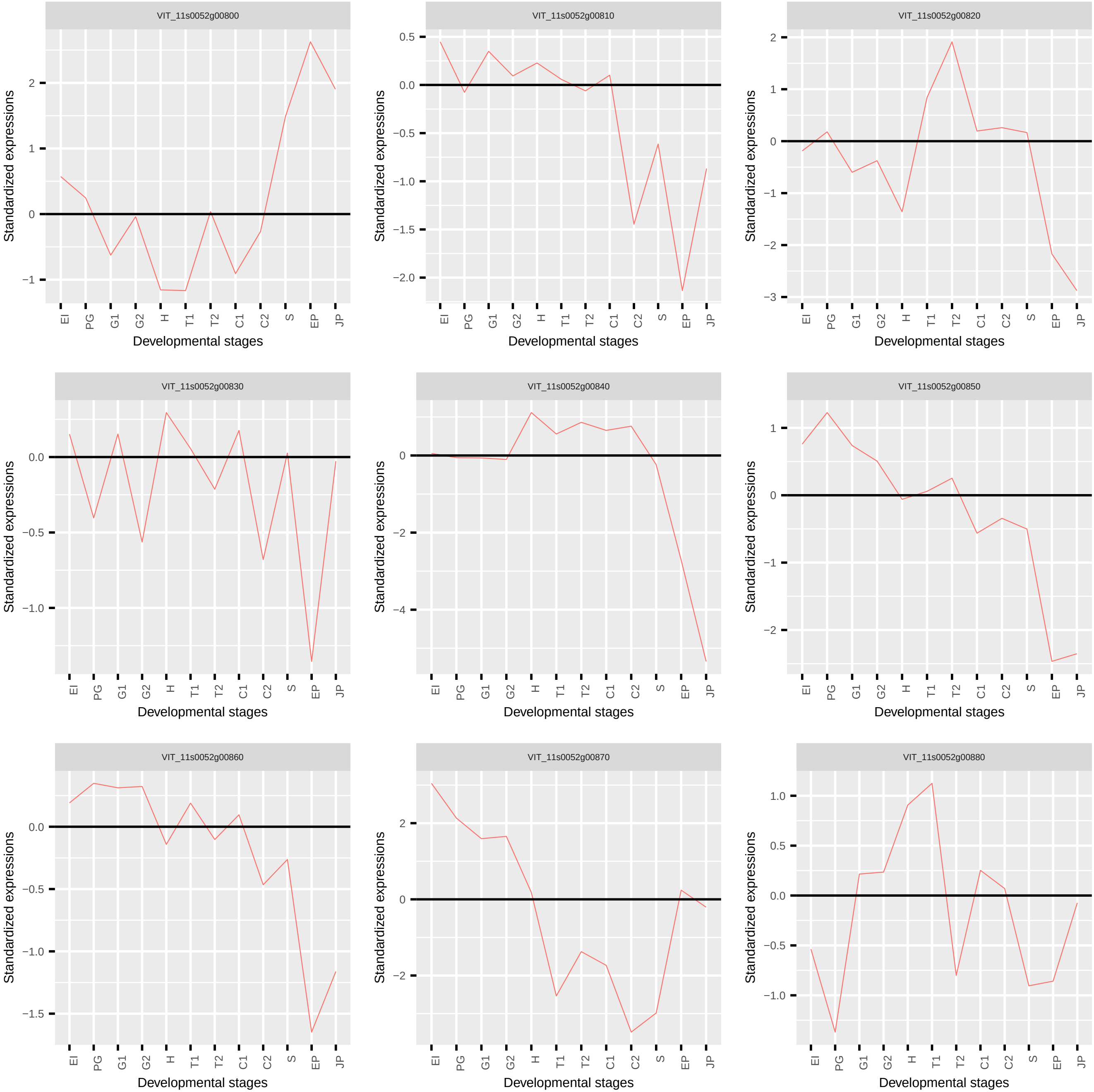

VIT_11s0052g00800
VIT_11s0052g00810
VIT_11s0052g00820
VIT_11s0052g00830
VIT_11s0052g00840
VIT_11s0052g00850
VIT_11s0052g00860
VIT_11s0052g00870
VIT_11s0052g00880
Standardized expressions
Developmental stages
EI PG G1 G2 H T1 T2 C1 C2 S EP JP

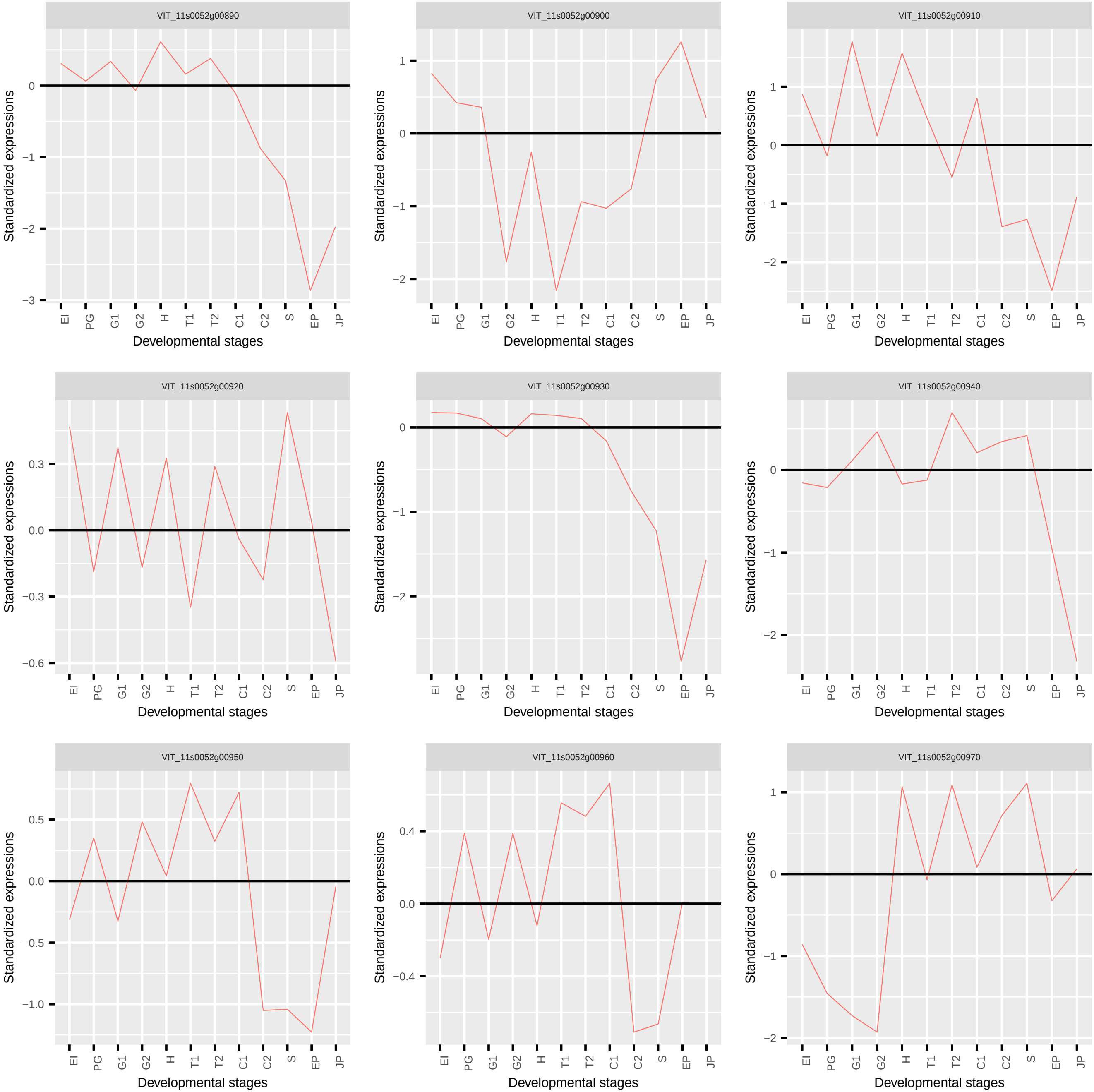
VIT_11s0052g00890
VIT_11s0052g00900
VIT_11s0052g00910
VIT_11s0052g00920
VIT_11s0052g00930
VIT_11s0052g00940
VIT_11s0052g00950
VIT_11s0052g00960
VIT_11s0052g00970
Standardized expressions
Developmental stages
EI PG G1 G2 H T1 T2 C1 C2 S EP JP

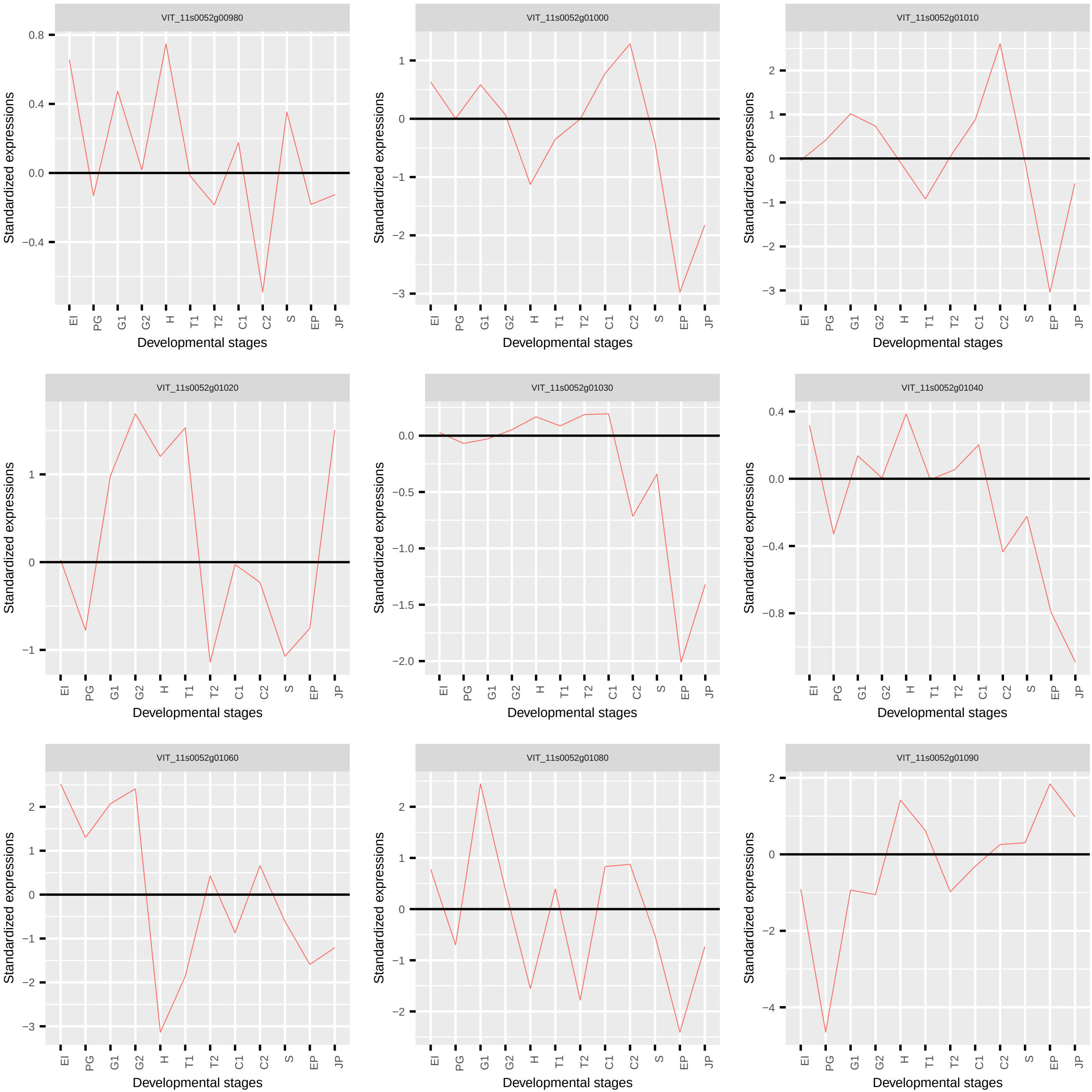
VIT_11s0052g00980
VIT_11s0052g01000
VIT_11s0052g01010
VIT_11s0052g01020
VIT_11s0052g01030
VIT_11s0052g01040
VIT_11s0052g01060
VIT_11s0052g01080
VIT_11s0052g01090
Standardized expressions
Developmental stages
EI PG G1 G2 H T1 T2 C1 C2 S EP JP

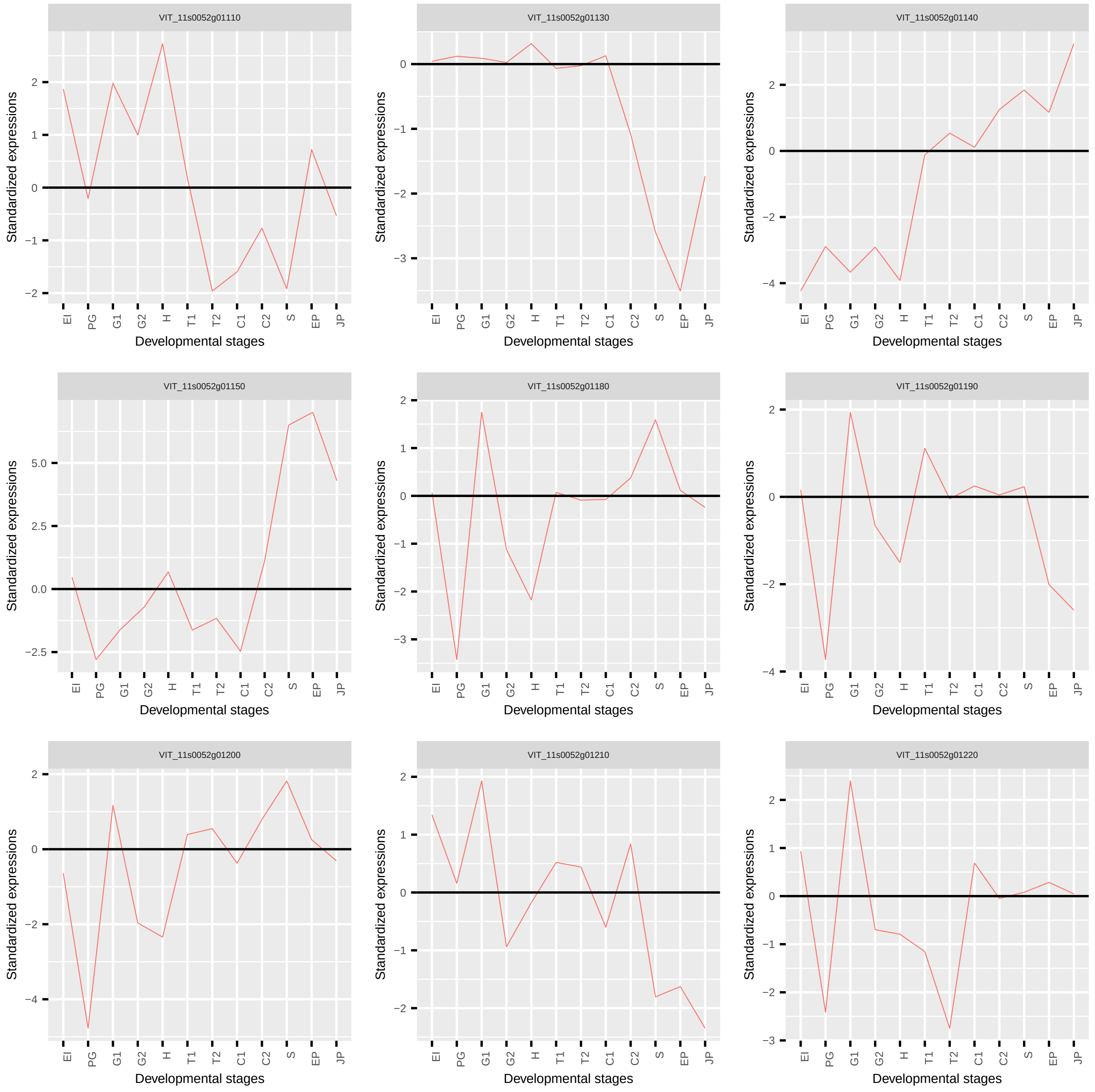
VIT_11s0052g01110
VIT_11s0052g01130
VIT_11s0052g01140
VIT_11s0052g01150
VIT_11s0052g01180
VIT_11s0052g01190
VIT_11s0052g01200
VIT_11s0052g01210
VIT_11s0052g01220
Standardized expressions
Developmental stages
EI PG G1 G2 H T1 T2 C1 C2 S EP JP

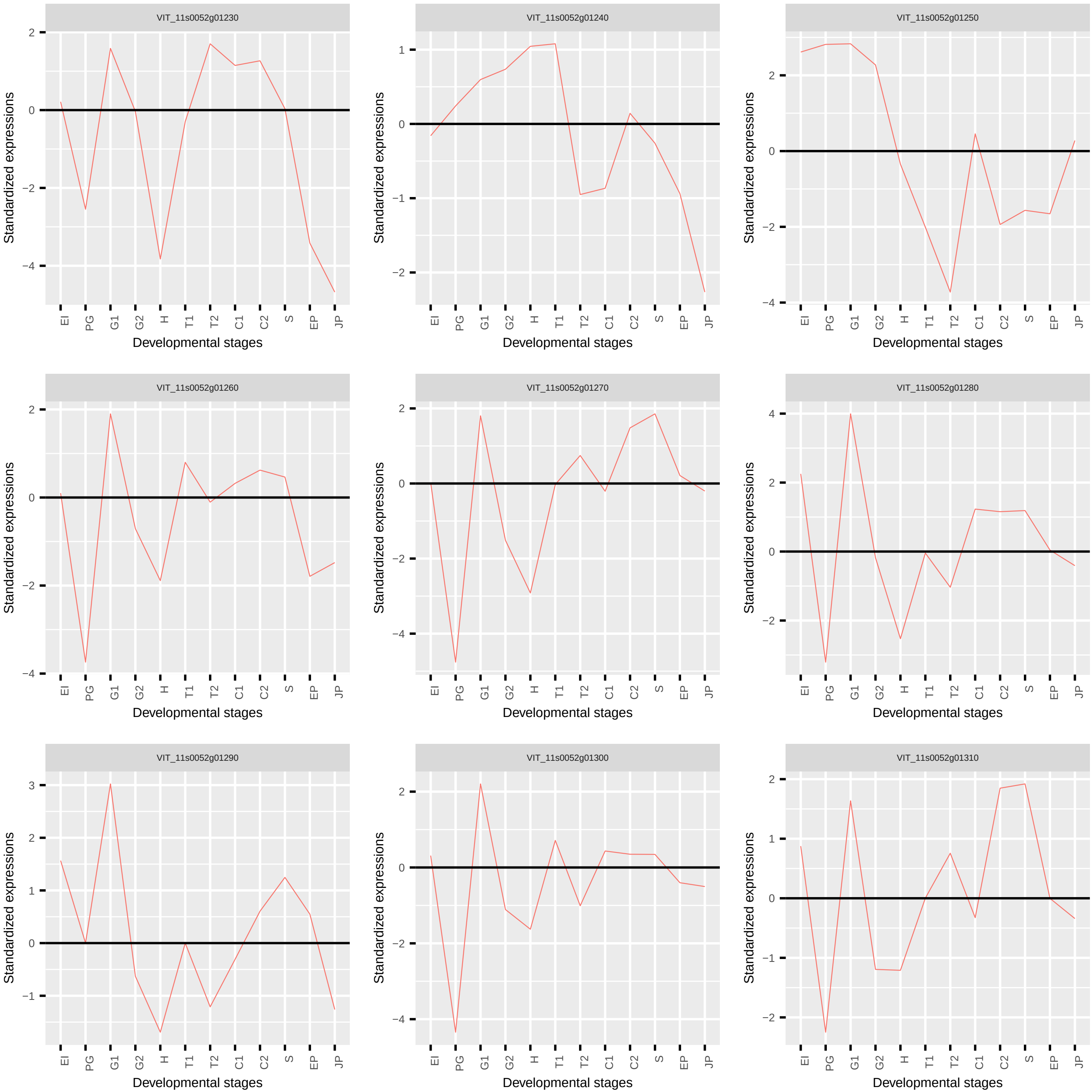
VIT_11s0052g01230
VIT_11s0052g01240
VIT_11s0052g01250
VIT_11s0052g01260
VIT_11s0052g01270
VIT_11s0052g01280
VIT_11s0052g01290
VIT_11s0052g01300
VIT_11s0052g01310
Standardized expressions
Developmental stages
EI
PG
G1
G2
H
T1
T2
C1
C2
S
EP
JP

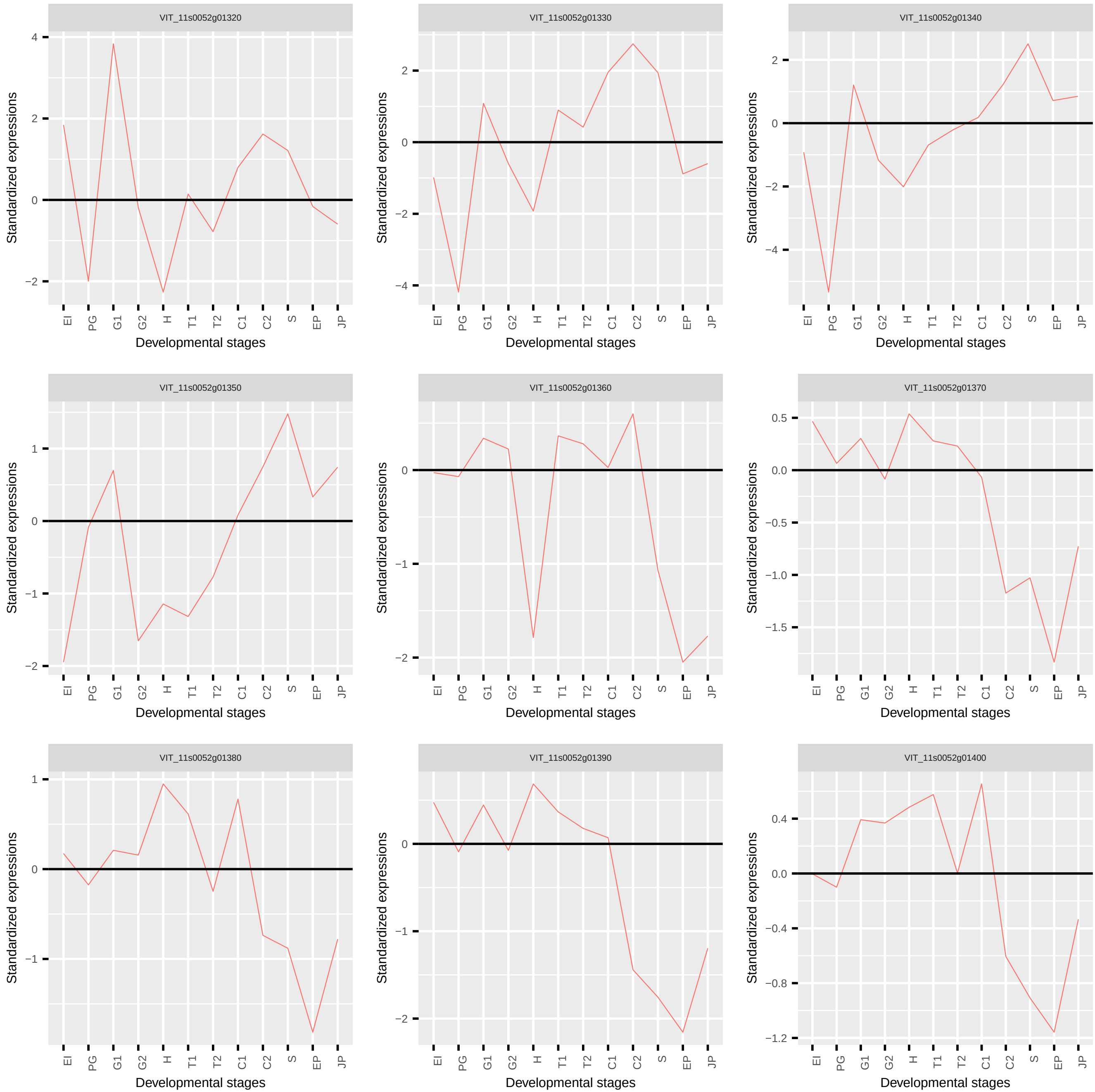
VIT_11s0052g01320
VIT_11s0052g01330
VIT_11s0052g01340
VIT_11s0052g01350
VIT_11s0052g01360
VIT_11s0052g01370
VIT_11s0052g01380
VIT_11s0052g01390
VIT_11s0052g01400
Standardized expressions
Developmental stages
EI
PG
G1
G2
H
T1
T2
C1
C2
S
EP
JP

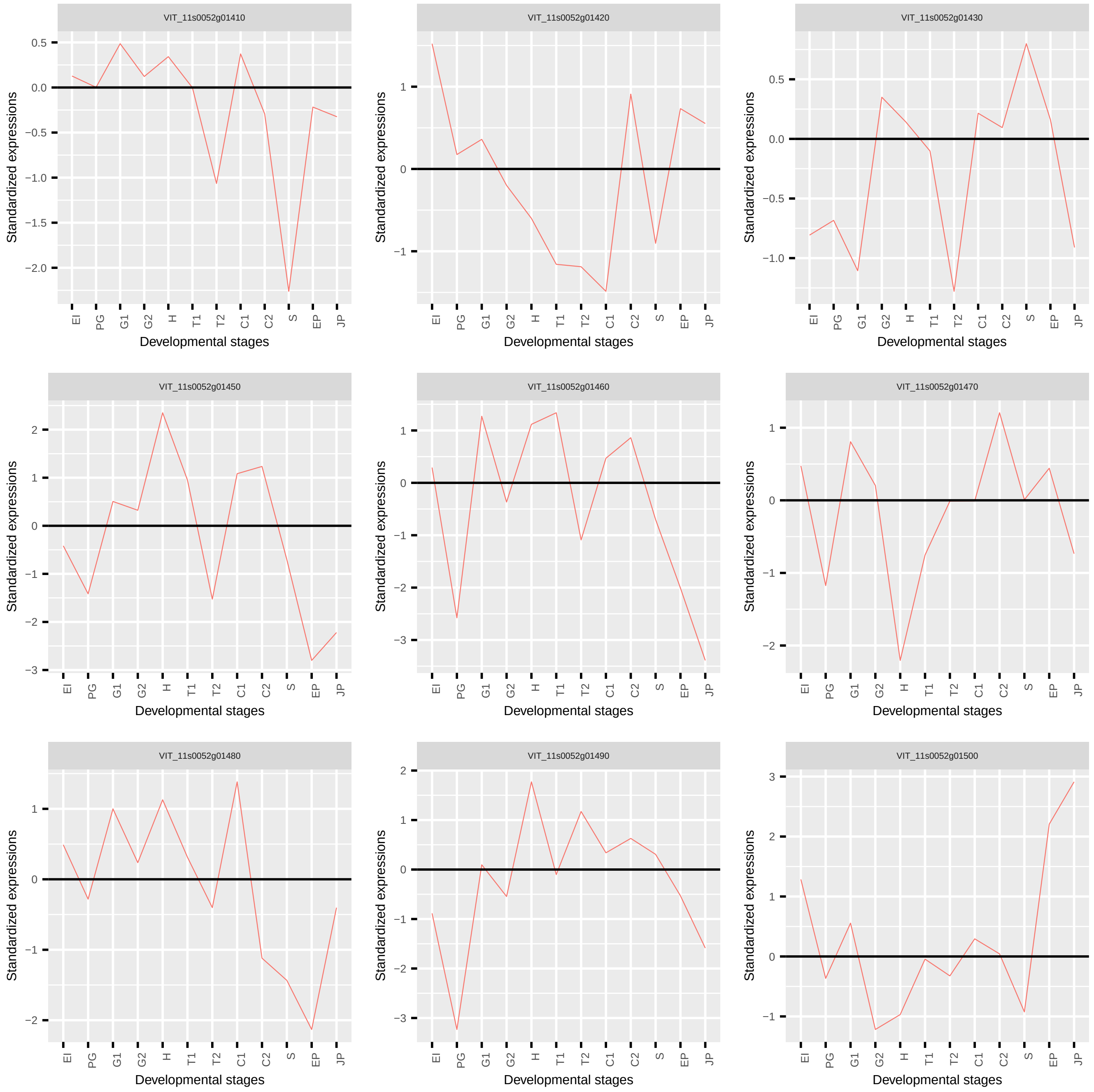

VIT_11s0052g01410
VIT_11s0052g01420
VIT_11s0052g01430
VIT_11s0052g01450
VIT_11s0052g01460
VIT_11s0052g01470
VIT_11s0052g01480
VIT_11s0052g01490
VIT_11s0052g01500
Standardized expressions
Developmental stages
EI PG G1 G2 H T1 T2 C1 C2 S EP JP

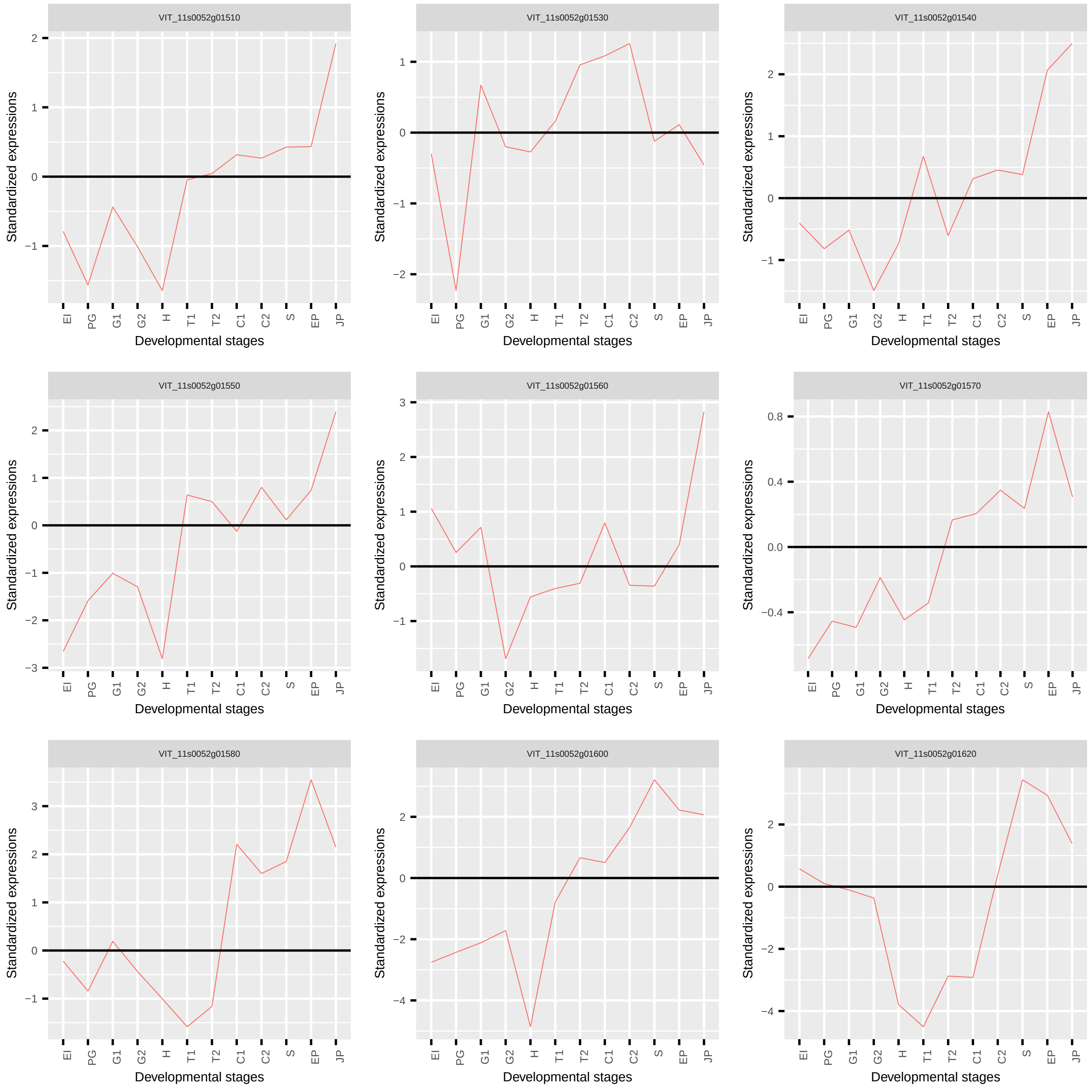
VIT_11s0052g01510
VIT_11s0052g01530
VIT_11s0052g01540
VIT_11s0052g01550
VIT_11s0052g01560
VIT_11s0052g01570
VIT_11s0052g01580
VIT_11s0052g01600
VIT_11s0052g01620
Standardized expressions
Developmental stages
EI
PG
G1
G2
H
T1
T2
C1
C2
S
EP
JP

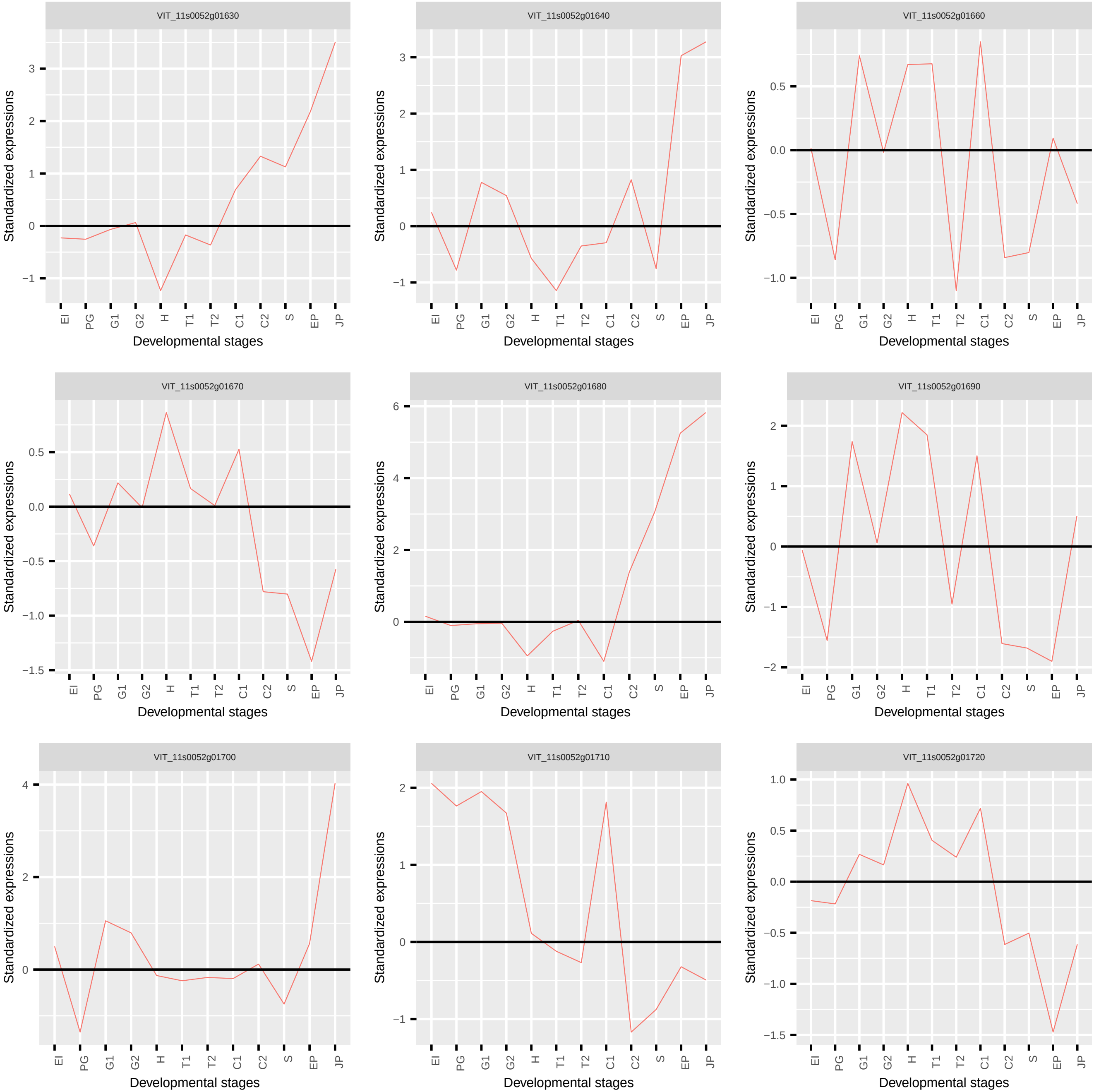
VIT_11s0052g01630
VIT_11s0052g01640
VIT_11s0052g01660
VIT_11s0052g01670
VIT_11s0052g01680
VIT_11s0052g01690
VIT_11s0052g01700
VIT_11s0052g01710
VIT_11s0052g01720
Standardized expressions
Developmental stages
EI
PG
G1
G2
H
T1
T2
C1
C2
S
EP
JP

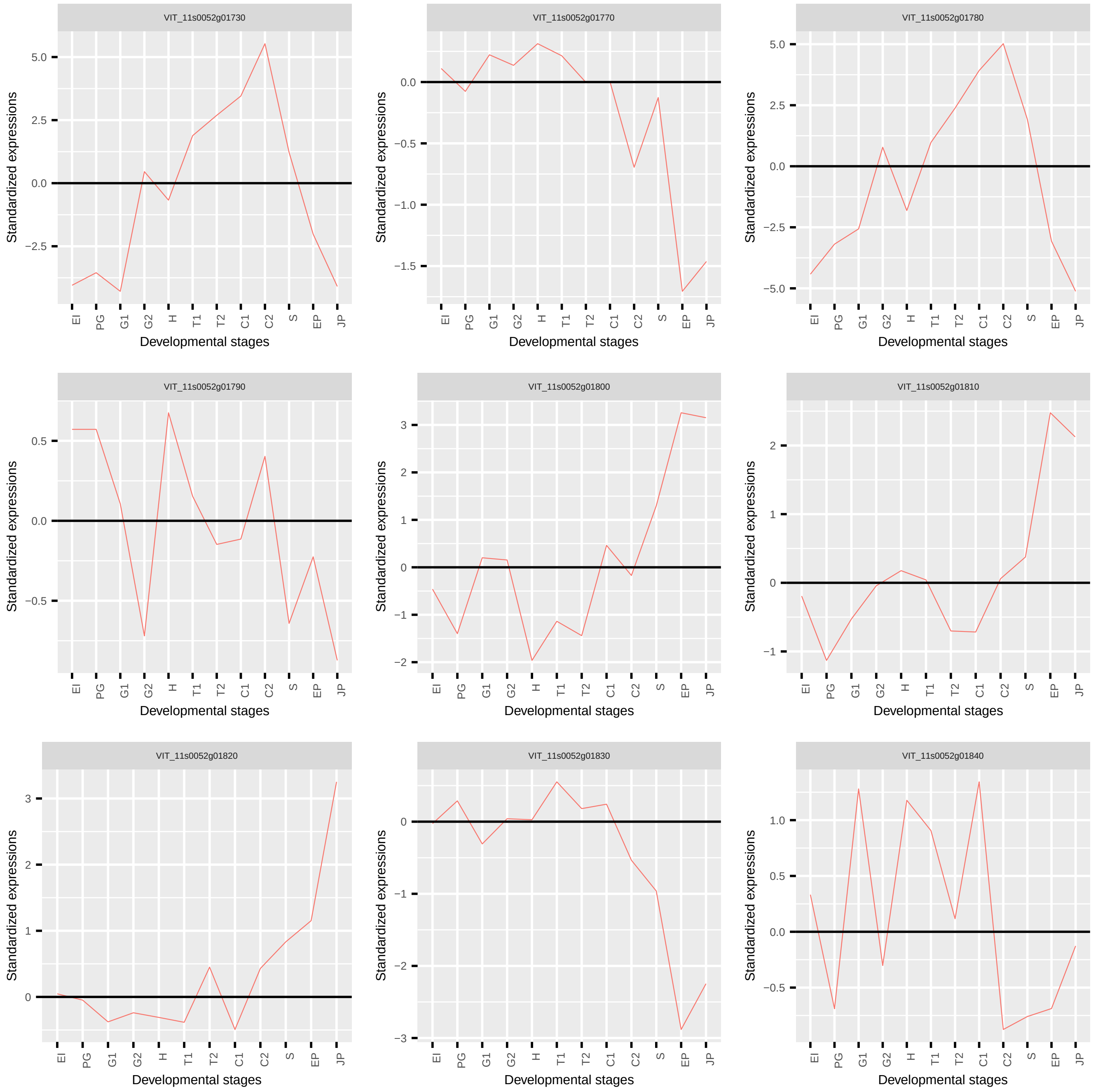
VIT_11s0052g01730
VIT_11s0052g01770
VIT_11s0052g01780
VIT_11s0052g01790
VIT_11s0052g01800
VIT_11s0052g01810
VIT_11s0052g01820
VIT_11s0052g01830
VIT_11s0052g01840
Standardized expressions
Developmental stages
EI
PG
G1
G2
H
T1
T2
C1
C2
S
EP
JP

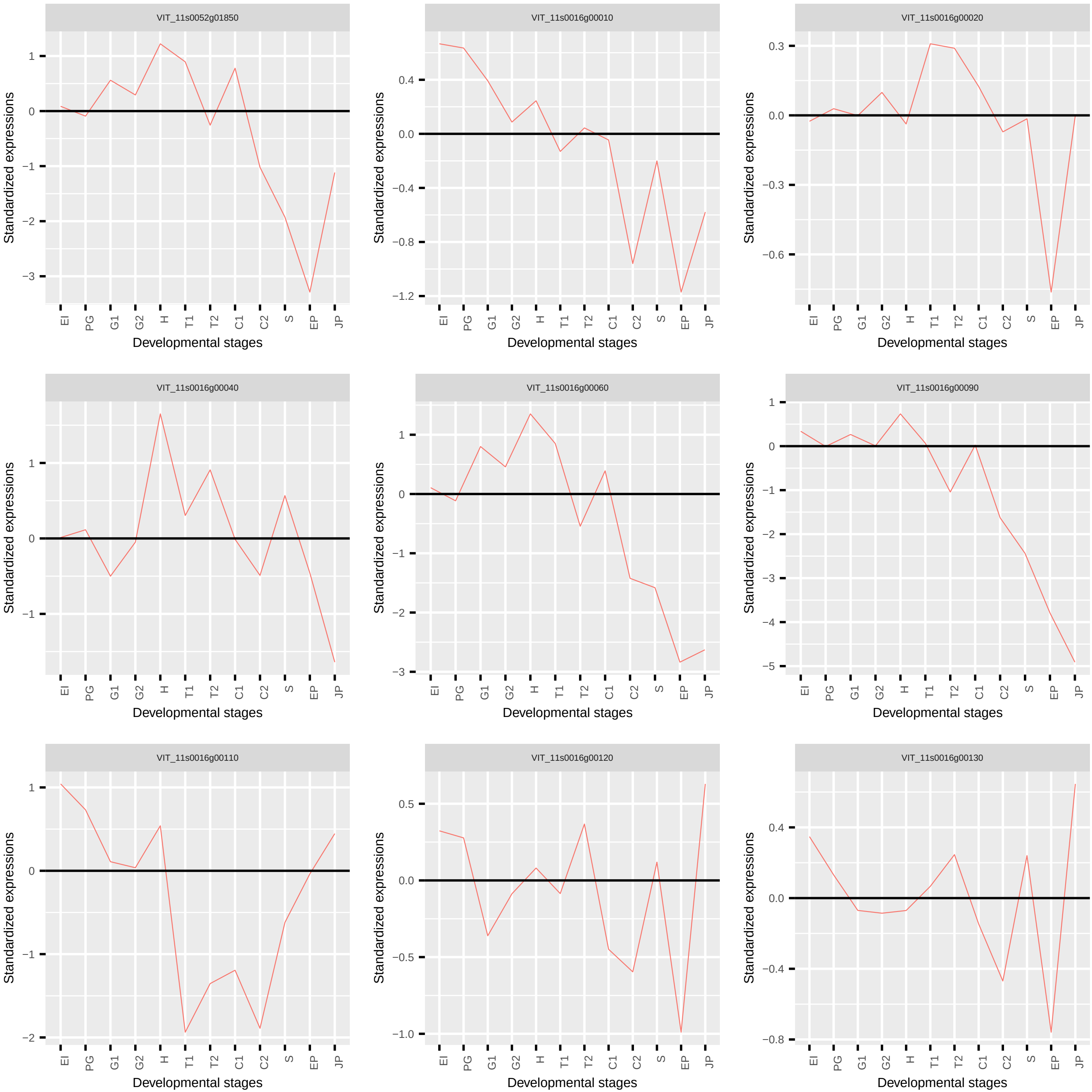
VIT_11s0052g01850
1
0
−1
−2
−3
Standardized expressions
EI PG G1 G2 H T1 T2 C1 C2 S EP JP
Developmental stages
VIT_11s0016g00010
0.4
0.0
−0.4
−0.8
−1.2
Standardized expressions
EI PG G1 G2 H T1 T2 C1 C2 S EP JP
Developmental stages
VIT_11s0016g00020
0.3
0.0
−0.3
−0.6
Standardized expressions
EI PG G1 G2 H T1 T2 C1 C2 S EP JP
Developmental stages
VIT_11s0016g00040
1
0
−1
Standardized expressions
EI PG G1 G2 H T1 T2 C1 C2 S EP JP
Developmental stages
VIT_11s0016g00060
1
0
−1
−2
−3
Standardized expressions
EI PG G1 G2 H T1 T2 C1 C2 S EP JP
Developmental stages
VIT_11s0016g00090
1
0
−1
−2
−3
−4
−5
Standardized expressions
EI PG G1 G2 H T1 T2 C1 C2 S EP JP
Developmental stages
VIT_11s0016g00110
1
0
−1
−2
Standardized expressions
EI PG G1 G2 H T1 T2 C1 C2 S EP JP
Developmental stages
VIT_11s0016g00120
0.5
0.0
−0.5
−1.0
Standardized expressions
EI PG G1 G2 H T1 T2 C1 C2 S EP JP
Developmental stages
VIT_11s0016g00130
0.4
0.0
−0.4
−0.8
Standardized expressions
EI PG G1 G2 H T1 T2 C1 C2 S EP JP
Developmental stages

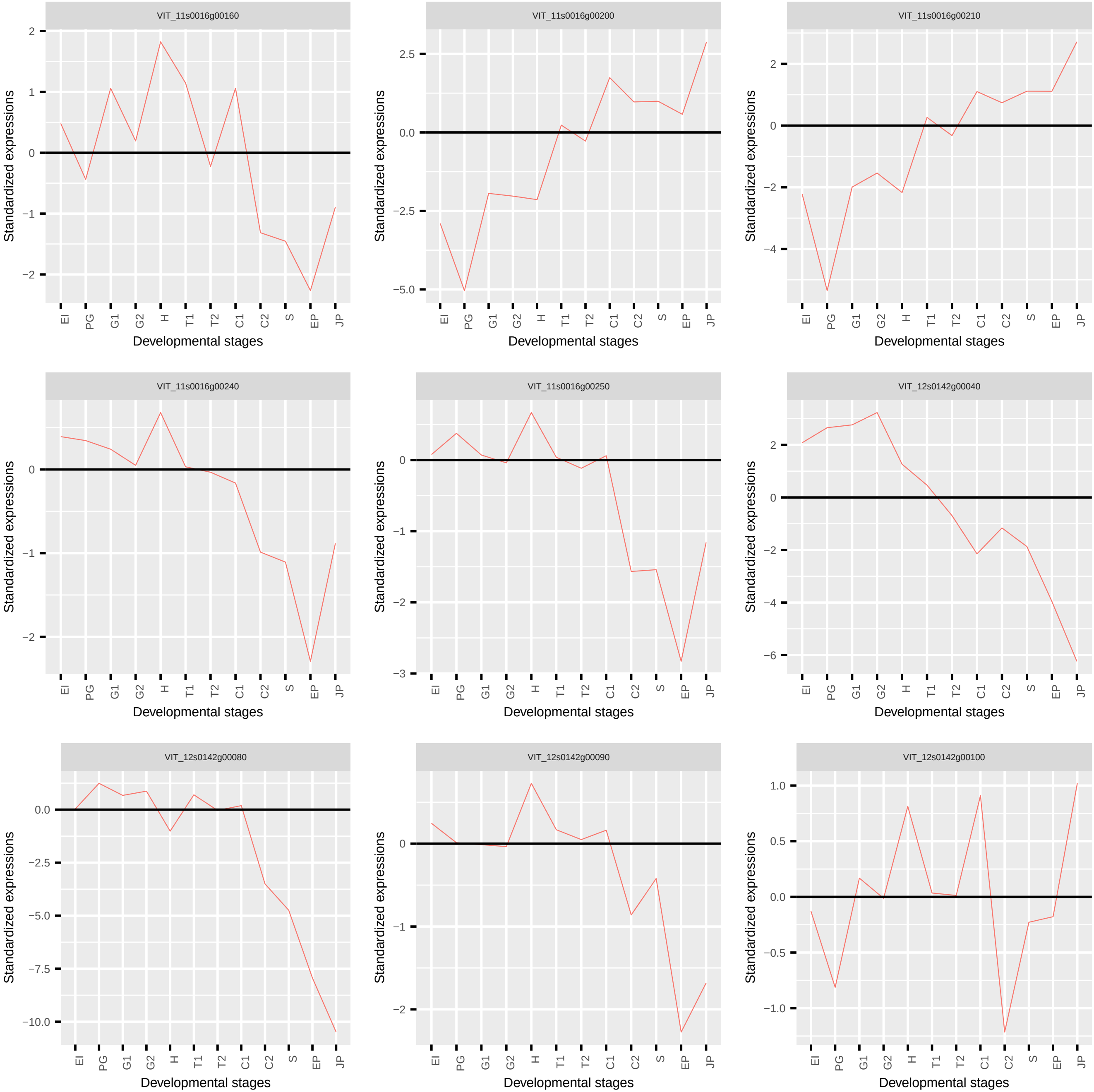
VIT_11s0016g00160
VIT_11s0016g00200
VIT_11s0016g00210
VIT_11s0016g00240
VIT_11s0016g00250
VIT_12s0142g00040
VIT_12s0142g00080
VIT_12s0142g00090
VIT_12s0142g00100
Standardized expressions
Developmental stages
EI
PG
G1
G2
H
T1
T2
C1
C2
S
EP
JP

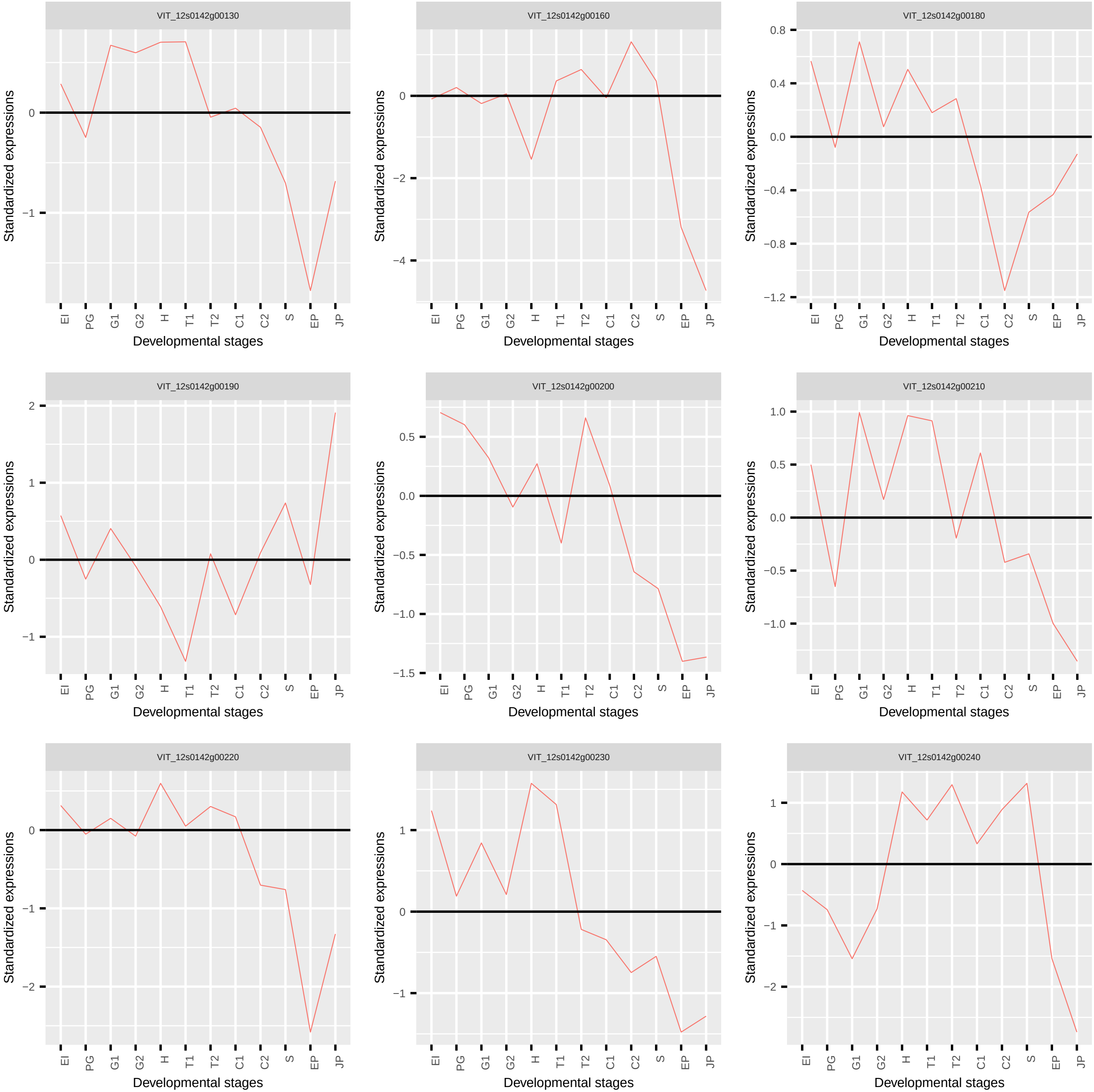
VIT_12s0142g00130
VIT_12s0142g00160
VIT_12s0142g00180
VIT_12s0142g00190
VIT_12s0142g00200
VIT_12s0142g00210
VIT_12s0142g00220
VIT_12s0142g00230
VIT_12s0142g00240
Standardized expressions
Developmental stages
EI PG G1 G2 H T1 T2 C1 C2 S EP JP

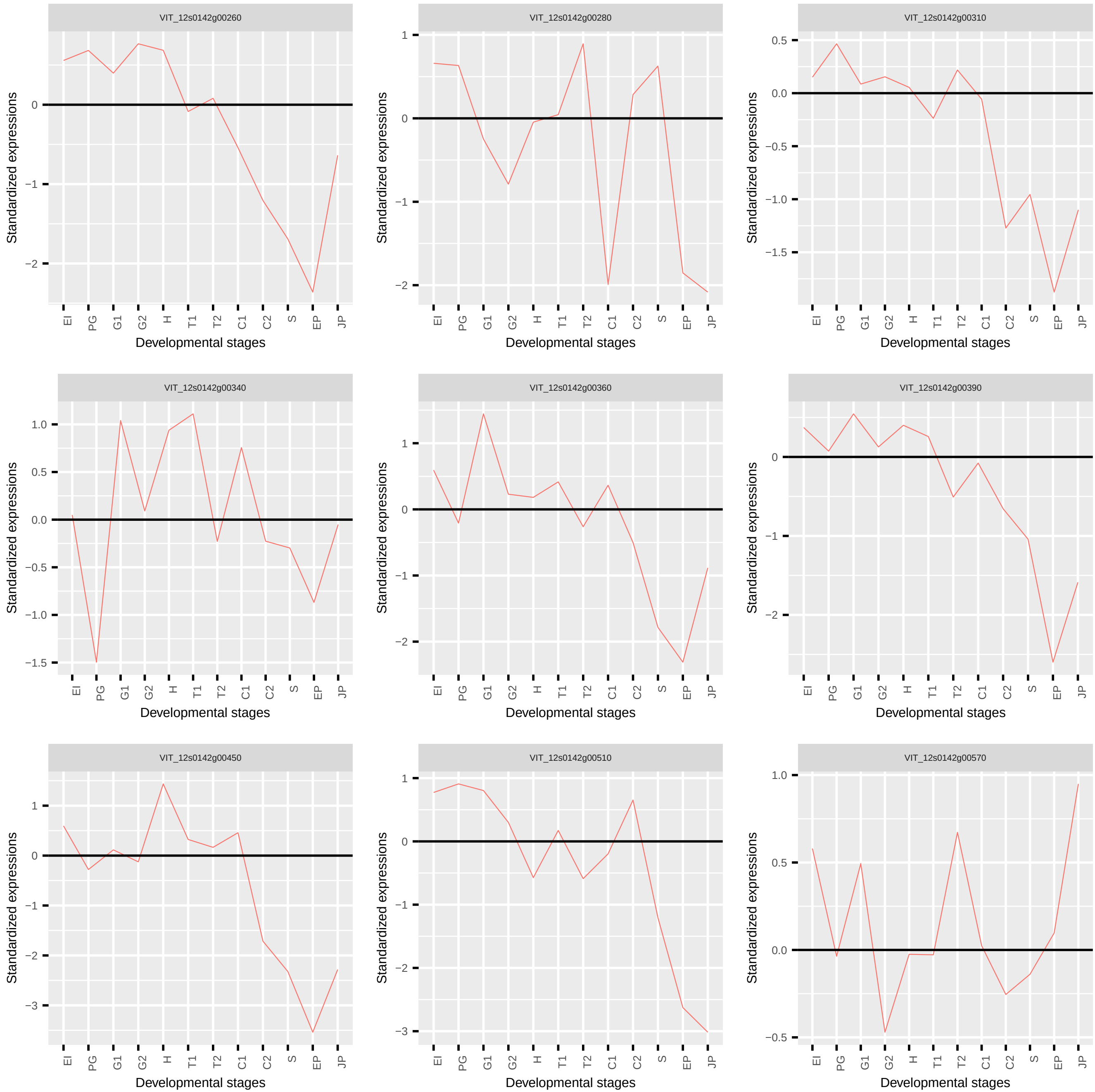
VIT_12s0142g00260
VIT_12s0142g00280
VIT_12s0142g00310
VIT_12s0142g00340
VIT_12s0142g00360
VIT_12s0142g00390
VIT_12s0142g00450
VIT_12s0142g00510
VIT_12s0142g00570
Standardized expressions
Developmental stages
EI PG G1 G2 H T1 T2 C1 C2 S EP JP

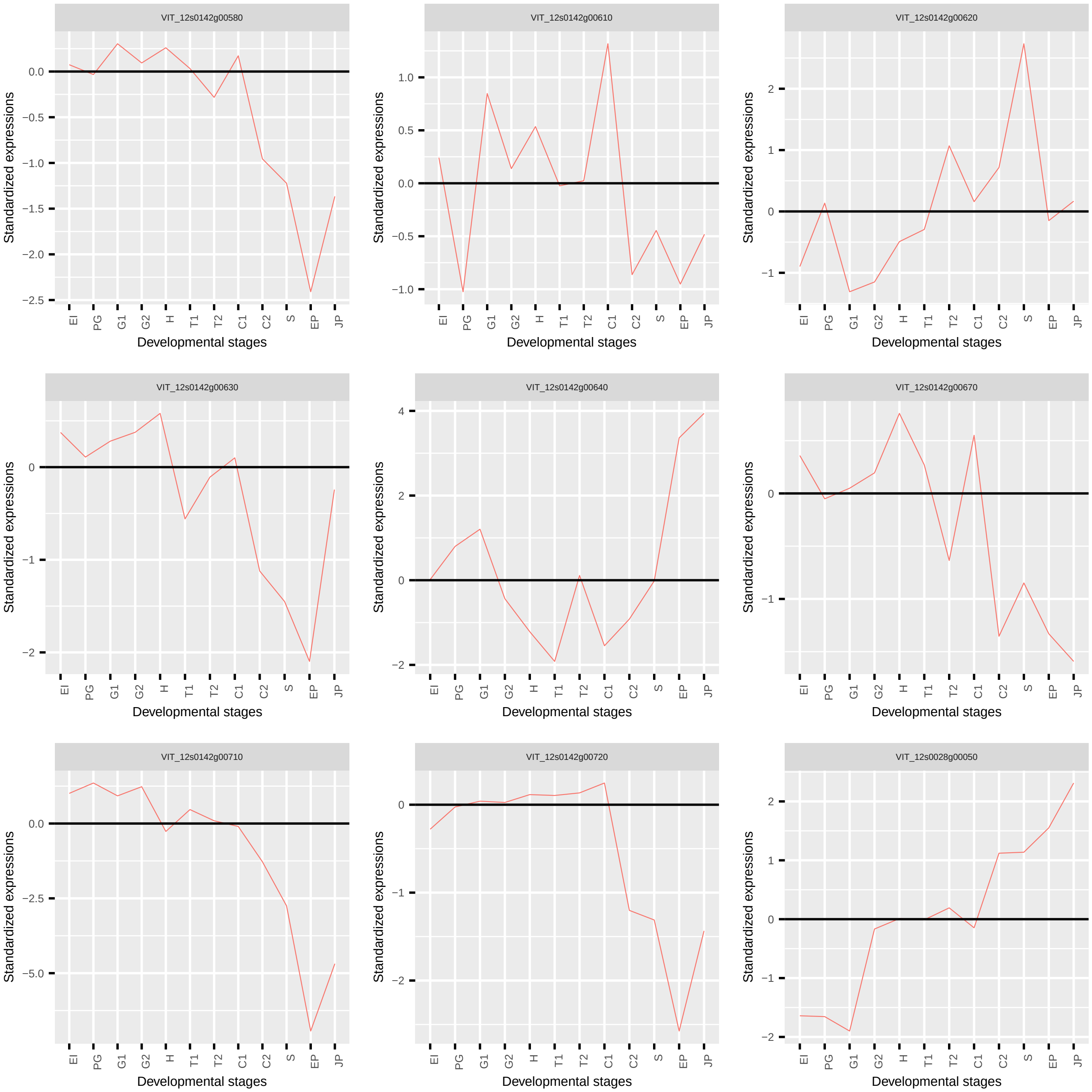
VIT_12s0142g00580
VIT_12s0142g00610
VIT_12s0142g00620
VIT_12s0142g00630
VIT_12s0142g00640
VIT_12s0142g00670
VIT_12s0142g00710
VIT_12s0142g00720
VIT_12s0028g00050
Standardized expressions
Developmental stages
EI
PG
G1
G2
H
T1
T2
C1
C2
S
EP
JP

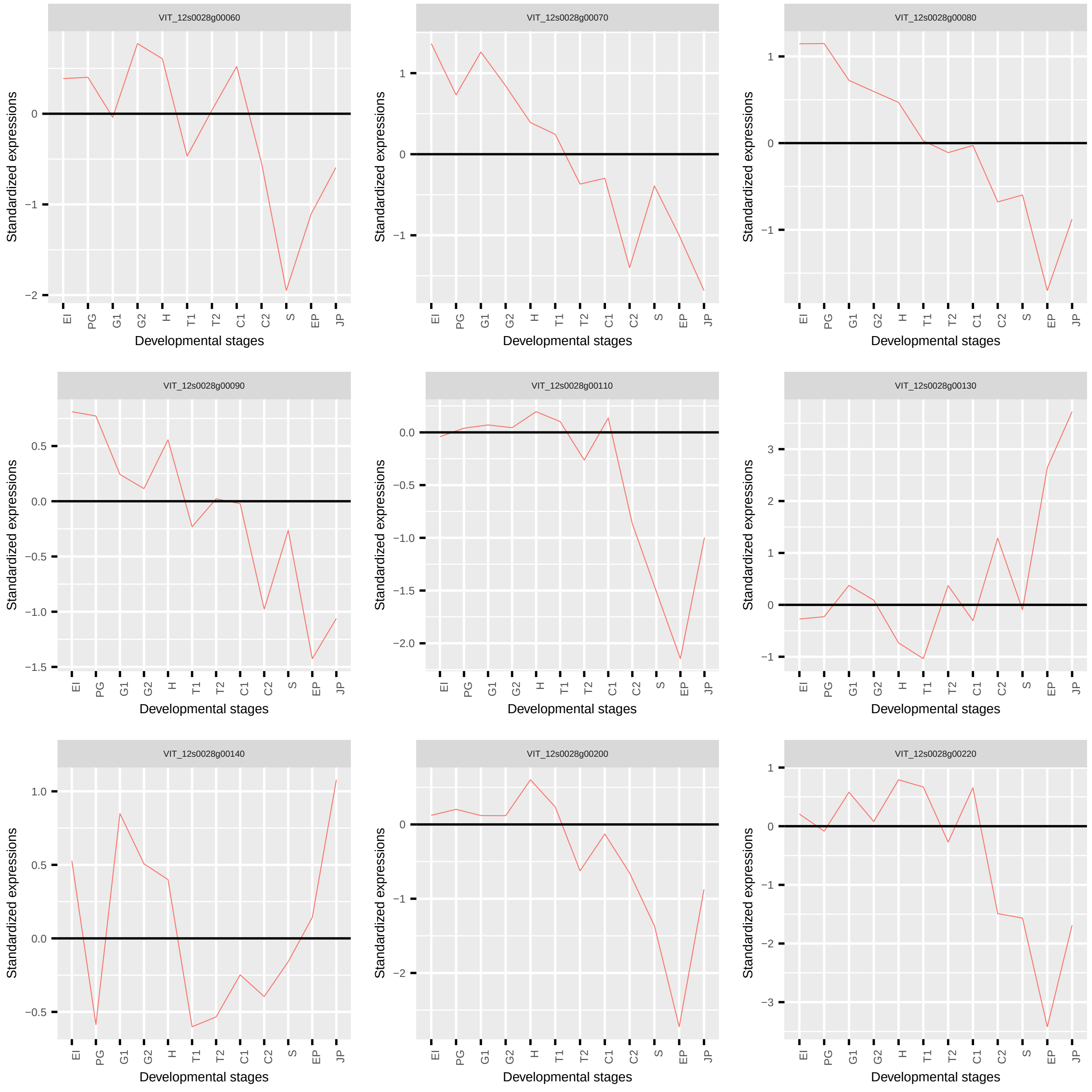
VIT_12s0028g00060
VIT_12s0028g00070
VIT_12s0028g00080
VIT_12s0028g00090
VIT_12s0028g00110
VIT_12s0028g00130
VIT_12s0028g00140
VIT_12s0028g00200
VIT_12s0028g00220
Standardized expressions
Developmental stages
EI
PG
G1
G2
H
T1
T2
C1
C2
S
EP
JP

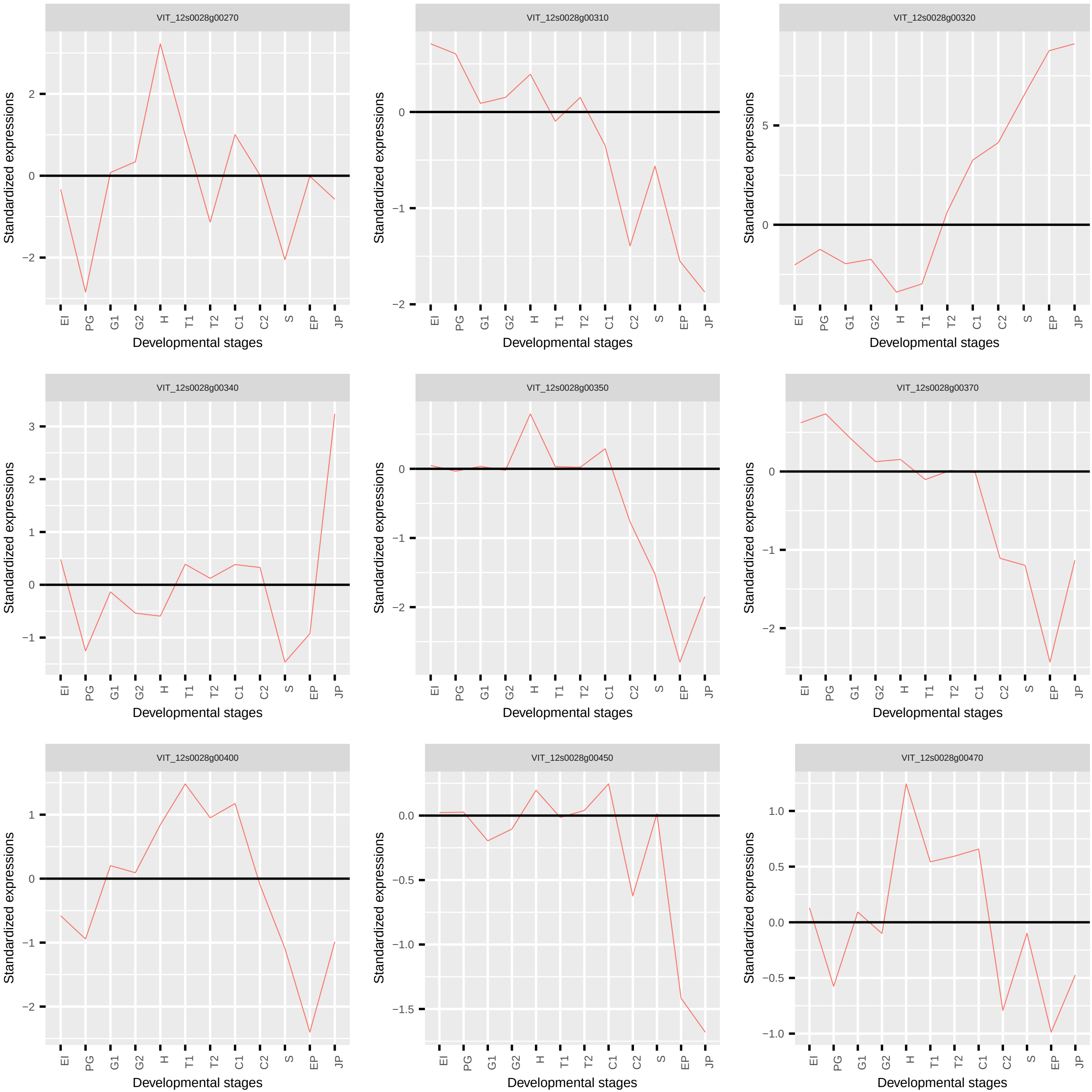
VIT_12s0028g00270
VIT_12s0028g00310
VIT_12s0028g00320
VIT_12s0028g00340
VIT_12s0028g00350
VIT_12s0028g00370
VIT_12s0028g00400
VIT_12s0028g00450
VIT_12s0028g00470
Standardized expressions
Developmental stages
EI PG G1 G2 H T1 T2 C1 C2 S EP JP

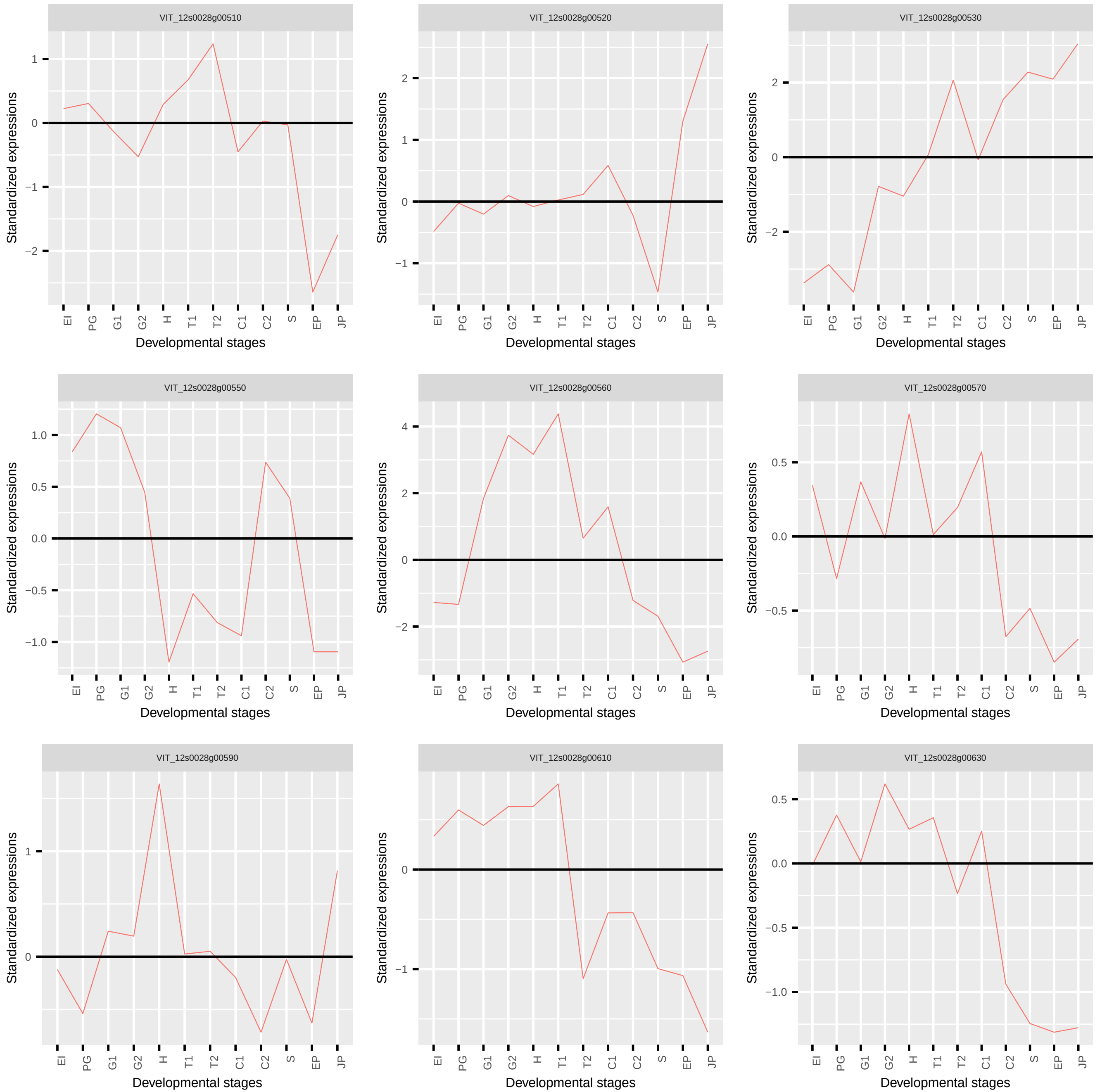
VIT_12s0028g00510
VIT_12s0028g00520
VIT_12s0028g00530
VIT_12s0028g00550
VIT_12s0028g00560
VIT_12s0028g00570
VIT_12s0028g00590
VIT_12s0028g00610
VIT_12s0028g00630
Standardized expressions
Developmental stages
EI PG G1 G2 H T1 T2 C1 C2 S EP JP

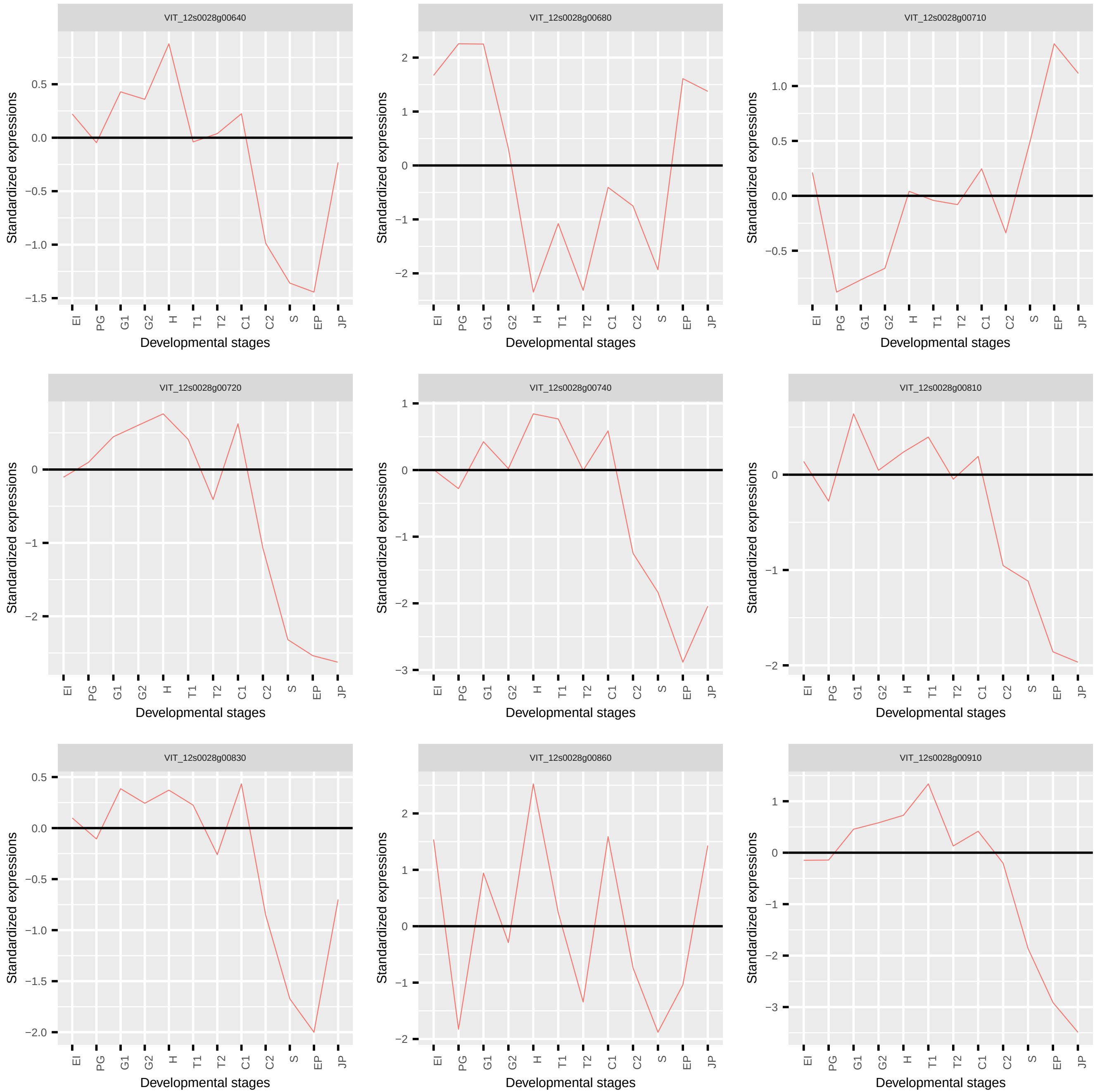
VIT_12s0028g00640
VIT_12s0028g00680
VIT_12s0028g00710
VIT_12s0028g00720
VIT_12s0028g00740
VIT_12s0028g00810
VIT_12s0028g00830
VIT_12s0028g00860
VIT_12s0028g00910
Standardized expressions
Developmental stages
EI PG G1 G2 H T1 T2 C1 C2 S EP JP

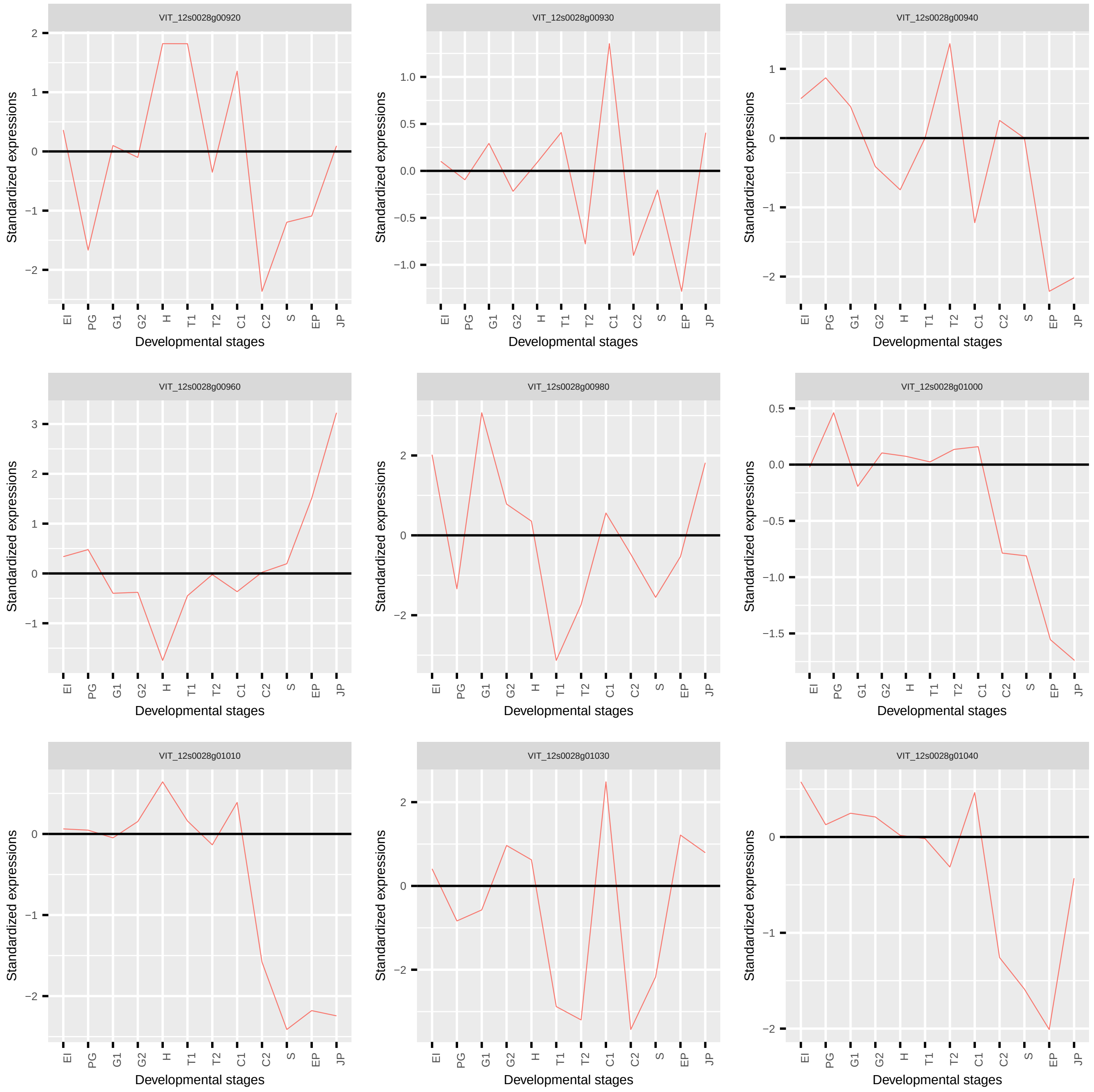

VIT_12s0028g00920
VIT_12s0028g00930
VIT_12s0028g00940
VIT_12s0028g00960
VIT_12s0028g00980
VIT_12s0028g01000
VIT_12s0028g01010
VIT_12s0028g01030
VIT_12s0028g01040
Standardized expressions
Developmental stages
EI PG G1 G2 H T1 T2 C1 C2 S EP JP

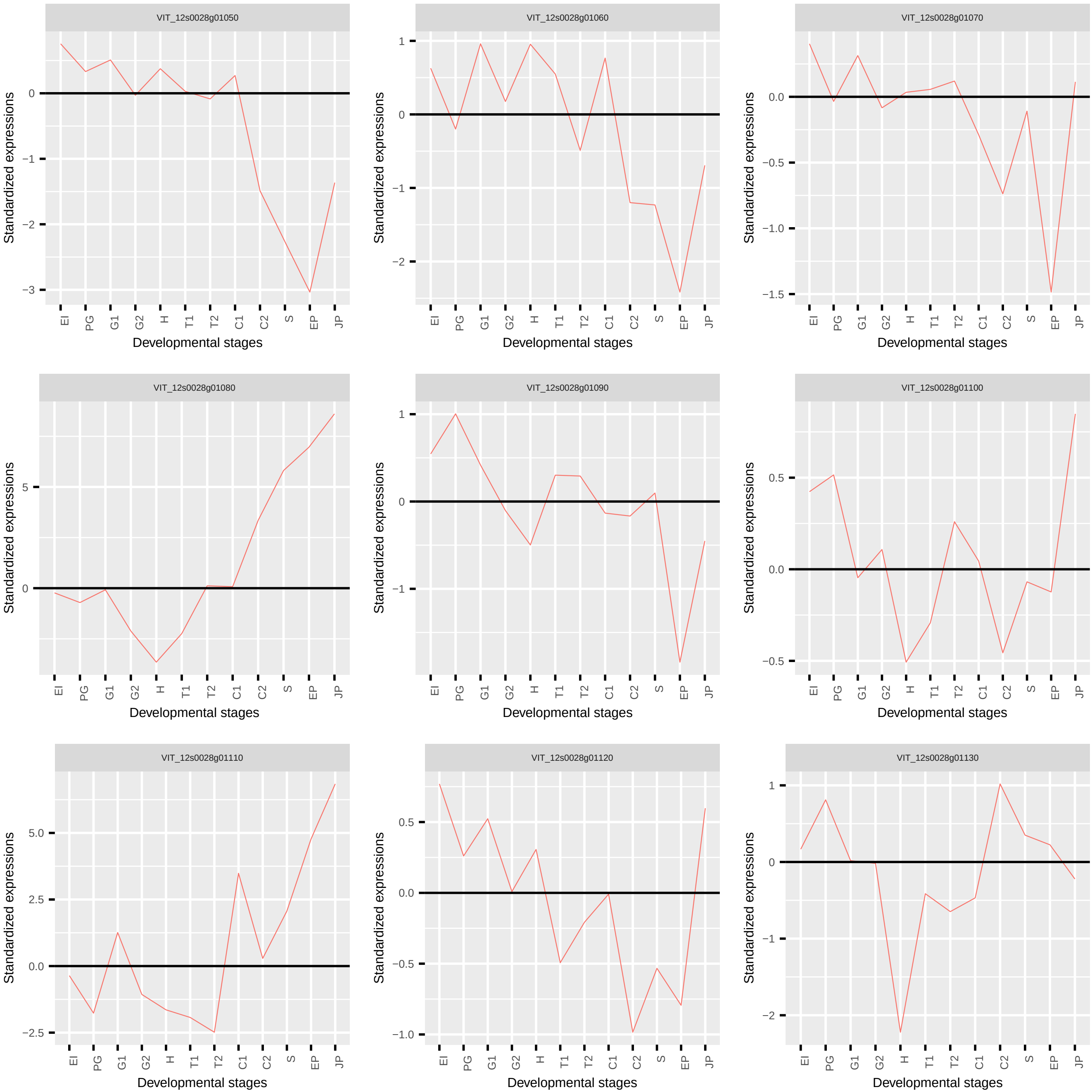
VIT_12s0028g01050
VIT_12s0028g01060
VIT_12s0028g01070
VIT_12s0028g01080
VIT_12s0028g01090
VIT_12s0028g01100
VIT_12s0028g01110
VIT_12s0028g01120
VIT_12s0028g01130
Standardized expressions
Developmental stages
EI PG G1 G2 H T1 T2 C1 C2 S EP JP

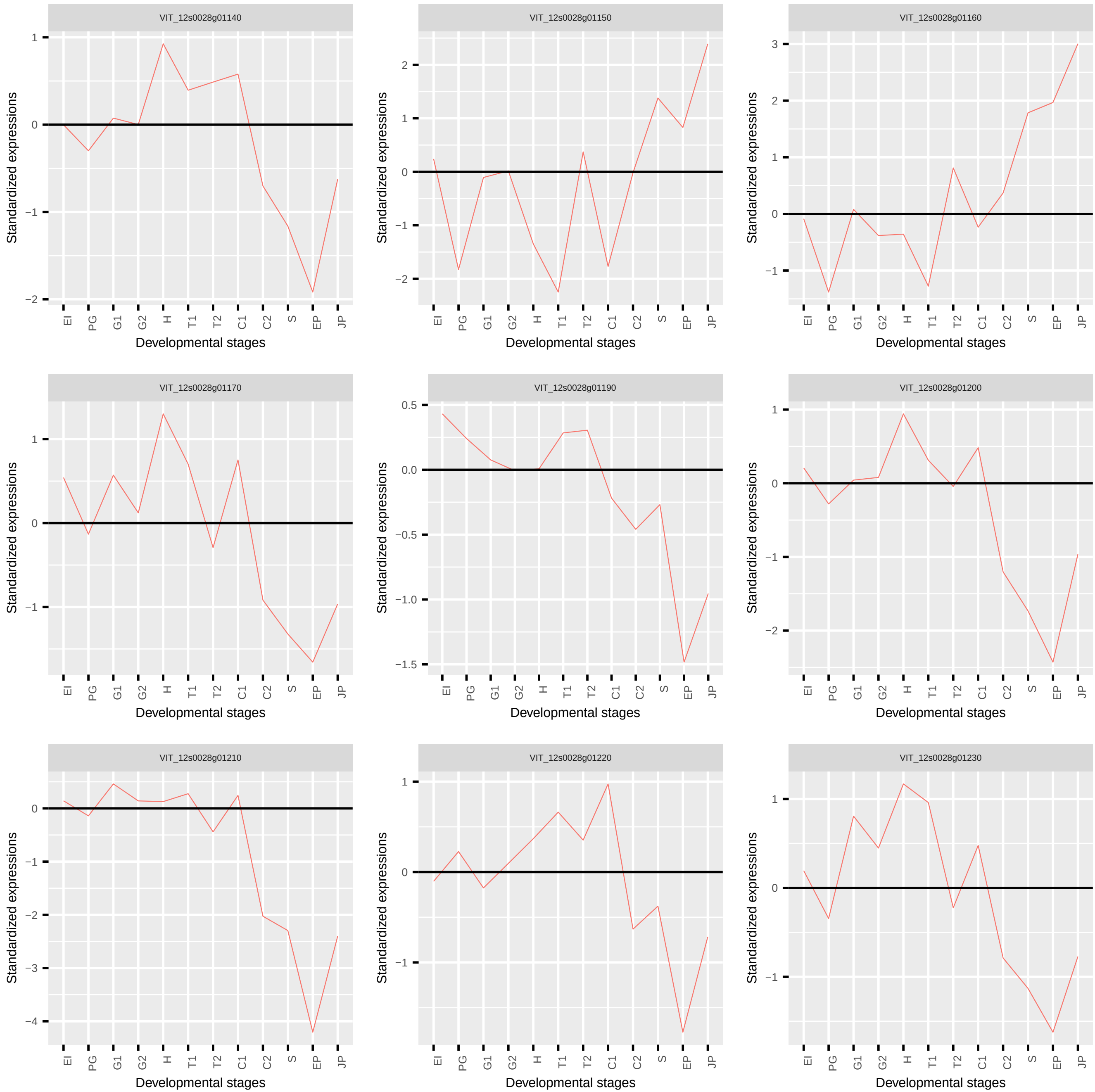

VIT_12s0028g01140
VIT_12s0028g01150
VIT_12s0028g01160
VIT_12s0028g01170
VIT_12s0028g01190
VIT_12s0028g01200
VIT_12s0028g01210
VIT_12s0028g01220
VIT_12s0028g01230
Standardized expressions
Developmental stages
EI PG G1 G2 H T1 T2 C1 C2 S EP JP

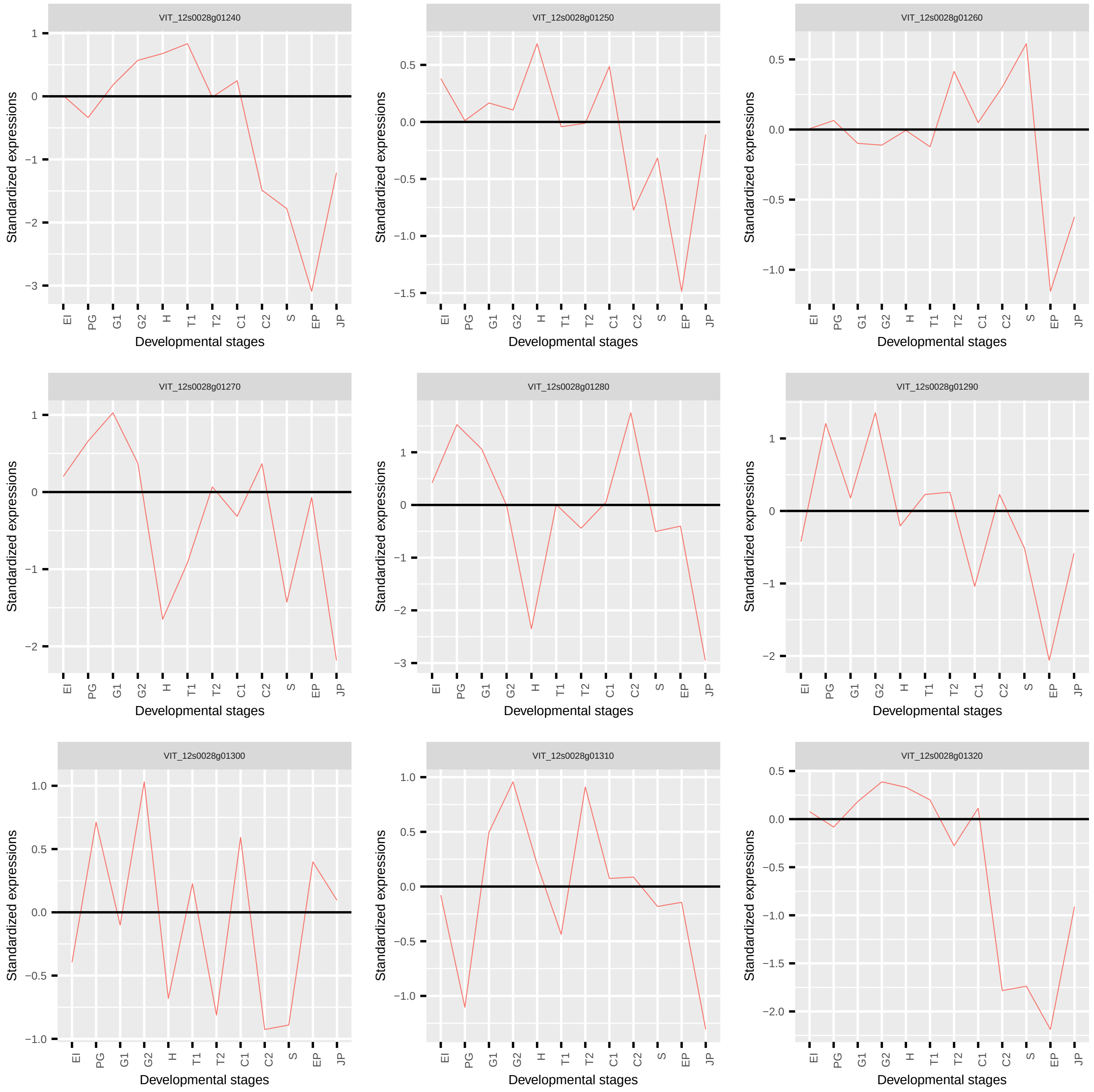

VIT_12s0028g01240
VIT_12s0028g01250
VIT_12s0028g01260
VIT_12s0028g01270
VIT_12s0028g01280
VIT_12s0028g01290
VIT_12s0028g01300
VIT_12s0028g01310
VIT_12s0028g01320
Standardized expressions
Developmental stages
EI PG G1 G2 H T1 T2 C1 C2 S EP JP

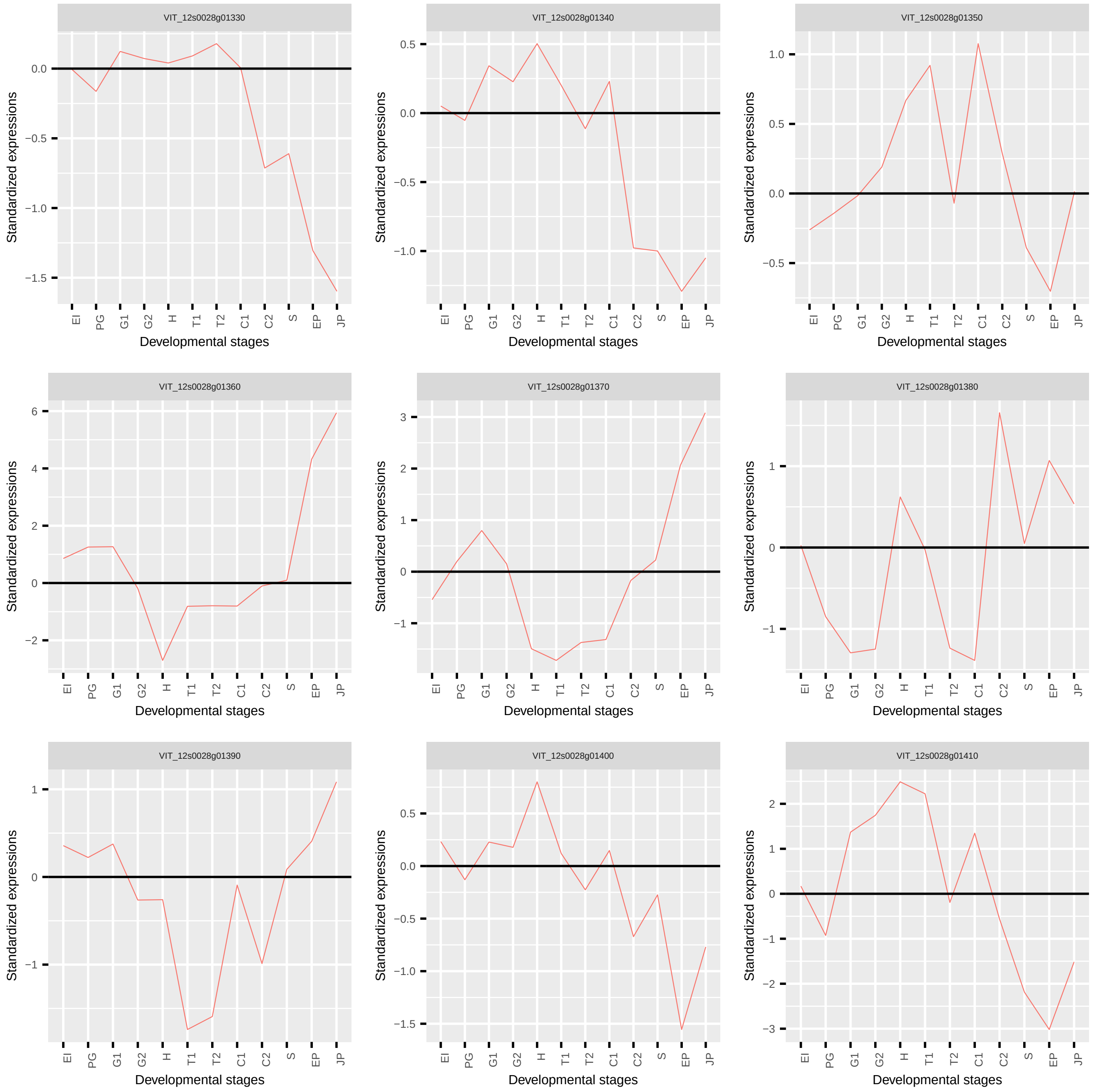
VIT_12s0028g01330
VIT_12s0028g01340
VIT_12s0028g01350
VIT_12s0028g01360
VIT_12s0028g01370
VIT_12s0028g01380
VIT_12s0028g01390
VIT_12s0028g01400
VIT_12s0028g01410
Standardized expressions
Developmental stages
EI
PG
G1
G2
H
T1
T2
C1
C2
S
EP
JP

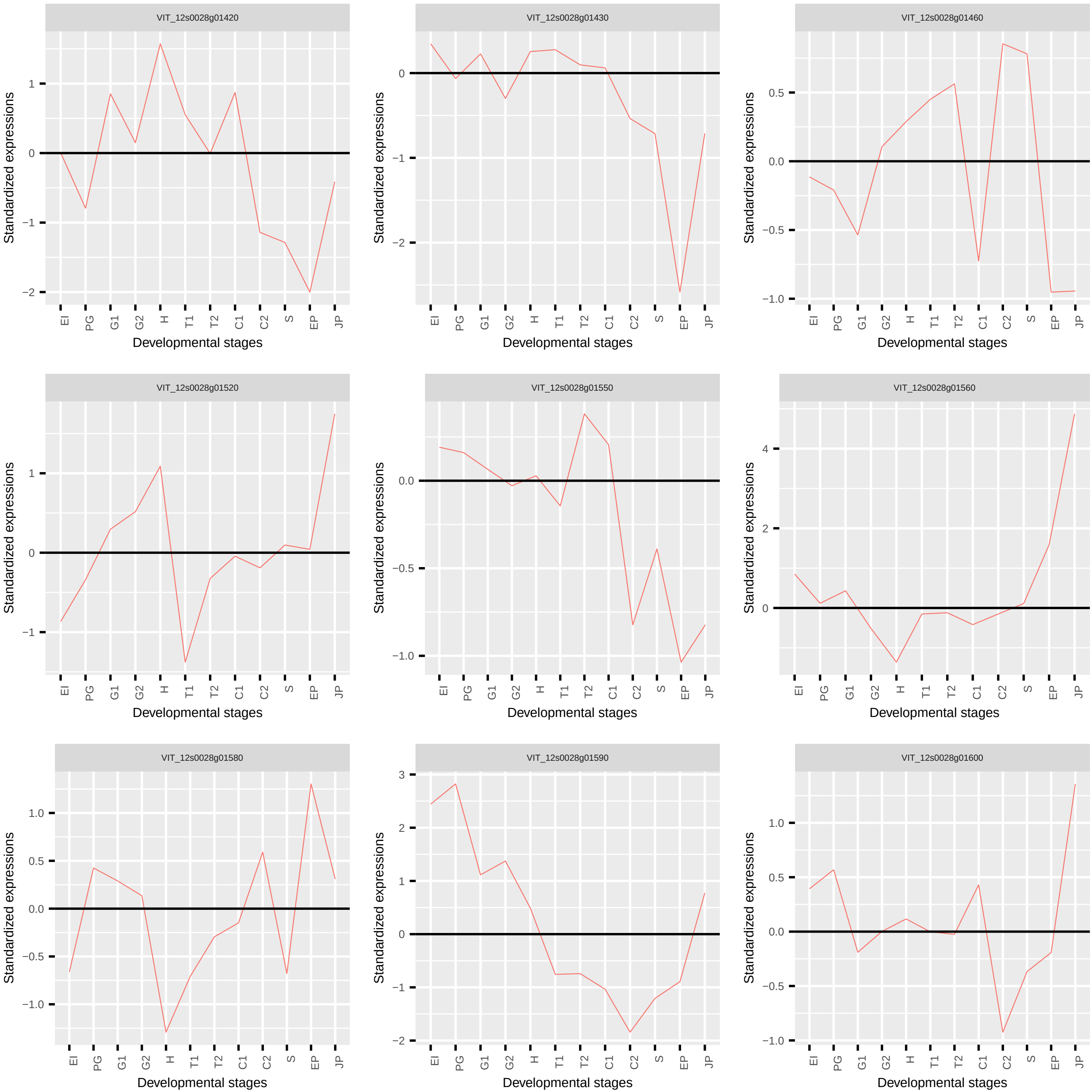

VIT_12s0028g01420
VIT_12s0028g01430
VIT_12s0028g01460
VIT_12s0028g01520
VIT_12s0028g01550
VIT_12s0028g01560
VIT_12s0028g01580
VIT_12s0028g01590
VIT_12s0028g01600
Standardized expressions
Developmental stages
EI PG G1 G2 H T1 T2 C1 C2 S EP JP

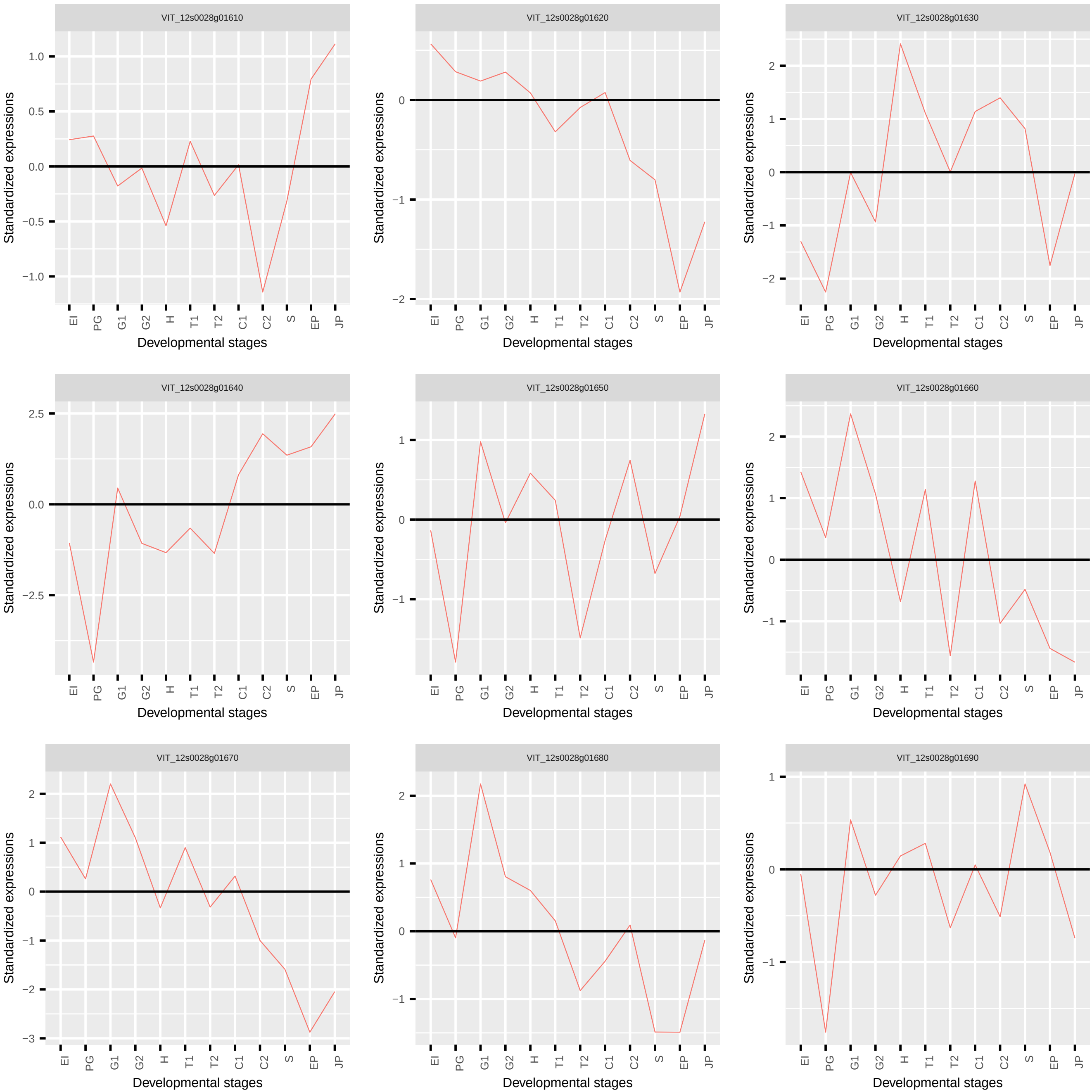

VIT_12s0028g01610
VIT_12s0028g01620
VIT_12s0028g01630
VIT_12s0028g01640
VIT_12s0028g01650
VIT_12s0028g01660
VIT_12s0028g01670
VIT_12s0028g01680
VIT_12s0028g01690
Standardized expressions
Developmental stages
EI PG G1 G2 H T1 T2 C1 C2 S EP JP

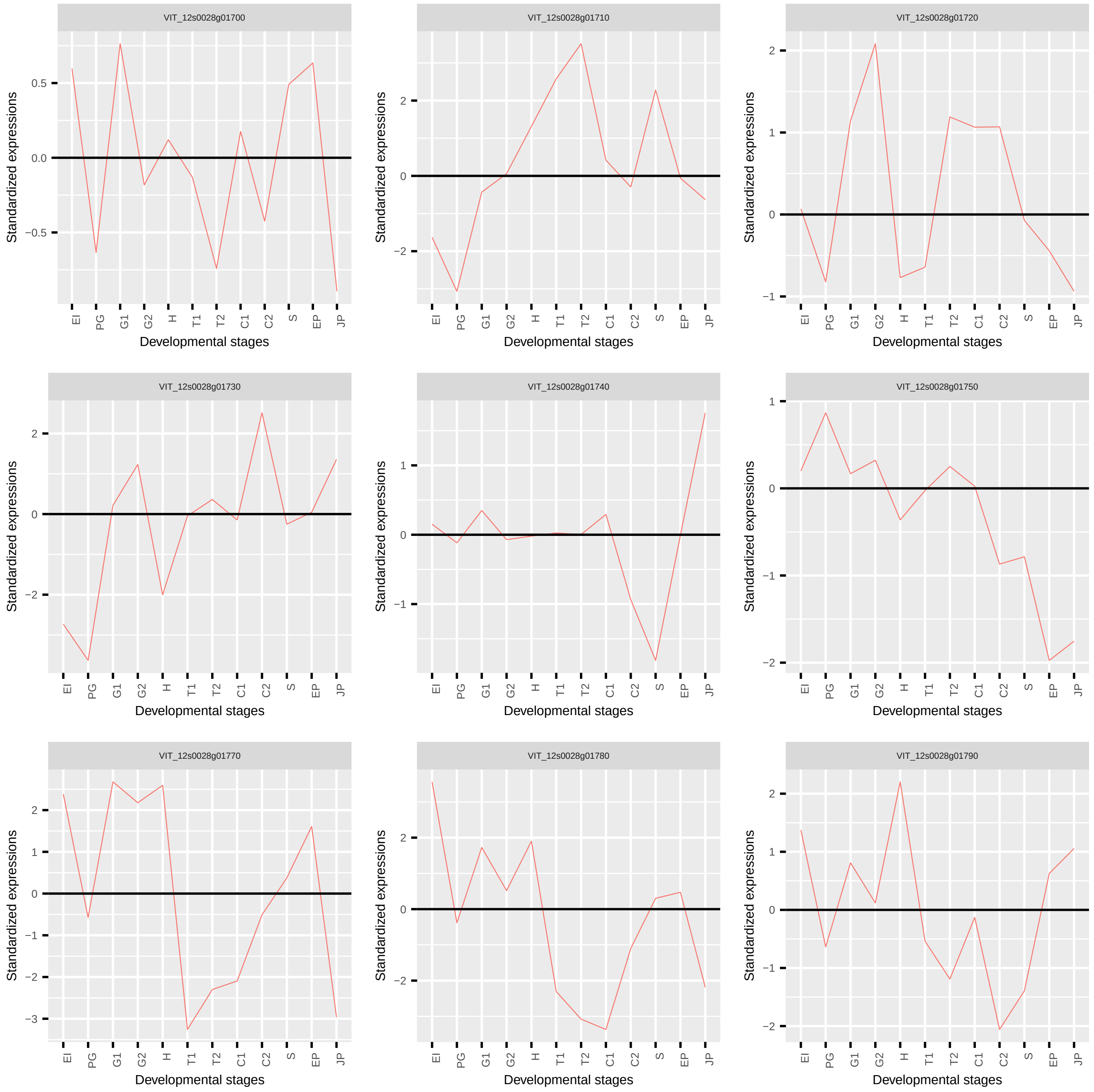
VIT_12s0028g01700
VIT_12s0028g01710
VIT_12s0028g01720
VIT_12s0028g01730
VIT_12s0028g01740
VIT_12s0028g01750
VIT_12s0028g01770
VIT_12s0028g01780
VIT_12s0028g01790
Standardized expressions
Developmental stages
EI PG G1 G2 H T1 T2 C1 C2 S EP JP

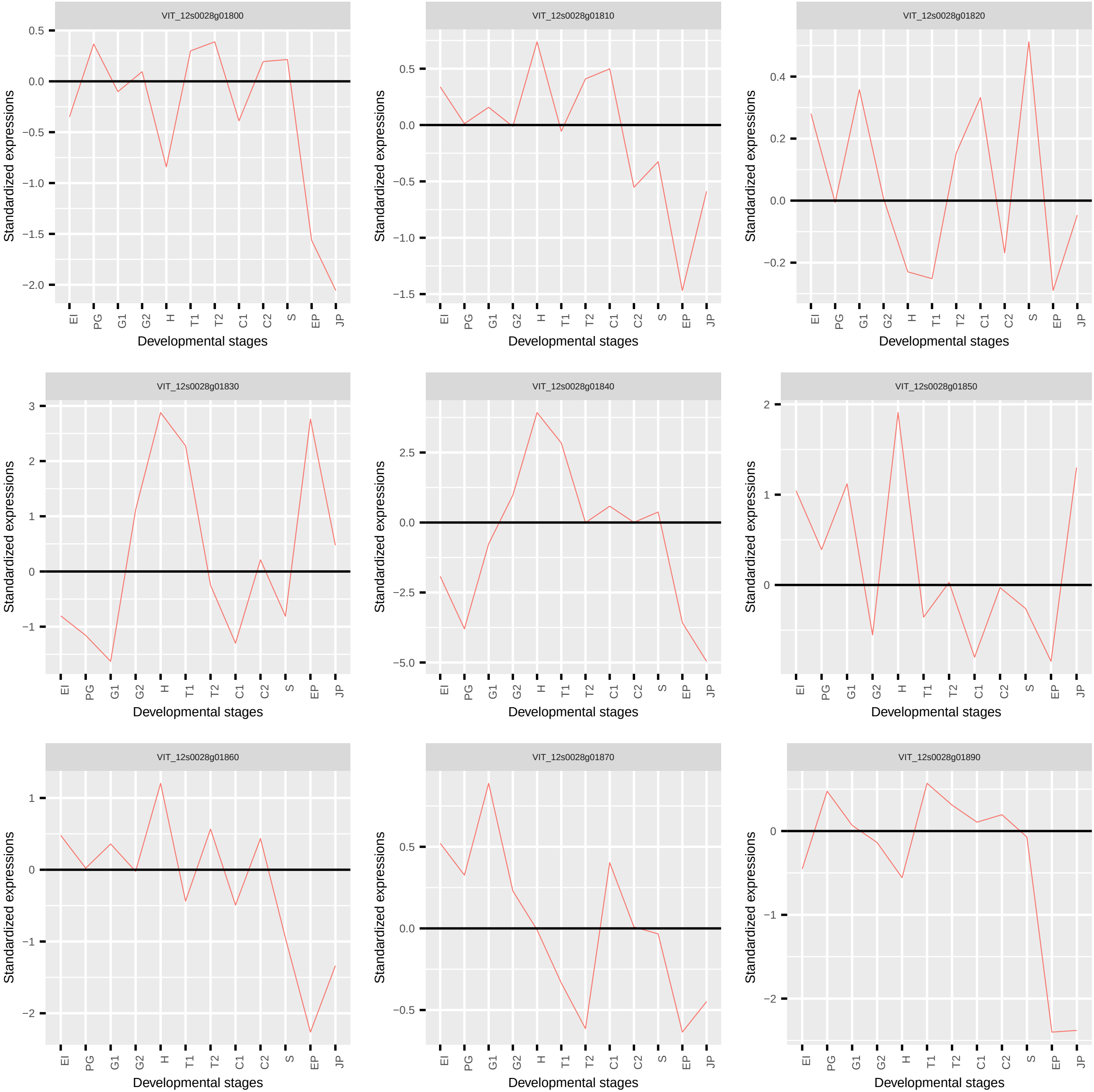

VIT_12s0028g01800
VIT_12s0028g01810
VIT_12s0028g01820
VIT_12s0028g01830
VIT_12s0028g01840
VIT_12s0028g01850
VIT_12s0028g01860
VIT_12s0028g01870
VIT_12s0028g01890
Standardized expressions
Developmental stages
EI
PG
G1
G2
H
T1
T2
C1
C2
S
EP
JP

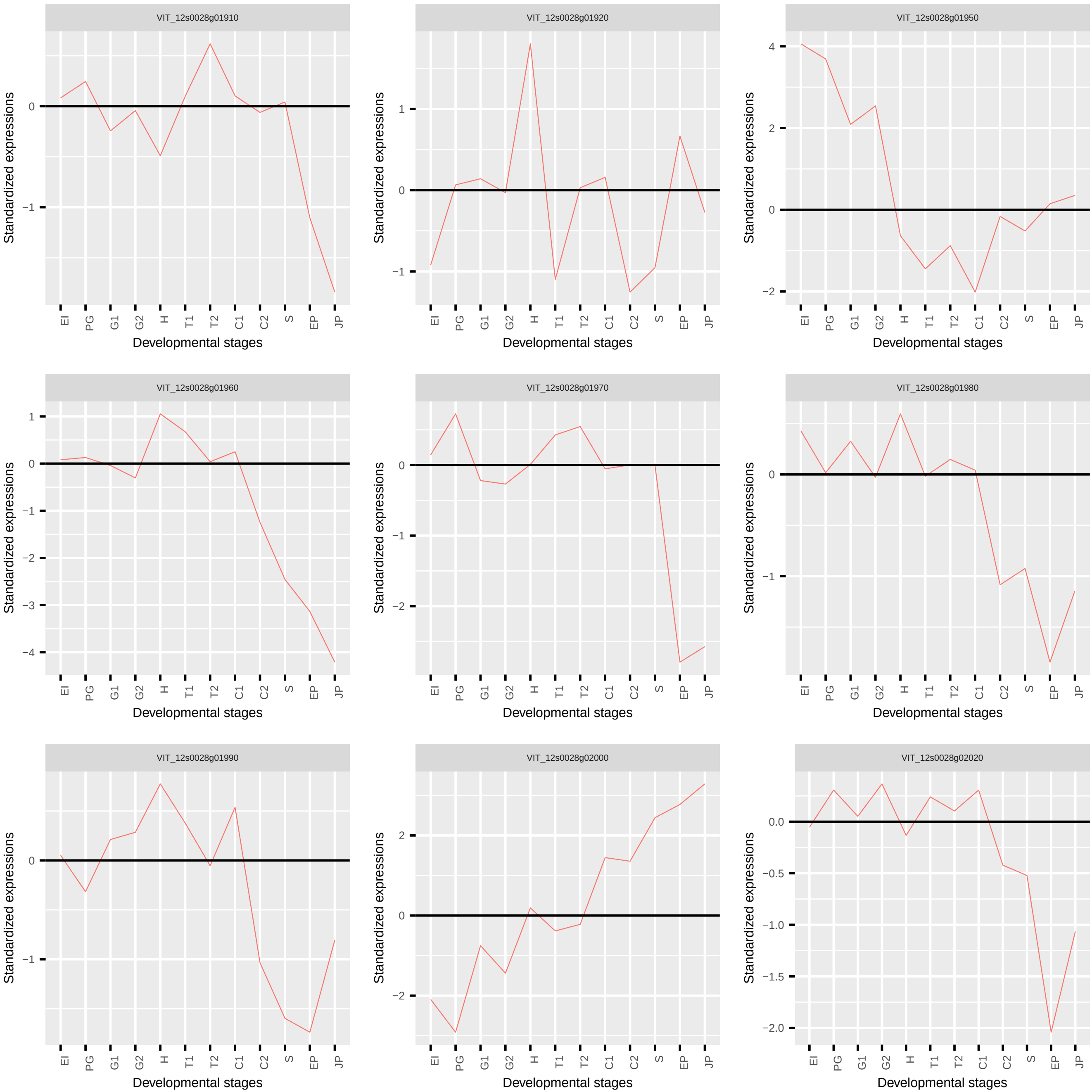
VIT_12s0028g01910
VIT_12s0028g01920
VIT_12s0028g01950
VIT_12s0028g01960
VIT_12s0028g01970
VIT_12s0028g01980
VIT_12s0028g01990
VIT_12s0028g02000
VIT_12s0028g02020
Standardized expressions
Developmental stages
EI
PG
G1
G2
H
T1
T2
C1
C2
S
EP
JP

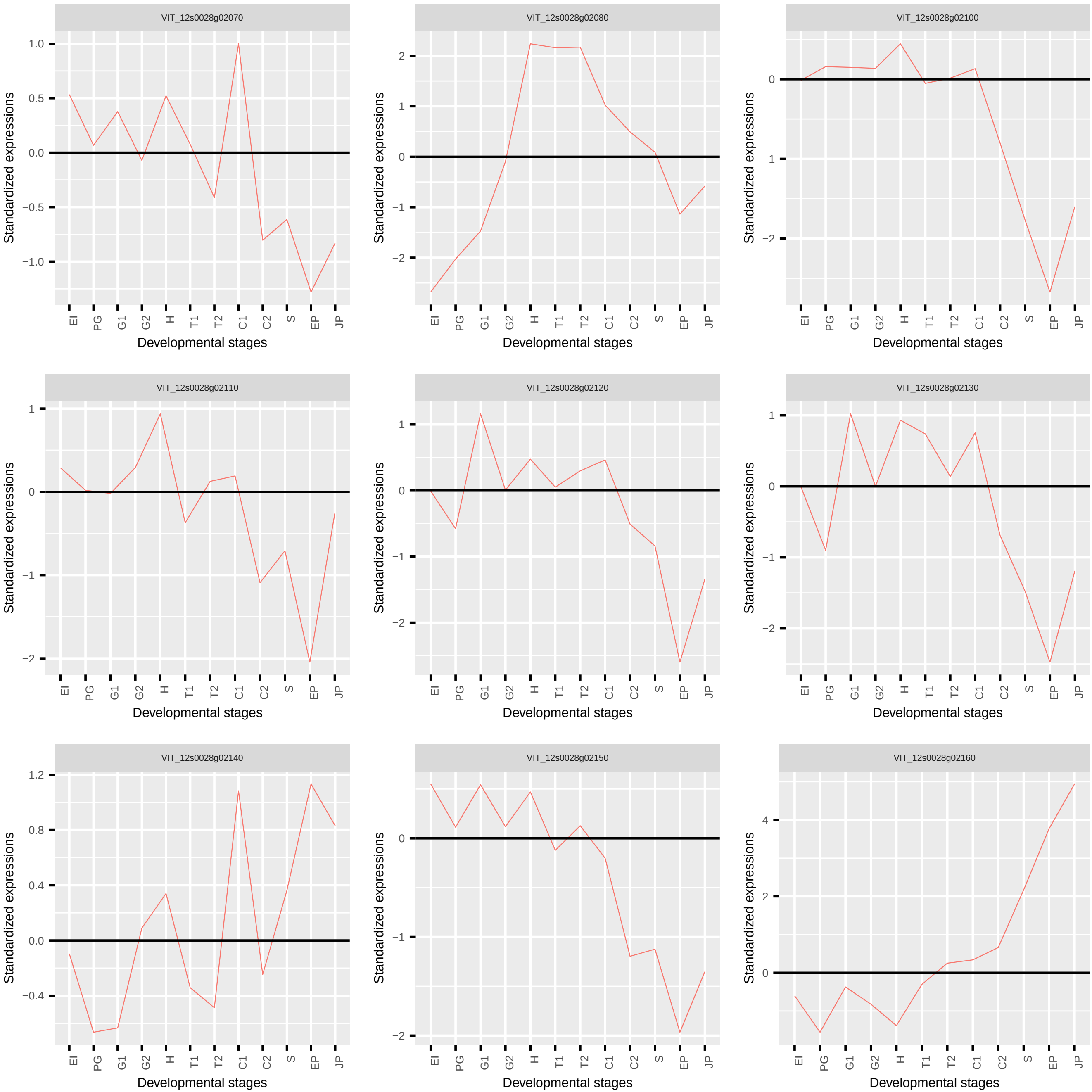
VIT_12s0028g02070
1.0
0.5
0.0
−0.5
−1.0
Standardized expressions
EI PG G1 G2 H T1 T2 C1 C2 S EP JP
Developmental stages
VIT_12s0028g02080
2
1
0
−1
−2
Standardized expressions
EI PG G1 G2 H T1 T2 C1 C2 S EP JP
Developmental stages
VIT_12s0028g02100
0
−1
−2
Standardized expressions
EI PG G1 G2 H T1 T2 C1 C2 S EP JP
Developmental stages
VIT_12s0028g02110
1
0
−1
−2
Standardized expressions
EI PG G1 G2 H T1 T2 C1 C2 S EP JP
Developmental stages
VIT_12s0028g02120
1
0
−1
−2
Standardized expressions
EI PG G1 G2 H T1 T2 C1 C2 S EP JP
Developmental stages
VIT_12s0028g02130
1
0
−1
−2
Standardized expressions
EI PG G1 G2 H T1 T2 C1 C2 S EP JP
Developmental stages
VIT_12s0028g02140
1.2
0.8
0.4
0.0
-0.4
Standardized expressions
EI PG G1 G2 H T1 T2 C1 C2 S EP JP
Developmental stages
VIT_12s0028g02150
0
-1
-2
Standardized expressions
EI PG G1 G2 H T1 T2 C1 C2 S EP JP
Developmental stages
VIT_12s0028g02160
4
2
0
Standardized expressions
EI PG G1 G2 H T1 T2 C1 C2 S EP JP
Developmental stages

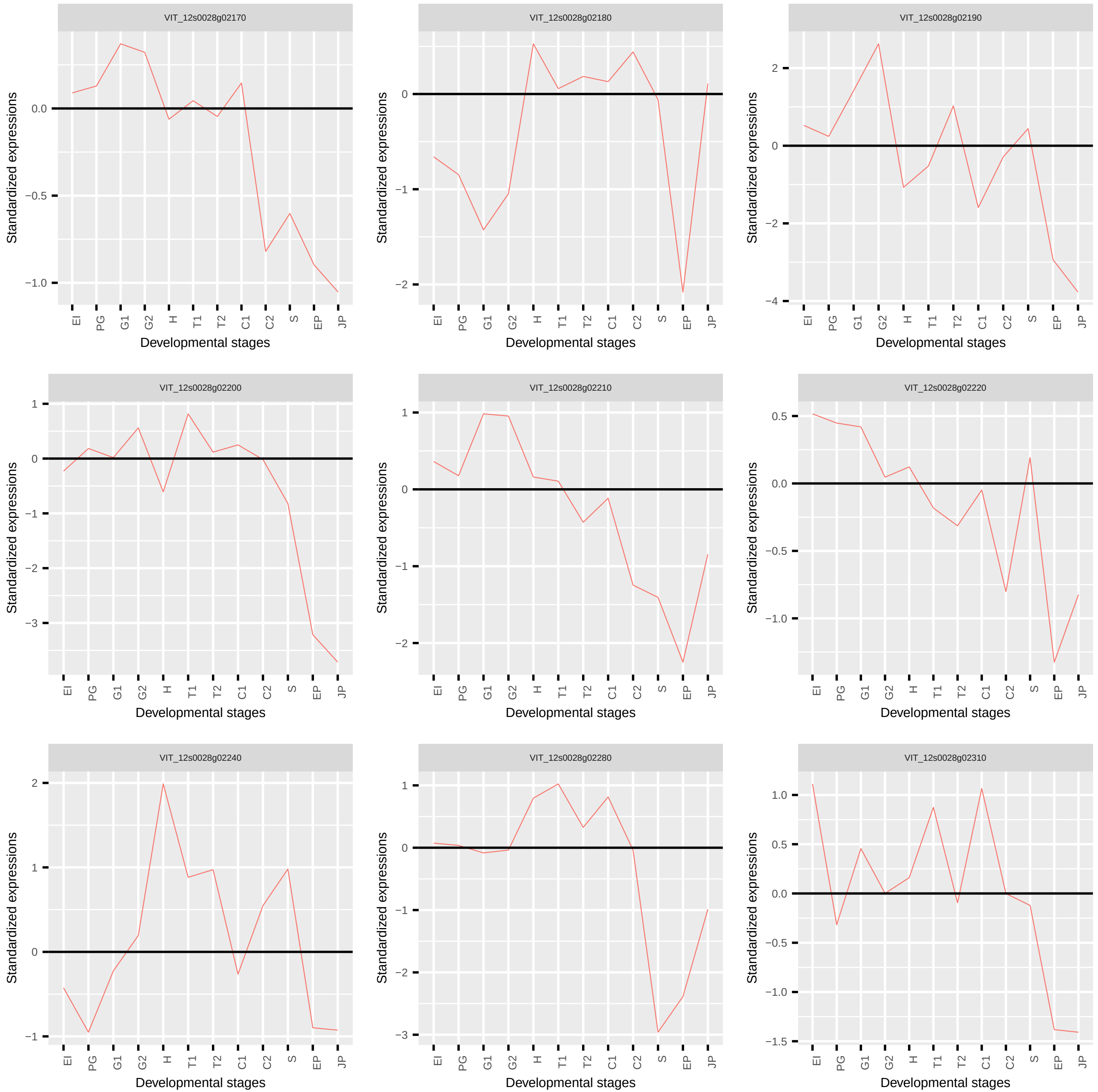
VIT_12s0028g02170
VIT_12s0028g02180
VIT_12s0028g02190
VIT_12s0028g02200
VIT_12s0028g02210
VIT_12s0028g02220
VIT_12s0028g02240
VIT_12s0028g02280
VIT_12s0028g02310
Standardized expressions
Developmental stages
EI PG G1 G2 H T1 T2 C1 C2 S EP JP

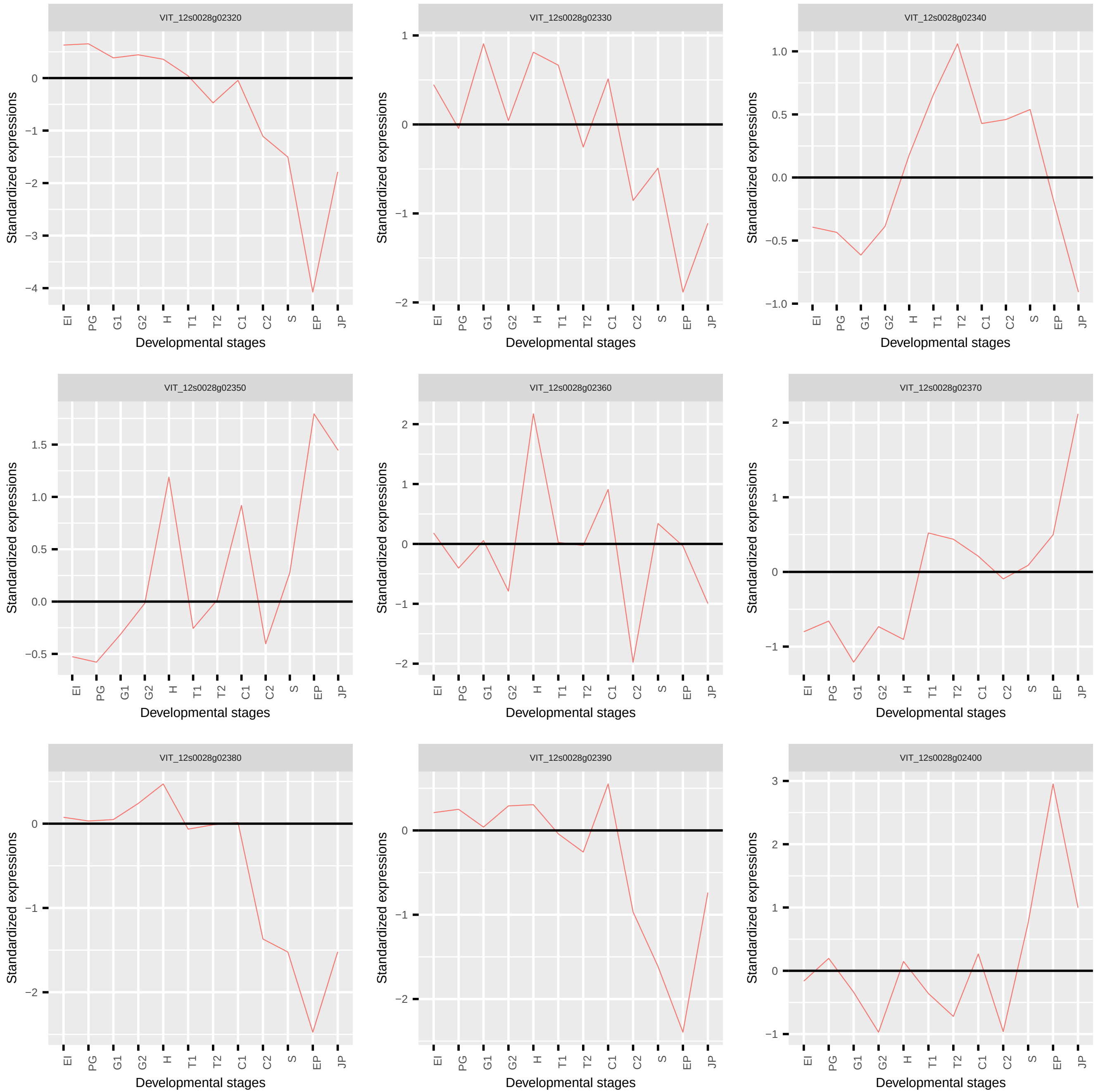
VIT_12s0028g02320
VIT_12s0028g02330
VIT_12s0028g02340
VIT_12s0028g02350
VIT_12s0028g02360
VIT_12s0028g02370
VIT_12s0028g02380
VIT_12s0028g02390
VIT_12s0028g02400
Standardized expressions
Developmental stages
EI PG G1 G2 H T1 T2 C1 C2 S EP JP

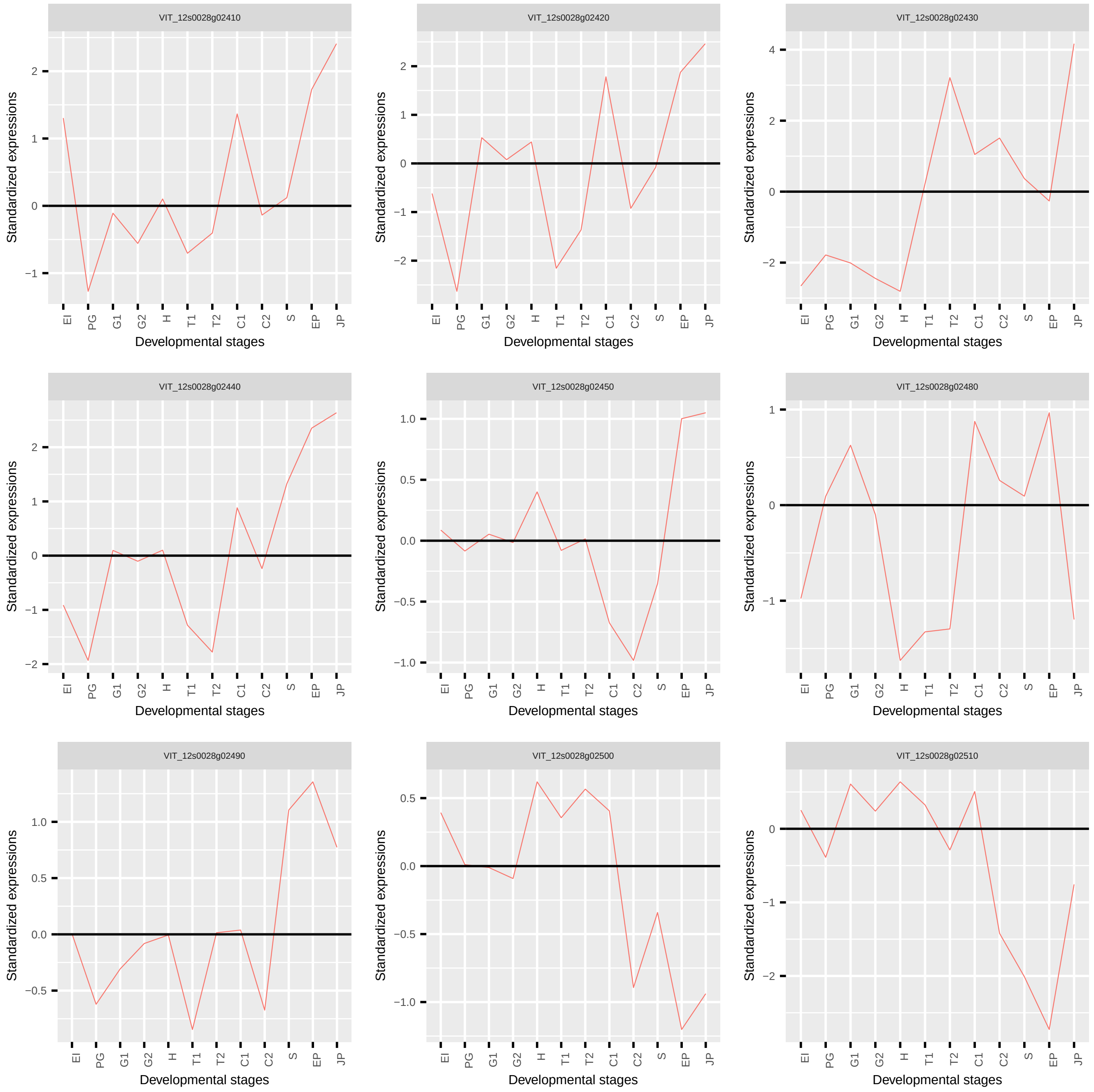
VIT_12s0028g02410
VIT_12s0028g02420
VIT_12s0028g02430
VIT_12s0028g02440
VIT_12s0028g02450
VIT_12s0028g02480
VIT_12s0028g02490
VIT_12s0028g02500
VIT_12s0028g02510
Standardized expressions
Developmental stages
EI PG G1 G2 H T1 T2 C1 C2 S EP JP

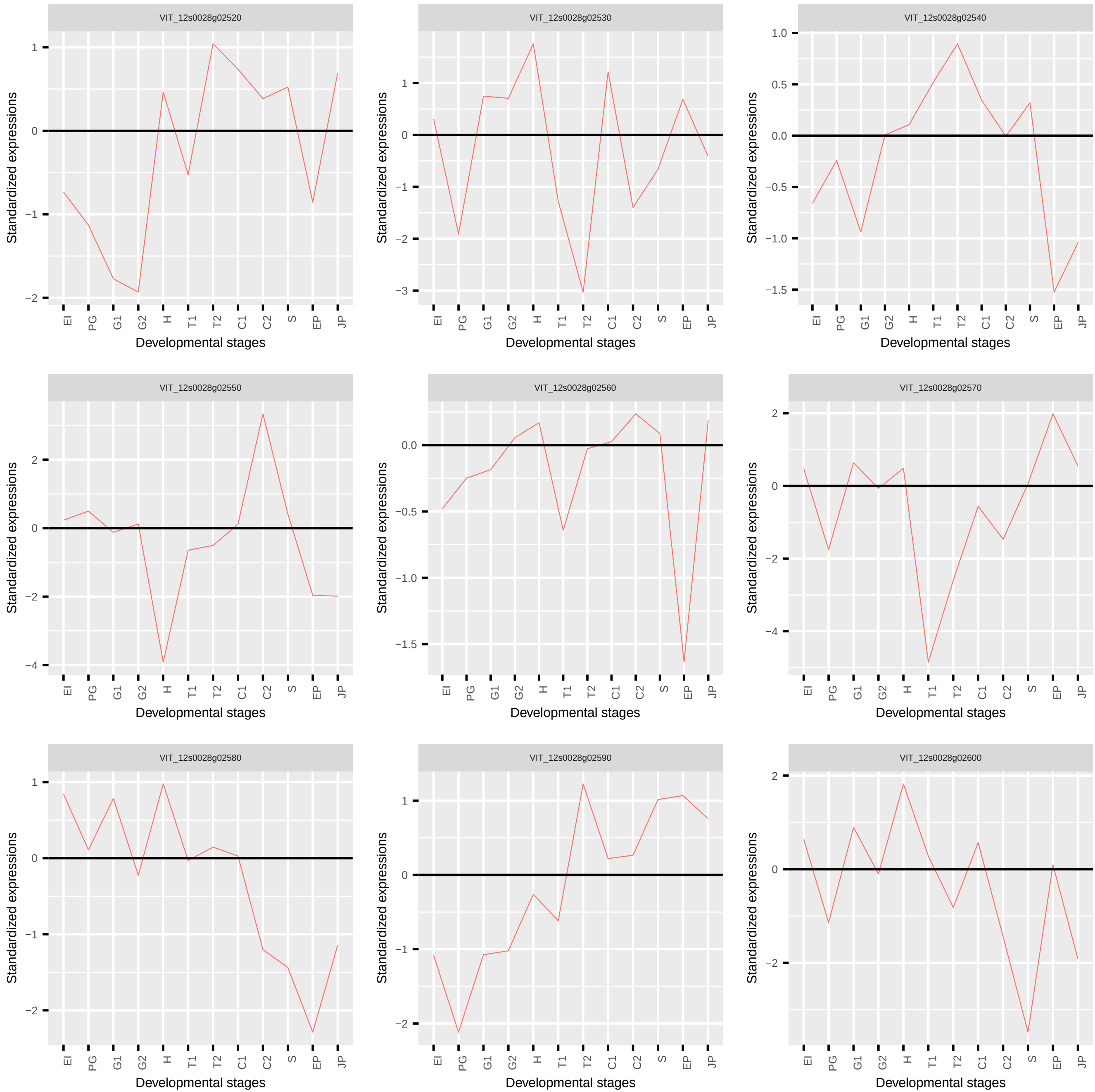

VIT_12s0028g02520
VIT_12s0028g02530
VIT_12s0028g02540
VIT_12s0028g02550
VIT_12s0028g02560
VIT_12s0028g02570
VIT_12s0028g02580
VIT_12s0028g02590
VIT_12s0028g02600
Standardized expressions
Developmental stages
EI PG G1 G2 H T1 T2 C1 C2 S EP JP

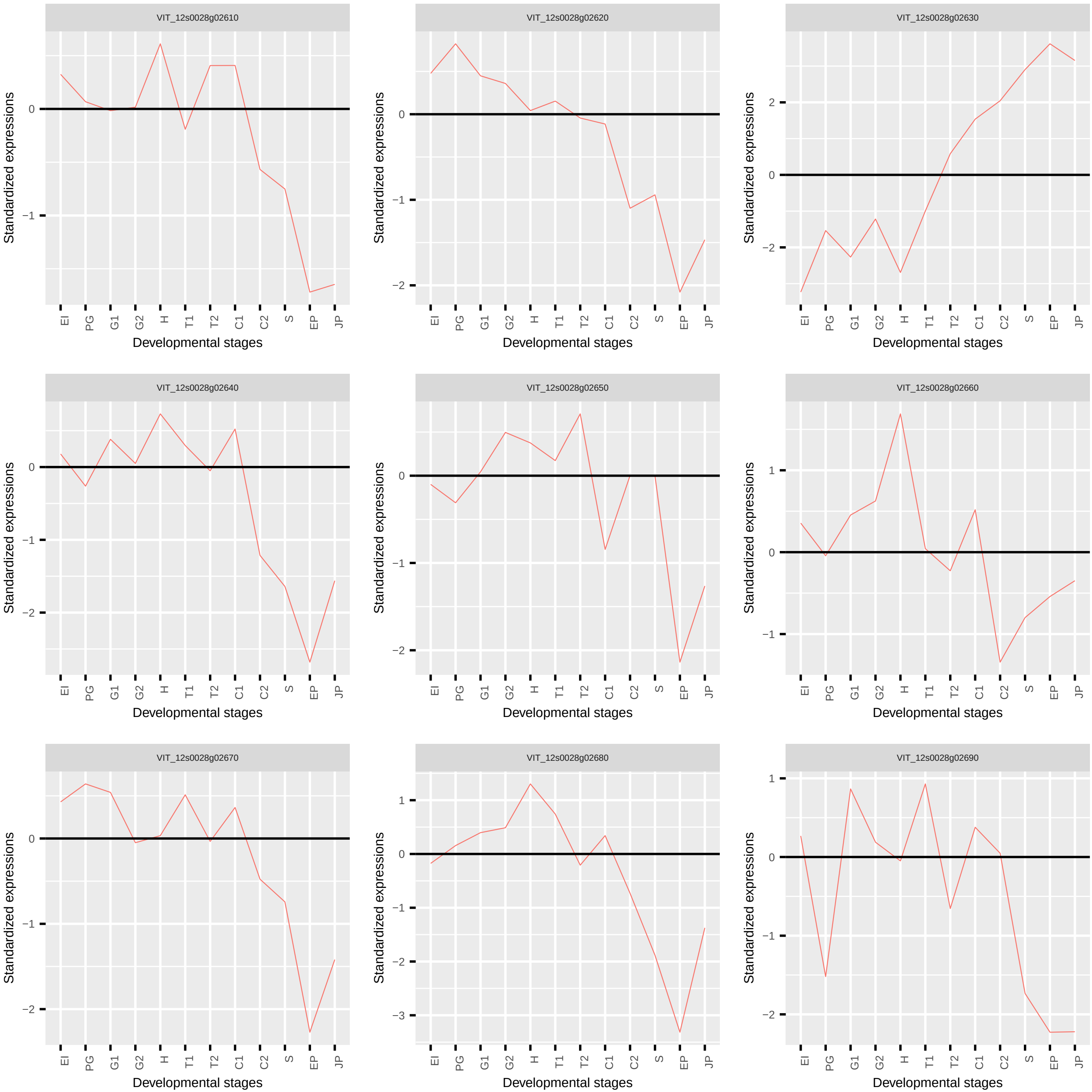
VIT_12s0028g02610
VIT_12s0028g02620
VIT_12s0028g02630
VIT_12s0028g02640
VIT_12s0028g02650
VIT_12s0028g02660
VIT_12s0028g02670
VIT_12s0028g02680
VIT_12s0028g02690
Standardized expressions
Developmental stages
EI
PG
G1
G2
H
T1
T2
C1
C2
S
EP
JP

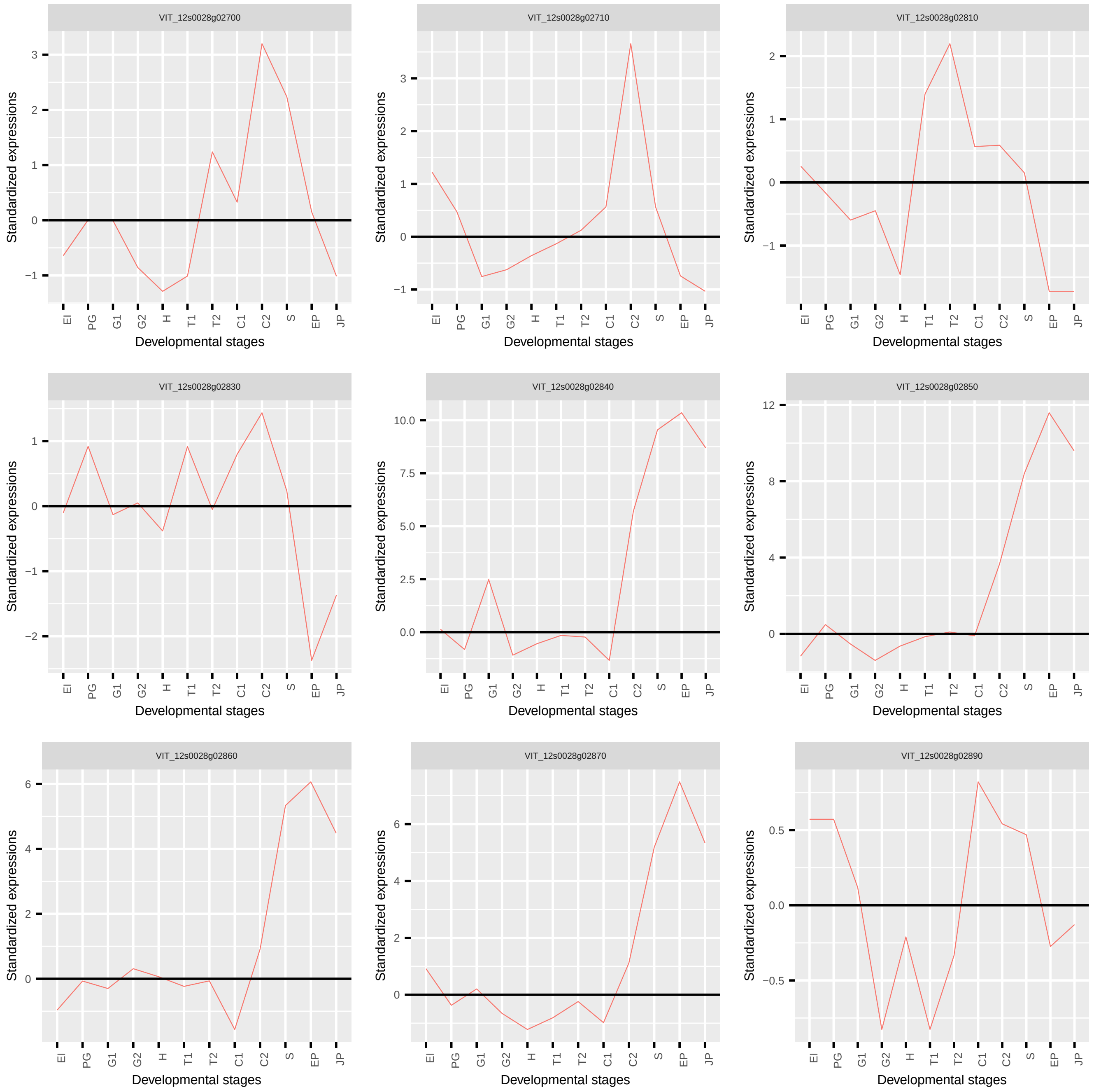
VIT_12s0028g02700
VIT_12s0028g02710
VIT_12s0028g02810
VIT_12s0028g02830
VIT_12s0028g02840
VIT_12s0028g02850
VIT_12s0028g02860
VIT_12s0028g02870
VIT_12s0028g02890
Standardized expressions
Developmental stages
EI PG G1 G2 H T1 T2 C1 C2 S EP JP

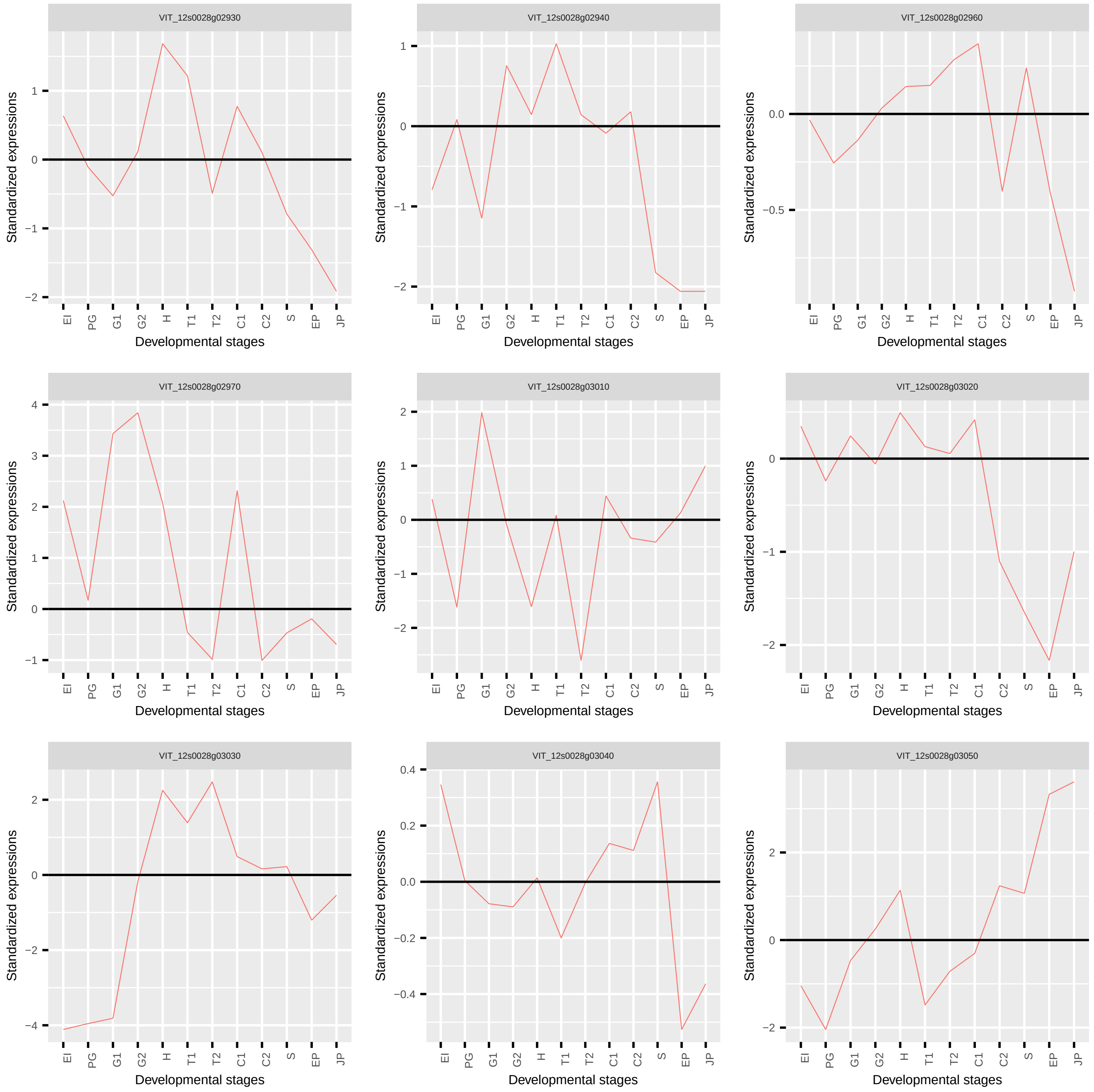

VIT_12s0028g02930
VIT_12s0028g02940
VIT_12s0028g02960
VIT_12s0028g02970
VIT_12s0028g03010
VIT_12s0028g03020
VIT_12s0028g03030
VIT_12s0028g03040
VIT_12s0028g03050
Standardized expressions
Developmental stages
EI
PG
G1
G2
H
T1
T2
C1
C2
S
EP
JP

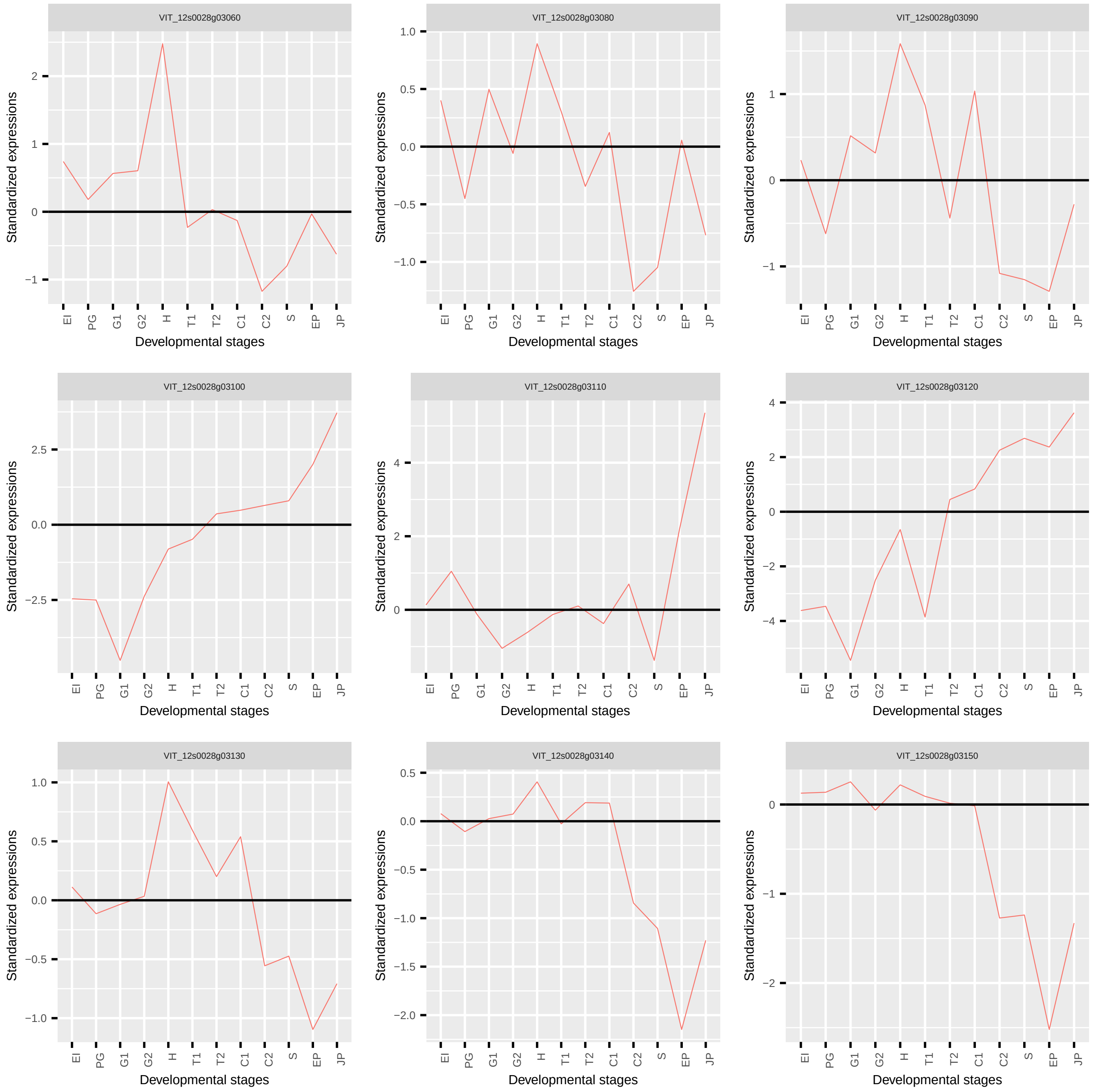
VIT_12s0028g03060
VIT_12s0028g03080
VIT_12s0028g03090
VIT_12s0028g03100
VIT_12s0028g03110
VIT_12s0028g03120
VIT_12s0028g03130
VIT_12s0028g03140
VIT_12s0028g03150
Standardized expressions
Developmental stages
EI
PG
G1
G2
H
T1
T2
C1
C2
S
EP
JP

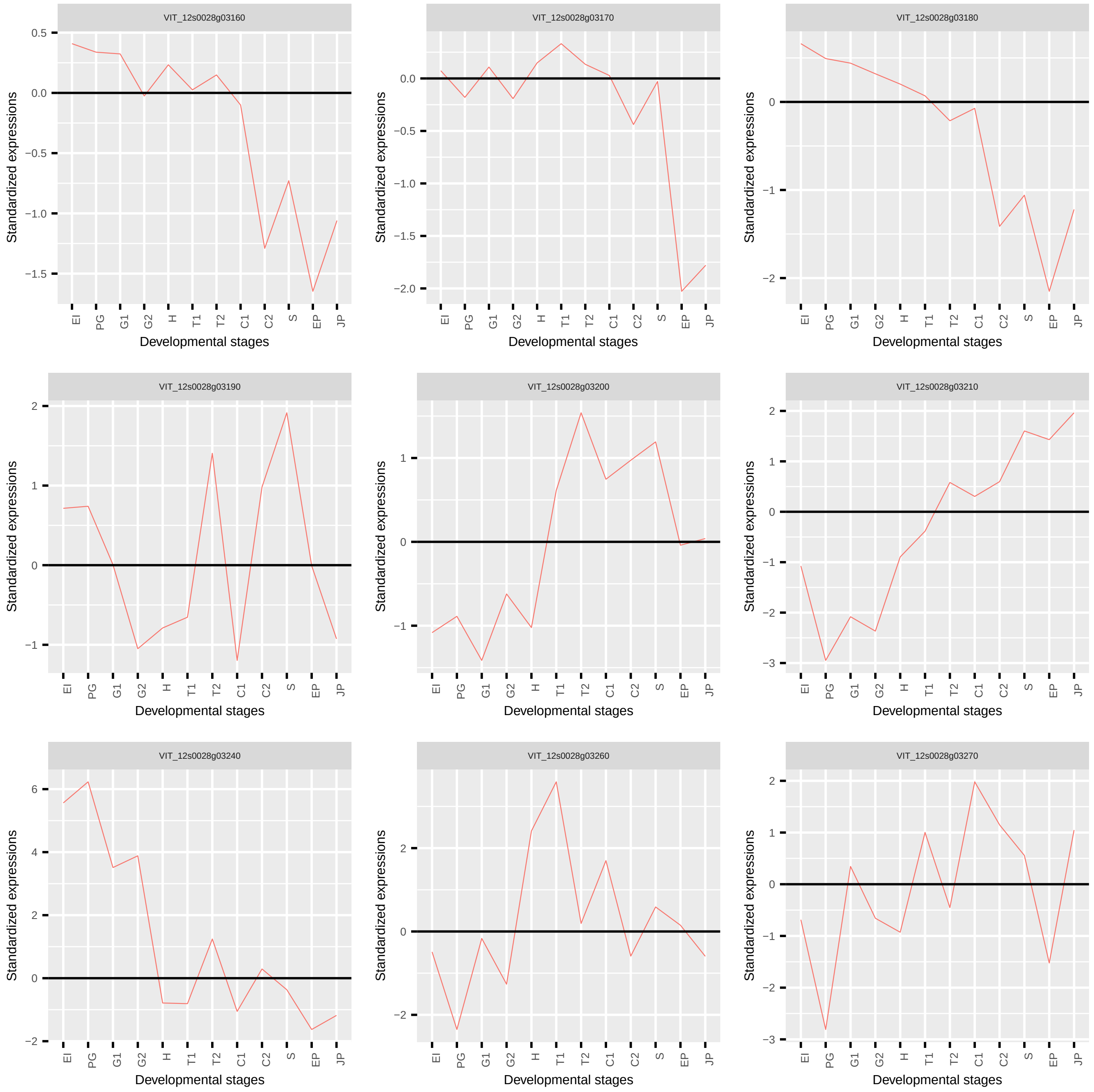

VIT_12s0028g03160
VIT_12s0028g03170
VIT_12s0028g03180
VIT_12s0028g03190
VIT_12s0028g03200
VIT_12s0028g03210
VIT_12s0028g03240
VIT_12s0028g03260
VIT_12s0028g03270
Standardized expressions
Developmental stages
EI
PG
G1
G2
H
T1
T2
C1
C2
S
EP
JP

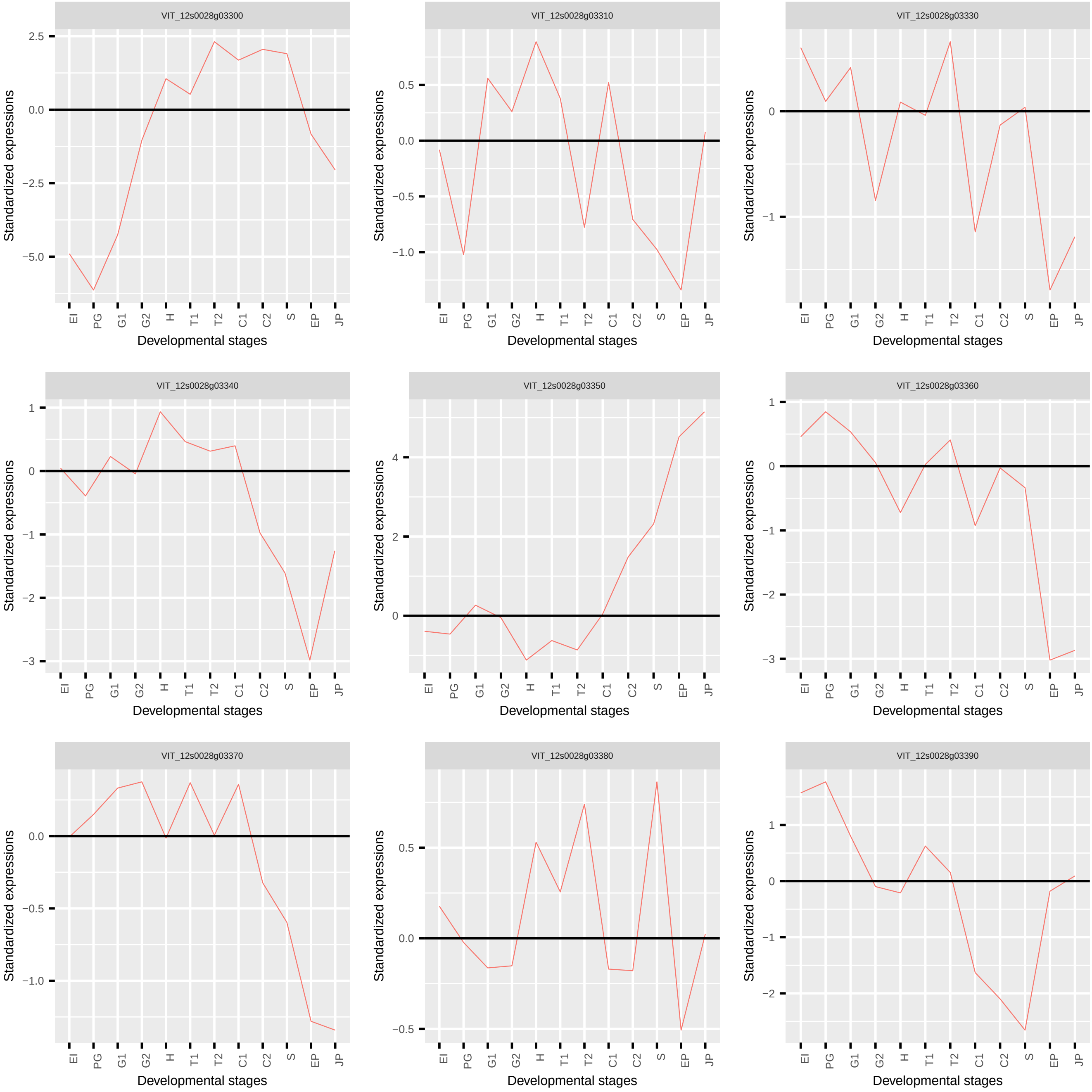
VIT_12s0028g03300
VIT_12s0028g03310
VIT_12s0028g03330
VIT_12s0028g03340
VIT_12s0028g03350
VIT_12s0028g03360
VIT_12s0028g03370
VIT_12s0028g03380
VIT_12s0028g03390
Standardized expressions
Developmental stages
EI PG G1 G2 H T1 T2 C1 C2 S EP JP
2.5
0.0
−2.5
−5.0
0.5
−0.5
−1.0
0
−1
1
−2
−3
4
2

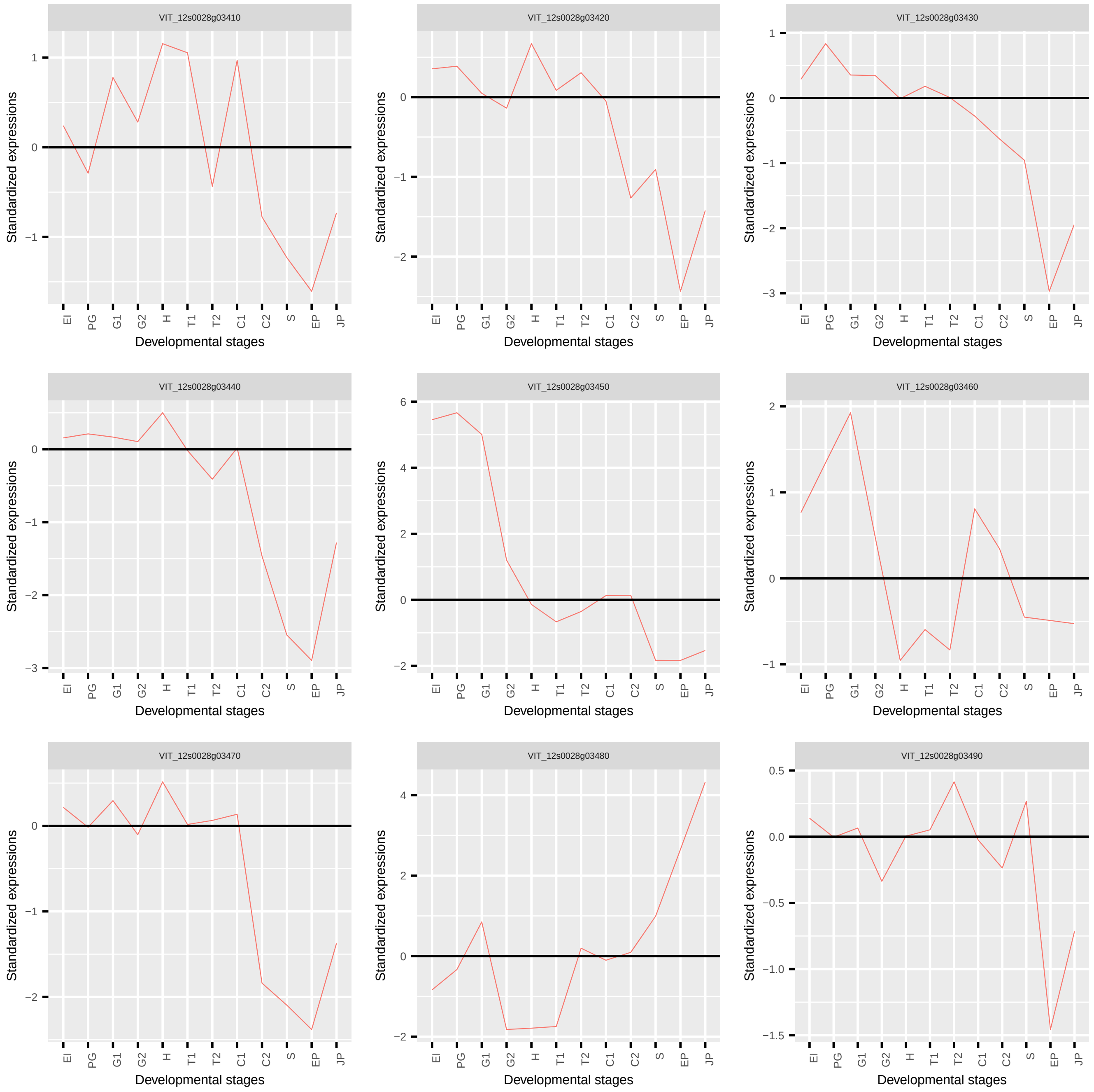

VIT_12s0028g03410
VIT_12s0028g03420
VIT_12s0028g03430
VIT_12s0028g03440
VIT_12s0028g03450
VIT_12s0028g03460
VIT_12s0028g03470
VIT_12s0028g03480
VIT_12s0028g03490
Standardized expressions
Developmental stages
EI PG G1 G2 H T1 T2 C1 C2 S EP JP

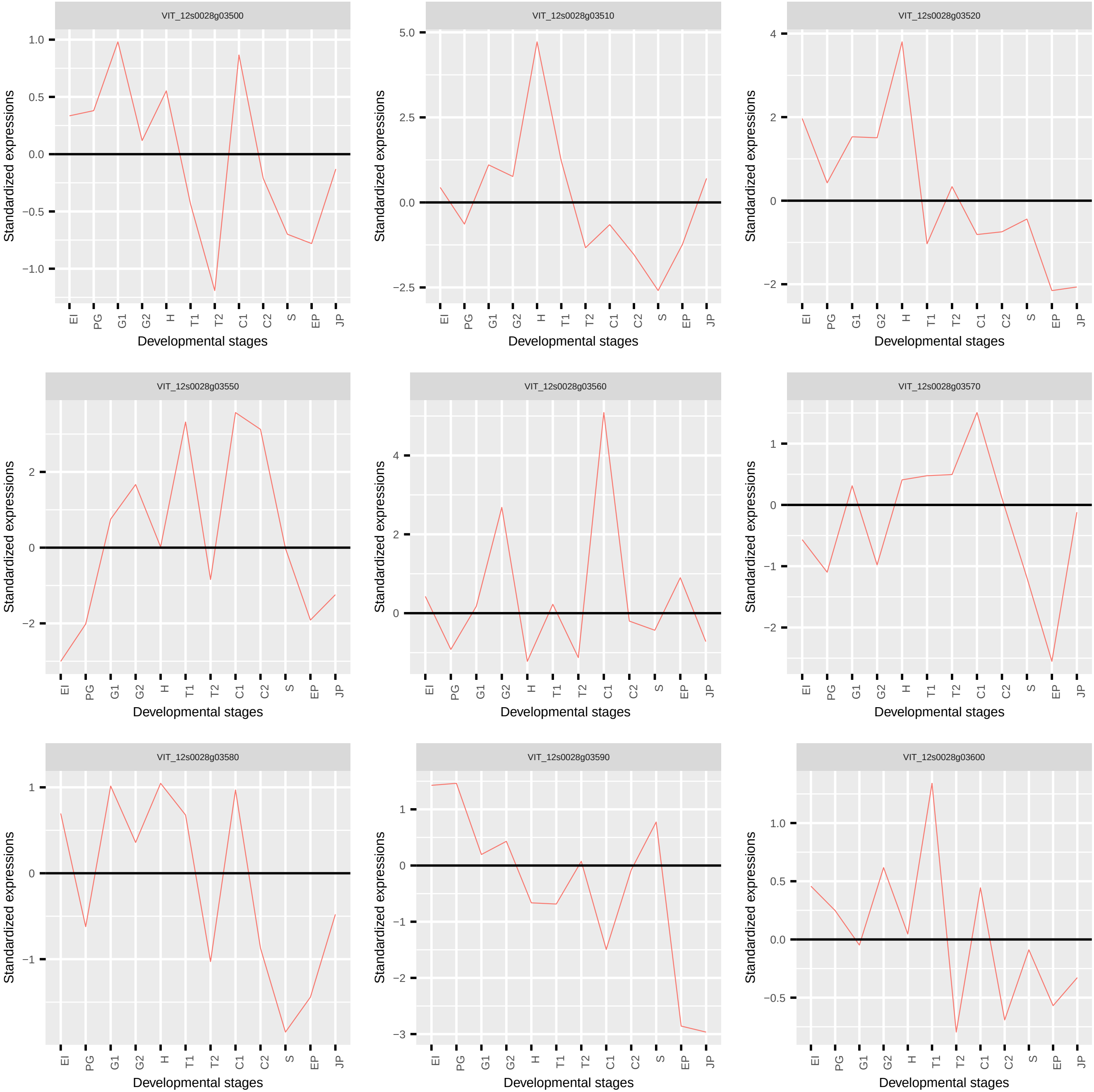
VIT_12s0028g03500
VIT_12s0028g03510
VIT_12s0028g03520
VIT_12s0028g03550
VIT_12s0028g03560
VIT_12s0028g03570
VIT_12s0028g03580
VIT_12s0028g03590
VIT_12s0028g03600
Standardized expressions
Developmental stages
EI PG G1 G2 H T1 T2 C1 C2 S EP JP

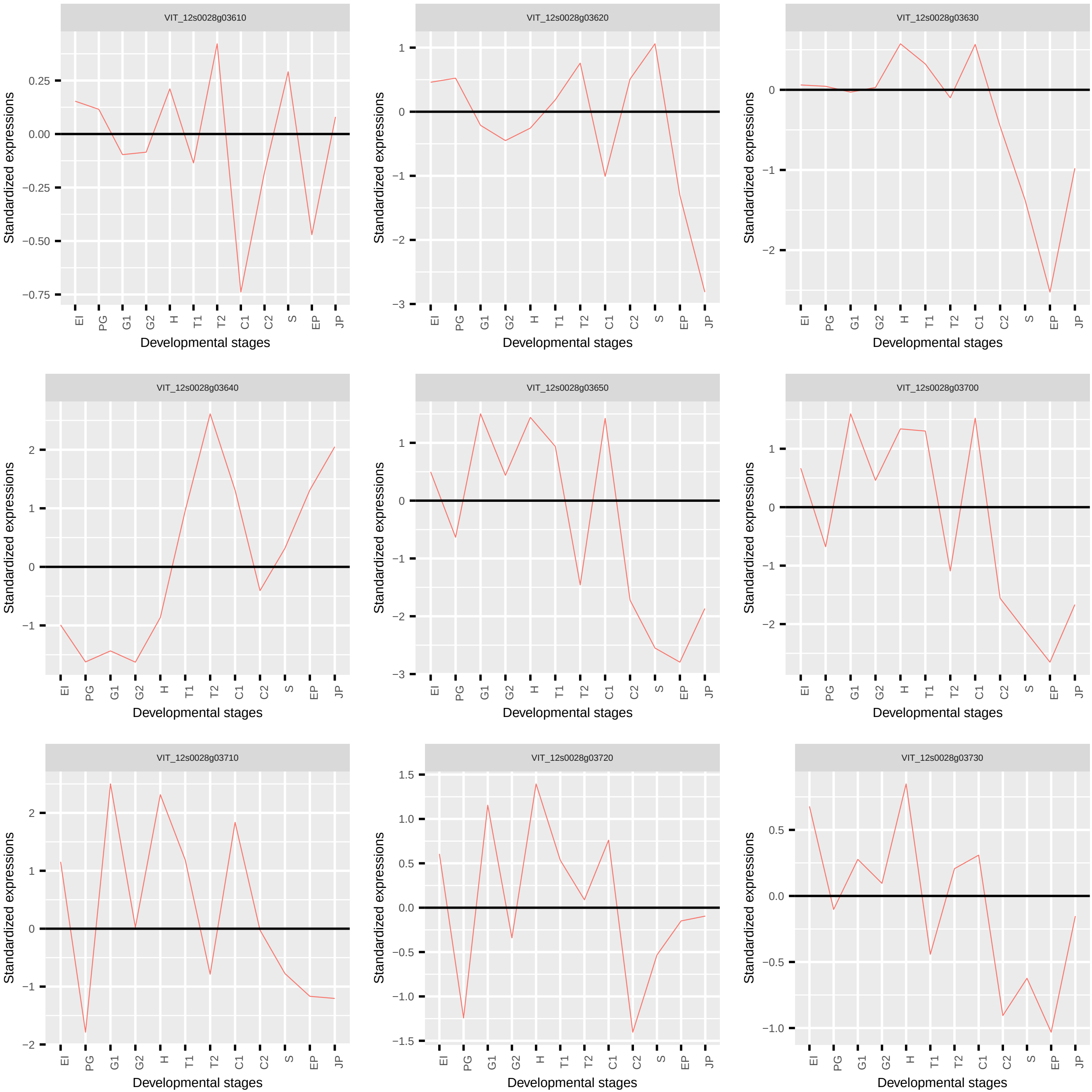
VIT_12s0028g03610
VIT_12s0028g03620
VIT_12s0028g03630
VIT_12s0028g03640
VIT_12s0028g03650
VIT_12s0028g03700
VIT_12s0028g03710
VIT_12s0028g03720
VIT_12s0028g03730
Standardized expressions
Developmental stages
EI
PG
G1
G2
H
T1
T2
C1
C2
S
EP
JP

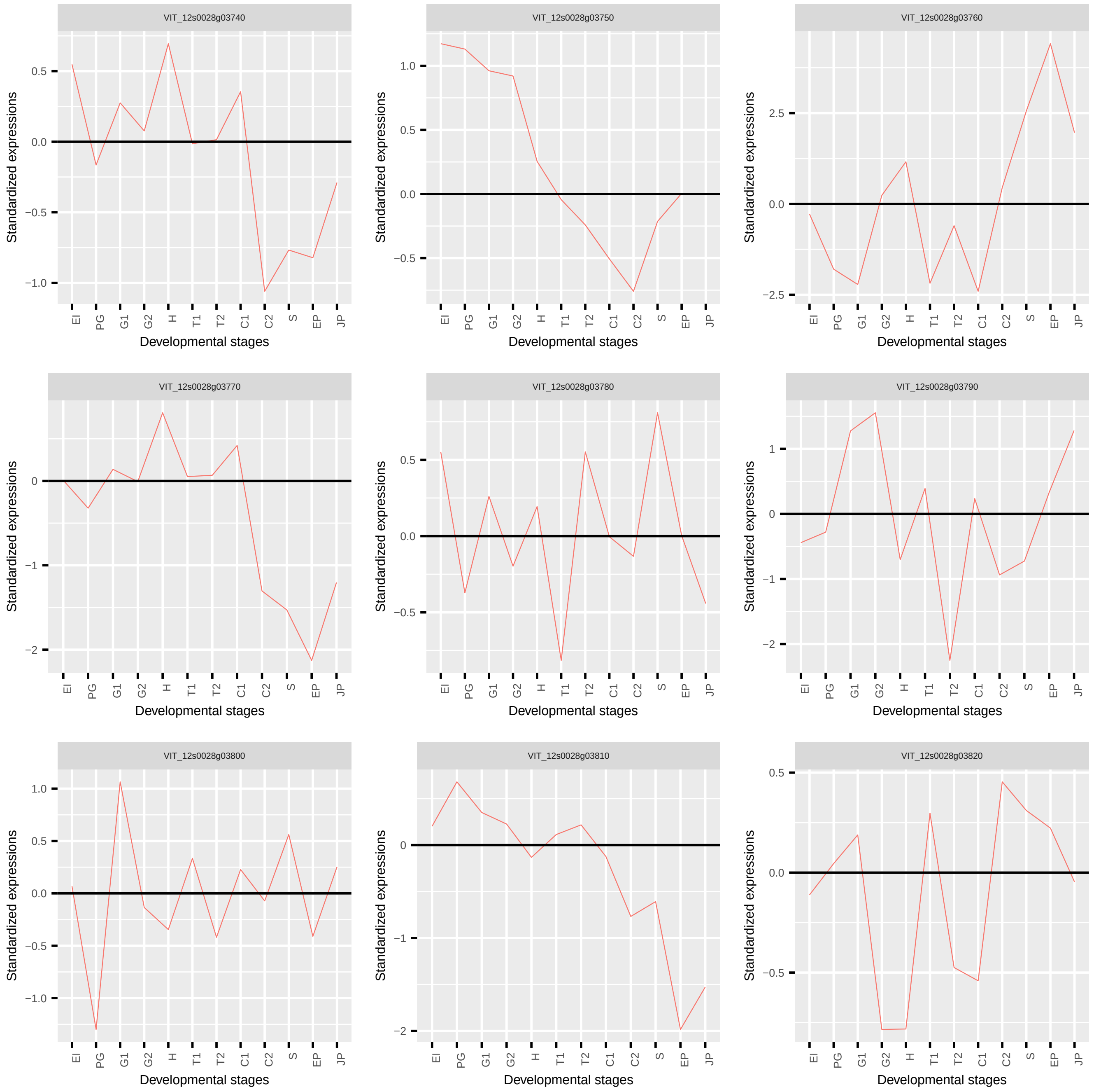
VIT_12s0028g03740
VIT_12s0028g03750
VIT_12s0028g03760
VIT_12s0028g03770
VIT_12s0028g03780
VIT_12s0028g03790
VIT_12s0028g03800
VIT_12s0028g03810
VIT_12s0028g03820
Standardized expressions
Developmental stages
EI PG G1 G2 H T1 T2 C1 C2 S EP JP

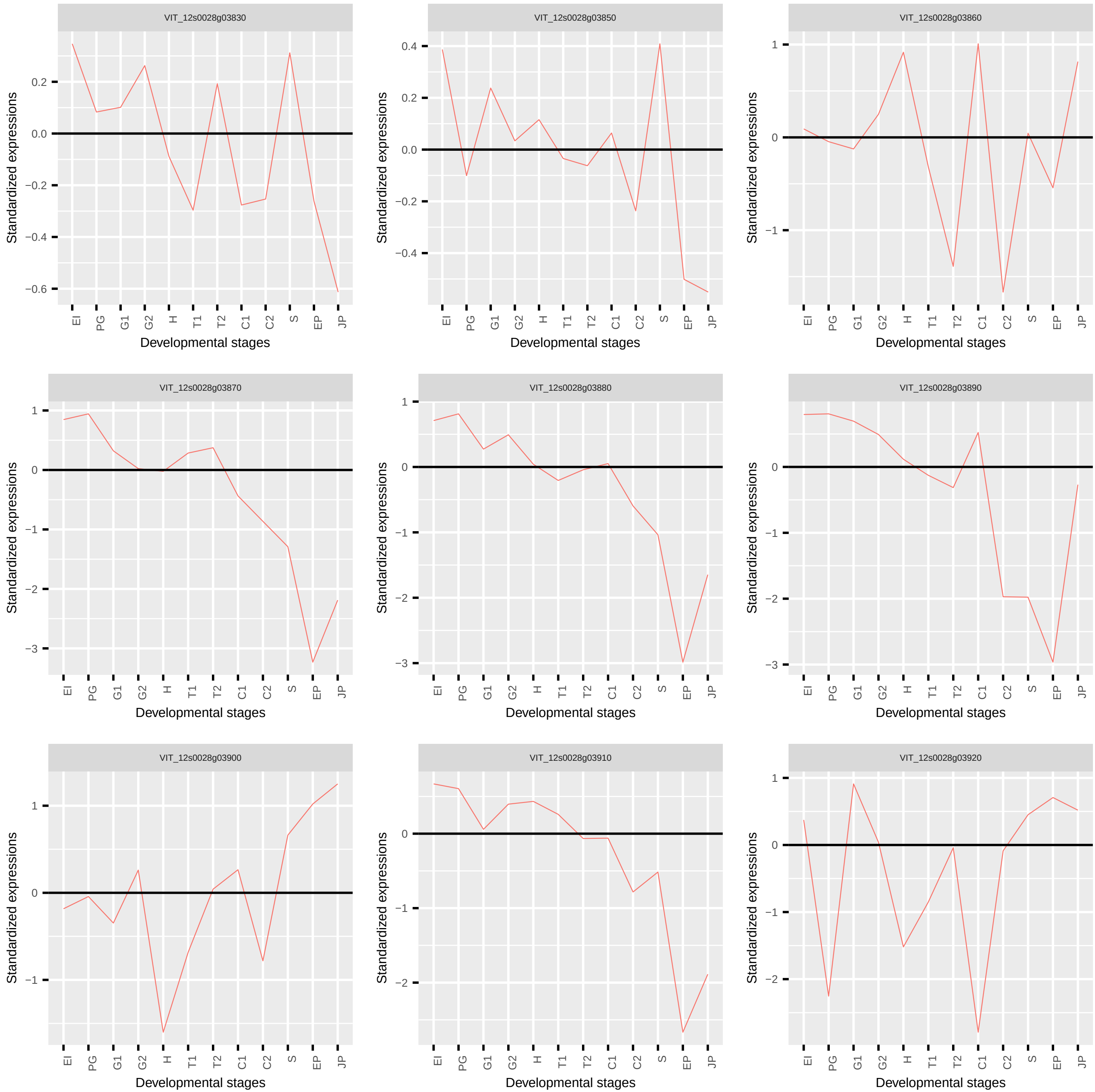
VIT_12s0028g03830
VIT_12s0028g03850
VIT_12s0028g03860
VIT_12s0028g03870
VIT_12s0028g03880
VIT_12s0028g03890
VIT_12s0028g03900
VIT_12s0028g03910
VIT_12s0028g03920
Standardized expressions
Developmental stages
EI
PG
G1
G2
H
T1
T2
C1
C2
S
EP
JP

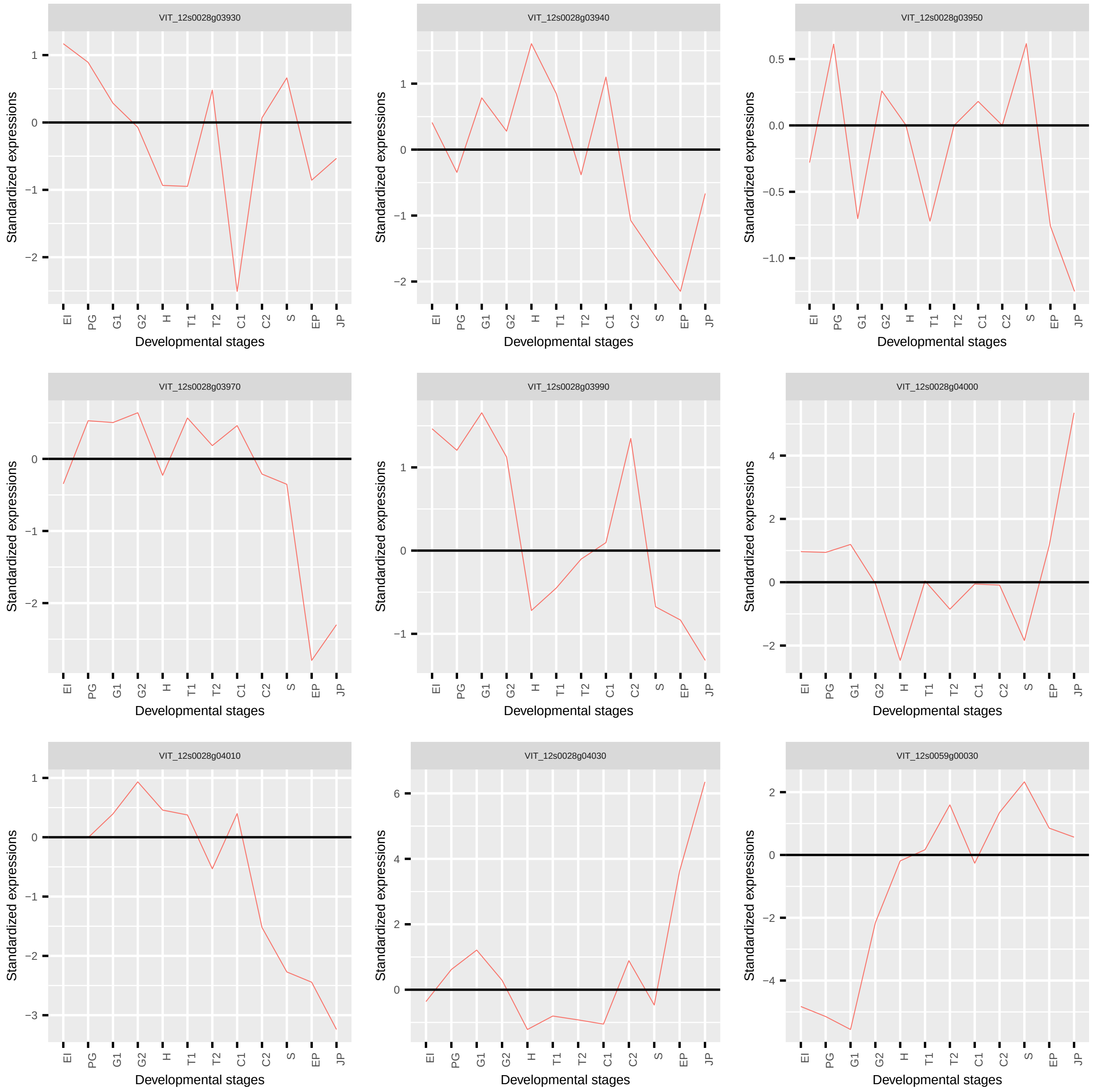

VIT_12s0028g03930
VIT_12s0028g03940
VIT_12s0028g03950
VIT_12s0028g03970
VIT_12s0028g03990
VIT_12s0028g04000
VIT_12s0028g04010
VIT_12s0028g04030
VIT_12s0059g00030
Standardized expressions
Developmental stages
EI PG G1 G2 H T1 T2 C1 C2 S EP JP
1 0 −1 −2
0.5 0.0 −0.5 −1.0
4 2 0 −2
−3
6 4 2 0
−4

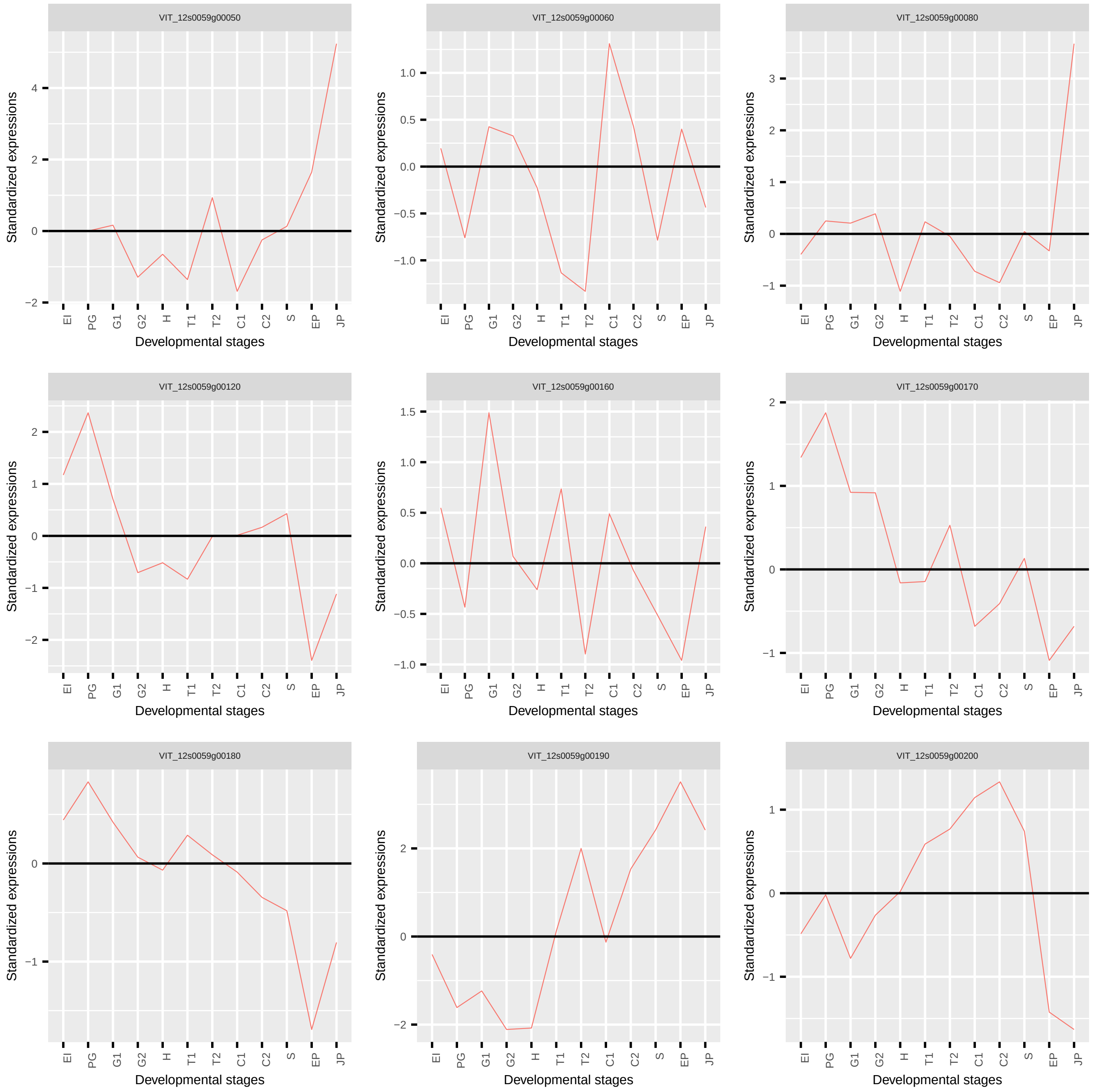
VIT_12s0059g00050
VIT_12s0059g00060
VIT_12s0059g00080
VIT_12s0059g00120
VIT_12s0059g00160
VIT_12s0059g00170
VIT_12s0059g00180
VIT_12s0059g00190
VIT_12s0059g00200
Standardized expressions
Developmental stages
EI PG G1 G2 H T1 T2 C1 C2 S EP JP

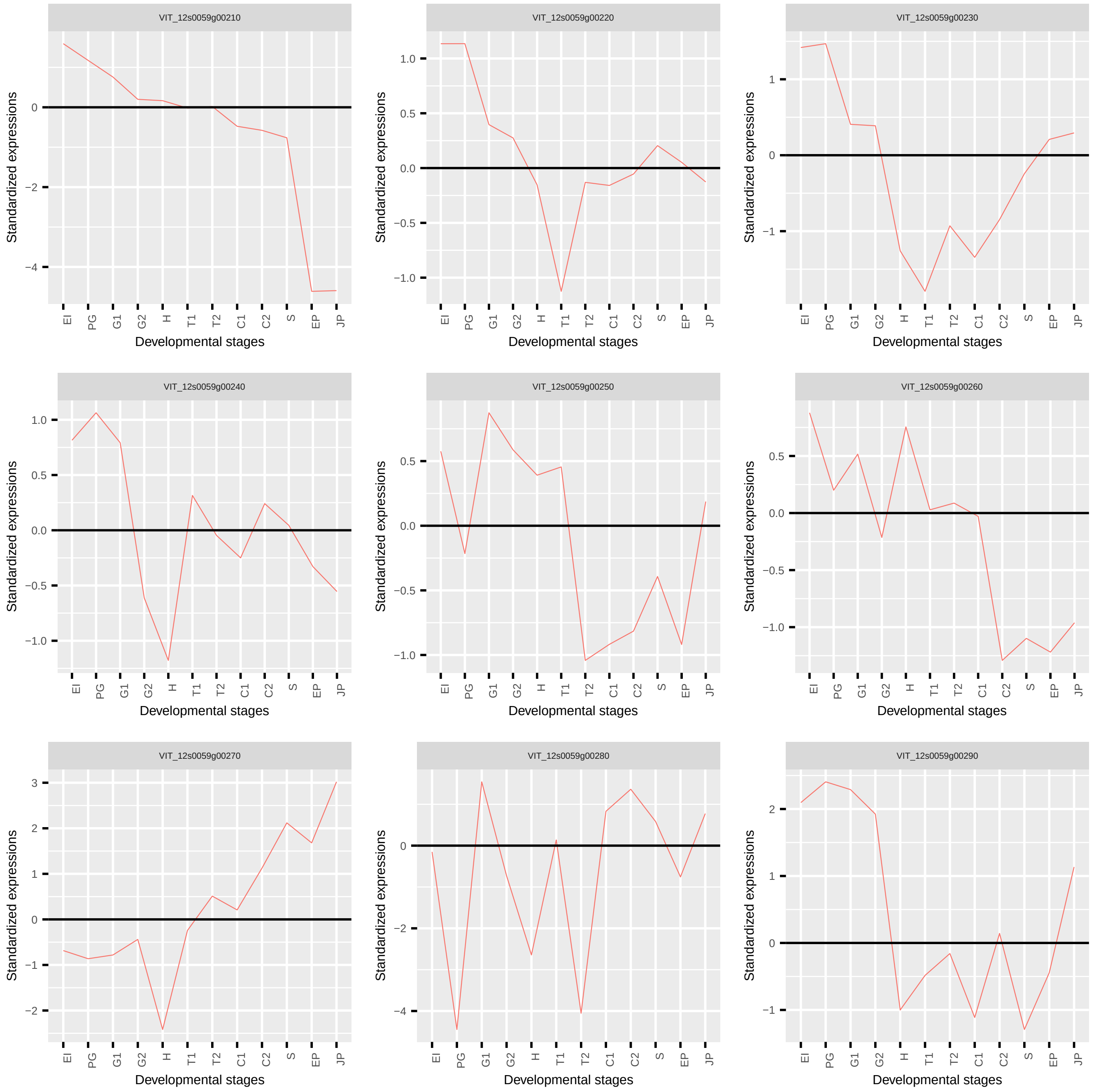
VIT_12s0059g00210
VIT_12s0059g00220
VIT_12s0059g00230
VIT_12s0059g00240
VIT_12s0059g00250
VIT_12s0059g00260
VIT_12s0059g00270
VIT_12s0059g00280
VIT_12s0059g00290
Standardized expressions
Developmental stages
EI
PG
G1
G2
H
T1
T2
C1
C2
S
EP
JP

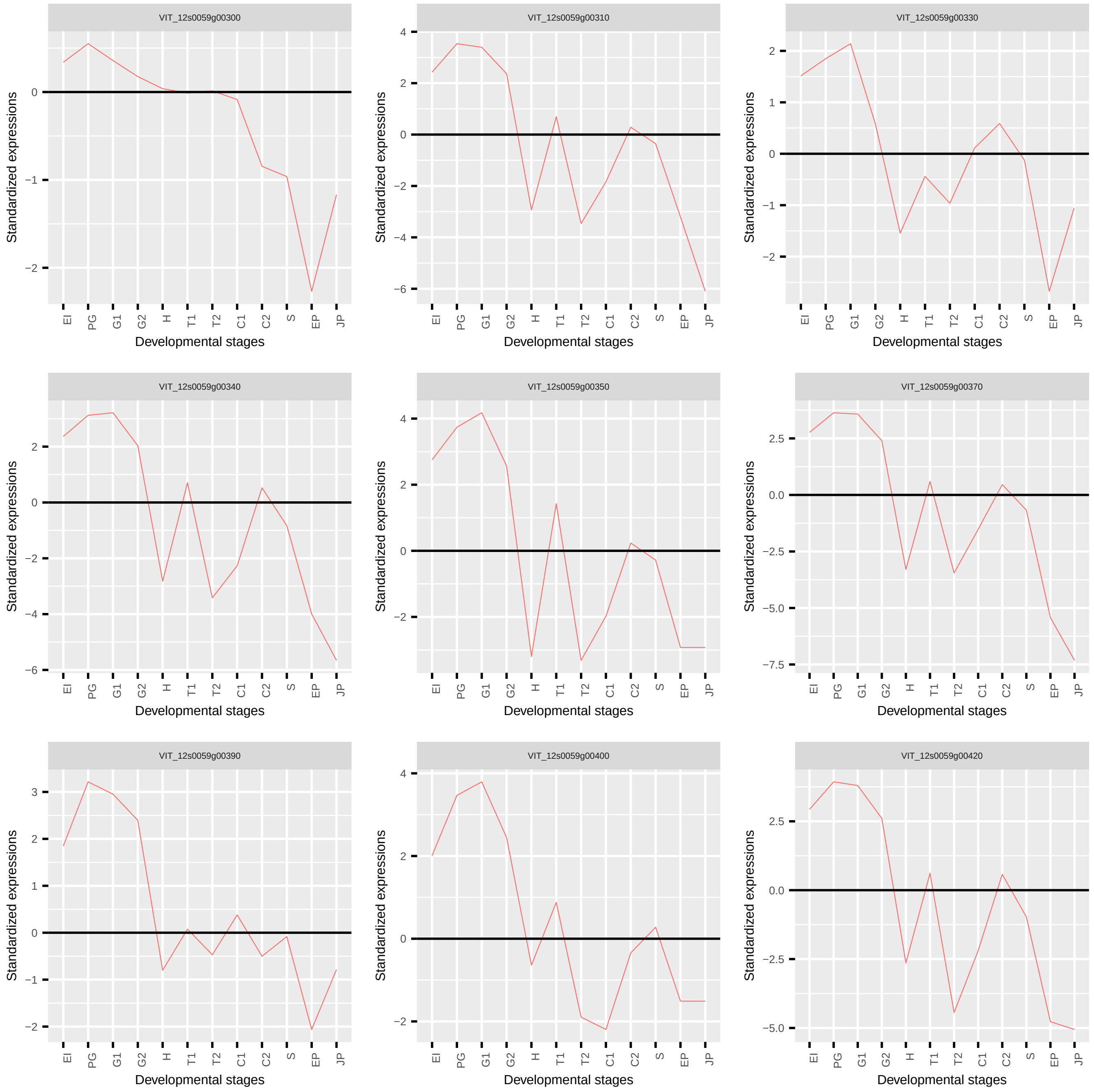
VIT_12s0059g00300
VIT_12s0059g00310
VIT_12s0059g00330
VIT_12s0059g00340
VIT_12s0059g00350
VIT_12s0059g00370
VIT_12s0059g00390
VIT_12s0059g00400
VIT_12s0059g00420
Standardized expressions
Developmental stages
EI PG G1 G2 H T1 T2 C1 C2 S EP JP

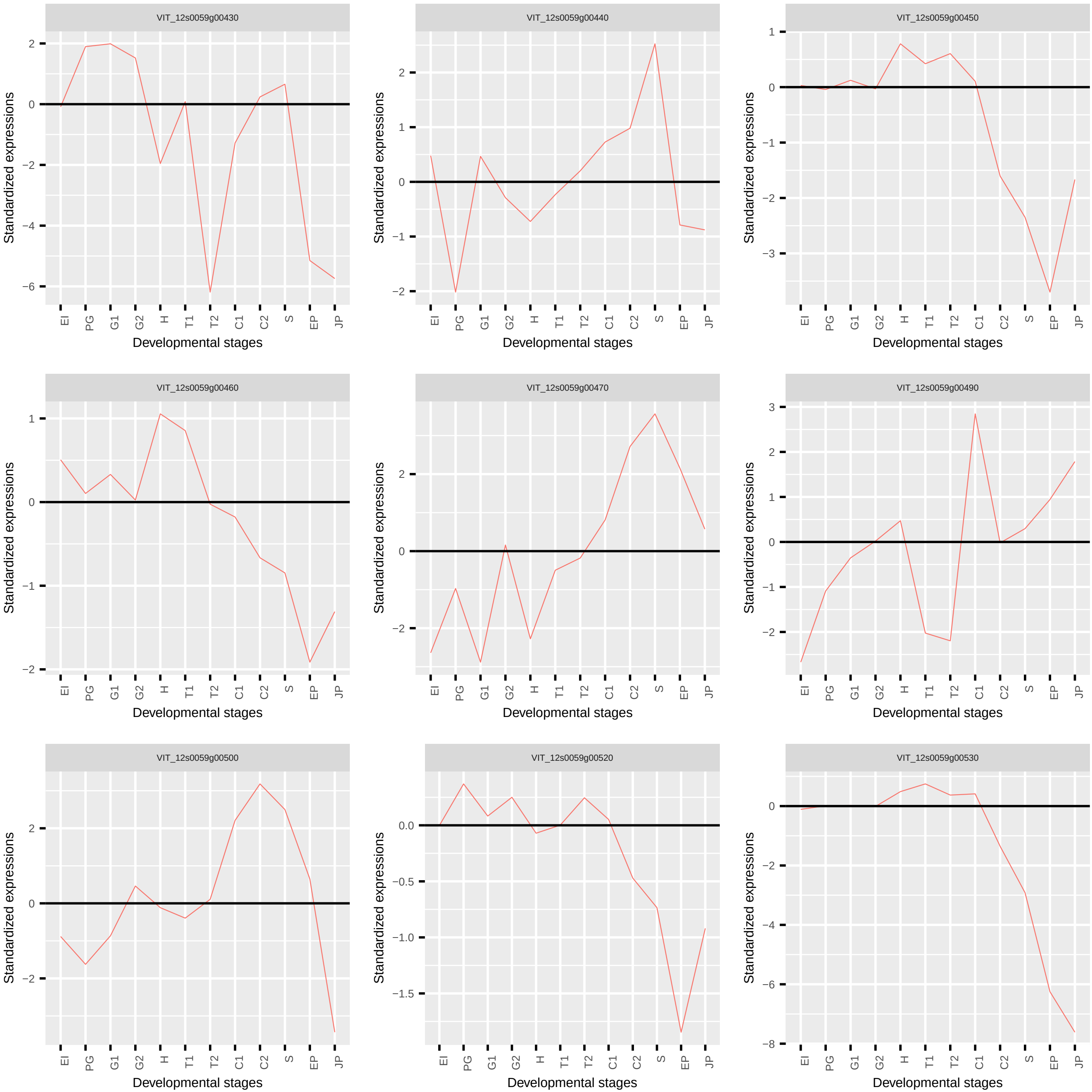
VIT_12s0059g00430
VIT_12s0059g00440
VIT_12s0059g00450
VIT_12s0059g00460
VIT_12s0059g00470
VIT_12s0059g00490
VIT_12s0059g00500
VIT_12s0059g00520
VIT_12s0059g00530
Standardized expressions
Developmental stages
EI
PG
G1
G2
H
T1
T2
C1
C2
S
EP
JP

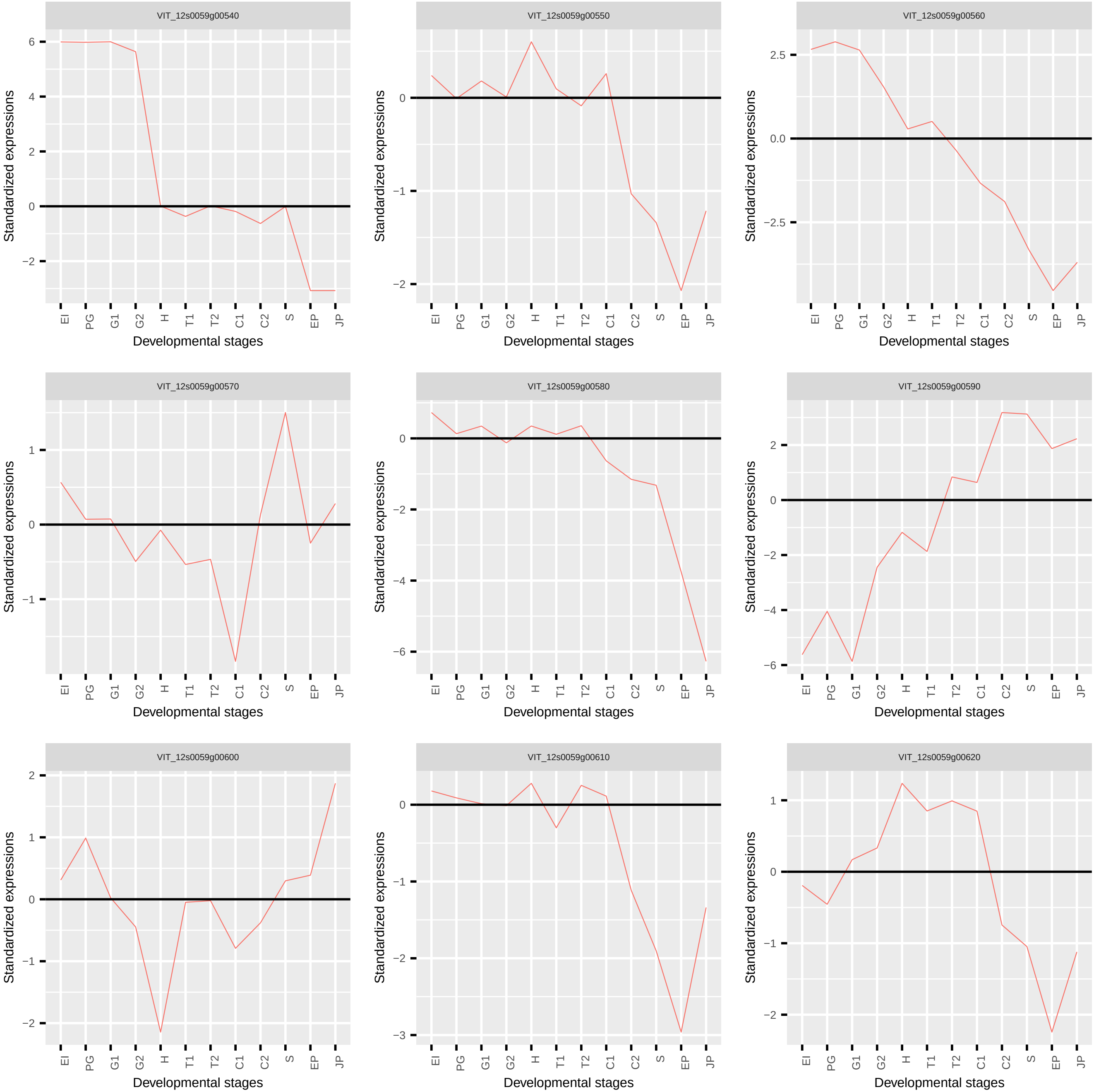

VIT_12s0059g00540
6
4
2
0
−2
Standardized expressions
EI PG G1 G2 H T1 T2 C1 C2 S EP JP
Developmental stages
VIT_12s0059g00550
0
−1
−2
Standardized expressions
EI PG G1 G2 H T1 T2 C1 C2 S EP JP
Developmental stages
VIT_12s0059g00560
2.5
0.0
−2.5
Standardized expressions
EI PG G1 G2 H T1 T2 C1 C2 S EP JP
Developmental stages
VIT_12s0059g00570
1
0
−1
Standardized expressions
EI PG G1 G2 H T1 T2 C1 C2 S EP JP
Developmental stages
VIT_12s0059g00580
0
−2
−4
−6
Standardized expressions
EI PG G1 G2 H T1 T2 C1 C2 S EP JP
Developmental stages
VIT_12s0059g00590
2
0
−2
−4
−6
Standardized expressions
EI PG G1 G2 H T1 T2 C1 C2 S EP JP
Developmental stages
VIT_12s0059g00600
2
1
0
−1
−2
Standardized expressions
EI PG G1 G2 H T1 T2 C1 C2 S EP JP
Developmental stages
VIT_12s0059g00610
0
−1
−2
−3
Standardized expressions
EI PG G1 G2 H T1 T2 C1 C2 S EP JP
Developmental stages
VIT_12s0059g00620
1
0
−1
−2
Standardized expressions
EI PG G1 G2 H T1 T2 C1 C2 S EP JP
Developmental stages

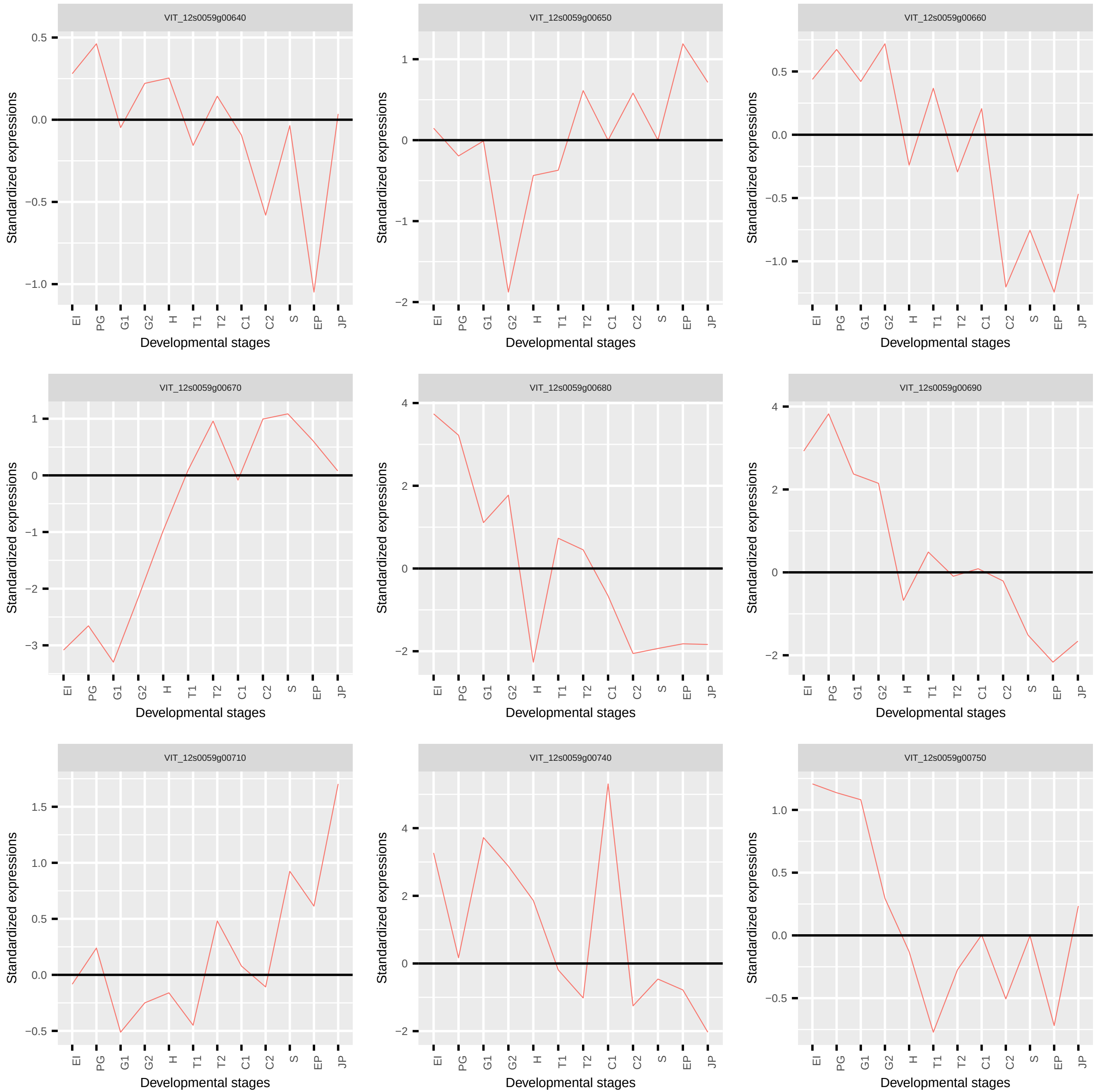
VIT_12s0059g00640
VIT_12s0059g00650
VIT_12s0059g00660
VIT_12s0059g00670
VIT_12s0059g00680
VIT_12s0059g00690
VIT_12s0059g00710
VIT_12s0059g00740
VIT_12s0059g00750
Standardized expressions
Developmental stages
EI PG G1 G2 H T1 T2 C1 C2 S EP JP

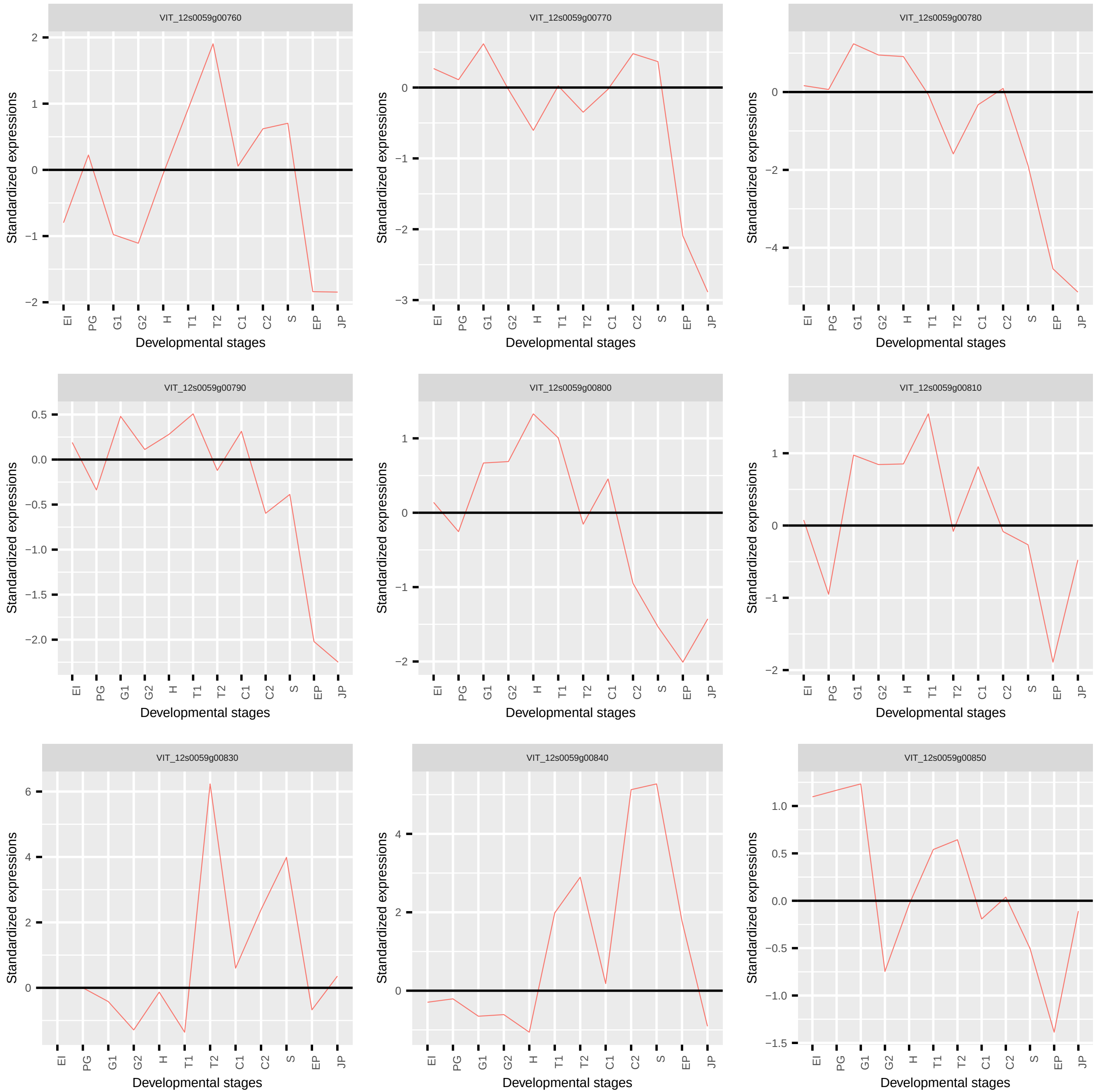
VIT_12s0059g00760
VIT_12s0059g00770
VIT_12s0059g00780
VIT_12s0059g00790
VIT_12s0059g00800
VIT_12s0059g00810
VIT_12s0059g00830
VIT_12s0059g00840
VIT_12s0059g00850
Standardized expressions
Developmental stages
EI
PG
G1
G2
H
T1
T2
C1
C2
S
EP
JP

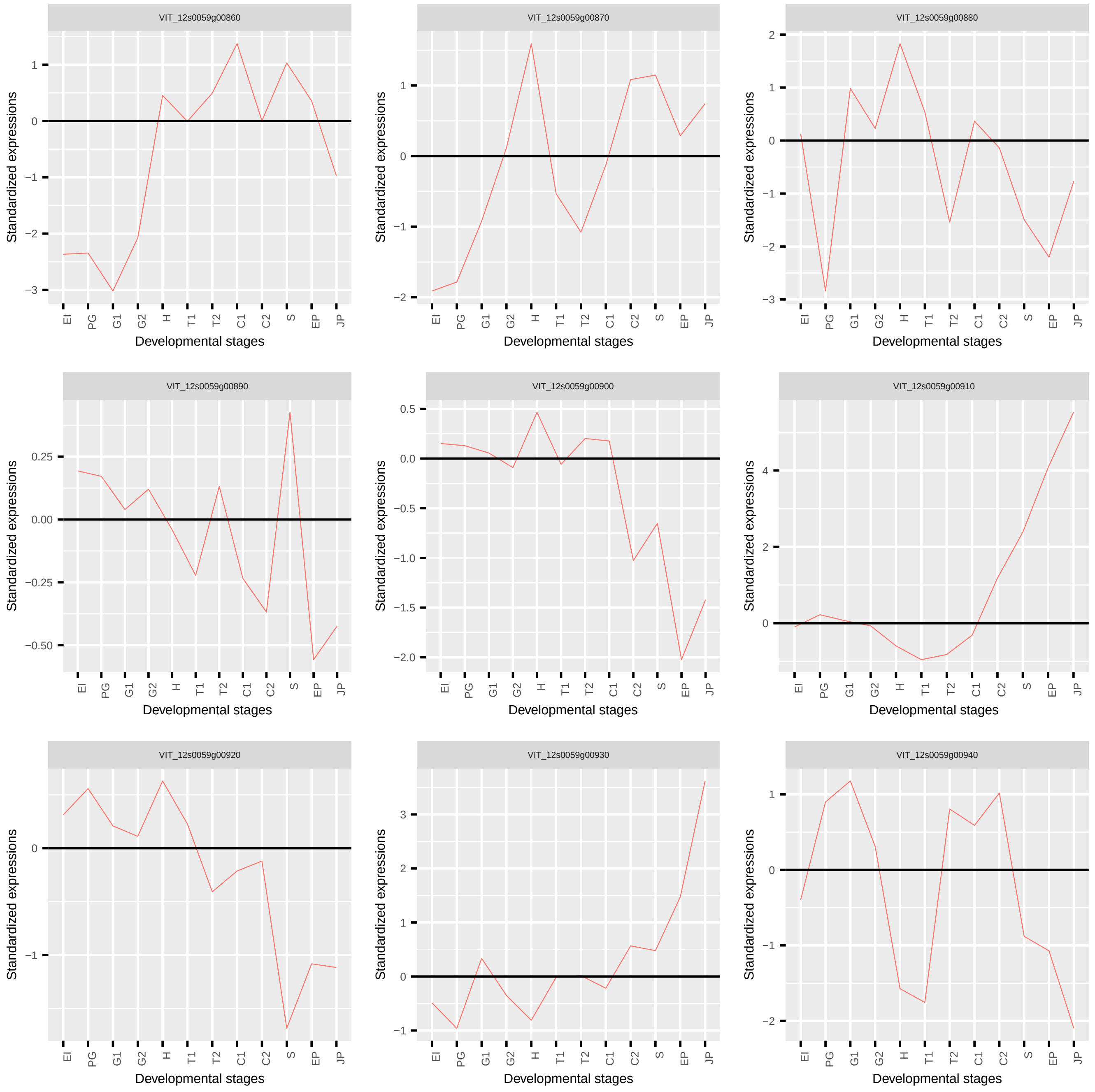
VIT_12s0059g00860
VIT_12s0059g00870
VIT_12s0059g00880
VIT_12s0059g00890
VIT_12s0059g00900
VIT_12s0059g00910
VIT_12s0059g00920
VIT_12s0059g00930
VIT_12s0059g00940
Standardized expressions
Developmental stages
EI
PG
G1
G2
H
T1
T2
C1
C2
S
EP
JP

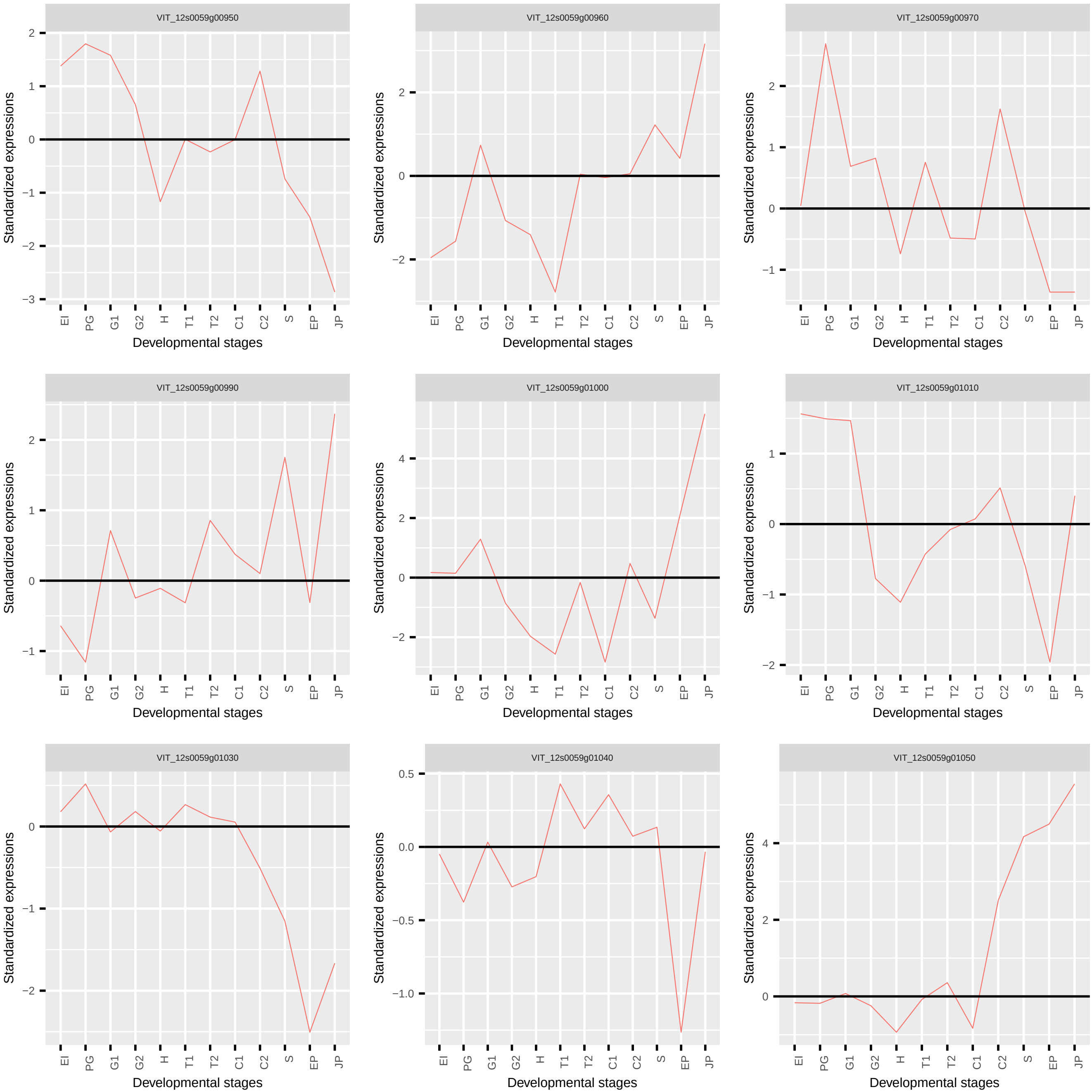
VIT_12s0059g00950
VIT_12s0059g00960
VIT_12s0059g00970
VIT_12s0059g00990
VIT_12s0059g01000
VIT_12s0059g01010
VIT_12s0059g01030
VIT_12s0059g01040
VIT_12s0059g01050
Standardized expressions
Developmental stages
EI PG G1 G2 H T1 T2 C1 C2 S EP JP

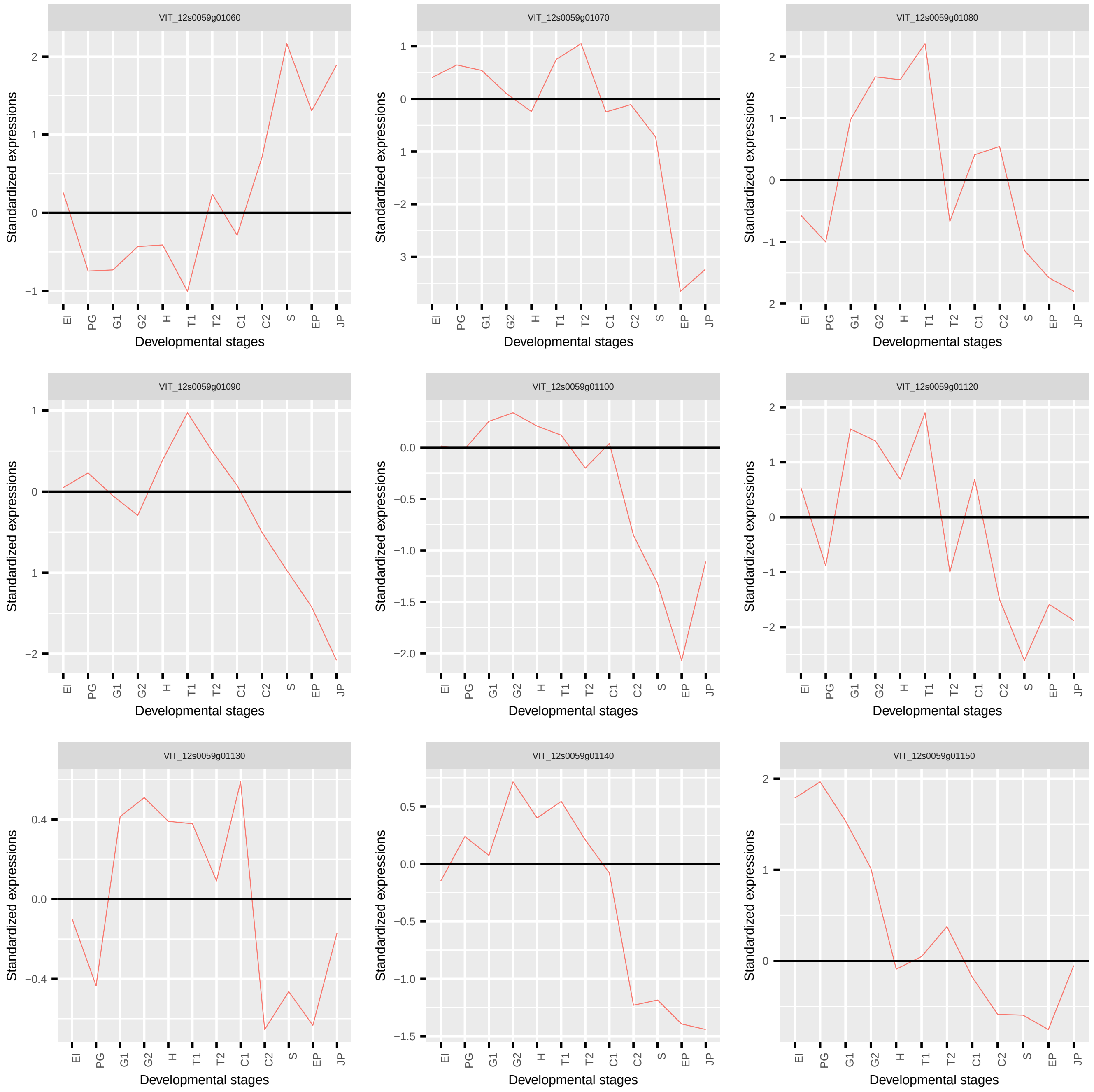
VIT_12s0059g01060
VIT_12s0059g01070
VIT_12s0059g01080
VIT_12s0059g01090
VIT_12s0059g01100
VIT_12s0059g01120
VIT_12s0059g01130
VIT_12s0059g01140
VIT_12s0059g01150
Standardized expressions
Developmental stages
EI PG G1 G2 H T1 T2 C1 C2 S EP JP

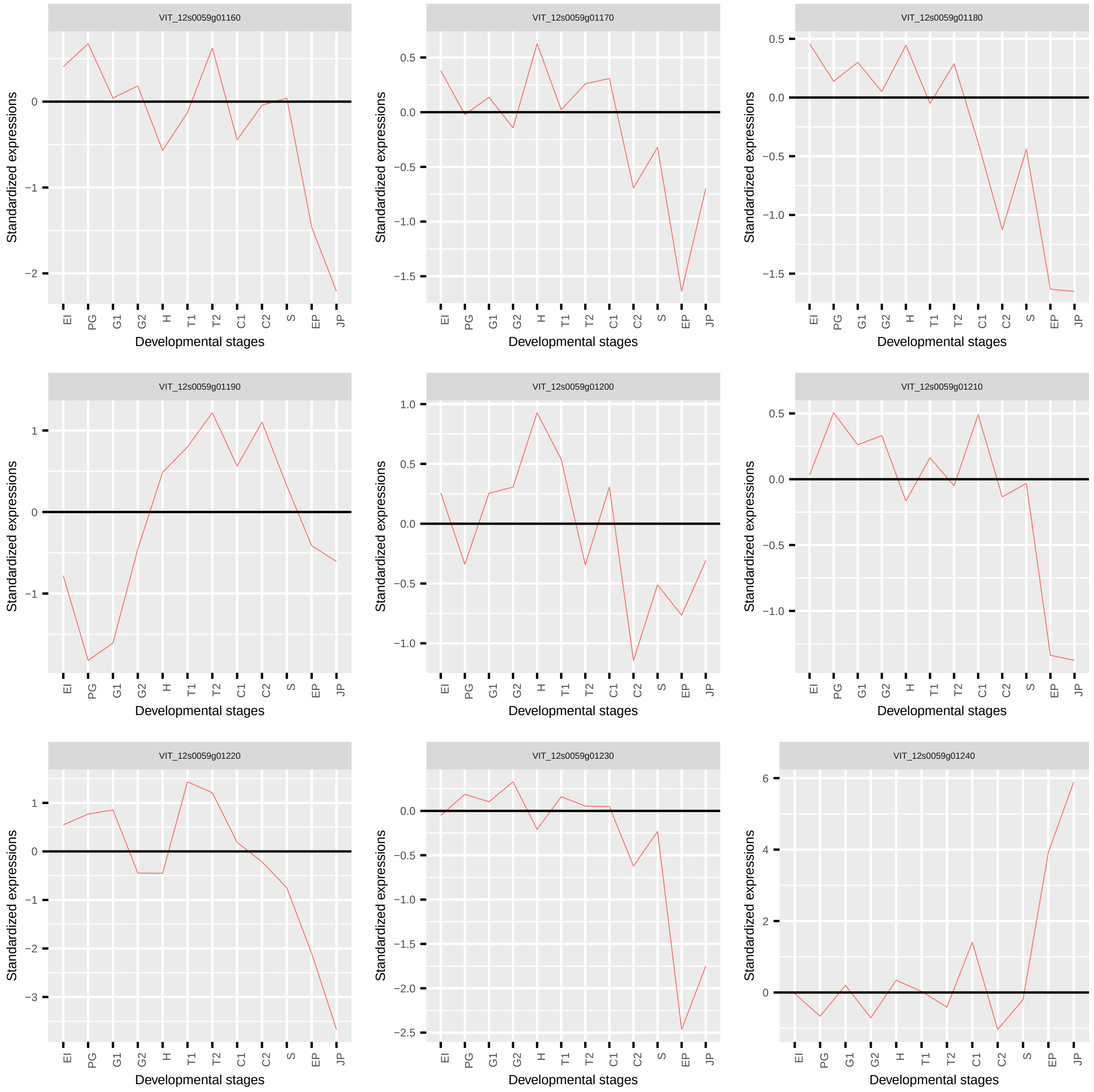
VIT_12s0059g01160
VIT_12s0059g01170
VIT_12s0059g01180
VIT_12s0059g01190
VIT_12s0059g01200
VIT_12s0059g01210
VIT_12s0059g01220
VIT_12s0059g01230
VIT_12s0059g01240
Standardized expressions
Developmental stages
EI PG G1 G2 H T1 T2 C1 C2 S EP JP
0
−1
−2
0.5
0.0
−0.5
−1.0
−1.5
1
−3
−2.0
−2.5
2
4
6

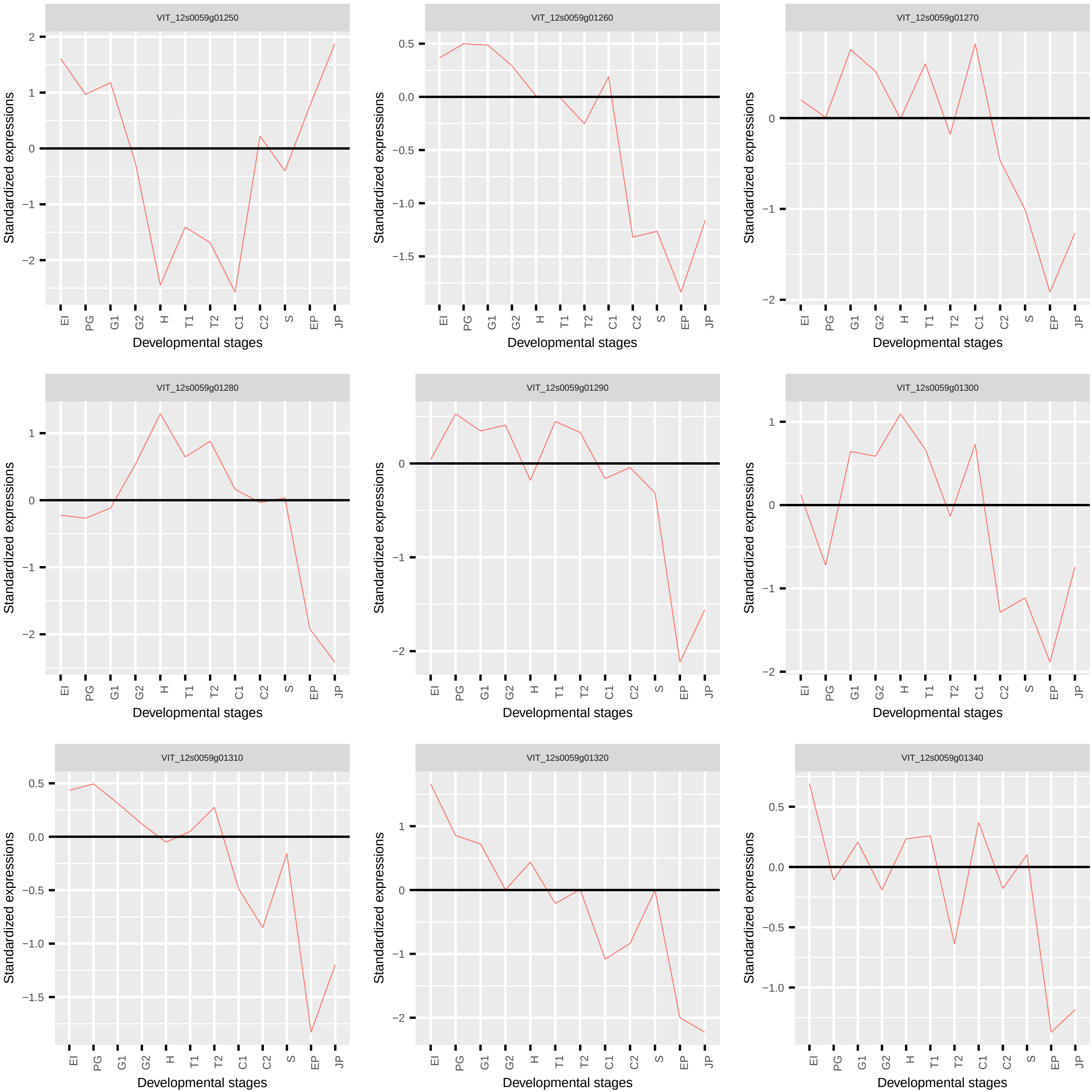

VIT_12s0059g01250
VIT_12s0059g01260
VIT_12s0059g01270
VIT_12s0059g01280
VIT_12s0059g01290
VIT_12s0059g01300
VIT_12s0059g01310
VIT_12s0059g01320
VIT_12s0059g01340
Standardized expressions
Developmental stages
EI PG G1 G2 H T1 T2 C1 C2 S EP JP

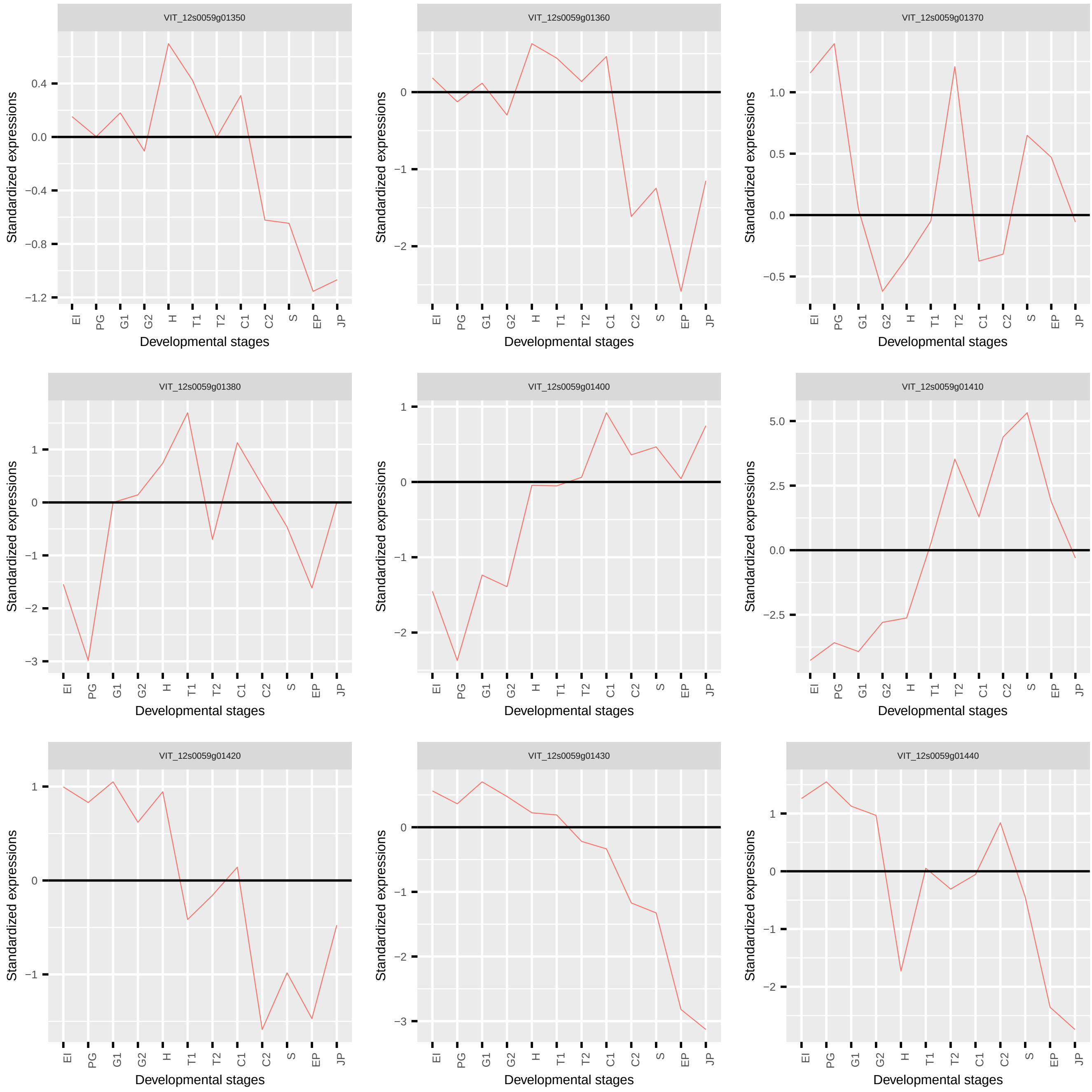
VIT_12s0059g01350
VIT_12s0059g01360
VIT_12s0059g01370
VIT_12s0059g01380
VIT_12s0059g01400
VIT_12s0059g01410
VIT_12s0059g01420
VIT_12s0059g01430
VIT_12s0059g01440
Standardized expressions
Developmental stages
EI PG G1 G2 H T1 T2 C1 C2 S EP JP

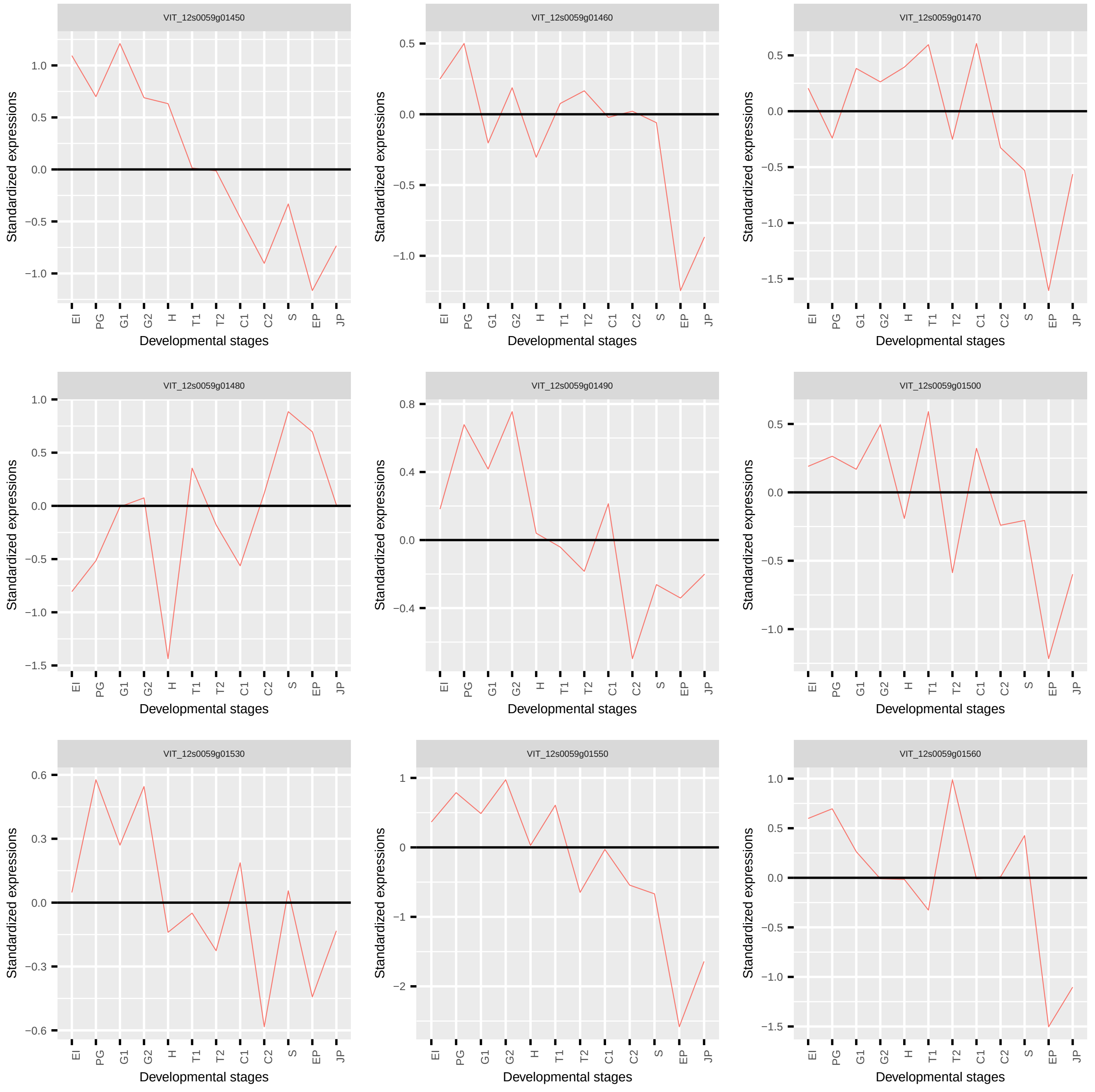

VIT_12s0059g01450
VIT_12s0059g01460
VIT_12s0059g01470
VIT_12s0059g01480
VIT_12s0059g01490
VIT_12s0059g01500
VIT_12s0059g01530
VIT_12s0059g01550
VIT_12s0059g01560
Standardized expressions
Developmental stages
EI
PG
G1
G2
H
T1
T2
C1
C2
S
EP
JP

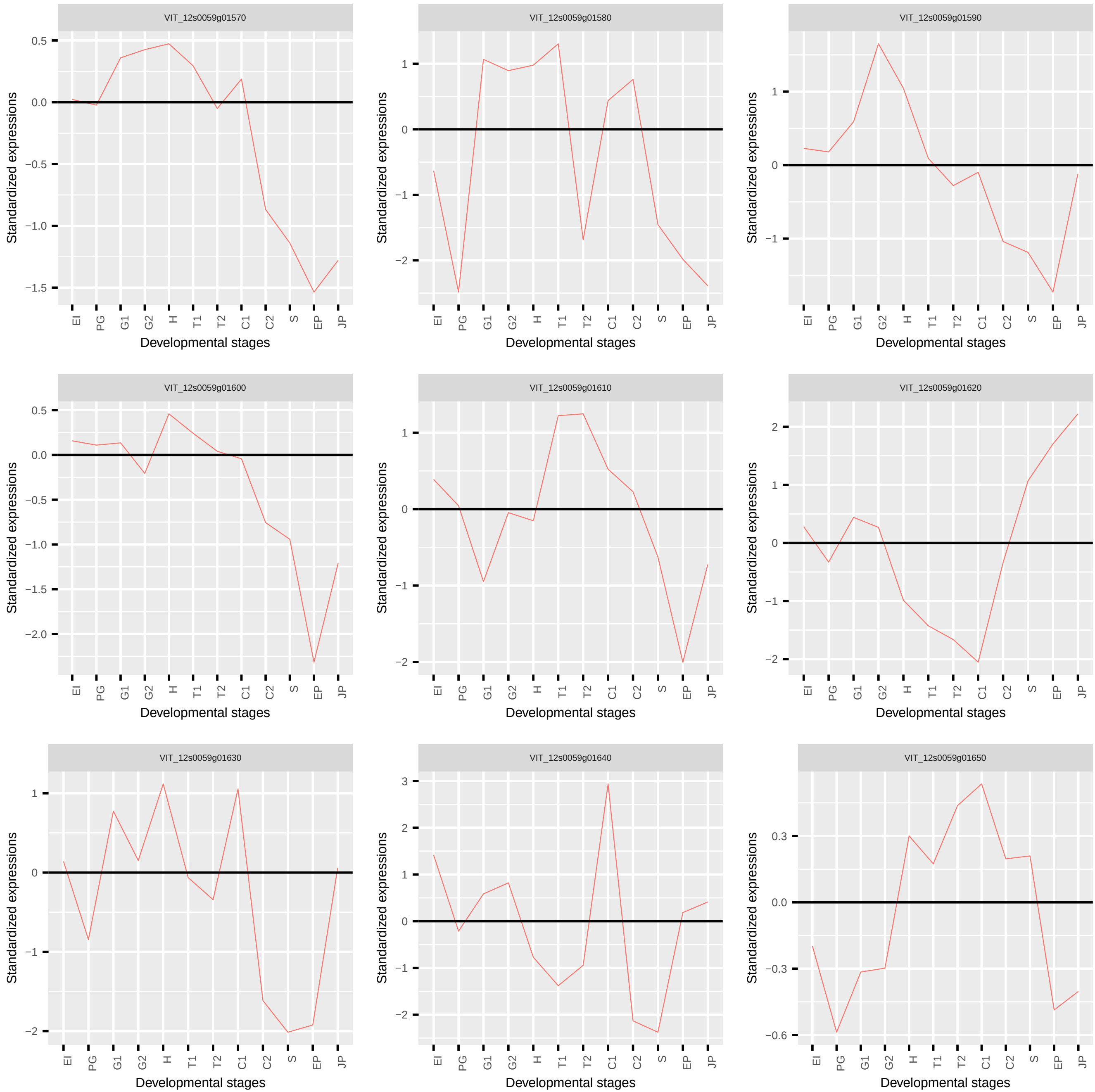

VIT_12s0059g01570
0.5
0.0
−0.5
−1.0
−1.5
Standardized expressions
EI PG G1 G2 H T1 T2 C1 C2 S EP JP
Developmental stages
VIT_12s0059g01580
1
0
−1
−2
Standardized expressions
EI PG G1 G2 H T1 T2 C1 C2 S EP JP
Developmental stages
VIT_12s0059g01590
1
0
−1
Standardized expressions
EI PG G1 G2 H T1 T2 C1 C2 S EP JP
Developmental stages
VIT_12s0059g01600
0.5
0.0
−0.5
−1.0
−1.5
−2.0
Standardized expressions
EI PG G1 G2 H T1 T2 C1 C2 S EP JP
Developmental stages
VIT_12s0059g01610
1
0
−1
−2
Standardized expressions
EI PG G1 G2 H T1 T2 C1 C2 S EP JP
Developmental stages
VIT_12s0059g01620
2
1
0
−1
−2
Standardized expressions
EI PG G1 G2 H T1 T2 C1 C2 S EP JP
Developmental stages
VIT_12s0059g01630
1
0
−1
−2
Standardized expressions
EI PG G1 G2 H T1 T2 C1 C2 S EP JP
Developmental stages
VIT_12s0059g01640
3
2
1
0
−1
−2
Standardized expressions
EI PG G1 G2 H T1 T2 C1 C2 S EP JP
Developmental stages
VIT_12s0059g01650
0.3
0.0
−0.3
−0.6
Standardized expressions
EI PG G1 G2 H T1 T2 C1 C2 S EP JP
Developmental stages

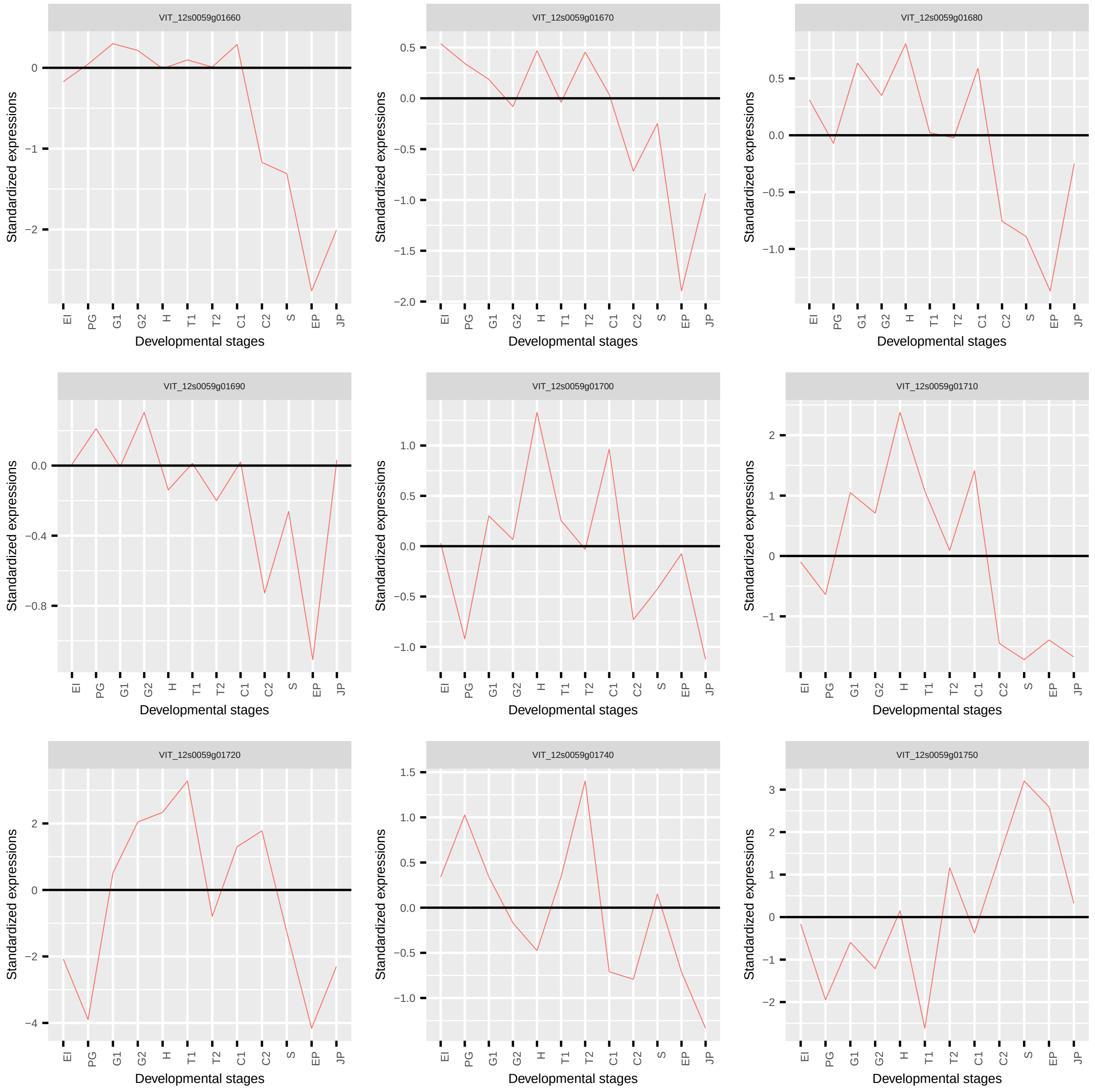
VIT_12s0059g01660
0
−1
−2
Standardized expressions
EI PG G1 G2 H T1 T2 C1 C2 S EP JP
Developmental stages
VIT_12s0059g01670
0.5
0.0
−0.5
−1.0
−1.5
−2.0
Standardized expressions
EI PG G1 G2 H T1 T2 C1 C2 S EP JP
Developmental stages
VIT_12s0059g01680
0.5
0.0
−0.5
−1.0
Standardized expressions
EI PG G1 G2 H T1 T2 C1 C2 S EP JP
Developmental stages
VIT_12s0059g01690
0.0
−0.4
−0.8
Standardized expressions
EI PG G1 G2 H T1 T2 C1 C2 S EP JP
Developmental stages
VIT_12s0059g01700
1.0
0.5
0.0
−0.5
−1.0
Standardized expressions
EI PG G1 G2 H T1 T2 C1 C2 S EP JP
Developmental stages
VIT_12s0059g01710
2
1
0
−1
Standardized expressions
EI PG G1 G2 H T1 T2 C1 C2 S EP JP
Developmental stages
VIT_12s0059g01720
2
0
−2
−4
Standardized expressions
EI PG G1 G2 H T1 T2 C1 C2 S EP JP
Developmental stages
VIT_12s0059g01740
1.5
1.0
0.5
0.0
−0.5
−1.0
Standardized expressions
EI PG G1 G2 H T1 T2 C1 C2 S EP JP
Developmental stages
VIT_12s0059g01750
3
2
1
0
−1
−2
Standardized expressions
EI PG G1 G2 H T1 T2 C1 C2 S EP JP
Developmental stages

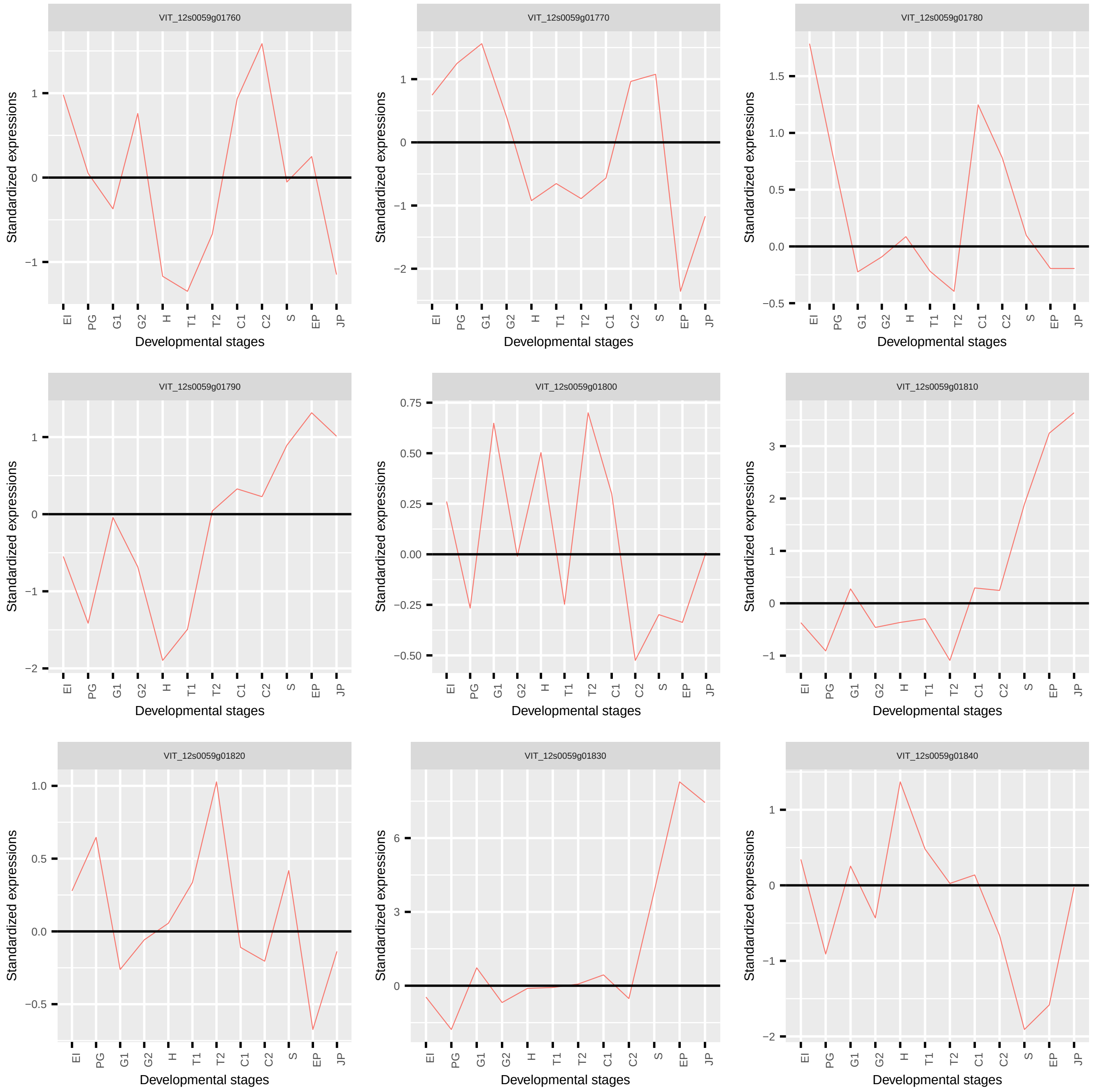
VIT_12s0059g01760
VIT_12s0059g01770
VIT_12s0059g01780
VIT_12s0059g01790
VIT_12s0059g01800
VIT_12s0059g01810
VIT_12s0059g01820
VIT_12s0059g01830
VIT_12s0059g01840
Standardized expressions
Developmental stages
EI PG G1 G2 H T1 T2 C1 C2 S EP JP

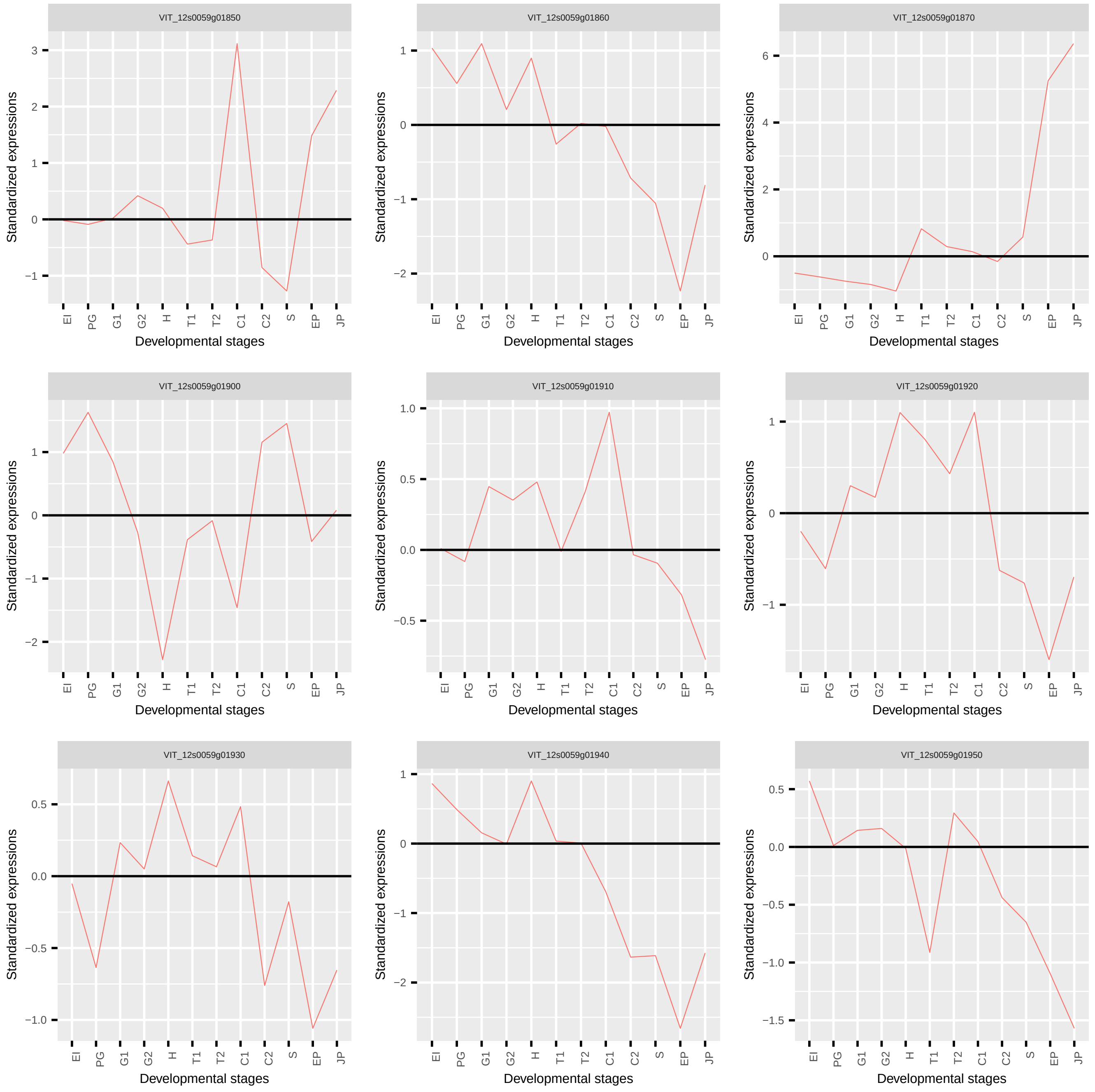
VIT_12s0059g01850
VIT_12s0059g01860
VIT_12s0059g01870
VIT_12s0059g01900
VIT_12s0059g01910
VIT_12s0059g01920
VIT_12s0059g01930
VIT_12s0059g01940
VIT_12s0059g01950
Standardized expressions
Developmental stages
EI PG G1 G2 H T1 T2 C1 C2 S EP JP

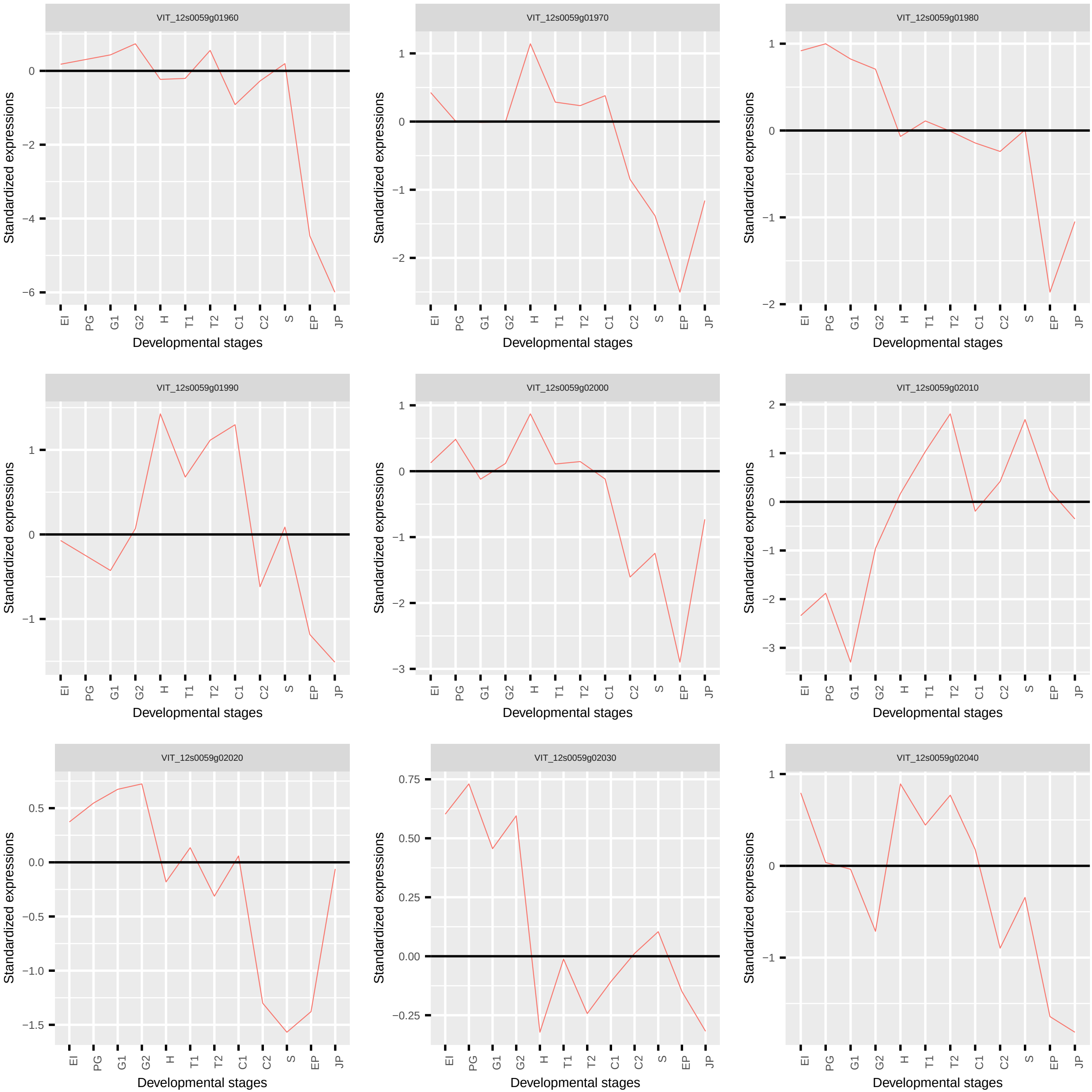
VIT_12s0059g01960
VIT_12s0059g01970
VIT_12s0059g01980
VIT_12s0059g01990
VIT_12s0059g02000
VIT_12s0059g02010
VIT_12s0059g02020
VIT_12s0059g02030
VIT_12s0059g02040
Standardized expressions
Developmental stages
EI PG G1 G2 H T1 T2 C1 C2 S EP JP

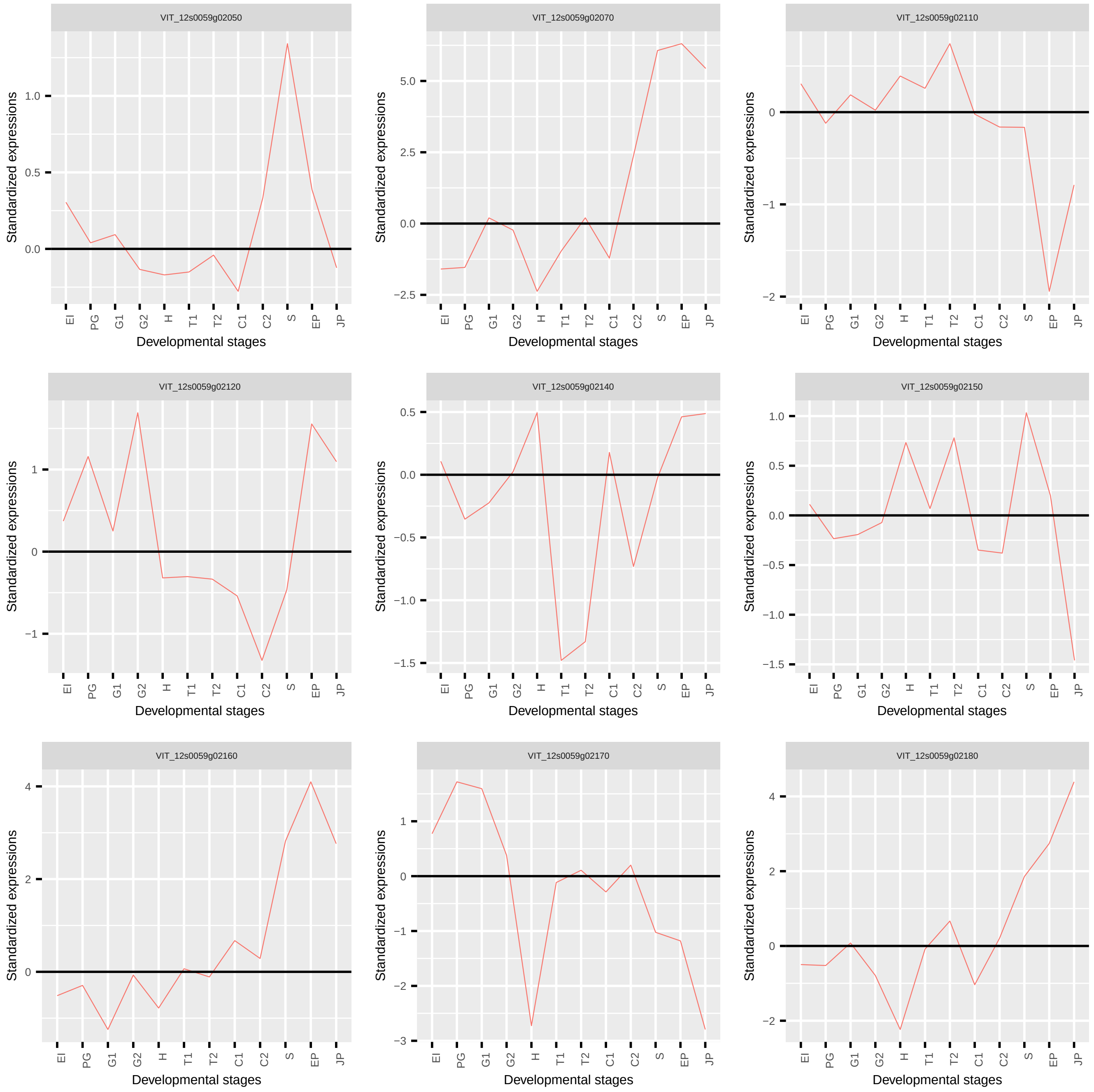

VIT_12s0059g02050
VIT_12s0059g02070
VIT_12s0059g02110
VIT_12s0059g02120
VIT_12s0059g02140
VIT_12s0059g02150
VIT_12s0059g02160
VIT_12s0059g02170
VIT_12s0059g02180
Standardized expressions
Developmental stages
EI
PG
G1
G2
H
T1
T2
C1
C2
S
EP
JP

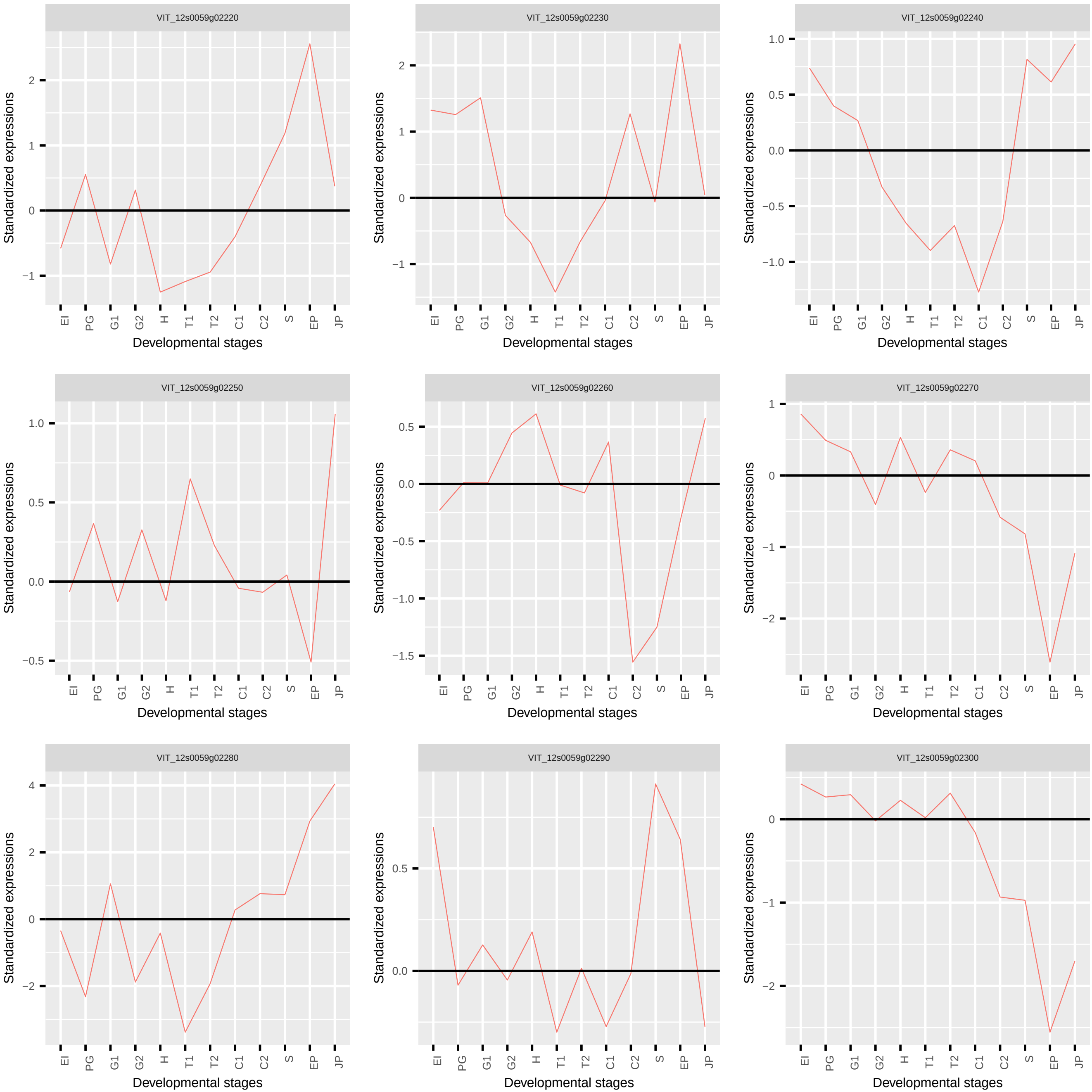
VIT_12s0059g02220
VIT_12s0059g02230
VIT_12s0059g02240
VIT_12s0059g02250
VIT_12s0059g02260
VIT_12s0059g02270
VIT_12s0059g02280
VIT_12s0059g02290
VIT_12s0059g02300
Standardized expressions
Developmental stages
EI PG G1 G2 H T1 T2 C1 C2 S EP JP

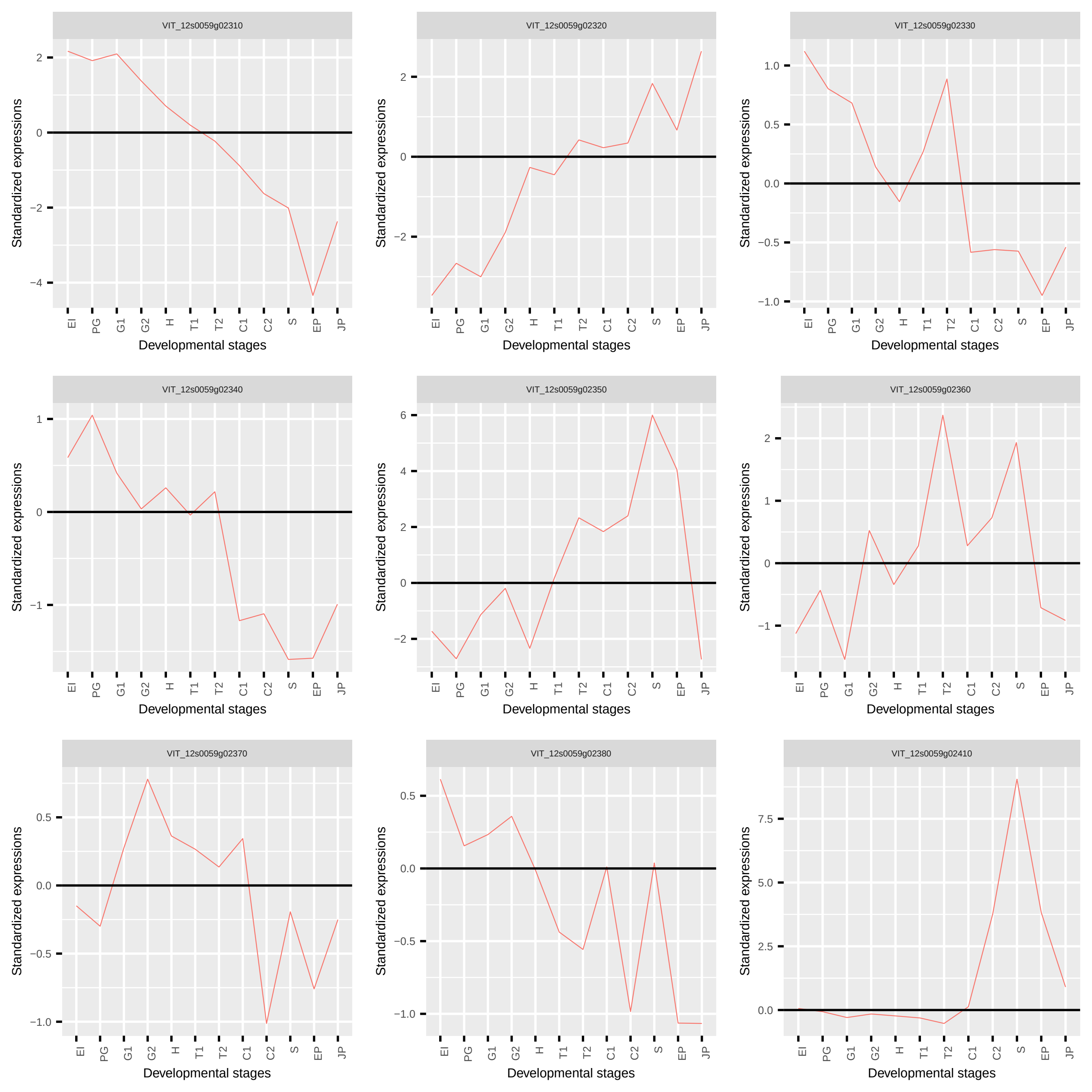
VIT_12s0059g02310
VIT_12s0059g02320
VIT_12s0059g02330
VIT_12s0059g02340
VIT_12s0059g02350
VIT_12s0059g02360
VIT_12s0059g02370
VIT_12s0059g02380
VIT_12s0059g02410
Standardized expressions
Developmental stages
EI PG G1 G2 H T1 T2 C1 C2 S EP JP

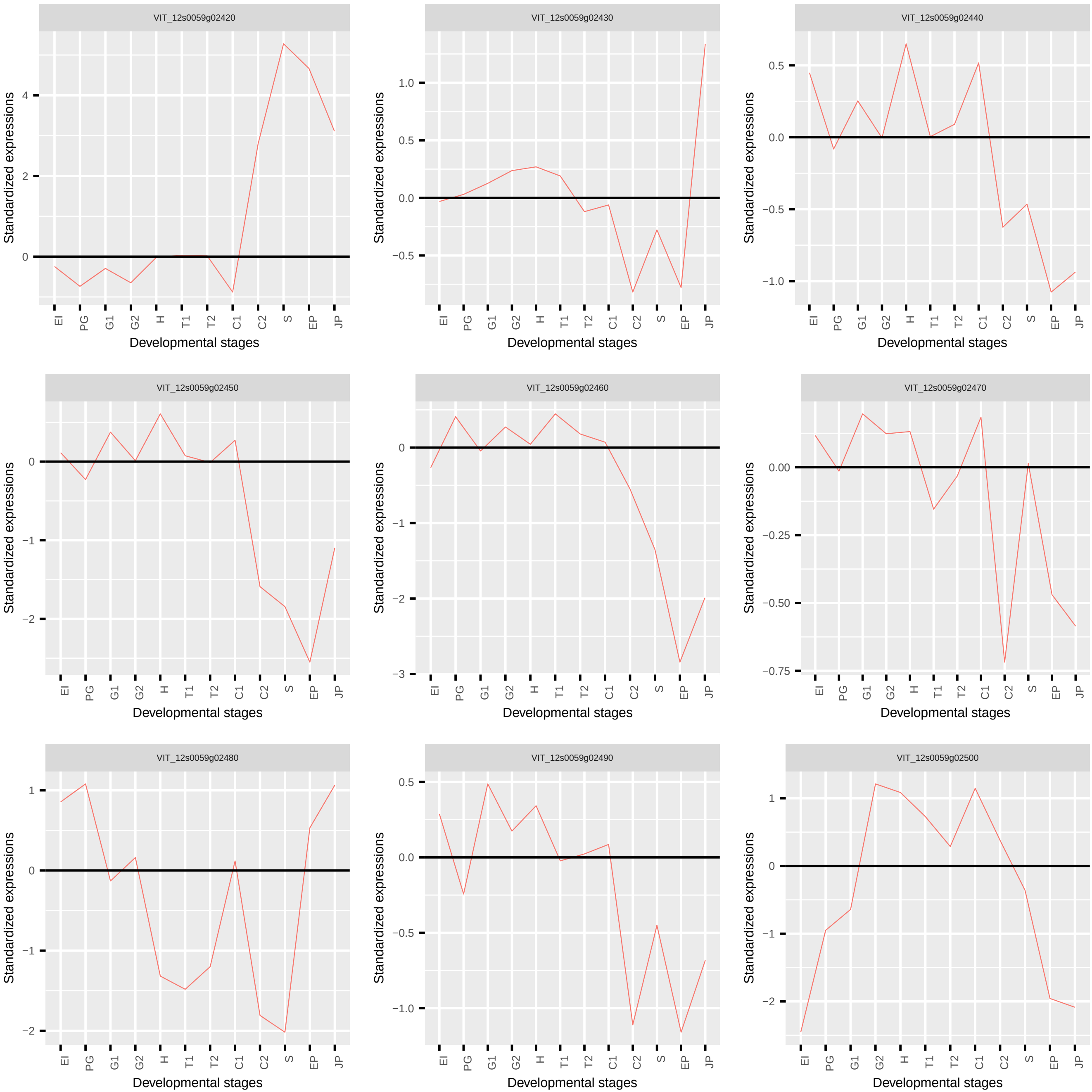
VIT_12s0059g02420
VIT_12s0059g02430
VIT_12s0059g02440
VIT_12s0059g02450
VIT_12s0059g02460
VIT_12s0059g02470
VIT_12s0059g02480
VIT_12s0059g02490
VIT_12s0059g02500
Standardized expressions
Developmental stages
EI PG G1 G2 H T1 T2 C1 C2 S EP JP

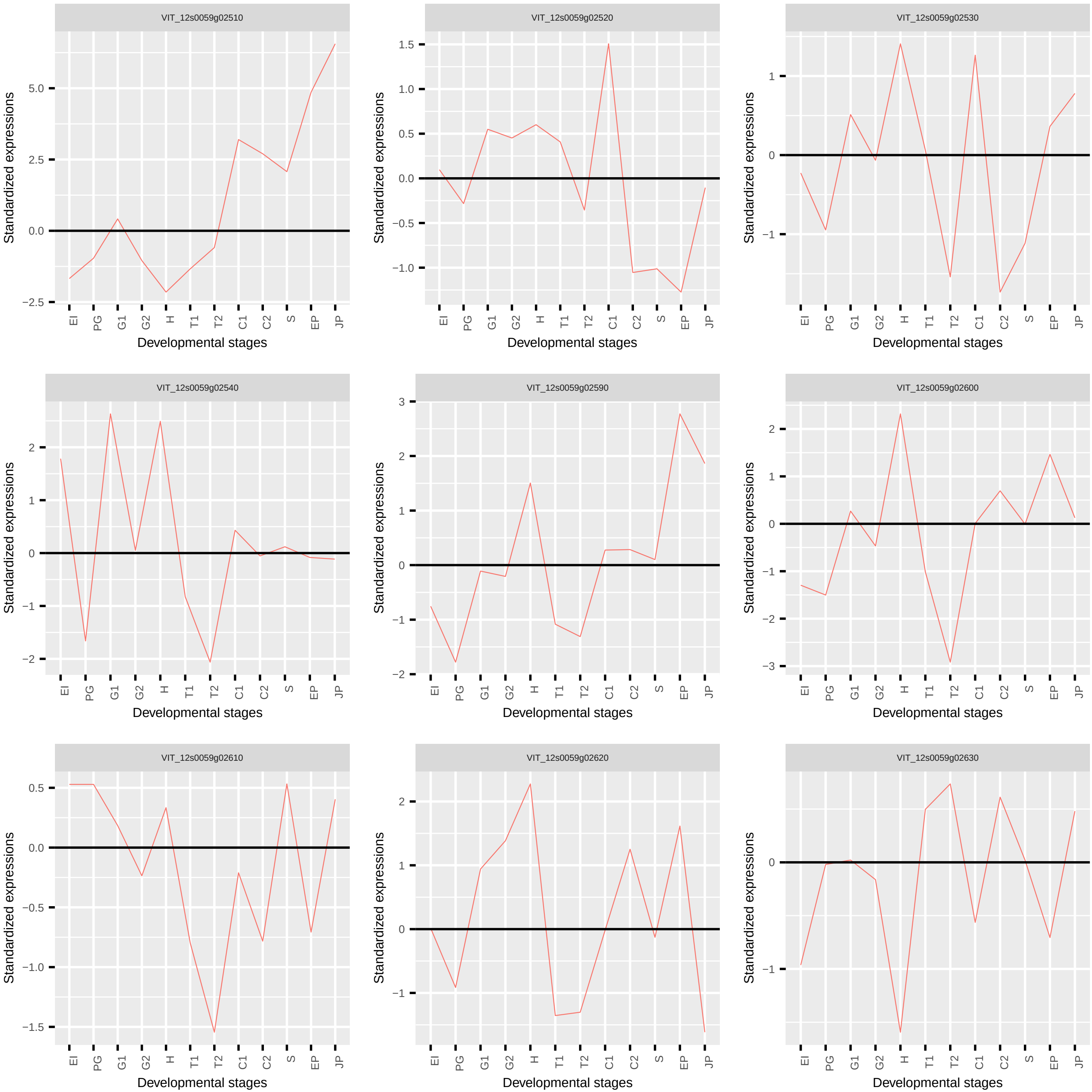
VIT_12s0059g02510
VIT_12s0059g02520
VIT_12s0059g02530
VIT_12s0059g02540
VIT_12s0059g02590
VIT_12s0059g02600
VIT_12s0059g02610
VIT_12s0059g02620
VIT_12s0059g02630
Standardized expressions
Developmental stages
EI PG G1 G2 H T1 T2 C1 C2 S EP JP
5.0
2.5
0.0
−2.5
1.5
1.0
0.5
−0.5
−1.0
−1.5
3
2
1
0
−1
−2
−3

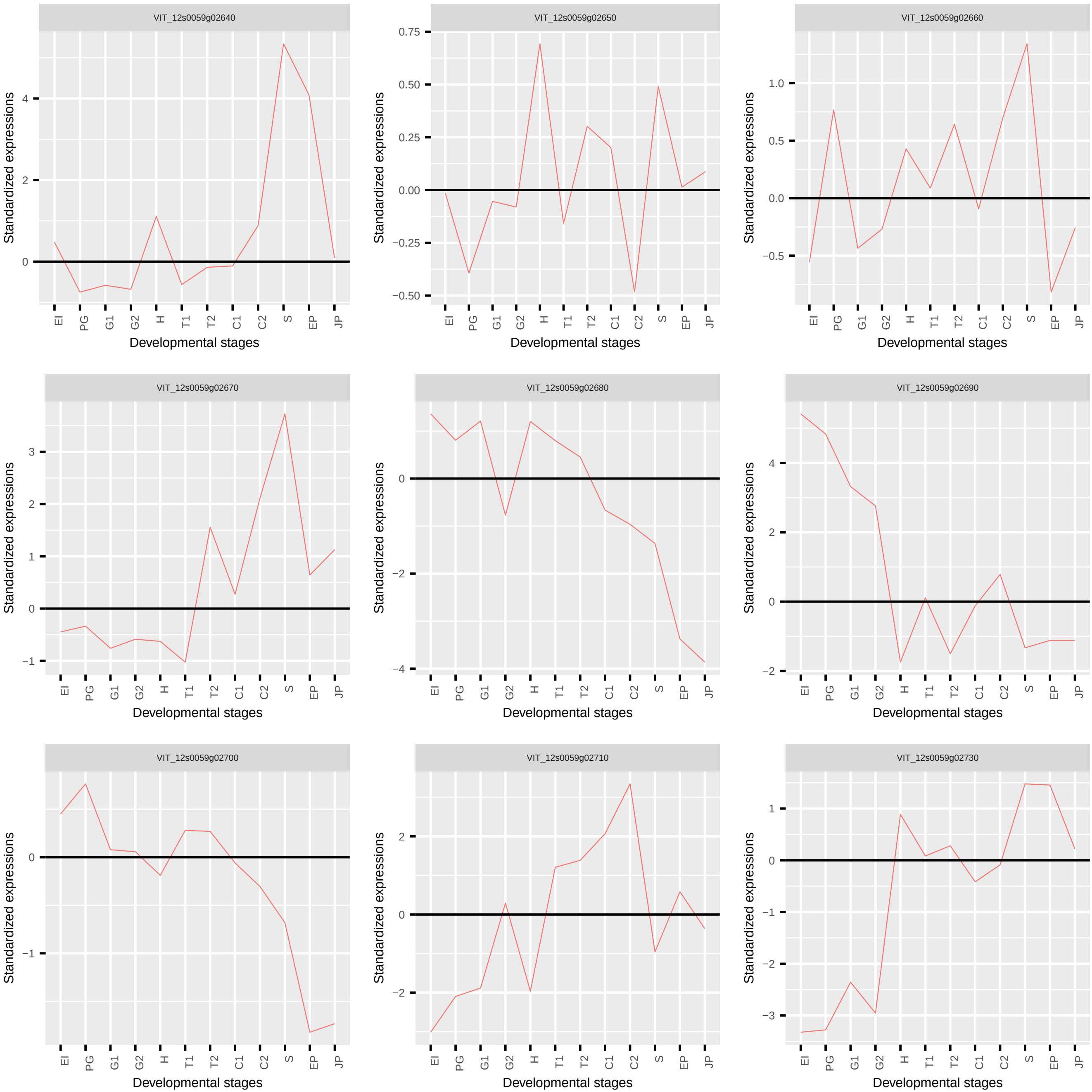

VIT_12s0059g02640
VIT_12s0059g02650
VIT_12s0059g02660
VIT_12s0059g02670
VIT_12s0059g02680
VIT_12s0059g02690
VIT_12s0059g02700
VIT_12s0059g02710
VIT_12s0059g02730
Standardized expressions
Developmental stages
EI PG G1 G2 H T1 T2 C1 C2 S EP JP

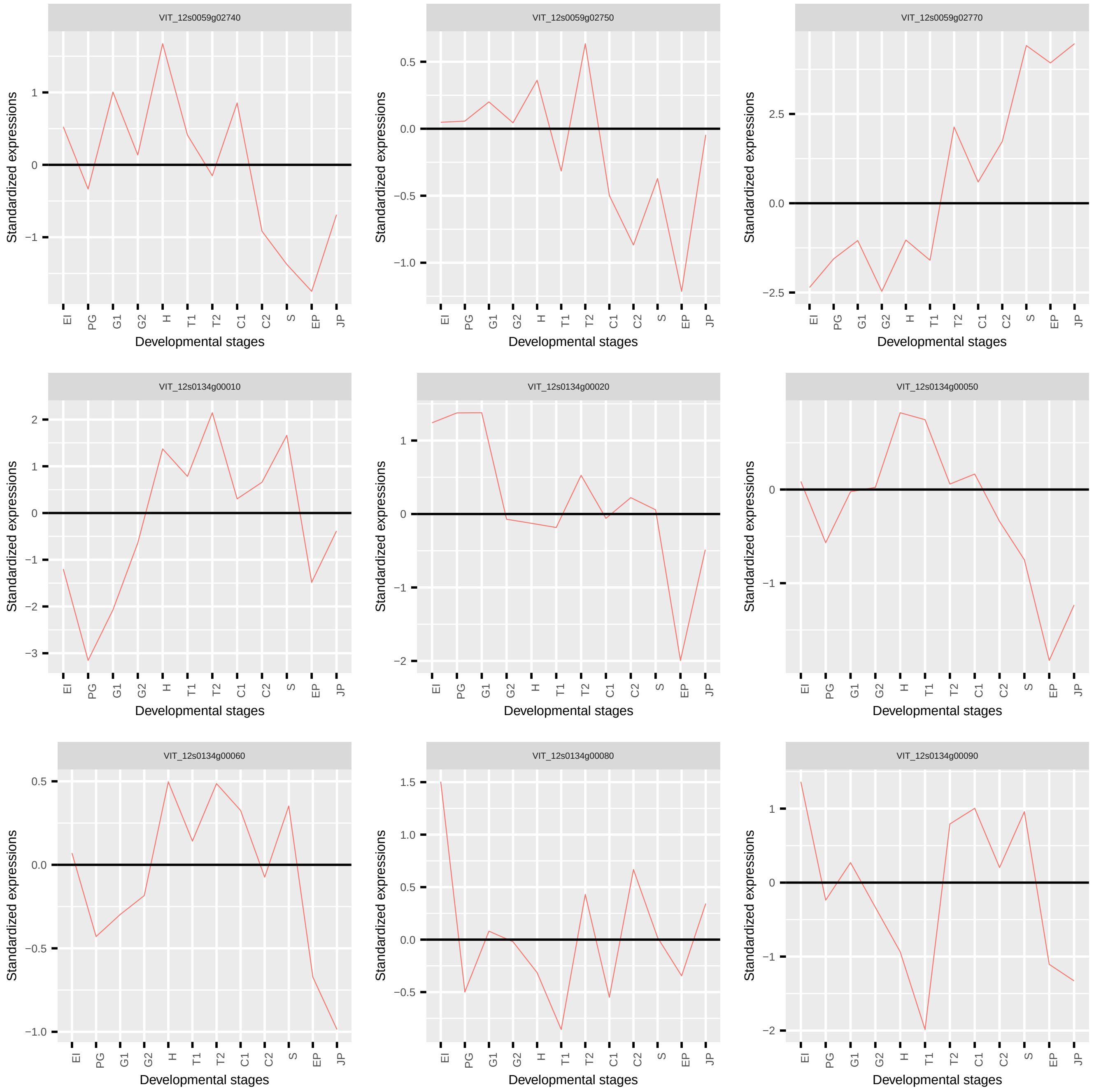
VIT_12s0059g02740
VIT_12s0059g02750
VIT_12s0059g02770
VIT_12s0134g00010
VIT_12s0134g00020
VIT_12s0134g00050
VIT_12s0134g00060
VIT_12s0134g00080
VIT_12s0134g00090
Standardized expressions
Developmental stages
EI PG G1 G2 H T1 T2 C1 C2 S EP JP

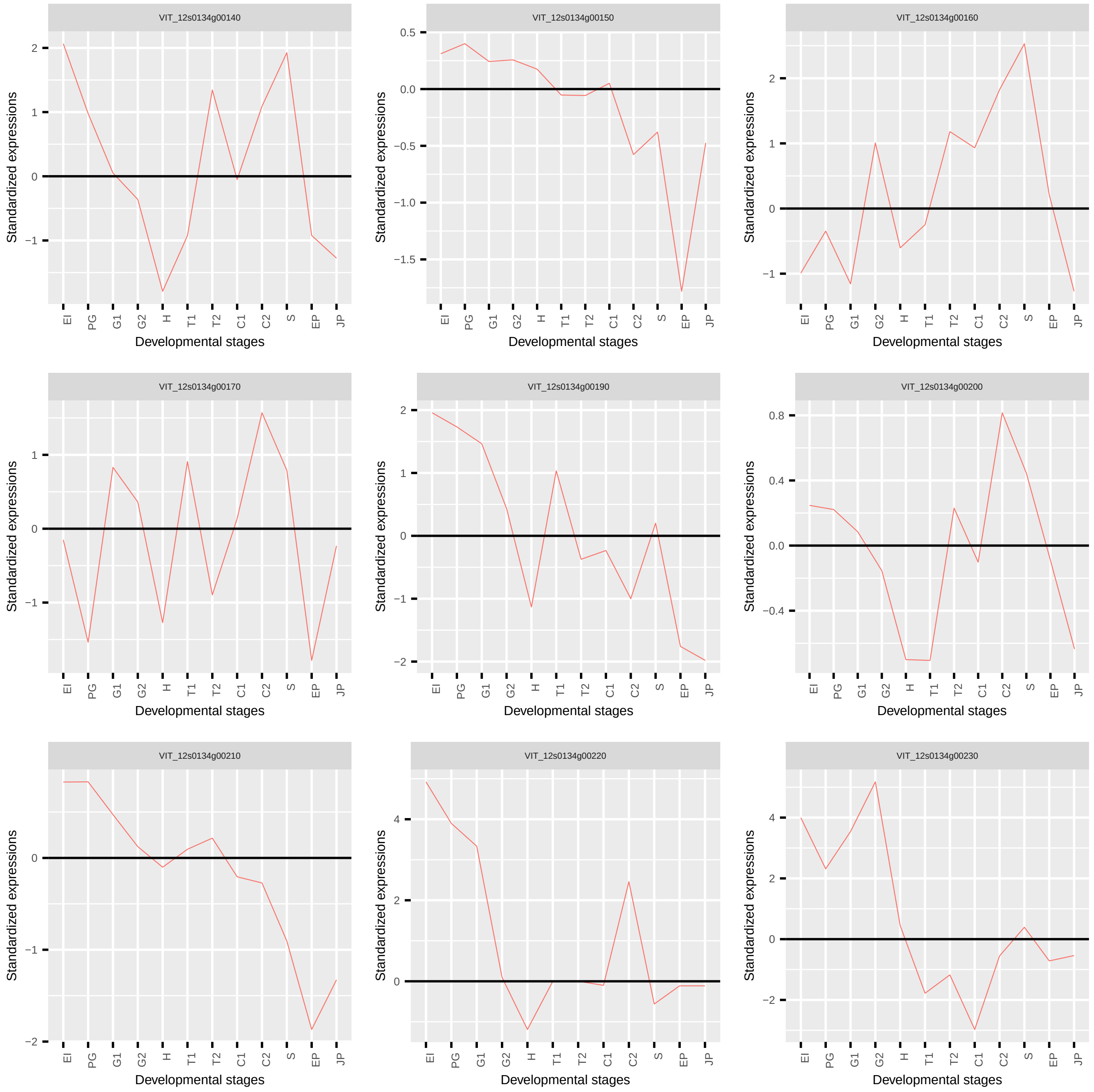
VIT_12s0134g00140
VIT_12s0134g00150
VIT_12s0134g00160
VIT_12s0134g00170
VIT_12s0134g00190
VIT_12s0134g00200
VIT_12s0134g00210
VIT_12s0134g00220
VIT_12s0134g00230
Standardized expressions
Developmental stages
EI
PG
G1
G2
H
T1
T2
C1
C2
S
EP
JP

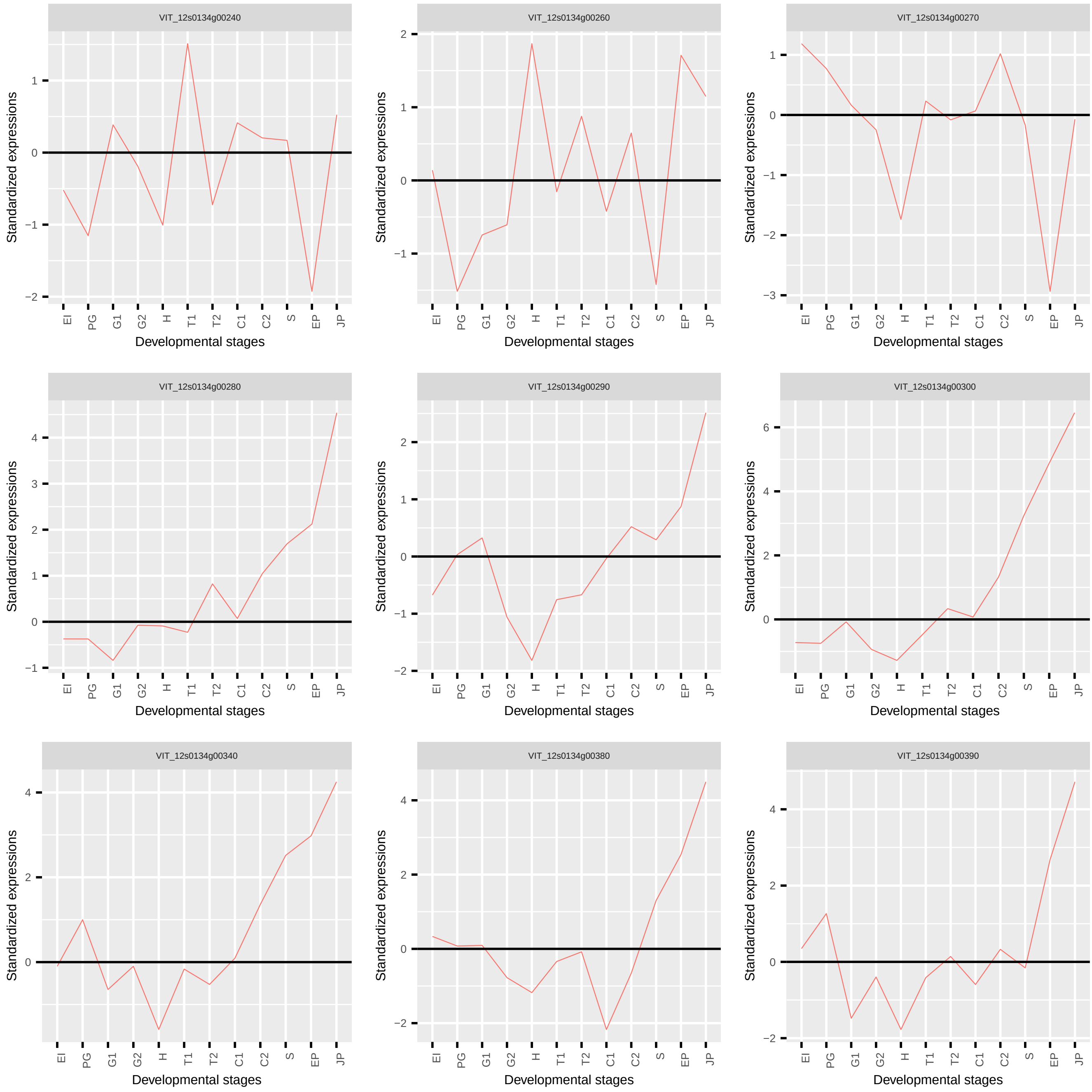
VIT_12s0134g00240
VIT_12s0134g00260
VIT_12s0134g00270
VIT_12s0134g00280
VIT_12s0134g00290
VIT_12s0134g00300
VIT_12s0134g00340
VIT_12s0134g00380
VIT_12s0134g00390
Standardized expressions
Developmental stages
EI
PG
G1
G2
H
T1
T2
C1
C2
S
EP
JP

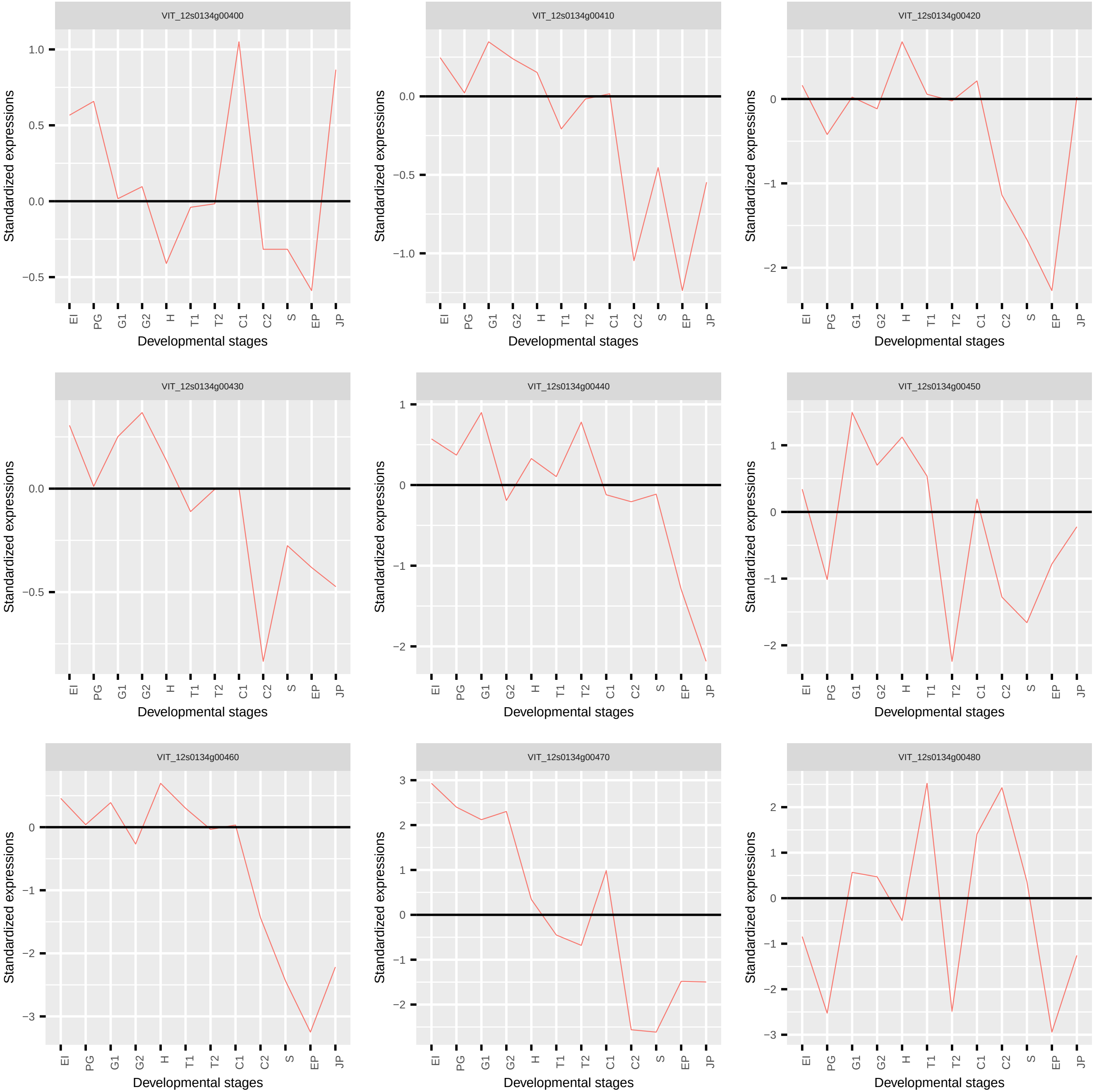
VIT_12s0134g00400
VIT_12s0134g00410
VIT_12s0134g00420
VIT_12s0134g00430
VIT_12s0134g00440
VIT_12s0134g00450
VIT_12s0134g00460
VIT_12s0134g00470
VIT_12s0134g00480
Standardized expressions
Developmental stages
EI PG G1 G2 H T1 T2 C1 C2 S EP JP

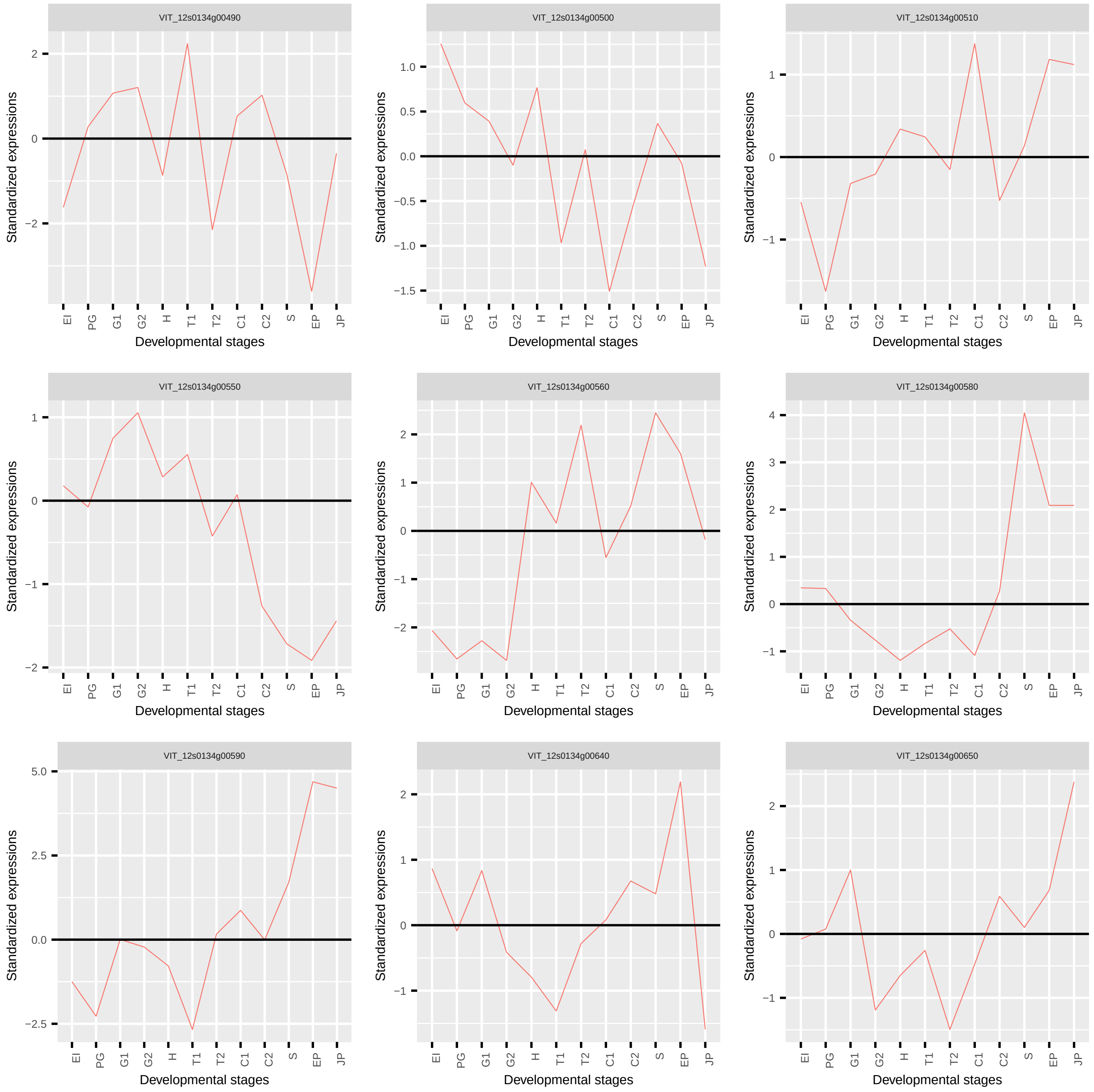
VIT_12s0134g00490
VIT_12s0134g00500
VIT_12s0134g00510
VIT_12s0134g00550
VIT_12s0134g00560
VIT_12s0134g00580
VIT_12s0134g00590
VIT_12s0134g00640
VIT_12s0134g00650
Standardized expressions
Developmental stages
EI
PG
G1
G2
H
T1
T2
C1
C2
S
EP
JP

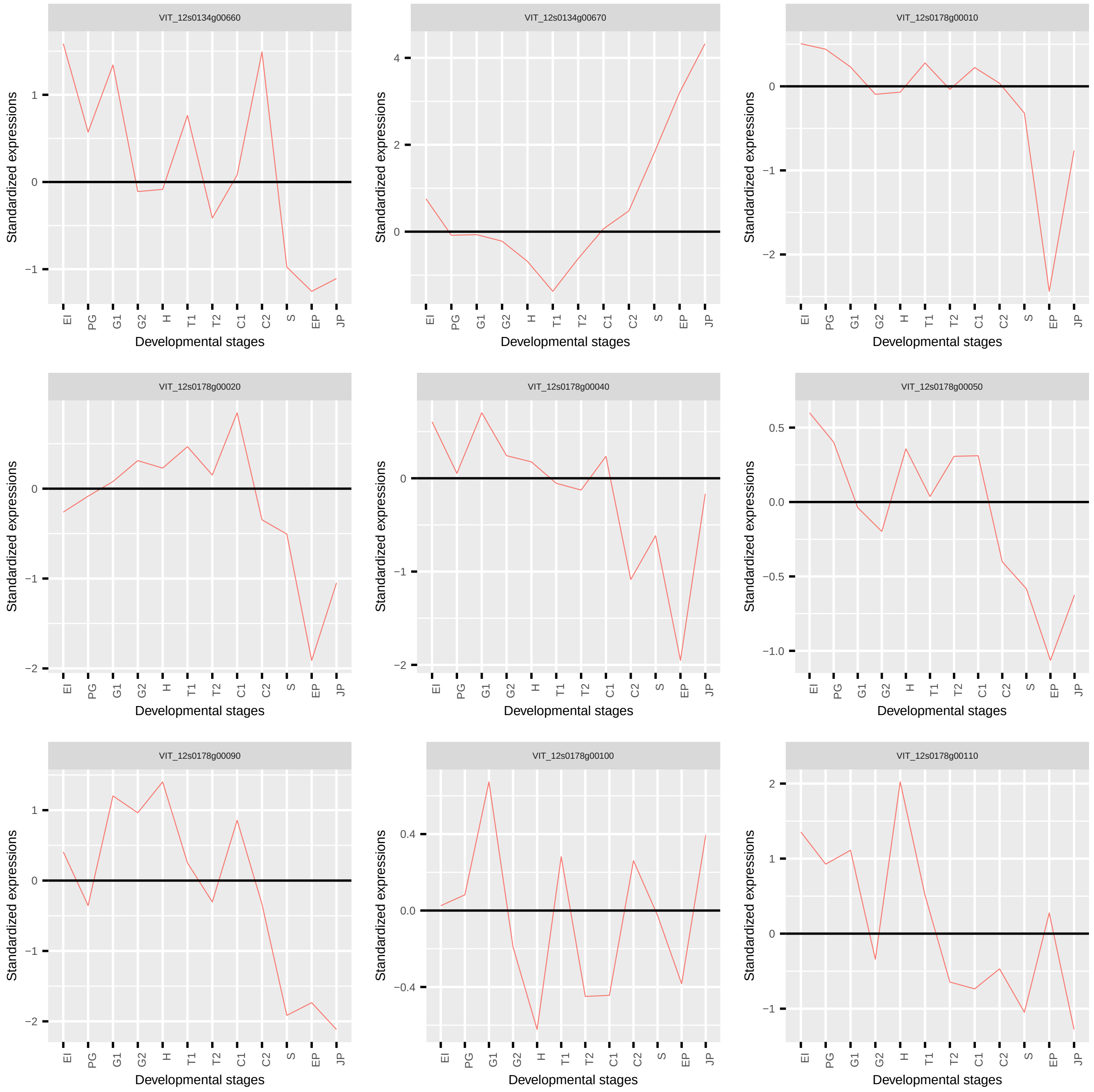
VIT_12s0134g00660
VIT_12s0134g00670
VIT_12s0178g00010
VIT_12s0178g00020
VIT_12s0178g00040
VIT_12s0178g00050
VIT_12s0178g00090
VIT_12s0178g00100
VIT_12s0178g00110
Standardized expressions
Developmental stages
EI PG G1 G2 H T1 T2 C1 C2 S EP JP

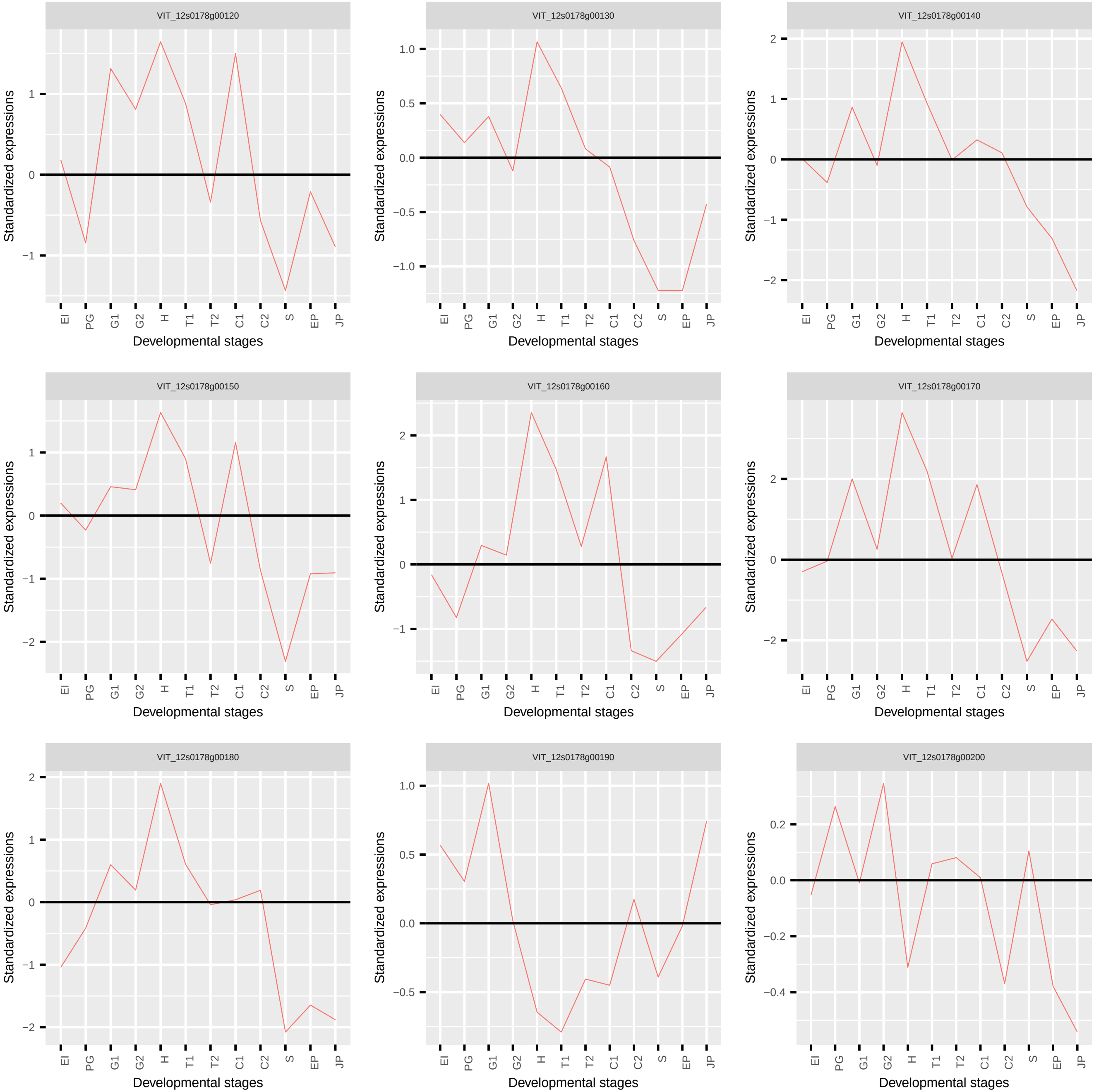
VIT_12s0178g00120
VIT_12s0178g00130
VIT_12s0178g00140
VIT_12s0178g00150
VIT_12s0178g00160
VIT_12s0178g00170
VIT_12s0178g00180
VIT_12s0178g00190
VIT_12s0178g00200
Standardized expressions
Developmental stages
EI PG G1 G2 H T1 T2 C1 C2 S EP JP
−1 0 1
−1.0 −0.5 0.0 0.5 1.0
−2 −1 0 1 2
−0.4 −0.2 0.0 0.2

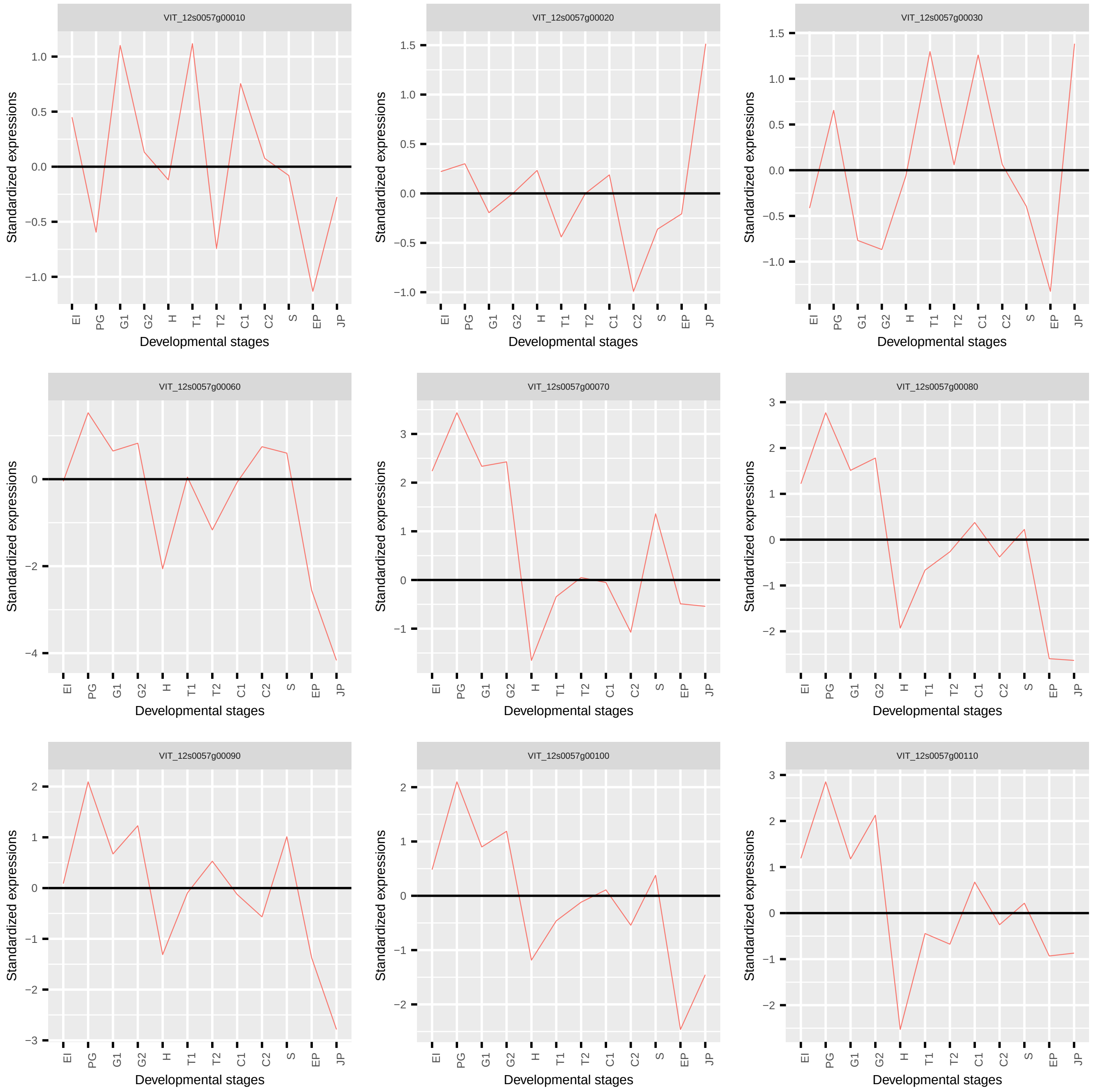

VIT_12s0057g00010
VIT_12s0057g00020
VIT_12s0057g00030
VIT_12s0057g00060
VIT_12s0057g00070
VIT_12s0057g00080
VIT_12s0057g00090
VIT_12s0057g00100
VIT_12s0057g00110
Standardized expressions
Developmental stages
EI PG G1 G2 H T1 T2 C1 C2 S EP JP

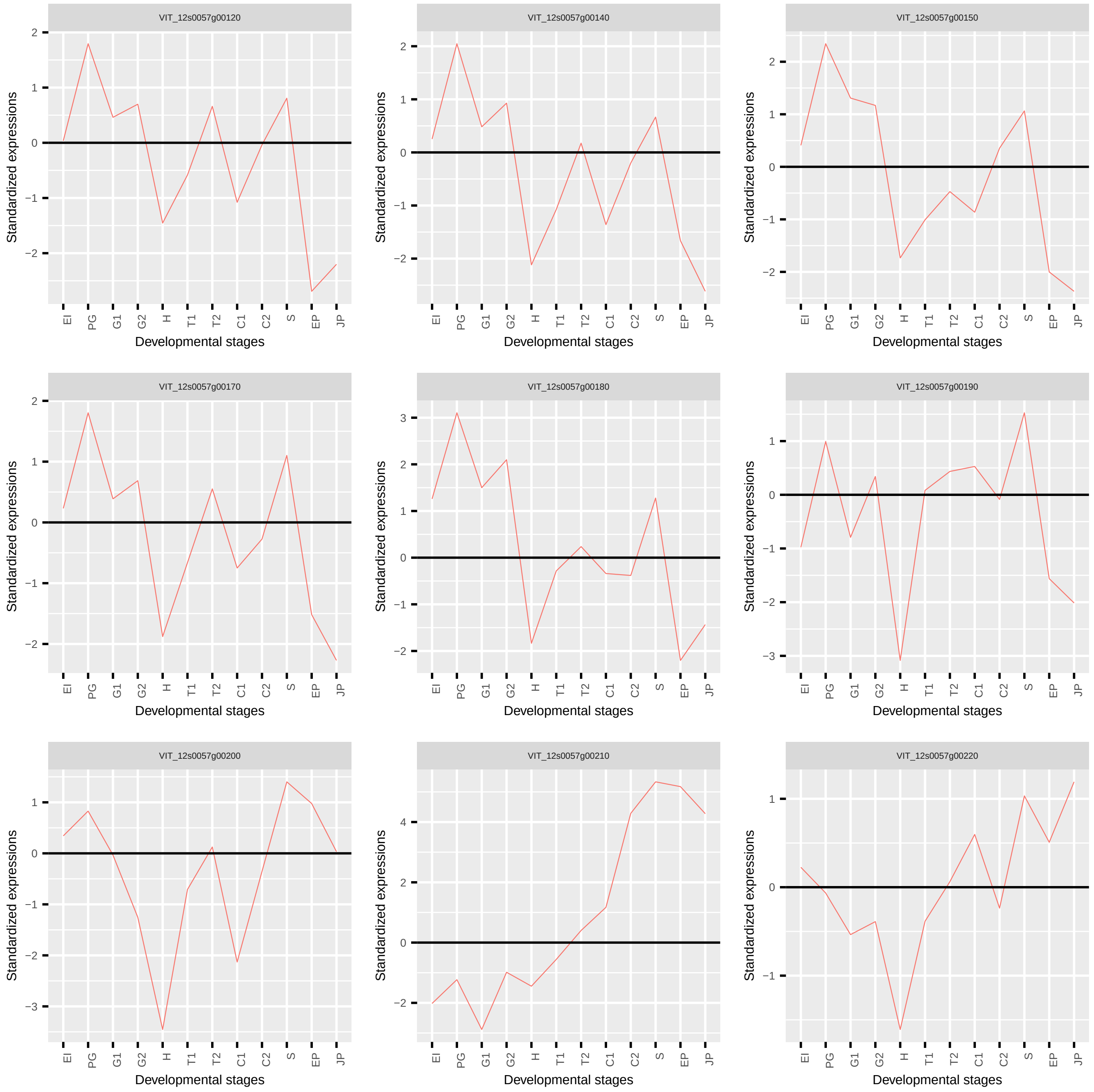

VIT_12s0057g00120
VIT_12s0057g00140
VIT_12s0057g00150
VIT_12s0057g00170
VIT_12s0057g00180
VIT_12s0057g00190
VIT_12s0057g00200
VIT_12s0057g00210
VIT_12s0057g00220
Standardized expressions
Developmental stages
EI
PG
G1
G2
H
T1
T2
C1
C2
S
EP
JP

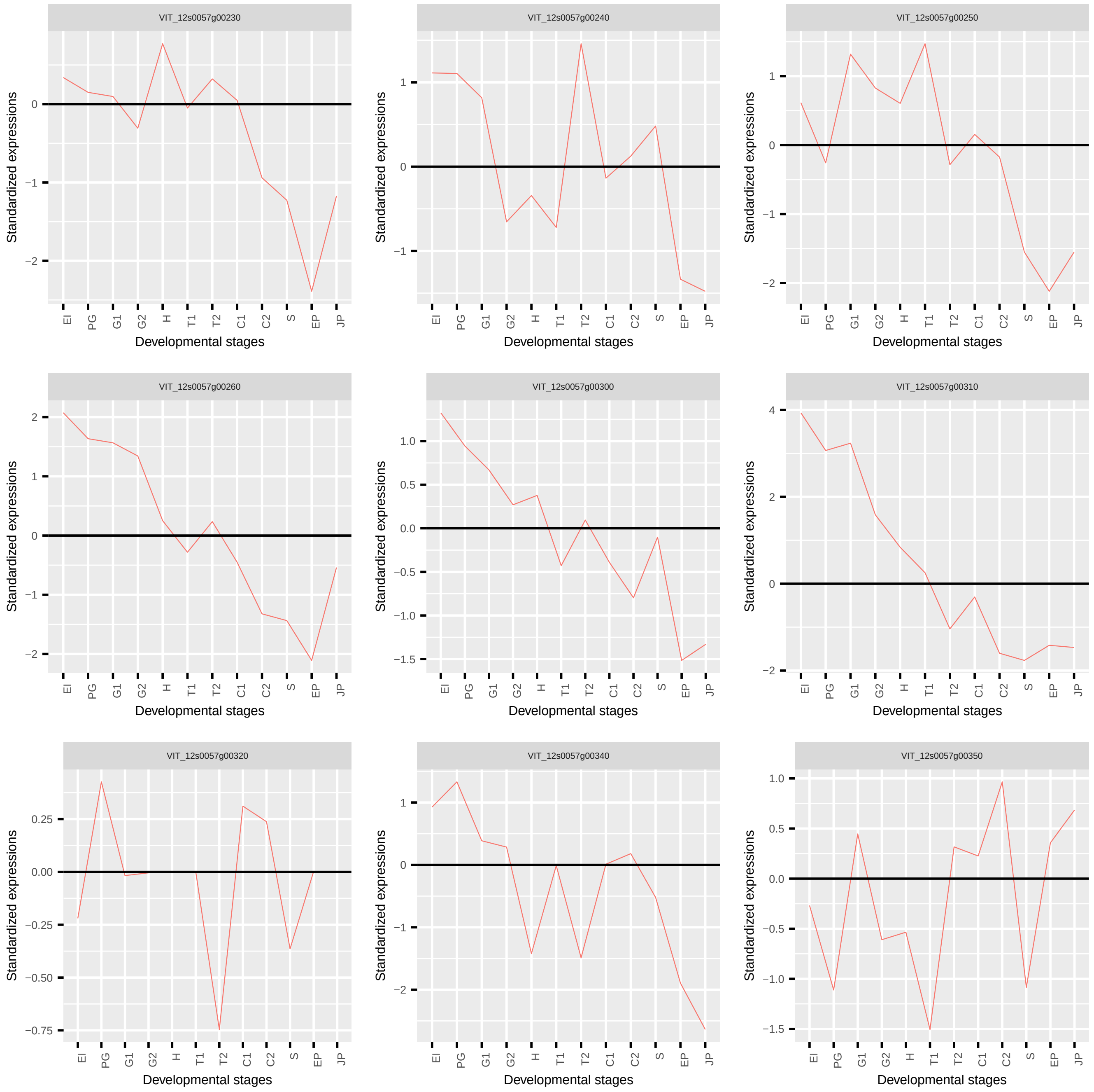
VIT_12s0057g00230
VIT_12s0057g00240
VIT_12s0057g00250
VIT_12s0057g00260
VIT_12s0057g00300
VIT_12s0057g00310
VIT_12s0057g00320
VIT_12s0057g00340
VIT_12s0057g00350
Standardized expressions
Developmental stages
EI PG G1 G2 H T1 T2 C1 C2 S EP JP

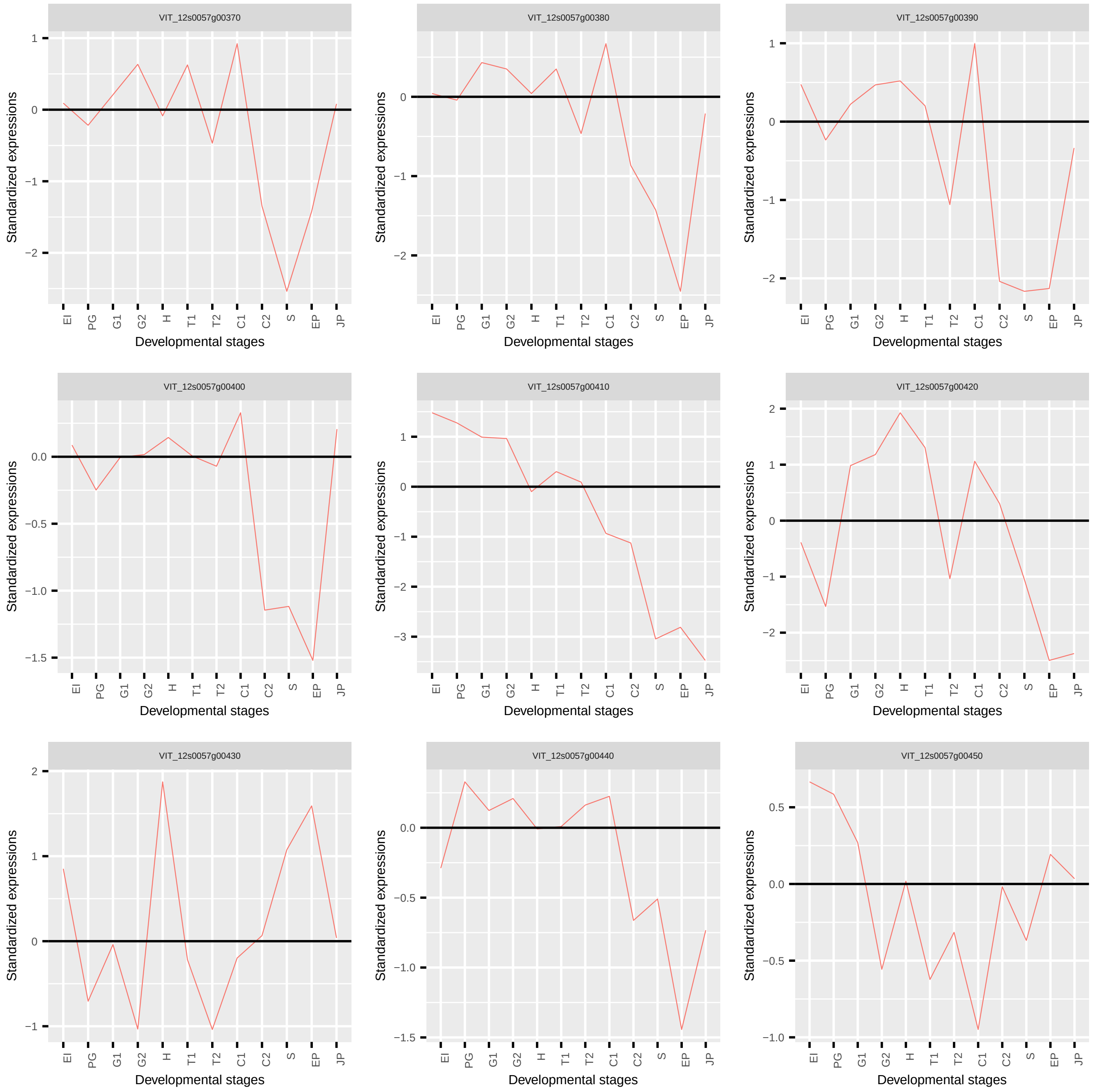

VIT_12s0057g00370
1
0
−1
−2
Standardized expressions
EI PG G1 G2 H T1 T2 C1 C2 S EP JP
Developmental stages
VIT_12s0057g00380
0
−1
−2
Standardized expressions
EI PG G1 G2 H T1 T2 C1 C2 S EP JP
Developmental stages
VIT_12s0057g00390
1
0
−1
−2
Standardized expressions
EI PG G1 G2 H T1 T2 C1 C2 S EP JP
Developmental stages
VIT_12s0057g00400
0.0
−0.5
−1.0
−1.5
Standardized expressions
EI PG G1 G2 H T1 T2 C1 C2 S EP JP
Developmental stages
VIT_12s0057g00410
1
0
−1
−2
−3
Standardized expressions
EI PG G1 G2 H T1 T2 C1 C2 S EP JP
Developmental stages
VIT_12s0057g00420
2
1
0
−1
−2
Standardized expressions
EI PG G1 G2 H T1 T2 C1 C2 S EP JP
Developmental stages
VIT_12s0057g00430
2
1
0
−1
Standardized expressions
EI PG G1 G2 H T1 T2 C1 C2 S EP JP
Developmental stages
VIT_12s0057g00440
0.0
−0.5
−1.0
−1.5
Standardized expressions
EI PG G1 G2 H T1 T2 C1 C2 S EP JP
Developmental stages
VIT_12s0057g00450
0.5
0.0
−0.5
−1.0
Standardized expressions
EI PG G1 G2 H T1 T2 C1 C2 S EP JP
Developmental stages

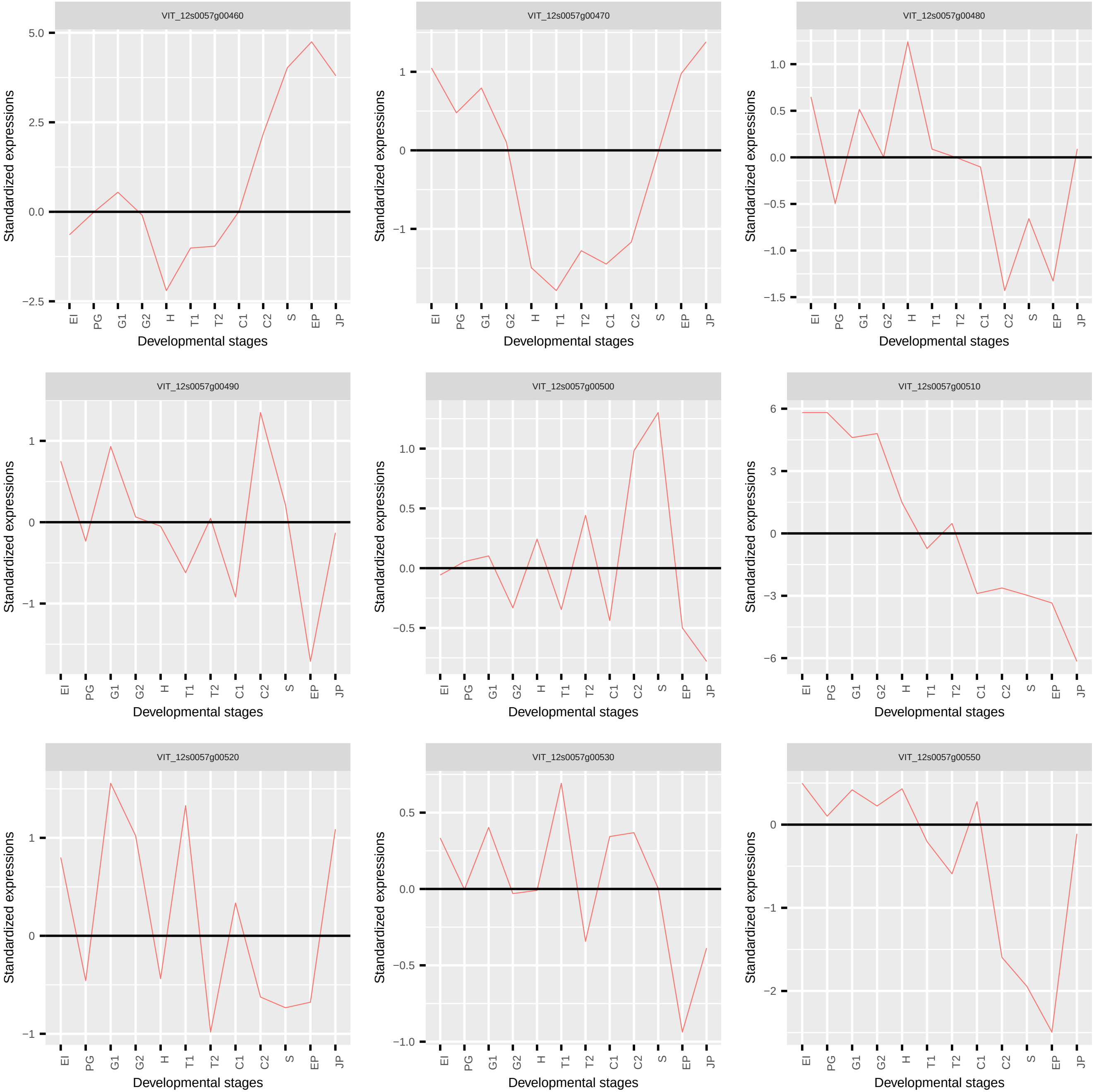
VIT_12s0057g00460
VIT_12s0057g00470
VIT_12s0057g00480
VIT_12s0057g00490
VIT_12s0057g00500
VIT_12s0057g00510
VIT_12s0057g00520
VIT_12s0057g00530
VIT_12s0057g00550
Standardized expressions
Developmental stages
EI
PG
G1
G2
H
T1
T2
C1
C2
S
EP
JP
5.0
2.5
0.0
−2.5
1
0
−1
1.0
0.5
0.0
−0.5
−1.0
−1.5
6
3
0
−3
−6
−2

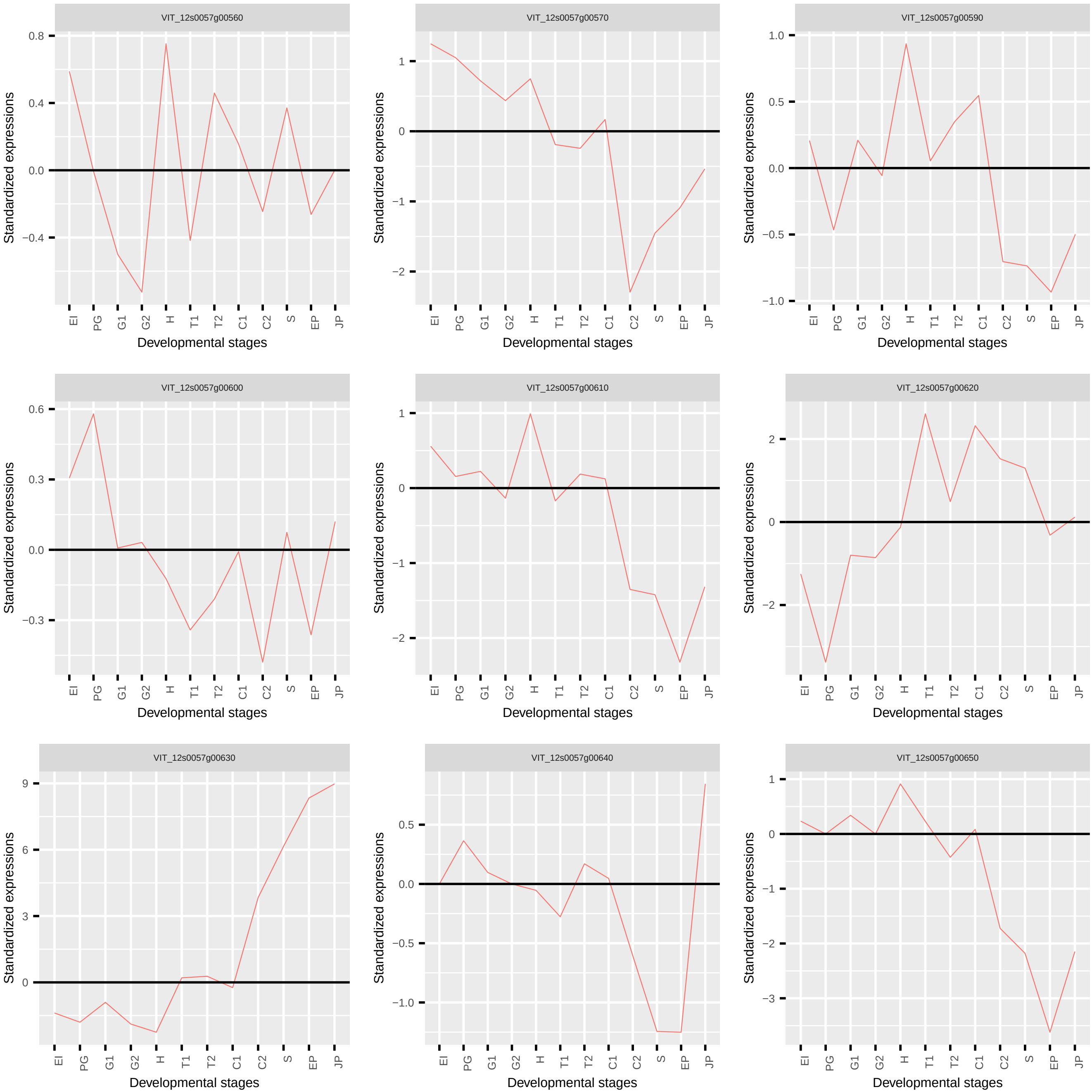

VIT_12s0057g00560
VIT_12s0057g00570
VIT_12s0057g00590
VIT_12s0057g00600
VIT_12s0057g00610
VIT_12s0057g00620
VIT_12s0057g00630
VIT_12s0057g00640
VIT_12s0057g00650
Standardized expressions
Developmental stages
EI
PG
G1
G2
H
T1
T2
C1
C2
S
EP
JP

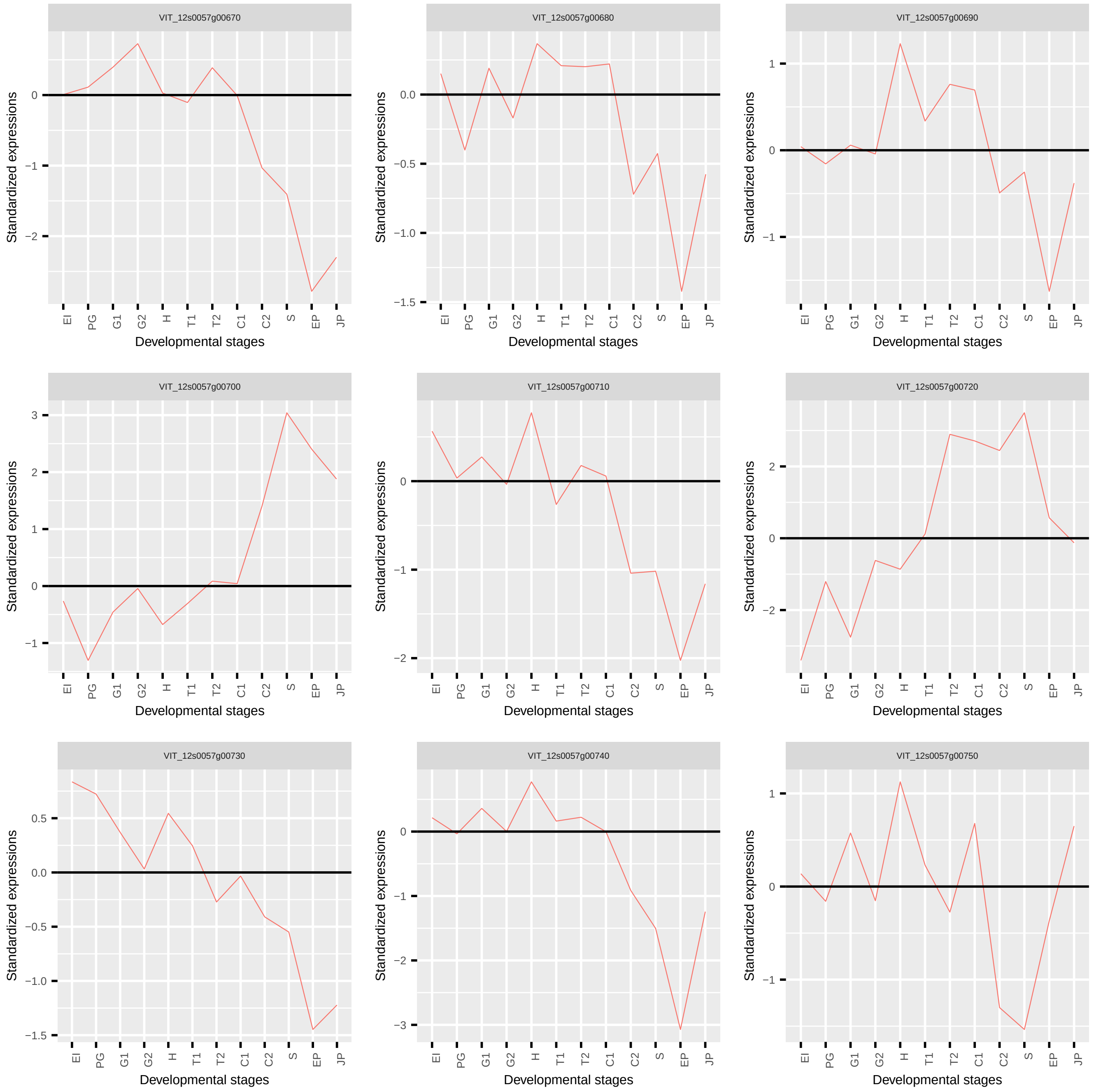
VIT_12s0057g00670
VIT_12s0057g00680
VIT_12s0057g00690
VIT_12s0057g00700
VIT_12s0057g00710
VIT_12s0057g00720
VIT_12s0057g00730
VIT_12s0057g00740
VIT_12s0057g00750
Standardized expressions
Developmental stages
EI PG G1 G2 H T1 T2 C1 C2 S EP JP

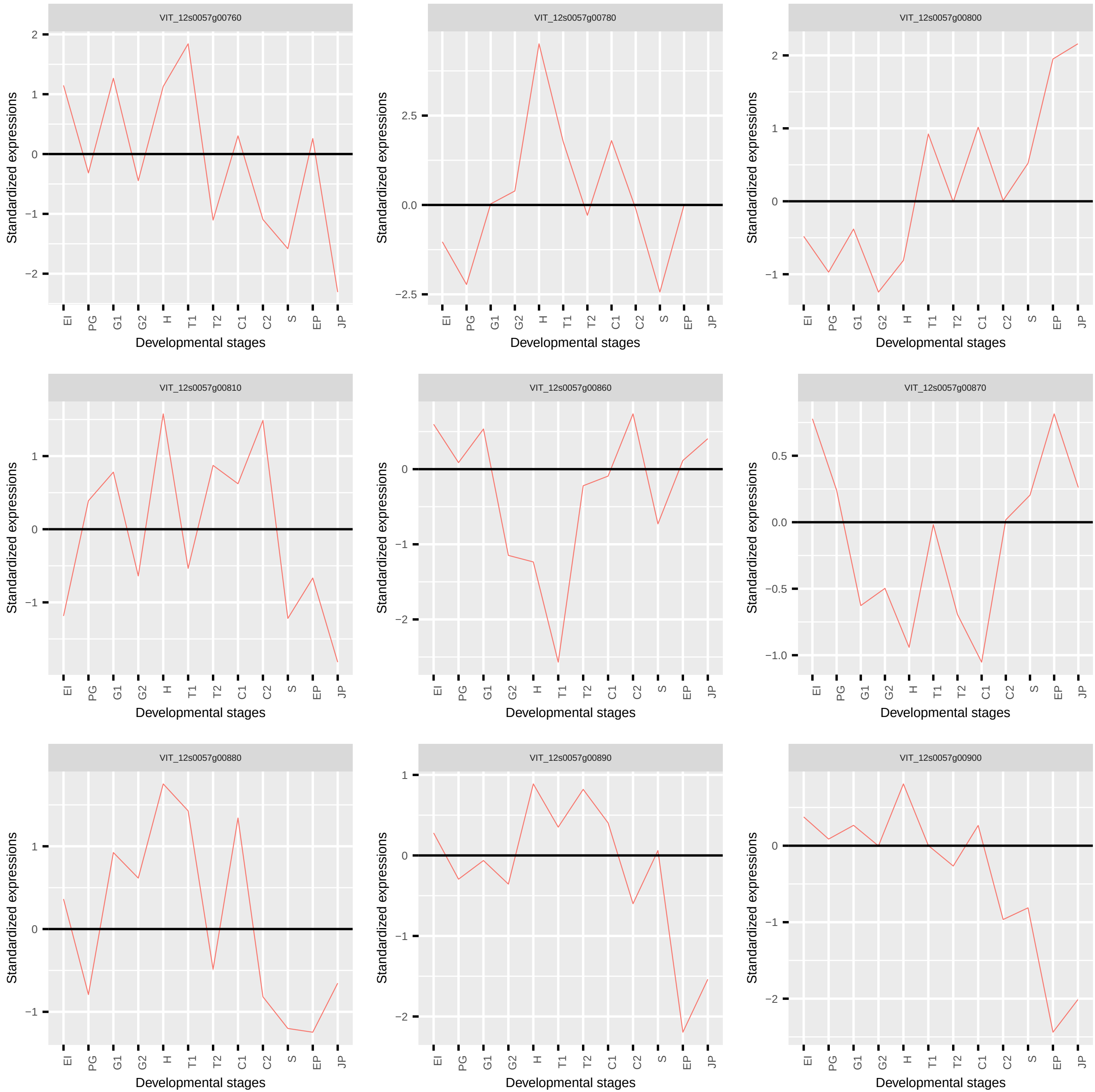
VIT_12s0057g00760
VIT_12s0057g00780
VIT_12s0057g00800
VIT_12s0057g00810
VIT_12s0057g00860
VIT_12s0057g00870
VIT_12s0057g00880
VIT_12s0057g00890
VIT_12s0057g00900
Standardized expressions
Developmental stages
EI PG G1 G2 H T1 T2 C1 C2 S EP JP

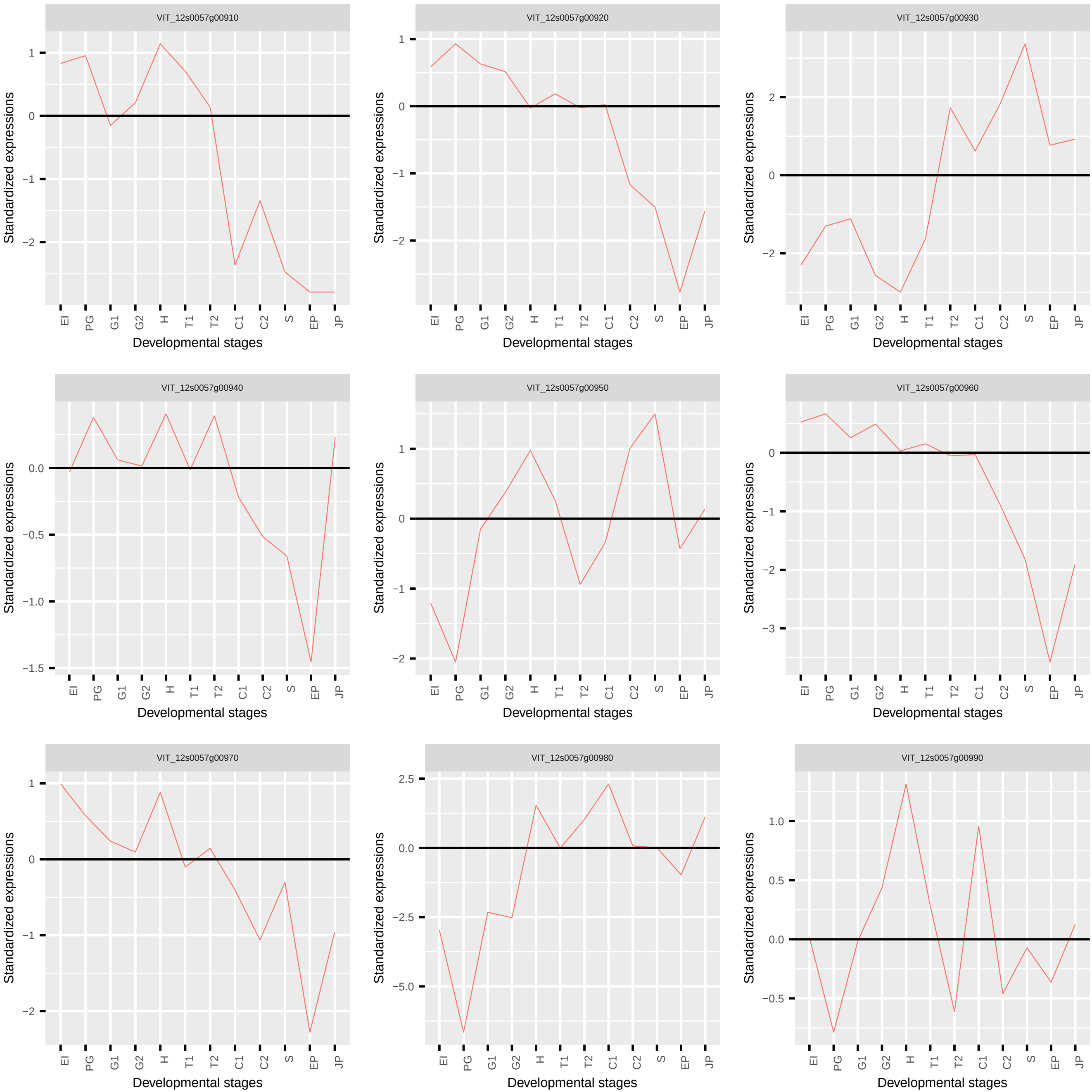
VIT_12s0057g00910
VIT_12s0057g00920
VIT_12s0057g00930
VIT_12s0057g00940
VIT_12s0057g00950
VIT_12s0057g00960
VIT_12s0057g00970
VIT_12s0057g00980
VIT_12s0057g00990
Standardized expressions
Developmental stages
EI
PG
G1
G2
H
T1
T2
C1
C2
S
EP
JP
1
0
−1
−2
2
0.0
−0.5
−1.0
−1.5
−3
2.5
−2.5
−5.0
1.0
0.5

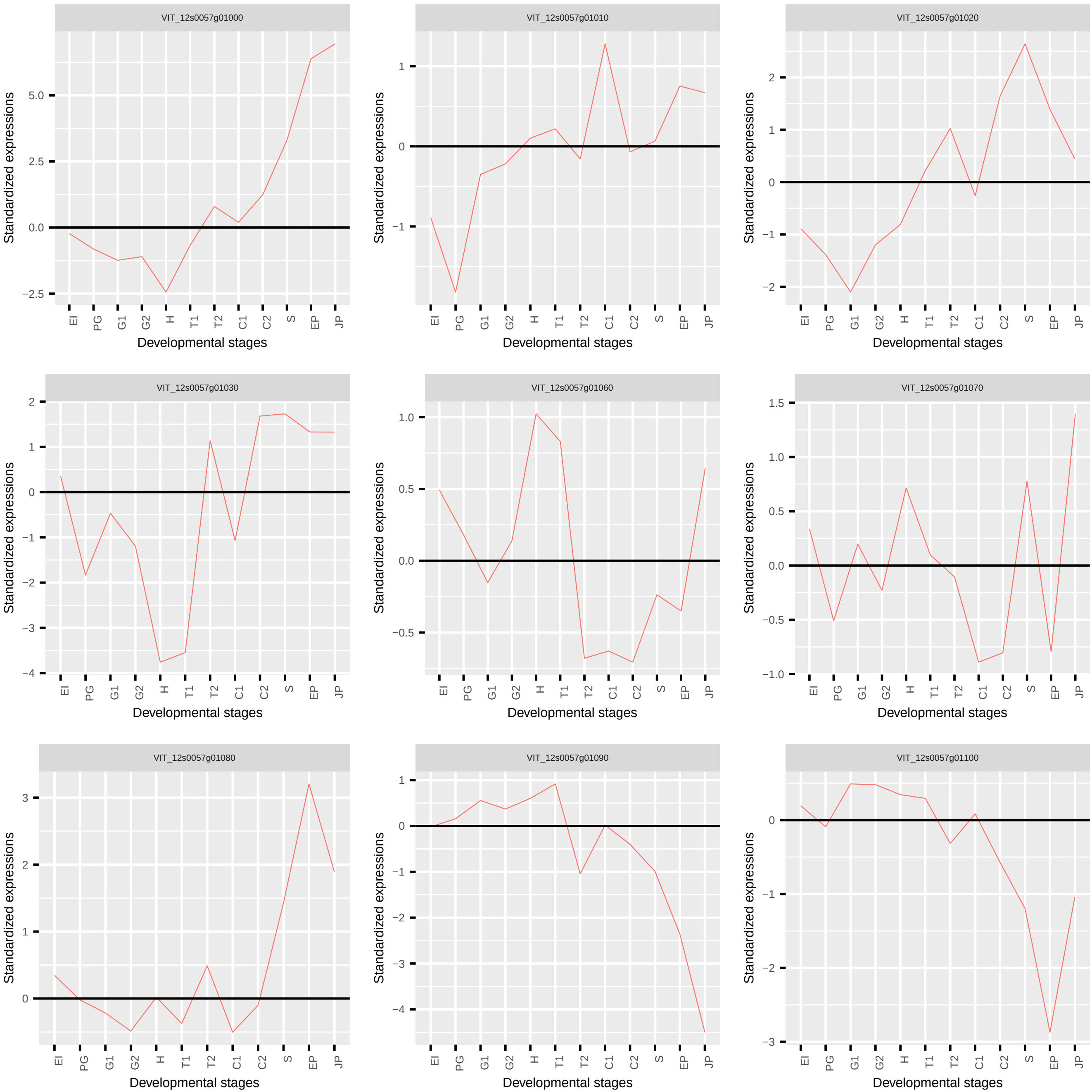
VIT_12s0057g01000
VIT_12s0057g01010
VIT_12s0057g01020
VIT_12s0057g01030
VIT_12s0057g01060
VIT_12s0057g01070
VIT_12s0057g01080
VIT_12s0057g01090
VIT_12s0057g01100
Standardized expressions
Developmental stages
EI
PG
G1
G2
H
T1
T2
C1
C2
S
EP
JP

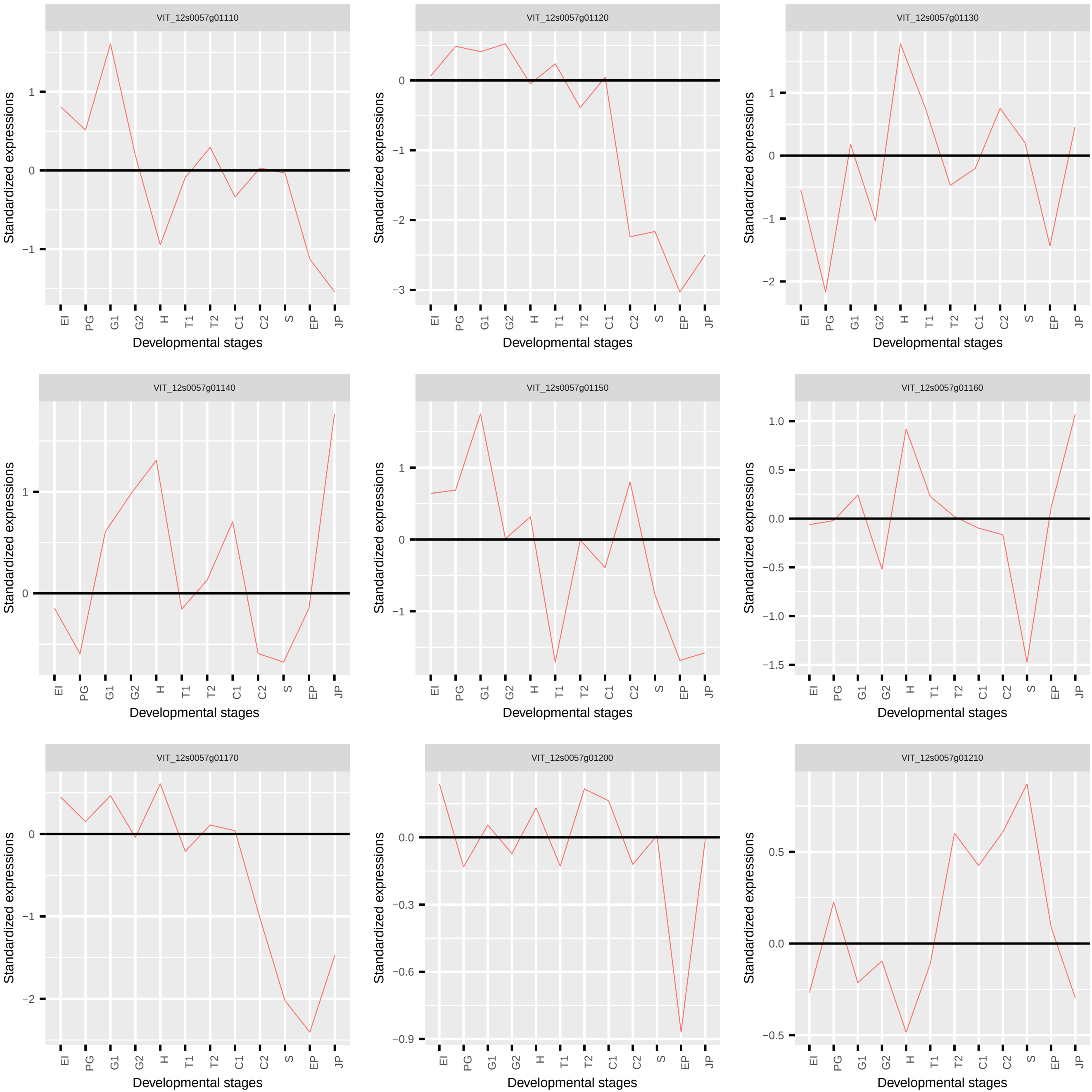
VIT_12s0057g01110
VIT_12s0057g01120
VIT_12s0057g01130
VIT_12s0057g01140
VIT_12s0057g01150
VIT_12s0057g01160
VIT_12s0057g01170
VIT_12s0057g01200
VIT_12s0057g01210
Standardized expressions
Developmental stages
EI PG G1 G2 H T1 T2 C1 C2 S EP JP
1
0
−1
−2
−3
1.0
0.5
0.0
−0.5
−1.0
−1.5
−0.3
−0.6
−0.9

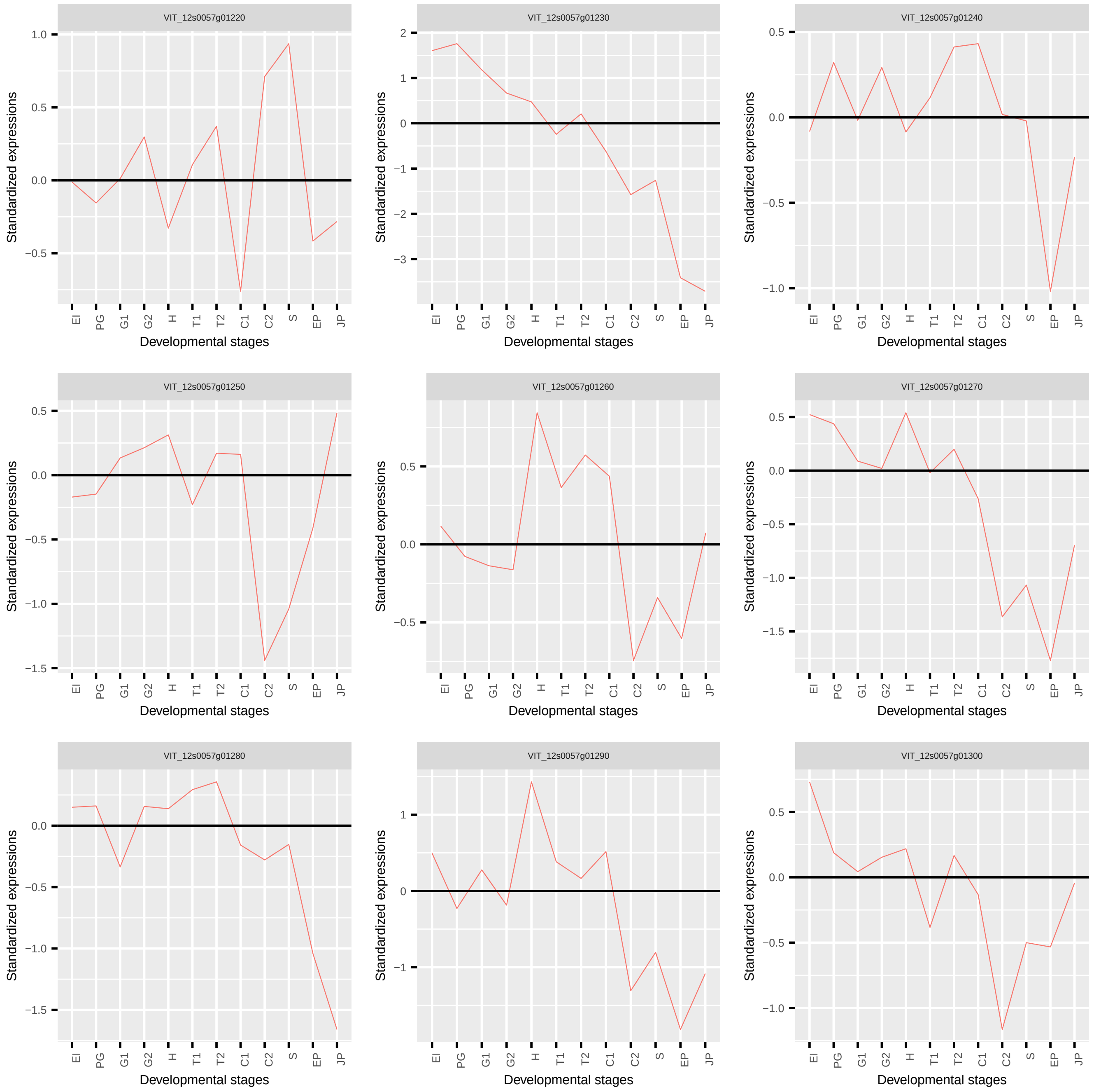
VIT_12s0057g01220
VIT_12s0057g01230
VIT_12s0057g01240
VIT_12s0057g01250
VIT_12s0057g01260
VIT_12s0057g01270
VIT_12s0057g01280
VIT_12s0057g01290
VIT_12s0057g01300
Standardized expressions
Developmental stages
EI PG G1 G2 H T1 T2 C1 C2 S EP JP

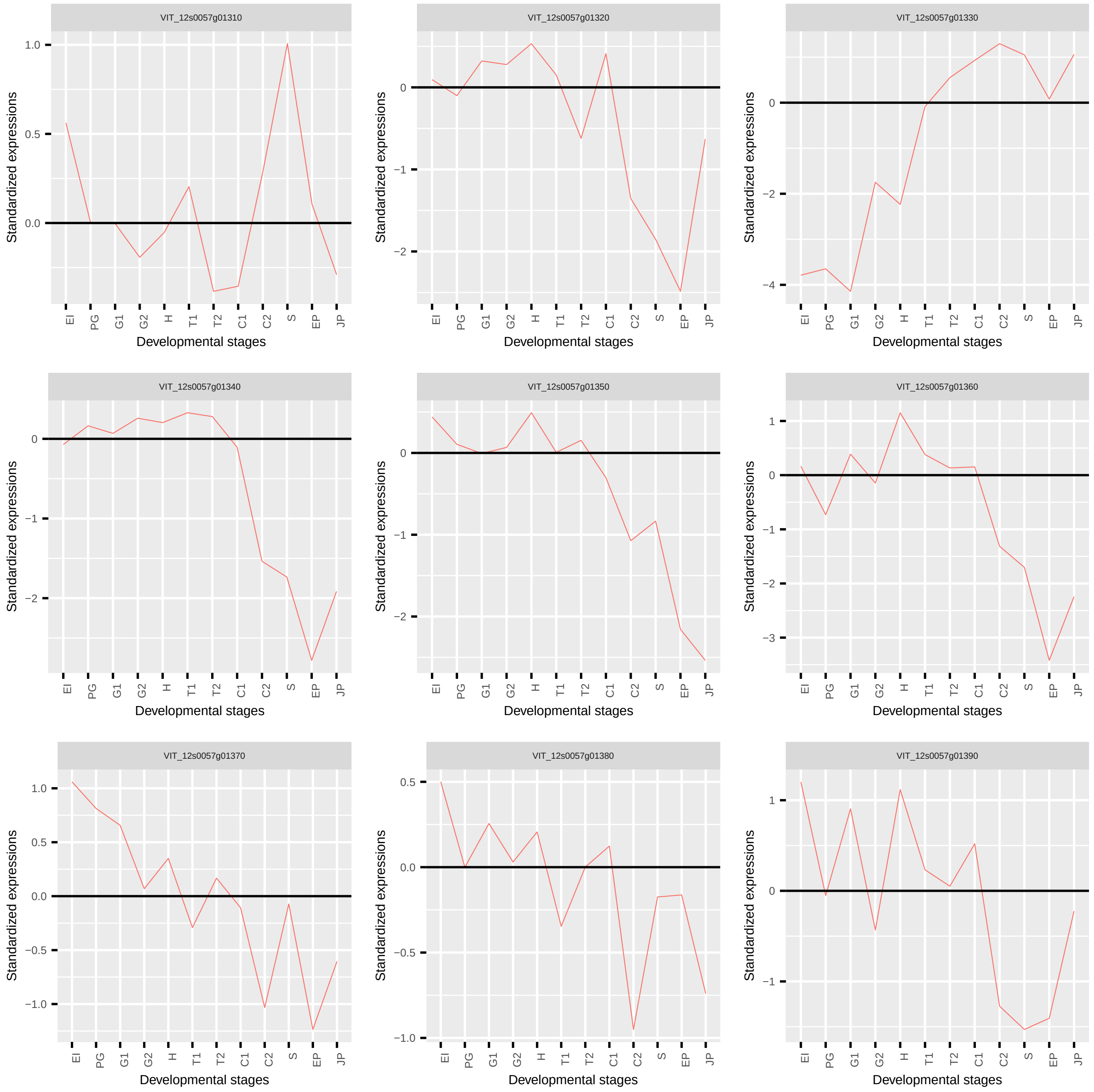

VIT_12s0057g01310
VIT_12s0057g01320
VIT_12s0057g01330
VIT_12s0057g01340
VIT_12s0057g01350
VIT_12s0057g01360
VIT_12s0057g01370
VIT_12s0057g01380
VIT_12s0057g01390
Standardized expressions
Developmental stages
EI PG G1 G2 H T1 T2 C1 C2 S EP JP

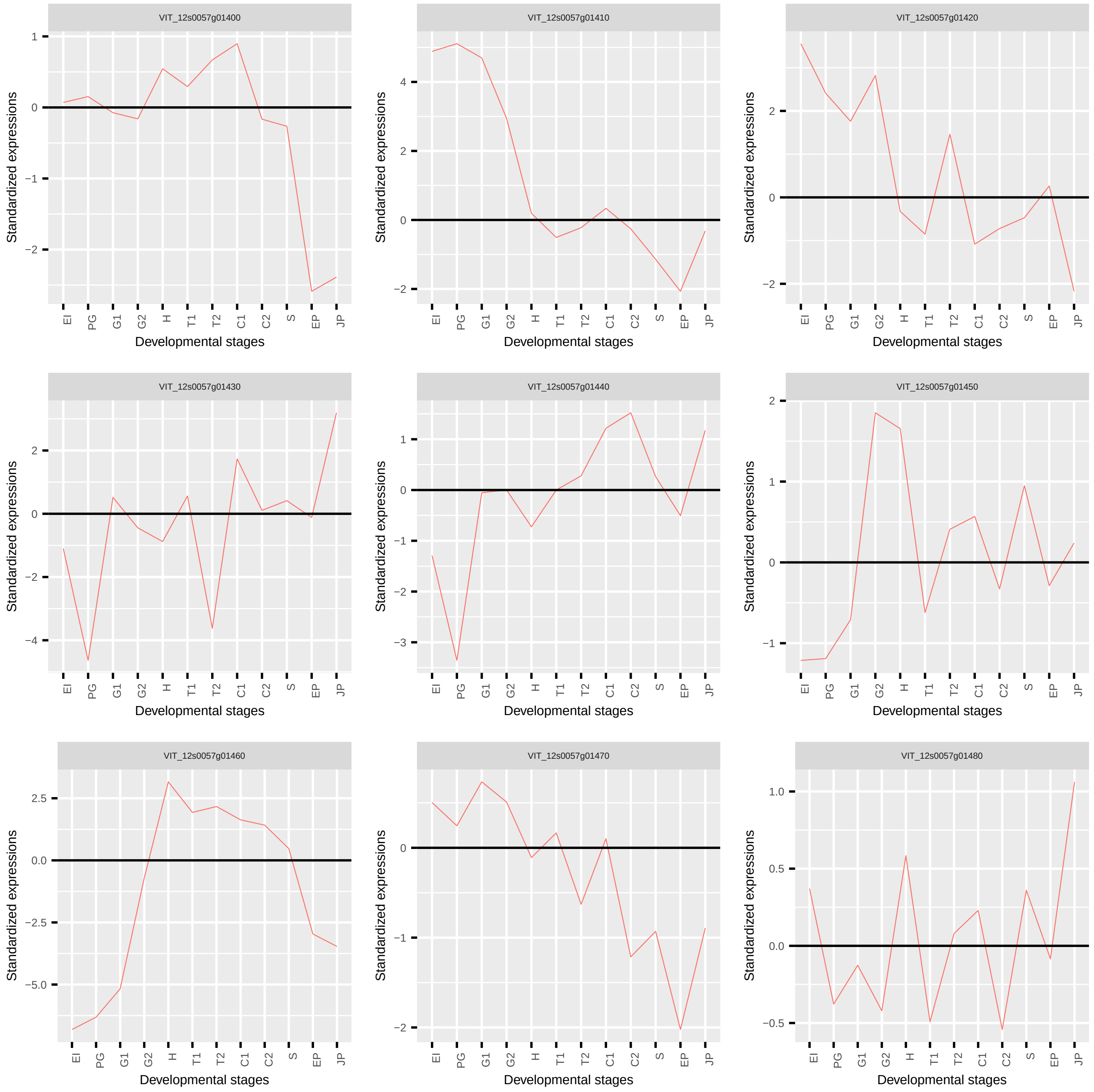
VIT_12s0057g01400
VIT_12s0057g01410
VIT_12s0057g01420
VIT_12s0057g01430
VIT_12s0057g01440
VIT_12s0057g01450
VIT_12s0057g01460
VIT_12s0057g01470
VIT_12s0057g01480
Standardized expressions
Developmental stages
EI
PG
G1
G2
H
T1
T2
C1
C2
S
EP
JP

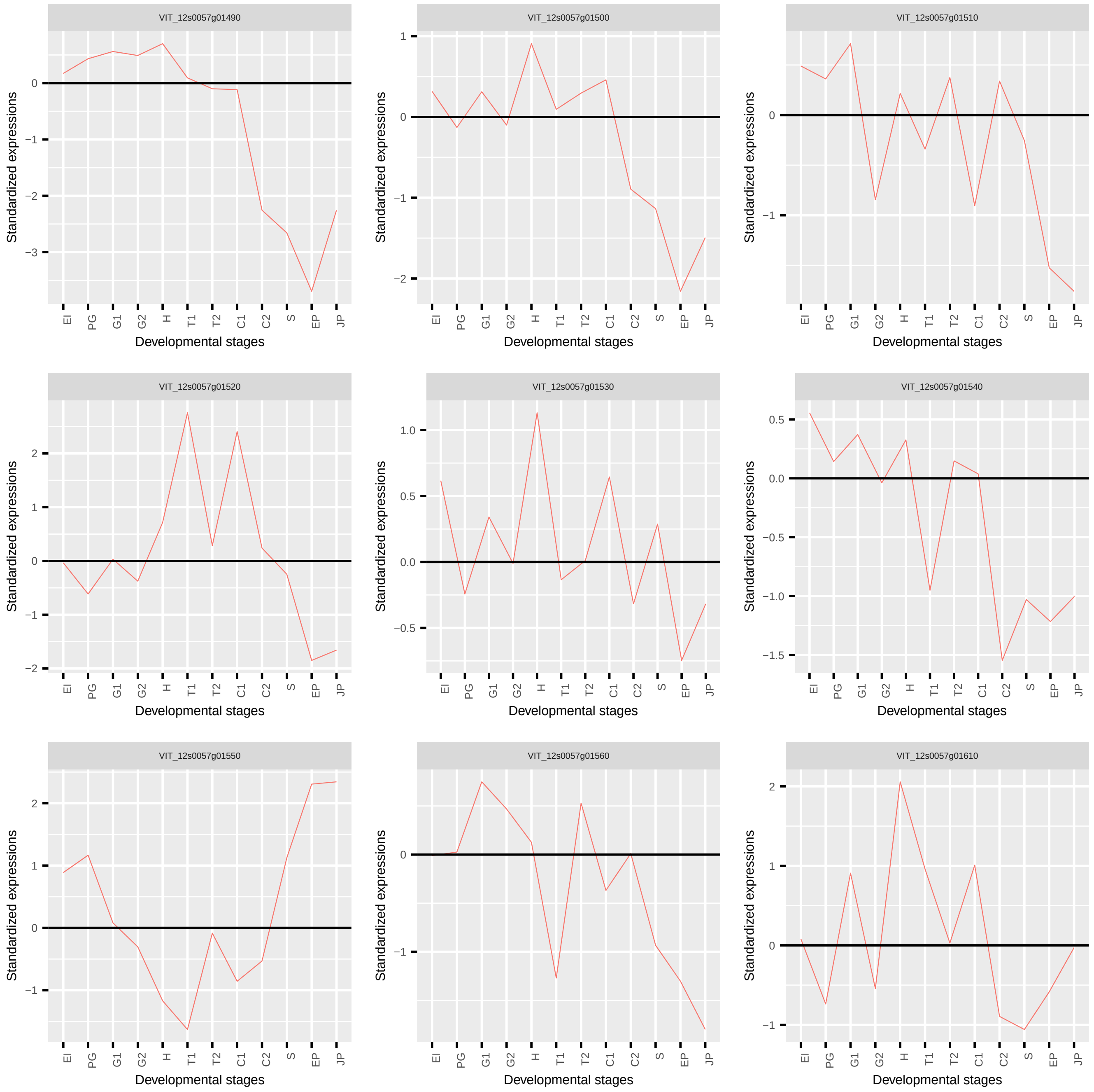
VIT_12s0057g01490
VIT_12s0057g01500
VIT_12s0057g01510
VIT_12s0057g01520
VIT_12s0057g01530
VIT_12s0057g01540
VIT_12s0057g01550
VIT_12s0057g01560
VIT_12s0057g01610
Standardized expressions
Developmental stages
EI PG G1 G2 H T1 T2 C1 C2 S EP JP

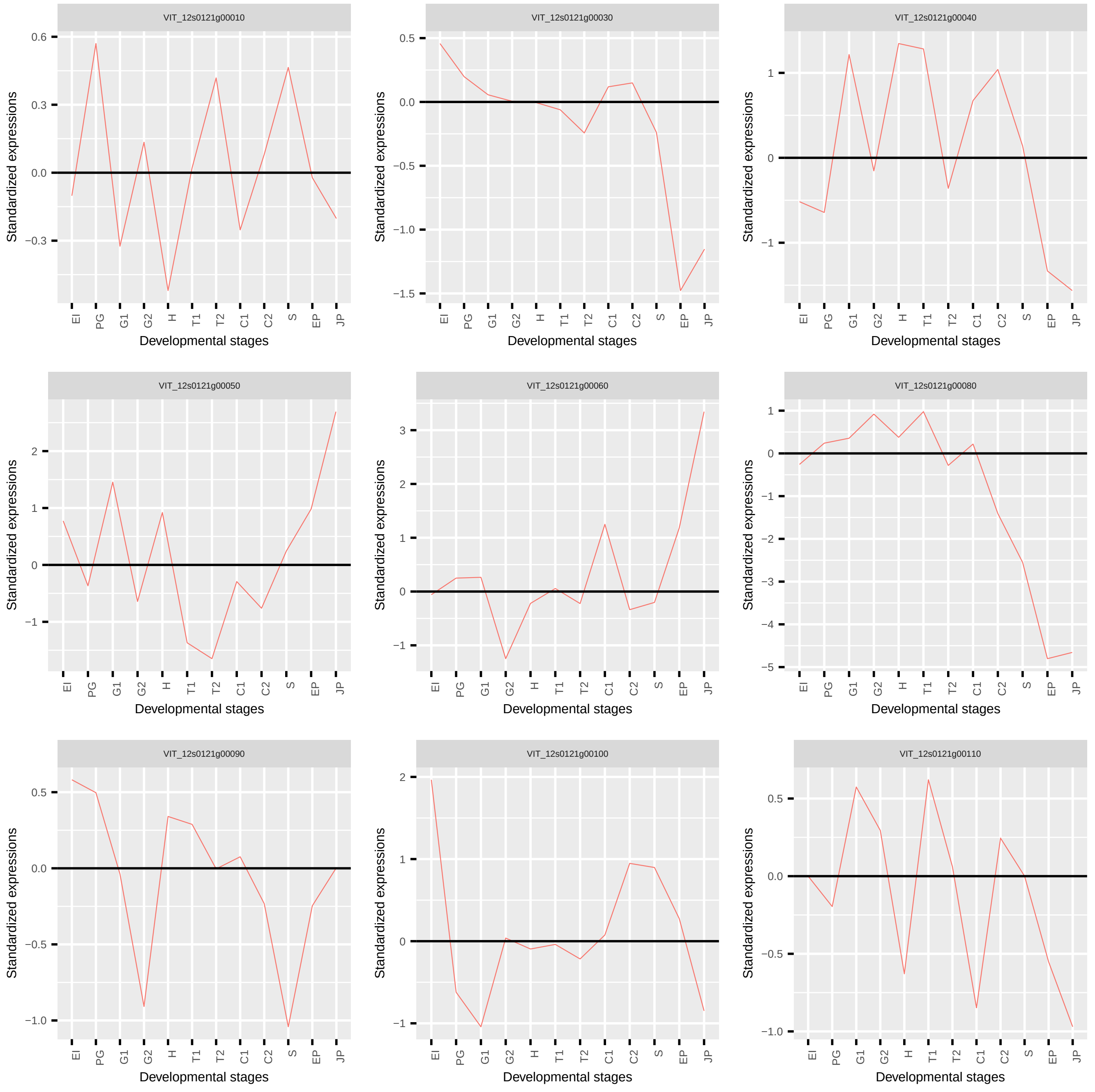

VIT_12s0121g00010
VIT_12s0121g00030
VIT_12s0121g00040
VIT_12s0121g00050
VIT_12s0121g00060
VIT_12s0121g00080
VIT_12s0121g00090
VIT_12s0121g00100
VIT_12s0121g00110
Standardized expressions
Developmental stages
EI PG G1 G2 H T1 T2 C1 C2 S EP JP

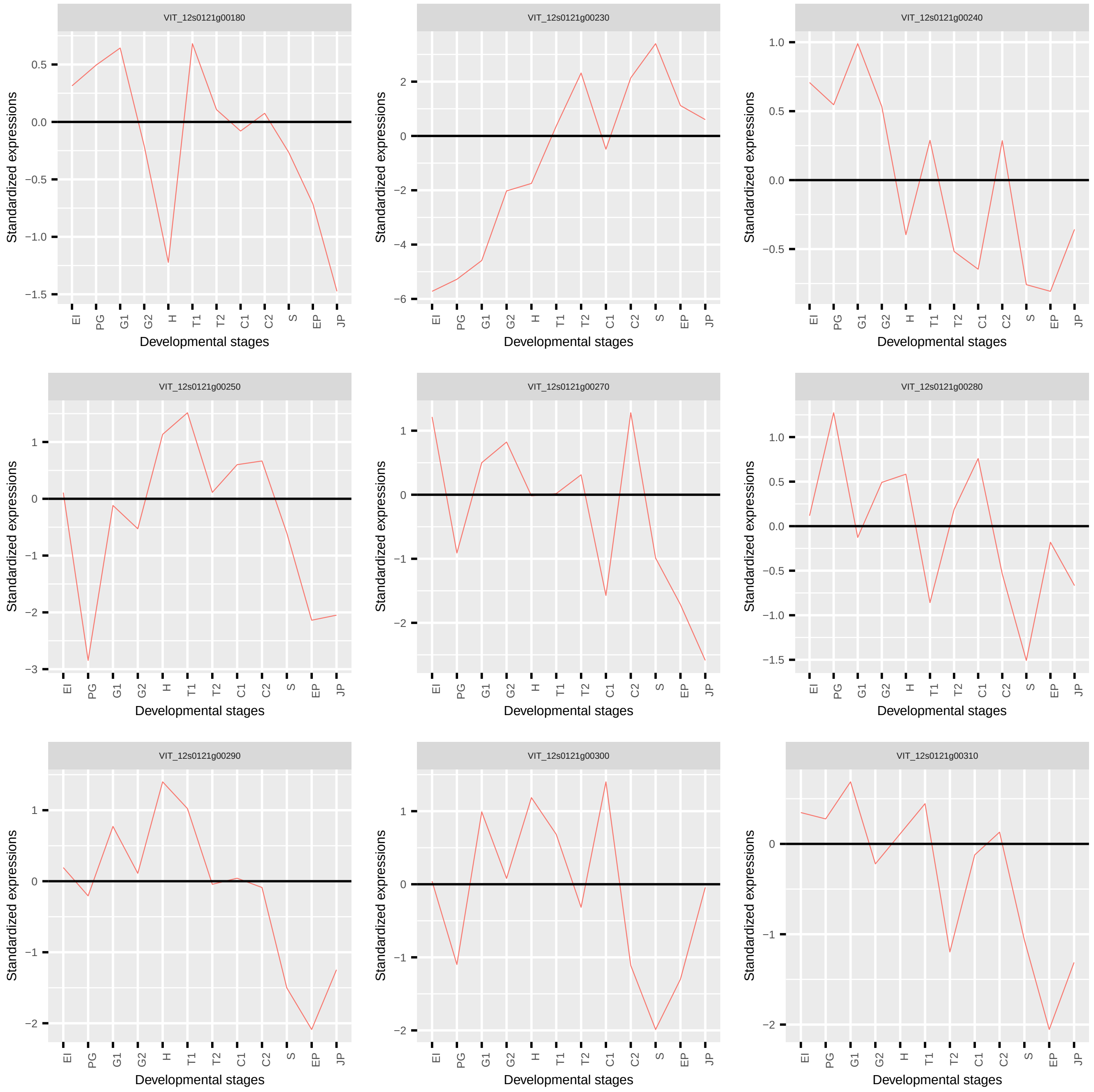
VIT_12s0121g00180
VIT_12s0121g00230
VIT_12s0121g00240
VIT_12s0121g00250
VIT_12s0121g00270
VIT_12s0121g00280
VIT_12s0121g00290
VIT_12s0121g00300
VIT_12s0121g00310
Standardized expressions
Developmental stages
EI
PG
G1
G2
H
T1
T2
C1
C2
S
EP
JP

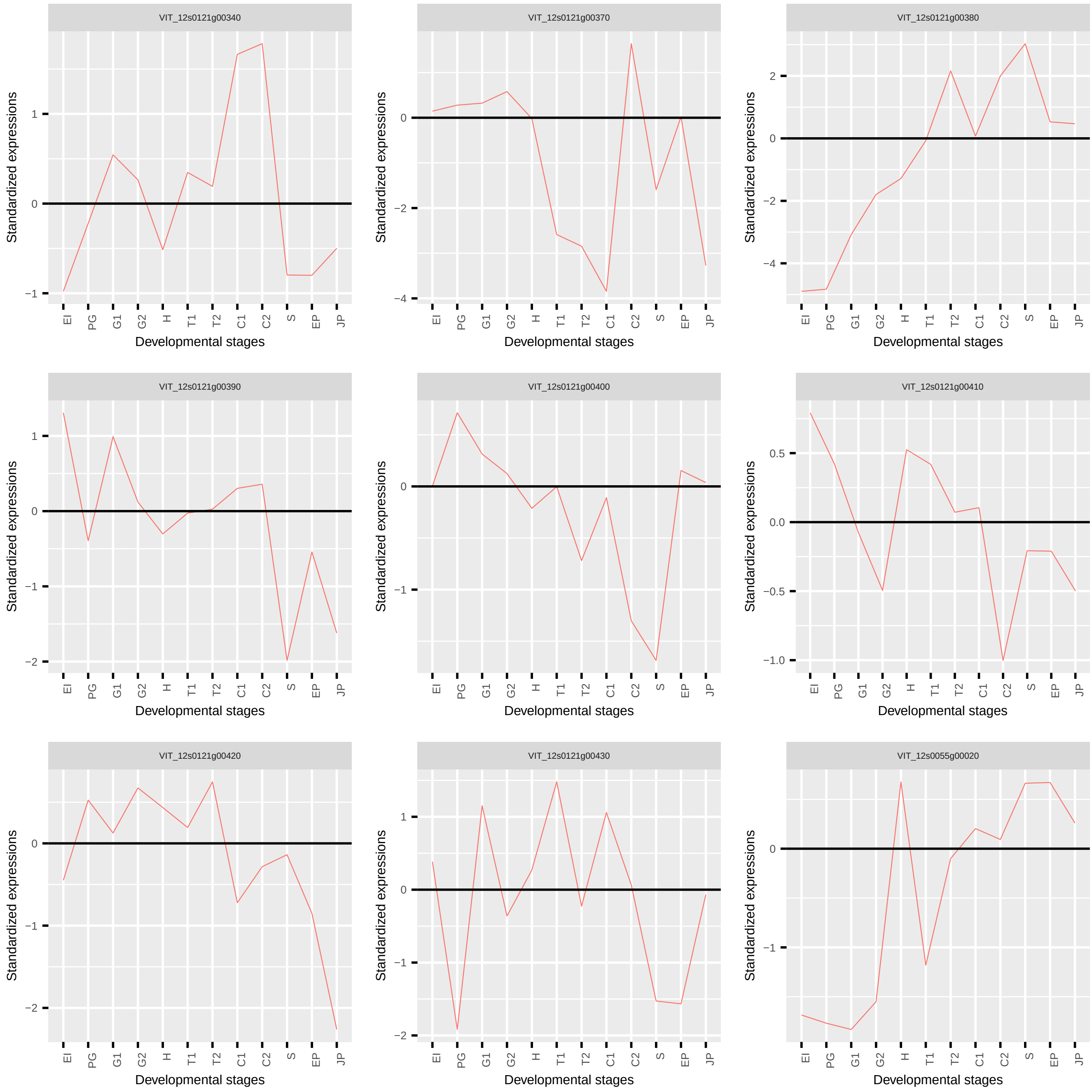
VIT_12s0121g00340
VIT_12s0121g00370
VIT_12s0121g00380
VIT_12s0121g00390
VIT_12s0121g00400
VIT_12s0121g00410
VIT_12s0121g00420
VIT_12s0121g00430
VIT_12s0055g00020
Standardized expressions
Developmental stages
EI PG G1 G2 H T1 T2 C1 C2 S EP JP

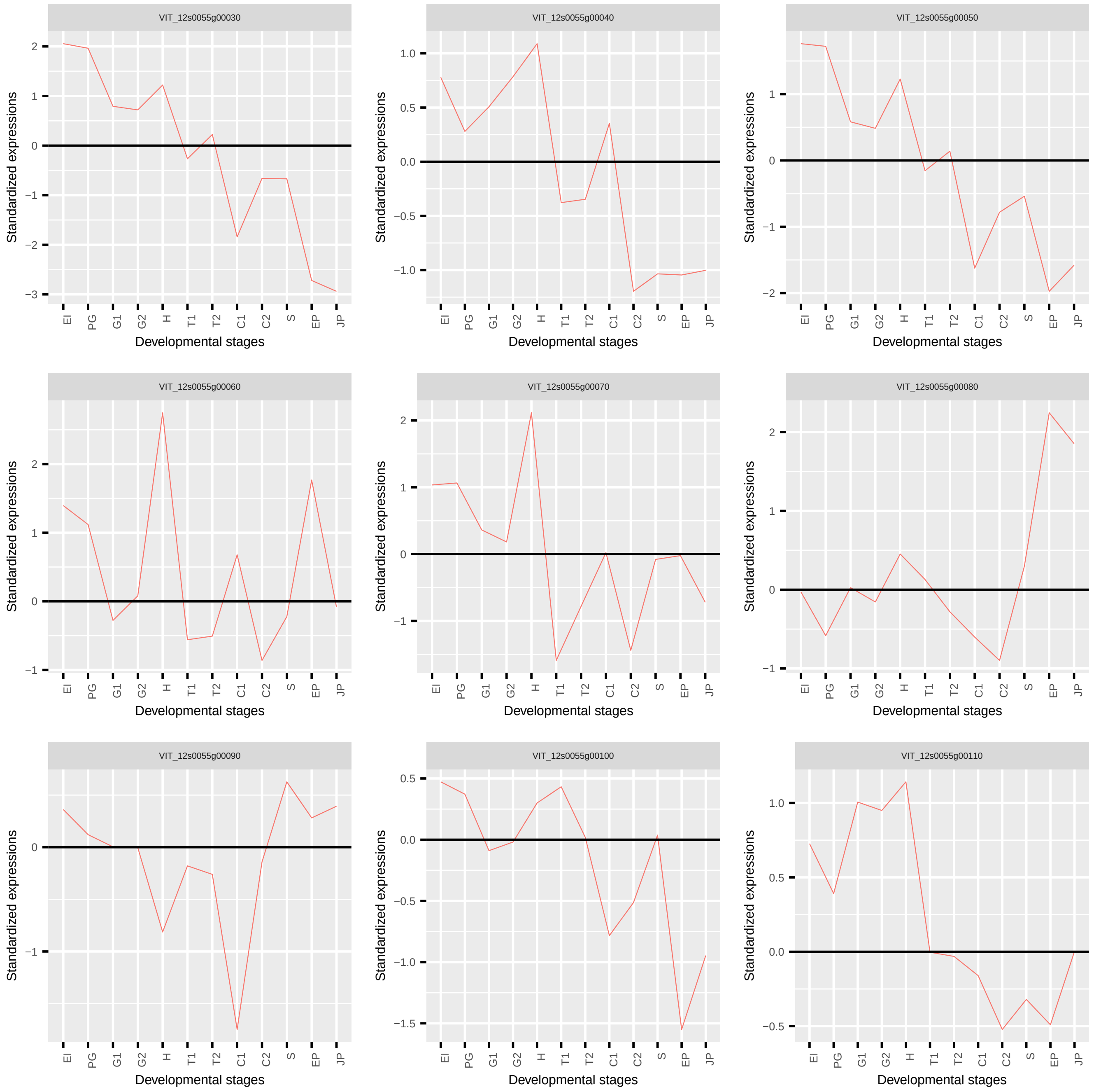

VIT_12s0055g00030
VIT_12s0055g00040
VIT_12s0055g00050
VIT_12s0055g00060
VIT_12s0055g00070
VIT_12s0055g00080
VIT_12s0055g00090
VIT_12s0055g00100
VIT_12s0055g00110
Standardized expressions
Developmental stages
EI
PG
G1
G2
H
T1
T2
C1
C2
S
EP
JP

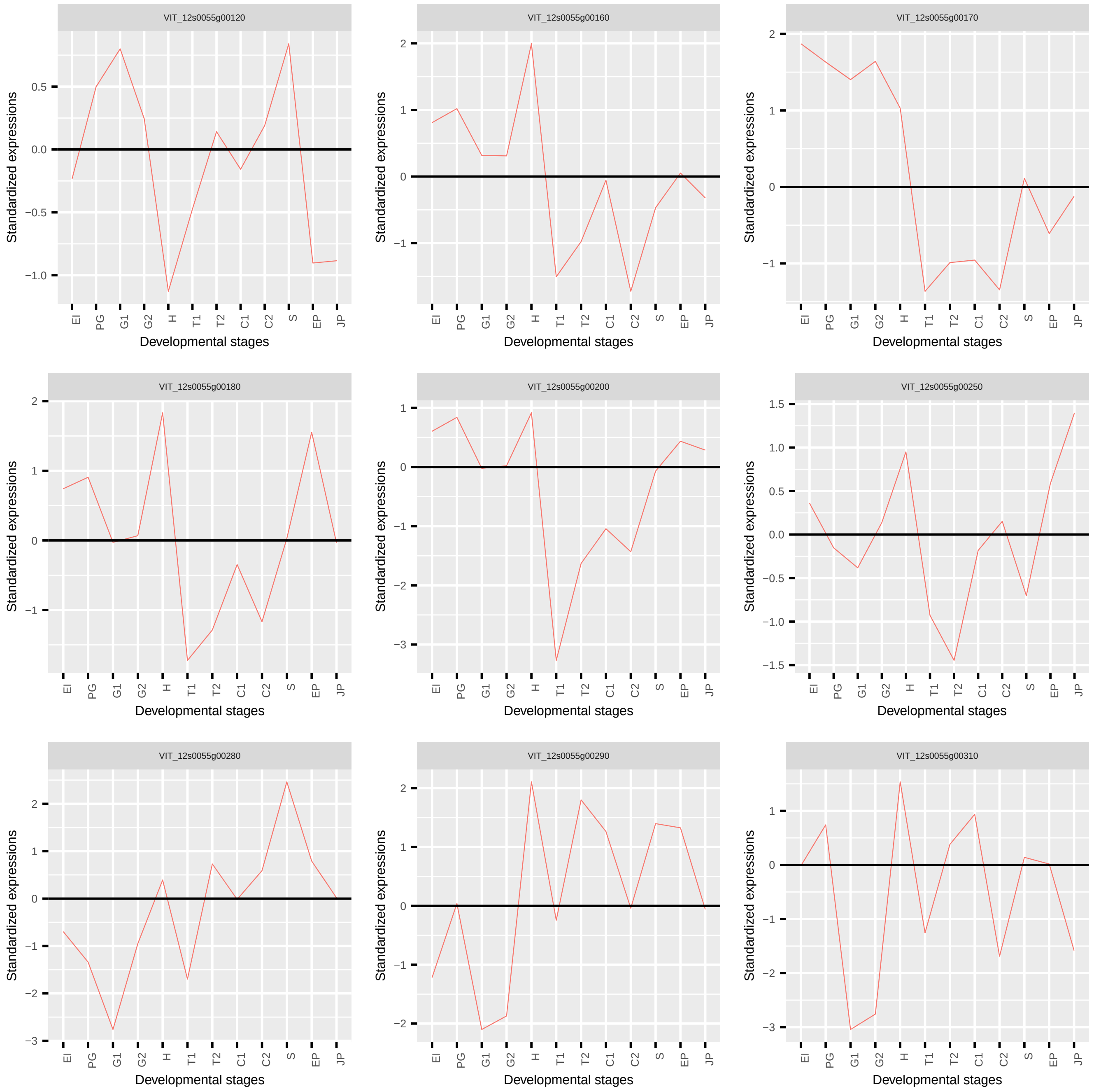
VIT_12s0055g00120
VIT_12s0055g00160
VIT_12s0055g00170
VIT_12s0055g00180
VIT_12s0055g00200
VIT_12s0055g00250
VIT_12s0055g00280
VIT_12s0055g00290
VIT_12s0055g00310
Standardized expressions
Developmental stages
EI PG G1 G2 H T1 T2 C1 C2 S EP JP

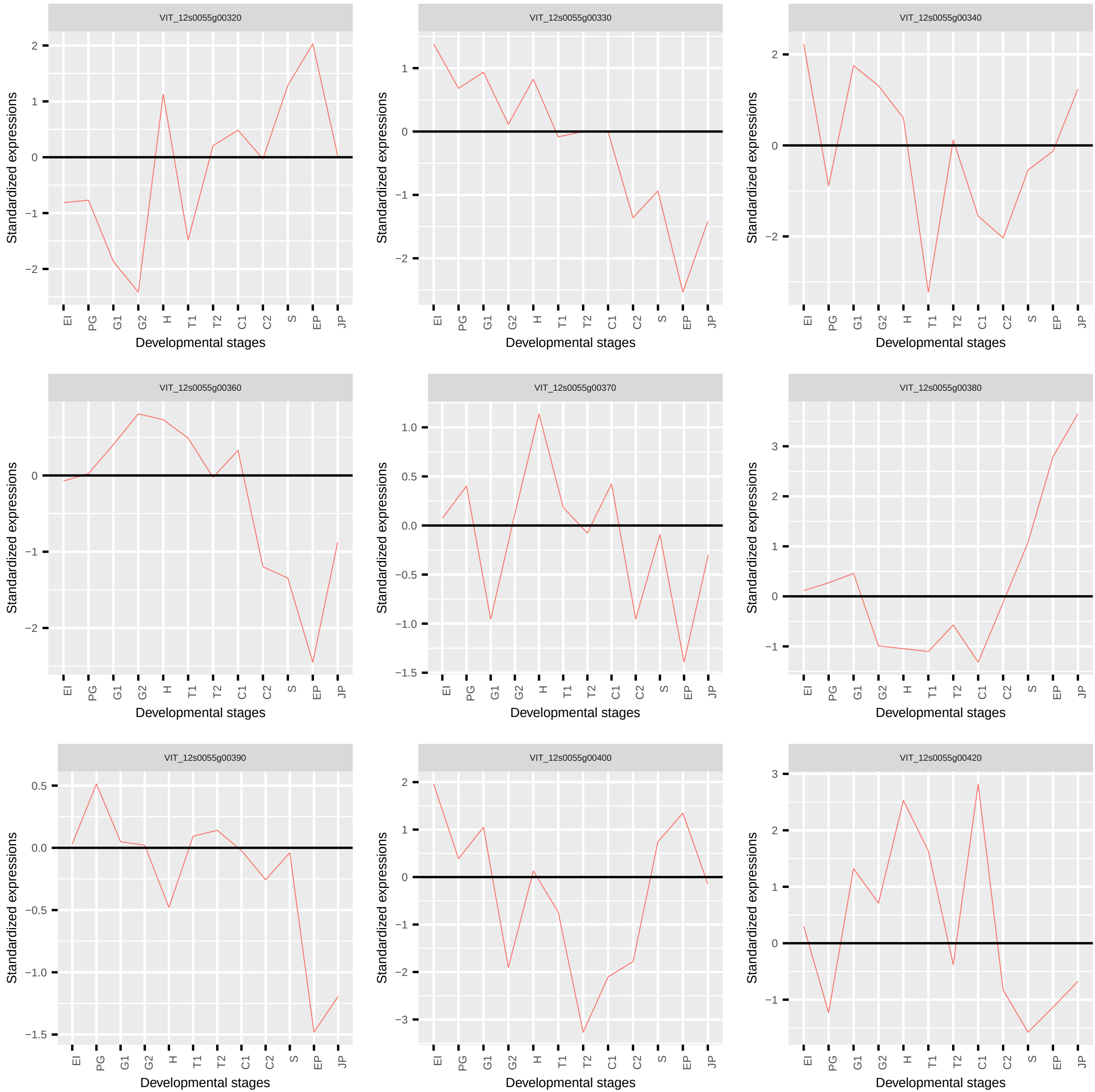
VIT_12s0055g00320
VIT_12s0055g00330
VIT_12s0055g00340
VIT_12s0055g00360
VIT_12s0055g00370
VIT_12s0055g00380
VIT_12s0055g00390
VIT_12s0055g00400
VIT_12s0055g00420
Standardized expressions
Developmental stages
EI
PG
G1
G2
H
T1
T2
C1
C2
S
EP
JP
2
1
0
−1
−2
−3
3
1.0
0.5
0.0
−0.5
−1.0
−1.5

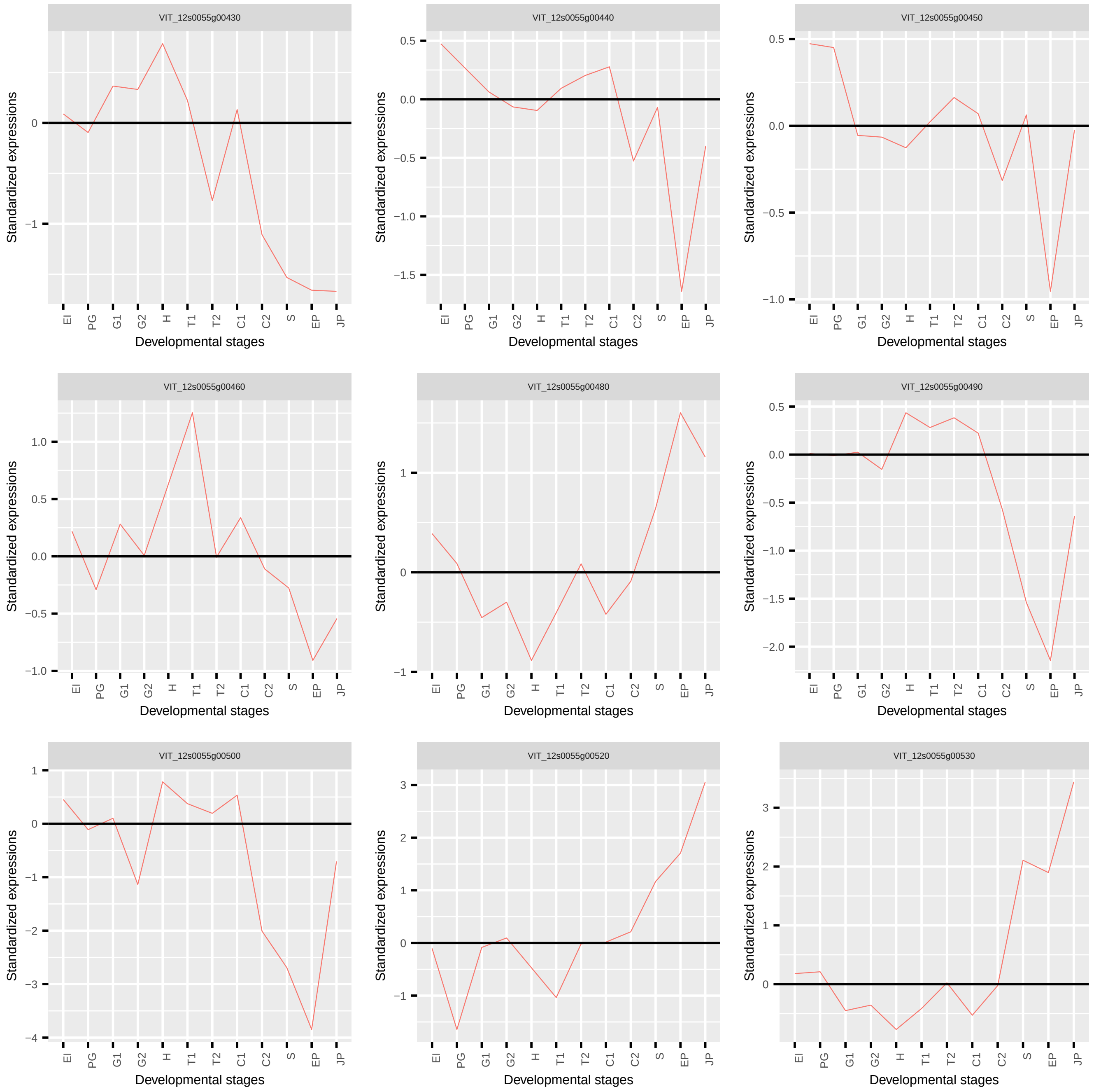

VIT_12s0055g00430
VIT_12s0055g00440
VIT_12s0055g00450
VIT_12s0055g00460
VIT_12s0055g00480
VIT_12s0055g00490
VIT_12s0055g00500
VIT_12s0055g00520
VIT_12s0055g00530
Standardized expressions
Developmental stages
EI PG G1 G2 H T1 T2 C1 C2 S EP JP

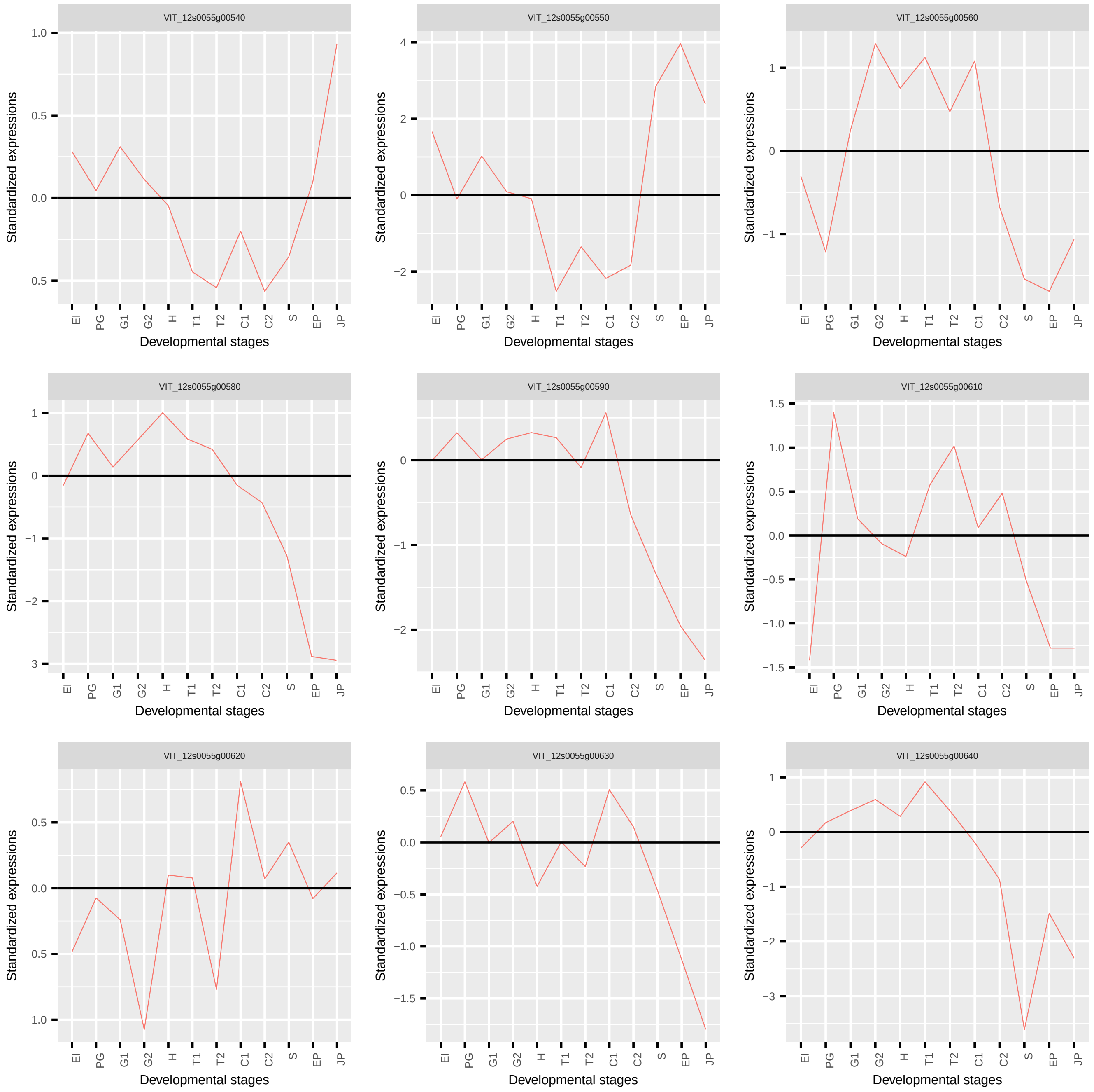
VIT_12s0055g00540
1.0
0.5
0.0
−0.5
Standardized expressions
EI PG G1 G2 H T1 T2 C1 C2 S EP JP
Developmental stages
VIT_12s0055g00550
4
2
0
−2
Standardized expressions
EI PG G1 G2 H T1 T2 C1 C2 S EP JP
Developmental stages
VIT_12s0055g00560
1
0
−1
Standardized expressions
EI PG G1 G2 H T1 T2 C1 C2 S EP JP
Developmental stages
VIT_12s0055g00580
1
0
−1
−2
−3
Standardized expressions
EI PG G1 G2 H T1 T2 C1 C2 S EP JP
Developmental stages
VIT_12s0055g00590
0
−1
−2
Standardized expressions
EI PG G1 G2 H T1 T2 C1 C2 S EP JP
Developmental stages
VIT_12s0055g00610
1.5
1.0
0.5
0.0
−0.5
−1.0
−1.5
Standardized expressions
EI PG G1 G2 H T1 T2 C1 C2 S EP JP
Developmental stages
VIT_12s0055g00620
0.5
0.0
−0.5
−1.0
Standardized expressions
EI PG G1 G2 H T1 T2 C1 C2 S EP JP
Developmental stages
VIT_12s0055g00630
0.5
0.0
−0.5
−1.0
−1.5
Standardized expressions
EI PG G1 G2 H T1 T2 C1 C2 S EP JP
Developmental stages
VIT_12s0055g00640
1
0
−1
−2
−3
Standardized expressions
EI PG G1 G2 H T1 T2 C1 C2 S EP JP
Developmental stages

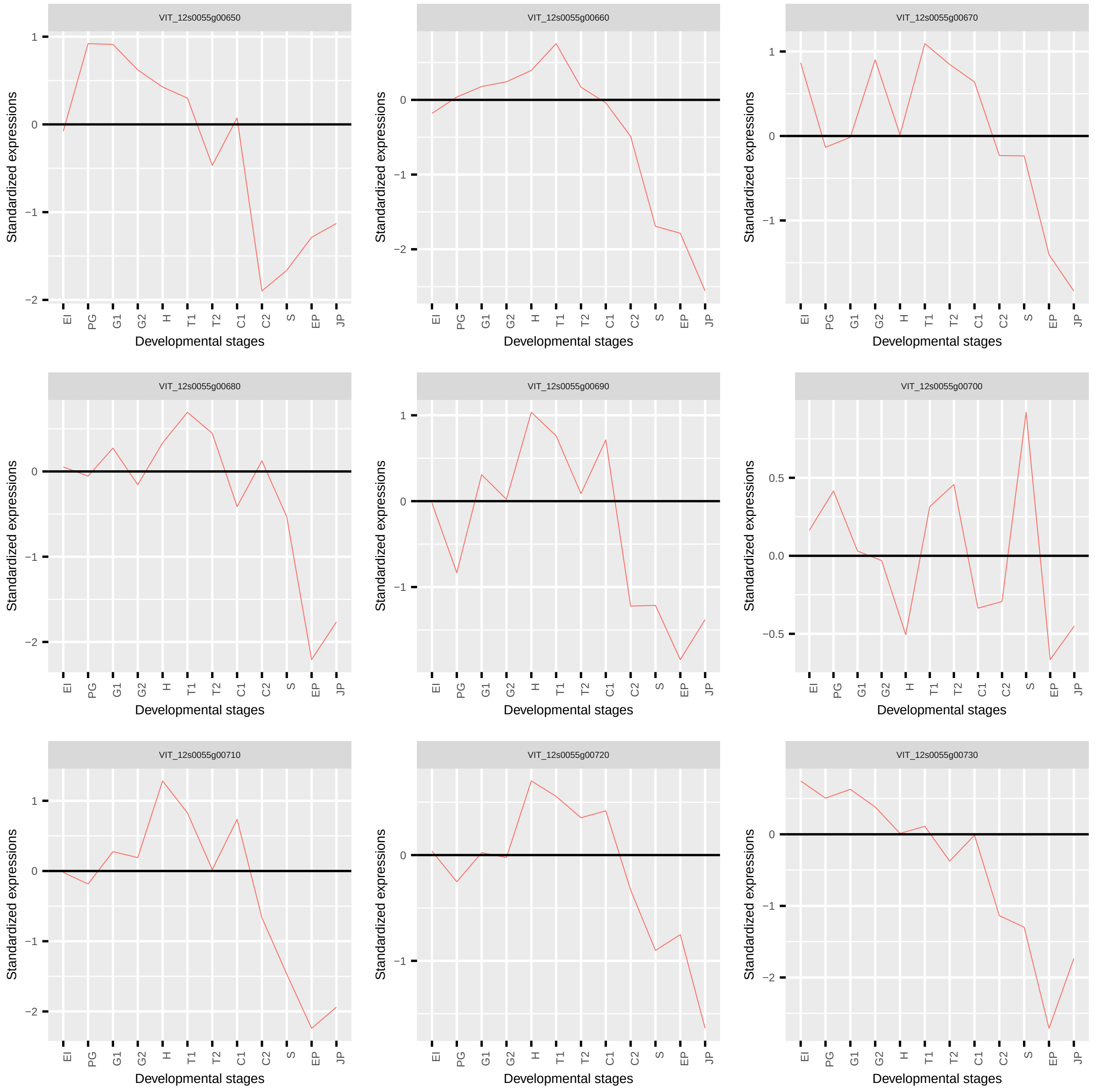
VIT_12s0055g00650
VIT_12s0055g00660
VIT_12s0055g00670
VIT_12s0055g00680
VIT_12s0055g00690
VIT_12s0055g00700
VIT_12s0055g00710
VIT_12s0055g00720
VIT_12s0055g00730
Standardized expressions
Developmental stages
EI
PG
G1
G2
H
T1
T2
C1
C2
S
EP
JP
1
0
−1
−2
0.5
0.0
−0.5

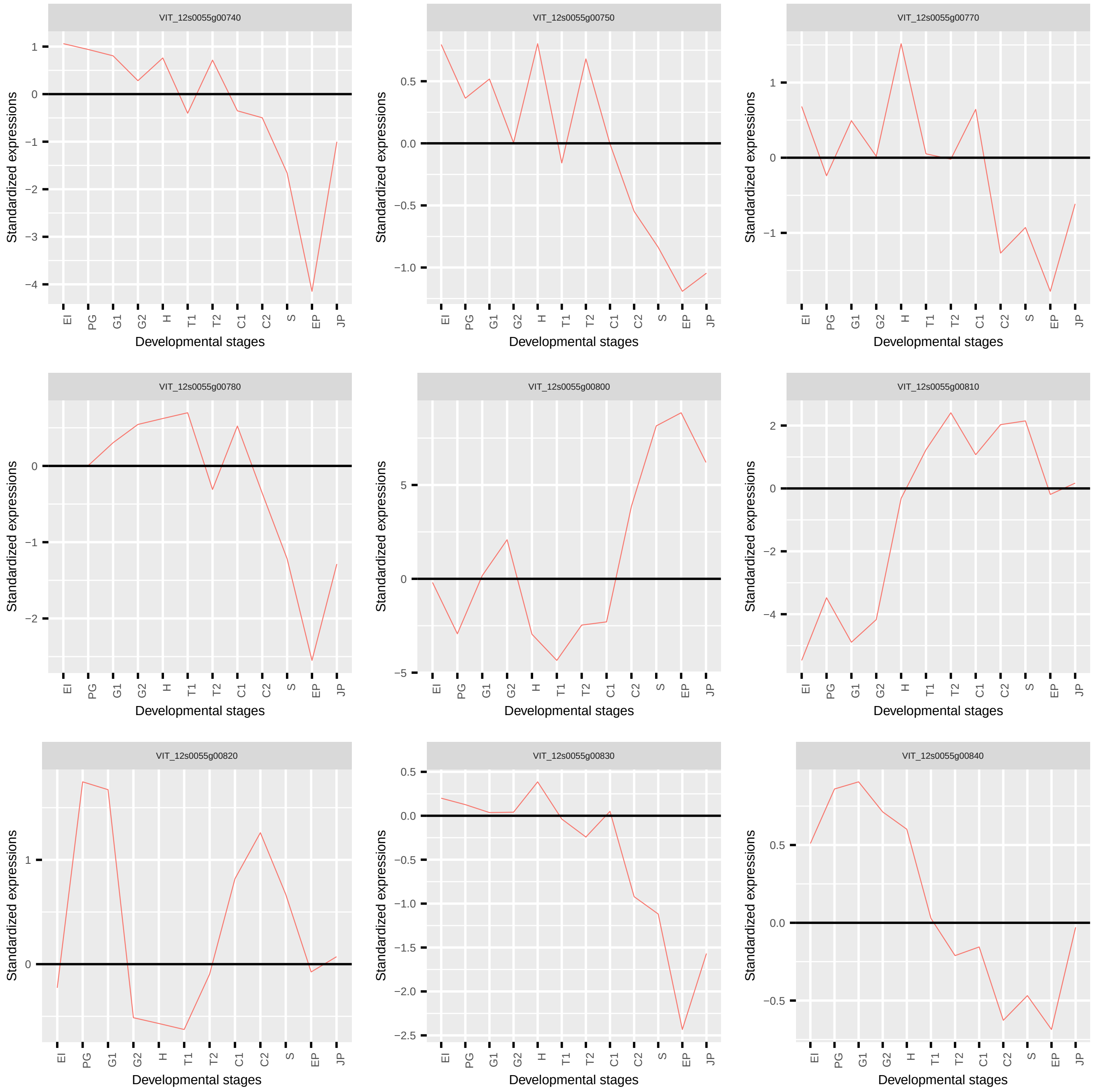
VIT_12s0055g00740
VIT_12s0055g00750
VIT_12s0055g00770
VIT_12s0055g00780
VIT_12s0055g00800
VIT_12s0055g00810
VIT_12s0055g00820
VIT_12s0055g00830
VIT_12s0055g00840
Standardized expressions
Developmental stages
EI
PG
G1
G2
H
T1
T2
C1
C2
S
EP
JP

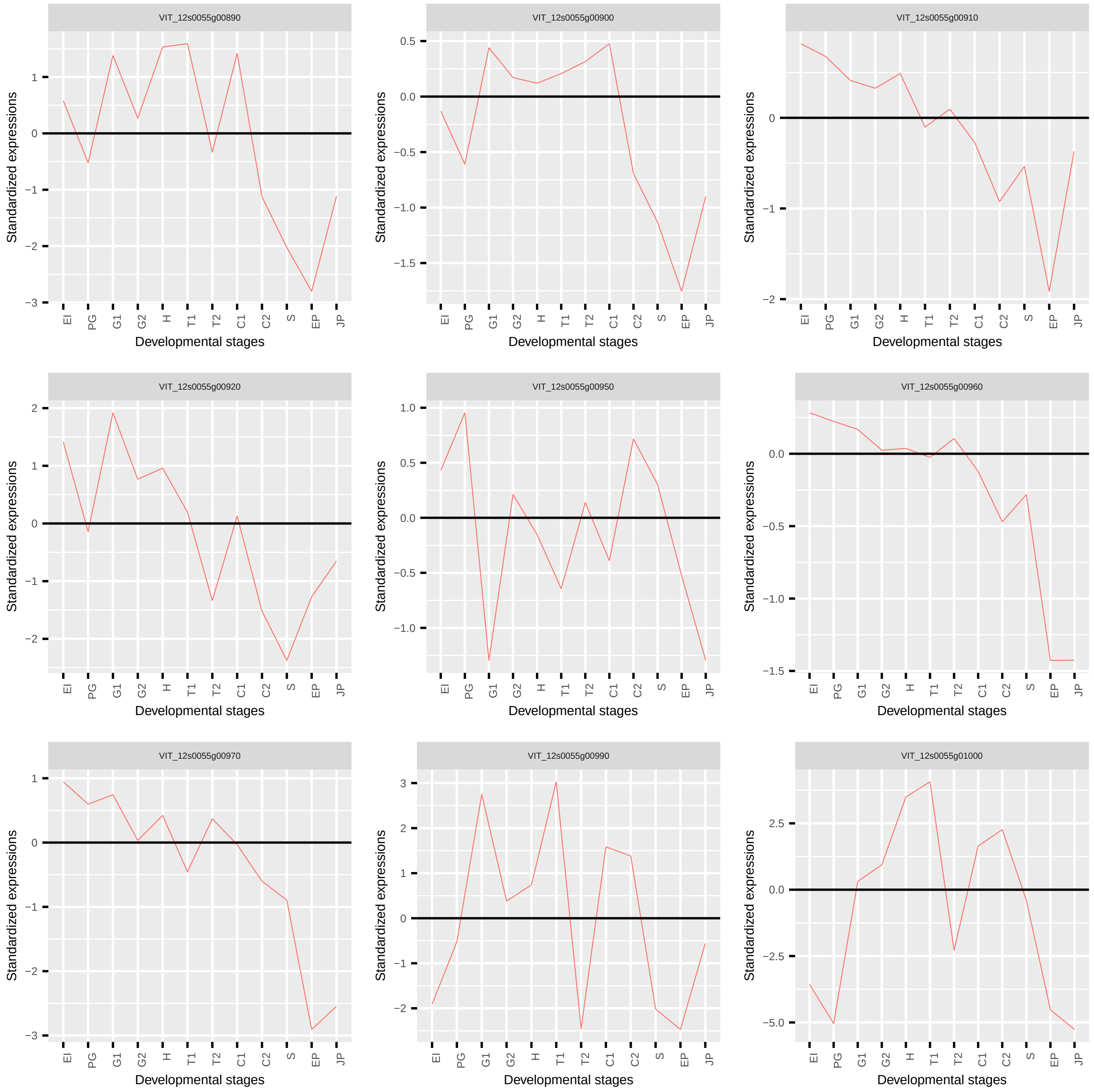
VIT_12s0055g00890
VIT_12s0055g00900
VIT_12s0055g00910
VIT_12s0055g00920
VIT_12s0055g00950
VIT_12s0055g00960
VIT_12s0055g00970
VIT_12s0055g00990
VIT_12s0055g01000
Standardized expressions
Developmental stages
EI PG G1 G2 H T1 T2 C1 C2 S EP JP

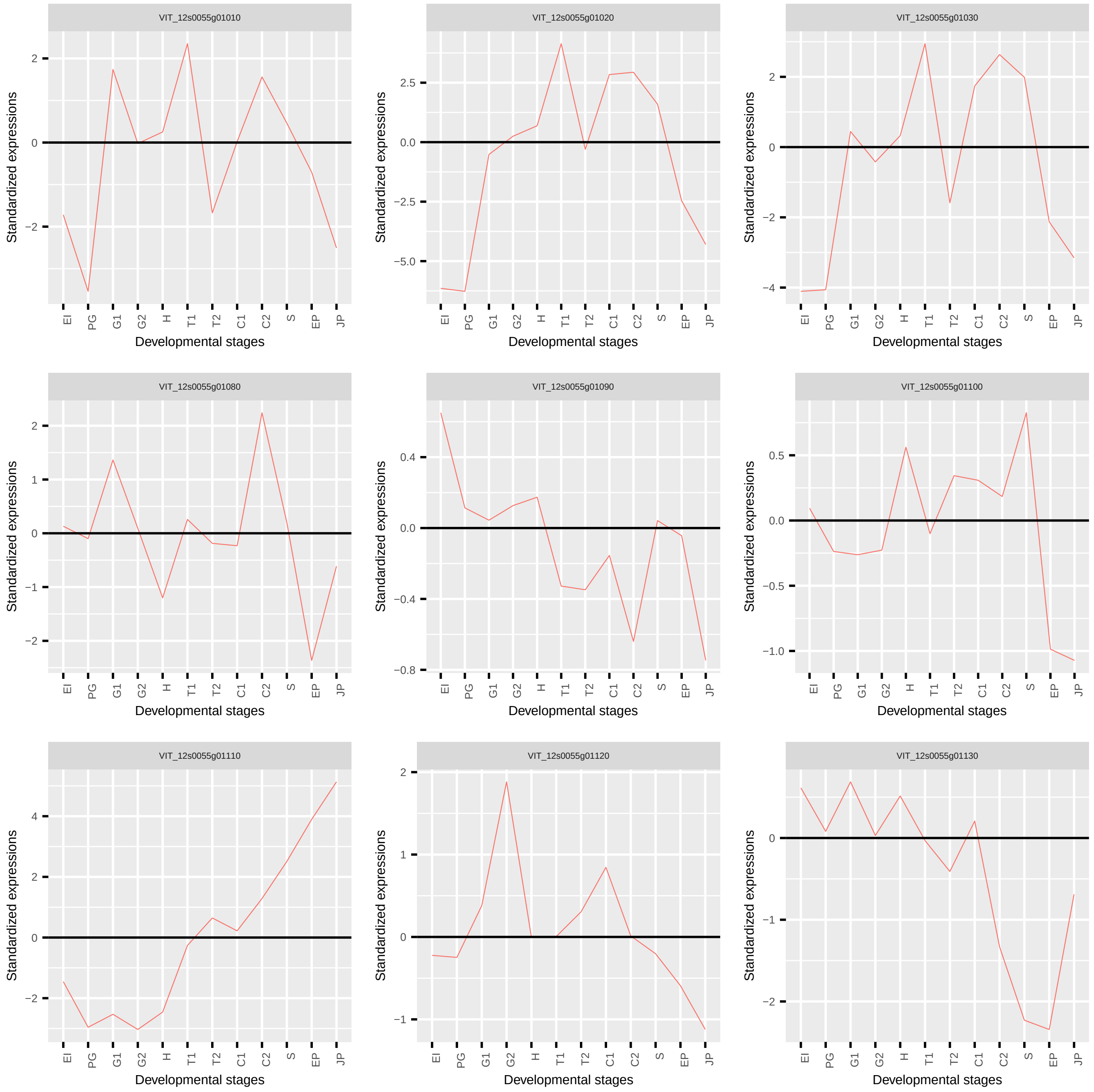
VIT_12s0055g01010
VIT_12s0055g01020
VIT_12s0055g01030
VIT_12s0055g01080
VIT_12s0055g01090
VIT_12s0055g01100
VIT_12s0055g01110
VIT_12s0055g01120
VIT_12s0055g01130
Standardized expressions
Developmental stages
EI PG G1 G2 H T1 T2 C1 C2 S EP JP

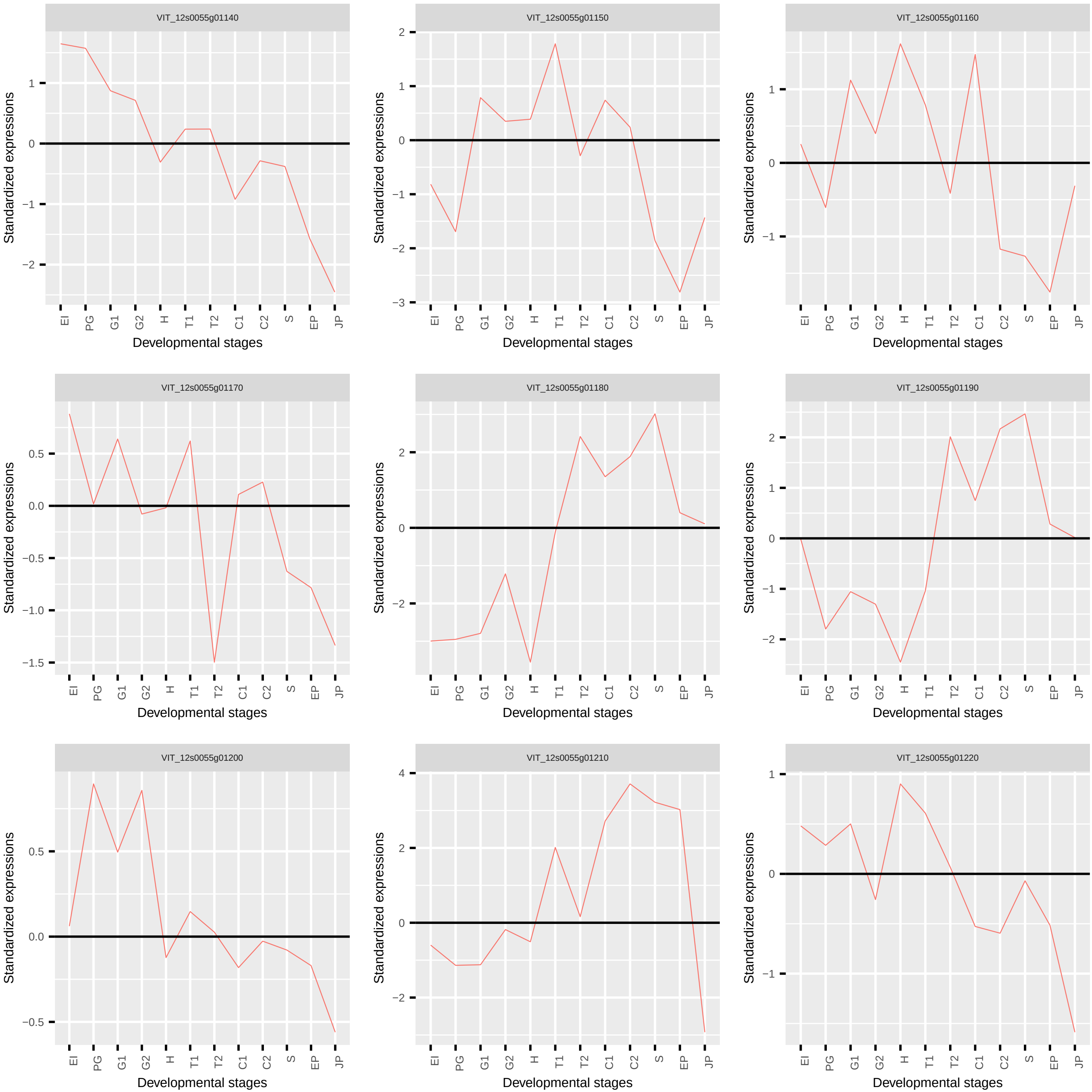
VIT_12s0055g01140
Standardized expressions
1
0
−1
−2
EI PG G1 G2 H T1 T2 C1 C2 S EP JP
Developmental stages
VIT_12s0055g01150
Standardized expressions
2
1
0
−1
−2
−3
EI PG G1 G2 H T1 T2 C1 C2 S EP JP
Developmental stages
VIT_12s0055g01160
Standardized expressions
1
0
−1
EI PG G1 G2 H T1 T2 C1 C2 S EP JP
Developmental stages
VIT_12s0055g01170
Standardized expressions
0.5
0.0
−0.5
−1.0
−1.5
EI PG G1 G2 H T1 T2 C1 C2 S EP JP
Developmental stages
VIT_12s0055g01180
Standardized expressions
2
0
−2
EI PG G1 G2 H T1 T2 C1 C2 S EP JP
Developmental stages
VIT_12s0055g01190
Standardized expressions
2
1
0
−1
−2
EI PG G1 G2 H T1 T2 C1 C2 S EP JP
Developmental stages
VIT_12s0055g01200
Standardized expressions
0.5
0.0
−0.5
EI PG G1 G2 H T1 T2 C1 C2 S EP JP
Developmental stages
VIT_12s0055g01210
Standardized expressions
4
2
0
−2
EI PG G1 G2 H T1 T2 C1 C2 S EP JP
Developmental stages
VIT_12s0055g01220
Standardized expressions
1
0
−1
EI PG G1 G2 H T1 T2 C1 C2 S EP JP
Developmental stages

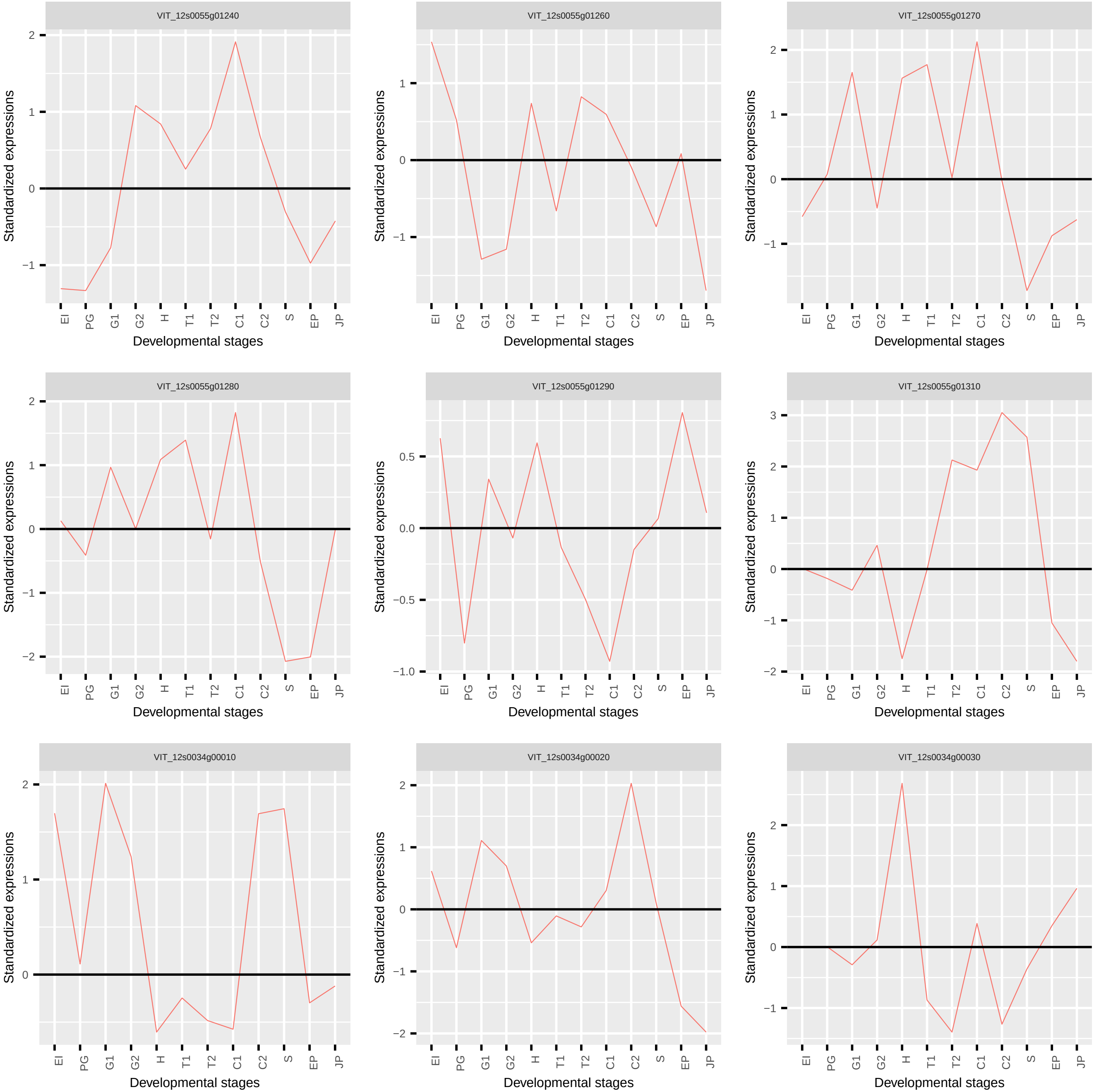

VIT_12s0055g01240
VIT_12s0055g01260
VIT_12s0055g01270
VIT_12s0055g01280
VIT_12s0055g01290
VIT_12s0055g01310
VIT_12s0034g00010
VIT_12s0034g00020
VIT_12s0034g00030
Standardized expressions
Developmental stages
EI PG G1 G2 H T1 T2 C1 C2 S EP JP

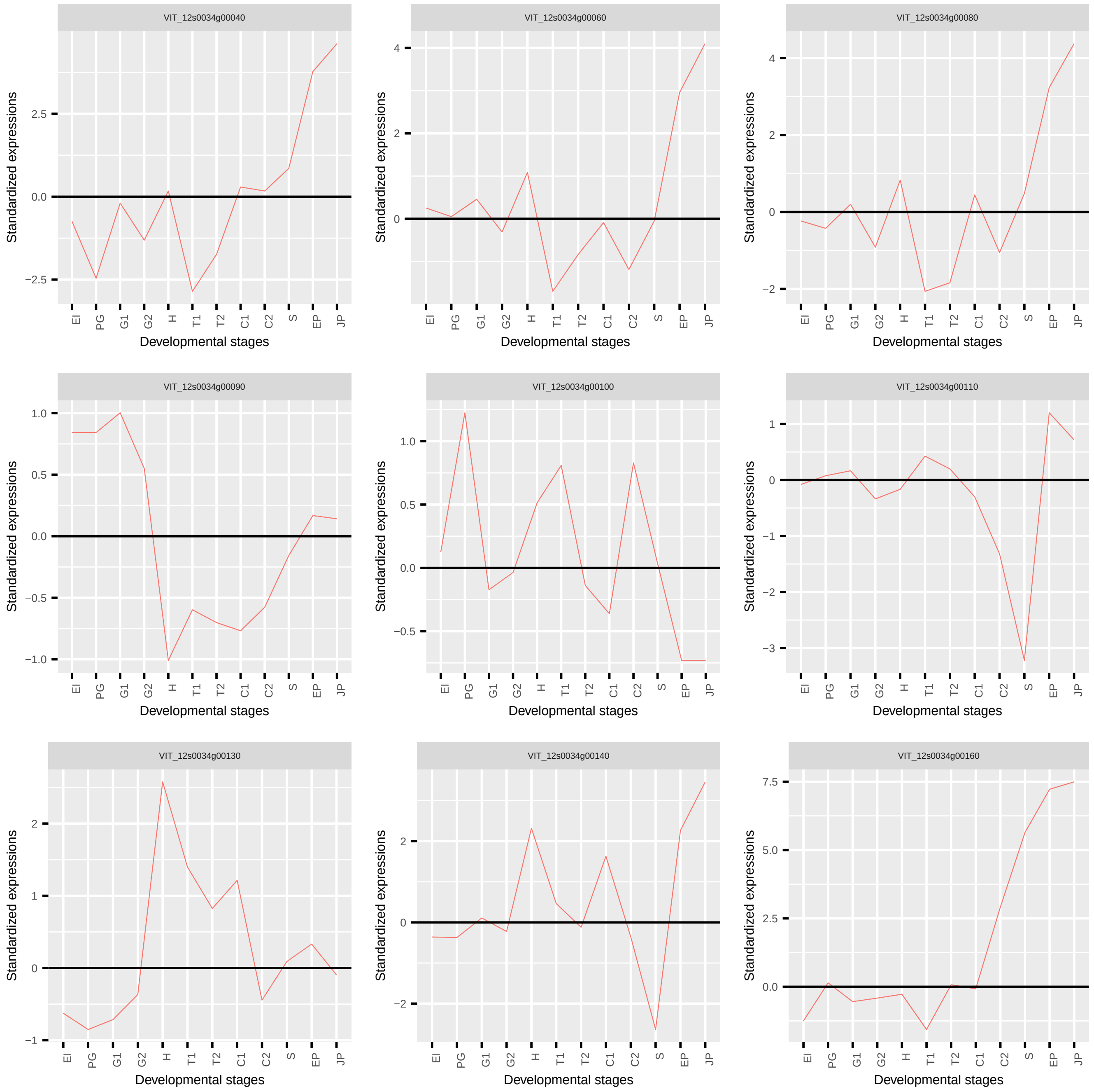
VIT_12s0034g00040
VIT_12s0034g00060
VIT_12s0034g00080
VIT_12s0034g00090
VIT_12s0034g00100
VIT_12s0034g00110
VIT_12s0034g00130
VIT_12s0034g00140
VIT_12s0034g00160
Standardized expressions
Developmental stages
EI PG G1 G2 H T1 T2 C1 C2 S EP JP

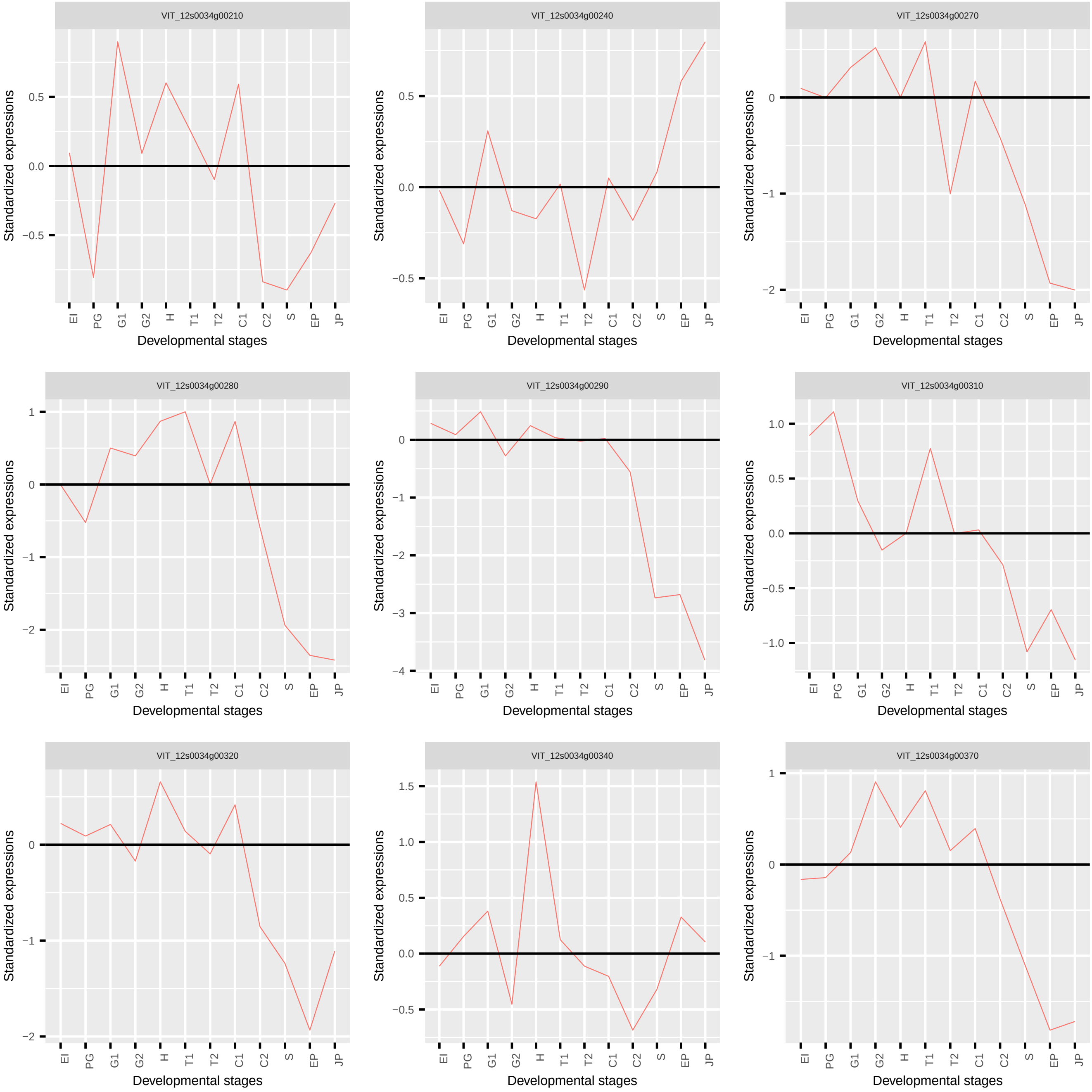

VIT_12s0034g00210
VIT_12s0034g00240
VIT_12s0034g00270
VIT_12s0034g00280
VIT_12s0034g00290
VIT_12s0034g00310
VIT_12s0034g00320
VIT_12s0034g00340
VIT_12s0034g00370
Standardized expressions
Developmental stages
EI
PG
G1
G2
H
T1
T2
C1
C2
S
EP
JP

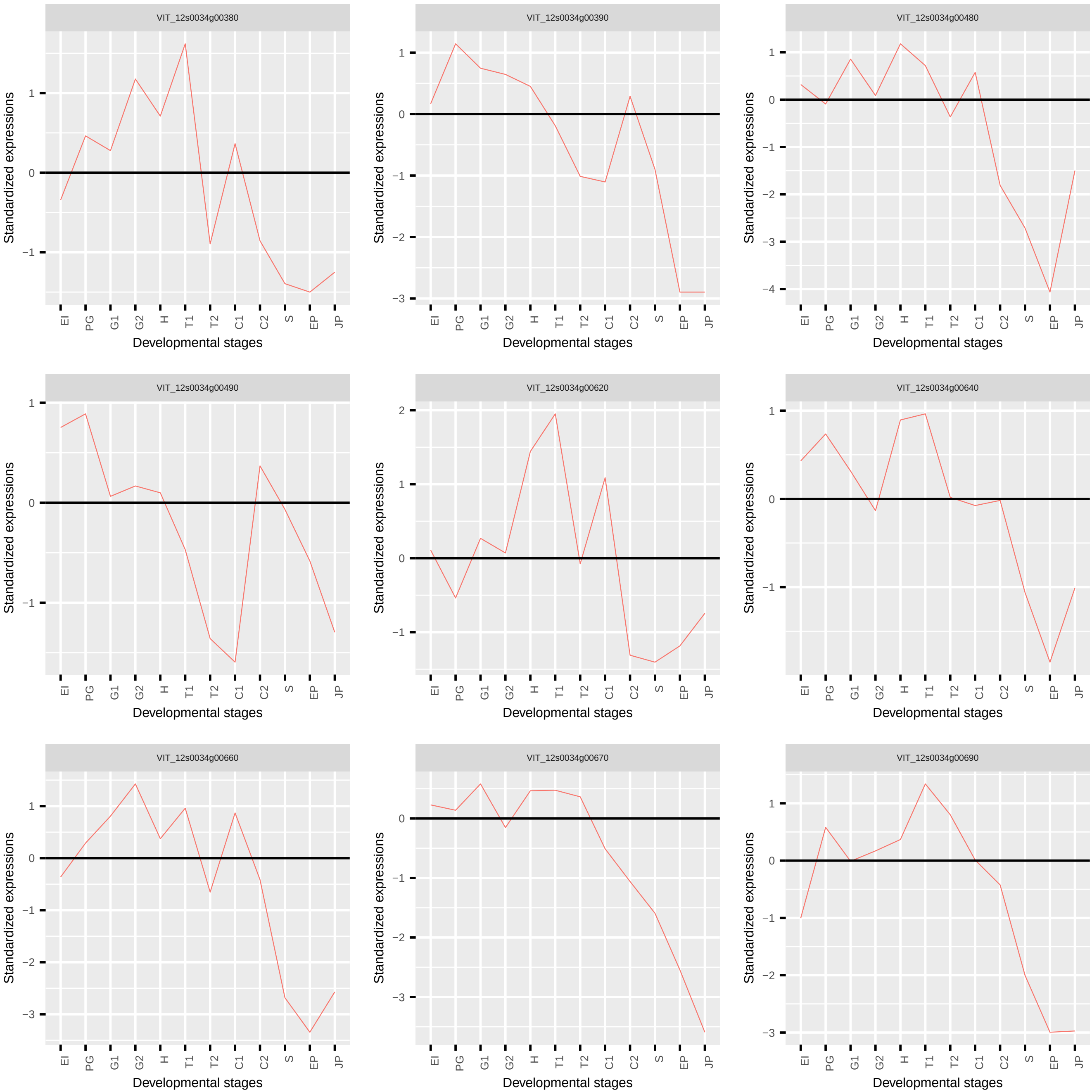

VIT_12s0034g00380
VIT_12s0034g00390
VIT_12s0034g00480
VIT_12s0034g00490
VIT_12s0034g00620
VIT_12s0034g00640
VIT_12s0034g00660
VIT_12s0034g00670
VIT_12s0034g00690
Standardized expressions
Developmental stages
EI PG G1 G2 H T1 T2 C1 C2 S EP JP

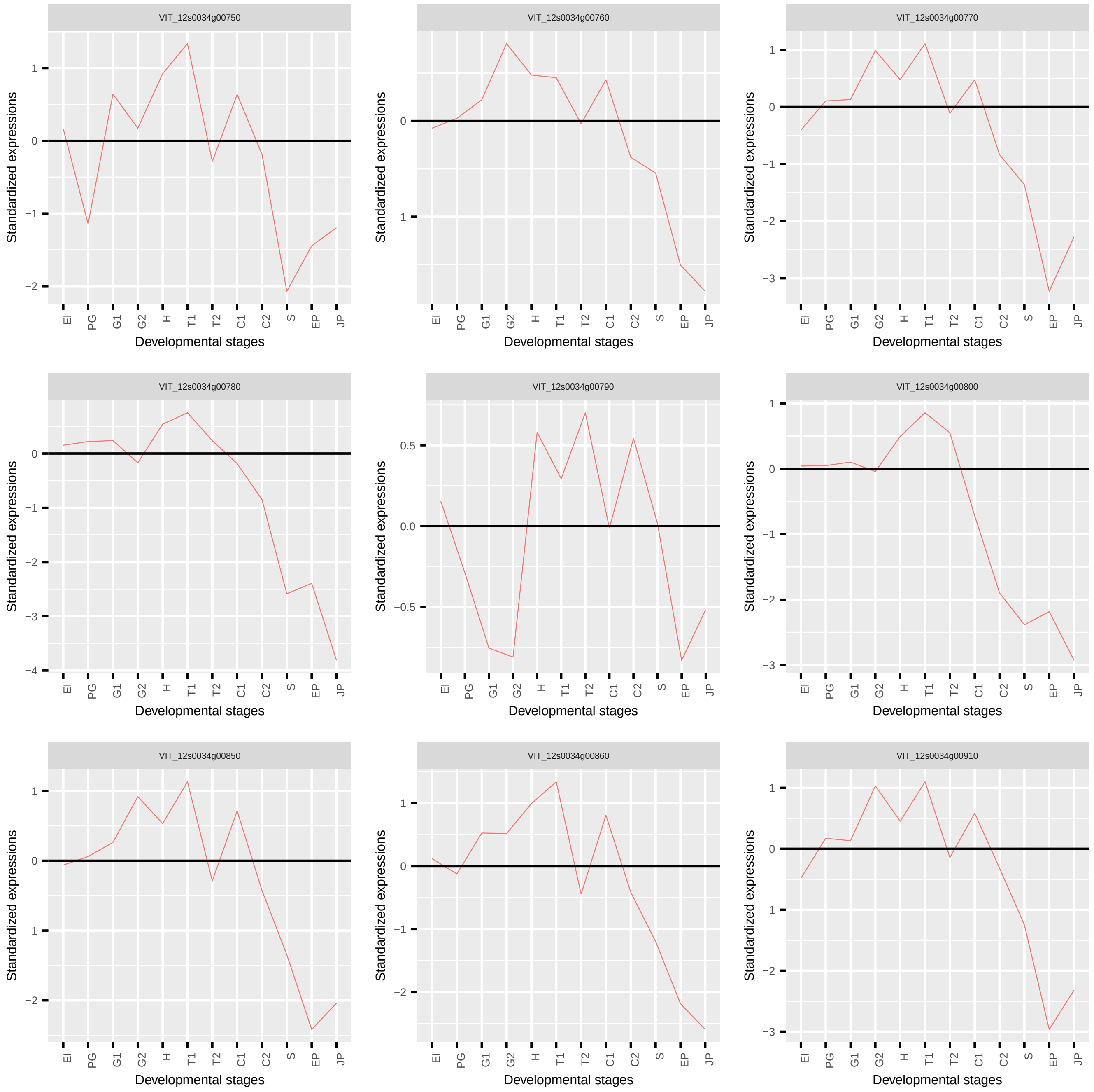

VIT_12s0034g00750
VIT_12s0034g00760
VIT_12s0034g00770
VIT_12s0034g00780
VIT_12s0034g00790
VIT_12s0034g00800
VIT_12s0034g00850
VIT_12s0034g00860
VIT_12s0034g00910
Standardized expressions
Developmental stages
EI PG G1 G2 H T1 T2 C1 C2 S EP JP

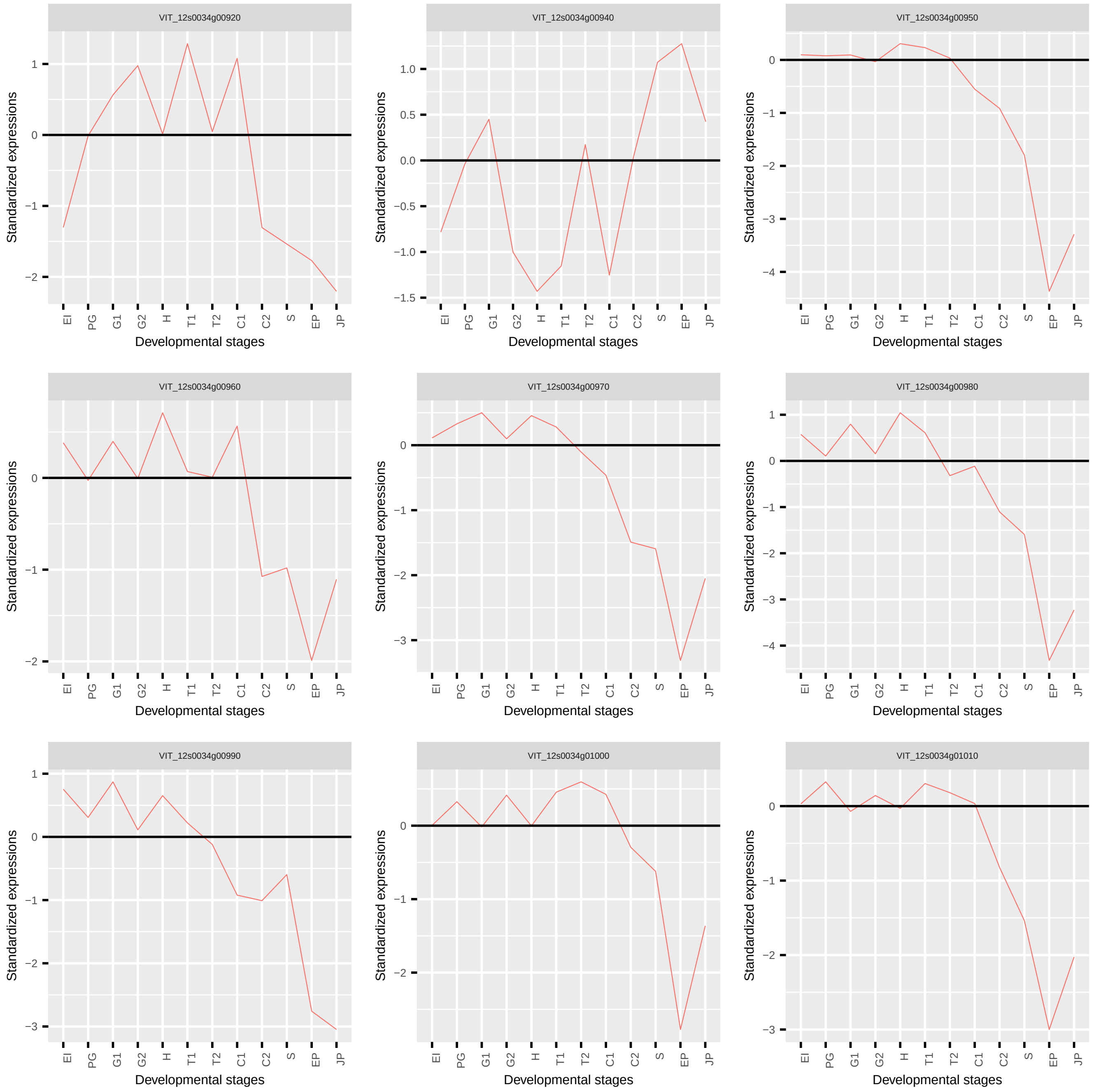

VIT_12s0034g00920
VIT_12s0034g00940
VIT_12s0034g00950
VIT_12s0034g00960
VIT_12s0034g00970
VIT_12s0034g00980
VIT_12s0034g00990
VIT_12s0034g01000
VIT_12s0034g01010
Standardized expressions
Developmental stages
EI
PG
G1
G2
H
T1
T2
C1
C2
S
EP
JP

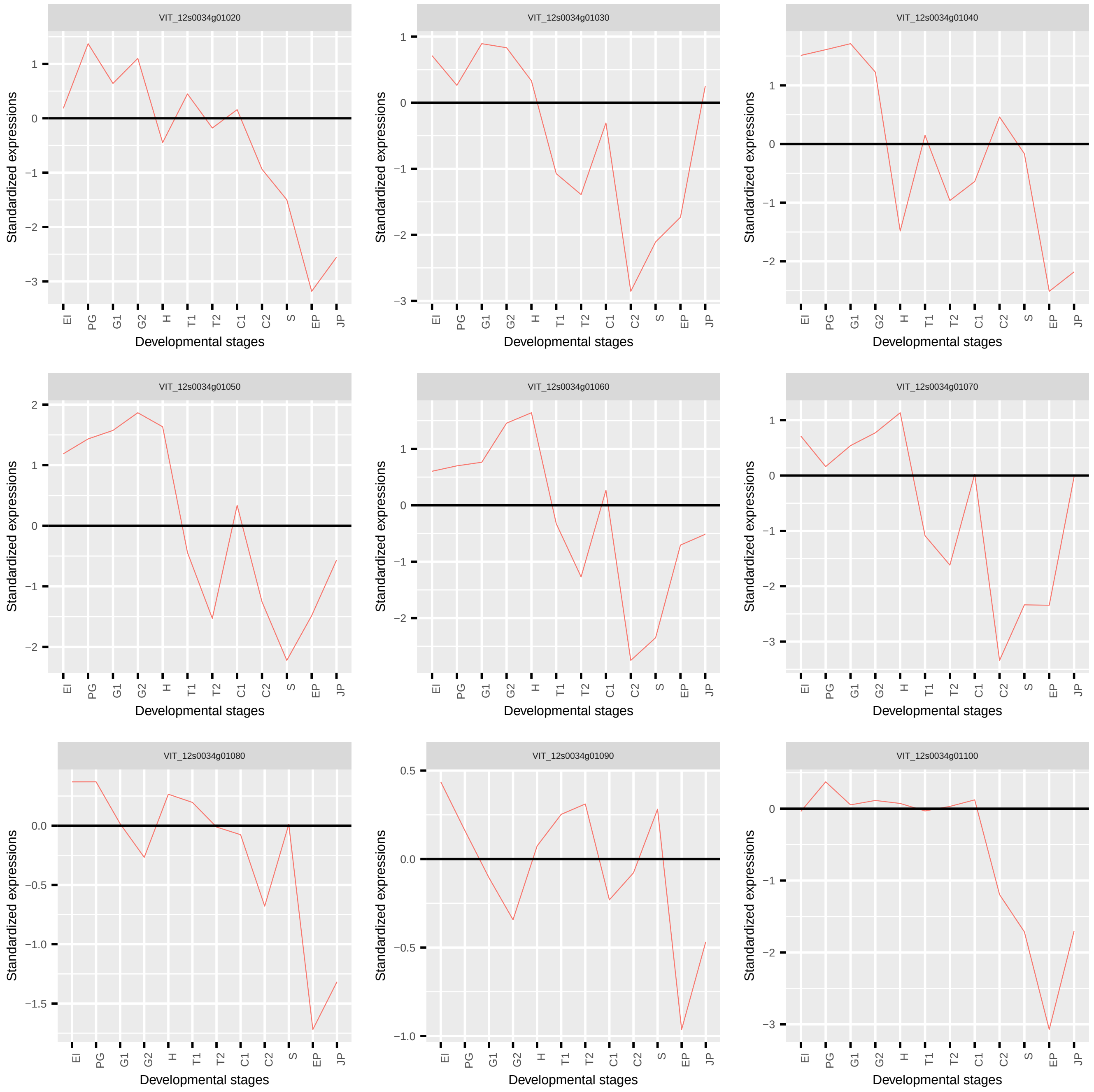
VIT_12s0034g01020
VIT_12s0034g01030
VIT_12s0034g01040
VIT_12s0034g01050
VIT_12s0034g01060
VIT_12s0034g01070
VIT_12s0034g01080
VIT_12s0034g01090
VIT_12s0034g01100
Standardized expressions
Developmental stages
EI
PG
G1
G2
H
T1
T2
C1
C2
S
EP
JP

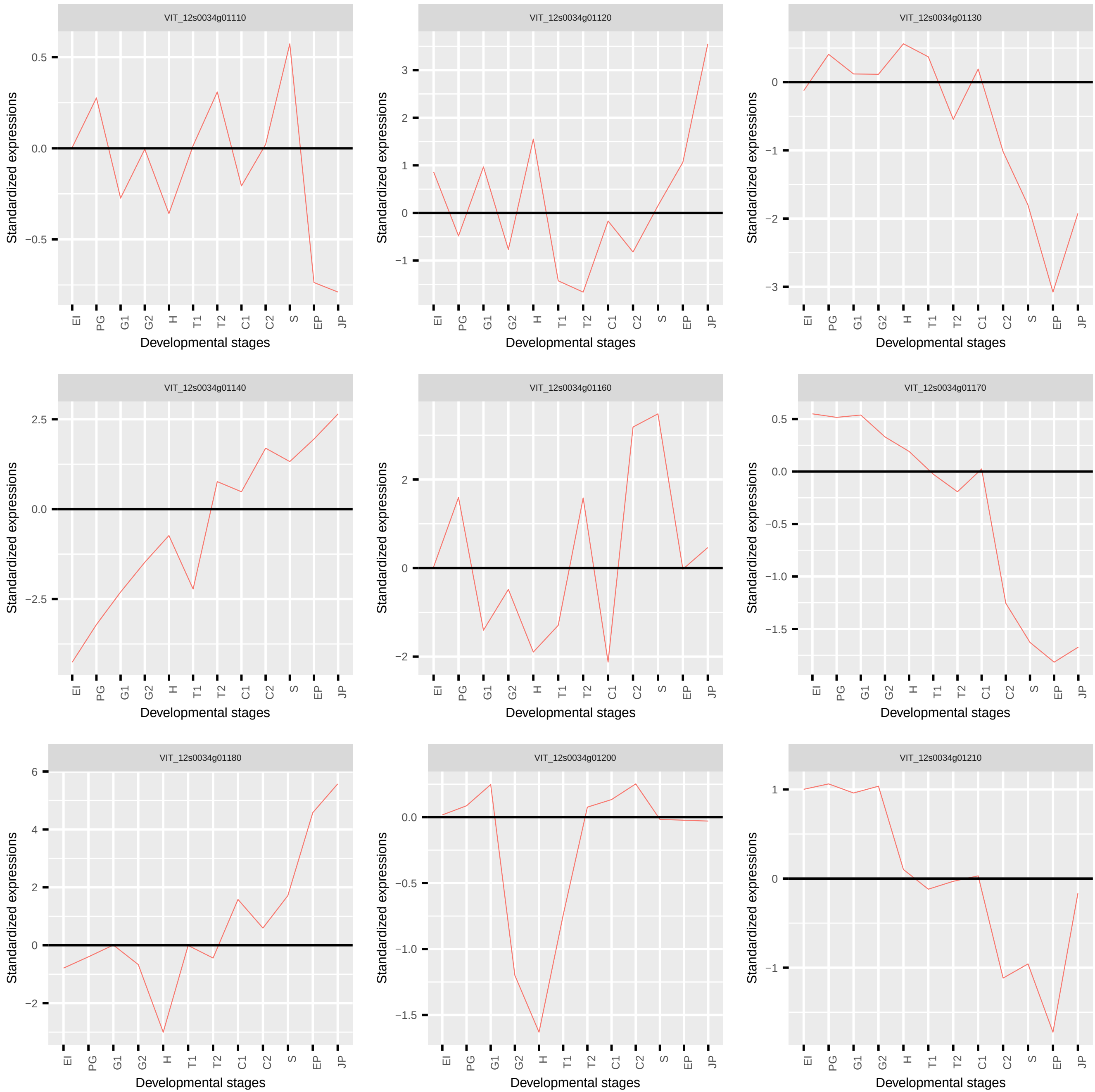
VIT_12s0034g01110
VIT_12s0034g01120
VIT_12s0034g01130
VIT_12s0034g01140
VIT_12s0034g01160
VIT_12s0034g01170
VIT_12s0034g01180
VIT_12s0034g01200
VIT_12s0034g01210
Standardized expressions
Developmental stages
EI PG G1 G2 H T1 T2 C1 C2 S EP JP

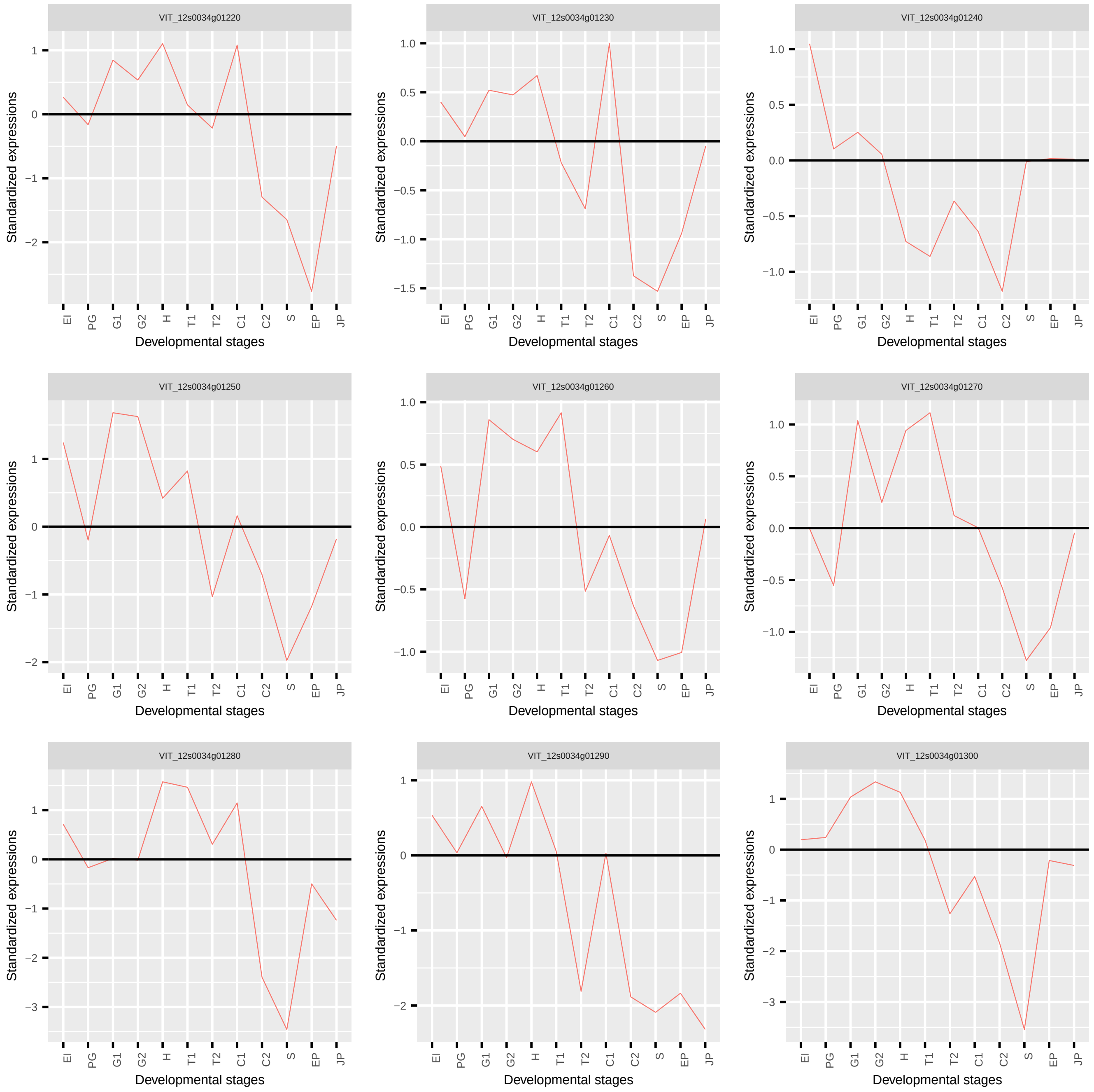
VIT_12s0034g01220
VIT_12s0034g01230
VIT_12s0034g01240
VIT_12s0034g01250
VIT_12s0034g01260
VIT_12s0034g01270
VIT_12s0034g01280
VIT_12s0034g01290
VIT_12s0034g01300
Standardized expressions
Developmental stages
EI PG G1 G2 H T1 T2 C1 C2 S EP JP

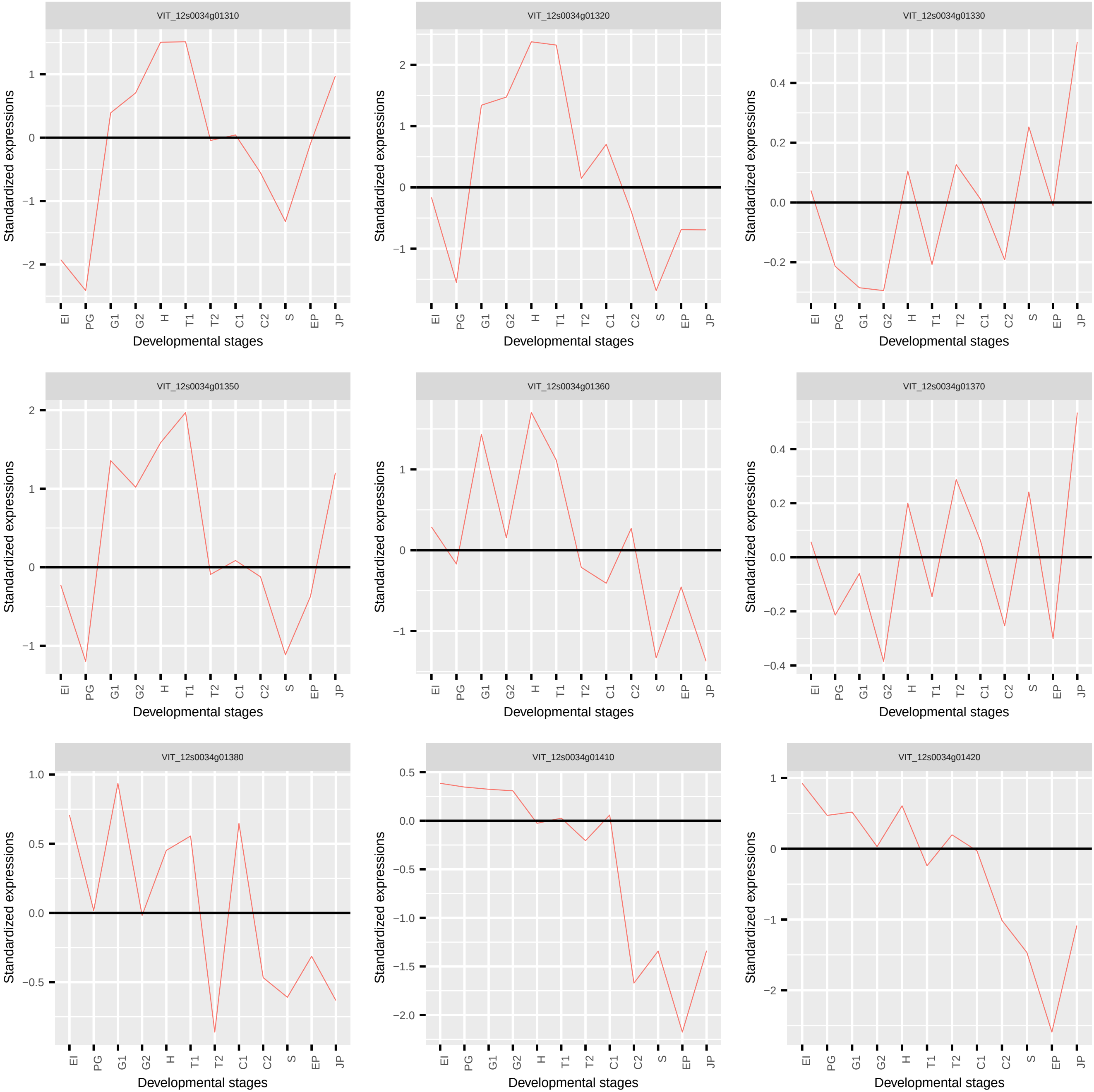

VIT_12s0034g01310
VIT_12s0034g01320
VIT_12s0034g01330
VIT_12s0034g01350
VIT_12s0034g01360
VIT_12s0034g01370
VIT_12s0034g01380
VIT_12s0034g01410
VIT_12s0034g01420
Standardized expressions
Developmental stages
EI PG G1 G2 H T1 T2 C1 C2 S EP JP

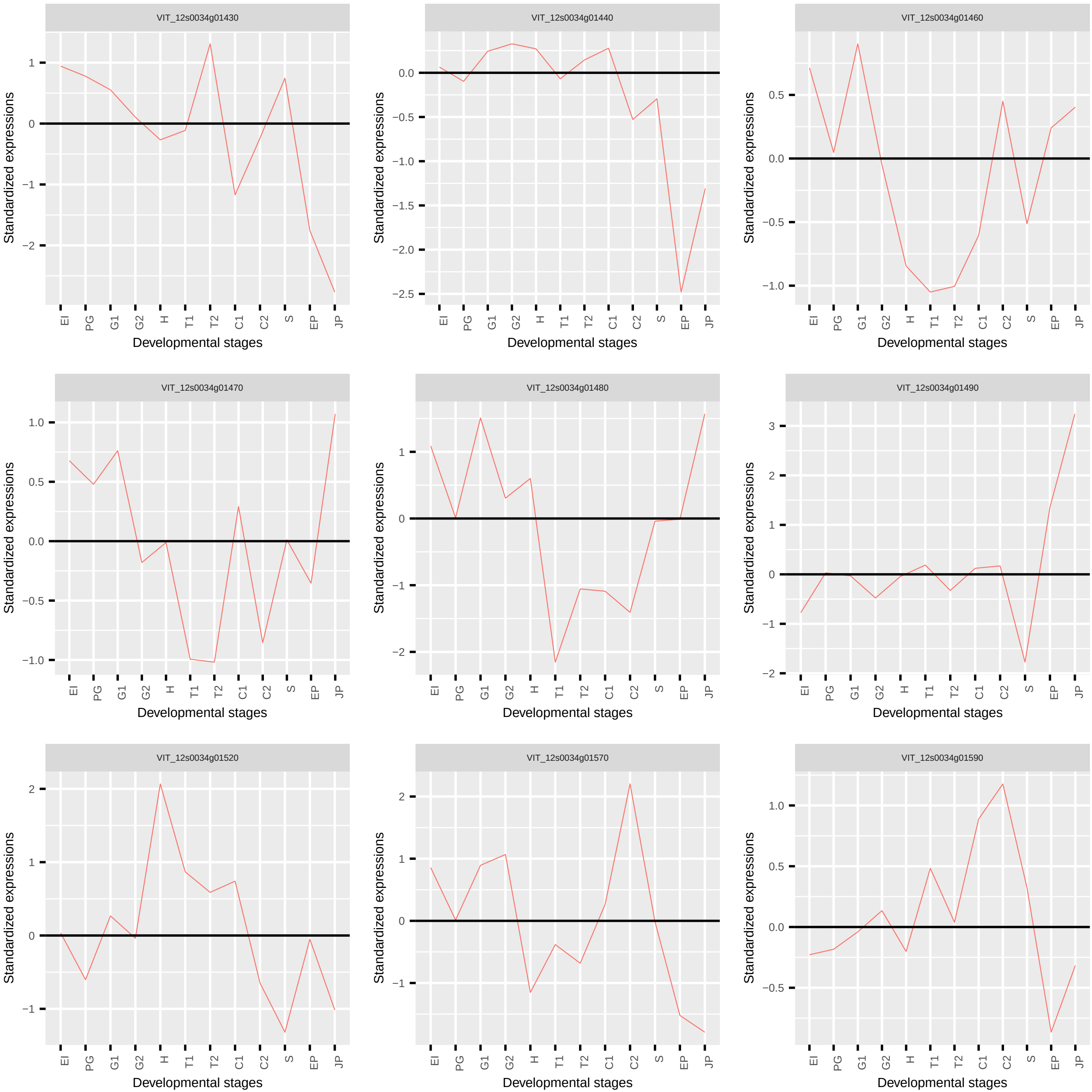
VIT_12s0034g01430
VIT_12s0034g01440
VIT_12s0034g01460
VIT_12s0034g01470
VIT_12s0034g01480
VIT_12s0034g01490
VIT_12s0034g01520
VIT_12s0034g01570
VIT_12s0034g01590
Standardized expressions
Developmental stages
EI PG G1 G2 H T1 T2 C1 C2 S EP JP

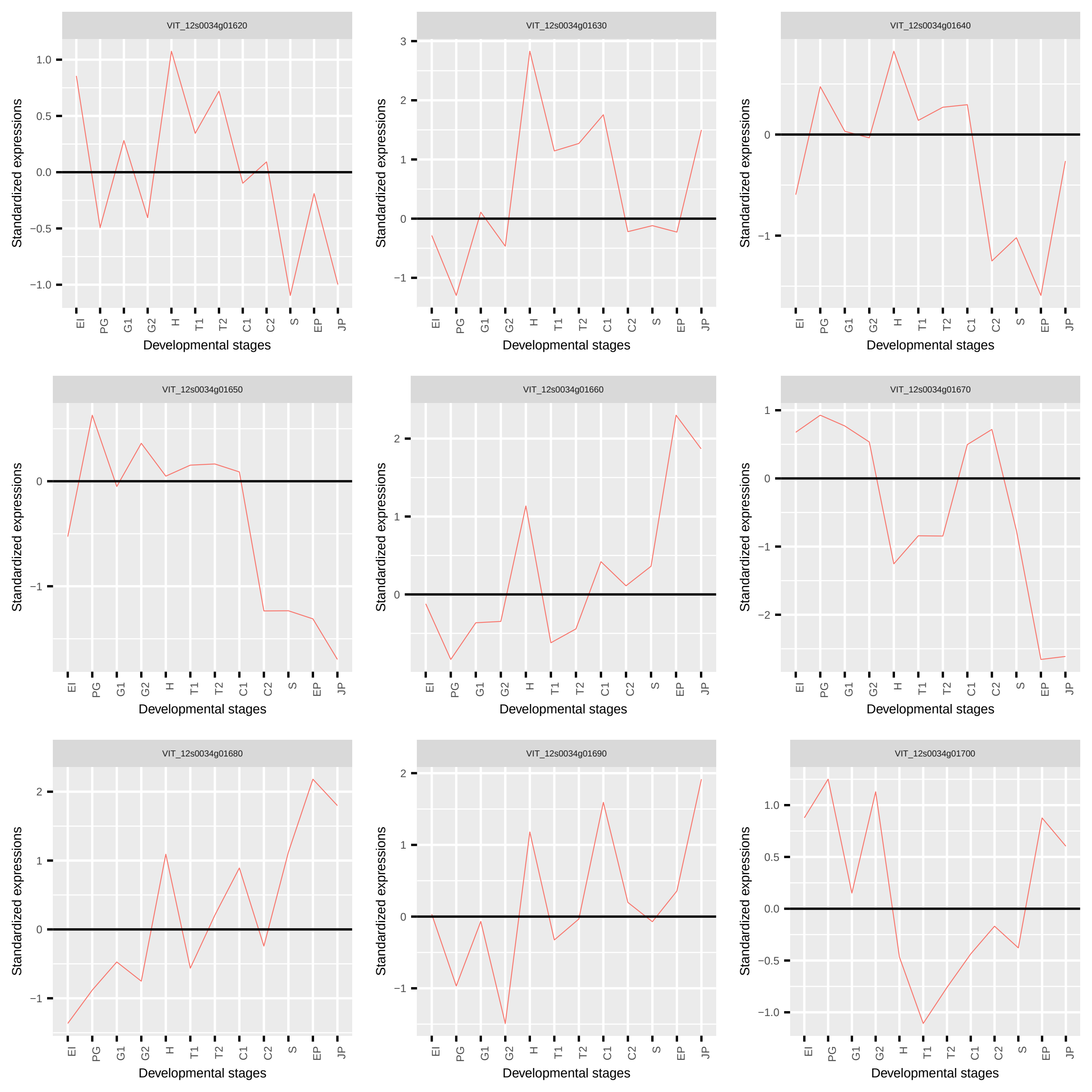
VIT_12s0034g01620
VIT_12s0034g01630
VIT_12s0034g01640
VIT_12s0034g01650
VIT_12s0034g01660
VIT_12s0034g01670
VIT_12s0034g01680
VIT_12s0034g01690
VIT_12s0034g01700
Standardized expressions
Developmental stages
EI PG G1 G2 H T1 T2 C1 C2 S EP JP

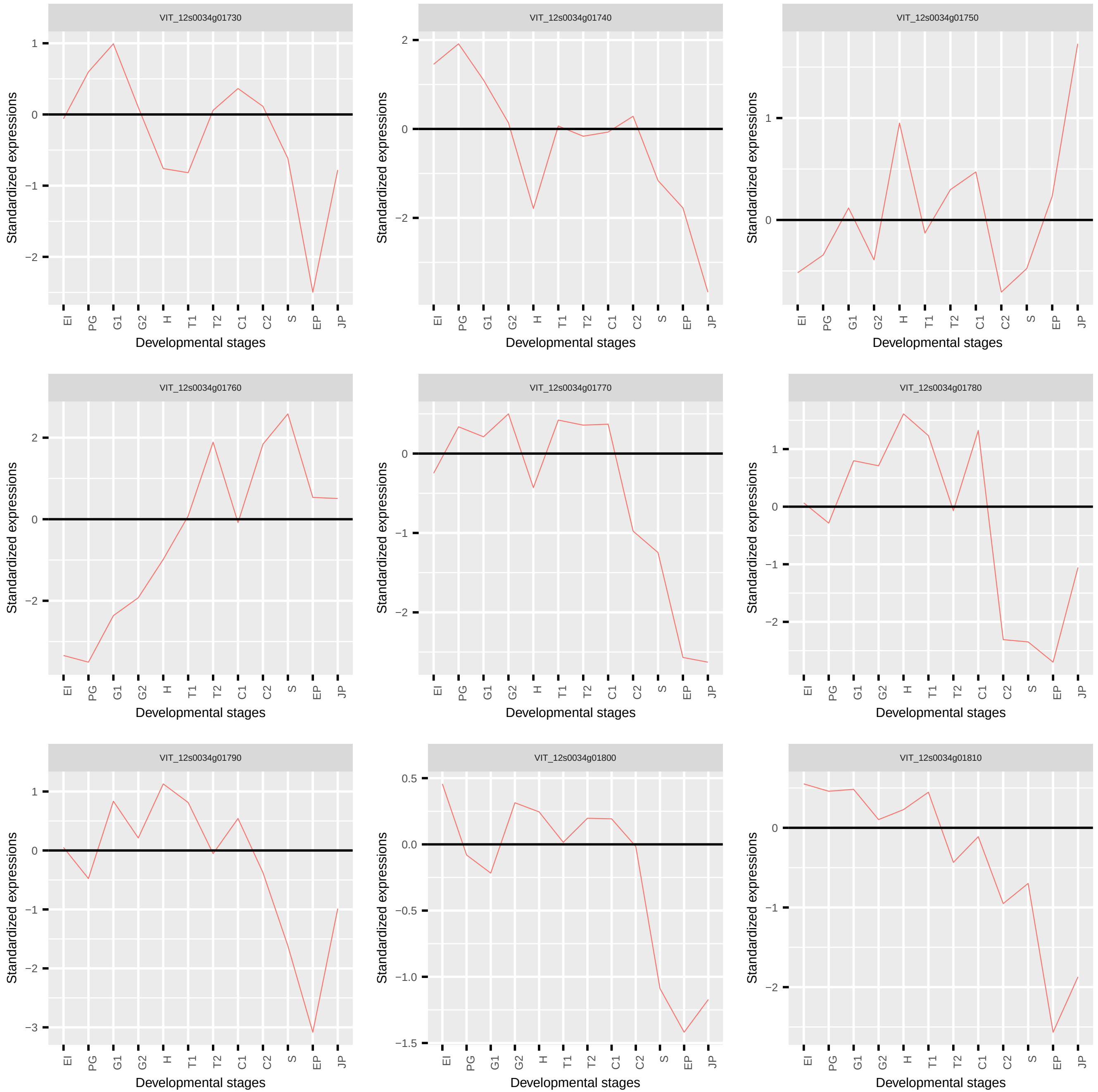
VIT_12s0034g01730
VIT_12s0034g01740
VIT_12s0034g01750
VIT_12s0034g01760
VIT_12s0034g01770
VIT_12s0034g01780
VIT_12s0034g01790
VIT_12s0034g01800
VIT_12s0034g01810
Standardized expressions
Developmental stages
EI PG G1 G2 H T1 T2 C1 C2 S EP JP

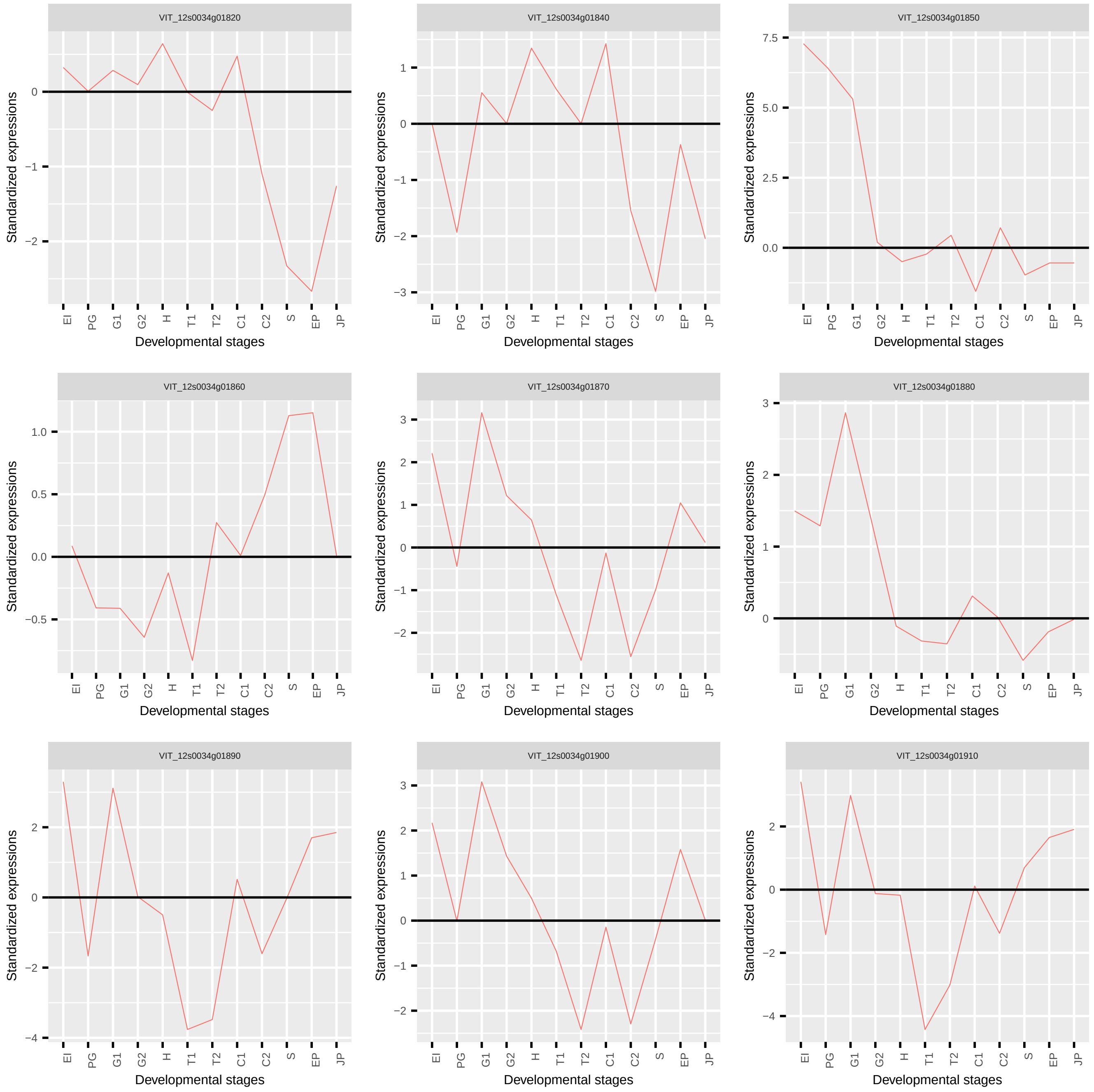

VIT_12s0034g01820
VIT_12s0034g01840
VIT_12s0034g01850
VIT_12s0034g01860
VIT_12s0034g01870
VIT_12s0034g01880
VIT_12s0034g01890
VIT_12s0034g01900
VIT_12s0034g01910
Standardized expressions
Developmental stages
EI
PG
G1
G2
H
T1
T2
C1
C2
S
EP
JP

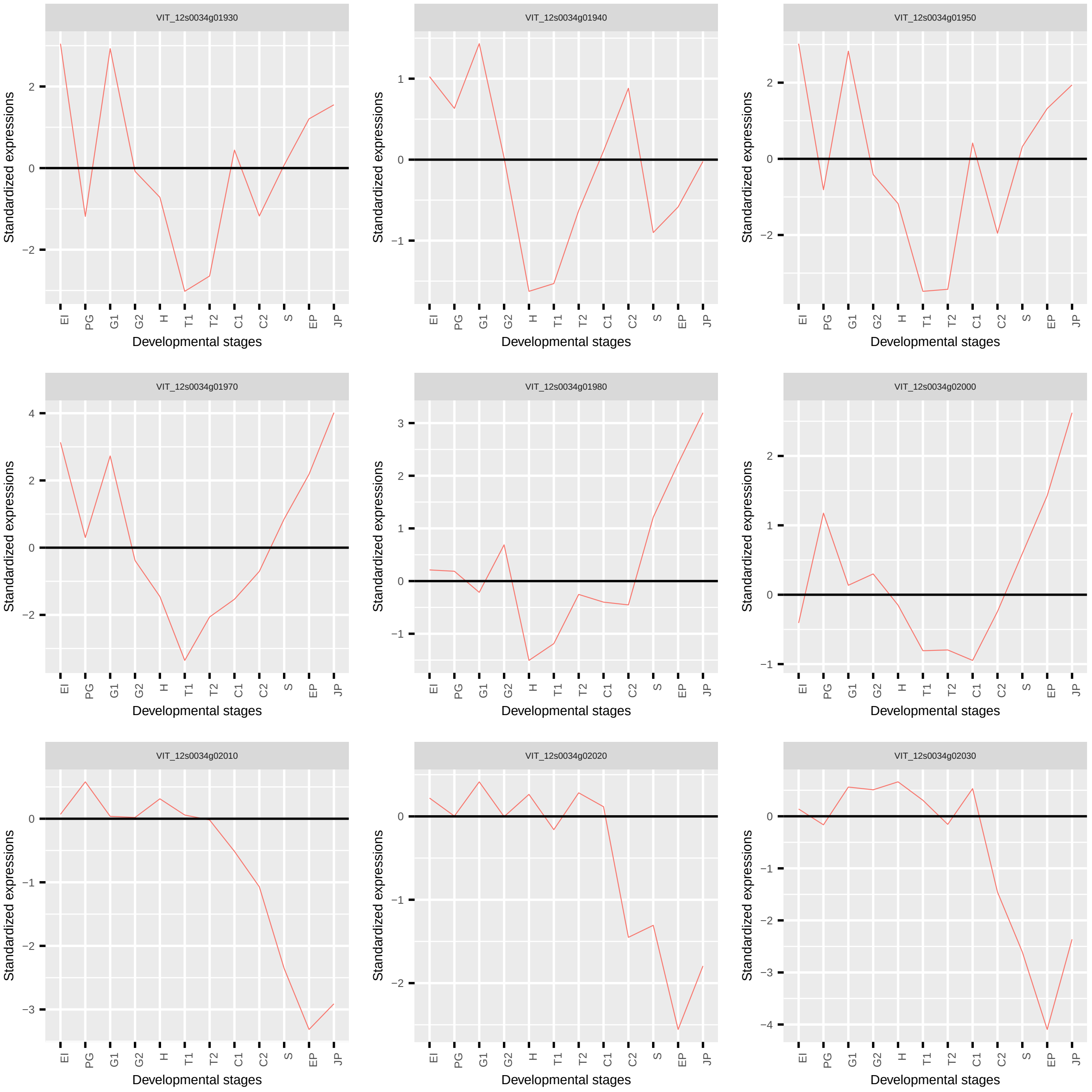

VIT_12s0034g01930
VIT_12s0034g01940
VIT_12s0034g01950
VIT_12s0034g01970
VIT_12s0034g01980
VIT_12s0034g02000
VIT_12s0034g02010
VIT_12s0034g02020
VIT_12s0034g02030
Standardized expressions
Developmental stages
EI PG G1 G2 H T1 T2 C1 C2 S EP JP

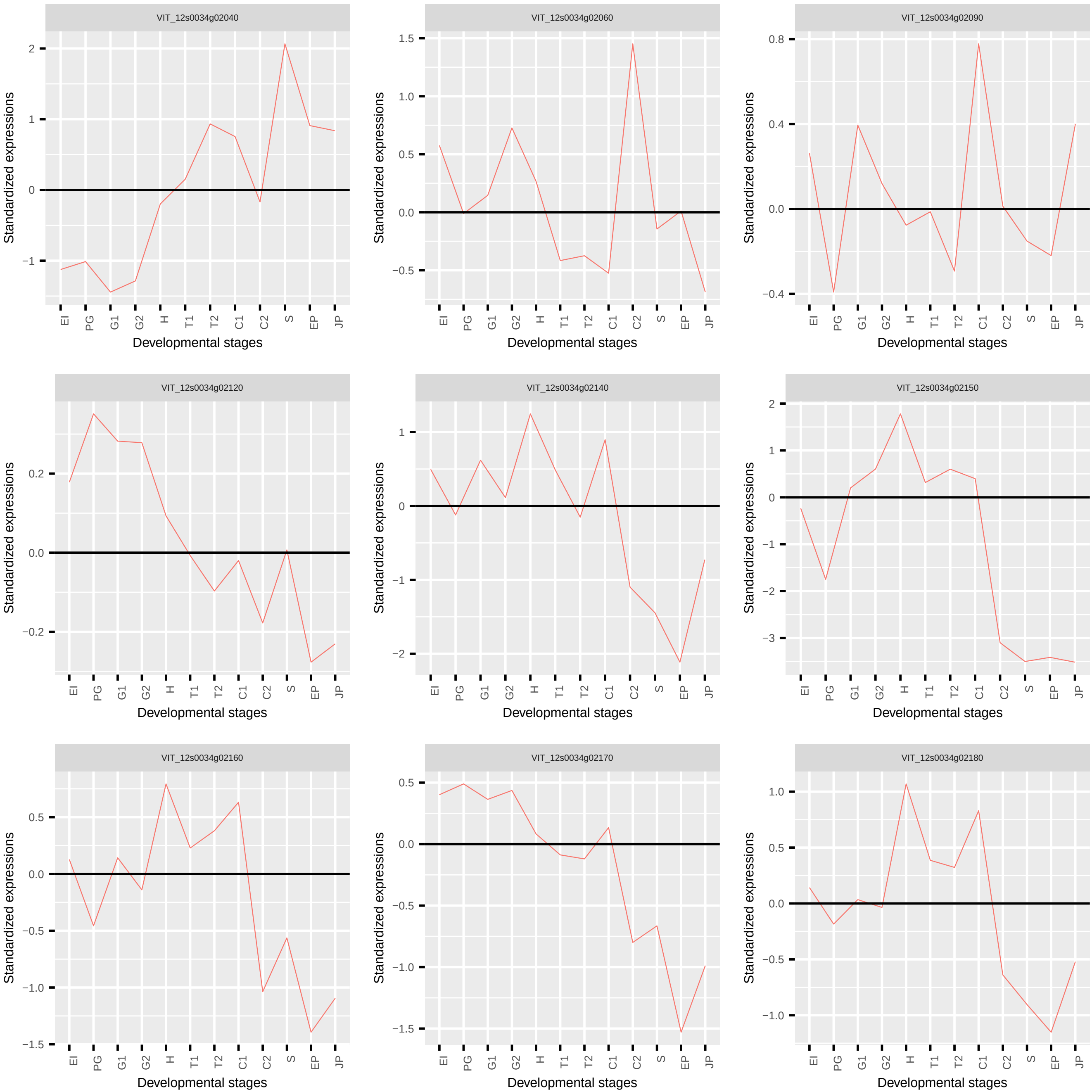

VIT_12s0034g02040
VIT_12s0034g02060
VIT_12s0034g02090
VIT_12s0034g02120
VIT_12s0034g02140
VIT_12s0034g02150
VIT_12s0034g02160
VIT_12s0034g02170
VIT_12s0034g02180
Standardized expressions
Developmental stages
EI PG G1 G2 H T1 T2 C1 C2 S EP JP

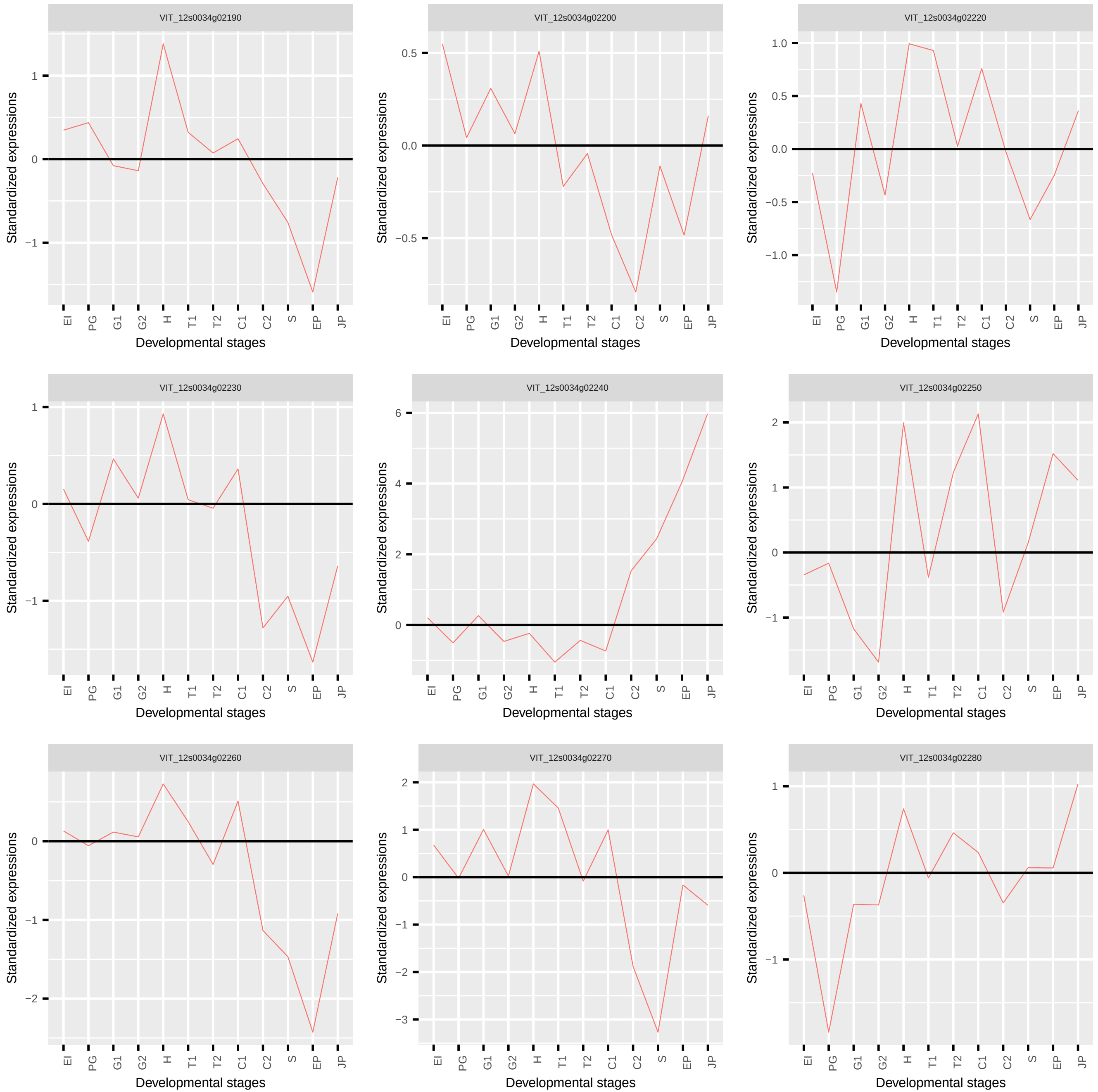
VIT_12s0034g02190
VIT_12s0034g02200
VIT_12s0034g02220
VIT_12s0034g02230
VIT_12s0034g02240
VIT_12s0034g02250
VIT_12s0034g02260
VIT_12s0034g02270
VIT_12s0034g02280
Standardized expressions
Developmental stages
EI PG G1 G2 H T1 T2 C1 C2 S EP JP

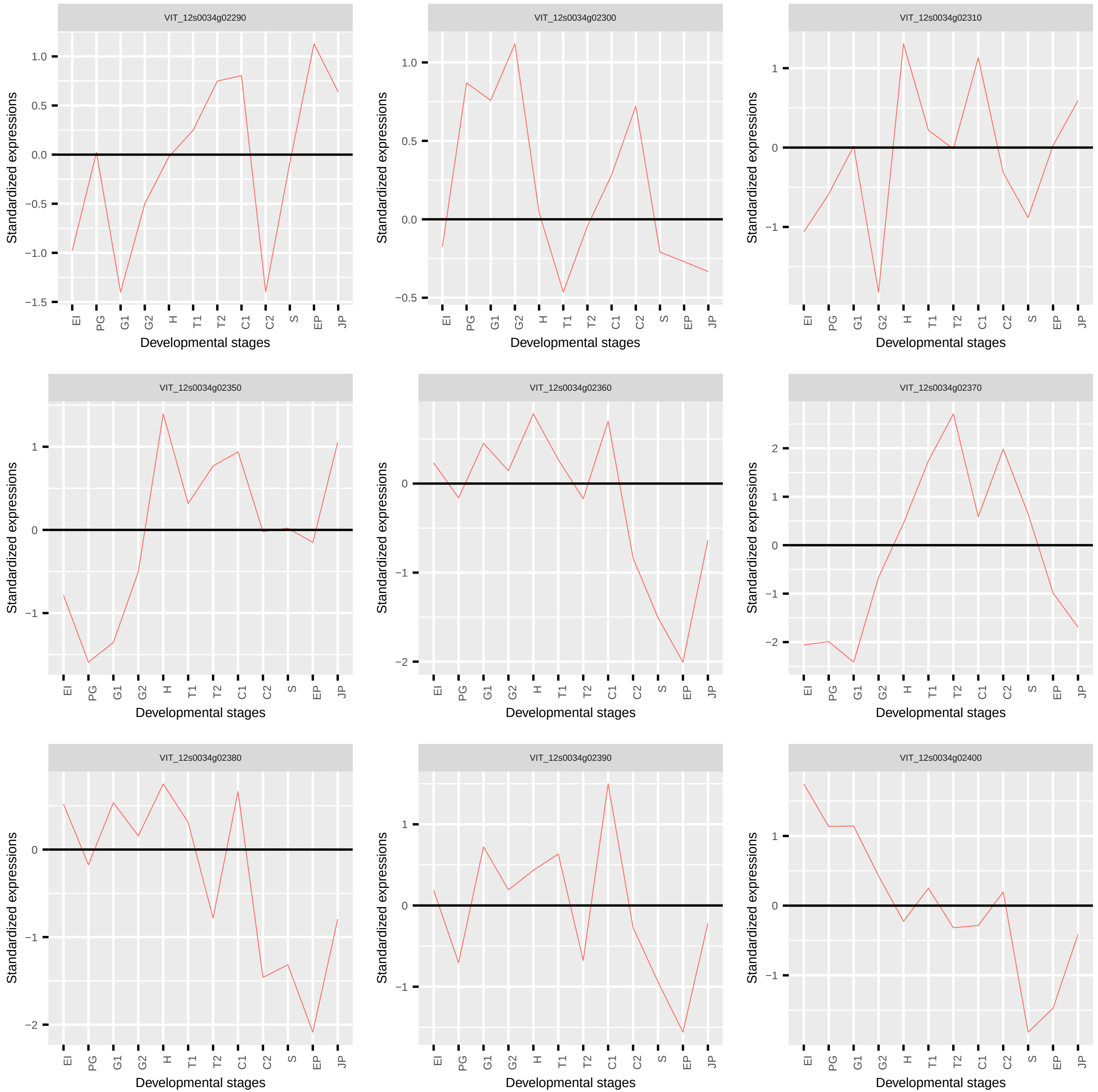
VIT_12s0034g02290
VIT_12s0034g02300
VIT_12s0034g02310
VIT_12s0034g02350
VIT_12s0034g02360
VIT_12s0034g02370
VIT_12s0034g02380
VIT_12s0034g02390
VIT_12s0034g02400
Standardized expressions
Developmental stages
EI PG G1 G2 H T1 T2 C1 C2 S EP JP

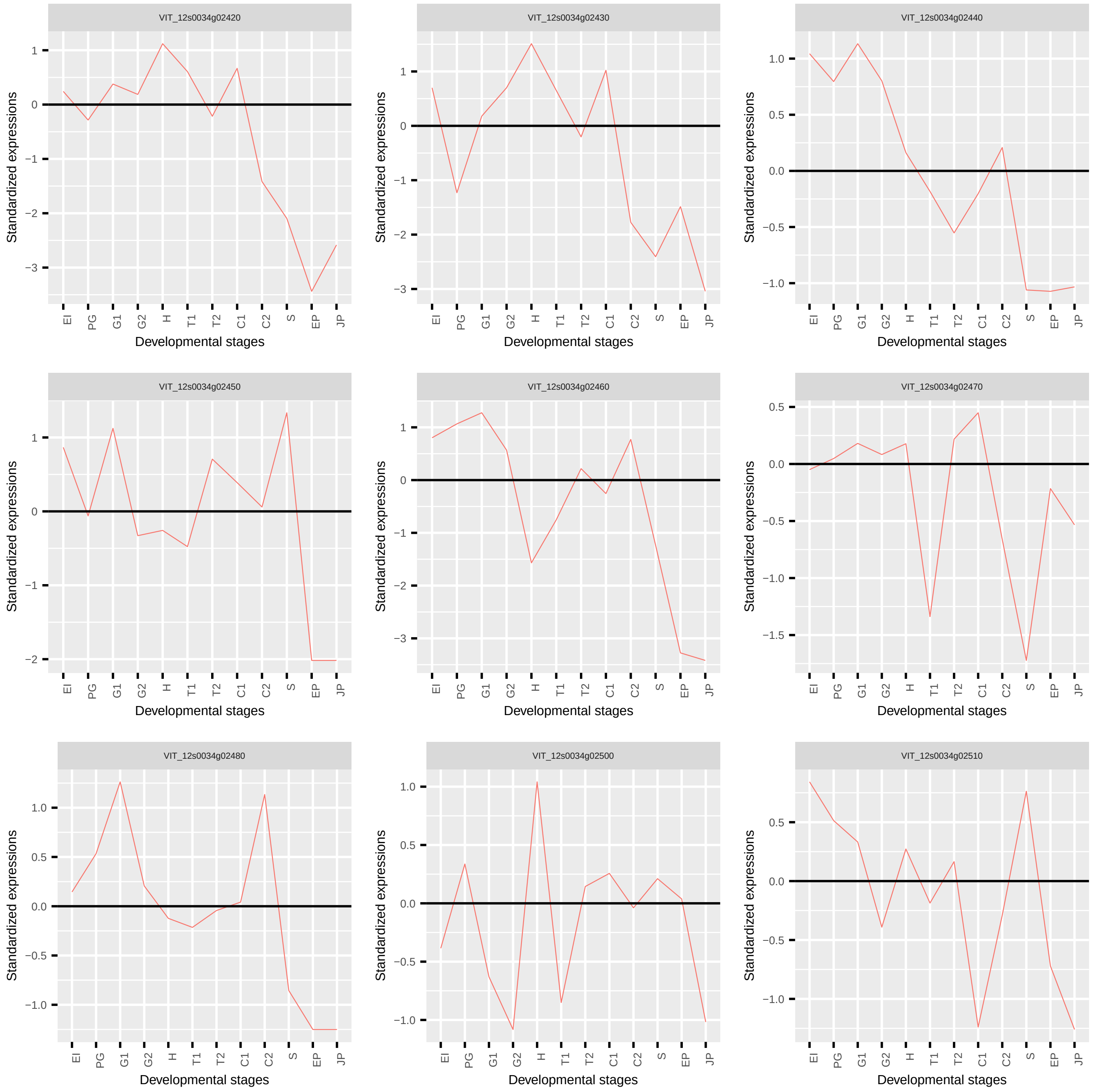

VIT_12s0034g02420
VIT_12s0034g02430
VIT_12s0034g02440
VIT_12s0034g02450
VIT_12s0034g02460
VIT_12s0034g02470
VIT_12s0034g02480
VIT_12s0034g02500
VIT_12s0034g02510
Standardized expressions
Developmental stages
EI PG G1 G2 H T1 T2 C1 C2 S EP JP

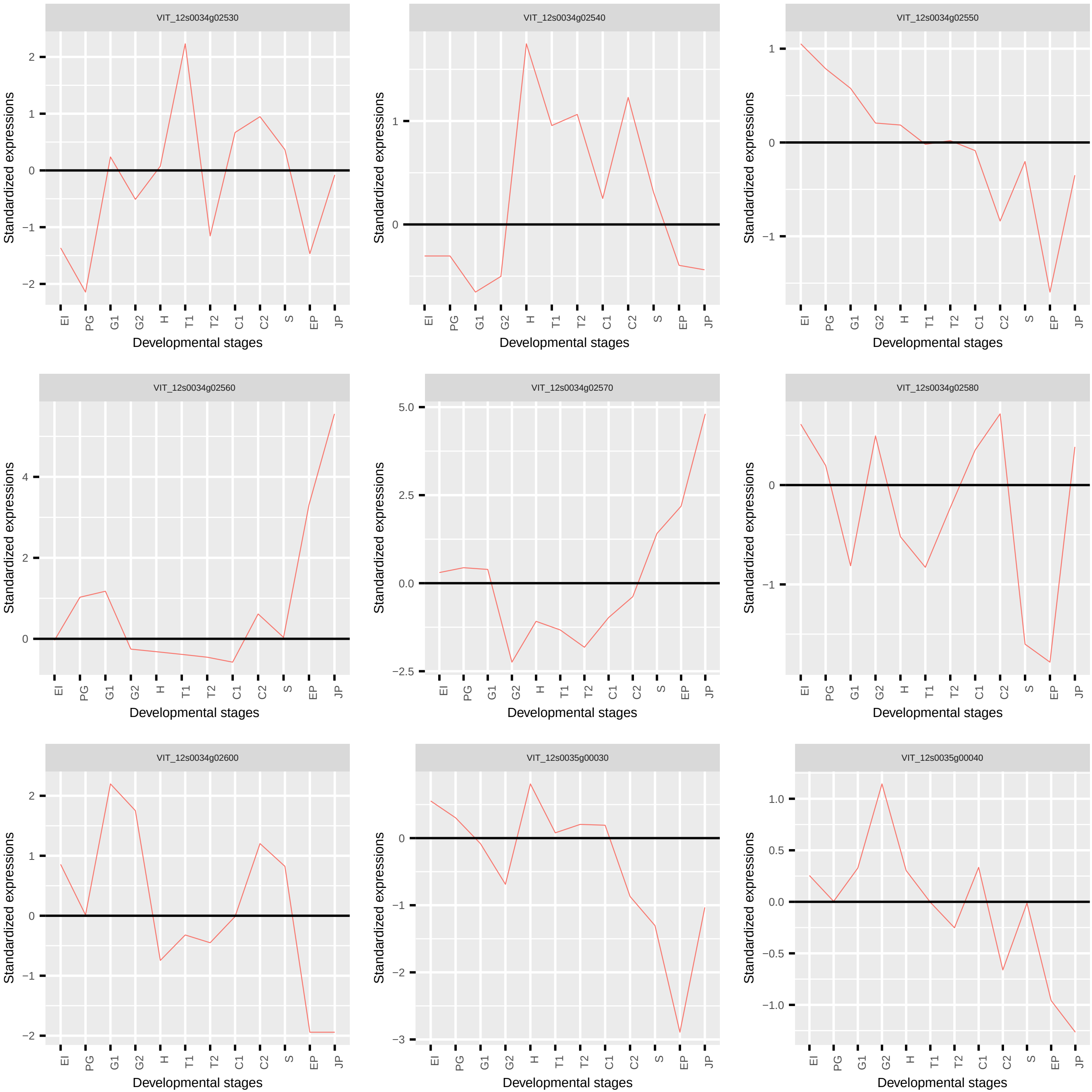
VIT_12s0034g02530
VIT_12s0034g02540
VIT_12s0034g02550
VIT_12s0034g02560
VIT_12s0034g02570
VIT_12s0034g02580
VIT_12s0034g02600
VIT_12s0035g00030
VIT_12s0035g00040
Standardized expressions
Developmental stages
EI PG G1 G2 H T1 T2 C1 C2 S EP JP

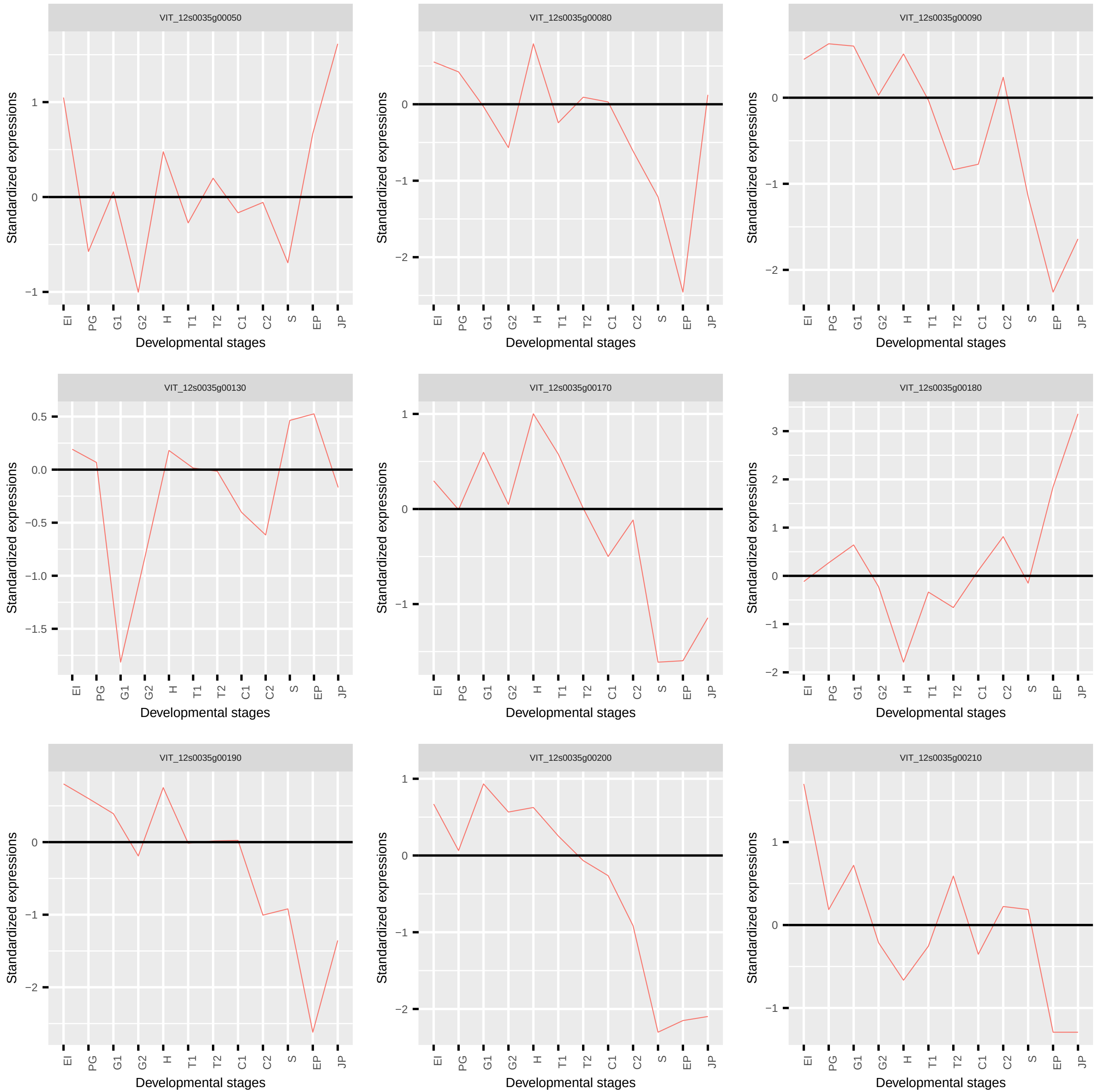
VIT_12s0035g00050
VIT_12s0035g00080
VIT_12s0035g00090
VIT_12s0035g00130
VIT_12s0035g00170
VIT_12s0035g00180
VIT_12s0035g00190
VIT_12s0035g00200
VIT_12s0035g00210
Standardized expressions
Developmental stages
EI PG G1 G2 H T1 T2 C1 C2 S EP JP

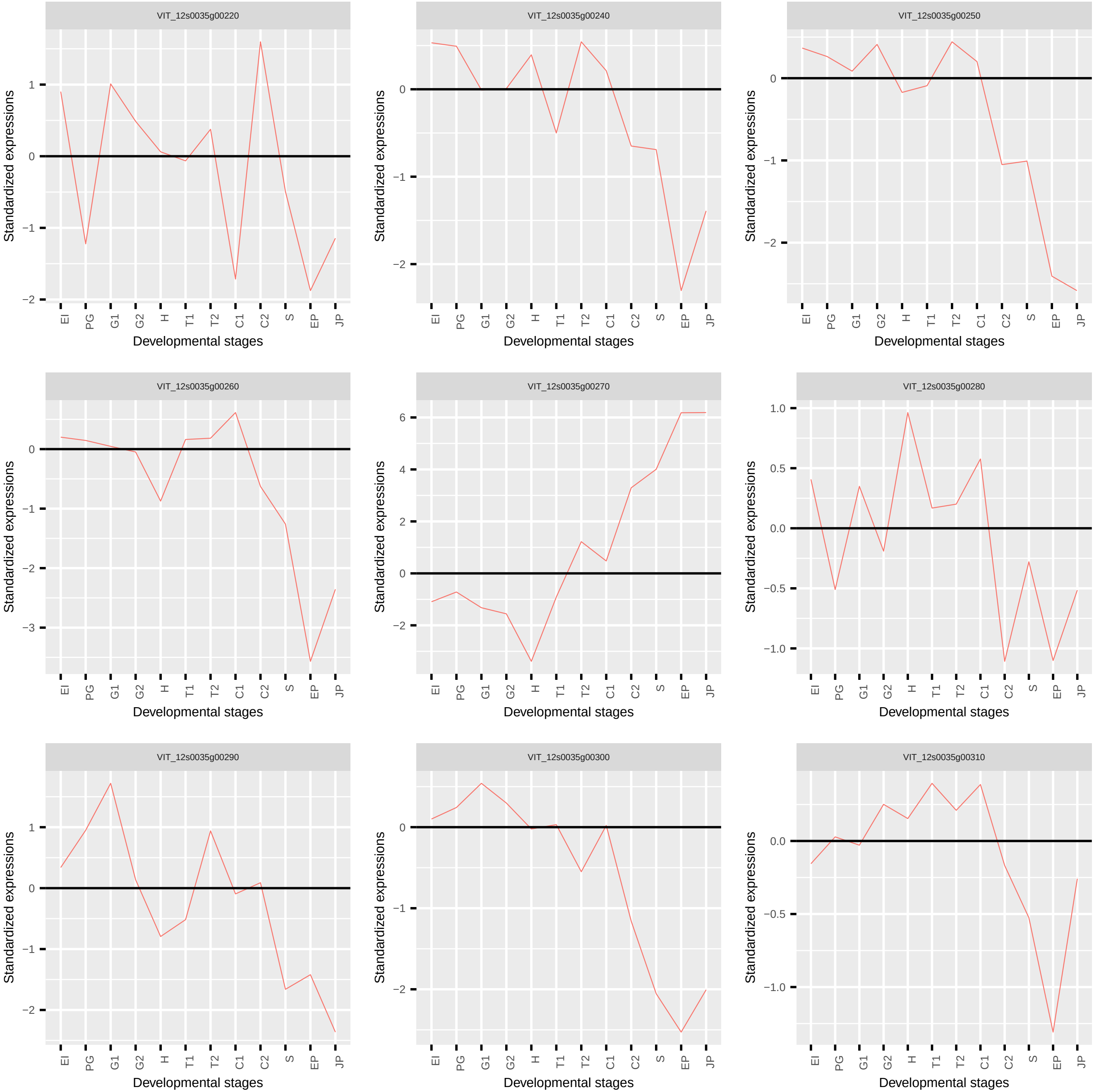

VIT_12s0035g00220
VIT_12s0035g00240
VIT_12s0035g00250
VIT_12s0035g00260
VIT_12s0035g00270
VIT_12s0035g00280
VIT_12s0035g00290
VIT_12s0035g00300
VIT_12s0035g00310
Standardized expressions
Developmental stages
EI PG G1 G2 H T1 T2 C1 C2 S EP JP

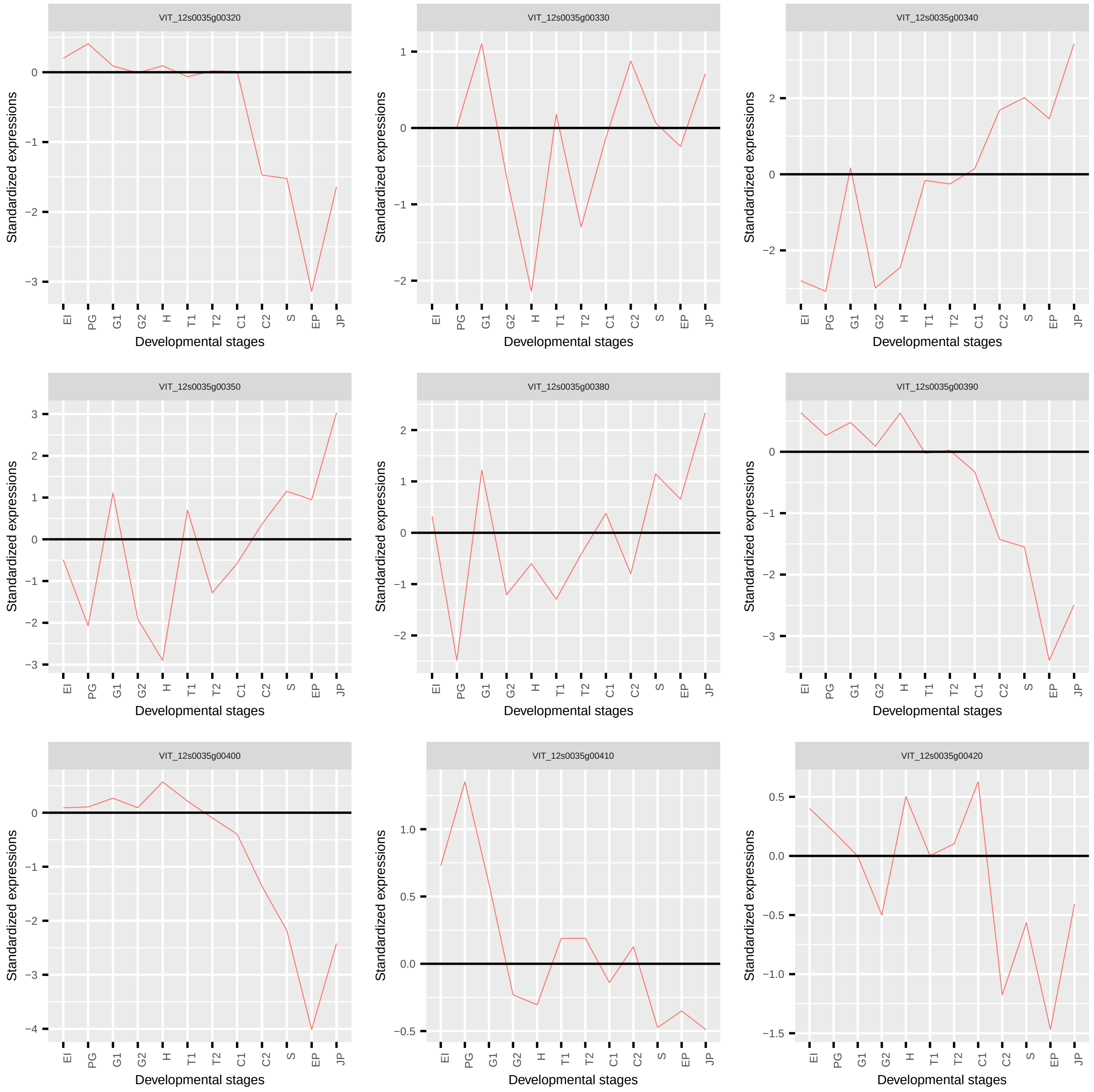
VIT_12s0035g00320
VIT_12s0035g00330
VIT_12s0035g00340
VIT_12s0035g00350
VIT_12s0035g00380
VIT_12s0035g00390
VIT_12s0035g00400
VIT_12s0035g00410
VIT_12s0035g00420
Standardized expressions
Developmental stages
EI PG G1 G2 H T1 T2 C1 C2 S EP JP

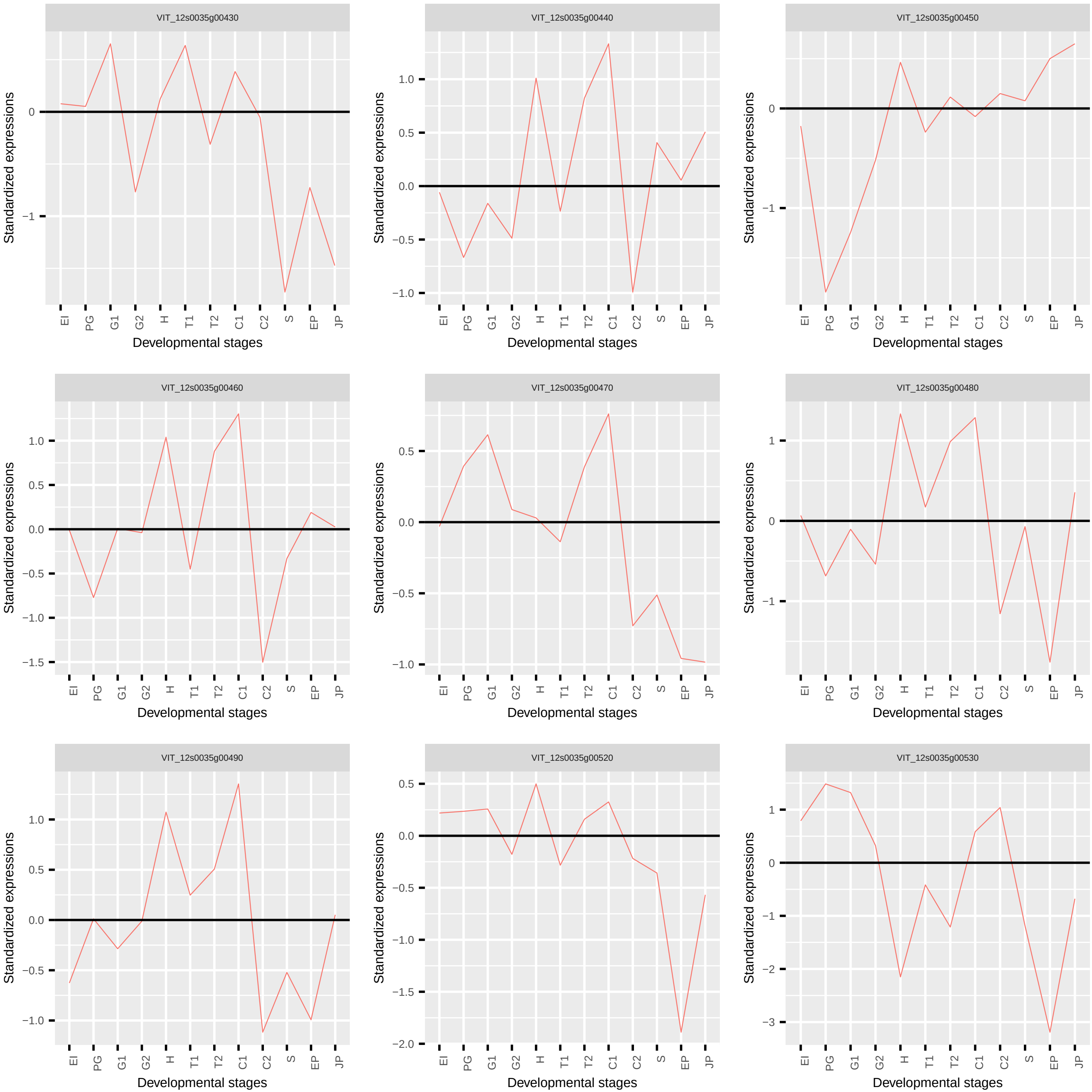
VIT_12s0035g00430
VIT_12s0035g00440
VIT_12s0035g00450
VIT_12s0035g00460
VIT_12s0035g00470
VIT_12s0035g00480
VIT_12s0035g00490
VIT_12s0035g00520
VIT_12s0035g00530
Standardized expressions
Developmental stages
EI PG G1 G2 H T1 T2 C1 C2 S EP JP

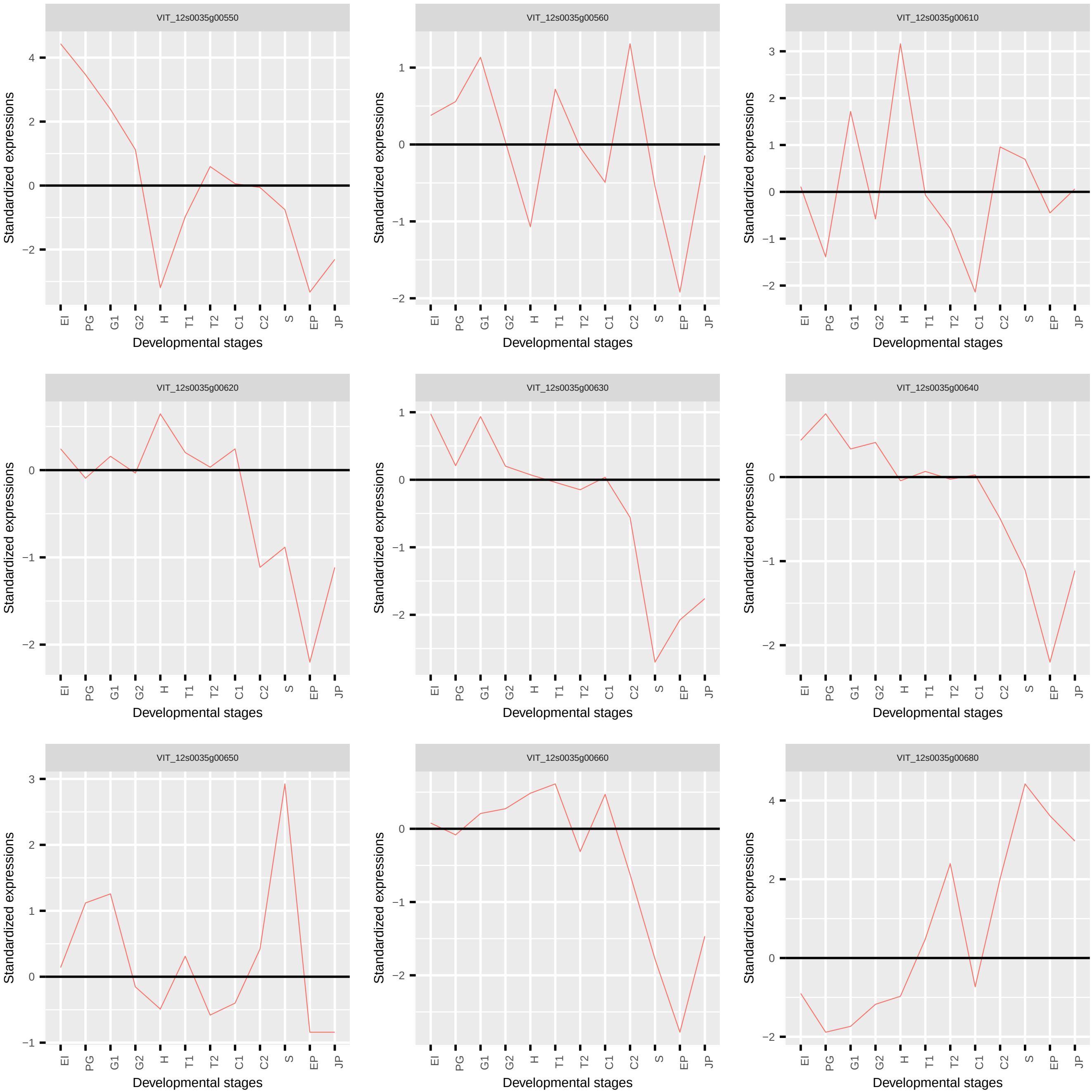
VIT_12s0035g00550
VIT_12s0035g00560
VIT_12s0035g00610
VIT_12s0035g00620
VIT_12s0035g00630
VIT_12s0035g00640
VIT_12s0035g00650
VIT_12s0035g00660
VIT_12s0035g00680
Standardized expressions
Developmental stages
EI PG G1 G2 H T1 T2 C1 C2 S EP JP

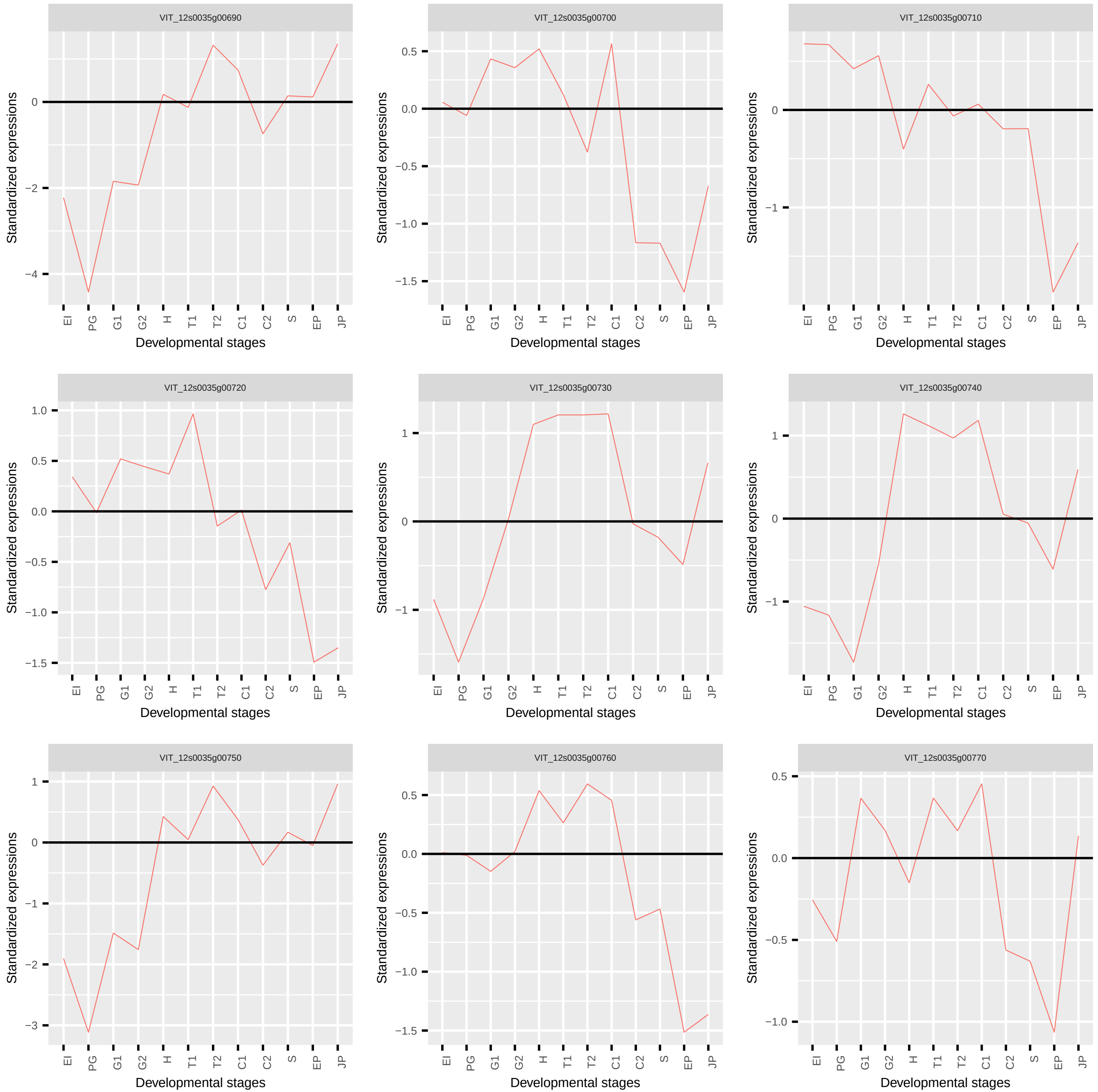
VIT_12s0035g00690
VIT_12s0035g00700
VIT_12s0035g00710
VIT_12s0035g00720
VIT_12s0035g00730
VIT_12s0035g00740
VIT_12s0035g00750
VIT_12s0035g00760
VIT_12s0035g00770
Standardized expressions
Developmental stages
EI PG G1 G2 H T1 T2 C1 C2 S EP JP

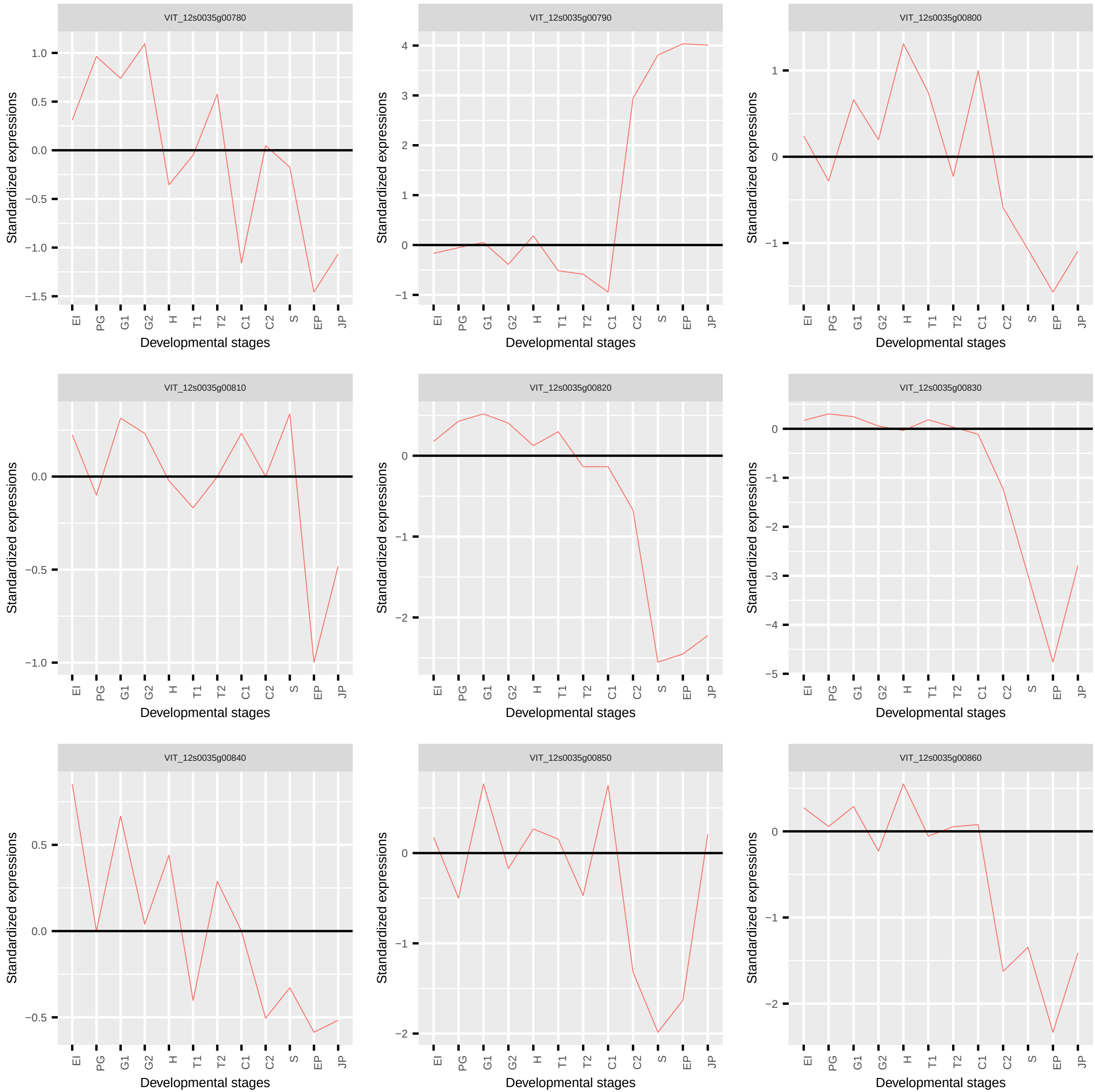
VIT_12s0035g00780
VIT_12s0035g00790
VIT_12s0035g00800
VIT_12s0035g00810
VIT_12s0035g00820
VIT_12s0035g00830
VIT_12s0035g00840
VIT_12s0035g00850
VIT_12s0035g00860
Standardized expressions
Developmental stages
EI PG G1 G2 H T1 T2 C1 C2 S EP JP

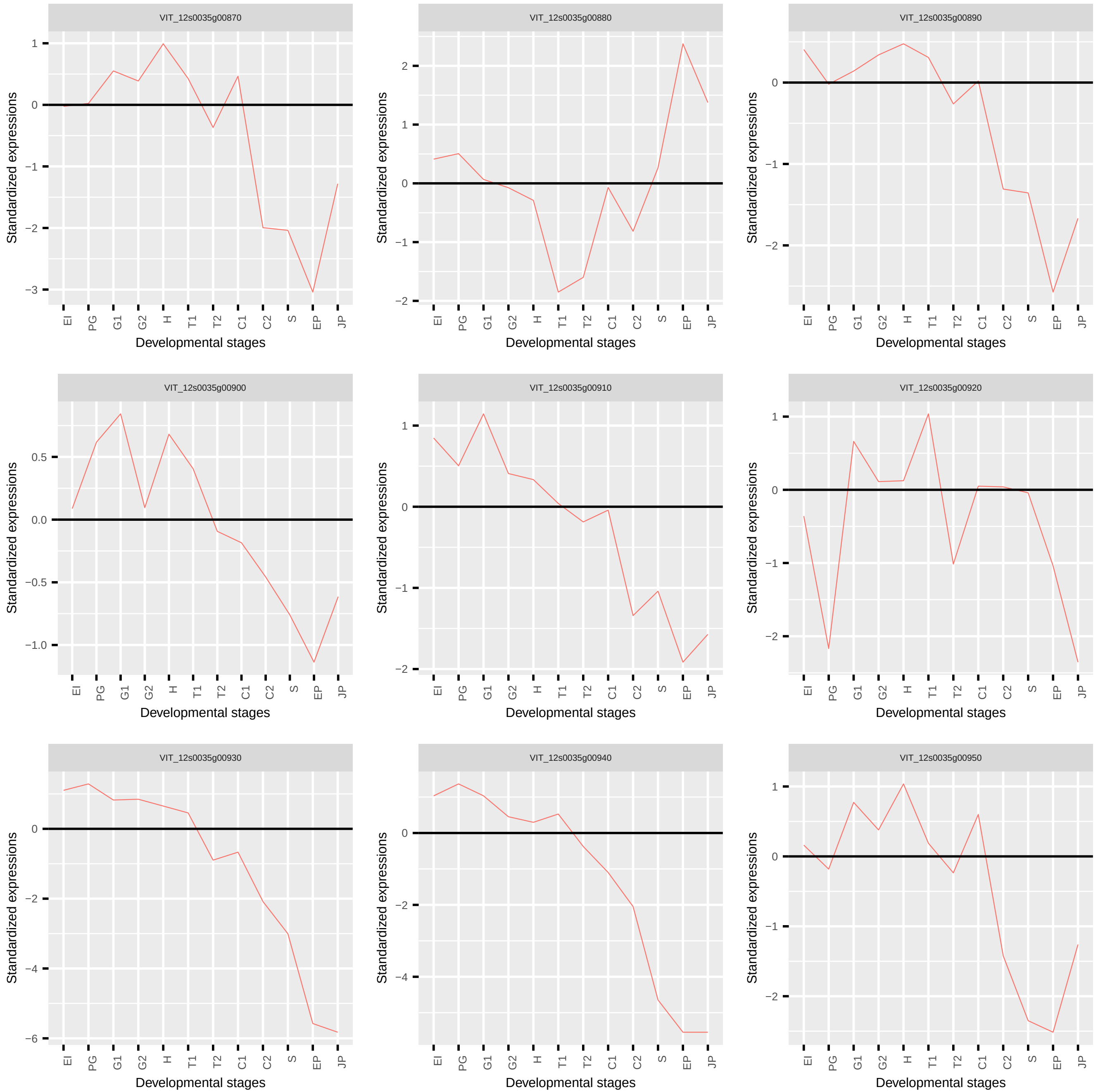
VIT_12s0035g00870
VIT_12s0035g00880
VIT_12s0035g00890
VIT_12s0035g00900
VIT_12s0035g00910
VIT_12s0035g00920
VIT_12s0035g00930
VIT_12s0035g00940
VIT_12s0035g00950
Standardized expressions
Developmental stages
EI PG G1 G2 H T1 T2 C1 C2 S EP JP

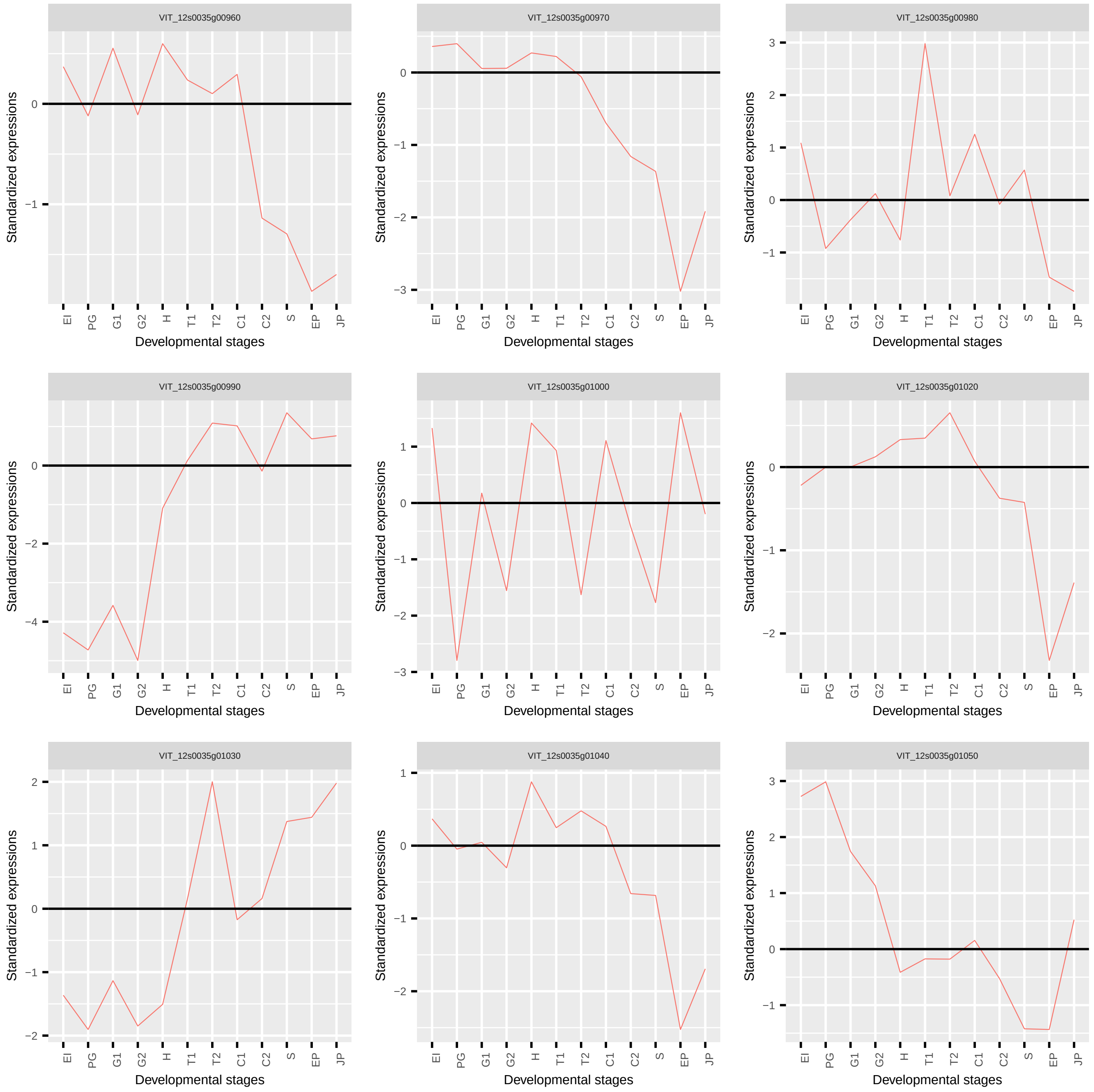
VIT_12s0035g00960
VIT_12s0035g00970
VIT_12s0035g00980
VIT_12s0035g00990
VIT_12s0035g01000
VIT_12s0035g01020
VIT_12s0035g01030
VIT_12s0035g01040
VIT_12s0035g01050
Standardized expressions
Developmental stages
EI
PG
G1
G2
H
T1
T2
C1
C2
S
EP
JP

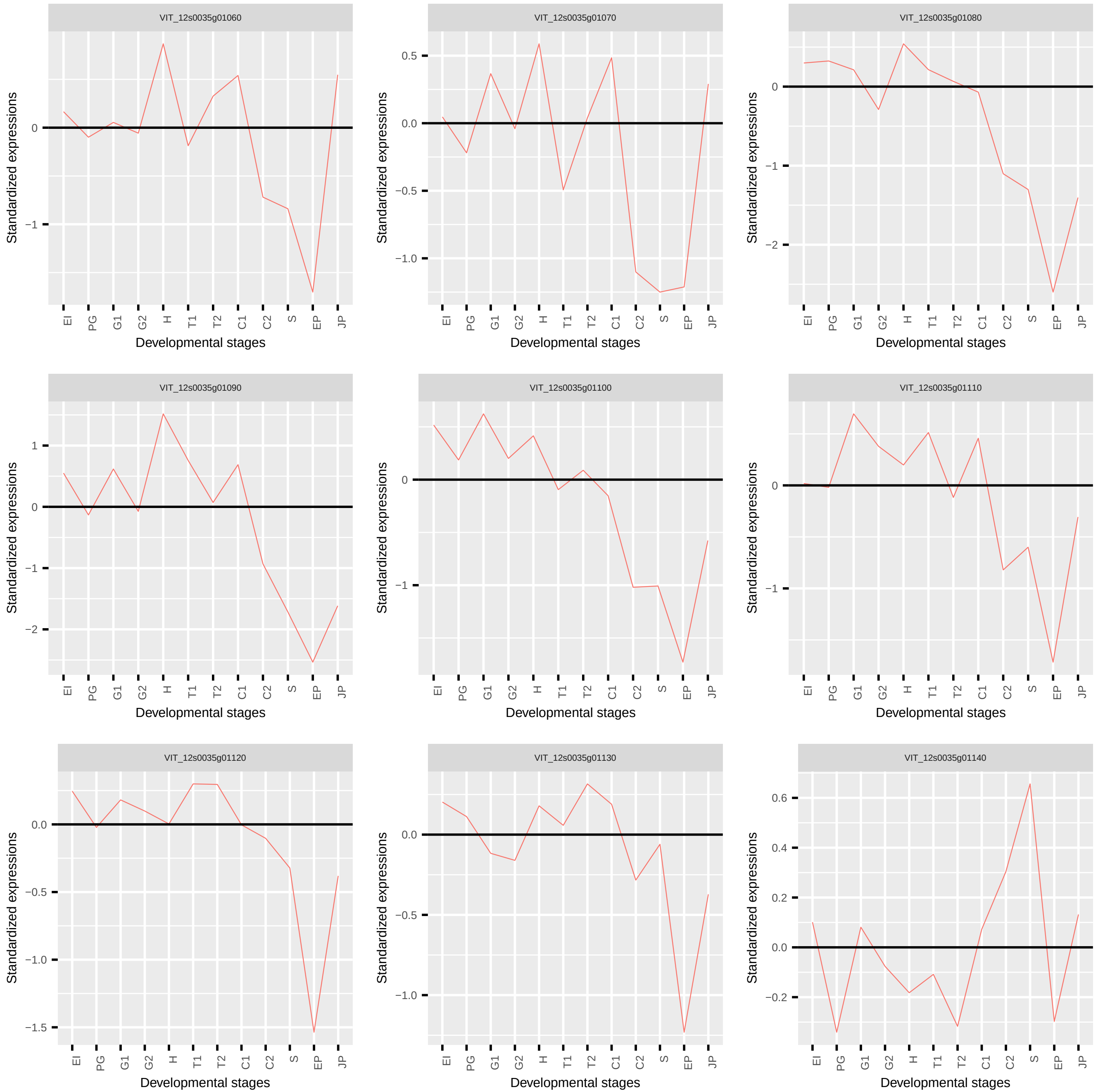
VIT_12s0035g01060
VIT_12s0035g01070
VIT_12s0035g01080
VIT_12s0035g01090
VIT_12s0035g01100
VIT_12s0035g01110
VIT_12s0035g01120
VIT_12s0035g01130
VIT_12s0035g01140
Standardized expressions
Developmental stages
EI PG G1 G2 H T1 T2 C1 C2 S EP JP
0
−1
0.5
0.0
−0.5
−1.0
−2
1
−1.5
0.6
0.4
0.2
−0.2

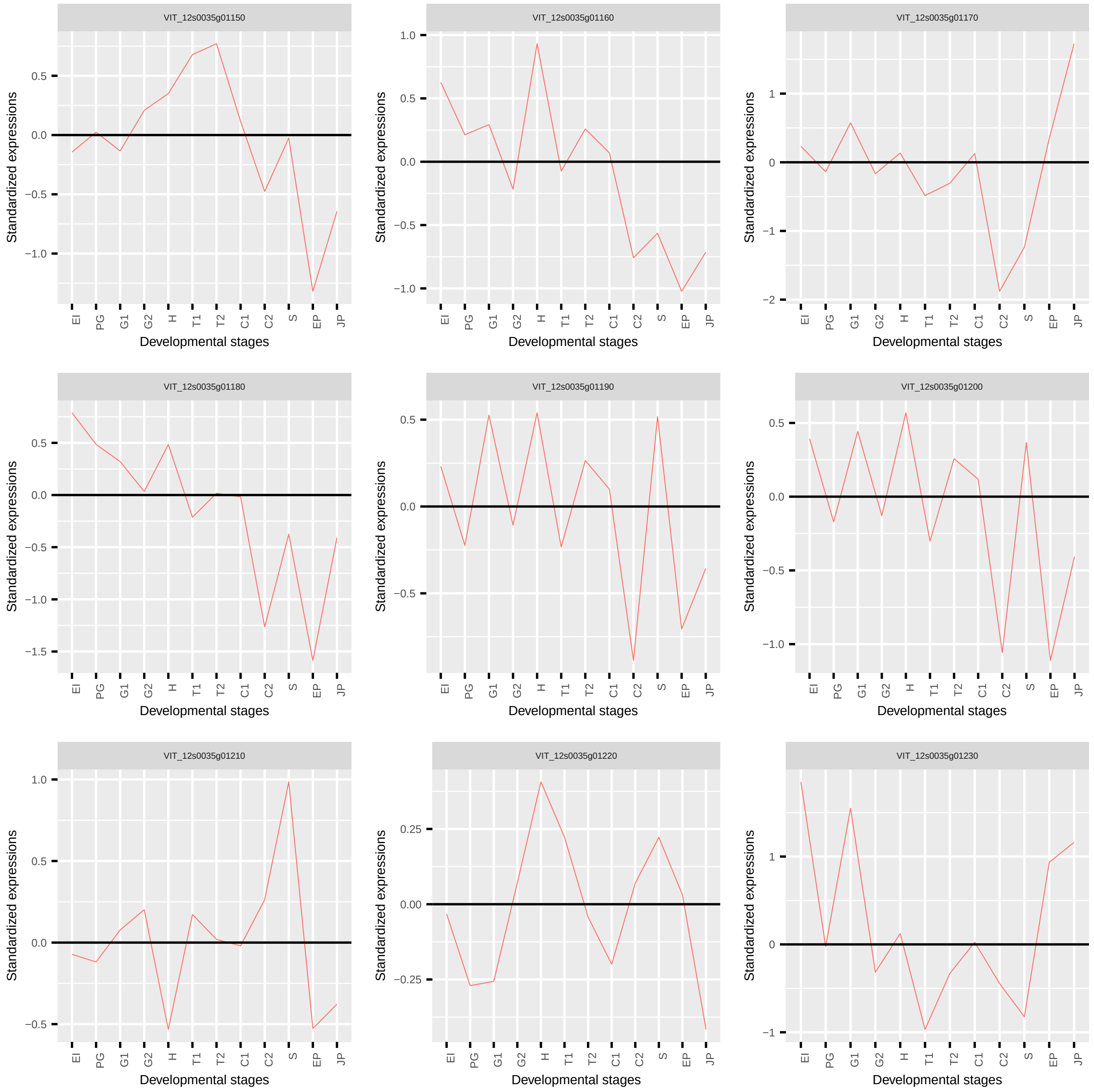
VIT_12s0035g01150
VIT_12s0035g01160
VIT_12s0035g01170
VIT_12s0035g01180
VIT_12s0035g01190
VIT_12s0035g01200
VIT_12s0035g01210
VIT_12s0035g01220
VIT_12s0035g01230
Standardized expressions
Developmental stages
EI PG G1 G2 H T1 T2 C1 C2 S EP JP

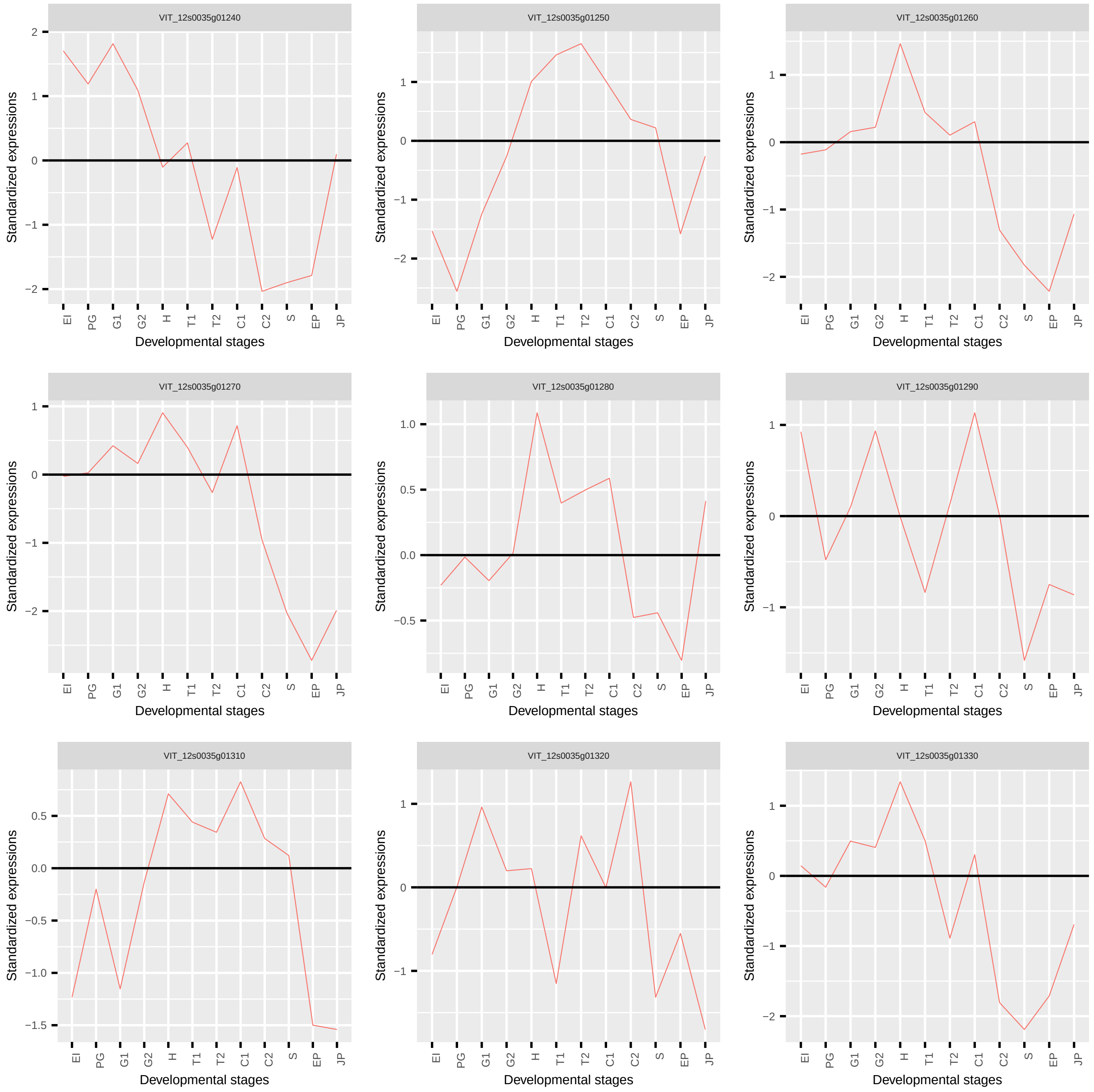
VIT_12s0035g01240
VIT_12s0035g01250
VIT_12s0035g01260
VIT_12s0035g01270
VIT_12s0035g01280
VIT_12s0035g01290
VIT_12s0035g01310
VIT_12s0035g01320
VIT_12s0035g01330
Standardized expressions
Developmental stages
EI
PG
G1
G2
H
T1
T2
C1
C2
S
EP
JP
2
1
0
−1
−2
1.0
0.5
0.0
−0.5
−1.0
−1.5

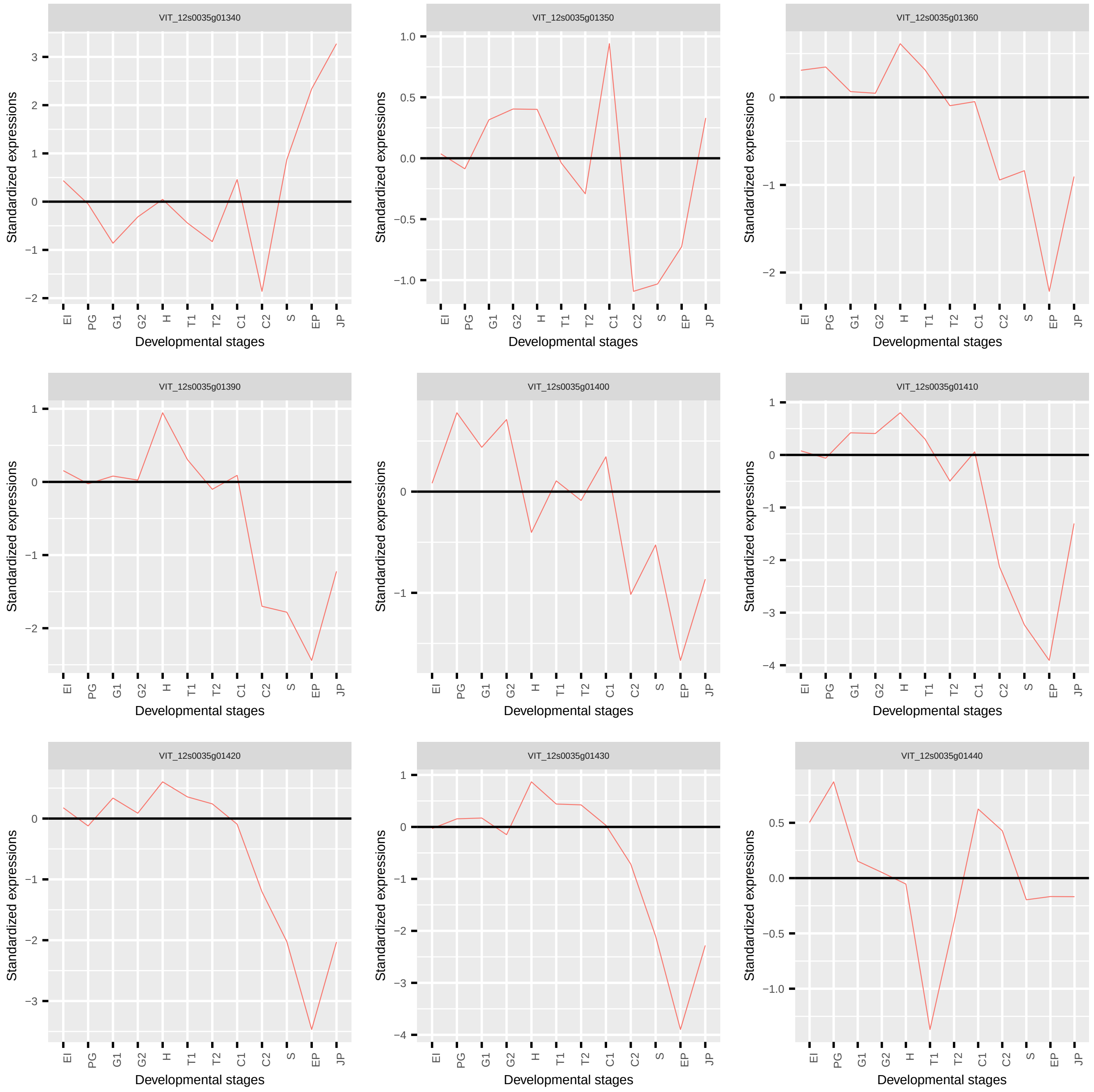
VIT_12s0035g01340
VIT_12s0035g01350
VIT_12s0035g01360
VIT_12s0035g01390
VIT_12s0035g01400
VIT_12s0035g01410
VIT_12s0035g01420
VIT_12s0035g01430
VIT_12s0035g01440
Standardized expressions
Developmental stages
EI PG G1 G2 H T1 T2 C1 C2 S EP JP

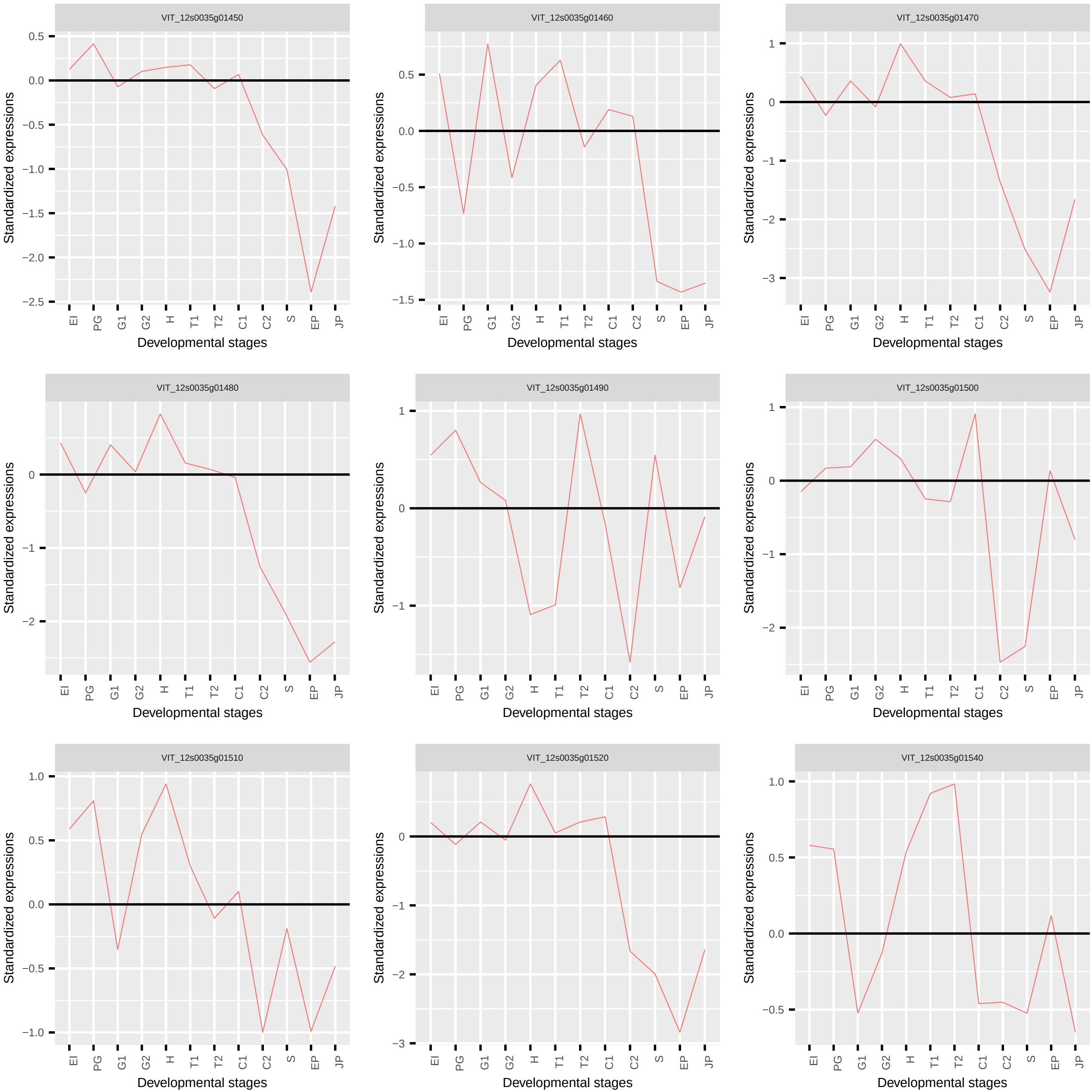
VIT_12s0035g01450
VIT_12s0035g01460
VIT_12s0035g01470
VIT_12s0035g01480
VIT_12s0035g01490
VIT_12s0035g01500
VIT_12s0035g01510
VIT_12s0035g01520
VIT_12s0035g01540
Standardized expressions
Developmental stages
EI PG G1 G2 H T1 T2 C1 C2 S EP JP

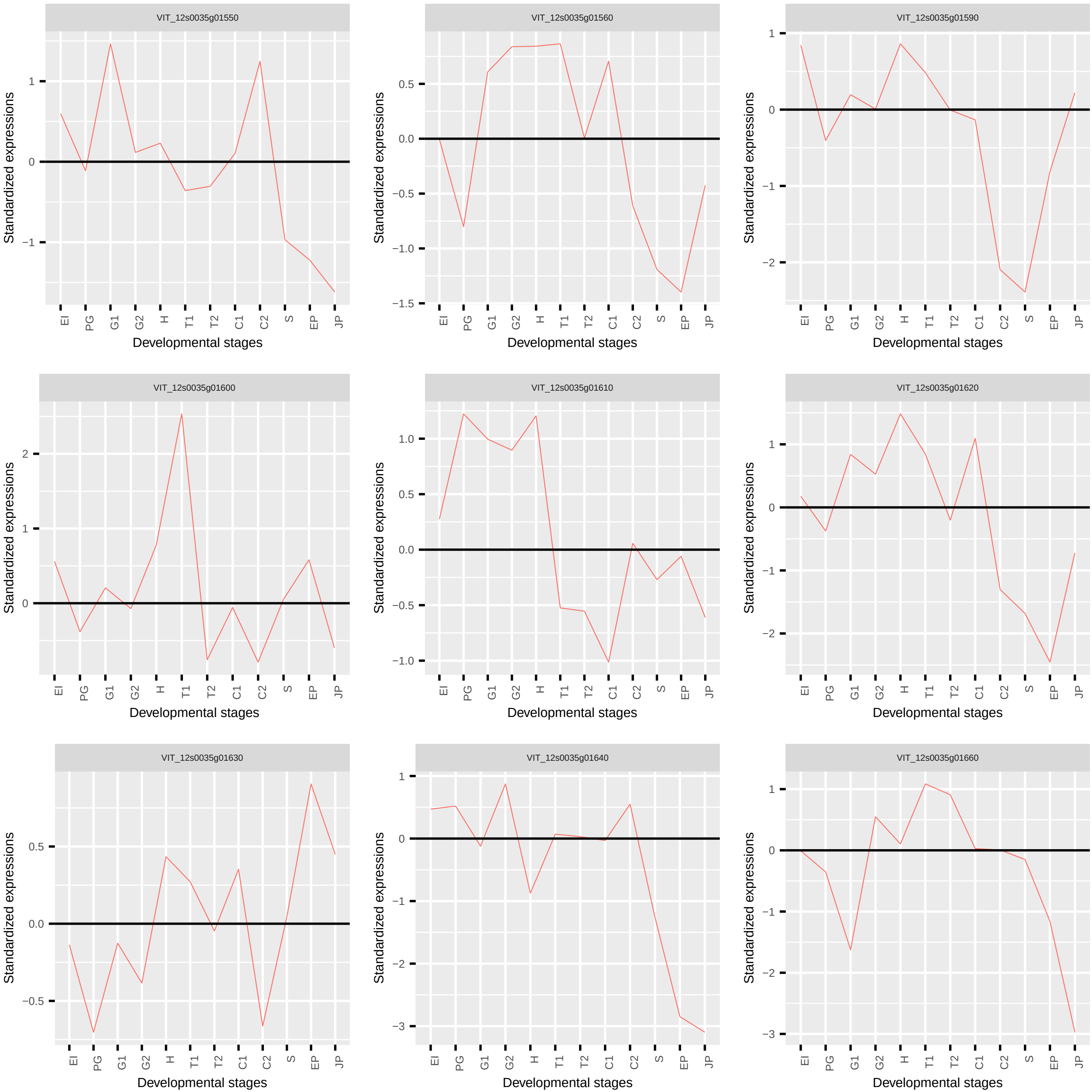
VIT_12s0035g01550
VIT_12s0035g01560
VIT_12s0035g01590
VIT_12s0035g01600
VIT_12s0035g01610
VIT_12s0035g01620
VIT_12s0035g01630
VIT_12s0035g01640
VIT_12s0035g01660
Standardized expressions
Developmental stages
EI PG G1 G2 H T1 T2 C1 C2 S EP JP

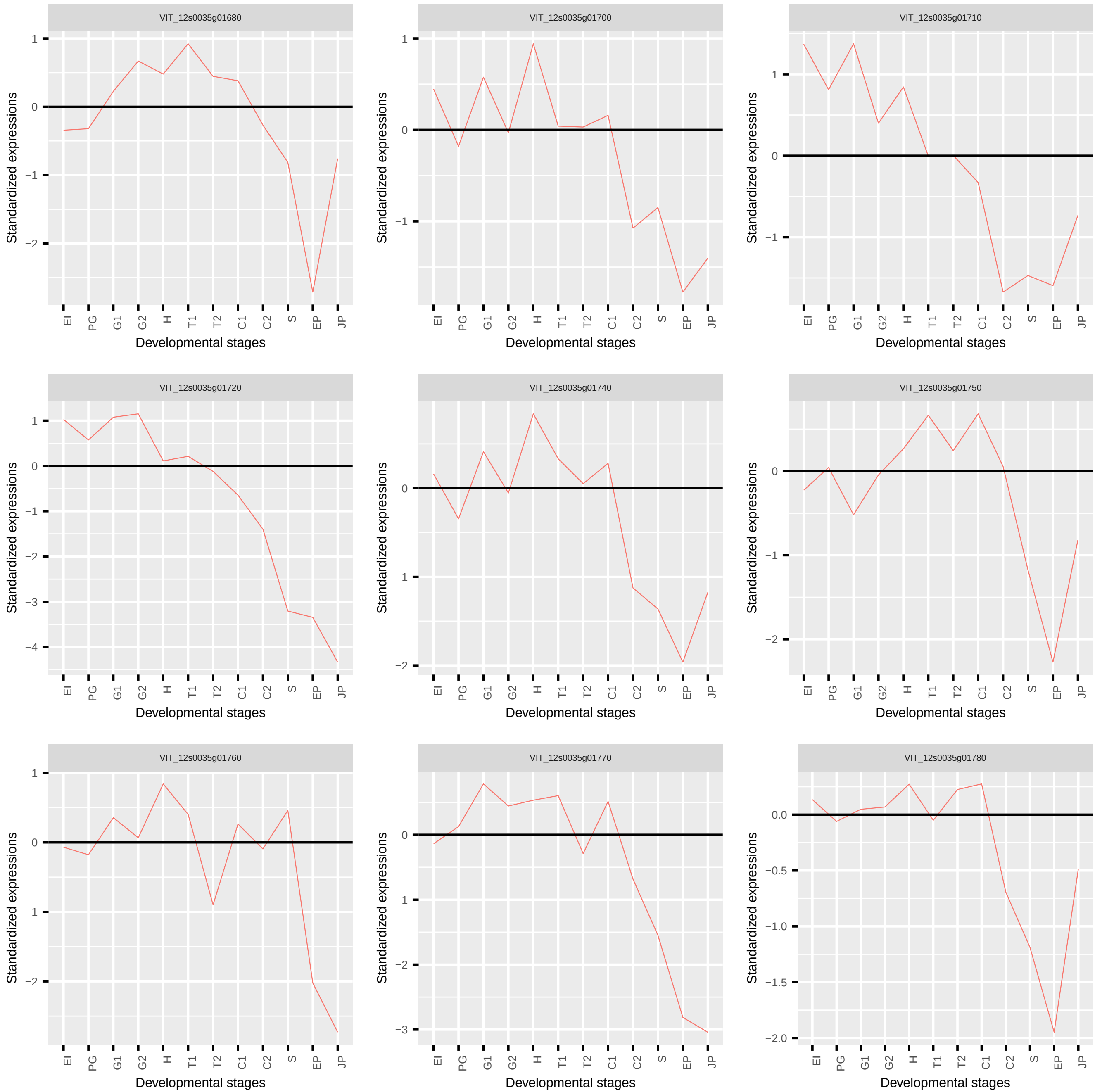
VIT_12s0035g01680
VIT_12s0035g01700
VIT_12s0035g01710
VIT_12s0035g01720
VIT_12s0035g01740
VIT_12s0035g01750
VIT_12s0035g01760
VIT_12s0035g01770
VIT_12s0035g01780
Standardized expressions
Developmental stages
EI
PG
G1
G2
H
T1
T2
C1
C2
S
EP
JP

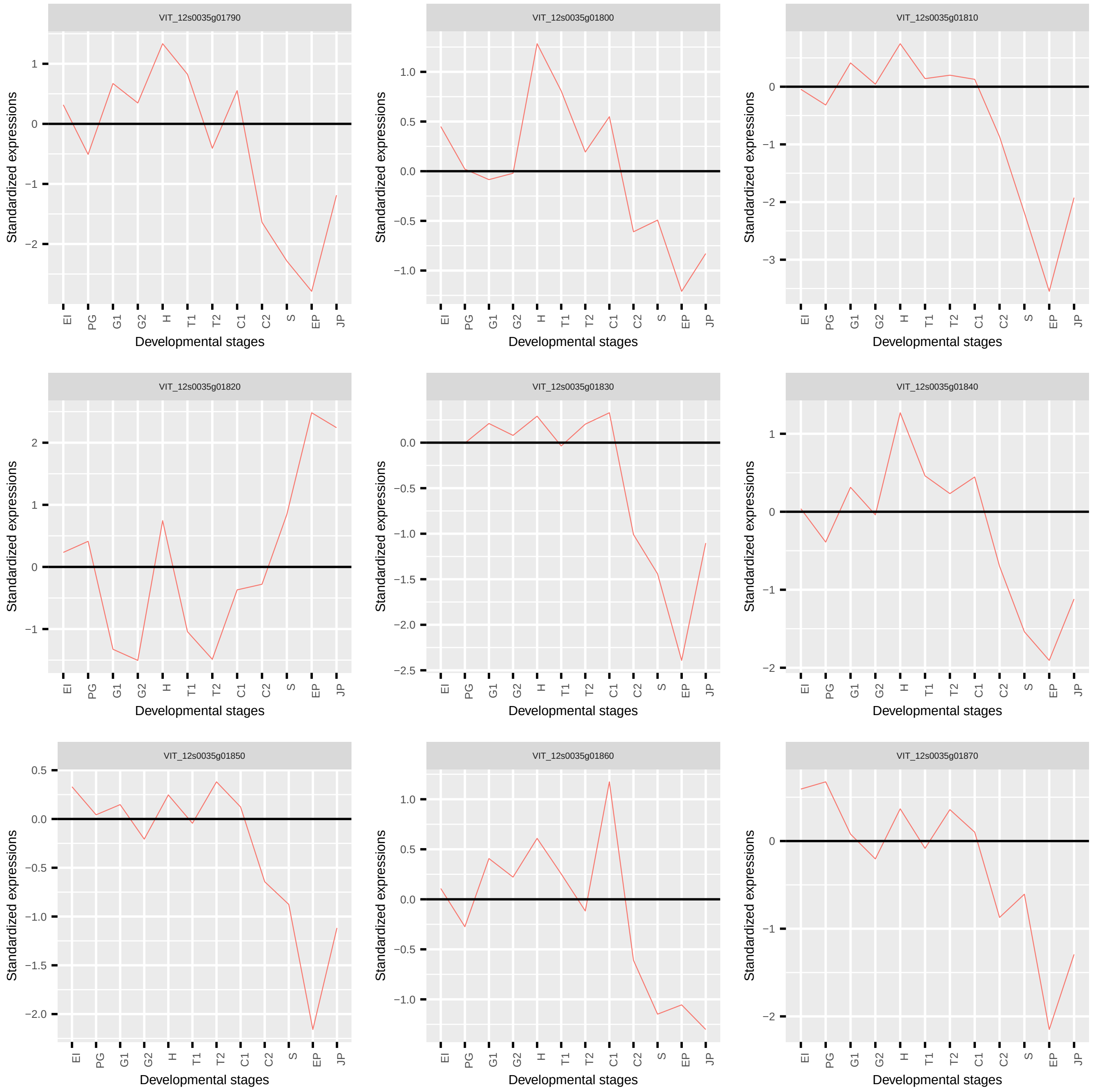
VIT_12s0035g01790
VIT_12s0035g01800
VIT_12s0035g01810
VIT_12s0035g01820
VIT_12s0035g01830
VIT_12s0035g01840
VIT_12s0035g01850
VIT_12s0035g01860
VIT_12s0035g01870
Standardized expressions
Developmental stages
EI PG G1 G2 H T1 T2 C1 C2 S EP JP

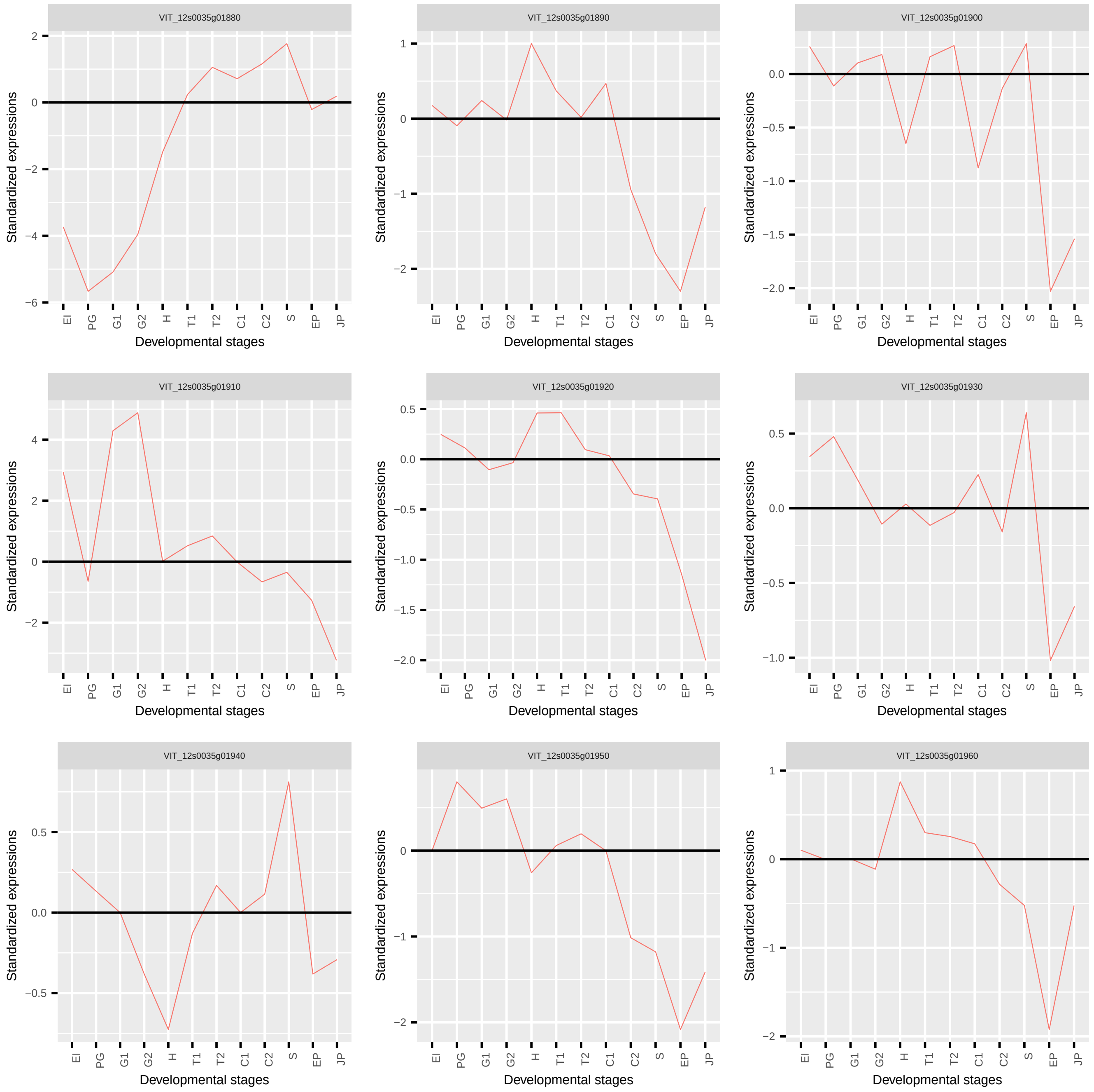
VIT_12s0035g01880
VIT_12s0035g01890
VIT_12s0035g01900
VIT_12s0035g01910
VIT_12s0035g01920
VIT_12s0035g01930
VIT_12s0035g01940
VIT_12s0035g01950
VIT_12s0035g01960
Standardized expressions
Developmental stages
EI PG G1 G2 H T1 T2 C1 C2 S EP JP

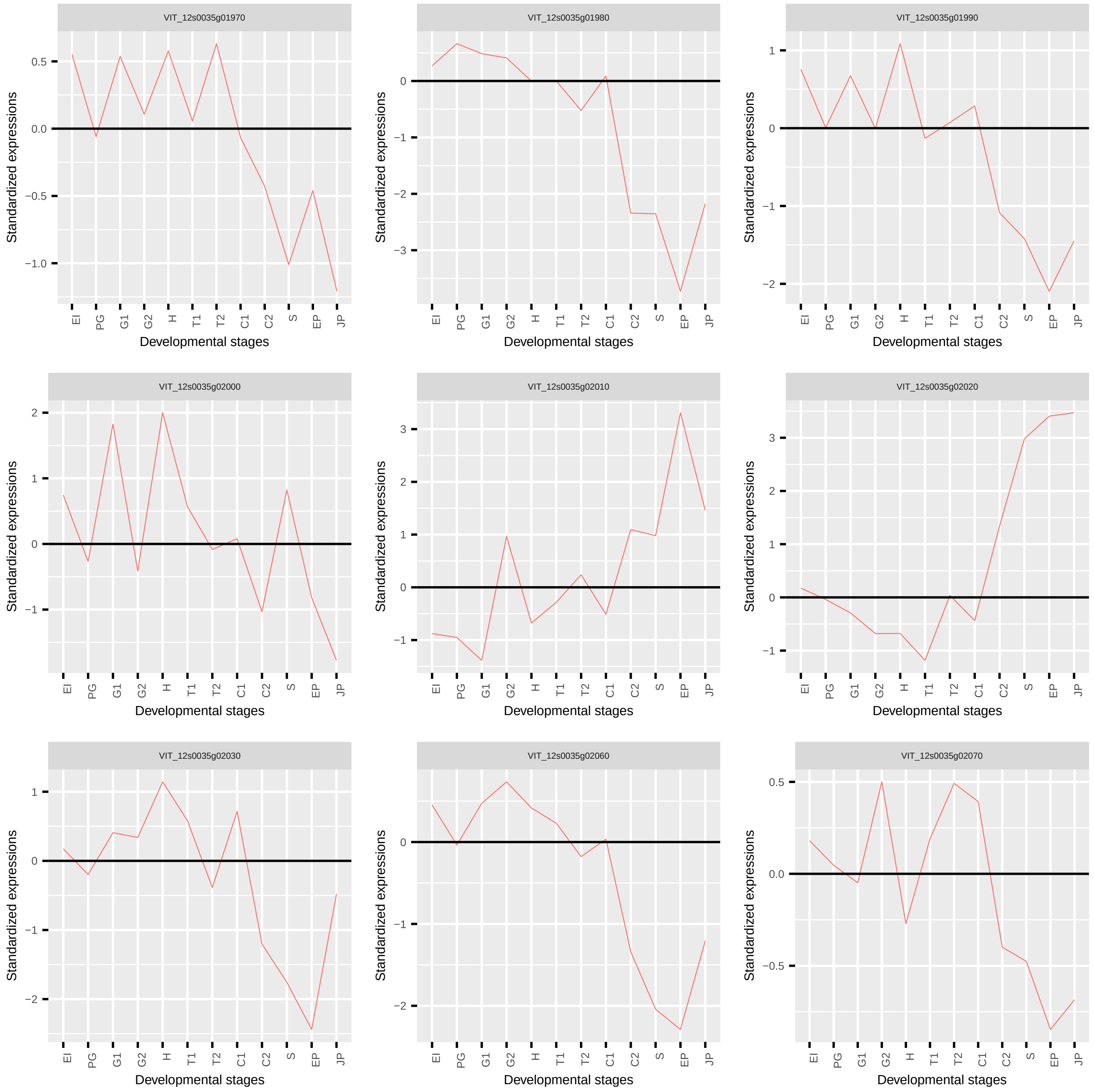
VIT_12s0035g01970
0.5
0.0
−0.5
−1.0
Standardized expressions
EI PG G1 G2 H T1 T2 C1 C2 S EP JP
Developmental stages
VIT_12s0035g01980
0
−1
−2
−3
Standardized expressions
EI PG G1 G2 H T1 T2 C1 C2 S EP JP
Developmental stages
VIT_12s0035g01990
1
0
−1
−2
Standardized expressions
EI PG G1 G2 H T1 T2 C1 C2 S EP JP
Developmental stages
VIT_12s0035g02000
2
1
0
−1
Standardized expressions
EI PG G1 G2 H T1 T2 C1 C2 S EP JP
Developmental stages
VIT_12s0035g02010
3
2
1
0
−1
Standardized expressions
EI PG G1 G2 H T1 T2 C1 C2 S EP JP
Developmental stages
VIT_12s0035g02020
3
2
1
0
−1
Standardized expressions
EI PG G1 G2 H T1 T2 C1 C2 S EP JP
Developmental stages
VIT_12s0035g02030
1
0
−1
−2
Standardized expressions
EI PG G1 G2 H T1 T2 C1 C2 S EP JP
Developmental stages
VIT_12s0035g02060
0
−1
−2
Standardized expressions
EI PG G1 G2 H T1 T2 C1 C2 S EP JP
Developmental stages
VIT_12s0035g02070
0.5
0.0
−0.5
Standardized expressions
EI PG G1 G2 H T1 T2 C1 C2 S EP JP
Developmental stages

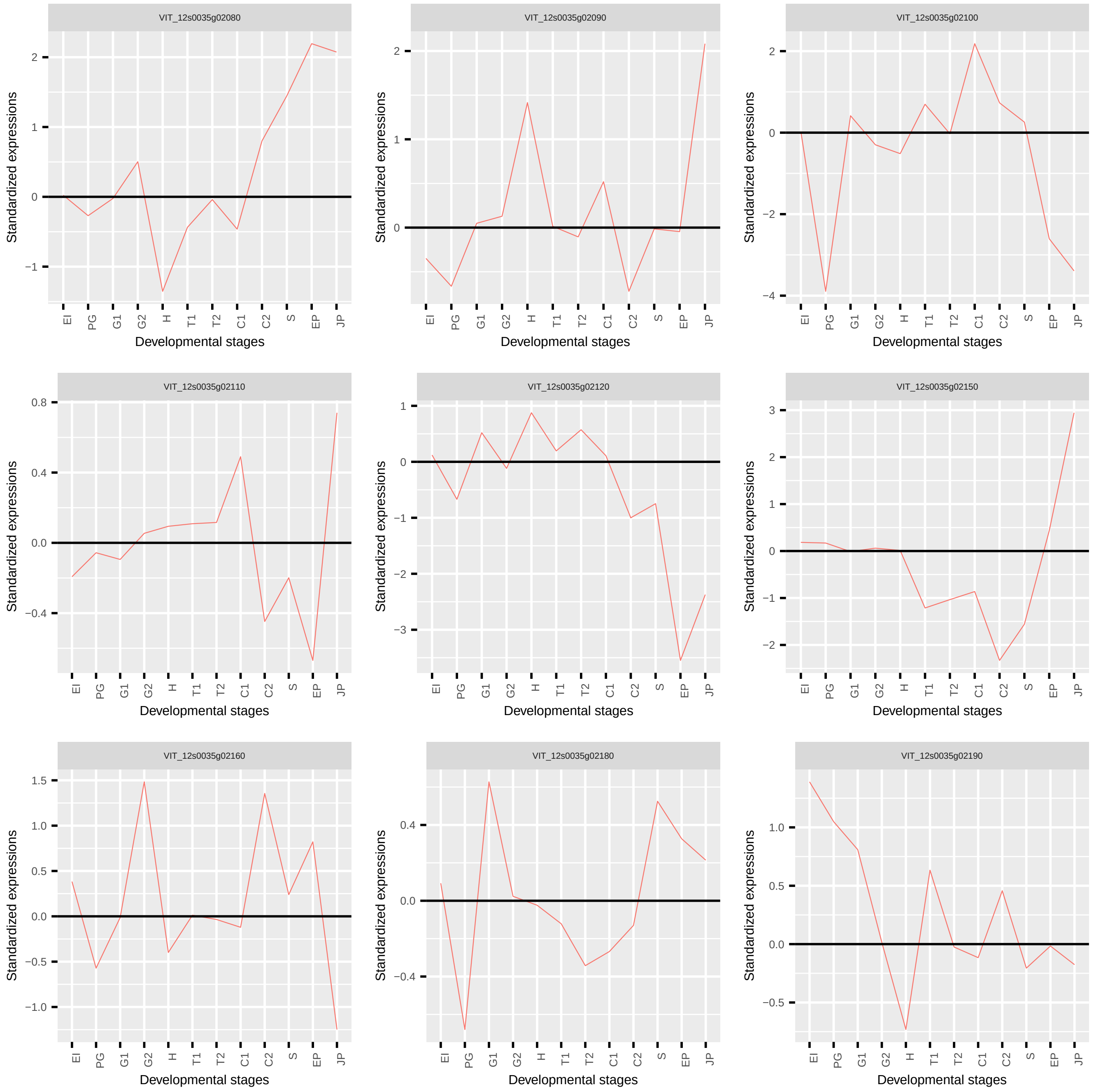

VIT_12s0035g02080
Standardized expressions
2
1
0
−1
EI
PG
G1
G2
H
T1
T2
C1
C2
S
EP
JP
Developmental stages
VIT_12s0035g02090
Standardized expressions
2
1
0
EI
PG
G1
G2
H
T1
T2
C1
C2
S
EP
JP
Developmental stages
VIT_12s0035g02100
Standardized expressions
2
0
−2
−4
EI
PG
G1
G2
H
T1
T2
C1
C2
S
EP
JP
Developmental stages
VIT_12s0035g02110
Standardized expressions
0.8
0.4
0.0
−0.4
EI
PG
G1
G2
H
T1
T2
C1
C2
S
EP
JP
Developmental stages
VIT_12s0035g02120
Standardized expressions
1
0
−1
−2
−3
EI
PG
G1
G2
H
T1
T2
C1
C2
S
EP
JP
Developmental stages
VIT_12s0035g02150
Standardized expressions
3
2
1
0
−1
−2
EI
PG
G1
G2
H
T1
T2
C1
C2
S
EP
JP
Developmental stages
VIT_12s0035g02160
Standardized expressions
1.5
1.0
0.5
0.0
−0.5
−1.0
EI
PG
G1
G2
H
T1
T2
C1
C2
S
EP
JP
Developmental stages
VIT_12s0035g02180
Standardized expressions
0.4
0.0
−0.4
EI
PG
G1
G2
H
T1
T2
C1
C2
S
EP
JP
Developmental stages
VIT_12s0035g02190
Standardized expressions
1.0
0.5
0.0
−0.5
EI
PG
G1
G2
H
T1
T2
C1
C2
S
EP
JP
Developmental stages

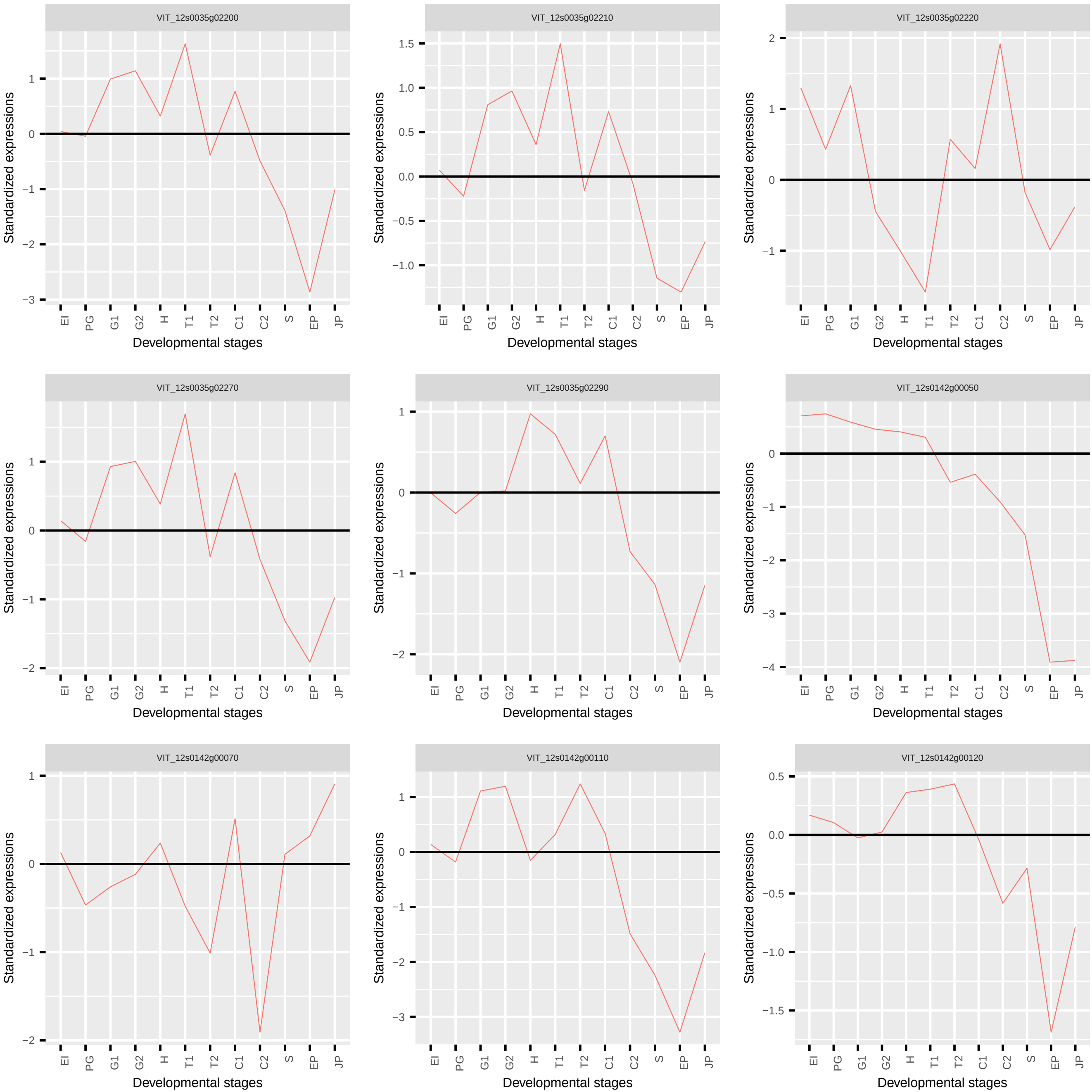
VIT_12s0035g02200
VIT_12s0035g02210
VIT_12s0035g02220
VIT_12s0035g02270
VIT_12s0035g02290
VIT_12s0142g00050
VIT_12s0142g00070
VIT_12s0142g00110
VIT_12s0142g00120
Standardized expressions
Developmental stages
EI
PG
G1
G2
H
T1
T2
C1
C2
S
EP
JP

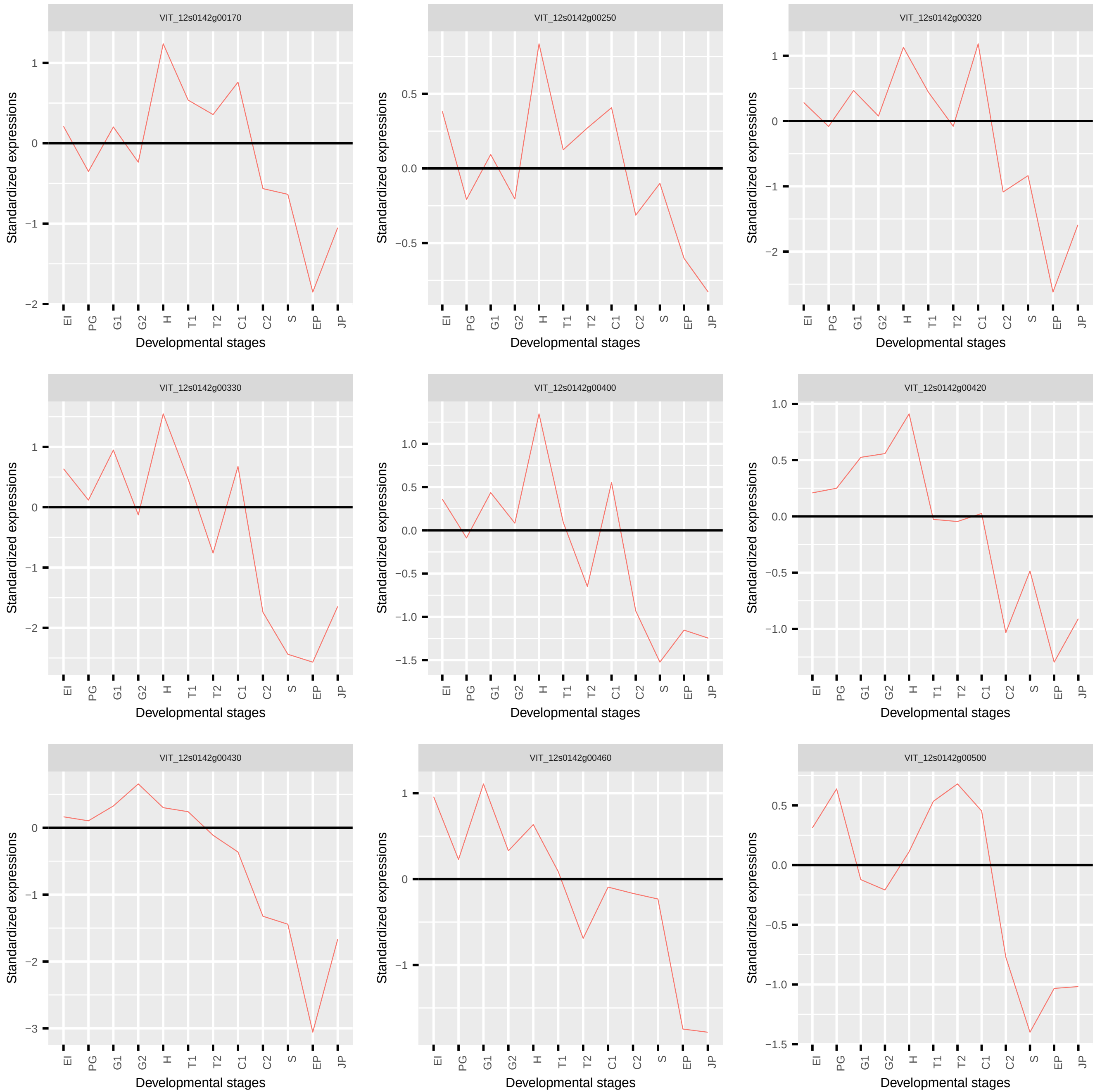
VIT_12s0142g00170
VIT_12s0142g00250
VIT_12s0142g00320
VIT_12s0142g00330
VIT_12s0142g00400
VIT_12s0142g00420
VIT_12s0142g00430
VIT_12s0142g00460
VIT_12s0142g00500
Standardized expressions
Developmental stages
EI
PG
G1
G2
H
T1
T2
C1
C2
S
EP
JP

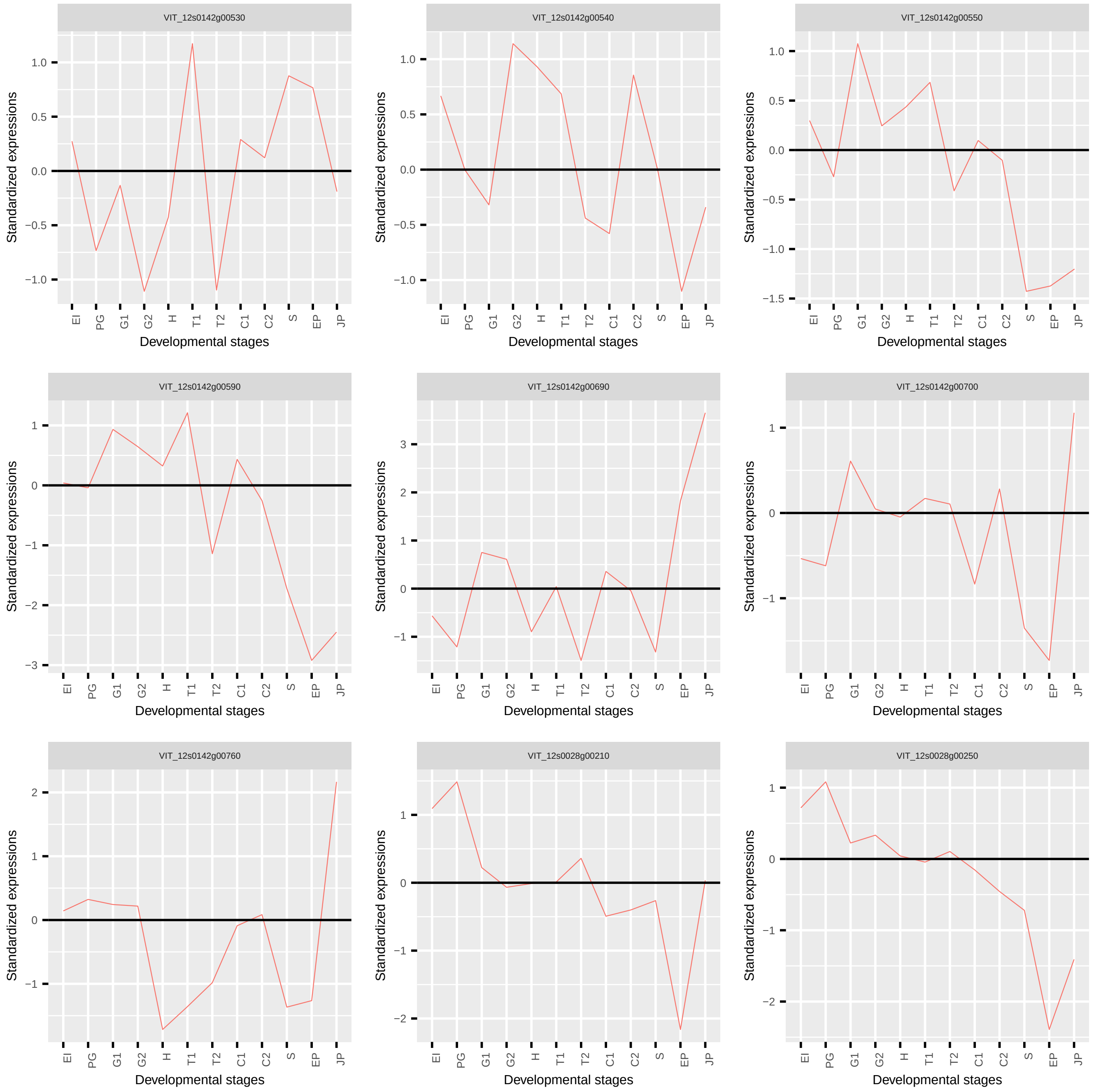
VIT_12s0142g00530
VIT_12s0142g00540
VIT_12s0142g00550
VIT_12s0142g00590
VIT_12s0142g00690
VIT_12s0142g00700
VIT_12s0142g00760
VIT_12s0028g00210
VIT_12s0028g00250
Standardized expressions
Developmental stages
EI PG G1 G2 H T1 T2 C1 C2 S EP JP

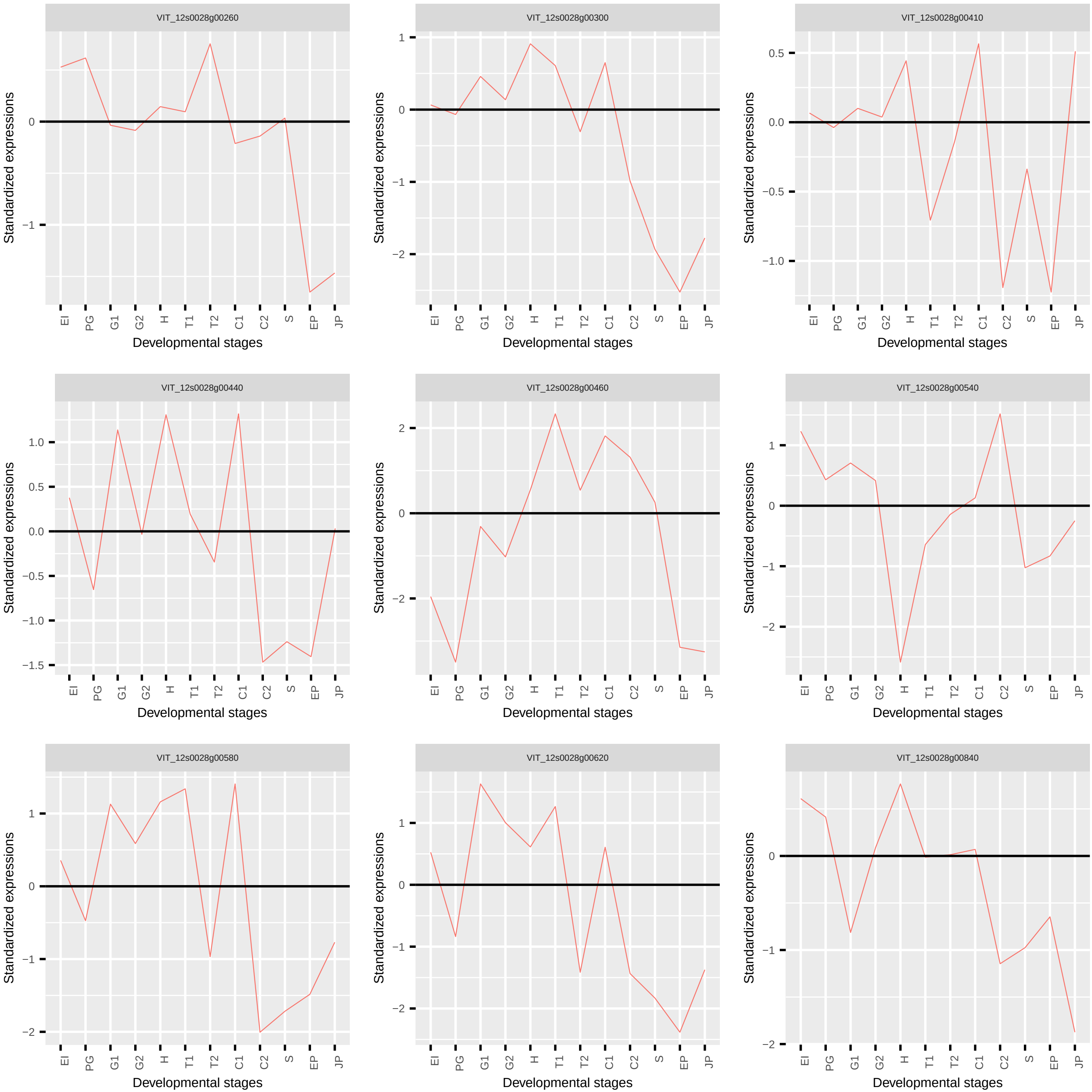
VIT_12s0028g00260
VIT_12s0028g00300
VIT_12s0028g00410
VIT_12s0028g00440
VIT_12s0028g00460
VIT_12s0028g00540
VIT_12s0028g00580
VIT_12s0028g00620
VIT_12s0028g00840
Standardized expressions
Developmental stages
EI PG G1 G2 H T1 T2 C1 C2 S EP JP

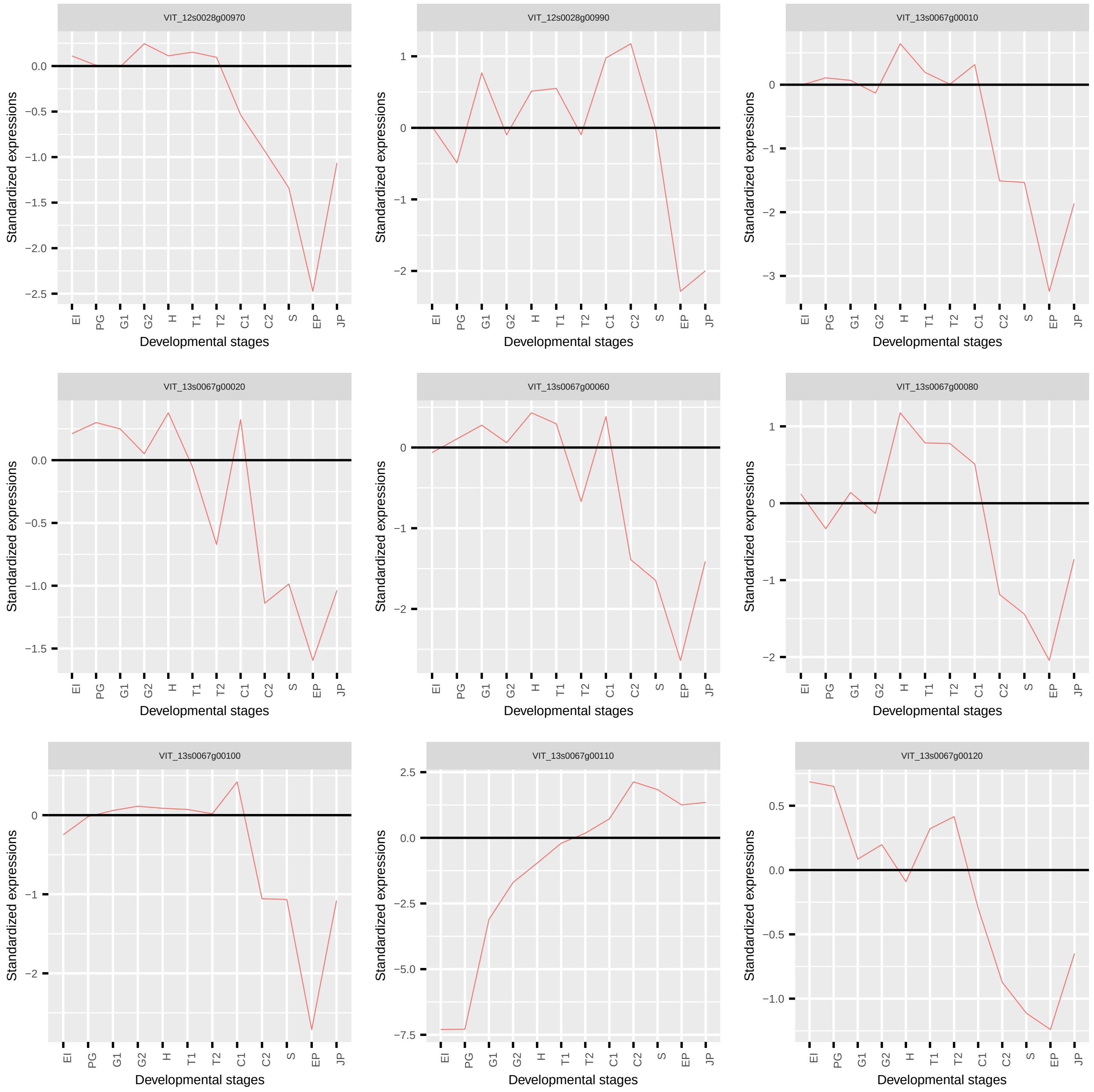
VIT_12s0028g00970
VIT_12s0028g00990
VIT_13s0067g00010
VIT_13s0067g00020
VIT_13s0067g00060
VIT_13s0067g00080
VIT_13s0067g00100
VIT_13s0067g00110
VIT_13s0067g00120
Standardized expressions
Developmental stages
EI PG G1 G2 H T1 T2 C1 C2 S EP JP

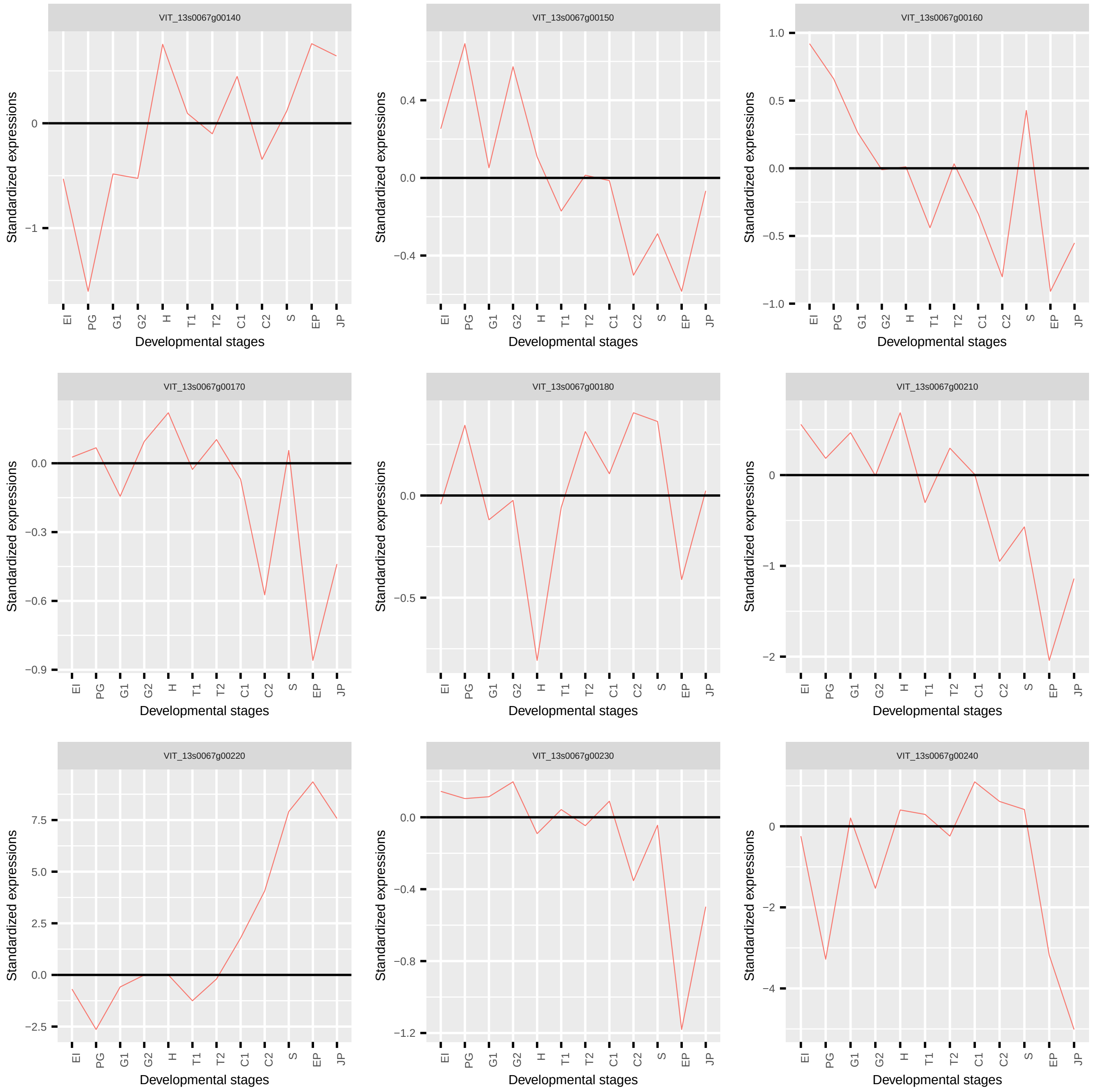
VIT_13s0067g00140
0
−1
Standardized expressions
EI PG G1 G2 H T1 T2 C1 C2 S EP JP
Developmental stages
VIT_13s0067g00150
0.4
0.0
−0.4
Standardized expressions
EI PG G1 G2 H T1 T2 C1 C2 S EP JP
Developmental stages
VIT_13s0067g00160
1.0
0.5
0.0
−0.5
−1.0
Standardized expressions
EI PG G1 G2 H T1 T2 C1 C2 S EP JP
Developmental stages
VIT_13s0067g00170
0.0
−0.3
−0.6
−0.9
Standardized expressions
EI PG G1 G2 H T1 T2 C1 C2 S EP JP
Developmental stages
VIT_13s0067g00180
0.0
−0.5
Standardized expressions
EI PG G1 G2 H T1 T2 C1 C2 S EP JP
Developmental stages
VIT_13s0067g00210
0
−1
−2
Standardized expressions
EI PG G1 G2 H T1 T2 C1 C2 S EP JP
Developmental stages
VIT_13s0067g00220
7.5
5.0
2.5
0.0
−2.5
Standardized expressions
EI PG G1 G2 H T1 T2 C1 C2 S EP JP
Developmental stages
VIT_13s0067g00230
0.0
−0.4
−0.8
−1.2
Standardized expressions
EI PG G1 G2 H T1 T2 C1 C2 S EP JP
Developmental stages
VIT_13s0067g00240
0
−2
−4
Standardized expressions
EI PG G1 G2 H T1 T2 C1 C2 S EP JP
Developmental stages

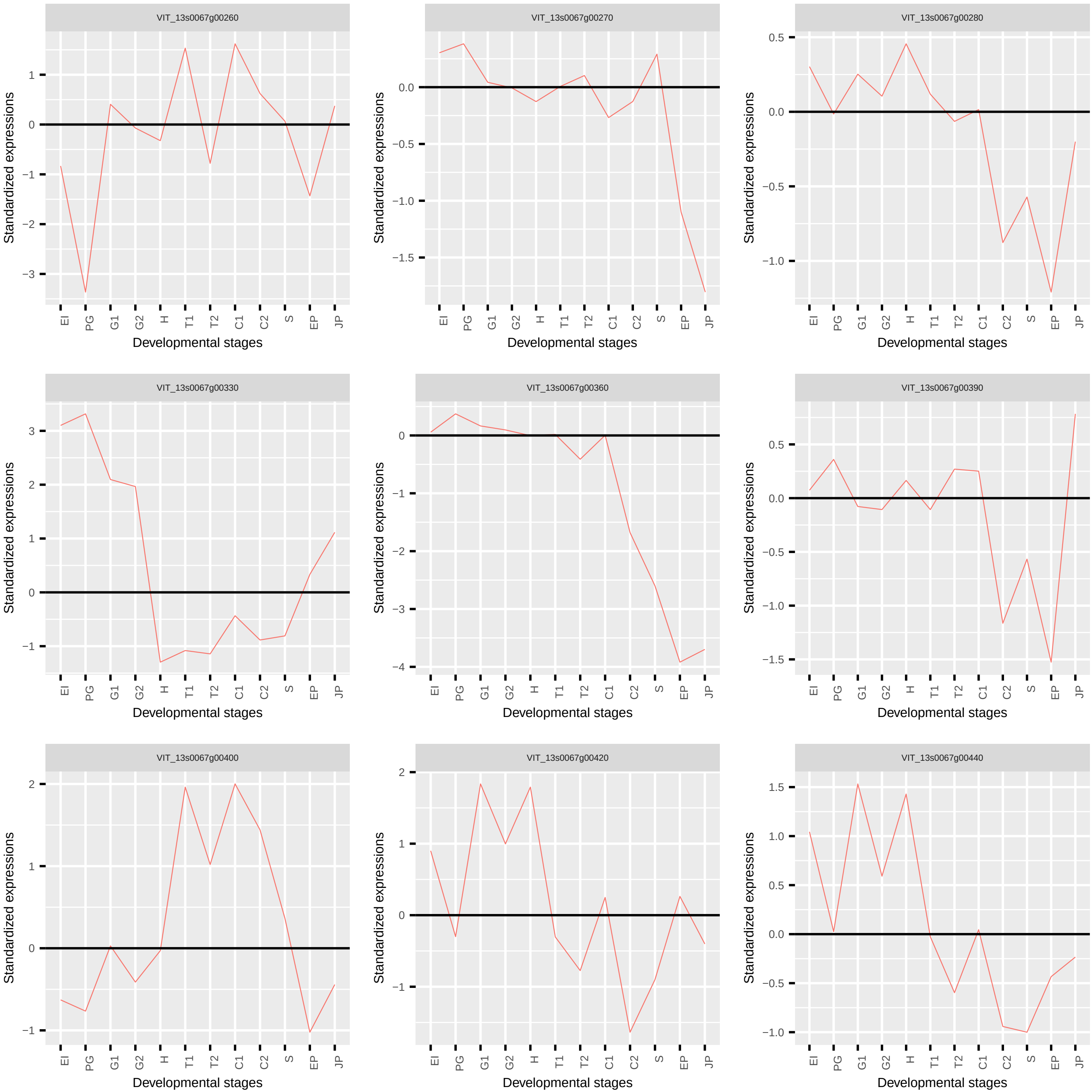
VIT_13s0067g00260
VIT_13s0067g00270
VIT_13s0067g00280
VIT_13s0067g00330
VIT_13s0067g00360
VIT_13s0067g00390
VIT_13s0067g00400
VIT_13s0067g00420
VIT_13s0067g00440
Standardized expressions
Developmental stages
EI PG G1 G2 H T1 T2 C1 C2 S EP JP

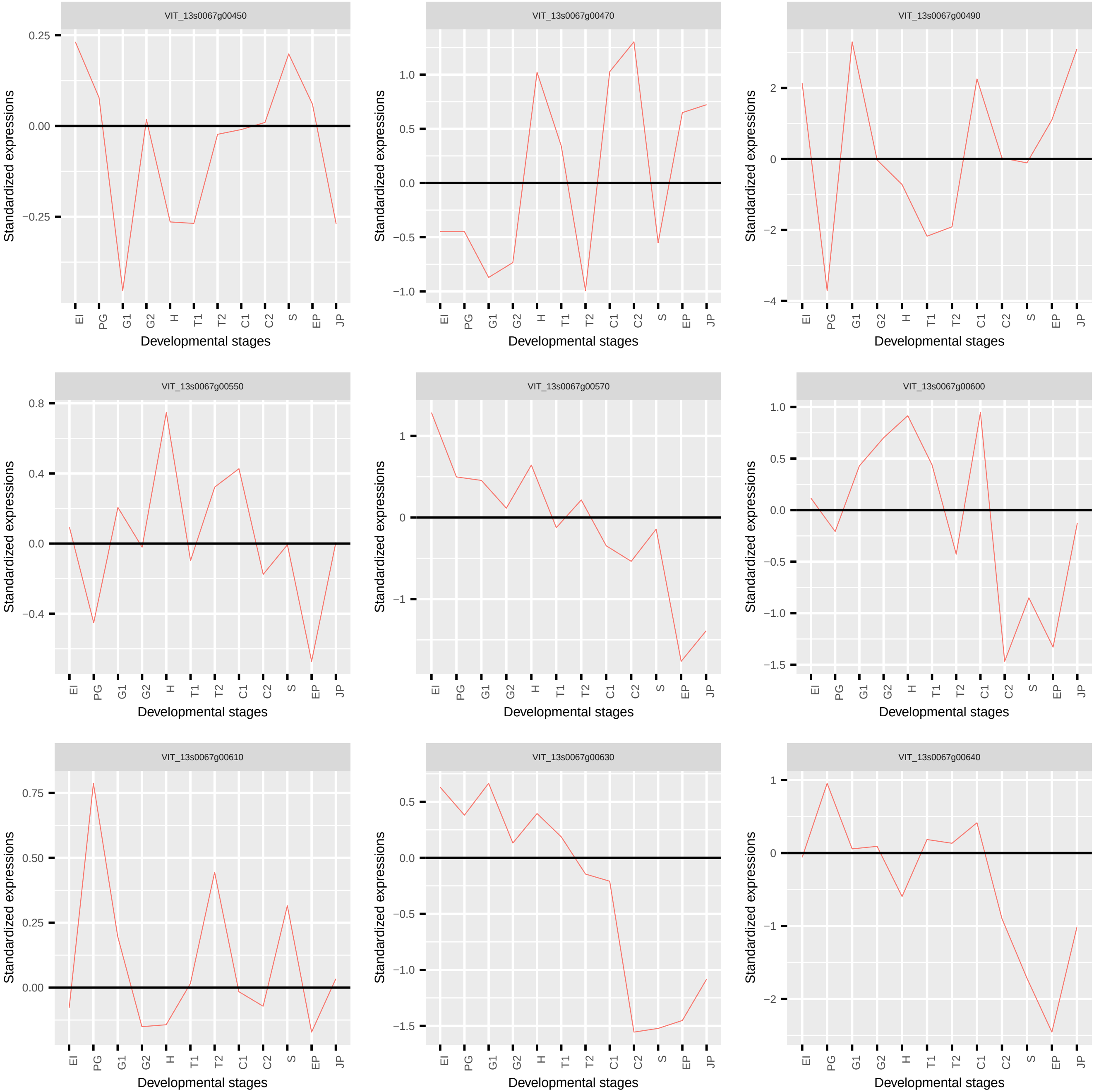
VIT_13s0067g00450
VIT_13s0067g00470
VIT_13s0067g00490
VIT_13s0067g00550
VIT_13s0067g00570
VIT_13s0067g00600
VIT_13s0067g00610
VIT_13s0067g00630
VIT_13s0067g00640
Standardized expressions
Developmental stages
EI PG G1 G2 H T1 T2 C1 C2 S EP JP

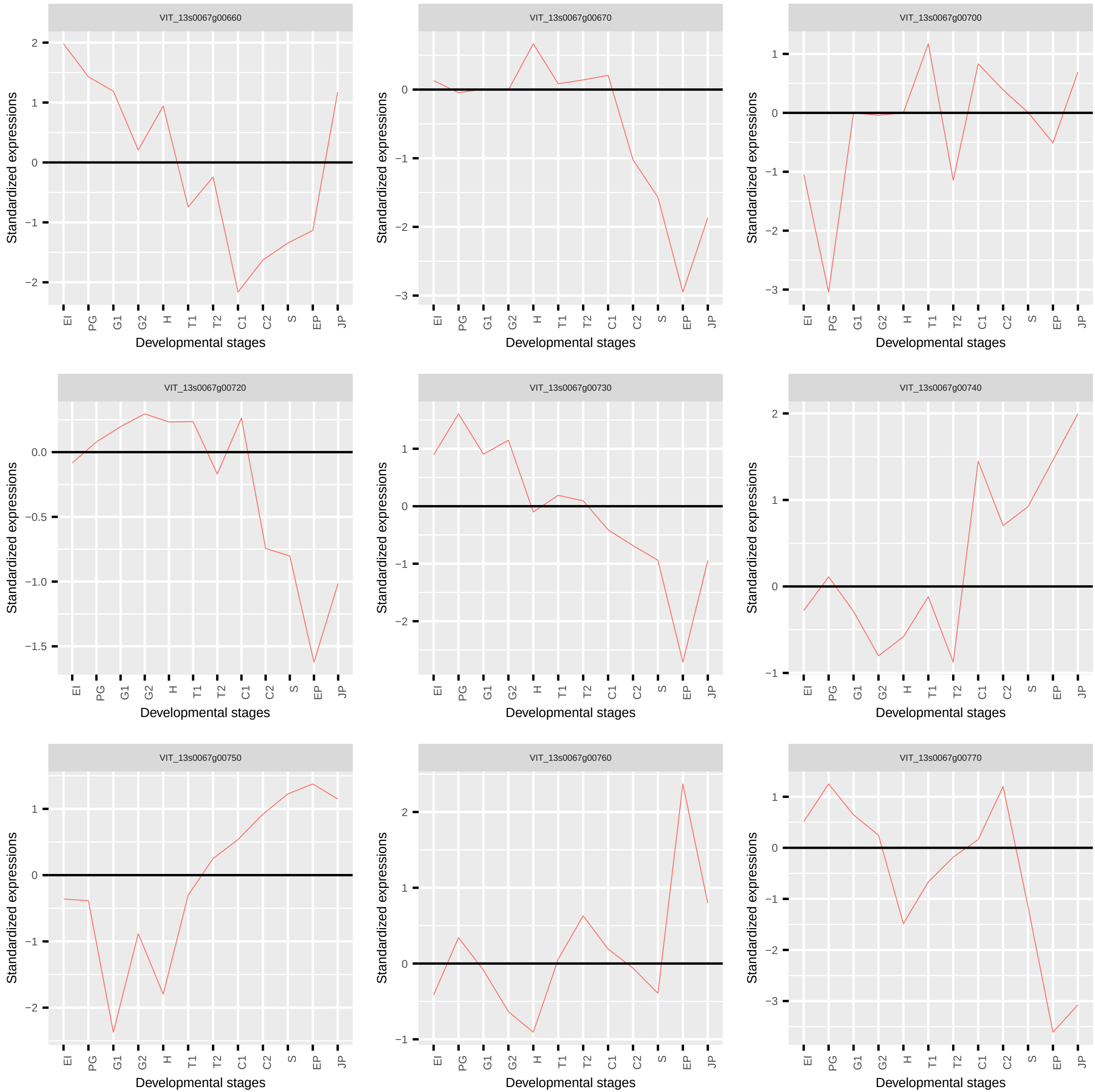
VIT_13s0067g00660
VIT_13s0067g00670
VIT_13s0067g00700
VIT_13s0067g00720
VIT_13s0067g00730
VIT_13s0067g00740
VIT_13s0067g00750
VIT_13s0067g00760
VIT_13s0067g00770
Standardized expressions
Developmental stages
EI
PG
G1
G2
H
T1
T2
C1
C2
S
EP
JP

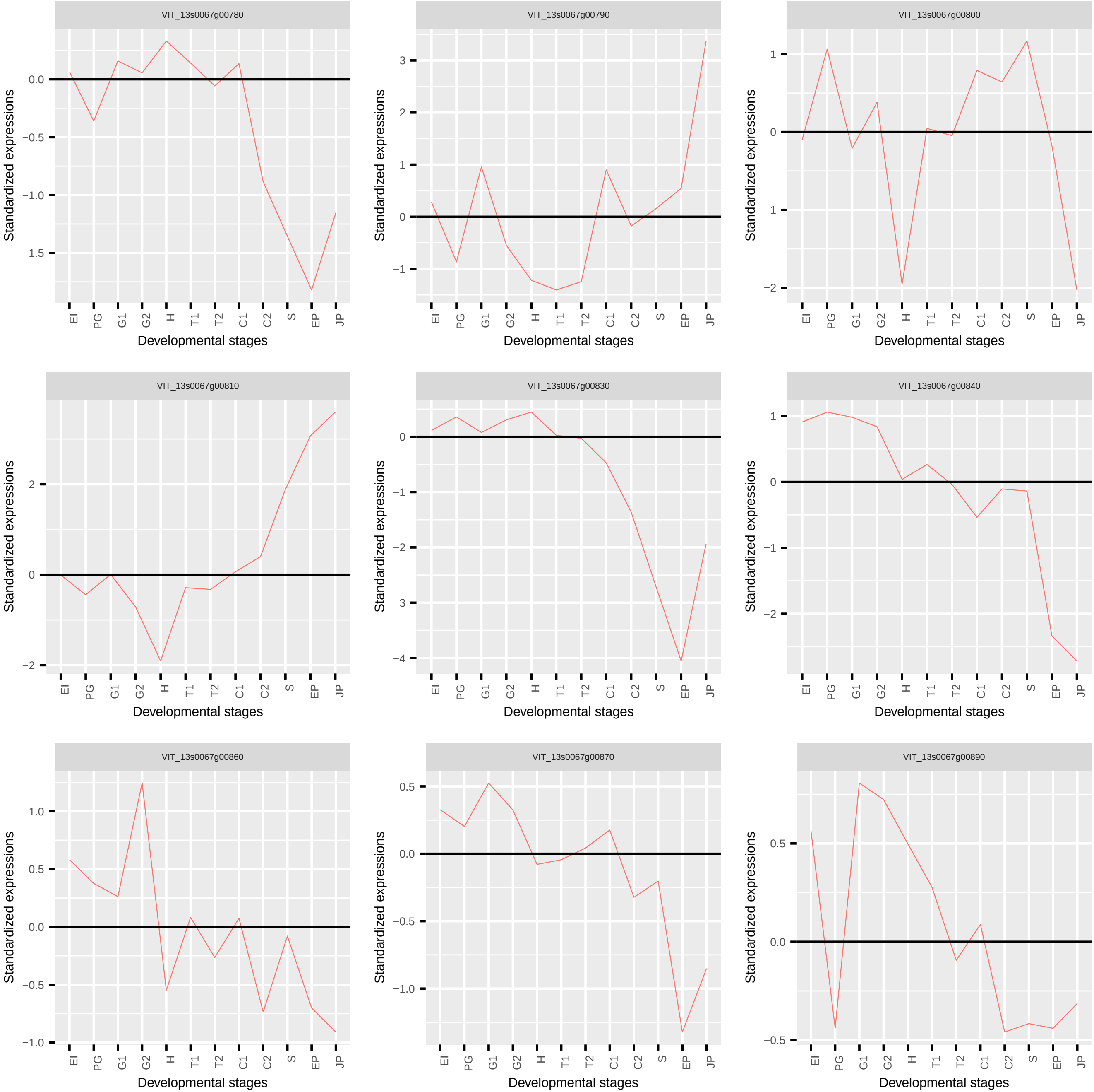

VIT_13s0067g00780
VIT_13s0067g00790
VIT_13s0067g00800
VIT_13s0067g00810
VIT_13s0067g00830
VIT_13s0067g00840
VIT_13s0067g00860
VIT_13s0067g00870
VIT_13s0067g00890
Standardized expressions
Developmental stages
EI
PG
G1
G2
H
T1
T2
C1
C2
S
EP
JP

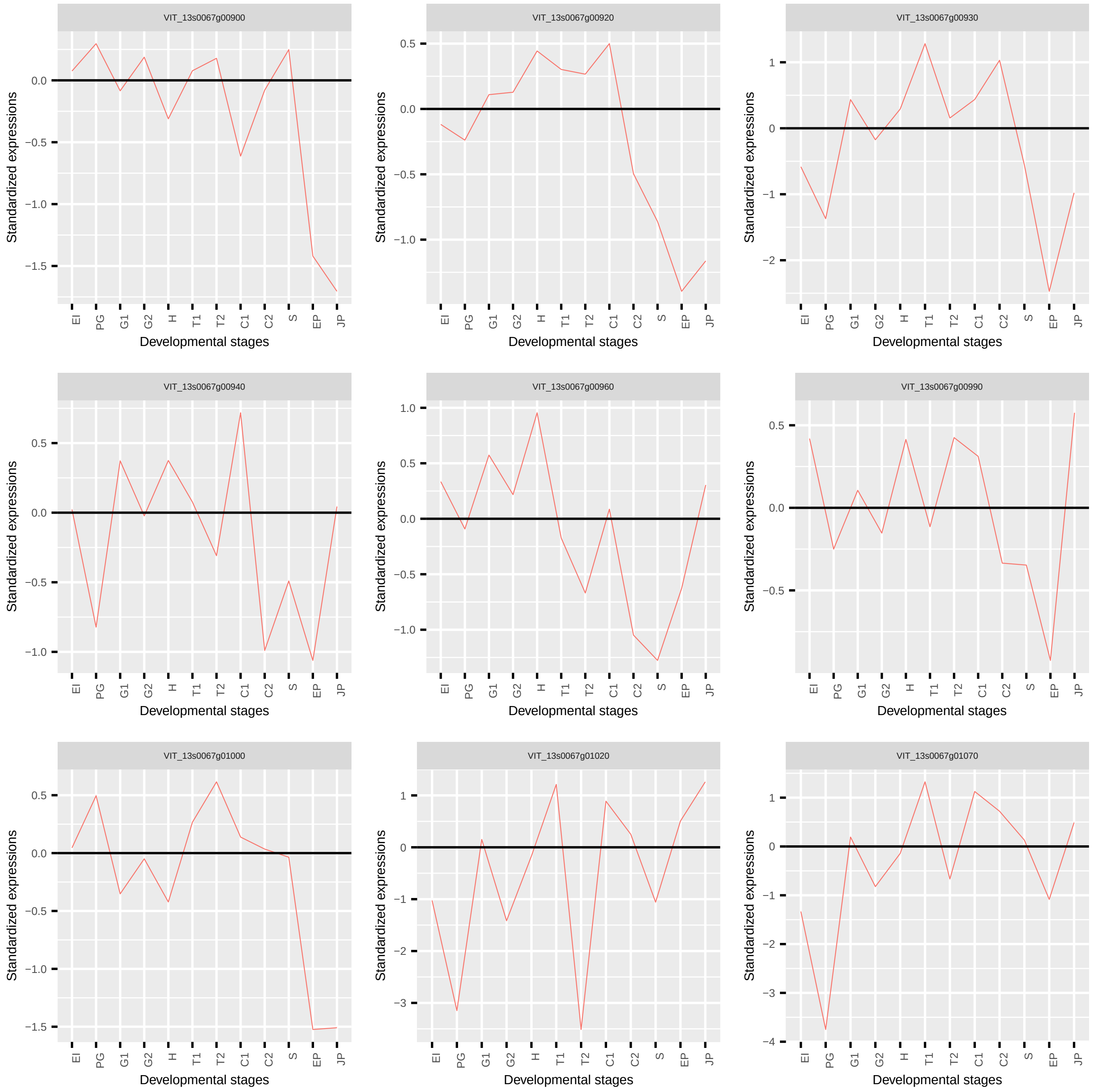
VIT_13s0067g00900
VIT_13s0067g00920
VIT_13s0067g00930
VIT_13s0067g00940
VIT_13s0067g00960
VIT_13s0067g00990
VIT_13s0067g01000
VIT_13s0067g01020
VIT_13s0067g01070
Standardized expressions
Developmental stages
EI PG G1 G2 H T1 T2 C1 C2 S EP JP

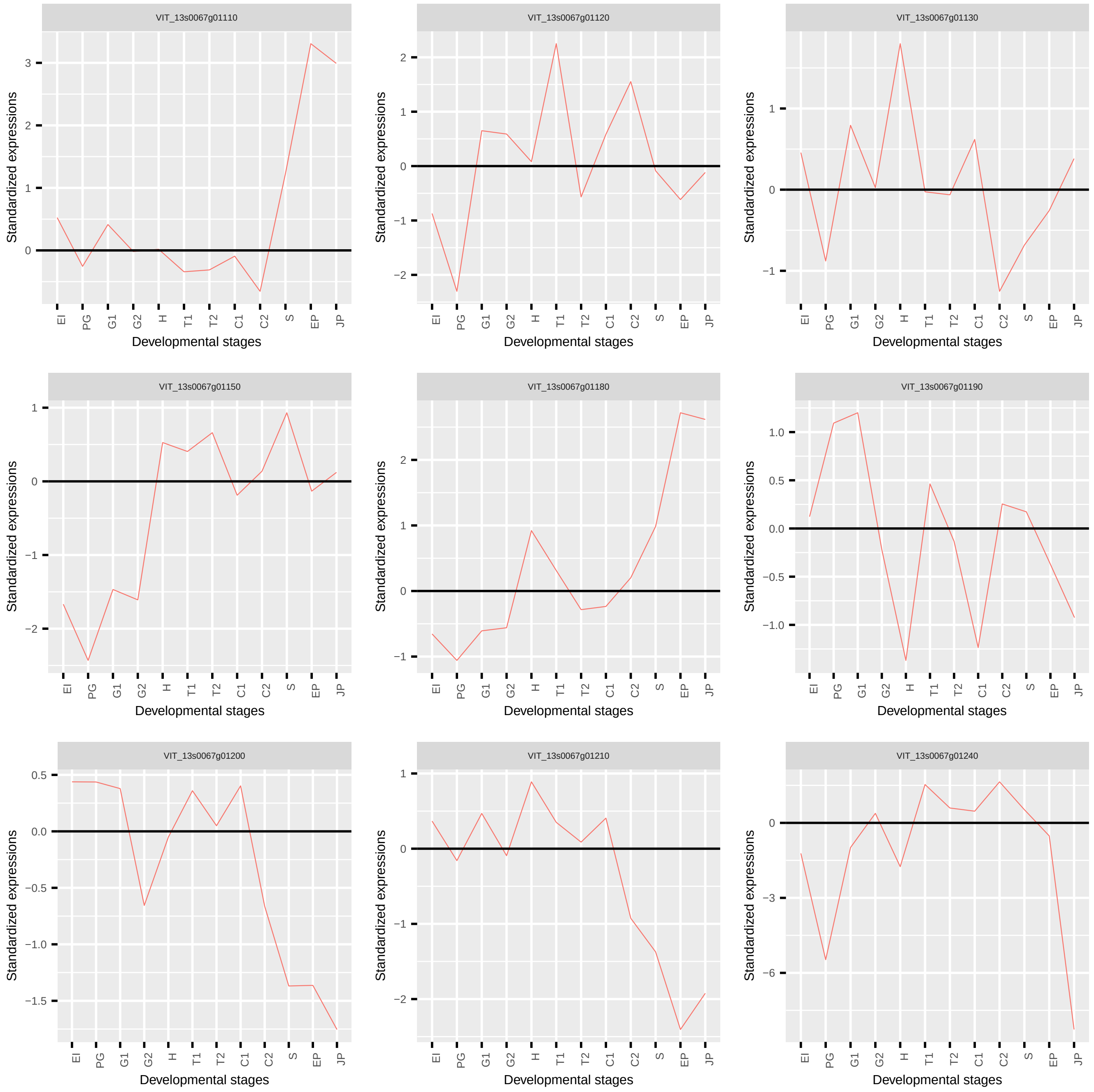
VIT_13s0067g01110
VIT_13s0067g01120
VIT_13s0067g01130
VIT_13s0067g01150
VIT_13s0067g01180
VIT_13s0067g01190
VIT_13s0067g01200
VIT_13s0067g01210
VIT_13s0067g01240
Standardized expressions
Developmental stages
EI
PG
G1
G2
H
T1
T2
C1
C2
S
EP
JP

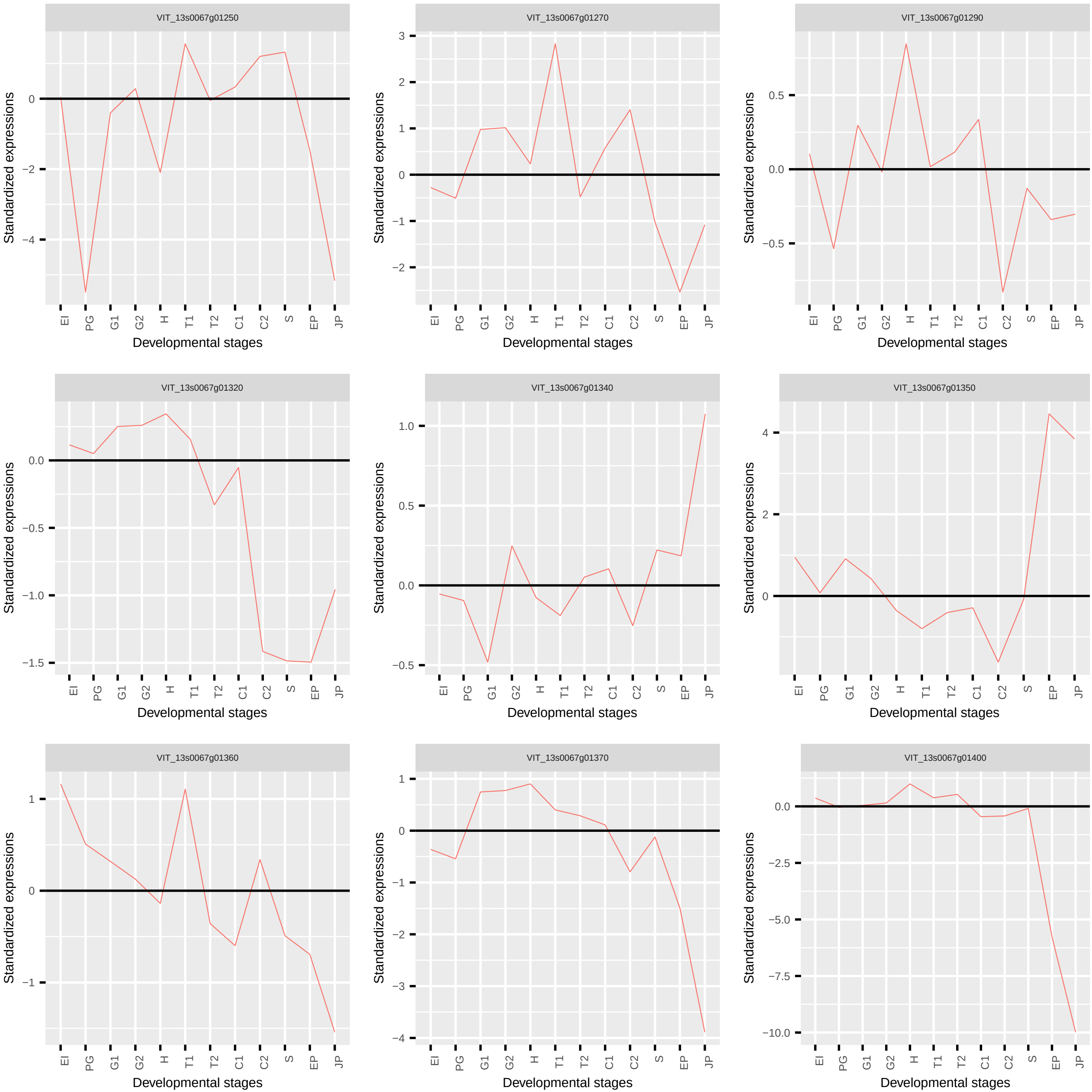
VIT_13s0067g01250
VIT_13s0067g01270
VIT_13s0067g01290
VIT_13s0067g01320
VIT_13s0067g01340
VIT_13s0067g01350
VIT_13s0067g01360
VIT_13s0067g01370
VIT_13s0067g01400
Standardized expressions
Developmental stages
EI PG G1 G2 H T1 T2 C1 C2 S EP JP

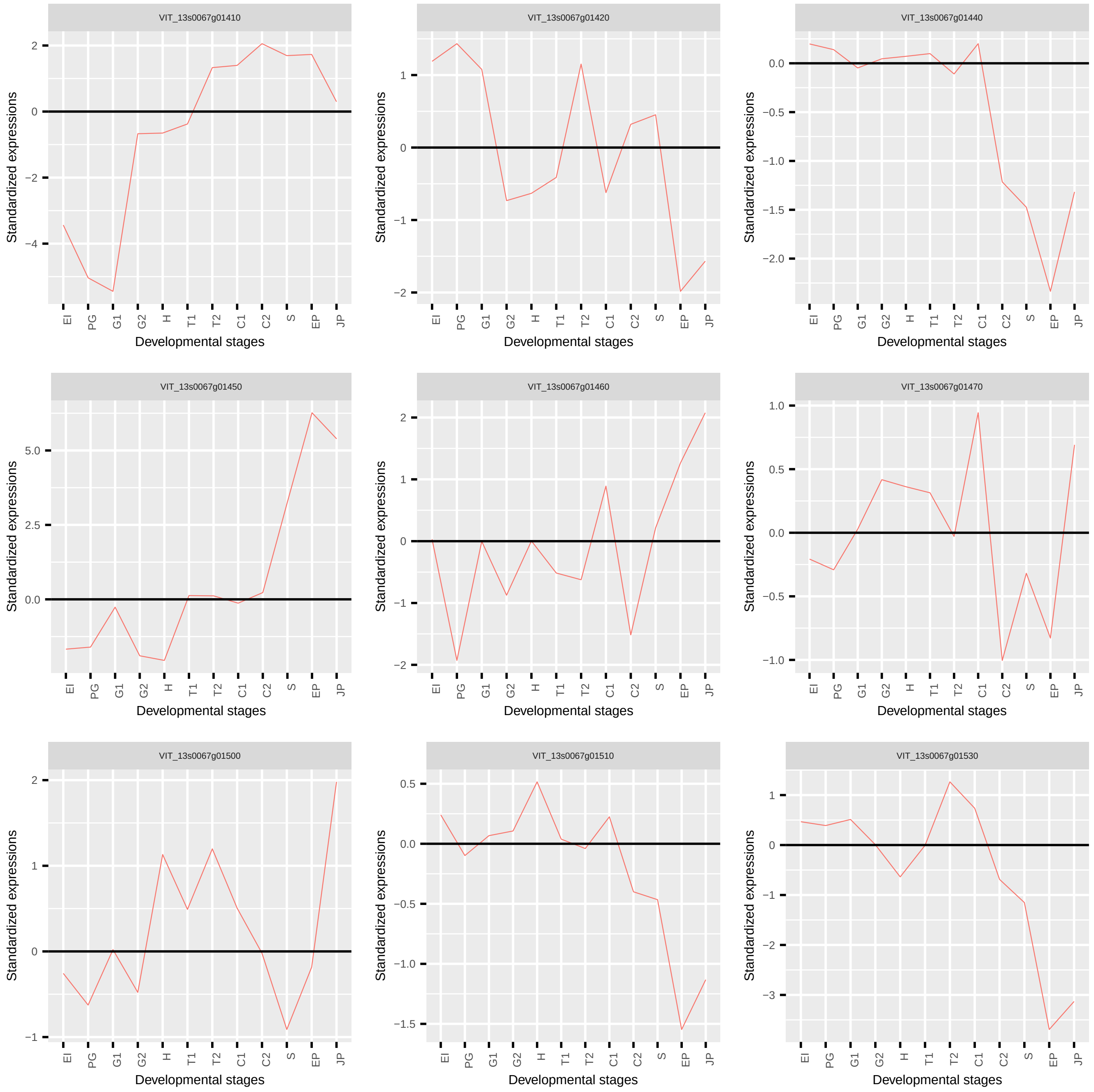

VIT_13s0067g01410
VIT_13s0067g01420
VIT_13s0067g01440
VIT_13s0067g01450
VIT_13s0067g01460
VIT_13s0067g01470
VIT_13s0067g01500
VIT_13s0067g01510
VIT_13s0067g01530
Standardized expressions
Developmental stages
EI PG G1 G2 H T1 T2 C1 C2 S EP JP
2 0 −2 −4
1 0 −1 −2
0.0 −0.5 −1.0 −1.5 −2.0
5.0 2.5 0.0
2 1 0 −1 −2
1.0 0.5 0.0 −0.5 −1.0
2 1 0 −1
0.5 0.0 −0.5 −1.0 −1.5
1 0 −1 −2 −3

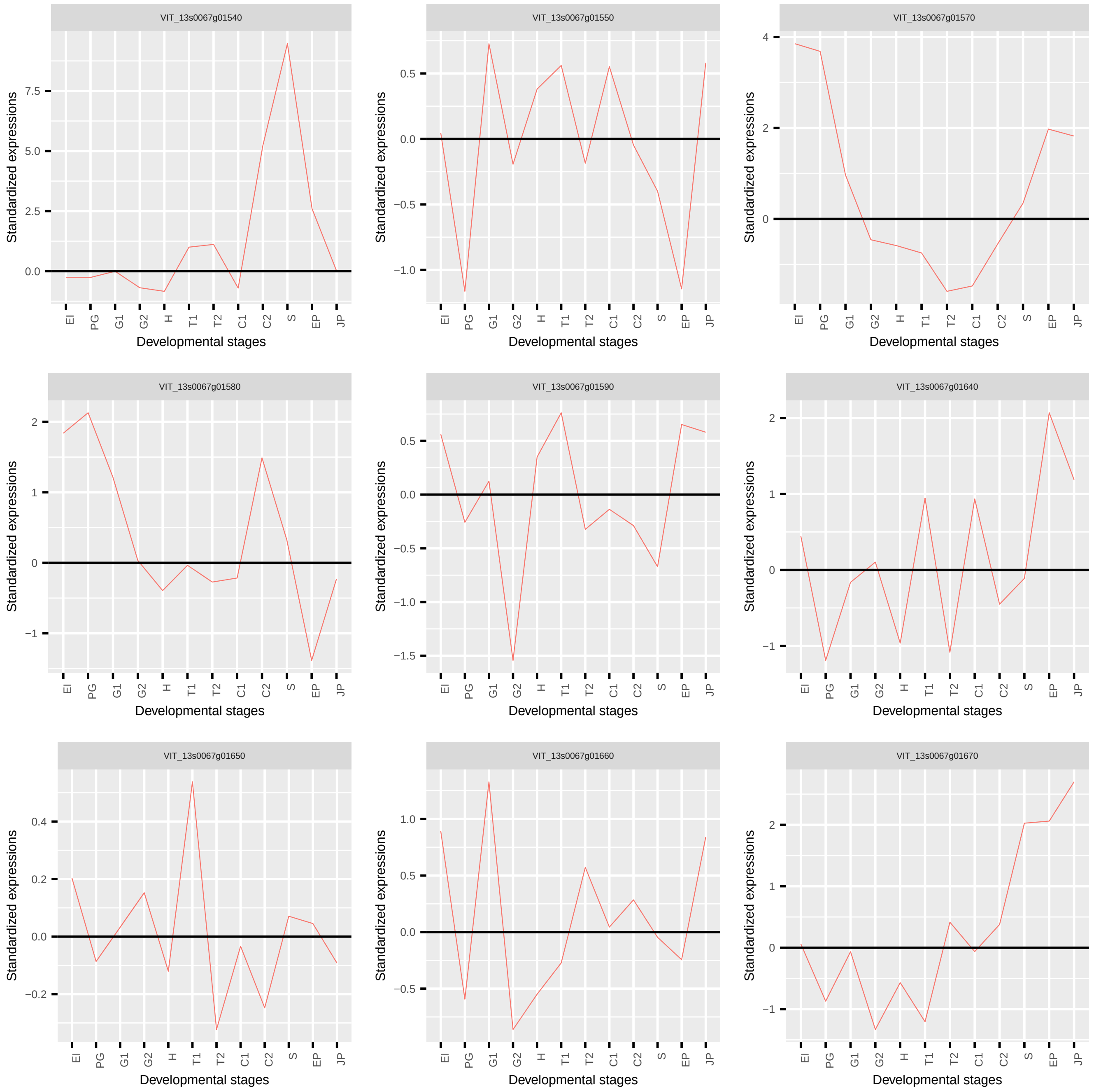

VIT_13s0067g01540
VIT_13s0067g01550
VIT_13s0067g01570
VIT_13s0067g01580
VIT_13s0067g01590
VIT_13s0067g01640
VIT_13s0067g01650
VIT_13s0067g01660
VIT_13s0067g01670
Standardized expressions
Developmental stages
EI
PG
G1
G2
H
T1
T2
C1
C2
S
EP
JP

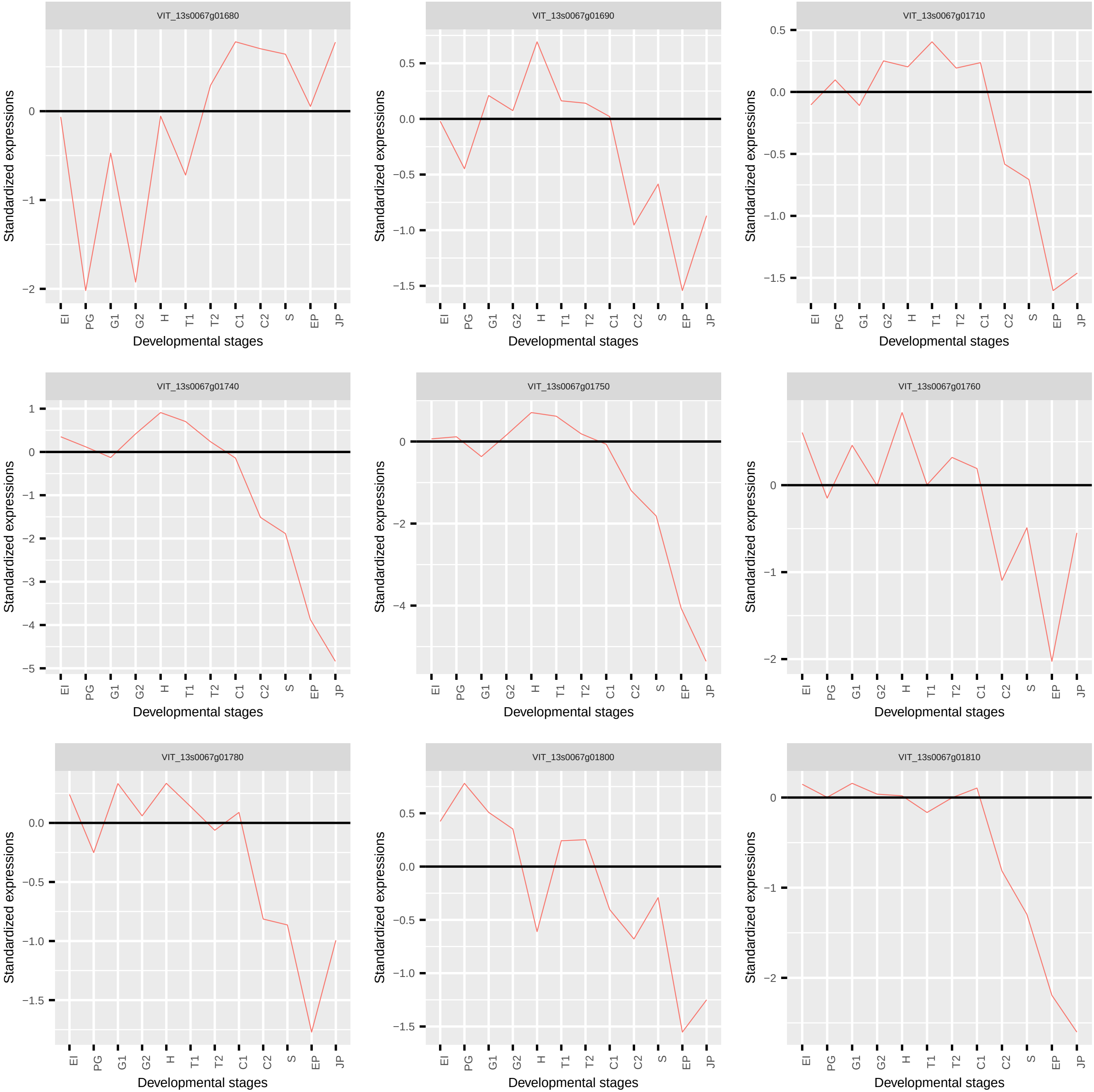

VIT_13s0067g01680
VIT_13s0067g01690
VIT_13s0067g01710
VIT_13s0067g01740
VIT_13s0067g01750
VIT_13s0067g01760
VIT_13s0067g01780
VIT_13s0067g01800
VIT_13s0067g01810
Standardized expressions
Developmental stages
EI
PG
G1
G2
H
T1
T2
C1
C2
S
EP
JP

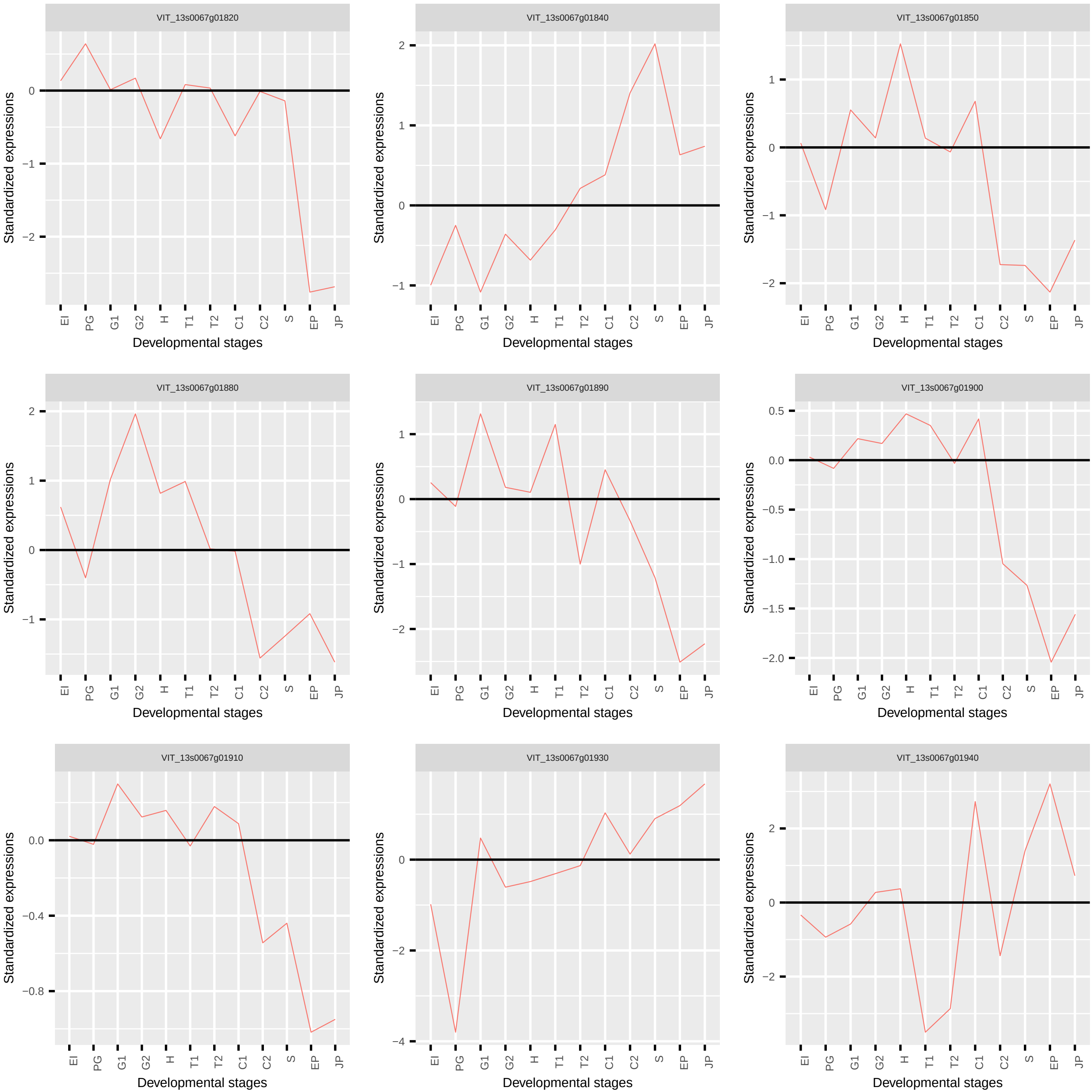
VIT_13s0067g01820
VIT_13s0067g01840
VIT_13s0067g01850
VIT_13s0067g01880
VIT_13s0067g01890
VIT_13s0067g01900
VIT_13s0067g01910
VIT_13s0067g01930
VIT_13s0067g01940
Standardized expressions
Developmental stages
EI PG G1 G2 H T1 T2 C1 C2 S EP JP

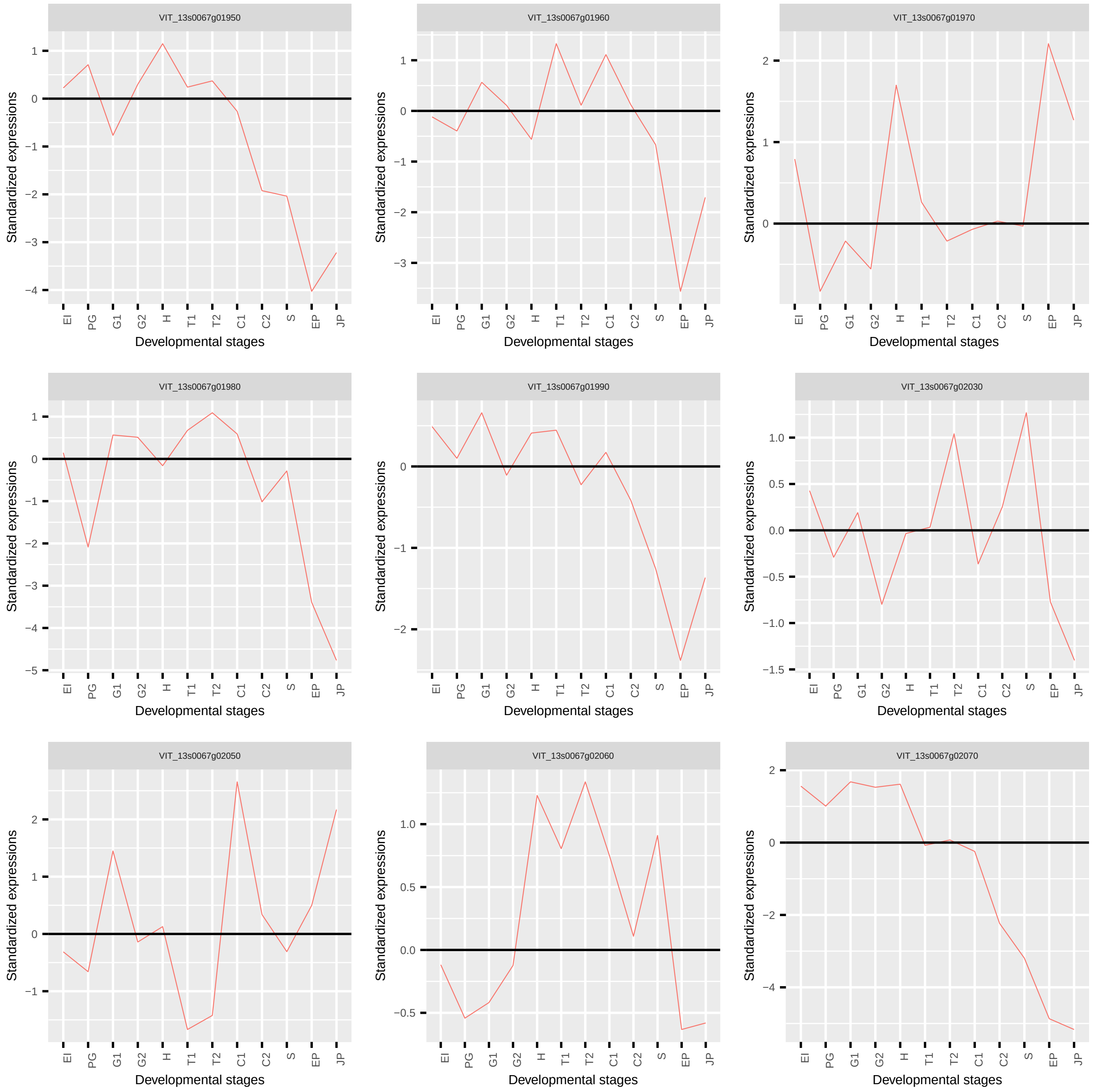
VIT_13s0067g01950
VIT_13s0067g01960
VIT_13s0067g01970
VIT_13s0067g01980
VIT_13s0067g01990
VIT_13s0067g02030
VIT_13s0067g02050
VIT_13s0067g02060
VIT_13s0067g02070
Standardized expressions
Developmental stages
EI PG G1 G2 H T1 T2 C1 C2 S EP JP

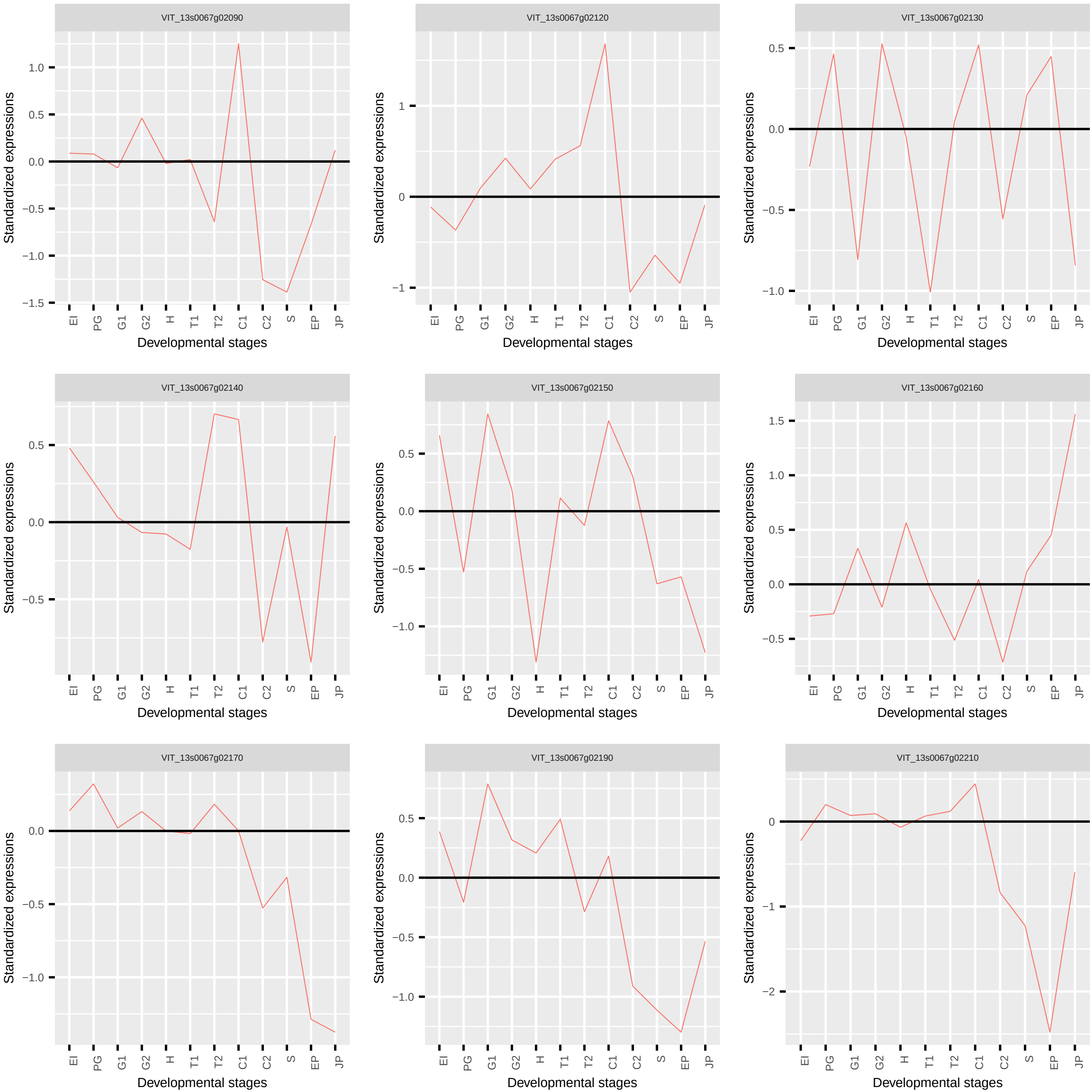
VIT_13s0067g02090
VIT_13s0067g02120
VIT_13s0067g02130
VIT_13s0067g02140
VIT_13s0067g02150
VIT_13s0067g02160
VIT_13s0067g02170
VIT_13s0067g02190
VIT_13s0067g02210
Standardized expressions
Developmental stages
EI PG G1 G2 H T1 T2 C1 C2 S EP JP

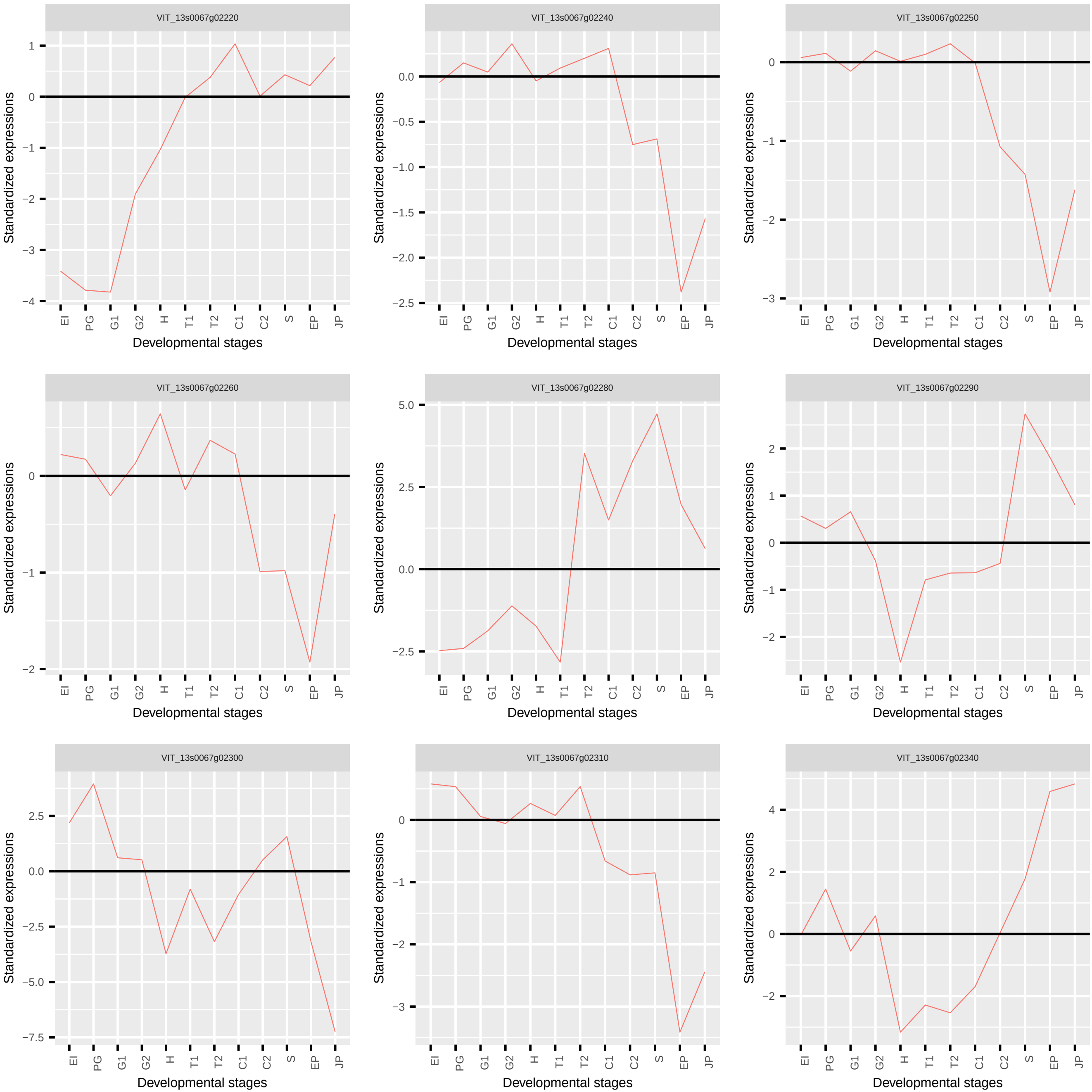
VIT_13s0067g02220
VIT_13s0067g02240
VIT_13s0067g02250
VIT_13s0067g02260
VIT_13s0067g02280
VIT_13s0067g02290
VIT_13s0067g02300
VIT_13s0067g02310
VIT_13s0067g02340
Standardized expressions
Developmental stages
EI PG G1 G2 H T1 T2 C1 C2 S EP JP

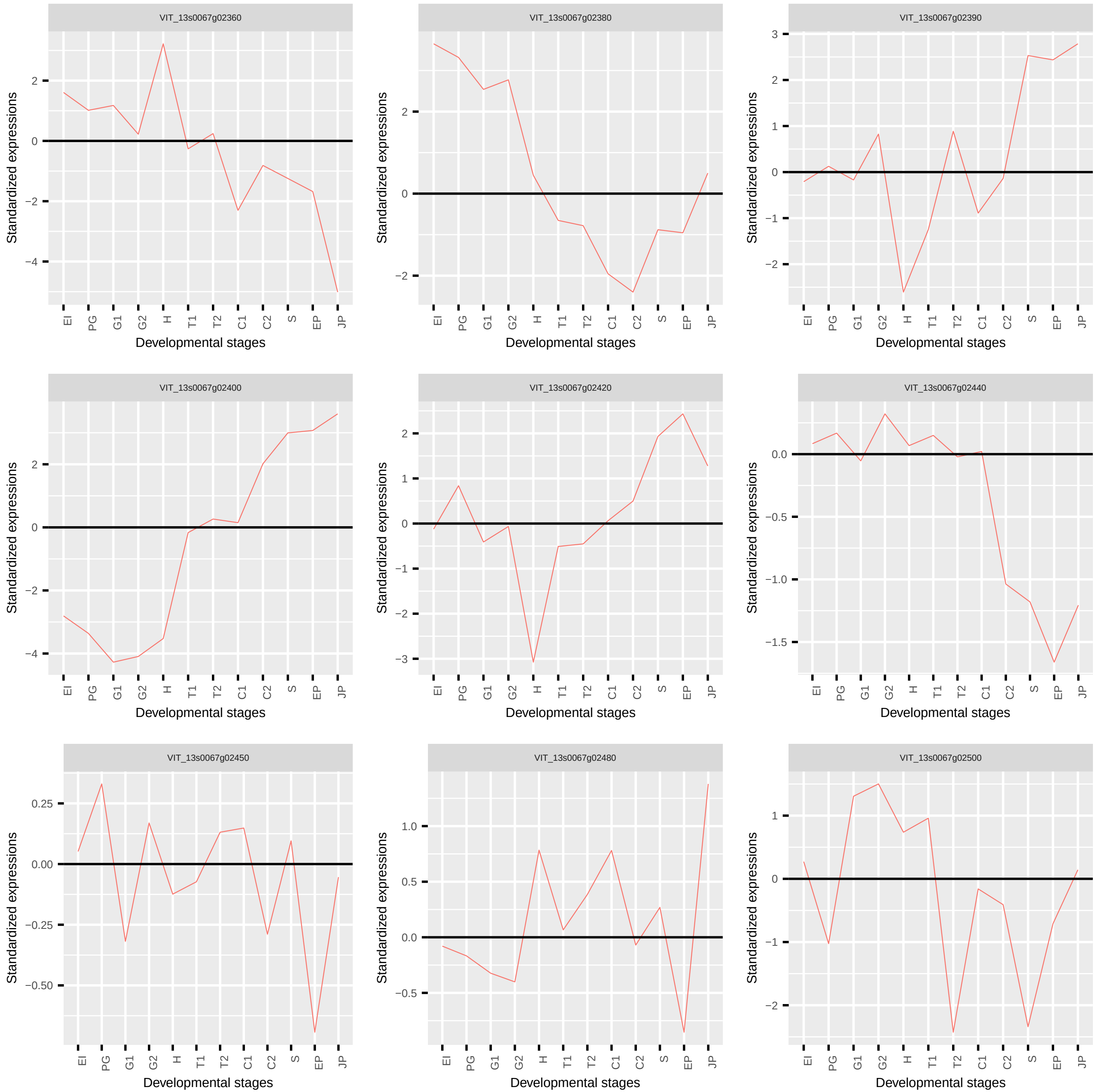
VIT_13s0067g02360
VIT_13s0067g02380
VIT_13s0067g02390
VIT_13s0067g02400
VIT_13s0067g02420
VIT_13s0067g02440
VIT_13s0067g02450
VIT_13s0067g02480
VIT_13s0067g02500
Standardized expressions
Developmental stages
EI
PG
G1
G2
H
T1
T2
C1
C2
S
EP
JP
2
0
−2
−4
3
1
−1
−3
0.0
−0.5
−1.0
−1.5
0.25
0.00
−0.25
−0.50
1.0
0.5

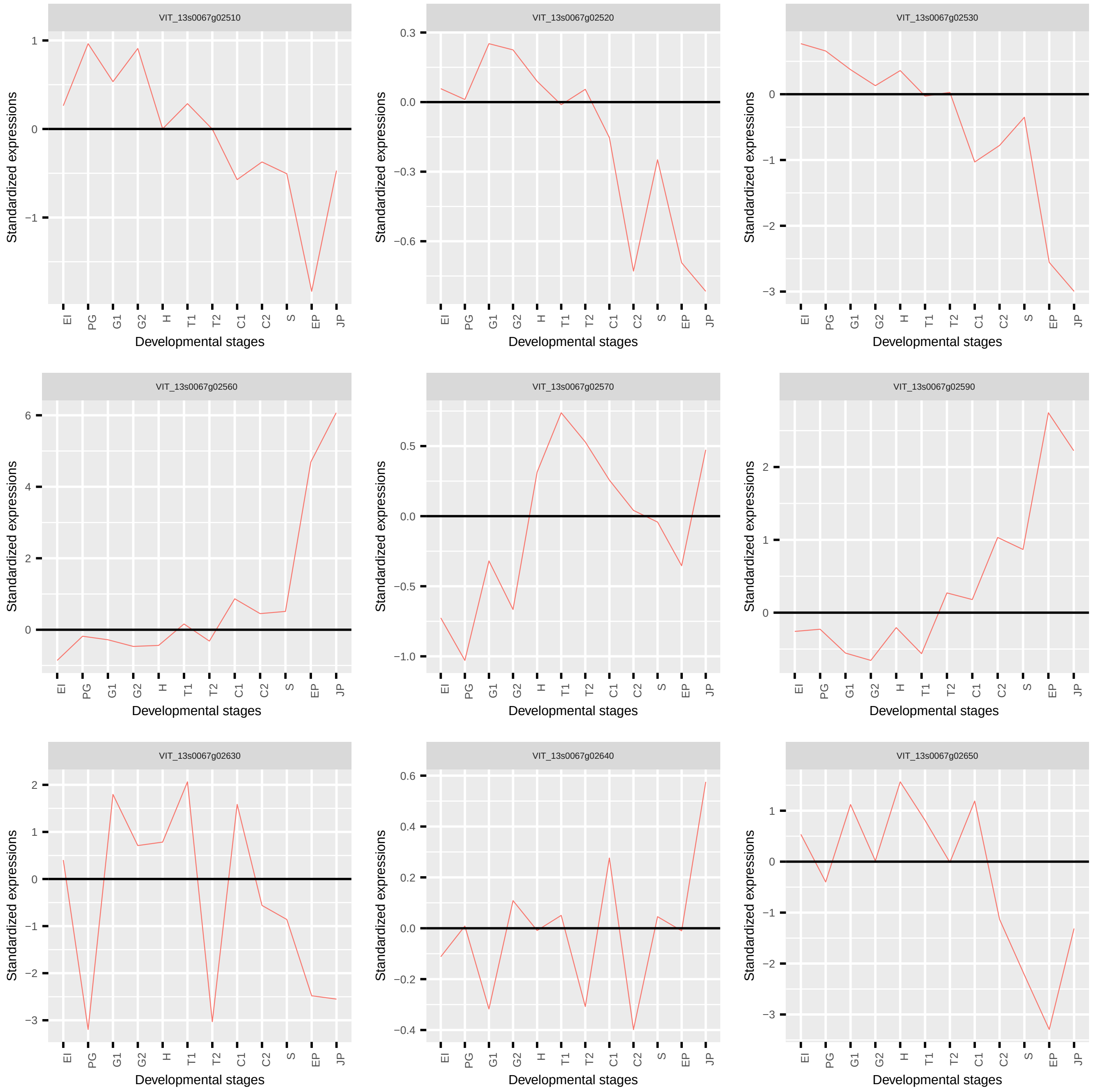
VIT_13s0067g02510
VIT_13s0067g02520
VIT_13s0067g02530
VIT_13s0067g02560
VIT_13s0067g02570
VIT_13s0067g02590
VIT_13s0067g02630
VIT_13s0067g02640
VIT_13s0067g02650
Standardized expressions
Developmental stages
EI
PG
G1
G2
H
T1
T2
C1
C2
S
EP
JP

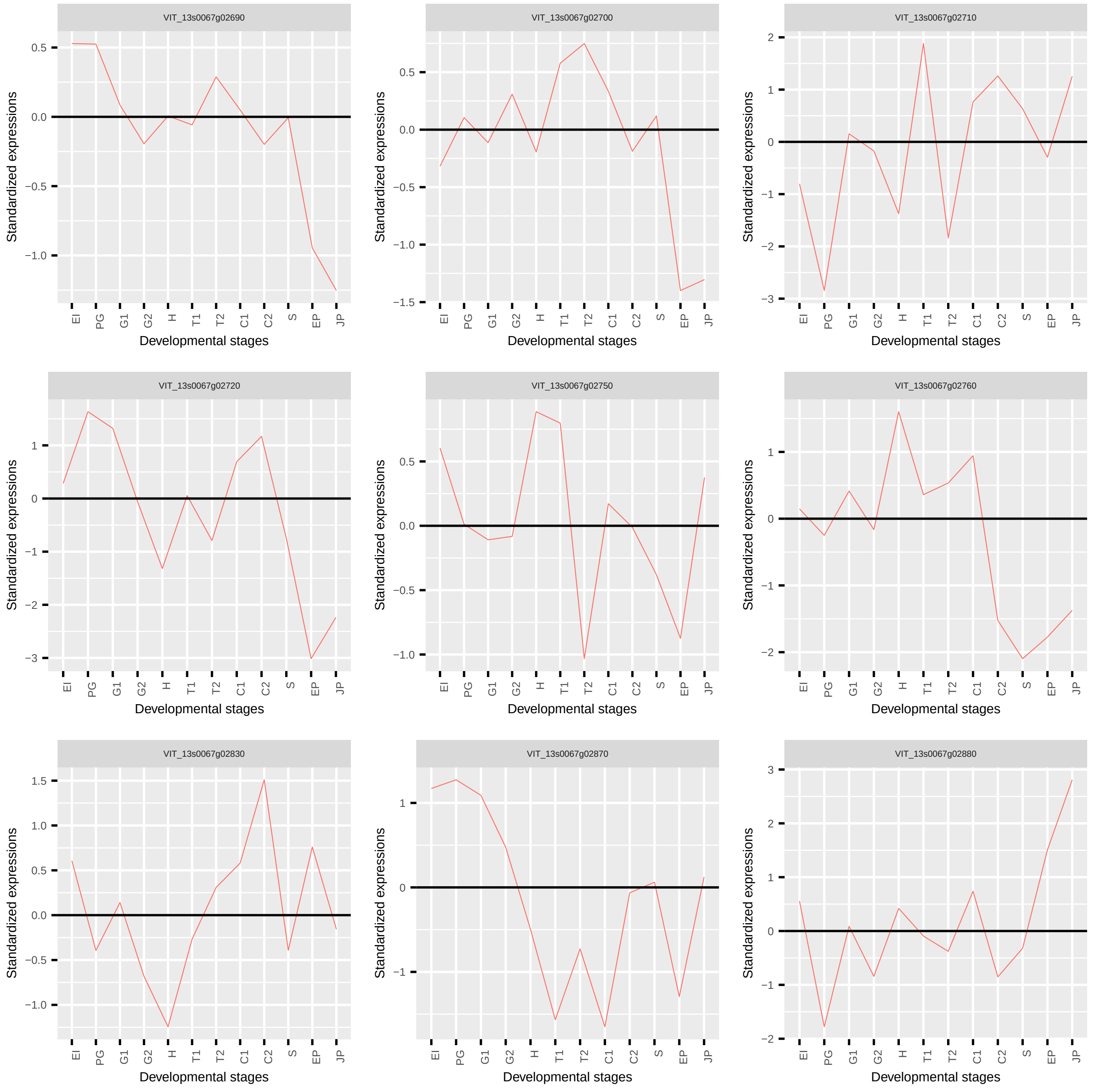

VIT_13s0067g02690
VIT_13s0067g02700
VIT_13s0067g02710
VIT_13s0067g02720
VIT_13s0067g02750
VIT_13s0067g02760
VIT_13s0067g02830
VIT_13s0067g02870
VIT_13s0067g02880
Standardized expressions
Developmental stages
EI PG G1 G2 H T1 T2 C1 C2 S EP JP

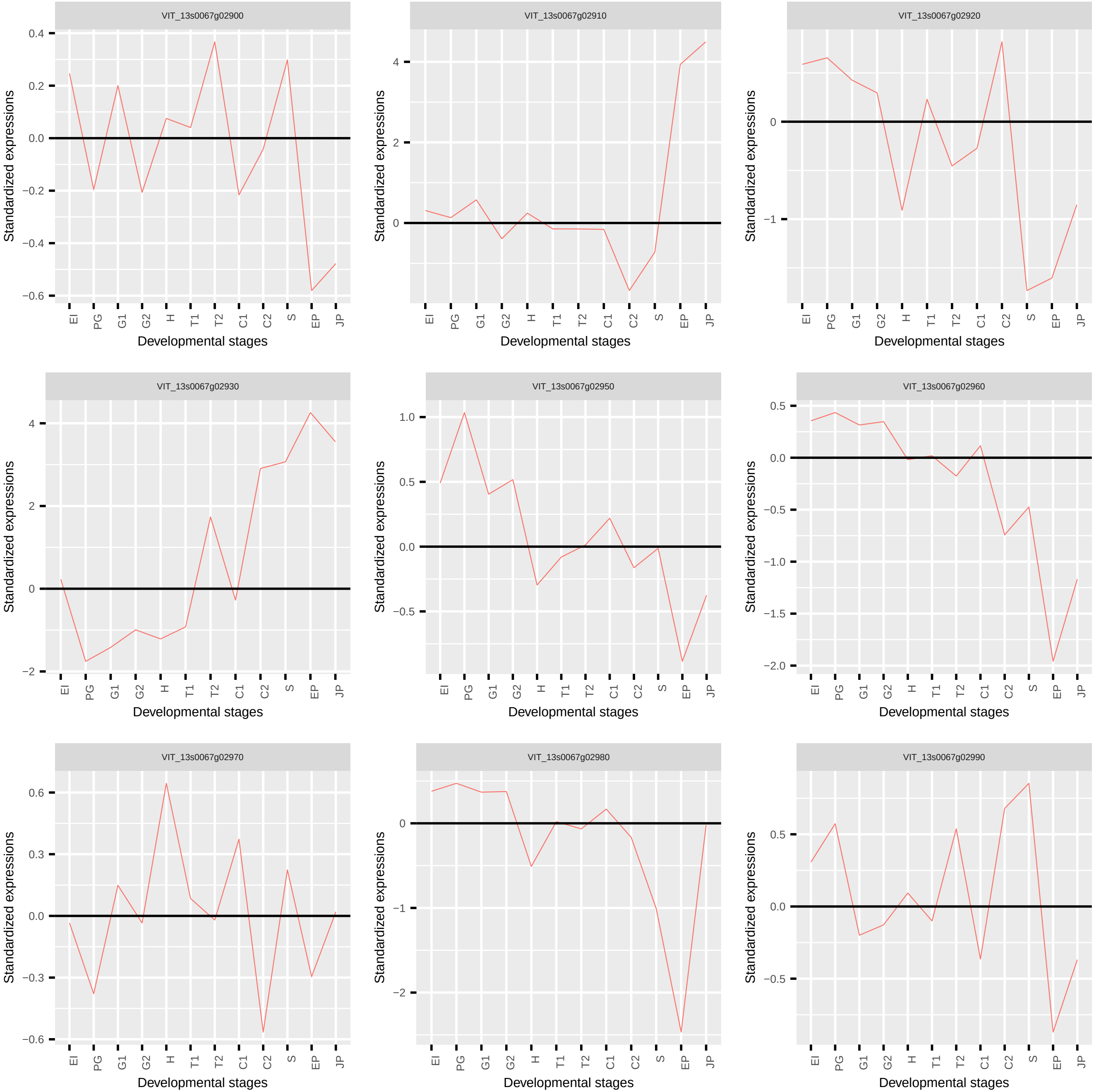
VIT_13s0067g02900
VIT_13s0067g02910
VIT_13s0067g02920
VIT_13s0067g02930
VIT_13s0067g02950
VIT_13s0067g02960
VIT_13s0067g02970
VIT_13s0067g02980
VIT_13s0067g02990
Standardized expressions
Developmental stages
EI PG G1 G2 H T1 T2 C1 C2 S EP JP

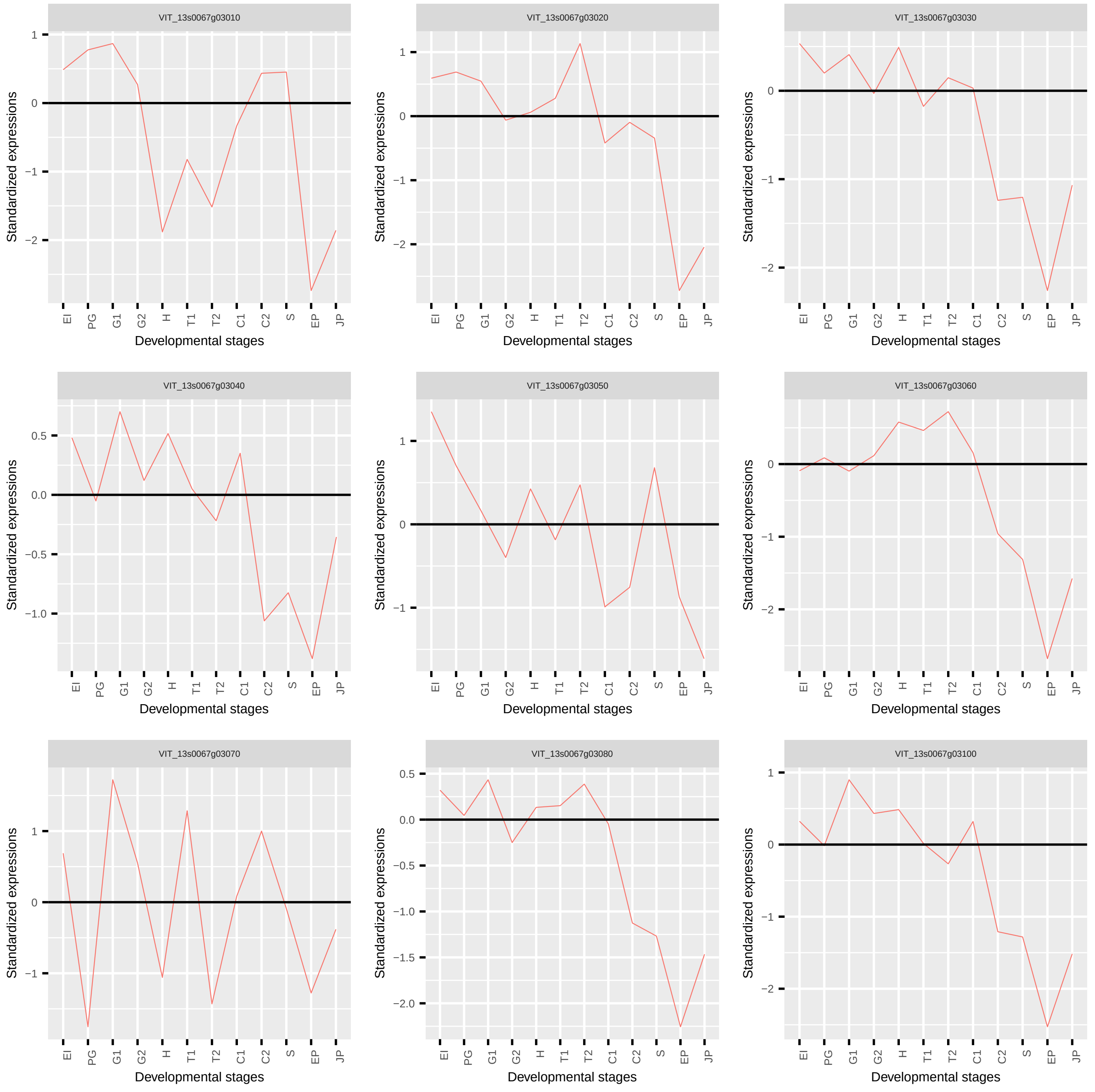
VIT_13s0067g03010
VIT_13s0067g03020
VIT_13s0067g03030
VIT_13s0067g03040
VIT_13s0067g03050
VIT_13s0067g03060
VIT_13s0067g03070
VIT_13s0067g03080
VIT_13s0067g03100
Standardized expressions
Developmental stages
EI PG G1 G2 H T1 T2 C1 C2 S EP JP

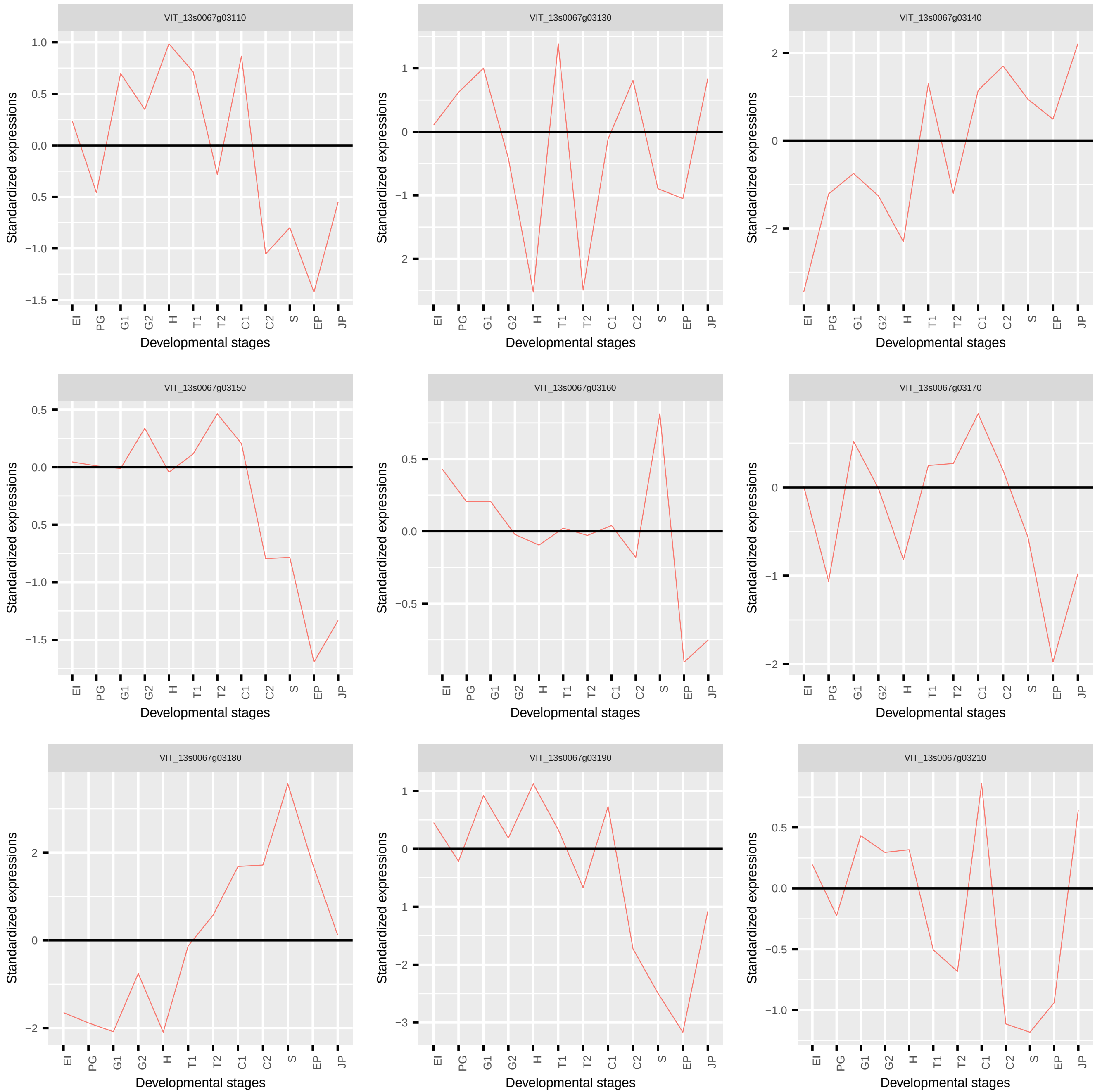
VIT_13s0067g03110
VIT_13s0067g03130
VIT_13s0067g03140
VIT_13s0067g03150
VIT_13s0067g03160
VIT_13s0067g03170
VIT_13s0067g03180
VIT_13s0067g03190
VIT_13s0067g03210
Standardized expressions
Developmental stages
EI PG G1 G2 H T1 T2 C1 C2 S EP JP
1.0
0.5
0.0
−0.5
−1.0
−1.5
2
1
0
−1
−2
−3

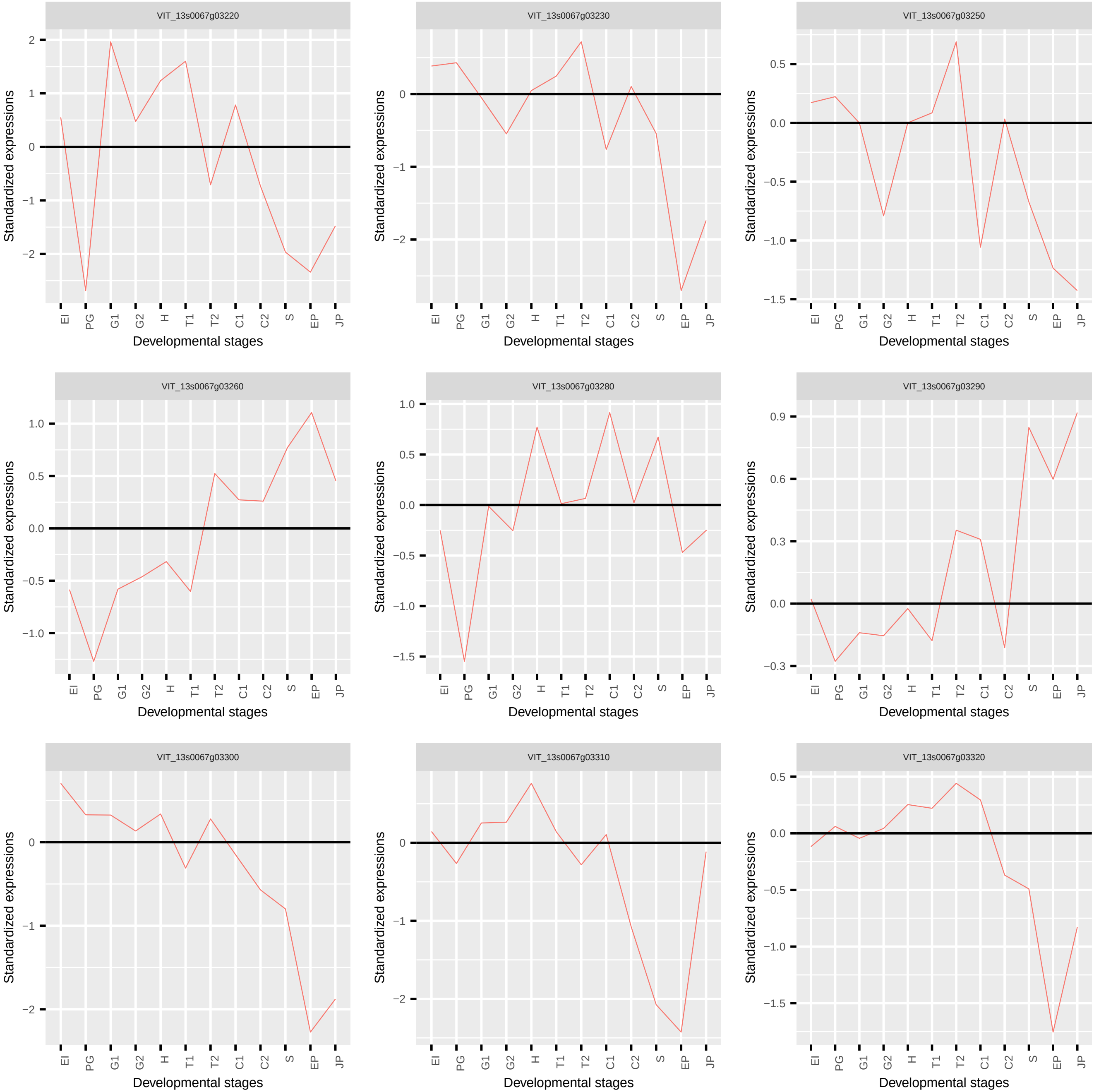
VIT_13s0067g03220
VIT_13s0067g03230
VIT_13s0067g03250
VIT_13s0067g03260
VIT_13s0067g03280
VIT_13s0067g03290
VIT_13s0067g03300
VIT_13s0067g03310
VIT_13s0067g03320
Standardized expressions
Developmental stages
EI PG G1 G2 H T1 T2 C1 C2 S EP JP

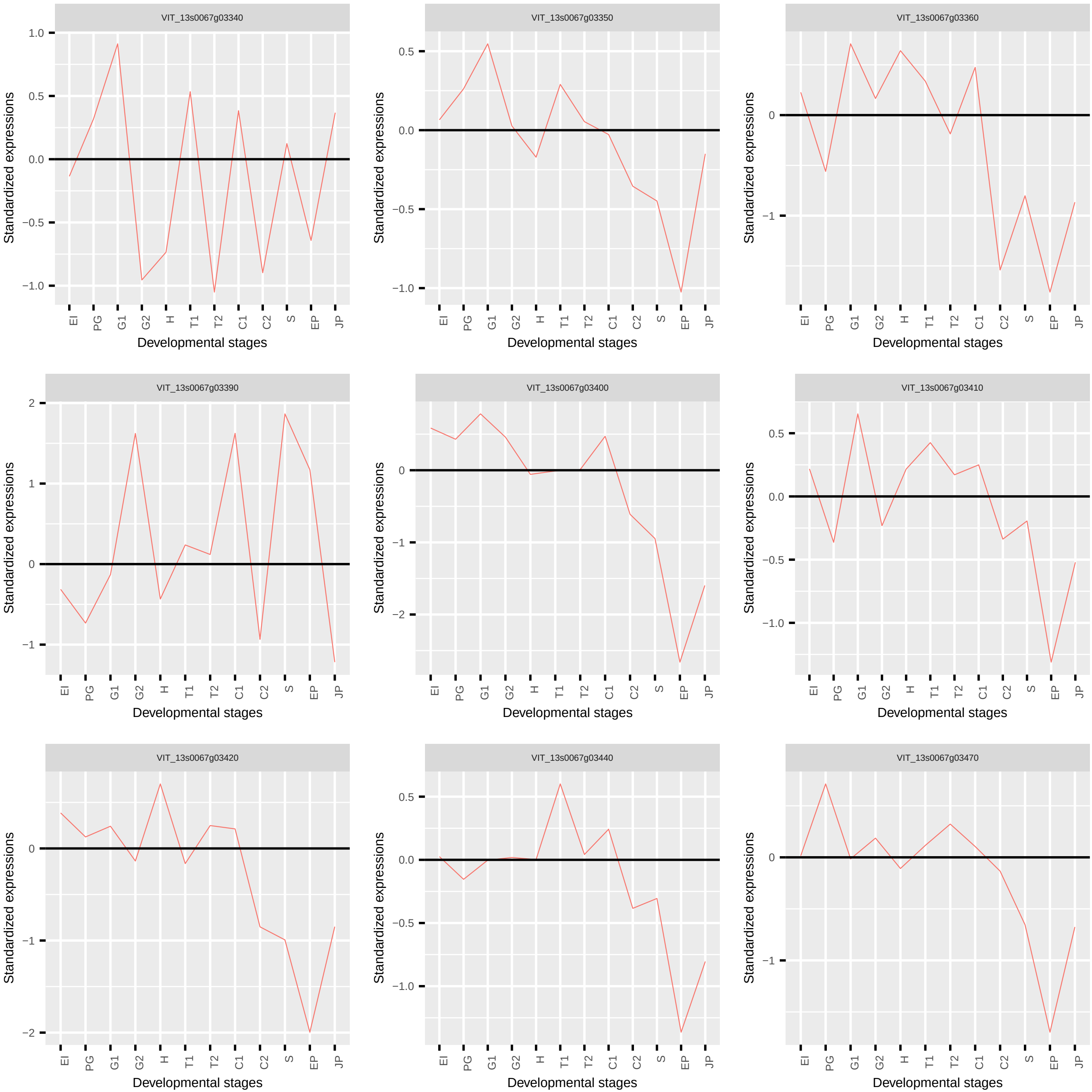

VIT_13s0067g03340
VIT_13s0067g03350
VIT_13s0067g03360
VIT_13s0067g03390
VIT_13s0067g03400
VIT_13s0067g03410
VIT_13s0067g03420
VIT_13s0067g03440
VIT_13s0067g03470
Standardized expressions
Developmental stages
EI
PG
G1
G2
H
T1
T2
C1
C2
S
EP
JP

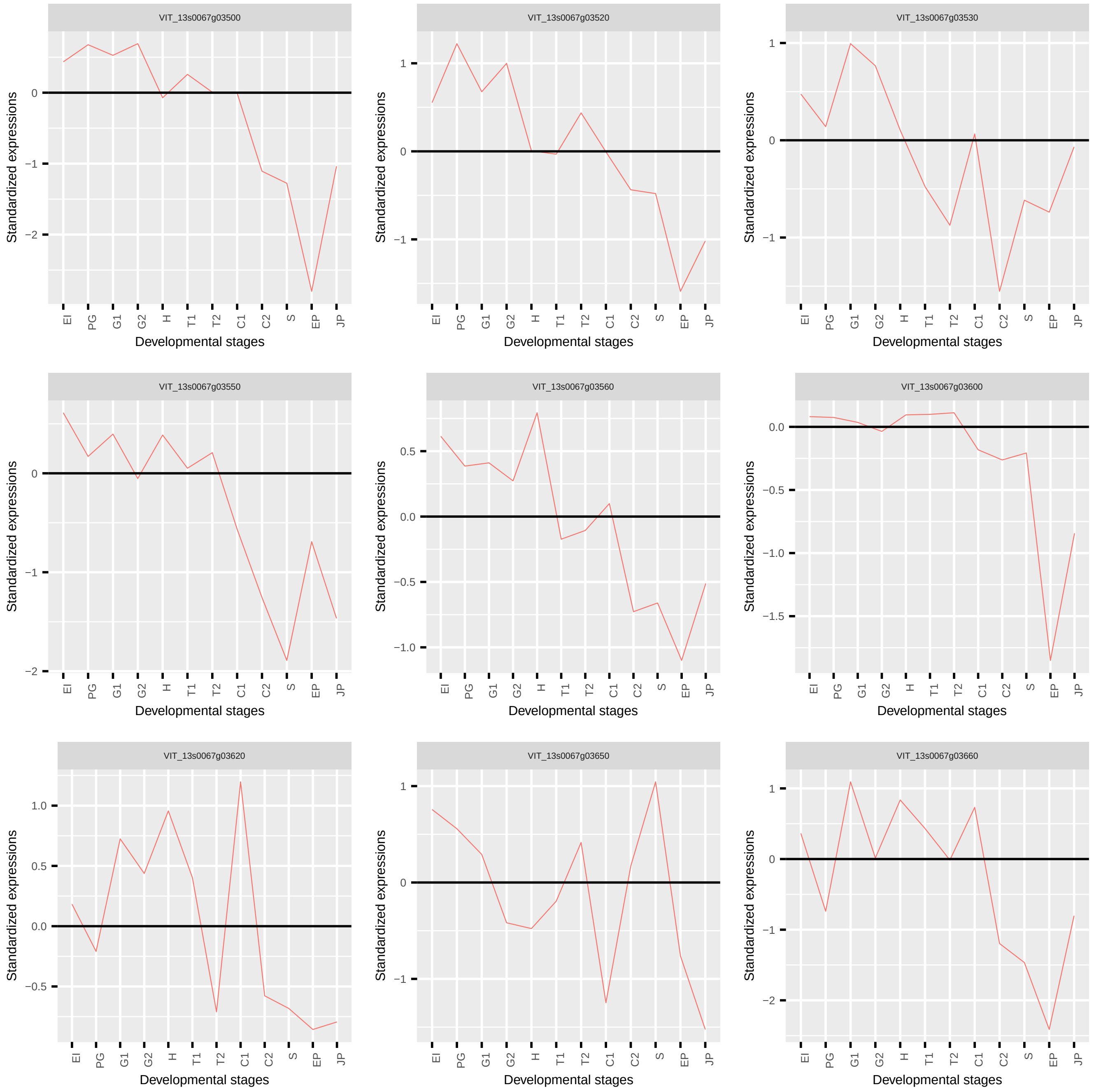
VIT_13s0067g03500
VIT_13s0067g03520
VIT_13s0067g03530
VIT_13s0067g03550
VIT_13s0067g03560
VIT_13s0067g03600
VIT_13s0067g03620
VIT_13s0067g03650
VIT_13s0067g03660
Standardized expressions
Developmental stages
EI PG G1 G2 H T1 T2 C1 C2 S EP JP

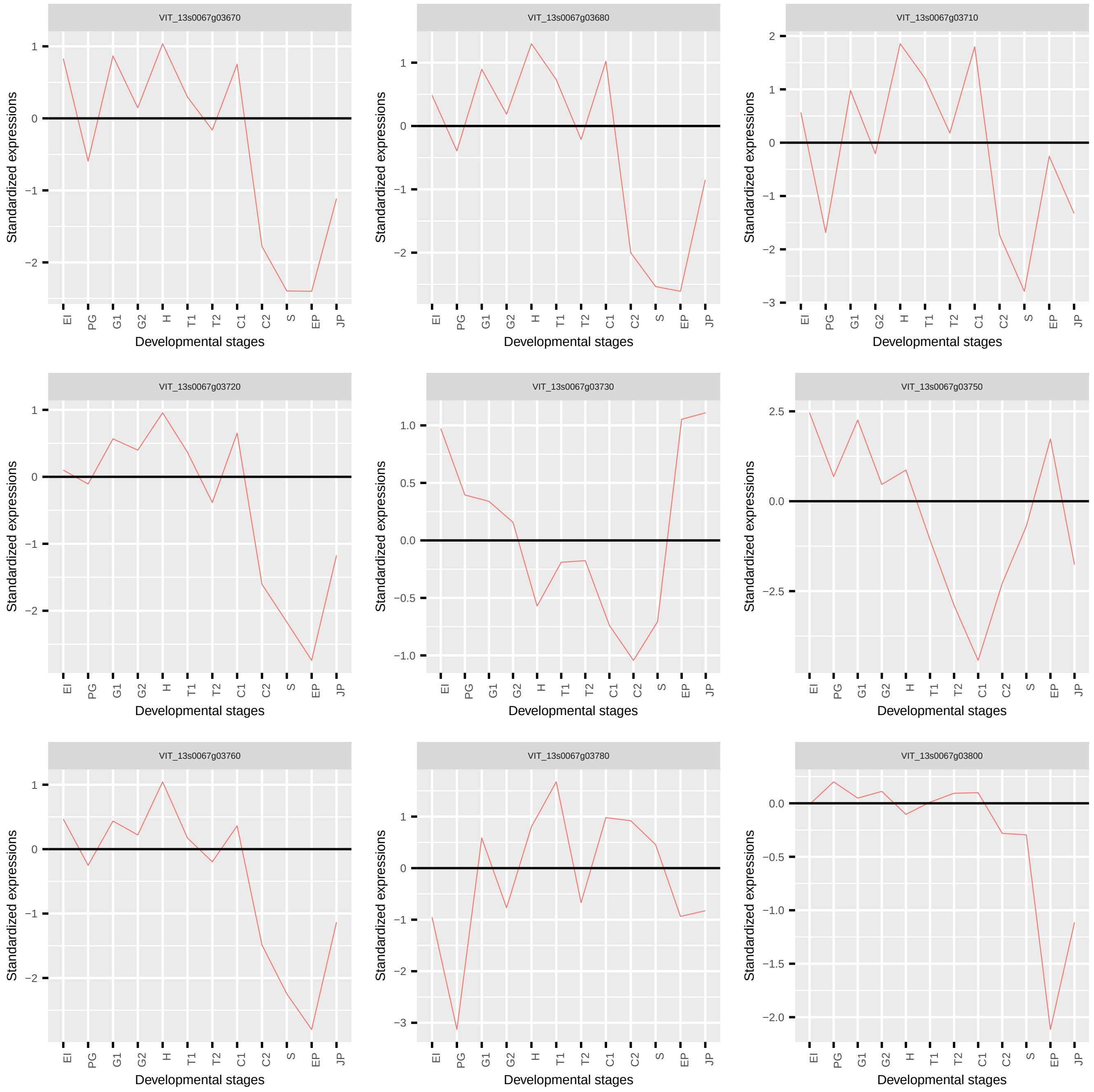

VIT_13s0067g03670
VIT_13s0067g03680
VIT_13s0067g03710
VIT_13s0067g03720
VIT_13s0067g03730
VIT_13s0067g03750
VIT_13s0067g03760
VIT_13s0067g03780
VIT_13s0067g03800
Standardized expressions
Developmental stages
EI PG G1 G2 H T1 T2 C1 C2 S EP JP

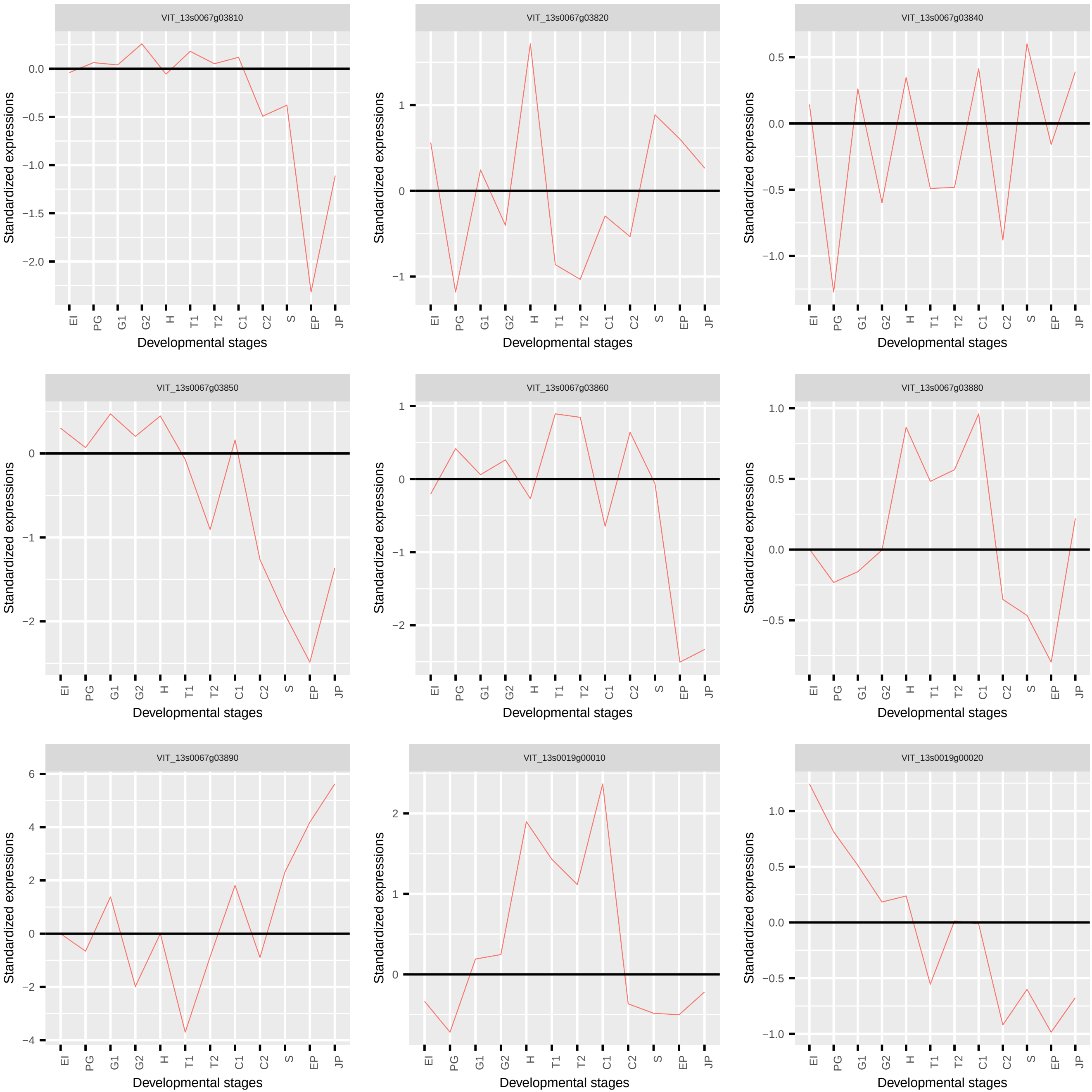
VIT_13s0067g03810
VIT_13s0067g03820
VIT_13s0067g03840
VIT_13s0067g03850
VIT_13s0067g03860
VIT_13s0067g03880
VIT_13s0067g03890
VIT_13s0019g00010
VIT_13s0019g00020
Standardized expressions
Developmental stages
EI PG G1 G2 H T1 T2 C1 C2 S EP JP

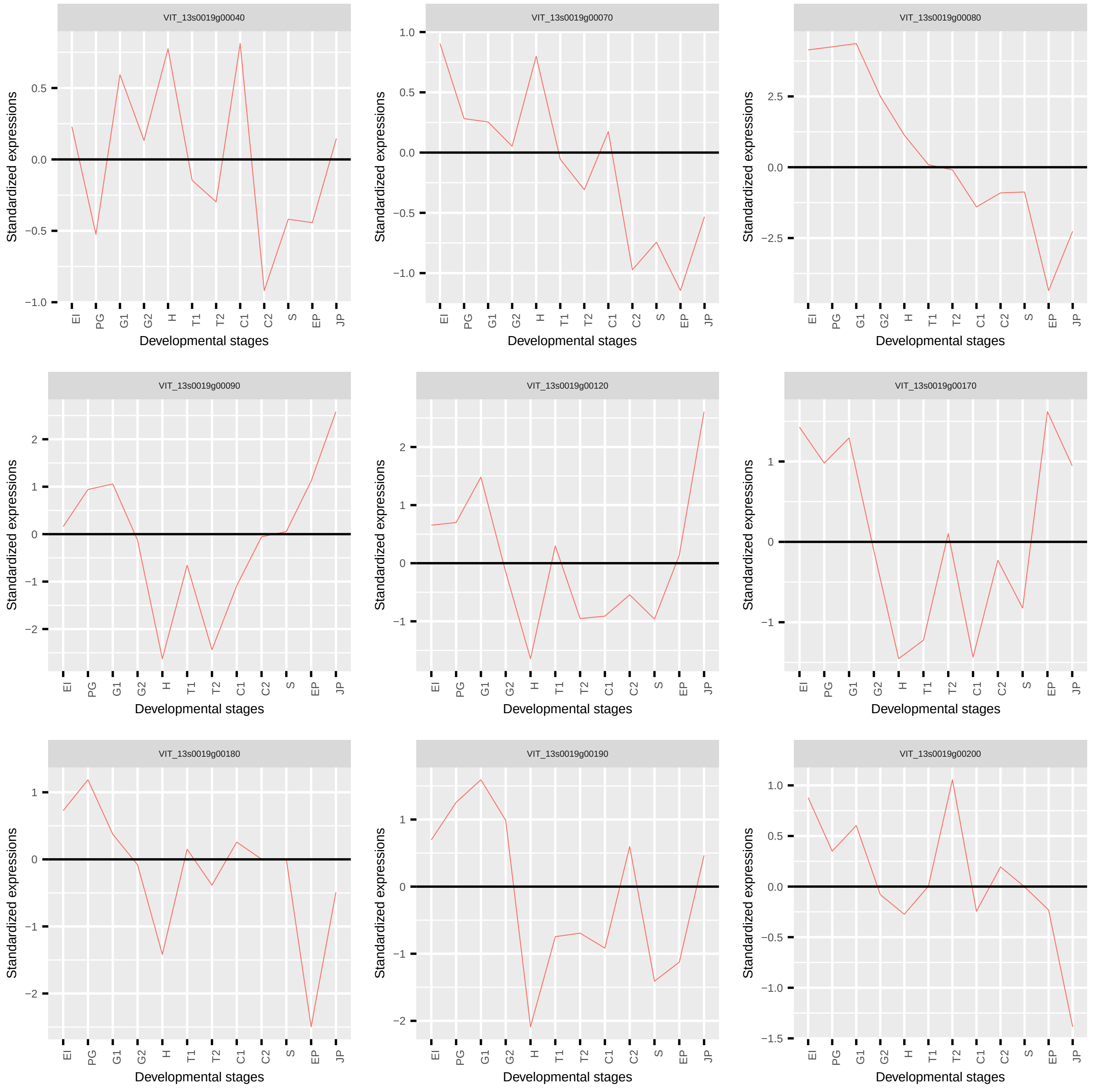
VIT_13s0019g00040
0.5
0.0
−0.5
−1.0
Standardized expressions
EI PG G1 G2 H T1 T2 C1 C2 S EP JP
Developmental stages
VIT_13s0019g00070
1.0
0.5
0.0
−0.5
−1.0
Standardized expressions
EI PG G1 G2 H T1 T2 C1 C2 S EP JP
Developmental stages
VIT_13s0019g00080
2.5
0.0
−2.5
Standardized expressions
EI PG G1 G2 H T1 T2 C1 C2 S EP JP
Developmental stages
VIT_13s0019g00090
2
1
0
−1
−2
Standardized expressions
EI PG G1 G2 H T1 T2 C1 C2 S EP JP
Developmental stages
VIT_13s0019g00120
2
1
0
−1
Standardized expressions
EI PG G1 G2 H T1 T2 C1 C2 S EP JP
Developmental stages
VIT_13s0019g00170
1
0
−1
Standardized expressions
EI PG G1 G2 H T1 T2 C1 C2 S EP JP
Developmental stages
VIT_13s0019g00180
1
0
−1
−2
Standardized expressions
EI PG G1 G2 H T1 T2 C1 C2 S EP JP
Developmental stages
VIT_13s0019g00190
1
0
−1
−2
Standardized expressions
EI PG G1 G2 H T1 T2 C1 C2 S EP JP
Developmental stages
VIT_13s0019g00200
1.0
0.5
0.0
−0.5
−1.0
−1.5
Standardized expressions
EI PG G1 G2 H T1 T2 C1 C2 S EP JP
Developmental stages

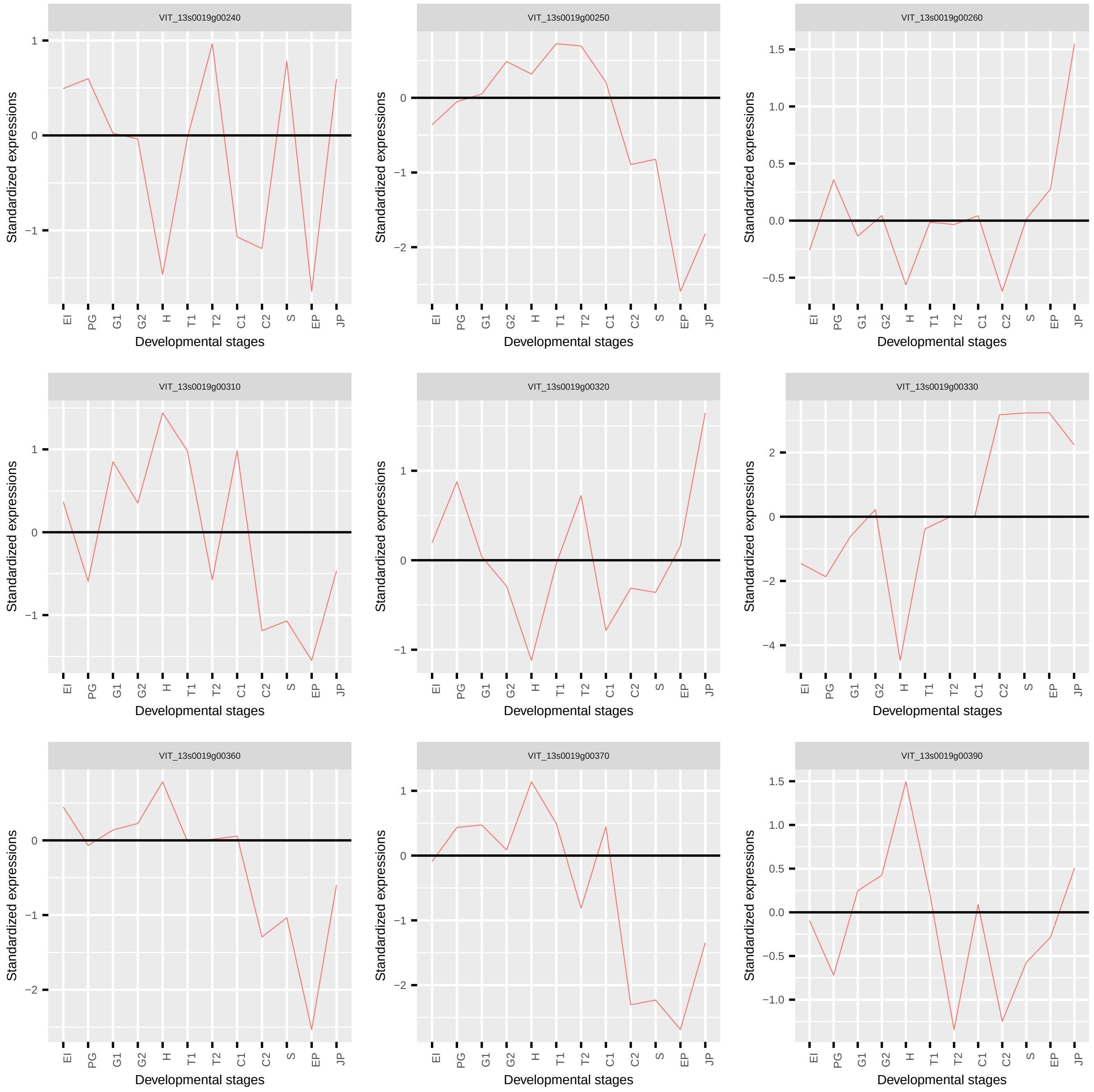
VIT_13s0019g00240
VIT_13s0019g00250
VIT_13s0019g00260
VIT_13s0019g00310
VIT_13s0019g00320
VIT_13s0019g00330
VIT_13s0019g00360
VIT_13s0019g00370
VIT_13s0019g00390
Standardized expressions
Developmental stages
EI
PG
G1
G2
H
T1
T2
C1
C2
S
EP
JP

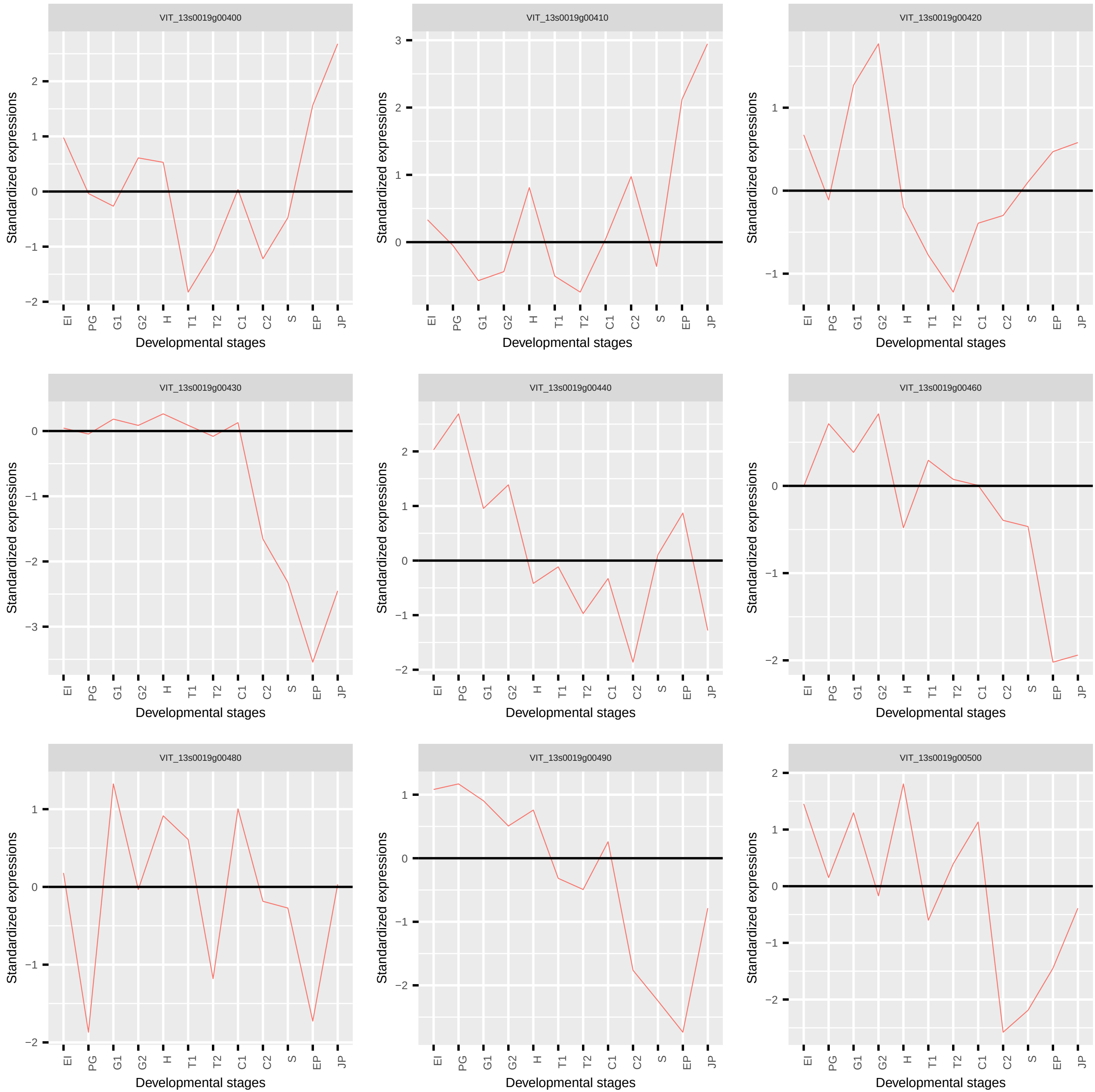
VIT_13s0019g00400
VIT_13s0019g00410
VIT_13s0019g00420
VIT_13s0019g00430
VIT_13s0019g00440
VIT_13s0019g00460
VIT_13s0019g00480
VIT_13s0019g00490
VIT_13s0019g00500
Standardized expressions
Developmental stages
EI PG G1 G2 H T1 T2 C1 C2 S EP JP

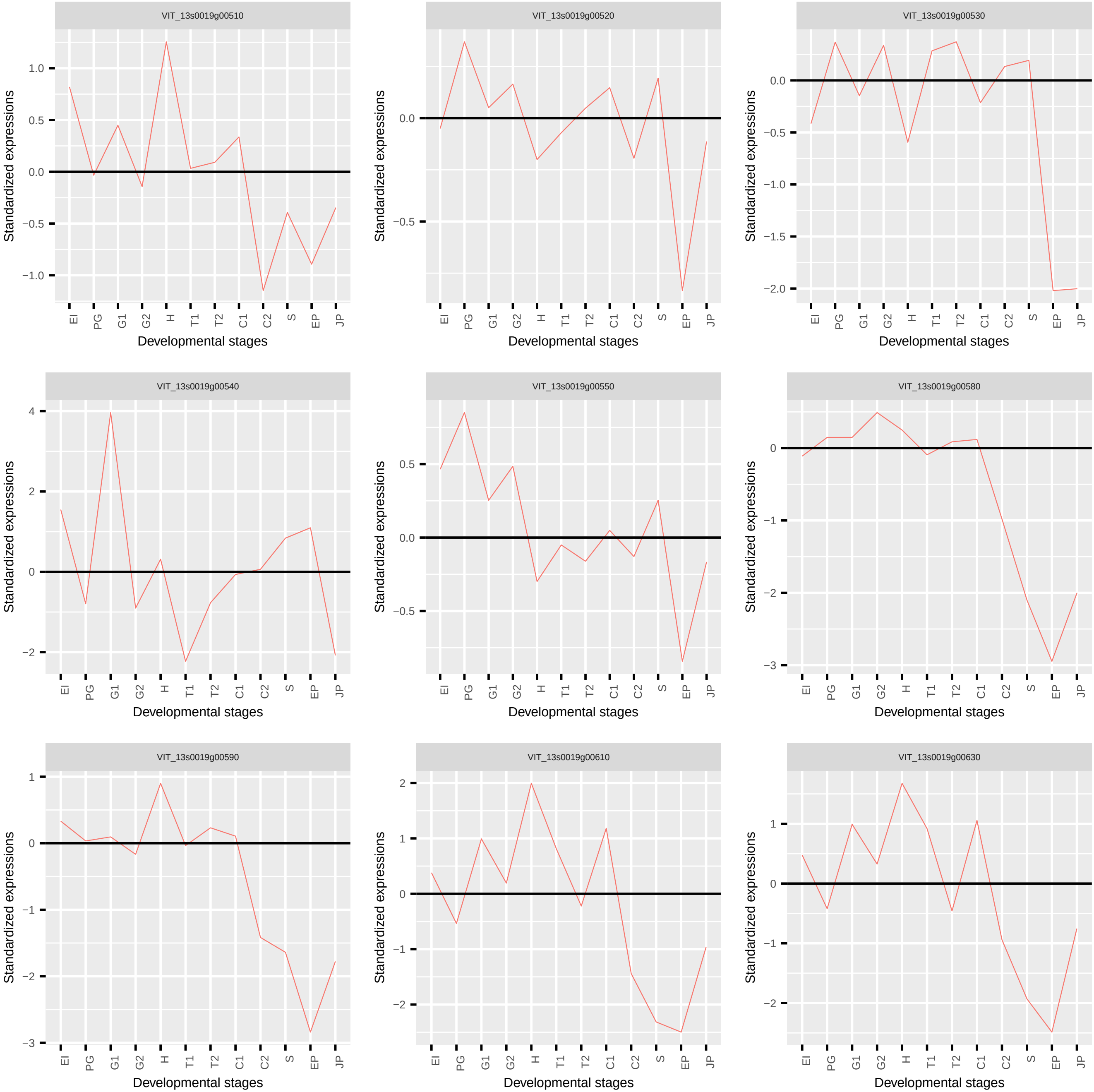
VIT_13s0019g00510
VIT_13s0019g00520
VIT_13s0019g00530
VIT_13s0019g00540
VIT_13s0019g00550
VIT_13s0019g00580
VIT_13s0019g00590
VIT_13s0019g00610
VIT_13s0019g00630
Standardized expressions
Developmental stages
EI
PG
G1
G2
H
T1
T2
C1
C2
S
EP
JP
1.0
0.5
0.0
−0.5
−1.0
−1.5
−2.0
4
2
0
−2
1
−1
−3

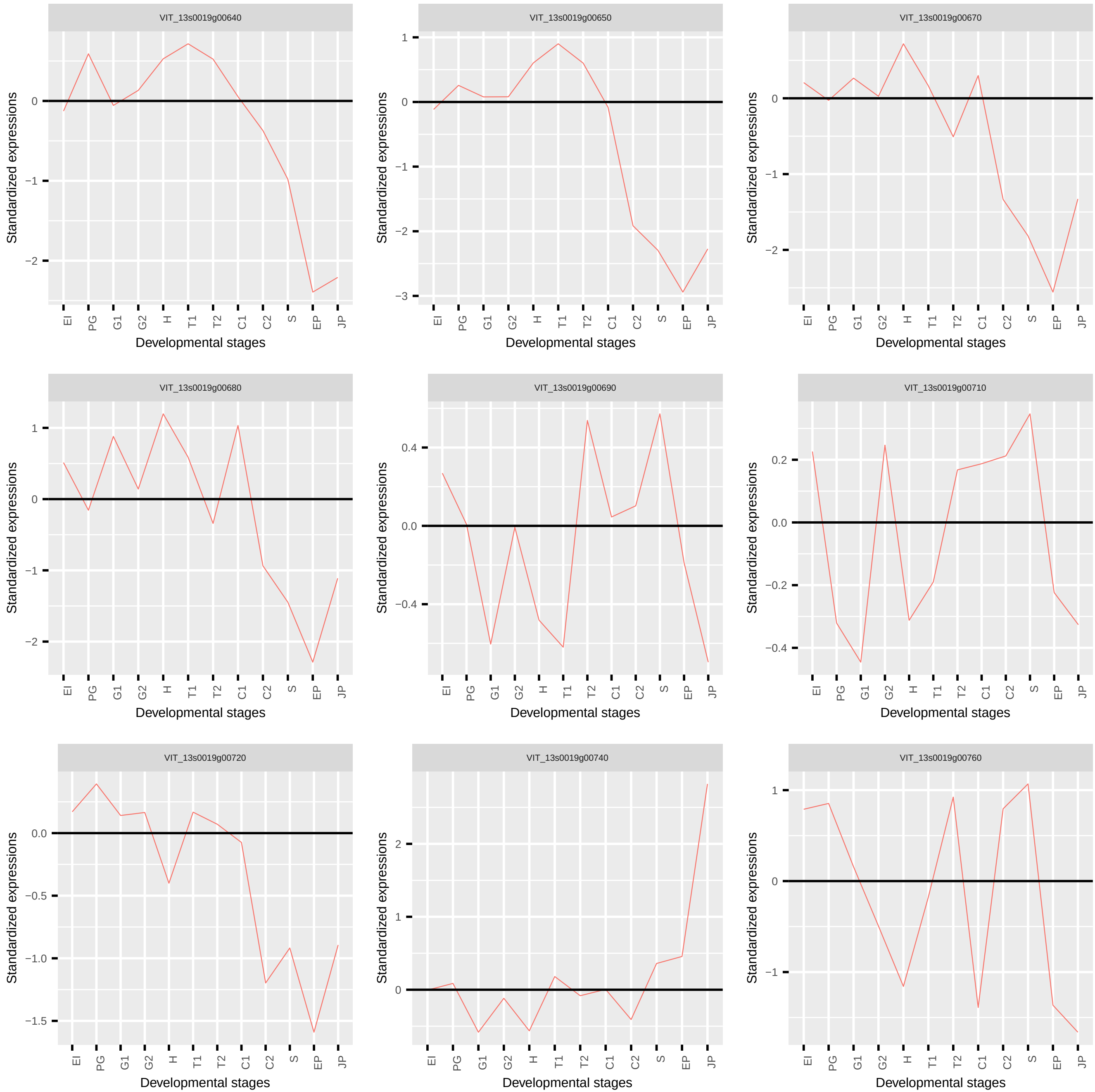
VIT_13s0019g00640
VIT_13s0019g00650
VIT_13s0019g00670
VIT_13s0019g00680
VIT_13s0019g00690
VIT_13s0019g00710
VIT_13s0019g00720
VIT_13s0019g00740
VIT_13s0019g00760
Standardized expressions
Developmental stages
EI PG G1 G2 H T1 T2 C1 C2 S EP JP

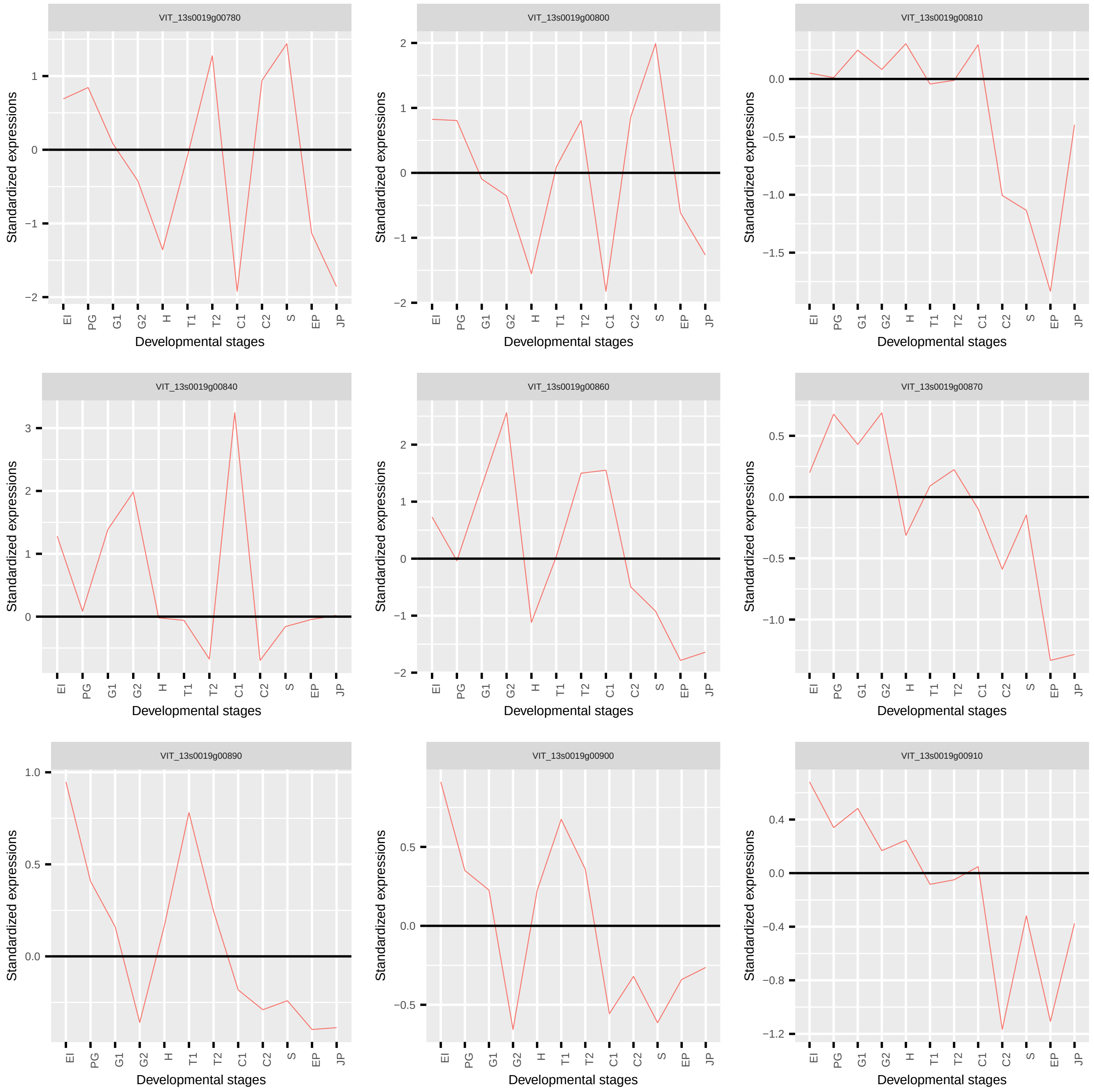
VIT_13s0019g00780
VIT_13s0019g00800
VIT_13s0019g00810
VIT_13s0019g00840
VIT_13s0019g00860
VIT_13s0019g00870
VIT_13s0019g00890
VIT_13s0019g00900
VIT_13s0019g00910
Standardized expressions
Developmental stages
EI PG G1 G2 H T1 T2 C1 C2 S EP JP

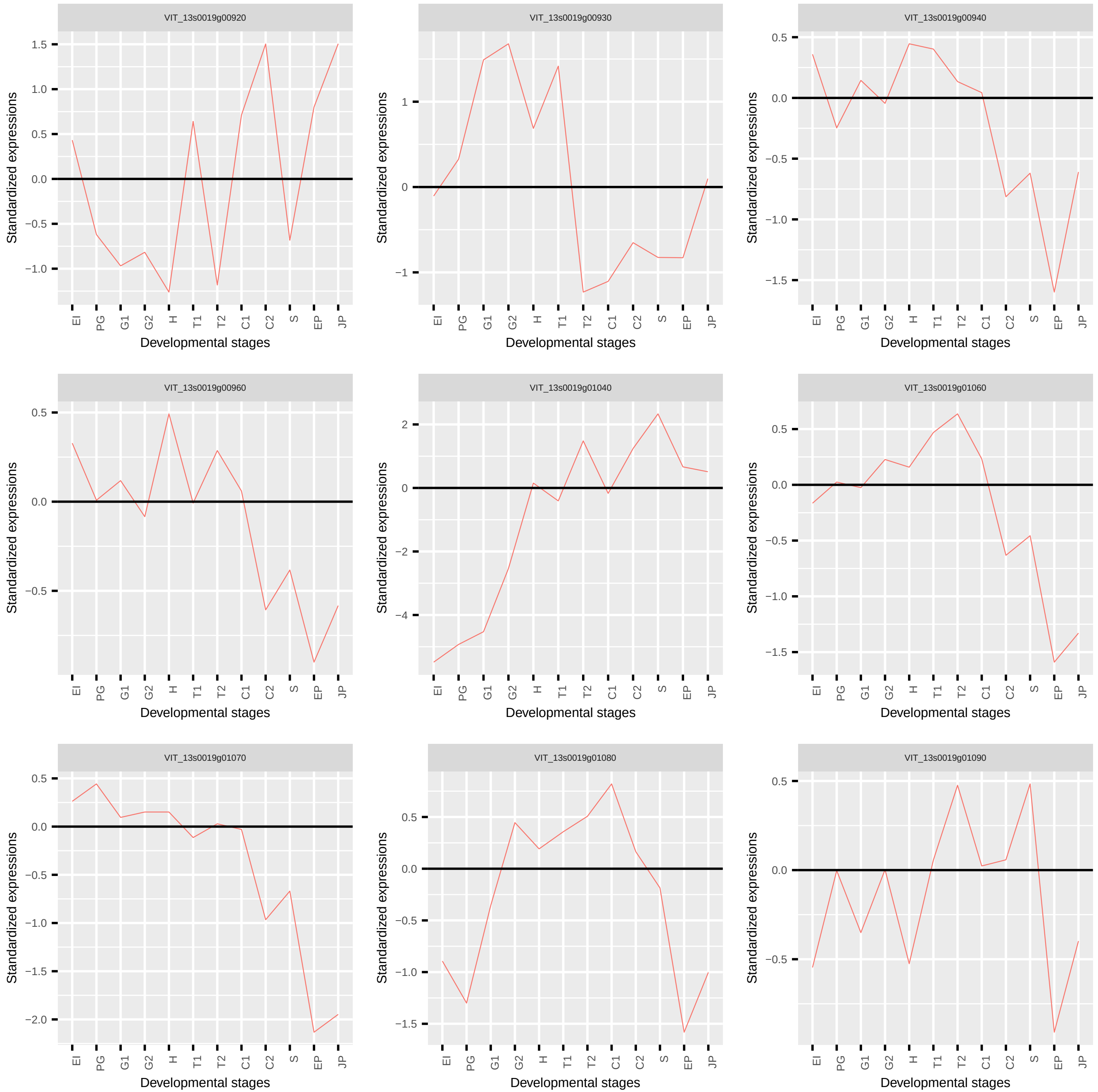
VIT_13s0019g00920
Standardized expressions
1.5
1.0
0.5
0.0
−0.5
−1.0
EI PG G1 G2 H T1 T2 C1 C2 S EP JP
Developmental stages
VIT_13s0019g00930
Standardized expressions
1
0
−1
EI PG G1 G2 H T1 T2 C1 C2 S EP JP
Developmental stages
VIT_13s0019g00940
Standardized expressions
0.5
0.0
−0.5
−1.0
−1.5
EI PG G1 G2 H T1 T2 C1 C2 S EP JP
Developmental stages
VIT_13s0019g00960
Standardized expressions
0.5
0.0
−0.5
EI PG G1 G2 H T1 T2 C1 C2 S EP JP
Developmental stages
VIT_13s0019g01040
Standardized expressions
2
0
−2
−4
EI PG G1 G2 H T1 T2 C1 C2 S EP JP
Developmental stages
VIT_13s0019g01060
Standardized expressions
0.5
0.0
−0.5
−1.0
−1.5
EI PG G1 G2 H T1 T2 C1 C2 S EP JP
Developmental stages
VIT_13s0019g01070
Standardized expressions
0.5
0.0
−0.5
−1.0
−1.5
−2.0
EI PG G1 G2 H T1 T2 C1 C2 S EP JP
Developmental stages
VIT_13s0019g01080
Standardized expressions
0.5
0.0
−0.5
−1.0
−1.5
EI PG G1 G2 H T1 T2 C1 C2 S EP JP
Developmental stages
VIT_13s0019g01090
Standardized expressions
0.5
0.0
−0.5
EI PG G1 G2 H T1 T2 C1 C2 S EP JP
Developmental stages

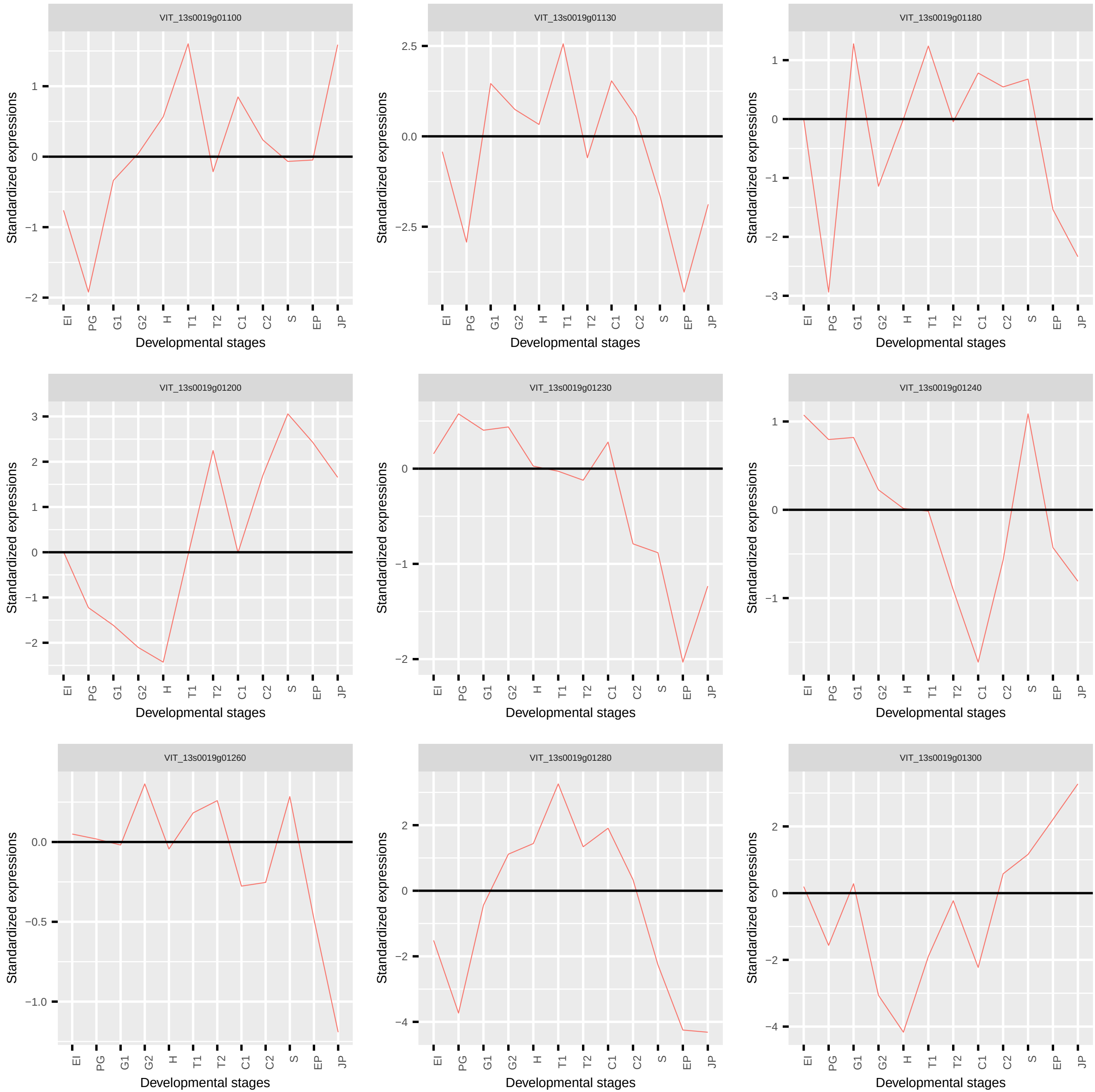
VIT_13s0019g01100
VIT_13s0019g01130
VIT_13s0019g01180
VIT_13s0019g01200
VIT_13s0019g01230
VIT_13s0019g01240
VIT_13s0019g01260
VIT_13s0019g01280
VIT_13s0019g01300
Standardized expressions
Developmental stages
EI
PG
G1
G2
H
T1
T2
C1
C2
S
EP
JP

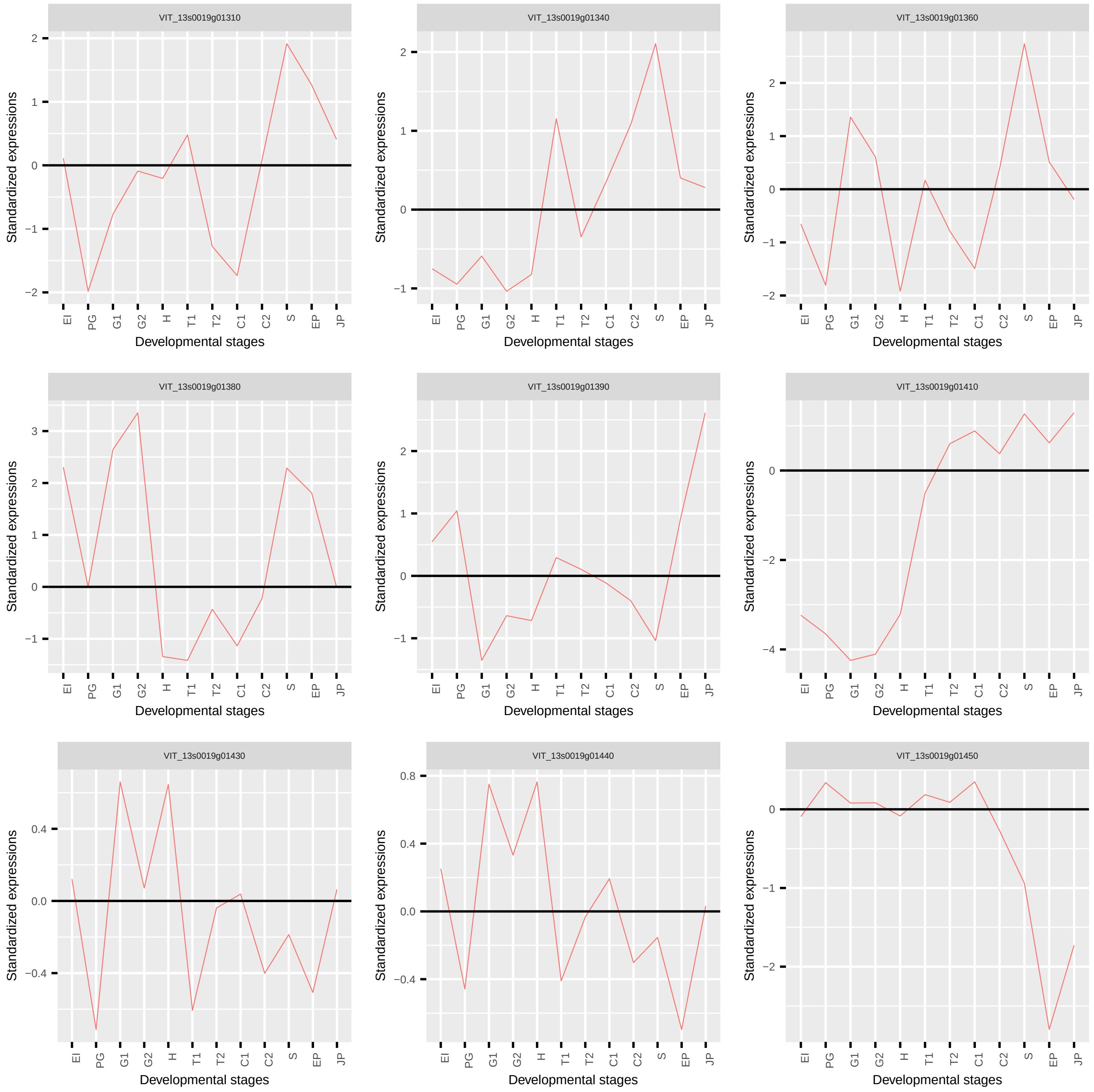
VIT_13s0019g01310
VIT_13s0019g01340
VIT_13s0019g01360
VIT_13s0019g01380
VIT_13s0019g01390
VIT_13s0019g01410
VIT_13s0019g01430
VIT_13s0019g01440
VIT_13s0019g01450
Standardized expressions
Developmental stages
EI PG G1 G2 H T1 T2 C1 C2 S EP JP

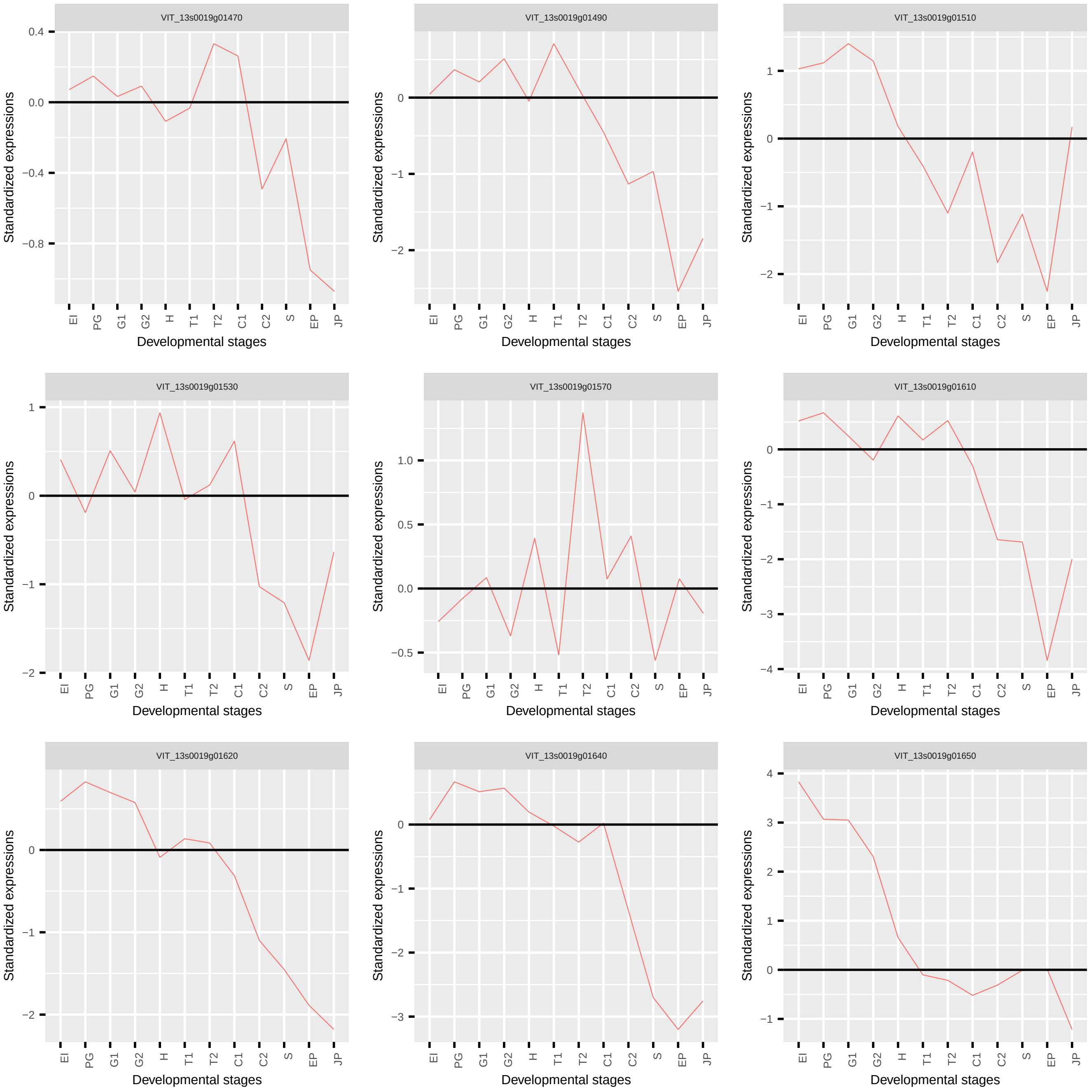

VIT_13s0019g01470
0.4
0.0
−0.4
−0.8
Standardized expressions
EI
PG
G1
G2
H
T1
T2
C1
C2
S
EP
JP
Developmental stages
VIT_13s0019g01490
0
−1
−2
Standardized expressions
EI
PG
G1
G2
H
T1
T2
C1
C2
S
EP
JP
Developmental stages
VIT_13s0019g01510
1
0
−1
−2
Standardized expressions
EI
PG
G1
G2
H
T1
T2
C1
C2
S
EP
JP
Developmental stages
VIT_13s0019g01530
1
0
−1
−2
Standardized expressions
EI
PG
G1
G2
H
T1
T2
C1
C2
S
EP
JP
Developmental stages
VIT_13s0019g01570
1.0
0.5
0.0
−0.5
Standardized expressions
EI
PG
G1
G2
H
T1
T2
C1
C2
S
EP
JP
Developmental stages
VIT_13s0019g01610
0
−1
−2
−3
−4
Standardized expressions
EI
PG
G1
G2
H
T1
T2
C1
C2
S
EP
JP
Developmental stages
VIT_13s0019g01620
0
−1
−2
Standardized expressions
EI
PG
G1
G2
H
T1
T2
C1
C2
S
EP
JP
Developmental stages
VIT_13s0019g01640
0
−1
−2
−3
Standardized expressions
EI
PG
G1
G2
H
T1
T2
C1
C2
S
EP
JP
Developmental stages
VIT_13s0019g01650
4
3
2
1
0
−1
Standardized expressions
EI
PG
G1
G2
H
T1
T2
C1
C2
S
EP
JP
Developmental stages

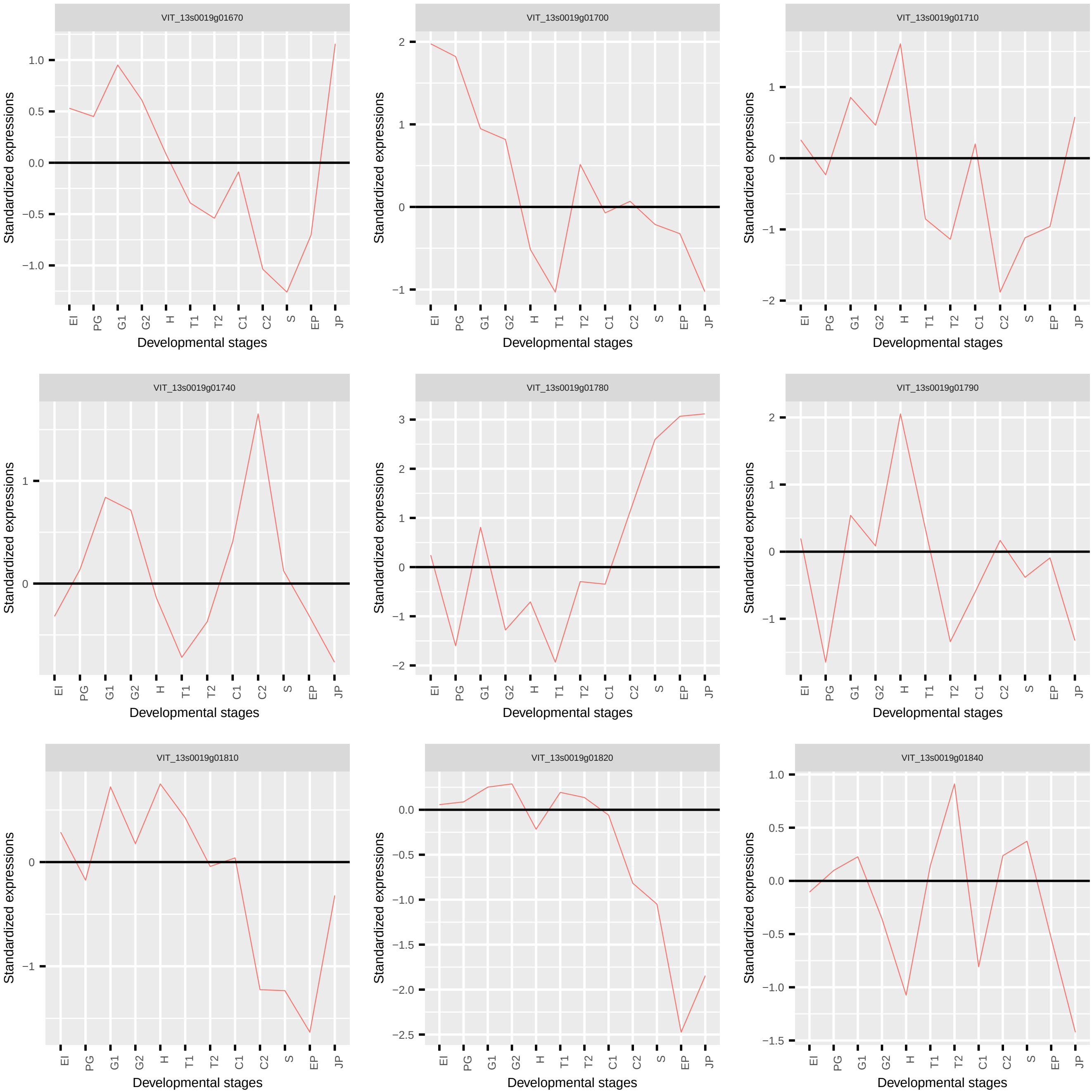

VIT_13s0019g01670
VIT_13s0019g01700
VIT_13s0019g01710
VIT_13s0019g01740
VIT_13s0019g01780
VIT_13s0019g01790
VIT_13s0019g01810
VIT_13s0019g01820
VIT_13s0019g01840
Standardized expressions
Developmental stages
EI PG G1 G2 H T1 T2 C1 C2 S EP JP

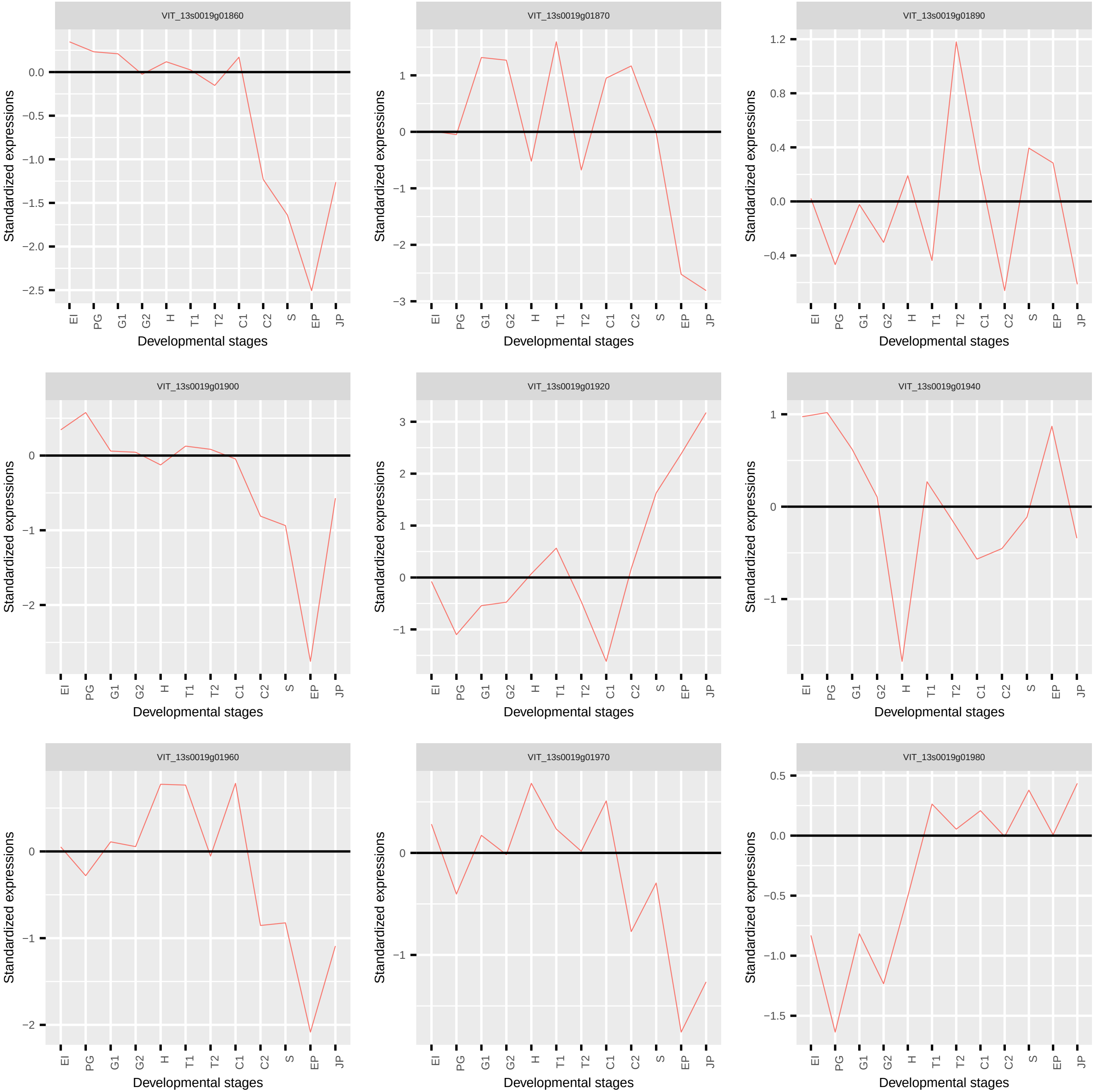
VIT_13s0019g01860
VIT_13s0019g01870
VIT_13s0019g01890
VIT_13s0019g01900
VIT_13s0019g01920
VIT_13s0019g01940
VIT_13s0019g01960
VIT_13s0019g01970
VIT_13s0019g01980
Standardized expressions
Developmental stages
EI PG G1 G2 H T1 T2 C1 C2 S EP JP

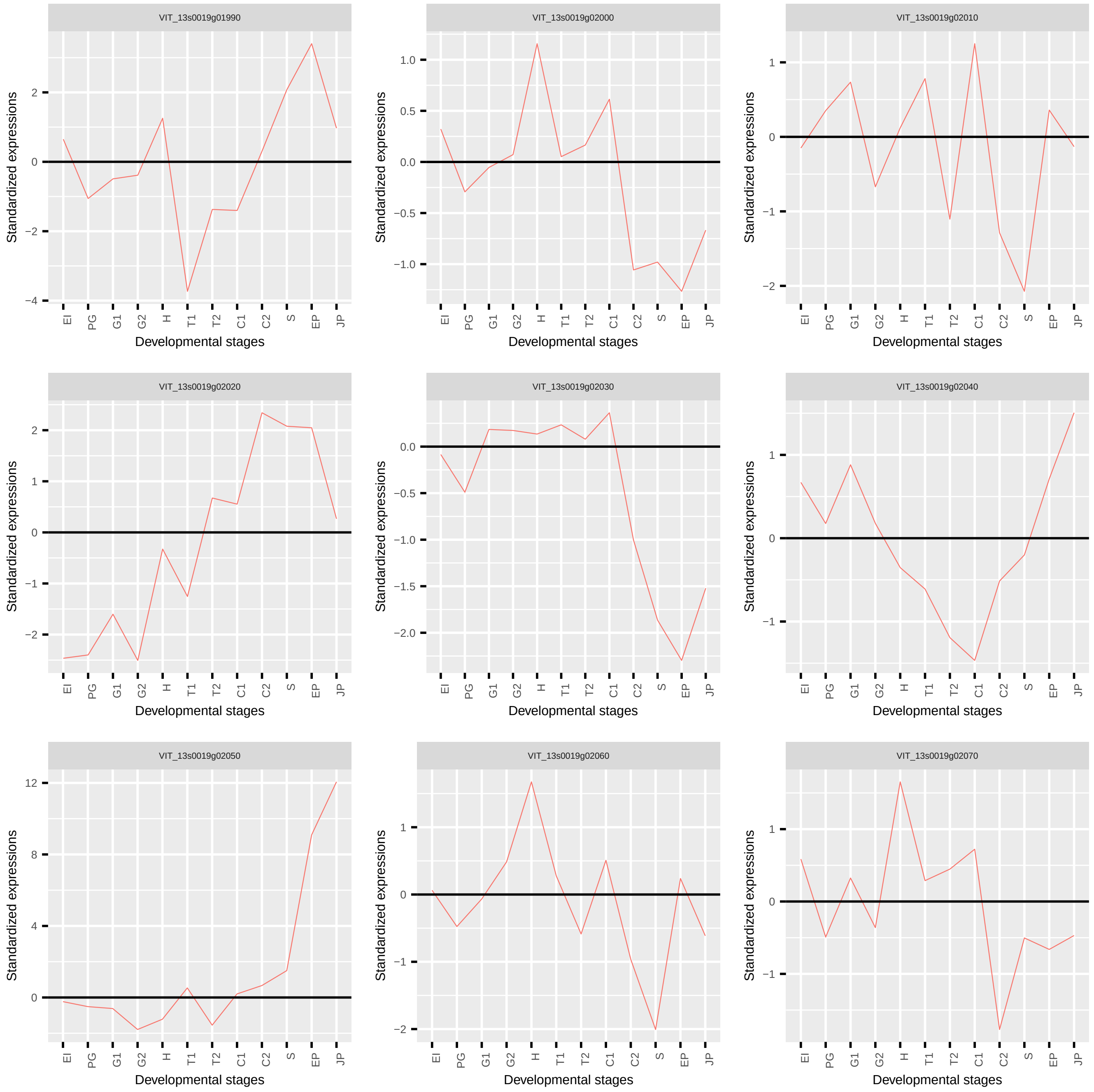

VIT_13s0019g01990
VIT_13s0019g02000
VIT_13s0019g02010
VIT_13s0019g02020
VIT_13s0019g02030
VIT_13s0019g02040
VIT_13s0019g02050
VIT_13s0019g02060
VIT_13s0019g02070
Standardized expressions
Developmental stages
EI PG G1 G2 H T1 T2 C1 C2 S EP JP

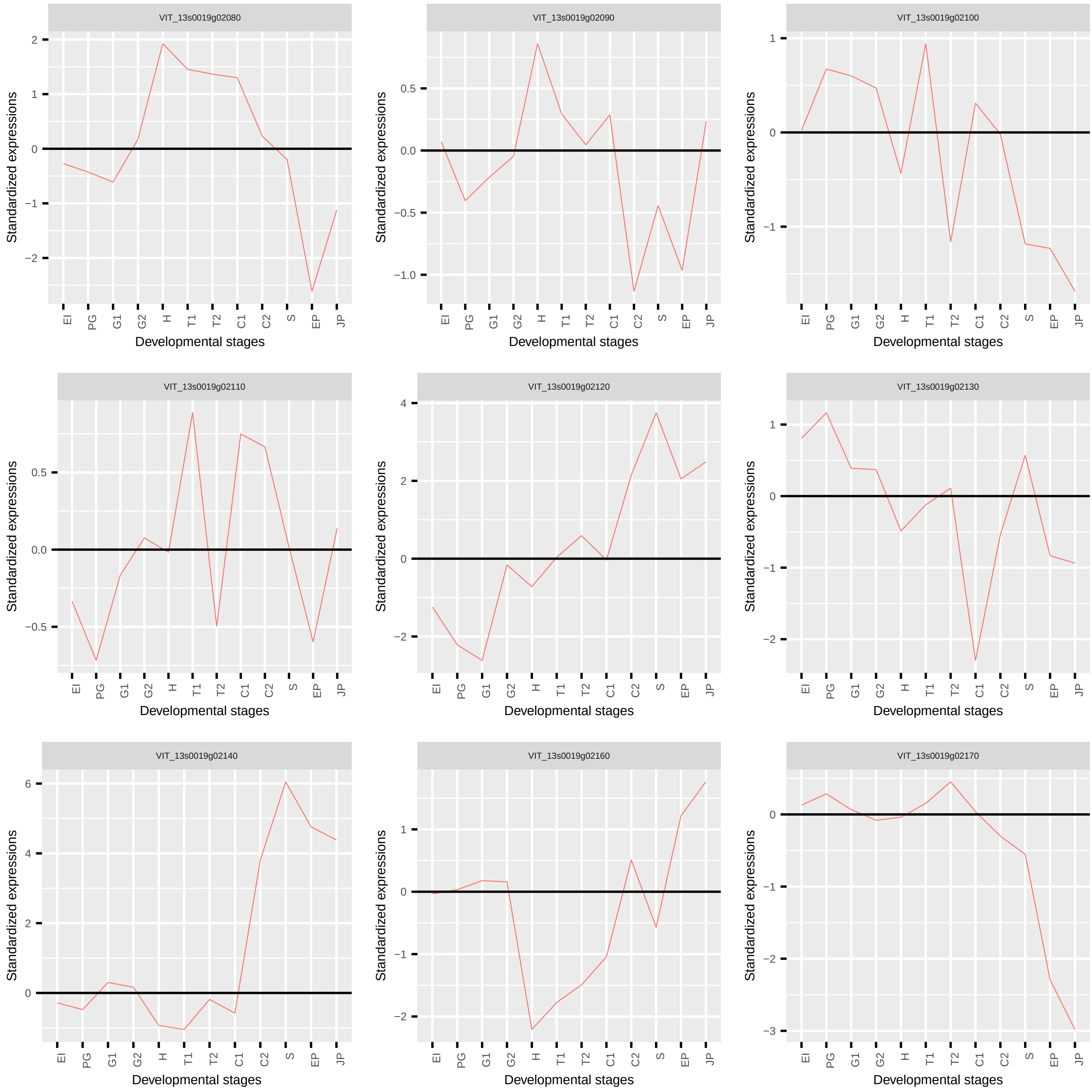
VIT_13s0019g02080
VIT_13s0019g02090
VIT_13s0019g02100
VIT_13s0019g02110
VIT_13s0019g02120
VIT_13s0019g02130
VIT_13s0019g02140
VIT_13s0019g02160
VIT_13s0019g02170
Standardized expressions
Developmental stages
EI PG G1 G2 H T1 T2 C1 C2 S EP JP

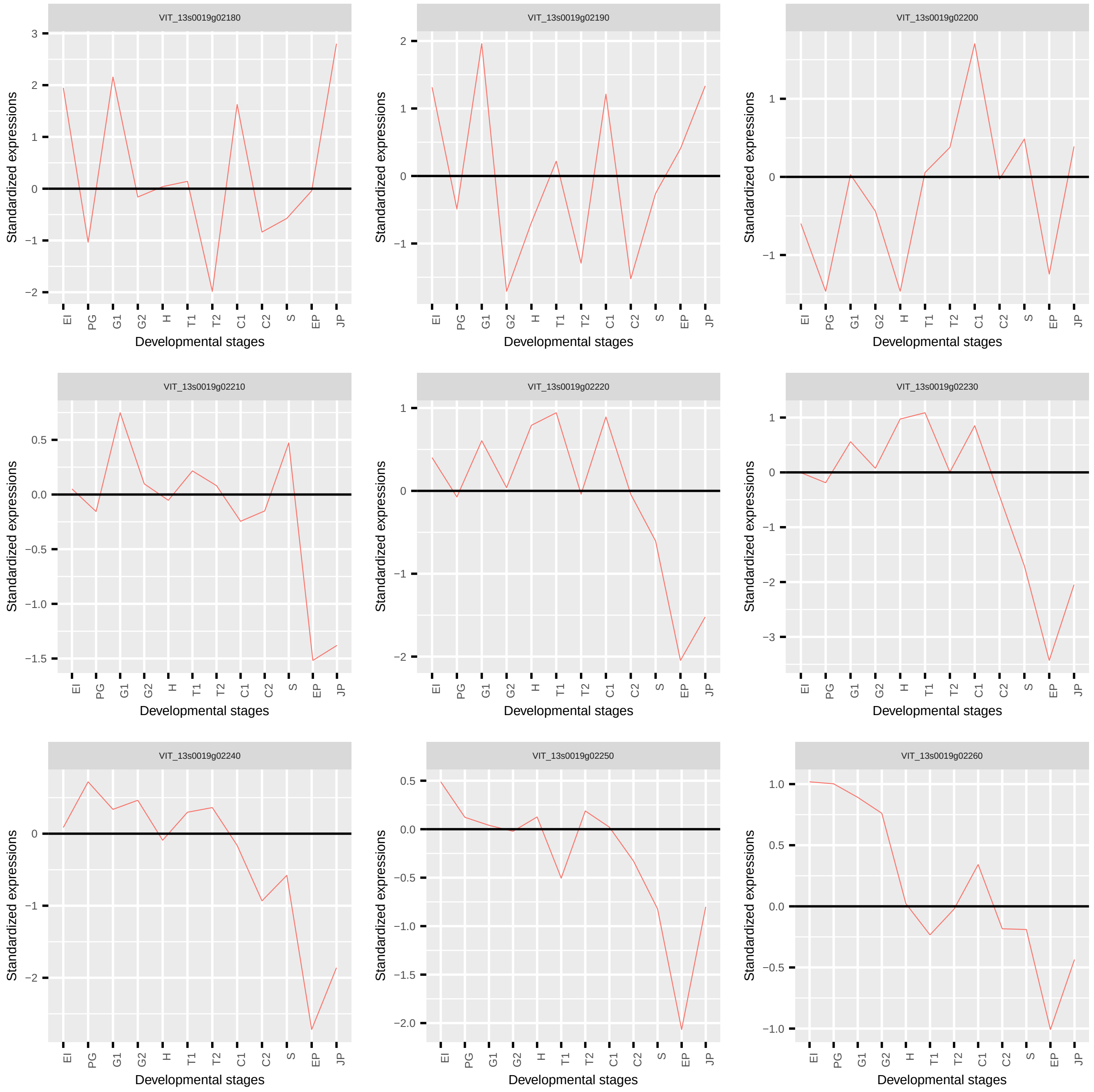
VIT_13s0019g02180
VIT_13s0019g02190
VIT_13s0019g02200
VIT_13s0019g02210
VIT_13s0019g02220
VIT_13s0019g02230
VIT_13s0019g02240
VIT_13s0019g02250
VIT_13s0019g02260
Standardized expressions
Developmental stages
EI PG G1 G2 H T1 T2 C1 C2 S EP JP

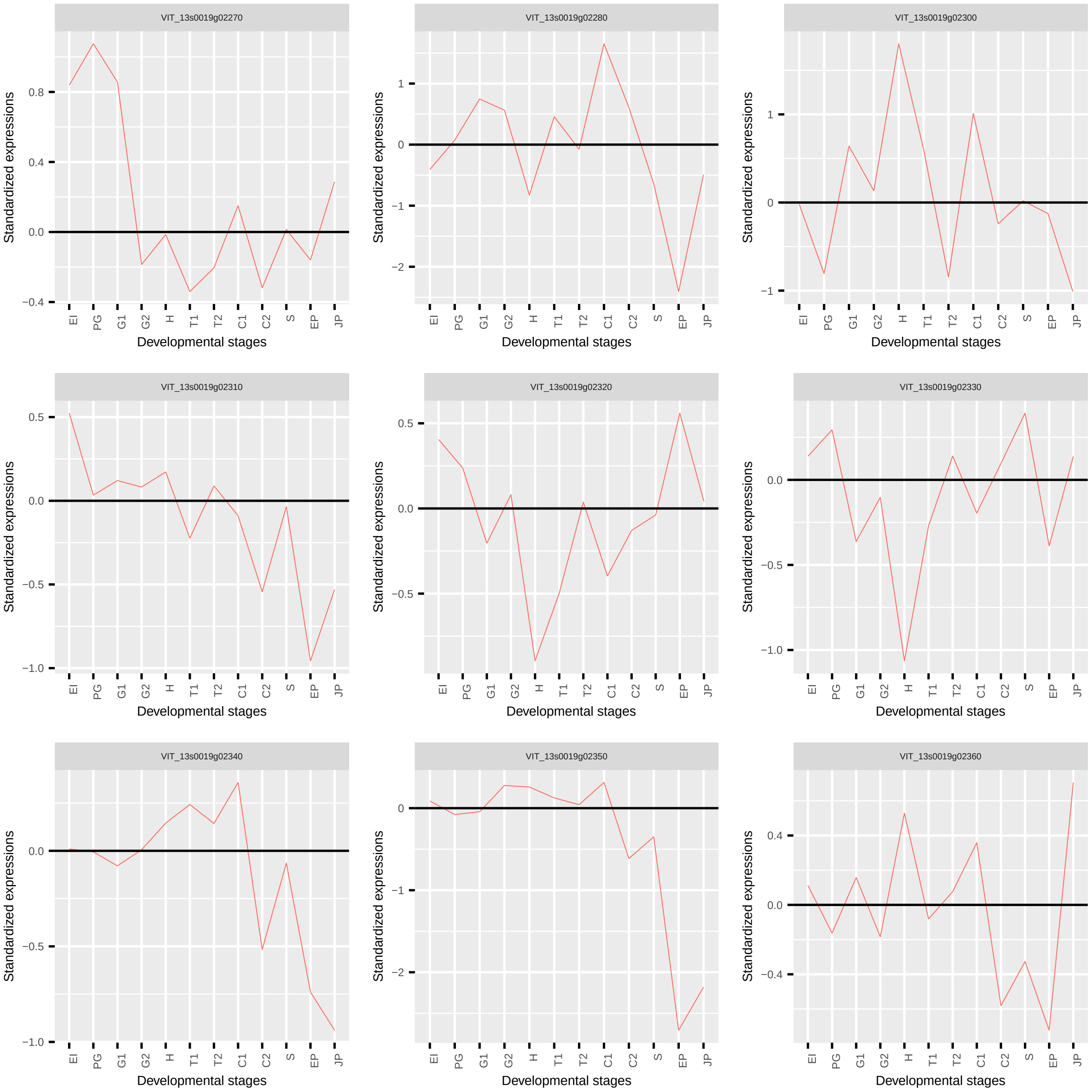
VIT_13s0019g02270
VIT_13s0019g02280
VIT_13s0019g02300
VIT_13s0019g02310
VIT_13s0019g02320
VIT_13s0019g02330
VIT_13s0019g02340
VIT_13s0019g02350
VIT_13s0019g02360
Standardized expressions
Developmental stages
EI PG G1 G2 H T1 T2 C1 C2 S EP JP

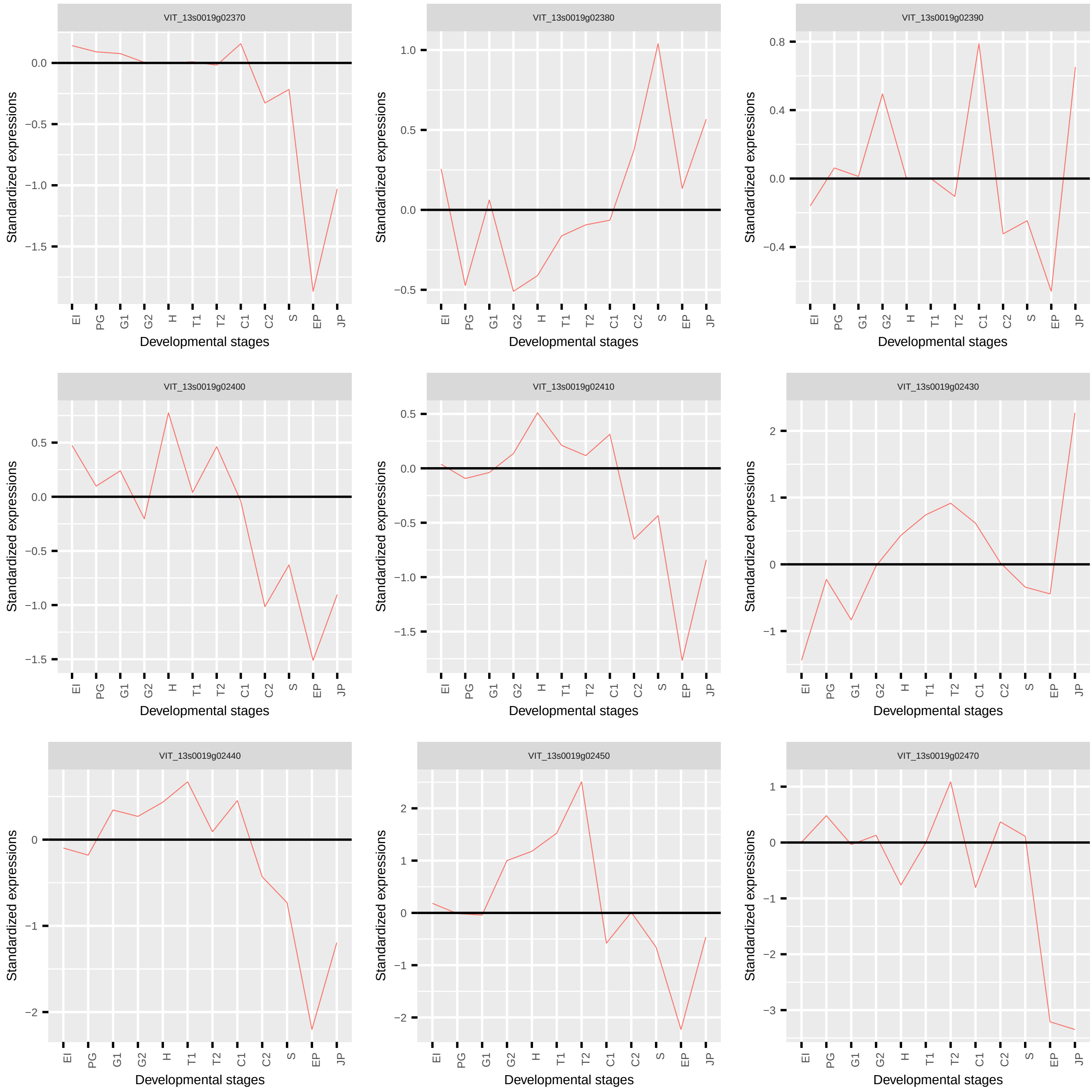
VIT_13s0019g02370
VIT_13s0019g02380
VIT_13s0019g02390
VIT_13s0019g02400
VIT_13s0019g02410
VIT_13s0019g02430
VIT_13s0019g02440
VIT_13s0019g02450
VIT_13s0019g02470
Standardized expressions
Developmental stages
EI PG G1 G2 H T1 T2 C1 C2 S EP JP

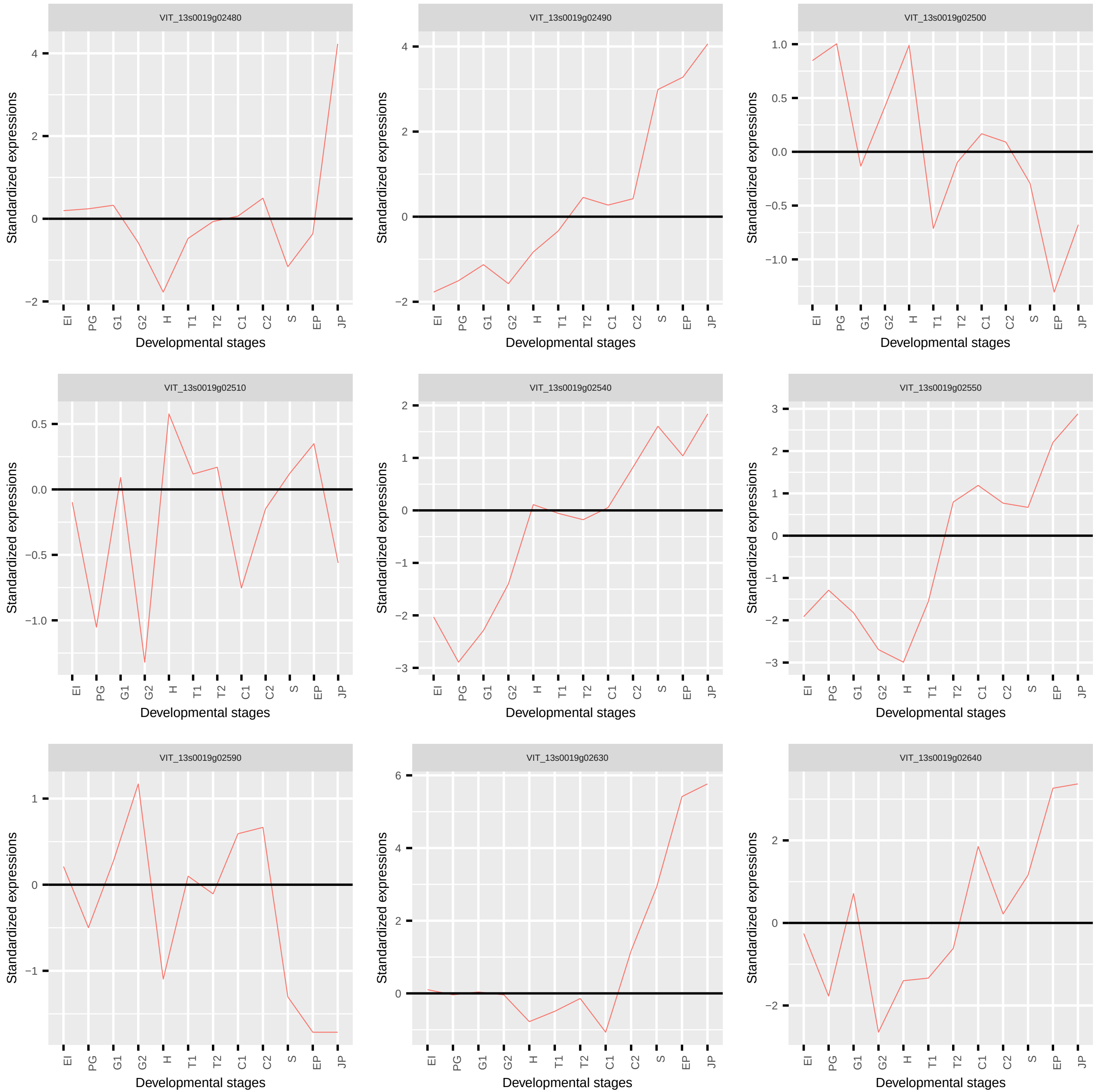
VIT_13s0019g02480
VIT_13s0019g02490
VIT_13s0019g02500
VIT_13s0019g02510
VIT_13s0019g02540
VIT_13s0019g02550
VIT_13s0019g02590
VIT_13s0019g02630
VIT_13s0019g02640
Standardized expressions
Developmental stages
EI
PG
G1
G2
H
T1
T2
C1
C2
S
EP
JP

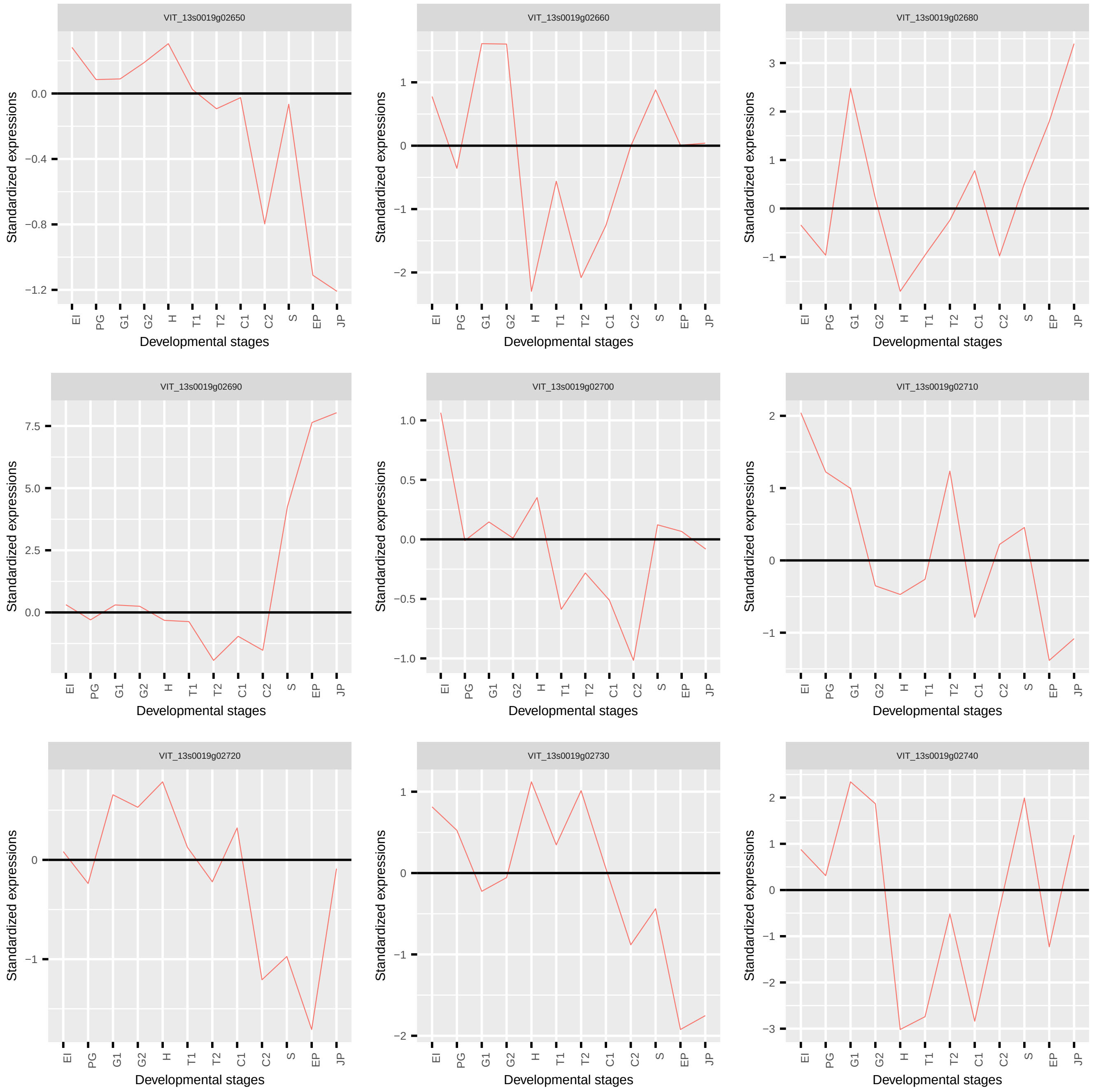

VIT_13s0019g02650
VIT_13s0019g02660
VIT_13s0019g02680
VIT_13s0019g02690
VIT_13s0019g02700
VIT_13s0019g02710
VIT_13s0019g02720
VIT_13s0019g02730
VIT_13s0019g02740
Standardized expressions
Developmental stages
EI PG G1 G2 H T1 T2 C1 C2 S EP JP

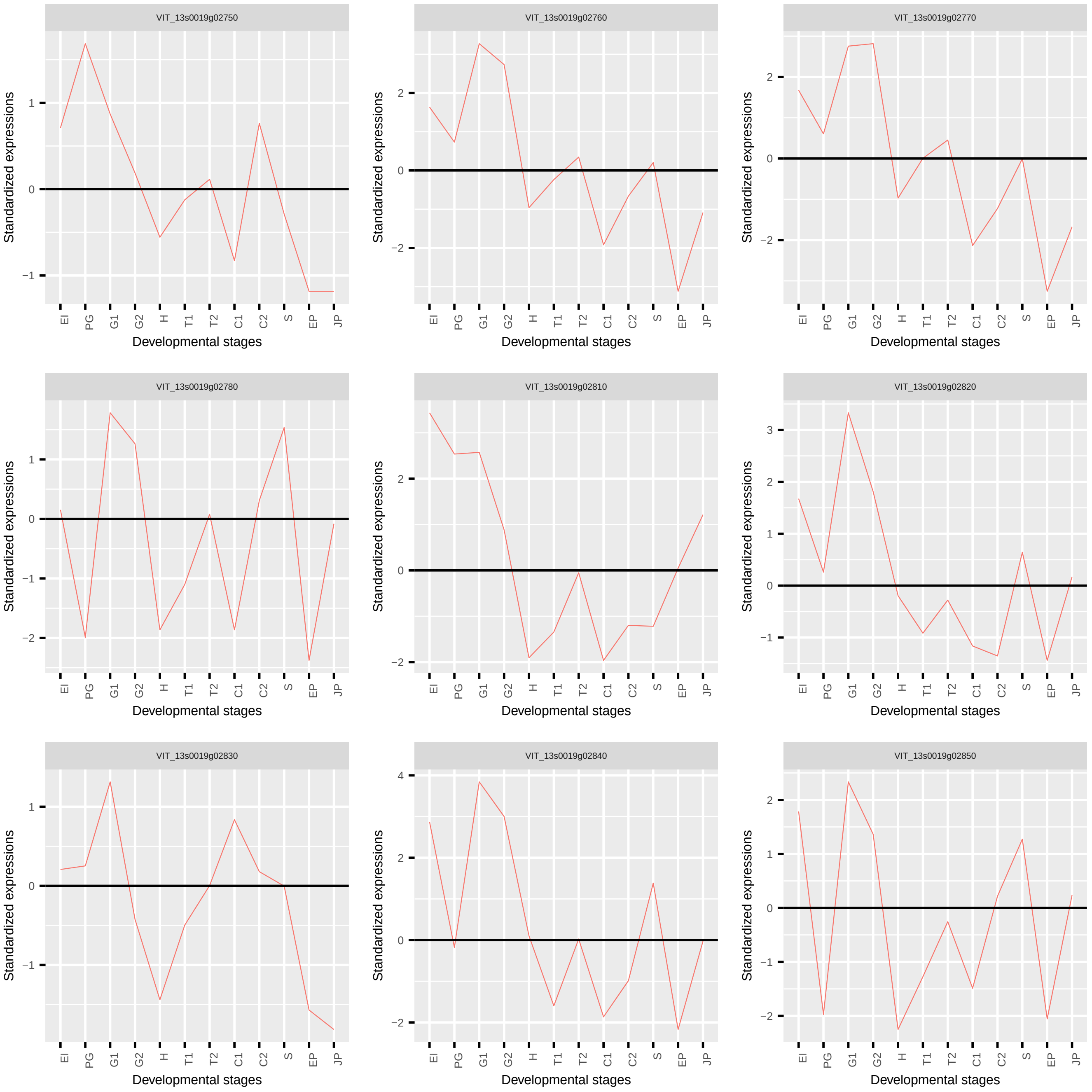

VIT_13s0019g02750
VIT_13s0019g02760
VIT_13s0019g02770
VIT_13s0019g02780
VIT_13s0019g02810
VIT_13s0019g02820
VIT_13s0019g02830
VIT_13s0019g02840
VIT_13s0019g02850
Standardized expressions
Developmental stages
EI PG G1 G2 H T1 T2 C1 C2 S EP JP

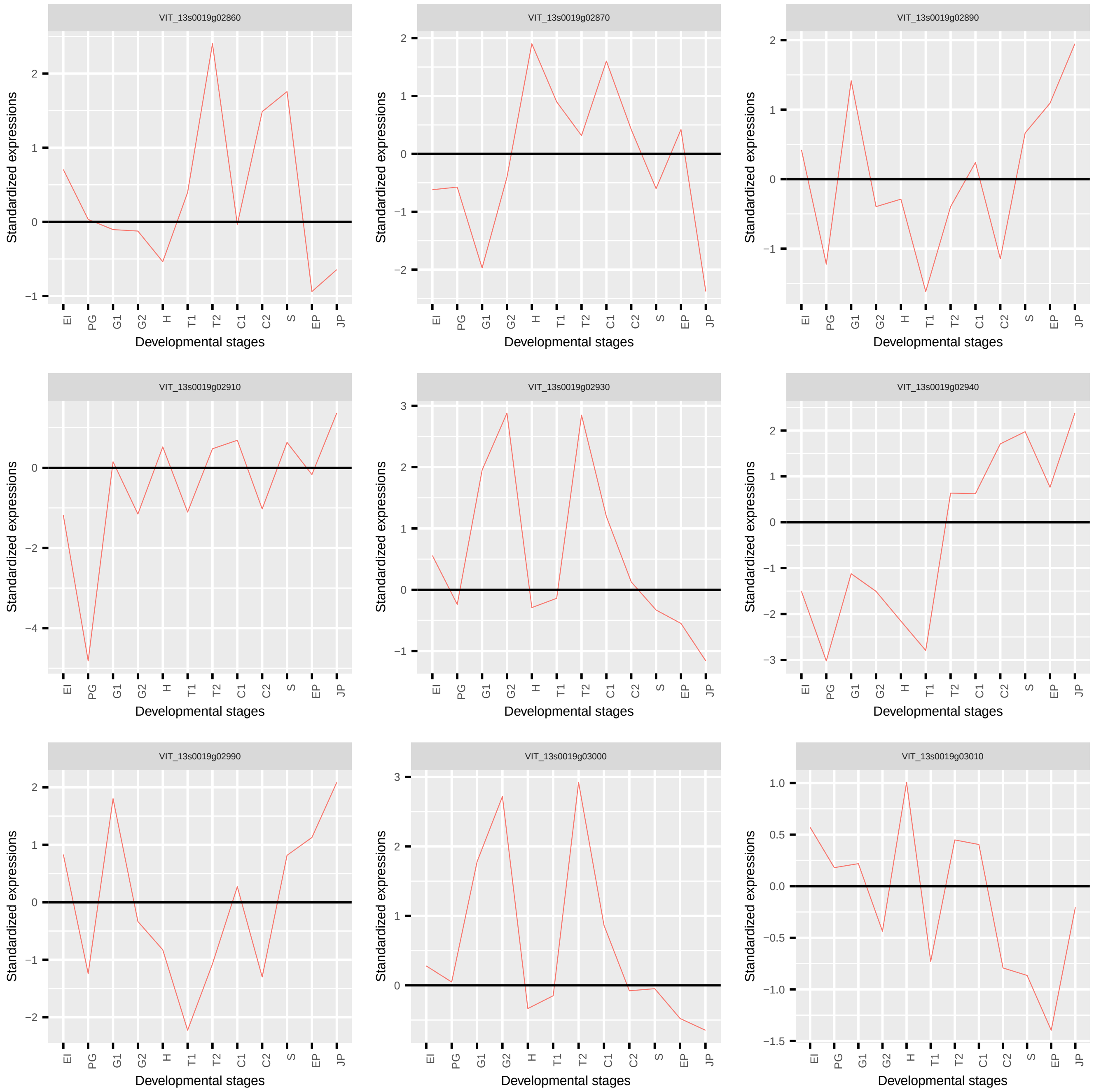
VIT_13s0019g02860
Standardized expressions
2
1
0
−1
EI PG G1 G2 H T1 T2 C1 C2 S EP JP
Developmental stages
VIT_13s0019g02870
Standardized expressions
2
1
0
−1
−2
EI PG G1 G2 H T1 T2 C1 C2 S EP JP
Developmental stages
VIT_13s0019g02890
Standardized expressions
2
1
0
−1
EI PG G1 G2 H T1 T2 C1 C2 S EP JP
Developmental stages
VIT_13s0019g02910
Standardized expressions
0
−2
−4
EI PG G1 G2 H T1 T2 C1 C2 S EP JP
Developmental stages
VIT_13s0019g02930
Standardized expressions
3
2
1
0
−1
EI PG G1 G2 H T1 T2 C1 C2 S EP JP
Developmental stages
VIT_13s0019g02940
Standardized expressions
2
1
0
−1
−2
−3
EI PG G1 G2 H T1 T2 C1 C2 S EP JP
Developmental stages
VIT_13s0019g02990
Standardized expressions
2
1
0
−1
−2
EI PG G1 G2 H T1 T2 C1 C2 S EP JP
Developmental stages
VIT_13s0019g03000
Standardized expressions
3
2
1
0
EI PG G1 G2 H T1 T2 C1 C2 S EP JP
Developmental stages
VIT_13s0019g03010
Standardized expressions
1.0
0.5
0.0
−0.5
−1.0
−1.5
EI PG G1 G2 H T1 T2 C1 C2 S EP JP
Developmental stages

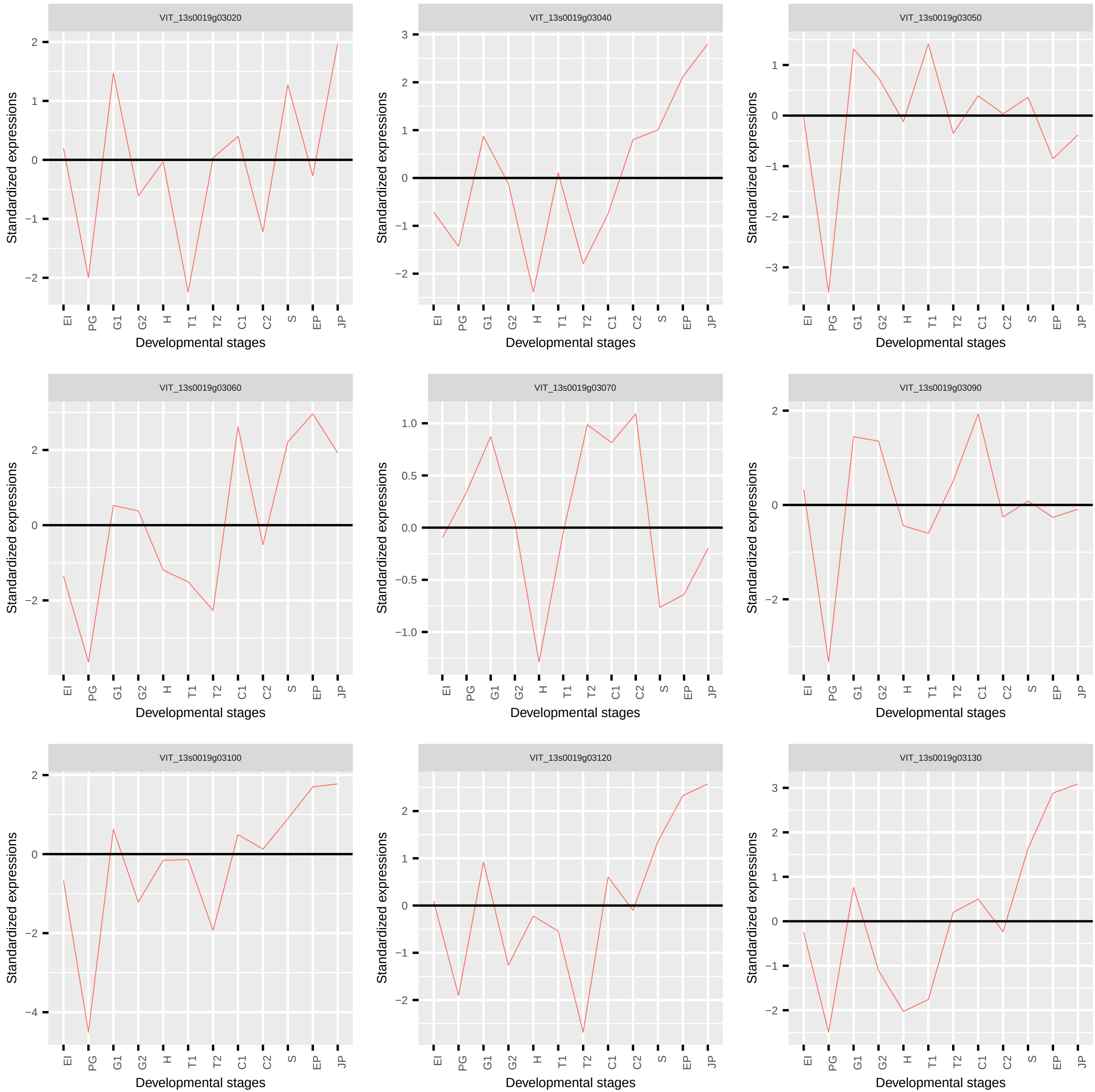
VIT_13s0019g03020
VIT_13s0019g03040
VIT_13s0019g03050
VIT_13s0019g03060
VIT_13s0019g03070
VIT_13s0019g03090
VIT_13s0019g03100
VIT_13s0019g03120
VIT_13s0019g03130
Standardized expressions
Developmental stages
EI
PG
G1
G2
H
T1
T2
C1
C2
S
EP
JP

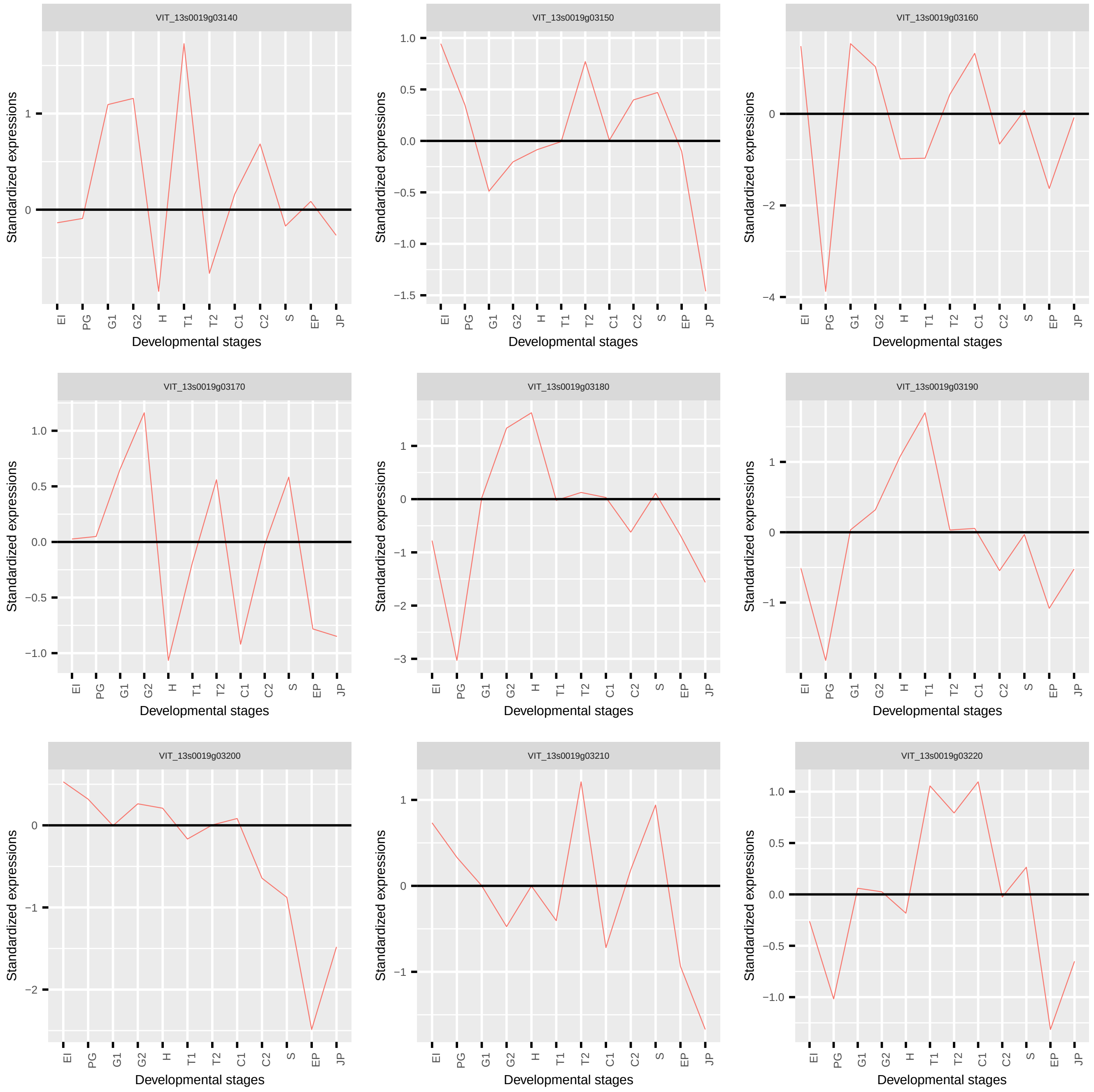
VIT_13s0019g03140
VIT_13s0019g03150
VIT_13s0019g03160
VIT_13s0019g03170
VIT_13s0019g03180
VIT_13s0019g03190
VIT_13s0019g03200
VIT_13s0019g03210
VIT_13s0019g03220
Standardized expressions
Developmental stages
EI PG G1 G2 H T1 T2 C1 C2 S EP JP

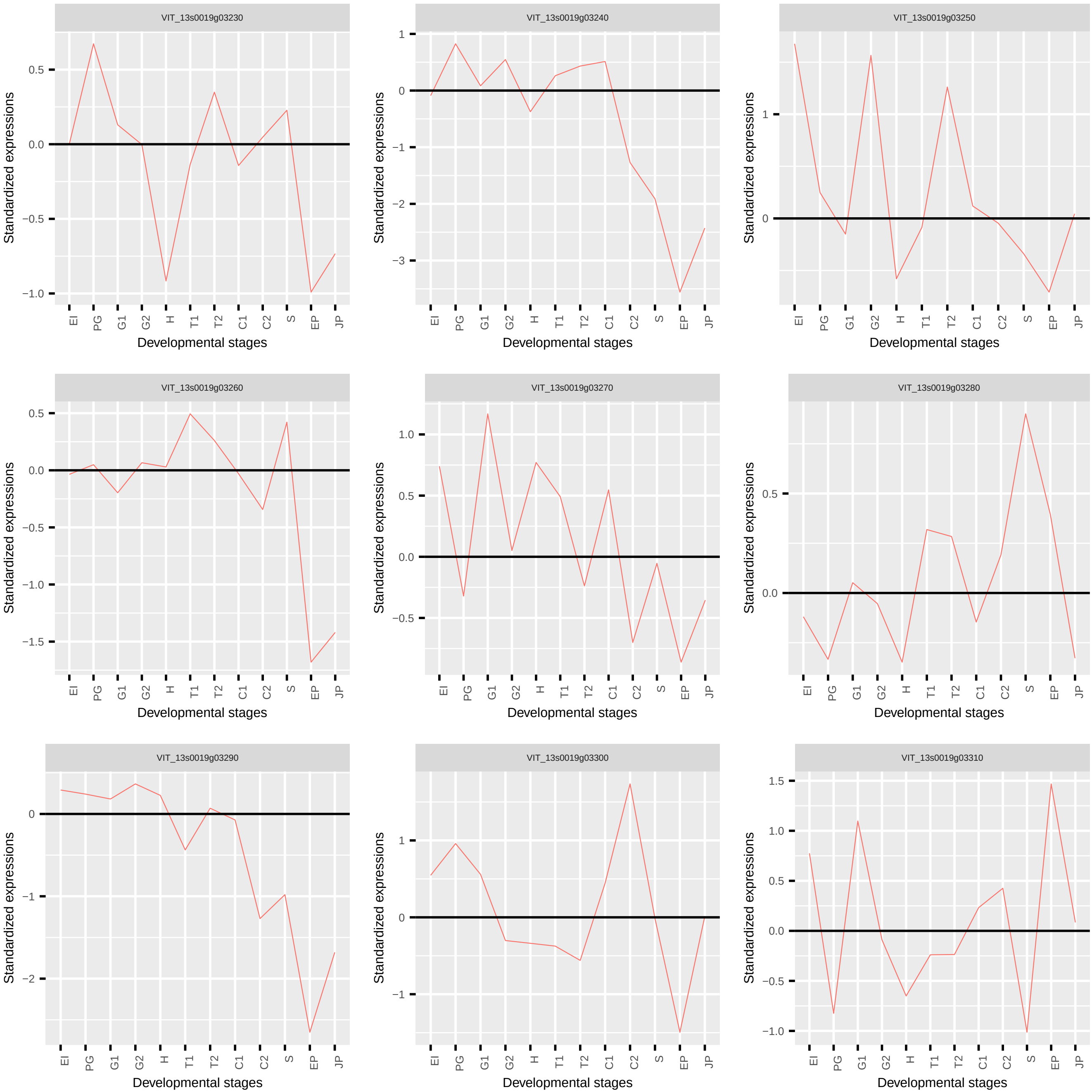

VIT_13s0019g03230
VIT_13s0019g03240
VIT_13s0019g03250
VIT_13s0019g03260
VIT_13s0019g03270
VIT_13s0019g03280
VIT_13s0019g03290
VIT_13s0019g03300
VIT_13s0019g03310
Standardized expressions
Developmental stages
EI
PG
G1
G2
H
T1
T2
C1
C2
S
EP
JP

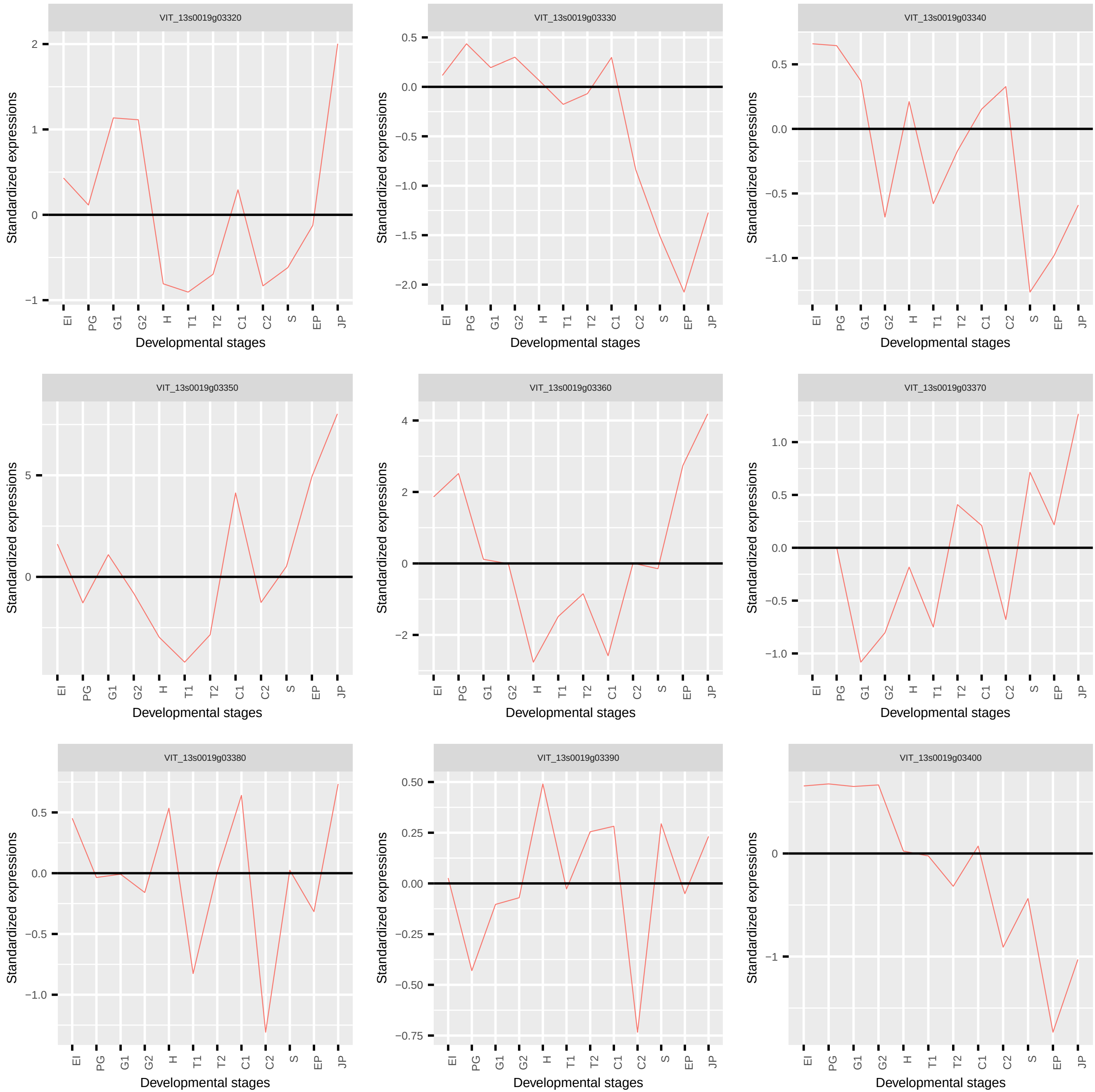
VIT_13s0019g03320
VIT_13s0019g03330
VIT_13s0019g03340
VIT_13s0019g03350
VIT_13s0019g03360
VIT_13s0019g03370
VIT_13s0019g03380
VIT_13s0019g03390
VIT_13s0019g03400
Standardized expressions
Developmental stages
EI PG G1 G2 H T1 T2 C1 C2 S EP JP
2
1
0
−1
0.5
0.0
−0.5
−1.0
−1.5
−2.0
5
4
−2
0.25
0.00
−0.25
−0.50
−0.75
0.50

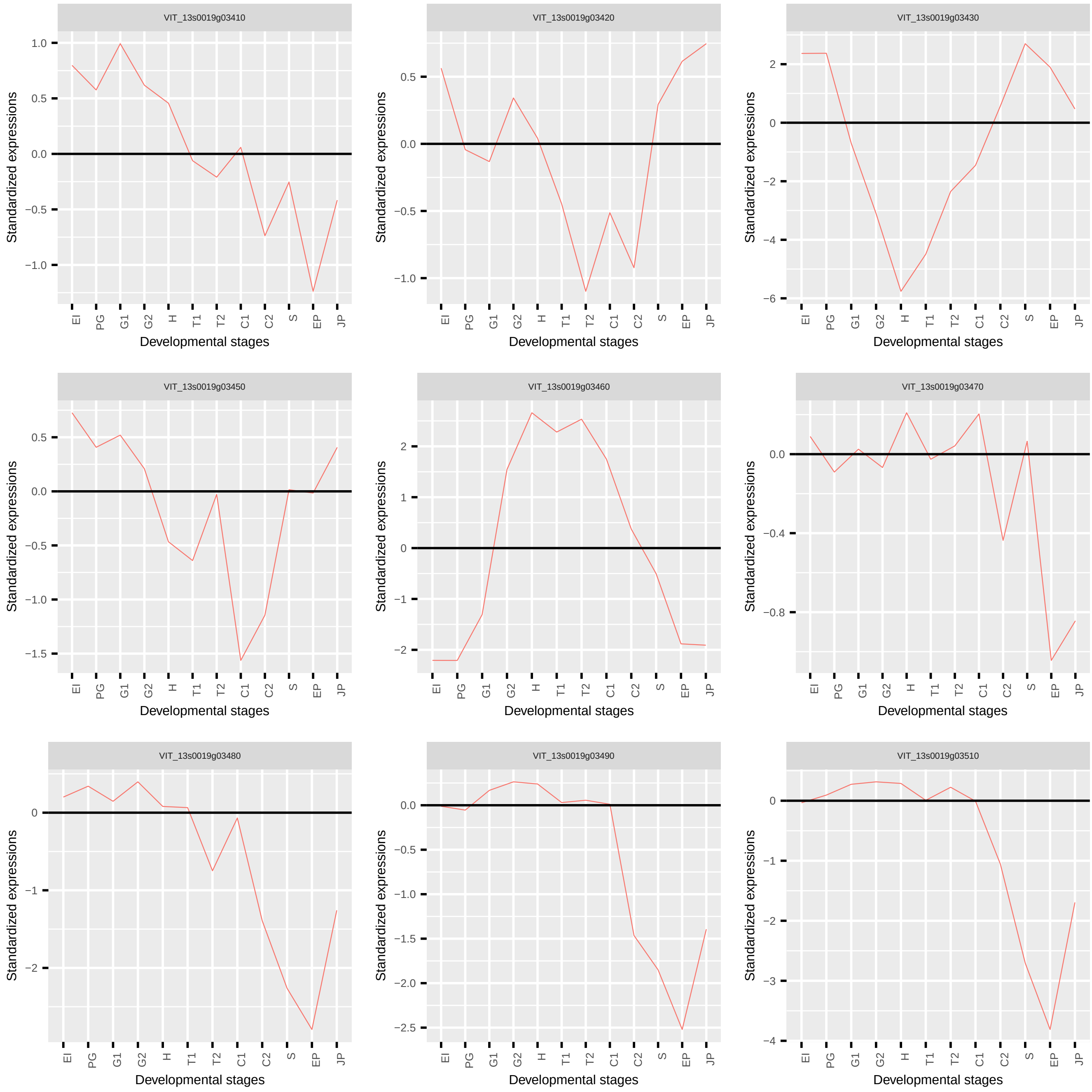
VIT_13s0019g03410
VIT_13s0019g03420
VIT_13s0019g03430
VIT_13s0019g03450
VIT_13s0019g03460
VIT_13s0019g03470
VIT_13s0019g03480
VIT_13s0019g03490
VIT_13s0019g03510
Standardized expressions
Developmental stages
EI PG G1 G2 H T1 T2 C1 C2 S EP JP

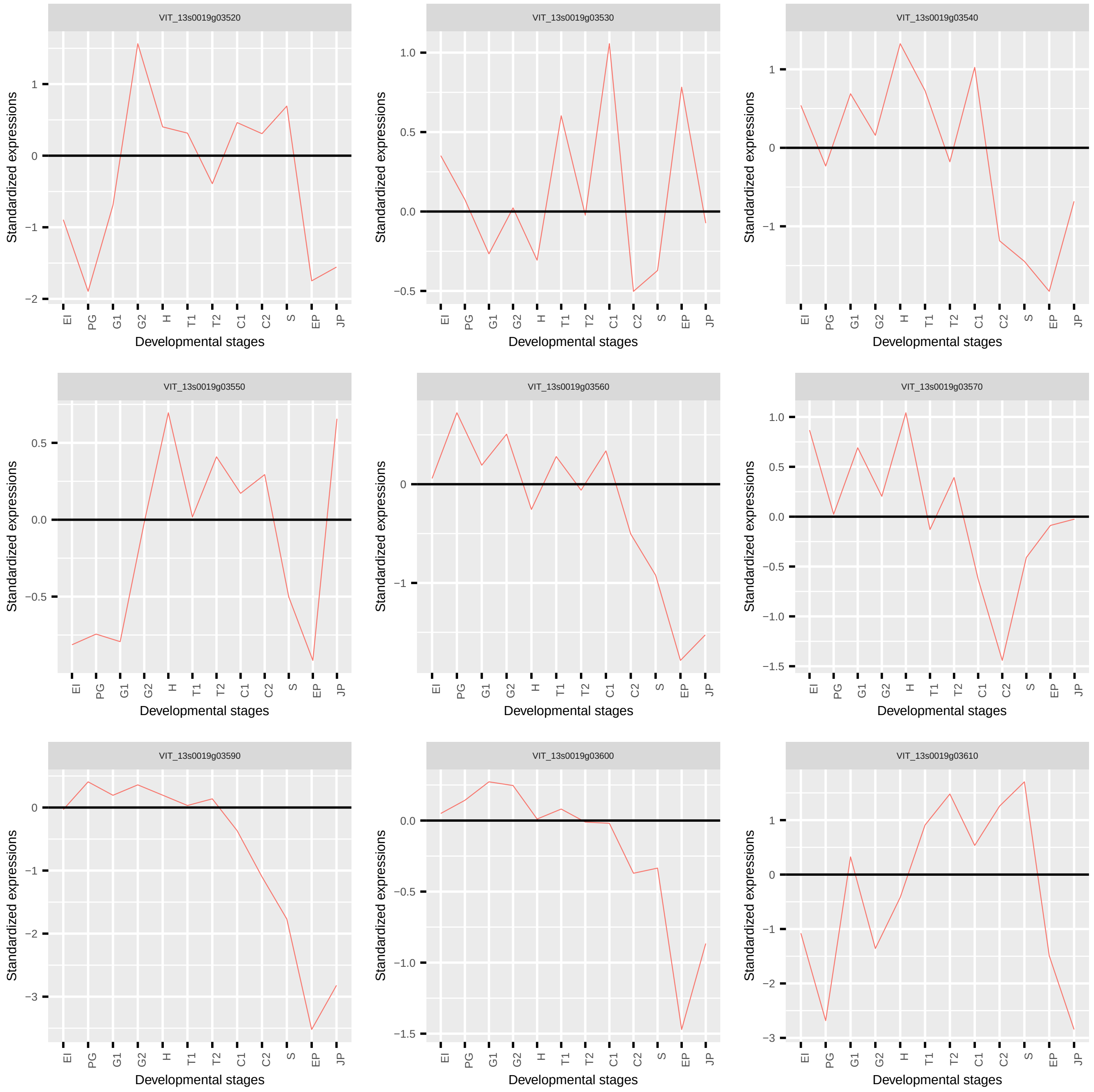

VIT_13s0019g03520
VIT_13s0019g03530
VIT_13s0019g03540
VIT_13s0019g03550
VIT_13s0019g03560
VIT_13s0019g03570
VIT_13s0019g03590
VIT_13s0019g03600
VIT_13s0019g03610
Standardized expressions
Developmental stages
EI PG G1 G2 H T1 T2 C1 C2 S EP JP

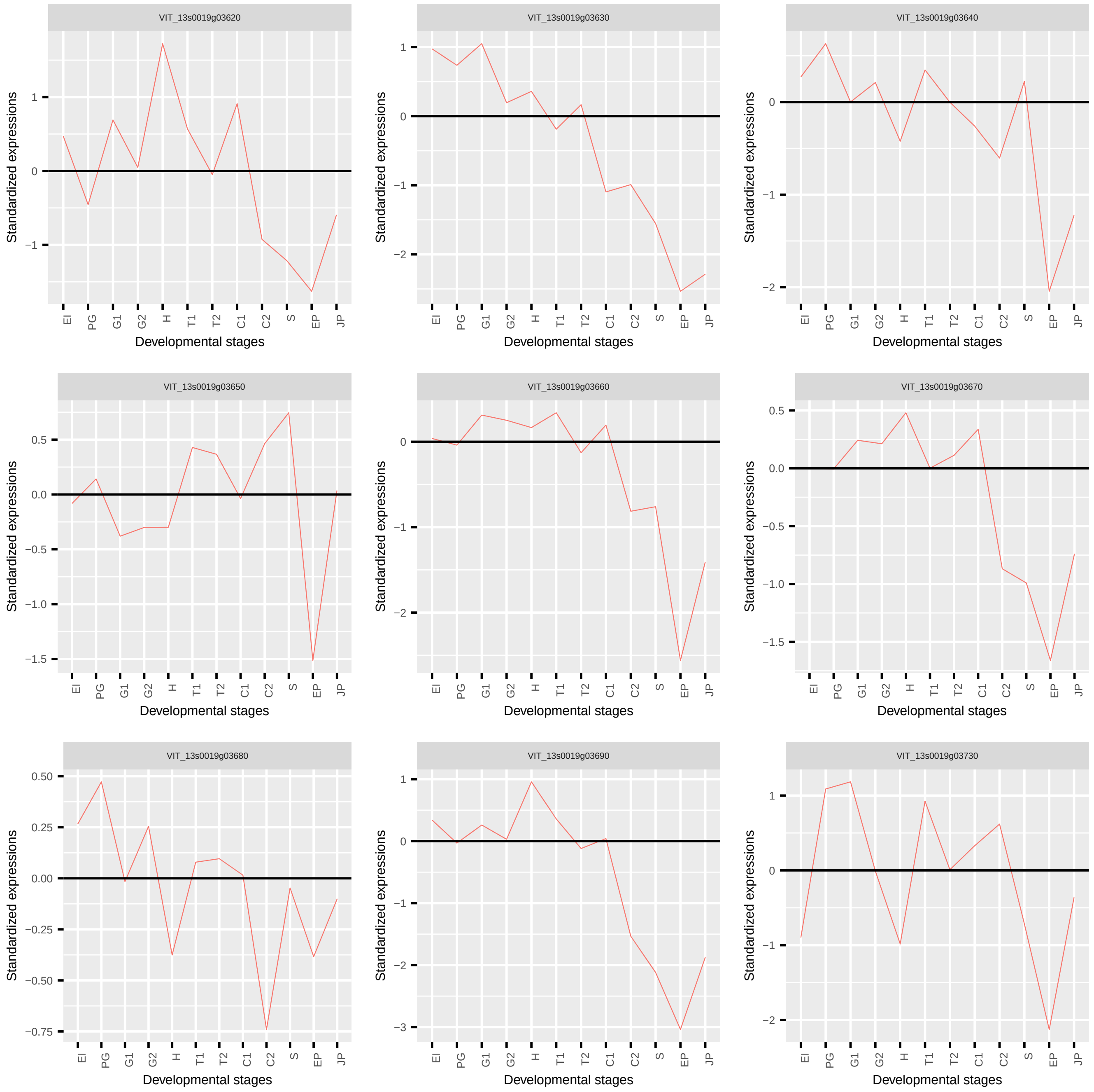
VIT_13s0019g03620
Standardized expressions
1
0
−1
EI PG G1 G2 H T1 T2 C1 C2 S EP JP
Developmental stages
VIT_13s0019g03630
Standardized expressions
1
0
−1
−2
EI PG G1 G2 H T1 T2 C1 C2 S EP JP
Developmental stages
VIT_13s0019g03640
Standardized expressions
0
−1
−2
EI PG G1 G2 H T1 T2 C1 C2 S EP JP
Developmental stages
VIT_13s0019g03650
Standardized expressions
0.5
0.0
−0.5
−1.0
−1.5
EI PG G1 G2 H T1 T2 C1 C2 S EP JP
Developmental stages
VIT_13s0019g03660
Standardized expressions
0
−1
−2
EI PG G1 G2 H T1 T2 C1 C2 S EP JP
Developmental stages
VIT_13s0019g03670
Standardized expressions
0.5
0.0
−0.5
−1.0
−1.5
EI PG G1 G2 H T1 T2 C1 C2 S EP JP
Developmental stages
VIT_13s0019g03680
Standardized expressions
0.50
0.25
0.00
−0.25
−0.50
−0.75
EI PG G1 G2 H T1 T2 C1 C2 S EP JP
Developmental stages
VIT_13s0019g03690
Standardized expressions
1
0
−1
−2
−3
EI PG G1 G2 H T1 T2 C1 C2 S EP JP
Developmental stages
VIT_13s0019g03730
Standardized expressions
1
0
−1
−2
EI PG G1 G2 H T1 T2 C1 C2 S EP JP
Developmental stages

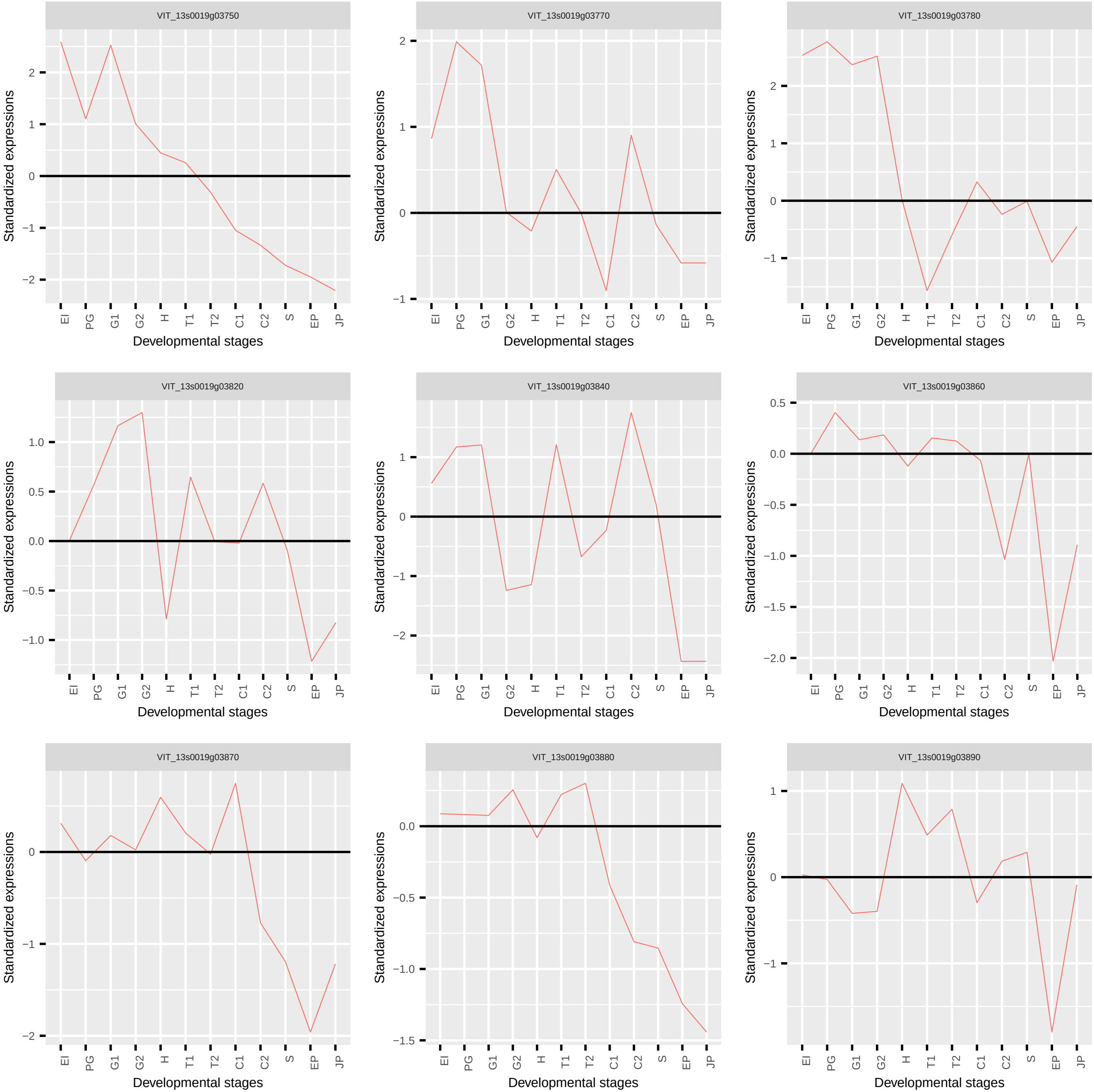
VIT_13s0019g03750
VIT_13s0019g03770
VIT_13s0019g03780
VIT_13s0019g03820
VIT_13s0019g03840
VIT_13s0019g03860
VIT_13s0019g03870
VIT_13s0019g03880
VIT_13s0019g03890
Standardized expressions
Developmental stages
EI PG G1 G2 H T1 T2 C1 C2 S EP JP

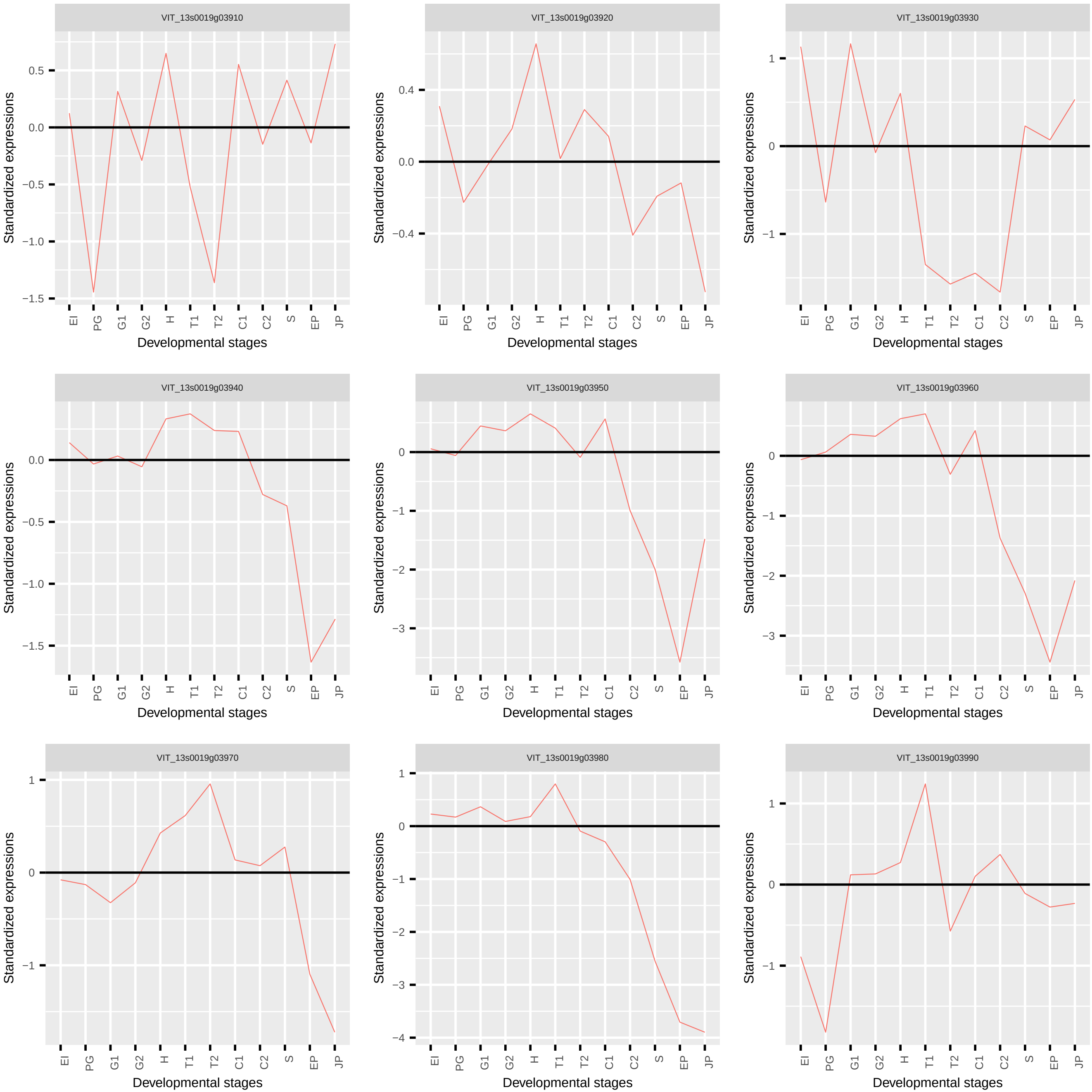
VIT_13s0019g03910
VIT_13s0019g03920
VIT_13s0019g03930
VIT_13s0019g03940
VIT_13s0019g03950
VIT_13s0019g03960
VIT_13s0019g03970
VIT_13s0019g03980
VIT_13s0019g03990
Standardized expressions
Developmental stages
EI PG G1 G2 H T1 T2 C1 C2 S EP JP

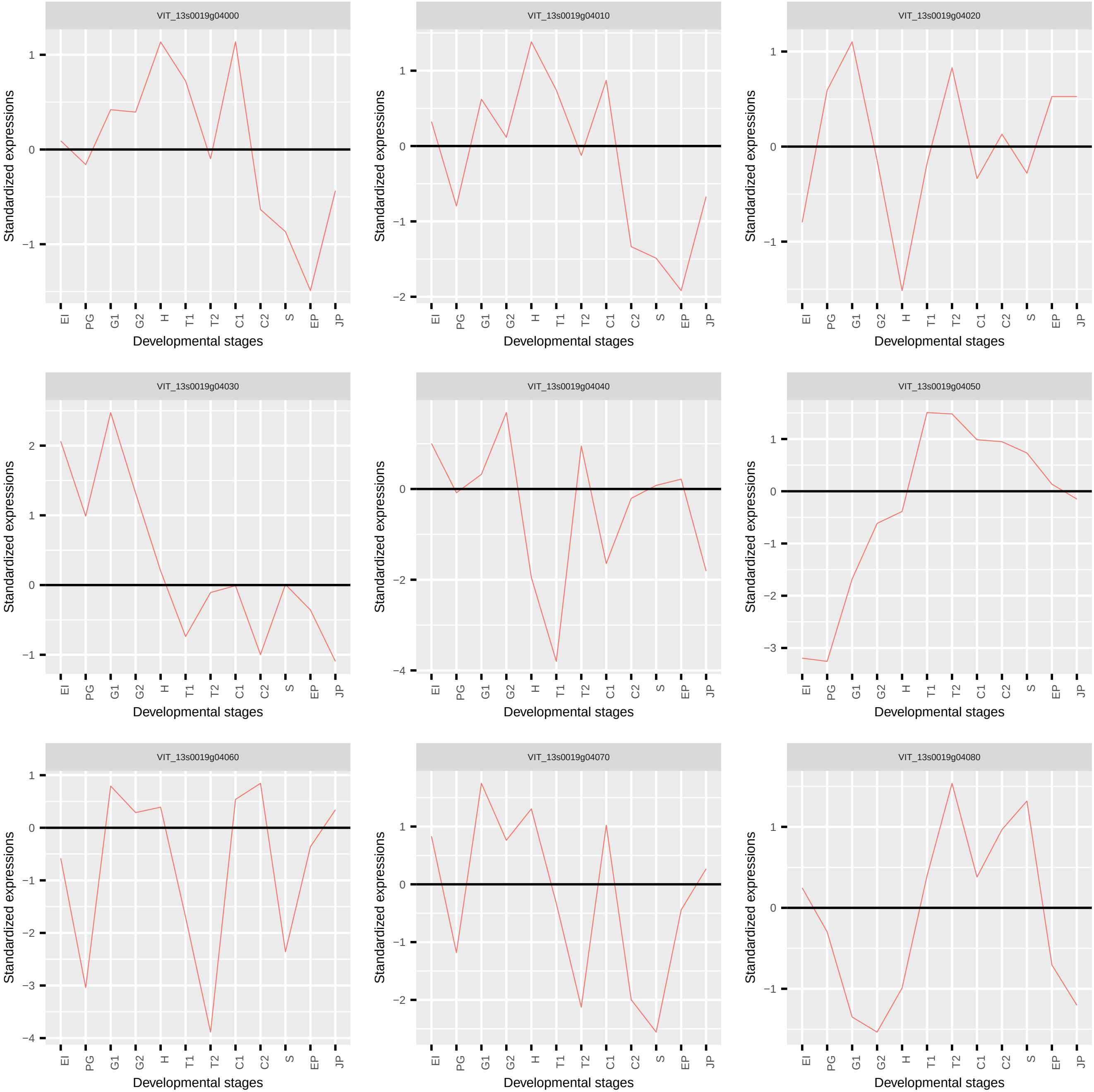
VIT_13s0019g04000
VIT_13s0019g04010
VIT_13s0019g04020
VIT_13s0019g04030
VIT_13s0019g04040
VIT_13s0019g04050
VIT_13s0019g04060
VIT_13s0019g04070
VIT_13s0019g04080
Standardized expressions
Developmental stages
EI PG G1 G2 H T1 T2 C1 C2 S EP JP

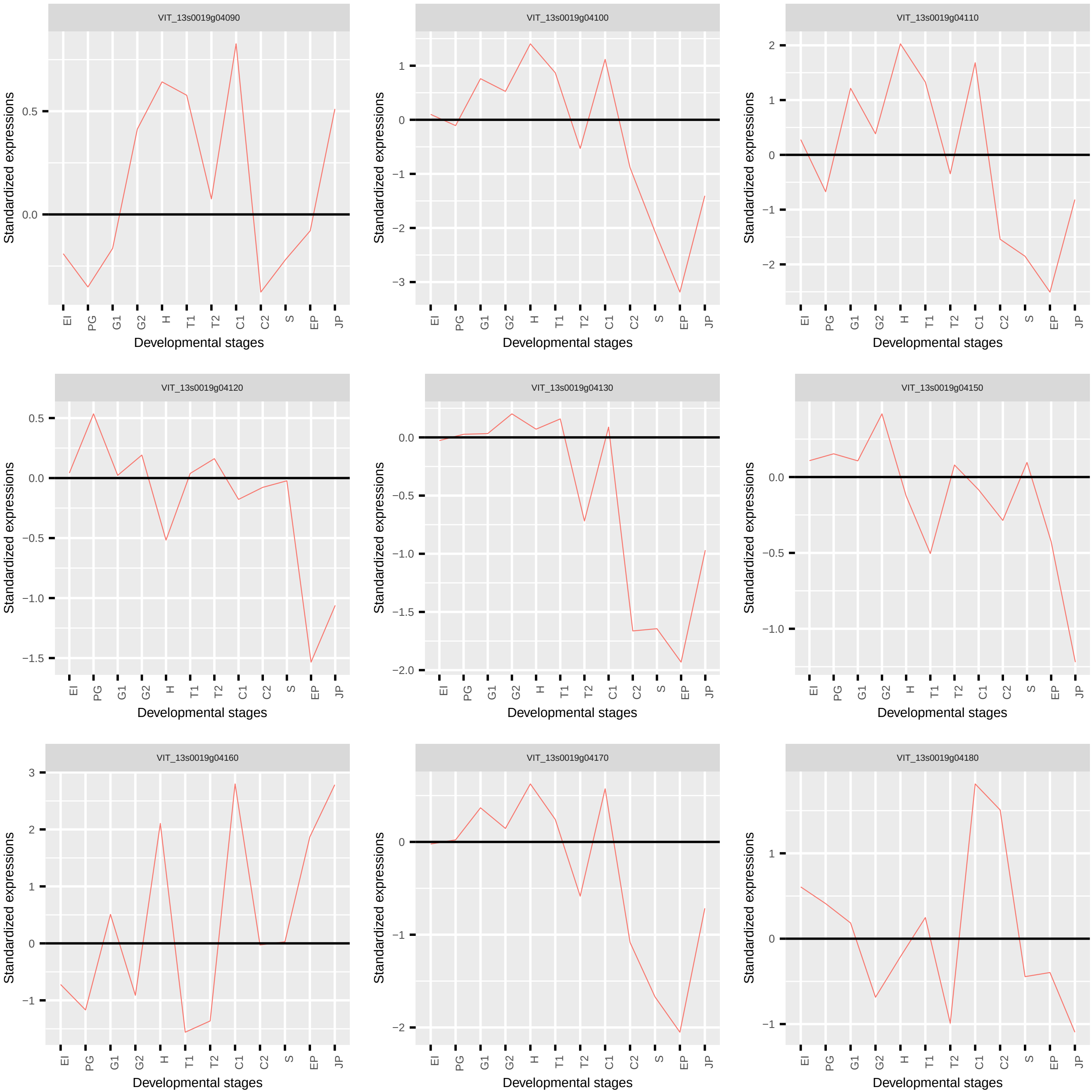
VIT_13s0019g04090
VIT_13s0019g04100
VIT_13s0019g04110
VIT_13s0019g04120
VIT_13s0019g04130
VIT_13s0019g04150
VIT_13s0019g04160
VIT_13s0019g04170
VIT_13s0019g04180
Standardized expressions
Developmental stages
EI PG G1 G2 H T1 T2 C1 C2 S EP JP

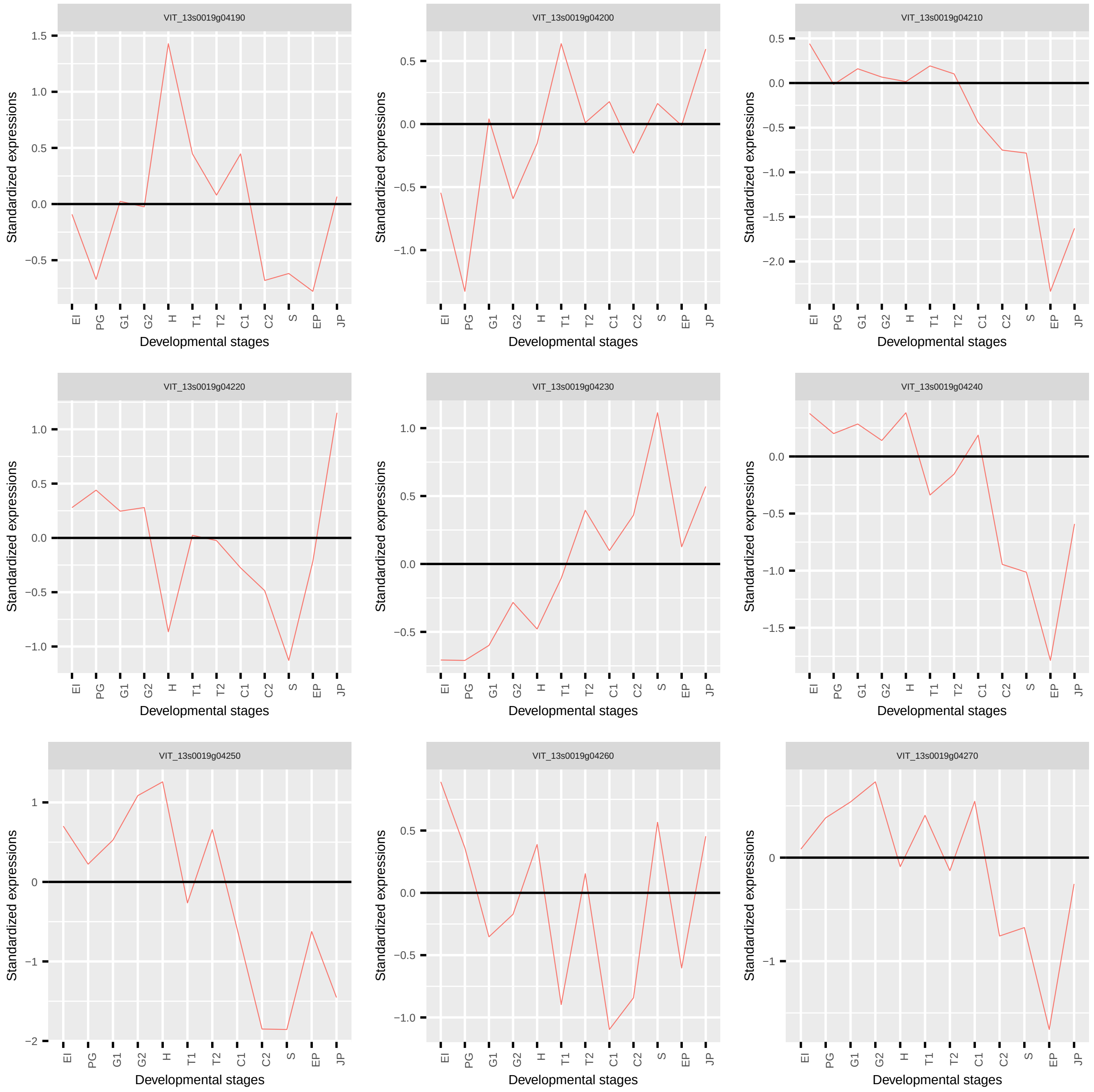
VIT_13s0019g04190
VIT_13s0019g04200
VIT_13s0019g04210
VIT_13s0019g04220
VIT_13s0019g04230
VIT_13s0019g04240
VIT_13s0019g04250
VIT_13s0019g04260
VIT_13s0019g04270
Standardized expressions
Developmental stages
EI PG G1 G2 H T1 T2 C1 C2 S EP JP

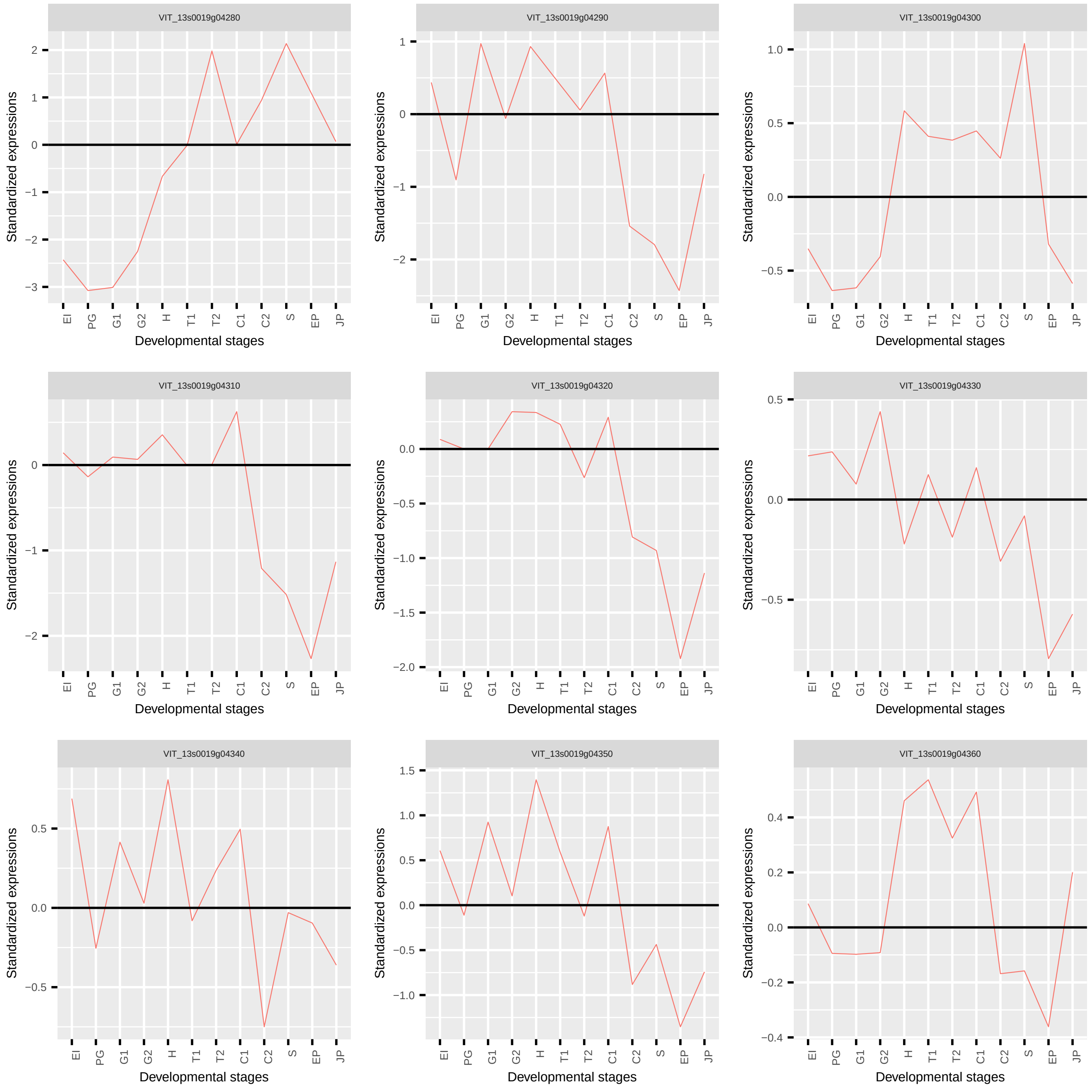

VIT_13s0019g04280
VIT_13s0019g04290
VIT_13s0019g04300
VIT_13s0019g04310
VIT_13s0019g04320
VIT_13s0019g04330
VIT_13s0019g04340
VIT_13s0019g04350
VIT_13s0019g04360
Standardized expressions
Developmental stages
EI PG G1 G2 H T1 T2 C1 C2 S EP JP

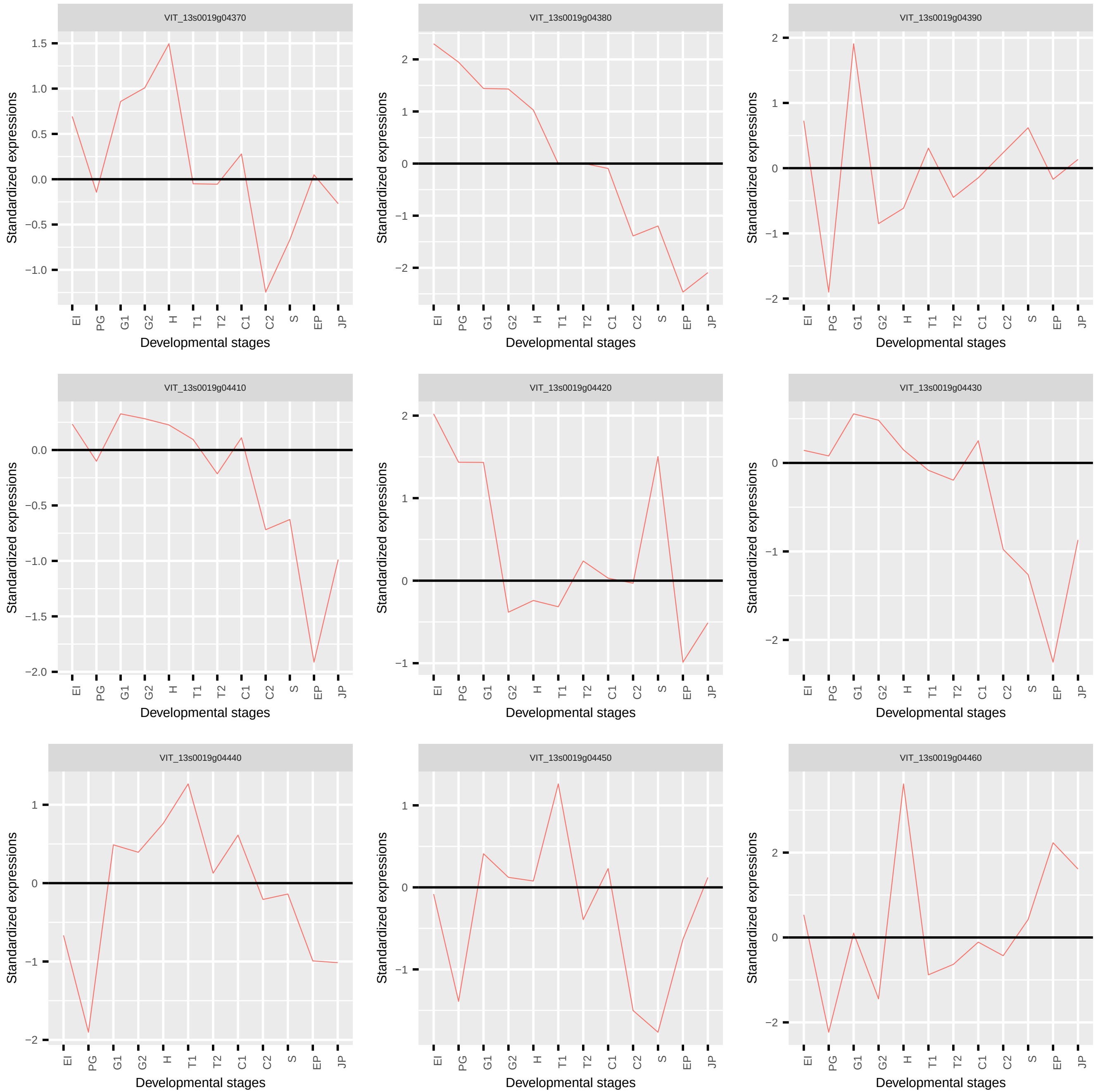
VIT_13s0019g04370
VIT_13s0019g04380
VIT_13s0019g04390
VIT_13s0019g04410
VIT_13s0019g04420
VIT_13s0019g04430
VIT_13s0019g04440
VIT_13s0019g04450
VIT_13s0019g04460
Standardized expressions
Developmental stages
EI PG G1 G2 H T1 T2 C1 C2 S EP JP

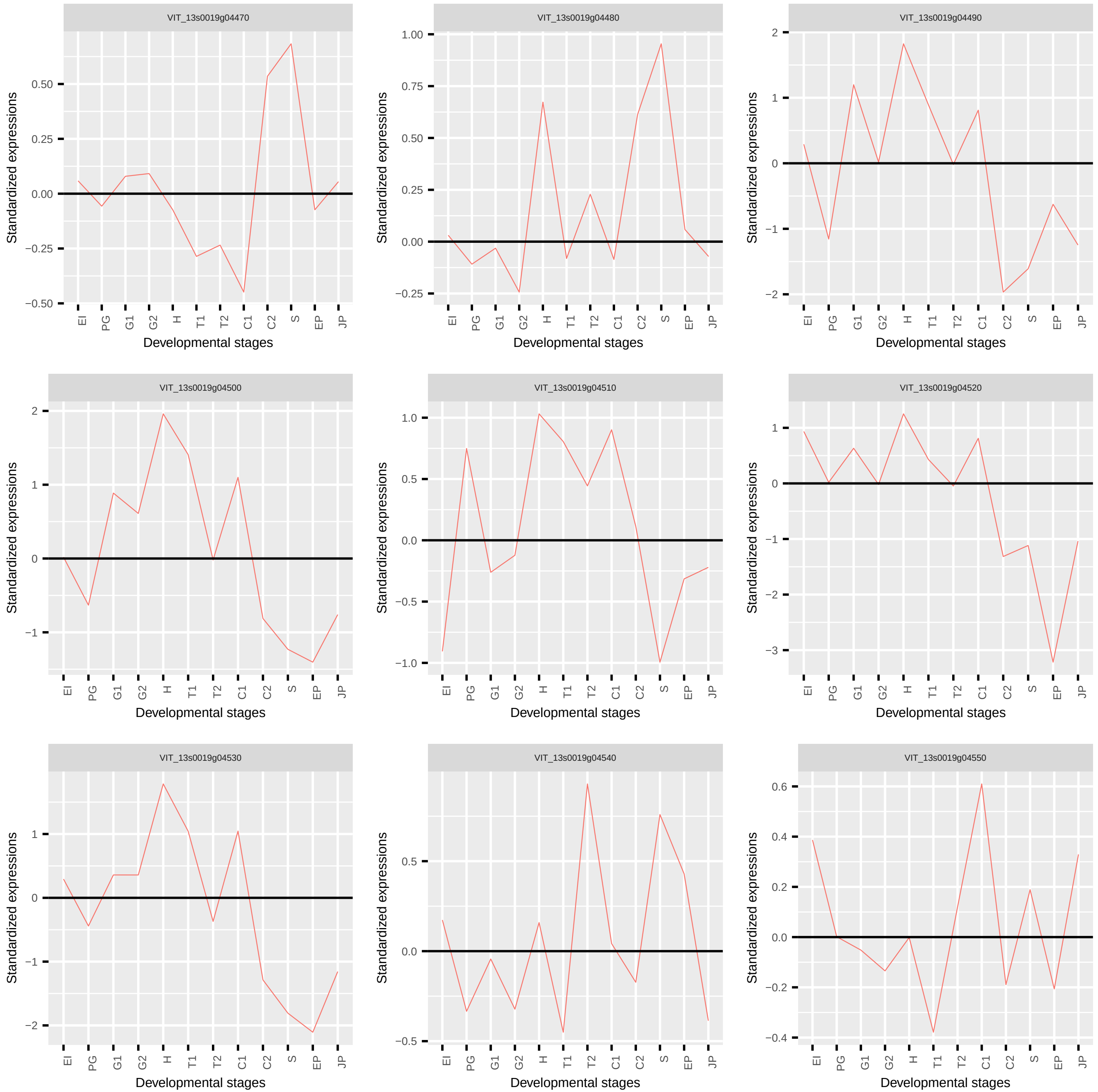
VIT_13s0019g04470
VIT_13s0019g04480
VIT_13s0019g04490
VIT_13s0019g04500
VIT_13s0019g04510
VIT_13s0019g04520
VIT_13s0019g04530
VIT_13s0019g04540
VIT_13s0019g04550
Standardized expressions
Developmental stages
EI PG G1 G2 H T1 T2 C1 C2 S EP JP

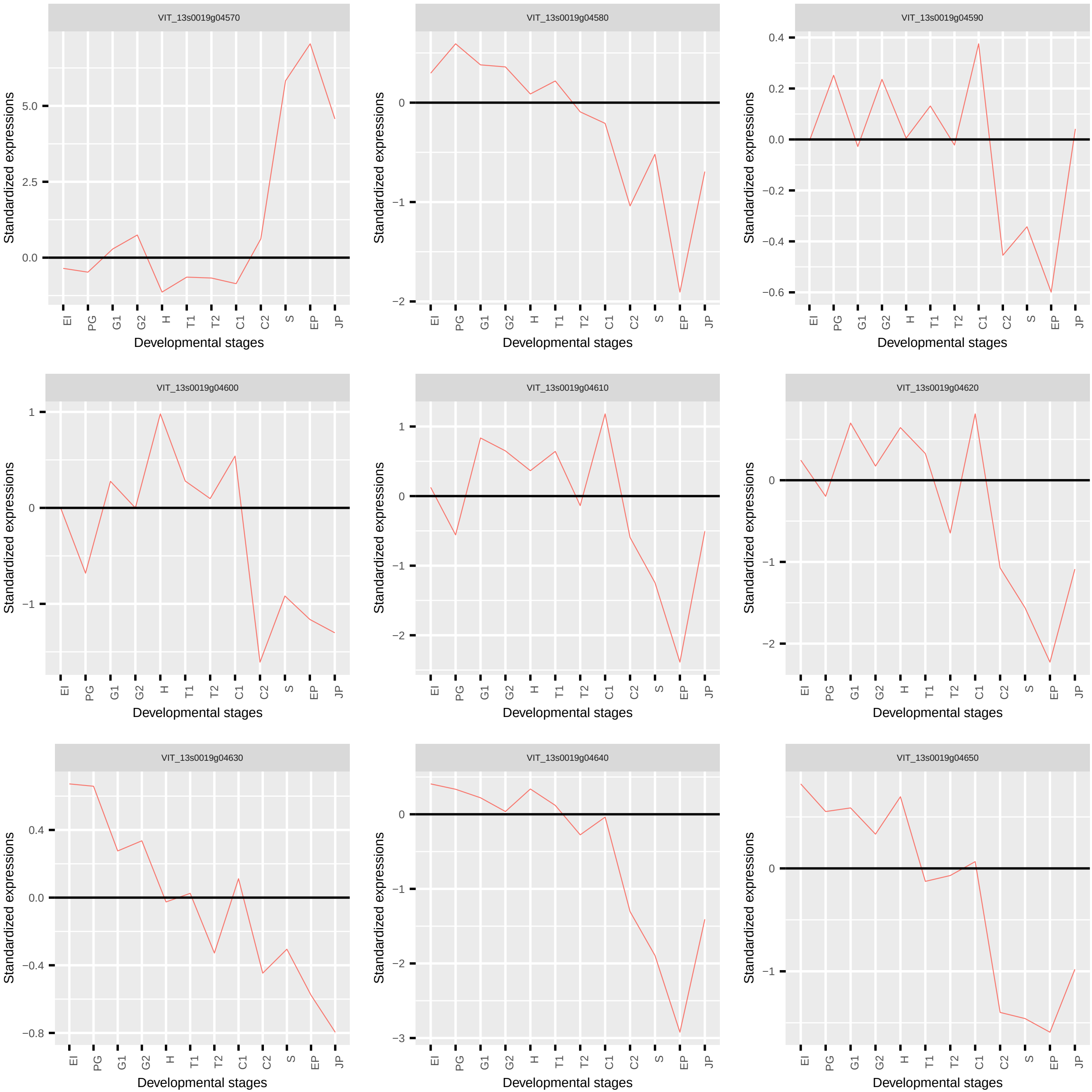
VIT_13s0019g04570
VIT_13s0019g04580
VIT_13s0019g04590
VIT_13s0019g04600
VIT_13s0019g04610
VIT_13s0019g04620
VIT_13s0019g04630
VIT_13s0019g04640
VIT_13s0019g04650
Standardized expressions
Developmental stages
EI PG G1 G2 H T1 T2 C1 C2 S EP JP

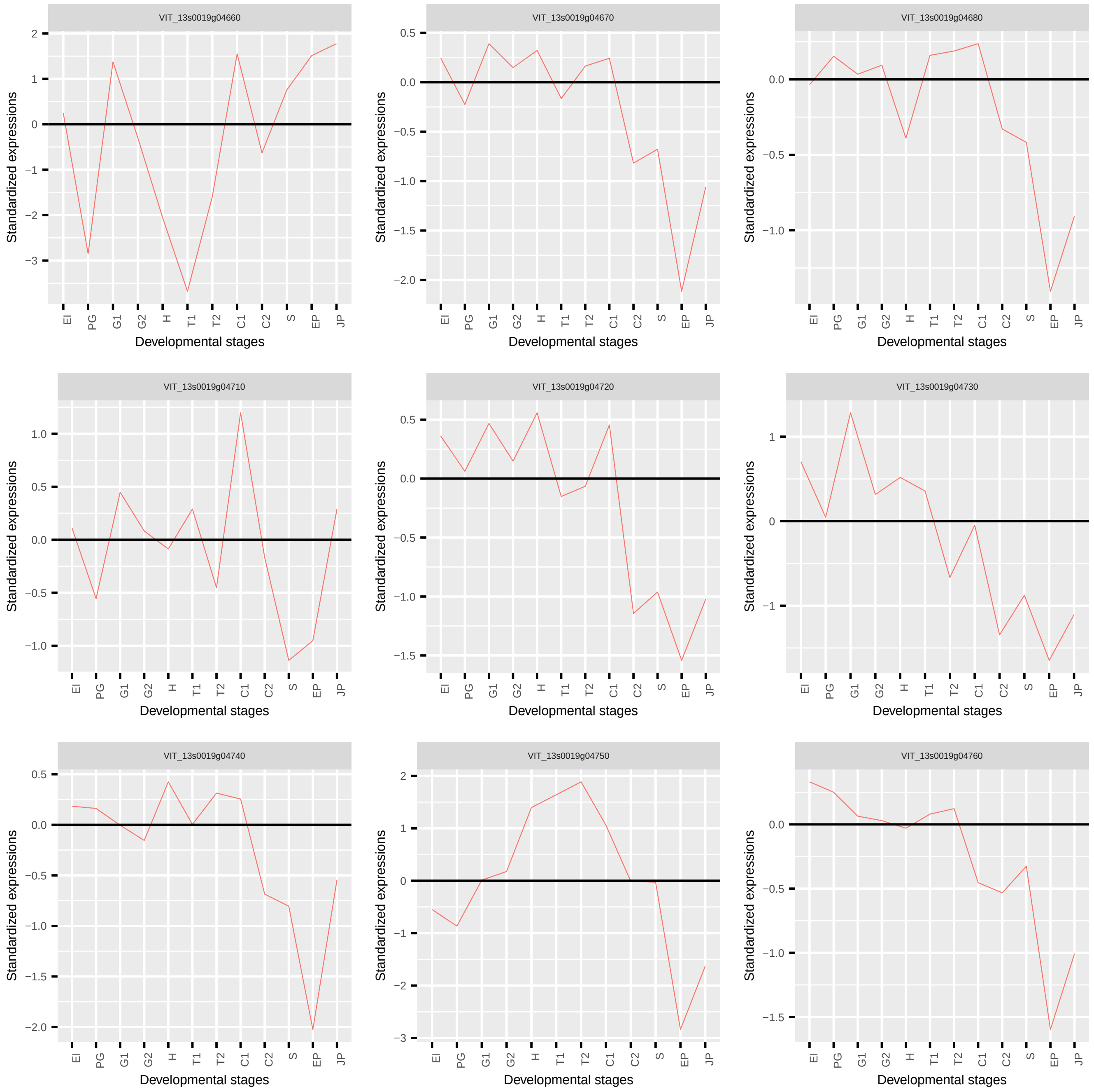
VIT_13s0019g04660
VIT_13s0019g04670
VIT_13s0019g04680
VIT_13s0019g04710
VIT_13s0019g04720
VIT_13s0019g04730
VIT_13s0019g04740
VIT_13s0019g04750
VIT_13s0019g04760
Standardized expressions
Developmental stages
EI PG G1 G2 H T1 T2 C1 C2 S EP JP

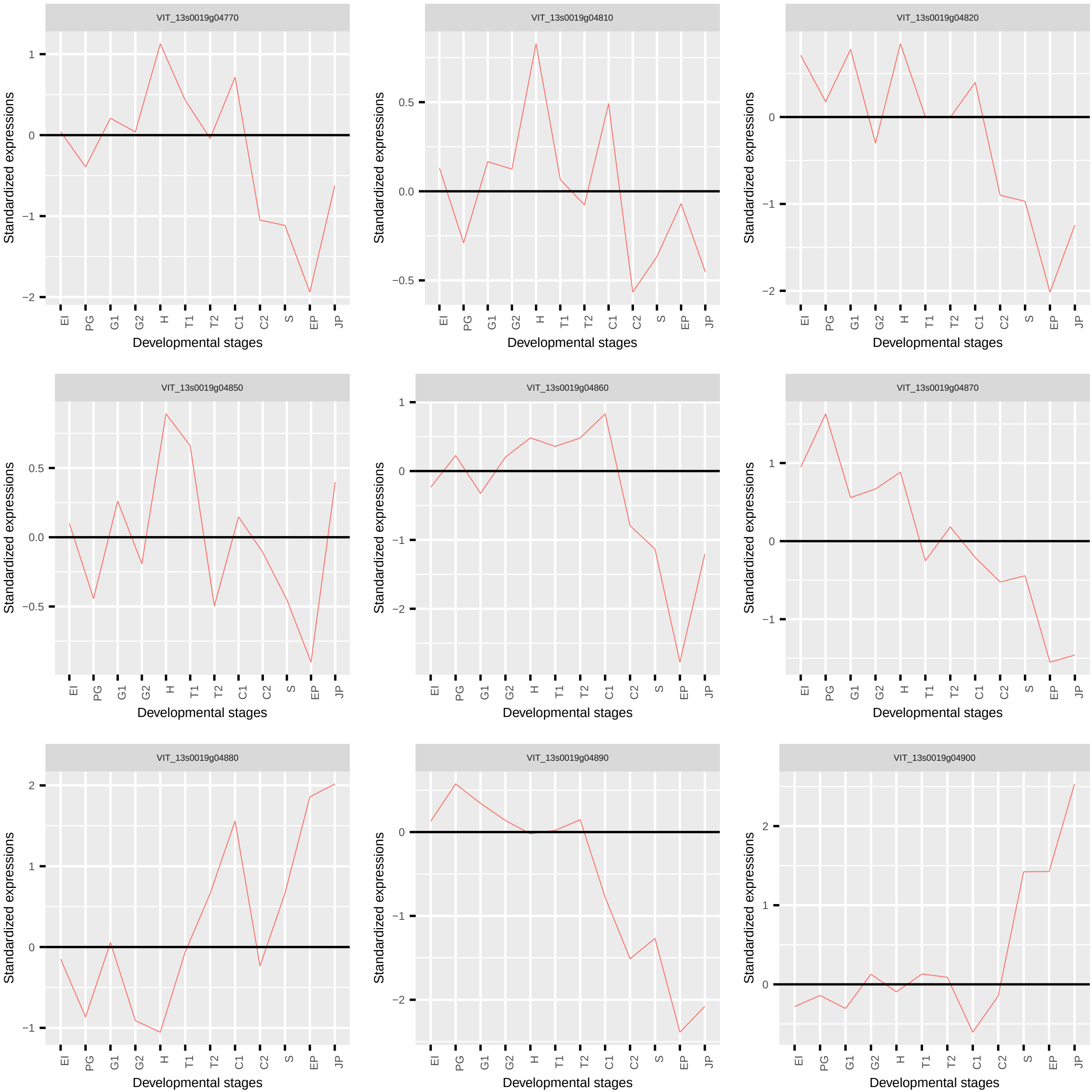
VIT_13s0019g04770
VIT_13s0019g04810
VIT_13s0019g04820
VIT_13s0019g04850
VIT_13s0019g04860
VIT_13s0019g04870
VIT_13s0019g04880
VIT_13s0019g04890
VIT_13s0019g04900
Standardized expressions
Developmental stages
EI
PG
G1
G2
H
T1
T2
C1
C2
S
EP
JP

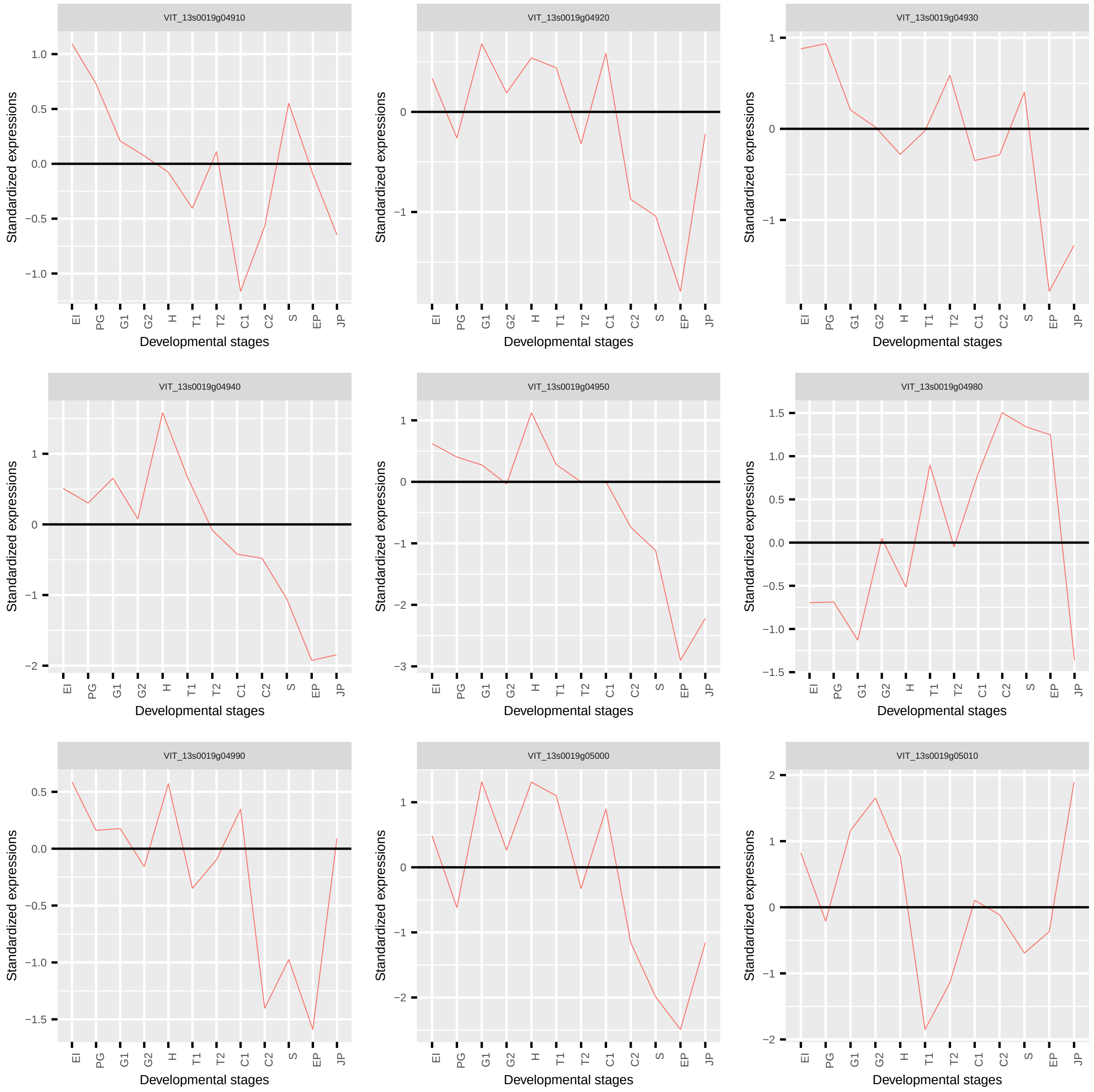

VIT_13s0019g04910
VIT_13s0019g04920
VIT_13s0019g04930
VIT_13s0019g04940
VIT_13s0019g04950
VIT_13s0019g04980
VIT_13s0019g04990
VIT_13s0019g05000
VIT_13s0019g05010
Standardized expressions
Developmental stages
EI PG G1 G2 H T1 T2 C1 C2 S EP JP

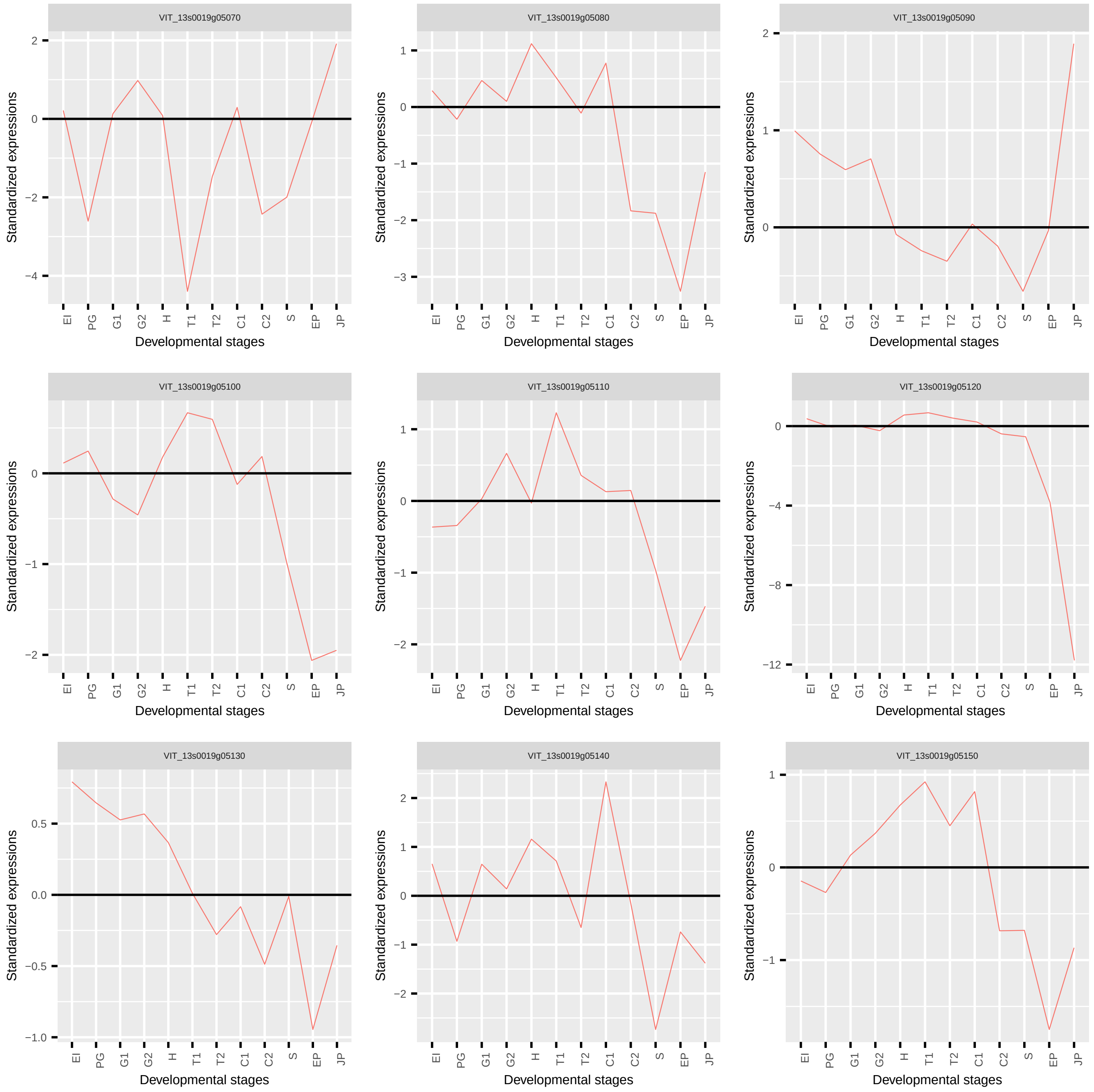
VIT_13s0019g05070
VIT_13s0019g05080
VIT_13s0019g05090
VIT_13s0019g05100
VIT_13s0019g05110
VIT_13s0019g05120
VIT_13s0019g05130
VIT_13s0019g05140
VIT_13s0019g05150
Standardized expressions
Developmental stages
EI PG G1 G2 H T1 T2 C1 C2 S EP JP

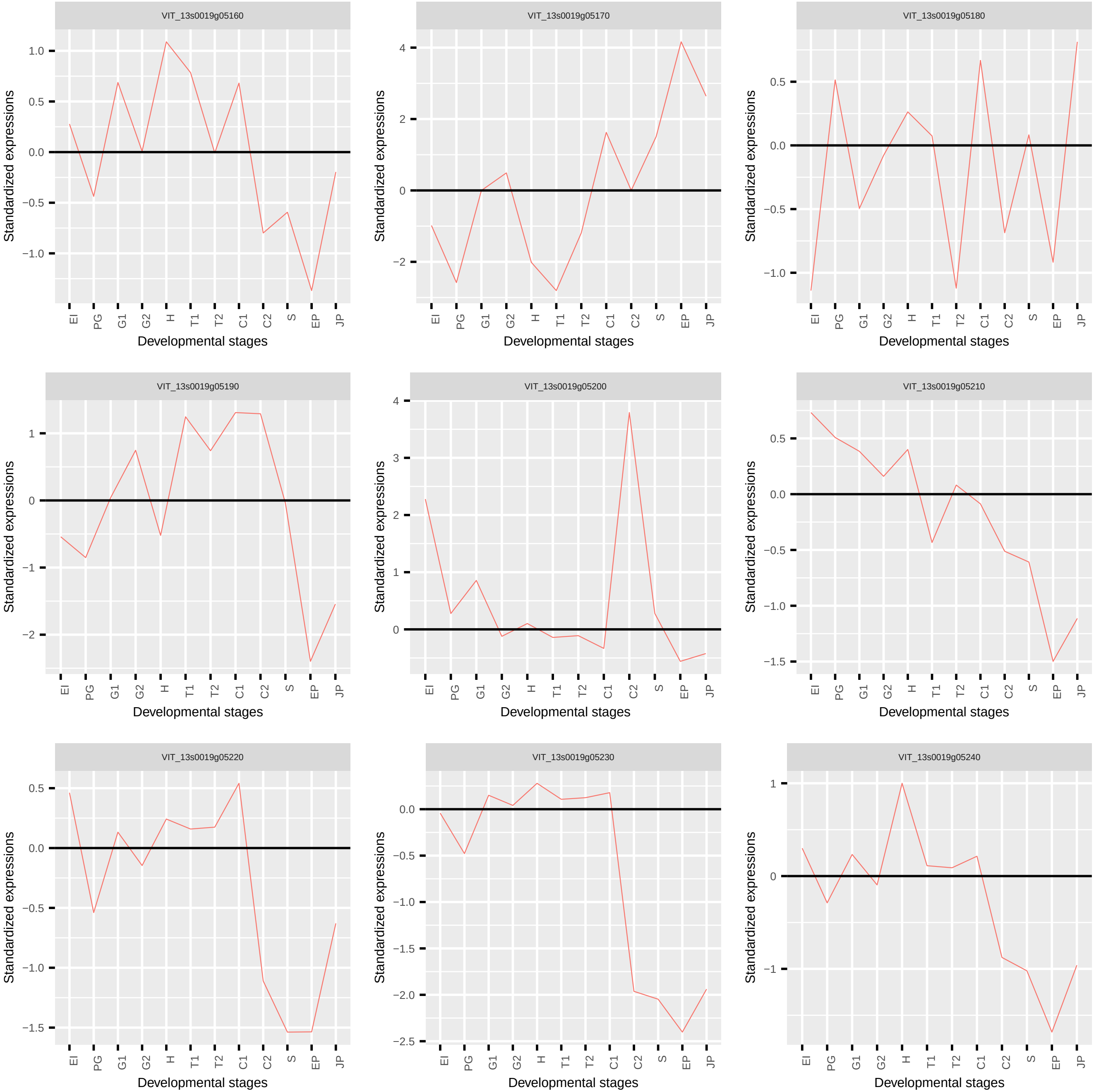
VIT_13s0019g05160
VIT_13s0019g05170
VIT_13s0019g05180
VIT_13s0019g05190
VIT_13s0019g05200
VIT_13s0019g05210
VIT_13s0019g05220
VIT_13s0019g05230
VIT_13s0019g05240
Standardized expressions
Developmental stages
EI PG G1 G2 H T1 T2 C1 C2 S EP JP

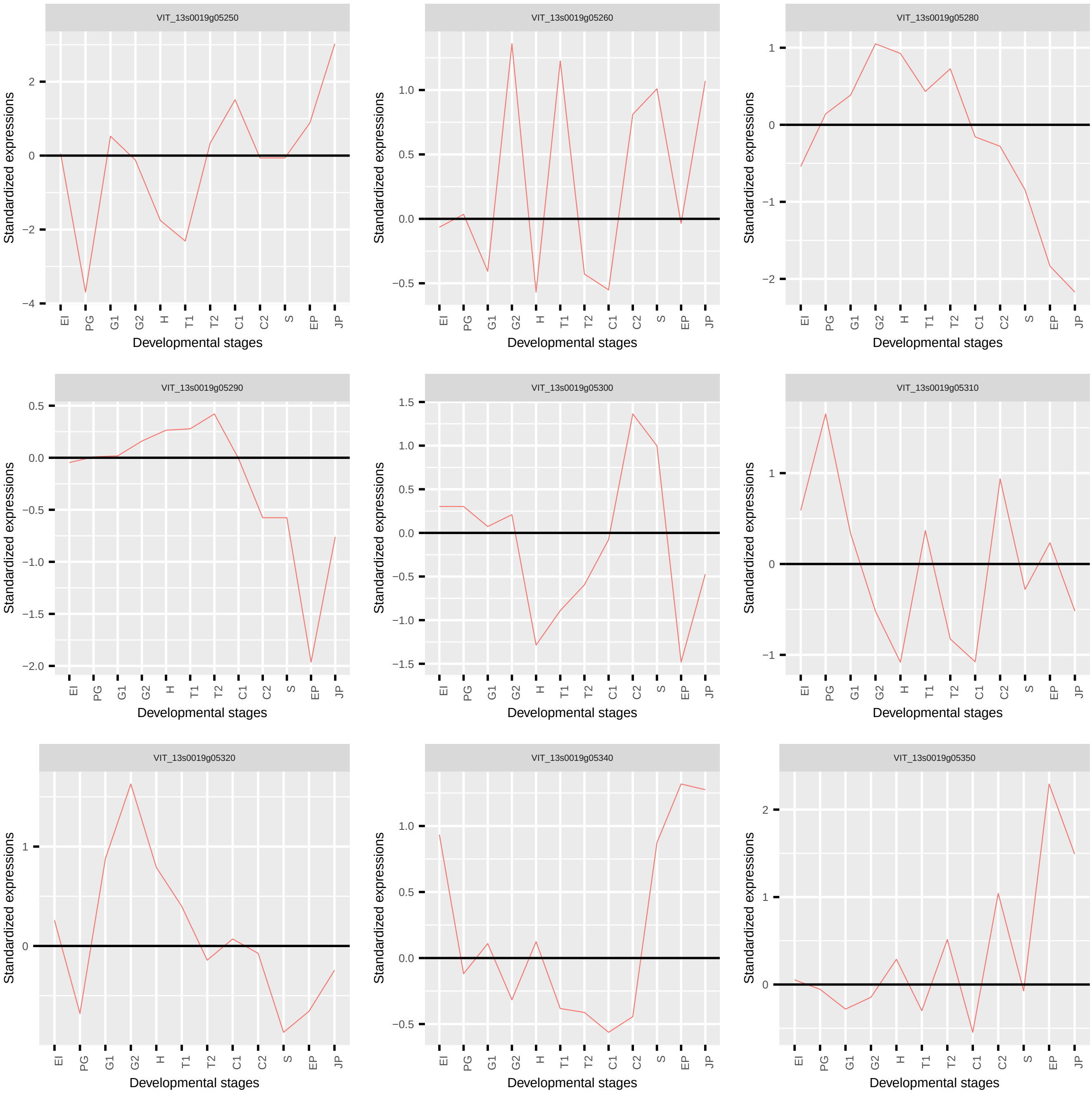
VIT_13s0019g05250
VIT_13s0019g05260
VIT_13s0019g05280
VIT_13s0019g05290
VIT_13s0019g05300
VIT_13s0019g05310
VIT_13s0019g05320
VIT_13s0019g05340
VIT_13s0019g05350
Standardized expressions
Developmental stages
EI PG G1 G2 H T1 T2 C1 C2 S EP JP

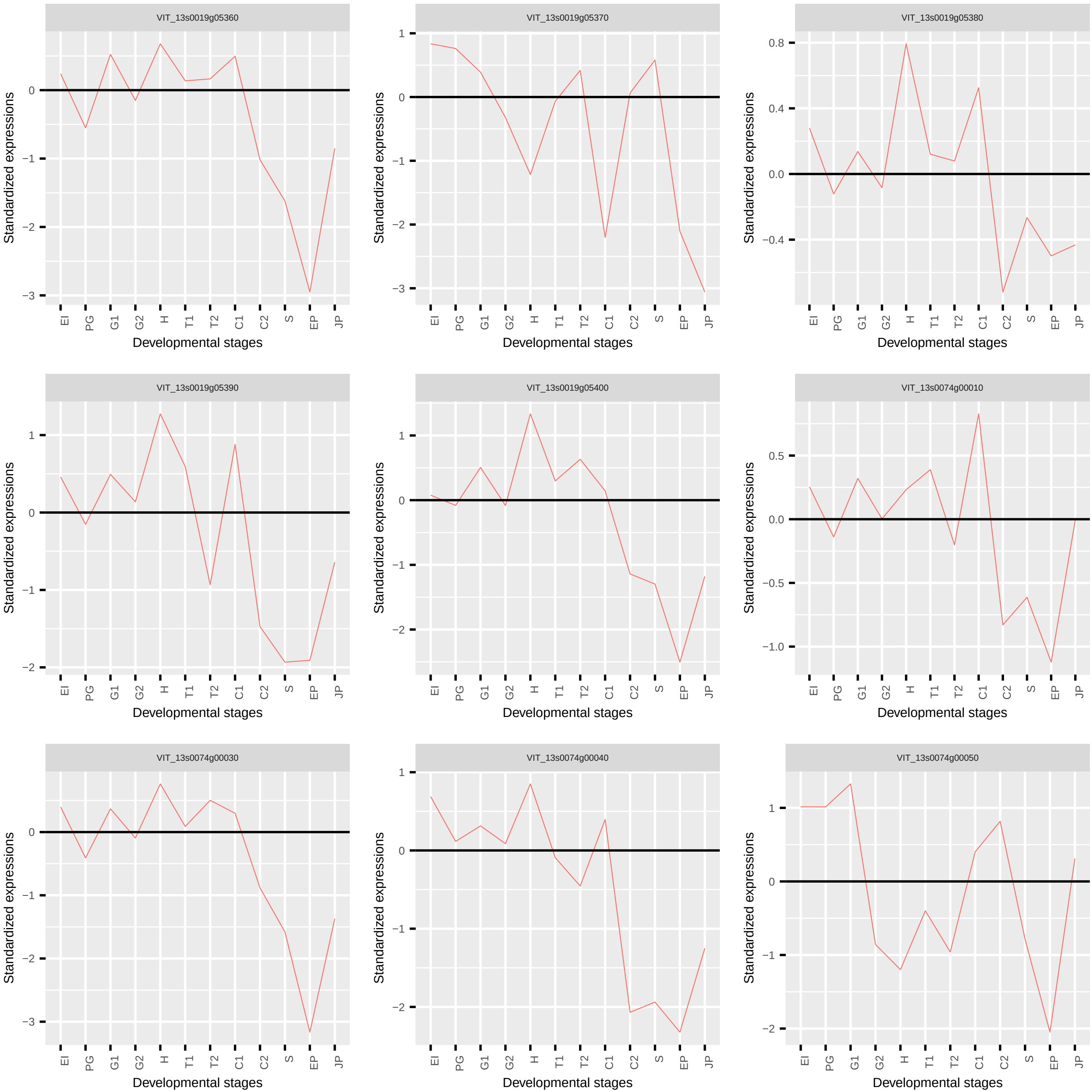
VIT_13s0019g05360
VIT_13s0019g05370
VIT_13s0019g05380
VIT_13s0019g05390
VIT_13s0019g05400
VIT_13s0074g00010
VIT_13s0074g00030
VIT_13s0074g00040
VIT_13s0074g00050
Standardized expressions
Developmental stages
EI
PG
G1
G2
H
T1
T2
C1
C2
S
EP
JP

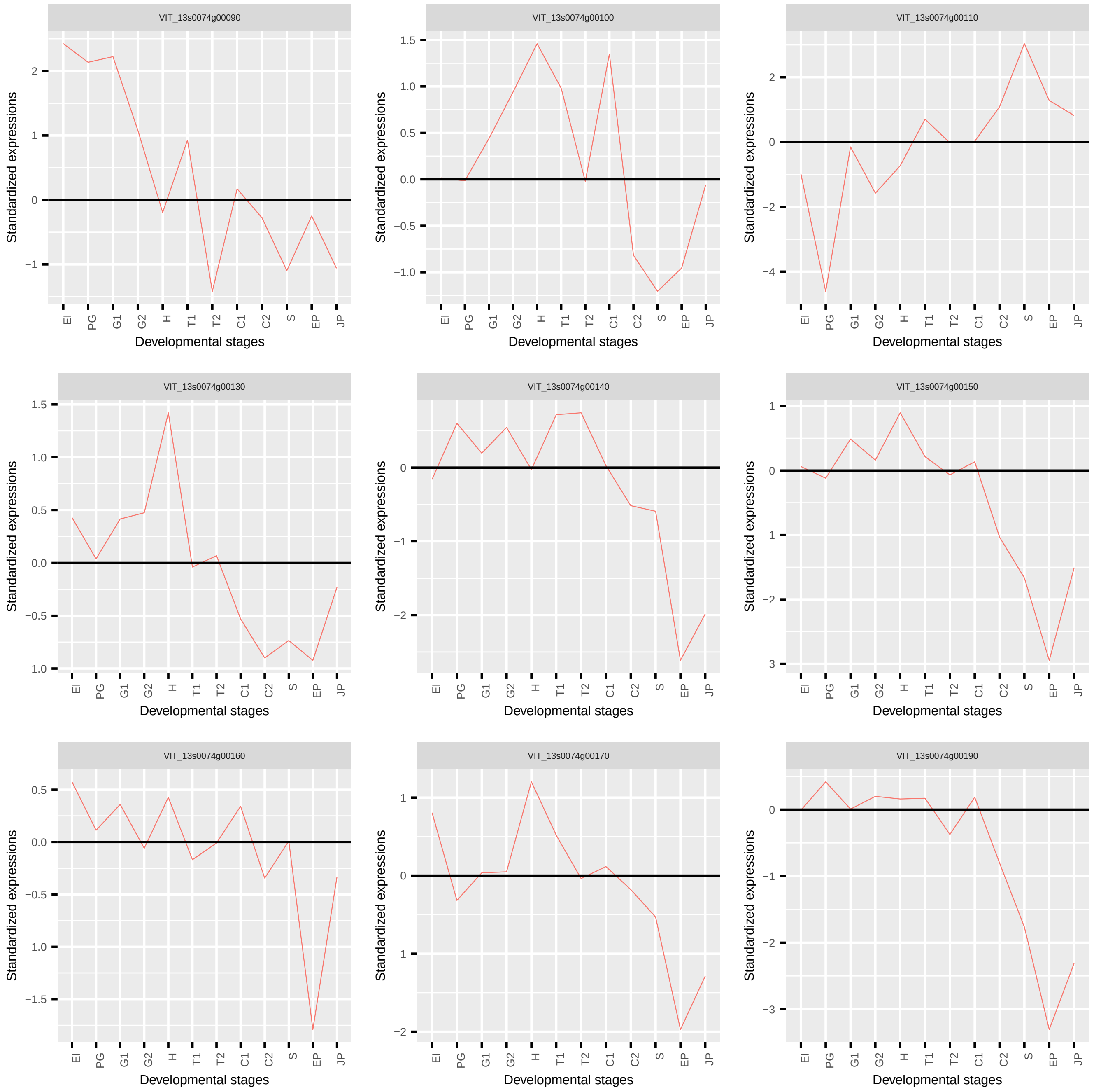
VIT_13s0074g00090
VIT_13s0074g00100
VIT_13s0074g00110
VIT_13s0074g00130
VIT_13s0074g00140
VIT_13s0074g00150
VIT_13s0074g00160
VIT_13s0074g00170
VIT_13s0074g00190
Standardized expressions
Developmental stages
EI PG G1 G2 H T1 T2 C1 C2 S EP JP

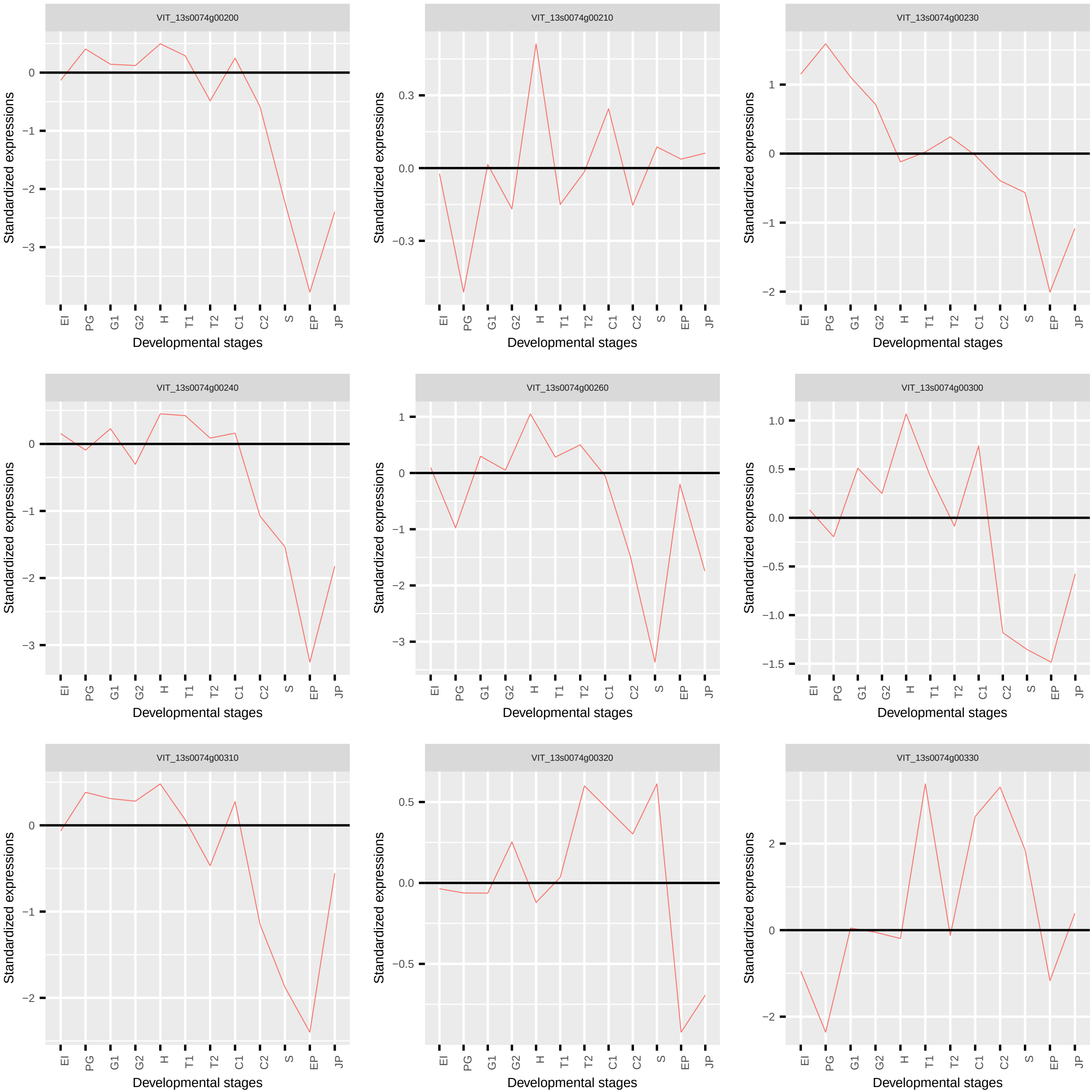
VIT_13s0074g00200
VIT_13s0074g00210
VIT_13s0074g00230
VIT_13s0074g00240
VIT_13s0074g00260
VIT_13s0074g00300
VIT_13s0074g00310
VIT_13s0074g00320
VIT_13s0074g00330
Standardized expressions
Developmental stages
EI
PG
G1
G2
H
T1
T2
C1
C2
S
EP
JP

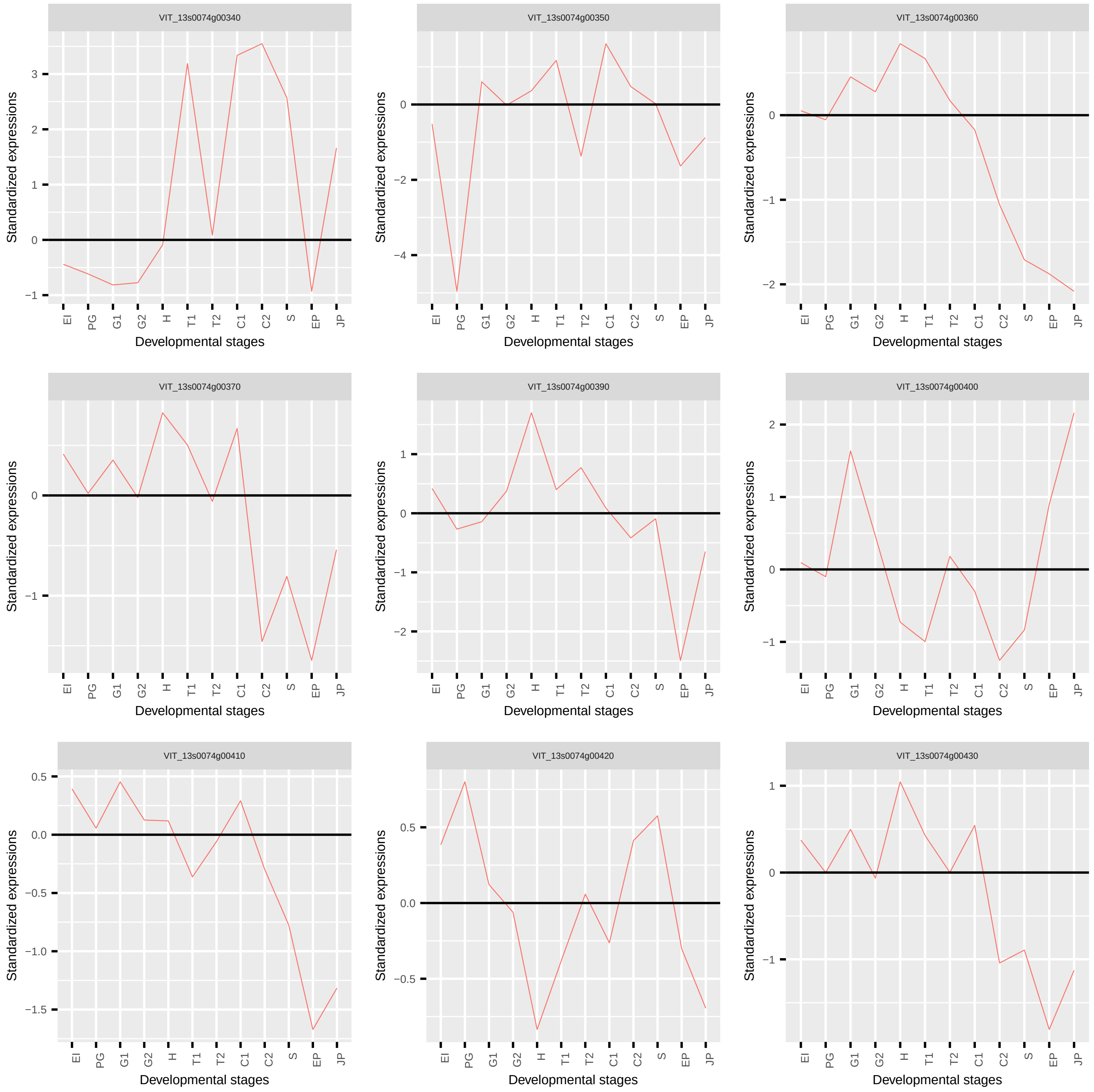
VIT_13s0074g00340
VIT_13s0074g00350
VIT_13s0074g00360
VIT_13s0074g00370
VIT_13s0074g00390
VIT_13s0074g00400
VIT_13s0074g00410
VIT_13s0074g00420
VIT_13s0074g00430
Standardized expressions
Developmental stages
EI PG G1 G2 H T1 T2 C1 C2 S EP JP

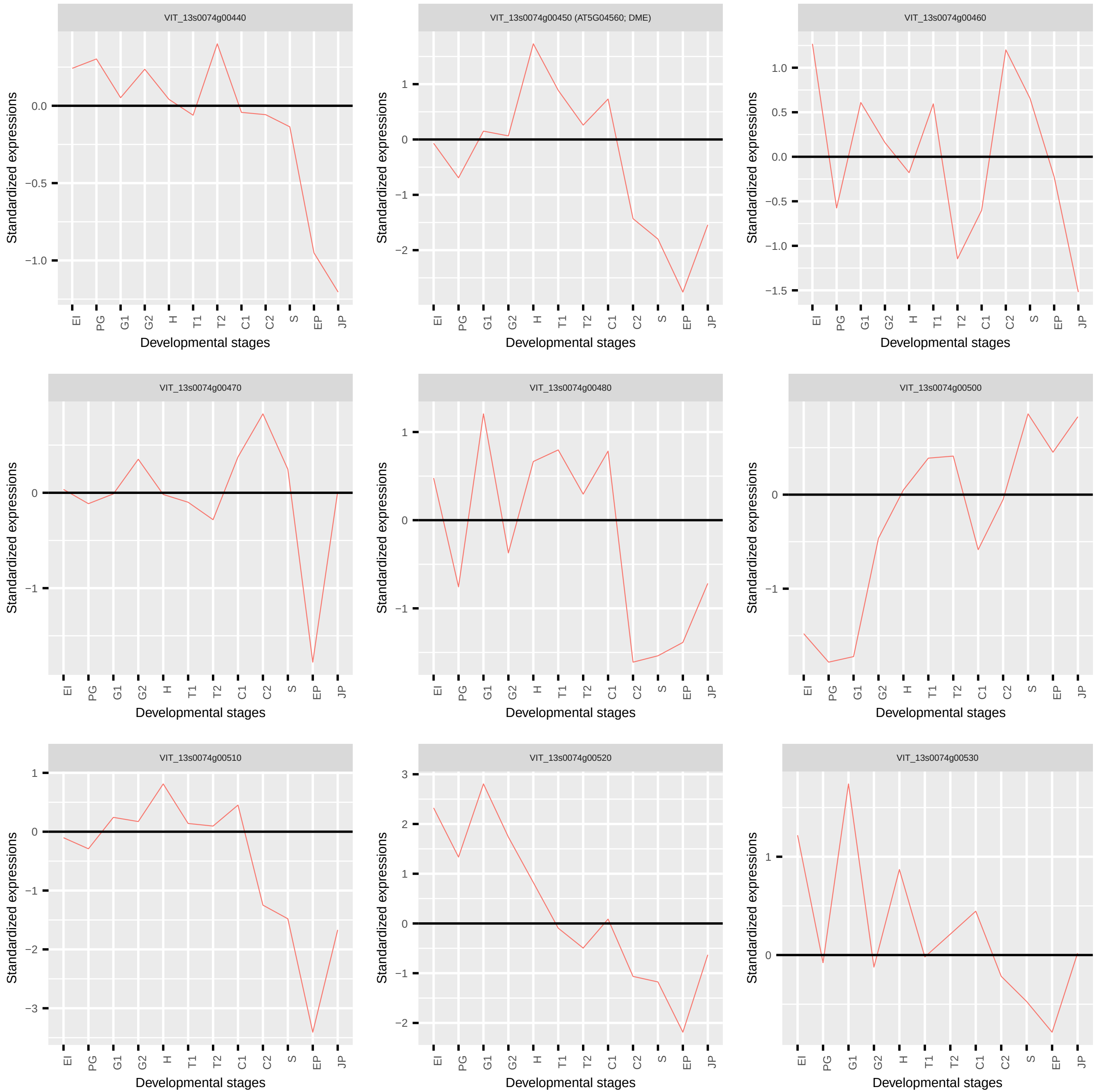
VIT_13s0074g00440
VIT_13s0074g00450 (AT5G04560; DME)
VIT_13s0074g00460
VIT_13s0074g00470
VIT_13s0074g00480
VIT_13s0074g00500
VIT_13s0074g00510
VIT_13s0074g00520
VIT_13s0074g00530
Standardized expressions
Developmental stages
EI
PG
G1
G2
H
T1
T2
C1
C2
S
EP
JP

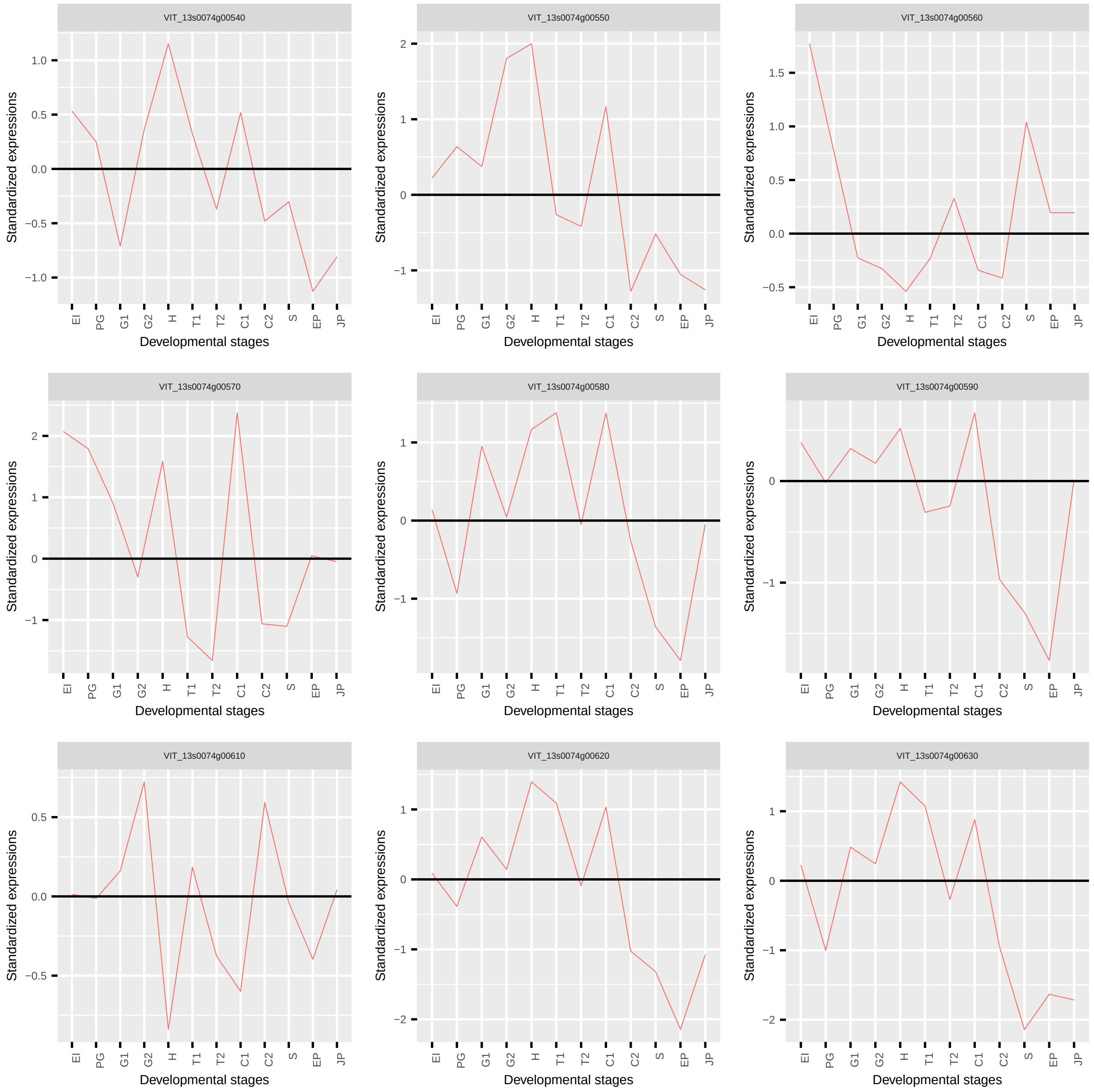
VIT_13s0074g00540
VIT_13s0074g00550
VIT_13s0074g00560
VIT_13s0074g00570
VIT_13s0074g00580
VIT_13s0074g00590
VIT_13s0074g00610
VIT_13s0074g00620
VIT_13s0074g00630
Standardized expressions
Developmental stages
EI PG G1 G2 H T1 T2 C1 C2 S EP JP

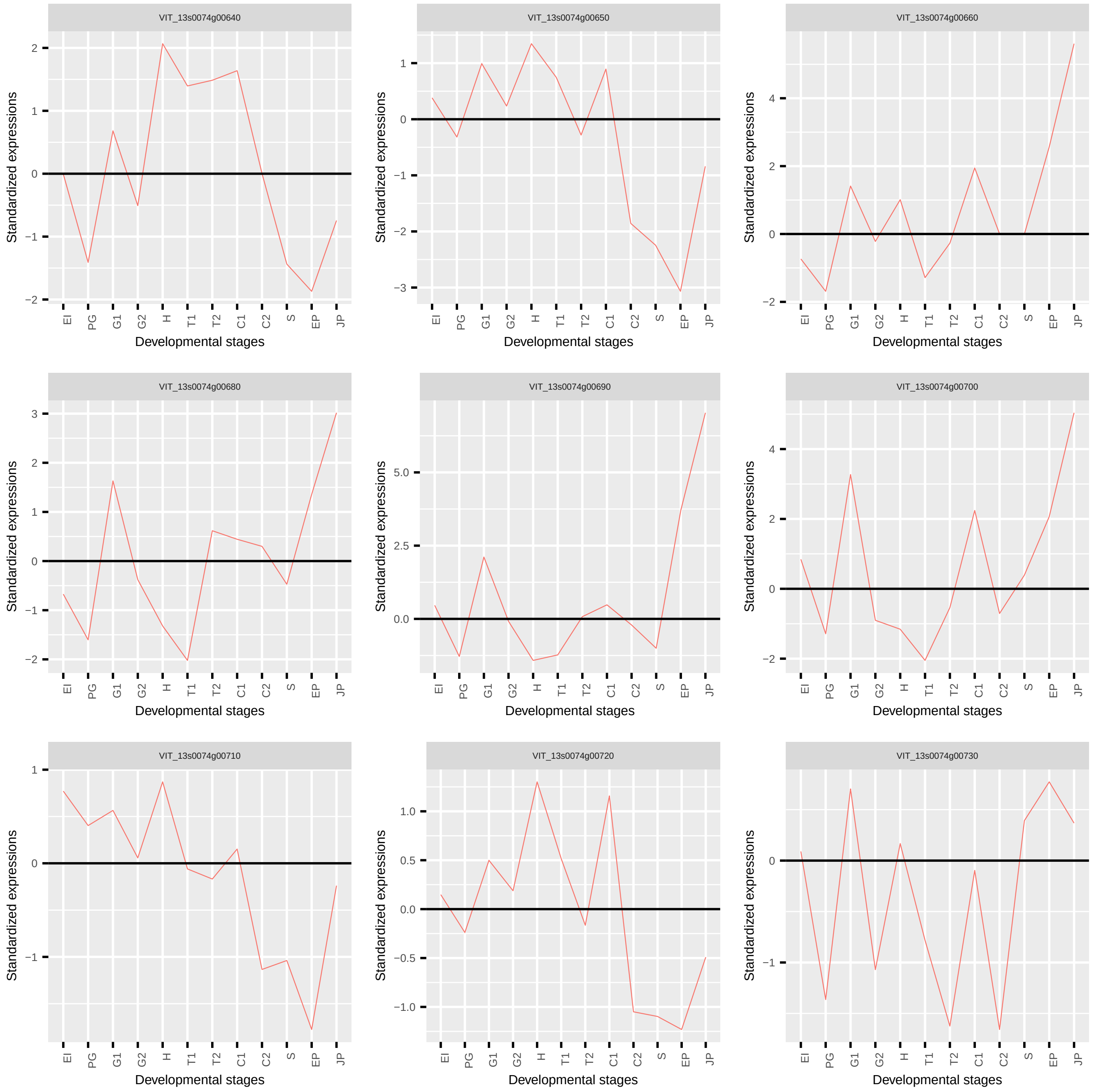
VIT_13s0074g00640
VIT_13s0074g00650
VIT_13s0074g00660
VIT_13s0074g00680
VIT_13s0074g00690
VIT_13s0074g00700
VIT_13s0074g00710
VIT_13s0074g00720
VIT_13s0074g00730
Standardized expressions
Developmental stages
EI PG G1 G2 H T1 T2 C1 C2 S EP JP

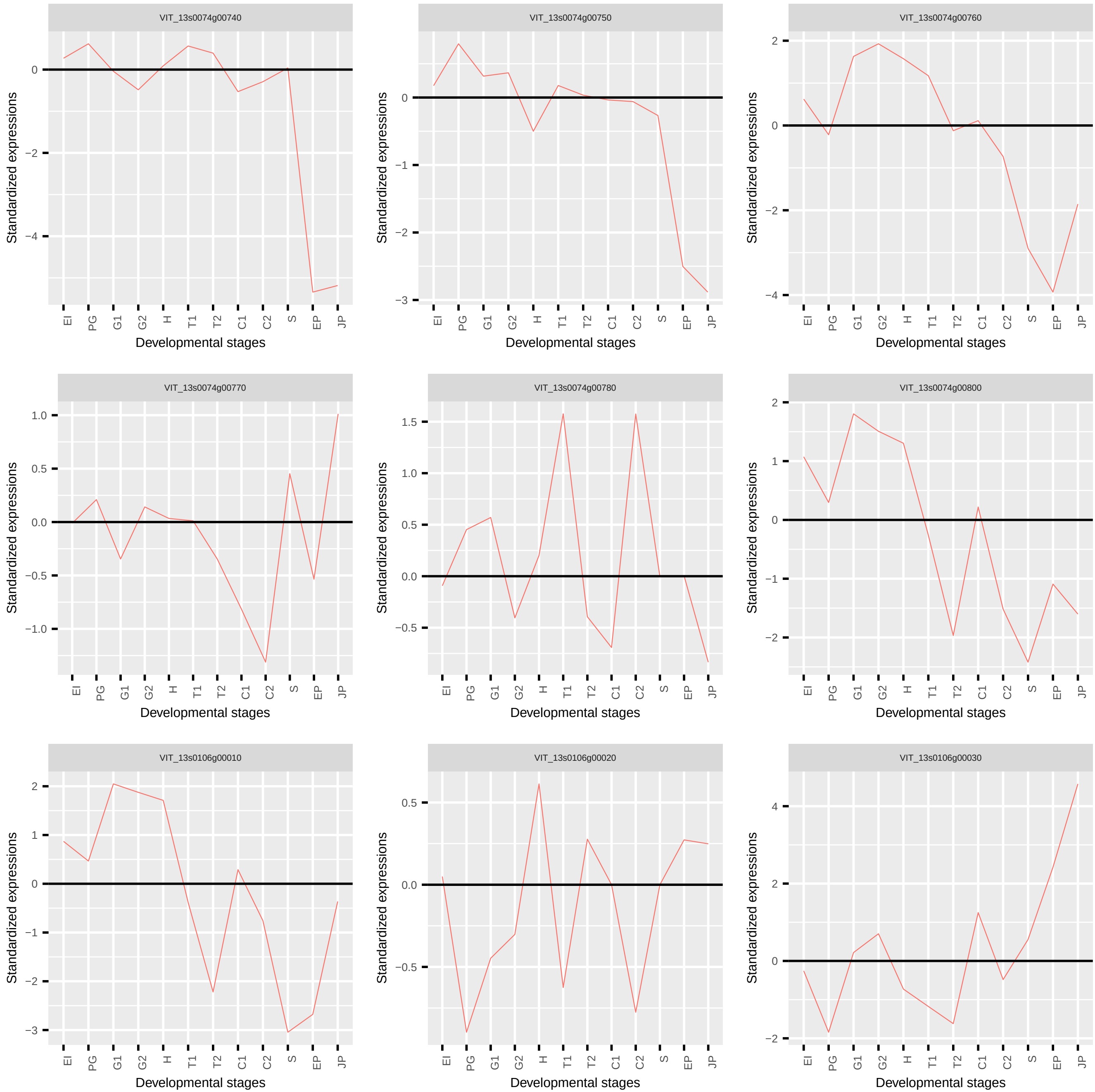

VIT_13s0074g00740
VIT_13s0074g00750
VIT_13s0074g00760
VIT_13s0074g00770
VIT_13s0074g00780
VIT_13s0074g00800
VIT_13s0106g00010
VIT_13s0106g00020
VIT_13s0106g00030
Standardized expressions
Developmental stages
EI
PG
G1
G2
H
T1
T2
C1
C2
S
EP
JP

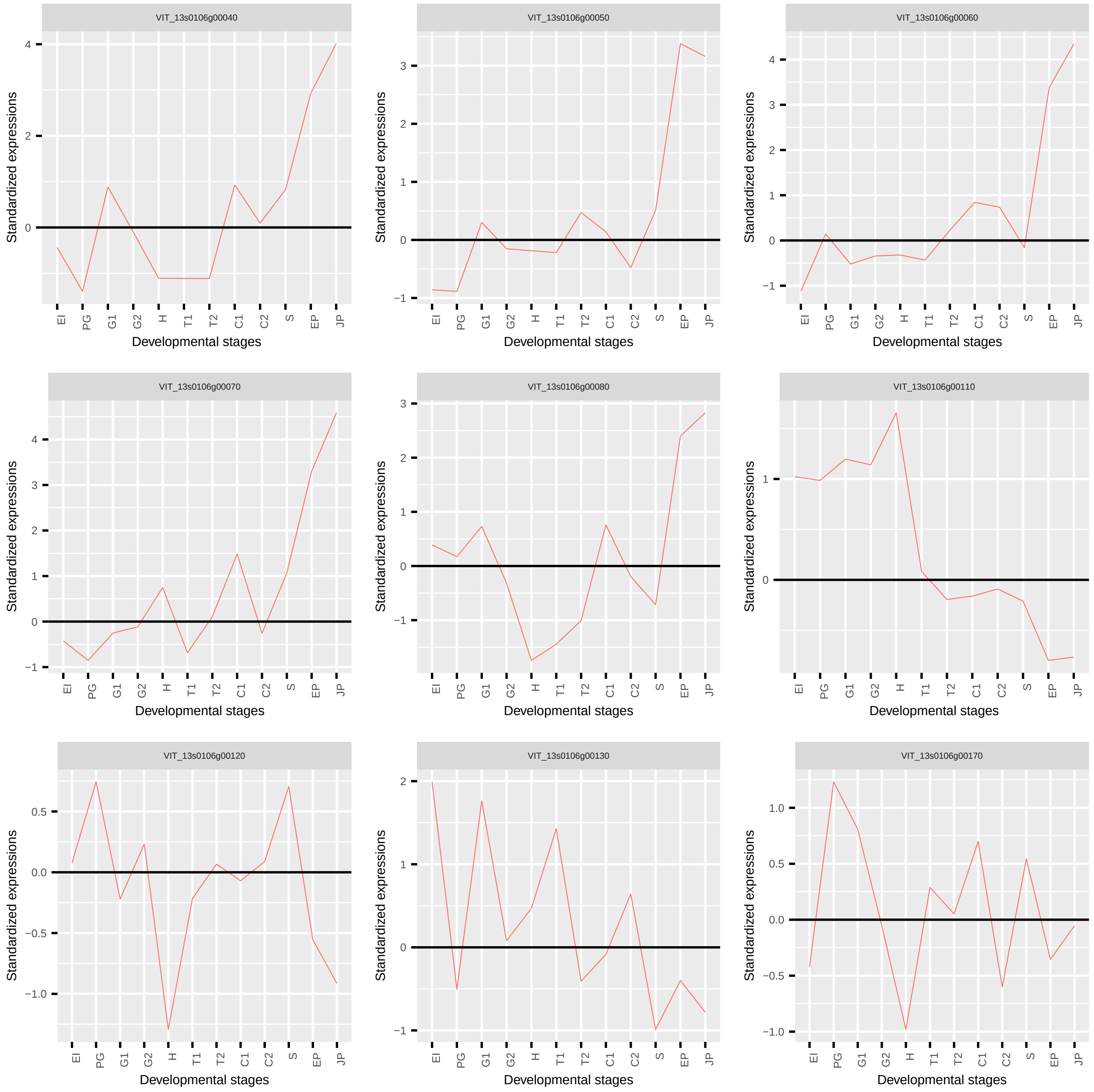
VIT_13s0106g00040
VIT_13s0106g00050
VIT_13s0106g00060
VIT_13s0106g00070
VIT_13s0106g00080
VIT_13s0106g00110
VIT_13s0106g00120
VIT_13s0106g00130
VIT_13s0106g00170
Standardized expressions
Developmental stages
EI PG G1 G2 H T1 T2 C1 C2 S EP JP

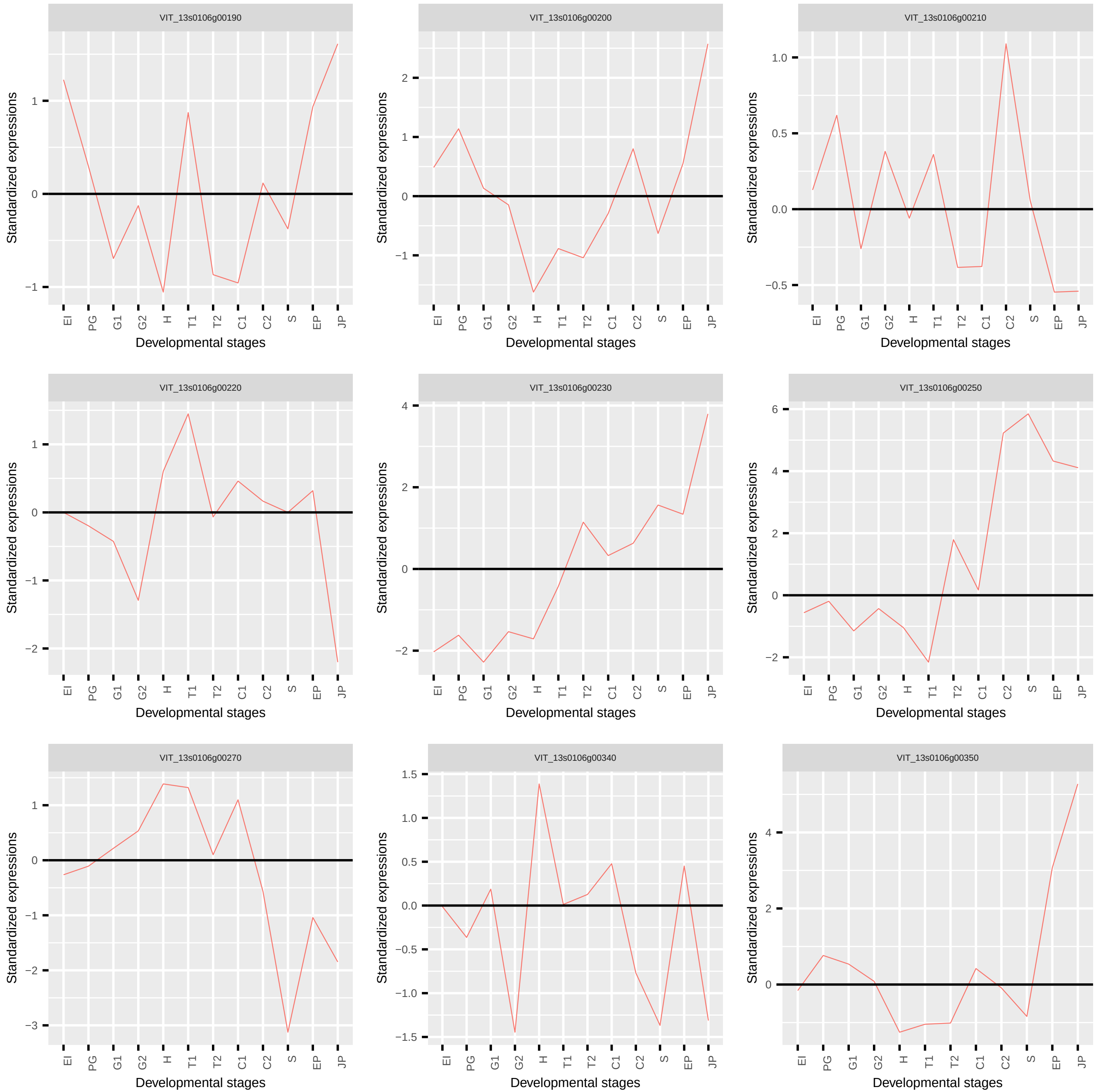
VIT_13s0106g00190
VIT_13s0106g00200
VIT_13s0106g00210
VIT_13s0106g00220
VIT_13s0106g00230
VIT_13s0106g00250
VIT_13s0106g00270
VIT_13s0106g00340
VIT_13s0106g00350
Standardized expressions
Developmental stages
EI PG G1 G2 H T1 T2 C1 C2 S EP JP

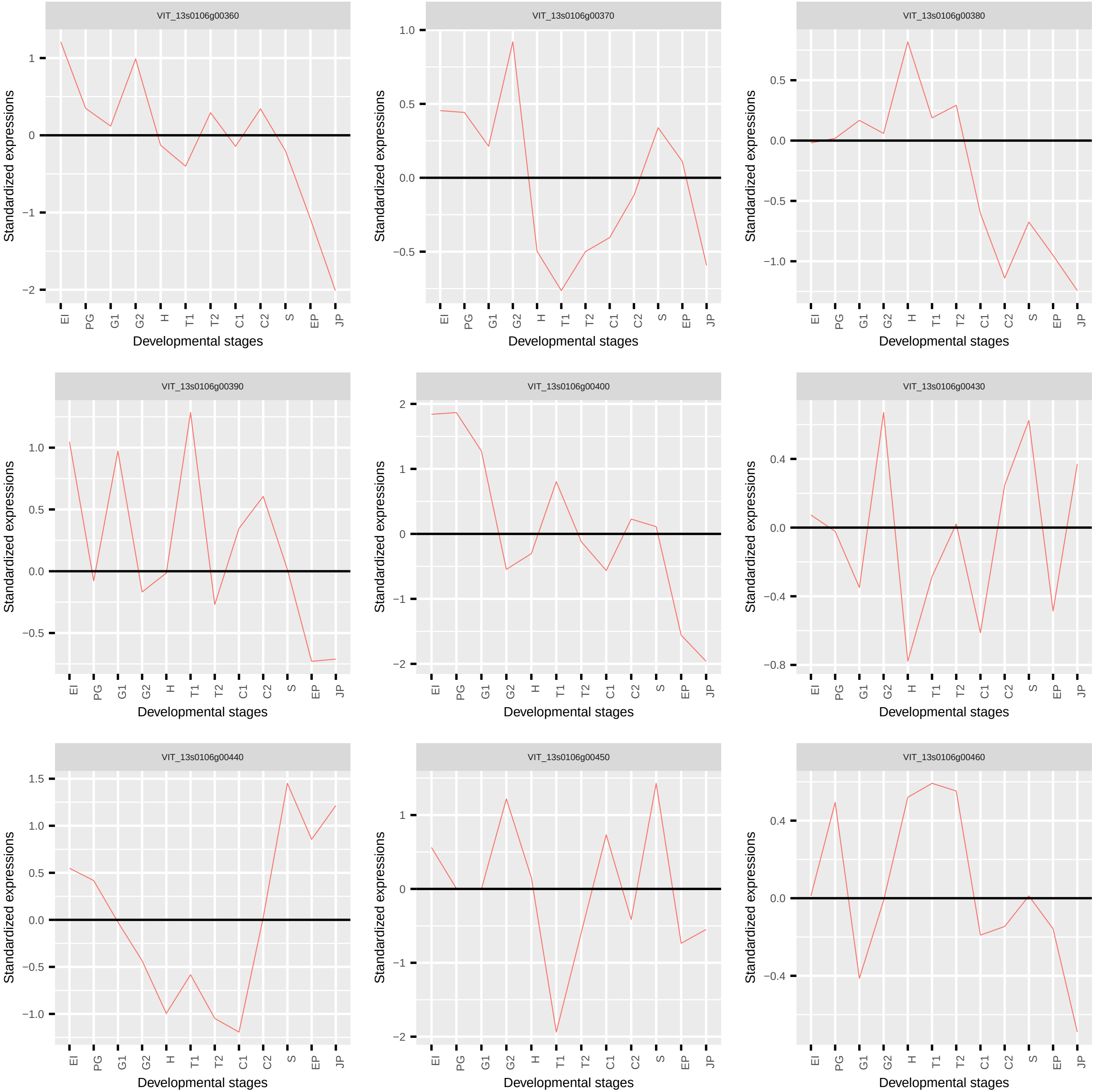
VIT_13s0106g00360
VIT_13s0106g00370
VIT_13s0106g00380
VIT_13s0106g00390
VIT_13s0106g00400
VIT_13s0106g00430
VIT_13s0106g00440
VIT_13s0106g00450
VIT_13s0106g00460
Standardized expressions
Developmental stages
EI PG G1 G2 H T1 T2 C1 C2 S EP JP

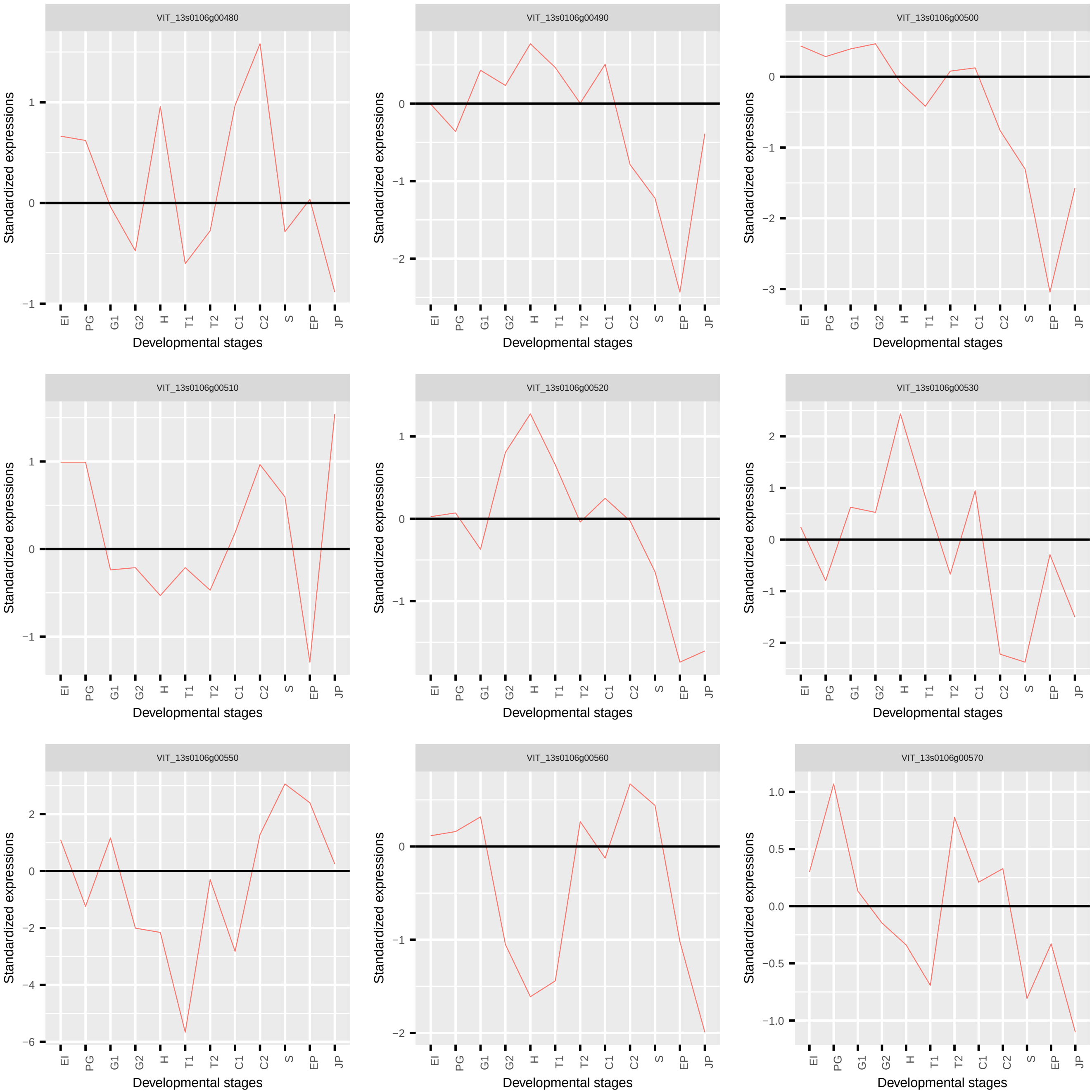
VIT_13s0106g00480
VIT_13s0106g00490
VIT_13s0106g00500
VIT_13s0106g00510
VIT_13s0106g00520
VIT_13s0106g00530
VIT_13s0106g00550
VIT_13s0106g00560
VIT_13s0106g00570
Standardized expressions
Developmental stages
EI PG G1 G2 H T1 T2 C1 C2 S EP JP

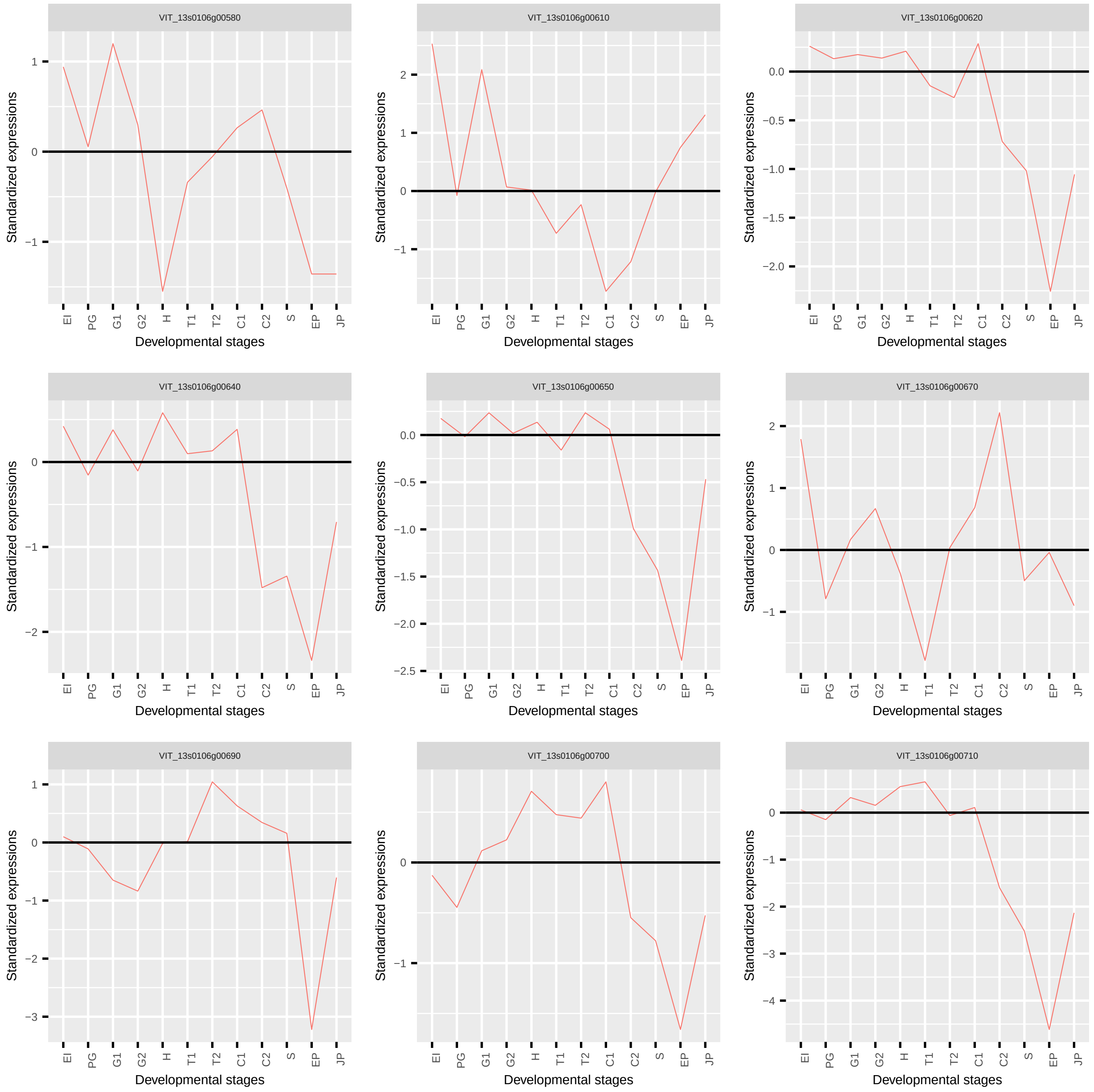
VIT_13s0106g00580
VIT_13s0106g00610
VIT_13s0106g00620
VIT_13s0106g00640
VIT_13s0106g00650
VIT_13s0106g00670
VIT_13s0106g00690
VIT_13s0106g00700
VIT_13s0106g00710
Standardized expressions
Developmental stages
EI
PG
G1
G2
H
T1
T2
C1
C2
S
EP
JP

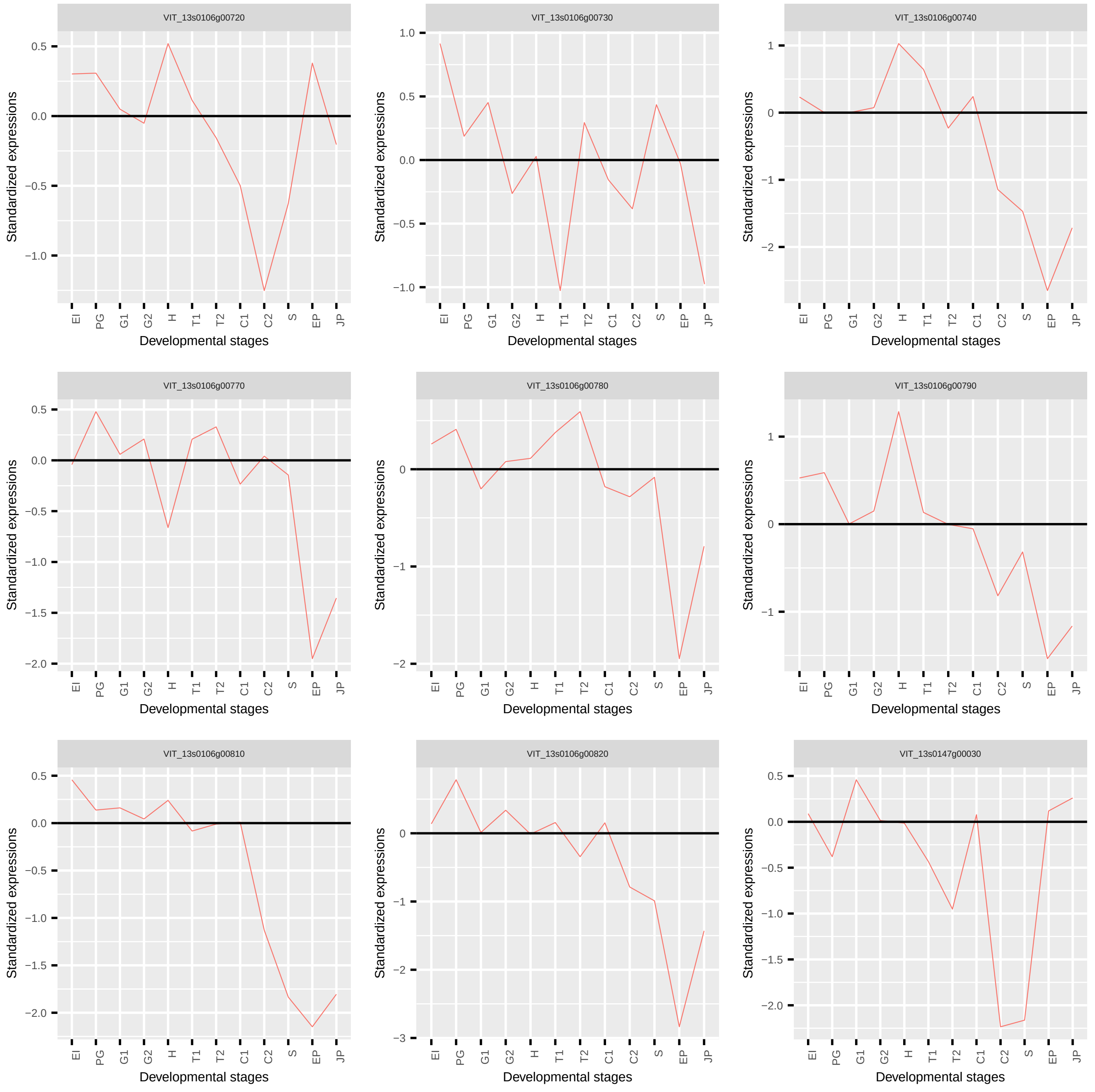

VIT_13s0106g00720
VIT_13s0106g00730
VIT_13s0106g00740
VIT_13s0106g00770
VIT_13s0106g00780
VIT_13s0106g00790
VIT_13s0106g00810
VIT_13s0106g00820
VIT_13s0147g00030
Standardized expressions
Developmental stages
EI
PG
G1
G2
H
T1
T2
C1
C2
S
EP
JP

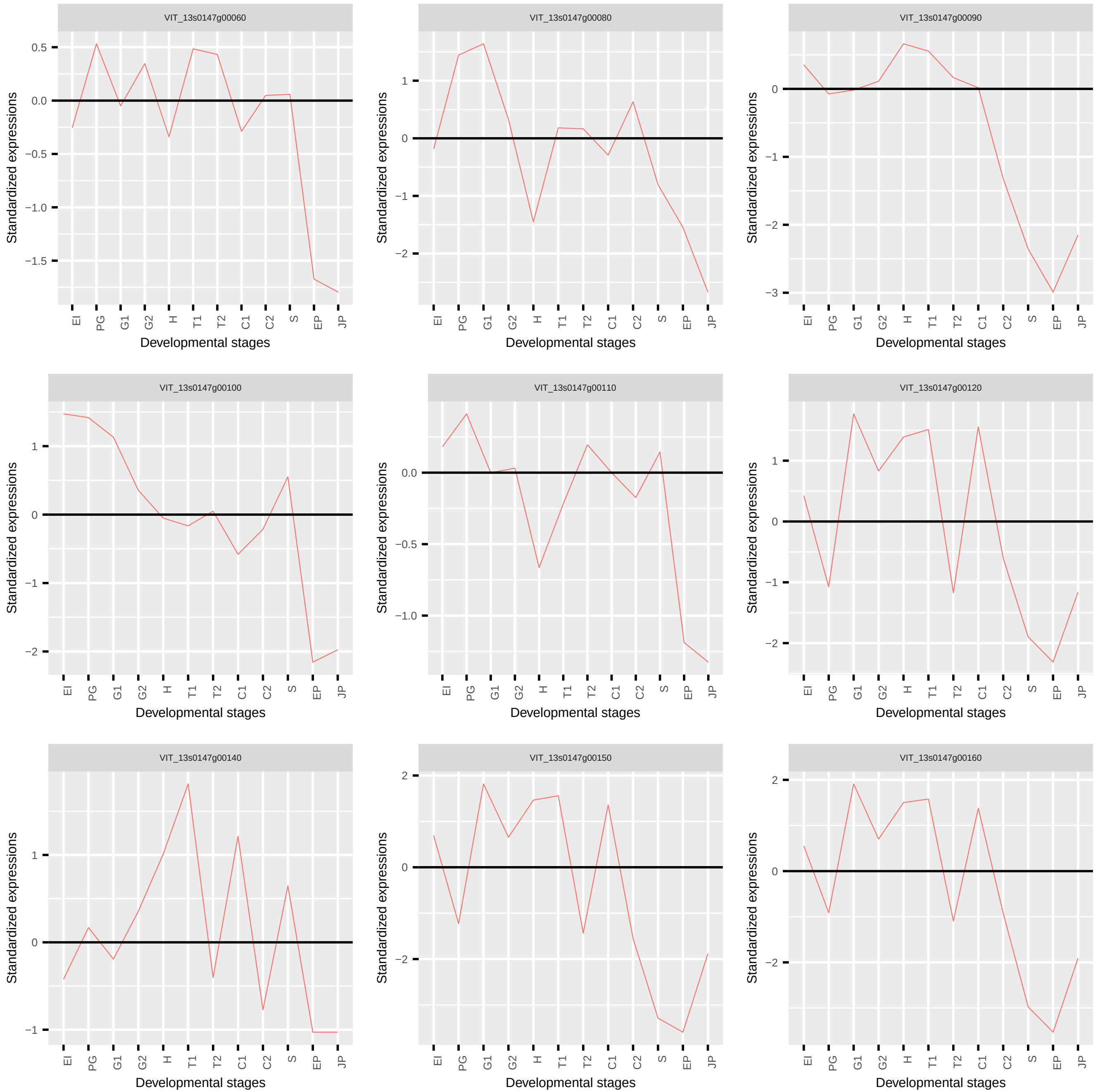

VIT_13s0147g00060
VIT_13s0147g00080
VIT_13s0147g00090
VIT_13s0147g00100
VIT_13s0147g00110
VIT_13s0147g00120
VIT_13s0147g00140
VIT_13s0147g00150
VIT_13s0147g00160
Standardized expressions
Developmental stages
EI
PG
G1
G2
H
T1
T2
C1
C2
S
EP
JP

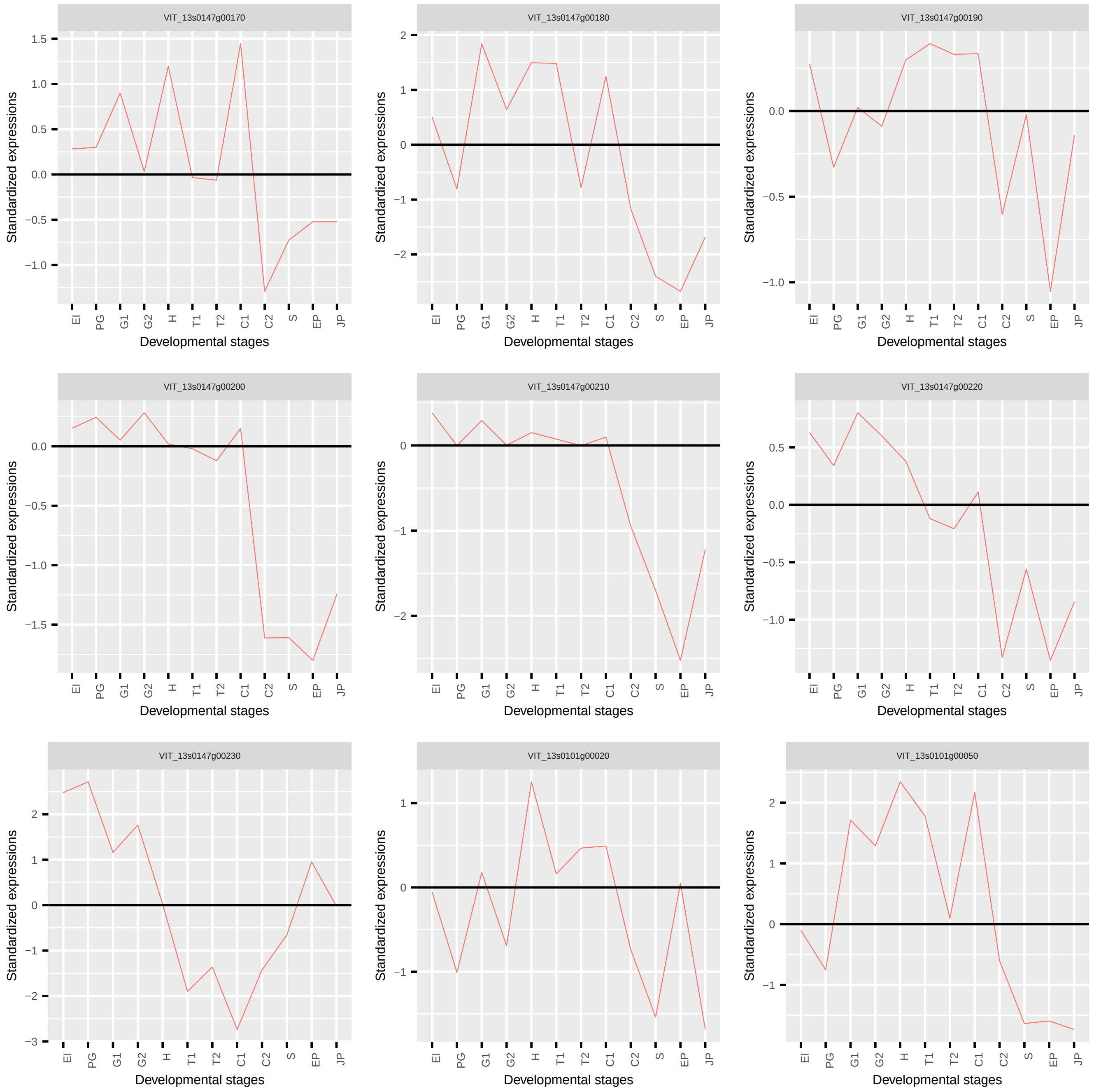

VIT_13s0147g00170
VIT_13s0147g00180
VIT_13s0147g00190
VIT_13s0147g00200
VIT_13s0147g00210
VIT_13s0147g00220
VIT_13s0147g00230
VIT_13s0101g00020
VIT_13s0101g00050
Standardized expressions
Developmental stages
EI
PG
G1
G2
H
T1
T2
C1
C2
S
EP
JP

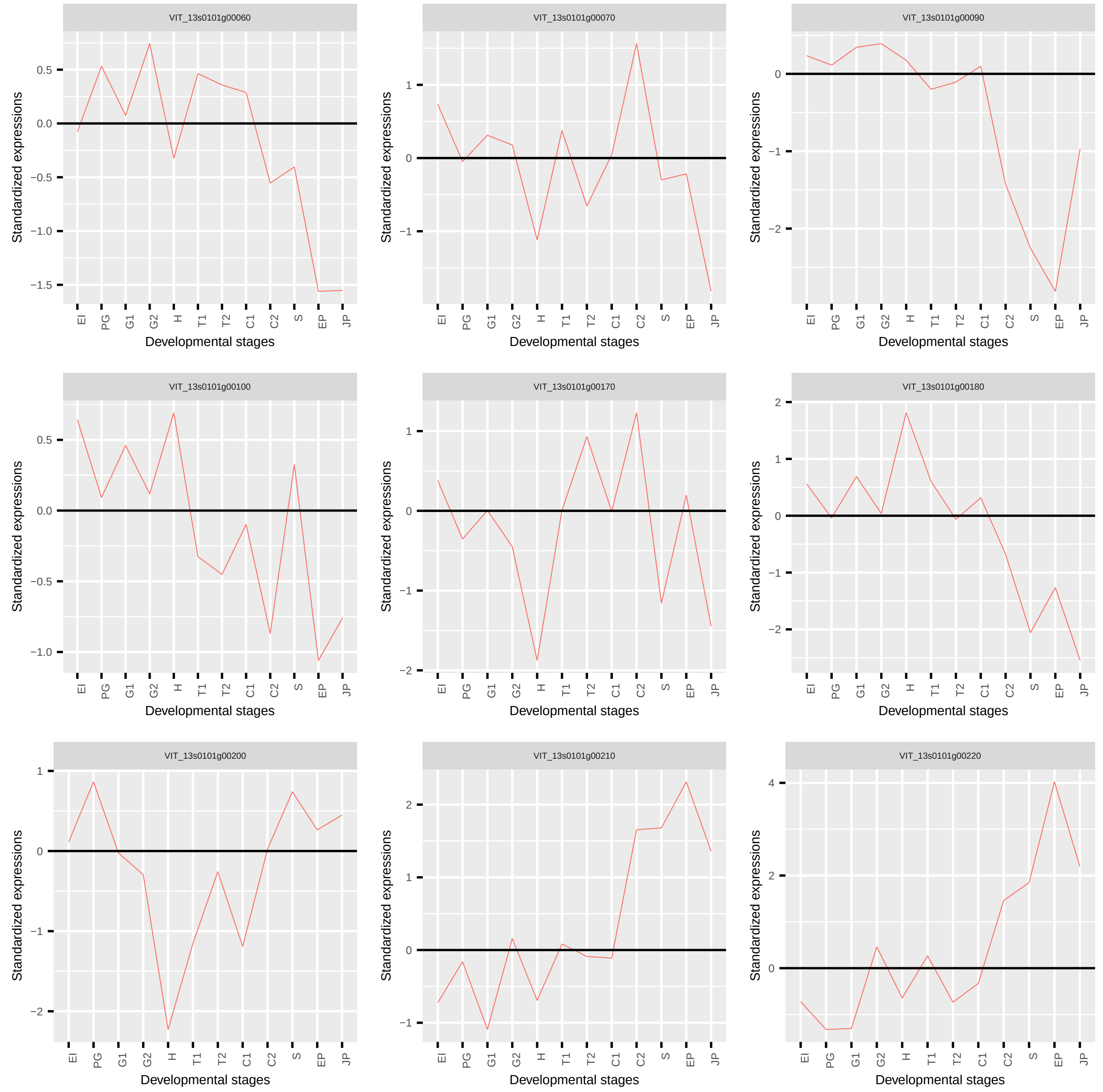
VIT_13s0101g00060
VIT_13s0101g00070
VIT_13s0101g00090
VIT_13s0101g00100
VIT_13s0101g00170
VIT_13s0101g00180
VIT_13s0101g00200
VIT_13s0101g00210
VIT_13s0101g00220
Standardized expressions
Developmental stages
EI PG G1 G2 H T1 T2 C1 C2 S EP JP

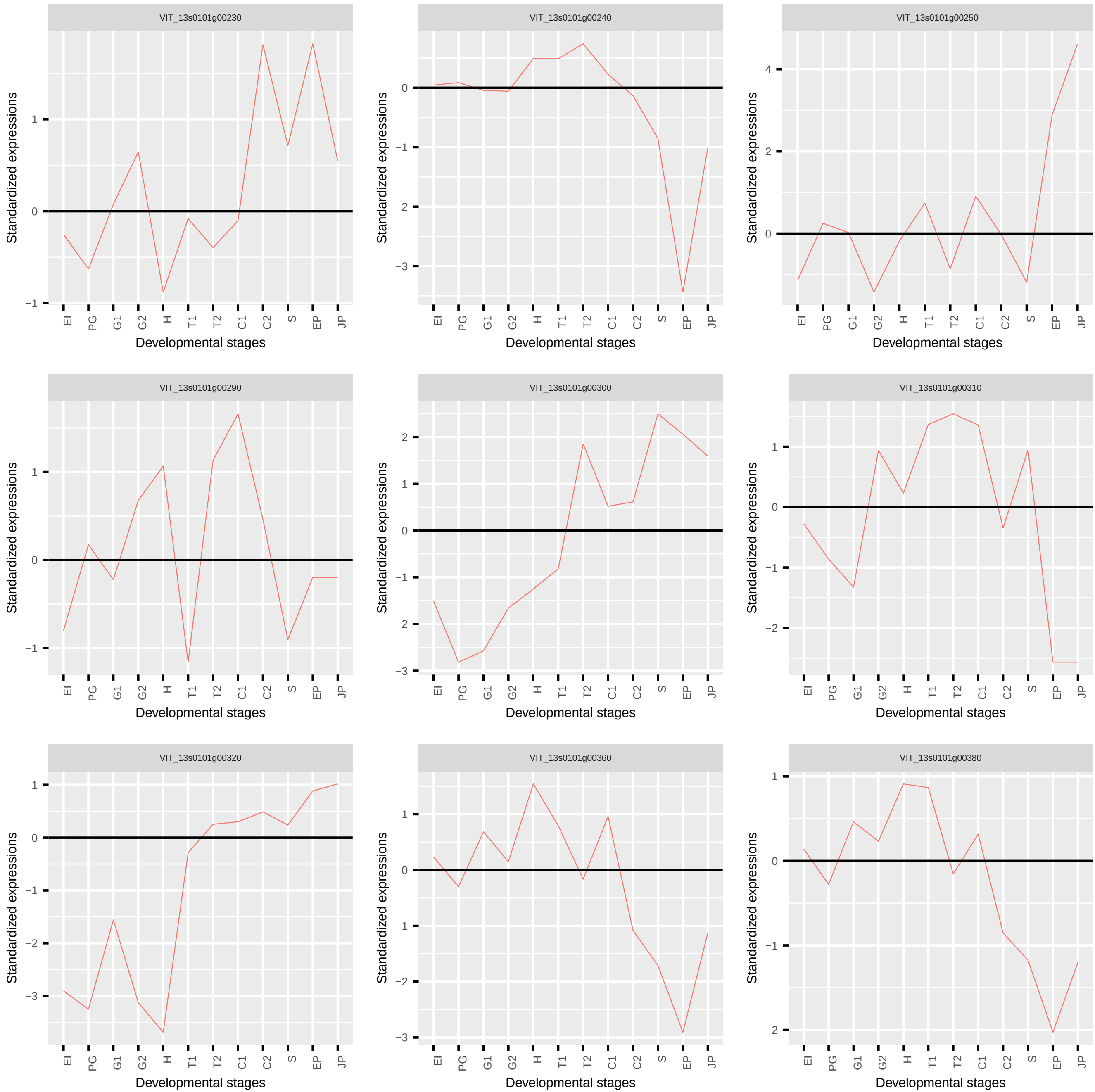
VIT_13s0101g00230
VIT_13s0101g00240
VIT_13s0101g00250
VIT_13s0101g00290
VIT_13s0101g00300
VIT_13s0101g00310
VIT_13s0101g00320
VIT_13s0101g00360
VIT_13s0101g00380
Standardized expressions
Developmental stages
EI PG G1 G2 H T1 T2 C1 C2 S EP JP

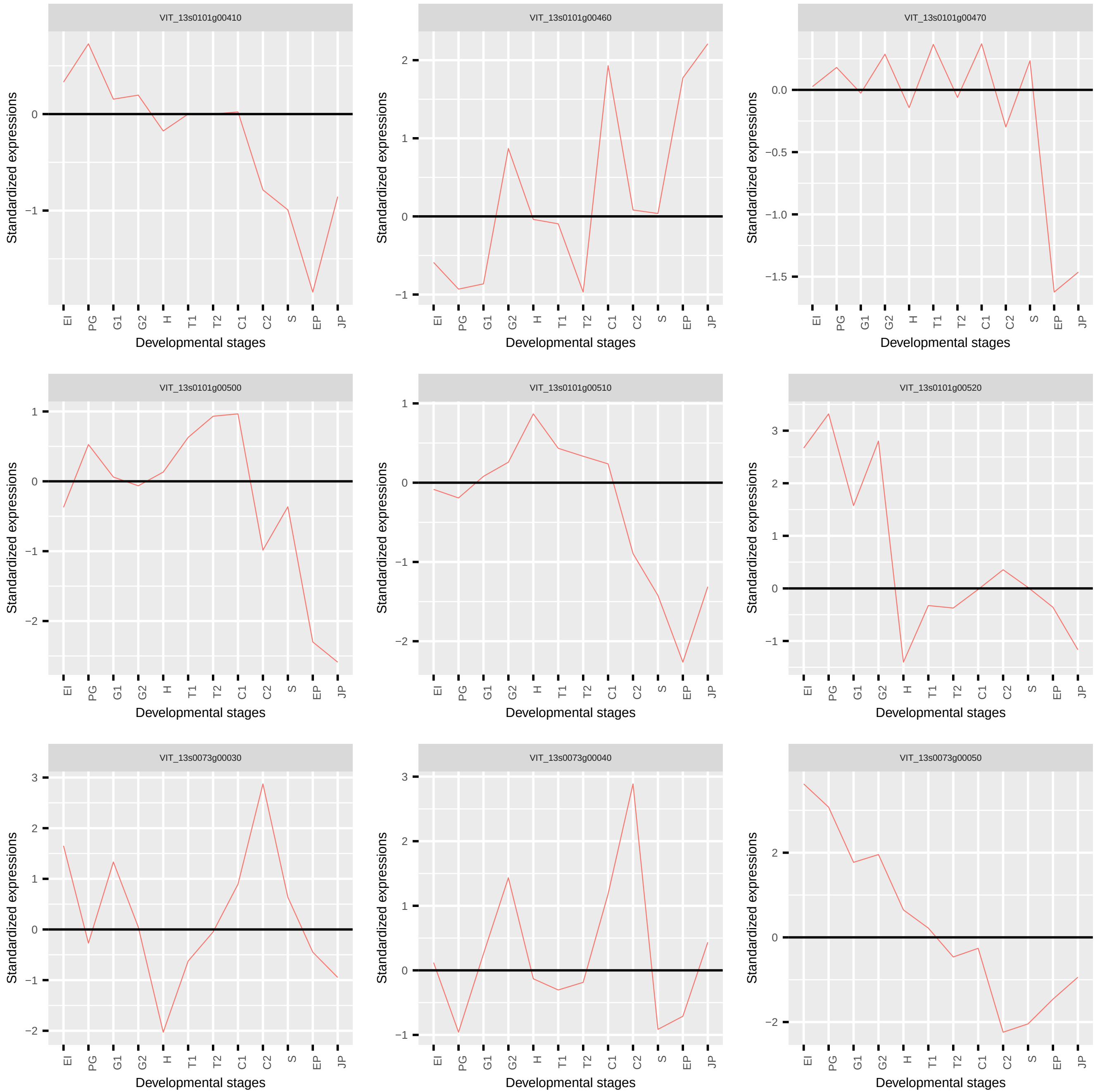
VIT_13s0101g00410
VIT_13s0101g00460
VIT_13s0101g00470
VIT_13s0101g00500
VIT_13s0101g00510
VIT_13s0101g00520
VIT_13s0073g00030
VIT_13s0073g00040
VIT_13s0073g00050
Standardized expressions
Developmental stages
EI PG G1 G2 H T1 T2 C1 C2 S EP JP

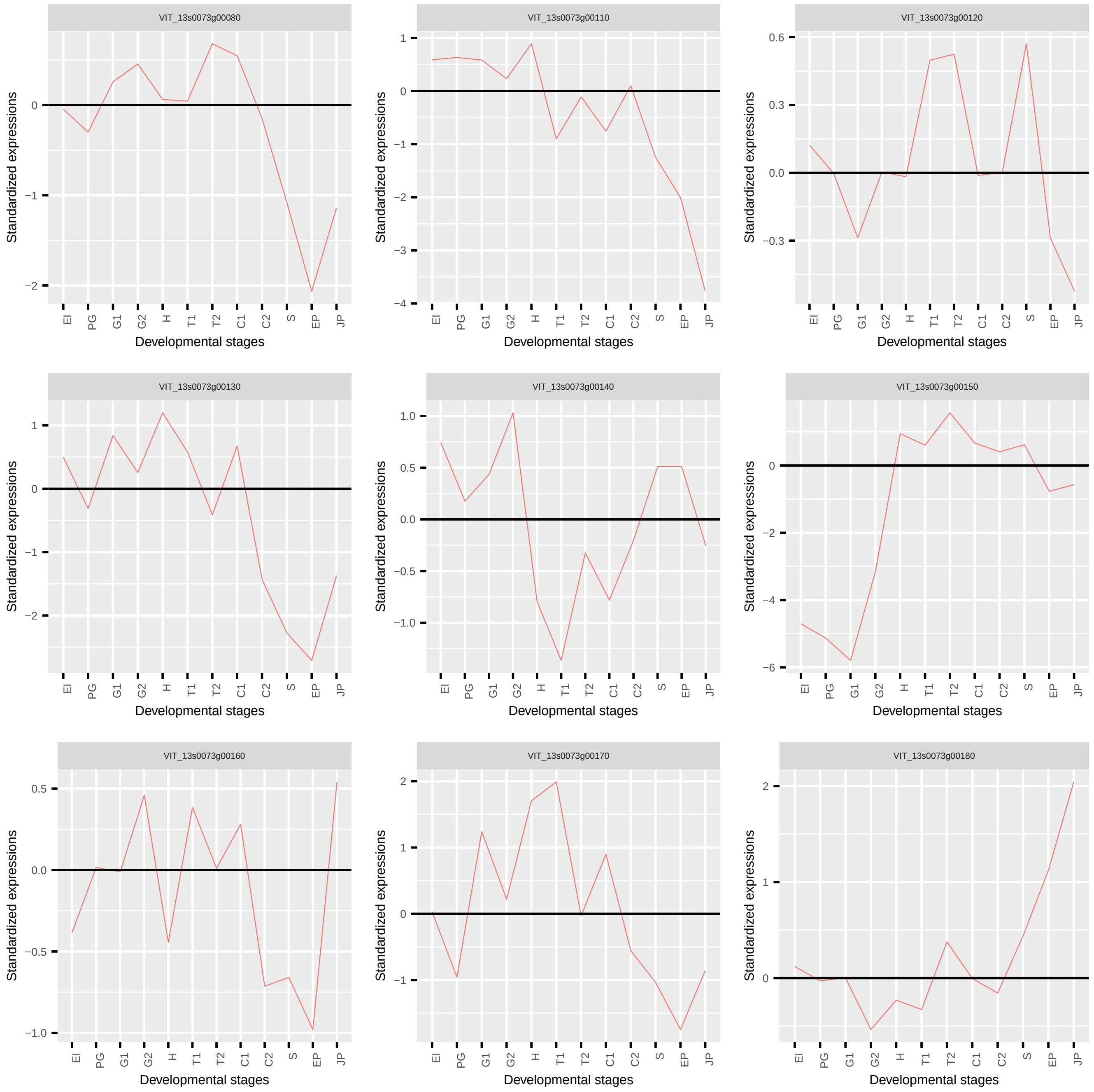
VIT_13s0073g00080
VIT_13s0073g00110
VIT_13s0073g00120
VIT_13s0073g00130
VIT_13s0073g00140
VIT_13s0073g00150
VIT_13s0073g00160
VIT_13s0073g00170
VIT_13s0073g00180
Standardized expressions
Developmental stages
EI
PG
G1
G2
H
T1
T2
C1
C2
S
EP
JP
0
−1
−2
1
−3
−4
0.6
0.3
0.0
−0.3
1.0
0.5
−0.5
−1.0
−6
−4
2

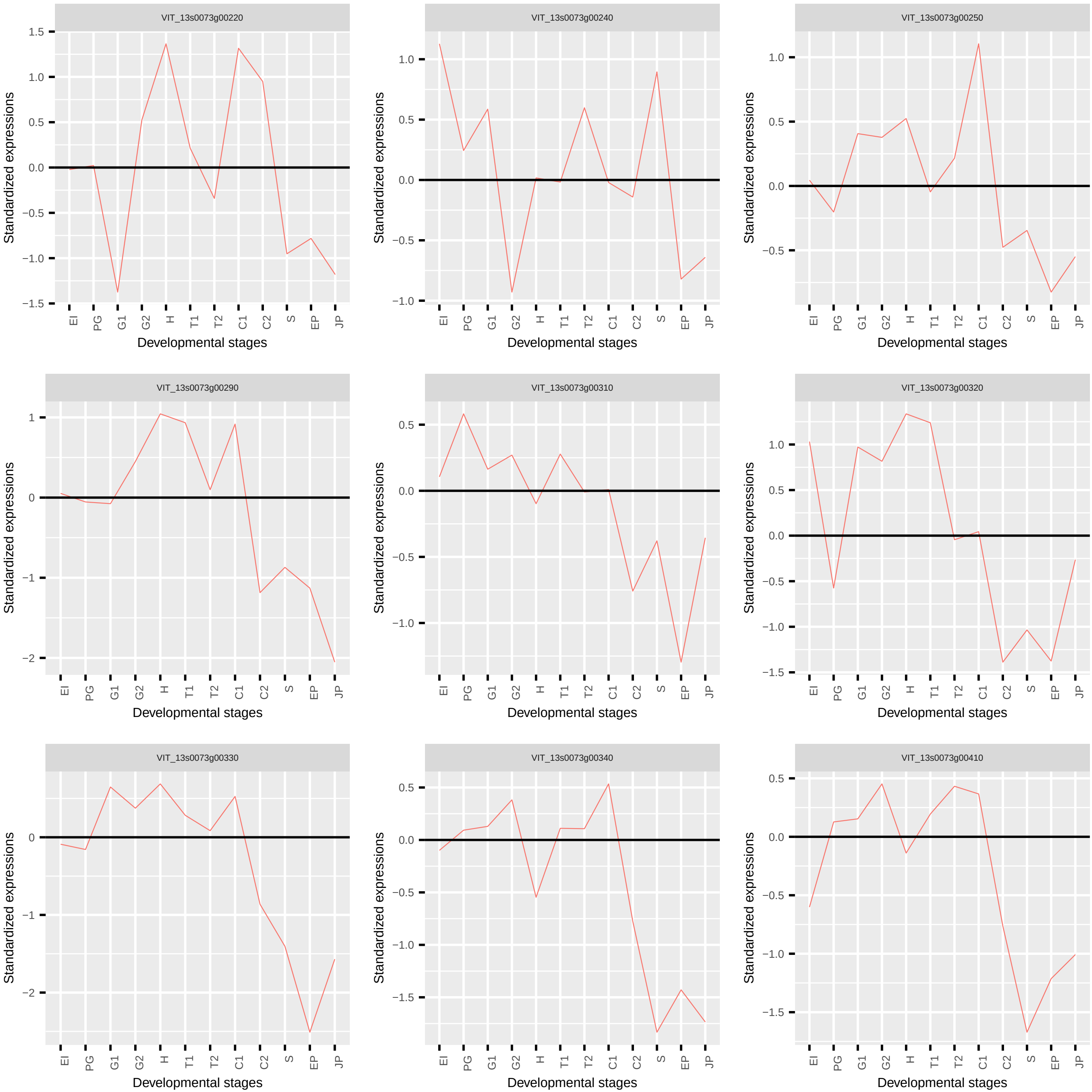
VIT_13s0073g00220
VIT_13s0073g00240
VIT_13s0073g00250
VIT_13s0073g00290
VIT_13s0073g00310
VIT_13s0073g00320
VIT_13s0073g00330
VIT_13s0073g00340
VIT_13s0073g00410
Standardized expressions
Developmental stages
EI
PG
G1
G2
H
T1
T2
C1
C2
S
EP
JP

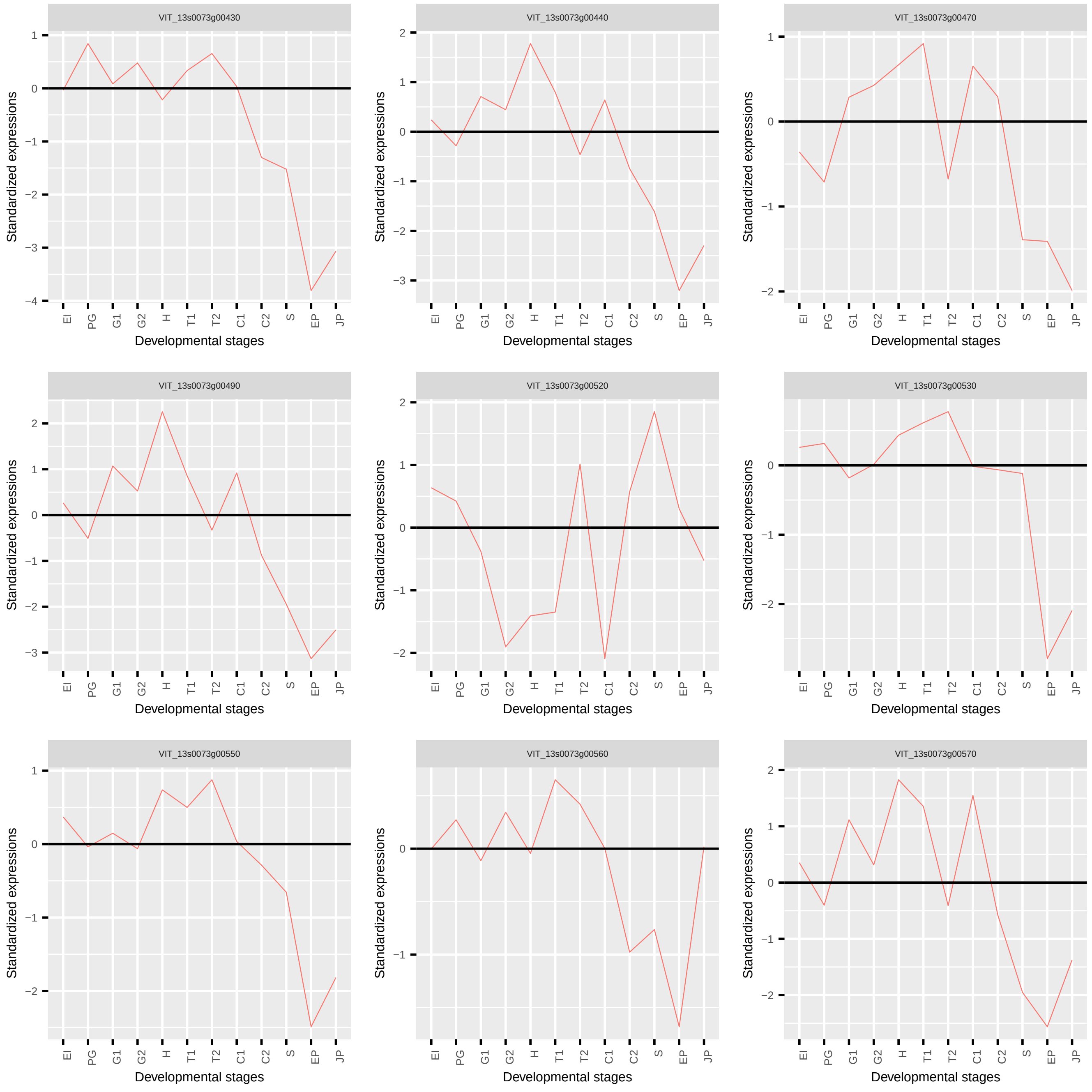
VIT_13s0073g00430
VIT_13s0073g00440
VIT_13s0073g00470
VIT_13s0073g00490
VIT_13s0073g00520
VIT_13s0073g00530
VIT_13s0073g00550
VIT_13s0073g00560
VIT_13s0073g00570
Standardized expressions
Developmental stages
EI PG G1 G2 H T1 T2 C1 C2 S EP JP

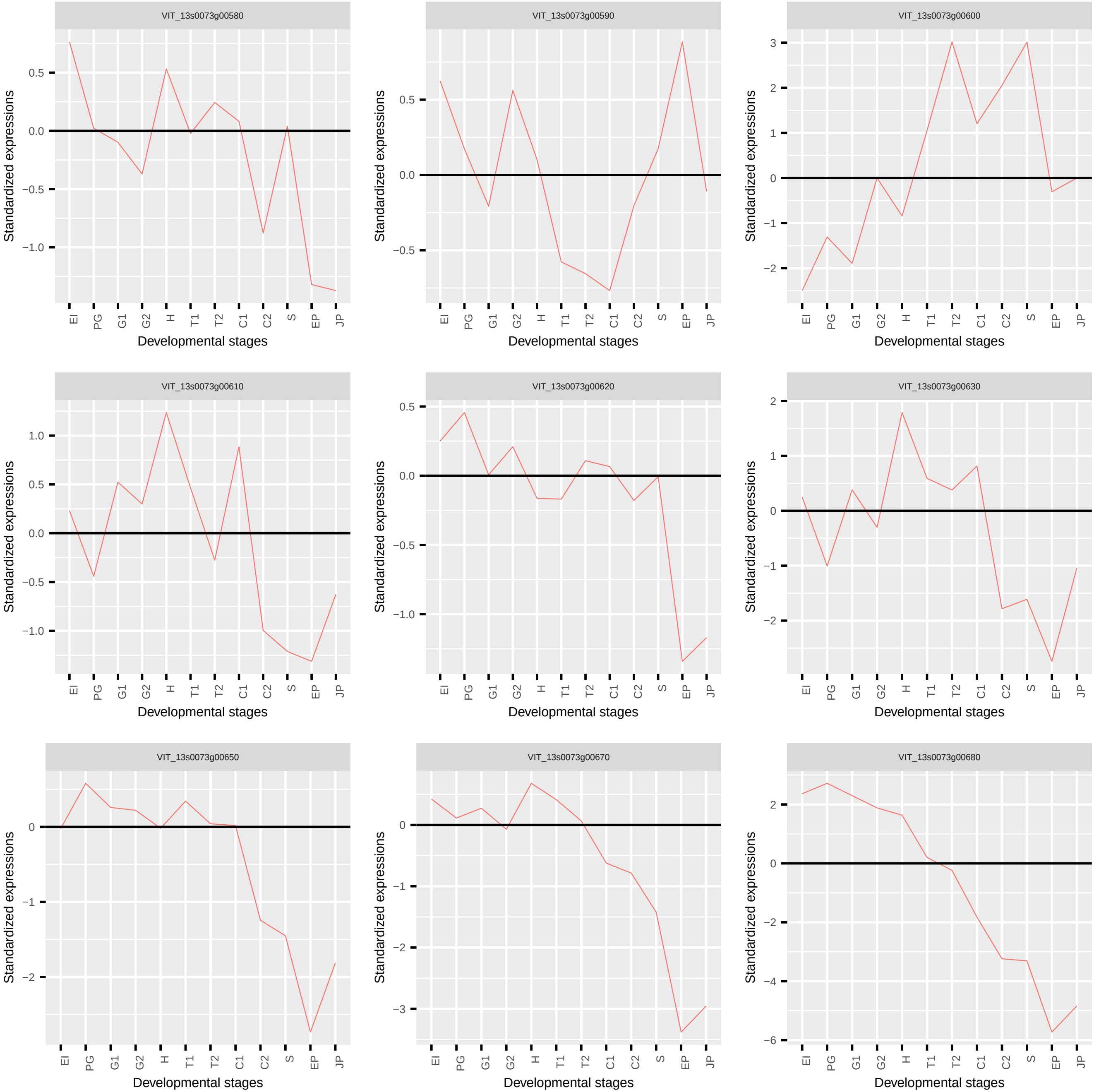
VIT_13s0073g00580
VIT_13s0073g00590
VIT_13s0073g00600
VIT_13s0073g00610
VIT_13s0073g00620
VIT_13s0073g00630
VIT_13s0073g00650
VIT_13s0073g00670
VIT_13s0073g00680
Standardized expressions
Developmental stages
EI PG G1 G2 H T1 T2 C1 C2 S EP JP

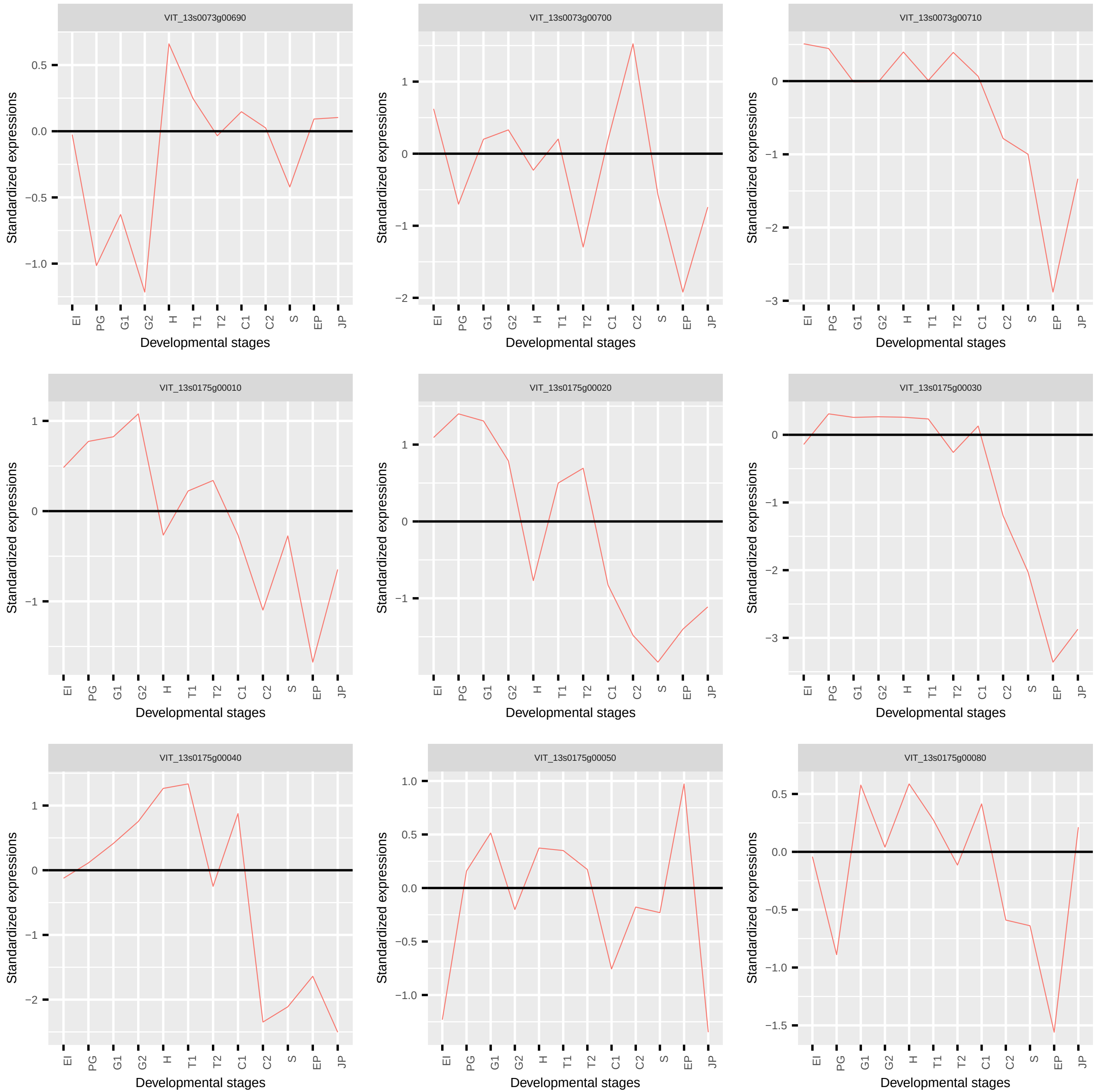
VIT_13s0073g00690
VIT_13s0073g00700
VIT_13s0073g00710
VIT_13s0175g00010
VIT_13s0175g00020
VIT_13s0175g00030
VIT_13s0175g00040
VIT_13s0175g00050
VIT_13s0175g00080
Standardized expressions
Developmental stages
EI PG G1 G2 H T1 T2 C1 C2 S EP JP
0.5 0.0 −0.5 −1.0
1 0 −1 −2
0 −1 −2 −3
1 0 −1
1 0 −1
0 −1 −2 −3
1 0 −1 −2
1.0 0.5 0.0 −0.5 −1.0
0.5 0.0 −0.5 −1.0 −1.5

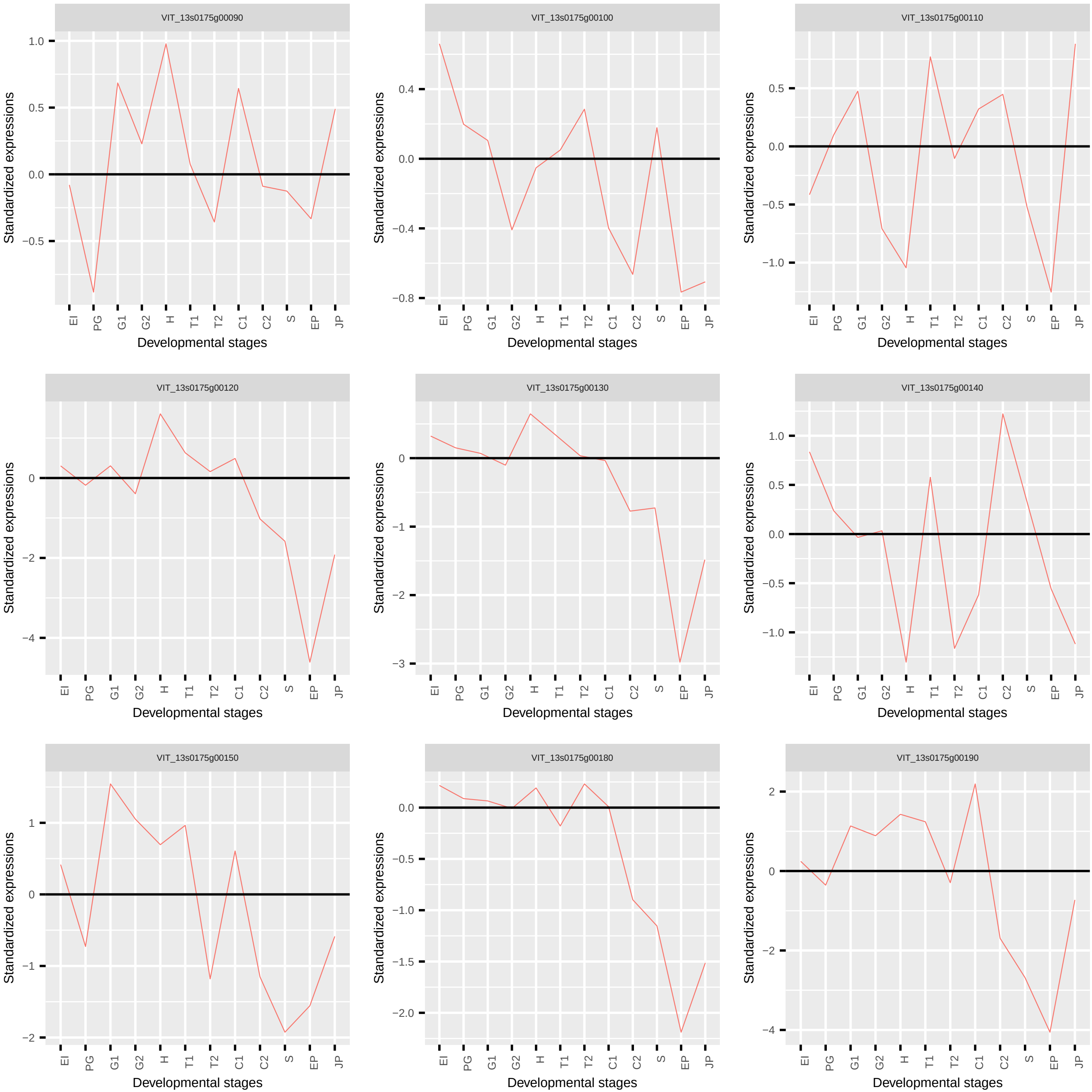
VIT_13s0175g00090
VIT_13s0175g00100
VIT_13s0175g00110
VIT_13s0175g00120
VIT_13s0175g00130
VIT_13s0175g00140
VIT_13s0175g00150
VIT_13s0175g00180
VIT_13s0175g00190
Standardized expressions
Developmental stages
EI PG G1 G2 H T1 T2 C1 C2 S EP JP

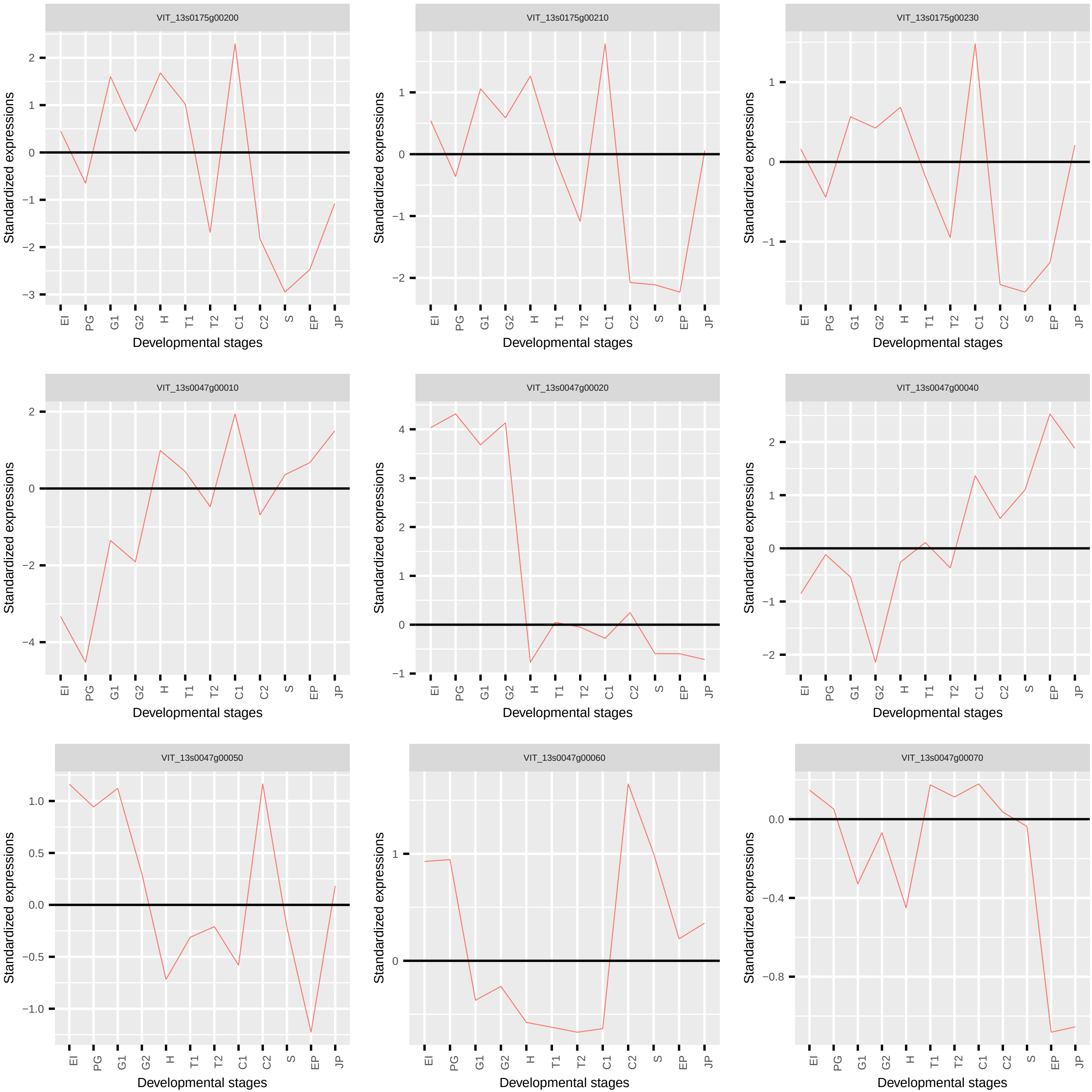
VIT_13s0175g00200
VIT_13s0175g00210
VIT_13s0175g00230
VIT_13s0047g00010
VIT_13s0047g00020
VIT_13s0047g00040
VIT_13s0047g00050
VIT_13s0047g00060
VIT_13s0047g00070
Standardized expressions
Developmental stages
EI PG G1 G2 H T1 T2 C1 C2 S EP JP

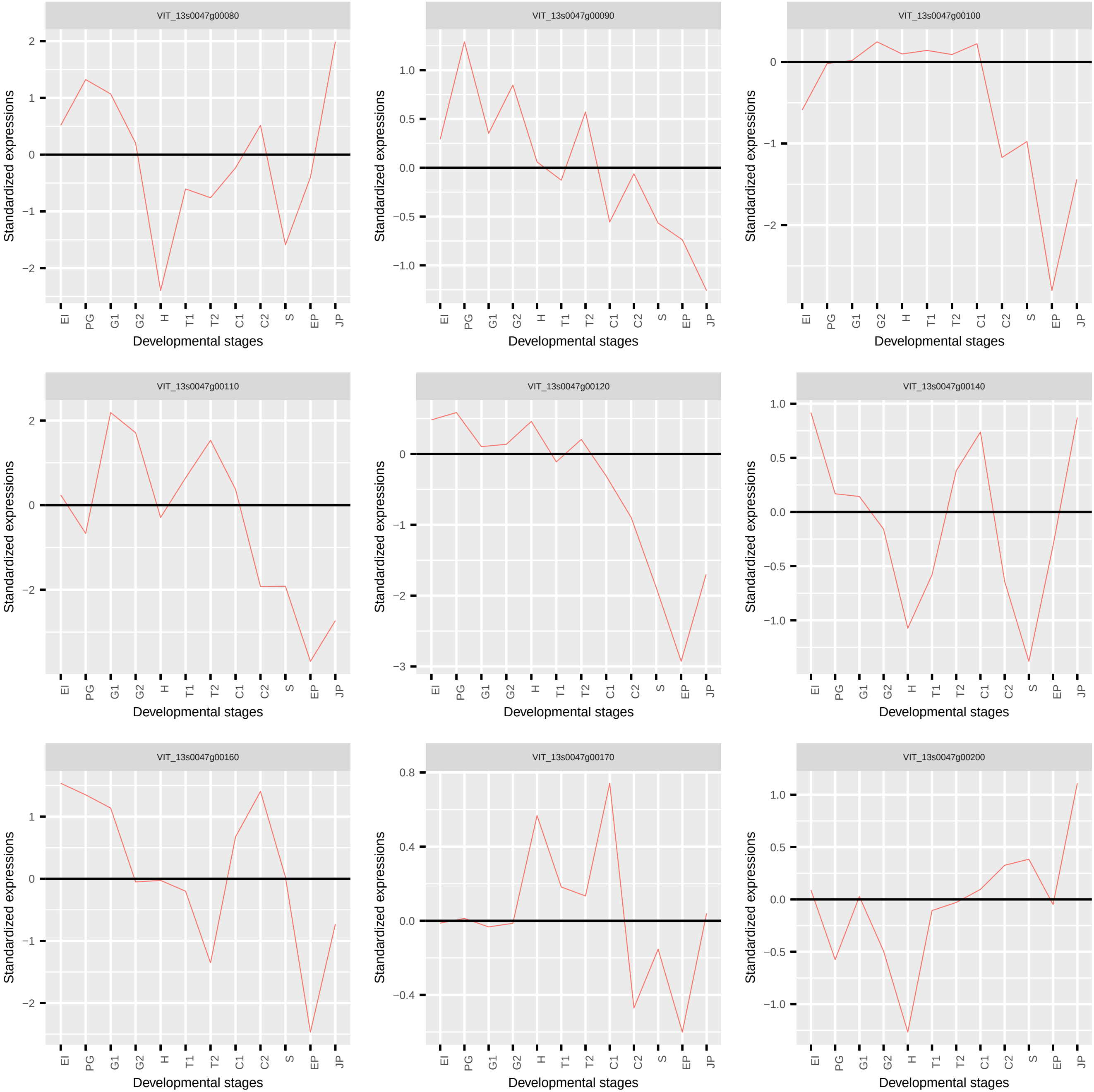
VIT_13s0047g00080
VIT_13s0047g00090
VIT_13s0047g00100
VIT_13s0047g00110
VIT_13s0047g00120
VIT_13s0047g00140
VIT_13s0047g00160
VIT_13s0047g00170
VIT_13s0047g00200
Standardized expressions
Developmental stages
EI
PG
G1
G2
H
T1
T2
C1
C2
S
EP
JP

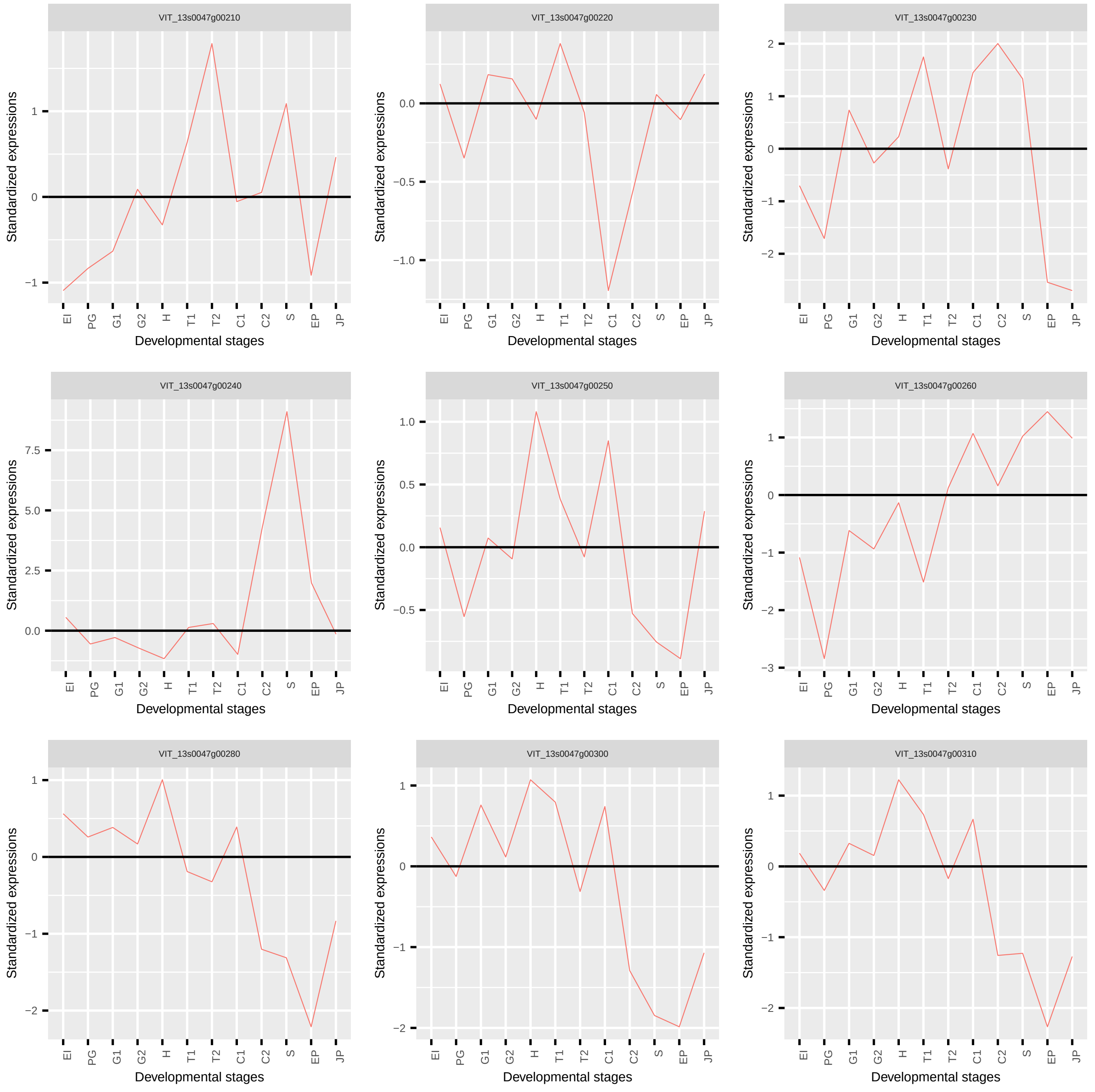

VIT_13s0047g00210
VIT_13s0047g00220
VIT_13s0047g00230
VIT_13s0047g00240
VIT_13s0047g00250
VIT_13s0047g00260
VIT_13s0047g00280
VIT_13s0047g00300
VIT_13s0047g00310
Standardized expressions
Developmental stages
EI
PG
G1
G2
H
T1
T2
C1
C2
S
EP
JP

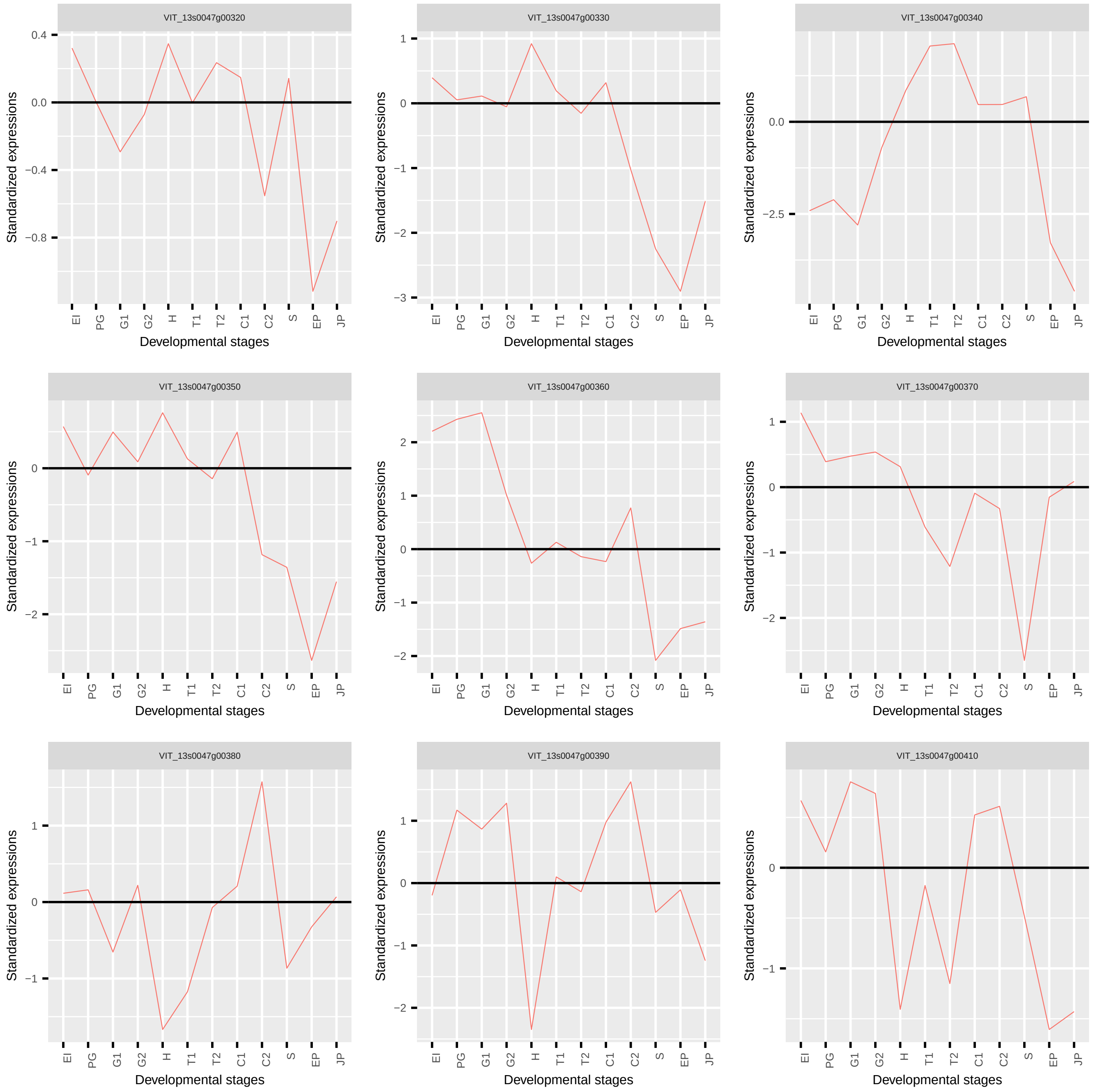
VIT_13s0047g00320
VIT_13s0047g00330
VIT_13s0047g00340
VIT_13s0047g00350
VIT_13s0047g00360
VIT_13s0047g00370
VIT_13s0047g00380
VIT_13s0047g00390
VIT_13s0047g00410
Standardized expressions
Developmental stages
EI PG G1 G2 H T1 T2 C1 C2 S EP JP
0.4
0.0
−0.4
−0.8
1
0
−1
−2
−3
0.0
−2.5
2

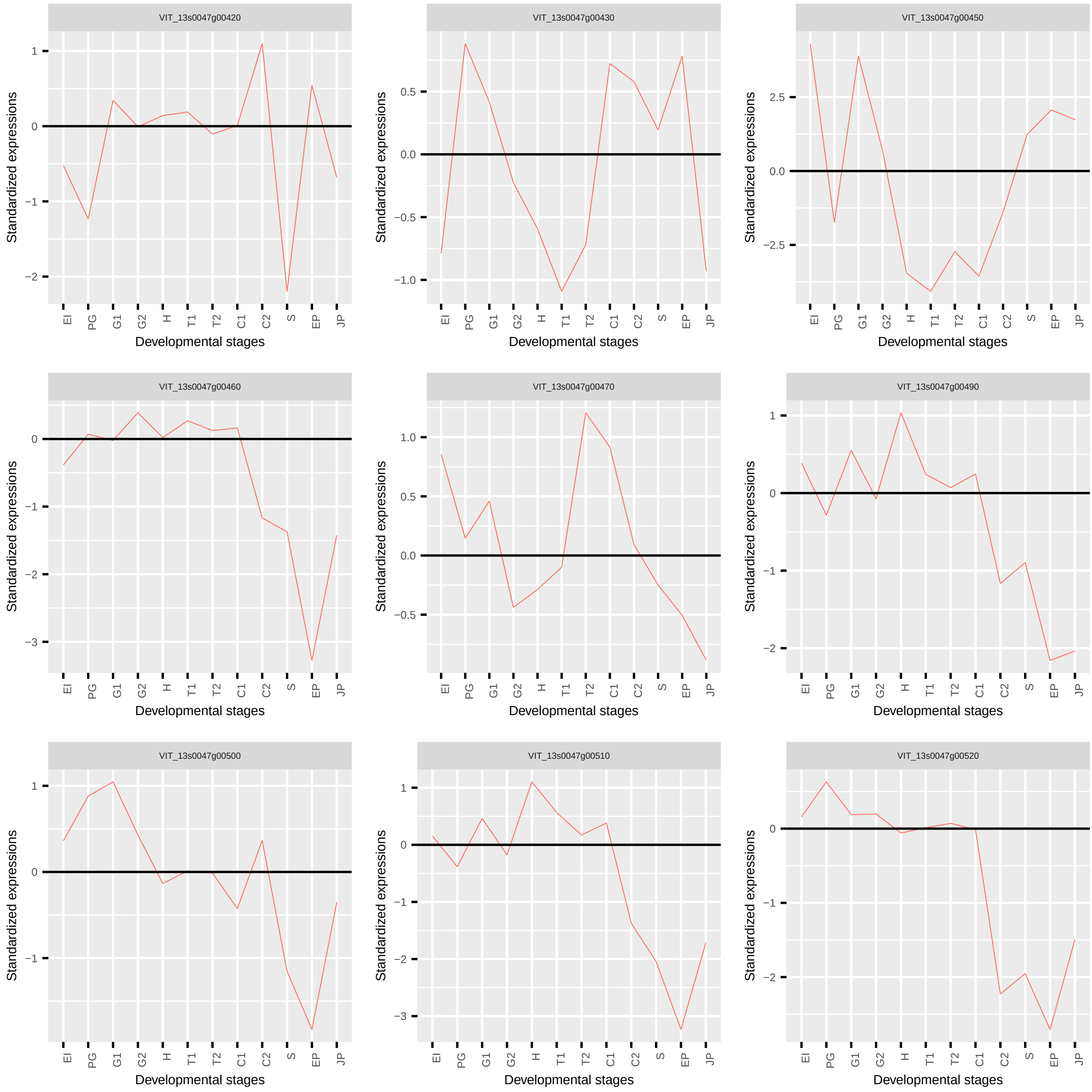
VIT_13s0047g00420
VIT_13s0047g00430
VIT_13s0047g00450
VIT_13s0047g00460
VIT_13s0047g00470
VIT_13s0047g00490
VIT_13s0047g00500
VIT_13s0047g00510
VIT_13s0047g00520
Standardized expressions
Developmental stages
EI PG G1 G2 H T1 T2 C1 C2 S EP JP

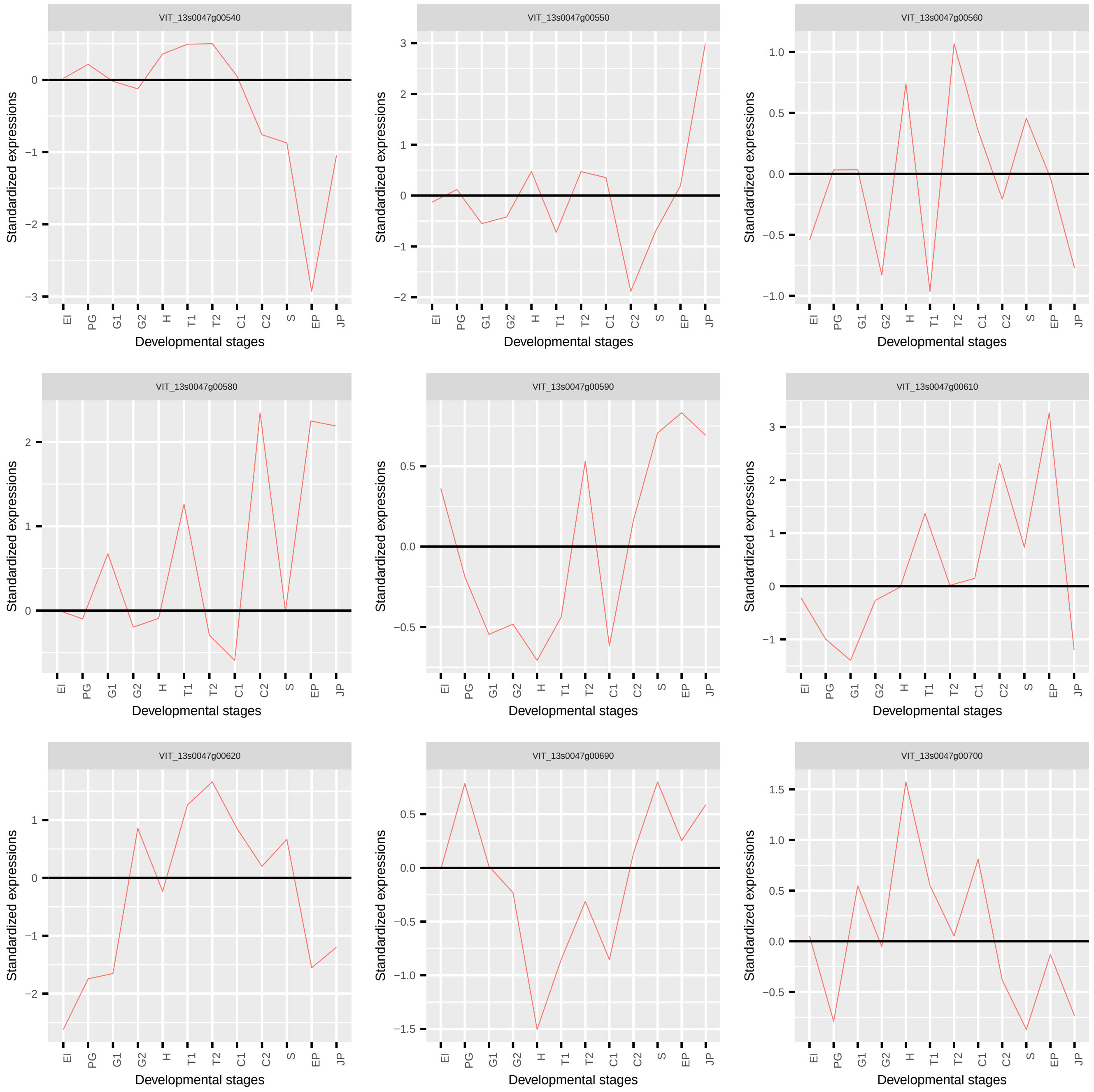
VIT_13s0047g00540
VIT_13s0047g00550
VIT_13s0047g00560
VIT_13s0047g00580
VIT_13s0047g00590
VIT_13s0047g00610
VIT_13s0047g00620
VIT_13s0047g00690
VIT_13s0047g00700
Standardized expressions
Developmental stages
EI PG G1 G2 H T1 T2 C1 C2 S EP JP

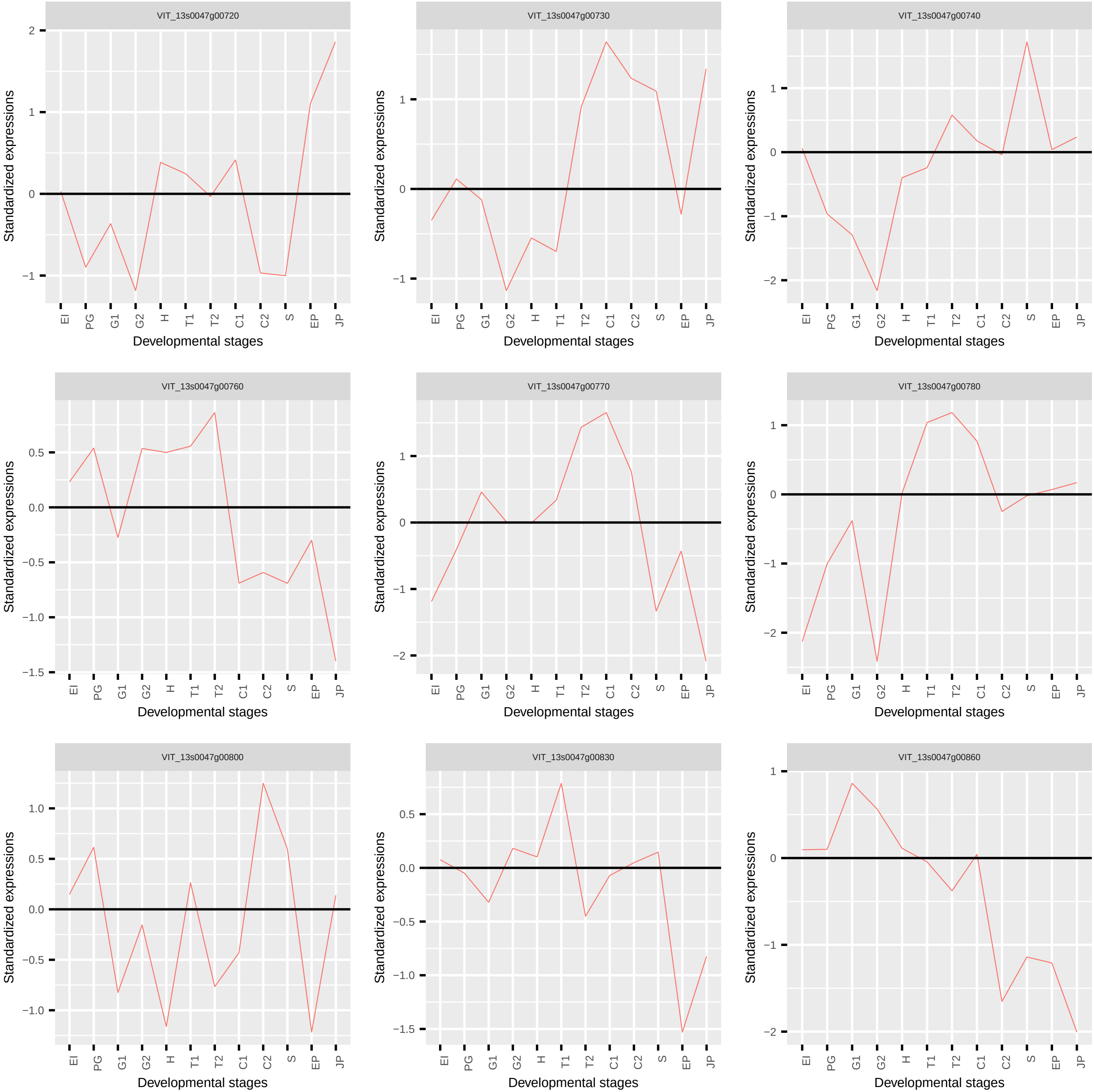
VIT_13s0047g00720
VIT_13s0047g00730
VIT_13s0047g00740
VIT_13s0047g00760
VIT_13s0047g00770
VIT_13s0047g00780
VIT_13s0047g00800
VIT_13s0047g00830
VIT_13s0047g00860
Standardized expressions
Developmental stages
EI PG G1 G2 H T1 T2 C1 C2 S EP JP

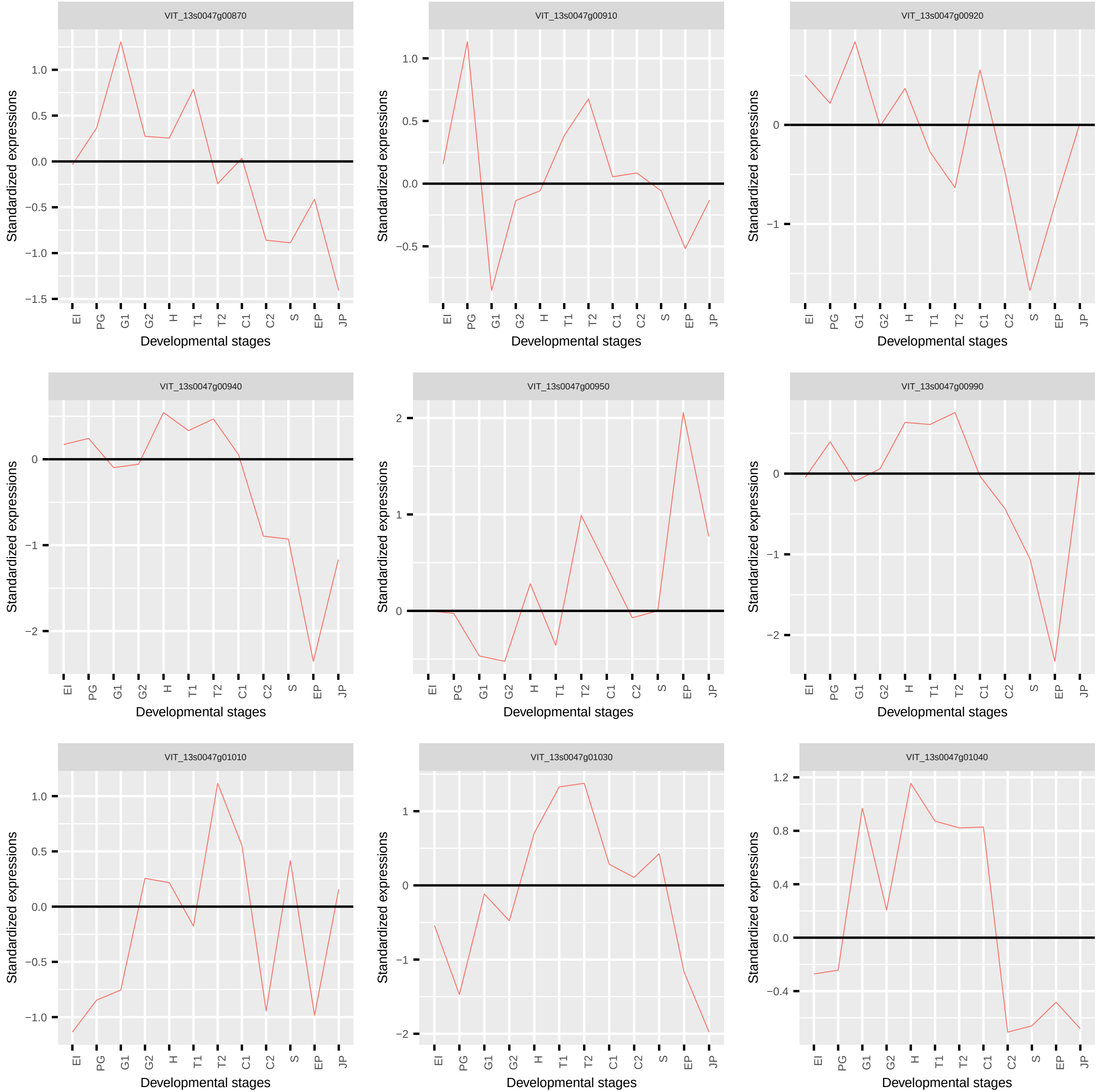
VIT_13s0047g00870
VIT_13s0047g00910
VIT_13s0047g00920
VIT_13s0047g00940
VIT_13s0047g00950
VIT_13s0047g00990
VIT_13s0047g01010
VIT_13s0047g01030
VIT_13s0047g01040
Standardized expressions
Developmental stages
EI
PG
G1
G2
H
T1
T2
C1
C2
S
EP
JP

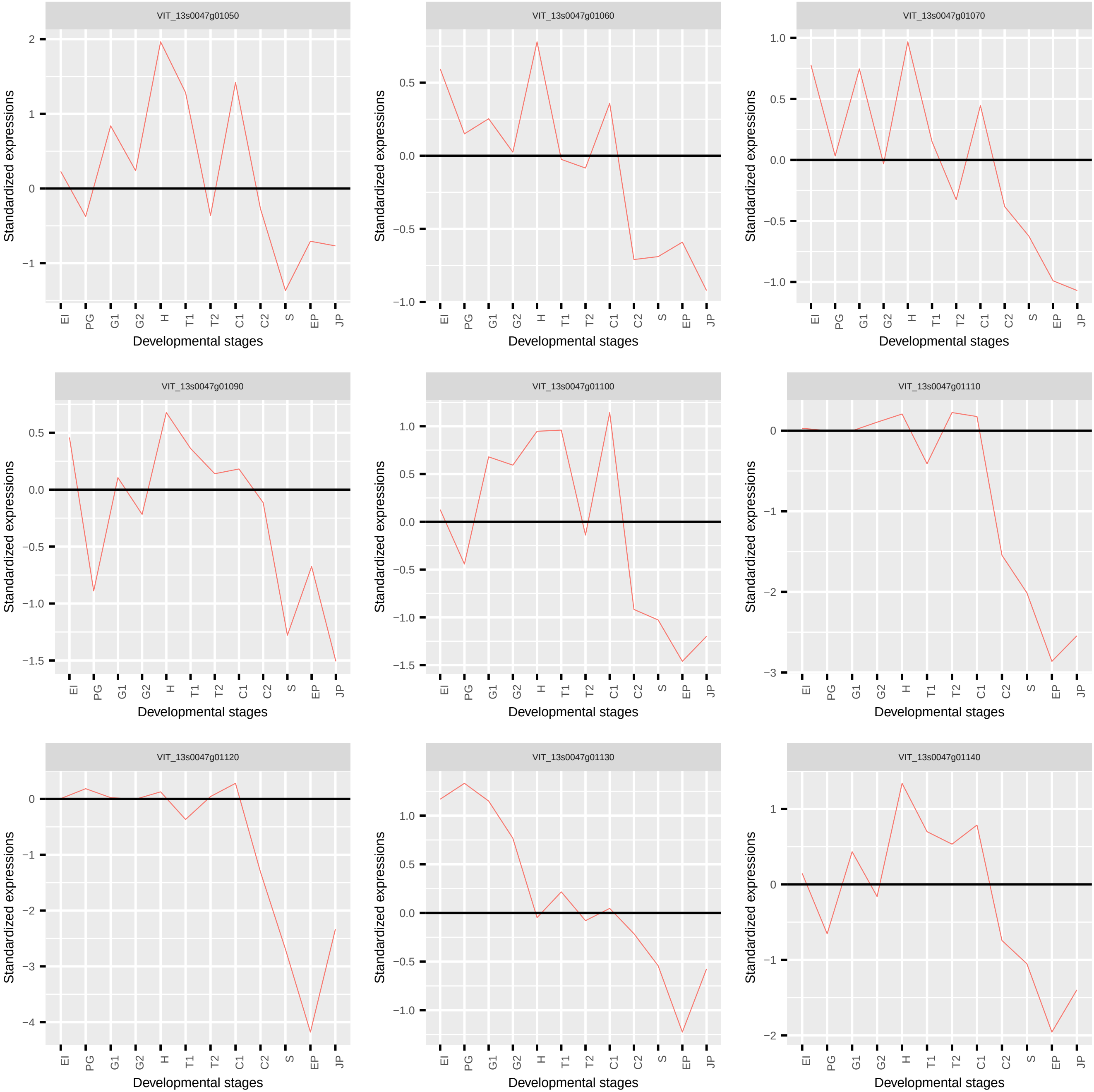

VIT_13s0047g01050
VIT_13s0047g01060
VIT_13s0047g01070
VIT_13s0047g01090
VIT_13s0047g01100
VIT_13s0047g01110
VIT_13s0047g01120
VIT_13s0047g01130
VIT_13s0047g01140
Standardized expressions
Developmental stages
EI
PG
G1
G2
H
T1
T2
C1
C2
S
EP
JP
2
1
0
−1
−2
−3
−4
1.0
0.5
0.0
−0.5
−1.0
−1.5

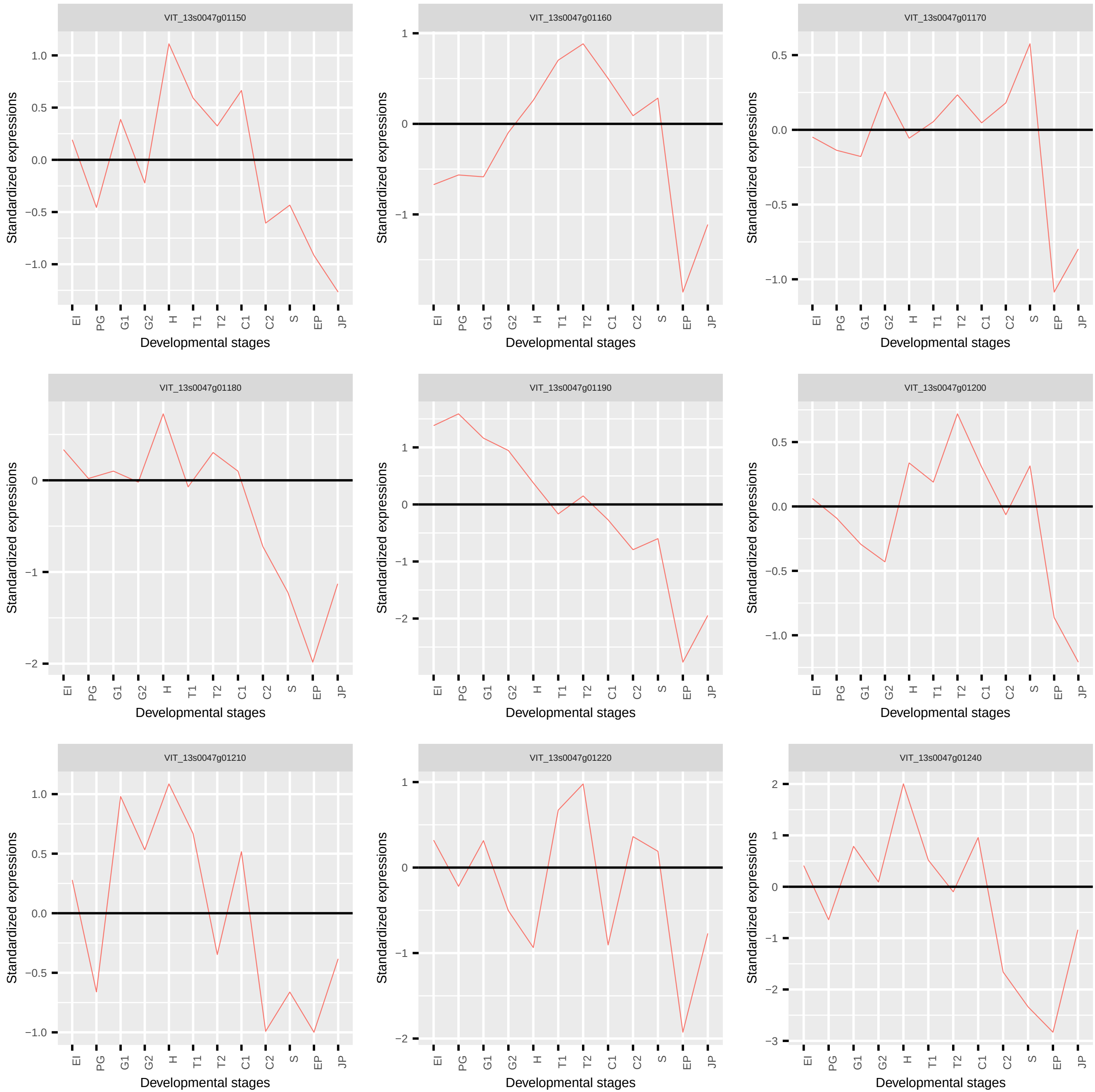
VIT_13s0047g01150
VIT_13s0047g01160
VIT_13s0047g01170
VIT_13s0047g01180
VIT_13s0047g01190
VIT_13s0047g01200
VIT_13s0047g01210
VIT_13s0047g01220
VIT_13s0047g01240
Standardized expressions
Developmental stages
EI PG G1 G2 H T1 T2 C1 C2 S EP JP

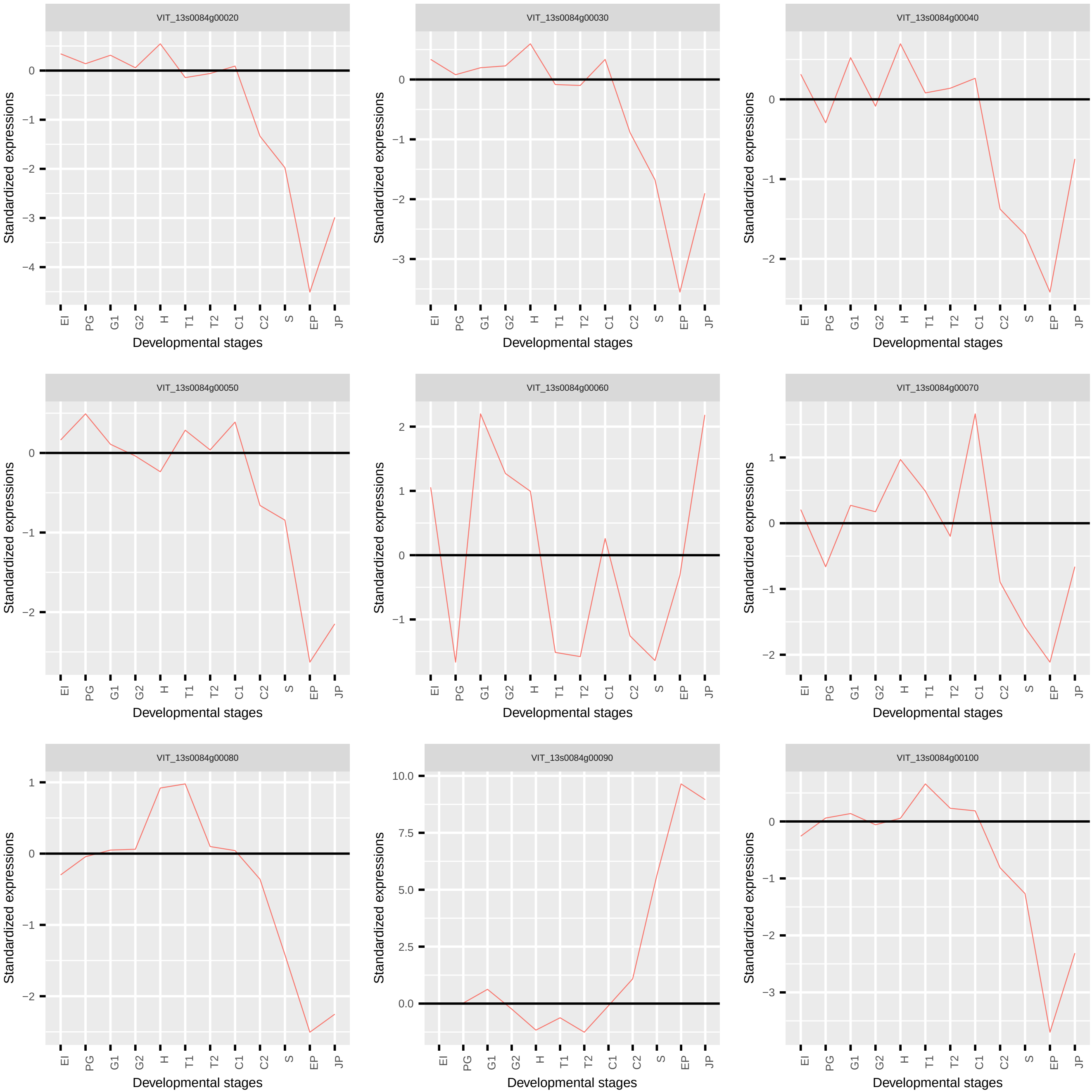
VIT_13s0084g00020
VIT_13s0084g00030
VIT_13s0084g00040
VIT_13s0084g00050
VIT_13s0084g00060
VIT_13s0084g00070
VIT_13s0084g00080
VIT_13s0084g00090
VIT_13s0084g00100
Standardized expressions
Developmental stages
EI PG G1 G2 H T1 T2 C1 C2 S EP JP
0 −1 −2 −3 −4
1 2
0.0 2.5 5.0 7.5 10.0

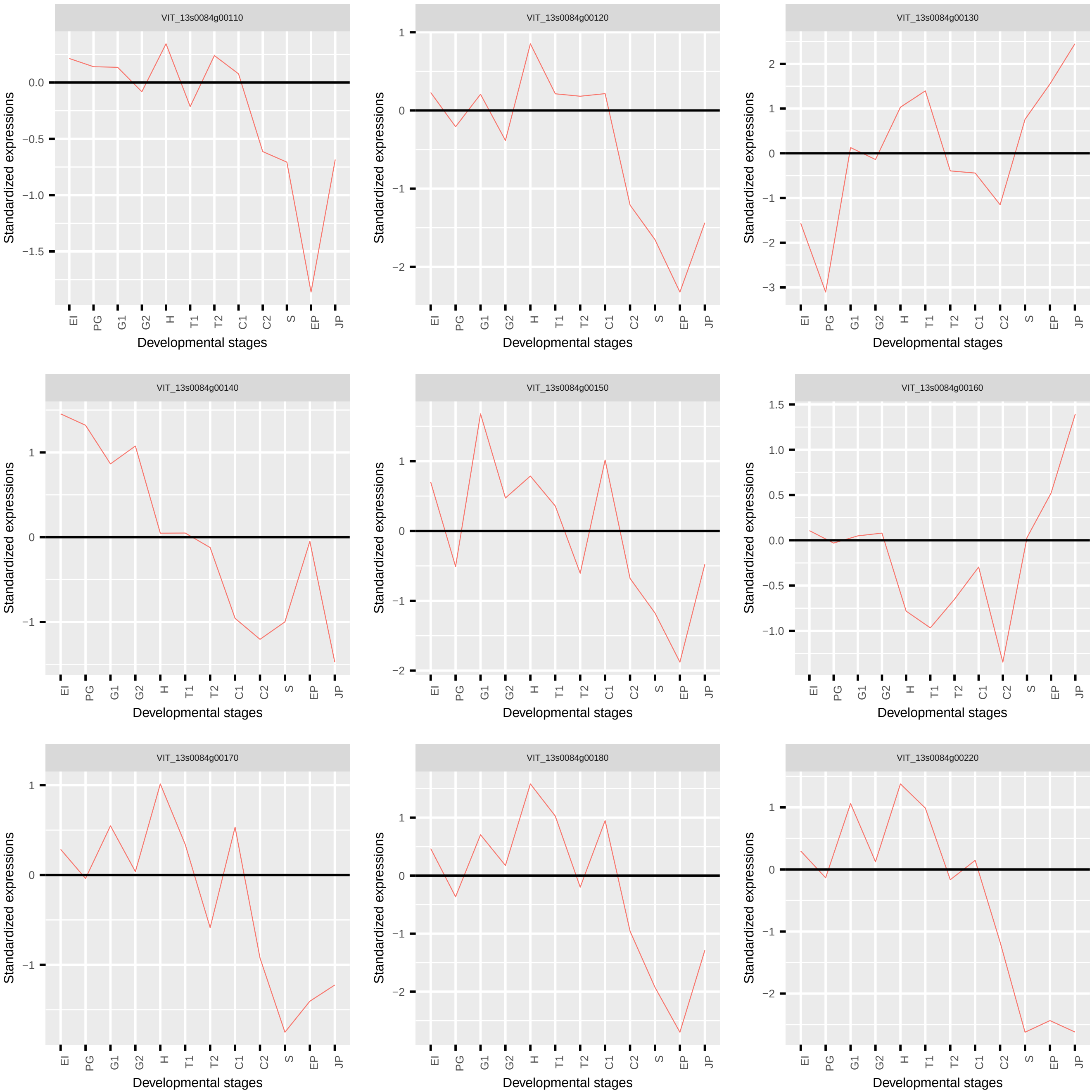

VIT_13s0084g00110
0.0
−0.5
−1.0
−1.5
VIT_13s0084g00120
1
0
−1
−2
VIT_13s0084g00130
2
1
0
−1
−2
−3
VIT_13s0084g00140
1
0
−1
VIT_13s0084g00150
1
0
−1
−2
VIT_13s0084g00160
1.5
1.0
0.5
0.0
−0.5
−1.0
VIT_13s0084g00170
1
0
−1
VIT_13s0084g00180
1
0
−1
−2
VIT_13s0084g00220
1
0
−1
−2
Standardized expressions
EI PG G1 G2 H T1 T2 C1 C2 S EP JP
Developmental stages

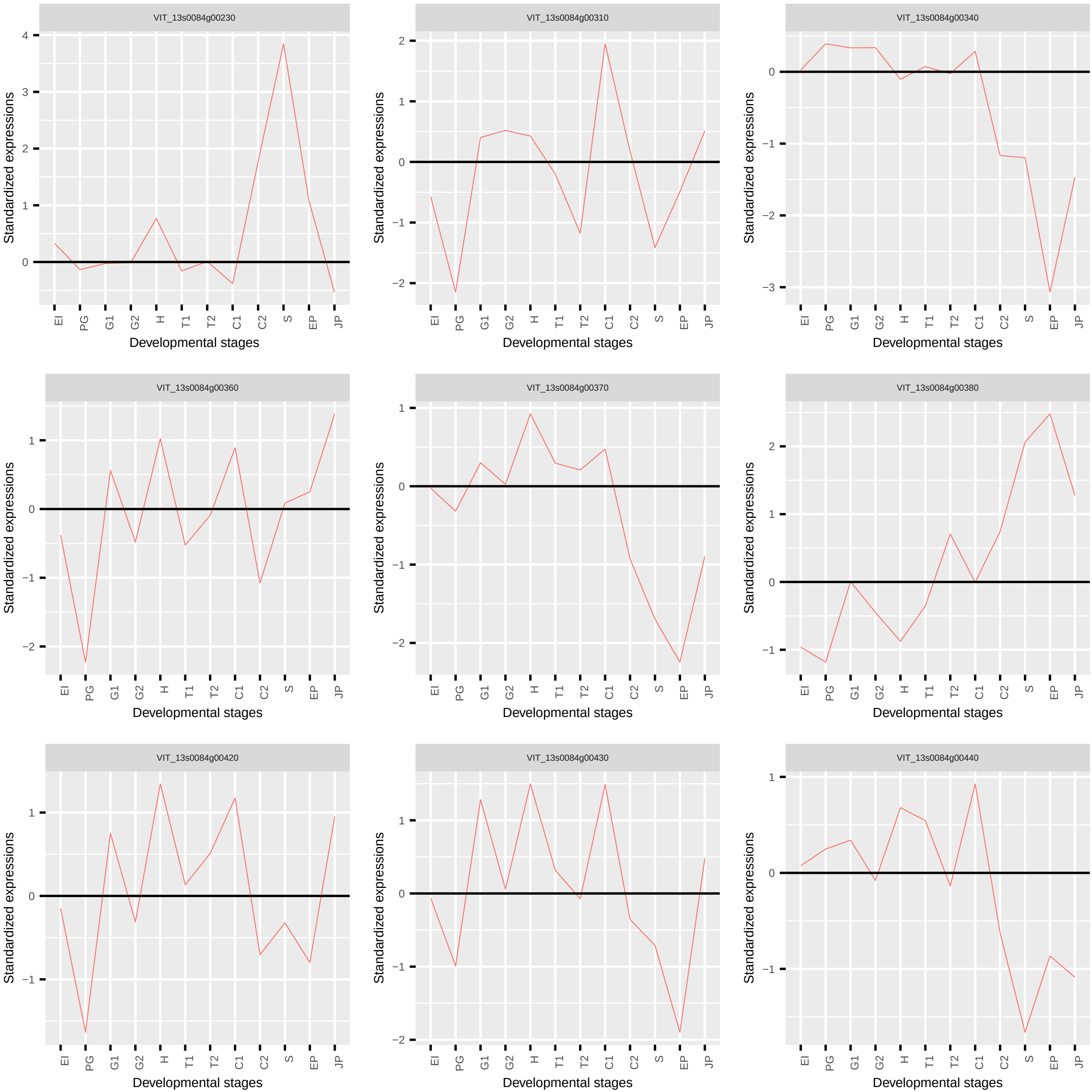
VIT_13s0084g00230
VIT_13s0084g00310
VIT_13s0084g00340
VIT_13s0084g00360
VIT_13s0084g00370
VIT_13s0084g00380
VIT_13s0084g00420
VIT_13s0084g00430
VIT_13s0084g00440
Standardized expressions
Developmental stages
EI PG G1 G2 H T1 T2 C1 C2 S EP JP

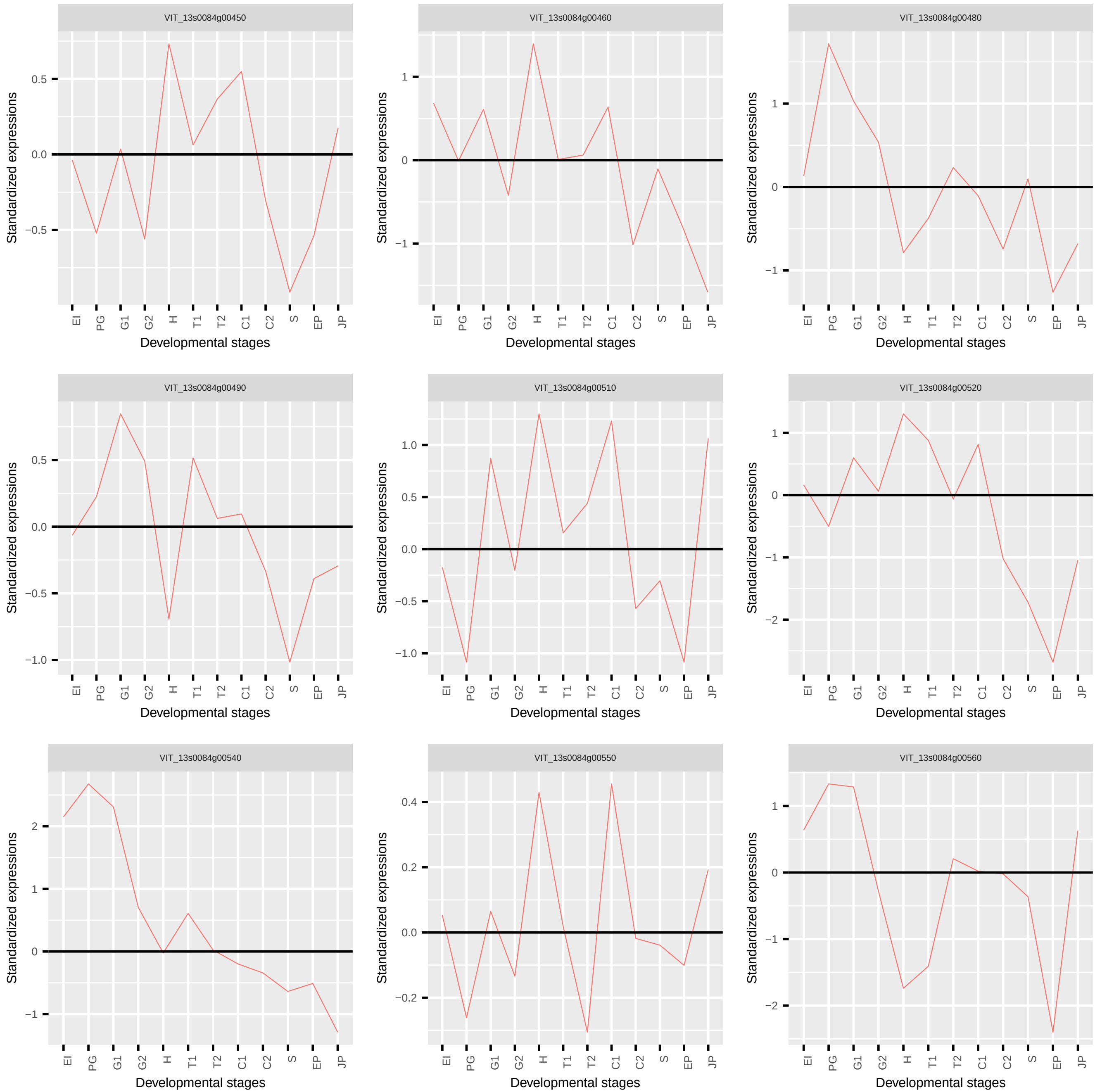

VIT_13s0084g00450
VIT_13s0084g00460
VIT_13s0084g00480
VIT_13s0084g00490
VIT_13s0084g00510
VIT_13s0084g00520
VIT_13s0084g00540
VIT_13s0084g00550
VIT_13s0084g00560
Standardized expressions
Developmental stages
EI PG G1 G2 H T1 T2 C1 C2 S EP JP

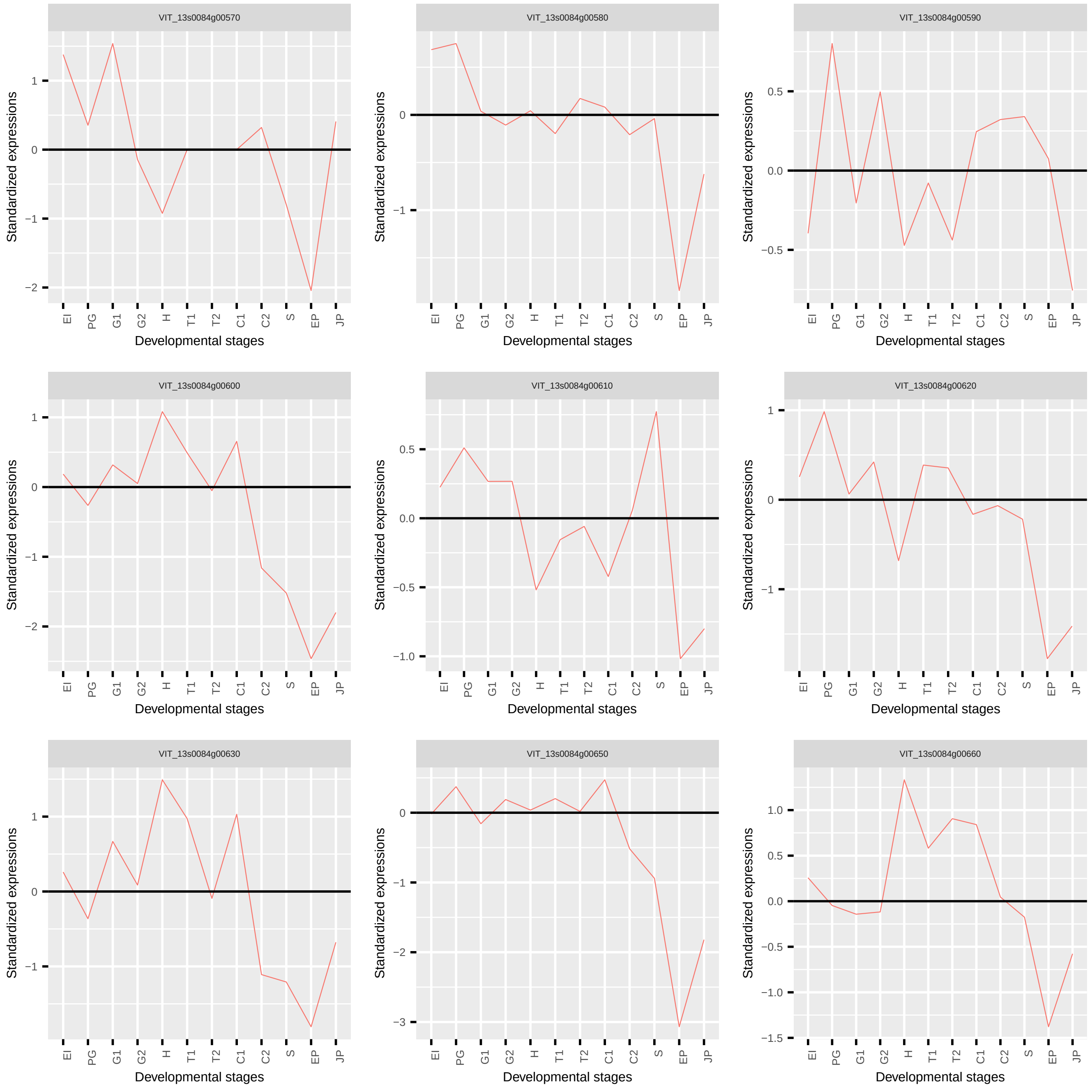
VIT_13s0084g00570
VIT_13s0084g00580
VIT_13s0084g00590
VIT_13s0084g00600
VIT_13s0084g00610
VIT_13s0084g00620
VIT_13s0084g00630
VIT_13s0084g00650
VIT_13s0084g00660
Standardized expressions
Developmental stages
EI
PG
G1
G2
H
T1
T2
C1
C2
S
EP
JP
1
0
−1
−2
−3
0.5
0.0
−0.5
−1.0
−1.5
1.0

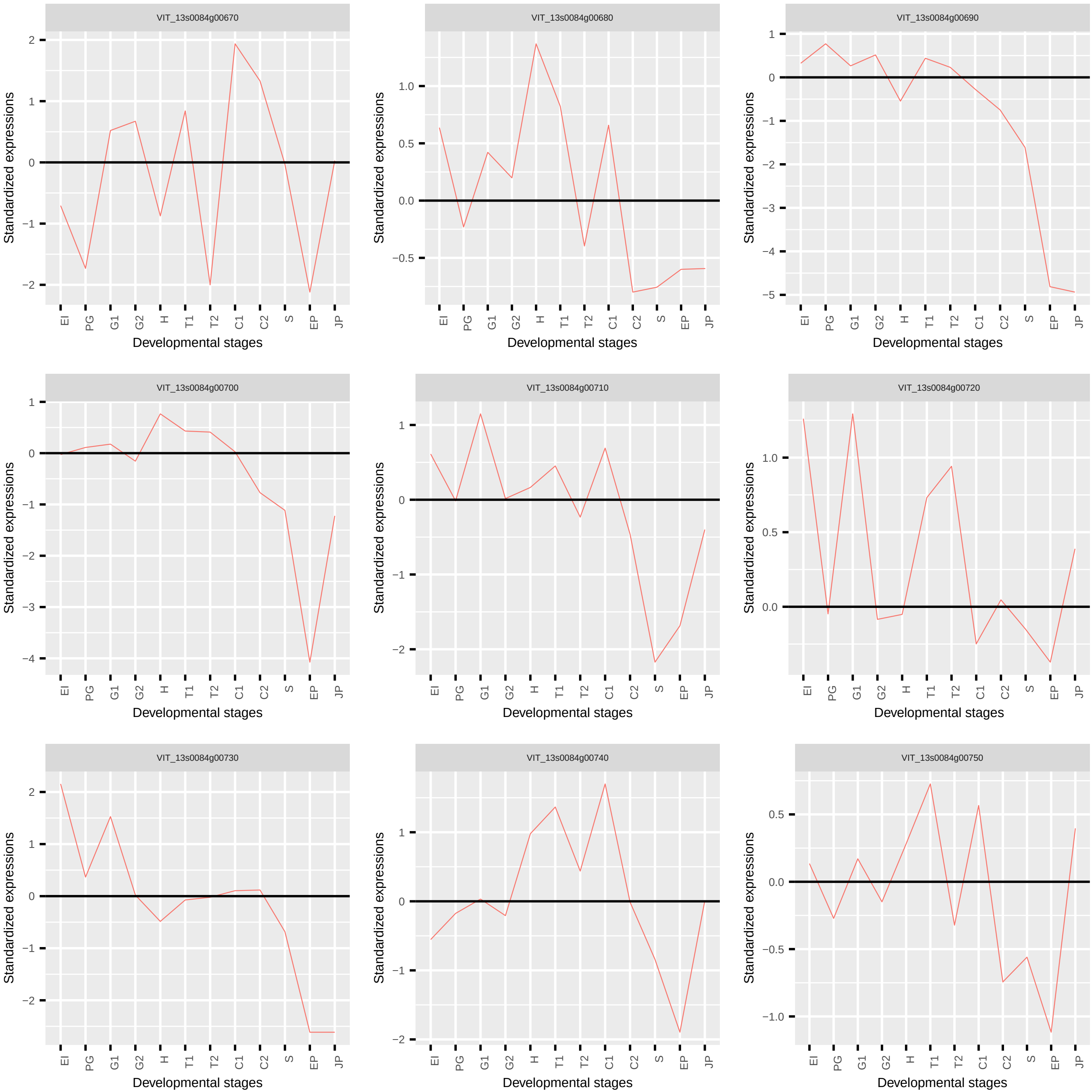
VIT_13s0084g00670
VIT_13s0084g00680
VIT_13s0084g00690
VIT_13s0084g00700
VIT_13s0084g00710
VIT_13s0084g00720
VIT_13s0084g00730
VIT_13s0084g00740
VIT_13s0084g00750
Standardized expressions
Developmental stages
EI PG G1 G2 H T1 T2 C1 C2 S EP JP

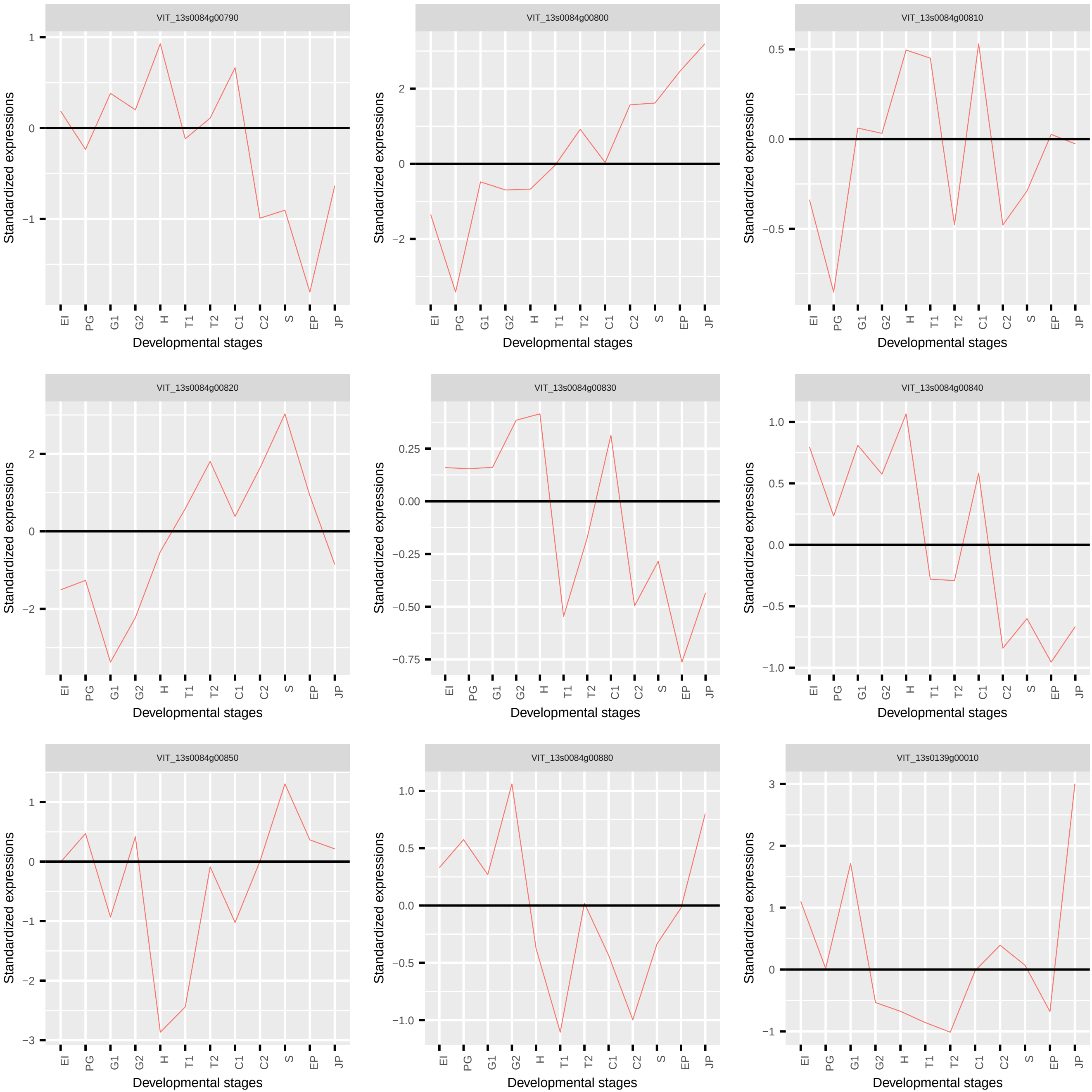
VIT_13s0084g00790
VIT_13s0084g00800
VIT_13s0084g00810
VIT_13s0084g00820
VIT_13s0084g00830
VIT_13s0084g00840
VIT_13s0084g00850
VIT_13s0084g00880
VIT_13s0139g00010
Standardized expressions
Developmental stages
EI PG G1 G2 H T1 T2 C1 C2 S EP JP

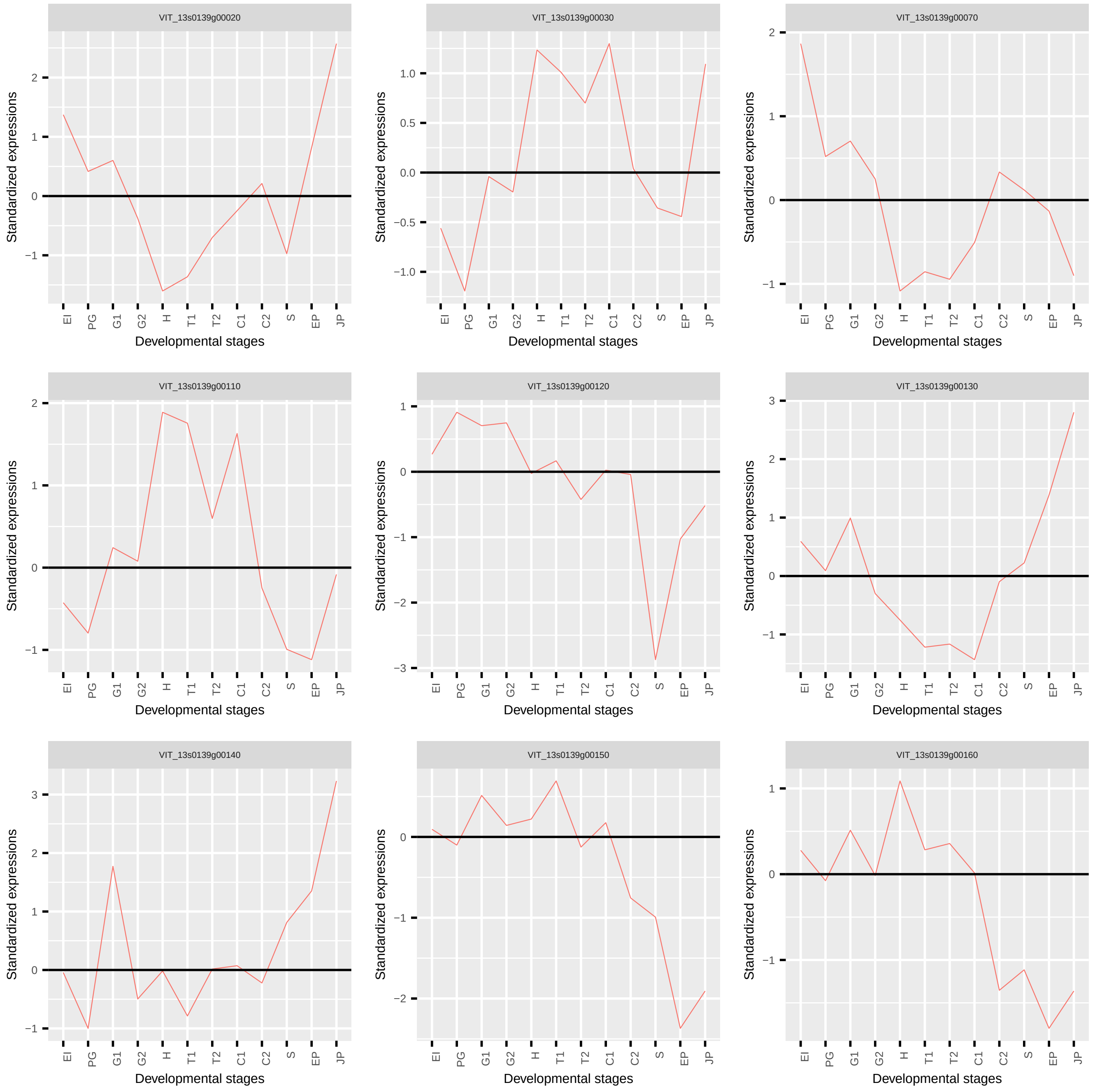
VIT_13s0139g00020
VIT_13s0139g00030
VIT_13s0139g00070
VIT_13s0139g00110
VIT_13s0139g00120
VIT_13s0139g00130
VIT_13s0139g00140
VIT_13s0139g00150
VIT_13s0139g00160
Standardized expressions
Developmental stages
EI PG G1 G2 H T1 T2 C1 C2 S EP JP

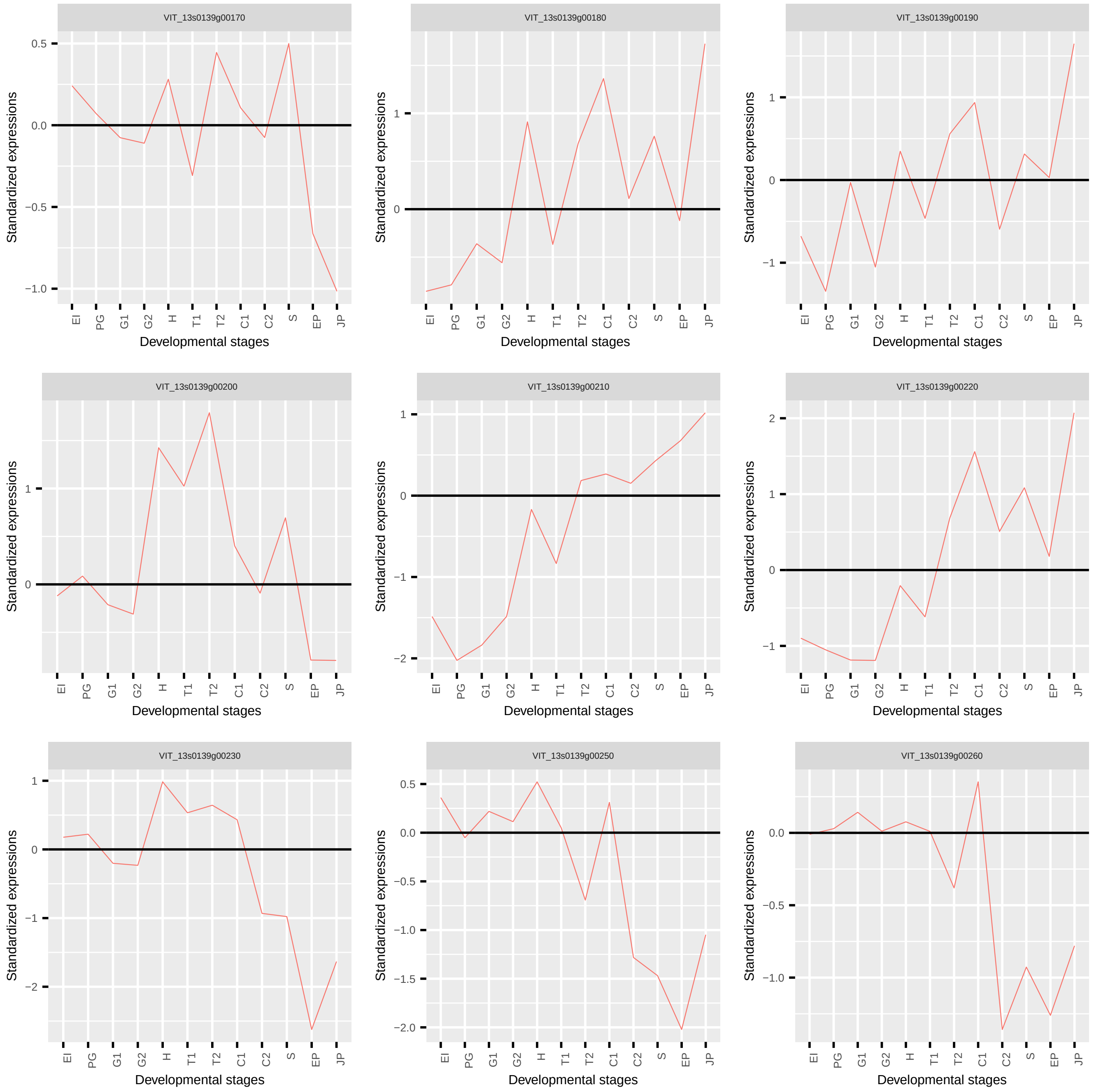

VIT_13s0139g00170
VIT_13s0139g00180
VIT_13s0139g00190
VIT_13s0139g00200
VIT_13s0139g00210
VIT_13s0139g00220
VIT_13s0139g00230
VIT_13s0139g00250
VIT_13s0139g00260
Standardized expressions
Developmental stages
EI PG G1 G2 H T1 T2 C1 C2 S EP JP

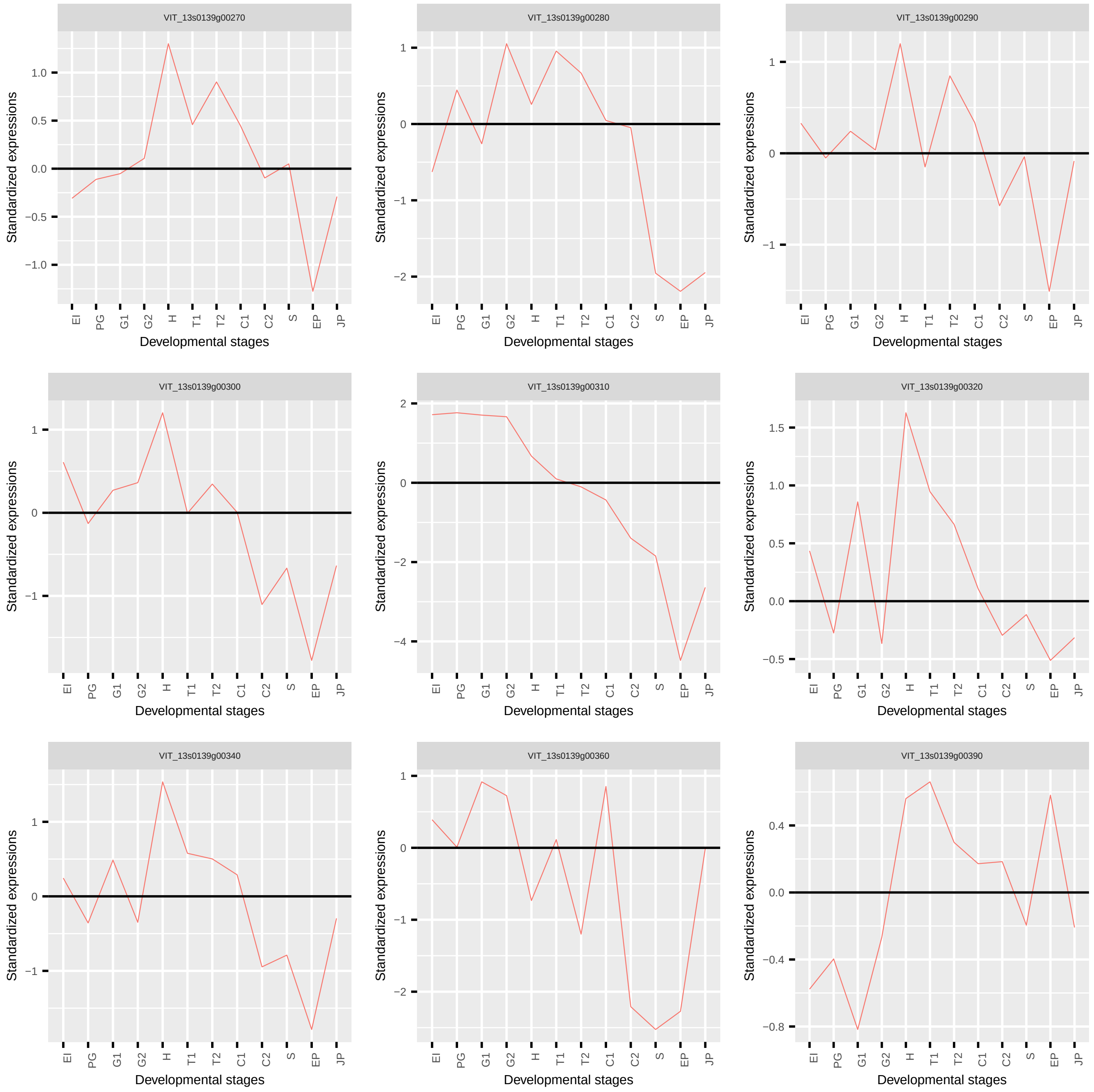

VIT_13s0139g00270
VIT_13s0139g00280
VIT_13s0139g00290
VIT_13s0139g00300
VIT_13s0139g00310
VIT_13s0139g00320
VIT_13s0139g00340
VIT_13s0139g00360
VIT_13s0139g00390
Standardized expressions
Developmental stages
EI PG G1 G2 H T1 T2 C1 C2 S EP JP

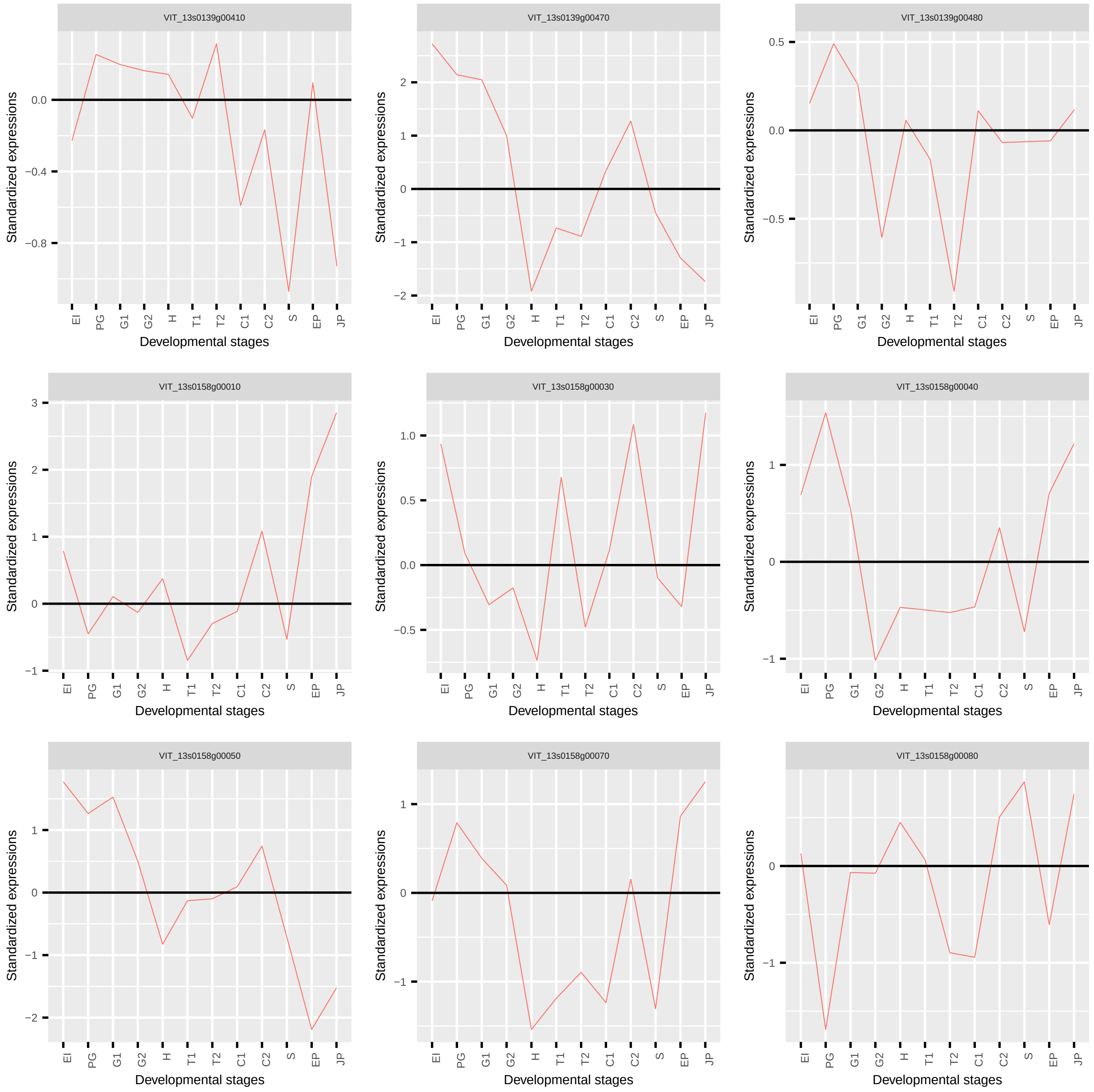
VIT_13s0139g00410
VIT_13s0139g00470
VIT_13s0139g00480
VIT_13s0158g00010
VIT_13s0158g00030
VIT_13s0158g00040
VIT_13s0158g00050
VIT_13s0158g00070
VIT_13s0158g00080
Standardized expressions
Developmental stages
EI PG G1 G2 H T1 T2 C1 C2 S EP JP

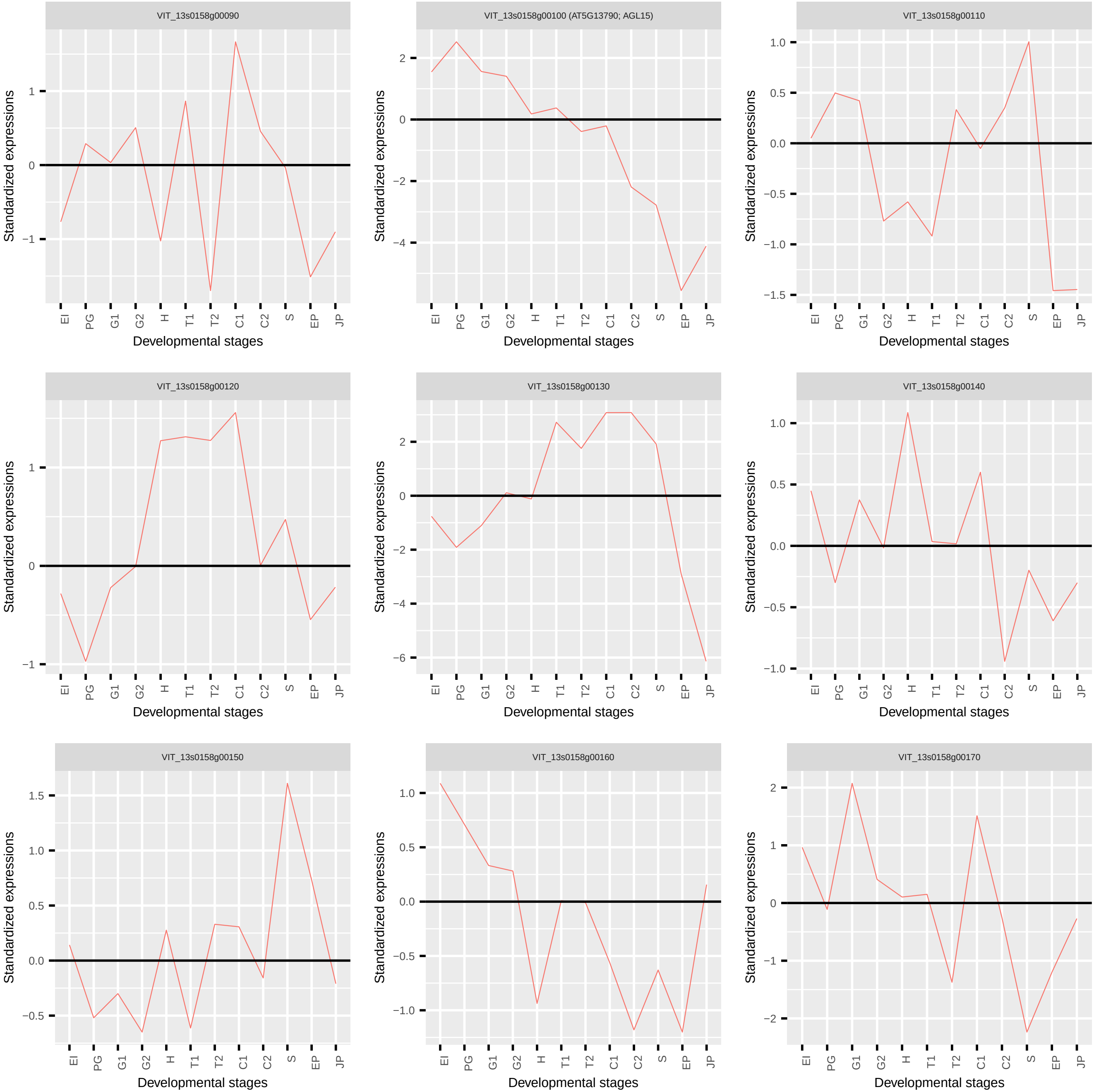
VIT_13s0158g00090
VIT_13s0158g00100 (AT5G13790; AGL15)
VIT_13s0158g00110
VIT_13s0158g00120
VIT_13s0158g00130
VIT_13s0158g00140
VIT_13s0158g00150
VIT_13s0158g00160
VIT_13s0158g00170
Standardized expressions
Developmental stages
EI PG G1 G2 H T1 T2 C1 C2 S EP JP

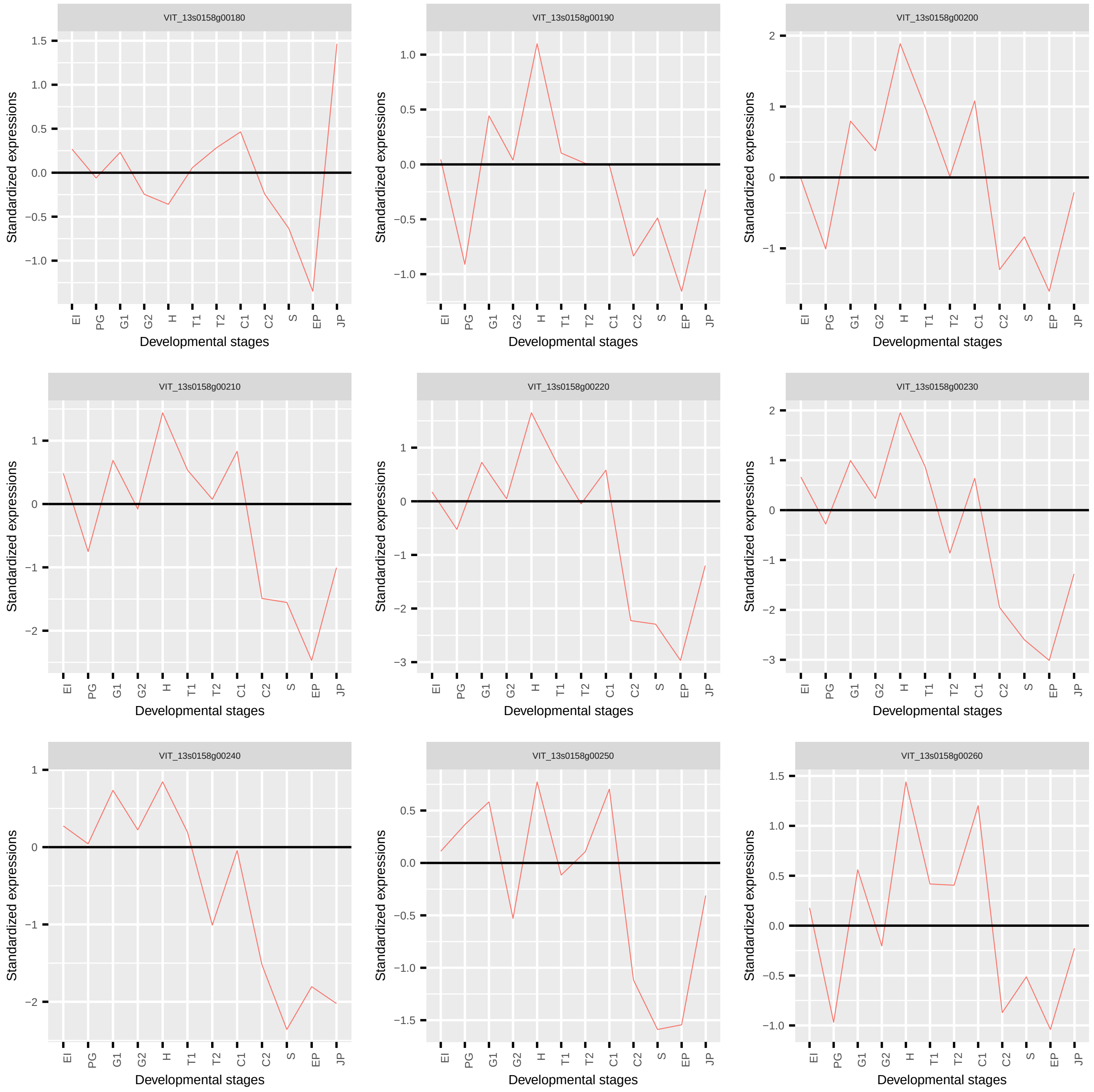
VIT_13s0158g00180
VIT_13s0158g00190
VIT_13s0158g00200
VIT_13s0158g00210
VIT_13s0158g00220
VIT_13s0158g00230
VIT_13s0158g00240
VIT_13s0158g00250
VIT_13s0158g00260
Standardized expressions
Developmental stages
EI PG G1 G2 H T1 T2 C1 C2 S EP JP

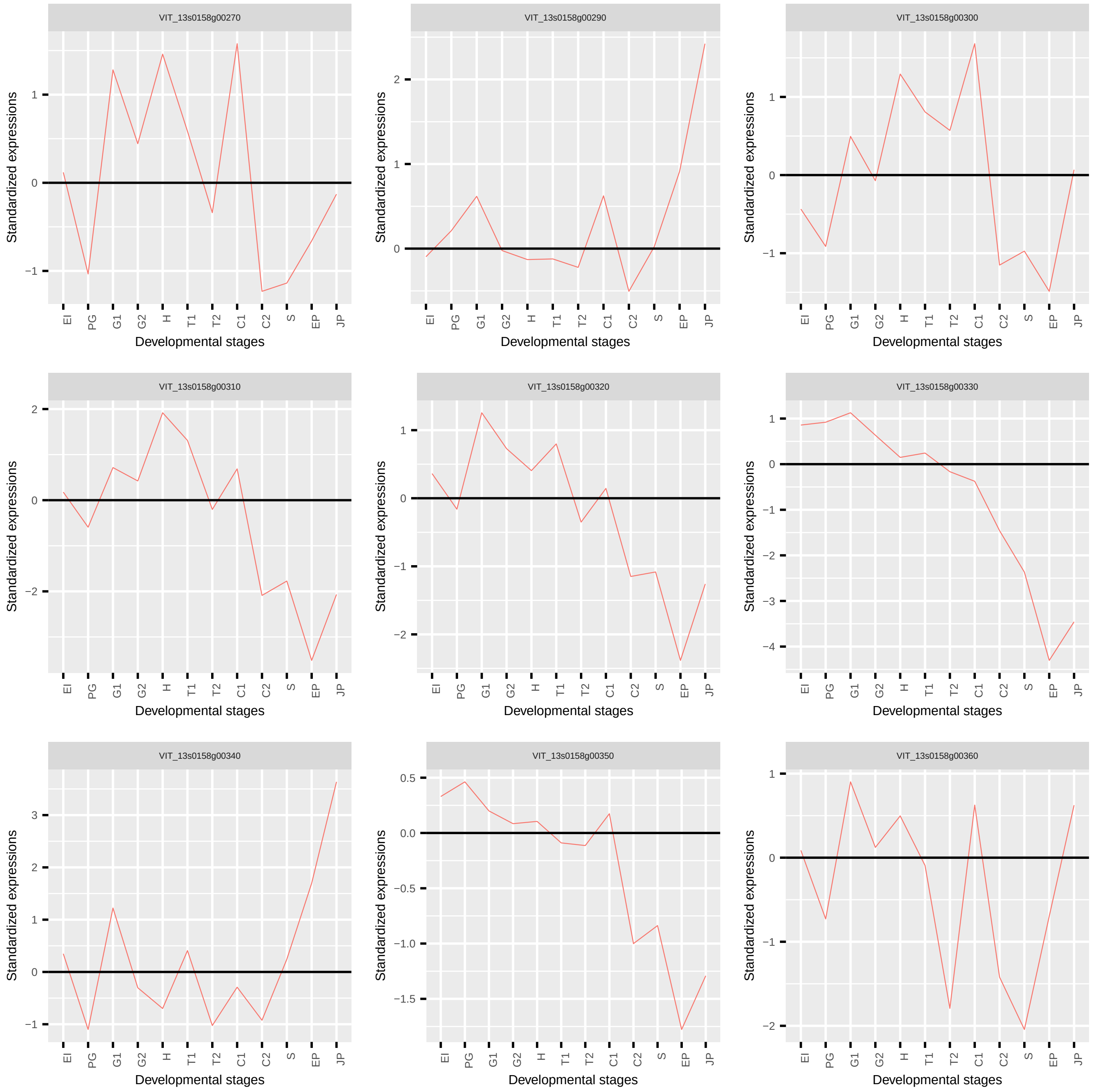
VIT_13s0158g00270
VIT_13s0158g00290
VIT_13s0158g00300
VIT_13s0158g00310
VIT_13s0158g00320
VIT_13s0158g00330
VIT_13s0158g00340
VIT_13s0158g00350
VIT_13s0158g00360
Standardized expressions
Developmental stages
EI PG G1 G2 H T1 T2 C1 C2 S EP JP

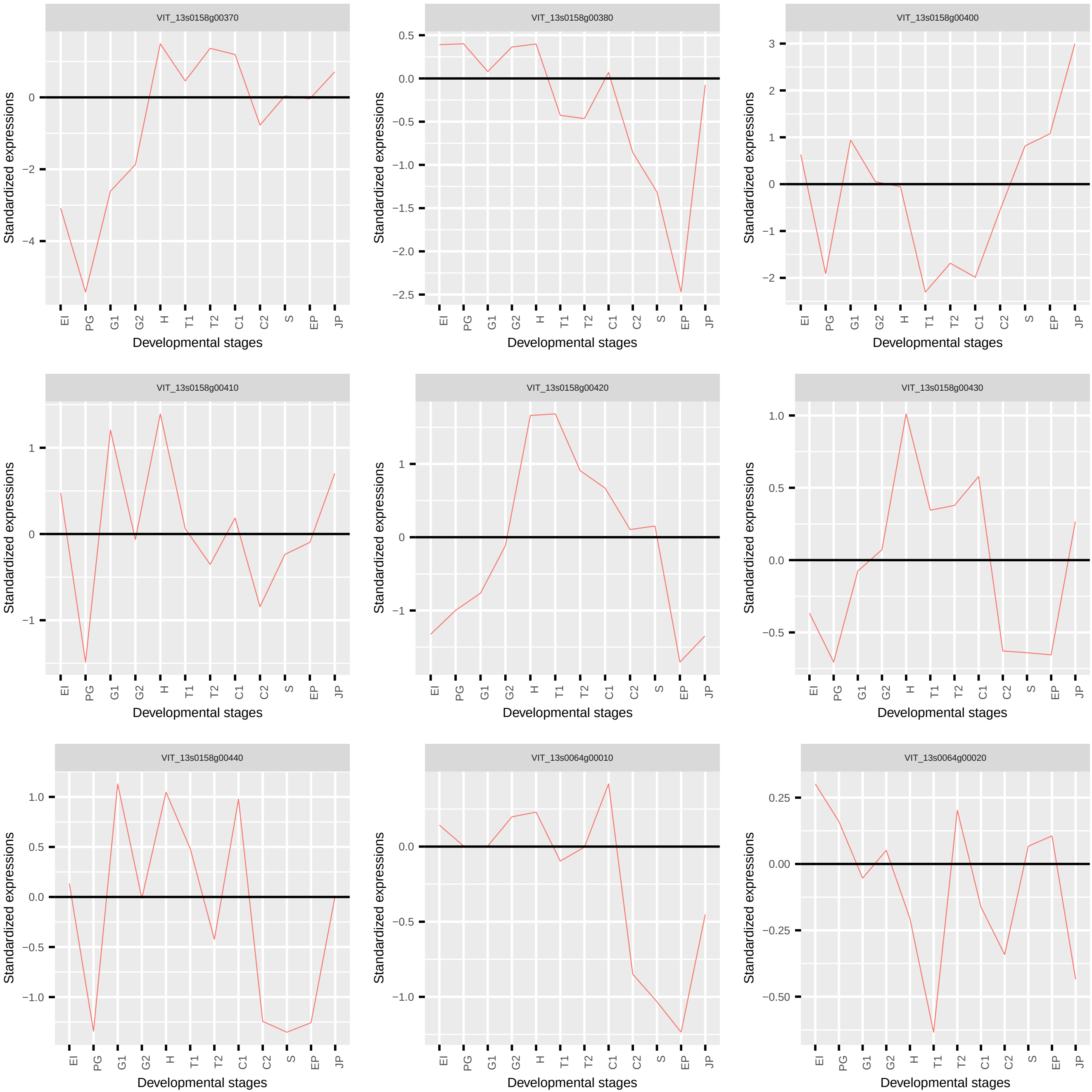
VIT_13s0158g00370
VIT_13s0158g00380
VIT_13s0158g00400
VIT_13s0158g00410
VIT_13s0158g00420
VIT_13s0158g00430
VIT_13s0158g00440
VIT_13s0064g00010
VIT_13s0064g00020
Standardized expressions
Developmental stages
EI
PG
G1
G2
H
T1
T2
C1
C2
S
EP
JP

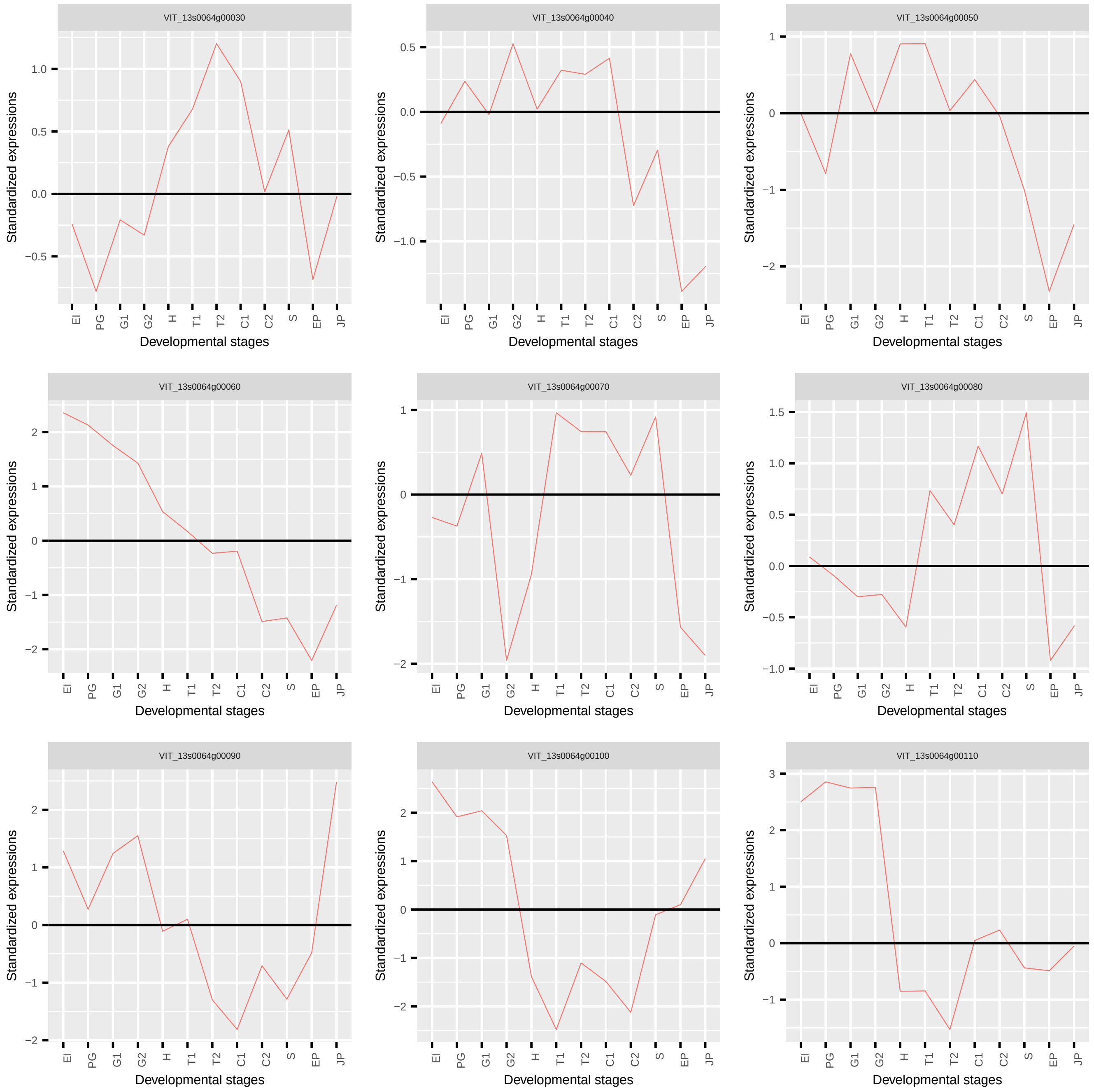
VIT_13s0064g00030
VIT_13s0064g00040
VIT_13s0064g00050
VIT_13s0064g00060
VIT_13s0064g00070
VIT_13s0064g00080
VIT_13s0064g00090
VIT_13s0064g00100
VIT_13s0064g00110
Standardized expressions
Developmental stages
EI
PG
G1
G2
H
T1
T2
C1
C2
S
EP
JP

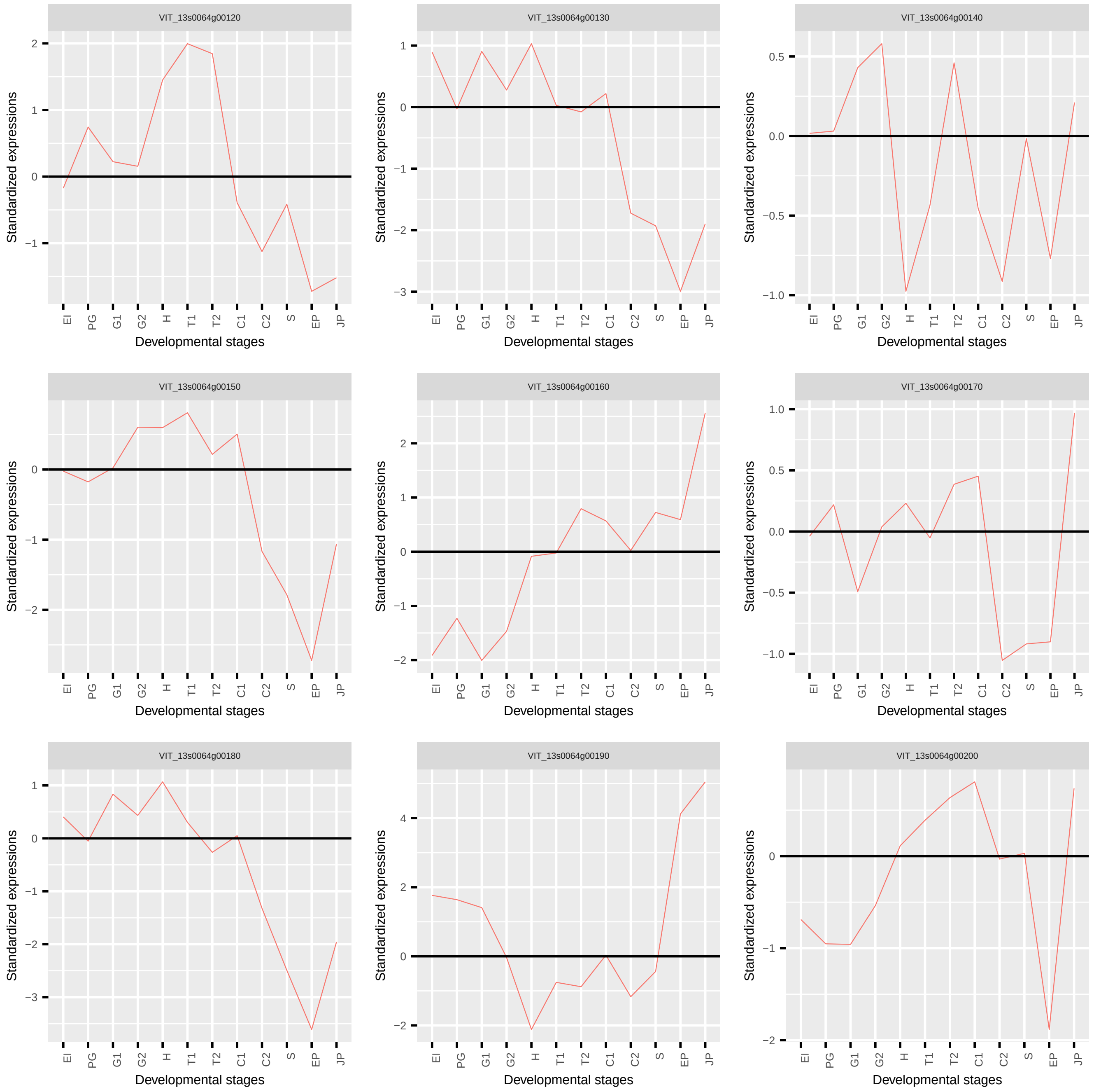
VIT_13s0064g00120
VIT_13s0064g00130
VIT_13s0064g00140
VIT_13s0064g00150
VIT_13s0064g00160
VIT_13s0064g00170
VIT_13s0064g00180
VIT_13s0064g00190
VIT_13s0064g00200
Standardized expressions
Developmental stages
EI PG G1 G2 H T1 T2 C1 C2 S EP JP

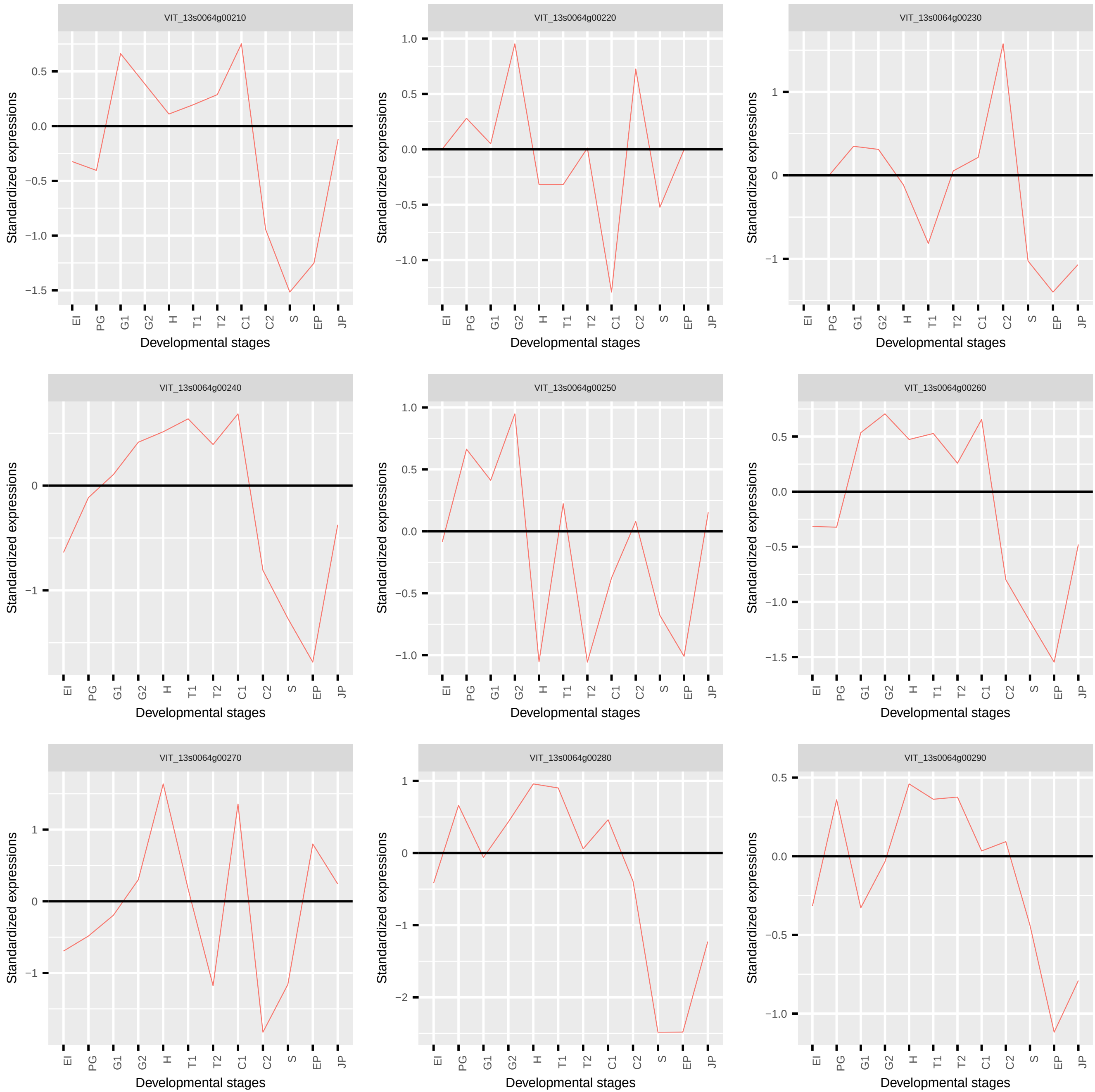
VIT_13s0064g00210
VIT_13s0064g00220
VIT_13s0064g00230
VIT_13s0064g00240
VIT_13s0064g00250
VIT_13s0064g00260
VIT_13s0064g00270
VIT_13s0064g00280
VIT_13s0064g00290
Standardized expressions
Developmental stages
EI PG G1 G2 H T1 T2 C1 C2 S EP JP

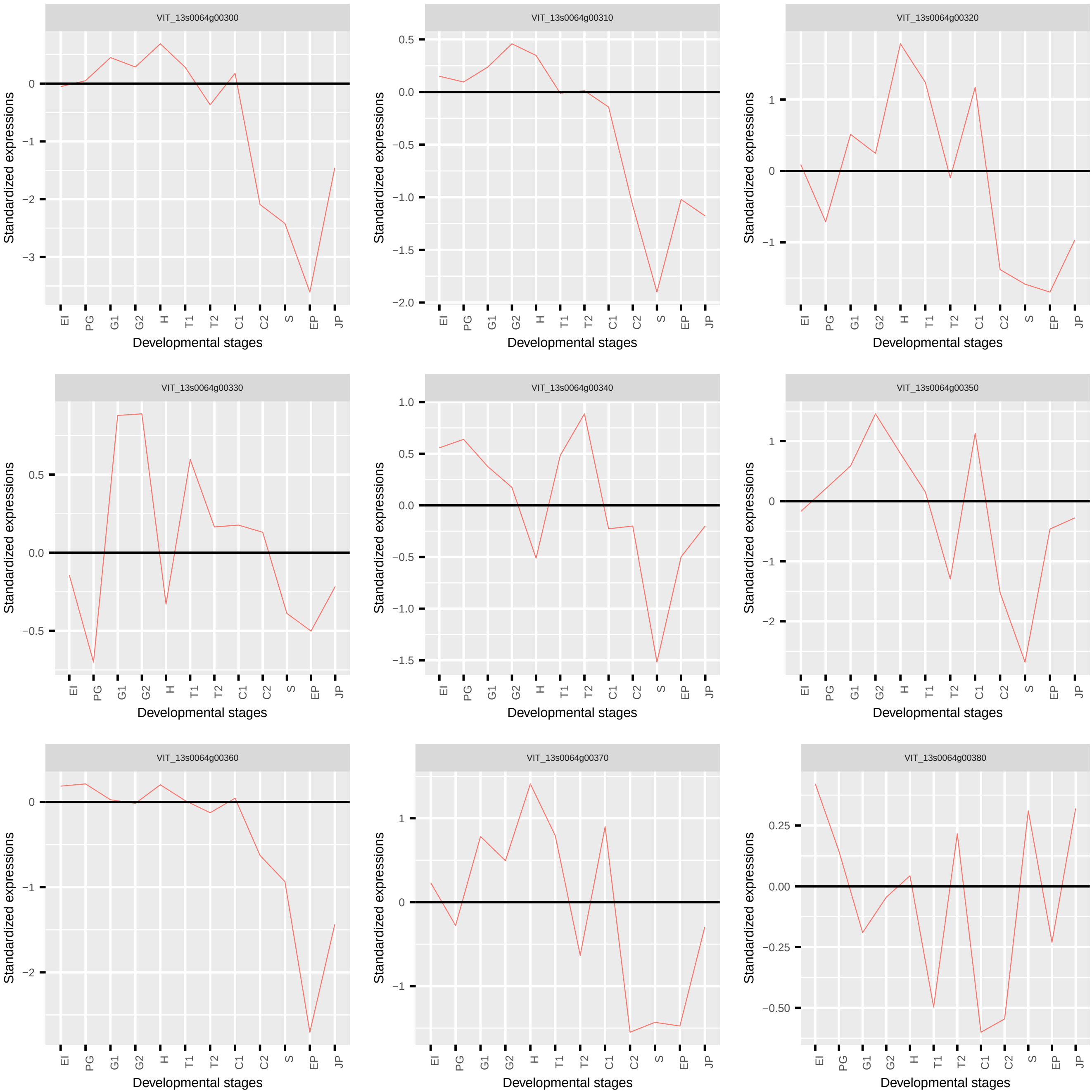
VIT_13s0064g00300
VIT_13s0064g00310
VIT_13s0064g00320
VIT_13s0064g00330
VIT_13s0064g00340
VIT_13s0064g00350
VIT_13s0064g00360
VIT_13s0064g00370
VIT_13s0064g00380
Standardized expressions
Developmental stages
EI PG G1 G2 H T1 T2 C1 C2 S EP JP

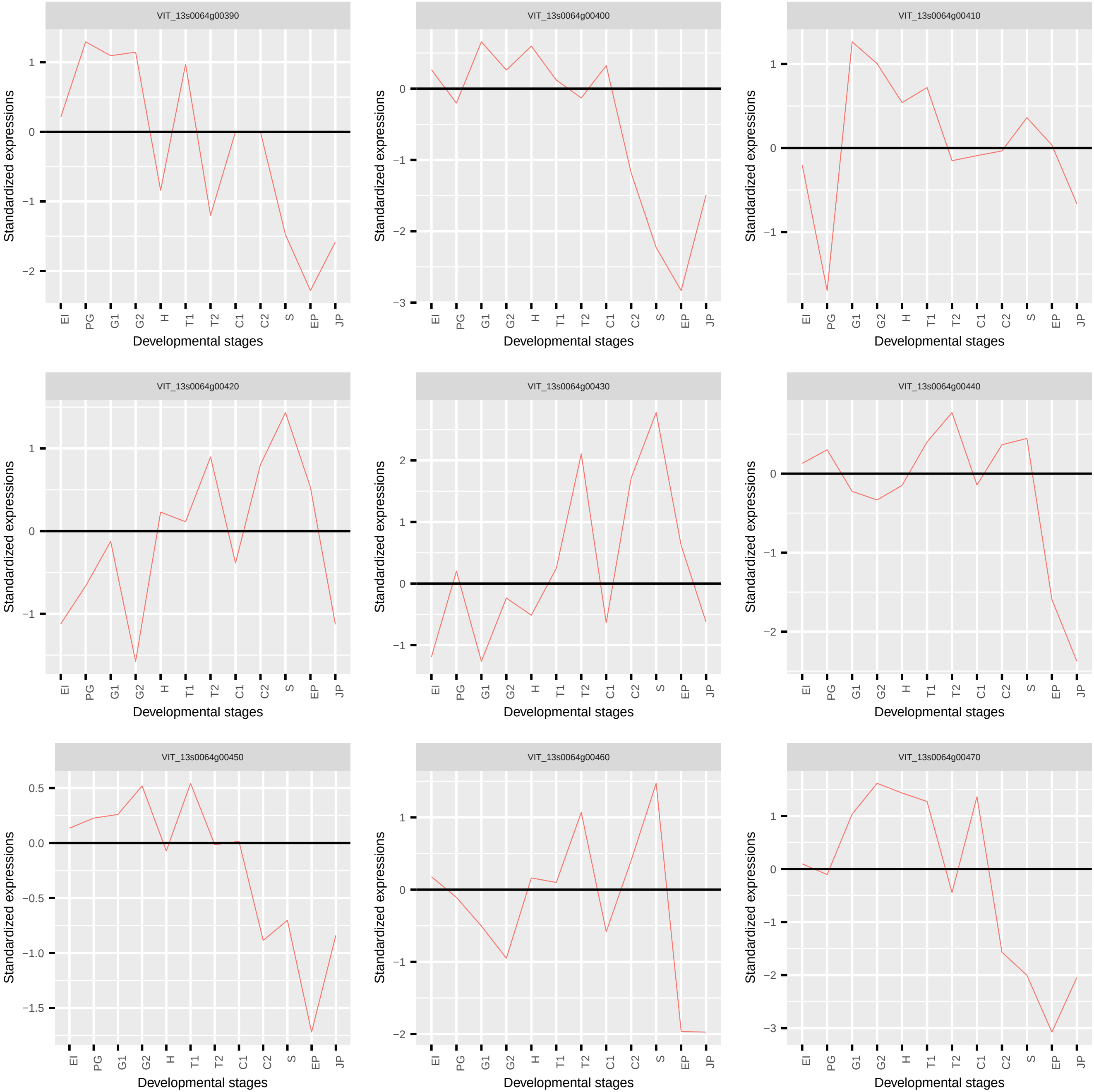
VIT_13s0064g00390
VIT_13s0064g00400
VIT_13s0064g00410
VIT_13s0064g00420
VIT_13s0064g00430
VIT_13s0064g00440
VIT_13s0064g00450
VIT_13s0064g00460
VIT_13s0064g00470
Standardized expressions
Developmental stages
EI PG G1 G2 H T1 T2 C1 C2 S EP JP

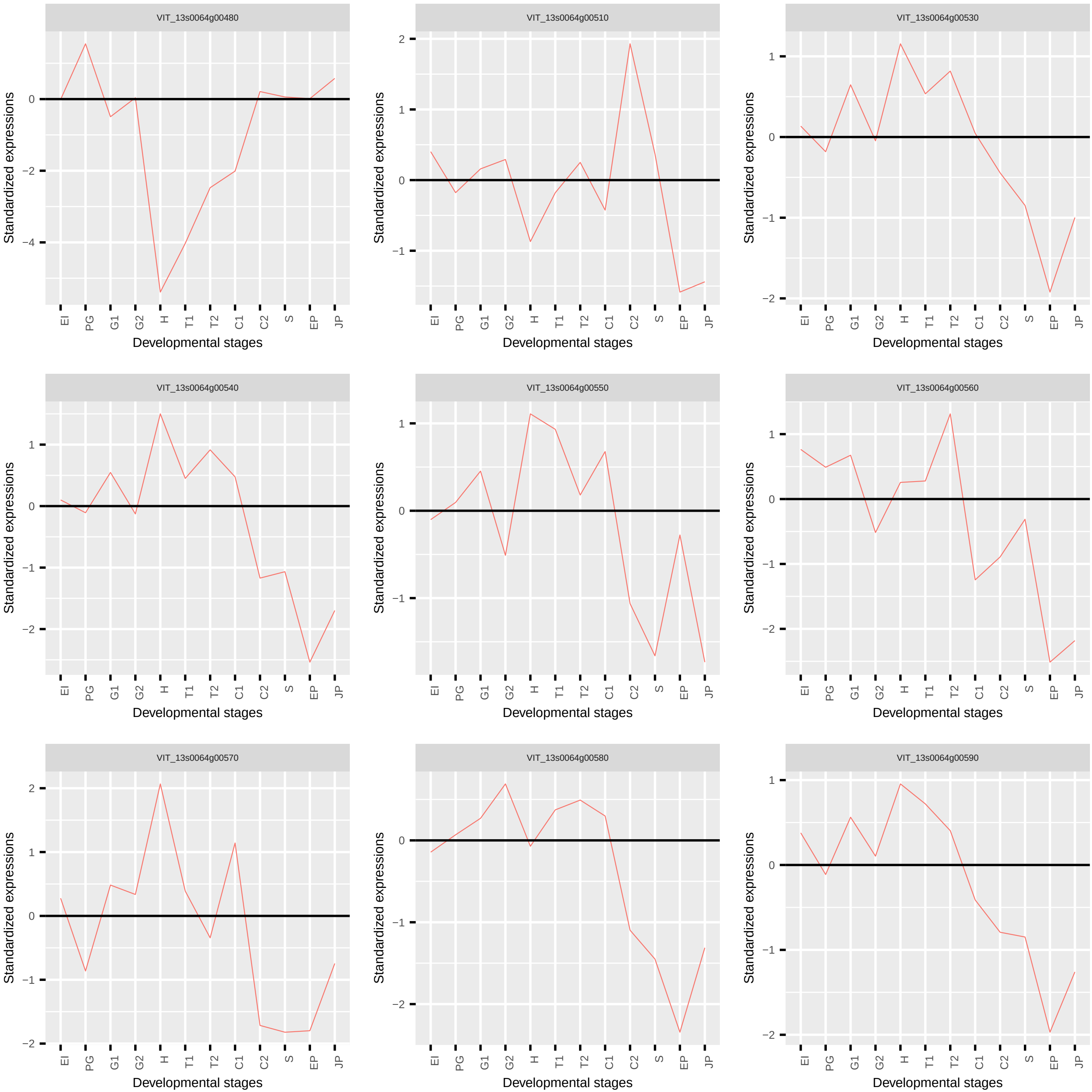
VIT_13s0064g00480
VIT_13s0064g00510
VIT_13s0064g00530
VIT_13s0064g00540
VIT_13s0064g00550
VIT_13s0064g00560
VIT_13s0064g00570
VIT_13s0064g00580
VIT_13s0064g00590
Standardized expressions
Developmental stages
EI PG G1 G2 H T1 T2 C1 C2 S EP JP

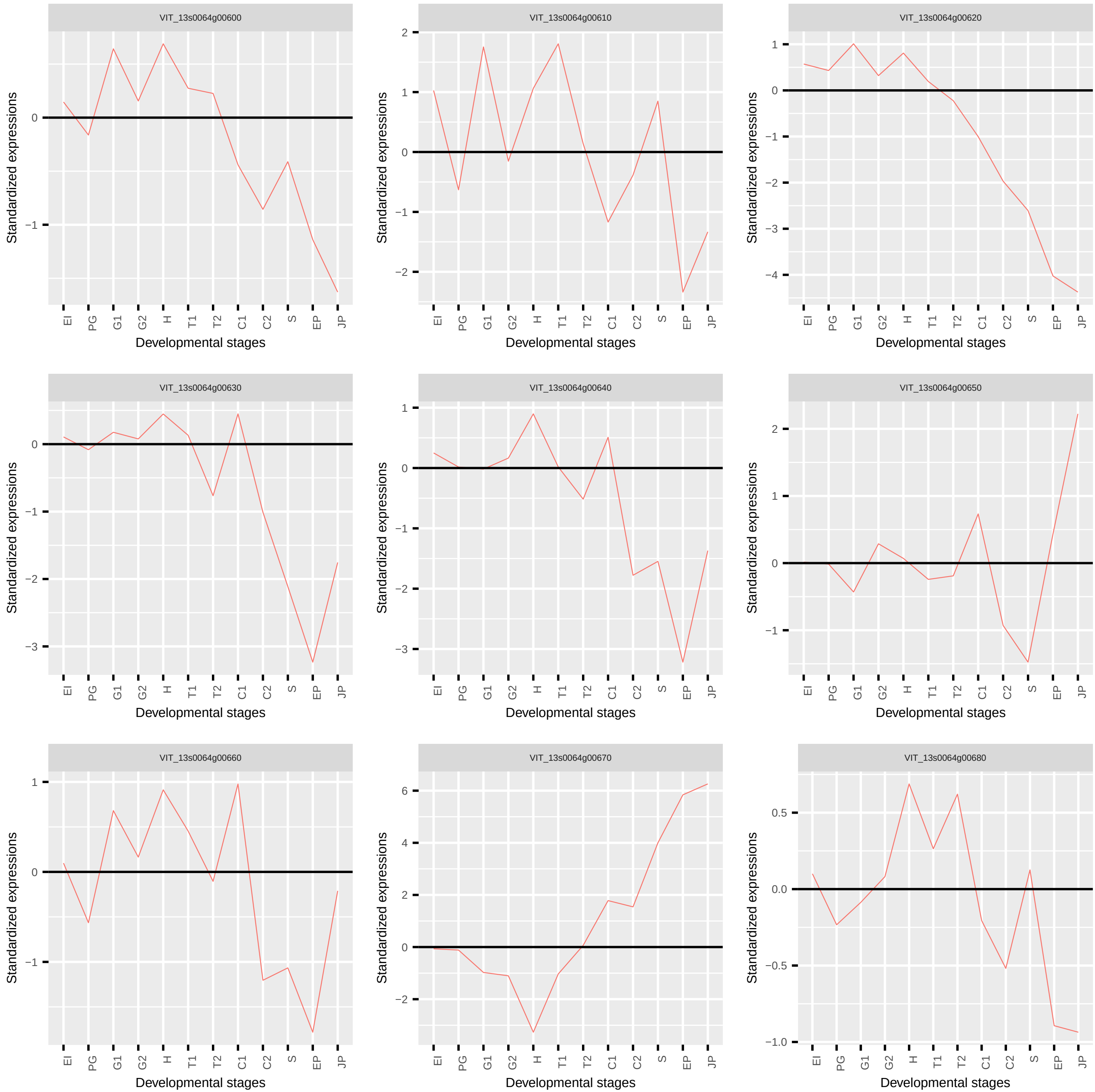

VIT_13s0064g00600
VIT_13s0064g00610
VIT_13s0064g00620
VIT_13s0064g00630
VIT_13s0064g00640
VIT_13s0064g00650
VIT_13s0064g00660
VIT_13s0064g00670
VIT_13s0064g00680
Standardized expressions
Developmental stages
EI PG G1 G2 H T1 T2 C1 C2 S EP JP

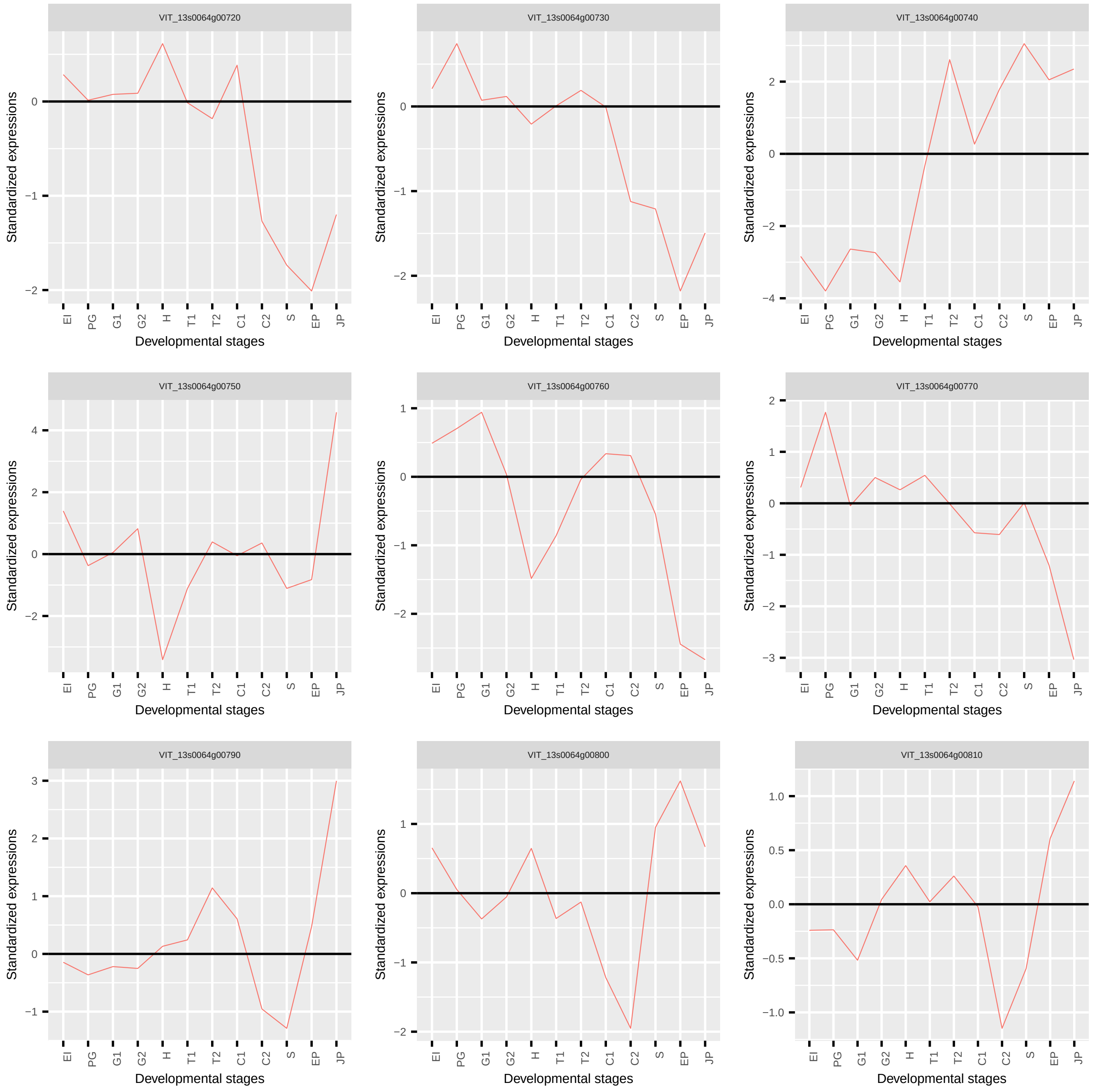
VIT_13s0064g00720
VIT_13s0064g00730
VIT_13s0064g00740
VIT_13s0064g00750
VIT_13s0064g00760
VIT_13s0064g00770
VIT_13s0064g00790
VIT_13s0064g00800
VIT_13s0064g00810
Standardized expressions
Developmental stages
EI PG G1 G2 H T1 T2 C1 C2 S EP JP

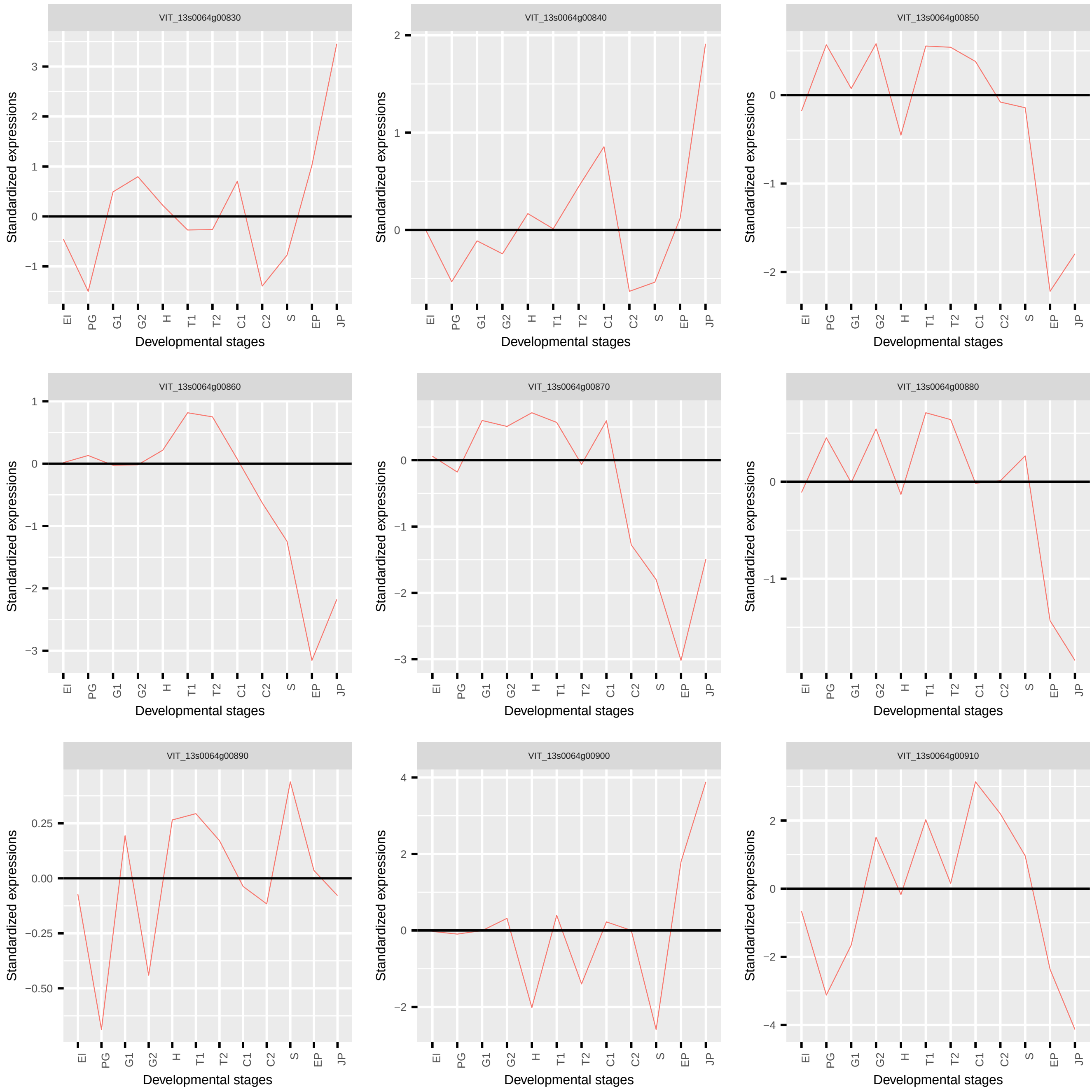
VIT_13s0064g00830
VIT_13s0064g00840
VIT_13s0064g00850
VIT_13s0064g00860
VIT_13s0064g00870
VIT_13s0064g00880
VIT_13s0064g00890
VIT_13s0064g00900
VIT_13s0064g00910
Standardized expressions
Developmental stages
EI PG G1 G2 H T1 T2 C1 C2 S EP JP

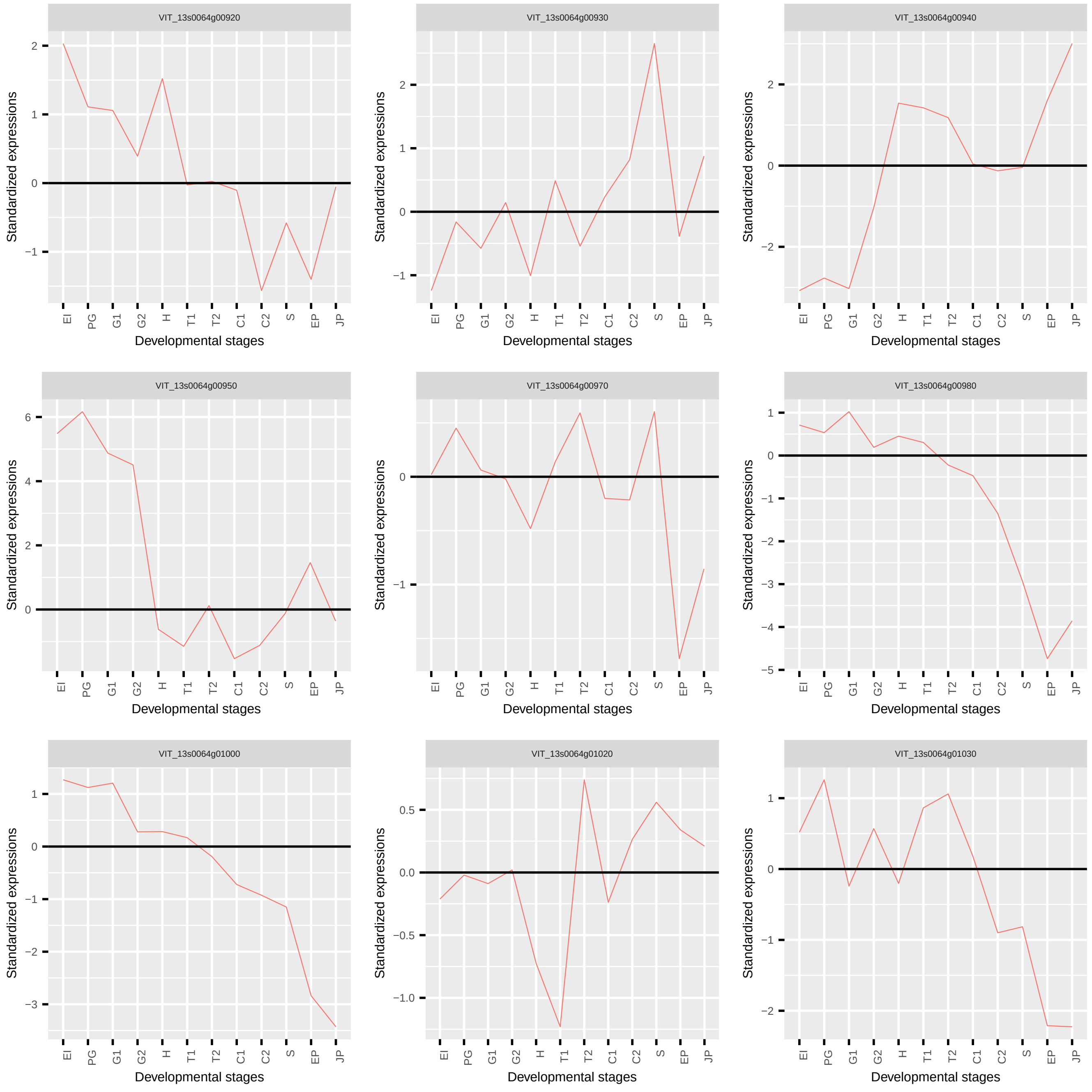
VIT_13s0064g00920
VIT_13s0064g00930
VIT_13s0064g00940
VIT_13s0064g00950
VIT_13s0064g00970
VIT_13s0064g00980
VIT_13s0064g01000
VIT_13s0064g01020
VIT_13s0064g01030
Standardized expressions
Developmental stages
EI PG G1 G2 H T1 T2 C1 C2 S EP JP

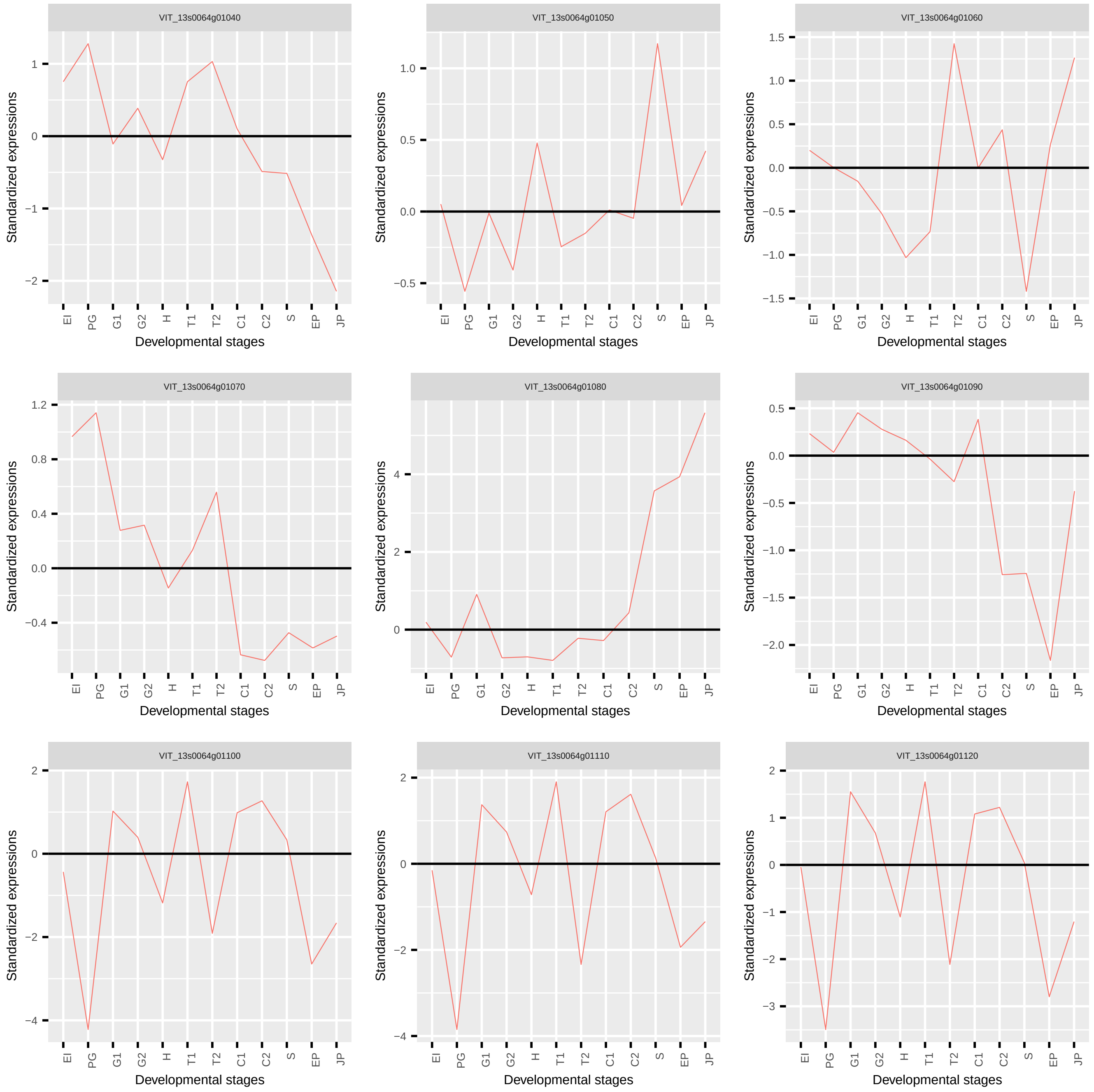
VIT_13s0064g01040
VIT_13s0064g01050
VIT_13s0064g01060
VIT_13s0064g01070
VIT_13s0064g01080
VIT_13s0064g01090
VIT_13s0064g01100
VIT_13s0064g01110
VIT_13s0064g01120
Standardized expressions
Developmental stages
EI
PG
G1
G2
H
T1
T2
C1
C2
S
EP
JP

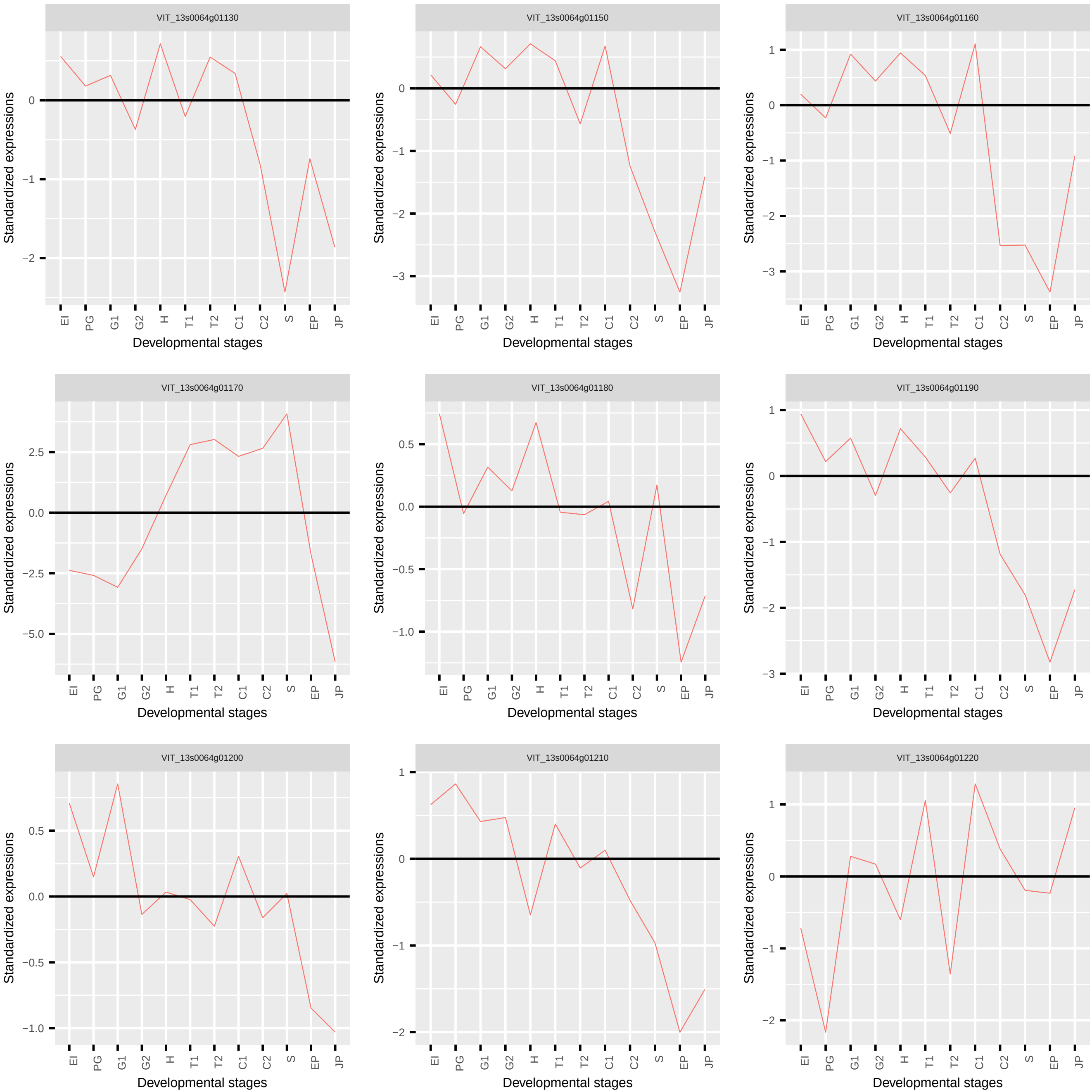
VIT_13s0064g01130
VIT_13s0064g01150
VIT_13s0064g01160
VIT_13s0064g01170
VIT_13s0064g01180
VIT_13s0064g01190
VIT_13s0064g01200
VIT_13s0064g01210
VIT_13s0064g01220
Standardized expressions
Developmental stages
EI
PG
G1
G2
H
T1
T2
C1
C2
S
EP
JP
1
0
−1
−2
−3
2.5
0.0
−2.5
−5.0
0.5
−0.5
−1.0

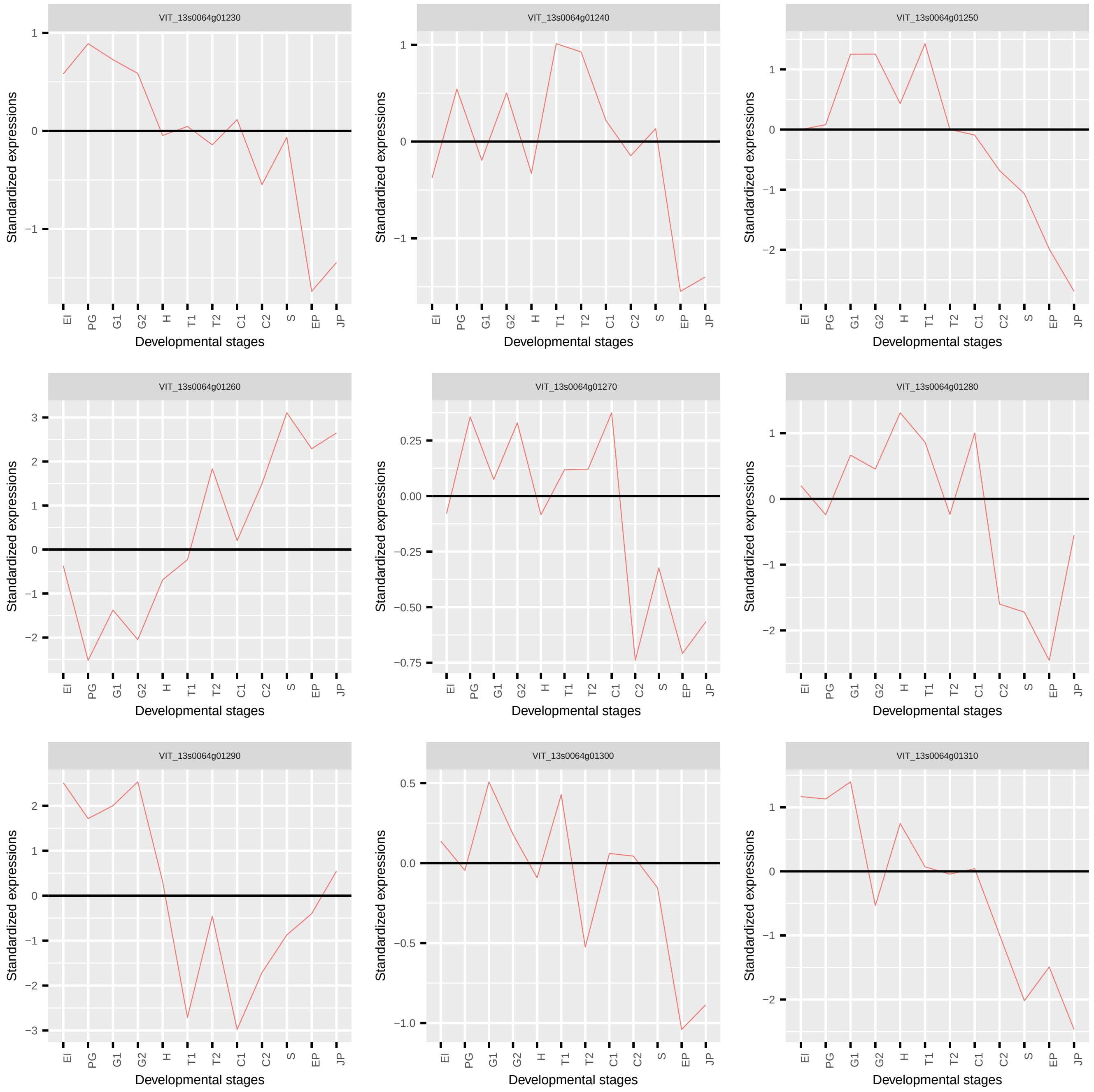
VIT_13s0064g01230
VIT_13s0064g01240
VIT_13s0064g01250
VIT_13s0064g01260
VIT_13s0064g01270
VIT_13s0064g01280
VIT_13s0064g01290
VIT_13s0064g01300
VIT_13s0064g01310
Standardized expressions
Developmental stages
EI PG G1 G2 H T1 T2 C1 C2 S EP JP

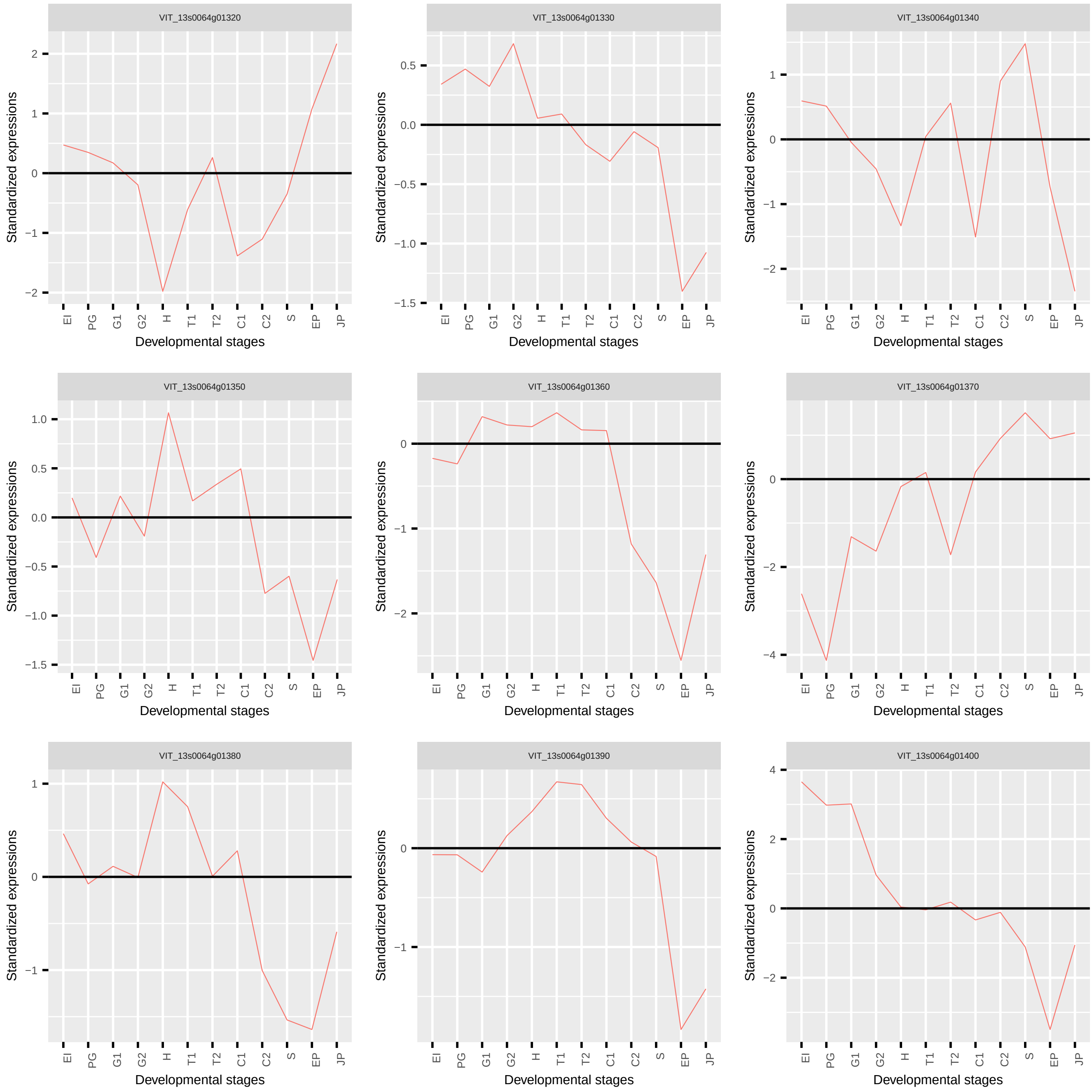

VIT_13s0064g01320
VIT_13s0064g01330
VIT_13s0064g01340
VIT_13s0064g01350
VIT_13s0064g01360
VIT_13s0064g01370
VIT_13s0064g01380
VIT_13s0064g01390
VIT_13s0064g01400
Standardized expressions
Developmental stages
EI
PG
G1
G2
H
T1
T2
C1
C2
S
EP
JP

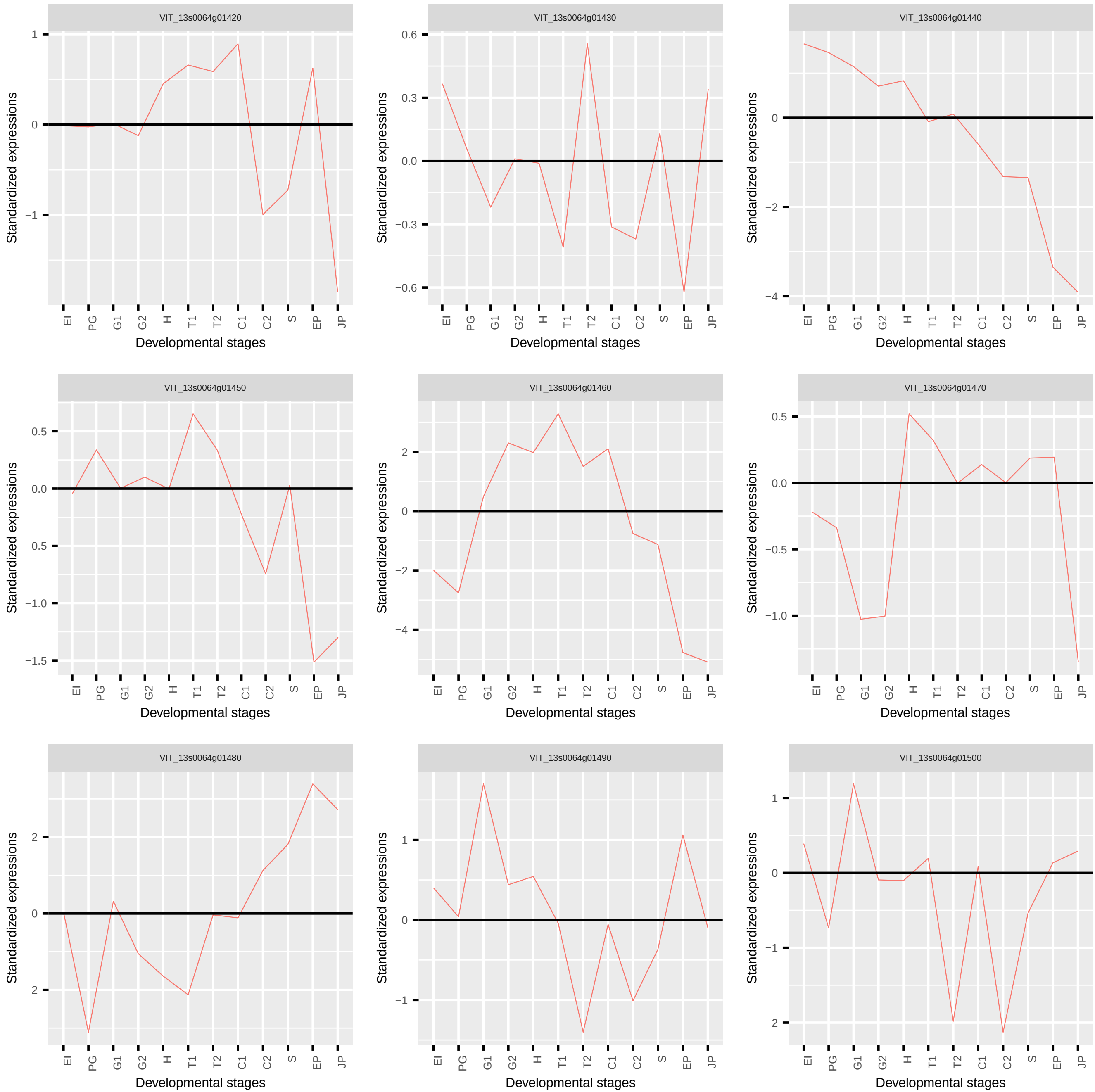

VIT_13s0064g01420
VIT_13s0064g01430
VIT_13s0064g01440
VIT_13s0064g01450
VIT_13s0064g01460
VIT_13s0064g01470
VIT_13s0064g01480
VIT_13s0064g01490
VIT_13s0064g01500
Standardized expressions
Developmental stages
EI PG G1 G2 H T1 T2 C1 C2 S EP JP

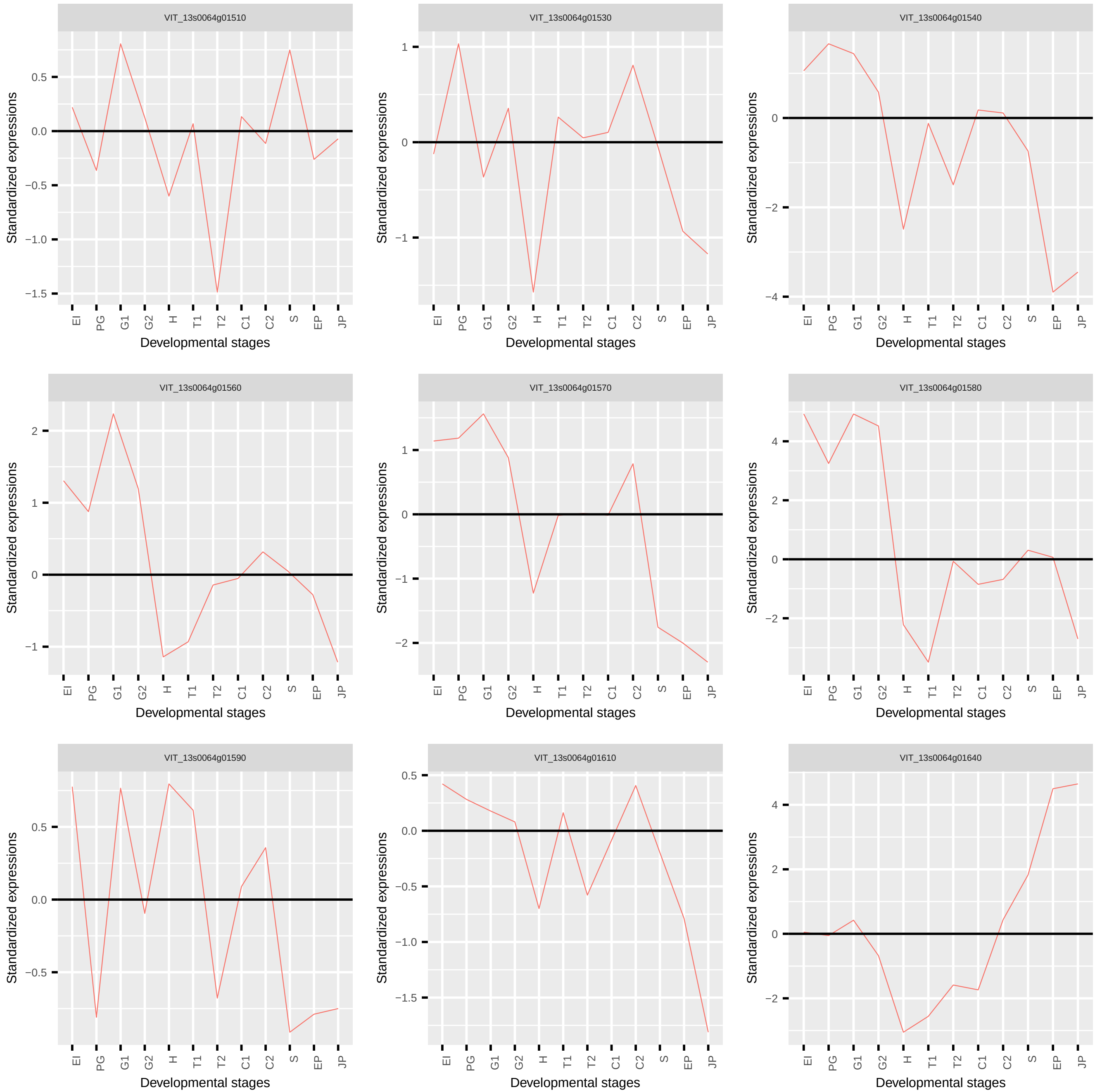
VIT_13s0064g01510
VIT_13s0064g01530
VIT_13s0064g01540
VIT_13s0064g01560
VIT_13s0064g01570
VIT_13s0064g01580
VIT_13s0064g01590
VIT_13s0064g01610
VIT_13s0064g01640
Standardized expressions
Developmental stages
EI
PG
G1
G2
H
T1
T2
C1
C2
S
EP
JP
0.5
0.0
−0.5
−1.0
−1.5
1
0
−1
−2
−4
2
4

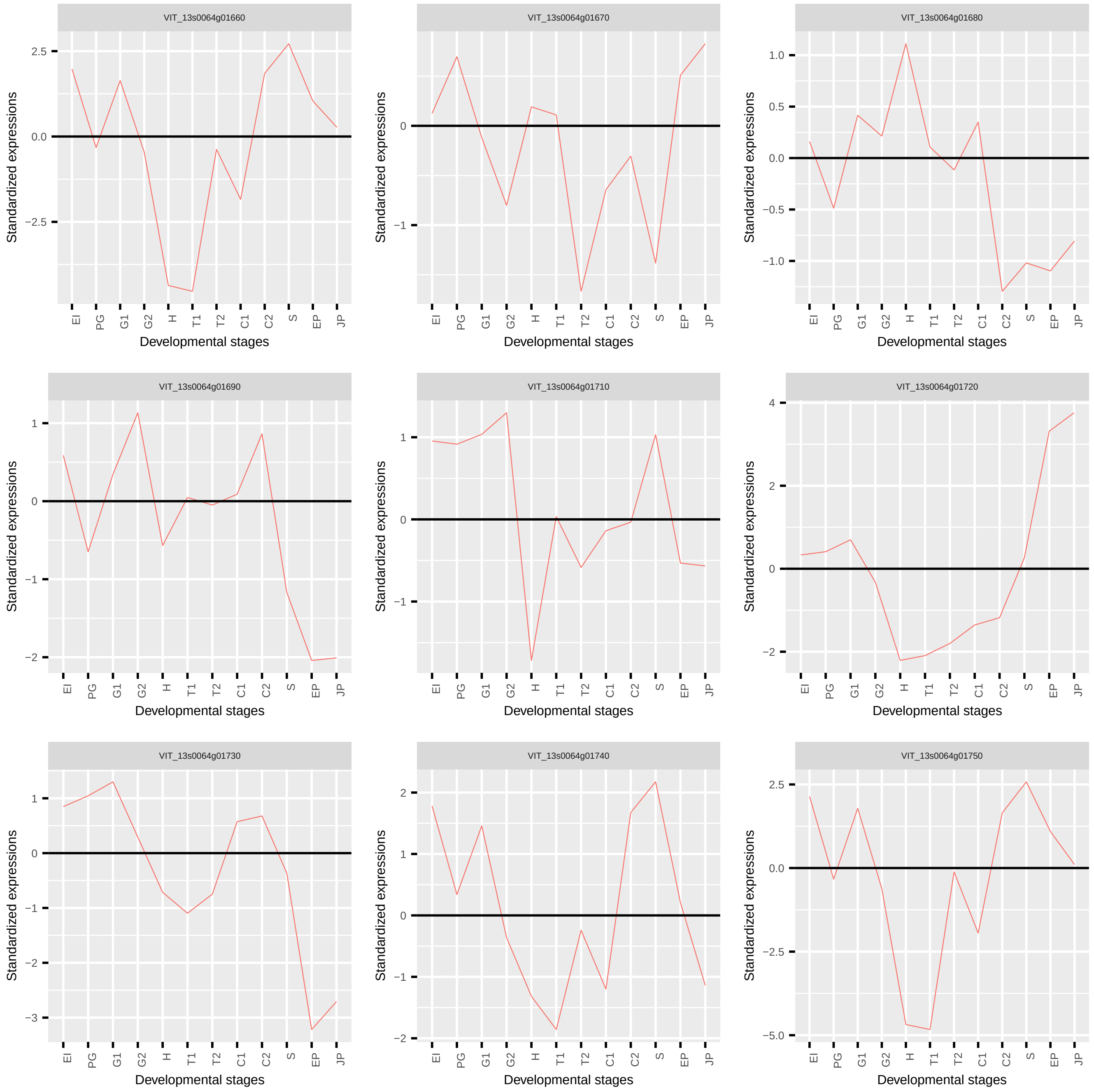

VIT_13s0064g01660
VIT_13s0064g01670
VIT_13s0064g01680
VIT_13s0064g01690
VIT_13s0064g01710
VIT_13s0064g01720
VIT_13s0064g01730
VIT_13s0064g01740
VIT_13s0064g01750
Standardized expressions
Developmental stages
EI PG G1 G2 H T1 T2 C1 C2 S EP JP

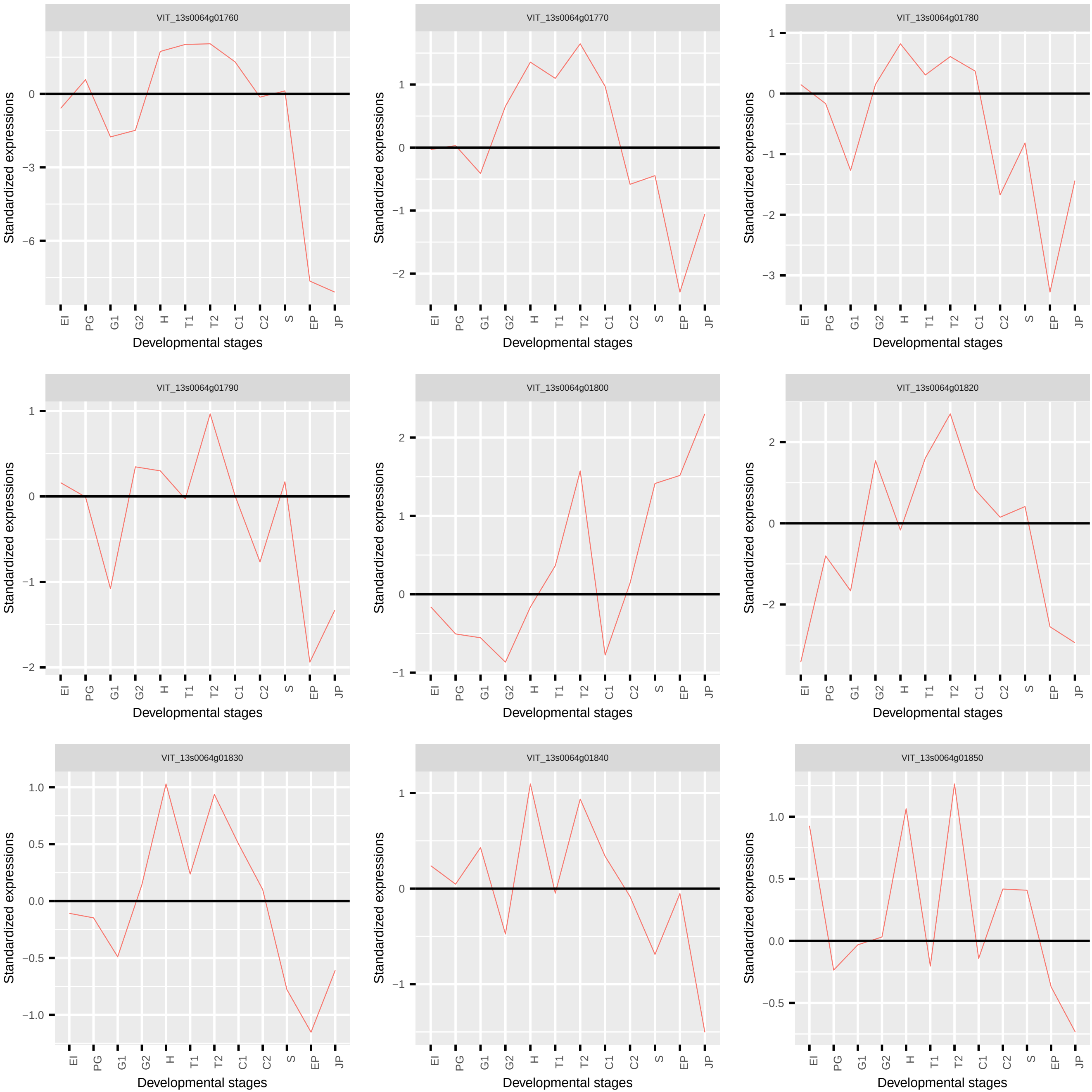
VIT_13s0064g01760
VIT_13s0064g01770
VIT_13s0064g01780
VIT_13s0064g01790
VIT_13s0064g01800
VIT_13s0064g01820
VIT_13s0064g01830
VIT_13s0064g01840
VIT_13s0064g01850
Standardized expressions
Developmental stages
EI
PG
G1
G2
H
T1
T2
C1
C2
S
EP
JP

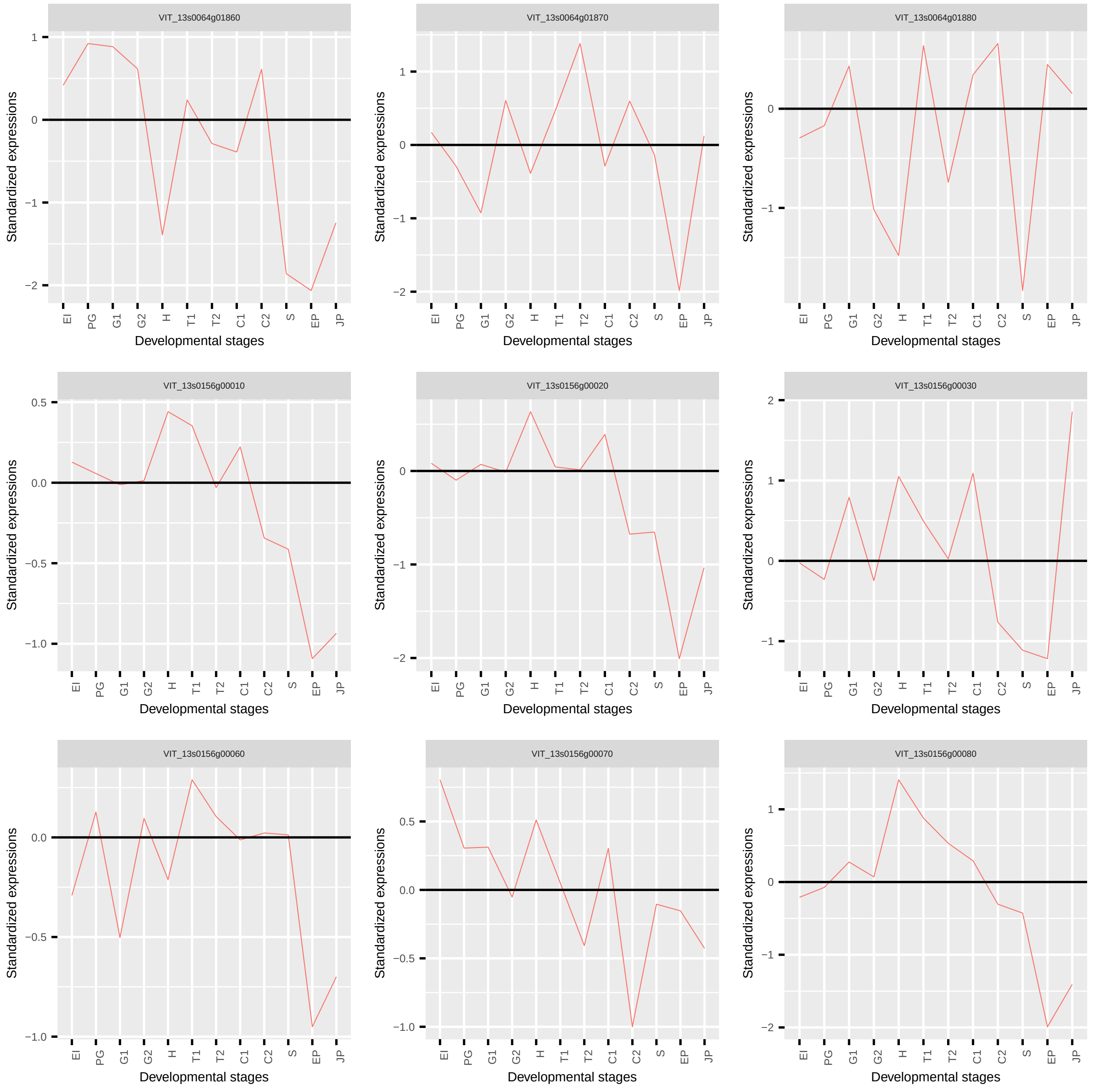
VIT_13s0064g01860
VIT_13s0064g01870
VIT_13s0064g01880
VIT_13s0156g00010
VIT_13s0156g00020
VIT_13s0156g00030
VIT_13s0156g00060
VIT_13s0156g00070
VIT_13s0156g00080
Standardized expressions
Developmental stages
EI
PG
G1
G2
H
T1
T2
C1
C2
S
EP
JP

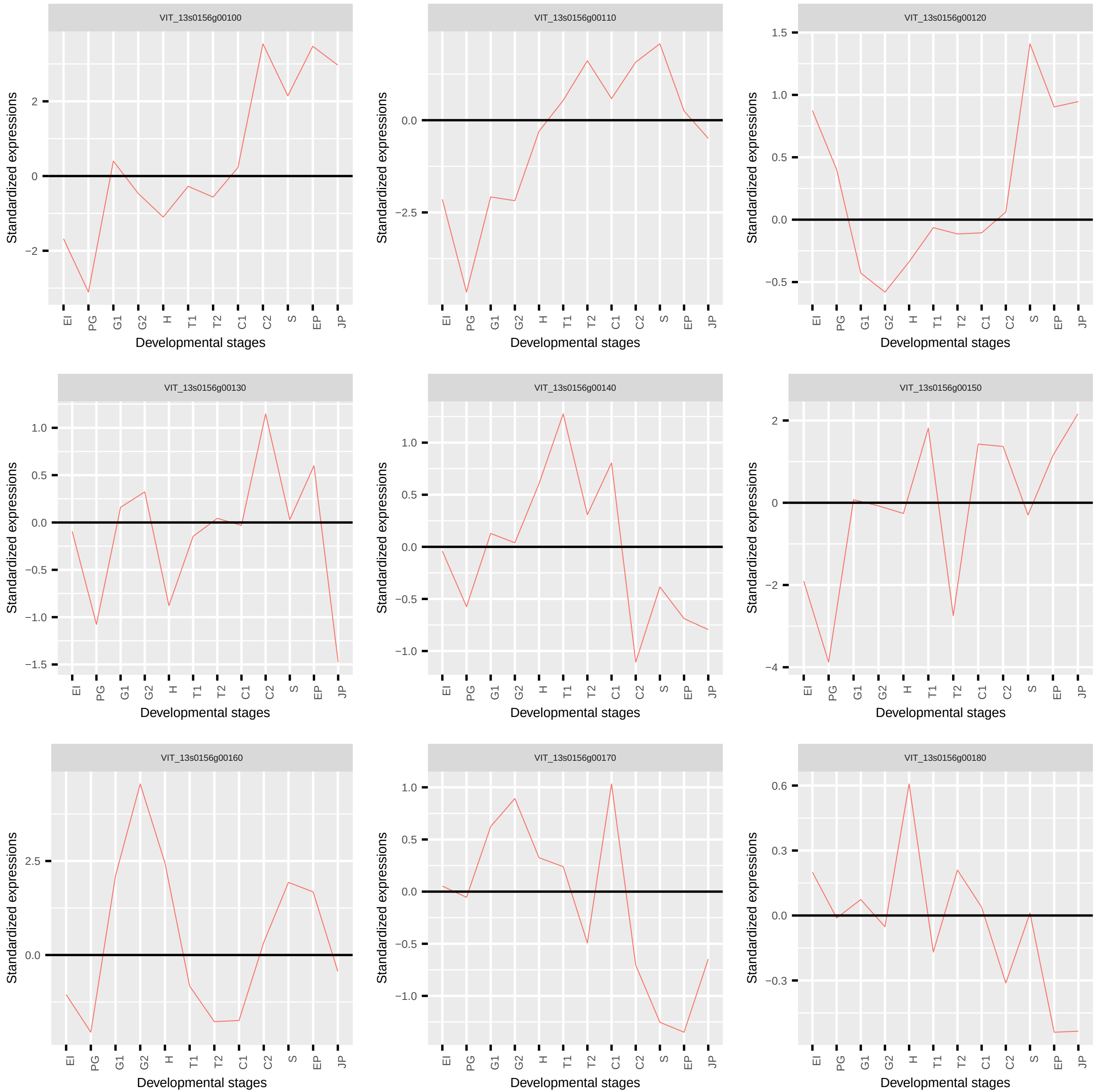
VIT_13s0156g00100
VIT_13s0156g00110
VIT_13s0156g00120
VIT_13s0156g00130
VIT_13s0156g00140
VIT_13s0156g00150
VIT_13s0156g00160
VIT_13s0156g00170
VIT_13s0156g00180
Standardized expressions
Developmental stages
EI
PG
G1
G2
H
T1
T2
C1
C2
S
EP
JP

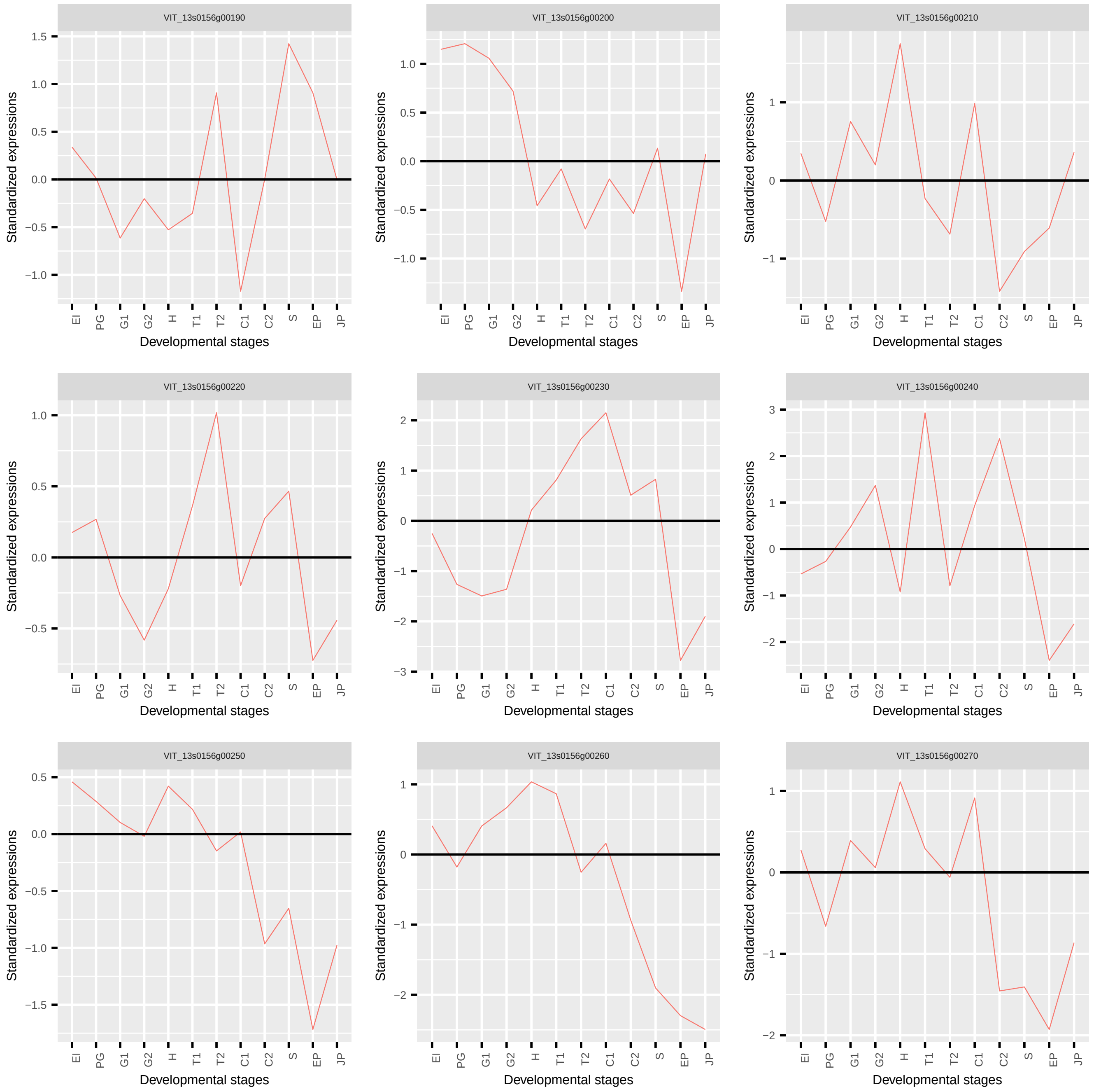
VIT_13s0156g00190
VIT_13s0156g00200
VIT_13s0156g00210
VIT_13s0156g00220
VIT_13s0156g00230
VIT_13s0156g00240
VIT_13s0156g00250
VIT_13s0156g00260
VIT_13s0156g00270
Standardized expressions
Developmental stages
EI
PG
G1
G2
H
T1
T2
C1
C2
S
EP
JP

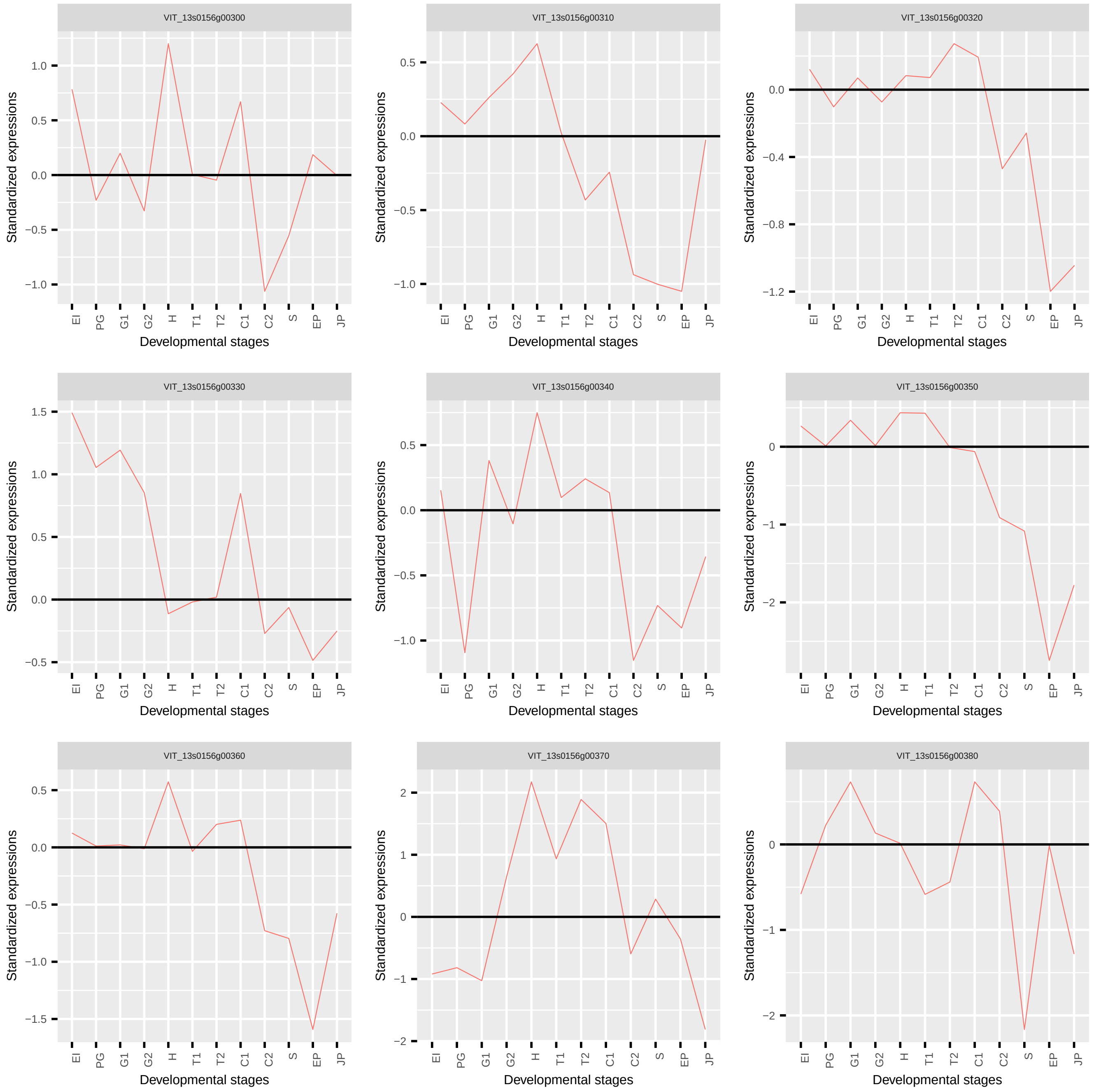
VIT_13s0156g00300
VIT_13s0156g00310
VIT_13s0156g00320
VIT_13s0156g00330
VIT_13s0156g00340
VIT_13s0156g00350
VIT_13s0156g00360
VIT_13s0156g00370
VIT_13s0156g00380
Standardized expressions
Developmental stages
EI
PG
G1
G2
H
T1
T2
C1
C2
S
EP
JP

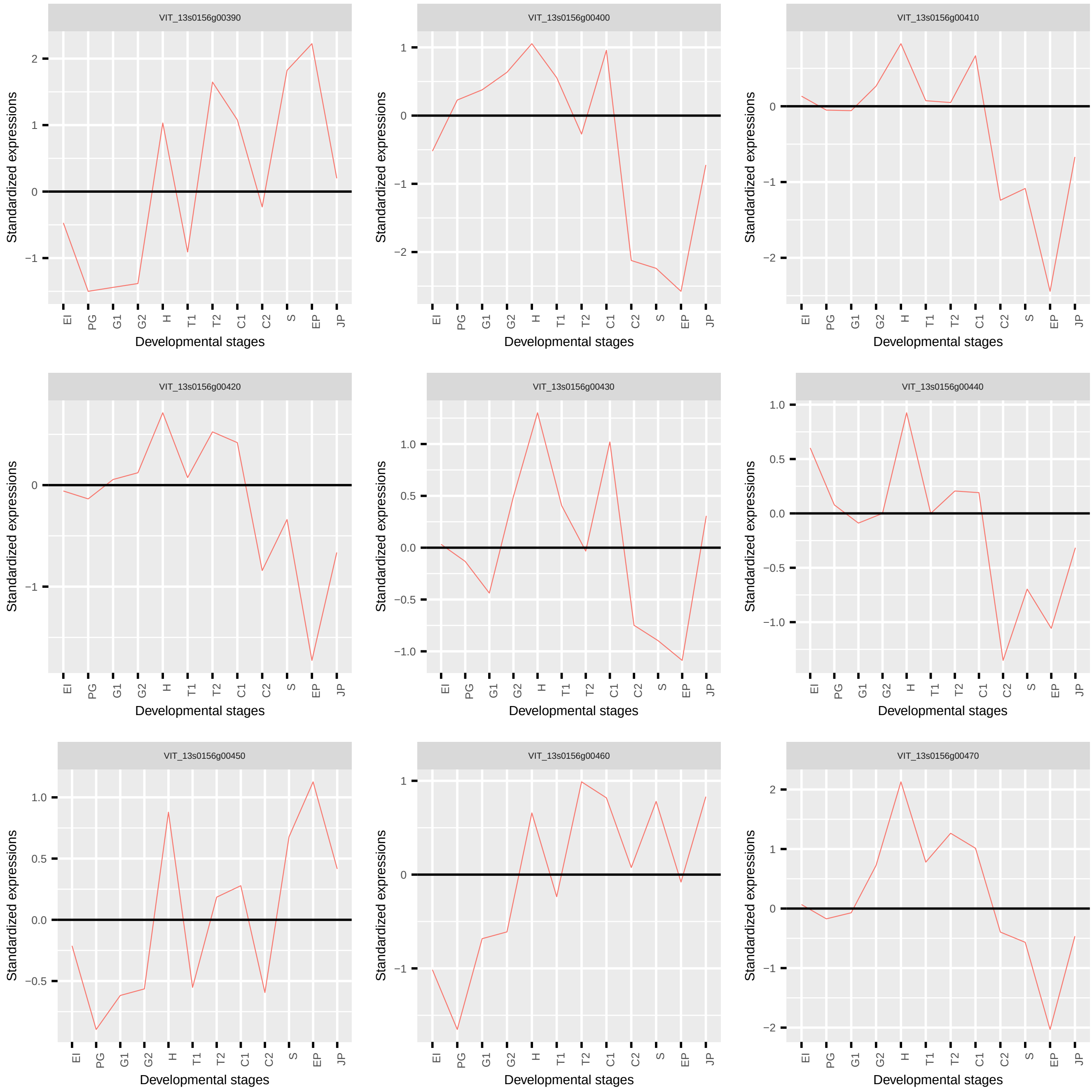

VIT_13s0156g00390
Standardized expressions
2
1
0
−1
EI PG G1 G2 H T1 T2 C1 C2 S EP JP
Developmental stages
VIT_13s0156g00400
Standardized expressions
1
0
−1
−2
EI PG G1 G2 H T1 T2 C1 C2 S EP JP
Developmental stages
VIT_13s0156g00410
Standardized expressions
0
−1
−2
EI PG G1 G2 H T1 T2 C1 C2 S EP JP
Developmental stages
VIT_13s0156g00420
Standardized expressions
0
−1
EI PG G1 G2 H T1 T2 C1 C2 S EP JP
Developmental stages
VIT_13s0156g00430
Standardized expressions
1.0
0.5
0.0
−0.5
−1.0
EI PG G1 G2 H T1 T2 C1 C2 S EP JP
Developmental stages
VIT_13s0156g00440
Standardized expressions
1.0
0.5
0.0
−0.5
−1.0
EI PG G1 G2 H T1 T2 C1 C2 S EP JP
Developmental stages
VIT_13s0156g00450
Standardized expressions
1.0
0.5
0.0
−0.5
EI PG G1 G2 H T1 T2 C1 C2 S EP JP
Developmental stages
VIT_13s0156g00460
Standardized expressions
1
0
−1
EI PG G1 G2 H T1 T2 C1 C2 S EP JP
Developmental stages
VIT_13s0156g00470
Standardized expressions
2
1
0
−1
−2
EI PG G1 G2 H T1 T2 C1 C2 S EP JP
Developmental stages

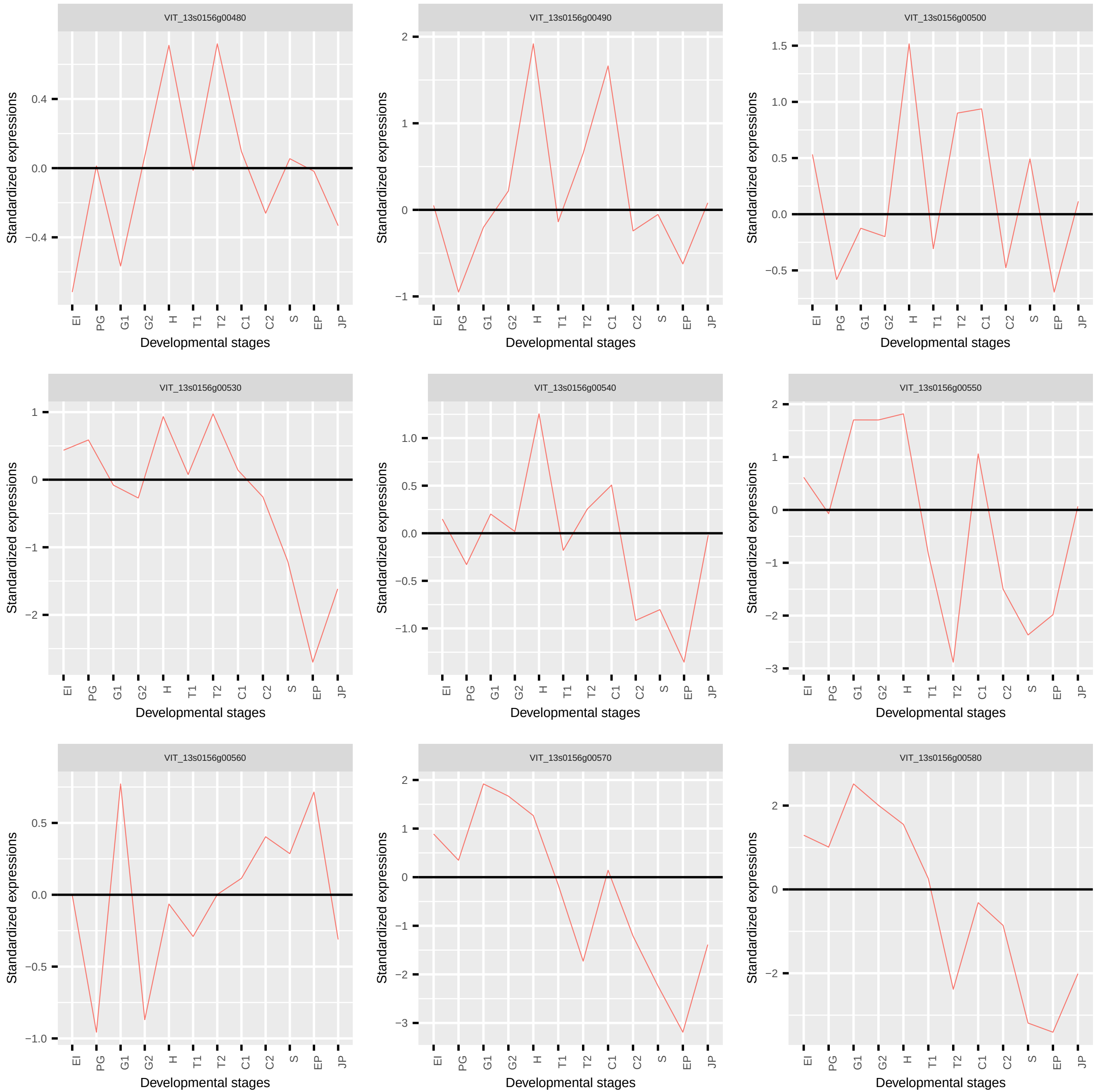
VIT_13s0156g00480
VIT_13s0156g00490
VIT_13s0156g00500
VIT_13s0156g00530
VIT_13s0156g00540
VIT_13s0156g00550
VIT_13s0156g00560
VIT_13s0156g00570
VIT_13s0156g00580
Standardized expressions
Developmental stages
EI
PG
G1
G2
H
T1
T2
C1
C2
S
EP
JP

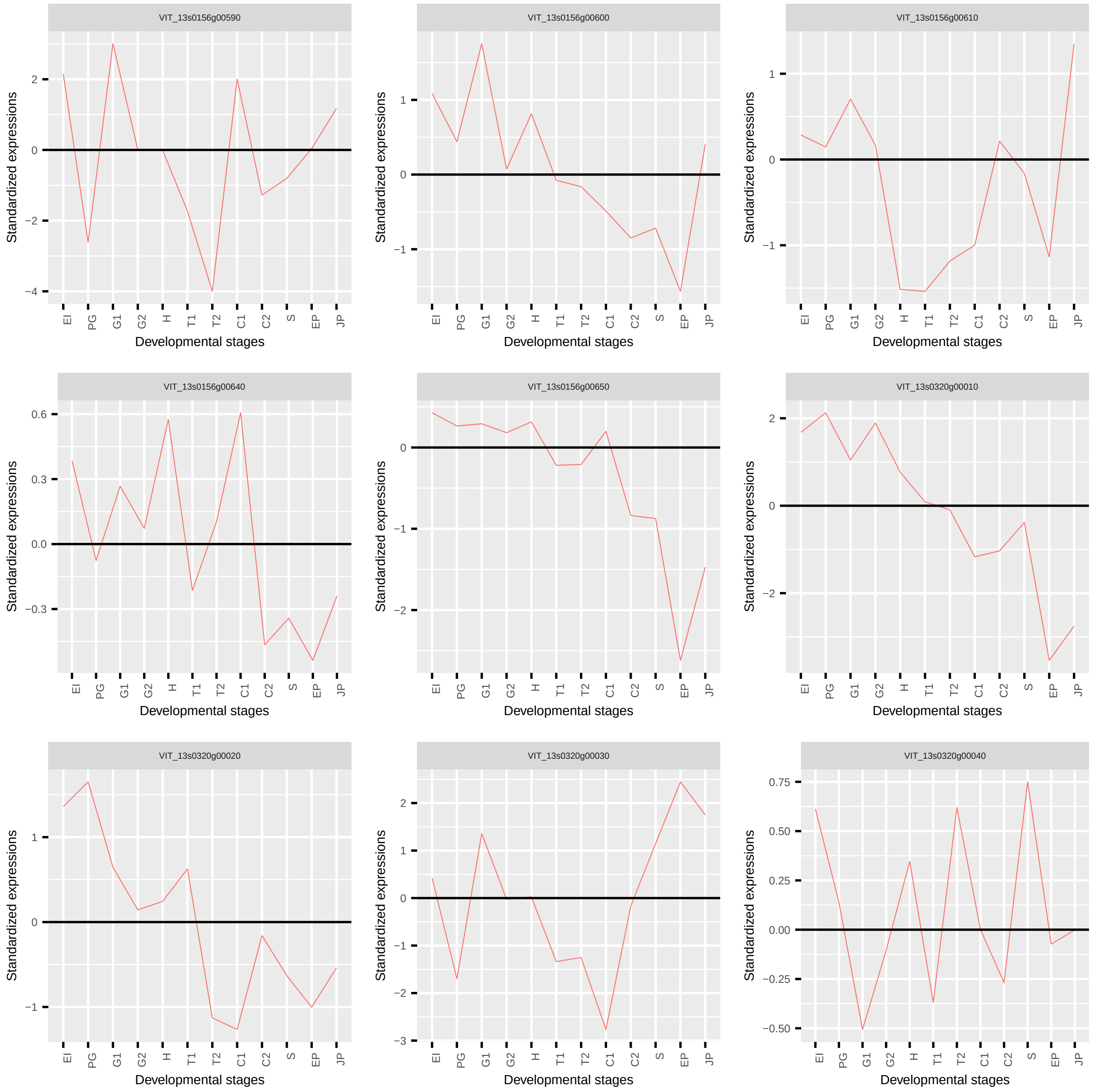
VIT_13s0156g00590
VIT_13s0156g00600
VIT_13s0156g00610
VIT_13s0156g00640
VIT_13s0156g00650
VIT_13s0320g00010
VIT_13s0320g00020
VIT_13s0320g00030
VIT_13s0320g00040
Standardized expressions
Developmental stages
EI
PG
G1
G2
H
T1
T2
C1
C2
S
EP
JP

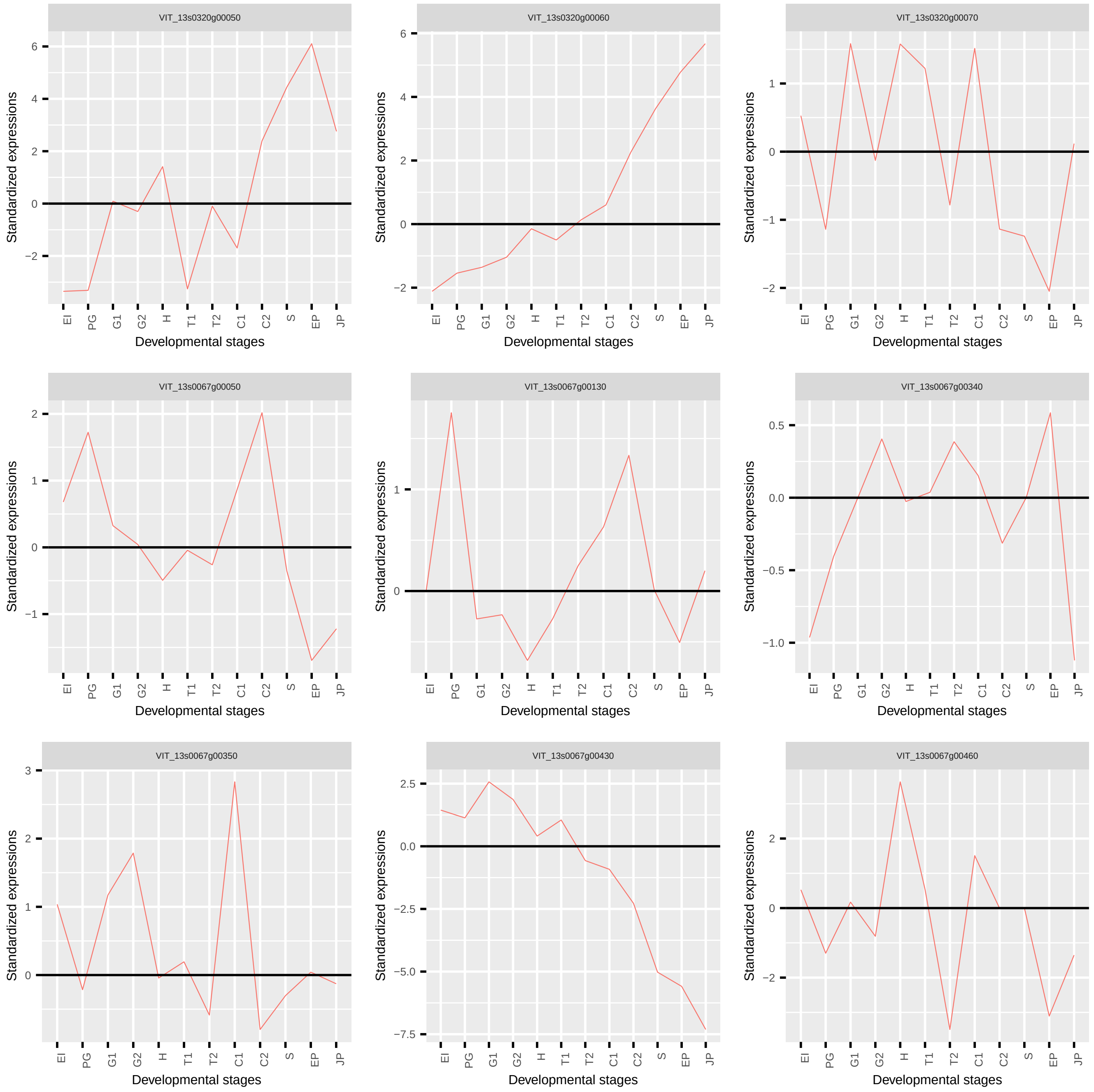

VIT_13s0320g00050
VIT_13s0320g00060
VIT_13s0320g00070
VIT_13s0067g00050
VIT_13s0067g00130
VIT_13s0067g00340
VIT_13s0067g00350
VIT_13s0067g00430
VIT_13s0067g00460
Standardized expressions
Developmental stages
EI PG G1 G2 H T1 T2 C1 C2 S EP JP

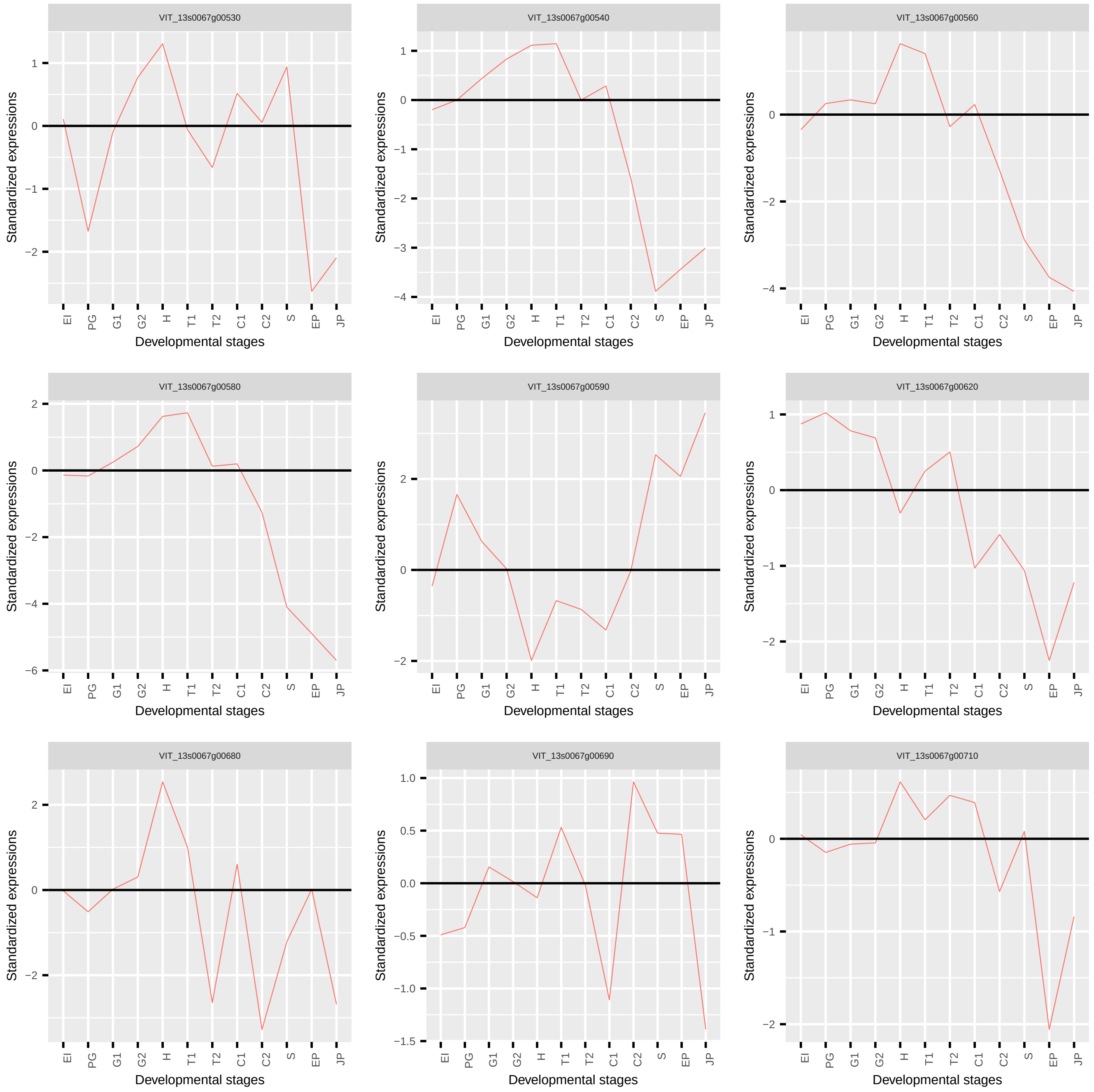
VIT_13s0067g00530
VIT_13s0067g00540
VIT_13s0067g00560
VIT_13s0067g00580
VIT_13s0067g00590
VIT_13s0067g00620
VIT_13s0067g00680
VIT_13s0067g00690
VIT_13s0067g00710
Standardized expressions
Developmental stages
EI
PG
G1
G2
H
T1
T2
C1
C2
S
EP
JP

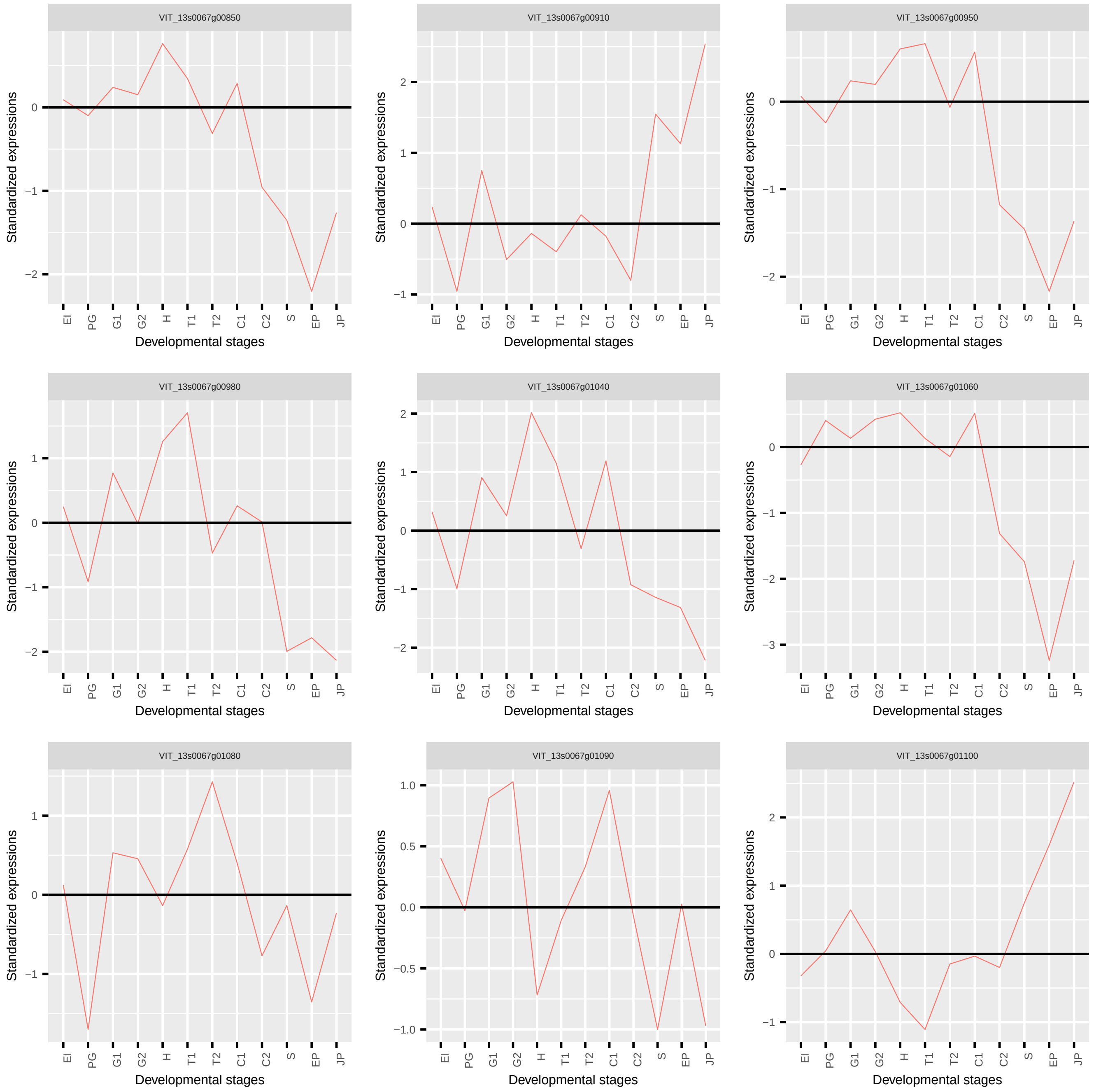
VIT_13s0067g00850
Standardized expressions
0
−1
−2
EI PG G1 G2 H T1 T2 C1 C2 S EP JP
Developmental stages
VIT_13s0067g00910
Standardized expressions
2
1
0
−1
EI PG G1 G2 H T1 T2 C1 C2 S EP JP
Developmental stages
VIT_13s0067g00950
Standardized expressions
0
−1
−2
EI PG G1 G2 H T1 T2 C1 C2 S EP JP
Developmental stages
VIT_13s0067g00980
Standardized expressions
1
0
−1
−2
EI PG G1 G2 H T1 T2 C1 C2 S EP JP
Developmental stages
VIT_13s0067g01040
Standardized expressions
2
1
0
−1
−2
EI PG G1 G2 H T1 T2 C1 C2 S EP JP
Developmental stages
VIT_13s0067g01060
Standardized expressions
0
−1
−2
−3
EI PG G1 G2 H T1 T2 C1 C2 S EP JP
Developmental stages
VIT_13s0067g01080
Standardized expressions
1
0
−1
EI PG G1 G2 H T1 T2 C1 C2 S EP JP
Developmental stages
VIT_13s0067g01090
Standardized expressions
1.0
0.5
0.0
−0.5
−1.0
EI PG G1 G2 H T1 T2 C1 C2 S EP JP
Developmental stages
VIT_13s0067g01100
Standardized expressions
2
1
0
−1
EI PG G1 G2 H T1 T2 C1 C2 S EP JP
Developmental stages

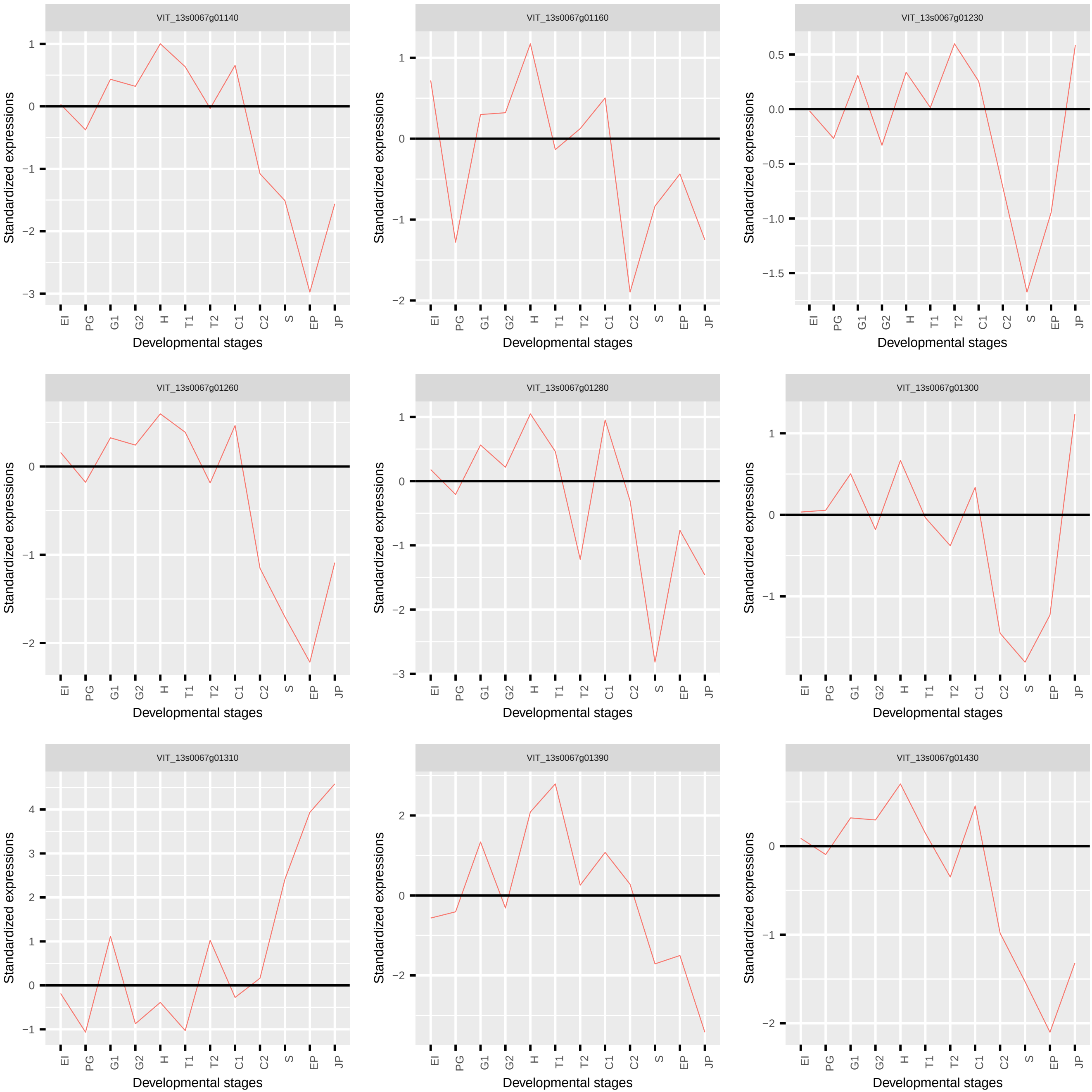
VIT_13s0067g01140
VIT_13s0067g01160
VIT_13s0067g01230
VIT_13s0067g01260
VIT_13s0067g01280
VIT_13s0067g01300
VIT_13s0067g01310
VIT_13s0067g01390
VIT_13s0067g01430
Standardized expressions
Developmental stages
EI
PG
G1
G2
H
T1
T2
C1
C2
S
EP
JP

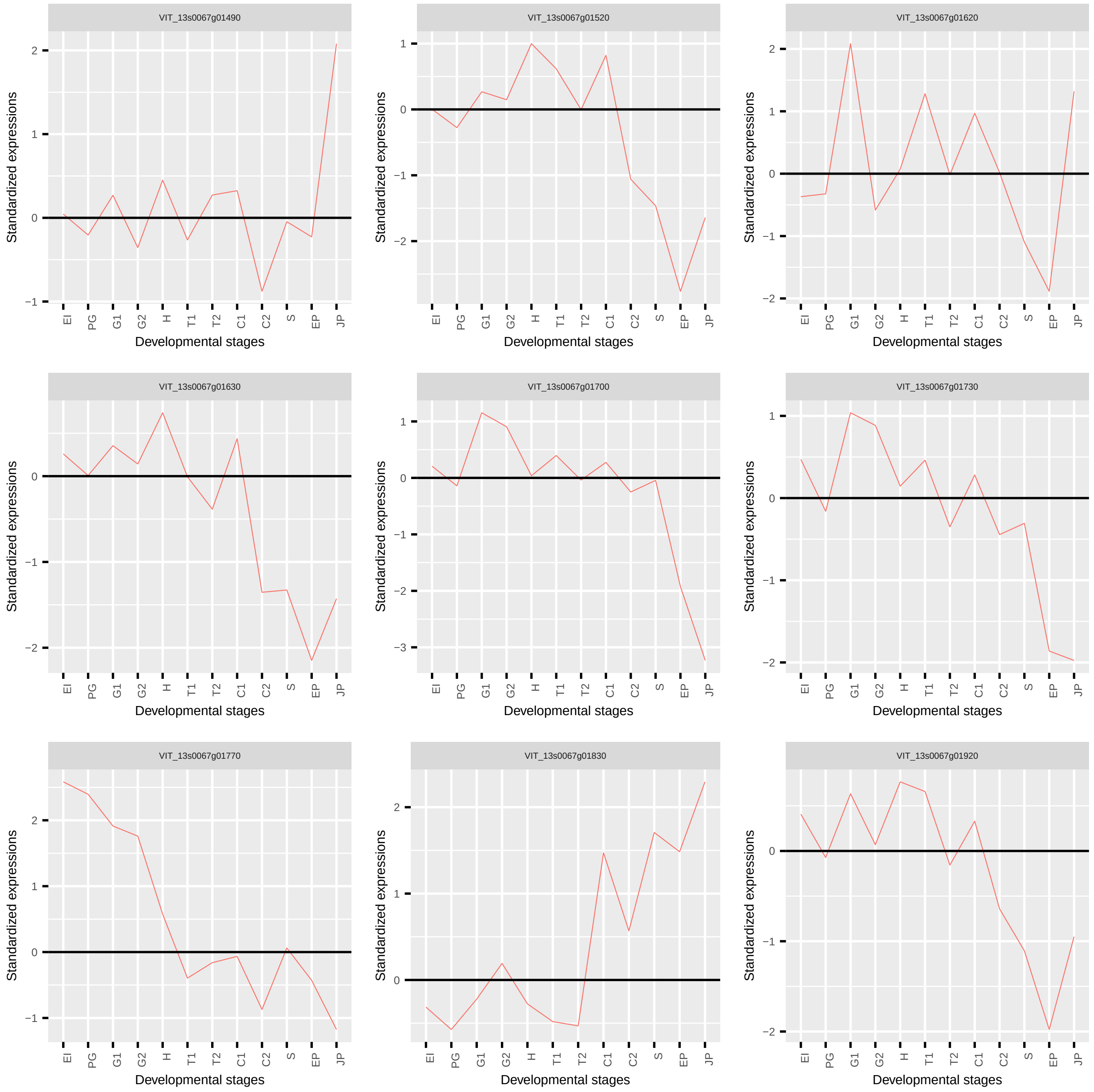
VIT_13s0067g01490
VIT_13s0067g01520
VIT_13s0067g01620
VIT_13s0067g01630
VIT_13s0067g01700
VIT_13s0067g01730
VIT_13s0067g01770
VIT_13s0067g01830
VIT_13s0067g01920
Standardized expressions
Developmental stages
EI PG G1 G2 H T1 T2 C1 C2 S EP JP

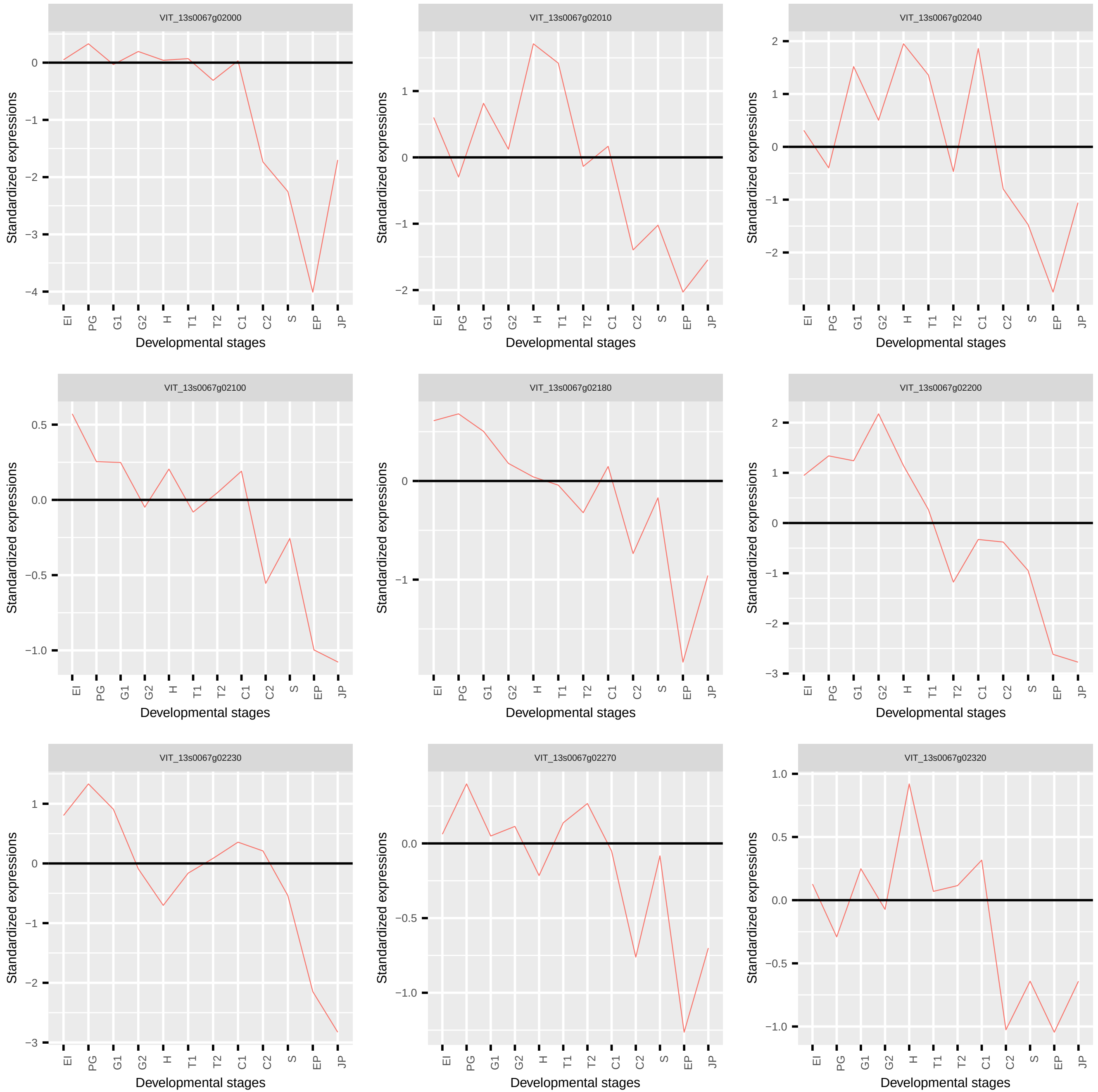
VIT_13s0067g02000
VIT_13s0067g02010
VIT_13s0067g02040
VIT_13s0067g02100
VIT_13s0067g02180
VIT_13s0067g02200
VIT_13s0067g02230
VIT_13s0067g02270
VIT_13s0067g02320
Standardized expressions
Developmental stages
EI PG G1 G2 H T1 T2 C1 C2 S EP JP

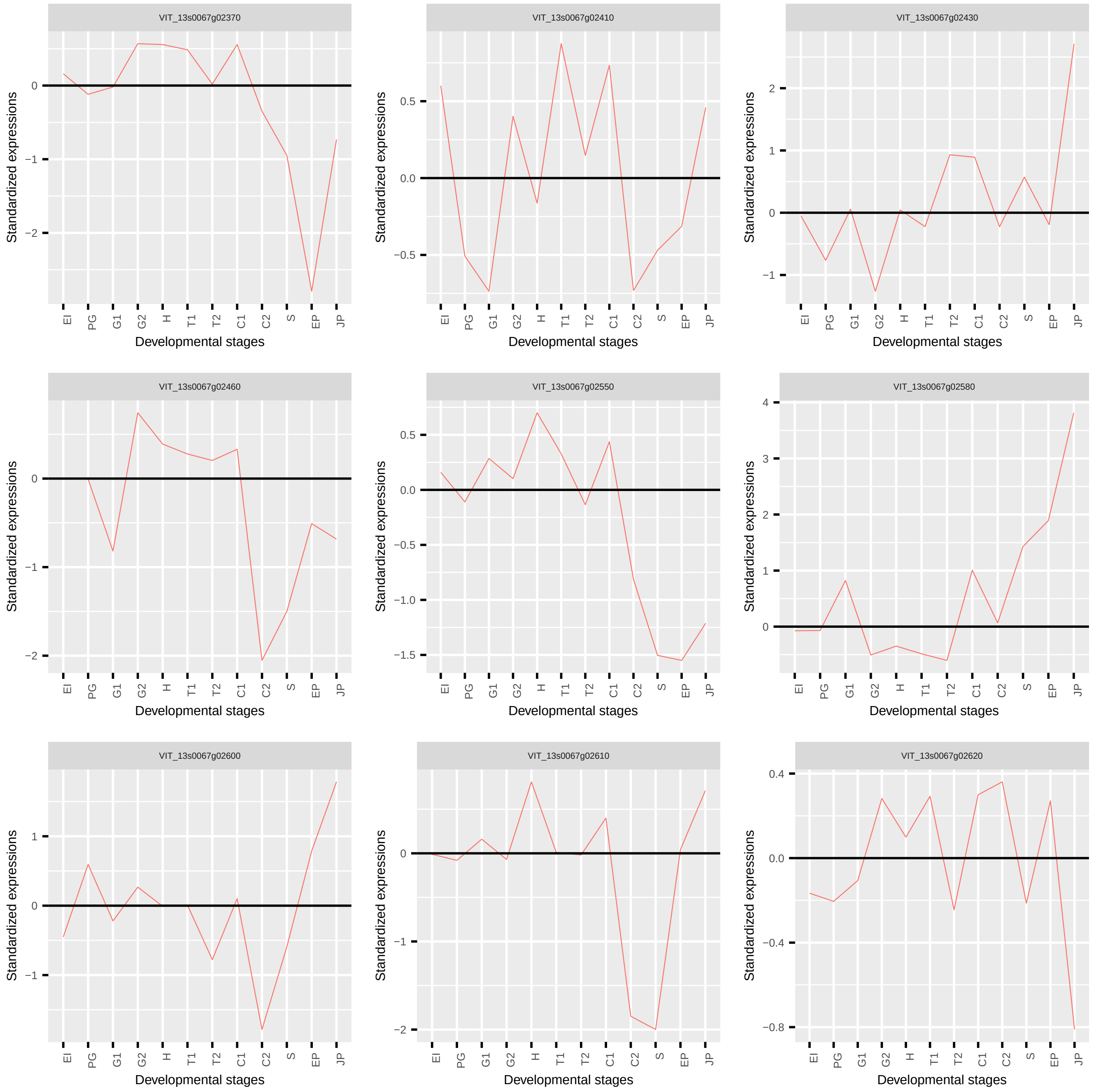
VIT_13s0067g02370
VIT_13s0067g02410
VIT_13s0067g02430
VIT_13s0067g02460
VIT_13s0067g02550
VIT_13s0067g02580
VIT_13s0067g02600
VIT_13s0067g02610
VIT_13s0067g02620
Standardized expressions
Developmental stages
EI PG G1 G2 H T1 T2 C1 C2 S EP JP

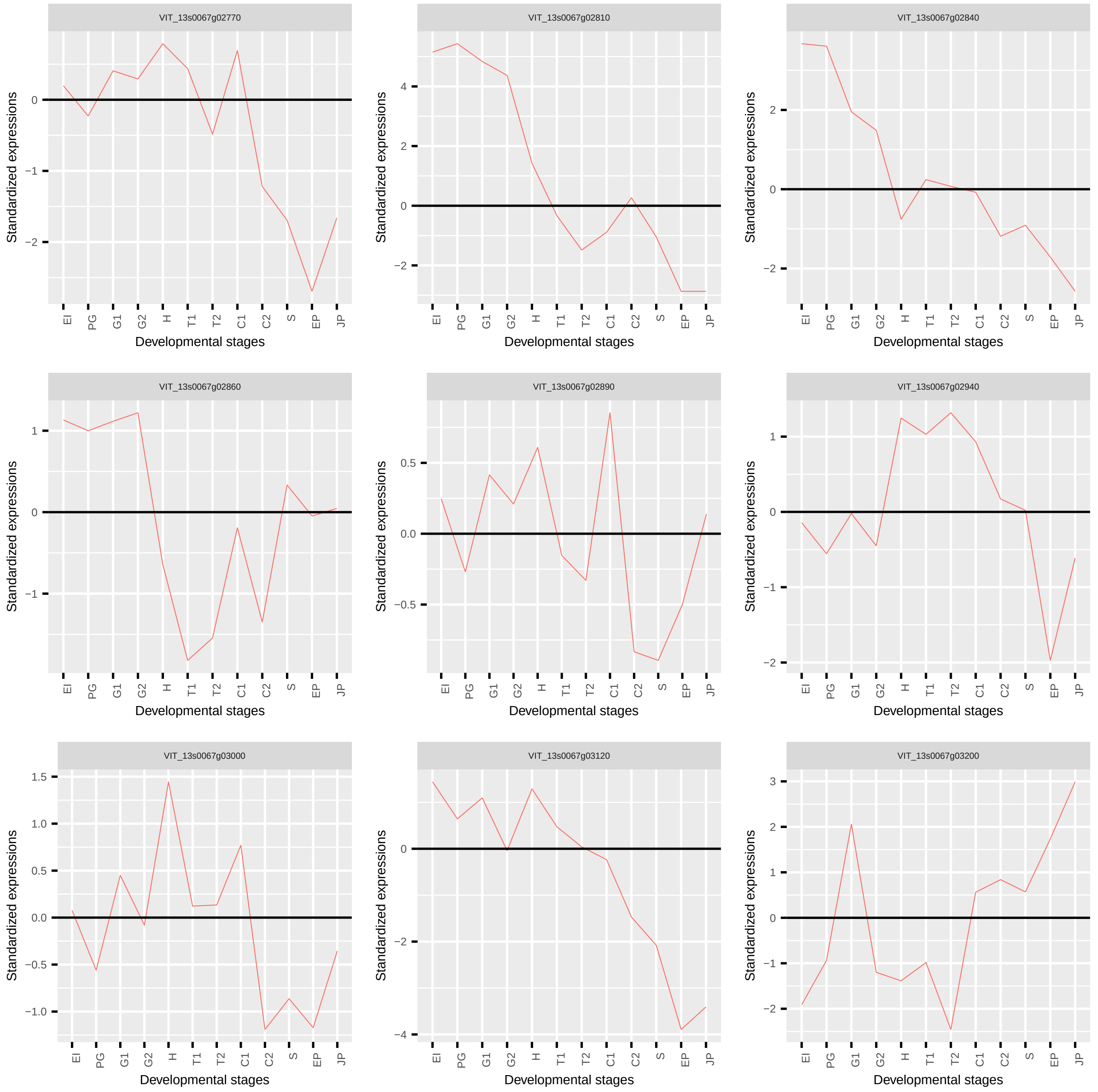
VIT_13s0067g02770
VIT_13s0067g02810
VIT_13s0067g02840
VIT_13s0067g02860
VIT_13s0067g02890
VIT_13s0067g02940
VIT_13s0067g03000
VIT_13s0067g03120
VIT_13s0067g03200
Standardized expressions
Developmental stages
EI PG G1 G2 H T1 T2 C1 C2 S EP JP

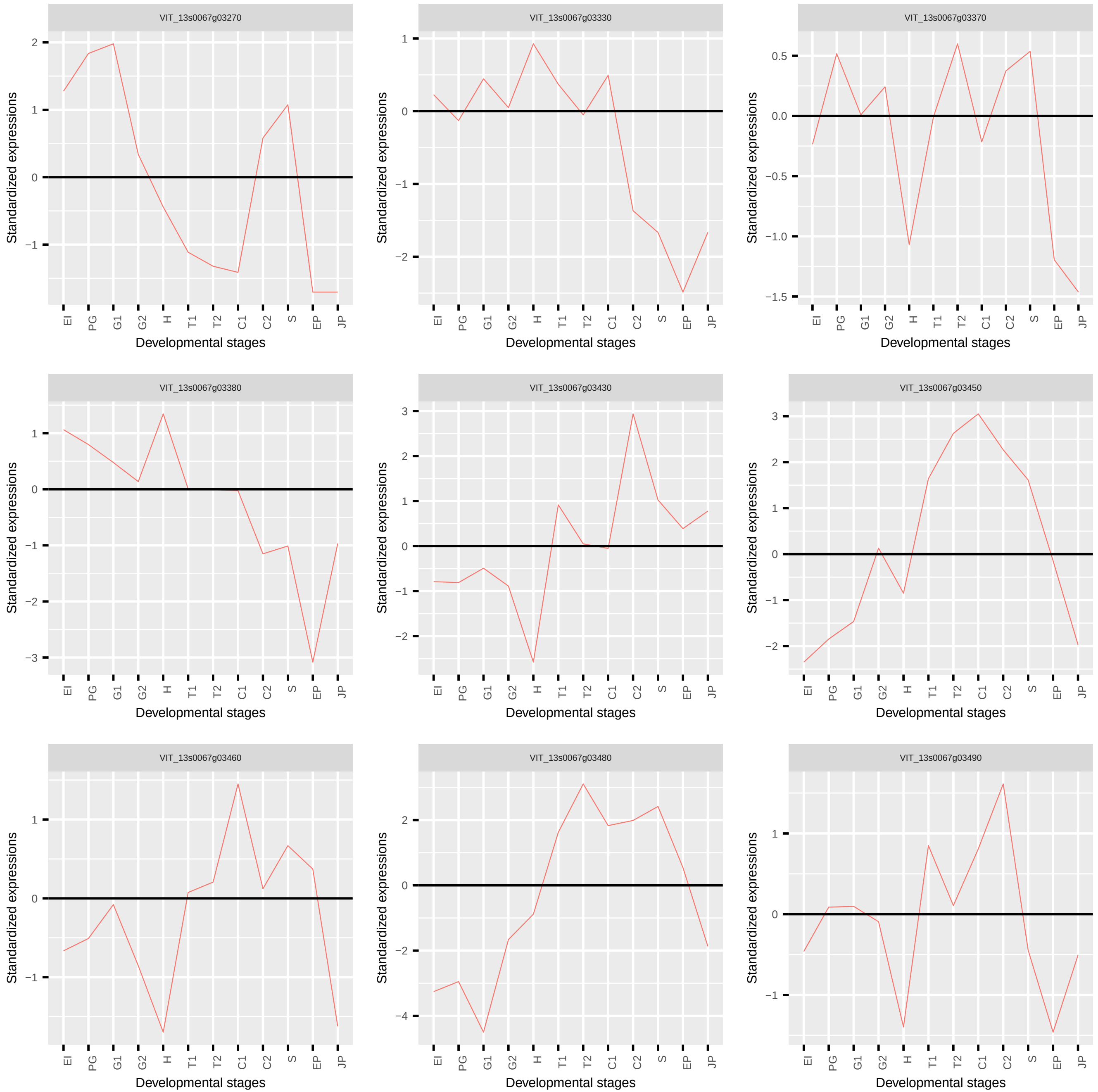
VIT_13s0067g03270
VIT_13s0067g03330
VIT_13s0067g03370
VIT_13s0067g03380
VIT_13s0067g03430
VIT_13s0067g03450
VIT_13s0067g03460
VIT_13s0067g03480
VIT_13s0067g03490
Standardized expressions
EI PG G1 G2 H T1 T2 C1 C2 S EP JP
Developmental stages

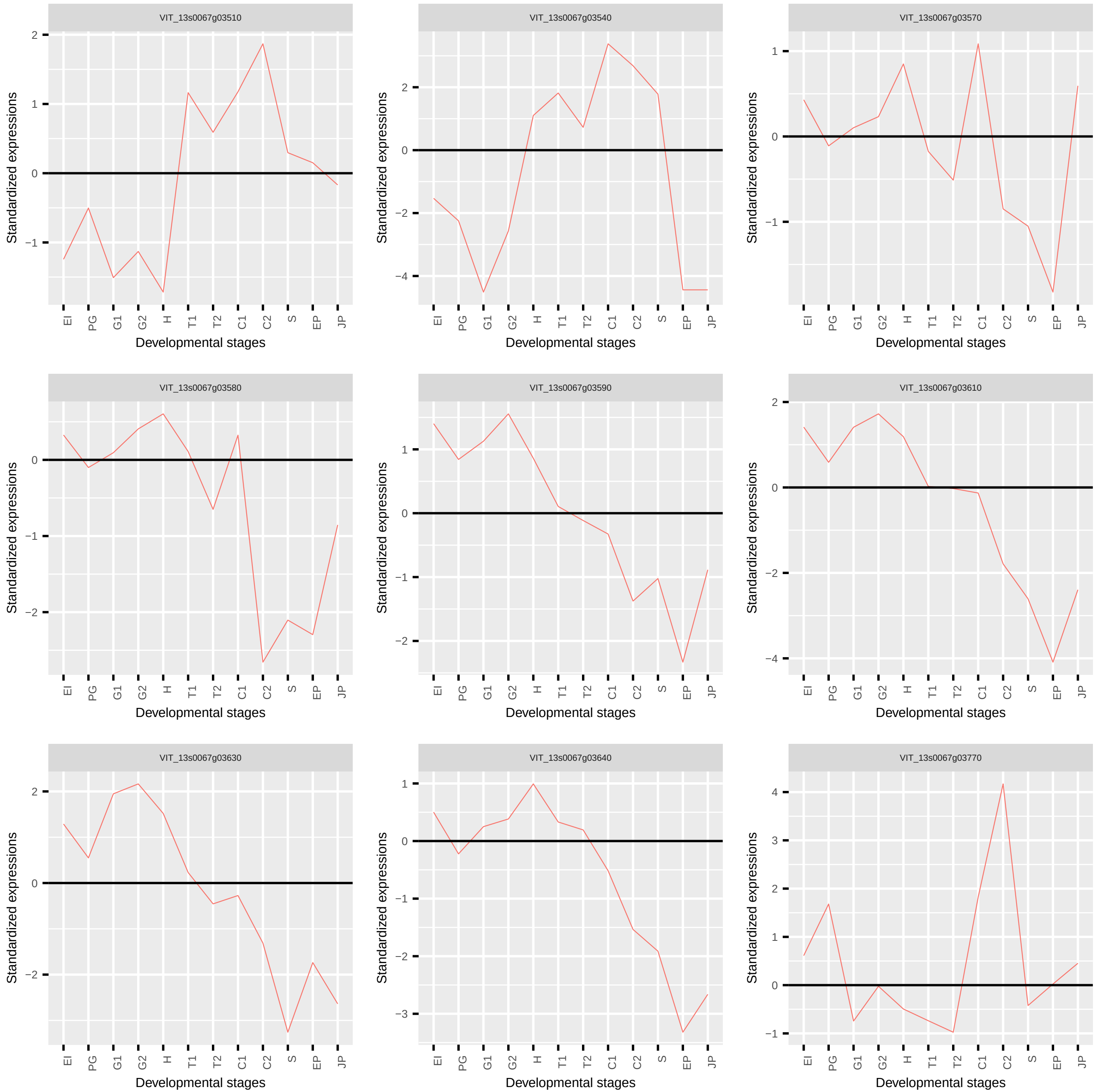
VIT_13s0067g03510
VIT_13s0067g03540
VIT_13s0067g03570
VIT_13s0067g03580
VIT_13s0067g03590
VIT_13s0067g03610
VIT_13s0067g03630
VIT_13s0067g03640
VIT_13s0067g03770
Standardized expressions
Developmental stages
EI
PG
G1
G2
H
T1
T2
C1
C2
S
EP
JP

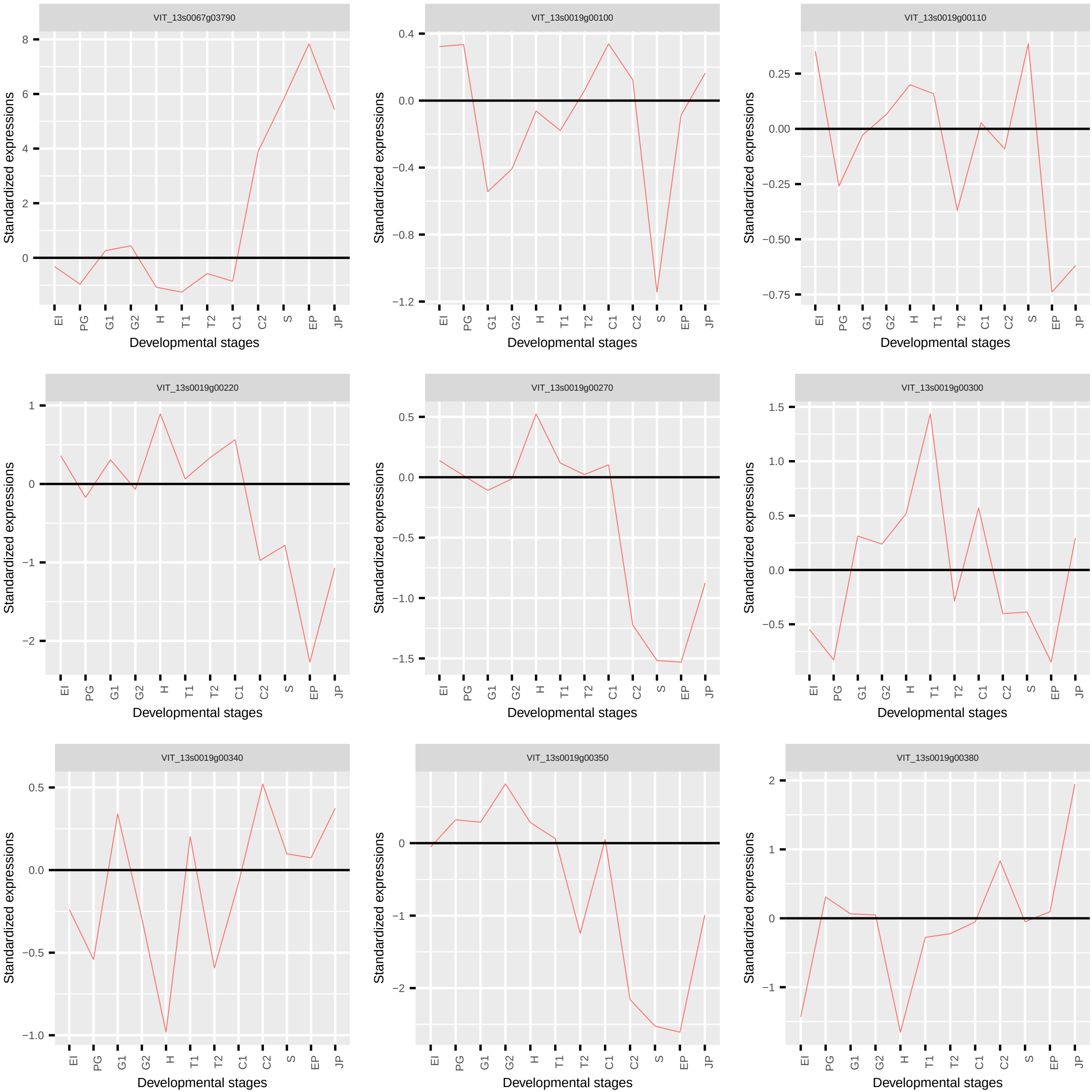
VIT_13s0067g03790
VIT_13s0019g00100
VIT_13s0019g00110
VIT_13s0019g00220
VIT_13s0019g00270
VIT_13s0019g00300
VIT_13s0019g00340
VIT_13s0019g00350
VIT_13s0019g00380
Standardized expressions
Developmental stages
EI PG G1 G2 H T1 T2 C1 C2 S EP JP

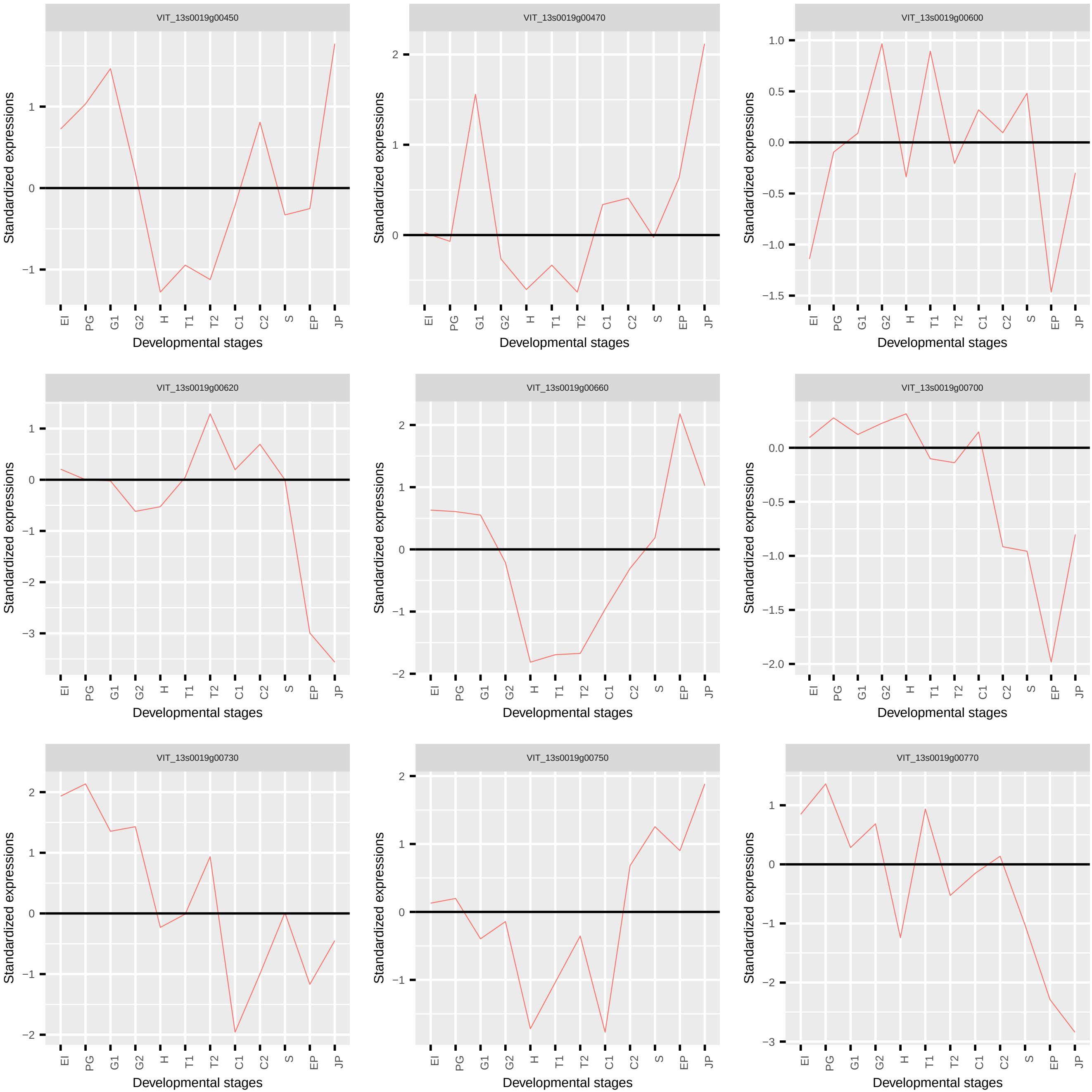
VIT_13s0019g00450
VIT_13s0019g00470
VIT_13s0019g00600
VIT_13s0019g00620
VIT_13s0019g00660
VIT_13s0019g00700
VIT_13s0019g00730
VIT_13s0019g00750
VIT_13s0019g00770
Standardized expressions
Developmental stages
EI PG G1 G2 H T1 T2 C1 C2 S EP JP

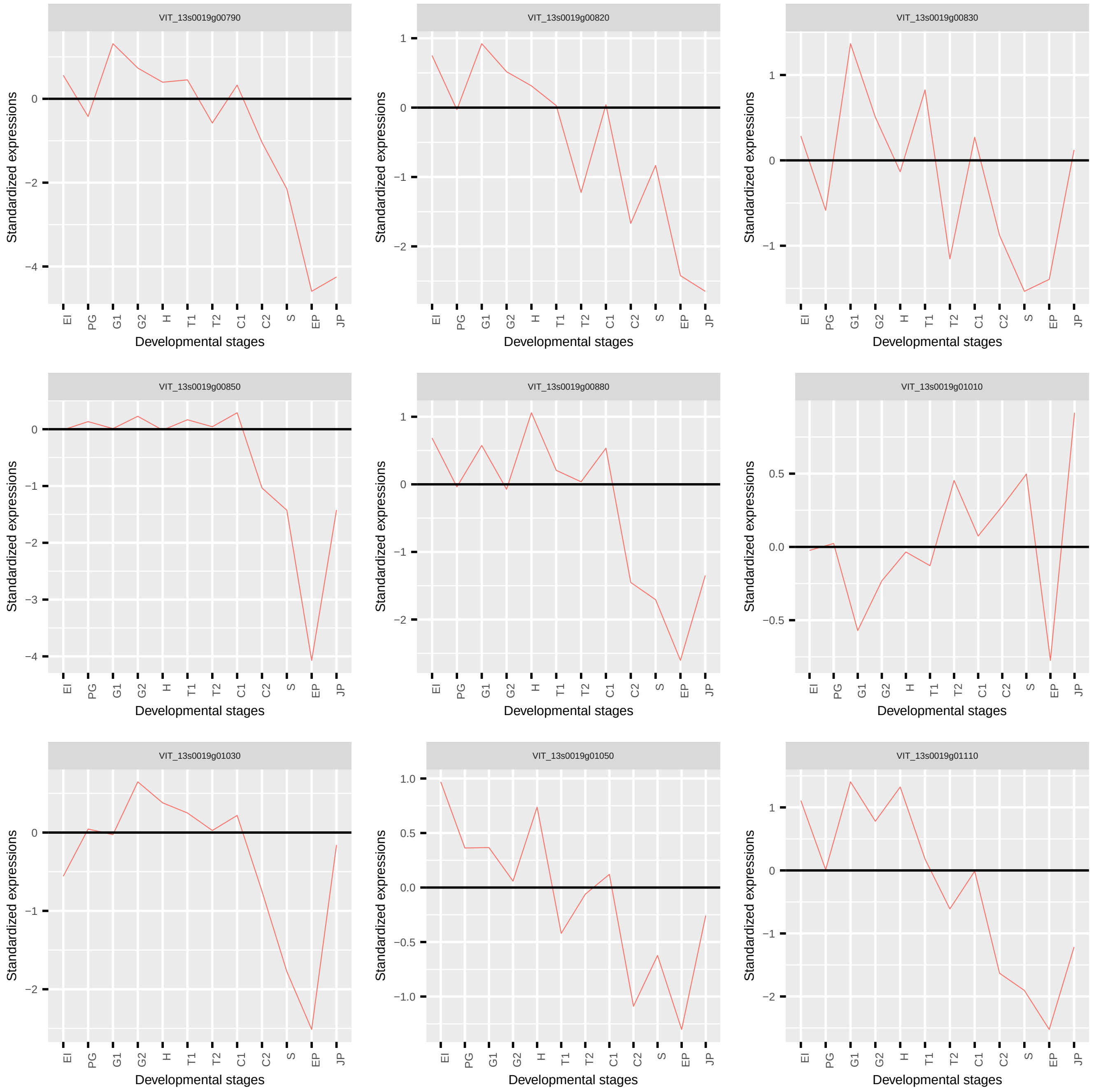
VIT_13s0019g00790
VIT_13s0019g00820
VIT_13s0019g00830
VIT_13s0019g00850
VIT_13s0019g00880
VIT_13s0019g01010
VIT_13s0019g01030
VIT_13s0019g01050
VIT_13s0019g01110
Standardized expressions
Developmental stages
EI PG G1 G2 H T1 T2 C1 C2 S EP JP

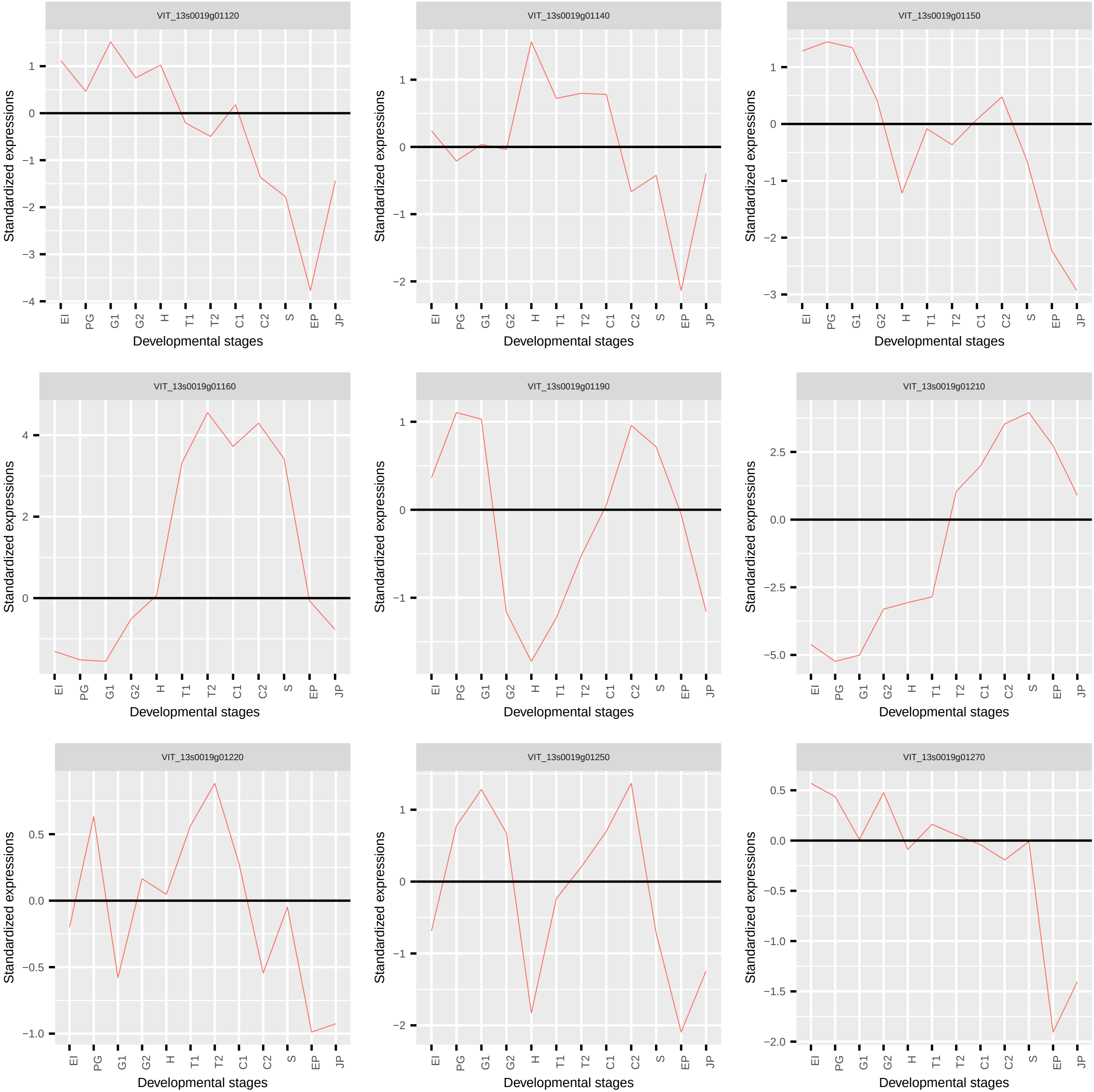
VIT_13s0019g01120
VIT_13s0019g01140
VIT_13s0019g01150
VIT_13s0019g01160
VIT_13s0019g01190
VIT_13s0019g01210
VIT_13s0019g01220
VIT_13s0019g01250
VIT_13s0019g01270
Standardized expressions
Developmental stages
EI PG G1 G2 H T1 T2 C1 C2 S EP JP

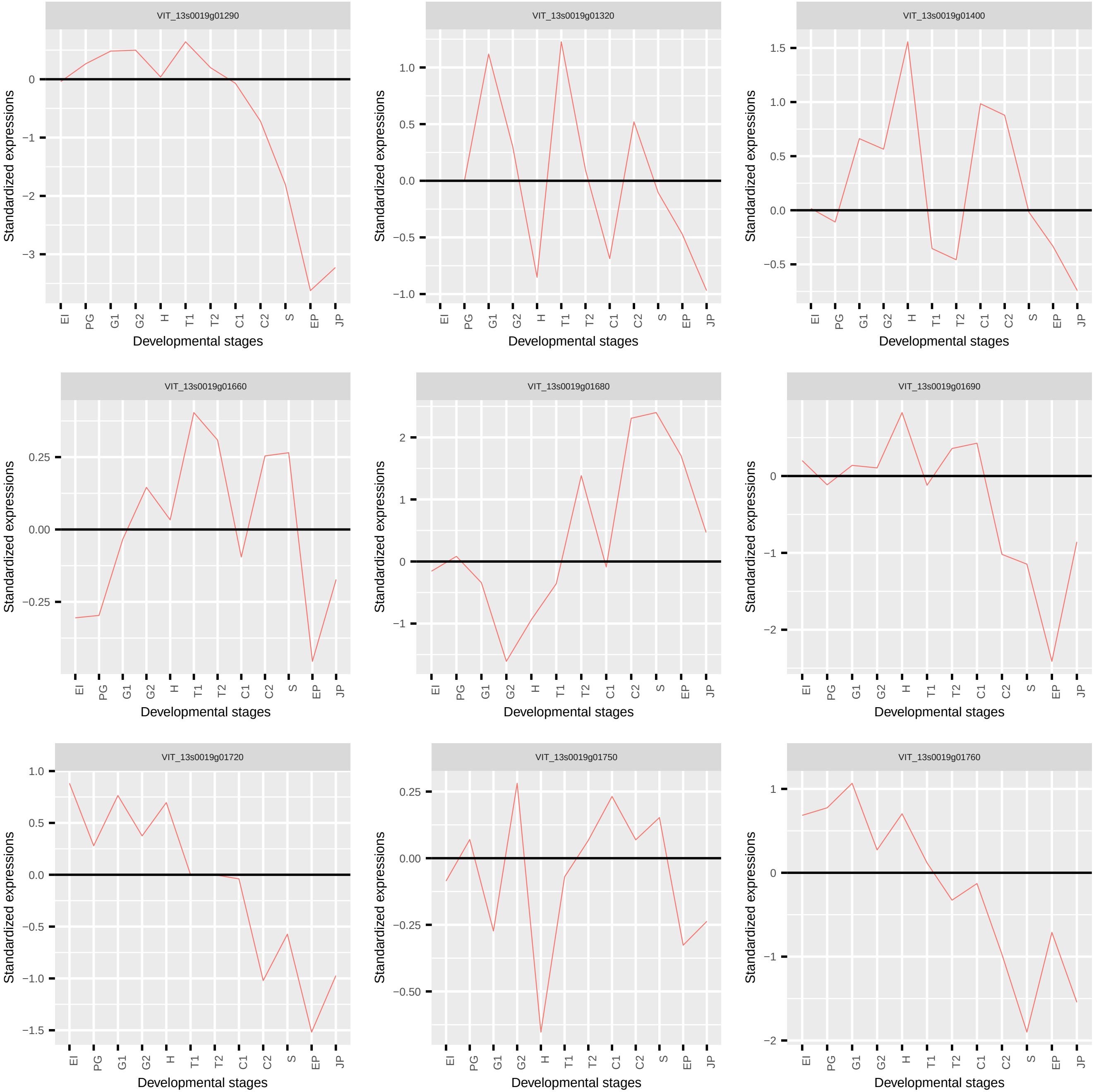

VIT_13s0019g01290
VIT_13s0019g01320
VIT_13s0019g01400
VIT_13s0019g01660
VIT_13s0019g01680
VIT_13s0019g01690
VIT_13s0019g01720
VIT_13s0019g01750
VIT_13s0019g01760
Standardized expressions
Developmental stages
EI PG G1 G2 H T1 T2 C1 C2 S EP JP

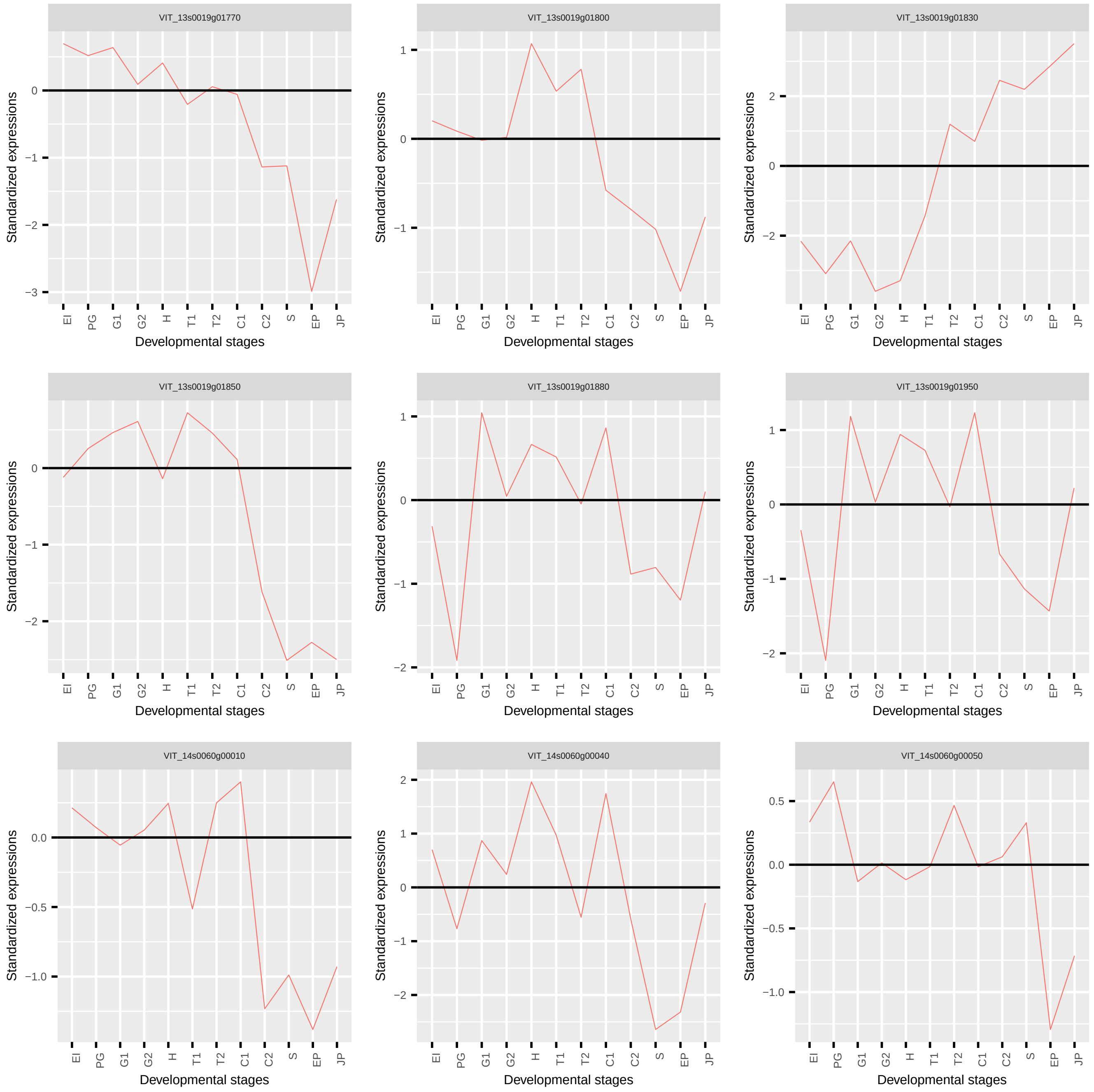
VIT_13s0019g01770
VIT_13s0019g01800
VIT_13s0019g01830
VIT_13s0019g01850
VIT_13s0019g01880
VIT_13s0019g01950
VIT_14s0060g00010
VIT_14s0060g00040
VIT_14s0060g00050
Standardized expressions
Developmental stages
EI PG G1 G2 H T1 T2 C1 C2 S EP JP

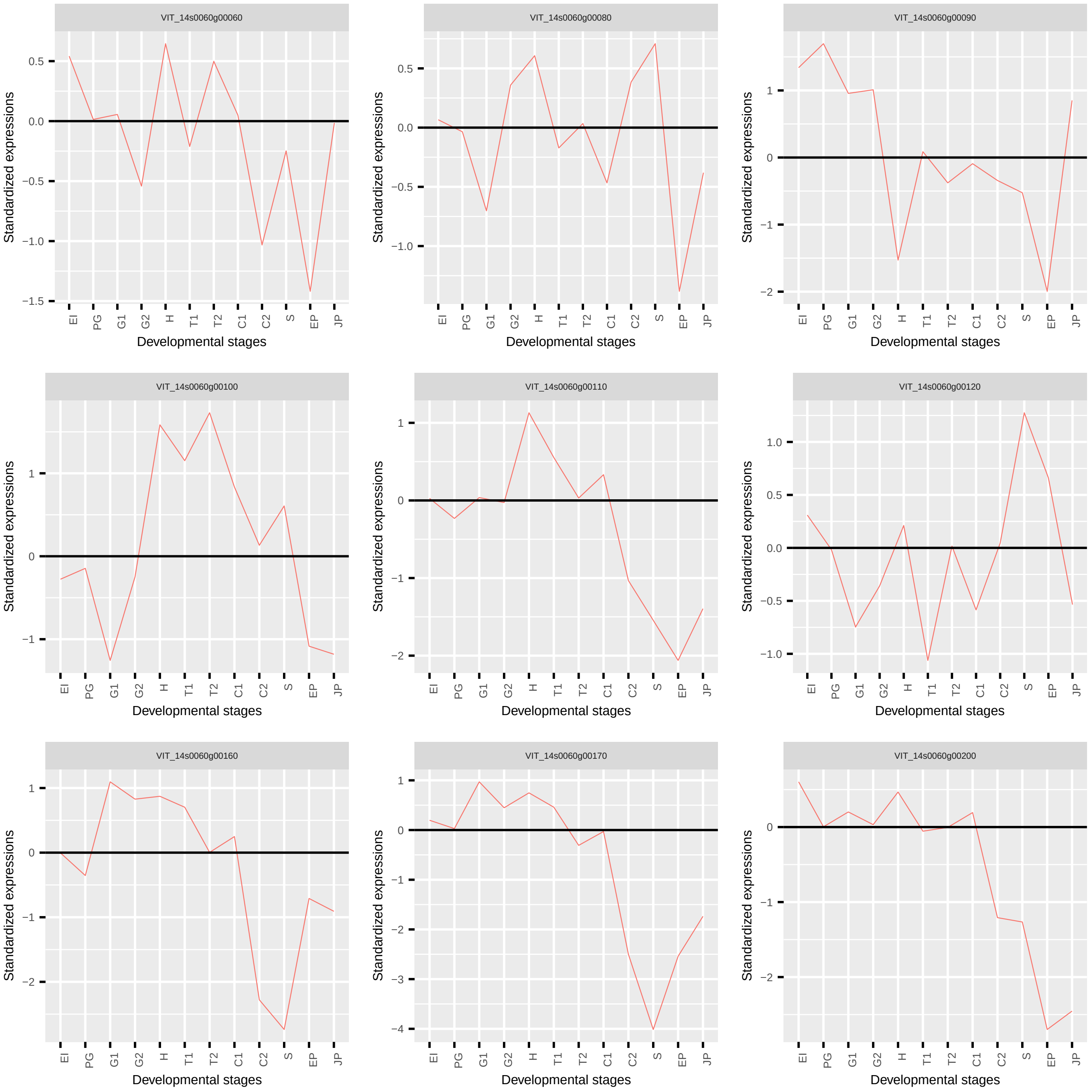
VIT_14s0060g00060
VIT_14s0060g00080
VIT_14s0060g00090
VIT_14s0060g00100
VIT_14s0060g00110
VIT_14s0060g00120
VIT_14s0060g00160
VIT_14s0060g00170
VIT_14s0060g00200
Standardized expressions
Developmental stages
EI
PG
G1
G2
H
T1
T2
C1
C2
S
EP
JP

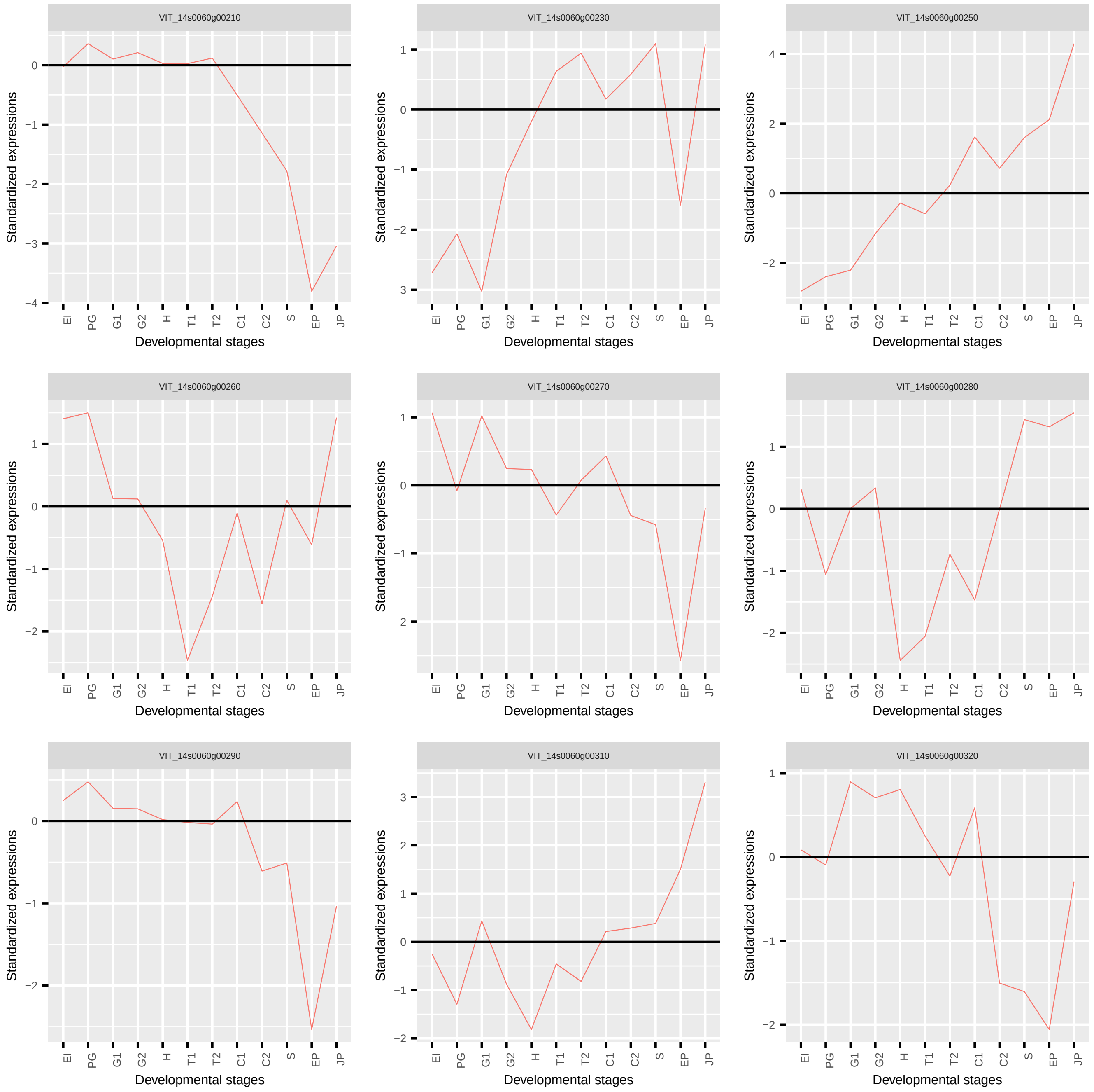

VIT_14s0060g00210
VIT_14s0060g00230
VIT_14s0060g00250
VIT_14s0060g00260
VIT_14s0060g00270
VIT_14s0060g00280
VIT_14s0060g00290
VIT_14s0060g00310
VIT_14s0060g00320
Standardized expressions
Developmental stages
EI
PG
G1
G2
H
T1
T2
C1
C2
S
EP
JP

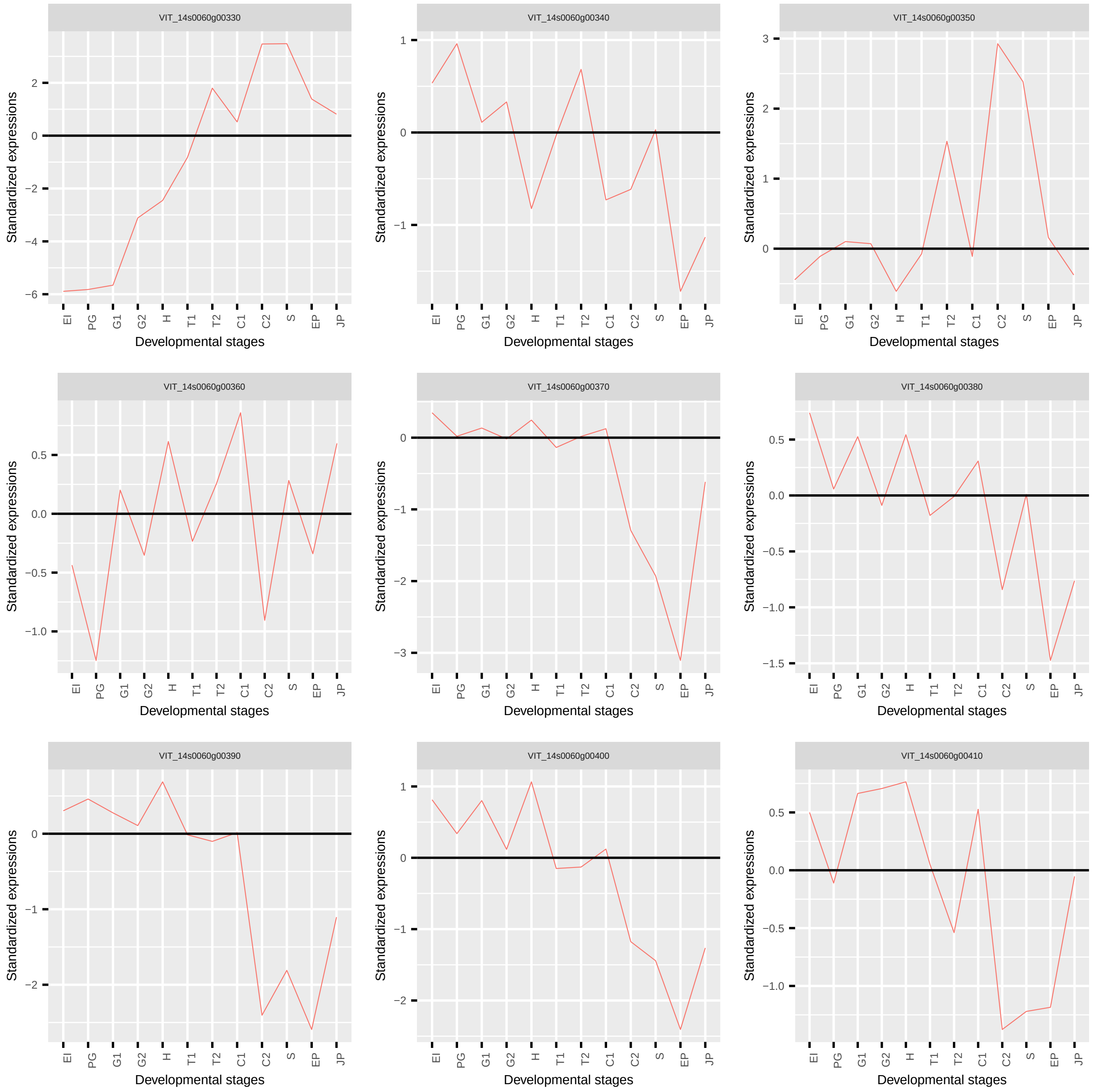
VIT_14s0060g00330
VIT_14s0060g00340
VIT_14s0060g00350
VIT_14s0060g00360
VIT_14s0060g00370
VIT_14s0060g00380
VIT_14s0060g00390
VIT_14s0060g00400
VIT_14s0060g00410
Standardized expressions
Developmental stages
EI PG G1 G2 H T1 T2 C1 C2 S EP JP

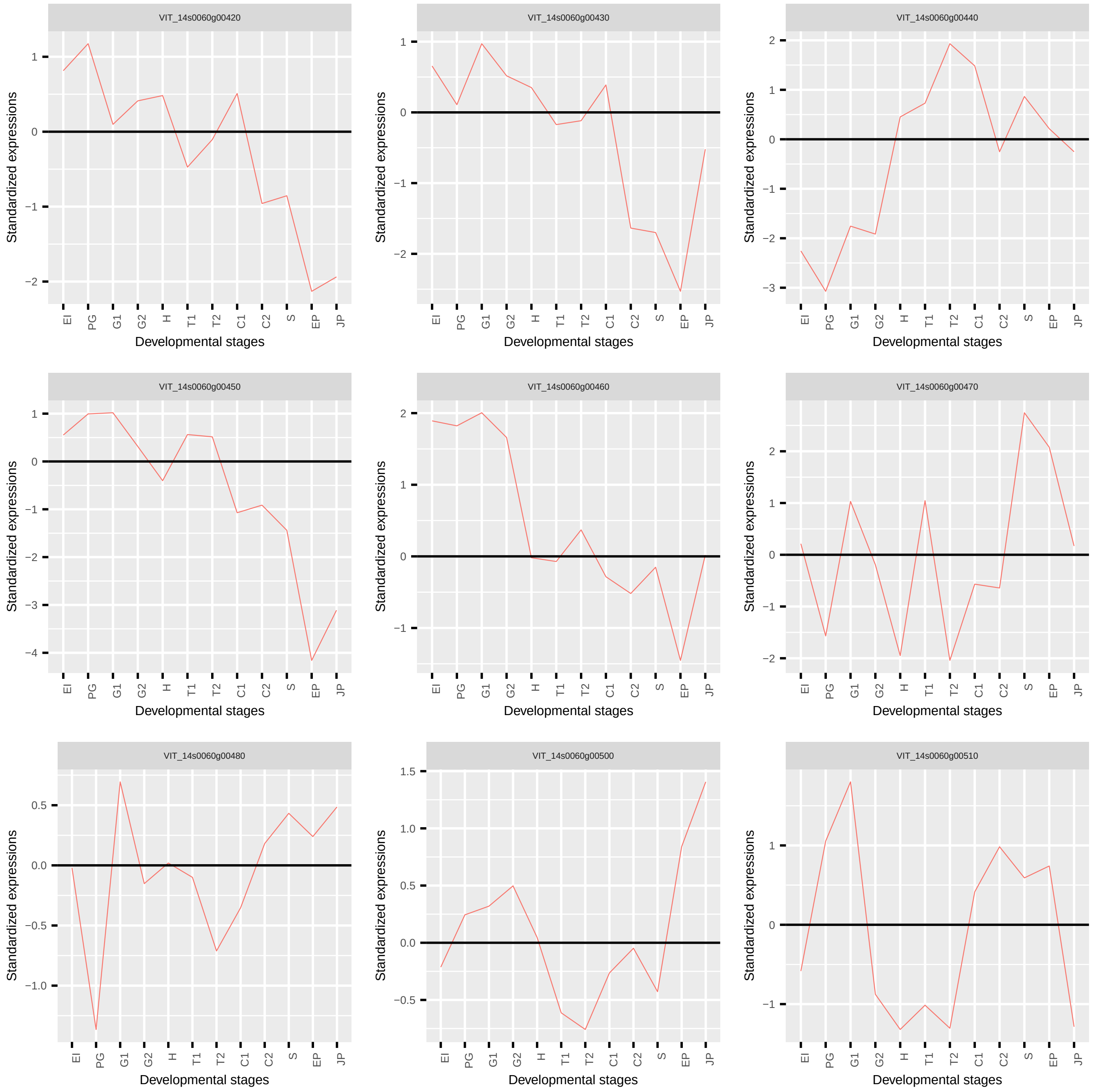
VIT_14s0060g00420
VIT_14s0060g00430
VIT_14s0060g00440
VIT_14s0060g00450
VIT_14s0060g00460
VIT_14s0060g00470
VIT_14s0060g00480
VIT_14s0060g00500
VIT_14s0060g00510
Standardized expressions
Developmental stages
EI PG G1 G2 H T1 T2 C1 C2 S EP JP

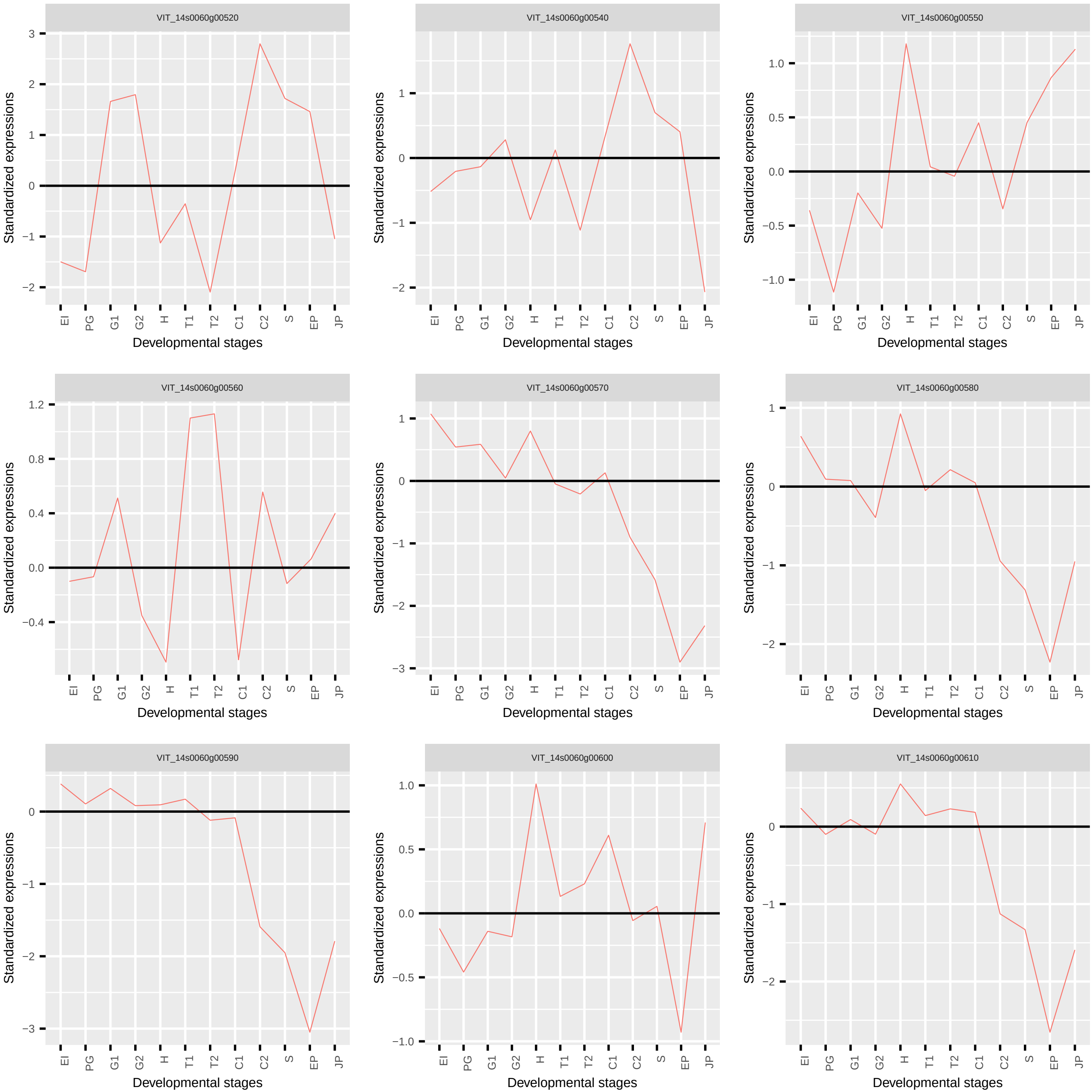
VIT_14s0060g00520
VIT_14s0060g00540
VIT_14s0060g00550
VIT_14s0060g00560
VIT_14s0060g00570
VIT_14s0060g00580
VIT_14s0060g00590
VIT_14s0060g00600
VIT_14s0060g00610
Standardized expressions
Developmental stages
EI PG G1 G2 H T1 T2 C1 C2 S EP JP

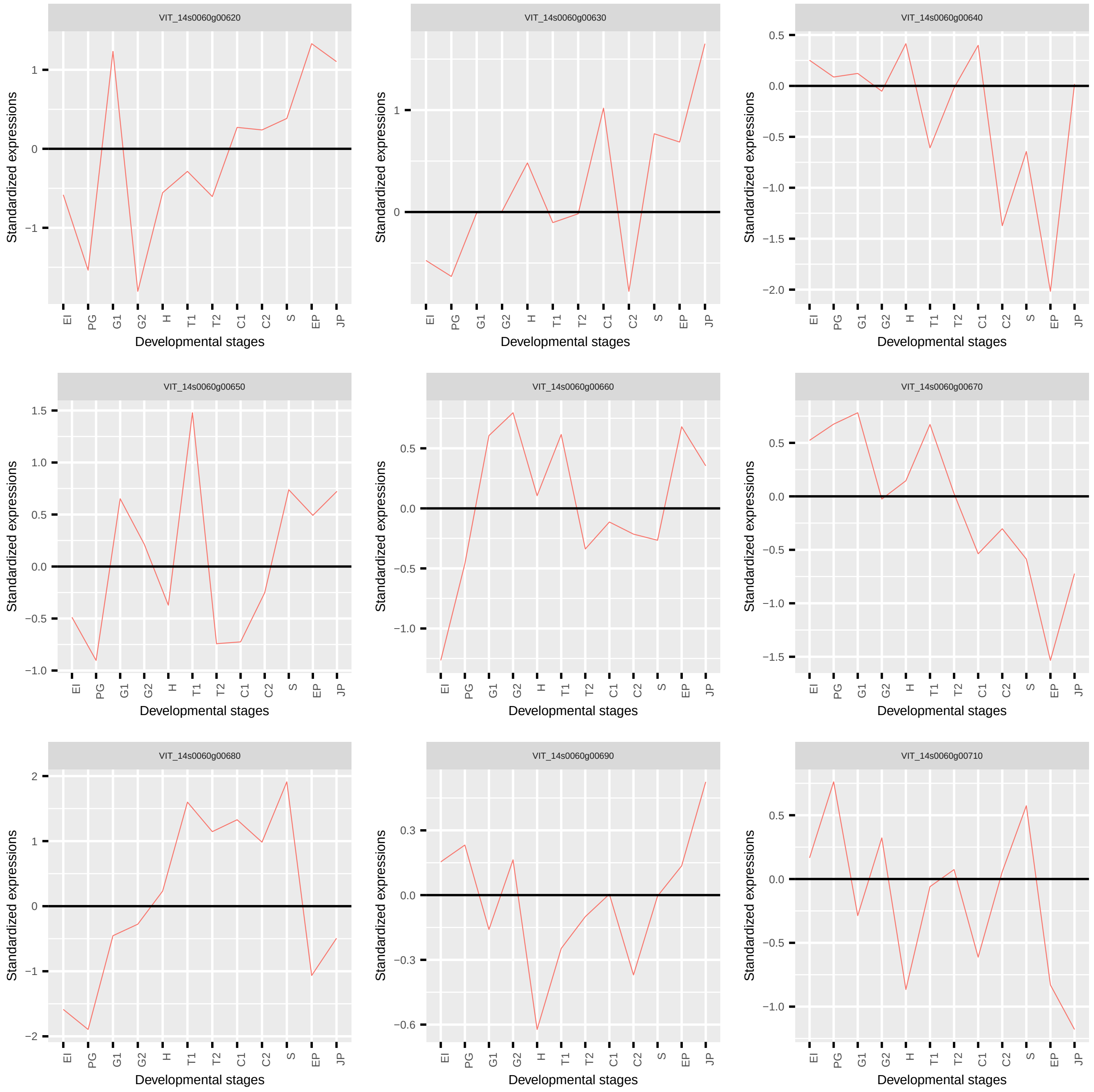
VIT_14s0060g00620
VIT_14s0060g00630
VIT_14s0060g00640
VIT_14s0060g00650
VIT_14s0060g00660
VIT_14s0060g00670
VIT_14s0060g00680
VIT_14s0060g00690
VIT_14s0060g00710
Standardized expressions
Developmental stages
EI
PG
G1
G2
H
T1
T2
C1
C2
S
EP
JP

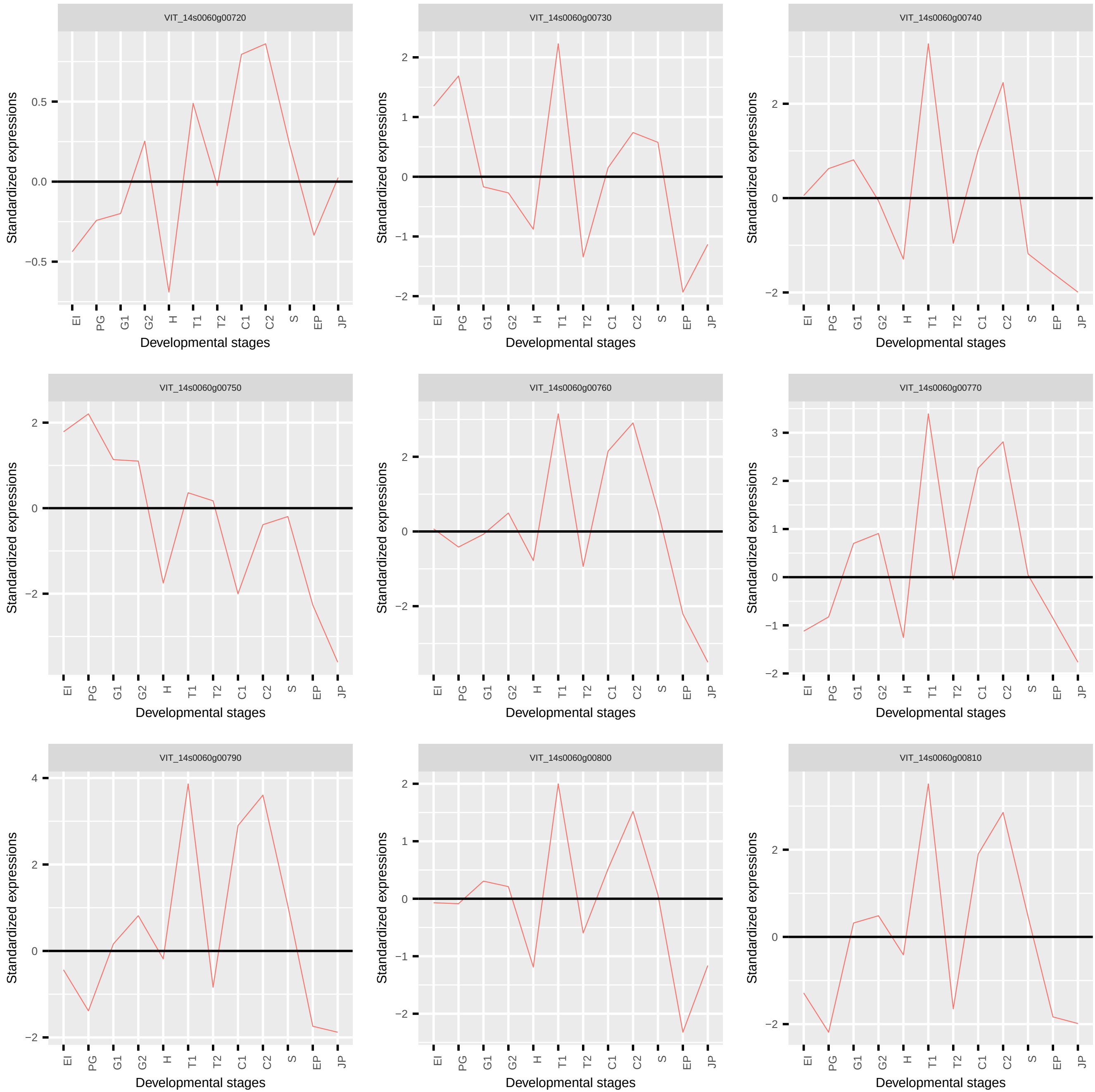

VIT_14s0060g00720
VIT_14s0060g00730
VIT_14s0060g00740
VIT_14s0060g00750
VIT_14s0060g00760
VIT_14s0060g00770
VIT_14s0060g00790
VIT_14s0060g00800
VIT_14s0060g00810
Standardized expressions
Developmental stages
EI
PG
G1
G2
H
T1
T2
C1
C2
S
EP
JP

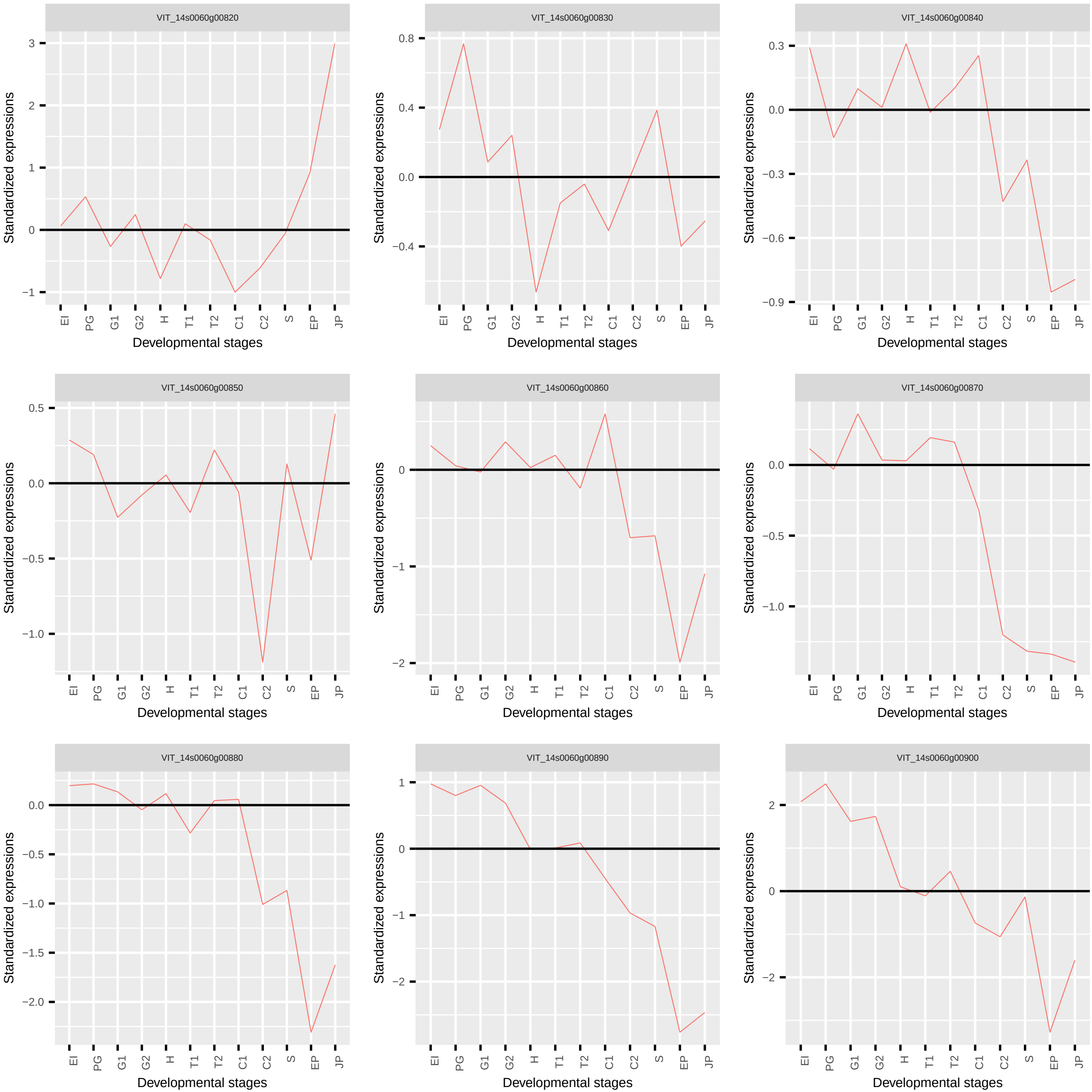

VIT_14s0060g00820
VIT_14s0060g00830
VIT_14s0060g00840
VIT_14s0060g00850
VIT_14s0060g00860
VIT_14s0060g00870
VIT_14s0060g00880
VIT_14s0060g00890
VIT_14s0060g00900
Standardized expressions
Developmental stages
EI PG G1 G2 H T1 T2 C1 C2 S EP JP

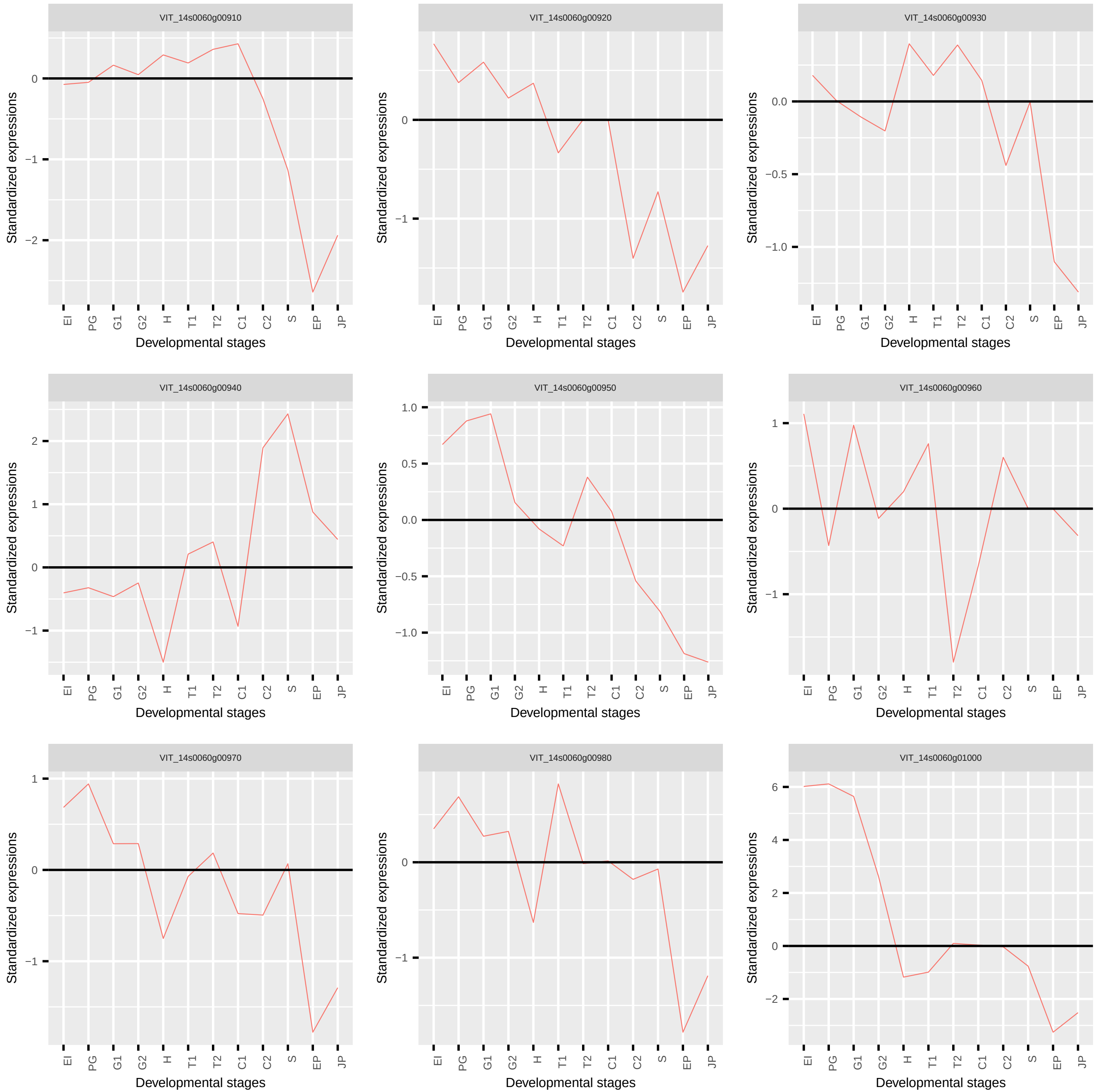
VIT_14s0060g00910
VIT_14s0060g00920
VIT_14s0060g00930
VIT_14s0060g00940
VIT_14s0060g00950
VIT_14s0060g00960
VIT_14s0060g00970
VIT_14s0060g00980
VIT_14s0060g01000
Standardized expressions
Developmental stages
EI PG G1 G2 H T1 T2 C1 C2 S EP JP

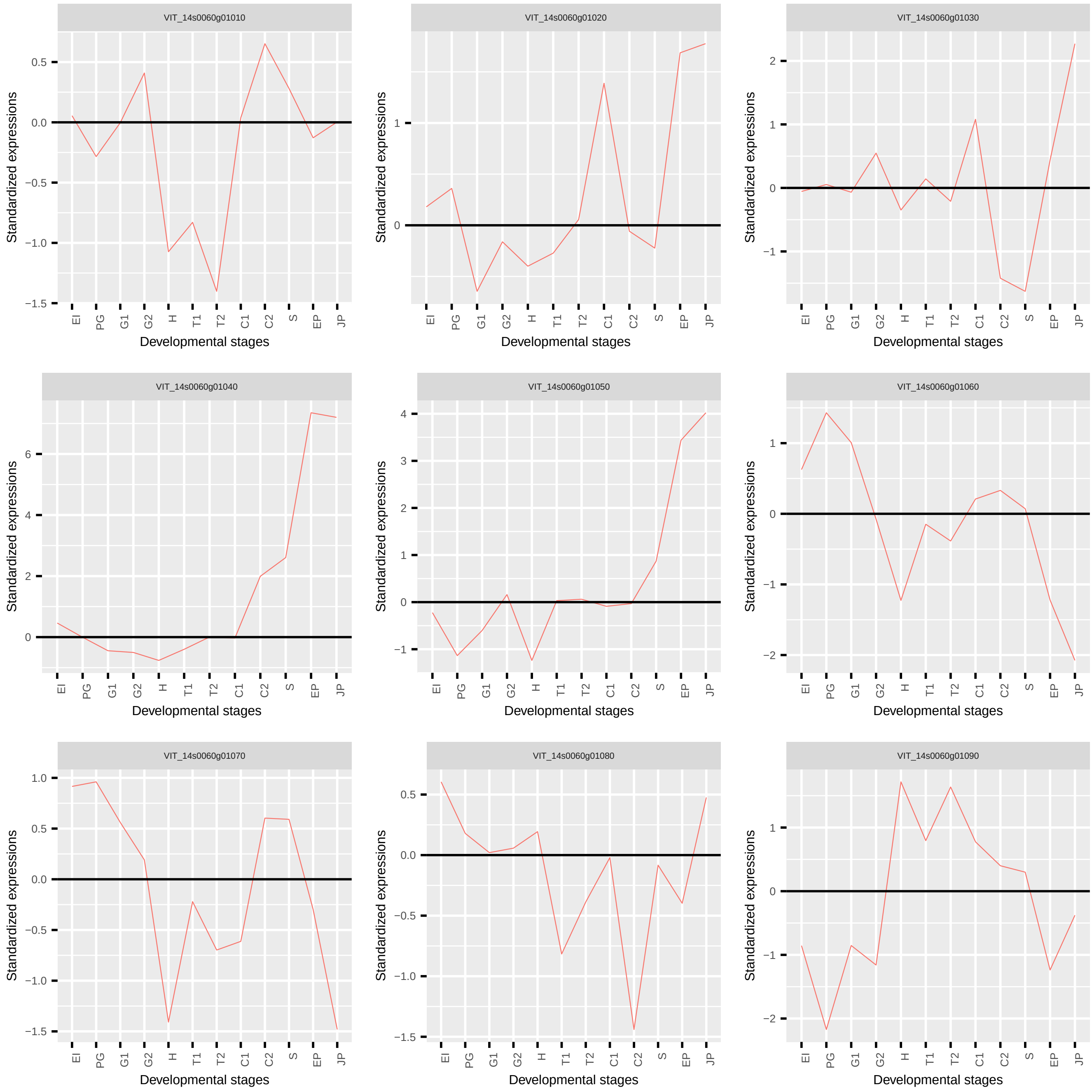

VIT_14s0060g01010
VIT_14s0060g01020
VIT_14s0060g01030
VIT_14s0060g01040
VIT_14s0060g01050
VIT_14s0060g01060
VIT_14s0060g01070
VIT_14s0060g01080
VIT_14s0060g01090
Standardized expressions
Developmental stages
EI
PG
G1
G2
H
T1
T2
C1
C2
S
EP
JP

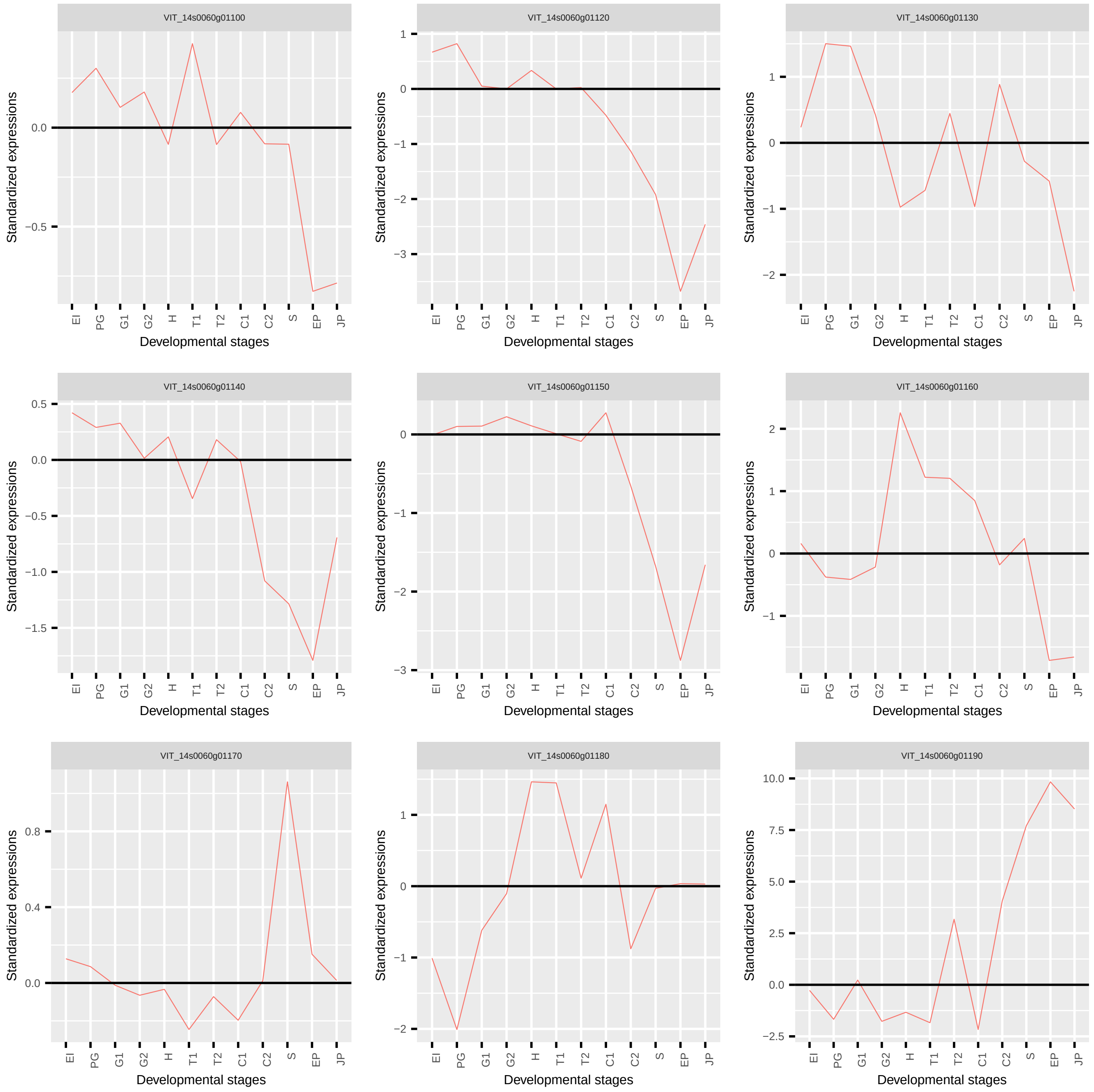
VIT_14s0060g01100
VIT_14s0060g01120
VIT_14s0060g01130
VIT_14s0060g01140
VIT_14s0060g01150
VIT_14s0060g01160
VIT_14s0060g01170
VIT_14s0060g01180
VIT_14s0060g01190
Standardized expressions
Developmental stages
EI PG G1 G2 H T1 T2 C1 C2 S EP JP
0.0
−0.5
1
0
−1
−2
−3
0.5
−1.0
−1.5
2
0.8
0.4
10.0
7.5
5.0
2.5
−2.5

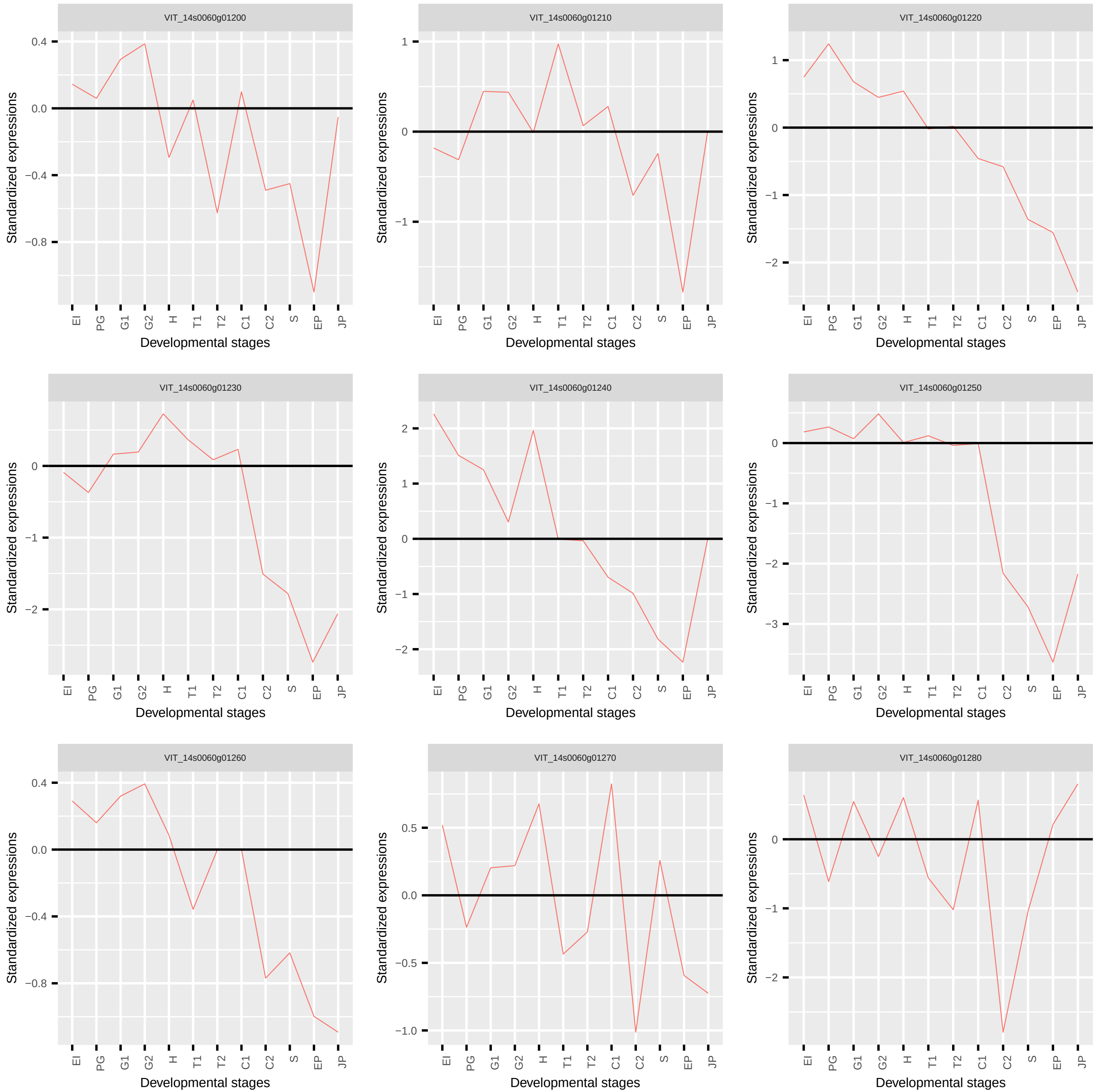
VIT_14s0060g01200
VIT_14s0060g01210
VIT_14s0060g01220
VIT_14s0060g01230
VIT_14s0060g01240
VIT_14s0060g01250
VIT_14s0060g01260
VIT_14s0060g01270
VIT_14s0060g01280
Standardized expressions
Developmental stages
EI
PG
G1
G2
H
T1
T2
C1
C2
S
EP
JP
0.4
0.0
−0.4
−0.8
1
0
−1
−2
−3
2
0.5
−0.5
−1.0

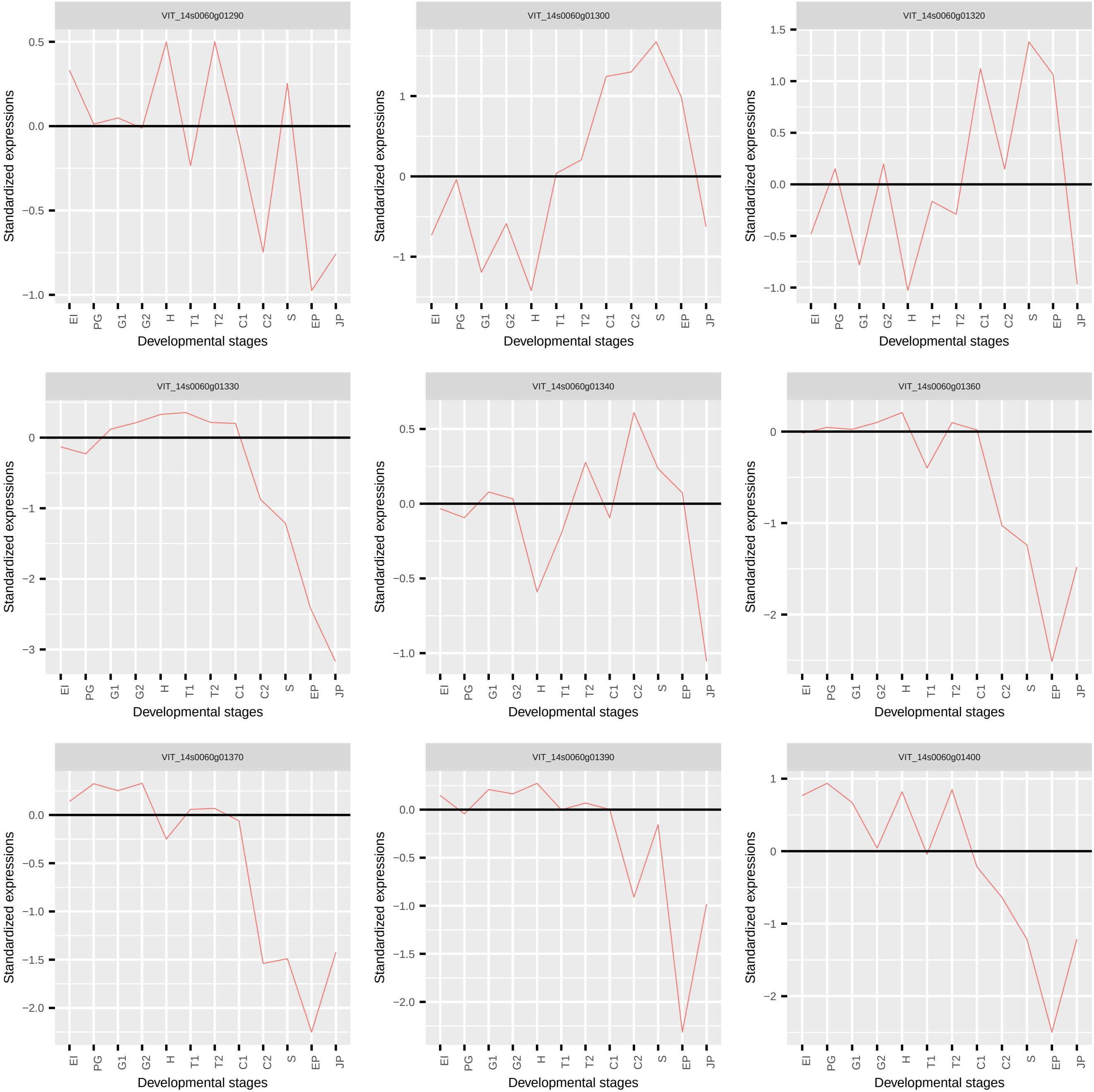
VIT_14s0060g01290
VIT_14s0060g01300
VIT_14s0060g01320
VIT_14s0060g01330
VIT_14s0060g01340
VIT_14s0060g01360
VIT_14s0060g01370
VIT_14s0060g01390
VIT_14s0060g01400
Standardized expressions
Developmental stages
EI PG G1 G2 H T1 T2 C1 C2 S EP JP

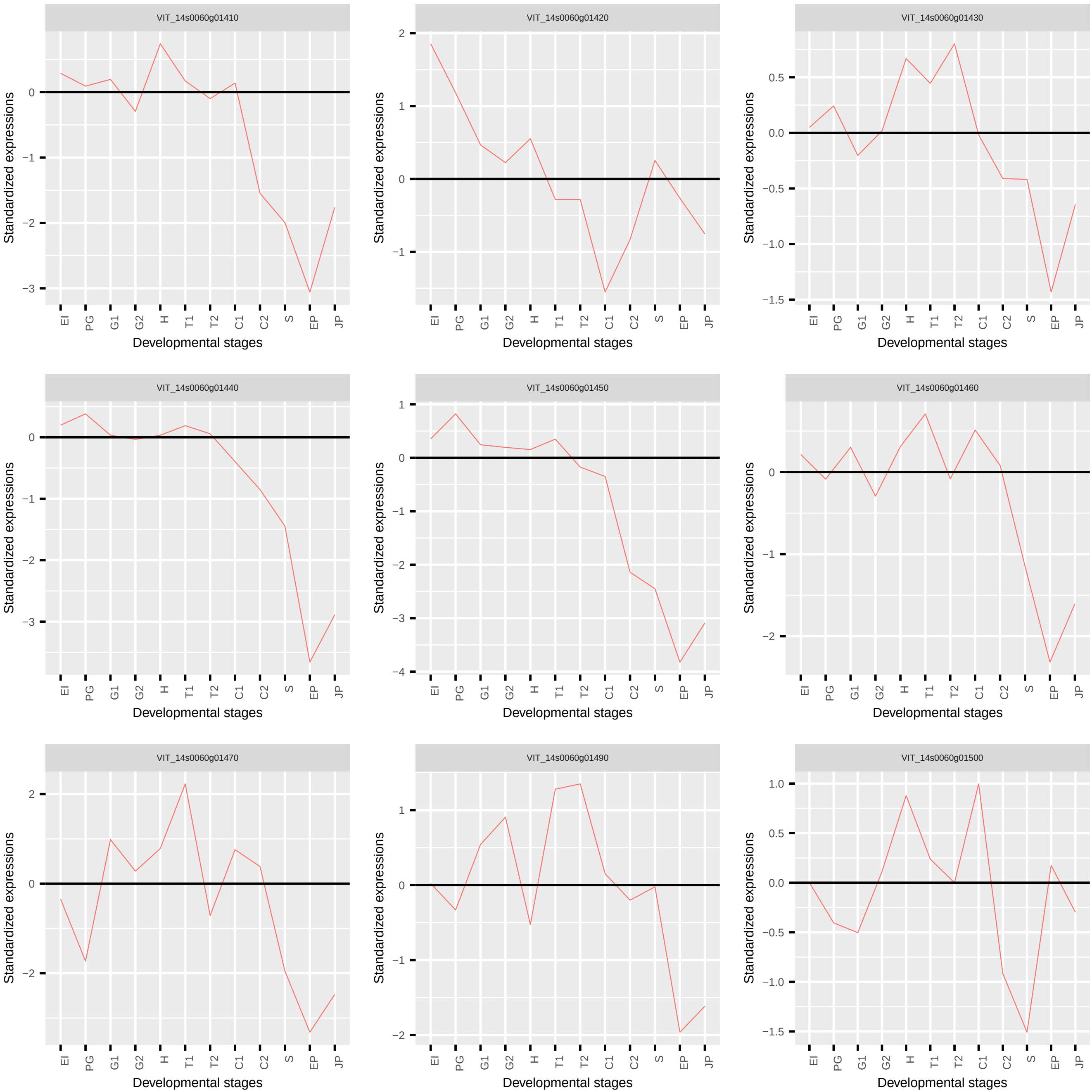
VIT_14s0060g01410
VIT_14s0060g01420
VIT_14s0060g01430
VIT_14s0060g01440
VIT_14s0060g01450
VIT_14s0060g01460
VIT_14s0060g01470
VIT_14s0060g01490
VIT_14s0060g01500
Standardized expressions
Developmental stages
EI PG G1 G2 H T1 T2 C1 C2 S EP JP

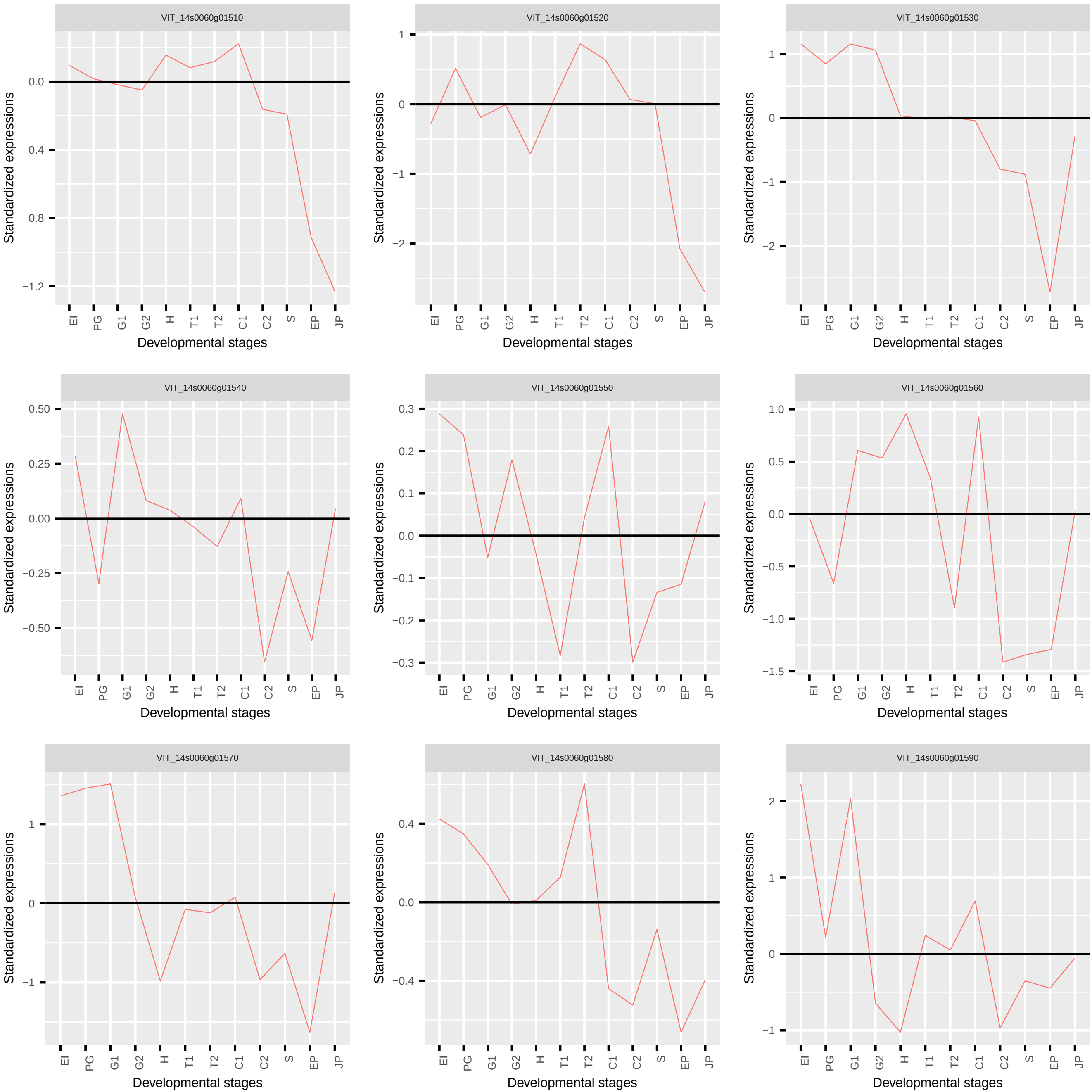
VIT_14s0060g01510
VIT_14s0060g01520
VIT_14s0060g01530
VIT_14s0060g01540
VIT_14s0060g01550
VIT_14s0060g01560
VIT_14s0060g01570
VIT_14s0060g01580
VIT_14s0060g01590
Standardized expressions
Developmental stages
EI PG G1 G2 H T1 T2 C1 C2 S EP JP

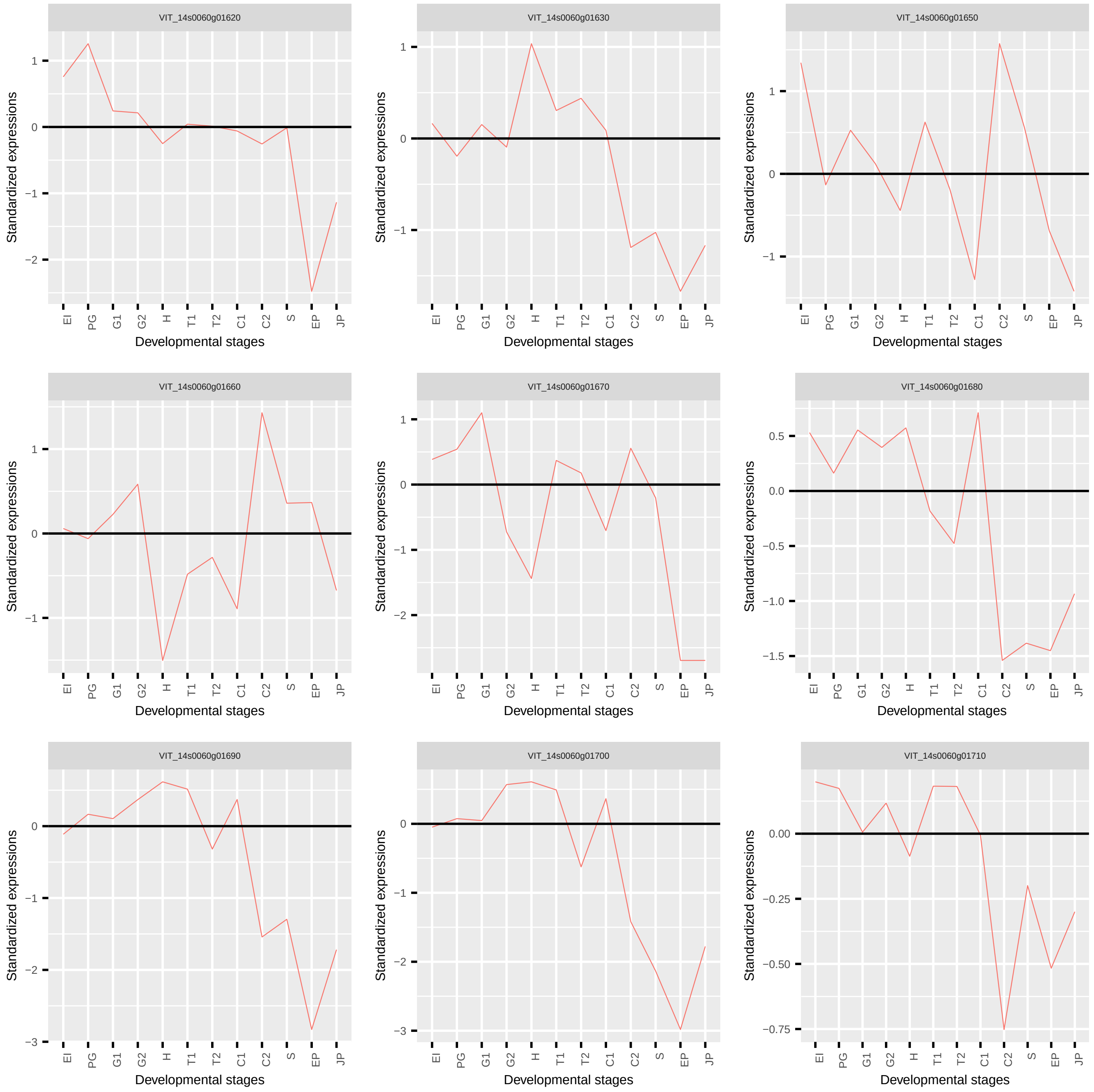

VIT_14s0060g01620
VIT_14s0060g01630
VIT_14s0060g01650
VIT_14s0060g01660
VIT_14s0060g01670
VIT_14s0060g01680
VIT_14s0060g01690
VIT_14s0060g01700
VIT_14s0060g01710
Standardized expressions
Developmental stages
EI
PG
G1
G2
H
T1
T2
C1
C2
S
EP
JP
1
0
−1
−2
−3
0.5
0.0
−0.5
−1.0
−1.5
0.00
−0.25
−0.50
−0.75

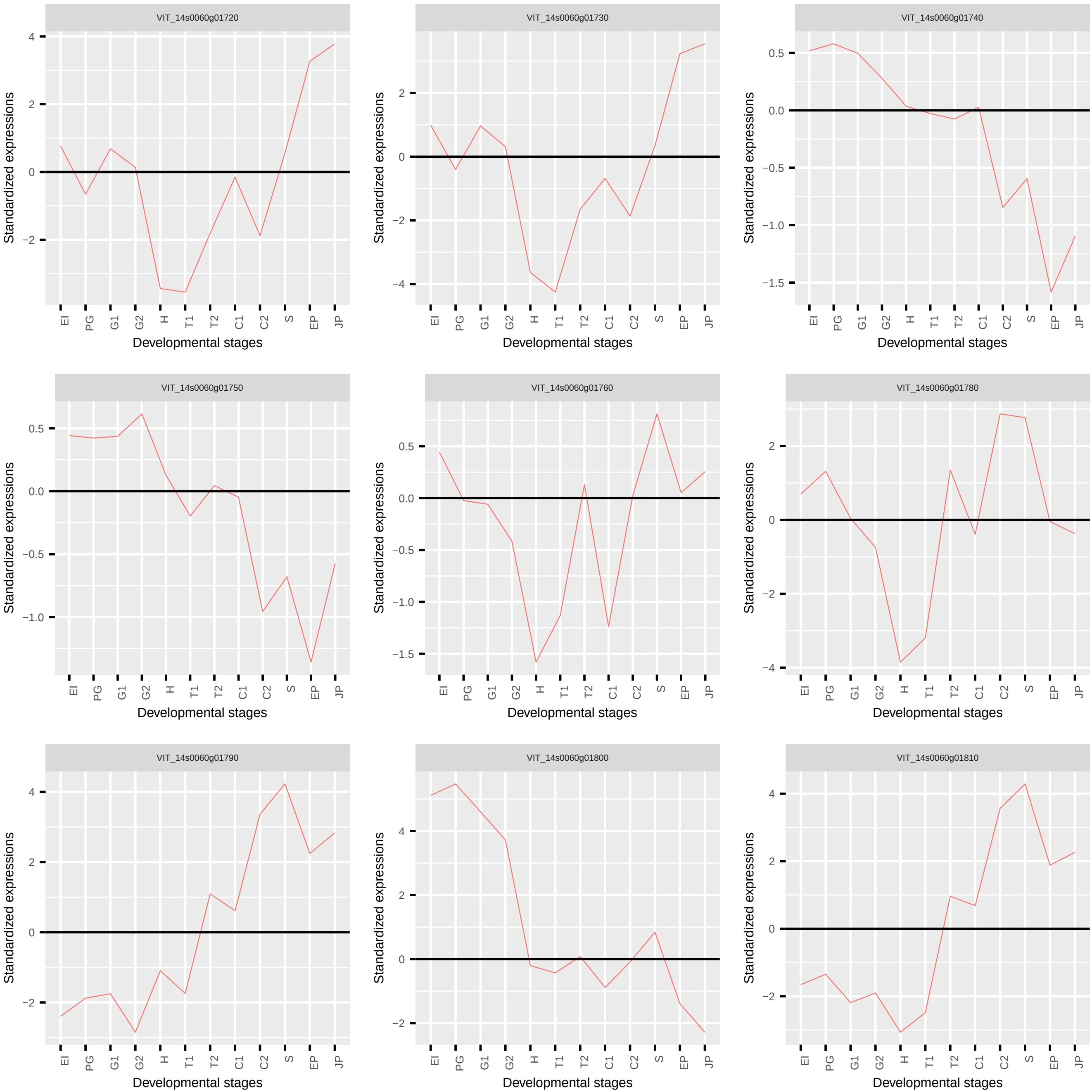
VIT_14s0060g01720
VIT_14s0060g01730
VIT_14s0060g01740
VIT_14s0060g01750
VIT_14s0060g01760
VIT_14s0060g01780
VIT_14s0060g01790
VIT_14s0060g01800
VIT_14s0060g01810
Standardized expressions
Developmental stages
EI PG G1 G2 H T1 T2 C1 C2 S EP JP

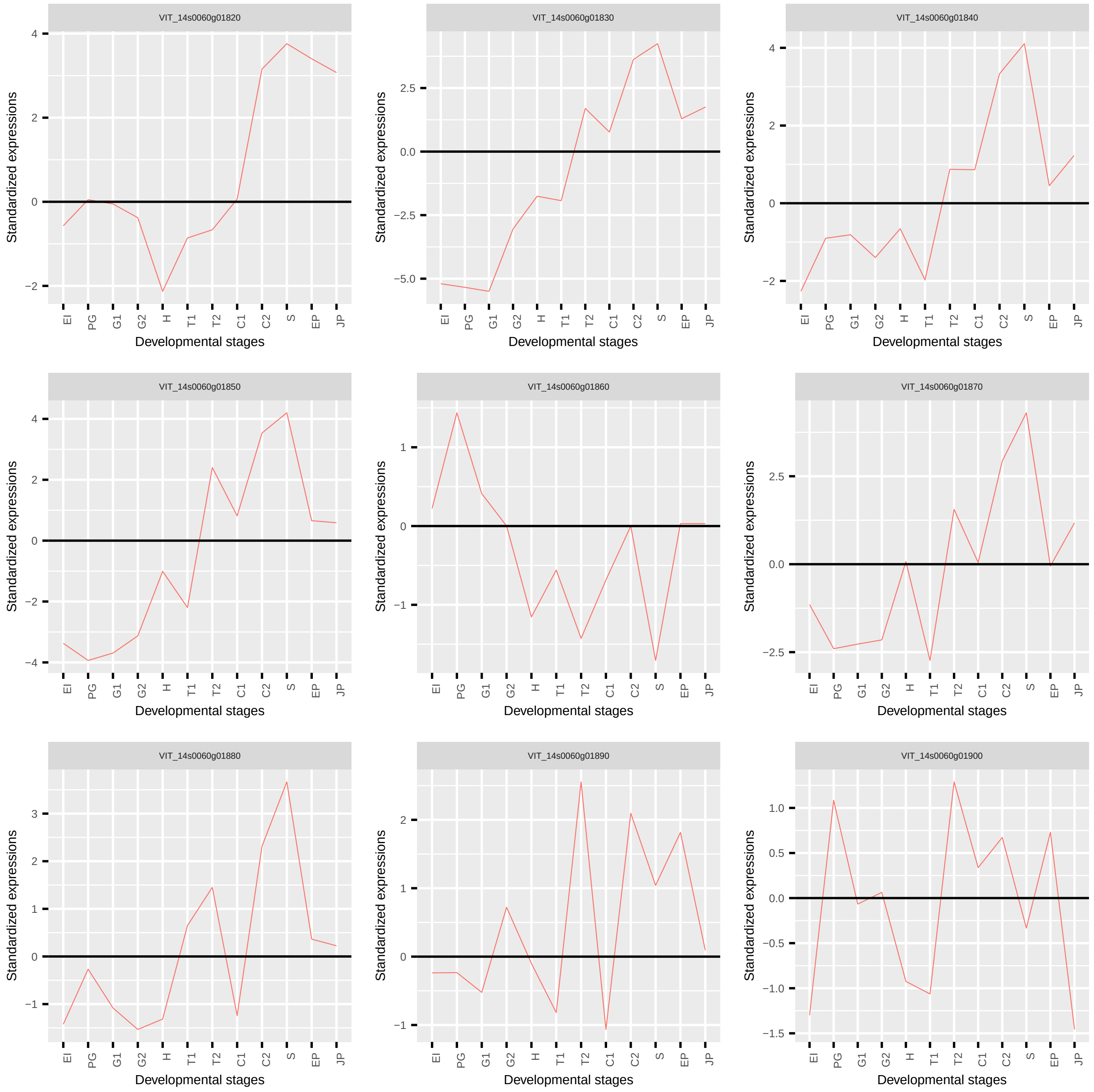
VIT_14s0060g01820
VIT_14s0060g01830
VIT_14s0060g01840
VIT_14s0060g01850
VIT_14s0060g01860
VIT_14s0060g01870
VIT_14s0060g01880
VIT_14s0060g01890
VIT_14s0060g01900
Standardized expressions
Developmental stages
EI
PG
G1
G2
H
T1
T2
C1
C2
S
EP
JP

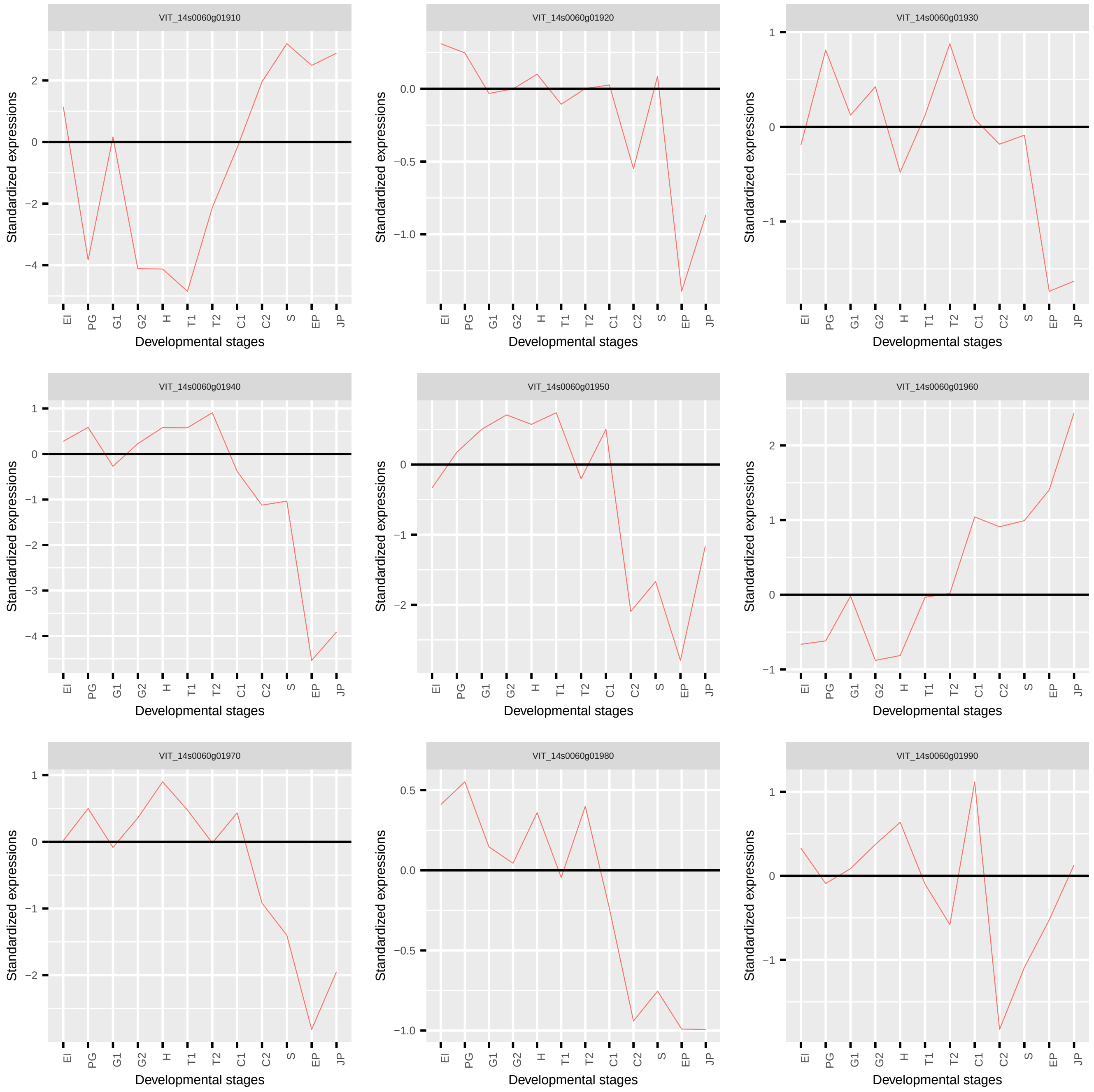
VIT_14s0060g01910
VIT_14s0060g01920
VIT_14s0060g01930
VIT_14s0060g01940
VIT_14s0060g01950
VIT_14s0060g01960
VIT_14s0060g01970
VIT_14s0060g01980
VIT_14s0060g01990
Standardized expressions
Developmental stages
EI PG G1 G2 H T1 T2 C1 C2 S EP JP

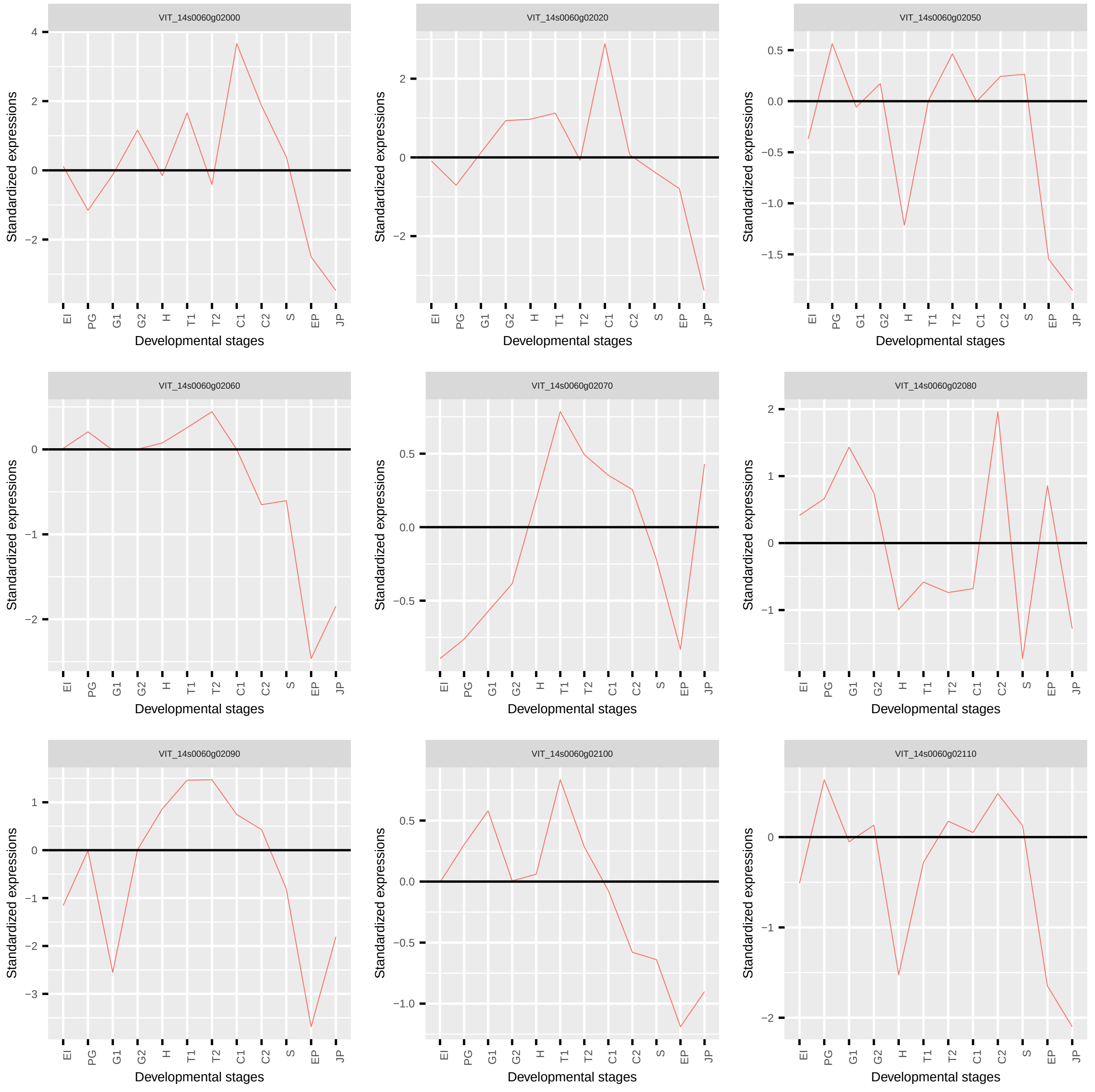
VIT_14s0060g02000
VIT_14s0060g02020
VIT_14s0060g02050
VIT_14s0060g02060
VIT_14s0060g02070
VIT_14s0060g02080
VIT_14s0060g02090
VIT_14s0060g02100
VIT_14s0060g02110
Standardized expressions
Developmental stages
EI PG G1 G2 H T1 T2 C1 C2 S EP JP

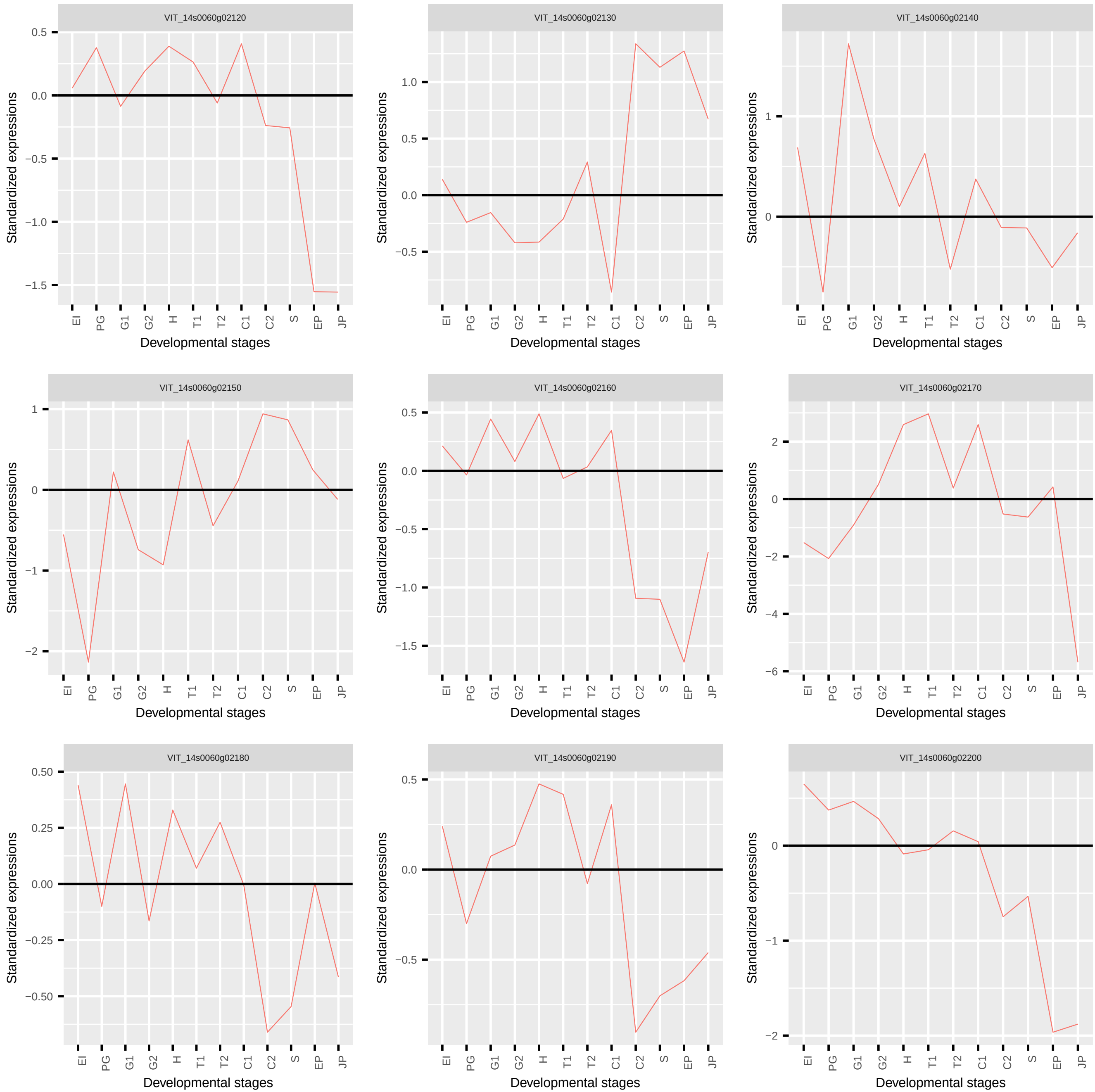
VIT_14s0060g02120
VIT_14s0060g02130
VIT_14s0060g02140
VIT_14s0060g02150
VIT_14s0060g02160
VIT_14s0060g02170
VIT_14s0060g02180
VIT_14s0060g02190
VIT_14s0060g02200
Standardized expressions
Developmental stages
EI PG G1 G2 H T1 T2 C1 C2 S EP JP

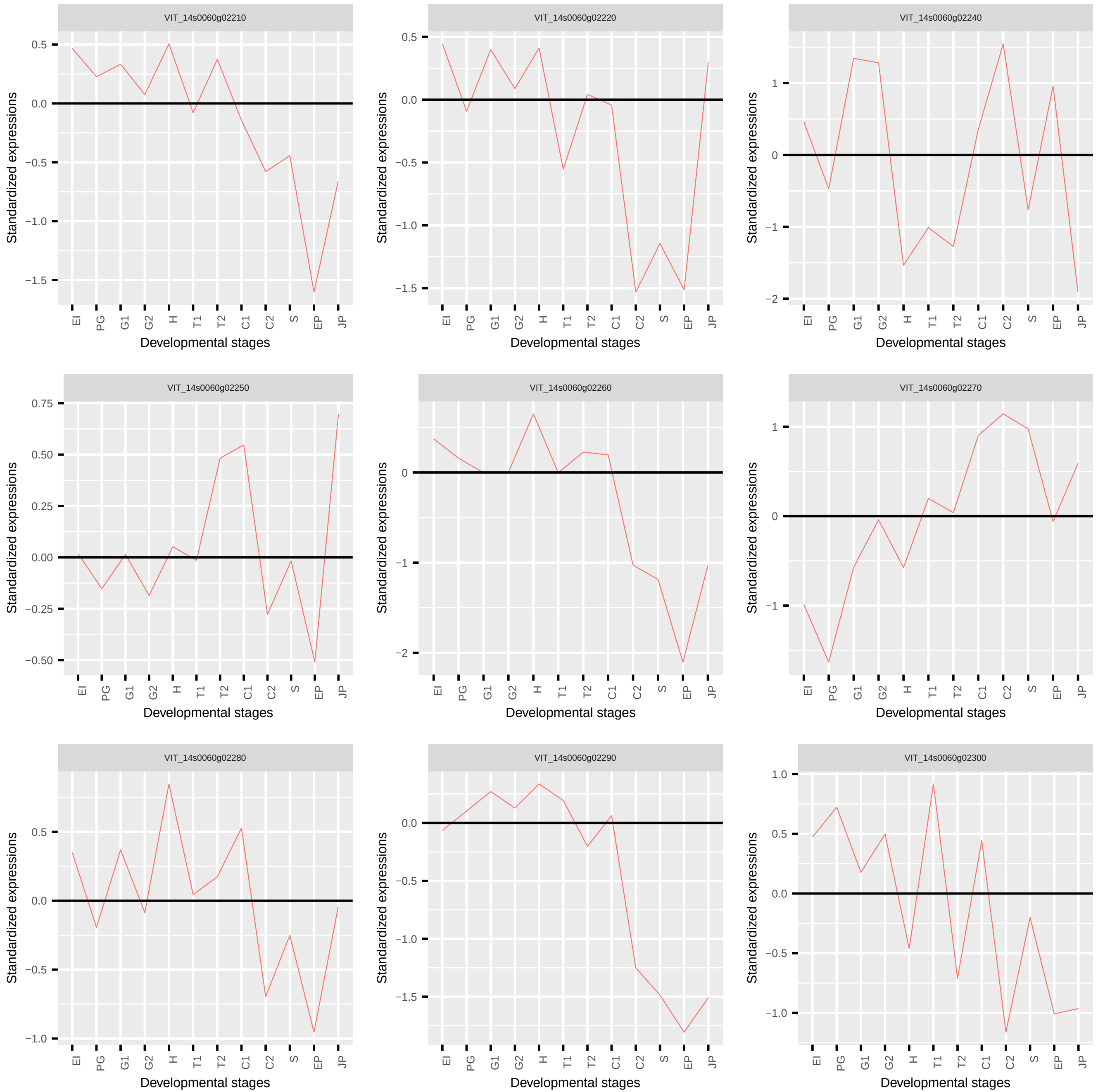
VIT_14s0060g02210
VIT_14s0060g02220
VIT_14s0060g02240
VIT_14s0060g02250
VIT_14s0060g02260
VIT_14s0060g02270
VIT_14s0060g02280
VIT_14s0060g02290
VIT_14s0060g02300
Standardized expressions
Developmental stages
EI PG G1 G2 H T1 T2 C1 C2 S EP JP

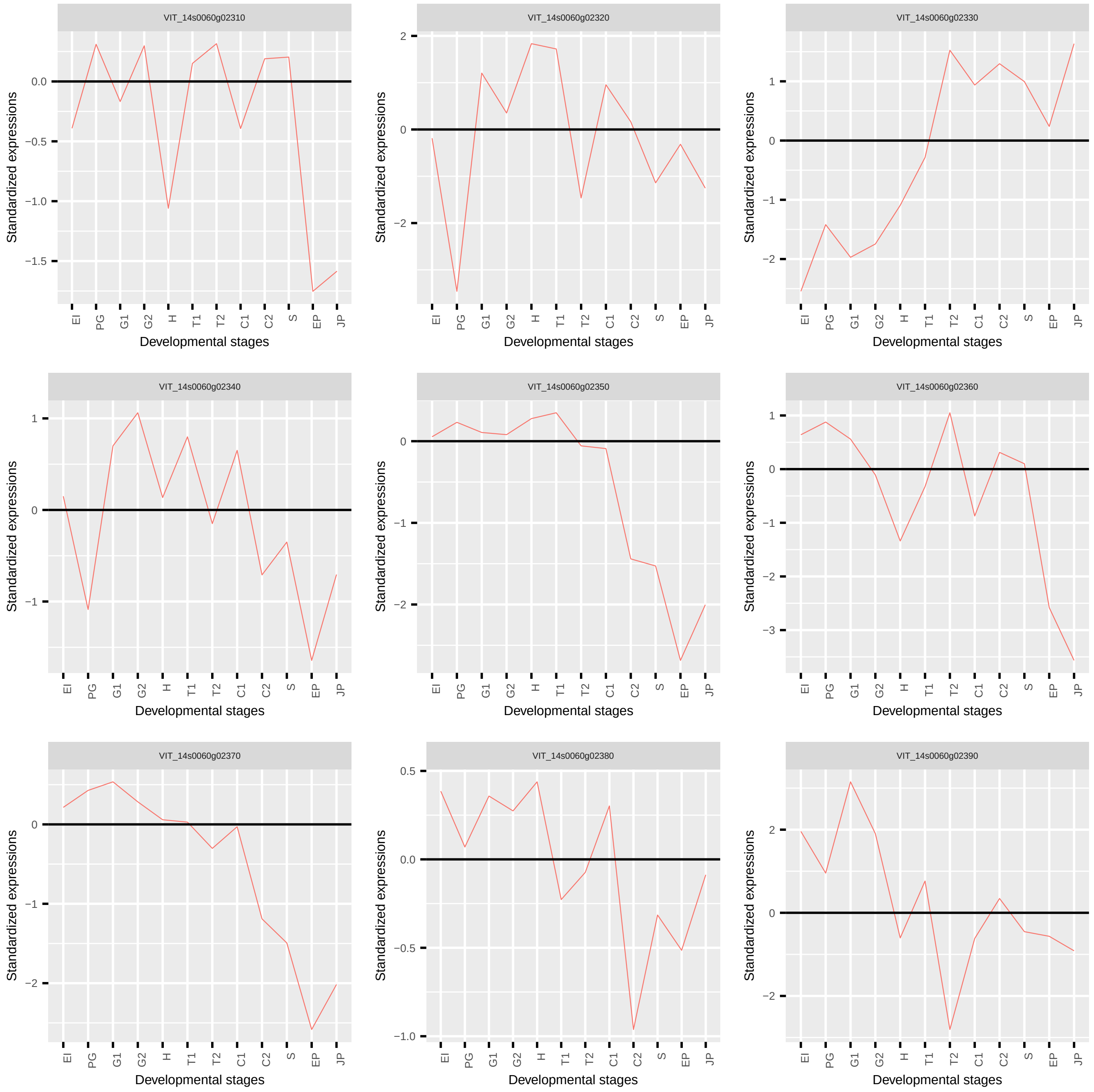
VIT_14s0060g02310
VIT_14s0060g02320
VIT_14s0060g02330
VIT_14s0060g02340
VIT_14s0060g02350
VIT_14s0060g02360
VIT_14s0060g02370
VIT_14s0060g02380
VIT_14s0060g02390
Standardized expressions
Developmental stages
EI
PG
G1
G2
H
T1
T2
C1
C2
S
EP
JP

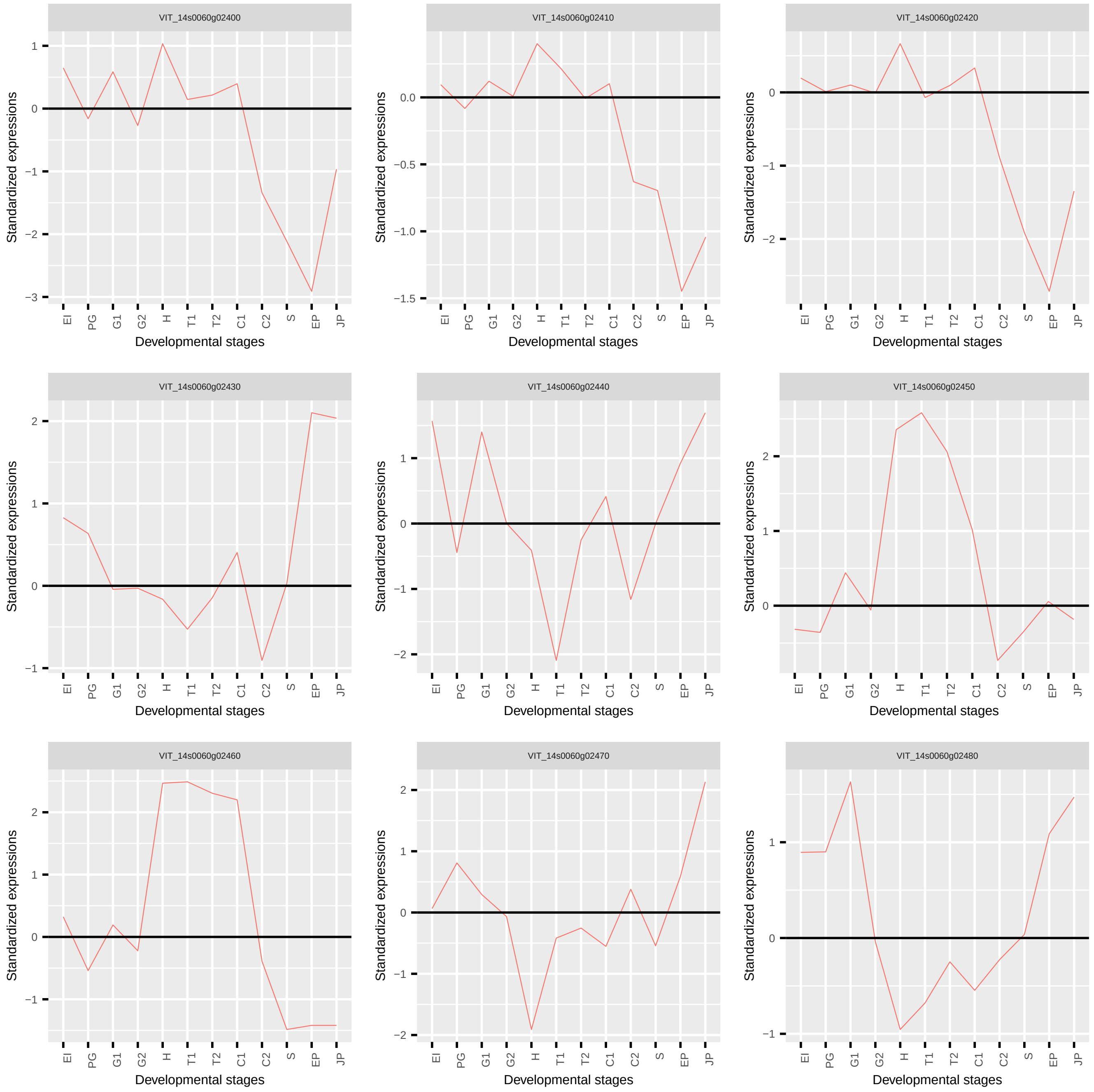
VIT_14s0060g02400
VIT_14s0060g02410
VIT_14s0060g02420
VIT_14s0060g02430
VIT_14s0060g02440
VIT_14s0060g02450
VIT_14s0060g02460
VIT_14s0060g02470
VIT_14s0060g02480
Standardized expressions
Developmental stages
EI PG G1 G2 H T1 T2 C1 C2 S EP JP

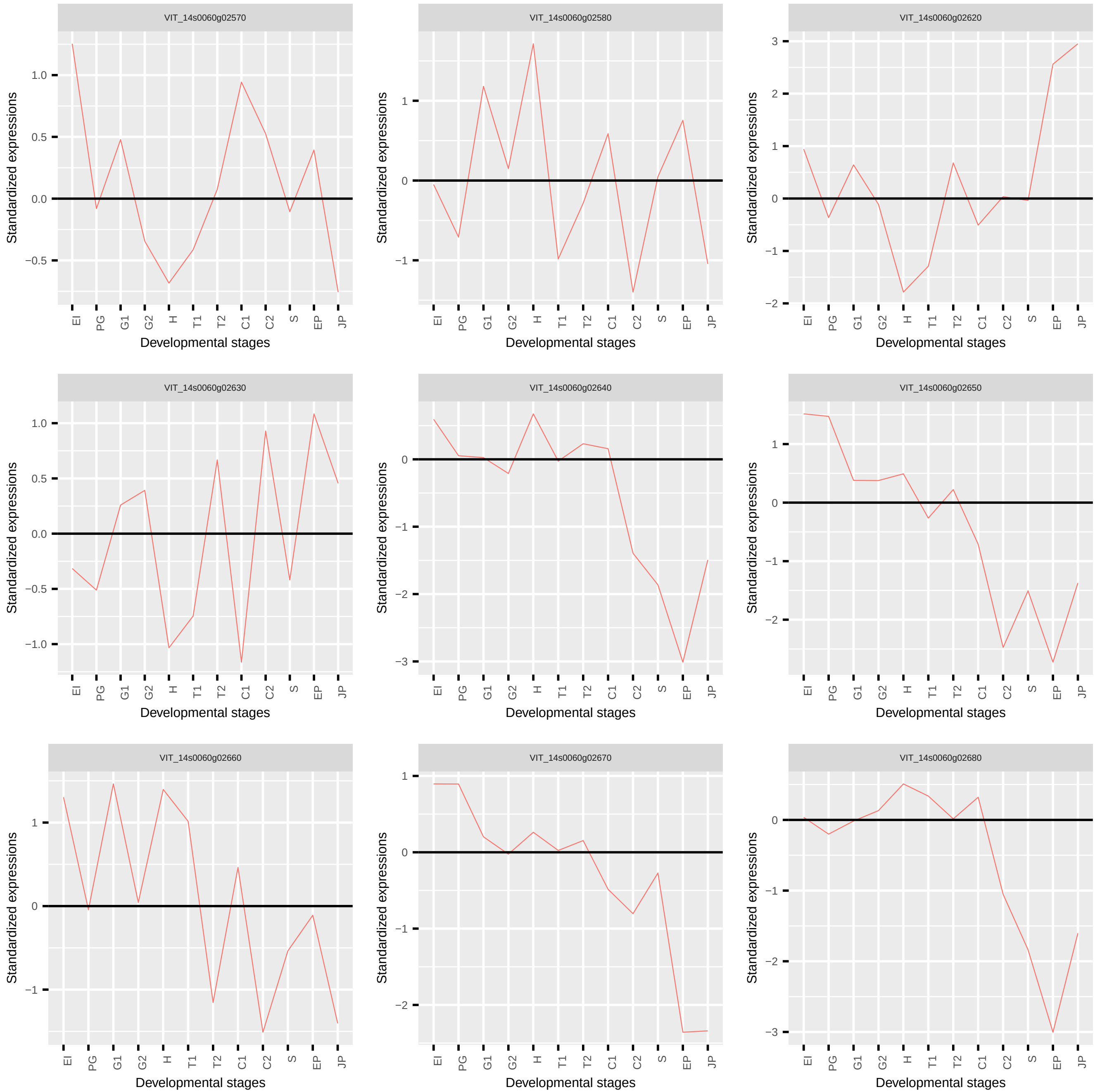
VIT_14s0060g02570
VIT_14s0060g02580
VIT_14s0060g02620
VIT_14s0060g02630
VIT_14s0060g02640
VIT_14s0060g02650
VIT_14s0060g02660
VIT_14s0060g02670
VIT_14s0060g02680
Standardized expressions
Developmental stages
EI
PG
G1
G2
H
T1
T2
C1
C2
S
EP
JP

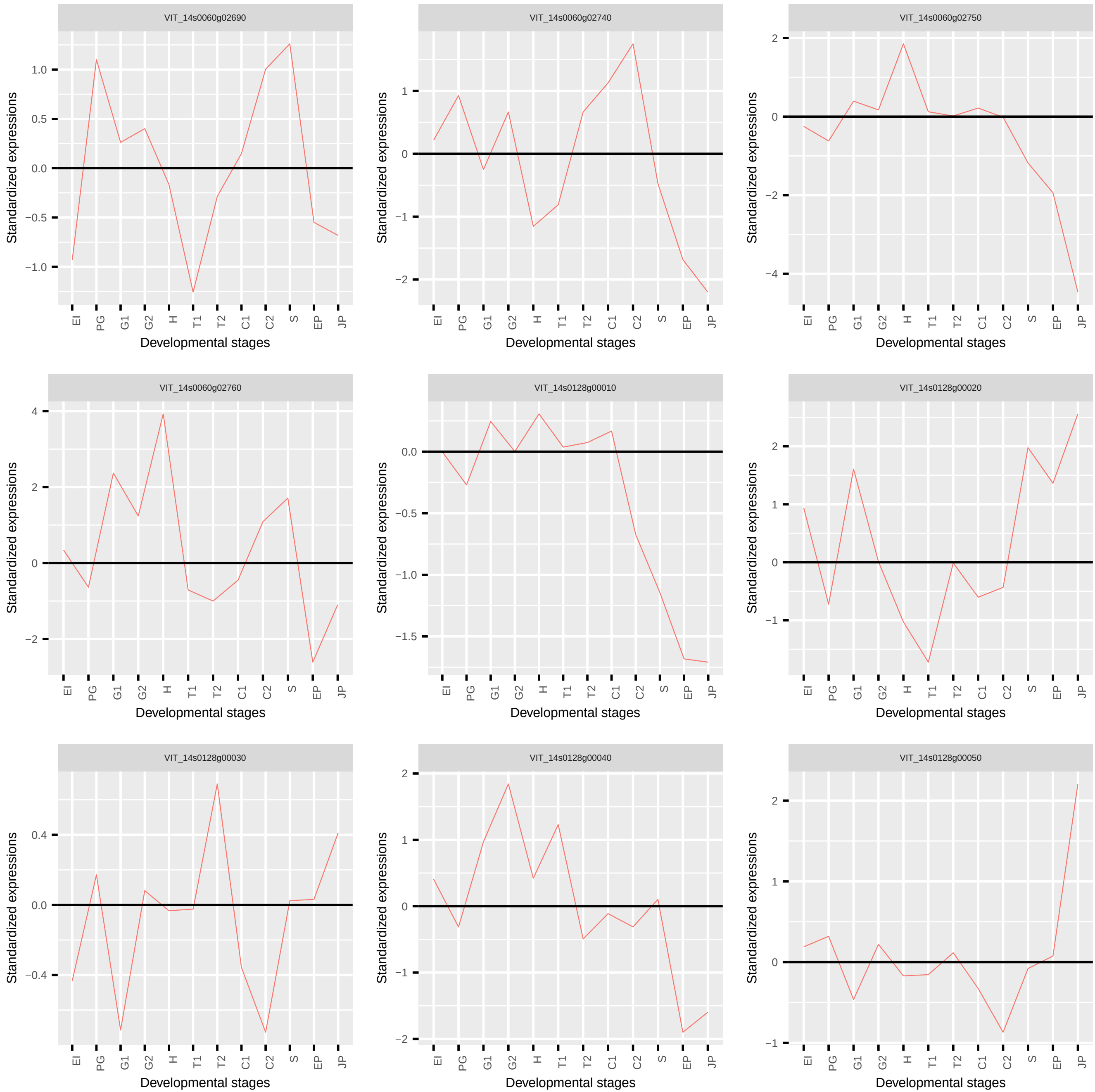
VIT_14s0060g02690
VIT_14s0060g02740
VIT_14s0060g02750
VIT_14s0060g02760
VIT_14s0128g00010
VIT_14s0128g00020
VIT_14s0128g00030
VIT_14s0128g00040
VIT_14s0128g00050
Standardized expressions
Developmental stages
EI
PG
G1
G2
H
T1
T2
C1
C2
S
EP
JP

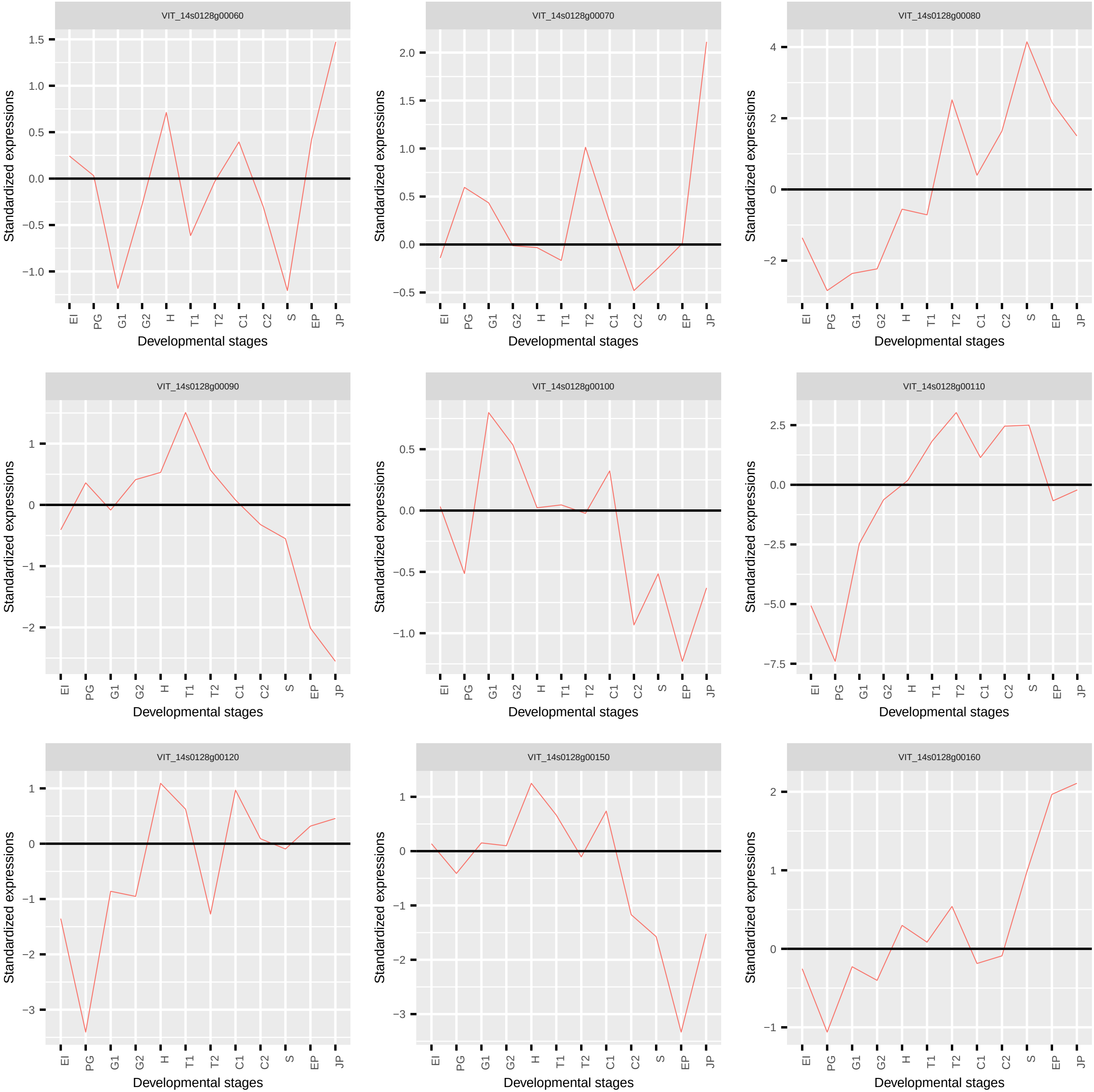
VIT_14s0128g00060
VIT_14s0128g00070
VIT_14s0128g00080
VIT_14s0128g00090
VIT_14s0128g00100
VIT_14s0128g00110
VIT_14s0128g00120
VIT_14s0128g00150
VIT_14s0128g00160
Standardized expressions
Developmental stages
EI PG G1 G2 H T1 T2 C1 C2 S EP JP

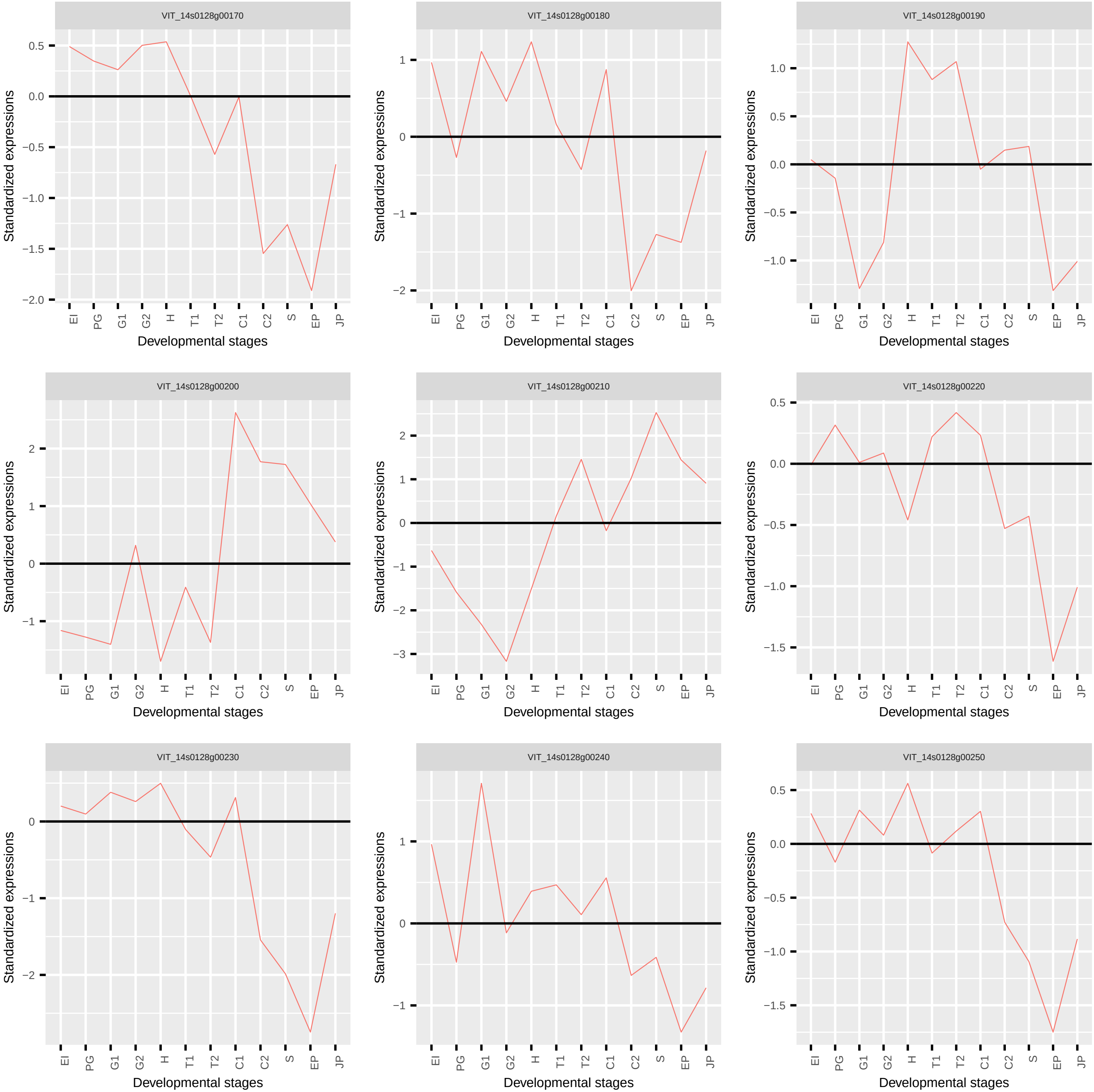
VIT_14s0128g00170
VIT_14s0128g00180
VIT_14s0128g00190
VIT_14s0128g00200
VIT_14s0128g00210
VIT_14s0128g00220
VIT_14s0128g00230
VIT_14s0128g00240
VIT_14s0128g00250
Standardized expressions
Developmental stages
EI PG G1 G2 H T1 T2 C1 C2 S EP JP

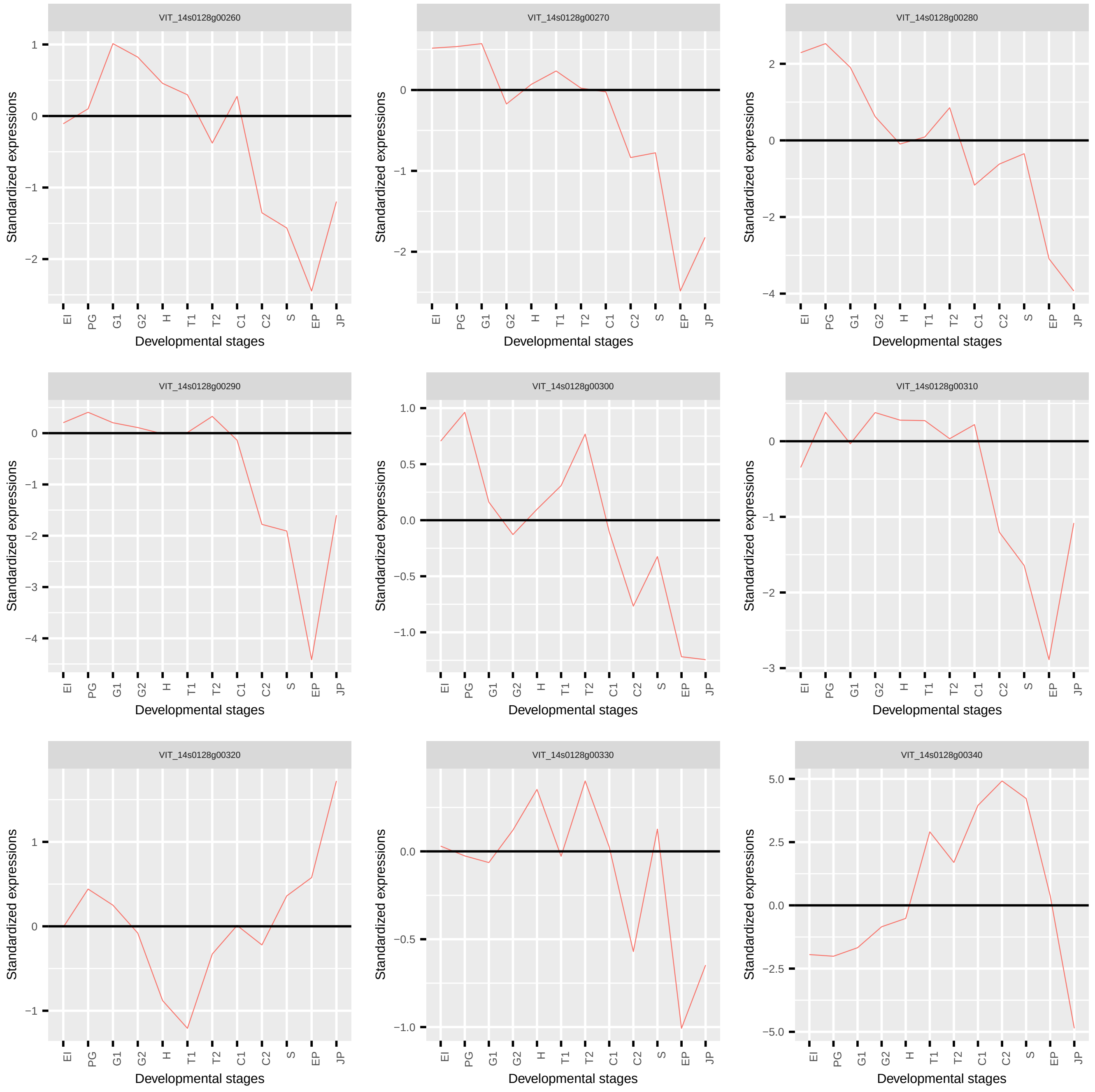

VIT_14s0128g00260
VIT_14s0128g00270
VIT_14s0128g00280
VIT_14s0128g00290
VIT_14s0128g00300
VIT_14s0128g00310
VIT_14s0128g00320
VIT_14s0128g00330
VIT_14s0128g00340
Standardized expressions
Developmental stages
EI PG G1 G2 H T1 T2 C1 C2 S EP JP

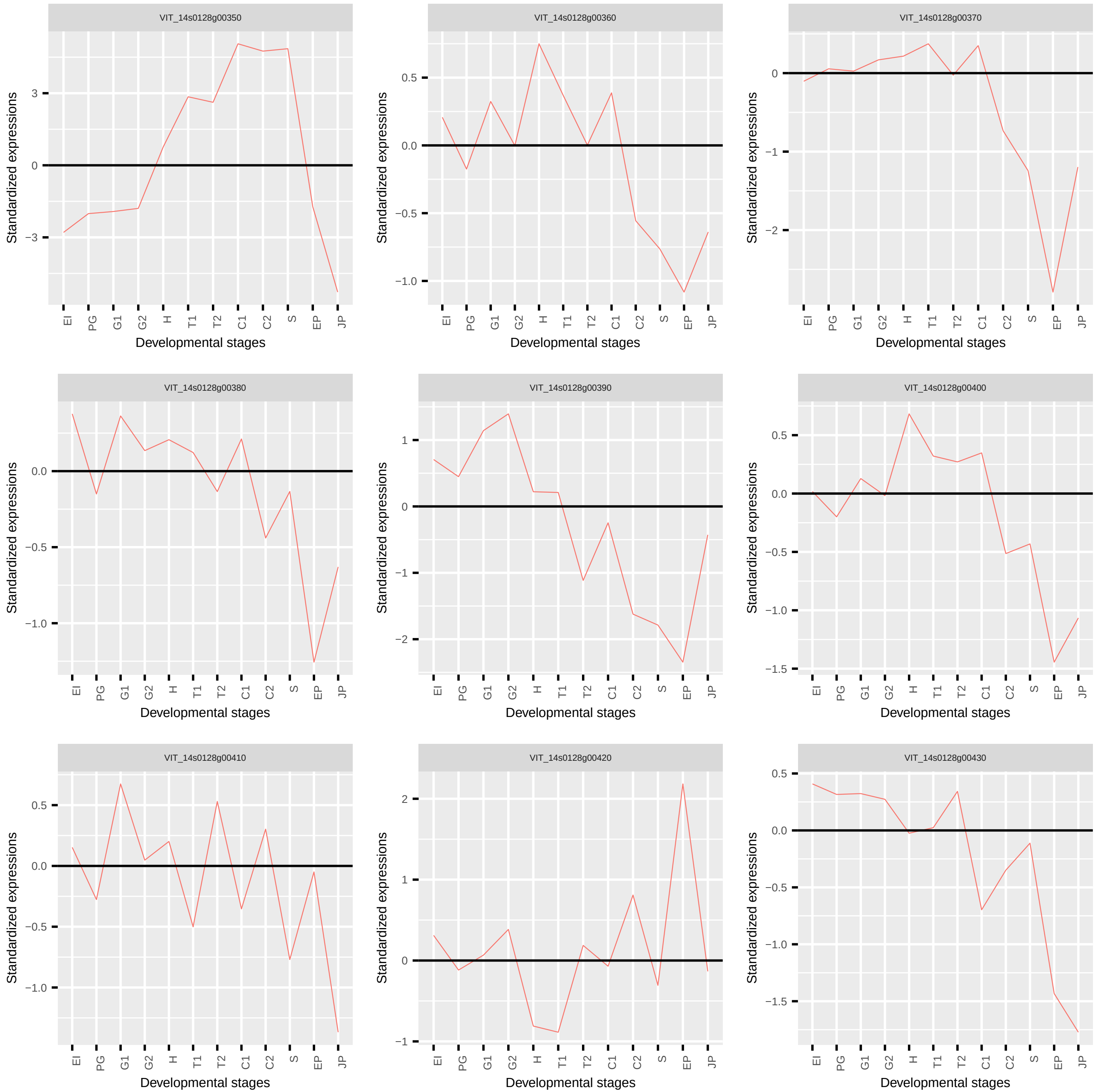
VIT_14s0128g00350
VIT_14s0128g00360
VIT_14s0128g00370
VIT_14s0128g00380
VIT_14s0128g00390
VIT_14s0128g00400
VIT_14s0128g00410
VIT_14s0128g00420
VIT_14s0128g00430
Standardized expressions
Developmental stages
EI
PG
G1
G2
H
T1
T2
C1
C2
S
EP
JP

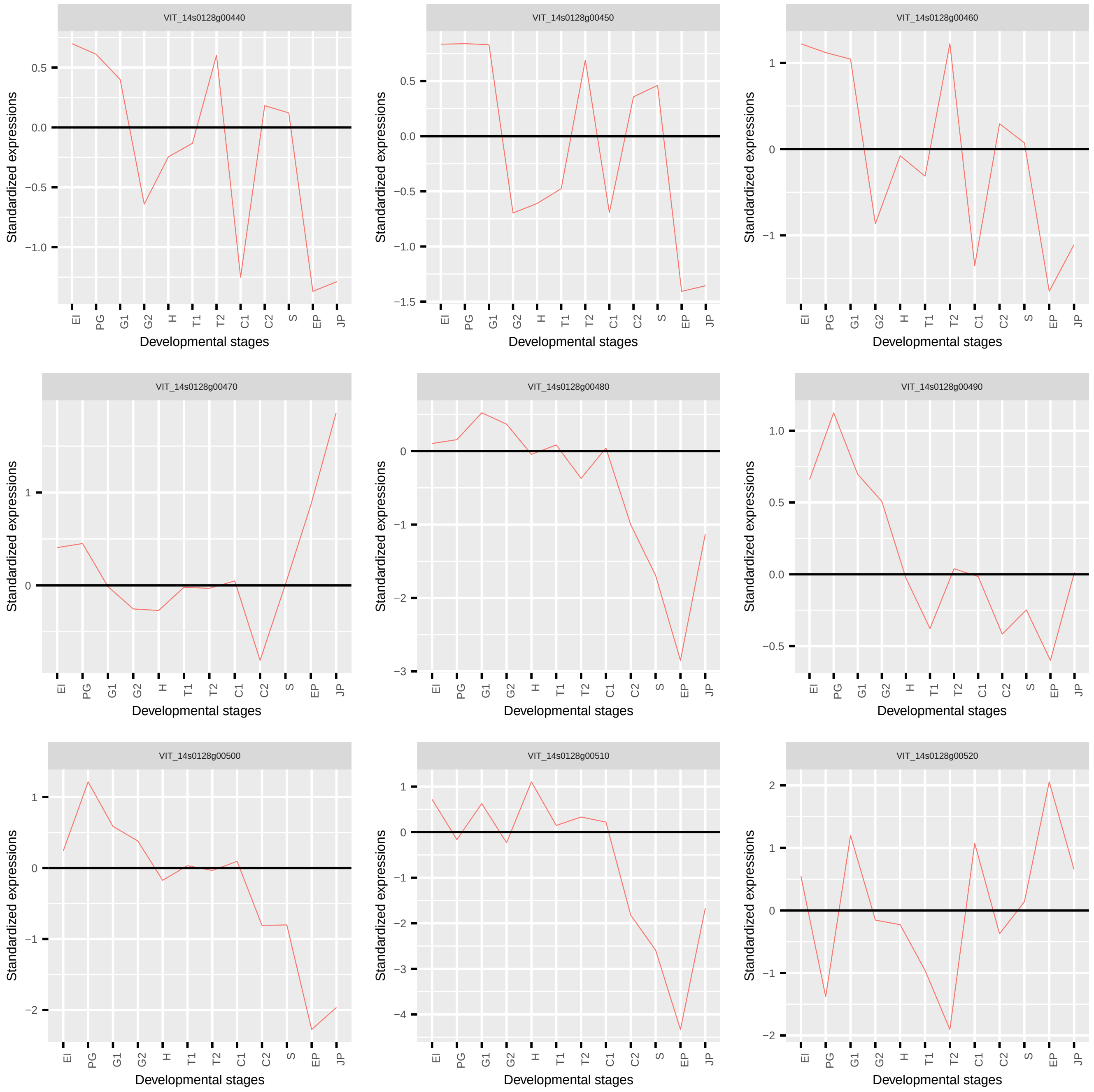
VIT_14s0128g00440
0.5
0.0
−0.5
−1.0
VIT_14s0128g00450
0.5
0.0
−0.5
−1.0
−1.5
VIT_14s0128g00460
1
0
−1
VIT_14s0128g00470
1
0
VIT_14s0128g00480
0
−1
−2
−3
VIT_14s0128g00490
1.0
0.5
0.0
−0.5
VIT_14s0128g00500
1
0
−1
−2
VIT_14s0128g00510
1
0
−1
−2
−3
−4
VIT_14s0128g00520
2
1
0
−1
−2
Standardized expressions
EI
PG
G1
G2
H
T1
T2
C1
C2
S
EP
JP
Developmental stages

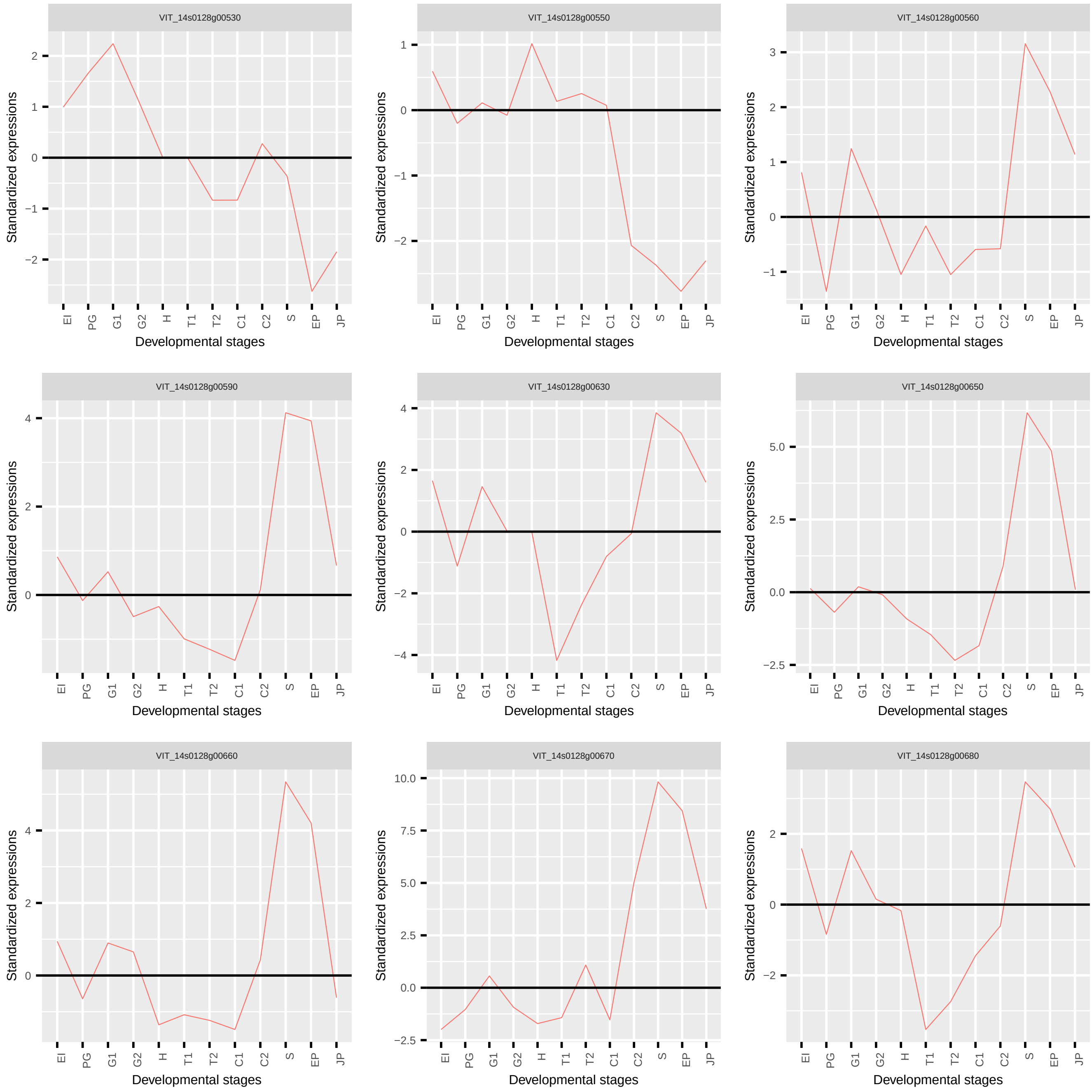
VIT_14s0128g00530
Standardized expressions
2
1
0
−1
−2
EI
PG
G1
G2
H
T1
T2
C1
C2
S
EP
JP
Developmental stages
VIT_14s0128g00550
Standardized expressions
1
0
−1
−2
EI
PG
G1
G2
H
T1
T2
C1
C2
S
EP
JP
Developmental stages
VIT_14s0128g00560
Standardized expressions
3
2
1
0
−1
EI
PG
G1
G2
H
T1
T2
C1
C2
S
EP
JP
Developmental stages
VIT_14s0128g00590
Standardized expressions
4
2
0
EI
PG
G1
G2
H
T1
T2
C1
C2
S
EP
JP
Developmental stages
VIT_14s0128g00630
Standardized expressions
4
2
0
−2
−4
EI
PG
G1
G2
H
T1
T2
C1
C2
S
EP
JP
Developmental stages
VIT_14s0128g00650
Standardized expressions
5.0
2.5
0.0
−2.5
EI
PG
G1
G2
H
T1
T2
C1
C2
S
EP
JP
Developmental stages
VIT_14s0128g00660
Standardized expressions
4
2
0
EI
PG
G1
G2
H
T1
T2
C1
C2
S
EP
JP
Developmental stages
VIT_14s0128g00670
Standardized expressions
10.0
7.5
5.0
2.5
0.0
−2.5
EI
PG
G1
G2
H
T1
T2
C1
C2
S
EP
JP
Developmental stages
VIT_14s0128g00680
Standardized expressions
2
0
−2
EI
PG
G1
G2
H
T1
T2
C1
C2
S
EP
JP
Developmental stages

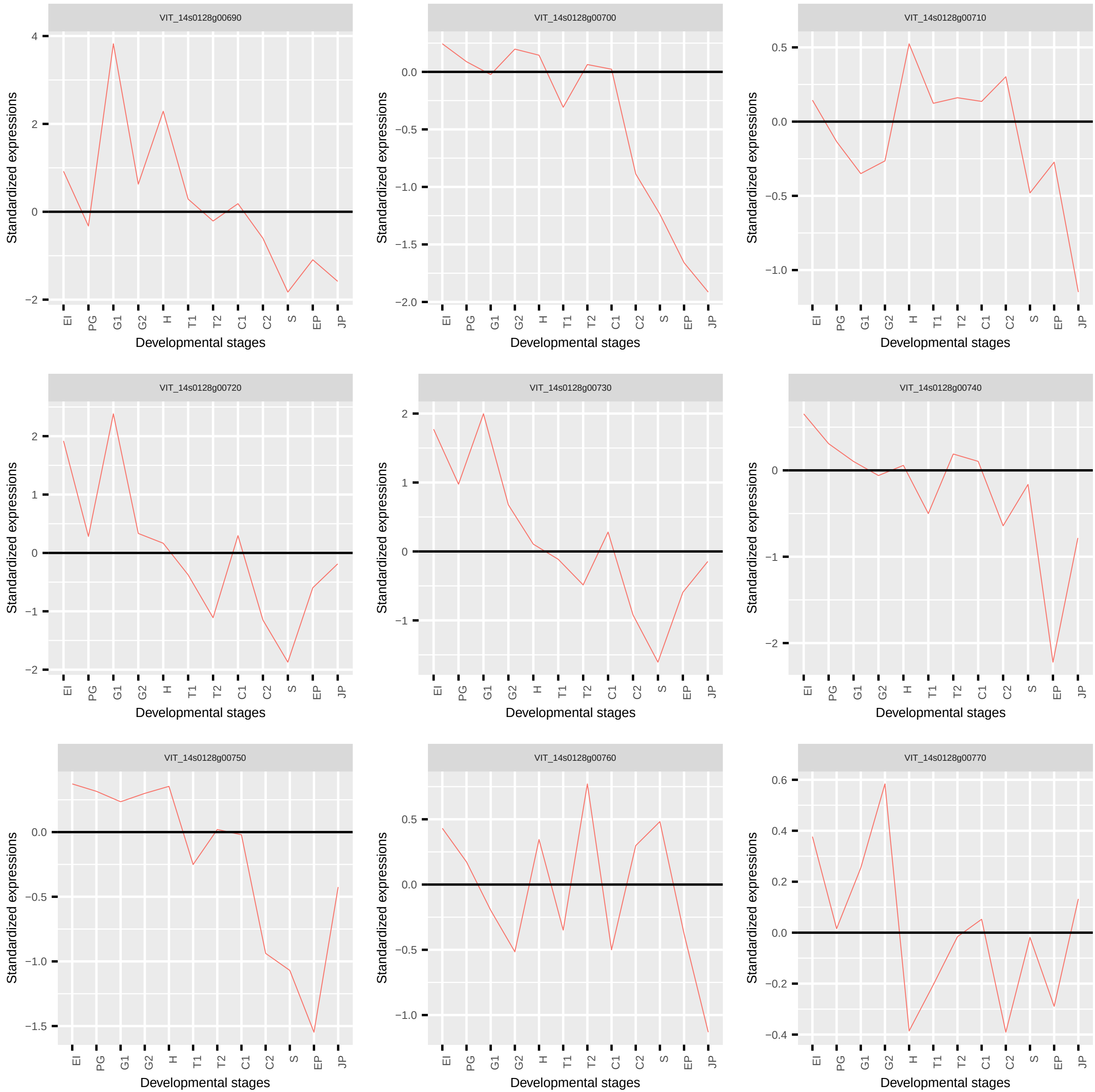
VIT_14s0128g00690
4
2
0
−2
Standardized expressions
EI PG G1 G2 H T1 T2 C1 C2 S EP JP
Developmental stages
VIT_14s0128g00700
0.0
−0.5
−1.0
−1.5
−2.0
Standardized expressions
EI PG G1 G2 H T1 T2 C1 C2 S EP JP
Developmental stages
VIT_14s0128g00710
0.5
0.0
−0.5
−1.0
Standardized expressions
EI PG G1 G2 H T1 T2 C1 C2 S EP JP
Developmental stages
VIT_14s0128g00720
2
1
0
−1
−2
Standardized expressions
EI PG G1 G2 H T1 T2 C1 C2 S EP JP
Developmental stages
VIT_14s0128g00730
2
1
0
−1
Standardized expressions
EI PG G1 G2 H T1 T2 C1 C2 S EP JP
Developmental stages
VIT_14s0128g00740
0
−1
−2
Standardized expressions
EI PG G1 G2 H T1 T2 C1 C2 S EP JP
Developmental stages
VIT_14s0128g00750
0.0
−0.5
−1.0
−1.5
Standardized expressions
EI PG G1 G2 H T1 T2 C1 C2 S EP JP
Developmental stages
VIT_14s0128g00760
0.5
0.0
−0.5
−1.0
Standardized expressions
EI PG G1 G2 H T1 T2 C1 C2 S EP JP
Developmental stages
VIT_14s0128g00770
0.6
0.4
0.2
0.0
−0.2
−0.4
Standardized expressions
EI PG G1 G2 H T1 T2 C1 C2 S EP JP
Developmental stages

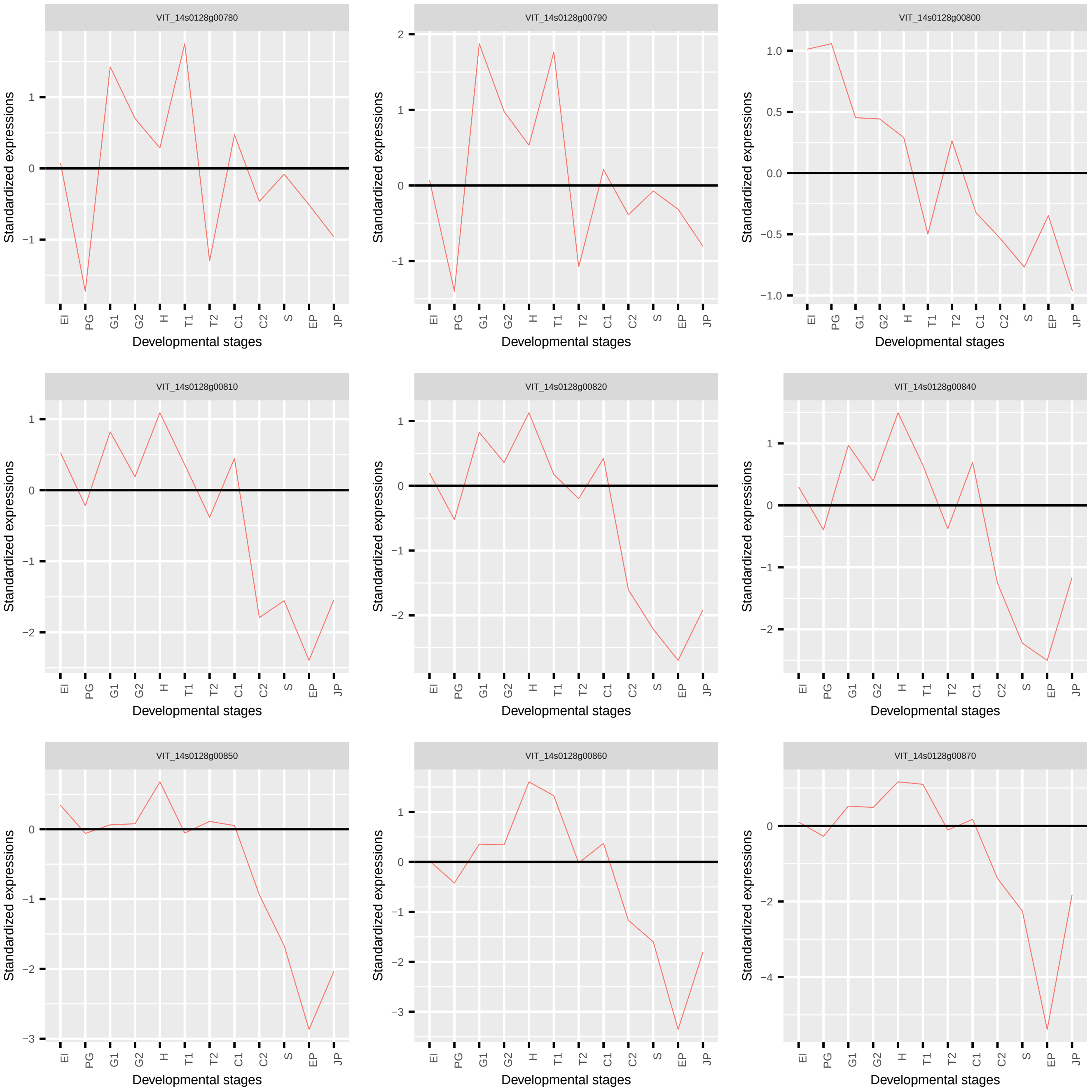
VIT_14s0128g00780
VIT_14s0128g00790
VIT_14s0128g00800
VIT_14s0128g00810
VIT_14s0128g00820
VIT_14s0128g00840
VIT_14s0128g00850
VIT_14s0128g00860
VIT_14s0128g00870
Standardized expressions
Developmental stages
EI PG G1 G2 H T1 T2 C1 C2 S EP JP

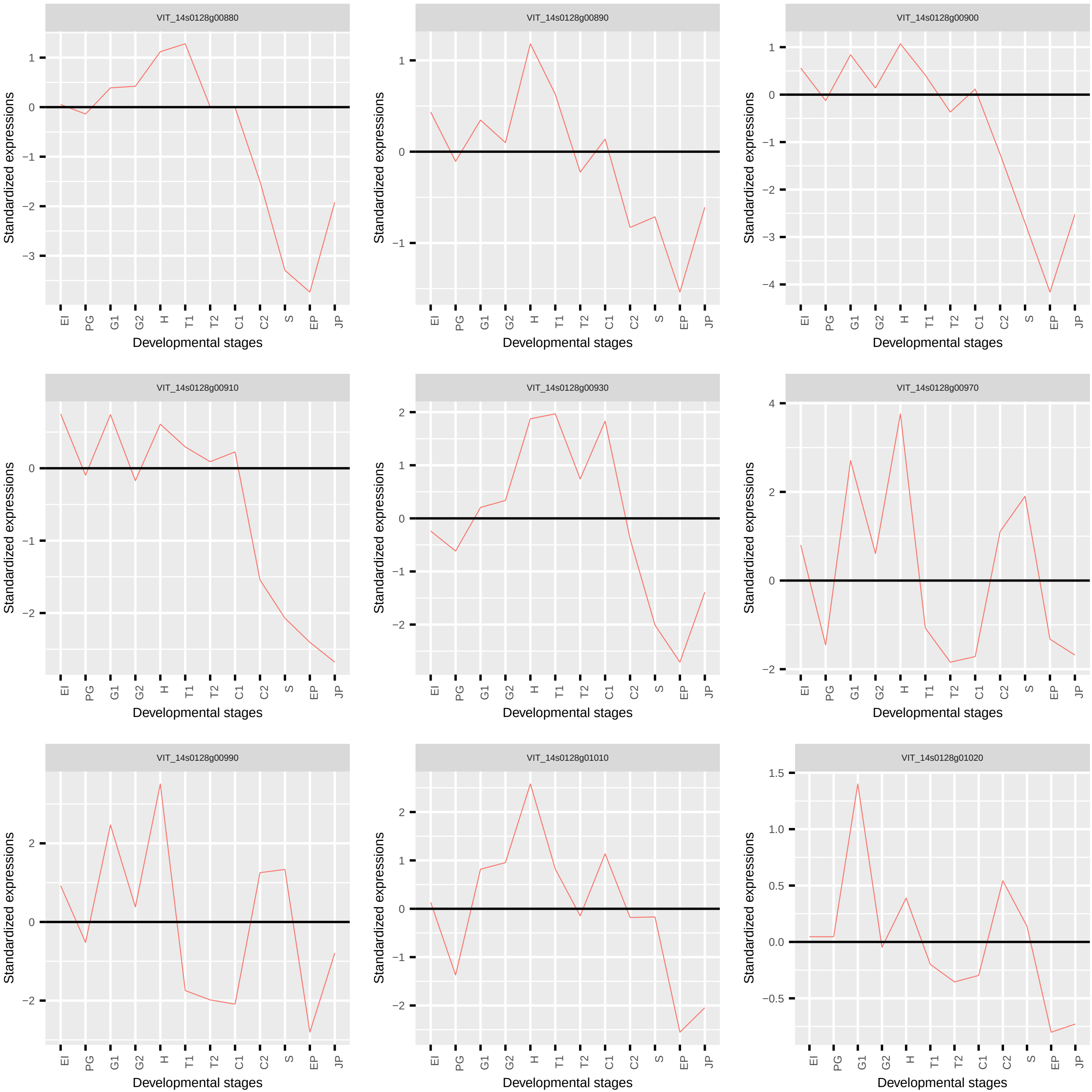
VIT_14s0128g00880
VIT_14s0128g00890
VIT_14s0128g00900
VIT_14s0128g00910
VIT_14s0128g00930
VIT_14s0128g00970
VIT_14s0128g00990
VIT_14s0128g01010
VIT_14s0128g01020
Standardized expressions
Developmental stages
EI PG G1 G2 H T1 T2 C1 C2 S EP JP

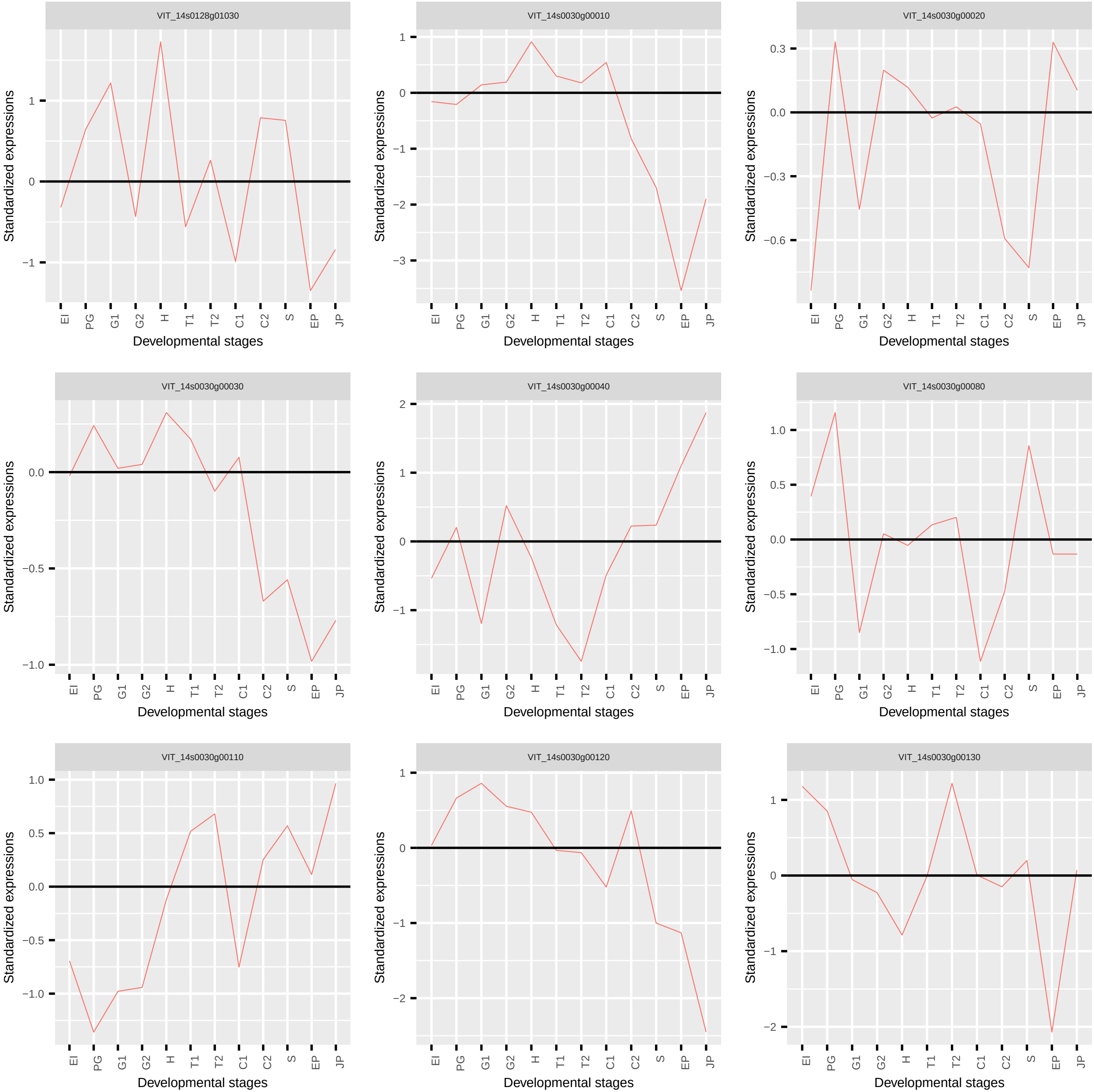
VIT_14s0128g01030
VIT_14s0030g00010
VIT_14s0030g00020
VIT_14s0030g00030
VIT_14s0030g00040
VIT_14s0030g00080
VIT_14s0030g00110
VIT_14s0030g00120
VIT_14s0030g00130
Standardized expressions
Developmental stages
EI PG G1 G2 H T1 T2 C1 C2 S EP JP

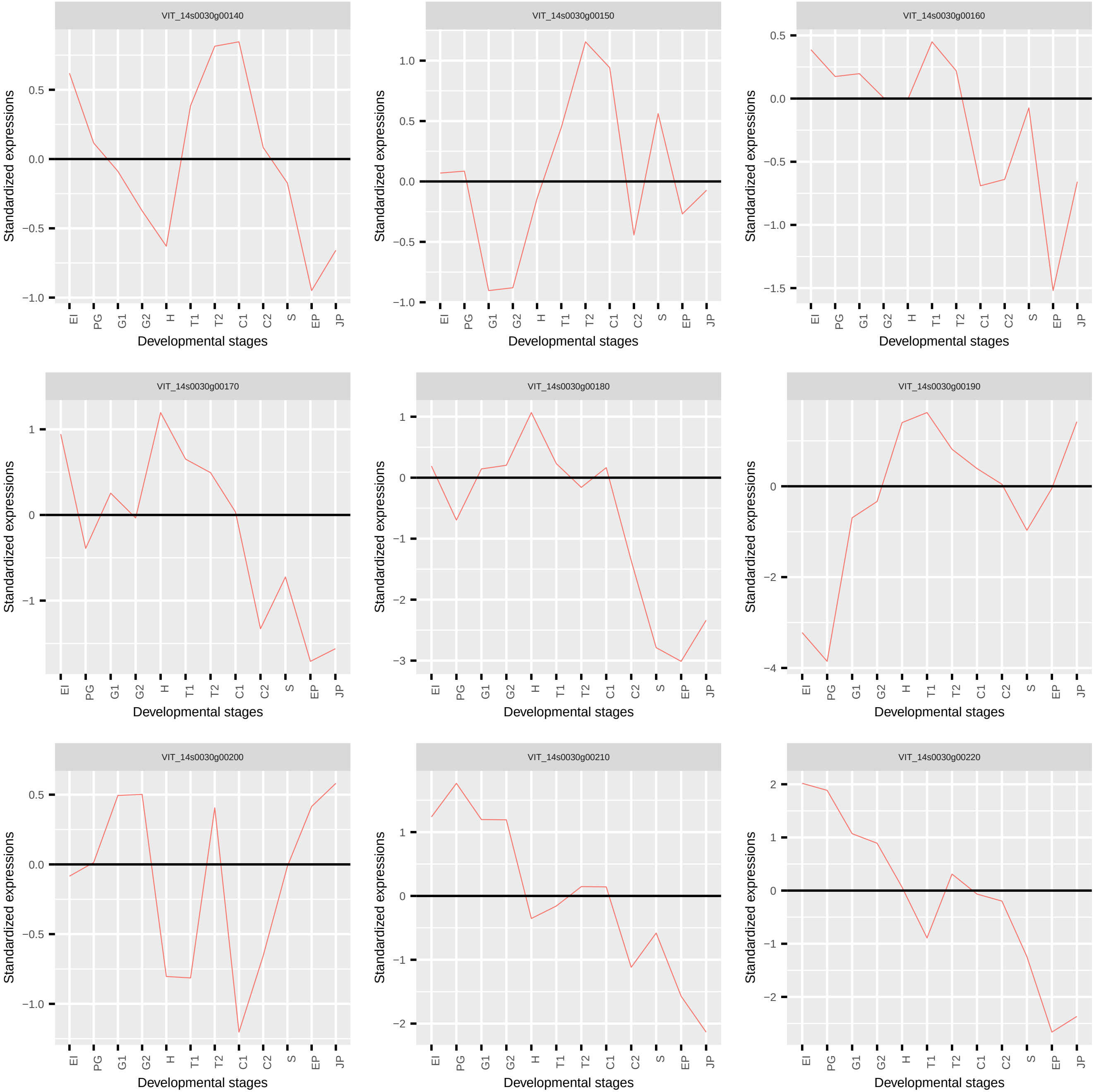
VIT_14s0030g00140
VIT_14s0030g00150
VIT_14s0030g00160
VIT_14s0030g00170
VIT_14s0030g00180
VIT_14s0030g00190
VIT_14s0030g00200
VIT_14s0030g00210
VIT_14s0030g00220
Standardized expressions
Developmental stages
EI PG G1 G2 H T1 T2 C1 C2 S EP JP

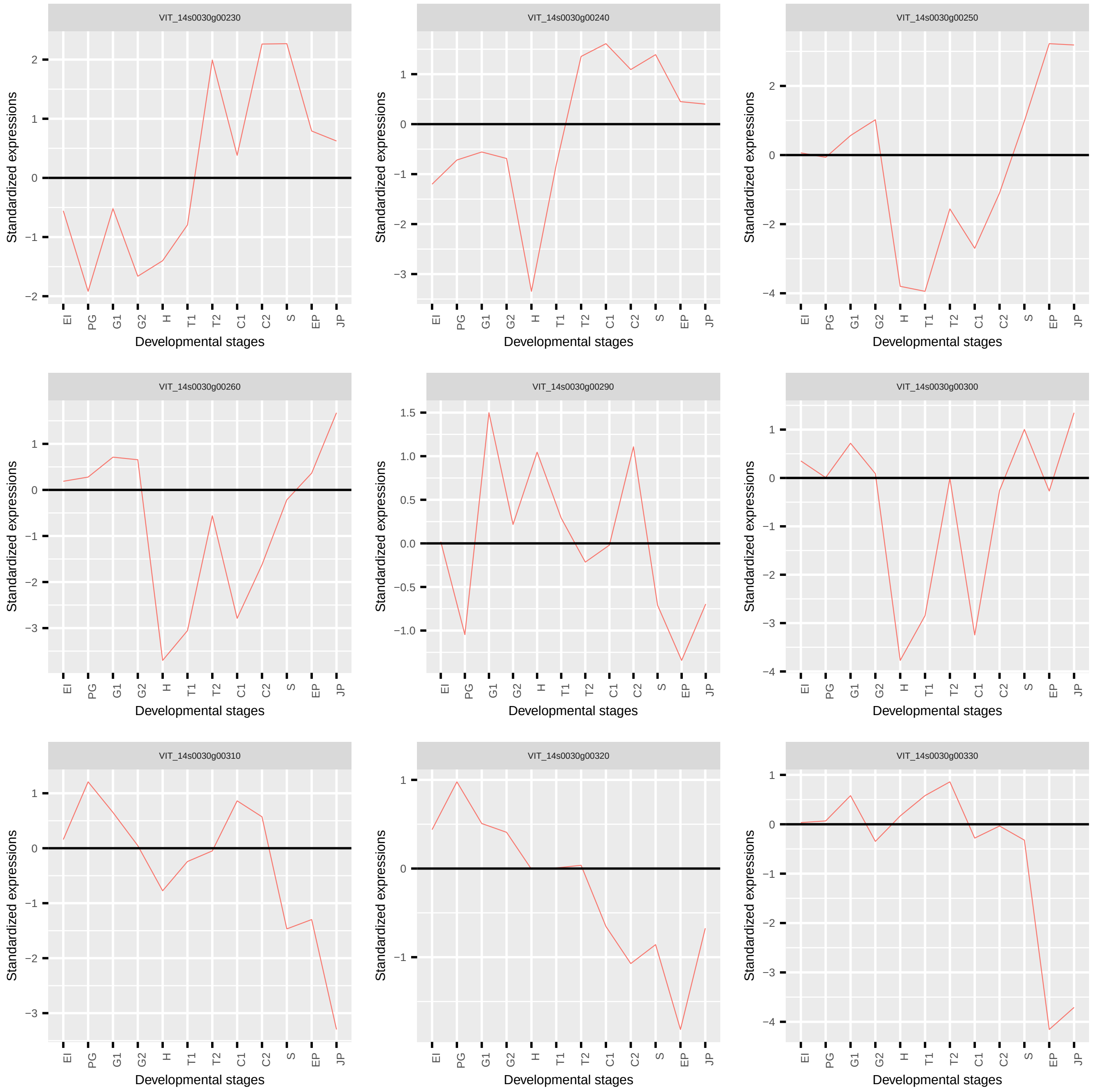

VIT_14s0030g00230
VIT_14s0030g00240
VIT_14s0030g00250
VIT_14s0030g00260
VIT_14s0030g00290
VIT_14s0030g00300
VIT_14s0030g00310
VIT_14s0030g00320
VIT_14s0030g00330
Standardized expressions
Developmental stages
EI PG G1 G2 H T1 T2 C1 C2 S EP JP

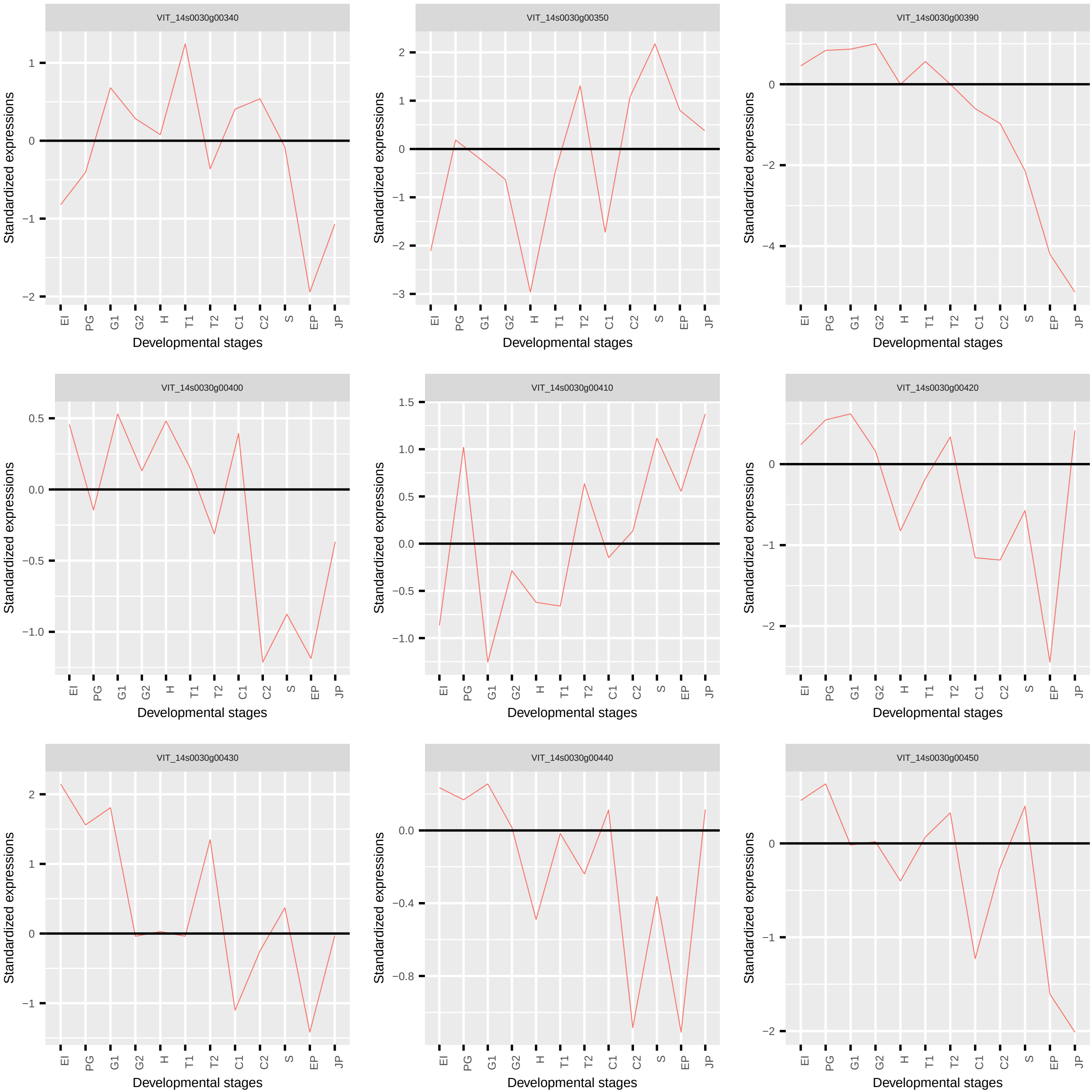
VIT_14s0030g00340
VIT_14s0030g00350
VIT_14s0030g00390
VIT_14s0030g00400
VIT_14s0030g00410
VIT_14s0030g00420
VIT_14s0030g00430
VIT_14s0030g00440
VIT_14s0030g00450
Standardized expressions
Developmental stages
EI
PG
G1
G2
H
T1
T2
C1
C2
S
EP
JP

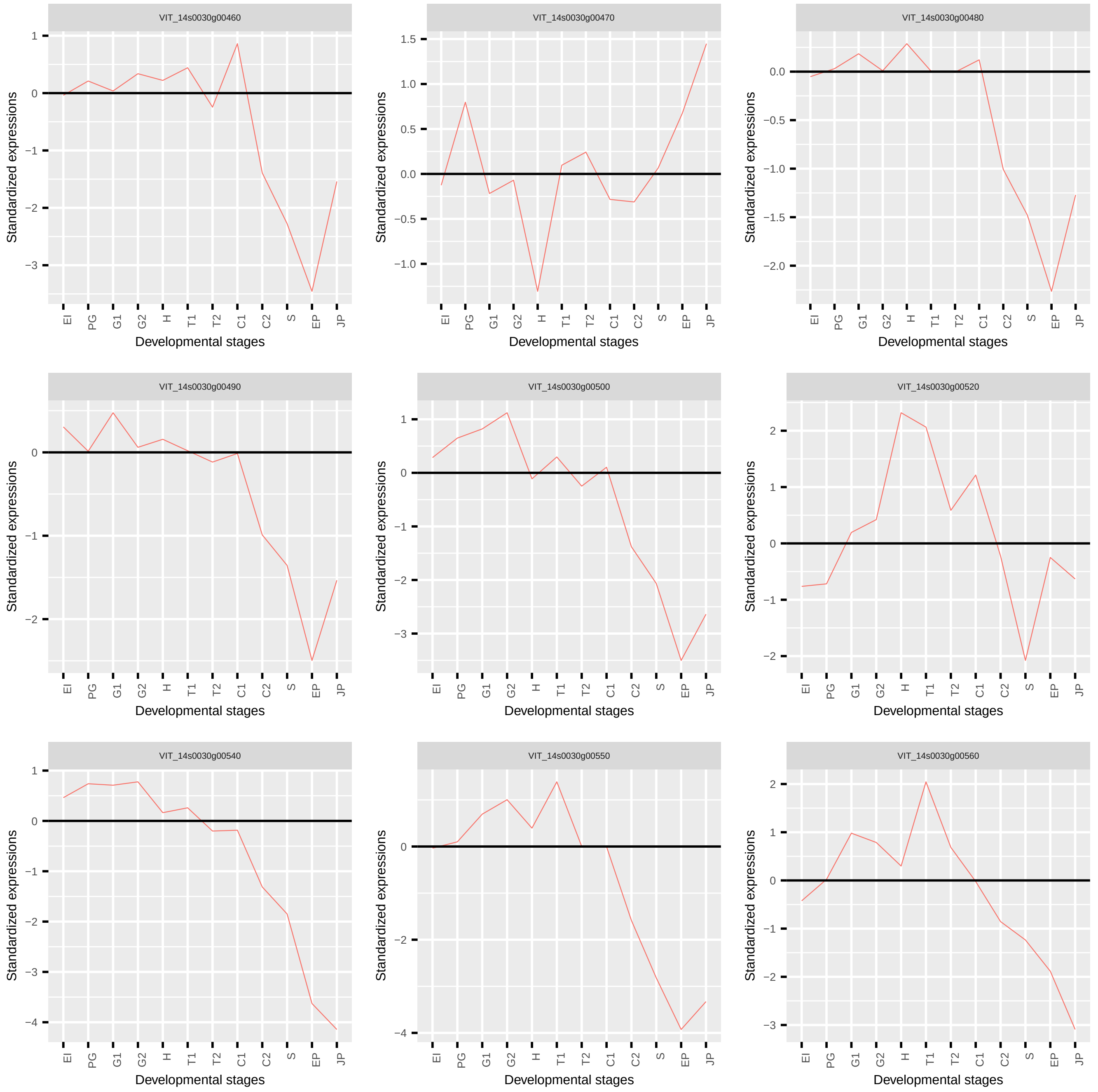
VIT_14s0030g00460
VIT_14s0030g00470
VIT_14s0030g00480
VIT_14s0030g00490
VIT_14s0030g00500
VIT_14s0030g00520
VIT_14s0030g00540
VIT_14s0030g00550
VIT_14s0030g00560
Standardized expressions
Developmental stages
EI PG G1 G2 H T1 T2 C1 C2 S EP JP

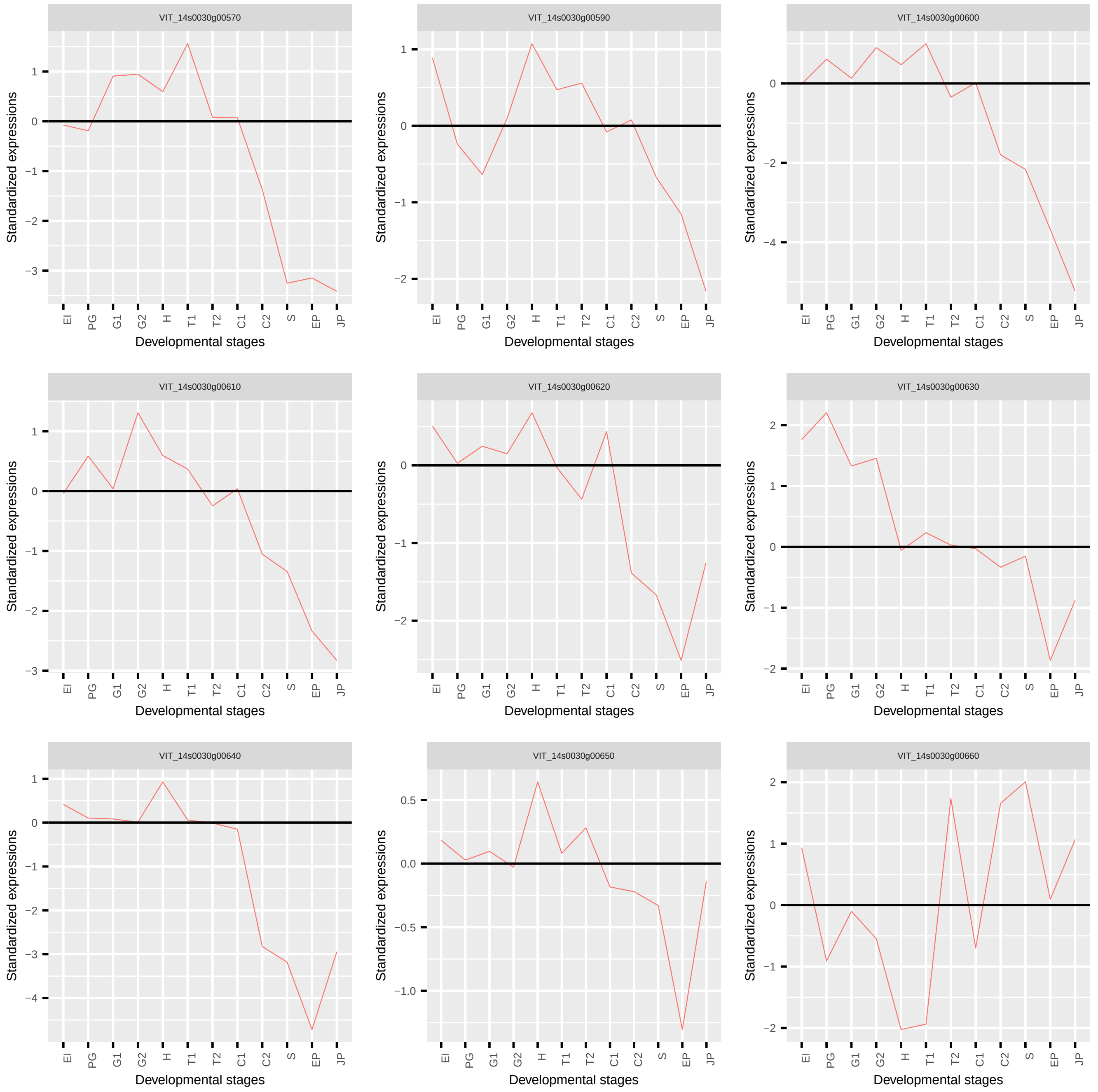
VIT_14s0030g00570
VIT_14s0030g00590
VIT_14s0030g00600
VIT_14s0030g00610
VIT_14s0030g00620
VIT_14s0030g00630
VIT_14s0030g00640
VIT_14s0030g00650
VIT_14s0030g00660
Standardized expressions
Developmental stages
EI
PG
G1
G2
H
T1
T2
C1
C2
S
EP
JP

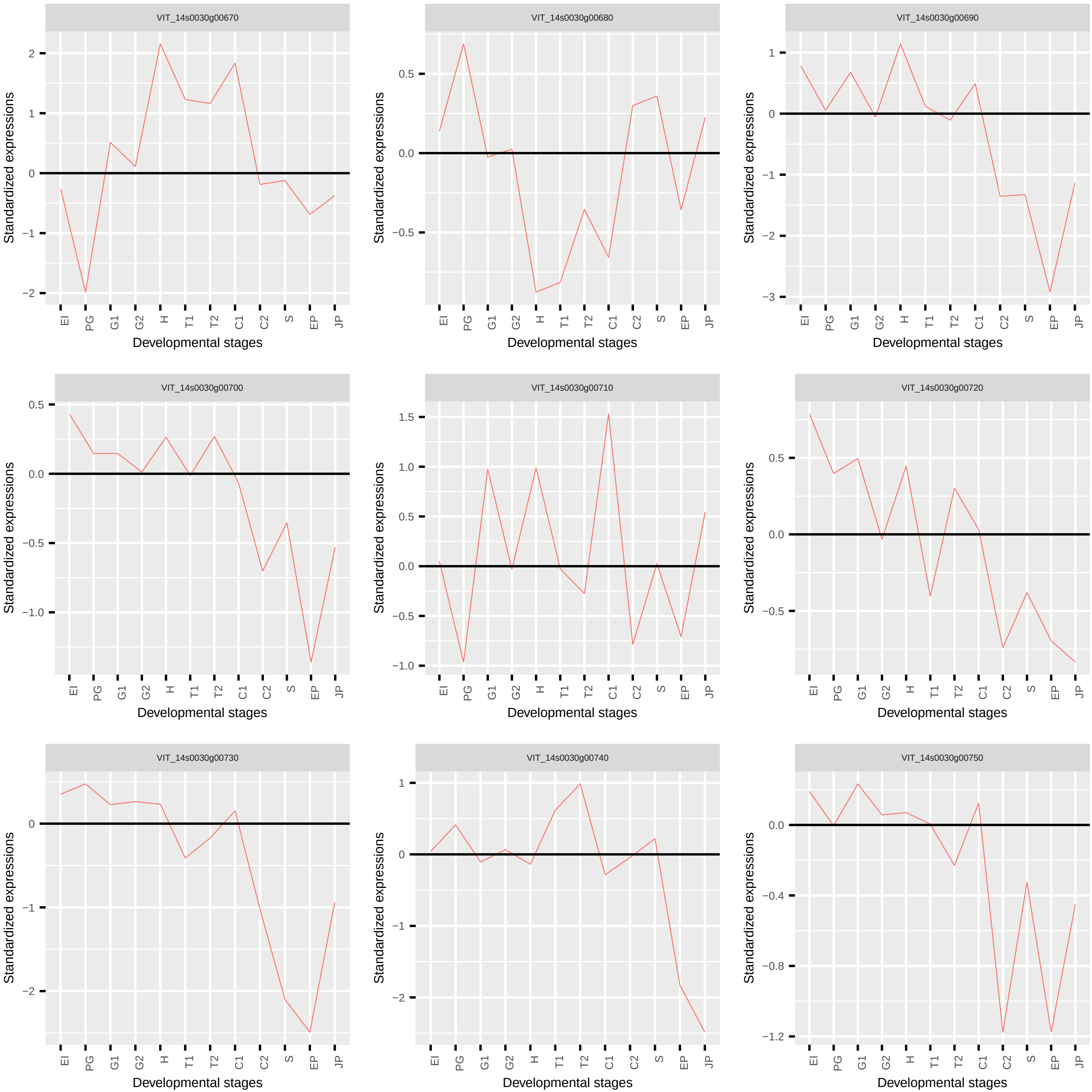
VIT_14s0030g00670
VIT_14s0030g00680
VIT_14s0030g00690
VIT_14s0030g00700
VIT_14s0030g00710
VIT_14s0030g00720
VIT_14s0030g00730
VIT_14s0030g00740
VIT_14s0030g00750
Standardized expressions
Developmental stages
EI PG G1 G2 H T1 T2 C1 C2 S EP JP

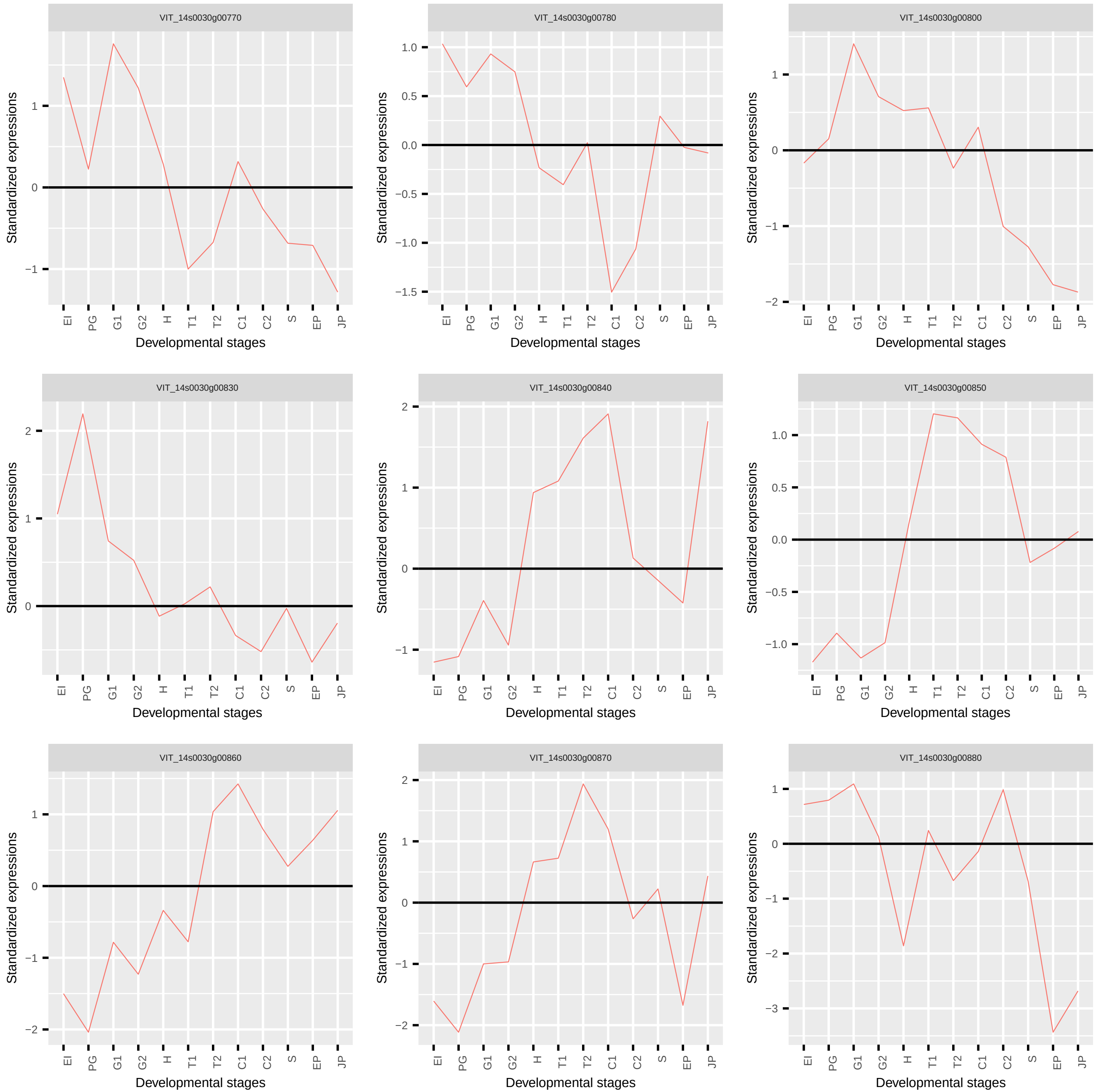
VIT_14s0030g00770
VIT_14s0030g00780
VIT_14s0030g00800
VIT_14s0030g00830
VIT_14s0030g00840
VIT_14s0030g00850
VIT_14s0030g00860
VIT_14s0030g00870
VIT_14s0030g00880
Standardized expressions
Developmental stages
EI PG G1 G2 H T1 T2 C1 C2 S EP JP

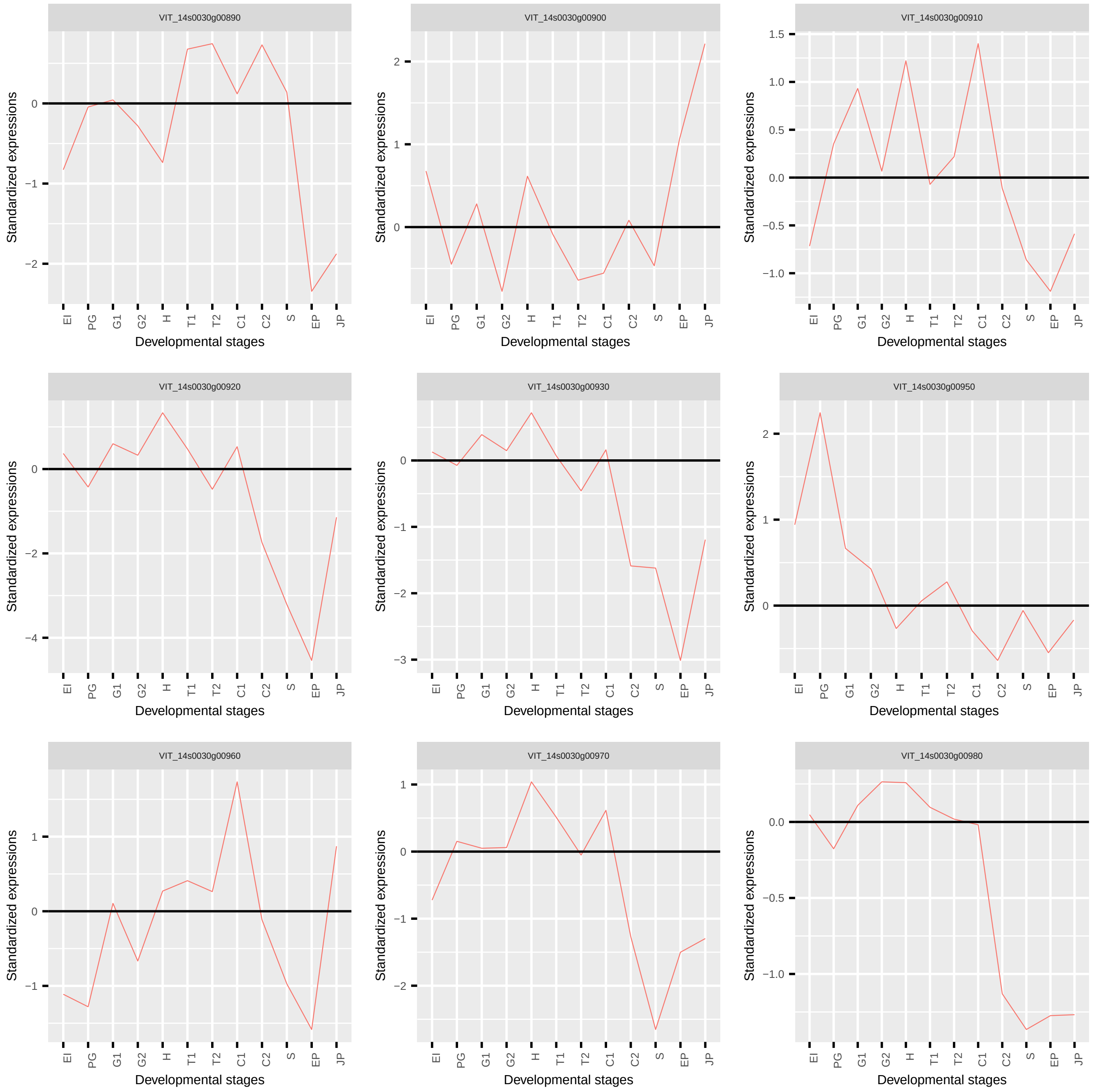
VIT_14s0030g00890
VIT_14s0030g00900
VIT_14s0030g00910
VIT_14s0030g00920
VIT_14s0030g00930
VIT_14s0030g00950
VIT_14s0030g00960
VIT_14s0030g00970
VIT_14s0030g00980
Standardized expressions
Developmental stages
EI
PG
G1
G2
H
T1
T2
C1
C2
S
EP
JP

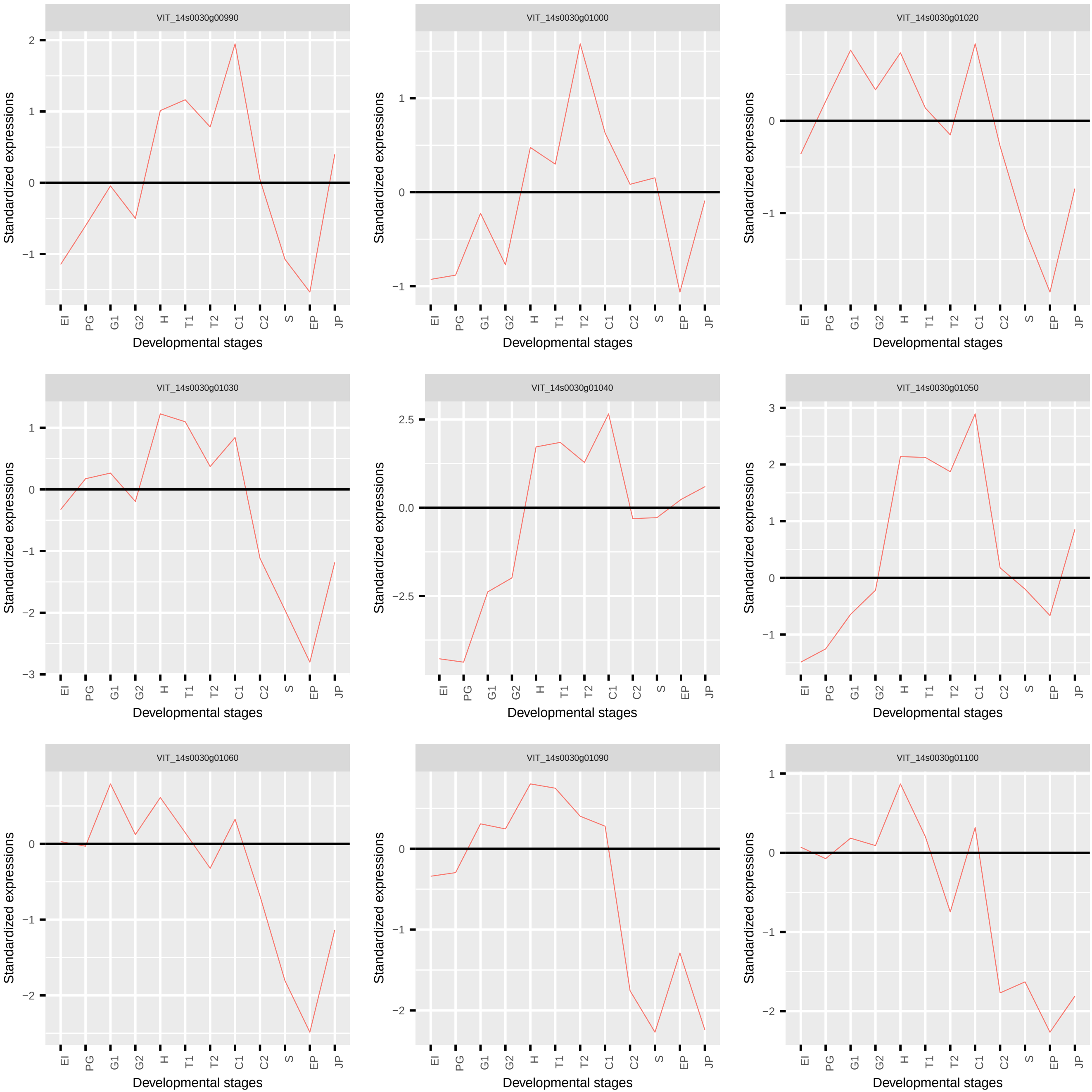
VIT_14s0030g00990
VIT_14s0030g01000
VIT_14s0030g01020
VIT_14s0030g01030
VIT_14s0030g01040
VIT_14s0030g01050
VIT_14s0030g01060
VIT_14s0030g01090
VIT_14s0030g01100
Standardized expressions
Developmental stages
EI PG G1 G2 H T1 T2 C1 C2 S EP JP

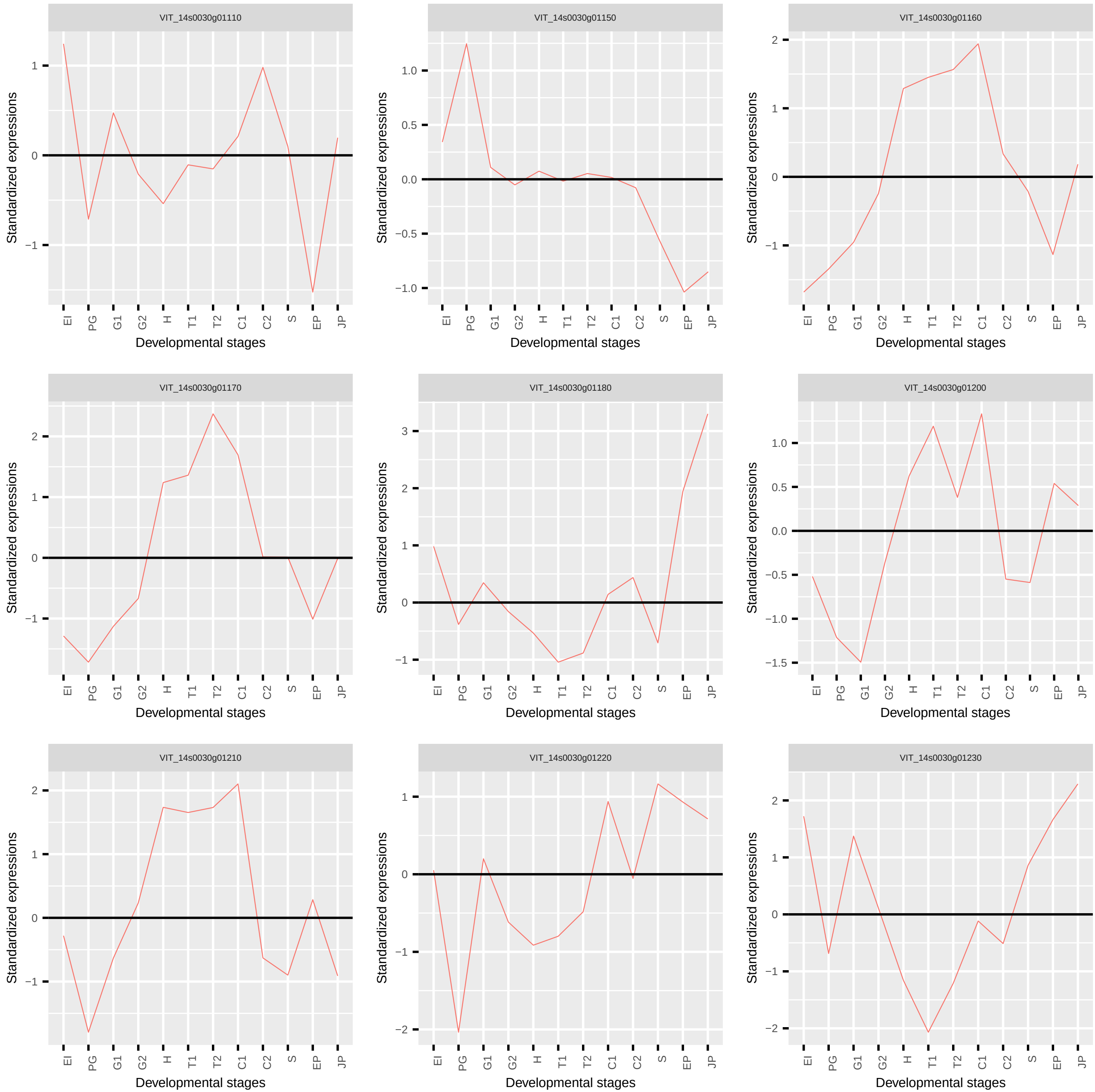
VIT_14s0030g01110
VIT_14s0030g01150
VIT_14s0030g01160
VIT_14s0030g01170
VIT_14s0030g01180
VIT_14s0030g01200
VIT_14s0030g01210
VIT_14s0030g01220
VIT_14s0030g01230
Standardized expressions
Developmental stages
EI PG G1 G2 H T1 T2 C1 C2 S EP JP

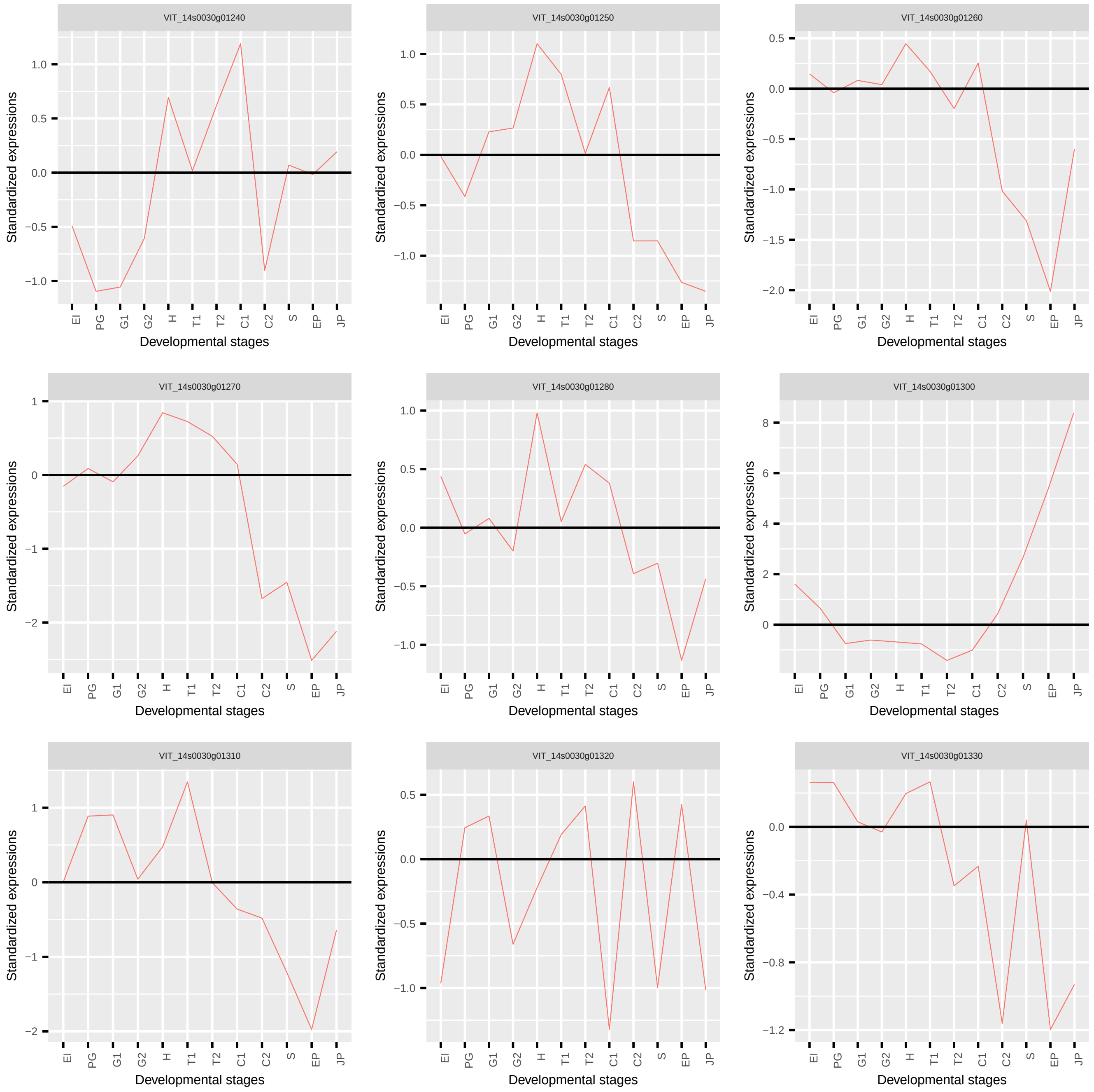
VIT_14s0030g01240
VIT_14s0030g01250
VIT_14s0030g01260
VIT_14s0030g01270
VIT_14s0030g01280
VIT_14s0030g01300
VIT_14s0030g01310
VIT_14s0030g01320
VIT_14s0030g01330
Standardized expressions
Developmental stages
EI
PG
G1
G2
H
T1
T2
C1
C2
S
EP
JP

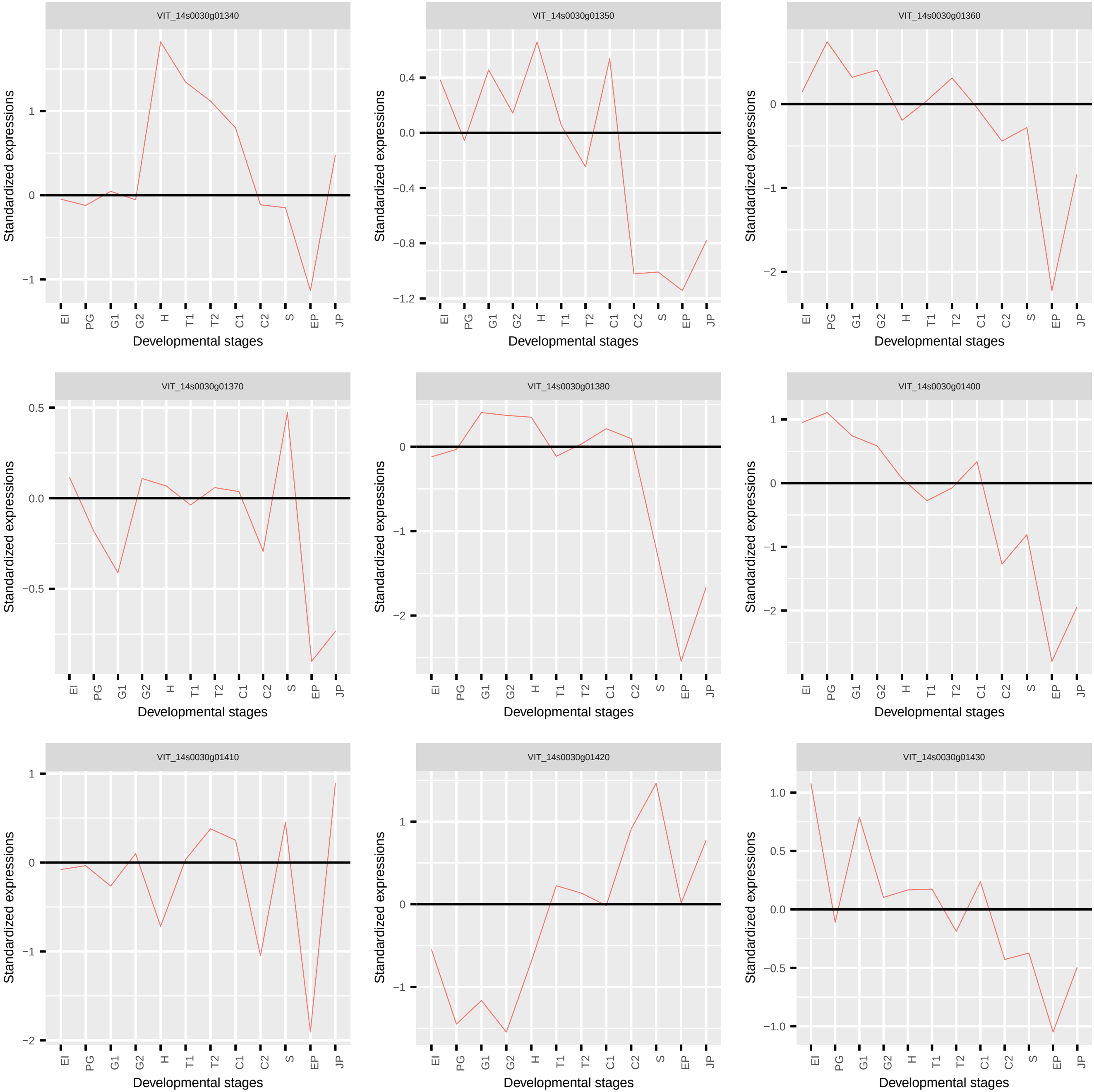
VIT_14s0030g01340
VIT_14s0030g01350
VIT_14s0030g01360
VIT_14s0030g01370
VIT_14s0030g01380
VIT_14s0030g01400
VIT_14s0030g01410
VIT_14s0030g01420
VIT_14s0030g01430
Standardized expressions
Developmental stages
EI
PG
G1
G2
H
T1
T2
C1
C2
S
EP
JP

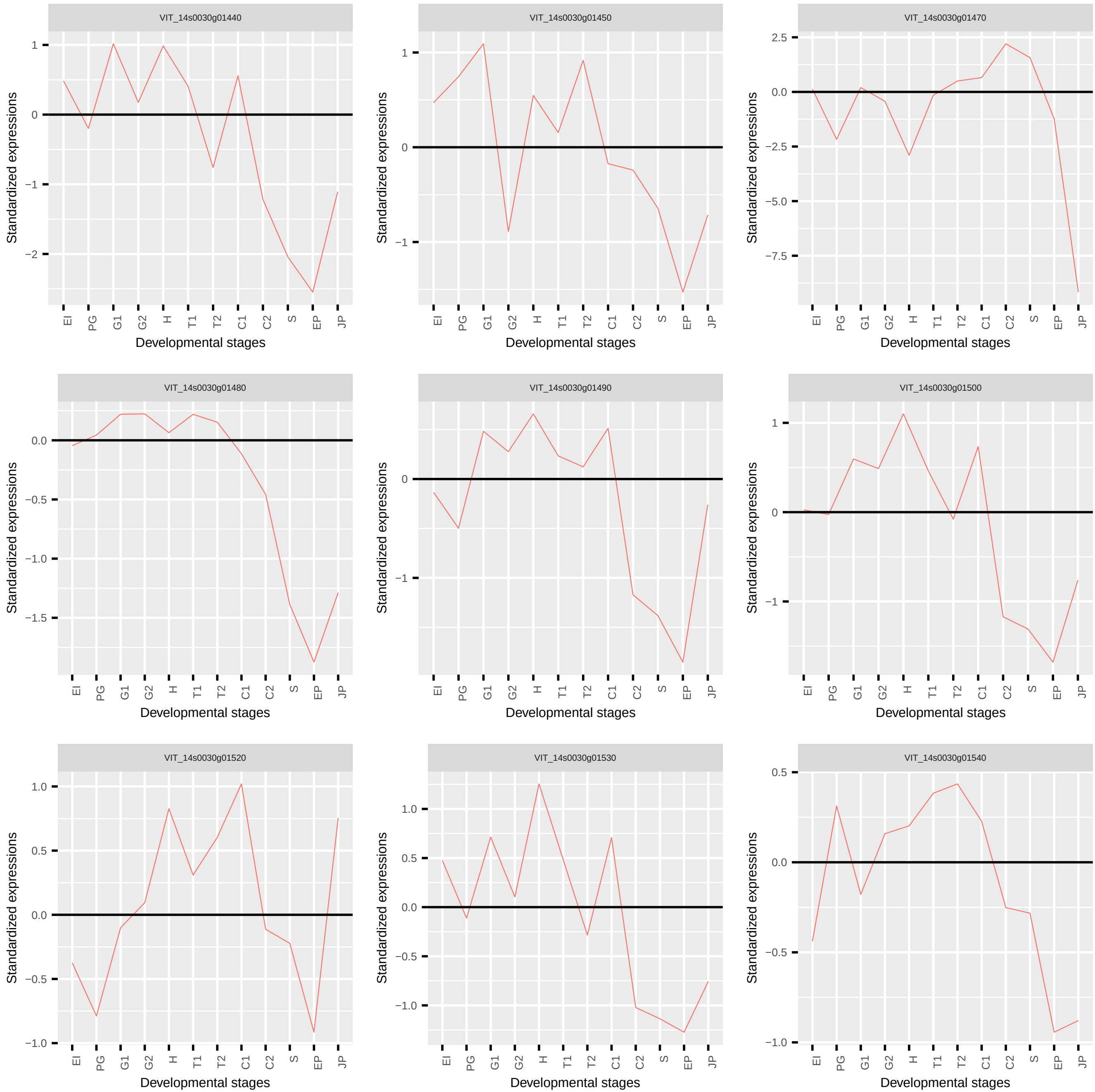

VIT_14s0030g01440
VIT_14s0030g01450
VIT_14s0030g01470
VIT_14s0030g01480
VIT_14s0030g01490
VIT_14s0030g01500
VIT_14s0030g01520
VIT_14s0030g01530
VIT_14s0030g01540
Standardized expressions
Developmental stages
EI PG G1 G2 H T1 T2 C1 C2 S EP JP

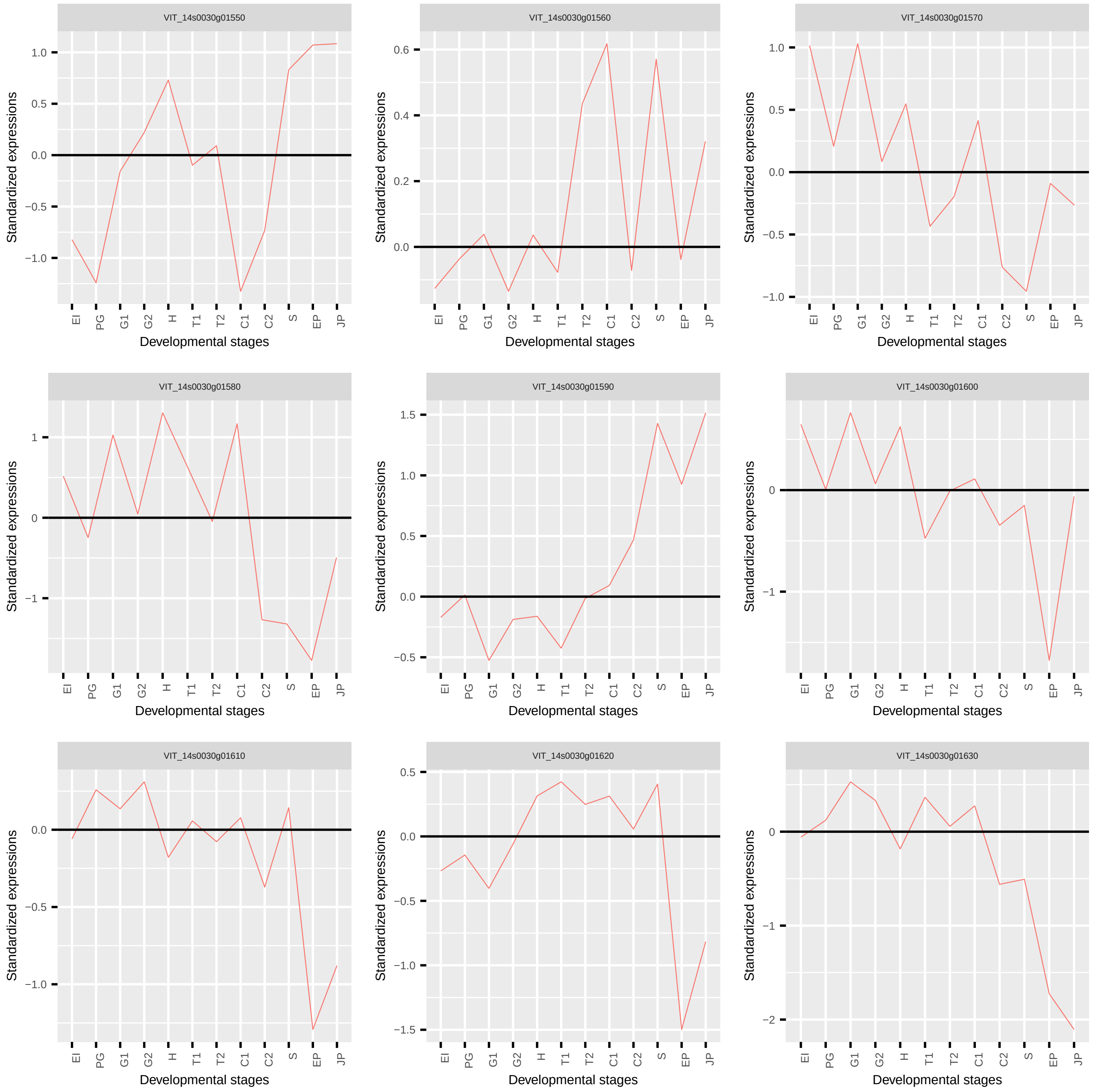
VIT_14s0030g01550
VIT_14s0030g01560
VIT_14s0030g01570
VIT_14s0030g01580
VIT_14s0030g01590
VIT_14s0030g01600
VIT_14s0030g01610
VIT_14s0030g01620
VIT_14s0030g01630
Standardized expressions
Developmental stages
EI
PG
G1
G2
H
T1
T2
C1
C2
S
EP
JP

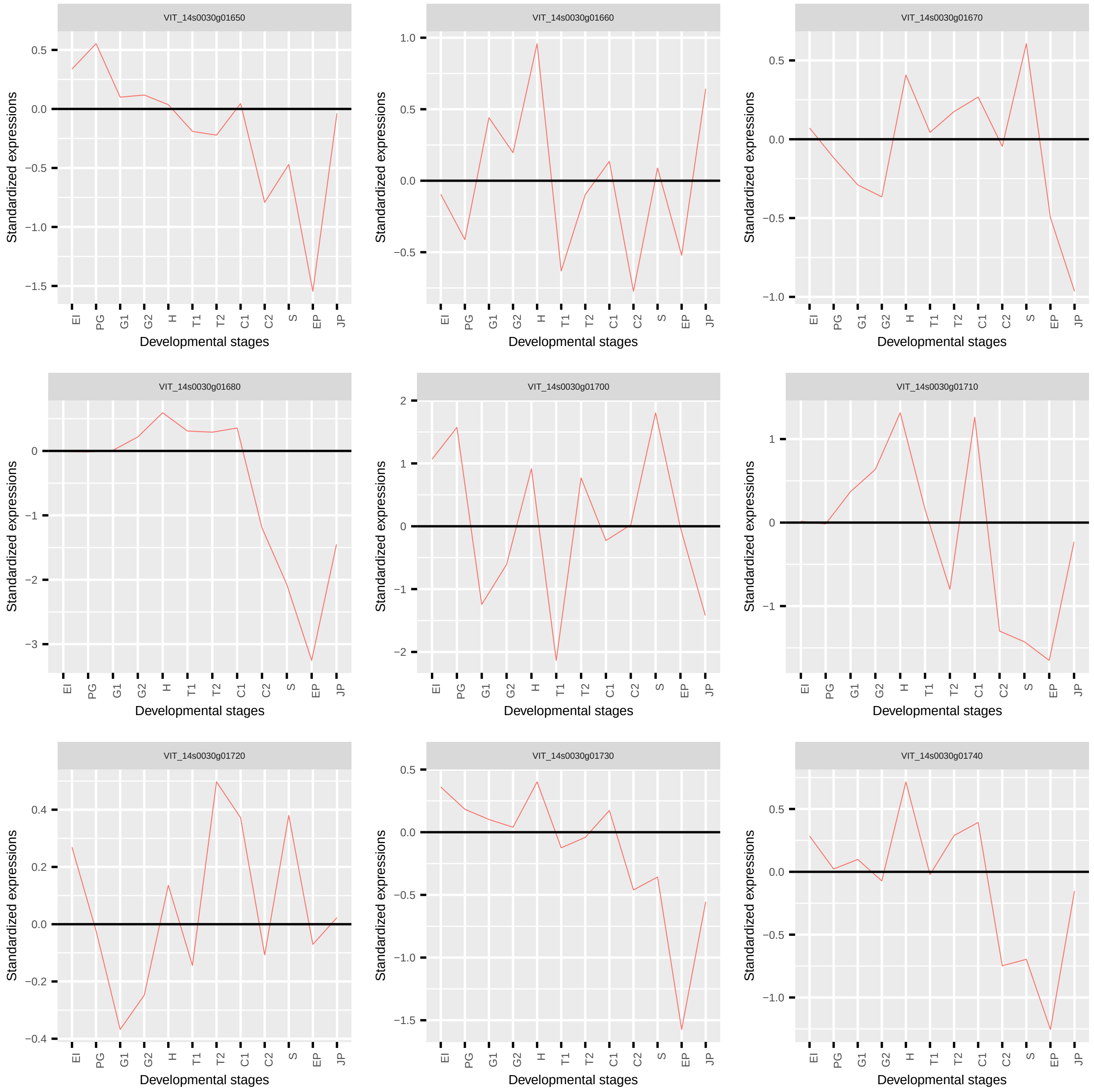
VIT_14s0030g01650
VIT_14s0030g01660
VIT_14s0030g01670
VIT_14s0030g01680
VIT_14s0030g01700
VIT_14s0030g01710
VIT_14s0030g01720
VIT_14s0030g01730
VIT_14s0030g01740
Standardized expressions
Developmental stages
EI PG G1 G2 H T1 T2 C1 C2 S EP JP

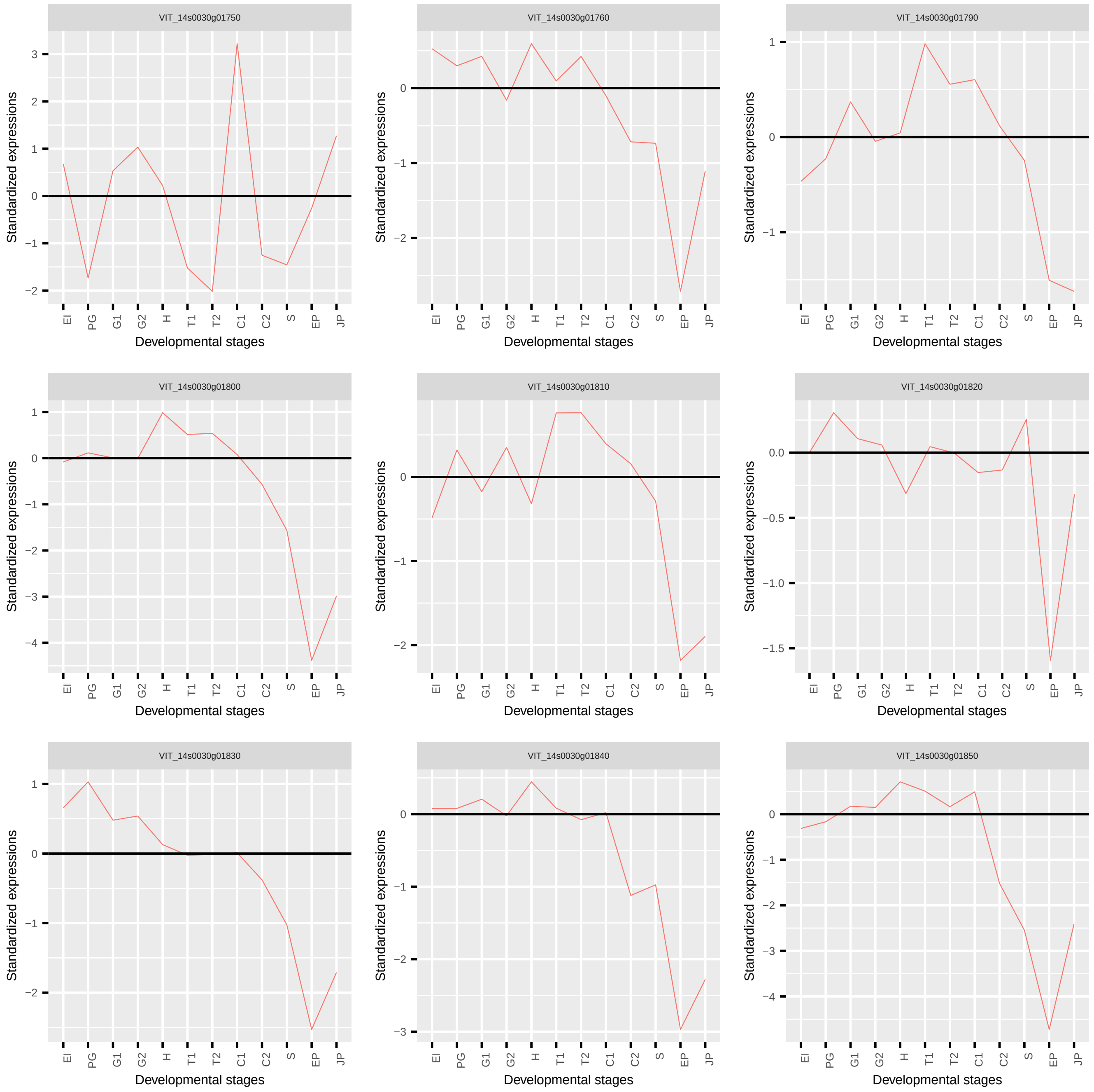
VIT_14s0030g01750
VIT_14s0030g01760
VIT_14s0030g01790
VIT_14s0030g01800
VIT_14s0030g01810
VIT_14s0030g01820
VIT_14s0030g01830
VIT_14s0030g01840
VIT_14s0030g01850
Standardized expressions
Developmental stages
EI PG G1 G2 H T1 T2 C1 C2 S EP JP

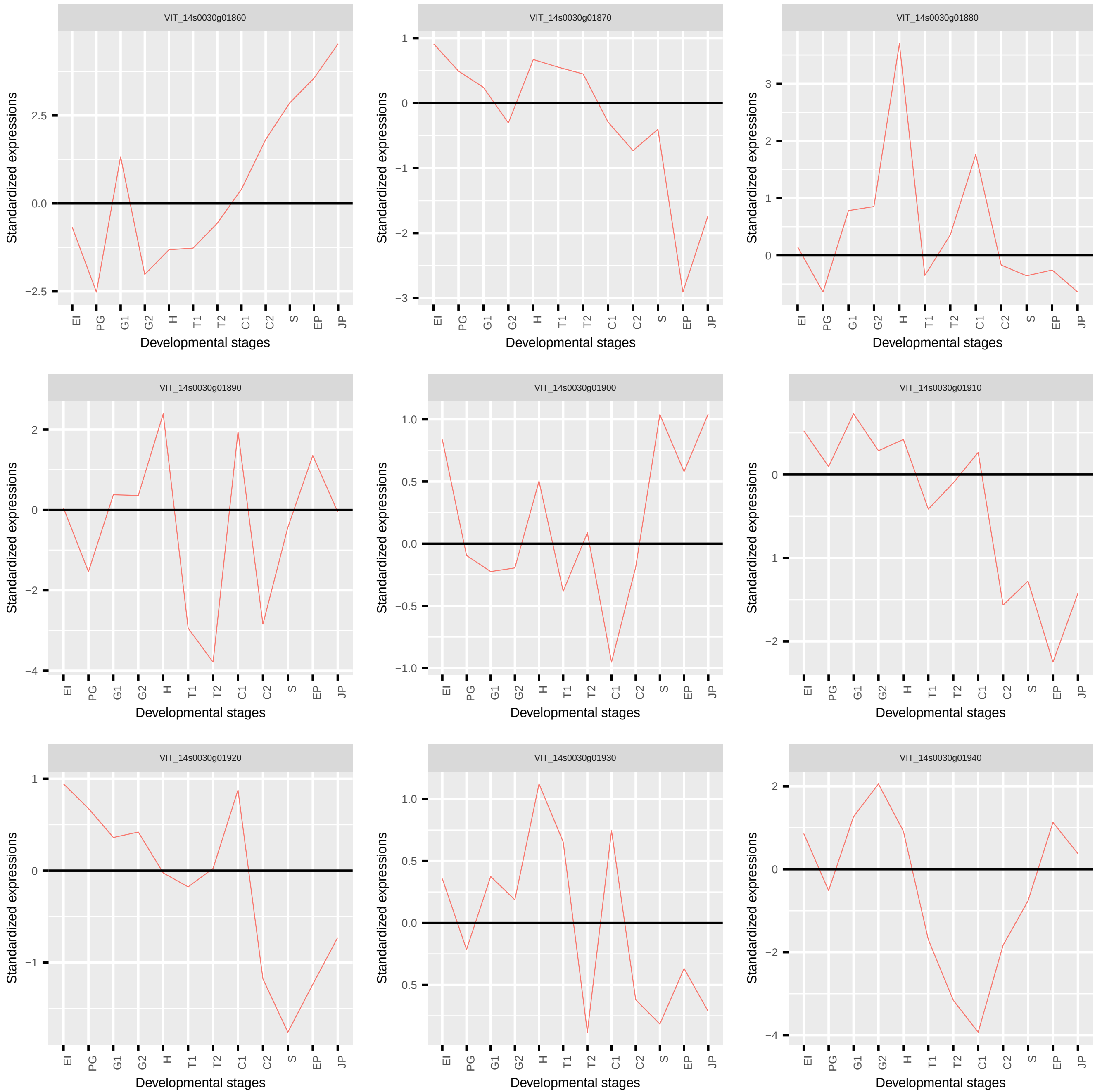

VIT_14s0030g01860
VIT_14s0030g01870
VIT_14s0030g01880
VIT_14s0030g01890
VIT_14s0030g01900
VIT_14s0030g01910
VIT_14s0030g01920
VIT_14s0030g01930
VIT_14s0030g01940
Standardized expressions
Developmental stages
EI
PG
G1
G2
H
T1
T2
C1
C2
S
EP
JP

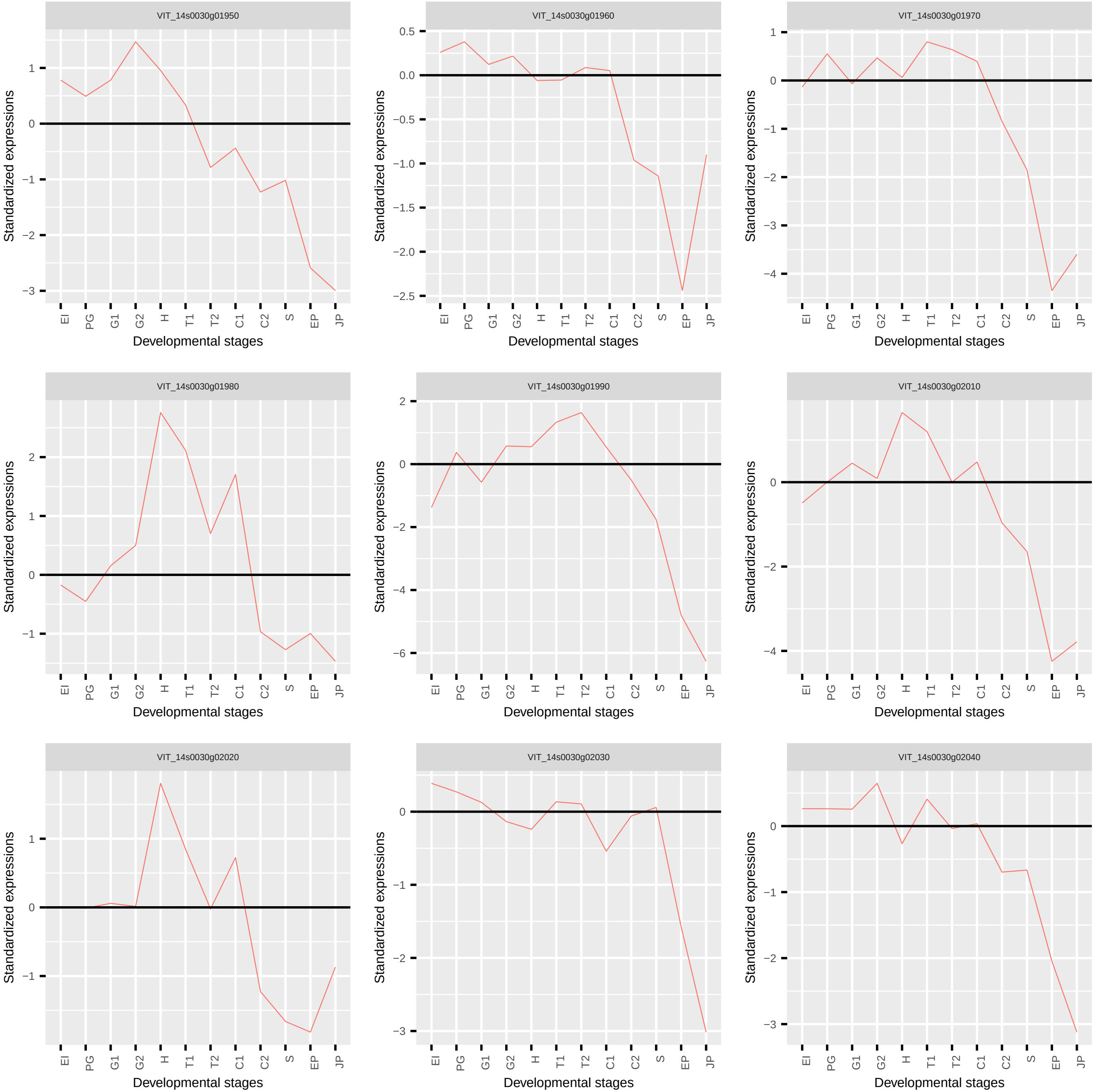
VIT_14s0030g01950
VIT_14s0030g01960
VIT_14s0030g01970
VIT_14s0030g01980
VIT_14s0030g01990
VIT_14s0030g02010
VIT_14s0030g02020
VIT_14s0030g02030
VIT_14s0030g02040
Standardized expressions
Developmental stages
EI
PG
G1
G2
H
T1
T2
C1
C2
S
EP
JP

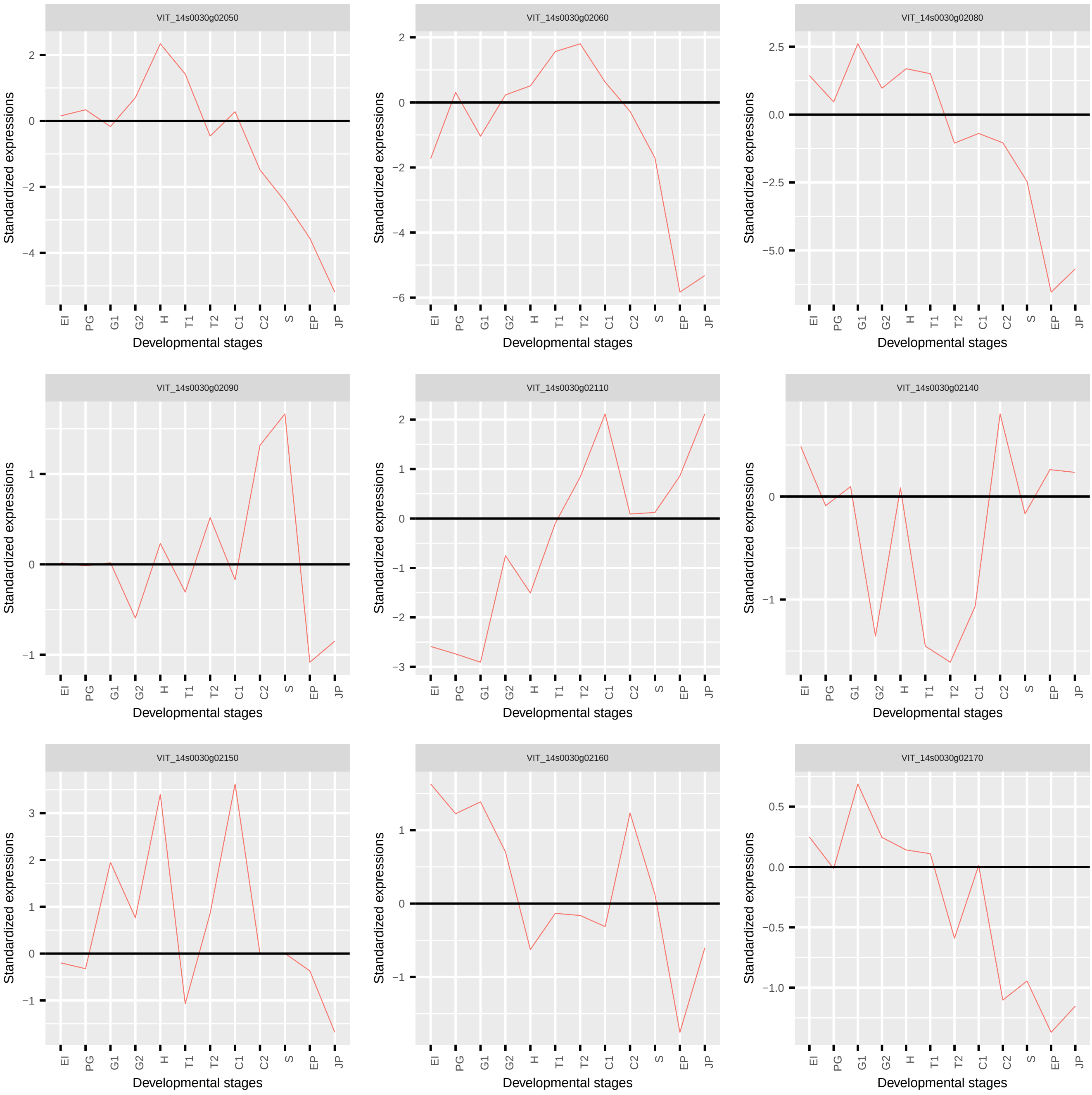

VIT_14s0030g02050
VIT_14s0030g02060
VIT_14s0030g02080
VIT_14s0030g02090
VIT_14s0030g02110
VIT_14s0030g02140
VIT_14s0030g02150
VIT_14s0030g02160
VIT_14s0030g02170
Standardized expressions
Developmental stages
EI PG G1 G2 H T1 T2 C1 C2 S EP JP

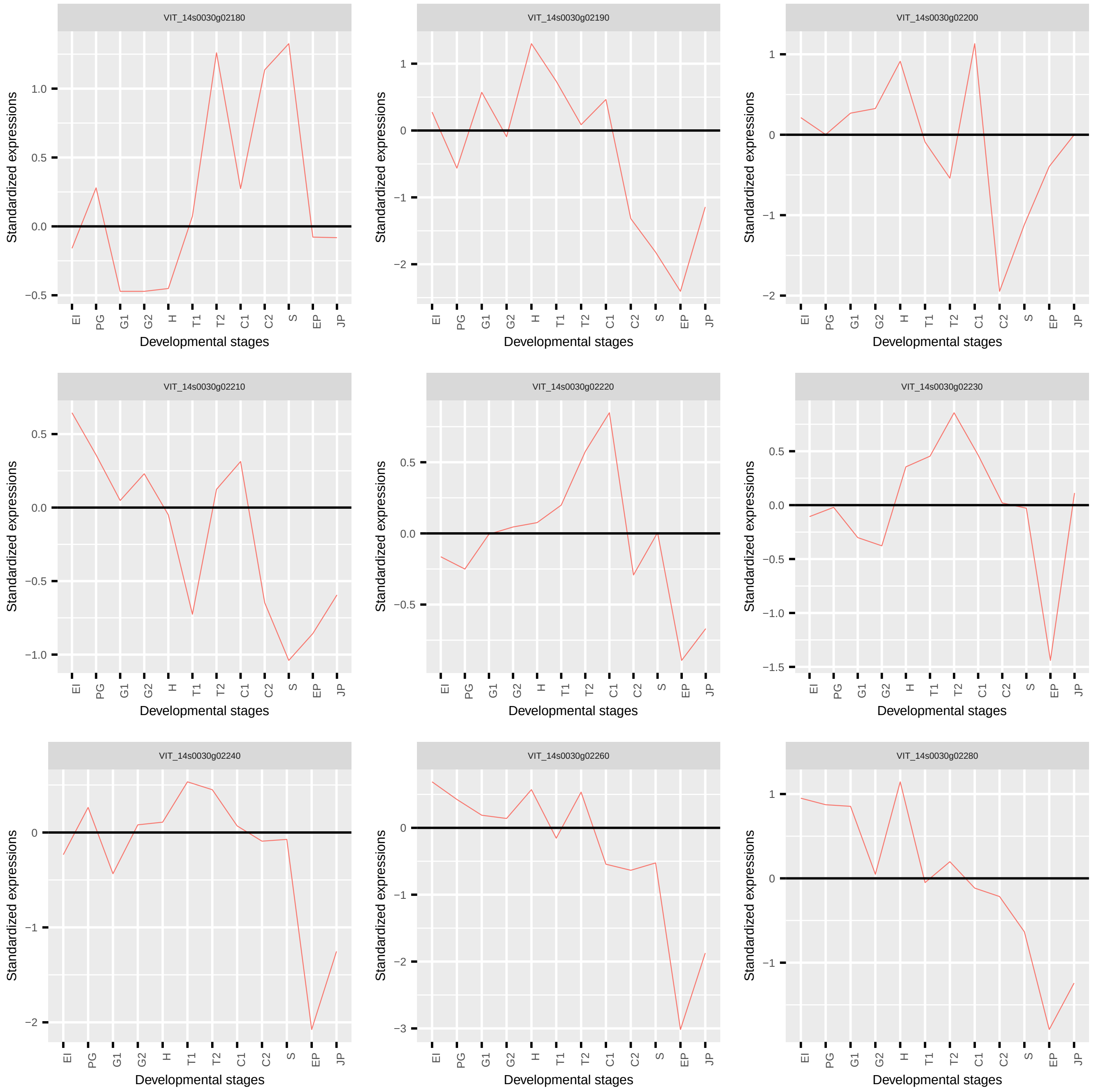
VIT_14s0030g02180
VIT_14s0030g02190
VIT_14s0030g02200
VIT_14s0030g02210
VIT_14s0030g02220
VIT_14s0030g02230
VIT_14s0030g02240
VIT_14s0030g02260
VIT_14s0030g02280
Standardized expressions
Developmental stages
EI PG G1 G2 H T1 T2 C1 C2 S EP JP

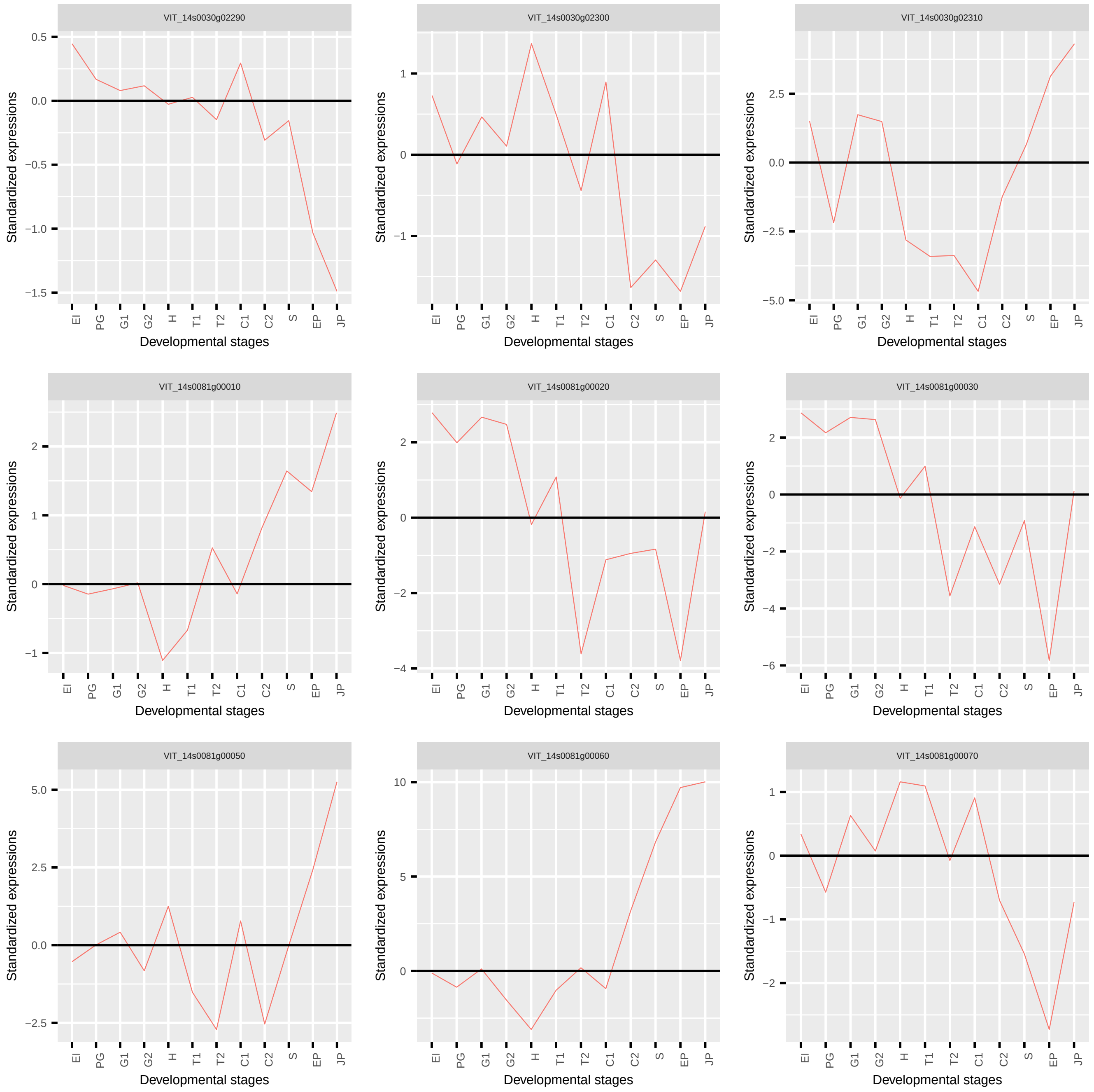
VIT_14s0030g02290
VIT_14s0030g02300
VIT_14s0030g02310
VIT_14s0081g00010
VIT_14s0081g00020
VIT_14s0081g00030
VIT_14s0081g00050
VIT_14s0081g00060
VIT_14s0081g00070
Standardized expressions
Developmental stages
EI
PG
G1
G2
H
T1
T2
C1
C2
S
EP
JP

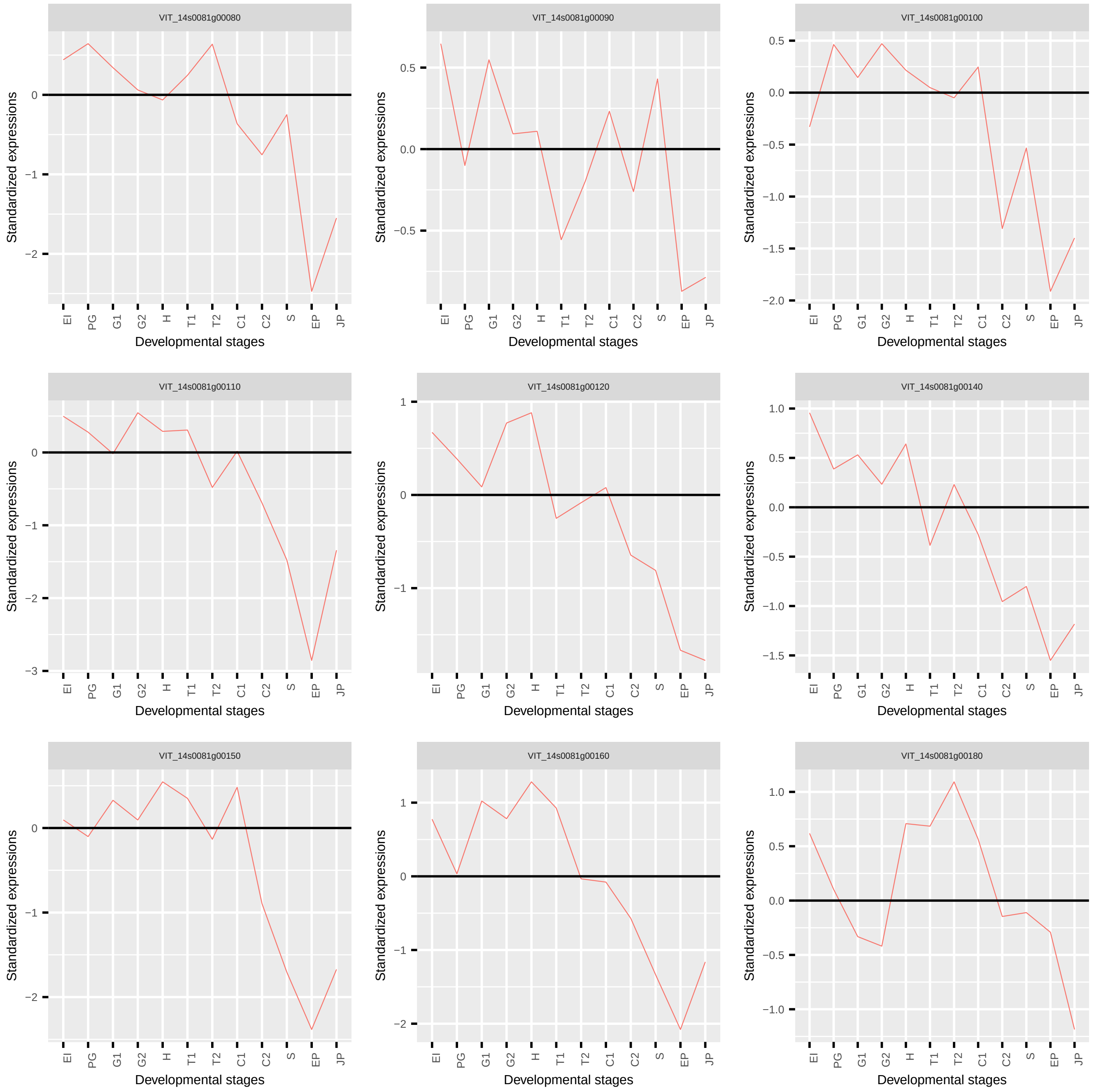
VIT_14s0081g00080
VIT_14s0081g00090
VIT_14s0081g00100
VIT_14s0081g00110
VIT_14s0081g00120
VIT_14s0081g00140
VIT_14s0081g00150
VIT_14s0081g00160
VIT_14s0081g00180
Standardized expressions
Developmental stages
EI PG G1 G2 H T1 T2 C1 C2 S EP JP

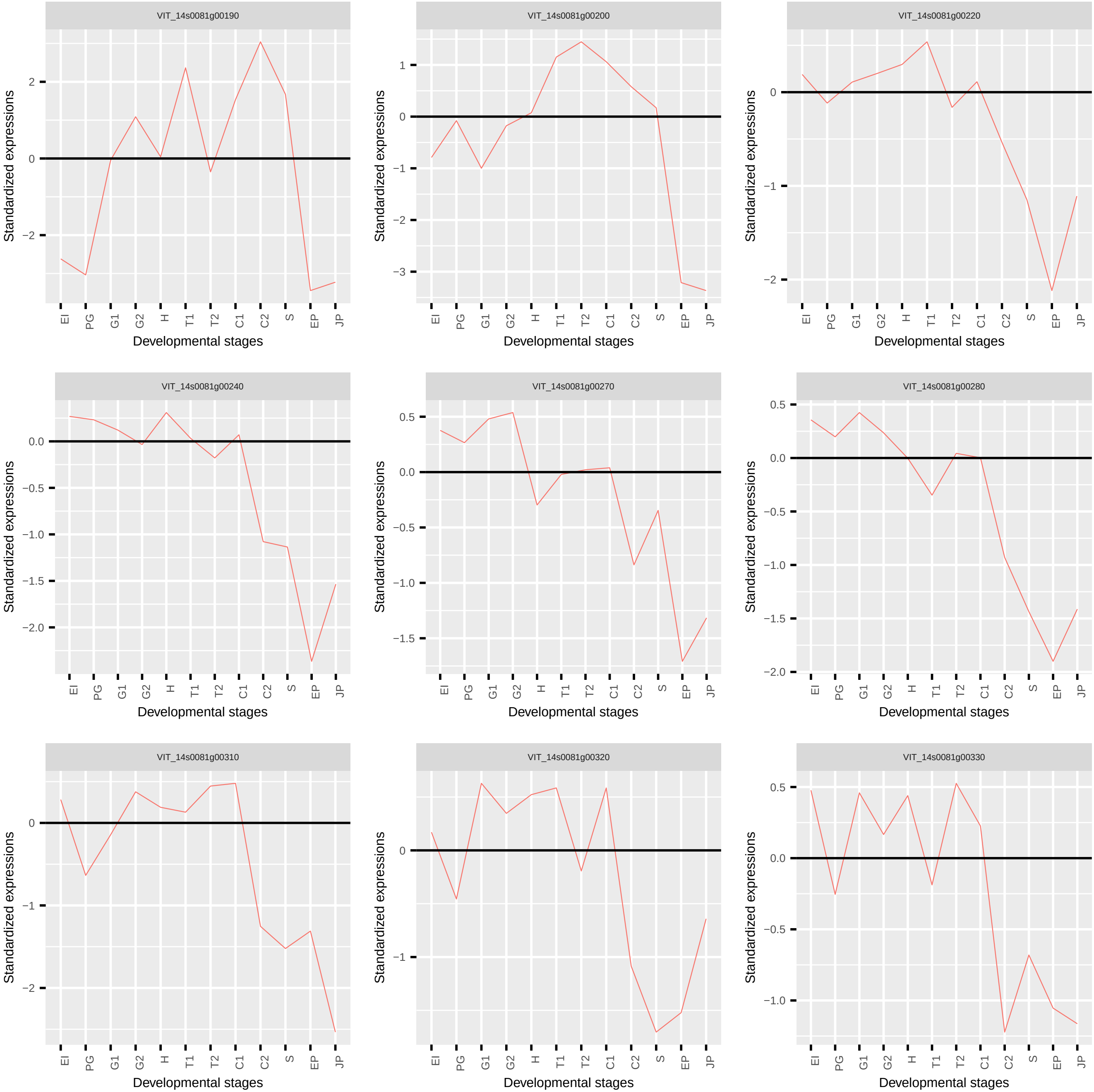
VIT_14s0081g00190
VIT_14s0081g00200
VIT_14s0081g00220
VIT_14s0081g00240
VIT_14s0081g00270
VIT_14s0081g00280
VIT_14s0081g00310
VIT_14s0081g00320
VIT_14s0081g00330
Standardized expressions
Developmental stages
EI PG G1 G2 H T1 T2 C1 C2 S EP JP

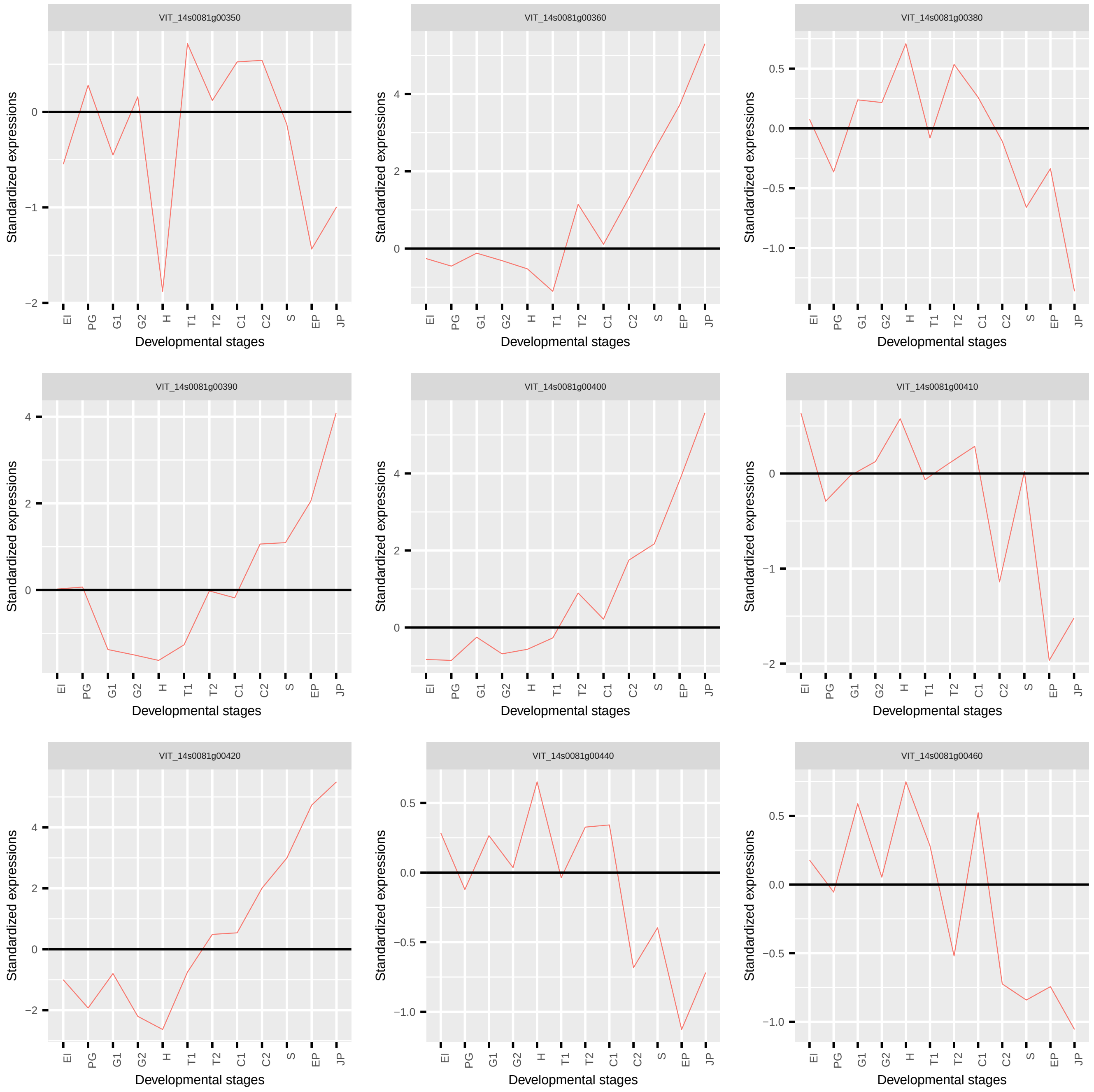
VIT_14s0081g00350
VIT_14s0081g00360
VIT_14s0081g00380
VIT_14s0081g00390
VIT_14s0081g00400
VIT_14s0081g00410
VIT_14s0081g00420
VIT_14s0081g00440
VIT_14s0081g00460
Standardized expressions
Developmental stages
EI PG G1 G2 H T1 T2 C1 C2 S EP JP
0
−1
−2
2
4
0.5
0.0
−0.5
−1.0

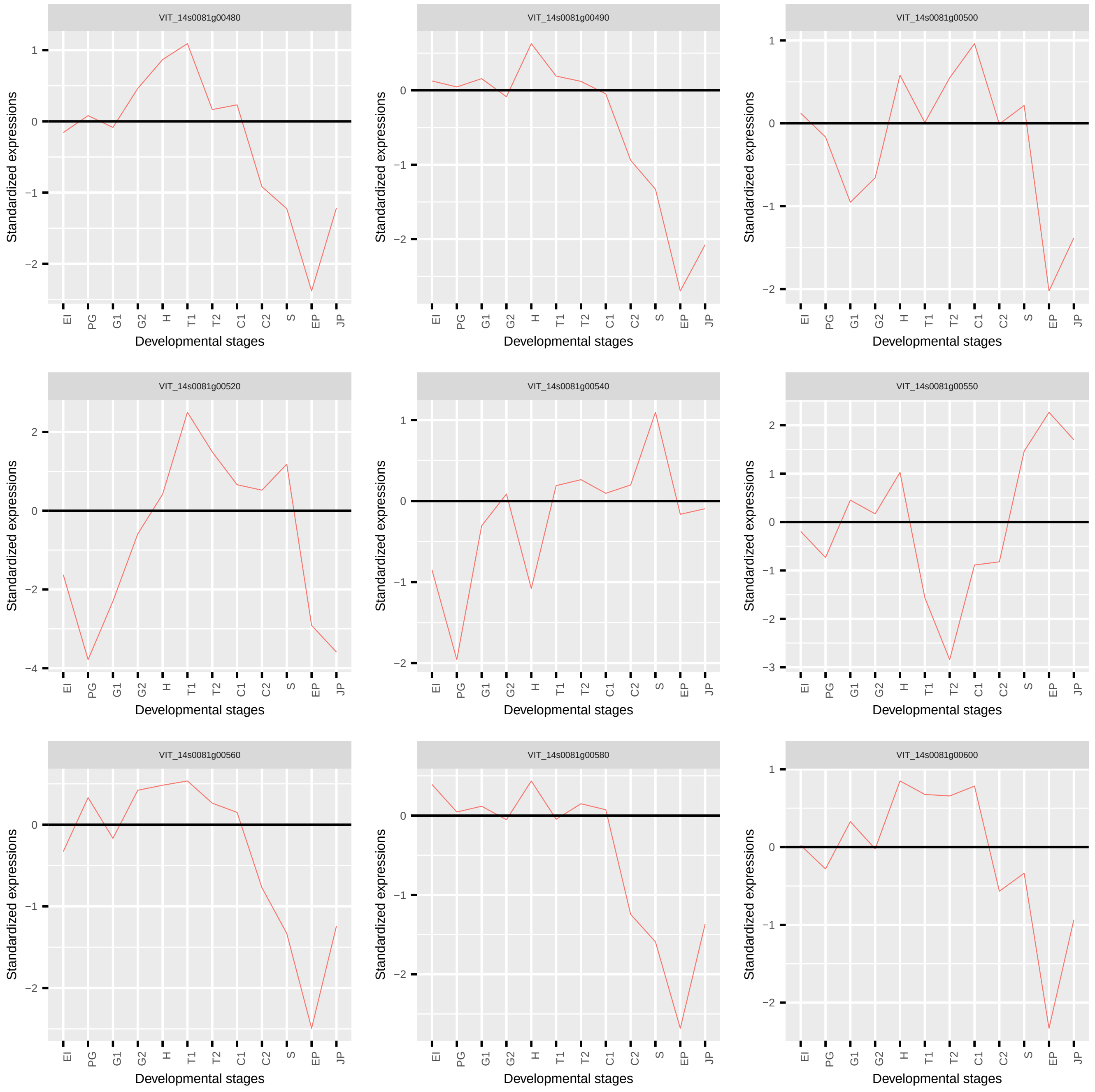
VIT_14s0081g00480
VIT_14s0081g00490
VIT_14s0081g00500
VIT_14s0081g00520
VIT_14s0081g00540
VIT_14s0081g00550
VIT_14s0081g00560
VIT_14s0081g00580
VIT_14s0081g00600
Standardized expressions
Developmental stages
EI
PG
G1
G2
H
T1
T2
C1
C2
S
EP
JP

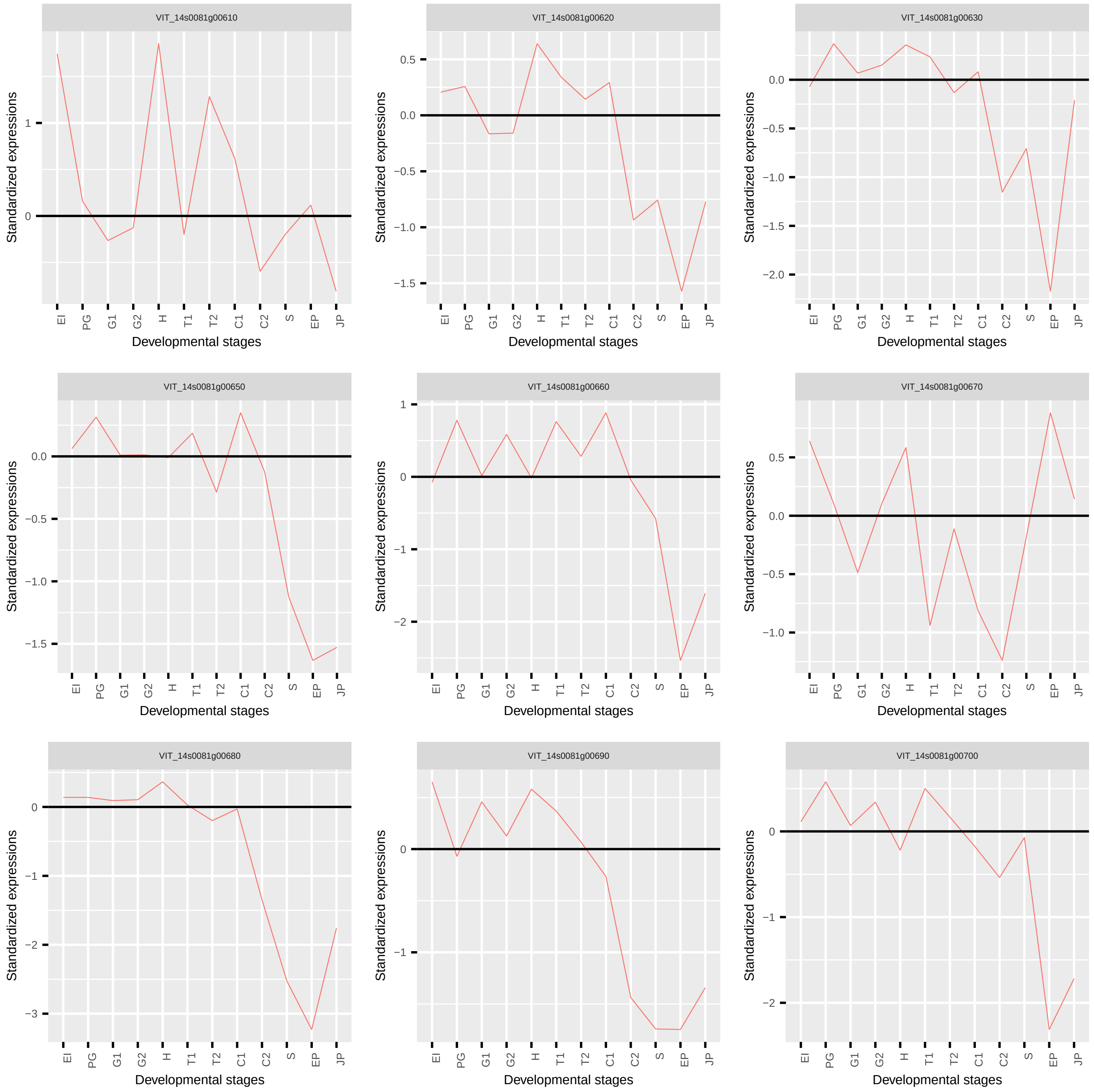

VIT_14s0081g00610
VIT_14s0081g00620
VIT_14s0081g00630
VIT_14s0081g00650
VIT_14s0081g00660
VIT_14s0081g00670
VIT_14s0081g00680
VIT_14s0081g00690
VIT_14s0081g00700
Standardized expressions
Developmental stages
EI
PG
G1
G2
H
T1
T2
C1
C2
S
EP
JP

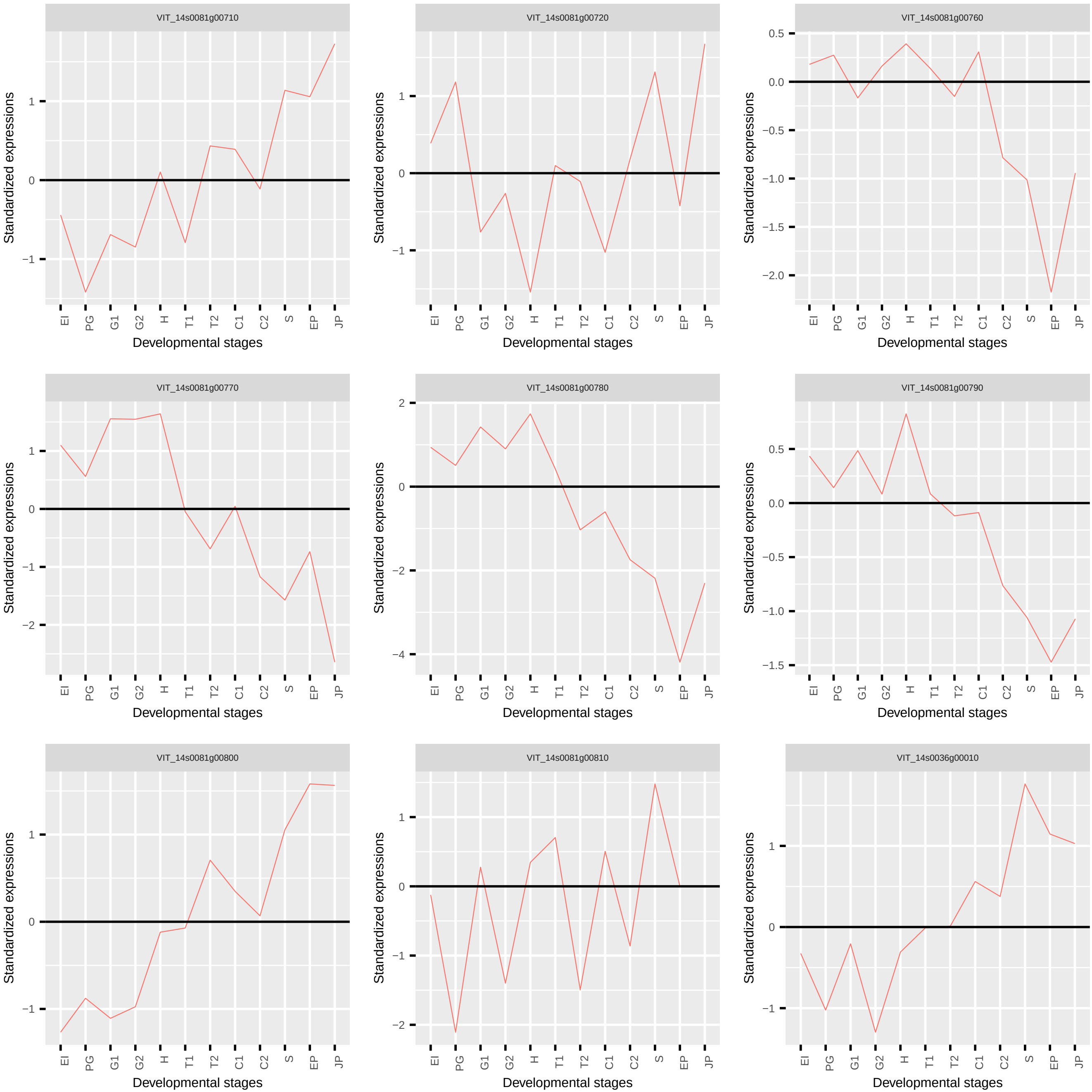
VIT_14s0081g00710
VIT_14s0081g00720
VIT_14s0081g00760
VIT_14s0081g00770
VIT_14s0081g00780
VIT_14s0081g00790
VIT_14s0081g00800
VIT_14s0081g00810
VIT_14s0036g00010
Standardized expressions
Developmental stages
EI PG G1 G2 H T1 T2 C1 C2 S EP JP

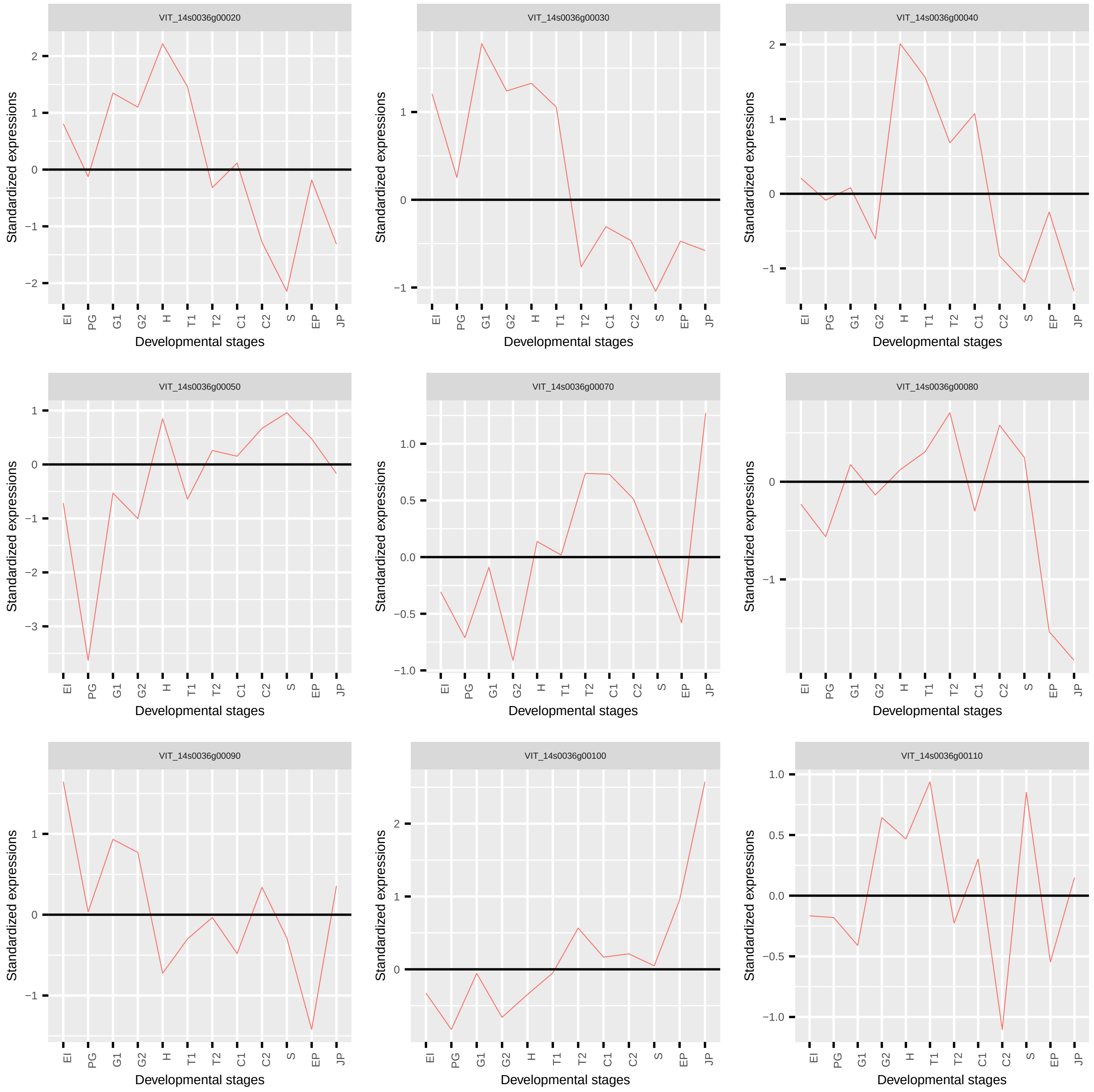
VIT_14s0036g00020
VIT_14s0036g00030
VIT_14s0036g00040
VIT_14s0036g00050
VIT_14s0036g00070
VIT_14s0036g00080
VIT_14s0036g00090
VIT_14s0036g00100
VIT_14s0036g00110
Standardized expressions
Developmental stages
EI PG G1 G2 H T1 T2 C1 C2 S EP JP

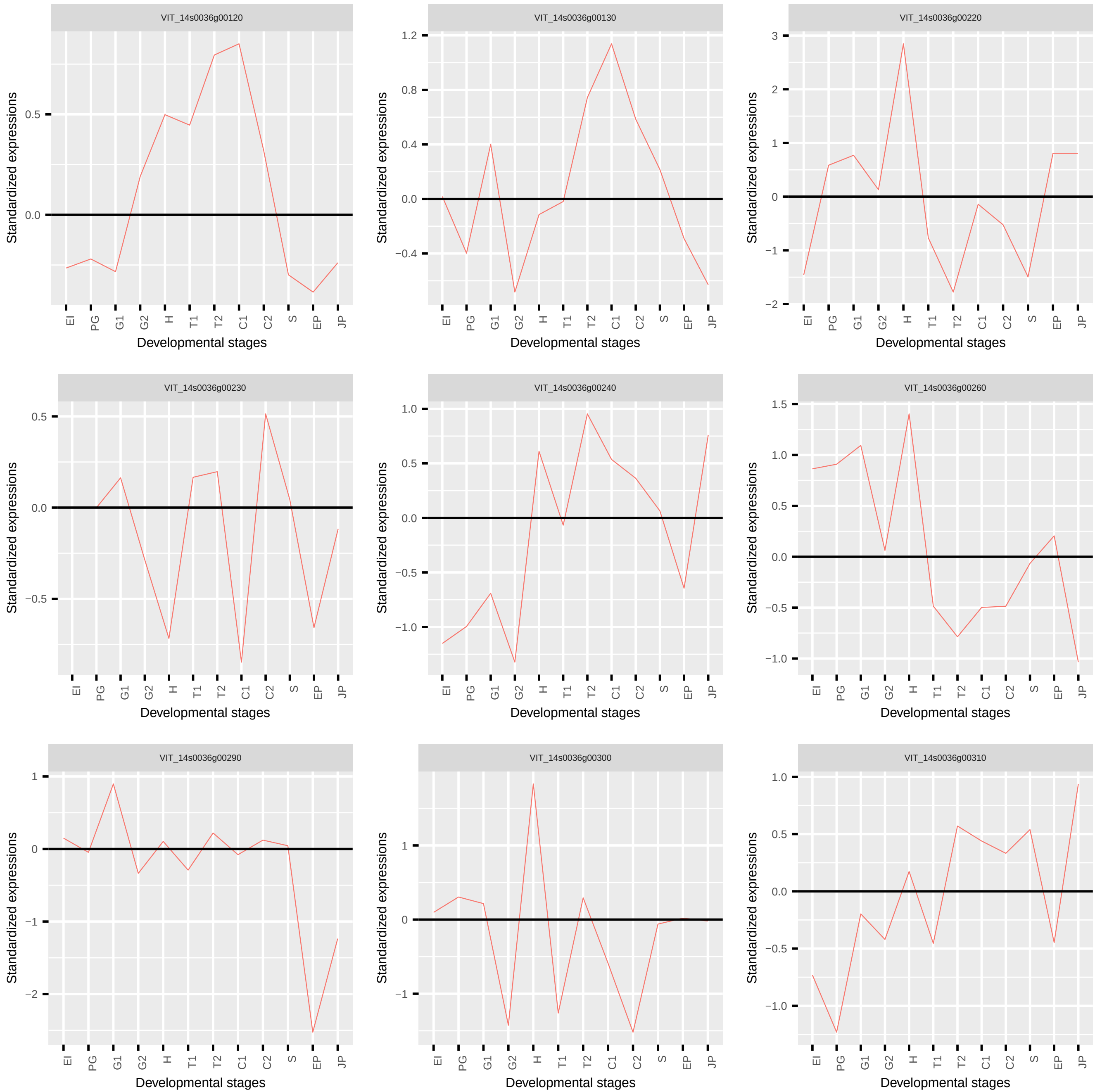

VIT_14s0036g00120
VIT_14s0036g00130
VIT_14s0036g00220
VIT_14s0036g00230
VIT_14s0036g00240
VIT_14s0036g00260
VIT_14s0036g00290
VIT_14s0036g00300
VIT_14s0036g00310
Standardized expressions
Developmental stages
EI
PG
G1
G2
H
T1
T2
C1
C2
S
EP
JP

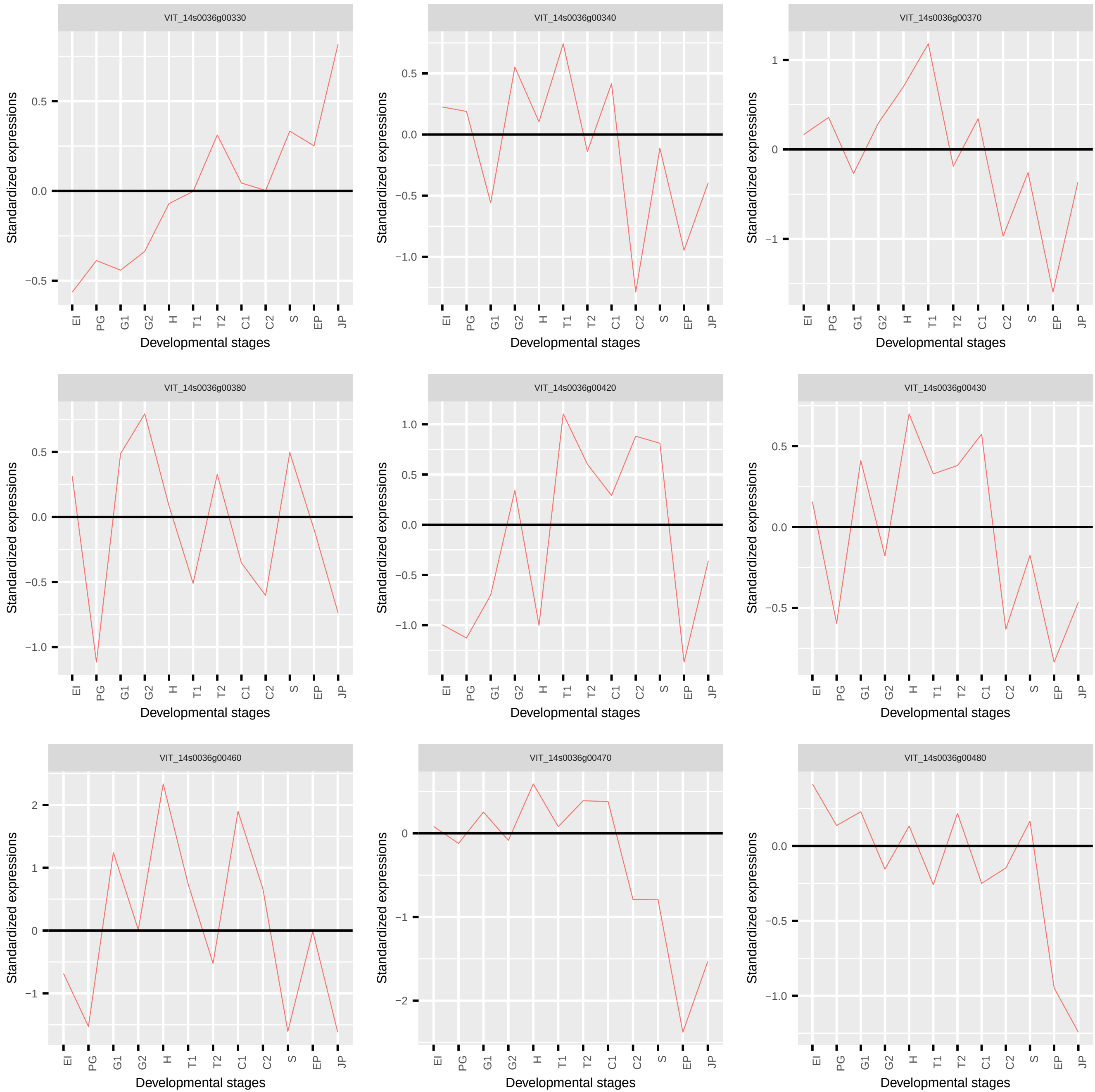

VIT_14s0036g00330
VIT_14s0036g00340
VIT_14s0036g00370
VIT_14s0036g00380
VIT_14s0036g00420
VIT_14s0036g00430
VIT_14s0036g00460
VIT_14s0036g00470
VIT_14s0036g00480
Standardized expressions
Developmental stages
EI
PG
G1
G2
H
T1
T2
C1
C2
S
EP
JP
0.5
0.0
−0.5
−1.0
1
0
−1
2
−2

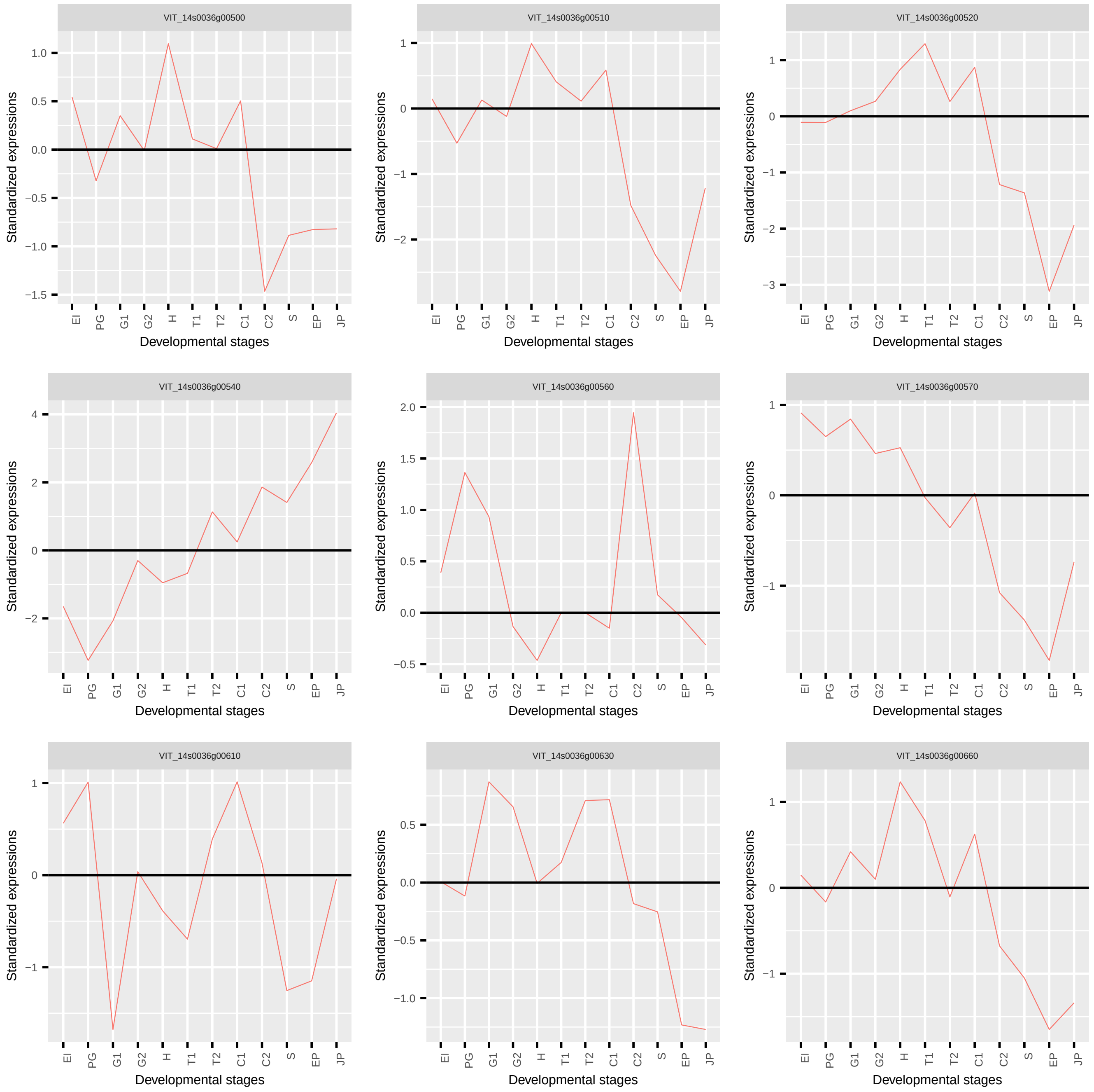
VIT_14s0036g00500
VIT_14s0036g00510
VIT_14s0036g00520
VIT_14s0036g00540
VIT_14s0036g00560
VIT_14s0036g00570
VIT_14s0036g00610
VIT_14s0036g00630
VIT_14s0036g00660
Standardized expressions
Developmental stages
EI PG G1 G2 H T1 T2 C1 C2 S EP JP

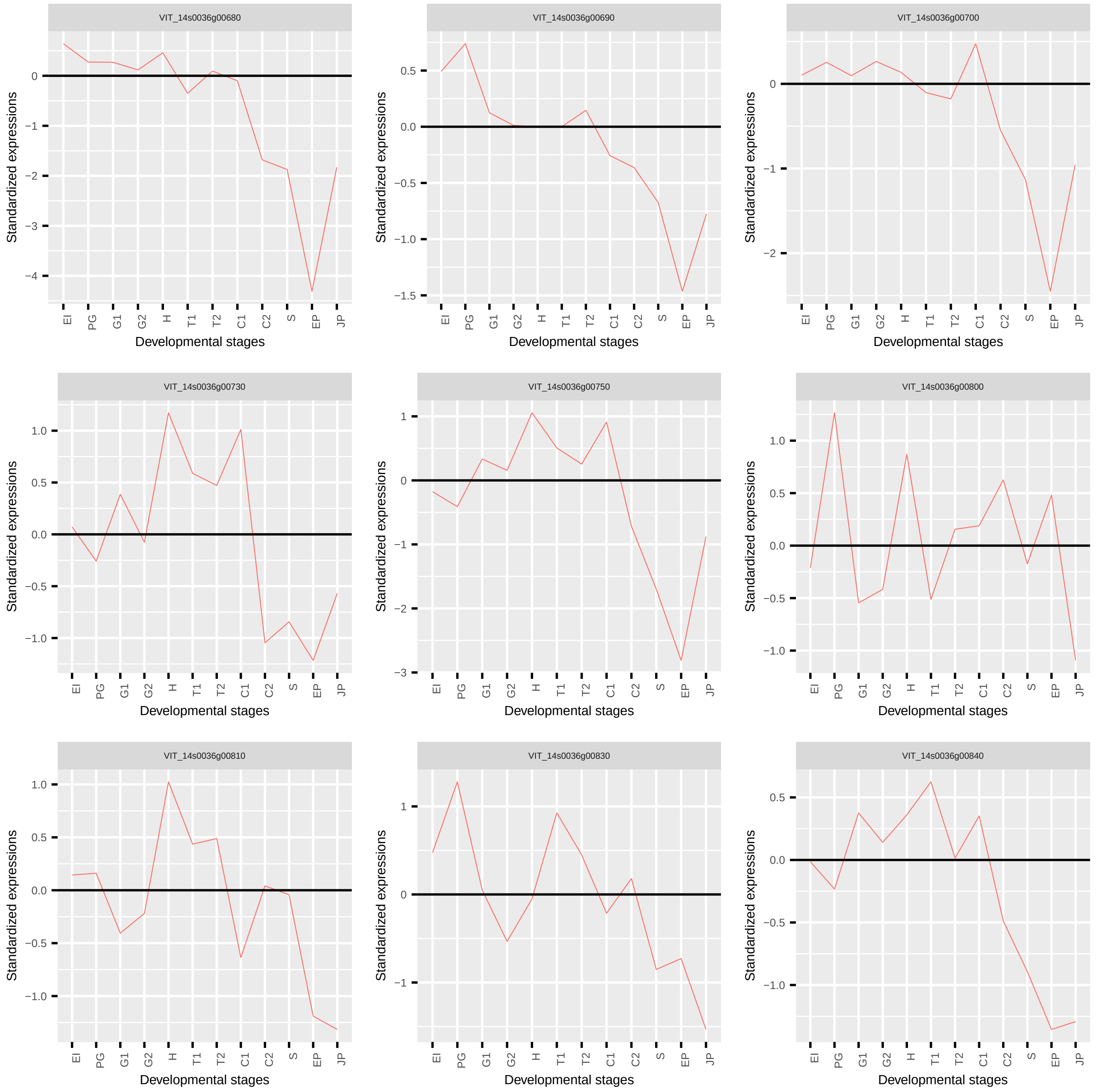
VIT_14s0036g00680
VIT_14s0036g00690
VIT_14s0036g00700
VIT_14s0036g00730
VIT_14s0036g00750
VIT_14s0036g00800
VIT_14s0036g00810
VIT_14s0036g00830
VIT_14s0036g00840
Standardized expressions
Developmental stages
EI
PG
G1
G2
H
T1
T2
C1
C2
S
EP
JP

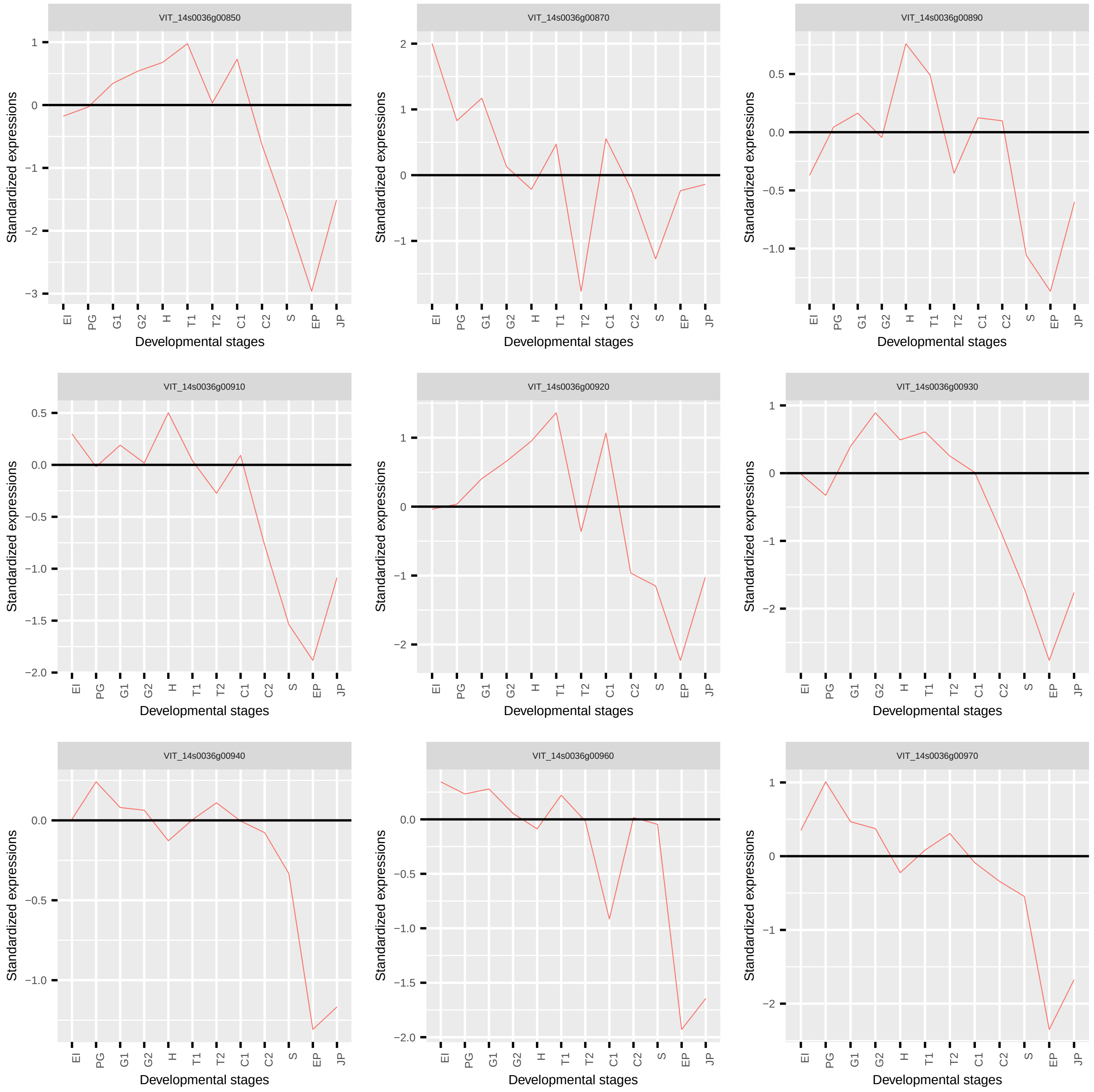
VIT_14s0036g00850
VIT_14s0036g00870
VIT_14s0036g00890
VIT_14s0036g00910
VIT_14s0036g00920
VIT_14s0036g00930
VIT_14s0036g00940
VIT_14s0036g00960
VIT_14s0036g00970
Standardized expressions
Developmental stages
EI PG G1 G2 H T1 T2 C1 C2 S EP JP

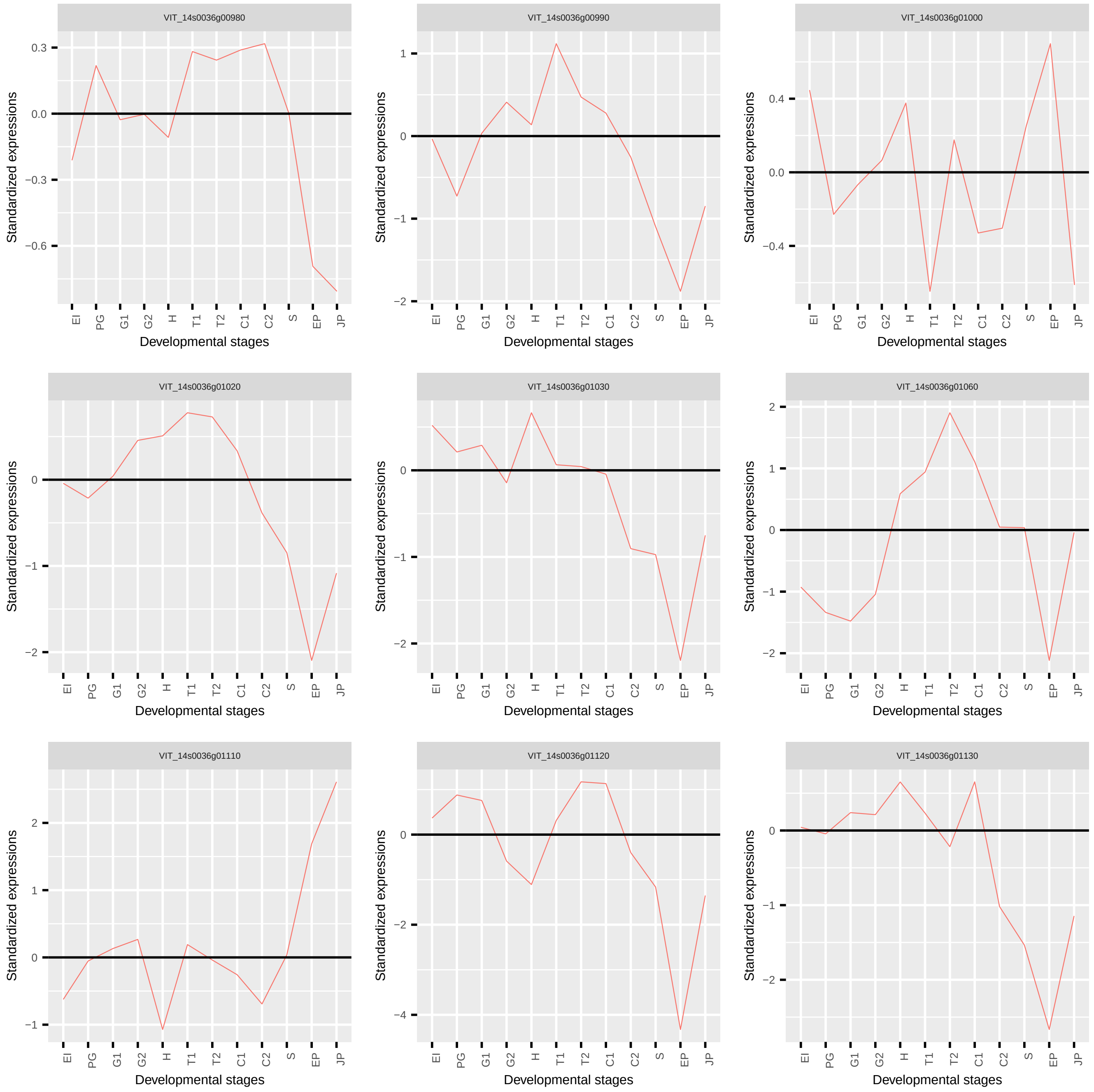
VIT_14s0036g00980
VIT_14s0036g00990
VIT_14s0036g01000
VIT_14s0036g01020
VIT_14s0036g01030
VIT_14s0036g01060
VIT_14s0036g01110
VIT_14s0036g01120
VIT_14s0036g01130
Standardized expressions
Developmental stages
EI
PG
G1
G2
H
T1
T2
C1
C2
S
EP
JP

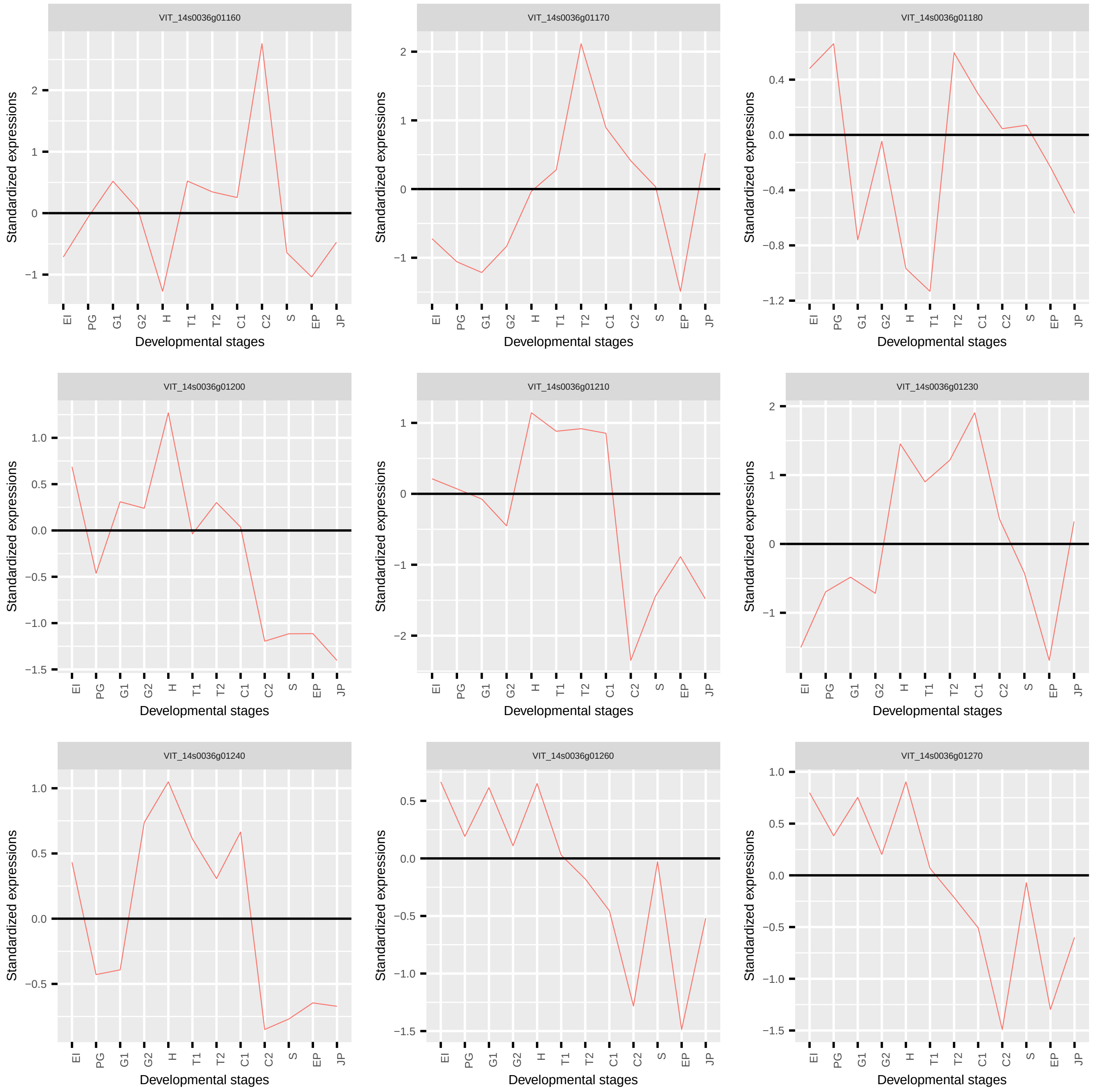

VIT_14s0036g01160
VIT_14s0036g01170
VIT_14s0036g01180
VIT_14s0036g01200
VIT_14s0036g01210
VIT_14s0036g01230
VIT_14s0036g01240
VIT_14s0036g01260
VIT_14s0036g01270
Standardized expressions
Developmental stages
EI
PG
G1
G2
H
T1
T2
C1
C2
S
EP
JP

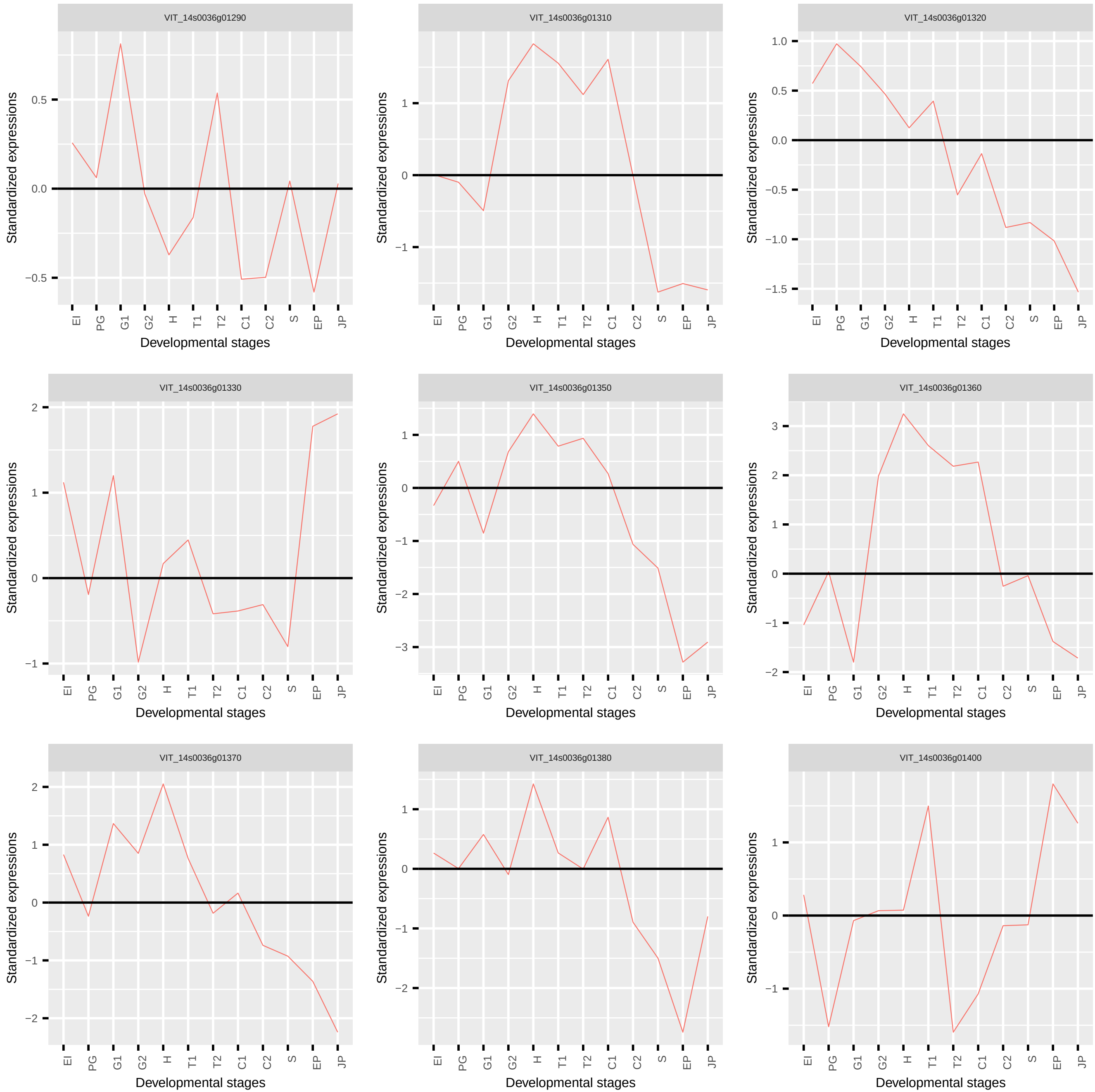
VIT_14s0036g01290
VIT_14s0036g01310
VIT_14s0036g01320
VIT_14s0036g01330
VIT_14s0036g01350
VIT_14s0036g01360
VIT_14s0036g01370
VIT_14s0036g01380
VIT_14s0036g01400
Standardized expressions
Developmental stages
EI
PG
G1
G2
H
T1
T2
C1
C2
S
EP
JP

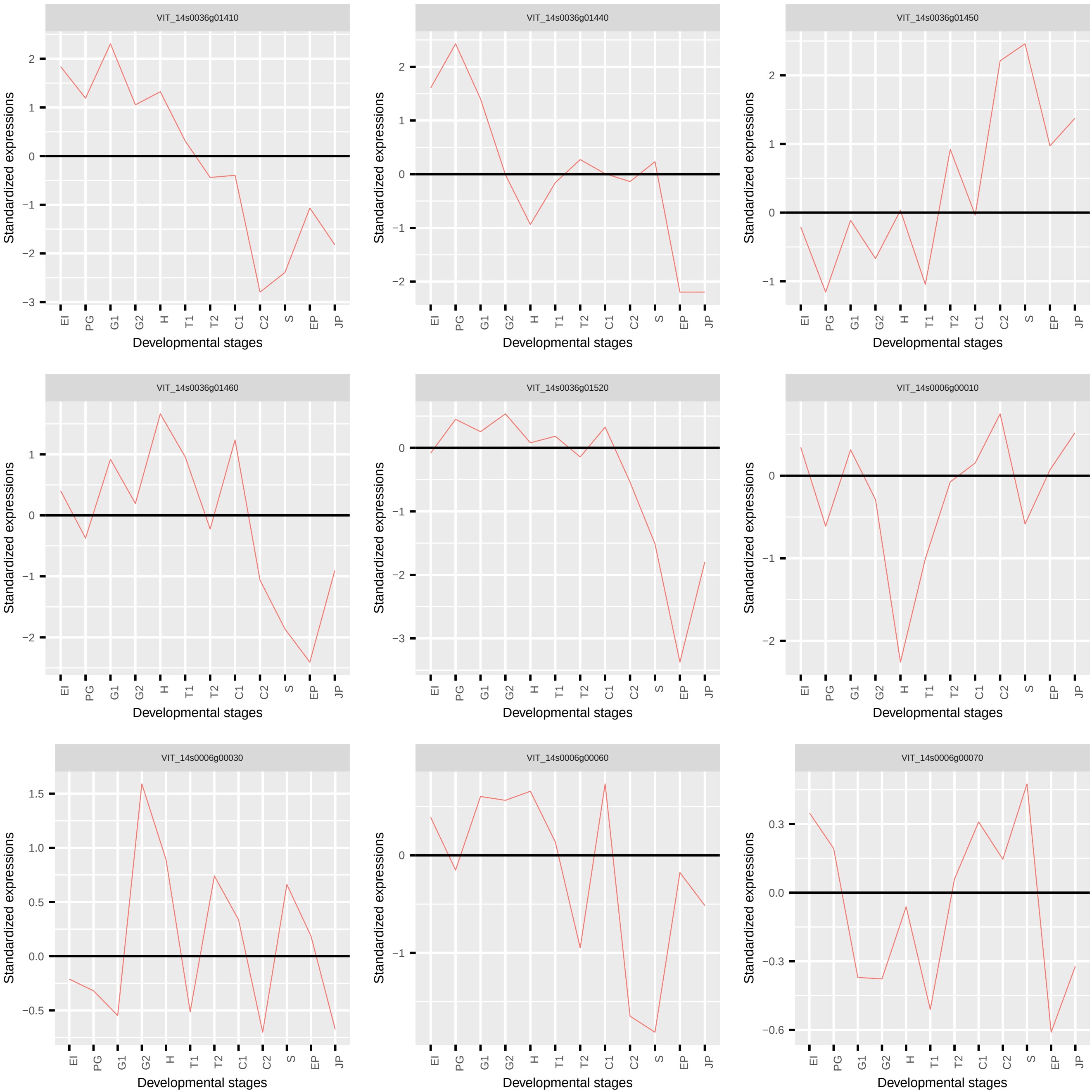

VIT_14s0036g01410
VIT_14s0036g01440
VIT_14s0036g01450
VIT_14s0036g01460
VIT_14s0036g01520
VIT_14s0006g00010
VIT_14s0006g00030
VIT_14s0006g00060
VIT_14s0006g00070
Standardized expressions
Developmental stages
EI
PG
G1
G2
H
T1
T2
C1
C2
S
EP
JP
2
1
0
−1
−2
−3
1.5
1.0
0.5
0.0
−0.5
0.3
−0.3
−0.6

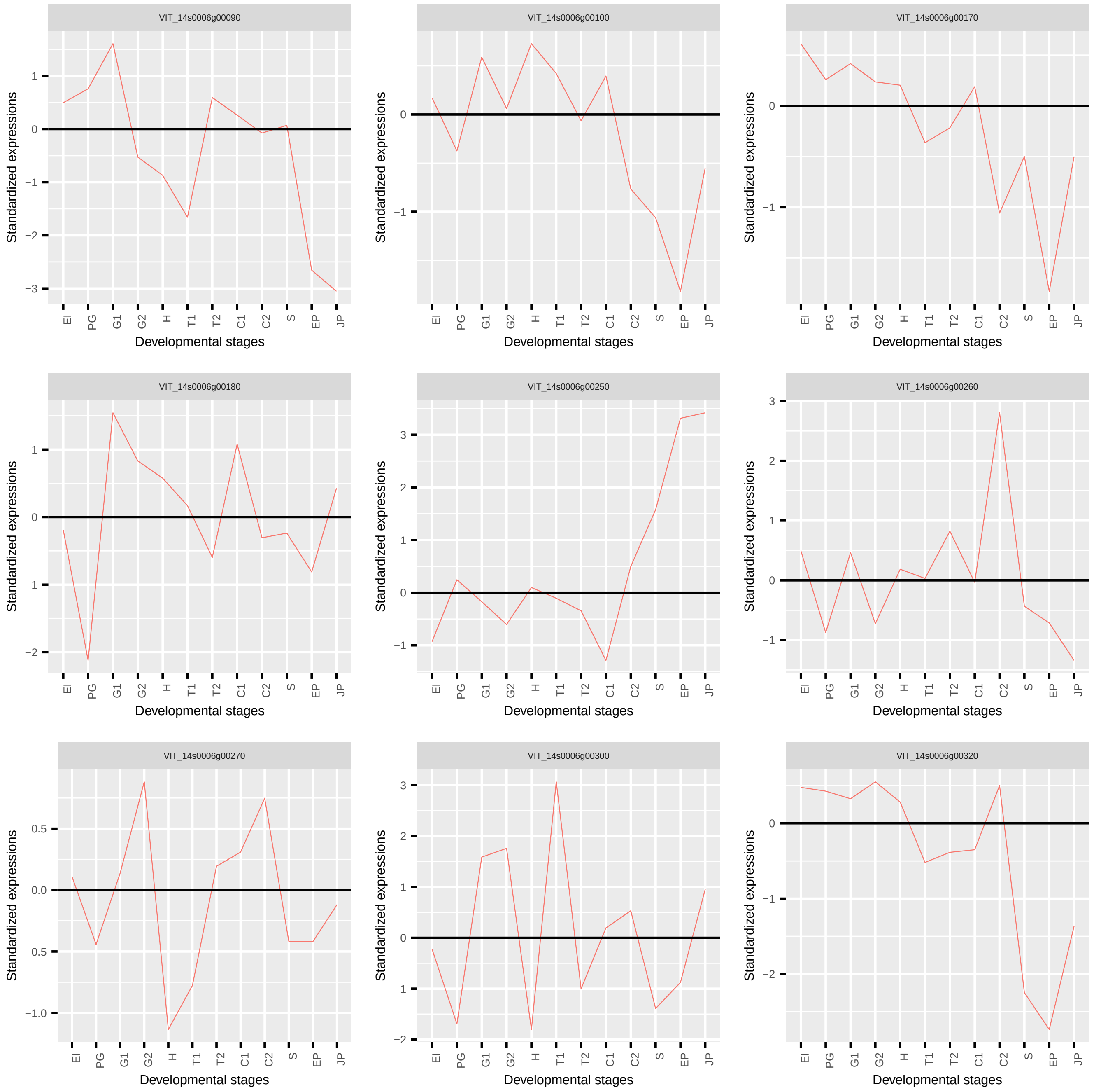

VIT_14s0006g00090
VIT_14s0006g00100
VIT_14s0006g00170
VIT_14s0006g00180
VIT_14s0006g00250
VIT_14s0006g00260
VIT_14s0006g00270
VIT_14s0006g00300
VIT_14s0006g00320
Standardized expressions
Developmental stages
EI
PG
G1
G2
H
T1
T2
C1
C2
S
EP
JP

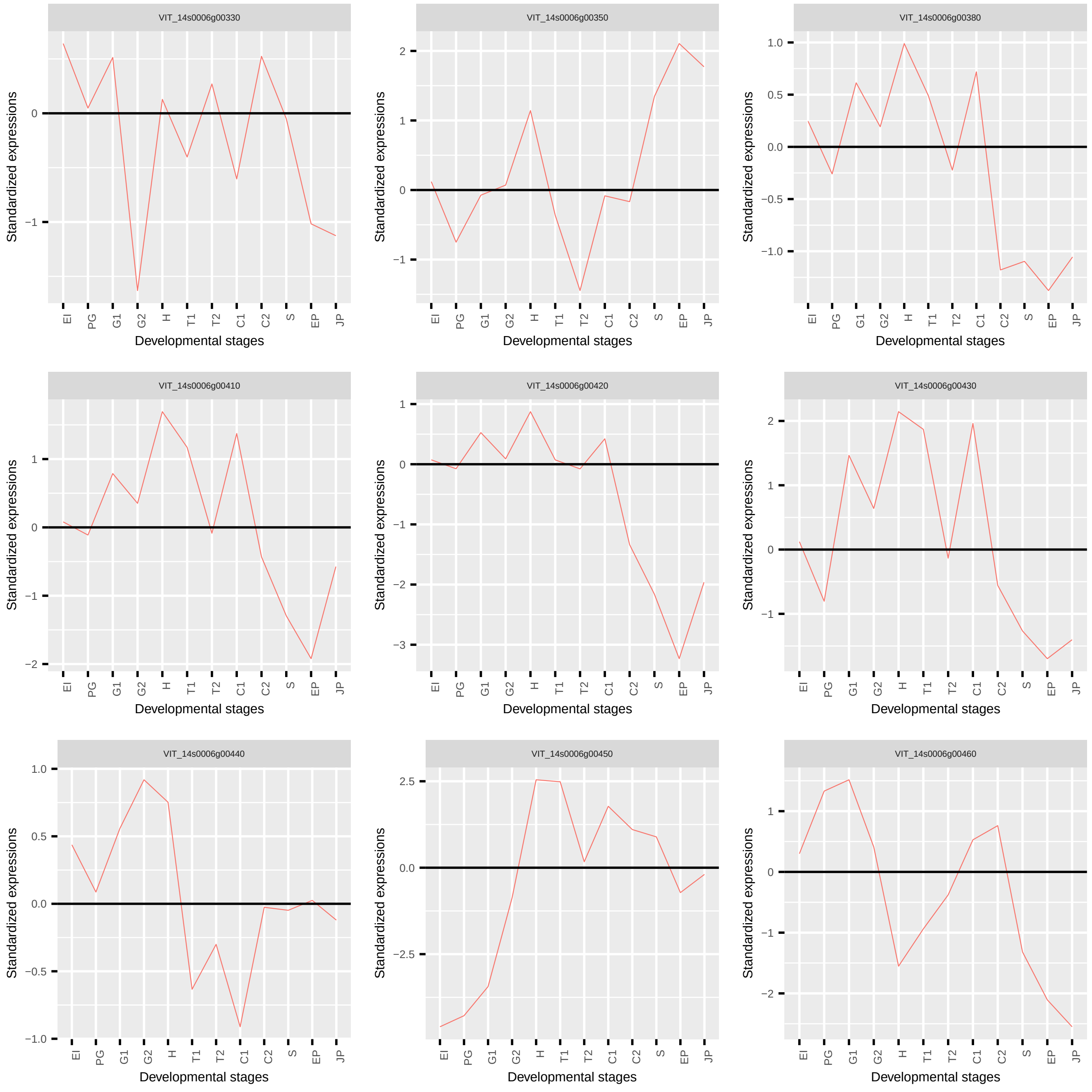
VIT_14s0006g00330
VIT_14s0006g00350
VIT_14s0006g00380
VIT_14s0006g00410
VIT_14s0006g00420
VIT_14s0006g00430
VIT_14s0006g00440
VIT_14s0006g00450
VIT_14s0006g00460
Standardized expressions
Developmental stages
EI PG G1 G2 H T1 T2 C1 C2 S EP JP

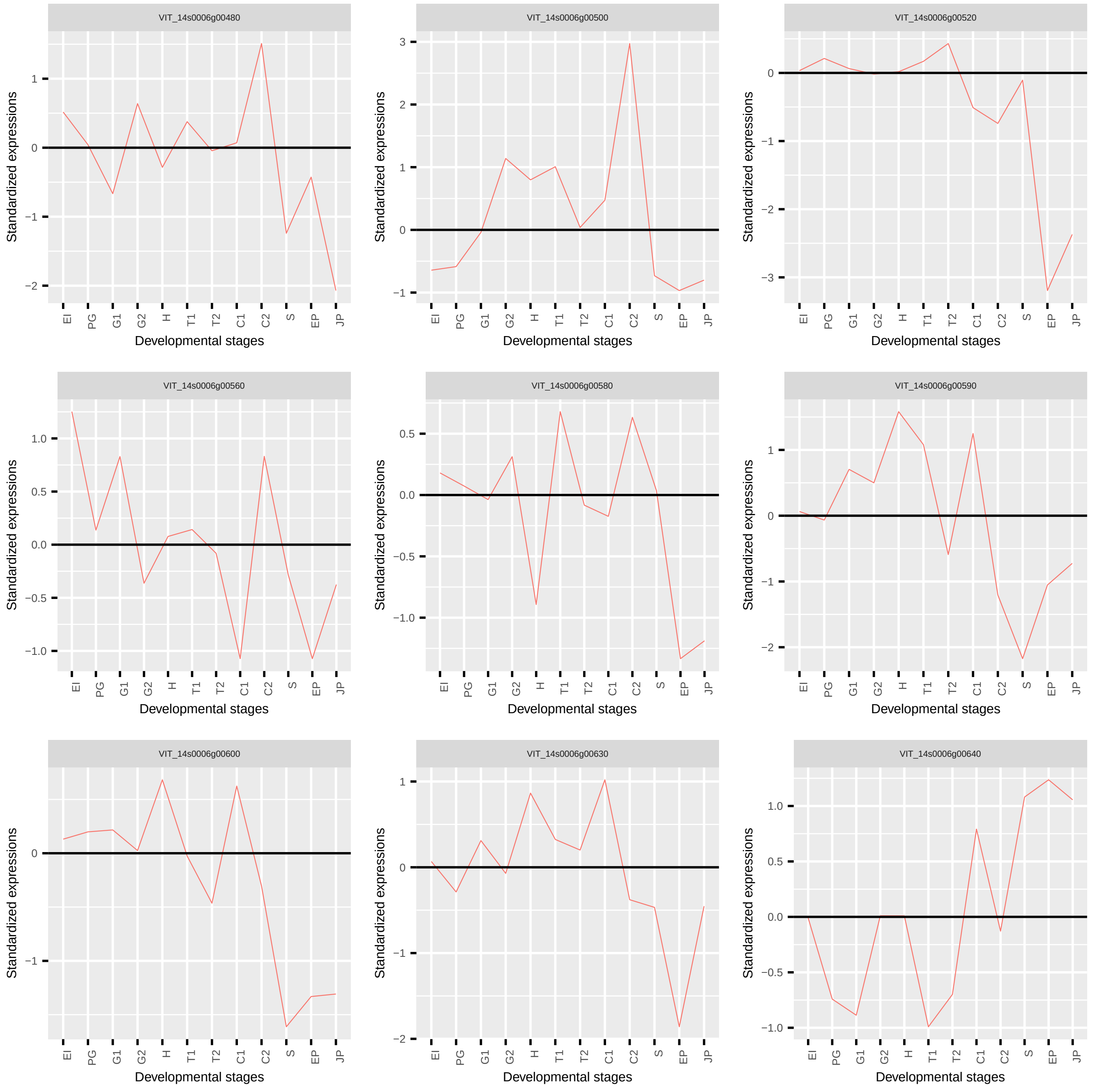
VIT_14s0006g00480
VIT_14s0006g00500
VIT_14s0006g00520
VIT_14s0006g00560
VIT_14s0006g00580
VIT_14s0006g00590
VIT_14s0006g00600
VIT_14s0006g00630
VIT_14s0006g00640
Standardized expressions
Developmental stages
EI PG G1 G2 H T1 T2 C1 C2 S EP JP

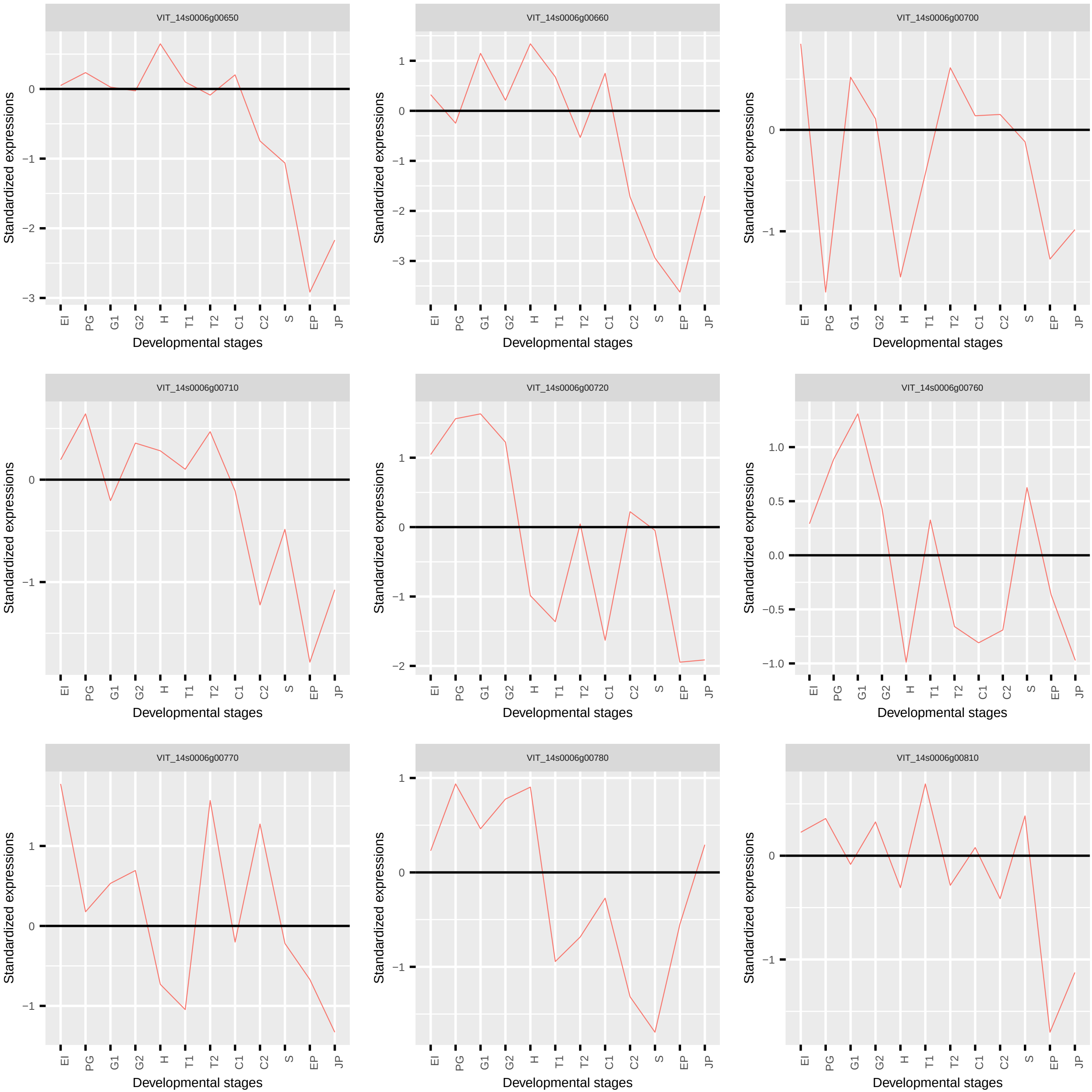
VIT_14s0006g00650
VIT_14s0006g00660
VIT_14s0006g00700
VIT_14s0006g00710
VIT_14s0006g00720
VIT_14s0006g00760
VIT_14s0006g00770
VIT_14s0006g00780
VIT_14s0006g00810
Standardized expressions
Developmental stages
EI PG G1 G2 H T1 T2 C1 C2 S EP JP
0
−1
−2
−3
1
1.0
0.5
0.0
−0.5
−1.0

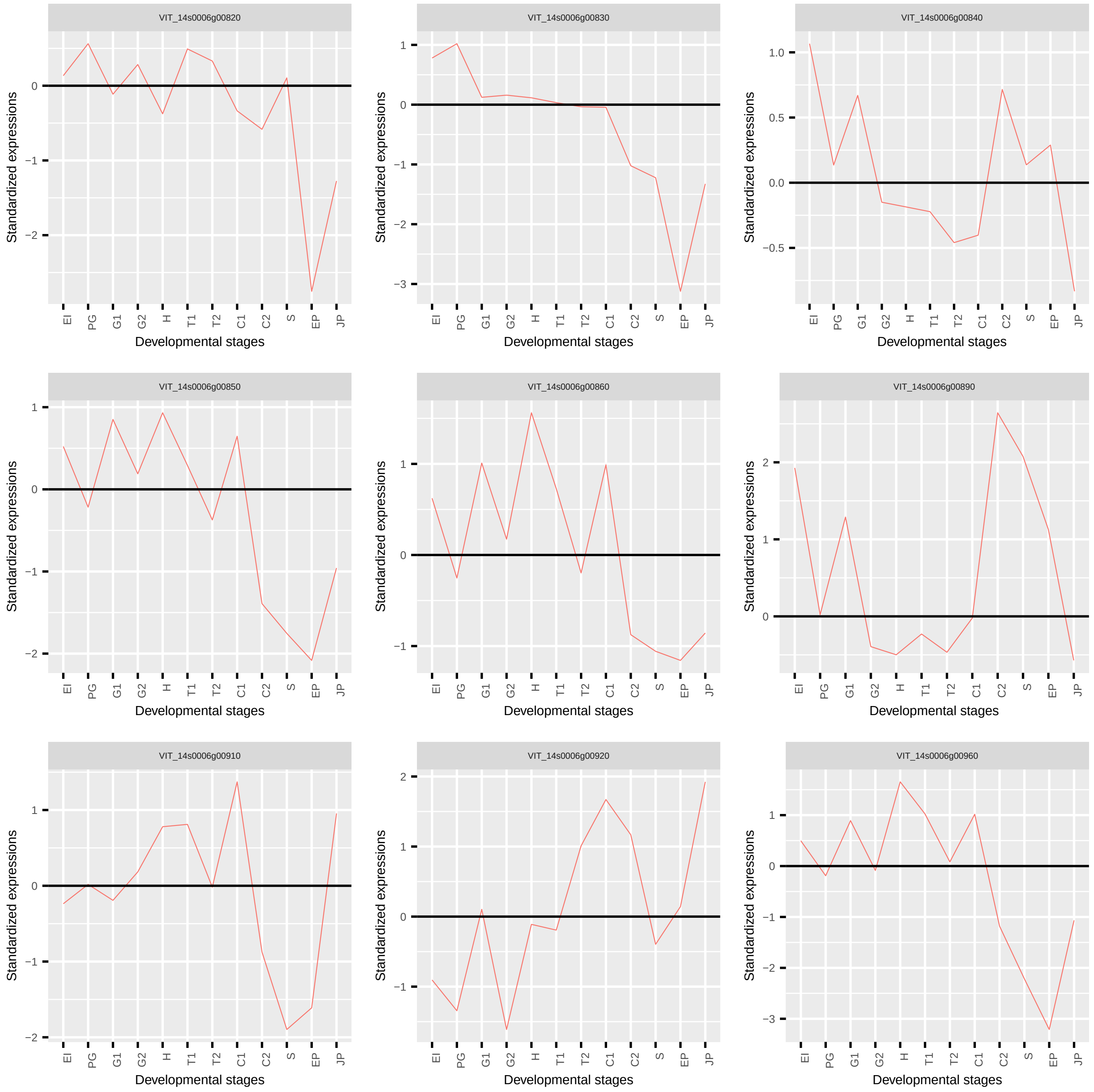
VIT_14s0006g00820
VIT_14s0006g00830
VIT_14s0006g00840
VIT_14s0006g00850
VIT_14s0006g00860
VIT_14s0006g00890
VIT_14s0006g00910
VIT_14s0006g00920
VIT_14s0006g00960
Standardized expressions
Developmental stages
EI PG G1 G2 H T1 T2 C1 C2 S EP JP

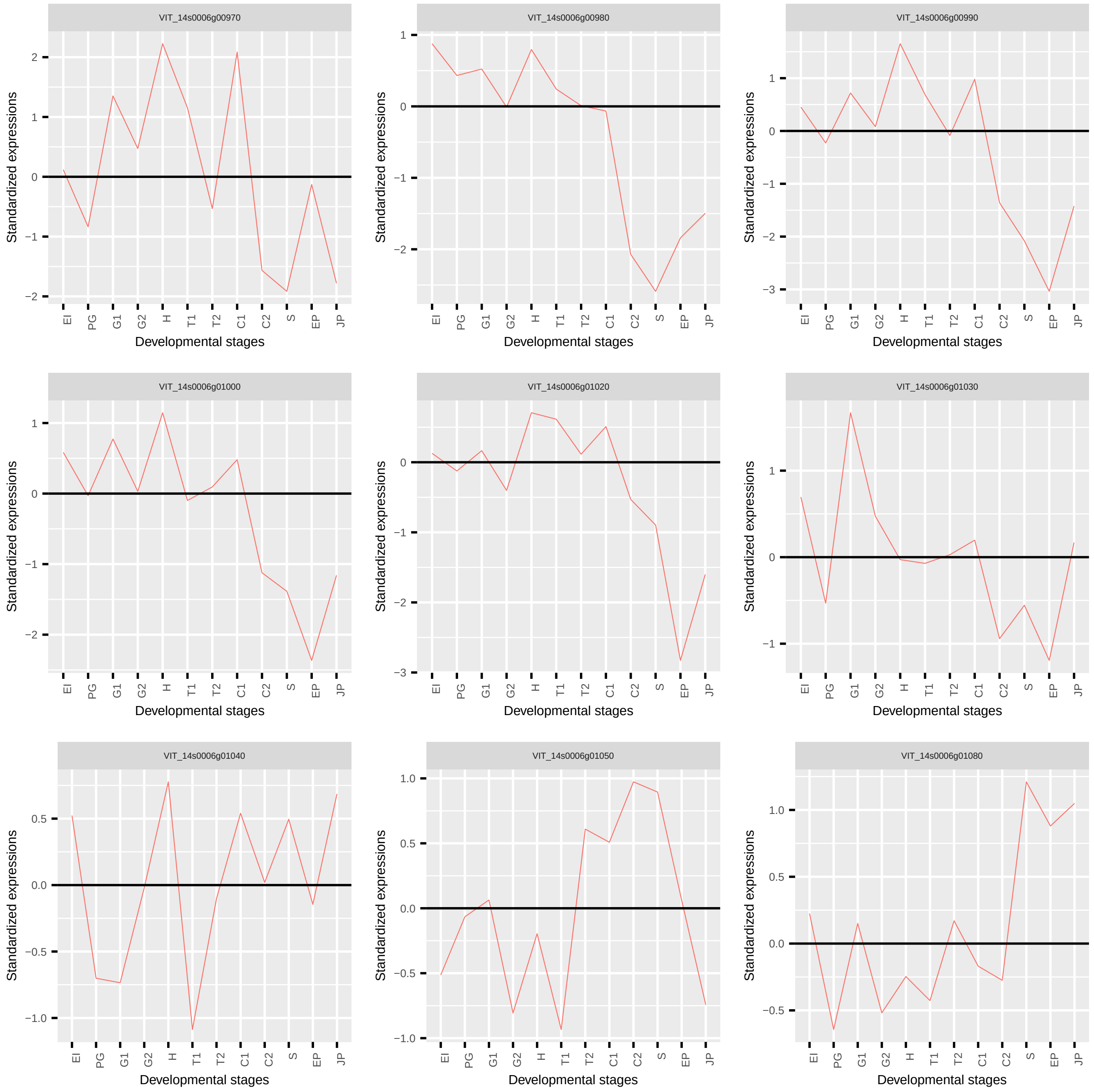

VIT_14s0006g00970
VIT_14s0006g00980
VIT_14s0006g00990
VIT_14s0006g01000
VIT_14s0006g01020
VIT_14s0006g01030
VIT_14s0006g01040
VIT_14s0006g01050
VIT_14s0006g01080
Standardized expressions
Developmental stages
EI
PG
G1
G2
H
T1
T2
C1
C2
S
EP
JP

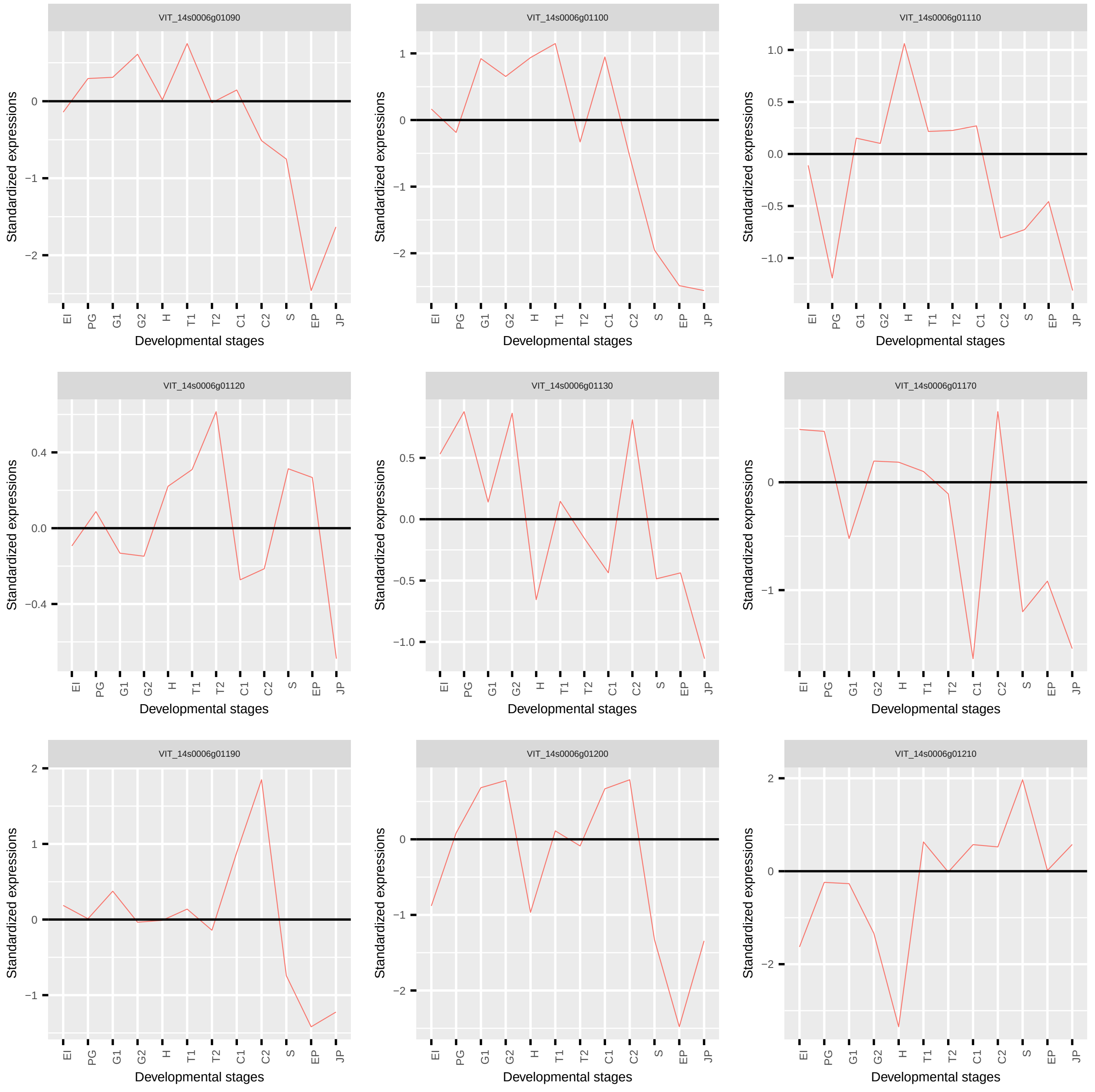

VIT_14s0006g01090
VIT_14s0006g01100
VIT_14s0006g01110
VIT_14s0006g01120
VIT_14s0006g01130
VIT_14s0006g01170
VIT_14s0006g01190
VIT_14s0006g01200
VIT_14s0006g01210
Standardized expressions
Developmental stages
EI
PG
G1
G2
H
T1
T2
C1
C2
S
EP
JP

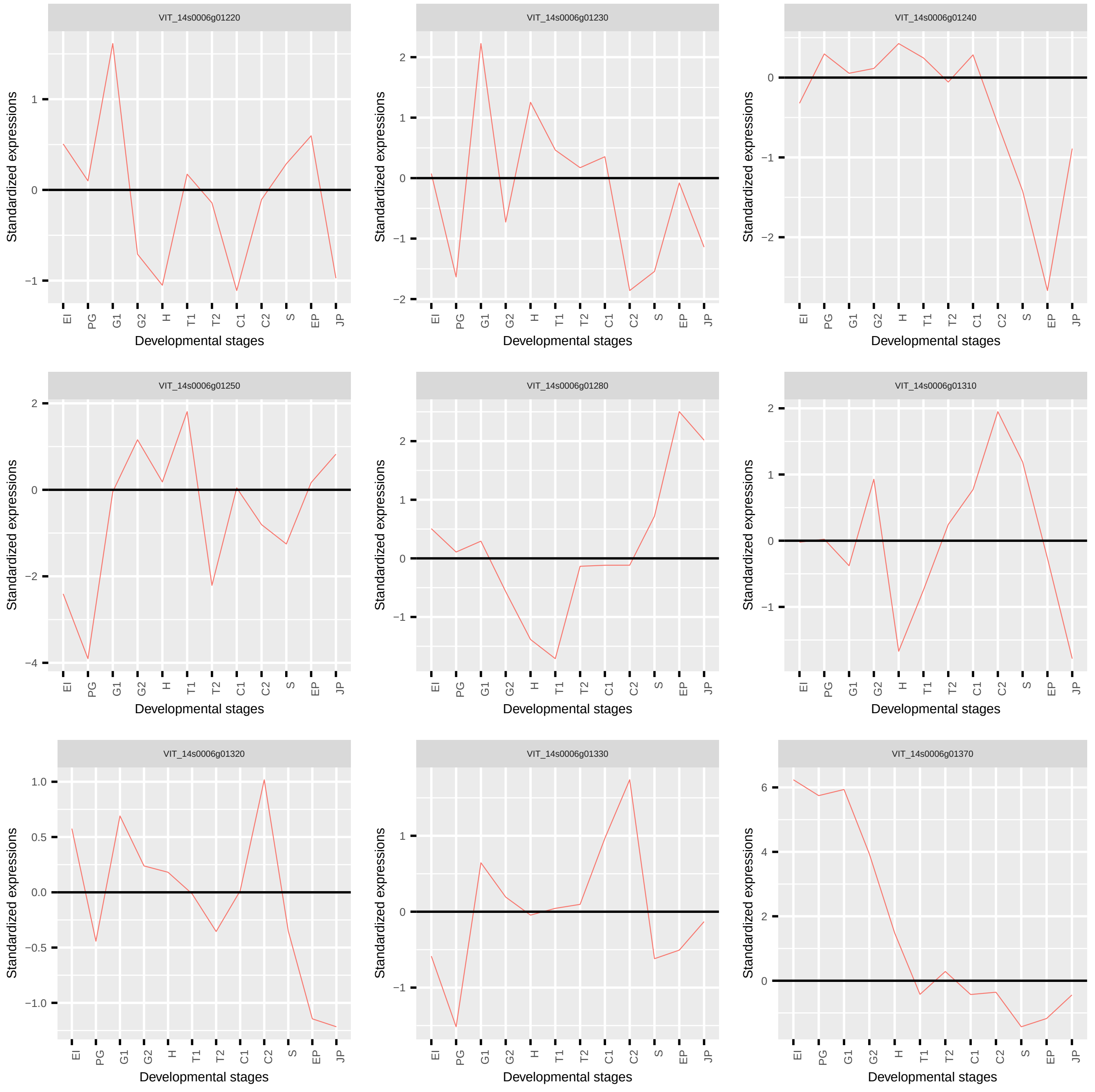
VIT_14s0006g01220
VIT_14s0006g01230
VIT_14s0006g01240
VIT_14s0006g01250
VIT_14s0006g01280
VIT_14s0006g01310
VIT_14s0006g01320
VIT_14s0006g01330
VIT_14s0006g01370
Standardized expressions
Developmental stages
EI
PG
G1
G2
H
T1
T2
C1
C2
S
EP
JP
−4
−2
−1
−0.5
−1.0
0
0.0
0.5
1
1.0
2
4
6

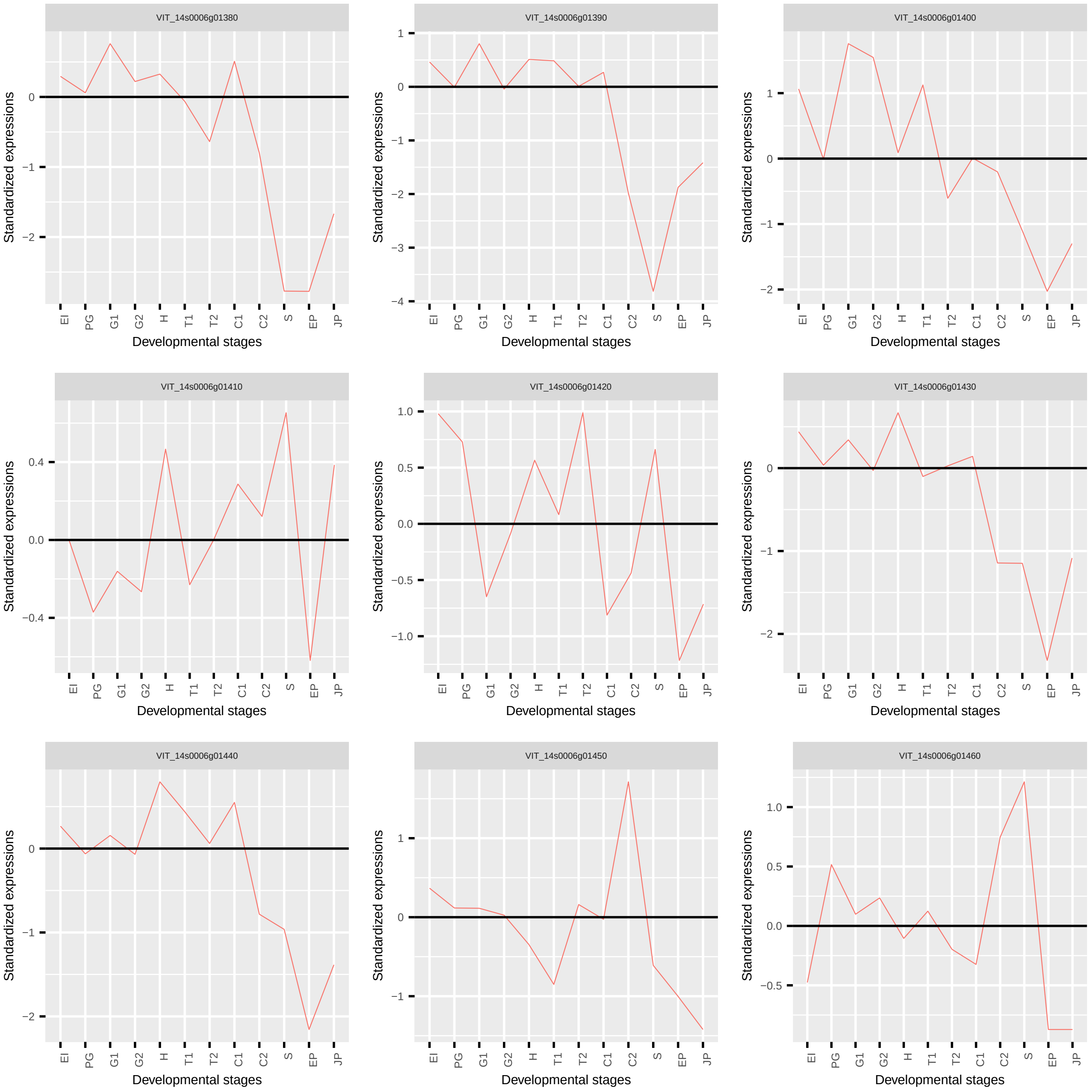
VIT_14s0006g01380
VIT_14s0006g01390
VIT_14s0006g01400
VIT_14s0006g01410
VIT_14s0006g01420
VIT_14s0006g01430
VIT_14s0006g01440
VIT_14s0006g01450
VIT_14s0006g01460
Standardized expressions
Developmental stages
EI PG G1 G2 H T1 T2 C1 C2 S EP JP
0 −1 −2
1 0 −1 −2 −3 −4
1 0 −1 −2
0.4 0.0 −0.4
1.0 0.5 0.0 −0.5 −1.0
0 −1 −2
0 −1 −2
1 0 −1
1.0 0.5 0.0 −0.5

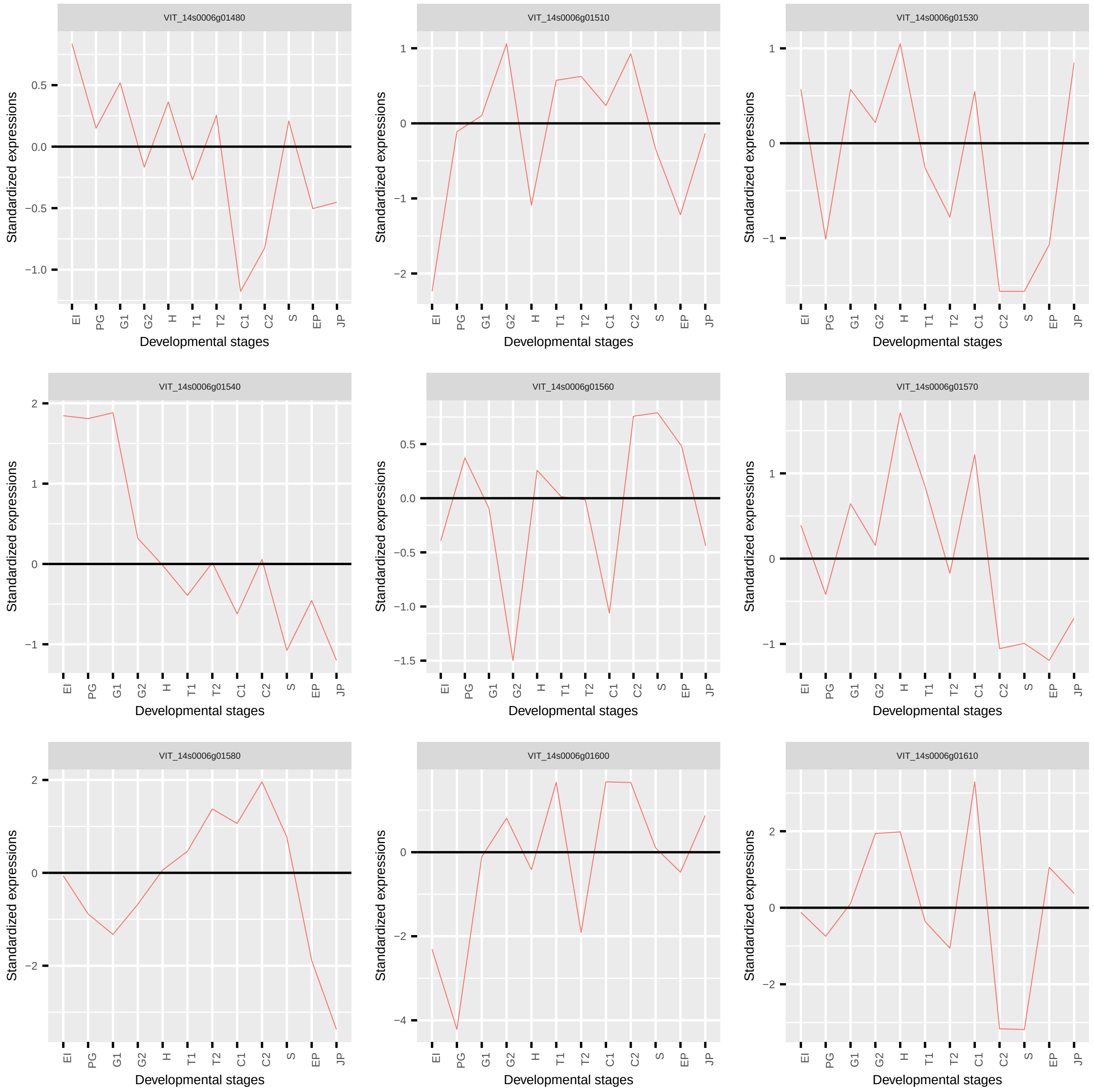
VIT_14s0006g01480
VIT_14s0006g01510
VIT_14s0006g01530
VIT_14s0006g01540
VIT_14s0006g01560
VIT_14s0006g01570
VIT_14s0006g01580
VIT_14s0006g01600
VIT_14s0006g01610
Standardized expressions
Developmental stages
EI PG G1 G2 H T1 T2 C1 C2 S EP JP

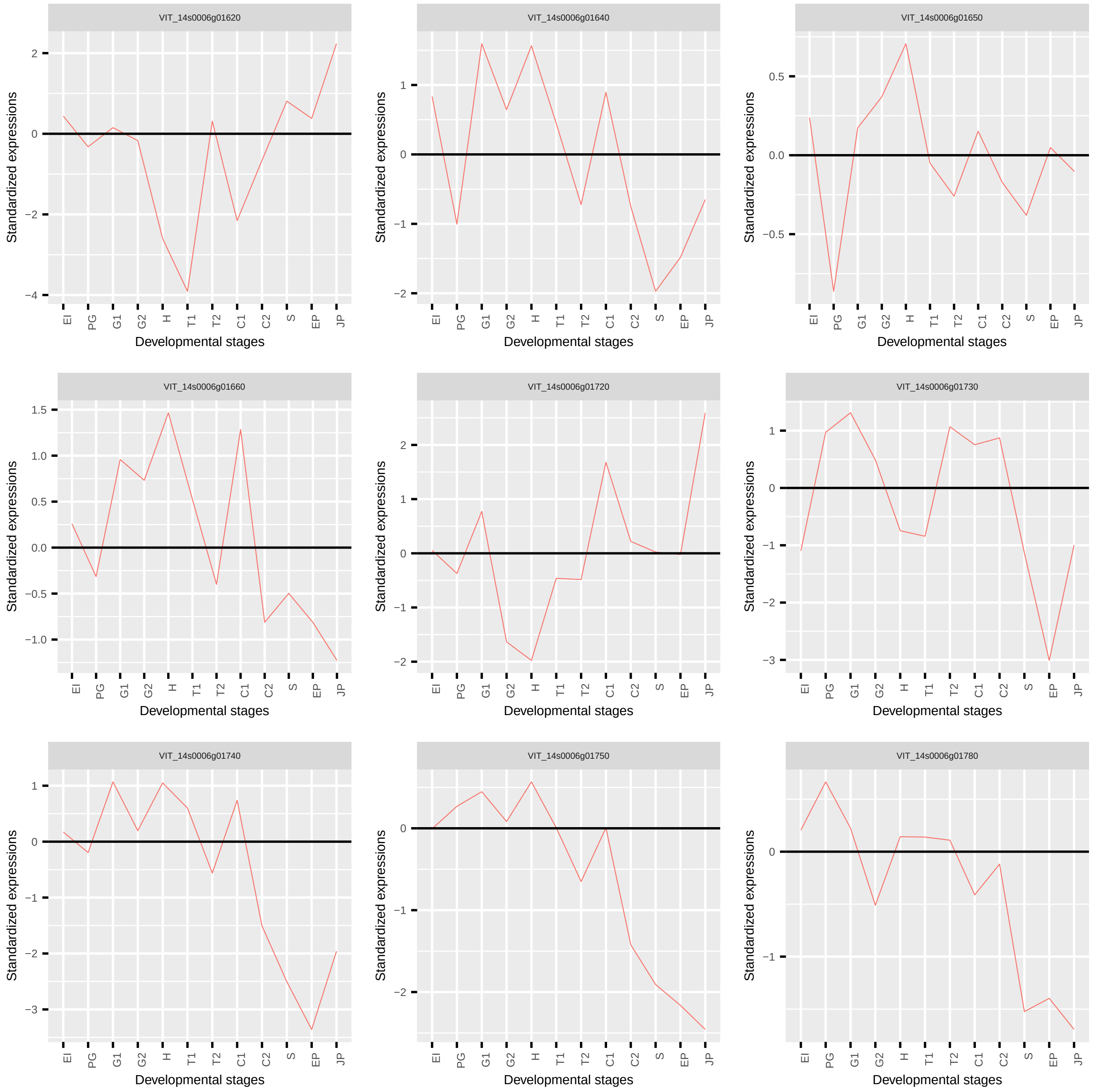

VIT_14s0006g01620
Standardized expressions
2
0
−2
−4
EI PG G1 G2 H T1 T2 C1 C2 S EP JP
Developmental stages
VIT_14s0006g01640
Standardized expressions
1
0
−1
−2
EI PG G1 G2 H T1 T2 C1 C2 S EP JP
Developmental stages
VIT_14s0006g01650
Standardized expressions
0.5
0.0
−0.5
EI PG G1 G2 H T1 T2 C1 C2 S EP JP
Developmental stages
VIT_14s0006g01660
Standardized expressions
1.5
1.0
0.5
0.0
−0.5
−1.0
EI PG G1 G2 H T1 T2 C1 C2 S EP JP
Developmental stages
VIT_14s0006g01720
Standardized expressions
2
1
0
−1
−2
EI PG G1 G2 H T1 T2 C1 C2 S EP JP
Developmental stages
VIT_14s0006g01730
Standardized expressions
1
0
−1
−2
−3
EI PG G1 G2 H T1 T2 C1 C2 S EP JP
Developmental stages
VIT_14s0006g01740
Standardized expressions
1
0
−1
−2
−3
EI PG G1 G2 H T1 T2 C1 C2 S EP JP
Developmental stages
VIT_14s0006g01750
Standardized expressions
0
−1
−2
EI PG G1 G2 H T1 T2 C1 C2 S EP JP
Developmental stages
VIT_14s0006g01780
Standardized expressions
0
−1
EI PG G1 G2 H T1 T2 C1 C2 S EP JP
Developmental stages

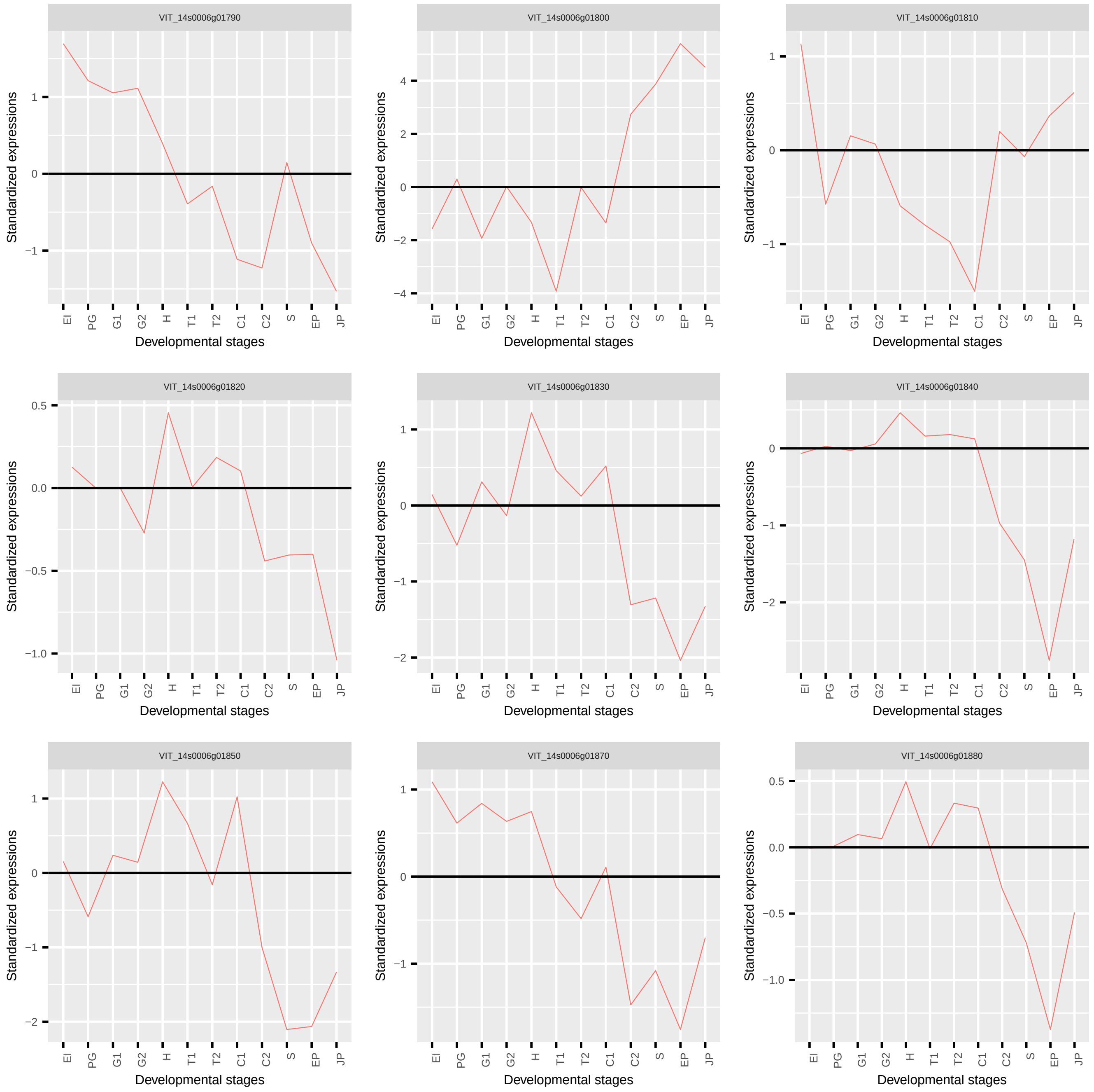
VIT_14s0006g01790
VIT_14s0006g01800
VIT_14s0006g01810
VIT_14s0006g01820
VIT_14s0006g01830
VIT_14s0006g01840
VIT_14s0006g01850
VIT_14s0006g01870
VIT_14s0006g01880
Standardized expressions
Developmental stages
EI PG G1 G2 H T1 T2 C1 C2 S EP JP

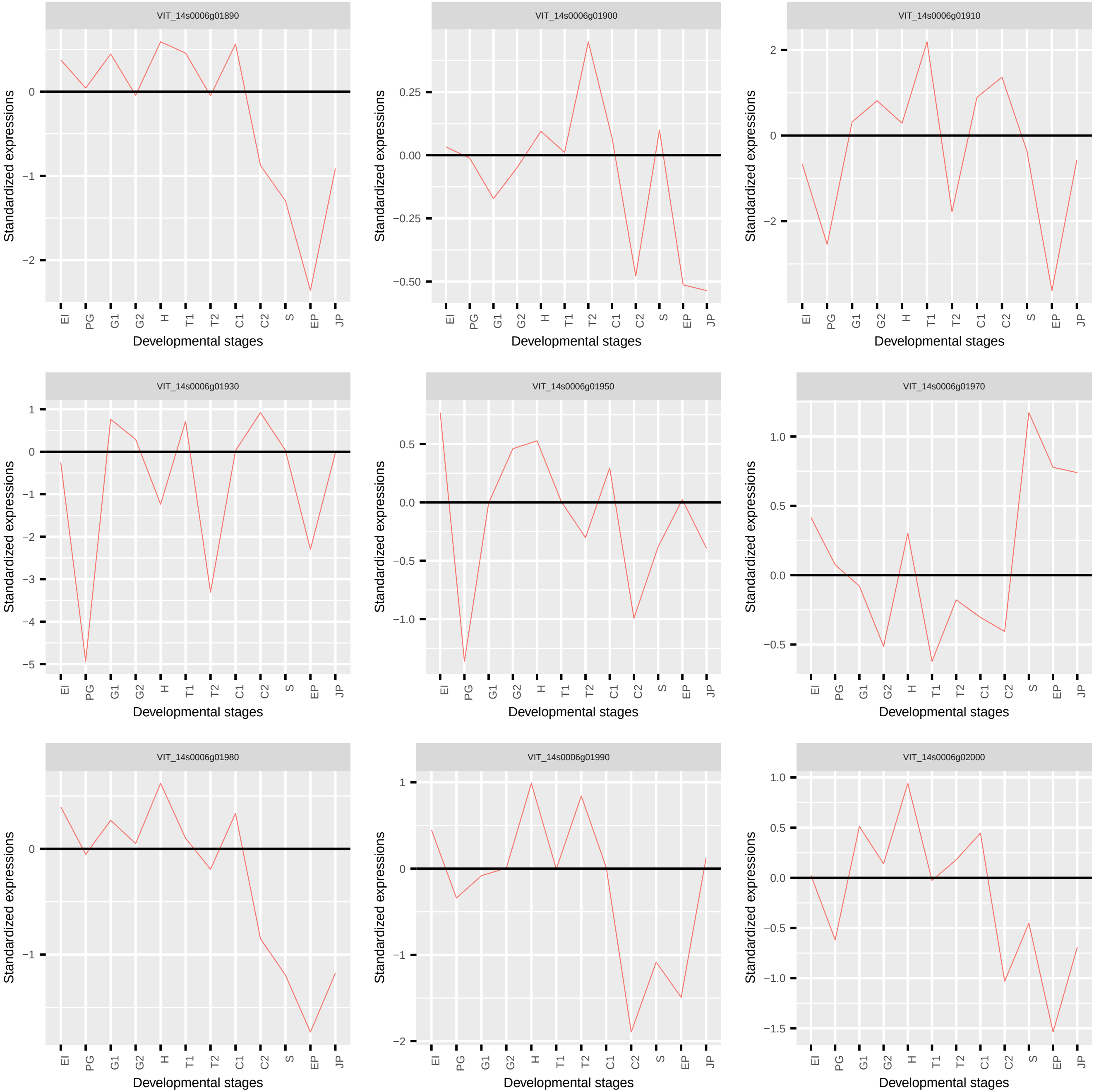
VIT_14s0006g01890
VIT_14s0006g01900
VIT_14s0006g01910
VIT_14s0006g01930
VIT_14s0006g01950
VIT_14s0006g01970
VIT_14s0006g01980
VIT_14s0006g01990
VIT_14s0006g02000
Standardized expressions
Developmental stages
EI
PG
G1
G2
H
T1
T2
C1
C2
S
EP
JP

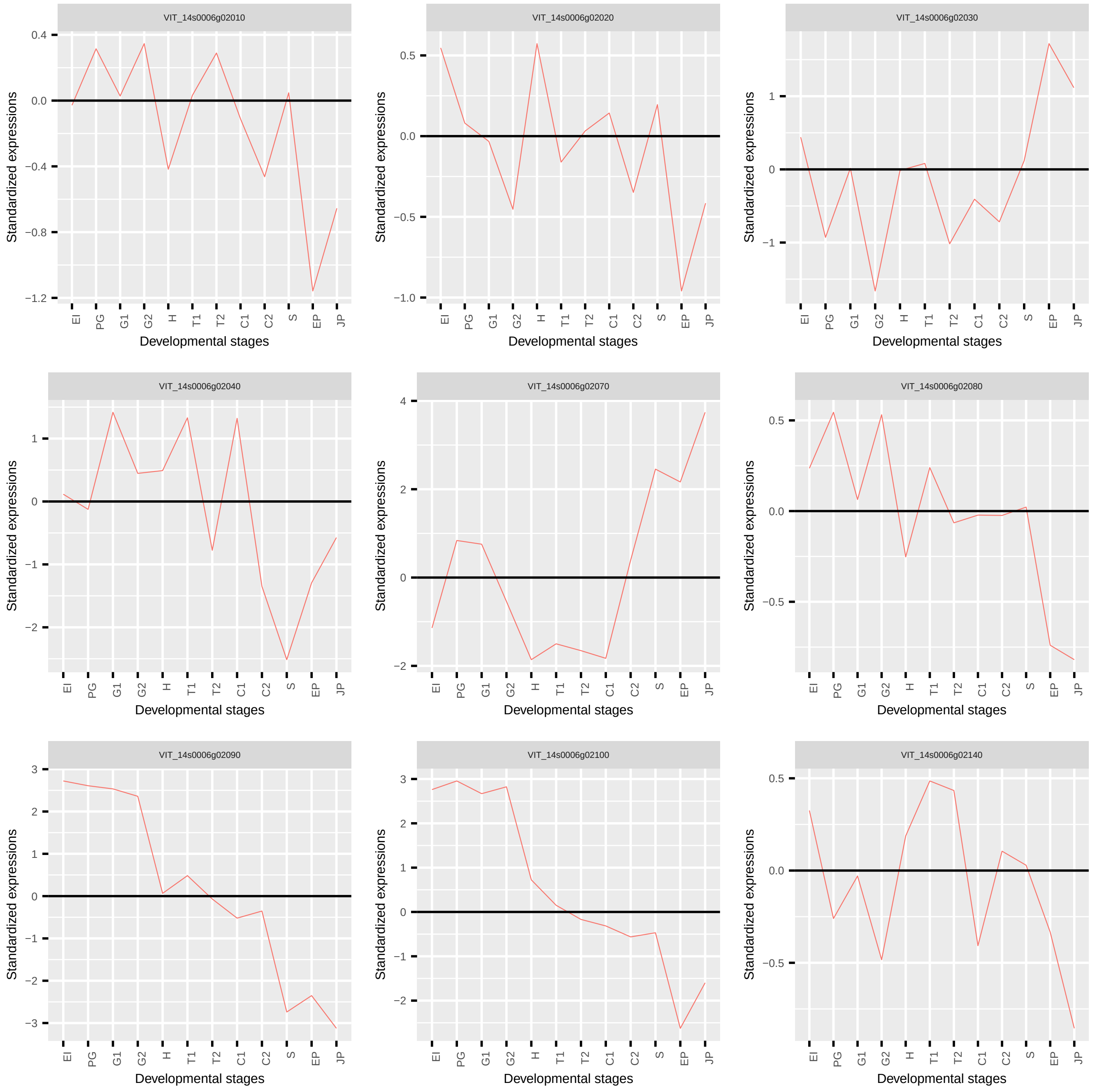
VIT_14s0006g02010
VIT_14s0006g02020
VIT_14s0006g02030
VIT_14s0006g02040
VIT_14s0006g02070
VIT_14s0006g02080
VIT_14s0006g02090
VIT_14s0006g02100
VIT_14s0006g02140
Standardized expressions
Developmental stages
EI PG G1 G2 H T1 T2 C1 C2 S EP JP

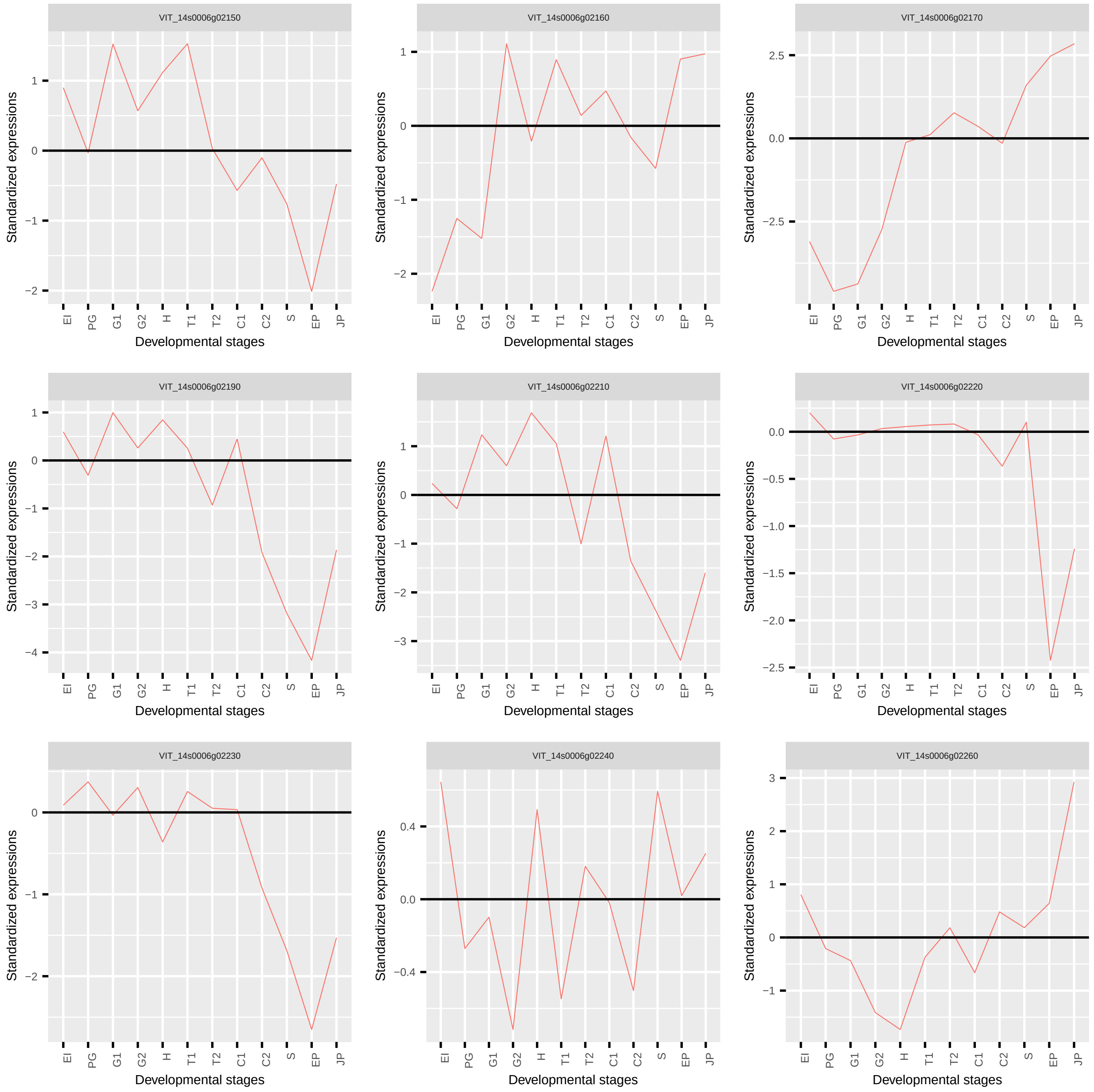

VIT_14s0006g02150
VIT_14s0006g02160
VIT_14s0006g02170
VIT_14s0006g02190
VIT_14s0006g02210
VIT_14s0006g02220
VIT_14s0006g02230
VIT_14s0006g02240
VIT_14s0006g02260
Standardized expressions
Developmental stages
EI PG G1 G2 H T1 T2 C1 C2 S EP JP

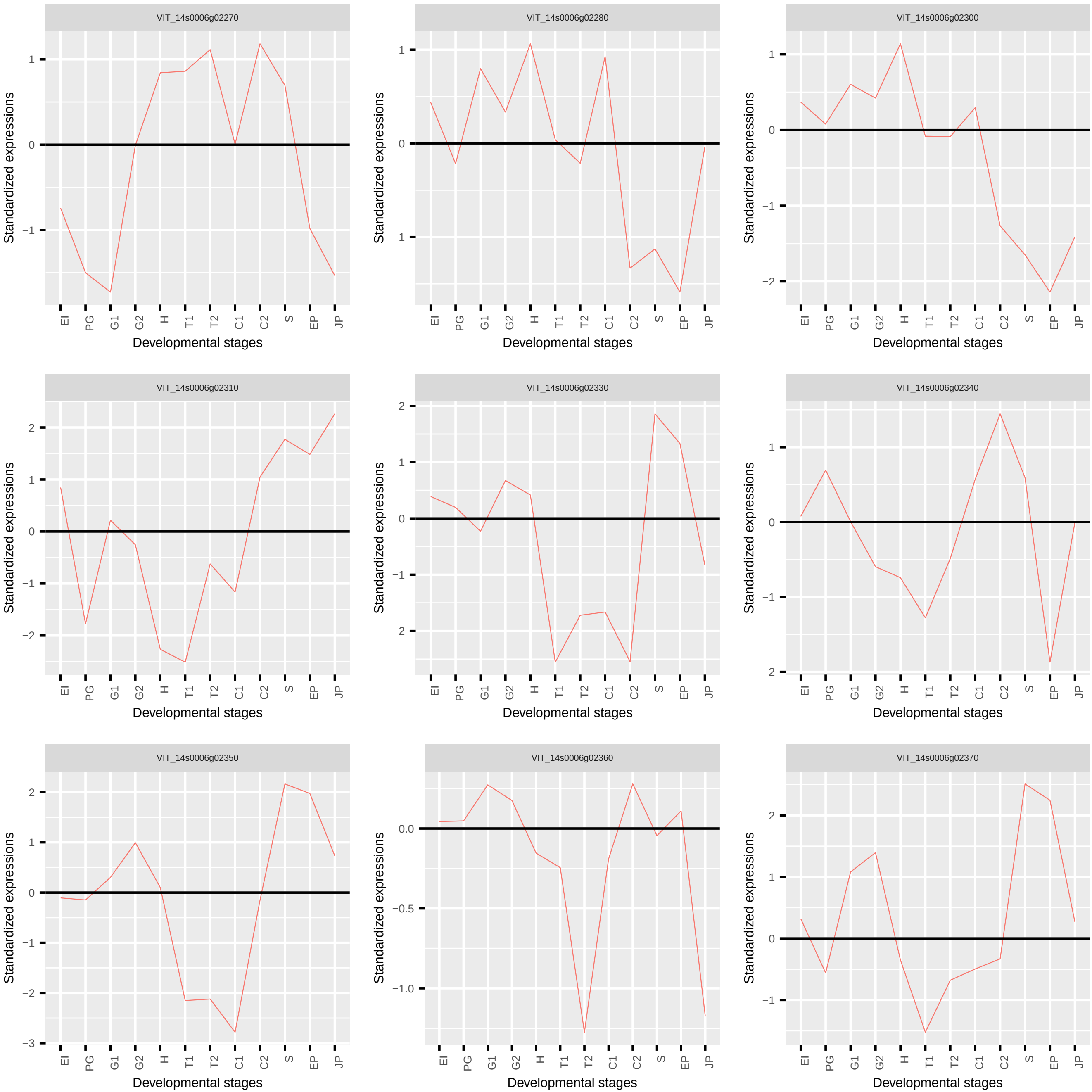

VIT_14s0006g02270
VIT_14s0006g02280
VIT_14s0006g02300
VIT_14s0006g02310
VIT_14s0006g02330
VIT_14s0006g02340
VIT_14s0006g02350
VIT_14s0006g02360
VIT_14s0006g02370
Standardized expressions
Developmental stages
EI
PG
G1
G2
H
T1
T2
C1
C2
S
EP
JP

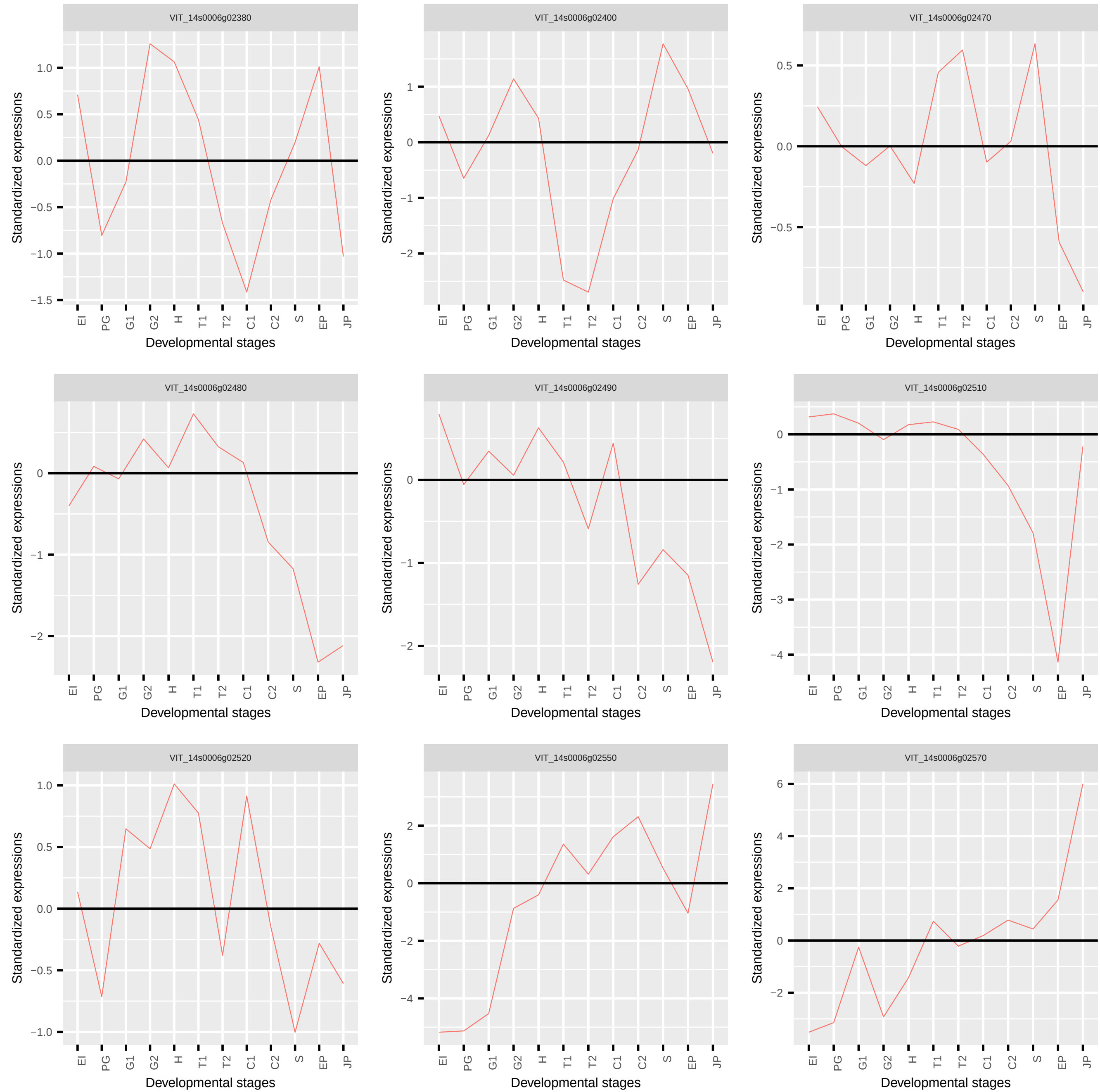
VIT_14s0006g02380
VIT_14s0006g02400
VIT_14s0006g02470
VIT_14s0006g02480
VIT_14s0006g02490
VIT_14s0006g02510
VIT_14s0006g02520
VIT_14s0006g02550
VIT_14s0006g02570
Standardized expressions
Developmental stages
EI PG G1 G2 H T1 T2 C1 C2 S EP JP

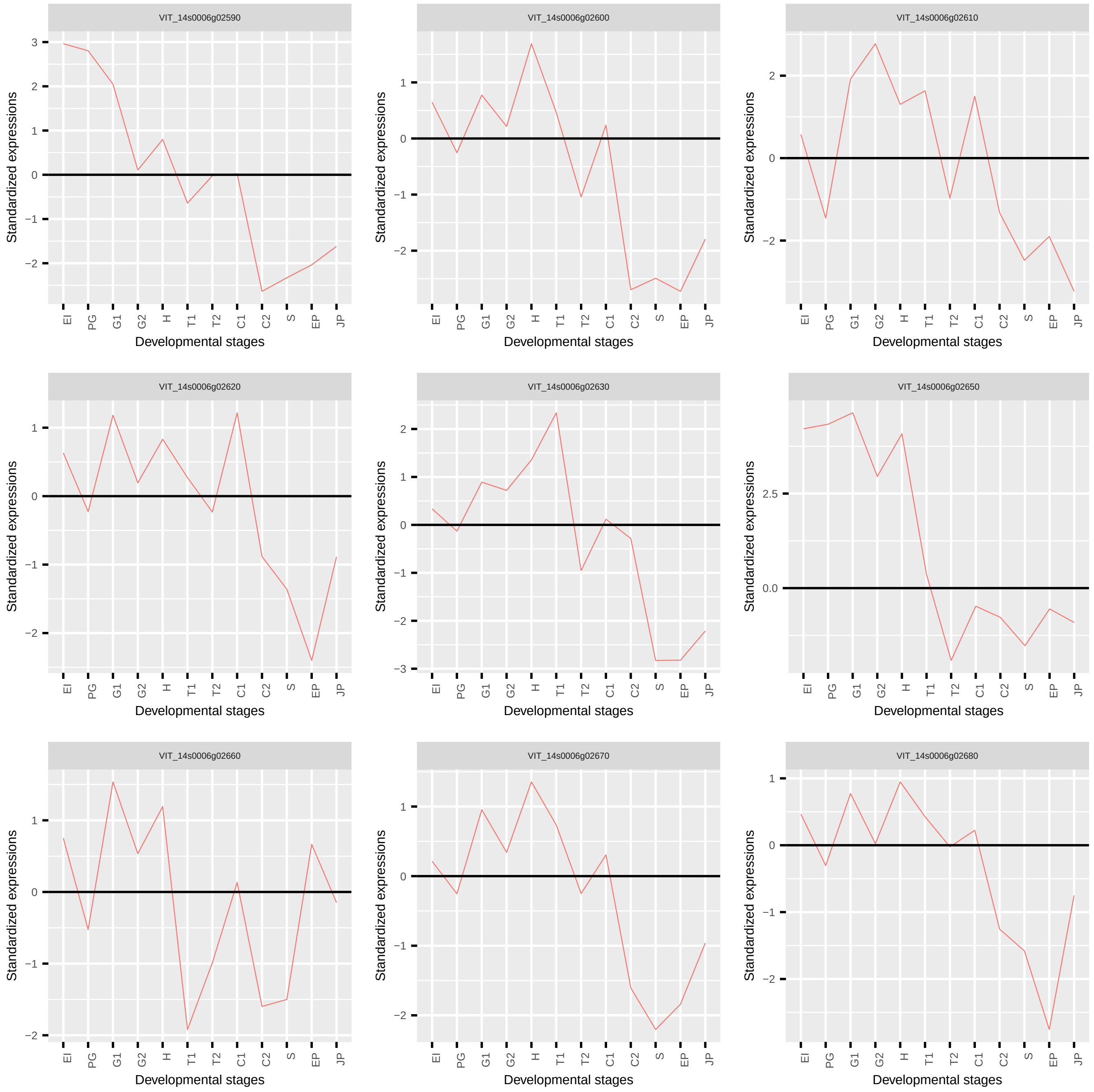
VIT_14s0006g02590
VIT_14s0006g02600
VIT_14s0006g02610
VIT_14s0006g02620
VIT_14s0006g02630
VIT_14s0006g02650
VIT_14s0006g02660
VIT_14s0006g02670
VIT_14s0006g02680
Standardized expressions
Developmental stages
EI PG G1 G2 H T1 T2 C1 C2 S EP JP

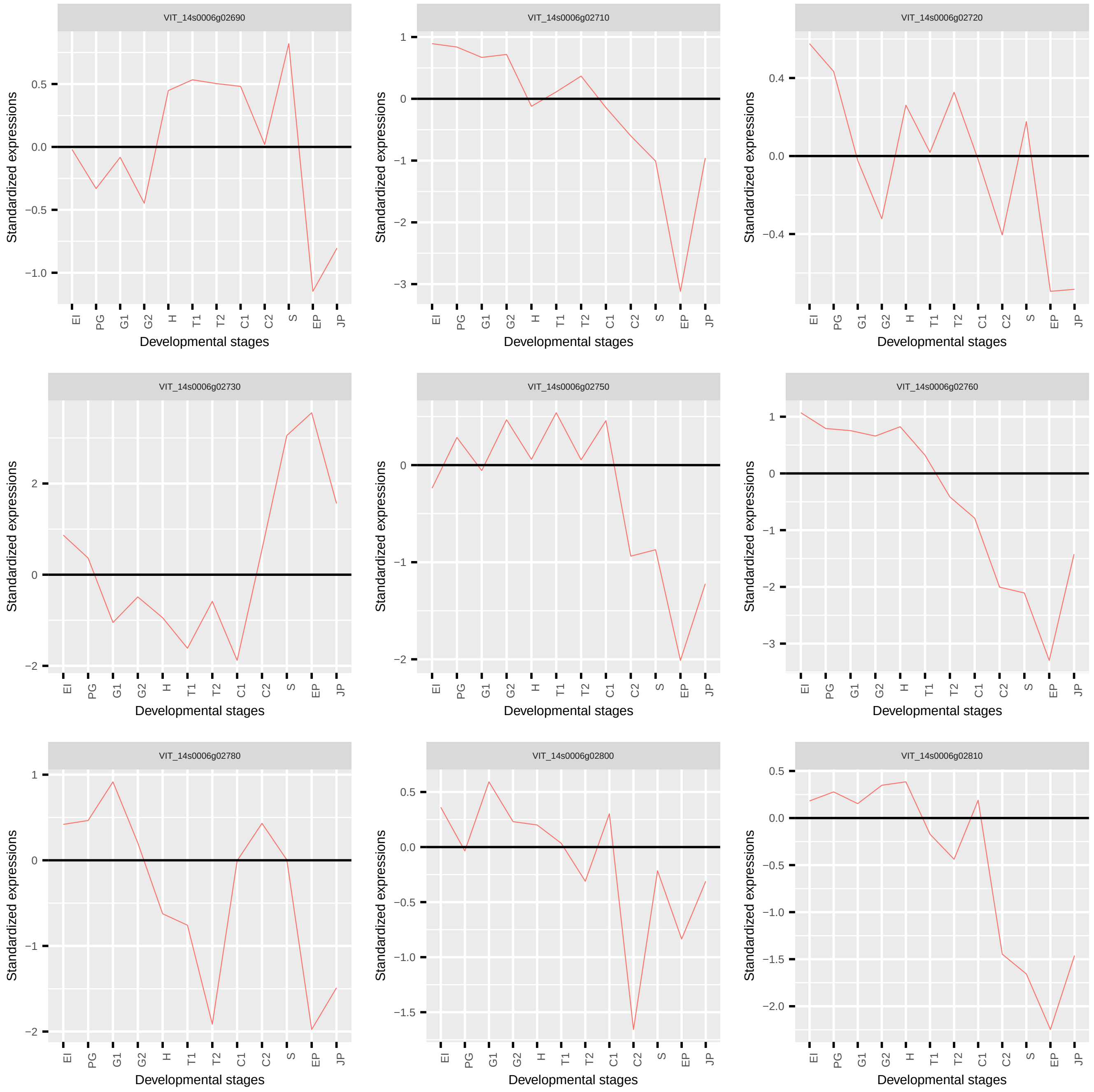
VIT_14s0006g02690
VIT_14s0006g02710
VIT_14s0006g02720
VIT_14s0006g02730
VIT_14s0006g02750
VIT_14s0006g02760
VIT_14s0006g02780
VIT_14s0006g02800
VIT_14s0006g02810
Standardized expressions
Developmental stages
EI
PG
G1
G2
H
T1
T2
C1
C2
S
EP
JP
0.5
0.0
−0.5
−1.0
−1.5
−2.0
1
0
−1
−2
−3
2
0.4
−0.4

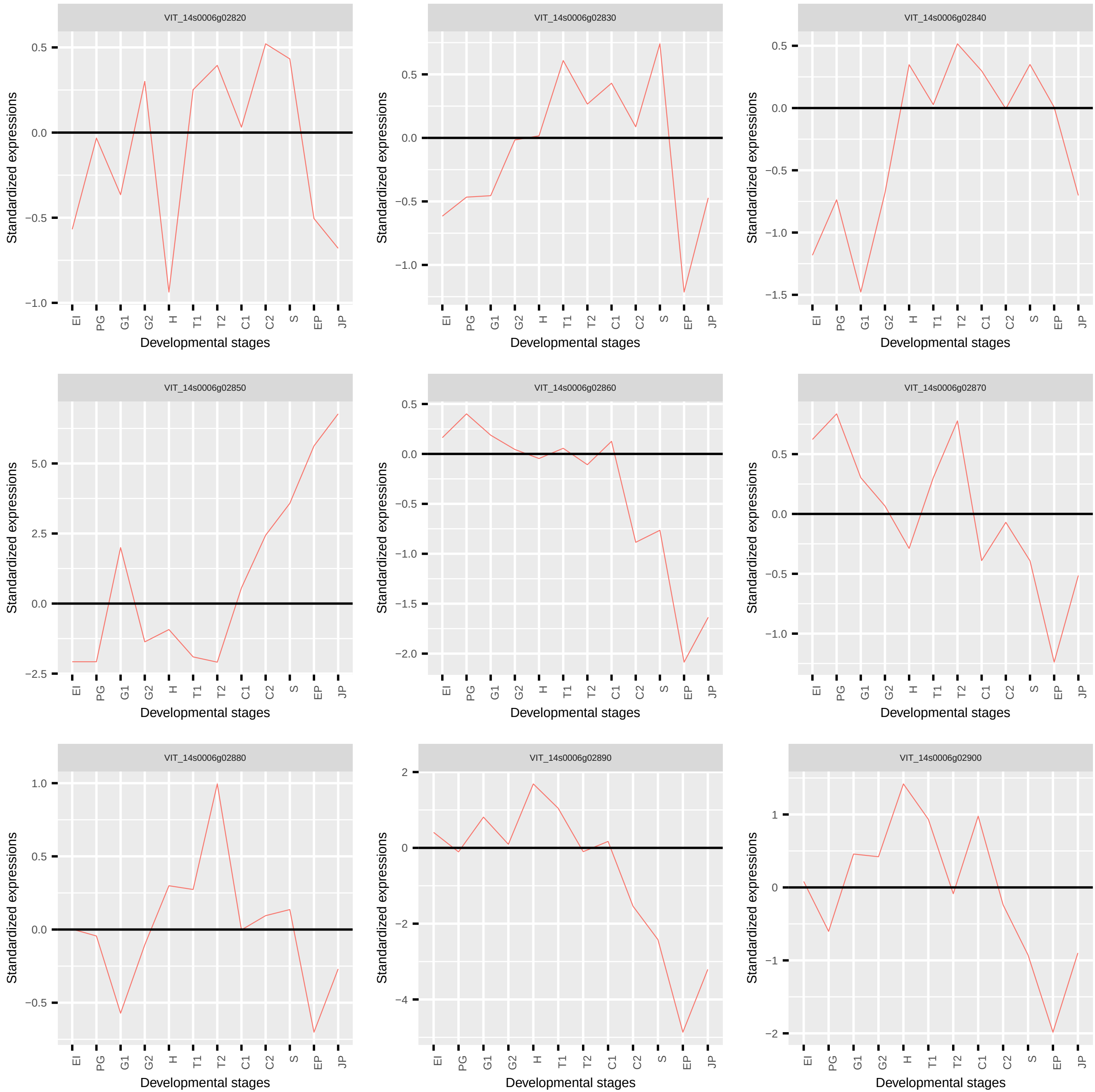
VIT_14s0006g02820
VIT_14s0006g02830
VIT_14s0006g02840
VIT_14s0006g02850
VIT_14s0006g02860
VIT_14s0006g02870
VIT_14s0006g02880
VIT_14s0006g02890
VIT_14s0006g02900
Standardized expressions
Developmental stages
EI PG G1 G2 H T1 T2 C1 C2 S EP JP

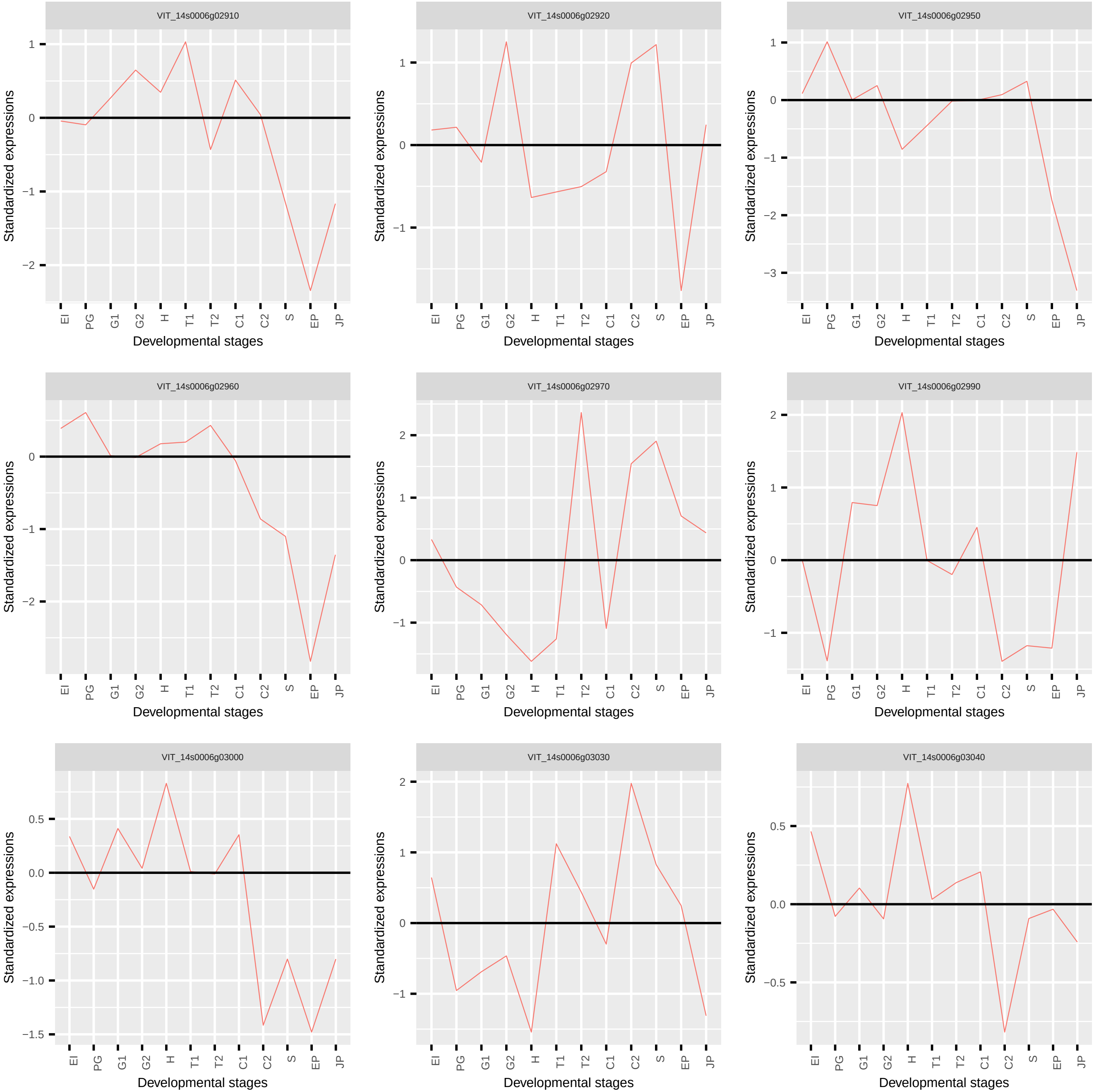

VIT_14s0006g02910
VIT_14s0006g02920
VIT_14s0006g02950
VIT_14s0006g02960
VIT_14s0006g02970
VIT_14s0006g02990
VIT_14s0006g03000
VIT_14s0006g03030
VIT_14s0006g03040
Standardized expressions
Developmental stages
EI PG G1 G2 H T1 T2 C1 C2 S EP JP

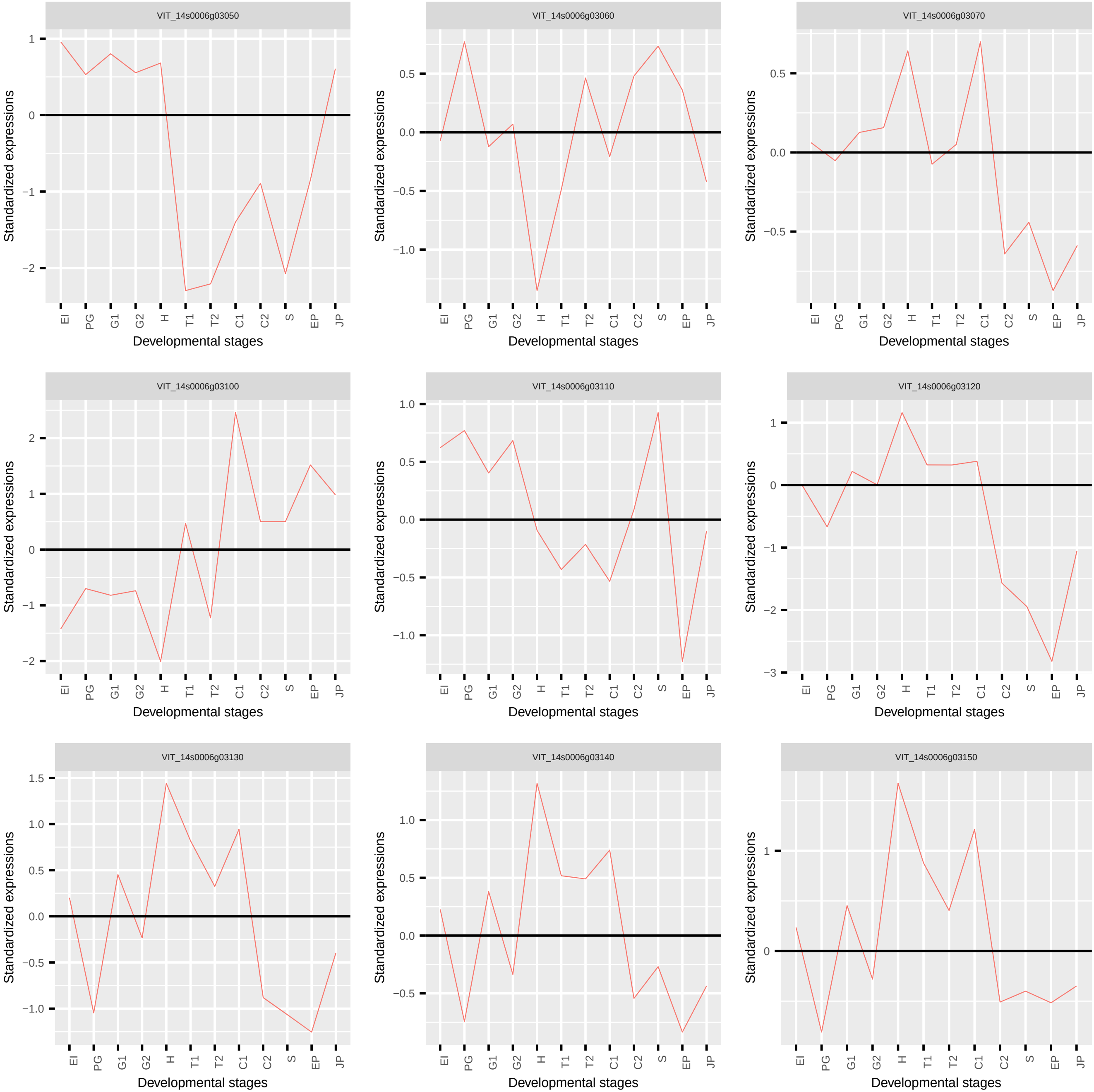
VIT_14s0006g03050
VIT_14s0006g03060
VIT_14s0006g03070
VIT_14s0006g03100
VIT_14s0006g03110
VIT_14s0006g03120
VIT_14s0006g03130
VIT_14s0006g03140
VIT_14s0006g03150
Standardized expressions
Developmental stages
EI
PG
G1
G2
H
T1
T2
C1
C2
S
EP
JP

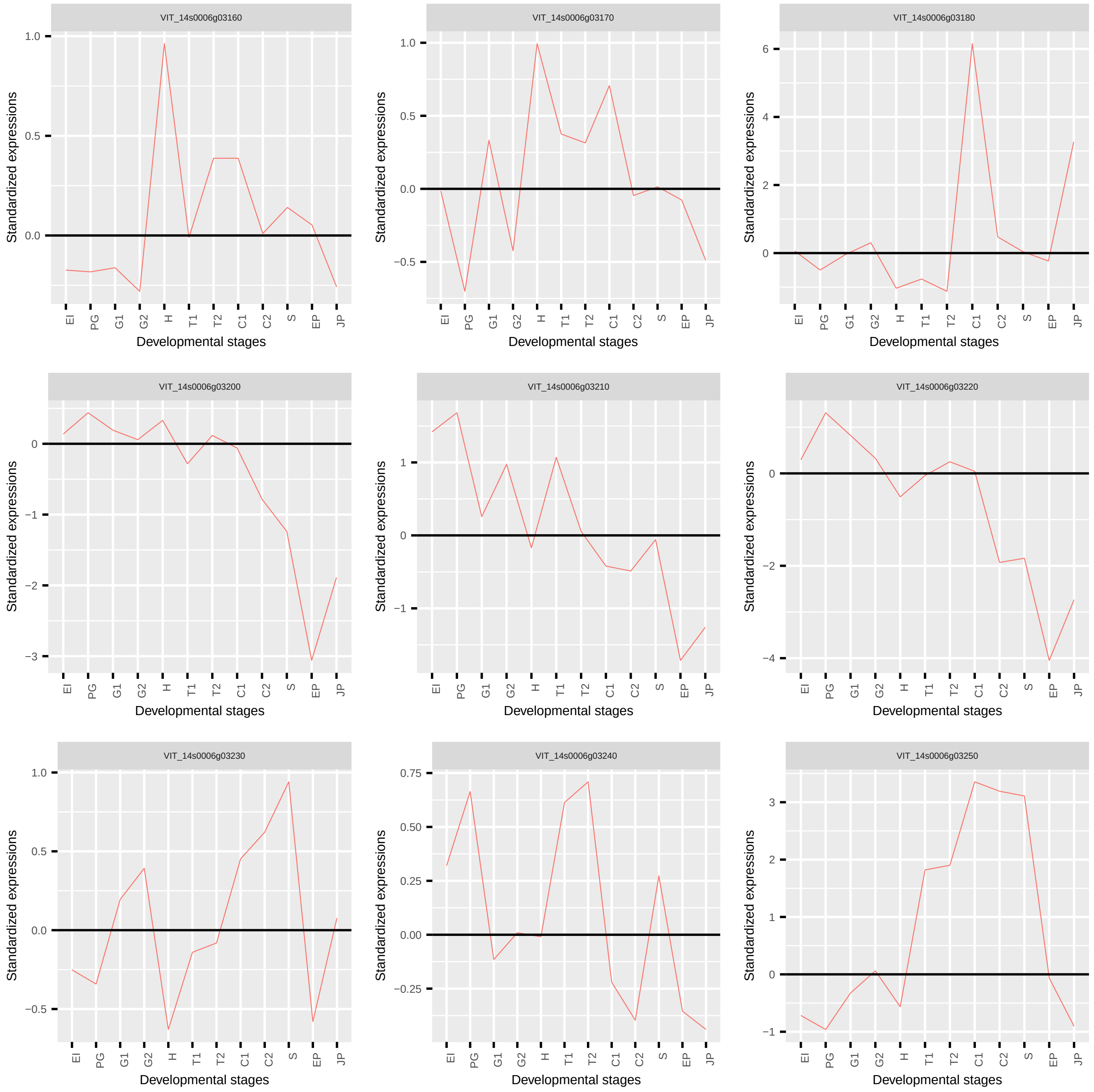

VIT_14s0006g03160
VIT_14s0006g03170
VIT_14s0006g03180
VIT_14s0006g03200
VIT_14s0006g03210
VIT_14s0006g03220
VIT_14s0006g03230
VIT_14s0006g03240
VIT_14s0006g03250
Standardized expressions
Developmental stages
EI
PG
G1
G2
H
T1
T2
C1
C2
S
EP
JP

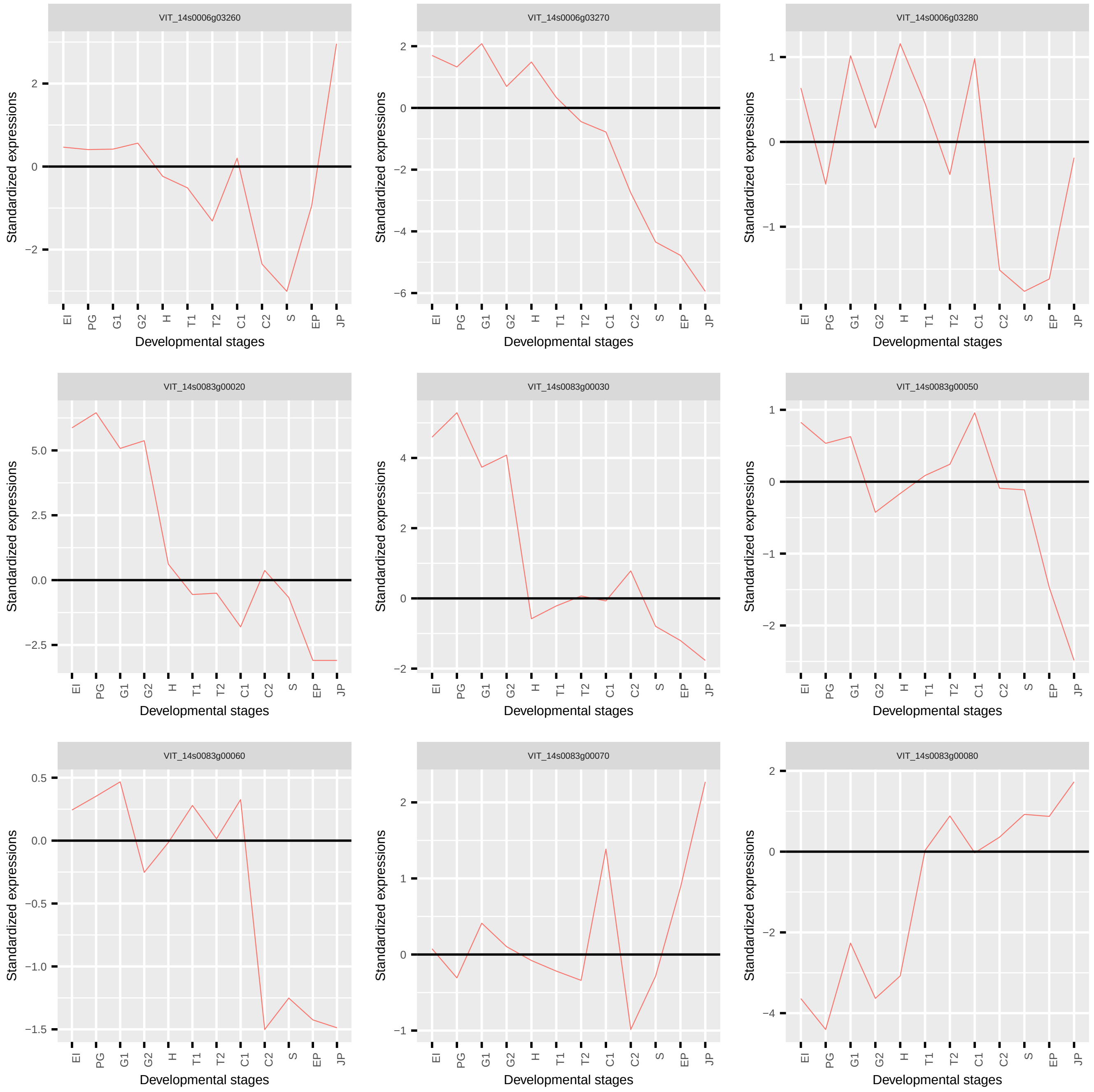
VIT_14s0006g03260
VIT_14s0006g03270
VIT_14s0006g03280
VIT_14s0083g00020
VIT_14s0083g00030
VIT_14s0083g00050
VIT_14s0083g00060
VIT_14s0083g00070
VIT_14s0083g00080
Standardized expressions
Developmental stages
EI PG G1 G2 H T1 T2 C1 C2 S EP JP

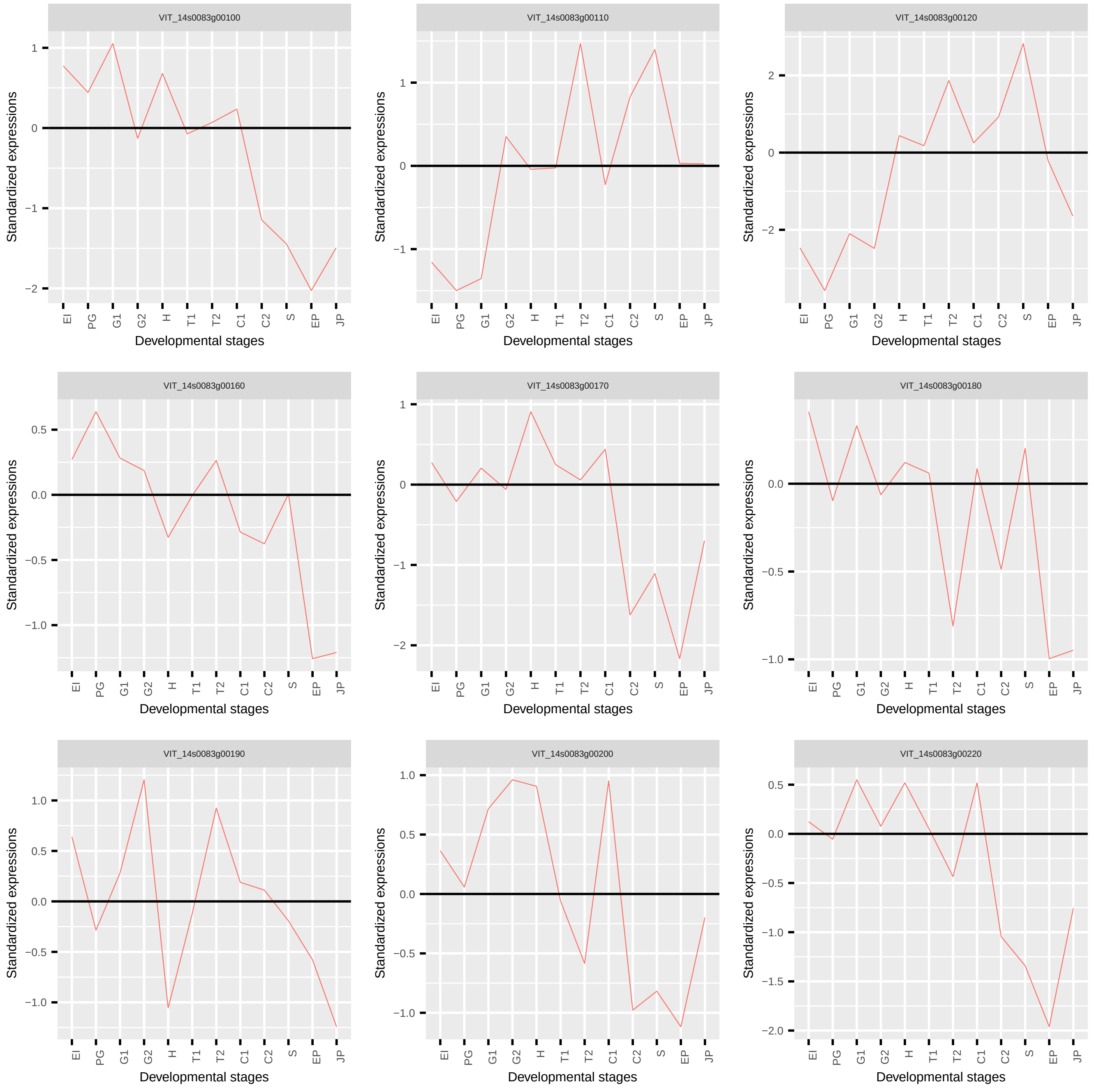
VIT_14s0083g00100
VIT_14s0083g00110
VIT_14s0083g00120
VIT_14s0083g00160
VIT_14s0083g00170
VIT_14s0083g00180
VIT_14s0083g00190
VIT_14s0083g00200
VIT_14s0083g00220
Standardized expressions
Developmental stages
EI PG G1 G2 H T1 T2 C1 C2 S EP JP

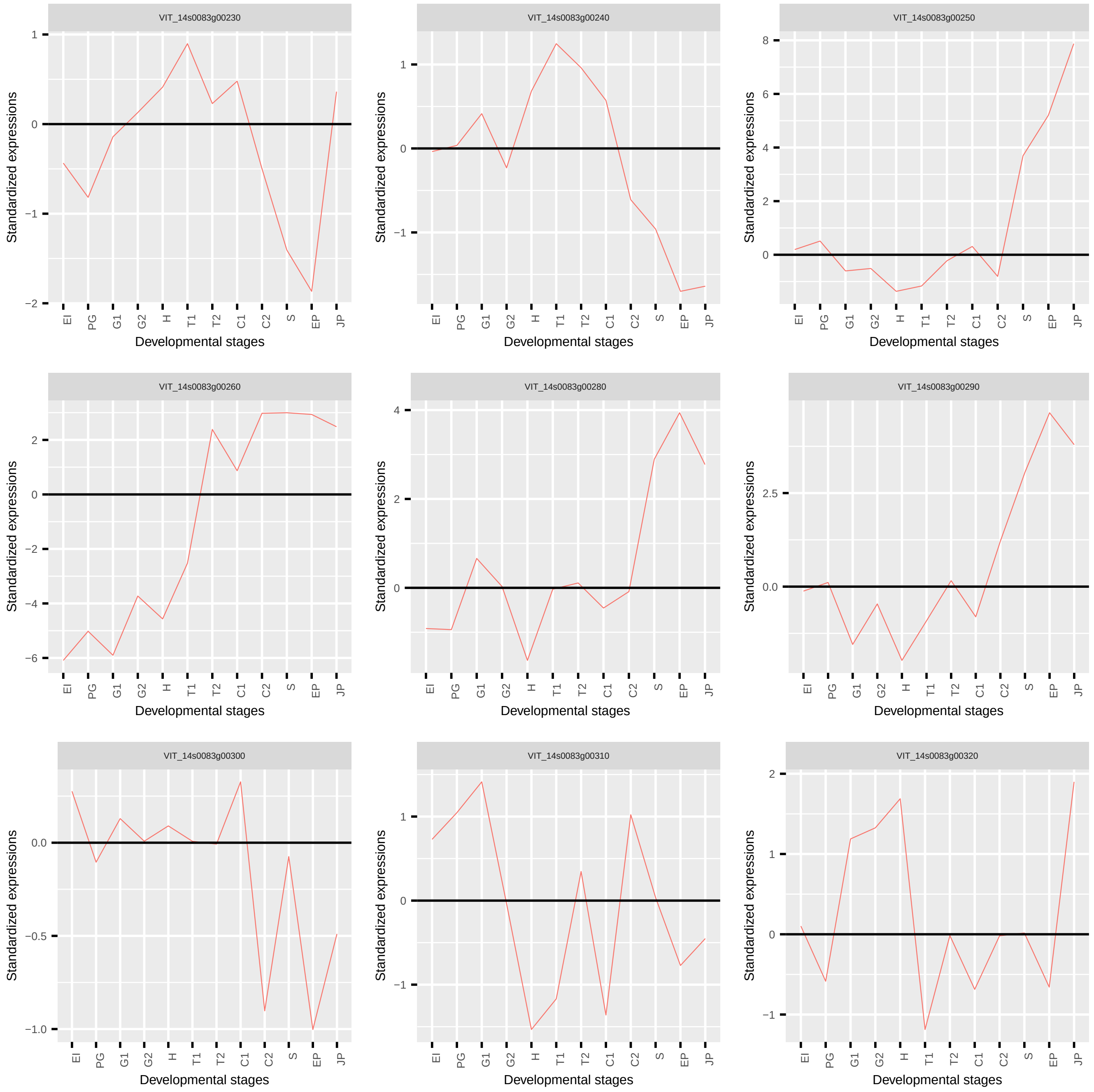
VIT_14s0083g00230
VIT_14s0083g00240
VIT_14s0083g00250
VIT_14s0083g00260
VIT_14s0083g00280
VIT_14s0083g00290
VIT_14s0083g00300
VIT_14s0083g00310
VIT_14s0083g00320
Standardized expressions
Developmental stages
EI PG G1 G2 H T1 T2 C1 C2 S EP JP

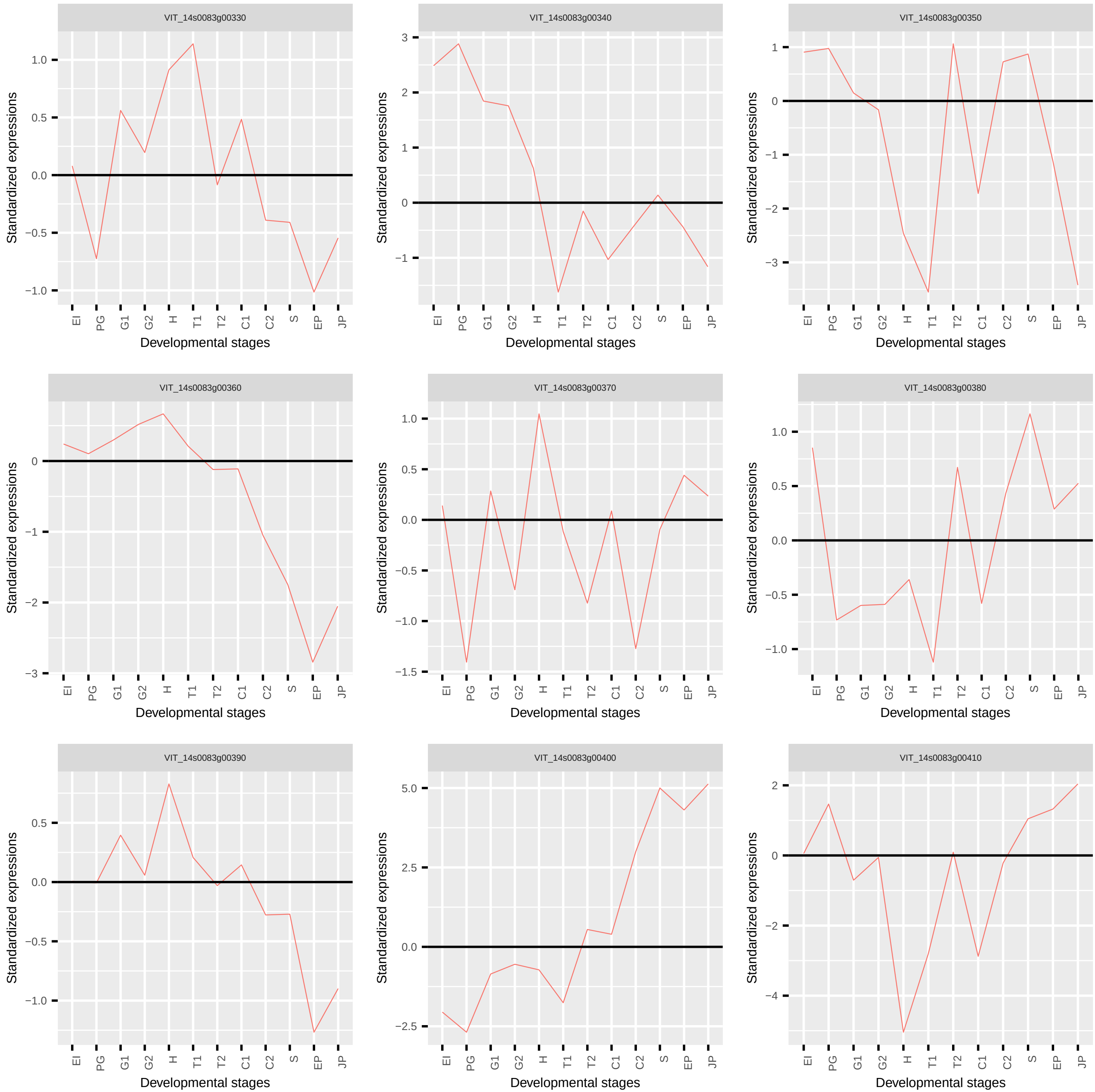

VIT_14s0083g00330
VIT_14s0083g00340
VIT_14s0083g00350
VIT_14s0083g00360
VIT_14s0083g00370
VIT_14s0083g00380
VIT_14s0083g00390
VIT_14s0083g00400
VIT_14s0083g00410
Standardized expressions
Developmental stages
EI
PG
G1
G2
H
T1
T2
C1
C2
S
EP
JP

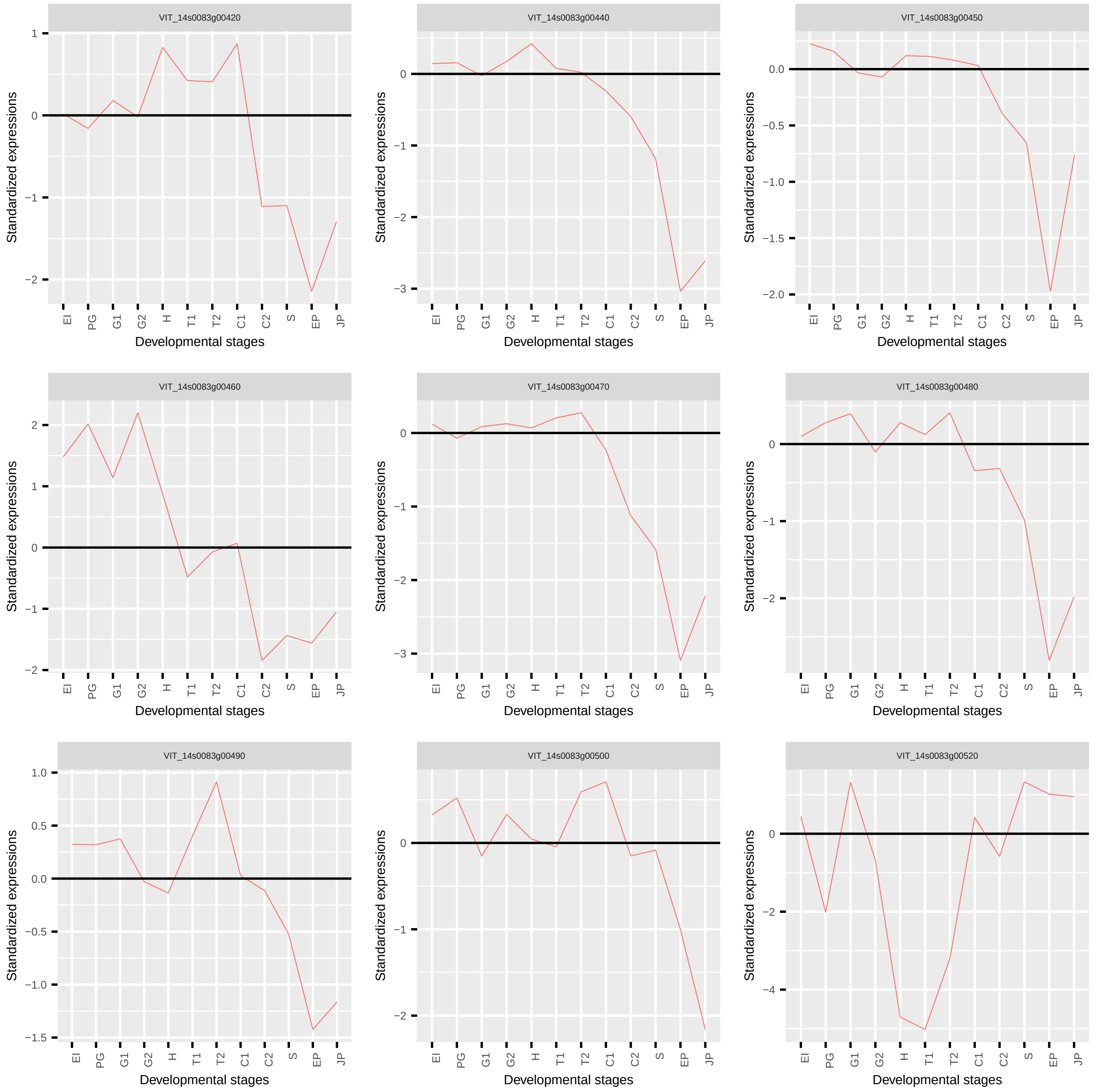

VIT_14s0083g00420
VIT_14s0083g00440
VIT_14s0083g00450
VIT_14s0083g00460
VIT_14s0083g00470
VIT_14s0083g00480
VIT_14s0083g00490
VIT_14s0083g00500
VIT_14s0083g00520
Standardized expressions
Developmental stages
EI PG G1 G2 H T1 T2 C1 C2 S EP JP

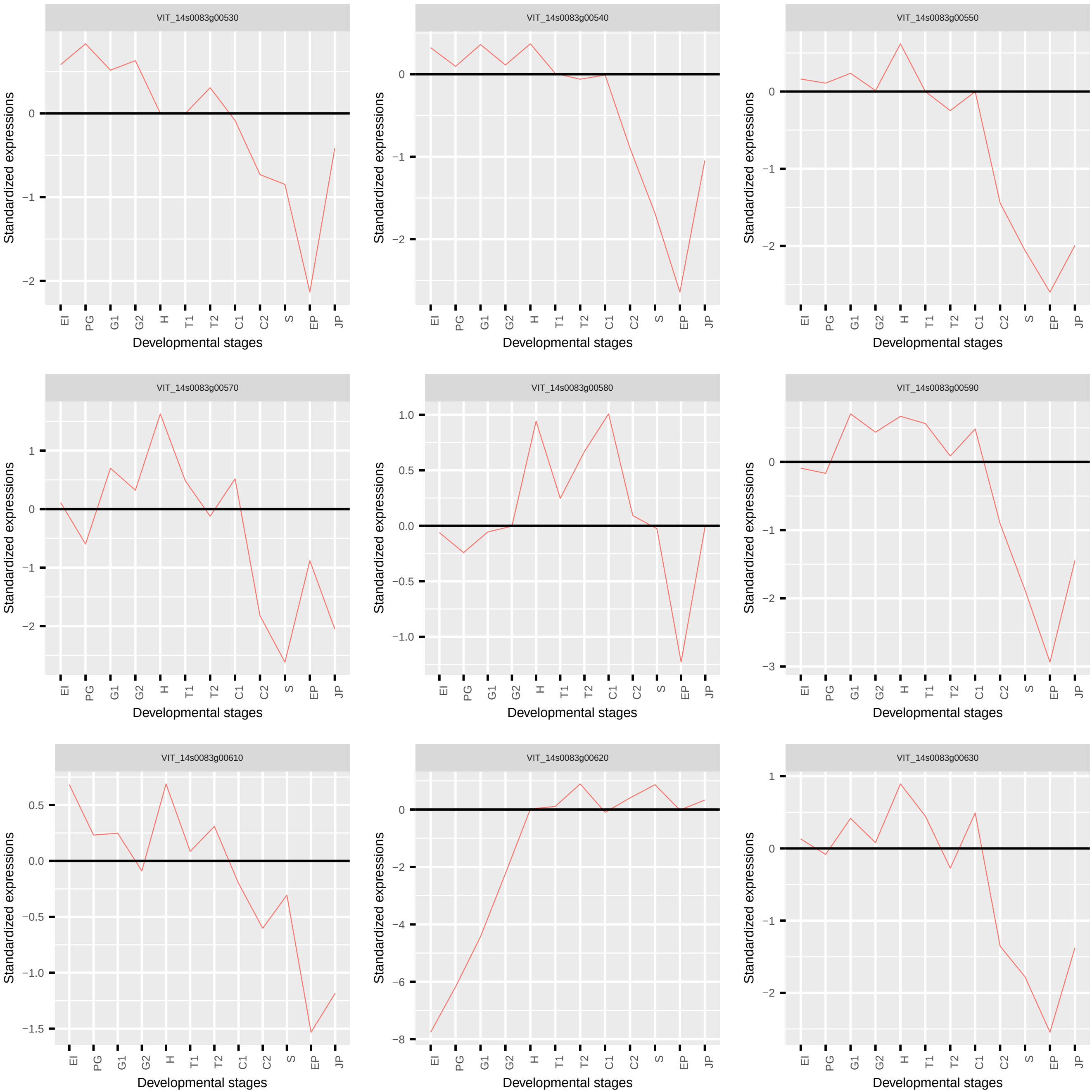
VIT_14s0083g00530
VIT_14s0083g00540
VIT_14s0083g00550
VIT_14s0083g00570
VIT_14s0083g00580
VIT_14s0083g00590
VIT_14s0083g00610
VIT_14s0083g00620
VIT_14s0083g00630
Standardized expressions
Developmental stages
EI PG G1 G2 H T1 T2 C1 C2 S EP JP

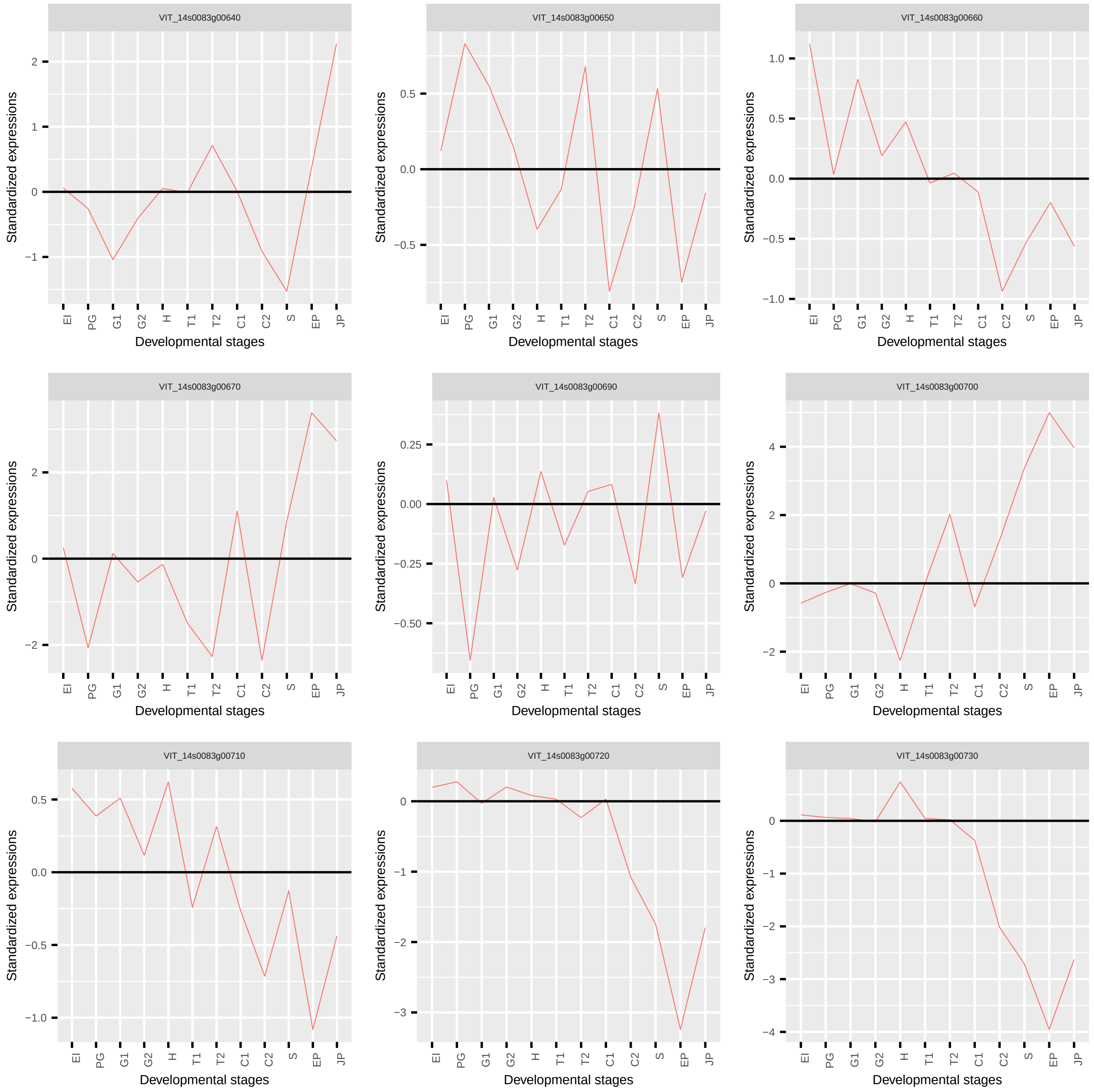
VIT_14s0083g00640
VIT_14s0083g00650
VIT_14s0083g00660
VIT_14s0083g00670
VIT_14s0083g00690
VIT_14s0083g00700
VIT_14s0083g00710
VIT_14s0083g00720
VIT_14s0083g00730
Standardized expressions
Developmental stages
EI PG G1 G2 H T1 T2 C1 C2 S EP JP

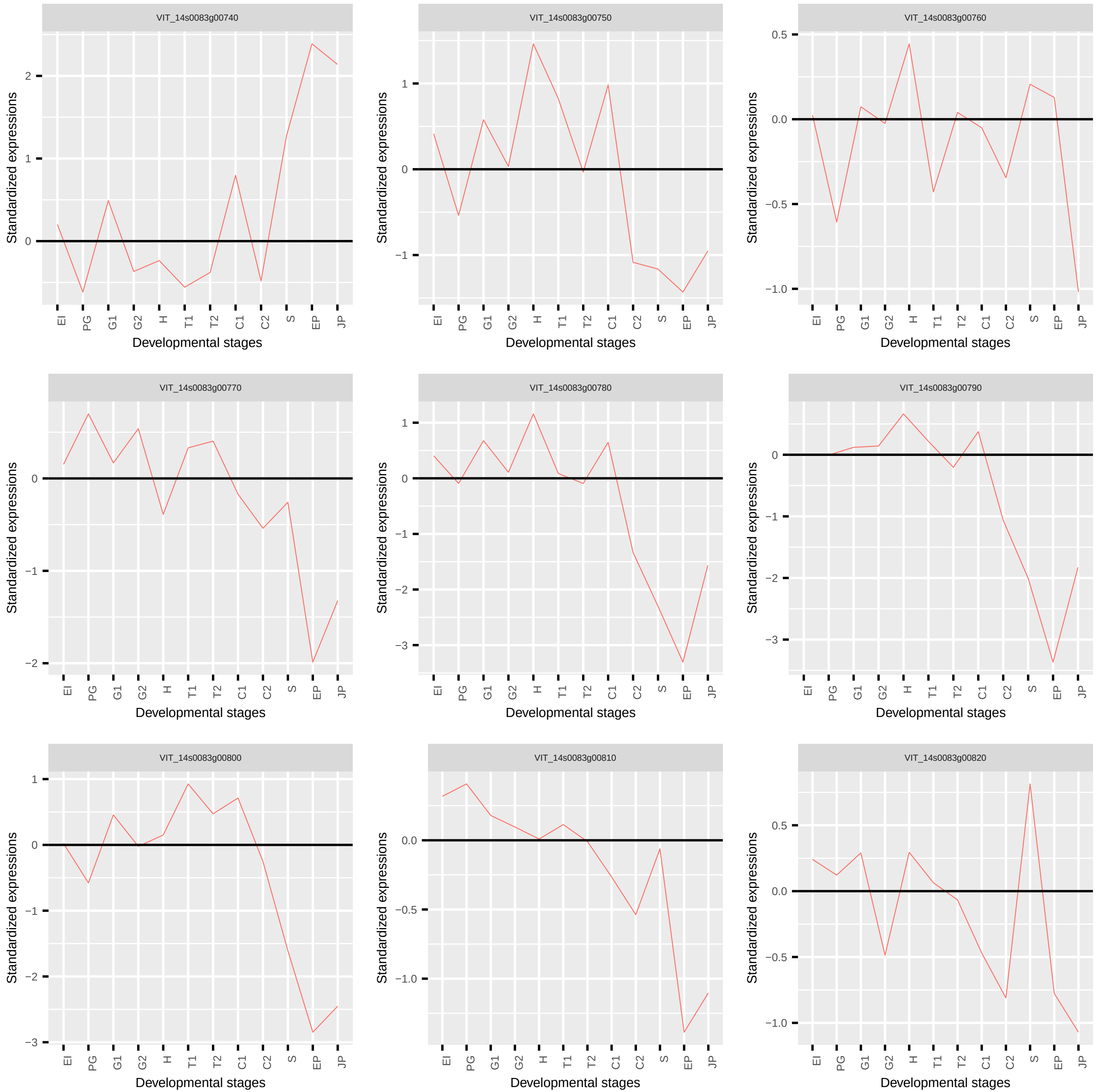
VIT_14s0083g00740
VIT_14s0083g00750
VIT_14s0083g00760
VIT_14s0083g00770
VIT_14s0083g00780
VIT_14s0083g00790
VIT_14s0083g00800
VIT_14s0083g00810
VIT_14s0083g00820
Standardized expressions
Developmental stages
EI PG G1 G2 H T1 T2 C1 C2 S EP JP

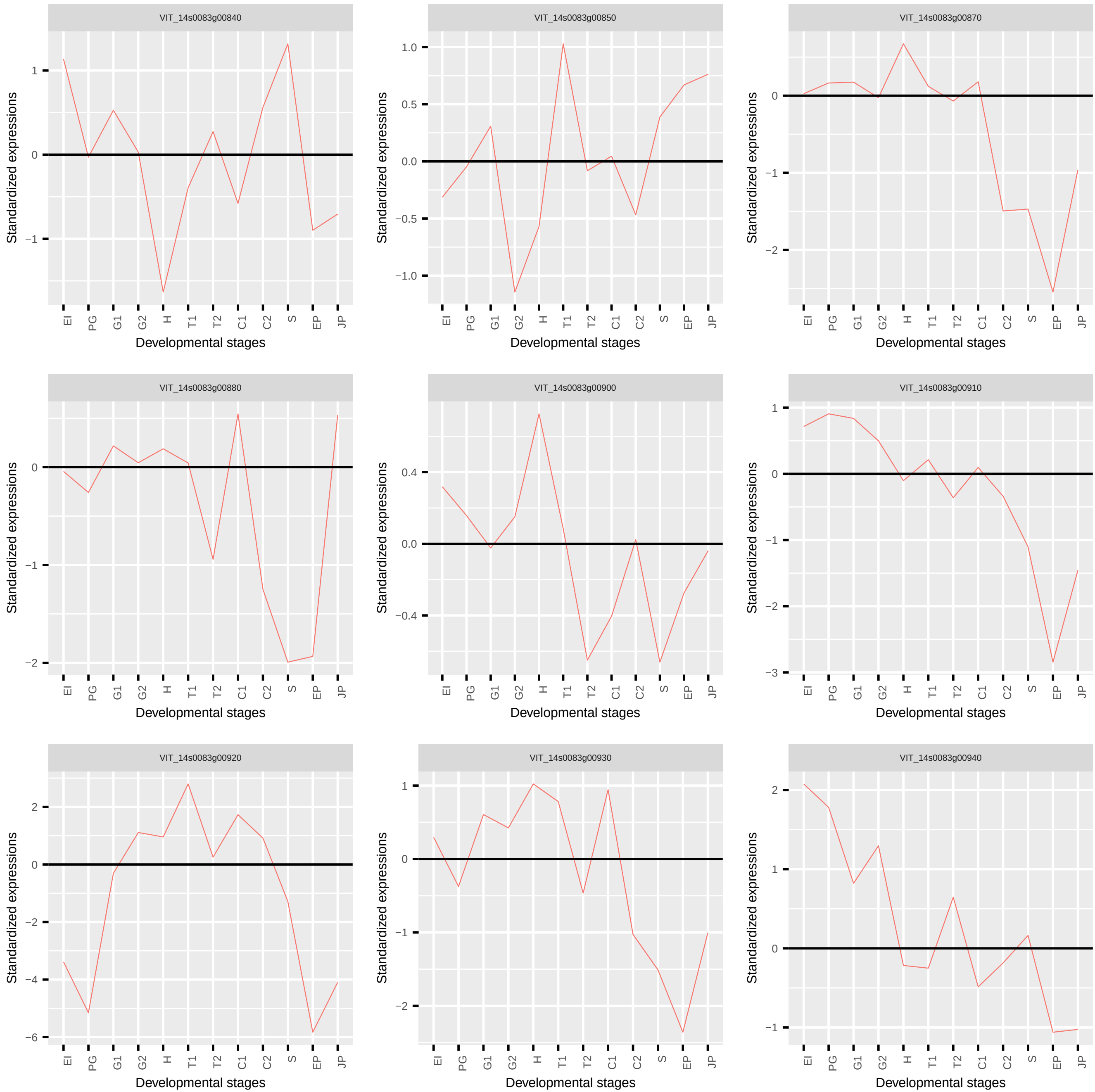
VIT_14s0083g00840
VIT_14s0083g00850
VIT_14s0083g00870
VIT_14s0083g00880
VIT_14s0083g00900
VIT_14s0083g00910
VIT_14s0083g00920
VIT_14s0083g00930
VIT_14s0083g00940
Standardized expressions
Developmental stages
EI PG G1 G2 H T1 T2 C1 C2 S EP JP

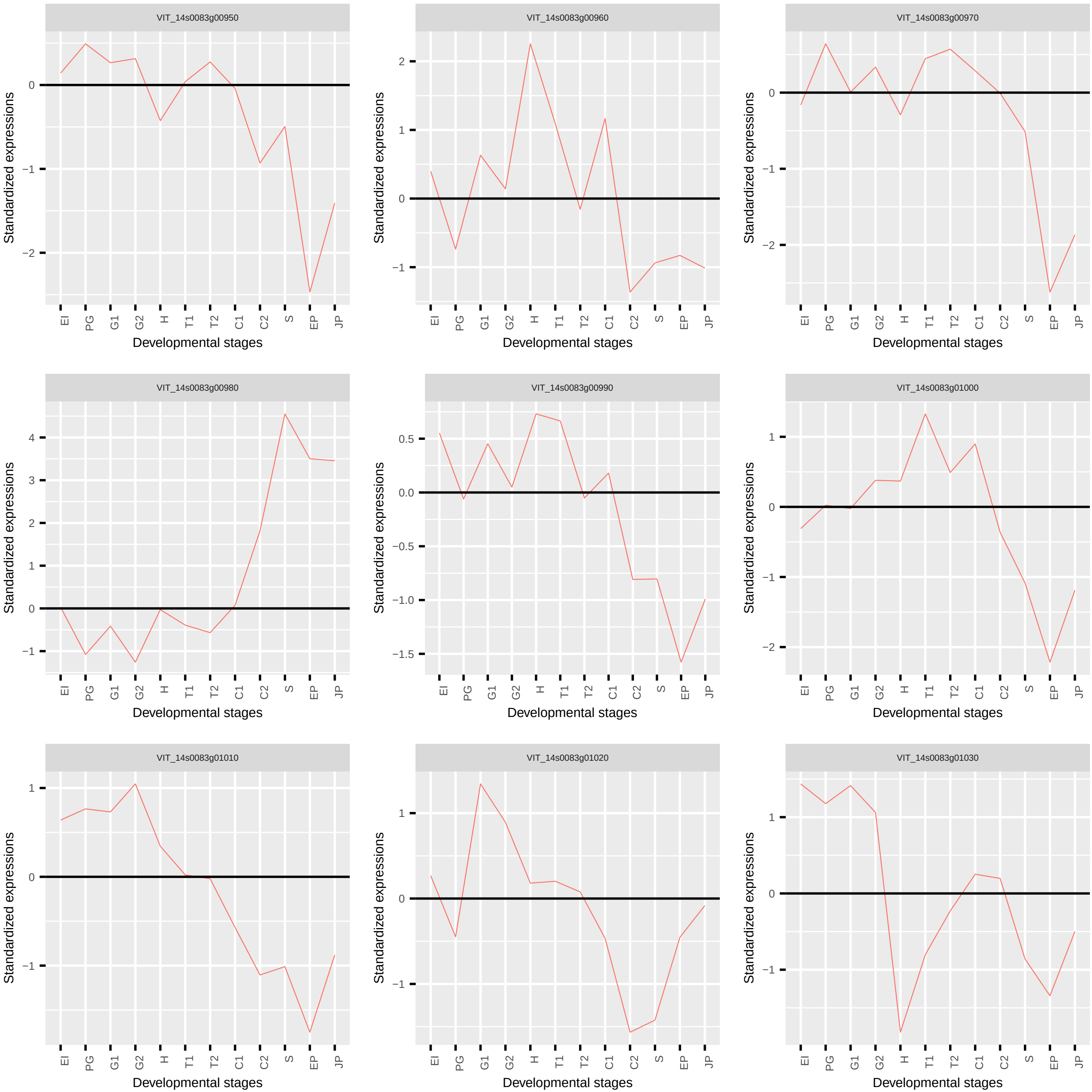
VIT_14s0083g00950
VIT_14s0083g00960
VIT_14s0083g00970
VIT_14s0083g00980
VIT_14s0083g00990
VIT_14s0083g01000
VIT_14s0083g01010
VIT_14s0083g01020
VIT_14s0083g01030
Standardized expressions
Developmental stages
EI PG G1 G2 H T1 T2 C1 C2 S EP JP

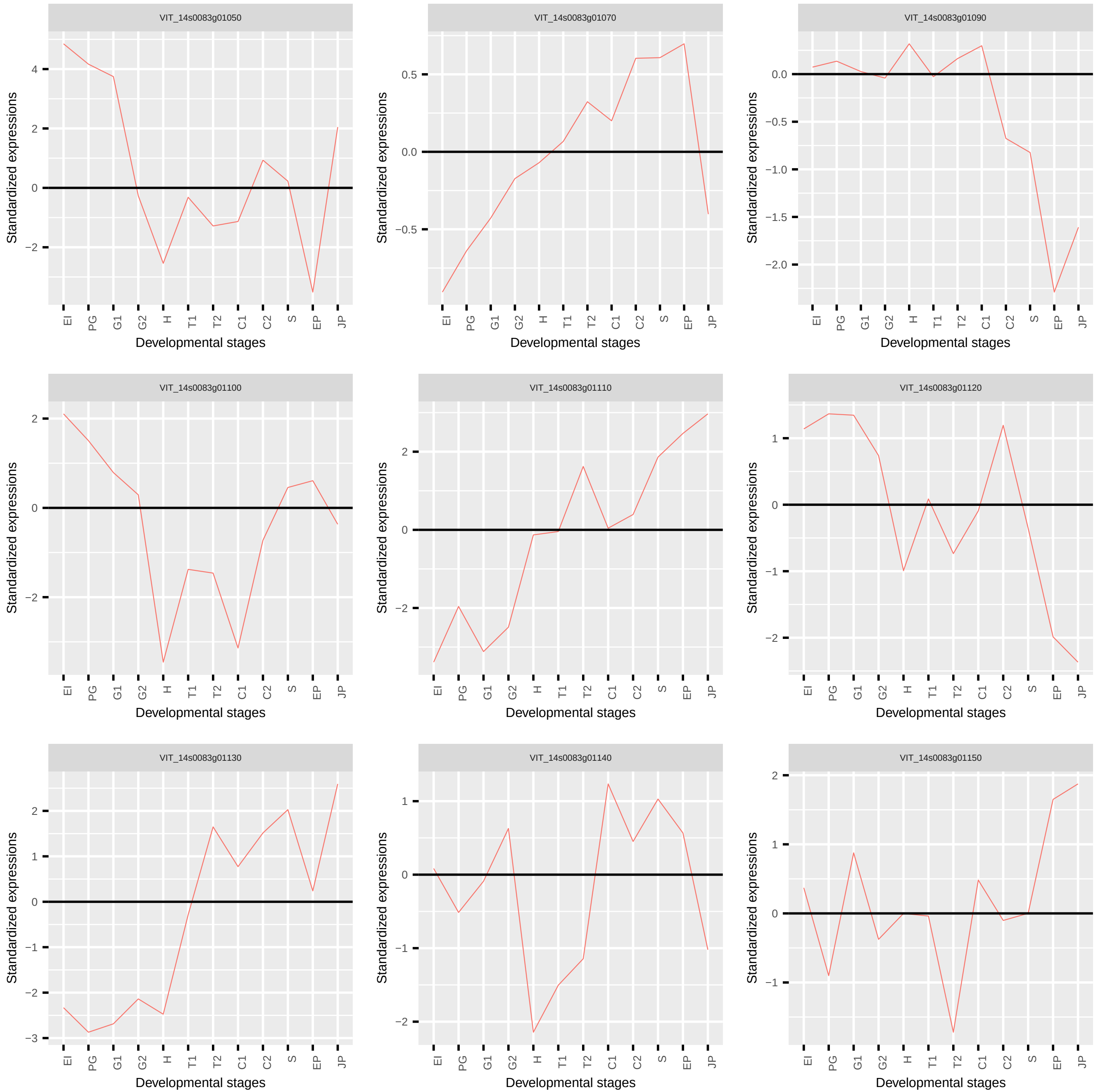

VIT_14s0083g01050
VIT_14s0083g01070
VIT_14s0083g01090
VIT_14s0083g01100
VIT_14s0083g01110
VIT_14s0083g01120
VIT_14s0083g01130
VIT_14s0083g01140
VIT_14s0083g01150
Standardized expressions
Developmental stages
EI PG G1 G2 H T1 T2 C1 C2 S EP JP

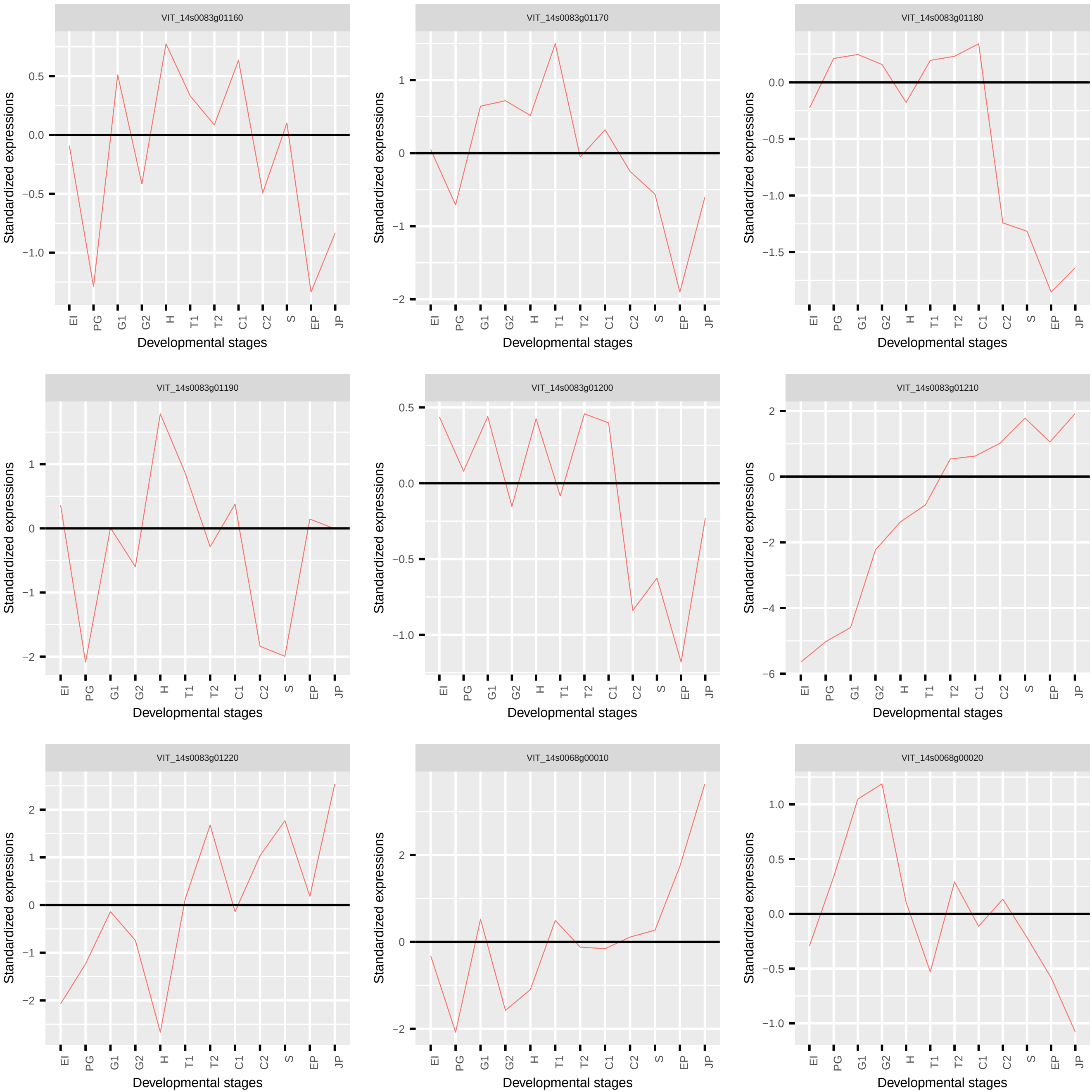
VIT_14s0083g01160
VIT_14s0083g01170
VIT_14s0083g01180
VIT_14s0083g01190
VIT_14s0083g01200
VIT_14s0083g01210
VIT_14s0083g01220
VIT_14s0068g00010
VIT_14s0068g00020
Standardized expressions
Developmental stages
EI PG G1 G2 H T1 T2 C1 C2 S EP JP

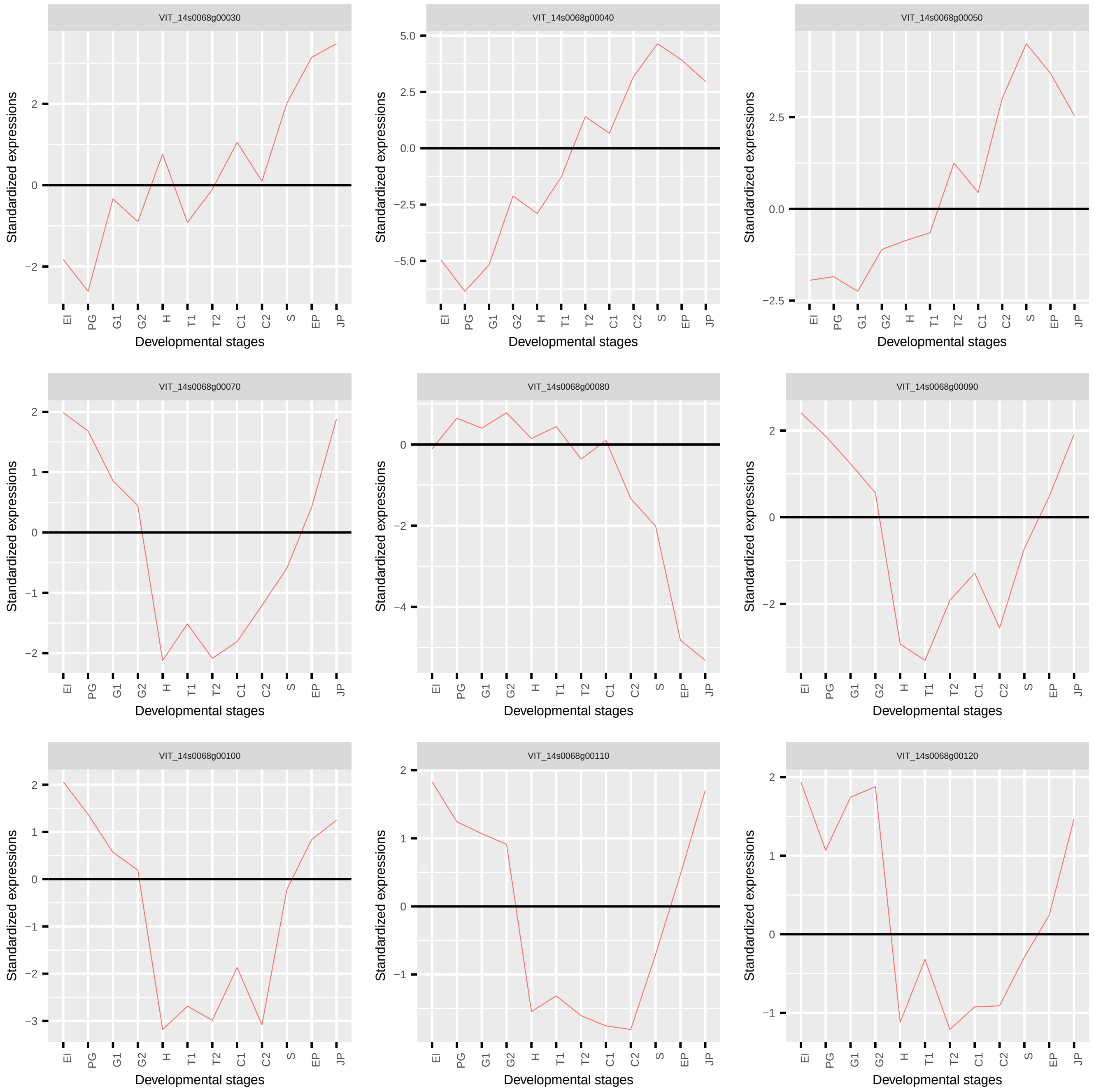

VIT_14s0068g00030
VIT_14s0068g00040
VIT_14s0068g00050
VIT_14s0068g00070
VIT_14s0068g00080
VIT_14s0068g00090
VIT_14s0068g00100
VIT_14s0068g00110
VIT_14s0068g00120
Standardized expressions
Developmental stages
EI
PG
G1
G2
H
T1
T2
C1
C2
S
EP
JP

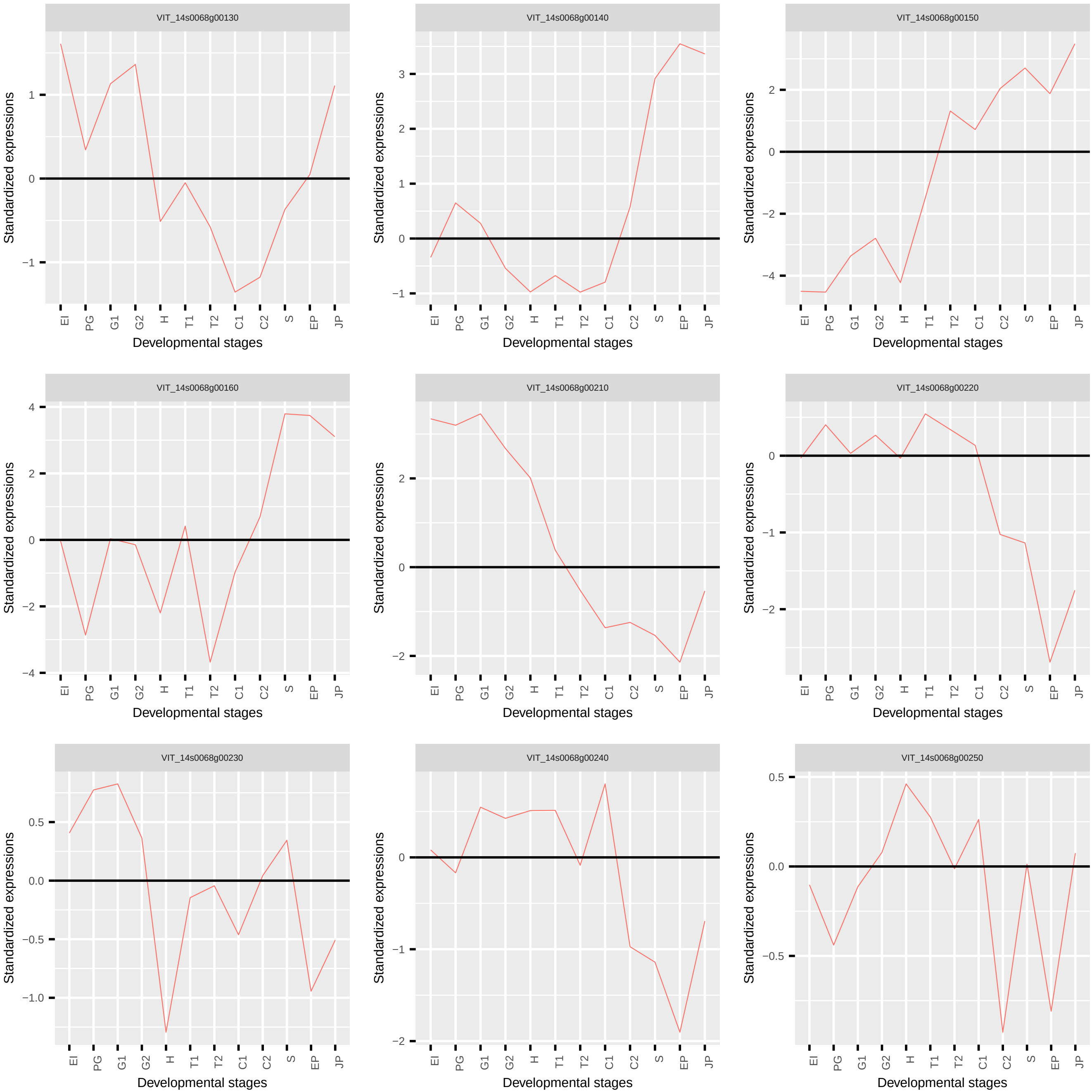
VIT_14s0068g00130
VIT_14s0068g00140
VIT_14s0068g00150
VIT_14s0068g00160
VIT_14s0068g00210
VIT_14s0068g00220
VIT_14s0068g00230
VIT_14s0068g00240
VIT_14s0068g00250
Standardized expressions
Developmental stages
EI
PG
G1
G2
H
T1
T2
C1
C2
S
EP
JP

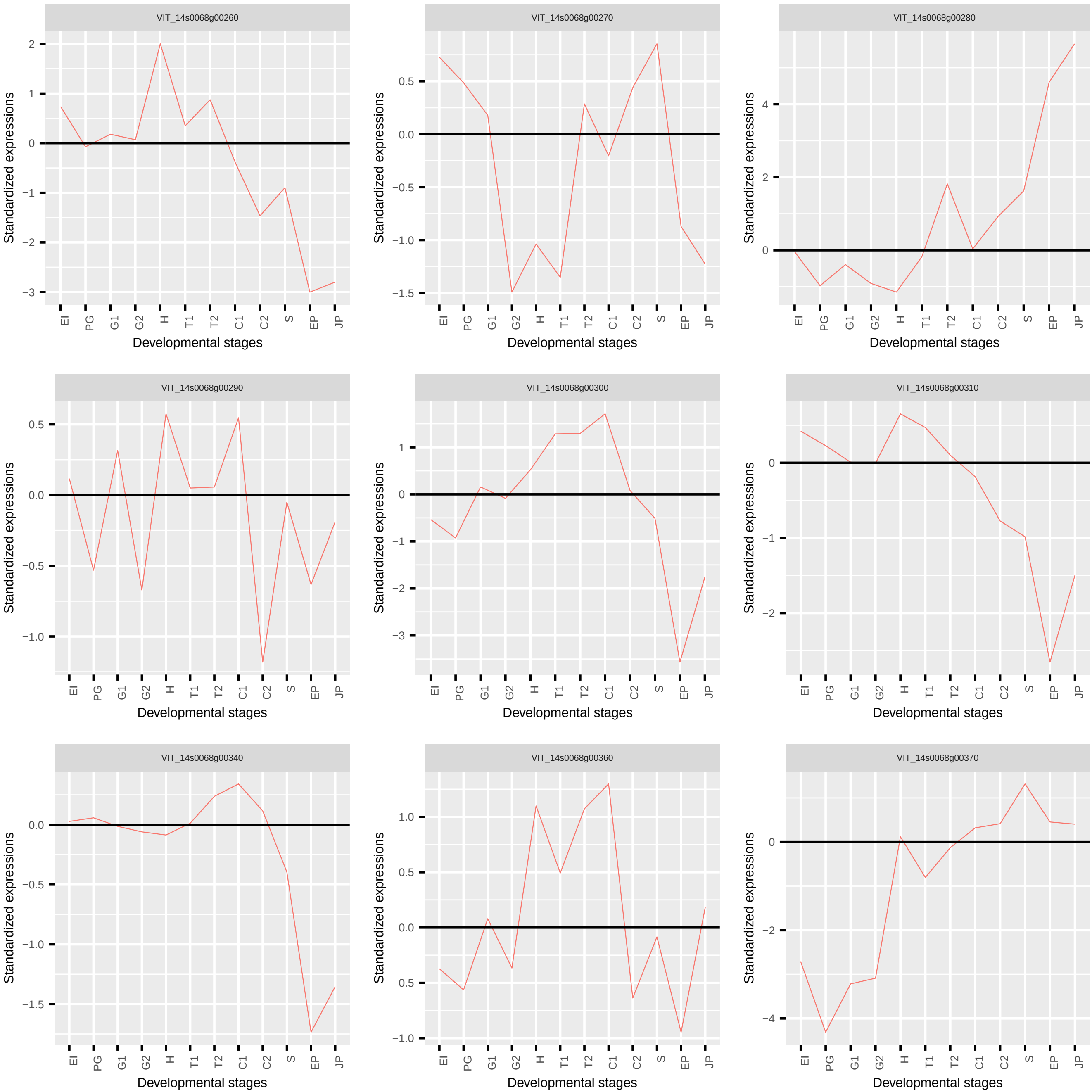
VIT_14s0068g00260
2
1
0
−1
−2
−3
Standardized expressions
EI PG G1 G2 H T1 T2 C1 C2 S EP JP
Developmental stages
VIT_14s0068g00270
0.5
0.0
−0.5
−1.0
−1.5
Standardized expressions
EI PG G1 G2 H T1 T2 C1 C2 S EP JP
Developmental stages
VIT_14s0068g00280
4
2
0
Standardized expressions
EI PG G1 G2 H T1 T2 C1 C2 S EP JP
Developmental stages
VIT_14s0068g00290
0.5
0.0
−0.5
−1.0
Standardized expressions
EI PG G1 G2 H T1 T2 C1 C2 S EP JP
Developmental stages
VIT_14s0068g00300
1
0
−1
−2
−3
Standardized expressions
EI PG G1 G2 H T1 T2 C1 C2 S EP JP
Developmental stages
VIT_14s0068g00310
0
−1
−2
Standardized expressions
EI PG G1 G2 H T1 T2 C1 C2 S EP JP
Developmental stages
VIT_14s0068g00340
0.0
−0.5
−1.0
−1.5
Standardized expressions
EI PG G1 G2 H T1 T2 C1 C2 S EP JP
Developmental stages
VIT_14s0068g00360
1.0
0.5
0.0
−0.5
−1.0
Standardized expressions
EI PG G1 G2 H T1 T2 C1 C2 S EP JP
Developmental stages
VIT_14s0068g00370
0
−2
−4
Standardized expressions
EI PG G1 G2 H T1 T2 C1 C2 S EP JP
Developmental stages

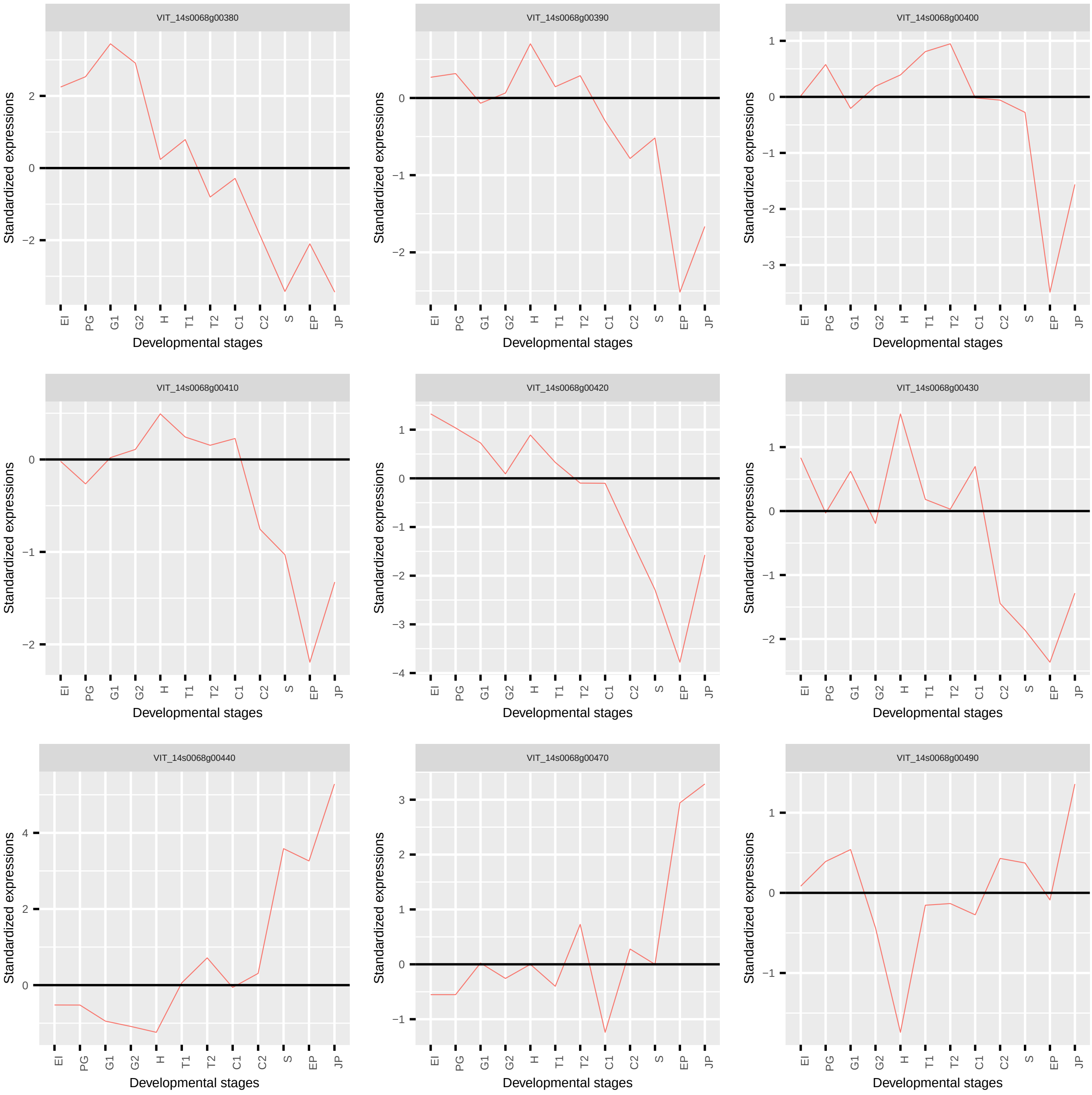
VIT_14s0068g00380
VIT_14s0068g00390
VIT_14s0068g00400
VIT_14s0068g00410
VIT_14s0068g00420
VIT_14s0068g00430
VIT_14s0068g00440
VIT_14s0068g00470
VIT_14s0068g00490
Standardized expressions
Developmental stages
EI
PG
G1
G2
H
T1
T2
C1
C2
S
EP
JP

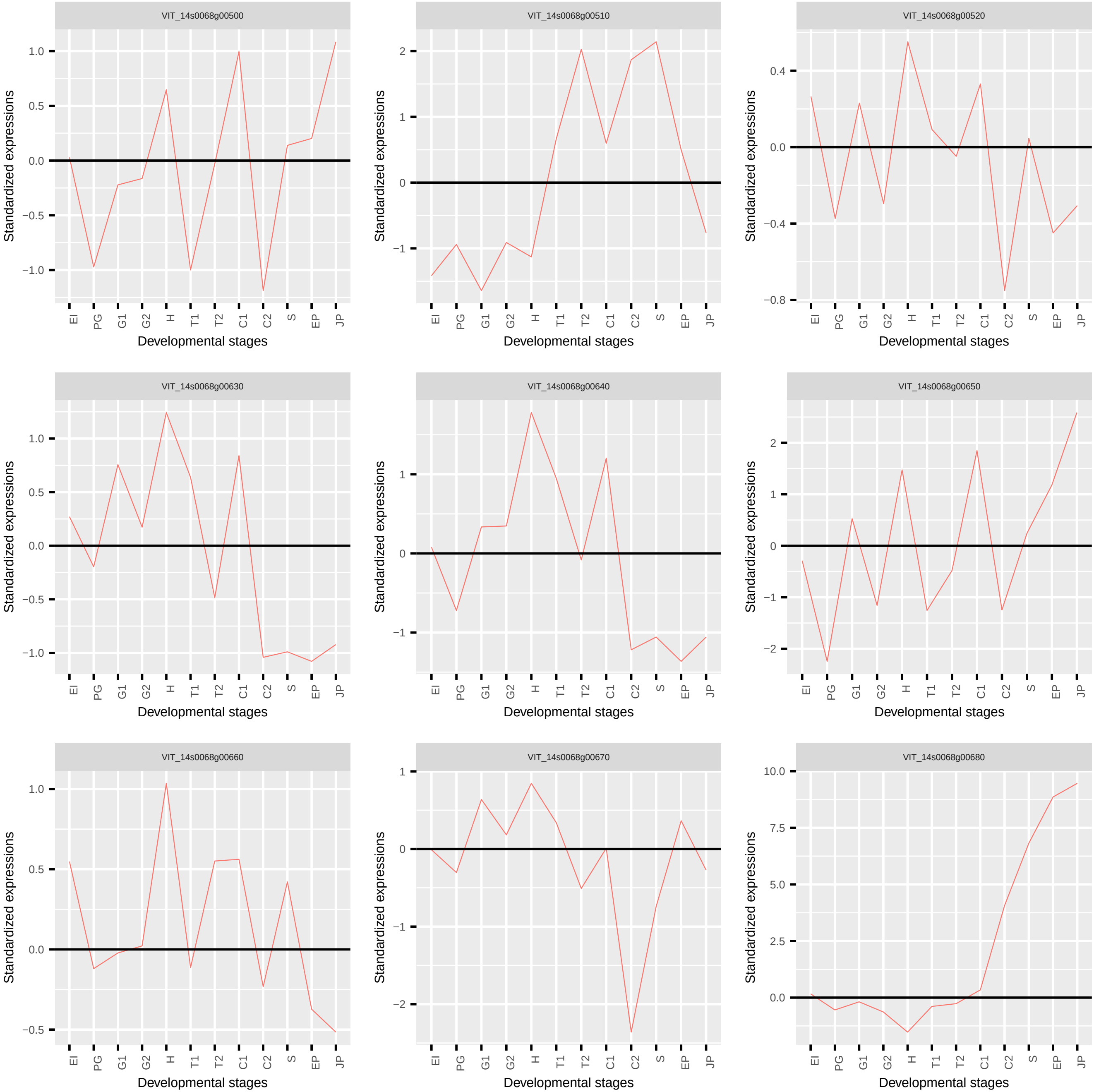

VIT_14s0068g00500
VIT_14s0068g00510
VIT_14s0068g00520
VIT_14s0068g00630
VIT_14s0068g00640
VIT_14s0068g00650
VIT_14s0068g00660
VIT_14s0068g00670
VIT_14s0068g00680
Standardized expressions
Developmental stages
EI PG G1 G2 H T1 T2 C1 C2 S EP JP

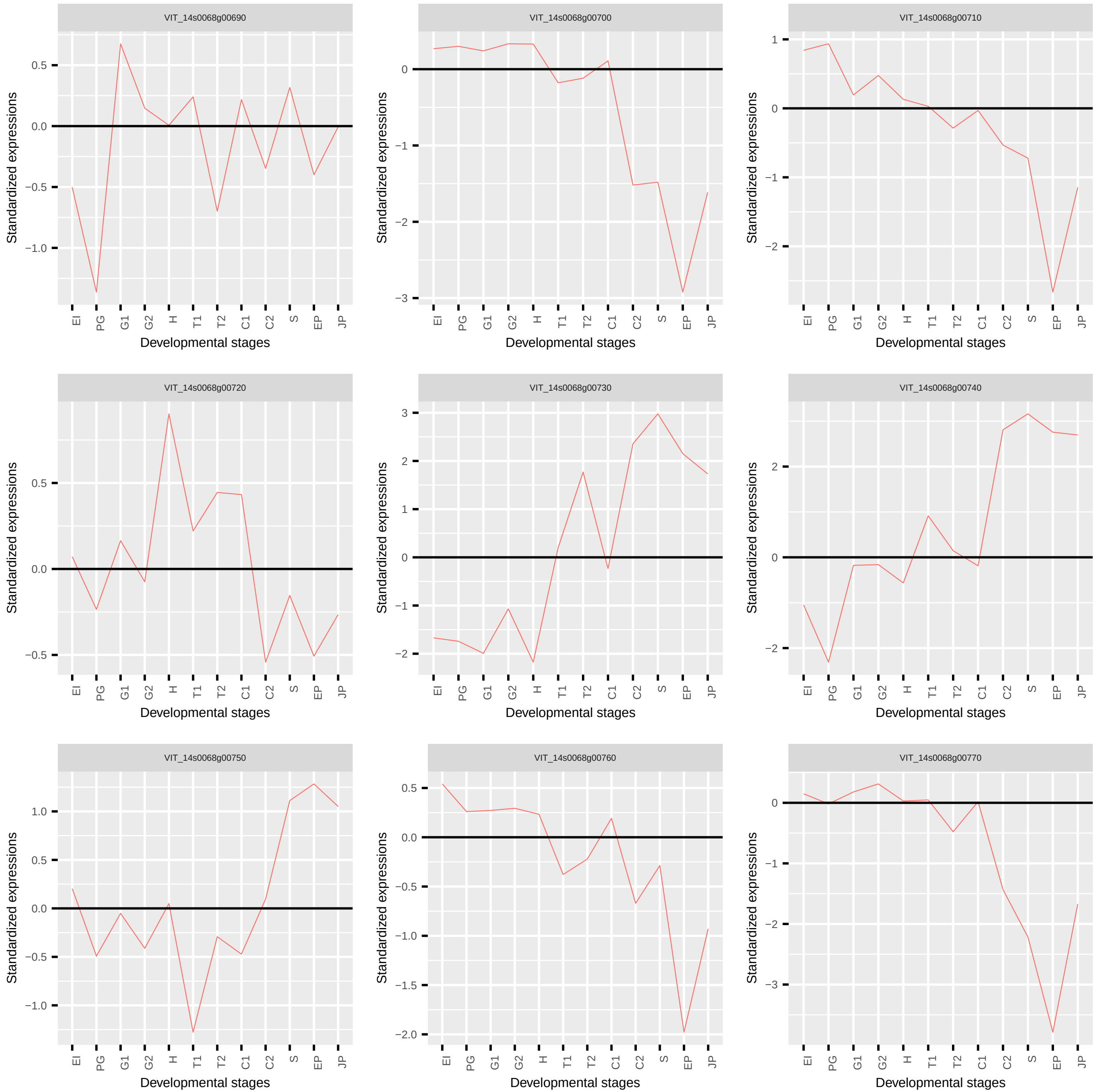
VIT_14s0068g00690
VIT_14s0068g00700
VIT_14s0068g00710
VIT_14s0068g00720
VIT_14s0068g00730
VIT_14s0068g00740
VIT_14s0068g00750
VIT_14s0068g00760
VIT_14s0068g00770
Standardized expressions
Developmental stages
EI
PG
G1
G2
H
T1
T2
C1
C2
S
EP
JP

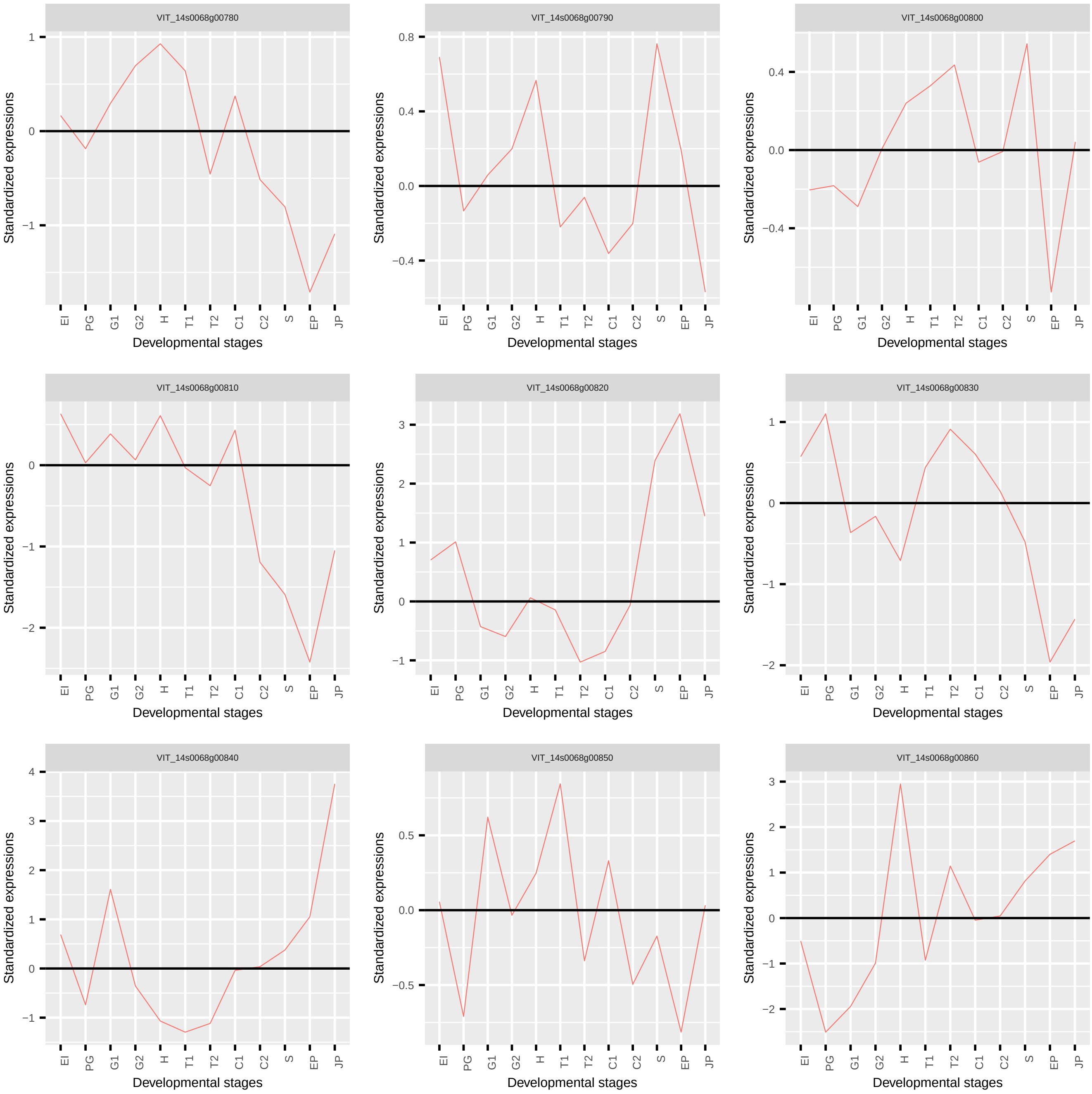
VIT_14s0068g00780
VIT_14s0068g00790
VIT_14s0068g00800
VIT_14s0068g00810
VIT_14s0068g00820
VIT_14s0068g00830
VIT_14s0068g00840
VIT_14s0068g00850
VIT_14s0068g00860
Standardized expressions
Developmental stages
EI
PG
G1
G2
H
T1
T2
C1
C2
S
EP
JP

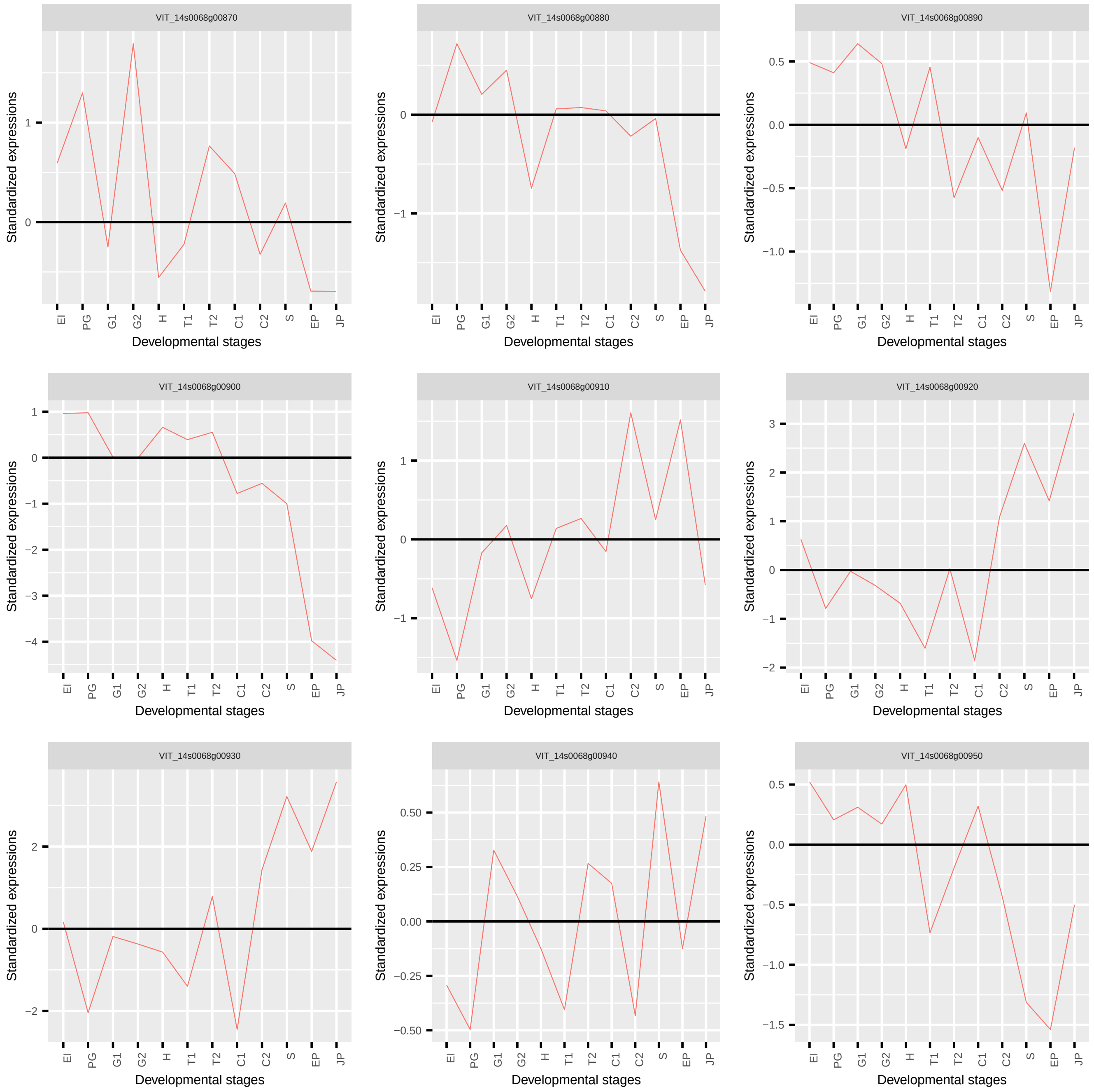
VIT_14s0068g00870
VIT_14s0068g00880
VIT_14s0068g00890
VIT_14s0068g00900
VIT_14s0068g00910
VIT_14s0068g00920
VIT_14s0068g00930
VIT_14s0068g00940
VIT_14s0068g00950
Standardized expressions
Developmental stages
EI PG G1 G2 H T1 T2 C1 C2 S EP JP

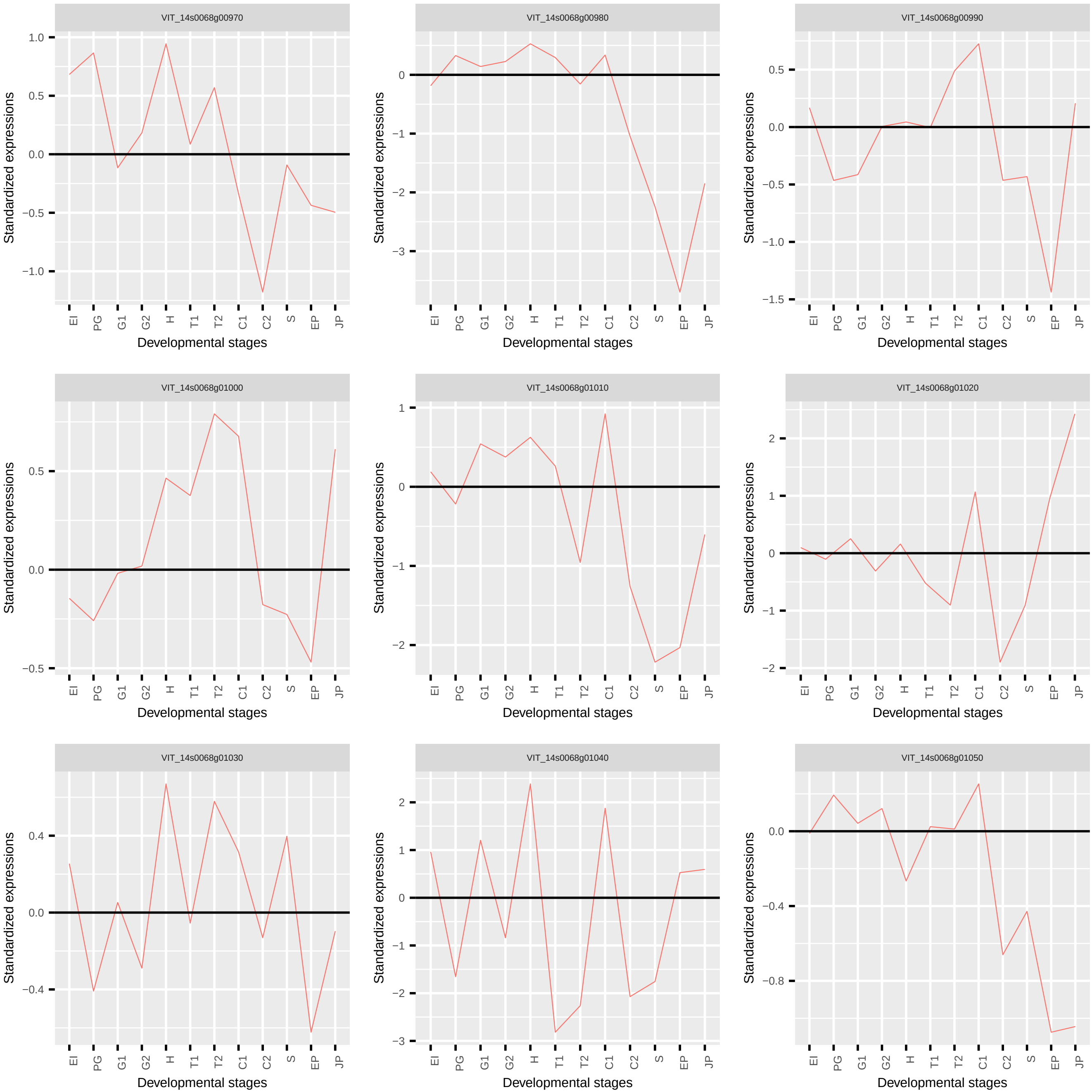
VIT_14s0068g00970
VIT_14s0068g00980
VIT_14s0068g00990
VIT_14s0068g01000
VIT_14s0068g01010
VIT_14s0068g01020
VIT_14s0068g01030
VIT_14s0068g01040
VIT_14s0068g01050
Standardized expressions
Developmental stages
EI PG G1 G2 H T1 T2 C1 C2 S EP JP

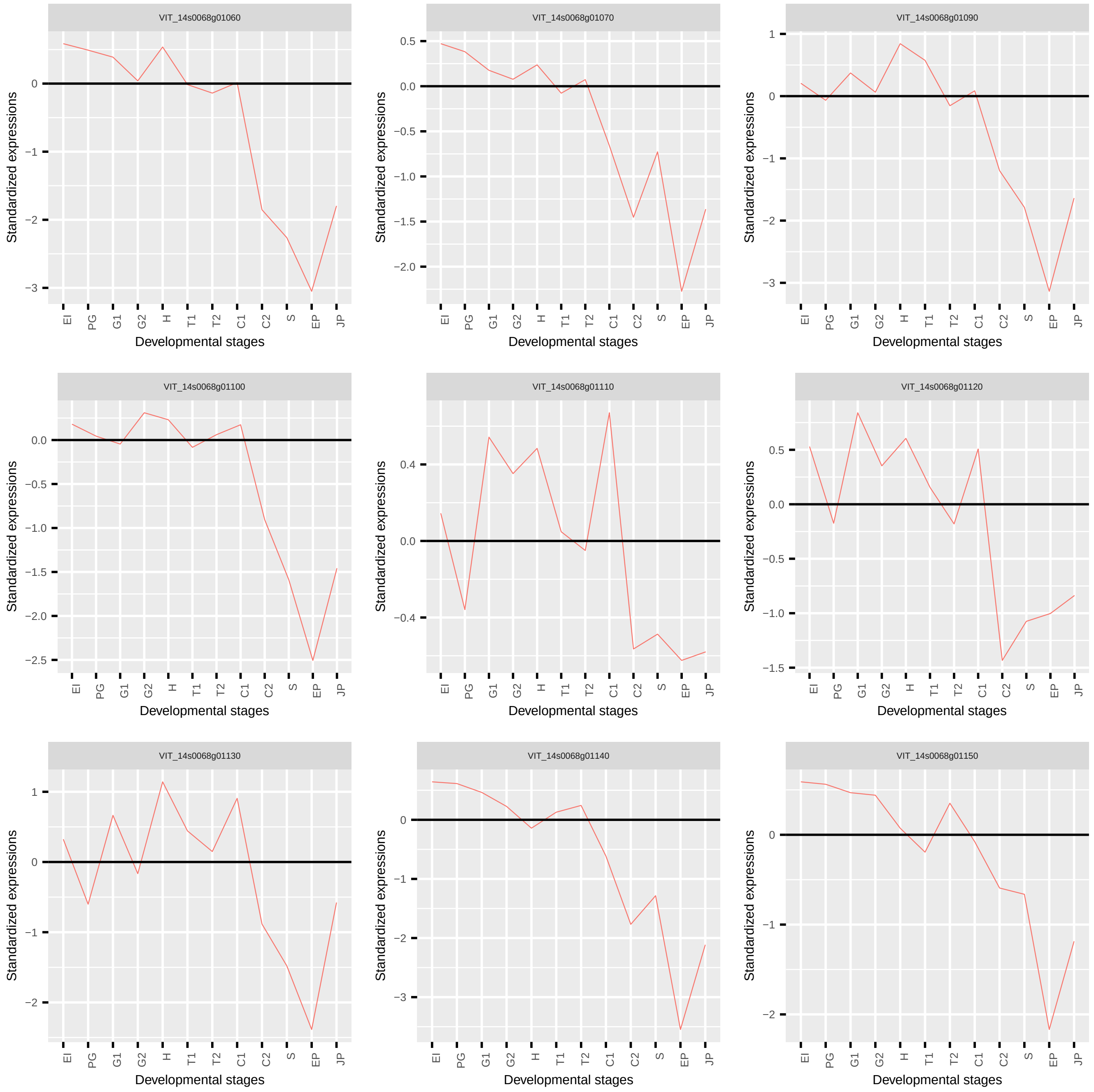
VIT_14s0068g01060
0
−1
−2
−3
Standardized expressions
EI PG G1 G2 H T1 T2 C1 C2 S EP JP
Developmental stages
VIT_14s0068g01070
0.5
0.0
−0.5
−1.0
−1.5
−2.0
Standardized expressions
EI PG G1 G2 H T1 T2 C1 C2 S EP JP
Developmental stages
VIT_14s0068g01090
1
0
−1
−2
−3
Standardized expressions
EI PG G1 G2 H T1 T2 C1 C2 S EP JP
Developmental stages
VIT_14s0068g01100
0.0
−0.5
−1.0
−1.5
−2.0
−2.5
Standardized expressions
EI PG G1 G2 H T1 T2 C1 C2 S EP JP
Developmental stages
VIT_14s0068g01110
0.4
0.0
−0.4
Standardized expressions
EI PG G1 G2 H T1 T2 C1 C2 S EP JP
Developmental stages
VIT_14s0068g01120
0.5
0.0
−0.5
−1.0
−1.5
Standardized expressions
EI PG G1 G2 H T1 T2 C1 C2 S EP JP
Developmental stages
VIT_14s0068g01130
1
0
−1
−2
Standardized expressions
EI PG G1 G2 H T1 T2 C1 C2 S EP JP
Developmental stages
VIT_14s0068g01140
0
−1
−2
−3
Standardized expressions
EI PG G1 G2 H T1 T2 C1 C2 S EP JP
Developmental stages
VIT_14s0068g01150
0
−1
−2
Standardized expressions
EI PG G1 G2 H T1 T2 C1 C2 S EP JP
Developmental stages

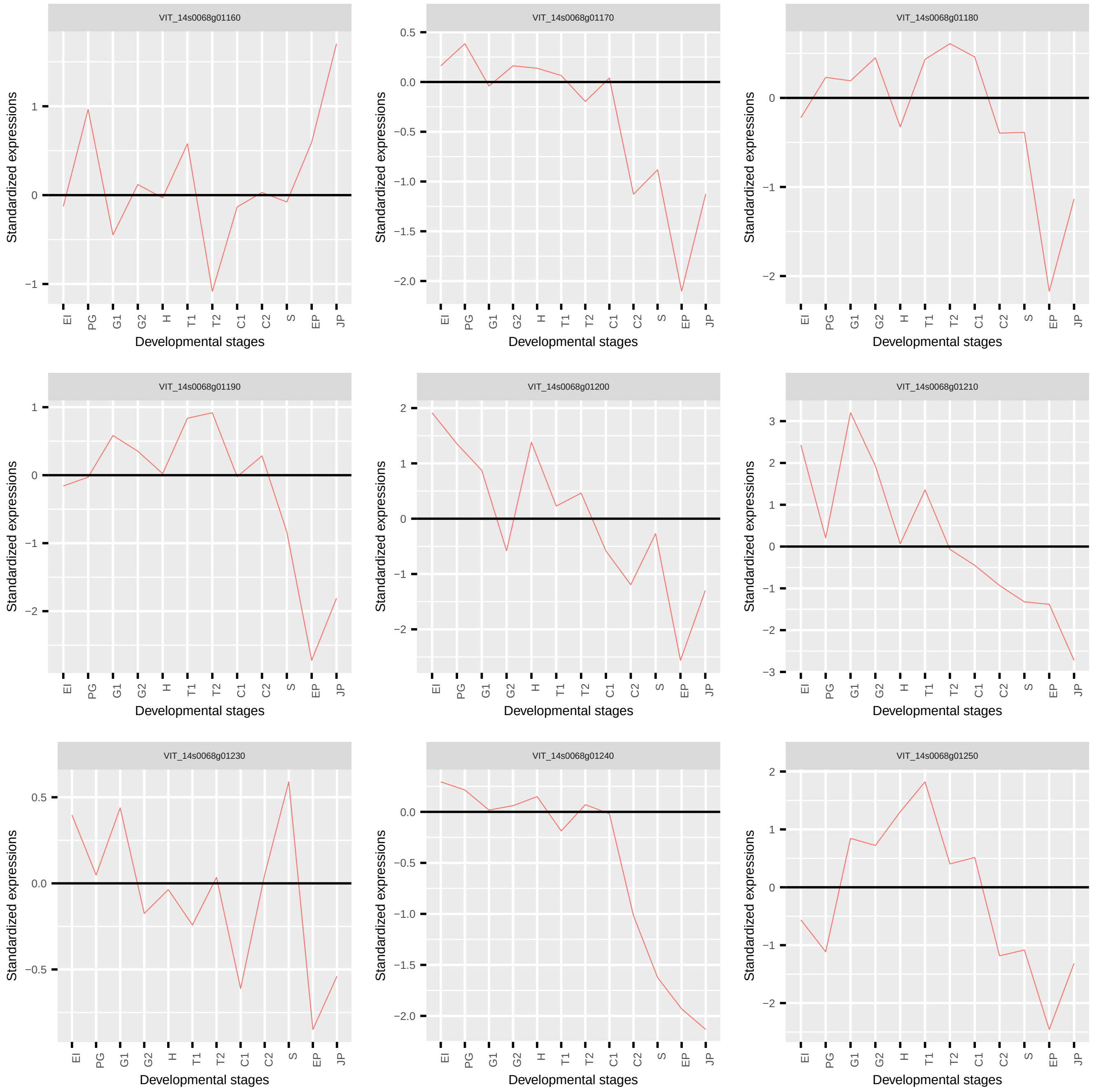
VIT_14s0068g01160
VIT_14s0068g01170
VIT_14s0068g01180
VIT_14s0068g01190
VIT_14s0068g01200
VIT_14s0068g01210
VIT_14s0068g01230
VIT_14s0068g01240
VIT_14s0068g01250
Standardized expressions
Developmental stages
EI PG G1 G2 H T1 T2 C1 C2 S EP JP

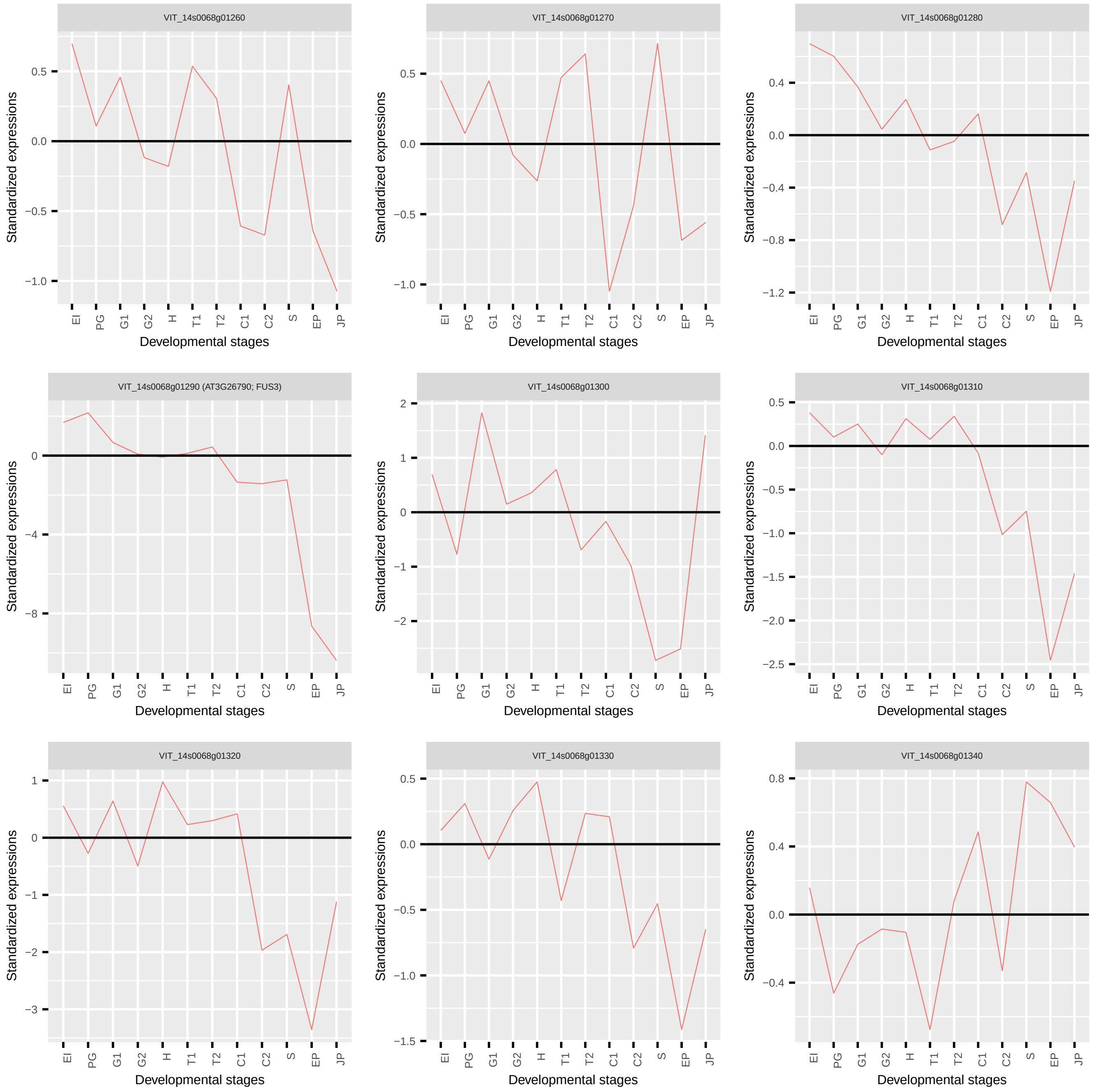
VIT_14s0068g01260
VIT_14s0068g01270
VIT_14s0068g01280
VIT_14s0068g01290 (AT3G26790; FUS3)
VIT_14s0068g01300
VIT_14s0068g01310
VIT_14s0068g01320
VIT_14s0068g01330
VIT_14s0068g01340
Standardized expressions
Developmental stages
EI PG G1 G2 H T1 T2 C1 C2 S EP JP

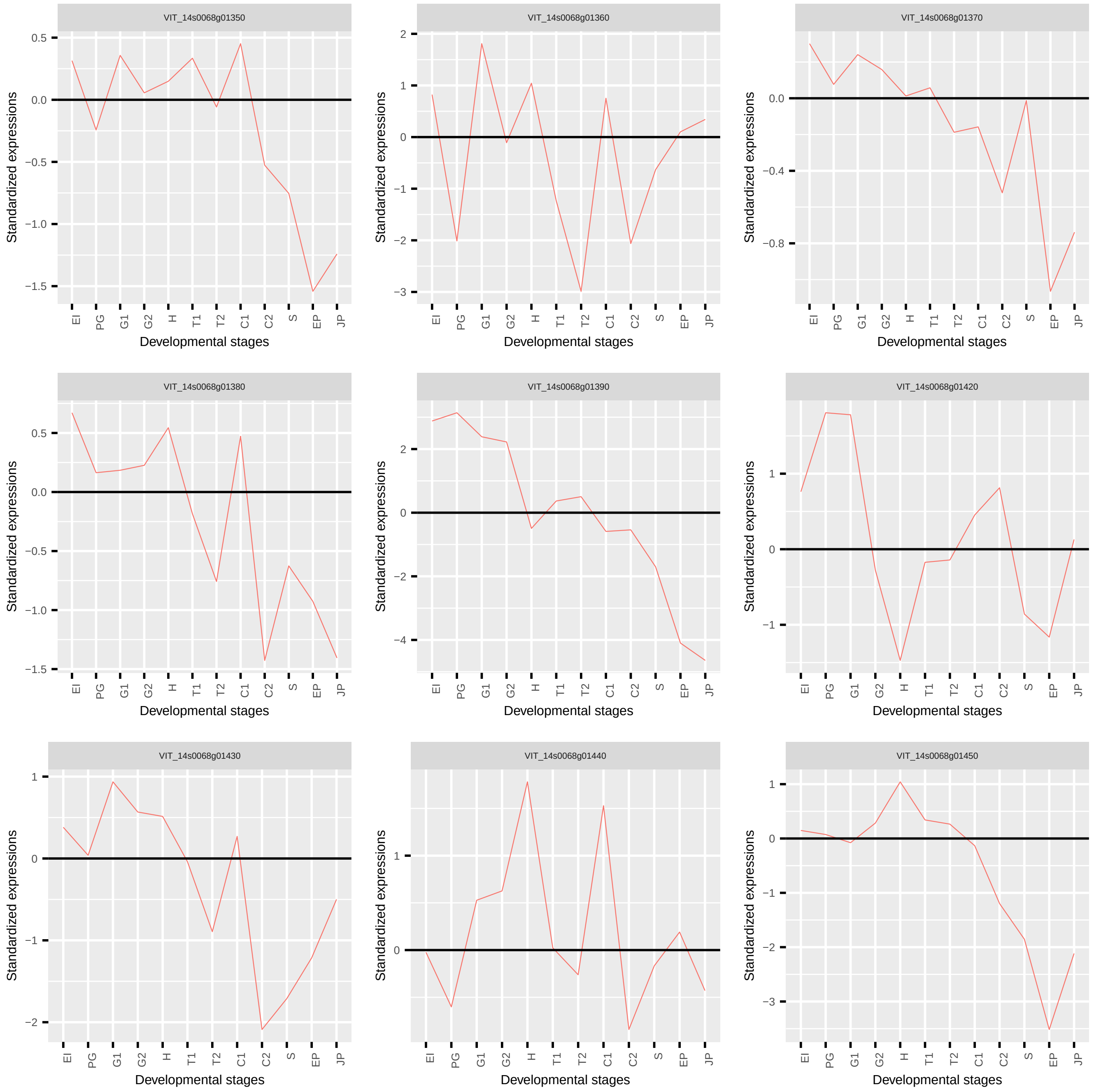
VIT_14s0068g01350
VIT_14s0068g01360
VIT_14s0068g01370
VIT_14s0068g01380
VIT_14s0068g01390
VIT_14s0068g01420
VIT_14s0068g01430
VIT_14s0068g01440
VIT_14s0068g01450
Standardized expressions
Developmental stages
EI PG G1 G2 H T1 T2 C1 C2 S EP JP

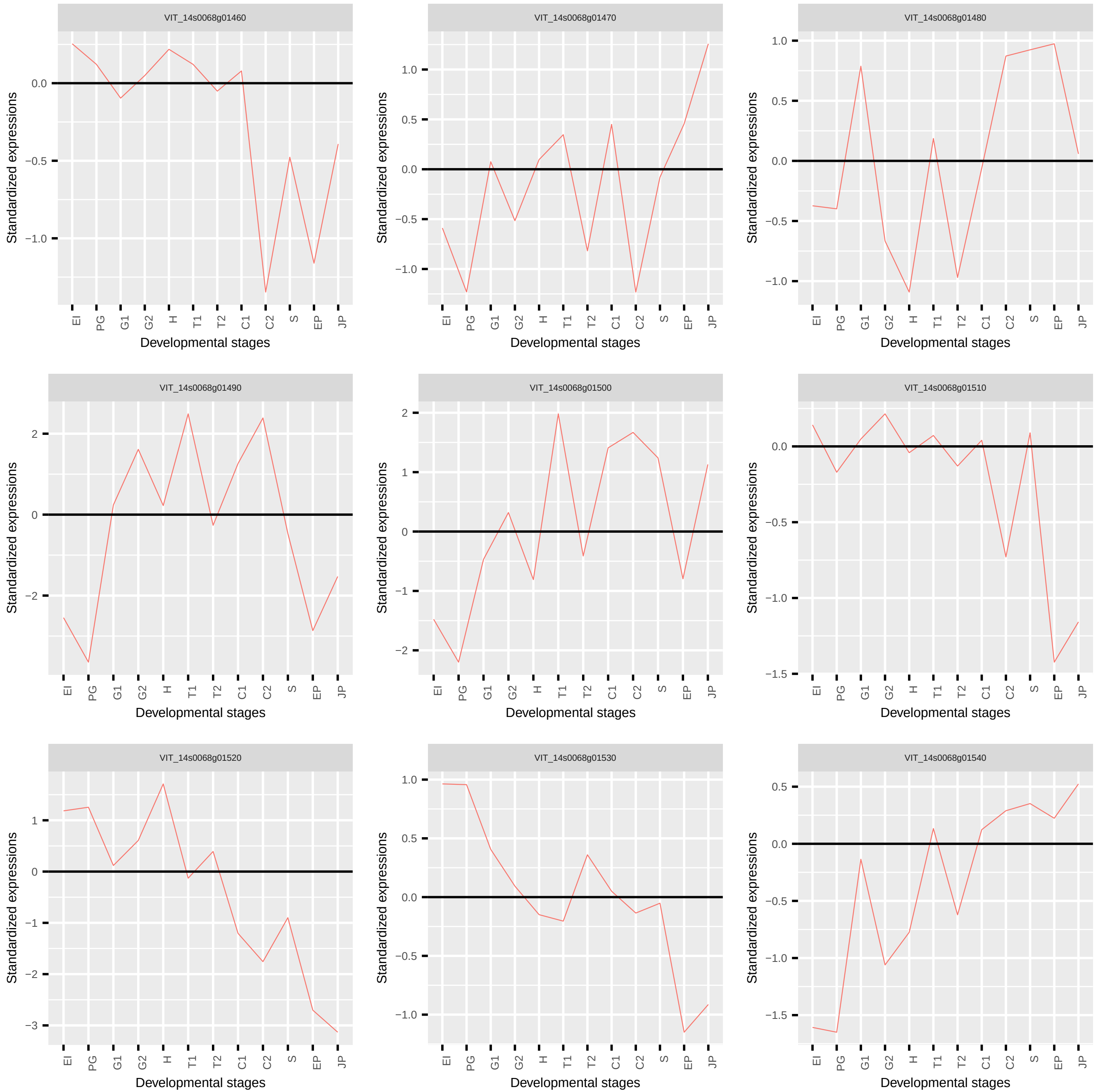
VIT_14s0068g01460
VIT_14s0068g01470
VIT_14s0068g01480
VIT_14s0068g01490
VIT_14s0068g01500
VIT_14s0068g01510
VIT_14s0068g01520
VIT_14s0068g01530
VIT_14s0068g01540
Standardized expressions
Developmental stages
EI
PG
G1
G2
H
T1
T2
C1
C2
S
EP
JP

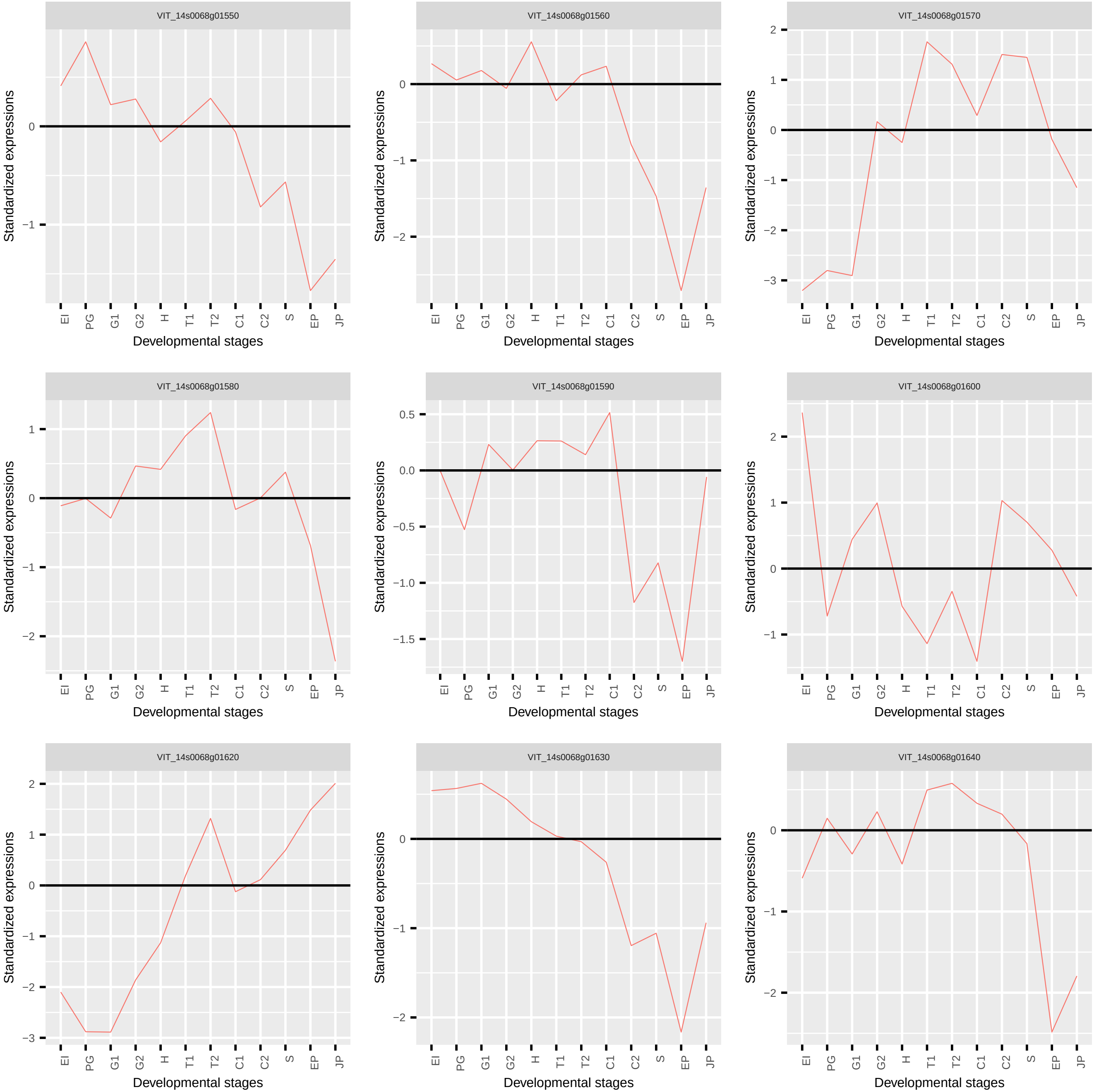

VIT_14s0068g01550
VIT_14s0068g01560
VIT_14s0068g01570
VIT_14s0068g01580
VIT_14s0068g01590
VIT_14s0068g01600
VIT_14s0068g01620
VIT_14s0068g01630
VIT_14s0068g01640
Standardized expressions
Developmental stages
EI PG G1 G2 H T1 T2 C1 C2 S EP JP

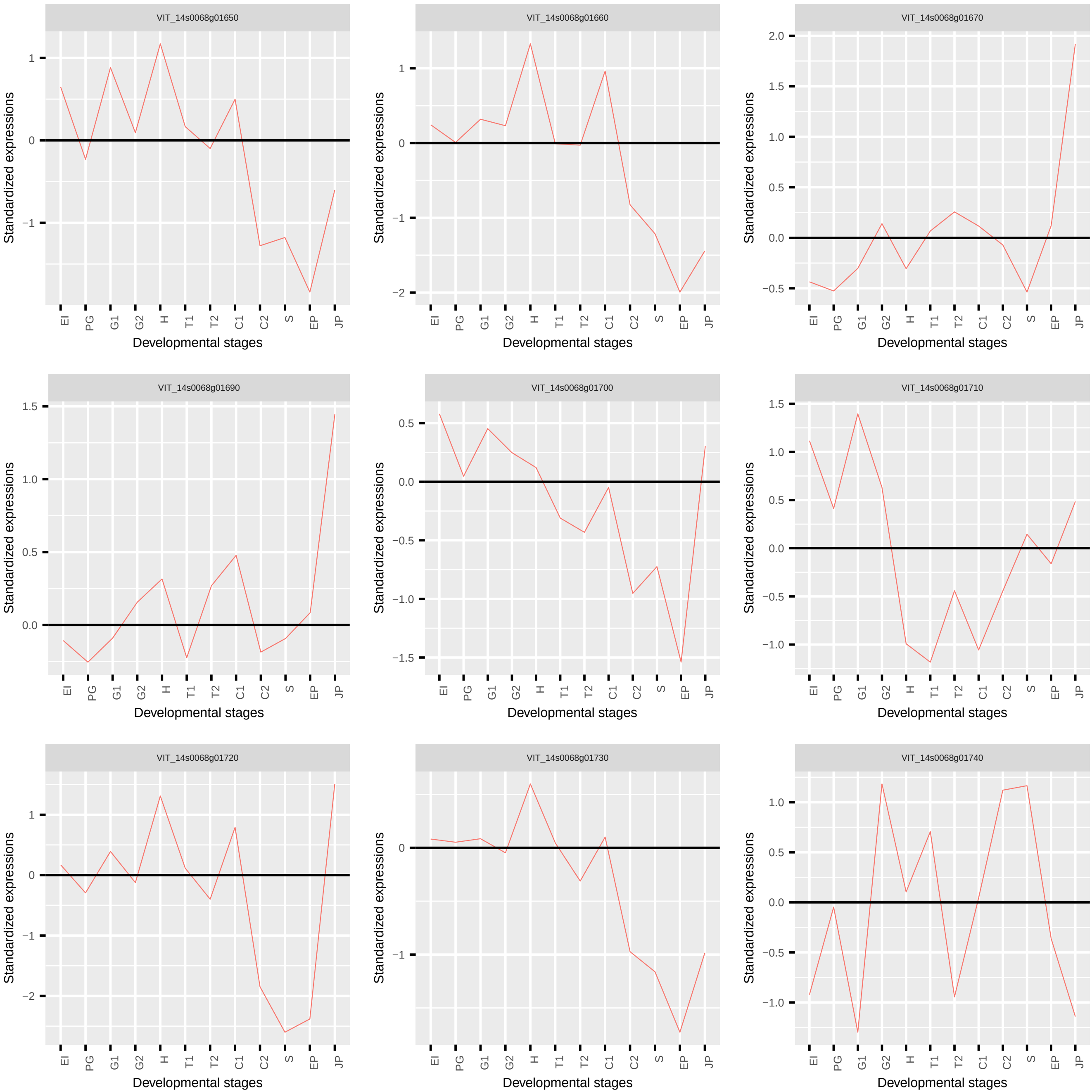
VIT_14s0068g01650
VIT_14s0068g01660
VIT_14s0068g01670
VIT_14s0068g01690
VIT_14s0068g01700
VIT_14s0068g01710
VIT_14s0068g01720
VIT_14s0068g01730
VIT_14s0068g01740
Standardized expressions
Developmental stages
EI PG G1 G2 H T1 T2 C1 C2 S EP JP

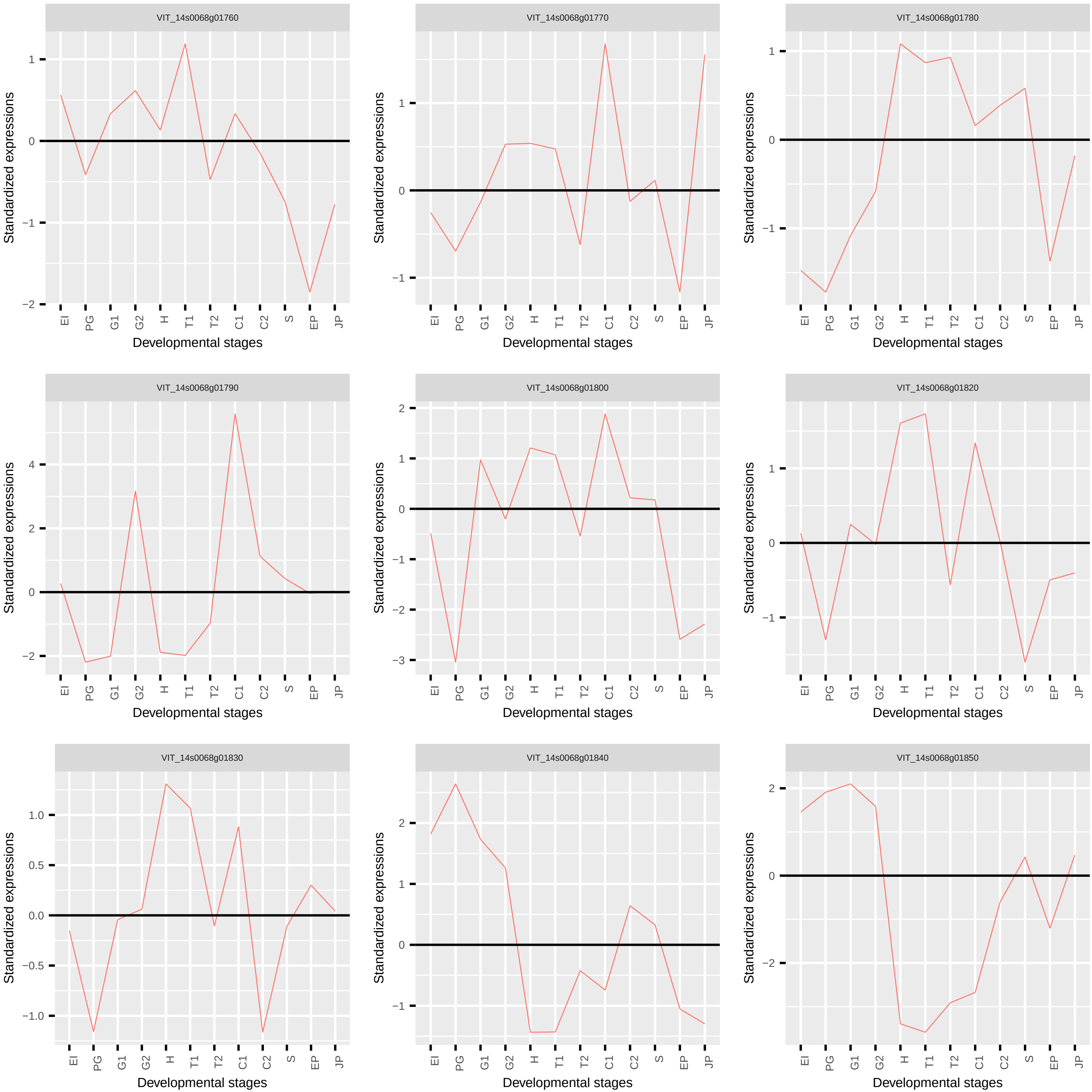
VIT_14s0068g01760
VIT_14s0068g01770
VIT_14s0068g01780
VIT_14s0068g01790
VIT_14s0068g01800
VIT_14s0068g01820
VIT_14s0068g01830
VIT_14s0068g01840
VIT_14s0068g01850
Standardized expressions
Developmental stages
EI PG G1 G2 H T1 T2 C1 C2 S EP JP

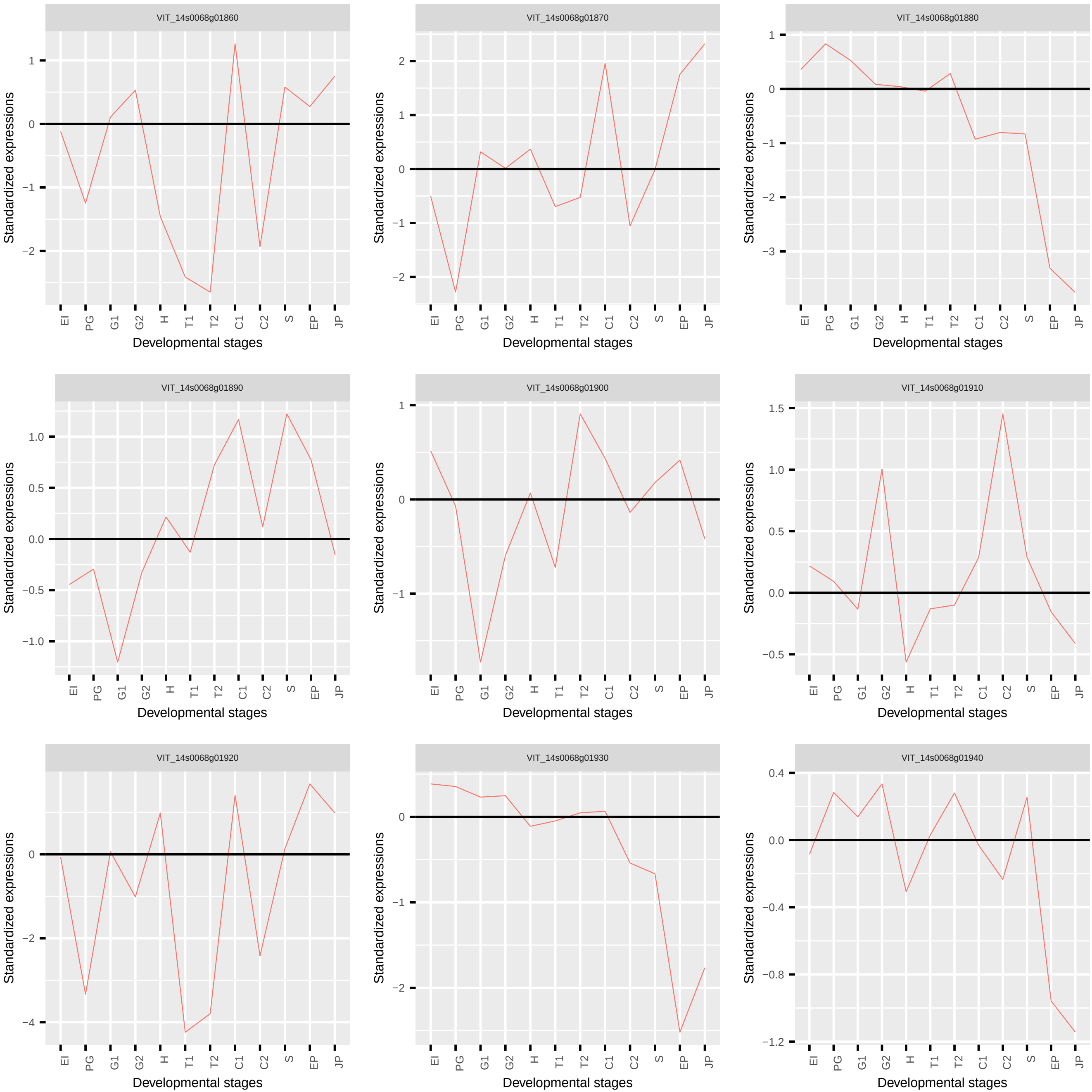
VIT_14s0068g01860
VIT_14s0068g01870
VIT_14s0068g01880
VIT_14s0068g01890
VIT_14s0068g01900
VIT_14s0068g01910
VIT_14s0068g01920
VIT_14s0068g01930
VIT_14s0068g01940
Standardized expressions
Developmental stages
EI PG G1 G2 H T1 T2 C1 C2 S EP JP

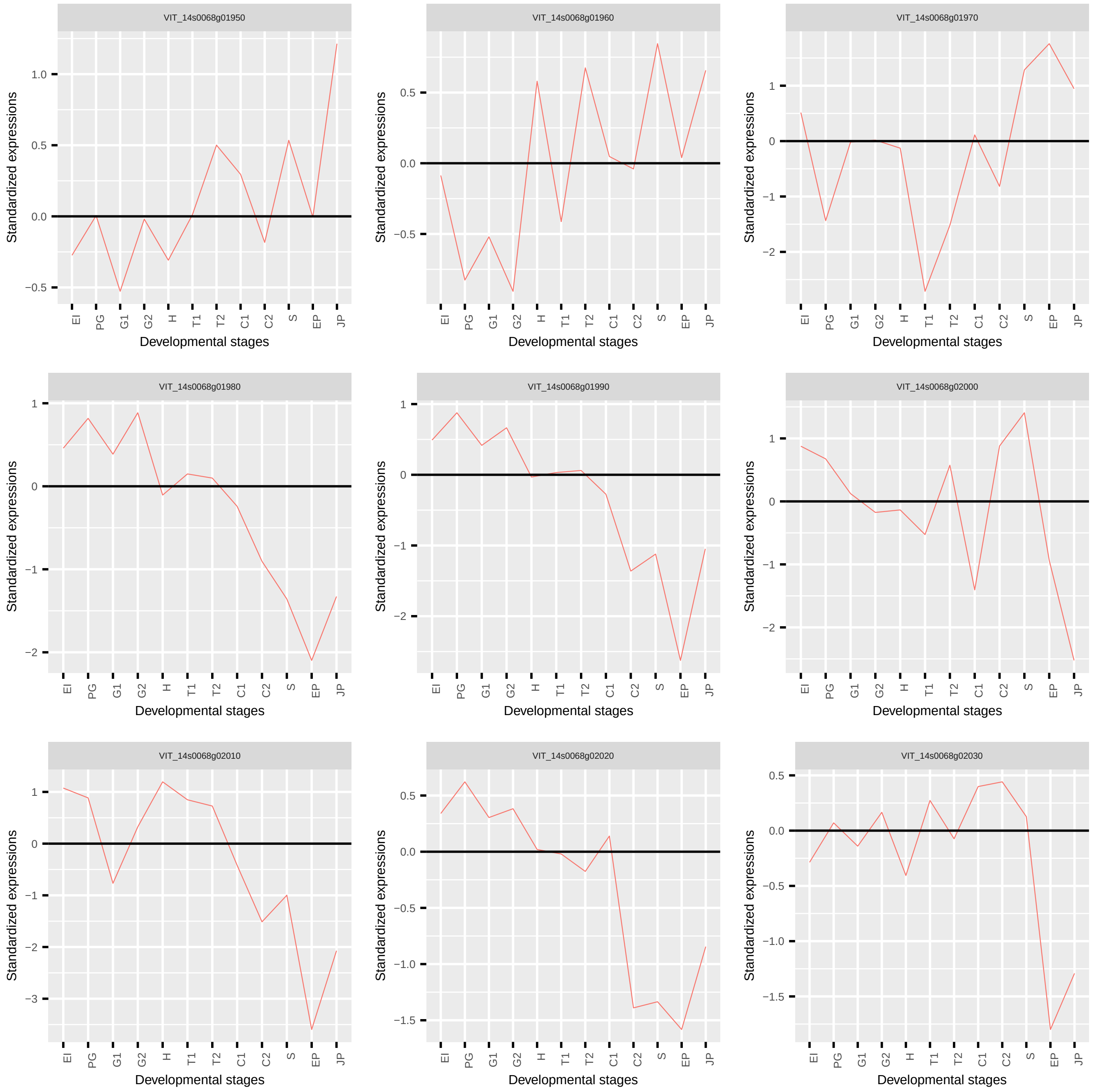

VIT_14s0068g01950
VIT_14s0068g01960
VIT_14s0068g01970
VIT_14s0068g01980
VIT_14s0068g01990
VIT_14s0068g02000
VIT_14s0068g02010
VIT_14s0068g02020
VIT_14s0068g02030
Standardized expressions
Developmental stages
EI PG G1 G2 H T1 T2 C1 C2 S EP JP
−1.5 −1.0 −0.5 0.0 0.5 1.0
−3 −2 −1 0 1

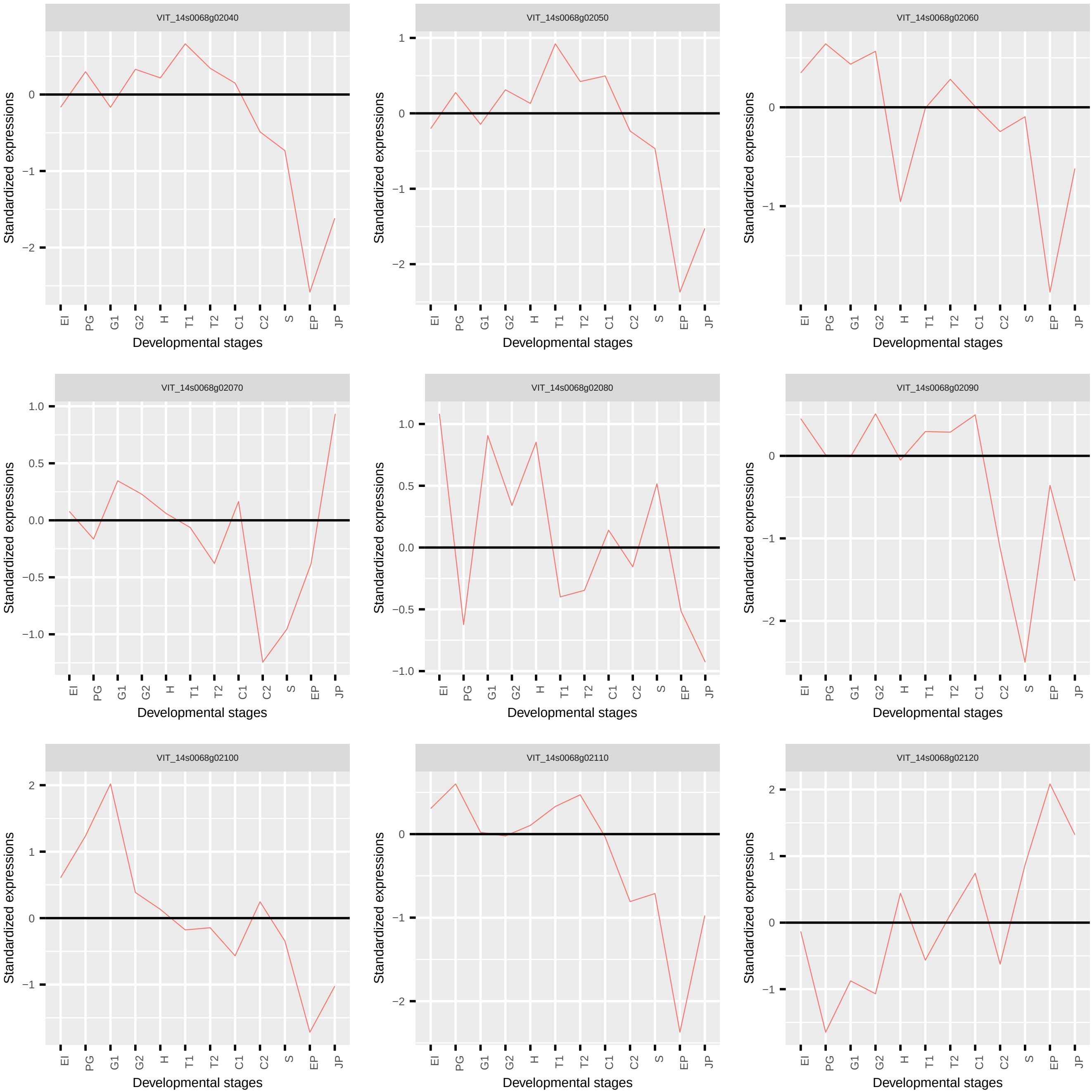

VIT_14s0068g02040
VIT_14s0068g02050
VIT_14s0068g02060
VIT_14s0068g02070
VIT_14s0068g02080
VIT_14s0068g02090
VIT_14s0068g02100
VIT_14s0068g02110
VIT_14s0068g02120
Standardized expressions
Developmental stages
EI PG G1 G2 H T1 T2 C1 C2 S EP JP

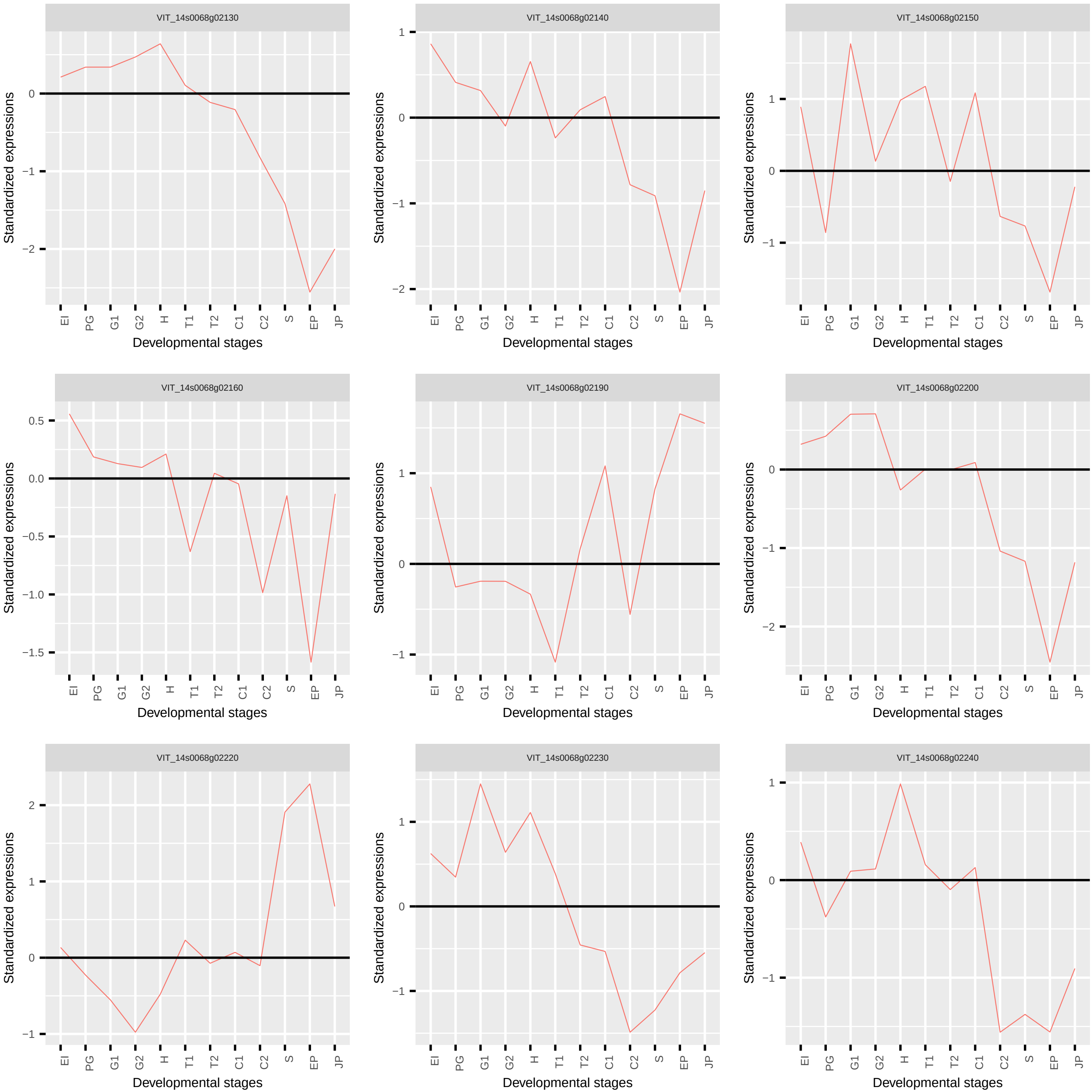
VIT_14s0068g02130
VIT_14s0068g02140
VIT_14s0068g02150
VIT_14s0068g02160
VIT_14s0068g02190
VIT_14s0068g02200
VIT_14s0068g02220
VIT_14s0068g02230
VIT_14s0068g02240
Standardized expressions
Developmental stages
EI PG G1 G2 H T1 T2 C1 C2 S EP JP

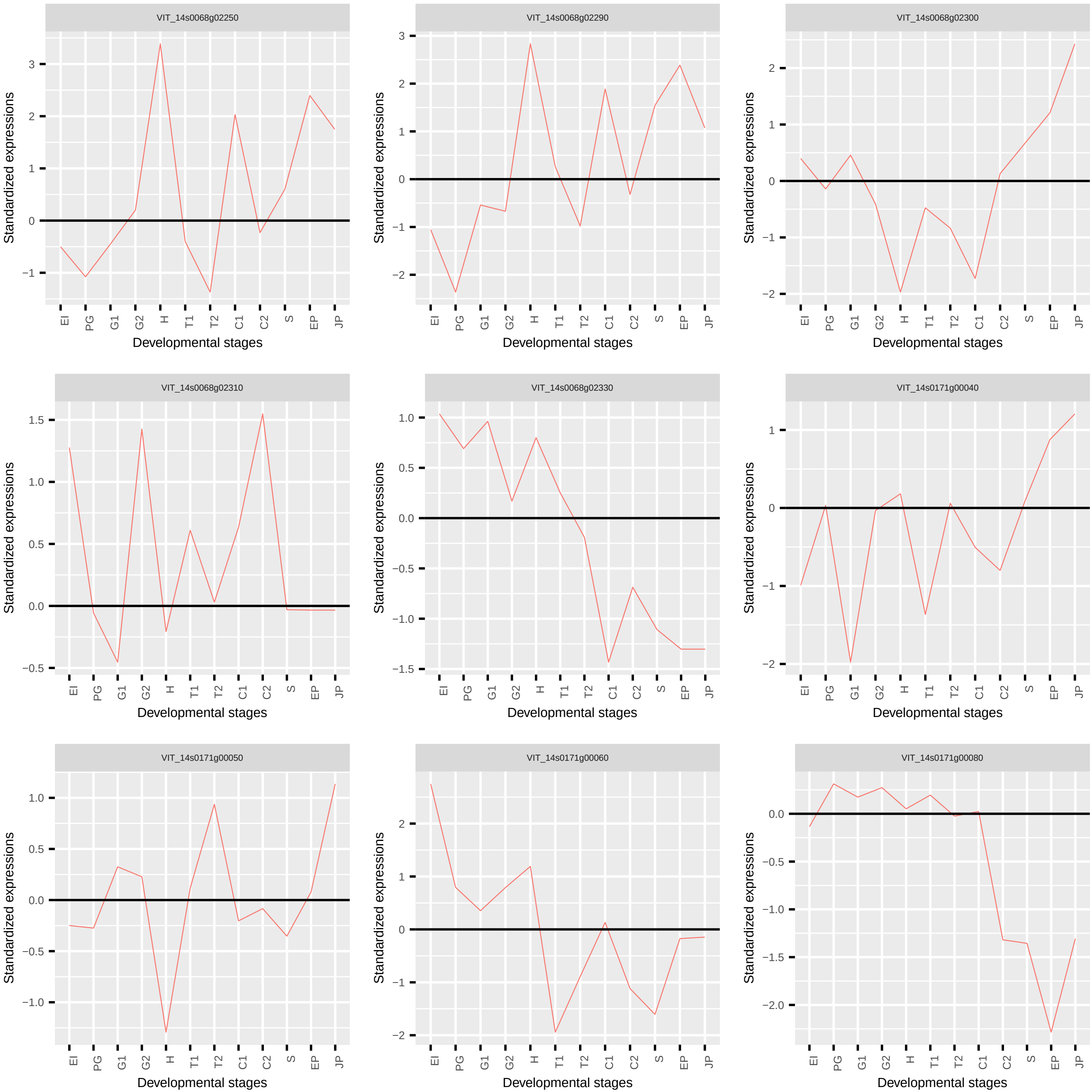
VIT_14s0068g02250
Standardized expressions
3
2
1
0
−1
EI
PG
G1
G2
H
T1
T2
C1
C2
S
EP
JP
Developmental stages
VIT_14s0068g02290
Standardized expressions
3
2
1
0
−1
−2
EI
PG
G1
G2
H
T1
T2
C1
C2
S
EP
JP
Developmental stages
VIT_14s0068g02300
Standardized expressions
2
1
0
−1
−2
EI
PG
G1
G2
H
T1
T2
C1
C2
S
EP
JP
Developmental stages
VIT_14s0068g02310
Standardized expressions
1.5
1.0
0.5
0.0
−0.5
EI
PG
G1
G2
H
T1
T2
C1
C2
S
EP
JP
Developmental stages
VIT_14s0068g02330
Standardized expressions
1.0
0.5
0.0
−0.5
−1.0
−1.5
EI
PG
G1
G2
H
T1
T2
C1
C2
S
EP
JP
Developmental stages
VIT_14s0171g00040
Standardized expressions
1
0
−1
−2
EI
PG
G1
G2
H
T1
T2
C1
C2
S
EP
JP
Developmental stages
VIT_14s0171g00050
Standardized expressions
1.0
0.5
0.0
−0.5
−1.0
EI
PG
G1
G2
H
T1
T2
C1
C2
S
EP
JP
Developmental stages
VIT_14s0171g00060
Standardized expressions
2
1
0
−1
−2
EI
PG
G1
G2
H
T1
T2
C1
C2
S
EP
JP
Developmental stages
VIT_14s0171g00080
Standardized expressions
0.0
−0.5
−1.0
−1.5
−2.0
EI
PG
G1
G2
H
T1
T2
C1
C2
S
EP
JP
Developmental stages

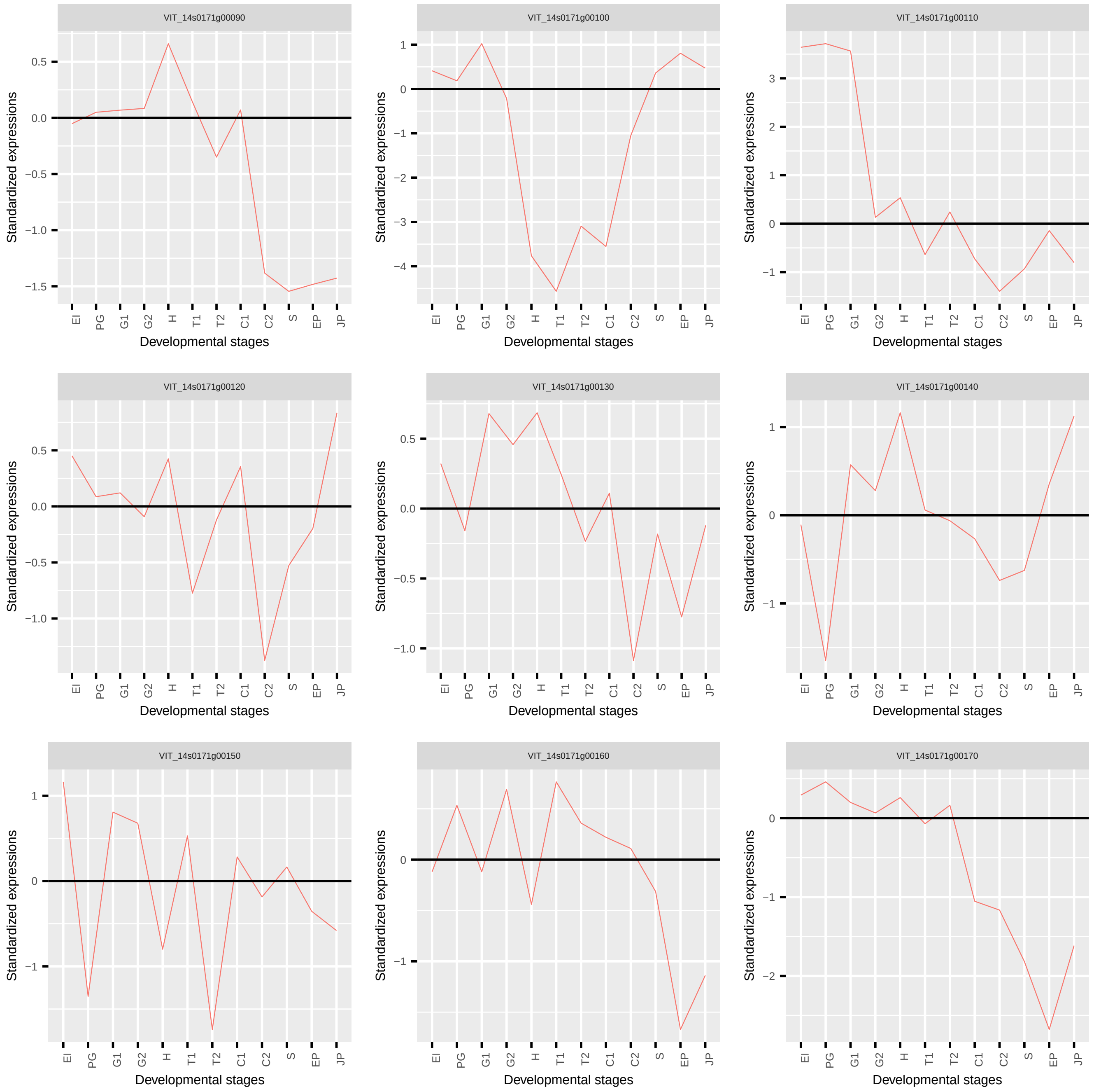
VIT_14s0171g00090
VIT_14s0171g00100
VIT_14s0171g00110
VIT_14s0171g00120
VIT_14s0171g00130
VIT_14s0171g00140
VIT_14s0171g00150
VIT_14s0171g00160
VIT_14s0171g00170
Standardized expressions
Developmental stages
EI PG G1 G2 H T1 T2 C1 C2 S EP JP

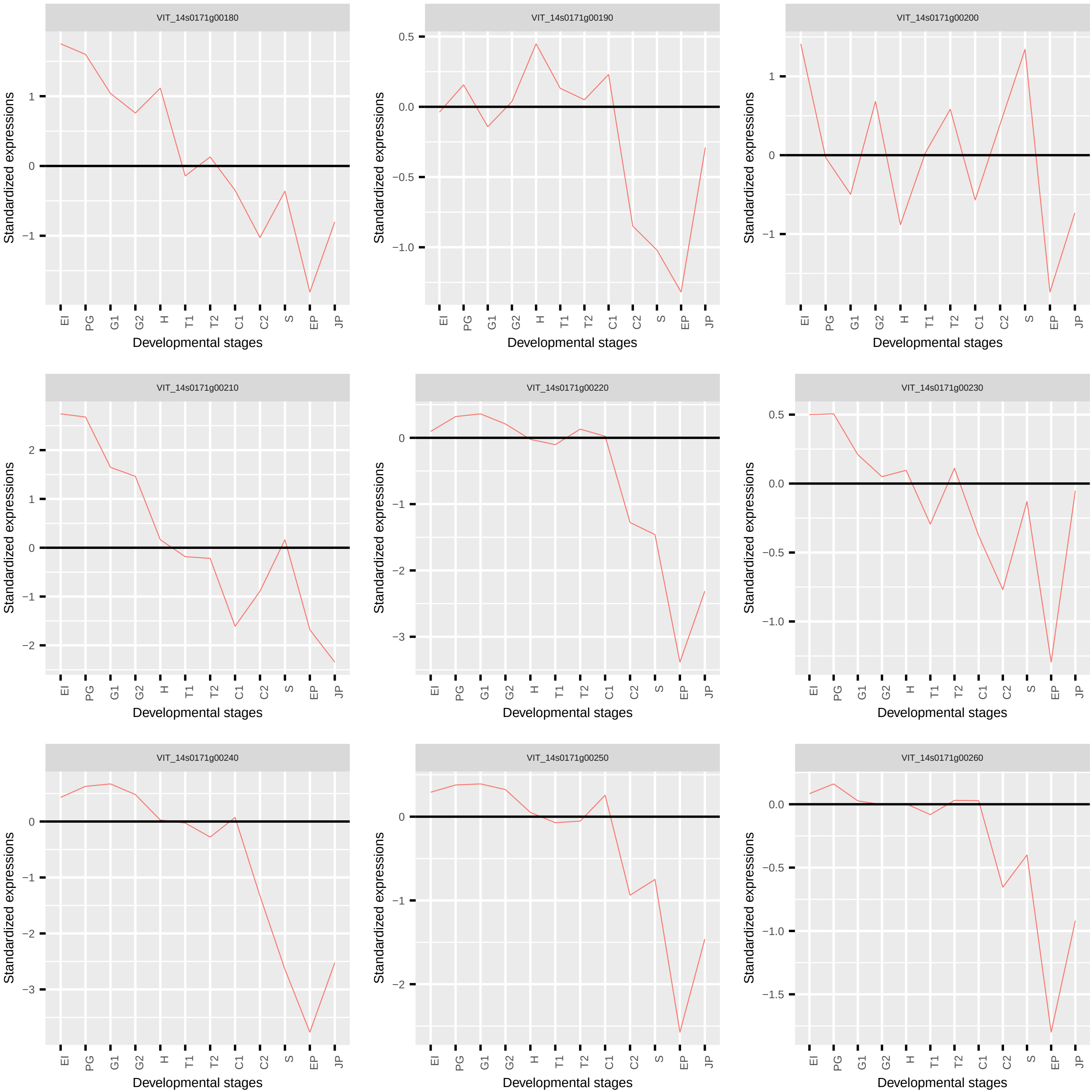
VIT_14s0171g00180
VIT_14s0171g00190
VIT_14s0171g00200
VIT_14s0171g00210
VIT_14s0171g00220
VIT_14s0171g00230
VIT_14s0171g00240
VIT_14s0171g00250
VIT_14s0171g00260
Standardized expressions
Developmental stages
EI
PG
G1
G2
H
T1
T2
C1
C2
S
EP
JP

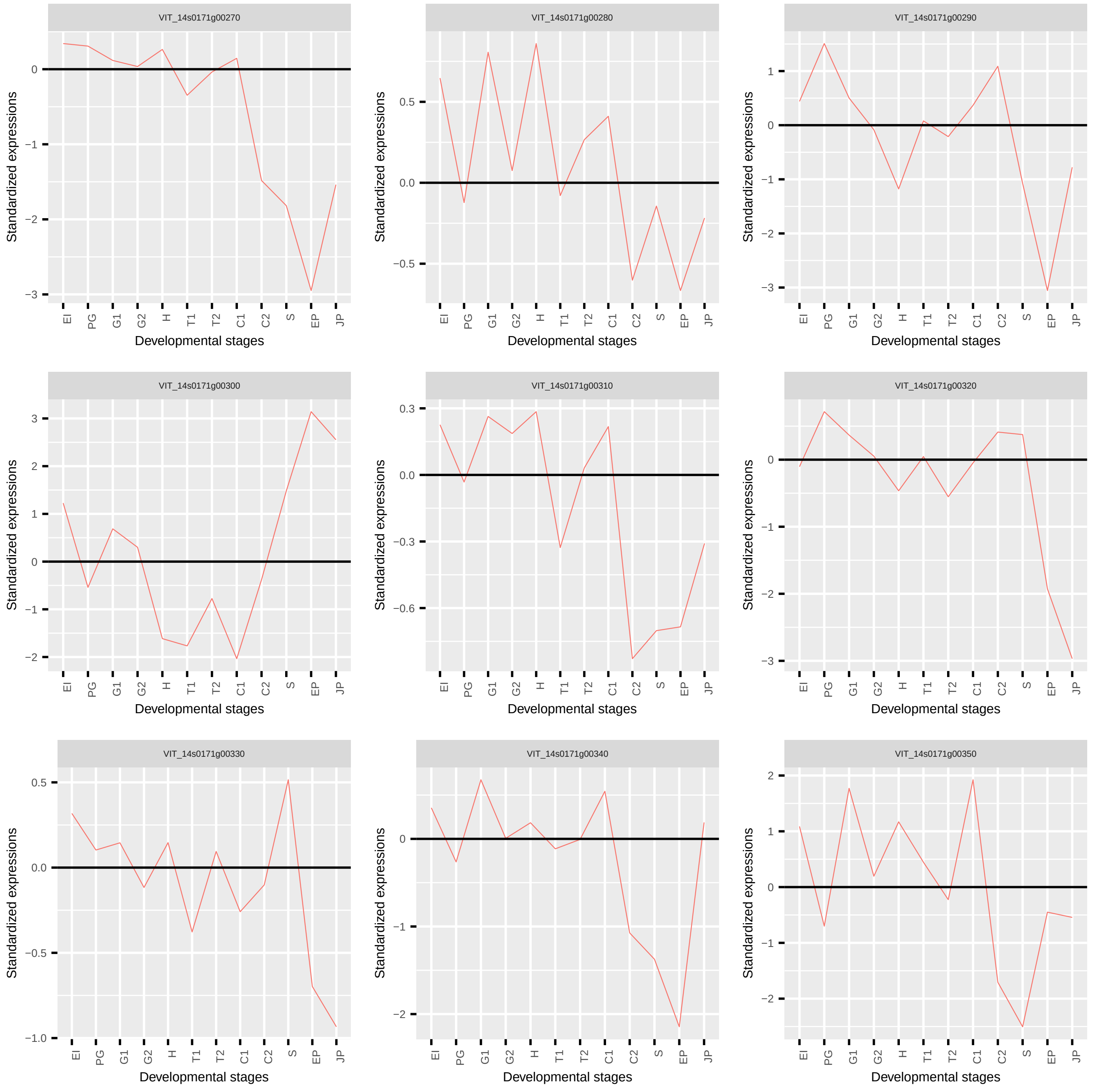

VIT_14s0171g00270
VIT_14s0171g00280
VIT_14s0171g00290
VIT_14s0171g00300
VIT_14s0171g00310
VIT_14s0171g00320
VIT_14s0171g00330
VIT_14s0171g00340
VIT_14s0171g00350
Standardized expressions
Developmental stages
EI
PG
G1
G2
H
T1
T2
C1
C2
S
EP
JP

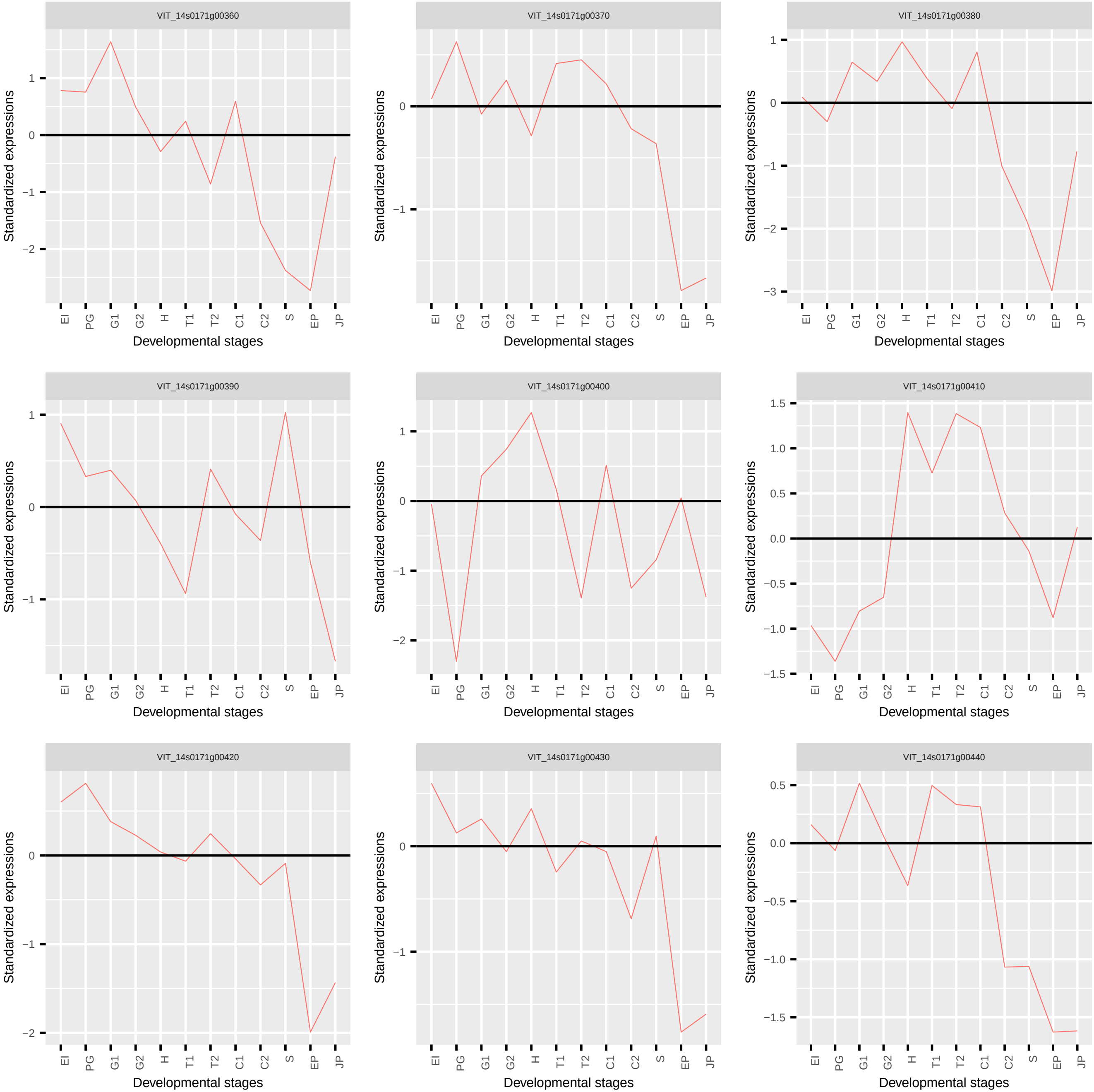
VIT_14s0171g00360
Standardized expressions
Developmental stages
VIT_14s0171g00370
Standardized expressions
Developmental stages
VIT_14s0171g00380
Standardized expressions
Developmental stages
VIT_14s0171g00390
Standardized expressions
Developmental stages
VIT_14s0171g00400
Standardized expressions
Developmental stages
VIT_14s0171g00410
Standardized expressions
Developmental stages
VIT_14s0171g00420
Standardized expressions
Developmental stages
VIT_14s0171g00430
Standardized expressions
Developmental stages
VIT_14s0171g00440
Standardized expressions
Developmental stages
EI
PG
G1
G2
H
T1
T2
C1
C2
S
EP
JP

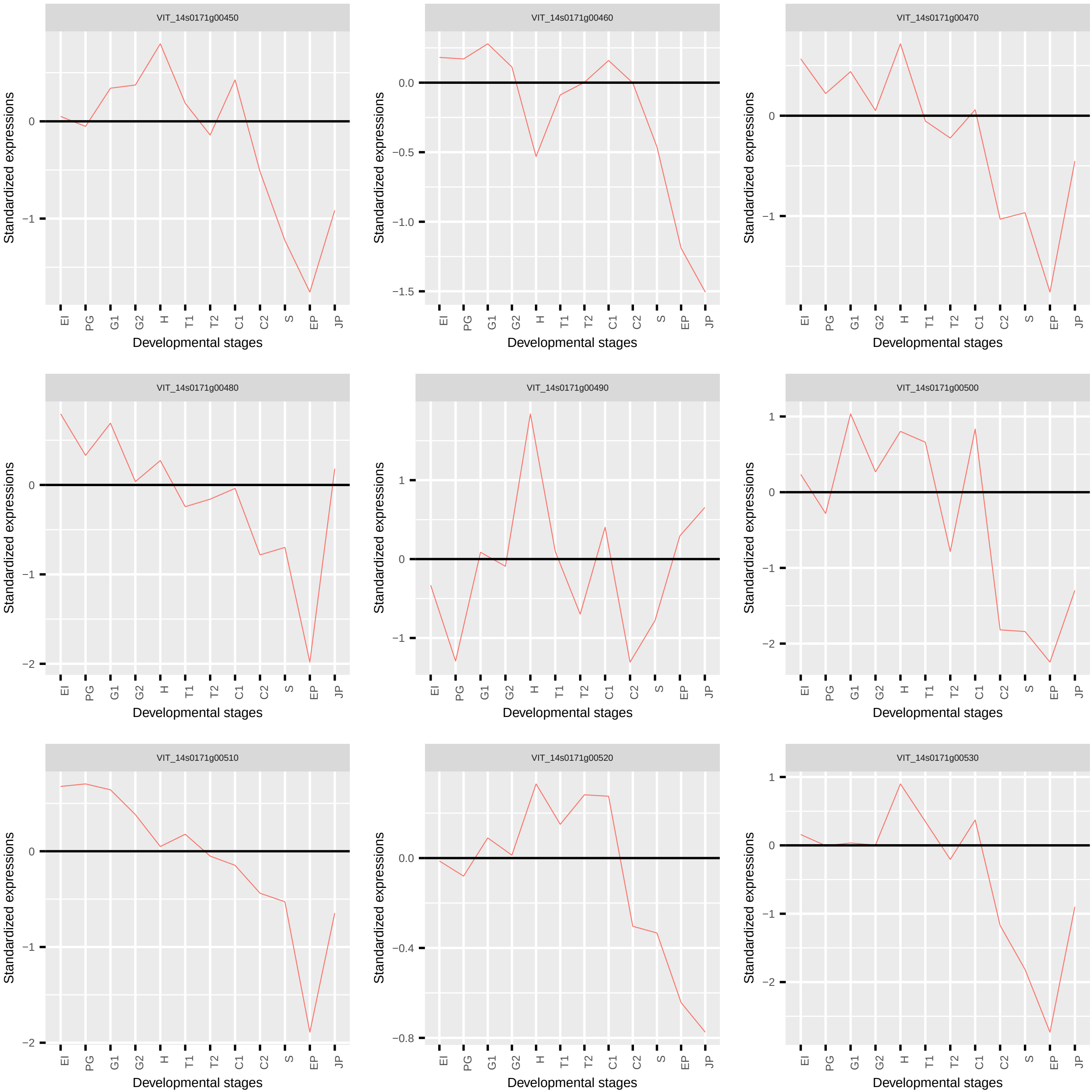
VIT_14s0171g00450
VIT_14s0171g00460
VIT_14s0171g00470
VIT_14s0171g00480
VIT_14s0171g00490
VIT_14s0171g00500
VIT_14s0171g00510
VIT_14s0171g00520
VIT_14s0171g00530
Standardized expressions
Developmental stages
EI
PG
G1
G2
H
T1
T2
C1
C2
S
EP
JP

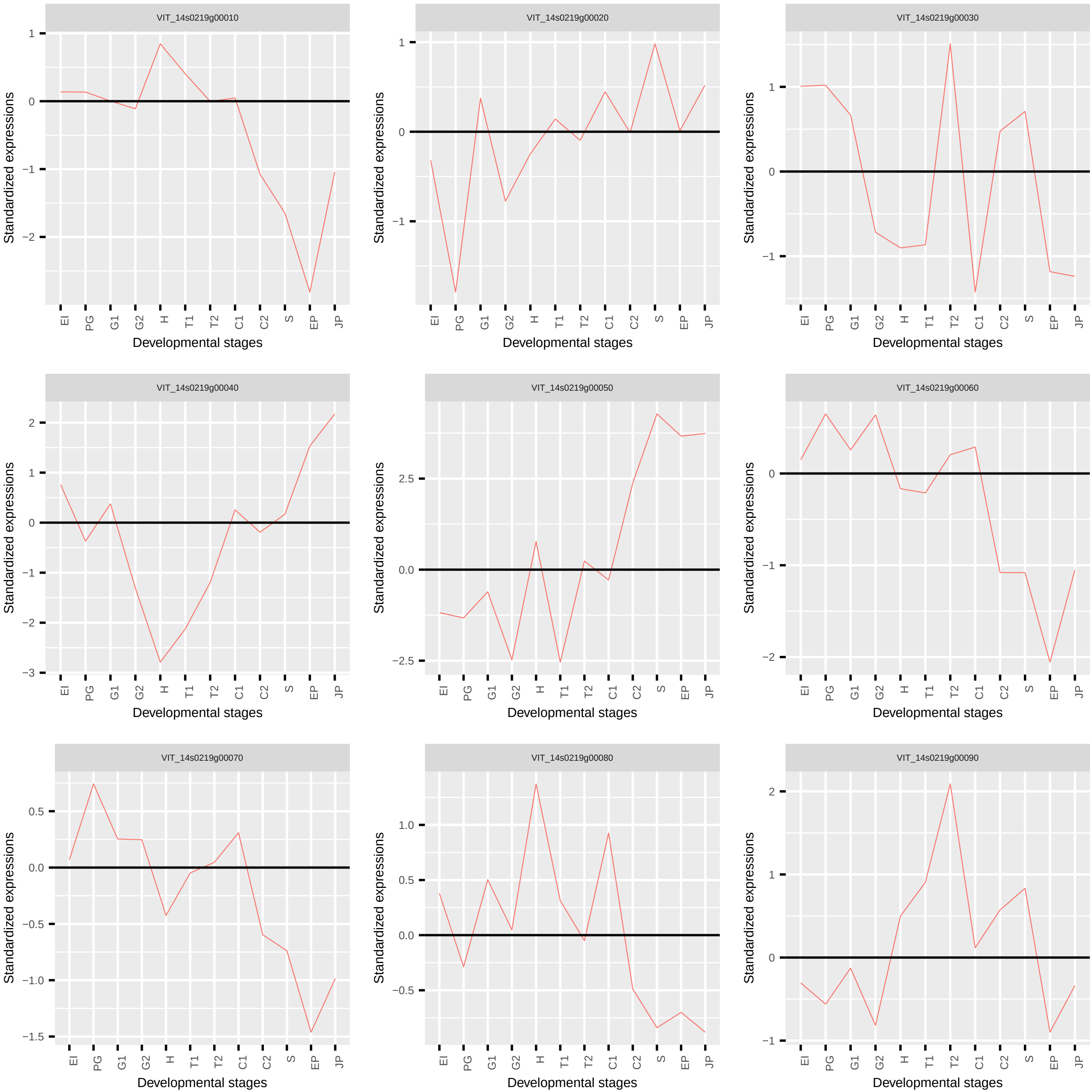
VIT_14s0219g00010
VIT_14s0219g00020
VIT_14s0219g00030
VIT_14s0219g00040
VIT_14s0219g00050
VIT_14s0219g00060
VIT_14s0219g00070
VIT_14s0219g00080
VIT_14s0219g00090
Standardized expressions
Developmental stages
EI PG G1 G2 H T1 T2 C1 C2 S EP JP
1 0 −1 −2
1 0 −1
1 0 −1
2 1 0 −1 −2 −3
2.5 0.0 −2.5
0 −1 −2
0.5 0.0 −0.5 −1.0 −1.5
1.0 0.5 0.0 −0.5
2 1 0 −1

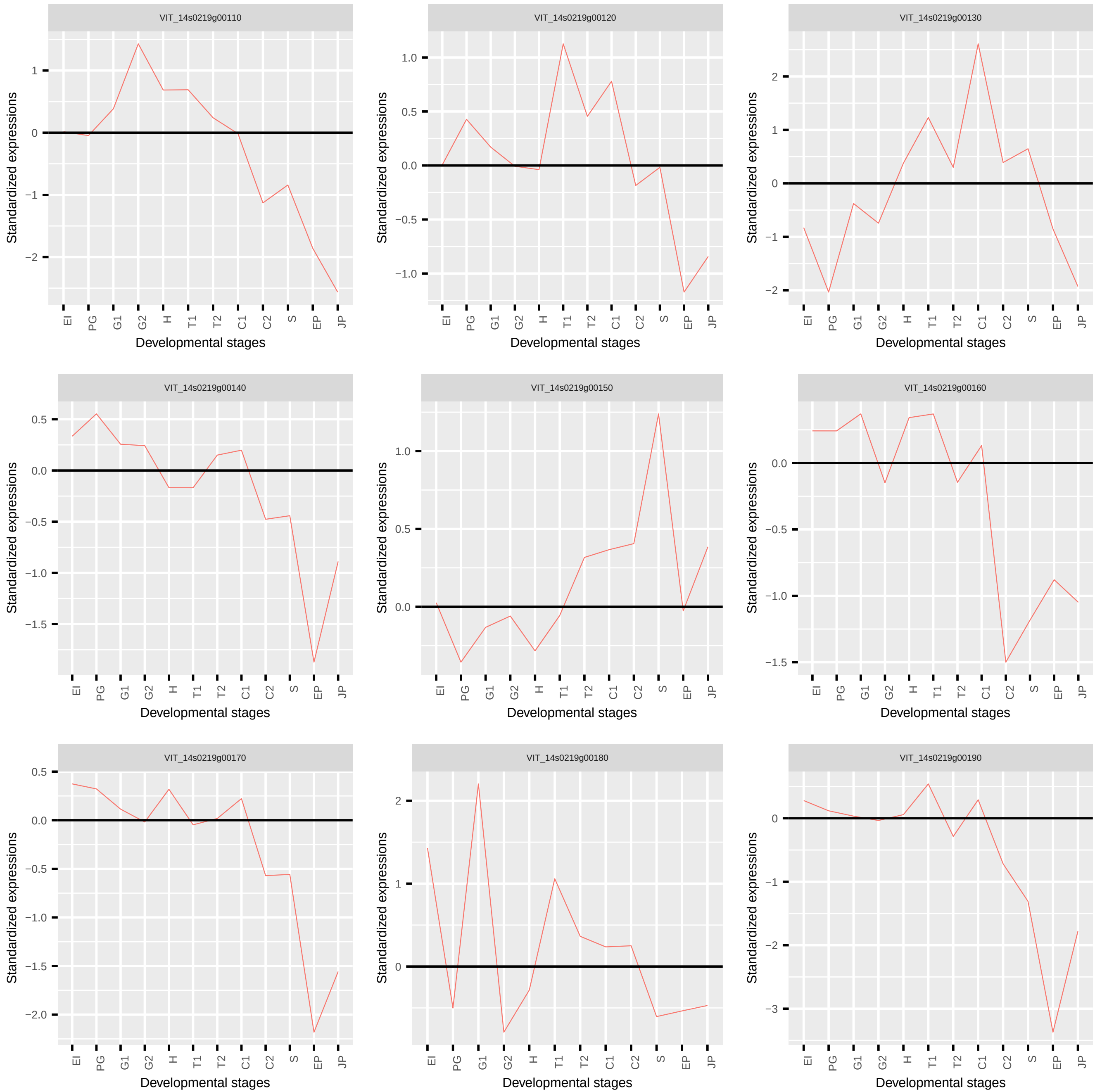

VIT_14s0219g00110
VIT_14s0219g00120
VIT_14s0219g00130
VIT_14s0219g00140
VIT_14s0219g00150
VIT_14s0219g00160
VIT_14s0219g00170
VIT_14s0219g00180
VIT_14s0219g00190
Standardized expressions
Developmental stages
EI PG G1 G2 H T1 T2 C1 C2 S EP JP

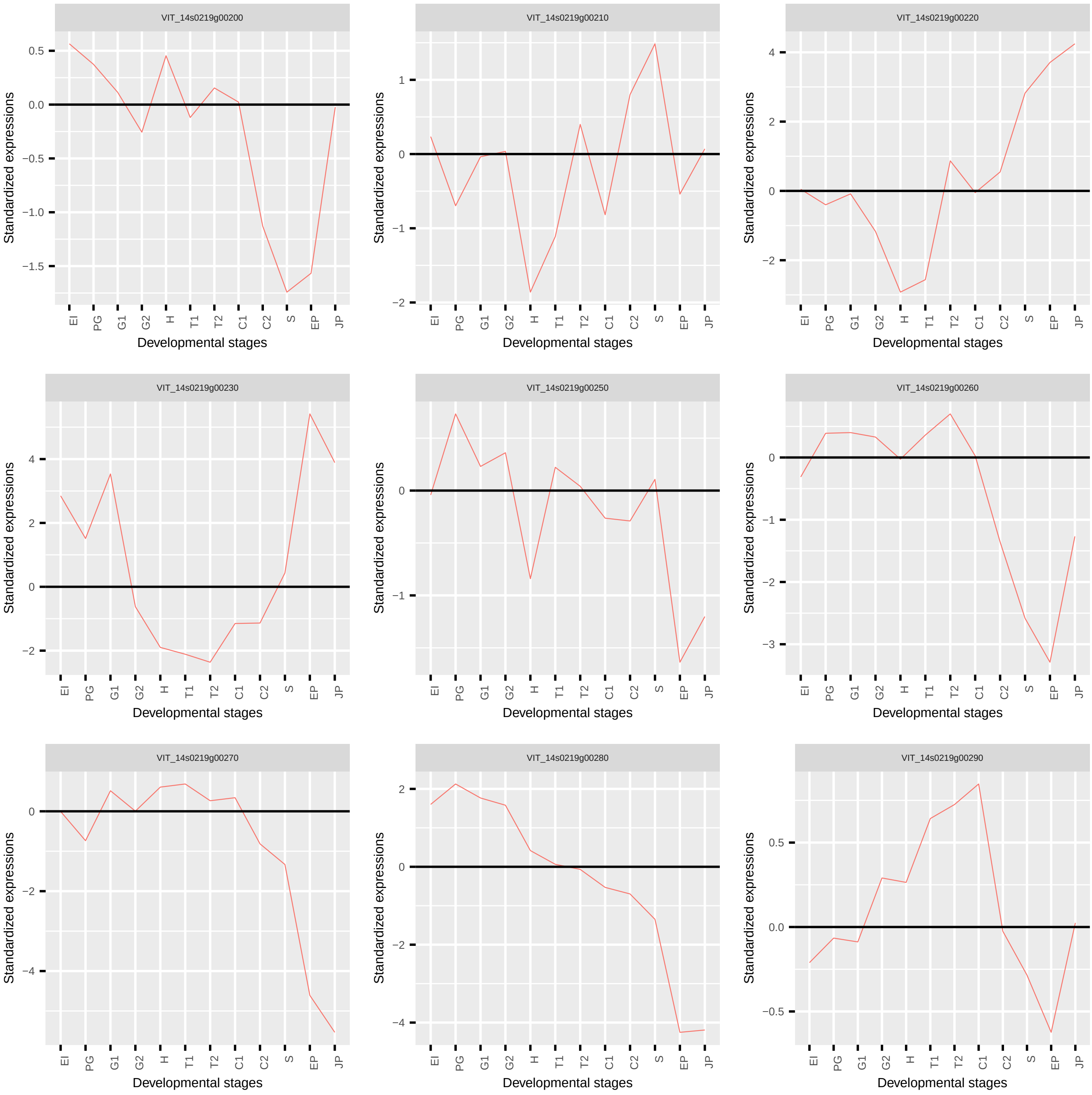
VIT_14s0219g00200
VIT_14s0219g00210
VIT_14s0219g00220
VIT_14s0219g00230
VIT_14s0219g00250
VIT_14s0219g00260
VIT_14s0219g00270
VIT_14s0219g00280
VIT_14s0219g00290
Standardized expressions
Developmental stages
EI PG G1 G2 H T1 T2 C1 C2 S EP JP

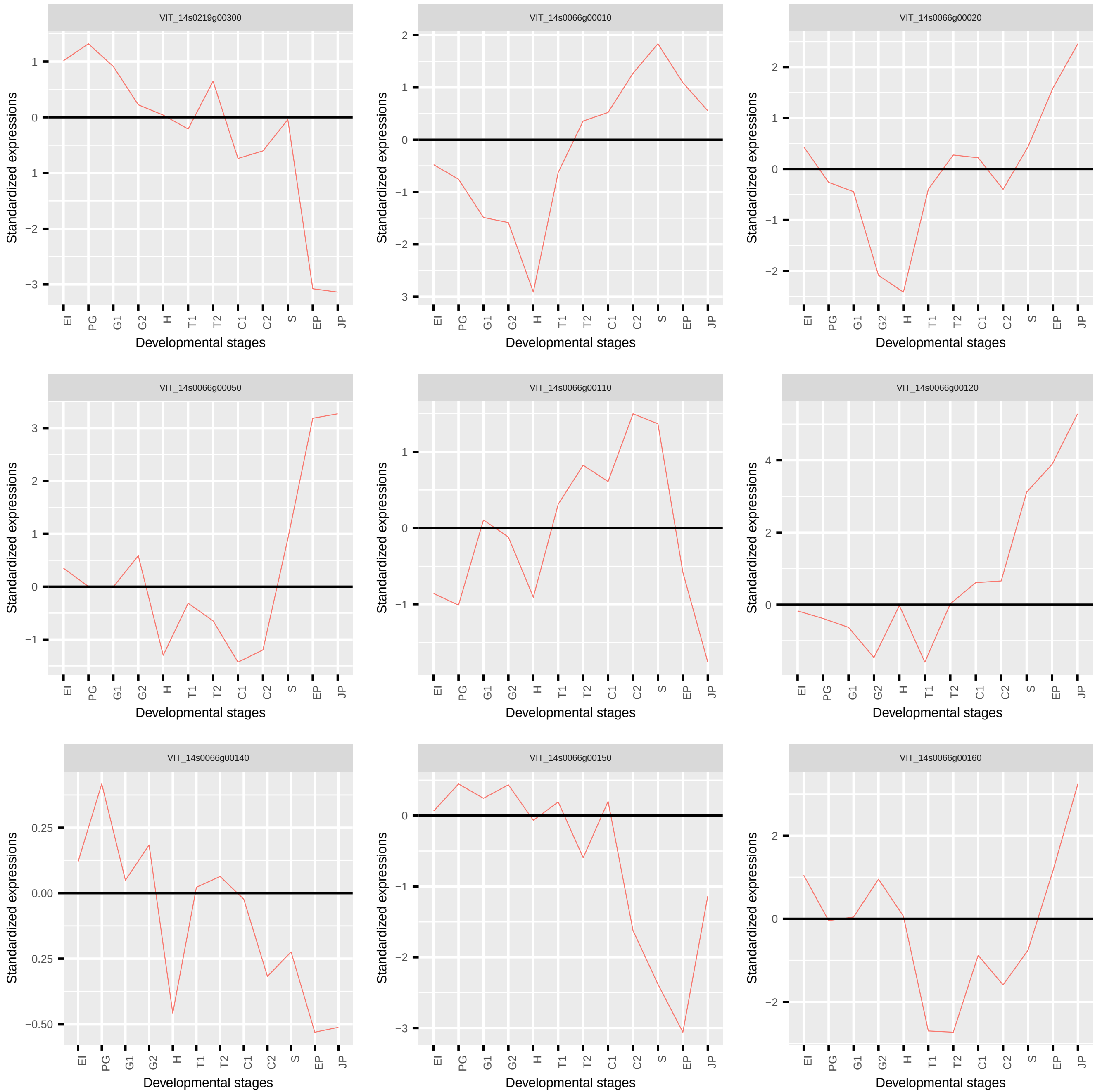
VIT_14s0219g00300
VIT_14s0066g00010
VIT_14s0066g00020
VIT_14s0066g00050
VIT_14s0066g00110
VIT_14s0066g00120
VIT_14s0066g00140
VIT_14s0066g00150
VIT_14s0066g00160
Standardized expressions
Developmental stages
EI
PG
G1
G2
H
T1
T2
C1
C2
S
EP
JP

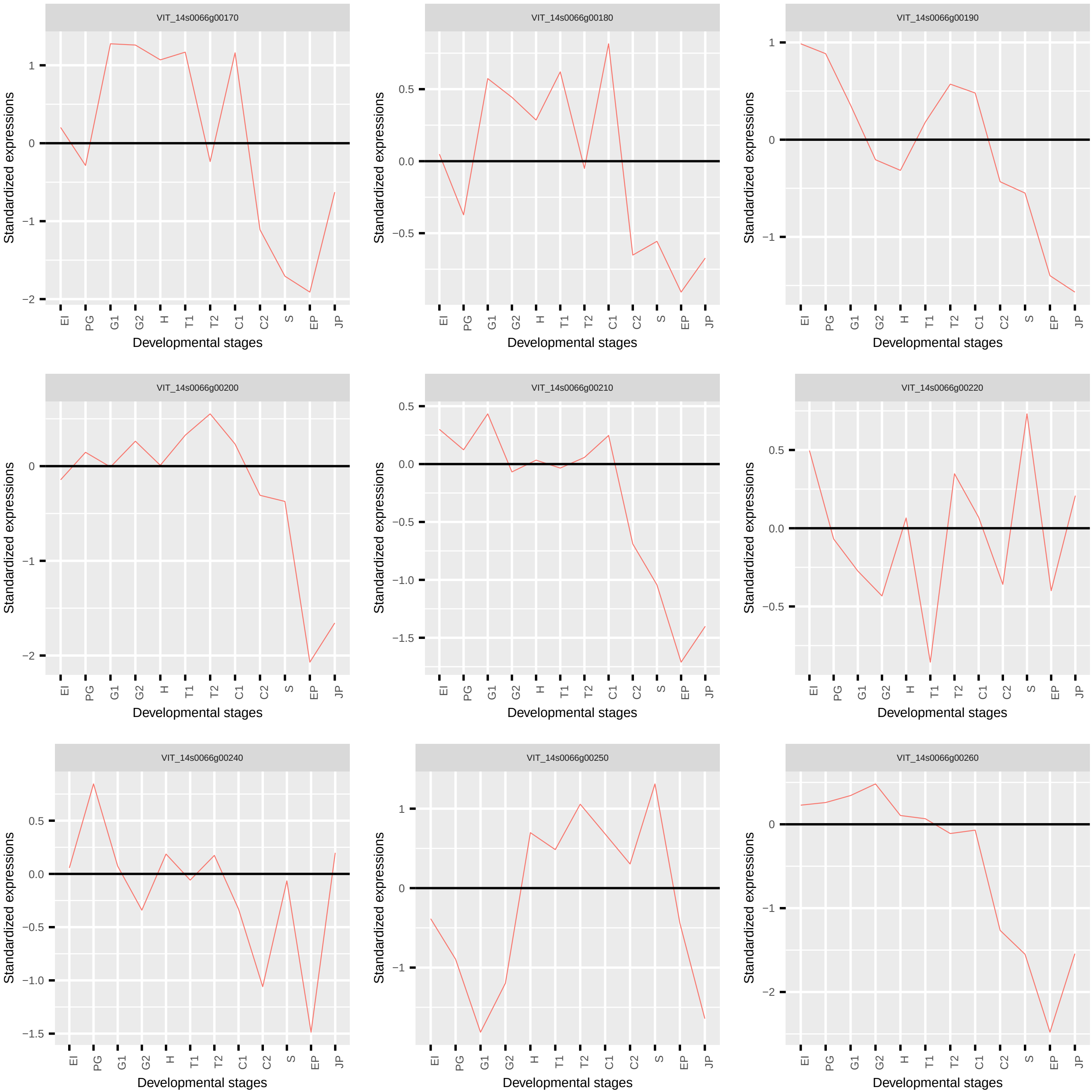
VIT_14s0066g00170
VIT_14s0066g00180
VIT_14s0066g00190
VIT_14s0066g00200
VIT_14s0066g00210
VIT_14s0066g00220
VIT_14s0066g00240
VIT_14s0066g00250
VIT_14s0066g00260
Standardized expressions
Developmental stages
EI
PG
G1
G2
H
T1
T2
C1
C2
S
EP
JP

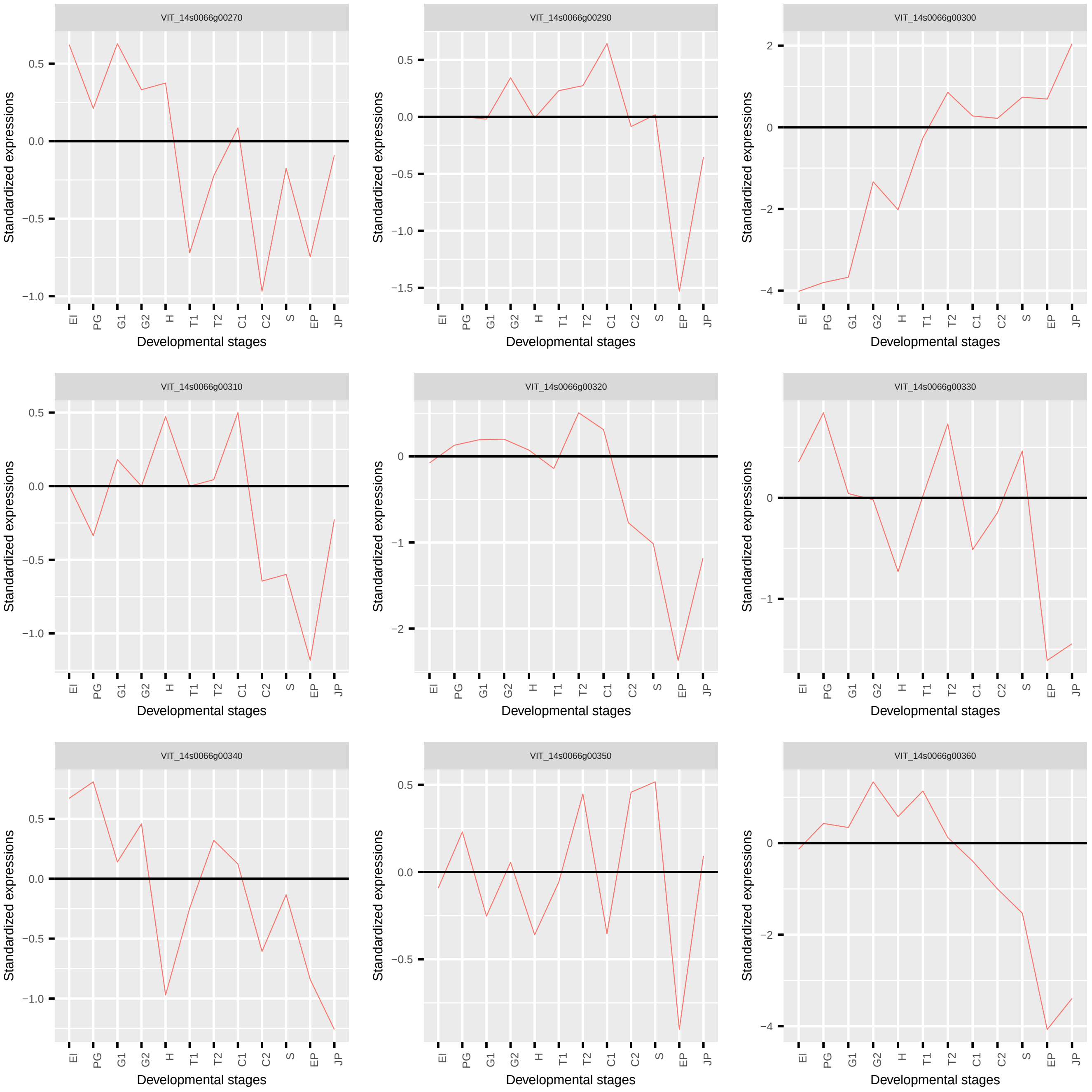
VIT_14s0066g00270
VIT_14s0066g00290
VIT_14s0066g00300
VIT_14s0066g00310
VIT_14s0066g00320
VIT_14s0066g00330
VIT_14s0066g00340
VIT_14s0066g00350
VIT_14s0066g00360
Standardized expressions
Developmental stages
EI PG G1 G2 H T1 T2 C1 C2 S EP JP

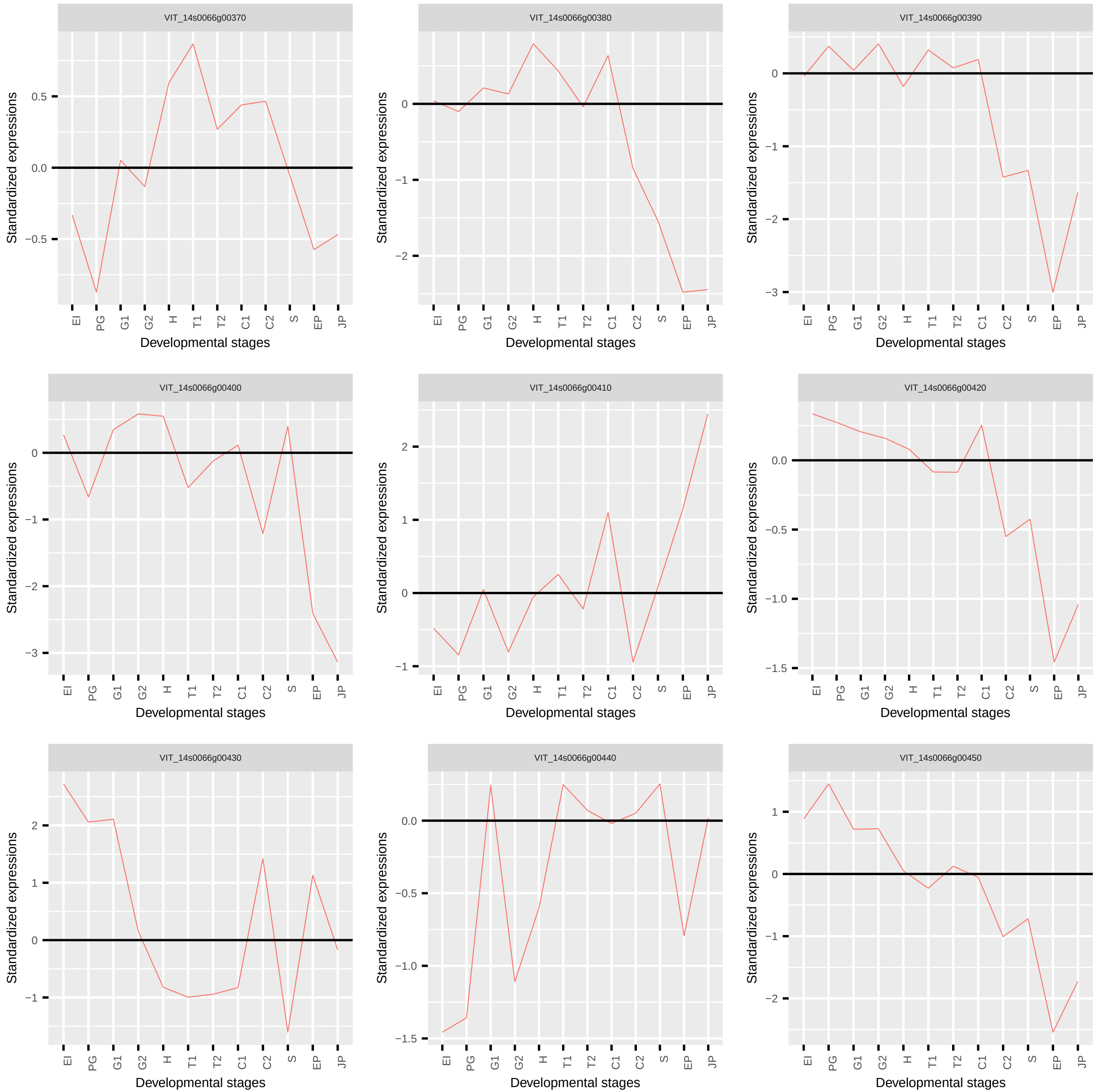
VIT_14s0066g00370
VIT_14s0066g00380
VIT_14s0066g00390
VIT_14s0066g00400
VIT_14s0066g00410
VIT_14s0066g00420
VIT_14s0066g00430
VIT_14s0066g00440
VIT_14s0066g00450
Standardized expressions
Developmental stages
EI PG G1 G2 H T1 T2 C1 C2 S EP JP

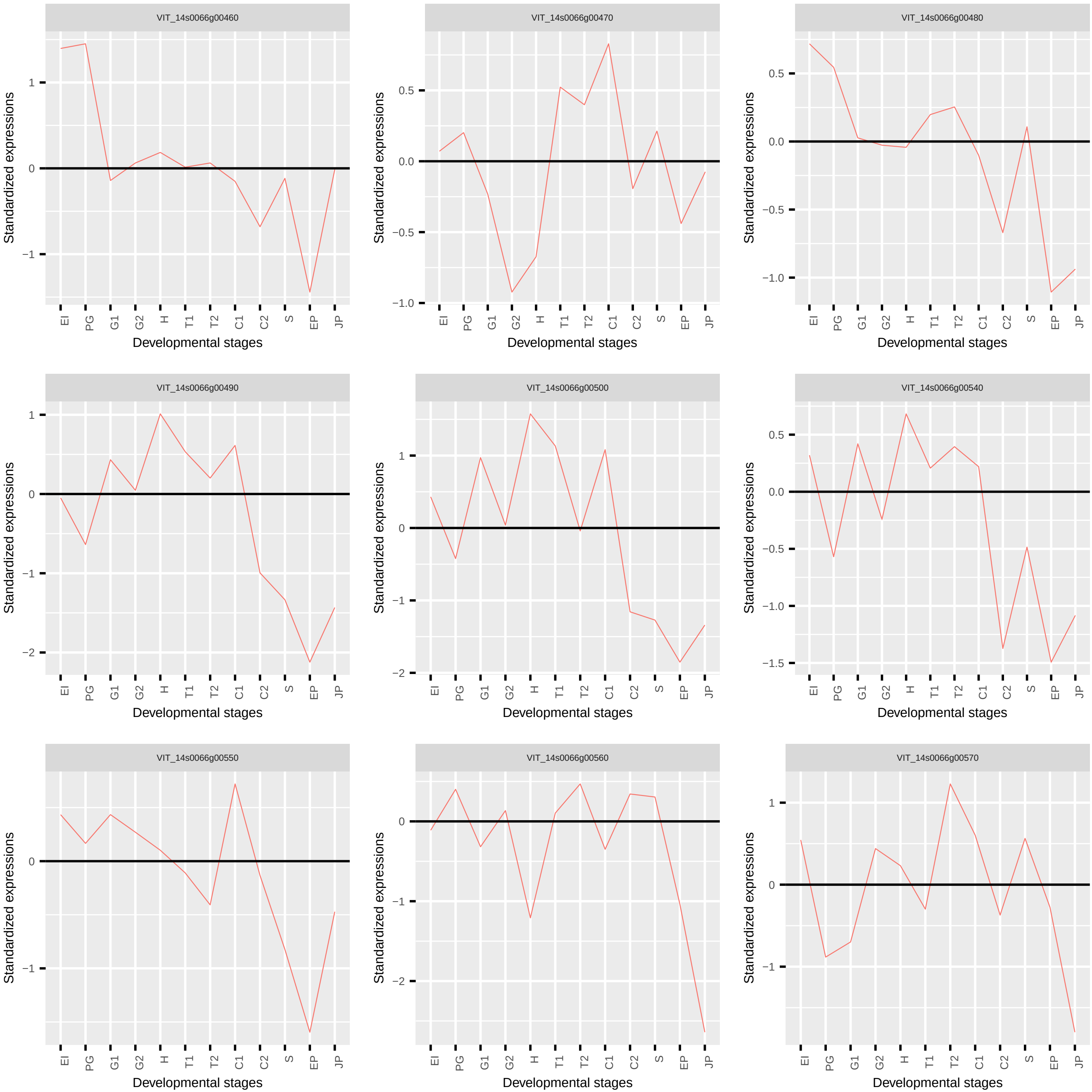
VIT_14s0066g00460
VIT_14s0066g00470
VIT_14s0066g00480
VIT_14s0066g00490
VIT_14s0066g00500
VIT_14s0066g00540
VIT_14s0066g00550
VIT_14s0066g00560
VIT_14s0066g00570
Standardized expressions
Developmental stages
EI
PG
G1
G2
H
T1
T2
C1
C2
S
EP
JP

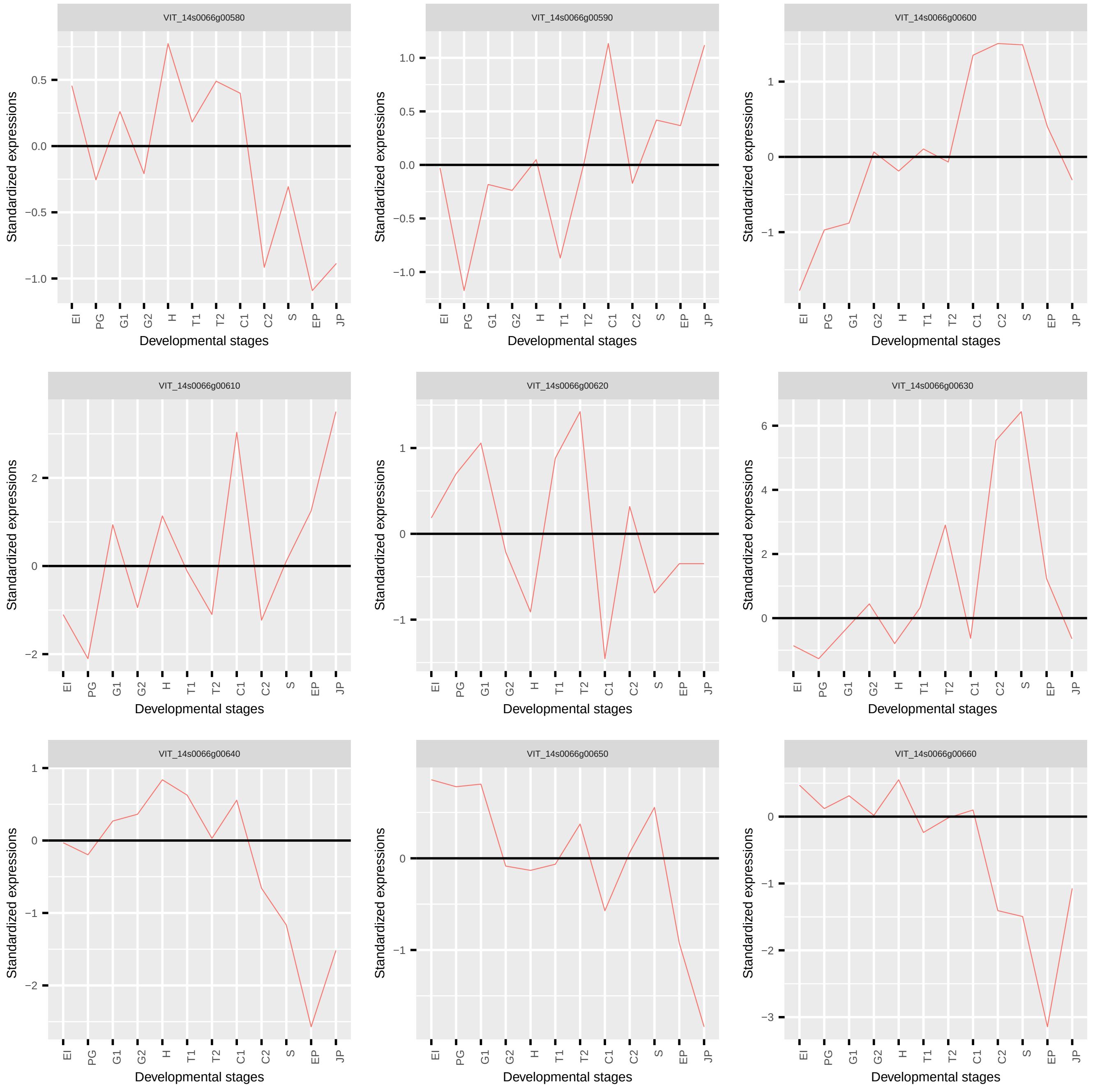
VIT_14s0066g00580
VIT_14s0066g00590
VIT_14s0066g00600
VIT_14s0066g00610
VIT_14s0066g00620
VIT_14s0066g00630
VIT_14s0066g00640
VIT_14s0066g00650
VIT_14s0066g00660
Standardized expressions
Developmental stages
EI PG G1 G2 H T1 T2 C1 C2 S EP JP

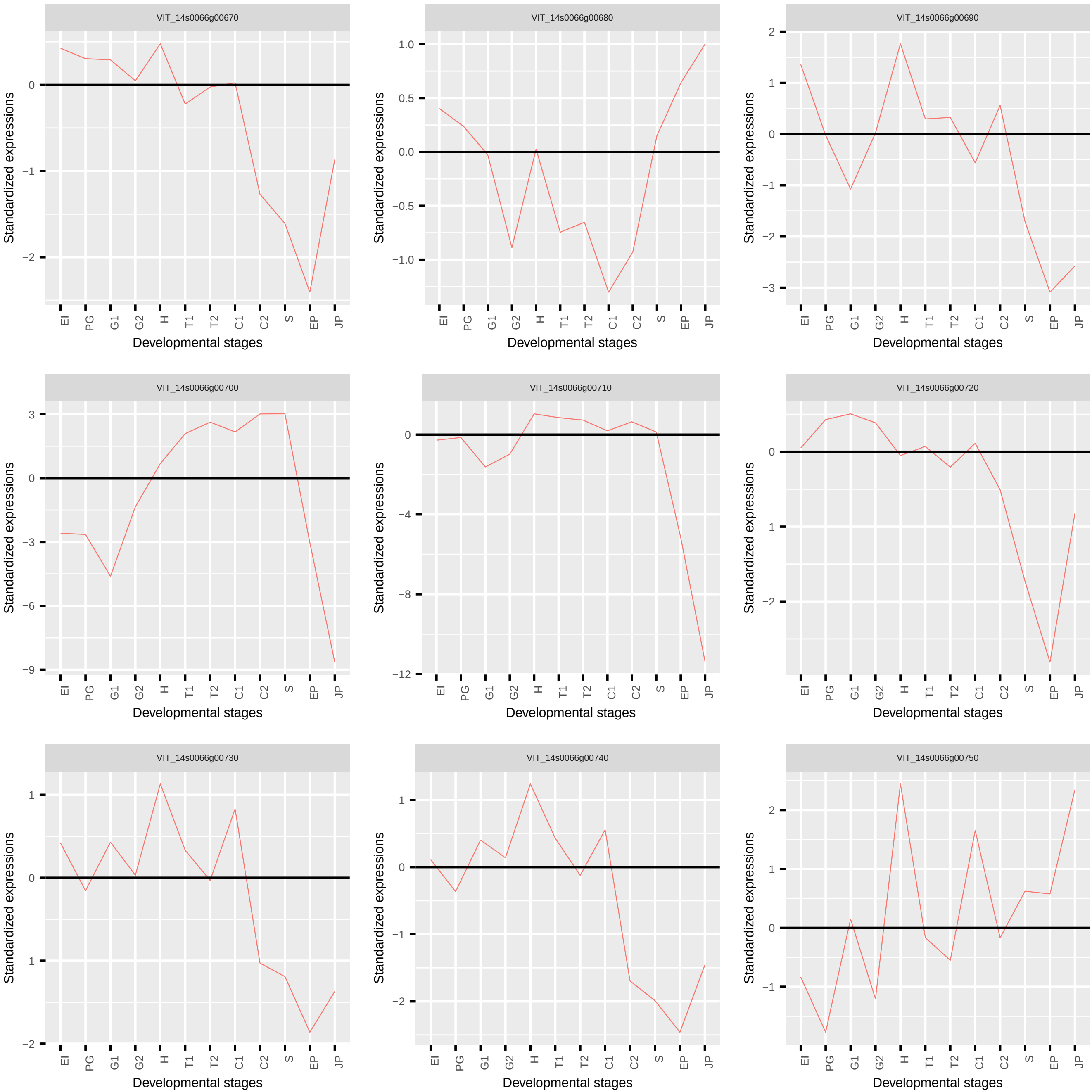

VIT_14s0066g00670
VIT_14s0066g00680
VIT_14s0066g00690
VIT_14s0066g00700
VIT_14s0066g00710
VIT_14s0066g00720
VIT_14s0066g00730
VIT_14s0066g00740
VIT_14s0066g00750
Standardized expressions
Developmental stages
EI
PG
G1
G2
H
T1
T2
C1
C2
S
EP
JP

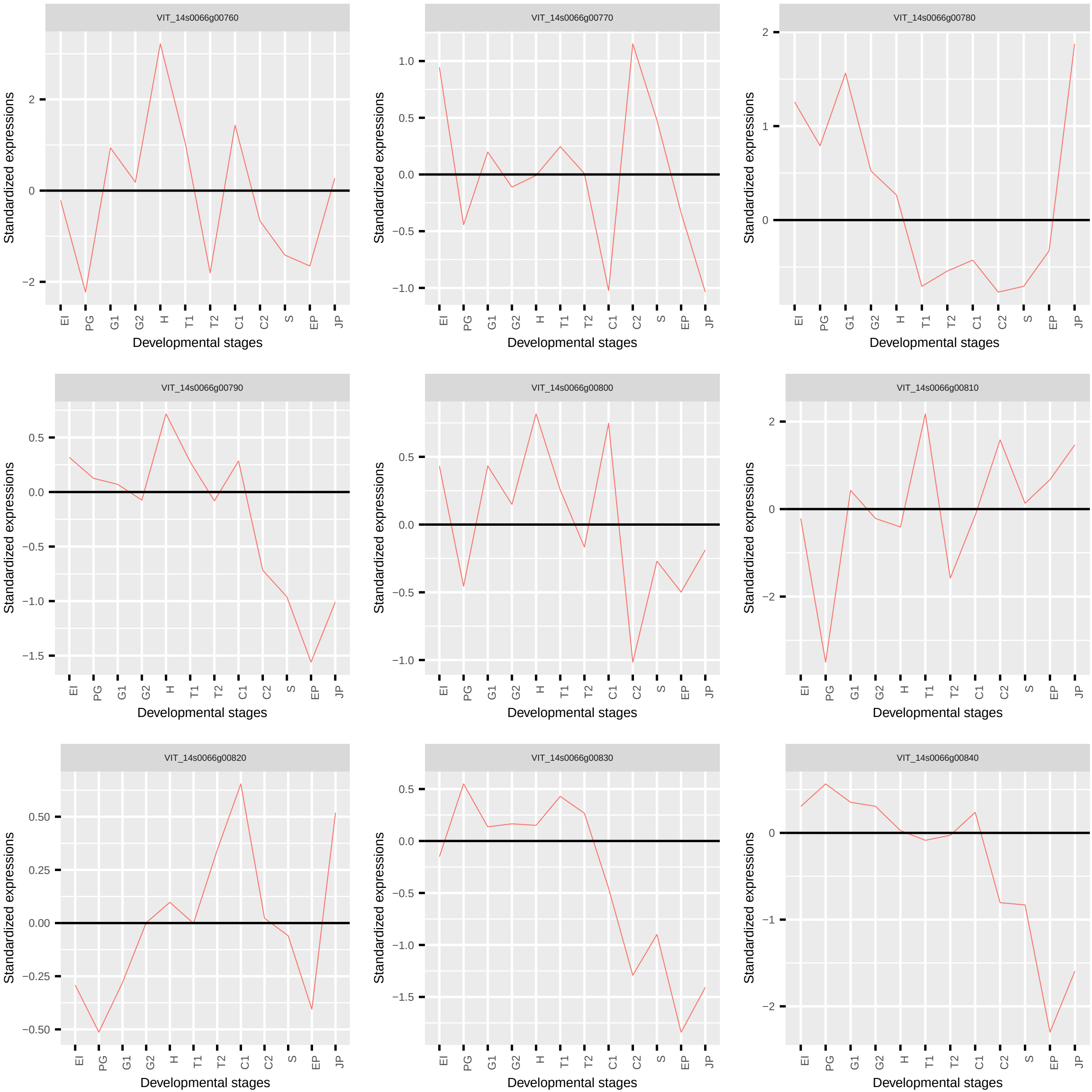
VIT_14s0066g00760
VIT_14s0066g00770
VIT_14s0066g00780
VIT_14s0066g00790
VIT_14s0066g00800
VIT_14s0066g00810
VIT_14s0066g00820
VIT_14s0066g00830
VIT_14s0066g00840
Standardized expressions
Developmental stages
EI PG G1 G2 H T1 T2 C1 C2 S EP JP

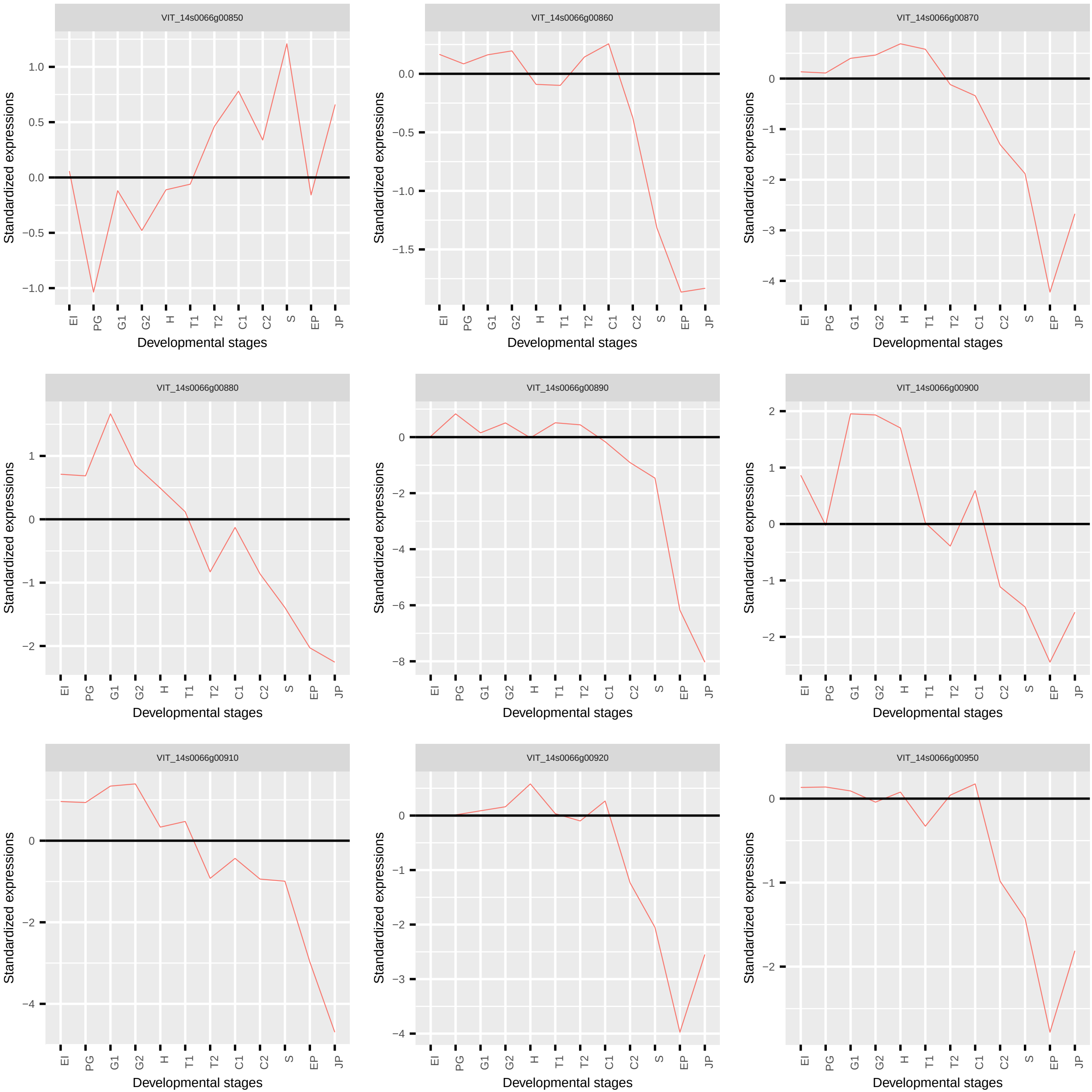
VIT_14s0066g00850
VIT_14s0066g00860
VIT_14s0066g00870
VIT_14s0066g00880
VIT_14s0066g00890
VIT_14s0066g00900
VIT_14s0066g00910
VIT_14s0066g00920
VIT_14s0066g00950
Standardized expressions
Developmental stages
EI PG G1 G2 H T1 T2 C1 C2 S EP JP

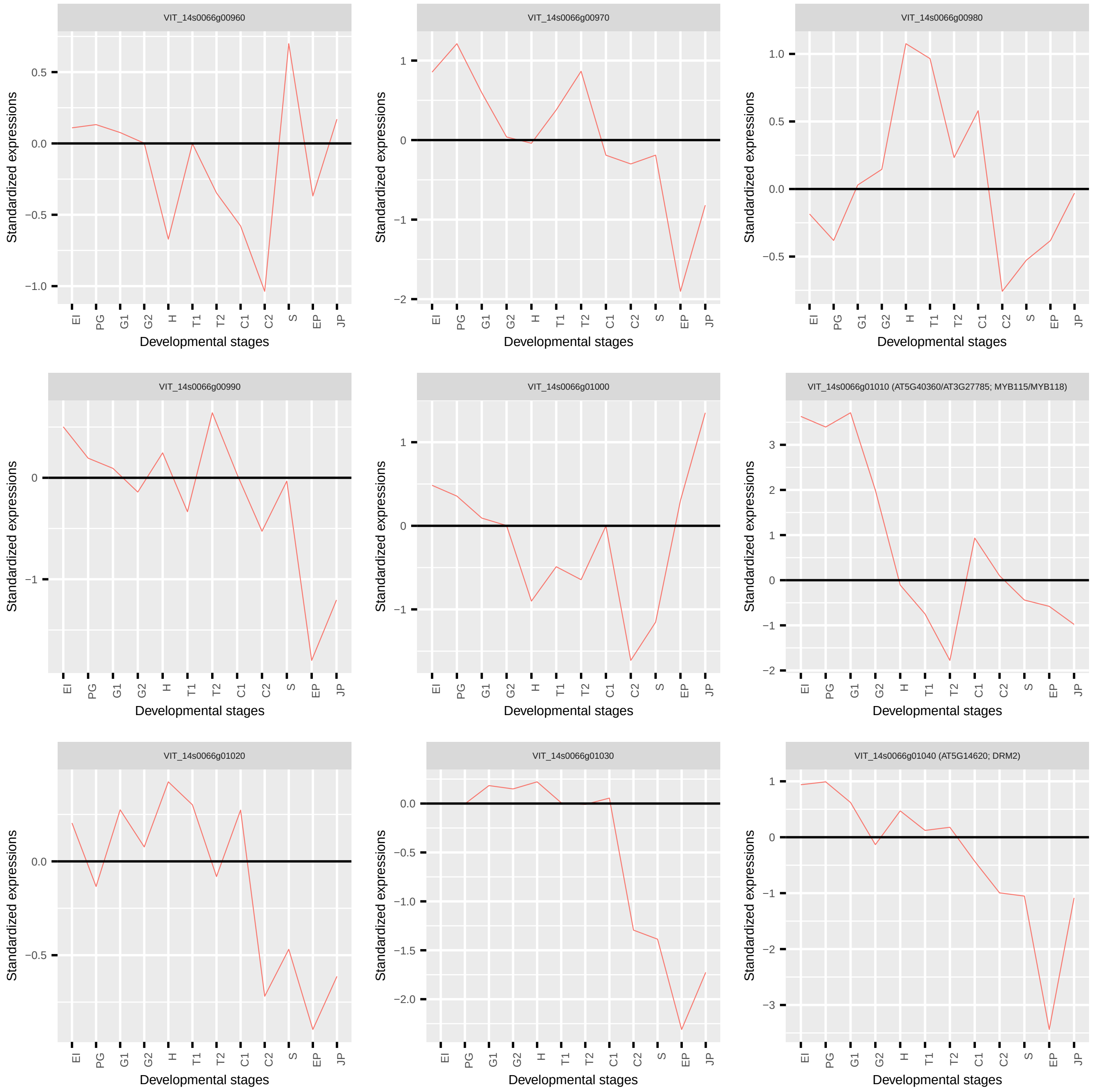
VIT_14s0066g00960
VIT_14s0066g00970
VIT_14s0066g00980
VIT_14s0066g00990
VIT_14s0066g01000
VIT_14s0066g01010 (AT5G40360/AT3G27785; MYB115/MYB118)
VIT_14s0066g01020
VIT_14s0066g01030
VIT_14s0066g01040 (AT5G14620; DRM2)
Standardized expressions
Developmental stages
EI
PG
G1
G2
H
T1
T2
C1
C2
S
EP
JP

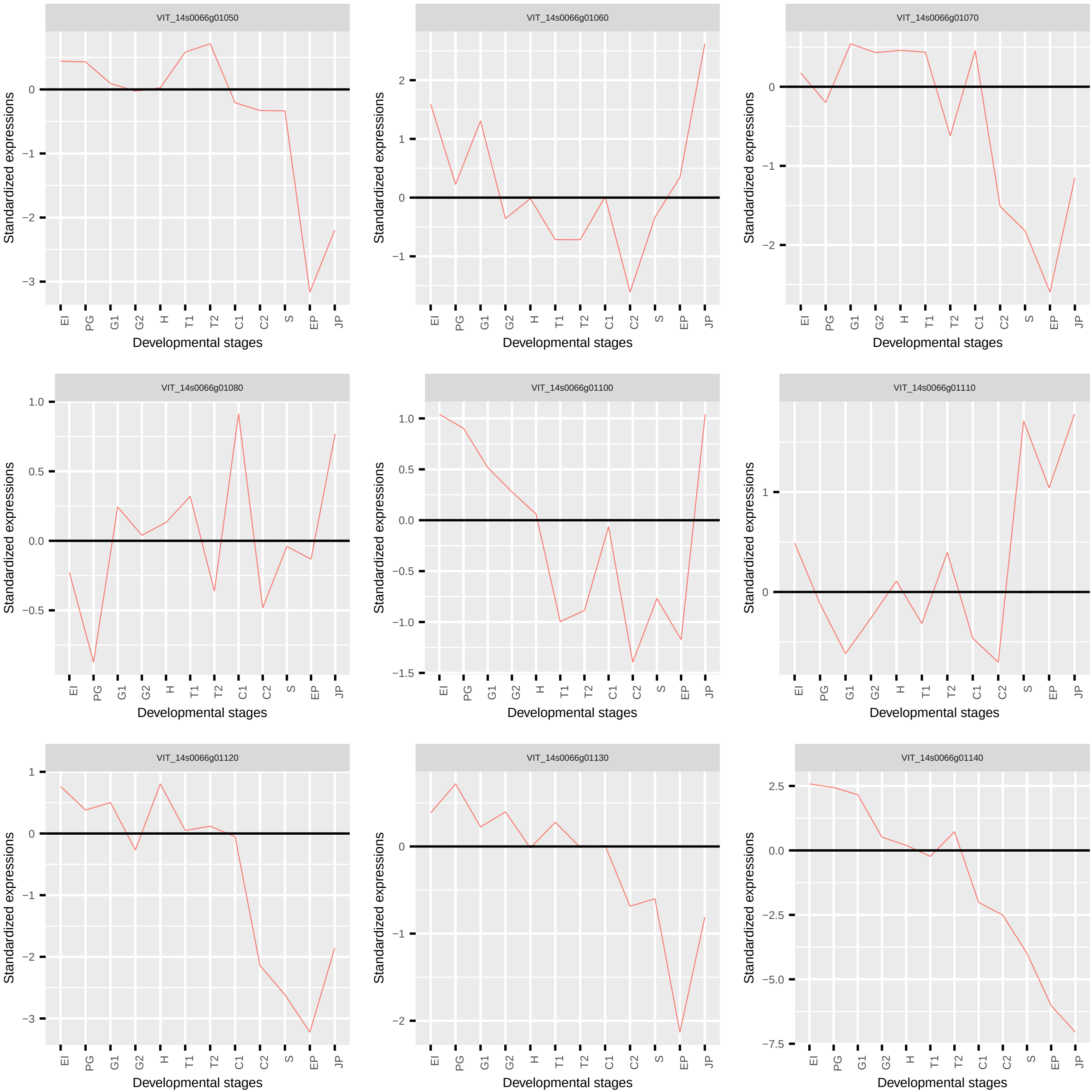

VIT_14s0066g01050
VIT_14s0066g01060
VIT_14s0066g01070
VIT_14s0066g01080
VIT_14s0066g01100
VIT_14s0066g01110
VIT_14s0066g01120
VIT_14s0066g01130
VIT_14s0066g01140
Standardized expressions
Developmental stages
EI PG G1 G2 H T1 T2 C1 C2 S EP JP
0 −1 −2 −3
2 1 0 −1
0 −1 −2
1.0 0.5 0.0 −0.5
1.0 0.5 0.0 −0.5 −1.0 −1.5
1 0
1 0 −1 −2 −3
0 −1 −2
2.5 0.0 −2.5 −5.0 −7.5

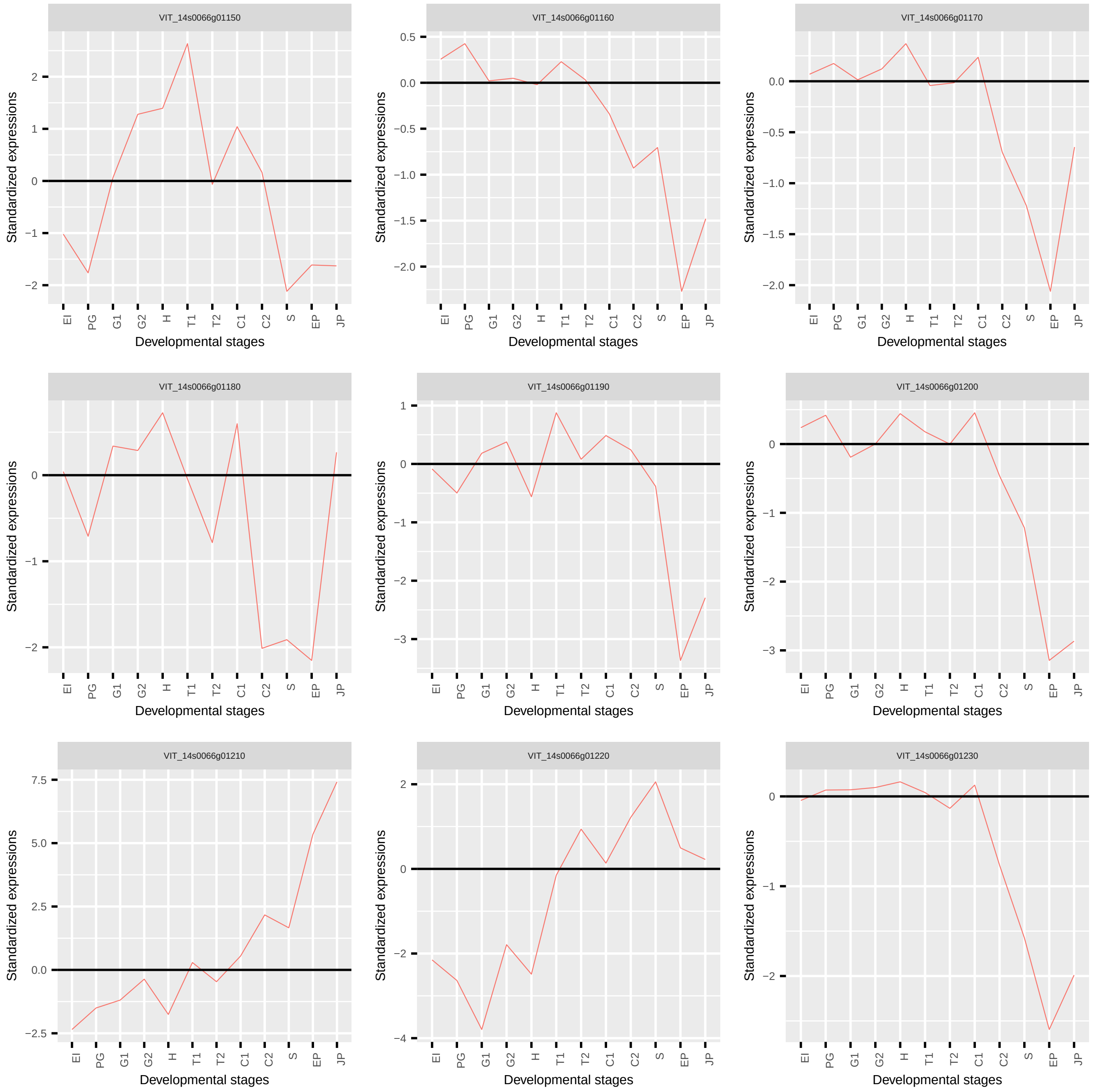
VIT_14s0066g01150
VIT_14s0066g01160
VIT_14s0066g01170
VIT_14s0066g01180
VIT_14s0066g01190
VIT_14s0066g01200
VIT_14s0066g01210
VIT_14s0066g01220
VIT_14s0066g01230
Standardized expressions
Developmental stages
EI PG G1 G2 H T1 T2 C1 C2 S EP JP

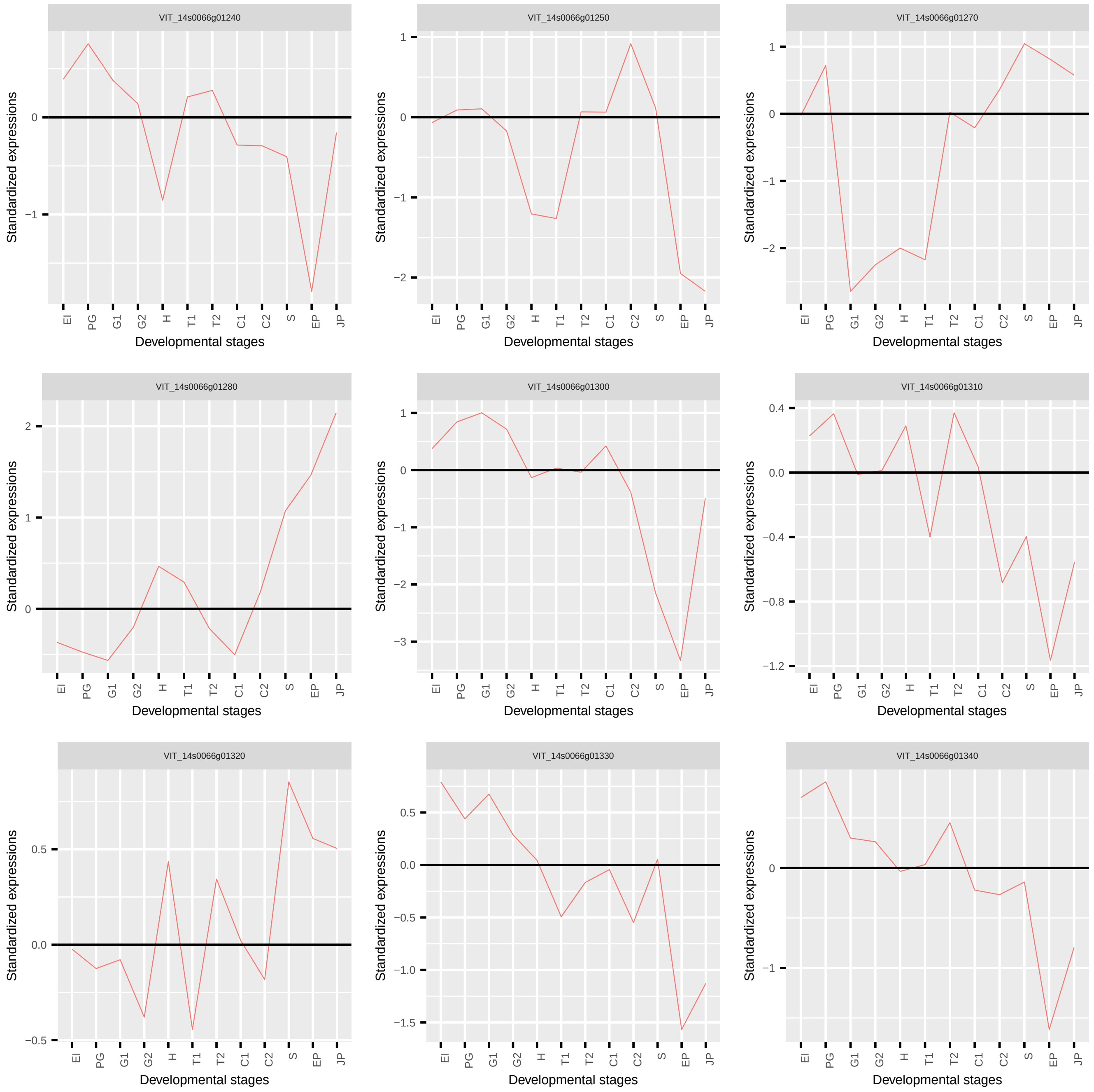
VIT_14s0066g01240
VIT_14s0066g01250
VIT_14s0066g01270
VIT_14s0066g01280
VIT_14s0066g01300
VIT_14s0066g01310
VIT_14s0066g01320
VIT_14s0066g01330
VIT_14s0066g01340
Standardized expressions
Developmental stages
EI PG G1 G2 H T1 T2 C1 C2 S EP JP

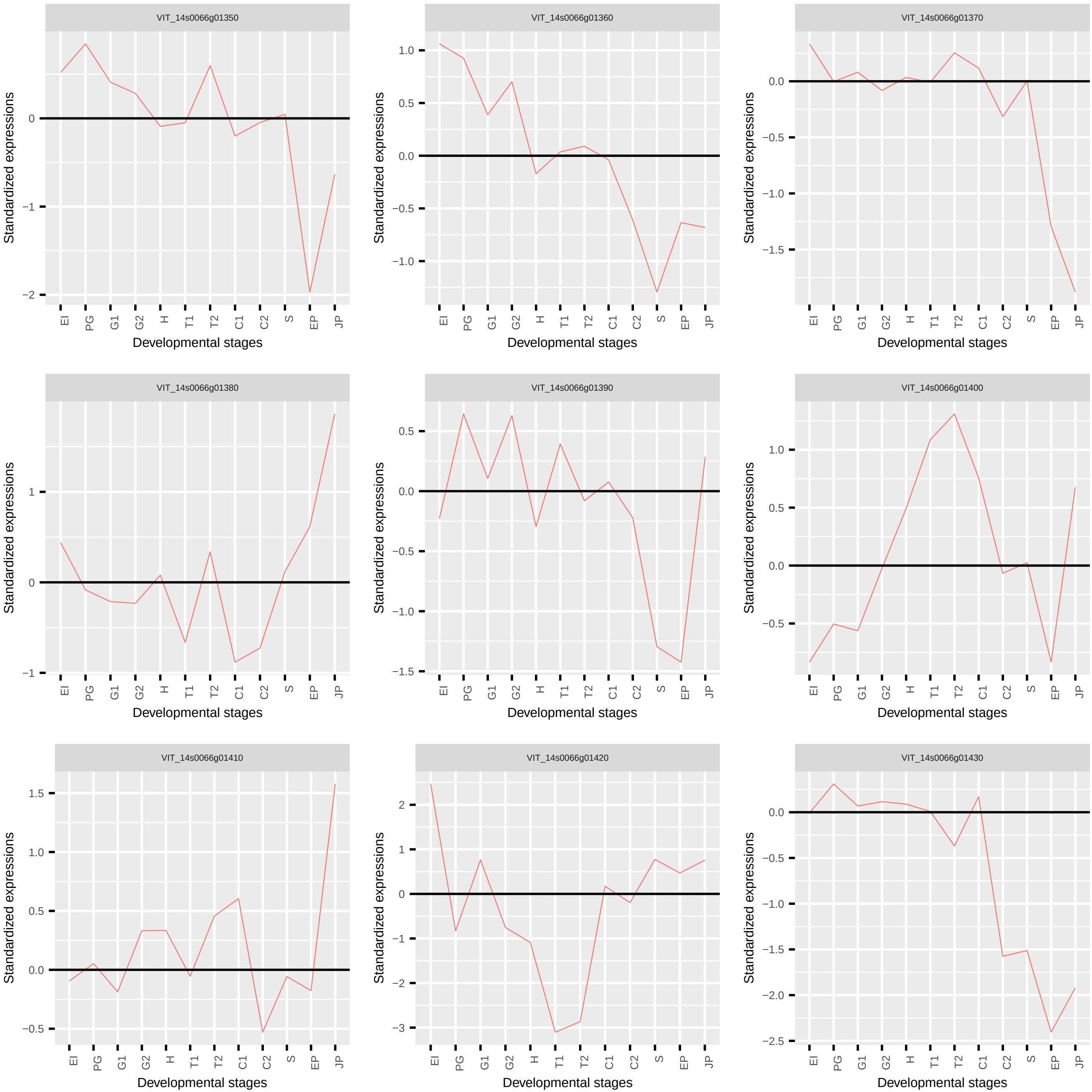
VIT_14s0066g01350
VIT_14s0066g01360
VIT_14s0066g01370
VIT_14s0066g01380
VIT_14s0066g01390
VIT_14s0066g01400
VIT_14s0066g01410
VIT_14s0066g01420
VIT_14s0066g01430
Standardized expressions
Developmental stages
EI PG G1 G2 H T1 T2 C1 C2 S EP JP

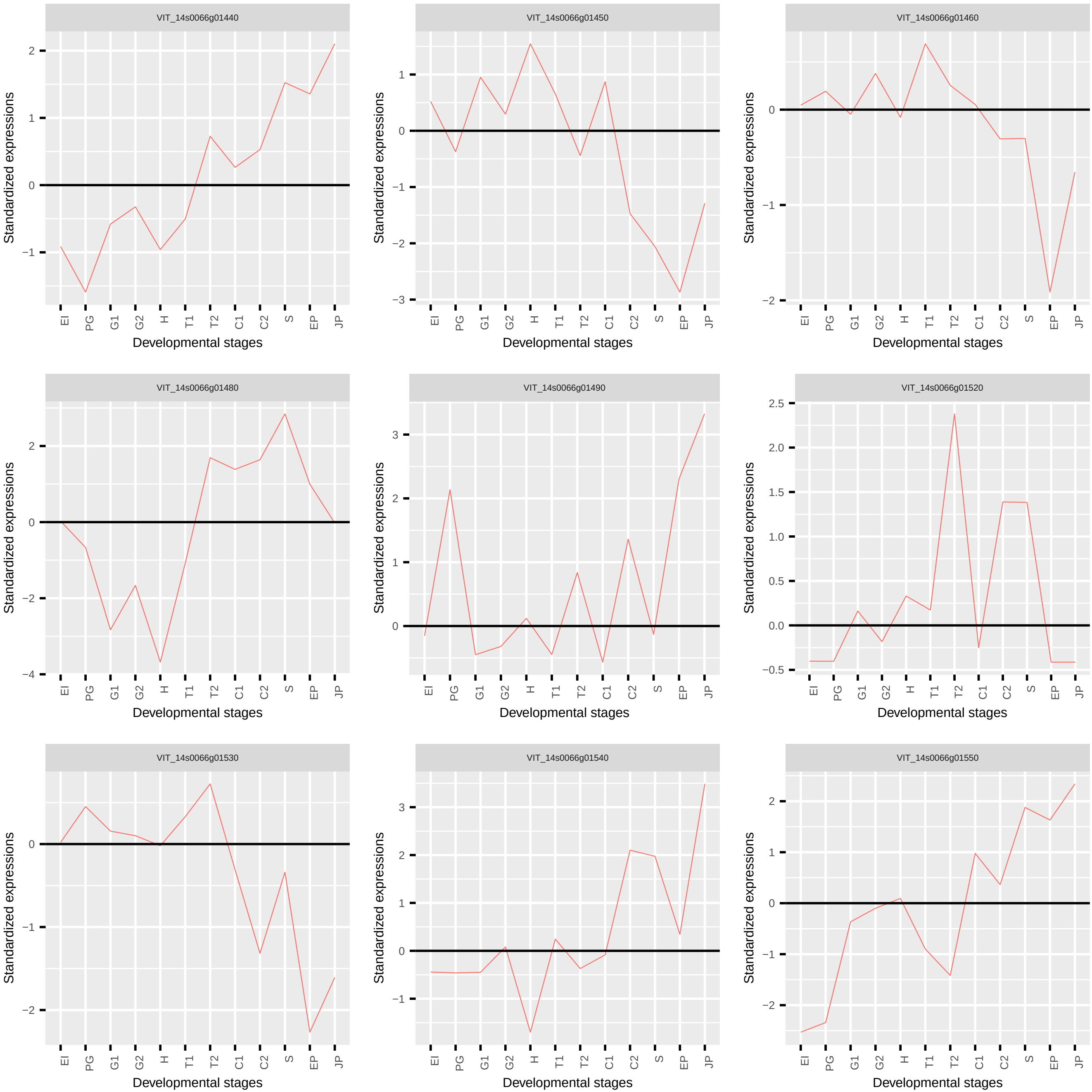

VIT_14s0066g01440
VIT_14s0066g01450
VIT_14s0066g01460
VIT_14s0066g01480
VIT_14s0066g01490
VIT_14s0066g01520
VIT_14s0066g01530
VIT_14s0066g01540
VIT_14s0066g01550
Standardized expressions
Developmental stages
EI
PG
G1
G2
H
T1
T2
C1
C2
S
EP
JP

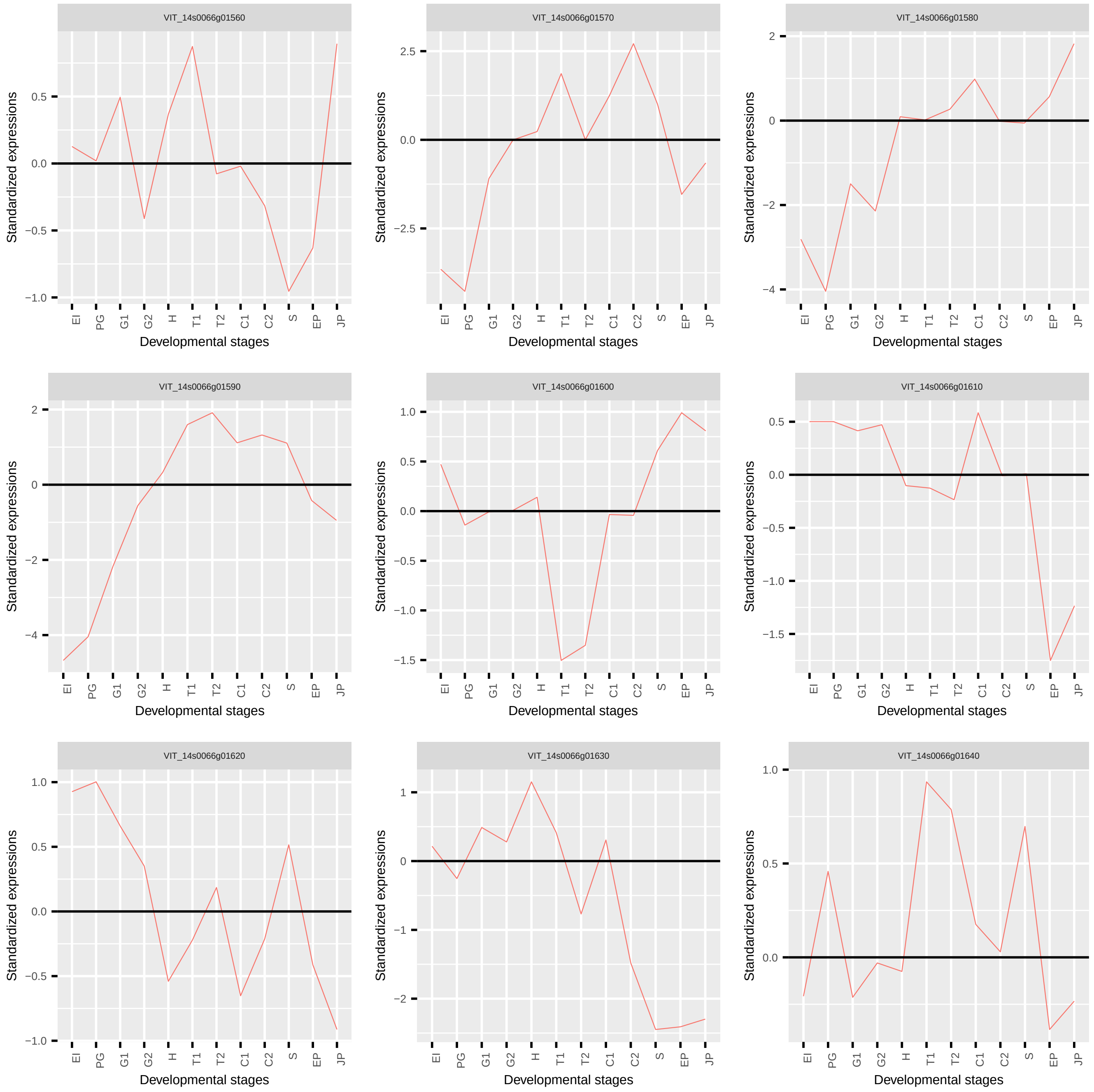
VIT_14s0066g01560
VIT_14s0066g01570
VIT_14s0066g01580
VIT_14s0066g01590
VIT_14s0066g01600
VIT_14s0066g01610
VIT_14s0066g01620
VIT_14s0066g01630
VIT_14s0066g01640
Standardized expressions
Developmental stages
EI PG G1 G2 H T1 T2 C1 C2 S EP JP

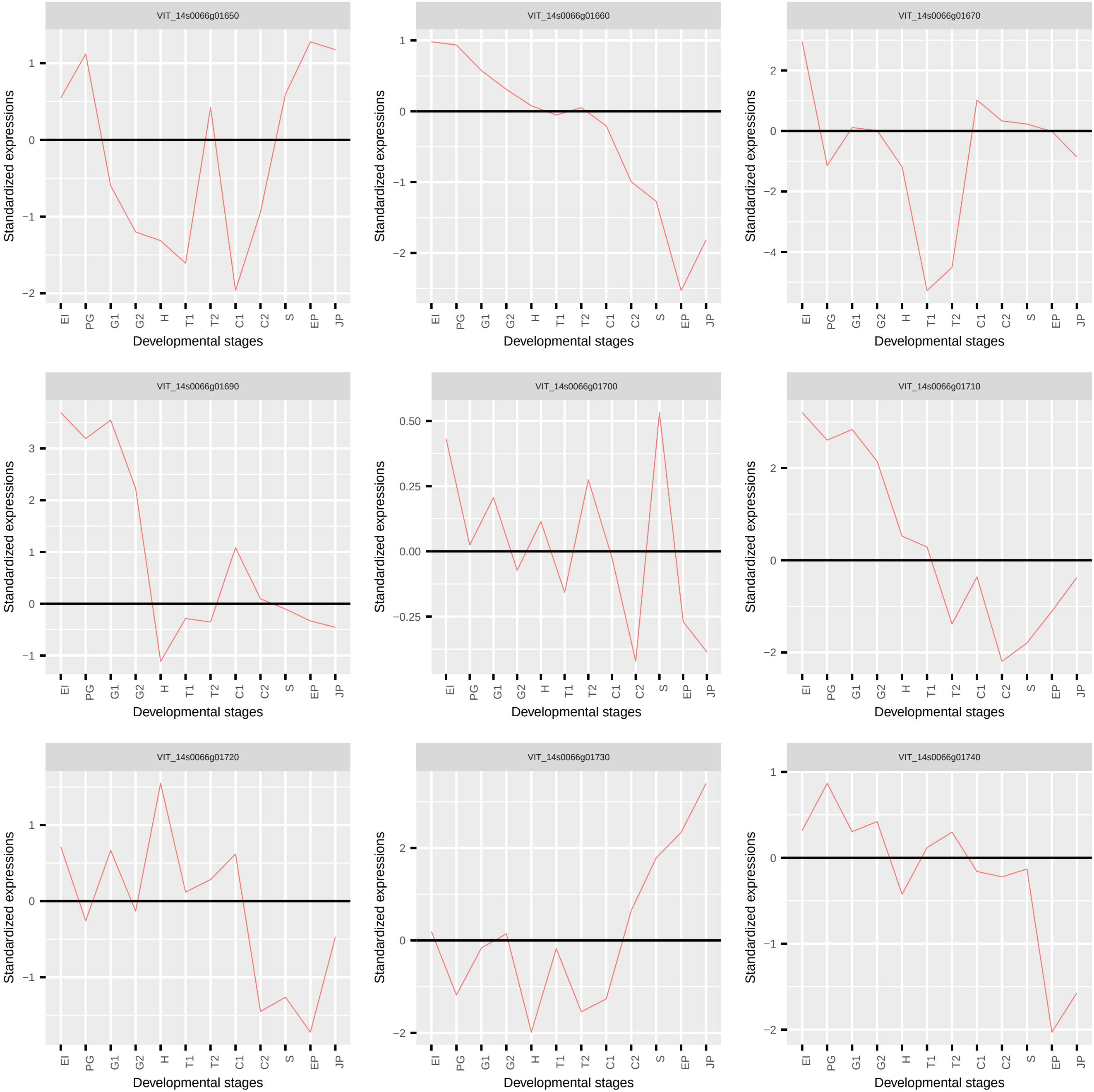

VIT_14s0066g01650
VIT_14s0066g01660
VIT_14s0066g01670
VIT_14s0066g01690
VIT_14s0066g01700
VIT_14s0066g01710
VIT_14s0066g01720
VIT_14s0066g01730
VIT_14s0066g01740
Standardized expressions
Developmental stages
EI
PG
G1
G2
H
T1
T2
C1
C2
S
EP
JP
1
0
−1
−2
2
−4
3
0.50
0.25
0.00
−0.25

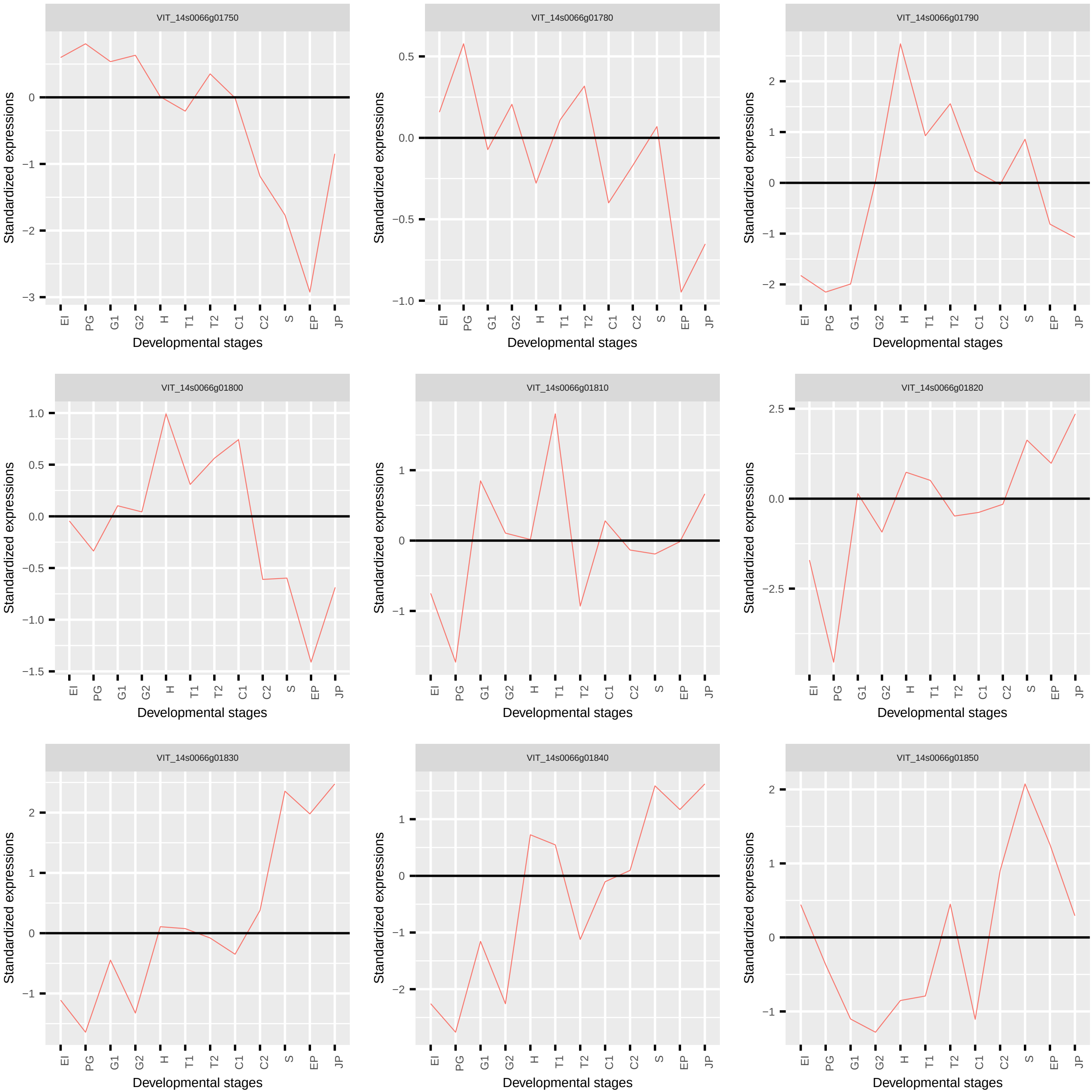
VIT_14s0066g01750
VIT_14s0066g01780
VIT_14s0066g01790
VIT_14s0066g01800
VIT_14s0066g01810
VIT_14s0066g01820
VIT_14s0066g01830
VIT_14s0066g01840
VIT_14s0066g01850
Standardized expressions
Developmental stages
EI PG G1 G2 H T1 T2 C1 C2 S EP JP

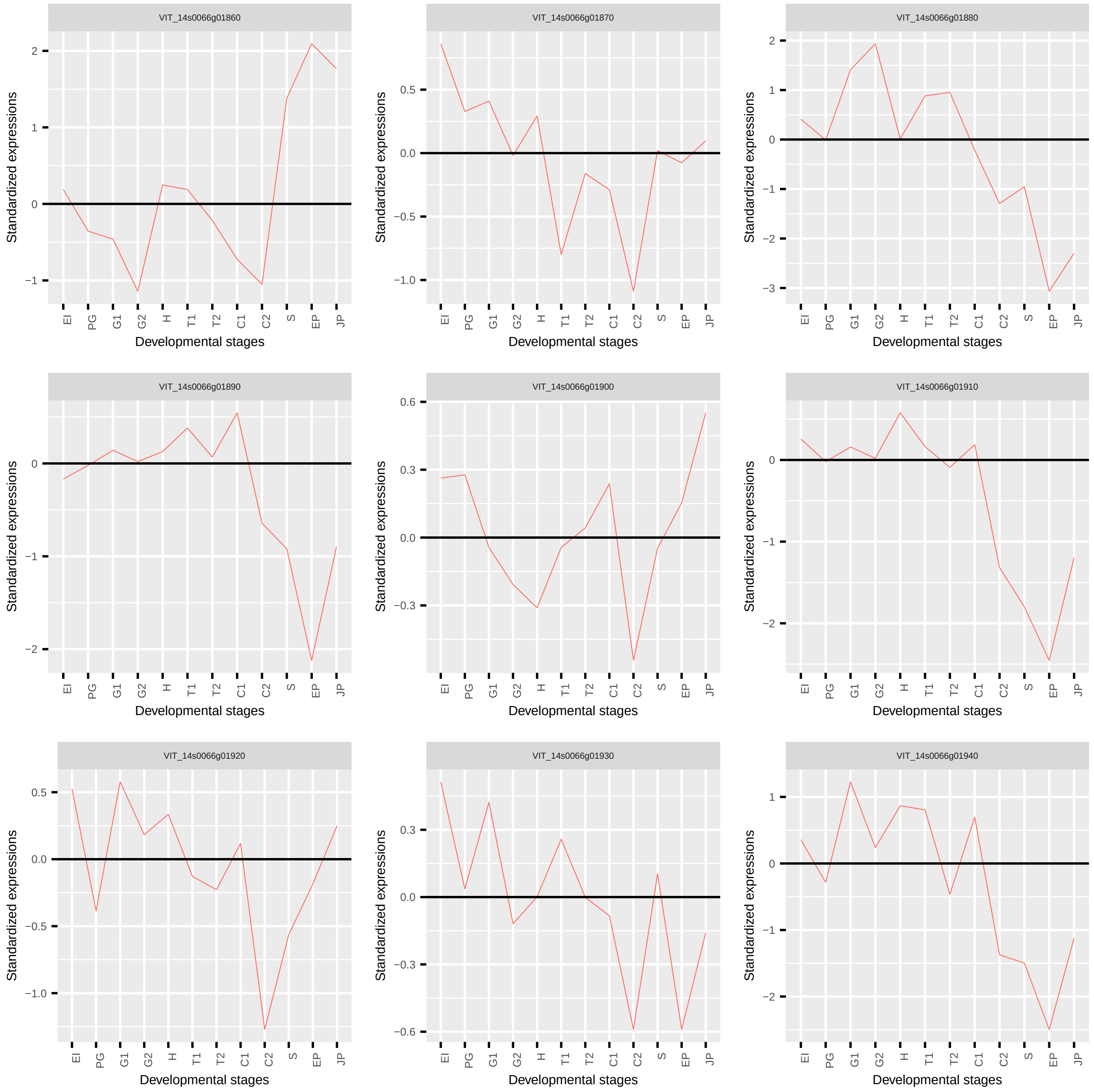
VIT_14s0066g01860
VIT_14s0066g01870
VIT_14s0066g01880
VIT_14s0066g01890
VIT_14s0066g01900
VIT_14s0066g01910
VIT_14s0066g01920
VIT_14s0066g01930
VIT_14s0066g01940
Standardized expressions
Developmental stages
EI PG G1 G2 H T1 T2 C1 C2 S EP JP

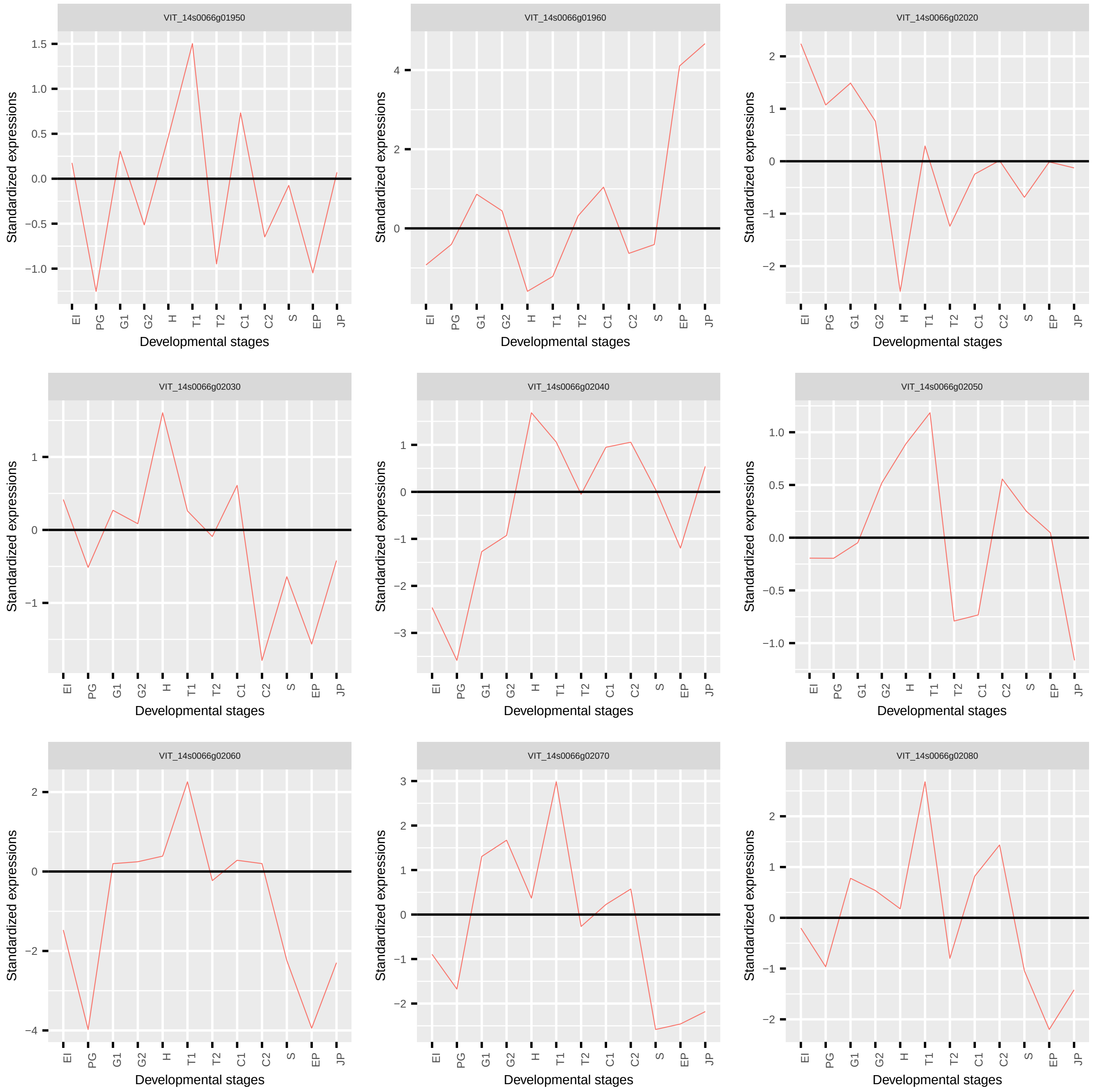
VIT_14s0066g01950
VIT_14s0066g01960
VIT_14s0066g02020
VIT_14s0066g02030
VIT_14s0066g02040
VIT_14s0066g02050
VIT_14s0066g02060
VIT_14s0066g02070
VIT_14s0066g02080
Standardized expressions
Developmental stages
EI PG G1 G2 H T1 T2 C1 C2 S EP JP

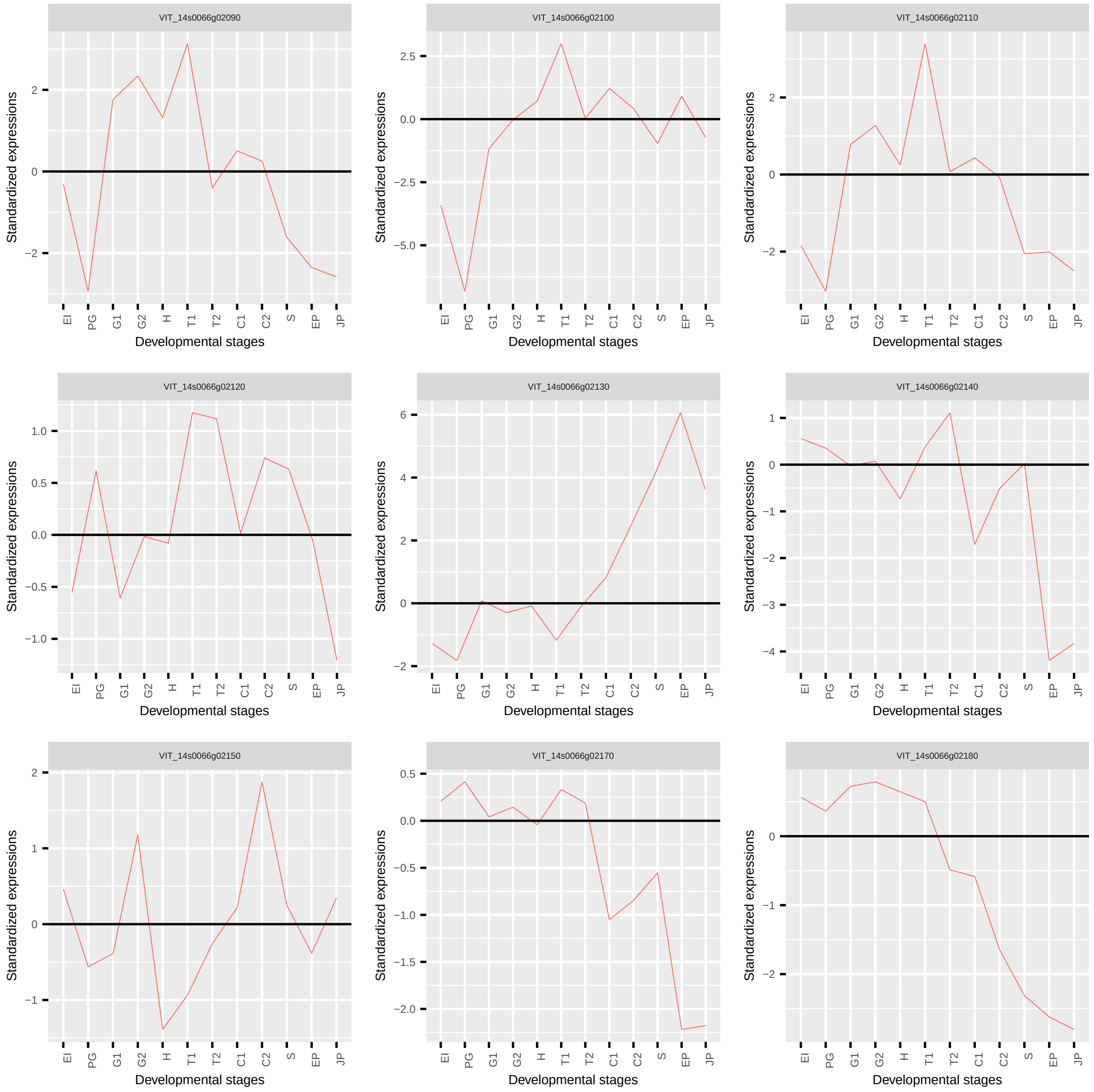
VIT_14s0066g02090
VIT_14s0066g02100
VIT_14s0066g02110
VIT_14s0066g02120
VIT_14s0066g02130
VIT_14s0066g02140
VIT_14s0066g02150
VIT_14s0066g02170
VIT_14s0066g02180
Standardized expressions
Developmental stages
EI PG G1 G2 H T1 T2 C1 C2 S EP JP

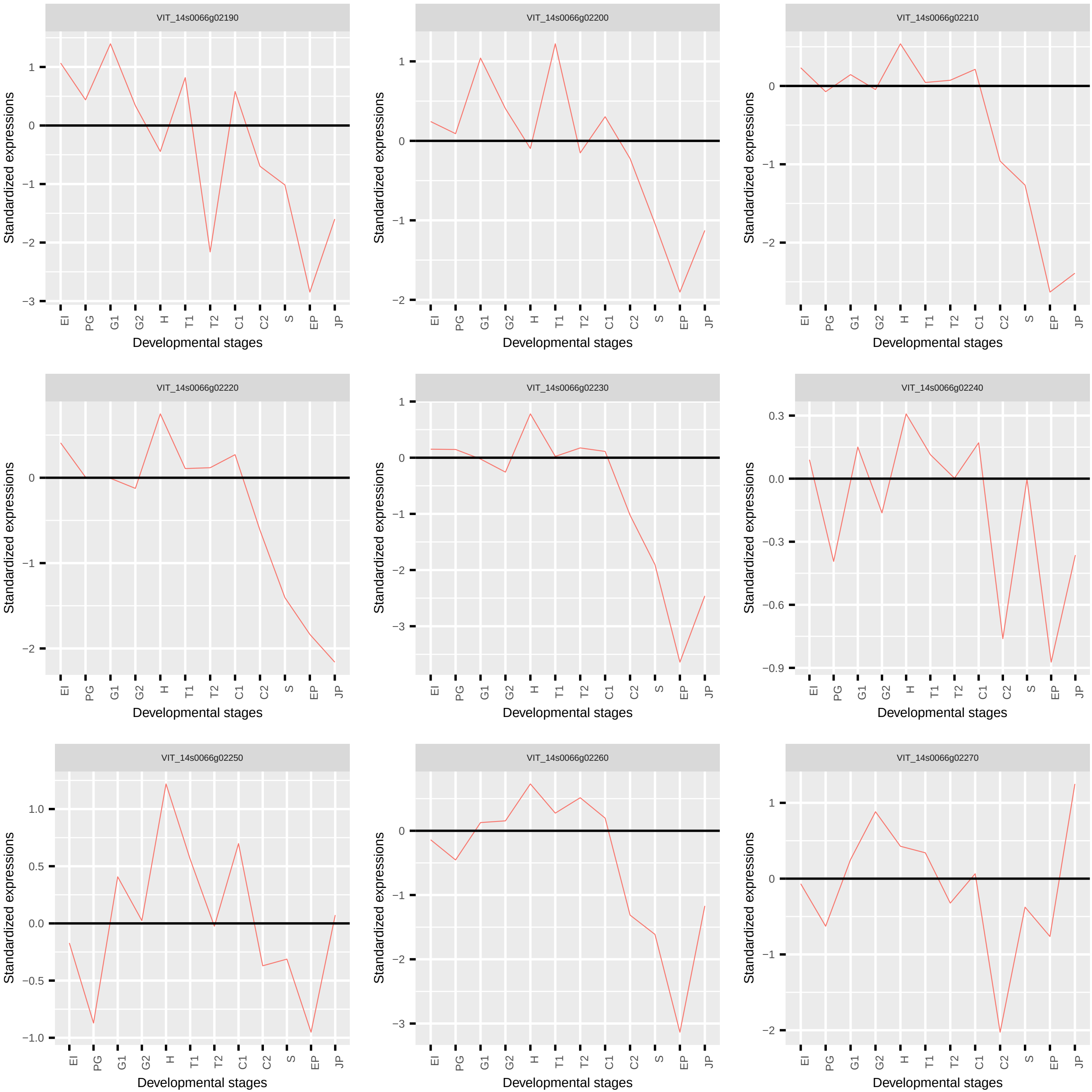
VIT_14s0066g02190
VIT_14s0066g02200
VIT_14s0066g02210
VIT_14s0066g02220
VIT_14s0066g02230
VIT_14s0066g02240
VIT_14s0066g02250
VIT_14s0066g02260
VIT_14s0066g02270
Standardized expressions
Developmental stages
EI PG G1 G2 H T1 T2 C1 C2 S EP JP

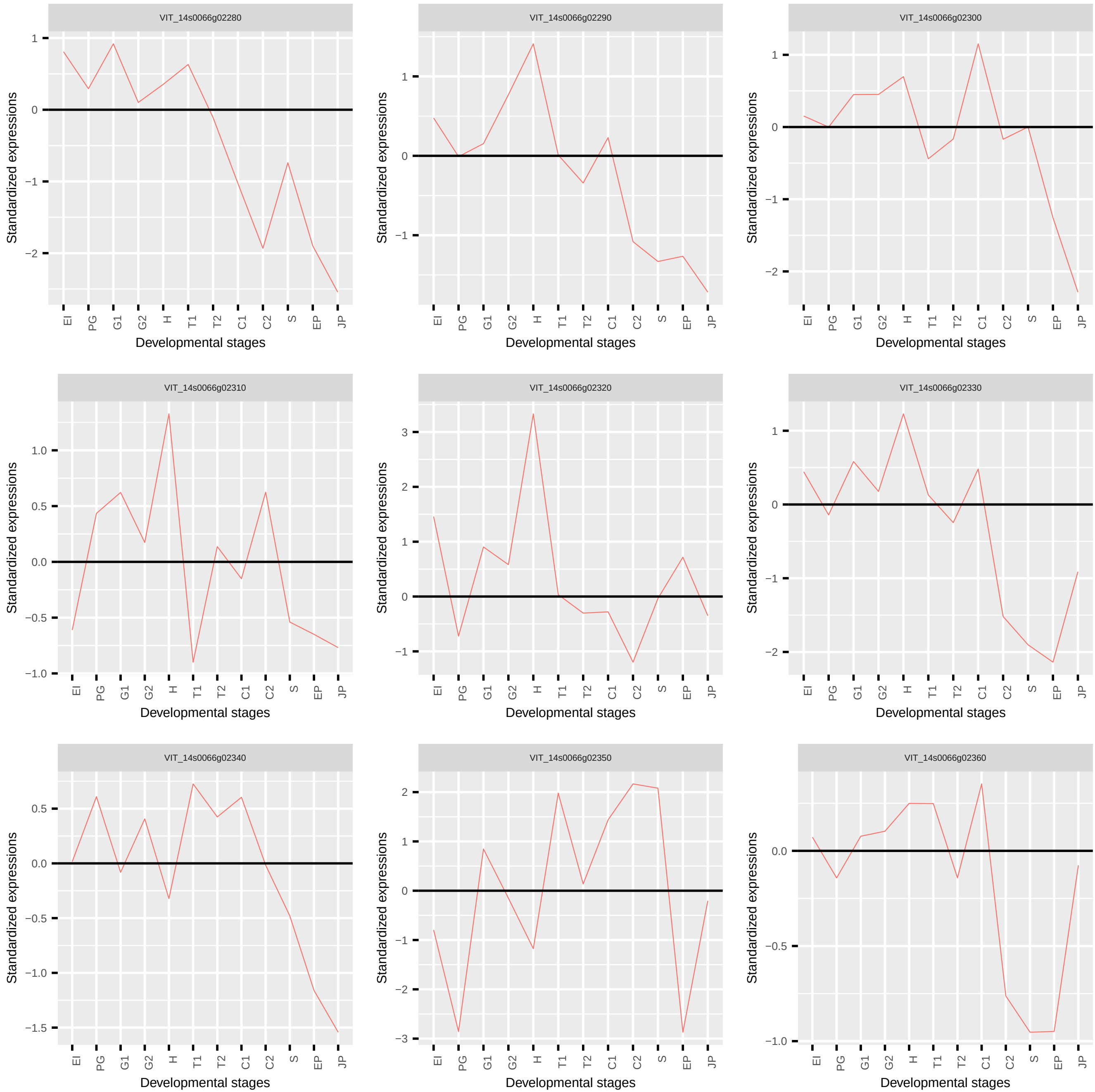
VIT_14s0066g02280
VIT_14s0066g02290
VIT_14s0066g02300
VIT_14s0066g02310
VIT_14s0066g02320
VIT_14s0066g02330
VIT_14s0066g02340
VIT_14s0066g02350
VIT_14s0066g02360
Standardized expressions
Developmental stages
EI PG G1 G2 H T1 T2 C1 C2 S EP JP

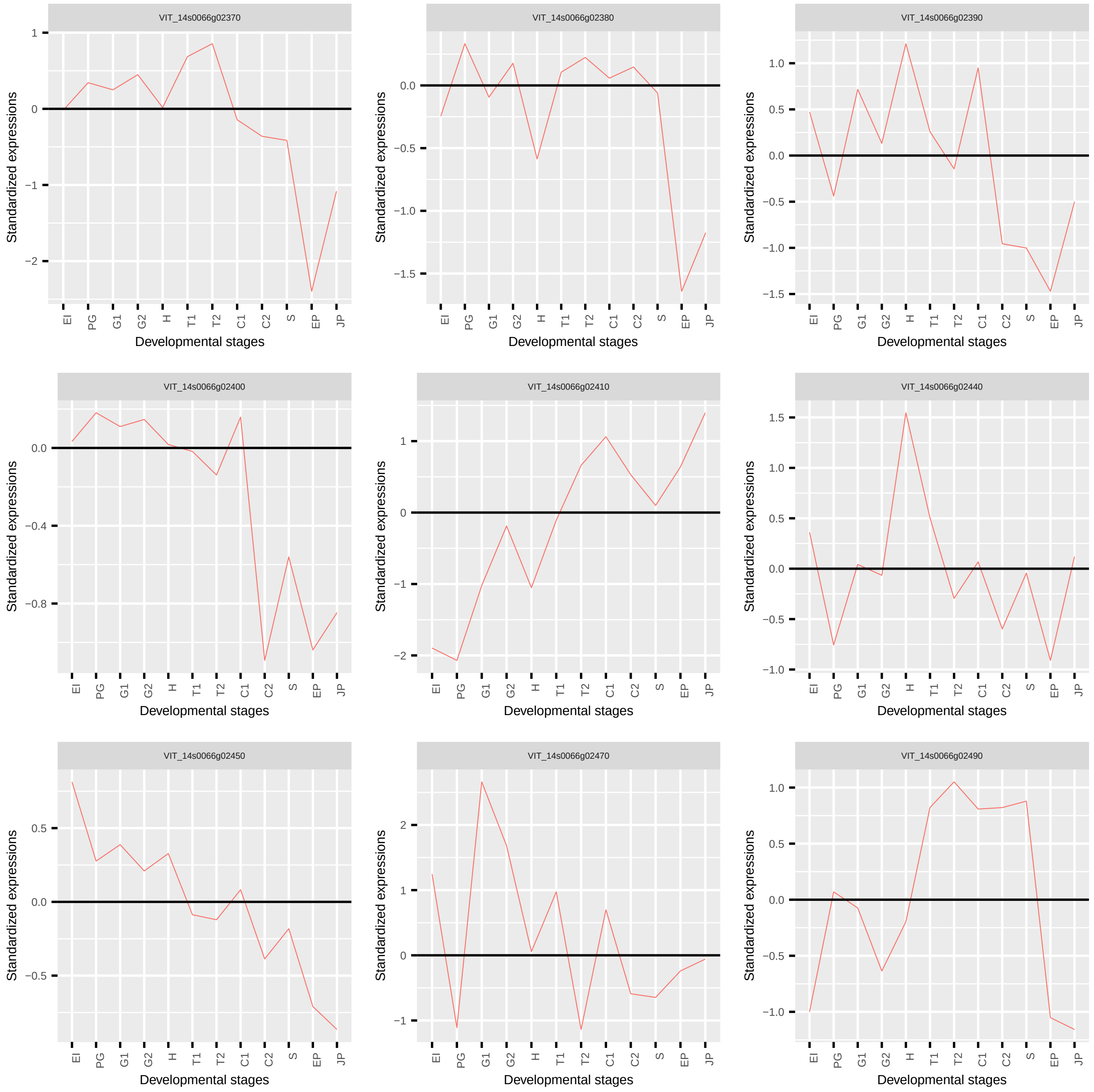
VIT_14s0066g02370
VIT_14s0066g02380
VIT_14s0066g02390
VIT_14s0066g02400
VIT_14s0066g02410
VIT_14s0066g02440
VIT_14s0066g02450
VIT_14s0066g02470
VIT_14s0066g02490
Standardized expressions
Developmental stages
EI
PG
G1
G2
H
T1
T2
C1
C2
S
EP
JP

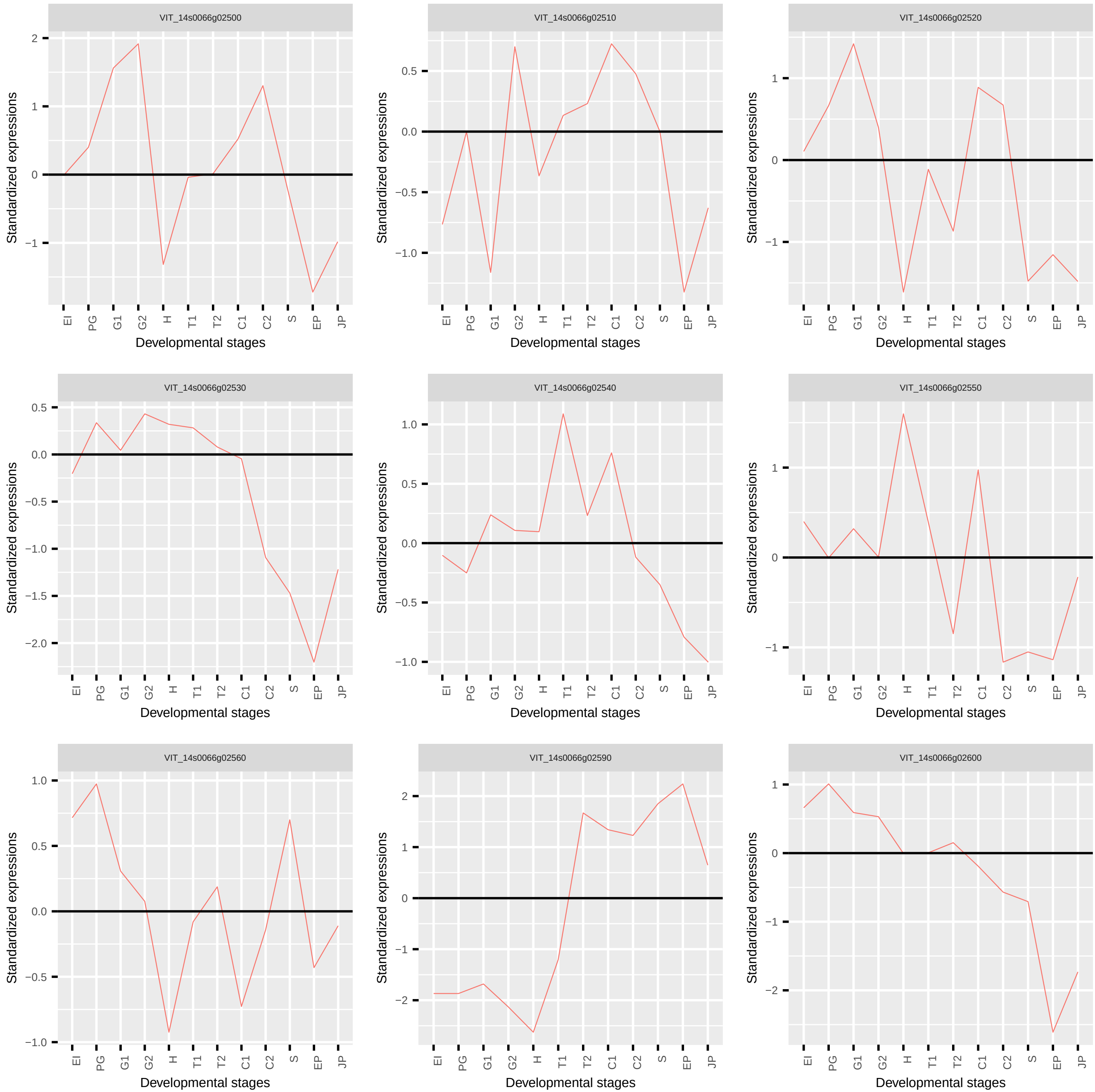
VIT_14s0066g02500
VIT_14s0066g02510
VIT_14s0066g02520
VIT_14s0066g02530
VIT_14s0066g02540
VIT_14s0066g02550
VIT_14s0066g02560
VIT_14s0066g02590
VIT_14s0066g02600
Standardized expressions
Developmental stages
EI
PG
G1
G2
H
T1
T2
C1
C2
S
EP
JP

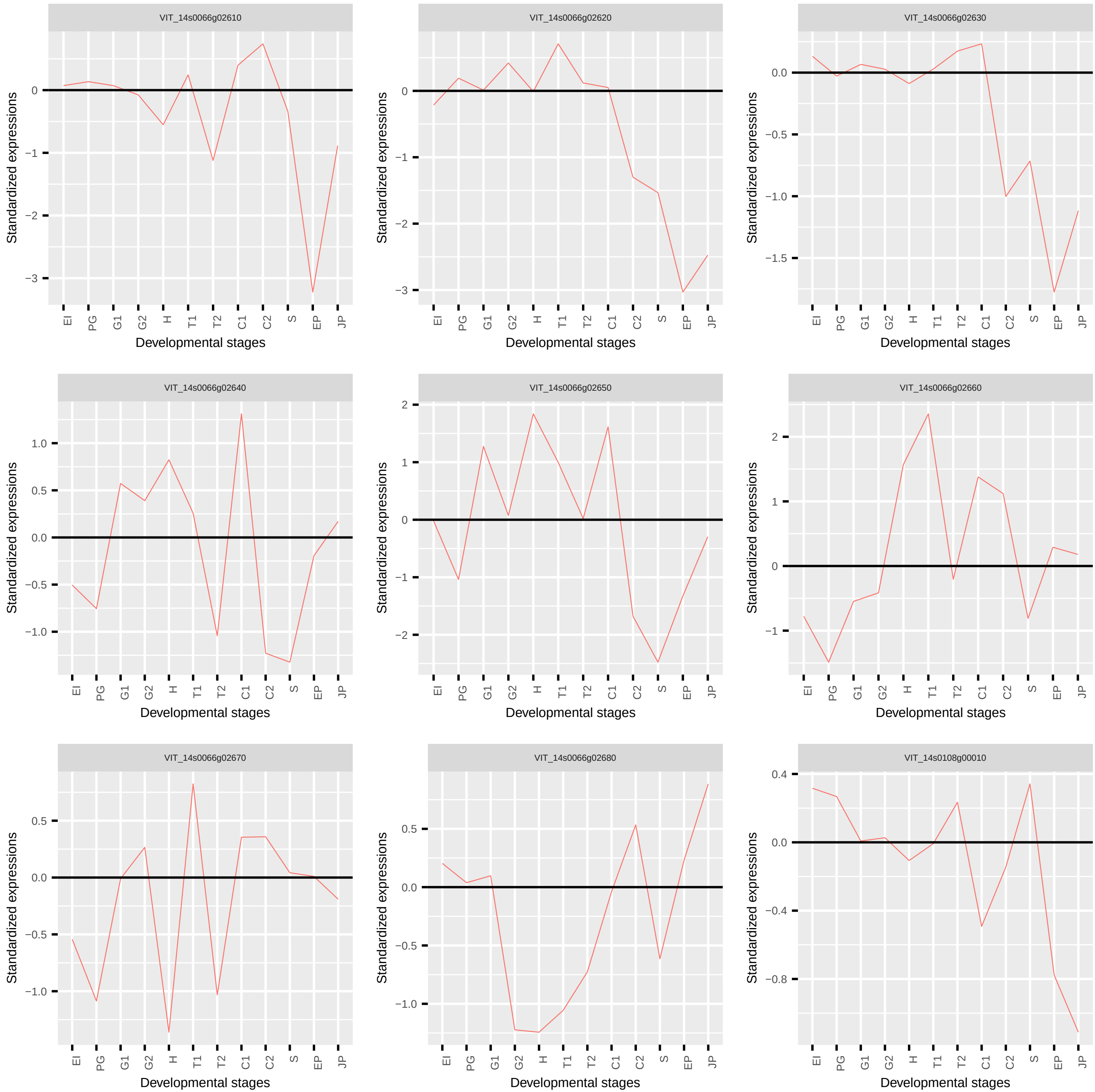

VIT_14s0066g02610
VIT_14s0066g02620
VIT_14s0066g02630
VIT_14s0066g02640
VIT_14s0066g02650
VIT_14s0066g02660
VIT_14s0066g02670
VIT_14s0066g02680
VIT_14s0108g00010
Standardized expressions
Developmental stages
EI
PG
G1
G2
H
T1
T2
C1
C2
S
EP
JP

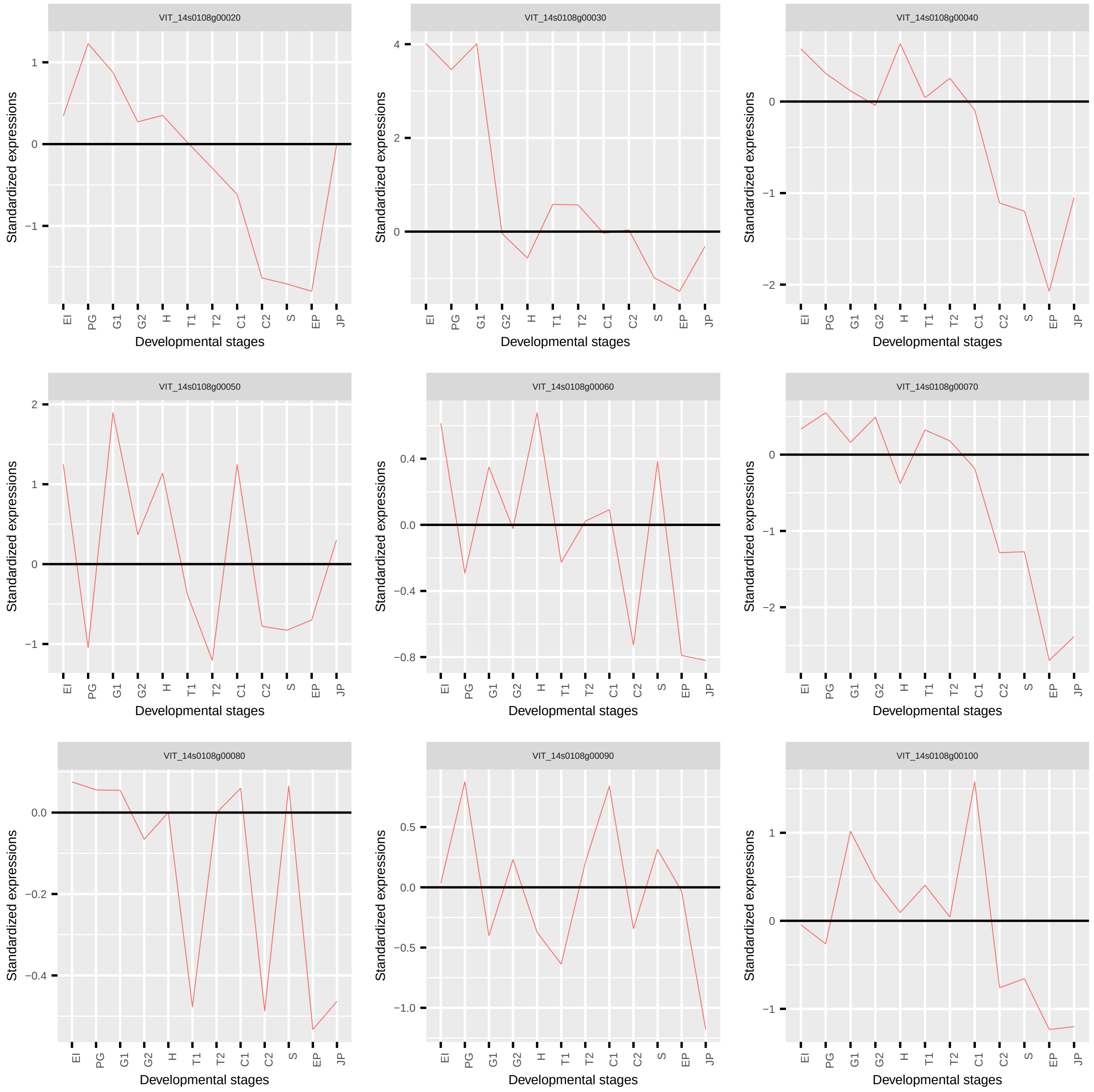

VIT_14s0108g00020
VIT_14s0108g00030
VIT_14s0108g00040
VIT_14s0108g00050
VIT_14s0108g00060
VIT_14s0108g00070
VIT_14s0108g00080
VIT_14s0108g00090
VIT_14s0108g00100
Standardized expressions
Developmental stages
EI PG G1 G2 H T1 T2 C1 C2 S EP JP

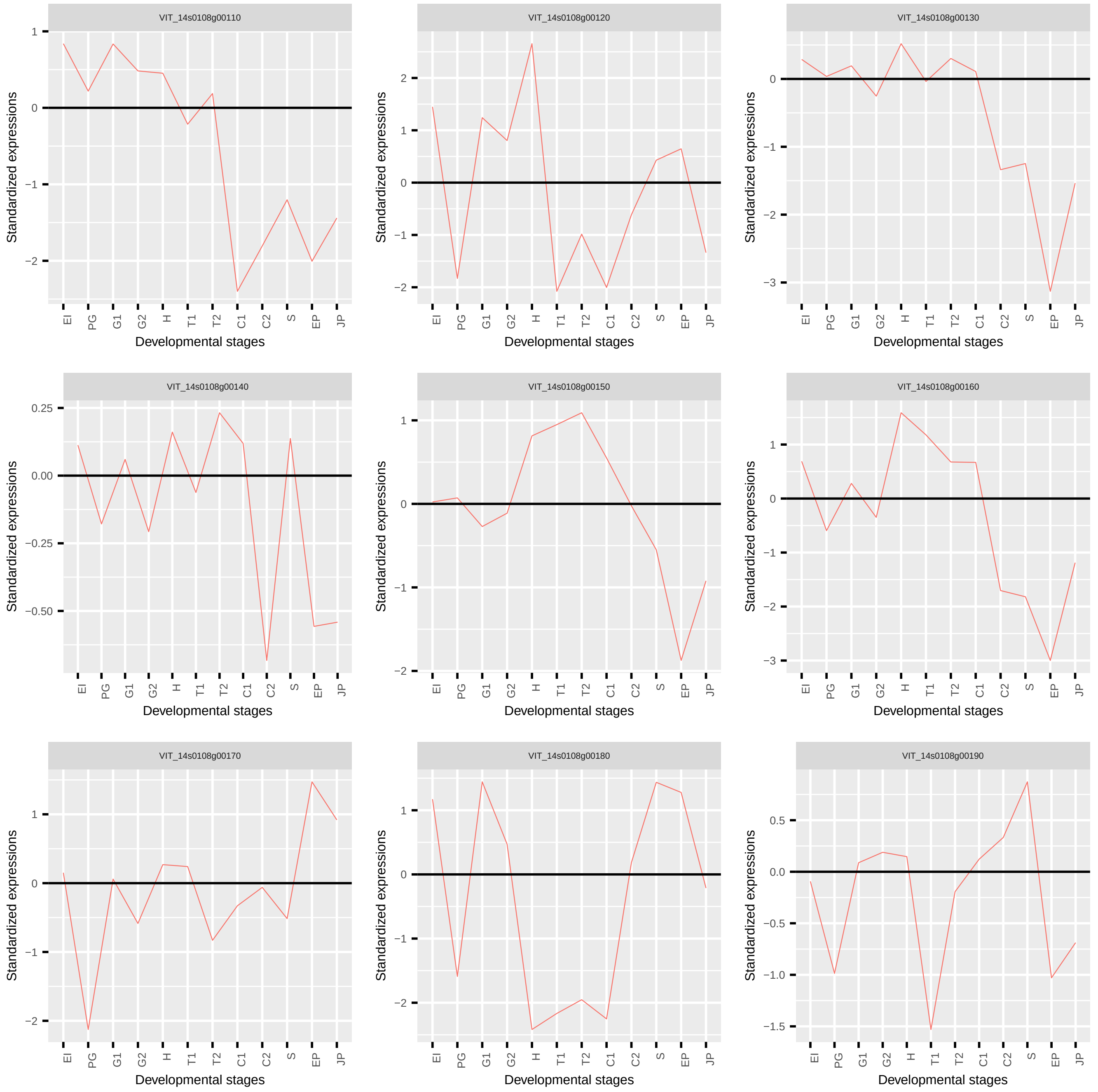
VIT_14s0108g00110
VIT_14s0108g00120
VIT_14s0108g00130
VIT_14s0108g00140
VIT_14s0108g00150
VIT_14s0108g00160
VIT_14s0108g00170
VIT_14s0108g00180
VIT_14s0108g00190
Standardized expressions
Developmental stages
EI PG G1 G2 H T1 T2 C1 C2 S EP JP

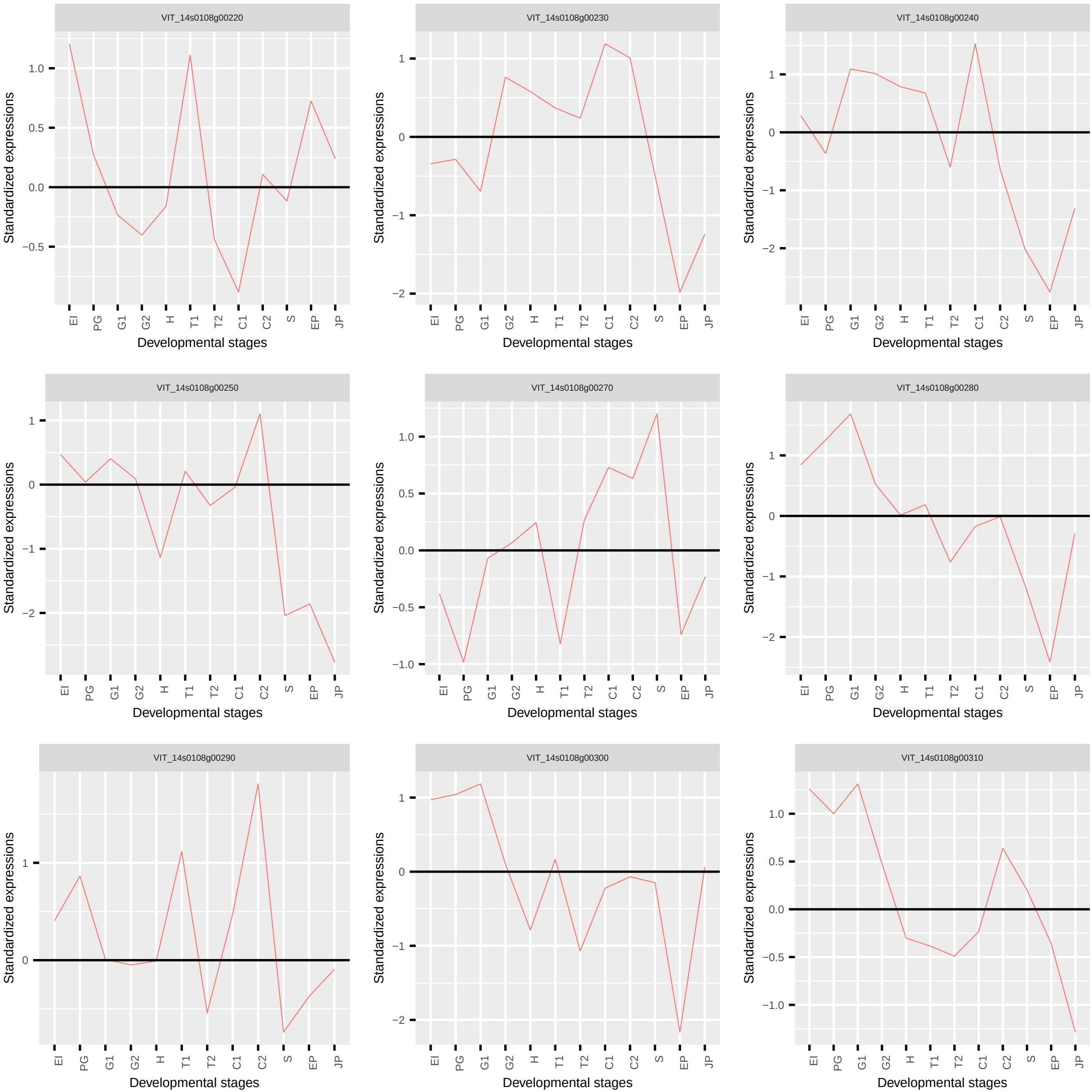
VIT_14s0108g00220
VIT_14s0108g00230
VIT_14s0108g00240
VIT_14s0108g00250
VIT_14s0108g00270
VIT_14s0108g00280
VIT_14s0108g00290
VIT_14s0108g00300
VIT_14s0108g00310
Standardized expressions
Developmental stages
EI PG G1 G2 H T1 T2 C1 C2 S EP JP
1.0 0.5 0.0 −0.5 −1.0
1 0 −1 −2

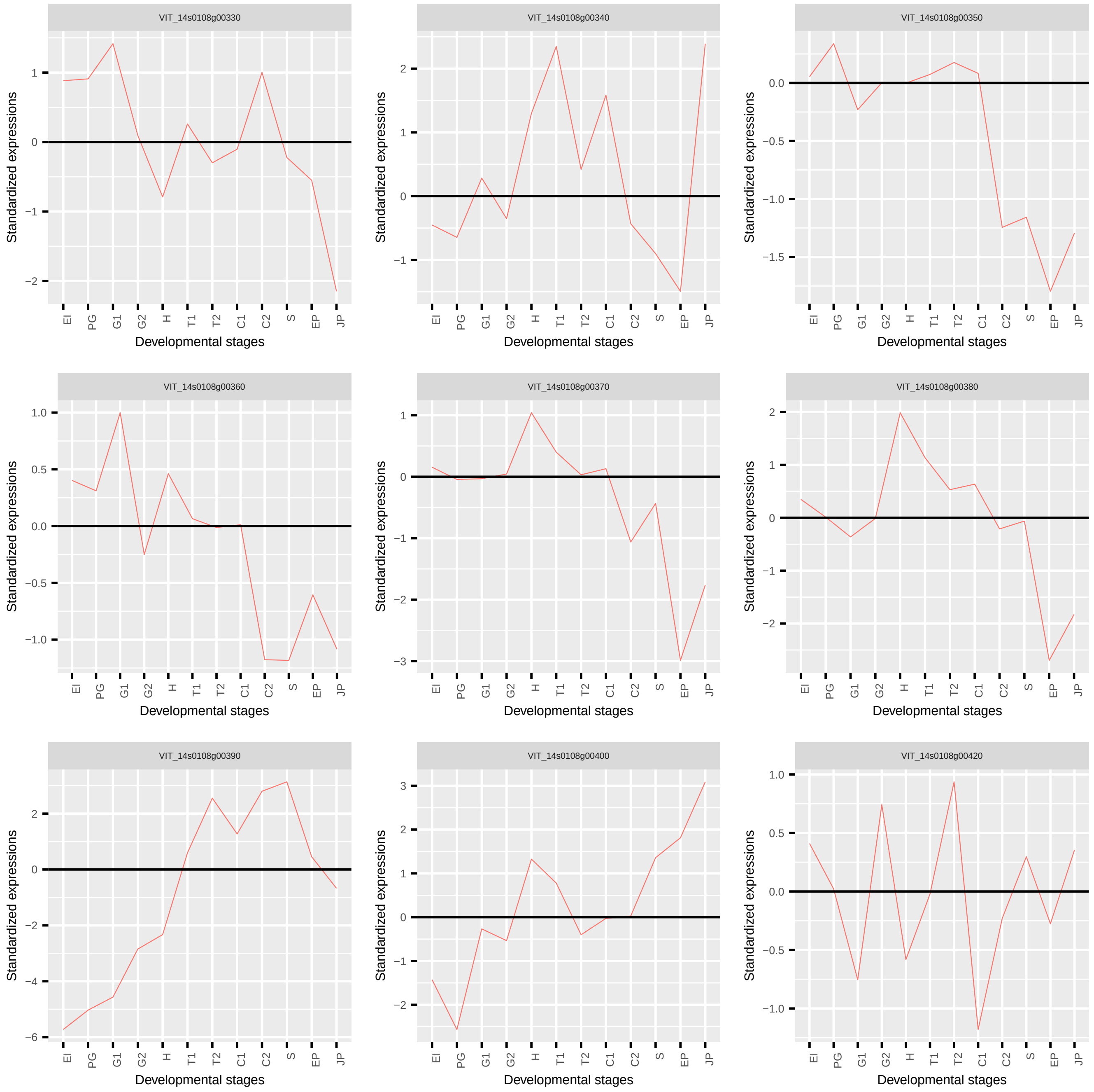

VIT_14s0108g00330
VIT_14s0108g00340
VIT_14s0108g00350
VIT_14s0108g00360
VIT_14s0108g00370
VIT_14s0108g00380
VIT_14s0108g00390
VIT_14s0108g00400
VIT_14s0108g00420
Standardized expressions
Developmental stages
EI PG G1 G2 H T1 T2 C1 C2 S EP JP

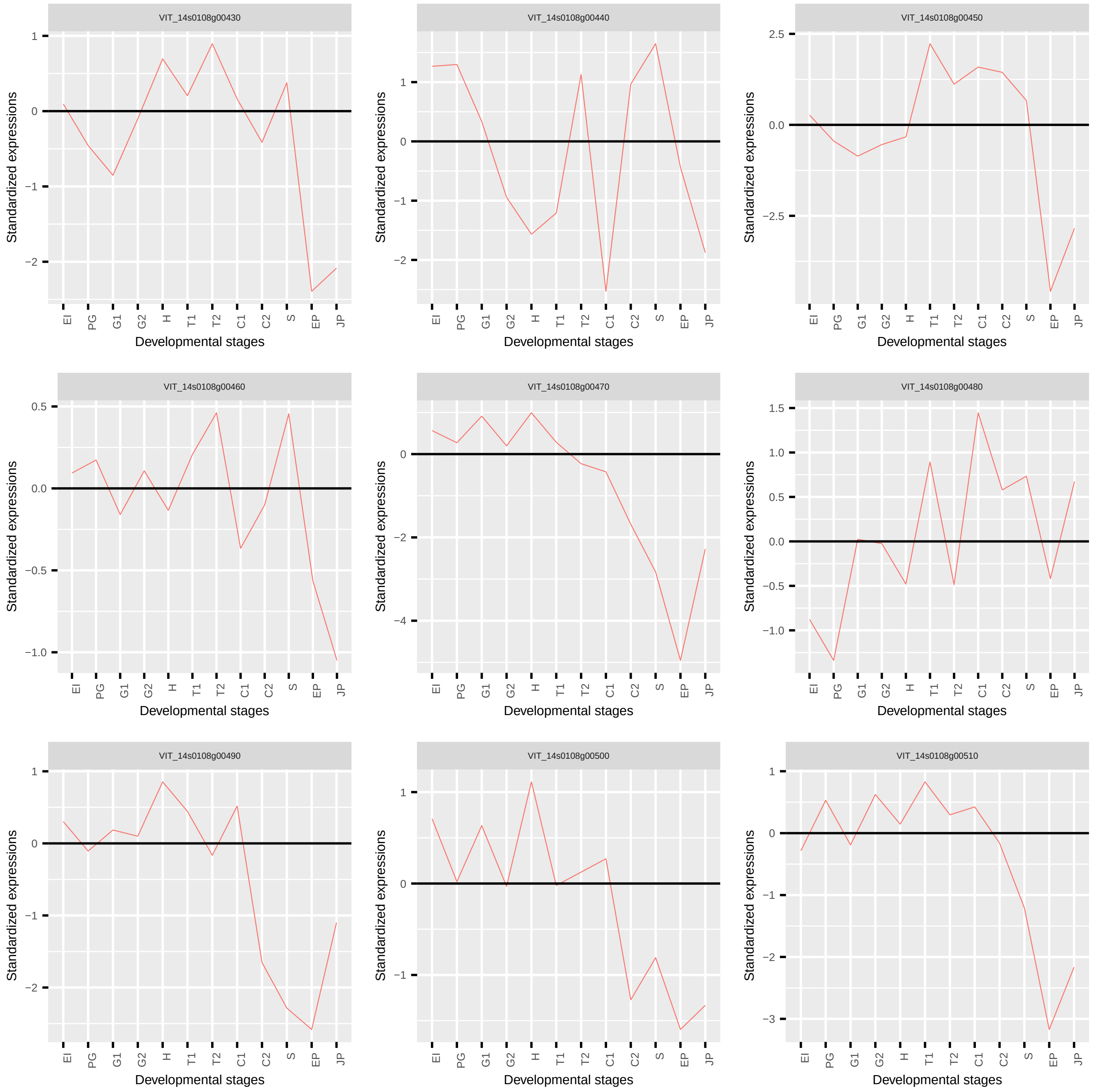
VIT_14s0108g00430
VIT_14s0108g00440
VIT_14s0108g00450
VIT_14s0108g00460
VIT_14s0108g00470
VIT_14s0108g00480
VIT_14s0108g00490
VIT_14s0108g00500
VIT_14s0108g00510
Standardized expressions
Developmental stages
EI PG G1 G2 H T1 T2 C1 C2 S EP JP
1
0
−1
−2
−3
2.5
0.0
−2.5
0.5
−0.5
−1.0
−4
1.5
1.0

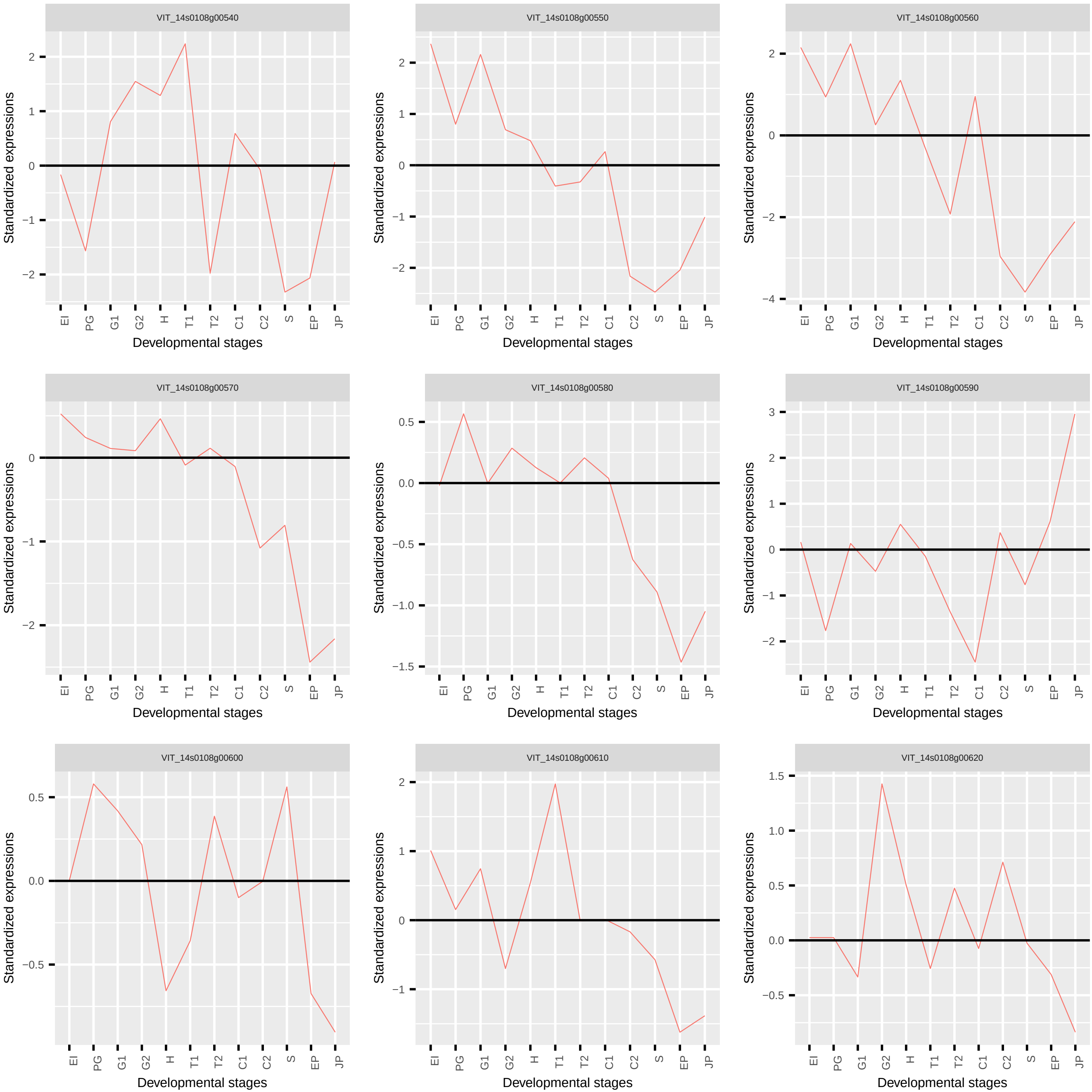
VIT_14s0108g00540
VIT_14s0108g00550
VIT_14s0108g00560
VIT_14s0108g00570
VIT_14s0108g00580
VIT_14s0108g00590
VIT_14s0108g00600
VIT_14s0108g00610
VIT_14s0108g00620
Standardized expressions
Developmental stages
EI PG G1 G2 H T1 T2 C1 C2 S EP JP

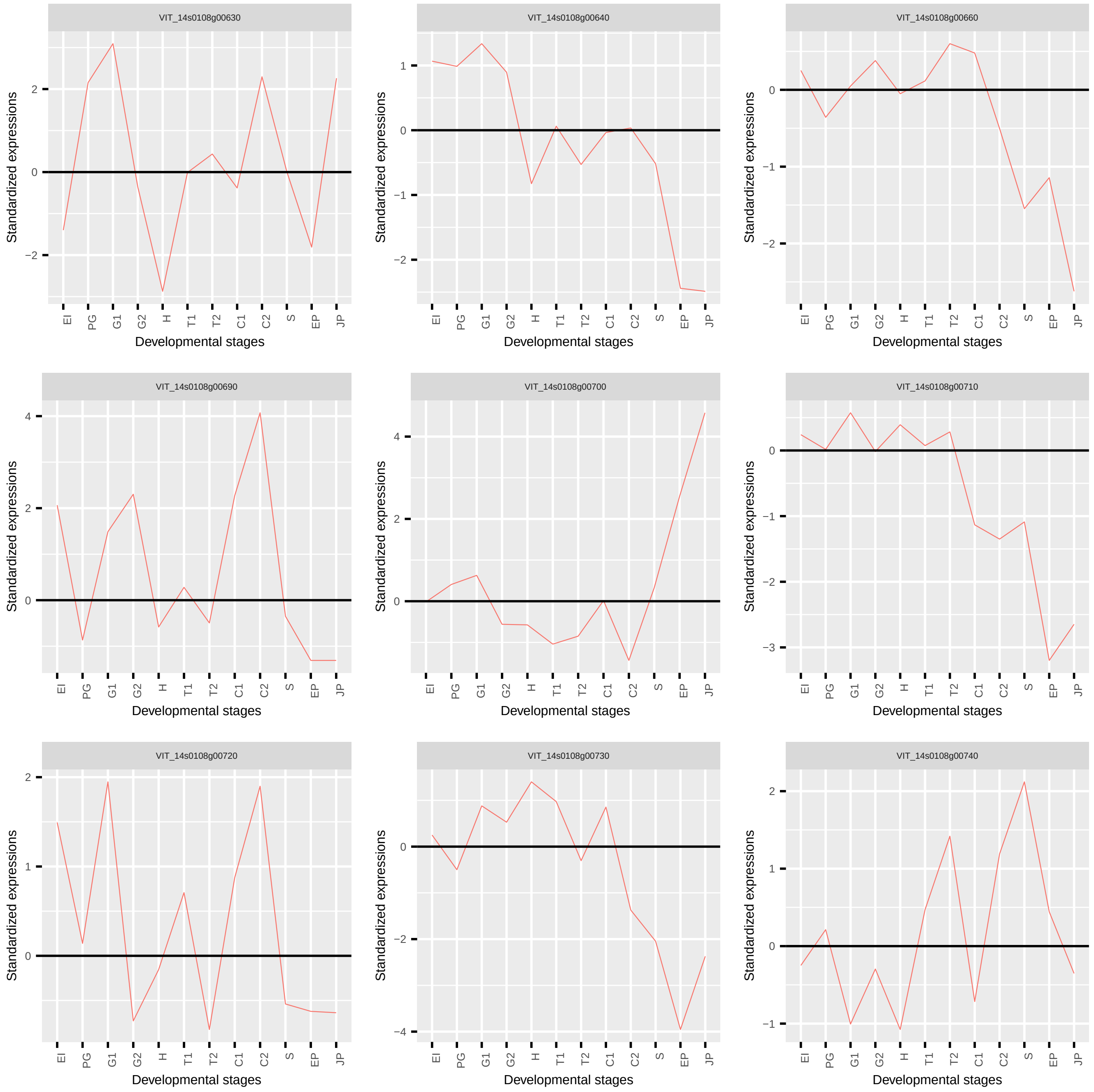

VIT_14s0108g00630
VIT_14s0108g00640
VIT_14s0108g00660
VIT_14s0108g00690
VIT_14s0108g00700
VIT_14s0108g00710
VIT_14s0108g00720
VIT_14s0108g00730
VIT_14s0108g00740
Standardized expressions
Developmental stages
EI PG G1 G2 H T1 T2 C1 C2 S EP JP

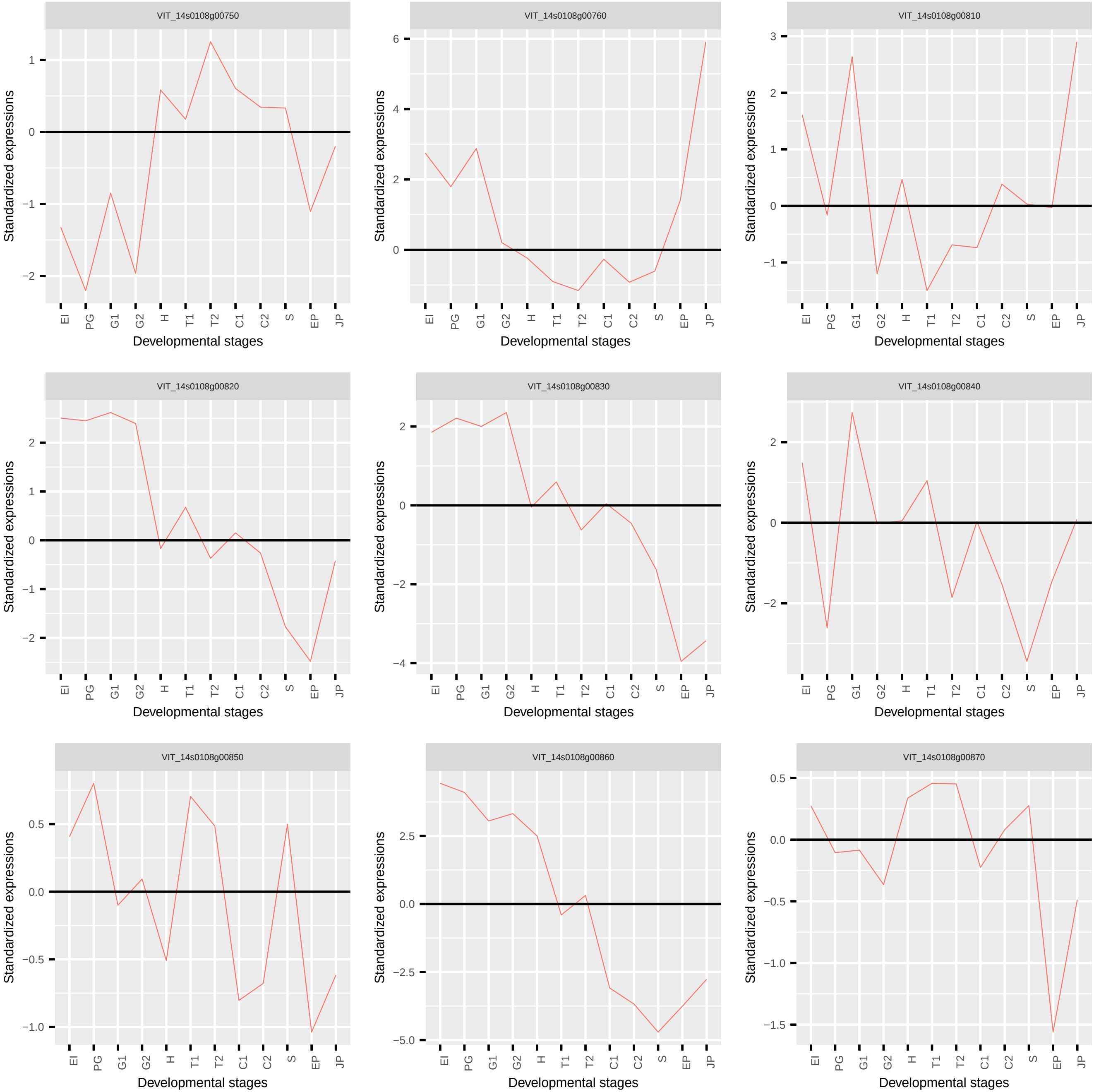
VIT_14s0108g00750
VIT_14s0108g00760
VIT_14s0108g00810
VIT_14s0108g00820
VIT_14s0108g00830
VIT_14s0108g00840
VIT_14s0108g00850
VIT_14s0108g00860
VIT_14s0108g00870
Standardized expressions
Developmental stages
EI PG G1 G2 H T1 T2 C1 C2 S EP JP

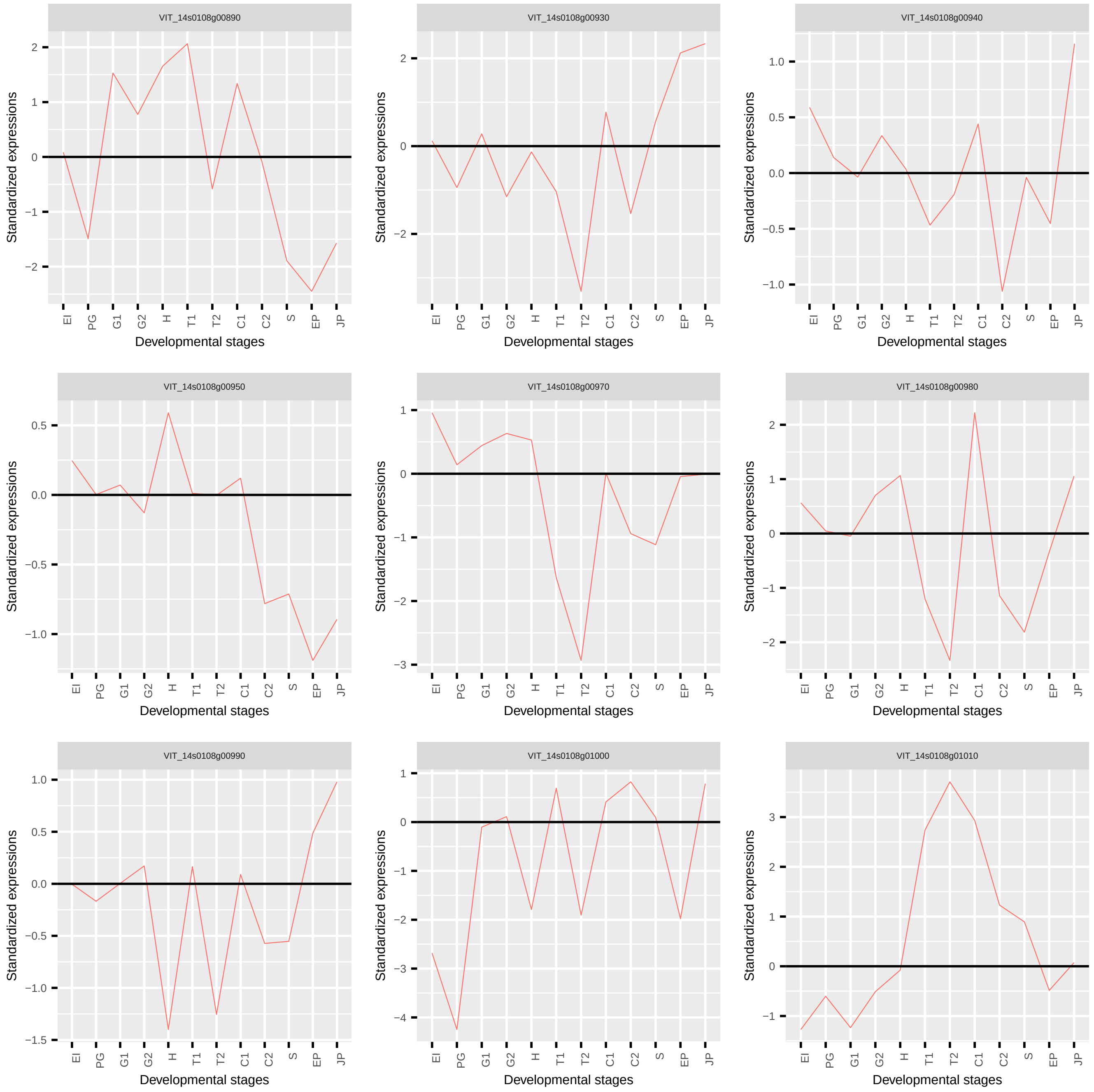
VIT_14s0108g00890
VIT_14s0108g00930
VIT_14s0108g00940
VIT_14s0108g00950
VIT_14s0108g00970
VIT_14s0108g00980
VIT_14s0108g00990
VIT_14s0108g01000
VIT_14s0108g01010
Standardized expressions
Developmental stages
EI PG G1 G2 H T1 T2 C1 C2 S EP JP

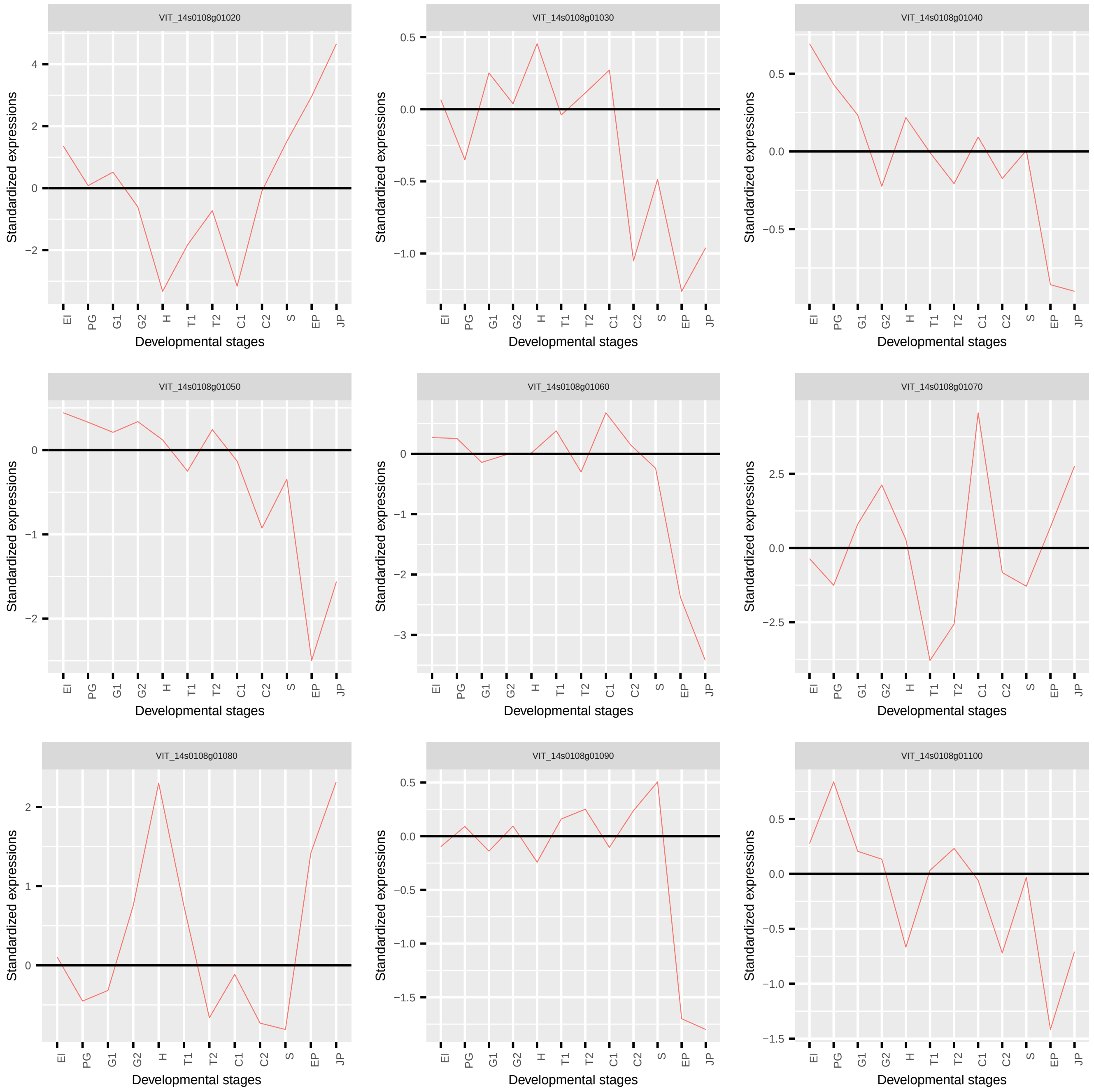
VIT_14s0108g01020
VIT_14s0108g01030
VIT_14s0108g01040
VIT_14s0108g01050
VIT_14s0108g01060
VIT_14s0108g01070
VIT_14s0108g01080
VIT_14s0108g01090
VIT_14s0108g01100
Standardized expressions
Developmental stages
EI PG G1 G2 H T1 T2 C1 C2 S EP JP

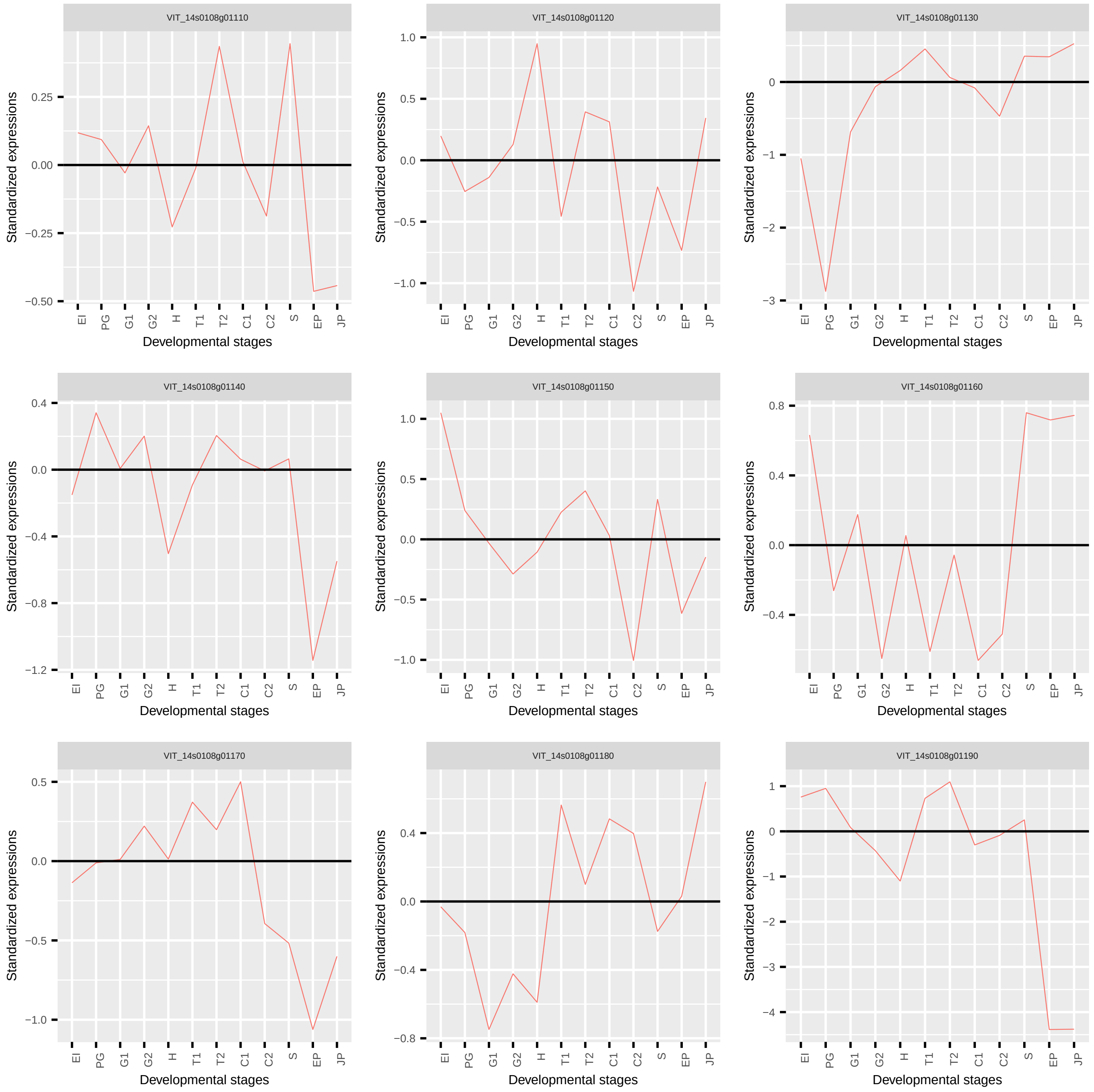
VIT_14s0108g01110
VIT_14s0108g01120
VIT_14s0108g01130
VIT_14s0108g01140
VIT_14s0108g01150
VIT_14s0108g01160
VIT_14s0108g01170
VIT_14s0108g01180
VIT_14s0108g01190
Standardized expressions
Developmental stages
EI
PG
G1
G2
H
T1
T2
C1
C2
S
EP
JP

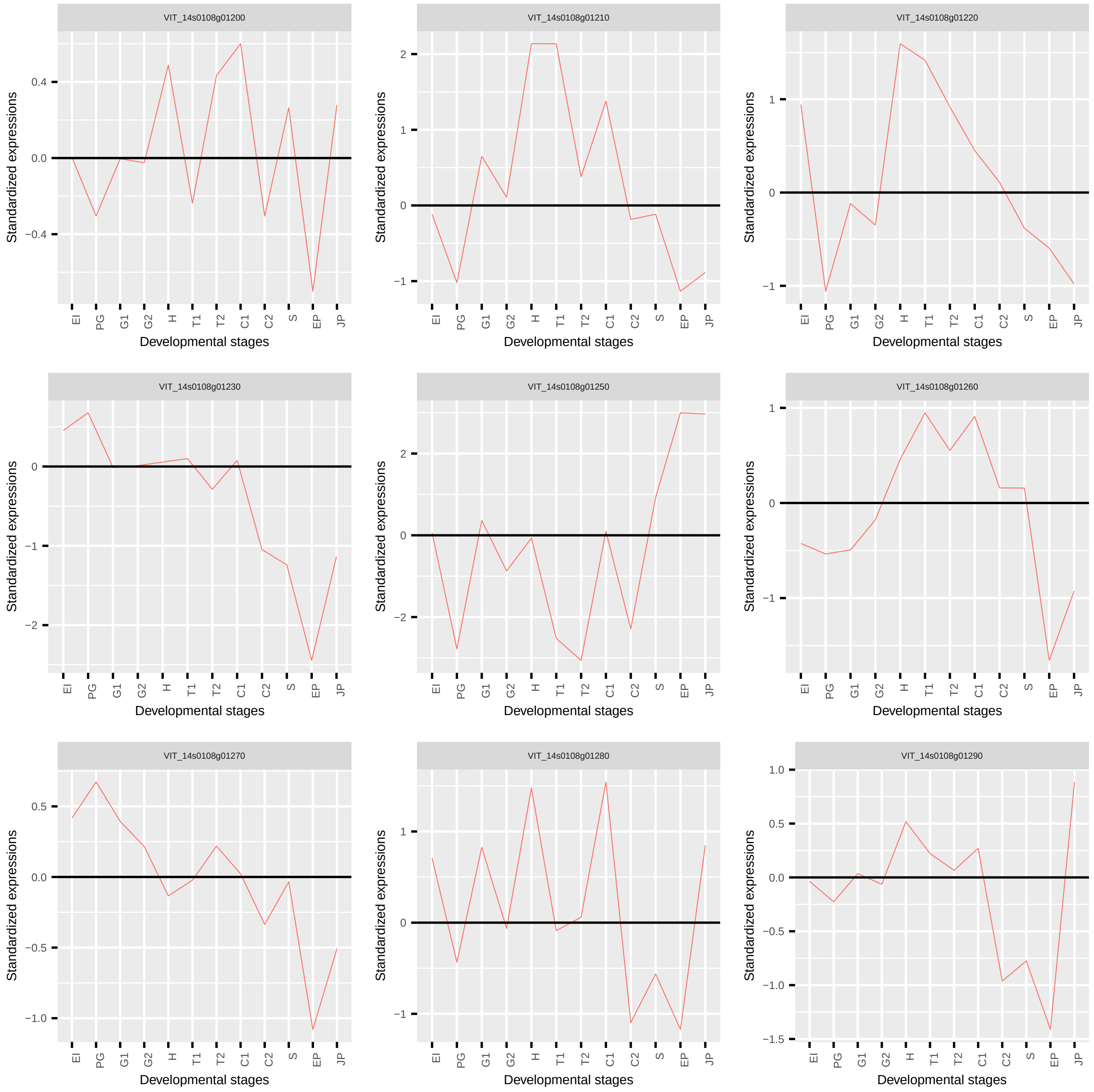
VIT_14s0108g01200
VIT_14s0108g01210
VIT_14s0108g01220
VIT_14s0108g01230
VIT_14s0108g01250
VIT_14s0108g01260
VIT_14s0108g01270
VIT_14s0108g01280
VIT_14s0108g01290
Standardized expressions
Developmental stages
EI
PG
G1
G2
H
T1
T2
C1
C2
S
EP
JP

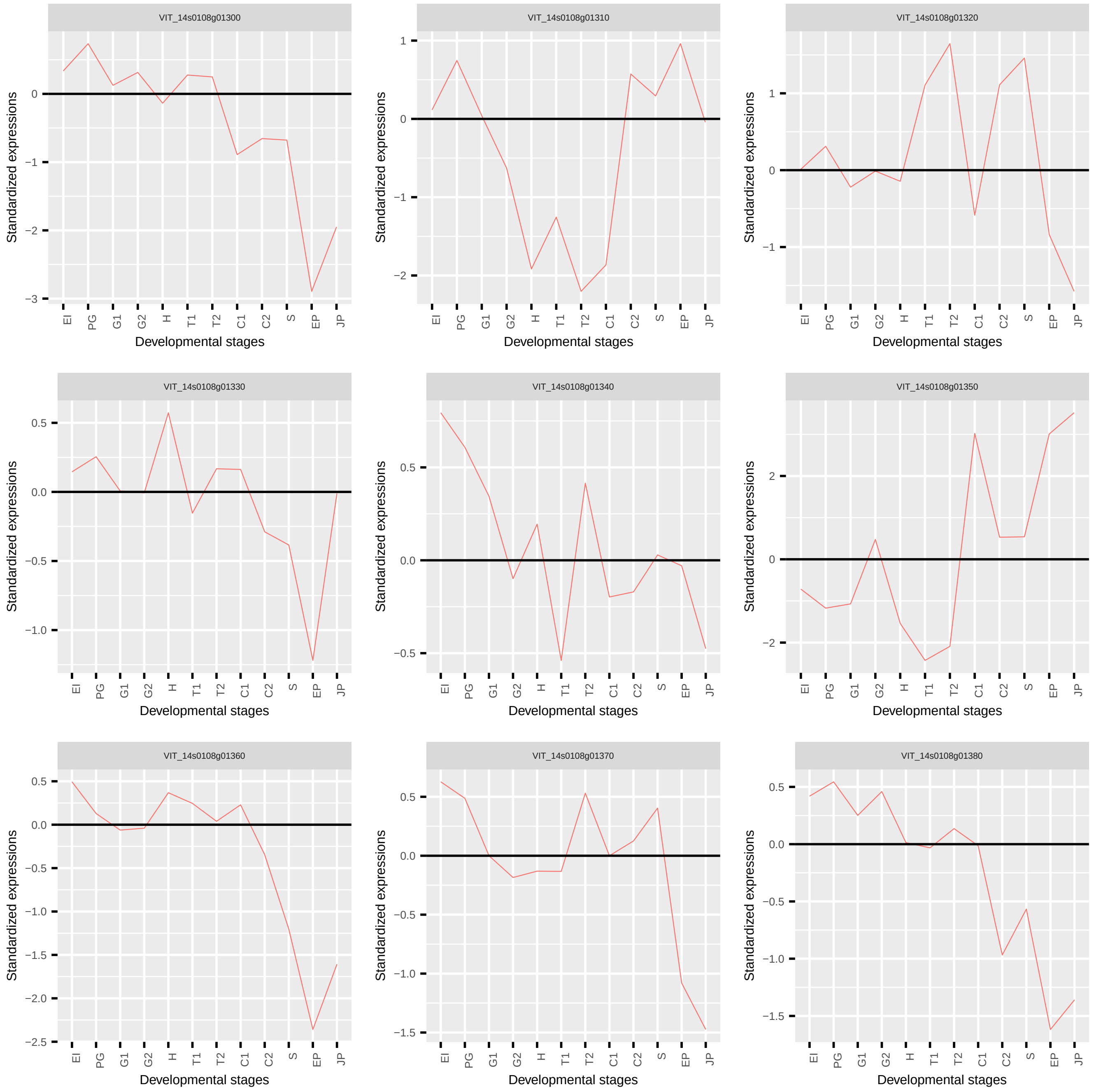

VIT_14s0108g01300
VIT_14s0108g01310
VIT_14s0108g01320
VIT_14s0108g01330
VIT_14s0108g01340
VIT_14s0108g01350
VIT_14s0108g01360
VIT_14s0108g01370
VIT_14s0108g01380
Standardized expressions
Developmental stages
EI PG G1 G2 H T1 T2 C1 C2 S EP JP

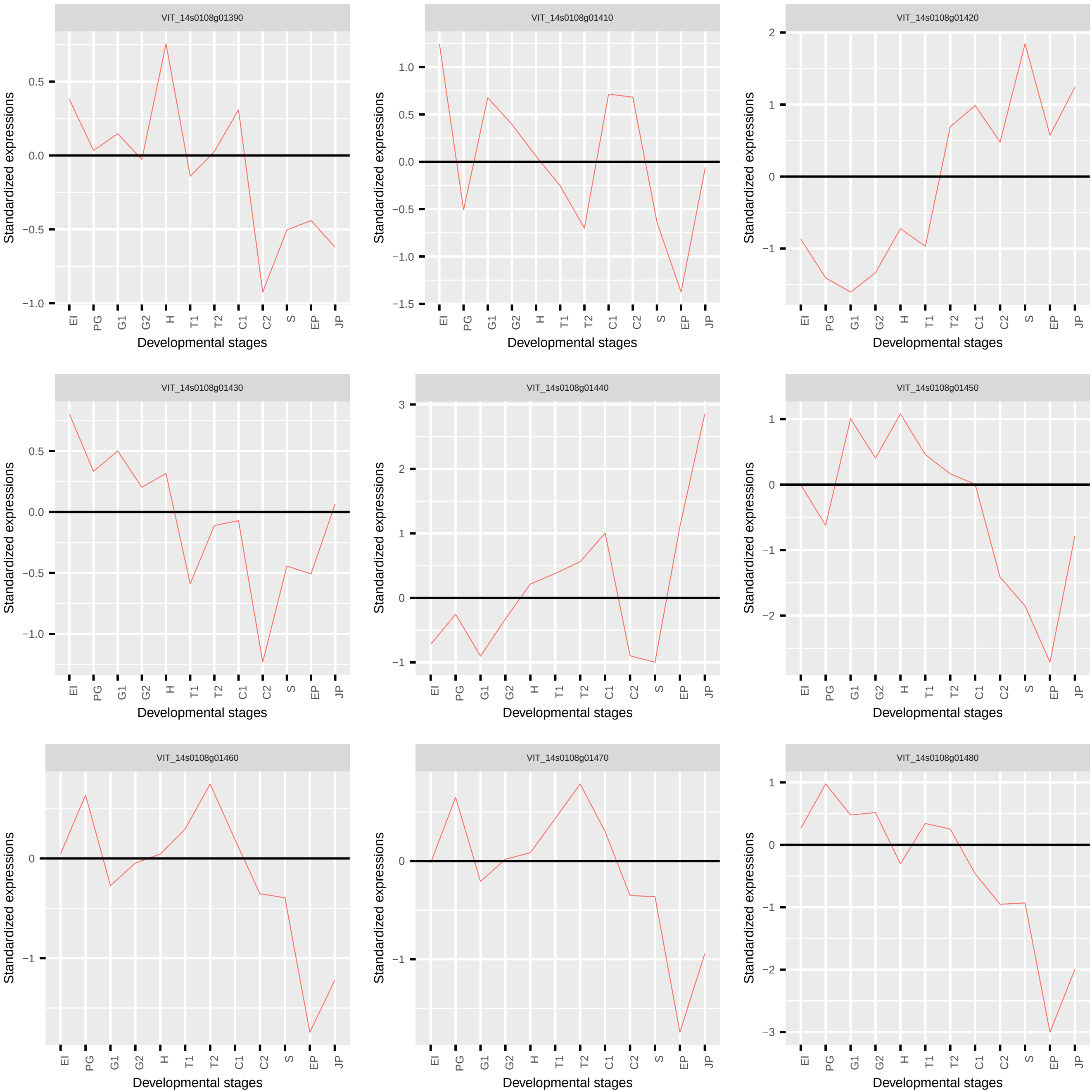
VIT_14s0108g01390
0.5
0.0
−0.5
−1.0
Standardized expressions
EI PG G1 G2 H T1 T2 C1 C2 S EP JP
Developmental stages
VIT_14s0108g01410
1.0
0.5
0.0
−0.5
−1.0
−1.5
Standardized expressions
EI PG G1 G2 H T1 T2 C1 C2 S EP JP
Developmental stages
VIT_14s0108g01420
2
1
0
−1
Standardized expressions
EI PG G1 G2 H T1 T2 C1 C2 S EP JP
Developmental stages
VIT_14s0108g01430
0.5
0.0
−0.5
−1.0
Standardized expressions
EI PG G1 G2 H T1 T2 C1 C2 S EP JP
Developmental stages
VIT_14s0108g01440
3
2
1
0
−1
Standardized expressions
EI PG G1 G2 H T1 T2 C1 C2 S EP JP
Developmental stages
VIT_14s0108g01450
1
0
−1
−2
Standardized expressions
EI PG G1 G2 H T1 T2 C1 C2 S EP JP
Developmental stages
VIT_14s0108g01460
0
−1
Standardized expressions
EI PG G1 G2 H T1 T2 C1 C2 S EP JP
Developmental stages
VIT_14s0108g01470
0
−1
Standardized expressions
EI PG G1 G2 H T1 T2 C1 C2 S EP JP
Developmental stages
VIT_14s0108g01480
1
0
−1
−2
−3
Standardized expressions
EI PG G1 G2 H T1 T2 C1 C2 S EP JP
Developmental stages

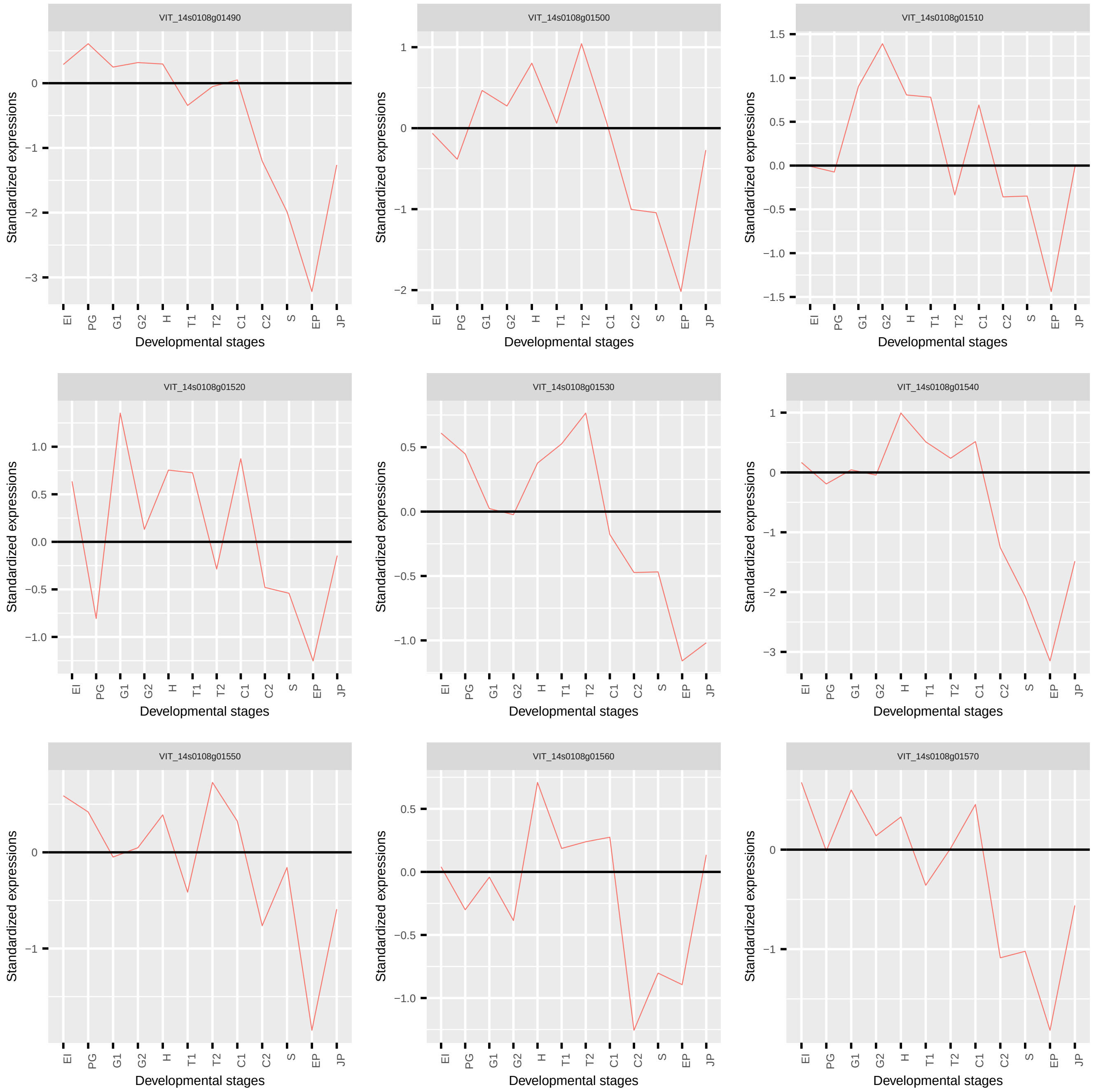
VIT_14s0108g01490
VIT_14s0108g01500
VIT_14s0108g01510
VIT_14s0108g01520
VIT_14s0108g01530
VIT_14s0108g01540
VIT_14s0108g01550
VIT_14s0108g01560
VIT_14s0108g01570
Standardized expressions
Developmental stages
EI PG G1 G2 H T1 T2 C1 C2 S EP JP

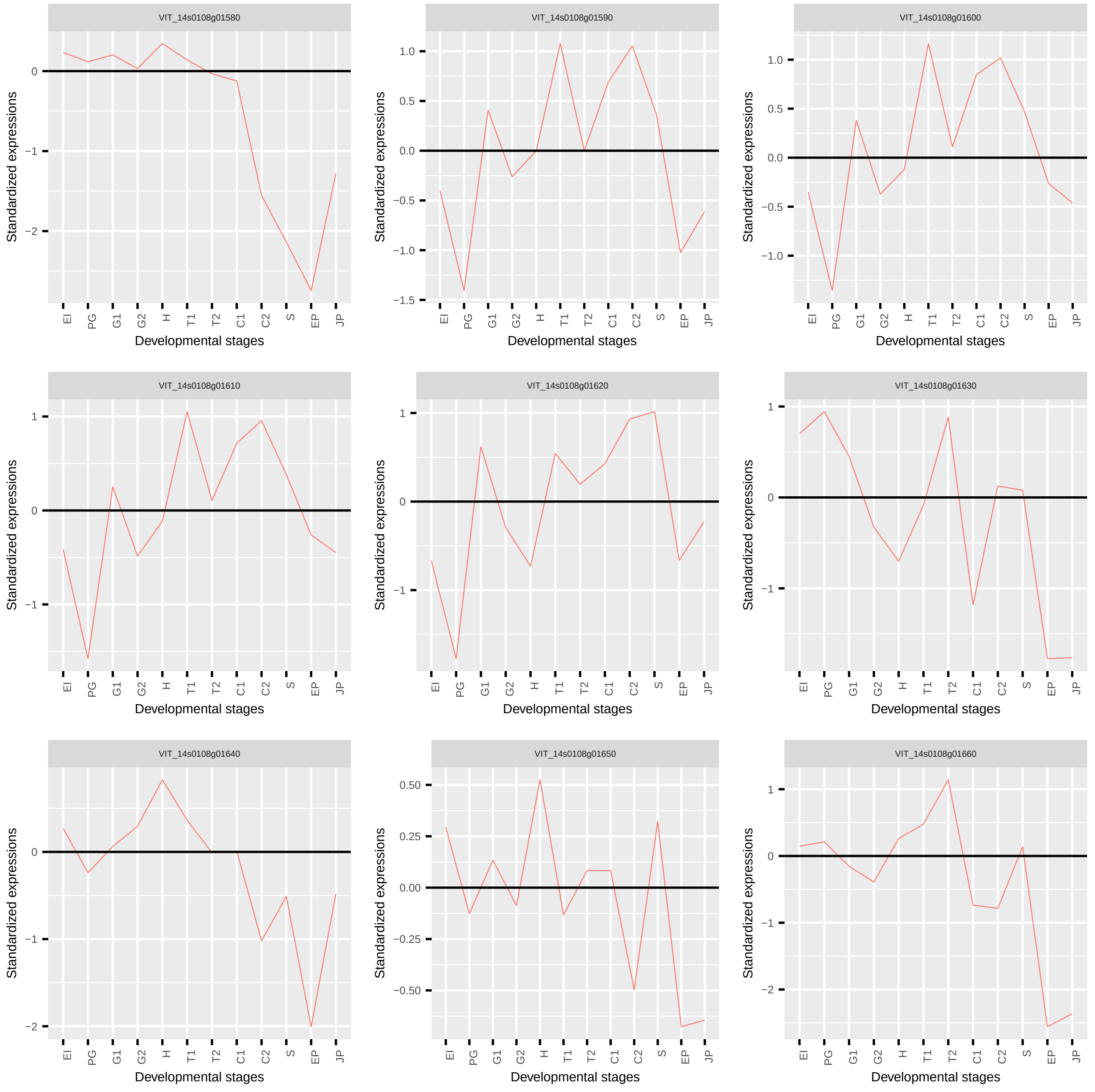
VIT_14s0108g01580
VIT_14s0108g01590
VIT_14s0108g01600
VIT_14s0108g01610
VIT_14s0108g01620
VIT_14s0108g01630
VIT_14s0108g01640
VIT_14s0108g01650
VIT_14s0108g01660
Standardized expressions
Developmental stages
EI PG G1 G2 H T1 T2 C1 C2 S EP JP

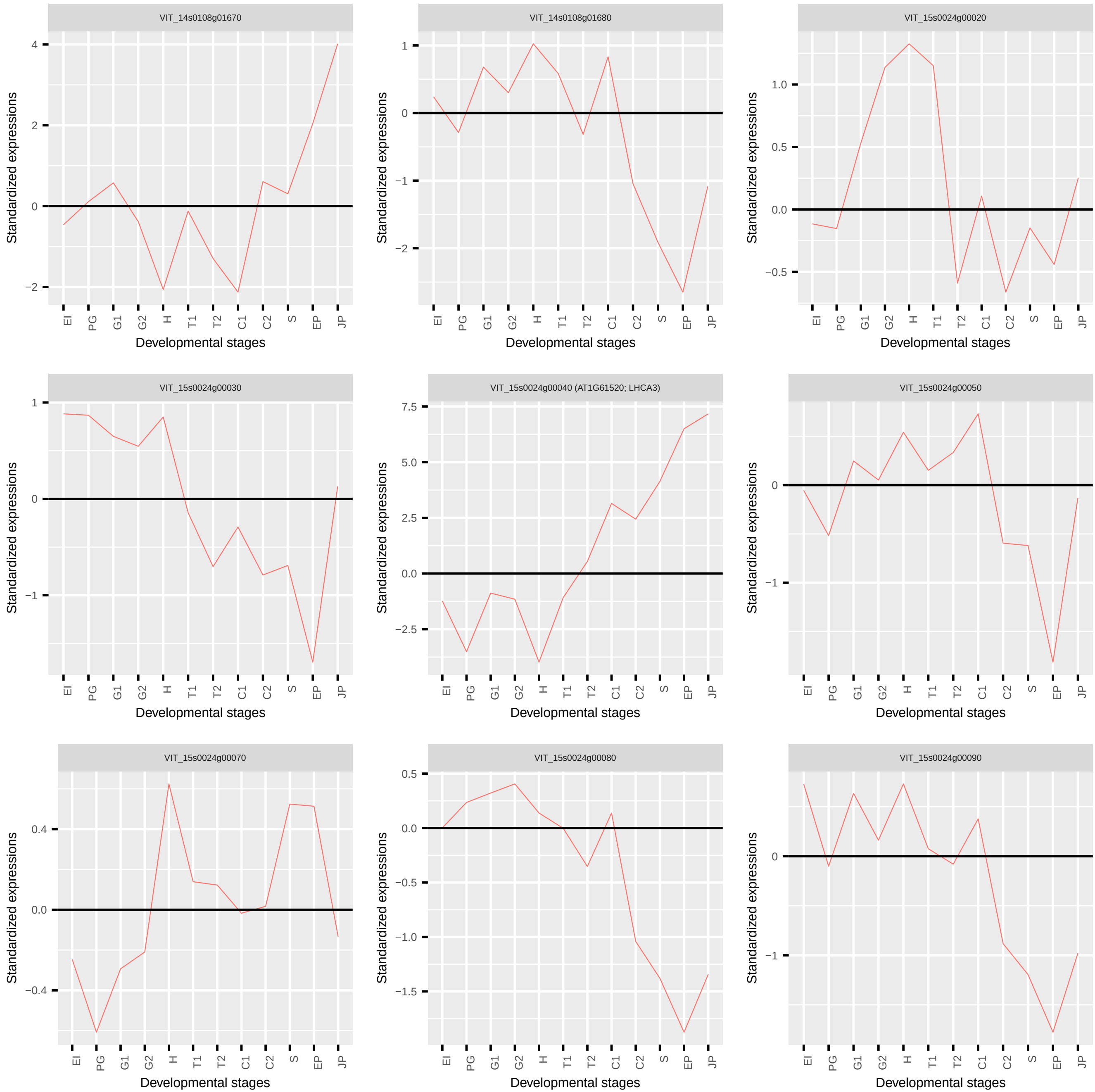
VIT_14s0108g01670
VIT_14s0108g01680
VIT_15s0024g00020
VIT_15s0024g00030
VIT_15s0024g00040 (AT1G61520; LHCA3)
VIT_15s0024g00050
VIT_15s0024g00070
VIT_15s0024g00080
VIT_15s0024g00090
Standardized expressions
Developmental stages
EI PG G1 G2 H T1 T2 C1 C2 S EP JP

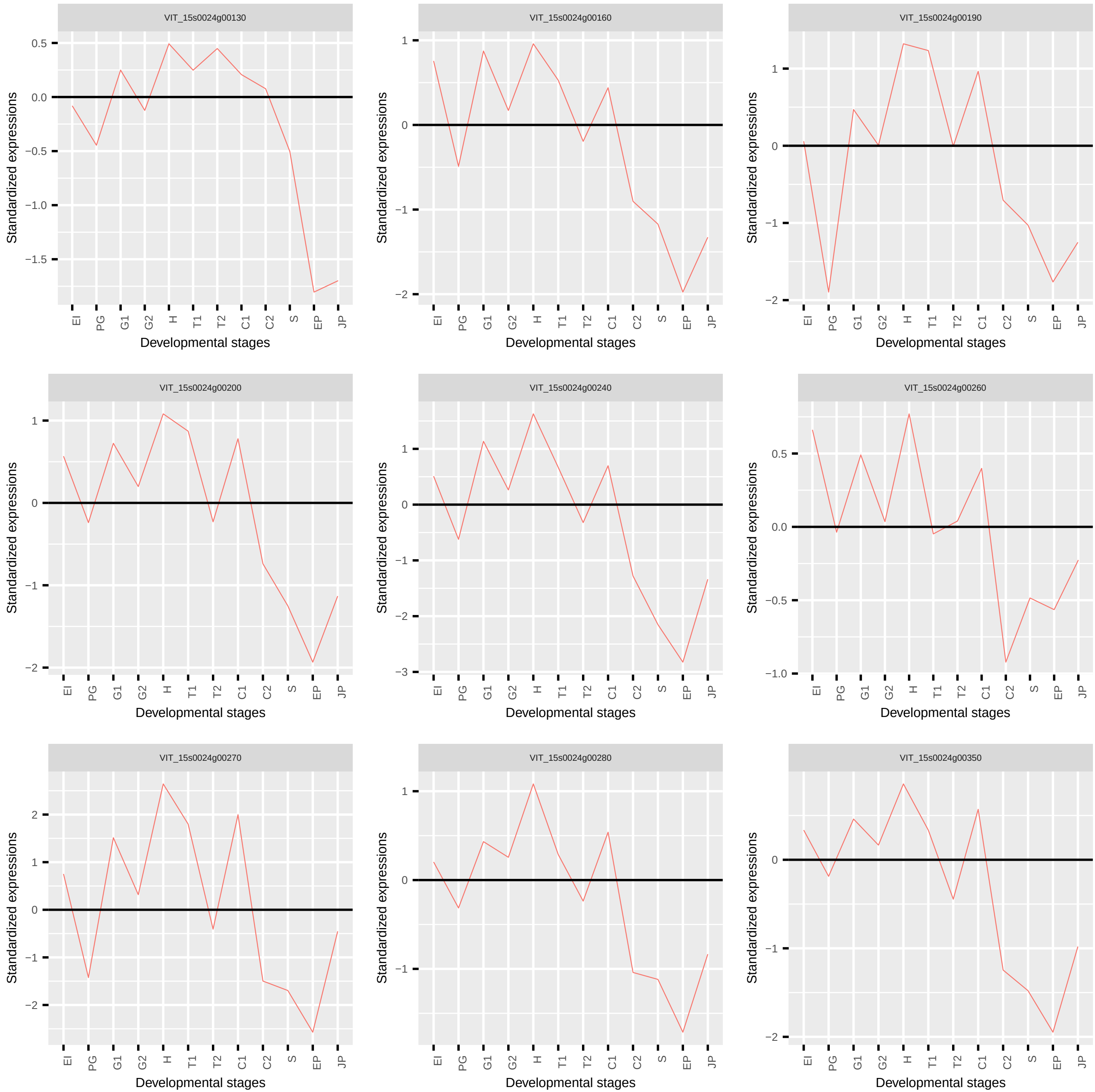
VIT_15s0024g00130
VIT_15s0024g00160
VIT_15s0024g00190
VIT_15s0024g00200
VIT_15s0024g00240
VIT_15s0024g00260
VIT_15s0024g00270
VIT_15s0024g00280
VIT_15s0024g00350
Standardized expressions
Developmental stages
EI PG G1 G2 H T1 T2 C1 C2 S EP JP

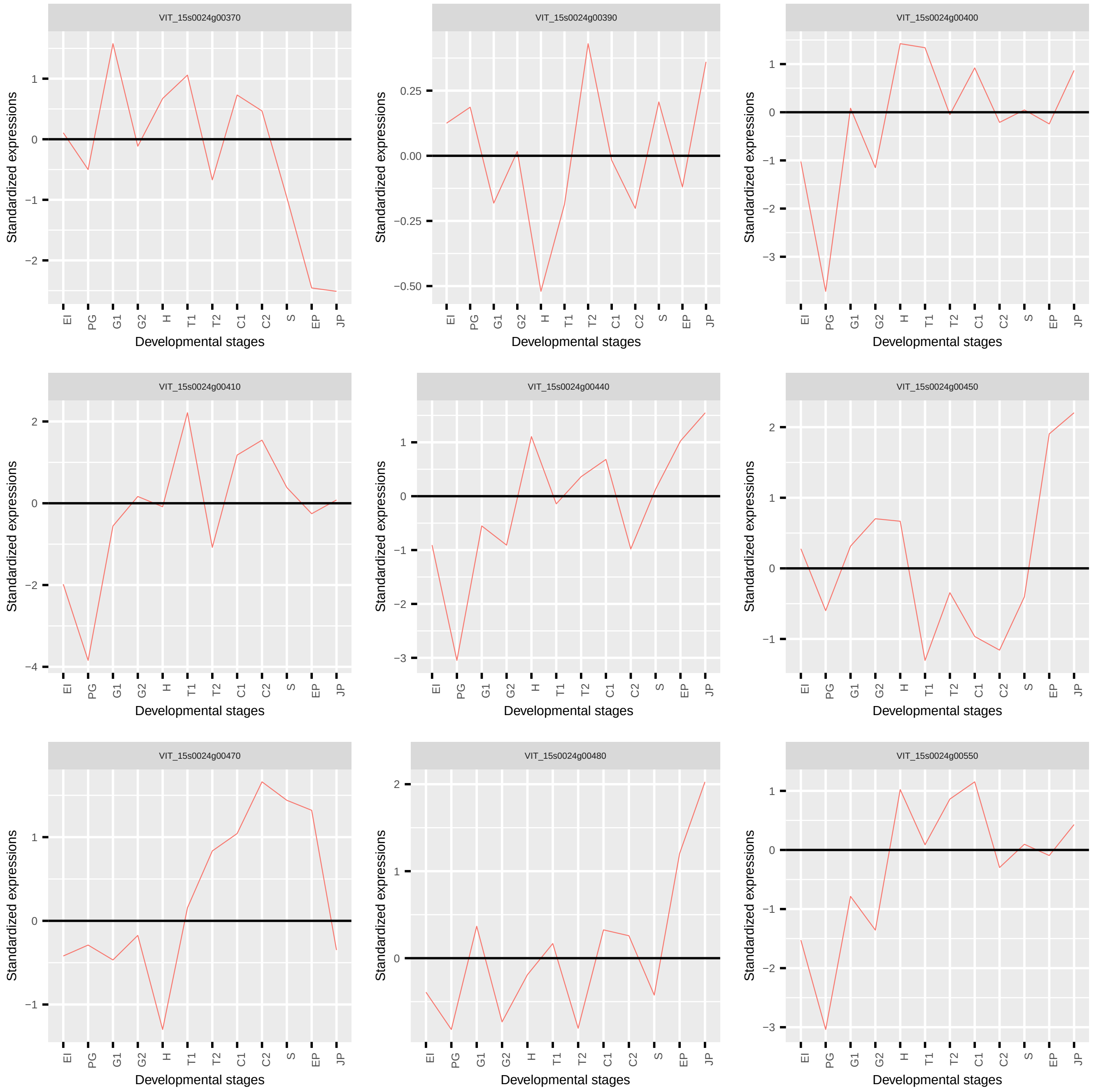
VIT_15s0024g00370
VIT_15s0024g00390
VIT_15s0024g00400
VIT_15s0024g00410
VIT_15s0024g00440
VIT_15s0024g00450
VIT_15s0024g00470
VIT_15s0024g00480
VIT_15s0024g00550
Standardized expressions
Developmental stages
EI PG G1 G2 H T1 T2 C1 C2 S EP JP

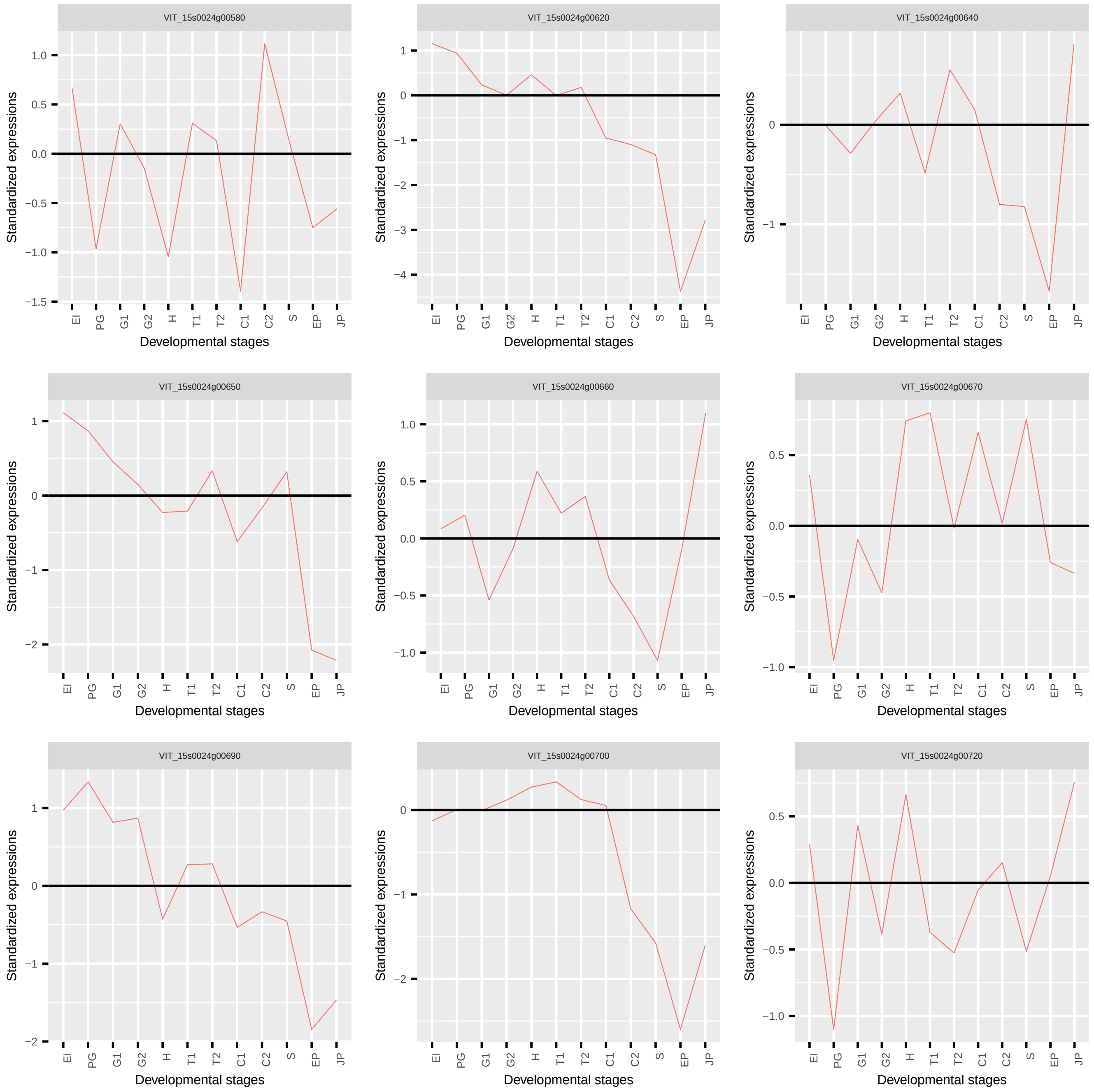
VIT_15s0024g00580
VIT_15s0024g00620
VIT_15s0024g00640
VIT_15s0024g00650
VIT_15s0024g00660
VIT_15s0024g00670
VIT_15s0024g00690
VIT_15s0024g00700
VIT_15s0024g00720
Standardized expressions
Developmental stages
EI PG G1 G2 H T1 T2 C1 C2 S EP JP

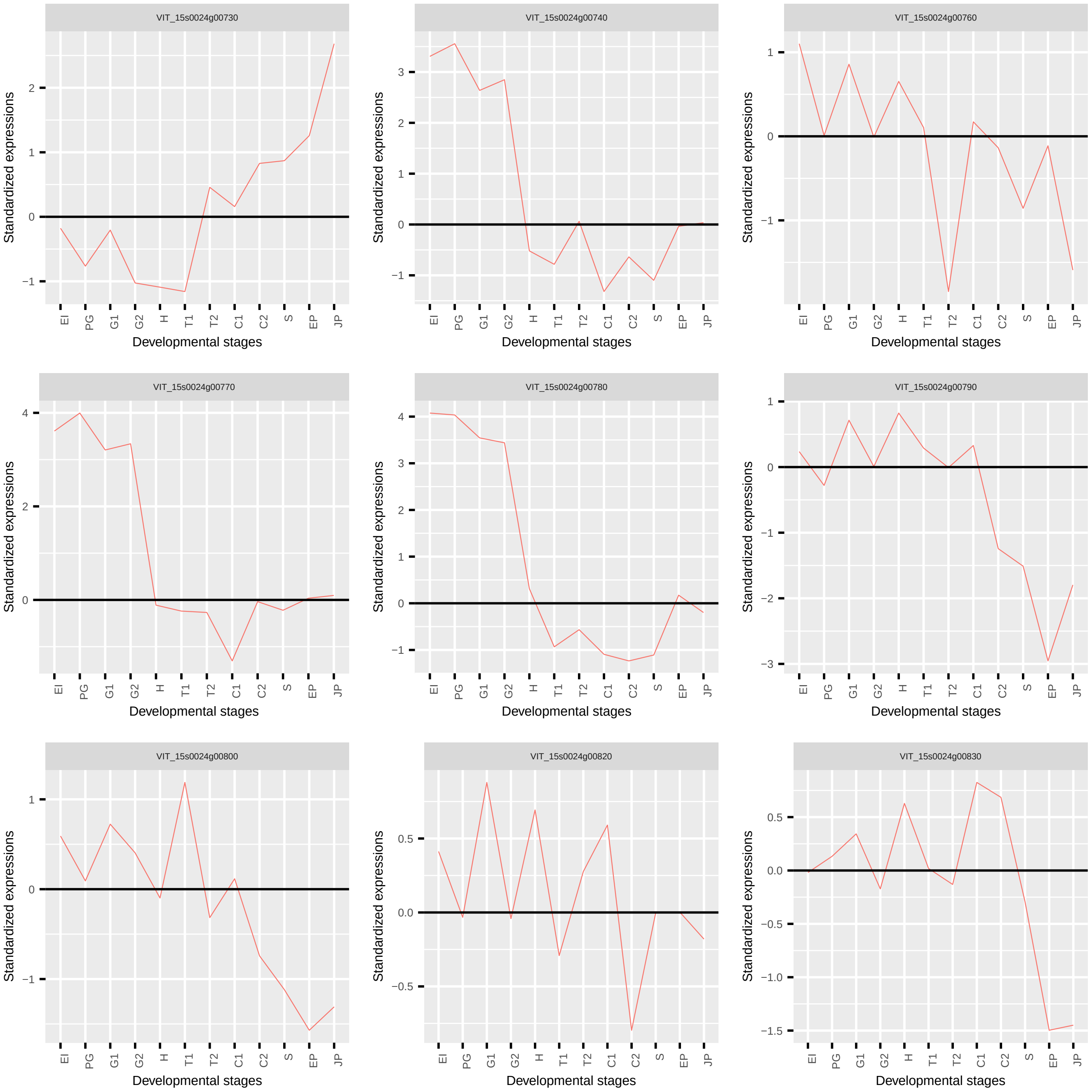

VIT_15s0024g00730
VIT_15s0024g00740
VIT_15s0024g00760
VIT_15s0024g00770
VIT_15s0024g00780
VIT_15s0024g00790
VIT_15s0024g00800
VIT_15s0024g00820
VIT_15s0024g00830
Standardized expressions
Developmental stages
EI PG G1 G2 H T1 T2 C1 C2 S EP JP

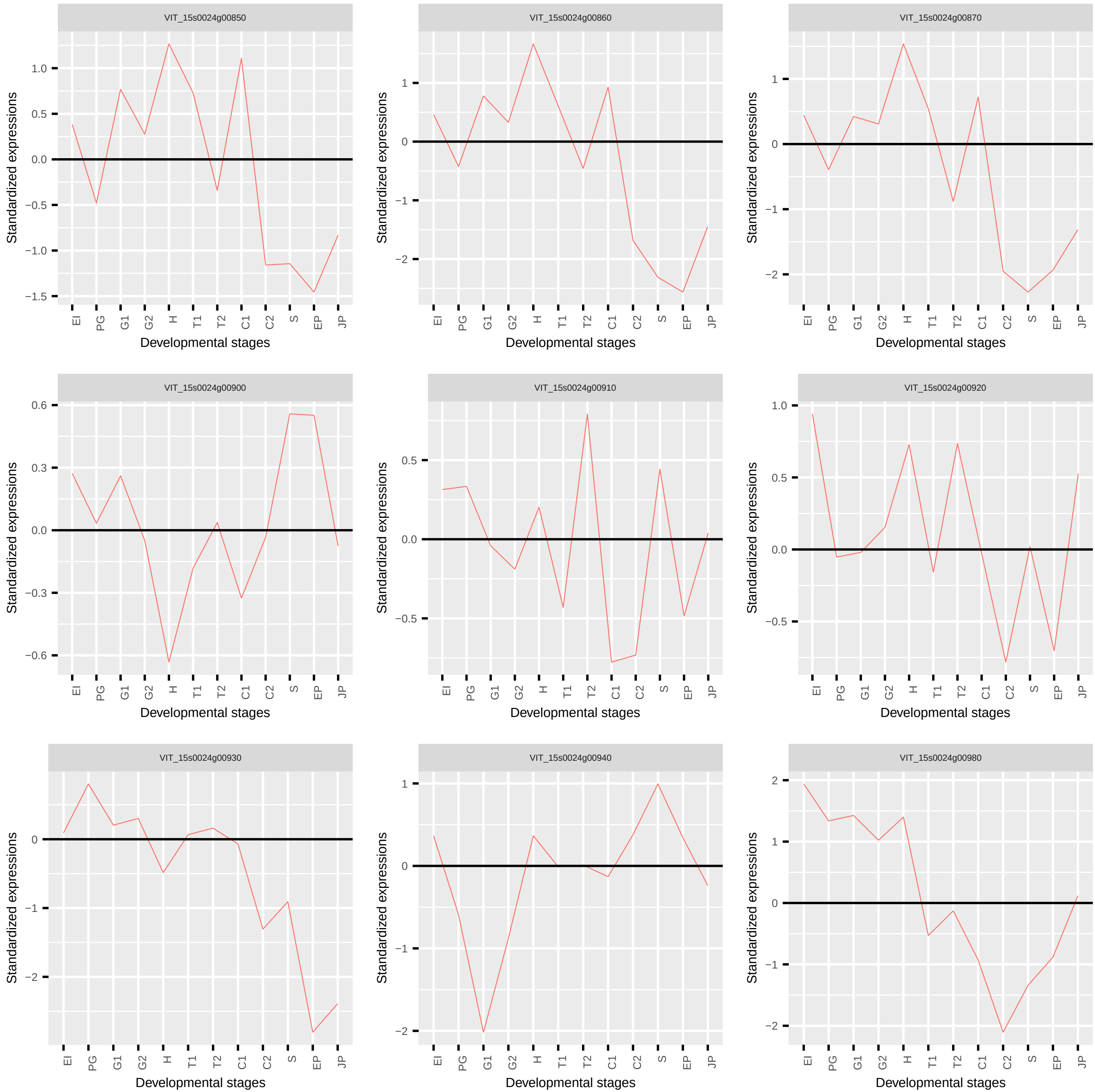
VIT_15s0024g00850
VIT_15s0024g00860
VIT_15s0024g00870
VIT_15s0024g00900
VIT_15s0024g00910
VIT_15s0024g00920
VIT_15s0024g00930
VIT_15s0024g00940
VIT_15s0024g00980
Standardized expressions
Developmental stages
EI PG G1 G2 H T1 T2 C1 C2 S EP JP

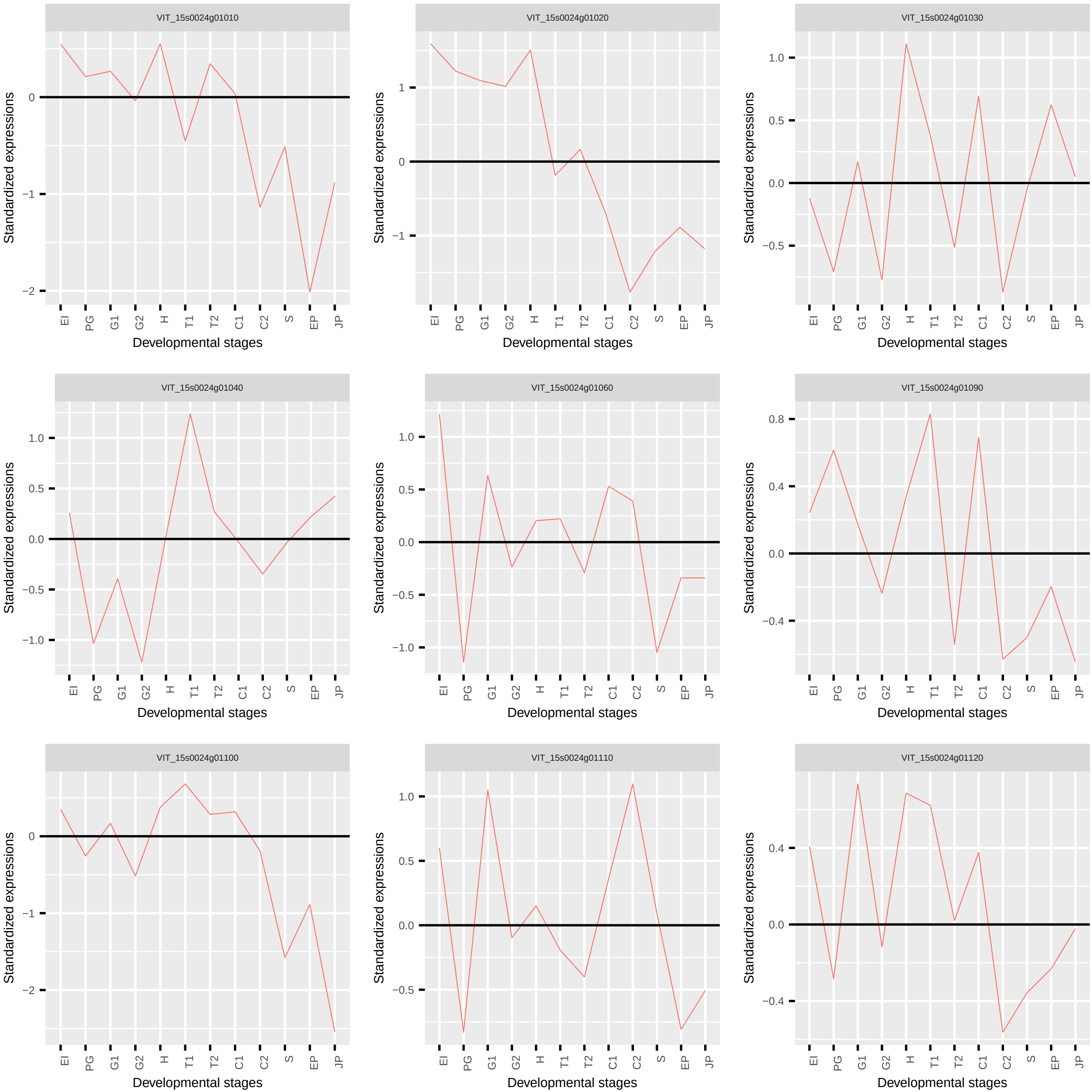
VIT_15s0024g01010
VIT_15s0024g01020
VIT_15s0024g01030
VIT_15s0024g01040
VIT_15s0024g01060
VIT_15s0024g01090
VIT_15s0024g01100
VIT_15s0024g01110
VIT_15s0024g01120
Standardized expressions
Developmental stages
EI PG G1 G2 H T1 T2 C1 C2 S EP JP

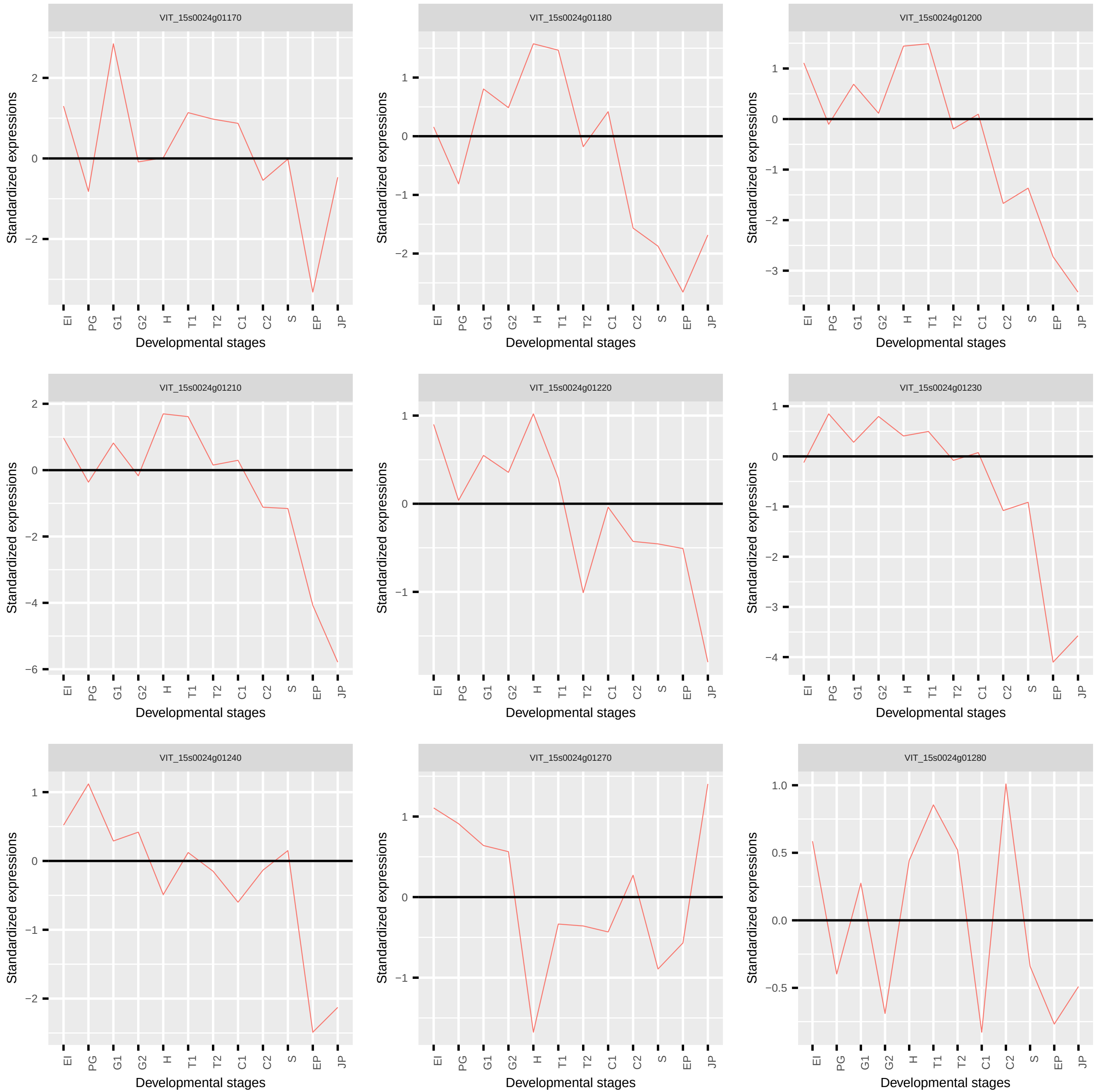

VIT_15s0024g01170
VIT_15s0024g01180
VIT_15s0024g01200
VIT_15s0024g01210
VIT_15s0024g01220
VIT_15s0024g01230
VIT_15s0024g01240
VIT_15s0024g01270
VIT_15s0024g01280
Standardized expressions
Developmental stages
EI
PG
G1
G2
H
T1
T2
C1
C2
S
EP
JP
2
0
−2
1
0
−1
−2
1
0
−1
−2
−3
2
0
−2
−4
−6
1
0
−1
1
0
−1
−2
−3
−4
1
0
−1
−2
1
0
−1
1.0
0.5
0.0
−0.5

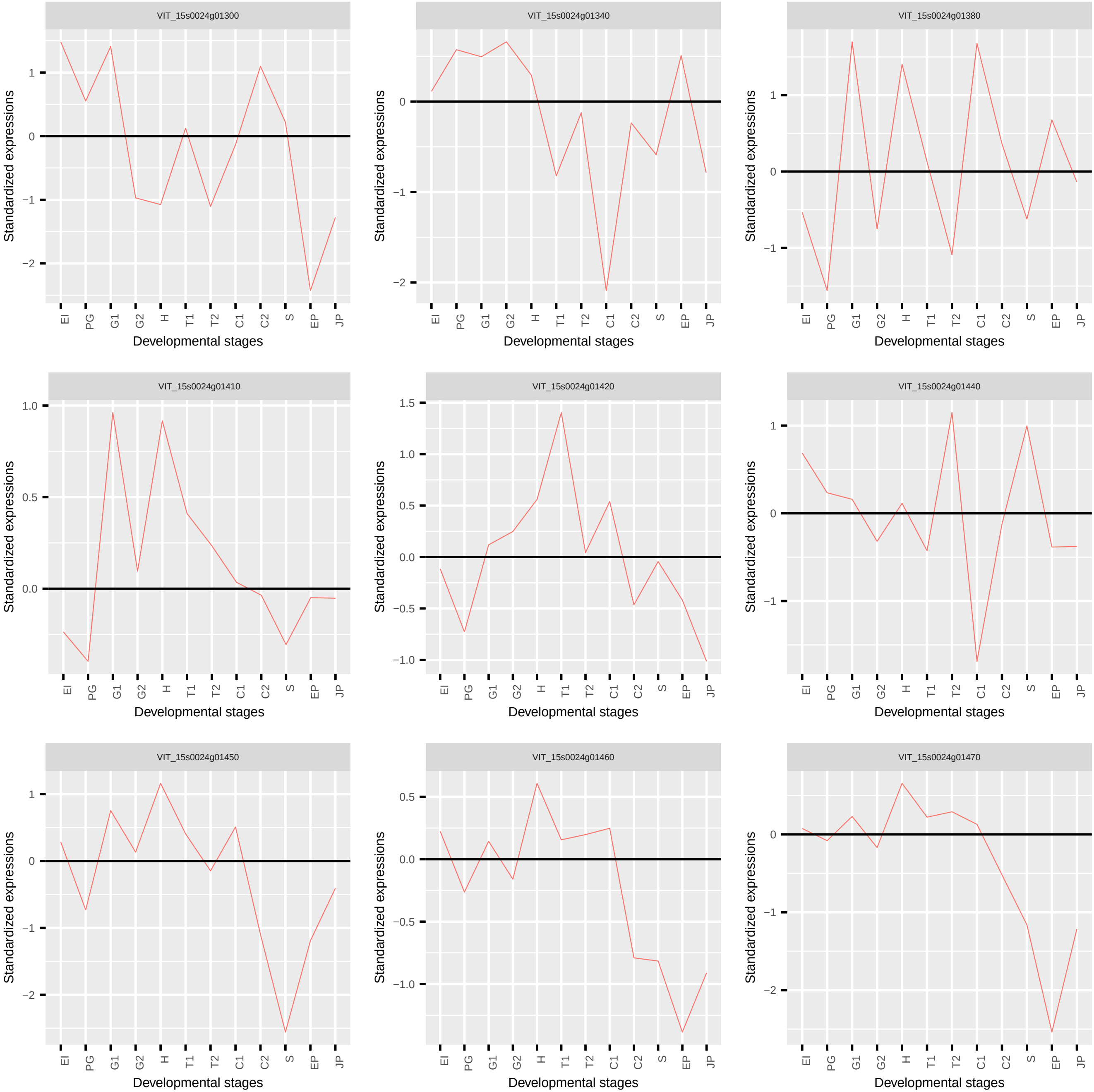
VIT_15s0024g01300
VIT_15s0024g01340
VIT_15s0024g01380
VIT_15s0024g01410
VIT_15s0024g01420
VIT_15s0024g01440
VIT_15s0024g01450
VIT_15s0024g01460
VIT_15s0024g01470
Standardized expressions
Developmental stages
EI
PG
G1
G2
H
T1
T2
C1
C2
S
EP
JP

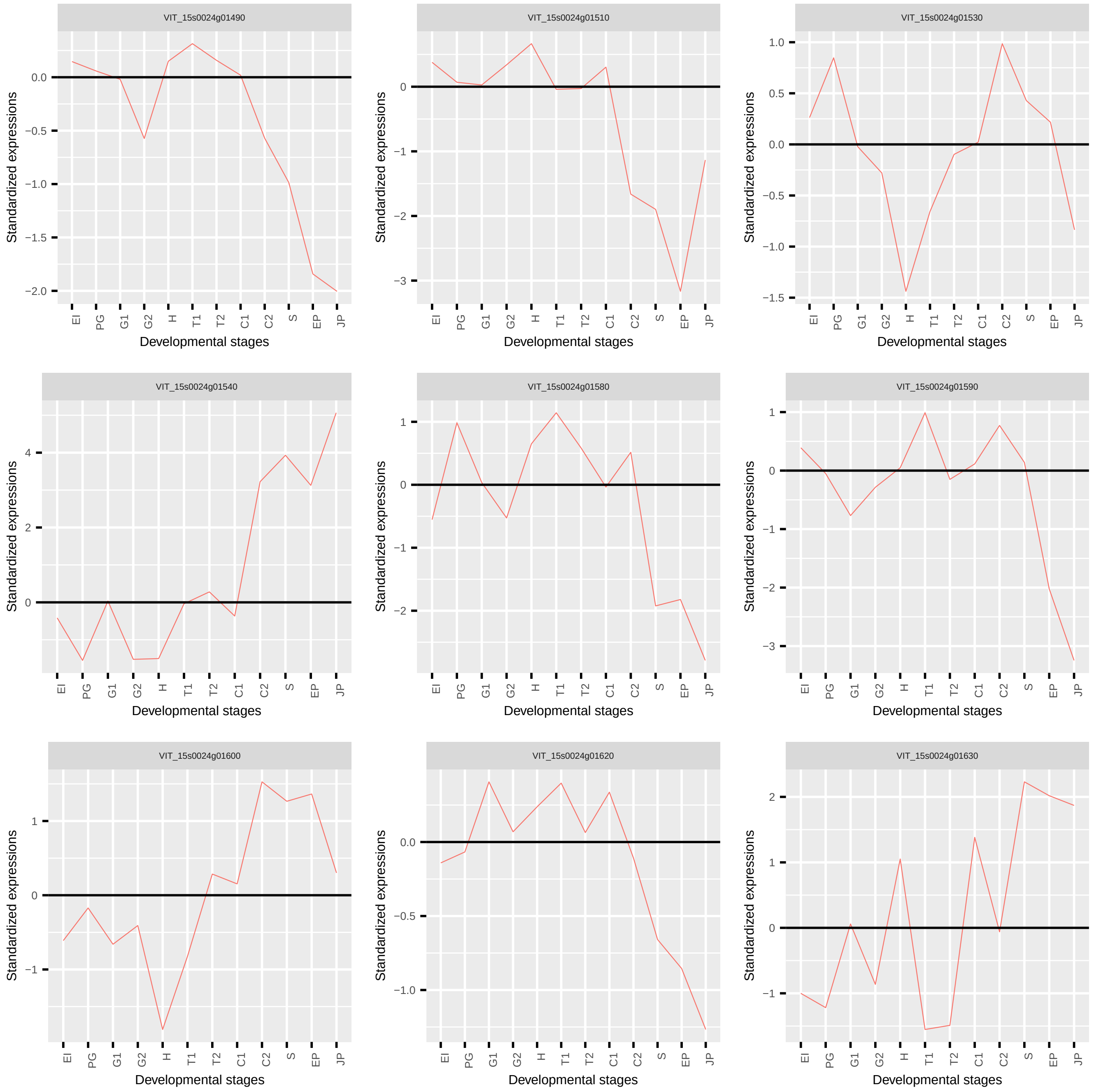
VIT_15s0024g01490
VIT_15s0024g01510
VIT_15s0024g01530
VIT_15s0024g01540
VIT_15s0024g01580
VIT_15s0024g01590
VIT_15s0024g01600
VIT_15s0024g01620
VIT_15s0024g01630
Standardized expressions
Developmental stages
EI PG G1 G2 H T1 T2 C1 C2 S EP JP

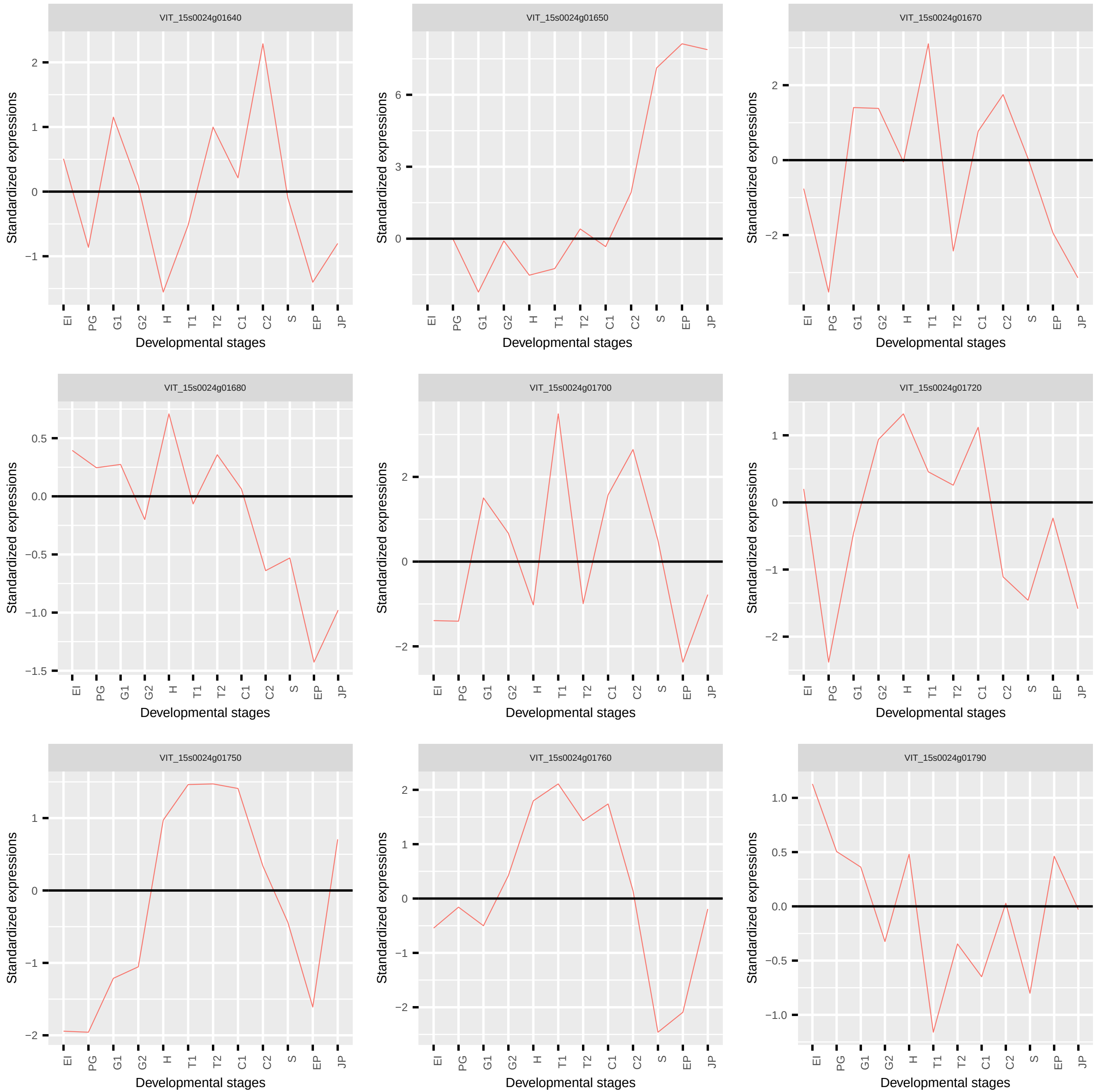
VIT_15s0024g01640
VIT_15s0024g01650
VIT_15s0024g01670
VIT_15s0024g01680
VIT_15s0024g01700
VIT_15s0024g01720
VIT_15s0024g01750
VIT_15s0024g01760
VIT_15s0024g01790
Standardized expressions
Developmental stages
EI PG G1 G2 H T1 T2 C1 C2 S EP JP

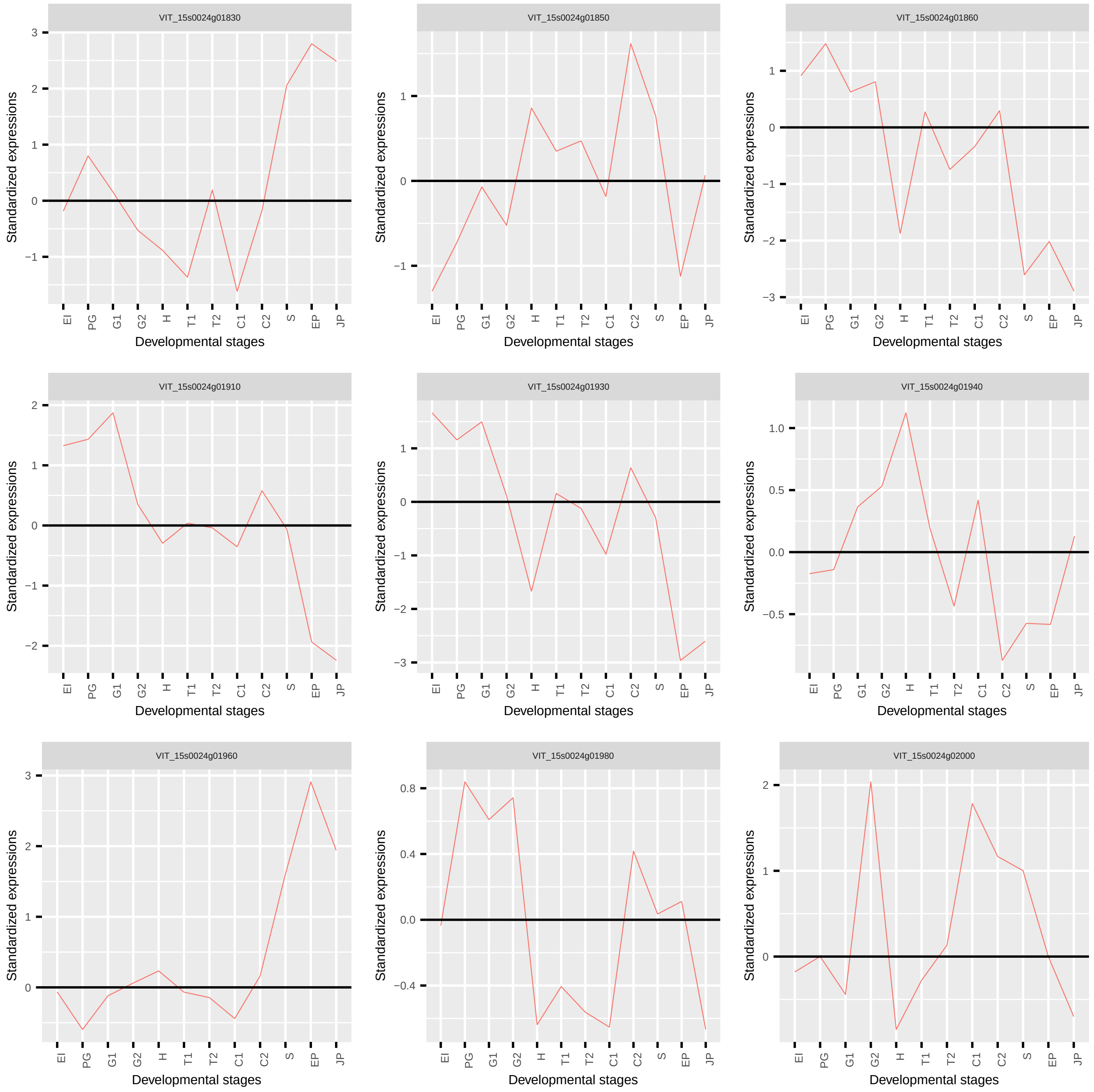
VIT_15s0024g01830
Standardized expressions
3
2
1
0
−1
EI PG G1 G2 H T1 T2 C1 C2 S EP JP
Developmental stages
VIT_15s0024g01850
Standardized expressions
1
0
−1
EI PG G1 G2 H T1 T2 C1 C2 S EP JP
Developmental stages
VIT_15s0024g01860
Standardized expressions
1
0
−1
−2
−3
EI PG G1 G2 H T1 T2 C1 C2 S EP JP
Developmental stages
VIT_15s0024g01910
Standardized expressions
2
1
0
−1
−2
EI PG G1 G2 H T1 T2 C1 C2 S EP JP
Developmental stages
VIT_15s0024g01930
Standardized expressions
1
0
−1
−2
−3
EI PG G1 G2 H T1 T2 C1 C2 S EP JP
Developmental stages
VIT_15s0024g01940
Standardized expressions
1.0
0.5
0.0
−0.5
EI PG G1 G2 H T1 T2 C1 C2 S EP JP
Developmental stages
VIT_15s0024g01960
Standardized expressions
3
2
1
0
EI PG G1 G2 H T1 T2 C1 C2 S EP JP
Developmental stages
VIT_15s0024g01980
Standardized expressions
0.8
0.4
0.0
−0.4
EI PG G1 G2 H T1 T2 C1 C2 S EP JP
Developmental stages
VIT_15s0024g02000
Standardized expressions
2
1
0
EI PG G1 G2 H T1 T2 C1 C2 S EP JP
Developmental stages

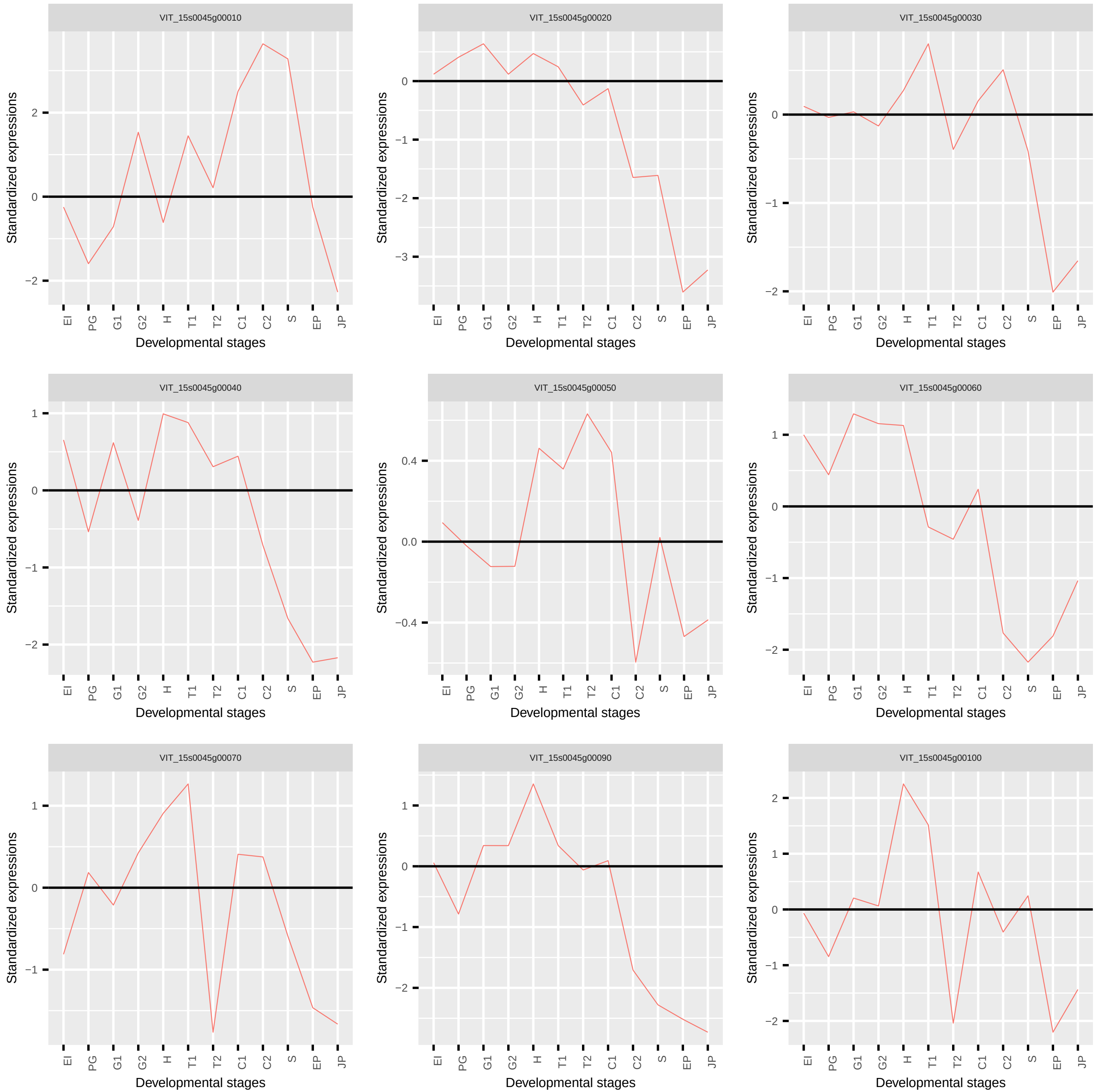

VIT_15s0045g00010
VIT_15s0045g00020
VIT_15s0045g00030
VIT_15s0045g00040
VIT_15s0045g00050
VIT_15s0045g00060
VIT_15s0045g00070
VIT_15s0045g00090
VIT_15s0045g00100
Standardized expressions
Developmental stages
EI
PG
G1
G2
H
T1
T2
C1
C2
S
EP
JP

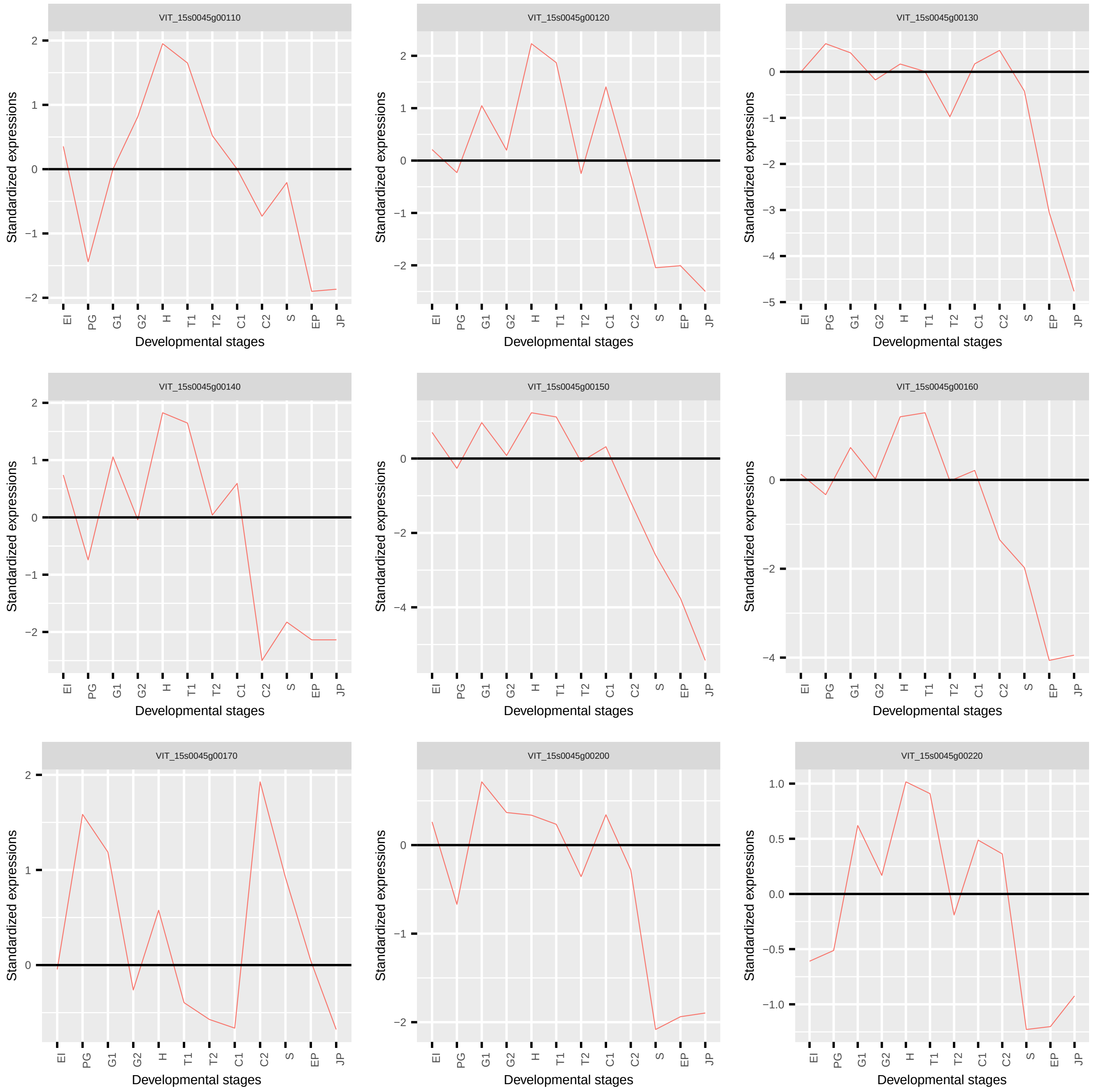
VIT_15s0045g00110
VIT_15s0045g00120
VIT_15s0045g00130
VIT_15s0045g00140
VIT_15s0045g00150
VIT_15s0045g00160
VIT_15s0045g00170
VIT_15s0045g00200
VIT_15s0045g00220
Standardized expressions
Developmental stages
EI
PG
G1
G2
H
T1
T2
C1
C2
S
EP
JP

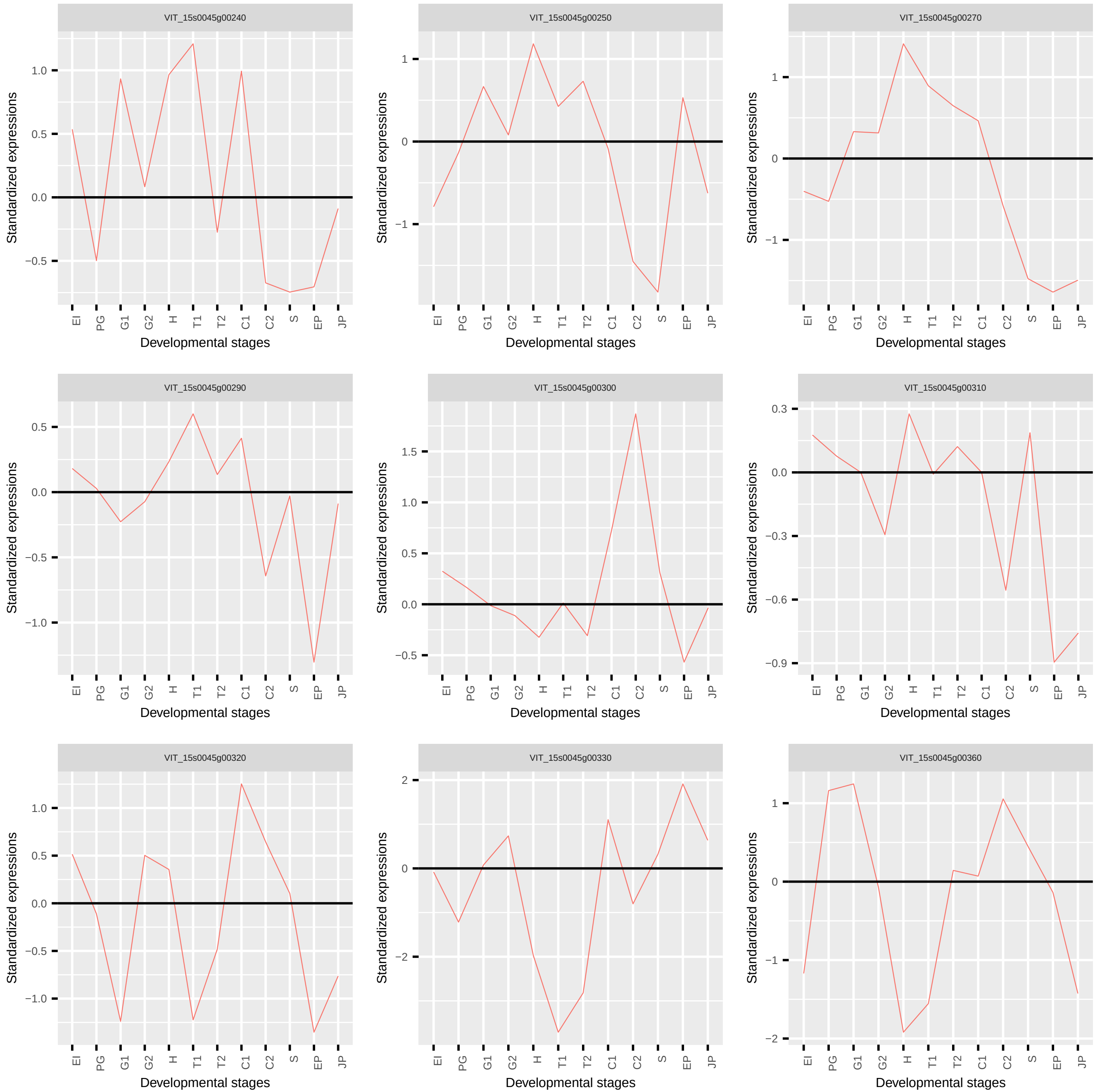
VIT_15s0045g00240
VIT_15s0045g00250
VIT_15s0045g00270
VIT_15s0045g00290
VIT_15s0045g00300
VIT_15s0045g00310
VIT_15s0045g00320
VIT_15s0045g00330
VIT_15s0045g00360
Standardized expressions
Developmental stages
EI
PG
G1
G2
H
T1
T2
C1
C2
S
EP
JP

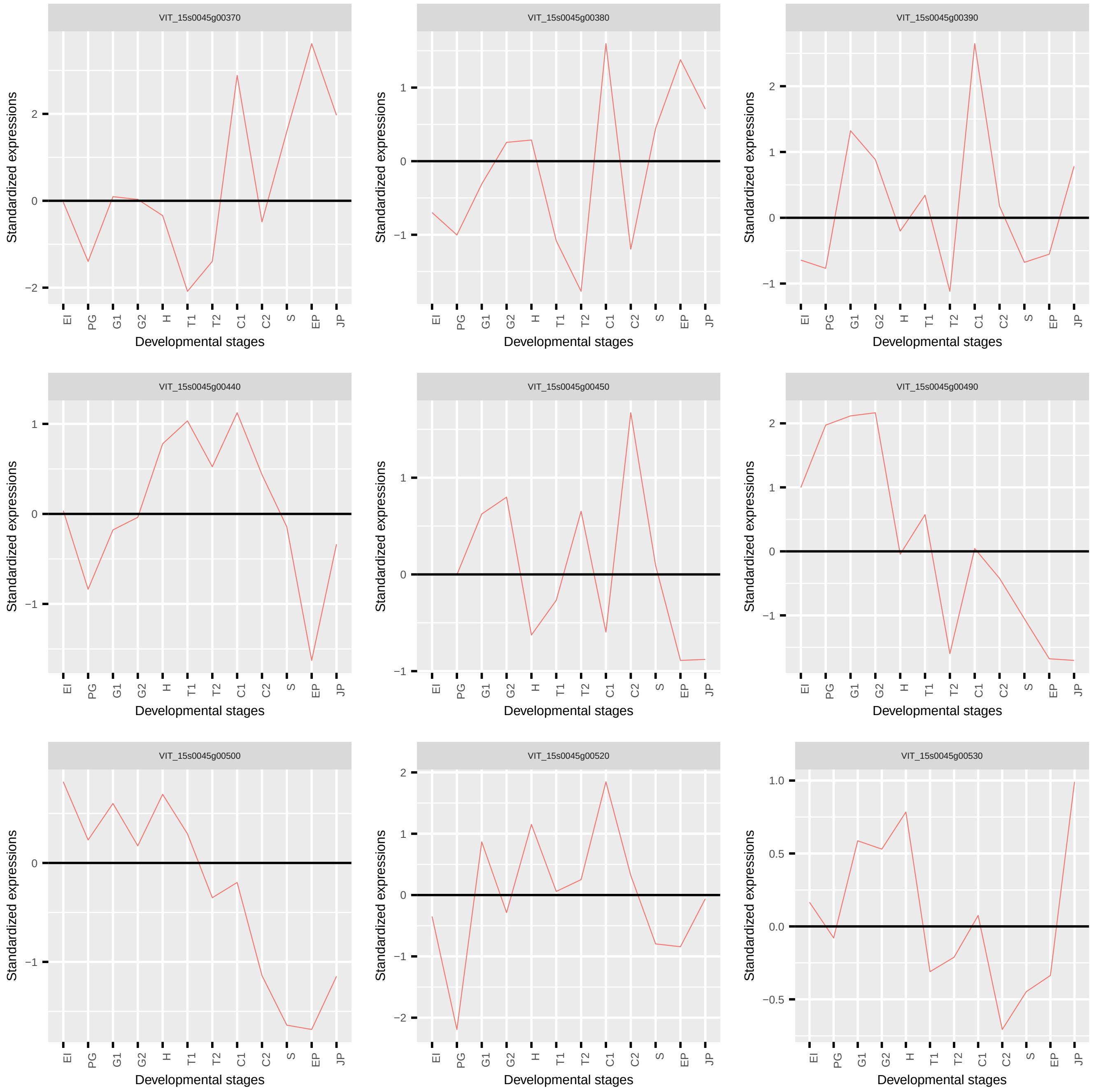

VIT_15s0045g00370
Standardized expressions
2
0
−2
EI PG G1 G2 H T1 T2 C1 C2 S EP JP
Developmental stages
VIT_15s0045g00380
Standardized expressions
1
0
−1
EI PG G1 G2 H T1 T2 C1 C2 S EP JP
Developmental stages
VIT_15s0045g00390
Standardized expressions
2
1
0
−1
EI PG G1 G2 H T1 T2 C1 C2 S EP JP
Developmental stages
VIT_15s0045g00440
Standardized expressions
1
0
−1
EI PG G1 G2 H T1 T2 C1 C2 S EP JP
Developmental stages
VIT_15s0045g00450
Standardized expressions
1
0
−1
EI PG G1 G2 H T1 T2 C1 C2 S EP JP
Developmental stages
VIT_15s0045g00490
Standardized expressions
2
1
0
−1
EI PG G1 G2 H T1 T2 C1 C2 S EP JP
Developmental stages
VIT_15s0045g00500
Standardized expressions
0
−1
EI PG G1 G2 H T1 T2 C1 C2 S EP JP
Developmental stages
VIT_15s0045g00520
Standardized expressions
2
1
0
−1
−2
EI PG G1 G2 H T1 T2 C1 C2 S EP JP
Developmental stages
VIT_15s0045g00530
Standardized expressions
1.0
0.5
0.0
−0.5
EI PG G1 G2 H T1 T2 C1 C2 S EP JP
Developmental stages

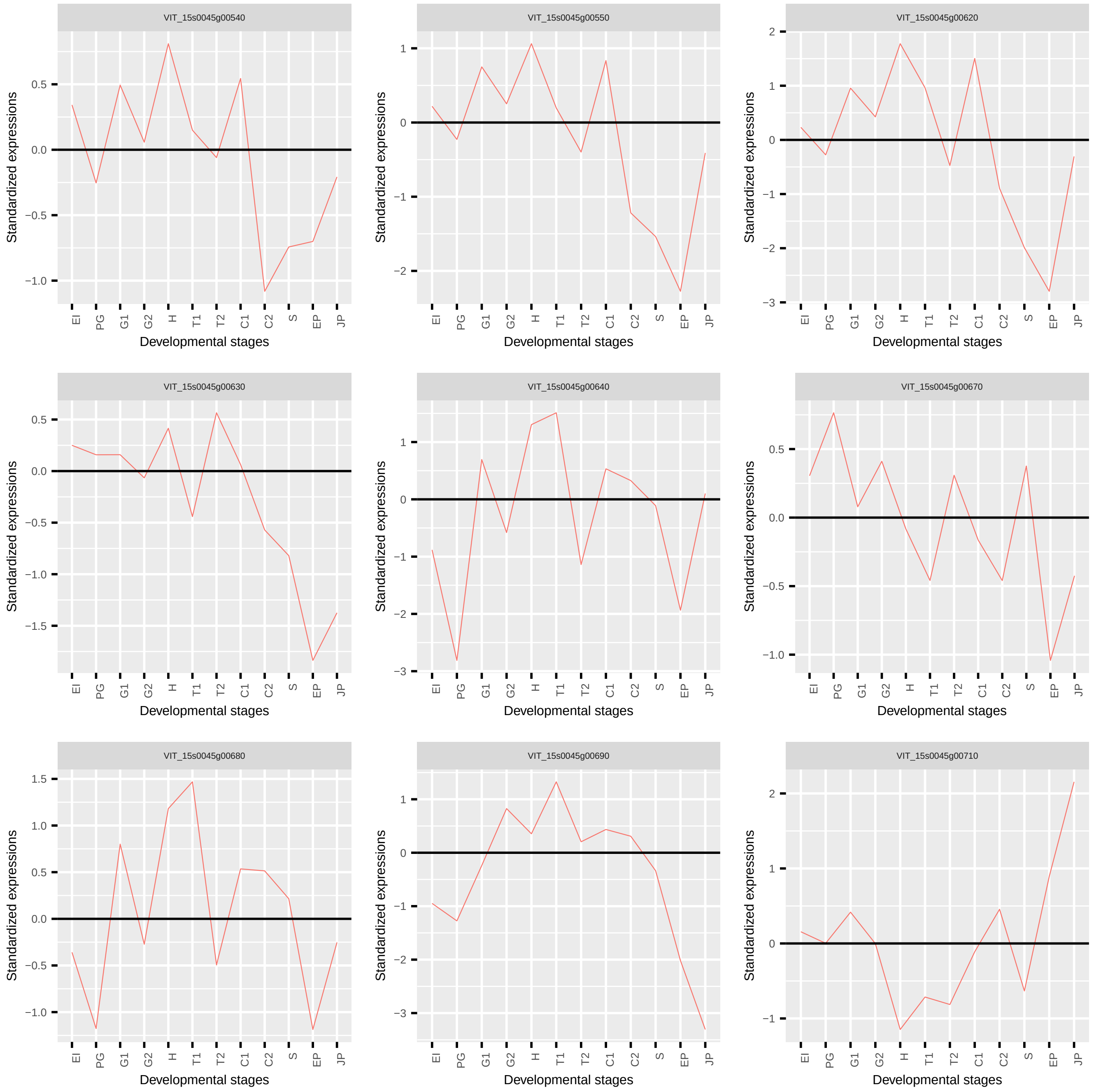
VIT_15s0045g00540
VIT_15s0045g00550
VIT_15s0045g00620
VIT_15s0045g00630
VIT_15s0045g00640
VIT_15s0045g00670
VIT_15s0045g00680
VIT_15s0045g00690
VIT_15s0045g00710
Standardized expressions
Developmental stages
EI PG G1 G2 H T1 T2 C1 C2 S EP JP

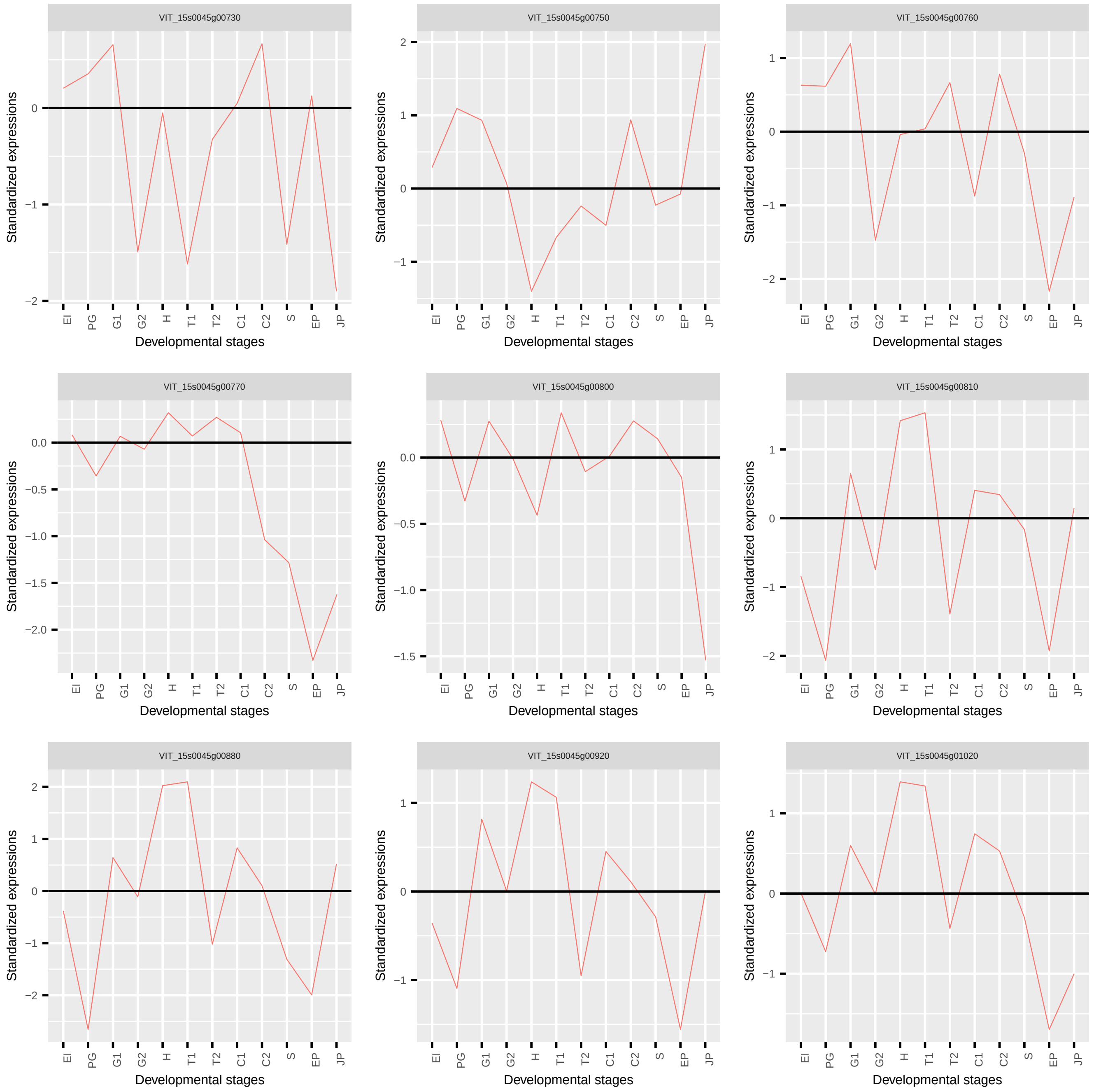

VIT_15s0045g00730
VIT_15s0045g00750
VIT_15s0045g00760
VIT_15s0045g00770
VIT_15s0045g00800
VIT_15s0045g00810
VIT_15s0045g00880
VIT_15s0045g00920
VIT_15s0045g01020
Standardized expressions
Developmental stages
EI PG G1 G2 H T1 T2 C1 C2 S EP JP

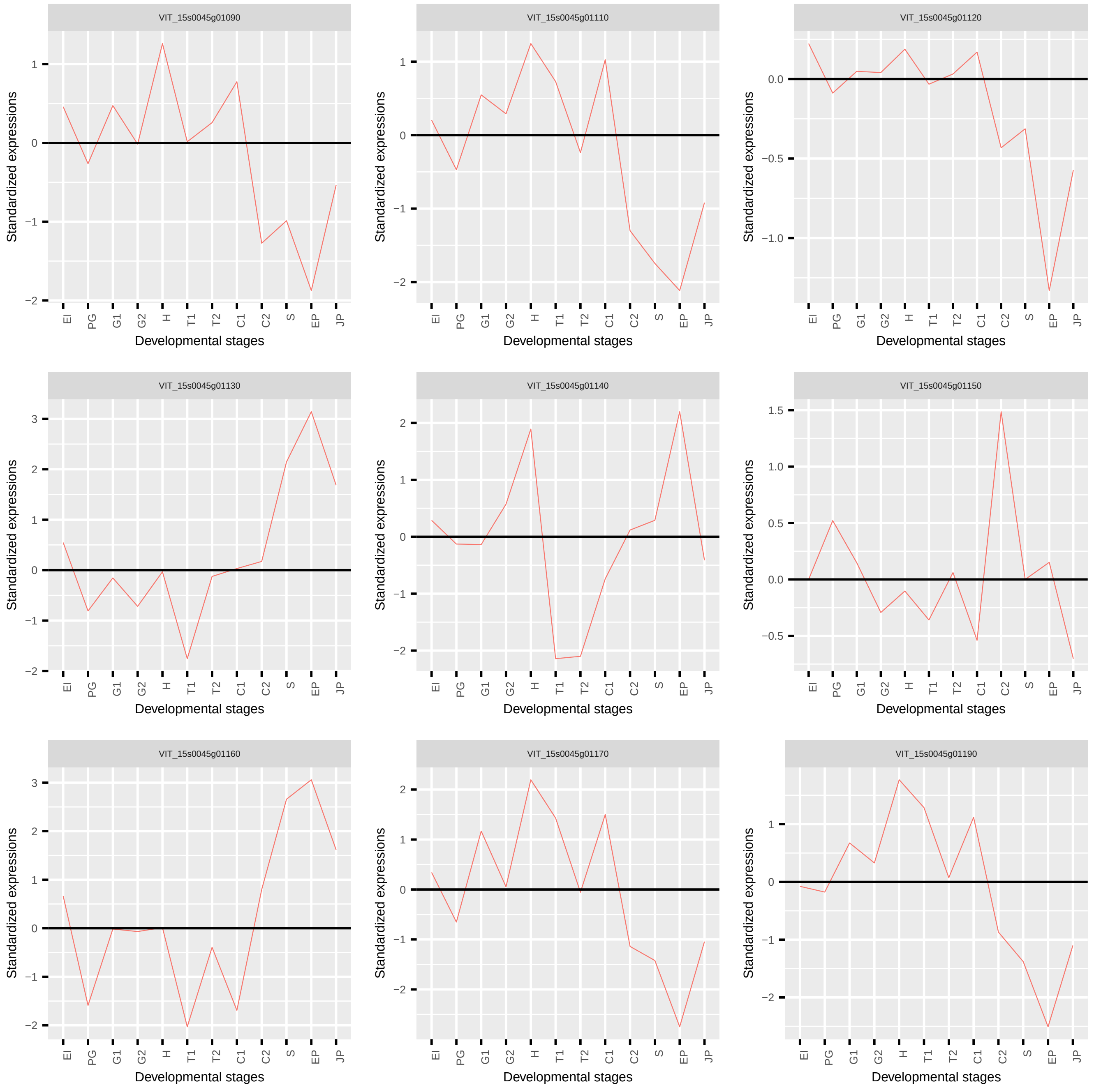
VIT_15s0045g01090
VIT_15s0045g01110
VIT_15s0045g01120
VIT_15s0045g01130
VIT_15s0045g01140
VIT_15s0045g01150
VIT_15s0045g01160
VIT_15s0045g01170
VIT_15s0045g01190
Standardized expressions
Developmental stages
EI PG G1 G2 H T1 T2 C1 C2 S EP JP

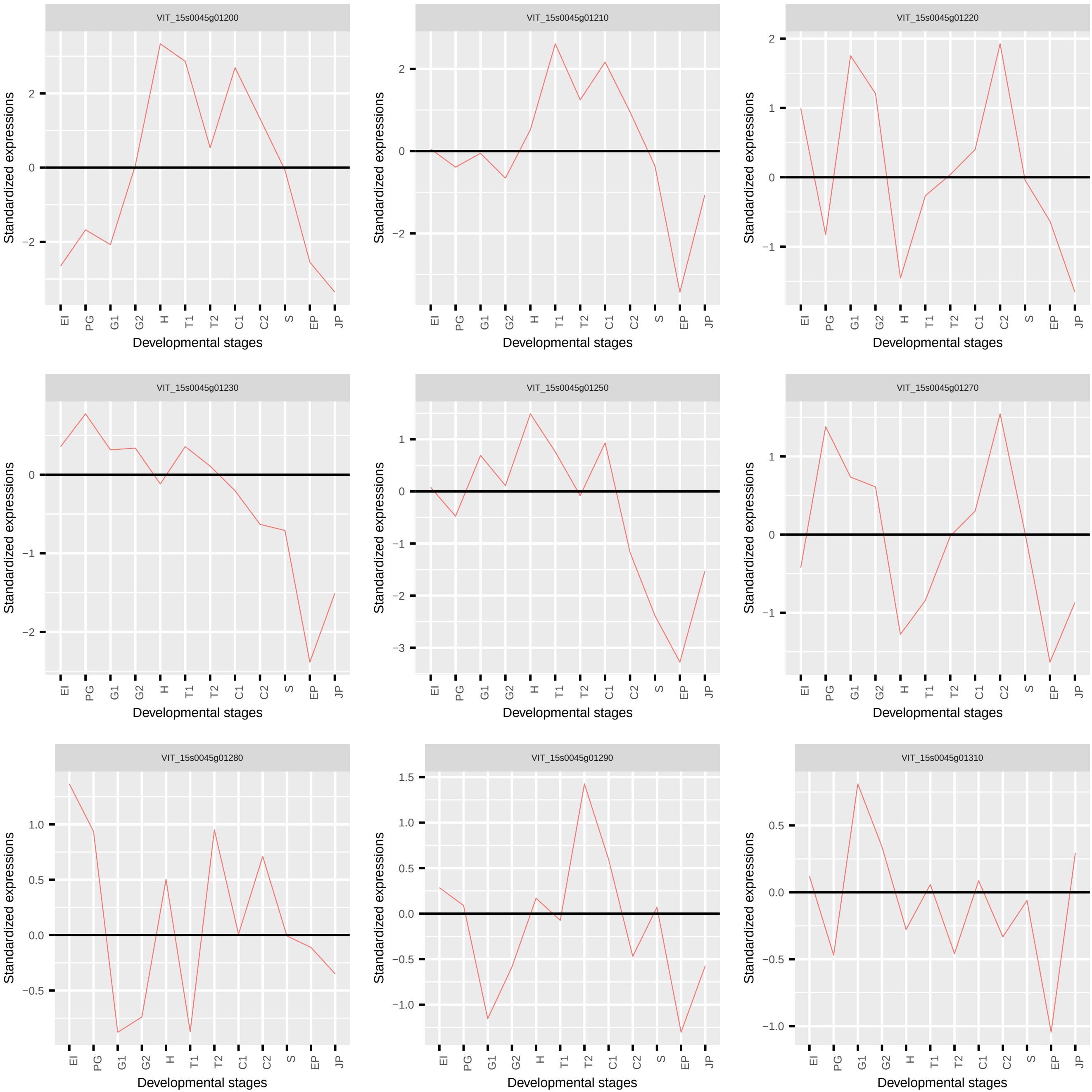
VIT_15s0045g01200
VIT_15s0045g01210
VIT_15s0045g01220
VIT_15s0045g01230
VIT_15s0045g01250
VIT_15s0045g01270
VIT_15s0045g01280
VIT_15s0045g01290
VIT_15s0045g01310
Standardized expressions
Developmental stages
EI PG G1 G2 H T1 T2 C1 C2 S EP JP

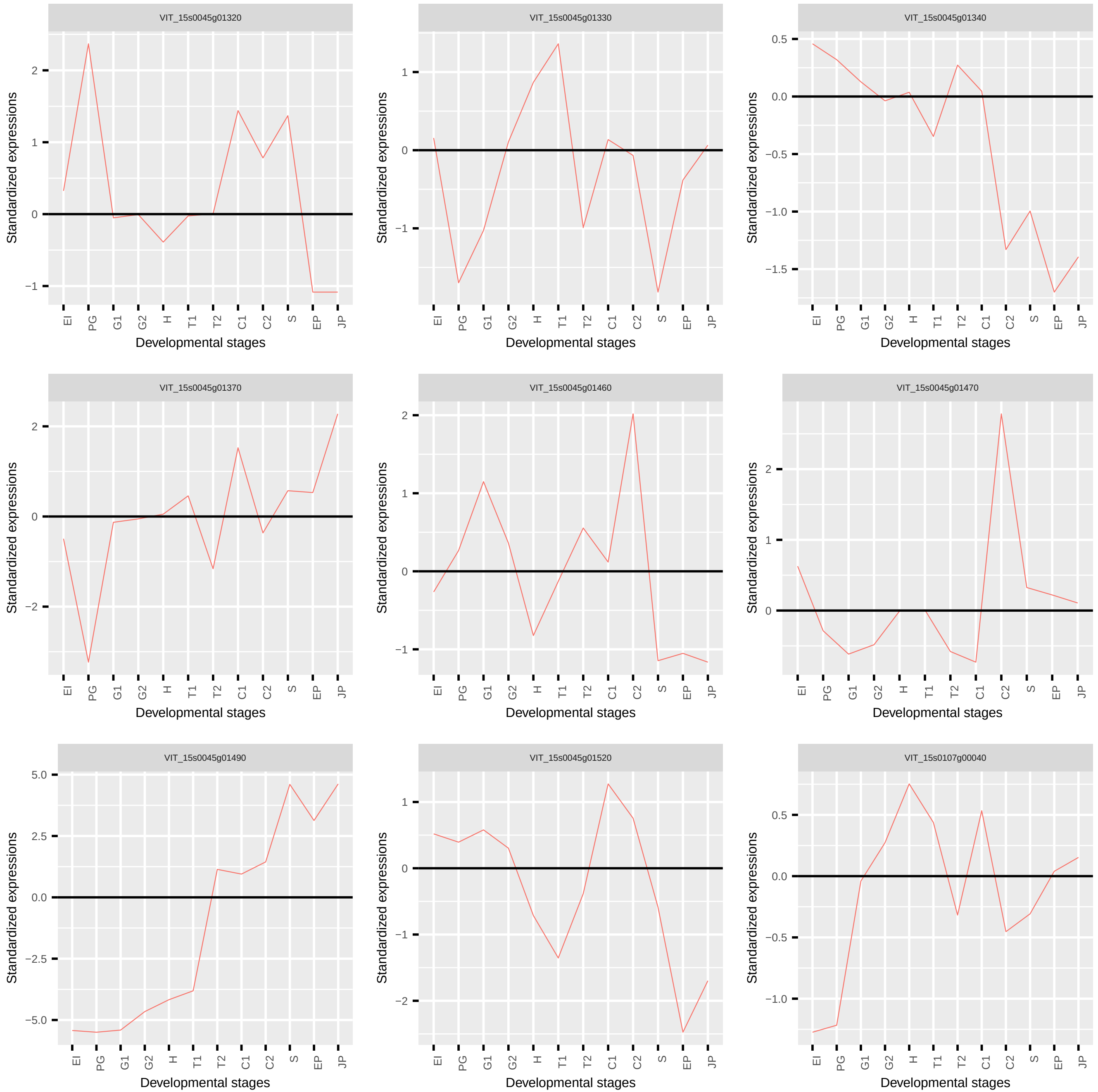

VIT_15s0045g01320
VIT_15s0045g01330
VIT_15s0045g01340
VIT_15s0045g01370
VIT_15s0045g01460
VIT_15s0045g01470
VIT_15s0045g01490
VIT_15s0045g01520
VIT_15s0107g00040
Standardized expressions
Developmental stages
EI PG G1 G2 H T1 T2 C1 C2 S EP JP

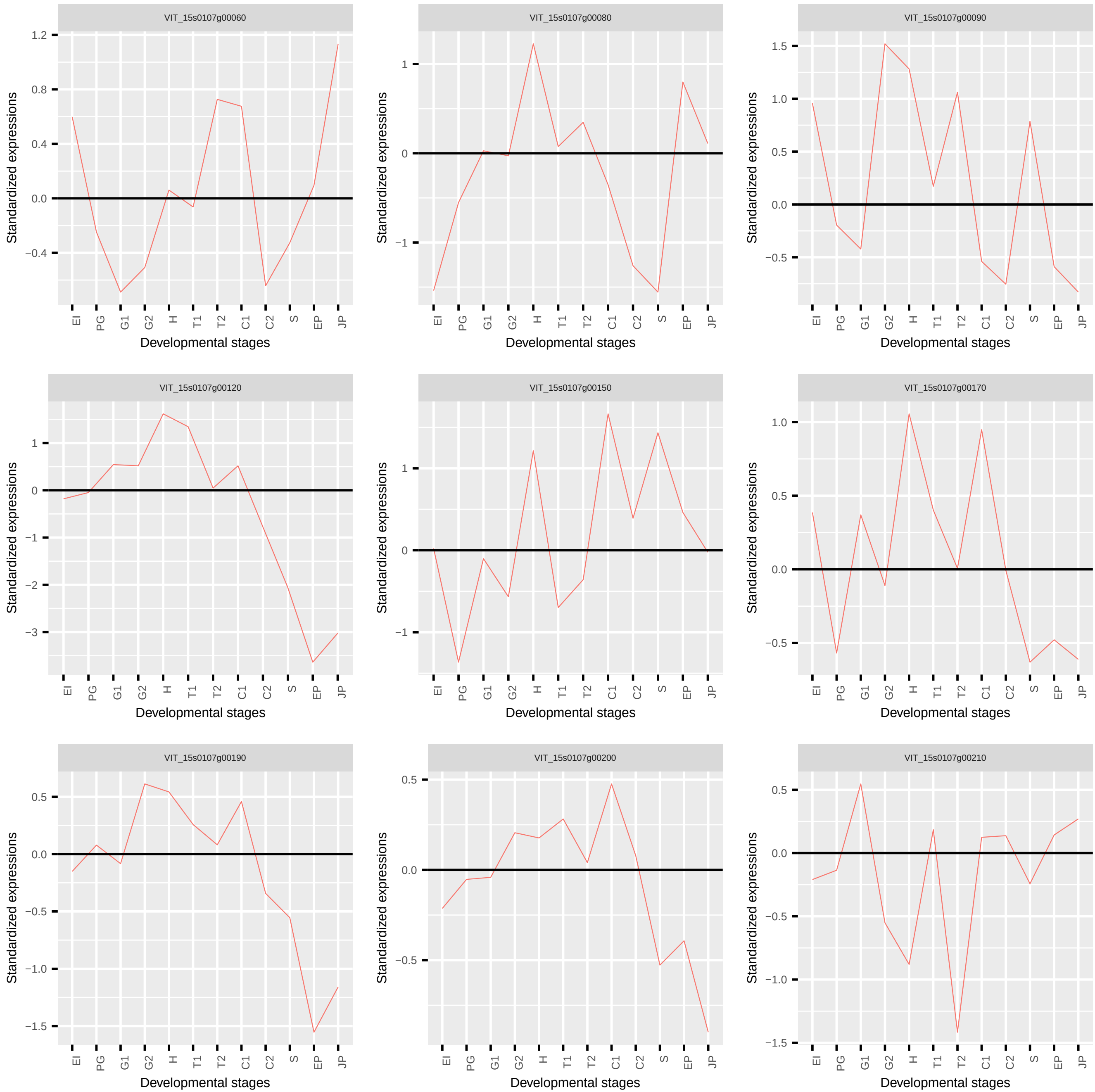
VIT_15s0107g00060
VIT_15s0107g00080
VIT_15s0107g00090
VIT_15s0107g00120
VIT_15s0107g00150
VIT_15s0107g00170
VIT_15s0107g00190
VIT_15s0107g00200
VIT_15s0107g00210
Standardized expressions
Developmental stages
EI PG G1 G2 H T1 T2 C1 C2 S EP JP

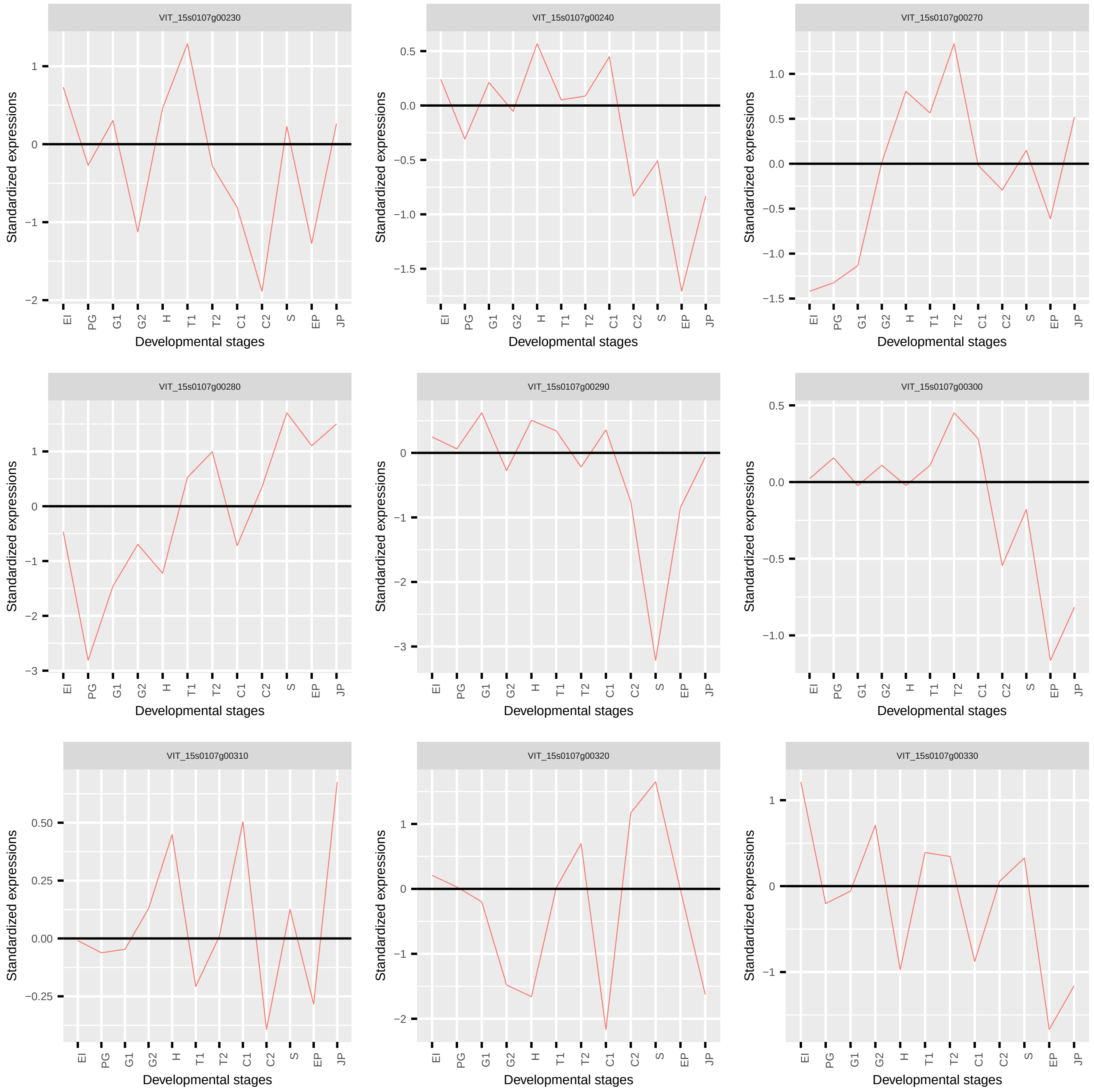
VIT_15s0107g00230
VIT_15s0107g00240
VIT_15s0107g00270
VIT_15s0107g00280
VIT_15s0107g00290
VIT_15s0107g00300
VIT_15s0107g00310
VIT_15s0107g00320
VIT_15s0107g00330
Standardized expressions
Developmental stages
EI
PG
G1
G2
H
T1
T2
C1
C2
S
EP
JP

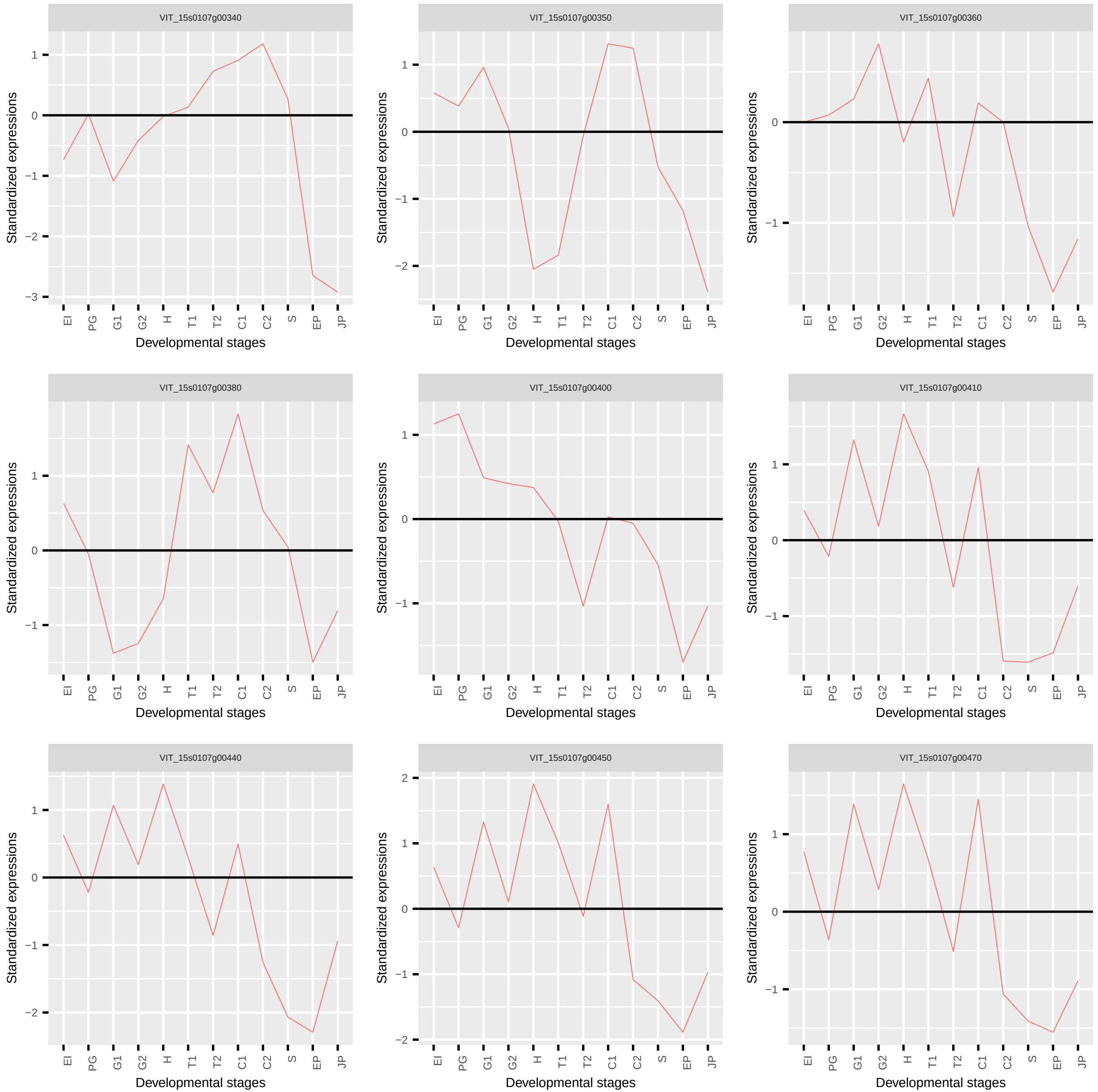

VIT_15s0107g00340
VIT_15s0107g00350
VIT_15s0107g00360
VIT_15s0107g00380
VIT_15s0107g00400
VIT_15s0107g00410
VIT_15s0107g00440
VIT_15s0107g00450
VIT_15s0107g00470
Standardized expressions
Developmental stages
EI
PG
G1
G2
H
T1
T2
C1
C2
S
EP
JP

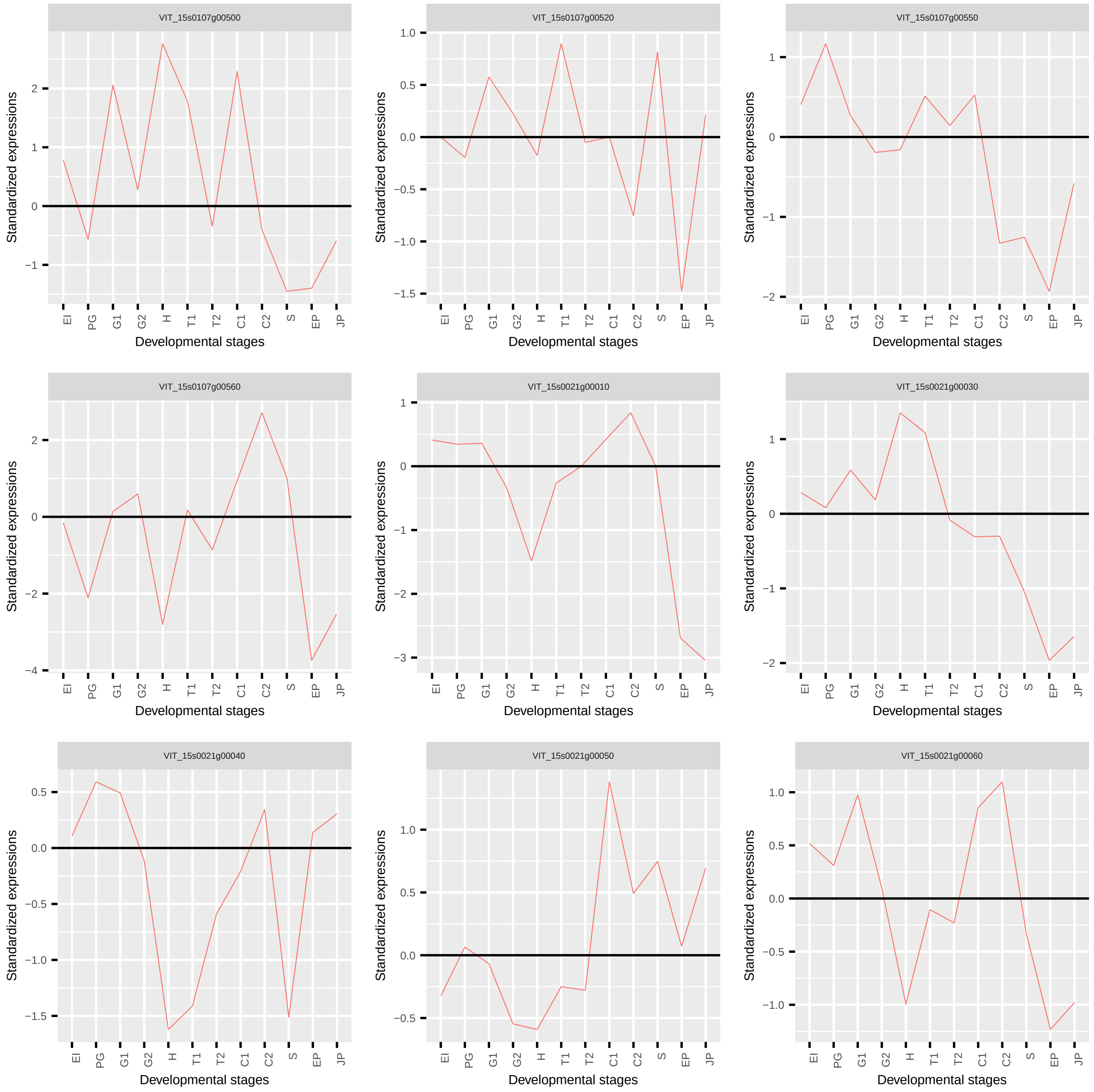
VIT_15s0107g00500
VIT_15s0107g00520
VIT_15s0107g00550
VIT_15s0107g00560
VIT_15s0021g00010
VIT_15s0021g00030
VIT_15s0021g00040
VIT_15s0021g00050
VIT_15s0021g00060
Standardized expressions
Developmental stages
EI PG G1 G2 H T1 T2 C1 C2 S EP JP

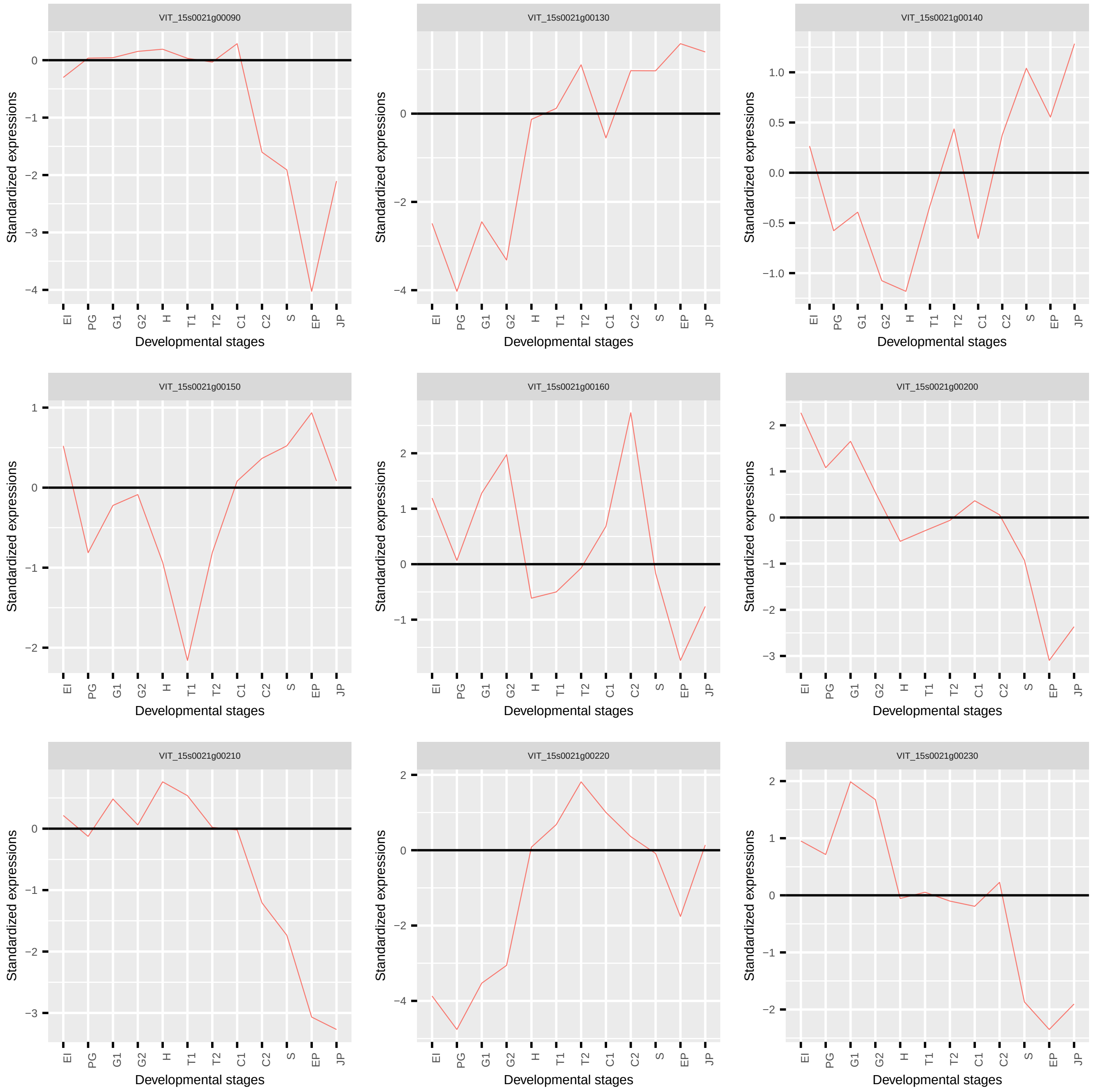

VIT_15s0021g00090
VIT_15s0021g00130
VIT_15s0021g00140
VIT_15s0021g00150
VIT_15s0021g00160
VIT_15s0021g00200
VIT_15s0021g00210
VIT_15s0021g00220
VIT_15s0021g00230
Standardized expressions
Developmental stages
EI PG G1 G2 H T1 T2 C1 C2 S EP JP

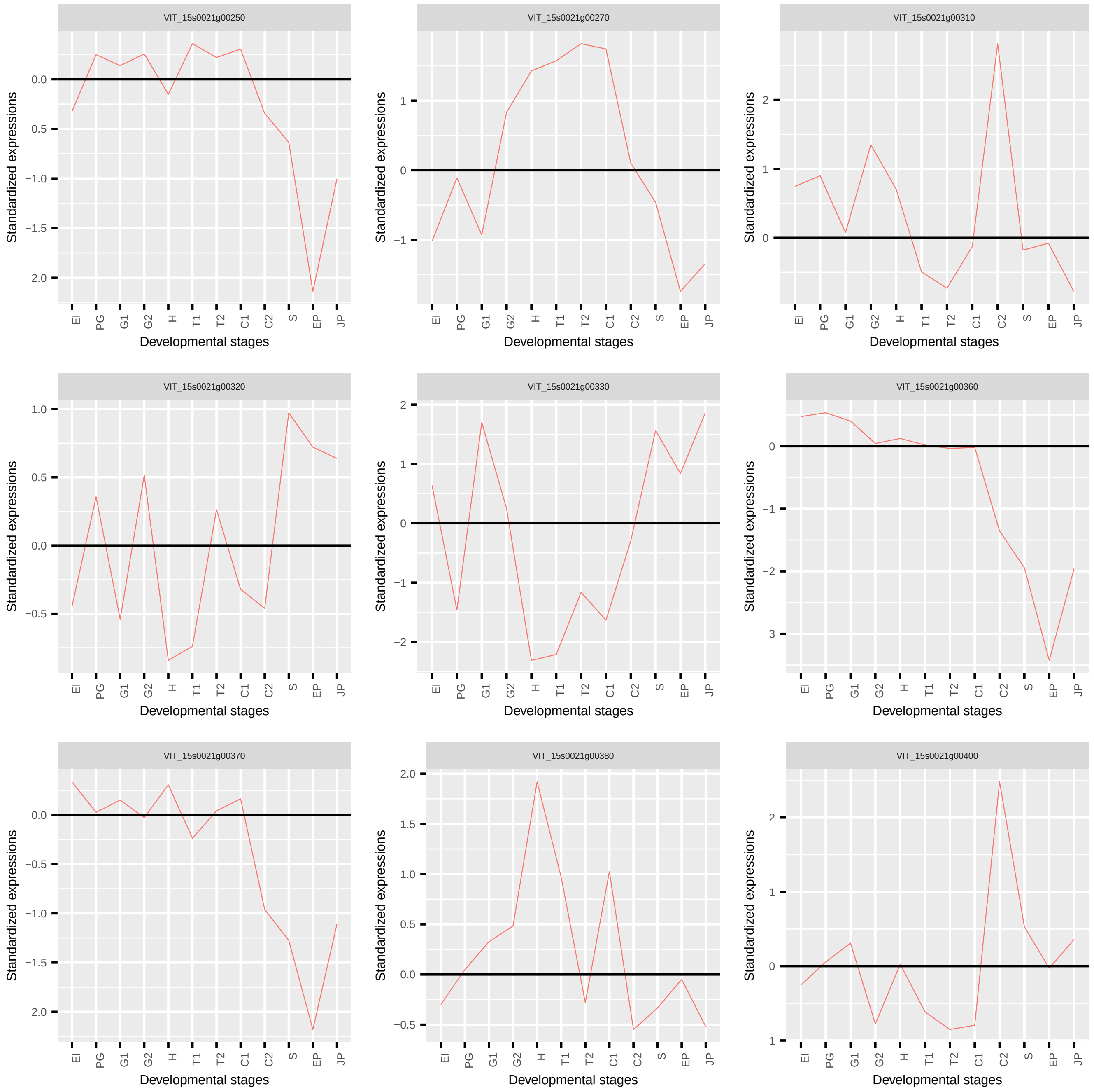

VIT_15s0021g00250
VIT_15s0021g00270
VIT_15s0021g00310
VIT_15s0021g00320
VIT_15s0021g00330
VIT_15s0021g00360
VIT_15s0021g00370
VIT_15s0021g00380
VIT_15s0021g00400
Standardized expressions
Developmental stages
EI PG G1 G2 H T1 T2 C1 C2 S EP JP

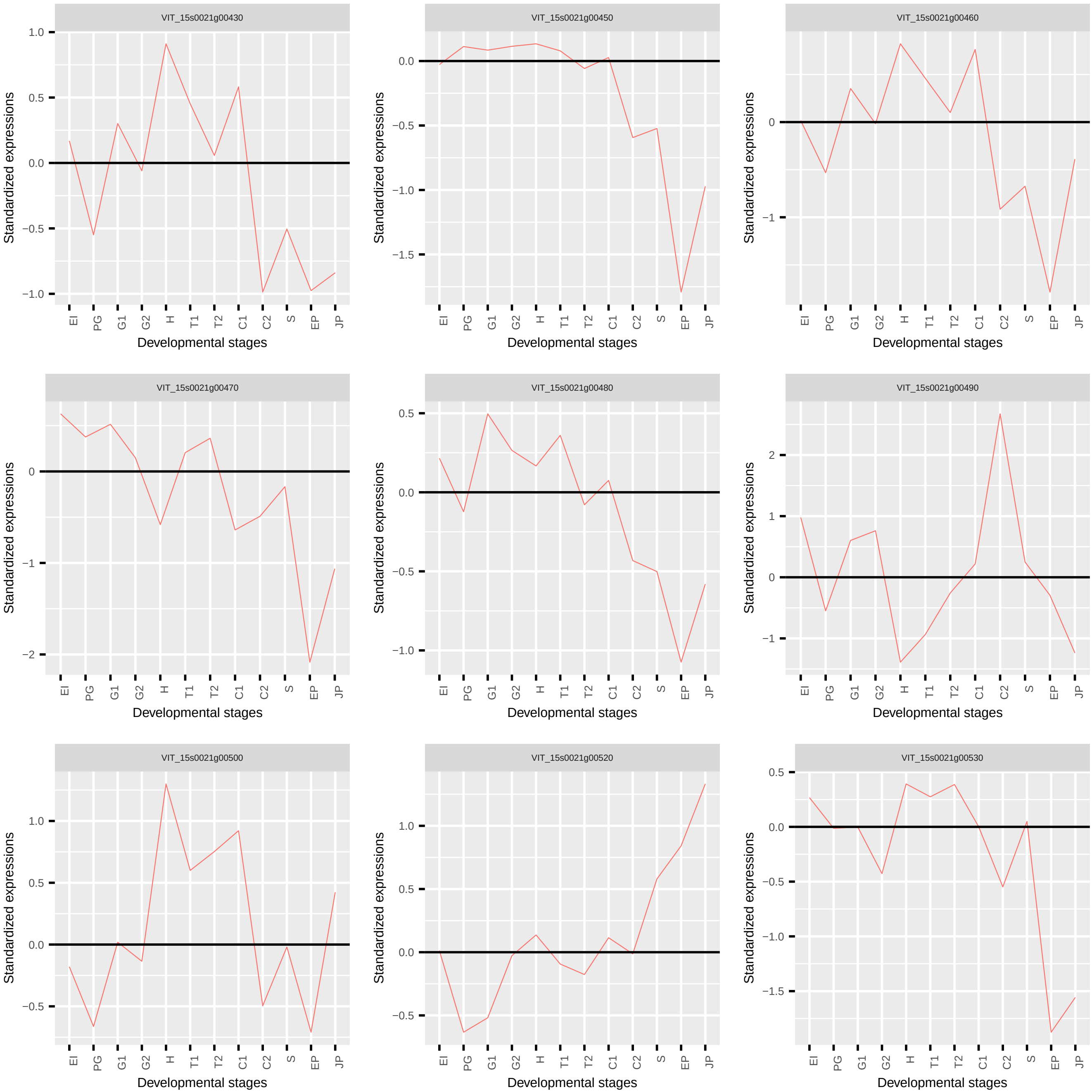
VIT_15s0021g00430
VIT_15s0021g00450
VIT_15s0021g00460
VIT_15s0021g00470
VIT_15s0021g00480
VIT_15s0021g00490
VIT_15s0021g00500
VIT_15s0021g00520
VIT_15s0021g00530
Standardized expressions
Developmental stages
EI PG G1 G2 H T1 T2 C1 C2 S EP JP
1.0 0.5 0.0 −0.5 −1.0 −1.5
2 1 0 −1 −2

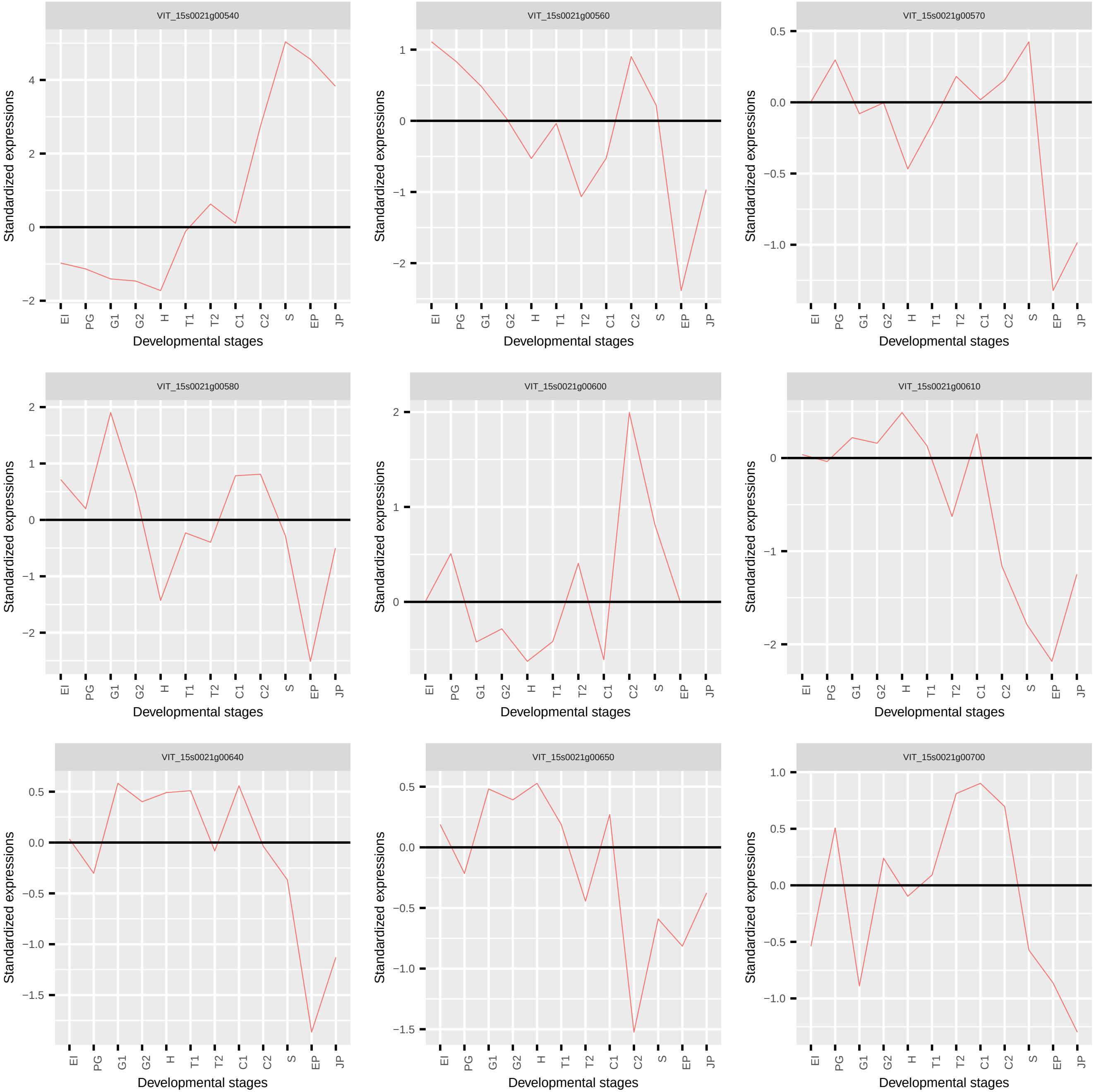
VIT_15s0021g00540
VIT_15s0021g00560
VIT_15s0021g00570
VIT_15s0021g00580
VIT_15s0021g00600
VIT_15s0021g00610
VIT_15s0021g00640
VIT_15s0021g00650
VIT_15s0021g00700
Standardized expressions
Developmental stages
EI PG G1 G2 H T1 T2 C1 C2 S EP JP

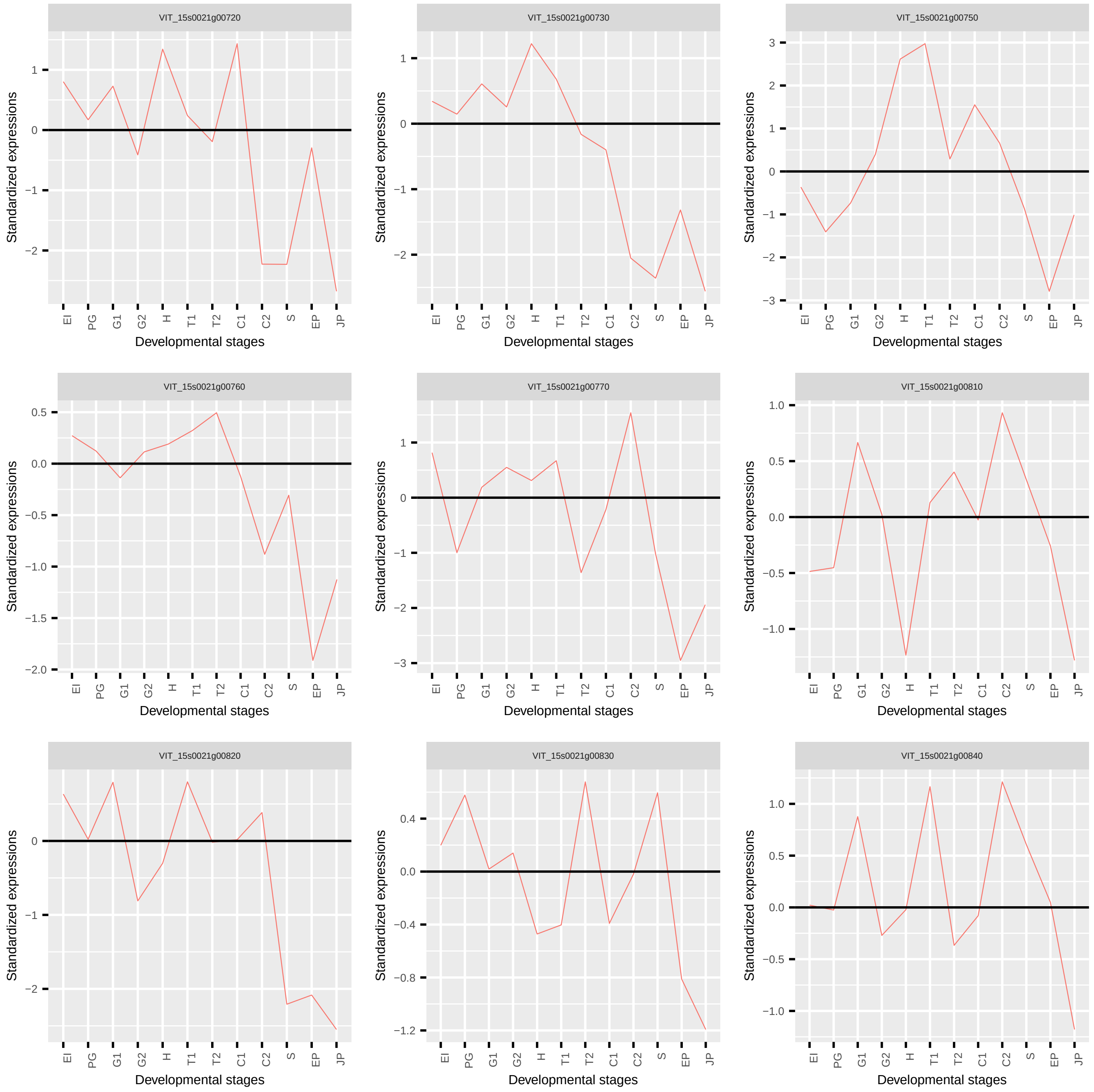
VIT_15s0021g00720
VIT_15s0021g00730
VIT_15s0021g00750
VIT_15s0021g00760
VIT_15s0021g00770
VIT_15s0021g00810
VIT_15s0021g00820
VIT_15s0021g00830
VIT_15s0021g00840
Standardized expressions
Developmental stages
EI
PG
G1
G2
H
T1
T2
C1
C2
S
EP
JP

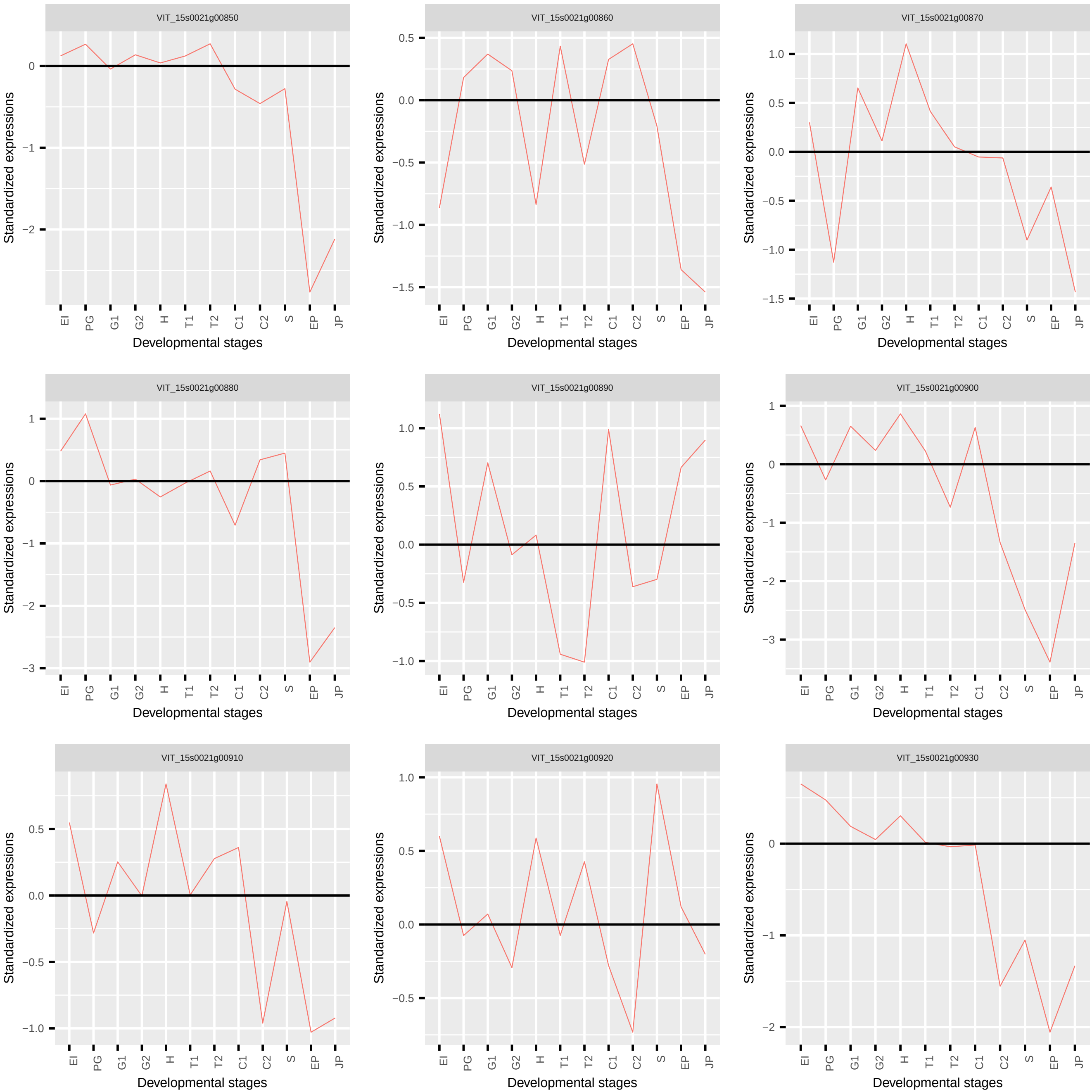
VIT_15s0021g00850
VIT_15s0021g00860
VIT_15s0021g00870
VIT_15s0021g00880
VIT_15s0021g00890
VIT_15s0021g00900
VIT_15s0021g00910
VIT_15s0021g00920
VIT_15s0021g00930
Standardized expressions
Developmental stages
EI PG G1 G2 H T1 T2 C1 C2 S EP JP

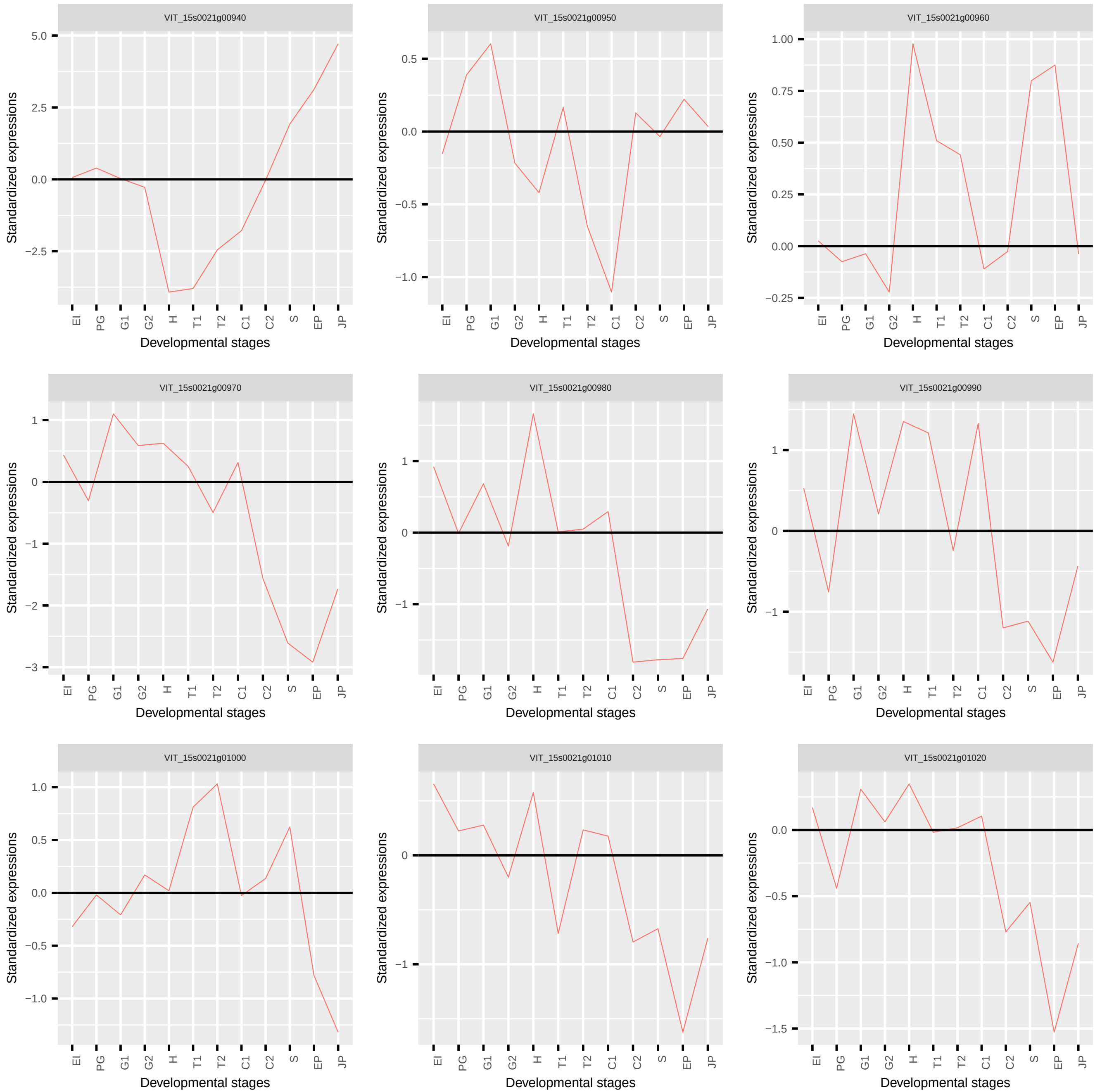
VIT_15s0021g00940
VIT_15s0021g00950
VIT_15s0021g00960
VIT_15s0021g00970
VIT_15s0021g00980
VIT_15s0021g00990
VIT_15s0021g01000
VIT_15s0021g01010
VIT_15s0021g01020
Standardized expressions
Developmental stages
EI PG G1 G2 H T1 T2 C1 C2 S EP JP

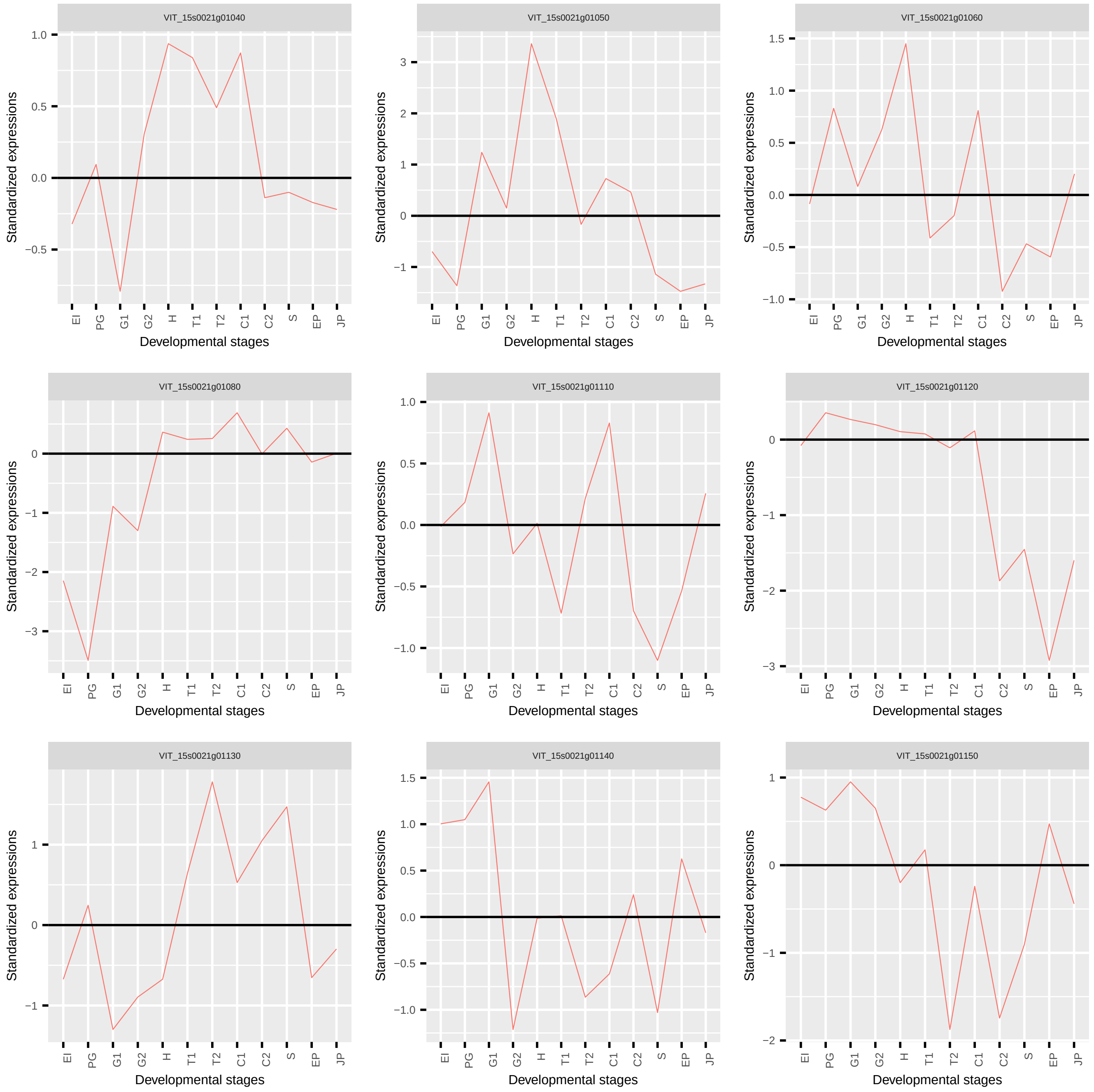
VIT_15s0021g01040
VIT_15s0021g01050
VIT_15s0021g01060
VIT_15s0021g01080
VIT_15s0021g01110
VIT_15s0021g01120
VIT_15s0021g01130
VIT_15s0021g01140
VIT_15s0021g01150
Standardized expressions
Developmental stages
EI
PG
G1
G2
H
T1
T2
C1
C2
S
EP
JP

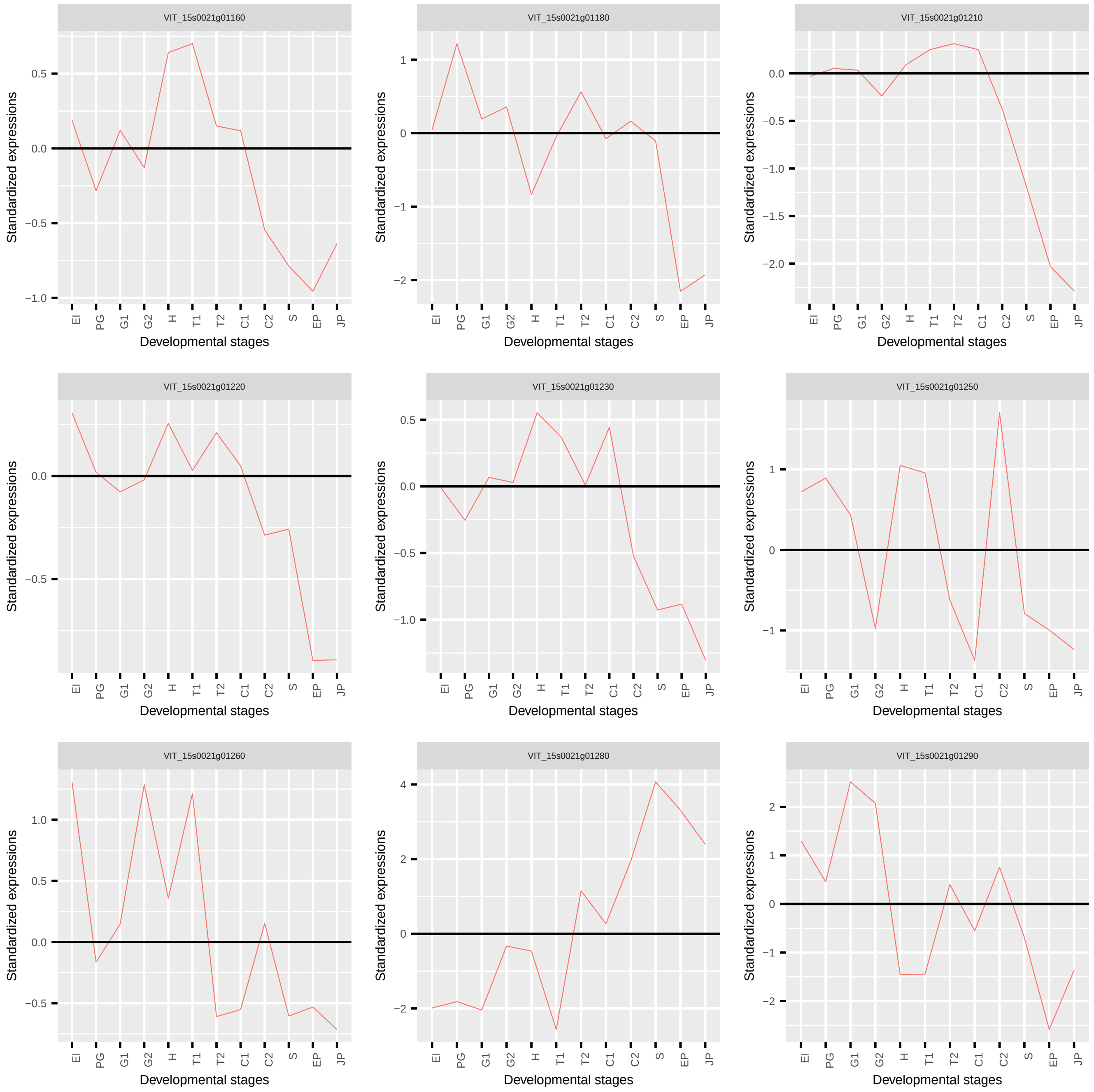
VIT_15s0021g01160
VIT_15s0021g01180
VIT_15s0021g01210
VIT_15s0021g01220
VIT_15s0021g01230
VIT_15s0021g01250
VIT_15s0021g01260
VIT_15s0021g01280
VIT_15s0021g01290
Standardized expressions
Developmental stages
EI PG G1 G2 H T1 T2 C1 C2 S EP JP

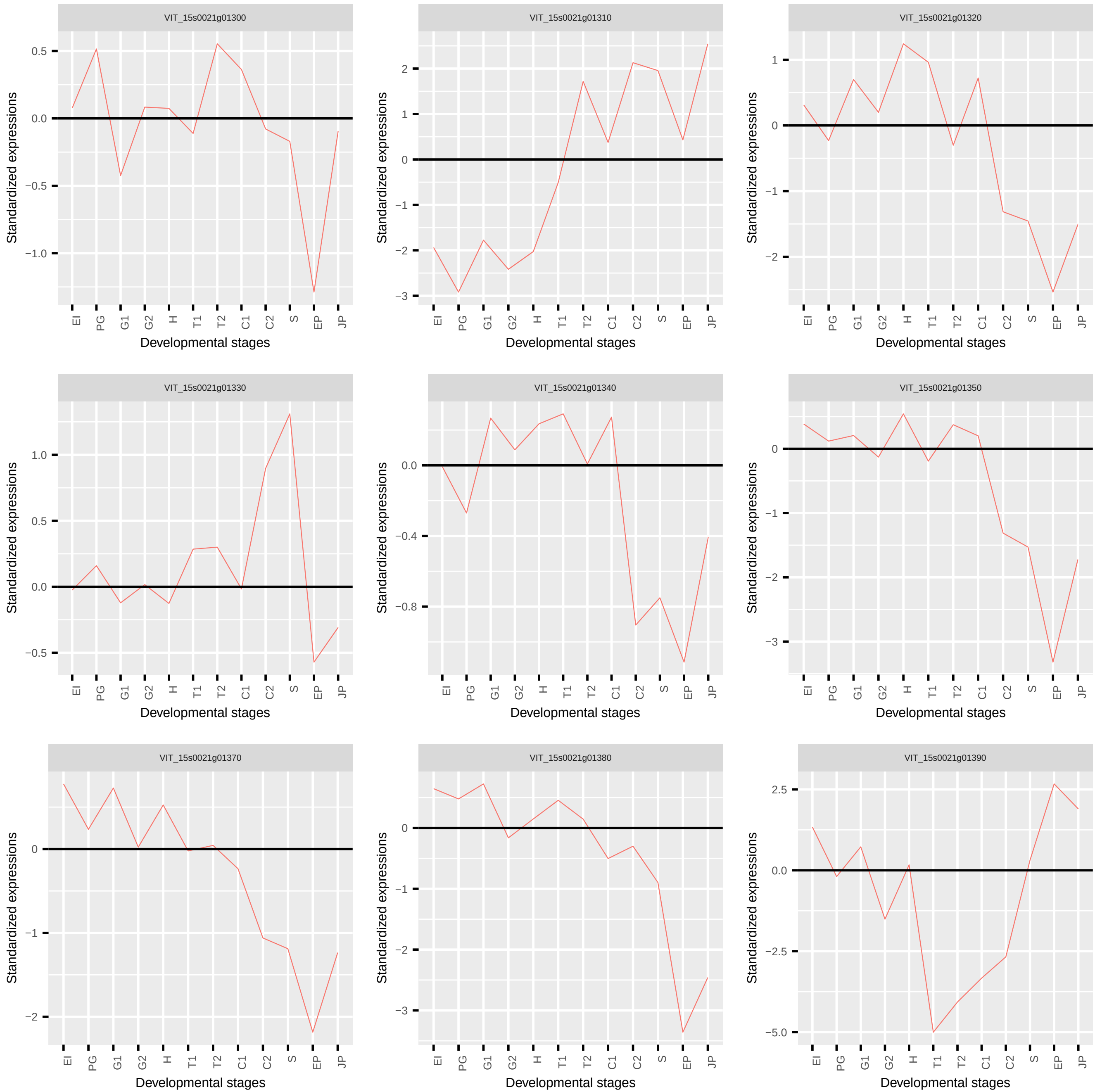
VIT_15s0021g01300
VIT_15s0021g01310
VIT_15s0021g01320
VIT_15s0021g01330
VIT_15s0021g01340
VIT_15s0021g01350
VIT_15s0021g01370
VIT_15s0021g01380
VIT_15s0021g01390
Standardized expressions
Developmental stages
EI
PG
G1
G2
H
T1
T2
C1
C2
S
EP
JP

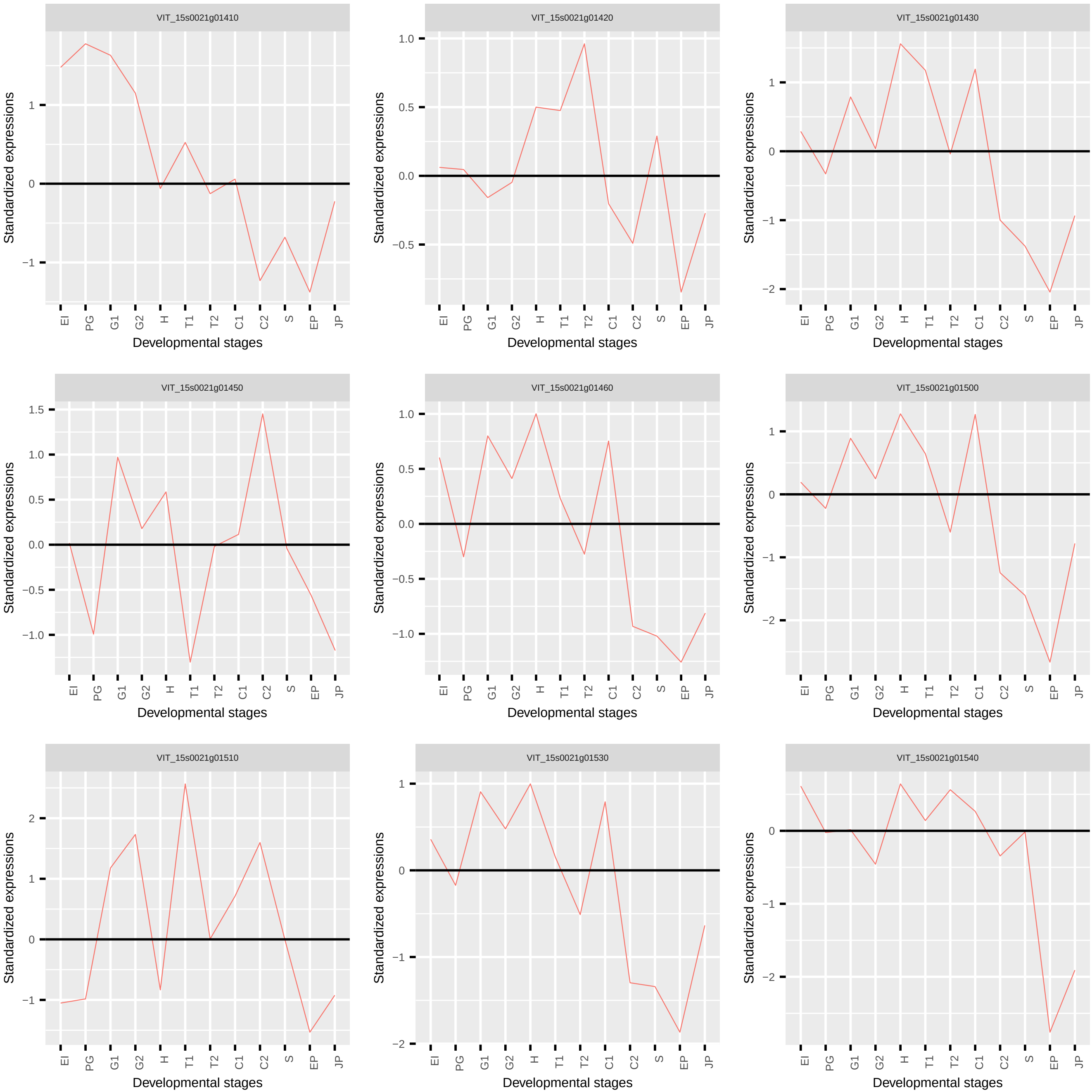
VIT_15s0021g01410
VIT_15s0021g01420
VIT_15s0021g01430
VIT_15s0021g01450
VIT_15s0021g01460
VIT_15s0021g01500
VIT_15s0021g01510
VIT_15s0021g01530
VIT_15s0021g01540
Standardized expressions
Developmental stages
EI
PG
G1
G2
H
T1
T2
C1
C2
S
EP
JP

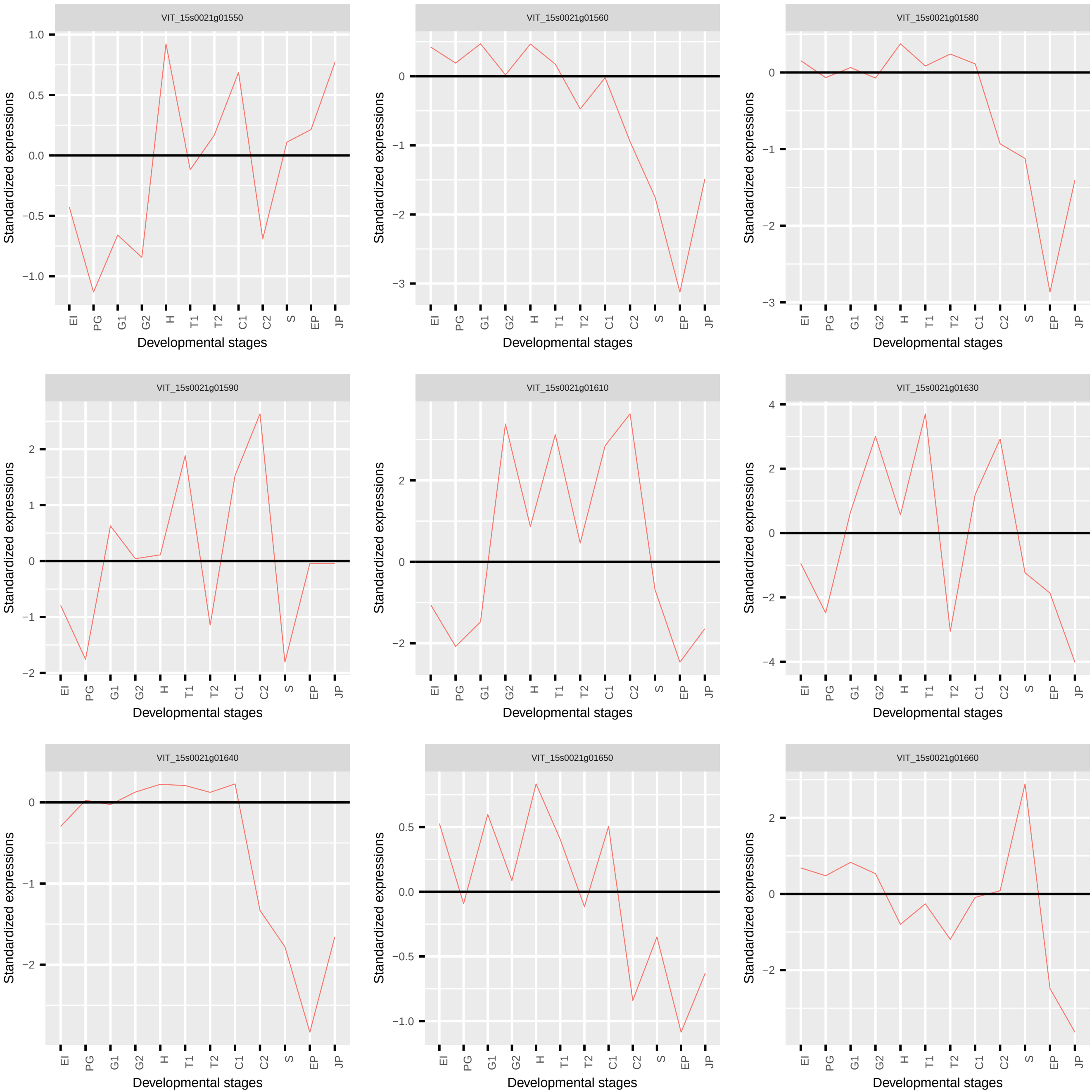
VIT_15s0021g01550
VIT_15s0021g01560
VIT_15s0021g01580
VIT_15s0021g01590
VIT_15s0021g01610
VIT_15s0021g01630
VIT_15s0021g01640
VIT_15s0021g01650
VIT_15s0021g01660
Standardized expressions
Developmental stages
EI
PG
G1
G2
H
T1
T2
C1
C2
S
EP
JP

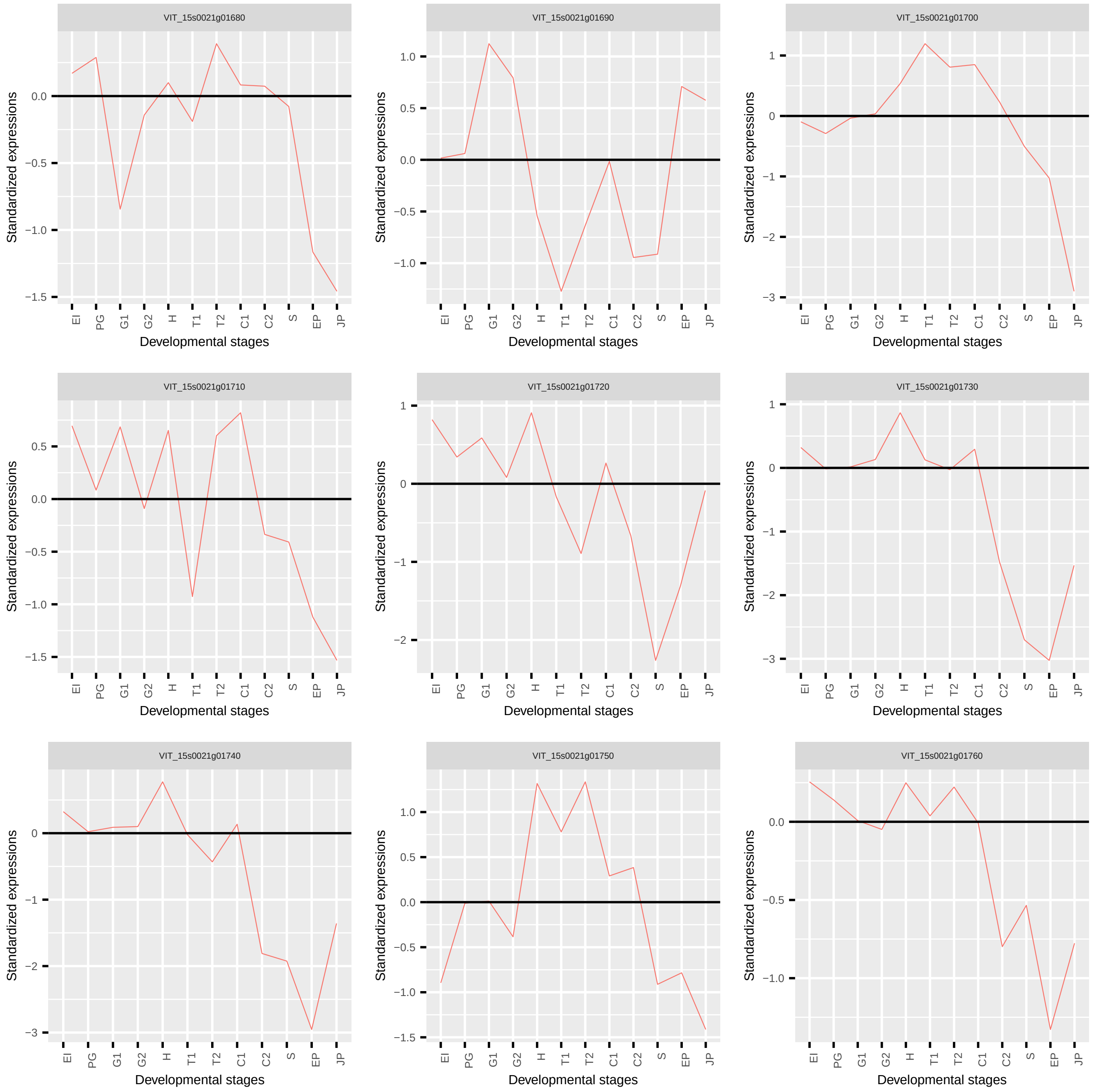

VIT_15s0021g01680
VIT_15s0021g01690
VIT_15s0021g01700
VIT_15s0021g01710
VIT_15s0021g01720
VIT_15s0021g01730
VIT_15s0021g01740
VIT_15s0021g01750
VIT_15s0021g01760
Standardized expressions
Developmental stages
EI
PG
G1
G2
H
T1
T2
C1
C2
S
EP
JP

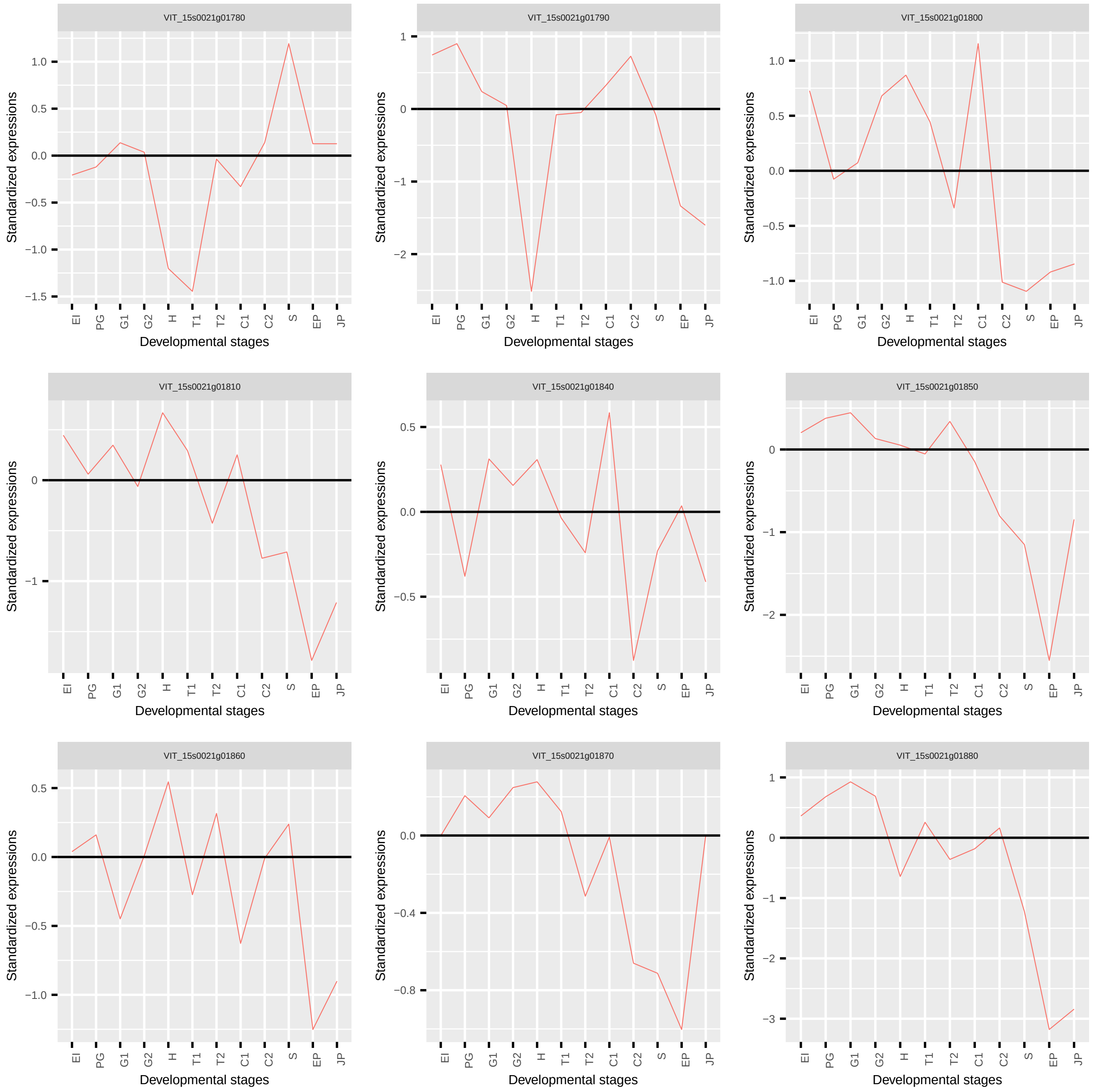

VIT_15s0021g01780
VIT_15s0021g01790
VIT_15s0021g01800
VIT_15s0021g01810
VIT_15s0021g01840
VIT_15s0021g01850
VIT_15s0021g01860
VIT_15s0021g01870
VIT_15s0021g01880
Standardized expressions
Developmental stages
EI
PG
G1
G2
H
T1
T2
C1
C2
S
EP
JP

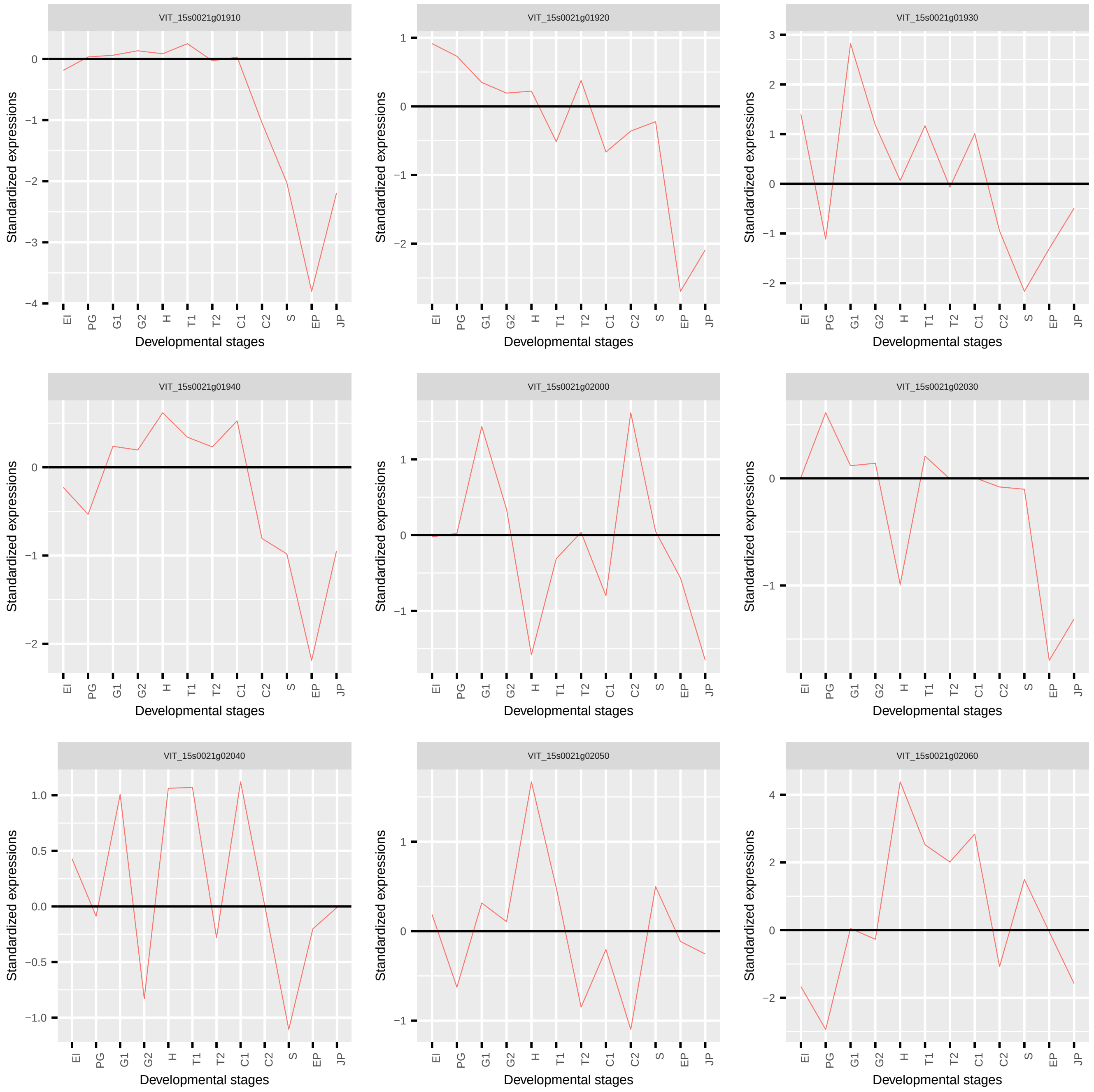
VIT_15s0021g01910
VIT_15s0021g01920
VIT_15s0021g01930
VIT_15s0021g01940
VIT_15s0021g02000
VIT_15s0021g02030
VIT_15s0021g02040
VIT_15s0021g02050
VIT_15s0021g02060
Standardized expressions
Developmental stages
EI PG G1 G2 H T1 T2 C1 C2 S EP JP

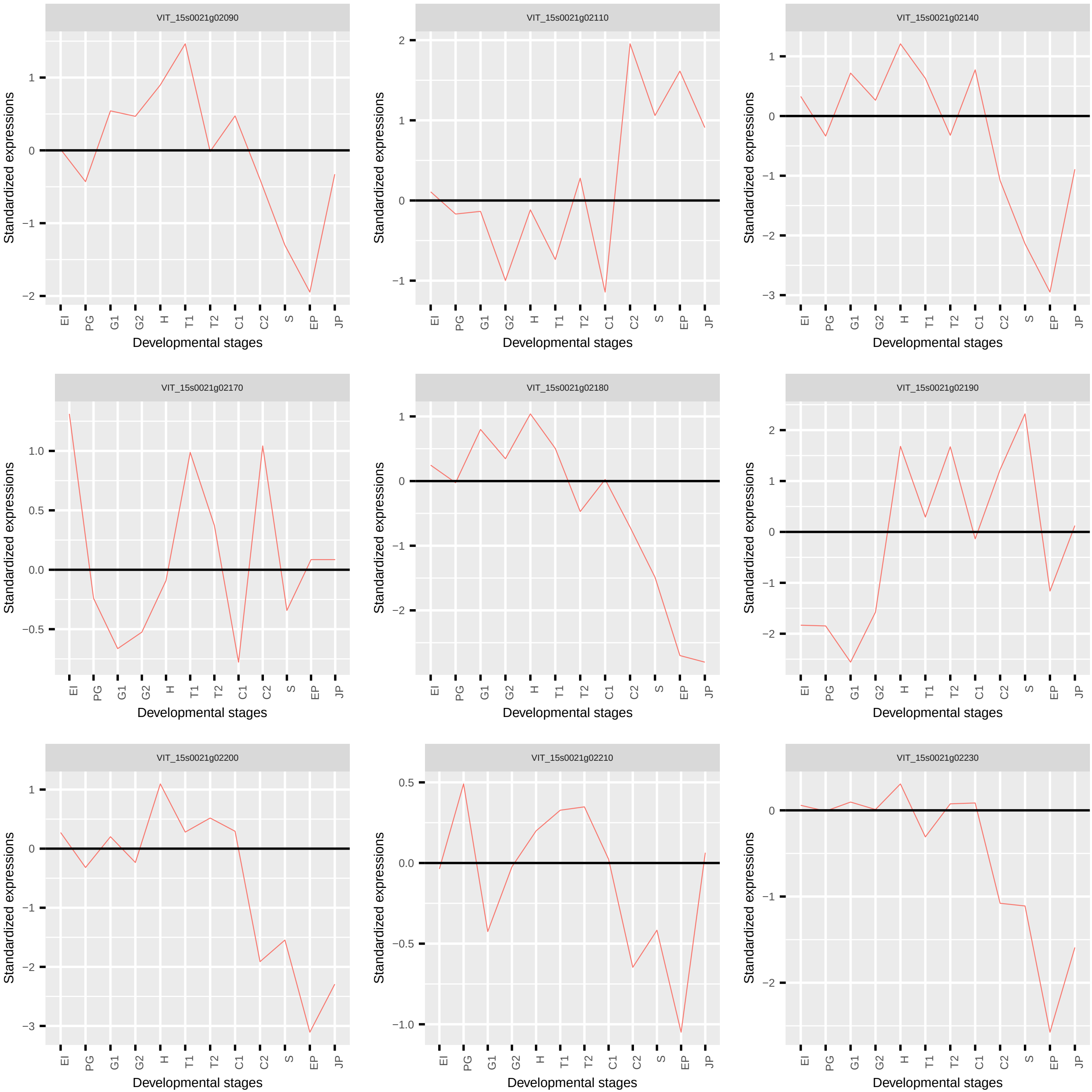

VIT_15s0021g02090
VIT_15s0021g02110
VIT_15s0021g02140
VIT_15s0021g02170
VIT_15s0021g02180
VIT_15s0021g02190
VIT_15s0021g02200
VIT_15s0021g02210
VIT_15s0021g02230
Standardized expressions
Developmental stages
EI
PG
G1
G2
H
T1
T2
C1
C2
S
EP
JP

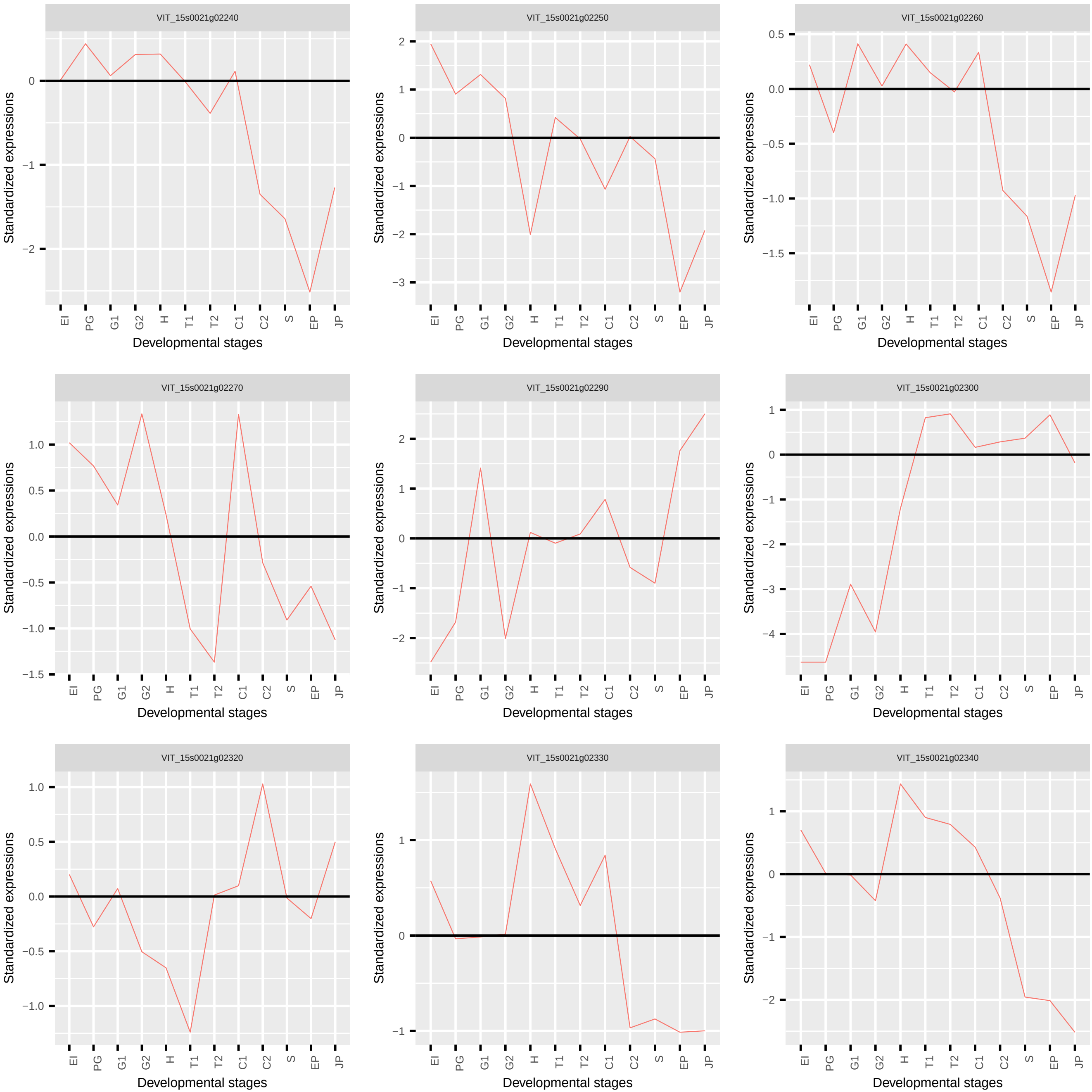
VIT_15s0021g02240
VIT_15s0021g02250
VIT_15s0021g02260
VIT_15s0021g02270
VIT_15s0021g02290
VIT_15s0021g02300
VIT_15s0021g02320
VIT_15s0021g02330
VIT_15s0021g02340
Standardized expressions
Developmental stages
EI PG G1 G2 H T1 T2 C1 C2 S EP JP

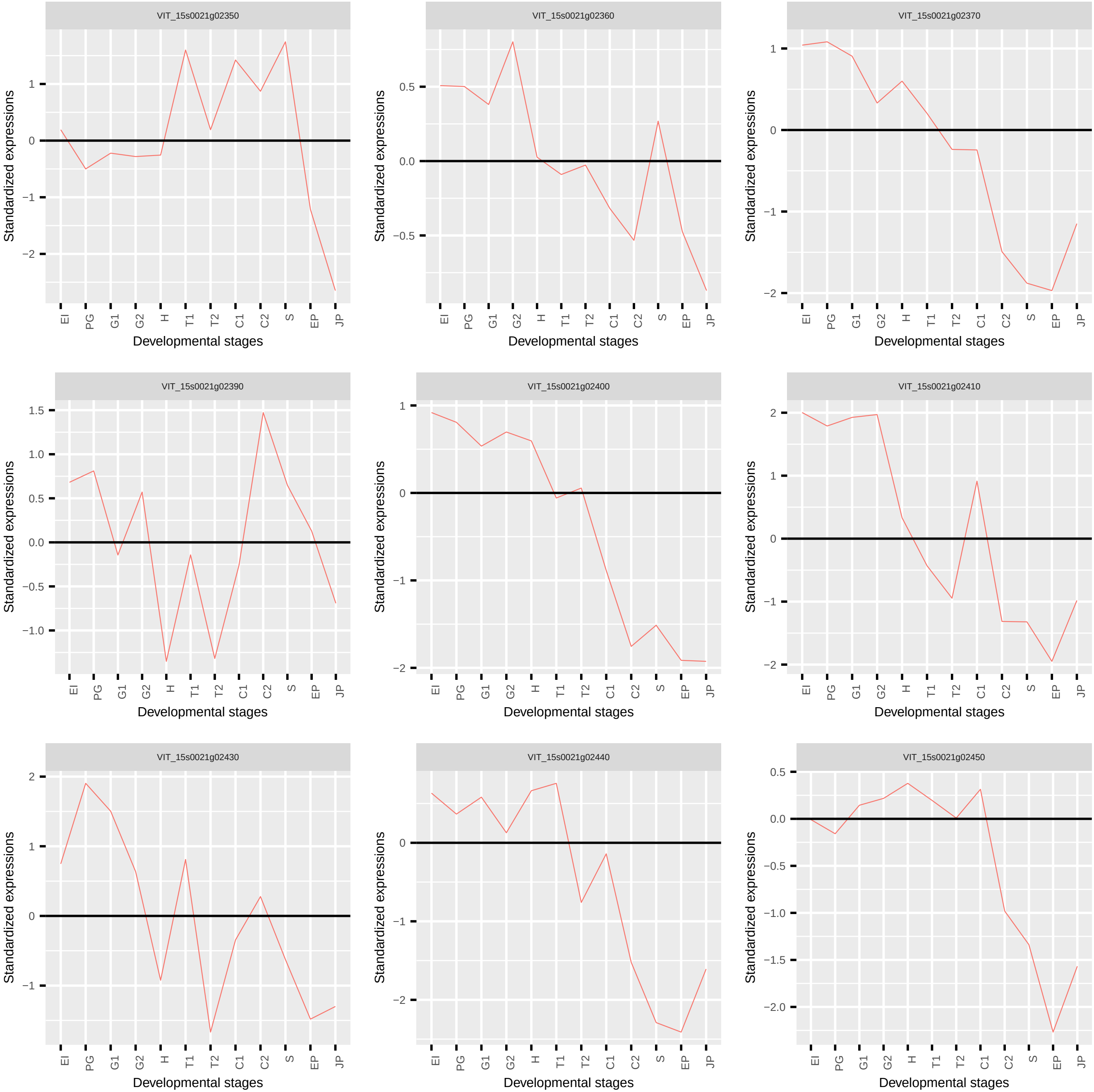

VIT_15s0021g02350
VIT_15s0021g02360
VIT_15s0021g02370
VIT_15s0021g02390
VIT_15s0021g02400
VIT_15s0021g02410
VIT_15s0021g02430
VIT_15s0021g02440
VIT_15s0021g02450
Standardized expressions
Developmental stages
EI PG G1 G2 H T1 T2 C1 C2 S EP JP

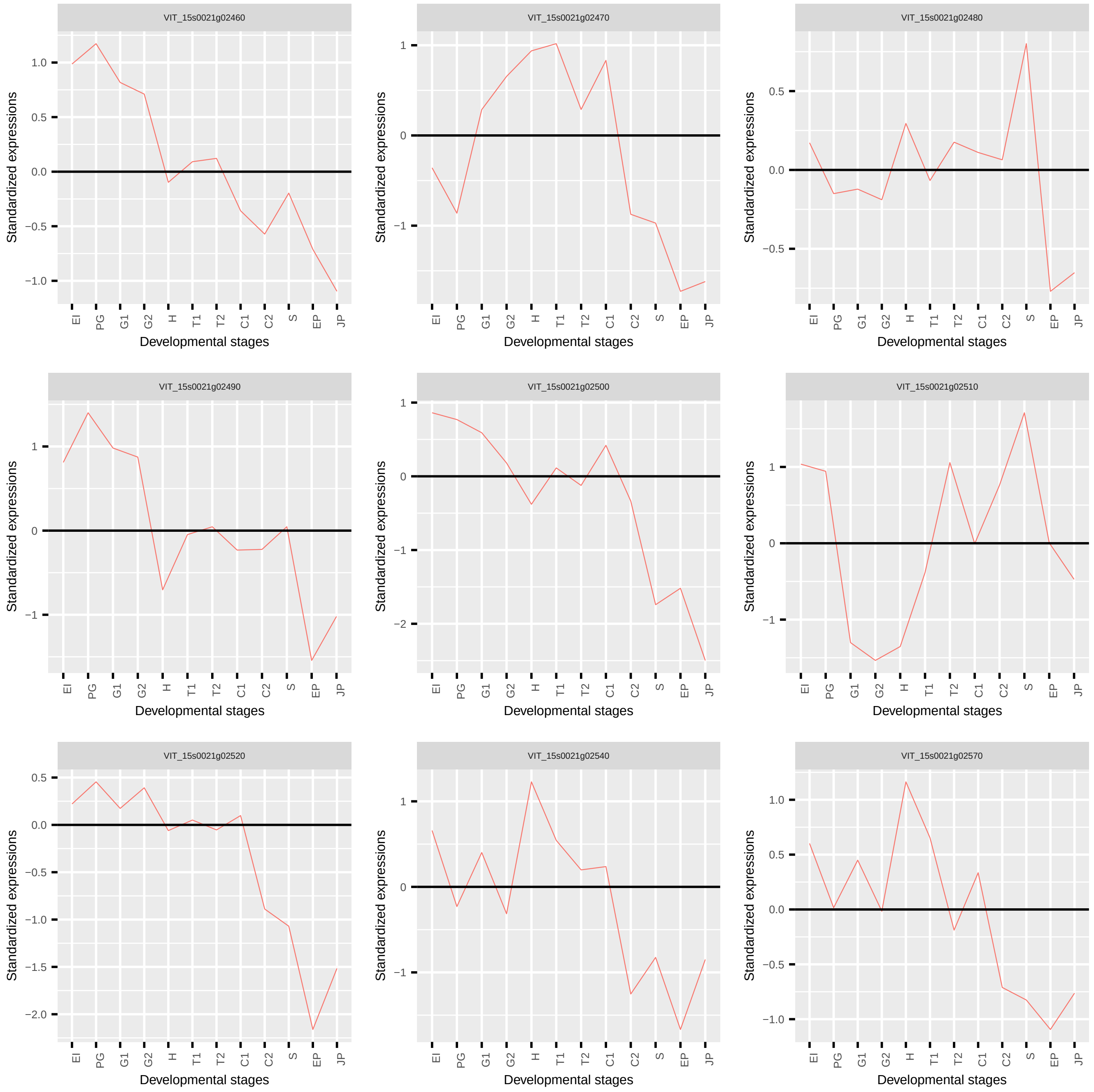

VIT_15s0021g02460
VIT_15s0021g02470
VIT_15s0021g02480
VIT_15s0021g02490
VIT_15s0021g02500
VIT_15s0021g02510
VIT_15s0021g02520
VIT_15s0021g02540
VIT_15s0021g02570
Standardized expressions
Developmental stages
EI PG G1 G2 H T1 T2 C1 C2 S EP JP

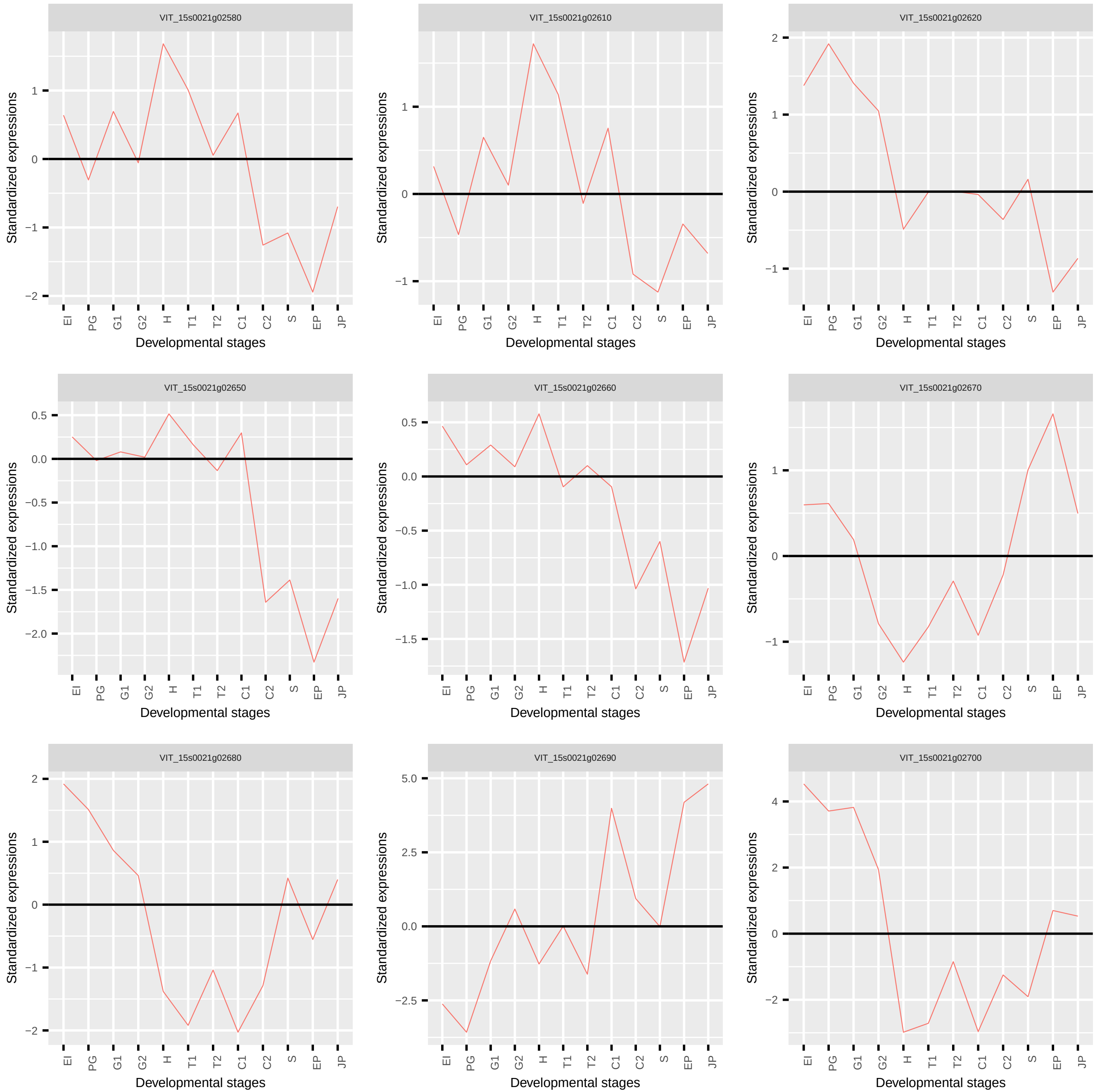
VIT_15s0021g02580
VIT_15s0021g02610
VIT_15s0021g02620
VIT_15s0021g02650
VIT_15s0021g02660
VIT_15s0021g02670
VIT_15s0021g02680
VIT_15s0021g02690
VIT_15s0021g02700
Standardized expressions
Developmental stages
EI PG G1 G2 H T1 T2 C1 C2 S EP JP

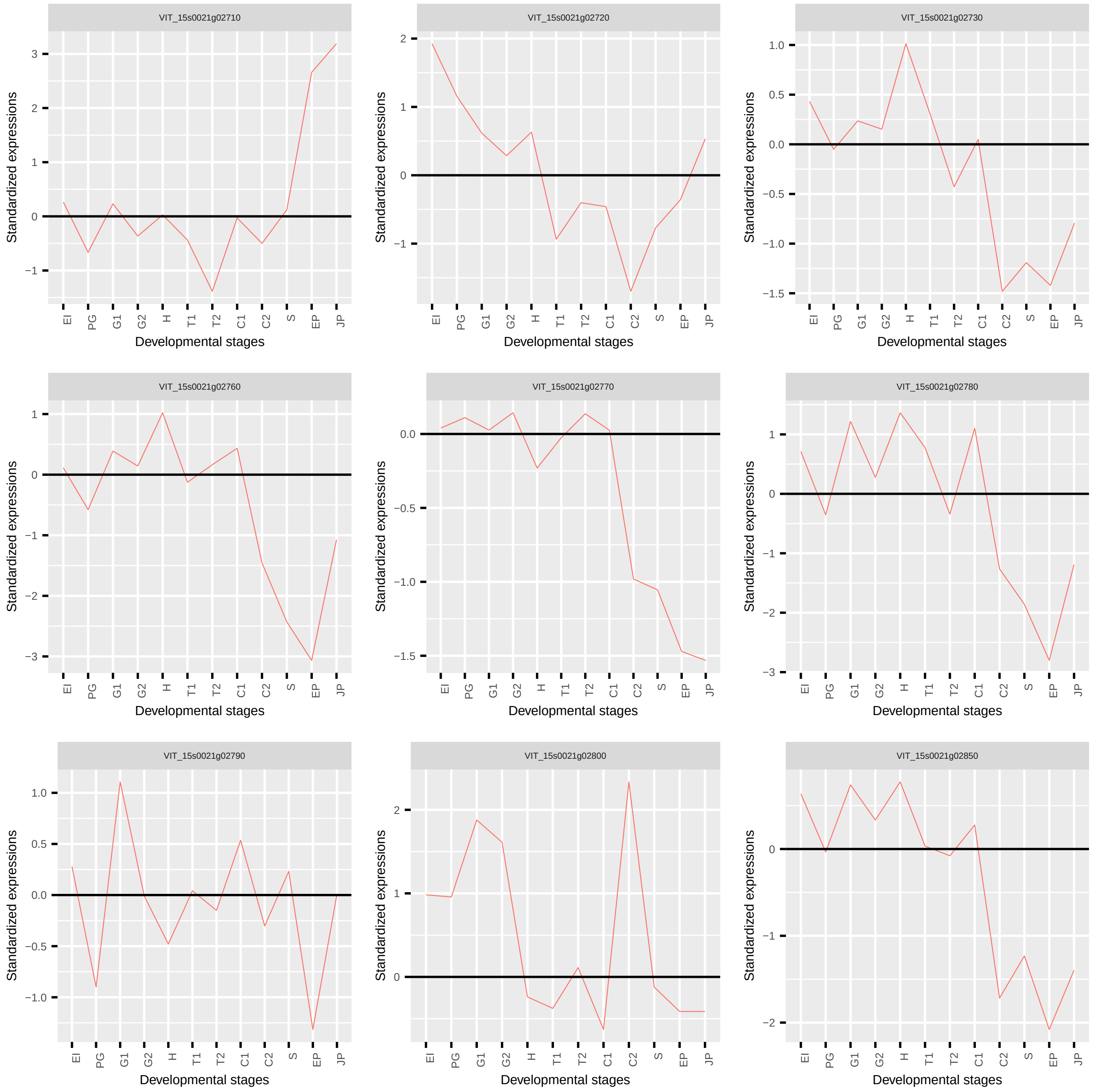
VIT_15s0021g02710
VIT_15s0021g02720
VIT_15s0021g02730
VIT_15s0021g02760
VIT_15s0021g02770
VIT_15s0021g02780
VIT_15s0021g02790
VIT_15s0021g02800
VIT_15s0021g02850
Standardized expressions
Developmental stages
EI PG G1 G2 H T1 T2 C1 C2 S EP JP

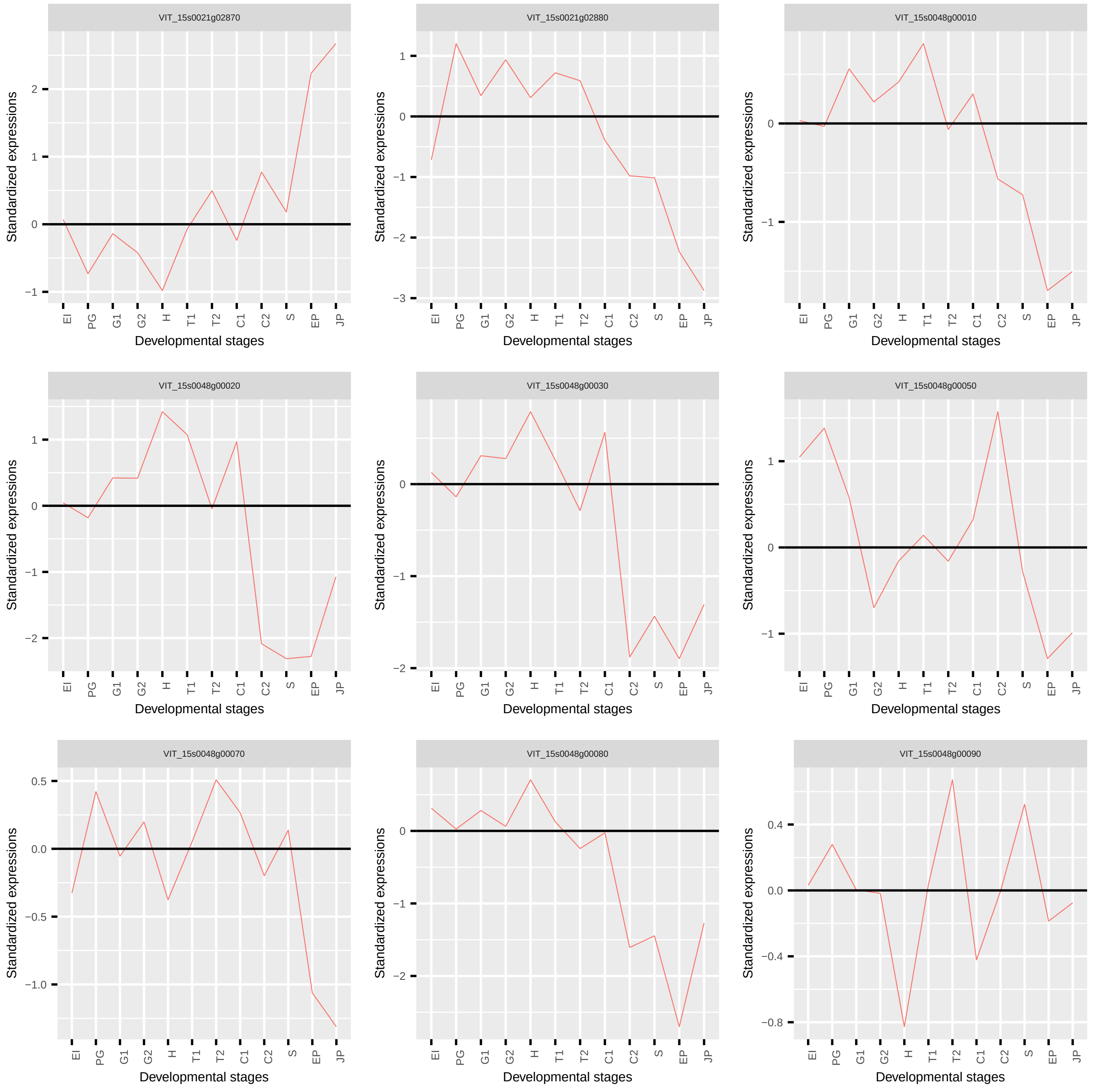
VIT_15s0021g02870
VIT_15s0021g02880
VIT_15s0048g00010
VIT_15s0048g00020
VIT_15s0048g00030
VIT_15s0048g00050
VIT_15s0048g00070
VIT_15s0048g00080
VIT_15s0048g00090
Standardized expressions
Developmental stages
EI PG G1 G2 H T1 T2 C1 C2 S EP JP

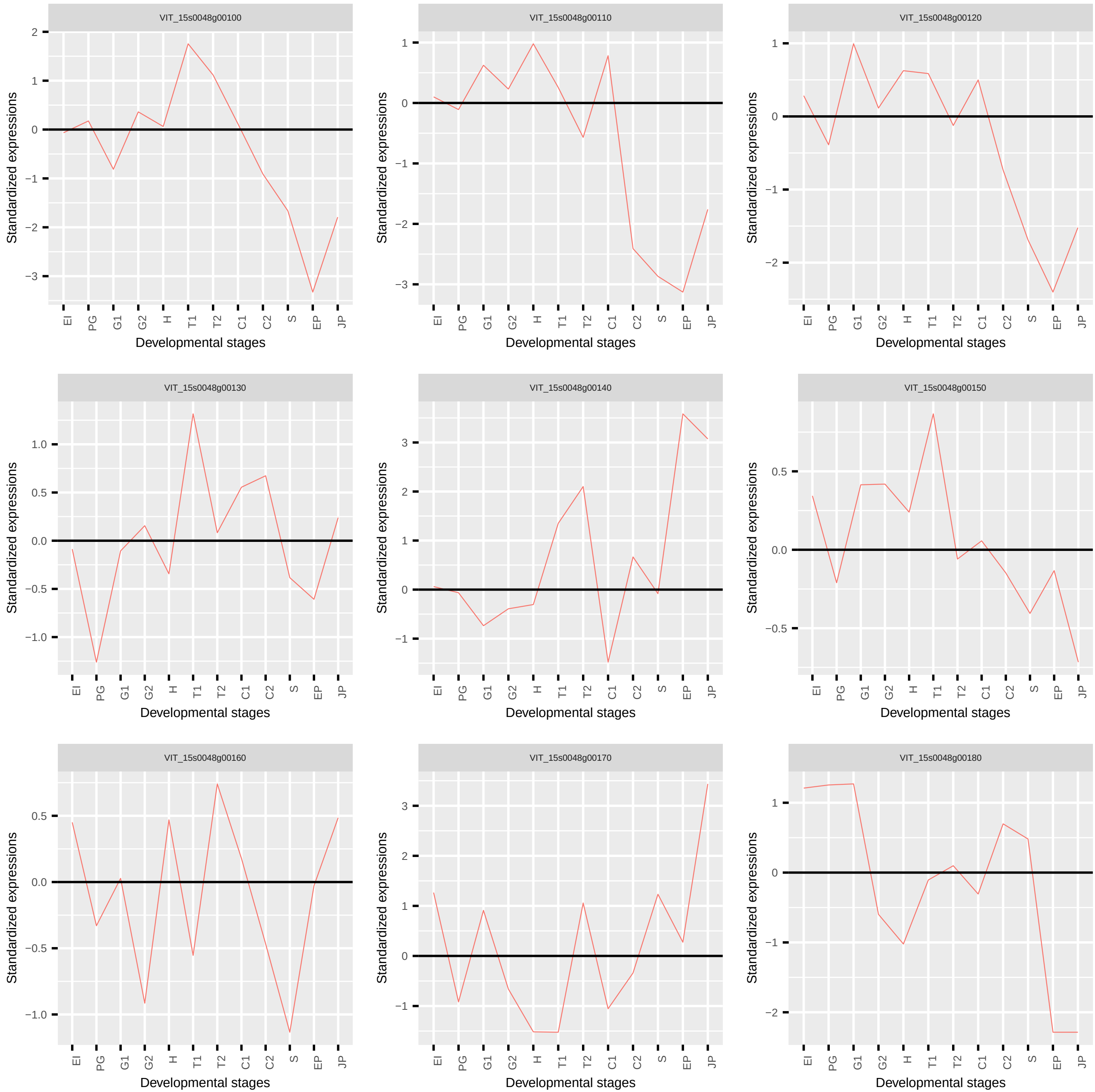
VIT_15s0048g00100
VIT_15s0048g00110
VIT_15s0048g00120
VIT_15s0048g00130
VIT_15s0048g00140
VIT_15s0048g00150
VIT_15s0048g00160
VIT_15s0048g00170
VIT_15s0048g00180
Standardized expressions
Developmental stages
EI
PG
G1
G2
H
T1
T2
C1
C2
S
EP
JP

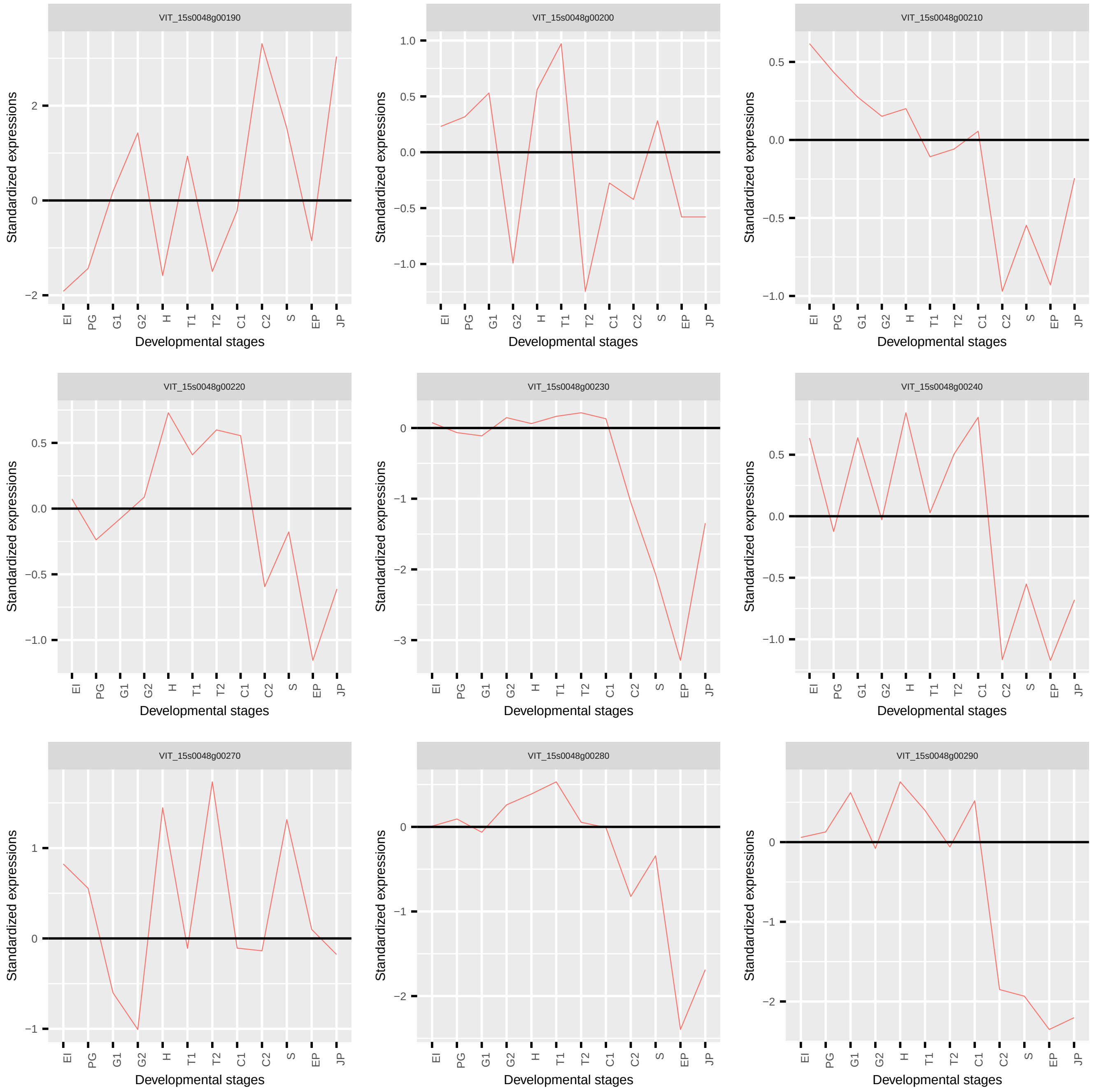

VIT_15s0048g00190
Standardized expressions
2
0
−2
EI
PG
G1
G2
H
T1
T2
C1
C2
S
EP
JP
Developmental stages
VIT_15s0048g00200
Standardized expressions
1.0
0.5
0.0
−0.5
−1.0
Developmental stages
VIT_15s0048g00210
Standardized expressions
0.5
0.0
−0.5
−1.0
Developmental stages
VIT_15s0048g00220
Standardized expressions
0.5
0.0
−0.5
−1.0
Developmental stages
VIT_15s0048g00230
Standardized expressions
0
−1
−2
−3
Developmental stages
VIT_15s0048g00240
Standardized expressions
0.5
0.0
−0.5
−1.0
Developmental stages
VIT_15s0048g00270
Standardized expressions
1
0
−1
Developmental stages
VIT_15s0048g00280
Standardized expressions
0
−1
−2
Developmental stages
VIT_15s0048g00290
Standardized expressions
0
−1
−2
Developmental stages

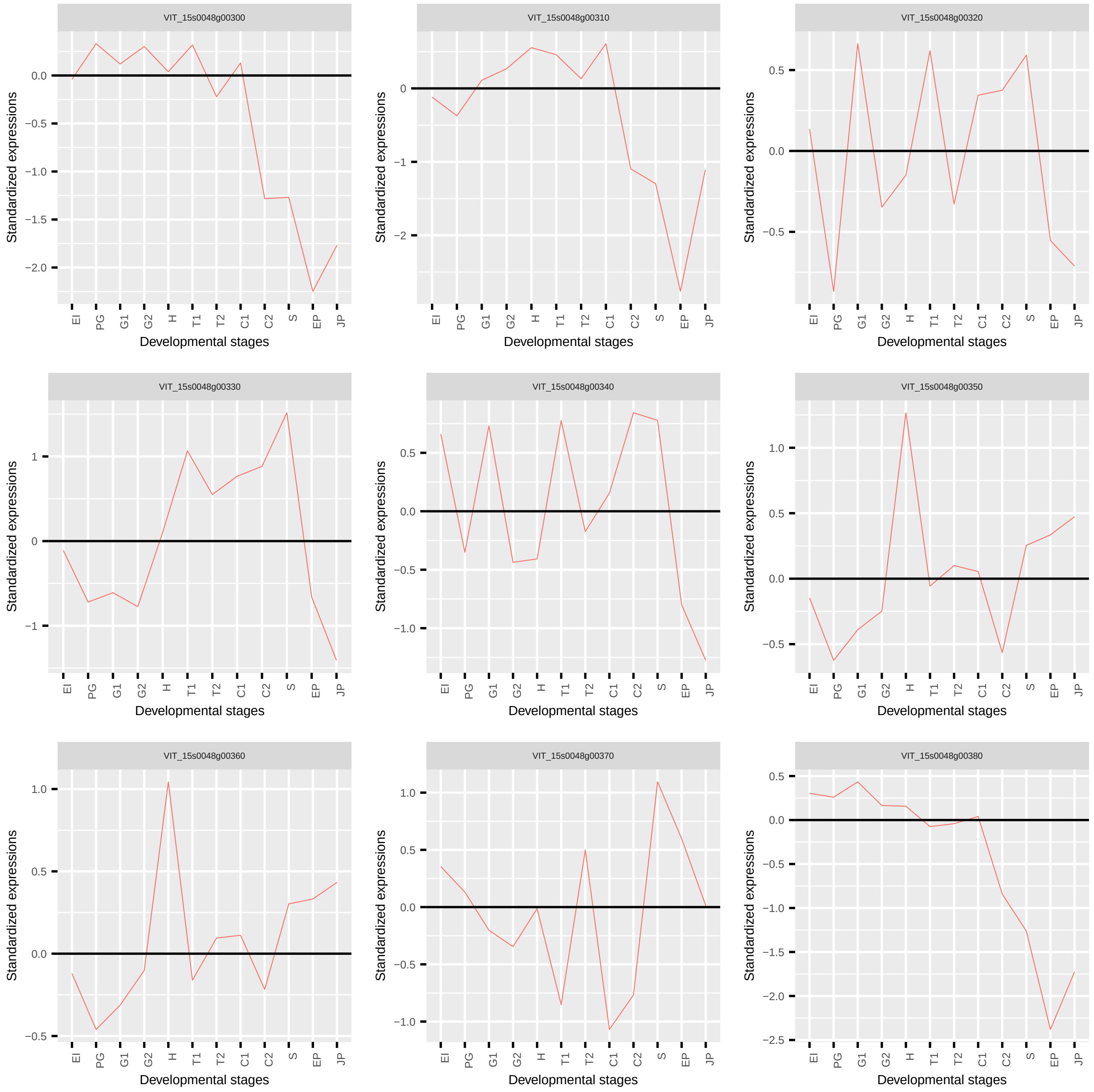
VIT_15s0048g00300
VIT_15s0048g00310
VIT_15s0048g00320
VIT_15s0048g00330
VIT_15s0048g00340
VIT_15s0048g00350
VIT_15s0048g00360
VIT_15s0048g00370
VIT_15s0048g00380
Standardized expressions
Developmental stages
EI PG G1 G2 H T1 T2 C1 C2 S EP JP

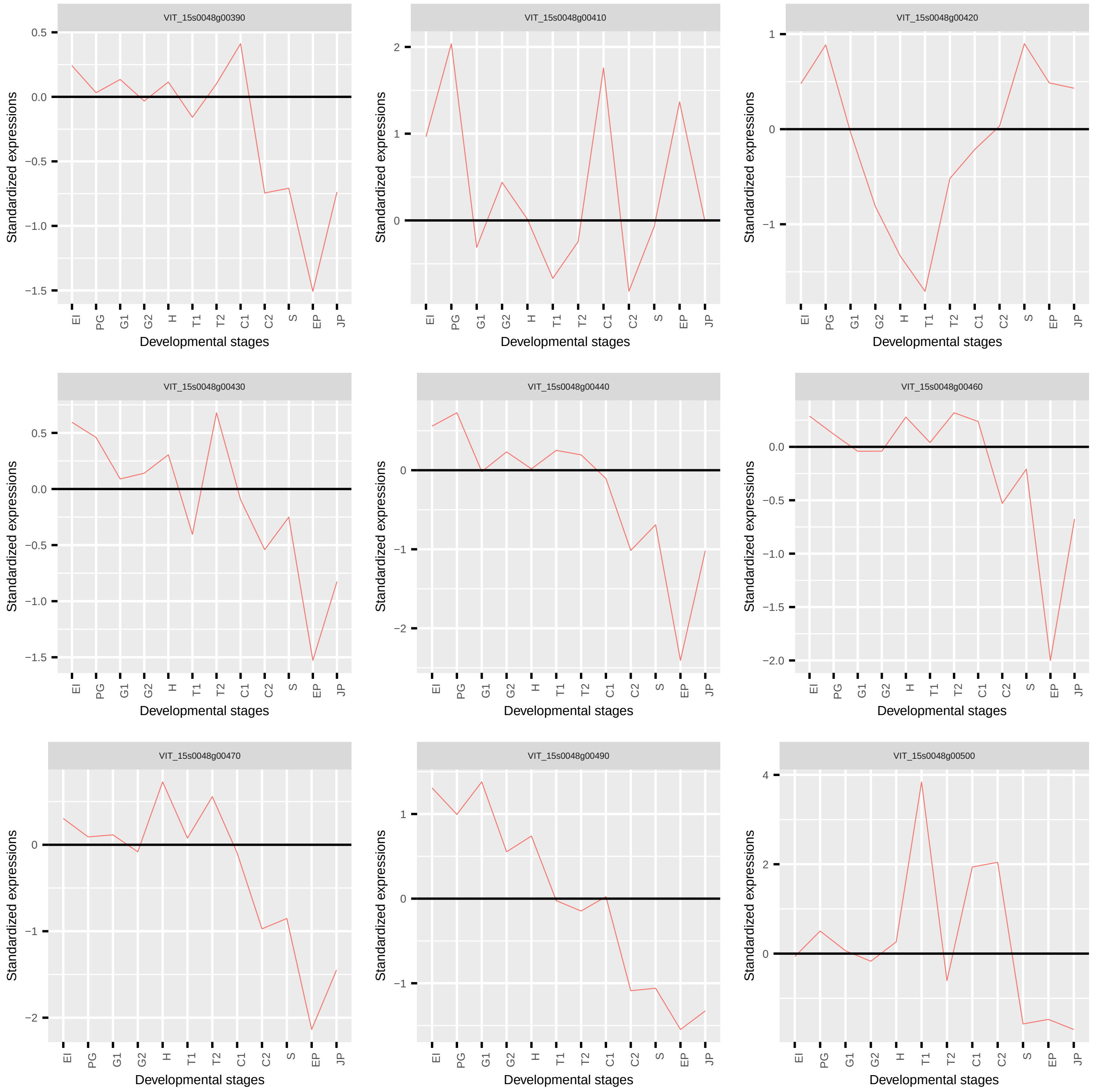
VIT_15s0048g00390
VIT_15s0048g00410
VIT_15s0048g00420
VIT_15s0048g00430
VIT_15s0048g00440
VIT_15s0048g00460
VIT_15s0048g00470
VIT_15s0048g00490
VIT_15s0048g00500
Standardized expressions
Developmental stages
EI PG G1 G2 H T1 T2 C1 C2 S EP JP

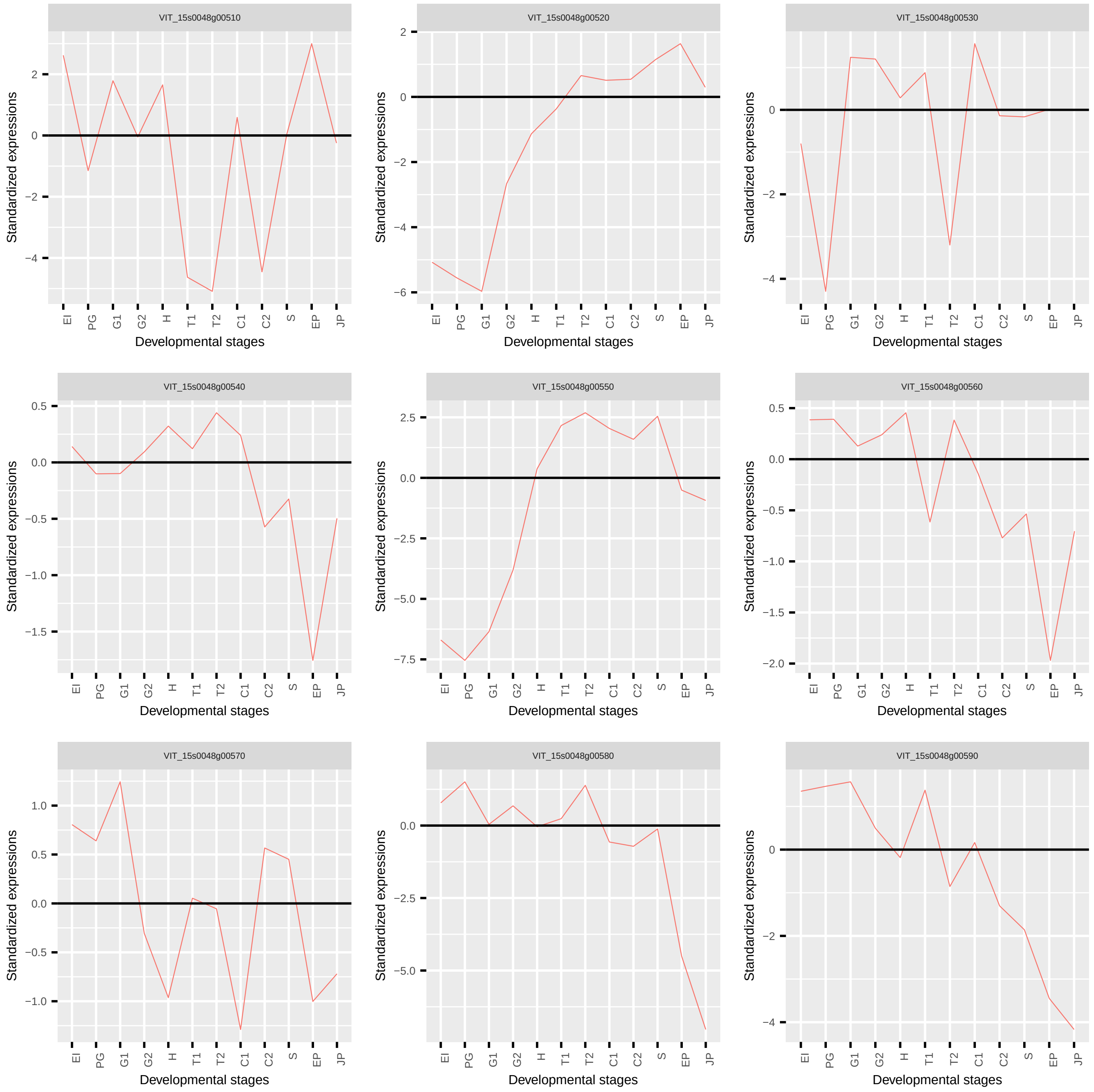

VIT_15s0048g00510
VIT_15s0048g00520
VIT_15s0048g00530
VIT_15s0048g00540
VIT_15s0048g00550
VIT_15s0048g00560
VIT_15s0048g00570
VIT_15s0048g00580
VIT_15s0048g00590
Standardized expressions
Developmental stages
EI PG G1 G2 H T1 T2 C1 C2 S EP JP

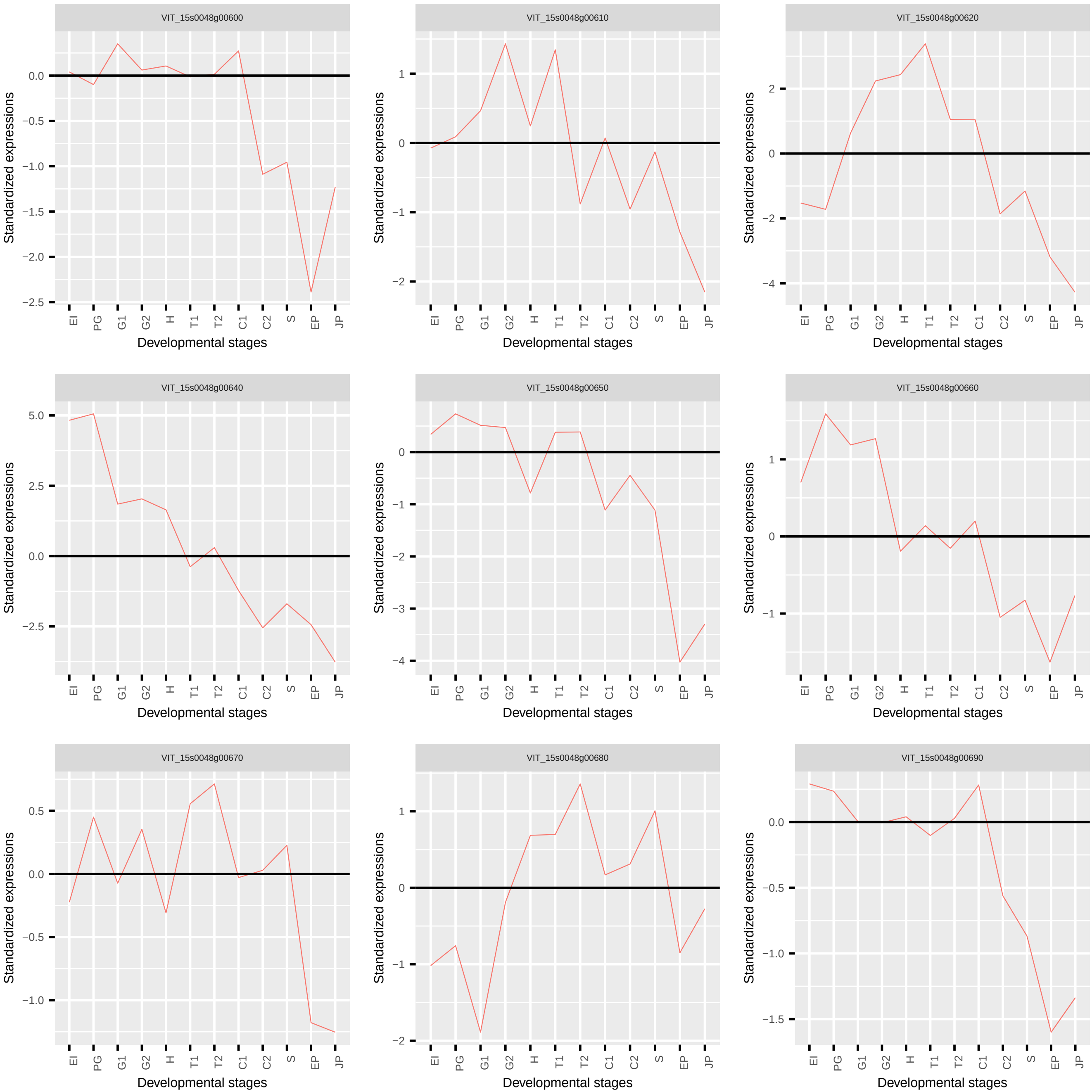
VIT_15s0048g00600
VIT_15s0048g00610
VIT_15s0048g00620
VIT_15s0048g00640
VIT_15s0048g00650
VIT_15s0048g00660
VIT_15s0048g00670
VIT_15s0048g00680
VIT_15s0048g00690
Standardized expressions
Developmental stages
EI PG G1 G2 H T1 T2 C1 C2 S EP JP

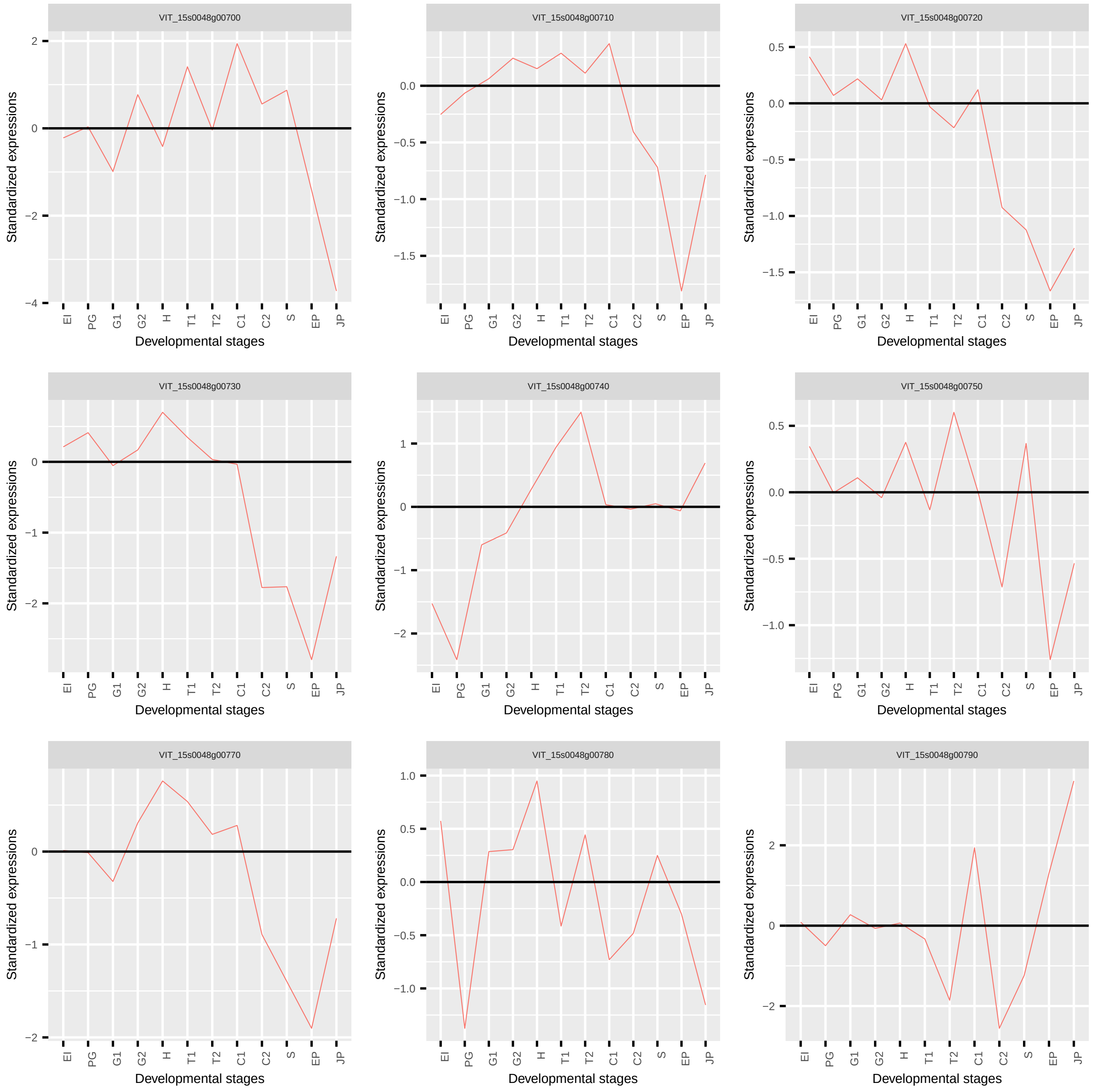

VIT_15s0048g00700
VIT_15s0048g00710
VIT_15s0048g00720
VIT_15s0048g00730
VIT_15s0048g00740
VIT_15s0048g00750
VIT_15s0048g00770
VIT_15s0048g00780
VIT_15s0048g00790
Standardized expressions
Developmental stages
EI PG G1 G2 H T1 T2 C1 C2 S EP JP

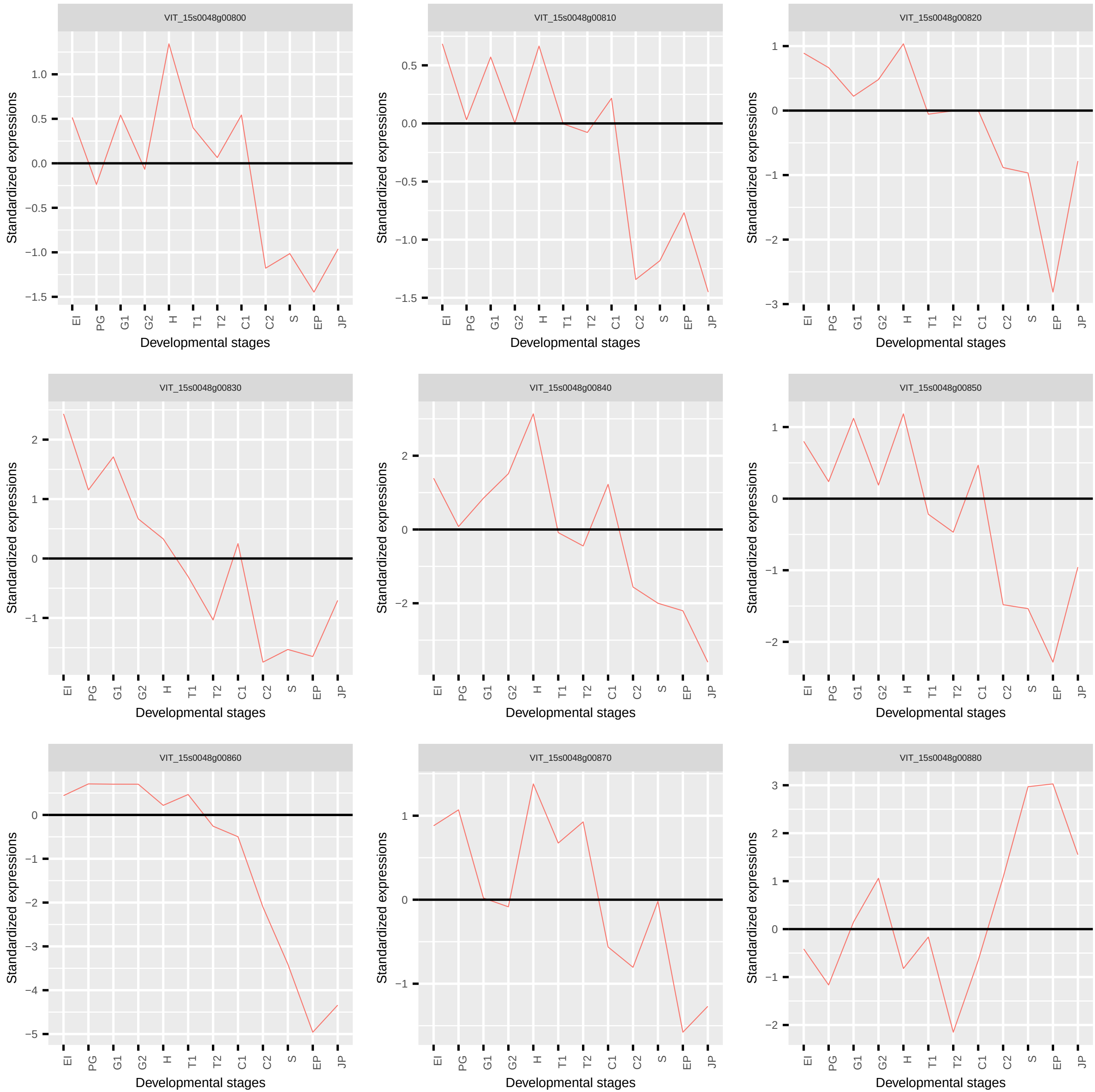

VIT_15s0048g00800
VIT_15s0048g00810
VIT_15s0048g00820
VIT_15s0048g00830
VIT_15s0048g00840
VIT_15s0048g00850
VIT_15s0048g00860
VIT_15s0048g00870
VIT_15s0048g00880
Standardized expressions
Developmental stages
EI
PG
G1
G2
H
T1
T2
C1
C2
S
EP
JP
1.0
0.5
0.0
−0.5
−1.0
−1.5
3
2
1
0
−1
−2
−3
−4
−5

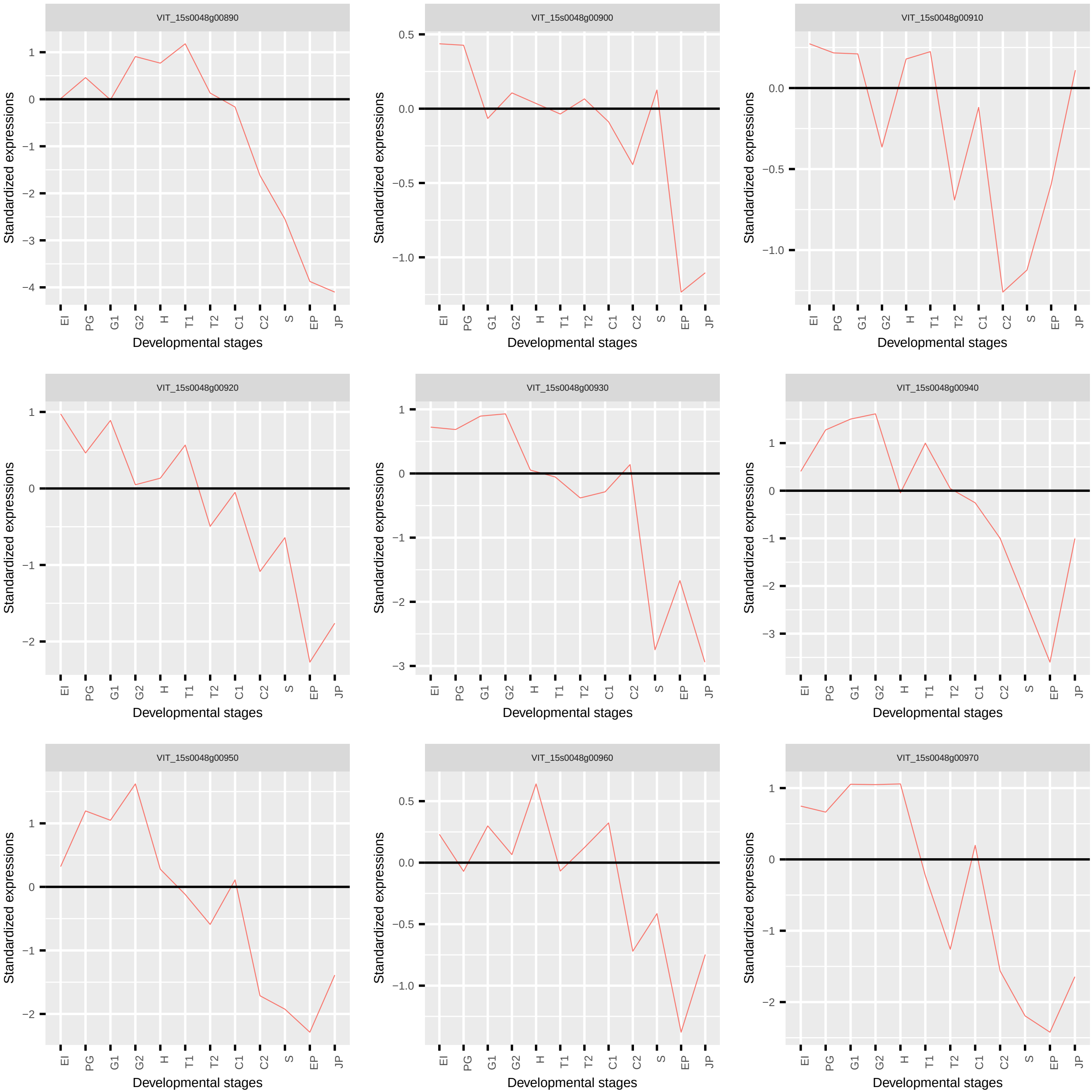
VIT_15s0048g00890
VIT_15s0048g00900
VIT_15s0048g00910
VIT_15s0048g00920
VIT_15s0048g00930
VIT_15s0048g00940
VIT_15s0048g00950
VIT_15s0048g00960
VIT_15s0048g00970
Standardized expressions
Developmental stages
EI
PG
G1
G2
H
T1
T2
C1
C2
S
EP
JP

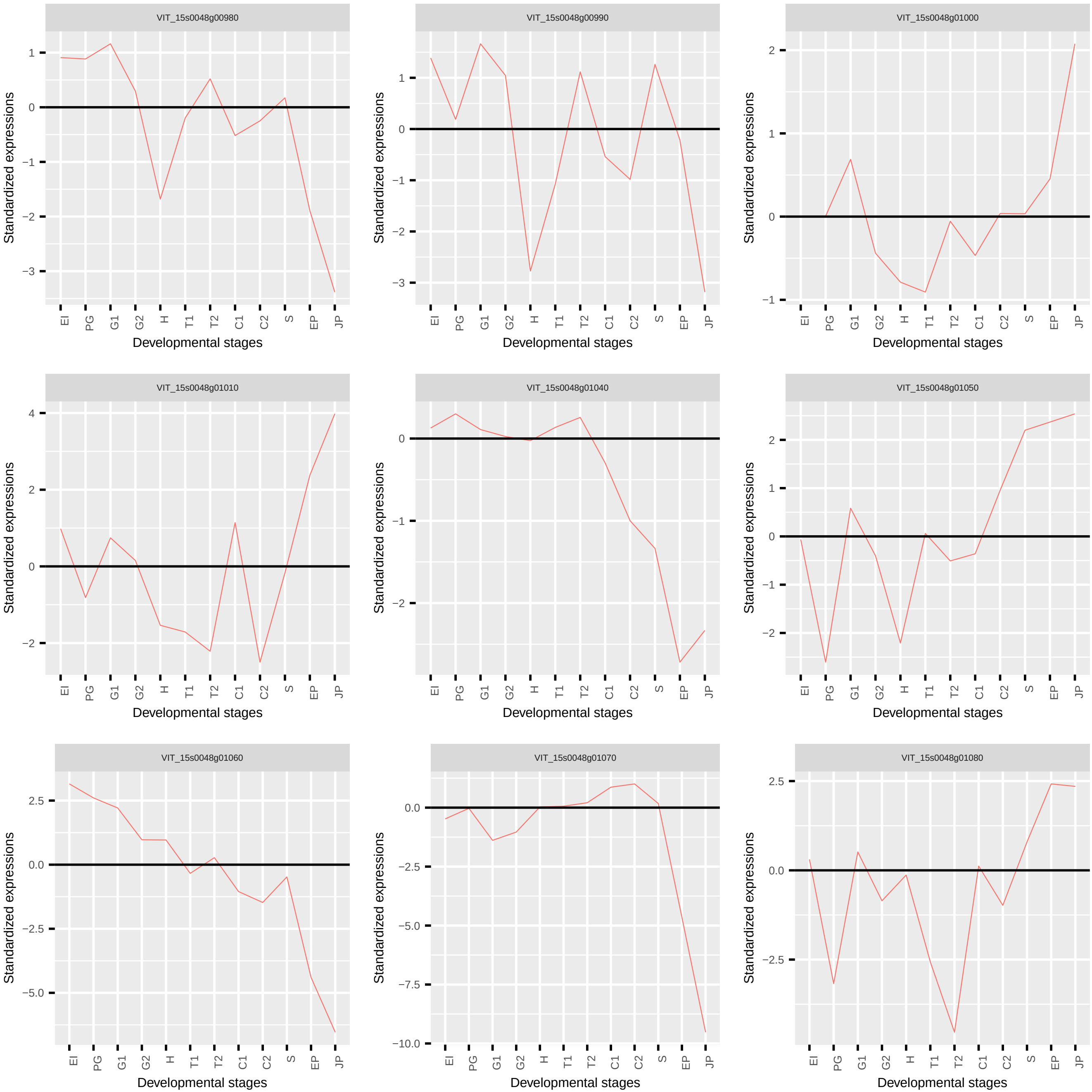
VIT_15s0048g00980
VIT_15s0048g00990
VIT_15s0048g01000
VIT_15s0048g01010
VIT_15s0048g01040
VIT_15s0048g01050
VIT_15s0048g01060
VIT_15s0048g01070
VIT_15s0048g01080
Standardized expressions
Developmental stages
EI
PG
G1
G2
H
T1
T2
C1
C2
S
EP
JP

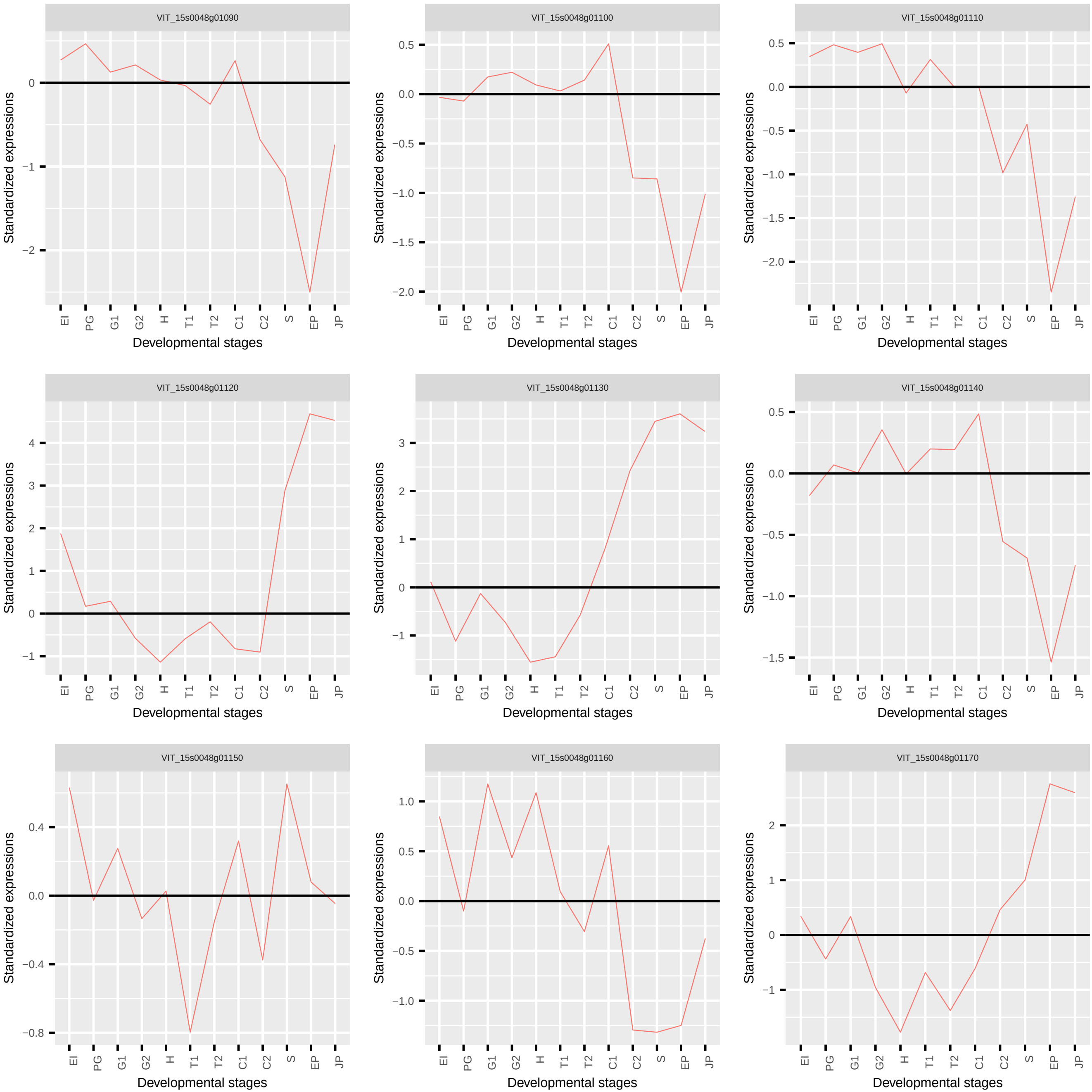

VIT_15s0048g01090
VIT_15s0048g01100
VIT_15s0048g01110
VIT_15s0048g01120
VIT_15s0048g01130
VIT_15s0048g01140
VIT_15s0048g01150
VIT_15s0048g01160
VIT_15s0048g01170
Standardized expressions
Developmental stages
EI PG G1 G2 H T1 T2 C1 C2 S EP JP
0 −1 −2
0.5 0.0 −0.5 −1.0 −1.5 −2.0
4 3 2 1 0 −1
3 2 1 0 −1
0.4 0.0 −0.4 −0.8
1.0 0.5 0.0 −0.5 −1.0
2 1 0 −1

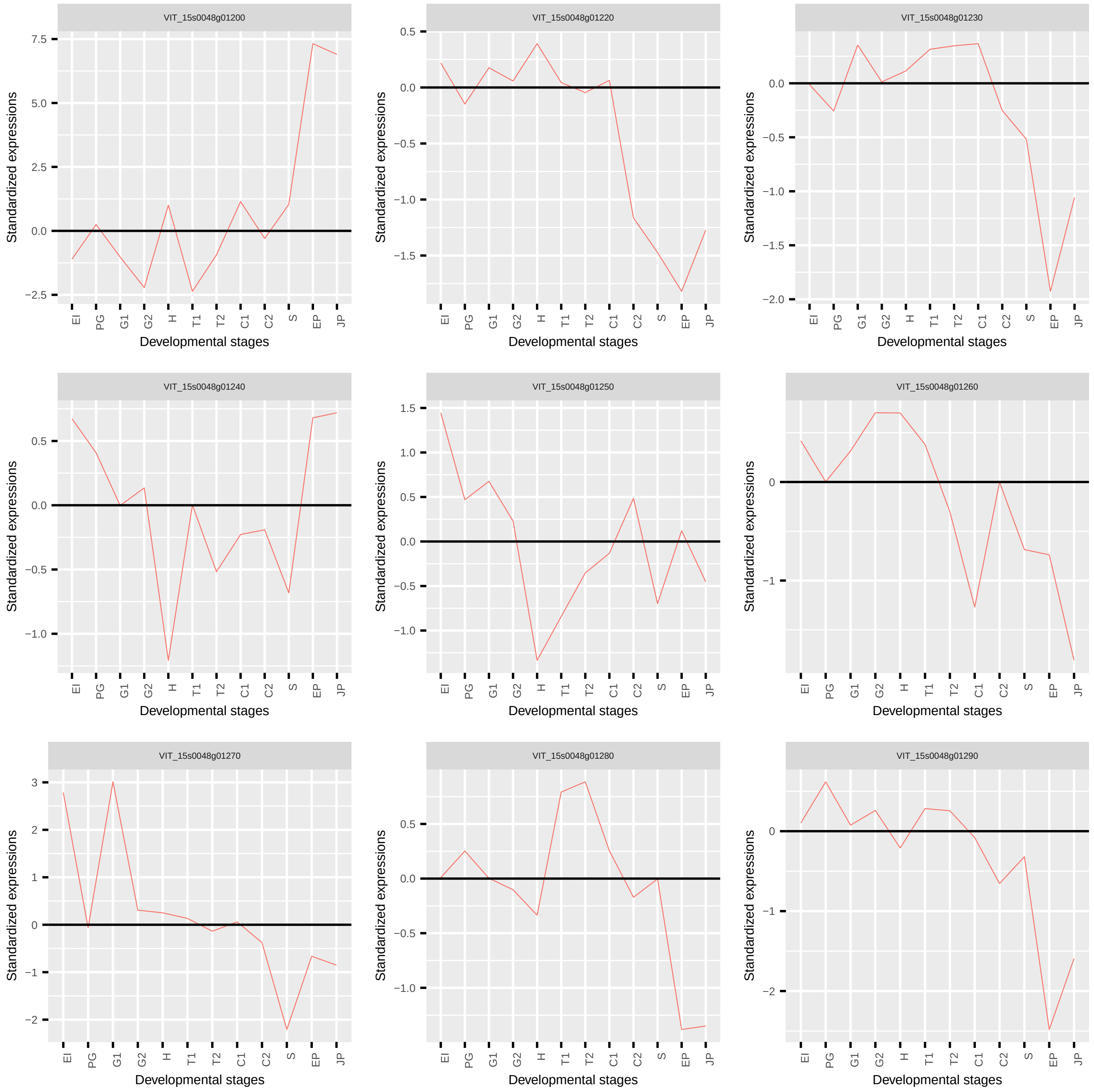
VIT_15s0048g01200
VIT_15s0048g01220
VIT_15s0048g01230
VIT_15s0048g01240
VIT_15s0048g01250
VIT_15s0048g01260
VIT_15s0048g01270
VIT_15s0048g01280
VIT_15s0048g01290
Standardized expressions
Developmental stages
EI
PG
G1
G2
H
T1
T2
C1
C2
S
EP
JP

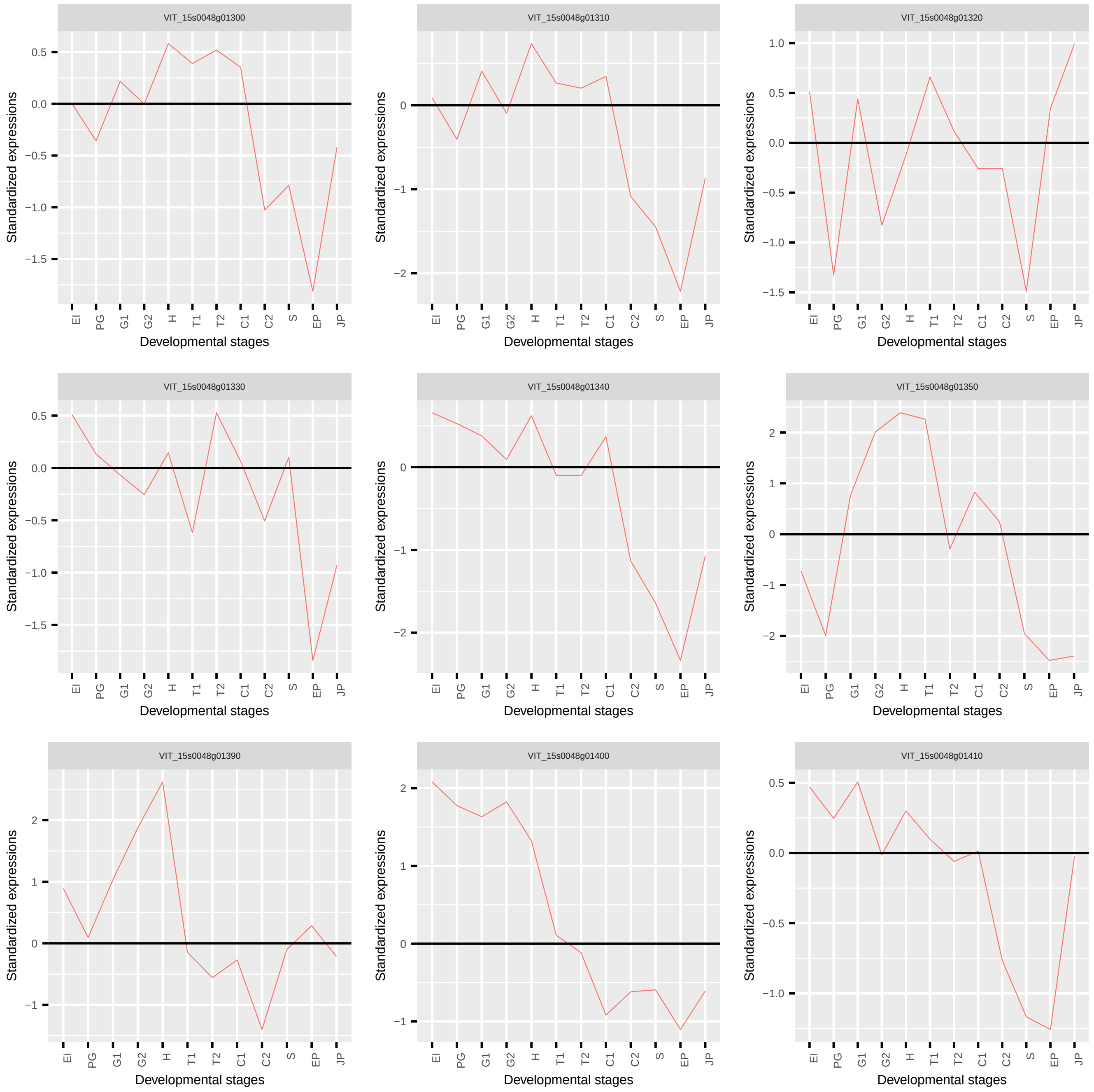

VIT_15s0048g01300
VIT_15s0048g01310
VIT_15s0048g01320
VIT_15s0048g01330
VIT_15s0048g01340
VIT_15s0048g01350
VIT_15s0048g01390
VIT_15s0048g01400
VIT_15s0048g01410
Standardized expressions
Developmental stages
EI PG G1 G2 H T1 T2 C1 C2 S EP JP

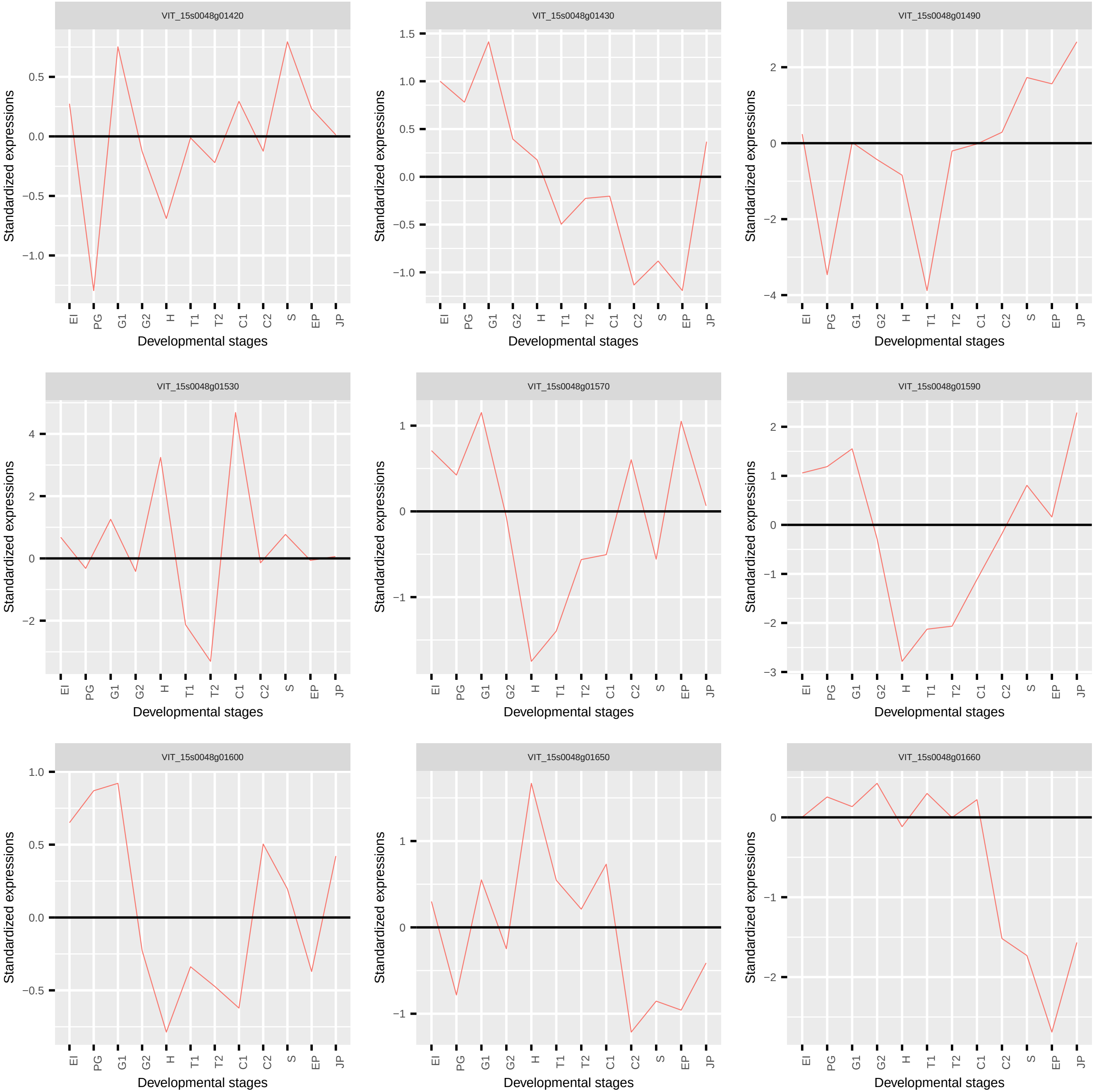

VIT_15s0048g01420
VIT_15s0048g01430
VIT_15s0048g01490
VIT_15s0048g01530
VIT_15s0048g01570
VIT_15s0048g01590
VIT_15s0048g01600
VIT_15s0048g01650
VIT_15s0048g01660
Standardized expressions
Developmental stages
EI PG G1 G2 H T1 T2 C1 C2 S EP JP

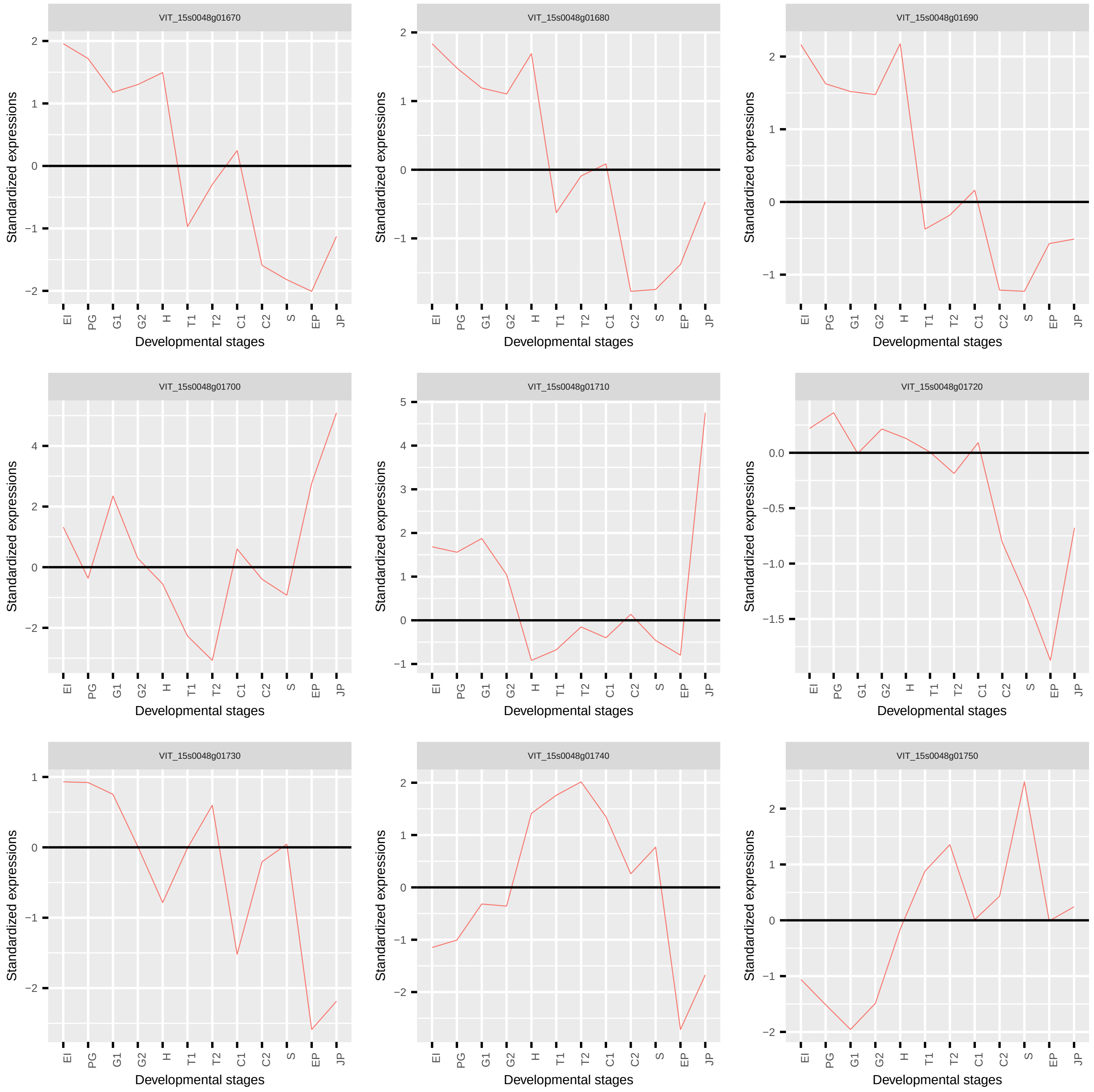
VIT_15s0048g01670
VIT_15s0048g01680
VIT_15s0048g01690
VIT_15s0048g01700
VIT_15s0048g01710
VIT_15s0048g01720
VIT_15s0048g01730
VIT_15s0048g01740
VIT_15s0048g01750
Standardized expressions
Developmental stages
EI PG G1 G2 H T1 T2 C1 C2 S EP JP

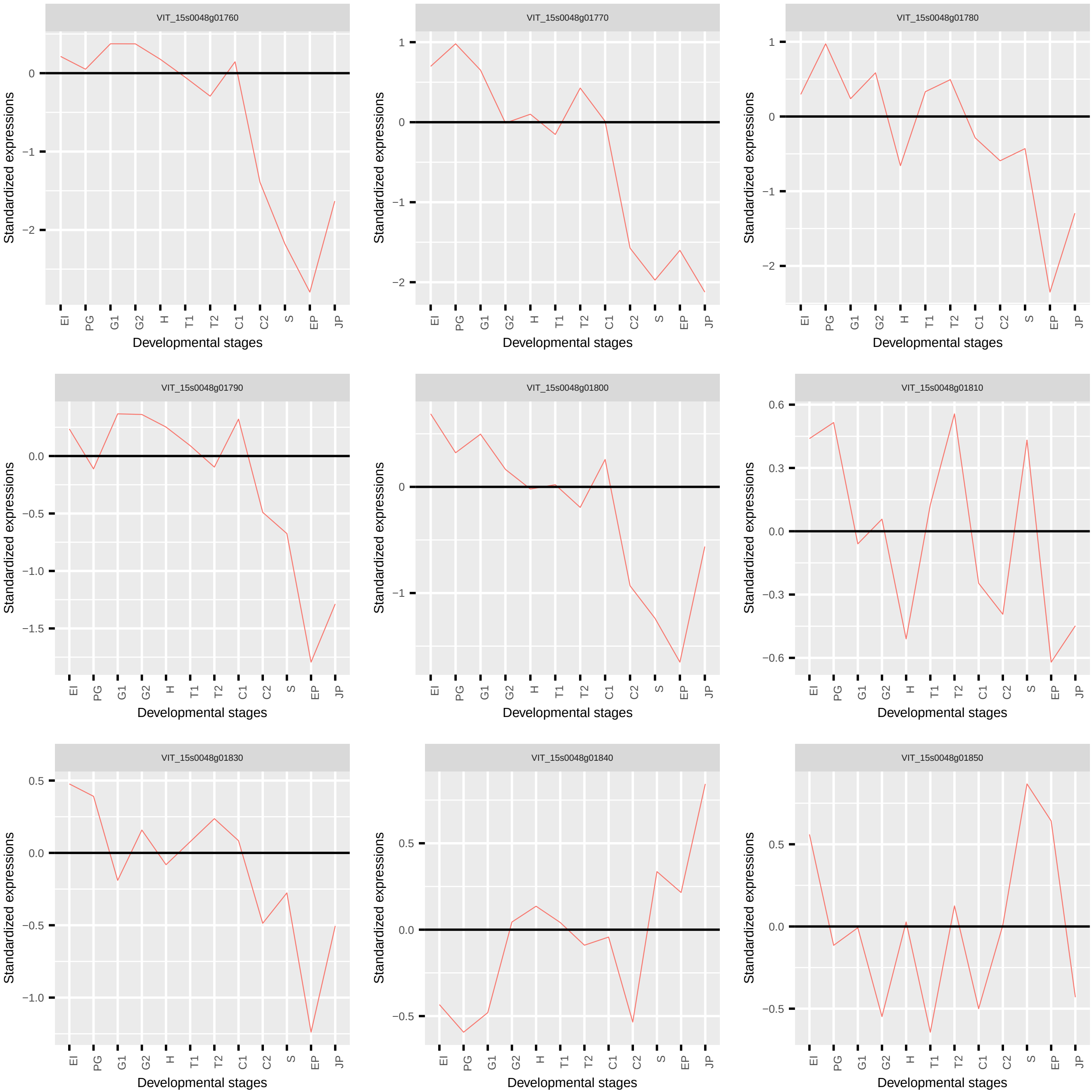
VIT_15s0048g01760
Standardized expressions
0
−1
−2
EI PG G1 G2 H T1 T2 C1 C2 S EP JP
Developmental stages
VIT_15s0048g01770
Standardized expressions
1
0
−1
−2
EI PG G1 G2 H T1 T2 C1 C2 S EP JP
Developmental stages
VIT_15s0048g01780
Standardized expressions
1
0
−1
−2
EI PG G1 G2 H T1 T2 C1 C2 S EP JP
Developmental stages
VIT_15s0048g01790
Standardized expressions
0.0
−0.5
−1.0
−1.5
EI PG G1 G2 H T1 T2 C1 C2 S EP JP
Developmental stages
VIT_15s0048g01800
Standardized expressions
0
−1
EI PG G1 G2 H T1 T2 C1 C2 S EP JP
Developmental stages
VIT_15s0048g01810
Standardized expressions
0.6
0.3
0.0
−0.3
−0.6
EI PG G1 G2 H T1 T2 C1 C2 S EP JP
Developmental stages
VIT_15s0048g01830
Standardized expressions
0.5
0.0
−0.5
−1.0
EI PG G1 G2 H T1 T2 C1 C2 S EP JP
Developmental stages
VIT_15s0048g01840
Standardized expressions
0.5
0.0
−0.5
EI PG G1 G2 H T1 T2 C1 C2 S EP JP
Developmental stages
VIT_15s0048g01850
Standardized expressions
0.5
0.0
−0.5
EI PG G1 G2 H T1 T2 C1 C2 S EP JP
Developmental stages

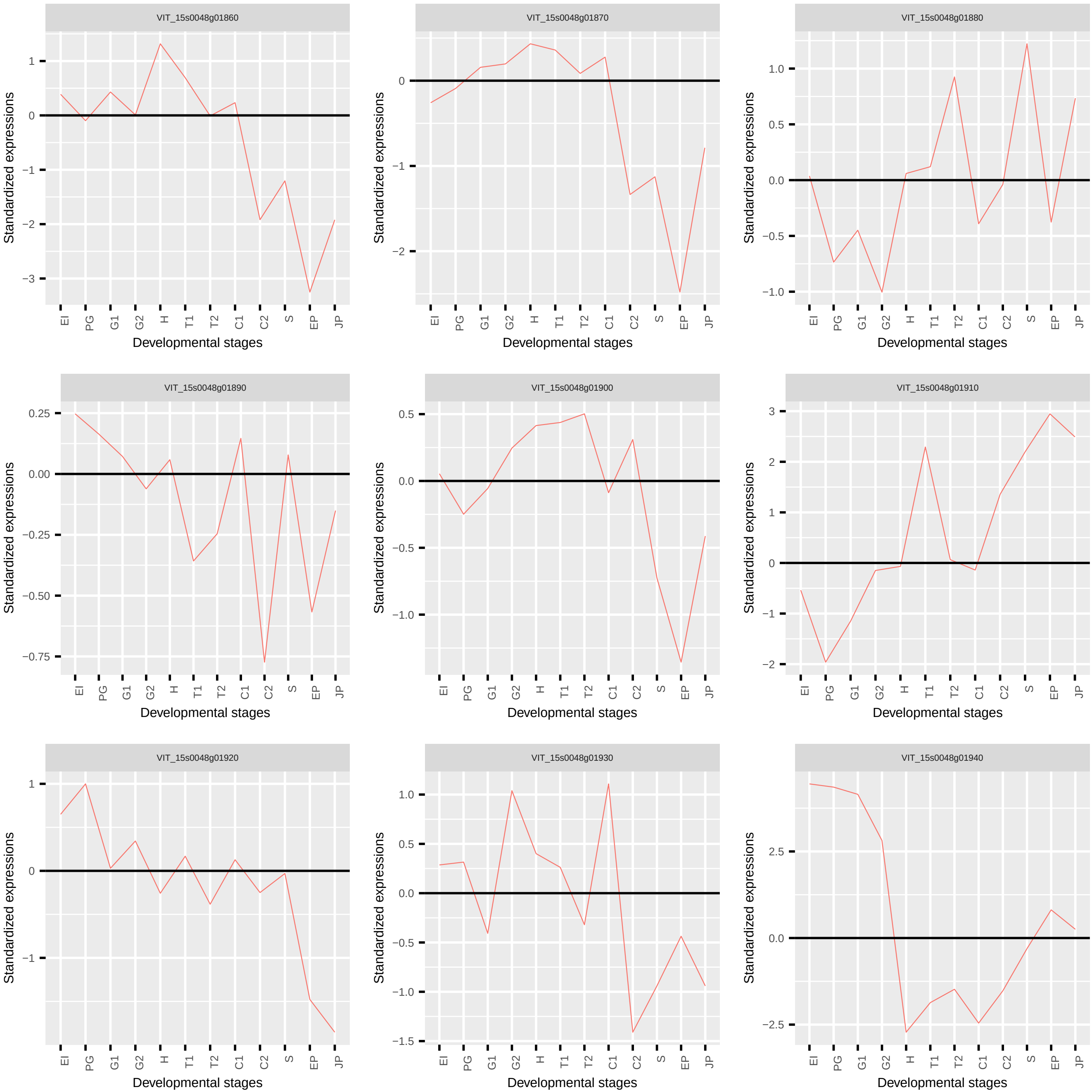
VIT_15s0048g01860
VIT_15s0048g01870
VIT_15s0048g01880
VIT_15s0048g01890
VIT_15s0048g01900
VIT_15s0048g01910
VIT_15s0048g01920
VIT_15s0048g01930
VIT_15s0048g01940
Standardized expressions
Developmental stages
EI PG G1 G2 H T1 T2 C1 C2 S EP JP

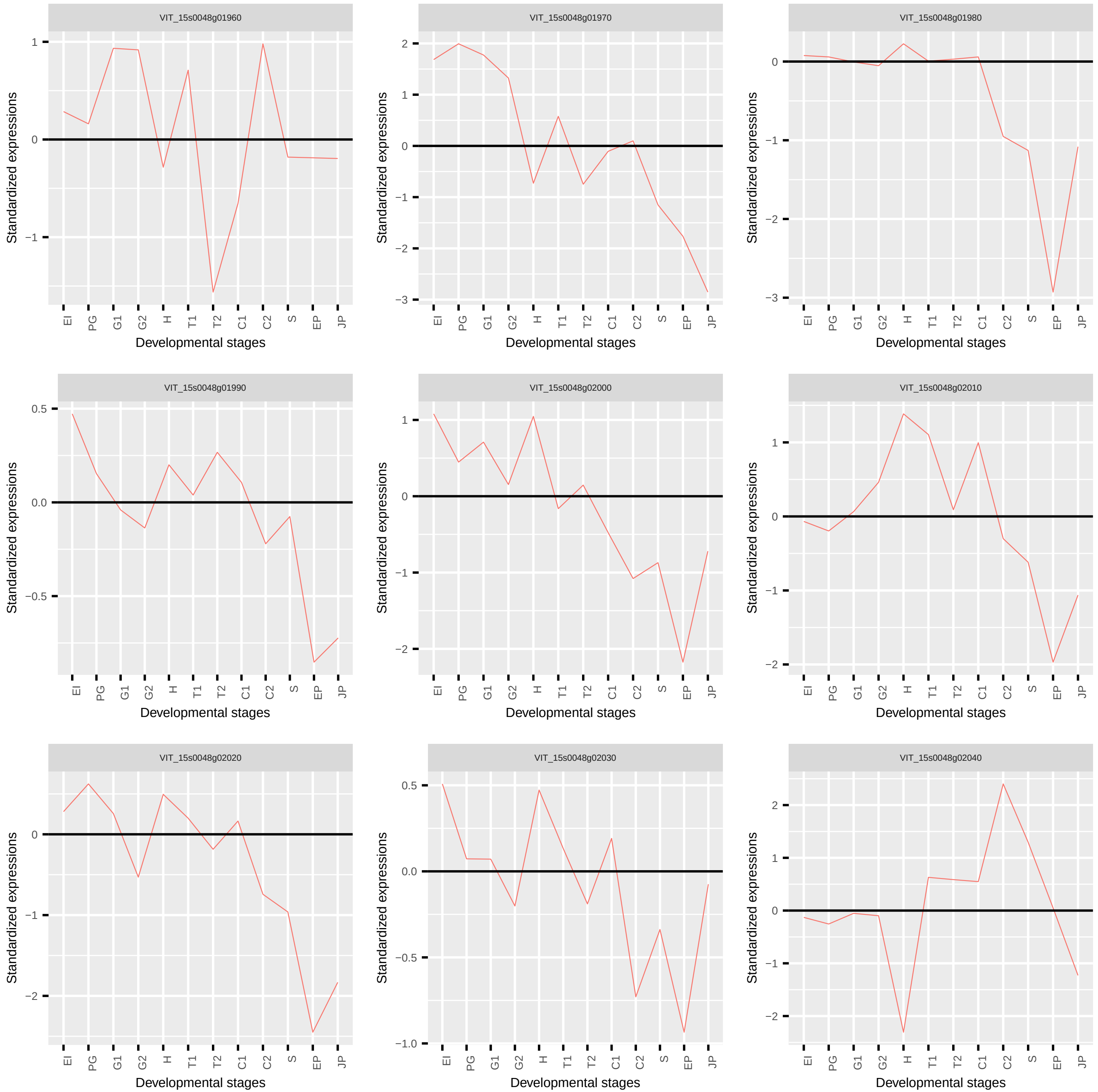

VIT_15s0048g01960
VIT_15s0048g01970
VIT_15s0048g01980
VIT_15s0048g01990
VIT_15s0048g02000
VIT_15s0048g02010
VIT_15s0048g02020
VIT_15s0048g02030
VIT_15s0048g02040
Standardized expressions
Developmental stages
EI
PG
G1
G2
H
T1
T2
C1
C2
S
EP
JP

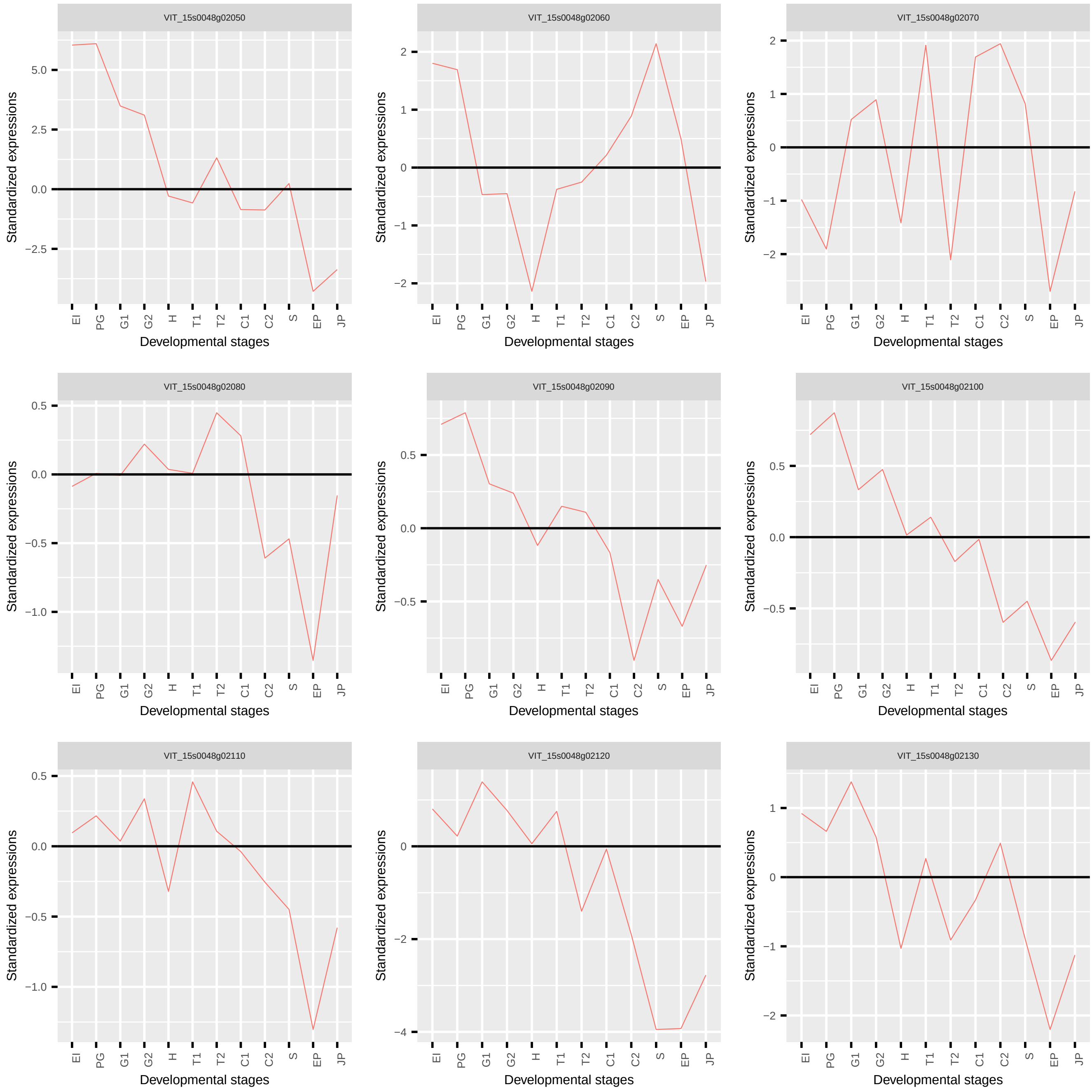
VIT_15s0048g02050
VIT_15s0048g02060
VIT_15s0048g02070
VIT_15s0048g02080
VIT_15s0048g02090
VIT_15s0048g02100
VIT_15s0048g02110
VIT_15s0048g02120
VIT_15s0048g02130
Standardized expressions
Developmental stages
EI PG G1 G2 H T1 T2 C1 C2 S EP JP

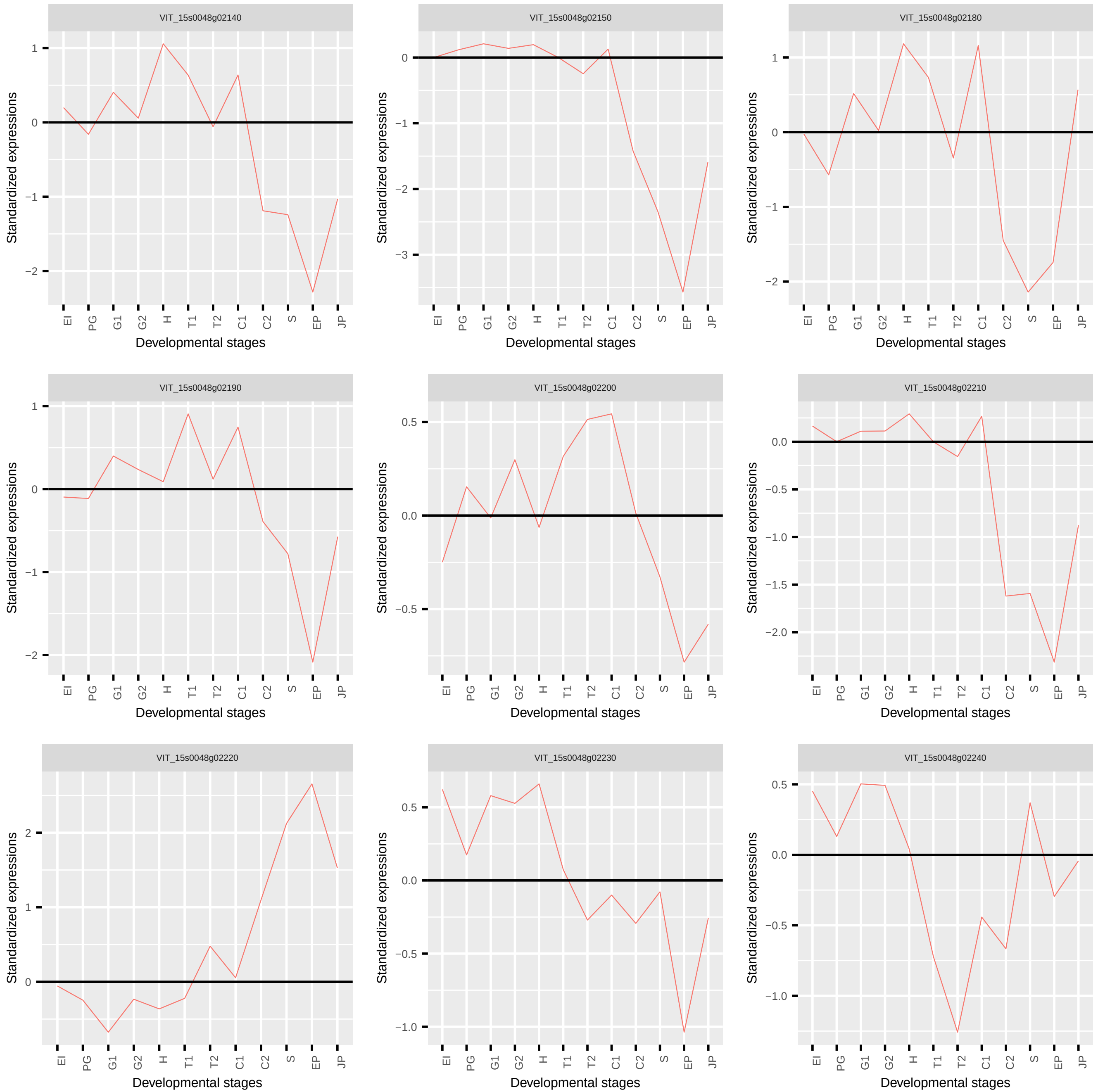

VIT_15s0048g02140
VIT_15s0048g02150
VIT_15s0048g02180
VIT_15s0048g02190
VIT_15s0048g02200
VIT_15s0048g02210
VIT_15s0048g02220
VIT_15s0048g02230
VIT_15s0048g02240
Standardized expressions
Developmental stages
EI
PG
G1
G2
H
T1
T2
C1
C2
S
EP
JP

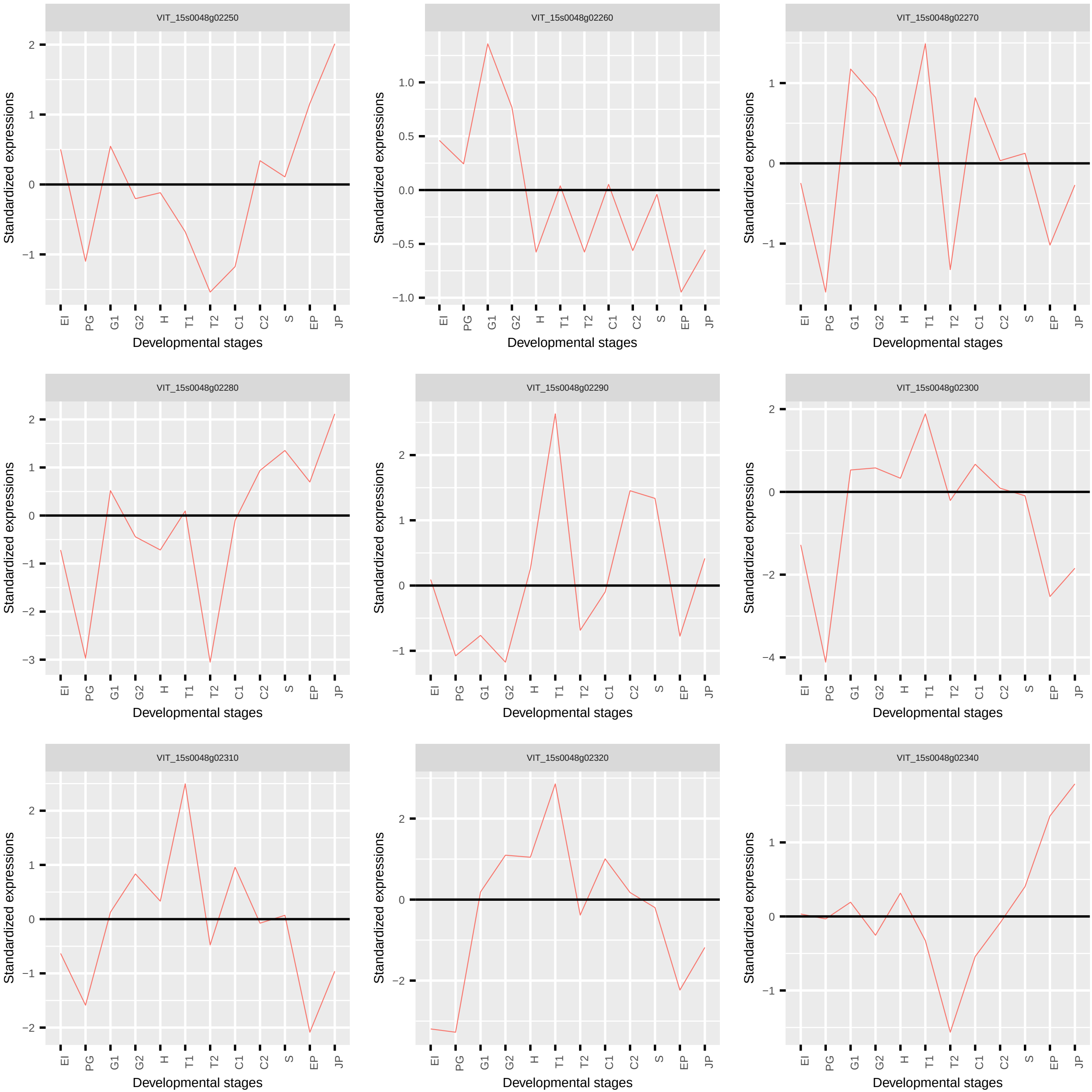
VIT_15s0048g02250
VIT_15s0048g02260
VIT_15s0048g02270
VIT_15s0048g02280
VIT_15s0048g02290
VIT_15s0048g02300
VIT_15s0048g02310
VIT_15s0048g02320
VIT_15s0048g02340
Standardized expressions
Developmental stages
EI PG G1 G2 H T1 T2 C1 C2 S EP JP

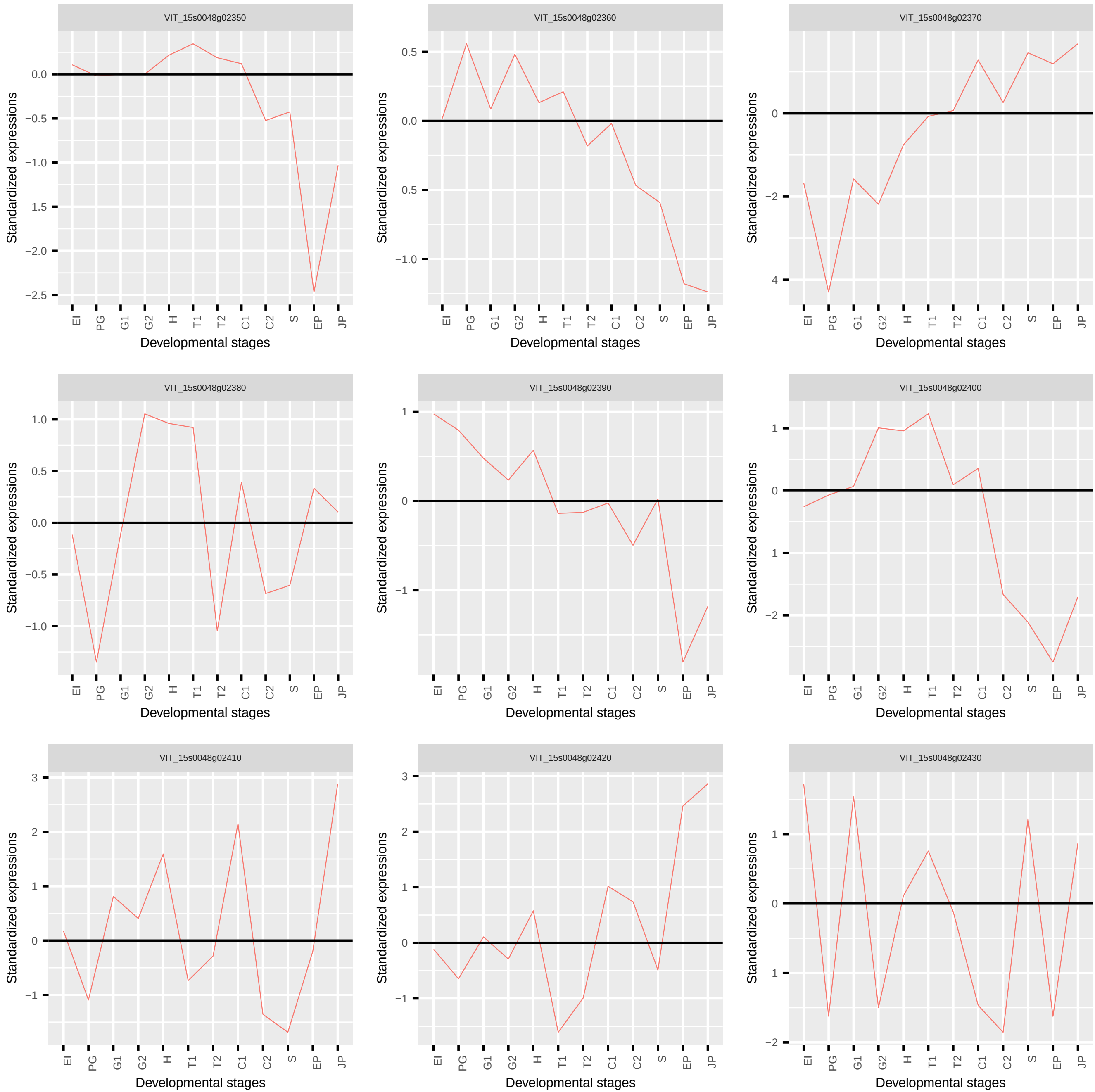
VIT_15s0048g02350
VIT_15s0048g02360
VIT_15s0048g02370
VIT_15s0048g02380
VIT_15s0048g02390
VIT_15s0048g02400
VIT_15s0048g02410
VIT_15s0048g02420
VIT_15s0048g02430
Standardized expressions
Developmental stages
EI PG G1 G2 H T1 T2 C1 C2 S EP JP

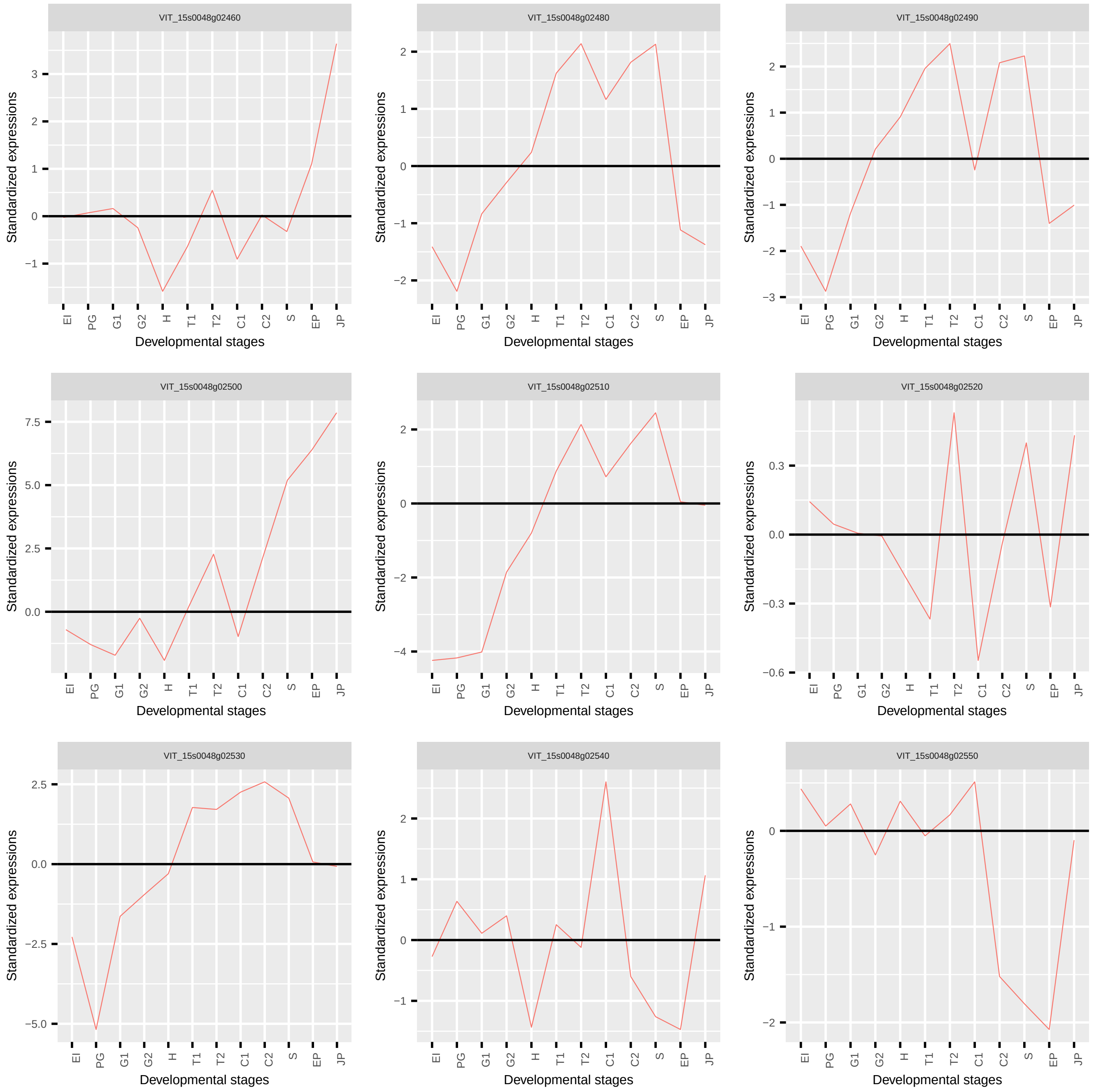

VIT_15s0048g02460
VIT_15s0048g02480
VIT_15s0048g02490
VIT_15s0048g02500
VIT_15s0048g02510
VIT_15s0048g02520
VIT_15s0048g02530
VIT_15s0048g02540
VIT_15s0048g02550
Standardized expressions
Developmental stages
EI PG G1 G2 H T1 T2 C1 C2 S EP JP

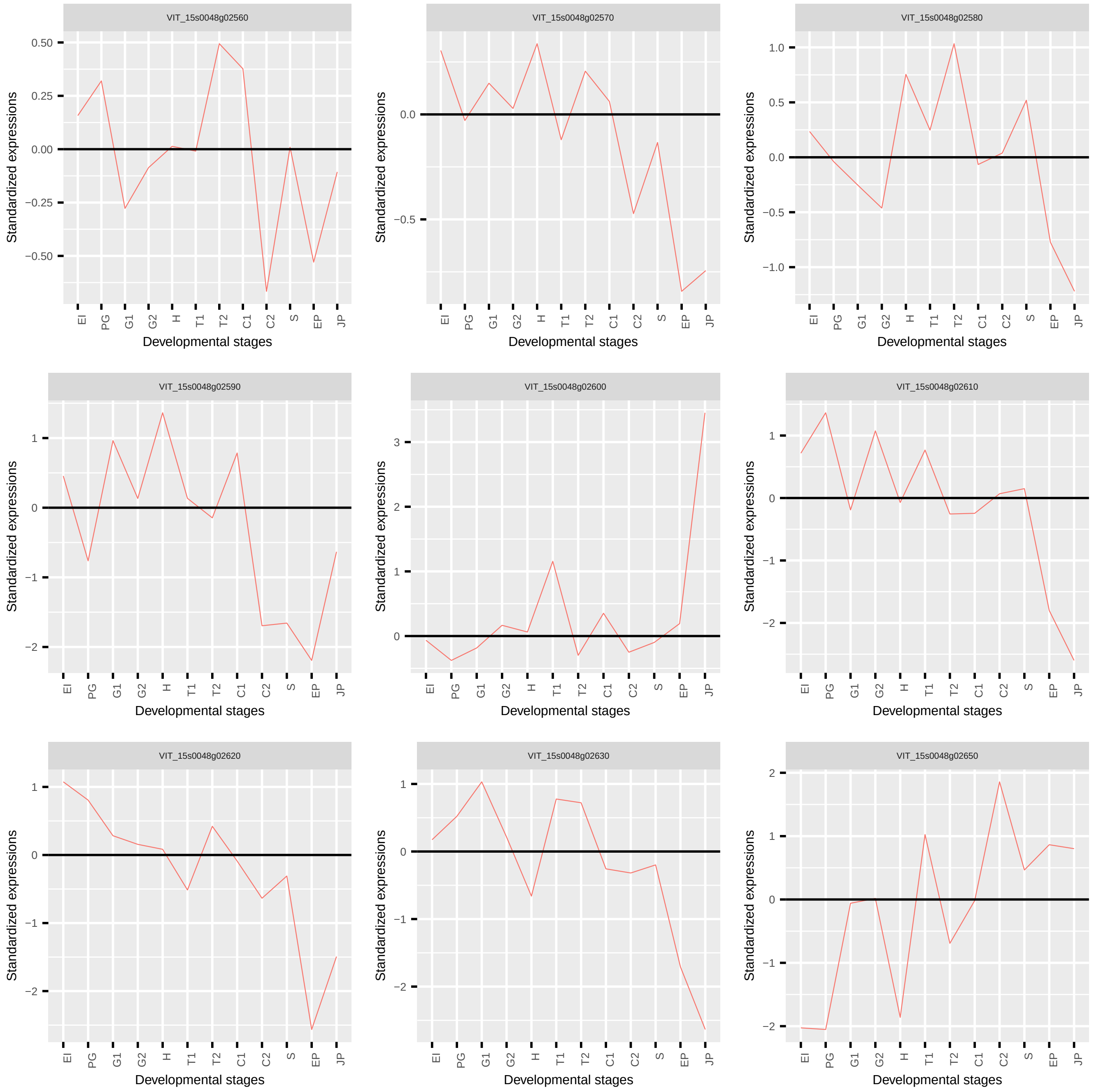

VIT_15s0048g02560
VIT_15s0048g02570
VIT_15s0048g02580
VIT_15s0048g02590
VIT_15s0048g02600
VIT_15s0048g02610
VIT_15s0048g02620
VIT_15s0048g02630
VIT_15s0048g02650
Standardized expressions
Developmental stages
EI
PG
G1
G2
H
T1
T2
C1
C2
S
EP
JP

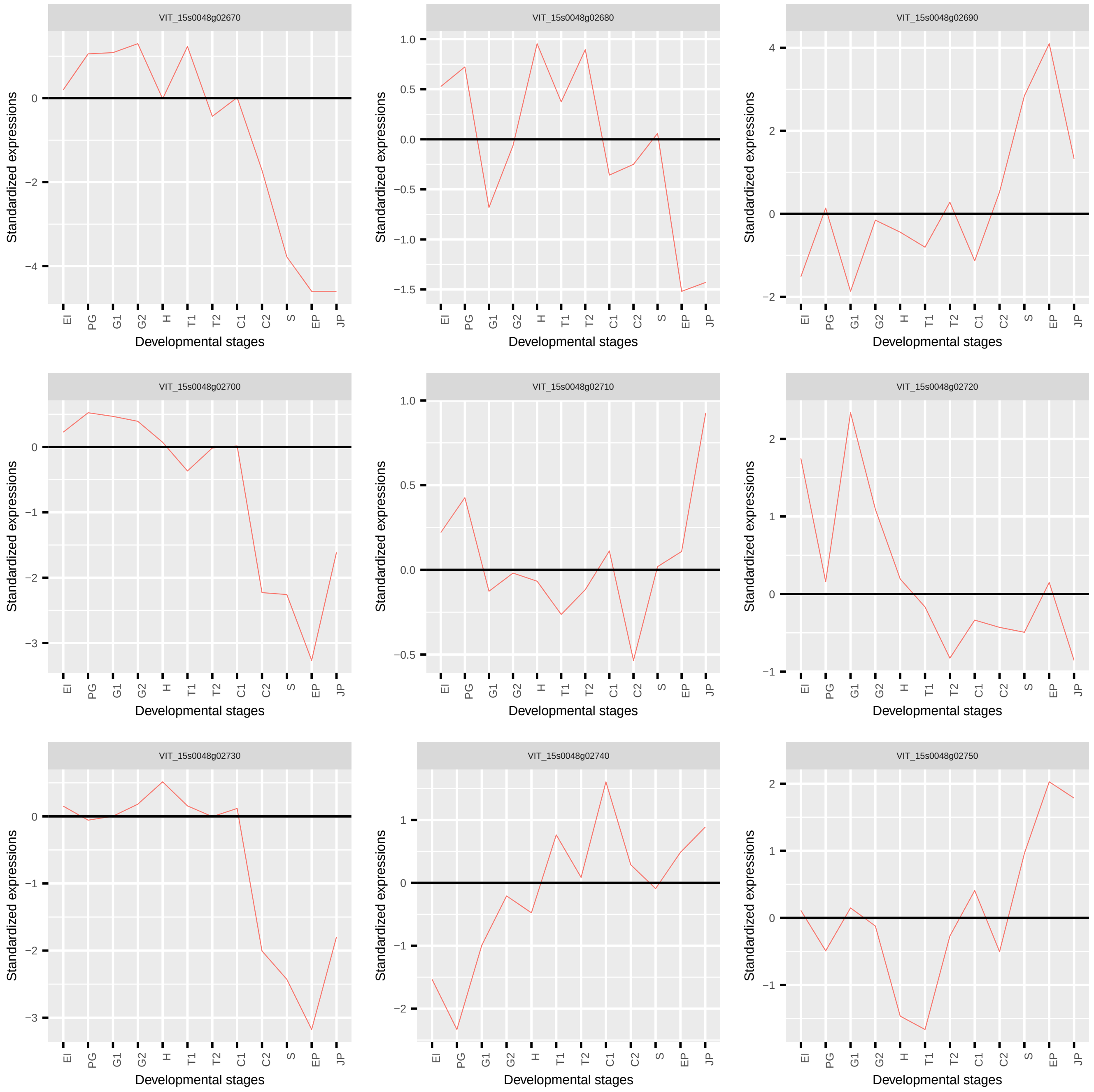

VIT_15s0048g02670
VIT_15s0048g02680
VIT_15s0048g02690
VIT_15s0048g02700
VIT_15s0048g02710
VIT_15s0048g02720
VIT_15s0048g02730
VIT_15s0048g02740
VIT_15s0048g02750
Standardized expressions
Developmental stages
EI PG G1 G2 H T1 T2 C1 C2 S EP JP

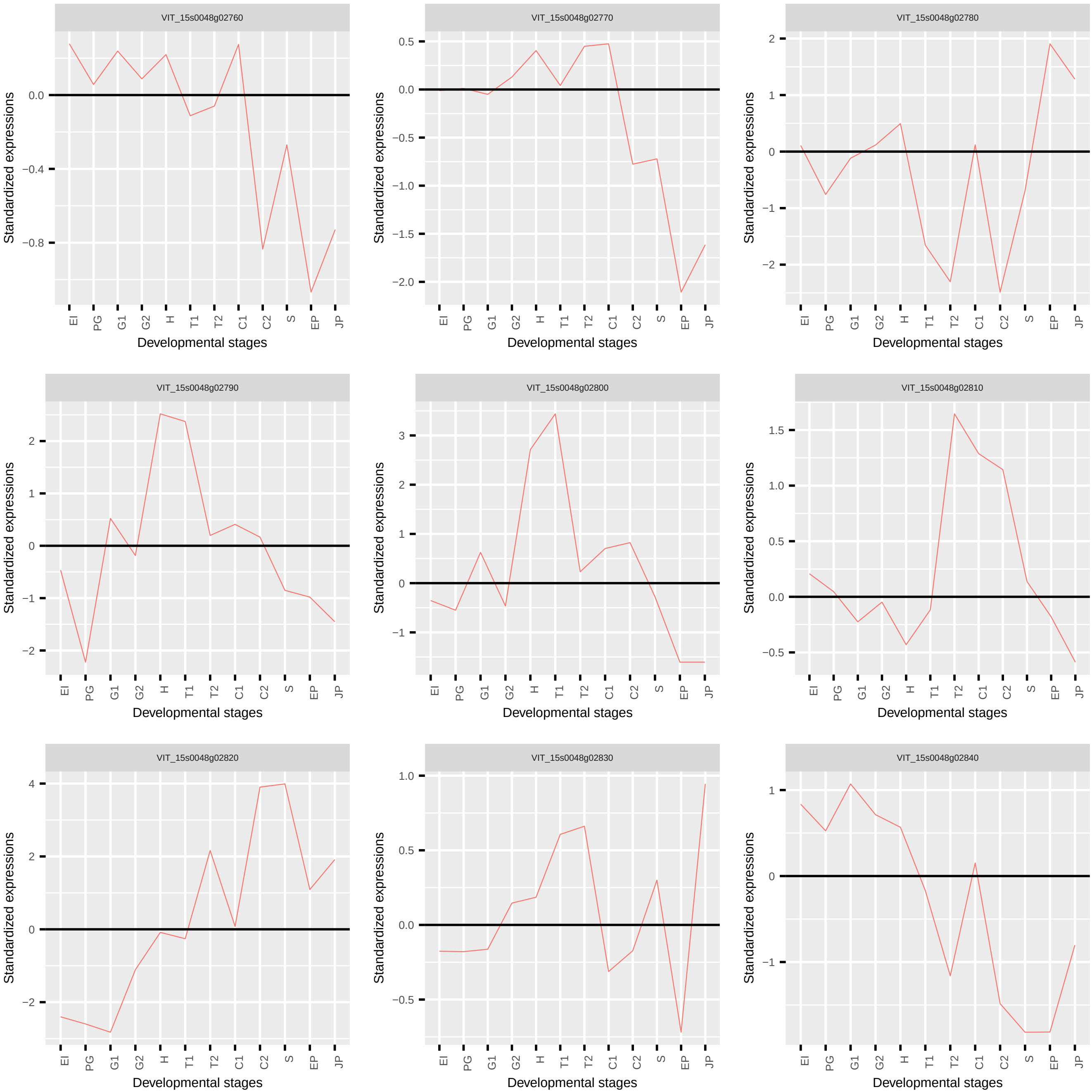
VIT_15s0048g02760
VIT_15s0048g02770
VIT_15s0048g02780
VIT_15s0048g02790
VIT_15s0048g02800
VIT_15s0048g02810
VIT_15s0048g02820
VIT_15s0048g02830
VIT_15s0048g02840
Standardized expressions
Developmental stages
EI PG G1 G2 H T1 T2 C1 C2 S EP JP
0.0 −0.4 −0.8
0.5 0.0 −0.5 −1.0 −1.5 −2.0
2 1 0 −1 −2
2 1 0 −1 −2
3 2 1 0 −1
1.5 1.0 0.5 0.0 −0.5
4 2 0 −2
1.0 0.5 0.0 −0.5
1 0 −1

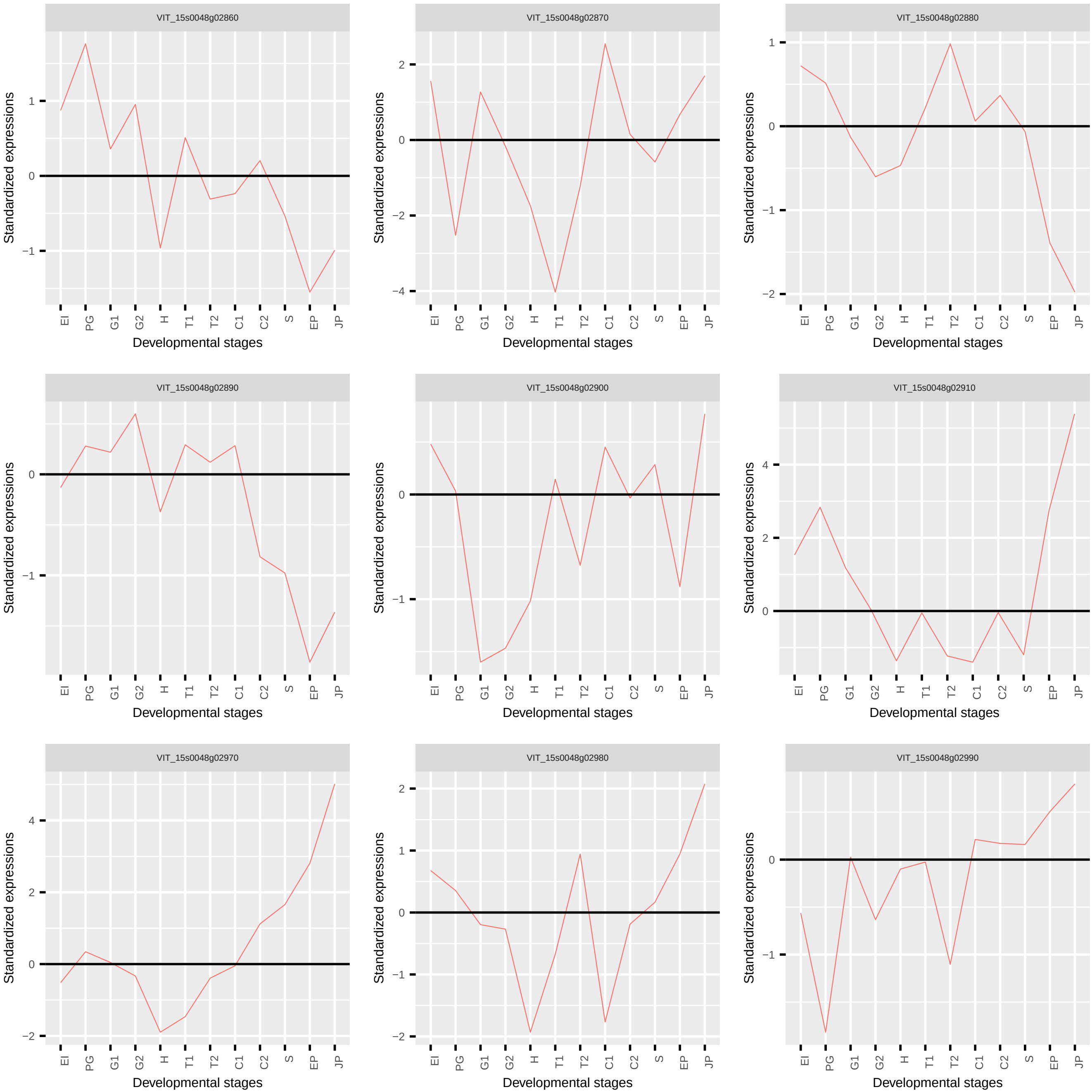
VIT_15s0048g02860
VIT_15s0048g02870
VIT_15s0048g02880
VIT_15s0048g02890
VIT_15s0048g02900
VIT_15s0048g02910
VIT_15s0048g02970
VIT_15s0048g02980
VIT_15s0048g02990
Standardized expressions
Developmental stages
EI
PG
G1
G2
H
T1
T2
C1
C2
S
EP
JP

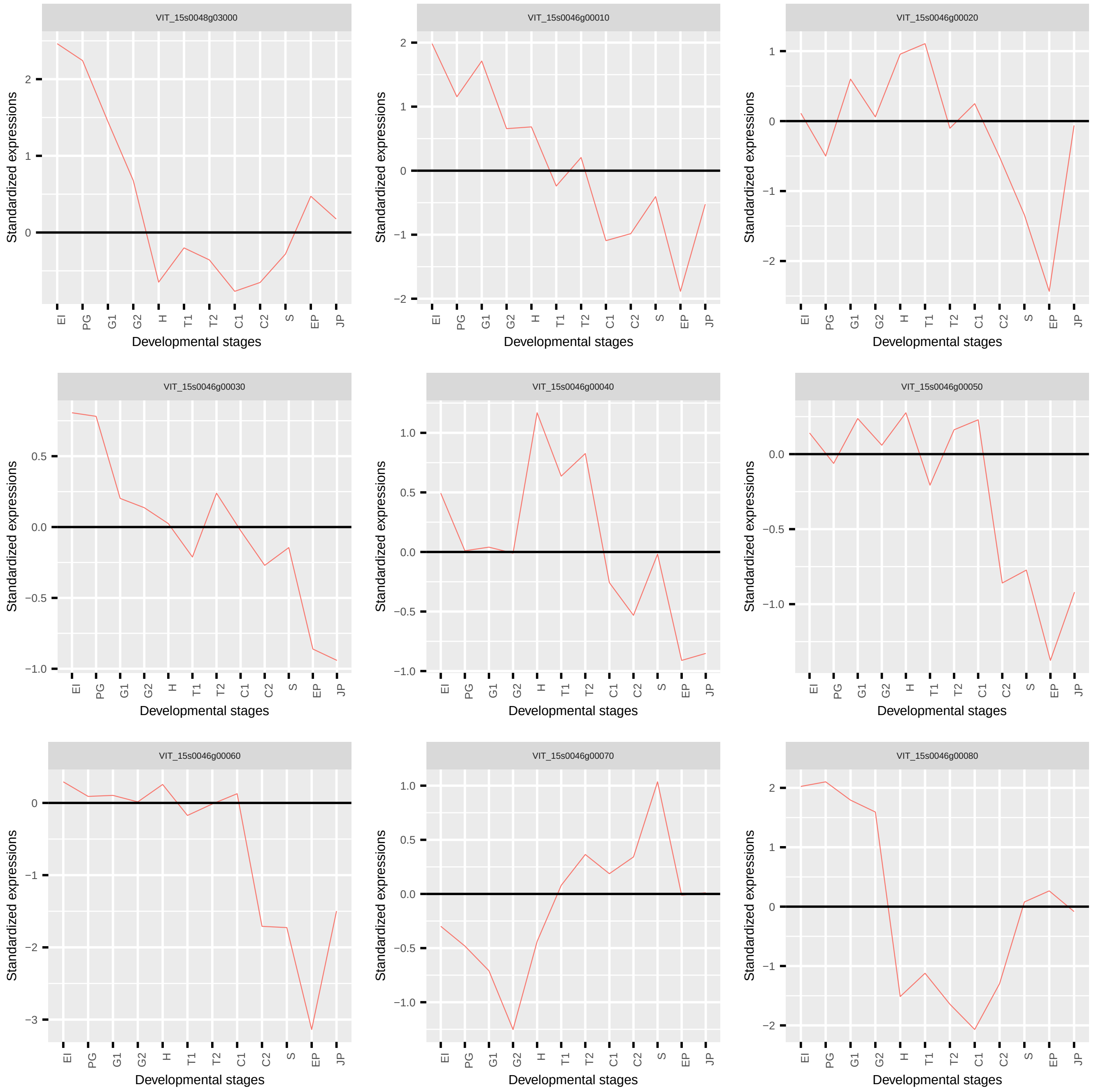
VIT_15s0048g03000
VIT_15s0046g00010
VIT_15s0046g00020
VIT_15s0046g00030
VIT_15s0046g00040
VIT_15s0046g00050
VIT_15s0046g00060
VIT_15s0046g00070
VIT_15s0046g00080
Standardized expressions
Developmental stages
EI PG G1 G2 H T1 T2 C1 C2 S EP JP

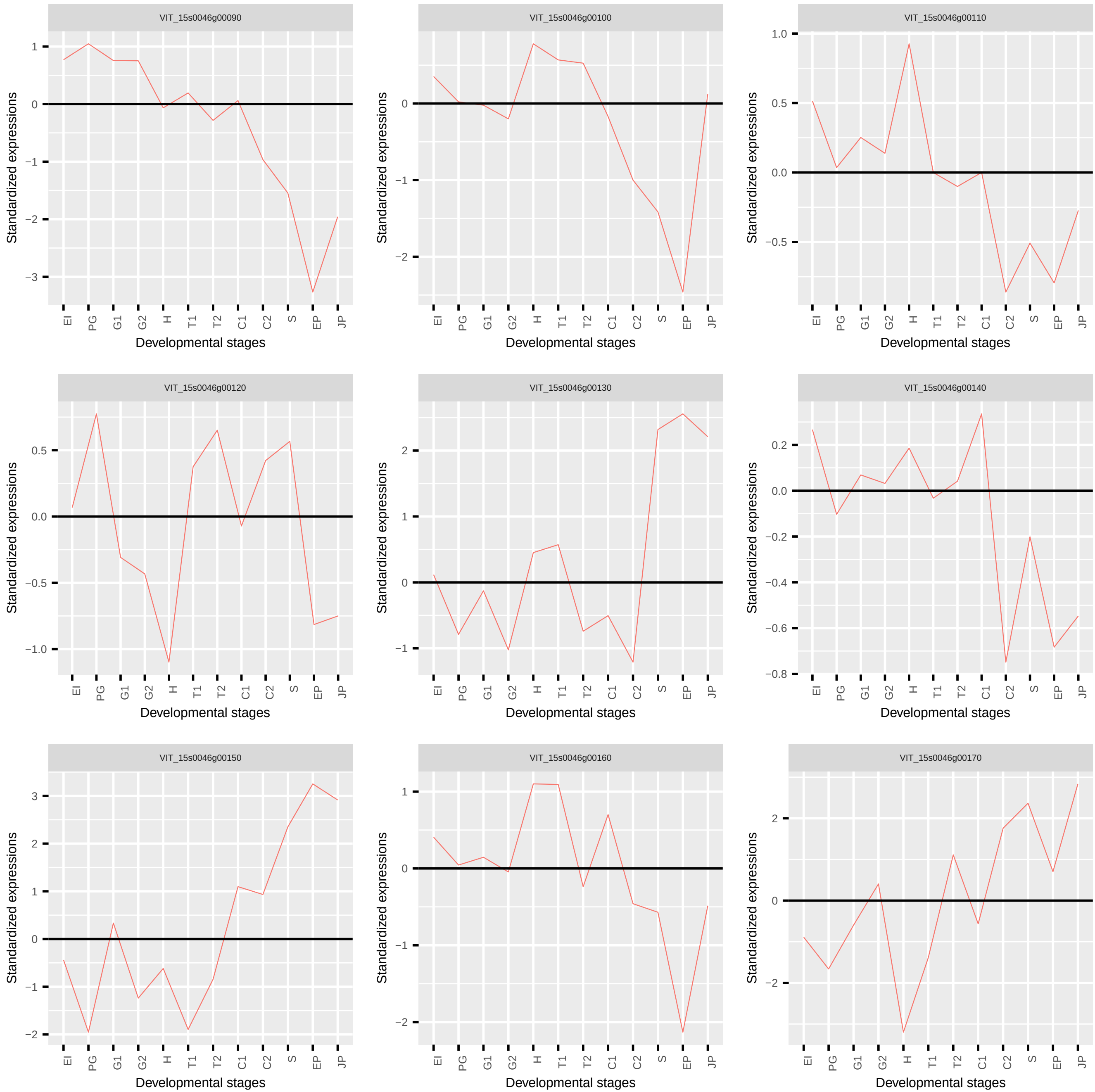
VIT_15s0046g00090
VIT_15s0046g00100
VIT_15s0046g00110
VIT_15s0046g00120
VIT_15s0046g00130
VIT_15s0046g00140
VIT_15s0046g00150
VIT_15s0046g00160
VIT_15s0046g00170
Standardized expressions
Developmental stages
EI PG G1 G2 H T1 T2 C1 C2 S EP JP

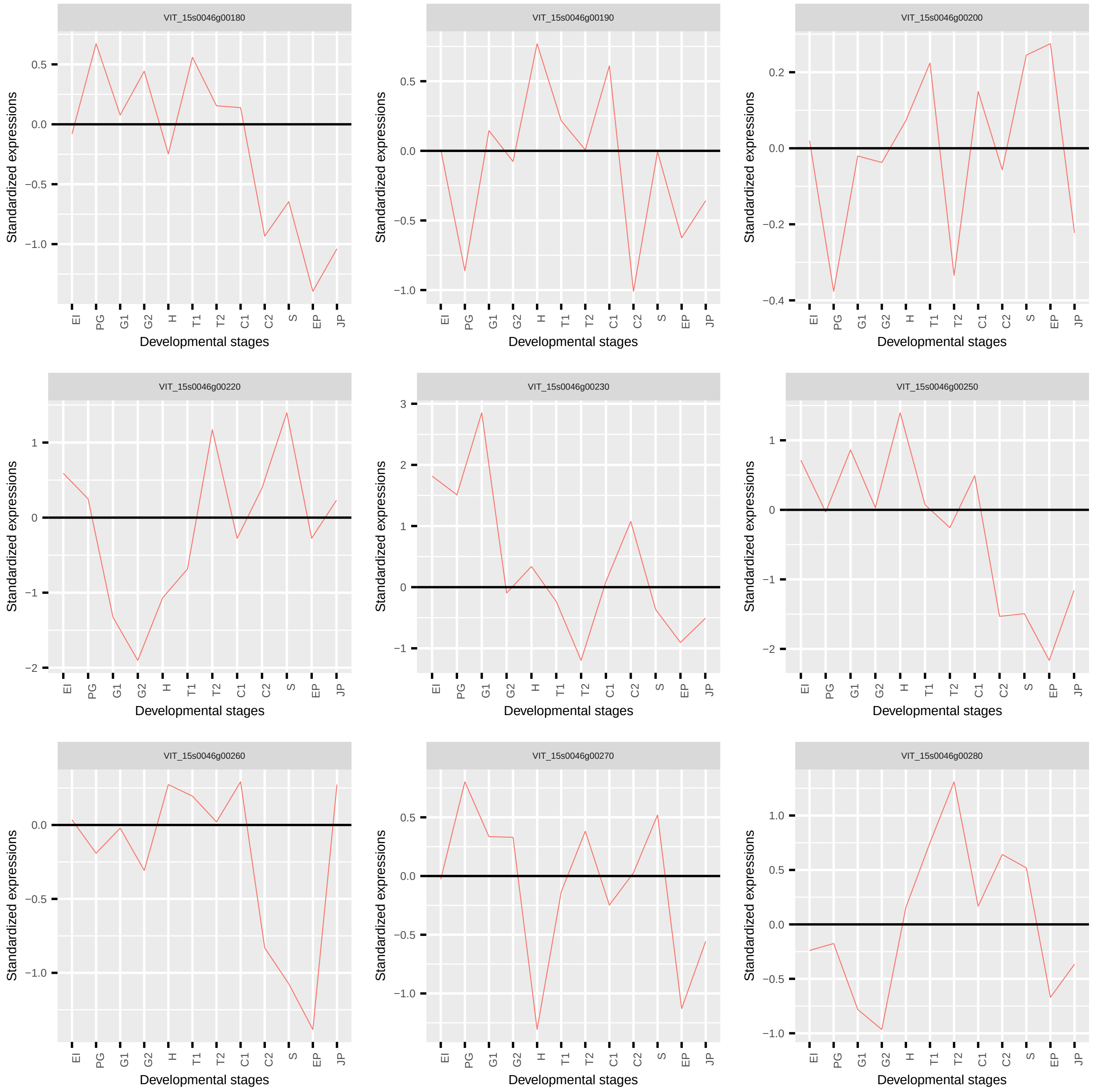
VIT_15s0046g00180
VIT_15s0046g00190
VIT_15s0046g00200
VIT_15s0046g00220
VIT_15s0046g00230
VIT_15s0046g00250
VIT_15s0046g00260
VIT_15s0046g00270
VIT_15s0046g00280
Standardized expressions
Developmental stages
EI
PG
G1
G2
H
T1
T2
C1
C2
S
EP
JP

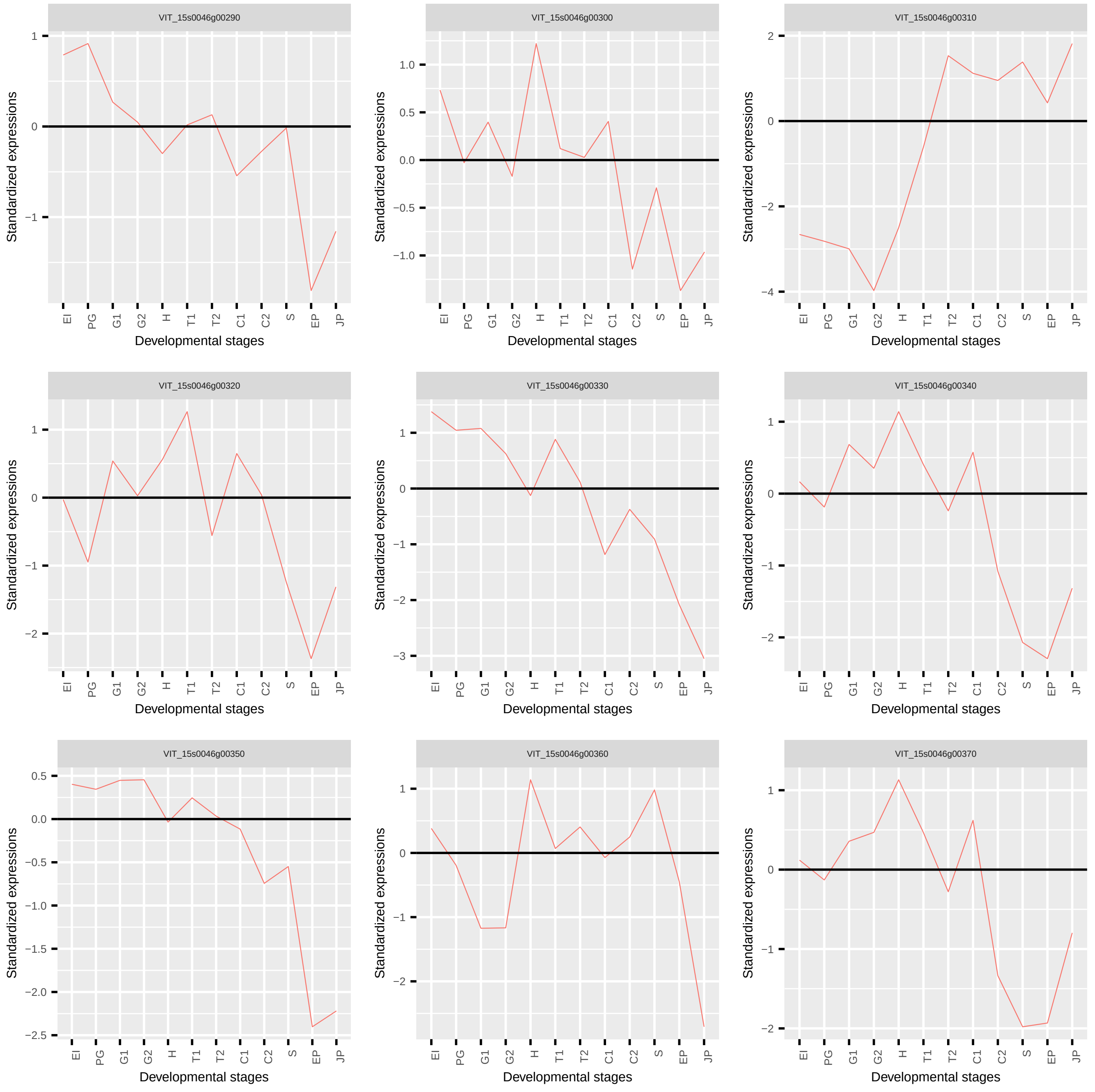

VIT_15s0046g00290
VIT_15s0046g00300
VIT_15s0046g00310
VIT_15s0046g00320
VIT_15s0046g00330
VIT_15s0046g00340
VIT_15s0046g00350
VIT_15s0046g00360
VIT_15s0046g00370
Standardized expressions
Developmental stages
EI PG G1 G2 H T1 T2 C1 C2 S EP JP

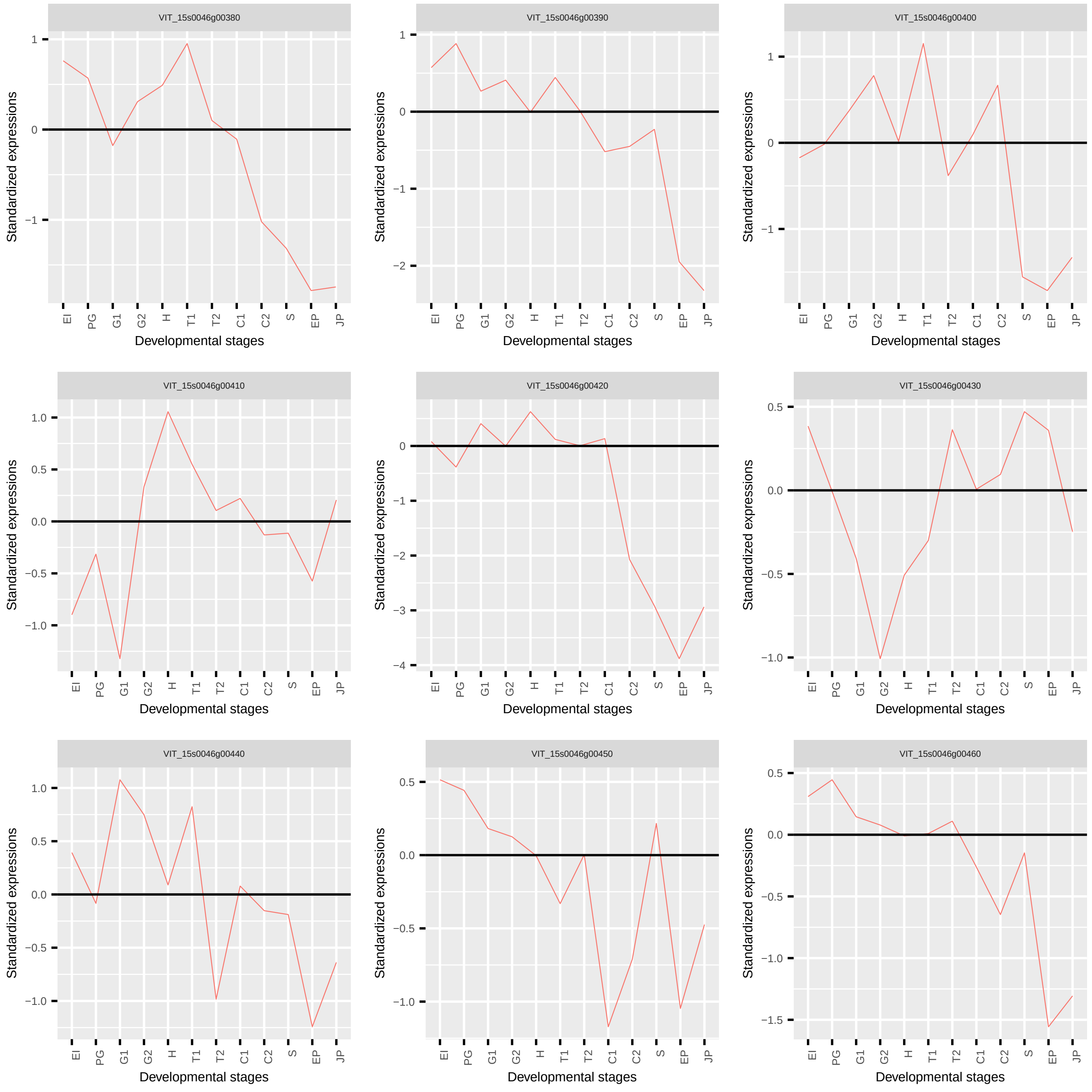
VIT_15s0046g00380
VIT_15s0046g00390
VIT_15s0046g00400
VIT_15s0046g00410
VIT_15s0046g00420
VIT_15s0046g00430
VIT_15s0046g00440
VIT_15s0046g00450
VIT_15s0046g00460
Standardized expressions
Developmental stages
EI PG G1 G2 H T1 T2 C1 C2 S EP JP

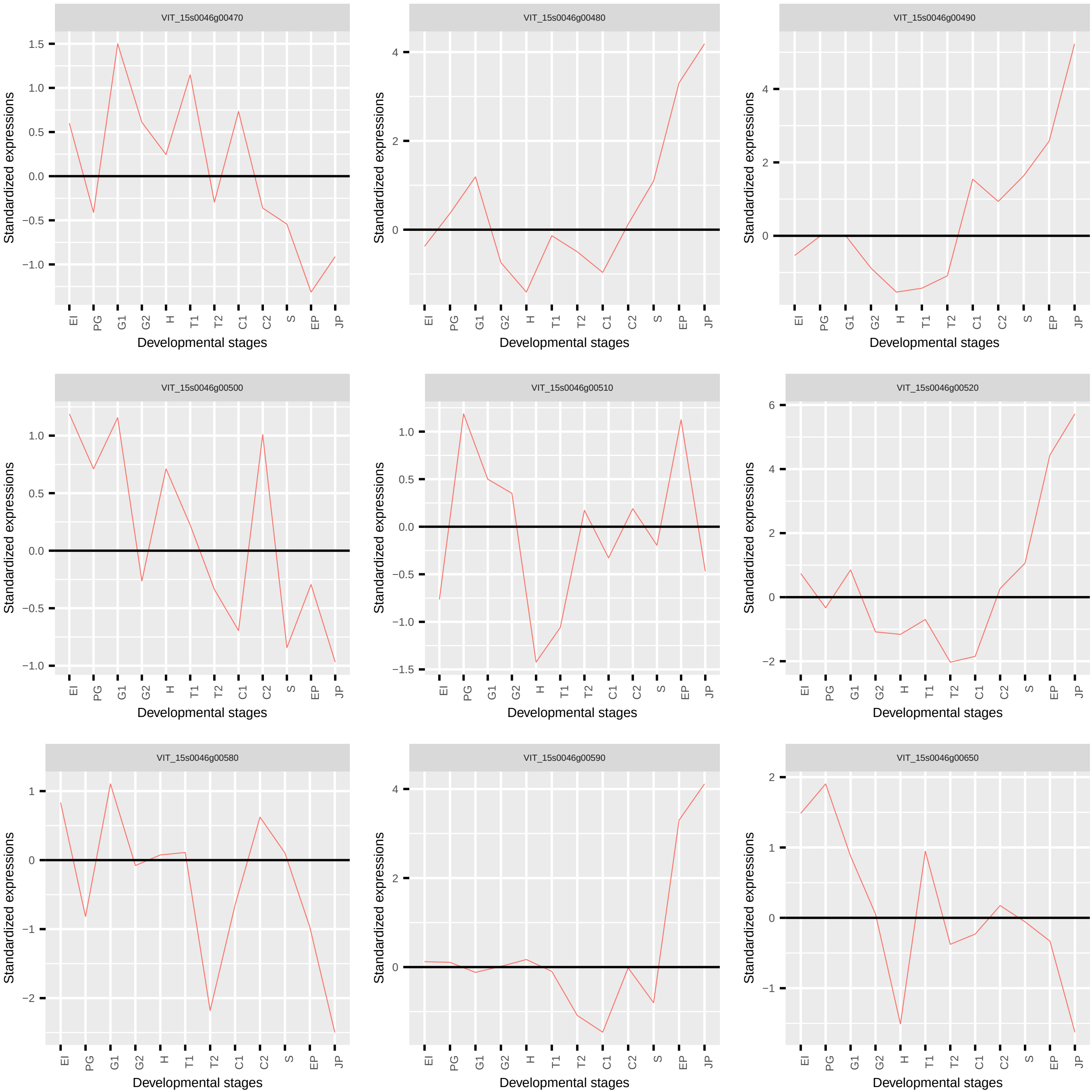
VIT_15s0046g00470
VIT_15s0046g00480
VIT_15s0046g00490
VIT_15s0046g00500
VIT_15s0046g00510
VIT_15s0046g00520
VIT_15s0046g00580
VIT_15s0046g00590
VIT_15s0046g00650
Standardized expressions
Developmental stages
EI
PG
G1
G2
H
T1
T2
C1
C2
S
EP
JP

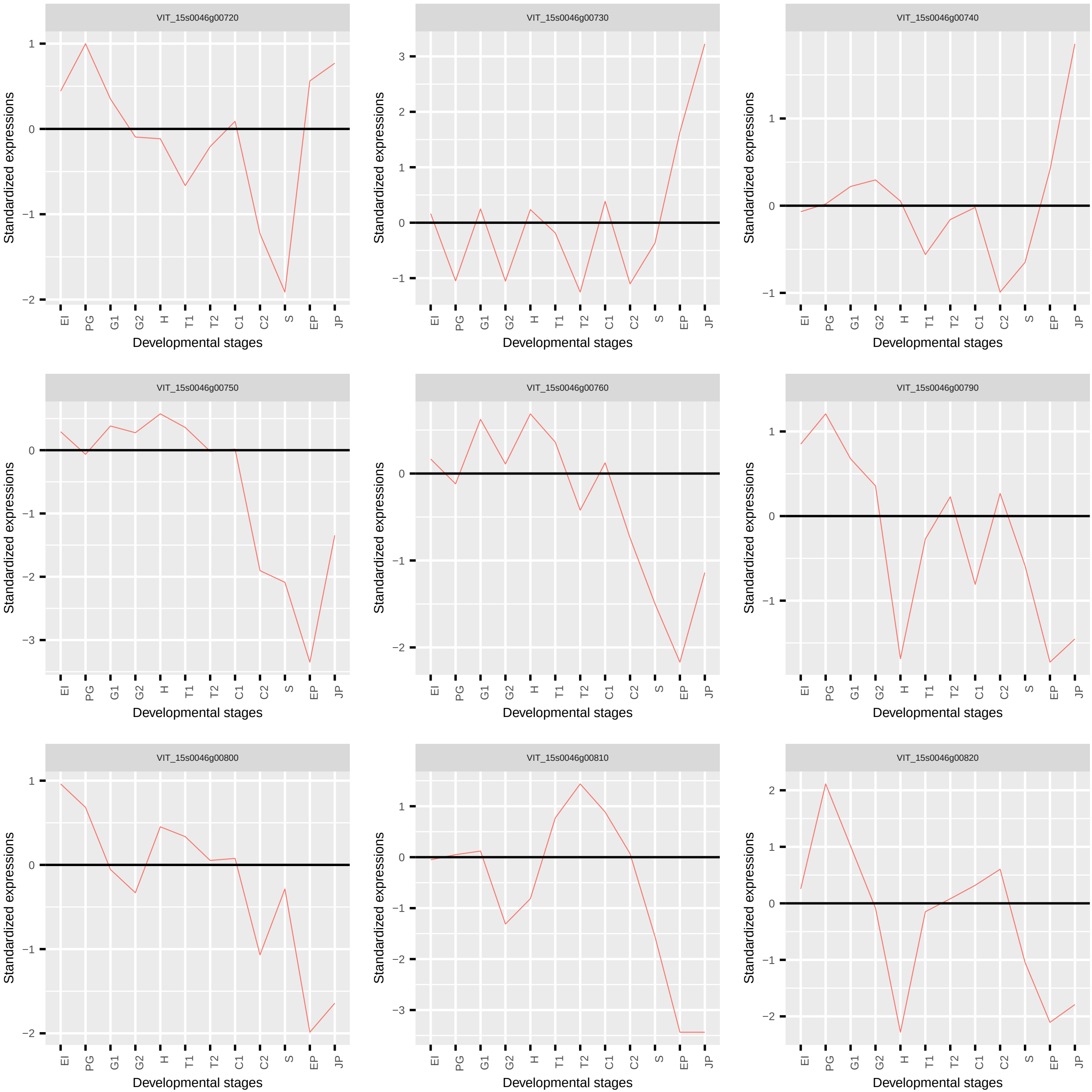
VIT_15s0046g00720
VIT_15s0046g00730
VIT_15s0046g00740
VIT_15s0046g00750
VIT_15s0046g00760
VIT_15s0046g00790
VIT_15s0046g00800
VIT_15s0046g00810
VIT_15s0046g00820
Standardized expressions
Developmental stages
EI
PG
G1
G2
H
T1
T2
C1
C2
S
EP
JP

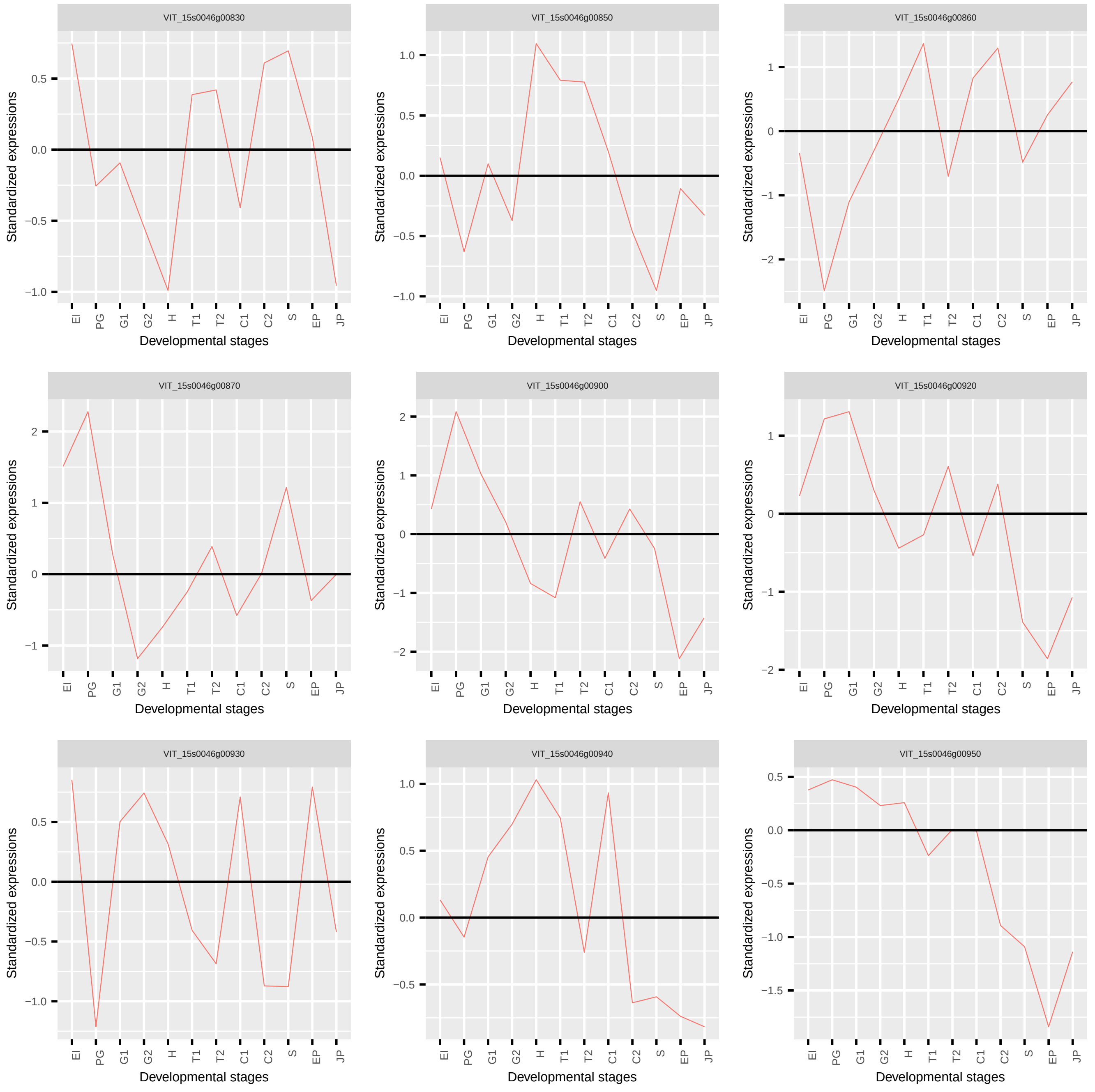
VIT_15s0046g00830
VIT_15s0046g00850
VIT_15s0046g00860
VIT_15s0046g00870
VIT_15s0046g00900
VIT_15s0046g00920
VIT_15s0046g00930
VIT_15s0046g00940
VIT_15s0046g00950
Standardized expressions
Developmental stages
EI PG G1 G2 H T1 T2 C1 C2 S EP JP

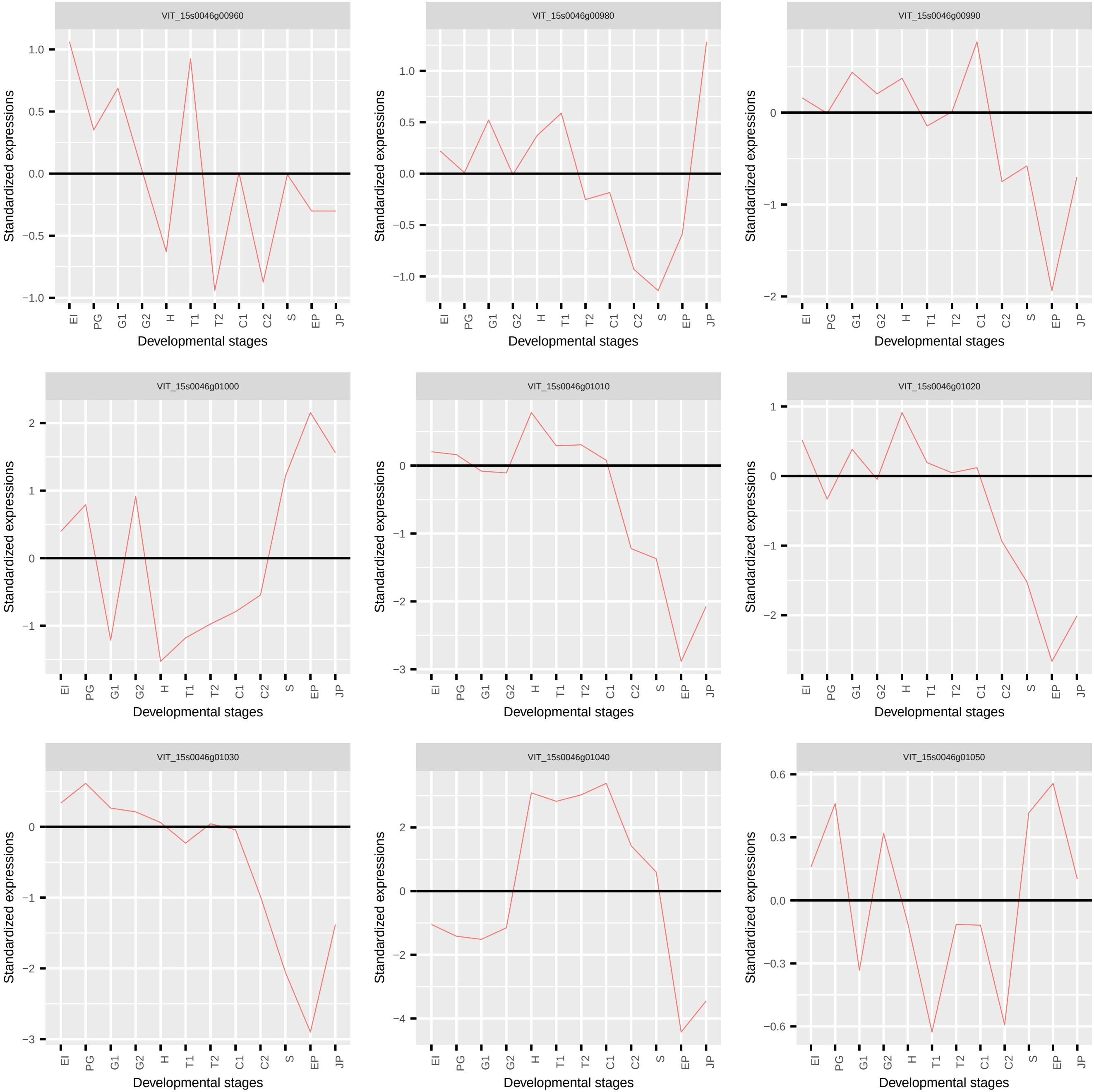

VIT_15s0046g00960
Standardized expressions
1.0
0.5
0.0
−0.5
−1.0
EI PG G1 G2 H T1 T2 C1 C2 S EP JP
Developmental stages
VIT_15s0046g00980
Standardized expressions
1.0
0.5
0.0
−0.5
−1.0
EI PG G1 G2 H T1 T2 C1 C2 S EP JP
Developmental stages
VIT_15s0046g00990
Standardized expressions
0
−1
−2
EI PG G1 G2 H T1 T2 C1 C2 S EP JP
Developmental stages
VIT_15s0046g01000
Standardized expressions
2
1
0
−1
EI PG G1 G2 H T1 T2 C1 C2 S EP JP
Developmental stages
VIT_15s0046g01010
Standardized expressions
0
−1
−2
−3
EI PG G1 G2 H T1 T2 C1 C2 S EP JP
Developmental stages
VIT_15s0046g01020
Standardized expressions
1
0
−1
−2
EI PG G1 G2 H T1 T2 C1 C2 S EP JP
Developmental stages
VIT_15s0046g01030
Standardized expressions
0
−1
−2
−3
EI PG G1 G2 H T1 T2 C1 C2 S EP JP
Developmental stages
VIT_15s0046g01040
Standardized expressions
2
0
−2
−4
EI PG G1 G2 H T1 T2 C1 C2 S EP JP
Developmental stages
VIT_15s0046g01050
Standardized expressions
0.6
0.3
0.0
−0.3
−0.6
EI PG G1 G2 H T1 T2 C1 C2 S EP JP
Developmental stages

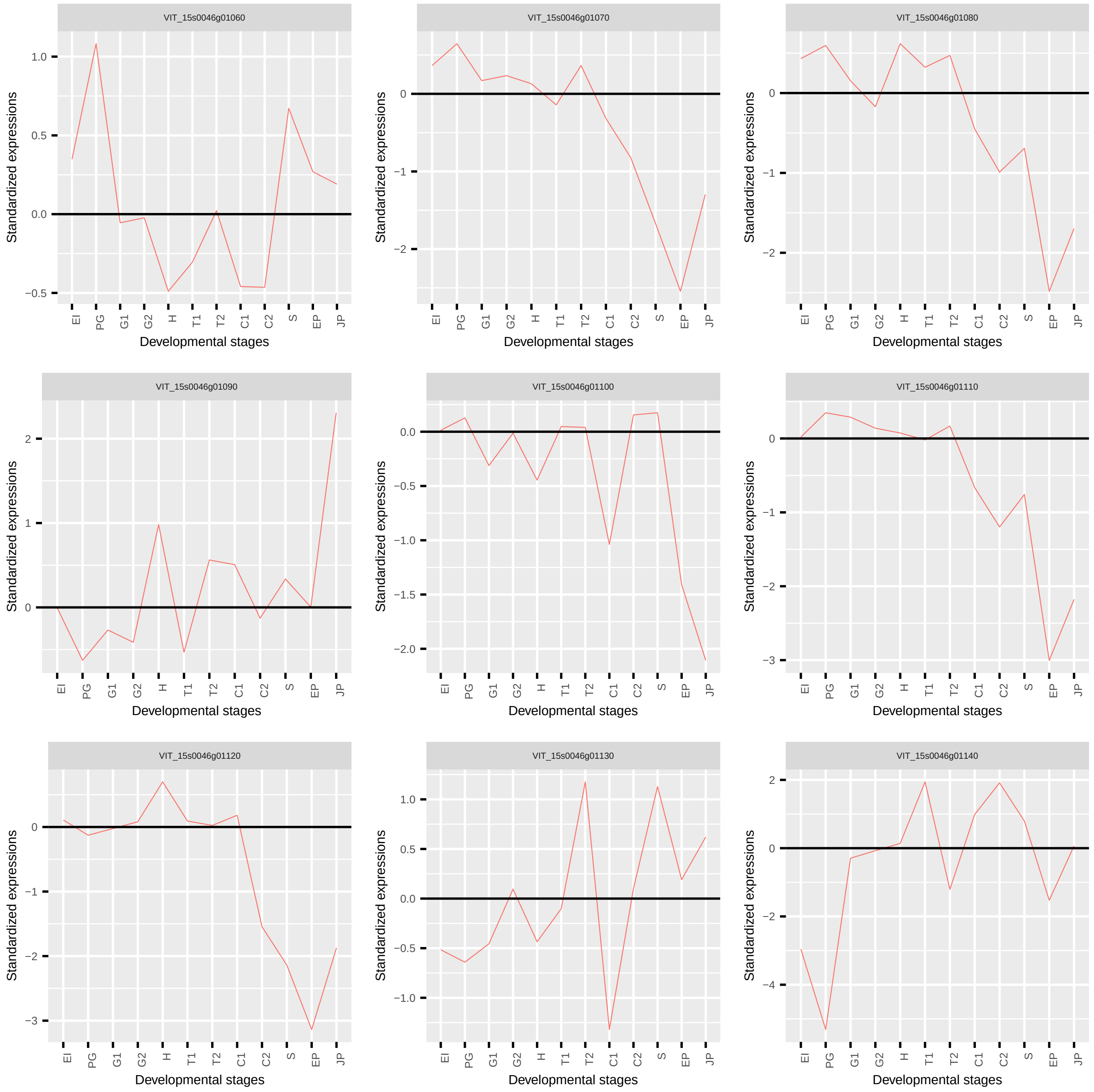

VIT_15s0046g01060
VIT_15s0046g01070
VIT_15s0046g01080
VIT_15s0046g01090
VIT_15s0046g01100
VIT_15s0046g01110
VIT_15s0046g01120
VIT_15s0046g01130
VIT_15s0046g01140
Standardized expressions
Developmental stages
EI PG G1 G2 H T1 T2 C1 C2 S EP JP

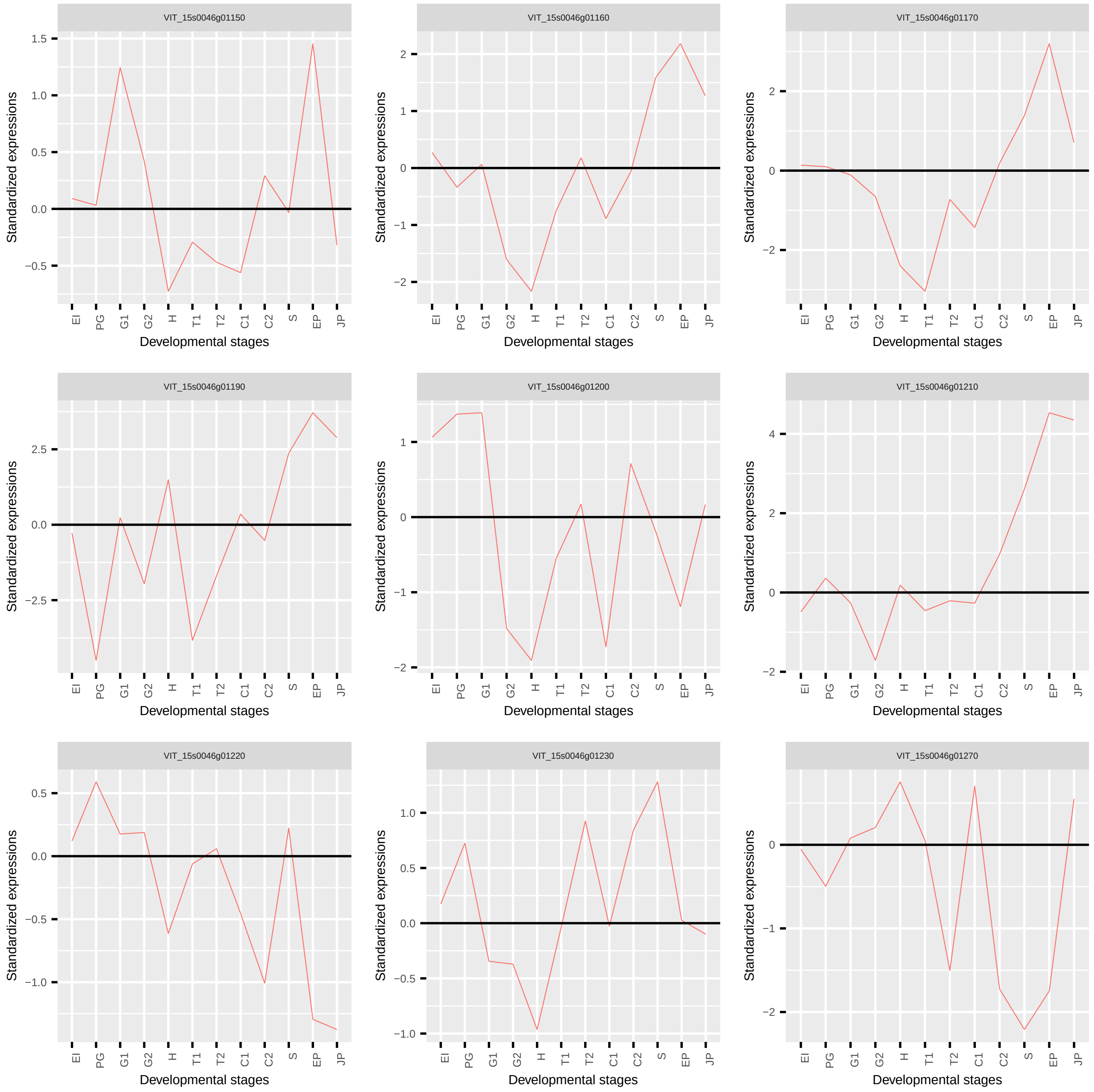

VIT_15s0046g01150
VIT_15s0046g01160
VIT_15s0046g01170
VIT_15s0046g01190
VIT_15s0046g01200
VIT_15s0046g01210
VIT_15s0046g01220
VIT_15s0046g01230
VIT_15s0046g01270
Standardized expressions
Developmental stages
EI PG G1 G2 H T1 T2 C1 C2 S EP JP

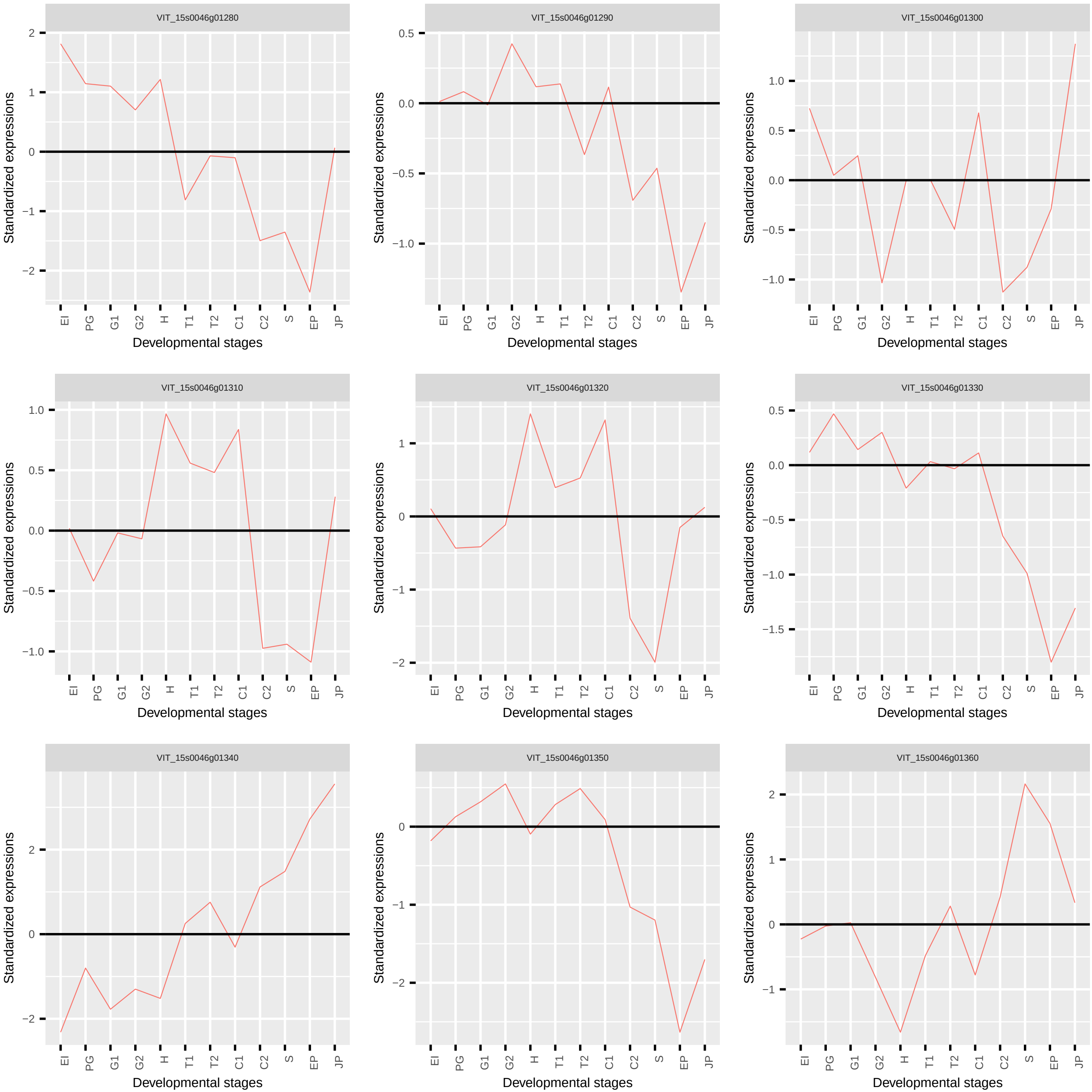
VIT_15s0046g01280
VIT_15s0046g01290
VIT_15s0046g01300
VIT_15s0046g01310
VIT_15s0046g01320
VIT_15s0046g01330
VIT_15s0046g01340
VIT_15s0046g01350
VIT_15s0046g01360
Standardized expressions
Developmental stages
EI PG G1 G2 H T1 T2 C1 C2 S EP JP

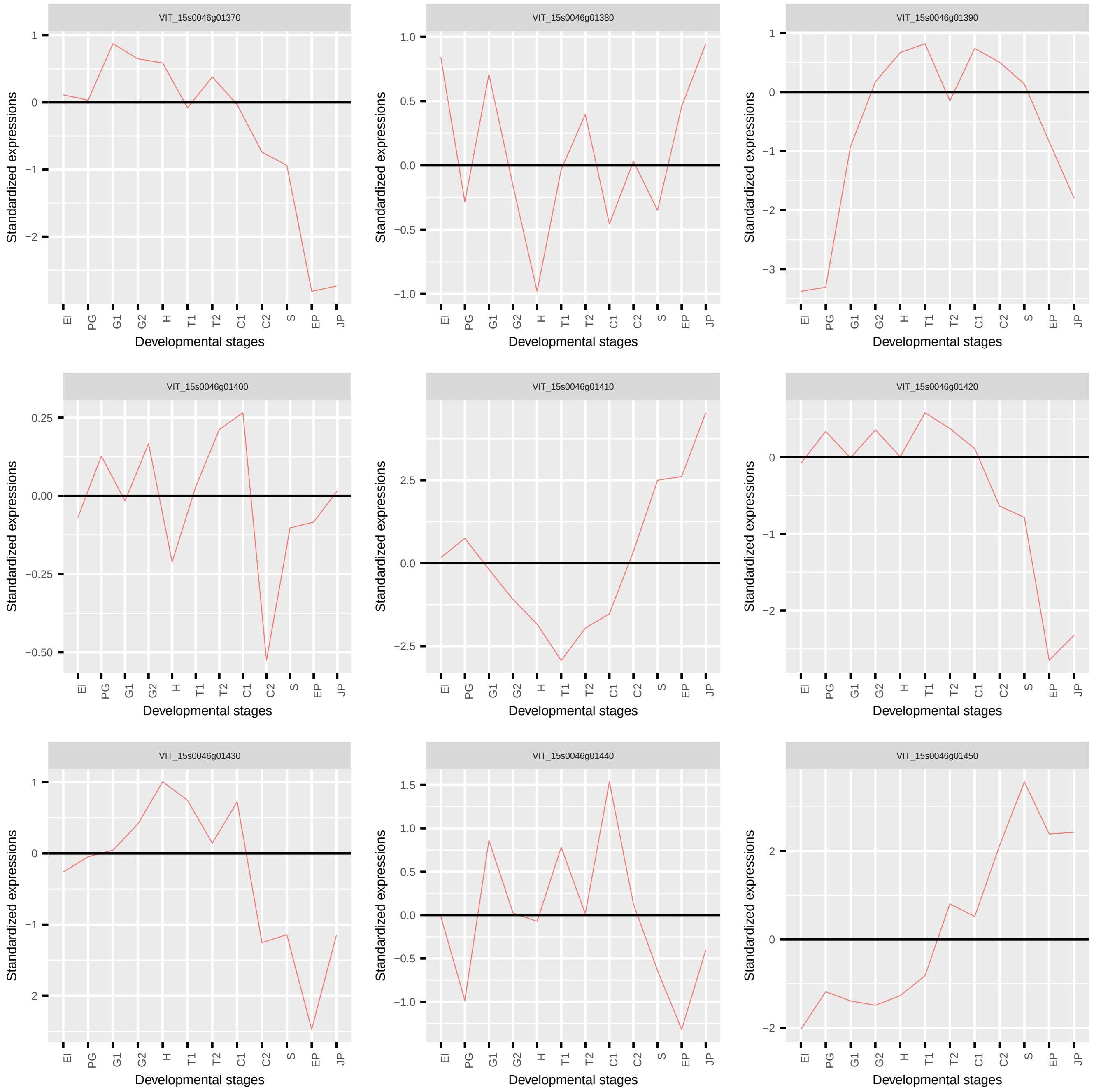

VIT_15s0046g01370
VIT_15s0046g01380
VIT_15s0046g01390
VIT_15s0046g01400
VIT_15s0046g01410
VIT_15s0046g01420
VIT_15s0046g01430
VIT_15s0046g01440
VIT_15s0046g01450
Standardized expressions
Developmental stages
EI PG G1 G2 H T1 T2 C1 C2 S EP JP

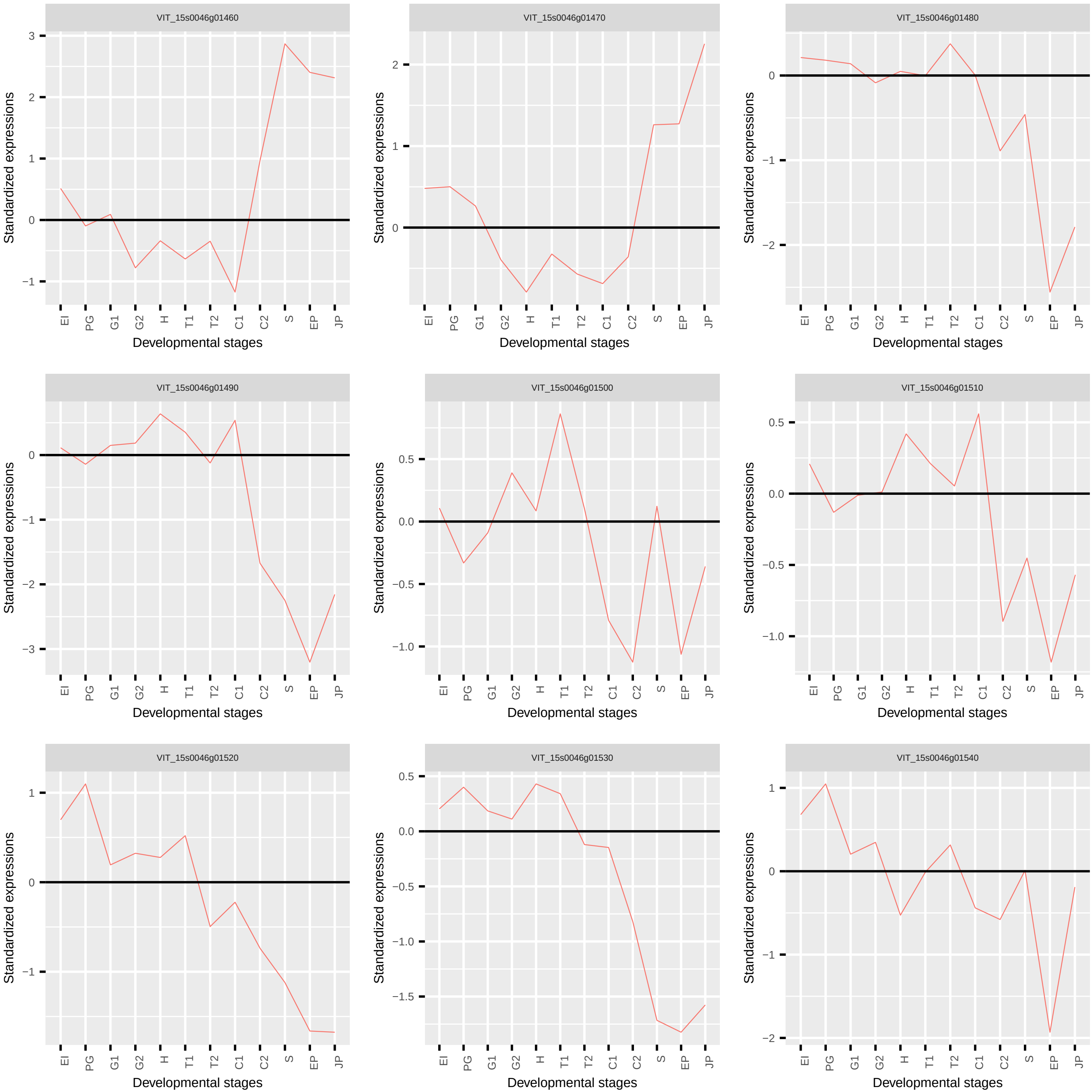
VIT_15s0046g01460
VIT_15s0046g01470
VIT_15s0046g01480
VIT_15s0046g01490
VIT_15s0046g01500
VIT_15s0046g01510
VIT_15s0046g01520
VIT_15s0046g01530
VIT_15s0046g01540
Standardized expressions
Developmental stages
EI PG G1 G2 H T1 T2 C1 C2 S EP JP

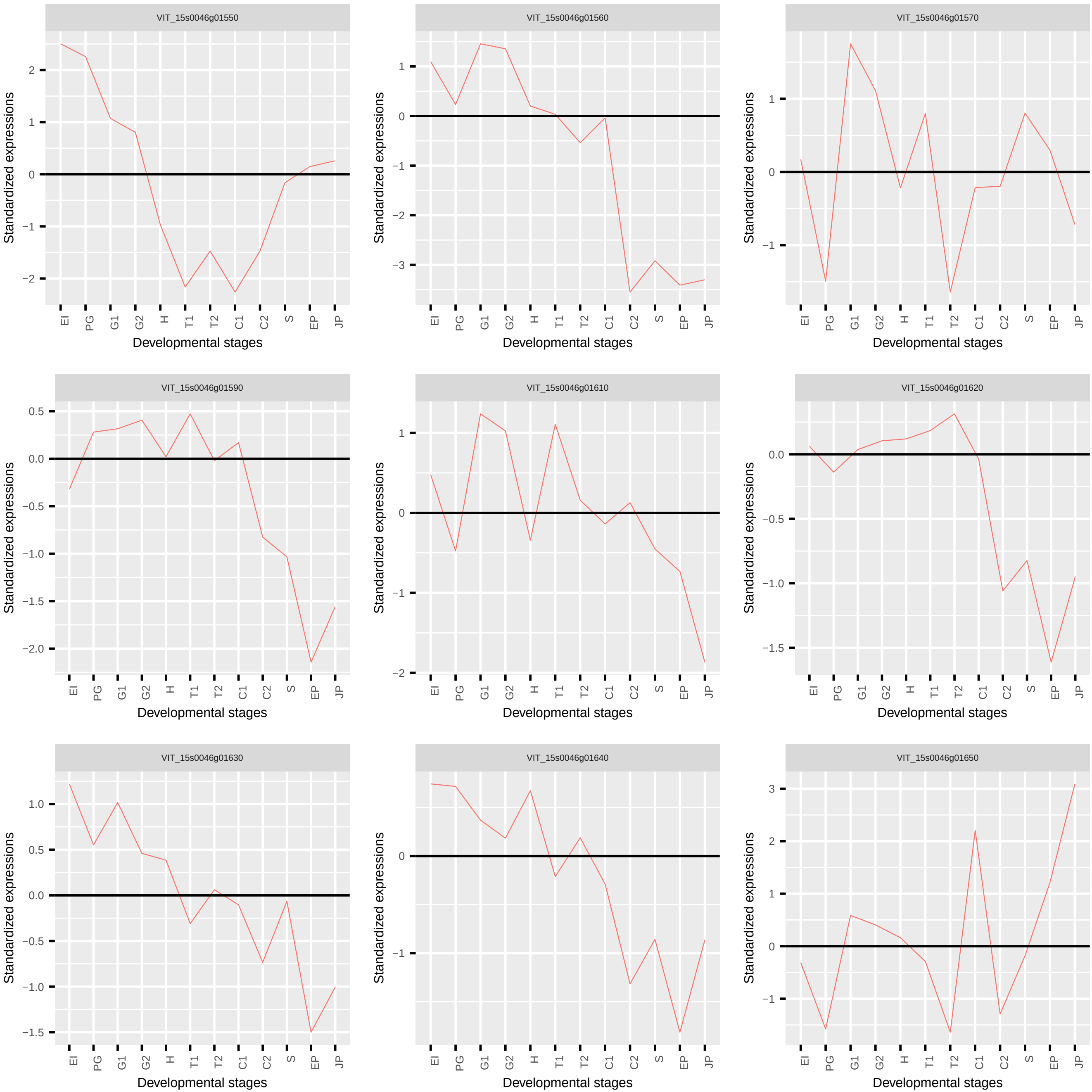
VIT_15s0046g01550
VIT_15s0046g01560
VIT_15s0046g01570
VIT_15s0046g01590
VIT_15s0046g01610
VIT_15s0046g01620
VIT_15s0046g01630
VIT_15s0046g01640
VIT_15s0046g01650
Standardized expressions
Developmental stages
EI PG G1 G2 H T1 T2 C1 C2 S EP JP

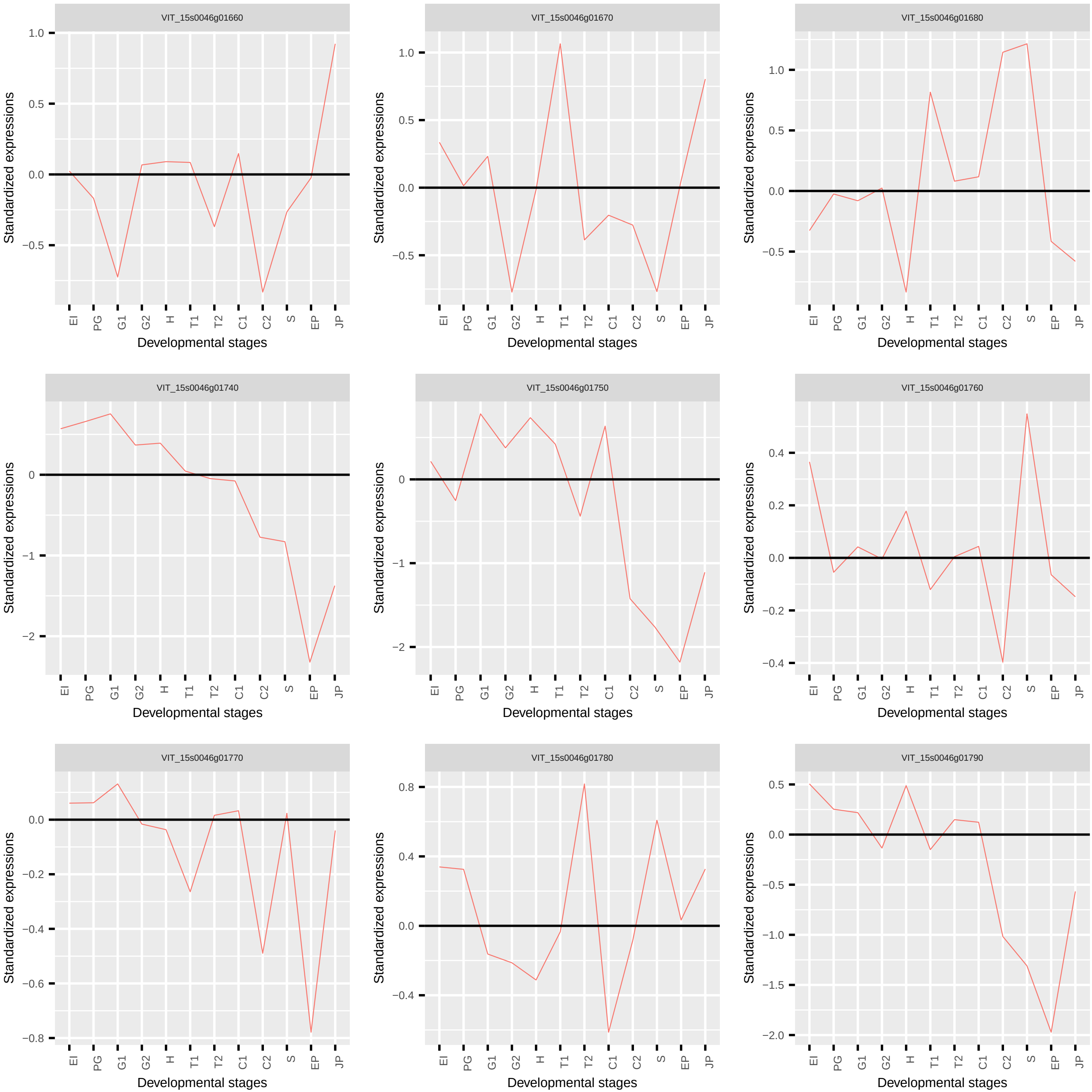
VIT_15s0046g01660
VIT_15s0046g01670
VIT_15s0046g01680
VIT_15s0046g01740
VIT_15s0046g01750
VIT_15s0046g01760
VIT_15s0046g01770
VIT_15s0046g01780
VIT_15s0046g01790
Standardized expressions
Developmental stages
EI
PG
G1
G2
H
T1
T2
C1
C2
S
EP
JP

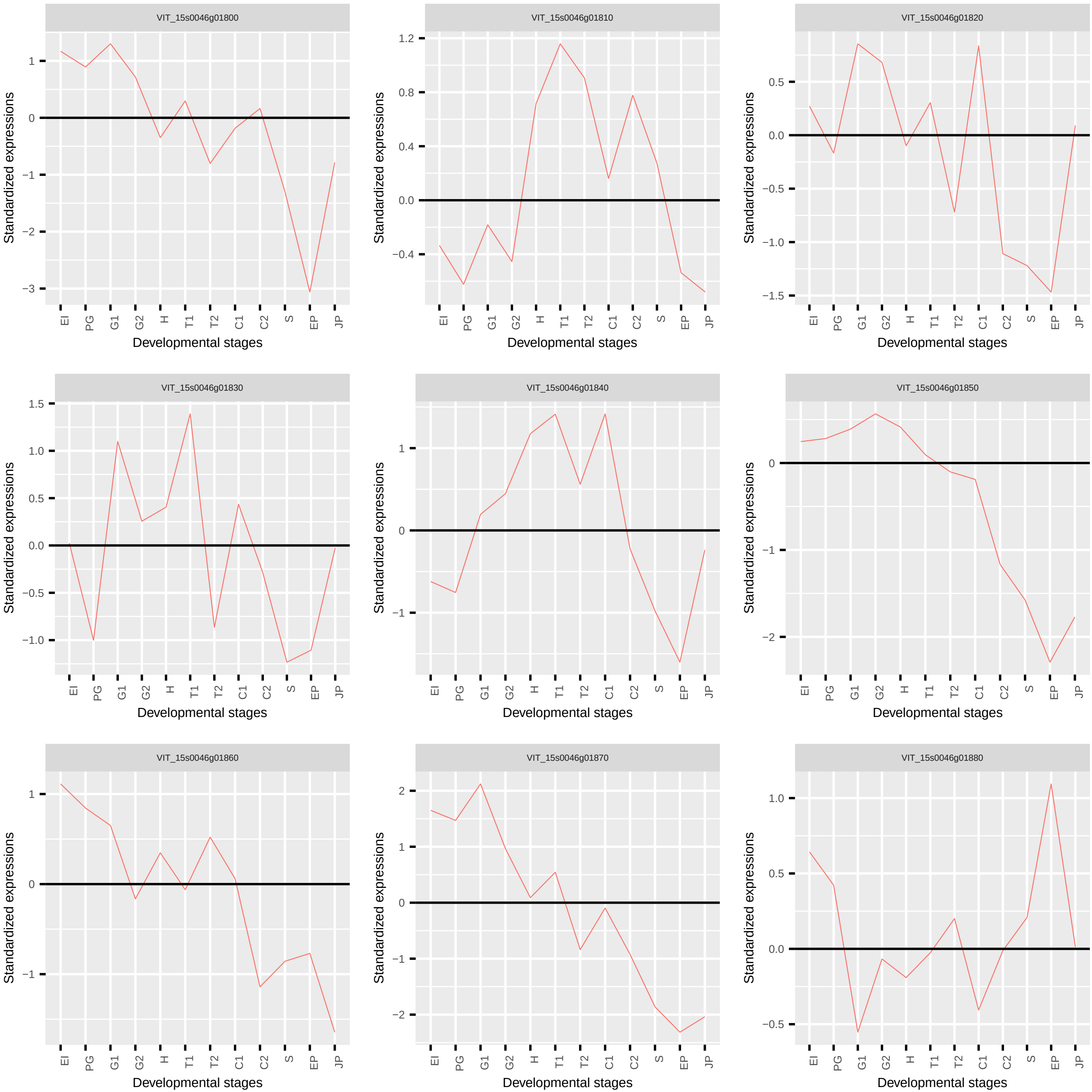
VIT_15s0046g01800
VIT_15s0046g01810
VIT_15s0046g01820
VIT_15s0046g01830
VIT_15s0046g01840
VIT_15s0046g01850
VIT_15s0046g01860
VIT_15s0046g01870
VIT_15s0046g01880
Standardized expressions
Developmental stages
EI PG G1 G2 H T1 T2 C1 C2 S EP JP

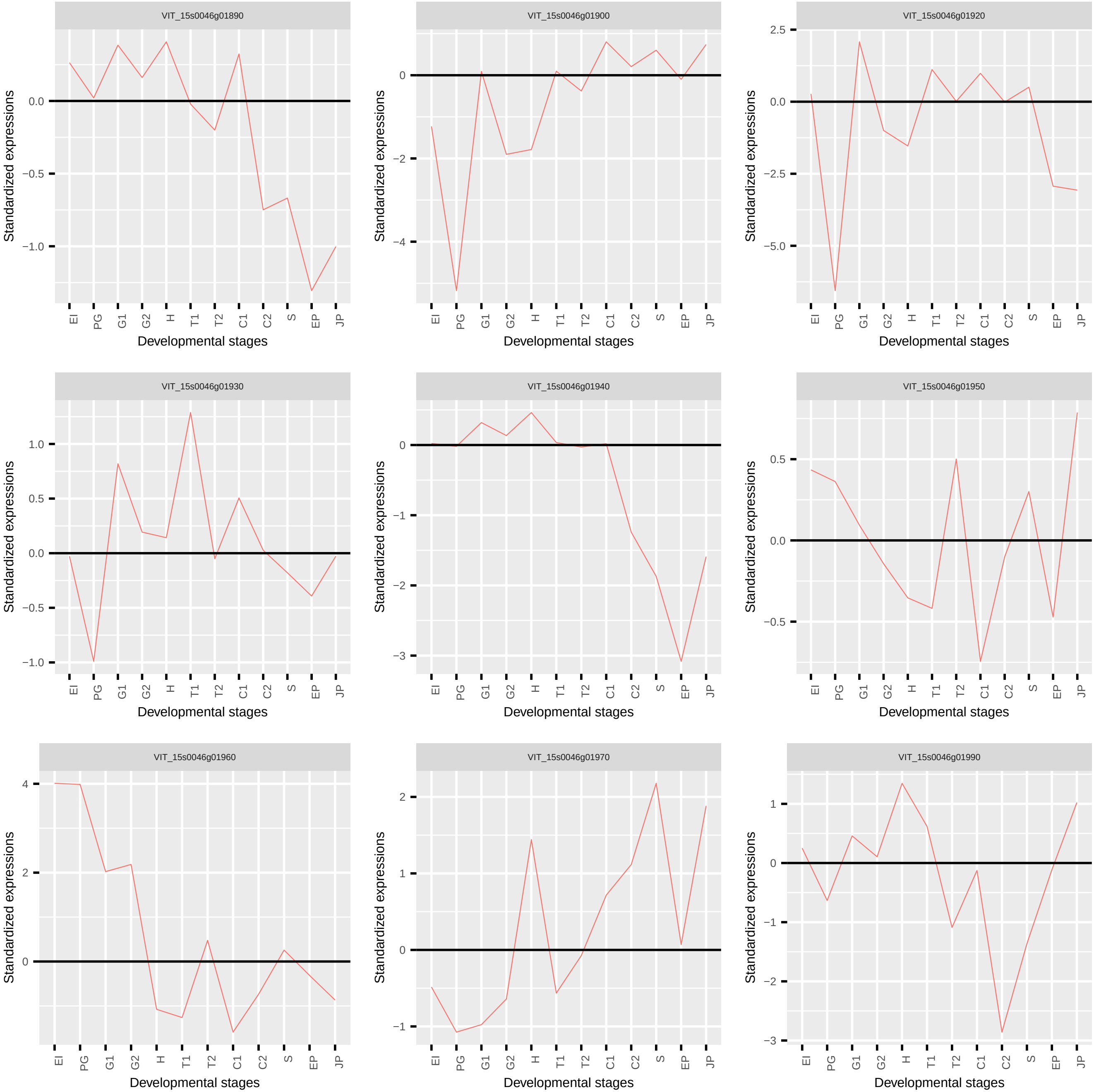
VIT_15s0046g01890
Standardized expressions
0.0
−0.5
−1.0
EI PG G1 G2 H T1 T2 C1 C2 S EP JP
Developmental stages
VIT_15s0046g01900
Standardized expressions
0
−2
−4
EI PG G1 G2 H T1 T2 C1 C2 S EP JP
Developmental stages
VIT_15s0046g01920
Standardized expressions
2.5
0.0
−2.5
−5.0
EI PG G1 G2 H T1 T2 C1 C2 S EP JP
Developmental stages
VIT_15s0046g01930
Standardized expressions
1.0
0.5
0.0
−0.5
−1.0
EI PG G1 G2 H T1 T2 C1 C2 S EP JP
Developmental stages
VIT_15s0046g01940
Standardized expressions
0
−1
−2
−3
EI PG G1 G2 H T1 T2 C1 C2 S EP JP
Developmental stages
VIT_15s0046g01950
Standardized expressions
0.5
0.0
−0.5
EI PG G1 G2 H T1 T2 C1 C2 S EP JP
Developmental stages
VIT_15s0046g01960
Standardized expressions
4
2
0
EI PG G1 G2 H T1 T2 C1 C2 S EP JP
Developmental stages
VIT_15s0046g01970
Standardized expressions
2
1
0
−1
EI PG G1 G2 H T1 T2 C1 C2 S EP JP
Developmental stages
VIT_15s0046g01990
Standardized expressions
1
0
−1
−2
−3
EI PG G1 G2 H T1 T2 C1 C2 S EP JP
Developmental stages

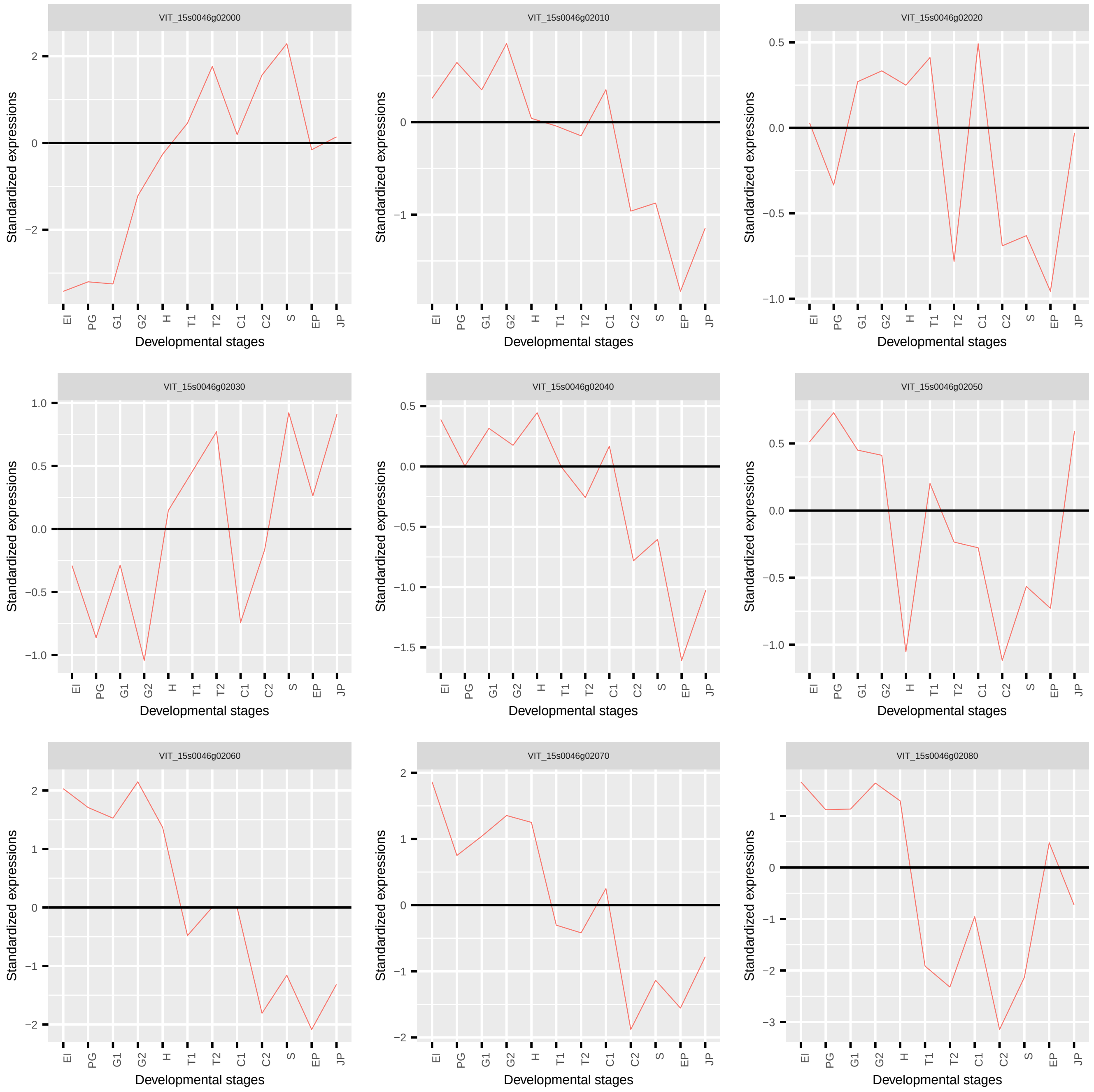
VIT_15s0046g02000
VIT_15s0046g02010
VIT_15s0046g02020
VIT_15s0046g02030
VIT_15s0046g02040
VIT_15s0046g02050
VIT_15s0046g02060
VIT_15s0046g02070
VIT_15s0046g02080
Standardized expressions
Developmental stages
EI PG G1 G2 H T1 T2 C1 C2 S EP JP

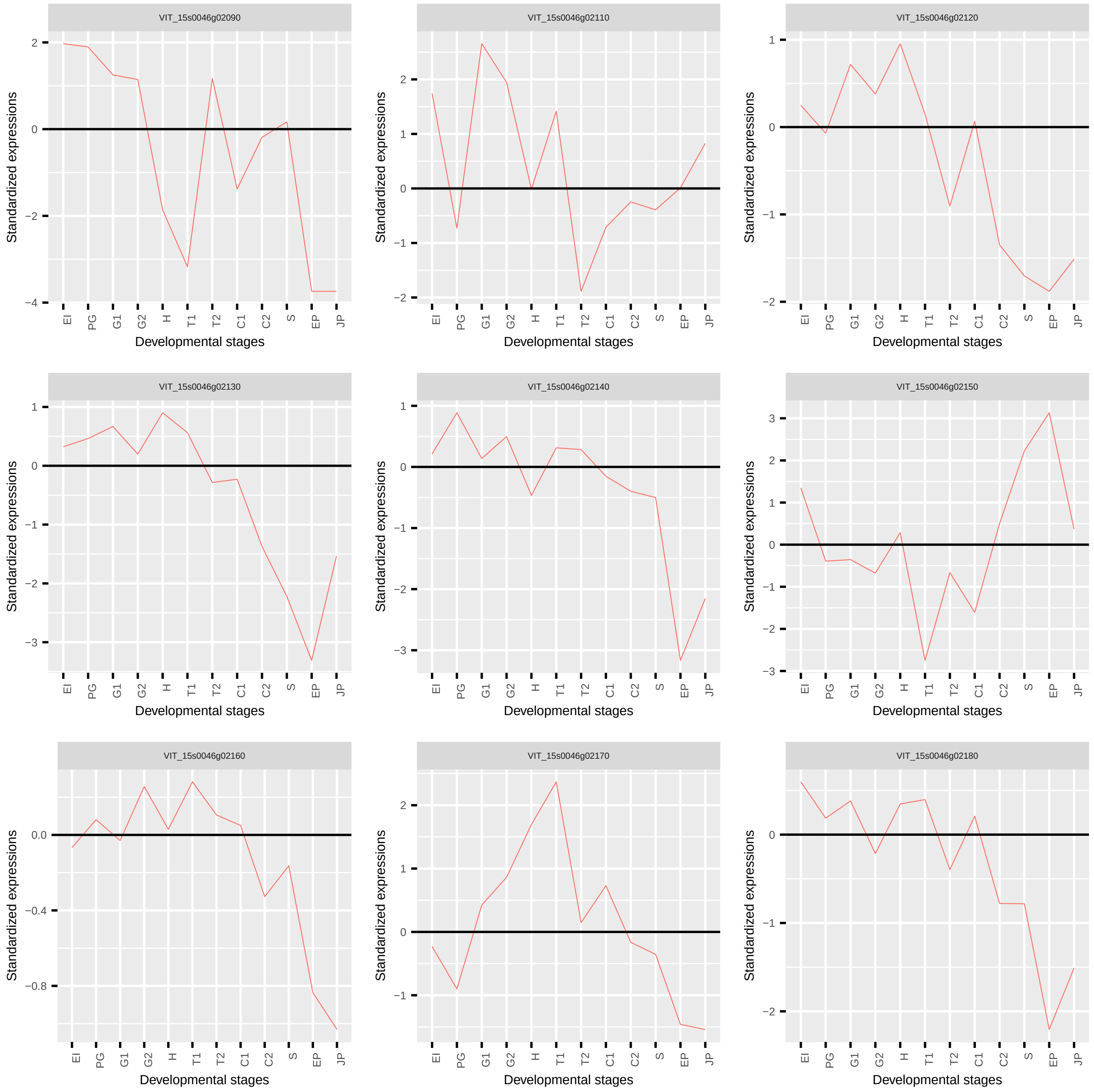
VIT_15s0046g02090
VIT_15s0046g02110
VIT_15s0046g02120
VIT_15s0046g02130
VIT_15s0046g02140
VIT_15s0046g02150
VIT_15s0046g02160
VIT_15s0046g02170
VIT_15s0046g02180
Standardized expressions
Developmental stages
EI PG G1 G2 H T1 T2 C1 C2 S EP JP

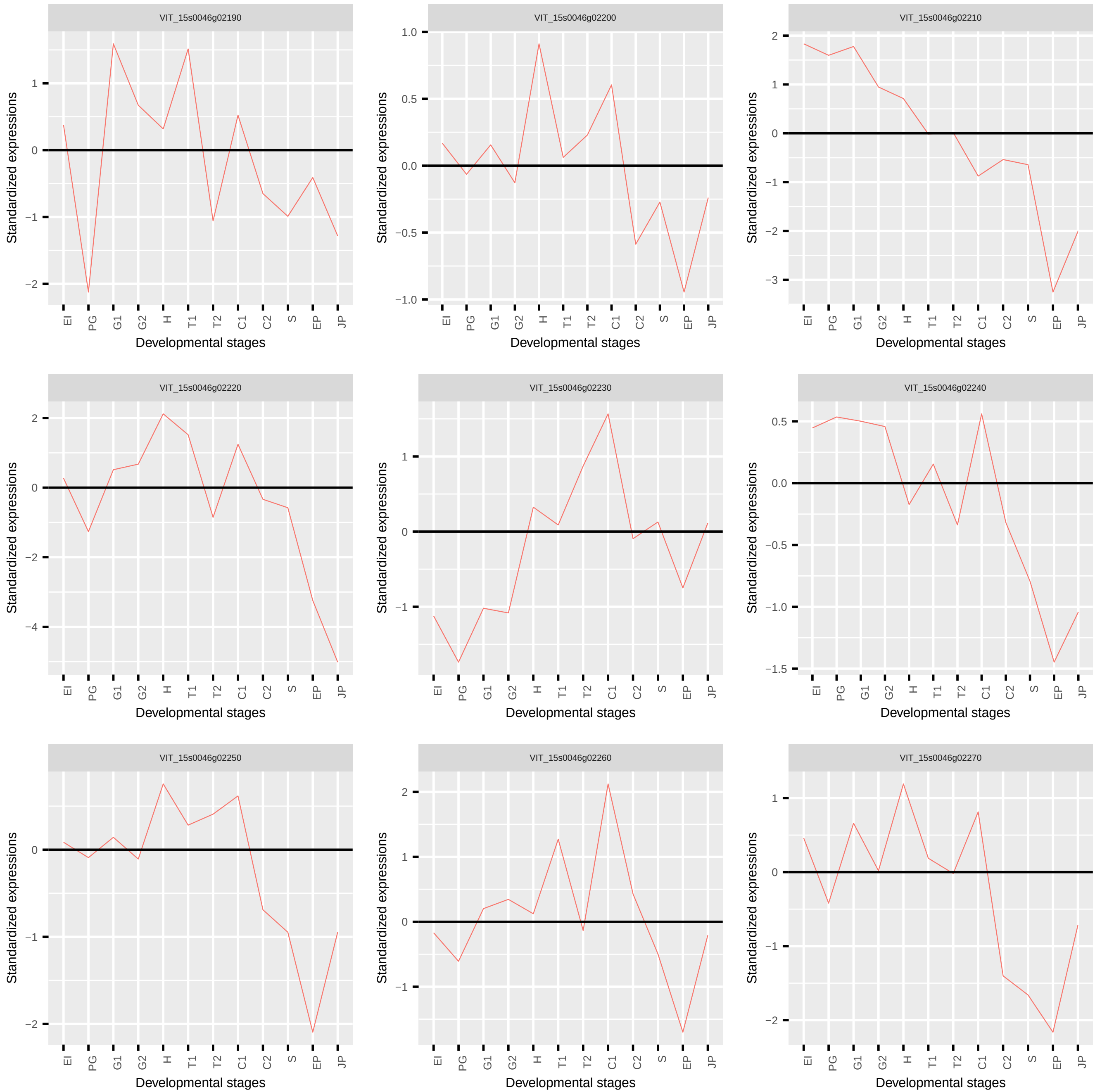
VIT_15s0046g02190
VIT_15s0046g02200
VIT_15s0046g02210
VIT_15s0046g02220
VIT_15s0046g02230
VIT_15s0046g02240
VIT_15s0046g02250
VIT_15s0046g02260
VIT_15s0046g02270
Standardized expressions
Developmental stages
EI PG G1 G2 H T1 T2 C1 C2 S EP JP

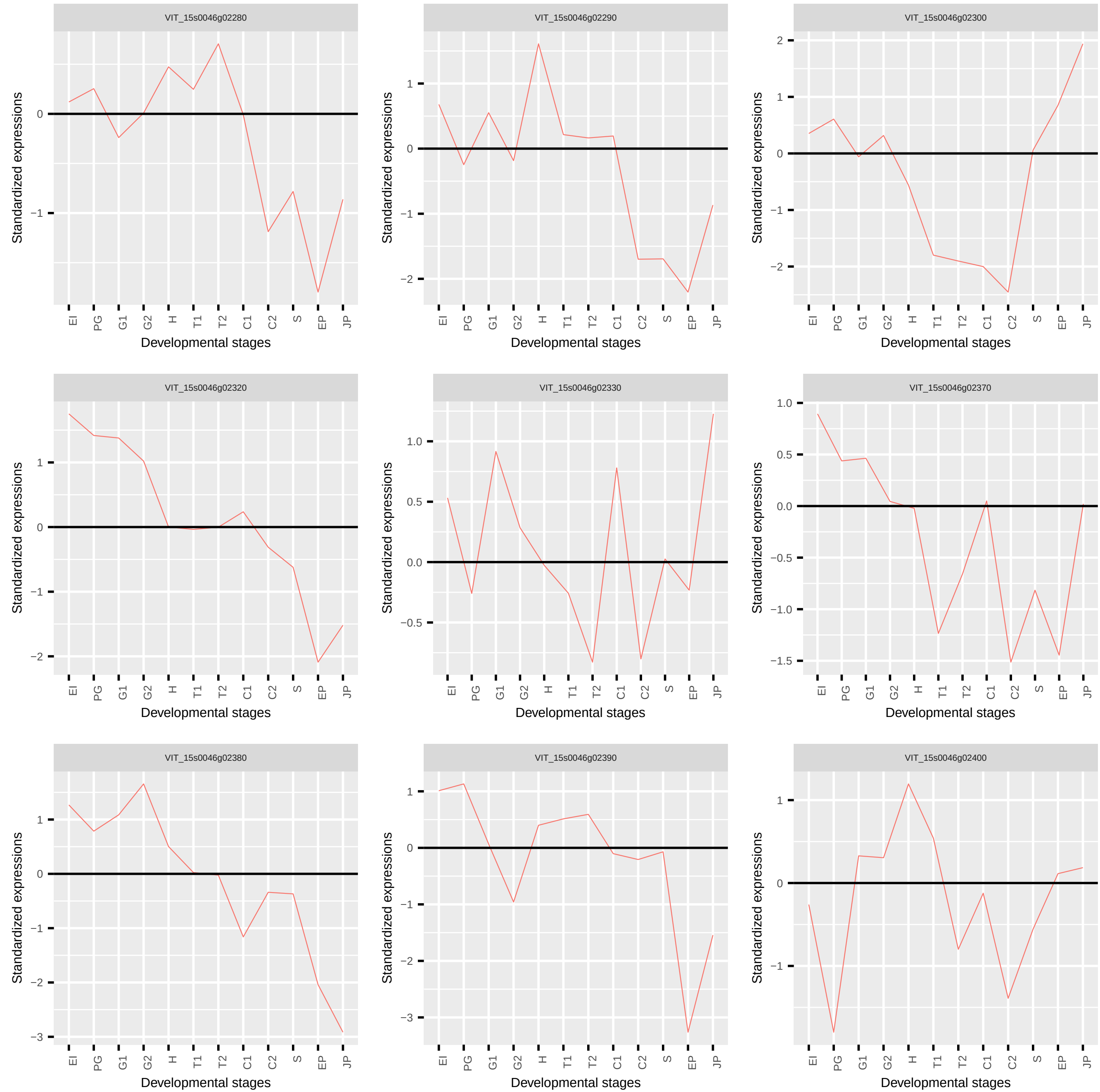
VIT_15s0046g02280
VIT_15s0046g02290
VIT_15s0046g02300
VIT_15s0046g02320
VIT_15s0046g02330
VIT_15s0046g02370
VIT_15s0046g02380
VIT_15s0046g02390
VIT_15s0046g02400
Standardized expressions
Developmental stages
EI PG G1 G2 H T1 T2 C1 C2 S EP JP
2
1
0
−1
−2
−3
1.0
0.5
0.0
−0.5
−1.0
−1.5

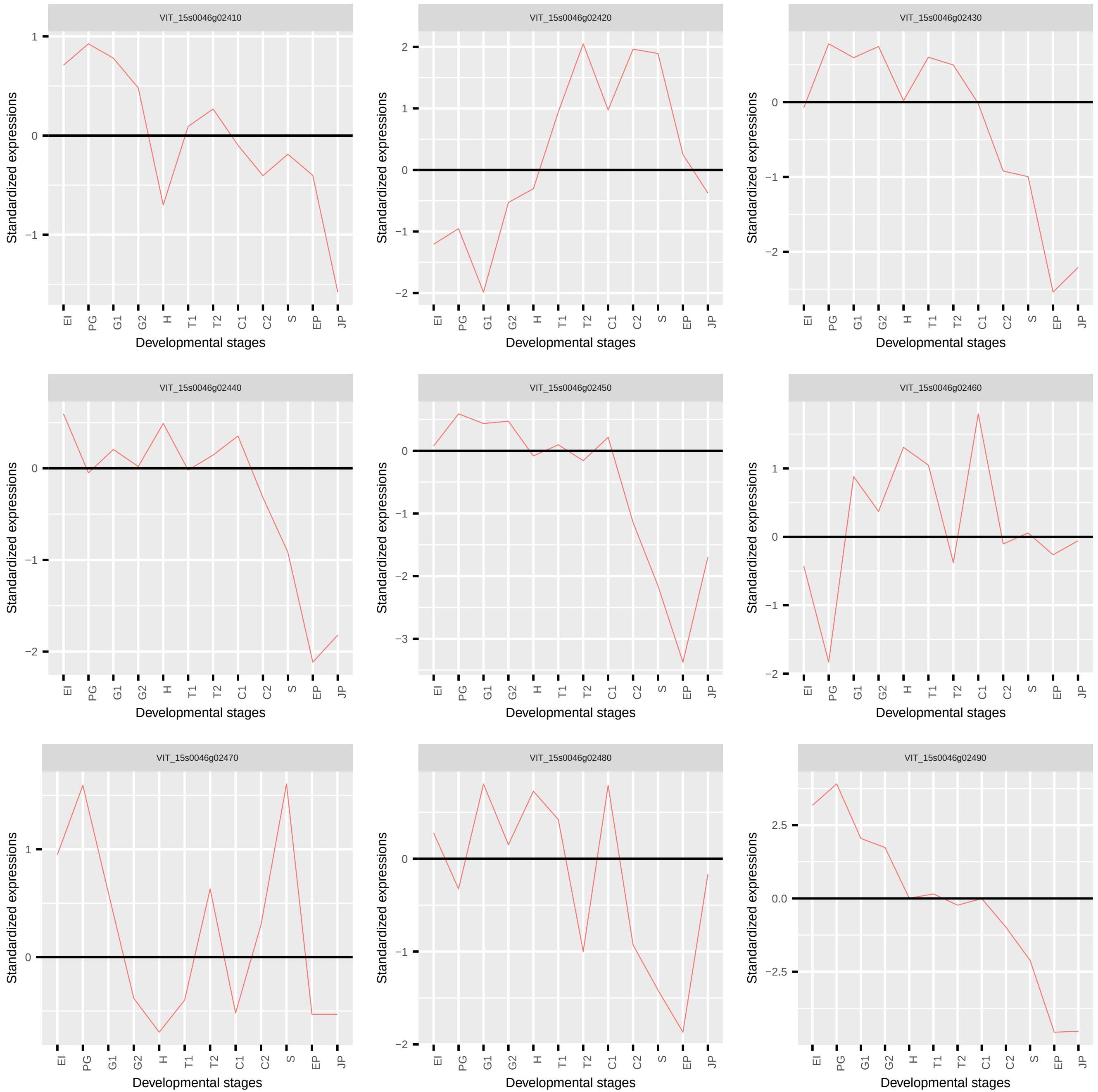
VIT_15s0046g02410
VIT_15s0046g02420
VIT_15s0046g02430
VIT_15s0046g02440
VIT_15s0046g02450
VIT_15s0046g02460
VIT_15s0046g02470
VIT_15s0046g02480
VIT_15s0046g02490
Standardized expressions
Developmental stages
EI PG G1 G2 H T1 T2 C1 C2 S EP JP

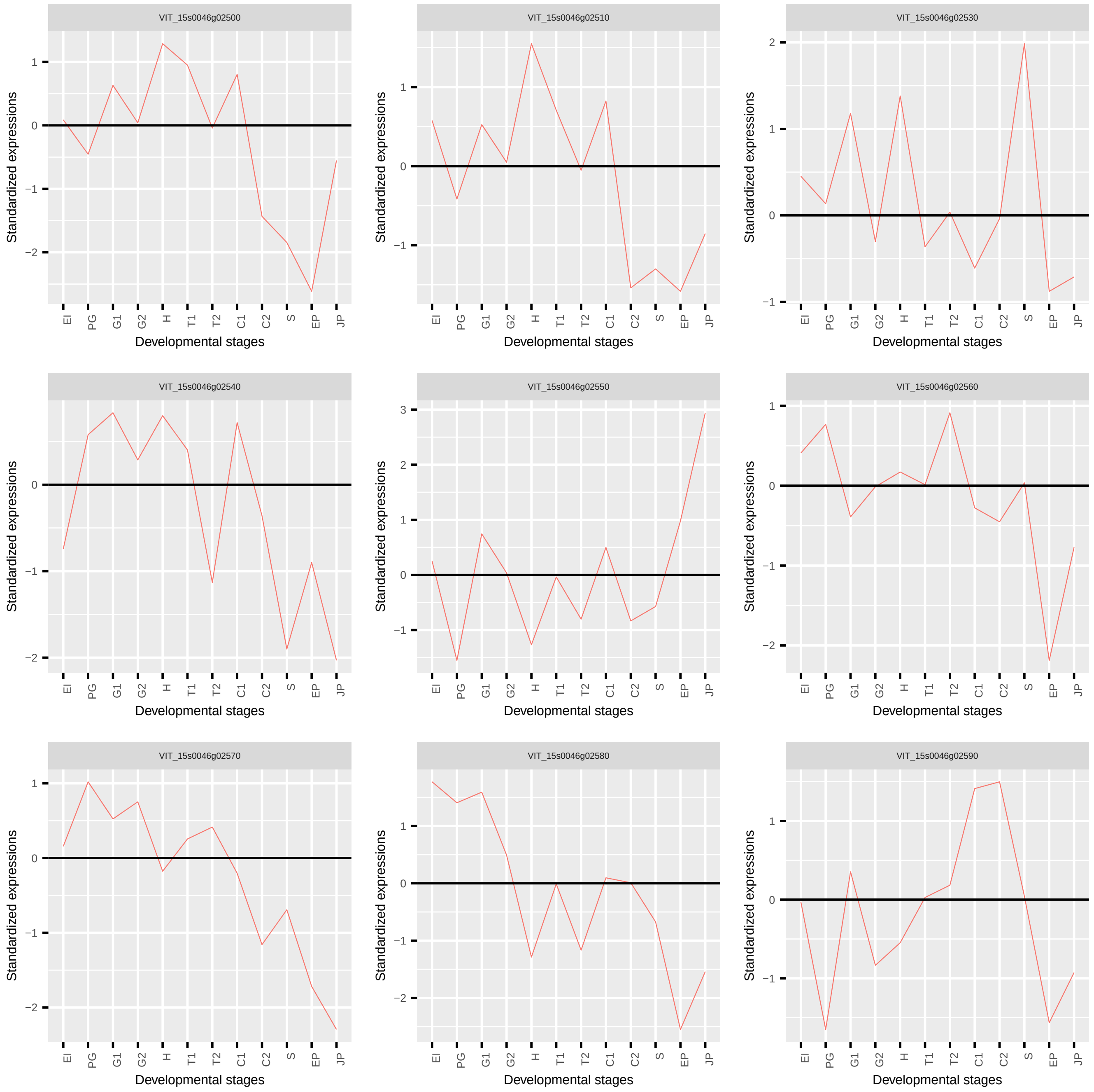

VIT_15s0046g02500
VIT_15s0046g02510
VIT_15s0046g02530
VIT_15s0046g02540
VIT_15s0046g02550
VIT_15s0046g02560
VIT_15s0046g02570
VIT_15s0046g02580
VIT_15s0046g02590
Standardized expressions
Developmental stages
EI
PG
G1
G2
H
T1
T2
C1
C2
S
EP
JP

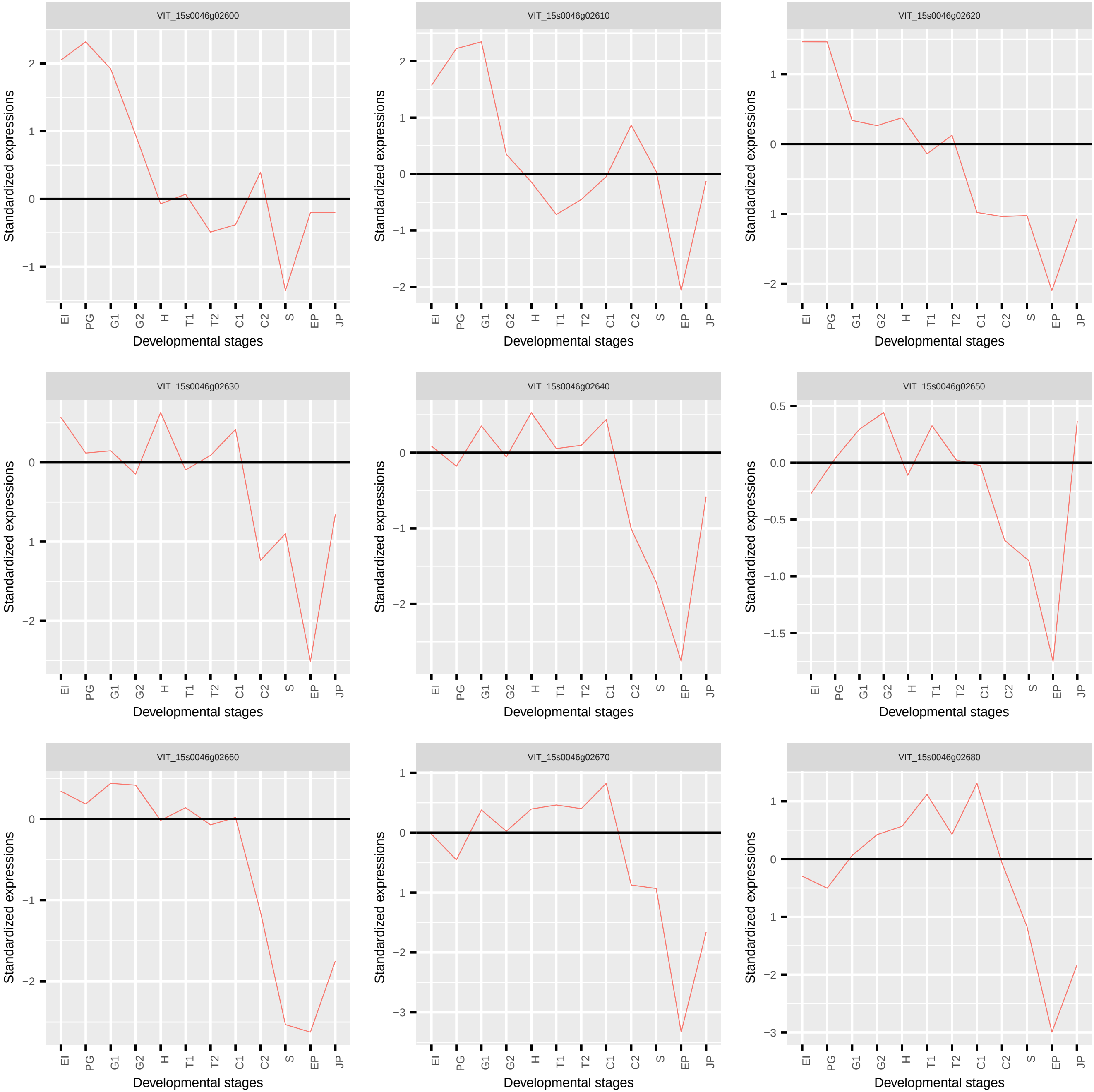
VIT_15s0046g02600
2
1
0
−1
Standardized expressions
EI PG G1 G2 H T1 T2 C1 C2 S EP JP
Developmental stages
VIT_15s0046g02610
2
1
0
−1
−2
Standardized expressions
EI PG G1 G2 H T1 T2 C1 C2 S EP JP
Developmental stages
VIT_15s0046g02620
1
0
−1
−2
Standardized expressions
EI PG G1 G2 H T1 T2 C1 C2 S EP JP
Developmental stages
VIT_15s0046g02630
0
−1
−2
Standardized expressions
EI PG G1 G2 H T1 T2 C1 C2 S EP JP
Developmental stages
VIT_15s0046g02640
0
−1
−2
Standardized expressions
EI PG G1 G2 H T1 T2 C1 C2 S EP JP
Developmental stages
VIT_15s0046g02650
0.5
0.0
−0.5
−1.0
−1.5
Standardized expressions
EI PG G1 G2 H T1 T2 C1 C2 S EP JP
Developmental stages
VIT_15s0046g02660
0
−1
−2
Standardized expressions
EI PG G1 G2 H T1 T2 C1 C2 S EP JP
Developmental stages
VIT_15s0046g02670
1
0
−1
−2
−3
Standardized expressions
EI PG G1 G2 H T1 T2 C1 C2 S EP JP
Developmental stages
VIT_15s0046g02680
1
0
−1
−2
−3
Standardized expressions
EI PG G1 G2 H T1 T2 C1 C2 S EP JP
Developmental stages

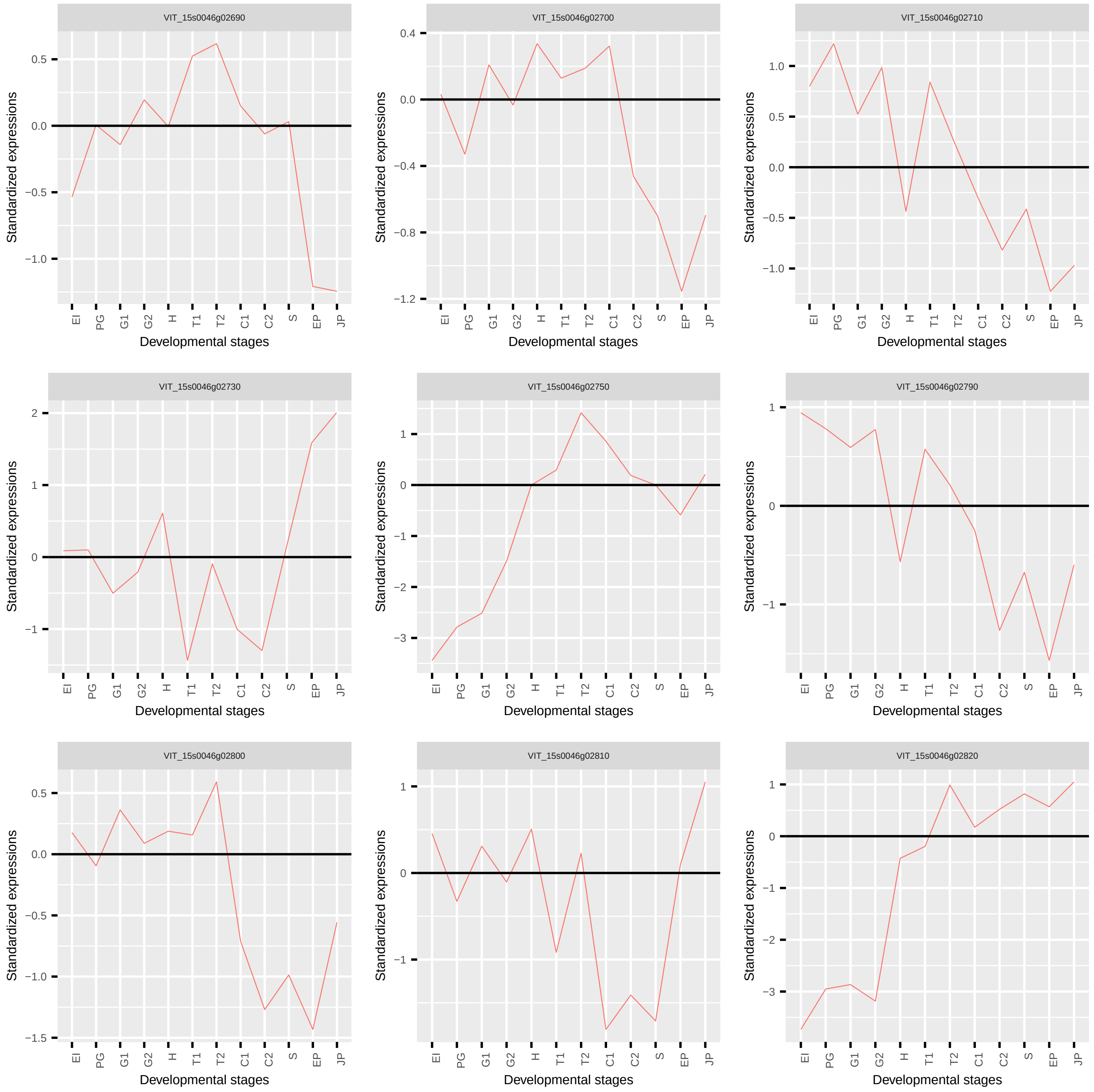

VIT_15s0046g02690
VIT_15s0046g02700
VIT_15s0046g02710
VIT_15s0046g02730
VIT_15s0046g02750
VIT_15s0046g02790
VIT_15s0046g02800
VIT_15s0046g02810
VIT_15s0046g02820
Standardized expressions
Developmental stages
EI
PG
G1
G2
H
T1
T2
C1
C2
S
EP
JP

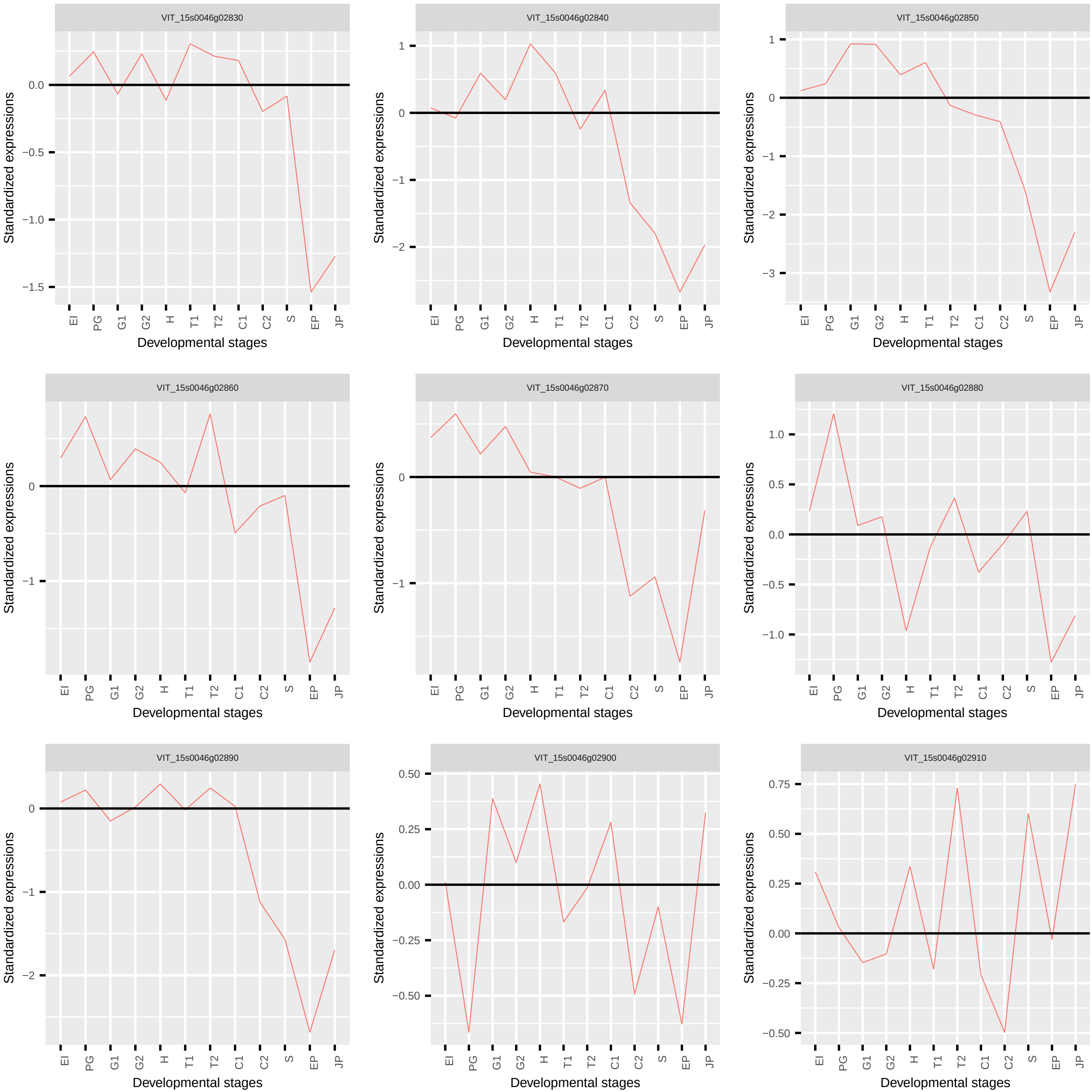

VIT_15s0046g02830
VIT_15s0046g02840
VIT_15s0046g02850
VIT_15s0046g02860
VIT_15s0046g02870
VIT_15s0046g02880
VIT_15s0046g02890
VIT_15s0046g02900
VIT_15s0046g02910
Standardized expressions
Developmental stages
EI PG G1 G2 H T1 T2 C1 C2 S EP JP
0.0 −0.5 −1.0 −1.5
1 0 −1 −2 −3
0.5 0.0 −0.5 −1.0
0.50 0.25 0.00 −0.25 −0.50
0.75 0.50 0.25 0.00 −0.25 −0.50

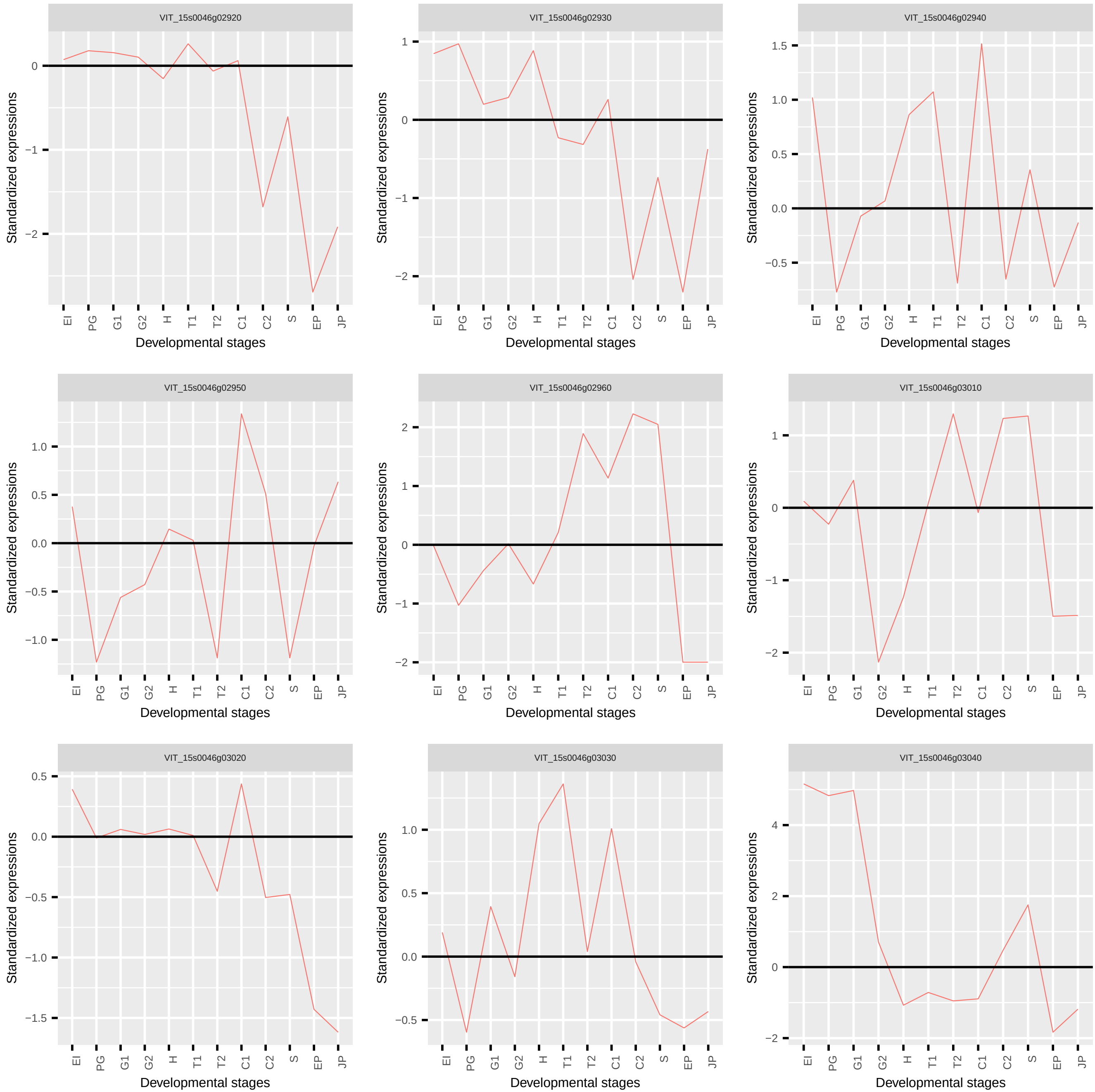

VIT_15s0046g02920
VIT_15s0046g02930
VIT_15s0046g02940
VIT_15s0046g02950
VIT_15s0046g02960
VIT_15s0046g03010
VIT_15s0046g03020
VIT_15s0046g03030
VIT_15s0046g03040
Standardized expressions
Developmental stages
EI
PG
G1
G2
H
T1
T2
C1
C2
S
EP
JP

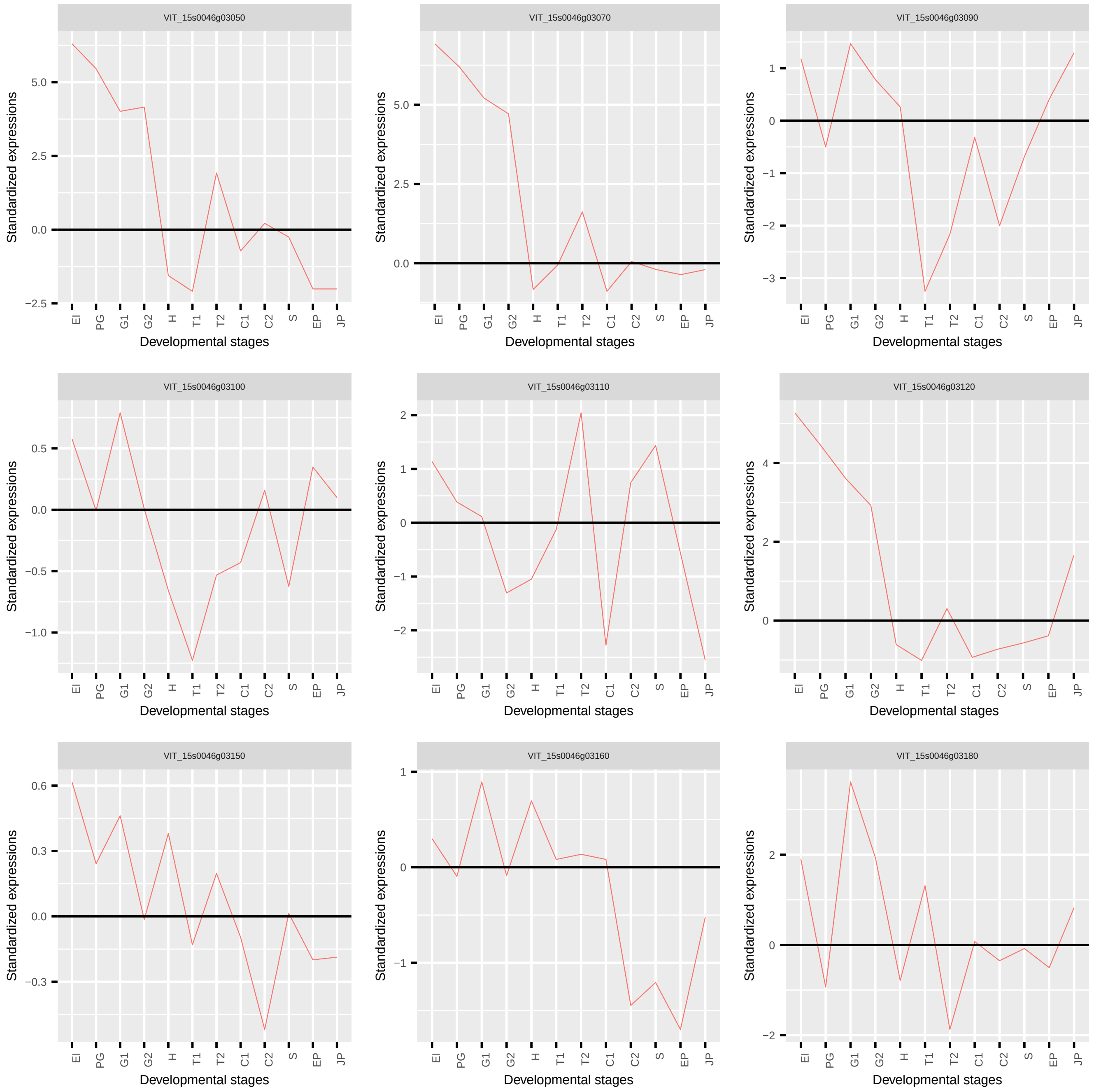
VIT_15s0046g03050
VIT_15s0046g03070
VIT_15s0046g03090
VIT_15s0046g03100
VIT_15s0046g03110
VIT_15s0046g03120
VIT_15s0046g03150
VIT_15s0046g03160
VIT_15s0046g03180
Standardized expressions
Developmental stages
EI PG G1 G2 H T1 T2 C1 C2 S EP JP

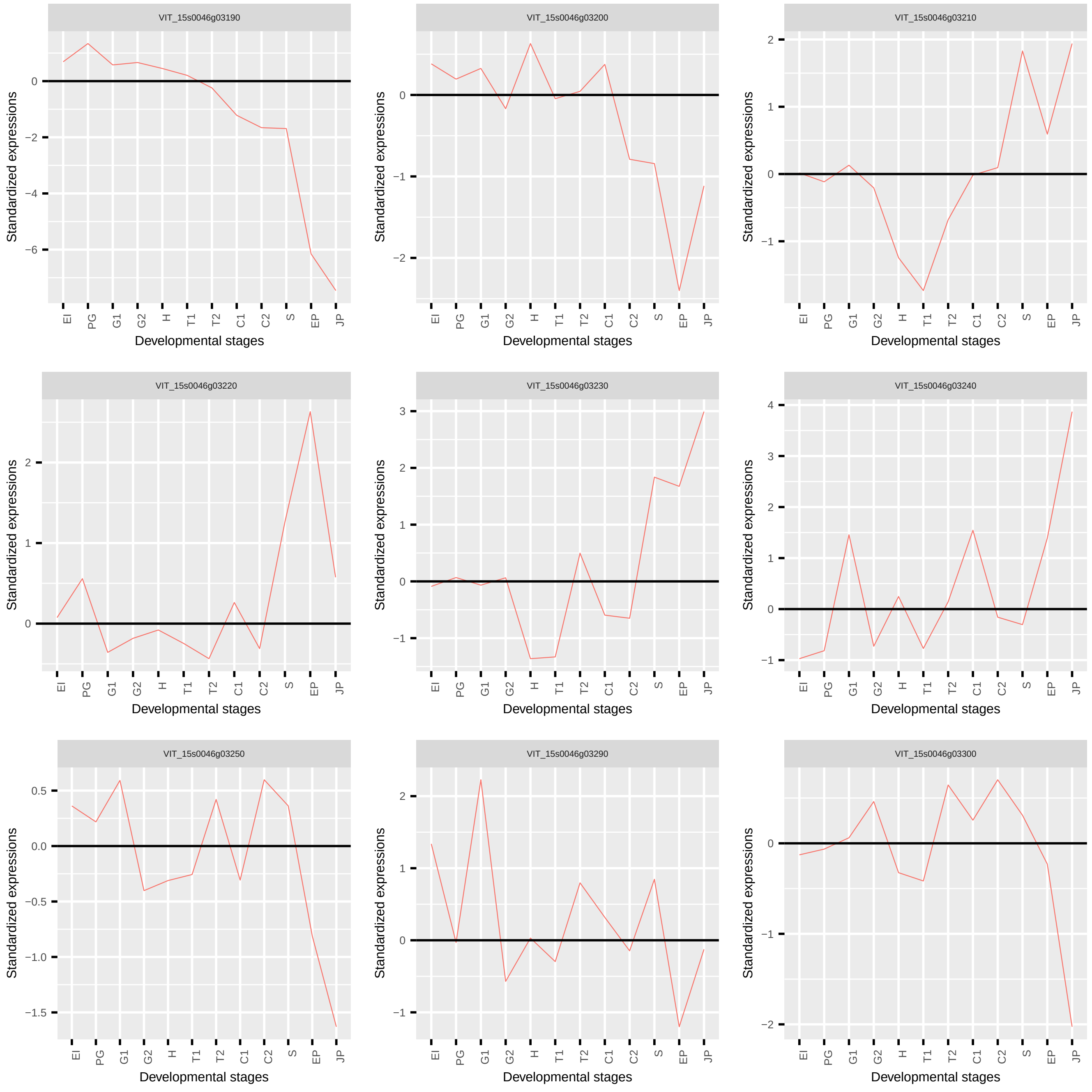
VIT_15s0046g03190
VIT_15s0046g03200
VIT_15s0046g03210
VIT_15s0046g03220
VIT_15s0046g03230
VIT_15s0046g03240
VIT_15s0046g03250
VIT_15s0046g03290
VIT_15s0046g03300
Standardized expressions
Developmental stages
EI
PG
G1
G2
H
T1
T2
C1
C2
S
EP
JP

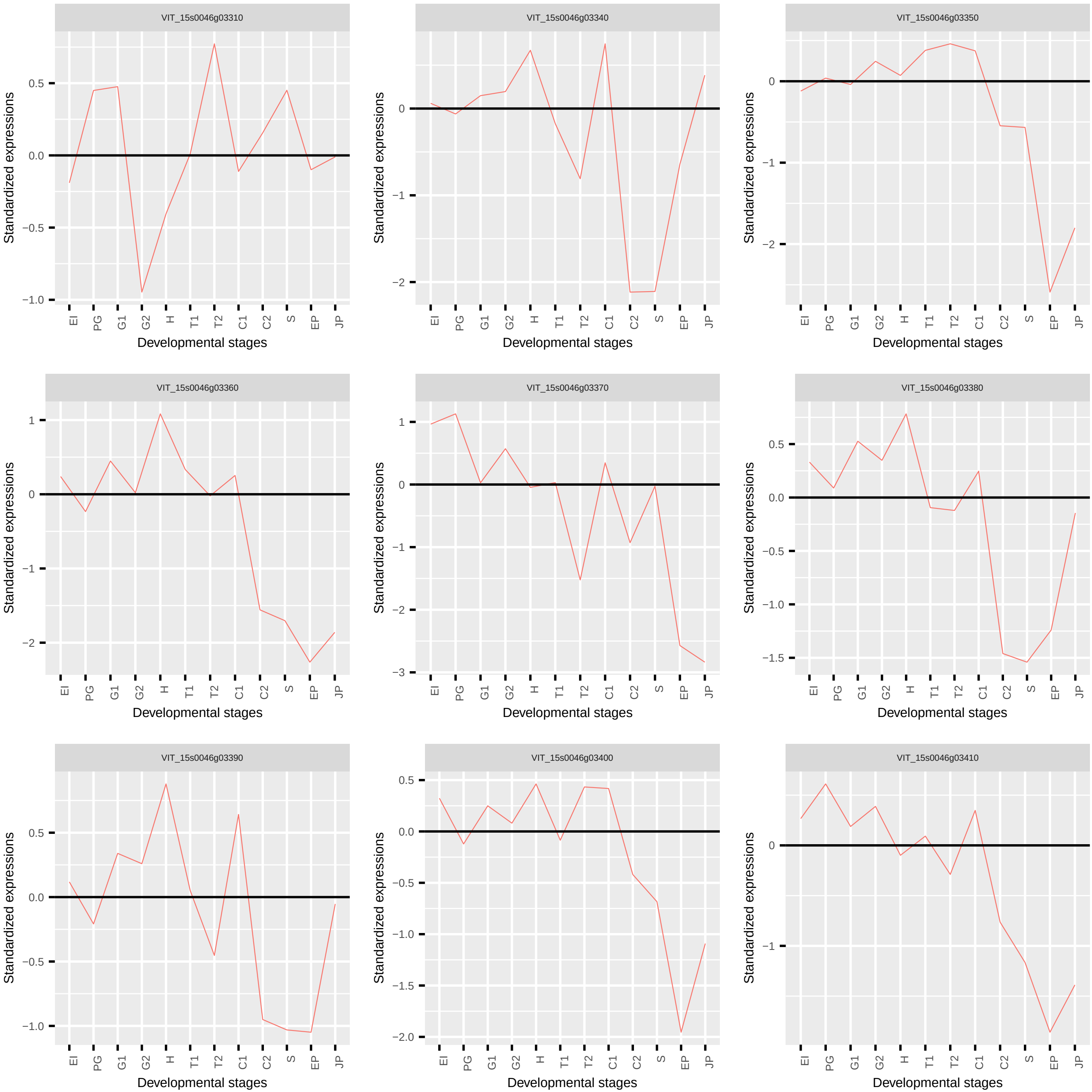
VIT_15s0046g03310
0.5
0.0
−0.5
−1.0
VIT_15s0046g03340
0
−1
−2
VIT_15s0046g03350
0
−1
−2
VIT_15s0046g03360
1
0
−1
−2
VIT_15s0046g03370
1
0
−1
−2
−3
VIT_15s0046g03380
0.5
0.0
−0.5
−1.0
−1.5
VIT_15s0046g03390
0.5
0.0
−0.5
−1.0
VIT_15s0046g03400
0.5
0.0
−0.5
−1.0
−1.5
−2.0
VIT_15s0046g03410
0
−1
Standardized expressions
EI PG G1 G2 H T1 T2 C1 C2 S EP JP
Developmental stages

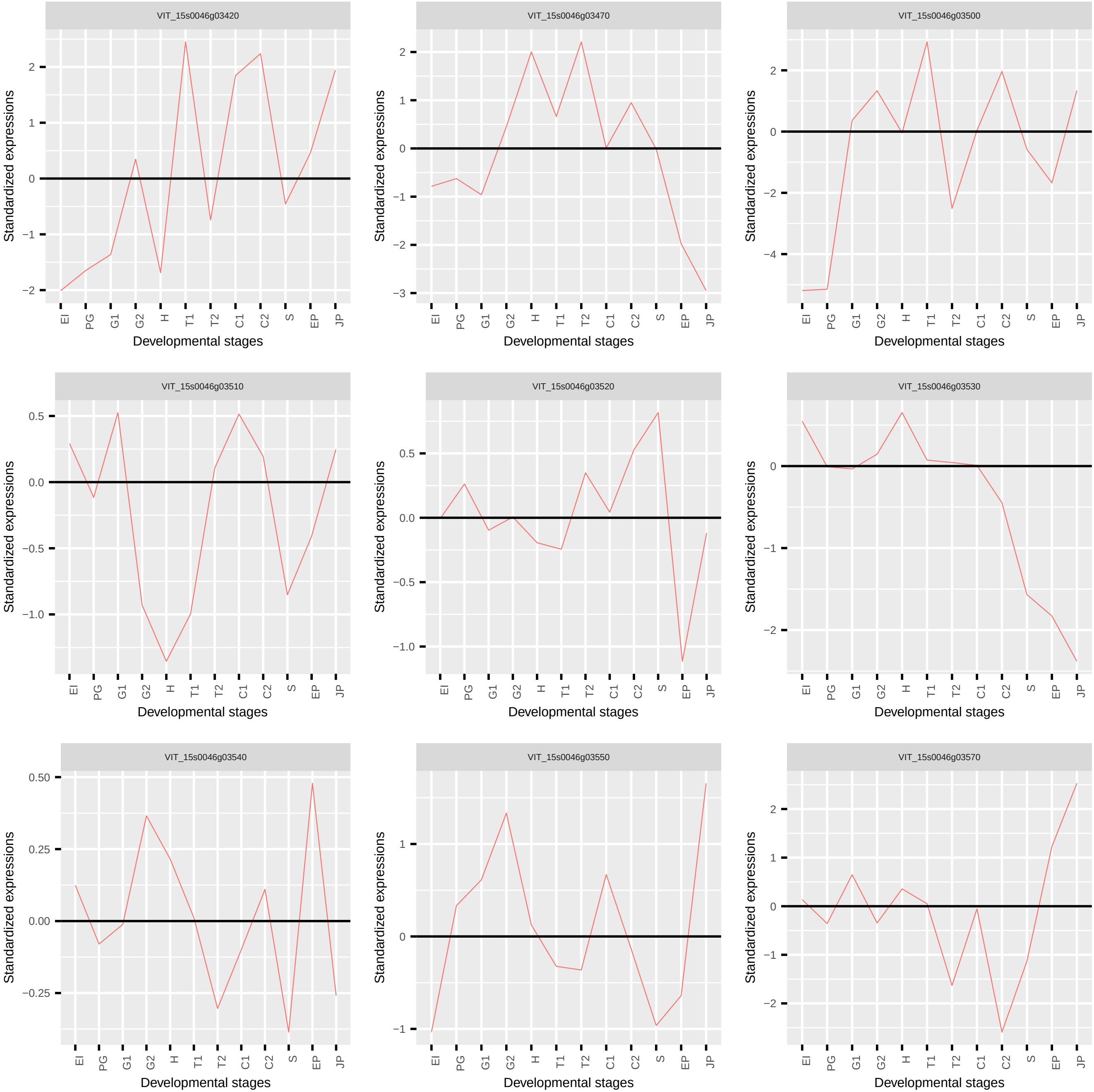
VIT_15s0046g03420
VIT_15s0046g03470
VIT_15s0046g03500
VIT_15s0046g03510
VIT_15s0046g03520
VIT_15s0046g03530
VIT_15s0046g03540
VIT_15s0046g03550
VIT_15s0046g03570
Standardized expressions
Developmental stages
EI PG G1 G2 H T1 T2 C1 C2 S EP JP

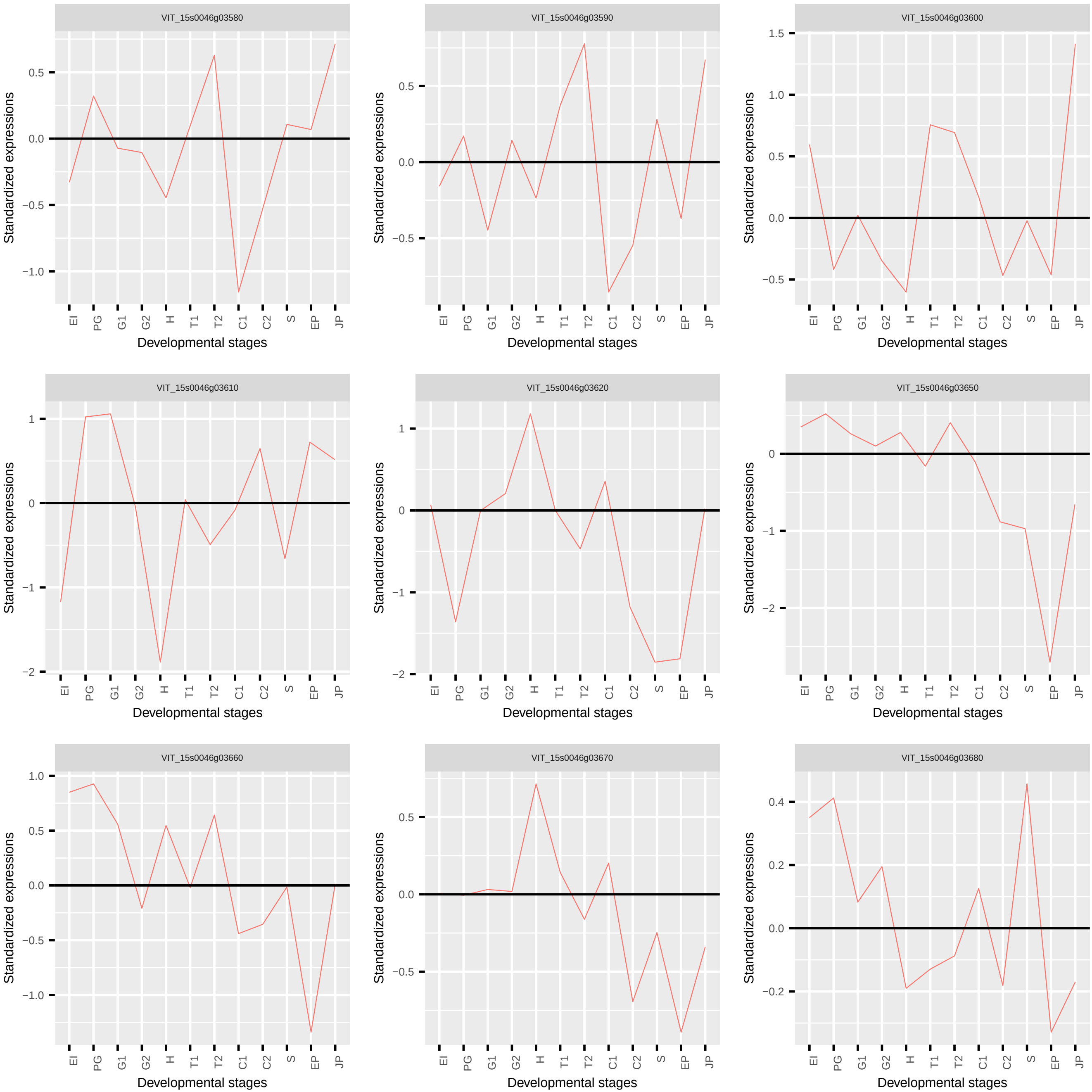
VIT_15s0046g03580
VIT_15s0046g03590
VIT_15s0046g03600
VIT_15s0046g03610
VIT_15s0046g03620
VIT_15s0046g03650
VIT_15s0046g03660
VIT_15s0046g03670
VIT_15s0046g03680
Standardized expressions
Developmental stages
EI PG G1 G2 H T1 T2 C1 C2 S EP JP

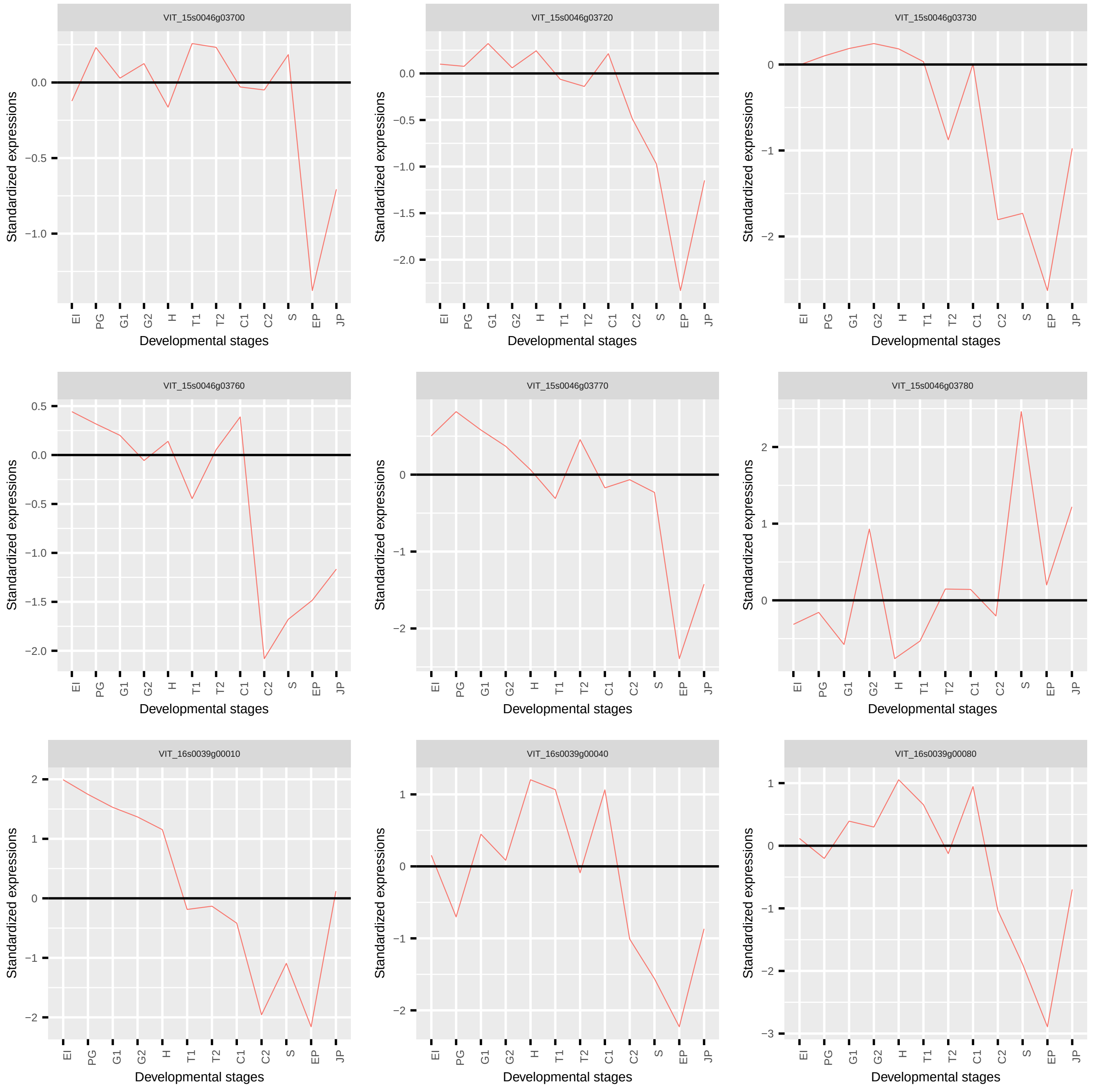
VIT_15s0046g03700
VIT_15s0046g03720
VIT_15s0046g03730
VIT_15s0046g03760
VIT_15s0046g03770
VIT_15s0046g03780
VIT_16s0039g00010
VIT_16s0039g00040
VIT_16s0039g00080
Standardized expressions
Developmental stages
EI PG G1 G2 H T1 T2 C1 C2 S EP JP
0.0
−0.5
−1.0
−1.5
−2.0
0.5
2
1
0
−1
−2
−3

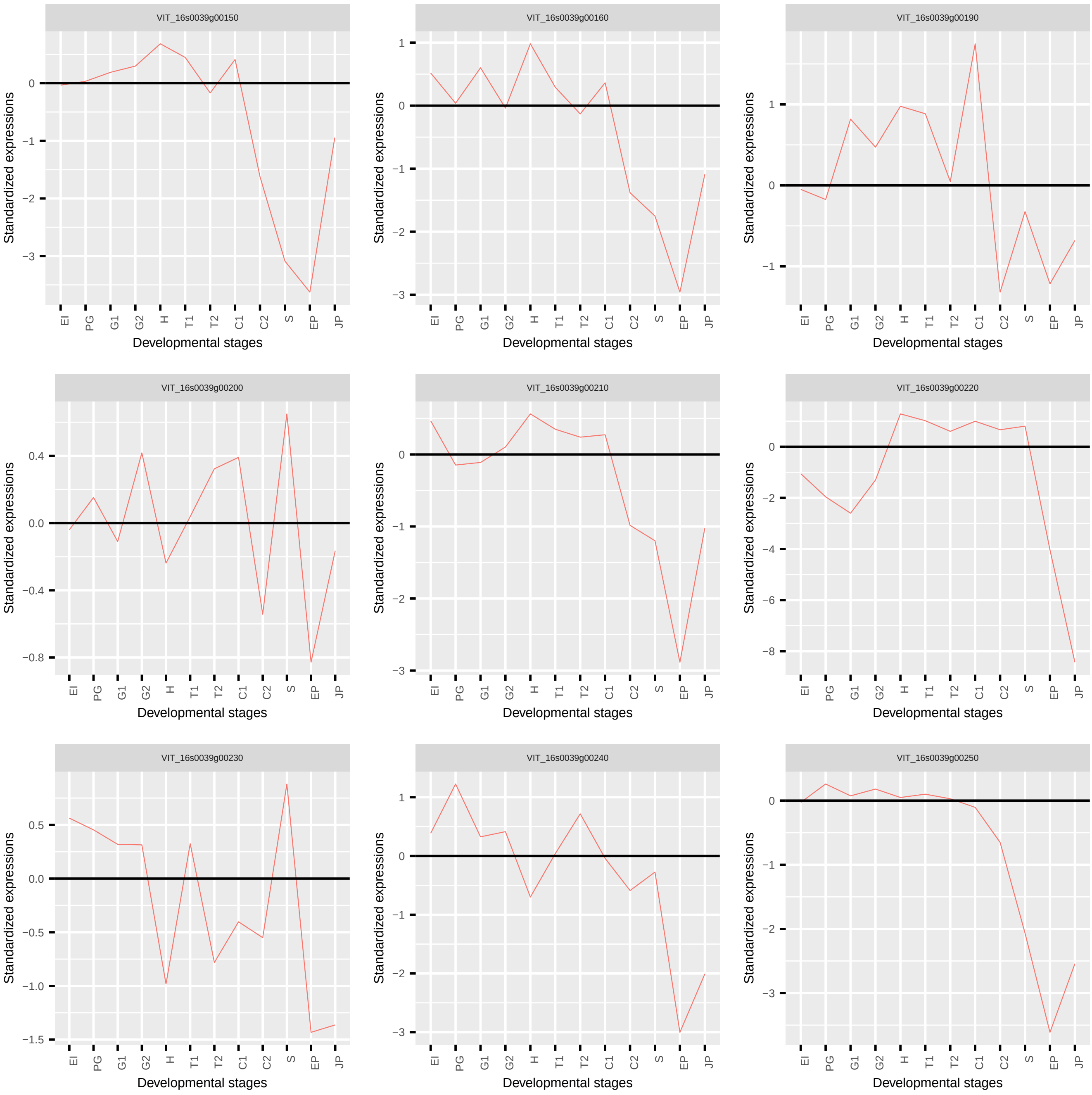
VIT_16s0039g00150
VIT_16s0039g00160
VIT_16s0039g00190
VIT_16s0039g00200
VIT_16s0039g00210
VIT_16s0039g00220
VIT_16s0039g00230
VIT_16s0039g00240
VIT_16s0039g00250
Standardized expressions
Developmental stages
EI PG G1 G2 H T1 T2 C1 C2 S EP JP

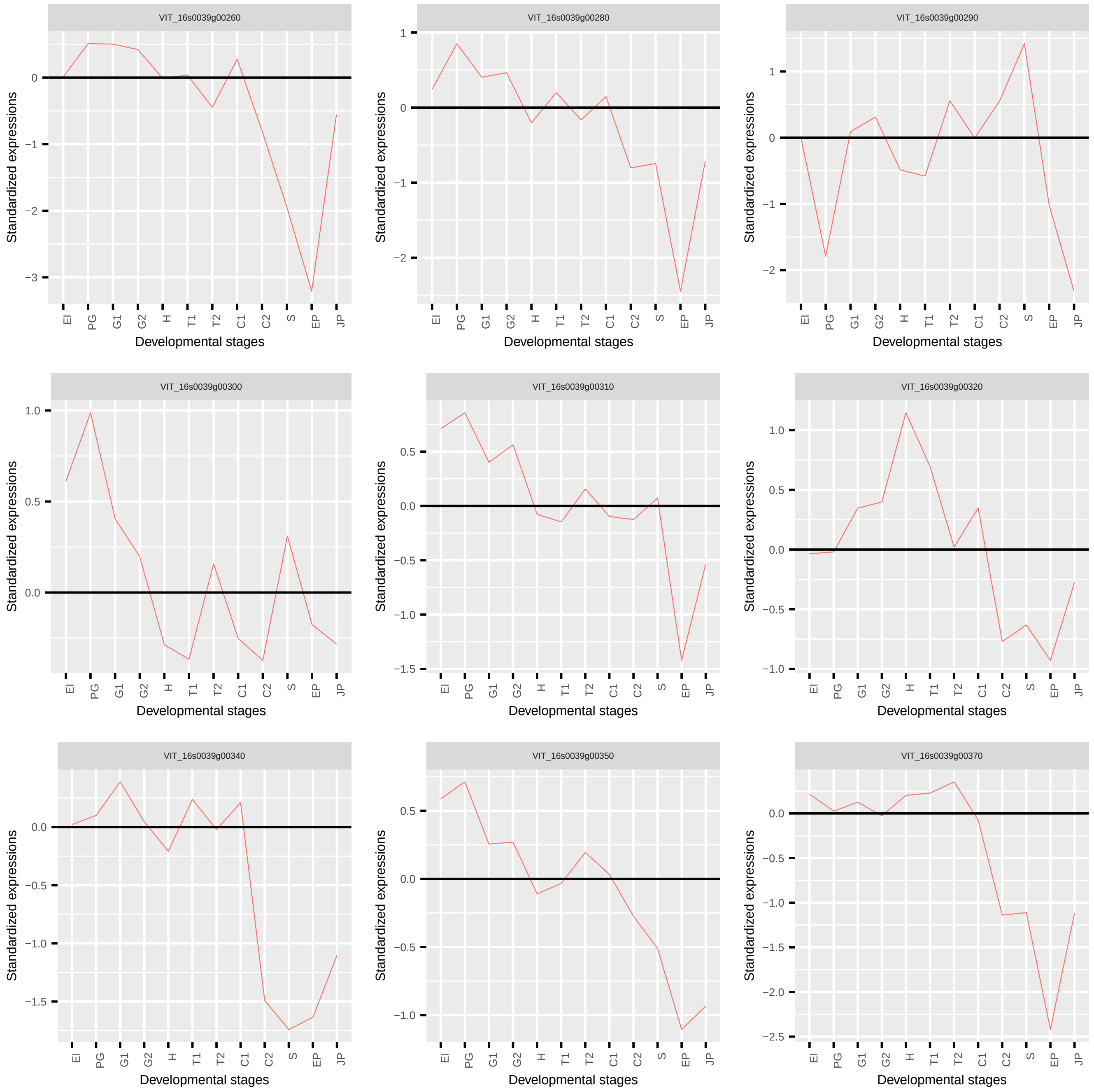
VIT_16s0039g00260
VIT_16s0039g00280
VIT_16s0039g00290
VIT_16s0039g00300
VIT_16s0039g00310
VIT_16s0039g00320
VIT_16s0039g00340
VIT_16s0039g00350
VIT_16s0039g00370
Standardized expressions
Developmental stages
EI
PG
G1
G2
H
T1
T2
C1
C2
S
EP
JP

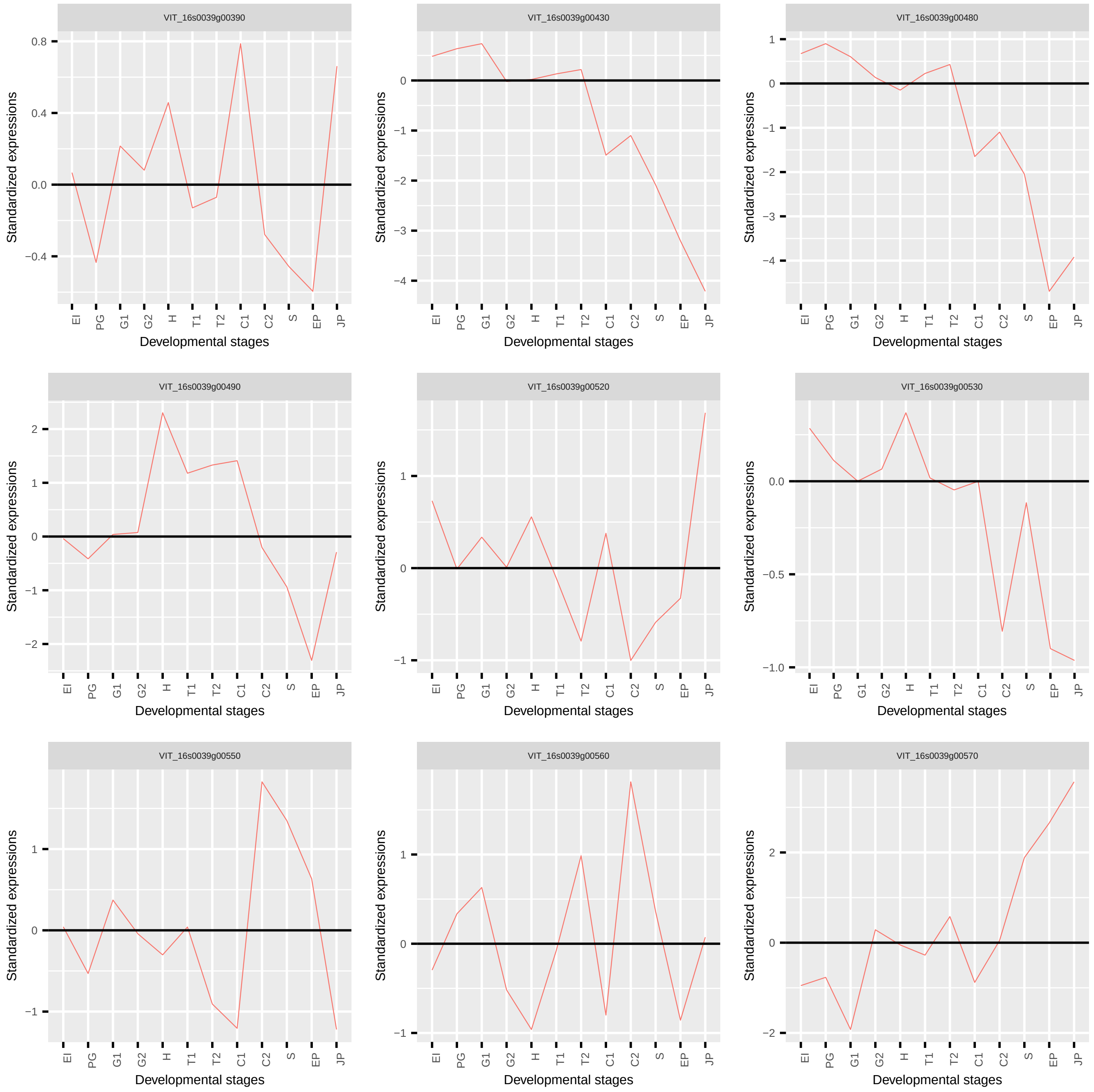

VIT_16s0039g00390
VIT_16s0039g00430
VIT_16s0039g00480
VIT_16s0039g00490
VIT_16s0039g00520
VIT_16s0039g00530
VIT_16s0039g00550
VIT_16s0039g00560
VIT_16s0039g00570
Standardized expressions
Developmental stages
EI
PG
G1
G2
H
T1
T2
C1
C2
S
EP
JP

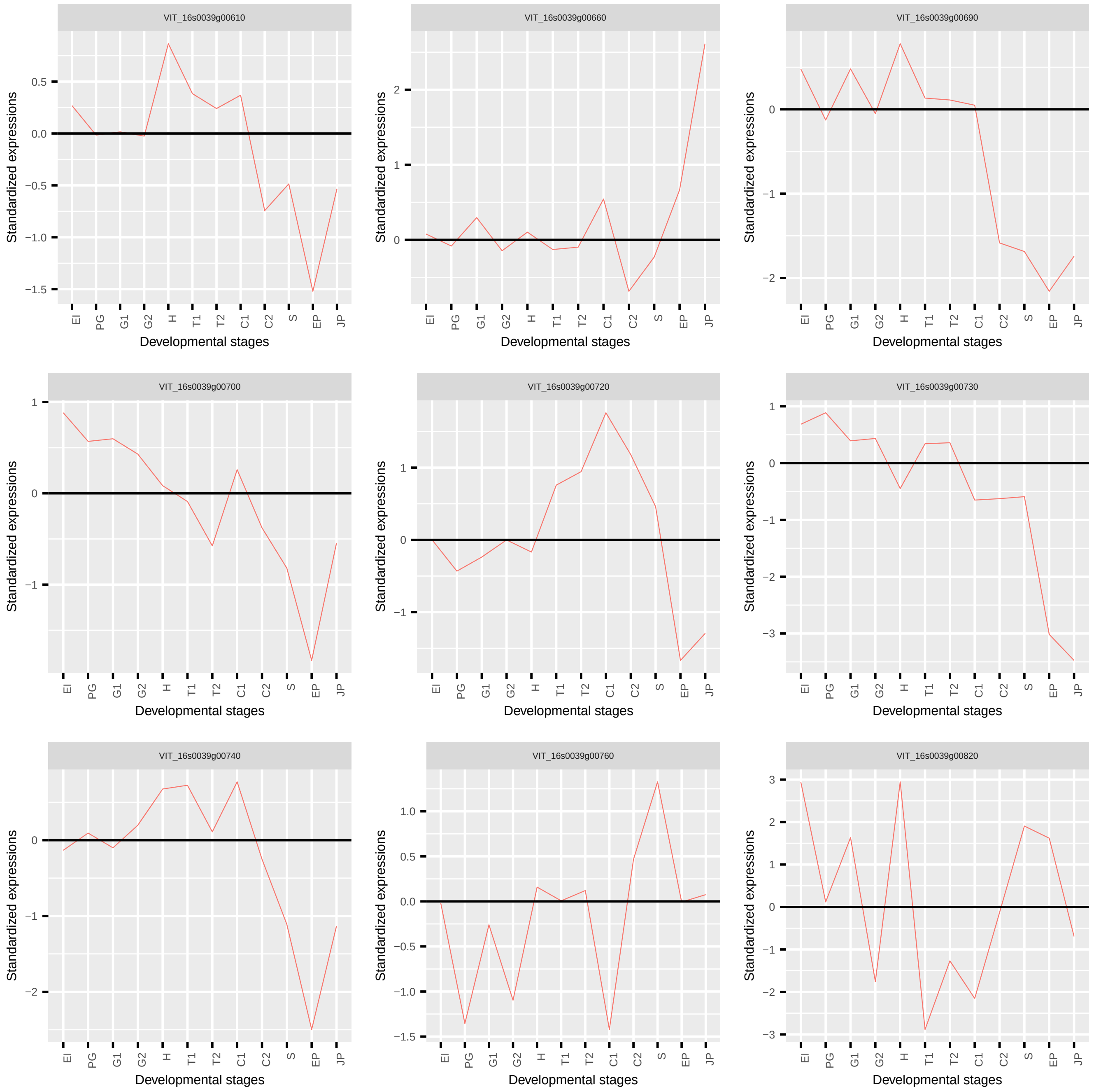
VIT_16s0039g00610
VIT_16s0039g00660
VIT_16s0039g00690
VIT_16s0039g00700
VIT_16s0039g00720
VIT_16s0039g00730
VIT_16s0039g00740
VIT_16s0039g00760
VIT_16s0039g00820
Standardized expressions
Developmental stages
EI PG G1 G2 H T1 T2 C1 C2 S EP JP

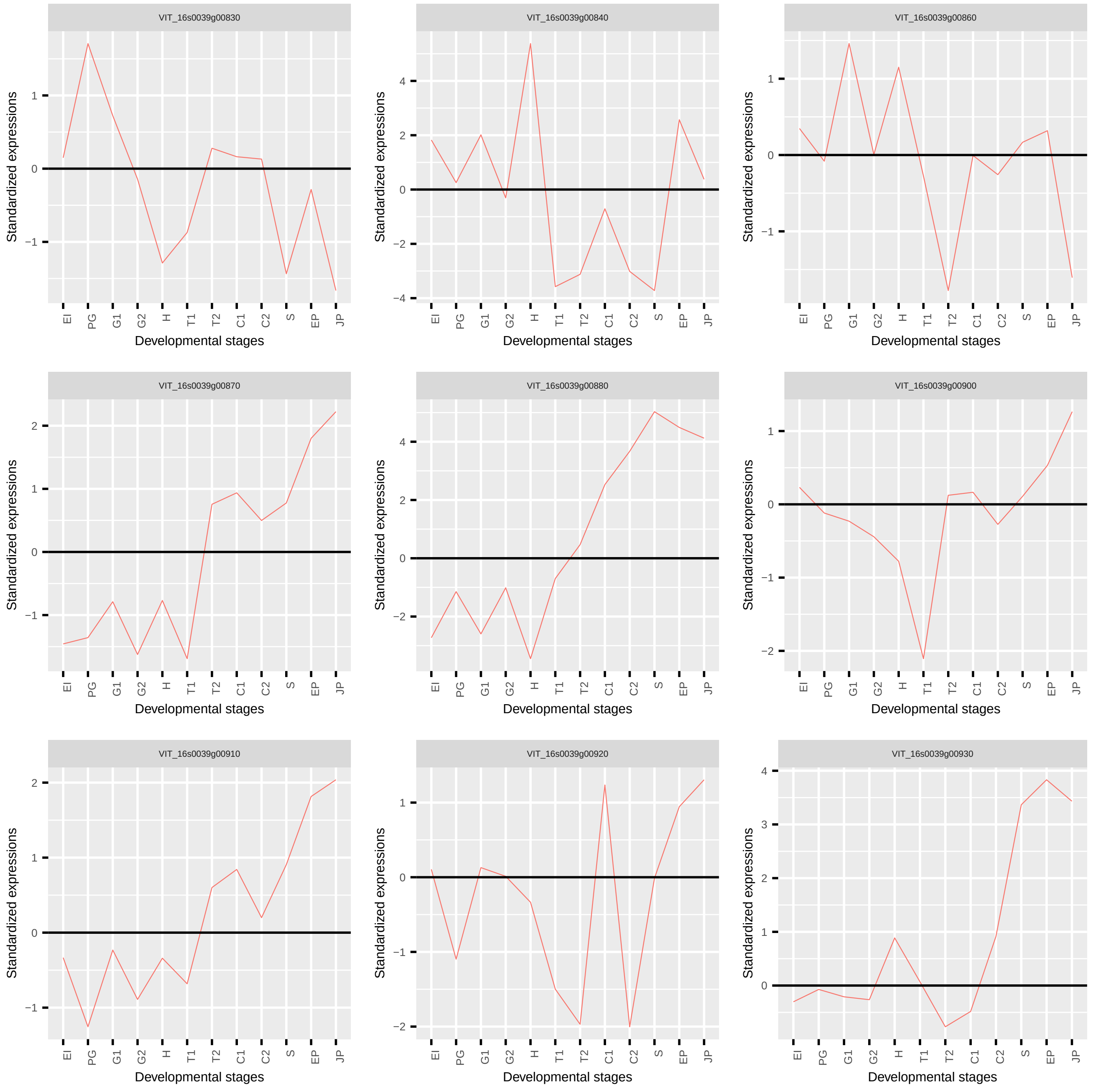
VIT_16s0039g00830
VIT_16s0039g00840
VIT_16s0039g00860
VIT_16s0039g00870
VIT_16s0039g00880
VIT_16s0039g00900
VIT_16s0039g00910
VIT_16s0039g00920
VIT_16s0039g00930
Standardized expressions
Developmental stages
EI
PG
G1
G2
H
T1
T2
C1
C2
S
EP
JP

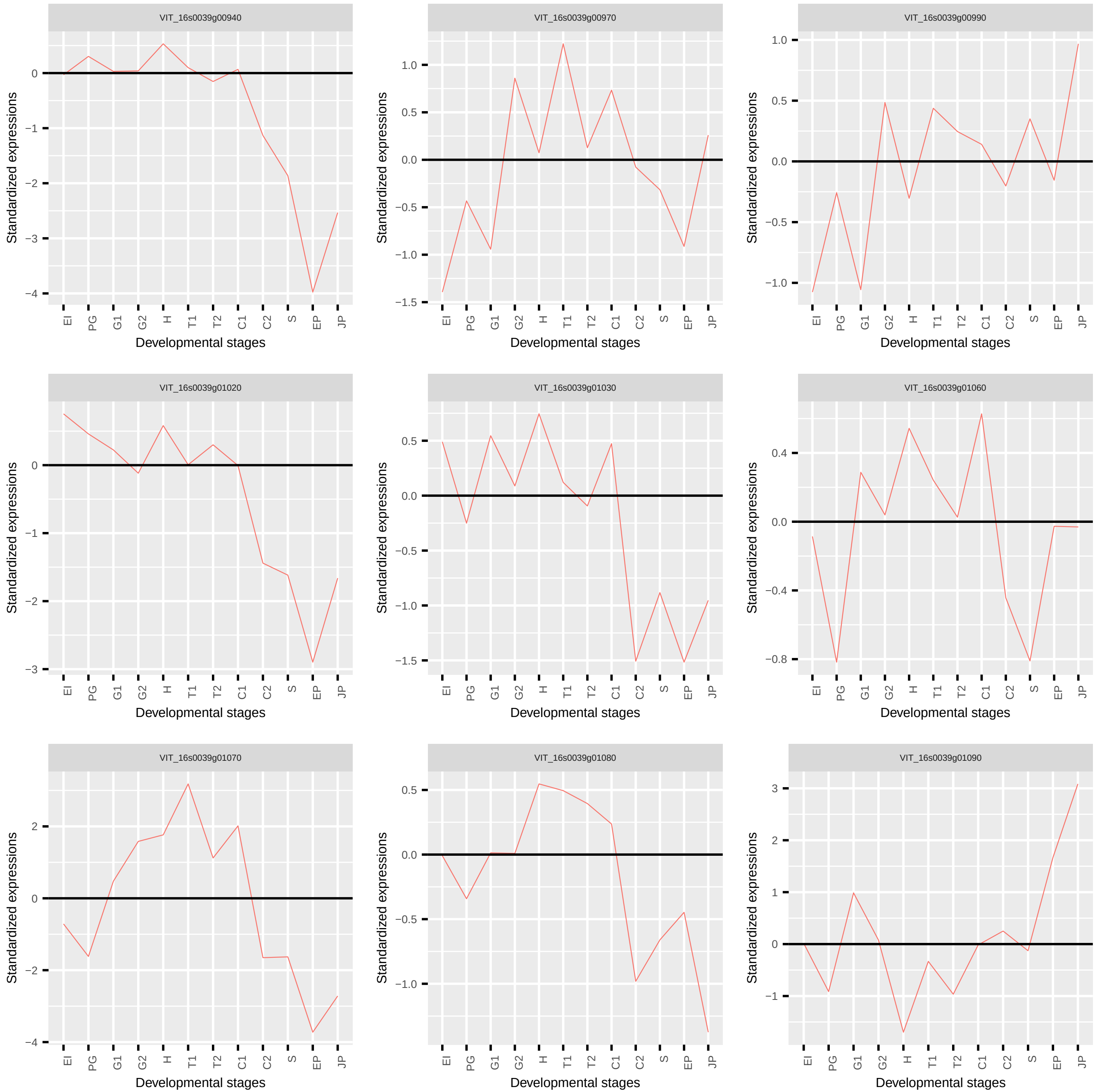
VIT_16s0039g00940
VIT_16s0039g00970
VIT_16s0039g00990
VIT_16s0039g01020
VIT_16s0039g01030
VIT_16s0039g01060
VIT_16s0039g01070
VIT_16s0039g01080
VIT_16s0039g01090
Standardized expressions
Developmental stages
EI PG G1 G2 H T1 T2 C1 C2 S EP JP

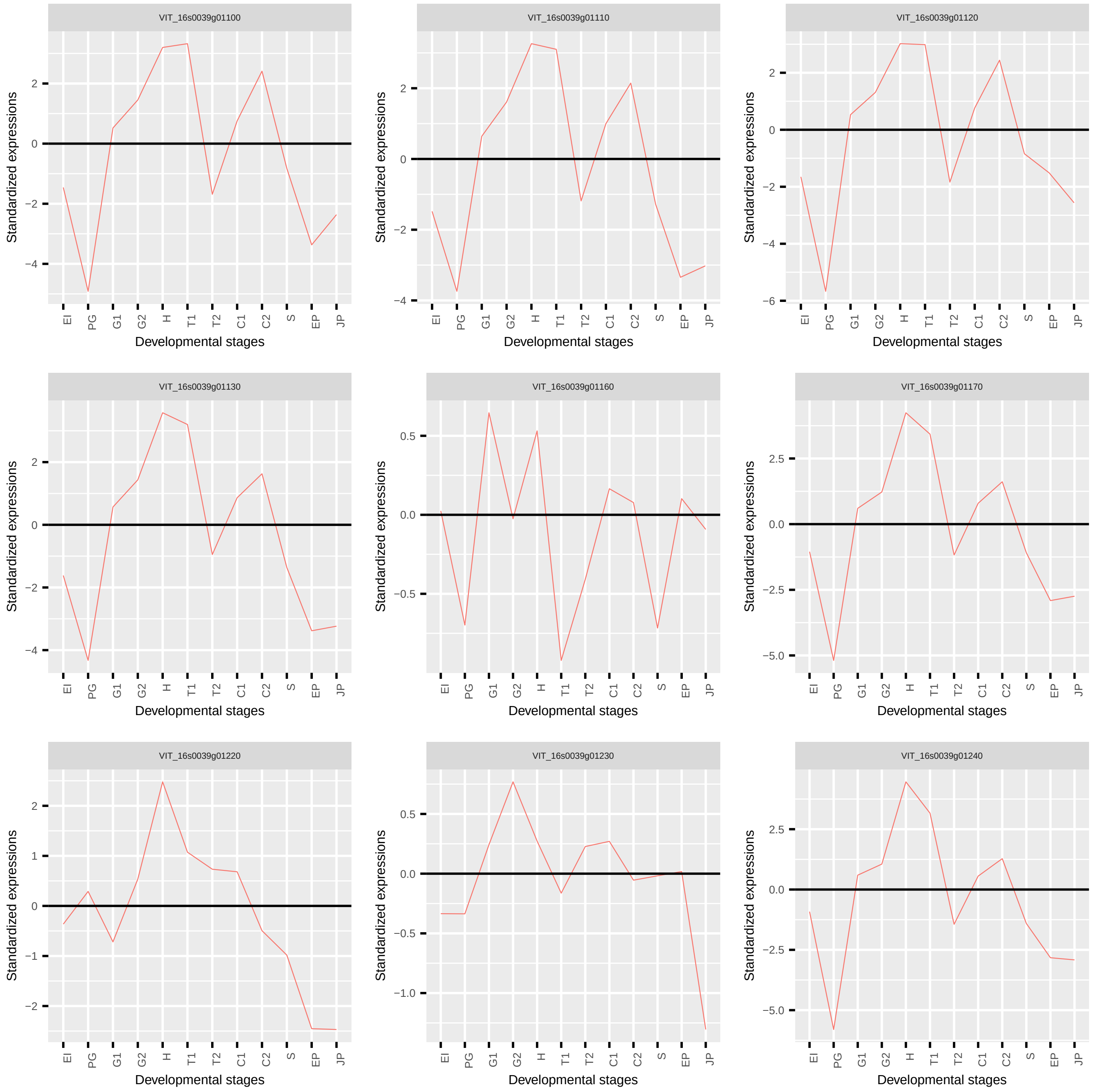
VIT_16s0039g01100
VIT_16s0039g01110
VIT_16s0039g01120
VIT_16s0039g01130
VIT_16s0039g01160
VIT_16s0039g01170
VIT_16s0039g01220
VIT_16s0039g01230
VIT_16s0039g01240
Standardized expressions
Developmental stages
EI
PG
G1
G2
H
T1
T2
C1
C2
S
EP
JP

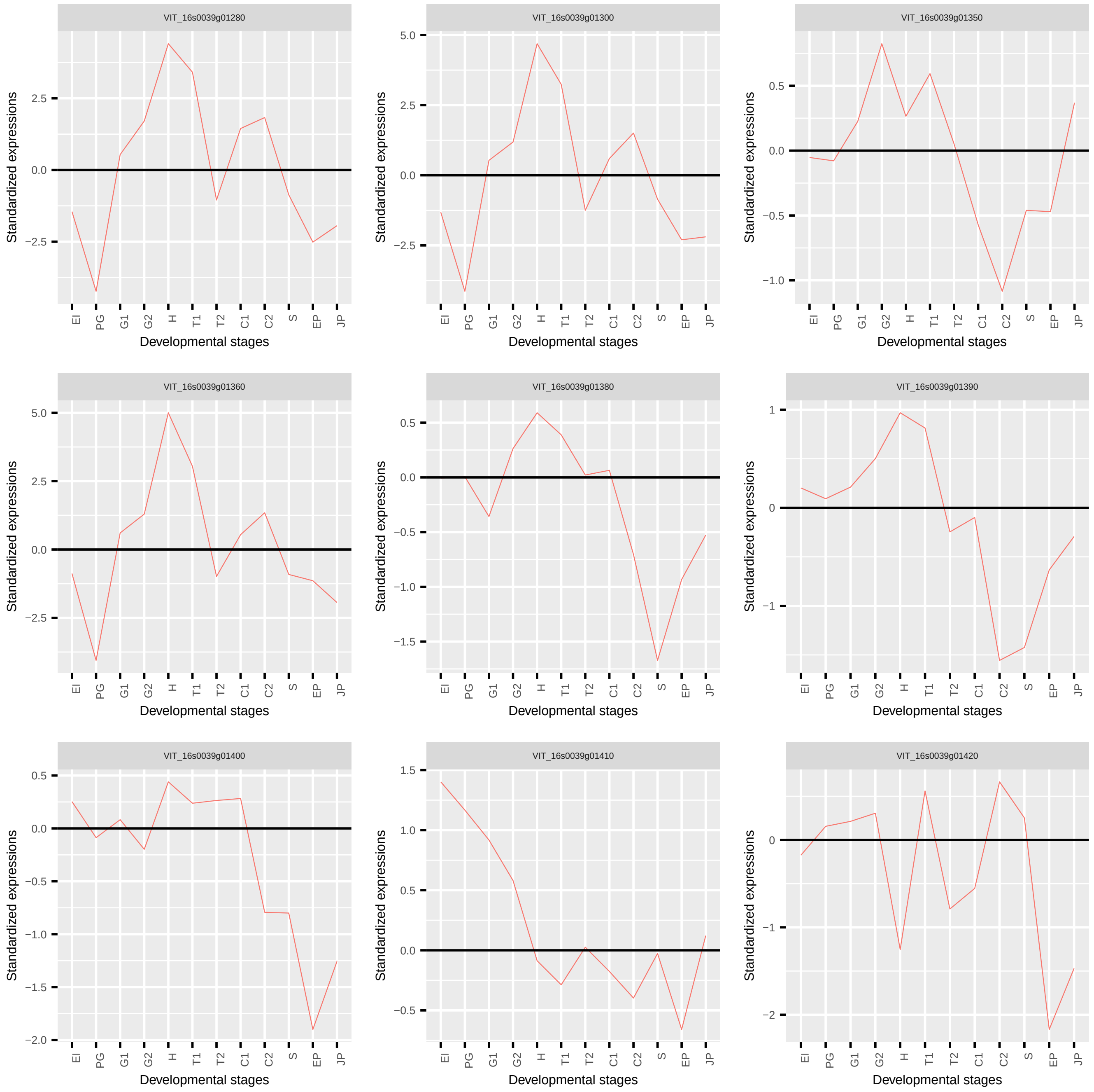

VIT_16s0039g01280
VIT_16s0039g01300
VIT_16s0039g01350
VIT_16s0039g01360
VIT_16s0039g01380
VIT_16s0039g01390
VIT_16s0039g01400
VIT_16s0039g01410
VIT_16s0039g01420
Standardized expressions
Developmental stages
EI
PG
G1
G2
H
T1
T2
C1
C2
S
EP
JP

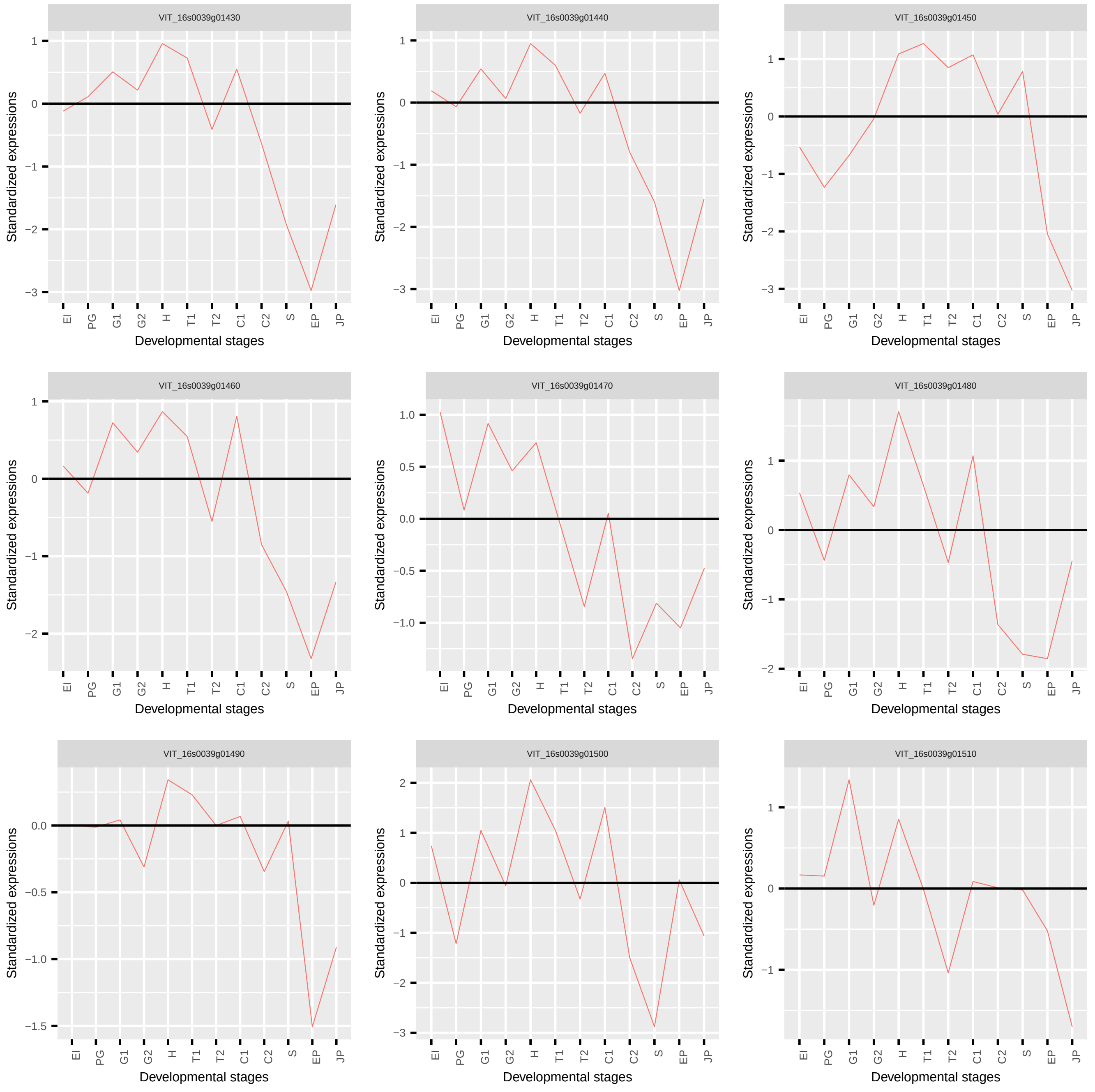
VIT_16s0039g01430
VIT_16s0039g01440
VIT_16s0039g01450
VIT_16s0039g01460
VIT_16s0039g01470
VIT_16s0039g01480
VIT_16s0039g01490
VIT_16s0039g01500
VIT_16s0039g01510
Standardized expressions
Developmental stages
EI PG G1 G2 H T1 T2 C1 C2 S EP JP

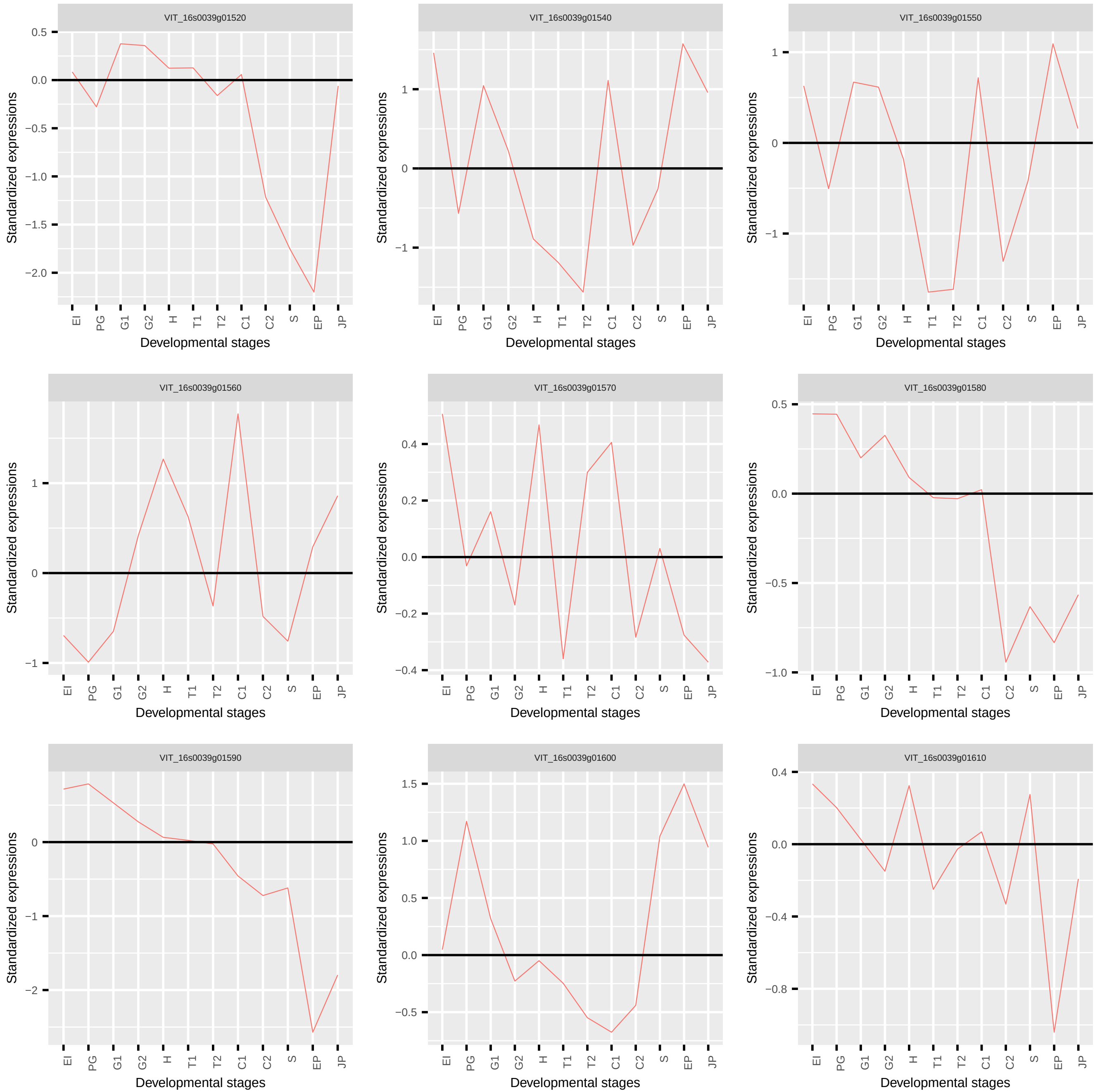
VIT_16s0039g01520
VIT_16s0039g01540
VIT_16s0039g01550
VIT_16s0039g01560
VIT_16s0039g01570
VIT_16s0039g01580
VIT_16s0039g01590
VIT_16s0039g01600
VIT_16s0039g01610
Standardized expressions
Developmental stages
EI PG G1 G2 H T1 T2 C1 C2 S EP JP

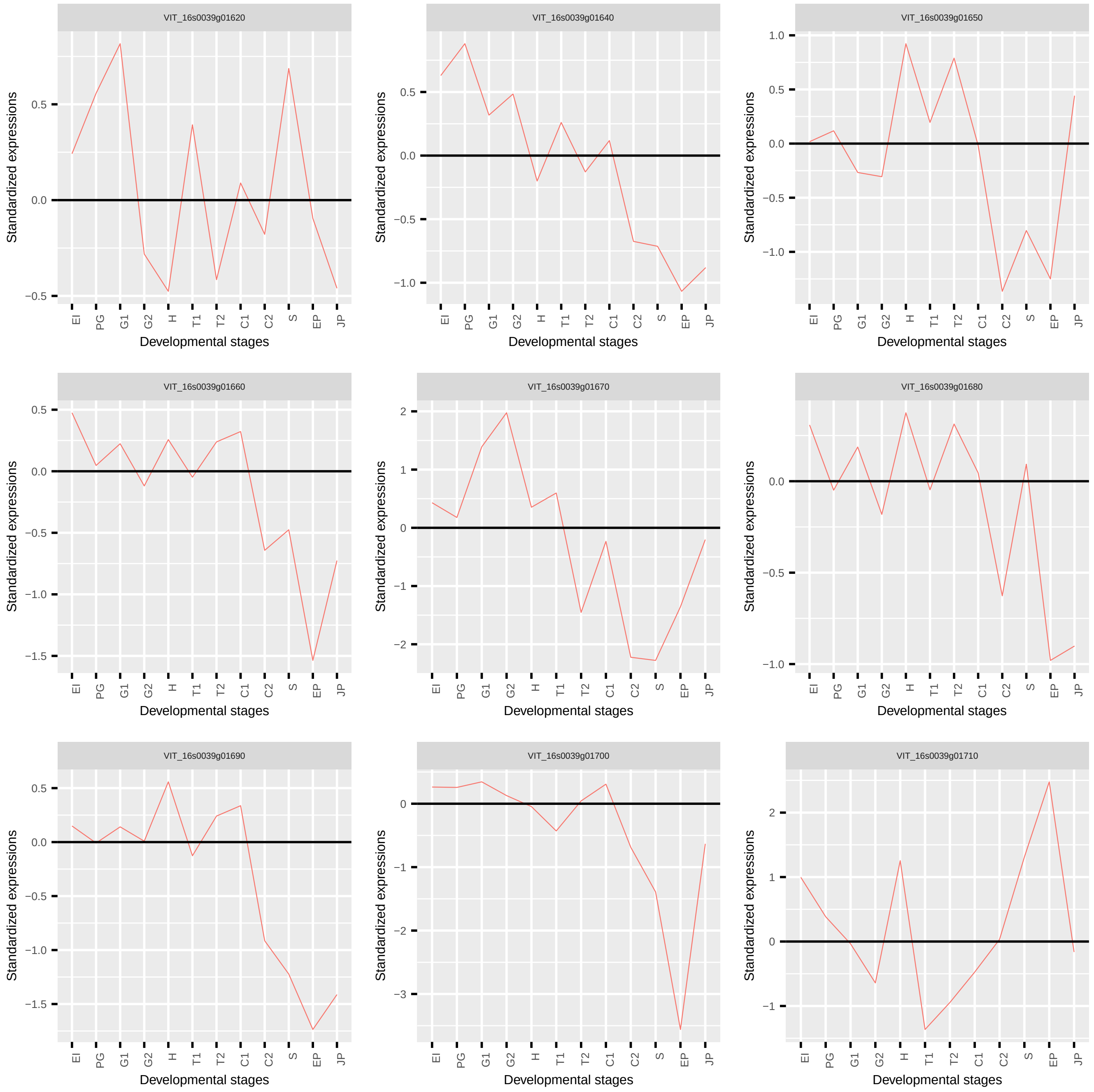
VIT_16s0039g01620
VIT_16s0039g01640
VIT_16s0039g01650
VIT_16s0039g01660
VIT_16s0039g01670
VIT_16s0039g01680
VIT_16s0039g01690
VIT_16s0039g01700
VIT_16s0039g01710
Standardized expressions
Developmental stages
EI PG G1 G2 H T1 T2 C1 C2 S EP JP

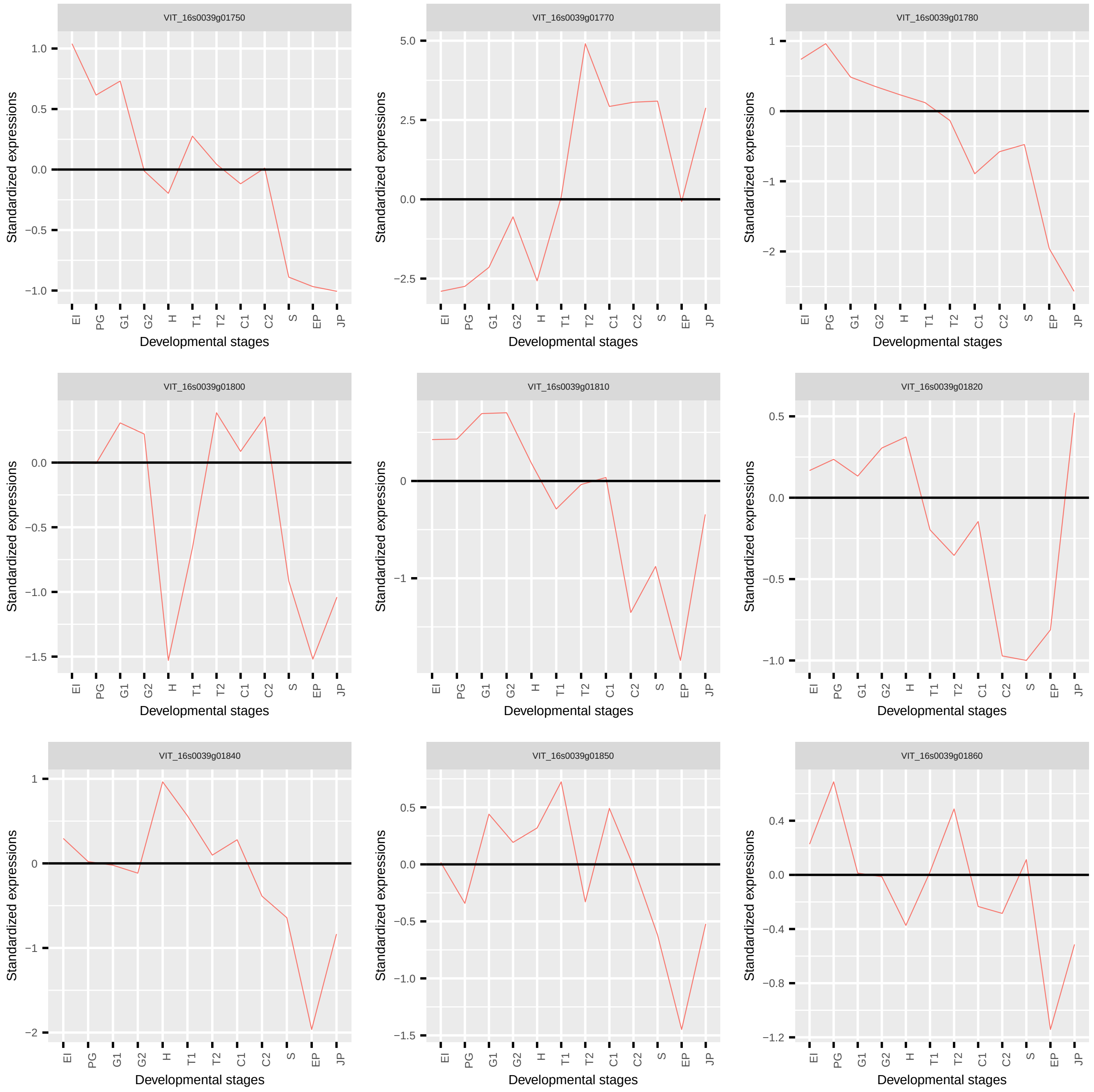
VIT_16s0039g01750
Standardized expressions
1.0
0.5
0.0
−0.5
−1.0
EI PG G1 G2 H T1 T2 C1 C2 S EP JP
Developmental stages
VIT_16s0039g01770
Standardized expressions
5.0
2.5
0.0
−2.5
EI PG G1 G2 H T1 T2 C1 C2 S EP JP
Developmental stages
VIT_16s0039g01780
Standardized expressions
1
0
−1
−2
EI PG G1 G2 H T1 T2 C1 C2 S EP JP
Developmental stages
VIT_16s0039g01800
Standardized expressions
0.0
−0.5
−1.0
−1.5
EI PG G1 G2 H T1 T2 C1 C2 S EP JP
Developmental stages
VIT_16s0039g01810
Standardized expressions
0
−1
EI PG G1 G2 H T1 T2 C1 C2 S EP JP
Developmental stages
VIT_16s0039g01820
Standardized expressions
0.5
0.0
−0.5
−1.0
EI PG G1 G2 H T1 T2 C1 C2 S EP JP
Developmental stages
VIT_16s0039g01840
Standardized expressions
1
0
−1
−2
EI PG G1 G2 H T1 T2 C1 C2 S EP JP
Developmental stages
VIT_16s0039g01850
Standardized expressions
0.5
0.0
−0.5
−1.0
−1.5
EI PG G1 G2 H T1 T2 C1 C2 S EP JP
Developmental stages
VIT_16s0039g01860
Standardized expressions
0.4
0.0
−0.4
−0.8
−1.2
EI PG G1 G2 H T1 T2 C1 C2 S EP JP
Developmental stages

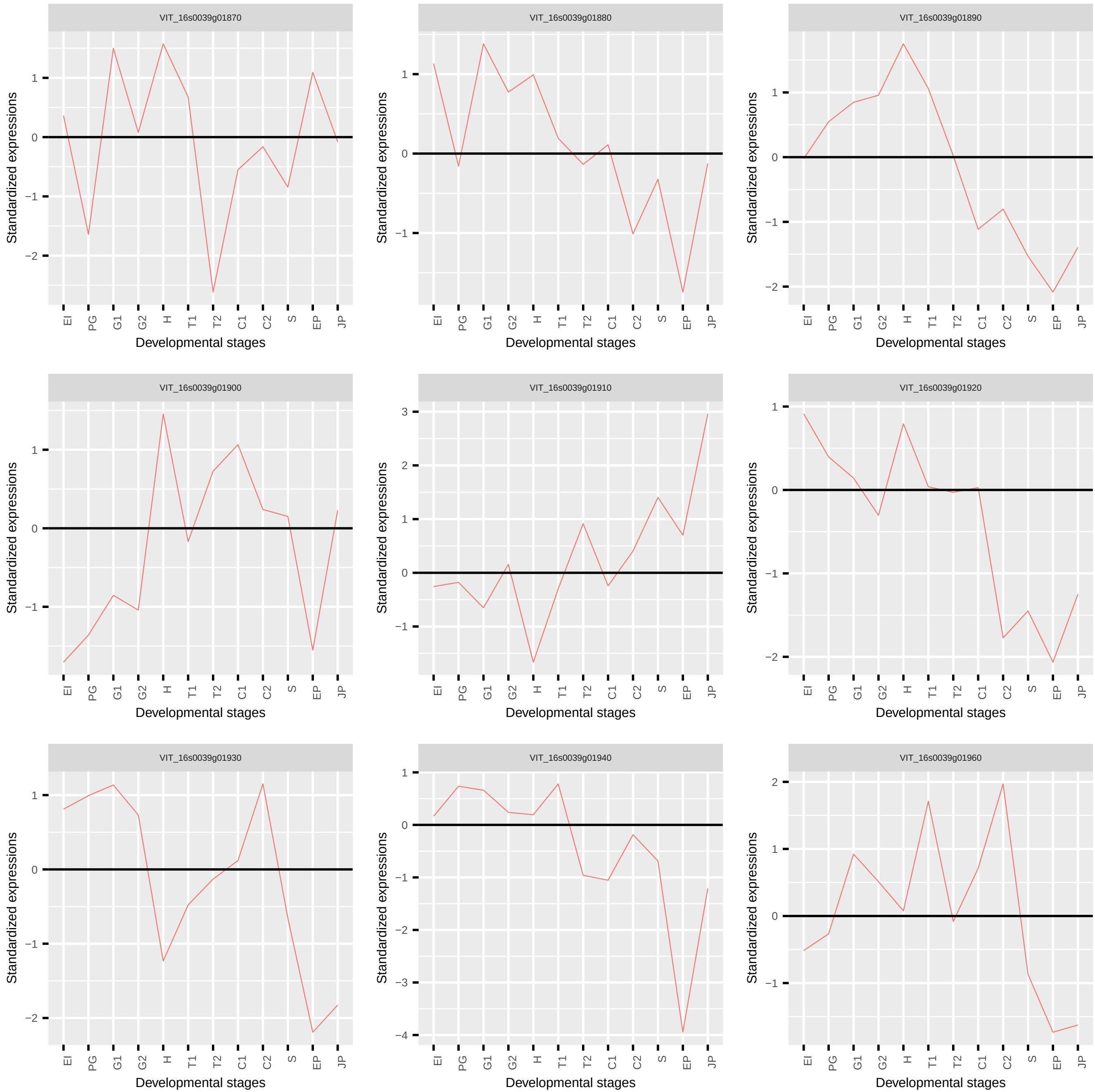
VIT_16s0039g01870
VIT_16s0039g01880
VIT_16s0039g01890
VIT_16s0039g01900
VIT_16s0039g01910
VIT_16s0039g01920
VIT_16s0039g01930
VIT_16s0039g01940
VIT_16s0039g01960
Standardized expressions
Developmental stages
EI PG G1 G2 H T1 T2 C1 C2 S EP JP

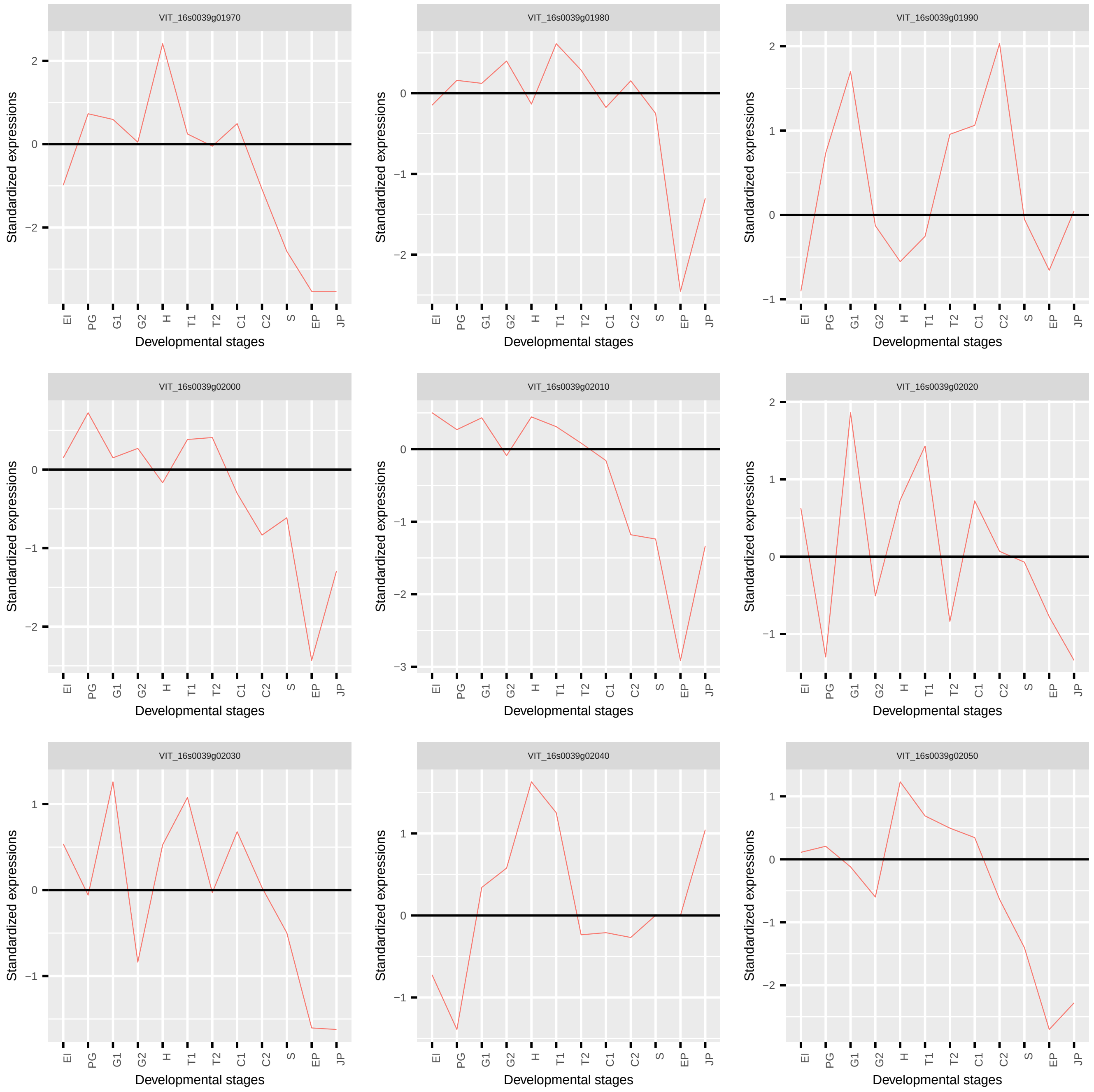
VIT_16s0039g01970
VIT_16s0039g01980
VIT_16s0039g01990
VIT_16s0039g02000
VIT_16s0039g02010
VIT_16s0039g02020
VIT_16s0039g02030
VIT_16s0039g02040
VIT_16s0039g02050
Standardized expressions
Developmental stages
EI PG G1 G2 H T1 T2 C1 C2 S EP JP

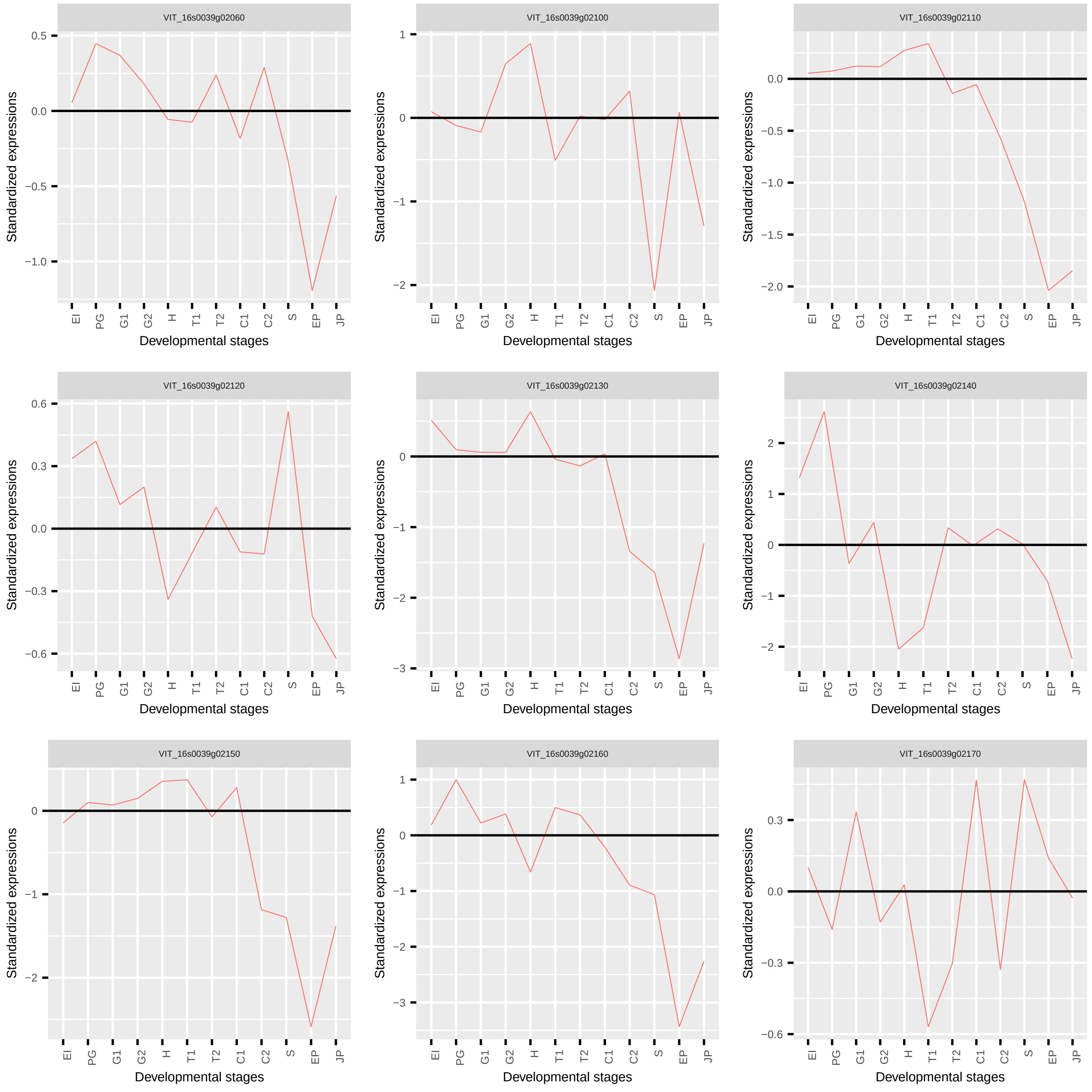
VIT_16s0039g02060
VIT_16s0039g02100
VIT_16s0039g02110
VIT_16s0039g02120
VIT_16s0039g02130
VIT_16s0039g02140
VIT_16s0039g02150
VIT_16s0039g02160
VIT_16s0039g02170
Standardized expressions
Developmental stages
EI
PG
G1
G2
H
T1
T2
C1
C2
S
EP
JP
0.5
0.0
−0.5
−1.0
−1.5
−2.0
1
0
−1
−2
−3
2
0.6
0.3
−0.3
−0.6

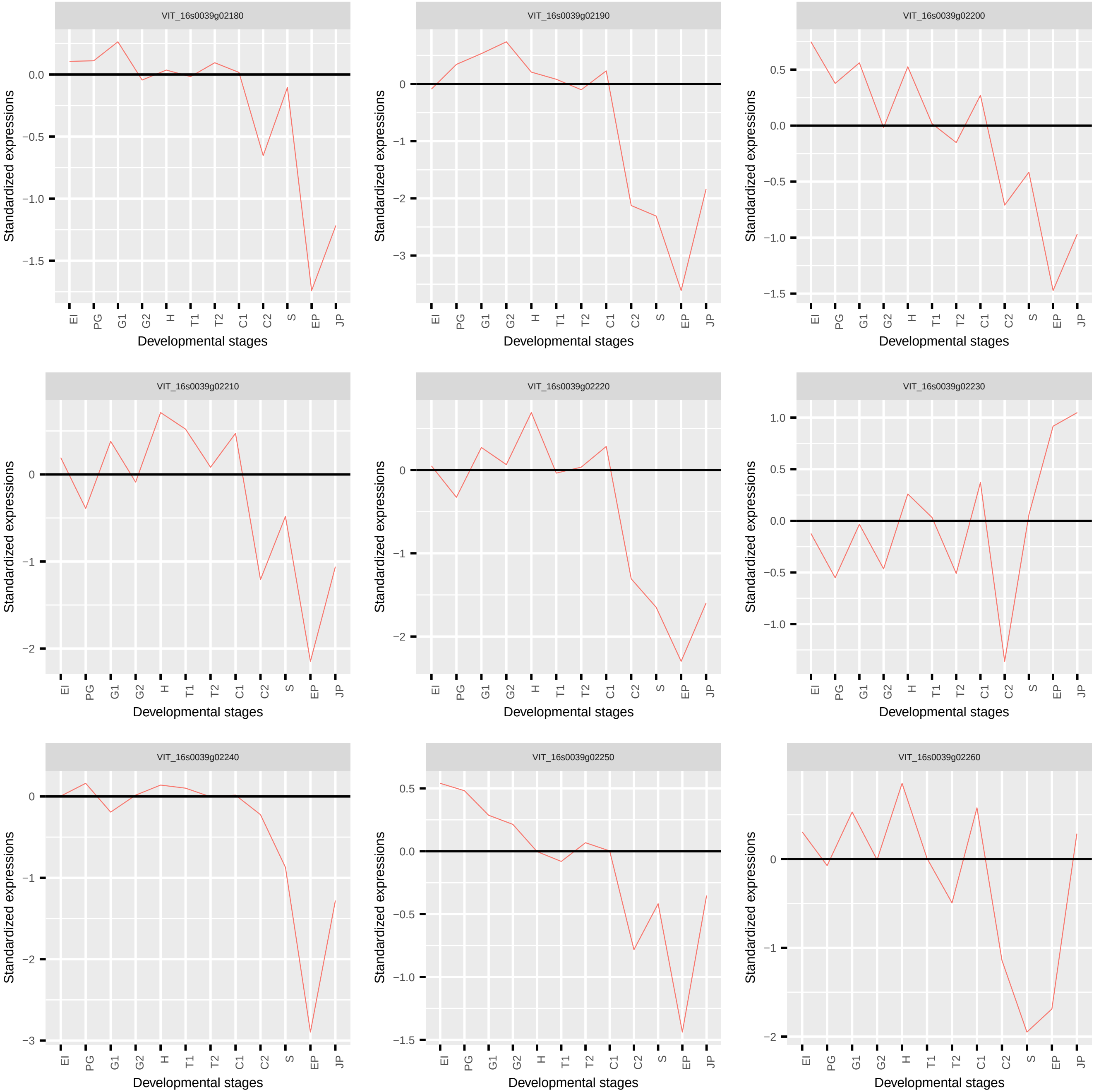
VIT_16s0039g02180
VIT_16s0039g02190
VIT_16s0039g02200
VIT_16s0039g02210
VIT_16s0039g02220
VIT_16s0039g02230
VIT_16s0039g02240
VIT_16s0039g02250
VIT_16s0039g02260
Standardized expressions
Developmental stages
EI
PG
G1
G2
H
T1
T2
C1
C2
S
EP
JP

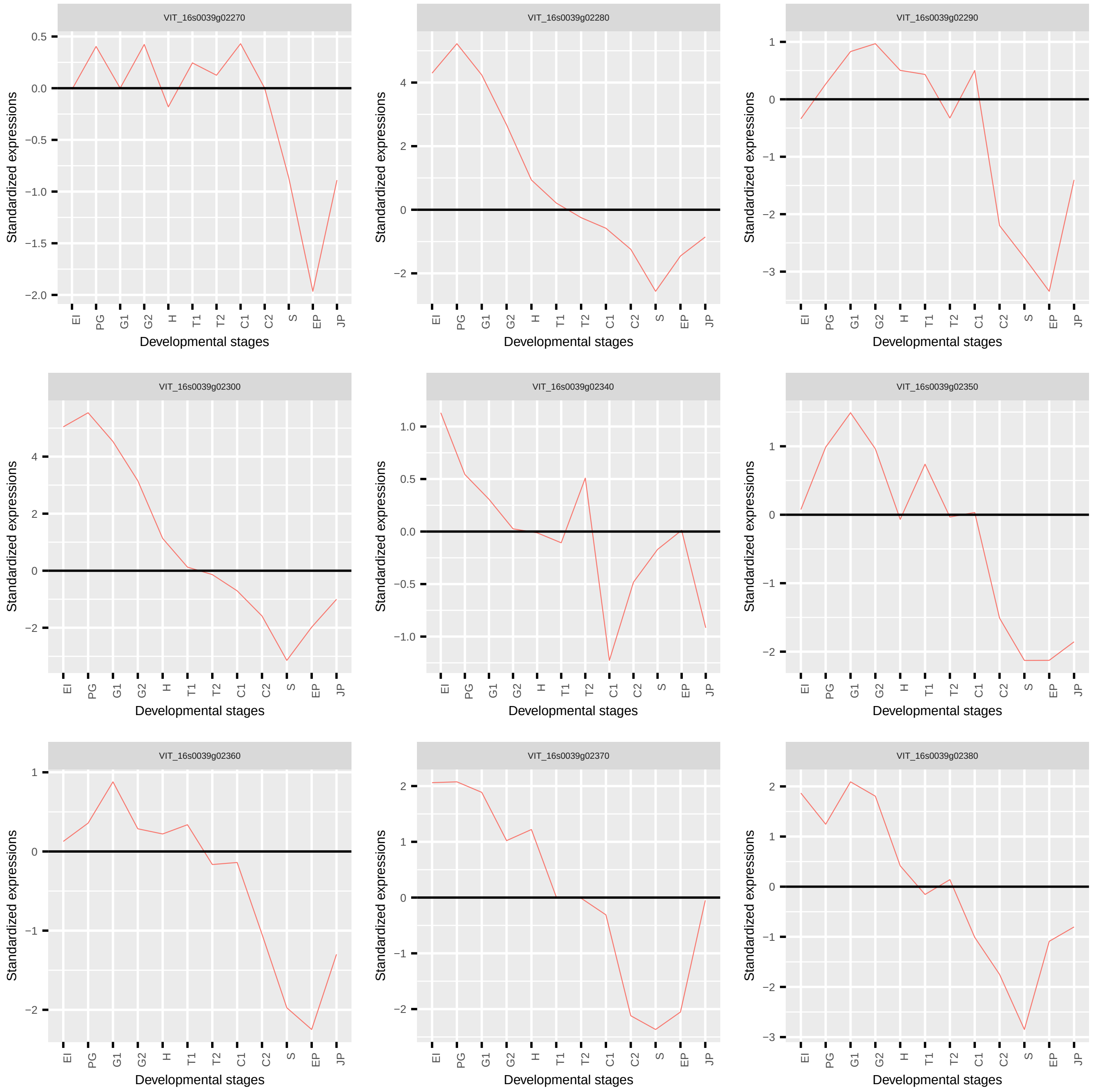
VIT_16s0039g02270
VIT_16s0039g02280
VIT_16s0039g02290
VIT_16s0039g02300
VIT_16s0039g02340
VIT_16s0039g02350
VIT_16s0039g02360
VIT_16s0039g02370
VIT_16s0039g02380
Standardized expressions
Developmental stages
EI PG G1 G2 H T1 T2 C1 C2 S EP JP

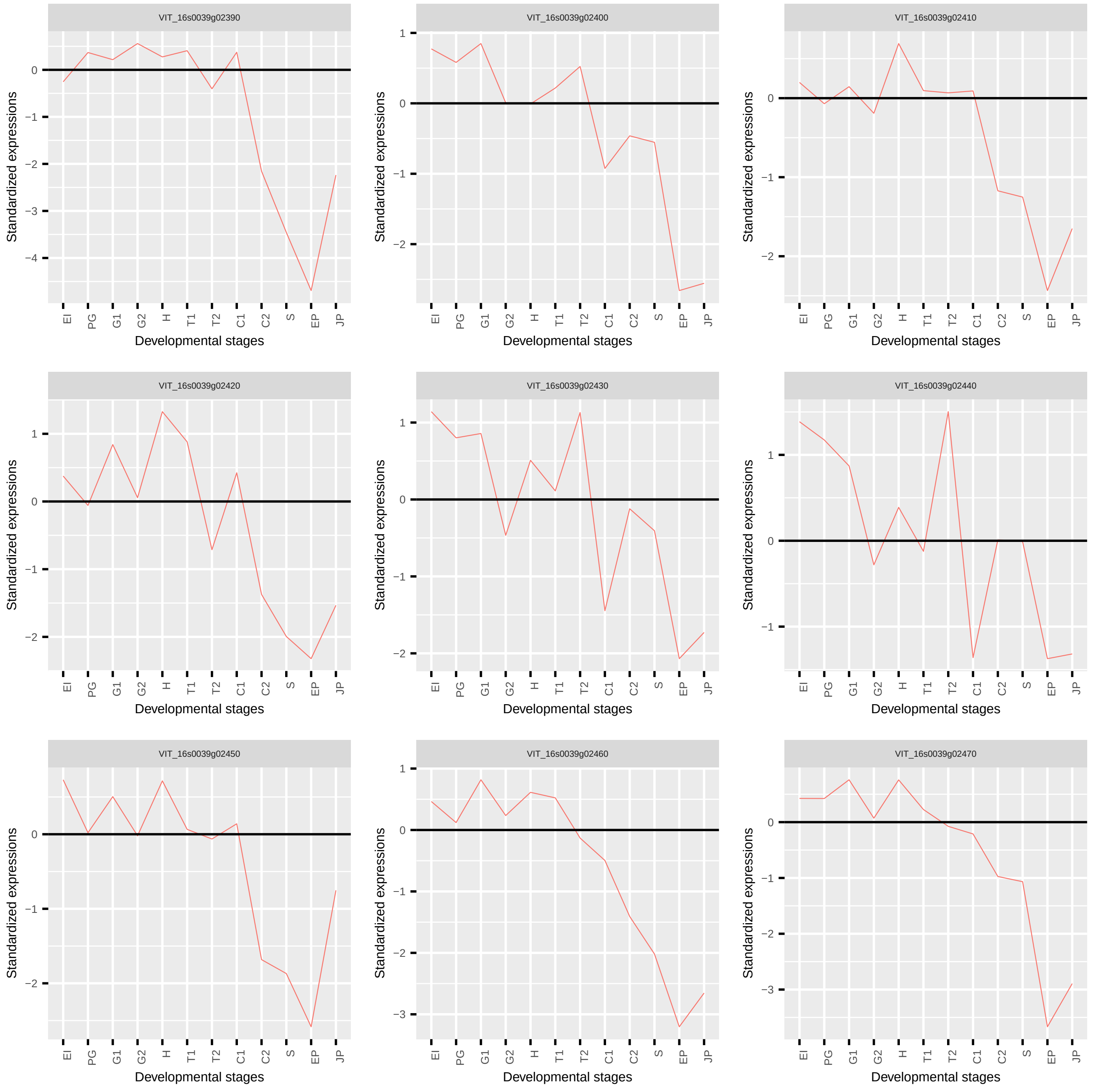
VIT_16s0039g02390
VIT_16s0039g02400
VIT_16s0039g02410
VIT_16s0039g02420
VIT_16s0039g02430
VIT_16s0039g02440
VIT_16s0039g02450
VIT_16s0039g02460
VIT_16s0039g02470
Standardized expressions
Developmental stages
EI PG G1 G2 H T1 T2 C1 C2 S EP JP

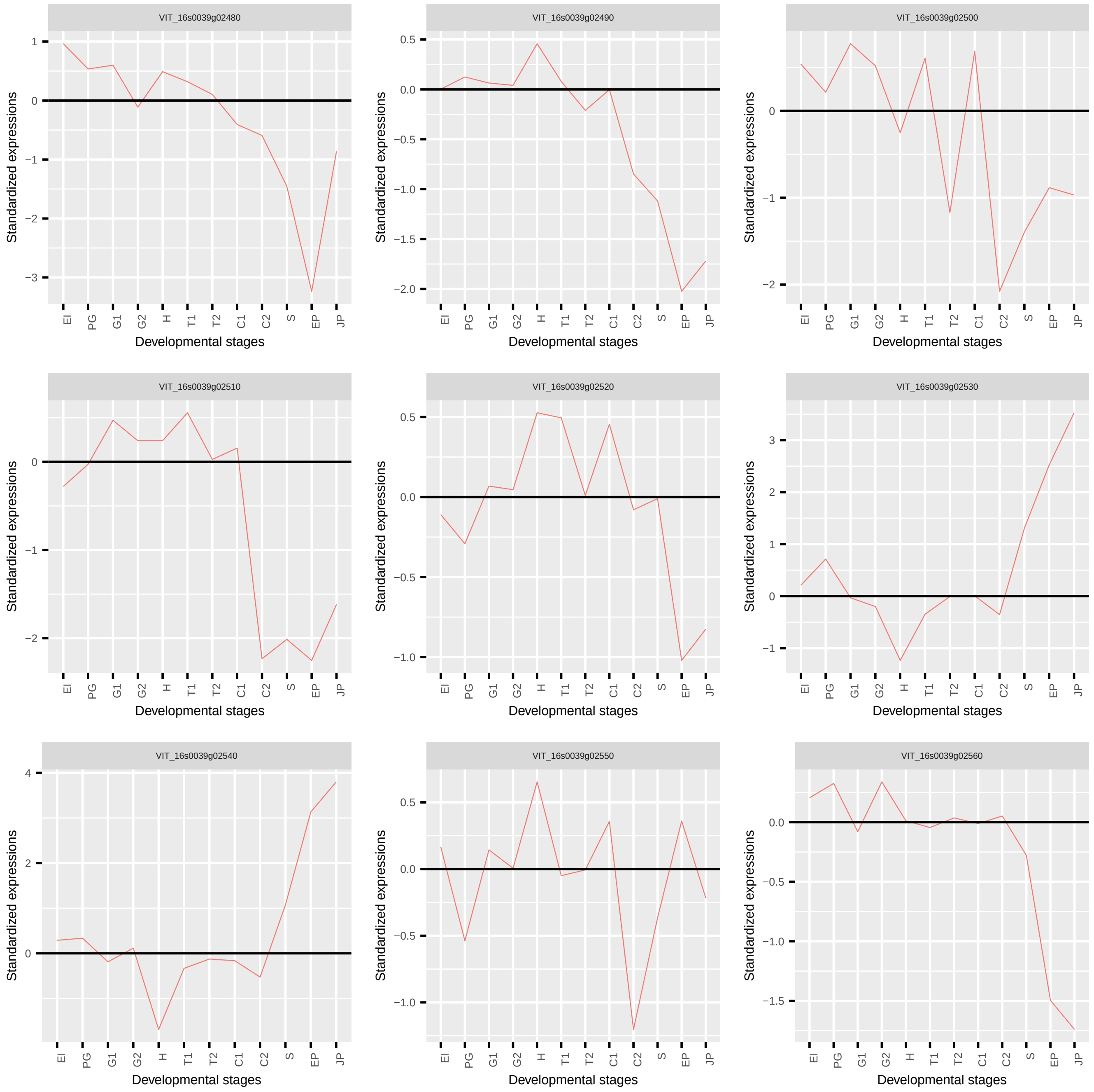
VIT_16s0039g02480
VIT_16s0039g02490
VIT_16s0039g02500
VIT_16s0039g02510
VIT_16s0039g02520
VIT_16s0039g02530
VIT_16s0039g02540
VIT_16s0039g02550
VIT_16s0039g02560
Standardized expressions
Developmental stages
EI PG G1 G2 H T1 T2 C1 C2 S EP JP

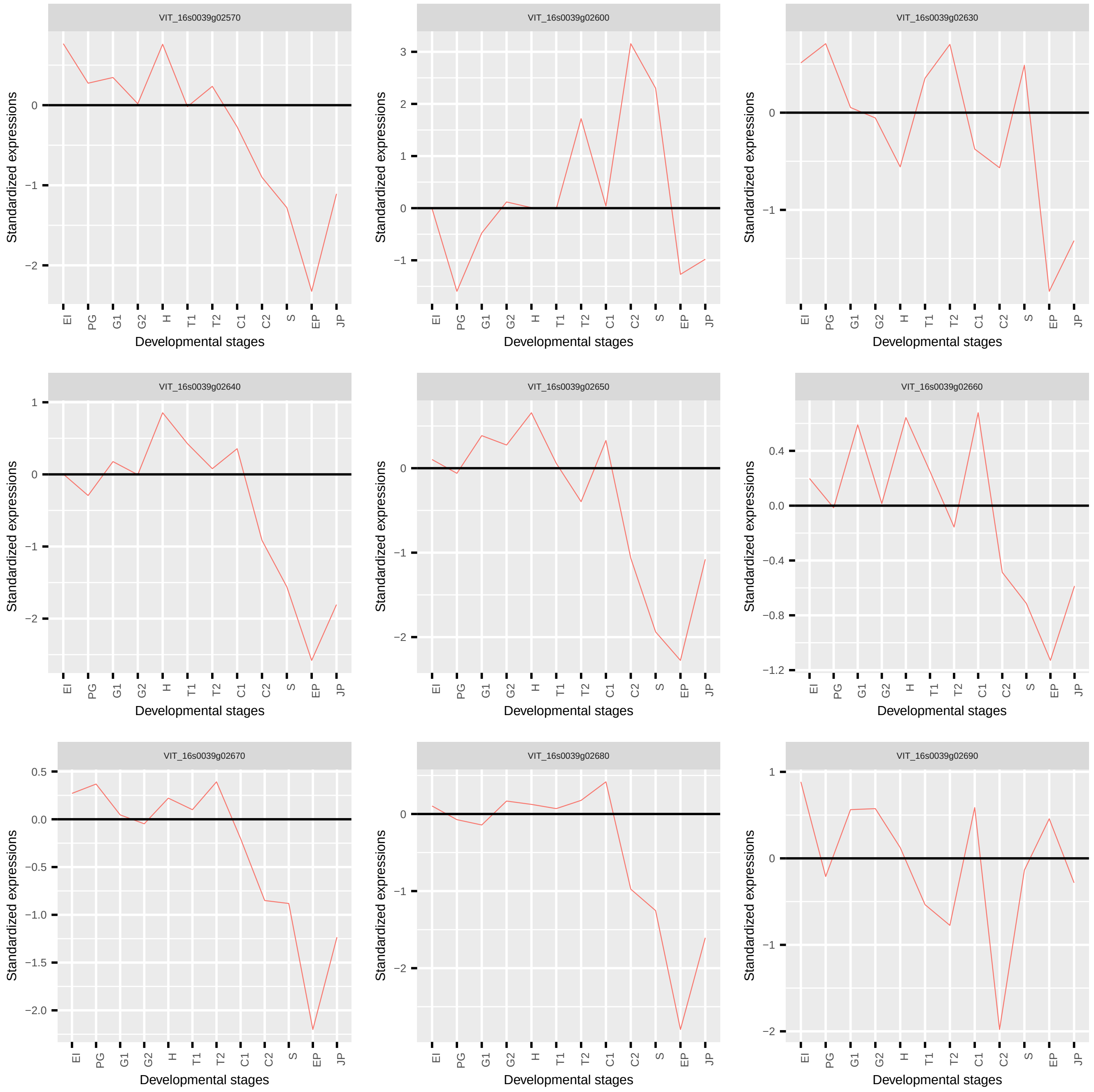

VIT_16s0039g02570
VIT_16s0039g02600
VIT_16s0039g02630
VIT_16s0039g02640
VIT_16s0039g02650
VIT_16s0039g02660
VIT_16s0039g02670
VIT_16s0039g02680
VIT_16s0039g02690
Standardized expressions
Developmental stages
EI PG G1 G2 H T1 T2 C1 C2 S EP JP

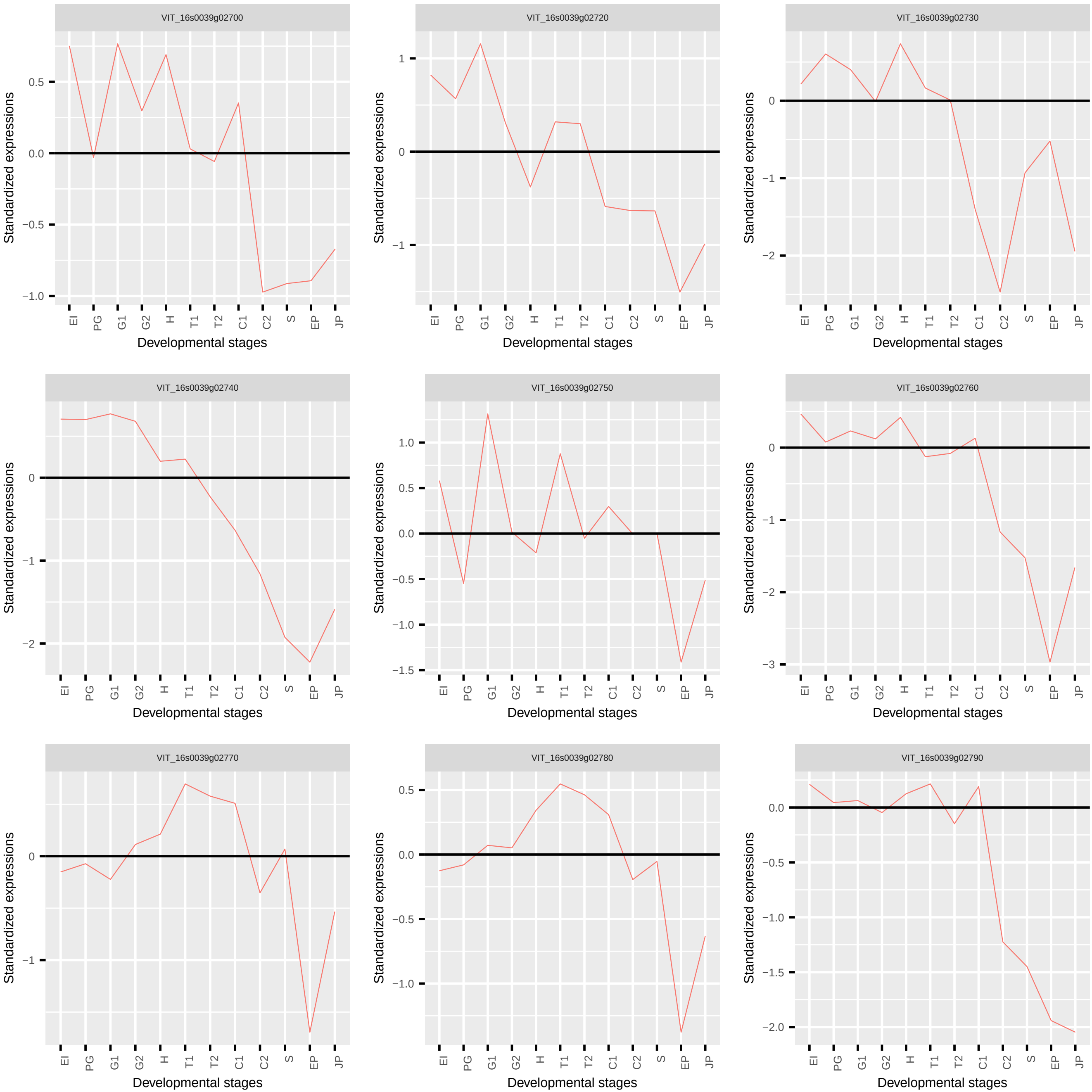
VIT_16s0039g02700
VIT_16s0039g02720
VIT_16s0039g02730
VIT_16s0039g02740
VIT_16s0039g02750
VIT_16s0039g02760
VIT_16s0039g02770
VIT_16s0039g02780
VIT_16s0039g02790
Standardized expressions
Developmental stages
EI PG G1 G2 H T1 T2 C1 C2 S EP JP
0.5 0.0 −0.5 −1.0
1 0 −1
0 −1 −2
0 −1 −2
1.0 0.5 0.0 −0.5 −1.0 −1.5
0 −1 −2 −3
0 −1
0.5 0.0 −0.5 −1.0
0.0 −0.5 −1.0 −1.5 −2.0

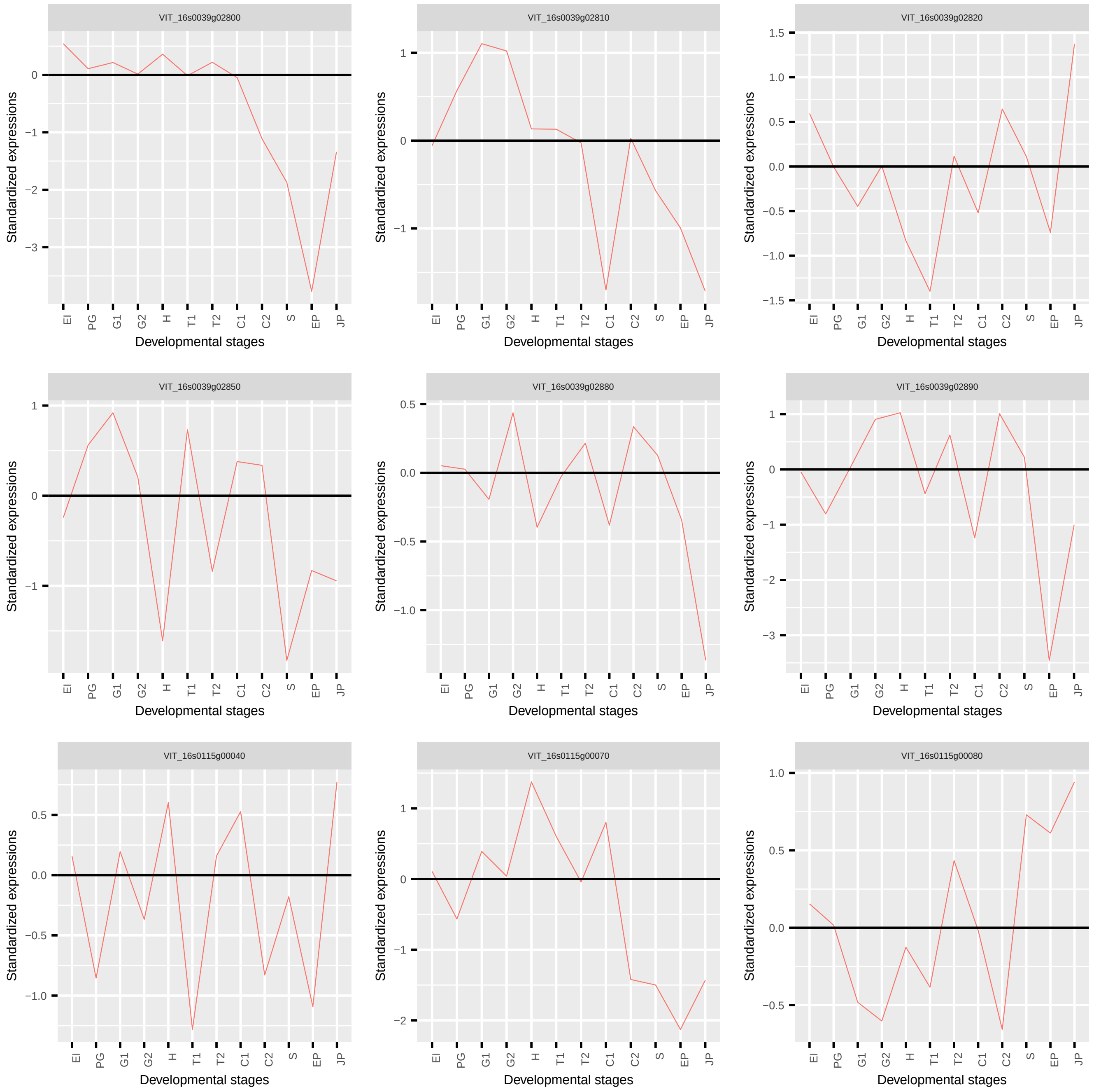
VIT_16s0039g02800
VIT_16s0039g02810
VIT_16s0039g02820
VIT_16s0039g02850
VIT_16s0039g02880
VIT_16s0039g02890
VIT_16s0115g00040
VIT_16s0115g00070
VIT_16s0115g00080
Standardized expressions
Developmental stages
EI
PG
G1
G2
H
T1
T2
C1
C2
S
EP
JP

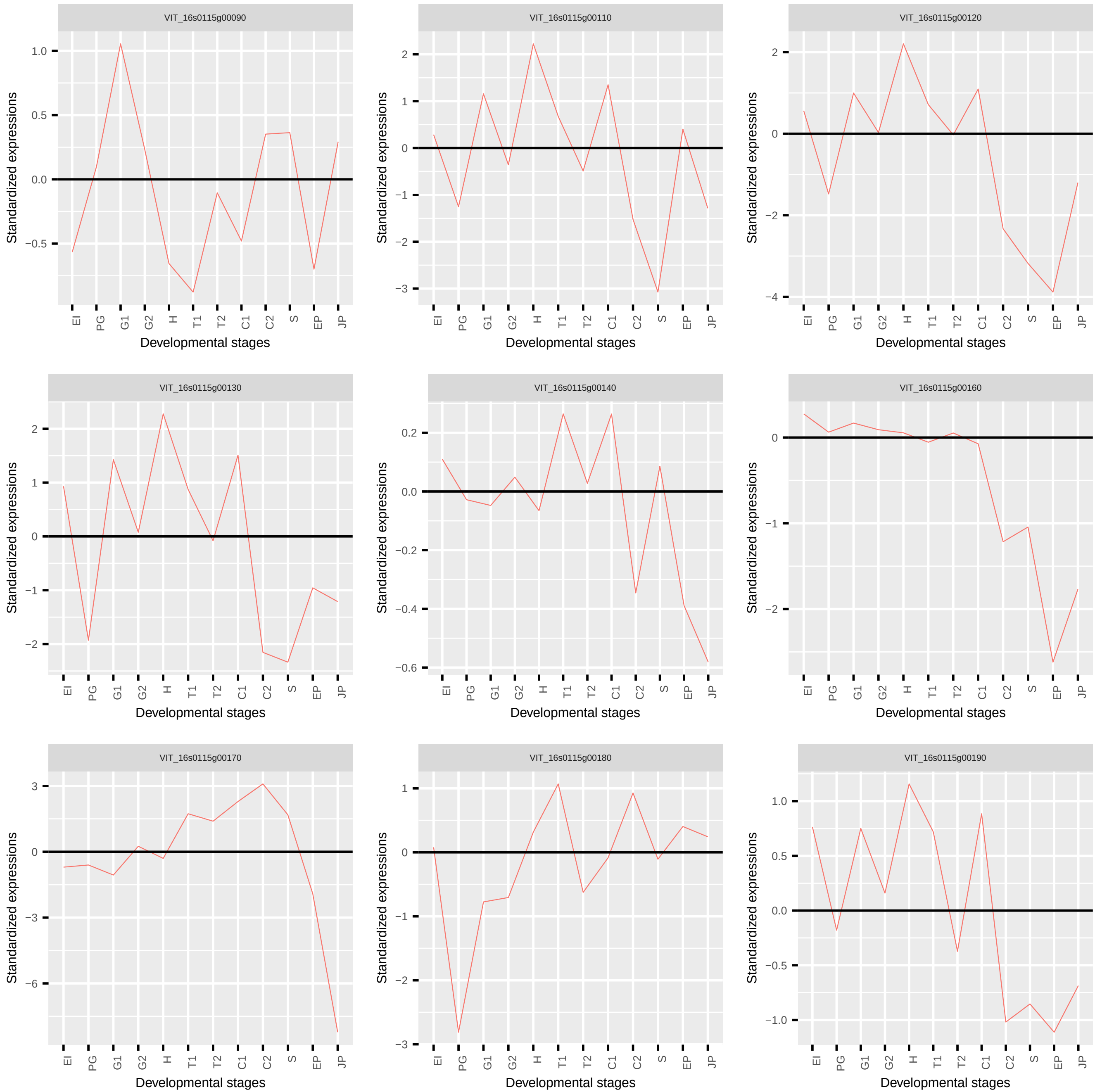
VIT_16s0115g00090
VIT_16s0115g00110
VIT_16s0115g00120
VIT_16s0115g00130
VIT_16s0115g00140
VIT_16s0115g00160
VIT_16s0115g00170
VIT_16s0115g00180
VIT_16s0115g00190
Standardized expressions
Developmental stages
EI
PG
G1
G2
H
T1
T2
C1
C2
S
EP
JP

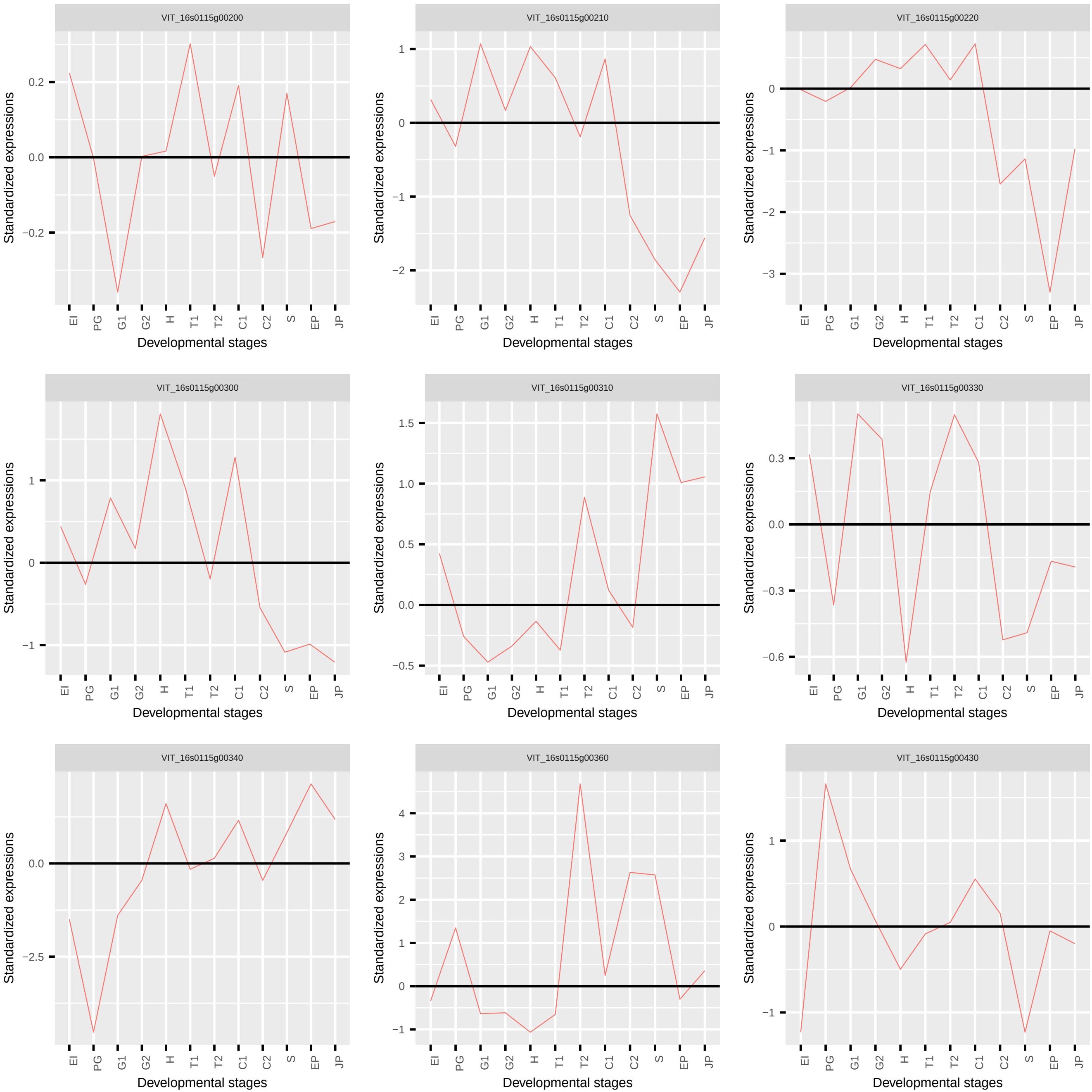
VIT_16s0115g00200
Standardized expressions
0.2
0.0
−0.2
EI PG G1 G2 H T1 T2 C1 C2 S EP JP
Developmental stages
VIT_16s0115g00210
Standardized expressions
1
0
−1
−2
EI PG G1 G2 H T1 T2 C1 C2 S EP JP
Developmental stages
VIT_16s0115g00220
Standardized expressions
0
−1
−2
−3
EI PG G1 G2 H T1 T2 C1 C2 S EP JP
Developmental stages
VIT_16s0115g00300
Standardized expressions
1
0
−1
EI PG G1 G2 H T1 T2 C1 C2 S EP JP
Developmental stages
VIT_16s0115g00310
Standardized expressions
1.5
1.0
0.5
0.0
−0.5
EI PG G1 G2 H T1 T2 C1 C2 S EP JP
Developmental stages
VIT_16s0115g00330
Standardized expressions
0.3
0.0
−0.3
−0.6
EI PG G1 G2 H T1 T2 C1 C2 S EP JP
Developmental stages
VIT_16s0115g00340
Standardized expressions
0.0
−2.5
EI PG G1 G2 H T1 T2 C1 C2 S EP JP
Developmental stages
VIT_16s0115g00360
Standardized expressions
4
3
2
1
0
−1
EI PG G1 G2 H T1 T2 C1 C2 S EP JP
Developmental stages
VIT_16s0115g00430
Standardized expressions
1
0
−1
EI PG G1 G2 H T1 T2 C1 C2 S EP JP
Developmental stages

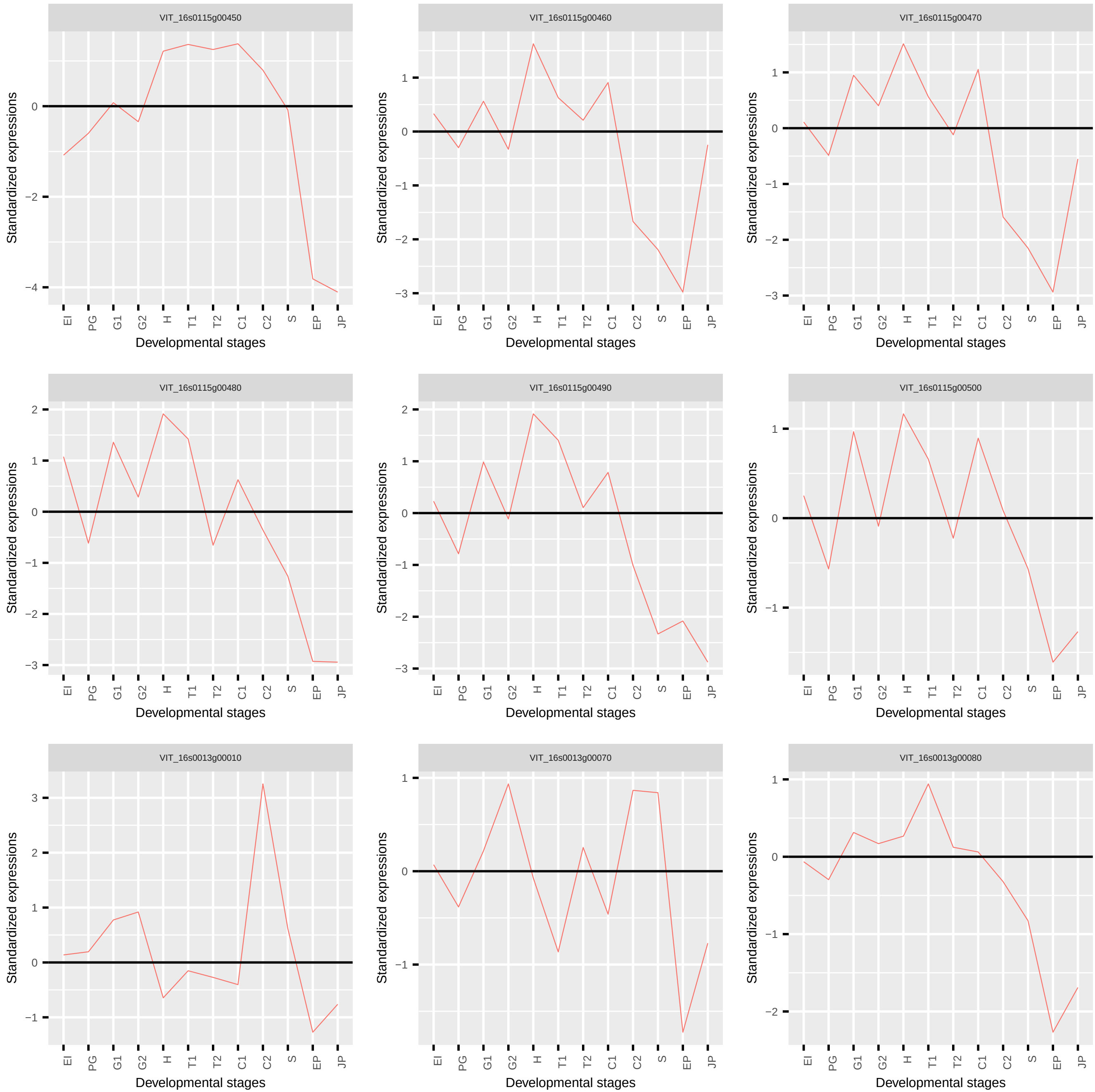

VIT_16s0115g00450
VIT_16s0115g00460
VIT_16s0115g00470
VIT_16s0115g00480
VIT_16s0115g00490
VIT_16s0115g00500
VIT_16s0013g00010
VIT_16s0013g00070
VIT_16s0013g00080
Standardized expressions
Developmental stages
EI PG G1 G2 H T1 T2 C1 C2 S EP JP

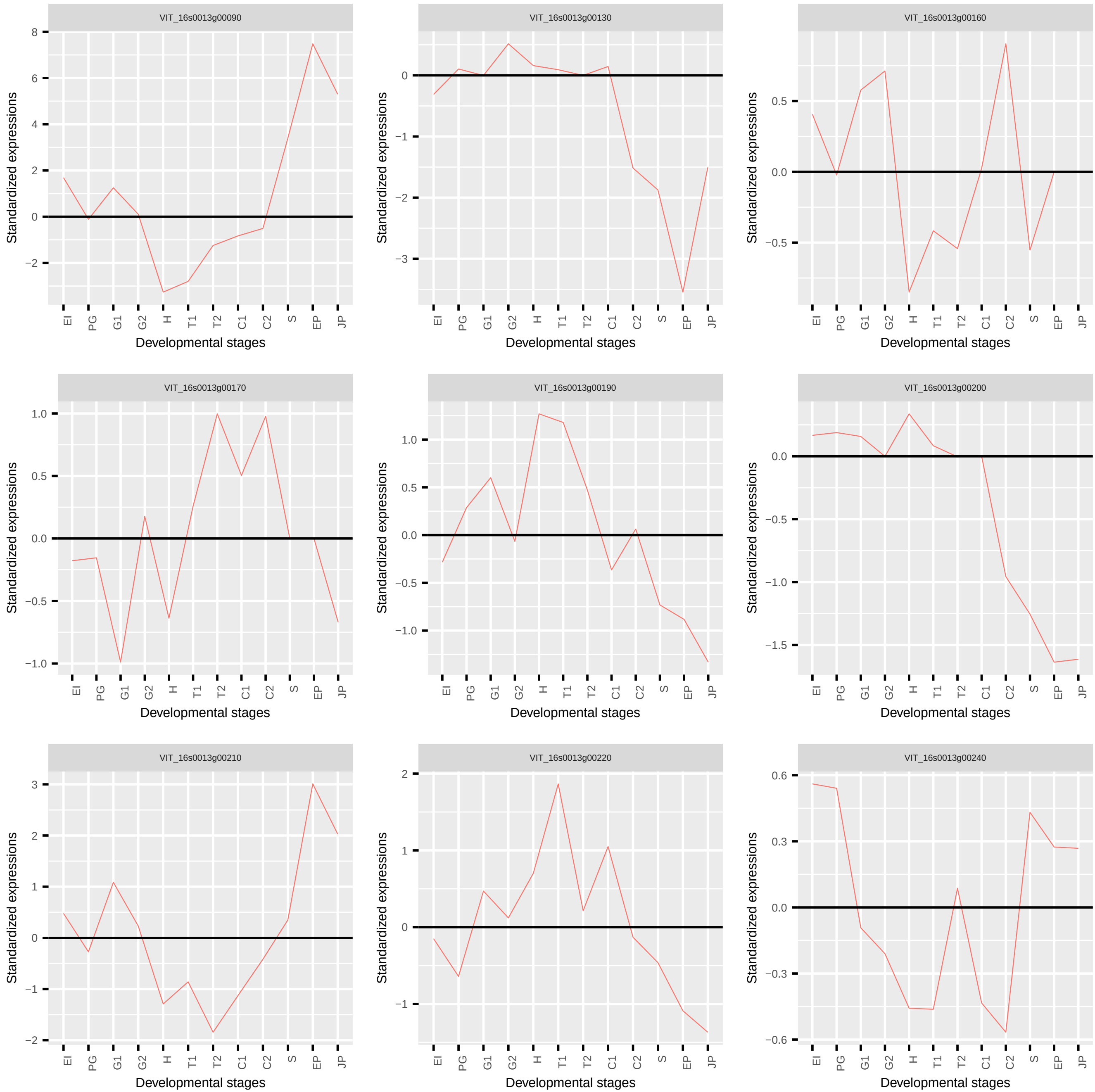
VIT_16s0013g00090
VIT_16s0013g00130
VIT_16s0013g00160
VIT_16s0013g00170
VIT_16s0013g00190
VIT_16s0013g00200
VIT_16s0013g00210
VIT_16s0013g00220
VIT_16s0013g00240
Standardized expressions
Developmental stages
EI PG G1 G2 H T1 T2 C1 C2 S EP JP

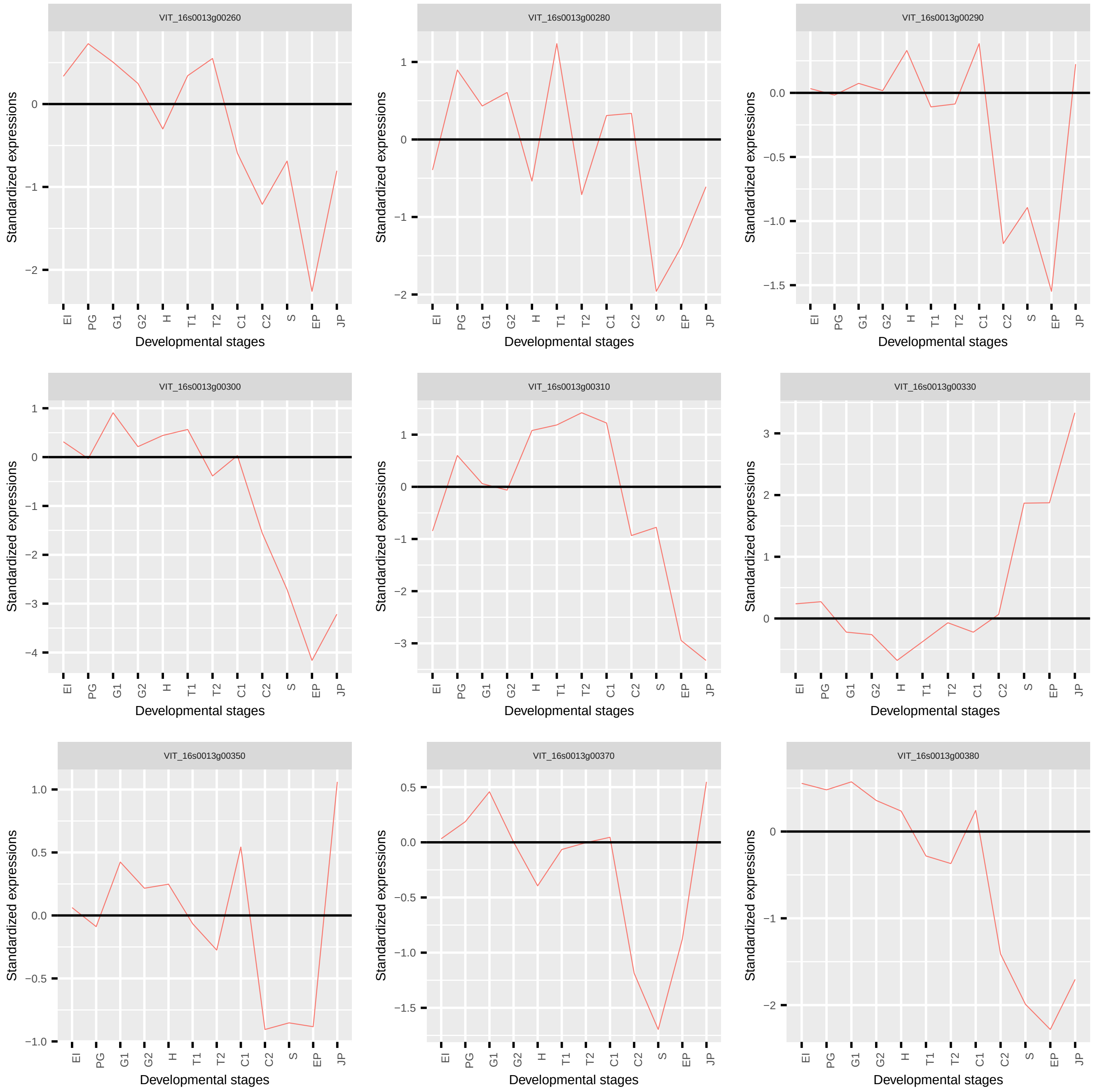

VIT_16s0013g00260
VIT_16s0013g00280
VIT_16s0013g00290
VIT_16s0013g00300
VIT_16s0013g00310
VIT_16s0013g00330
VIT_16s0013g00350
VIT_16s0013g00370
VIT_16s0013g00380
Standardized expressions
Developmental stages
EI PG G1 G2 H T1 T2 C1 C2 S EP JP

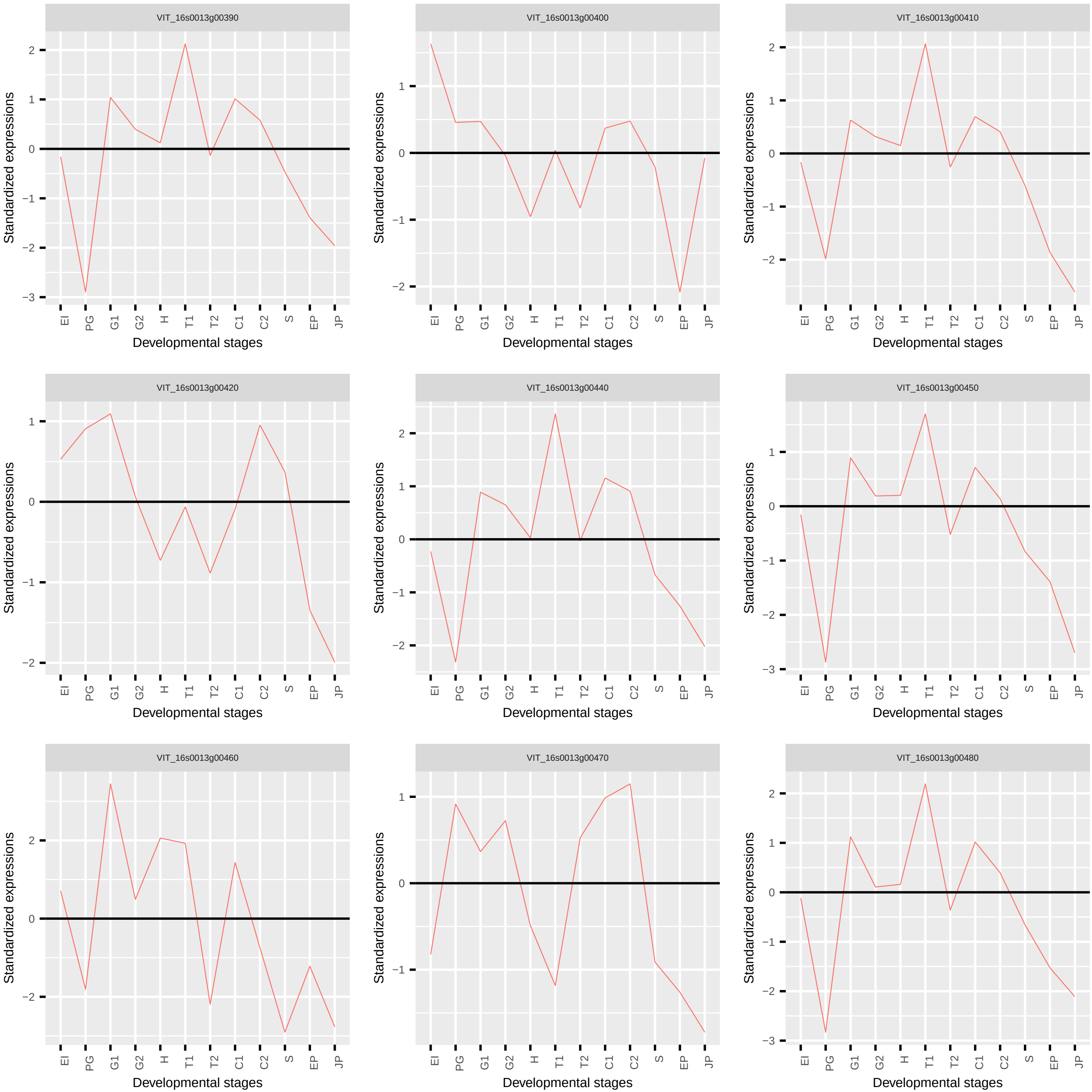
VIT_16s0013g00390
VIT_16s0013g00400
VIT_16s0013g00410
VIT_16s0013g00420
VIT_16s0013g00440
VIT_16s0013g00450
VIT_16s0013g00460
VIT_16s0013g00470
VIT_16s0013g00480
Standardized expressions
Developmental stages
EI PG G1 G2 H T1 T2 C1 C2 S EP JP

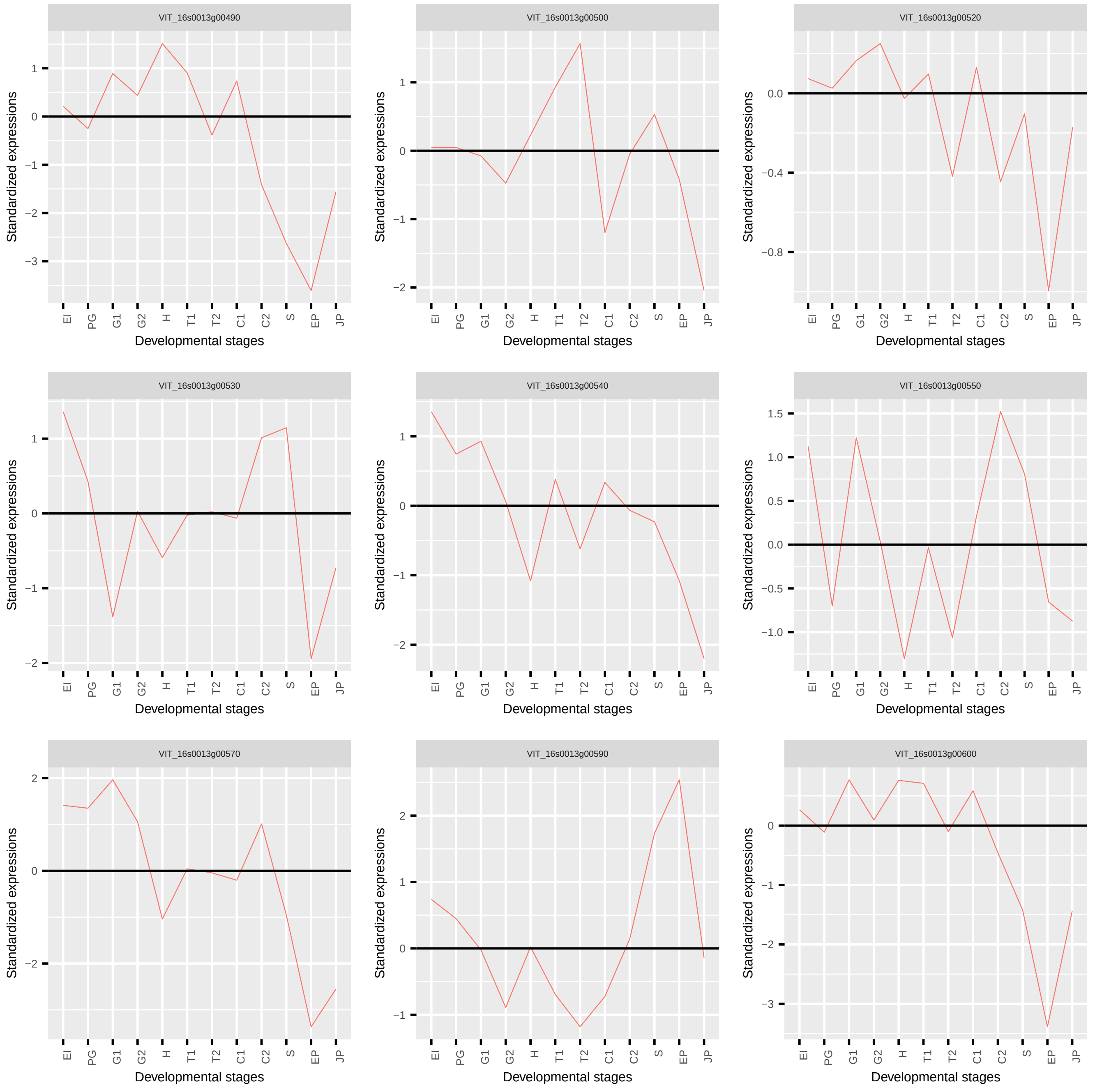

VIT_16s0013g00490
VIT_16s0013g00500
VIT_16s0013g00520
VIT_16s0013g00530
VIT_16s0013g00540
VIT_16s0013g00550
VIT_16s0013g00570
VIT_16s0013g00590
VIT_16s0013g00600
Standardized expressions
Developmental stages
EI
PG
G1
G2
H
T1
T2
C1
C2
S
EP
JP

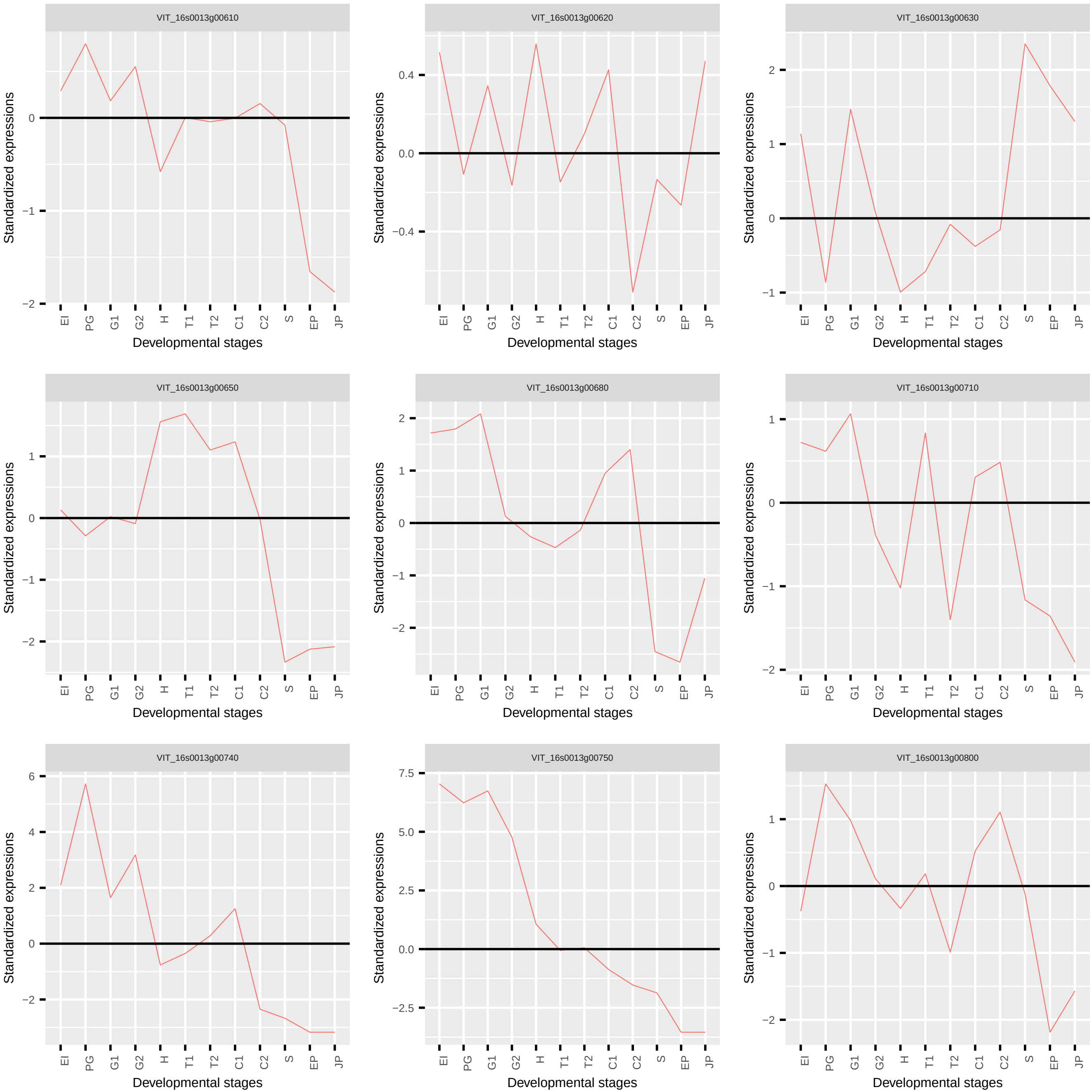

VIT_16s0013g00610
Standardized expressions
0
−1
−2
EI PG G1 G2 H T1 T2 C1 C2 S EP JP
Developmental stages
VIT_16s0013g00620
Standardized expressions
0.4
0.0
−0.4
EI PG G1 G2 H T1 T2 C1 C2 S EP JP
Developmental stages
VIT_16s0013g00630
Standardized expressions
2
1
0
−1
EI PG G1 G2 H T1 T2 C1 C2 S EP JP
Developmental stages
VIT_16s0013g00650
Standardized expressions
1
0
−1
−2
EI PG G1 G2 H T1 T2 C1 C2 S EP JP
Developmental stages
VIT_16s0013g00680
Standardized expressions
2
1
0
−1
−2
EI PG G1 G2 H T1 T2 C1 C2 S EP JP
Developmental stages
VIT_16s0013g00710
Standardized expressions
1
0
−1
−2
EI PG G1 G2 H T1 T2 C1 C2 S EP JP
Developmental stages
VIT_16s0013g00740
Standardized expressions
6
4
2
0
−2
EI PG G1 G2 H T1 T2 C1 C2 S EP JP
Developmental stages
VIT_16s0013g00750
Standardized expressions
7.5
5.0
2.5
0.0
−2.5
EI PG G1 G2 H T1 T2 C1 C2 S EP JP
Developmental stages
VIT_16s0013g00800
Standardized expressions
1
0
−1
−2
EI PG G1 G2 H T1 T2 C1 C2 S EP JP
Developmental stages

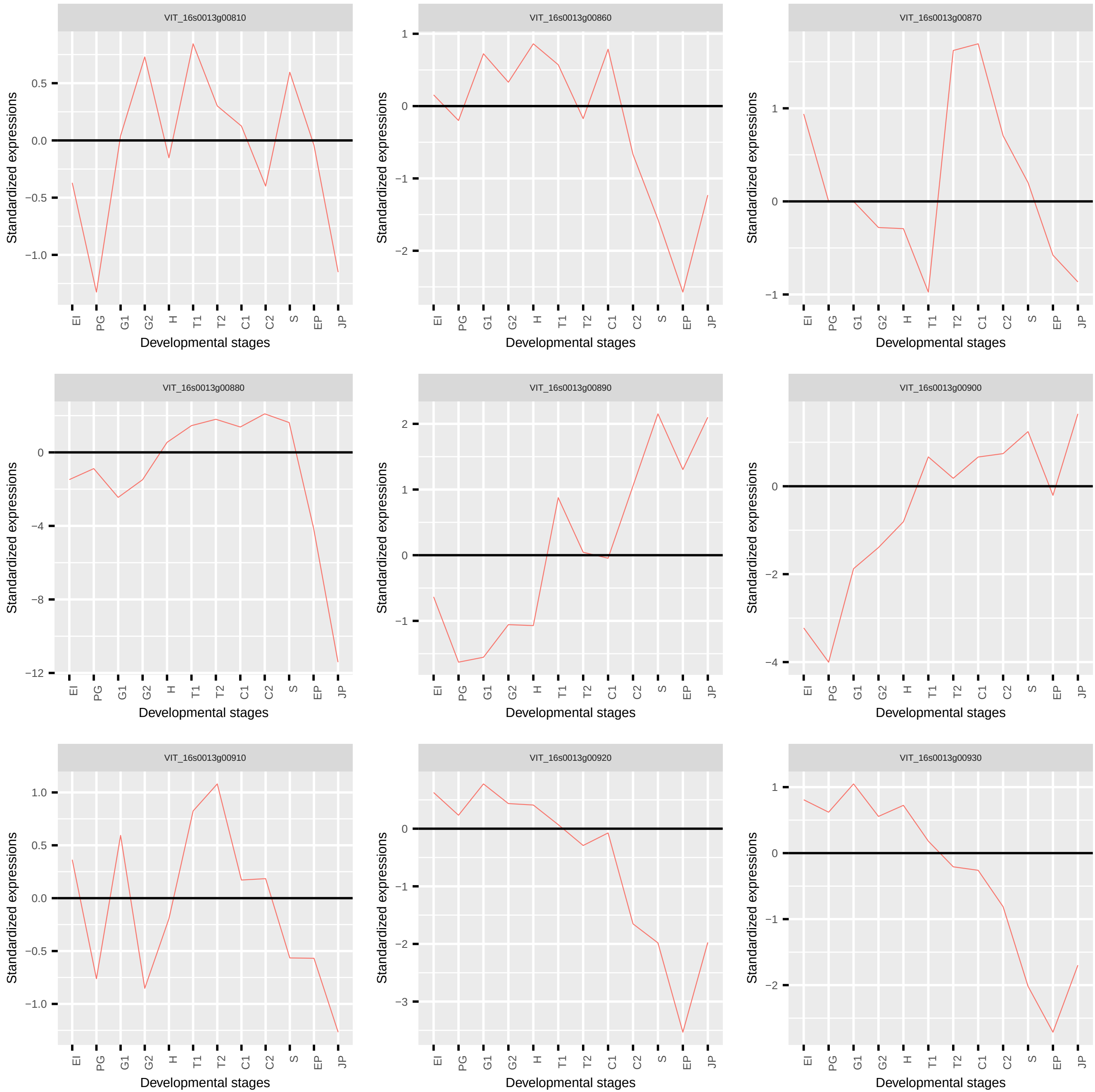
VIT_16s0013g00810
Standardized expressions
0.5
0.0
−0.5
−1.0
EI PG G1 G2 H T1 T2 C1 C2 S EP JP
Developmental stages
VIT_16s0013g00860
Standardized expressions
1
0
−1
−2
EI PG G1 G2 H T1 T2 C1 C2 S EP JP
Developmental stages
VIT_16s0013g00870
Standardized expressions
1
0
−1
EI PG G1 G2 H T1 T2 C1 C2 S EP JP
Developmental stages
VIT_16s0013g00880
Standardized expressions
0
−4
−8
−12
EI PG G1 G2 H T1 T2 C1 C2 S EP JP
Developmental stages
VIT_16s0013g00890
Standardized expressions
2
1
0
−1
EI PG G1 G2 H T1 T2 C1 C2 S EP JP
Developmental stages
VIT_16s0013g00900
Standardized expressions
0
−2
−4
EI PG G1 G2 H T1 T2 C1 C2 S EP JP
Developmental stages
VIT_16s0013g00910
Standardized expressions
1.0
0.5
0.0
−0.5
−1.0
EI PG G1 G2 H T1 T2 C1 C2 S EP JP
Developmental stages
VIT_16s0013g00920
Standardized expressions
0
−1
−2
−3
EI PG G1 G2 H T1 T2 C1 C2 S EP JP
Developmental stages
VIT_16s0013g00930
Standardized expressions
1
0
−1
−2
EI PG G1 G2 H T1 T2 C1 C2 S EP JP
Developmental stages

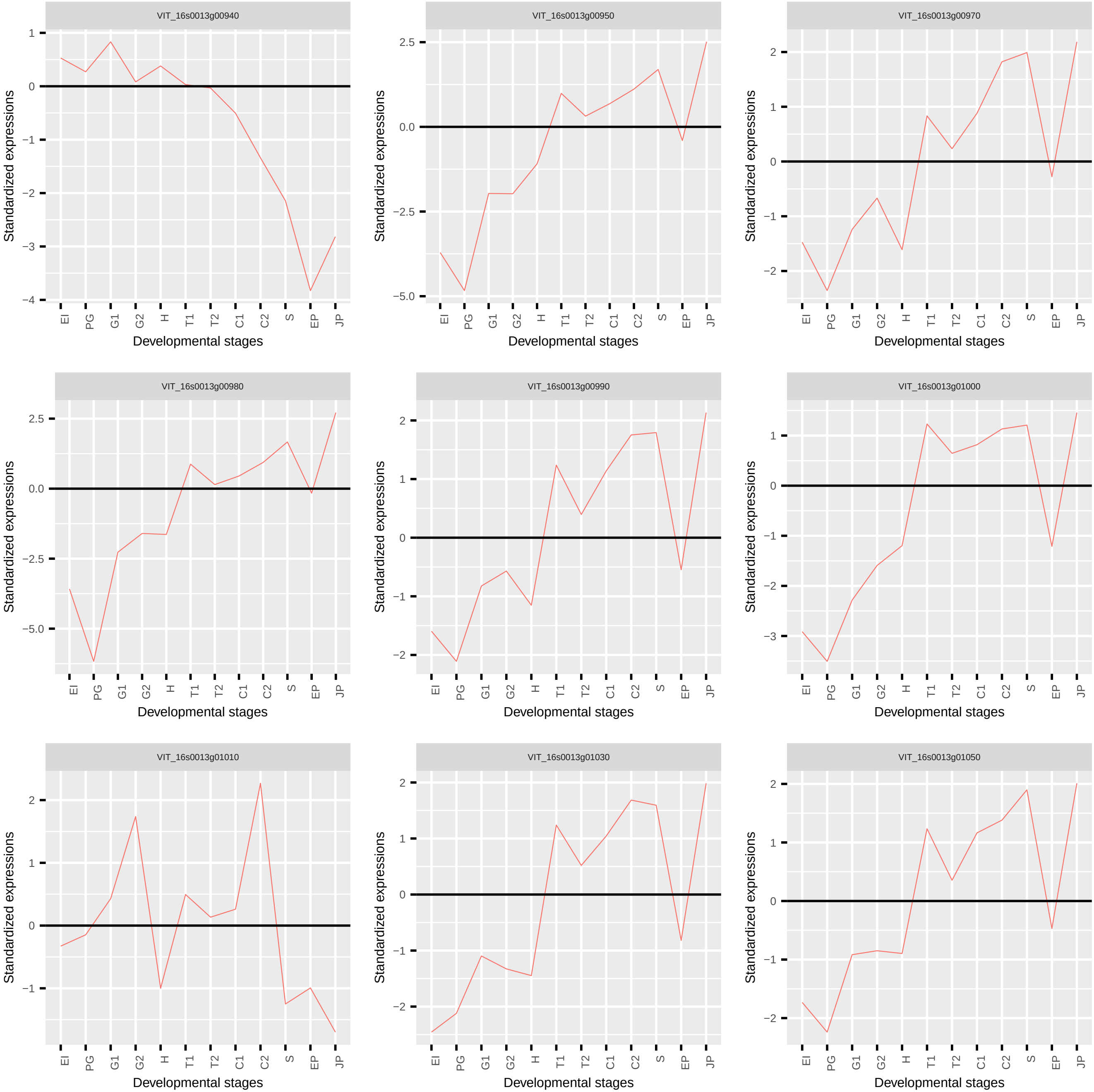
VIT_16s0013g00940
VIT_16s0013g00950
VIT_16s0013g00970
VIT_16s0013g00980
VIT_16s0013g00990
VIT_16s0013g01000
VIT_16s0013g01010
VIT_16s0013g01030
VIT_16s0013g01050
Standardized expressions
Developmental stages
EI
PG
G1
G2
H
T1
T2
C1
C2
S
EP
JP

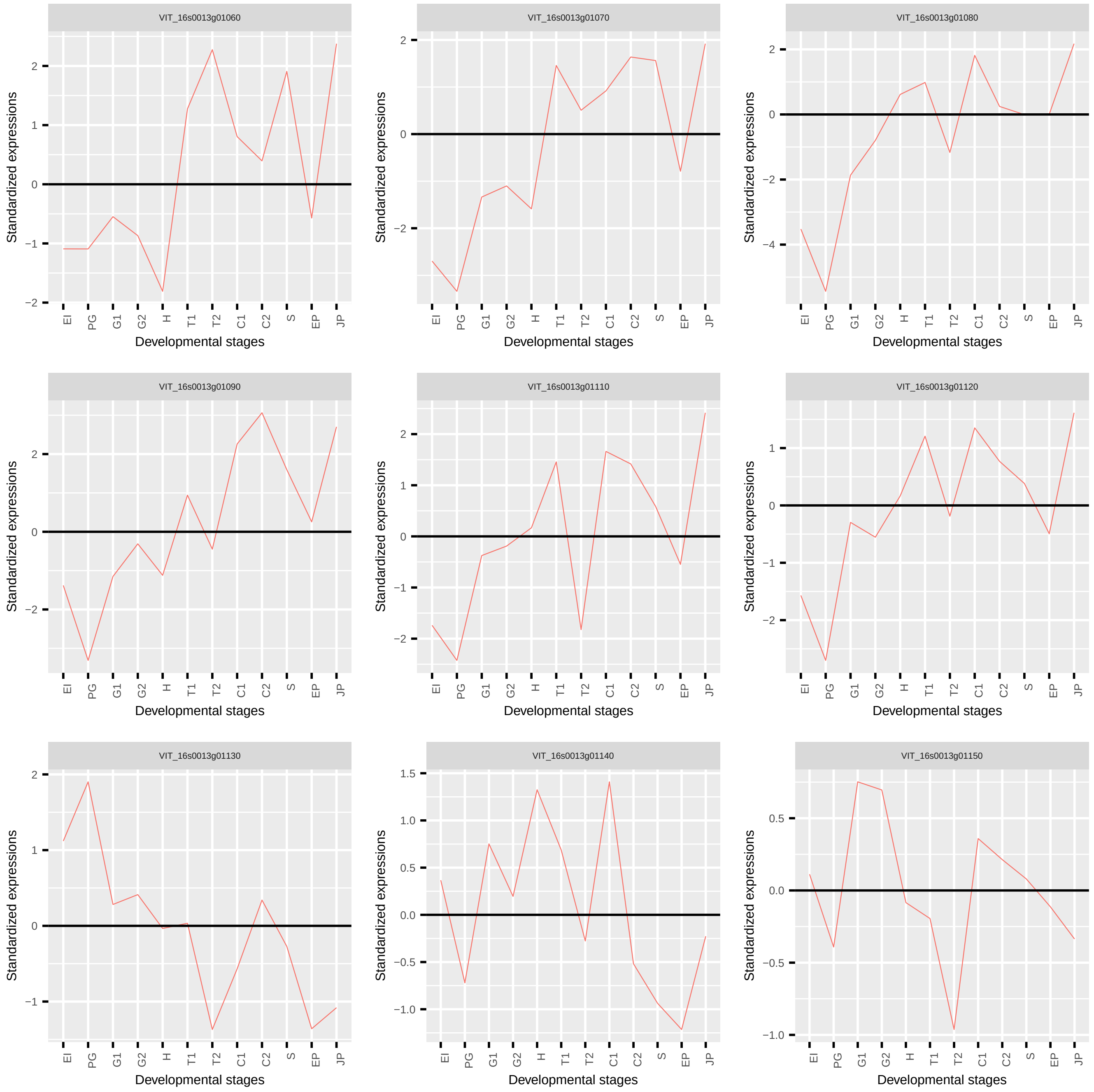
VIT_16s0013g01060
VIT_16s0013g01070
VIT_16s0013g01080
VIT_16s0013g01090
VIT_16s0013g01110
VIT_16s0013g01120
VIT_16s0013g01130
VIT_16s0013g01140
VIT_16s0013g01150
Standardized expressions
Developmental stages
EI PG G1 G2 H T1 T2 C1 C2 S EP JP

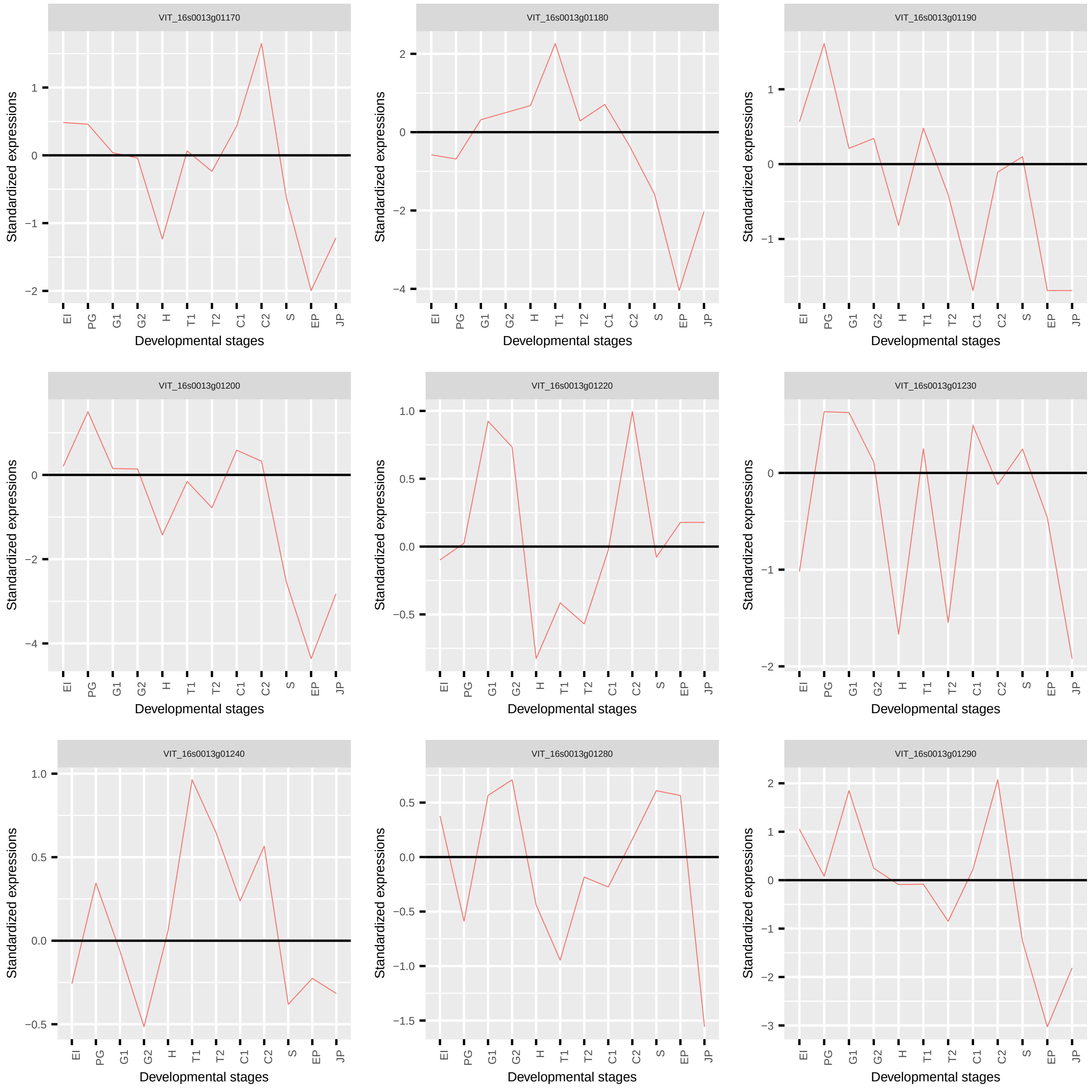

VIT_16s0013g01170
VIT_16s0013g01180
VIT_16s0013g01190
VIT_16s0013g01200
VIT_16s0013g01220
VIT_16s0013g01230
VIT_16s0013g01240
VIT_16s0013g01280
VIT_16s0013g01290
Standardized expressions
Developmental stages
EI PG G1 G2 H T1 T2 C1 C2 S EP JP

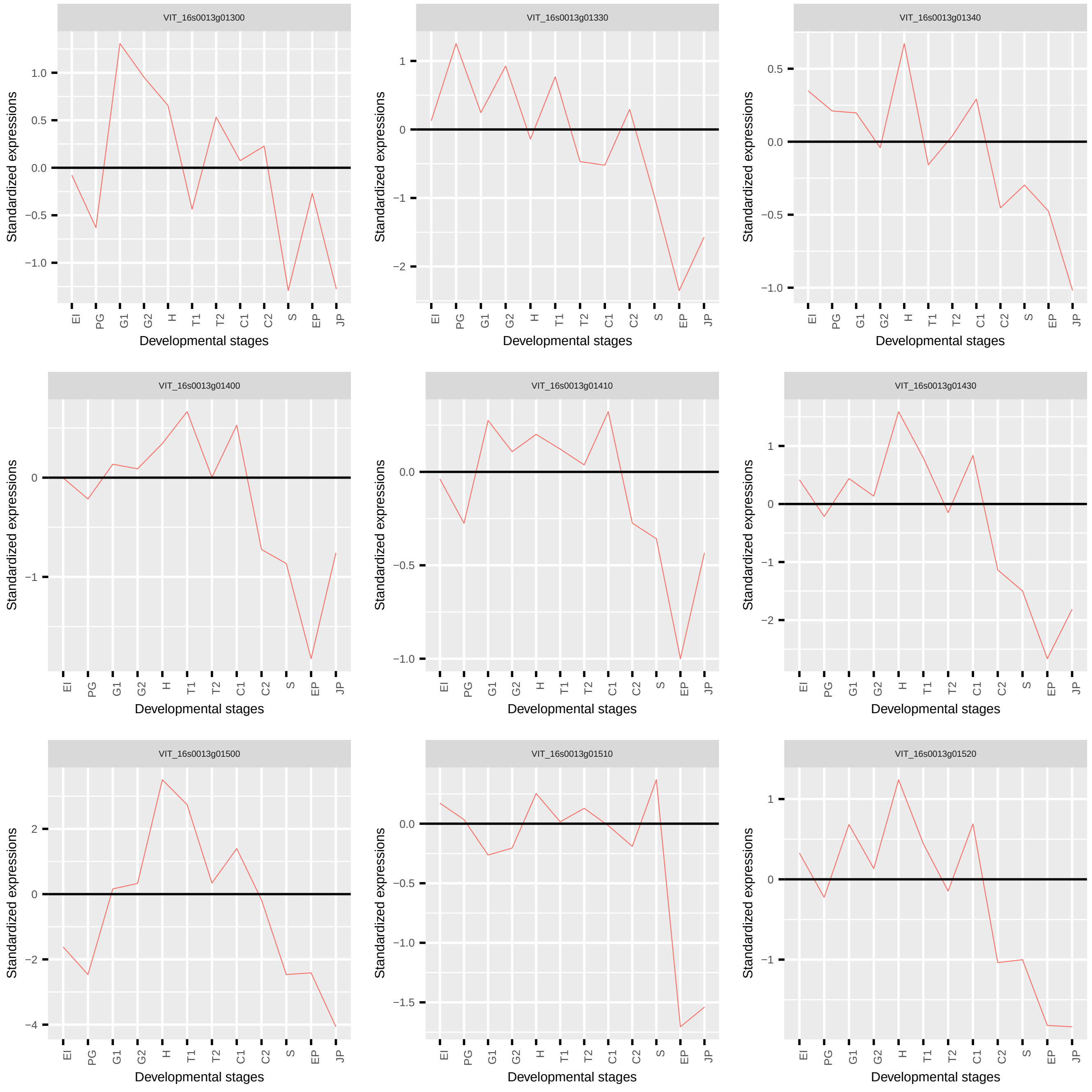
VIT_16s0013g01300
VIT_16s0013g01330
VIT_16s0013g01340
VIT_16s0013g01400
VIT_16s0013g01410
VIT_16s0013g01430
VIT_16s0013g01500
VIT_16s0013g01510
VIT_16s0013g01520
Standardized expressions
Developmental stages
EI
PG
G1
G2
H
T1
T2
C1
C2
S
EP
JP

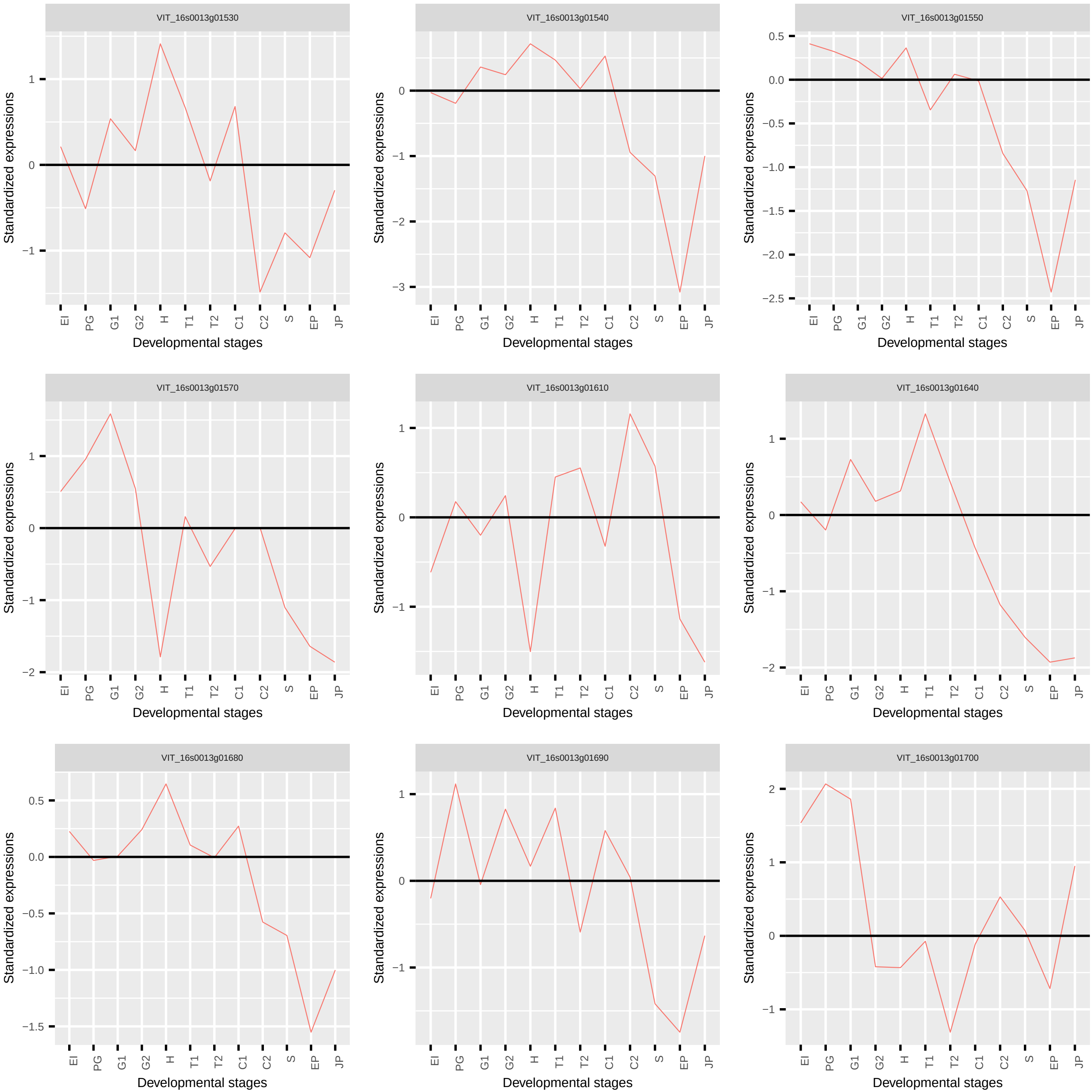
VIT_16s0013g01530
VIT_16s0013g01540
VIT_16s0013g01550
VIT_16s0013g01570
VIT_16s0013g01610
VIT_16s0013g01640
VIT_16s0013g01680
VIT_16s0013g01690
VIT_16s0013g01700
Standardized expressions
Developmental stages
EI
PG
G1
G2
H
T1
T2
C1
C2
S
EP
JP

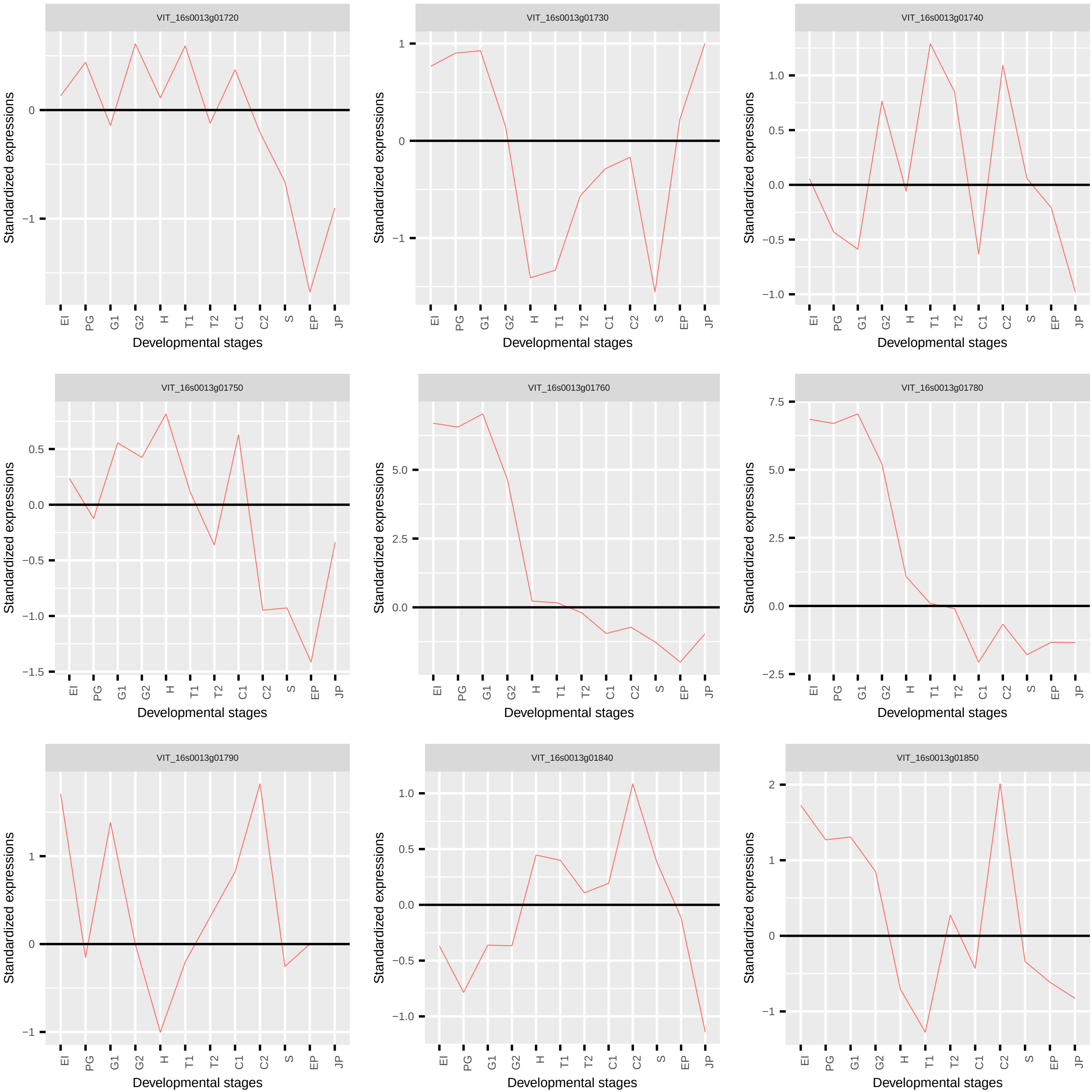
VIT_16s0013g01720
VIT_16s0013g01730
VIT_16s0013g01740
VIT_16s0013g01750
VIT_16s0013g01760
VIT_16s0013g01780
VIT_16s0013g01790
VIT_16s0013g01840
VIT_16s0013g01850
Standardized expressions
Developmental stages
EI PG G1 G2 H T1 T2 C1 C2 S EP JP

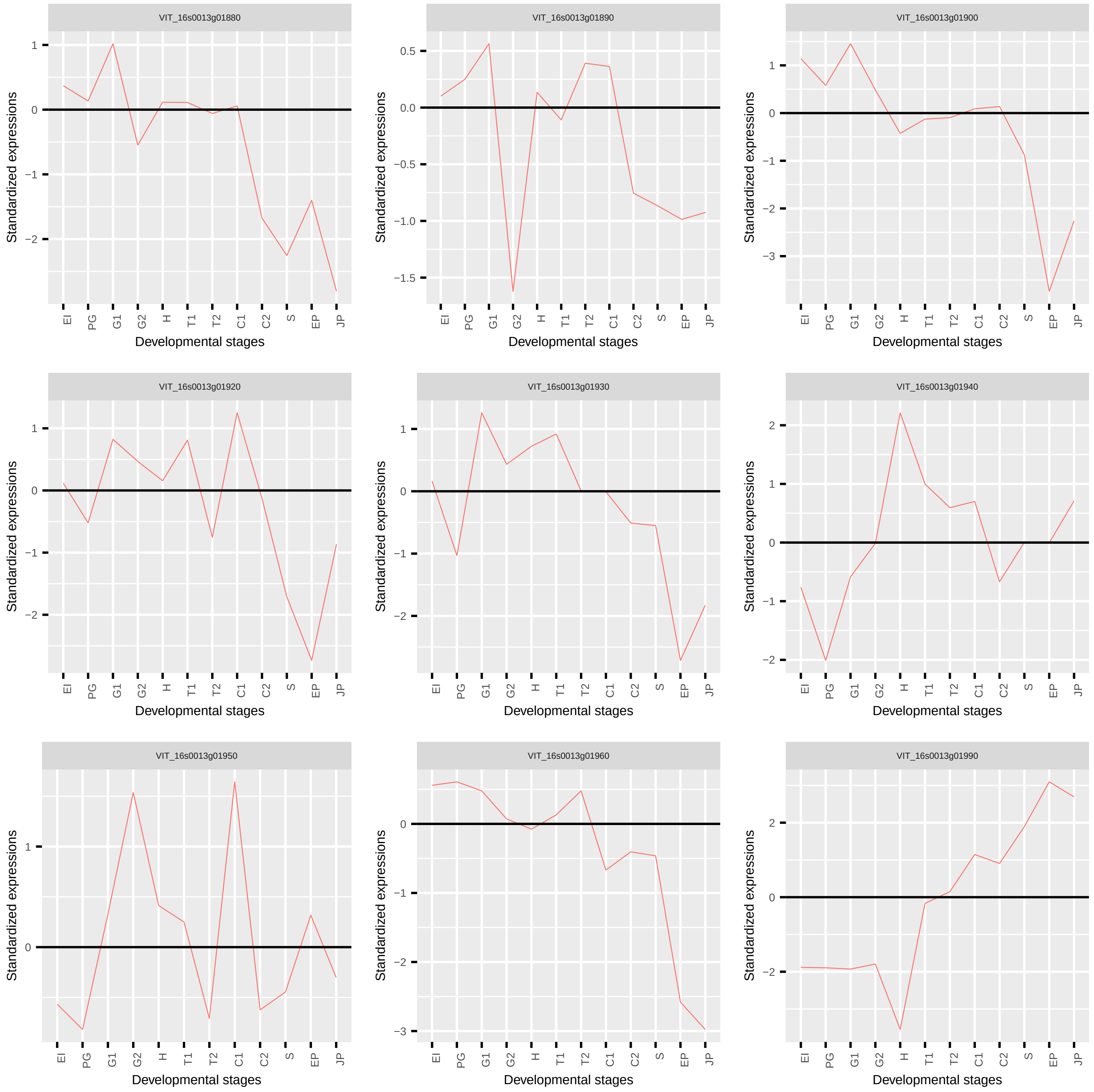
VIT_16s0013g01880
VIT_16s0013g01890
VIT_16s0013g01900
VIT_16s0013g01920
VIT_16s0013g01930
VIT_16s0013g01940
VIT_16s0013g01950
VIT_16s0013g01960
VIT_16s0013g01990
Standardized expressions
Developmental stages
EI PG G1 G2 H T1 T2 C1 C2 S EP JP
1
0
−1
−2
−3
0.5
0.0
−0.5
−1.0
−1.5
2

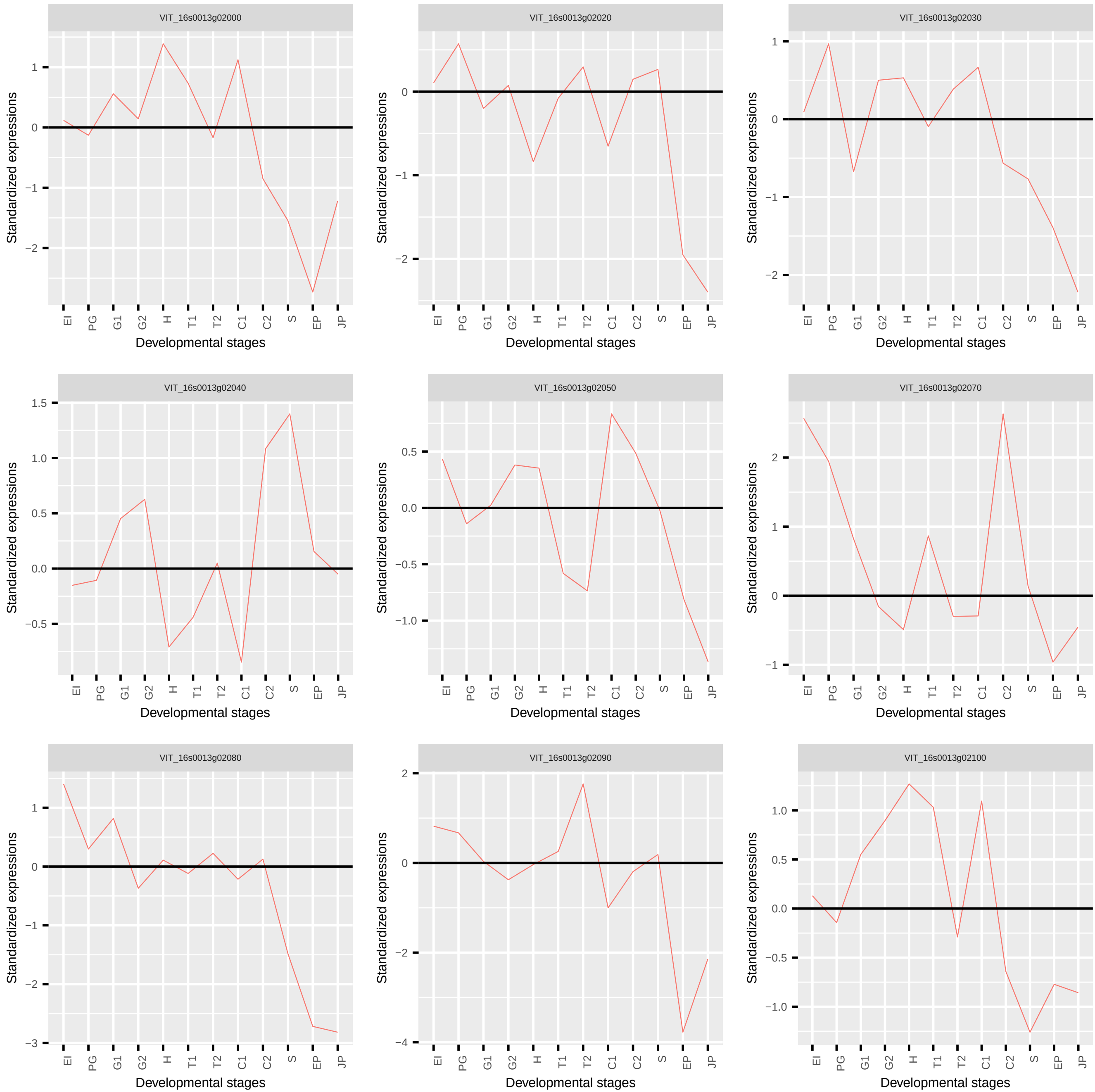
VIT_16s0013g02000
VIT_16s0013g02020
VIT_16s0013g02030
VIT_16s0013g02040
VIT_16s0013g02050
VIT_16s0013g02070
VIT_16s0013g02080
VIT_16s0013g02090
VIT_16s0013g02100
Standardized expressions
Developmental stages
EI PG G1 G2 H T1 T2 C1 C2 S EP JP

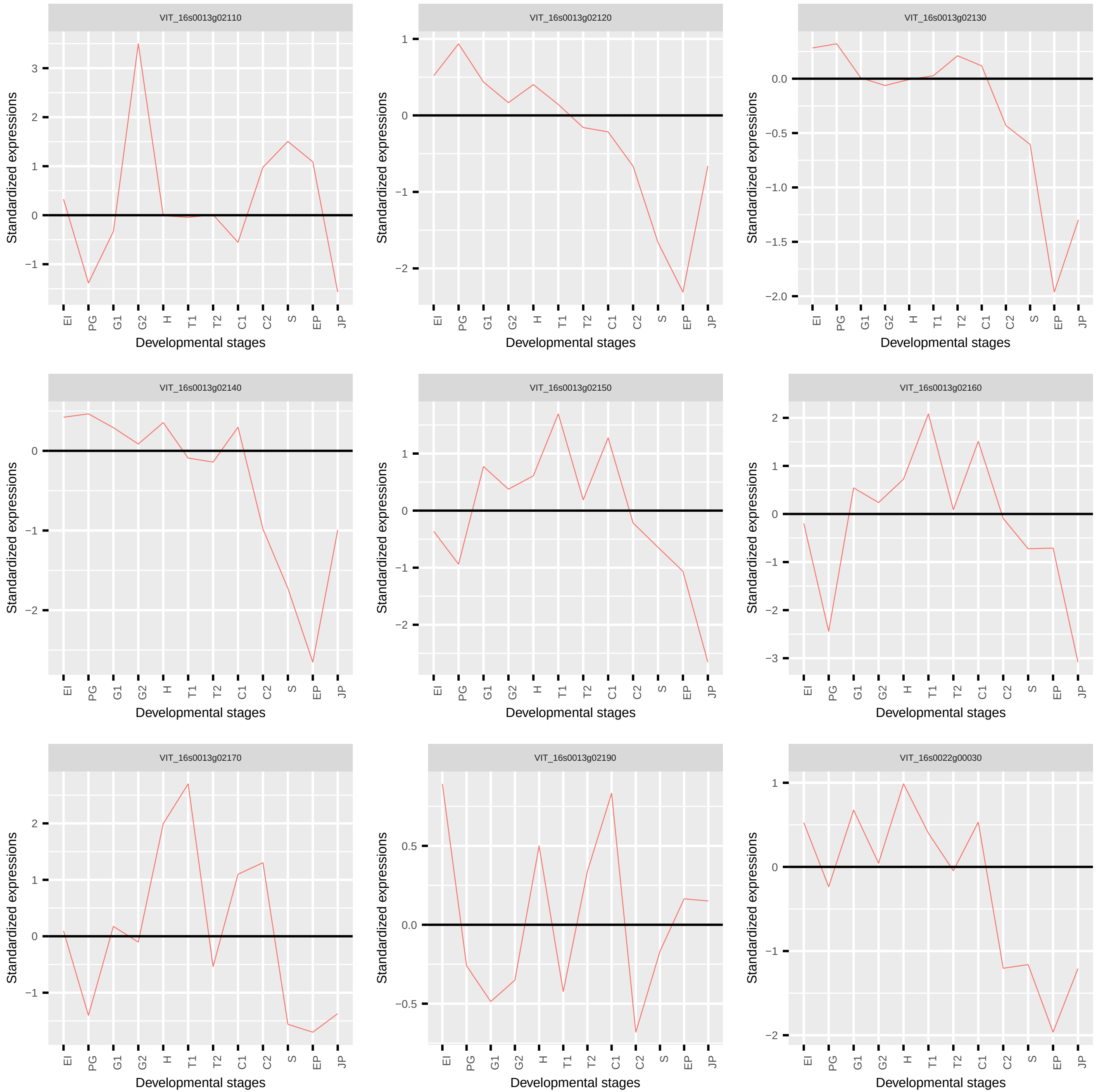
VIT_16s0013g02110
VIT_16s0013g02120
VIT_16s0013g02130
VIT_16s0013g02140
VIT_16s0013g02150
VIT_16s0013g02160
VIT_16s0013g02170
VIT_16s0013g02190
VIT_16s0022g00030
Standardized expressions
Developmental stages
EI PG G1 G2 H T1 T2 C1 C2 S EP JP

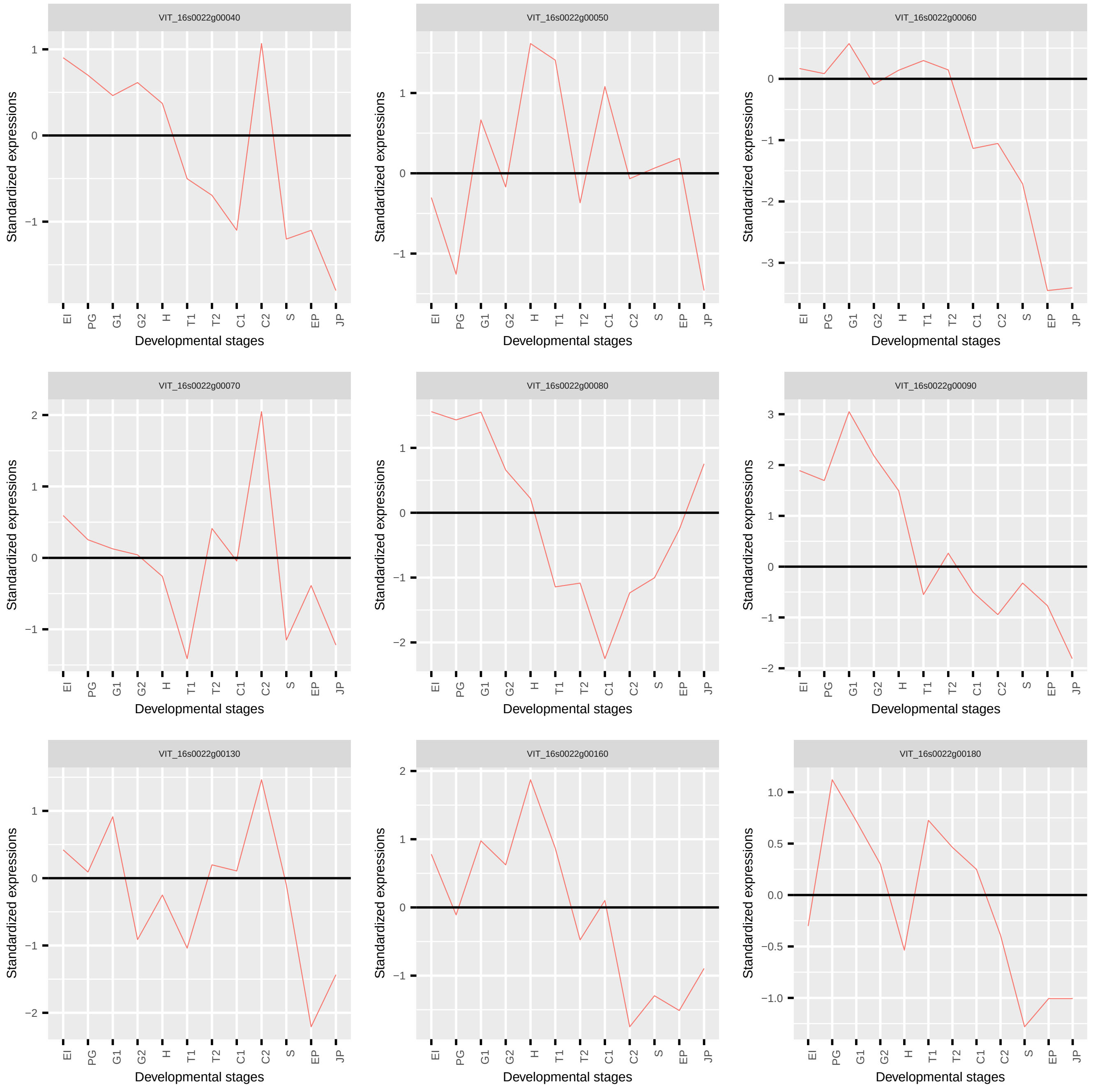
VIT_16s0022g00040
VIT_16s0022g00050
VIT_16s0022g00060
VIT_16s0022g00070
VIT_16s0022g00080
VIT_16s0022g00090
VIT_16s0022g00130
VIT_16s0022g00160
VIT_16s0022g00180
Standardized expressions
Developmental stages
EI PG G1 G2 H T1 T2 C1 C2 S EP JP

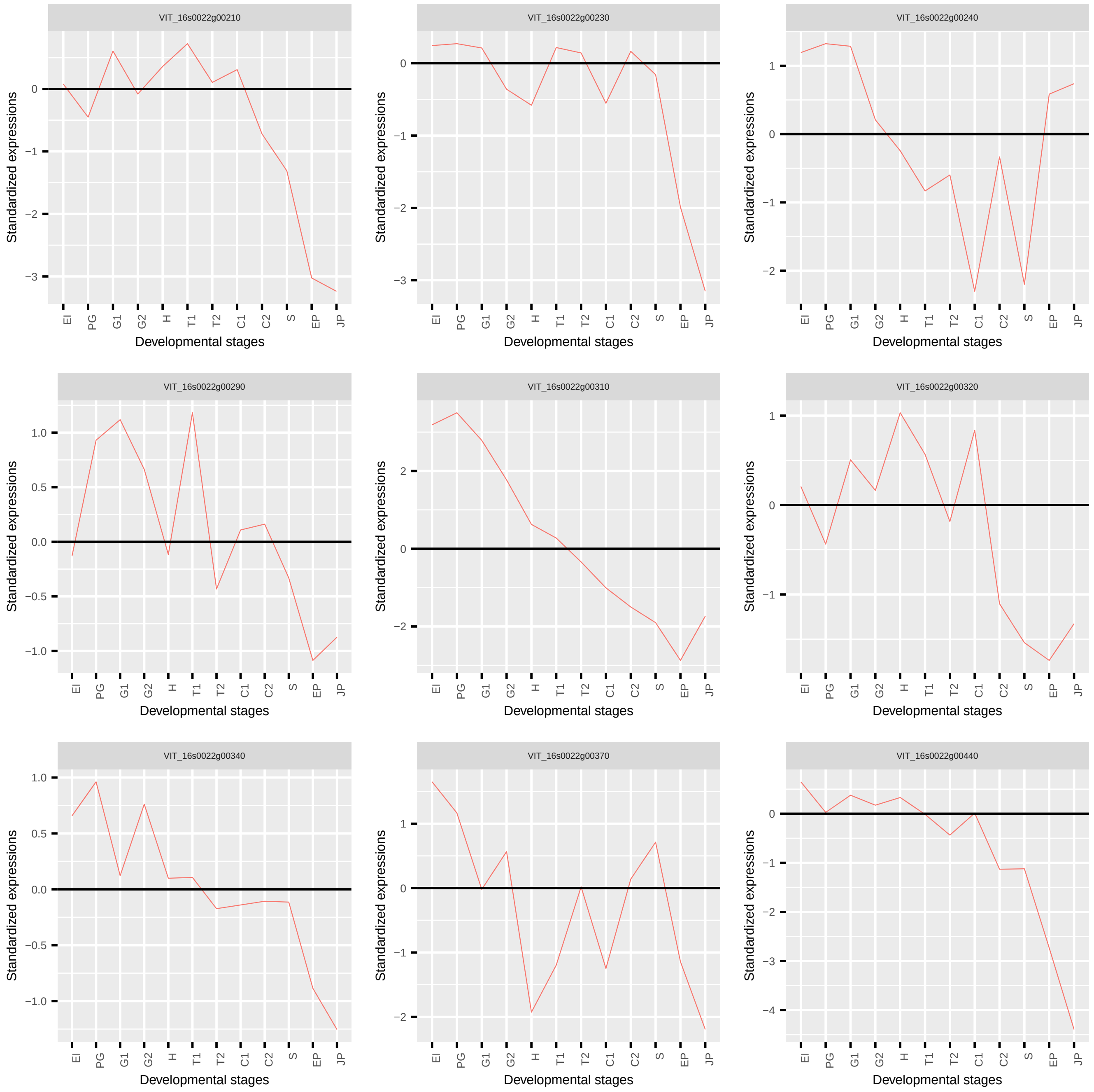
VIT_16s0022g00210
VIT_16s0022g00230
VIT_16s0022g00240
VIT_16s0022g00290
VIT_16s0022g00310
VIT_16s0022g00320
VIT_16s0022g00340
VIT_16s0022g00370
VIT_16s0022g00440
Standardized expressions
Developmental stages
EI
PG
G1
G2
H
T1
T2
C1
C2
S
EP
JP

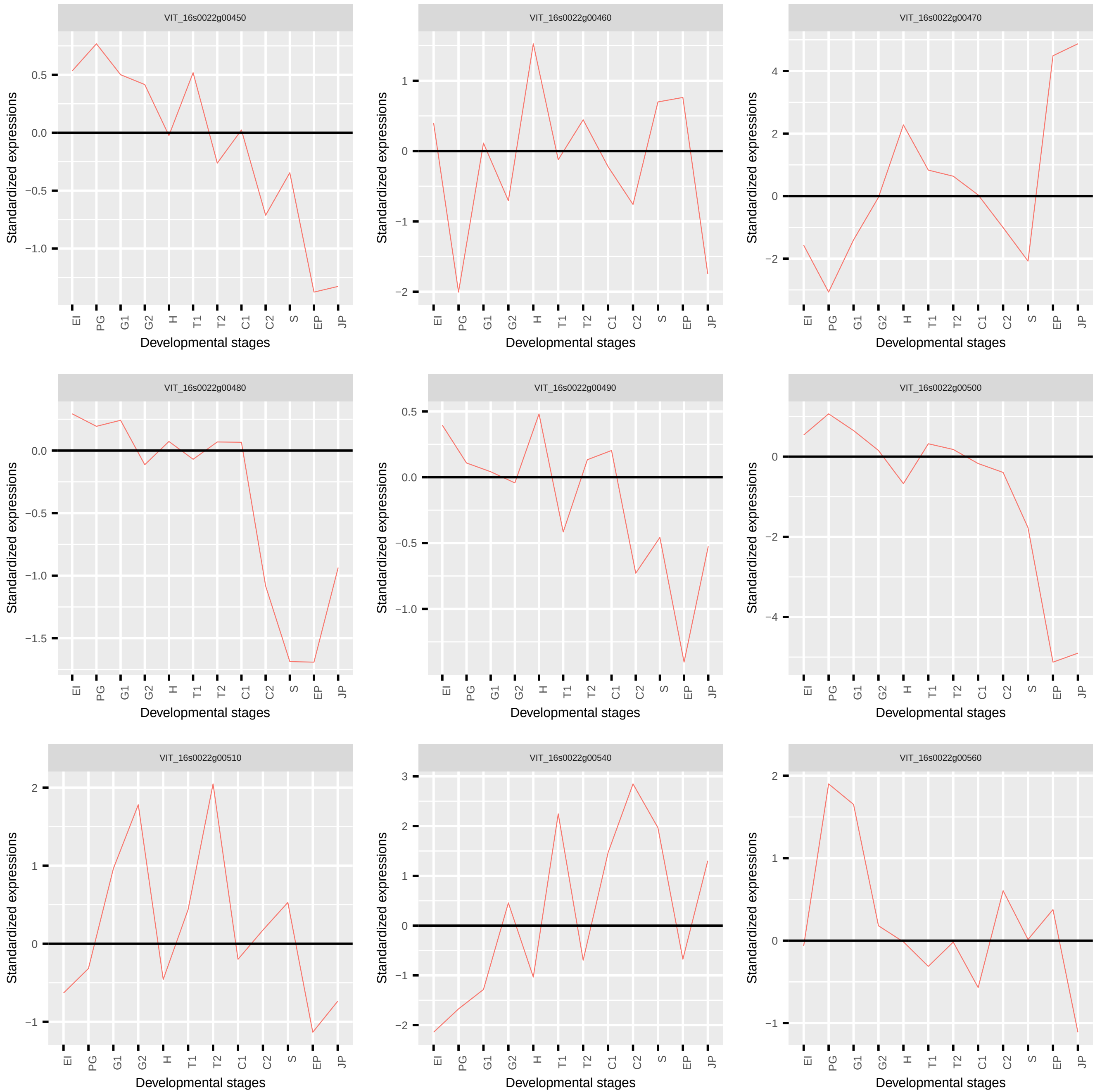
VIT_16s0022g00450
VIT_16s0022g00460
VIT_16s0022g00470
VIT_16s0022g00480
VIT_16s0022g00490
VIT_16s0022g00500
VIT_16s0022g00510
VIT_16s0022g00540
VIT_16s0022g00560
Standardized expressions
Developmental stages
EI PG G1 G2 H T1 T2 C1 C2 S EP JP

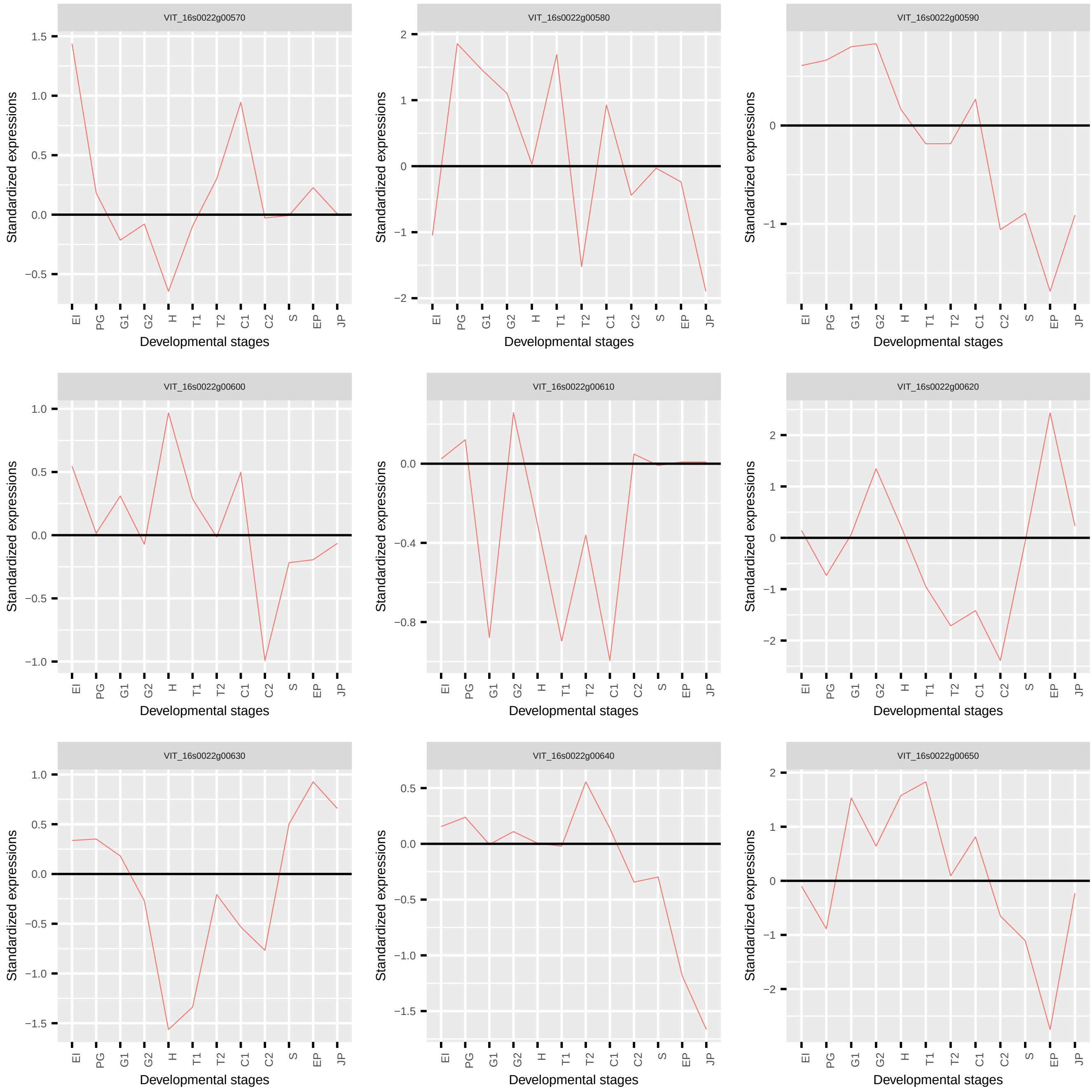

VIT_16s0022g00570
VIT_16s0022g00580
VIT_16s0022g00590
VIT_16s0022g00600
VIT_16s0022g00610
VIT_16s0022g00620
VIT_16s0022g00630
VIT_16s0022g00640
VIT_16s0022g00650
Standardized expressions
Developmental stages
EI PG G1 G2 H T1 T2 C1 C2 S EP JP

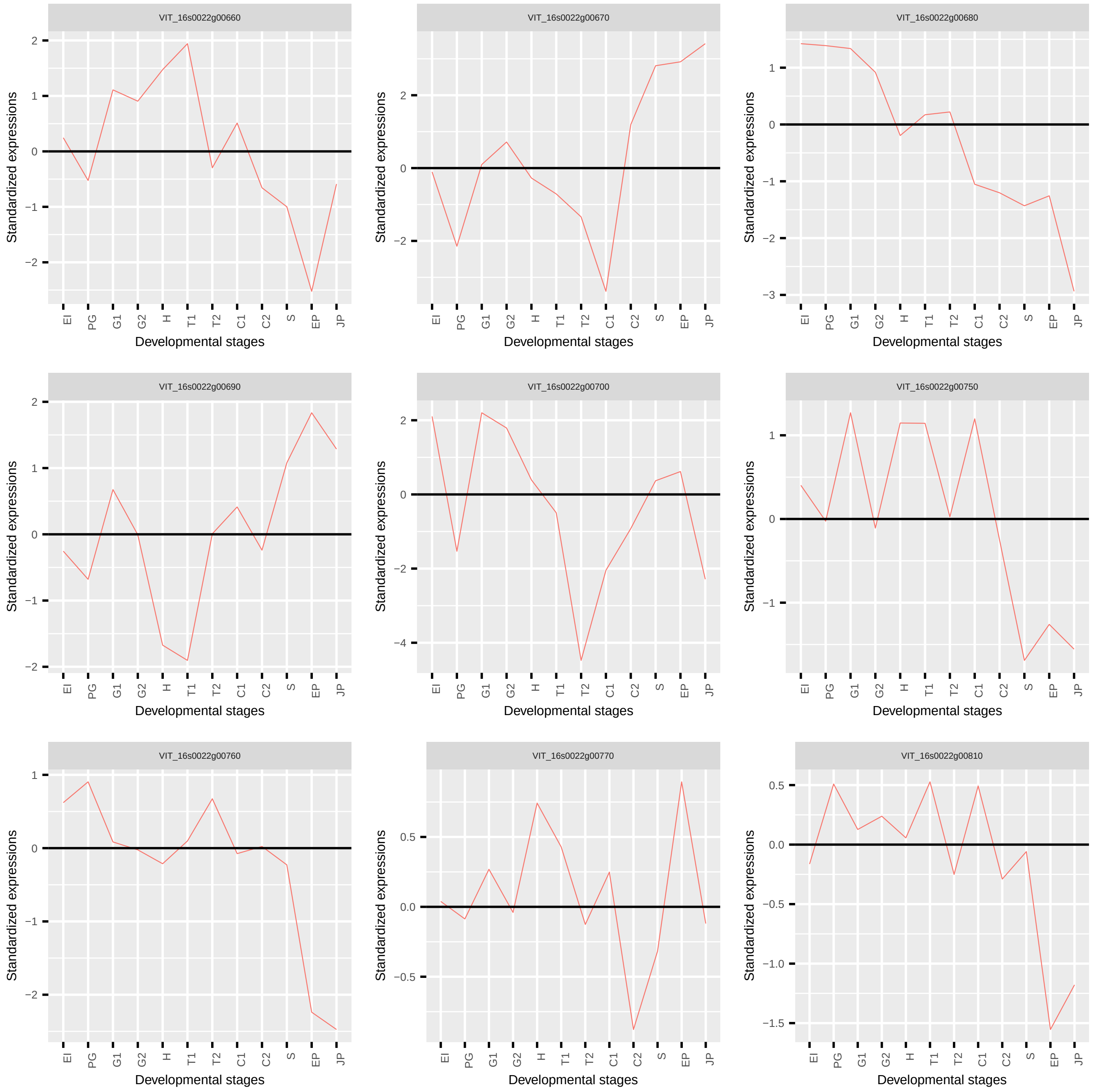

VIT_16s0022g00660
VIT_16s0022g00670
VIT_16s0022g00680
VIT_16s0022g00690
VIT_16s0022g00700
VIT_16s0022g00750
VIT_16s0022g00760
VIT_16s0022g00770
VIT_16s0022g00810
Standardized expressions
Developmental stages
EI PG G1 G2 H T1 T2 C1 C2 S EP JP

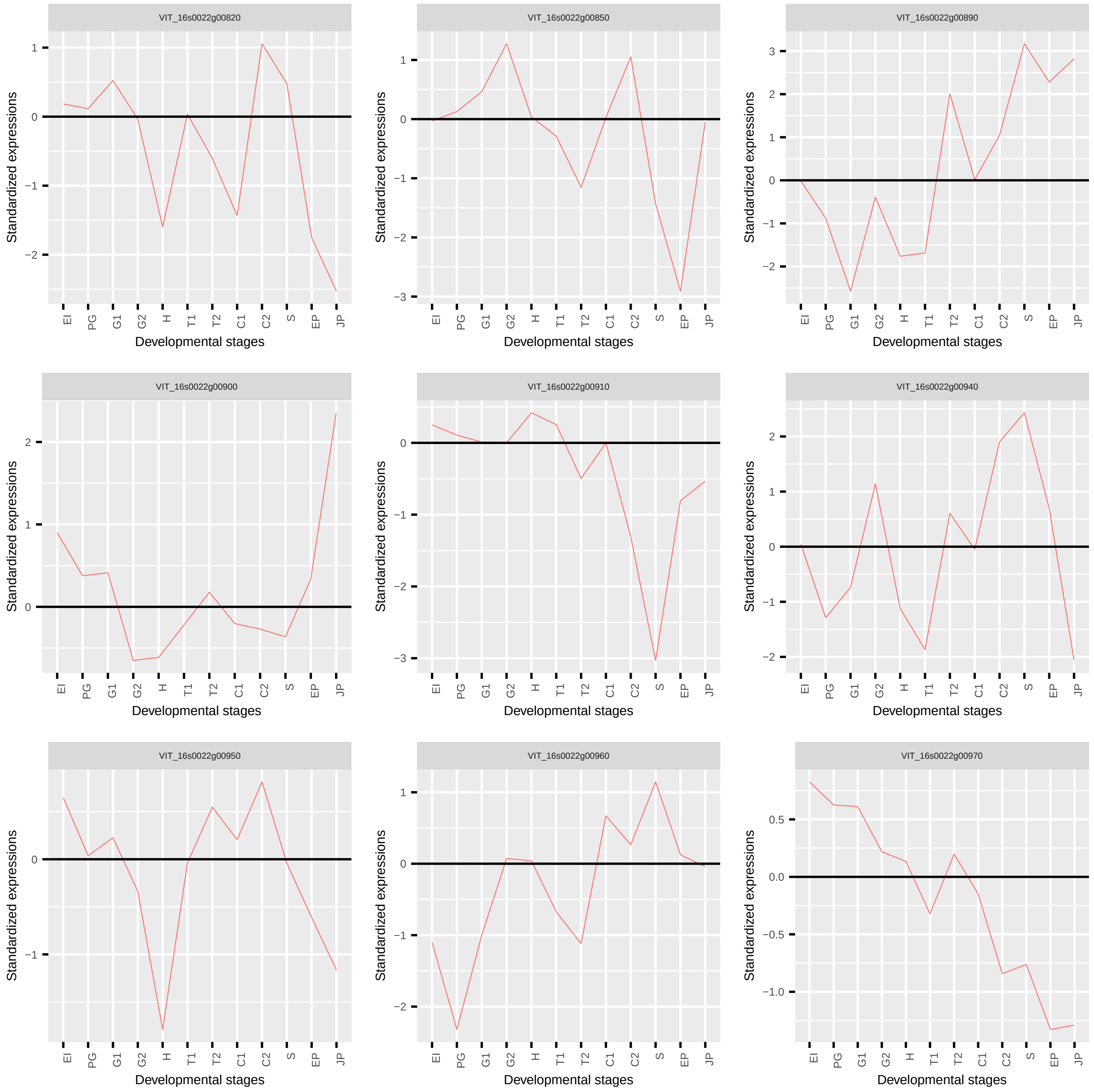

VIT_16s0022g00820
VIT_16s0022g00850
VIT_16s0022g00890
VIT_16s0022g00900
VIT_16s0022g00910
VIT_16s0022g00940
VIT_16s0022g00950
VIT_16s0022g00960
VIT_16s0022g00970
Standardized expressions
Developmental stages
EI PG G1 G2 H T1 T2 C1 C2 S EP JP

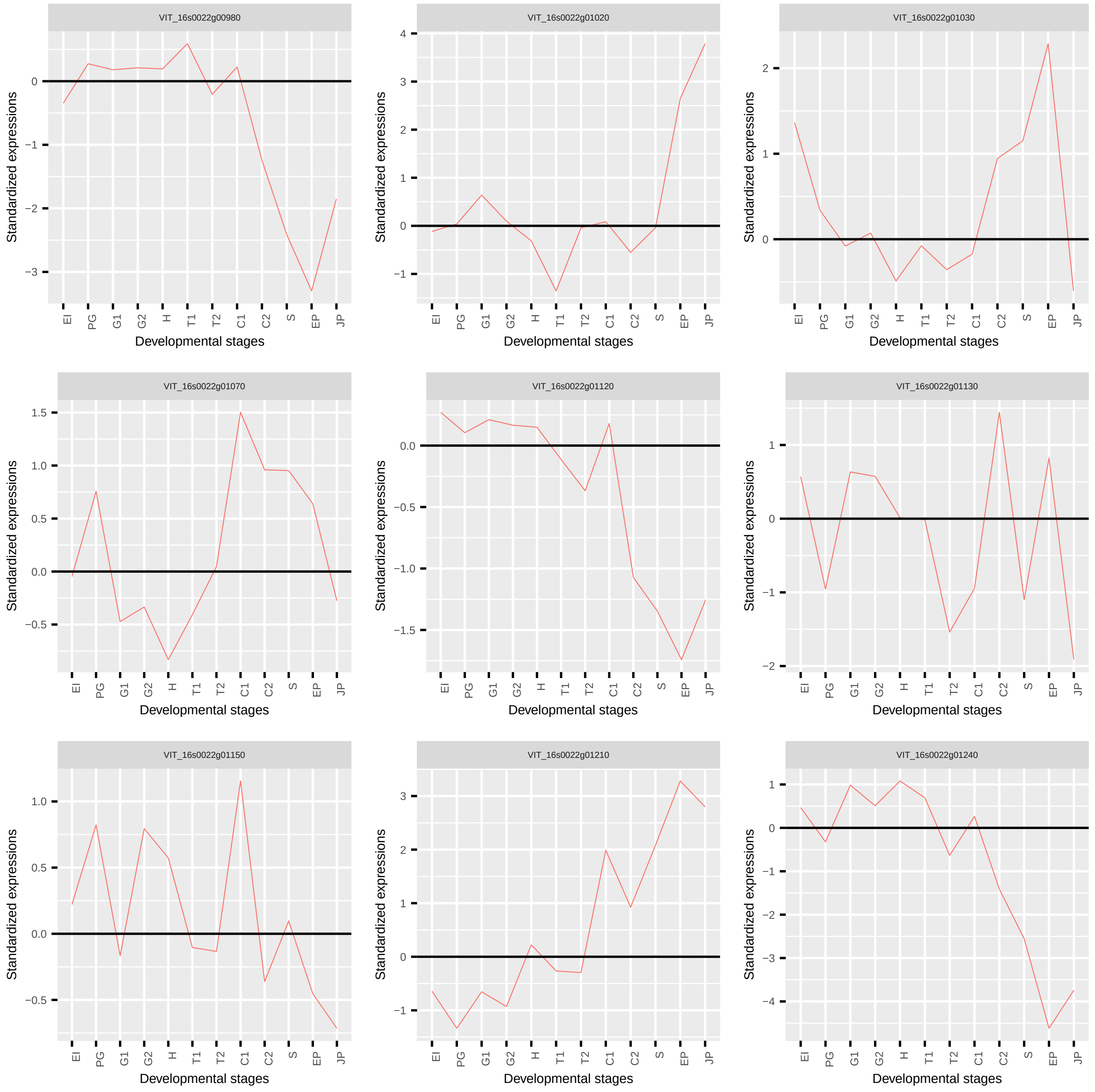
VIT_16s0022g00980
VIT_16s0022g01020
VIT_16s0022g01030
VIT_16s0022g01070
VIT_16s0022g01120
VIT_16s0022g01130
VIT_16s0022g01150
VIT_16s0022g01210
VIT_16s0022g01240
Standardized expressions
Developmental stages
EI PG G1 G2 H T1 T2 C1 C2 S EP JP

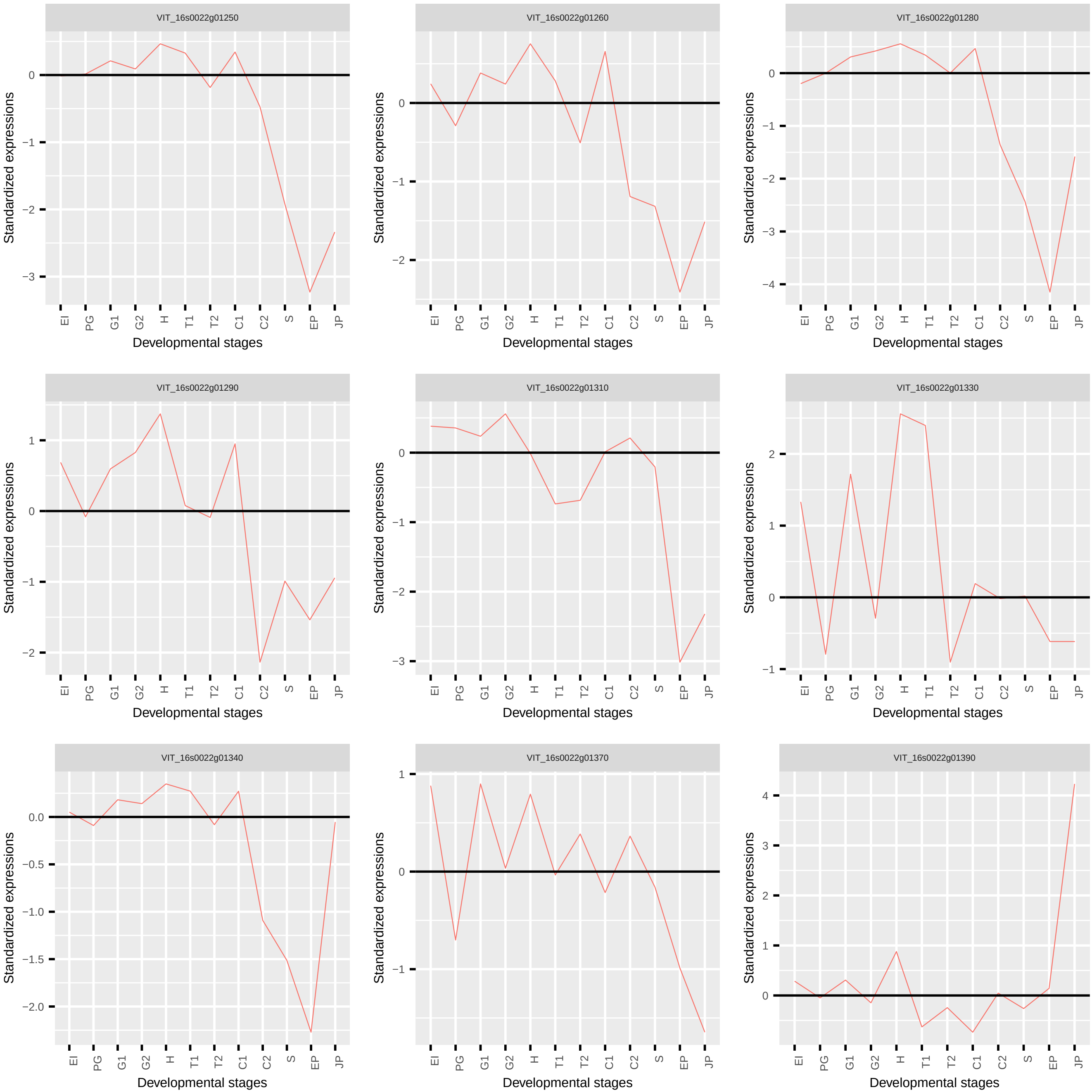
VIT_16s0022g01250
VIT_16s0022g01260
VIT_16s0022g01280
VIT_16s0022g01290
VIT_16s0022g01310
VIT_16s0022g01330
VIT_16s0022g01340
VIT_16s0022g01370
VIT_16s0022g01390
Standardized expressions
Developmental stages
EI PG G1 G2 H T1 T2 C1 C2 S EP JP
0 −1 −2 −3 −4
1 2 3 4
0.0 −0.5 −1.0 −1.5 −2.0

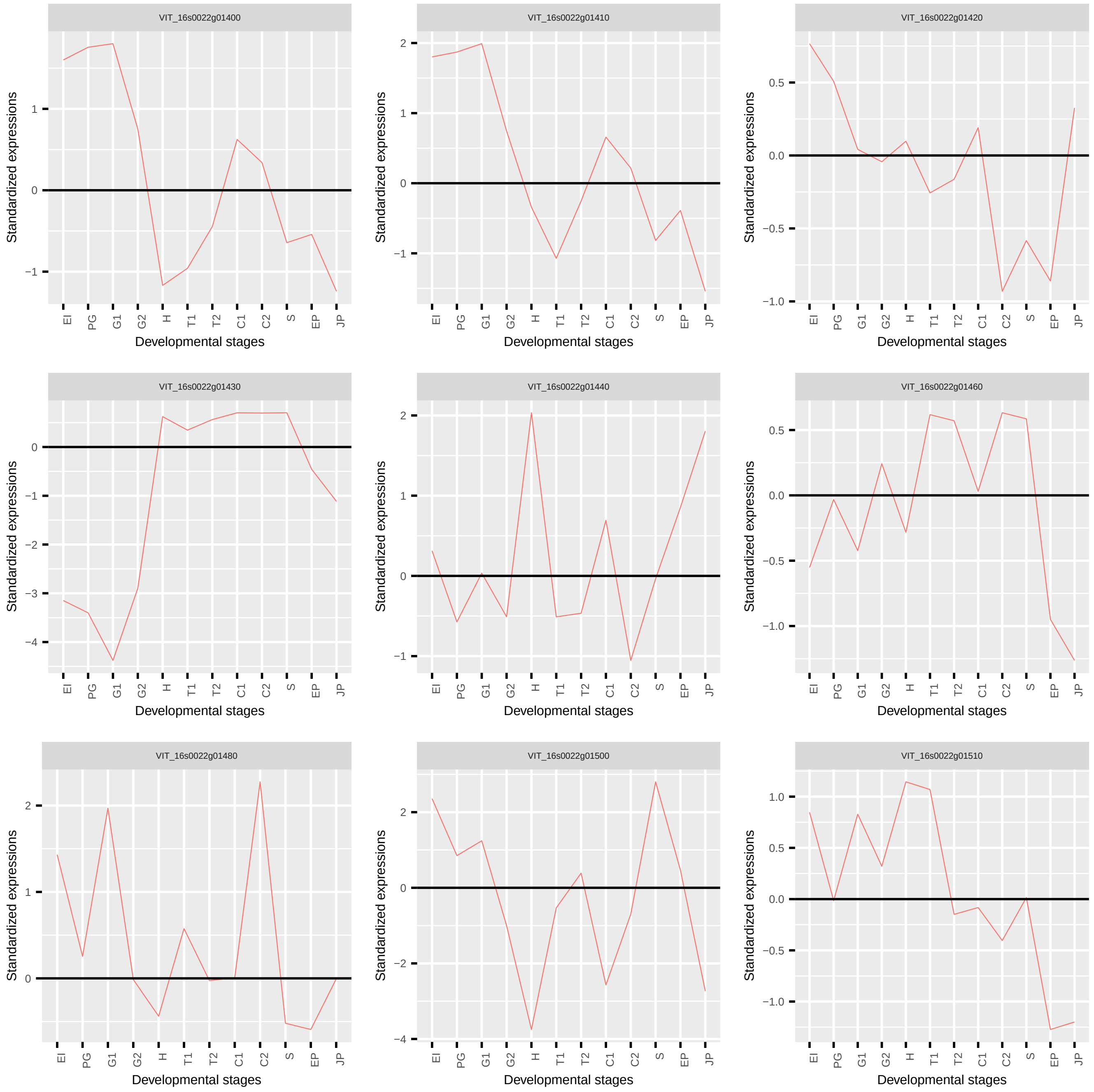
VIT_16s0022g01400
Standardized expressions
1
0
−1
EI
PG
G1
G2
H
T1
T2
C1
C2
S
EP
JP
Developmental stages
VIT_16s0022g01410
Standardized expressions
2
1
0
−1
EI
PG
G1
G2
H
T1
T2
C1
C2
S
EP
JP
Developmental stages
VIT_16s0022g01420
Standardized expressions
0.5
0.0
−0.5
−1.0
EI
PG
G1
G2
H
T1
T2
C1
C2
S
EP
JP
Developmental stages
VIT_16s0022g01430
Standardized expressions
0
−1
−2
−3
−4
EI
PG
G1
G2
H
T1
T2
C1
C2
S
EP
JP
Developmental stages
VIT_16s0022g01440
Standardized expressions
2
1
0
−1
EI
PG
G1
G2
H
T1
T2
C1
C2
S
EP
JP
Developmental stages
VIT_16s0022g01460
Standardized expressions
0.5
0.0
−0.5
−1.0
EI
PG
G1
G2
H
T1
T2
C1
C2
S
EP
JP
Developmental stages
VIT_16s0022g01480
Standardized expressions
2
1
0
EI
PG
G1
G2
H
T1
T2
C1
C2
S
EP
JP
Developmental stages
VIT_16s0022g01500
Standardized expressions
2
0
−2
−4
EI
PG
G1
G2
H
T1
T2
C1
C2
S
EP
JP
Developmental stages
VIT_16s0022g01510
Standardized expressions
1.0
0.5
0.0
−0.5
−1.0
EI
PG
G1
G2
H
T1
T2
C1
C2
S
EP
JP
Developmental stages

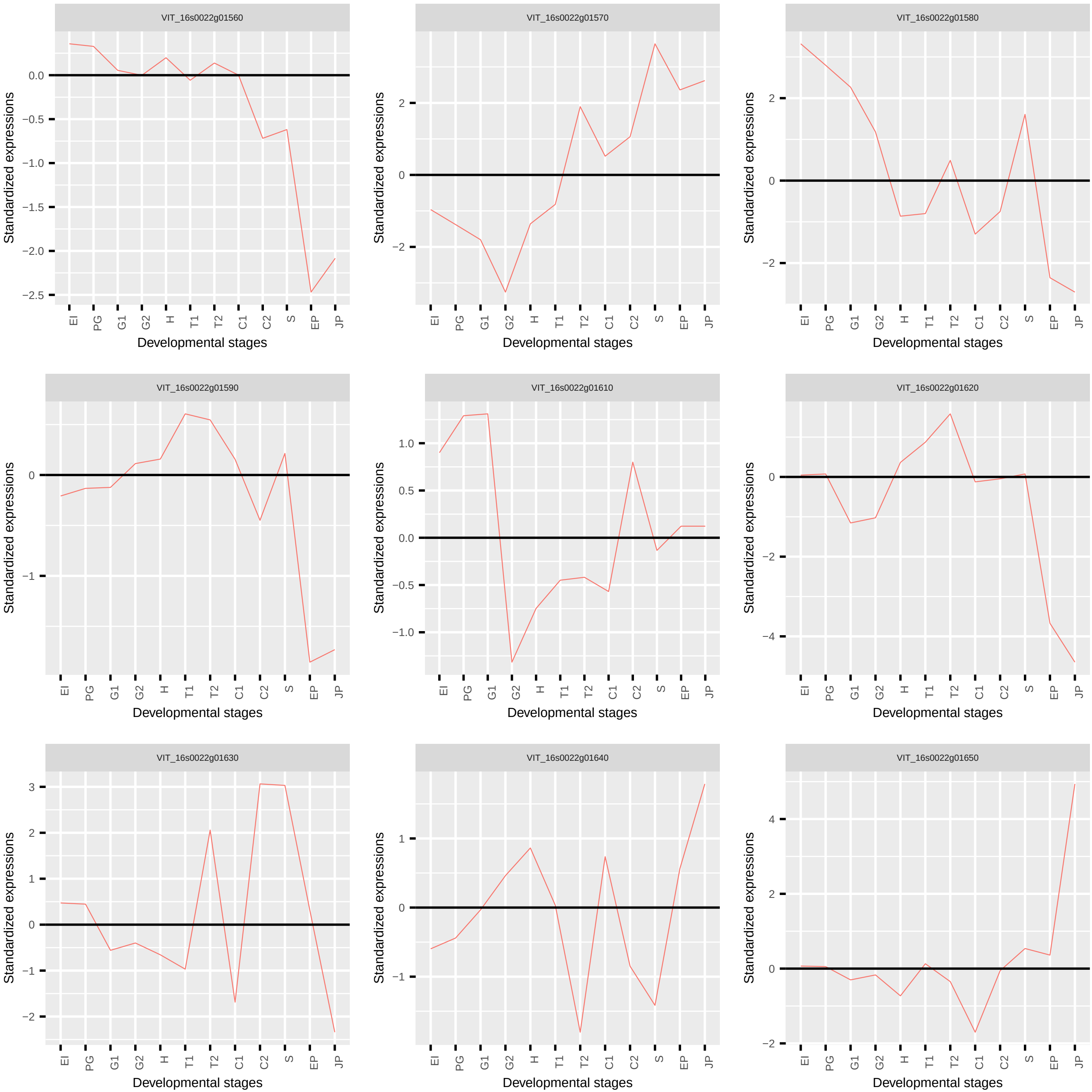

VIT_16s0022g01560
VIT_16s0022g01570
VIT_16s0022g01580
VIT_16s0022g01590
VIT_16s0022g01610
VIT_16s0022g01620
VIT_16s0022g01630
VIT_16s0022g01640
VIT_16s0022g01650
Standardized expressions
Developmental stages
EI PG G1 G2 H T1 T2 C1 C2 S EP JP

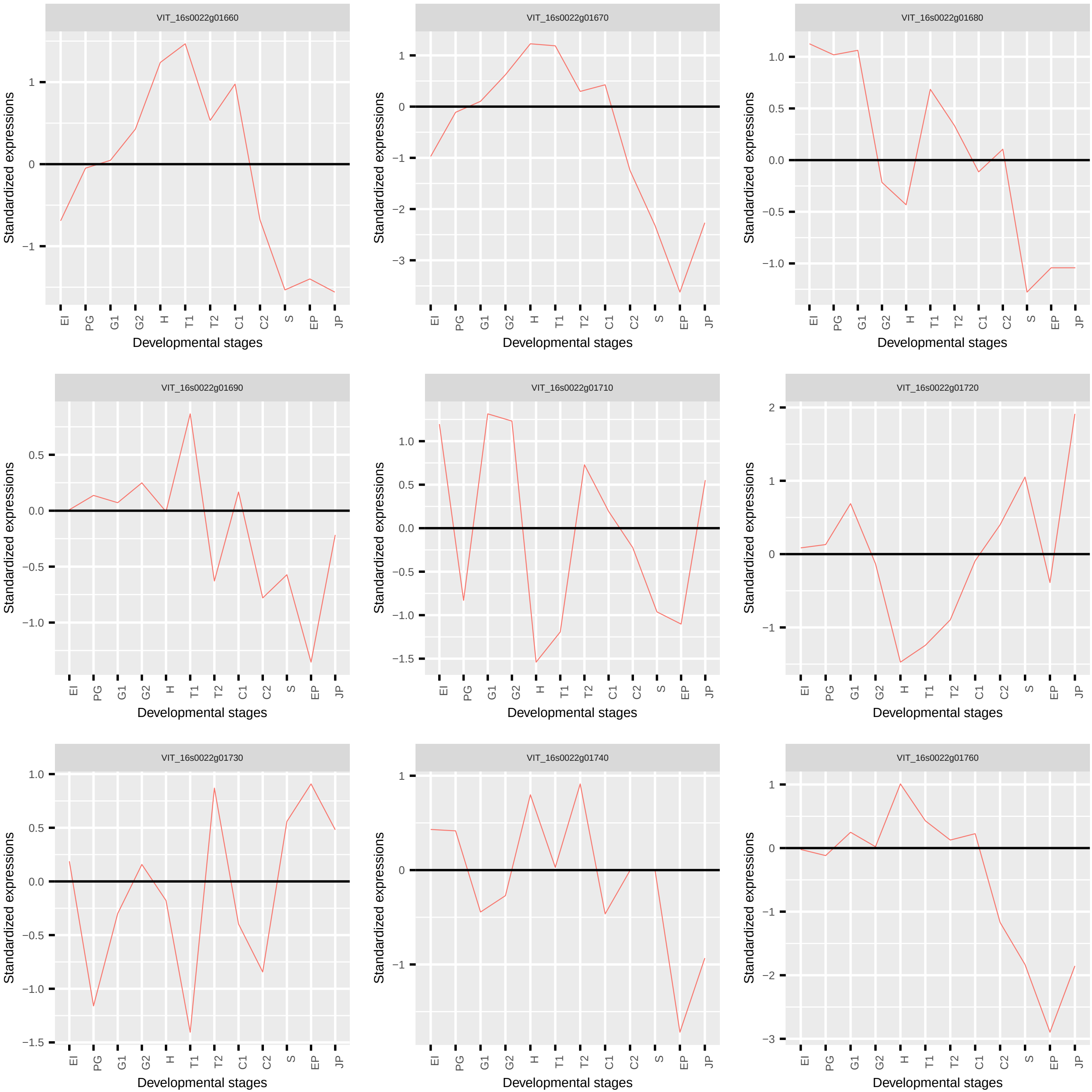

VIT_16s0022g01660
VIT_16s0022g01670
VIT_16s0022g01680
VIT_16s0022g01690
VIT_16s0022g01710
VIT_16s0022g01720
VIT_16s0022g01730
VIT_16s0022g01740
VIT_16s0022g01760
Standardized expressions
Developmental stages
EI
PG
G1
G2
H
T1
T2
C1
C2
S
EP
JP

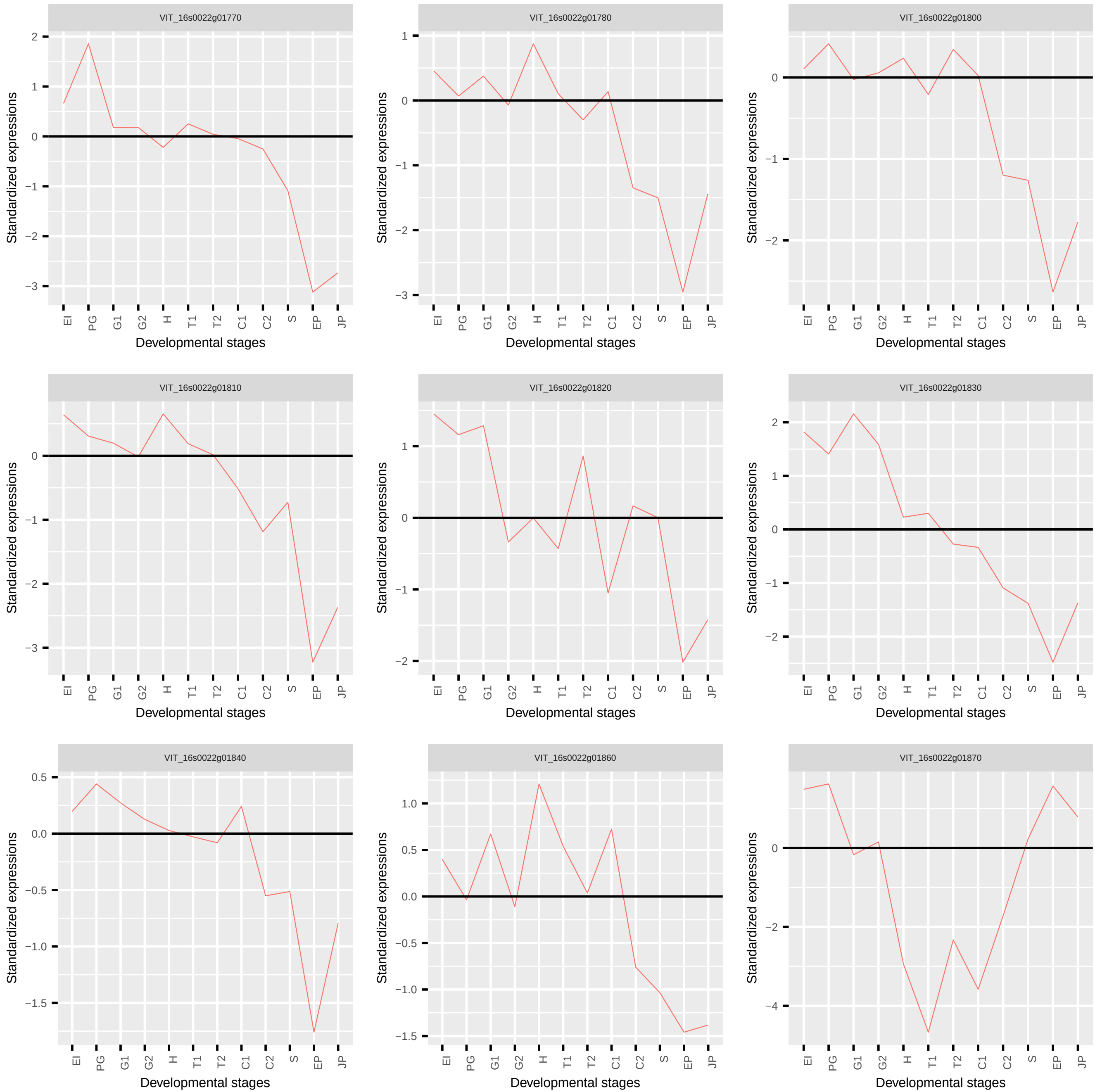
VIT_16s0022g01770
VIT_16s0022g01780
VIT_16s0022g01800
VIT_16s0022g01810
VIT_16s0022g01820
VIT_16s0022g01830
VIT_16s0022g01840
VIT_16s0022g01860
VIT_16s0022g01870
Standardized expressions
Developmental stages
EI PG G1 G2 H T1 T2 C1 C2 S EP JP

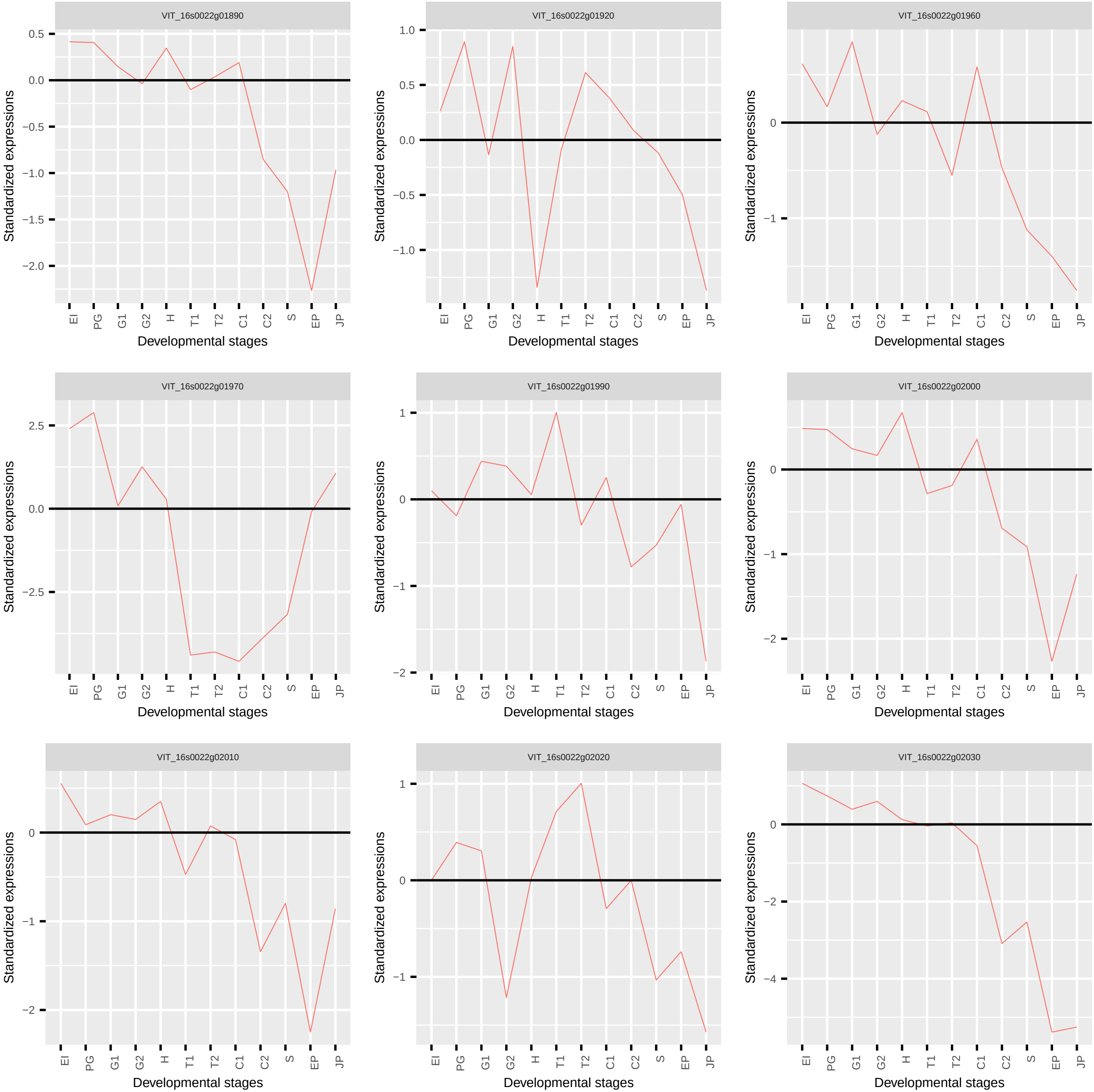
VIT_16s0022g01890
0.5
0.0
−0.5
−1.0
−1.5
−2.0
Standardized expressions
EI PG G1 G2 H T1 T2 C1 C2 S EP JP
Developmental stages
VIT_16s0022g01920
1.0
0.5
0.0
−0.5
−1.0
Standardized expressions
EI PG G1 G2 H T1 T2 C1 C2 S EP JP
Developmental stages
VIT_16s0022g01960
0
−1
Standardized expressions
EI PG G1 G2 H T1 T2 C1 C2 S EP JP
Developmental stages
VIT_16s0022g01970
2.5
0.0
−2.5
Standardized expressions
EI PG G1 G2 H T1 T2 C1 C2 S EP JP
Developmental stages
VIT_16s0022g01990
1
0
−1
−2
Standardized expressions
EI PG G1 G2 H T1 T2 C1 C2 S EP JP
Developmental stages
VIT_16s0022g02000
0
−1
−2
Standardized expressions
EI PG G1 G2 H T1 T2 C1 C2 S EP JP
Developmental stages
VIT_16s0022g02010
0
−1
−2
Standardized expressions
EI PG G1 G2 H T1 T2 C1 C2 S EP JP
Developmental stages
VIT_16s0022g02020
1
0
−1
Standardized expressions
EI PG G1 G2 H T1 T2 C1 C2 S EP JP
Developmental stages
VIT_16s0022g02030
0
−2
−4
Standardized expressions
EI PG G1 G2 H T1 T2 C1 C2 S EP JP
Developmental stages

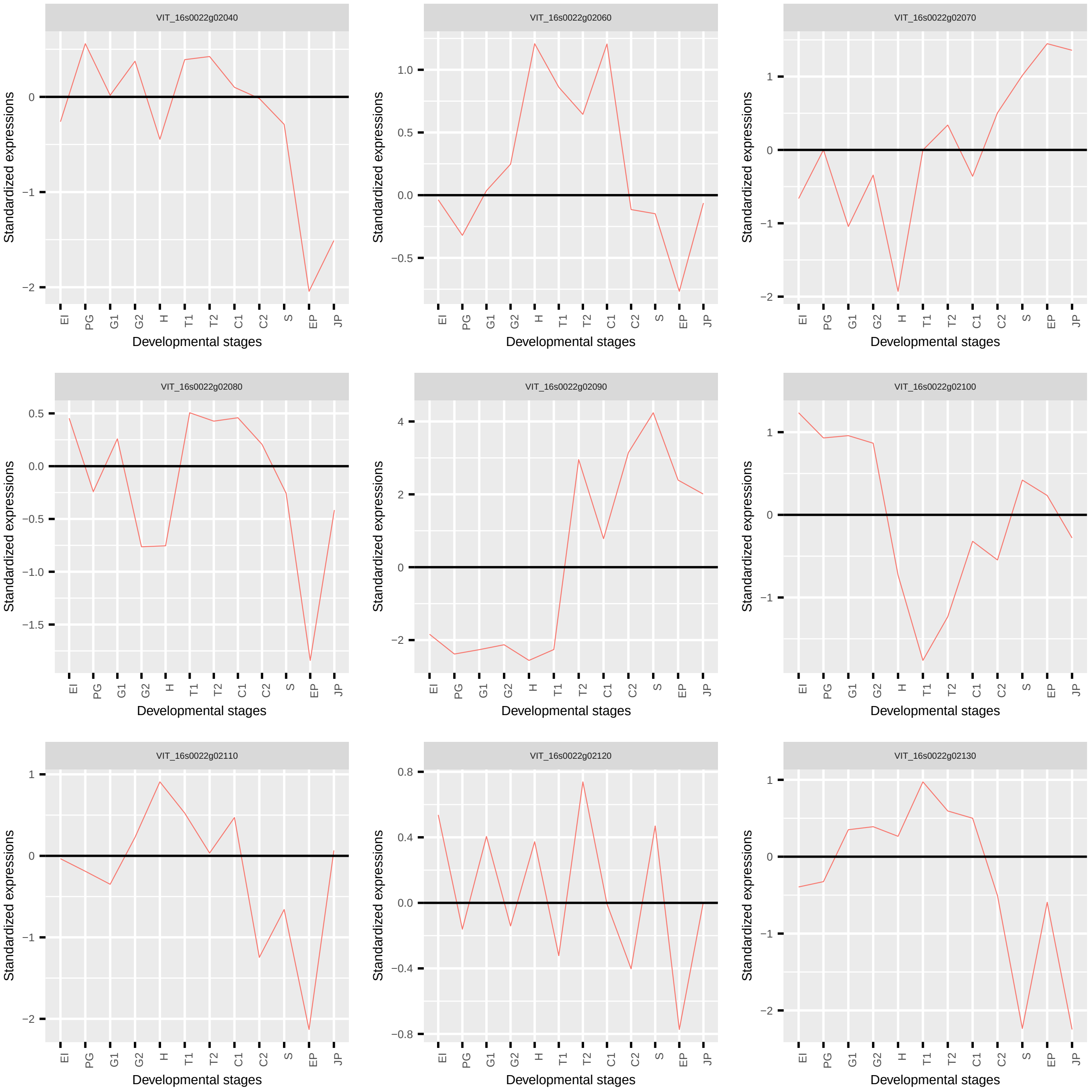
VIT_16s0022g02040
VIT_16s0022g02060
VIT_16s0022g02070
VIT_16s0022g02080
VIT_16s0022g02090
VIT_16s0022g02100
VIT_16s0022g02110
VIT_16s0022g02120
VIT_16s0022g02130
Standardized expressions
Developmental stages
EI PG G1 G2 H T1 T2 C1 C2 S EP JP

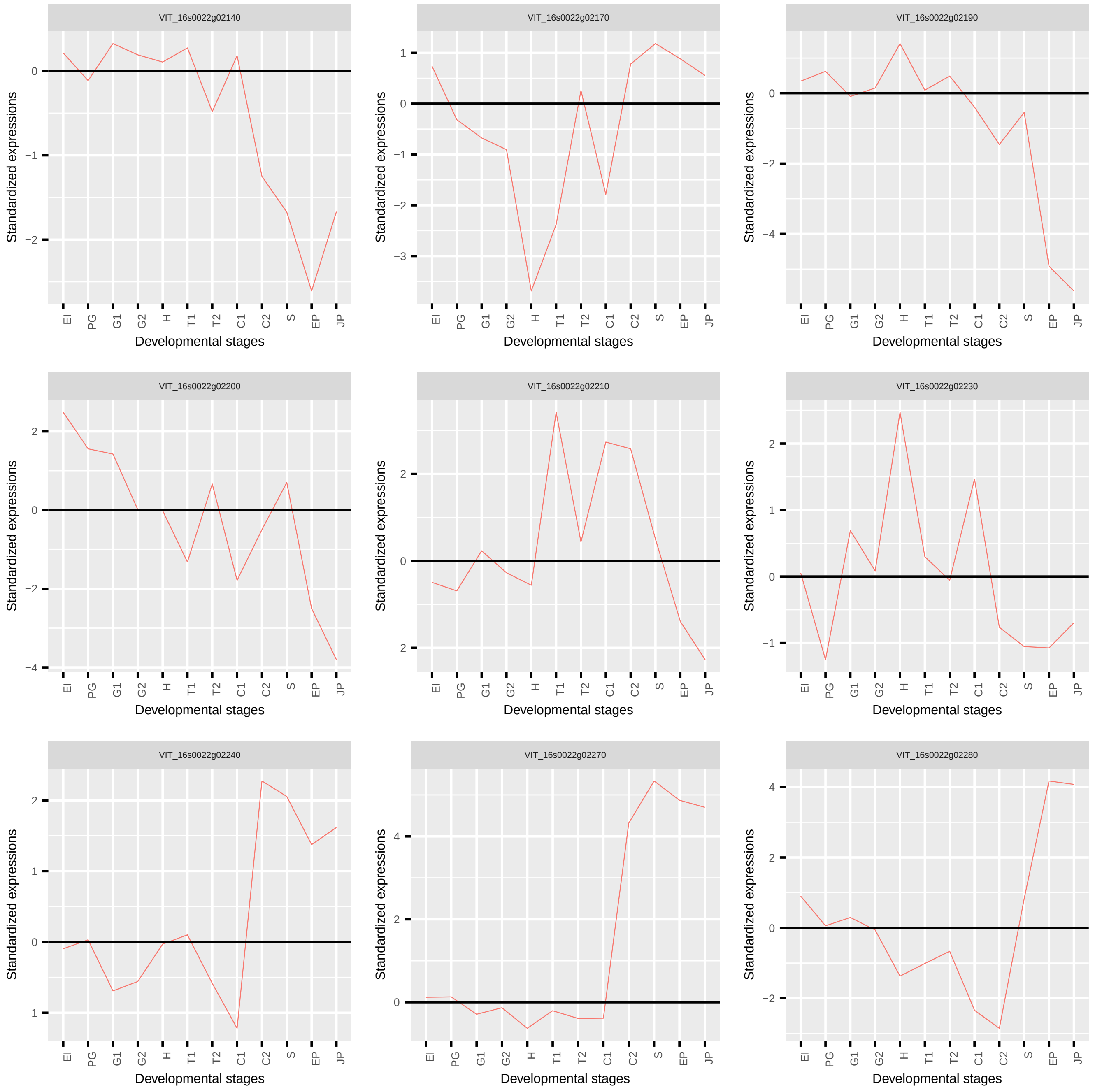
VIT_16s0022g02140
VIT_16s0022g02170
VIT_16s0022g02190
VIT_16s0022g02200
VIT_16s0022g02210
VIT_16s0022g02230
VIT_16s0022g02240
VIT_16s0022g02270
VIT_16s0022g02280
Standardized expressions
Developmental stages
EI PG G1 G2 H T1 T2 C1 C2 S EP JP

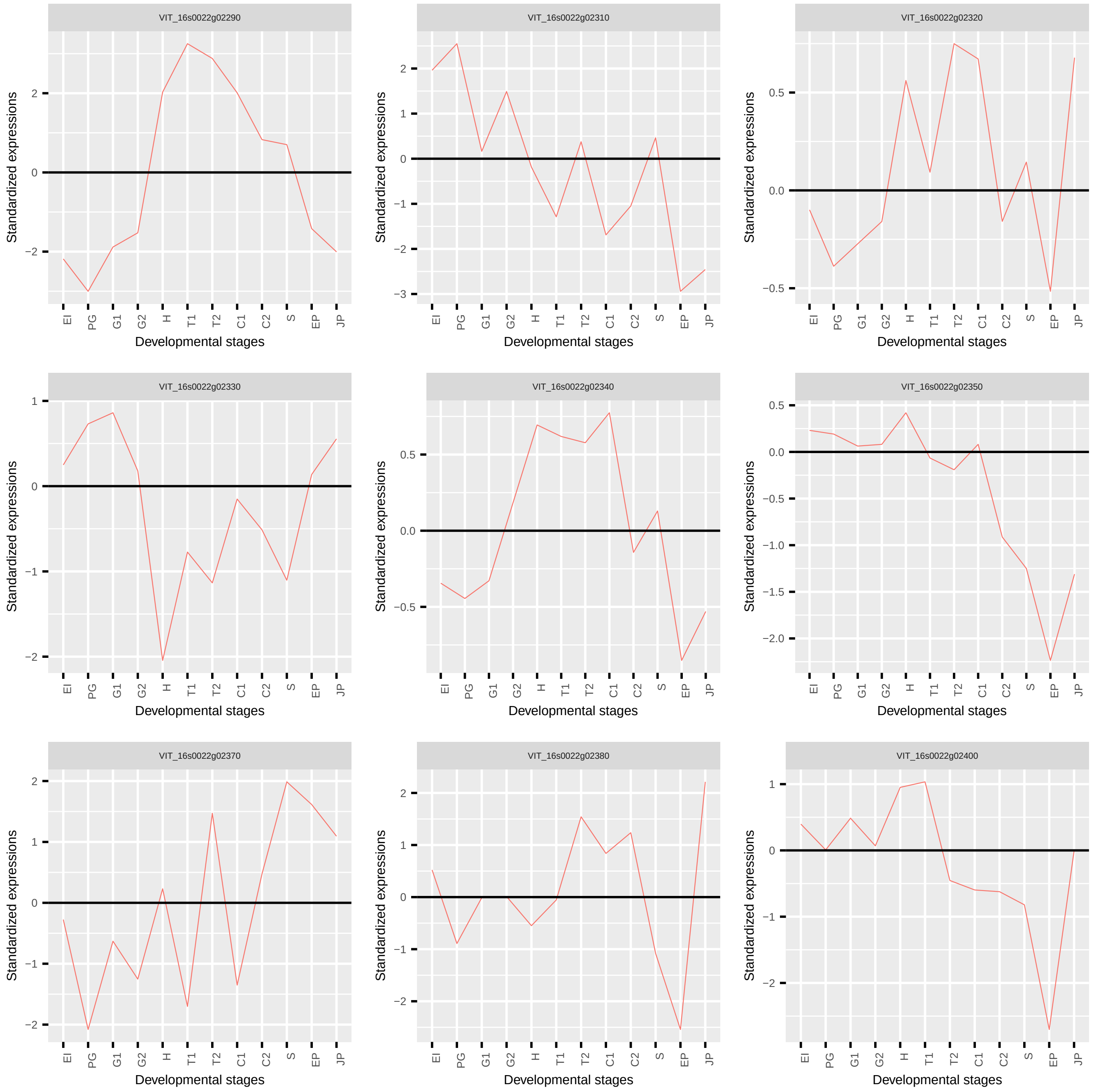

VIT_16s0022g02290
VIT_16s0022g02310
VIT_16s0022g02320
VIT_16s0022g02330
VIT_16s0022g02340
VIT_16s0022g02350
VIT_16s0022g02370
VIT_16s0022g02380
VIT_16s0022g02400
Standardized expressions
Developmental stages
EI PG G1 G2 H T1 T2 C1 C2 S EP JP

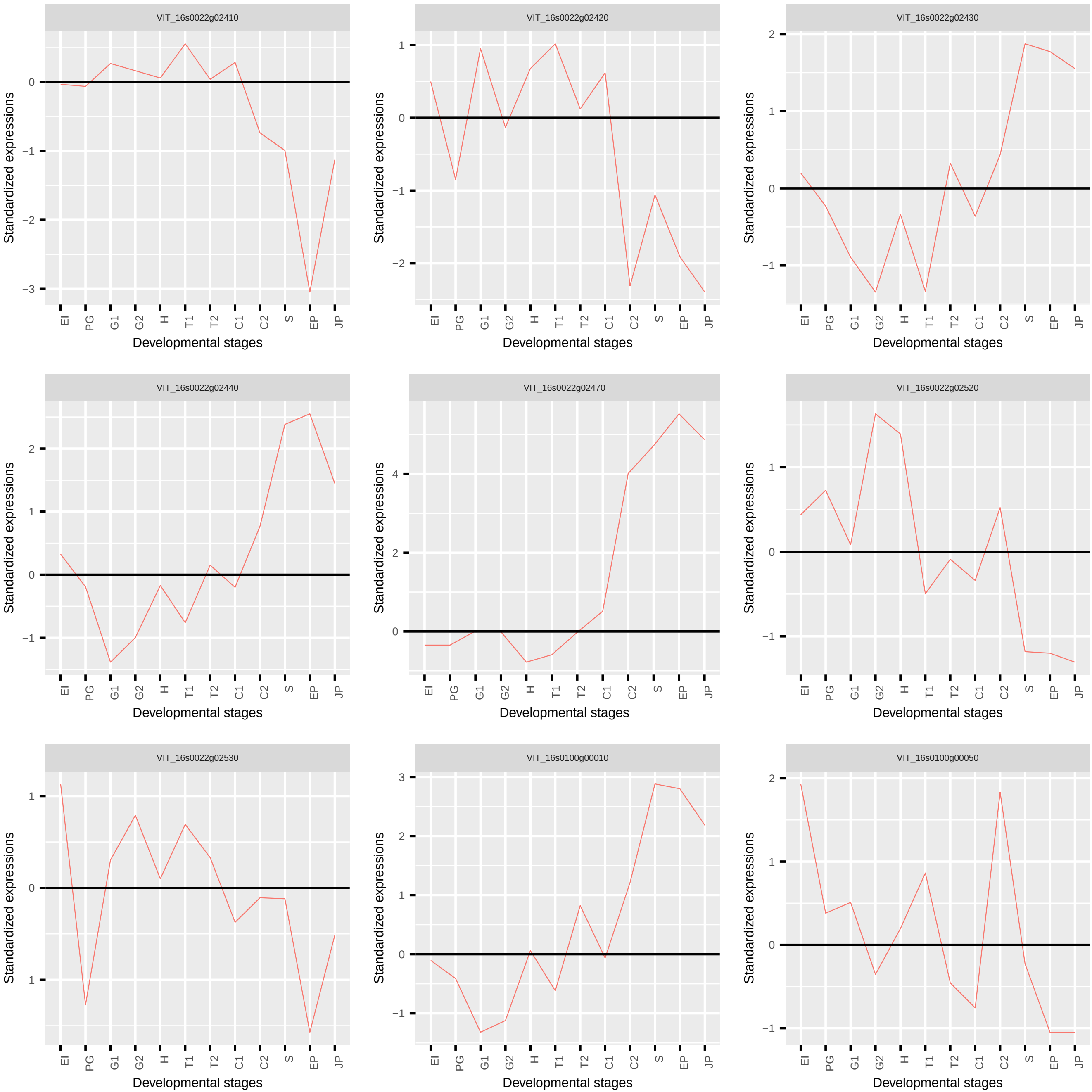
VIT_16s0022g02410
VIT_16s0022g02420
VIT_16s0022g02430
VIT_16s0022g02440
VIT_16s0022g02470
VIT_16s0022g02520
VIT_16s0022g02530
VIT_16s0100g00010
VIT_16s0100g00050
Standardized expressions
Developmental stages
EI
PG
G1
G2
H
T1
T2
C1
C2
S
EP
JP

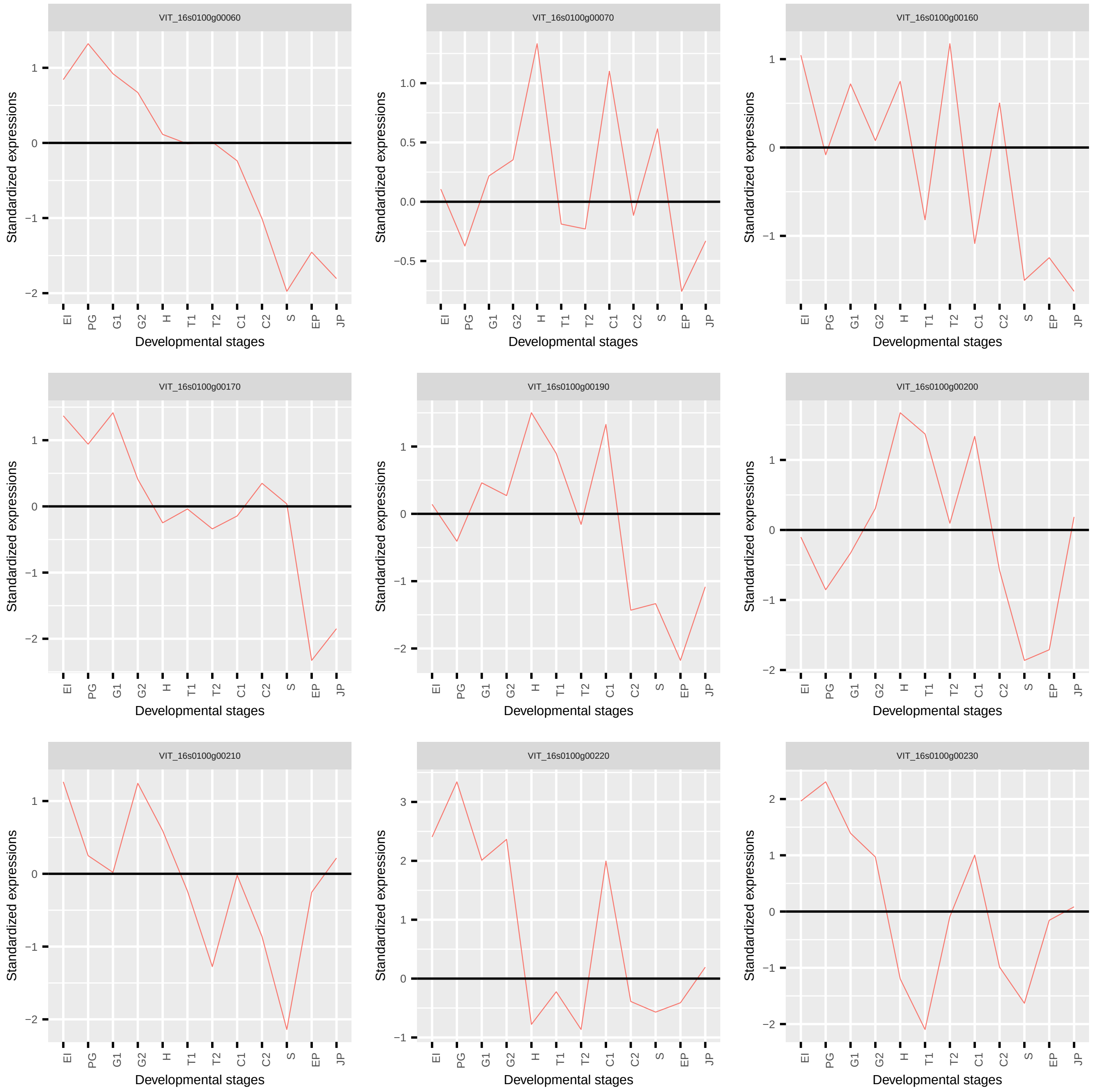

VIT_16s0100g00060
VIT_16s0100g00070
VIT_16s0100g00160
VIT_16s0100g00170
VIT_16s0100g00190
VIT_16s0100g00200
VIT_16s0100g00210
VIT_16s0100g00220
VIT_16s0100g00230
Standardized expressions
Developmental stages
EI PG G1 G2 H T1 T2 C1 C2 S EP JP

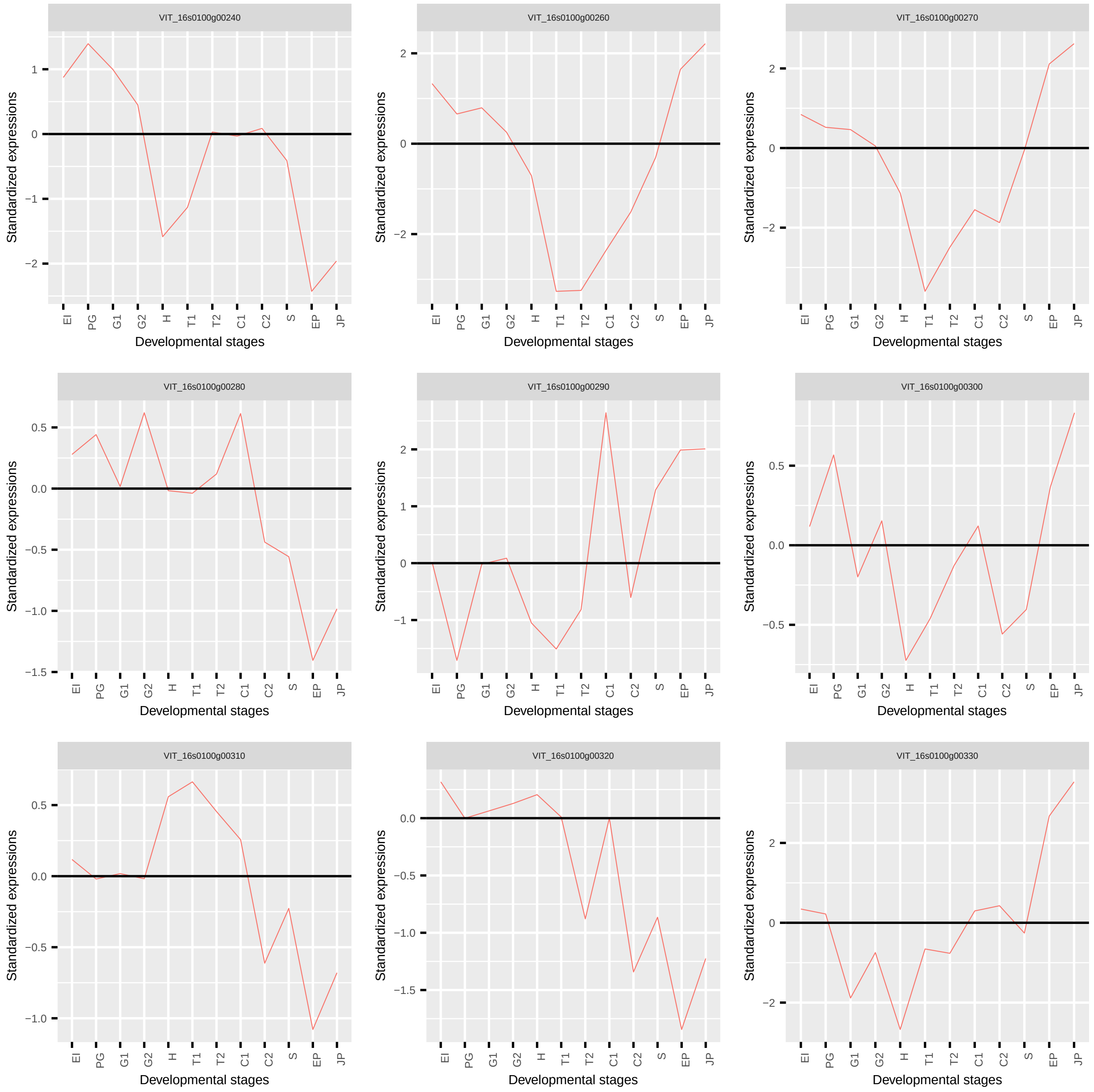

VIT_16s0100g00240
VIT_16s0100g00260
VIT_16s0100g00270
VIT_16s0100g00280
VIT_16s0100g00290
VIT_16s0100g00300
VIT_16s0100g00310
VIT_16s0100g00320
VIT_16s0100g00330
Standardized expressions
Developmental stages
EI PG G1 G2 H T1 T2 C1 C2 S EP JP

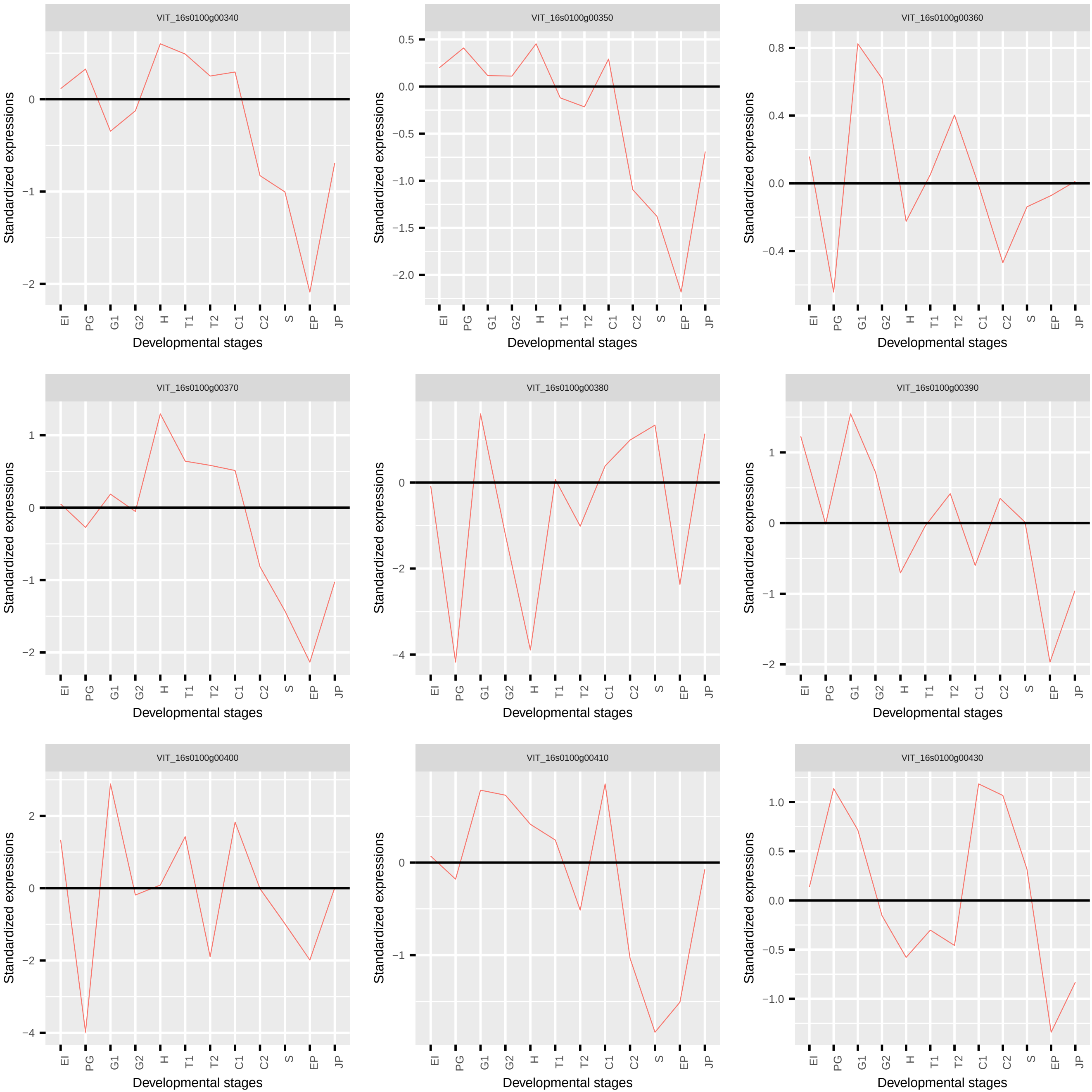
VIT_16s0100g00340
VIT_16s0100g00350
VIT_16s0100g00360
VIT_16s0100g00370
VIT_16s0100g00380
VIT_16s0100g00390
VIT_16s0100g00400
VIT_16s0100g00410
VIT_16s0100g00430
Standardized expressions
Developmental stages
EI PG G1 G2 H T1 T2 C1 C2 S EP JP

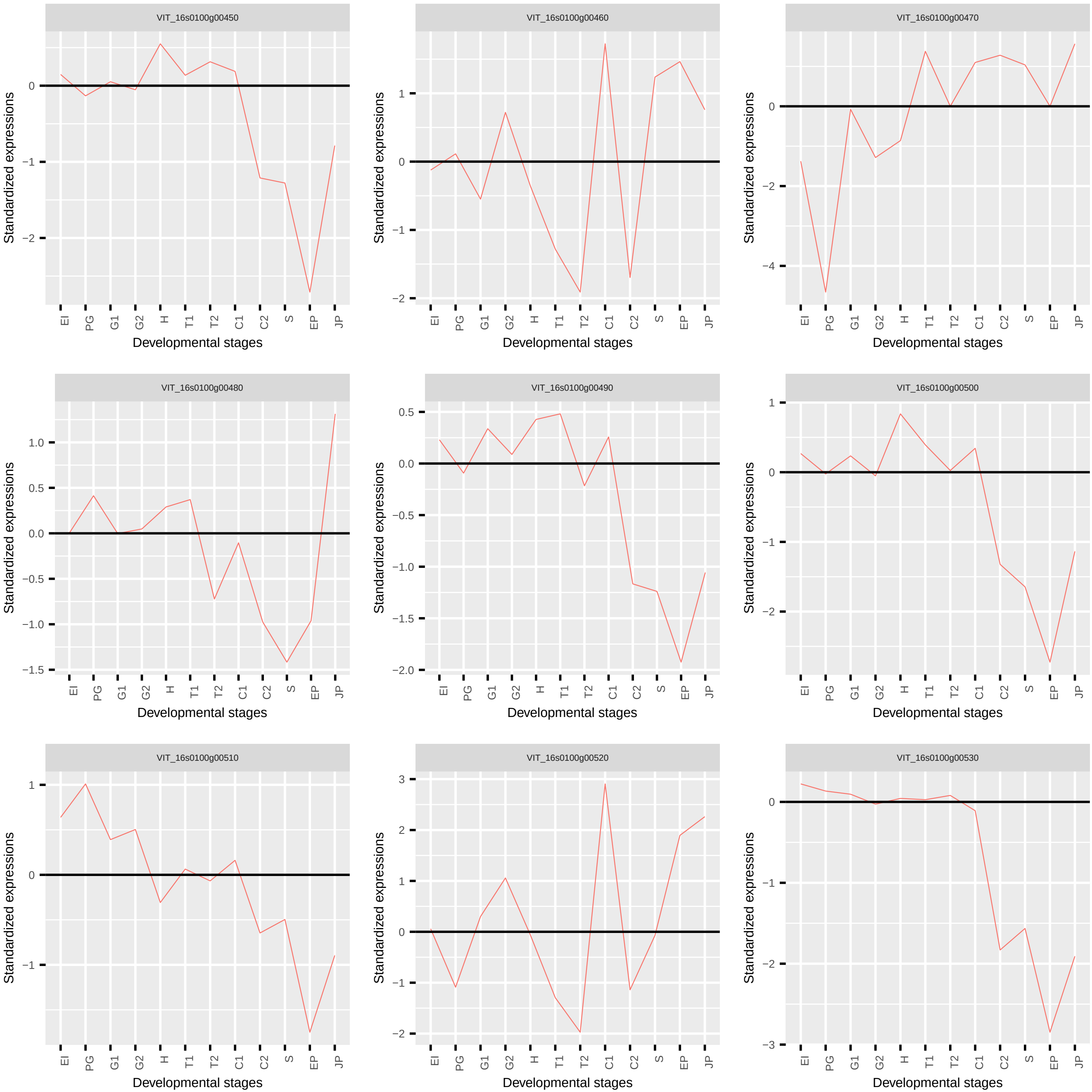

VIT_16s0100g00450
0
−1
−2
Standardized expressions
EI PG G1 G2 H T1 T2 C1 C2 S EP JP
Developmental stages
VIT_16s0100g00460
1
0
−1
−2
Standardized expressions
EI PG G1 G2 H T1 T2 C1 C2 S EP JP
Developmental stages
VIT_16s0100g00470
0
−2
−4
Standardized expressions
EI PG G1 G2 H T1 T2 C1 C2 S EP JP
Developmental stages
VIT_16s0100g00480
1.0
0.5
0.0
−0.5
−1.0
−1.5
Standardized expressions
EI PG G1 G2 H T1 T2 C1 C2 S EP JP
Developmental stages
VIT_16s0100g00490
0.5
0.0
−0.5
−1.0
−1.5
−2.0
Standardized expressions
EI PG G1 G2 H T1 T2 C1 C2 S EP JP
Developmental stages
VIT_16s0100g00500
1
0
−1
−2
Standardized expressions
EI PG G1 G2 H T1 T2 C1 C2 S EP JP
Developmental stages
VIT_16s0100g00510
1
0
−1
Standardized expressions
EI PG G1 G2 H T1 T2 C1 C2 S EP JP
Developmental stages
VIT_16s0100g00520
3
2
1
0
−1
−2
Standardized expressions
EI PG G1 G2 H T1 T2 C1 C2 S EP JP
Developmental stages
VIT_16s0100g00530
0
−1
−2
−3
Standardized expressions
EI PG G1 G2 H T1 T2 C1 C2 S EP JP
Developmental stages

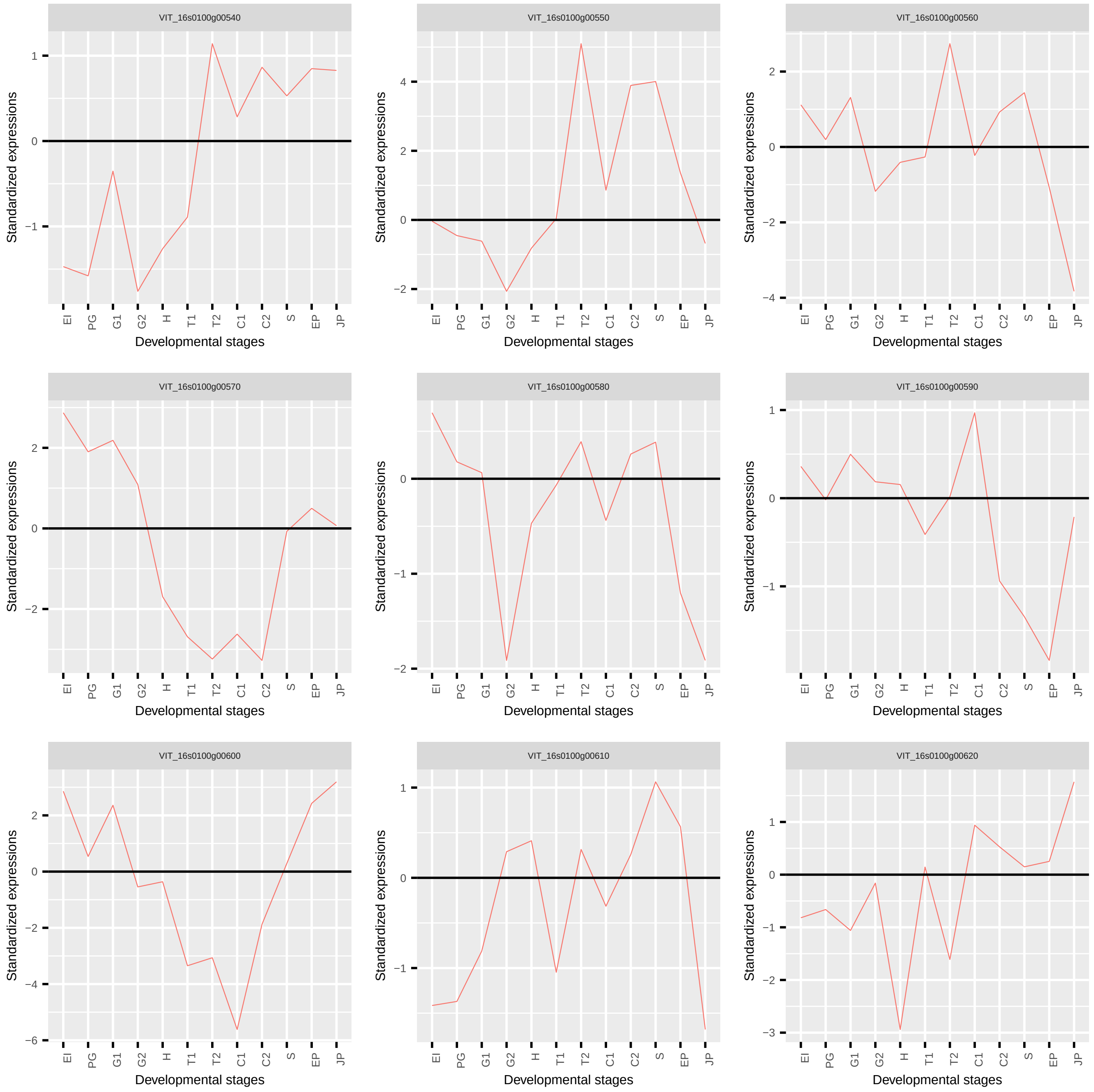
VIT_16s0100g00540
VIT_16s0100g00550
VIT_16s0100g00560
VIT_16s0100g00570
VIT_16s0100g00580
VIT_16s0100g00590
VIT_16s0100g00600
VIT_16s0100g00610
VIT_16s0100g00620
Standardized expressions
Developmental stages
EI
PG
G1
G2
H
T1
T2
C1
C2
S
EP
JP

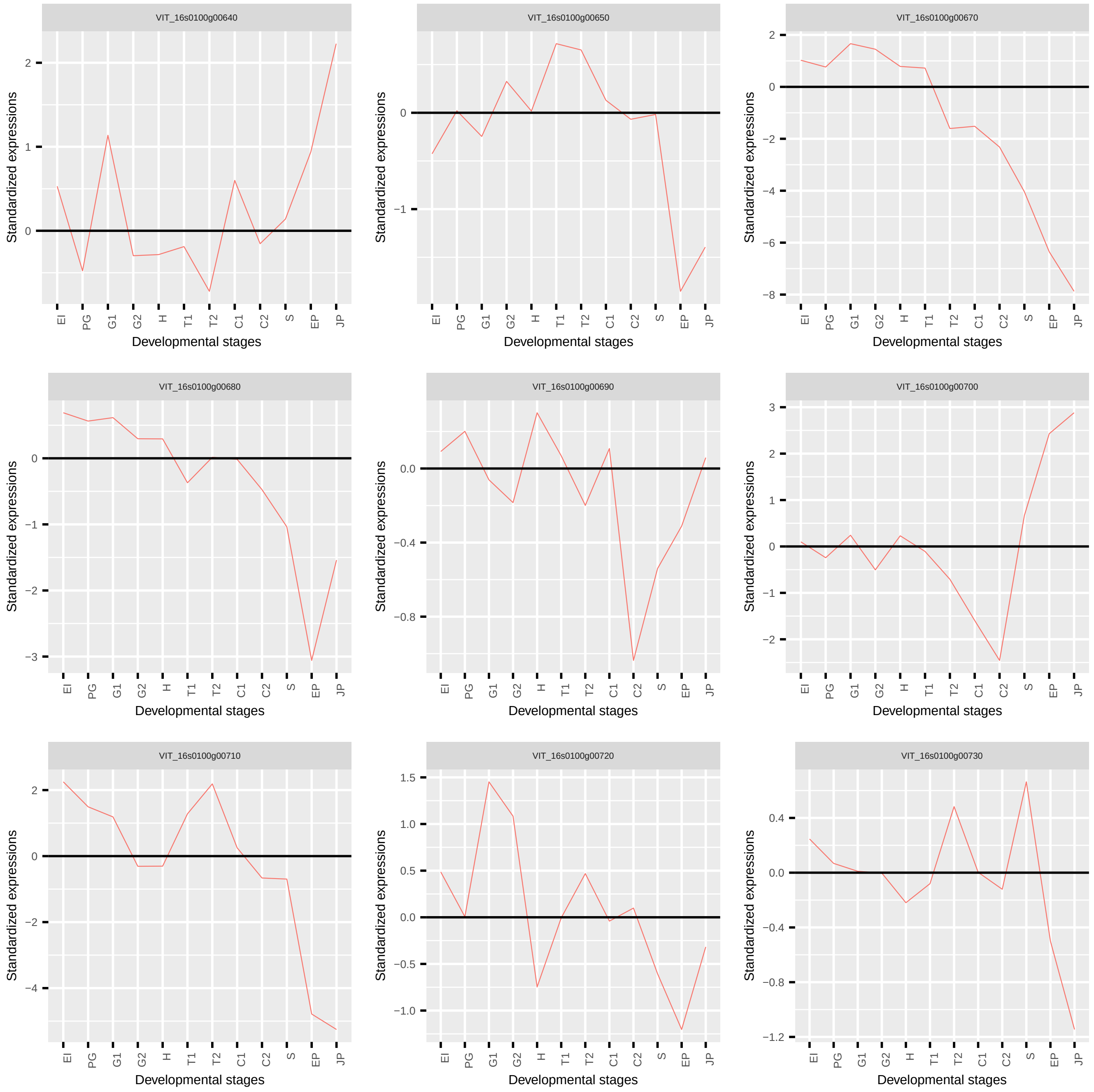
VIT_16s0100g00640
VIT_16s0100g00650
VIT_16s0100g00670
VIT_16s0100g00680
VIT_16s0100g00690
VIT_16s0100g00700
VIT_16s0100g00710
VIT_16s0100g00720
VIT_16s0100g00730
Standardized expressions
Developmental stages
EI PG G1 G2 H T1 T2 C1 C2 S EP JP

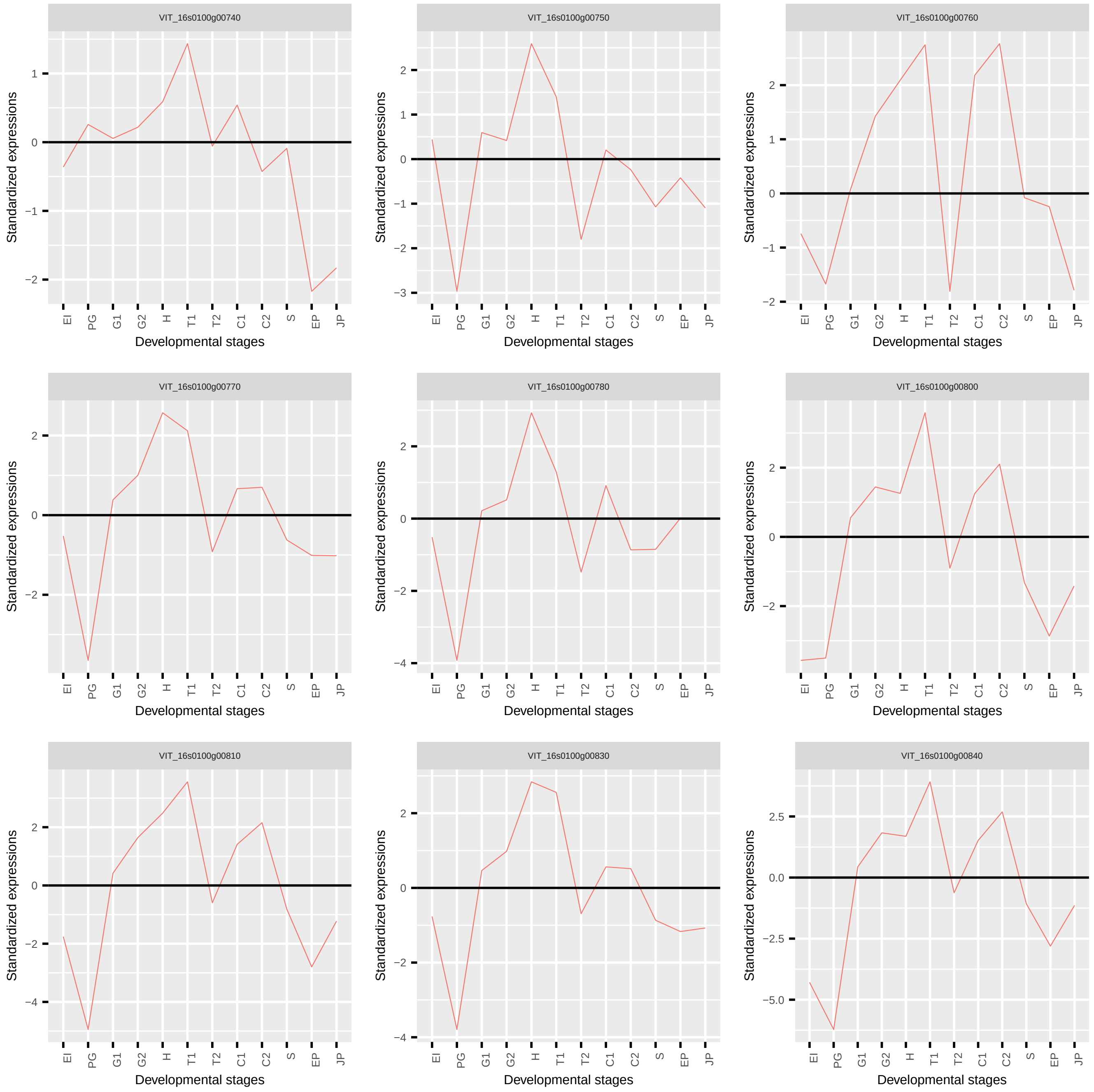
VIT_16s0100g00740
VIT_16s0100g00750
VIT_16s0100g00760
VIT_16s0100g00770
VIT_16s0100g00780
VIT_16s0100g00800
VIT_16s0100g00810
VIT_16s0100g00830
VIT_16s0100g00840
Standardized expressions
Developmental stages
EI PG G1 G2 H T1 T2 C1 C2 S EP JP

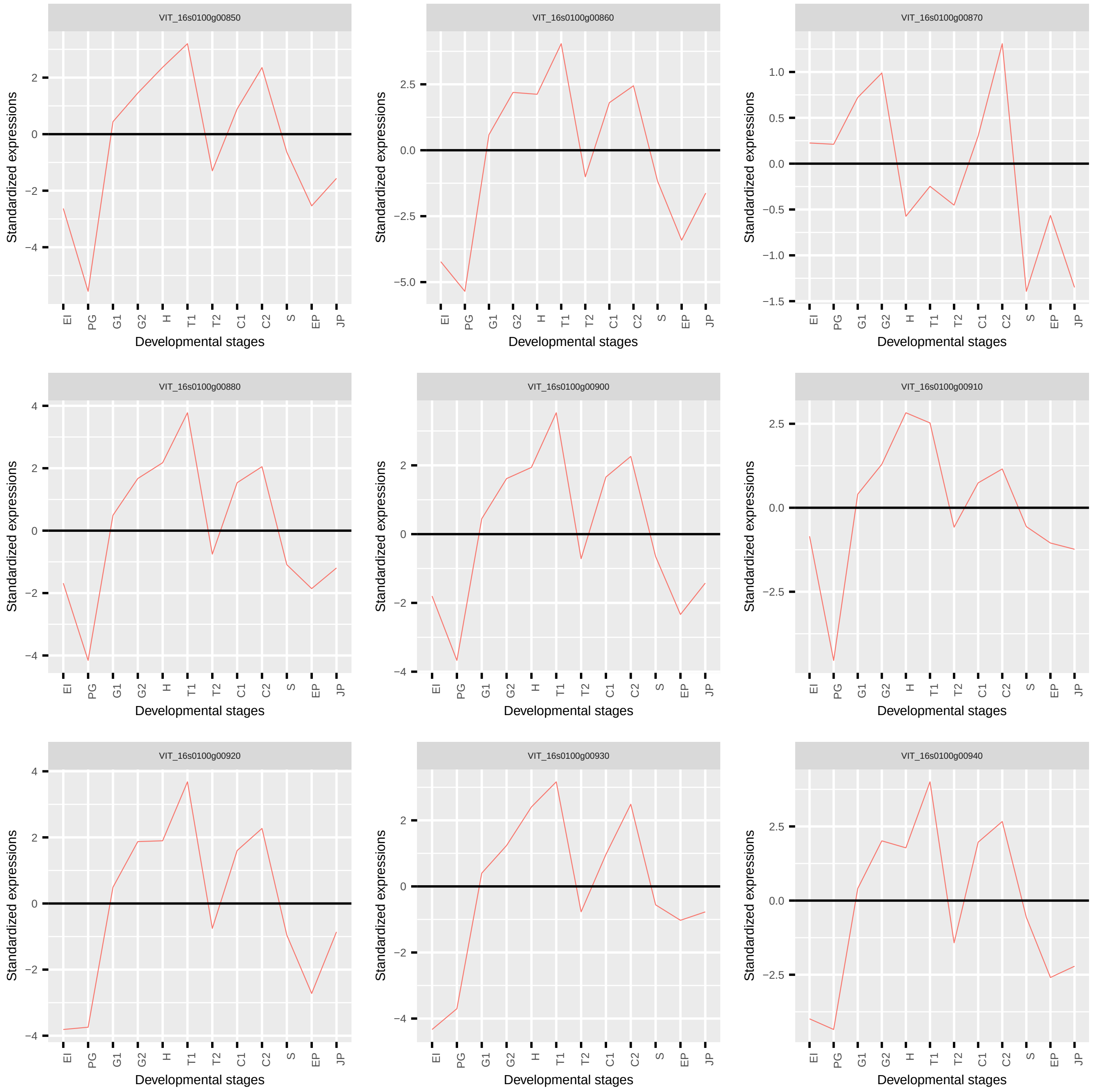

VIT_16s0100g00850
VIT_16s0100g00860
VIT_16s0100g00870
VIT_16s0100g00880
VIT_16s0100g00900
VIT_16s0100g00910
VIT_16s0100g00920
VIT_16s0100g00930
VIT_16s0100g00940
Standardized expressions
Developmental stages
EI PG G1 G2 H T1 T2 C1 C2 S EP JP

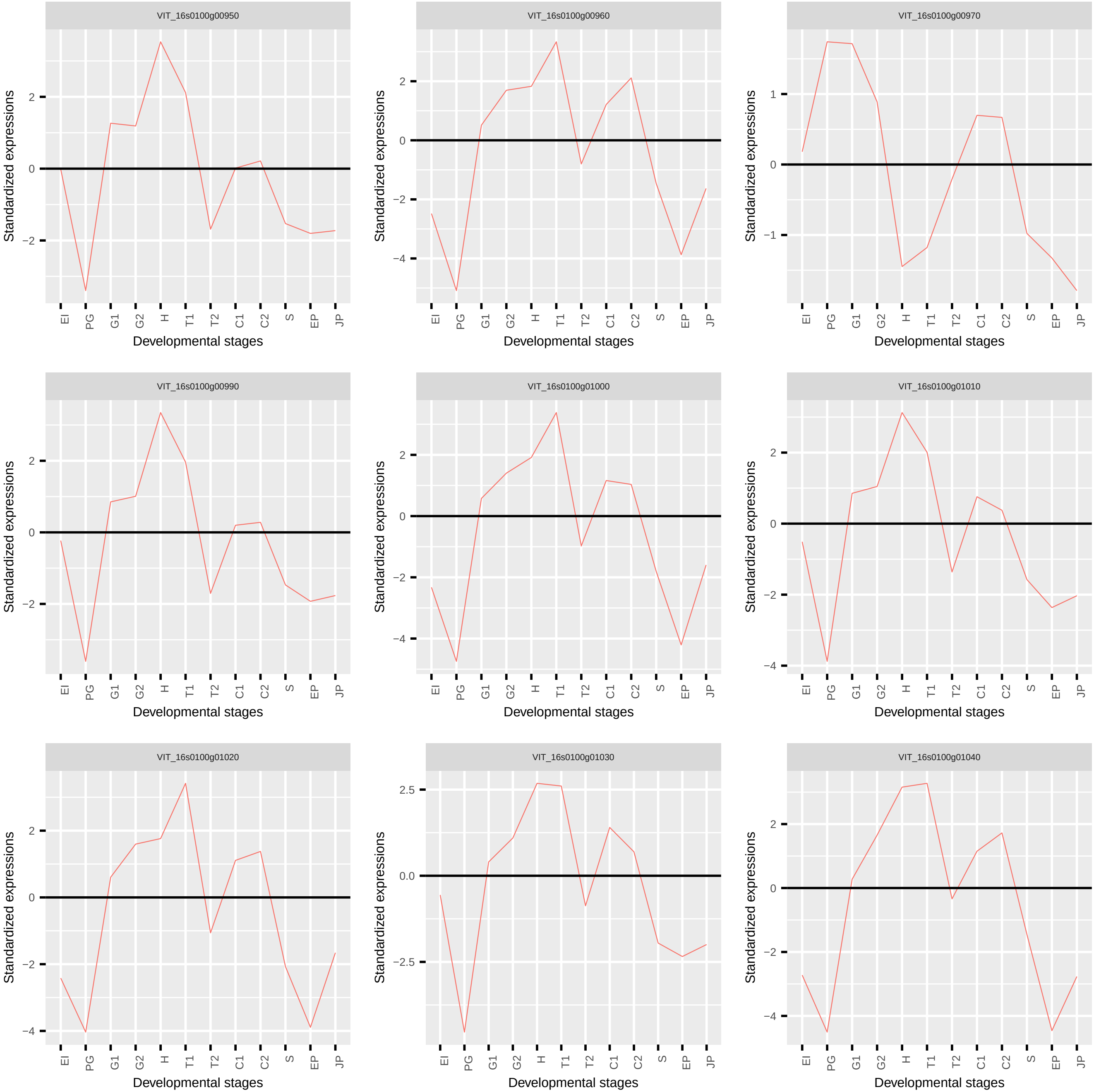
VIT_16s0100g00950
VIT_16s0100g00960
VIT_16s0100g00970
VIT_16s0100g00990
VIT_16s0100g01000
VIT_16s0100g01010
VIT_16s0100g01020
VIT_16s0100g01030
VIT_16s0100g01040
Standardized expressions
Developmental stages
EI PG G1 G2 H T1 T2 C1 C2 S EP JP
2
0
−2
−4
1
−1
2.5
0.0
−2.5

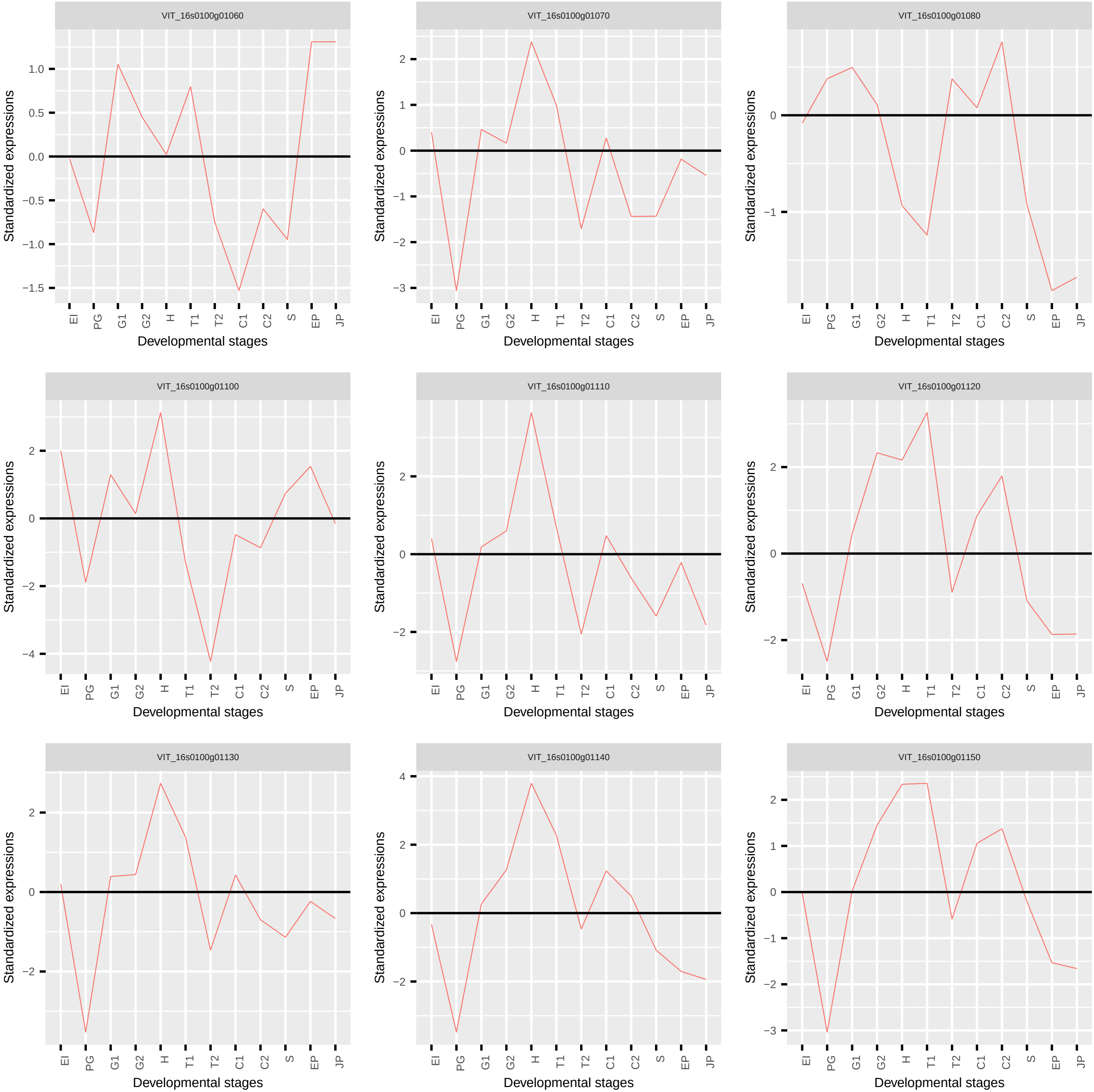
VIT_16s0100g01060
VIT_16s0100g01070
VIT_16s0100g01080
VIT_16s0100g01100
VIT_16s0100g01110
VIT_16s0100g01120
VIT_16s0100g01130
VIT_16s0100g01140
VIT_16s0100g01150
Standardized expressions
Developmental stages
EI PG G1 G2 H T1 T2 C1 C2 S EP JP
1.0
0.5
0.0
−0.5
−1.0
−1.5
4
3
2
1
0
−1
−2
−3
−4

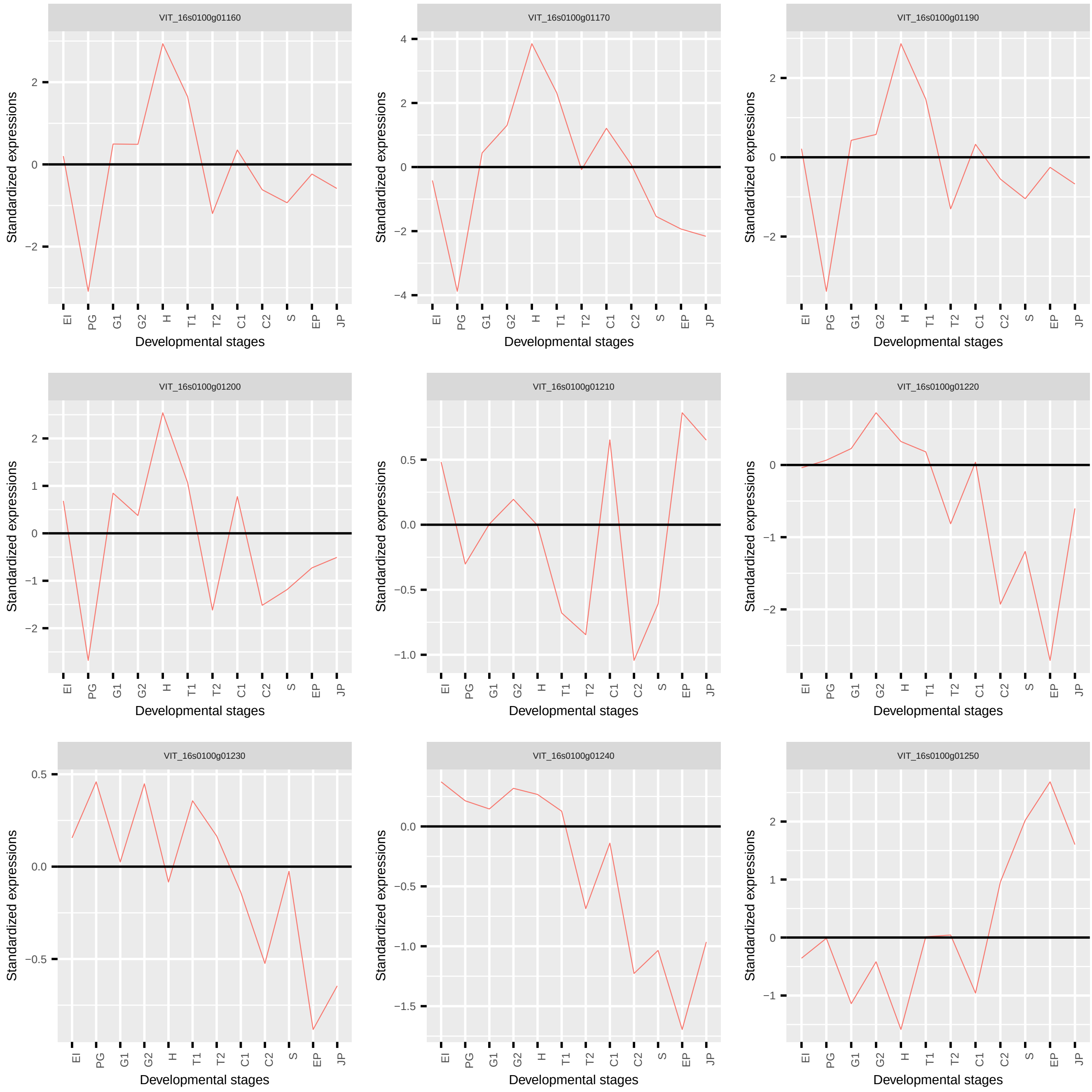

VIT_16s0100g01160
VIT_16s0100g01170
VIT_16s0100g01190
VIT_16s0100g01200
VIT_16s0100g01210
VIT_16s0100g01220
VIT_16s0100g01230
VIT_16s0100g01240
VIT_16s0100g01250
Standardized expressions
Developmental stages
EI
PG
G1
G2
H
T1
T2
C1
C2
S
EP
JP

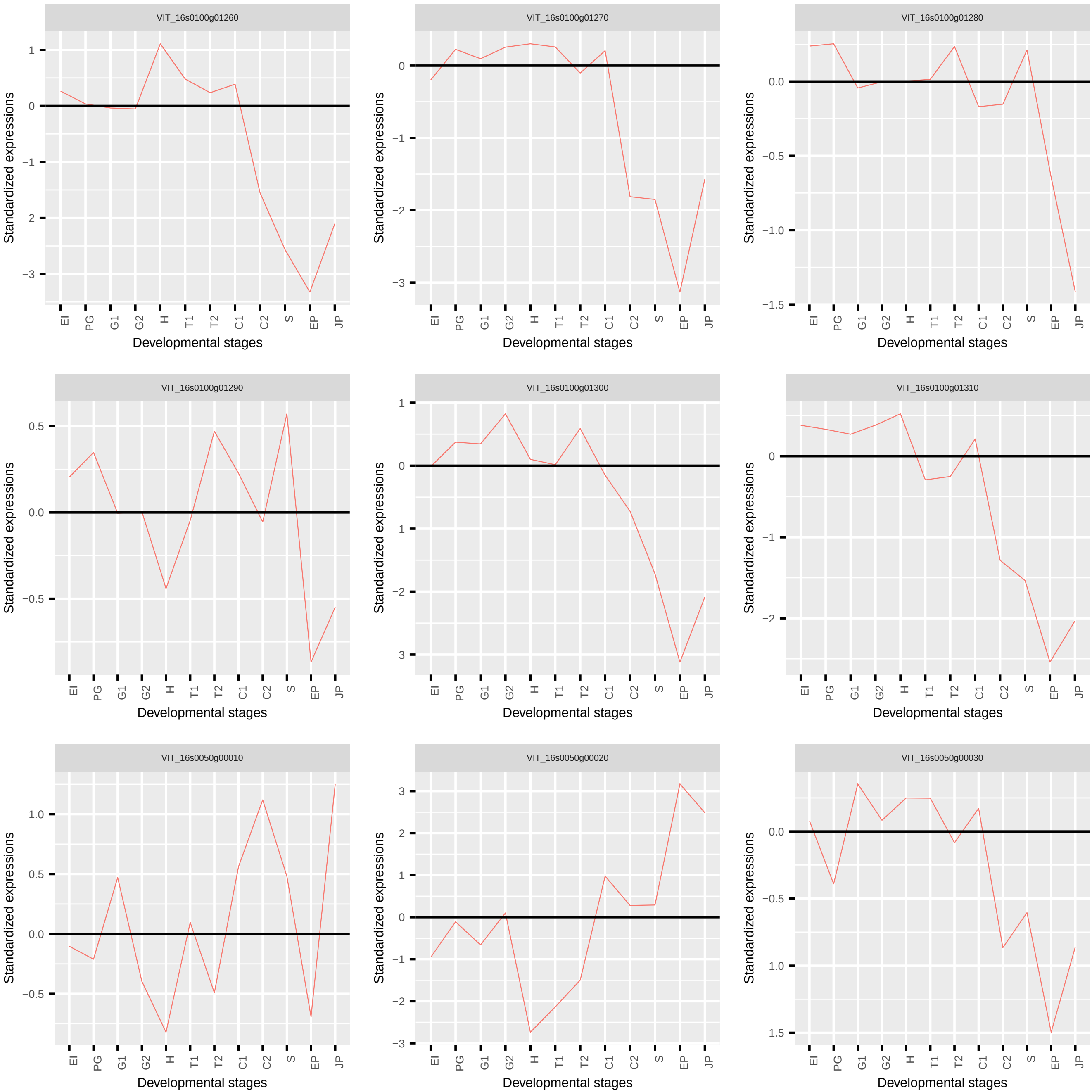
VIT_16s0100g01260
VIT_16s0100g01270
VIT_16s0100g01280
VIT_16s0100g01290
VIT_16s0100g01300
VIT_16s0100g01310
VIT_16s0050g00010
VIT_16s0050g00020
VIT_16s0050g00030
Standardized expressions
Developmental stages
EI PG G1 G2 H T1 T2 C1 C2 S EP JP

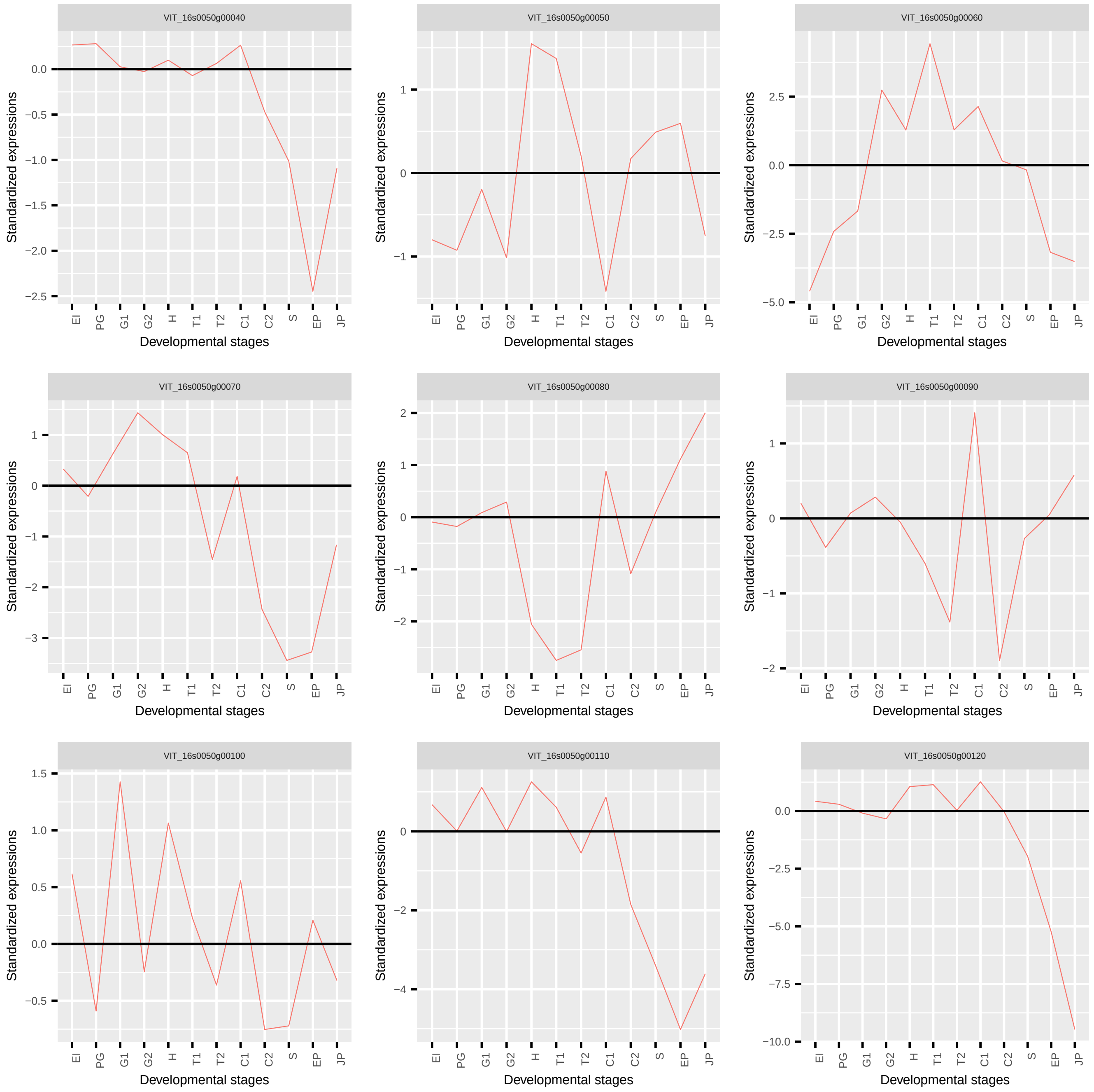
VIT_16s0050g00040
VIT_16s0050g00050
VIT_16s0050g00060
VIT_16s0050g00070
VIT_16s0050g00080
VIT_16s0050g00090
VIT_16s0050g00100
VIT_16s0050g00110
VIT_16s0050g00120
Standardized expressions
Developmental stages
EI
PG
G1
G2
H
T1
T2
C1
C2
S
EP
JP

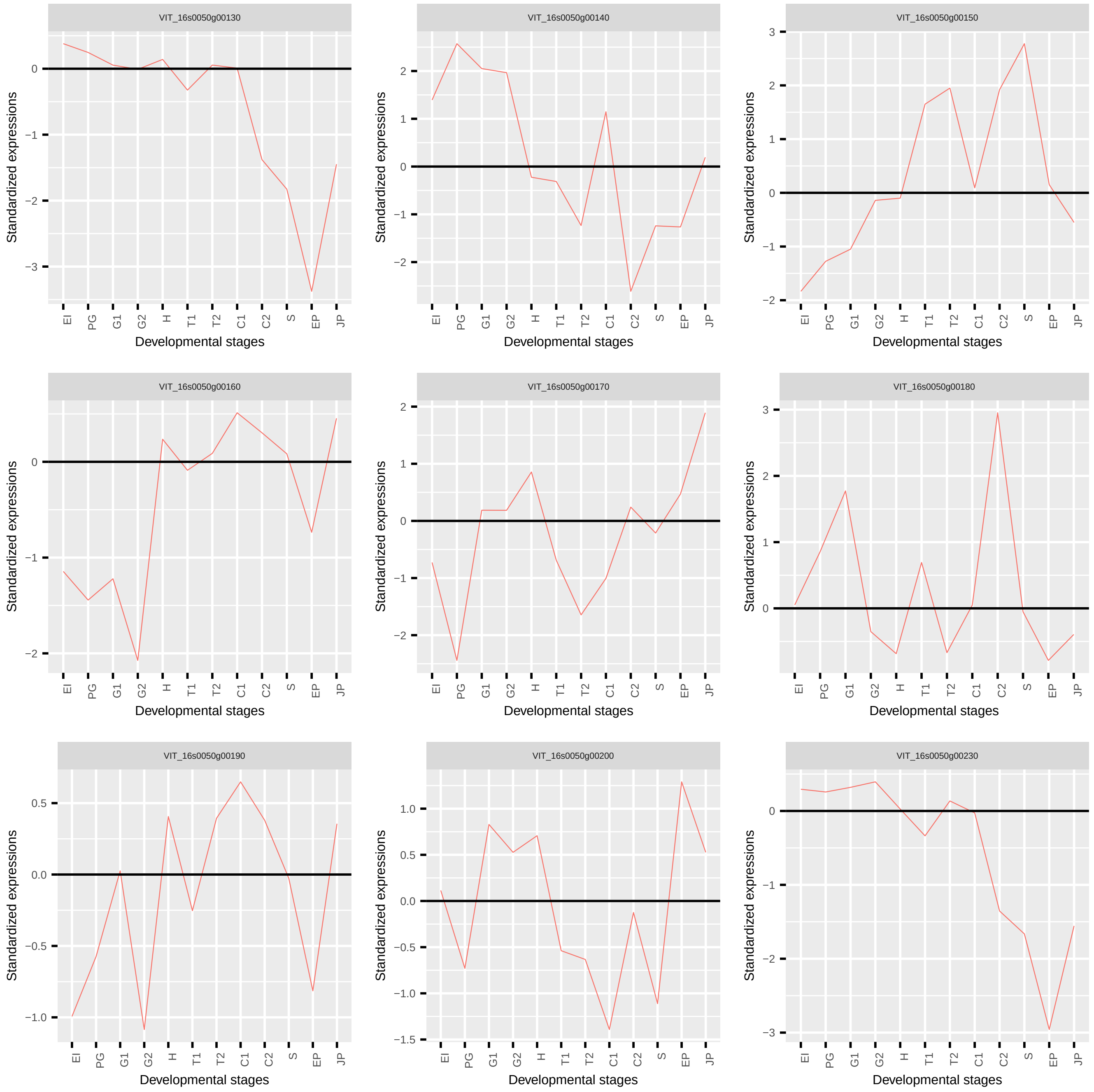

VIT_16s0050g00130
VIT_16s0050g00140
VIT_16s0050g00150
VIT_16s0050g00160
VIT_16s0050g00170
VIT_16s0050g00180
VIT_16s0050g00190
VIT_16s0050g00200
VIT_16s0050g00230
Standardized expressions
Developmental stages
EI
PG
G1
G2
H
T1
T2
C1
C2
S
EP
JP

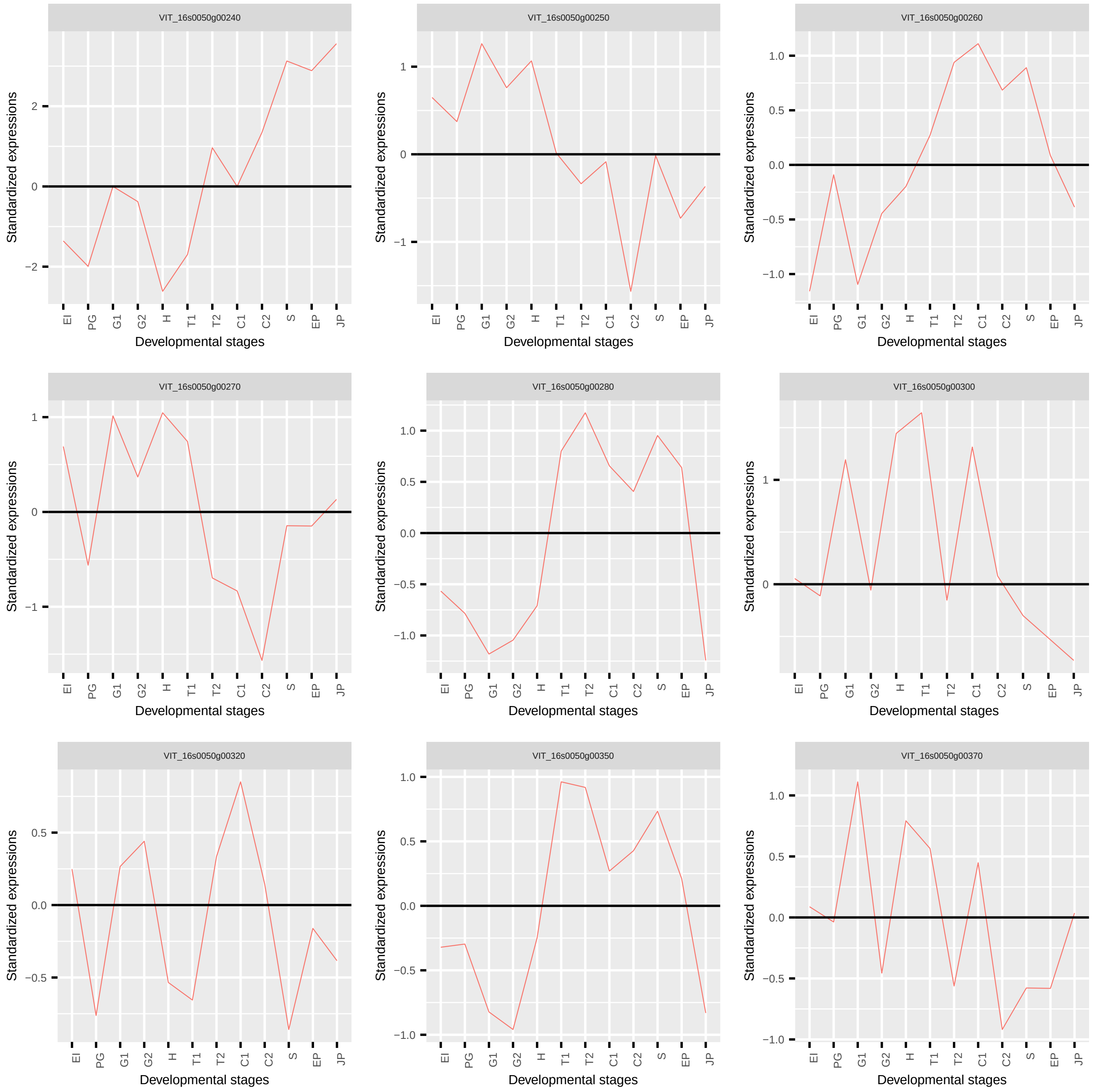
VIT_16s0050g00240
VIT_16s0050g00250
VIT_16s0050g00260
VIT_16s0050g00270
VIT_16s0050g00280
VIT_16s0050g00300
VIT_16s0050g00320
VIT_16s0050g00350
VIT_16s0050g00370
Standardized expressions
Developmental stages
EI PG G1 G2 H T1 T2 C1 C2 S EP JP

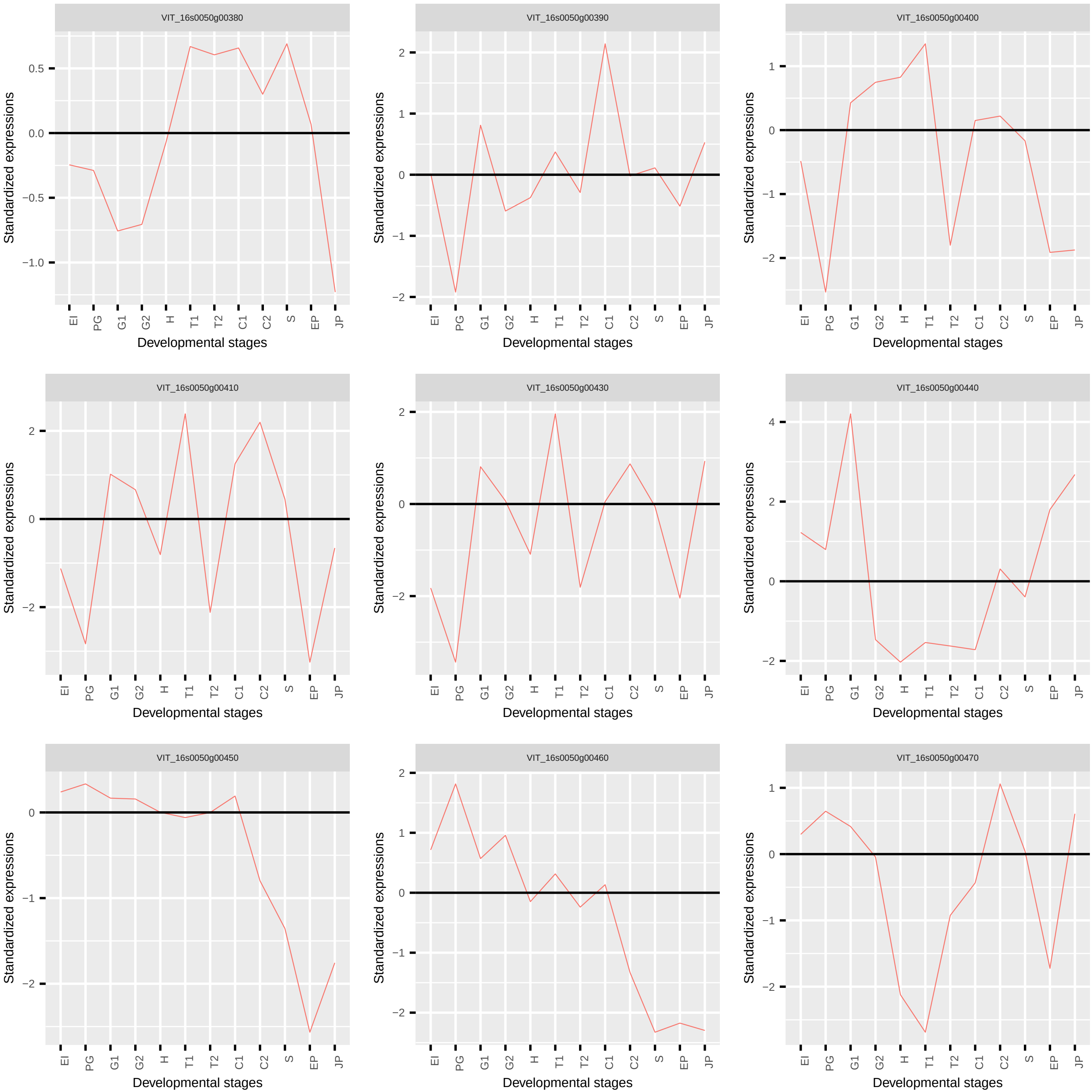
VIT_16s0050g00380
VIT_16s0050g00390
VIT_16s0050g00400
VIT_16s0050g00410
VIT_16s0050g00430
VIT_16s0050g00440
VIT_16s0050g00450
VIT_16s0050g00460
VIT_16s0050g00470
Standardized expressions
Developmental stages
EI PG G1 G2 H T1 T2 C1 C2 S EP JP

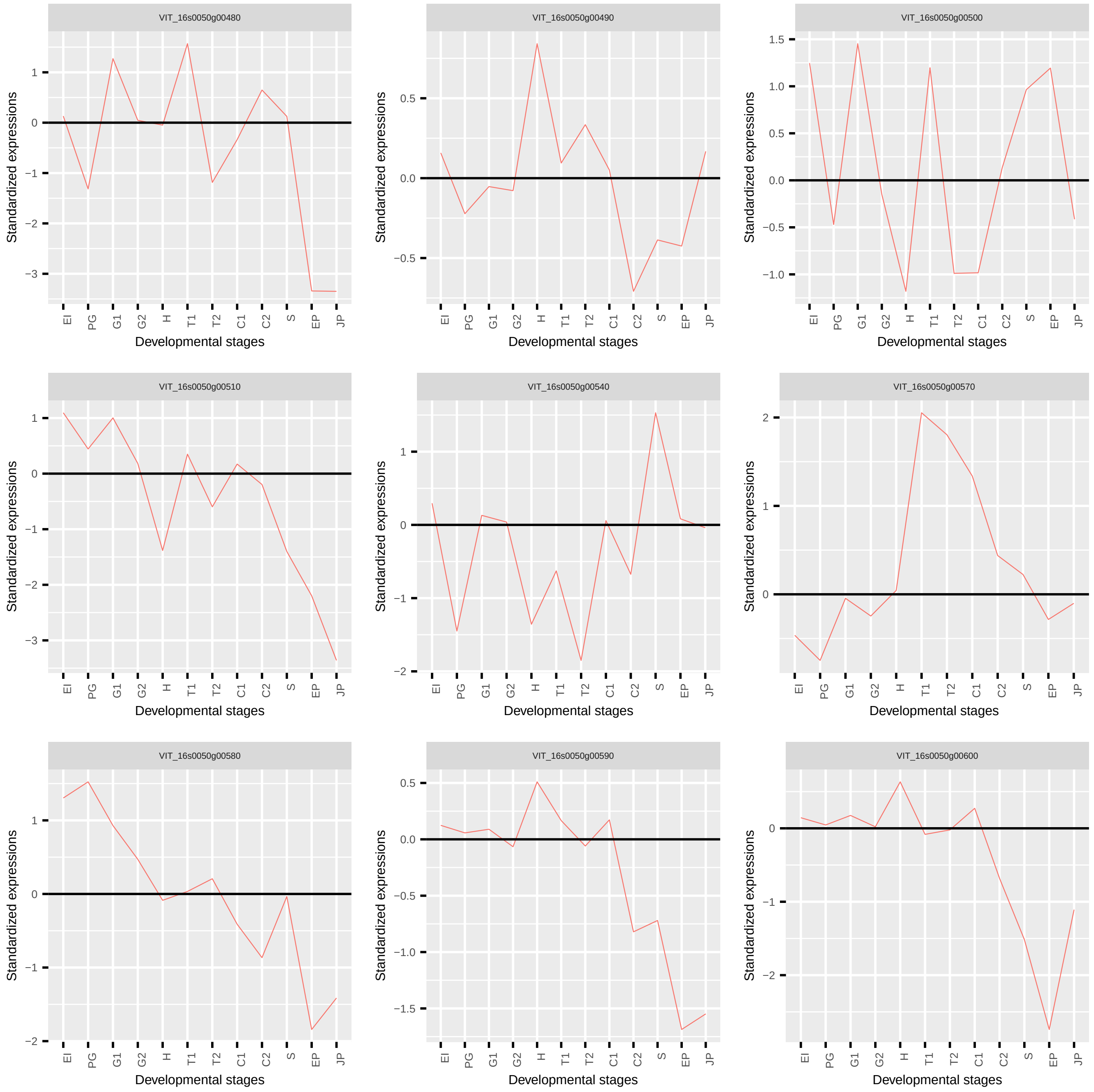

VIT_16s0050g00480
VIT_16s0050g00490
VIT_16s0050g00500
VIT_16s0050g00510
VIT_16s0050g00540
VIT_16s0050g00570
VIT_16s0050g00580
VIT_16s0050g00590
VIT_16s0050g00600
Standardized expressions
Developmental stages
EI PG G1 G2 H T1 T2 C1 C2 S EP JP

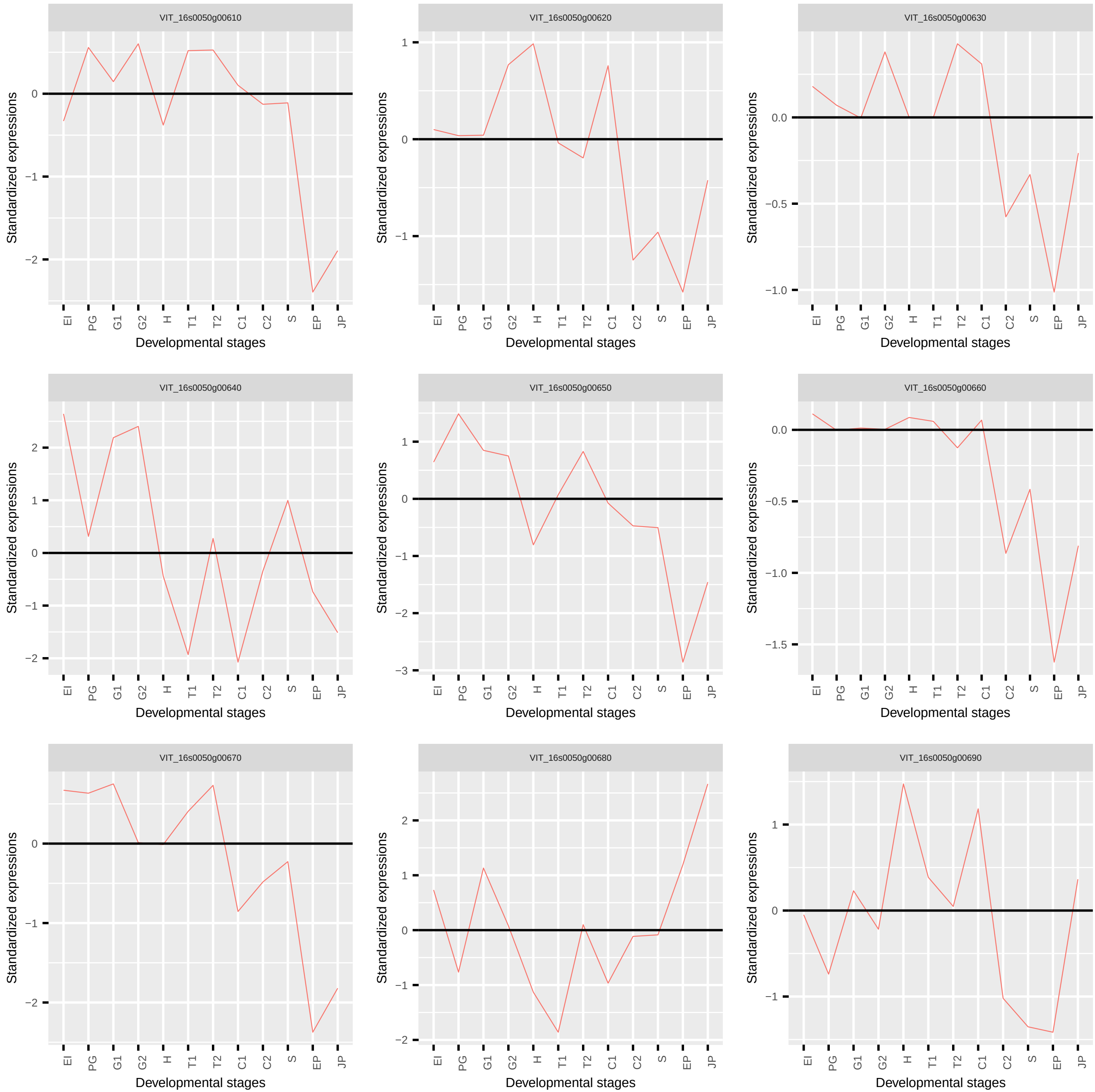

VIT_16s0050g00610
VIT_16s0050g00620
VIT_16s0050g00630
VIT_16s0050g00640
VIT_16s0050g00650
VIT_16s0050g00660
VIT_16s0050g00670
VIT_16s0050g00680
VIT_16s0050g00690
Standardized expressions
Developmental stages
EI PG G1 G2 H T1 T2 C1 C2 S EP JP

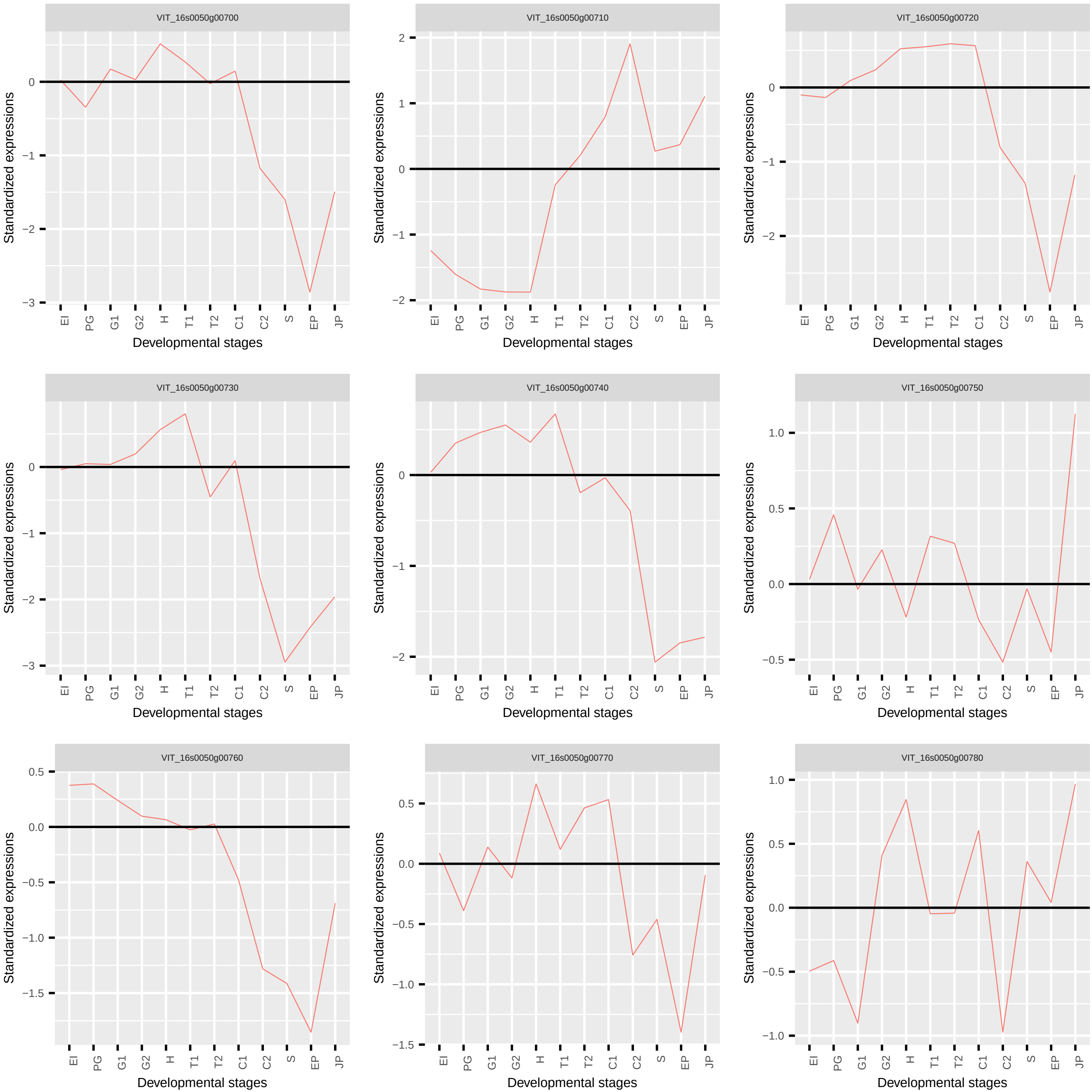
VIT_16s0050g00700
VIT_16s0050g00710
VIT_16s0050g00720
VIT_16s0050g00730
VIT_16s0050g00740
VIT_16s0050g00750
VIT_16s0050g00760
VIT_16s0050g00770
VIT_16s0050g00780
Standardized expressions
Developmental stages
EI PG G1 G2 H T1 T2 C1 C2 S EP JP
2 1 0 −1 −2 −3
1.0 0.5 0.0 −0.5 −1.0 −1.5

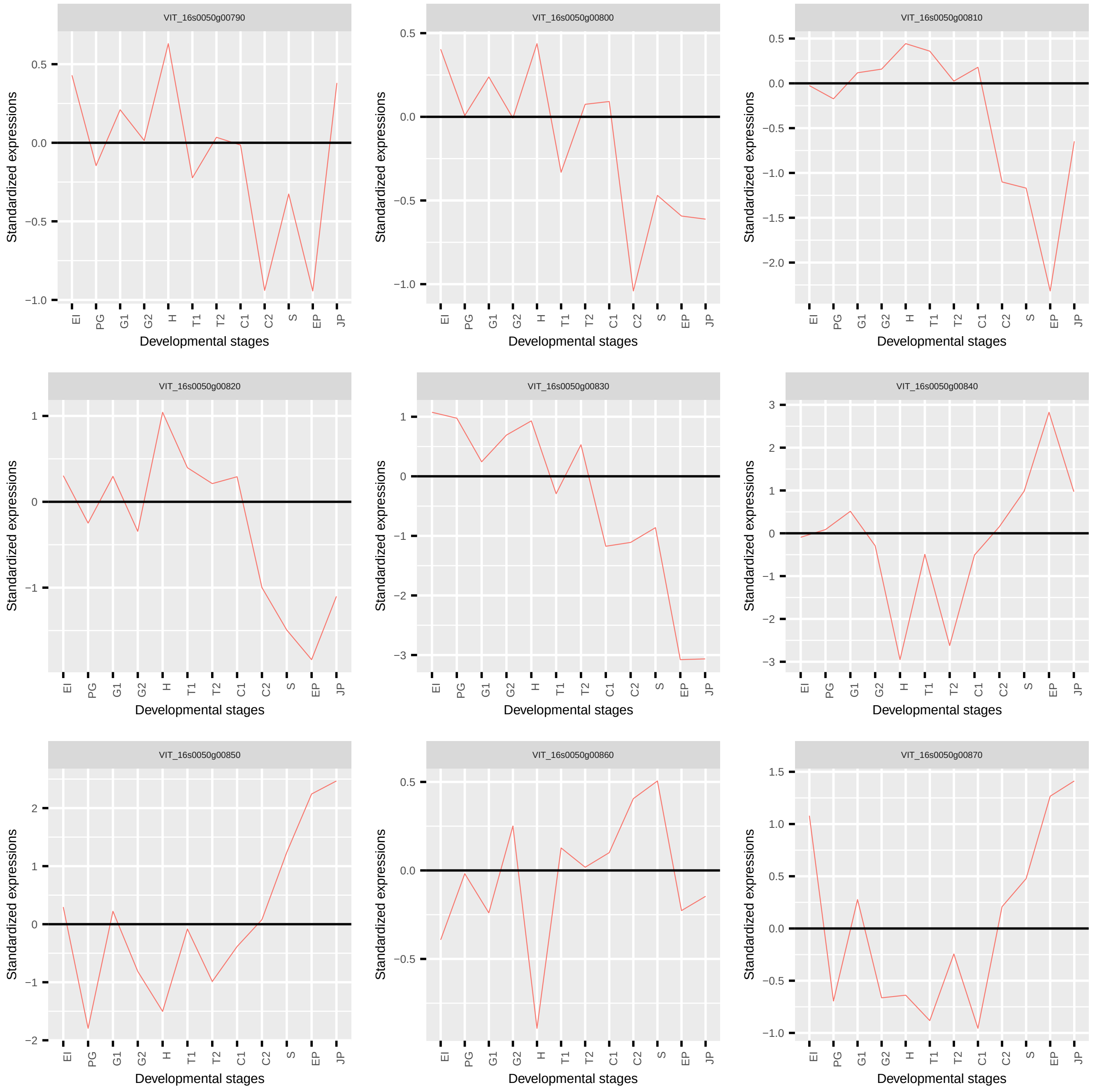
VIT_16s0050g00790
VIT_16s0050g00800
VIT_16s0050g00810
VIT_16s0050g00820
VIT_16s0050g00830
VIT_16s0050g00840
VIT_16s0050g00850
VIT_16s0050g00860
VIT_16s0050g00870
Standardized expressions
Developmental stages
EI PG G1 G2 H T1 T2 C1 C2 S EP JP

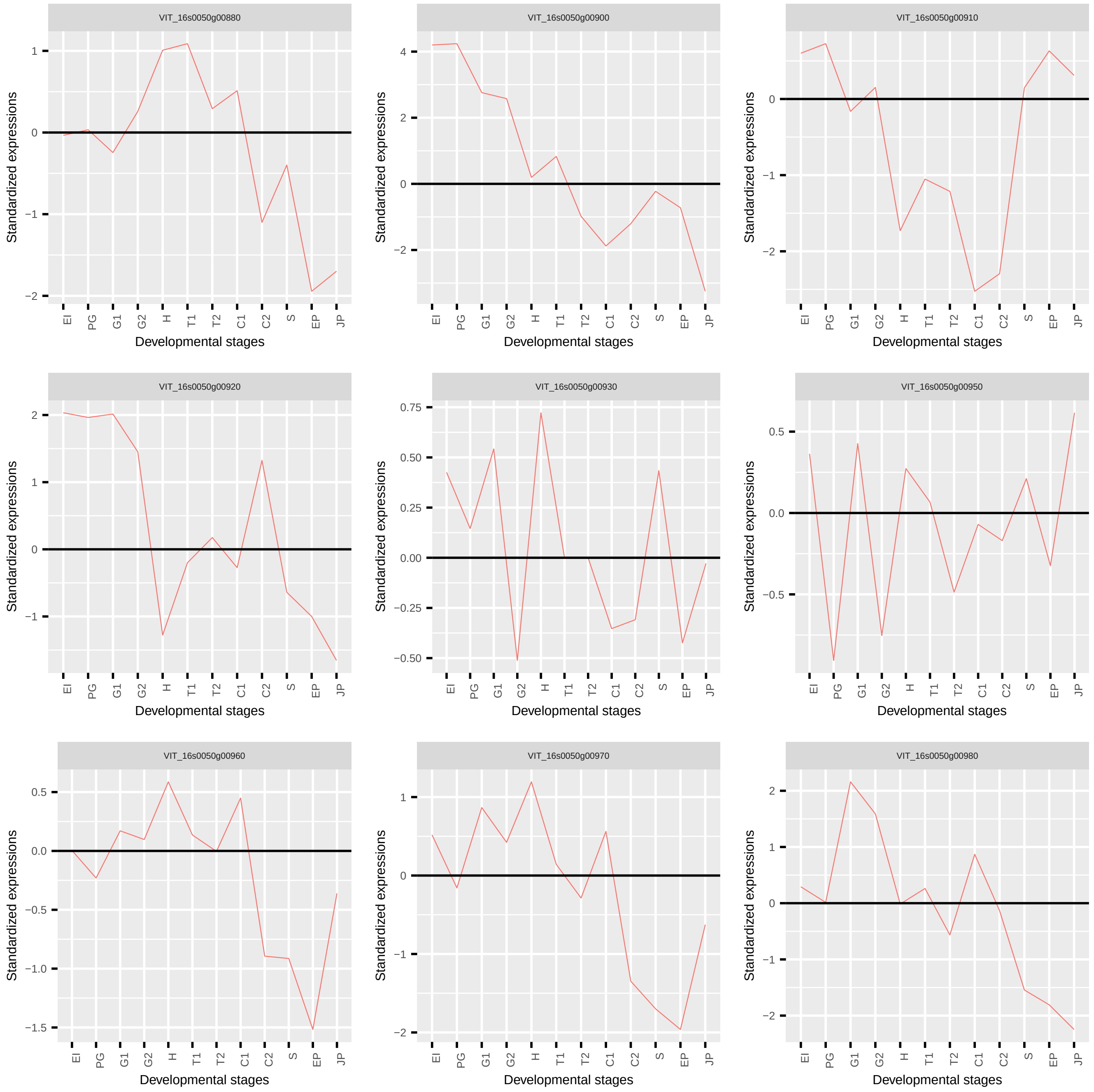
VIT_16s0050g00880
VIT_16s0050g00900
VIT_16s0050g00910
VIT_16s0050g00920
VIT_16s0050g00930
VIT_16s0050g00950
VIT_16s0050g00960
VIT_16s0050g00970
VIT_16s0050g00980
Standardized expressions
Developmental stages
EI PG G1 G2 H T1 T2 C1 C2 S EP JP

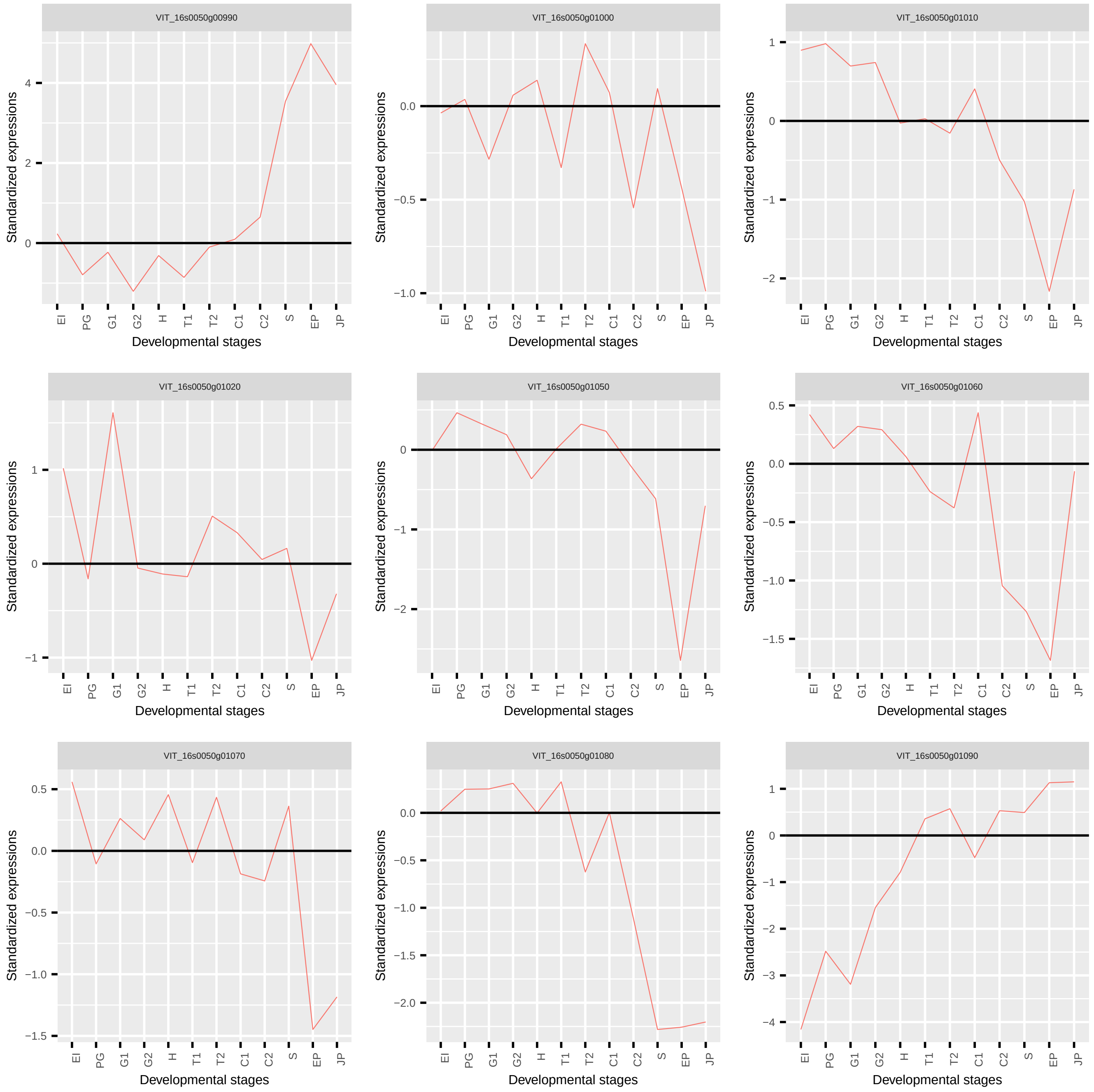
VIT_16s0050g00990
Standardized expressions
0
2
4
EI PG G1 G2 H T1 T2 C1 C2 S EP JP
Developmental stages
VIT_16s0050g01000
Standardized expressions
0.0
−0.5
−1.0
EI PG G1 G2 H T1 T2 C1 C2 S EP JP
Developmental stages
VIT_16s0050g01010
Standardized expressions
1
0
−1
−2
EI PG G1 G2 H T1 T2 C1 C2 S EP JP
Developmental stages
VIT_16s0050g01020
Standardized expressions
1
0
−1
EI PG G1 G2 H T1 T2 C1 C2 S EP JP
Developmental stages
VIT_16s0050g01050
Standardized expressions
0
−1
−2
EI PG G1 G2 H T1 T2 C1 C2 S EP JP
Developmental stages
VIT_16s0050g01060
Standardized expressions
0.5
0.0
−0.5
−1.0
−1.5
EI PG G1 G2 H T1 T2 C1 C2 S EP JP
Developmental stages
VIT_16s0050g01070
Standardized expressions
0.5
0.0
−0.5
−1.0
−1.5
EI PG G1 G2 H T1 T2 C1 C2 S EP JP
Developmental stages
VIT_16s0050g01080
Standardized expressions
0.0
−0.5
−1.0
−1.5
−2.0
EI PG G1 G2 H T1 T2 C1 C2 S EP JP
Developmental stages
VIT_16s0050g01090
Standardized expressions
1
0
−1
−2
−3
−4
EI PG G1 G2 H T1 T2 C1 C2 S EP JP
Developmental stages

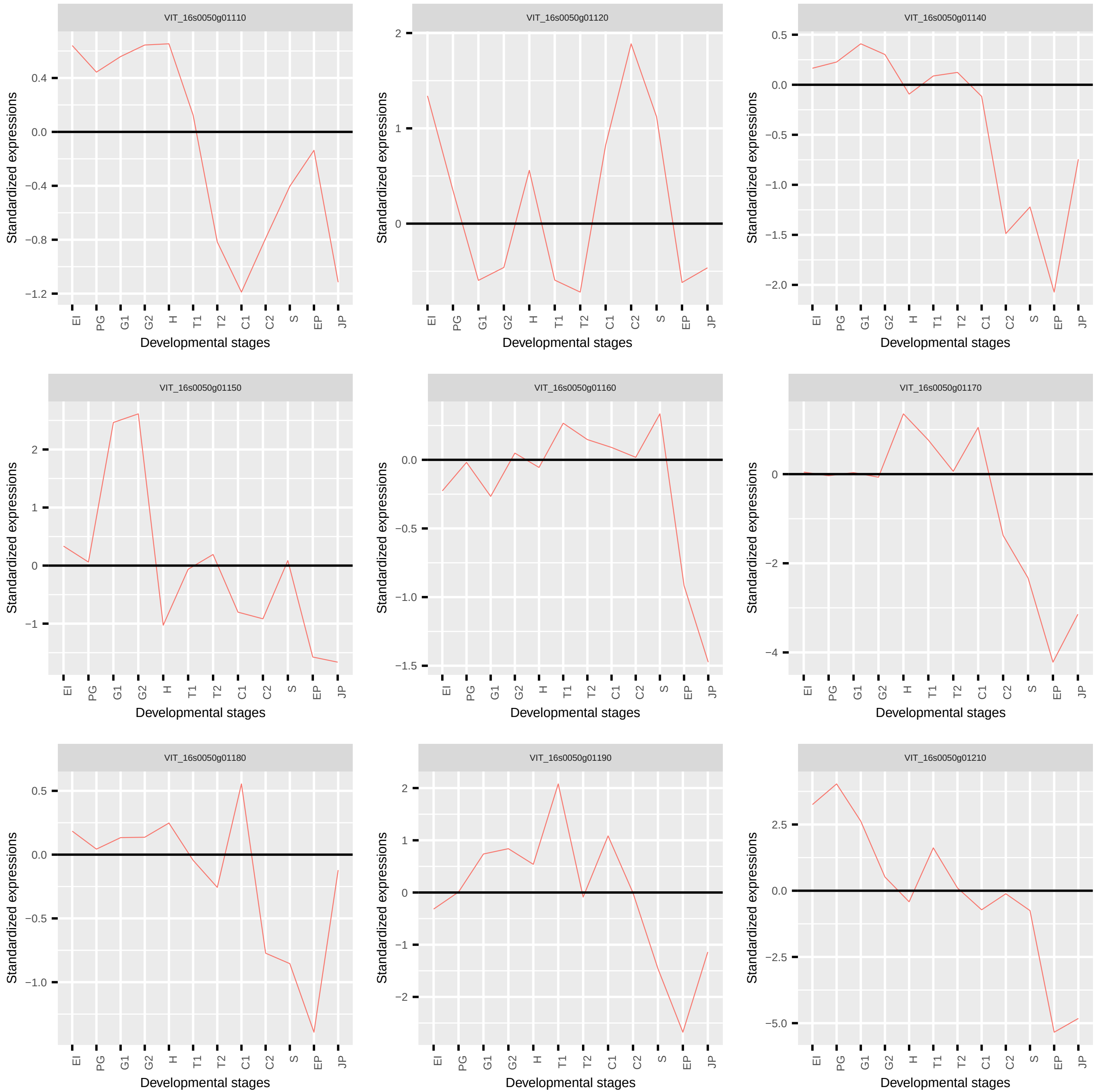
VIT_16s0050g01110
VIT_16s0050g01120
VIT_16s0050g01140
VIT_16s0050g01150
VIT_16s0050g01160
VIT_16s0050g01170
VIT_16s0050g01180
VIT_16s0050g01190
VIT_16s0050g01210
Standardized expressions
Developmental stages
EI
PG
G1
G2
H
T1
T2
C1
C2
S
EP
JP
0.4
0.0
−0.4
−0.8
−1.2
2
1
0
0.5
−0.5
−1.0
−1.5
−2.0
−1
−2
−4
2.5
−2.5
−5.0

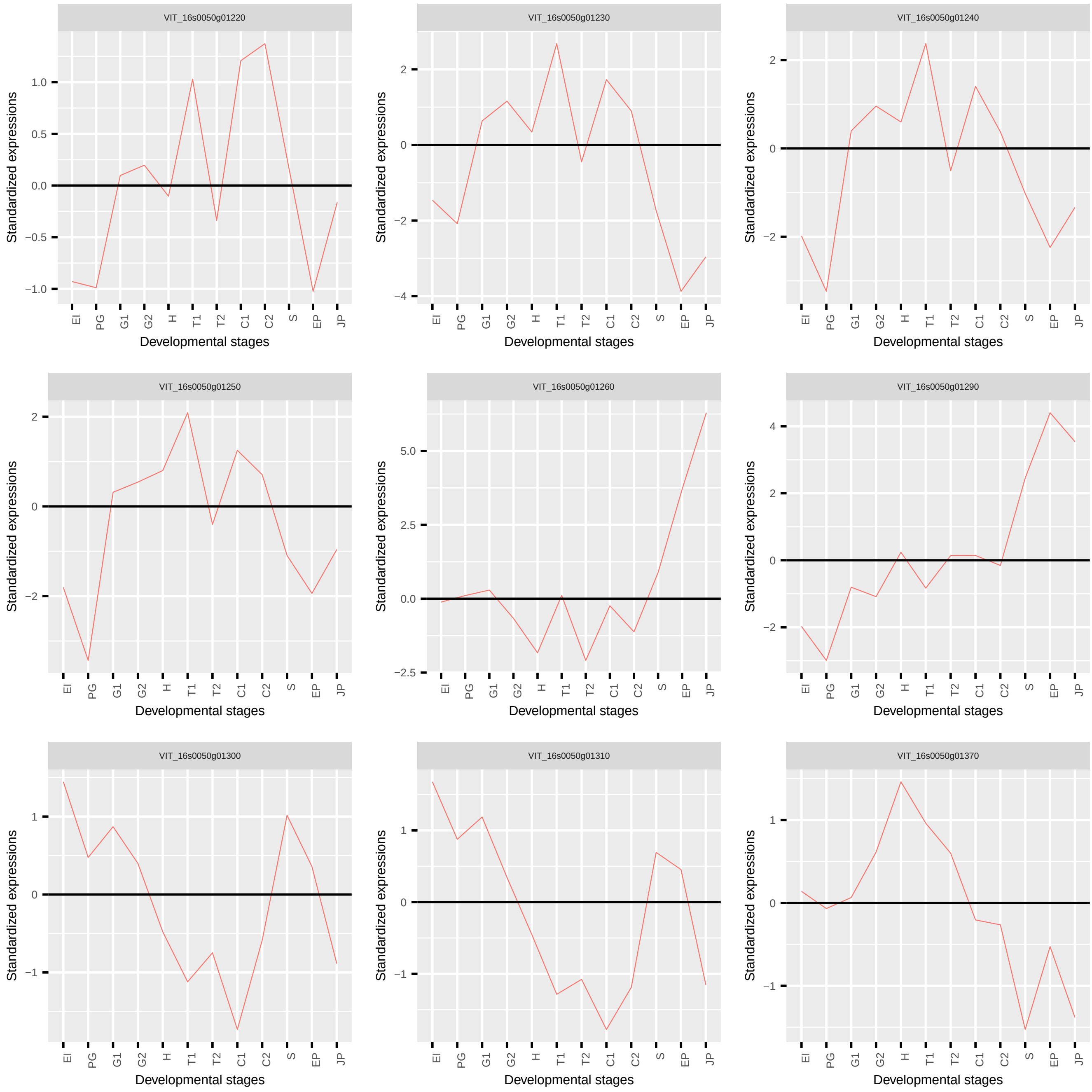
VIT_16s0050g01220
VIT_16s0050g01230
VIT_16s0050g01240
VIT_16s0050g01250
VIT_16s0050g01260
VIT_16s0050g01290
VIT_16s0050g01300
VIT_16s0050g01310
VIT_16s0050g01370
Standardized expressions
Developmental stages
EI PG G1 G2 H T1 T2 C1 C2 S EP JP

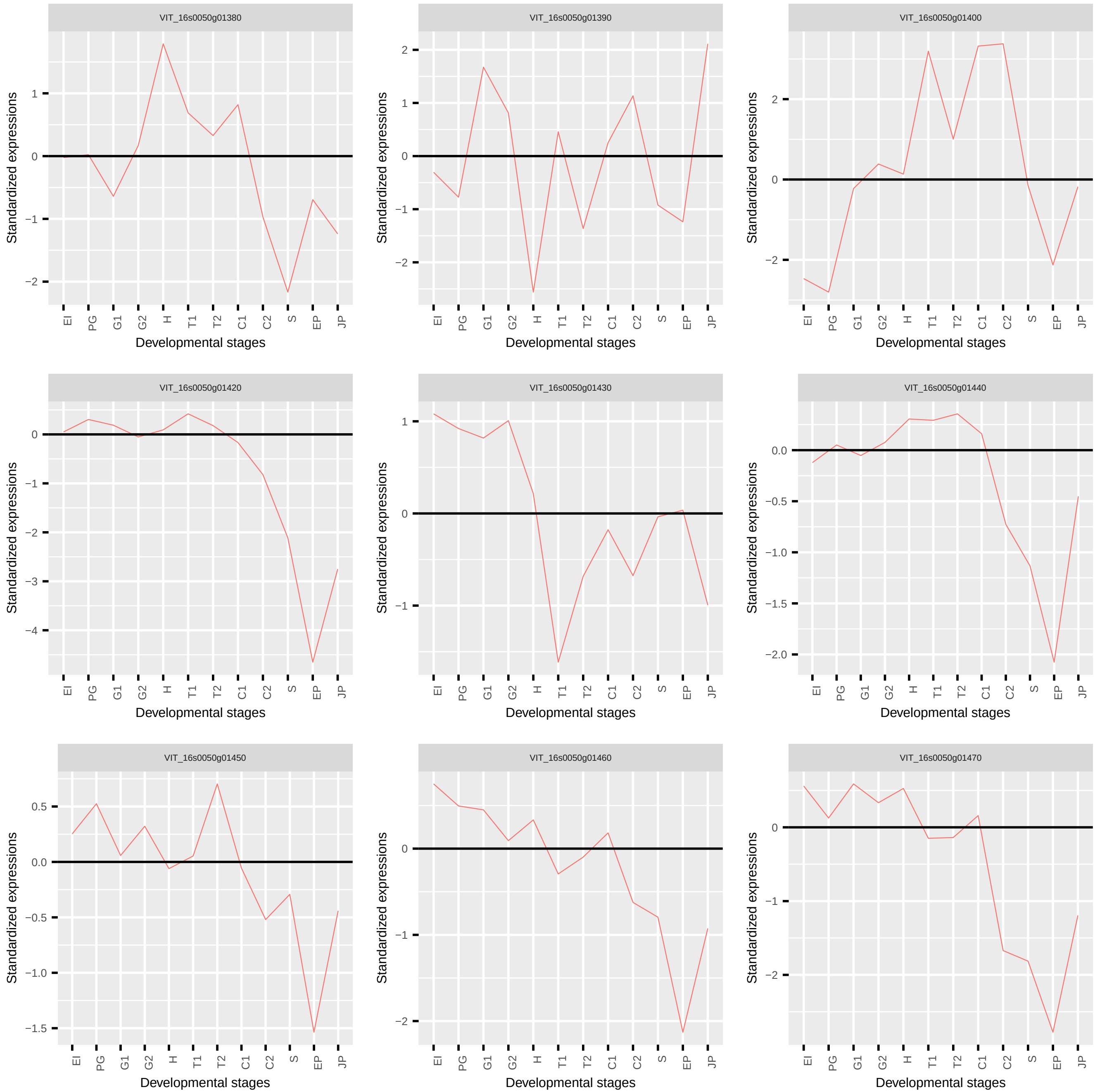

VIT_16s0050g01380
VIT_16s0050g01390
VIT_16s0050g01400
VIT_16s0050g01420
VIT_16s0050g01430
VIT_16s0050g01440
VIT_16s0050g01450
VIT_16s0050g01460
VIT_16s0050g01470
Standardized expressions
Developmental stages
EI PG G1 G2 H T1 T2 C1 C2 S EP JP

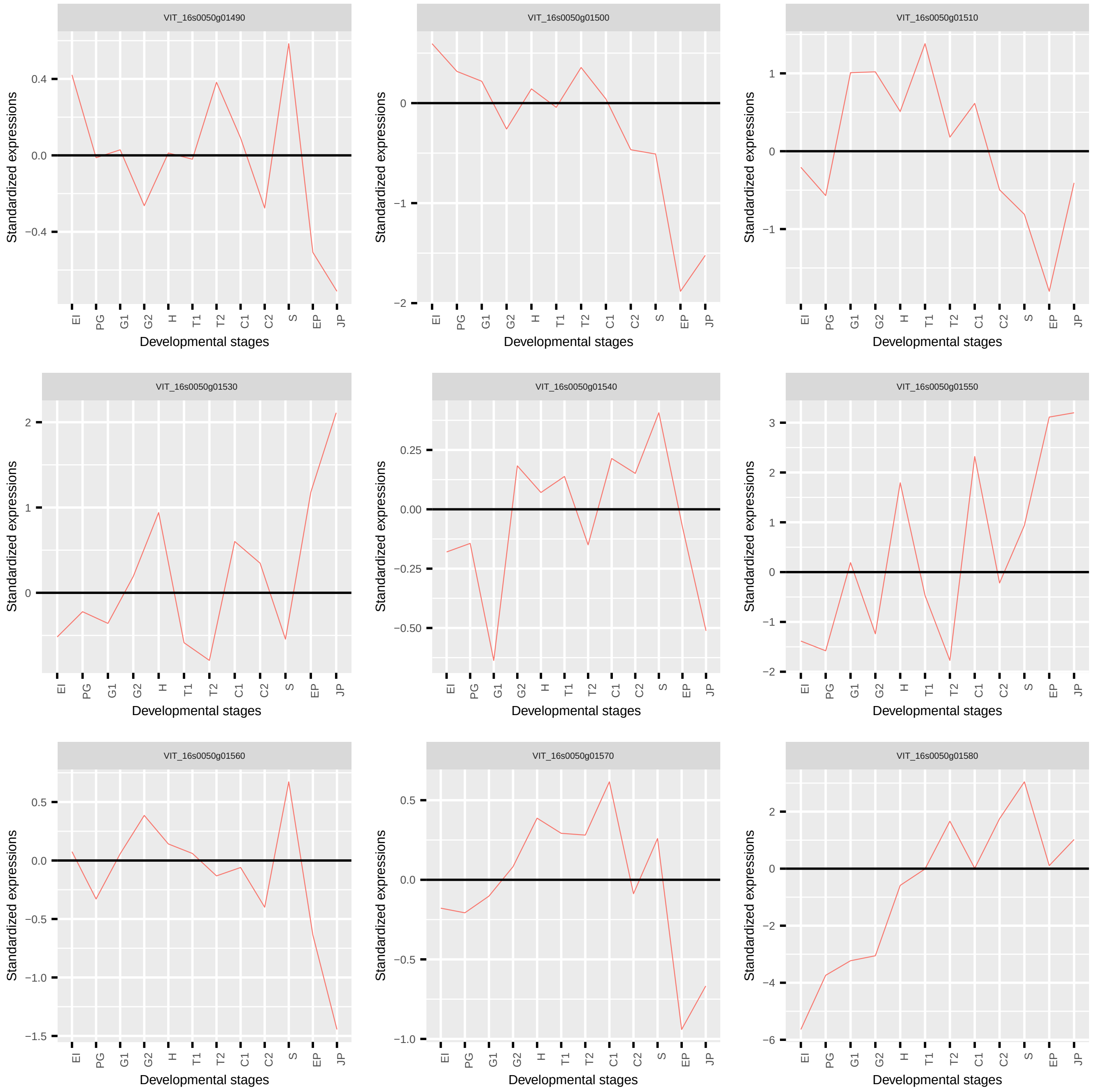
VIT_16s0050g01490
VIT_16s0050g01500
VIT_16s0050g01510
VIT_16s0050g01530
VIT_16s0050g01540
VIT_16s0050g01550
VIT_16s0050g01560
VIT_16s0050g01570
VIT_16s0050g01580
Standardized expressions
Developmental stages
EI
PG
G1
G2
H
T1
T2
C1
C2
S
EP
JP

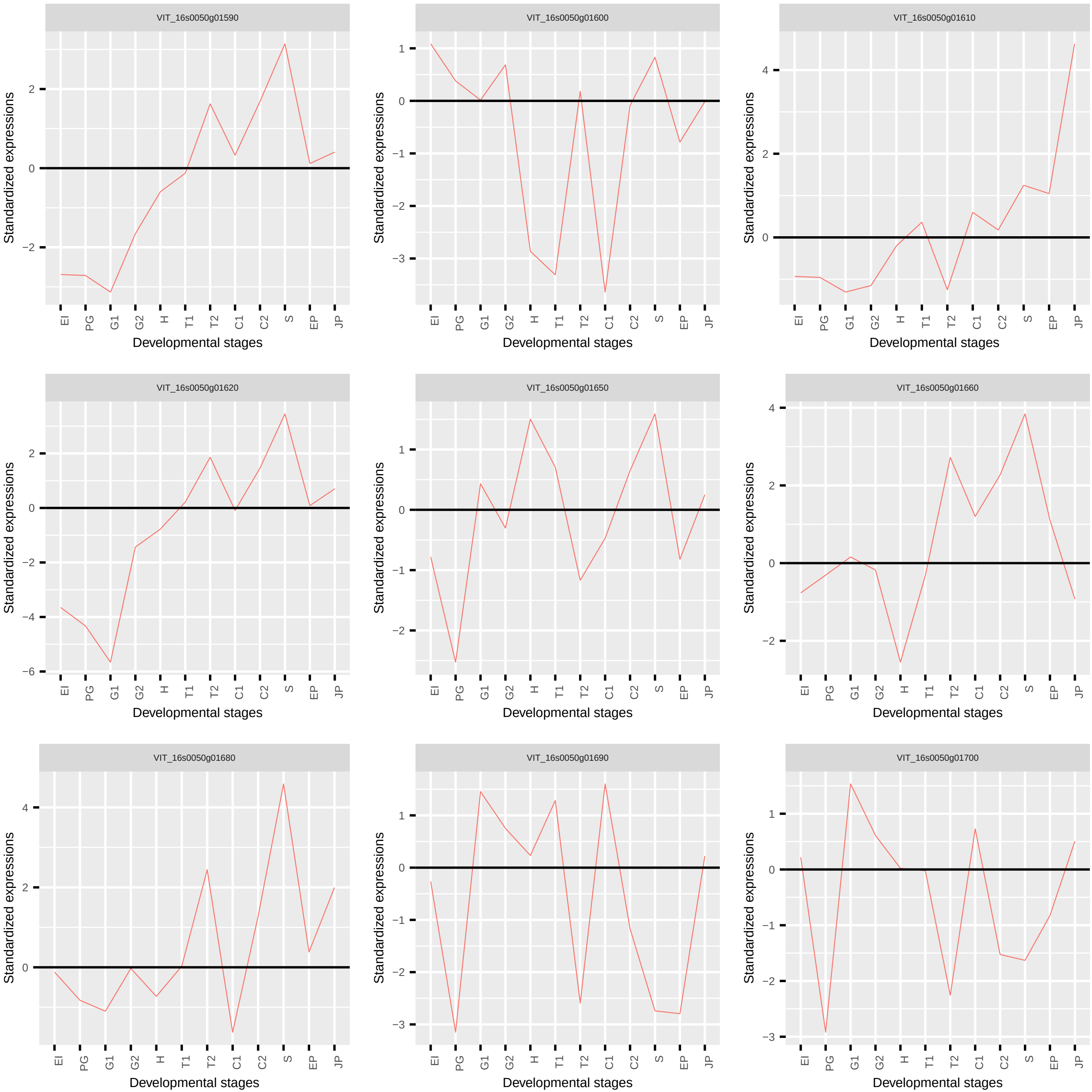
VIT_16s0050g01590
VIT_16s0050g01600
VIT_16s0050g01610
VIT_16s0050g01620
VIT_16s0050g01650
VIT_16s0050g01660
VIT_16s0050g01680
VIT_16s0050g01690
VIT_16s0050g01700
Standardized expressions
Developmental stages
EI PG G1 G2 H T1 T2 C1 C2 S EP JP

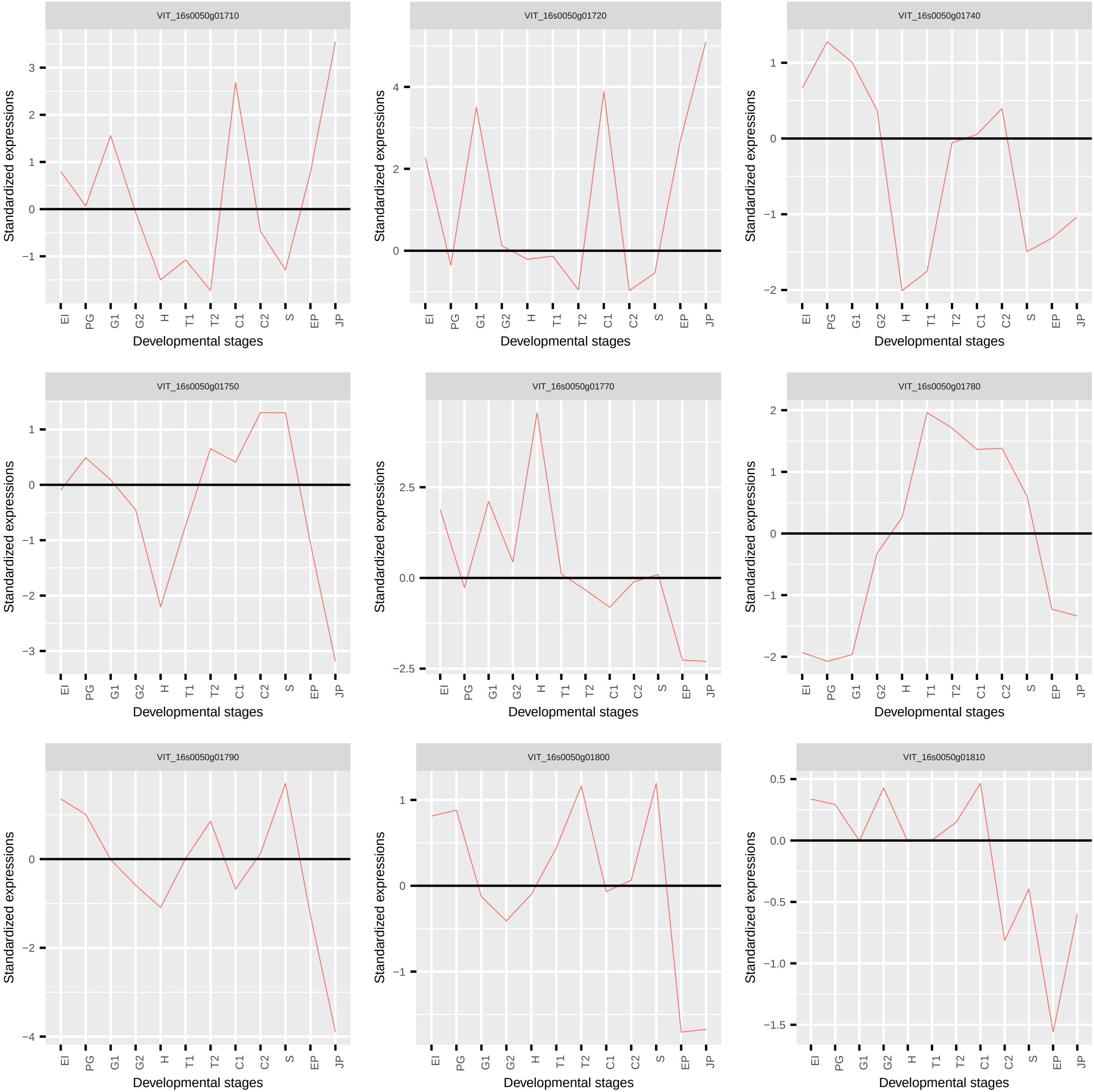
VIT_16s0050g01710
VIT_16s0050g01720
VIT_16s0050g01740
VIT_16s0050g01750
VIT_16s0050g01770
VIT_16s0050g01780
VIT_16s0050g01790
VIT_16s0050g01800
VIT_16s0050g01810
Standardized expressions
Developmental stages
EI PG G1 G2 H T1 T2 C1 C2 S EP JP

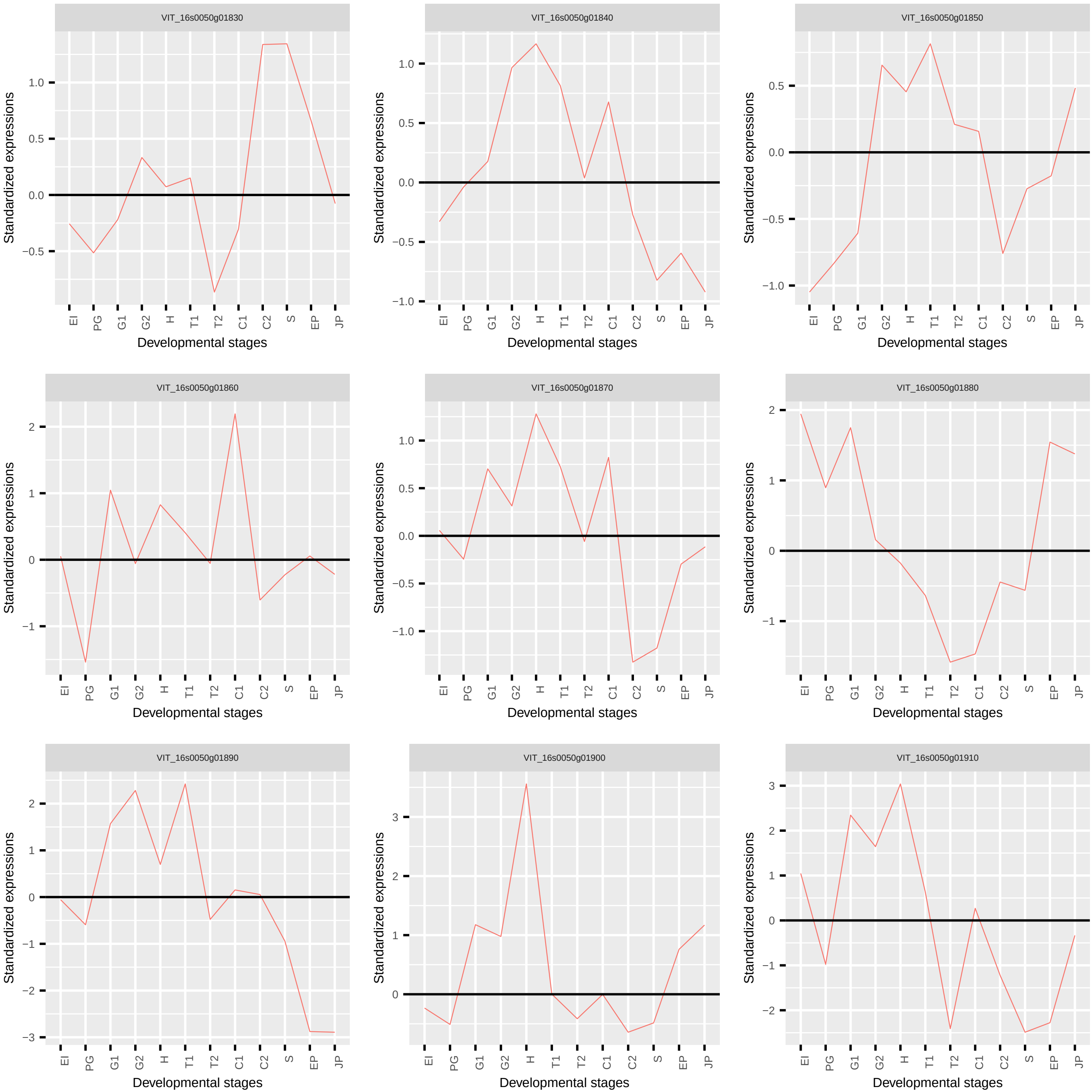
VIT_16s0050g01830
VIT_16s0050g01840
VIT_16s0050g01850
VIT_16s0050g01860
VIT_16s0050g01870
VIT_16s0050g01880
VIT_16s0050g01890
VIT_16s0050g01900
VIT_16s0050g01910
Standardized expressions
Developmental stages
EI
PG
G1
G2
H
T1
T2
C1
C2
S
EP
JP

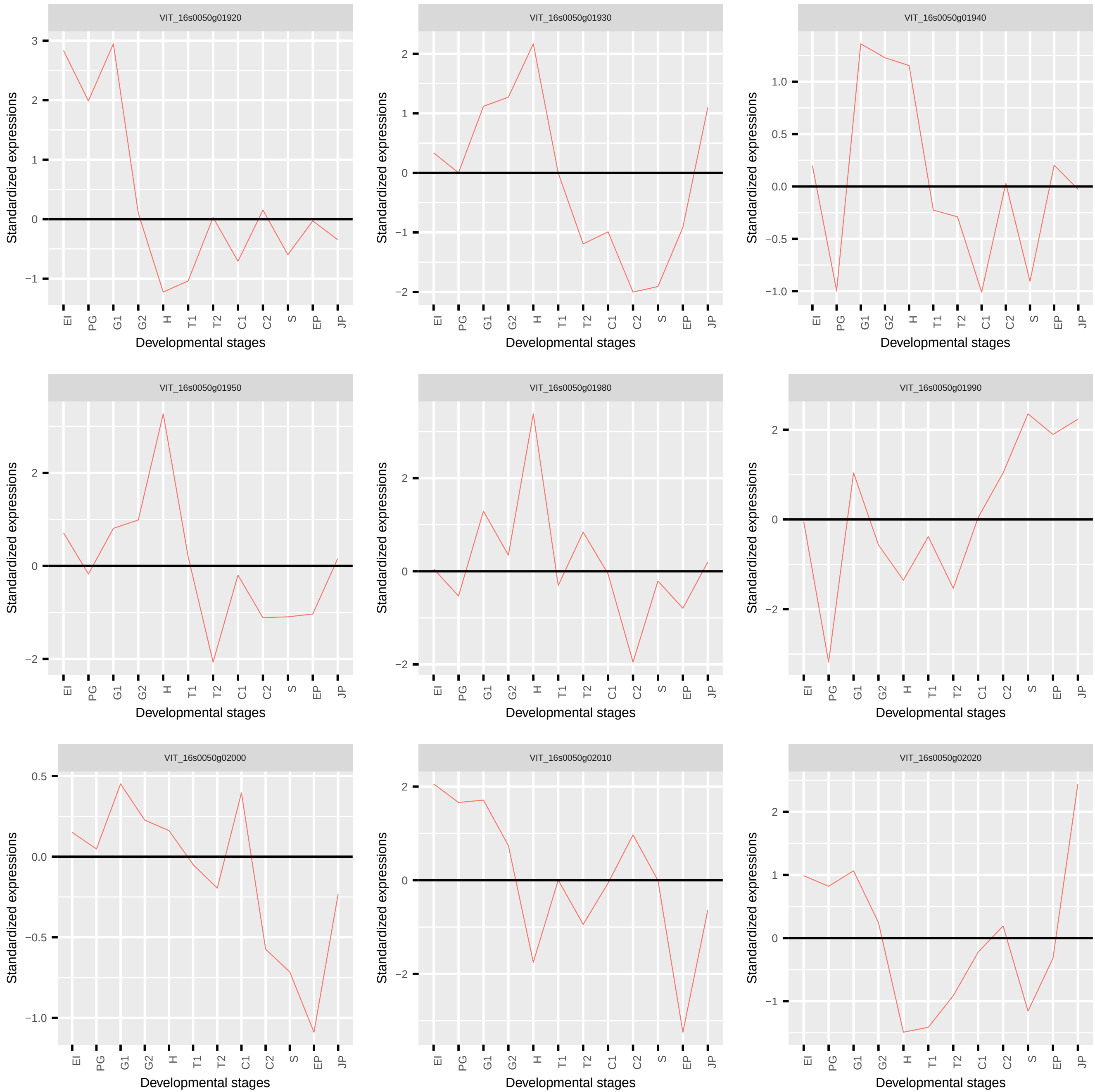

VIT_16s0050g01920
VIT_16s0050g01930
VIT_16s0050g01940
VIT_16s0050g01950
VIT_16s0050g01980
VIT_16s0050g01990
VIT_16s0050g02000
VIT_16s0050g02010
VIT_16s0050g02020
Standardized expressions
Developmental stages
EI PG G1 G2 H T1 T2 C1 C2 S EP JP

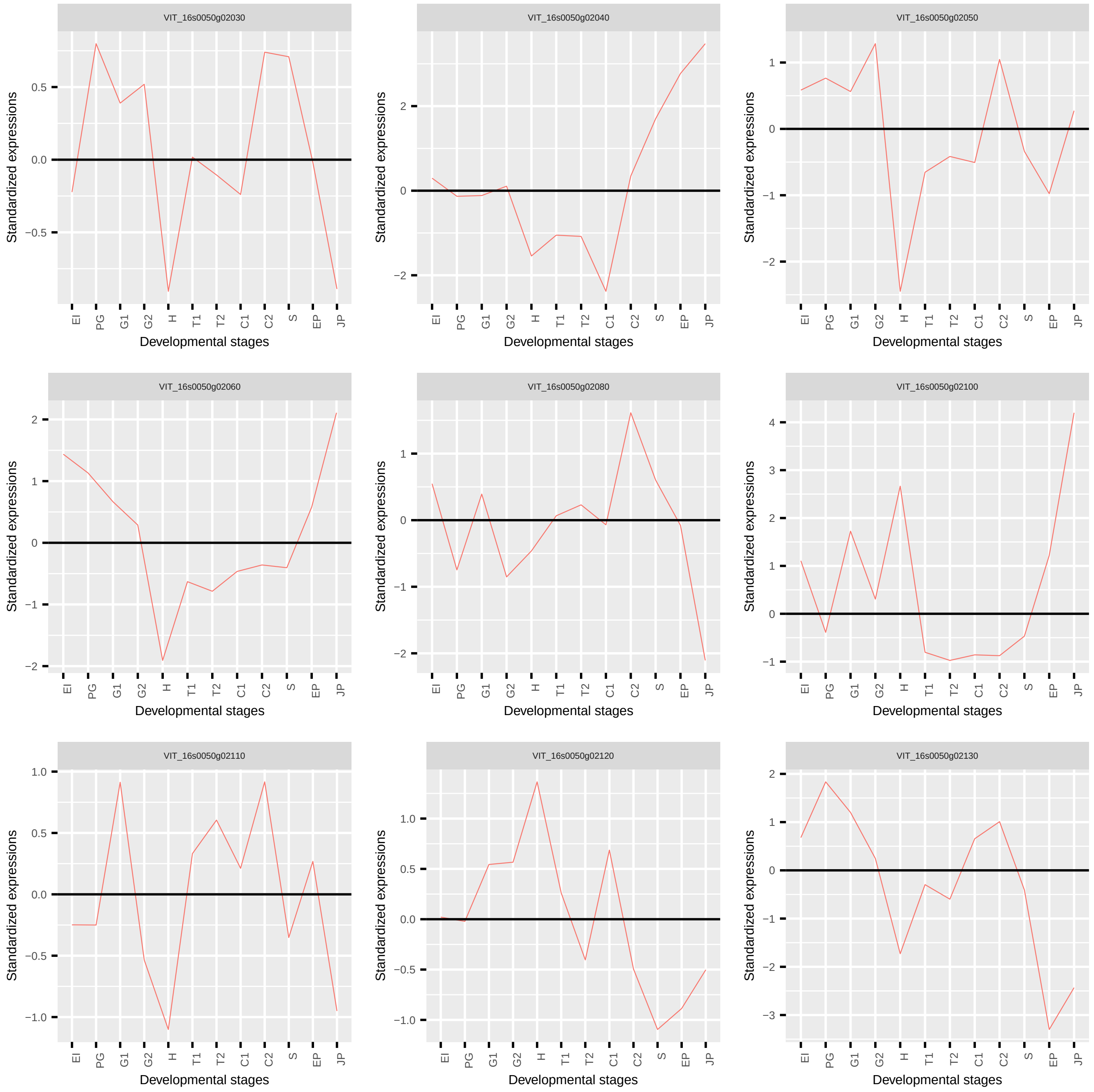

VIT_16s0050g02030
VIT_16s0050g02040
VIT_16s0050g02050
VIT_16s0050g02060
VIT_16s0050g02080
VIT_16s0050g02100
VIT_16s0050g02110
VIT_16s0050g02120
VIT_16s0050g02130
Standardized expressions
Developmental stages
EI
PG
G1
G2
H
T1
T2
C1
C2
S
EP
JP

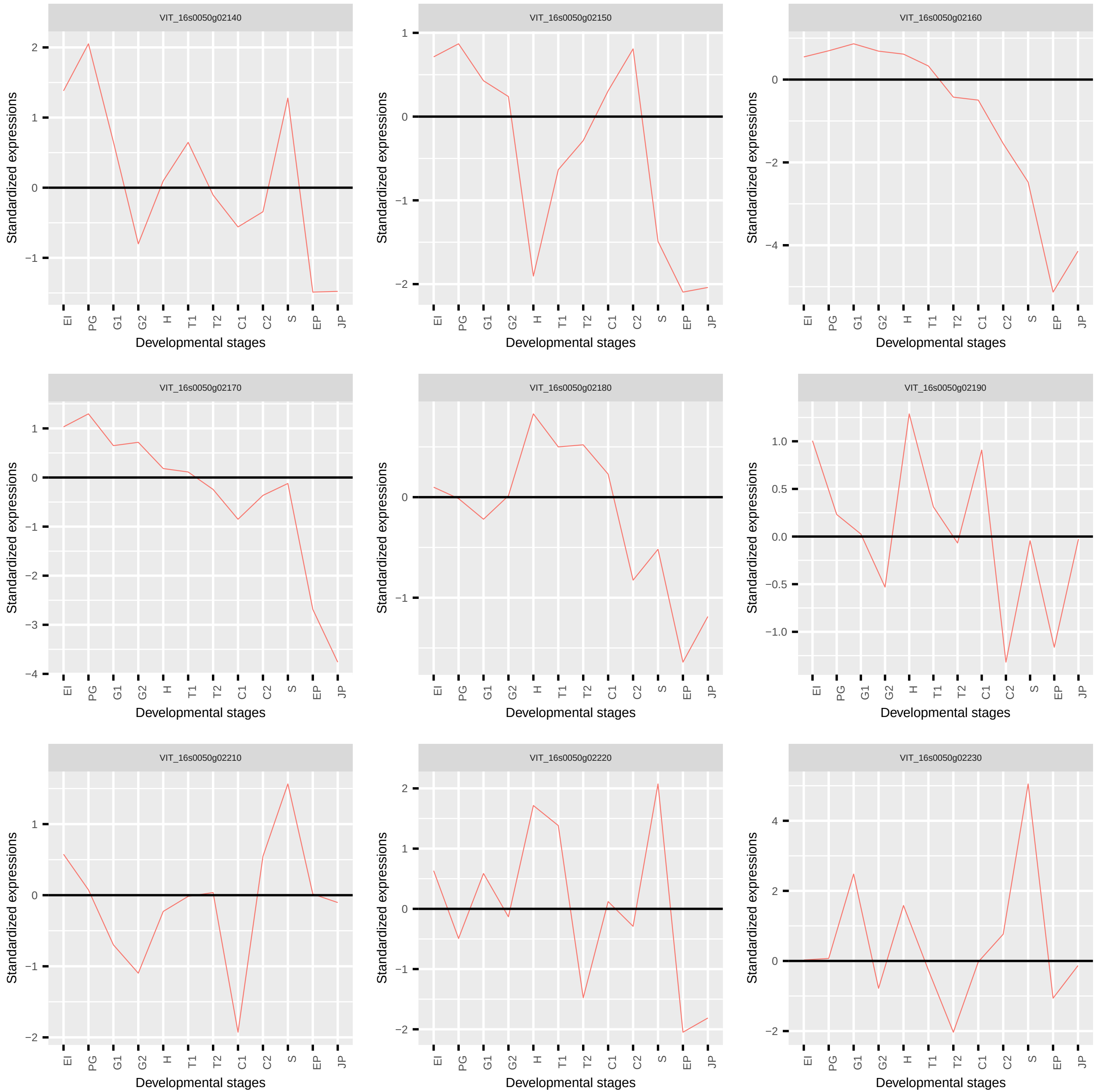
VIT_16s0050g02140
VIT_16s0050g02150
VIT_16s0050g02160
VIT_16s0050g02170
VIT_16s0050g02180
VIT_16s0050g02190
VIT_16s0050g02210
VIT_16s0050g02220
VIT_16s0050g02230
Standardized expressions
Developmental stages
EI PG G1 G2 H T1 T2 C1 C2 S EP JP

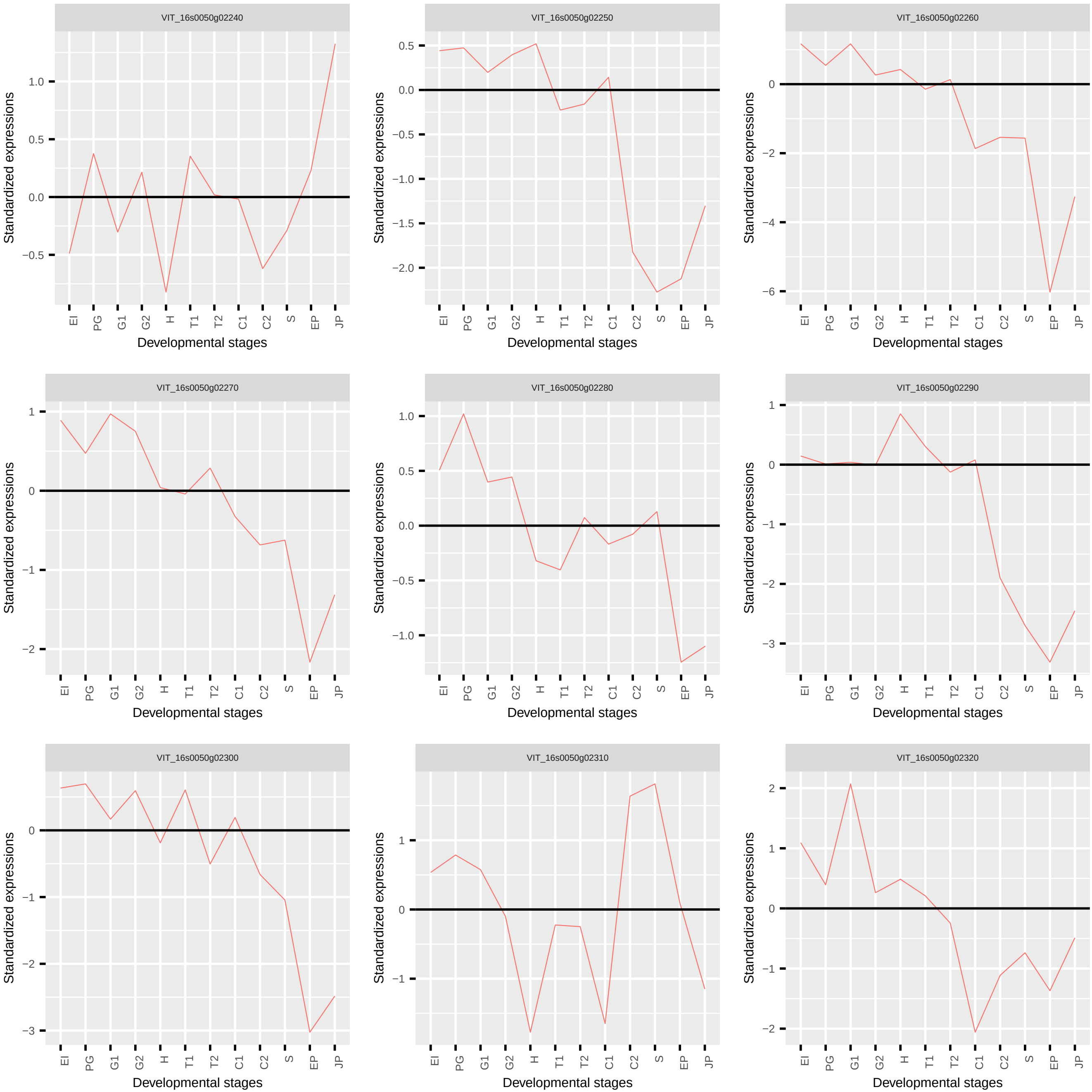
VIT_16s0050g02240
VIT_16s0050g02250
VIT_16s0050g02260
VIT_16s0050g02270
VIT_16s0050g02280
VIT_16s0050g02290
VIT_16s0050g02300
VIT_16s0050g02310
VIT_16s0050g02320
Standardized expressions
Developmental stages
EI PG G1 G2 H T1 T2 C1 C2 S EP JP

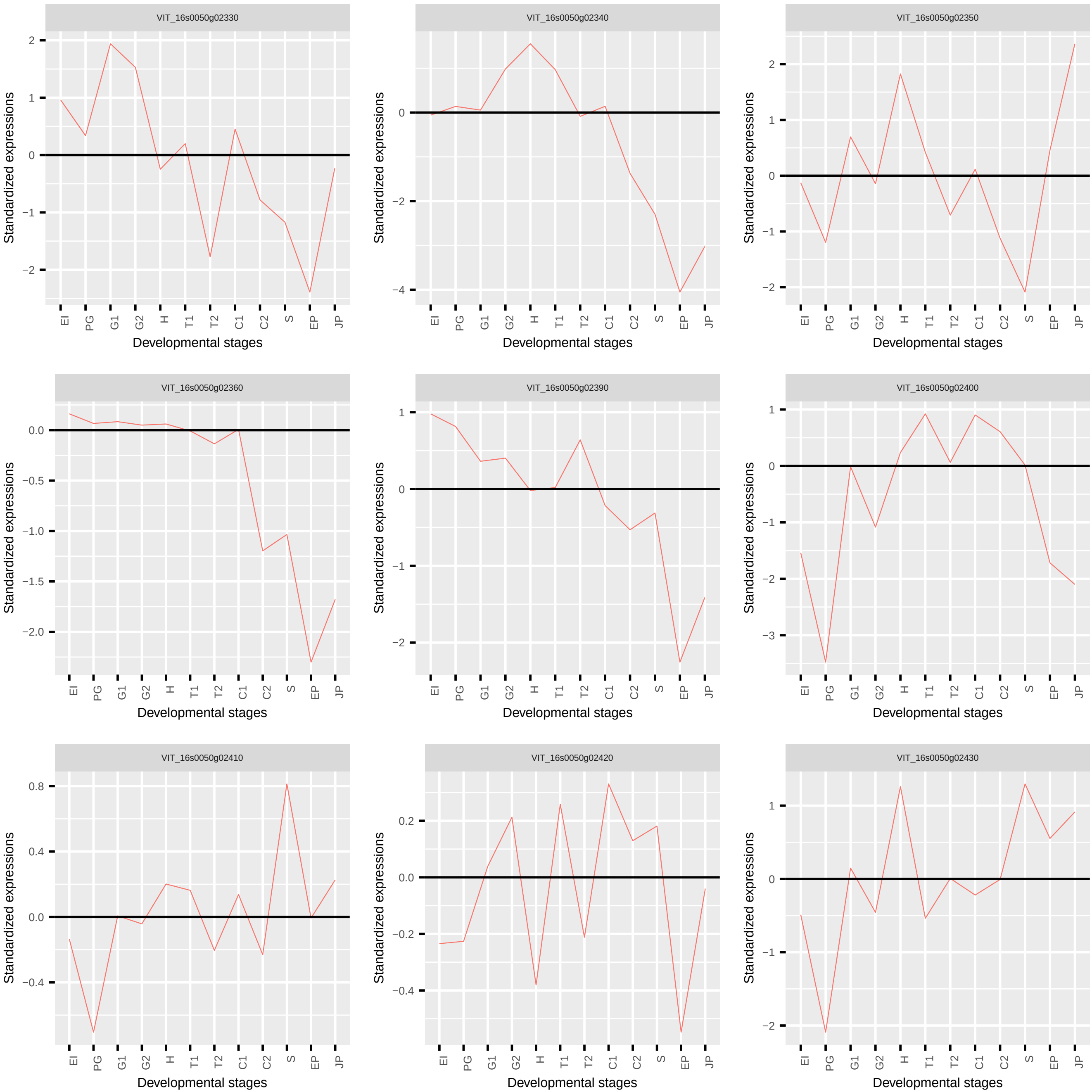
VIT_16s0050g02330
VIT_16s0050g02340
VIT_16s0050g02350
VIT_16s0050g02360
VIT_16s0050g02390
VIT_16s0050g02400
VIT_16s0050g02410
VIT_16s0050g02420
VIT_16s0050g02430
Standardized expressions
Developmental stages
EI PG G1 G2 H T1 T2 C1 C2 S EP JP

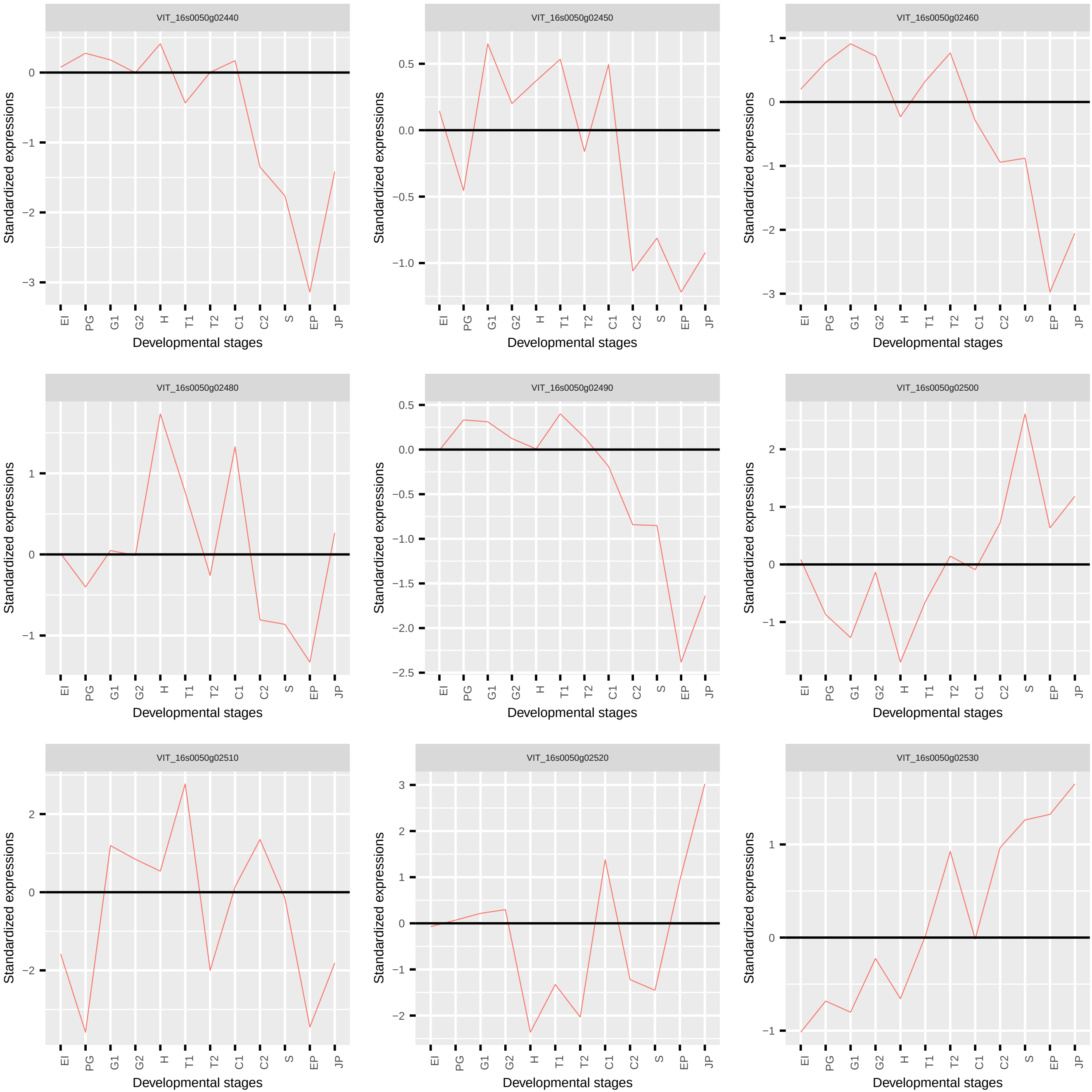
VIT_16s0050g02440
VIT_16s0050g02450
VIT_16s0050g02460
VIT_16s0050g02480
VIT_16s0050g02490
VIT_16s0050g02500
VIT_16s0050g02510
VIT_16s0050g02520
VIT_16s0050g02530
Standardized expressions
Developmental stages
EI PG G1 G2 H T1 T2 C1 C2 S EP JP

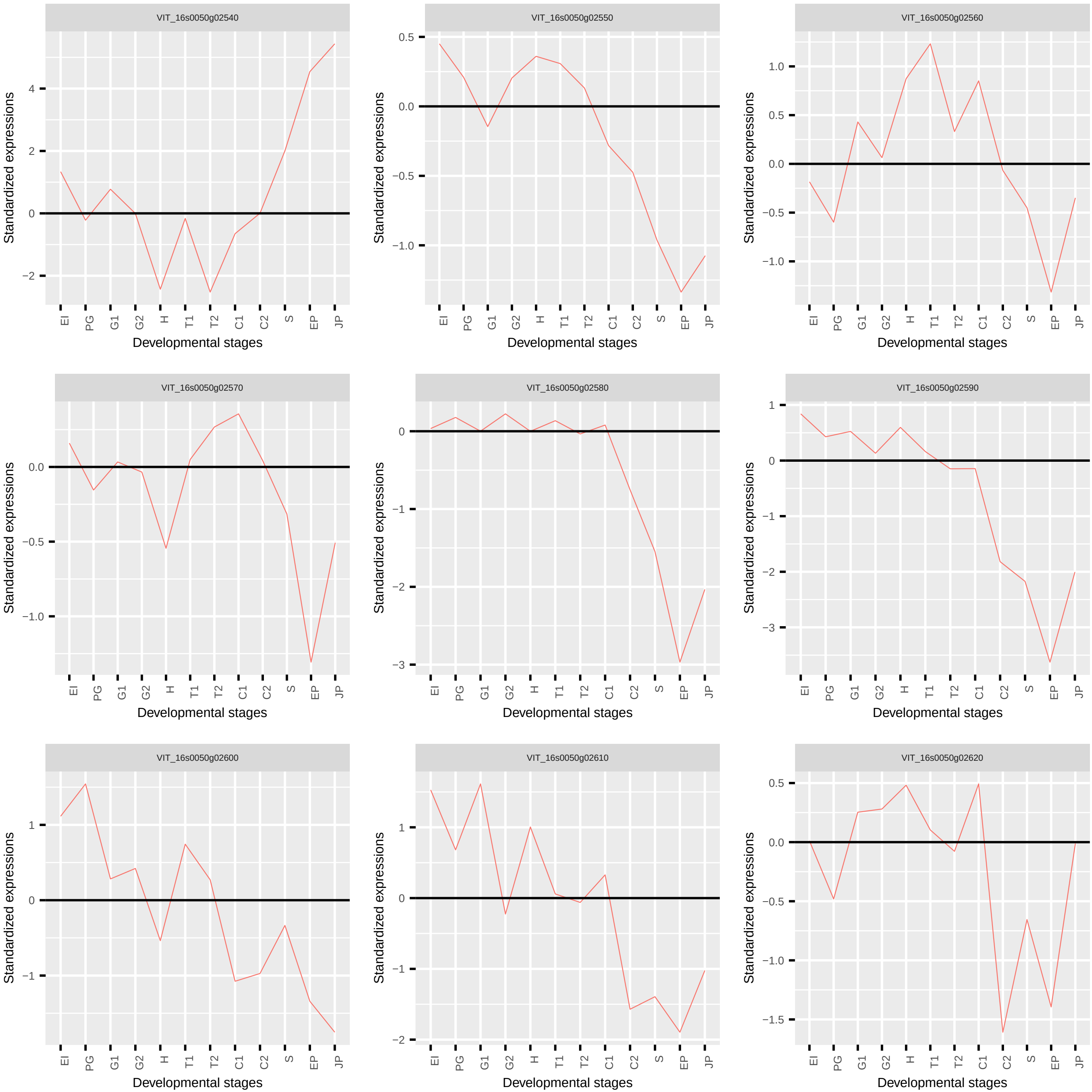

VIT_16s0050g02540
VIT_16s0050g02550
VIT_16s0050g02560
VIT_16s0050g02570
VIT_16s0050g02580
VIT_16s0050g02590
VIT_16s0050g02600
VIT_16s0050g02610
VIT_16s0050g02620
Standardized expressions
Developmental stages
EI PG G1 G2 H T1 T2 C1 C2 S EP JP

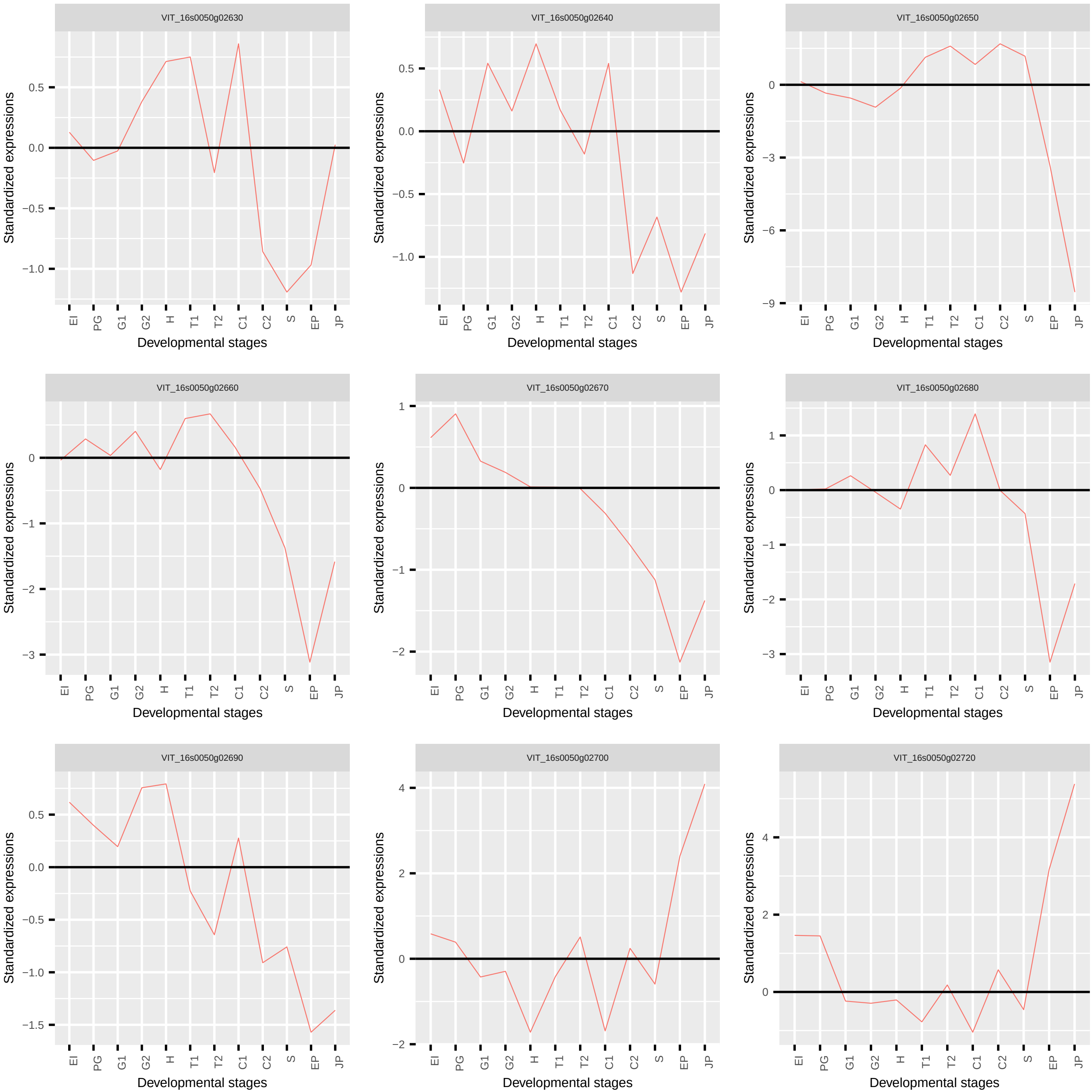
VIT_16s0050g02630
VIT_16s0050g02640
VIT_16s0050g02650
VIT_16s0050g02660
VIT_16s0050g02670
VIT_16s0050g02680
VIT_16s0050g02690
VIT_16s0050g02700
VIT_16s0050g02720
Standardized expressions
Developmental stages
EI PG G1 G2 H T1 T2 C1 C2 S EP JP

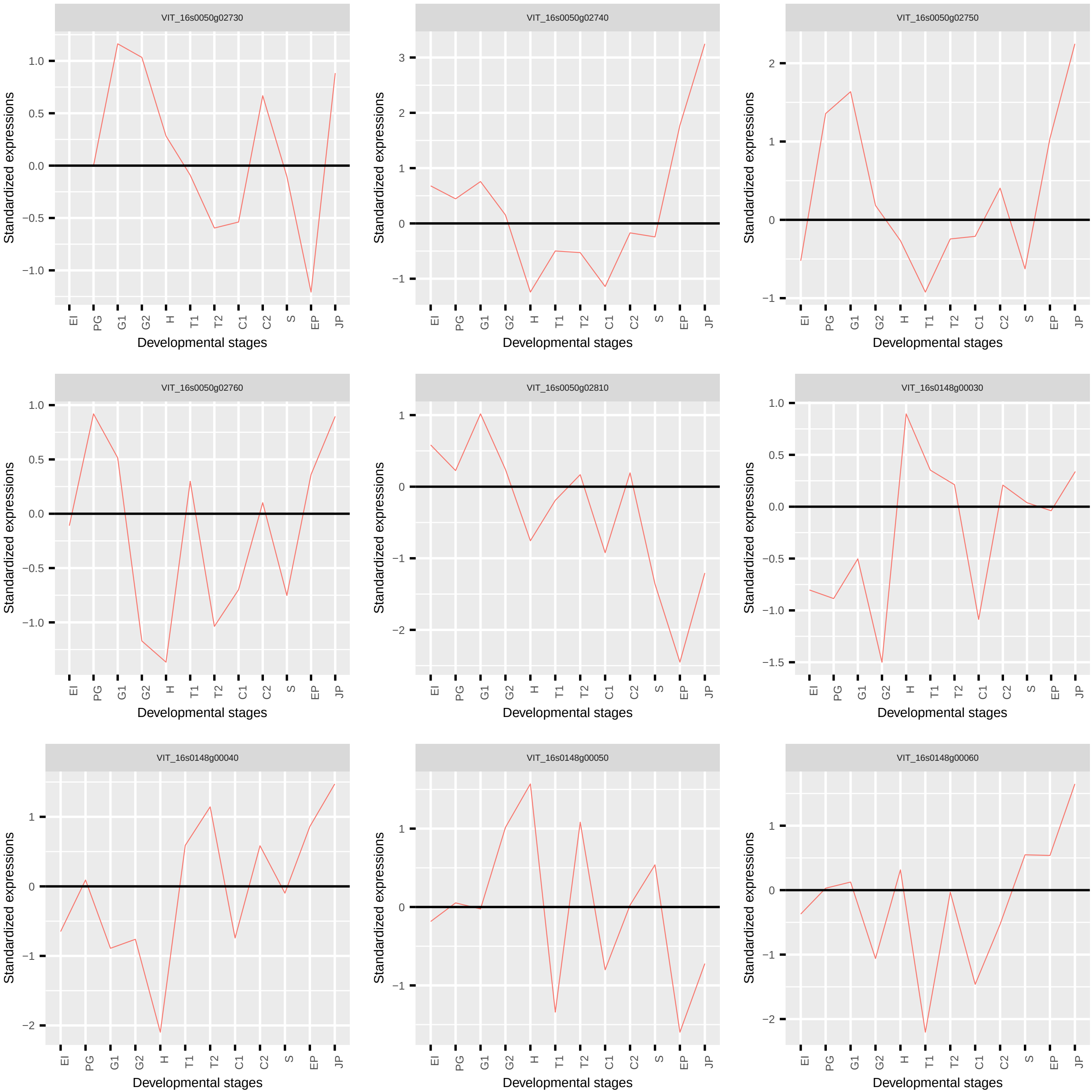
VIT_16s0050g02730
VIT_16s0050g02740
VIT_16s0050g02750
VIT_16s0050g02760
VIT_16s0050g02810
VIT_16s0148g00030
VIT_16s0148g00040
VIT_16s0148g00050
VIT_16s0148g00060
Standardized expressions
Developmental stages
EI PG G1 G2 H T1 T2 C1 C2 S EP JP

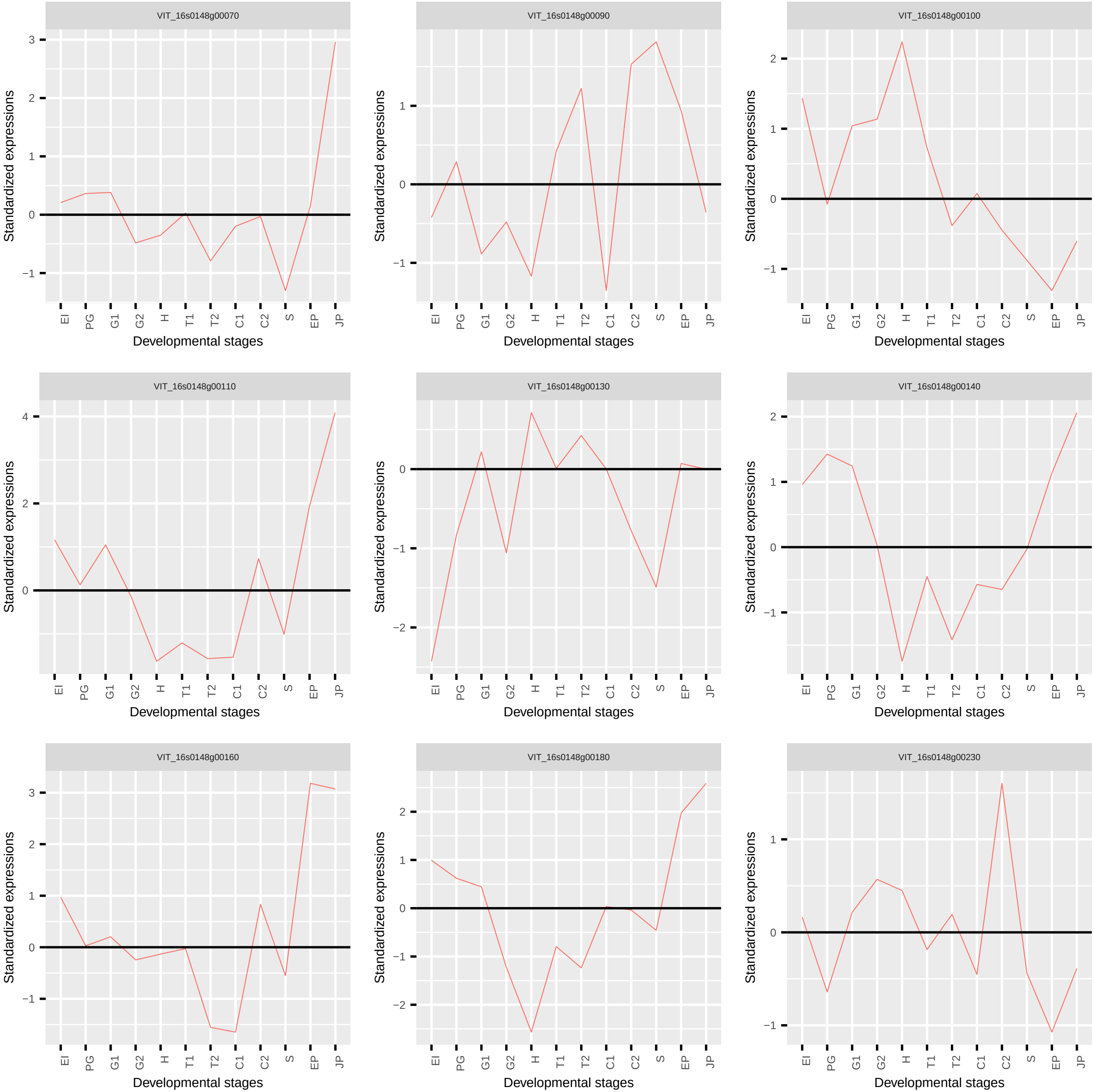
VIT_16s0148g00070
Standardized expressions
3
2
1
0
−1
EI PG G1 G2 H T1 T2 C1 C2 S EP JP
Developmental stages
VIT_16s0148g00090
Standardized expressions
1
0
−1
EI PG G1 G2 H T1 T2 C1 C2 S EP JP
Developmental stages
VIT_16s0148g00100
Standardized expressions
2
1
0
−1
EI PG G1 G2 H T1 T2 C1 C2 S EP JP
Developmental stages
VIT_16s0148g00110
Standardized expressions
4
2
0
EI PG G1 G2 H T1 T2 C1 C2 S EP JP
Developmental stages
VIT_16s0148g00130
Standardized expressions
0
−1
−2
EI PG G1 G2 H T1 T2 C1 C2 S EP JP
Developmental stages
VIT_16s0148g00140
Standardized expressions
2
1
0
−1
EI PG G1 G2 H T1 T2 C1 C2 S EP JP
Developmental stages
VIT_16s0148g00160
Standardized expressions
3
2
1
0
−1
EI PG G1 G2 H T1 T2 C1 C2 S EP JP
Developmental stages
VIT_16s0148g00180
Standardized expressions
2
1
0
−1
−2
EI PG G1 G2 H T1 T2 C1 C2 S EP JP
Developmental stages
VIT_16s0148g00230
Standardized expressions
1
0
−1
EI PG G1 G2 H T1 T2 C1 C2 S EP JP
Developmental stages

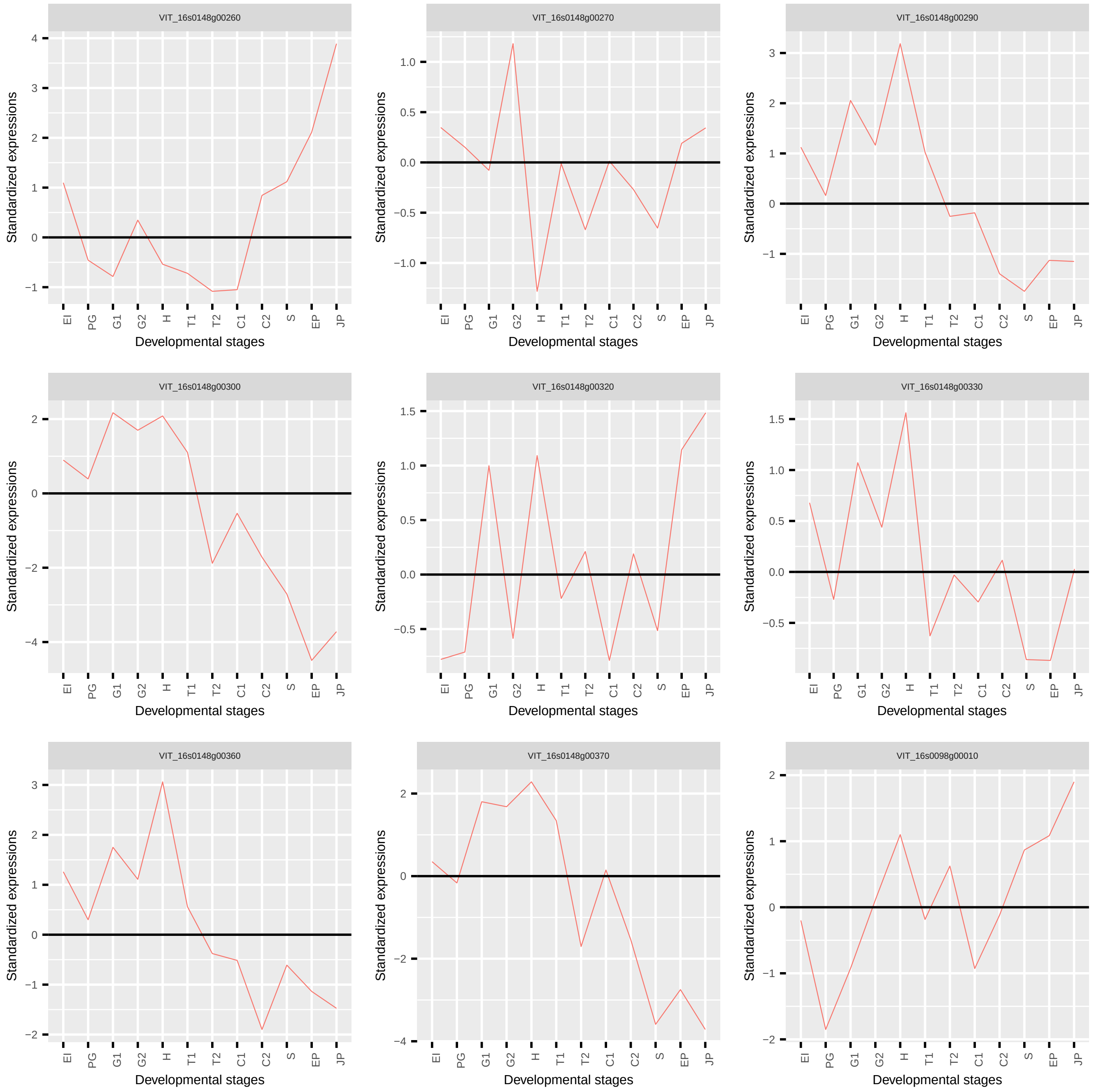
VIT_16s0148g00260
VIT_16s0148g00270
VIT_16s0148g00290
VIT_16s0148g00300
VIT_16s0148g00320
VIT_16s0148g00330
VIT_16s0148g00360
VIT_16s0148g00370
VIT_16s0098g00010
Standardized expressions
Developmental stages
EI PG G1 G2 H T1 T2 C1 C2 S EP JP

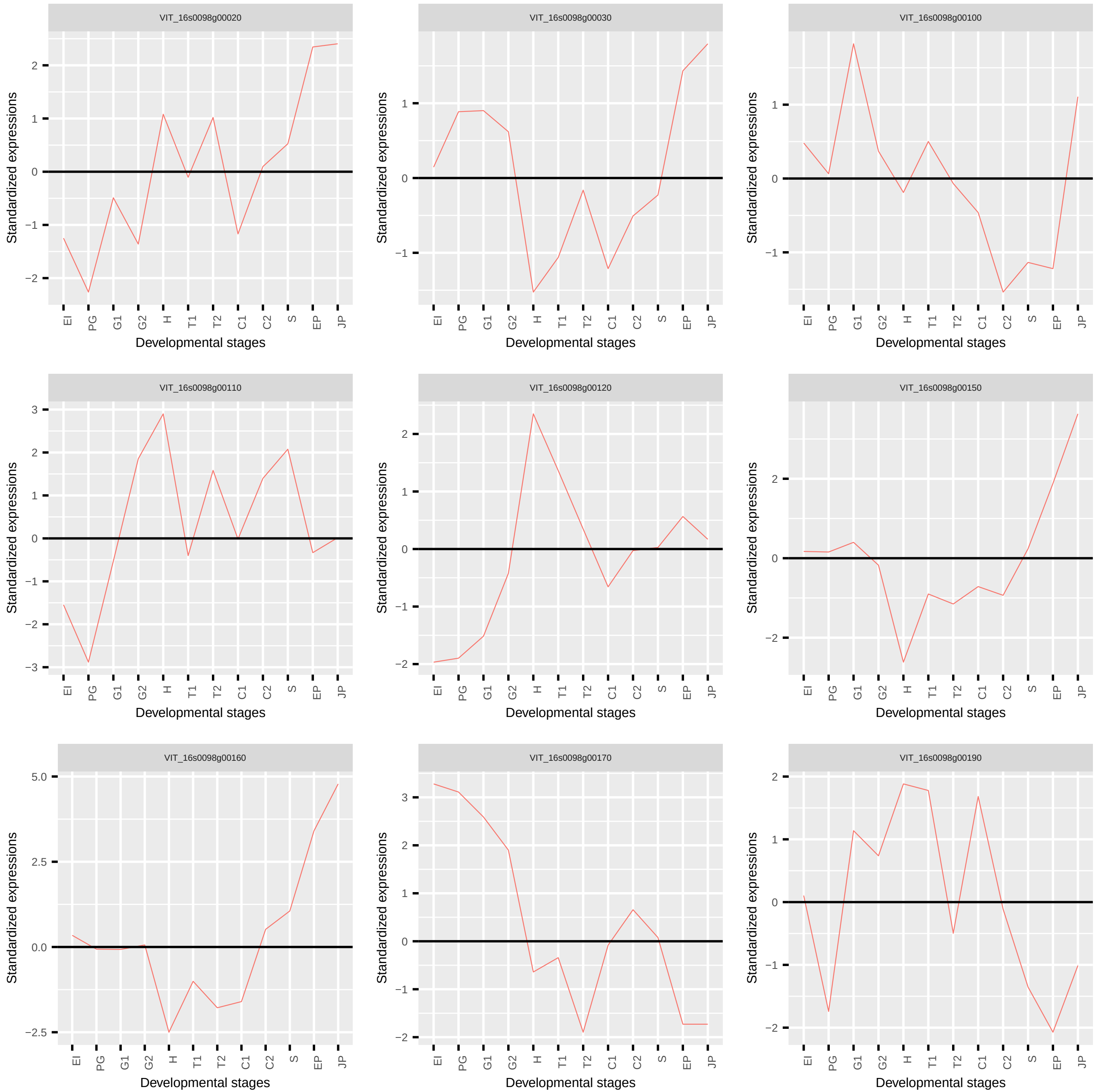
VIT_16s0098g00020
VIT_16s0098g00030
VIT_16s0098g00100
VIT_16s0098g00110
VIT_16s0098g00120
VIT_16s0098g00150
VIT_16s0098g00160
VIT_16s0098g00170
VIT_16s0098g00190
Standardized expressions
Developmental stages
EI PG G1 G2 H T1 T2 C1 C2 S EP JP

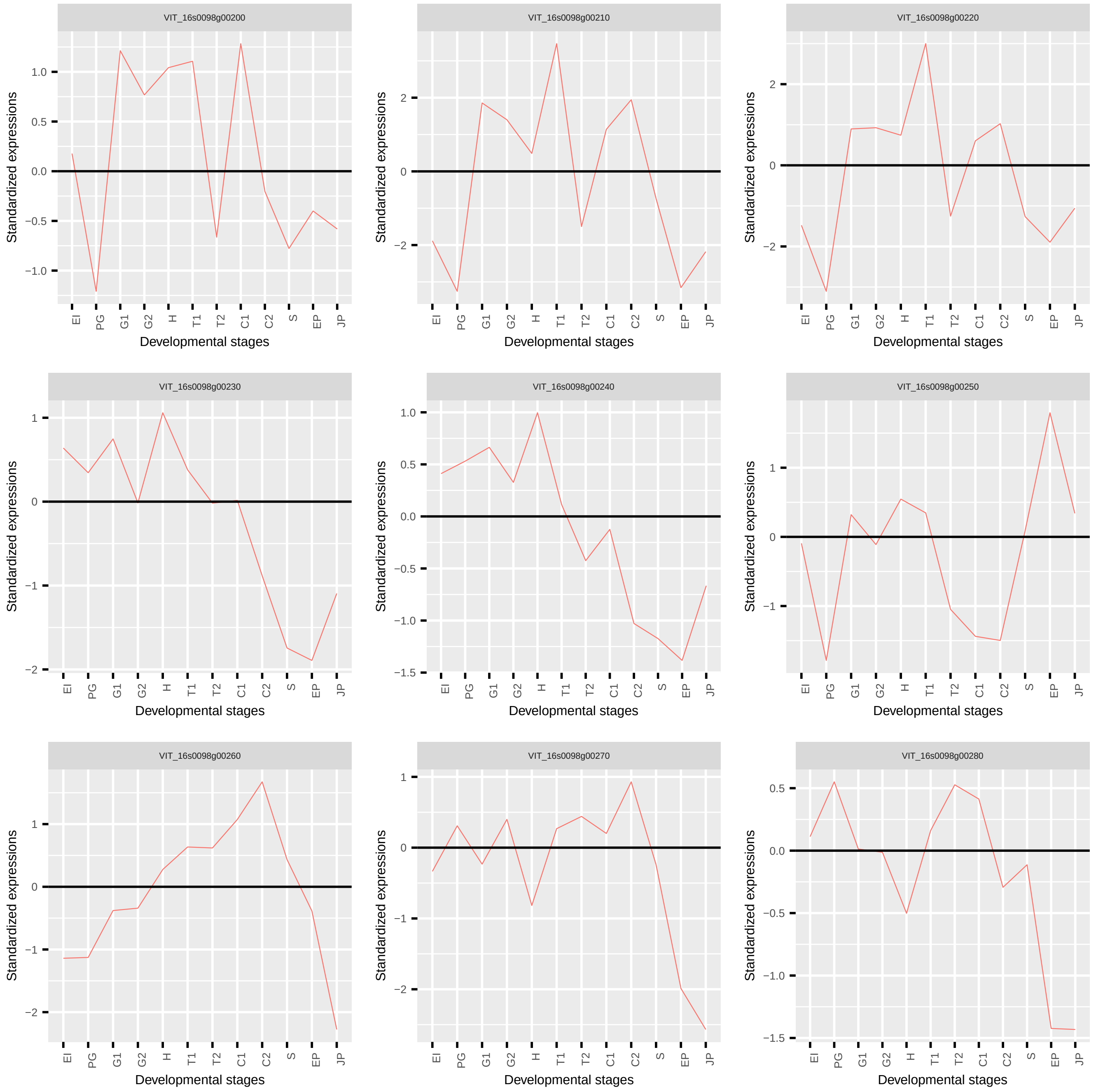
VIT_16s0098g00200
VIT_16s0098g00210
VIT_16s0098g00220
VIT_16s0098g00230
VIT_16s0098g00240
VIT_16s0098g00250
VIT_16s0098g00260
VIT_16s0098g00270
VIT_16s0098g00280
Standardized expressions
Developmental stages
EI PG G1 G2 H T1 T2 C1 C2 S EP JP

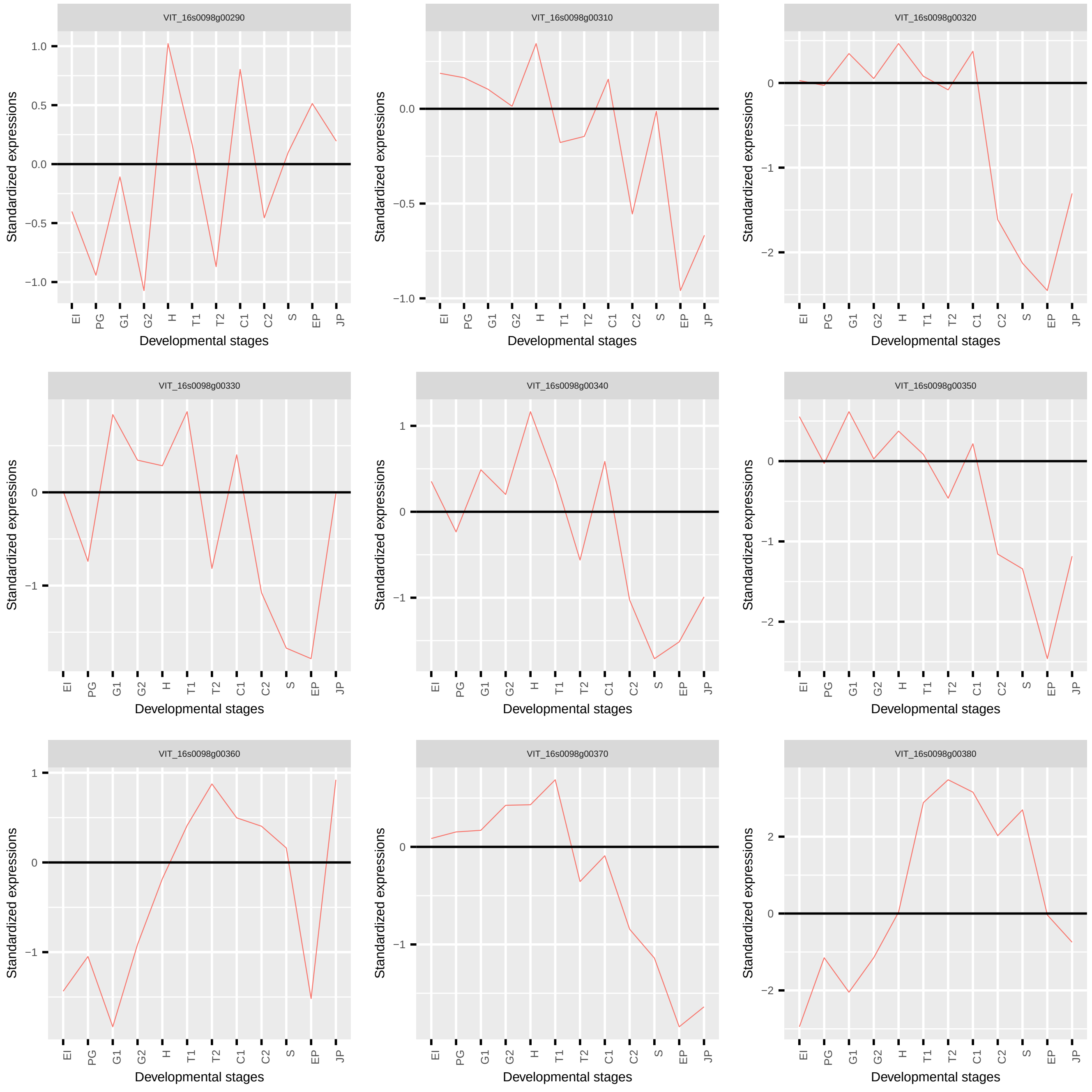
VIT_16s0098g00290
VIT_16s0098g00310
VIT_16s0098g00320
VIT_16s0098g00330
VIT_16s0098g00340
VIT_16s0098g00350
VIT_16s0098g00360
VIT_16s0098g00370
VIT_16s0098g00380
Standardized expressions
Developmental stages
EI
PG
G1
G2
H
T1
T2
C1
C2
S
EP
JP

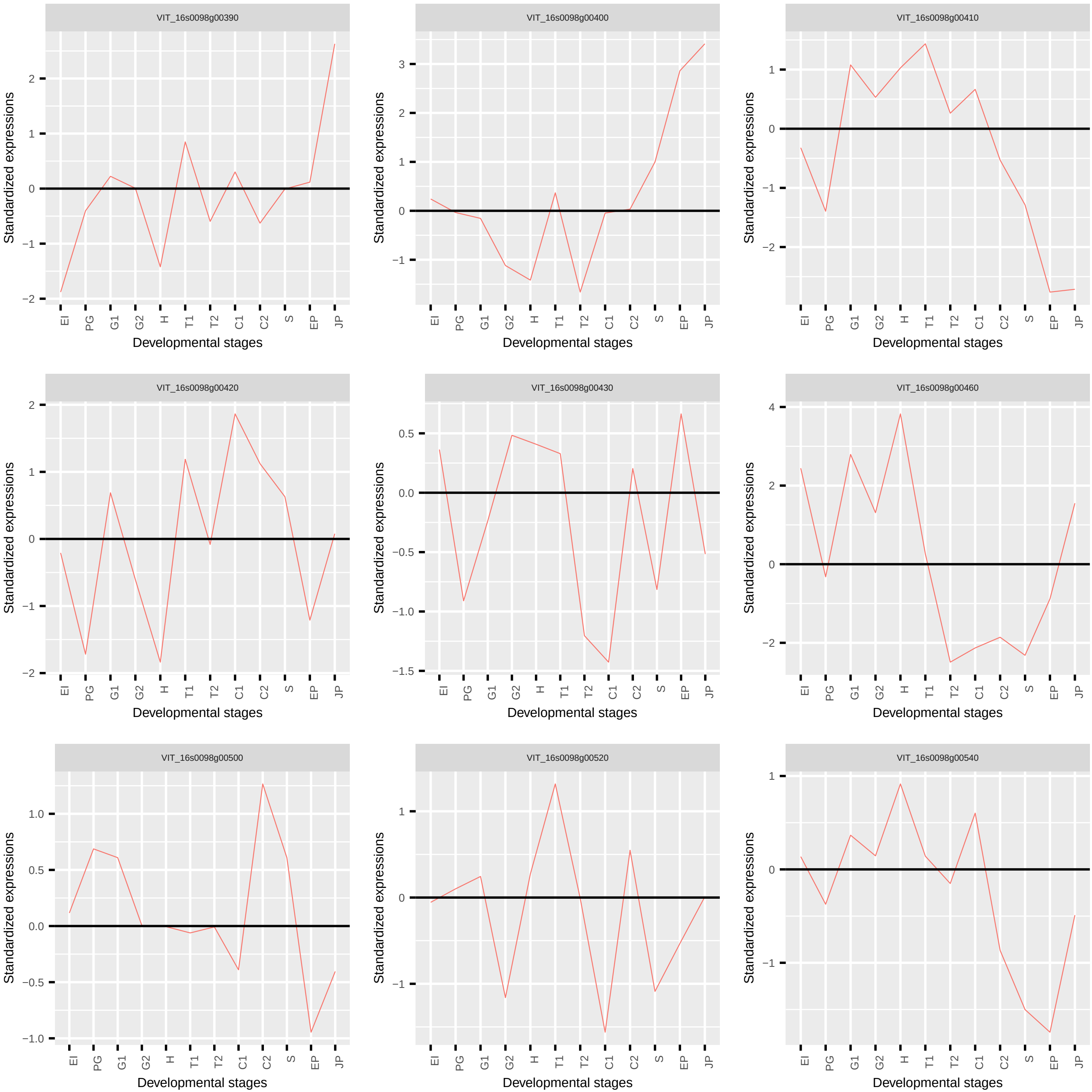
VIT_16s0098g00390
VIT_16s0098g00400
VIT_16s0098g00410
VIT_16s0098g00420
VIT_16s0098g00430
VIT_16s0098g00460
VIT_16s0098g00500
VIT_16s0098g00520
VIT_16s0098g00540
Standardized expressions
Developmental stages
EI PG G1 G2 H T1 T2 C1 C2 S EP JP

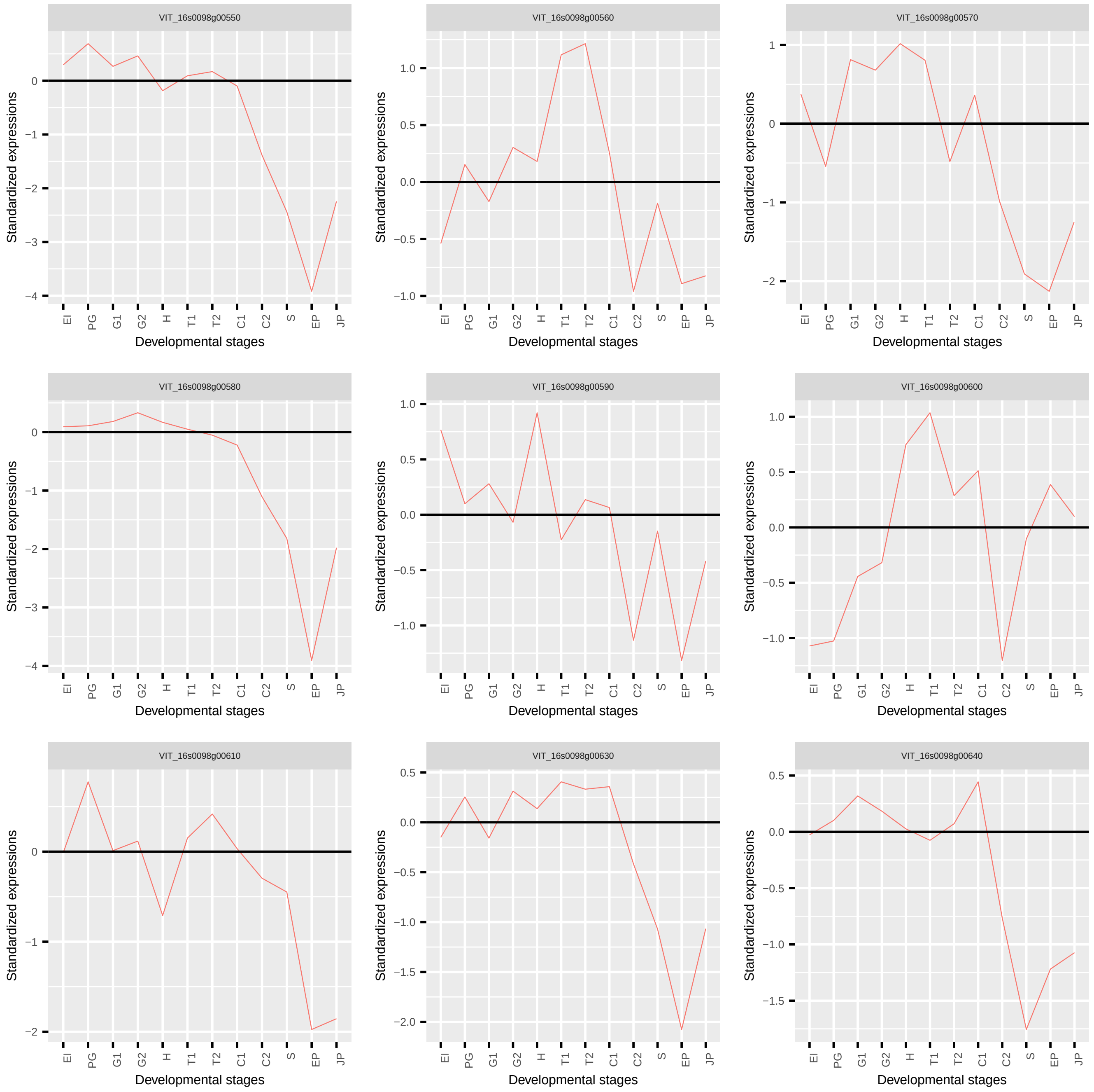
VIT_16s0098g00550
VIT_16s0098g00560
VIT_16s0098g00570
VIT_16s0098g00580
VIT_16s0098g00590
VIT_16s0098g00600
VIT_16s0098g00610
VIT_16s0098g00630
VIT_16s0098g00640
Standardized expressions
Developmental stages
EI PG G1 G2 H T1 T2 C1 C2 S EP JP

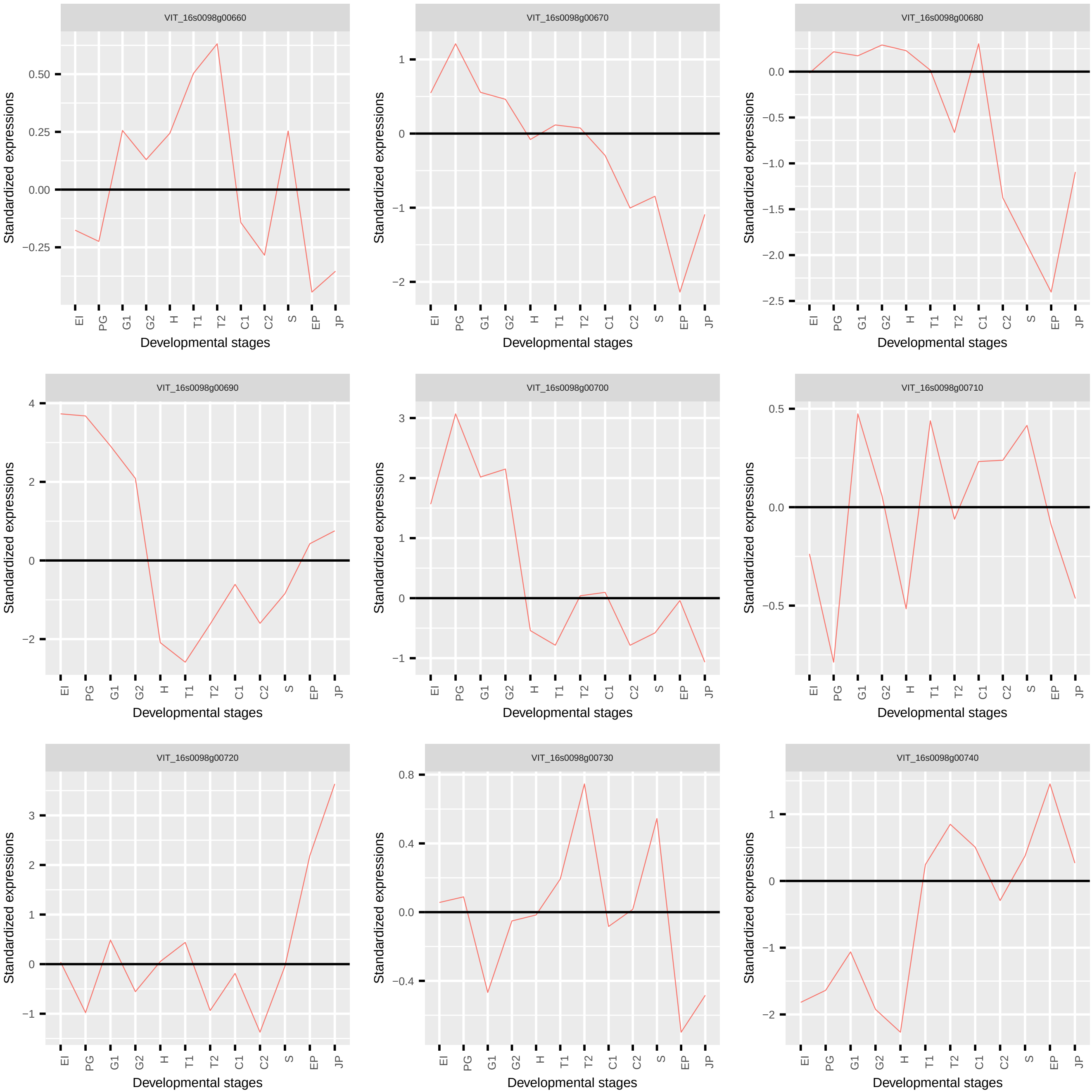

VIT_16s0098g00660
0.50
0.25
0.00
−0.25
VIT_16s0098g00670
1
0
−1
−2
VIT_16s0098g00680
0.0
−0.5
−1.0
−1.5
−2.0
−2.5
VIT_16s0098g00690
4
2
0
−2
VIT_16s0098g00700
3
2
1
0
−1
VIT_16s0098g00710
0.5
0.0
−0.5
VIT_16s0098g00720
3
2
1
0
−1
VIT_16s0098g00730
0.8
0.4
0.0
−0.4
VIT_16s0098g00740
1
0
−1
−2
Standardized expressions
EI PG G1 G2 H T1 T2 C1 C2 S EP JP
Developmental stages

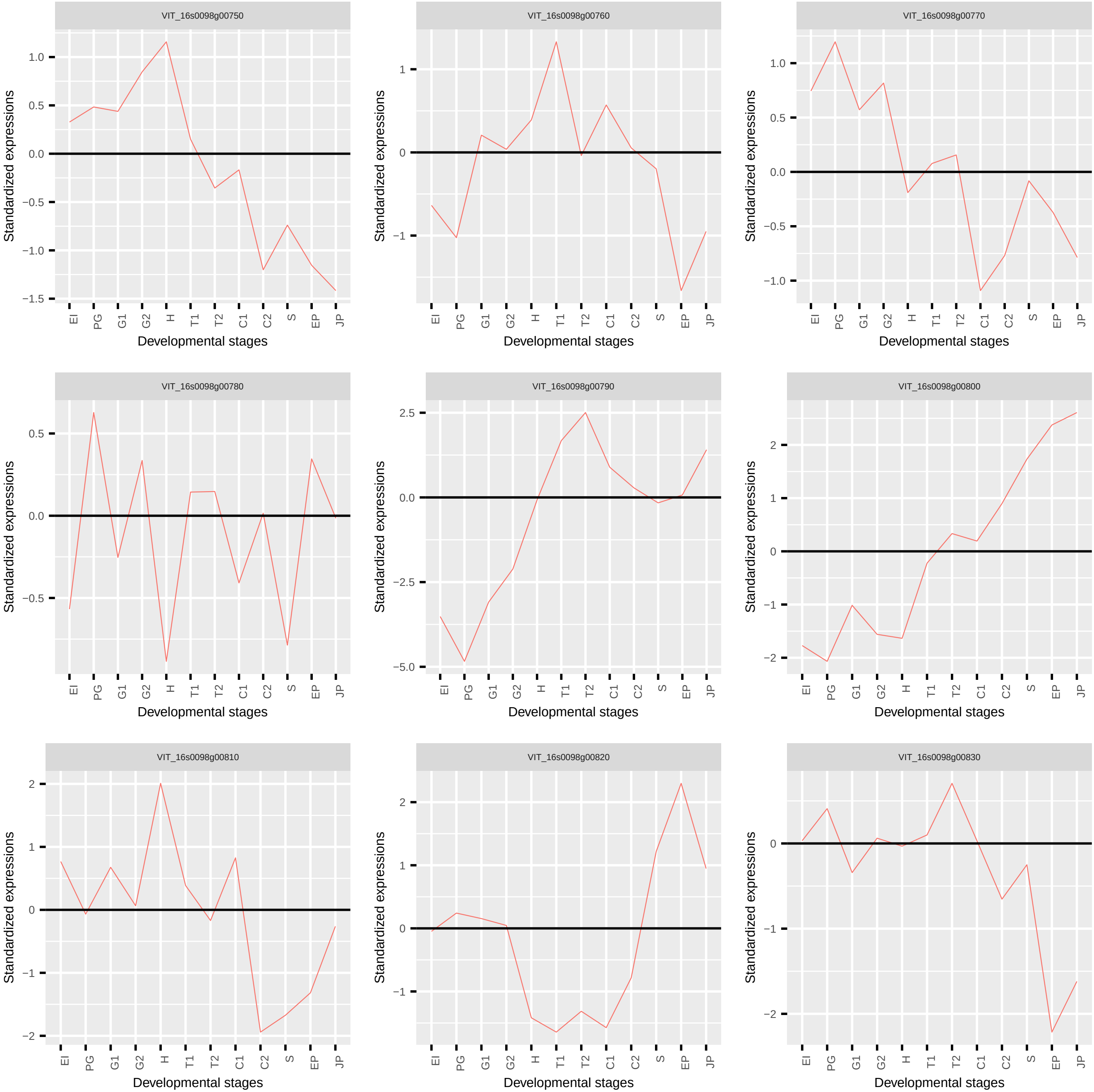
VIT_16s0098g00750
1.0
0.5
0.0
−0.5
−1.0
−1.5
VIT_16s0098g00760
1
0
−1
VIT_16s0098g00770
1.0
0.5
0.0
−0.5
−1.0
VIT_16s0098g00780
0.5
0.0
−0.5
VIT_16s0098g00790
2.5
0.0
−2.5
−5.0
VIT_16s0098g00800
2
1
0
−1
−2
VIT_16s0098g00810
2
1
0
−1
−2
VIT_16s0098g00820
2
1
0
−1
VIT_16s0098g00830
0
−1
−2
Standardized expressions
EI
PG
G1
G2
H
T1
T2
C1
C2
S
EP
JP
Developmental stages

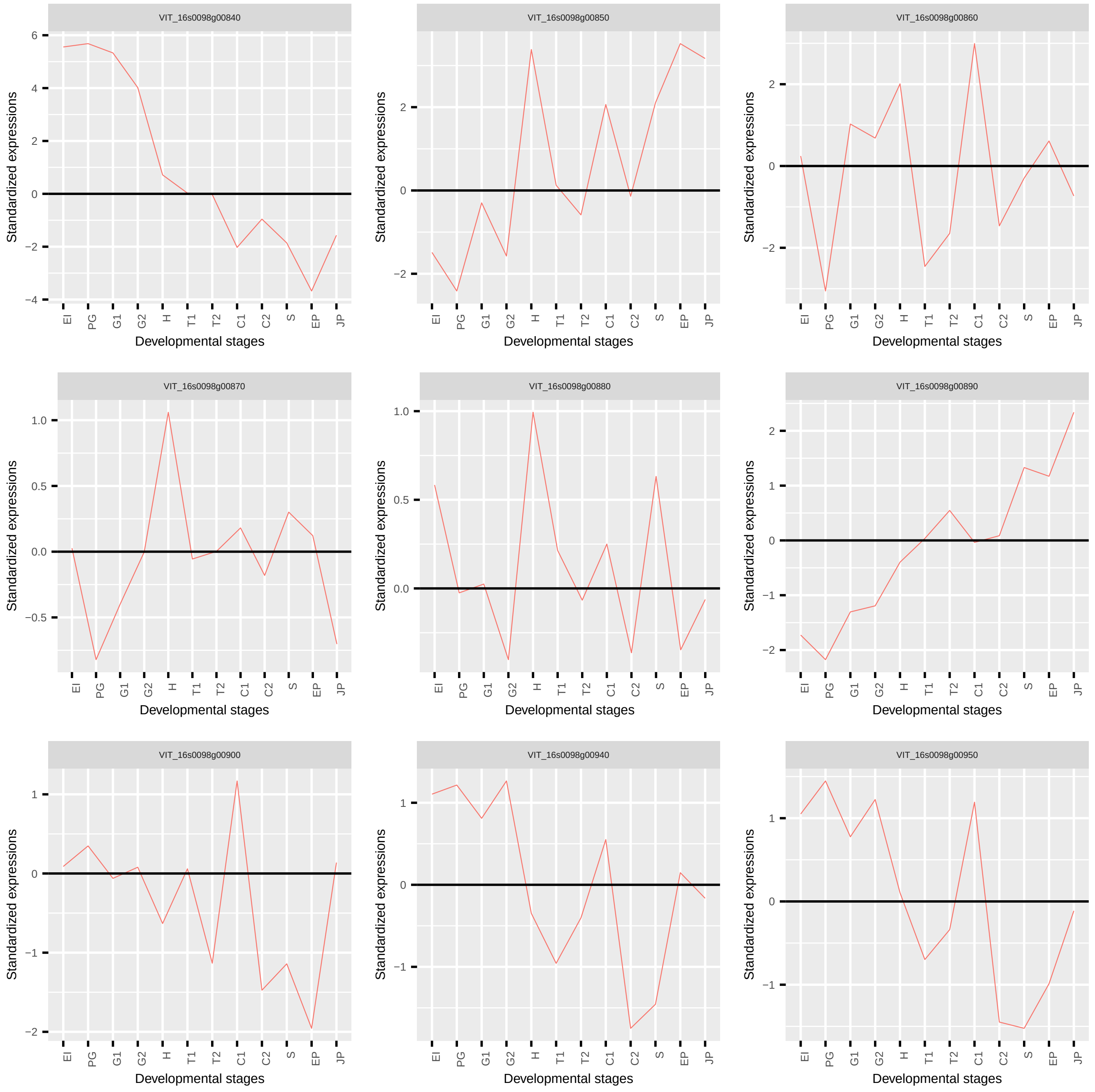
VIT_16s0098g00840
VIT_16s0098g00850
VIT_16s0098g00860
VIT_16s0098g00870
VIT_16s0098g00880
VIT_16s0098g00890
VIT_16s0098g00900
VIT_16s0098g00940
VIT_16s0098g00950
Standardized expressions
Developmental stages
EI
PG
G1
G2
H
T1
T2
C1
C2
S
EP
JP

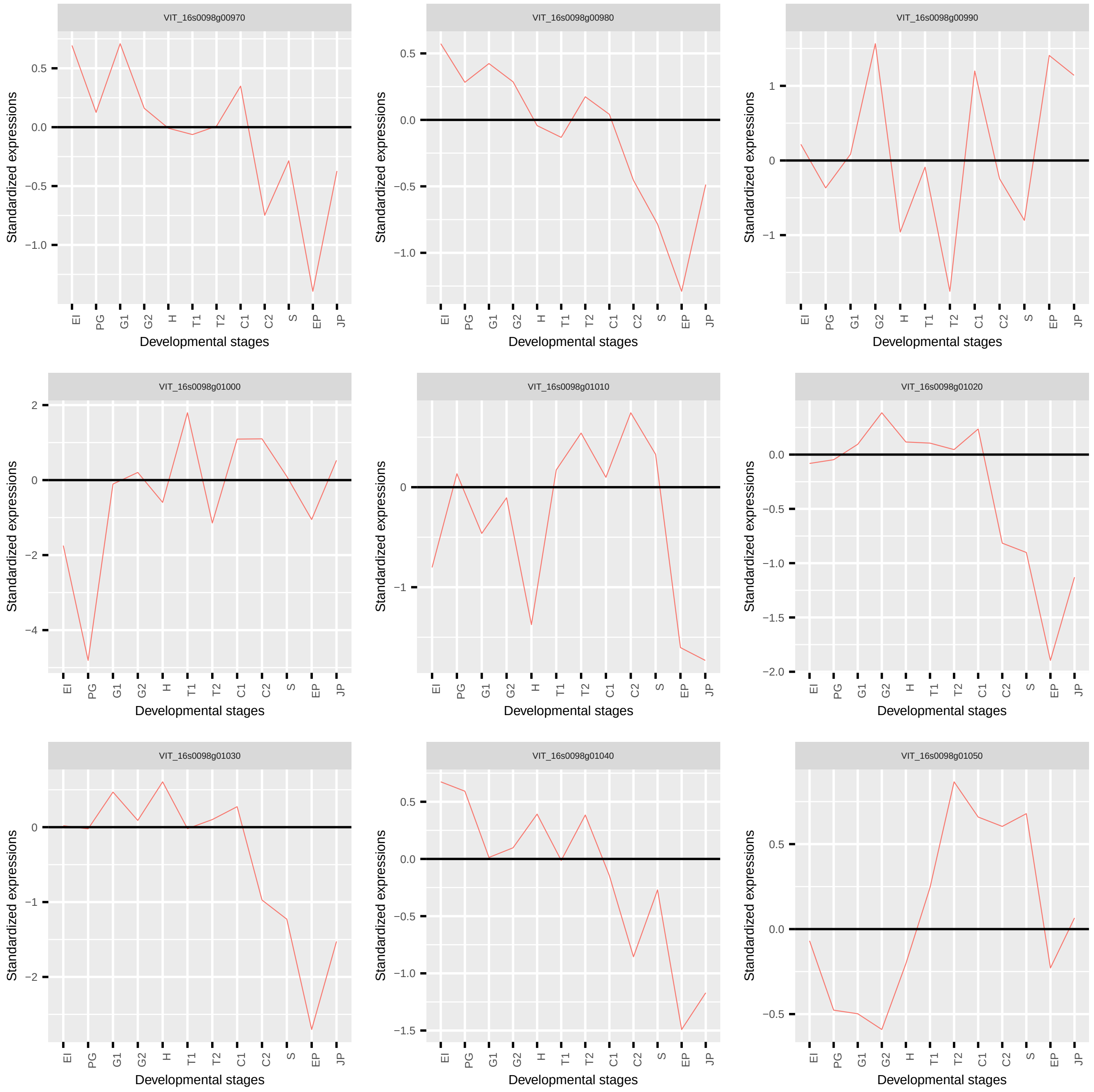
VIT_16s0098g00970
VIT_16s0098g00980
VIT_16s0098g00990
VIT_16s0098g01000
VIT_16s0098g01010
VIT_16s0098g01020
VIT_16s0098g01030
VIT_16s0098g01040
VIT_16s0098g01050
Standardized expressions
Developmental stages
EI PG G1 G2 H T1 T2 C1 C2 S EP JP

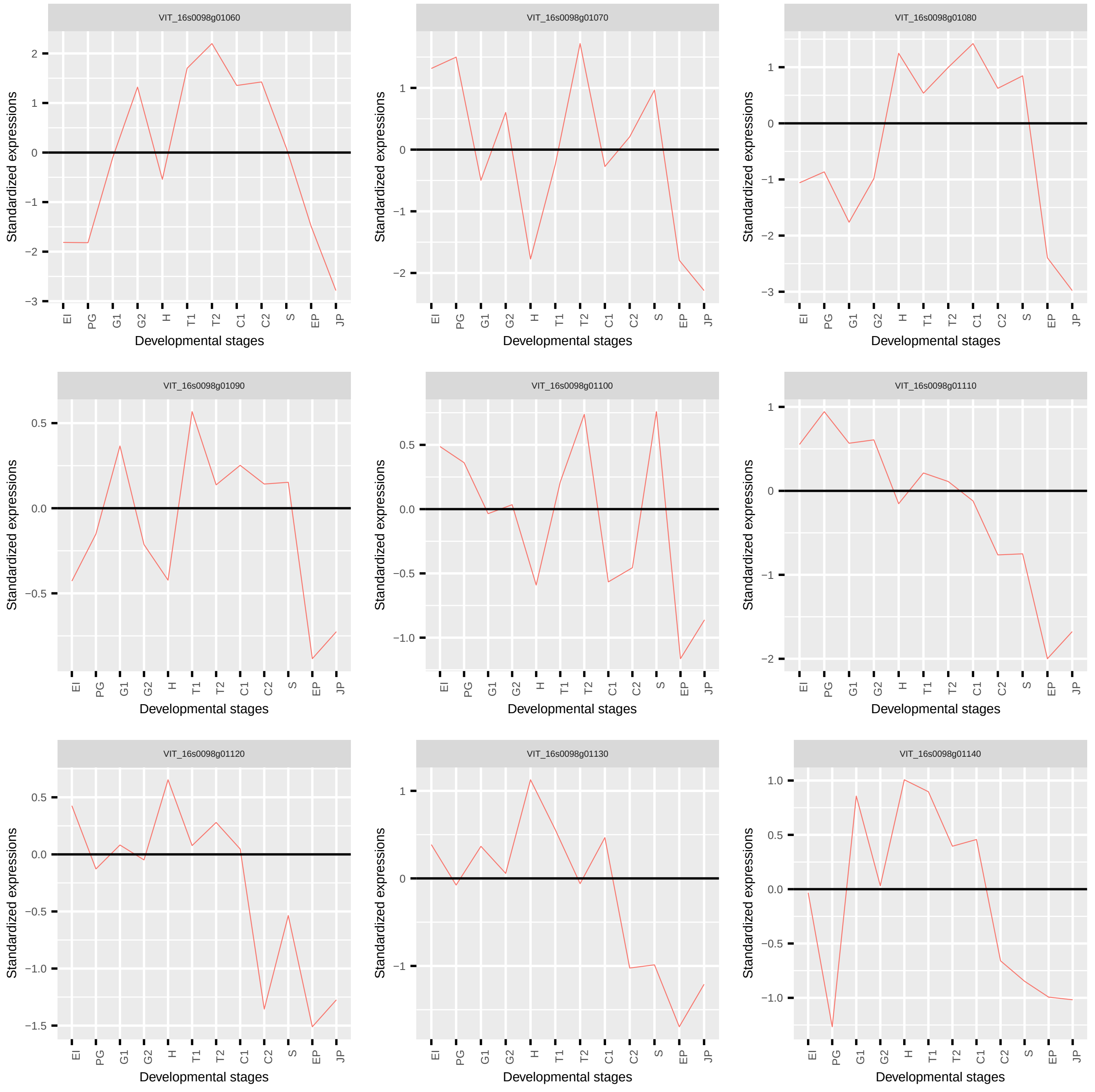
VIT_16s0098g01060
VIT_16s0098g01070
VIT_16s0098g01080
VIT_16s0098g01090
VIT_16s0098g01100
VIT_16s0098g01110
VIT_16s0098g01120
VIT_16s0098g01130
VIT_16s0098g01140
Standardized expressions
Developmental stages
EI
PG
G1
G2
H
T1
T2
C1
C2
S
EP
JP

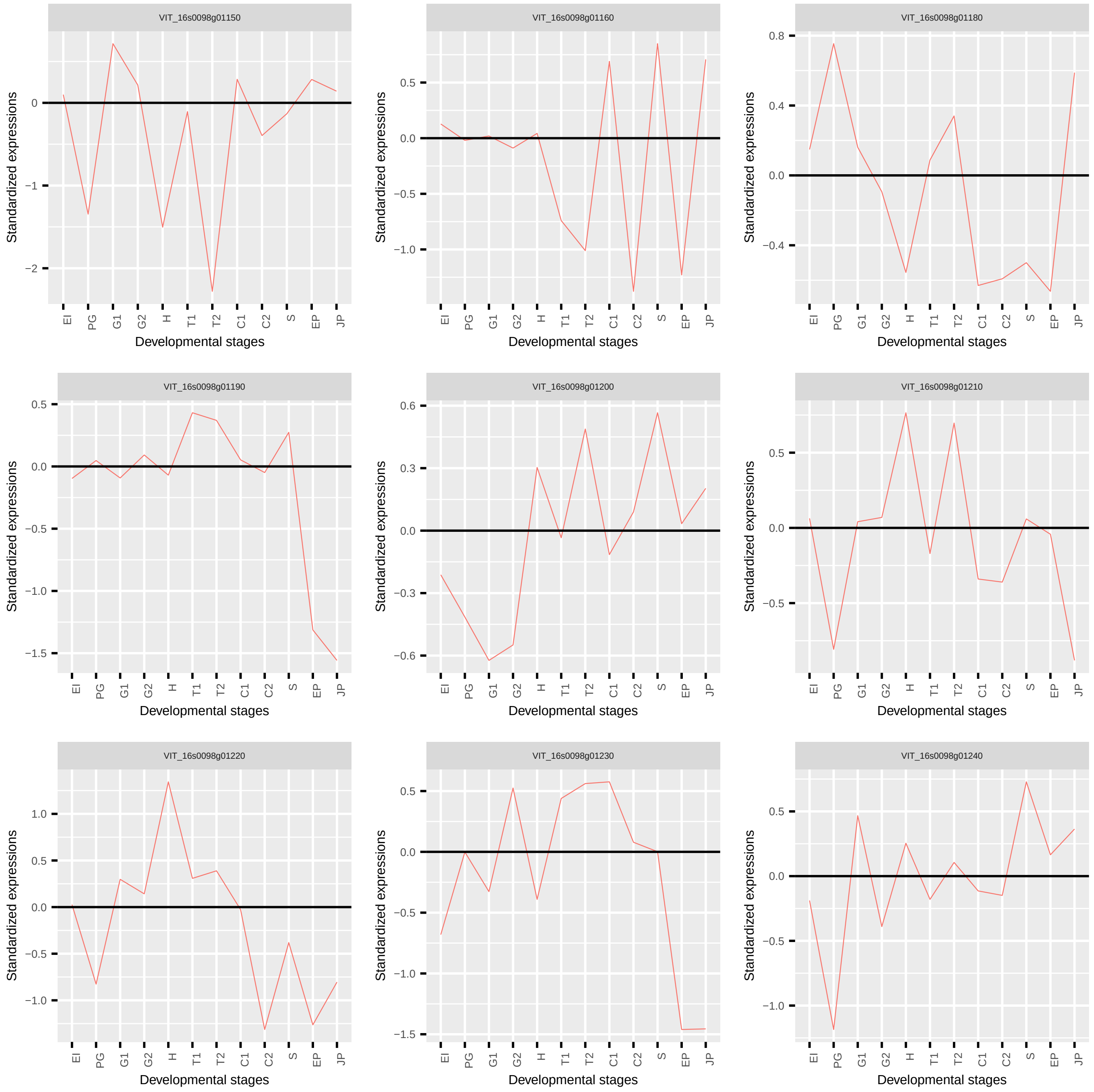
VIT_16s0098g01150
VIT_16s0098g01160
VIT_16s0098g01180
VIT_16s0098g01190
VIT_16s0098g01200
VIT_16s0098g01210
VIT_16s0098g01220
VIT_16s0098g01230
VIT_16s0098g01240
Standardized expressions
Developmental stages
EI PG G1 G2 H T1 T2 C1 C2 S EP JP

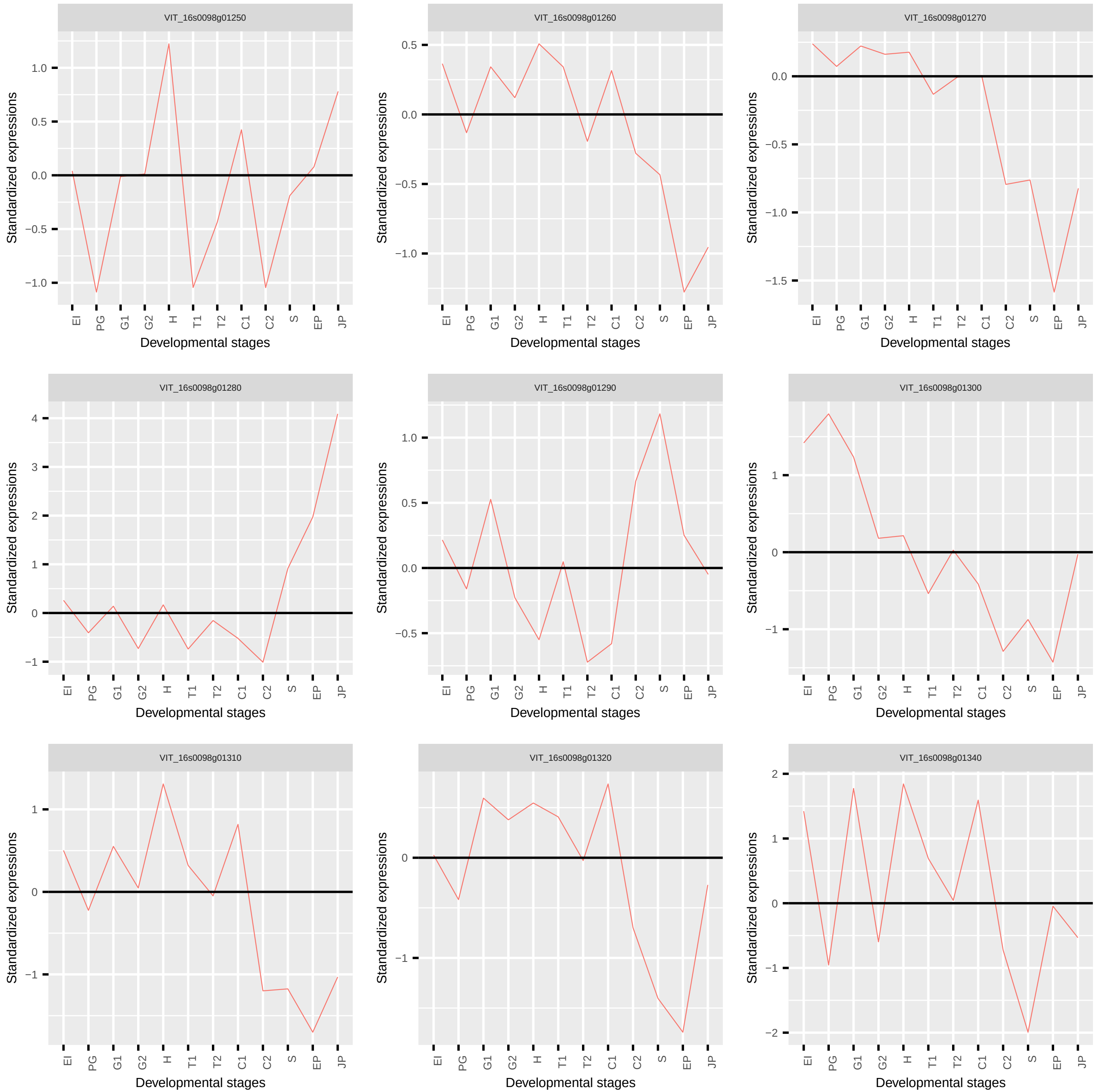

VIT_16s0098g01250
VIT_16s0098g01260
VIT_16s0098g01270
VIT_16s0098g01280
VIT_16s0098g01290
VIT_16s0098g01300
VIT_16s0098g01310
VIT_16s0098g01320
VIT_16s0098g01340
Standardized expressions
Developmental stages
EI PG G1 G2 H T1 T2 C1 C2 S EP JP

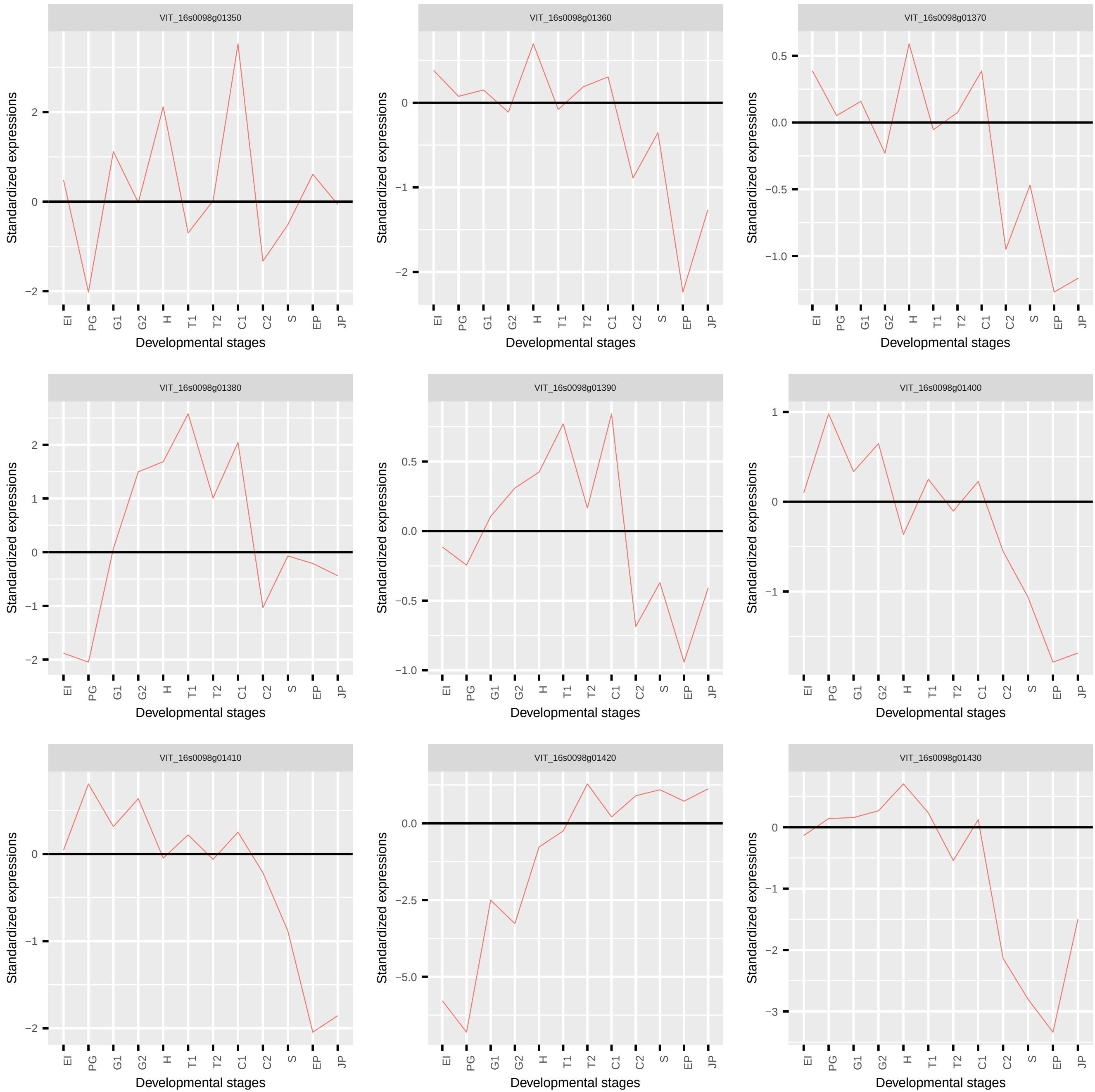
VIT_16s0098g01350
VIT_16s0098g01360
VIT_16s0098g01370
VIT_16s0098g01380
VIT_16s0098g01390
VIT_16s0098g01400
VIT_16s0098g01410
VIT_16s0098g01420
VIT_16s0098g01430
Standardized expressions
Developmental stages
EI PG G1 G2 H T1 T2 C1 C2 S EP JP

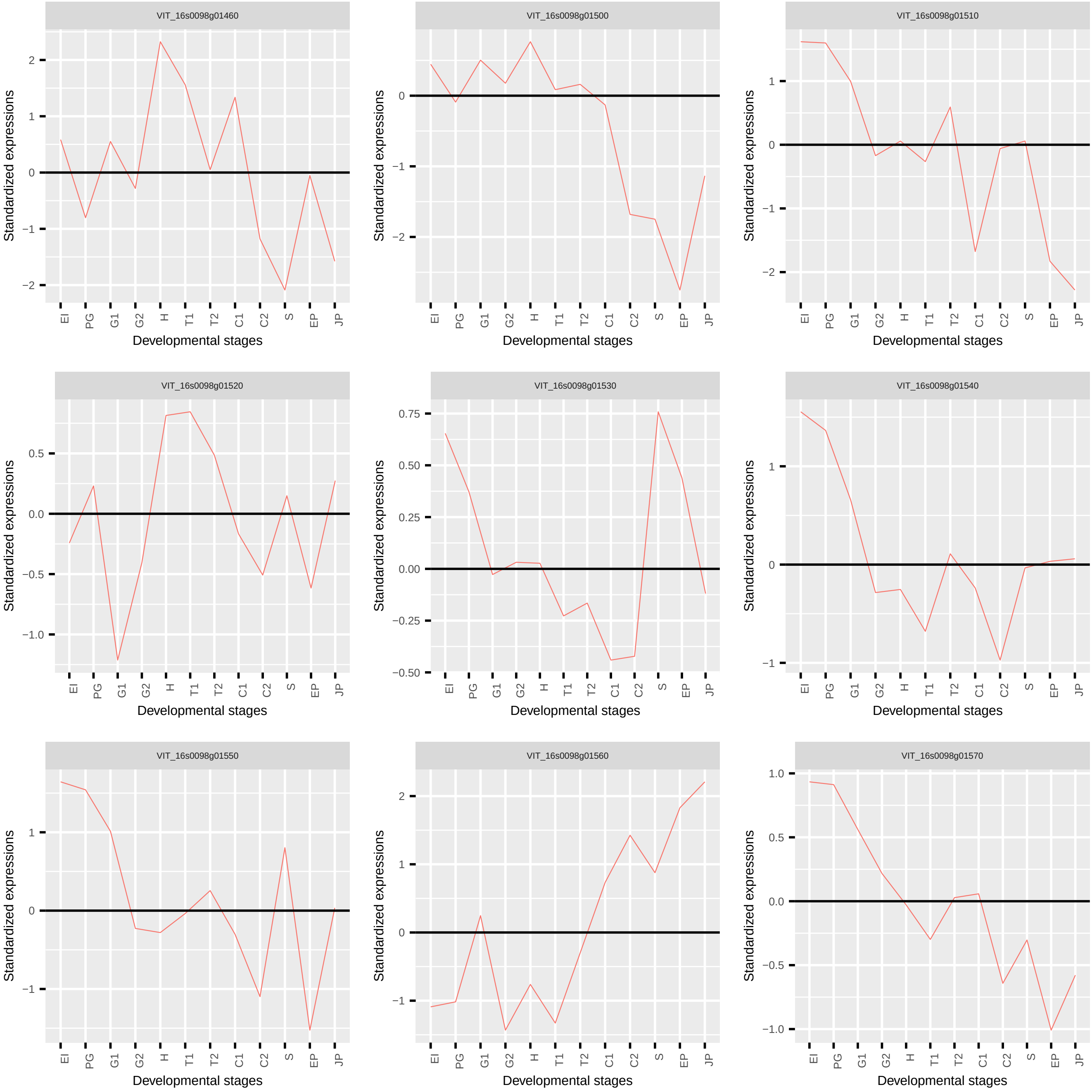
VIT_16s0098g01460
VIT_16s0098g01500
VIT_16s0098g01510
VIT_16s0098g01520
VIT_16s0098g01530
VIT_16s0098g01540
VIT_16s0098g01550
VIT_16s0098g01560
VIT_16s0098g01570
Standardized expressions
Developmental stages
EI
PG
G1
G2
H
T1
T2
C1
C2
S
EP
JP

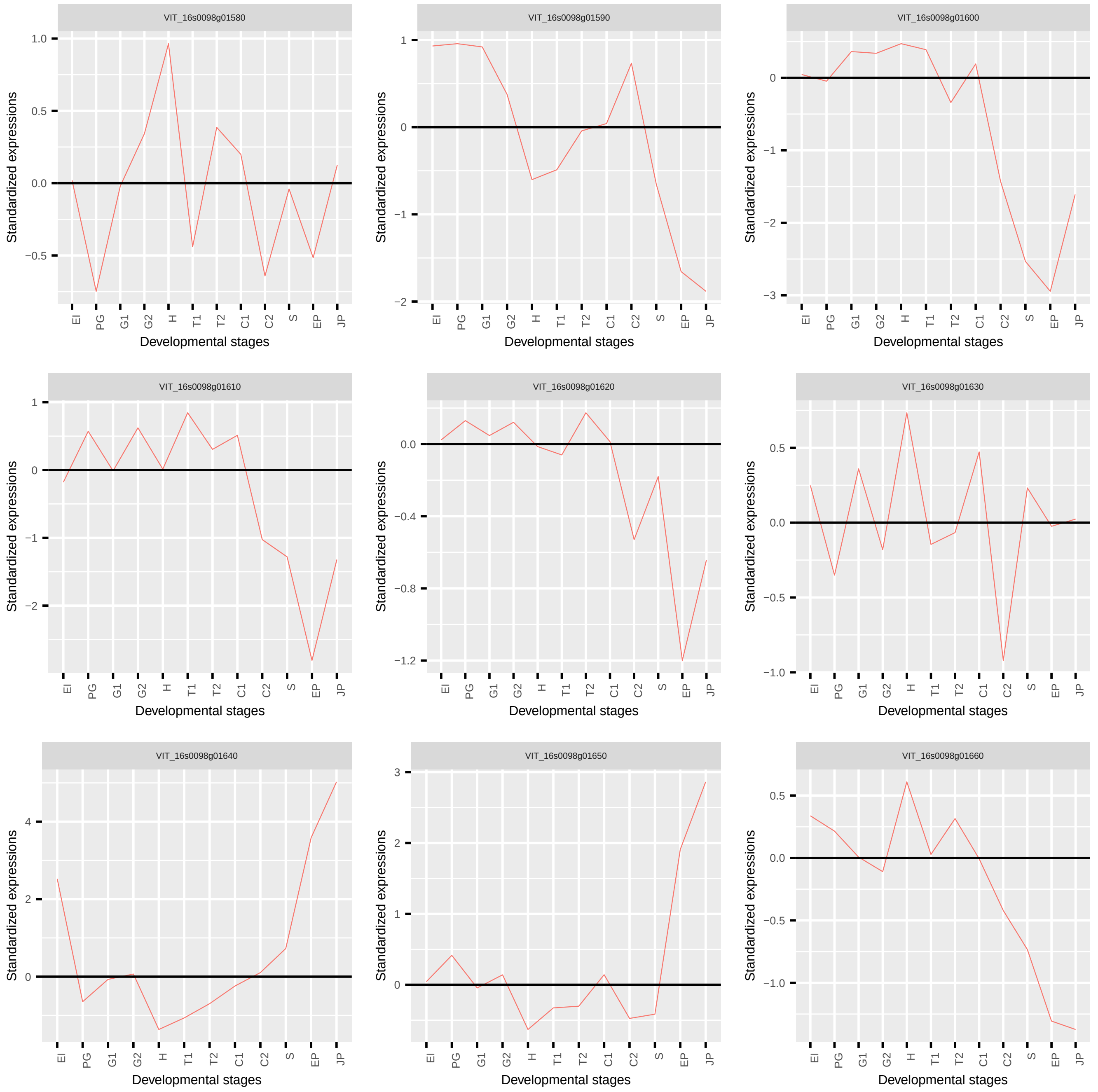

VIT_16s0098g01580
VIT_16s0098g01590
VIT_16s0098g01600
VIT_16s0098g01610
VIT_16s0098g01620
VIT_16s0098g01630
VIT_16s0098g01640
VIT_16s0098g01650
VIT_16s0098g01660
Standardized expressions
Developmental stages
EI PG G1 G2 H T1 T2 C1 C2 S EP JP

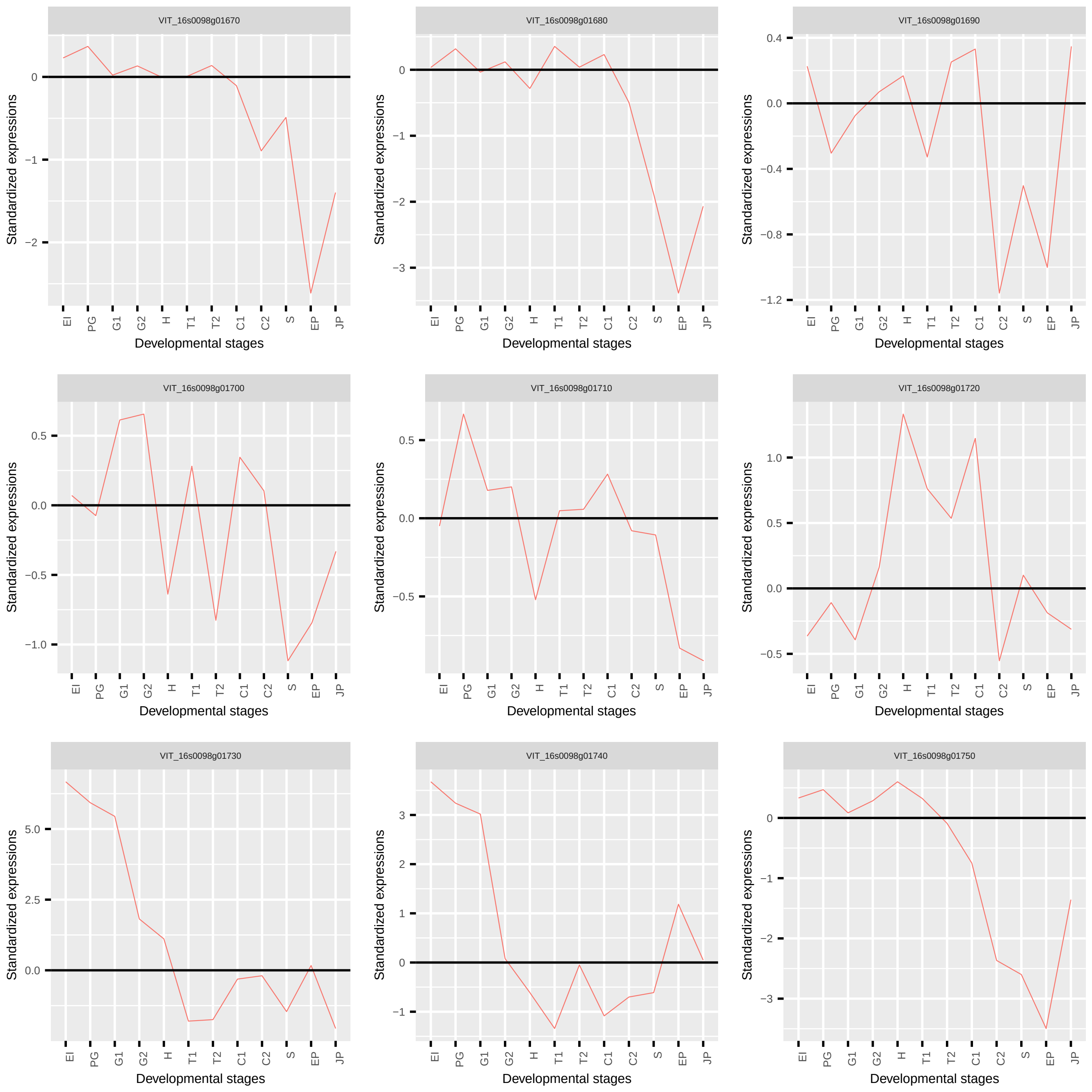

VIT_16s0098g01670
VIT_16s0098g01680
VIT_16s0098g01690
VIT_16s0098g01700
VIT_16s0098g01710
VIT_16s0098g01720
VIT_16s0098g01730
VIT_16s0098g01740
VIT_16s0098g01750
Standardized expressions
Developmental stages
EI PG G1 G2 H T1 T2 C1 C2 S EP JP

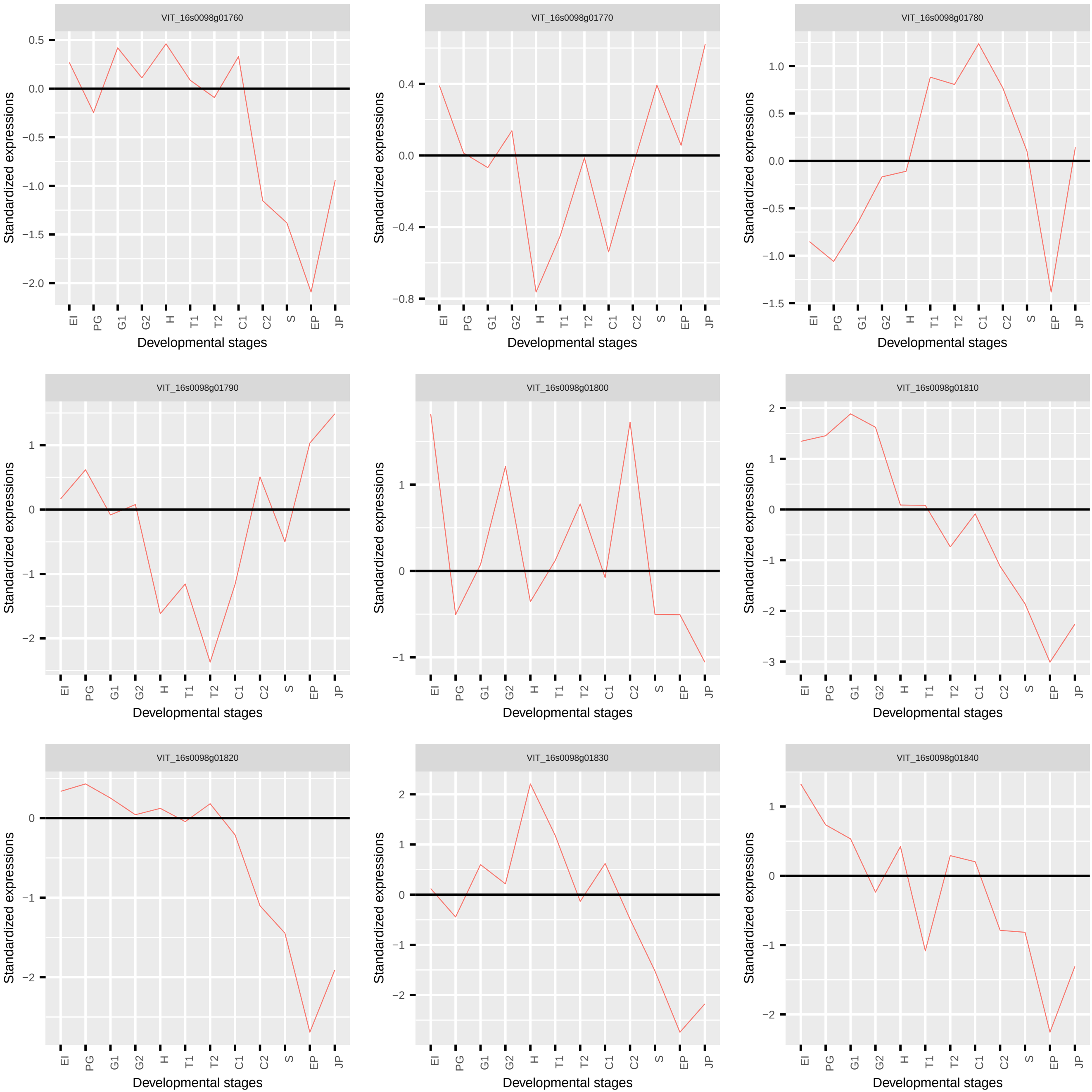

VIT_16s0098g01760
0.5
0.0
−0.5
−1.0
−1.5
−2.0
VIT_16s0098g01770
0.4
0.0
−0.4
−0.8
VIT_16s0098g01780
1.0
0.5
0.0
−0.5
−1.0
−1.5
VIT_16s0098g01790
1
0
−1
−2
VIT_16s0098g01800
1
0
−1
VIT_16s0098g01810
2
1
0
−1
−2
−3
VIT_16s0098g01820
0
−1
−2
VIT_16s0098g01830
2
1
0
−1
−2
VIT_16s0098g01840
1
0
−1
−2
Standardized expressions
EI PG G1 G2 H T1 T2 C1 C2 S EP JP
Developmental stages

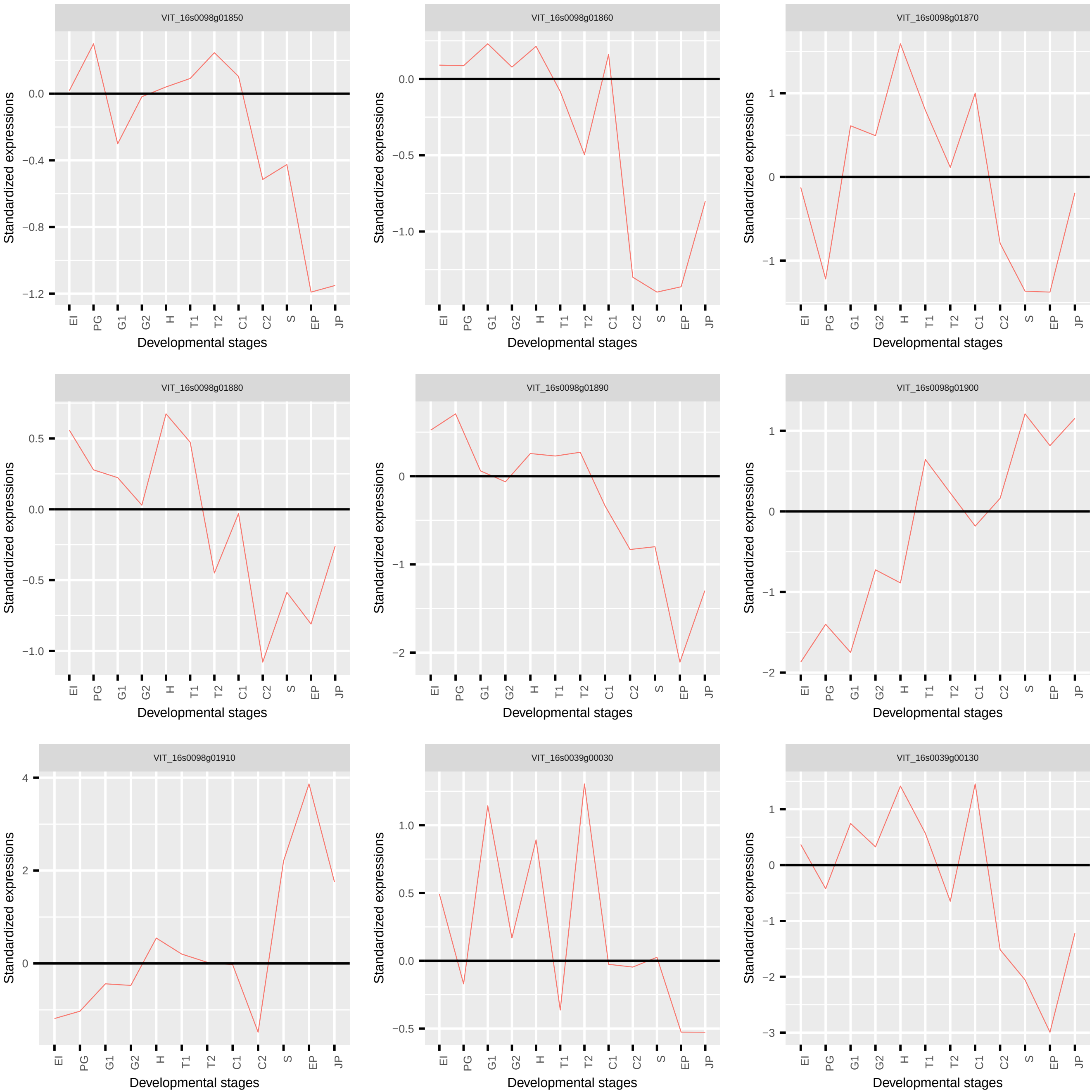
VIT_16s0098g01850
VIT_16s0098g01860
VIT_16s0098g01870
VIT_16s0098g01880
VIT_16s0098g01890
VIT_16s0098g01900
VIT_16s0098g01910
VIT_16s0039g00030
VIT_16s0039g00130
Standardized expressions
Developmental stages
EI PG G1 G2 H T1 T2 C1 C2 S EP JP
0.0
−0.4
−0.8
−1.2
−0.5
−1.0
1
0
−1
0.5
−2
−3
4
2
1.0

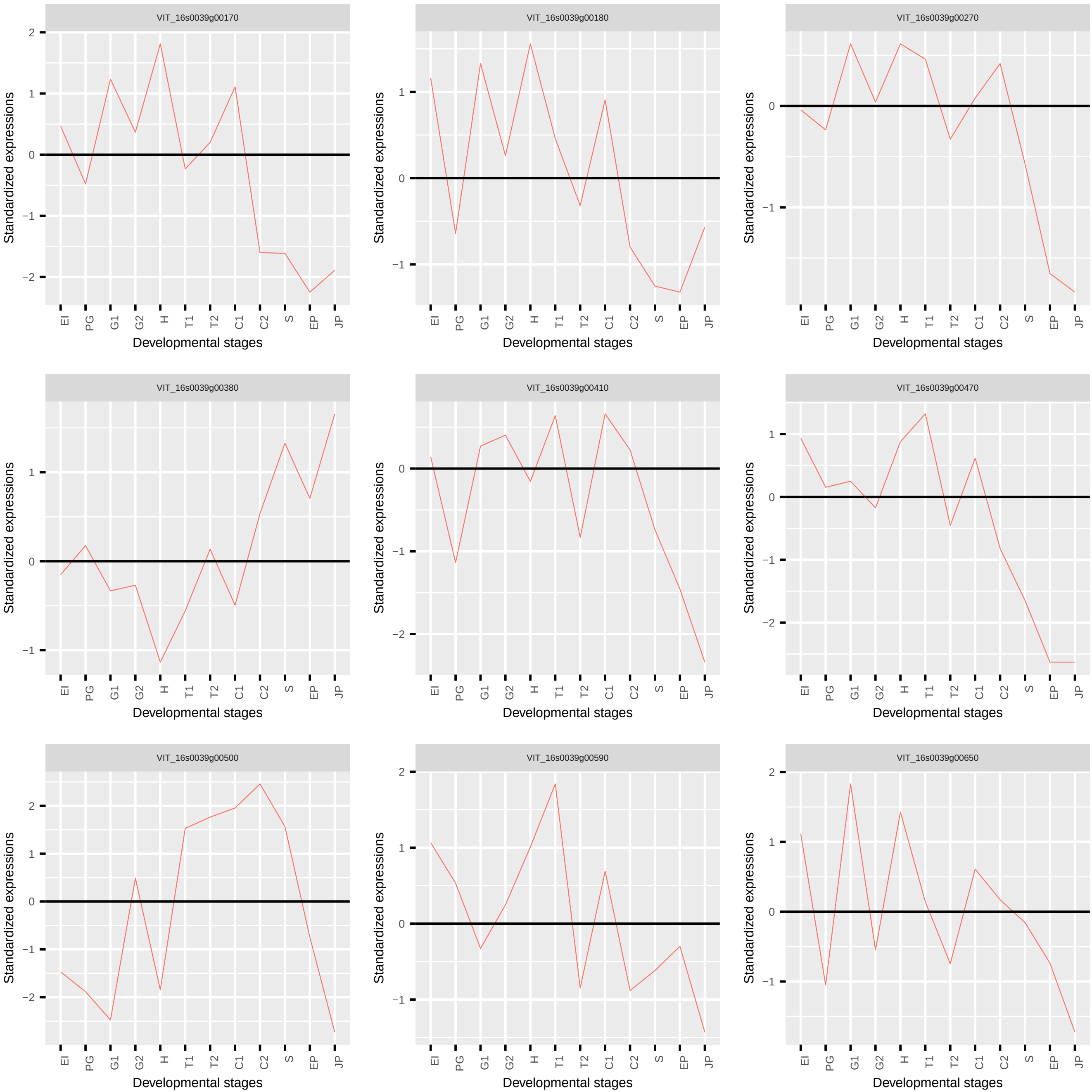

VIT_16s0039g00170
VIT_16s0039g00180
VIT_16s0039g00270
VIT_16s0039g00380
VIT_16s0039g00410
VIT_16s0039g00470
VIT_16s0039g00500
VIT_16s0039g00590
VIT_16s0039g00650
Standardized expressions
Developmental stages
EI PG G1 G2 H T1 T2 C1 C2 S EP JP

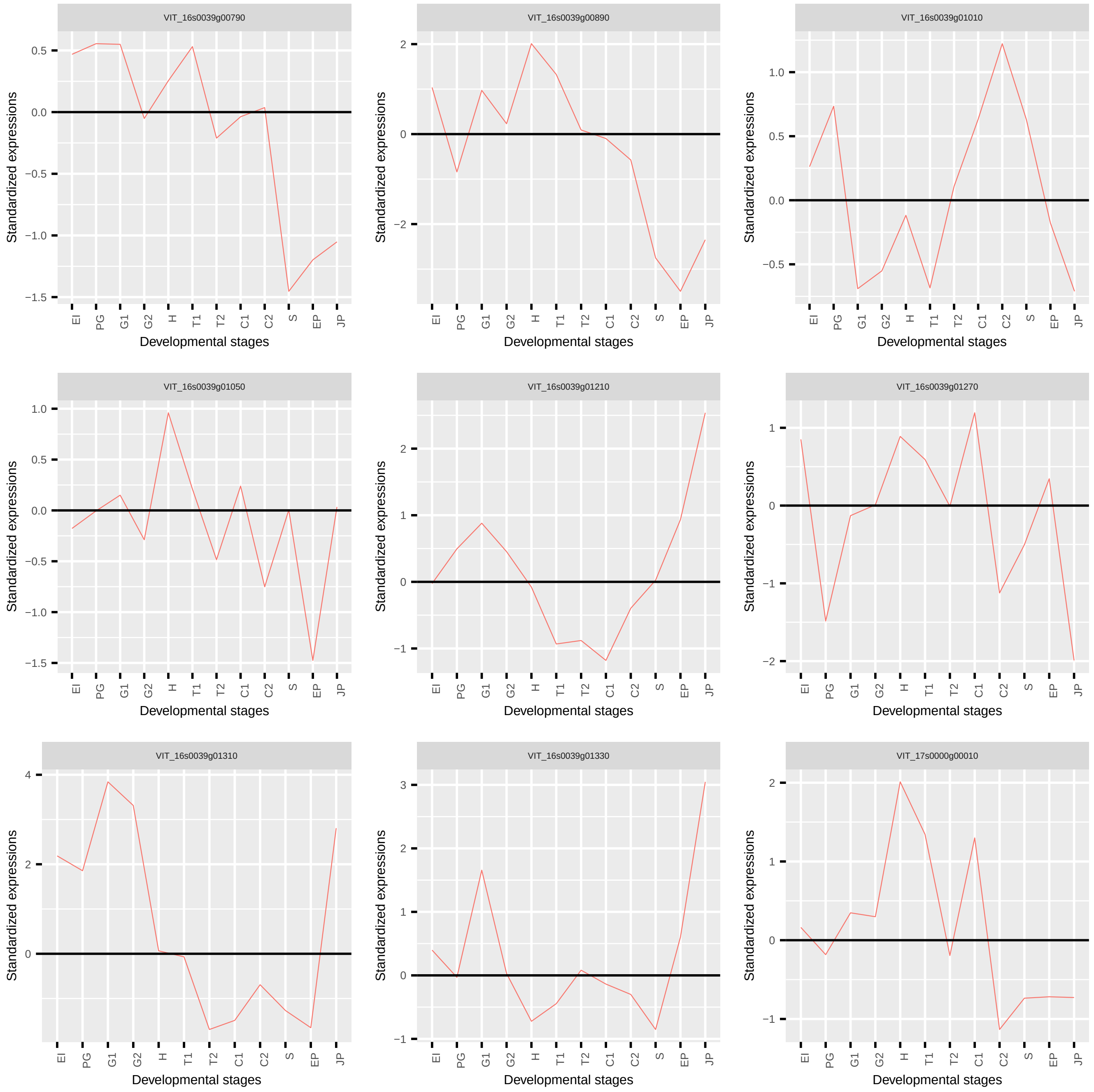

VIT_16s0039g00790
0.5
0.0
−0.5
−1.0
−1.5
Standardized expressions
EI PG G1 G2 H T1 T2 C1 C2 S EP JP
Developmental stages
VIT_16s0039g00890
2
0
−2
Standardized expressions
EI PG G1 G2 H T1 T2 C1 C2 S EP JP
Developmental stages
VIT_16s0039g01010
1.0
0.5
0.0
−0.5
Standardized expressions
EI PG G1 G2 H T1 T2 C1 C2 S EP JP
Developmental stages
VIT_16s0039g01050
1.0
0.5
0.0
−0.5
−1.0
−1.5
Standardized expressions
EI PG G1 G2 H T1 T2 C1 C2 S EP JP
Developmental stages
VIT_16s0039g01210
2
1
0
−1
Standardized expressions
EI PG G1 G2 H T1 T2 C1 C2 S EP JP
Developmental stages
VIT_16s0039g01270
1
0
−1
−2
Standardized expressions
EI PG G1 G2 H T1 T2 C1 C2 S EP JP
Developmental stages
VIT_16s0039g01310
4
2
0
Standardized expressions
EI PG G1 G2 H T1 T2 C1 C2 S EP JP
Developmental stages
VIT_16s0039g01330
3
2
1
0
−1
Standardized expressions
EI PG G1 G2 H T1 T2 C1 C2 S EP JP
Developmental stages
VIT_17s0000g00010
2
1
0
−1
Standardized expressions
EI PG G1 G2 H T1 T2 C1 C2 S EP JP
Developmental stages

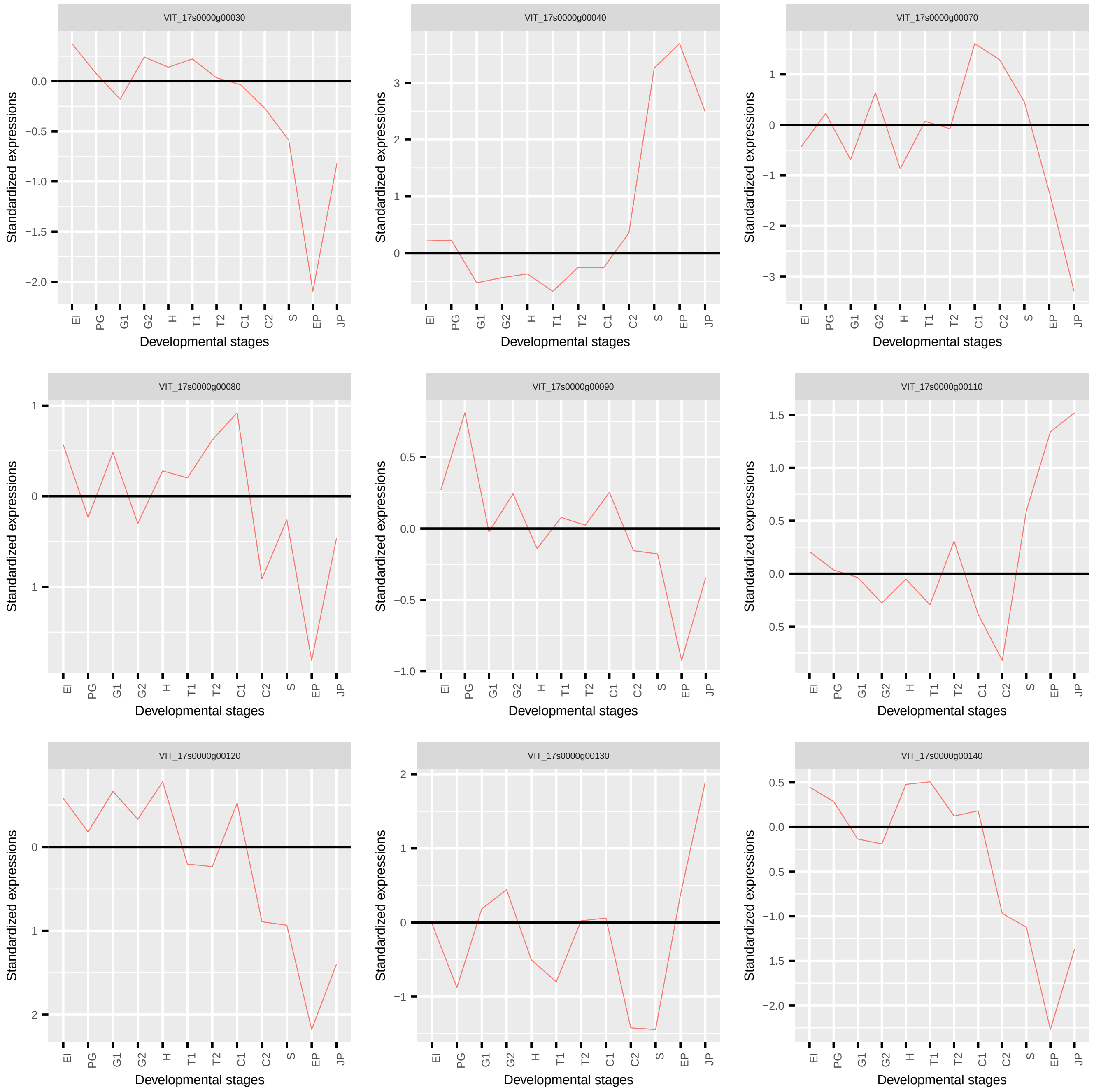

VIT_17s0000g00030
VIT_17s0000g00040
VIT_17s0000g00070
VIT_17s0000g00080
VIT_17s0000g00090
VIT_17s0000g00110
VIT_17s0000g00120
VIT_17s0000g00130
VIT_17s0000g00140
Standardized expressions
Developmental stages
EI PG G1 G2 H T1 T2 C1 C2 S EP JP

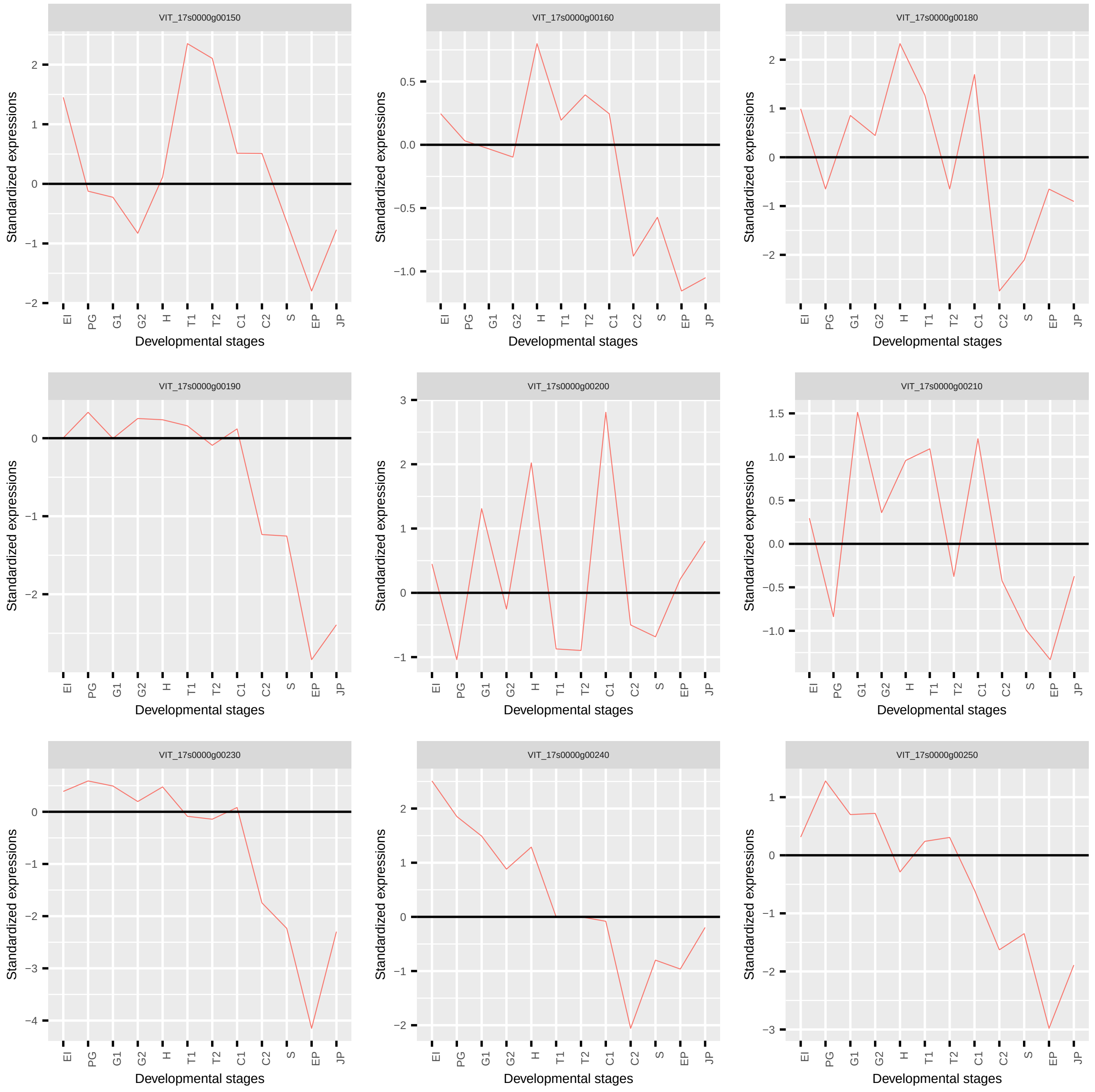

VIT_17s0000g00150
VIT_17s0000g00160
VIT_17s0000g00180
VIT_17s0000g00190
VIT_17s0000g00200
VIT_17s0000g00210
VIT_17s0000g00230
VIT_17s0000g00240
VIT_17s0000g00250
Standardized expressions
Developmental stages
EI PG G1 G2 H T1 T2 C1 C2 S EP JP

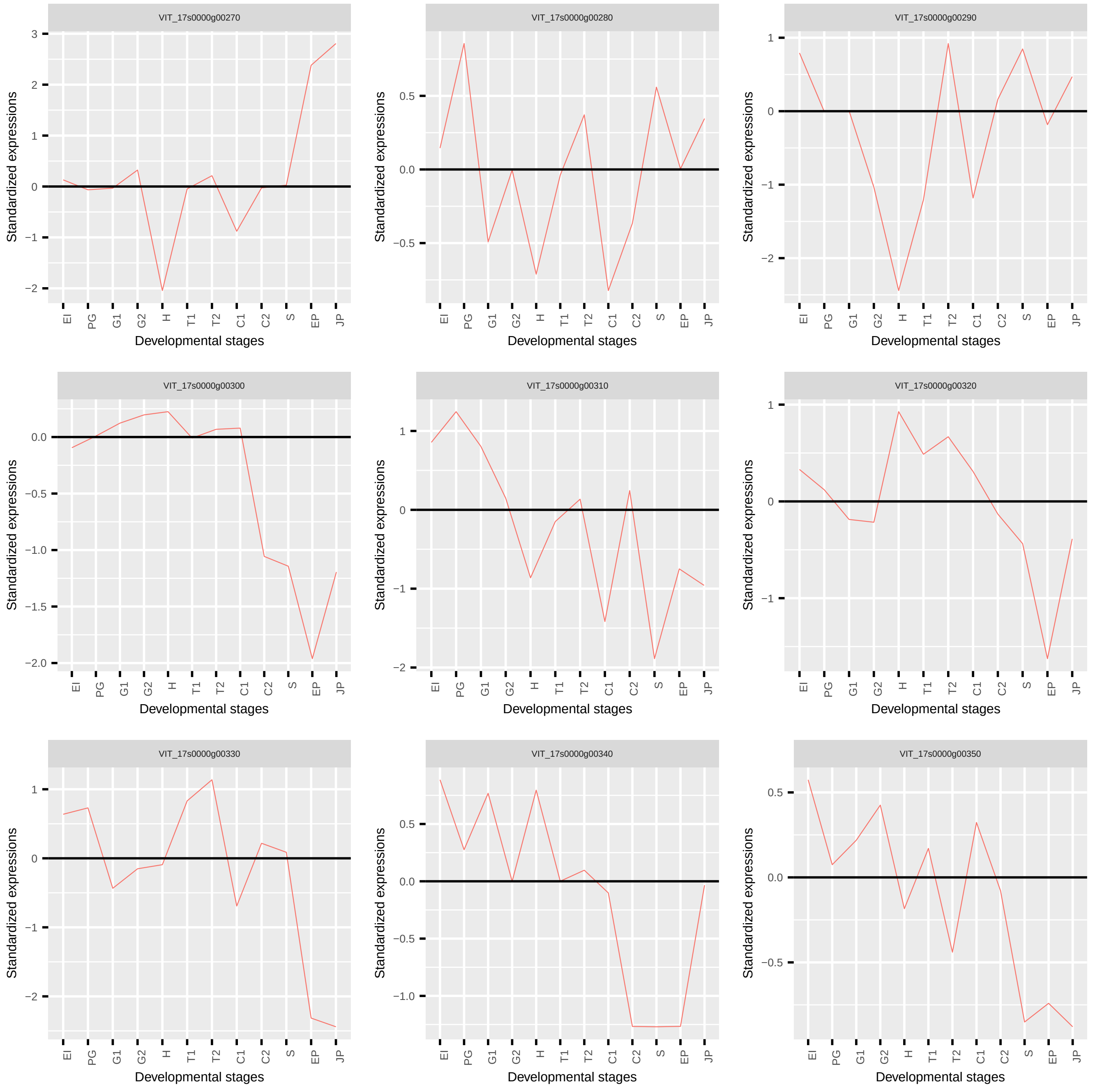

VIT_17s0000g00270
VIT_17s0000g00280
VIT_17s0000g00290
VIT_17s0000g00300
VIT_17s0000g00310
VIT_17s0000g00320
VIT_17s0000g00330
VIT_17s0000g00340
VIT_17s0000g00350
Standardized expressions
Developmental stages
EI PG G1 G2 H T1 T2 C1 C2 S EP JP

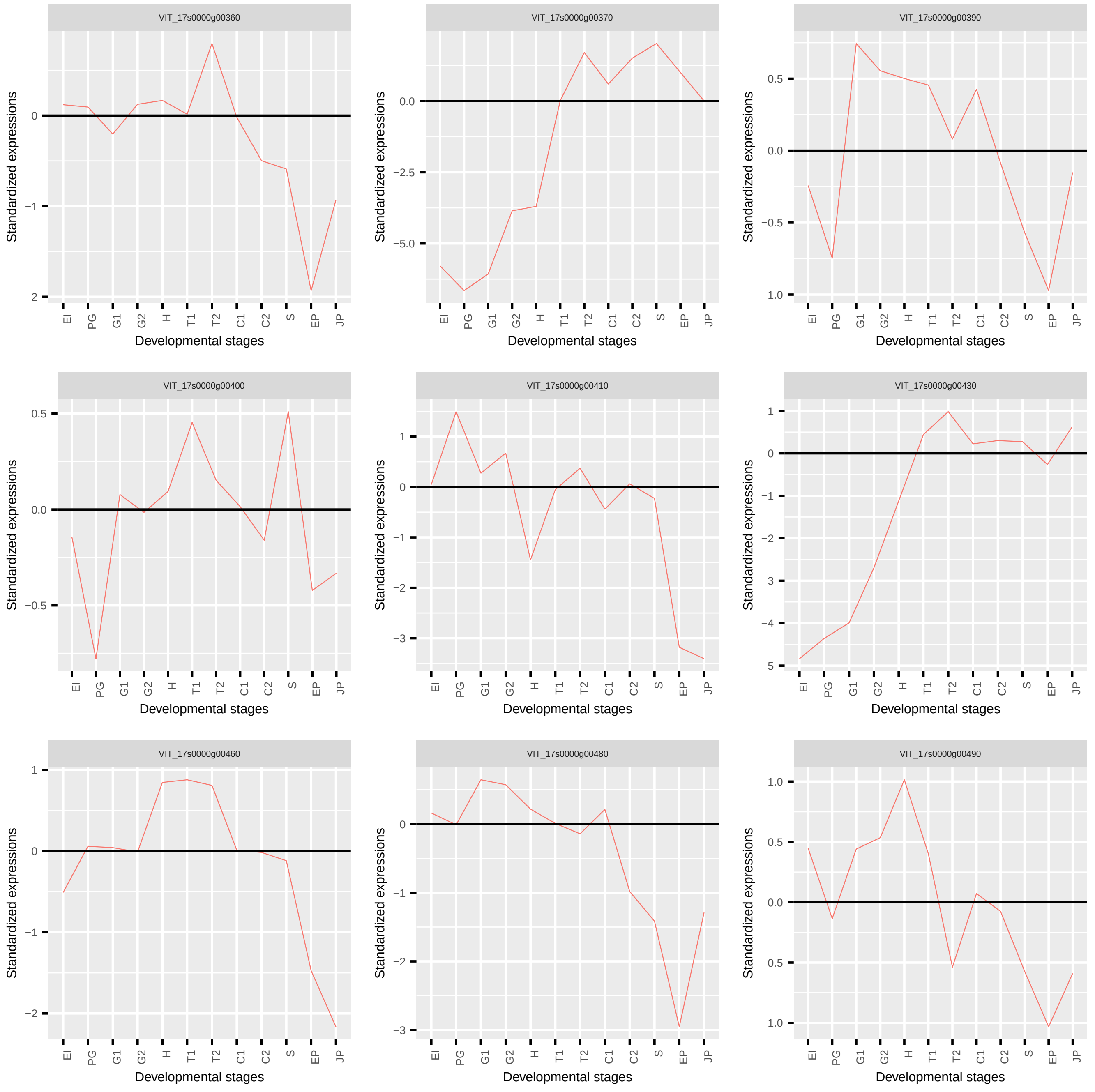

VIT_17s0000g00360
VIT_17s0000g00370
VIT_17s0000g00390
VIT_17s0000g00400
VIT_17s0000g00410
VIT_17s0000g00430
VIT_17s0000g00460
VIT_17s0000g00480
VIT_17s0000g00490
Standardized expressions
Developmental stages
EI PG G1 G2 H T1 T2 C1 C2 S EP JP

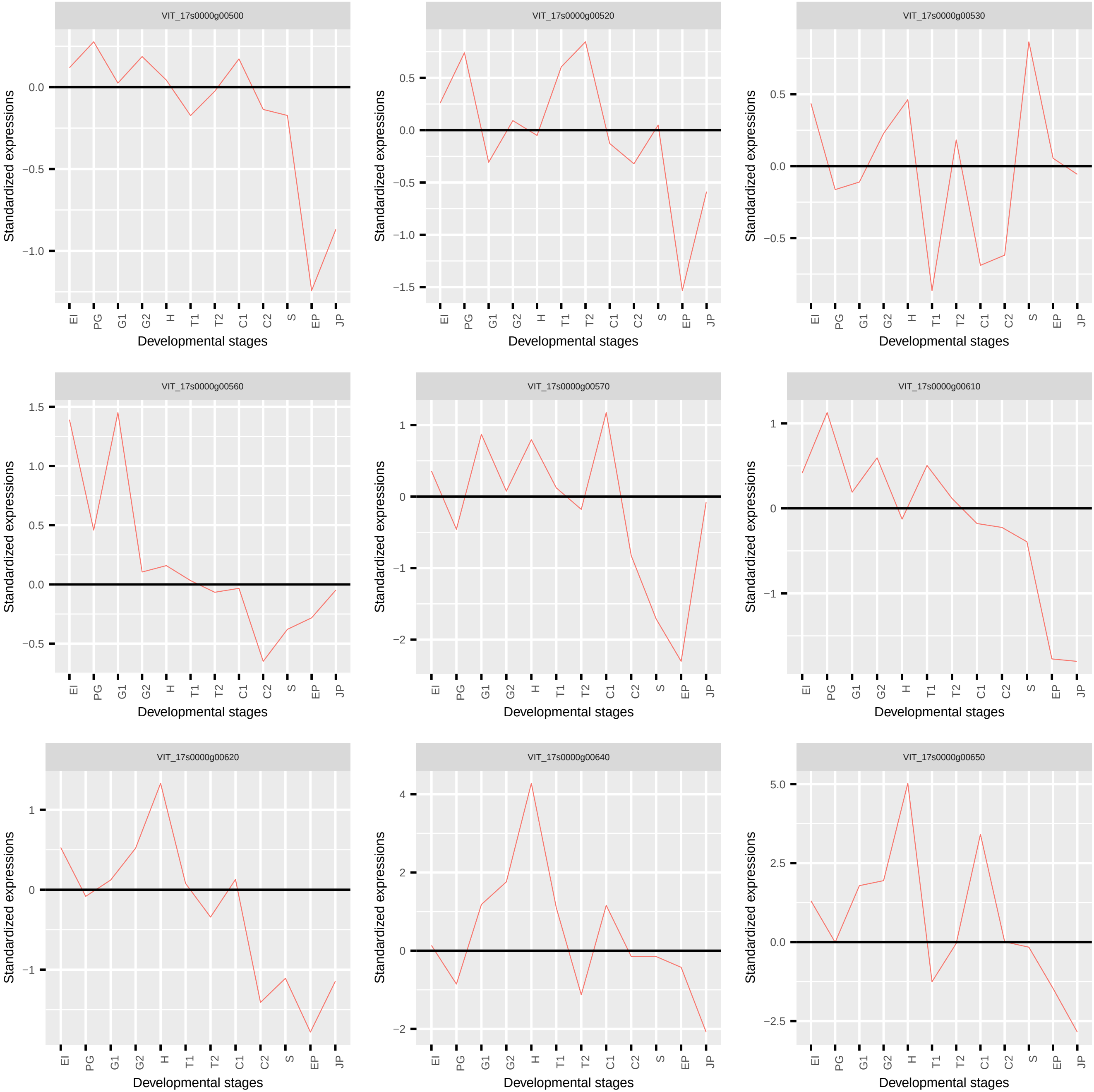
VIT_17s0000g00500
VIT_17s0000g00520
VIT_17s0000g00530
VIT_17s0000g00560
VIT_17s0000g00570
VIT_17s0000g00610
VIT_17s0000g00620
VIT_17s0000g00640
VIT_17s0000g00650
Standardized expressions
Developmental stages
EI
PG
G1
G2
H
T1
T2
C1
C2
S
EP
JP

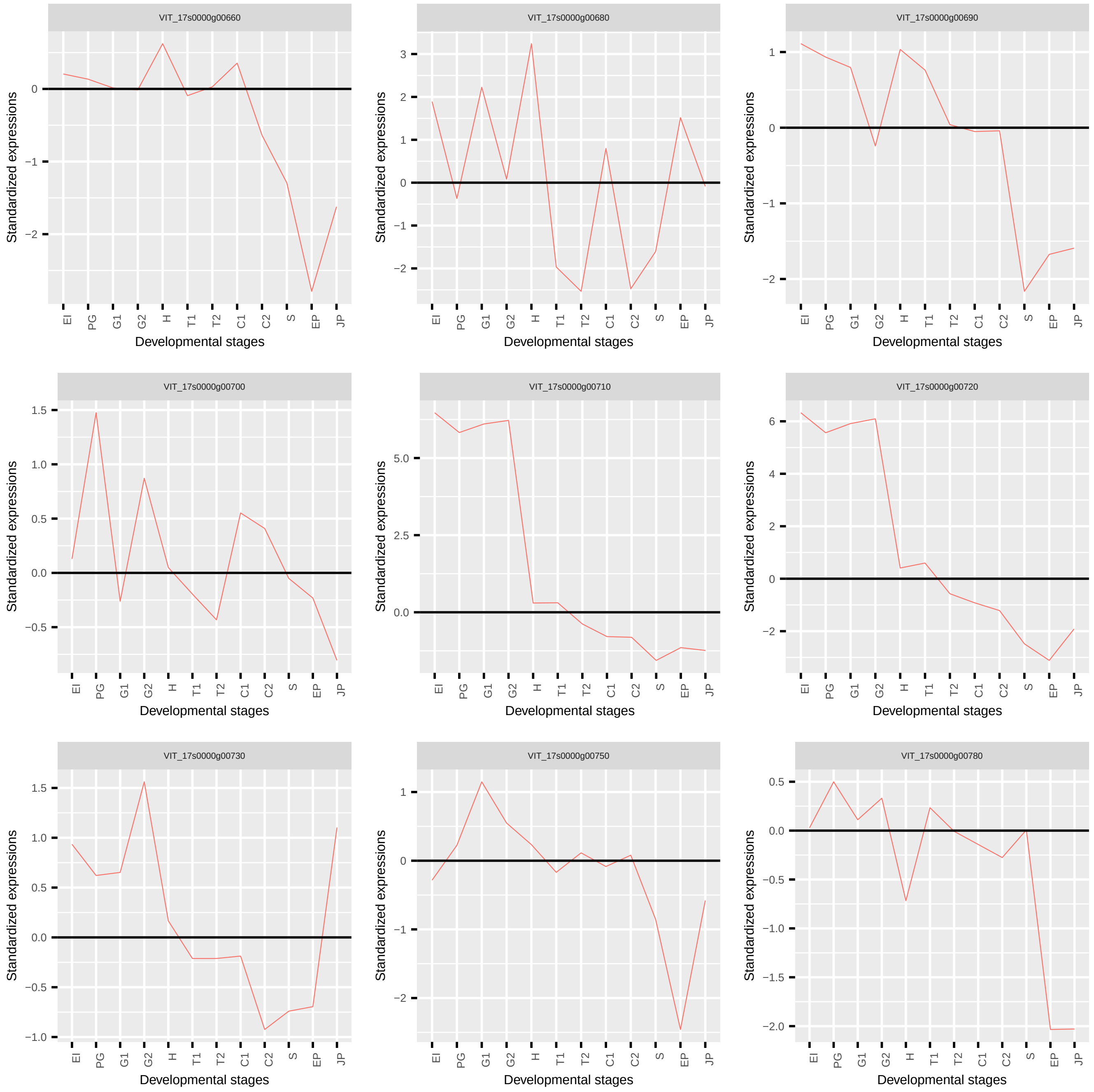
VIT_17s0000g00660
VIT_17s0000g00680
VIT_17s0000g00690
VIT_17s0000g00700
VIT_17s0000g00710
VIT_17s0000g00720
VIT_17s0000g00730
VIT_17s0000g00750
VIT_17s0000g00780
Standardized expressions
Developmental stages
EI PG G1 G2 H T1 T2 C1 C2 S EP JP

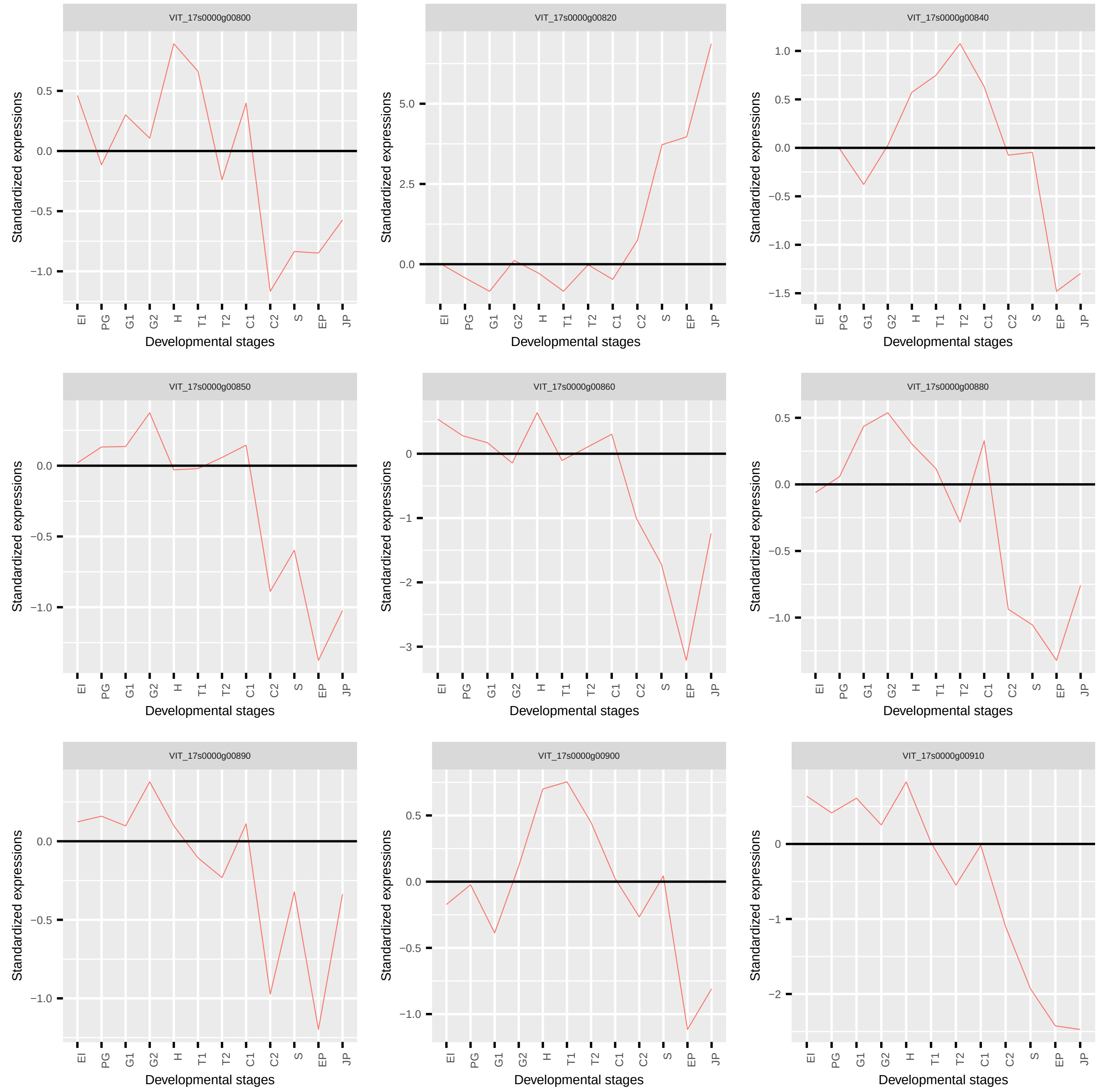
VIT_17s0000g00800
VIT_17s0000g00820
VIT_17s0000g00840
VIT_17s0000g00850
VIT_17s0000g00860
VIT_17s0000g00880
VIT_17s0000g00890
VIT_17s0000g00900
VIT_17s0000g00910
Standardized expressions
Developmental stages
EI PG G1 G2 H T1 T2 C1 C2 S EP JP
0.5 0.0 −0.5 −1.0
5.0 2.5 0.0
1.0 0.5 0.0 −0.5 −1.0 −1.5
0.0 −0.5 −1.0
0 −1 −2 −3
0.5 0.0 −0.5 −1.0
0.0 −0.5 −1.0
0.5 0.0 −0.5 −1.0
0 −1 −2

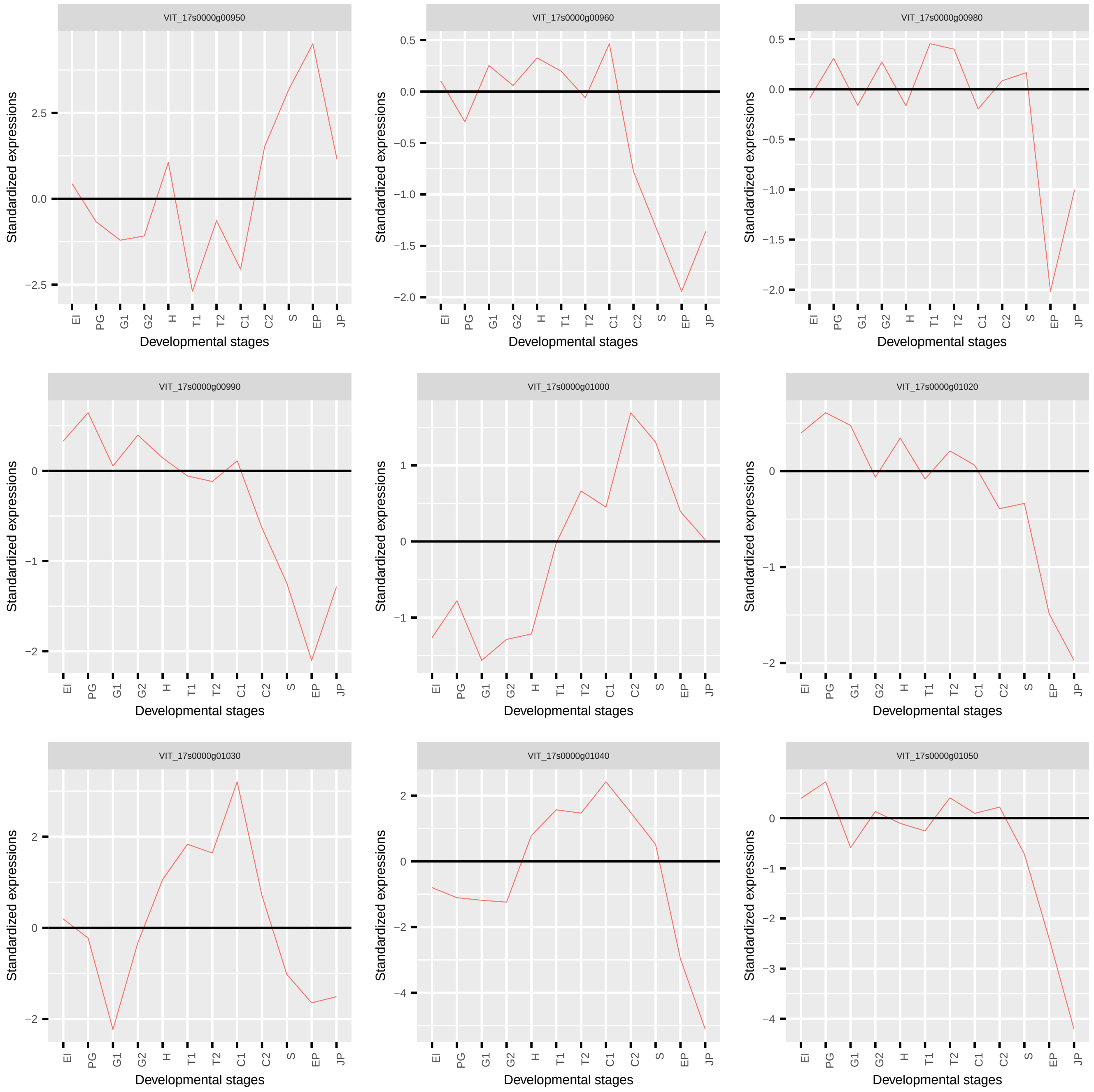
VIT_17s0000g00950
VIT_17s0000g00960
VIT_17s0000g00980
VIT_17s0000g00990
VIT_17s0000g01000
VIT_17s0000g01020
VIT_17s0000g01030
VIT_17s0000g01040
VIT_17s0000g01050
Standardized expressions
Developmental stages
EI PG G1 G2 H T1 T2 C1 C2 S EP JP

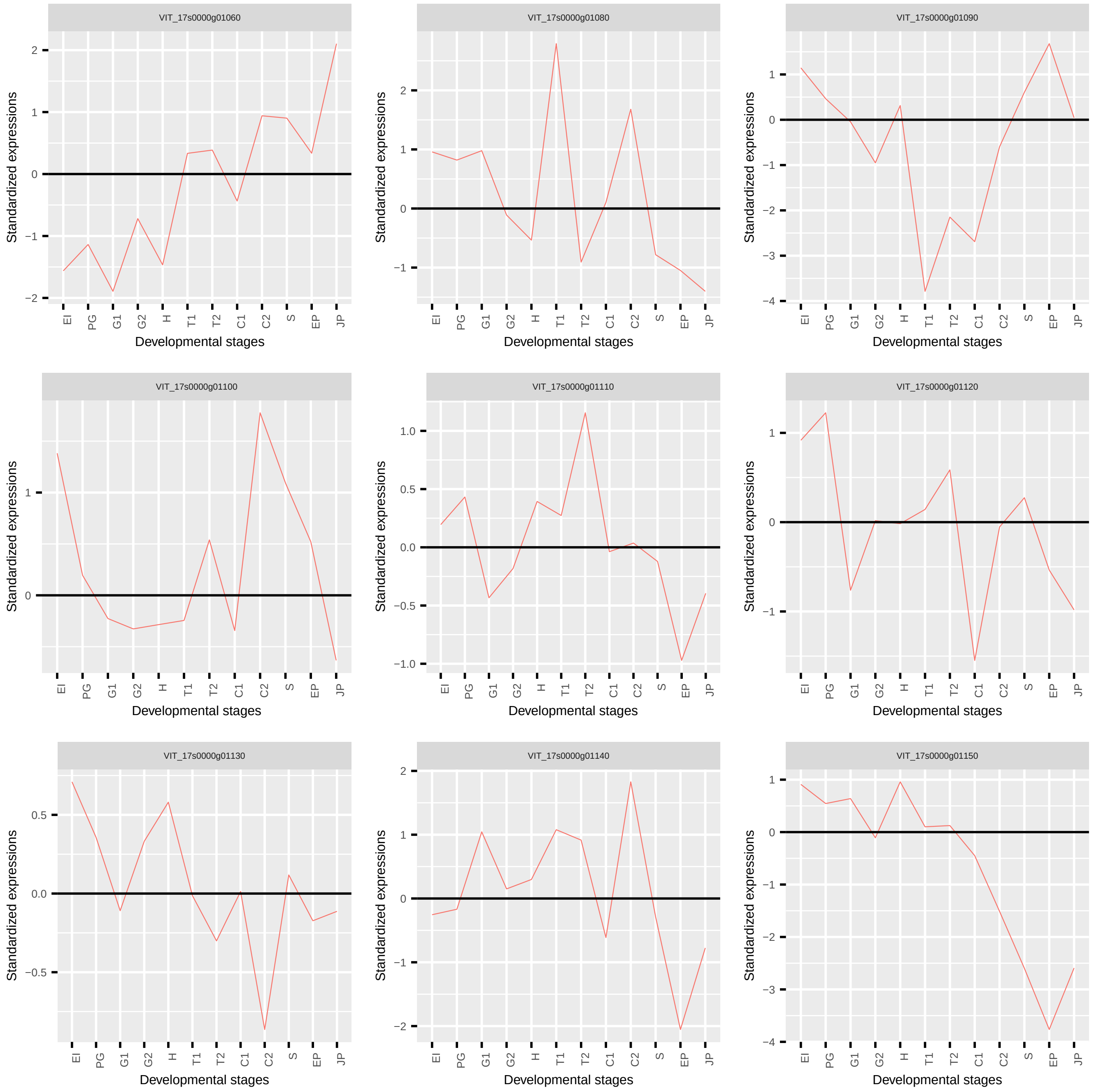

VIT_17s0000g01060
VIT_17s0000g01080
VIT_17s0000g01090
VIT_17s0000g01100
VIT_17s0000g01110
VIT_17s0000g01120
VIT_17s0000g01130
VIT_17s0000g01140
VIT_17s0000g01150
Standardized expressions
Developmental stages
EI PG G1 G2 H T1 T2 C1 C2 S EP JP

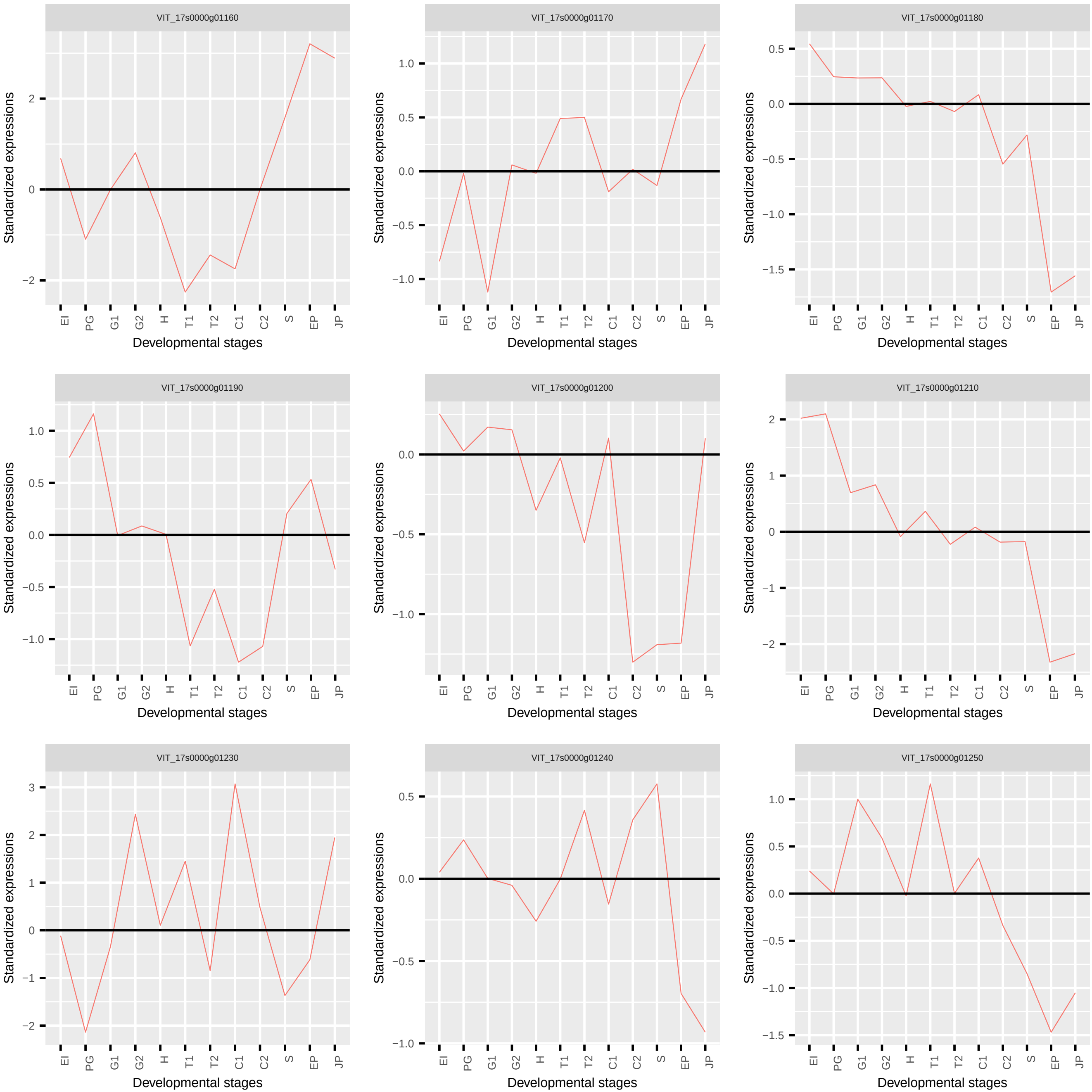
VIT_17s0000g01160
VIT_17s0000g01170
VIT_17s0000g01180
VIT_17s0000g01190
VIT_17s0000g01200
VIT_17s0000g01210
VIT_17s0000g01230
VIT_17s0000g01240
VIT_17s0000g01250
Standardized expressions
Developmental stages
EI PG G1 G2 H T1 T2 C1 C2 S EP JP

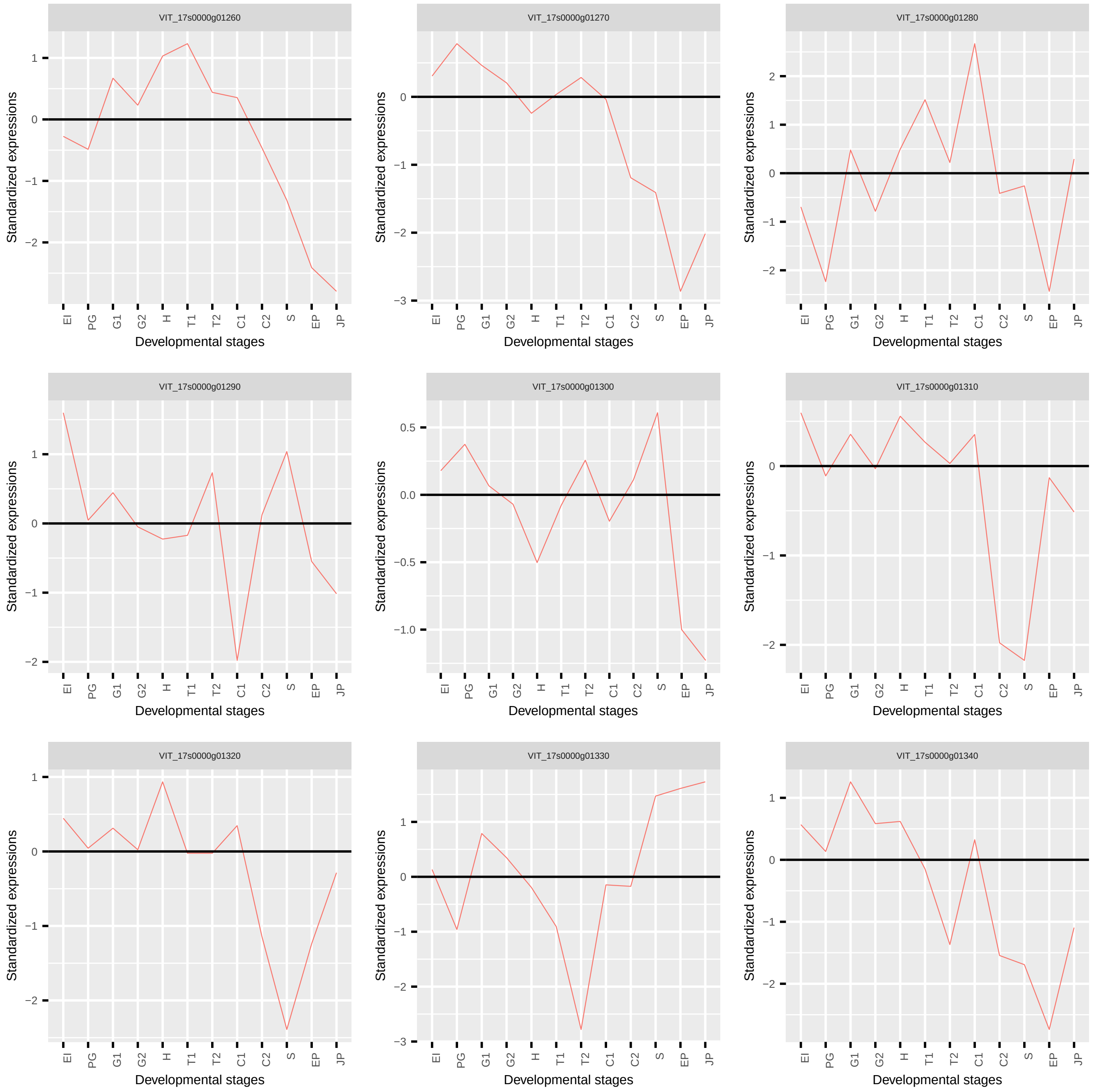
VIT_17s0000g01260
VIT_17s0000g01270
VIT_17s0000g01280
VIT_17s0000g01290
VIT_17s0000g01300
VIT_17s0000g01310
VIT_17s0000g01320
VIT_17s0000g01330
VIT_17s0000g01340
Standardized expressions
Developmental stages
EI PG G1 G2 H T1 T2 C1 C2 S EP JP

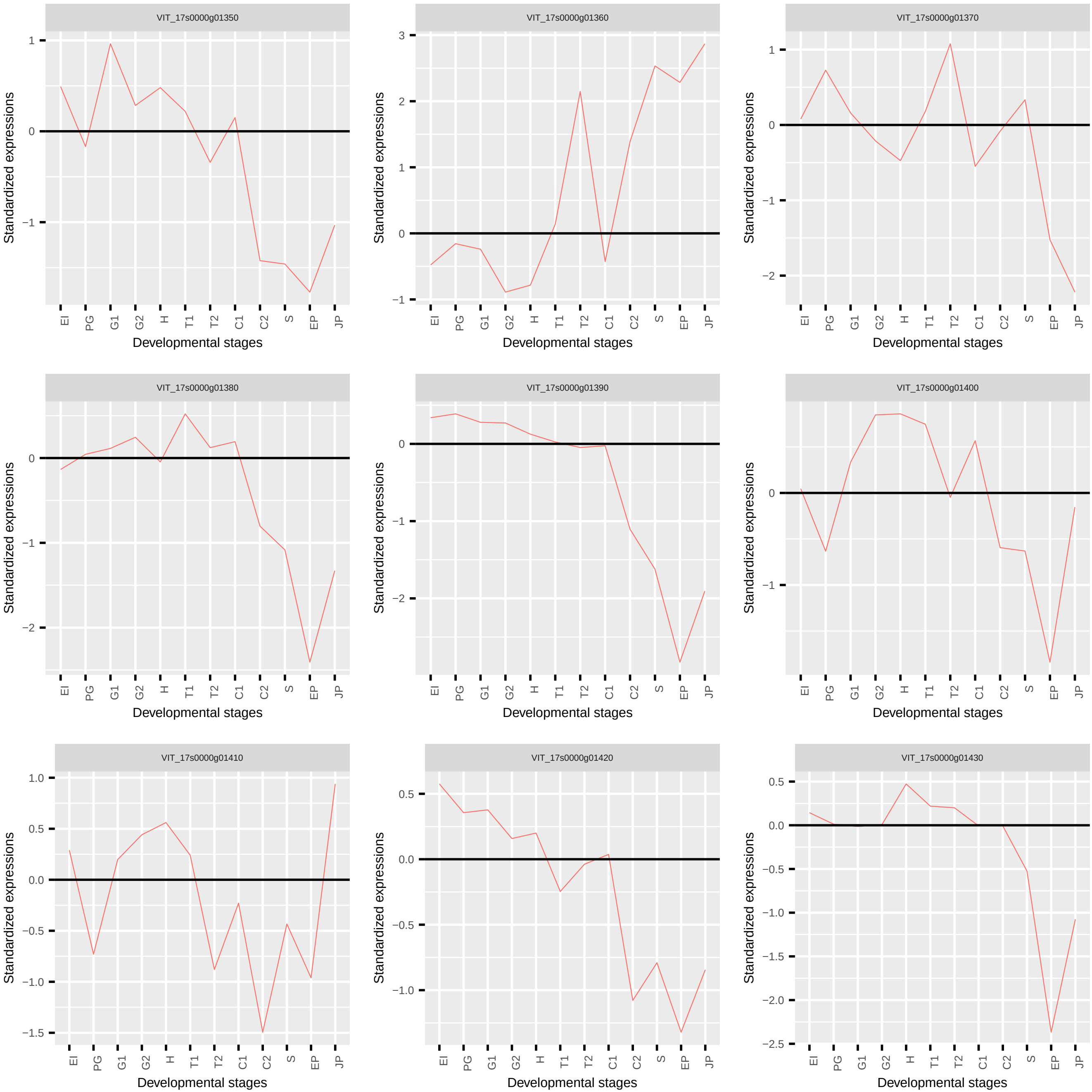
VIT_17s0000g01350
VIT_17s0000g01360
VIT_17s0000g01370
VIT_17s0000g01380
VIT_17s0000g01390
VIT_17s0000g01400
VIT_17s0000g01410
VIT_17s0000g01420
VIT_17s0000g01430
Standardized expressions
Developmental stages
EI PG G1 G2 H T1 T2 C1 C2 S EP JP
1 0 −1
3 2 1 0 −1
1 0 −1 −2
0 −1 −2
0 −1 −2
0 −1
1.0 0.5 0.0 −0.5 −1.0 −1.5
0.5 0.0 −0.5 −1.0
0.5 0.0 −0.5 −1.0 −1.5 −2.0 −2.5

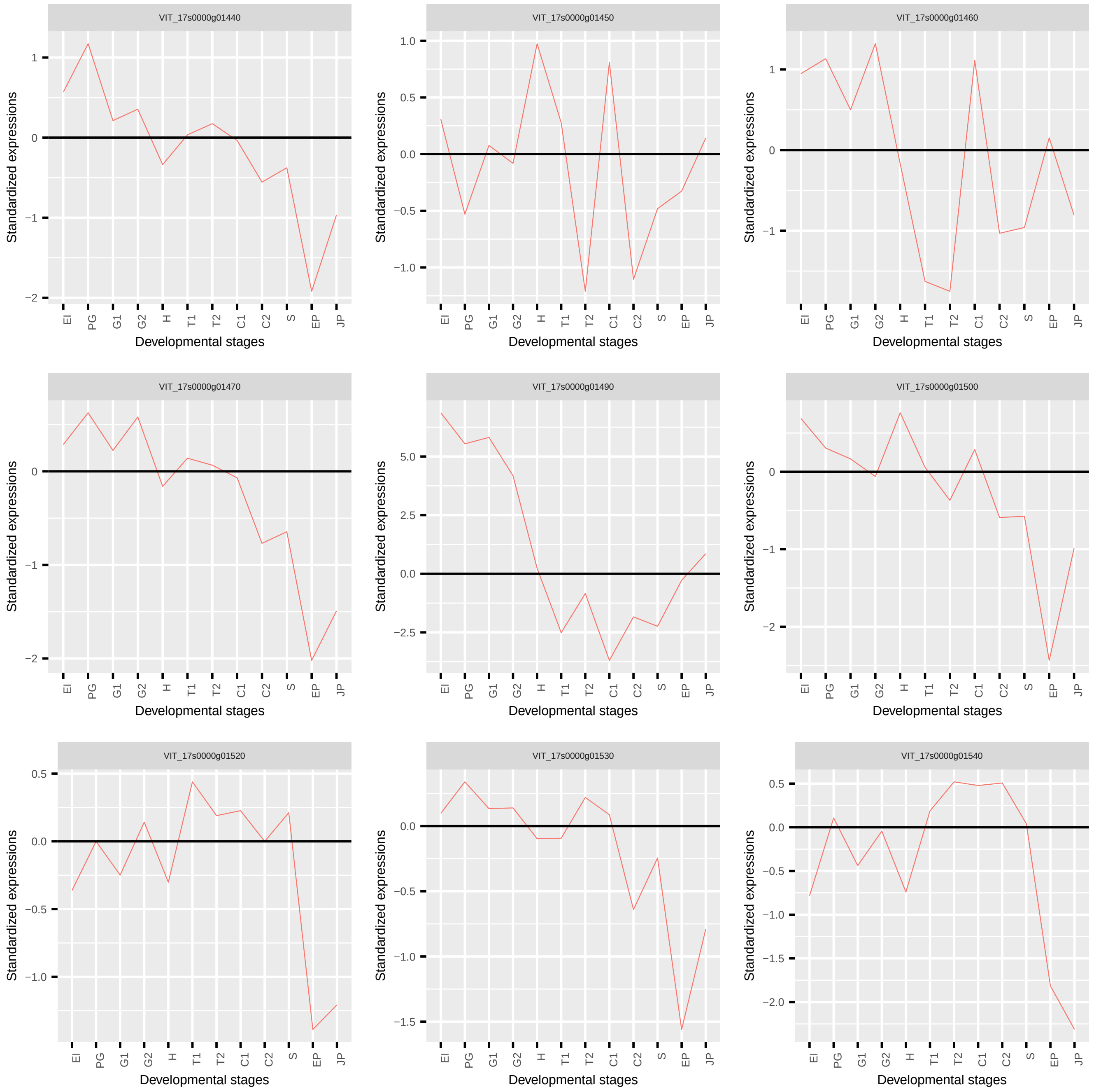
VIT_17s0000g01440
VIT_17s0000g01450
VIT_17s0000g01460
VIT_17s0000g01470
VIT_17s0000g01490
VIT_17s0000g01500
VIT_17s0000g01520
VIT_17s0000g01530
VIT_17s0000g01540
Standardized expressions
Developmental stages
EI PG G1 G2 H T1 T2 C1 C2 S EP JP

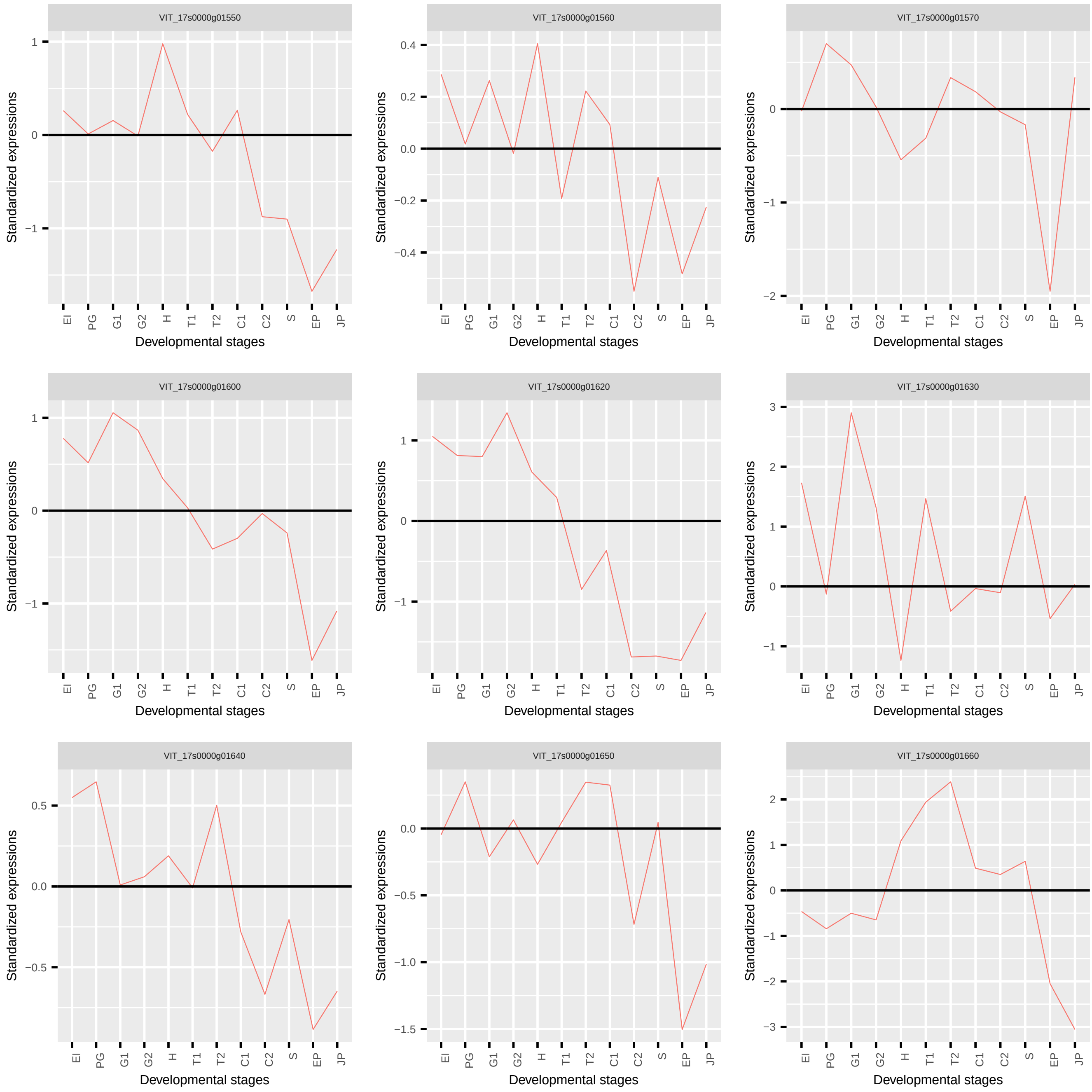

VIT_17s0000g01550
VIT_17s0000g01560
VIT_17s0000g01570
VIT_17s0000g01600
VIT_17s0000g01620
VIT_17s0000g01630
VIT_17s0000g01640
VIT_17s0000g01650
VIT_17s0000g01660
Standardized expressions
Developmental stages
EI PG G1 G2 H T1 T2 C1 C2 S EP JP

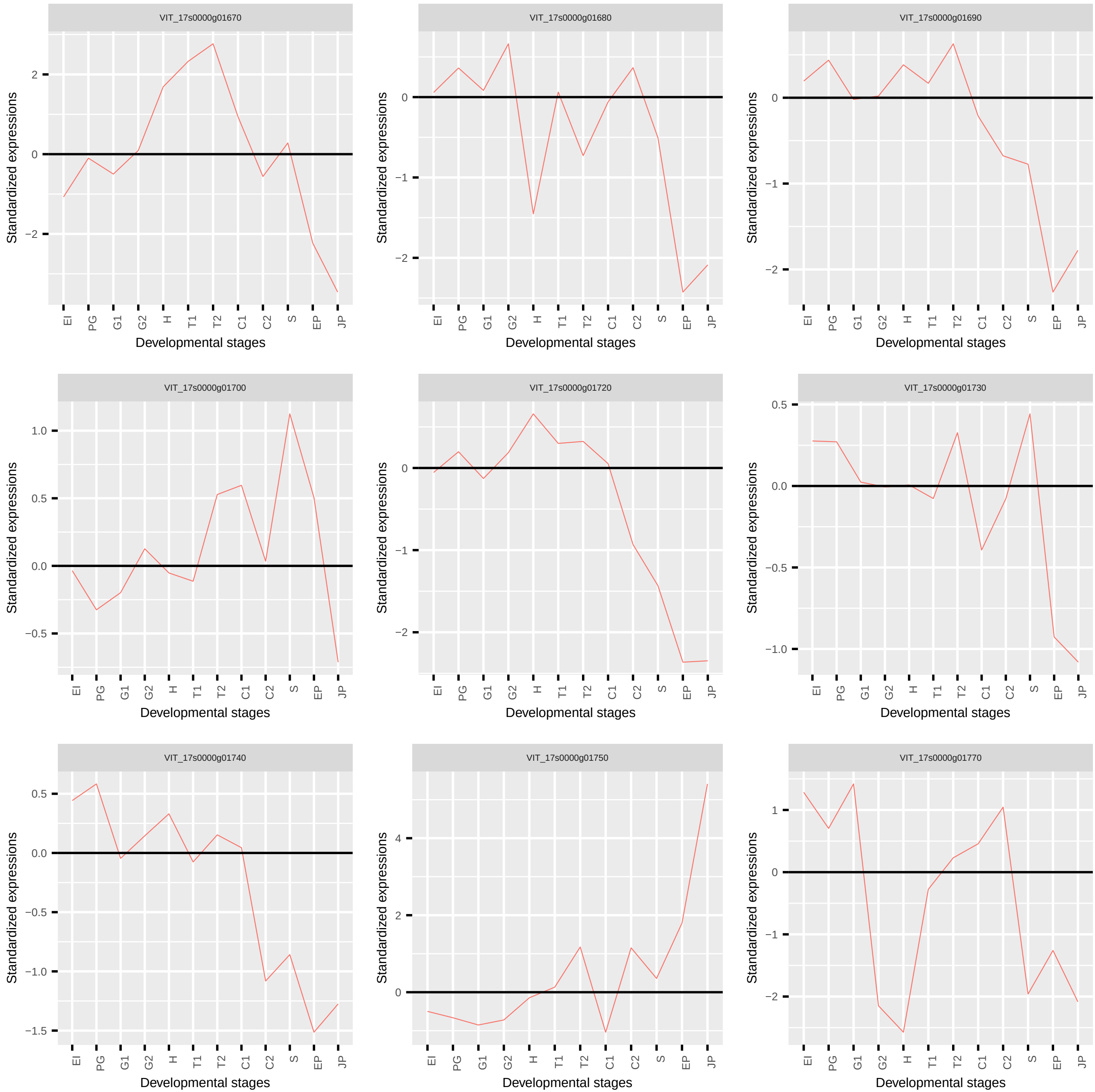
VIT_17s0000g01670
VIT_17s0000g01680
VIT_17s0000g01690
VIT_17s0000g01700
VIT_17s0000g01720
VIT_17s0000g01730
VIT_17s0000g01740
VIT_17s0000g01750
VIT_17s0000g01770
Standardized expressions
Developmental stages
EI PG G1 G2 H T1 T2 C1 C2 S EP JP

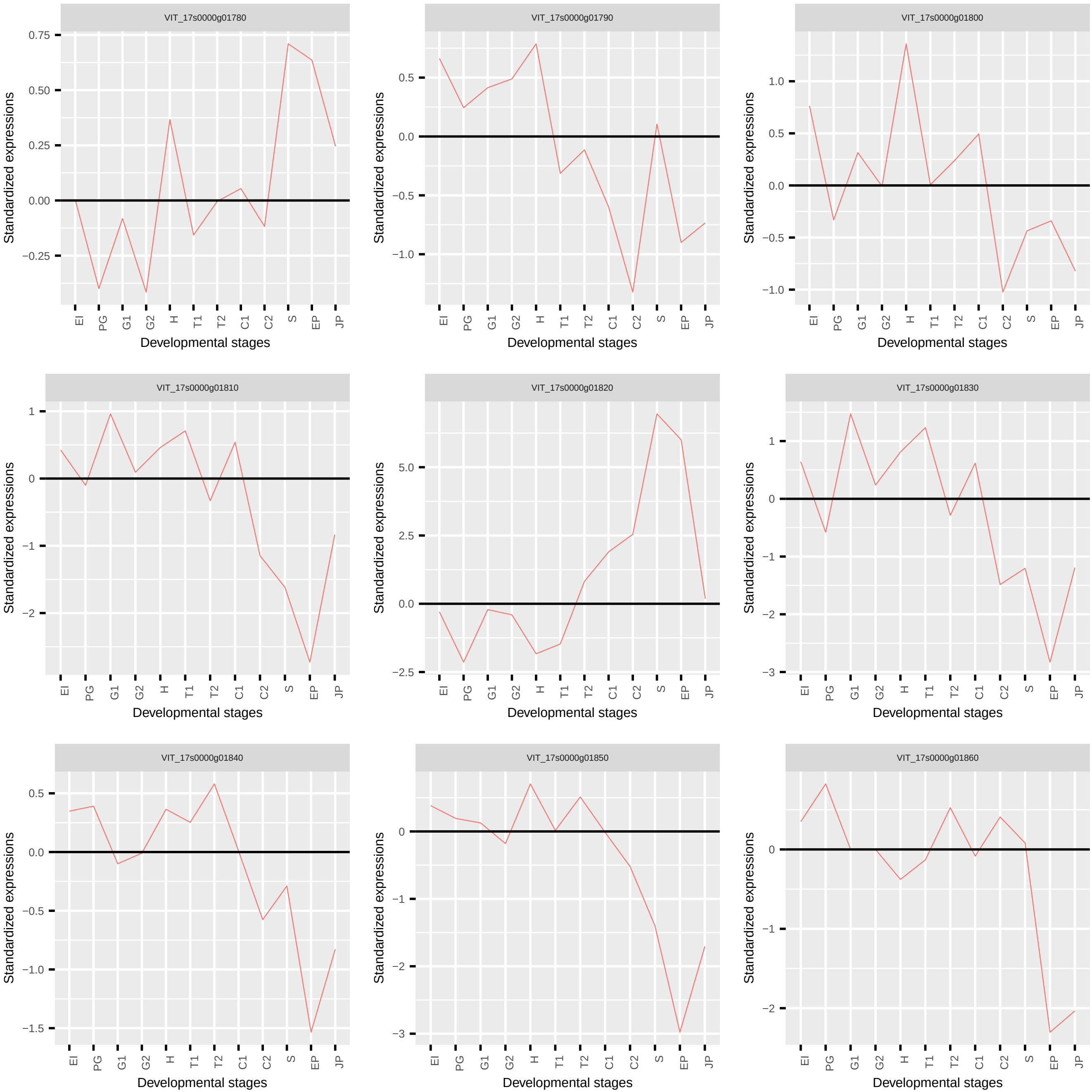
VIT_17s0000g01780
VIT_17s0000g01790
VIT_17s0000g01800
VIT_17s0000g01810
VIT_17s0000g01820
VIT_17s0000g01830
VIT_17s0000g01840
VIT_17s0000g01850
VIT_17s0000g01860
Standardized expressions
Developmental stages
EI
PG
G1
G2
H
T1
T2
C1
C2
S
EP
JP
0.75
0.50
0.25
0.00
−0.25
0.5
0.0
−0.5
−1.0
1.0
1
0
−1
−2
−3
5.0
2.5
−2.5
−1.5

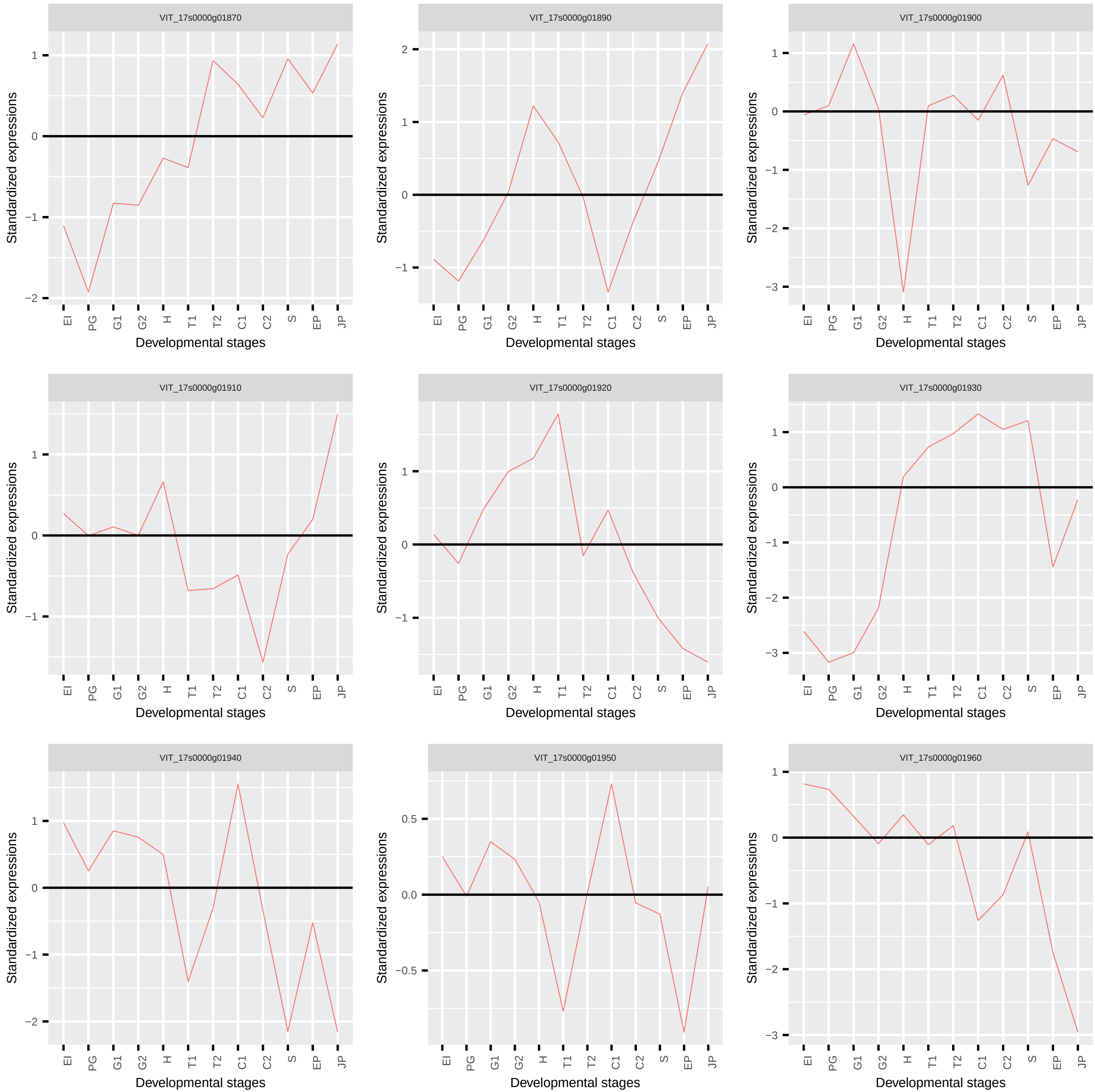
VIT_17s0000g01870
VIT_17s0000g01890
VIT_17s0000g01900
VIT_17s0000g01910
VIT_17s0000g01920
VIT_17s0000g01930
VIT_17s0000g01940
VIT_17s0000g01950
VIT_17s0000g01960
Standardized expressions
Developmental stages
EI PG G1 G2 H T1 T2 C1 C2 S EP JP

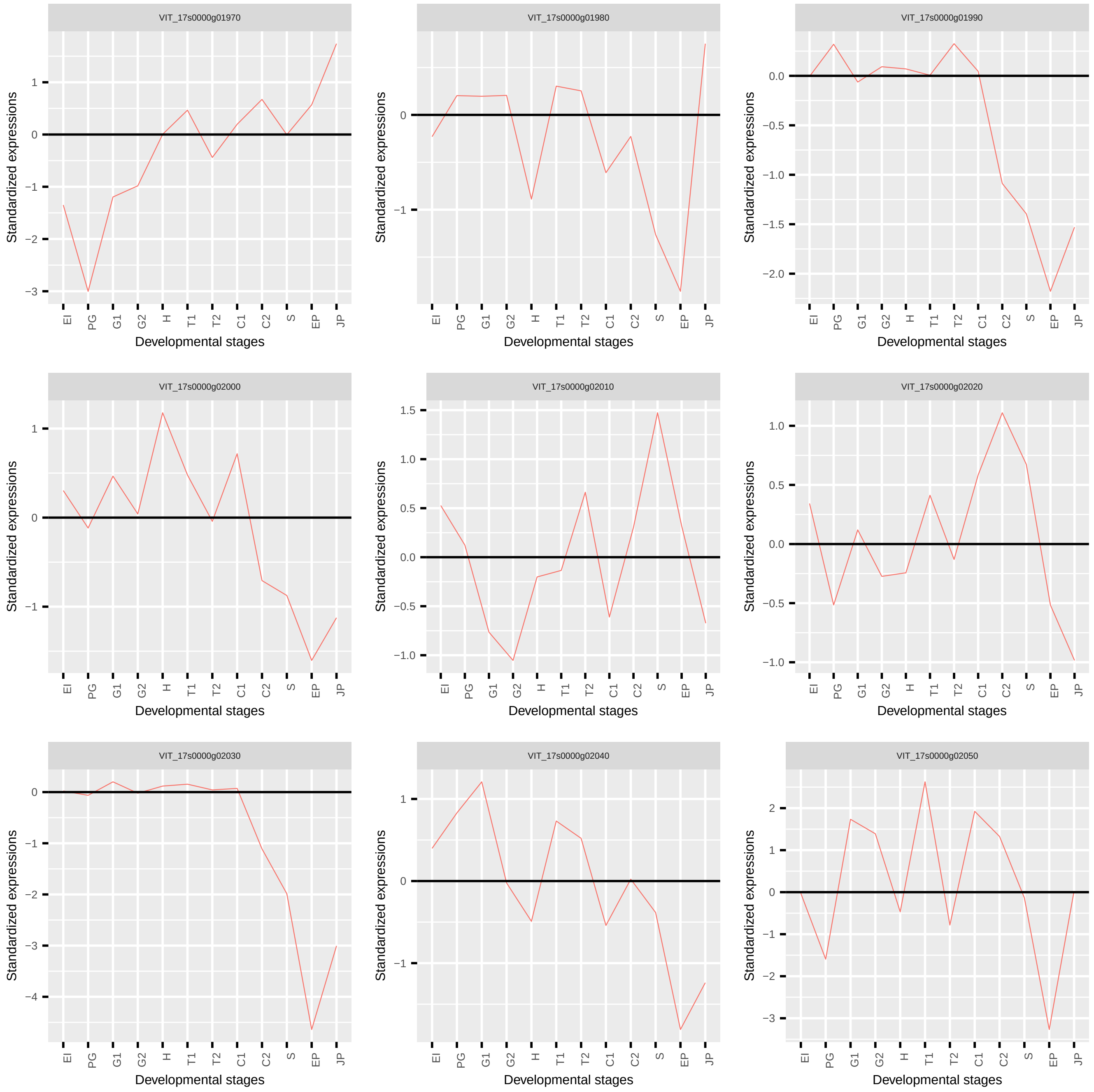
VIT_17s0000g01970
VIT_17s0000g01980
VIT_17s0000g01990
VIT_17s0000g02000
VIT_17s0000g02010
VIT_17s0000g02020
VIT_17s0000g02030
VIT_17s0000g02040
VIT_17s0000g02050
Standardized expressions
EI PG G1 G2 H T1 T2 C1 C2 S EP JP
Developmental stages

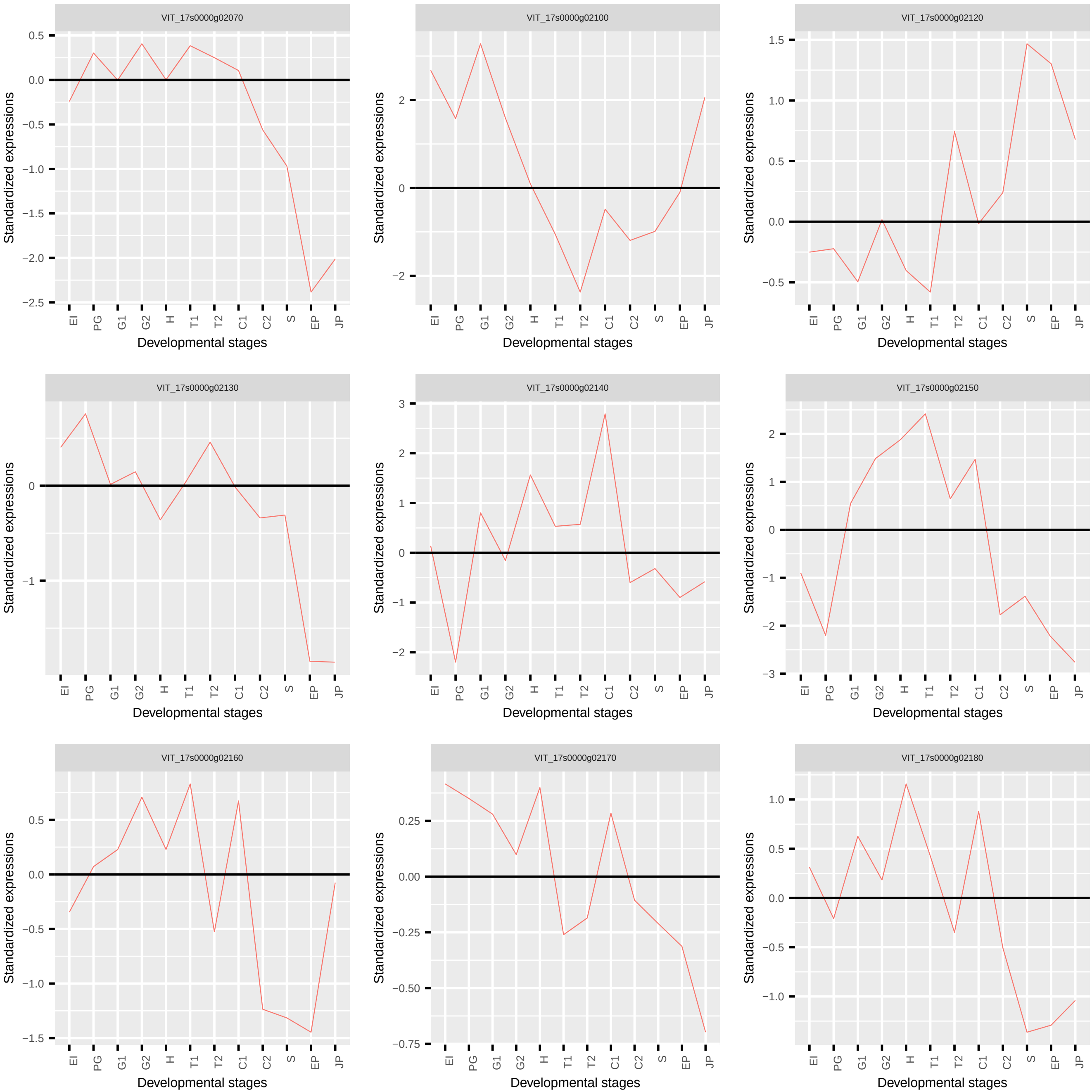
VIT_17s0000g02070
VIT_17s0000g02100
VIT_17s0000g02120
VIT_17s0000g02130
VIT_17s0000g02140
VIT_17s0000g02150
VIT_17s0000g02160
VIT_17s0000g02170
VIT_17s0000g02180
Standardized expressions
Developmental stages
EI PG G1 G2 H T1 T2 C1 C2 S EP JP

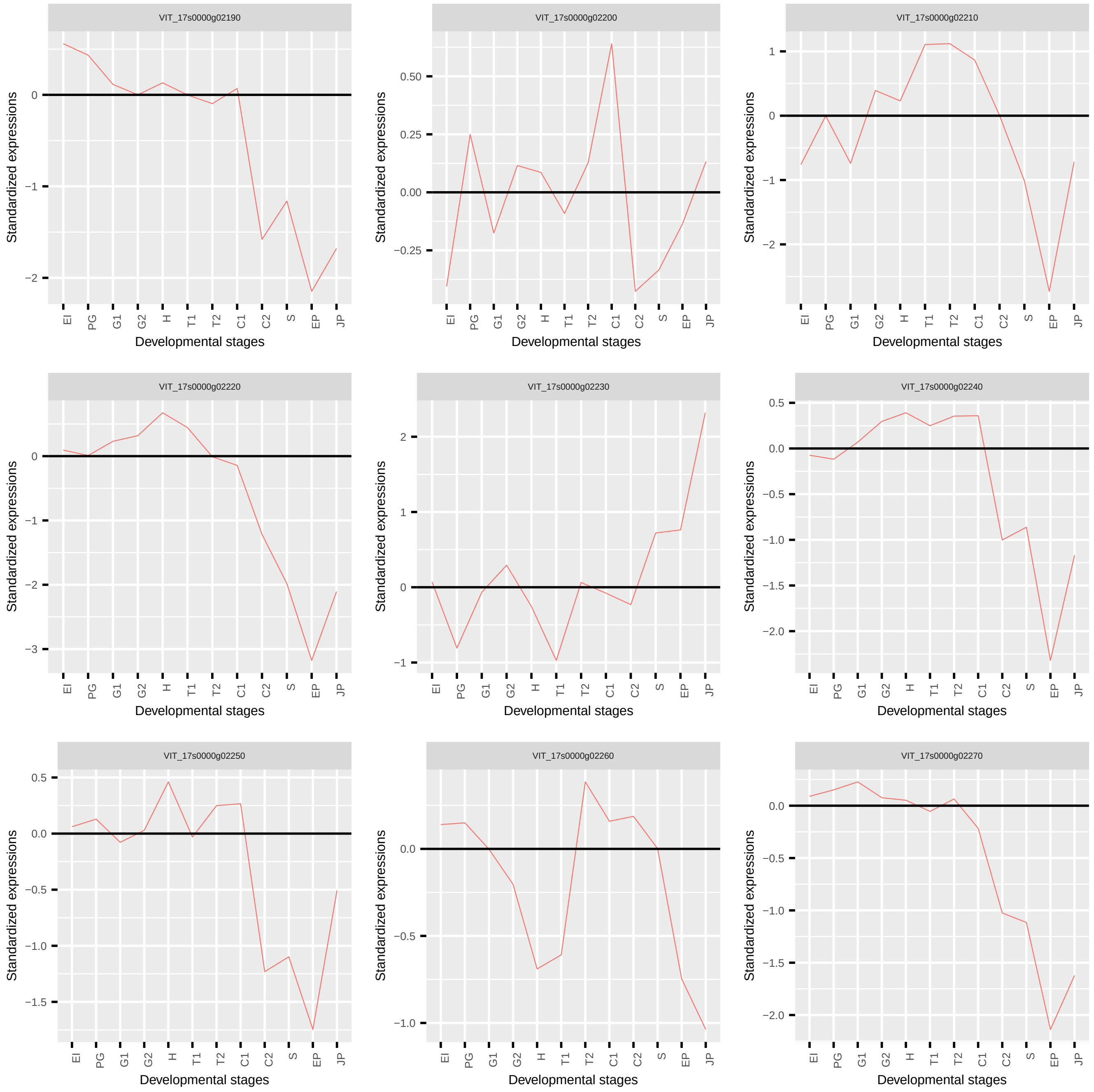
VIT_17s0000g02190
Standardized expressions
0
−1
−2
EI PG G1 G2 H T1 T2 C1 C2 S EP JP
Developmental stages
VIT_17s0000g02200
Standardized expressions
0.50
0.25
0.00
−0.25
EI PG G1 G2 H T1 T2 C1 C2 S EP JP
Developmental stages
VIT_17s0000g02210
Standardized expressions
1
0
−1
−2
EI PG G1 G2 H T1 T2 C1 C2 S EP JP
Developmental stages
VIT_17s0000g02220
Standardized expressions
0
−1
−2
−3
EI PG G1 G2 H T1 T2 C1 C2 S EP JP
Developmental stages
VIT_17s0000g02230
Standardized expressions
2
1
0
−1
EI PG G1 G2 H T1 T2 C1 C2 S EP JP
Developmental stages
VIT_17s0000g02240
Standardized expressions
0.5
0.0
−0.5
−1.0
−1.5
−2.0
EI PG G1 G2 H T1 T2 C1 C2 S EP JP
Developmental stages
VIT_17s0000g02250
Standardized expressions
0.5
0.0
−0.5
−1.0
−1.5
EI PG G1 G2 H T1 T2 C1 C2 S EP JP
Developmental stages
VIT_17s0000g02260
Standardized expressions
0.0
−0.5
−1.0
EI PG G1 G2 H T1 T2 C1 C2 S EP JP
Developmental stages
VIT_17s0000g02270
Standardized expressions
0.0
−0.5
−1.0
−1.5
−2.0
EI PG G1 G2 H T1 T2 C1 C2 S EP JP
Developmental stages

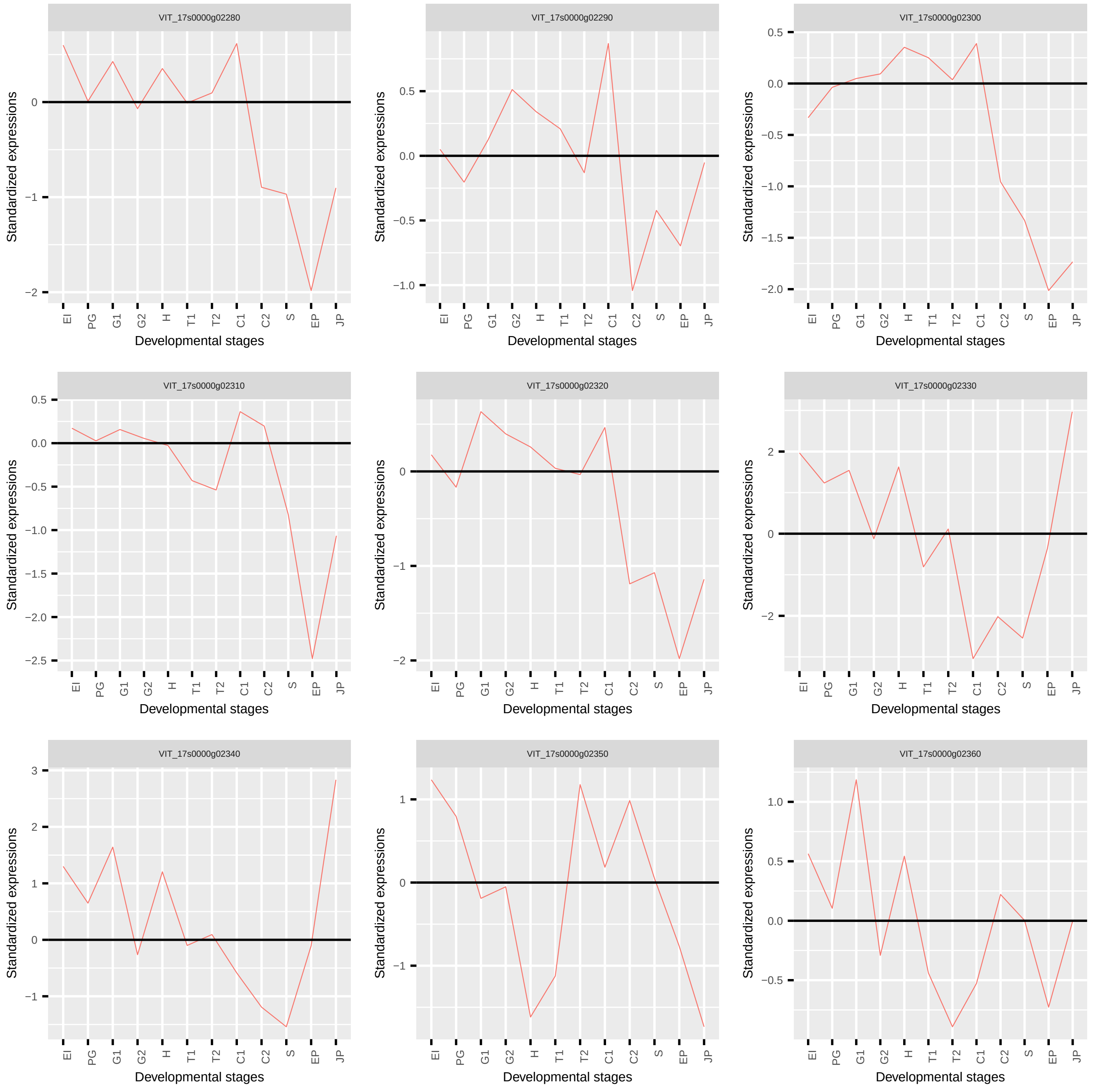
VIT_17s0000g02280
VIT_17s0000g02290
VIT_17s0000g02300
VIT_17s0000g02310
VIT_17s0000g02320
VIT_17s0000g02330
VIT_17s0000g02340
VIT_17s0000g02350
VIT_17s0000g02360
Standardized expressions
Developmental stages
EI
PG
G1
G2
H
T1
T2
C1
C2
S
EP
JP

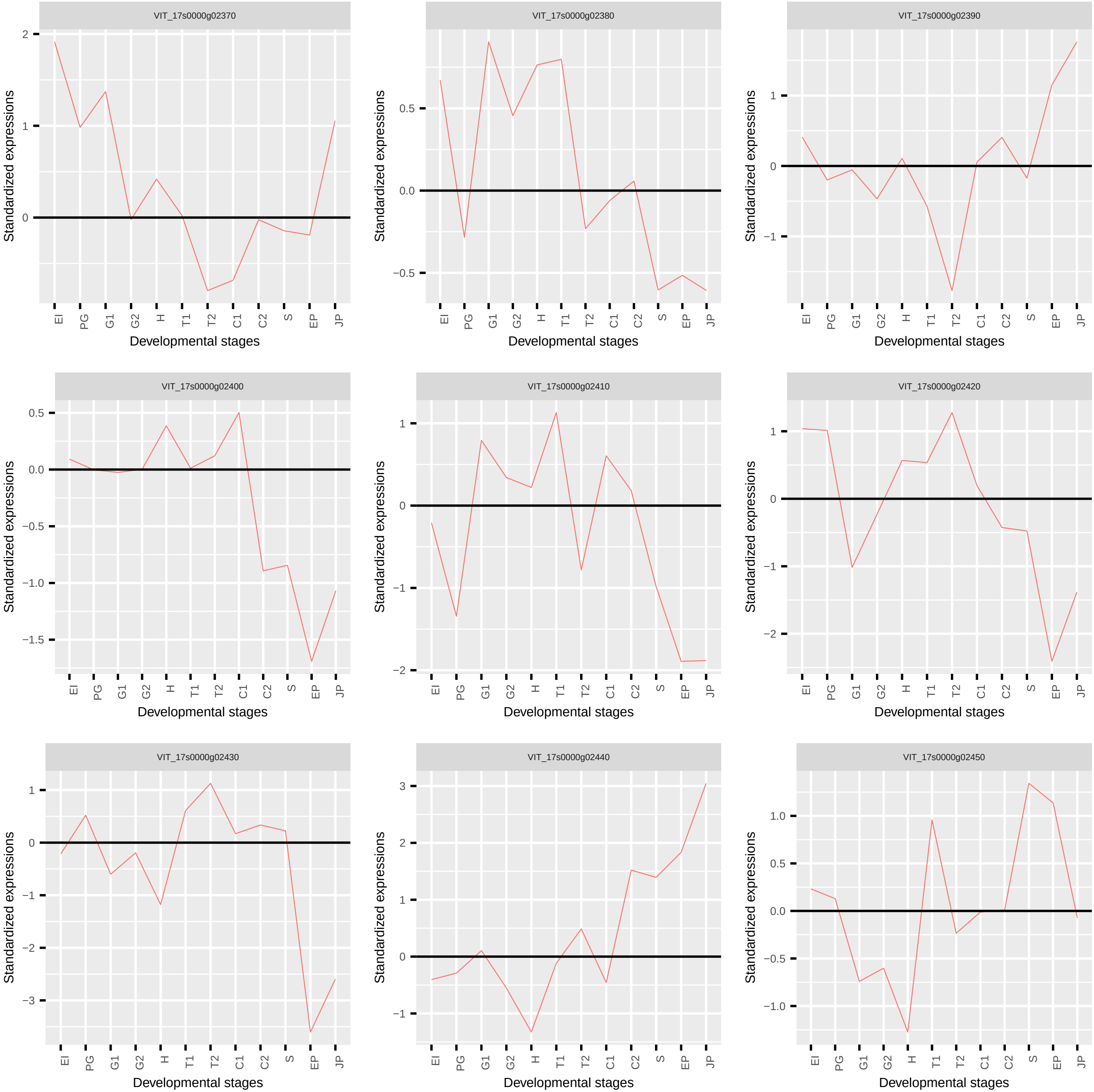
VIT_17s0000g02370
2
1
0
Standardized expressions
EI PG G1 G2 H T1 T2 C1 C2 S EP JP
Developmental stages
VIT_17s0000g02380
0.5
0.0
−0.5
Standardized expressions
EI PG G1 G2 H T1 T2 C1 C2 S EP JP
Developmental stages
VIT_17s0000g02390
1
0
−1
Standardized expressions
EI PG G1 G2 H T1 T2 C1 C2 S EP JP
Developmental stages
VIT_17s0000g02400
0.5
0.0
−0.5
−1.0
−1.5
Standardized expressions
EI PG G1 G2 H T1 T2 C1 C2 S EP JP
Developmental stages
VIT_17s0000g02410
1
0
−1
−2
Standardized expressions
EI PG G1 G2 H T1 T2 C1 C2 S EP JP
Developmental stages
VIT_17s0000g02420
1
0
−1
−2
Standardized expressions
EI PG G1 G2 H T1 T2 C1 C2 S EP JP
Developmental stages
VIT_17s0000g02430
1
0
−1
−2
−3
Standardized expressions
EI PG G1 G2 H T1 T2 C1 C2 S EP JP
Developmental stages
VIT_17s0000g02440
3
2
1
0
−1
Standardized expressions
EI PG G1 G2 H T1 T2 C1 C2 S EP JP
Developmental stages
VIT_17s0000g02450
1.0
0.5
0.0
−0.5
−1.0
Standardized expressions
EI PG G1 G2 H T1 T2 C1 C2 S EP JP
Developmental stages

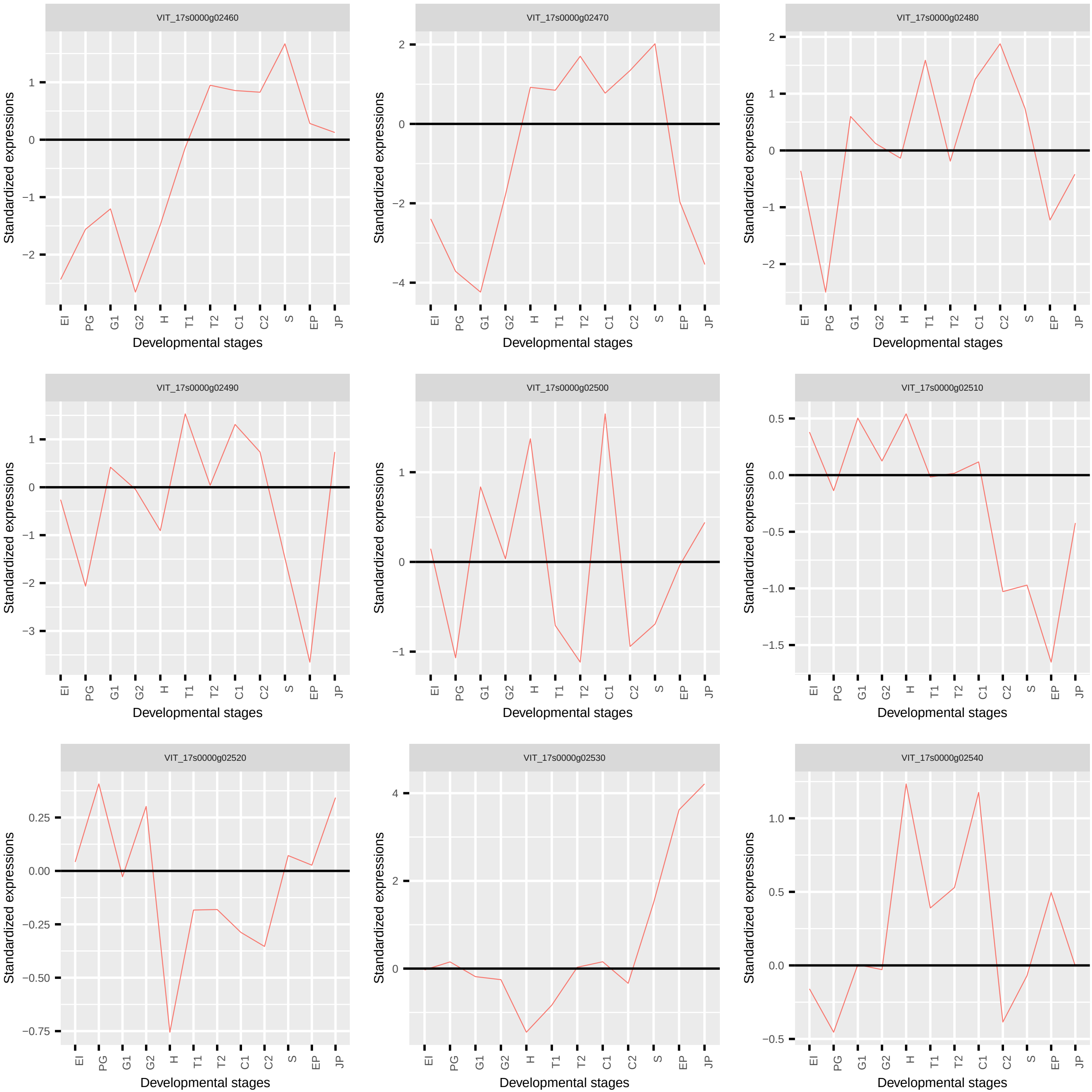
VIT_17s0000g02460
VIT_17s0000g02470
VIT_17s0000g02480
VIT_17s0000g02490
VIT_17s0000g02500
VIT_17s0000g02510
VIT_17s0000g02520
VIT_17s0000g02530
VIT_17s0000g02540
Standardized expressions
Developmental stages
EI PG G1 G2 H T1 T2 C1 C2 S EP JP

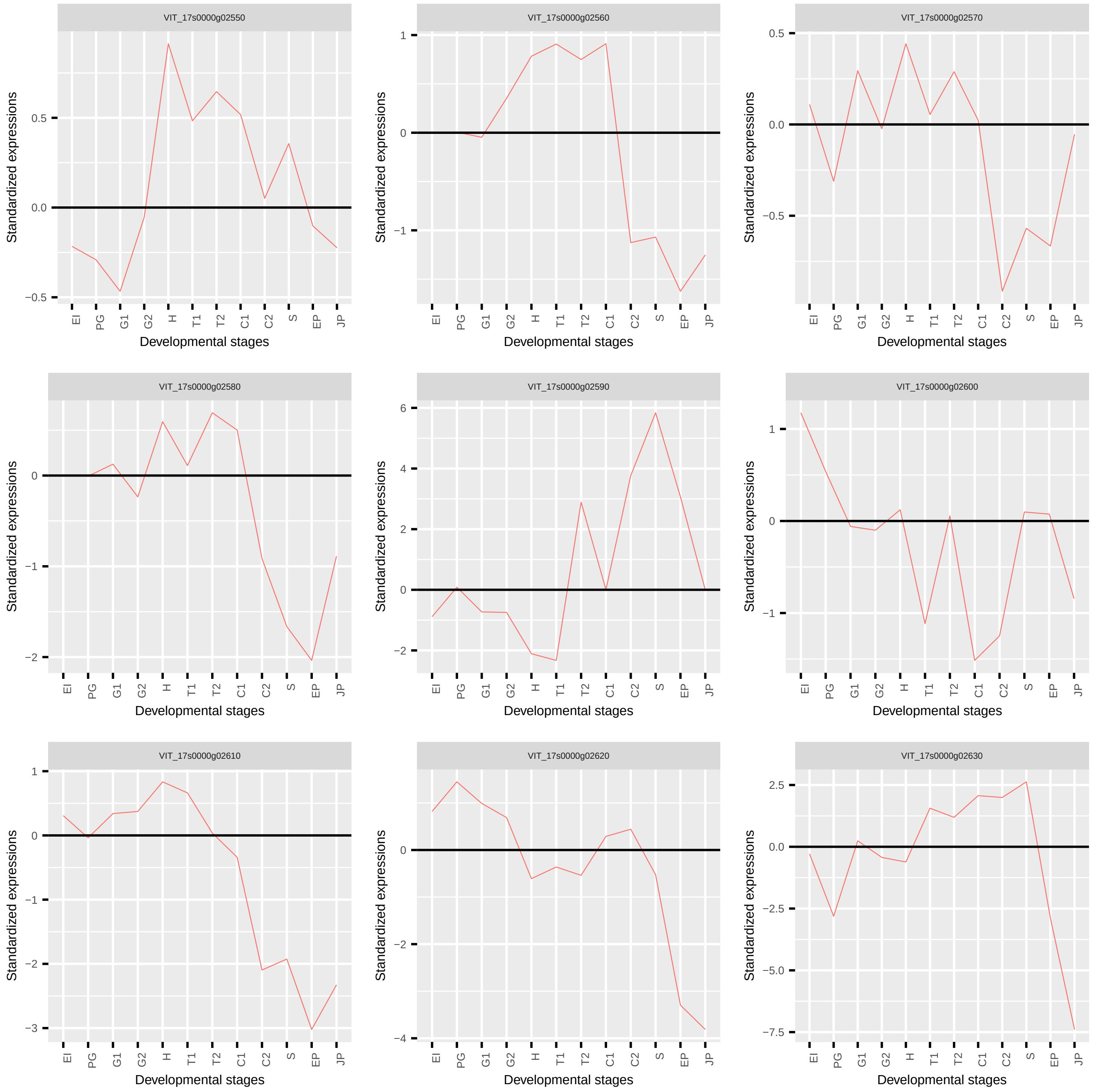
VIT_17s0000g02550
VIT_17s0000g02560
VIT_17s0000g02570
VIT_17s0000g02580
VIT_17s0000g02590
VIT_17s0000g02600
VIT_17s0000g02610
VIT_17s0000g02620
VIT_17s0000g02630
Standardized expressions
Developmental stages
EI PG G1 G2 H T1 T2 C1 C2 S EP JP

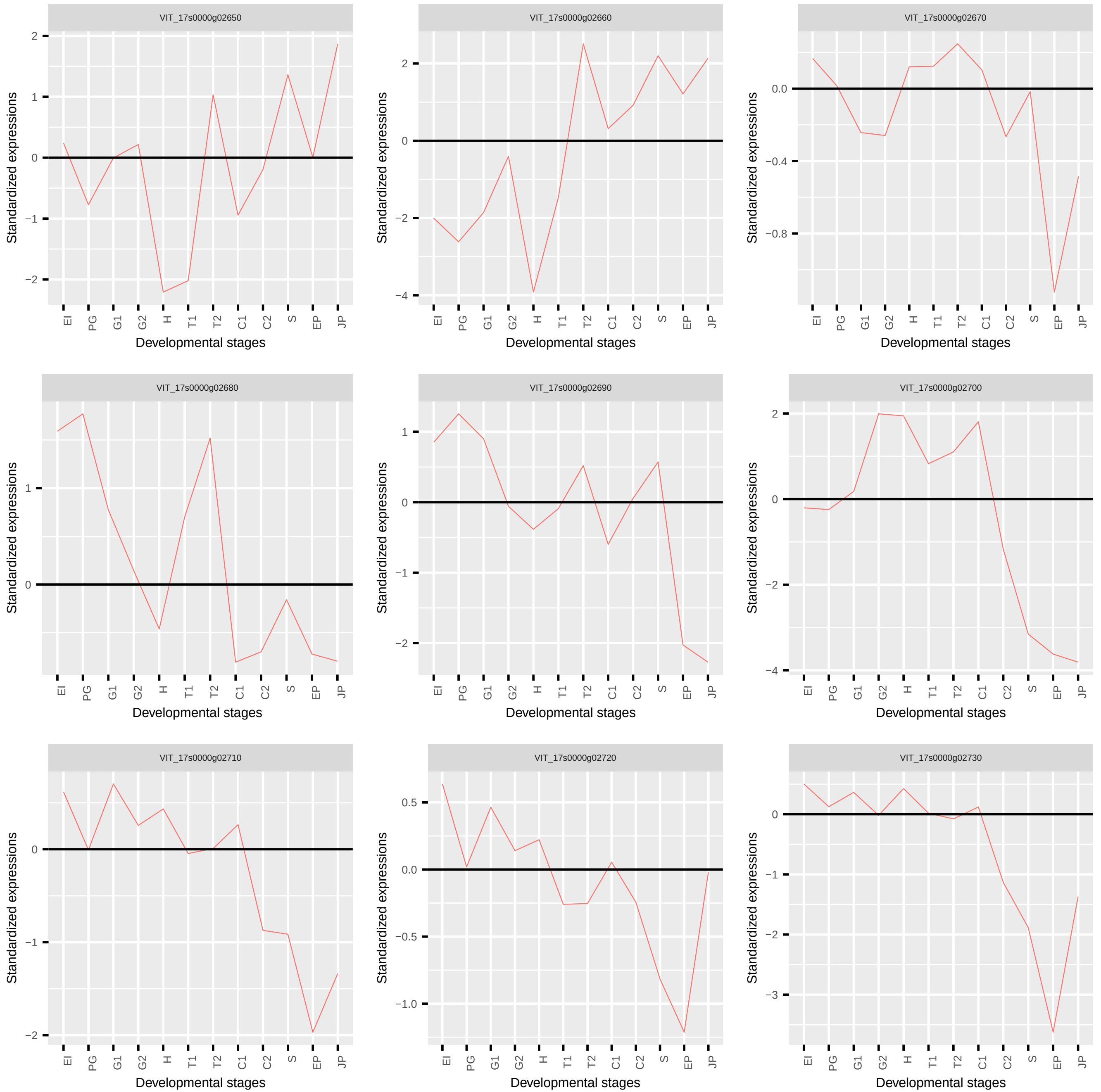

VIT_17s0000g02650
VIT_17s0000g02660
VIT_17s0000g02670
VIT_17s0000g02680
VIT_17s0000g02690
VIT_17s0000g02700
VIT_17s0000g02710
VIT_17s0000g02720
VIT_17s0000g02730
Standardized expressions
Developmental stages
EI PG G1 G2 H T1 T2 C1 C2 S EP JP

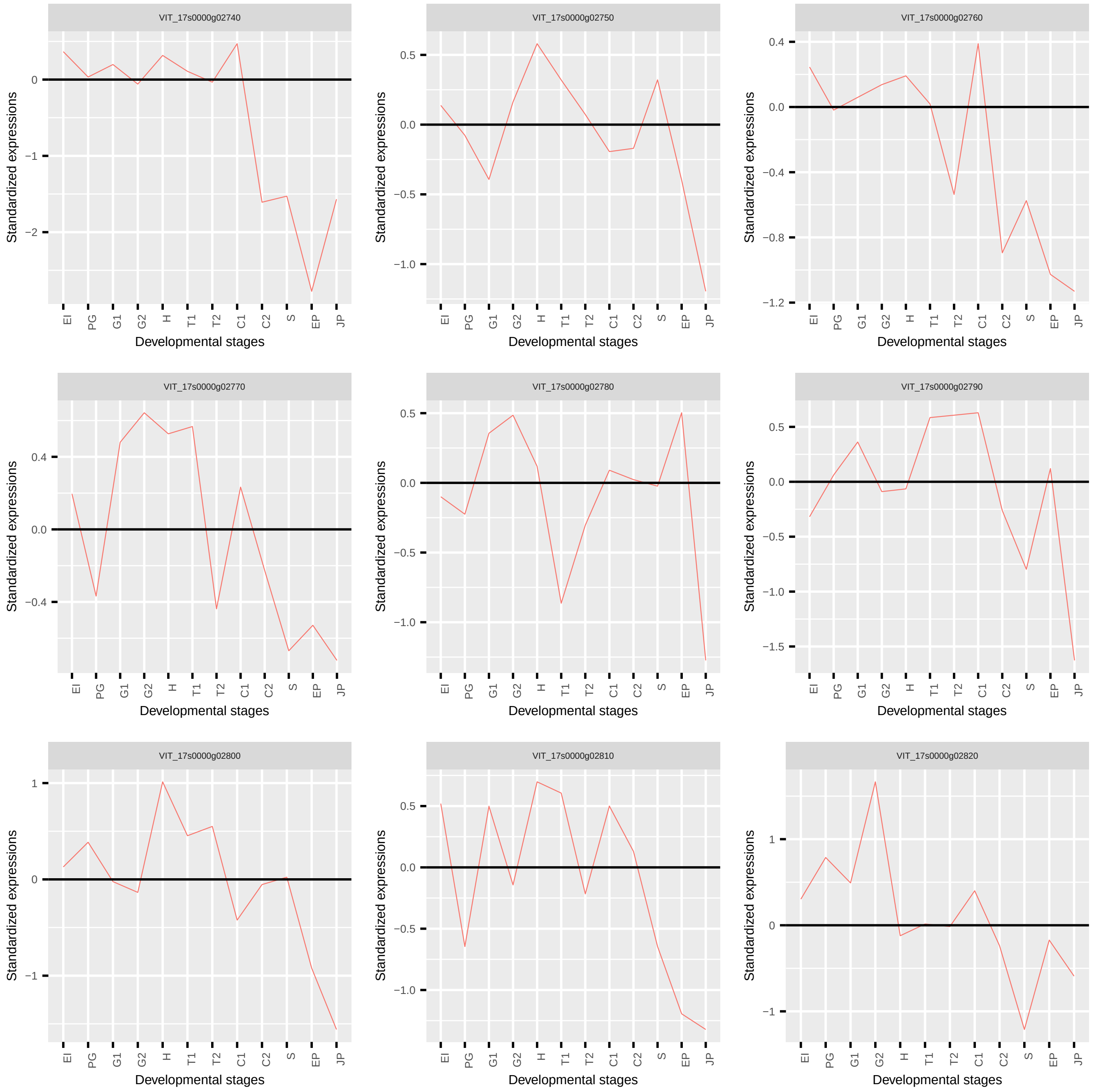
VIT_17s0000g02740
VIT_17s0000g02750
VIT_17s0000g02760
VIT_17s0000g02770
VIT_17s0000g02780
VIT_17s0000g02790
VIT_17s0000g02800
VIT_17s0000g02810
VIT_17s0000g02820
Standardized expressions
Developmental stages
EI
PG
G1
G2
H
T1
T2
C1
C2
S
EP
JP

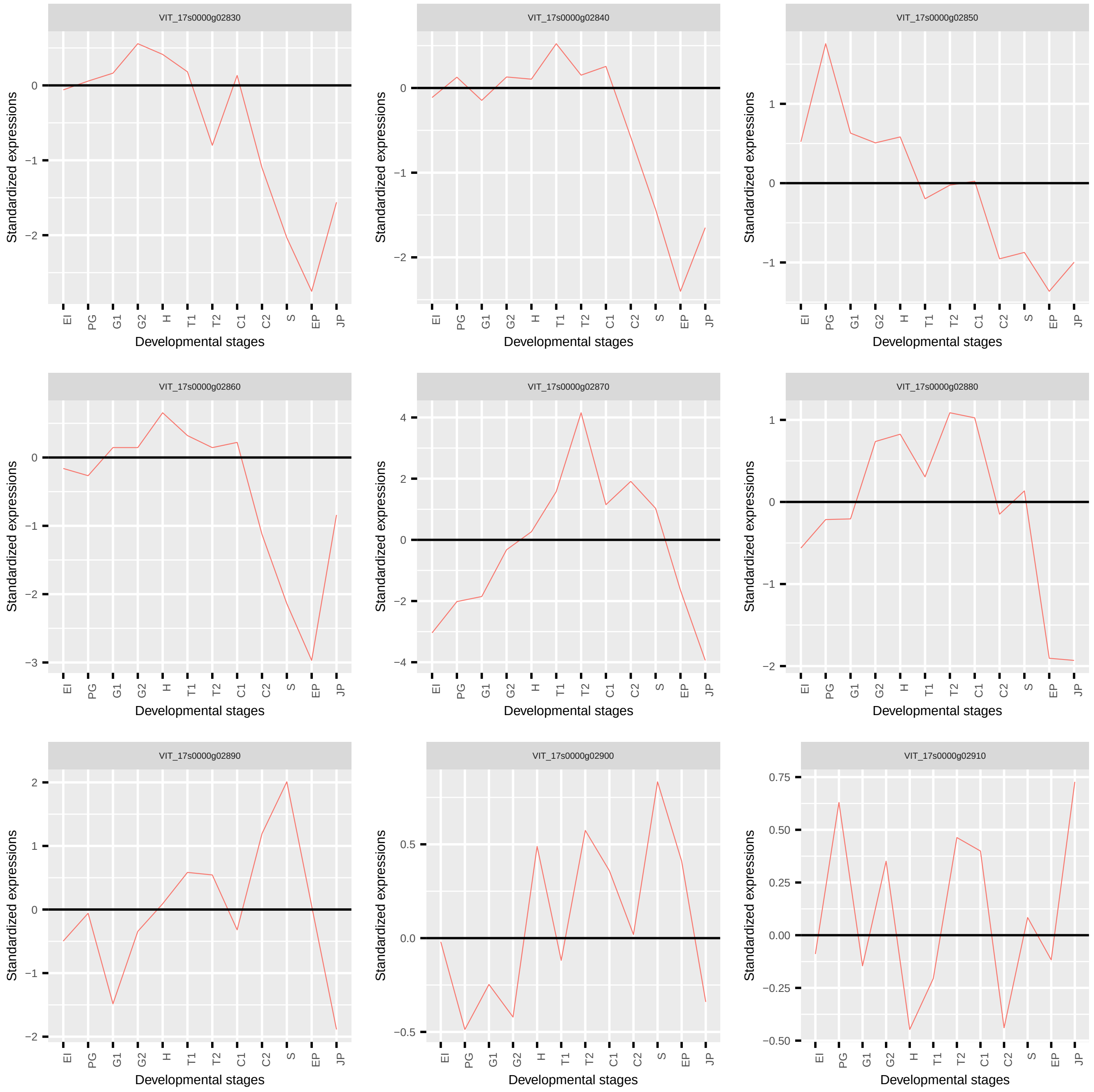
VIT_17s0000g02830
VIT_17s0000g02840
VIT_17s0000g02850
VIT_17s0000g02860
VIT_17s0000g02870
VIT_17s0000g02880
VIT_17s0000g02890
VIT_17s0000g02900
VIT_17s0000g02910
Standardized expressions
Developmental stages
EI PG G1 G2 H T1 T2 C1 C2 S EP JP

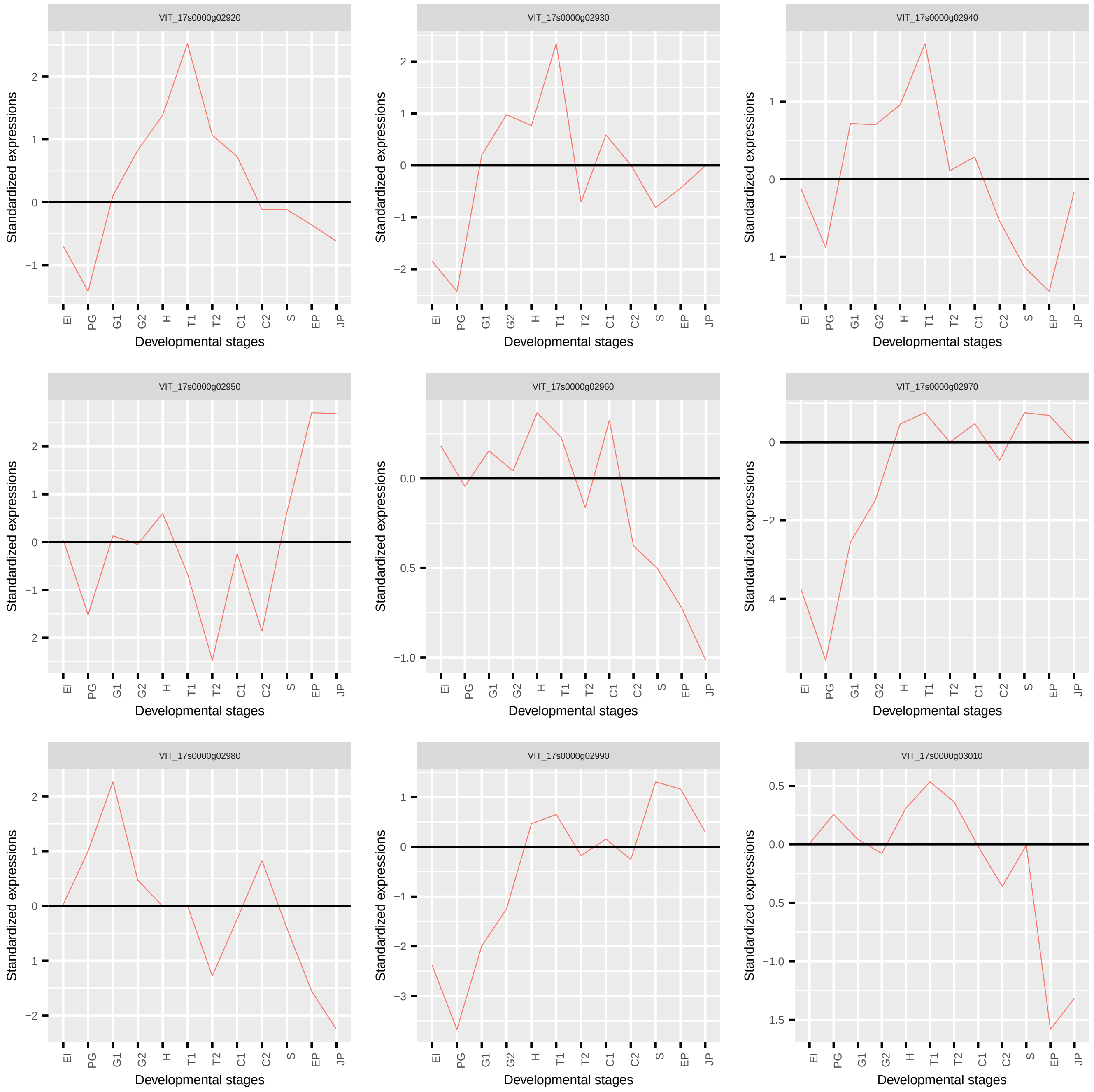

VIT_17s0000g02920
VIT_17s0000g02930
VIT_17s0000g02940
VIT_17s0000g02950
VIT_17s0000g02960
VIT_17s0000g02970
VIT_17s0000g02980
VIT_17s0000g02990
VIT_17s0000g03010
Standardized expressions
Developmental stages
EI PG G1 G2 H T1 T2 C1 C2 S EP JP

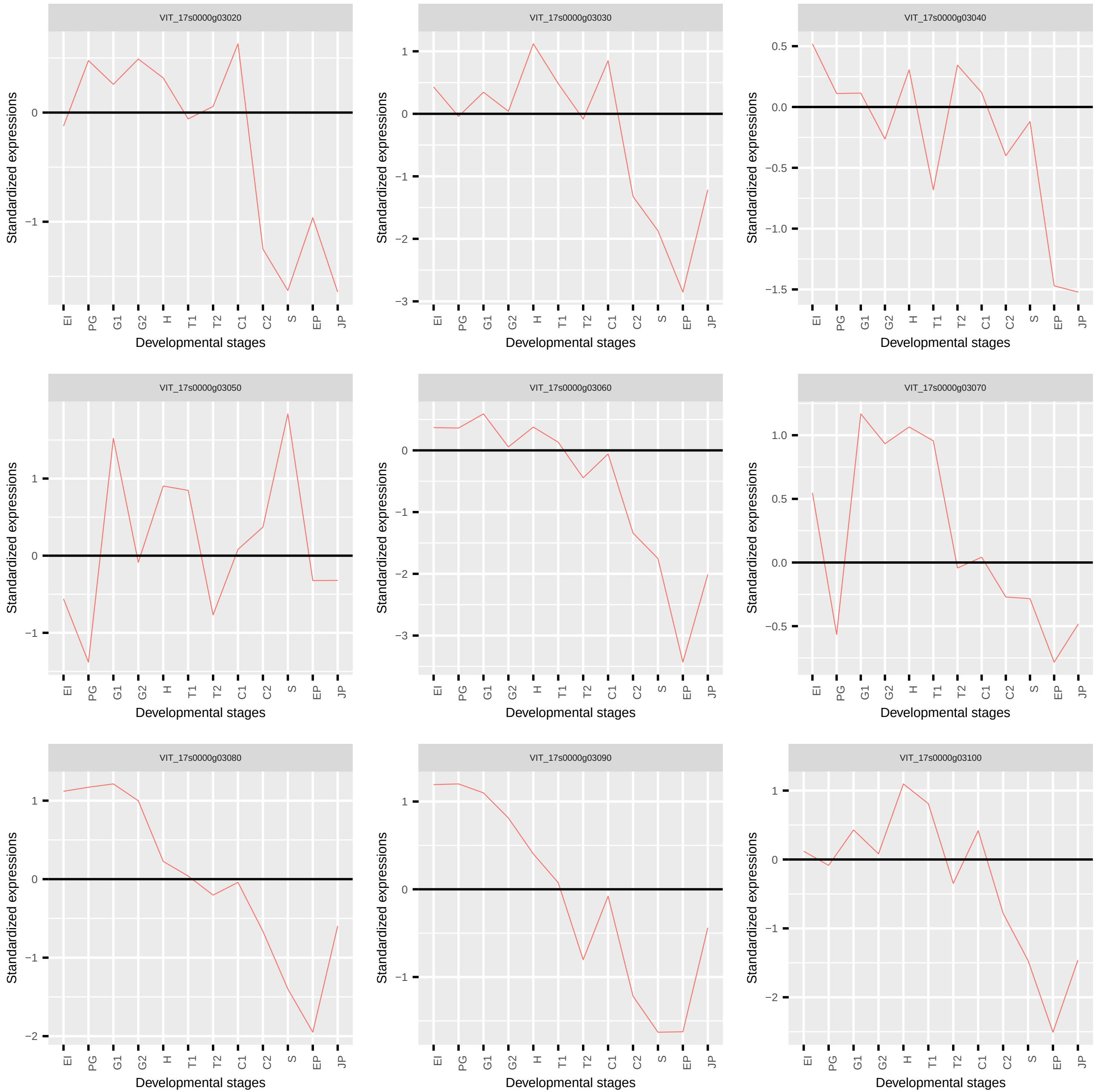
VIT_17s0000g03020
VIT_17s0000g03030
VIT_17s0000g03040
VIT_17s0000g03050
VIT_17s0000g03060
VIT_17s0000g03070
VIT_17s0000g03080
VIT_17s0000g03090
VIT_17s0000g03100
Standardized expressions
Developmental stages
EI PG G1 G2 H T1 T2 C1 C2 S EP JP

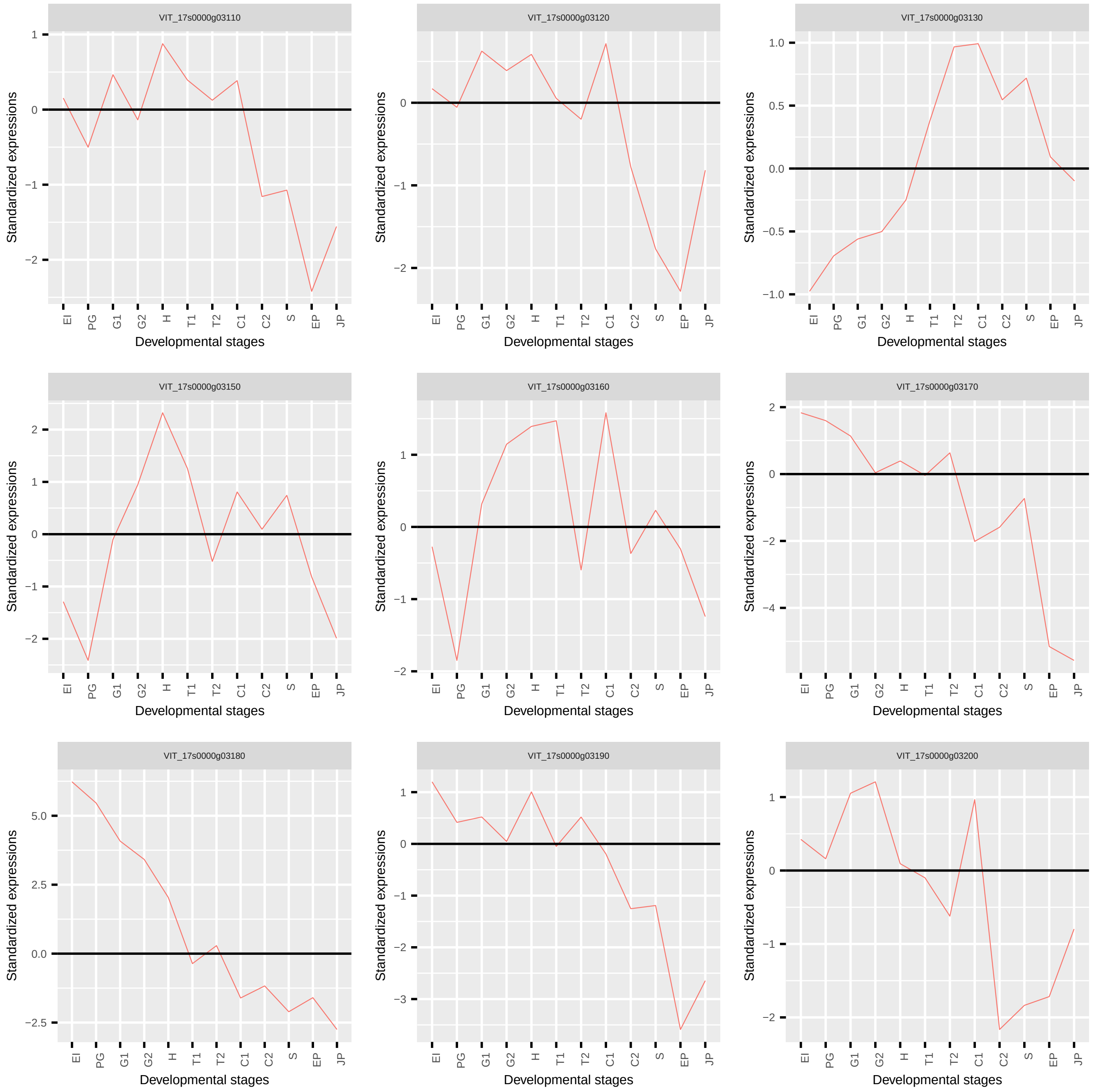
VIT_17s0000g03110
VIT_17s0000g03120
VIT_17s0000g03130
VIT_17s0000g03150
VIT_17s0000g03160
VIT_17s0000g03170
VIT_17s0000g03180
VIT_17s0000g03190
VIT_17s0000g03200
Standardized expressions
Developmental stages
EI
PG
G1
G2
H
T1
T2
C1
C2
S
EP
JP

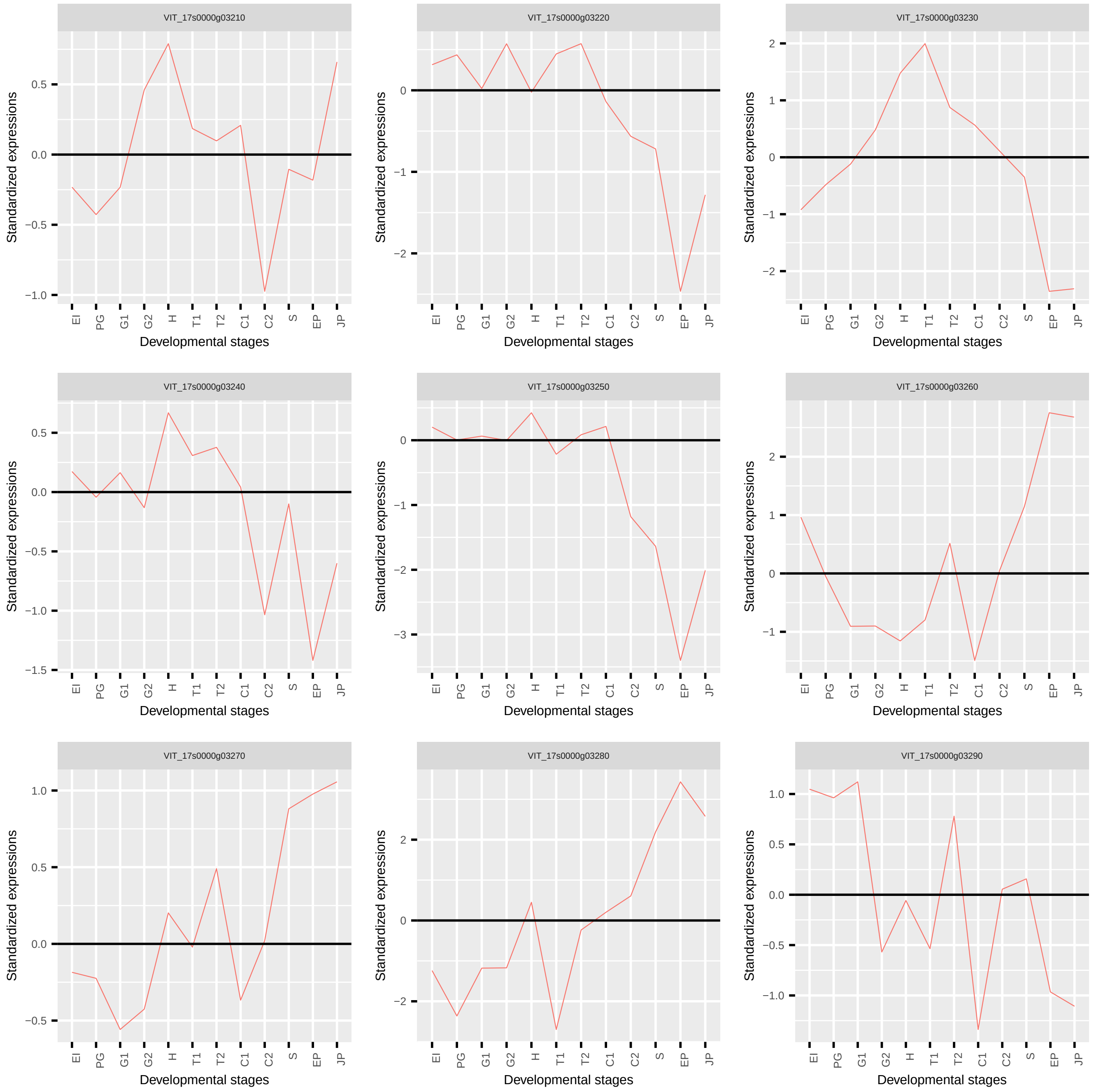

VIT_17s0000g03210
VIT_17s0000g03220
VIT_17s0000g03230
VIT_17s0000g03240
VIT_17s0000g03250
VIT_17s0000g03260
VIT_17s0000g03270
VIT_17s0000g03280
VIT_17s0000g03290
Standardized expressions
Developmental stages
EI PG G1 G2 H T1 T2 C1 C2 S EP JP

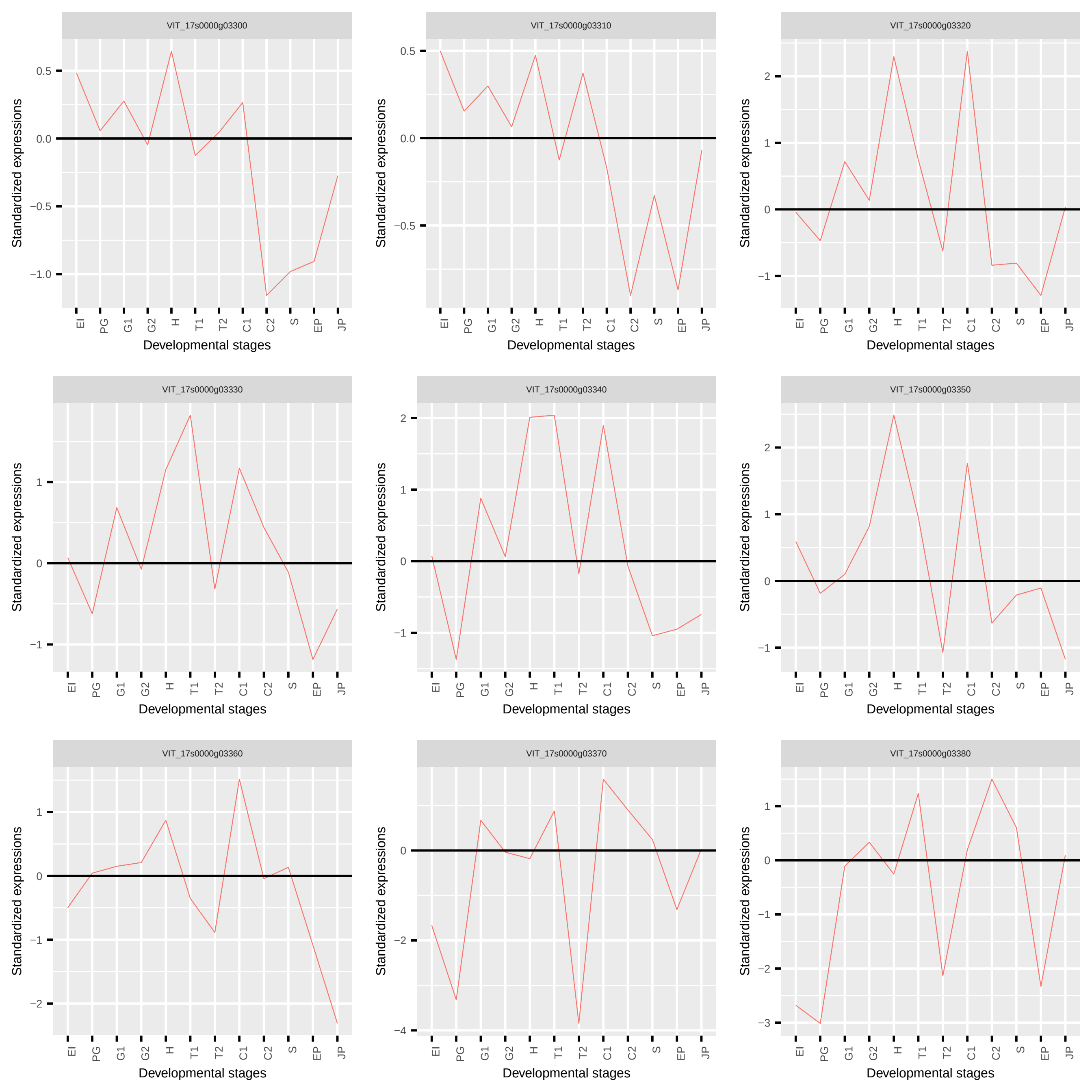

VIT_17s0000g03300
VIT_17s0000g03310
VIT_17s0000g03320
VIT_17s0000g03330
VIT_17s0000g03340
VIT_17s0000g03350
VIT_17s0000g03360
VIT_17s0000g03370
VIT_17s0000g03380
Standardized expressions
Developmental stages
EI
PG
G1
G2
H
T1
T2
C1
C2
S
EP
JP

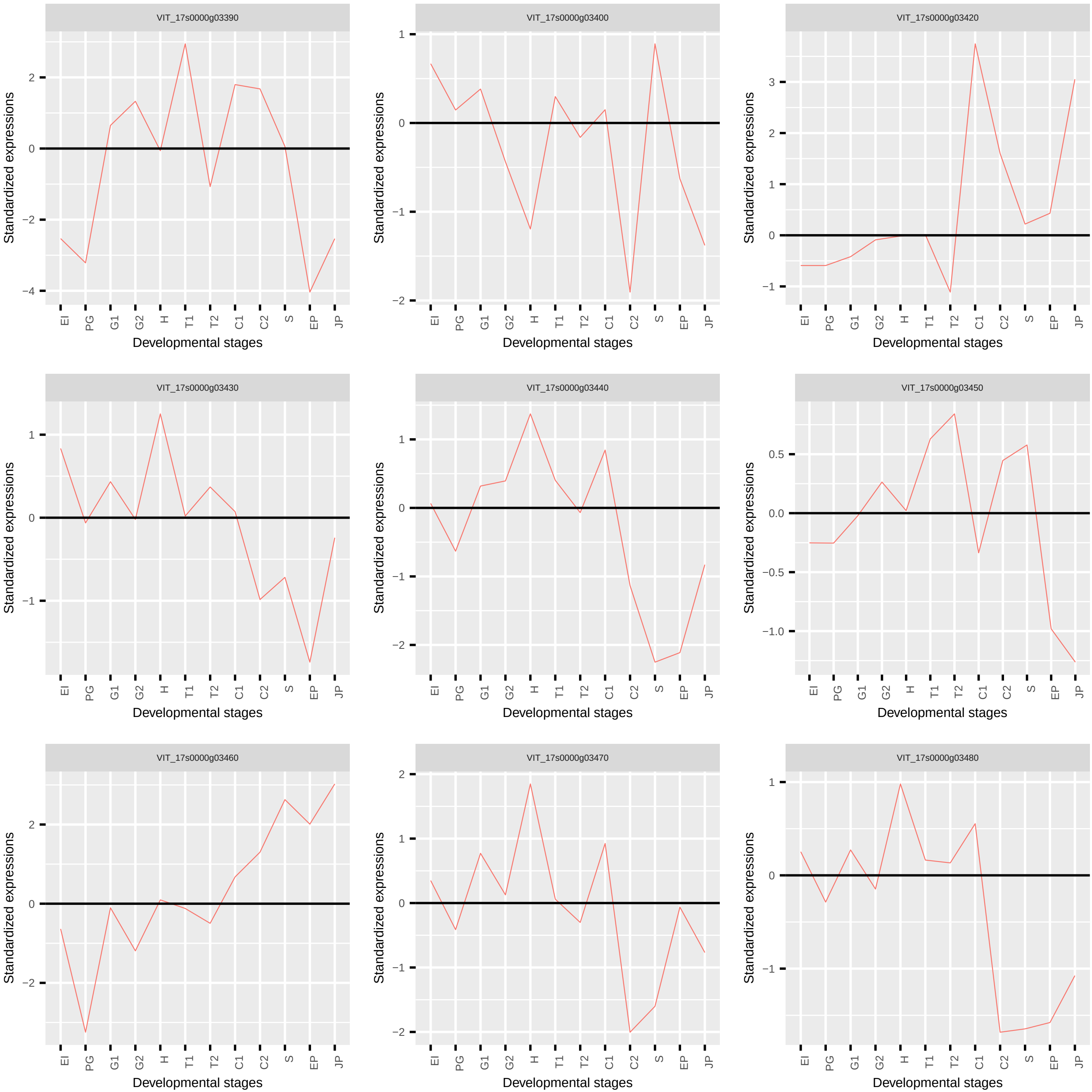
VIT_17s0000g03390
VIT_17s0000g03400
VIT_17s0000g03420
VIT_17s0000g03430
VIT_17s0000g03440
VIT_17s0000g03450
VIT_17s0000g03460
VIT_17s0000g03470
VIT_17s0000g03480
Standardized expressions
Developmental stages
EI PG G1 G2 H T1 T2 C1 C2 S EP JP

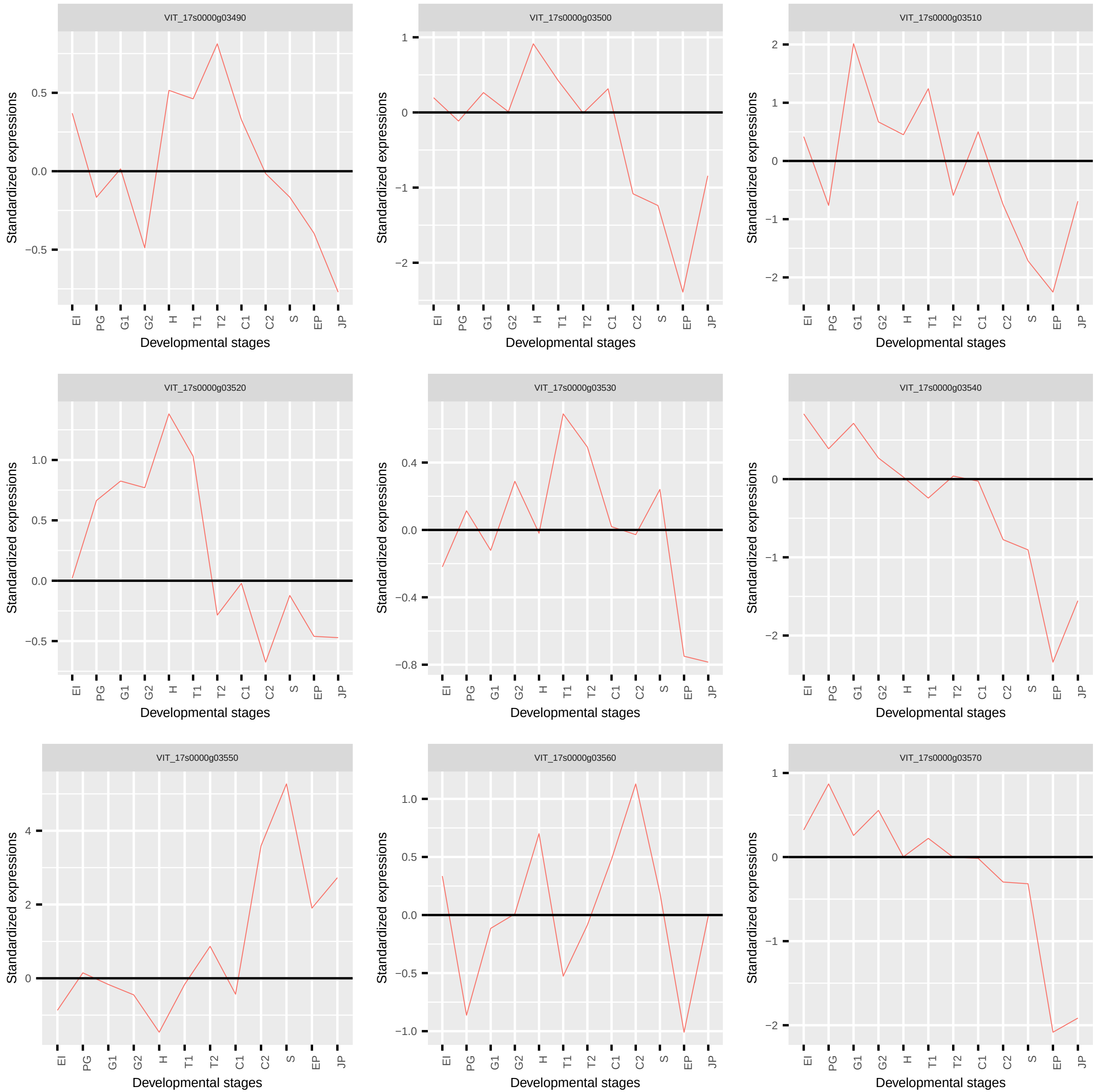
VIT_17s0000g03490
Standardized expressions
0.5
0.0
−0.5
EI PG G1 G2 H T1 T2 C1 C2 S EP JP
Developmental stages
VIT_17s0000g03500
Standardized expressions
1
0
−1
−2
EI PG G1 G2 H T1 T2 C1 C2 S EP JP
Developmental stages
VIT_17s0000g03510
Standardized expressions
2
1
0
−1
−2
EI PG G1 G2 H T1 T2 C1 C2 S EP JP
Developmental stages
VIT_17s0000g03520
Standardized expressions
1.0
0.5
0.0
−0.5
EI PG G1 G2 H T1 T2 C1 C2 S EP JP
Developmental stages
VIT_17s0000g03530
Standardized expressions
0.4
0.0
−0.4
−0.8
EI PG G1 G2 H T1 T2 C1 C2 S EP JP
Developmental stages
VIT_17s0000g03540
Standardized expressions
0
−1
−2
EI PG G1 G2 H T1 T2 C1 C2 S EP JP
Developmental stages
VIT_17s0000g03550
Standardized expressions
4
2
0
EI PG G1 G2 H T1 T2 C1 C2 S EP JP
Developmental stages
VIT_17s0000g03560
Standardized expressions
1.0
0.5
0.0
−0.5
−1.0
EI PG G1 G2 H T1 T2 C1 C2 S EP JP
Developmental stages
VIT_17s0000g03570
Standardized expressions
1
0
−1
−2
EI PG G1 G2 H T1 T2 C1 C2 S EP JP
Developmental stages

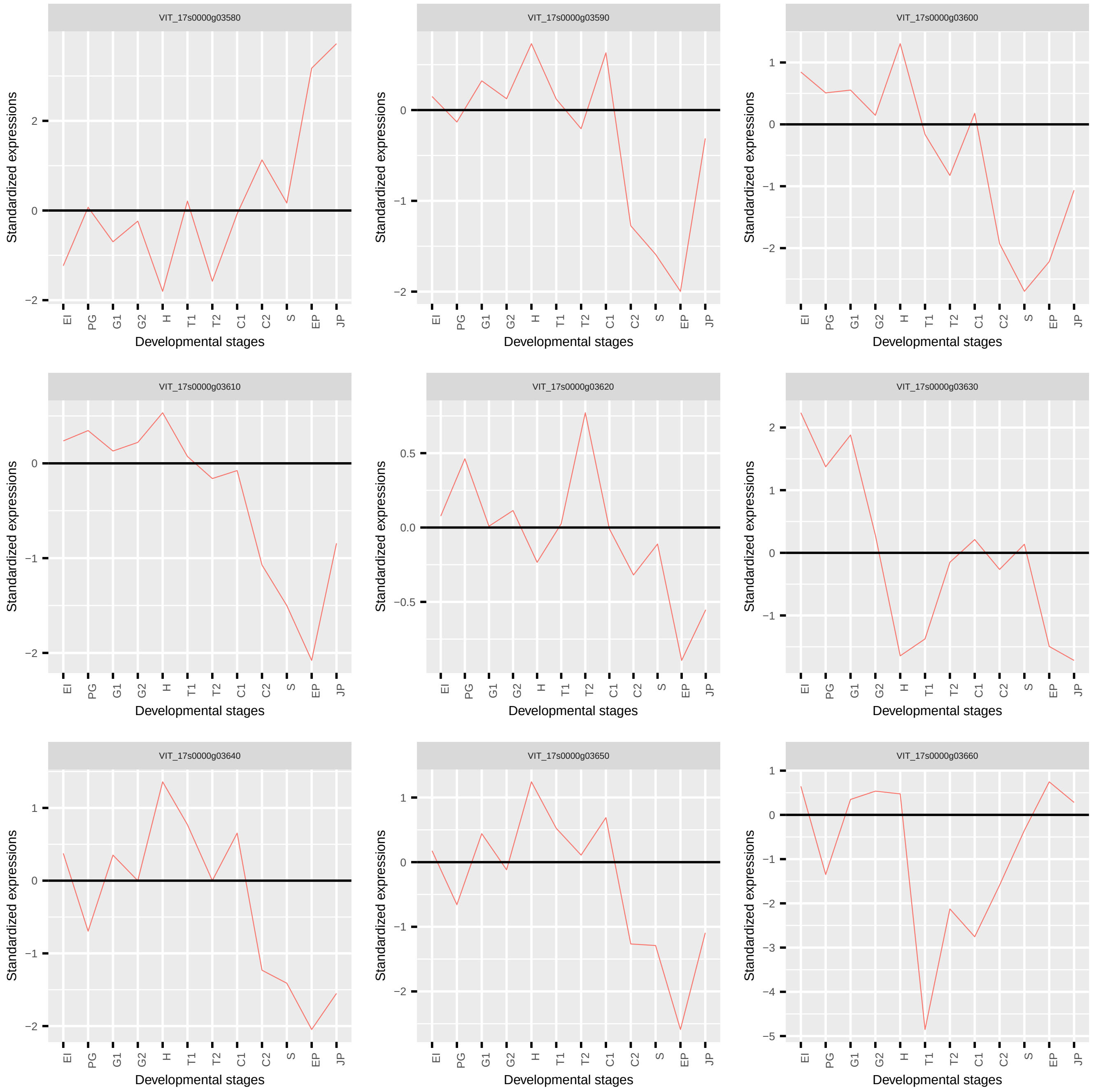
VIT_17s0000g03580
VIT_17s0000g03590
VIT_17s0000g03600
VIT_17s0000g03610
VIT_17s0000g03620
VIT_17s0000g03630
VIT_17s0000g03640
VIT_17s0000g03650
VIT_17s0000g03660
Standardized expressions
Developmental stages
EI
PG
G1
G2
H
T1
T2
C1
C2
S
EP
JP

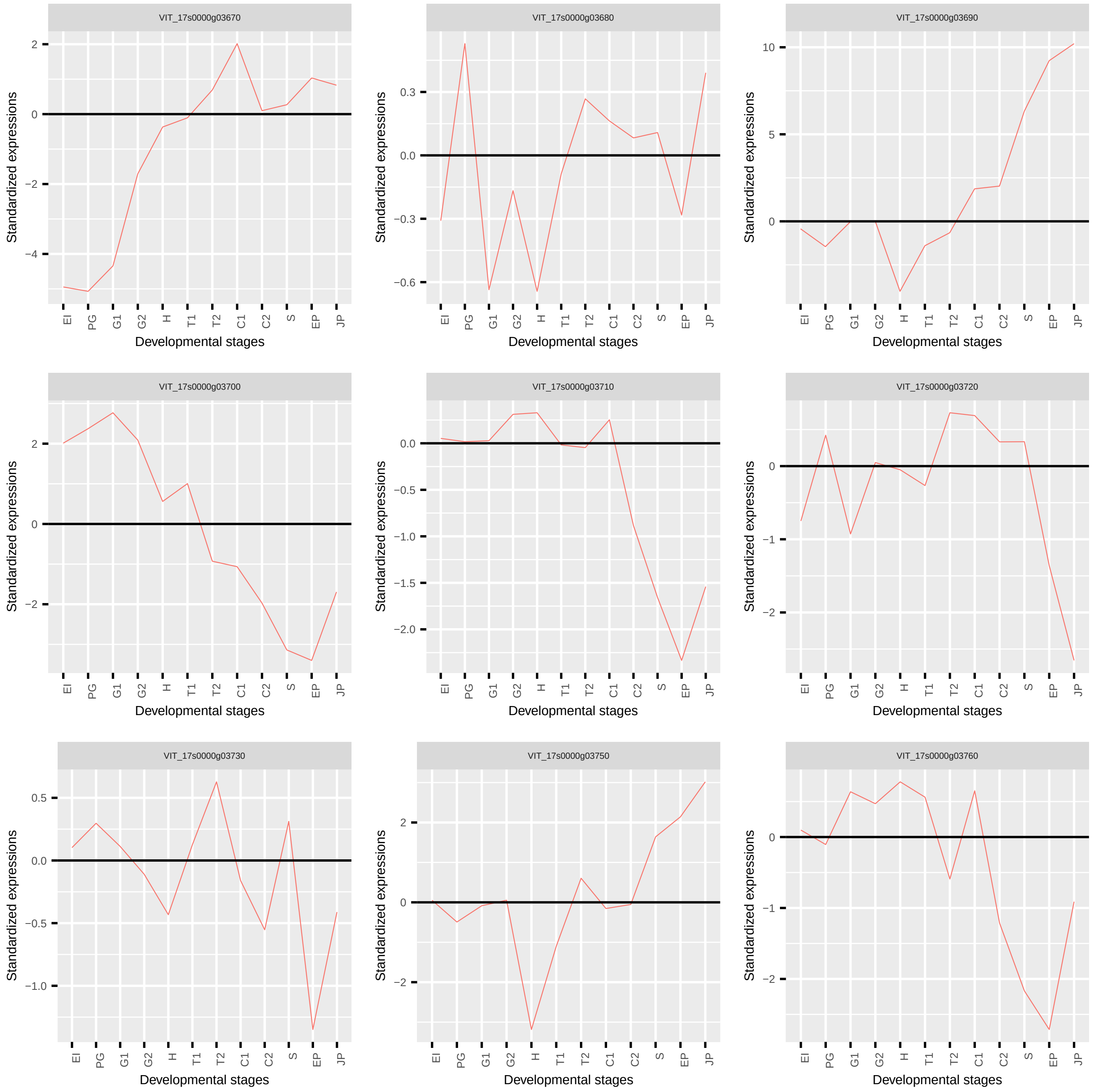
VIT_17s0000g03670
2
0
−2
−4
Standardized expressions
EI PG G1 G2 H T1 T2 C1 C2 S EP JP
Developmental stages
VIT_17s0000g03680
0.3
0.0
−0.3
−0.6
Standardized expressions
EI PG G1 G2 H T1 T2 C1 C2 S EP JP
Developmental stages
VIT_17s0000g03690
10
5
0
Standardized expressions
EI PG G1 G2 H T1 T2 C1 C2 S EP JP
Developmental stages
VIT_17s0000g03700
2
0
−2
Standardized expressions
EI PG G1 G2 H T1 T2 C1 C2 S EP JP
Developmental stages
VIT_17s0000g03710
0.0
−0.5
−1.0
−1.5
−2.0
Standardized expressions
EI PG G1 G2 H T1 T2 C1 C2 S EP JP
Developmental stages
VIT_17s0000g03720
0
−1
−2
Standardized expressions
EI PG G1 G2 H T1 T2 C1 C2 S EP JP
Developmental stages
VIT_17s0000g03730
0.5
0.0
−0.5
−1.0
Standardized expressions
EI PG G1 G2 H T1 T2 C1 C2 S EP JP
Developmental stages
VIT_17s0000g03750
2
0
−2
Standardized expressions
EI PG G1 G2 H T1 T2 C1 C2 S EP JP
Developmental stages
VIT_17s0000g03760
0
−1
−2
Standardized expressions
EI PG G1 G2 H T1 T2 C1 C2 S EP JP
Developmental stages

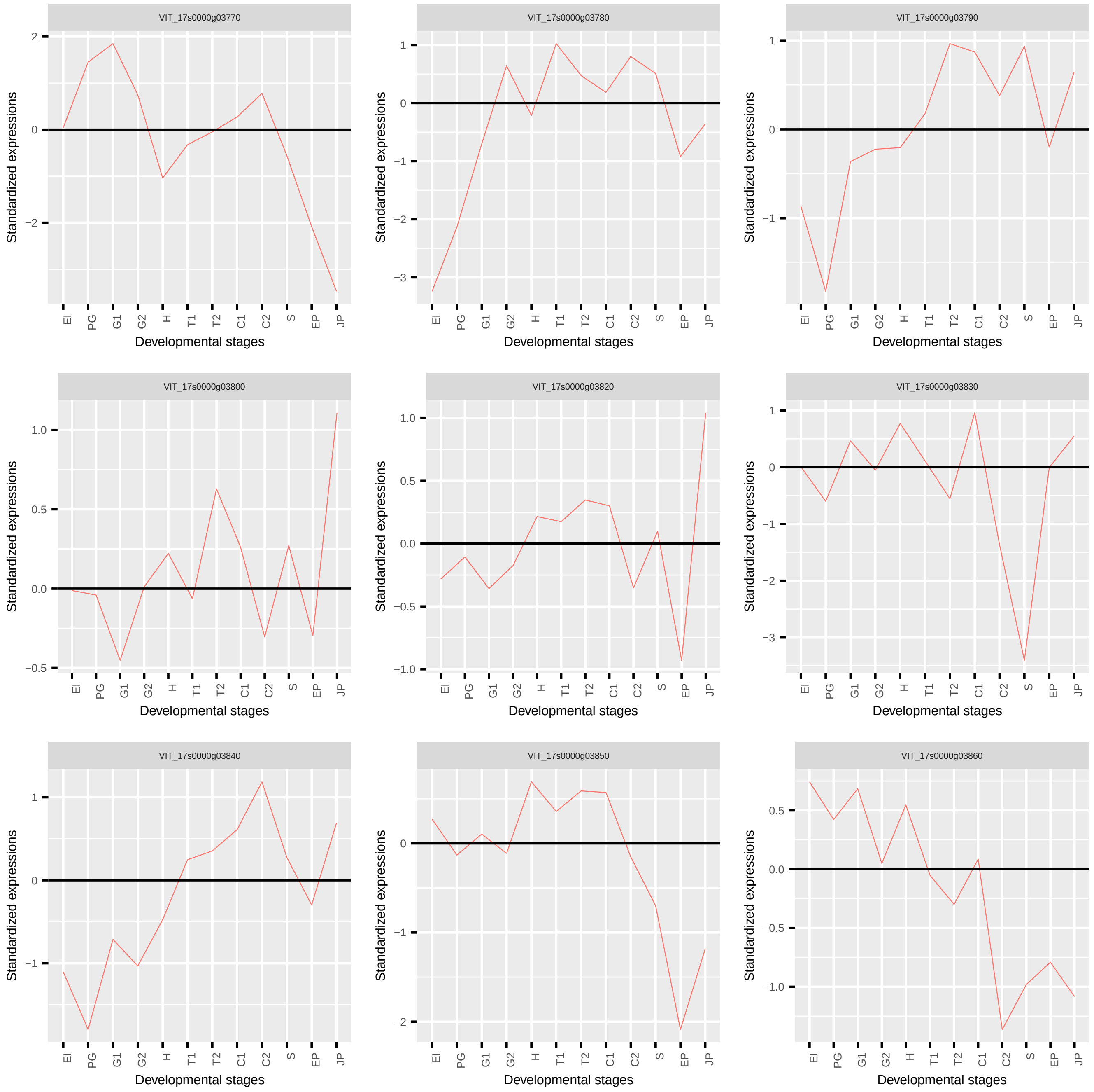

VIT_17s0000g03770
VIT_17s0000g03780
VIT_17s0000g03790
VIT_17s0000g03800
VIT_17s0000g03820
VIT_17s0000g03830
VIT_17s0000g03840
VIT_17s0000g03850
VIT_17s0000g03860
Standardized expressions
Developmental stages
EI PG G1 G2 H T1 T2 C1 C2 S EP JP

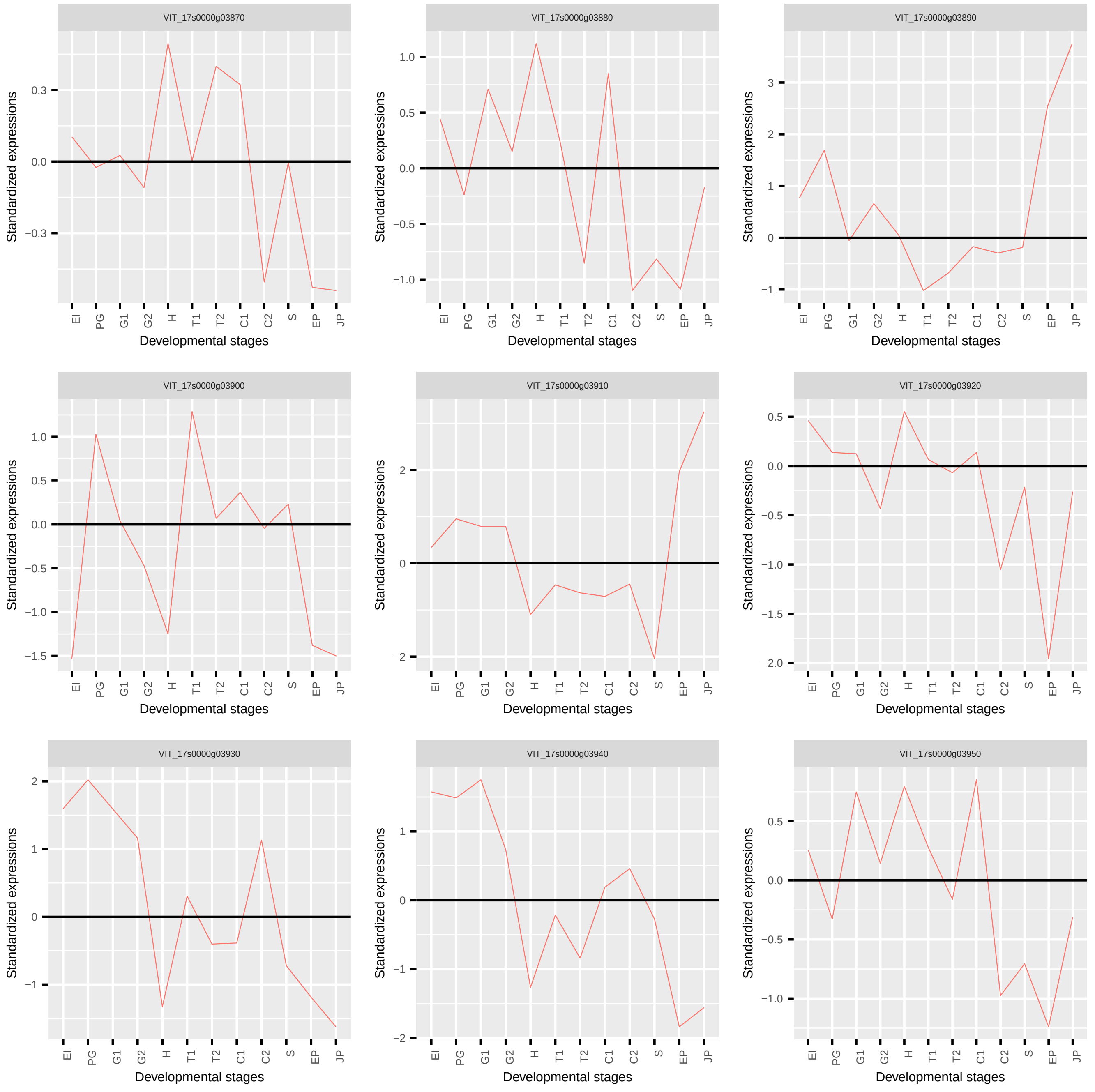
VIT_17s0000g03870
VIT_17s0000g03880
VIT_17s0000g03890
VIT_17s0000g03900
VIT_17s0000g03910
VIT_17s0000g03920
VIT_17s0000g03930
VIT_17s0000g03940
VIT_17s0000g03950
Standardized expressions
Developmental stages
EI
PG
G1
G2
H
T1
T2
C1
C2
S
EP
JP

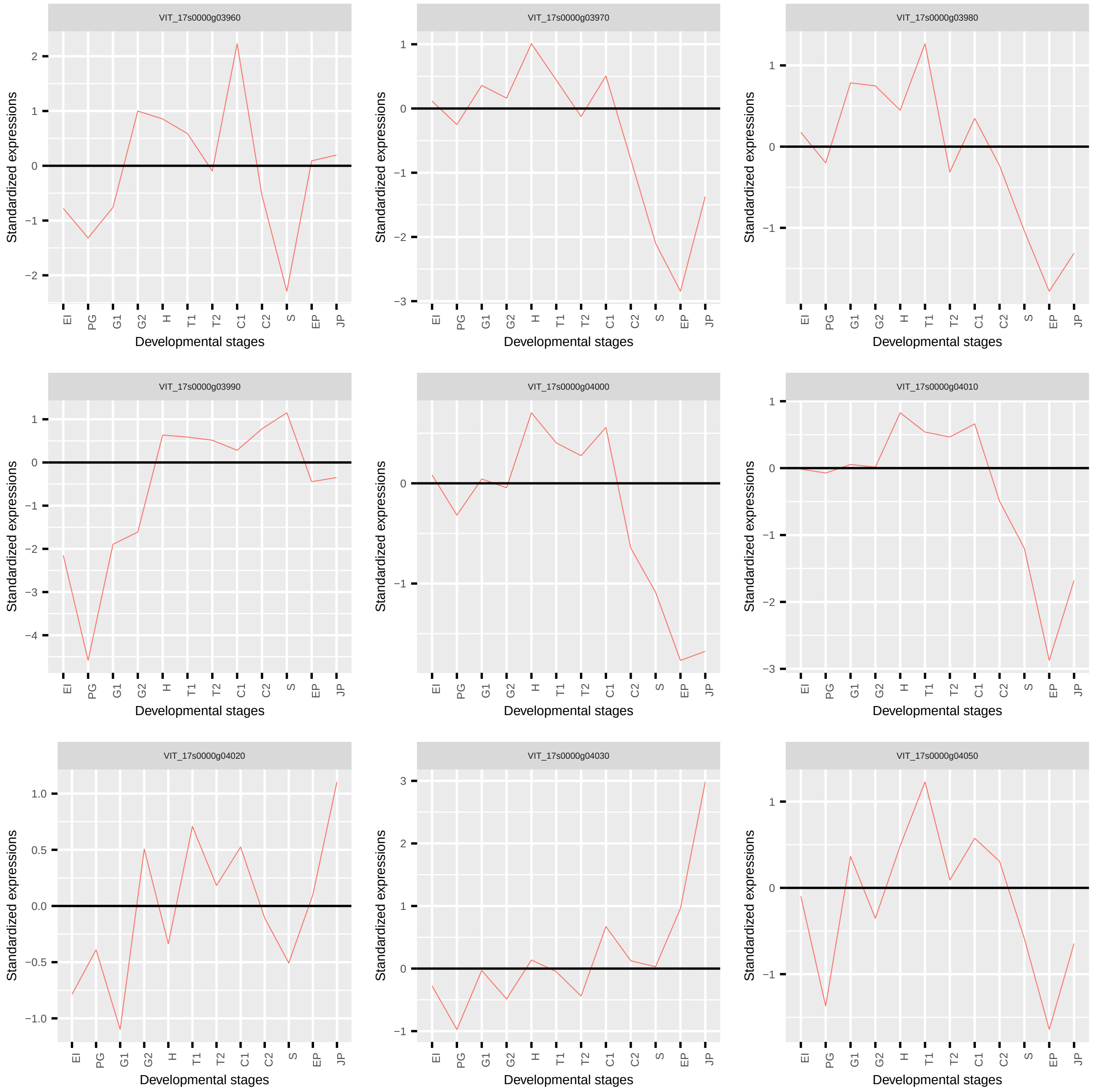
VIT_17s0000g03960
VIT_17s0000g03970
VIT_17s0000g03980
VIT_17s0000g03990
VIT_17s0000g04000
VIT_17s0000g04010
VIT_17s0000g04020
VIT_17s0000g04030
VIT_17s0000g04050
Standardized expressions
Developmental stages
EI PG G1 G2 H T1 T2 C1 C2 S EP JP

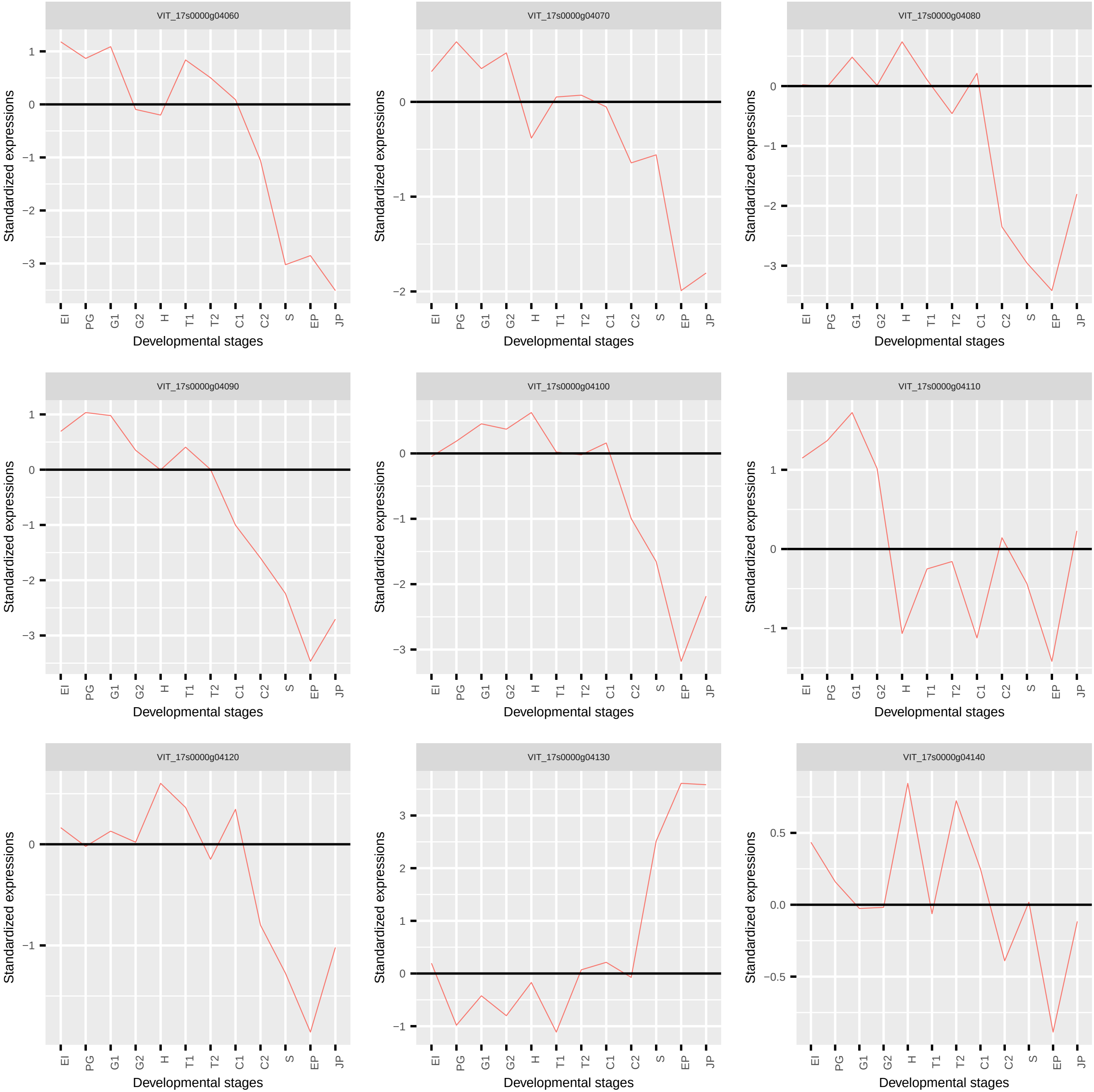
VIT_17s0000g04060
VIT_17s0000g04070
VIT_17s0000g04080
VIT_17s0000g04090
VIT_17s0000g04100
VIT_17s0000g04110
VIT_17s0000g04120
VIT_17s0000g04130
VIT_17s0000g04140
Standardized expressions
Developmental stages
EI PG G1 G2 H T1 T2 C1 C2 S EP JP
1 0 −1 −2 −3
0 −1 −2
0 −1 −2 −3
1 0 −1 −2 −3
0 −1 −2 −3
1 0 −1
0 −1
3 2 1 0 −1
0.5 0.0 −0.5

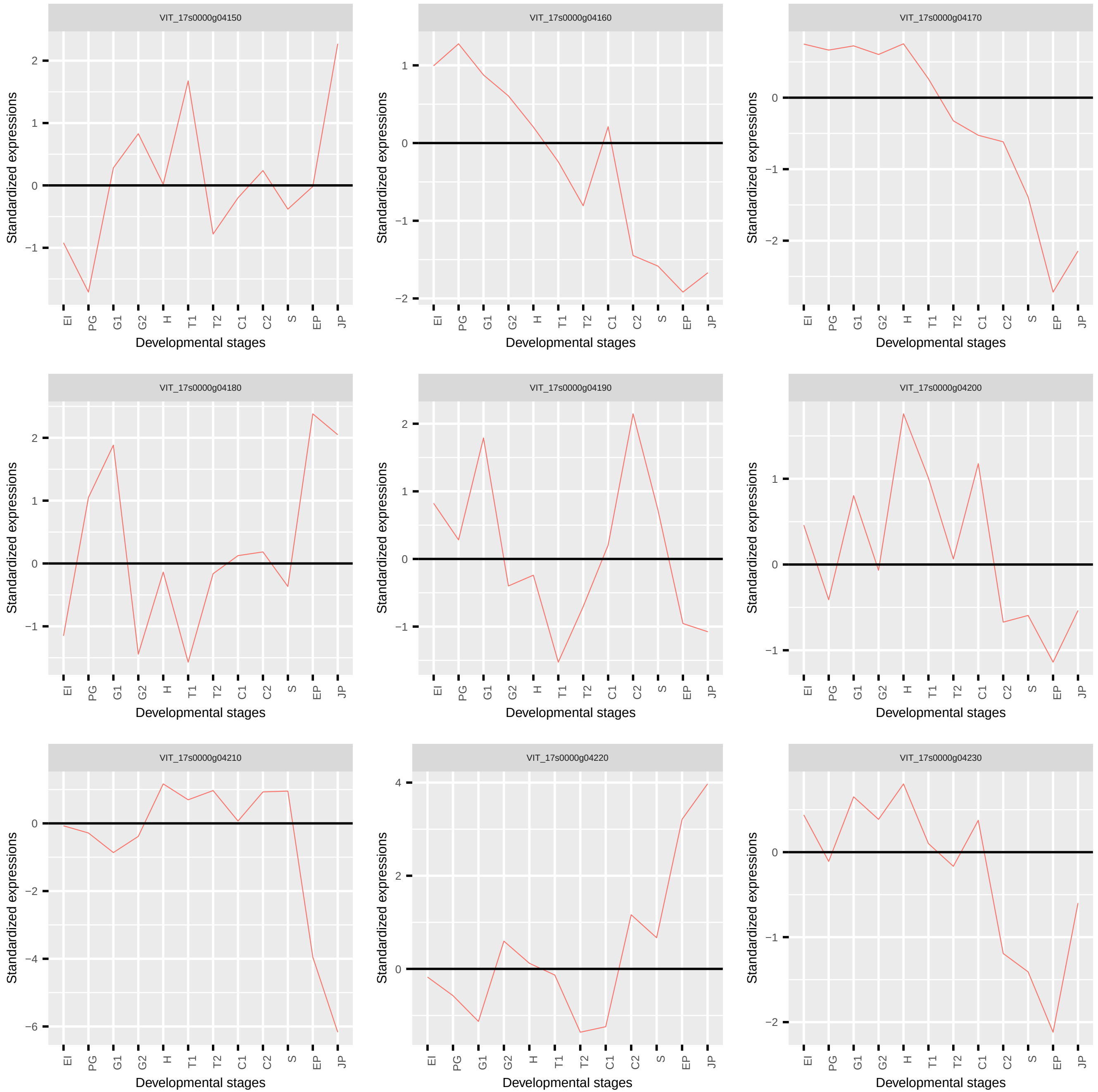

VIT_17s0000g04150
VIT_17s0000g04160
VIT_17s0000g04170
VIT_17s0000g04180
VIT_17s0000g04190
VIT_17s0000g04200
VIT_17s0000g04210
VIT_17s0000g04220
VIT_17s0000g04230
Standardized expressions
Developmental stages
EI PG G1 G2 H T1 T2 C1 C2 S EP JP

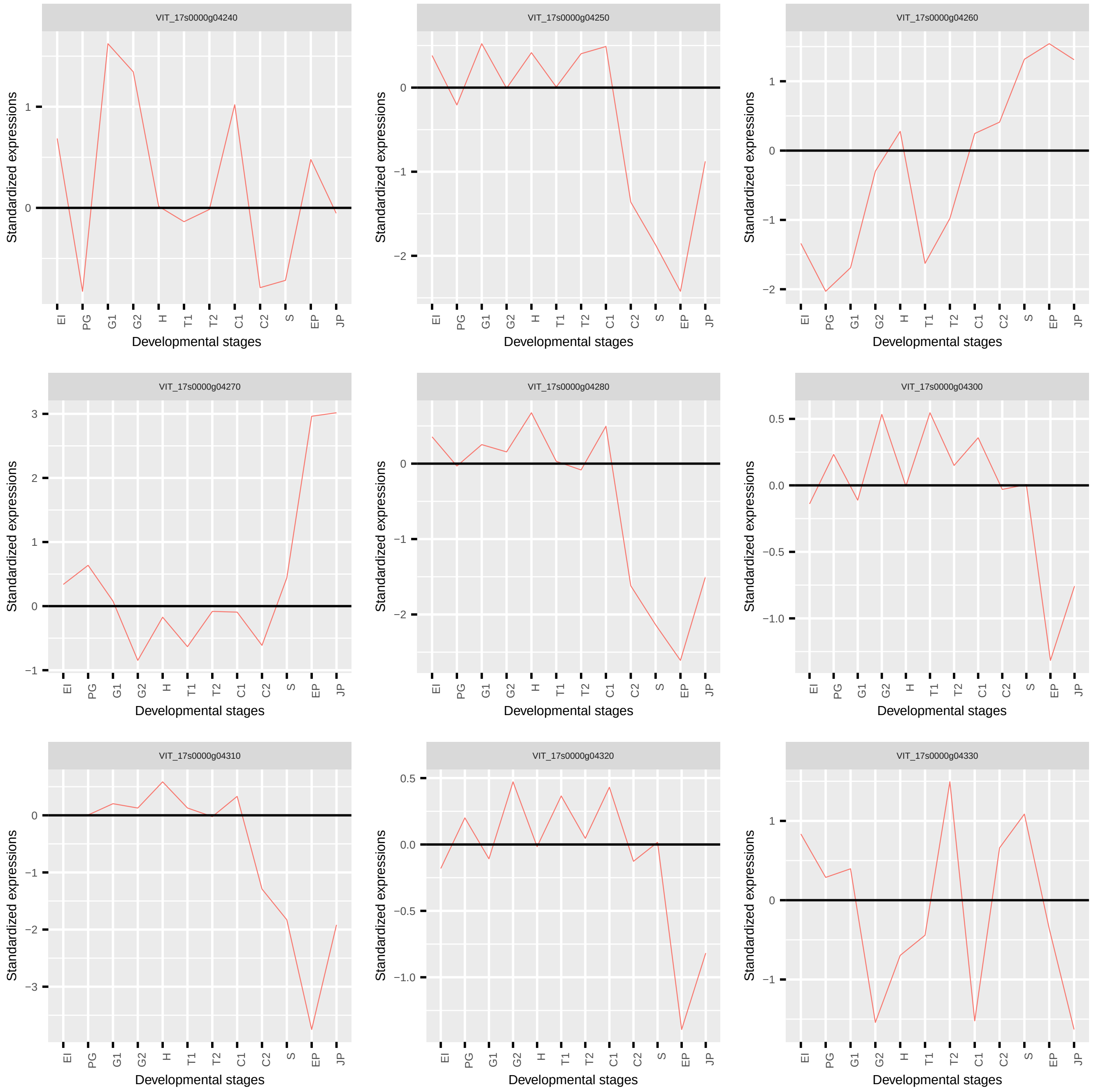
VIT_17s0000g04240
VIT_17s0000g04250
VIT_17s0000g04260
VIT_17s0000g04270
VIT_17s0000g04280
VIT_17s0000g04300
VIT_17s0000g04310
VIT_17s0000g04320
VIT_17s0000g04330
Standardized expressions
Developmental stages
EI
PG
G1
G2
H
T1
T2
C1
C2
S
EP
JP

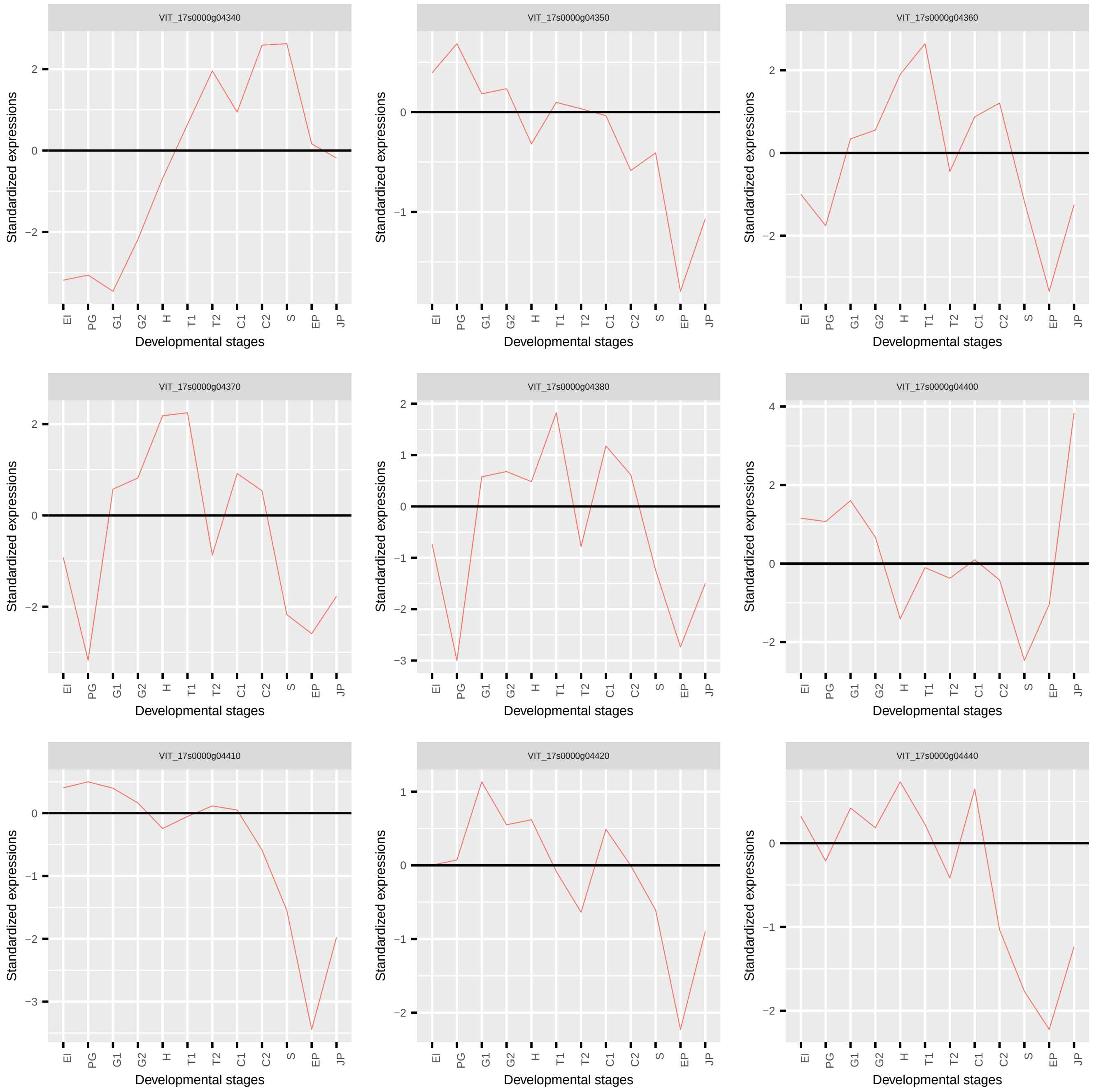
VIT_17s0000g04340
VIT_17s0000g04350
VIT_17s0000g04360
VIT_17s0000g04370
VIT_17s0000g04380
VIT_17s0000g04400
VIT_17s0000g04410
VIT_17s0000g04420
VIT_17s0000g04440
Standardized expressions
Developmental stages
EI PG G1 G2 H T1 T2 C1 C2 S EP JP

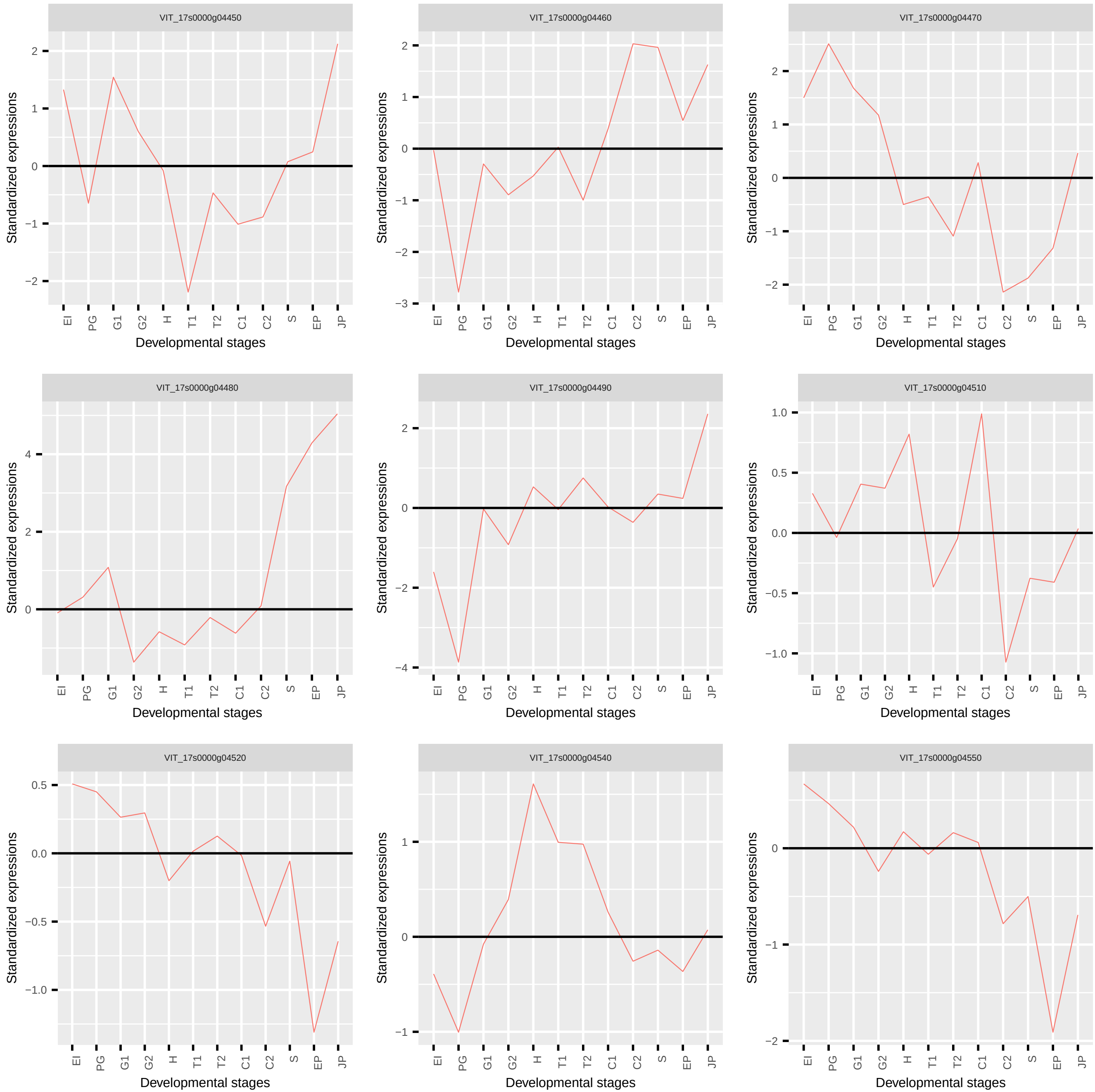

VIT_17s0000g04450
VIT_17s0000g04460
VIT_17s0000g04470
VIT_17s0000g04480
VIT_17s0000g04490
VIT_17s0000g04510
VIT_17s0000g04520
VIT_17s0000g04540
VIT_17s0000g04550
Standardized expressions
Developmental stages
EI PG G1 G2 H T1 T2 C1 C2 S EP JP

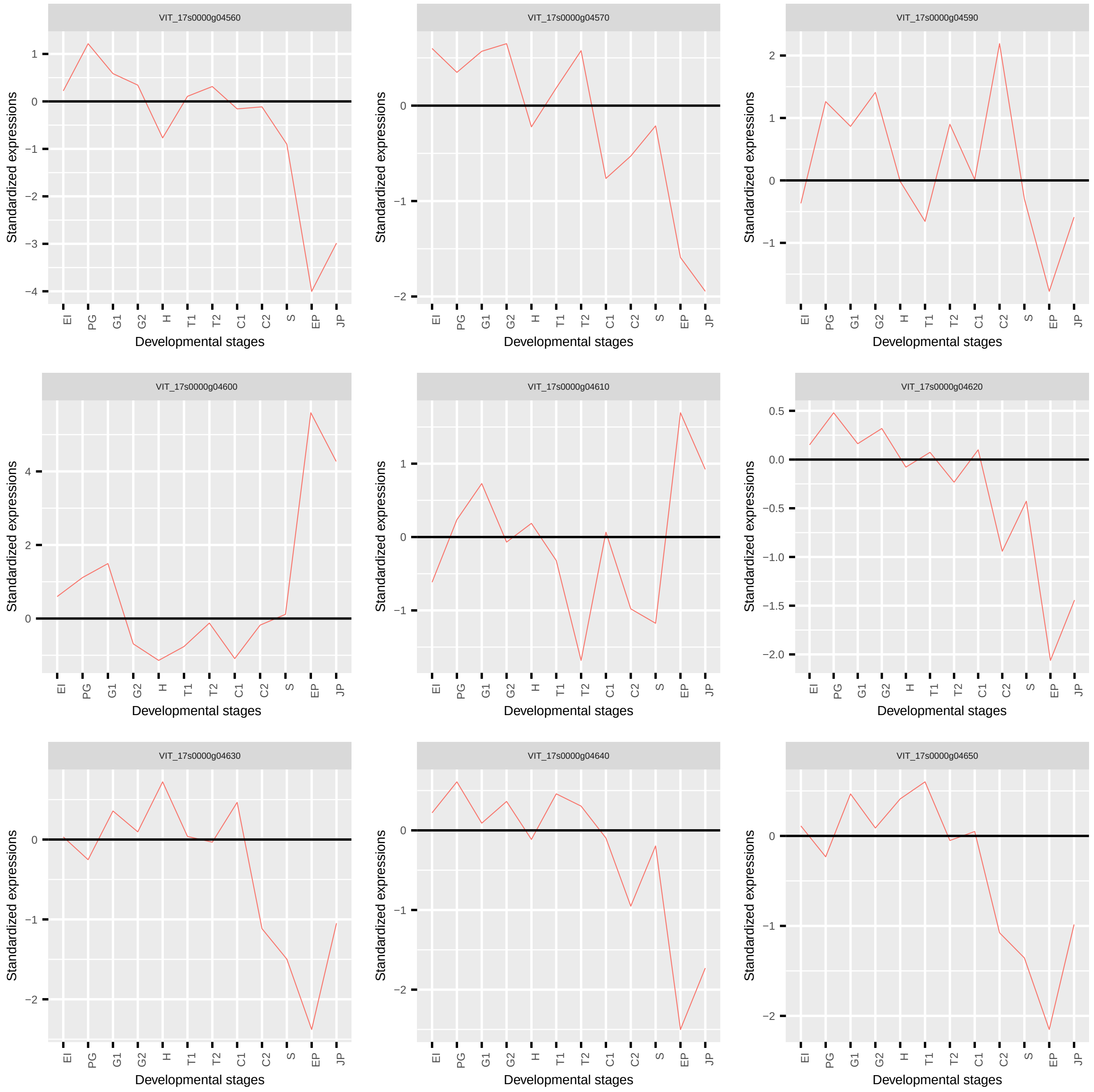
VIT_17s0000g04560
VIT_17s0000g04570
VIT_17s0000g04590
VIT_17s0000g04600
VIT_17s0000g04610
VIT_17s0000g04620
VIT_17s0000g04630
VIT_17s0000g04640
VIT_17s0000g04650
Standardized expressions
Developmental stages
EI
PG
G1
G2
H
T1
T2
C1
C2
S
EP
JP

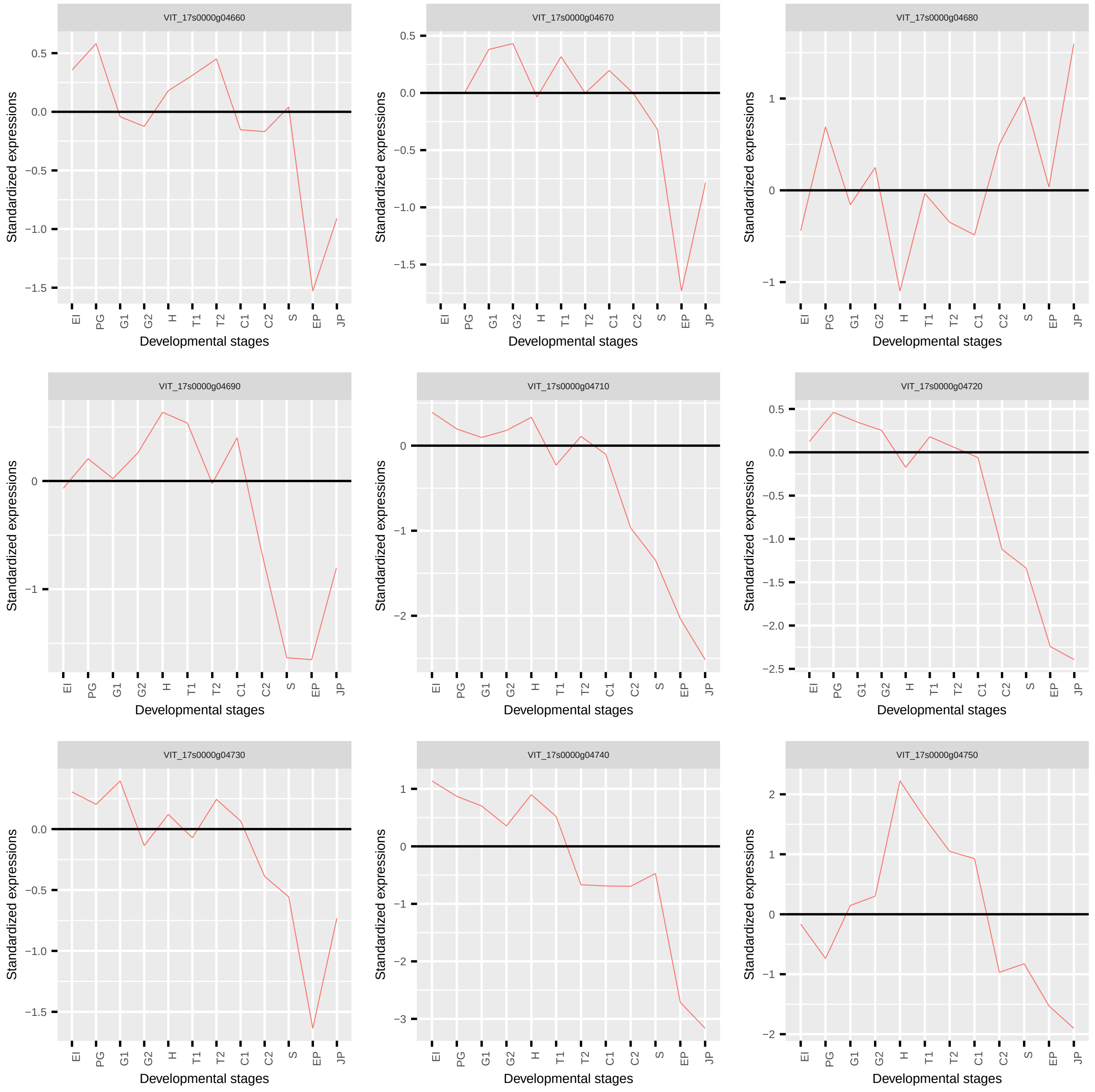

VIT_17s0000g04660
VIT_17s0000g04670
VIT_17s0000g04680
VIT_17s0000g04690
VIT_17s0000g04710
VIT_17s0000g04720
VIT_17s0000g04730
VIT_17s0000g04740
VIT_17s0000g04750
Standardized expressions
Developmental stages
EI PG G1 G2 H T1 T2 C1 C2 S EP JP

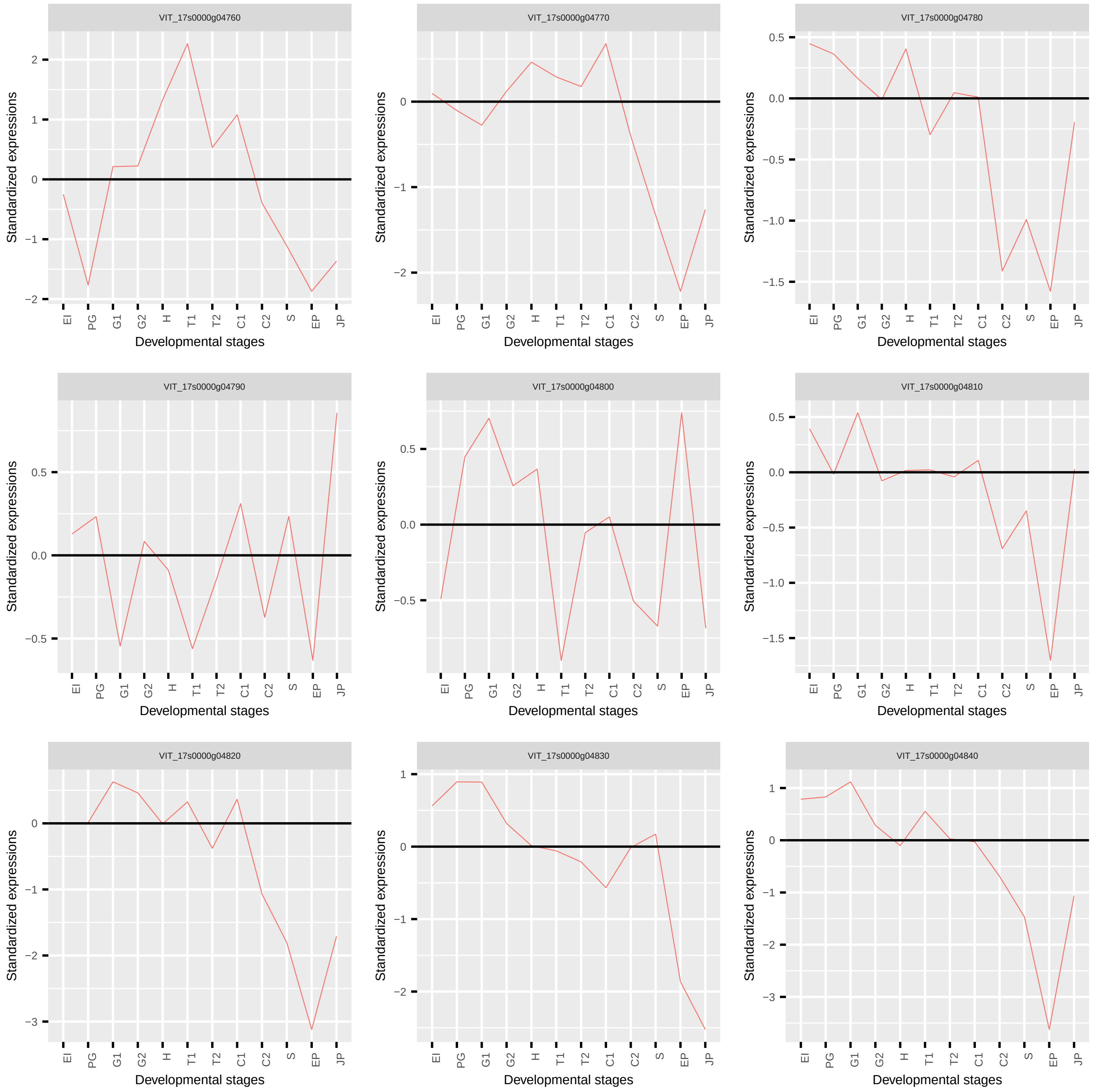
VIT_17s0000g04760
VIT_17s0000g04770
VIT_17s0000g04780
VIT_17s0000g04790
VIT_17s0000g04800
VIT_17s0000g04810
VIT_17s0000g04820
VIT_17s0000g04830
VIT_17s0000g04840
Standardized expressions
Developmental stages
EI PG G1 G2 H T1 T2 C1 C2 S EP JP

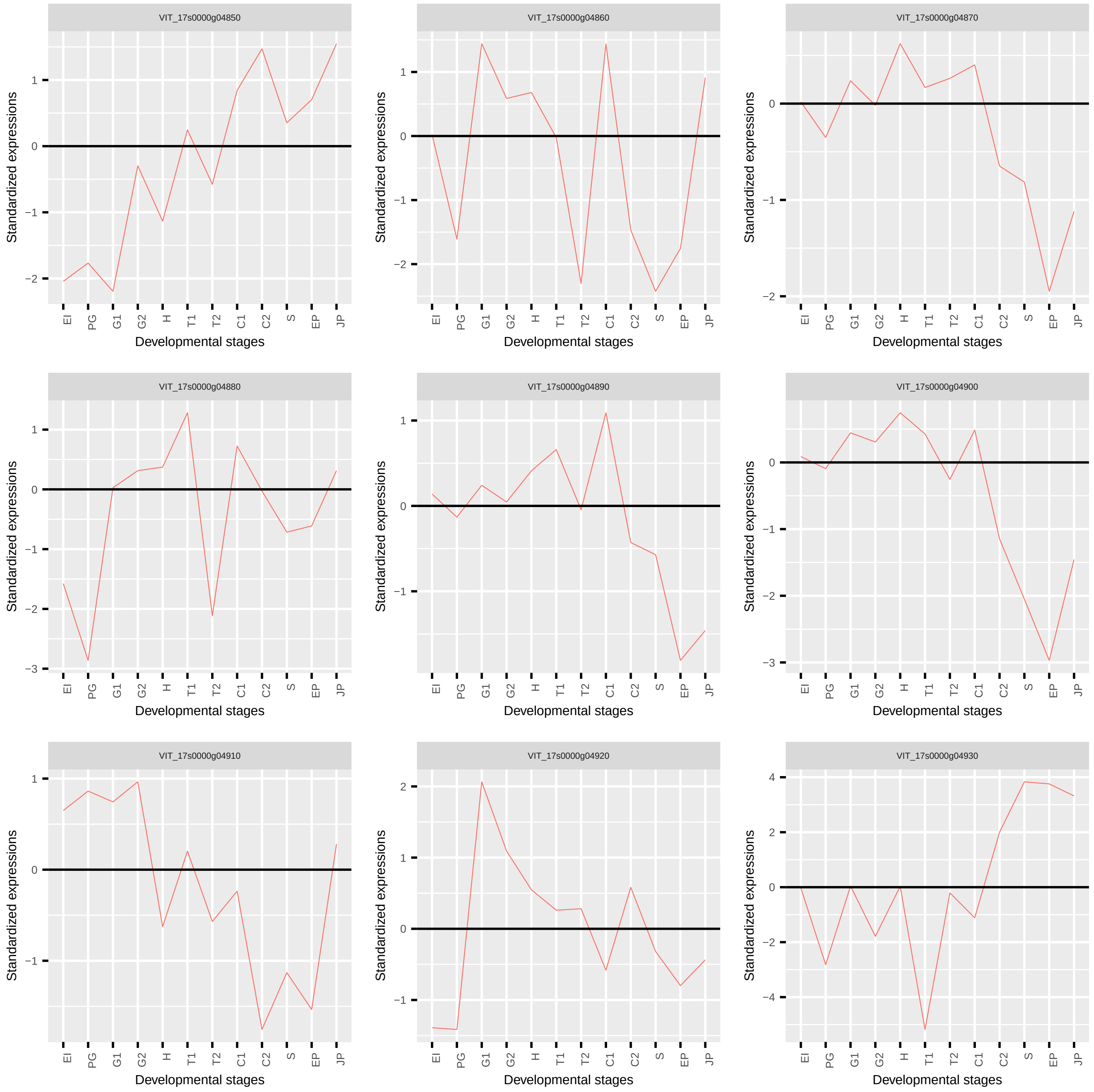

VIT_17s0000g04850
VIT_17s0000g04860
VIT_17s0000g04870
VIT_17s0000g04880
VIT_17s0000g04890
VIT_17s0000g04900
VIT_17s0000g04910
VIT_17s0000g04920
VIT_17s0000g04930
Standardized expressions
Developmental stages
EI PG G1 G2 H T1 T2 C1 C2 S EP JP

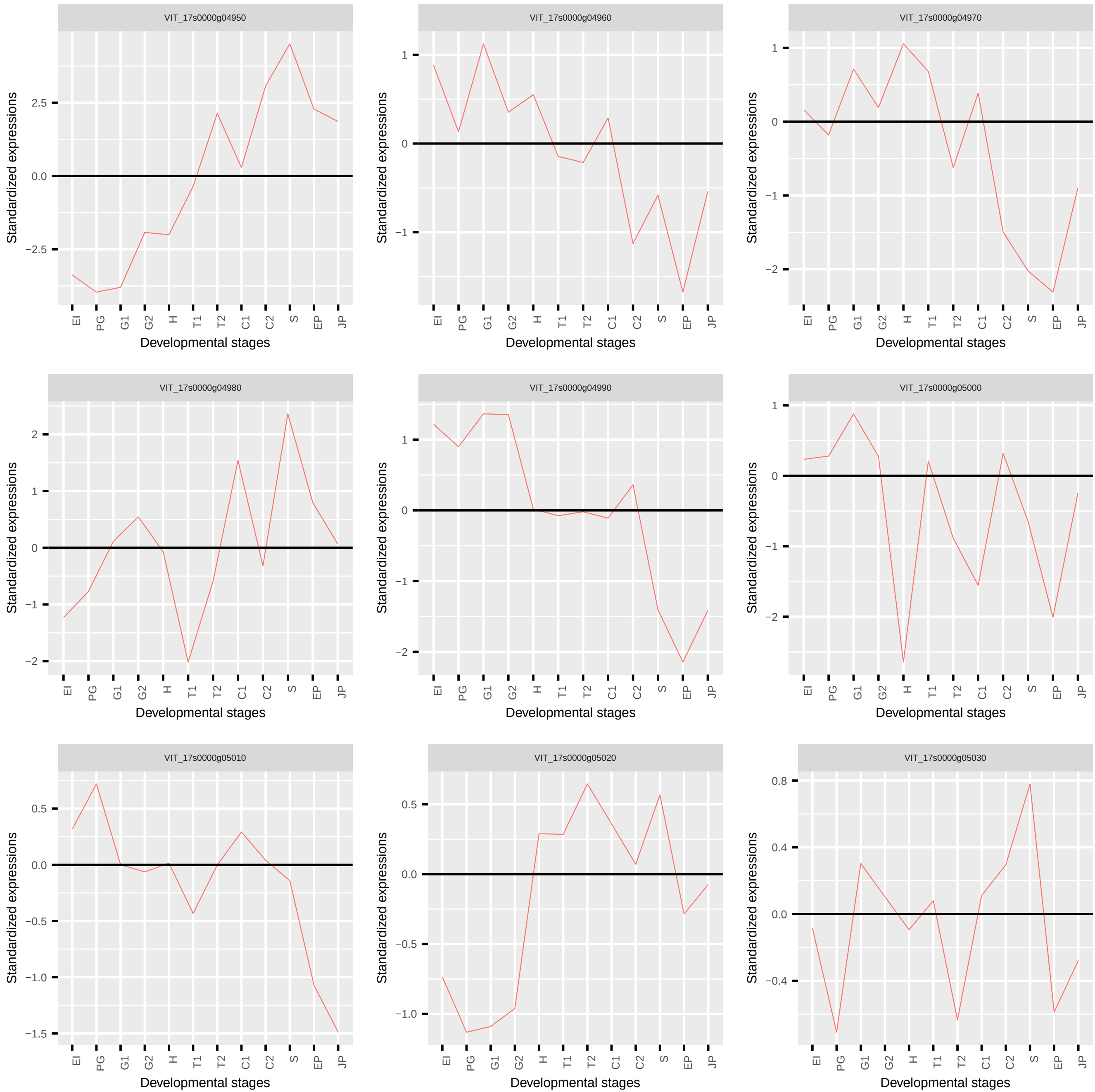
VIT_17s0000g04950
VIT_17s0000g04960
VIT_17s0000g04970
VIT_17s0000g04980
VIT_17s0000g04990
VIT_17s0000g05000
VIT_17s0000g05010
VIT_17s0000g05020
VIT_17s0000g05030
Standardized expressions
Developmental stages
EI PG G1 G2 H T1 T2 C1 C2 S EP JP

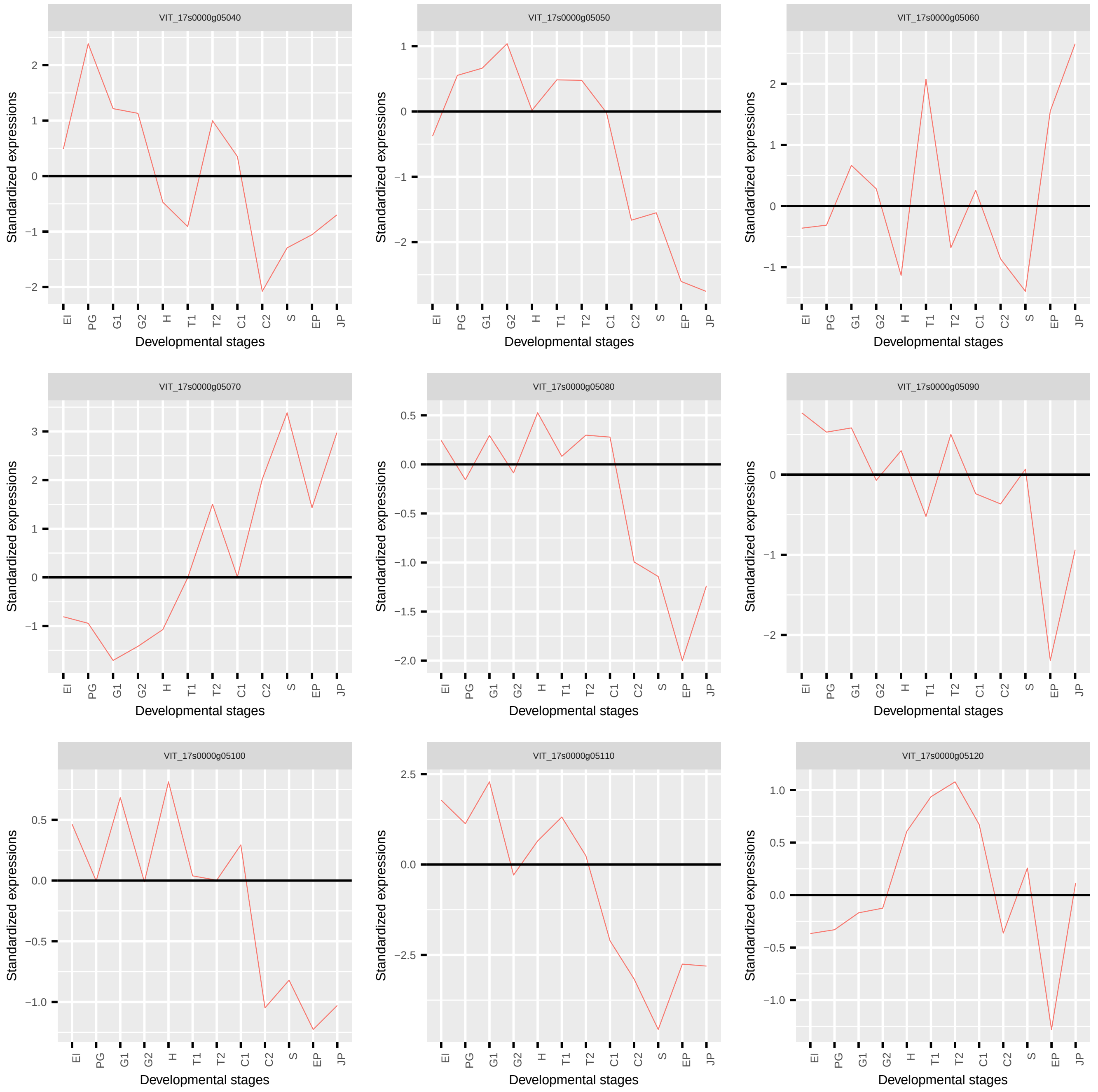
VIT_17s0000g05040
VIT_17s0000g05050
VIT_17s0000g05060
VIT_17s0000g05070
VIT_17s0000g05080
VIT_17s0000g05090
VIT_17s0000g05100
VIT_17s0000g05110
VIT_17s0000g05120
Standardized expressions
Developmental stages
EI PG G1 G2 H T1 T2 C1 C2 S EP JP

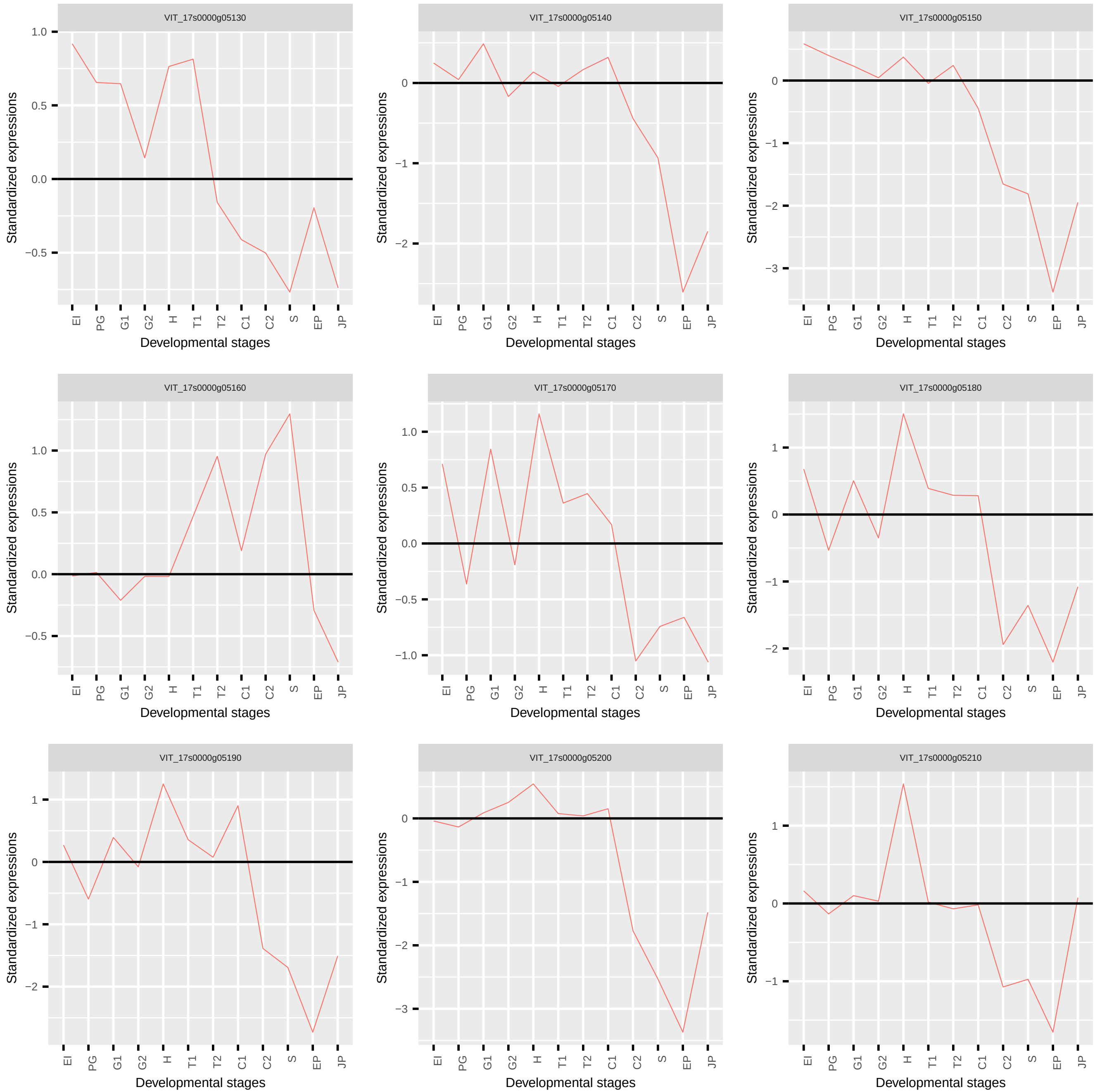
VIT_17s0000g05130
VIT_17s0000g05140
VIT_17s0000g05150
VIT_17s0000g05160
VIT_17s0000g05170
VIT_17s0000g05180
VIT_17s0000g05190
VIT_17s0000g05200
VIT_17s0000g05210
Standardized expressions
Developmental stages
EI PG G1 G2 H T1 T2 C1 C2 S EP JP

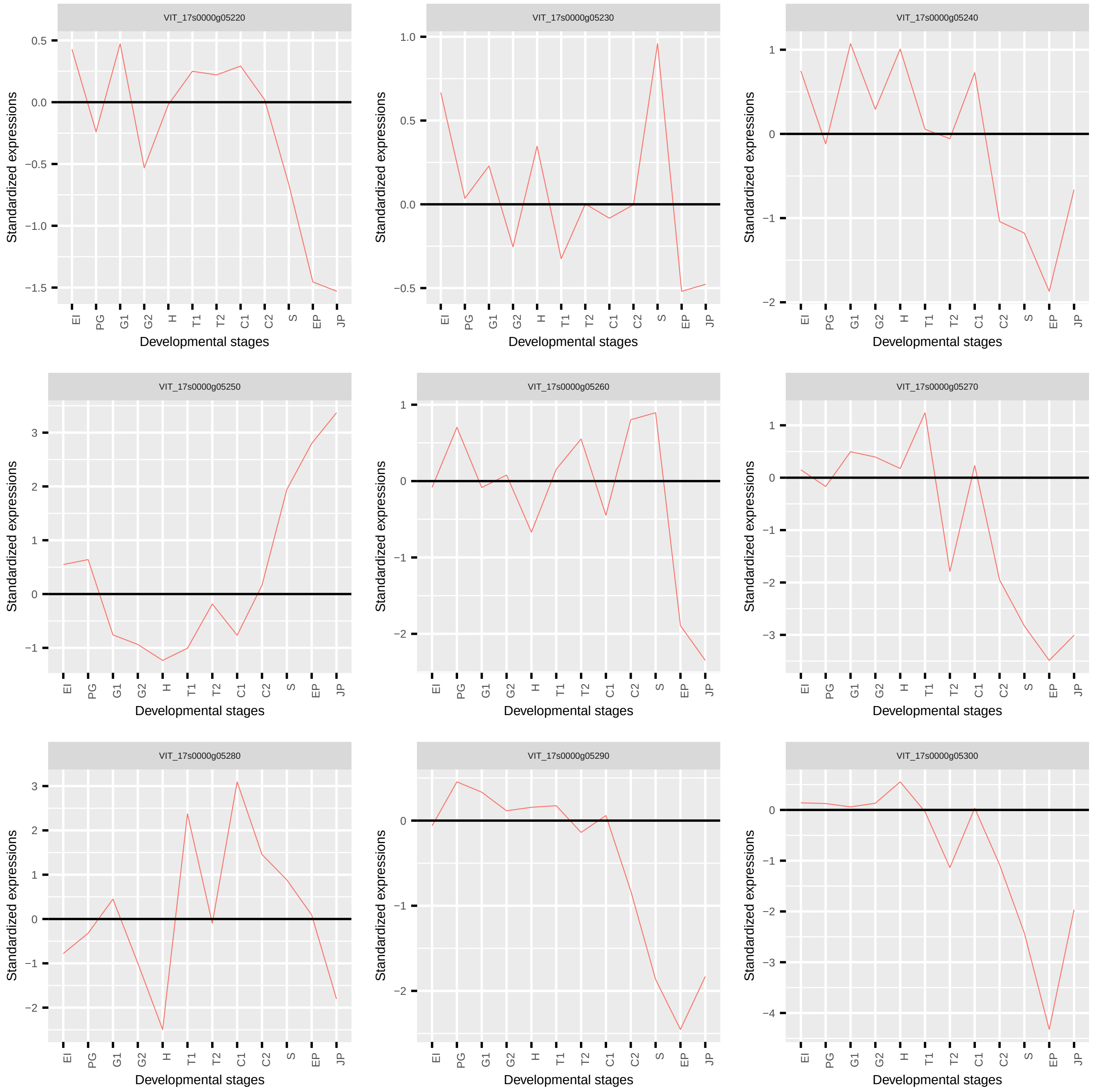
VIT_17s0000g05220
VIT_17s0000g05230
VIT_17s0000g05240
VIT_17s0000g05250
VIT_17s0000g05260
VIT_17s0000g05270
VIT_17s0000g05280
VIT_17s0000g05290
VIT_17s0000g05300
Standardized expressions
Developmental stages
EI PG G1 G2 H T1 T2 C1 C2 S EP JP

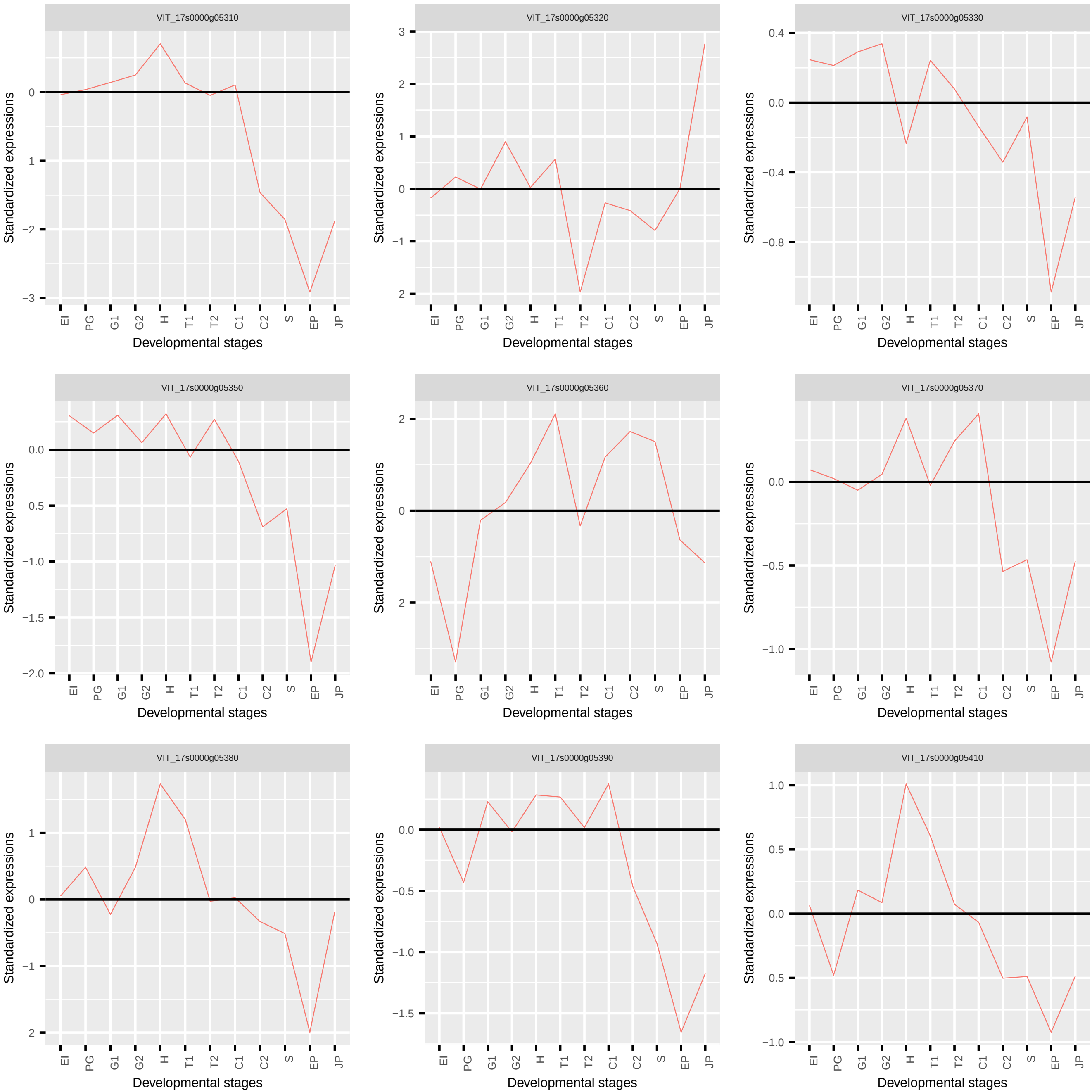

VIT_17s0000g05310
VIT_17s0000g05320
VIT_17s0000g05330
VIT_17s0000g05350
VIT_17s0000g05360
VIT_17s0000g05370
VIT_17s0000g05380
VIT_17s0000g05390
VIT_17s0000g05410
Standardized expressions
Developmental stages
EI PG G1 G2 H T1 T2 C1 C2 S EP JP

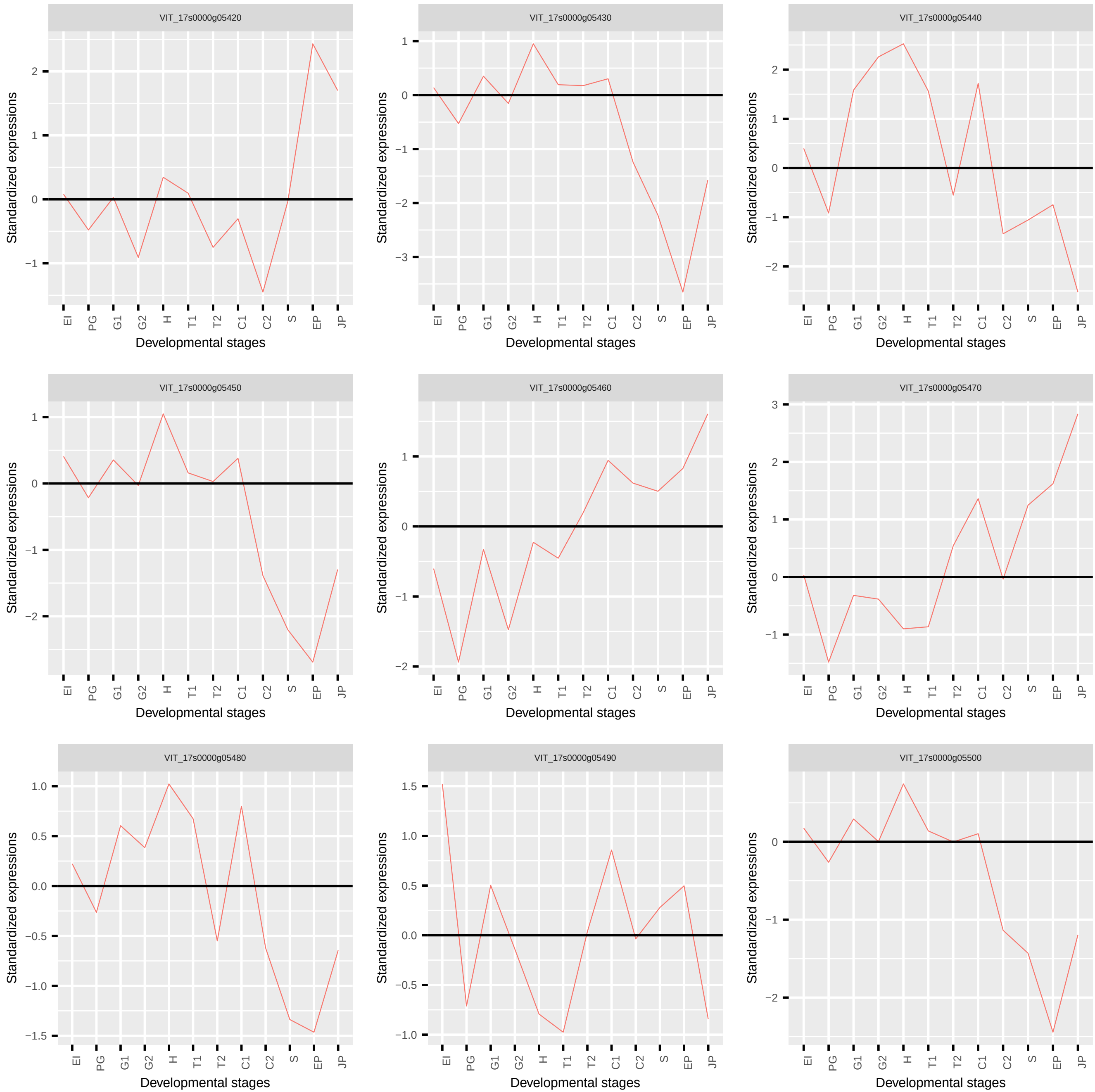
VIT_17s0000g05420
VIT_17s0000g05430
VIT_17s0000g05440
VIT_17s0000g05450
VIT_17s0000g05460
VIT_17s0000g05470
VIT_17s0000g05480
VIT_17s0000g05490
VIT_17s0000g05500
Standardized expressions
Developmental stages
EI PG G1 G2 H T1 T2 C1 C2 S EP JP

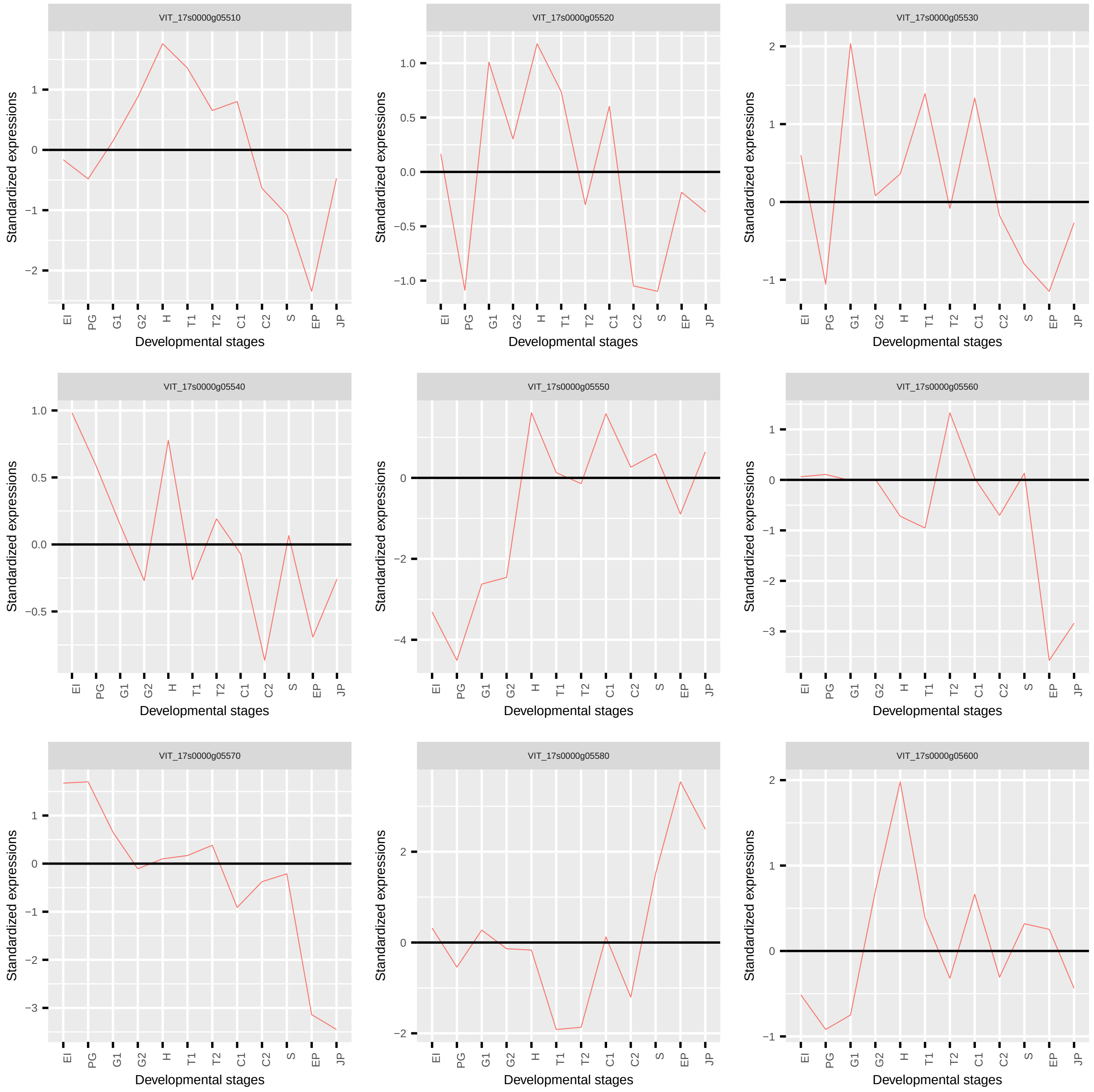
VIT_17s0000g05510
VIT_17s0000g05520
VIT_17s0000g05530
VIT_17s0000g05540
VIT_17s0000g05550
VIT_17s0000g05560
VIT_17s0000g05570
VIT_17s0000g05580
VIT_17s0000g05600
Standardized expressions
Developmental stages
EI
PG
G1
G2
H
T1
T2
C1
C2
S
EP
JP

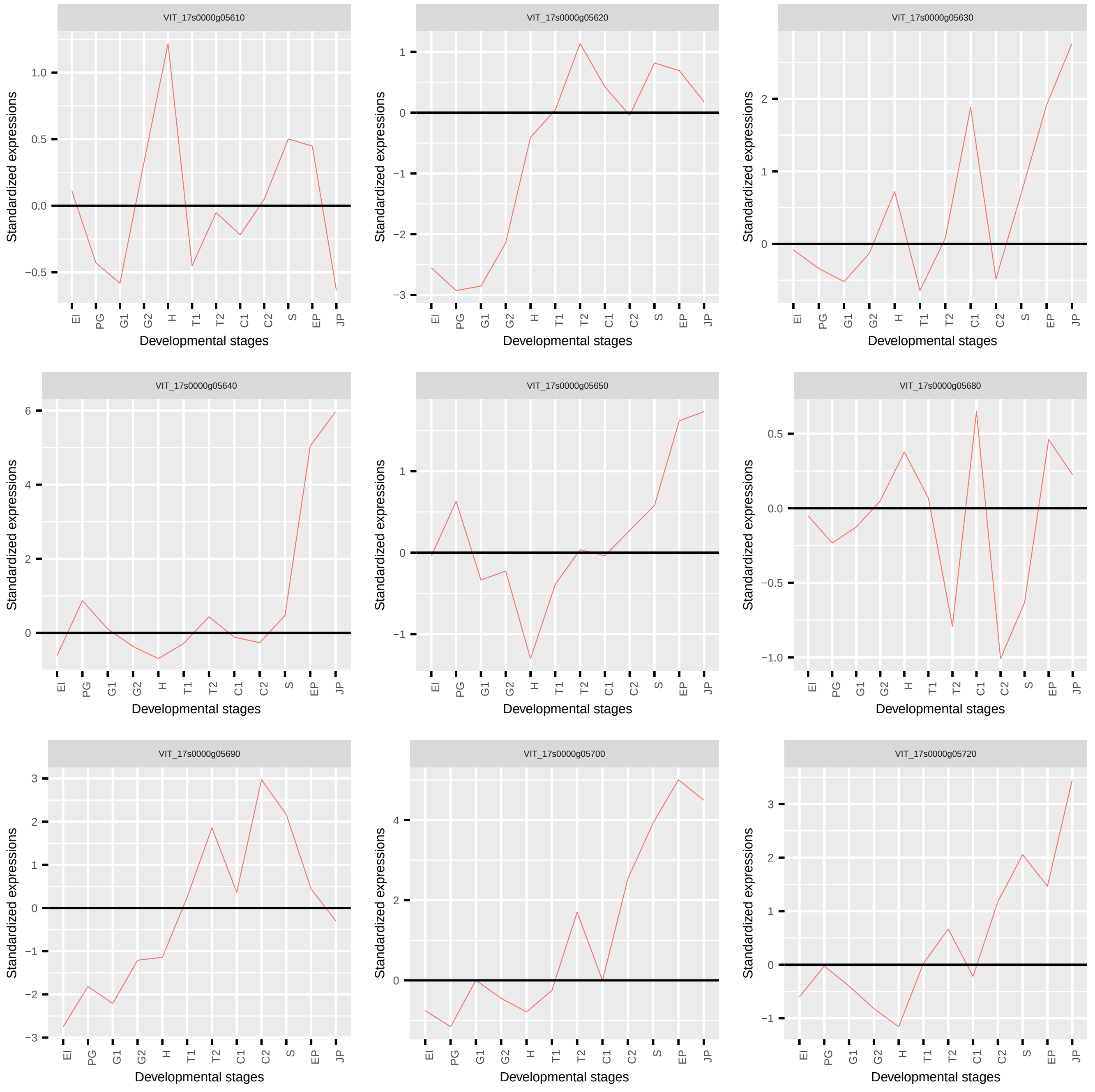
VIT_17s0000g05610
1.0
0.5
0.0
−0.5
Standardized expressions
EI PG G1 G2 H T1 T2 C1 C2 S EP JP
Developmental stages
VIT_17s0000g05620
1
0
−1
−2
−3
Standardized expressions
EI PG G1 G2 H T1 T2 C1 C2 S EP JP
Developmental stages
VIT_17s0000g05630
2
1
0
Standardized expressions
EI PG G1 G2 H T1 T2 C1 C2 S EP JP
Developmental stages
VIT_17s0000g05640
6
4
2
0
Standardized expressions
EI PG G1 G2 H T1 T2 C1 C2 S EP JP
Developmental stages
VIT_17s0000g05650
1
0
−1
Standardized expressions
EI PG G1 G2 H T1 T2 C1 C2 S EP JP
Developmental stages
VIT_17s0000g05680
0.5
0.0
−0.5
−1.0
Standardized expressions
EI PG G1 G2 H T1 T2 C1 C2 S EP JP
Developmental stages
VIT_17s0000g05690
3
2
1
0
−1
−2
−3
Standardized expressions
EI PG G1 G2 H T1 T2 C1 C2 S EP JP
Developmental stages
VIT_17s0000g05700
4
2
0
Standardized expressions
EI PG G1 G2 H T1 T2 C1 C2 S EP JP
Developmental stages
VIT_17s0000g05720
3
2
1
0
−1
Standardized expressions
EI PG G1 G2 H T1 T2 C1 C2 S EP JP
Developmental stages

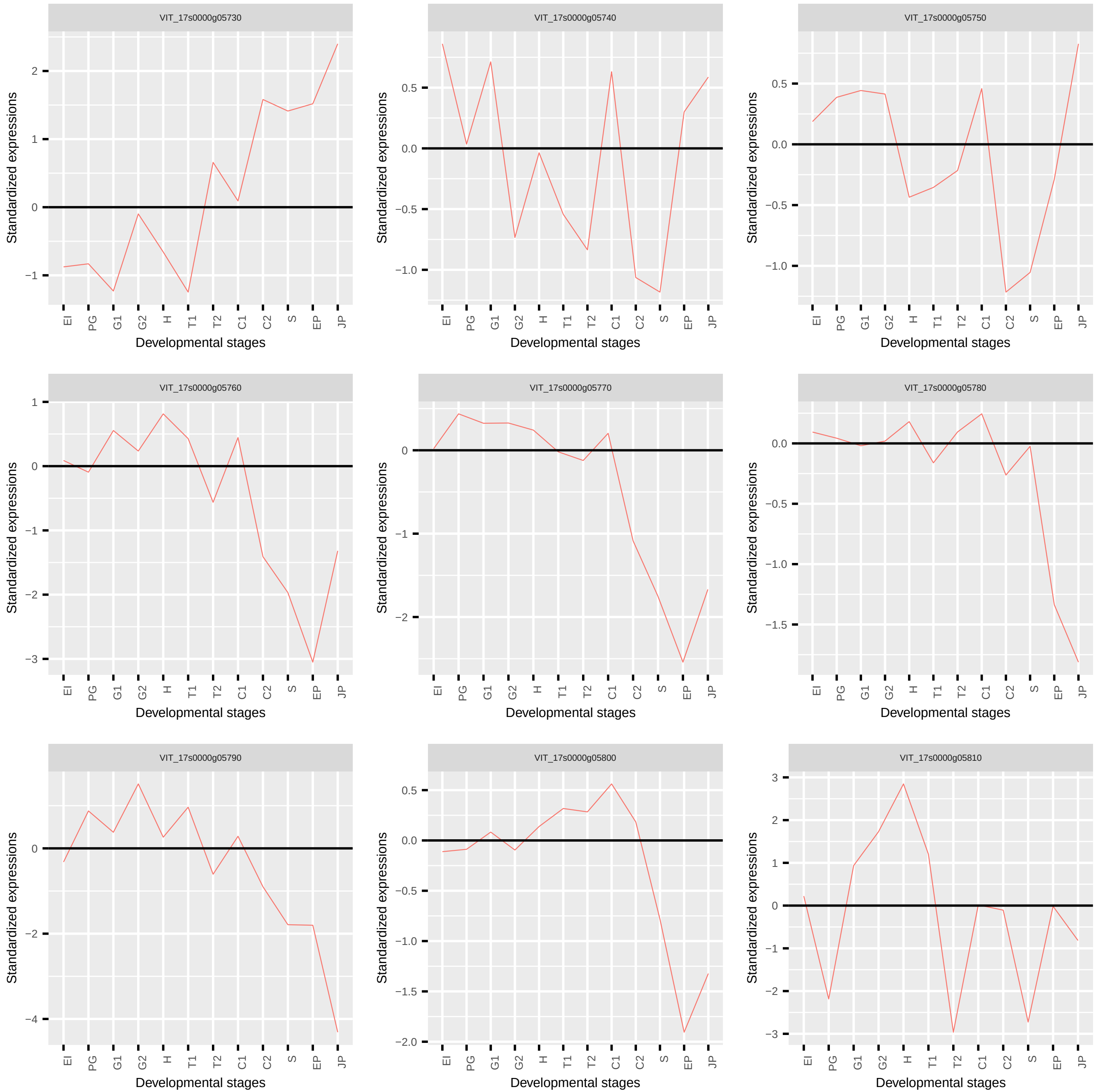
VIT_17s0000g05730
VIT_17s0000g05740
VIT_17s0000g05750
VIT_17s0000g05760
VIT_17s0000g05770
VIT_17s0000g05780
VIT_17s0000g05790
VIT_17s0000g05800
VIT_17s0000g05810
Standardized expressions
Developmental stages
EI PG G1 G2 H T1 T2 C1 C2 S EP JP

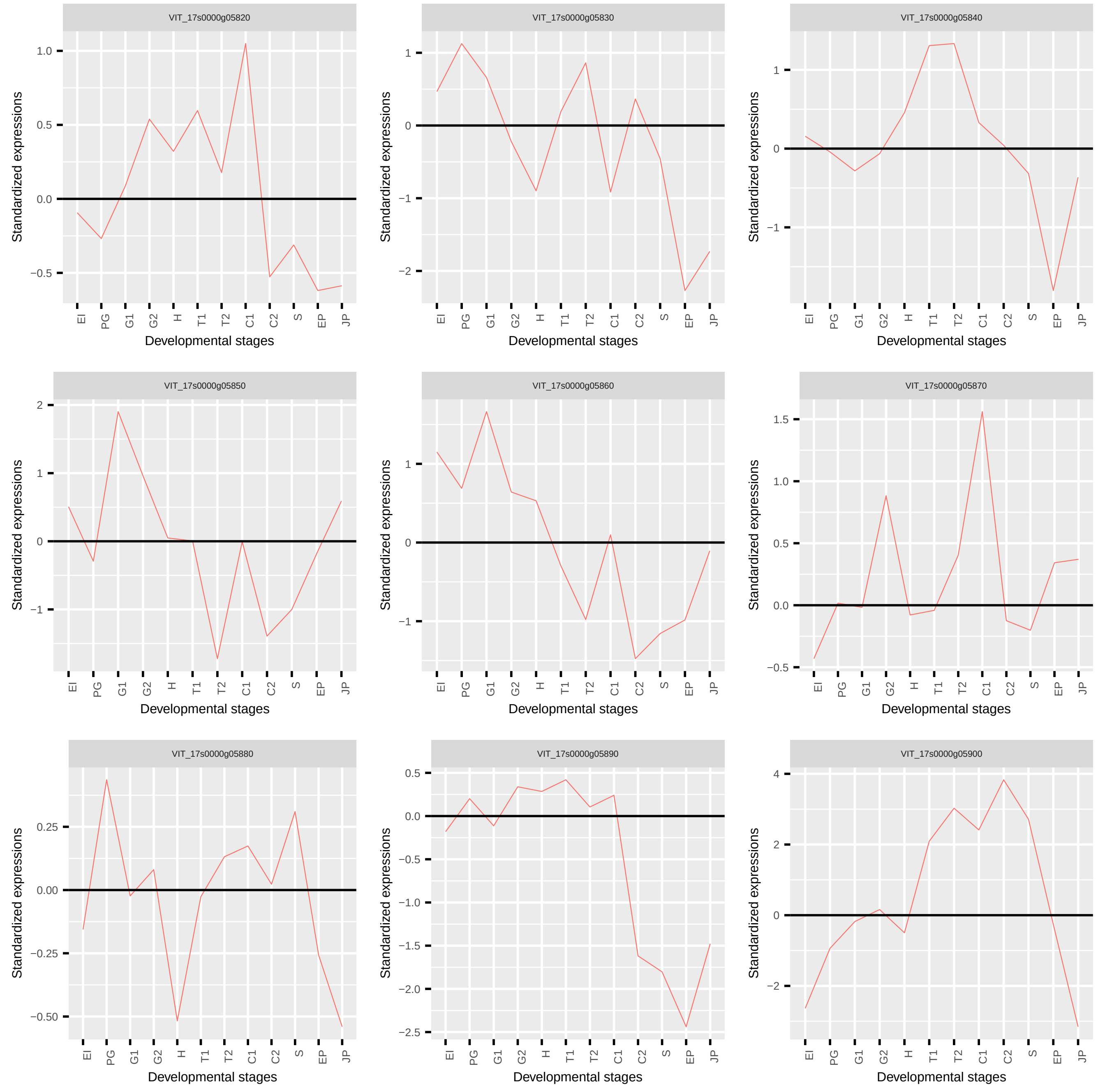
VIT_17s0000g05820
VIT_17s0000g05830
VIT_17s0000g05840
VIT_17s0000g05850
VIT_17s0000g05860
VIT_17s0000g05870
VIT_17s0000g05880
VIT_17s0000g05890
VIT_17s0000g05900
Standardized expressions
Developmental stages
EI PG G1 G2 H T1 T2 C1 C2 S EP JP

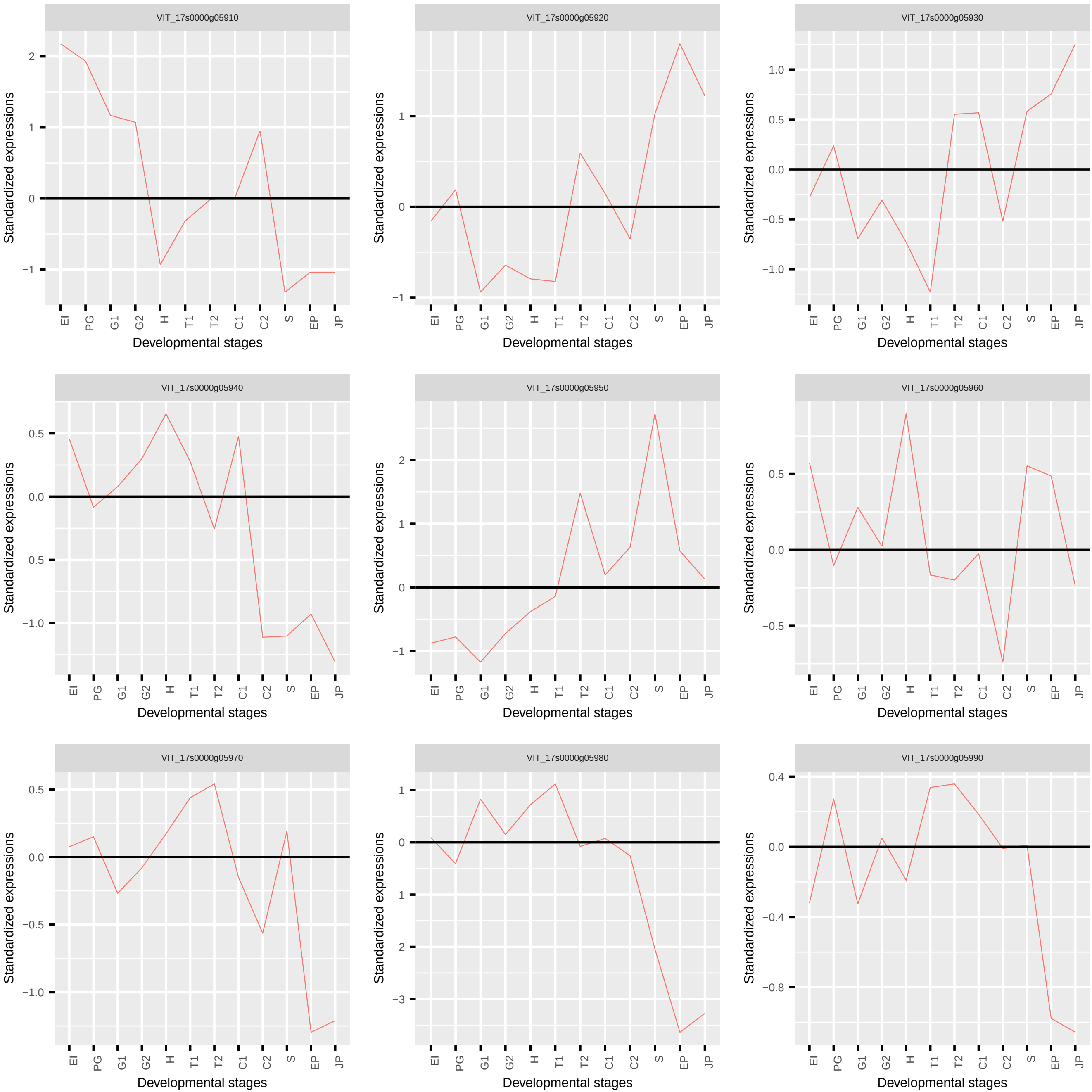
VIT_17s0000g05910
VIT_17s0000g05920
VIT_17s0000g05930
VIT_17s0000g05940
VIT_17s0000g05950
VIT_17s0000g05960
VIT_17s0000g05970
VIT_17s0000g05980
VIT_17s0000g05990
Standardized expressions
Developmental stages
EI PG G1 G2 H T1 T2 C1 C2 S EP JP

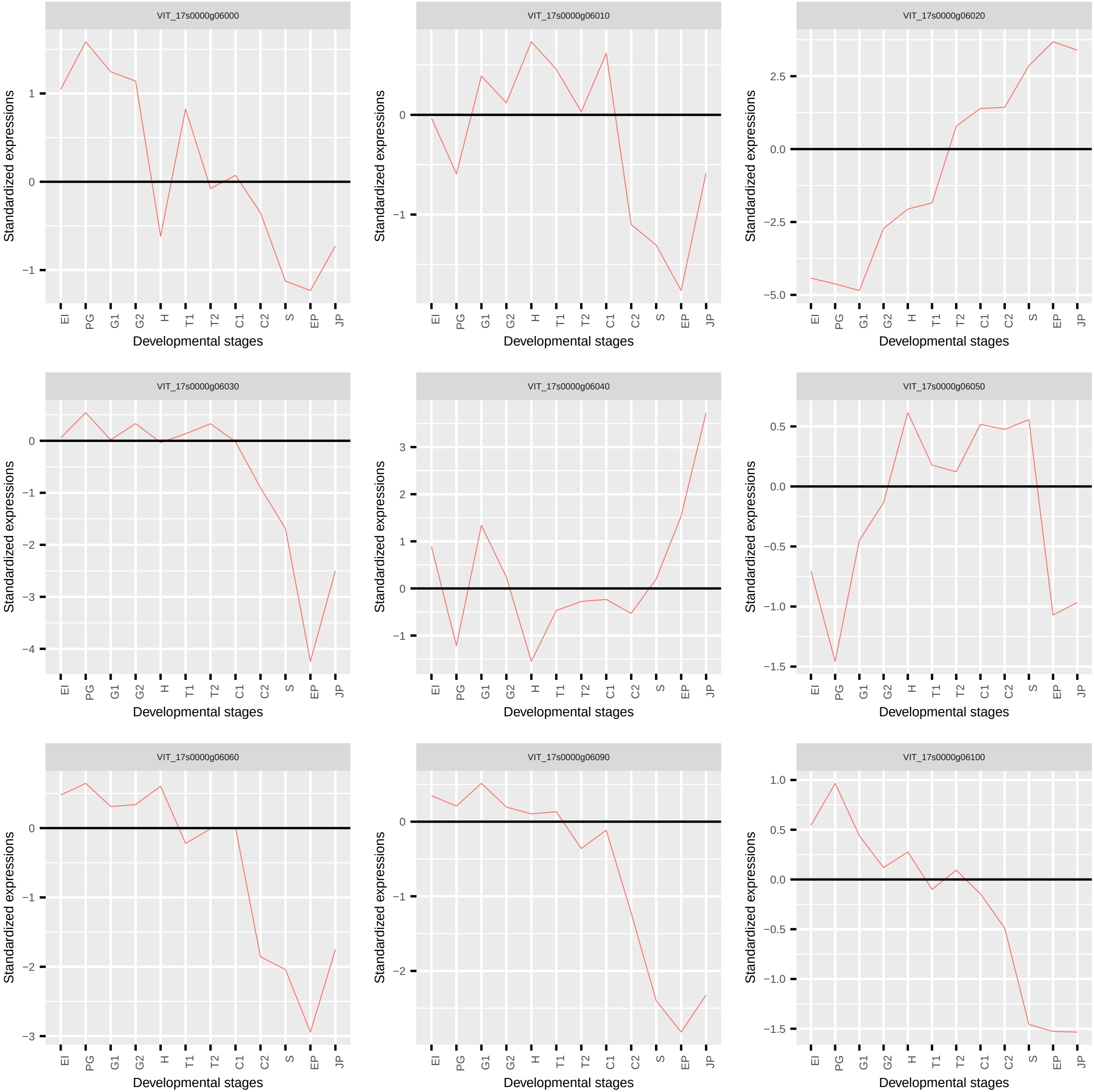

VIT_17s0000g06000
VIT_17s0000g06010
VIT_17s0000g06020
VIT_17s0000g06030
VIT_17s0000g06040
VIT_17s0000g06050
VIT_17s0000g06060
VIT_17s0000g06090
VIT_17s0000g06100
Standardized expressions
Developmental stages
EI PG G1 G2 H T1 T2 C1 C2 S EP JP

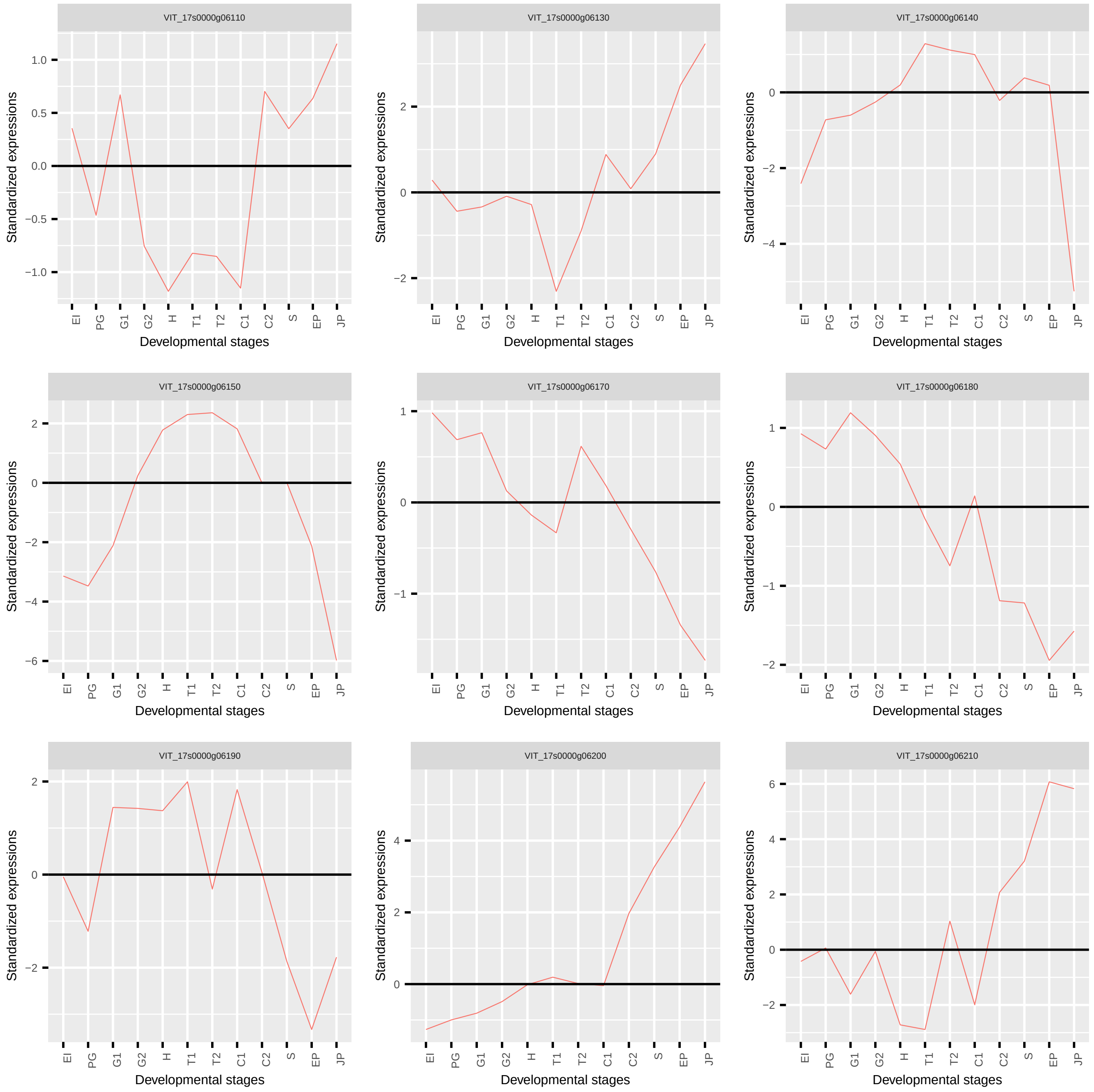
VIT_17s0000g06110
VIT_17s0000g06130
VIT_17s0000g06140
VIT_17s0000g06150
VIT_17s0000g06170
VIT_17s0000g06180
VIT_17s0000g06190
VIT_17s0000g06200
VIT_17s0000g06210
Standardized expressions
Developmental stages
EI PG G1 G2 H T1 T2 C1 C2 S EP JP

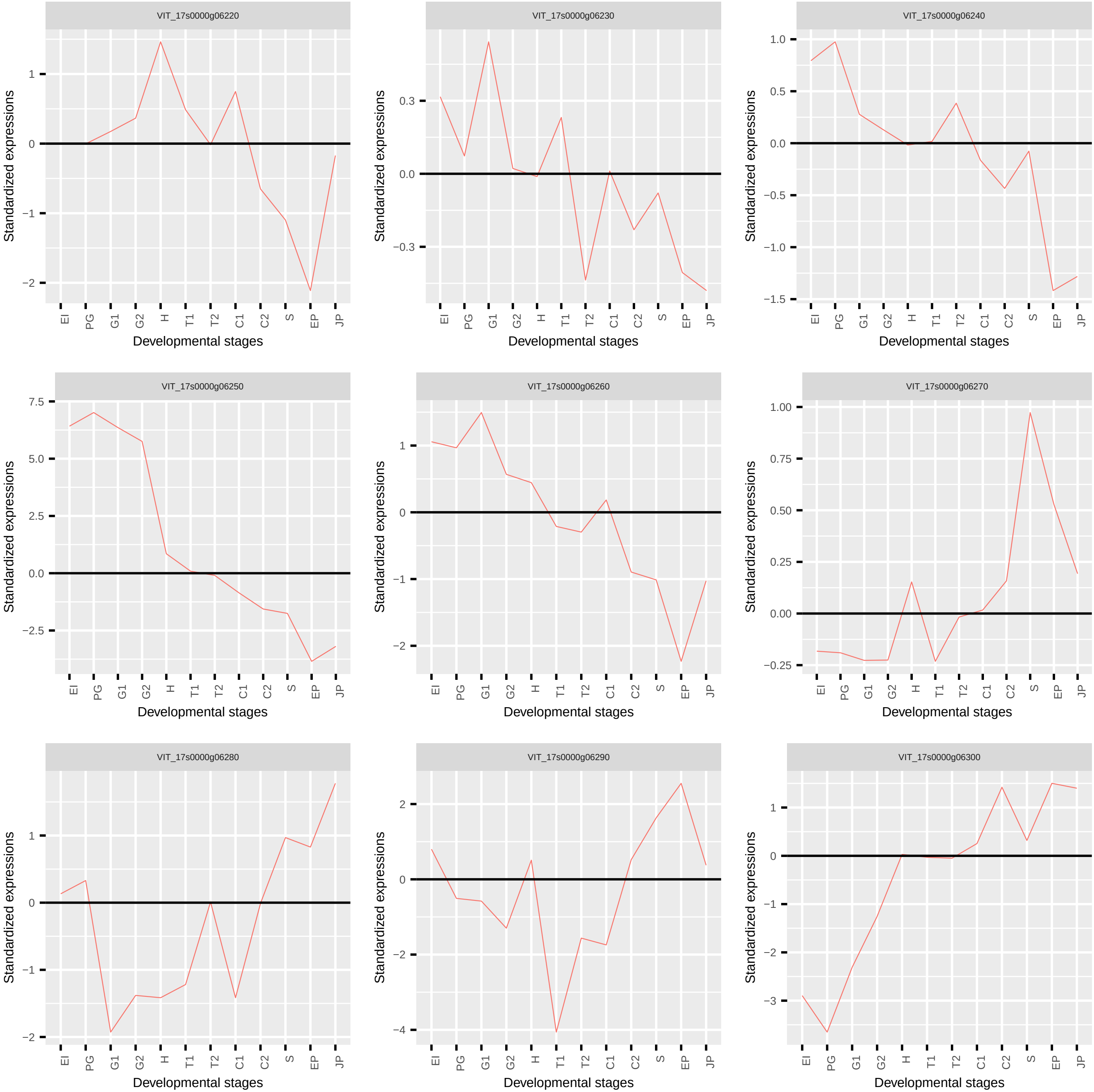
VIT_17s0000g06220
VIT_17s0000g06230
VIT_17s0000g06240
VIT_17s0000g06250
VIT_17s0000g06260
VIT_17s0000g06270
VIT_17s0000g06280
VIT_17s0000g06290
VIT_17s0000g06300
Standardized expressions
Developmental stages
EI
PG
G1
G2
H
T1
T2
C1
C2
S
EP
JP

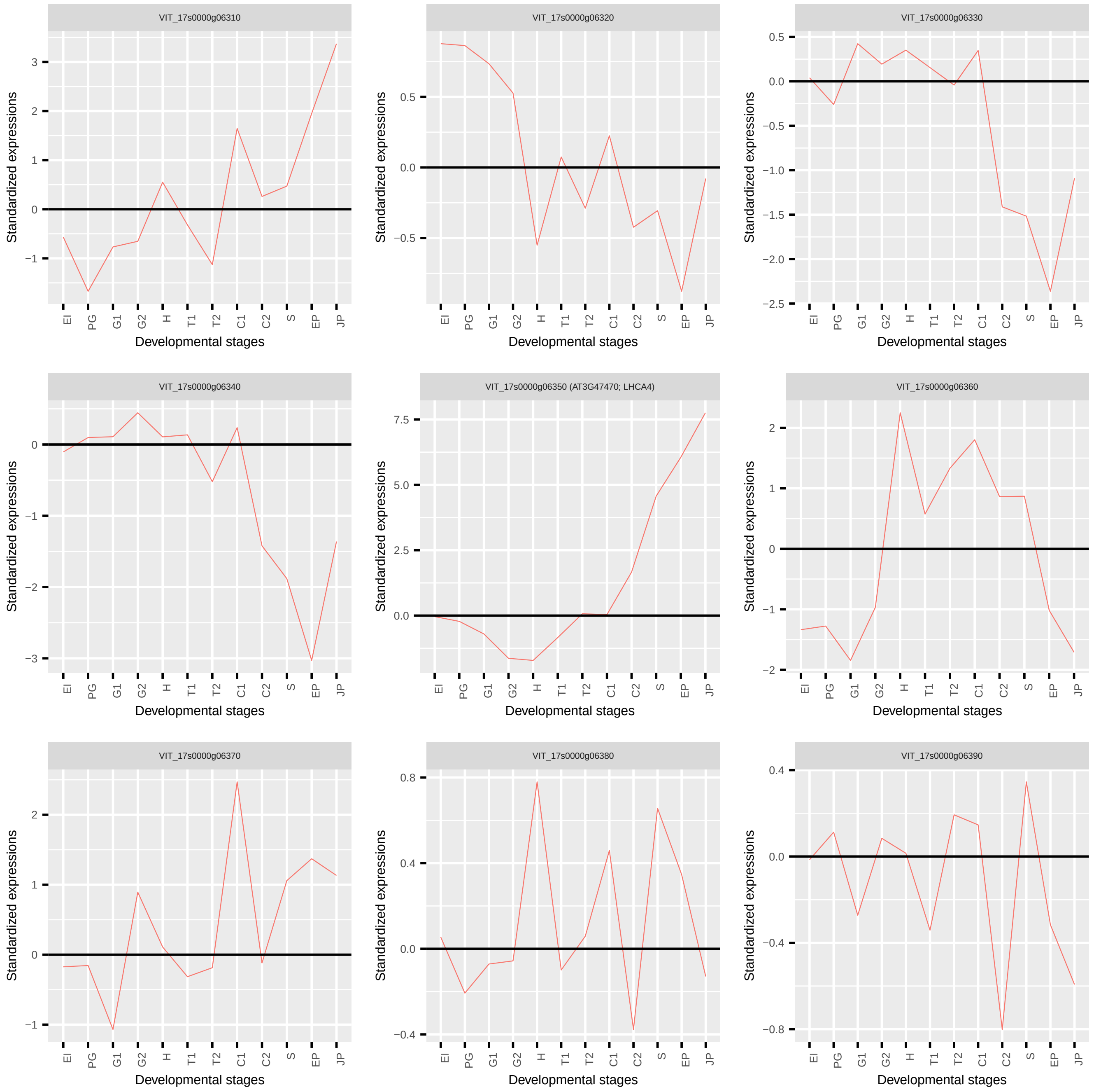
VIT_17s0000g06310
VIT_17s0000g06320
VIT_17s0000g06330
VIT_17s0000g06340
VIT_17s0000g06350 (AT3G47470; LHCA4)
VIT_17s0000g06360
VIT_17s0000g06370
VIT_17s0000g06380
VIT_17s0000g06390
Standardized expressions
Developmental stages
EI PG G1 G2 H T1 T2 C1 C2 S EP JP
3 2 1 0 −1
0.5 0.0 −0.5
0.5 0.0 −0.5 −1.0 −1.5 −2.0 −2.5
0 −1 −2 −3
7.5 5.0 2.5 0.0
2 1 0 −1 −2
2 1 0 −1
0.8 0.4 0.0 −0.4
0.4 0.0 −0.4 −0.8

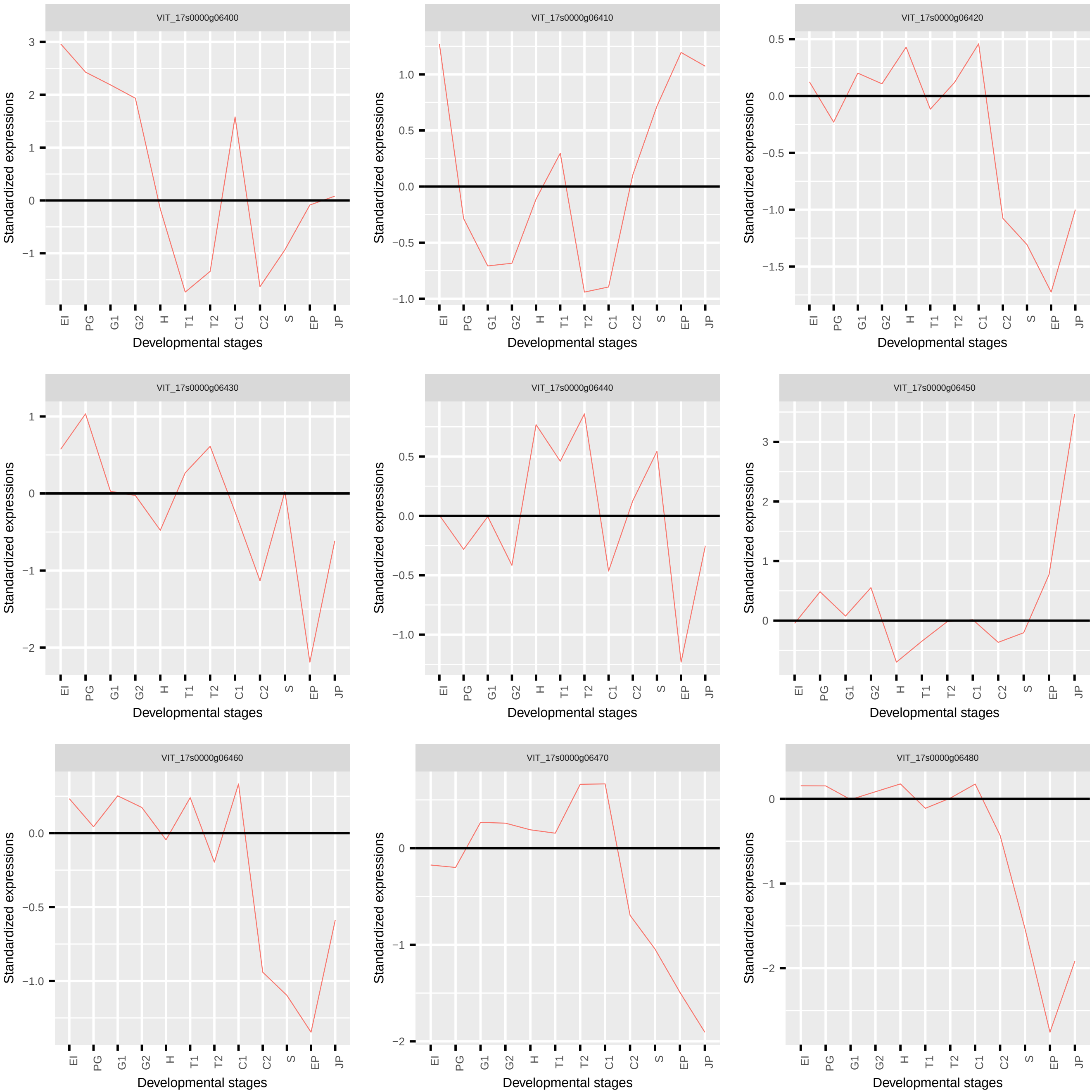
VIT_17s0000g06400
VIT_17s0000g06410
VIT_17s0000g06420
VIT_17s0000g06430
VIT_17s0000g06440
VIT_17s0000g06450
VIT_17s0000g06460
VIT_17s0000g06470
VIT_17s0000g06480
Standardized expressions
Developmental stages
EI PG G1 G2 H T1 T2 C1 C2 S EP JP

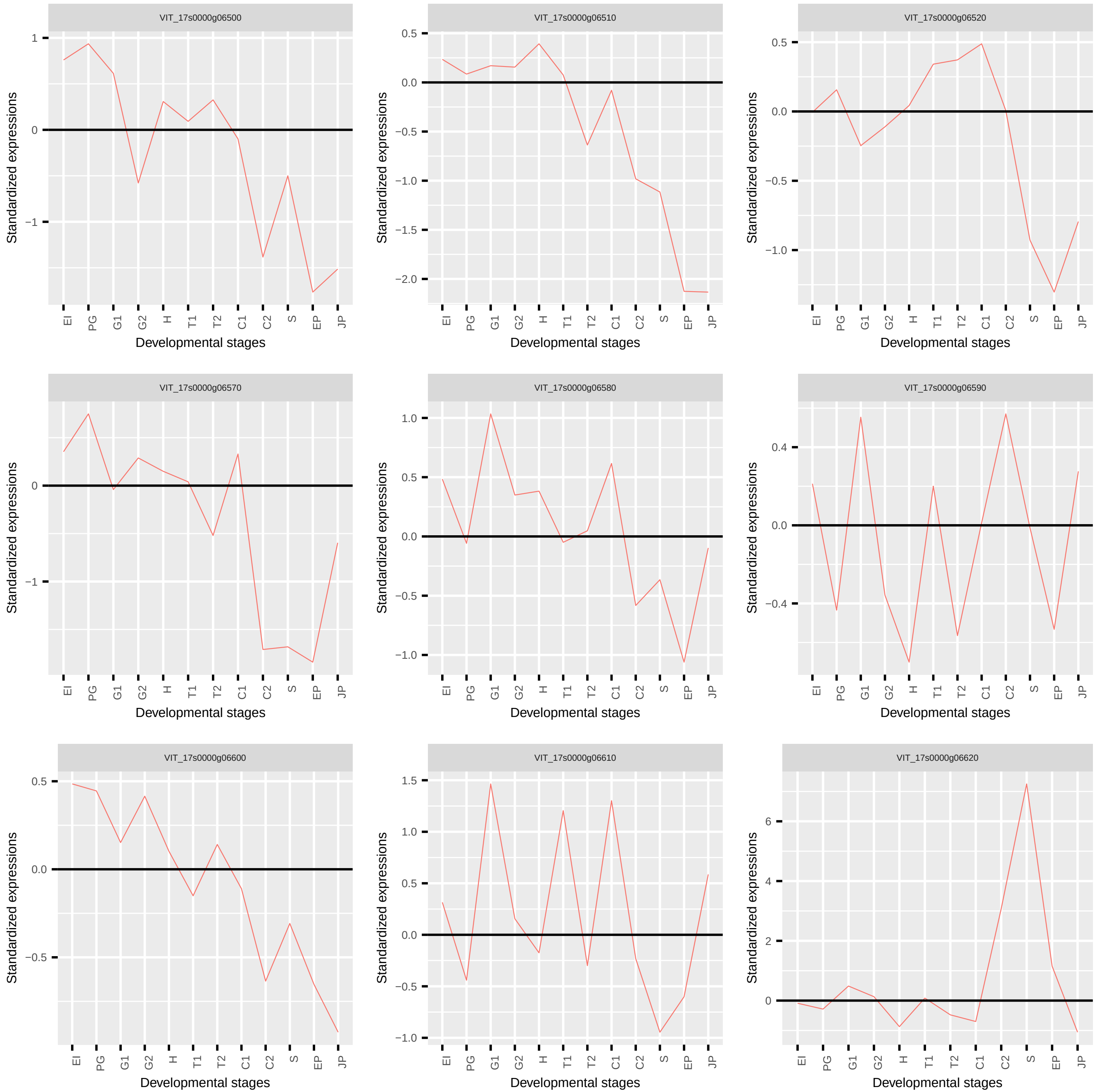

VIT_17s0000g06500
VIT_17s0000g06510
VIT_17s0000g06520
VIT_17s0000g06570
VIT_17s0000g06580
VIT_17s0000g06590
VIT_17s0000g06600
VIT_17s0000g06610
VIT_17s0000g06620
Standardized expressions
Developmental stages
EI
PG
G1
G2
H
T1
T2
C1
C2
S
EP
JP

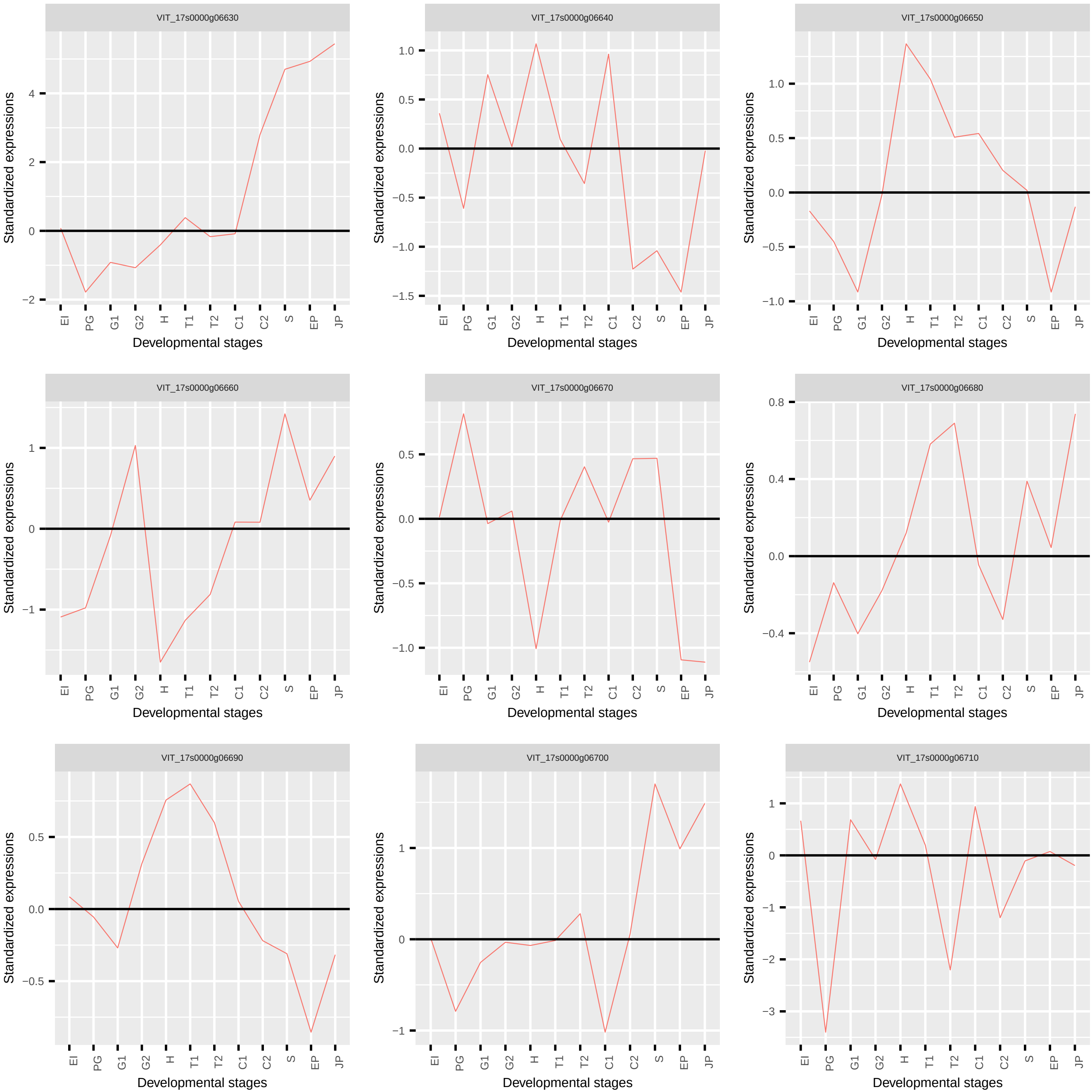

VIT_17s0000g06630
VIT_17s0000g06640
VIT_17s0000g06650
VIT_17s0000g06660
VIT_17s0000g06670
VIT_17s0000g06680
VIT_17s0000g06690
VIT_17s0000g06700
VIT_17s0000g06710
Standardized expressions
Developmental stages
EI PG G1 G2 H T1 T2 C1 C2 S EP JP

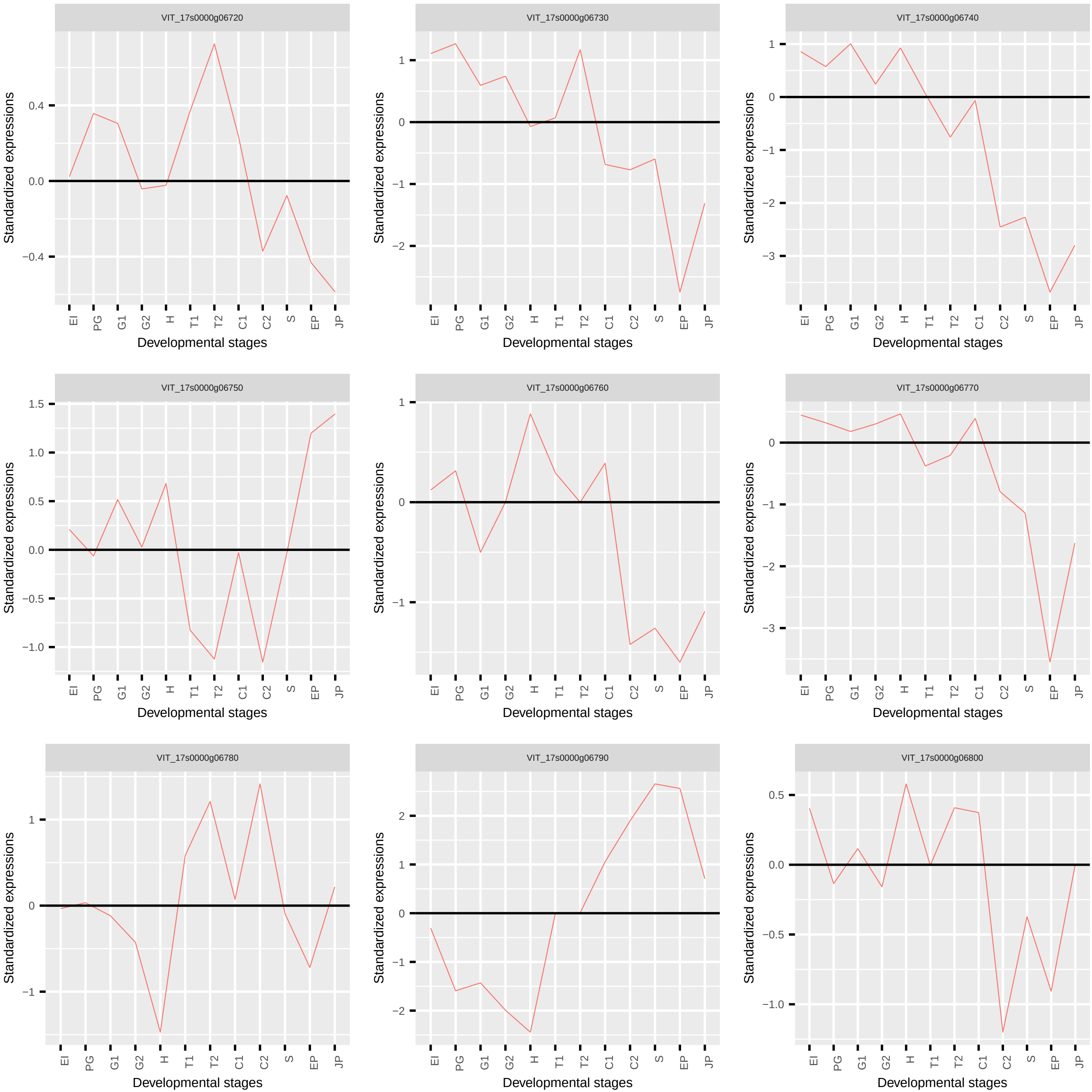
VIT_17s0000g06720
VIT_17s0000g06730
VIT_17s0000g06740
VIT_17s0000g06750
VIT_17s0000g06760
VIT_17s0000g06770
VIT_17s0000g06780
VIT_17s0000g06790
VIT_17s0000g06800
Standardized expressions
Developmental stages
EI PG G1 G2 H T1 T2 C1 C2 S EP JP

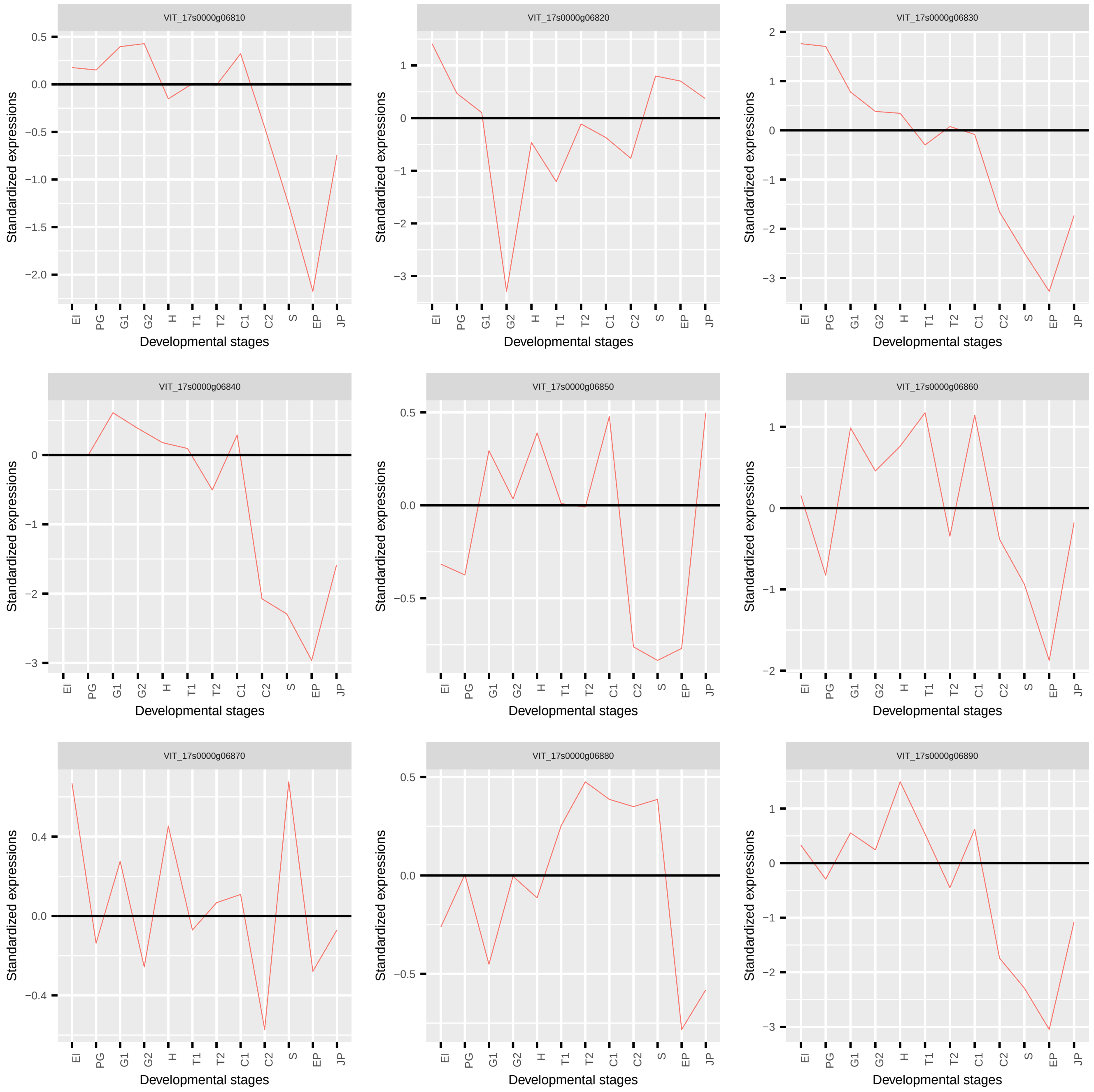
VIT_17s0000g06810
VIT_17s0000g06820
VIT_17s0000g06830
VIT_17s0000g06840
VIT_17s0000g06850
VIT_17s0000g06860
VIT_17s0000g06870
VIT_17s0000g06880
VIT_17s0000g06890
Standardized expressions
Developmental stages
EI PG G1 G2 H T1 T2 C1 C2 S EP JP

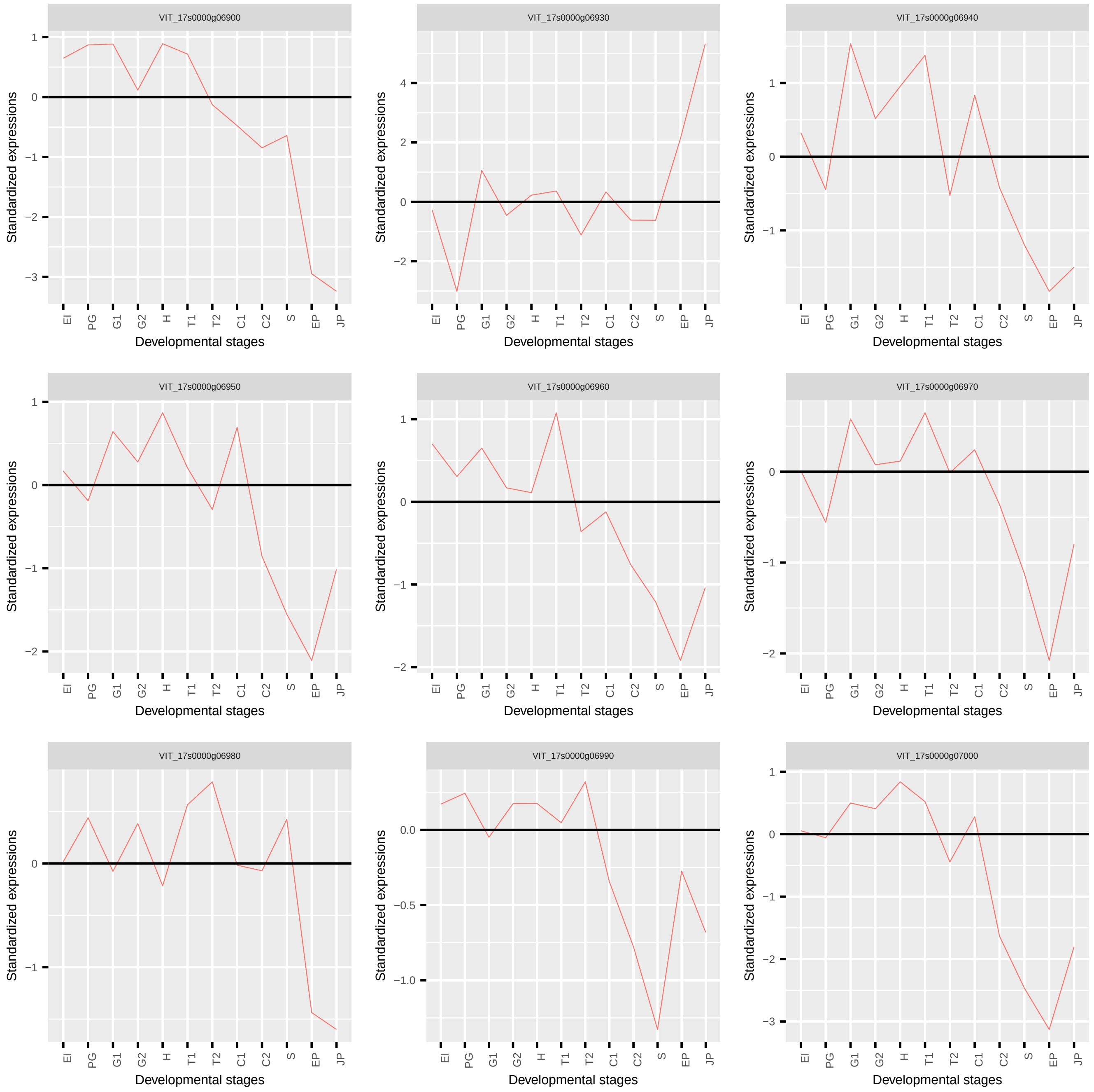
VIT_17s0000g06900
VIT_17s0000g06930
VIT_17s0000g06940
VIT_17s0000g06950
VIT_17s0000g06960
VIT_17s0000g06970
VIT_17s0000g06980
VIT_17s0000g06990
VIT_17s0000g07000
Standardized expressions
Developmental stages
EI PG G1 G2 H T1 T2 C1 C2 S EP JP

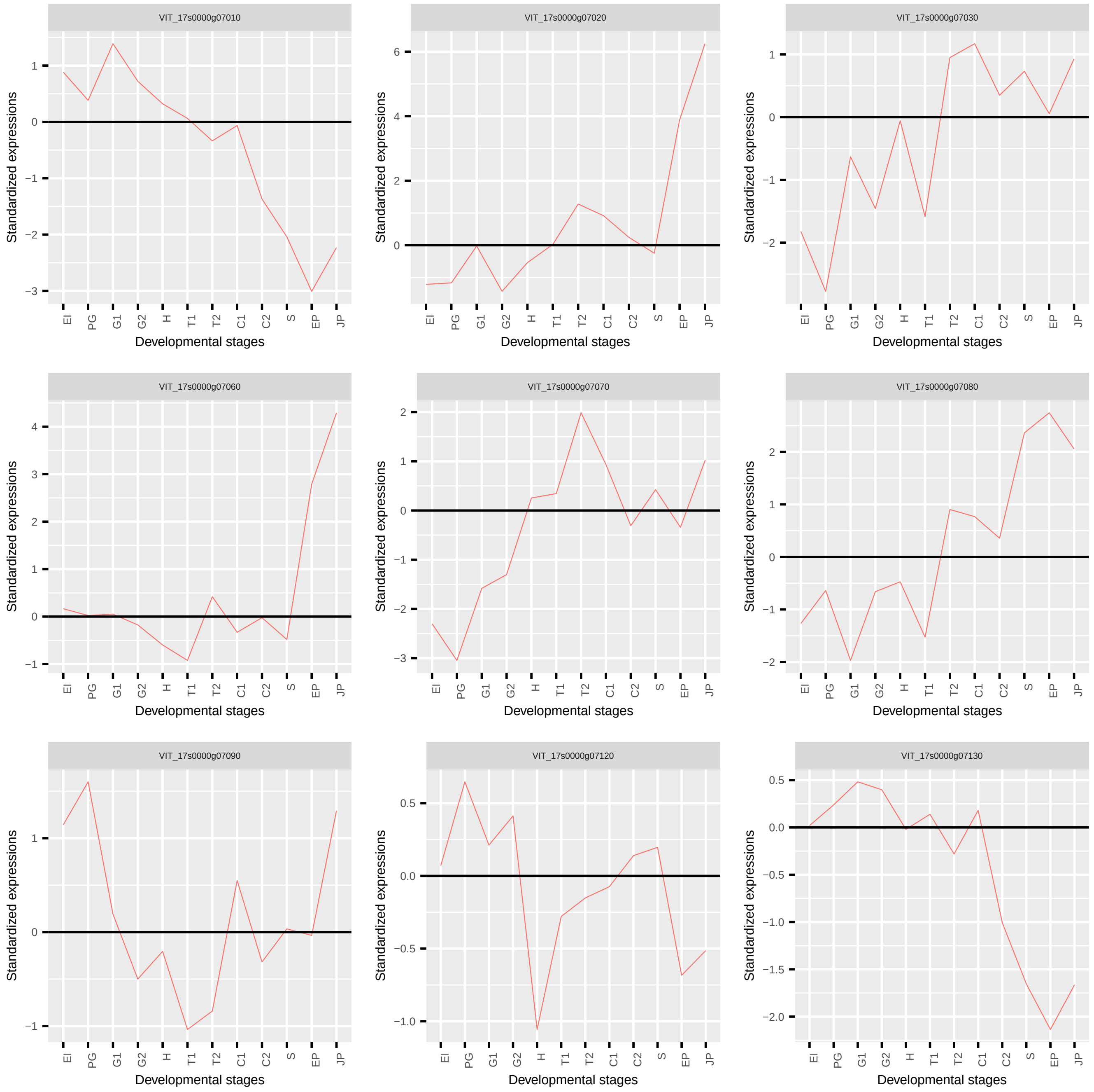
VIT_17s0000g07010
VIT_17s0000g07020
VIT_17s0000g07030
VIT_17s0000g07060
VIT_17s0000g07070
VIT_17s0000g07080
VIT_17s0000g07090
VIT_17s0000g07120
VIT_17s0000g07130
Standardized expressions
Developmental stages
EI PG G1 G2 H T1 T2 C1 C2 S EP JP

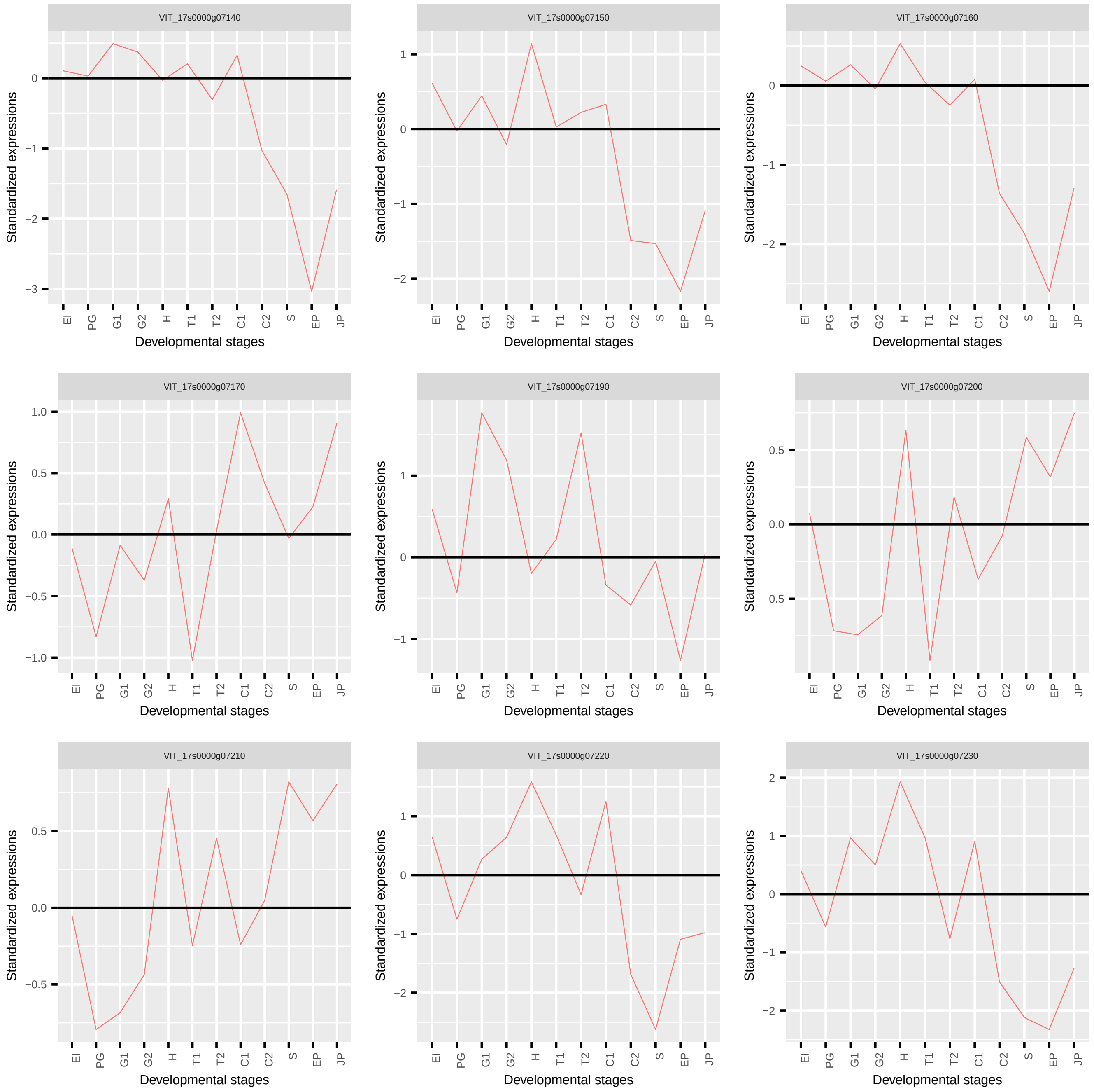

VIT_17s0000g07140
VIT_17s0000g07150
VIT_17s0000g07160
VIT_17s0000g07170
VIT_17s0000g07190
VIT_17s0000g07200
VIT_17s0000g07210
VIT_17s0000g07220
VIT_17s0000g07230
Standardized expressions
Developmental stages
EI
PG
G1
G2
H
T1
T2
C1
C2
S
EP
JP

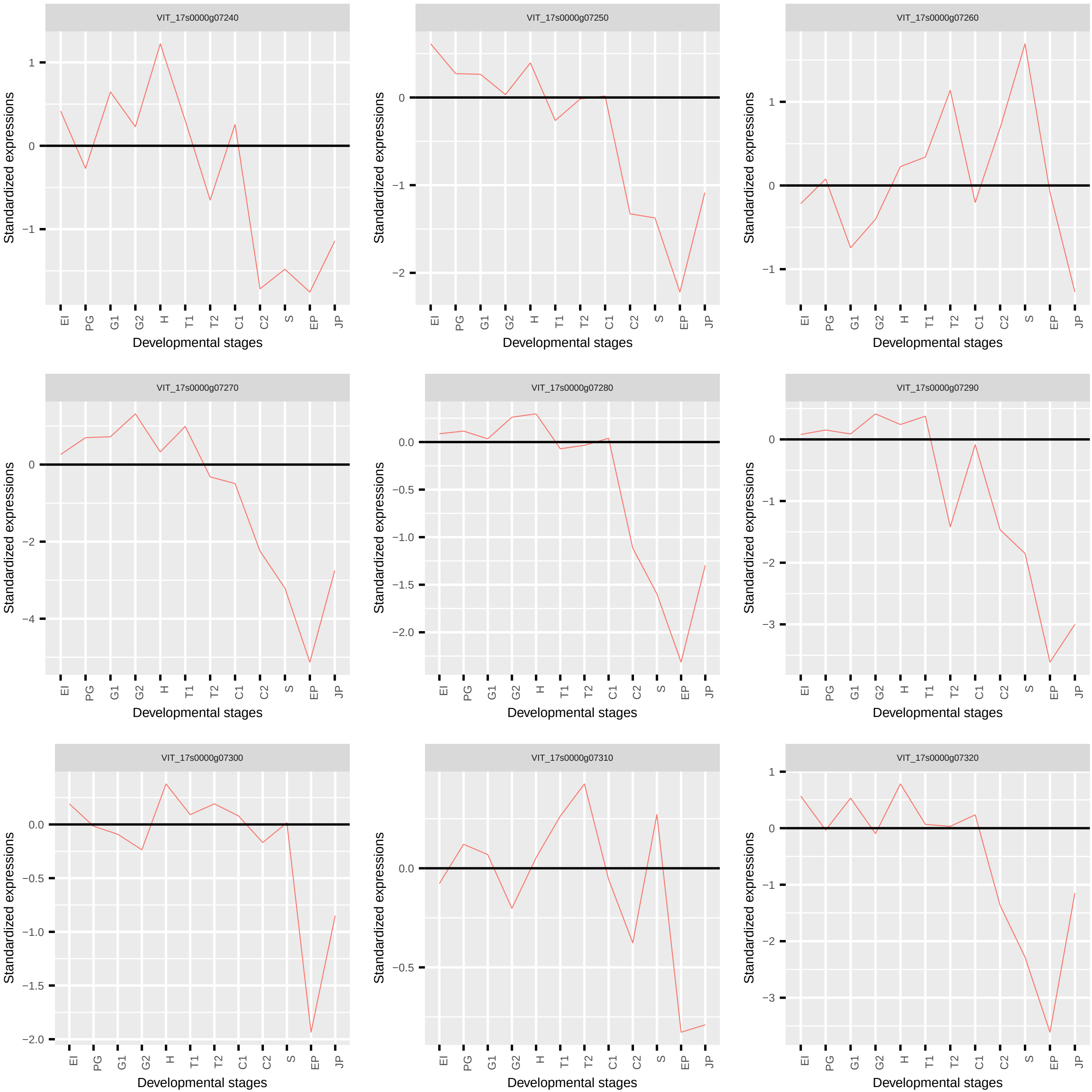

VIT_17s0000g07240
VIT_17s0000g07250
VIT_17s0000g07260
VIT_17s0000g07270
VIT_17s0000g07280
VIT_17s0000g07290
VIT_17s0000g07300
VIT_17s0000g07310
VIT_17s0000g07320
Standardized expressions
Developmental stages
EI
PG
G1
G2
H
T1
T2
C1
C2
S
EP
JP

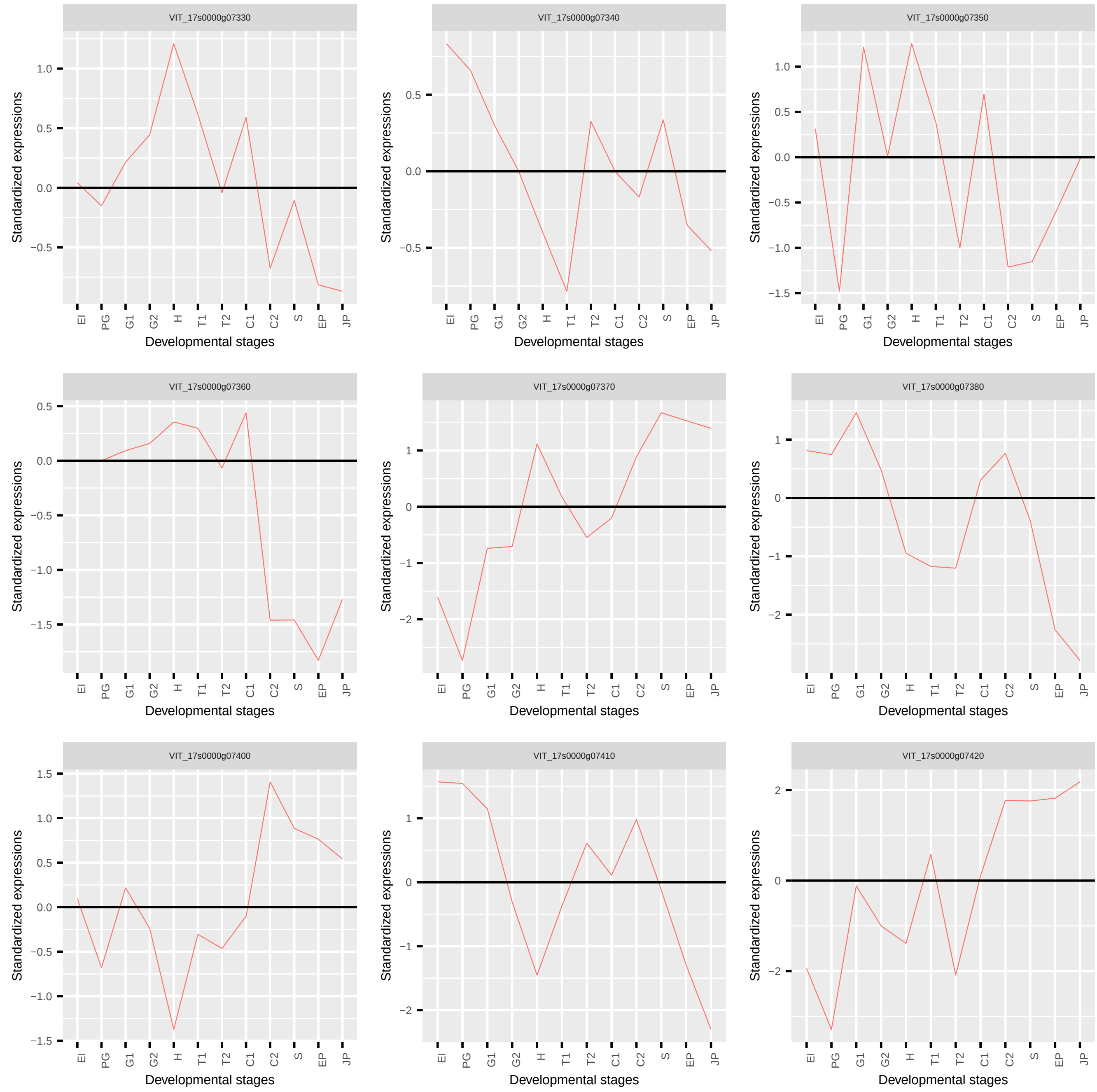

VIT_17s0000g07330
VIT_17s0000g07340
VIT_17s0000g07350
VIT_17s0000g07360
VIT_17s0000g07370
VIT_17s0000g07380
VIT_17s0000g07400
VIT_17s0000g07410
VIT_17s0000g07420
Standardized expressions
Developmental stages
EI PG G1 G2 H T1 T2 C1 C2 S EP JP

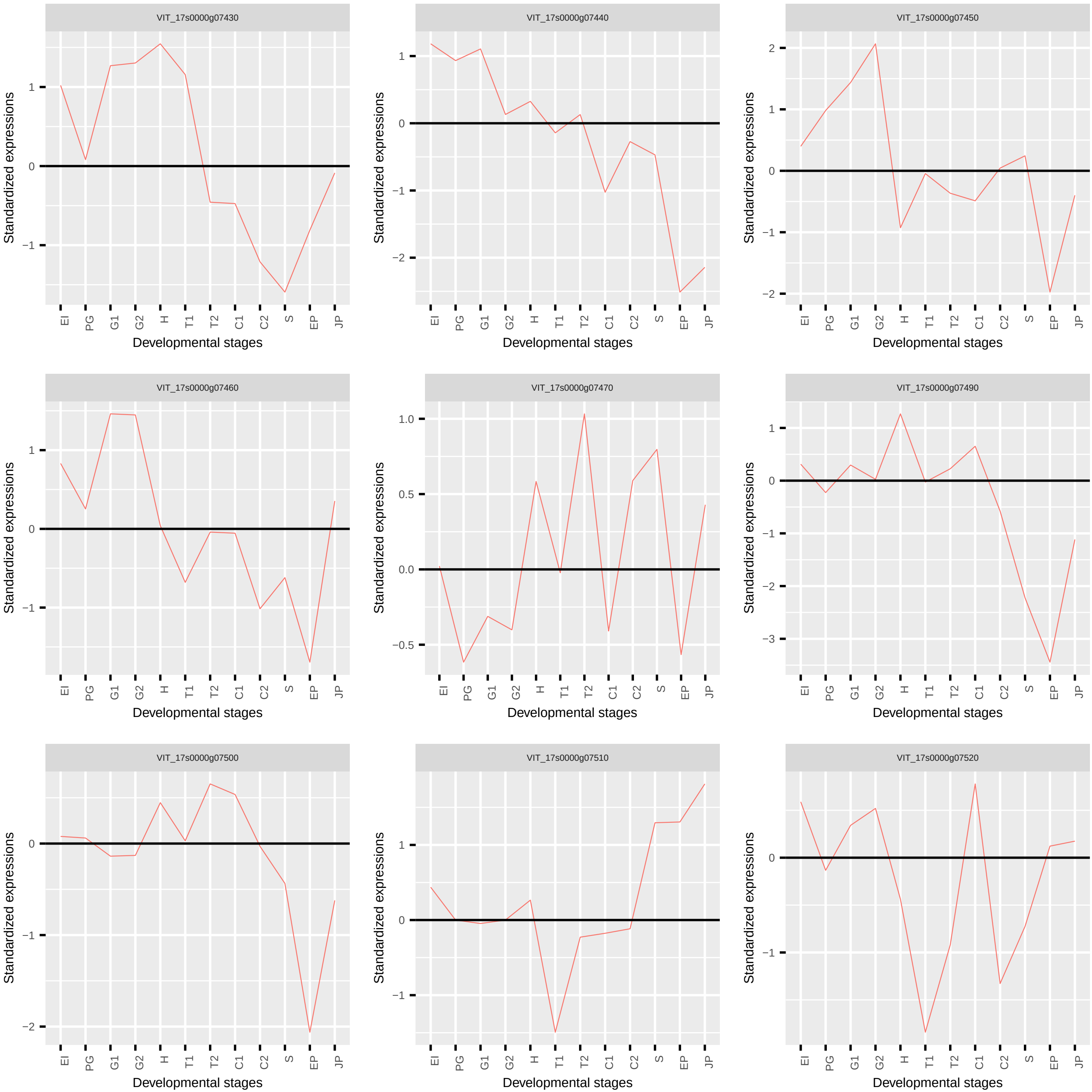
VIT_17s0000g07430
Standardized expressions
1
0
−1
EI PG G1 G2 H T1 T2 C1 C2 S EP JP
Developmental stages
VIT_17s0000g07440
Standardized expressions
1
0
−1
−2
EI PG G1 G2 H T1 T2 C1 C2 S EP JP
Developmental stages
VIT_17s0000g07450
Standardized expressions
2
1
0
−1
−2
EI PG G1 G2 H T1 T2 C1 C2 S EP JP
Developmental stages
VIT_17s0000g07460
Standardized expressions
1
0
−1
EI PG G1 G2 H T1 T2 C1 C2 S EP JP
Developmental stages
VIT_17s0000g07470
Standardized expressions
1.0
0.5
0.0
−0.5
EI PG G1 G2 H T1 T2 C1 C2 S EP JP
Developmental stages
VIT_17s0000g07490
Standardized expressions
1
0
−1
−2
−3
EI PG G1 G2 H T1 T2 C1 C2 S EP JP
Developmental stages
VIT_17s0000g07500
Standardized expressions
0
−1
−2
EI PG G1 G2 H T1 T2 C1 C2 S EP JP
Developmental stages
VIT_17s0000g07510
Standardized expressions
1
0
−1
EI PG G1 G2 H T1 T2 C1 C2 S EP JP
Developmental stages
VIT_17s0000g07520
Standardized expressions
0
−1
EI PG G1 G2 H T1 T2 C1 C2 S EP JP
Developmental stages

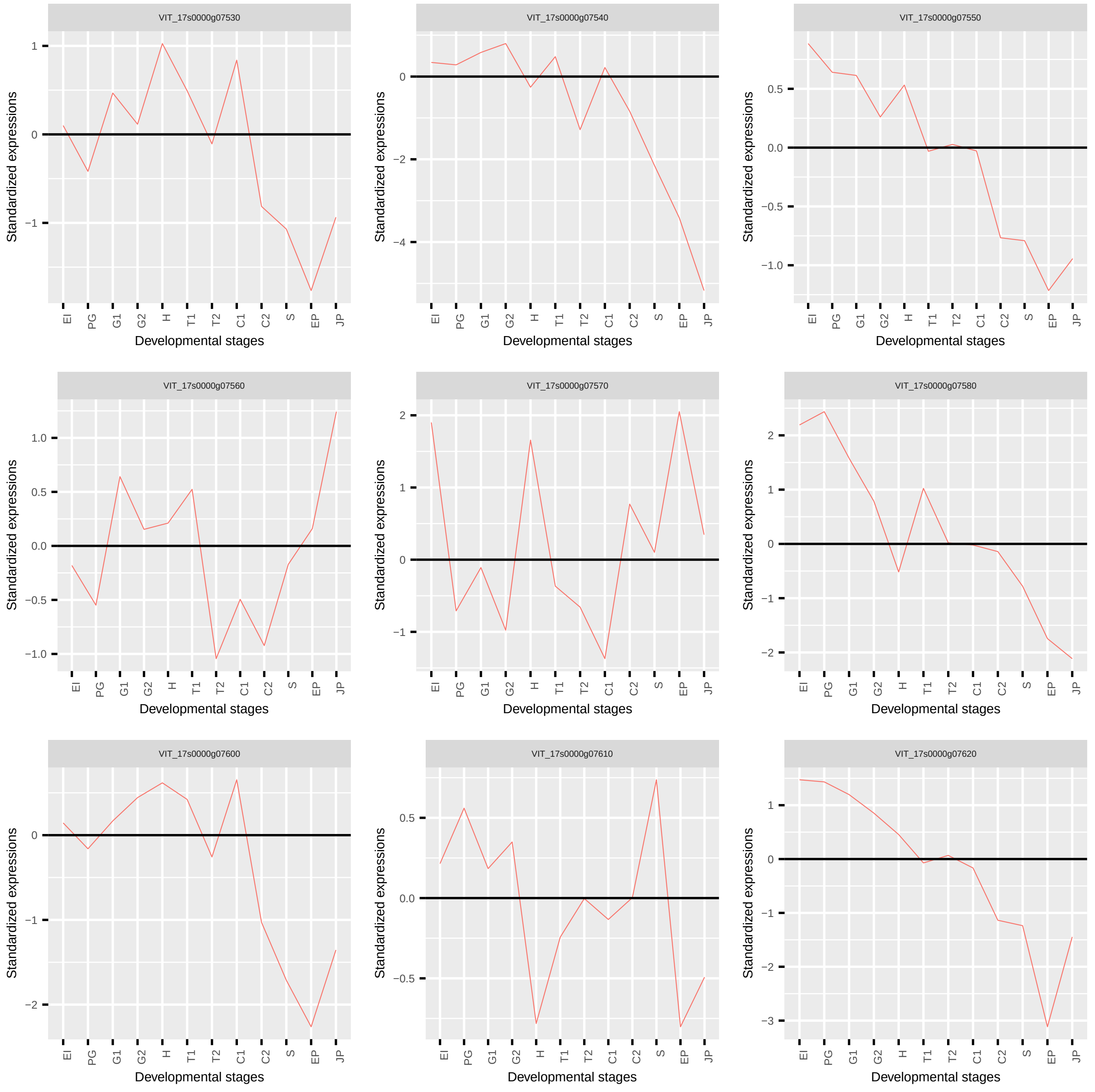
VIT_17s0000g07530
VIT_17s0000g07540
VIT_17s0000g07550
VIT_17s0000g07560
VIT_17s0000g07570
VIT_17s0000g07580
VIT_17s0000g07600
VIT_17s0000g07610
VIT_17s0000g07620
Standardized expressions
Developmental stages
EI
PG
G1
G2
H
T1
T2
C1
C2
S
EP
JP

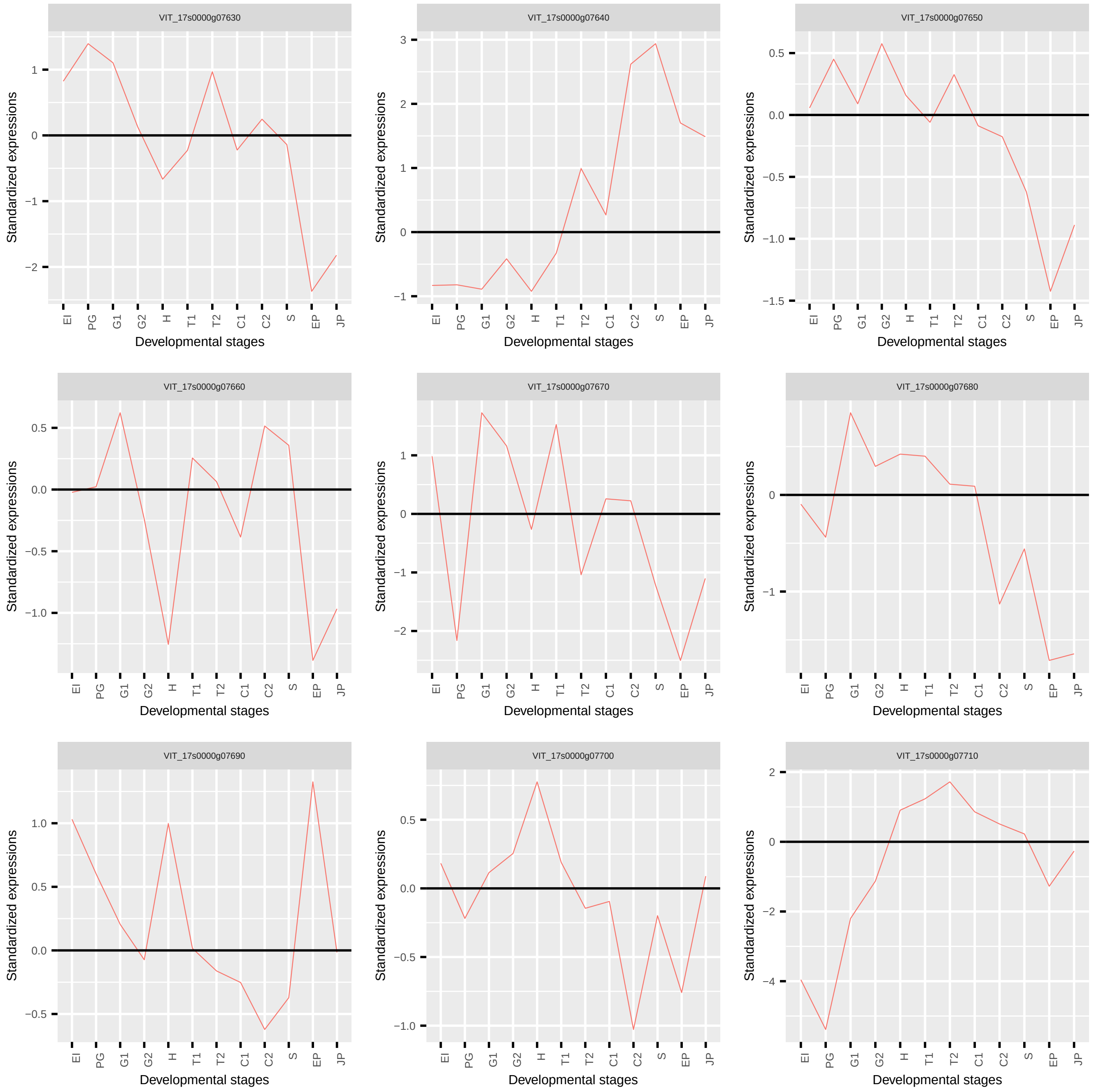
VIT_17s0000g07630
VIT_17s0000g07640
VIT_17s0000g07650
VIT_17s0000g07660
VIT_17s0000g07670
VIT_17s0000g07680
VIT_17s0000g07690
VIT_17s0000g07700
VIT_17s0000g07710
Standardized expressions
Developmental stages
EI
PG
G1
G2
H
T1
T2
C1
C2
S
EP
JP

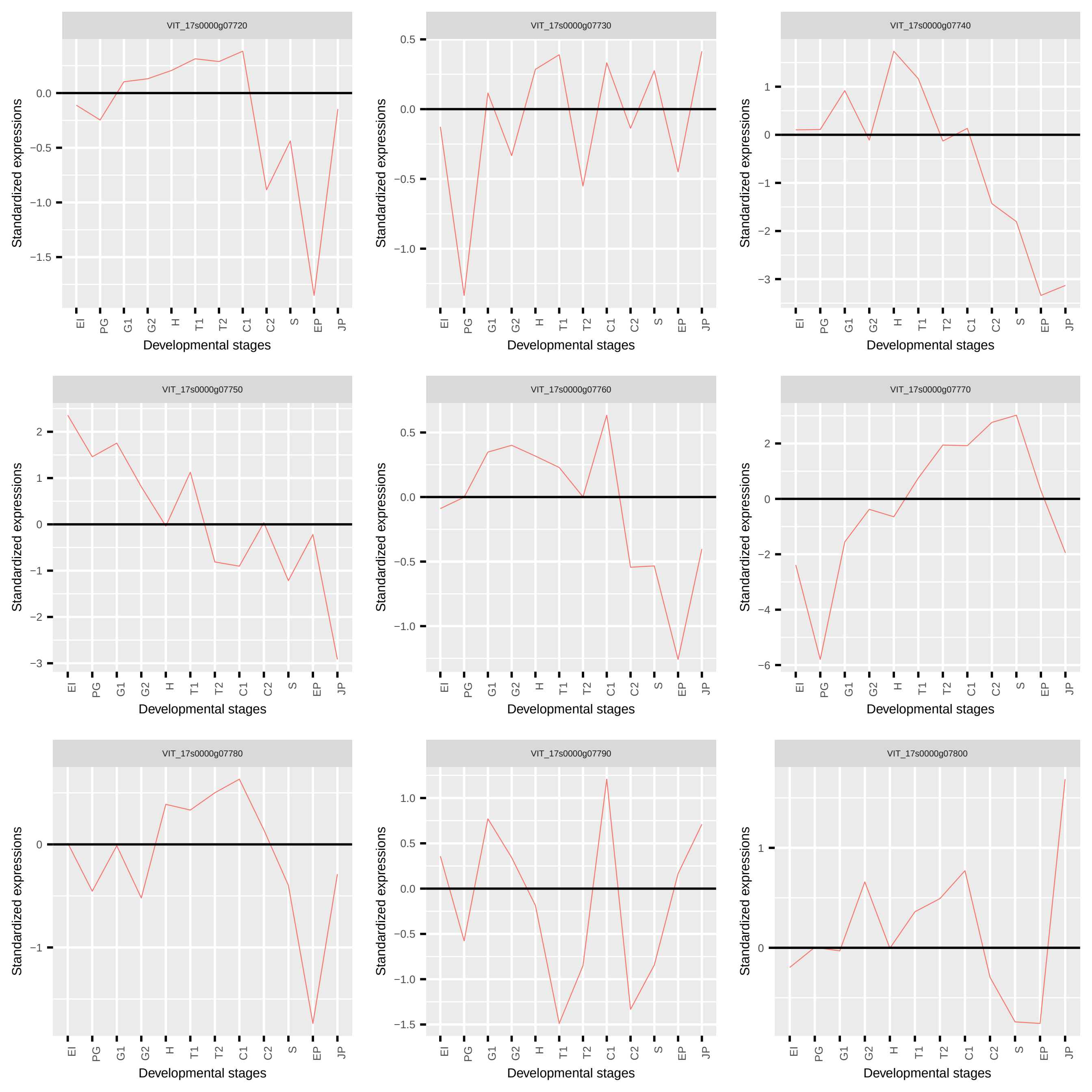

VIT_17s0000g07720
VIT_17s0000g07730
VIT_17s0000g07740
VIT_17s0000g07750
VIT_17s0000g07760
VIT_17s0000g07770
VIT_17s0000g07780
VIT_17s0000g07790
VIT_17s0000g07800
Standardized expressions
Developmental stages
EI
PG
G1
G2
H
T1
T2
C1
C2
S
EP
JP

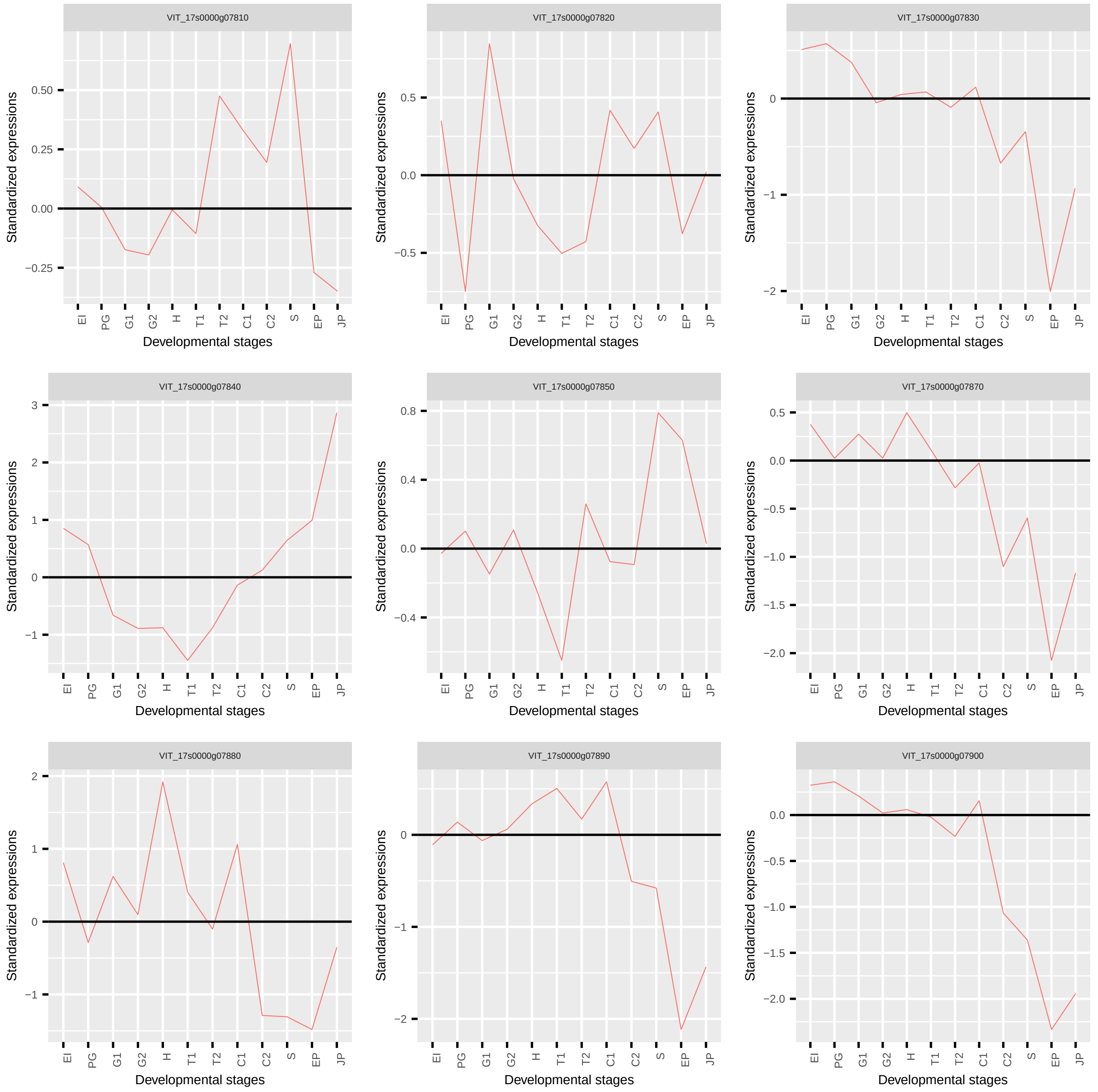
VIT_17s0000g07810
VIT_17s0000g07820
VIT_17s0000g07830
VIT_17s0000g07840
VIT_17s0000g07850
VIT_17s0000g07870
VIT_17s0000g07880
VIT_17s0000g07890
VIT_17s0000g07900
Standardized expressions
Developmental stages
EI PG G1 G2 H T1 T2 C1 C2 S EP JP

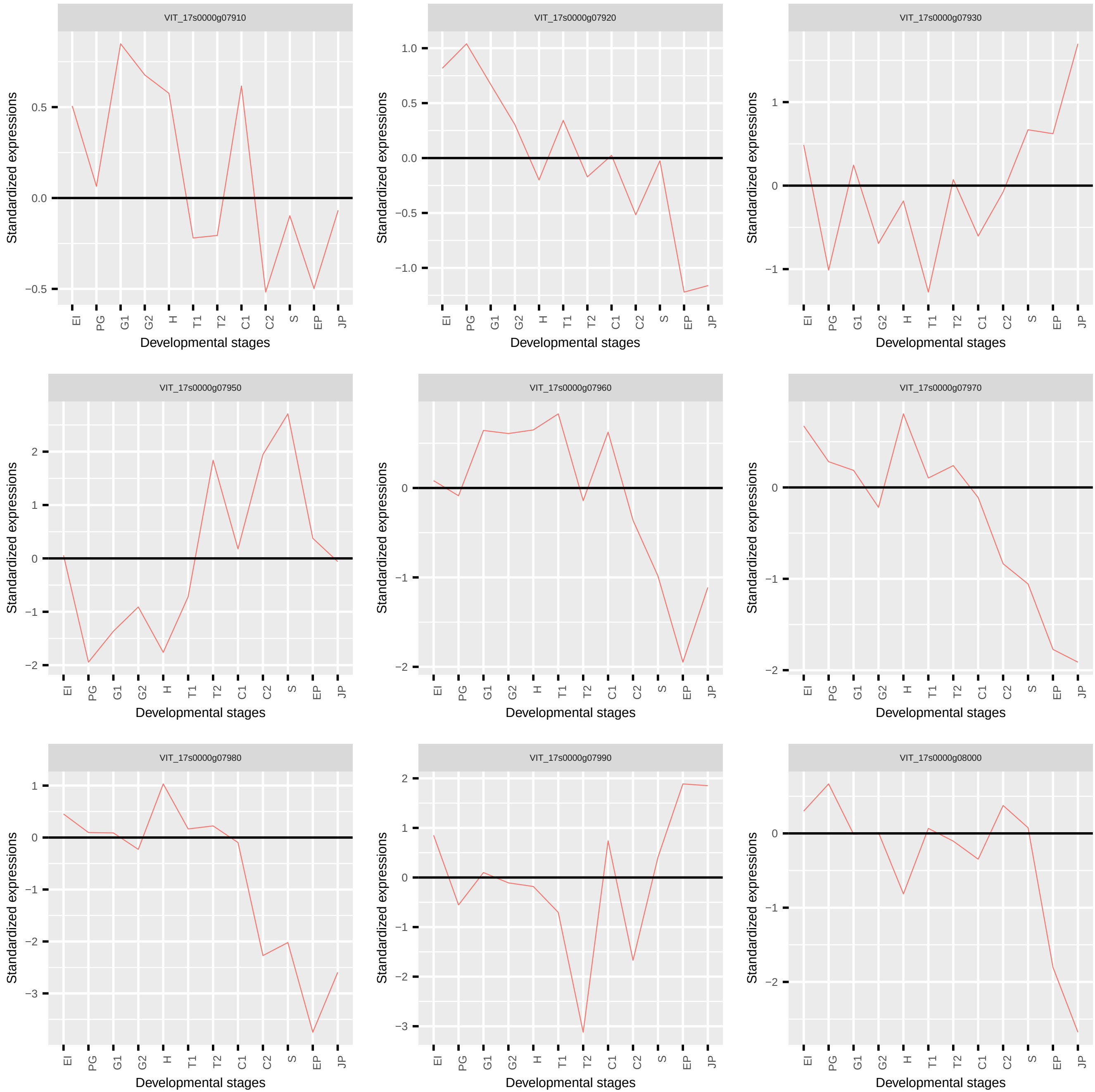

VIT_17s0000g07910
VIT_17s0000g07920
VIT_17s0000g07930
VIT_17s0000g07950
VIT_17s0000g07960
VIT_17s0000g07970
VIT_17s0000g07980
VIT_17s0000g07990
VIT_17s0000g08000
Standardized expressions
Developmental stages
EI PG G1 G2 H T1 T2 C1 C2 S EP JP

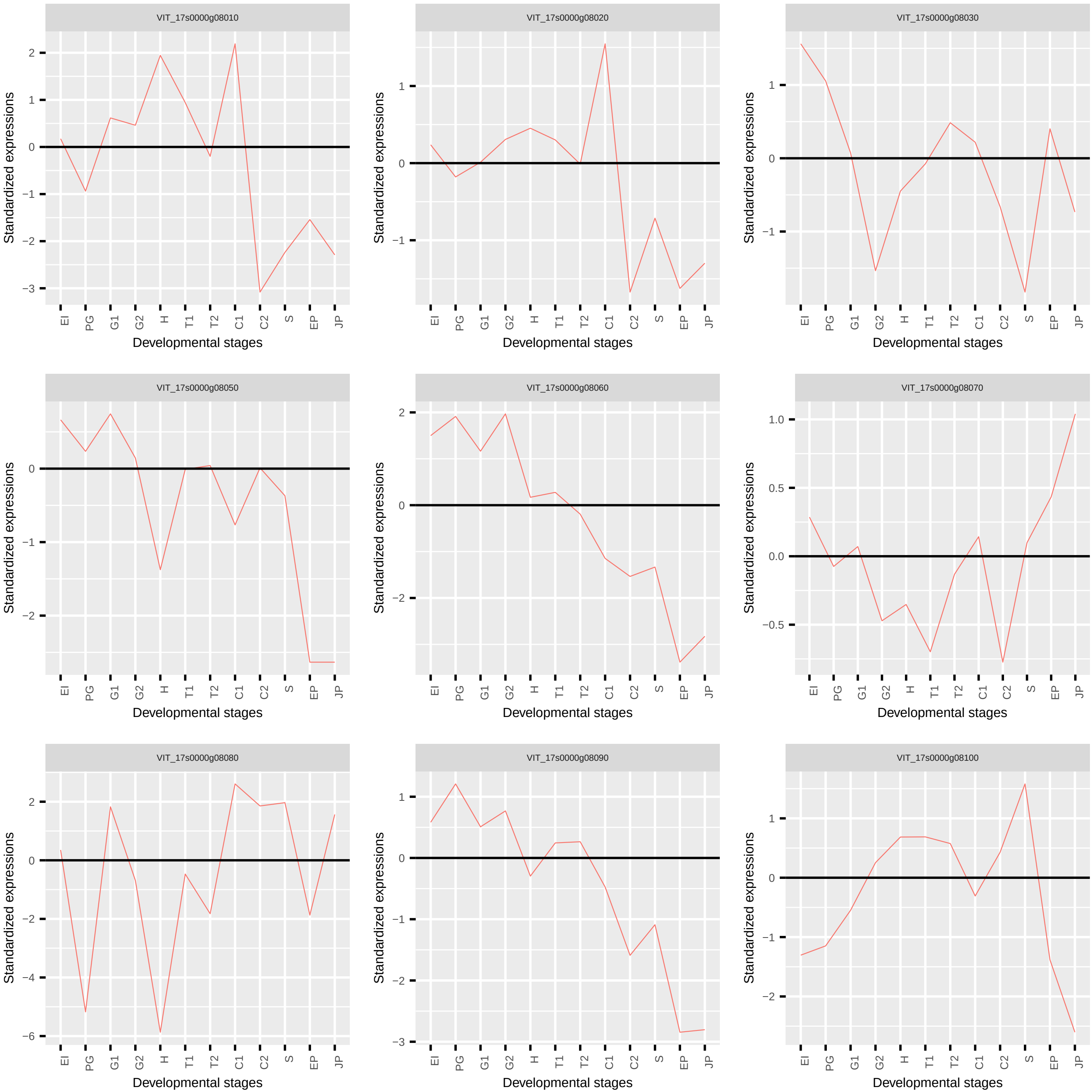
VIT_17s0000g08010
VIT_17s0000g08020
VIT_17s0000g08030
VIT_17s0000g08050
VIT_17s0000g08060
VIT_17s0000g08070
VIT_17s0000g08080
VIT_17s0000g08090
VIT_17s0000g08100
Standardized expressions
Developmental stages
EI PG G1 G2 H T1 T2 C1 C2 S EP JP

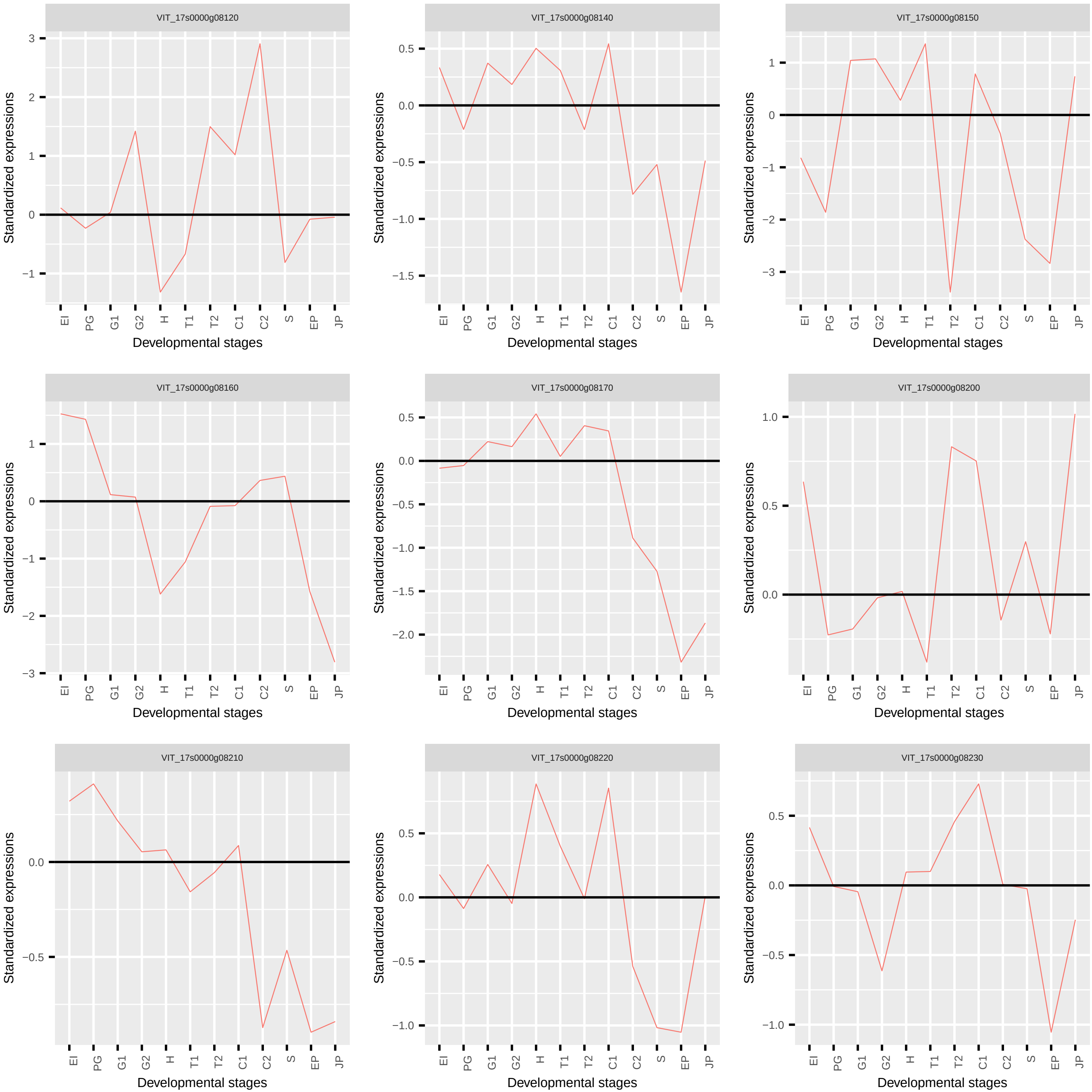
VIT_17s0000g08120
3
2
1
0
−1
Standardized expressions
EI
PG
G1
G2
H
T1
T2
C1
C2
S
EP
JP
Developmental stages
VIT_17s0000g08140
0.5
0.0
−0.5
−1.0
−1.5
Standardized expressions
EI
PG
G1
G2
H
T1
T2
C1
C2
S
EP
JP
Developmental stages
VIT_17s0000g08150
1
0
−1
−2
−3
Standardized expressions
EI
PG
G1
G2
H
T1
T2
C1
C2
S
EP
JP
Developmental stages
VIT_17s0000g08160
1
0
−1
−2
−3
Standardized expressions
EI
PG
G1
G2
H
T1
T2
C1
C2
S
EP
JP
Developmental stages
VIT_17s0000g08170
0.5
0.0
−0.5
−1.0
−1.5
−2.0
Standardized expressions
EI
PG
G1
G2
H
T1
T2
C1
C2
S
EP
JP
Developmental stages
VIT_17s0000g08200
1.0
0.5
0.0
Standardized expressions
EI
PG
G1
G2
H
T1
T2
C1
C2
S
EP
JP
Developmental stages
VIT_17s0000g08210
0.0
−0.5
Standardized expressions
EI
PG
G1
G2
H
T1
T2
C1
C2
S
EP
JP
Developmental stages
VIT_17s0000g08220
0.5
0.0
−0.5
−1.0
Standardized expressions
EI
PG
G1
G2
H
T1
T2
C1
C2
S
EP
JP
Developmental stages
VIT_17s0000g08230
0.5
0.0
−0.5
−1.0
Standardized expressions
EI
PG
G1
G2
H
T1
T2
C1
C2
S
EP
JP
Developmental stages

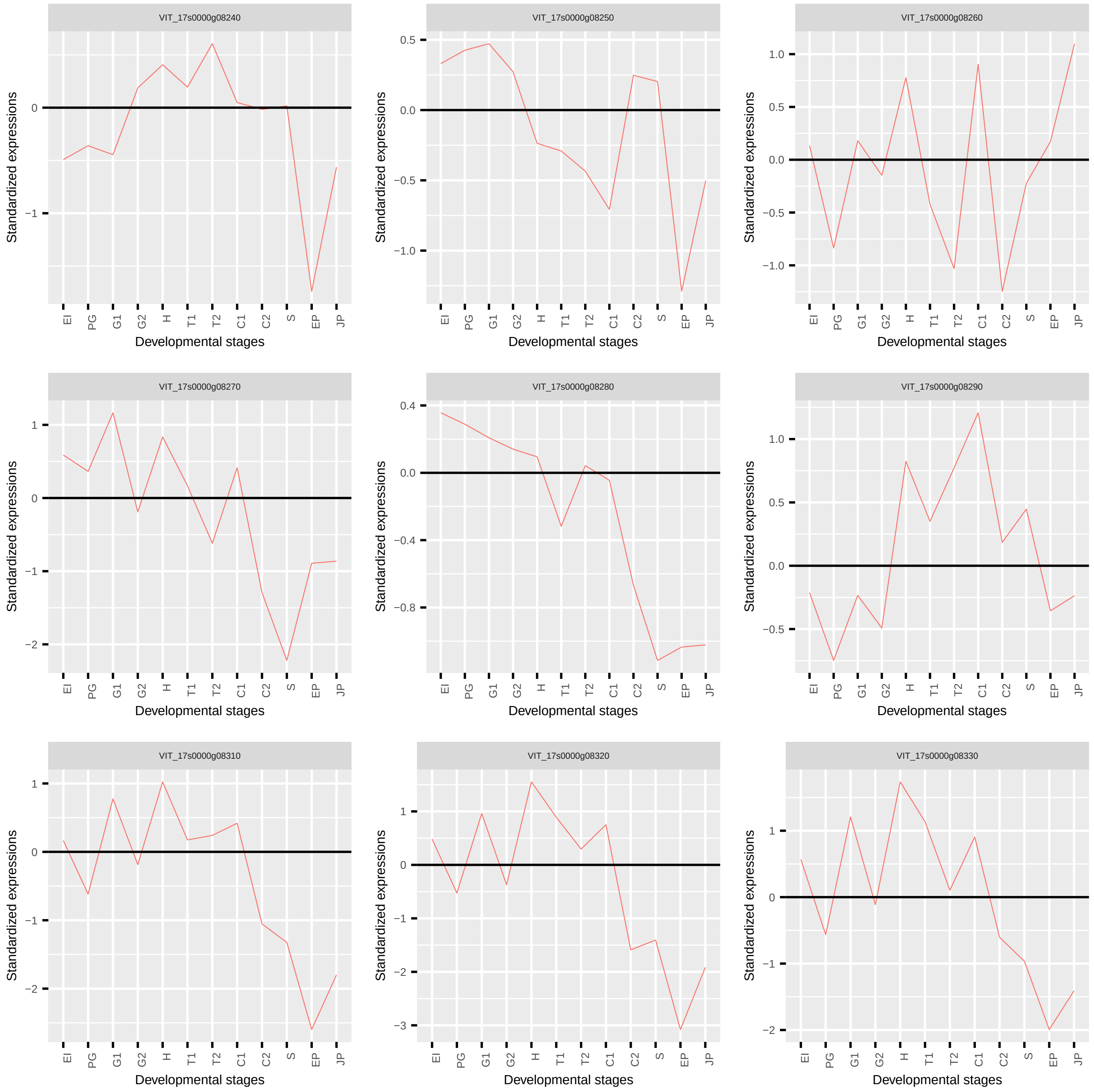
VIT_17s0000g08240
VIT_17s0000g08250
VIT_17s0000g08260
VIT_17s0000g08270
VIT_17s0000g08280
VIT_17s0000g08290
VIT_17s0000g08310
VIT_17s0000g08320
VIT_17s0000g08330
Standardized expressions
Developmental stages
EI PG G1 G2 H T1 T2 C1 C2 S EP JP

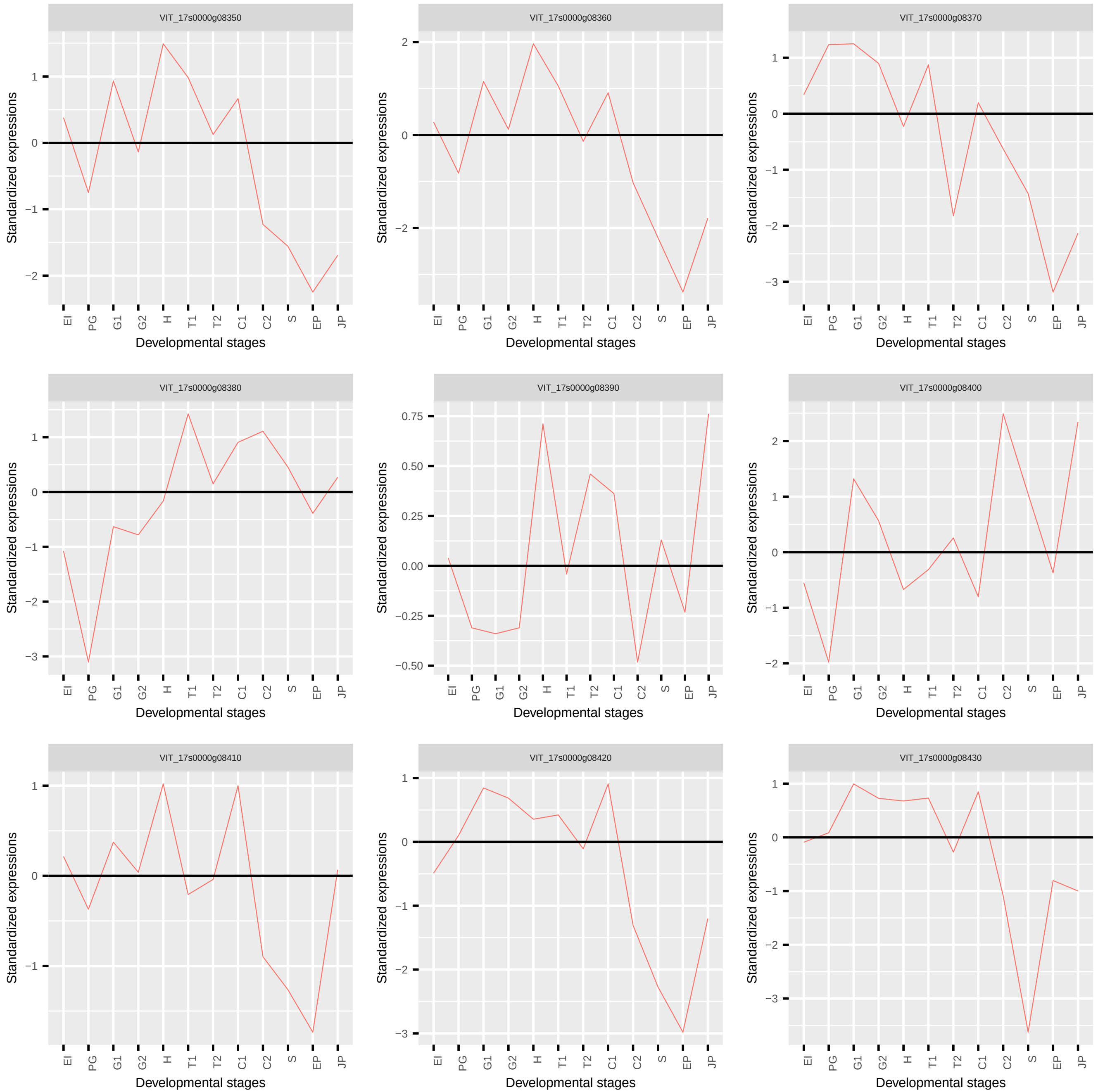
VIT_17s0000g08350
VIT_17s0000g08360
VIT_17s0000g08370
VIT_17s0000g08380
VIT_17s0000g08390
VIT_17s0000g08400
VIT_17s0000g08410
VIT_17s0000g08420
VIT_17s0000g08430
Standardized expressions
Developmental stages
EI
PG
G1
G2
H
T1
T2
C1
C2
S
EP
JP
2
1
0
−1
−2
−3
0.75
0.50
0.25
0.00
−0.25
−0.50

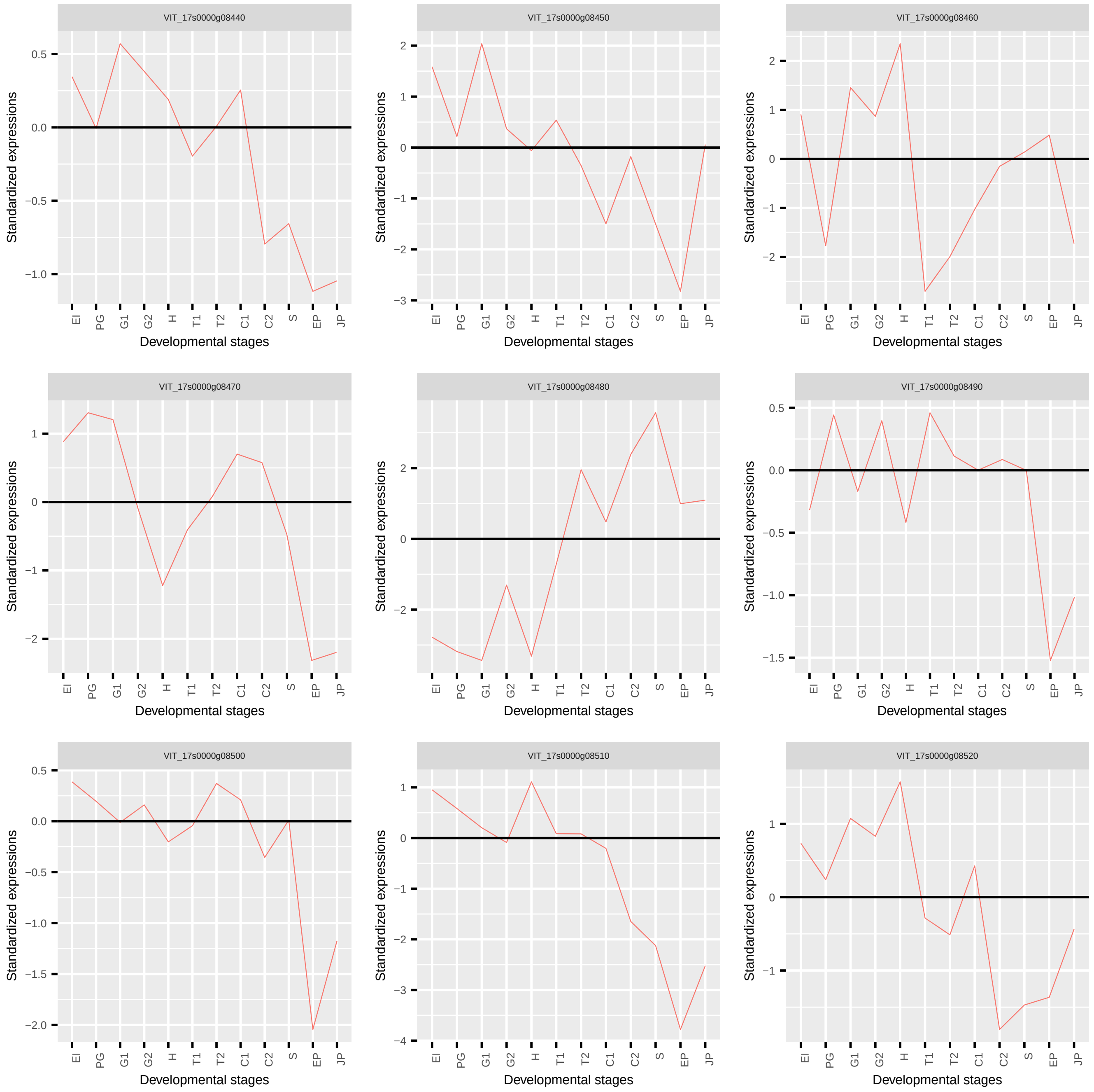
VIT_17s0000g08440
VIT_17s0000g08450
VIT_17s0000g08460
VIT_17s0000g08470
VIT_17s0000g08480
VIT_17s0000g08490
VIT_17s0000g08500
VIT_17s0000g08510
VIT_17s0000g08520
Standardized expressions
Developmental stages
EI PG G1 G2 H T1 T2 C1 C2 S EP JP

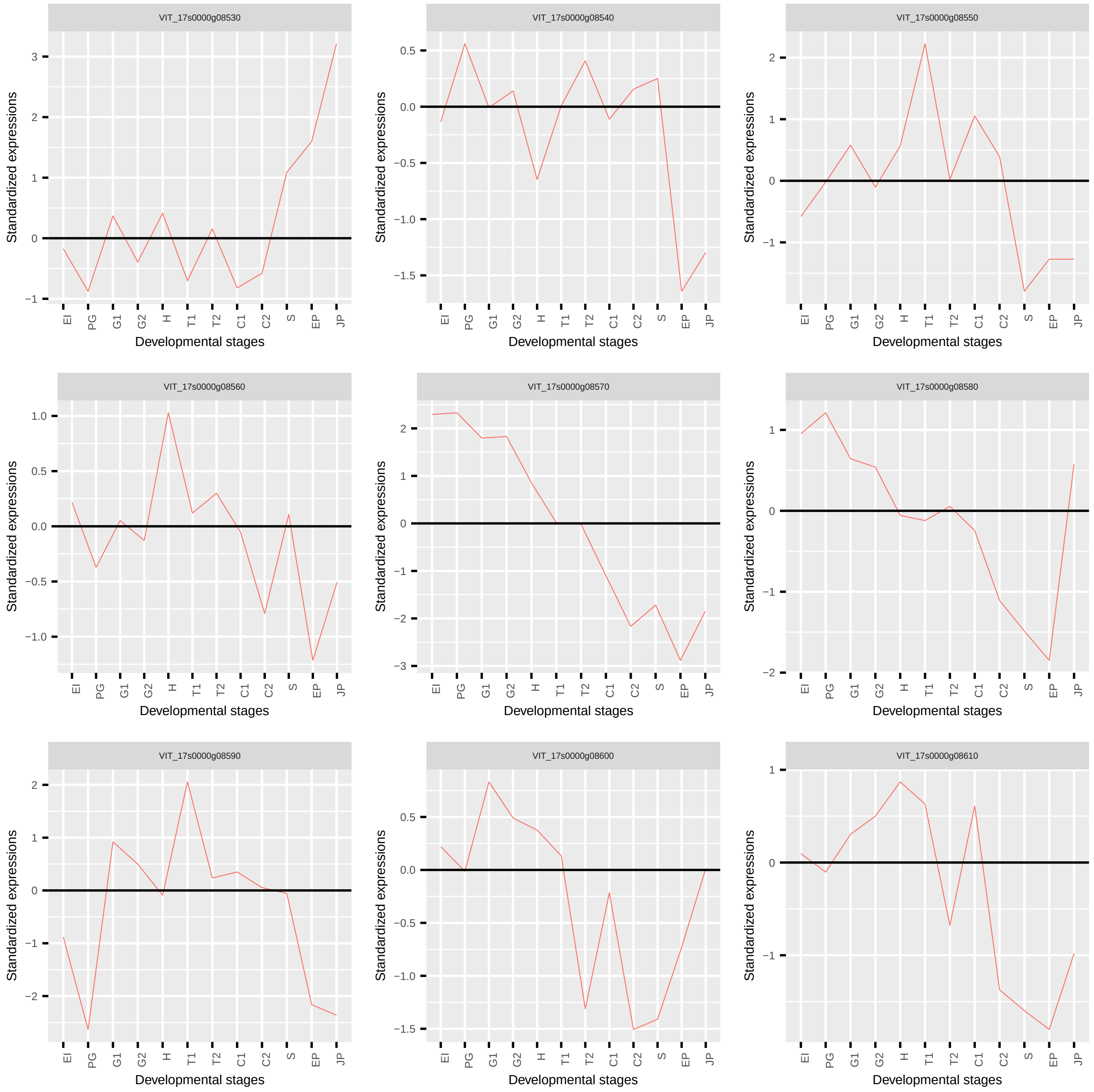
VIT_17s0000g08530
Standardized expressions
3
2
1
0
−1
EI PG G1 G2 H T1 T2 C1 C2 S EP JP
Developmental stages
VIT_17s0000g08540
Standardized expressions
0.5
0.0
−0.5
−1.0
−1.5
EI PG G1 G2 H T1 T2 C1 C2 S EP JP
Developmental stages
VIT_17s0000g08550
Standardized expressions
2
1
0
−1
EI PG G1 G2 H T1 T2 C1 C2 S EP JP
Developmental stages
VIT_17s0000g08560
Standardized expressions
1.0
0.5
0.0
−0.5
−1.0
EI PG G1 G2 H T1 T2 C1 C2 S EP JP
Developmental stages
VIT_17s0000g08570
Standardized expressions
2
1
0
−1
−2
−3
EI PG G1 G2 H T1 T2 C1 C2 S EP JP
Developmental stages
VIT_17s0000g08580
Standardized expressions
1
0
−1
−2
EI PG G1 G2 H T1 T2 C1 C2 S EP JP
Developmental stages
VIT_17s0000g08590
Standardized expressions
2
1
0
−1
−2
EI PG G1 G2 H T1 T2 C1 C2 S EP JP
Developmental stages
VIT_17s0000g08600
Standardized expressions
0.5
0.0
−0.5
−1.0
−1.5
EI PG G1 G2 H T1 T2 C1 C2 S EP JP
Developmental stages
VIT_17s0000g08610
Standardized expressions
1
0
−1
EI PG G1 G2 H T1 T2 C1 C2 S EP JP
Developmental stages

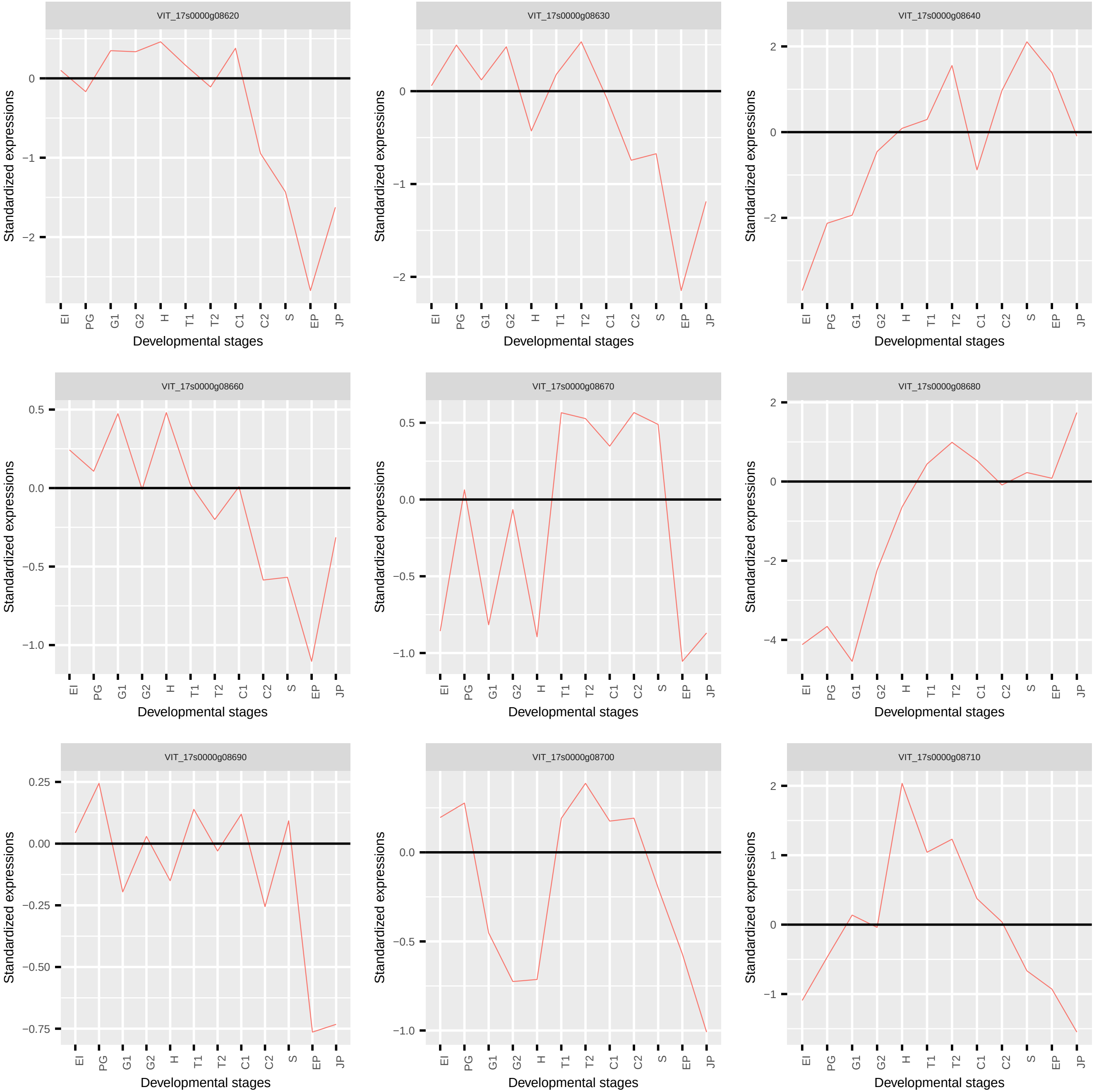
VIT_17s0000g08620
Standardized expressions
0
−1
−2
EI PG G1 G2 H T1 T2 C1 C2 S EP JP
Developmental stages
VIT_17s0000g08630
Standardized expressions
0
−1
−2
EI PG G1 G2 H T1 T2 C1 C2 S EP JP
Developmental stages
VIT_17s0000g08640
Standardized expressions
2
0
−2
EI PG G1 G2 H T1 T2 C1 C2 S EP JP
Developmental stages
VIT_17s0000g08660
Standardized expressions
0.5
0.0
−0.5
−1.0
EI PG G1 G2 H T1 T2 C1 C2 S EP JP
Developmental stages
VIT_17s0000g08670
Standardized expressions
0.5
0.0
−0.5
−1.0
EI PG G1 G2 H T1 T2 C1 C2 S EP JP
Developmental stages
VIT_17s0000g08680
Standardized expressions
2
0
−2
−4
EI PG G1 G2 H T1 T2 C1 C2 S EP JP
Developmental stages
VIT_17s0000g08690
Standardized expressions
0.25
0.00
−0.25
−0.50
−0.75
EI PG G1 G2 H T1 T2 C1 C2 S EP JP
Developmental stages
VIT_17s0000g08700
Standardized expressions
0.0
−0.5
−1.0
EI PG G1 G2 H T1 T2 C1 C2 S EP JP
Developmental stages
VIT_17s0000g08710
Standardized expressions
2
1
0
−1
EI PG G1 G2 H T1 T2 C1 C2 S EP JP
Developmental stages

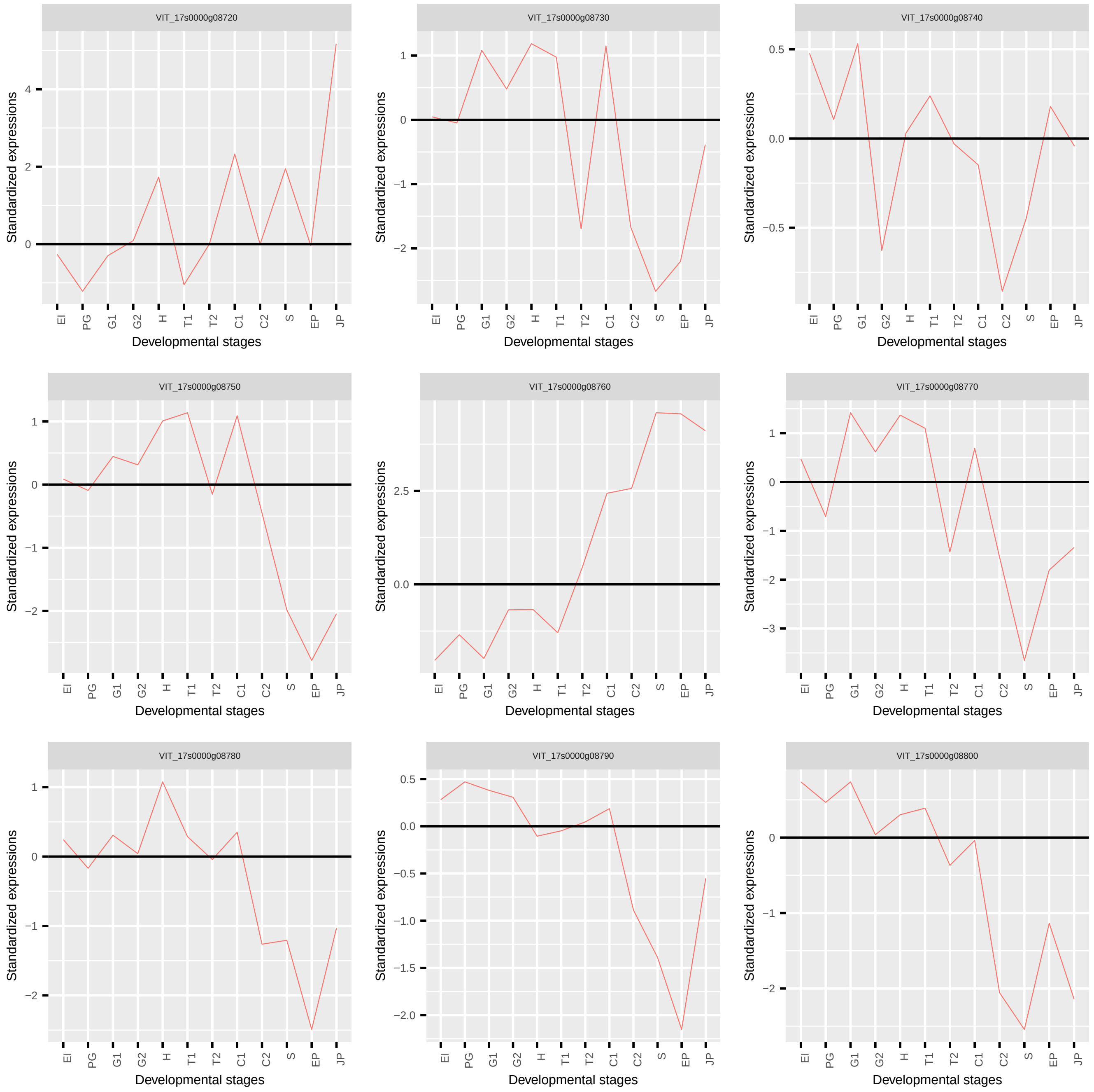
VIT_17s0000g08720
VIT_17s0000g08730
VIT_17s0000g08740
VIT_17s0000g08750
VIT_17s0000g08760
VIT_17s0000g08770
VIT_17s0000g08780
VIT_17s0000g08790
VIT_17s0000g08800
Standardized expressions
Developmental stages
EI PG G1 G2 H T1 T2 C1 C2 S EP JP

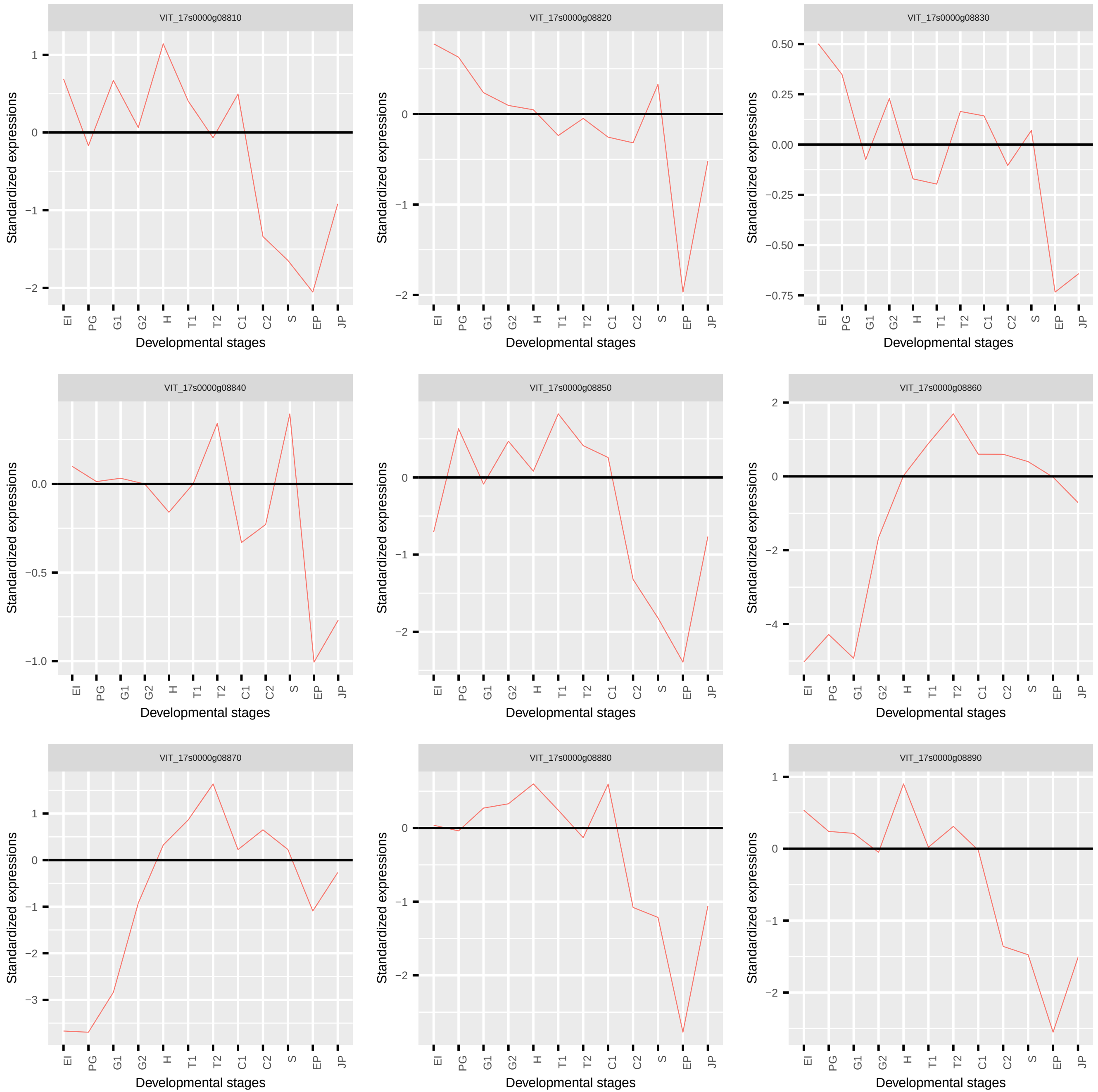
VIT_17s0000g08810
VIT_17s0000g08820
VIT_17s0000g08830
VIT_17s0000g08840
VIT_17s0000g08850
VIT_17s0000g08860
VIT_17s0000g08870
VIT_17s0000g08880
VIT_17s0000g08890
Standardized expressions
Developmental stages
EI PG G1 G2 H T1 T2 C1 C2 S EP JP

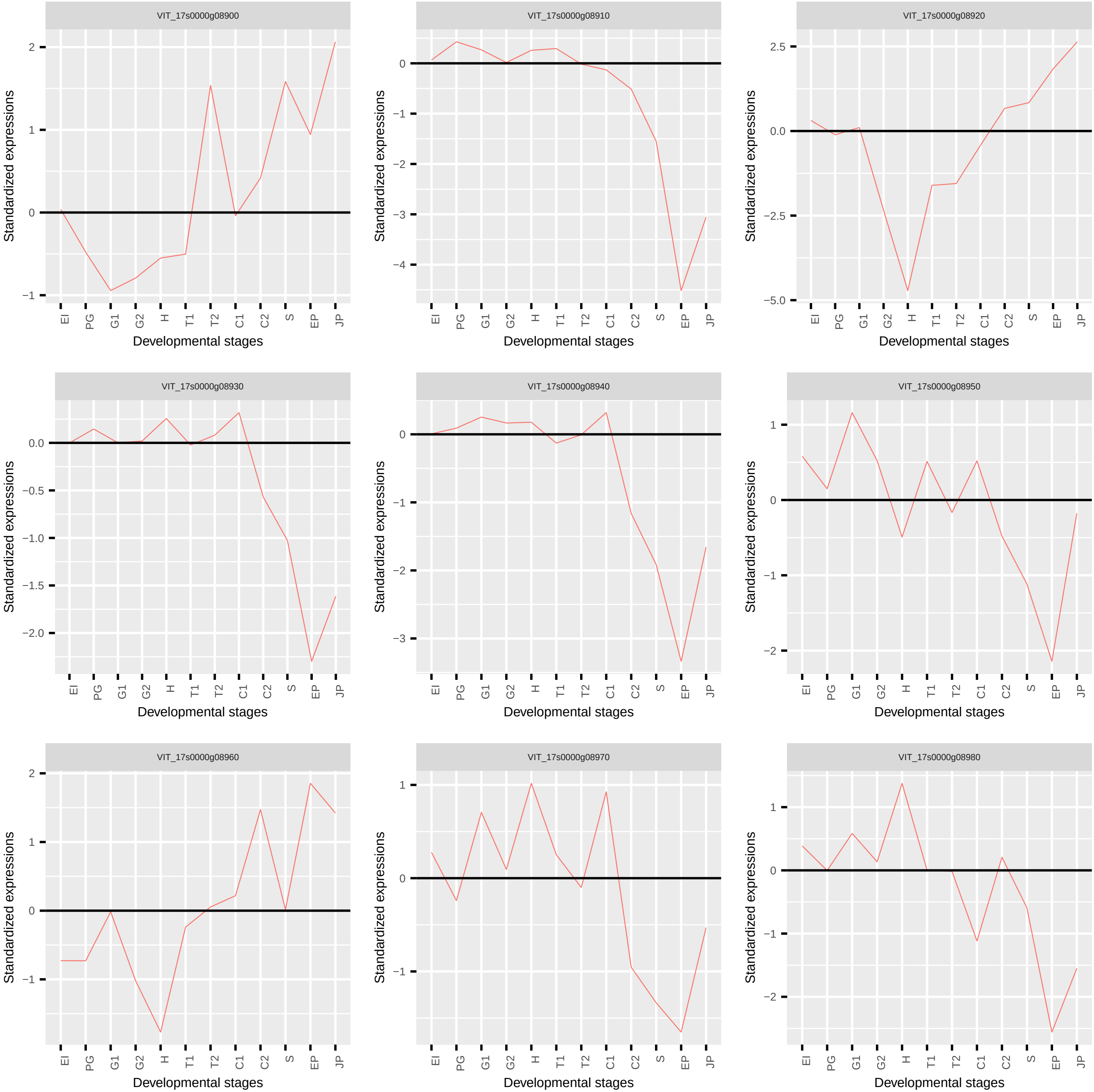
VIT_17s0000g08900
Standardized expressions
2
1
0
−1
EI PG G1 G2 H T1 T2 C1 C2 S EP JP
Developmental stages
VIT_17s0000g08910
Standardized expressions
0
−1
−2
−3
−4
EI PG G1 G2 H T1 T2 C1 C2 S EP JP
Developmental stages
VIT_17s0000g08920
Standardized expressions
2.5
0.0
−2.5
−5.0
EI PG G1 G2 H T1 T2 C1 C2 S EP JP
Developmental stages
VIT_17s0000g08930
Standardized expressions
0.0
−0.5
−1.0
−1.5
−2.0
EI PG G1 G2 H T1 T2 C1 C2 S EP JP
Developmental stages
VIT_17s0000g08940
Standardized expressions
0
−1
−2
−3
EI PG G1 G2 H T1 T2 C1 C2 S EP JP
Developmental stages
VIT_17s0000g08950
Standardized expressions
1
0
−1
−2
EI PG G1 G2 H T1 T2 C1 C2 S EP JP
Developmental stages
VIT_17s0000g08960
Standardized expressions
2
1
0
−1
EI PG G1 G2 H T1 T2 C1 C2 S EP JP
Developmental stages
VIT_17s0000g08970
Standardized expressions
1
0
−1
EI PG G1 G2 H T1 T2 C1 C2 S EP JP
Developmental stages
VIT_17s0000g08980
Standardized expressions
1
0
−1
−2
EI PG G1 G2 H T1 T2 C1 C2 S EP JP
Developmental stages

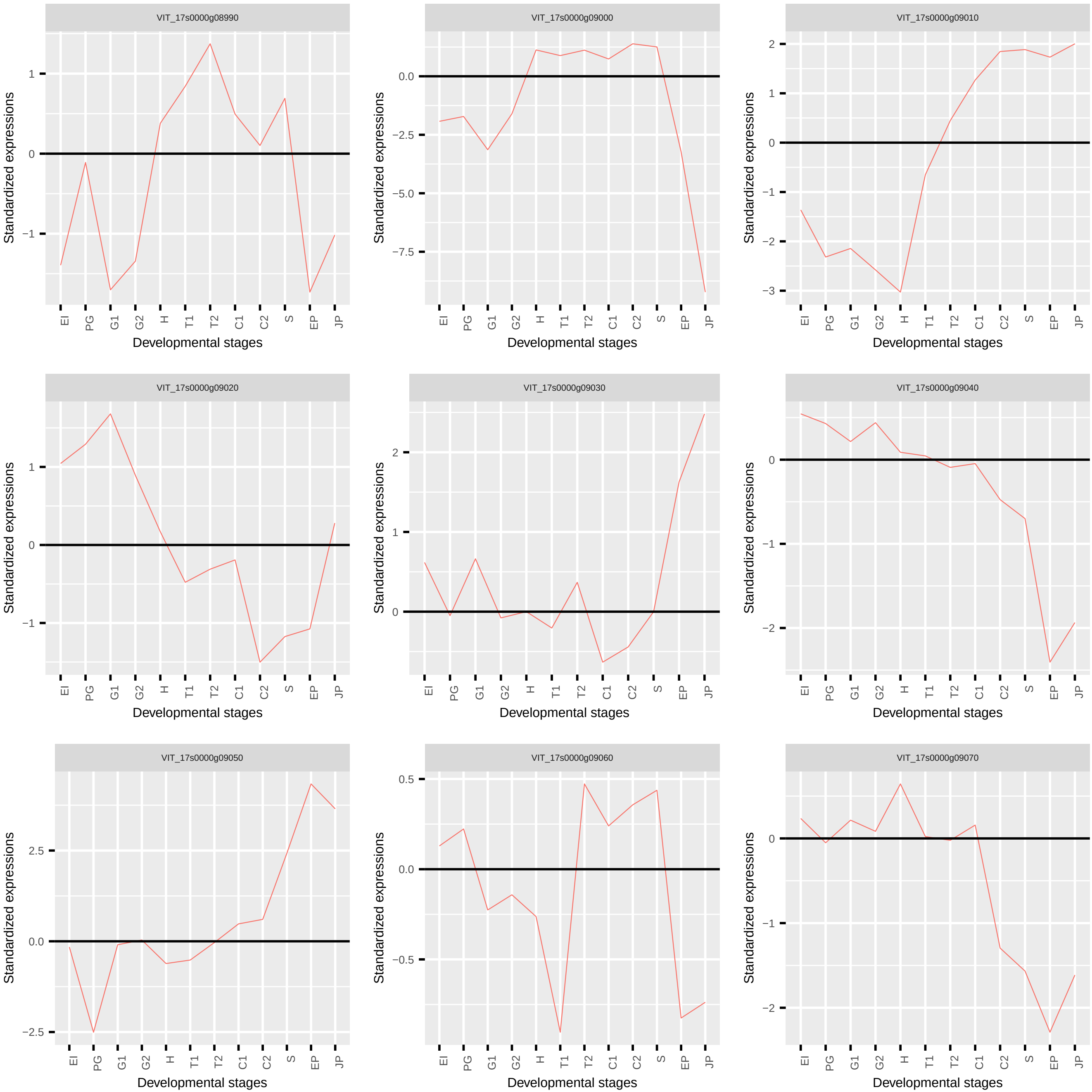
VIT_17s0000g08990
VIT_17s0000g09000
VIT_17s0000g09010
VIT_17s0000g09020
VIT_17s0000g09030
VIT_17s0000g09040
VIT_17s0000g09050
VIT_17s0000g09060
VIT_17s0000g09070
Standardized expressions
Developmental stages
EI PG G1 G2 H T1 T2 C1 C2 S EP JP

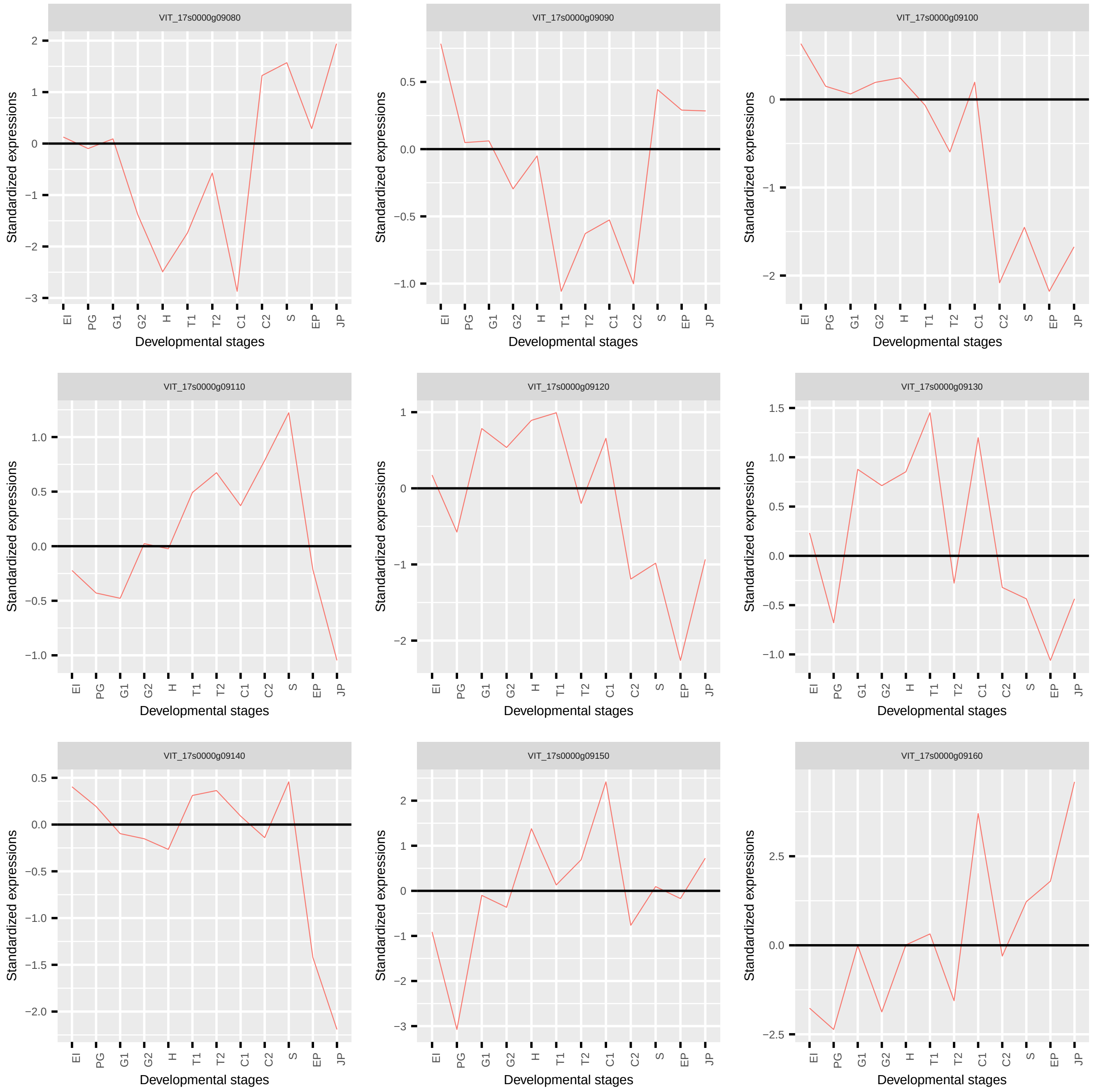
VIT_17s0000g09080
VIT_17s0000g09090
VIT_17s0000g09100
VIT_17s0000g09110
VIT_17s0000g09120
VIT_17s0000g09130
VIT_17s0000g09140
VIT_17s0000g09150
VIT_17s0000g09160
Standardized expressions
Developmental stages
EI PG G1 G2 H T1 T2 C1 C2 S EP JP

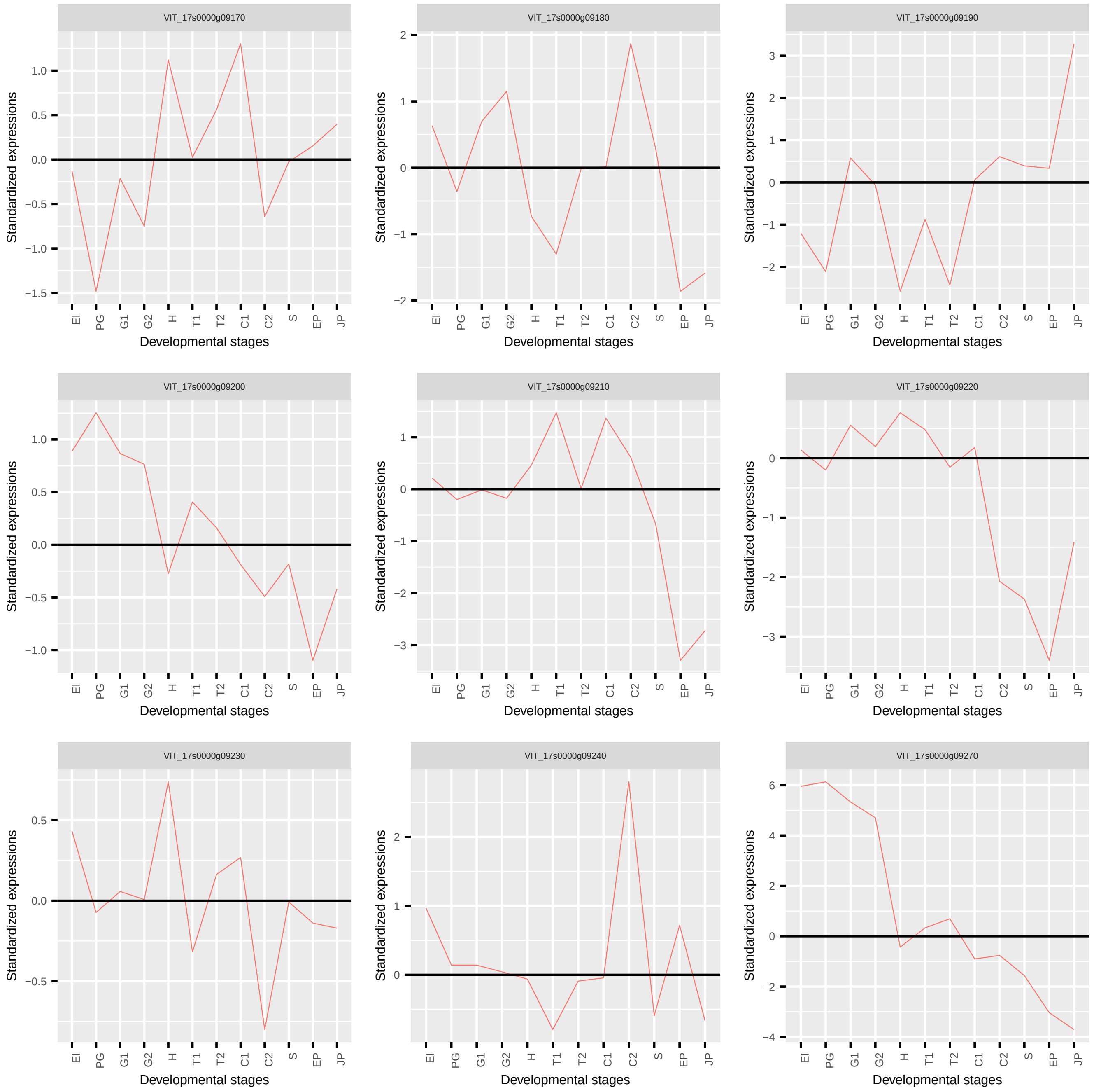
VIT_17s0000g09170
VIT_17s0000g09180
VIT_17s0000g09190
VIT_17s0000g09200
VIT_17s0000g09210
VIT_17s0000g09220
VIT_17s0000g09230
VIT_17s0000g09240
VIT_17s0000g09270
Standardized expressions
Developmental stages
EI PG G1 G2 H T1 T2 C1 C2 S EP JP

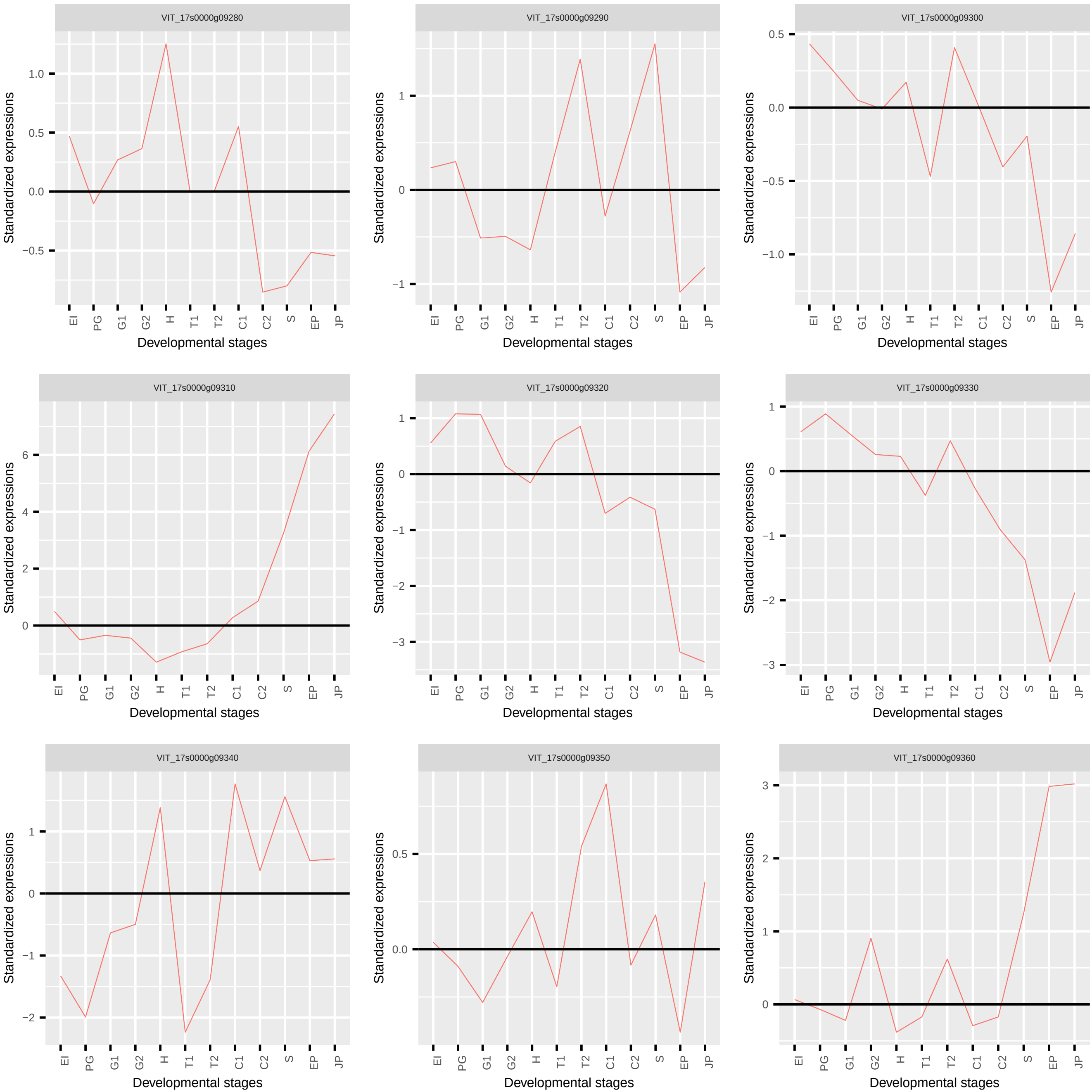
VIT_17s0000g09280
1.0
0.5
0.0
−0.5
Standardized expressions
EI PG G1 G2 H T1 T2 C1 C2 S EP JP
Developmental stages
VIT_17s0000g09290
1
0
−1
Standardized expressions
EI PG G1 G2 H T1 T2 C1 C2 S EP JP
Developmental stages
VIT_17s0000g09300
0.5
0.0
−0.5
−1.0
Standardized expressions
EI PG G1 G2 H T1 T2 C1 C2 S EP JP
Developmental stages
VIT_17s0000g09310
6
4
2
0
Standardized expressions
EI PG G1 G2 H T1 T2 C1 C2 S EP JP
Developmental stages
VIT_17s0000g09320
1
0
−1
−2
−3
Standardized expressions
EI PG G1 G2 H T1 T2 C1 C2 S EP JP
Developmental stages
VIT_17s0000g09330
1
0
−1
−2
−3
Standardized expressions
EI PG G1 G2 H T1 T2 C1 C2 S EP JP
Developmental stages
VIT_17s0000g09340
1
0
−1
−2
Standardized expressions
EI PG G1 G2 H T1 T2 C1 C2 S EP JP
Developmental stages
VIT_17s0000g09350
0.5
0.0
Standardized expressions
EI PG G1 G2 H T1 T2 C1 C2 S EP JP
Developmental stages
VIT_17s0000g09360
3
2
1
0
Standardized expressions
EI PG G1 G2 H T1 T2 C1 C2 S EP JP
Developmental stages

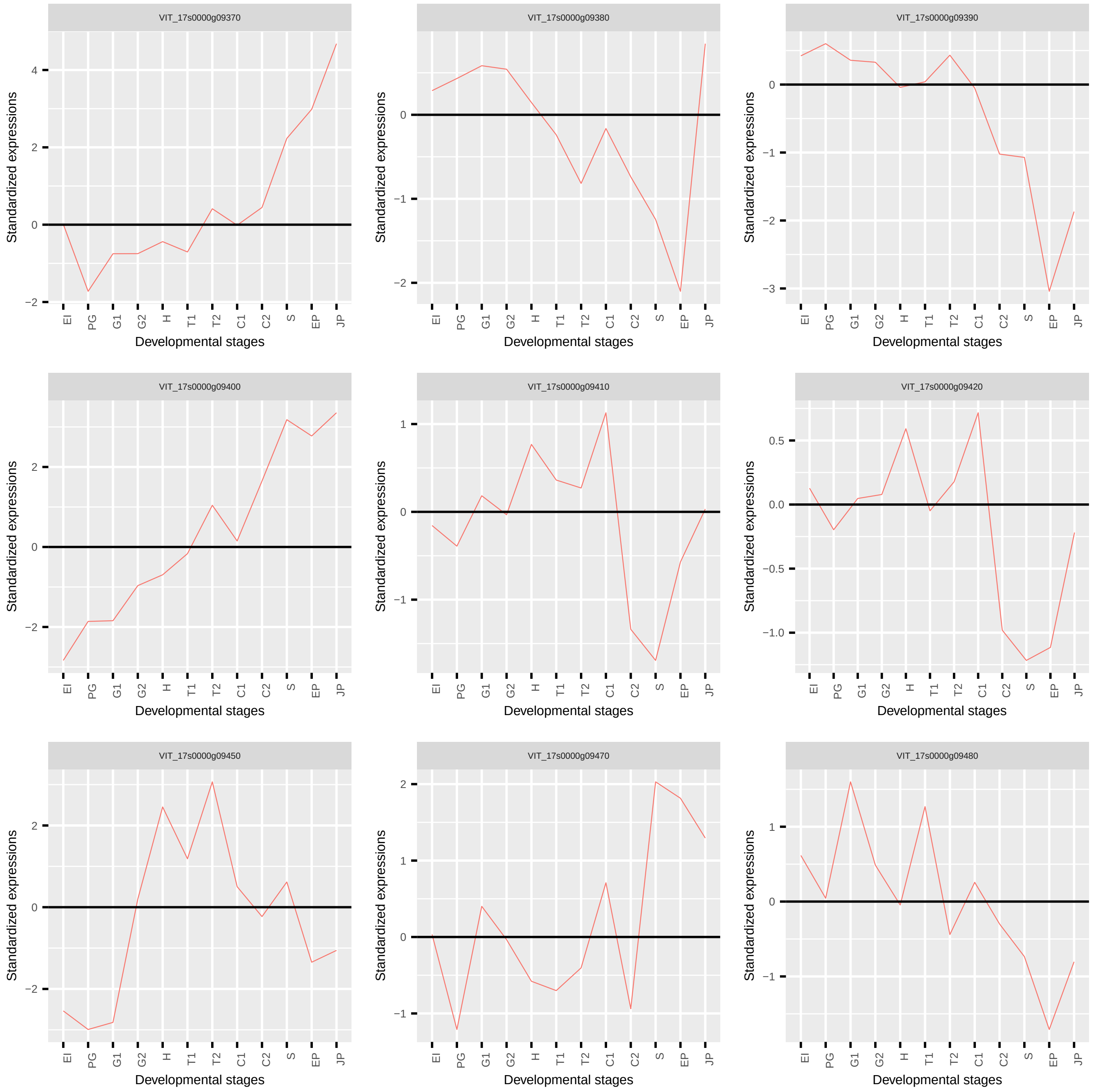
VIT_17s0000g09370
VIT_17s0000g09380
VIT_17s0000g09390
VIT_17s0000g09400
VIT_17s0000g09410
VIT_17s0000g09420
VIT_17s0000g09450
VIT_17s0000g09470
VIT_17s0000g09480
Standardized expressions
Developmental stages
EI
PG
G1
G2
H
T1
T2
C1
C2
S
EP
JP

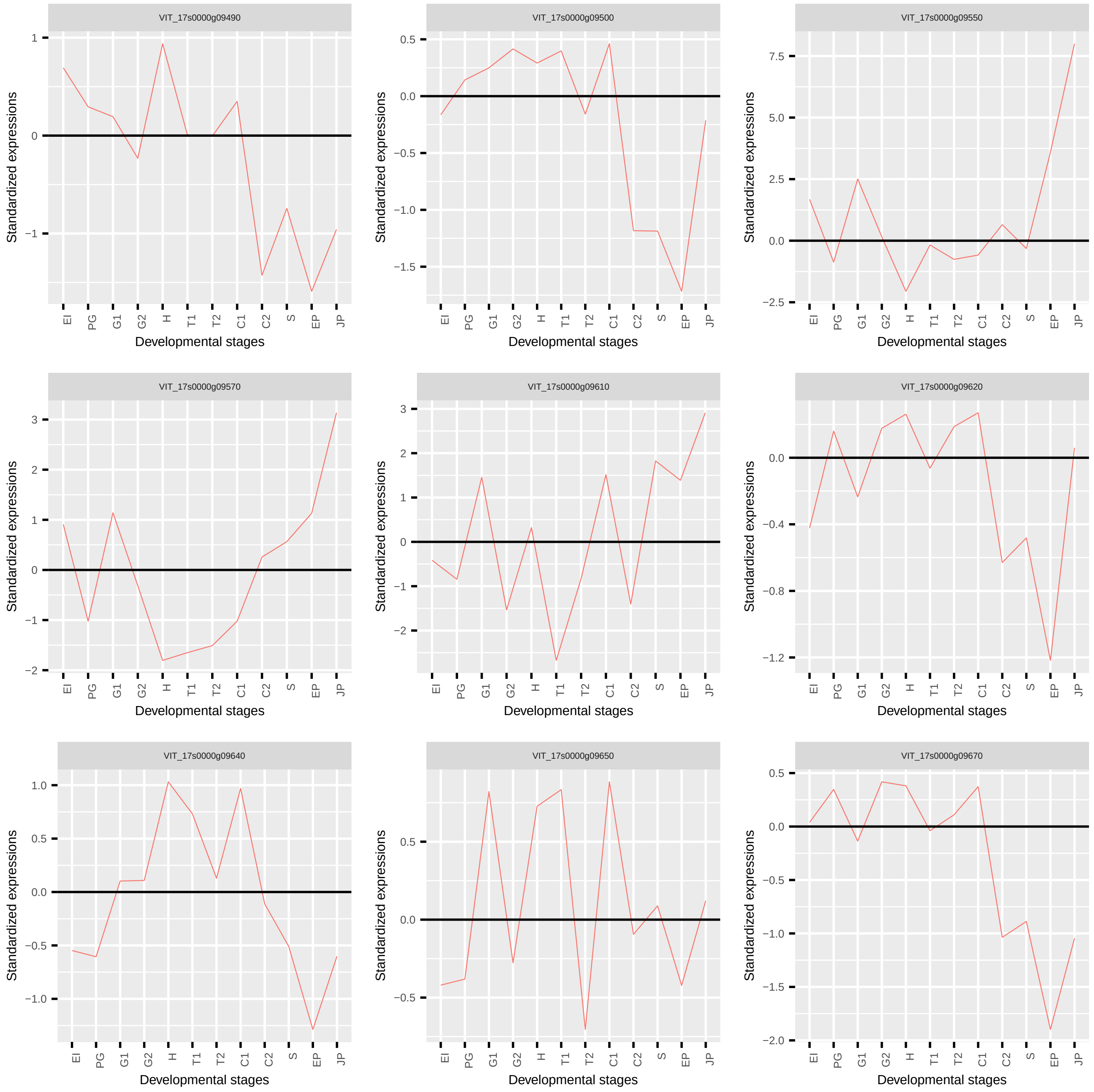
VIT_17s0000g09490
VIT_17s0000g09500
VIT_17s0000g09550
VIT_17s0000g09570
VIT_17s0000g09610
VIT_17s0000g09620
VIT_17s0000g09640
VIT_17s0000g09650
VIT_17s0000g09670
Standardized expressions
Developmental stages
EI PG G1 G2 H T1 T2 C1 C2 S EP JP

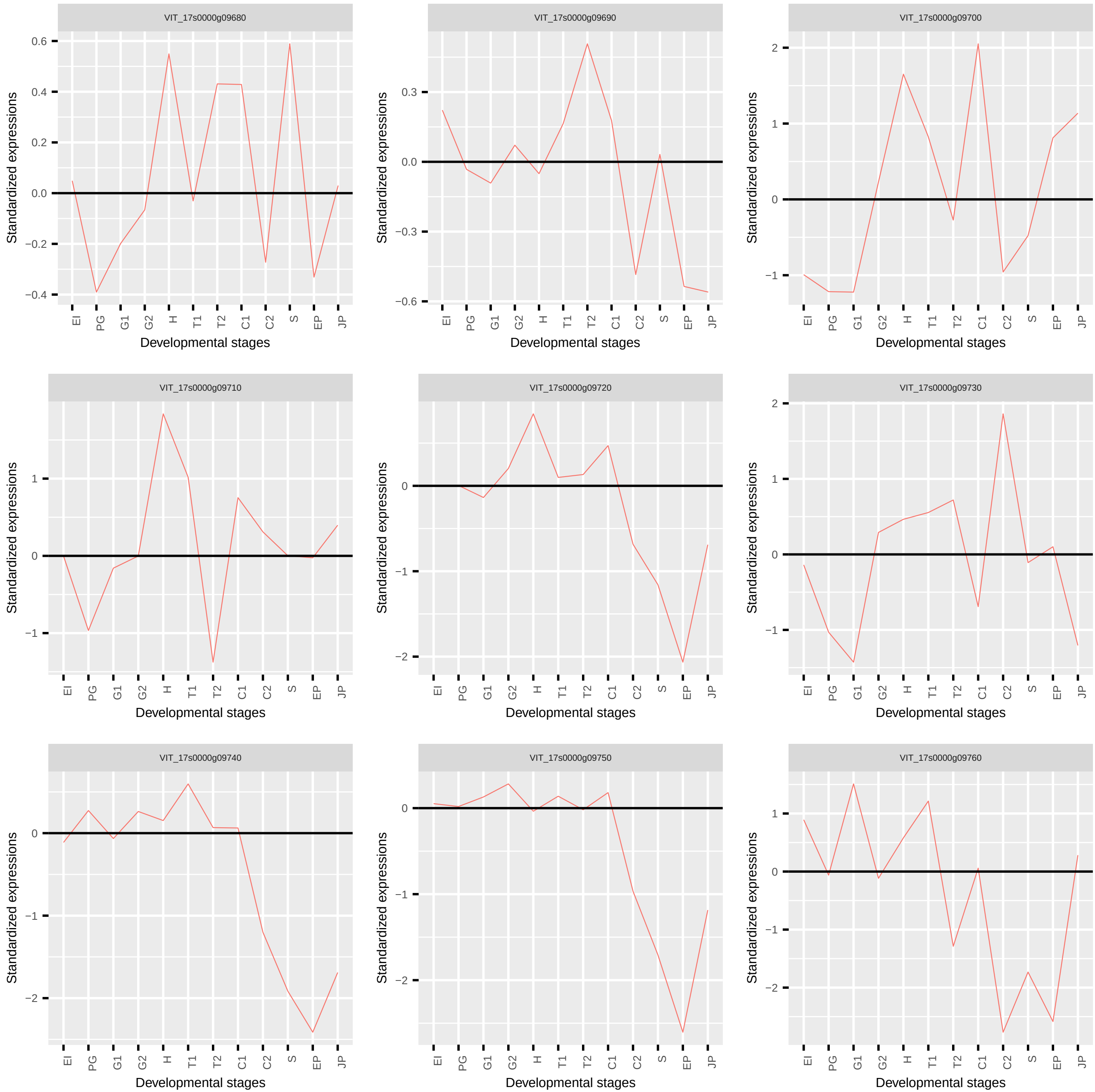

VIT_17s0000g09680
VIT_17s0000g09690
VIT_17s0000g09700
VIT_17s0000g09710
VIT_17s0000g09720
VIT_17s0000g09730
VIT_17s0000g09740
VIT_17s0000g09750
VIT_17s0000g09760
Standardized expressions
Developmental stages
EI PG G1 G2 H T1 T2 C1 C2 S EP JP

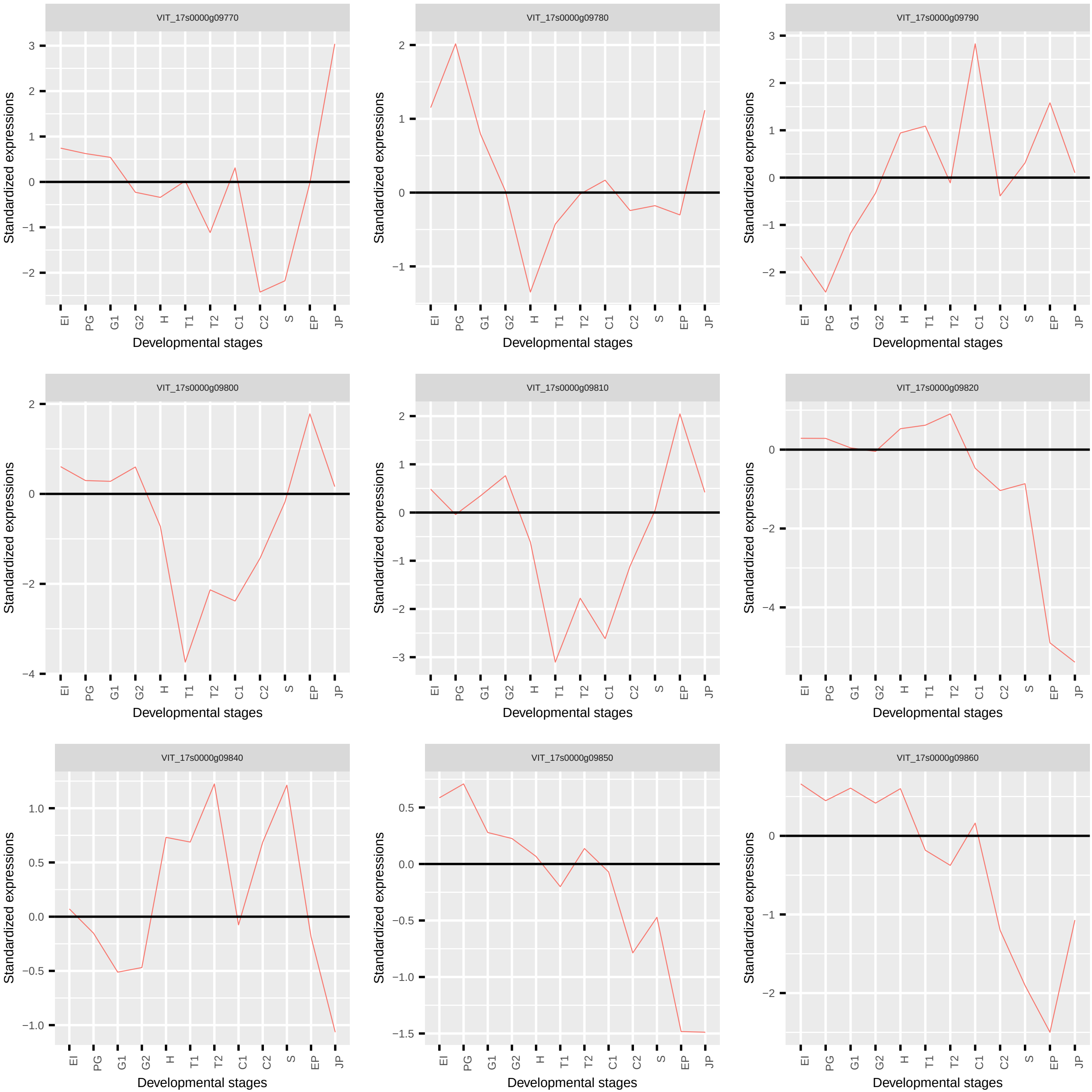

VIT_17s0000g09770
VIT_17s0000g09780
VIT_17s0000g09790
VIT_17s0000g09800
VIT_17s0000g09810
VIT_17s0000g09820
VIT_17s0000g09840
VIT_17s0000g09850
VIT_17s0000g09860
Standardized expressions
Developmental stages
EI PG G1 G2 H T1 T2 C1 C2 S EP JP

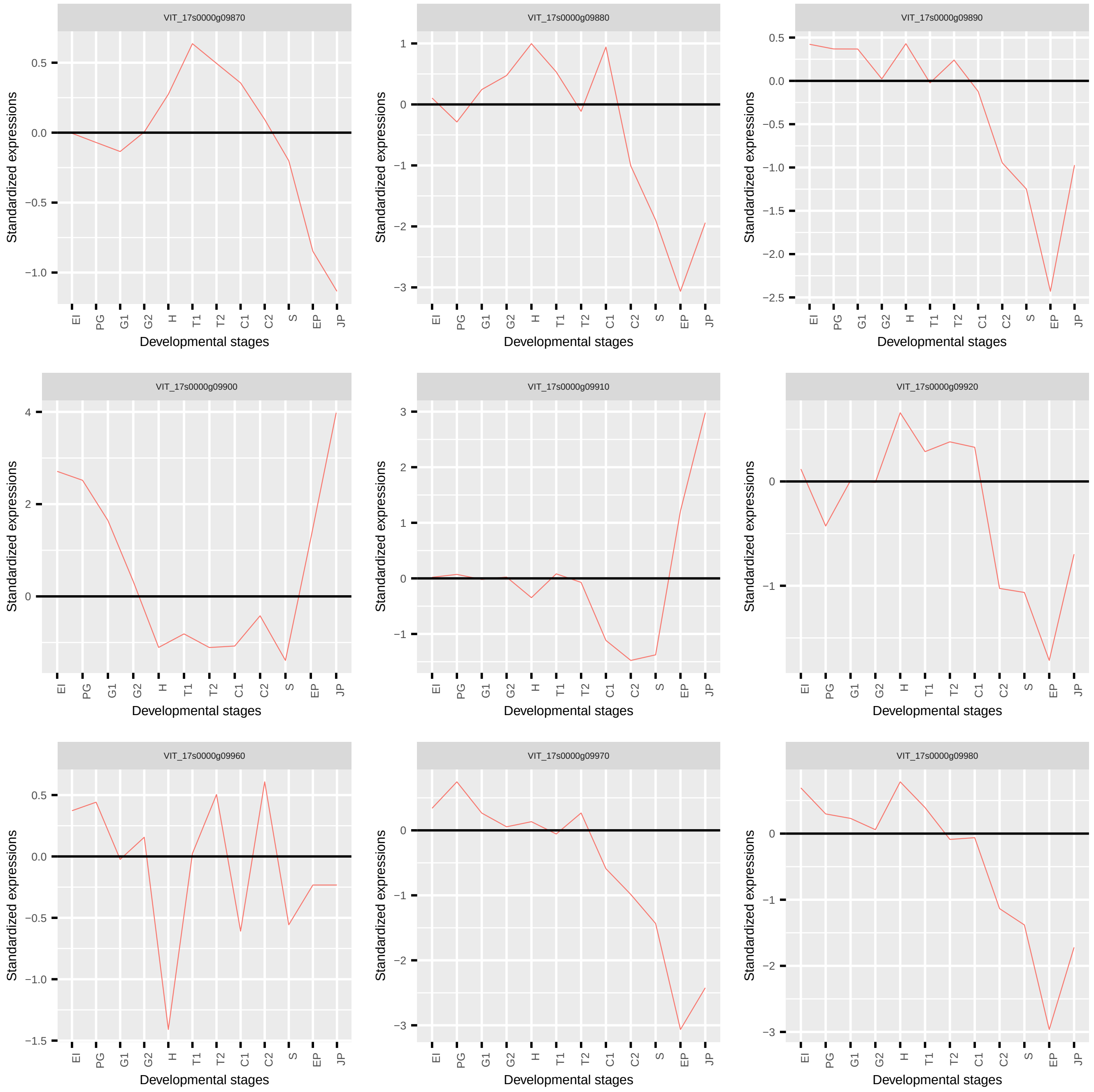

VIT_17s0000g09870
VIT_17s0000g09880
VIT_17s0000g09890
VIT_17s0000g09900
VIT_17s0000g09910
VIT_17s0000g09920
VIT_17s0000g09960
VIT_17s0000g09970
VIT_17s0000g09980
Standardized expressions
Developmental stages
EI PG G1 G2 H T1 T2 C1 C2 S EP JP

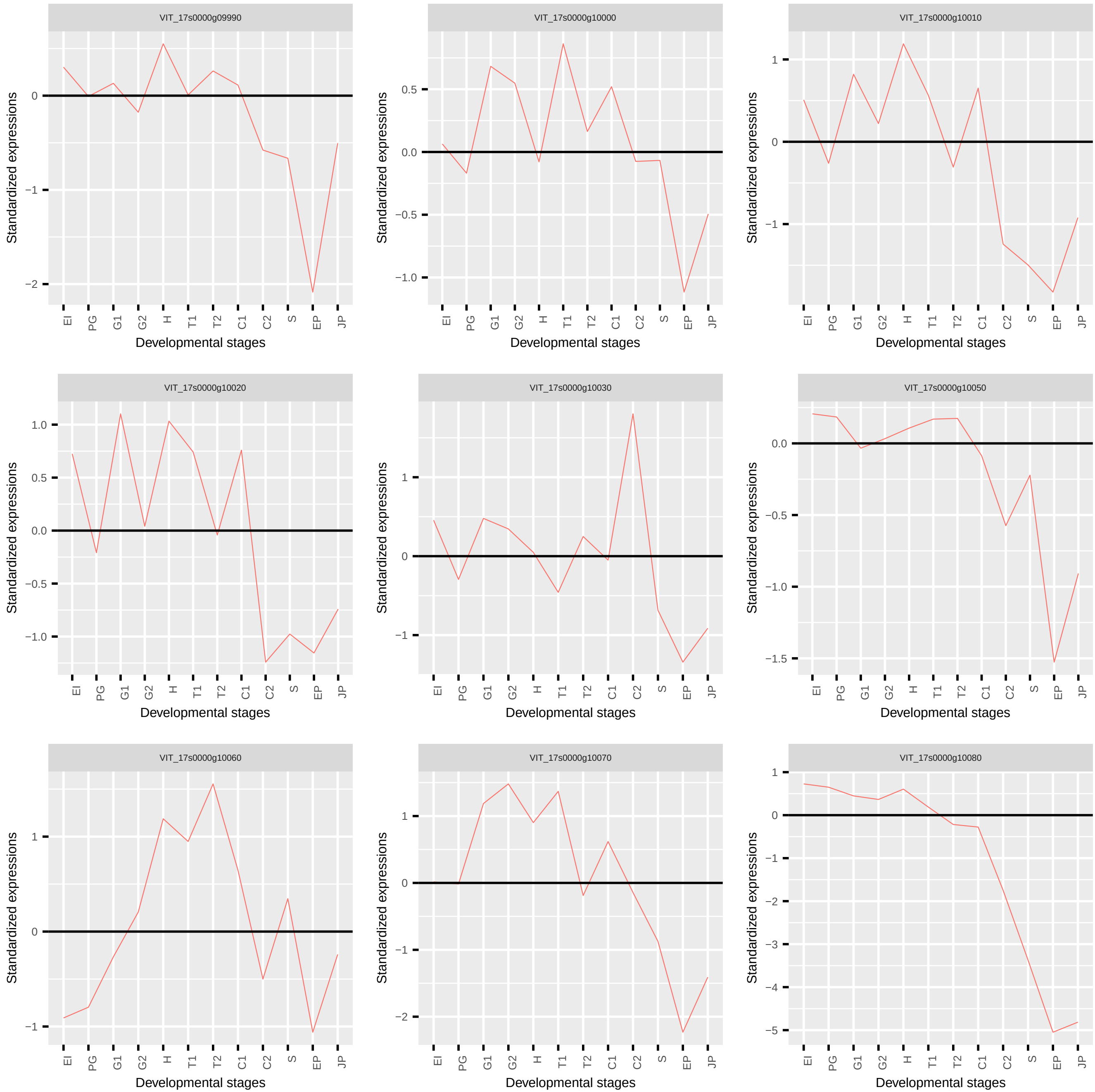
VIT_17s0000g09990
VIT_17s0000g10000
VIT_17s0000g10010
VIT_17s0000g10020
VIT_17s0000g10030
VIT_17s0000g10050
VIT_17s0000g10060
VIT_17s0000g10070
VIT_17s0000g10080
Standardized expressions
Developmental stages
EI PG G1 G2 H T1 T2 C1 C2 S EP JP
0 −1 −2
0.5 0.0 −0.5 −1.0
1 0 −1
1.0 0.5 0.0 −0.5 −1.0
1 0 −1
0.0 −0.5 −1.0 −1.5
1 0 −1
1 0 −1 −2
1 0 −1 −2 −3 −4 −5

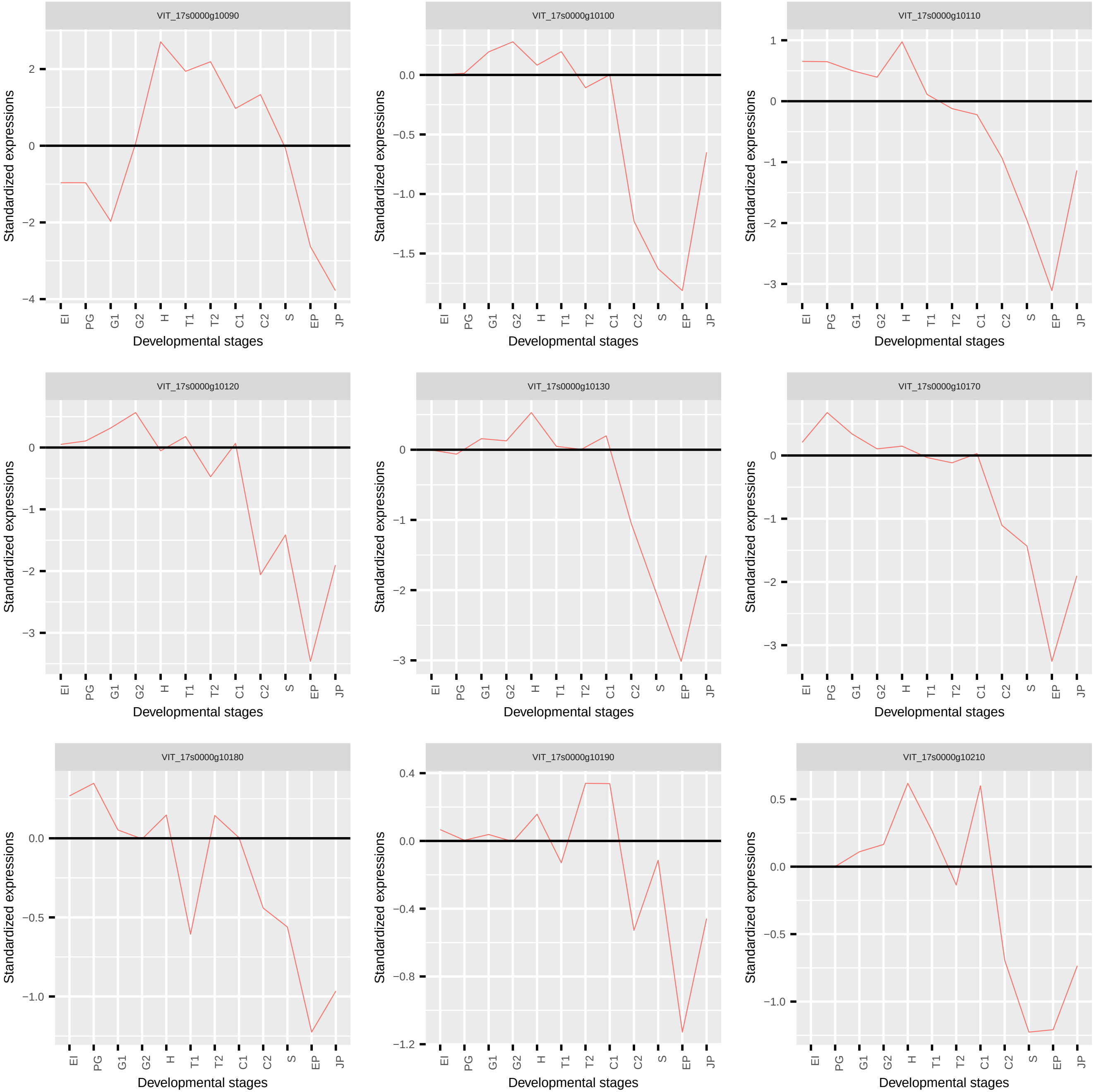
VIT_17s0000g10090
VIT_17s0000g10100
VIT_17s0000g10110
VIT_17s0000g10120
VIT_17s0000g10130
VIT_17s0000g10170
VIT_17s0000g10180
VIT_17s0000g10190
VIT_17s0000g10210
Standardized expressions
Developmental stages
EI PG G1 G2 H T1 T2 C1 C2 S EP JP
2
0
−2
−4
0.0
−0.5
−1.0
−1.5
1
−1
−3
0.4
−0.4
−0.8
−1.2
0.5

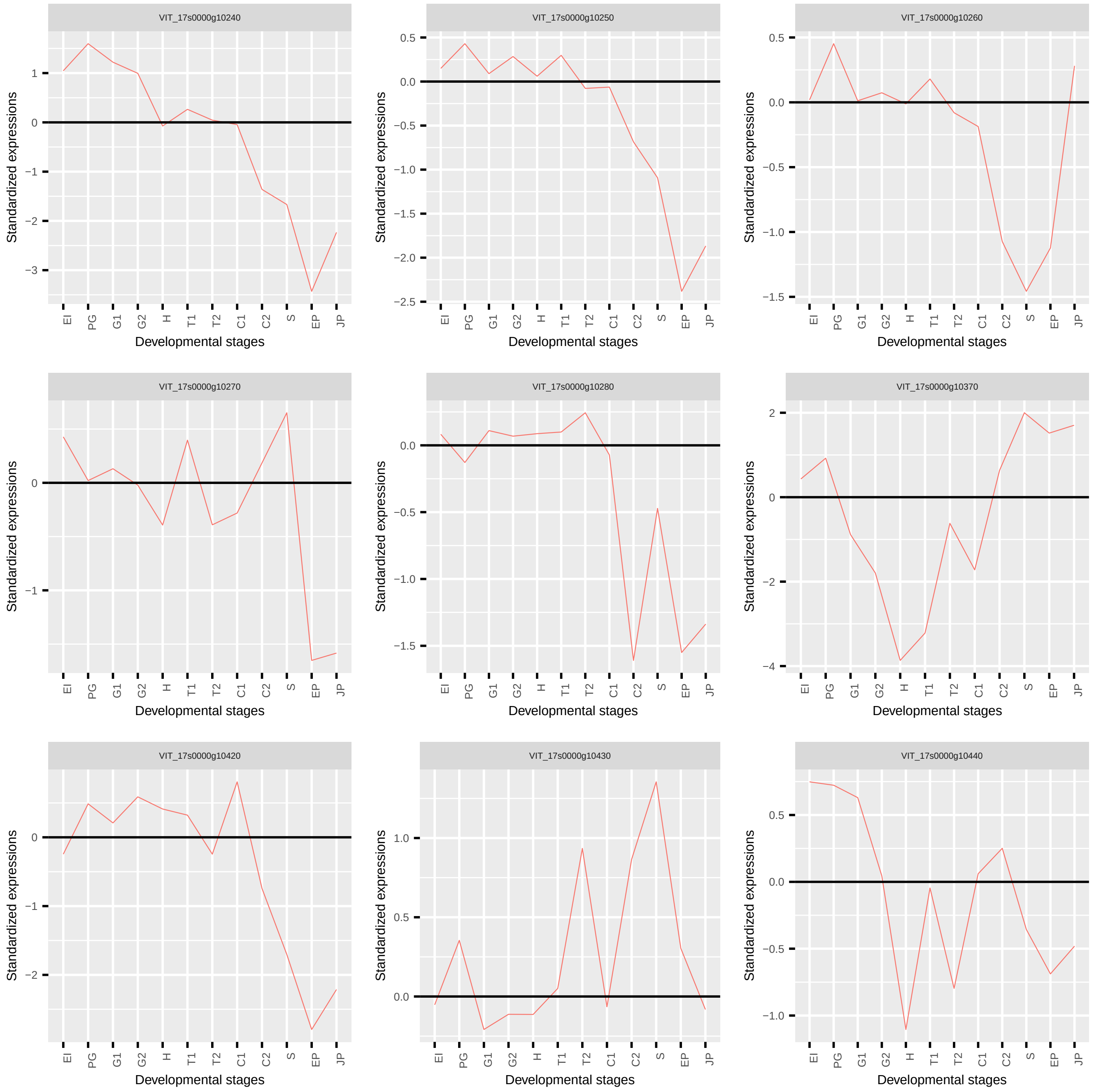
VIT_17s0000g10240
VIT_17s0000g10250
VIT_17s0000g10260
VIT_17s0000g10270
VIT_17s0000g10280
VIT_17s0000g10370
VIT_17s0000g10420
VIT_17s0000g10430
VIT_17s0000g10440
Standardized expressions
Developmental stages
EI PG G1 G2 H T1 T2 C1 C2 S EP JP

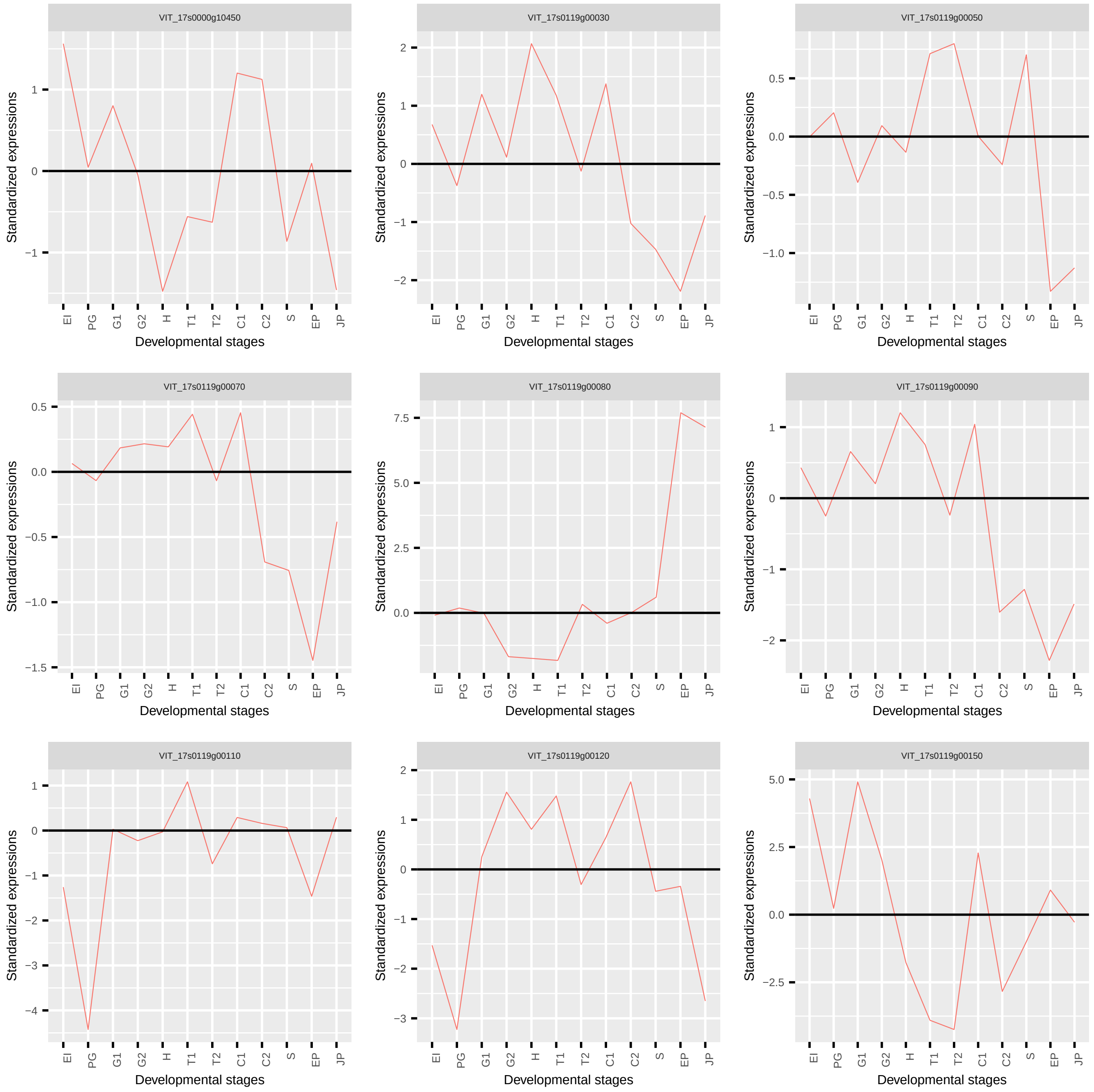
VIT_17s0000g10450
VIT_17s0119g00030
VIT_17s0119g00050
VIT_17s0119g00070
VIT_17s0119g00080
VIT_17s0119g00090
VIT_17s0119g00110
VIT_17s0119g00120
VIT_17s0119g00150
Standardized expressions
Developmental stages
EI
PG
G1
G2
H
T1
T2
C1
C2
S
EP
JP

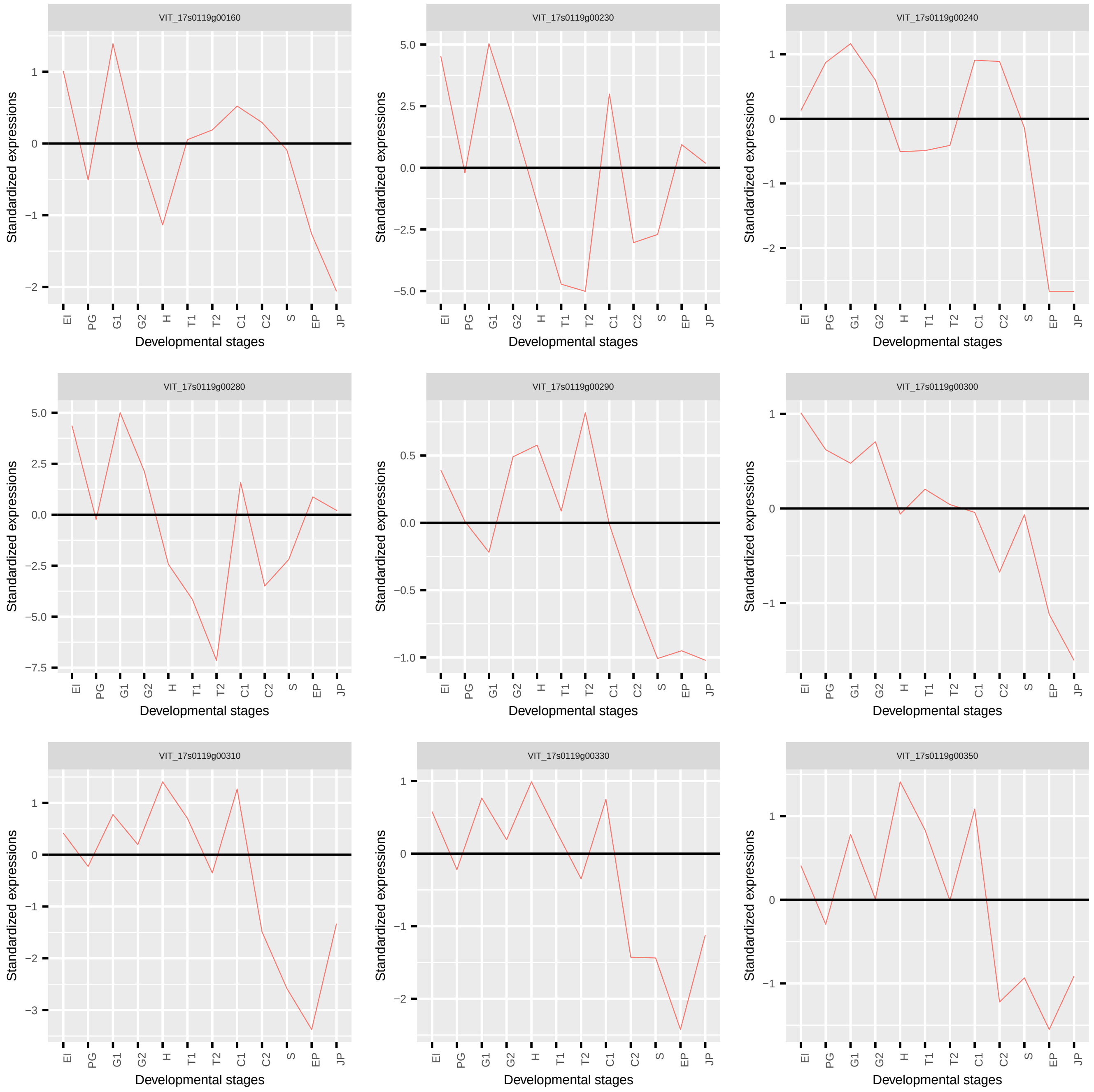

VIT_17s0119g00160
VIT_17s0119g00230
VIT_17s0119g00240
VIT_17s0119g00280
VIT_17s0119g00290
VIT_17s0119g00300
VIT_17s0119g00310
VIT_17s0119g00330
VIT_17s0119g00350
Standardized expressions
Developmental stages
EI PG G1 G2 H T1 T2 C1 C2 S EP JP
1 0 −1 −2
5.0 2.5 0.0 −2.5 −5.0
1 0 −1 −2
5.0 2.5 0.0 −2.5 −5.0 −7.5
0.5 0.0 −0.5 −1.0
1 0 −1
1 0 −1 −2 −3
1 0 −1 −2
1 0 −1

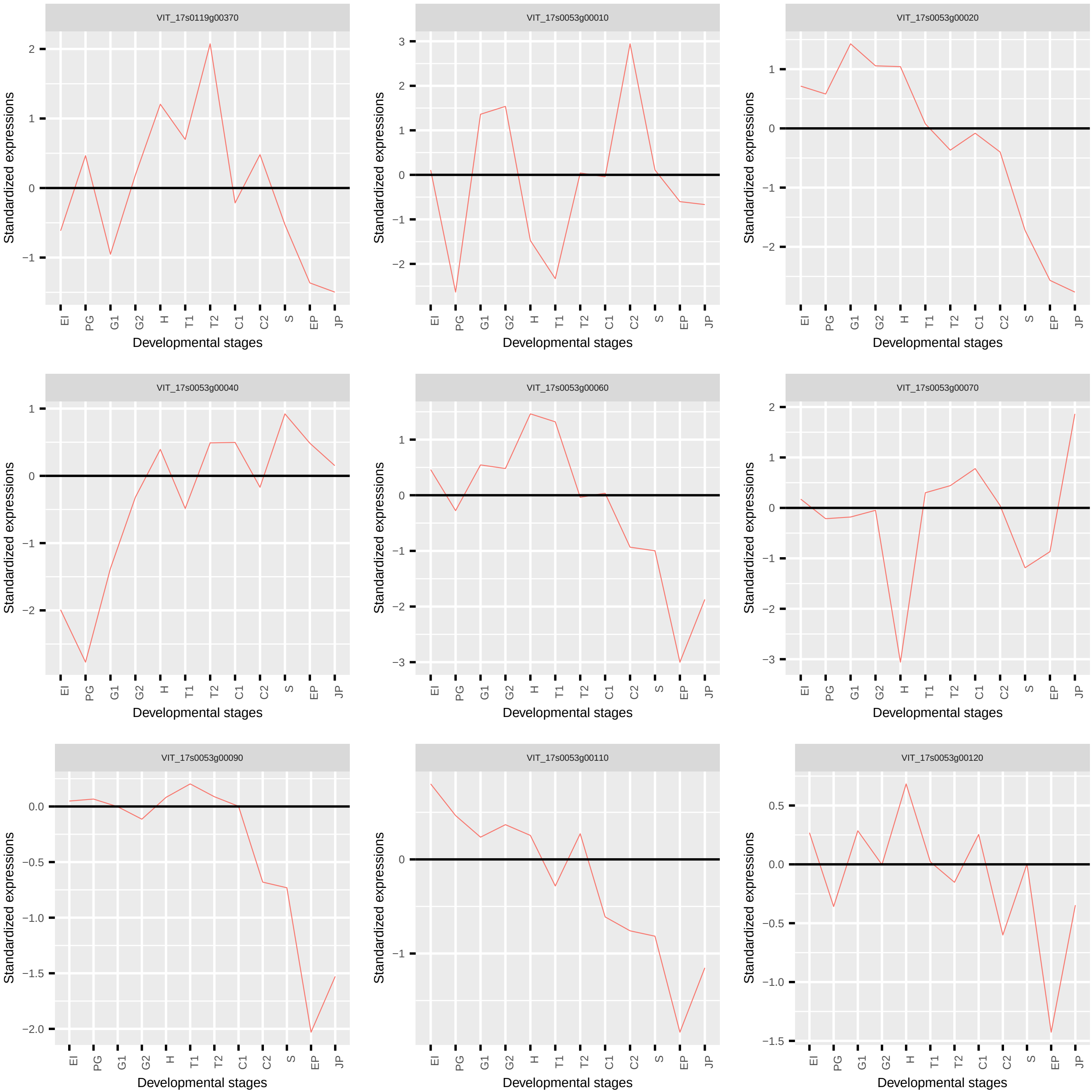
VIT_17s0119g00370
VIT_17s0053g00010
VIT_17s0053g00020
VIT_17s0053g00040
VIT_17s0053g00060
VIT_17s0053g00070
VIT_17s0053g00090
VIT_17s0053g00110
VIT_17s0053g00120
Standardized expressions
Developmental stages
EI
PG
G1
G2
H
T1
T2
C1
C2
S
EP
JP

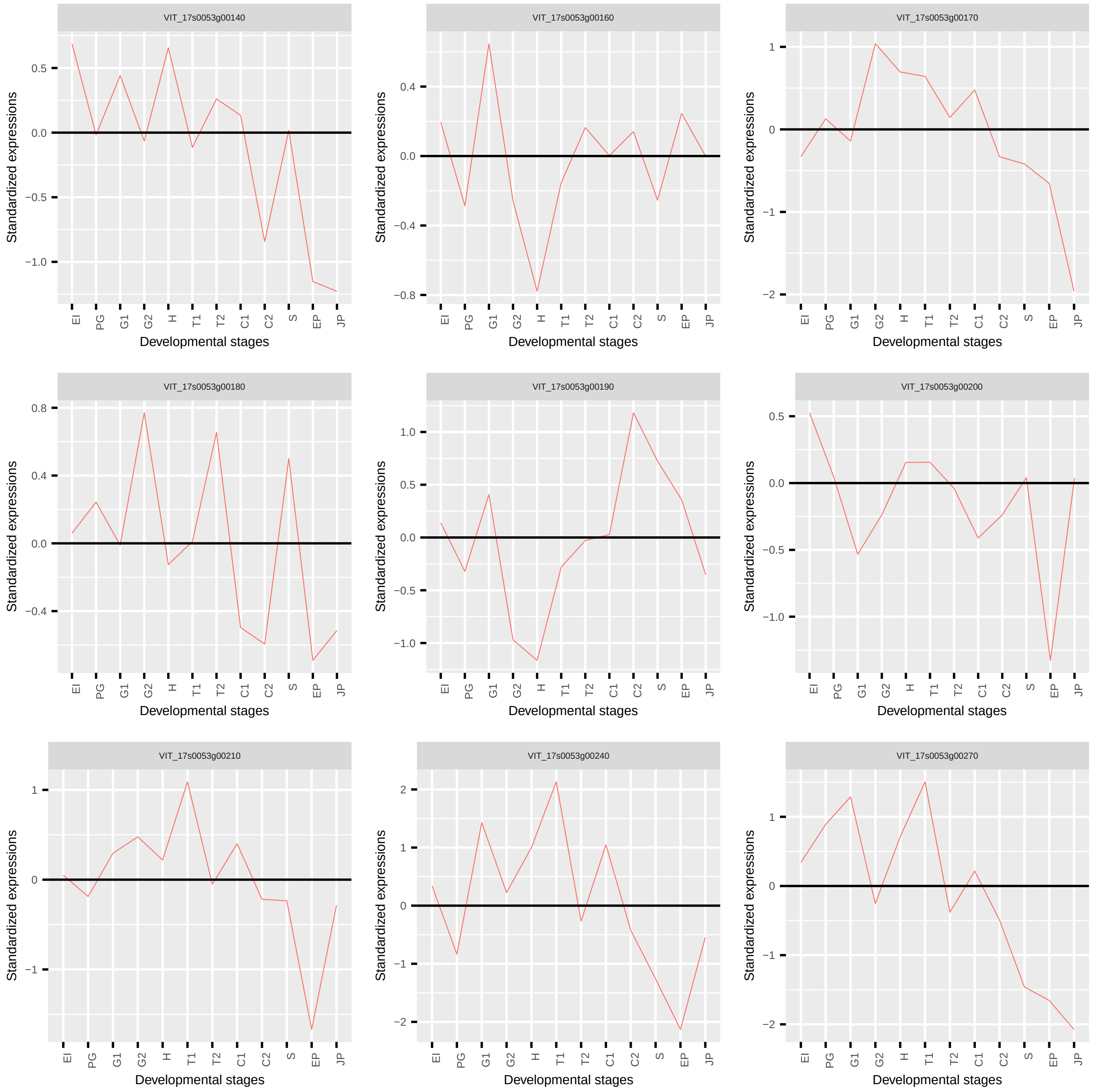
VIT_17s0053g00140
0.5
0.0
−0.5
−1.0
VIT_17s0053g00160
0.4
0.0
−0.4
−0.8
VIT_17s0053g00170
1
0
−1
−2
VIT_17s0053g00180
0.8
0.4
0.0
−0.4
VIT_17s0053g00190
1.0
0.5
0.0
−0.5
−1.0
VIT_17s0053g00200
0.5
0.0
−0.5
−1.0
VIT_17s0053g00210
1
0
−1
VIT_17s0053g00240
2
1
0
−1
−2
VIT_17s0053g00270
1
0
−1
−2
Standardized expressions
EI PG G1 G2 H T1 T2 C1 C2 S EP JP
Developmental stages

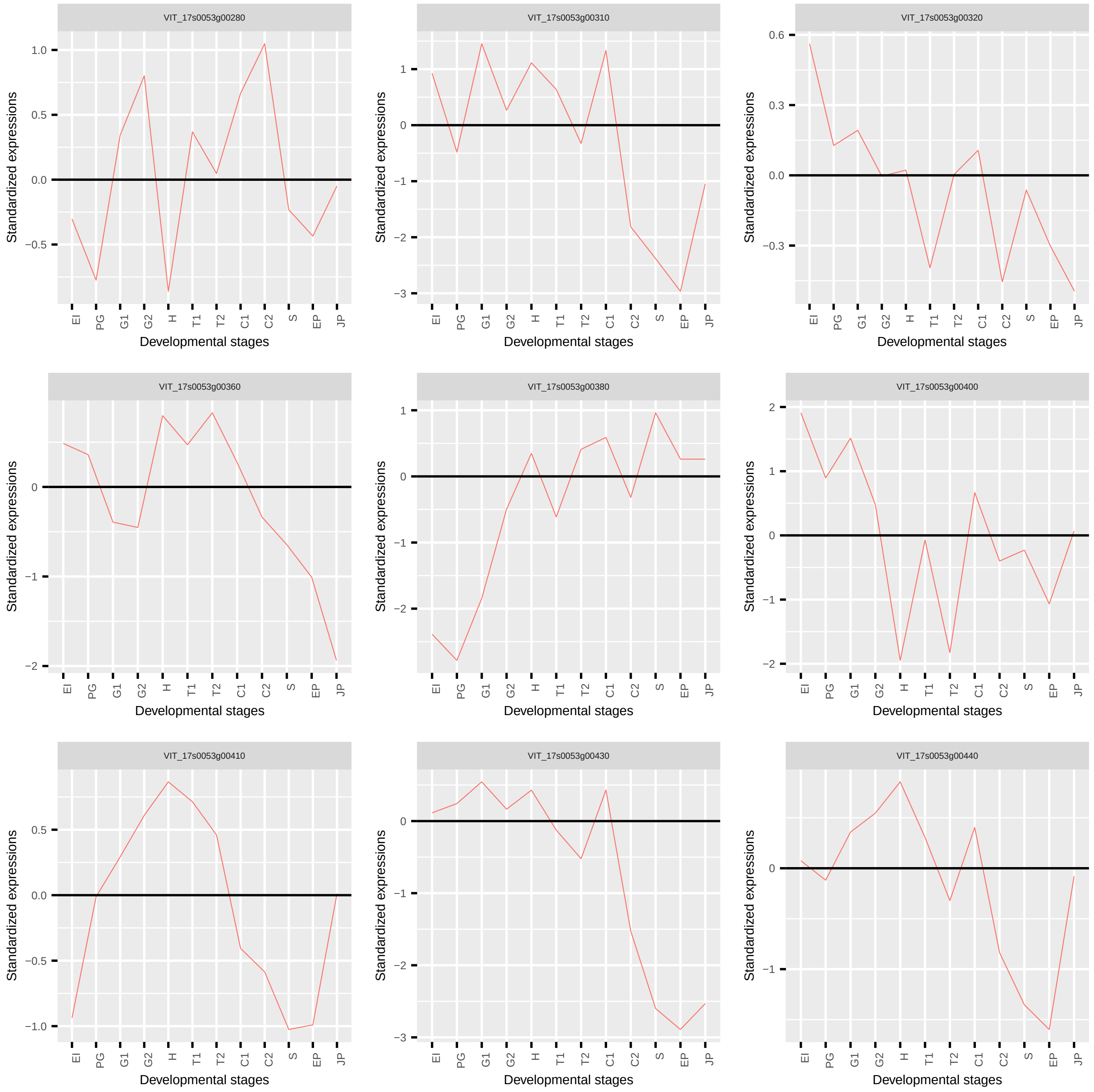
VIT_17s0053g00280
VIT_17s0053g00310
VIT_17s0053g00320
VIT_17s0053g00360
VIT_17s0053g00380
VIT_17s0053g00400
VIT_17s0053g00410
VIT_17s0053g00430
VIT_17s0053g00440
Standardized expressions
Developmental stages
EI PG G1 G2 H T1 T2 C1 C2 S EP JP

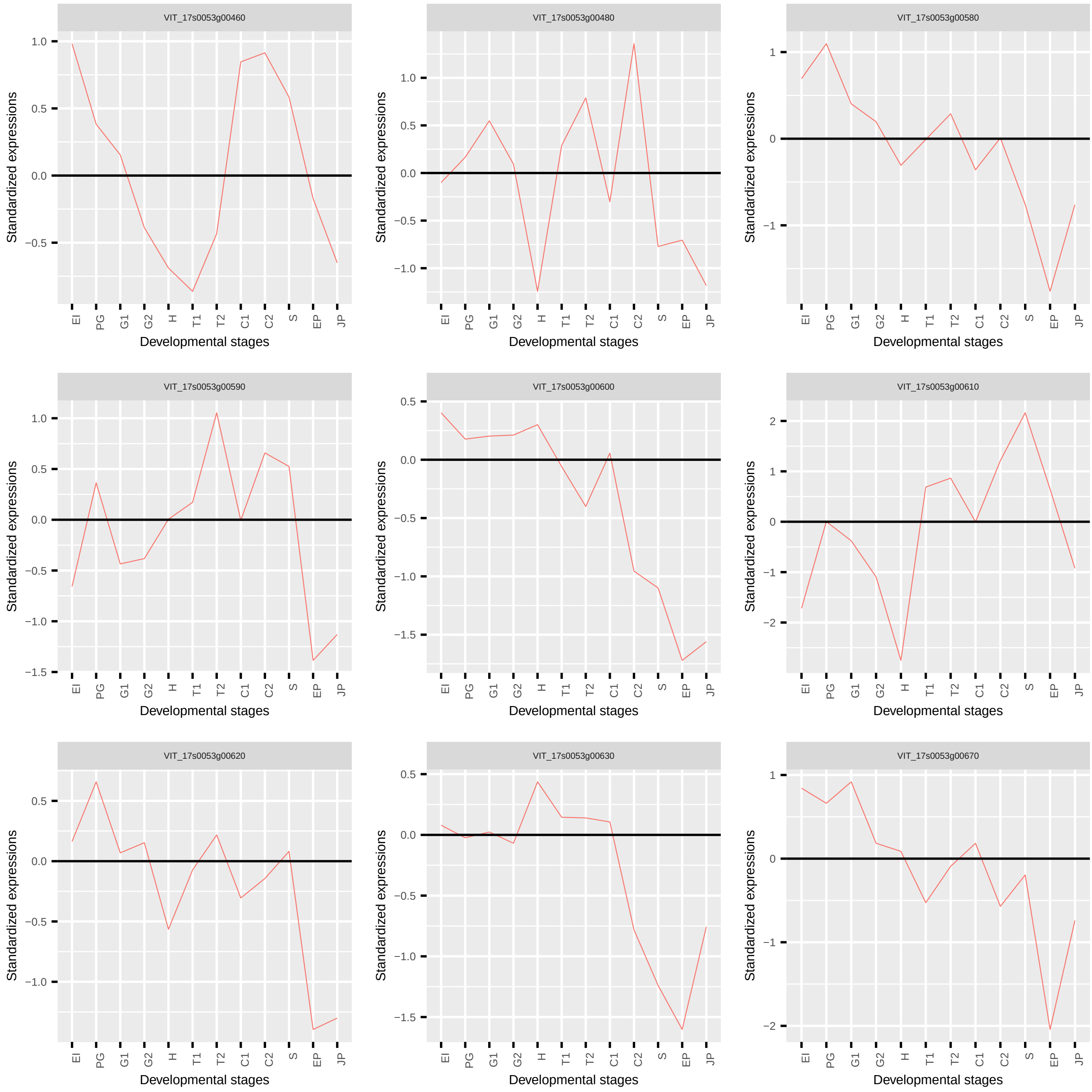
VIT_17s0053g00460
VIT_17s0053g00480
VIT_17s0053g00580
VIT_17s0053g00590
VIT_17s0053g00600
VIT_17s0053g00610
VIT_17s0053g00620
VIT_17s0053g00630
VIT_17s0053g00670
Standardized expressions
Developmental stages
EI PG G1 G2 H T1 T2 C1 C2 S EP JP

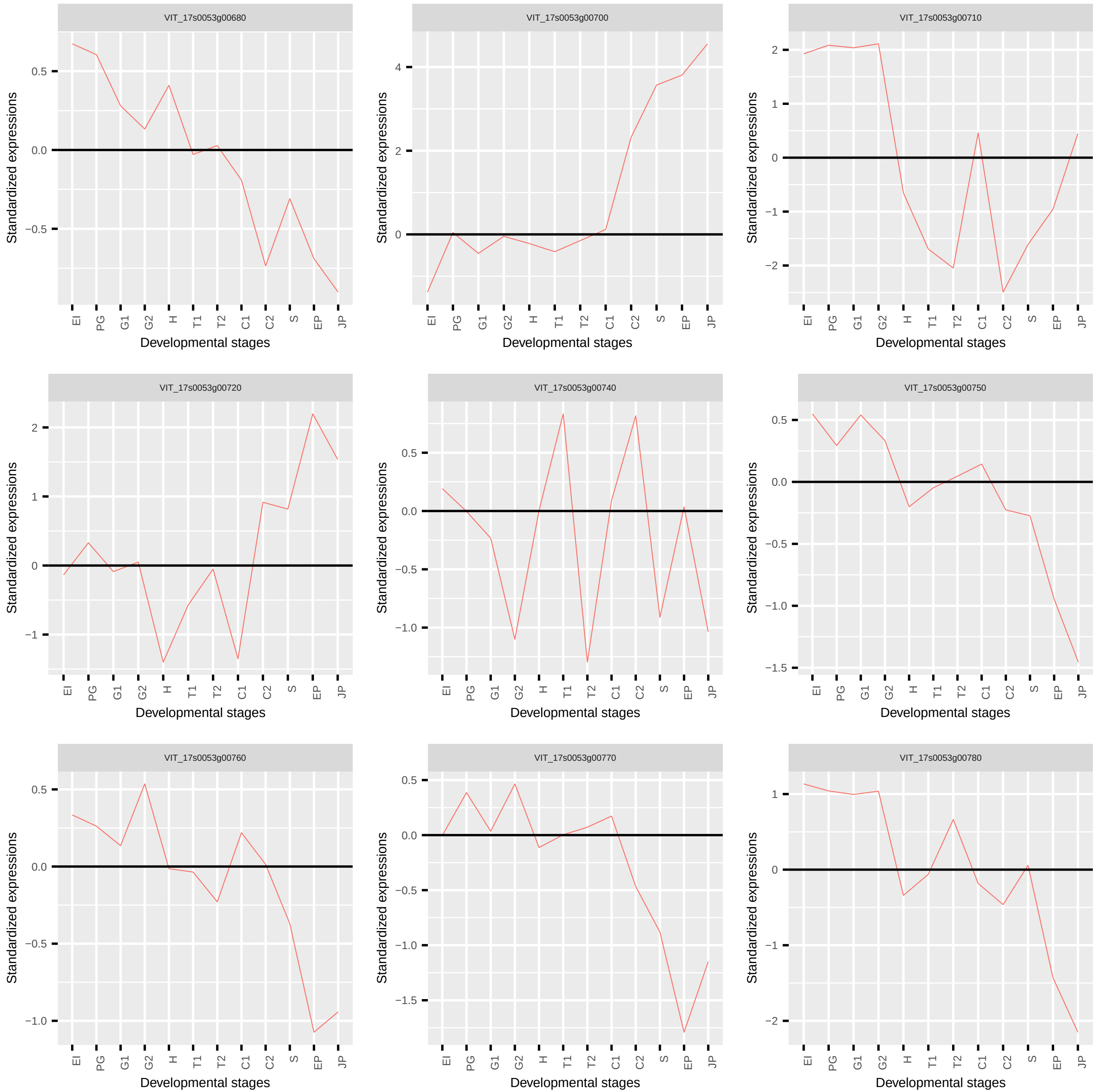

VIT_17s0053g00680
VIT_17s0053g00700
VIT_17s0053g00710
VIT_17s0053g00720
VIT_17s0053g00740
VIT_17s0053g00750
VIT_17s0053g00760
VIT_17s0053g00770
VIT_17s0053g00780
Standardized expressions
Developmental stages
EI PG G1 G2 H T1 T2 C1 C2 S EP JP

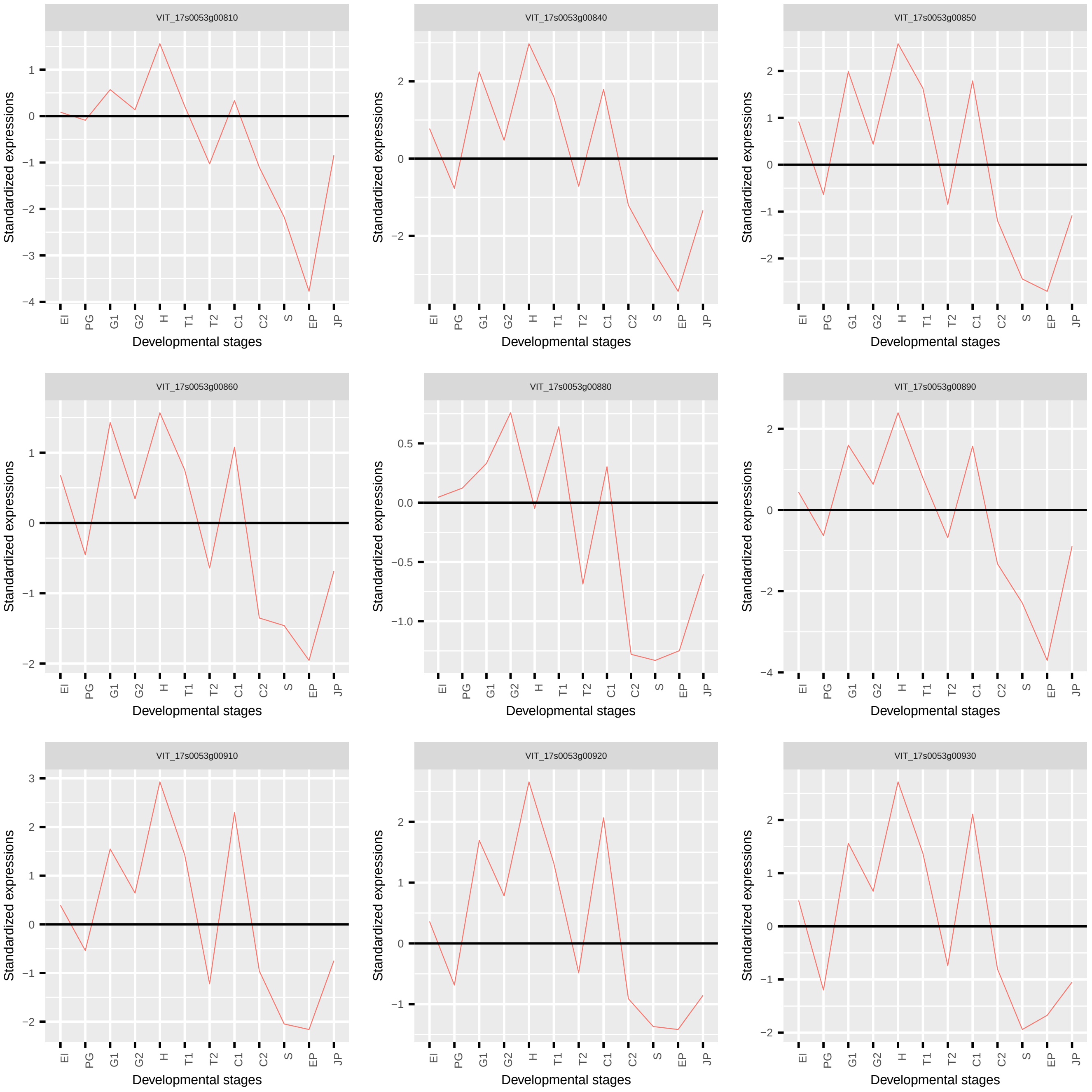
VIT_17s0053g00810
Standardized expressions
1
0
−1
−2
−3
−4
EI PG G1 G2 H T1 T2 C1 C2 S EP JP
Developmental stages
VIT_17s0053g00840
Standardized expressions
2
0
−2
EI PG G1 G2 H T1 T2 C1 C2 S EP JP
Developmental stages
VIT_17s0053g00850
Standardized expressions
2
1
0
−1
−2
EI PG G1 G2 H T1 T2 C1 C2 S EP JP
Developmental stages
VIT_17s0053g00860
Standardized expressions
1
0
−1
−2
EI PG G1 G2 H T1 T2 C1 C2 S EP JP
Developmental stages
VIT_17s0053g00880
Standardized expressions
0.5
0.0
−0.5
−1.0
EI PG G1 G2 H T1 T2 C1 C2 S EP JP
Developmental stages
VIT_17s0053g00890
Standardized expressions
2
0
−2
−4
EI PG G1 G2 H T1 T2 C1 C2 S EP JP
Developmental stages
VIT_17s0053g00910
Standardized expressions
3
2
1
0
−1
−2
EI PG G1 G2 H T1 T2 C1 C2 S EP JP
Developmental stages
VIT_17s0053g00920
Standardized expressions
2
1
0
−1
EI PG G1 G2 H T1 T2 C1 C2 S EP JP
Developmental stages
VIT_17s0053g00930
Standardized expressions
2
1
0
−1
−2
EI PG G1 G2 H T1 T2 C1 C2 S EP JP
Developmental stages

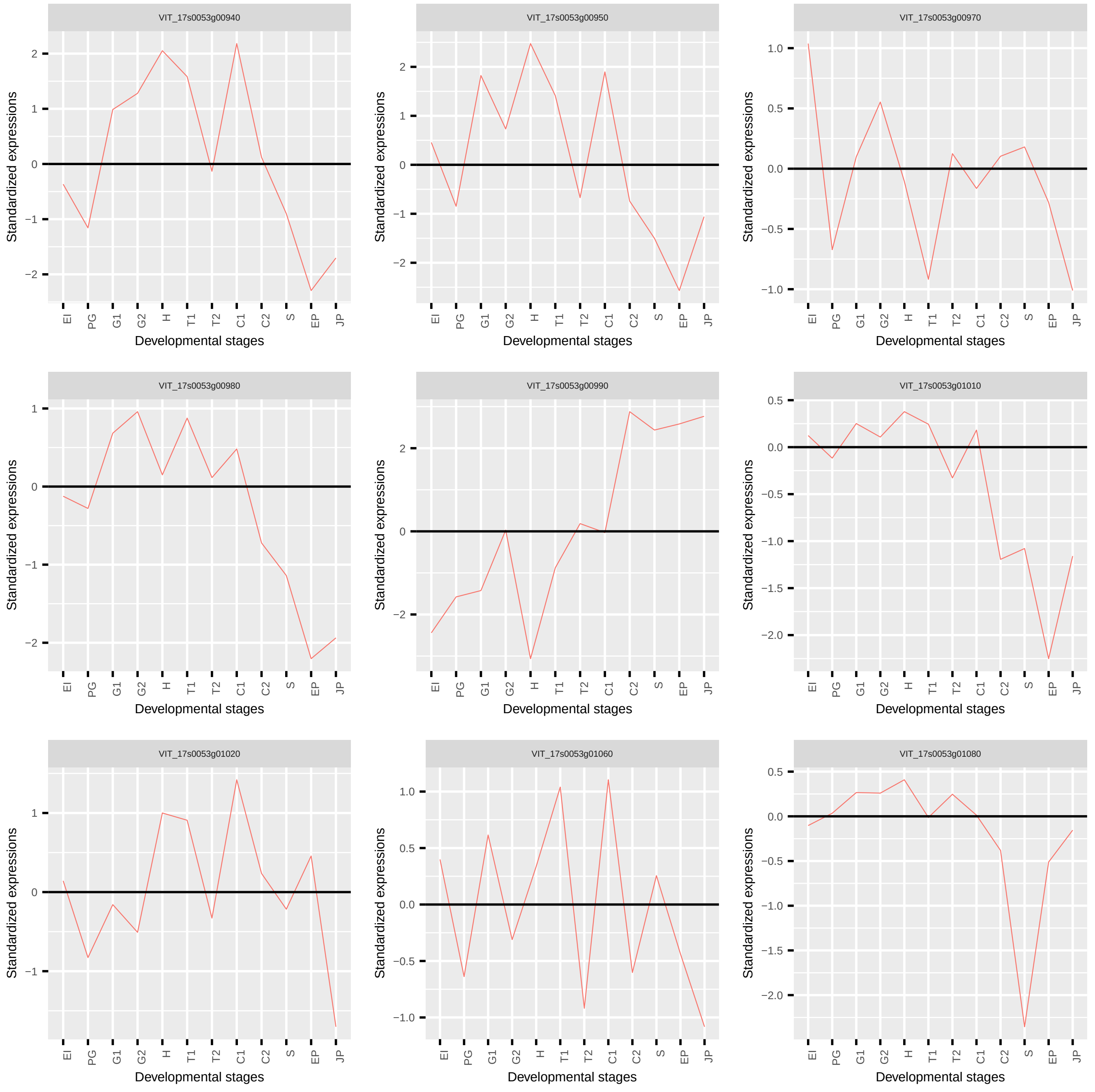

VIT_17s0053g00940
VIT_17s0053g00950
VIT_17s0053g00970
VIT_17s0053g00980
VIT_17s0053g00990
VIT_17s0053g01010
VIT_17s0053g01020
VIT_17s0053g01060
VIT_17s0053g01080
Standardized expressions
Developmental stages
EI PG G1 G2 H T1 T2 C1 C2 S EP JP

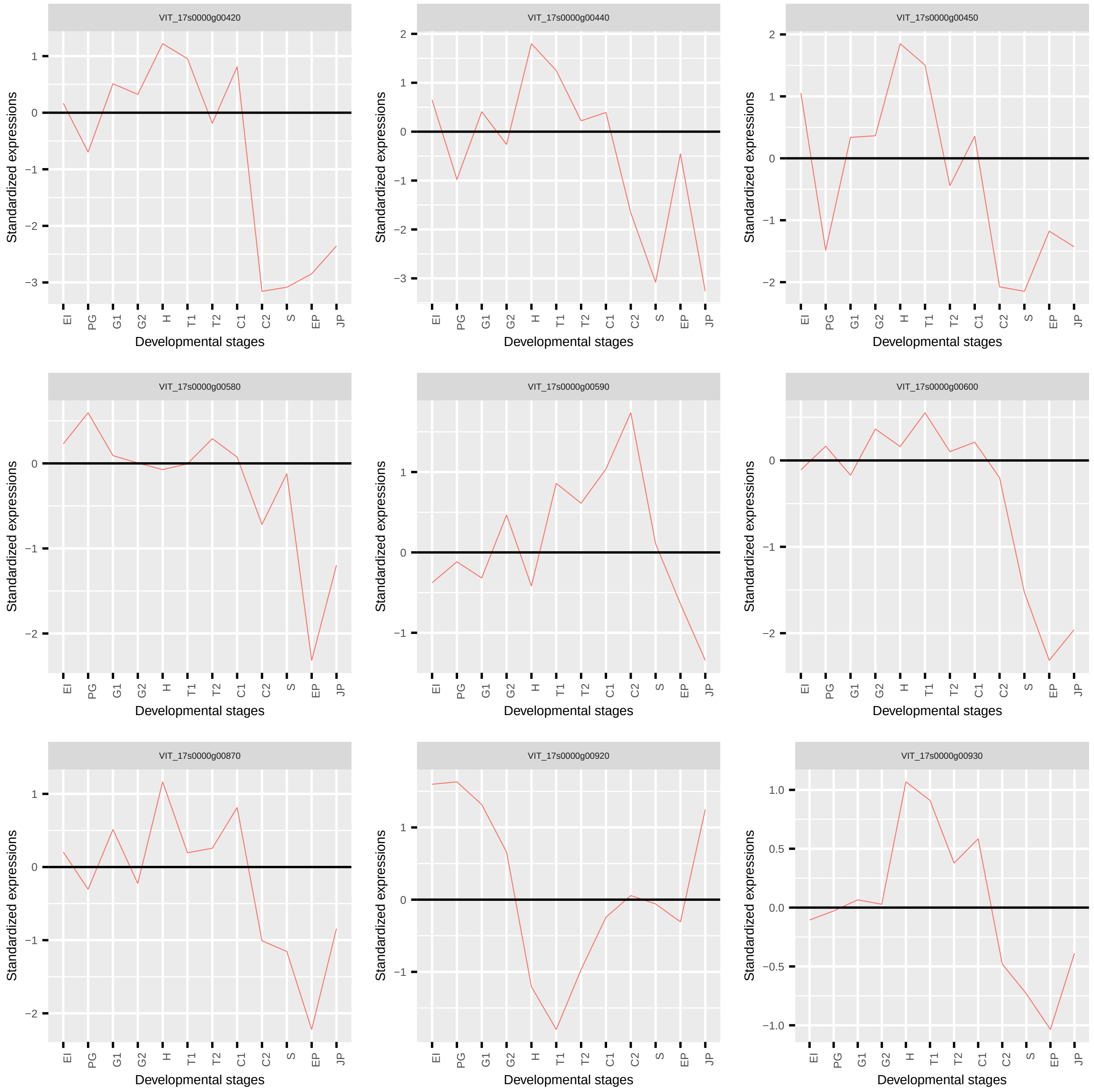
VIT_17s0000g00420
VIT_17s0000g00440
VIT_17s0000g00450
VIT_17s0000g00580
VIT_17s0000g00590
VIT_17s0000g00600
VIT_17s0000g00870
VIT_17s0000g00920
VIT_17s0000g00930
Standardized expressions
Developmental stages
EI PG G1 G2 H T1 T2 C1 C2 S EP JP

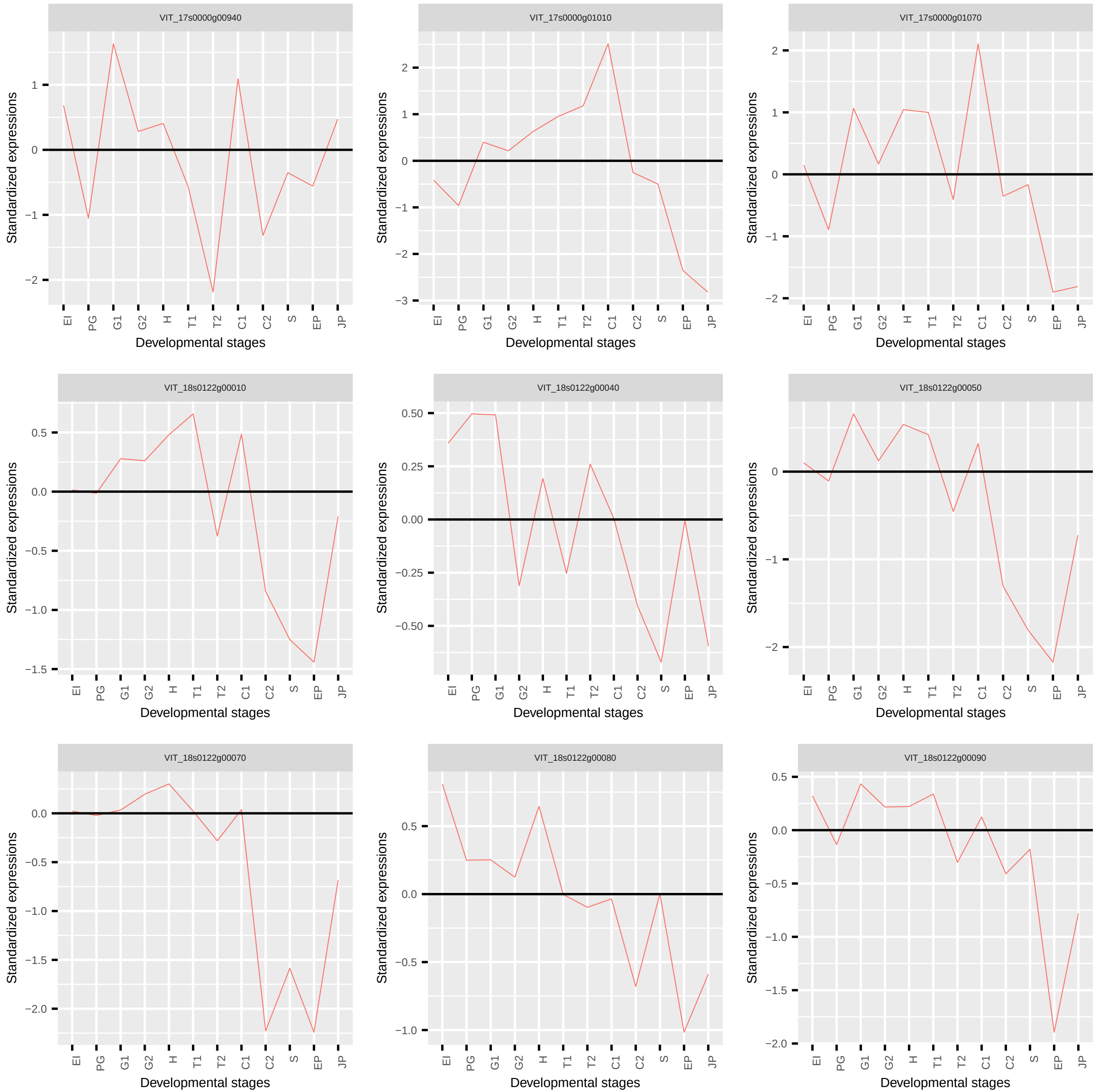
VIT_17s0000g00940
VIT_17s0000g01010
VIT_17s0000g01070
VIT_18s0122g00010
VIT_18s0122g00040
VIT_18s0122g00050
VIT_18s0122g00070
VIT_18s0122g00080
VIT_18s0122g00090
Standardized expressions
Developmental stages
EI PG G1 G2 H T1 T2 C1 C2 S EP JP

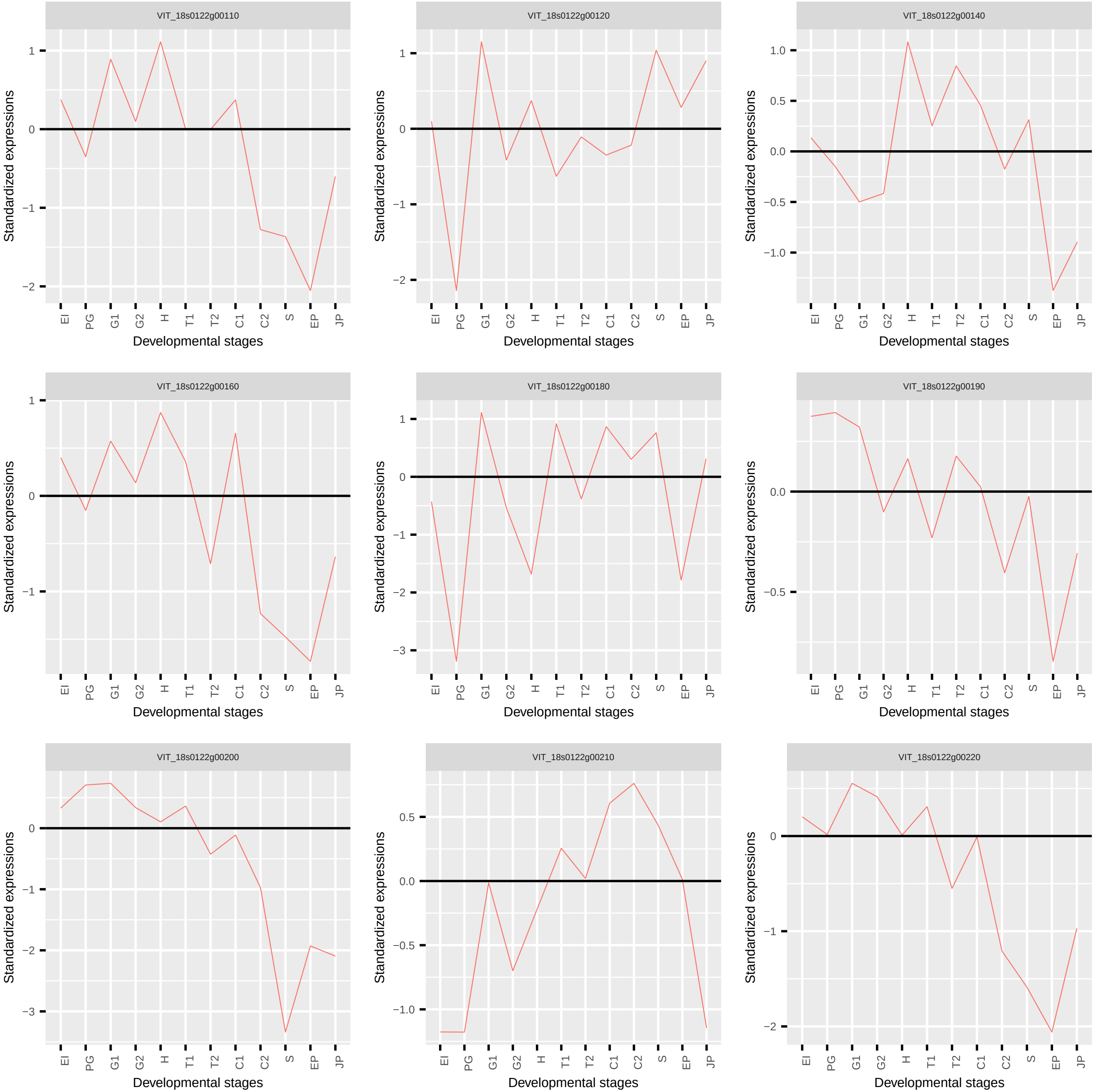
VIT_18s0122g00110
VIT_18s0122g00120
VIT_18s0122g00140
VIT_18s0122g00160
VIT_18s0122g00180
VIT_18s0122g00190
VIT_18s0122g00200
VIT_18s0122g00210
VIT_18s0122g00220
Standardized expressions
Developmental stages
EI PG G1 G2 H T1 T2 C1 C2 S EP JP

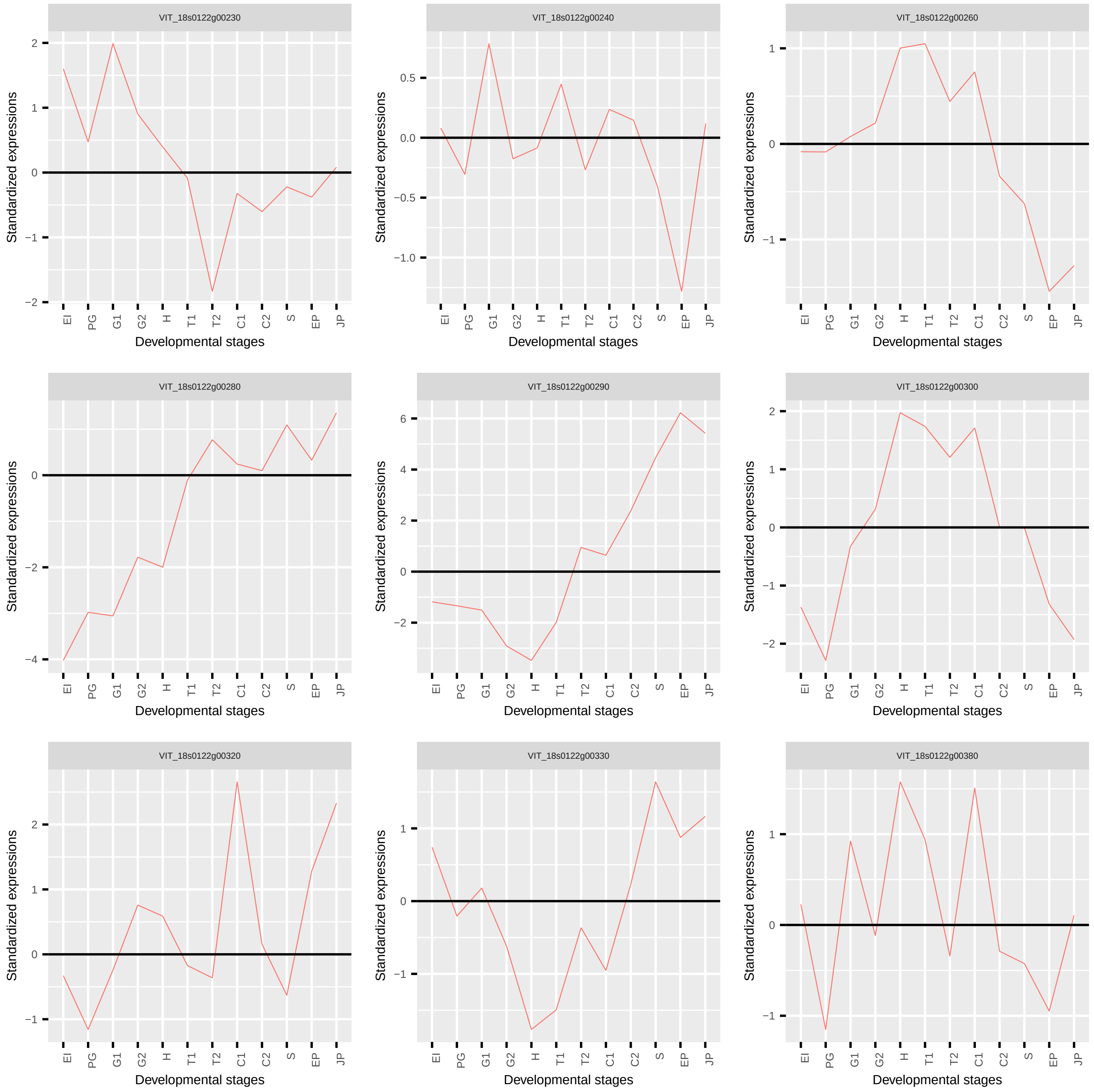

VIT_18s0122g00230
Standardized expressions
2
1
0
−1
−2
EI
PG
G1
G2
H
T1
T2
C1
C2
S
EP
JP
Developmental stages
VIT_18s0122g00240
Standardized expressions
0.5
0.0
−0.5
−1.0
EI
PG
G1
G2
H
T1
T2
C1
C2
S
EP
JP
Developmental stages
VIT_18s0122g00260
Standardized expressions
1
0
−1
EI
PG
G1
G2
H
T1
T2
C1
C2
S
EP
JP
Developmental stages
VIT_18s0122g00280
Standardized expressions
0
−2
−4
EI
PG
G1
G2
H
T1
T2
C1
C2
S
EP
JP
Developmental stages
VIT_18s0122g00290
Standardized expressions
6
4
2
0
−2
EI
PG
G1
G2
H
T1
T2
C1
C2
S
EP
JP
Developmental stages
VIT_18s0122g00300
Standardized expressions
2
1
0
−1
−2
EI
PG
G1
G2
H
T1
T2
C1
C2
S
EP
JP
Developmental stages
VIT_18s0122g00320
Standardized expressions
2
1
0
−1
EI
PG
G1
G2
H
T1
T2
C1
C2
S
EP
JP
Developmental stages
VIT_18s0122g00330
Standardized expressions
1
0
−1
EI
PG
G1
G2
H
T1
T2
C1
C2
S
EP
JP
Developmental stages
VIT_18s0122g00380
Standardized expressions
1
0
−1
EI
PG
G1
G2
H
T1
T2
C1
C2
S
EP
JP
Developmental stages

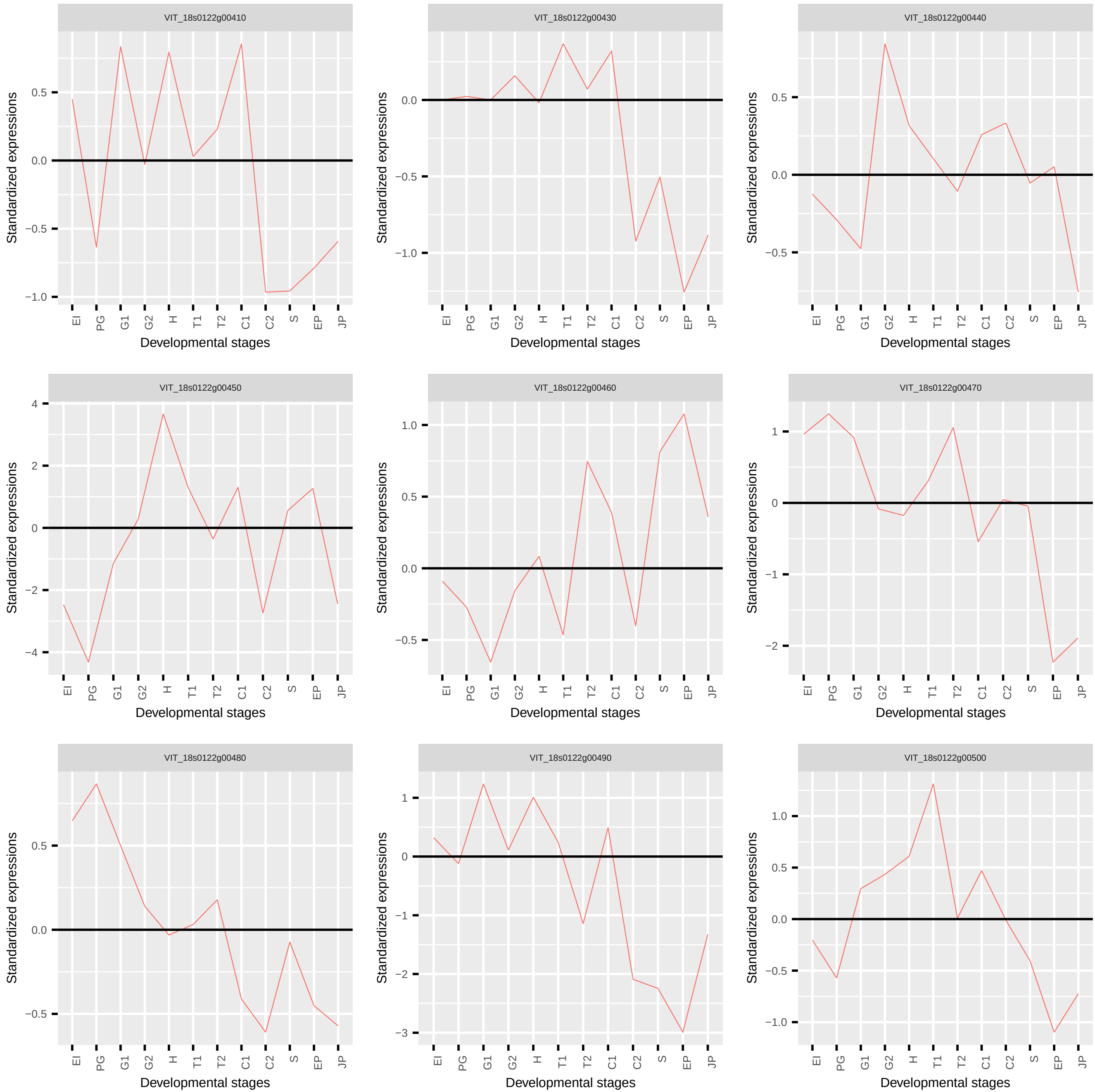

VIT_18s0122g00410
VIT_18s0122g00430
VIT_18s0122g00440
VIT_18s0122g00450
VIT_18s0122g00460
VIT_18s0122g00470
VIT_18s0122g00480
VIT_18s0122g00490
VIT_18s0122g00500
Standardized expressions
Developmental stages
EI PG G1 G2 H T1 T2 C1 C2 S EP JP

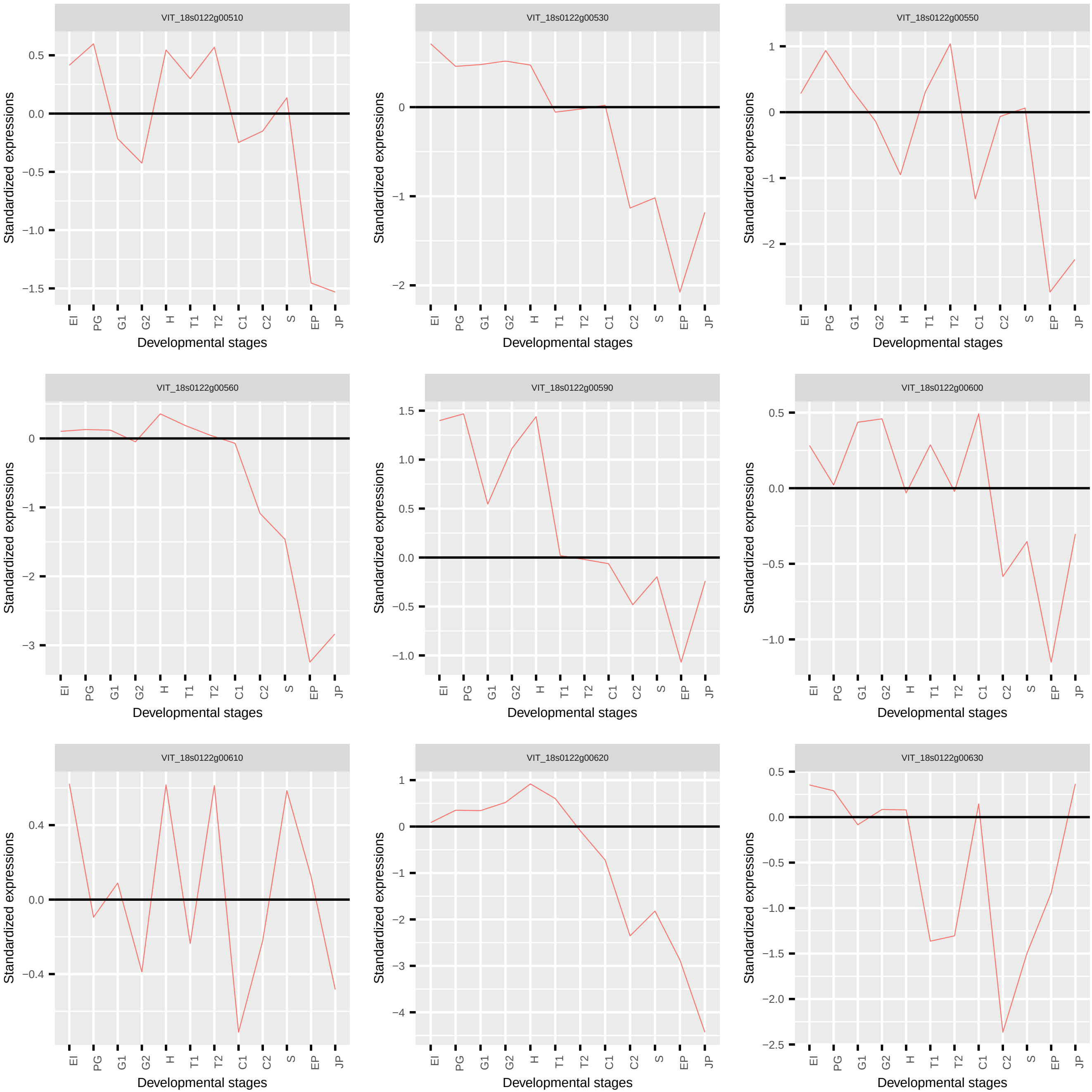
VIT_18s0122g00510
VIT_18s0122g00530
VIT_18s0122g00550
VIT_18s0122g00560
VIT_18s0122g00590
VIT_18s0122g00600
VIT_18s0122g00610
VIT_18s0122g00620
VIT_18s0122g00630
Standardized expressions
Developmental stages
EI PG G1 G2 H T1 T2 C1 C2 S EP JP

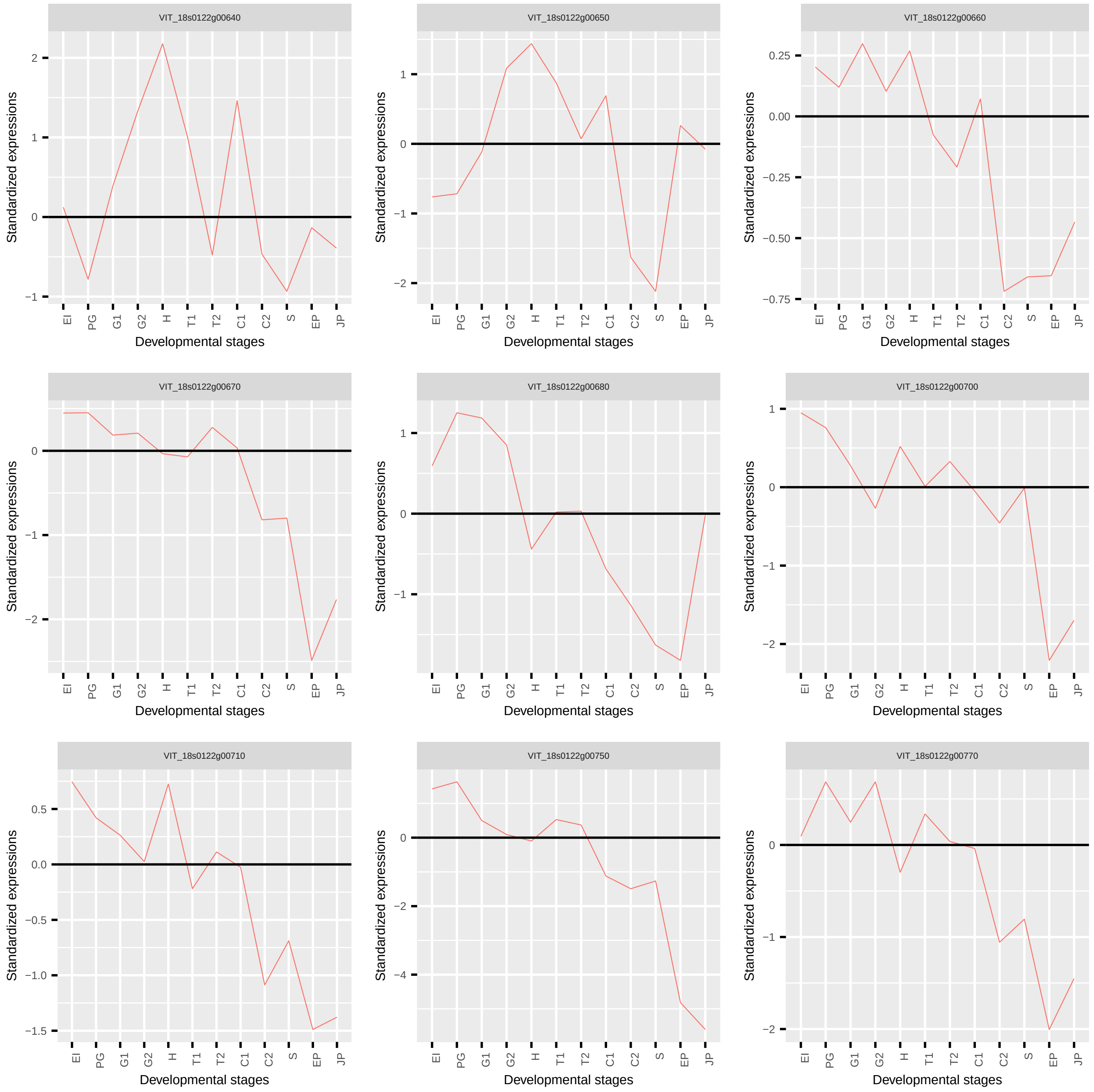
VIT_18s0122g00640
VIT_18s0122g00650
VIT_18s0122g00660
VIT_18s0122g00670
VIT_18s0122g00680
VIT_18s0122g00700
VIT_18s0122g00710
VIT_18s0122g00750
VIT_18s0122g00770
Standardized expressions
Developmental stages
EI
PG
G1
G2
H
T1
T2
C1
C2
S
EP
JP

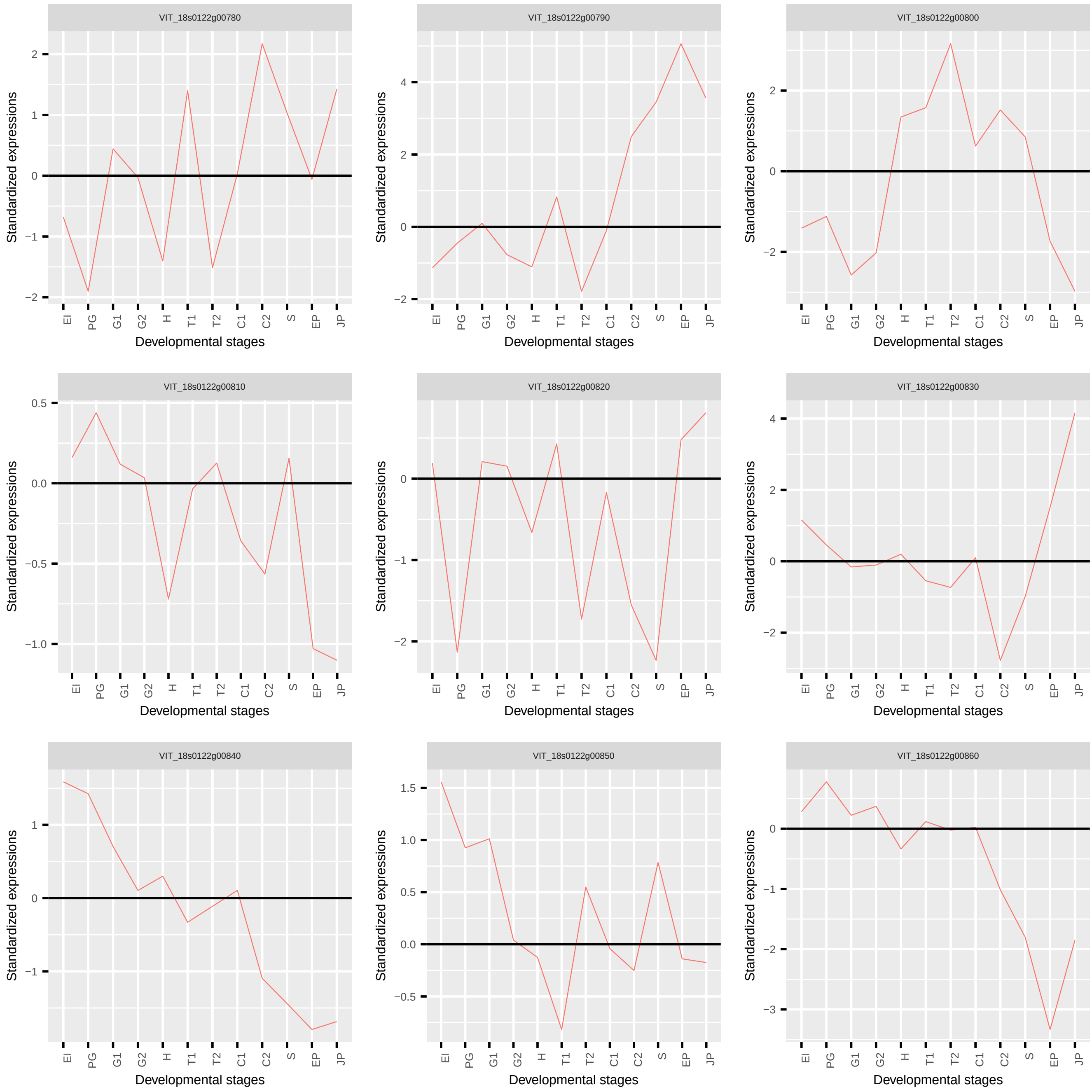
VIT_18s0122g00780
VIT_18s0122g00790
VIT_18s0122g00800
VIT_18s0122g00810
VIT_18s0122g00820
VIT_18s0122g00830
VIT_18s0122g00840
VIT_18s0122g00850
VIT_18s0122g00860
Standardized expressions
Developmental stages
EI PG G1 G2 H T1 T2 C1 C2 S EP JP

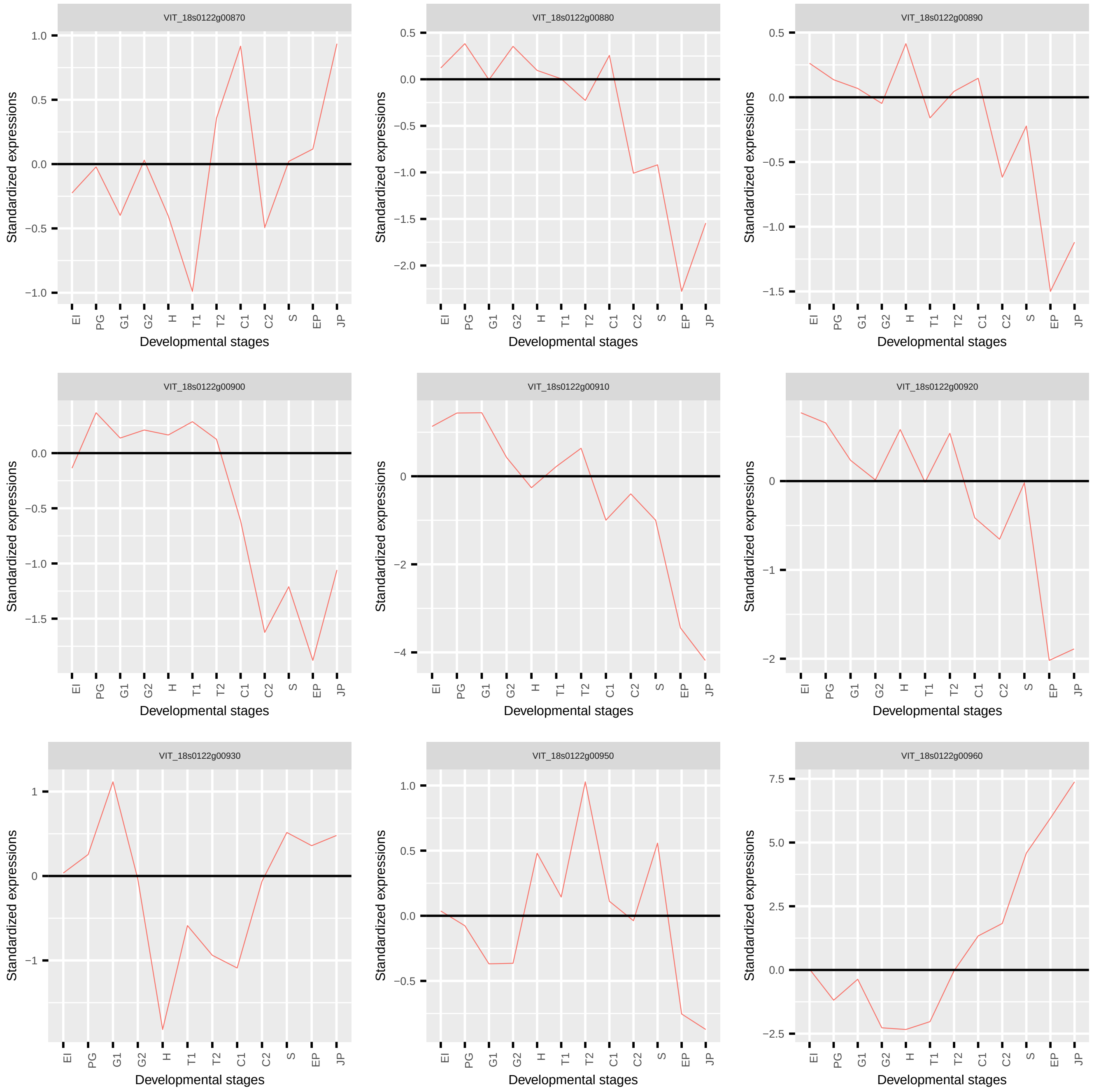
VIT_18s0122g00870
VIT_18s0122g00880
VIT_18s0122g00890
VIT_18s0122g00900
VIT_18s0122g00910
VIT_18s0122g00920
VIT_18s0122g00930
VIT_18s0122g00950
VIT_18s0122g00960
Standardized expressions
Developmental stages
EI PG G1 G2 H T1 T2 C1 C2 S EP JP

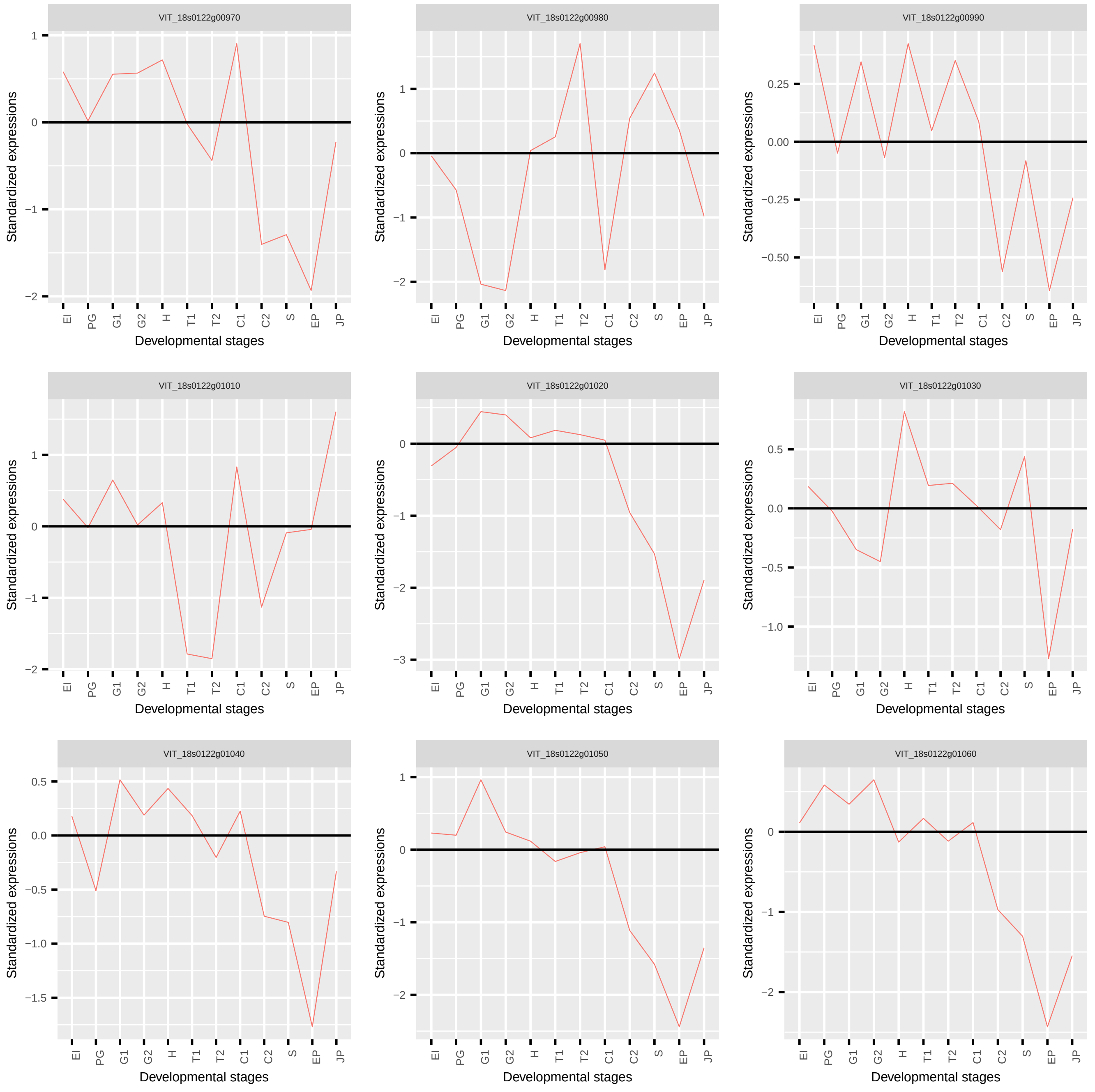

VIT_18s0122g00970
VIT_18s0122g00980
VIT_18s0122g00990
VIT_18s0122g01010
VIT_18s0122g01020
VIT_18s0122g01030
VIT_18s0122g01040
VIT_18s0122g01050
VIT_18s0122g01060
Standardized expressions
Developmental stages
EI PG G1 G2 H T1 T2 C1 C2 S EP JP

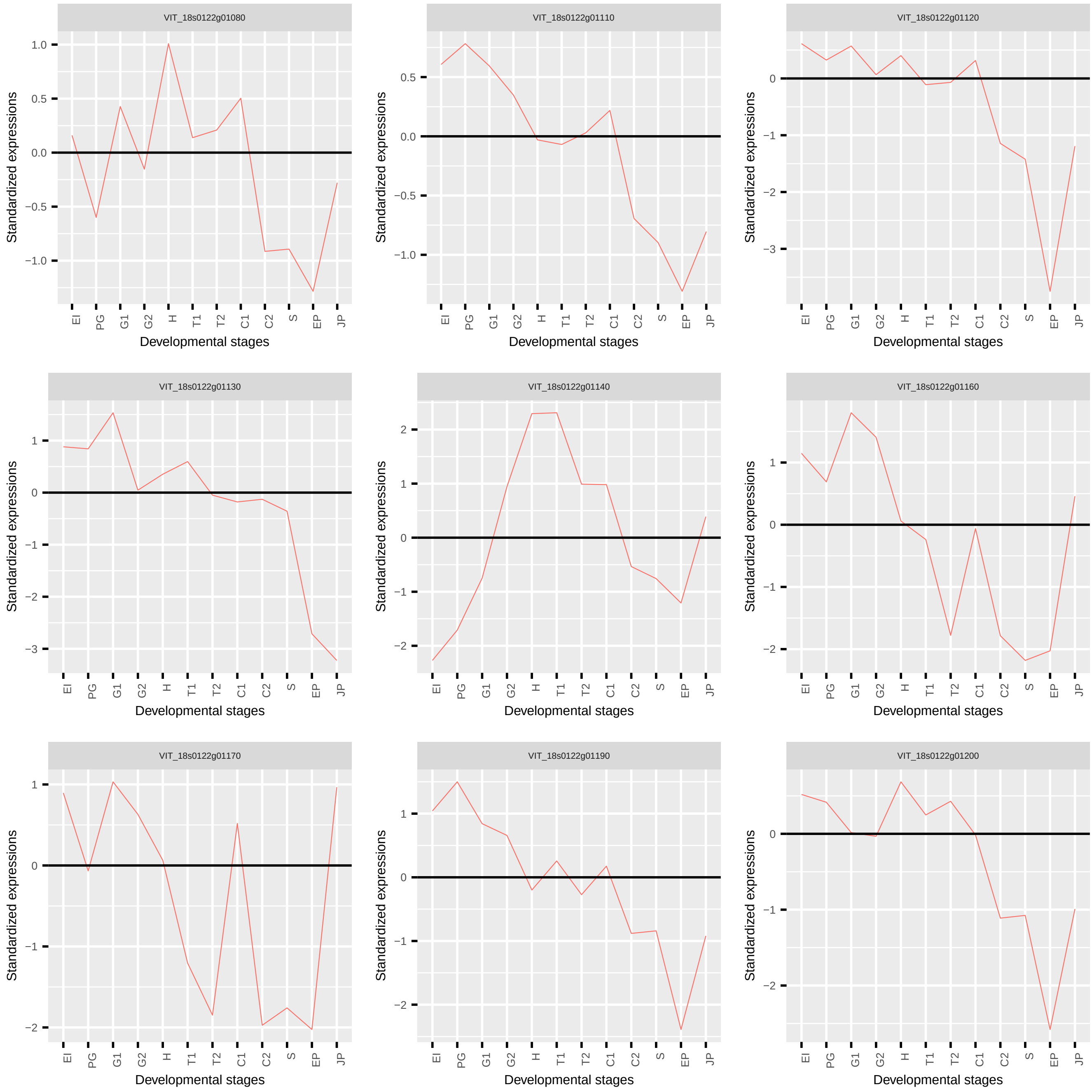

VIT_18s0122g01080
VIT_18s0122g01110
VIT_18s0122g01120
VIT_18s0122g01130
VIT_18s0122g01140
VIT_18s0122g01160
VIT_18s0122g01170
VIT_18s0122g01190
VIT_18s0122g01200
Standardized expressions
Developmental stages
EI PG G1 G2 H T1 T2 C1 C2 S EP JP

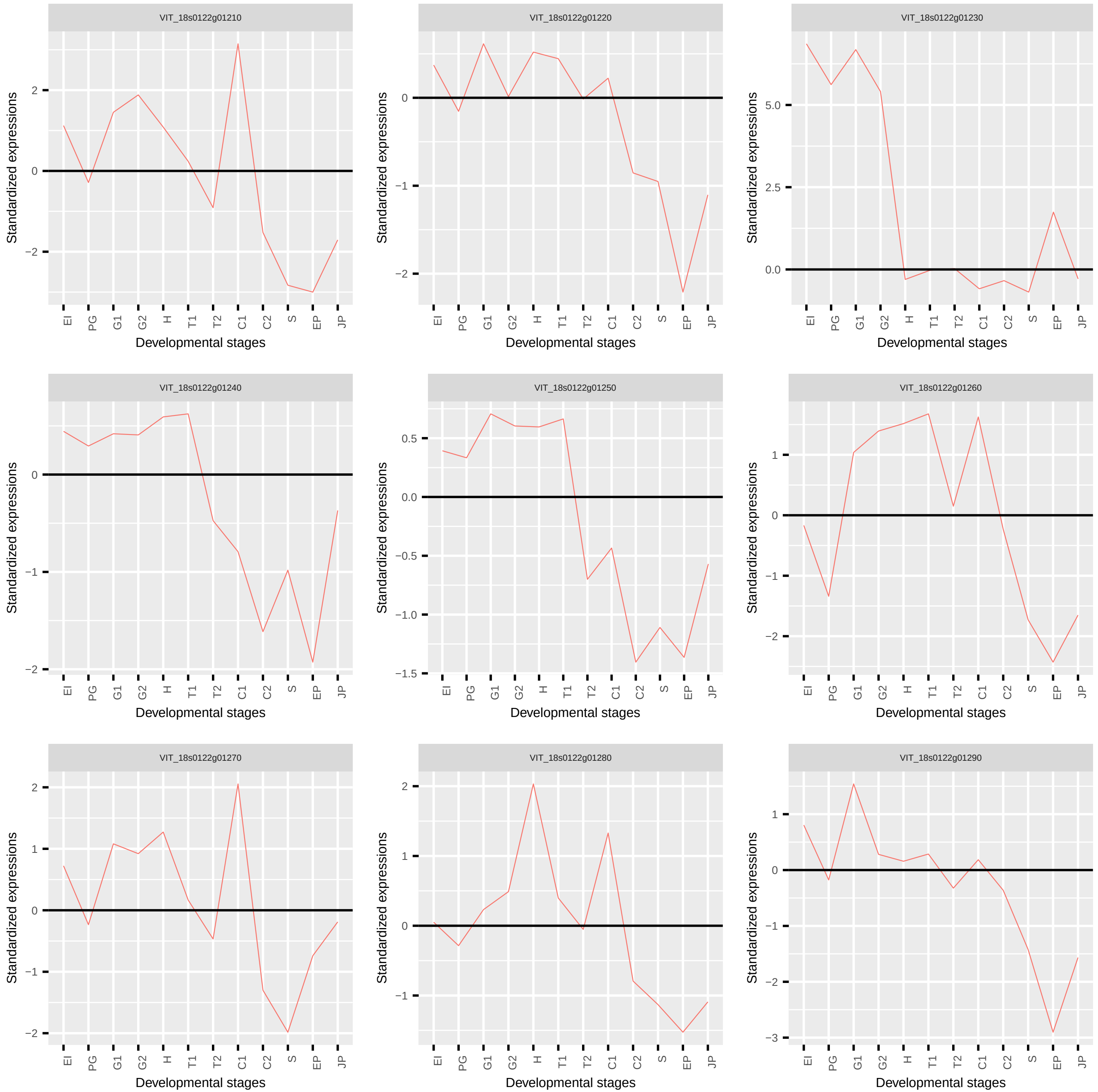

VIT_18s0122g01210
VIT_18s0122g01220
VIT_18s0122g01230
VIT_18s0122g01240
VIT_18s0122g01250
VIT_18s0122g01260
VIT_18s0122g01270
VIT_18s0122g01280
VIT_18s0122g01290
Standardized expressions
Developmental stages
EI PG G1 G2 H T1 T2 C1 C2 S EP JP

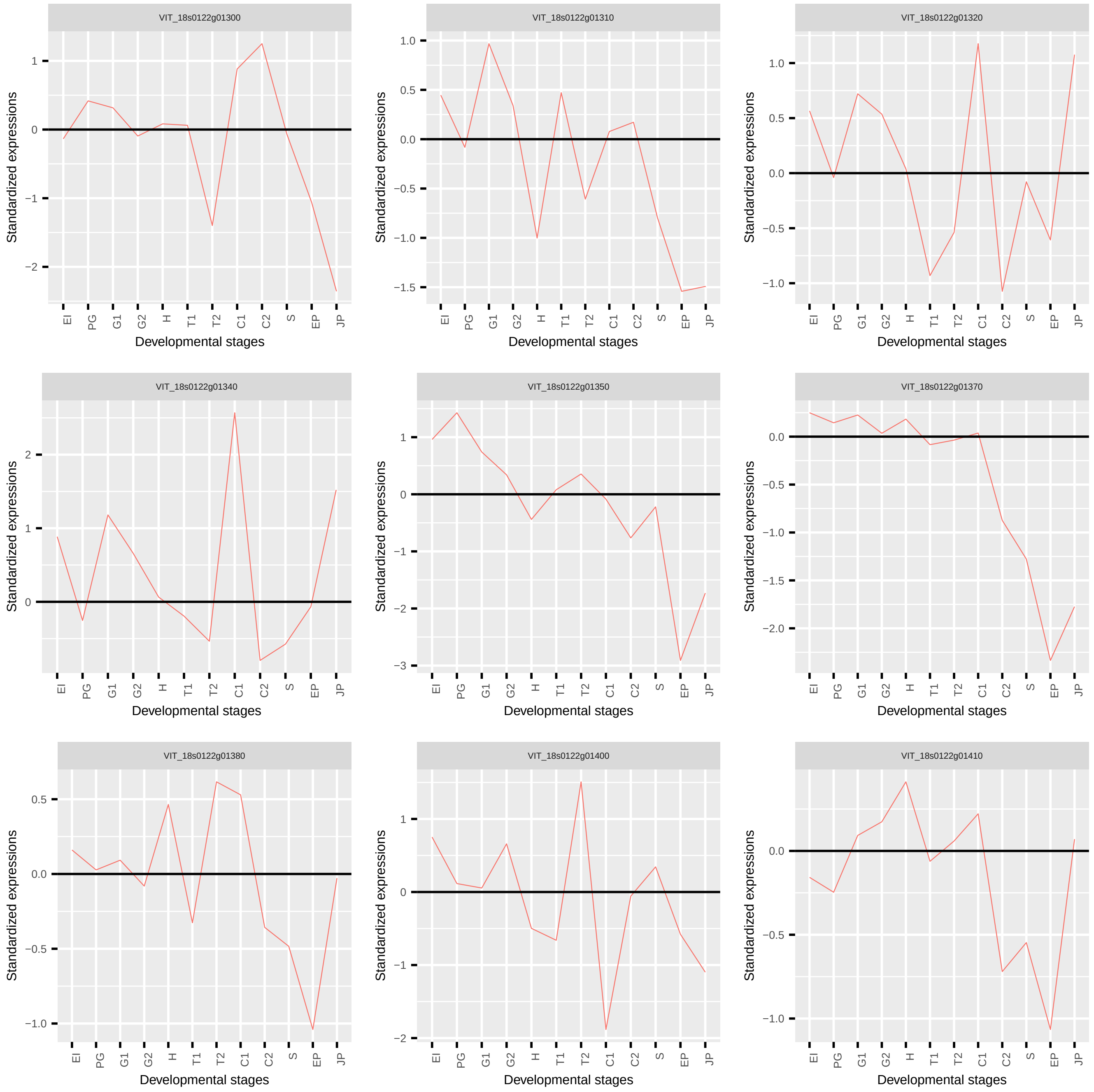
VIT_18s0122g01300
VIT_18s0122g01310
VIT_18s0122g01320
VIT_18s0122g01340
VIT_18s0122g01350
VIT_18s0122g01370
VIT_18s0122g01380
VIT_18s0122g01400
VIT_18s0122g01410
Standardized expressions
Developmental stages
EI
PG
G1
G2
H
T1
T2
C1
C2
S
EP
JP

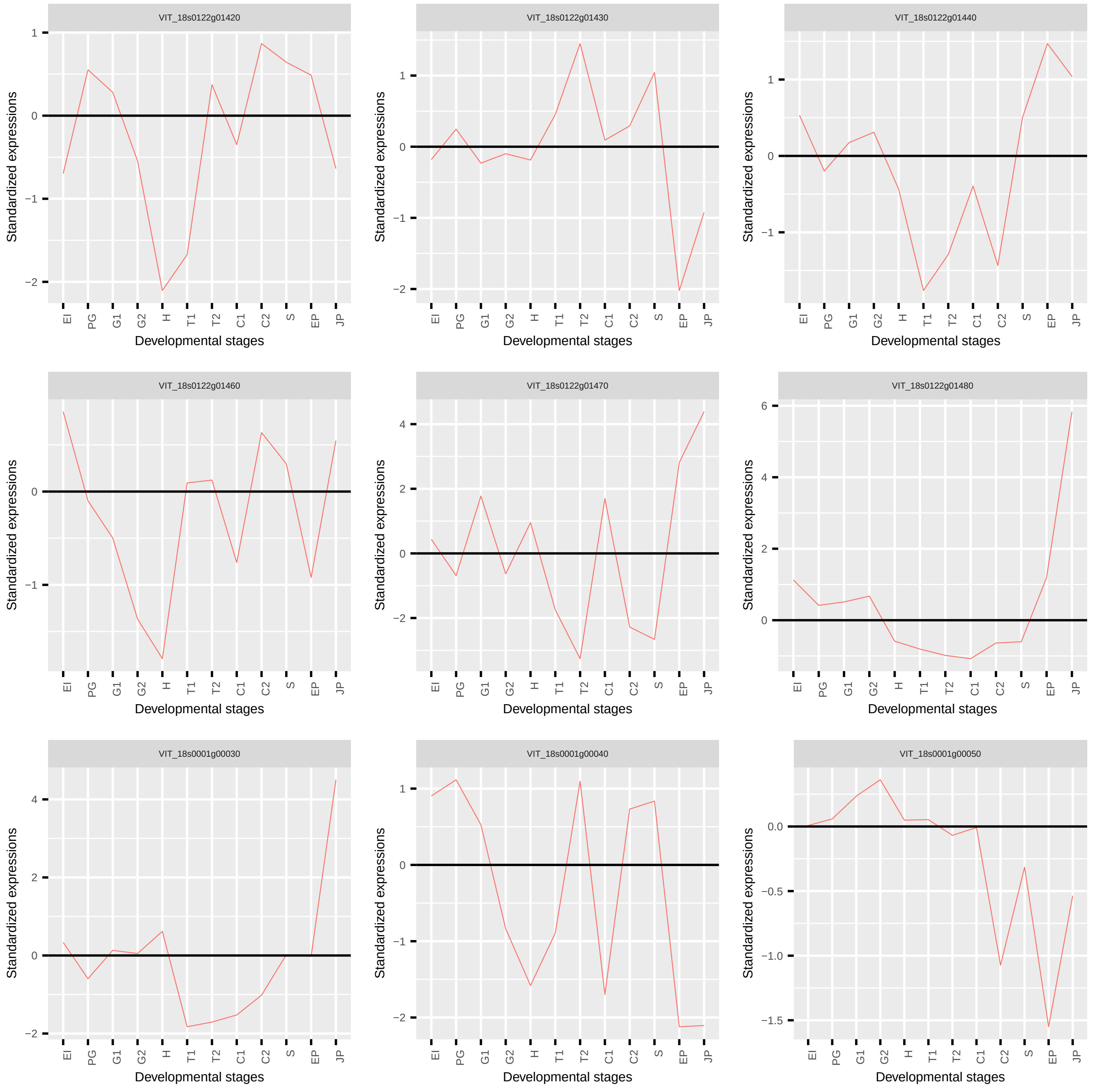
VIT_18s0122g01420
VIT_18s0122g01430
VIT_18s0122g01440
VIT_18s0122g01460
VIT_18s0122g01470
VIT_18s0122g01480
VIT_18s0001g00030
VIT_18s0001g00040
VIT_18s0001g00050
Standardized expressions
Developmental stages
EI PG G1 G2 H T1 T2 C1 C2 S EP JP

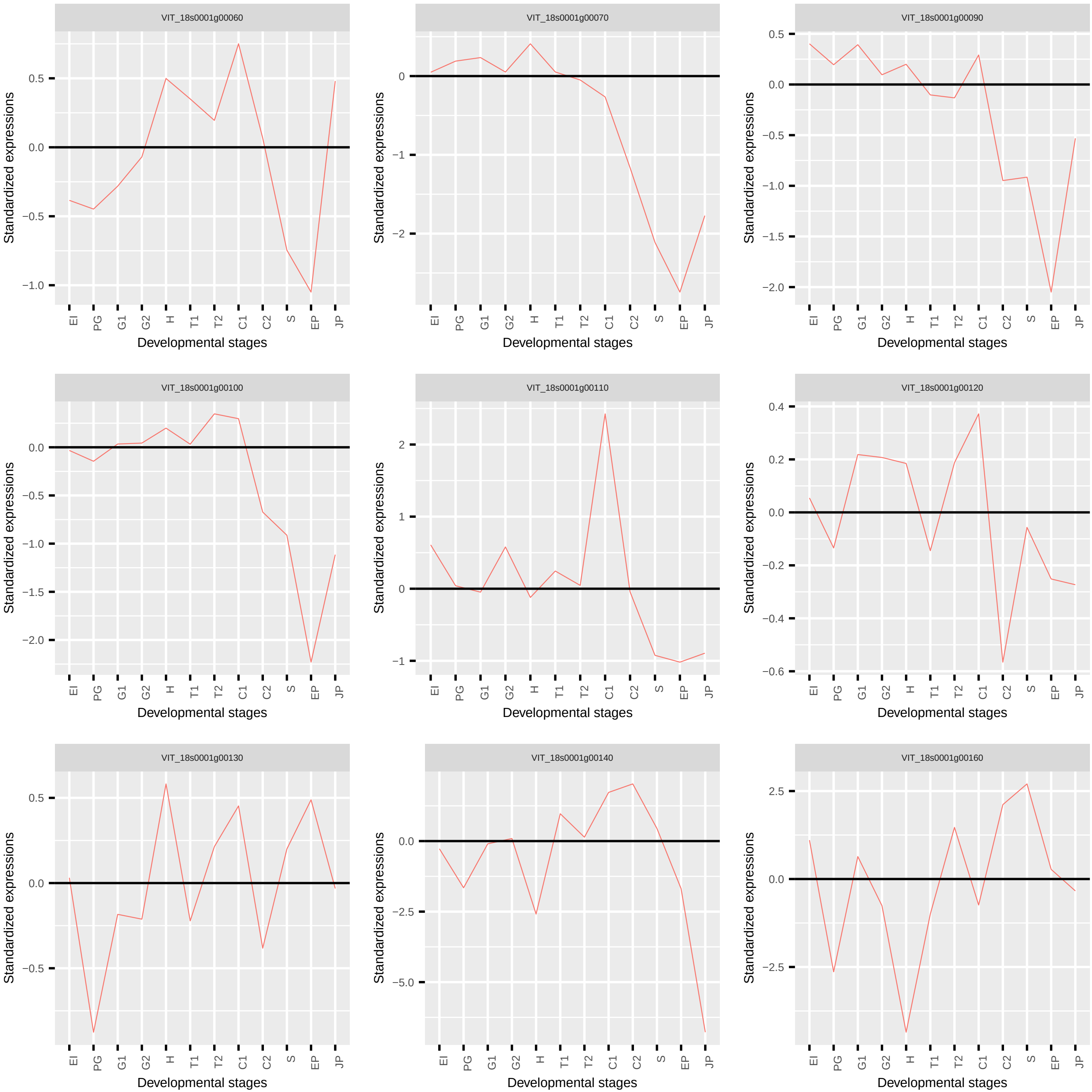
VIT_18s0001g00060
VIT_18s0001g00070
VIT_18s0001g00090
VIT_18s0001g00100
VIT_18s0001g00110
VIT_18s0001g00120
VIT_18s0001g00130
VIT_18s0001g00140
VIT_18s0001g00160
Standardized expressions
Developmental stages
EI PG G1 G2 H T1 T2 C1 C2 S EP JP

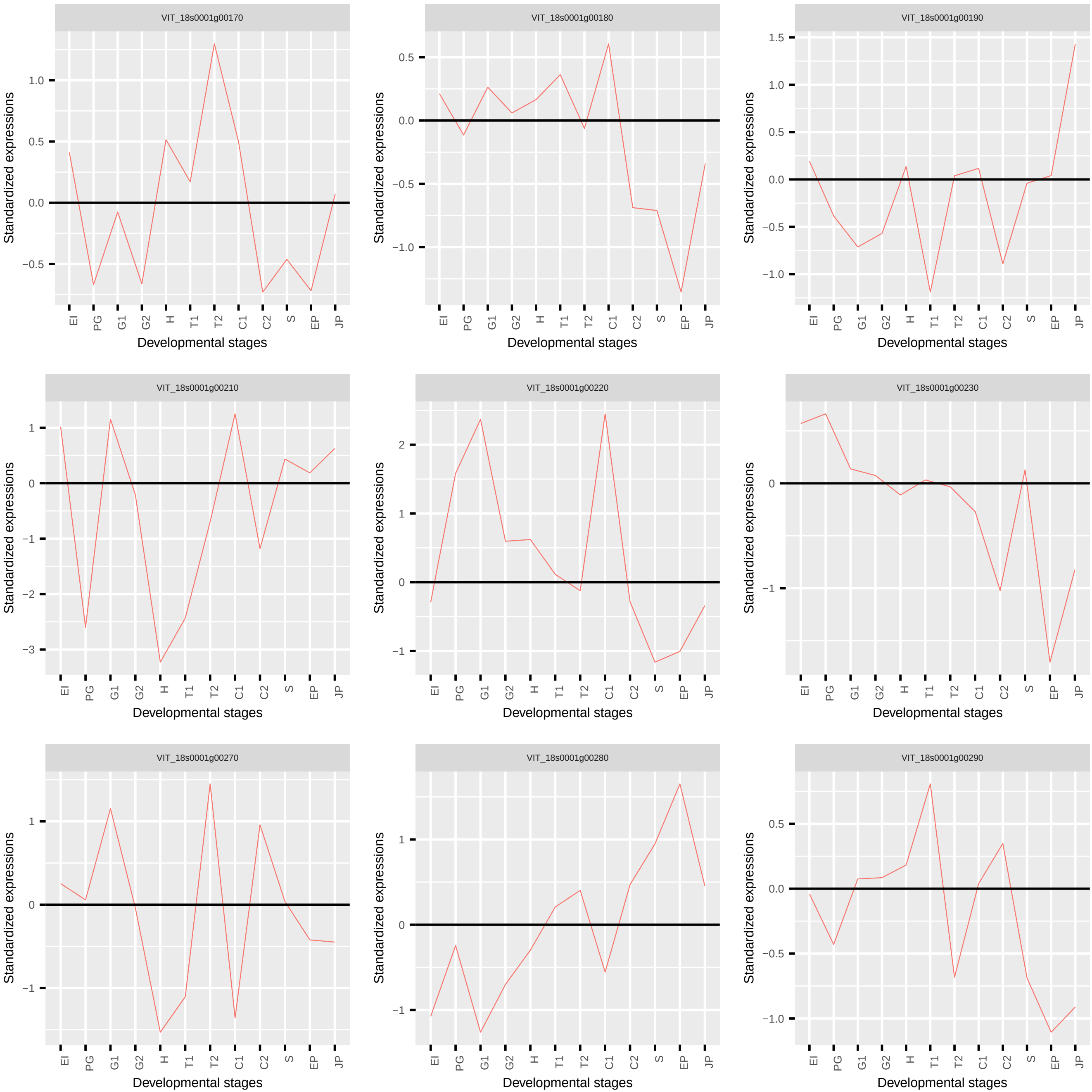
VIT_18s0001g00170
VIT_18s0001g00180
VIT_18s0001g00190
VIT_18s0001g00210
VIT_18s0001g00220
VIT_18s0001g00230
VIT_18s0001g00270
VIT_18s0001g00280
VIT_18s0001g00290
Standardized expressions
Developmental stages
EI PG G1 G2 H T1 T2 C1 C2 S EP JP

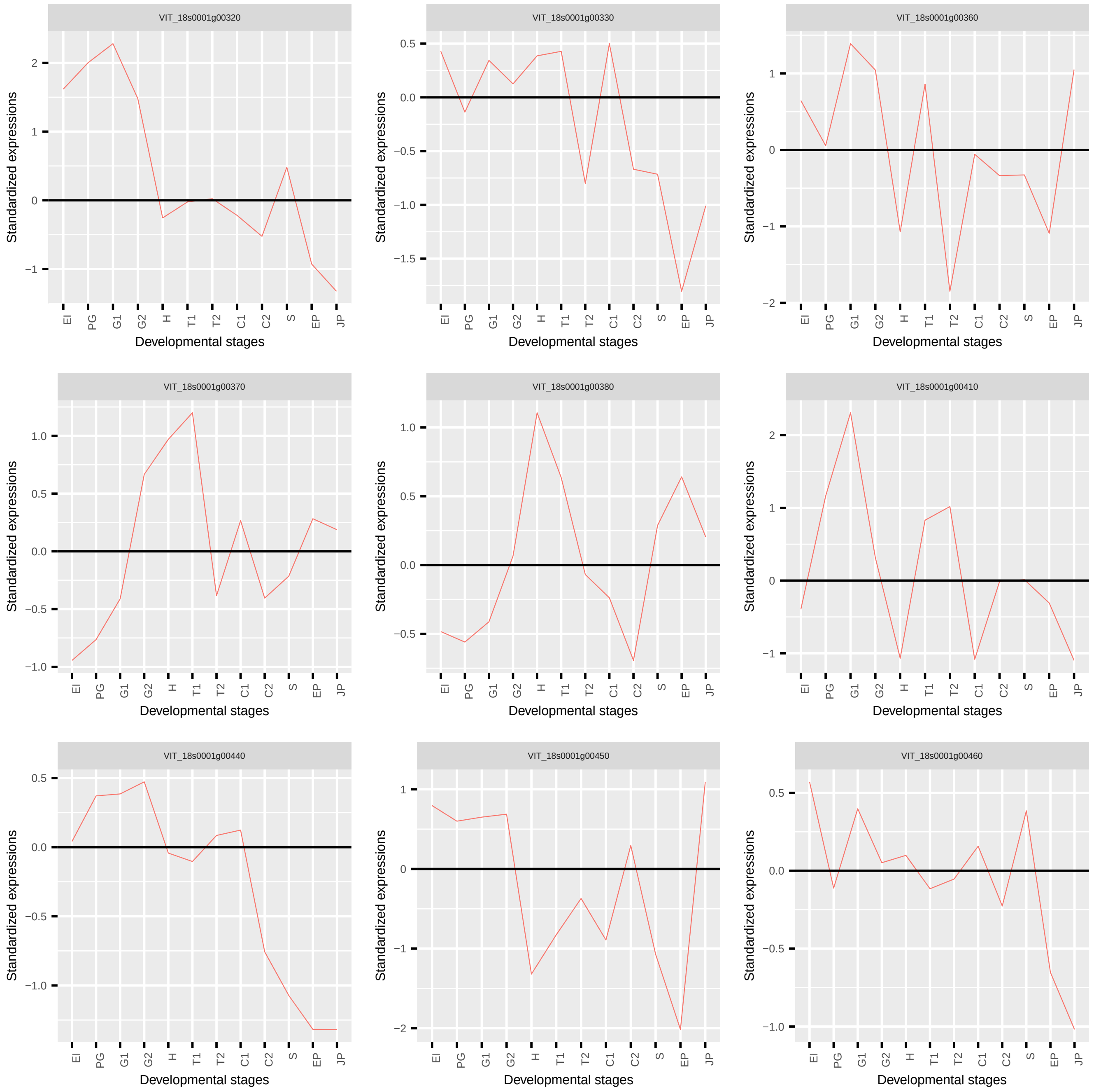
VIT_18s0001g00320
VIT_18s0001g00330
VIT_18s0001g00360
VIT_18s0001g00370
VIT_18s0001g00380
VIT_18s0001g00410
VIT_18s0001g00440
VIT_18s0001g00450
VIT_18s0001g00460
Standardized expressions
Developmental stages
EI
PG
G1
G2
H
T1
T2
C1
C2
S
EP
JP

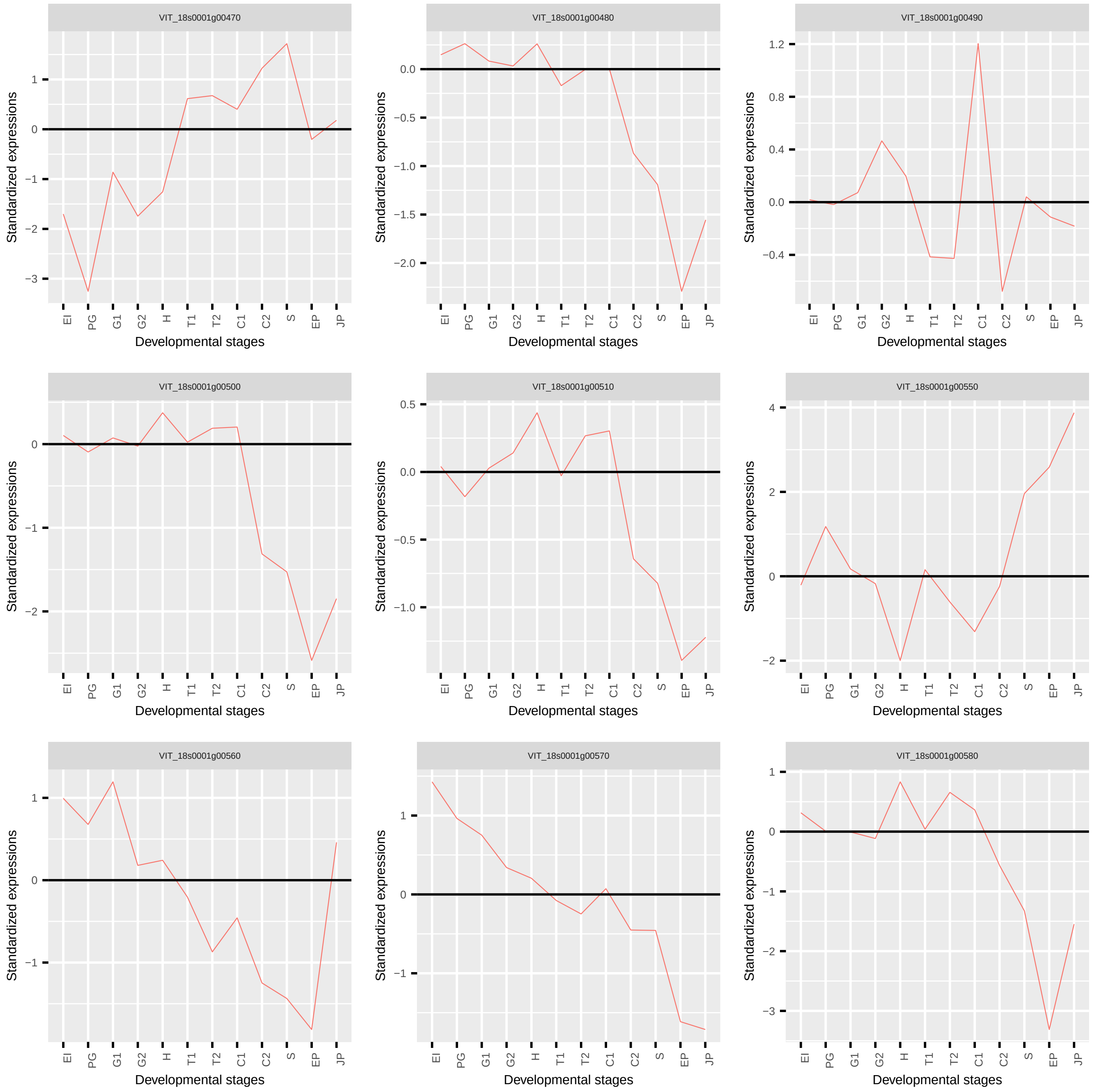
VIT_18s0001g00470
VIT_18s0001g00480
VIT_18s0001g00490
VIT_18s0001g00500
VIT_18s0001g00510
VIT_18s0001g00550
VIT_18s0001g00560
VIT_18s0001g00570
VIT_18s0001g00580
Standardized expressions
Developmental stages
EI PG G1 G2 H T1 T2 C1 C2 S EP JP

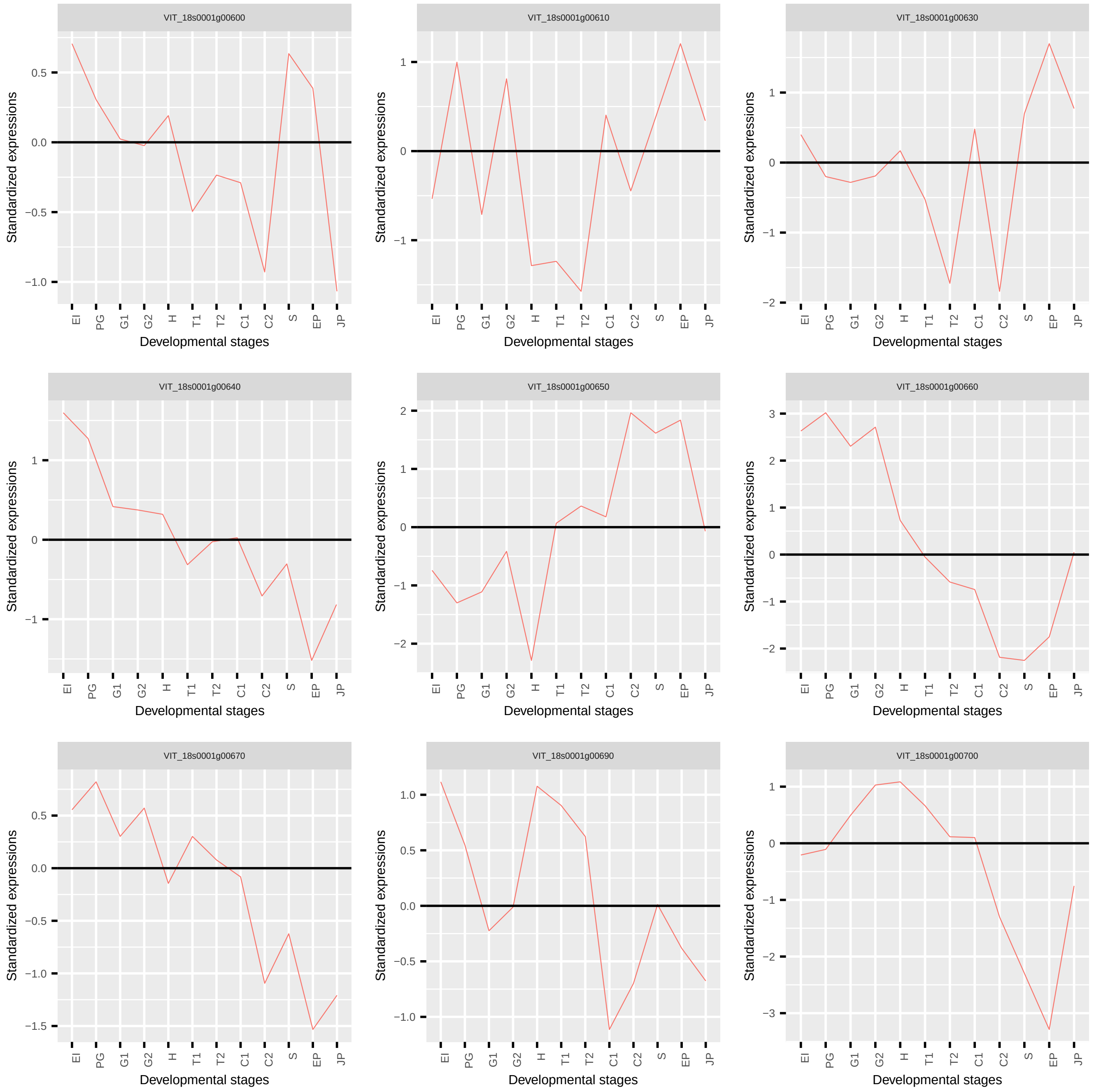
VIT_18s0001g00600
VIT_18s0001g00610
VIT_18s0001g00630
VIT_18s0001g00640
VIT_18s0001g00650
VIT_18s0001g00660
VIT_18s0001g00670
VIT_18s0001g00690
VIT_18s0001g00700
Standardized expressions
Developmental stages
EI PG G1 G2 H T1 T2 C1 C2 S EP JP

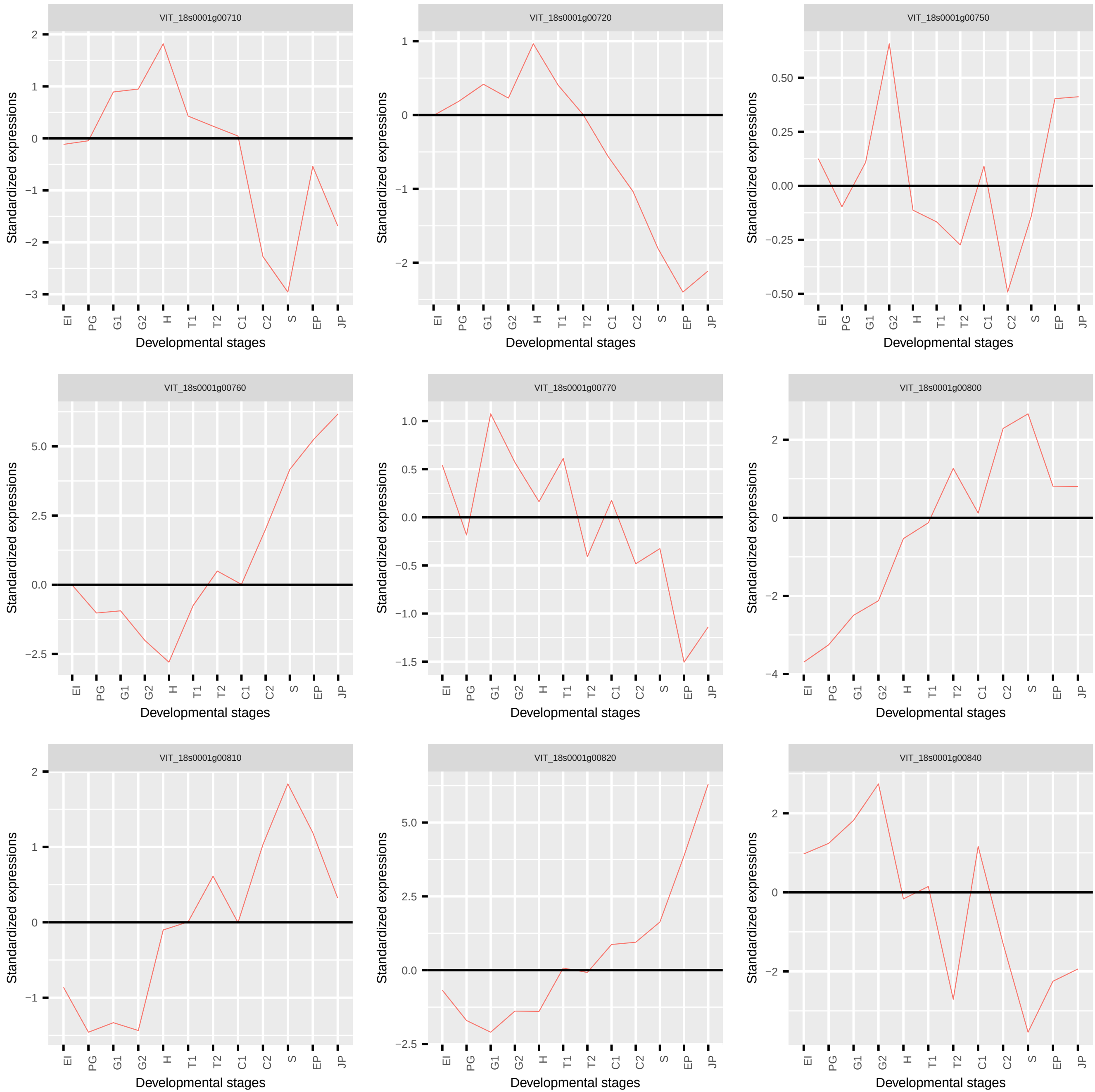
VIT_18s0001g00710
VIT_18s0001g00720
VIT_18s0001g00750
VIT_18s0001g00760
VIT_18s0001g00770
VIT_18s0001g00800
VIT_18s0001g00810
VIT_18s0001g00820
VIT_18s0001g00840
Standardized expressions
Developmental stages
EI PG G1 G2 H T1 T2 C1 C2 S EP JP

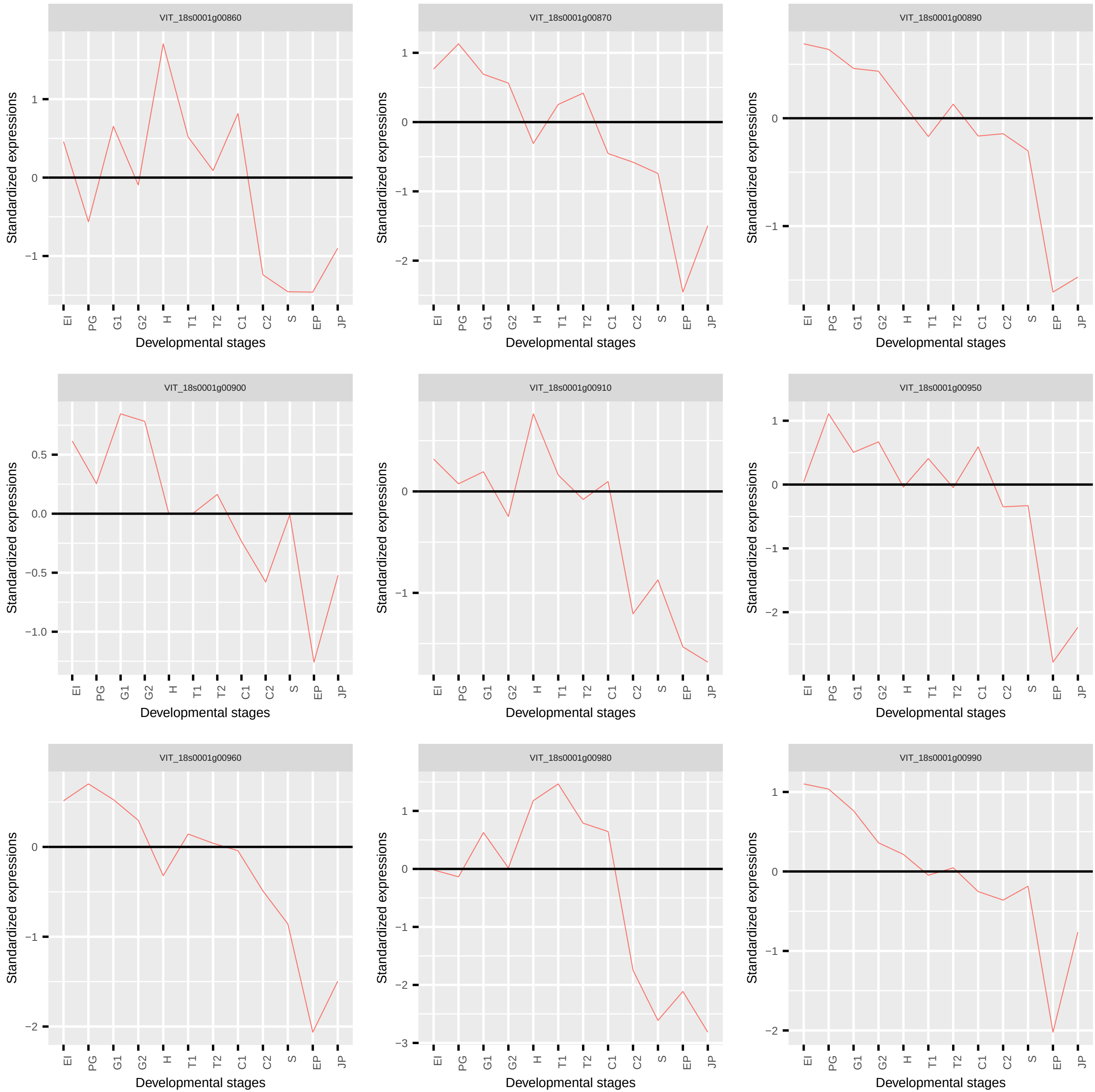
VIT_18s0001g00860
VIT_18s0001g00870
VIT_18s0001g00890
VIT_18s0001g00900
VIT_18s0001g00910
VIT_18s0001g00950
VIT_18s0001g00960
VIT_18s0001g00980
VIT_18s0001g00990
Standardized expressions
Developmental stages
EI
PG
G1
G2
H
T1
T2
C1
C2
S
EP
JP
1
0
−1
−2
−3
0.5
0.0
−0.5
−1.0

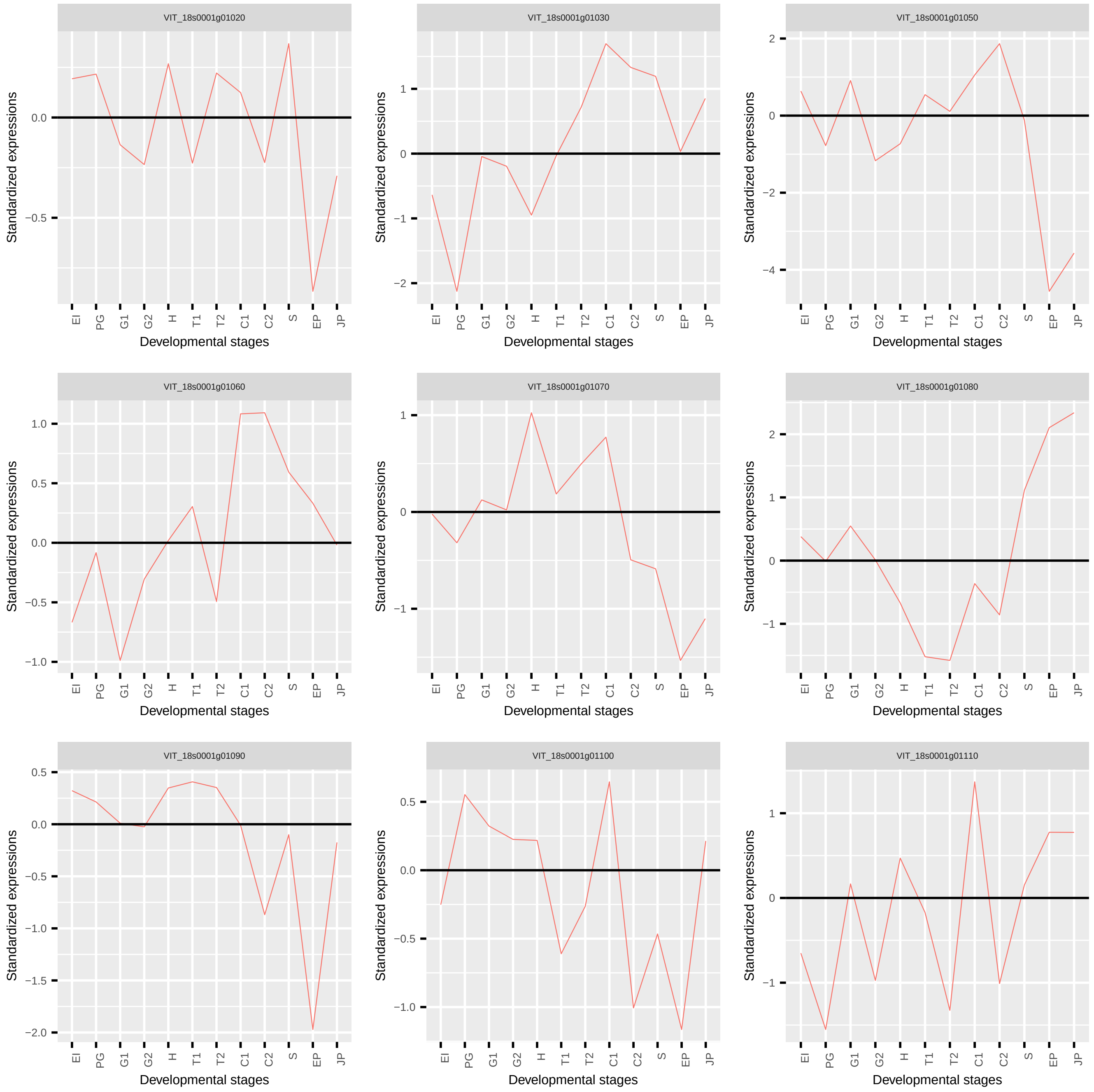
VIT_18s0001g01020
VIT_18s0001g01030
VIT_18s0001g01050
VIT_18s0001g01060
VIT_18s0001g01070
VIT_18s0001g01080
VIT_18s0001g01090
VIT_18s0001g01100
VIT_18s0001g01110
Standardized expressions
Developmental stages
EI PG G1 G2 H T1 T2 C1 C2 S EP JP

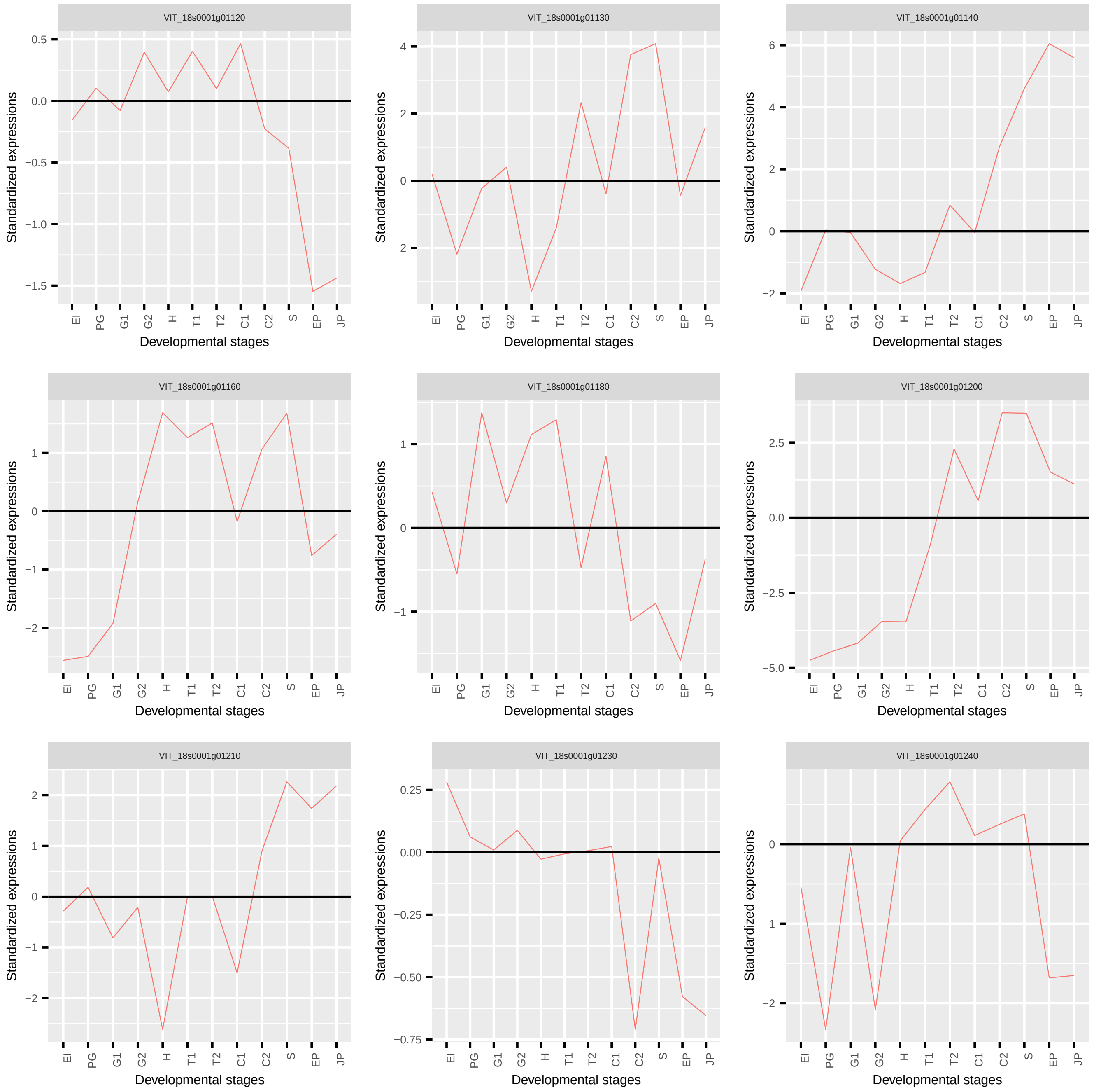
VIT_18s0001g01120
0.5
0.0
−0.5
−1.0
−1.5
Standardized expressions
EI PG G1 G2 H T1 T2 C1 C2 S EP JP
Developmental stages
VIT_18s0001g01130
4
2
0
−2
Standardized expressions
EI PG G1 G2 H T1 T2 C1 C2 S EP JP
Developmental stages
VIT_18s0001g01140
6
4
2
0
−2
Standardized expressions
EI PG G1 G2 H T1 T2 C1 C2 S EP JP
Developmental stages
VIT_18s0001g01160
1
0
−1
−2
Standardized expressions
EI PG G1 G2 H T1 T2 C1 C2 S EP JP
Developmental stages
VIT_18s0001g01180
1
0
−1
Standardized expressions
EI PG G1 G2 H T1 T2 C1 C2 S EP JP
Developmental stages
VIT_18s0001g01200
2.5
0.0
−2.5
−5.0
Standardized expressions
EI PG G1 G2 H T1 T2 C1 C2 S EP JP
Developmental stages
VIT_18s0001g01210
2
1
0
−1
−2
Standardized expressions
EI PG G1 G2 H T1 T2 C1 C2 S EP JP
Developmental stages
VIT_18s0001g01230
0.25
0.00
−0.25
−0.50
−0.75
Standardized expressions
EI PG G1 G2 H T1 T2 C1 C2 S EP JP
Developmental stages
VIT_18s0001g01240
0
−1
−2
Standardized expressions
EI PG G1 G2 H T1 T2 C1 C2 S EP JP
Developmental stages

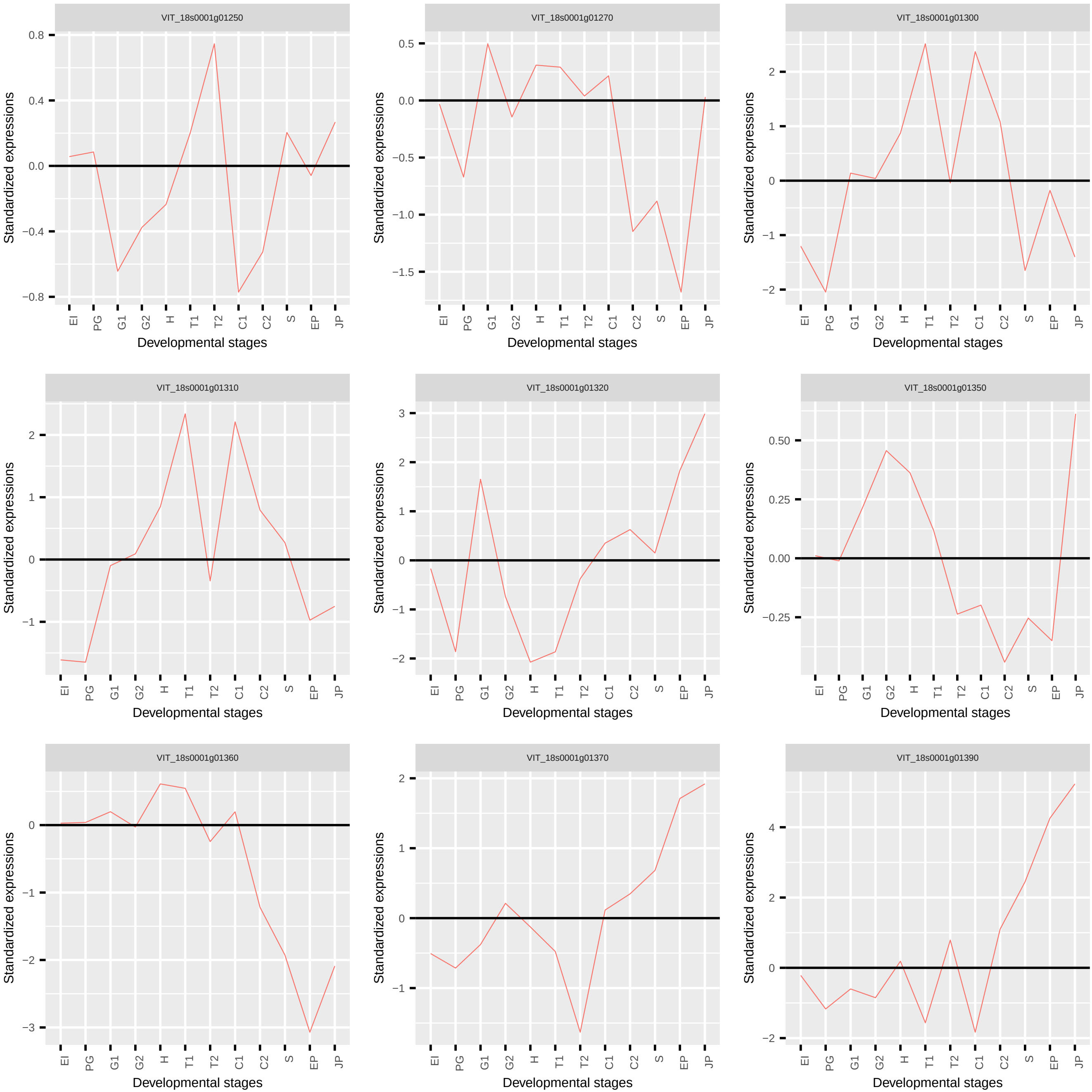
VIT_18s0001g01250
VIT_18s0001g01270
VIT_18s0001g01300
VIT_18s0001g01310
VIT_18s0001g01320
VIT_18s0001g01350
VIT_18s0001g01360
VIT_18s0001g01370
VIT_18s0001g01390
Standardized expressions
Developmental stages
EI
PG
G1
G2
H
T1
T2
C1
C2
S
EP
JP
0.8
0.4
0.0
−0.4
−0.8
0.5
−0.5
−1.0
−1.5
0.50
0.25
0.00
−0.25
4
3
2
1
0
−1
−2
−3

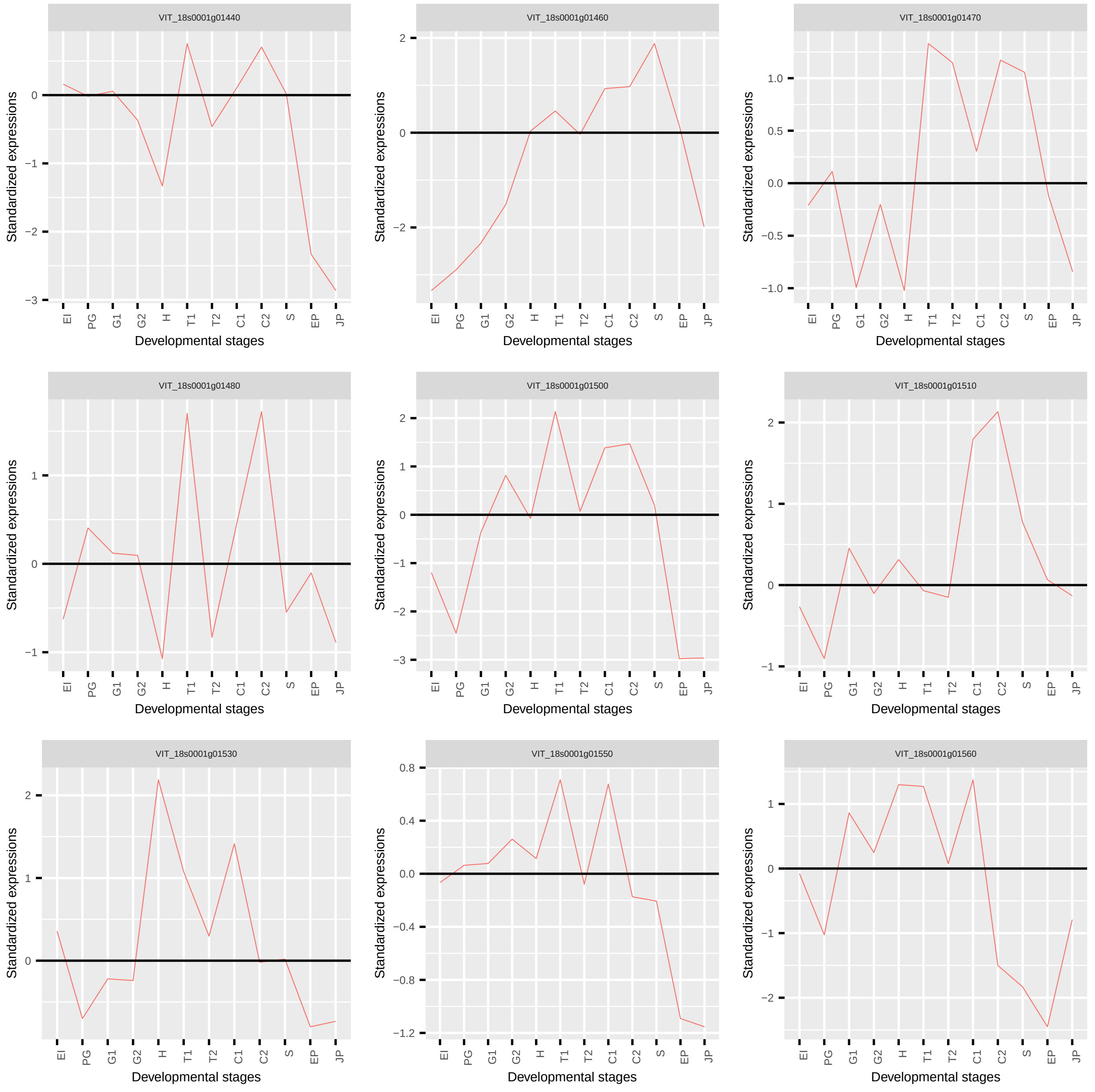
VIT_18s0001g01440
VIT_18s0001g01460
VIT_18s0001g01470
VIT_18s0001g01480
VIT_18s0001g01500
VIT_18s0001g01510
VIT_18s0001g01530
VIT_18s0001g01550
VIT_18s0001g01560
Standardized expressions
Developmental stages
EI PG G1 G2 H T1 T2 C1 C2 S EP JP

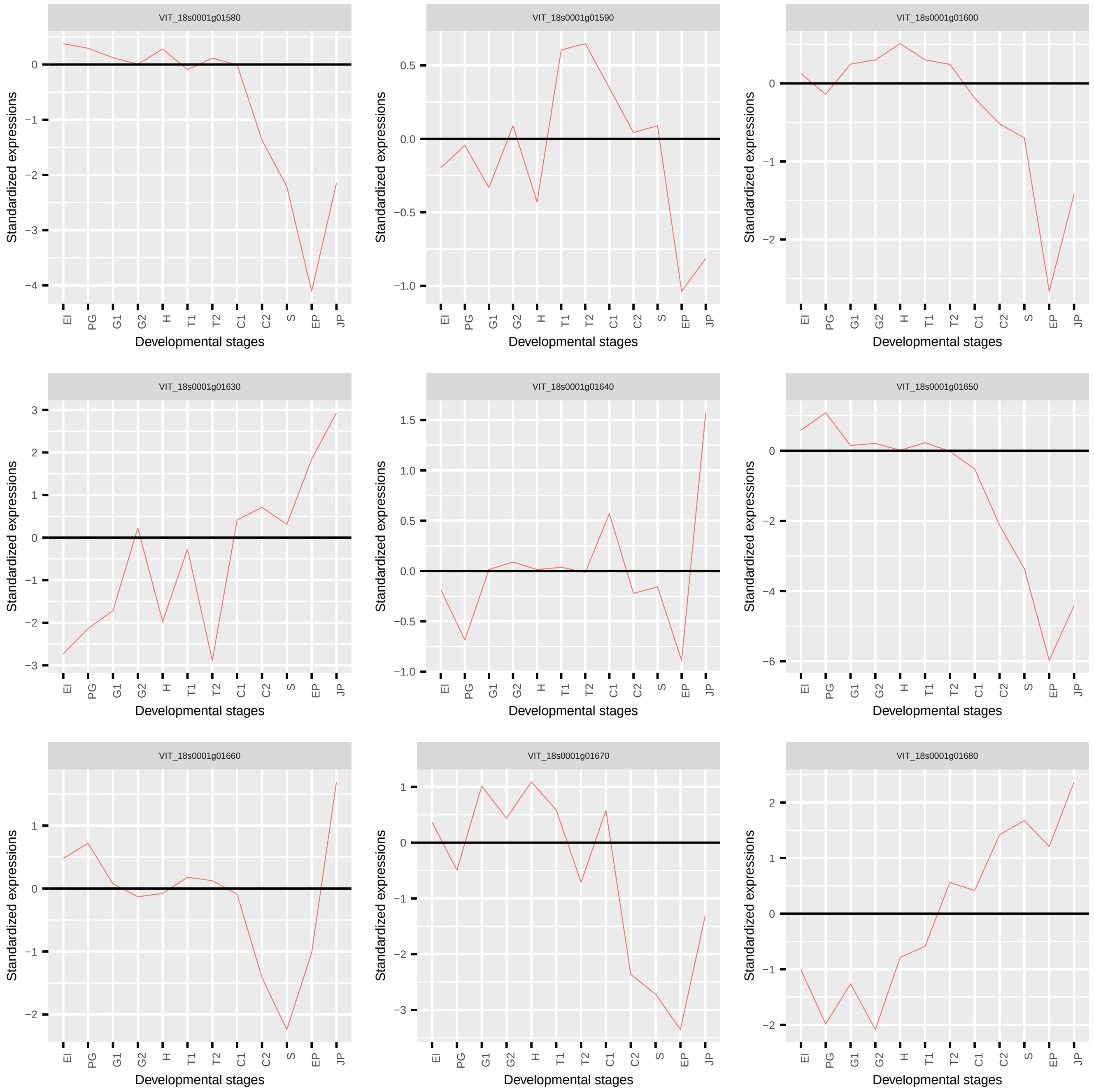
VIT_18s0001g01580
VIT_18s0001g01590
VIT_18s0001g01600
VIT_18s0001g01630
VIT_18s0001g01640
VIT_18s0001g01650
VIT_18s0001g01660
VIT_18s0001g01670
VIT_18s0001g01680
Standardized expressions
Developmental stages
EI PG G1 G2 H T1 T2 C1 C2 S EP JP

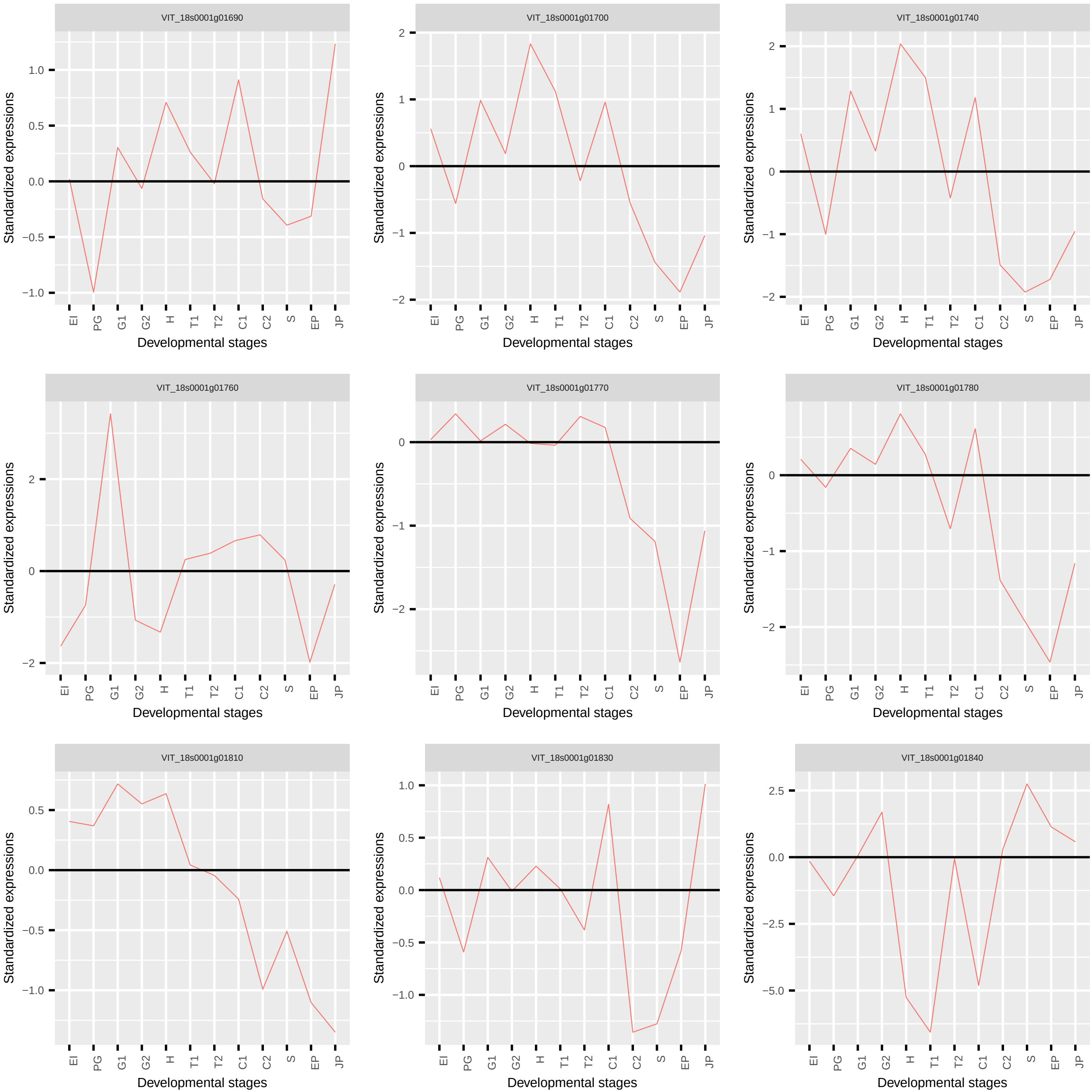
VIT_18s0001g01690
Standardized expressions
1.0
0.5
0.0
−0.5
−1.0
EI PG G1 G2 H T1 T2 C1 C2 S EP JP
Developmental stages
VIT_18s0001g01700
Standardized expressions
2
1
0
−1
−2
EI PG G1 G2 H T1 T2 C1 C2 S EP JP
Developmental stages
VIT_18s0001g01740
Standardized expressions
2
1
0
−1
−2
EI PG G1 G2 H T1 T2 C1 C2 S EP JP
Developmental stages
VIT_18s0001g01760
Standardized expressions
2
0
−2
EI PG G1 G2 H T1 T2 C1 C2 S EP JP
Developmental stages
VIT_18s0001g01770
Standardized expressions
0
−1
−2
EI PG G1 G2 H T1 T2 C1 C2 S EP JP
Developmental stages
VIT_18s0001g01780
Standardized expressions
0
−1
−2
EI PG G1 G2 H T1 T2 C1 C2 S EP JP
Developmental stages
VIT_18s0001g01810
Standardized expressions
0.5
0.0
−0.5
−1.0
EI PG G1 G2 H T1 T2 C1 C2 S EP JP
Developmental stages
VIT_18s0001g01830
Standardized expressions
1.0
0.5
0.0
−0.5
−1.0
EI PG G1 G2 H T1 T2 C1 C2 S EP JP
Developmental stages
VIT_18s0001g01840
Standardized expressions
2.5
0.0
−2.5
−5.0
EI PG G1 G2 H T1 T2 C1 C2 S EP JP
Developmental stages

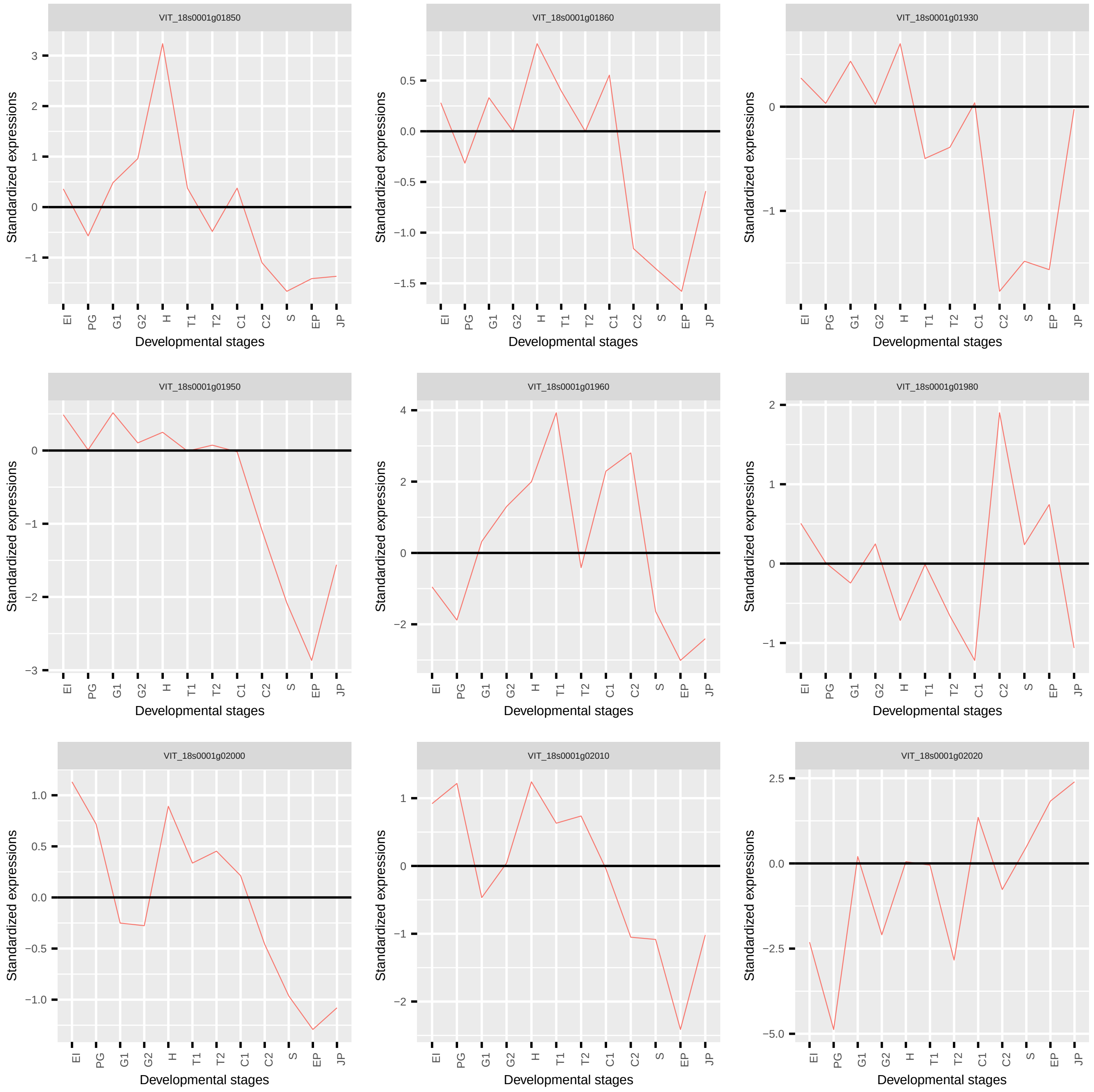
VIT_18s0001g01850
VIT_18s0001g01860
VIT_18s0001g01930
VIT_18s0001g01950
VIT_18s0001g01960
VIT_18s0001g01980
VIT_18s0001g02000
VIT_18s0001g02010
VIT_18s0001g02020
Standardized expressions
Developmental stages
EI PG G1 G2 H T1 T2 C1 C2 S EP JP

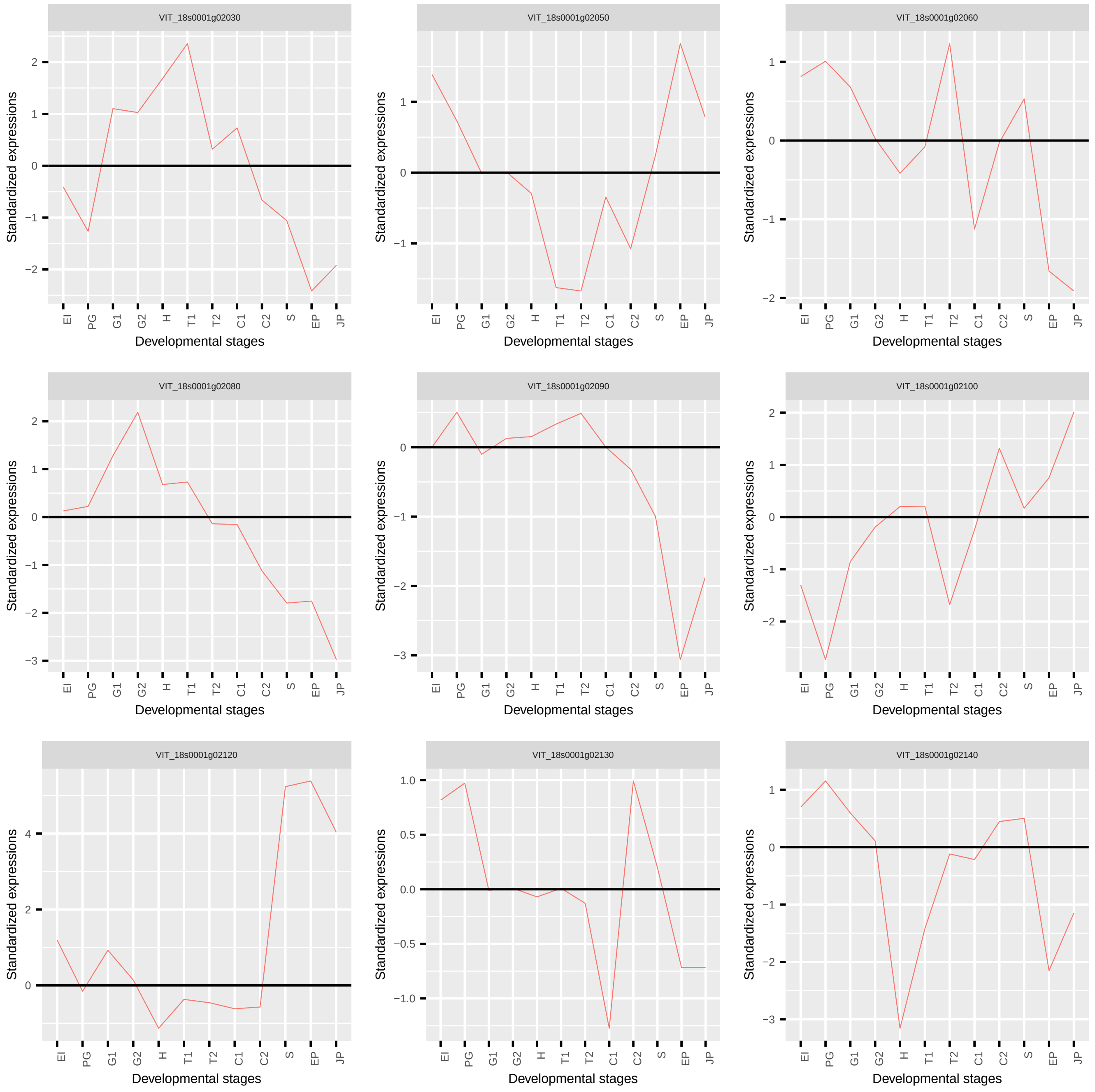
VIT_18s0001g02030
VIT_18s0001g02050
VIT_18s0001g02060
VIT_18s0001g02080
VIT_18s0001g02090
VIT_18s0001g02100
VIT_18s0001g02120
VIT_18s0001g02130
VIT_18s0001g02140
Standardized expressions
Developmental stages
EI PG G1 G2 H T1 T2 C1 C2 S EP JP

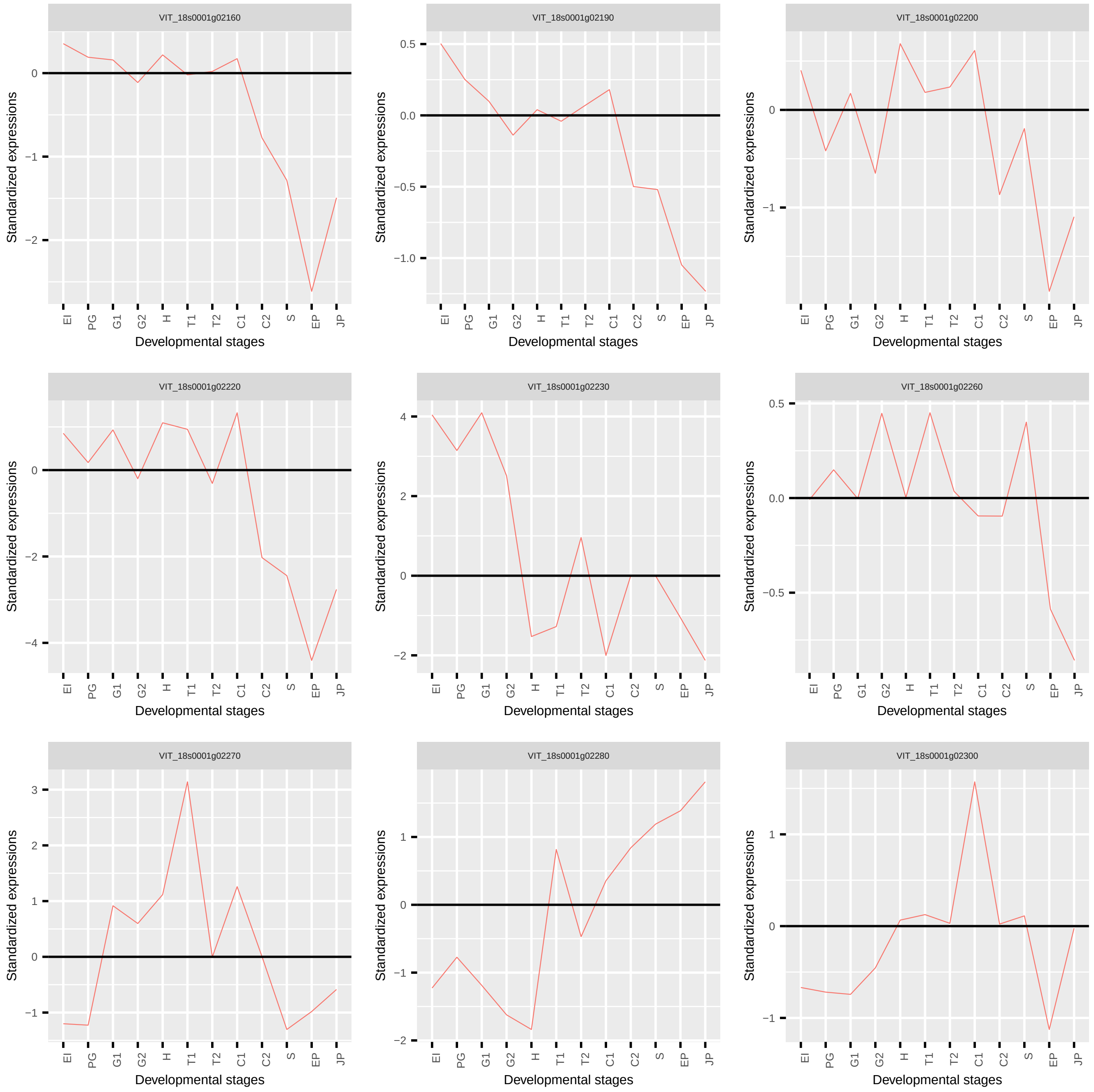
VIT_18s0001g02160
VIT_18s0001g02190
VIT_18s0001g02200
VIT_18s0001g02220
VIT_18s0001g02230
VIT_18s0001g02260
VIT_18s0001g02270
VIT_18s0001g02280
VIT_18s0001g02300
Standardized expressions
Developmental stages
EI PG G1 G2 H T1 T2 C1 C2 S EP JP

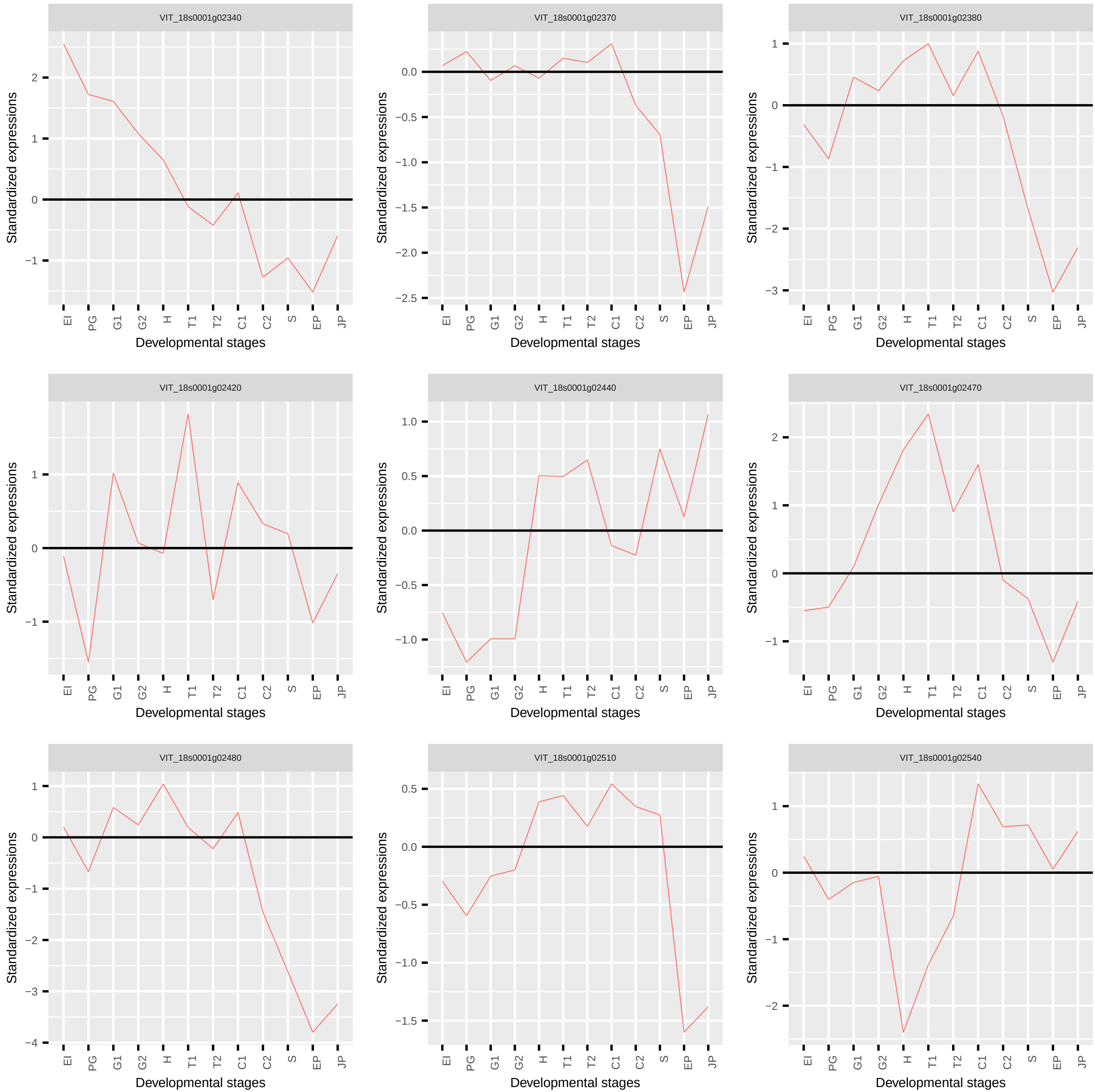
VIT_18s0001g02340
VIT_18s0001g02370
VIT_18s0001g02380
VIT_18s0001g02420
VIT_18s0001g02440
VIT_18s0001g02470
VIT_18s0001g02480
VIT_18s0001g02510
VIT_18s0001g02540
Standardized expressions
Developmental stages
EI PG G1 G2 H T1 T2 C1 C2 S EP JP
2 1 0 −1
0.0 −0.5 −1.0 −1.5 −2.0 −2.5
1 0 −1 −2 −3
1 0 −1
1.0 0.5 0.0 −0.5 −1.0
2 1 0 −1
1 0 −1 −2 −3 −4
0.5 0.0 −0.5 −1.0 −1.5
1 0 −1 −2

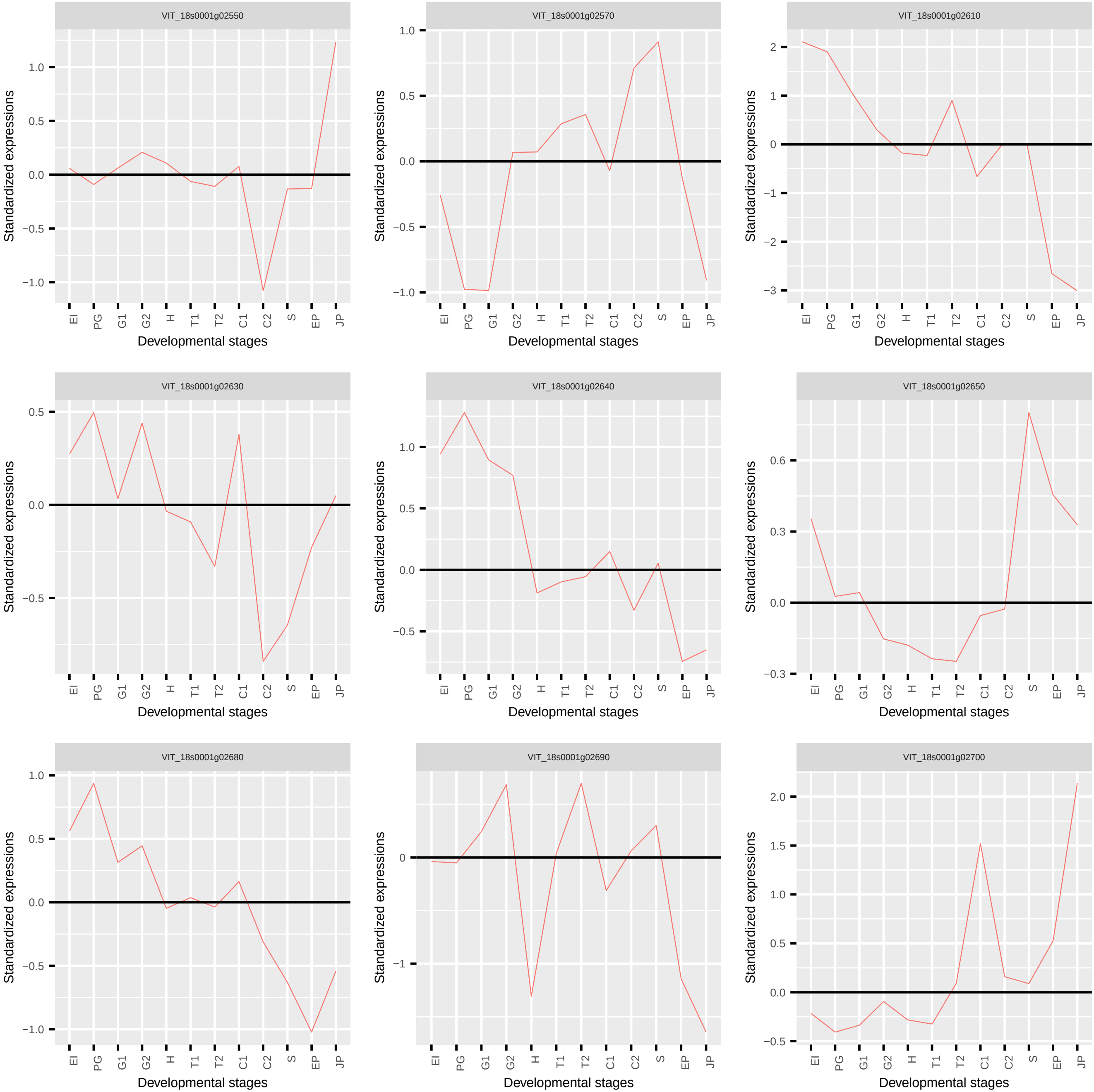
VIT_18s0001g02550
VIT_18s0001g02570
VIT_18s0001g02610
VIT_18s0001g02630
VIT_18s0001g02640
VIT_18s0001g02650
VIT_18s0001g02680
VIT_18s0001g02690
VIT_18s0001g02700
Standardized expressions
Developmental stages
EI
PG
G1
G2
H
T1
T2
C1
C2
S
EP
JP

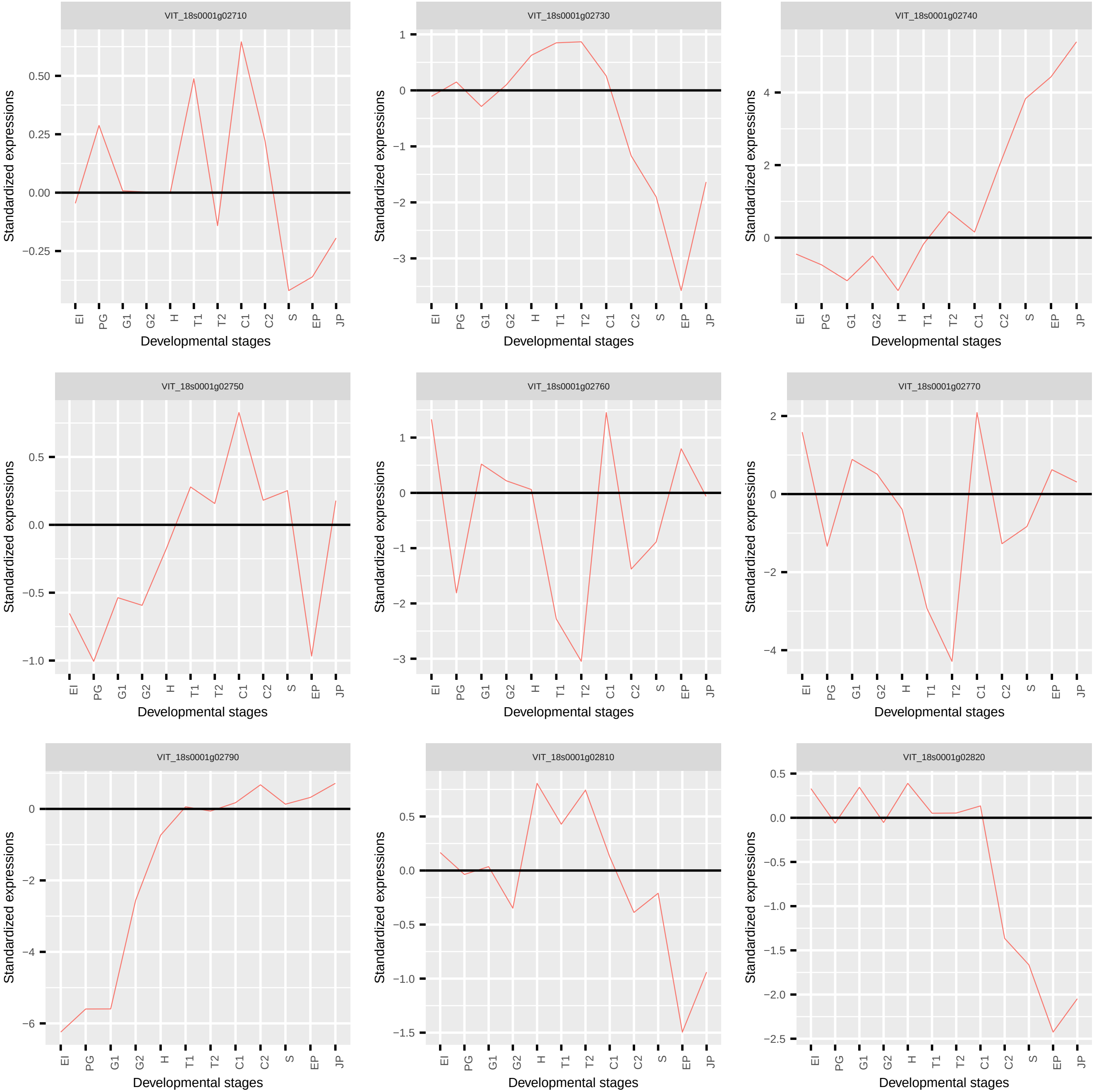

VIT_18s0001g02710
Standardized expressions
0.50
0.25
0.00
−0.25
EI PG G1 G2 H T1 T2 C1 C2 S EP JP
Developmental stages
VIT_18s0001g02730
Standardized expressions
1
0
−1
−2
−3
EI PG G1 G2 H T1 T2 C1 C2 S EP JP
Developmental stages
VIT_18s0001g02740
Standardized expressions
4
2
0
EI PG G1 G2 H T1 T2 C1 C2 S EP JP
Developmental stages
VIT_18s0001g02750
Standardized expressions
0.5
0.0
−0.5
−1.0
EI PG G1 G2 H T1 T2 C1 C2 S EP JP
Developmental stages
VIT_18s0001g02760
Standardized expressions
1
0
−1
−2
−3
EI PG G1 G2 H T1 T2 C1 C2 S EP JP
Developmental stages
VIT_18s0001g02770
Standardized expressions
2
0
−2
−4
EI PG G1 G2 H T1 T2 C1 C2 S EP JP
Developmental stages
VIT_18s0001g02790
Standardized expressions
0
−2
−4
−6
EI PG G1 G2 H T1 T2 C1 C2 S EP JP
Developmental stages
VIT_18s0001g02810
Standardized expressions
0.5
0.0
−0.5
−1.0
−1.5
EI PG G1 G2 H T1 T2 C1 C2 S EP JP
Developmental stages
VIT_18s0001g02820
Standardized expressions
0.5
0.0
−0.5
−1.0
−1.5
−2.0
−2.5
EI PG G1 G2 H T1 T2 C1 C2 S EP JP
Developmental stages

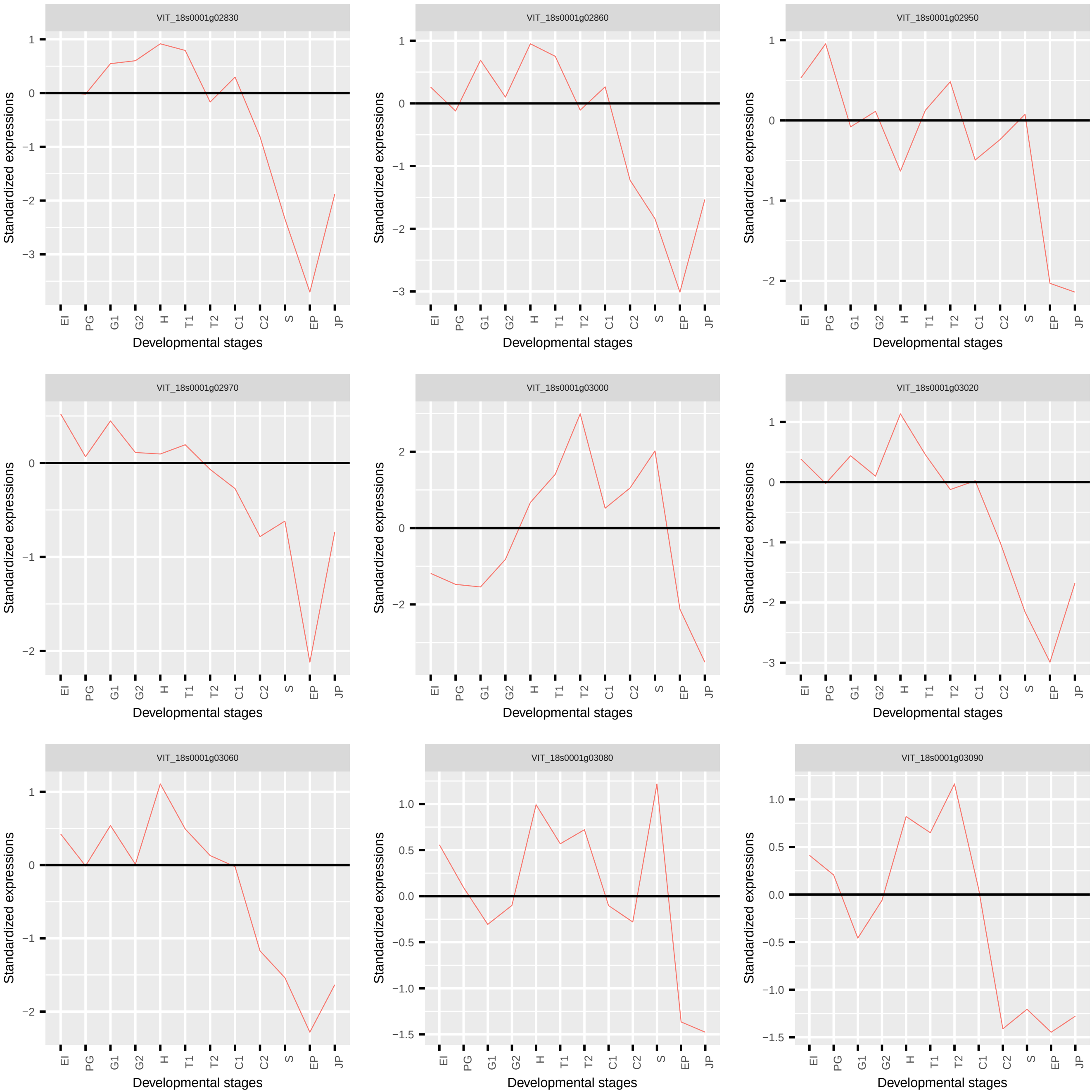
VIT_18s0001g02830
VIT_18s0001g02860
VIT_18s0001g02950
VIT_18s0001g02970
VIT_18s0001g03000
VIT_18s0001g03020
VIT_18s0001g03060
VIT_18s0001g03080
VIT_18s0001g03090
Standardized expressions
Developmental stages
EI
PG
G1
G2
H
T1
T2
C1
C2
S
EP
JP

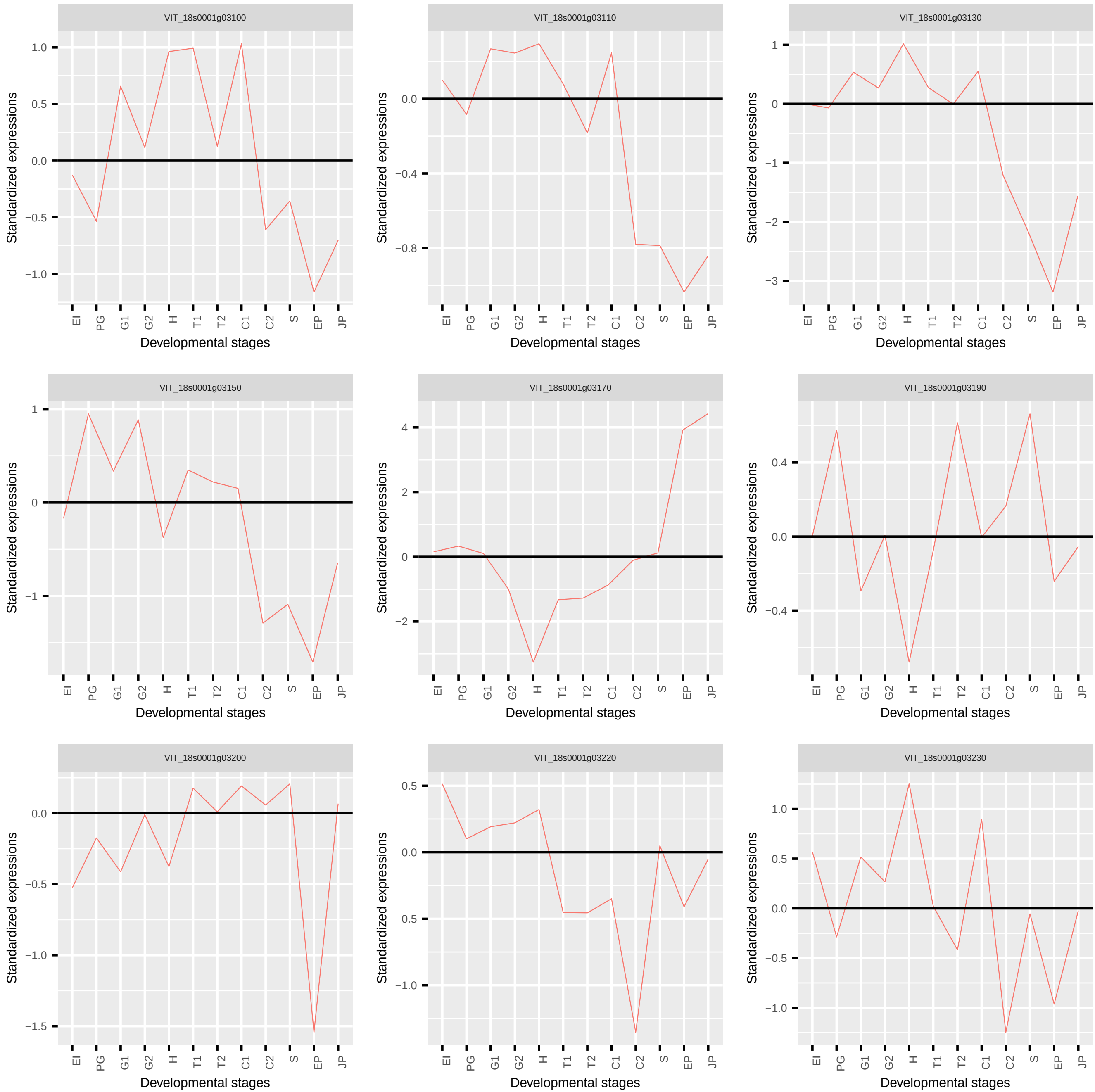

VIT_18s0001g03100
VIT_18s0001g03110
VIT_18s0001g03130
VIT_18s0001g03150
VIT_18s0001g03170
VIT_18s0001g03190
VIT_18s0001g03200
VIT_18s0001g03220
VIT_18s0001g03230
Standardized expressions
Developmental stages
EI
PG
G1
G2
H
T1
T2
C1
C2
S
EP
JP

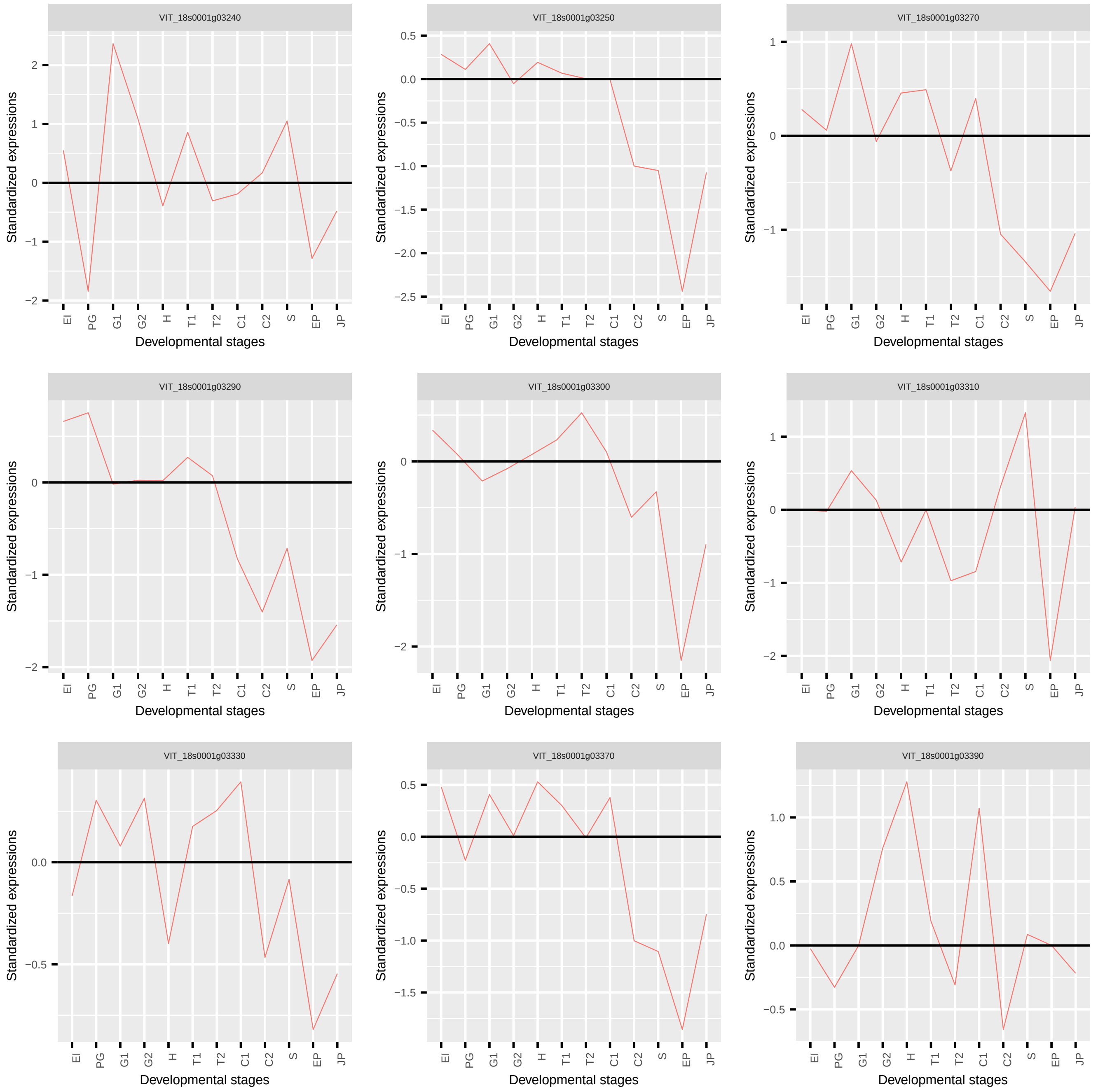
VIT_18s0001g03240
VIT_18s0001g03250
VIT_18s0001g03270
VIT_18s0001g03290
VIT_18s0001g03300
VIT_18s0001g03310
VIT_18s0001g03330
VIT_18s0001g03370
VIT_18s0001g03390
Standardized expressions
Developmental stages
EI PG G1 G2 H T1 T2 C1 C2 S EP JP
2
1
0
−1
−2
0.5
0.0
−0.5
−1.0
−1.5
−2.0
−2.5

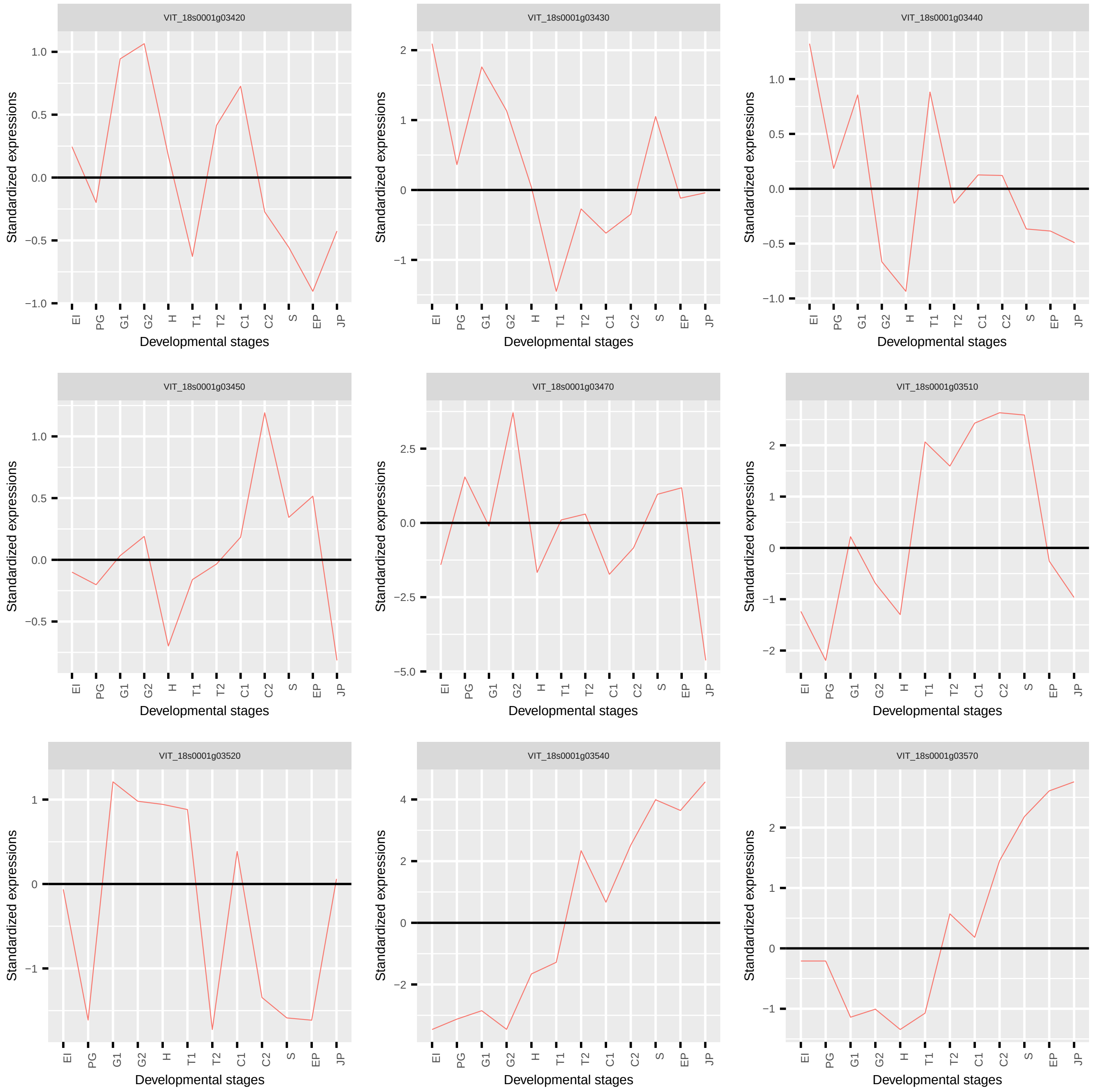
VIT_18s0001g03420
VIT_18s0001g03430
VIT_18s0001g03440
VIT_18s0001g03450
VIT_18s0001g03470
VIT_18s0001g03510
VIT_18s0001g03520
VIT_18s0001g03540
VIT_18s0001g03570
Standardized expressions
Developmental stages
EI PG G1 G2 H T1 T2 C1 C2 S EP JP

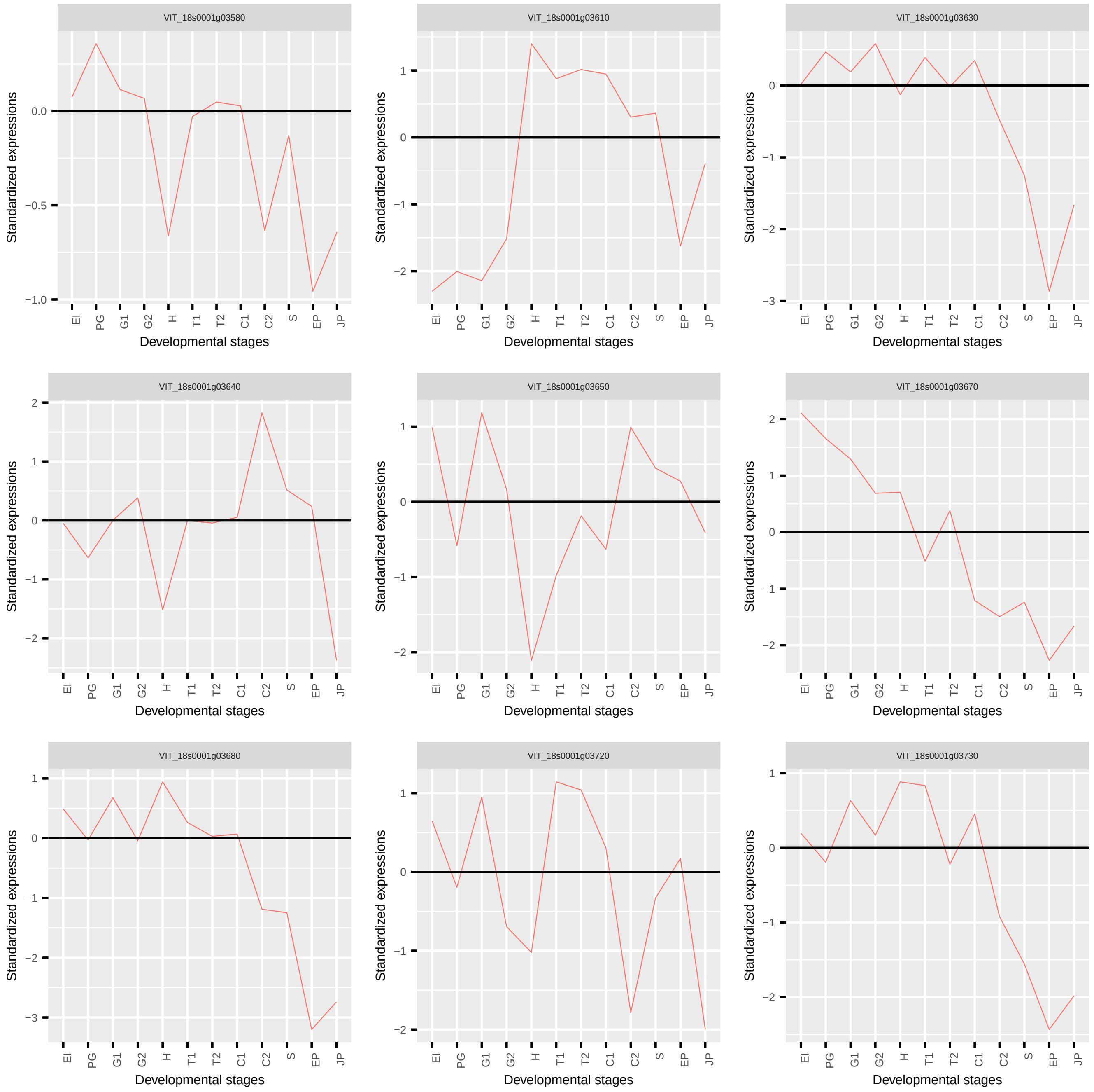
VIT_18s0001g03580
VIT_18s0001g03610
VIT_18s0001g03630
VIT_18s0001g03640
VIT_18s0001g03650
VIT_18s0001g03670
VIT_18s0001g03680
VIT_18s0001g03720
VIT_18s0001g03730
Standardized expressions
Developmental stages
EI
PG
G1
G2
H
T1
T2
C1
C2
S
EP
JP

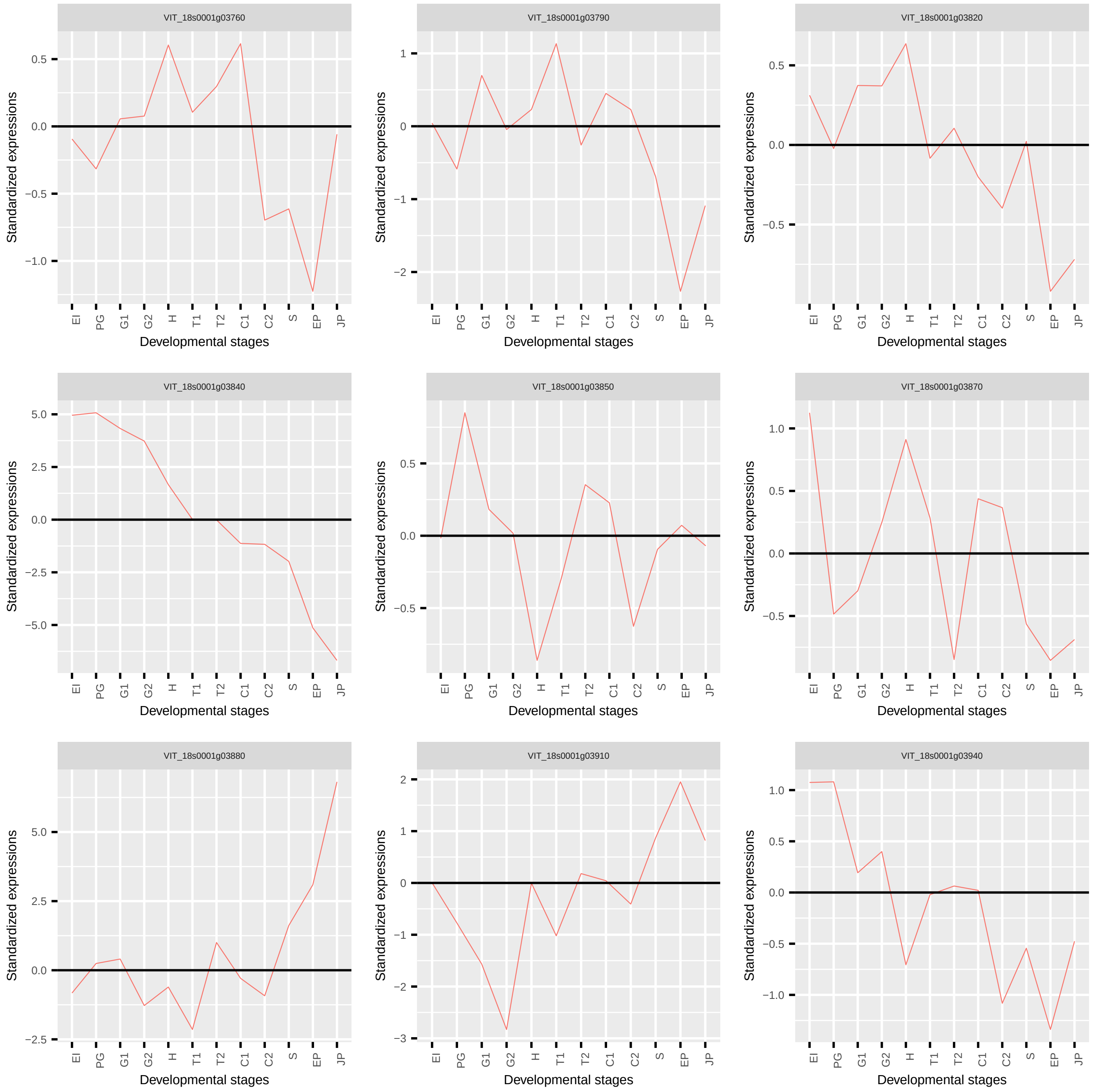
VIT_18s0001g03760
Standardized expressions
0.5
0.0
−0.5
−1.0
EI PG G1 G2 H T1 T2 C1 C2 S EP JP
Developmental stages
VIT_18s0001g03790
Standardized expressions
1
0
−1
−2
EI PG G1 G2 H T1 T2 C1 C2 S EP JP
Developmental stages
VIT_18s0001g03820
Standardized expressions
0.5
0.0
−0.5
EI PG G1 G2 H T1 T2 C1 C2 S EP JP
Developmental stages
VIT_18s0001g03840
Standardized expressions
5.0
2.5
0.0
−2.5
−5.0
EI PG G1 G2 H T1 T2 C1 C2 S EP JP
Developmental stages
VIT_18s0001g03850
Standardized expressions
0.5
0.0
−0.5
EI PG G1 G2 H T1 T2 C1 C2 S EP JP
Developmental stages
VIT_18s0001g03870
Standardized expressions
1.0
0.5
0.0
−0.5
EI PG G1 G2 H T1 T2 C1 C2 S EP JP
Developmental stages
VIT_18s0001g03880
Standardized expressions
5.0
2.5
0.0
−2.5
EI PG G1 G2 H T1 T2 C1 C2 S EP JP
Developmental stages
VIT_18s0001g03910
Standardized expressions
2
1
0
−1
−2
−3
EI PG G1 G2 H T1 T2 C1 C2 S EP JP
Developmental stages
VIT_18s0001g03940
Standardized expressions
1.0
0.5
0.0
−0.5
−1.0
EI PG G1 G2 H T1 T2 C1 C2 S EP JP
Developmental stages

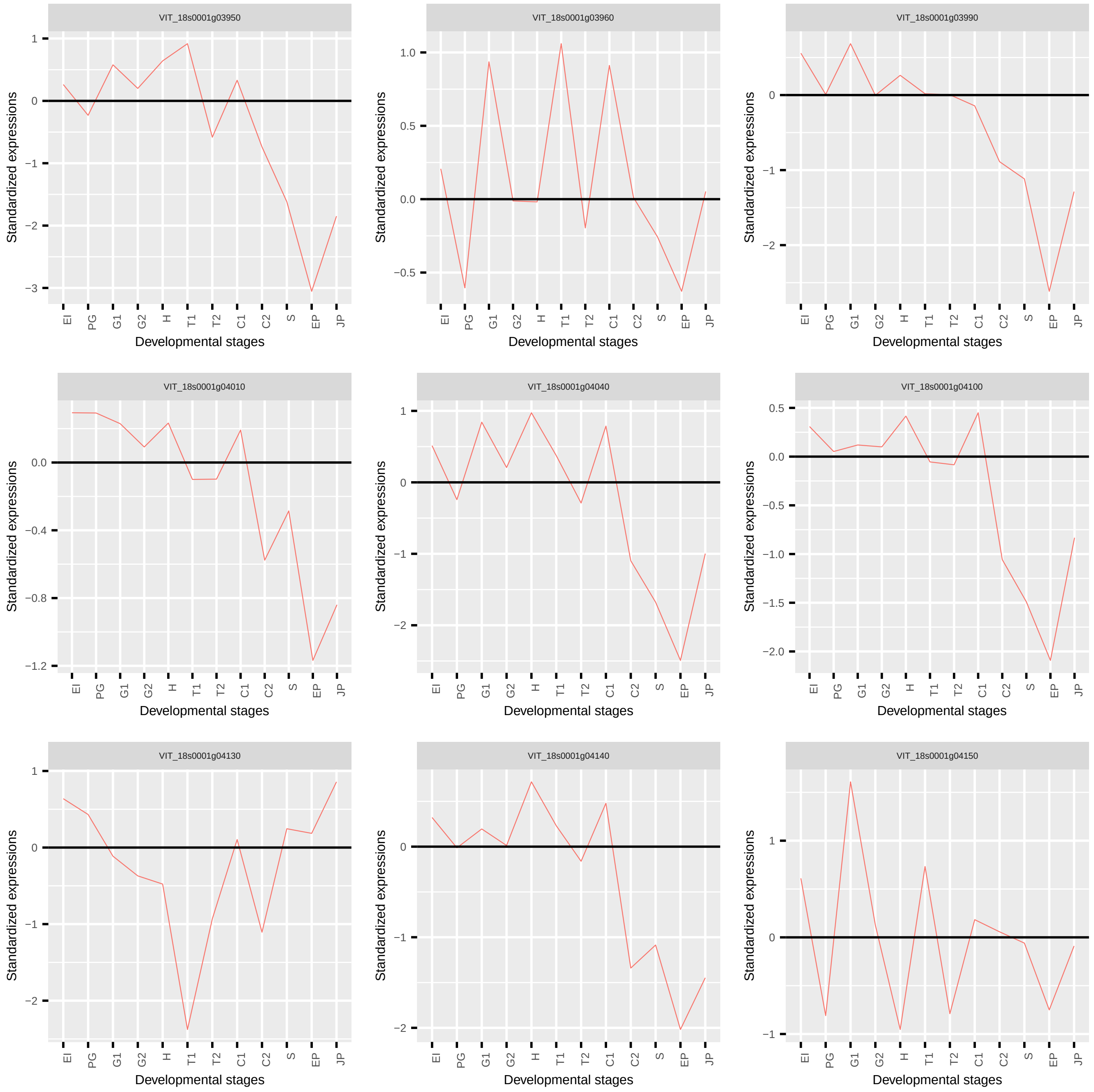
VIT_18s0001g03950
1
0
−1
−2
−3
Standardized expressions
EI PG G1 G2 H T1 T2 C1 C2 S EP JP
Developmental stages
VIT_18s0001g03960
1.0
0.5
0.0
−0.5
Standardized expressions
EI PG G1 G2 H T1 T2 C1 C2 S EP JP
Developmental stages
VIT_18s0001g03990
0
−1
−2
Standardized expressions
EI PG G1 G2 H T1 T2 C1 C2 S EP JP
Developmental stages
VIT_18s0001g04010
0.0
−0.4
−0.8
−1.2
Standardized expressions
EI PG G1 G2 H T1 T2 C1 C2 S EP JP
Developmental stages
VIT_18s0001g04040
1
0
−1
−2
Standardized expressions
EI PG G1 G2 H T1 T2 C1 C2 S EP JP
Developmental stages
VIT_18s0001g04100
0.5
0.0
−0.5
−1.0
−1.5
−2.0
Standardized expressions
EI PG G1 G2 H T1 T2 C1 C2 S EP JP
Developmental stages
VIT_18s0001g04130
1
0
−1
−2
Standardized expressions
EI PG G1 G2 H T1 T2 C1 C2 S EP JP
Developmental stages
VIT_18s0001g04140
0
−1
−2
Standardized expressions
EI PG G1 G2 H T1 T2 C1 C2 S EP JP
Developmental stages
VIT_18s0001g04150
1
0
−1
Standardized expressions
EI PG G1 G2 H T1 T2 C1 C2 S EP JP
Developmental stages

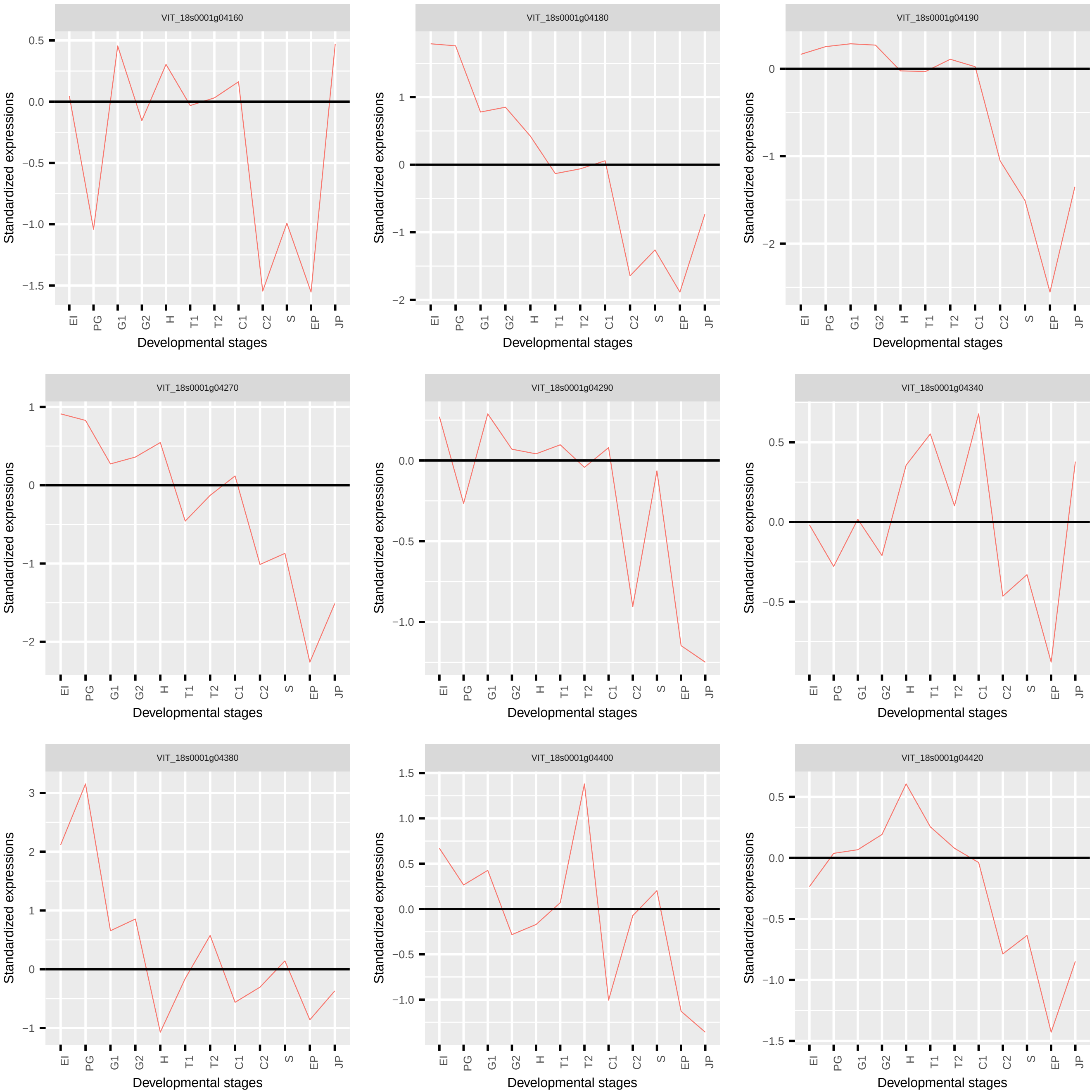
VIT_18s0001g04160
VIT_18s0001g04180
VIT_18s0001g04190
VIT_18s0001g04270
VIT_18s0001g04290
VIT_18s0001g04340
VIT_18s0001g04380
VIT_18s0001g04400
VIT_18s0001g04420
Standardized expressions
Developmental stages
EI PG G1 G2 H T1 T2 C1 C2 S EP JP

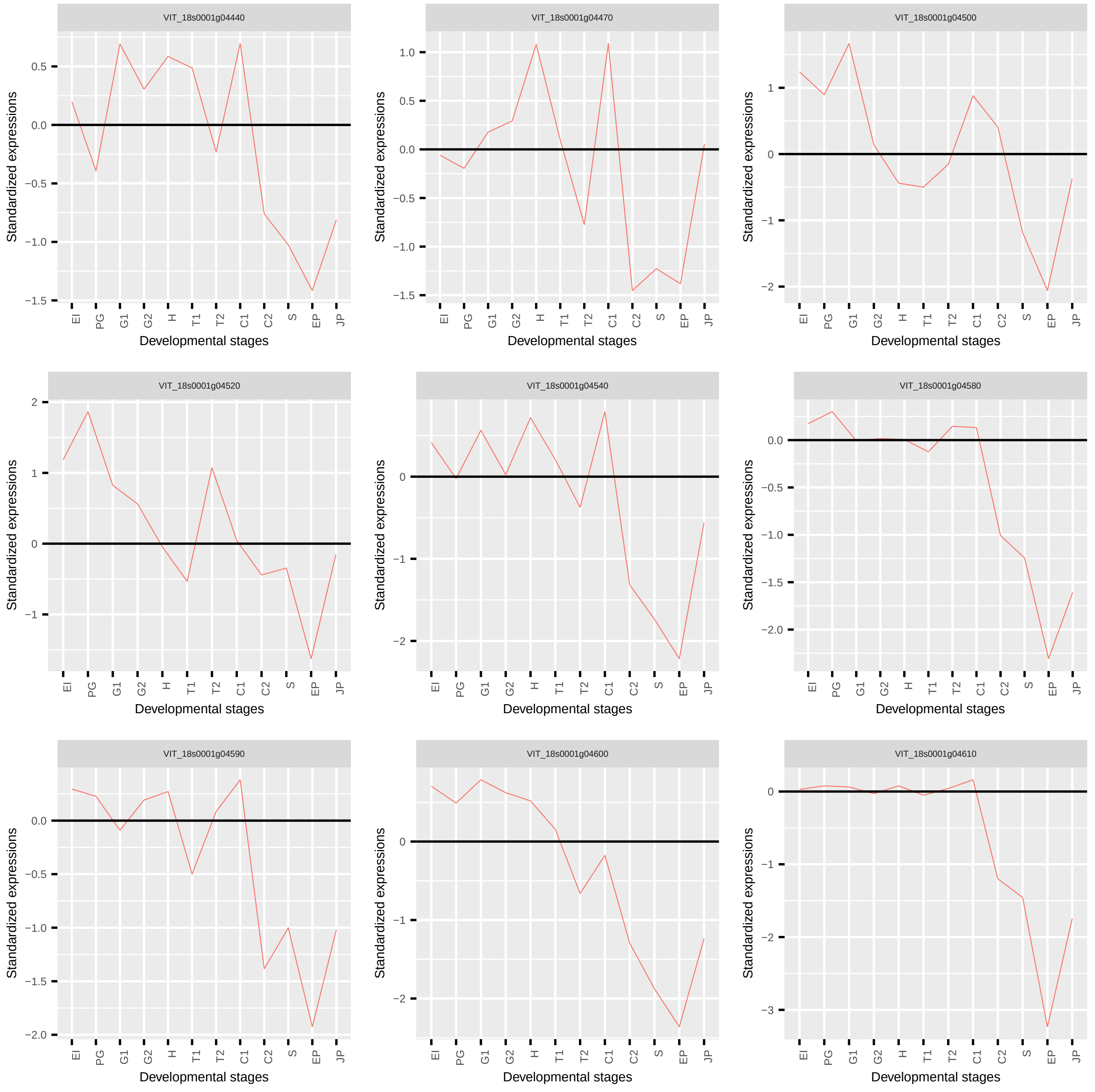
VIT_18s0001g04440
VIT_18s0001g04470
VIT_18s0001g04500
VIT_18s0001g04520
VIT_18s0001g04540
VIT_18s0001g04580
VIT_18s0001g04590
VIT_18s0001g04600
VIT_18s0001g04610
Standardized expressions
Developmental stages
EI PG G1 G2 H T1 T2 C1 C2 S EP JP

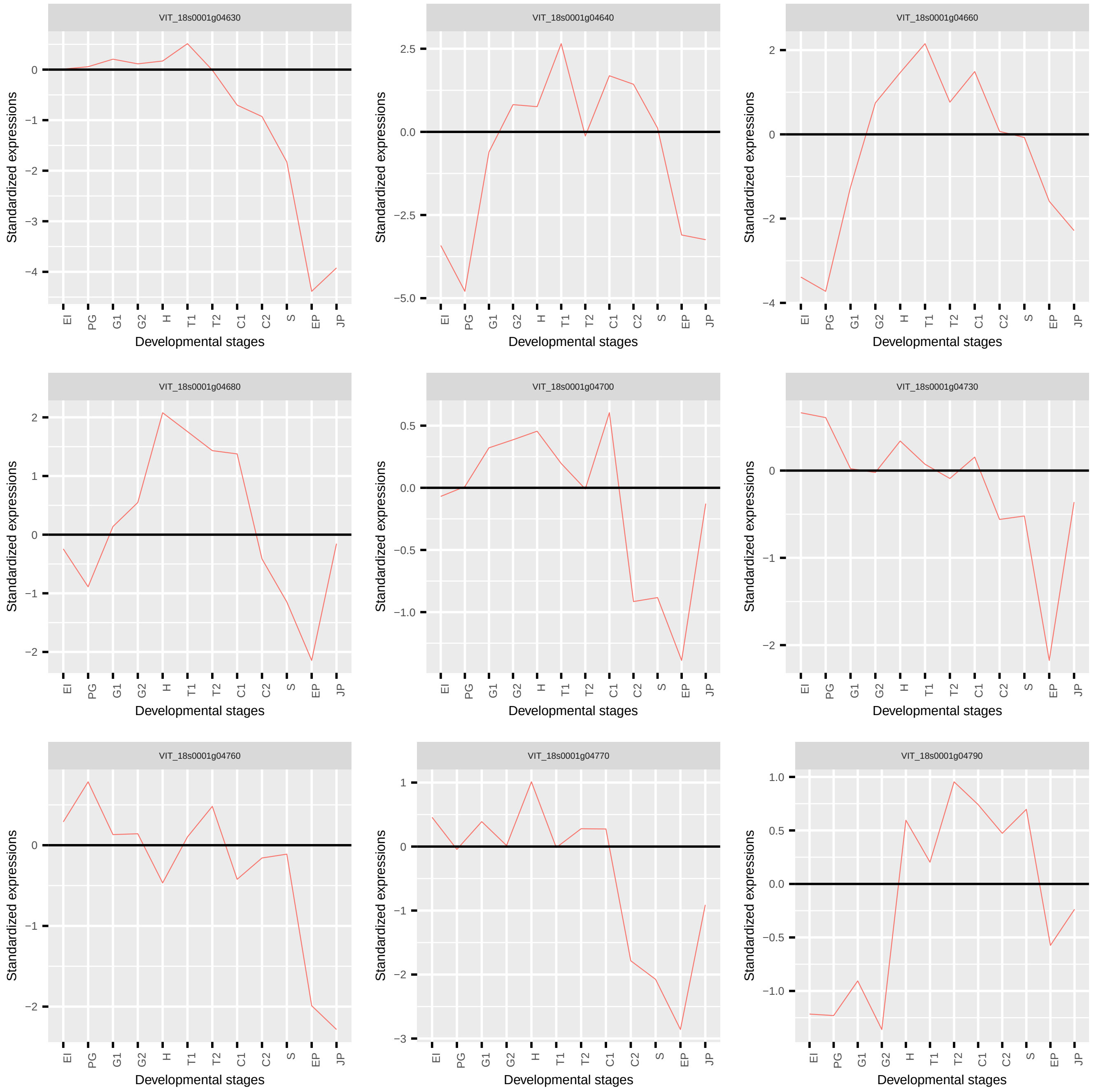
VIT_18s0001g04630
VIT_18s0001g04640
VIT_18s0001g04660
VIT_18s0001g04680
VIT_18s0001g04700
VIT_18s0001g04730
VIT_18s0001g04760
VIT_18s0001g04770
VIT_18s0001g04790
Standardized expressions
Developmental stages
EI PG G1 G2 H T1 T2 C1 C2 S EP JP

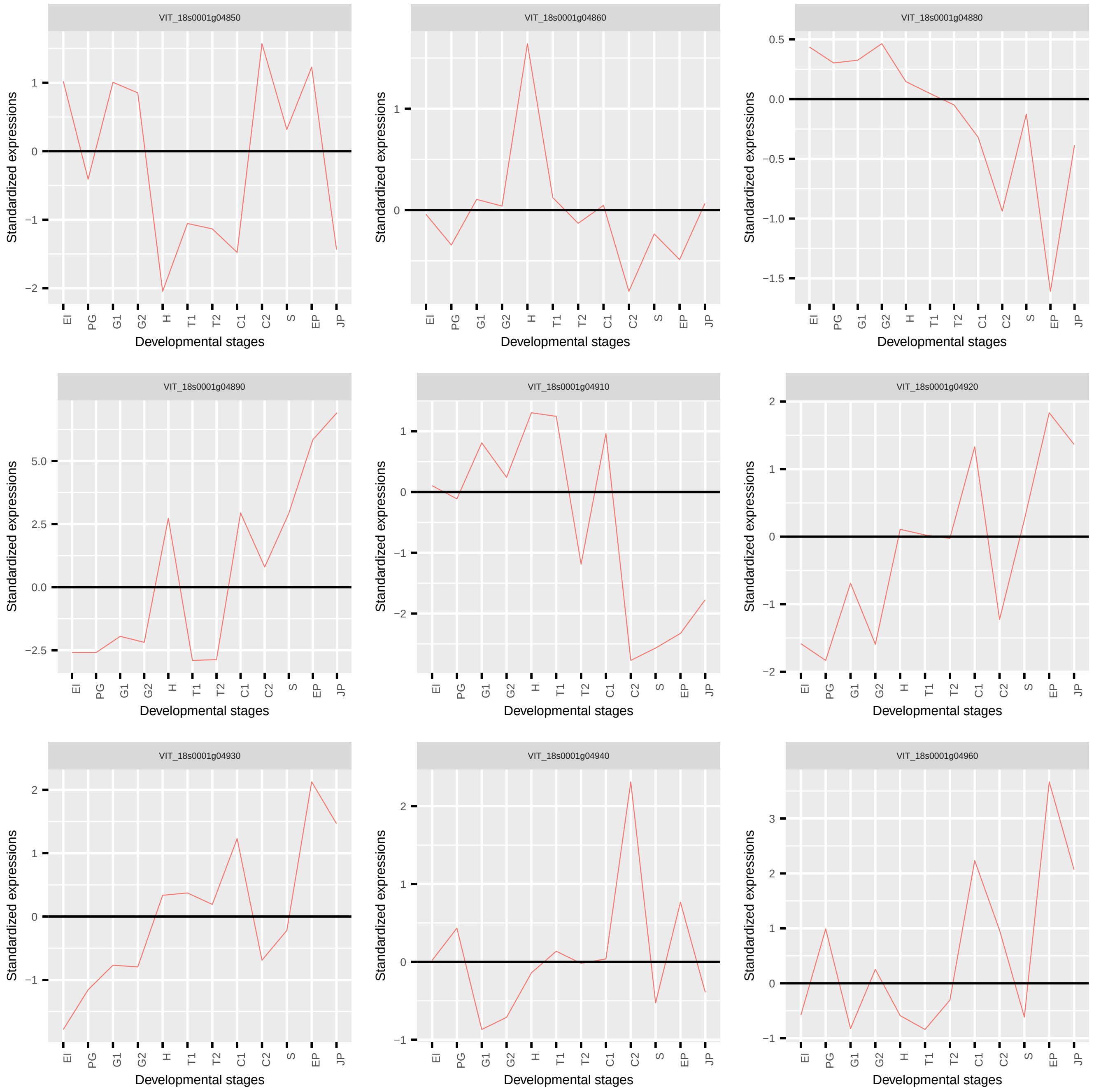
VIT_18s0001g04850
VIT_18s0001g04860
VIT_18s0001g04880
VIT_18s0001g04890
VIT_18s0001g04910
VIT_18s0001g04920
VIT_18s0001g04930
VIT_18s0001g04940
VIT_18s0001g04960
Standardized expressions
Developmental stages
EI PG G1 G2 H T1 T2 C1 C2 S EP JP

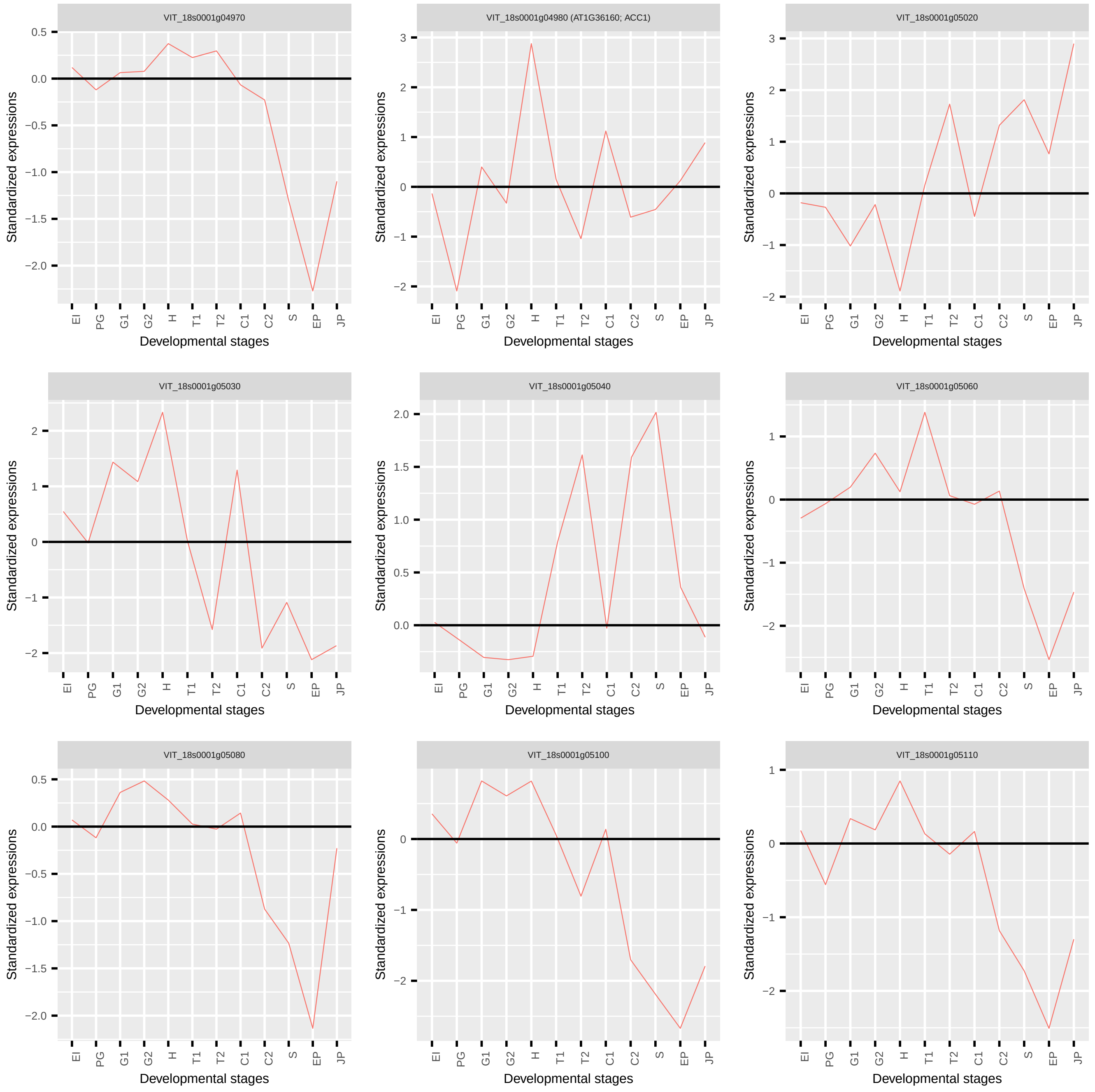
VIT_18s0001g04970
VIT_18s0001g04980 (AT1G36160; ACC1)
VIT_18s0001g05020
VIT_18s0001g05030
VIT_18s0001g05040
VIT_18s0001g05060
VIT_18s0001g05080
VIT_18s0001g05100
VIT_18s0001g05110
Standardized expressions
Developmental stages
EI PG G1 G2 H T1 T2 C1 C2 S EP JP

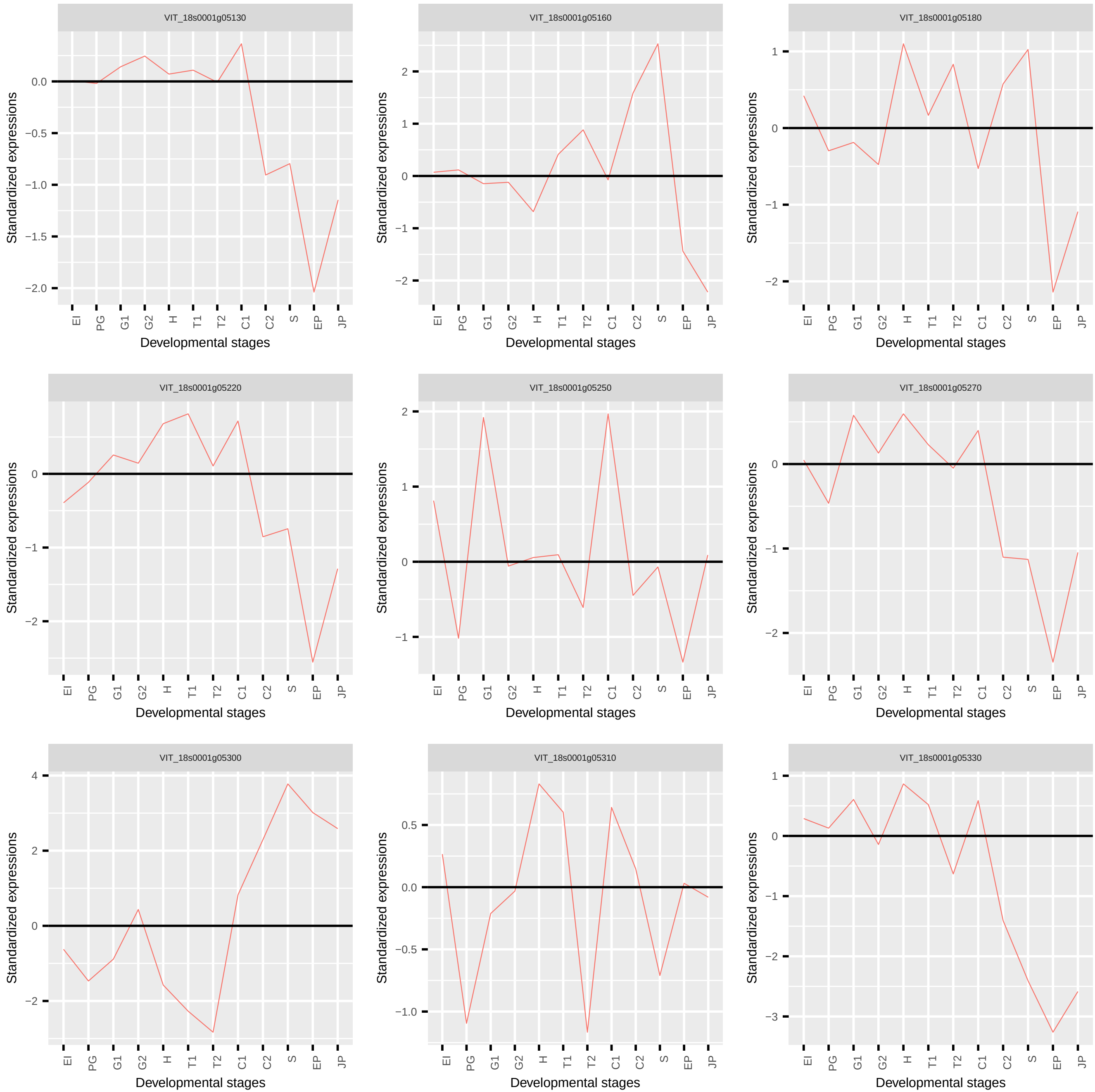
VIT_18s0001g05130
VIT_18s0001g05160
VIT_18s0001g05180
VIT_18s0001g05220
VIT_18s0001g05250
VIT_18s0001g05270
VIT_18s0001g05300
VIT_18s0001g05310
VIT_18s0001g05330
Standardized expressions
Developmental stages
EI PG G1 G2 H T1 T2 C1 C2 S EP JP

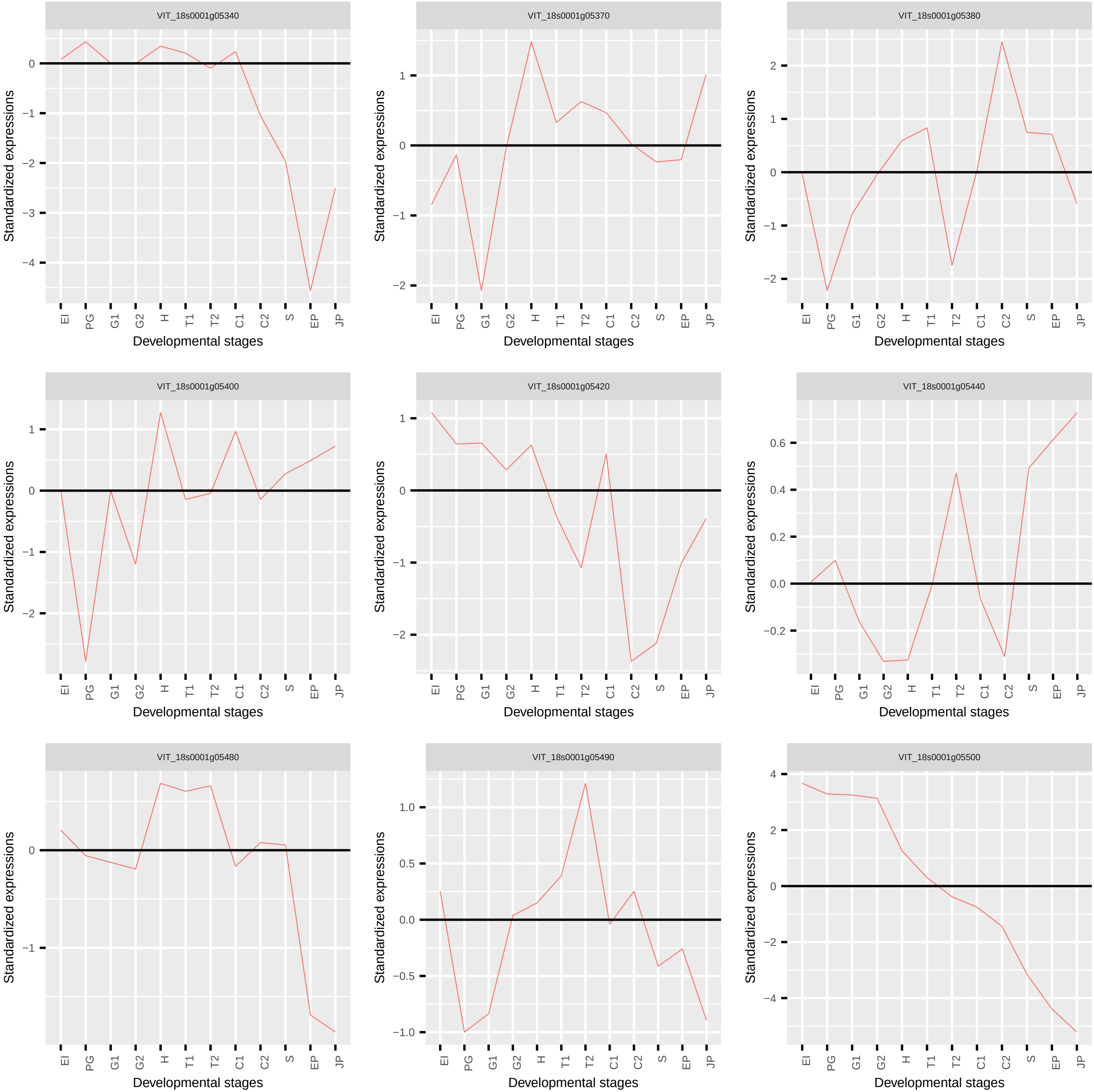
VIT_18s0001g05340
VIT_18s0001g05370
VIT_18s0001g05380
VIT_18s0001g05400
VIT_18s0001g05420
VIT_18s0001g05440
VIT_18s0001g05480
VIT_18s0001g05490
VIT_18s0001g05500
Standardized expressions
Developmental stages
EI
PG
G1
G2
H
T1
T2
C1
C2
S
EP
JP

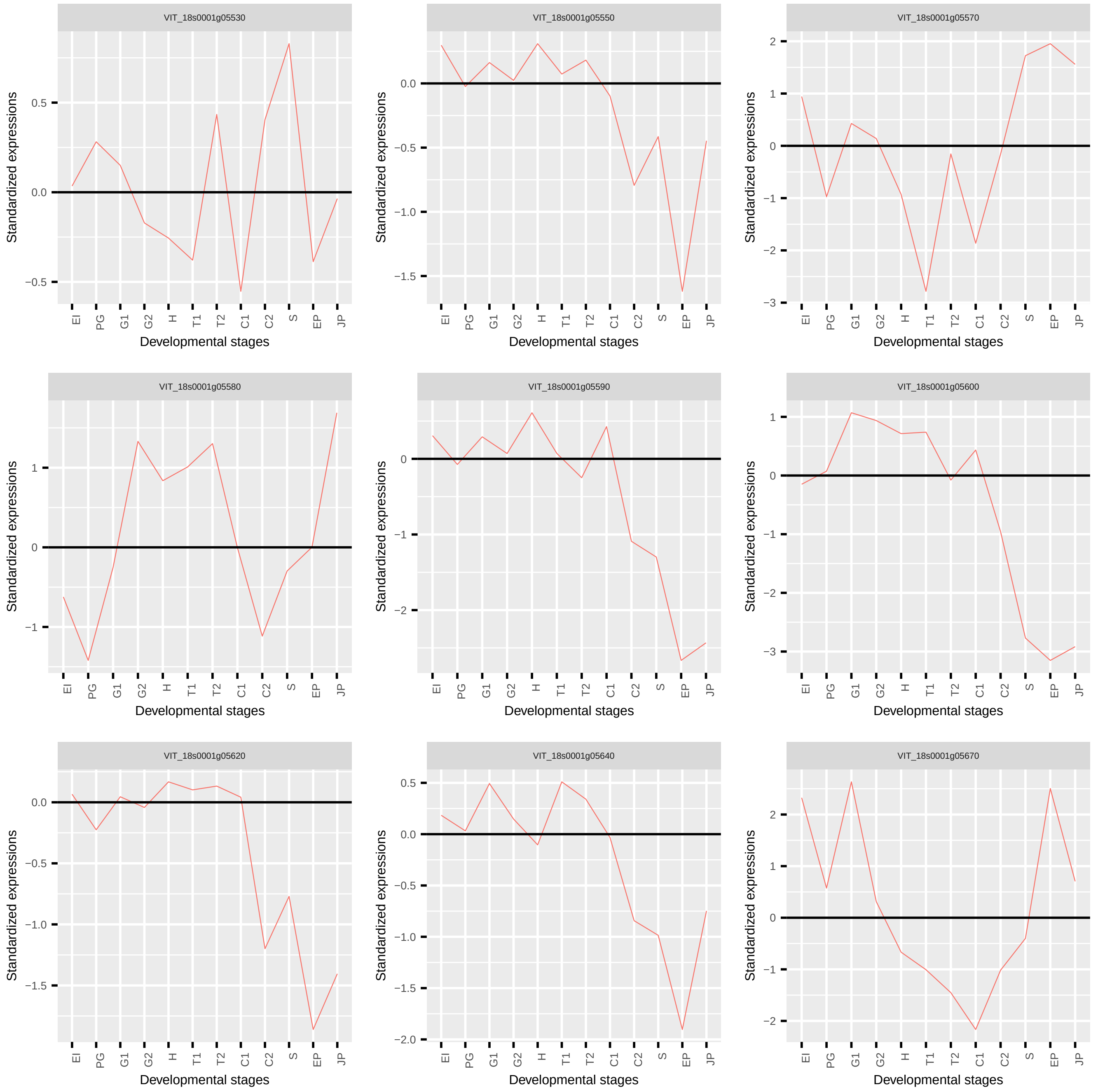

VIT_18s0001g05530
VIT_18s0001g05550
VIT_18s0001g05570
VIT_18s0001g05580
VIT_18s0001g05590
VIT_18s0001g05600
VIT_18s0001g05620
VIT_18s0001g05640
VIT_18s0001g05670
Standardized expressions
Developmental stages
EI
PG
G1
G2
H
T1
T2
C1
C2
S
EP
JP

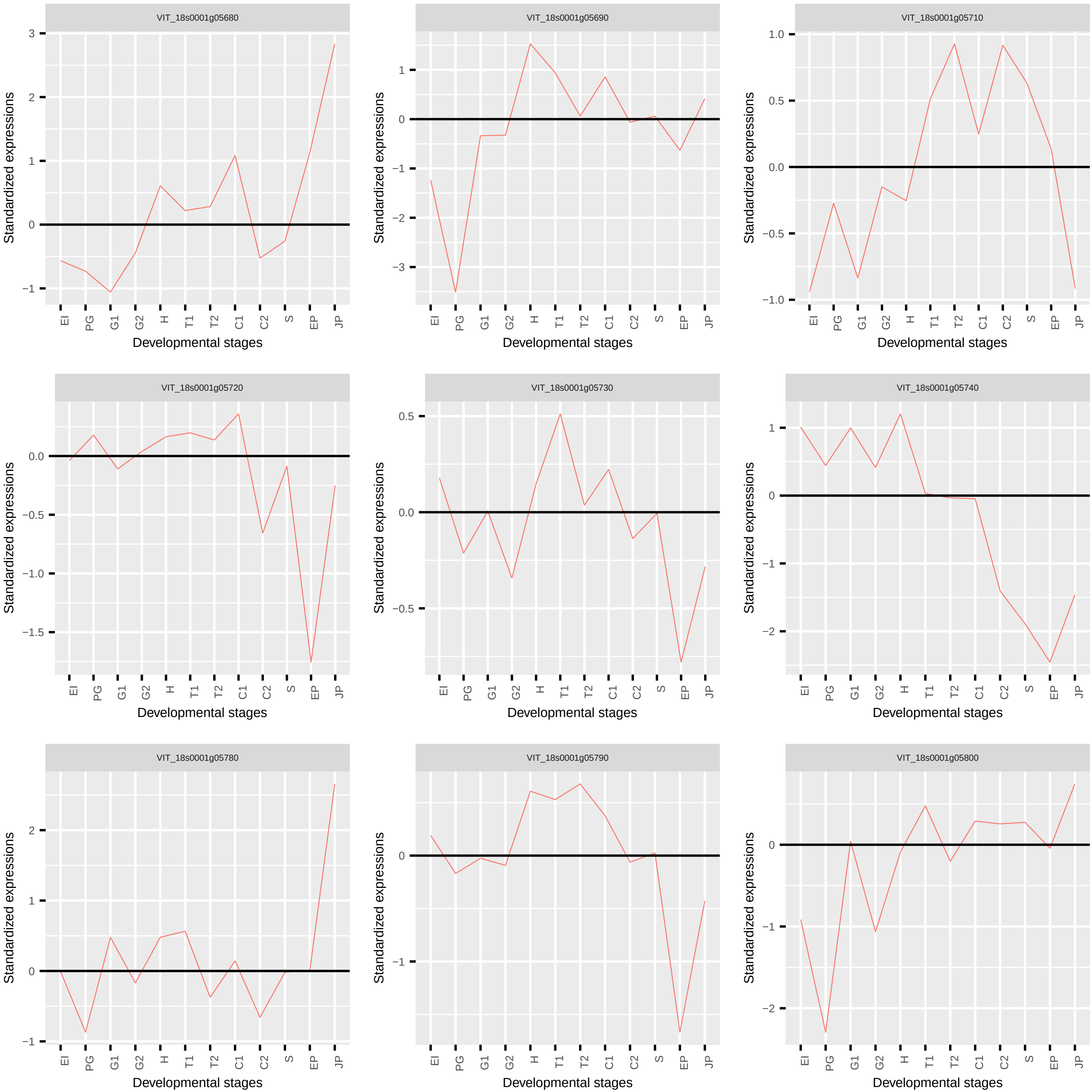
VIT_18s0001g05680
VIT_18s0001g05690
VIT_18s0001g05710
VIT_18s0001g05720
VIT_18s0001g05730
VIT_18s0001g05740
VIT_18s0001g05780
VIT_18s0001g05790
VIT_18s0001g05800
Standardized expressions
Developmental stages
EI
PG
G1
G2
H
T1
T2
C1
C2
S
EP
JP

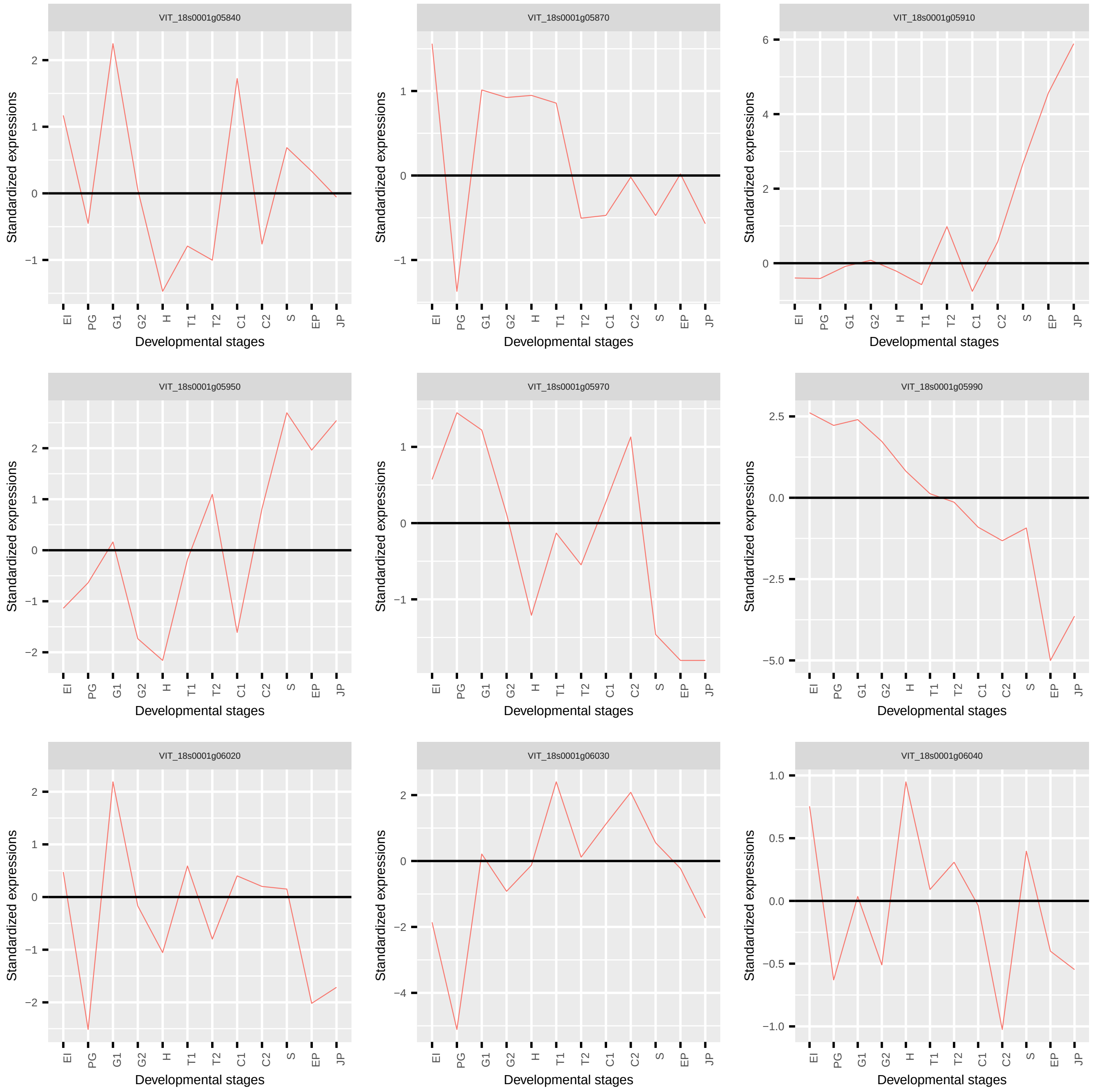
VIT_18s0001g05840
−1 0 1 2
Standardized expressions
EI PG G1 G2 H T1 T2 C1 C2 S EP JP
Developmental stages
VIT_18s0001g05870
−1 0 1
Standardized expressions
EI PG G1 G2 H T1 T2 C1 C2 S EP JP
Developmental stages
VIT_18s0001g05910
0 2 4 6
Standardized expressions
EI PG G1 G2 H T1 T2 C1 C2 S EP JP
Developmental stages
VIT_18s0001g05950
−2 −1 0 1 2
Standardized expressions
EI PG G1 G2 H T1 T2 C1 C2 S EP JP
Developmental stages
VIT_18s0001g05970
−1 0 1
Standardized expressions
EI PG G1 G2 H T1 T2 C1 C2 S EP JP
Developmental stages
VIT_18s0001g05990
−5.0 −2.5 0.0 2.5
Standardized expressions
EI PG G1 G2 H T1 T2 C1 C2 S EP JP
Developmental stages
VIT_18s0001g06020
−2 −1 0 1 2
Standardized expressions
EI PG G1 G2 H T1 T2 C1 C2 S EP JP
Developmental stages
VIT_18s0001g06030
−4 −2 0 2
Standardized expressions
EI PG G1 G2 H T1 T2 C1 C2 S EP JP
Developmental stages
VIT_18s0001g06040
−1.0 −0.5 0.0 0.5 1.0
Standardized expressions
EI PG G1 G2 H T1 T2 C1 C2 S EP JP
Developmental stages

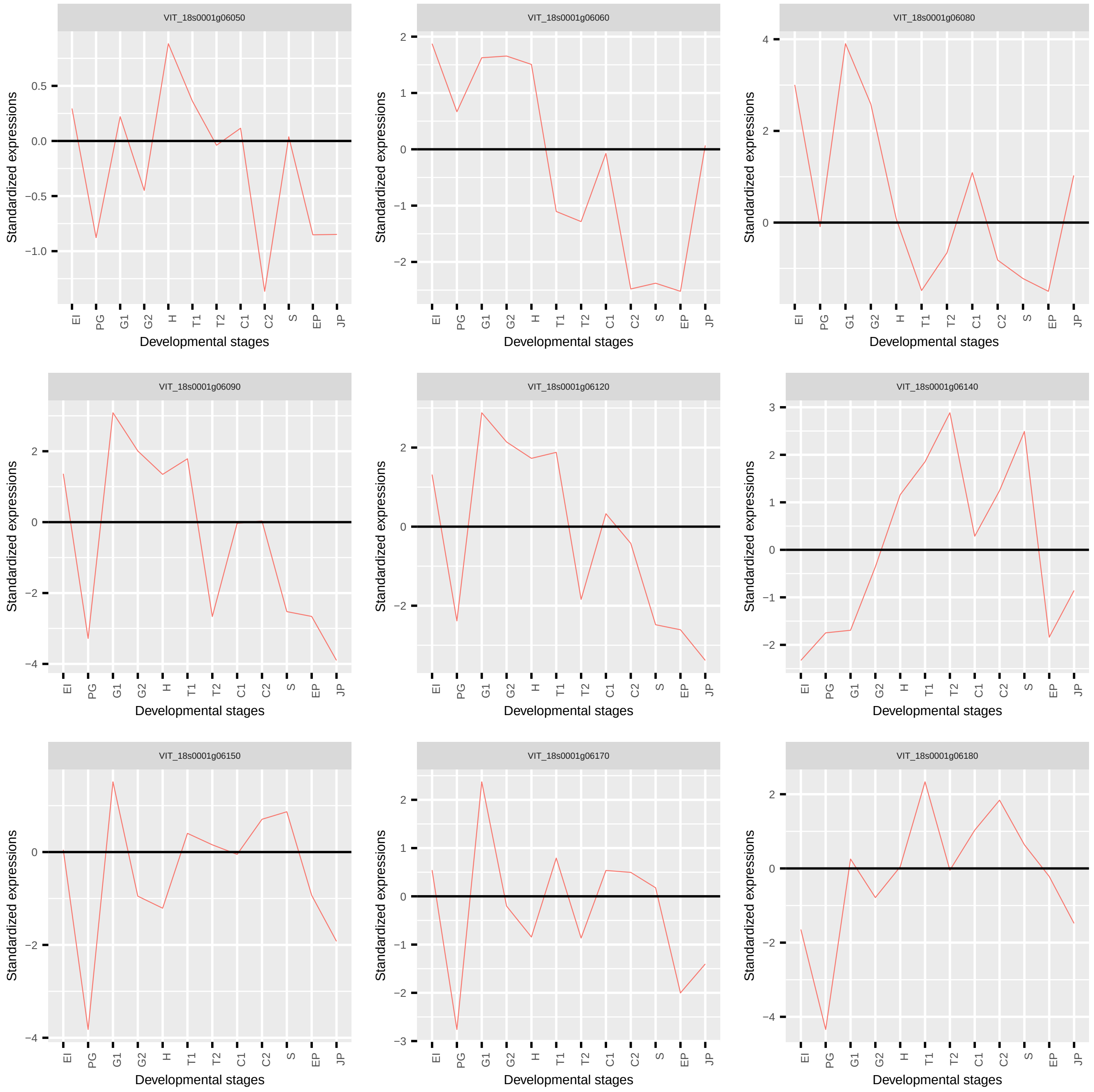
VIT_18s0001g06050
VIT_18s0001g06060
VIT_18s0001g06080
VIT_18s0001g06090
VIT_18s0001g06120
VIT_18s0001g06140
VIT_18s0001g06150
VIT_18s0001g06170
VIT_18s0001g06180
Standardized expressions
Developmental stages
EI PG G1 G2 H T1 T2 C1 C2 S EP JP

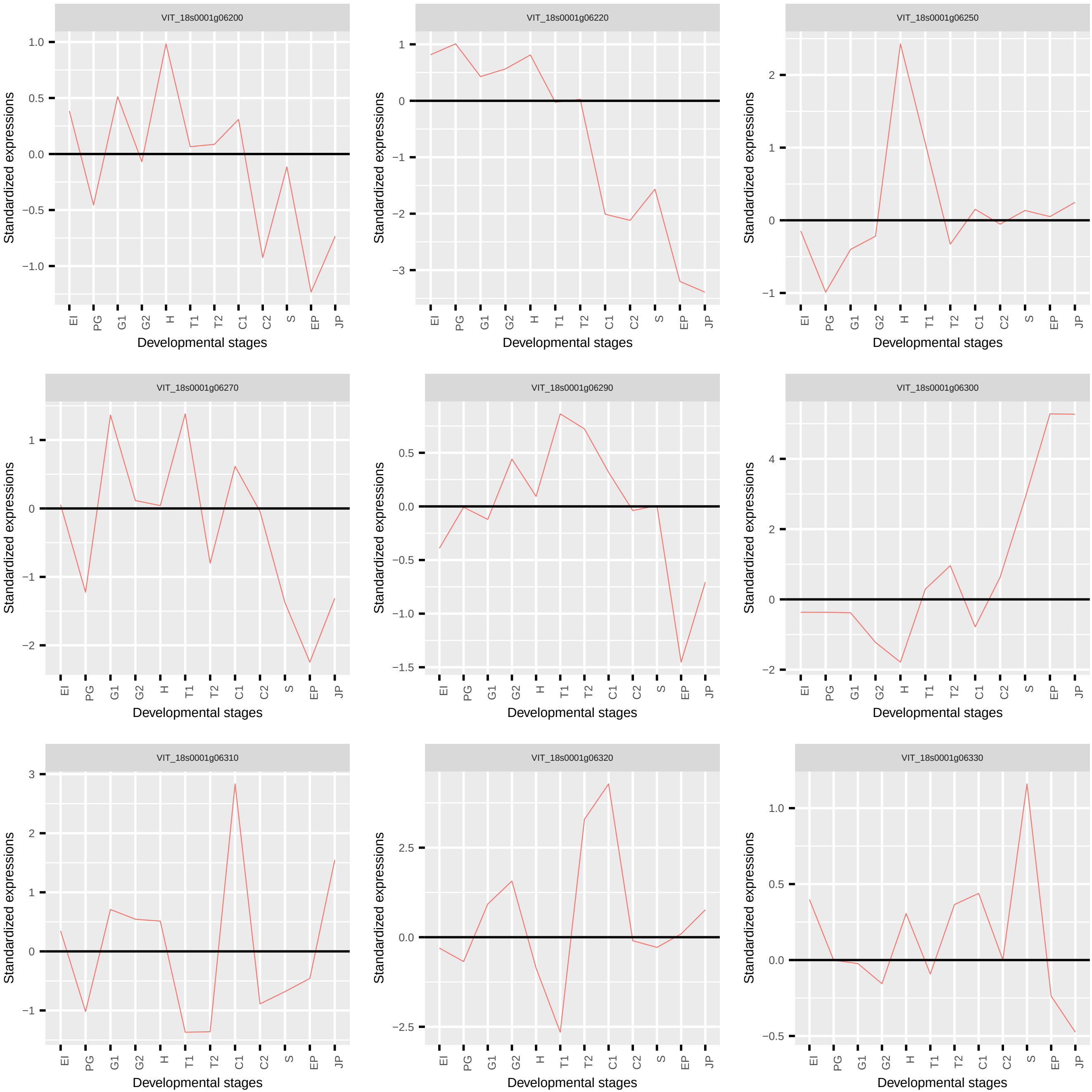
VIT_18s0001g06200
VIT_18s0001g06220
VIT_18s0001g06250
VIT_18s0001g06270
VIT_18s0001g06290
VIT_18s0001g06300
VIT_18s0001g06310
VIT_18s0001g06320
VIT_18s0001g06330
Standardized expressions
Developmental stages
EI PG G1 G2 H T1 T2 C1 C2 S EP JP

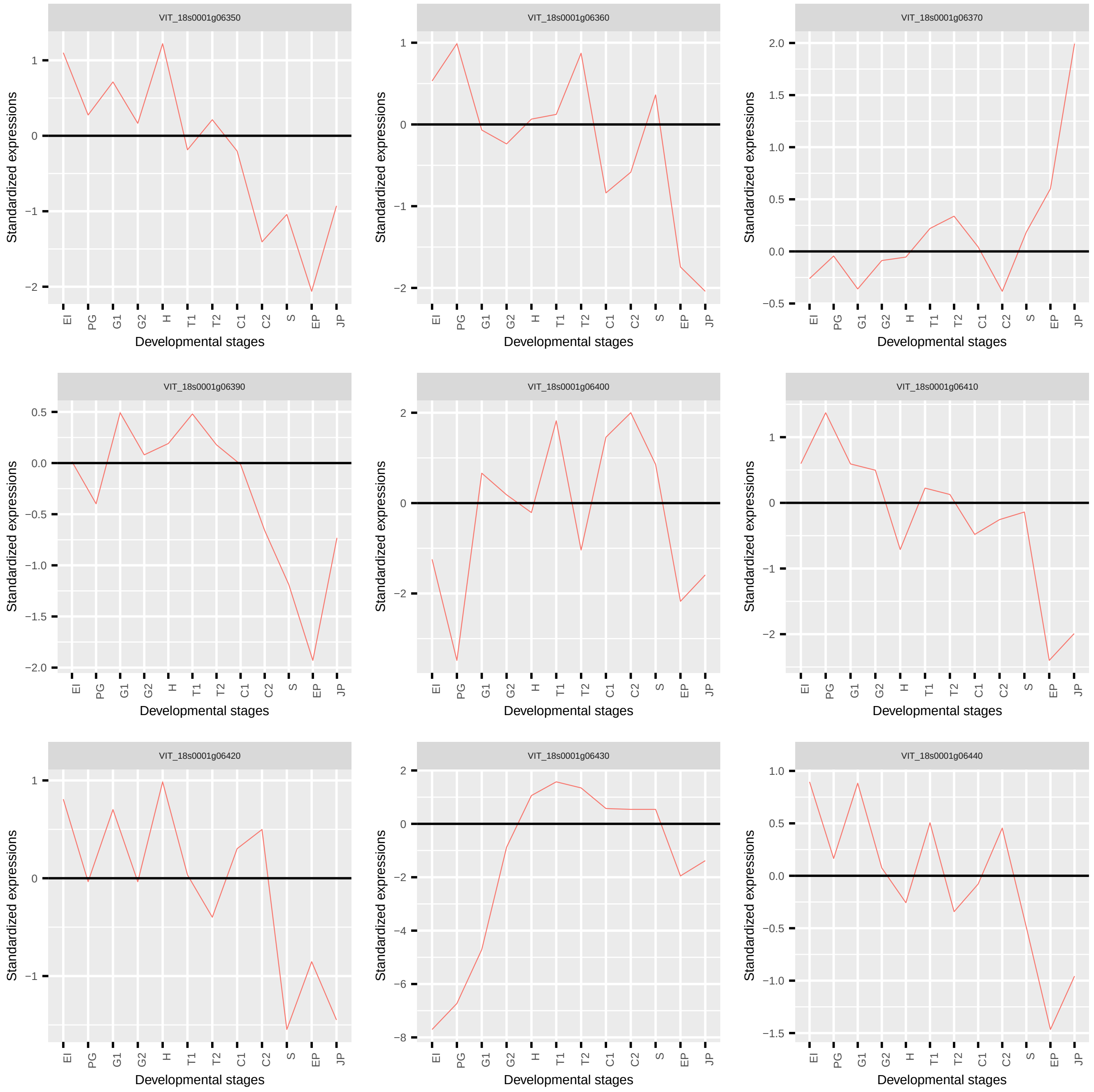
VIT_18s0001g06350
VIT_18s0001g06360
VIT_18s0001g06370
VIT_18s0001g06390
VIT_18s0001g06400
VIT_18s0001g06410
VIT_18s0001g06420
VIT_18s0001g06430
VIT_18s0001g06440
Standardized expressions
Developmental stages
EI PG G1 G2 H T1 T2 C1 C2 S EP JP

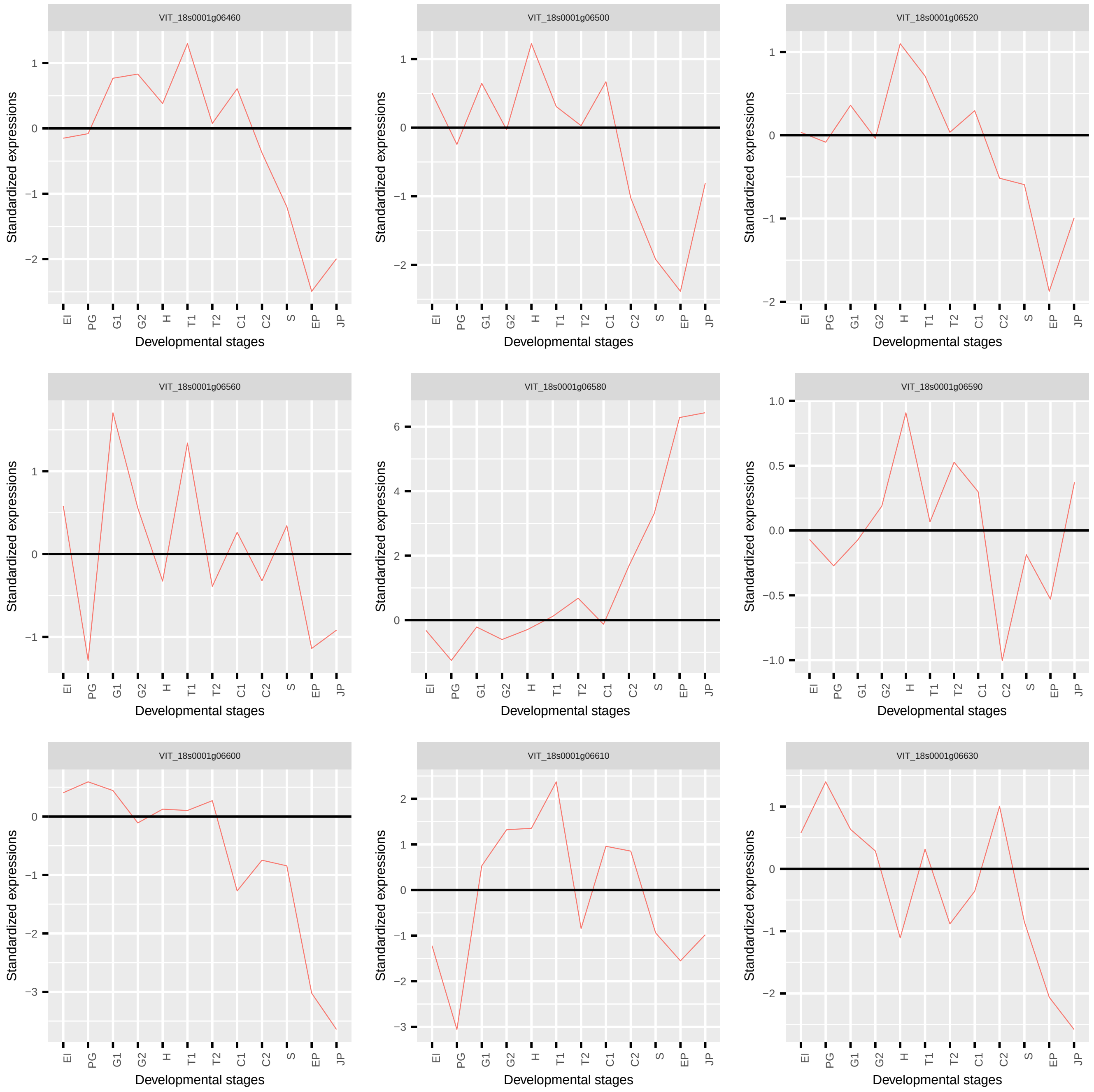

VIT_18s0001g06460
VIT_18s0001g06500
VIT_18s0001g06520
VIT_18s0001g06560
VIT_18s0001g06580
VIT_18s0001g06590
VIT_18s0001g06600
VIT_18s0001g06610
VIT_18s0001g06630
Standardized expressions
Developmental stages
EI
PG
G1
G2
H
T1
T2
C1
C2
S
EP
JP

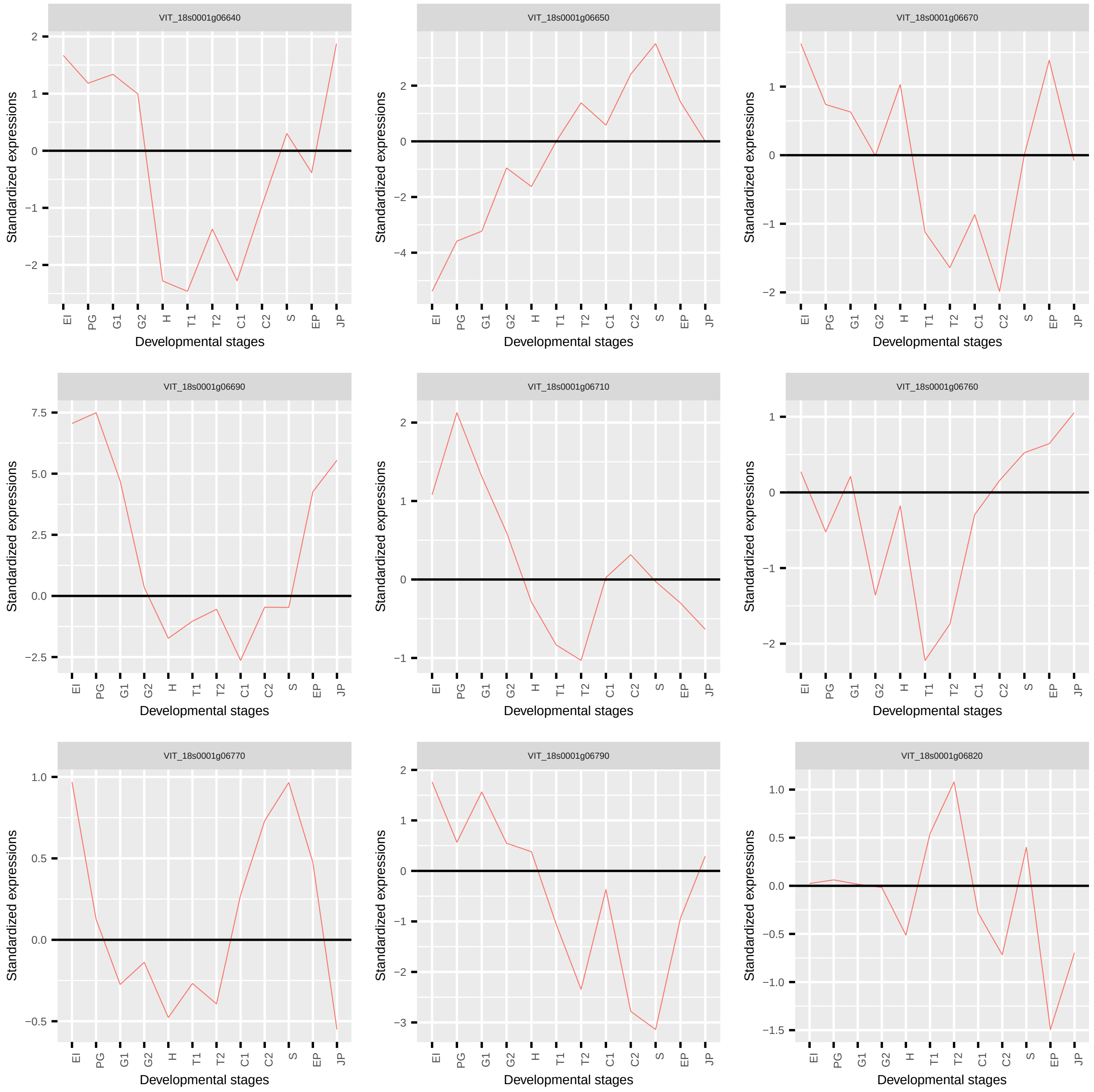

VIT_18s0001g06640
VIT_18s0001g06650
VIT_18s0001g06670
VIT_18s0001g06690
VIT_18s0001g06710
VIT_18s0001g06760
VIT_18s0001g06770
VIT_18s0001g06790
VIT_18s0001g06820
Standardized expressions
Developmental stages
EI PG G1 G2 H T1 T2 C1 C2 S EP JP

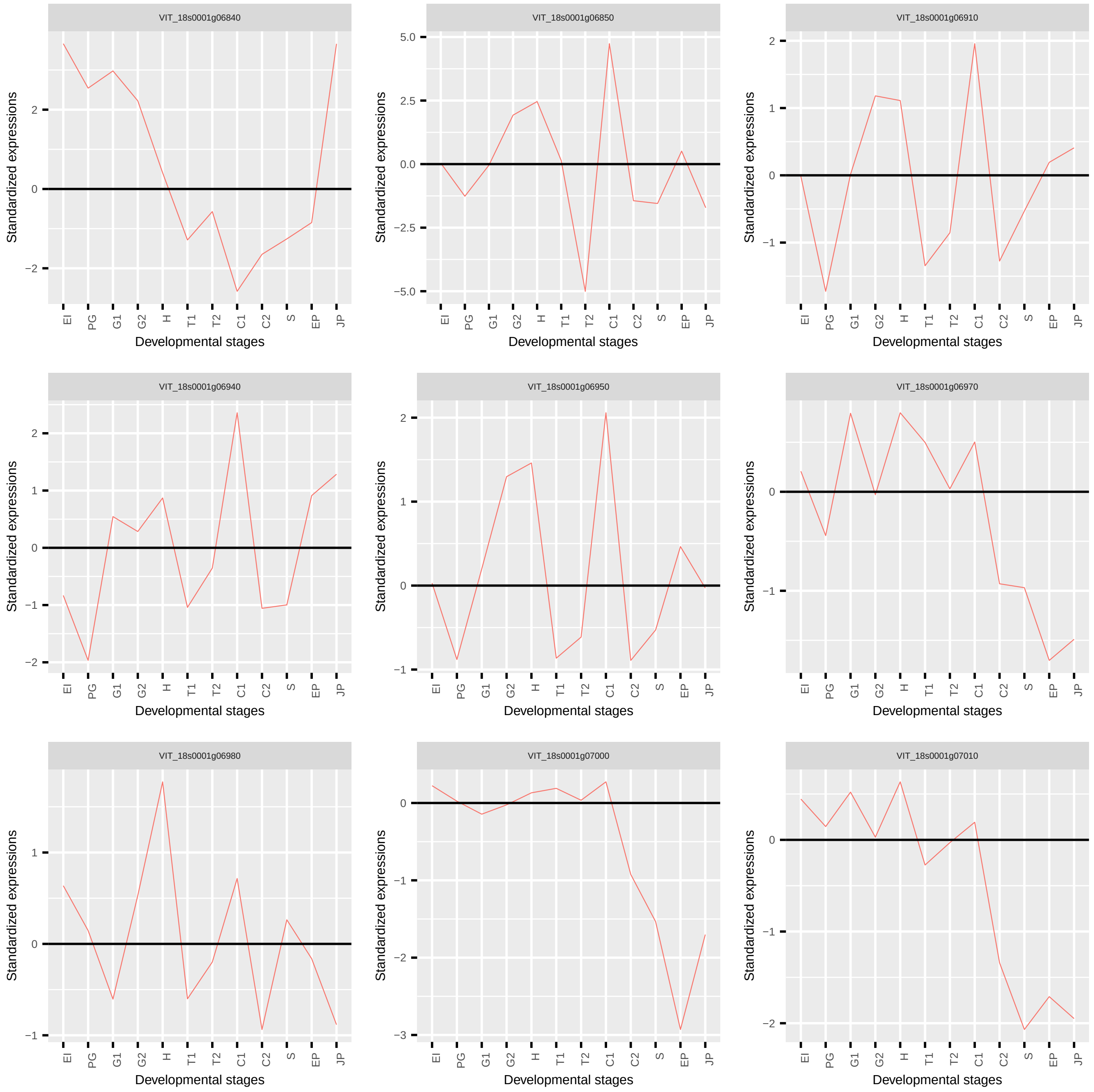
VIT_18s0001g06840
VIT_18s0001g06850
VIT_18s0001g06910
VIT_18s0001g06940
VIT_18s0001g06950
VIT_18s0001g06970
VIT_18s0001g06980
VIT_18s0001g07000
VIT_18s0001g07010
Standardized expressions
Developmental stages
EI
PG
G1
G2
H
T1
T2
C1
C2
S
EP
JP
5.0
2.5
0.0
−2.5
−5.0
2
1
0
−1
−2
−3

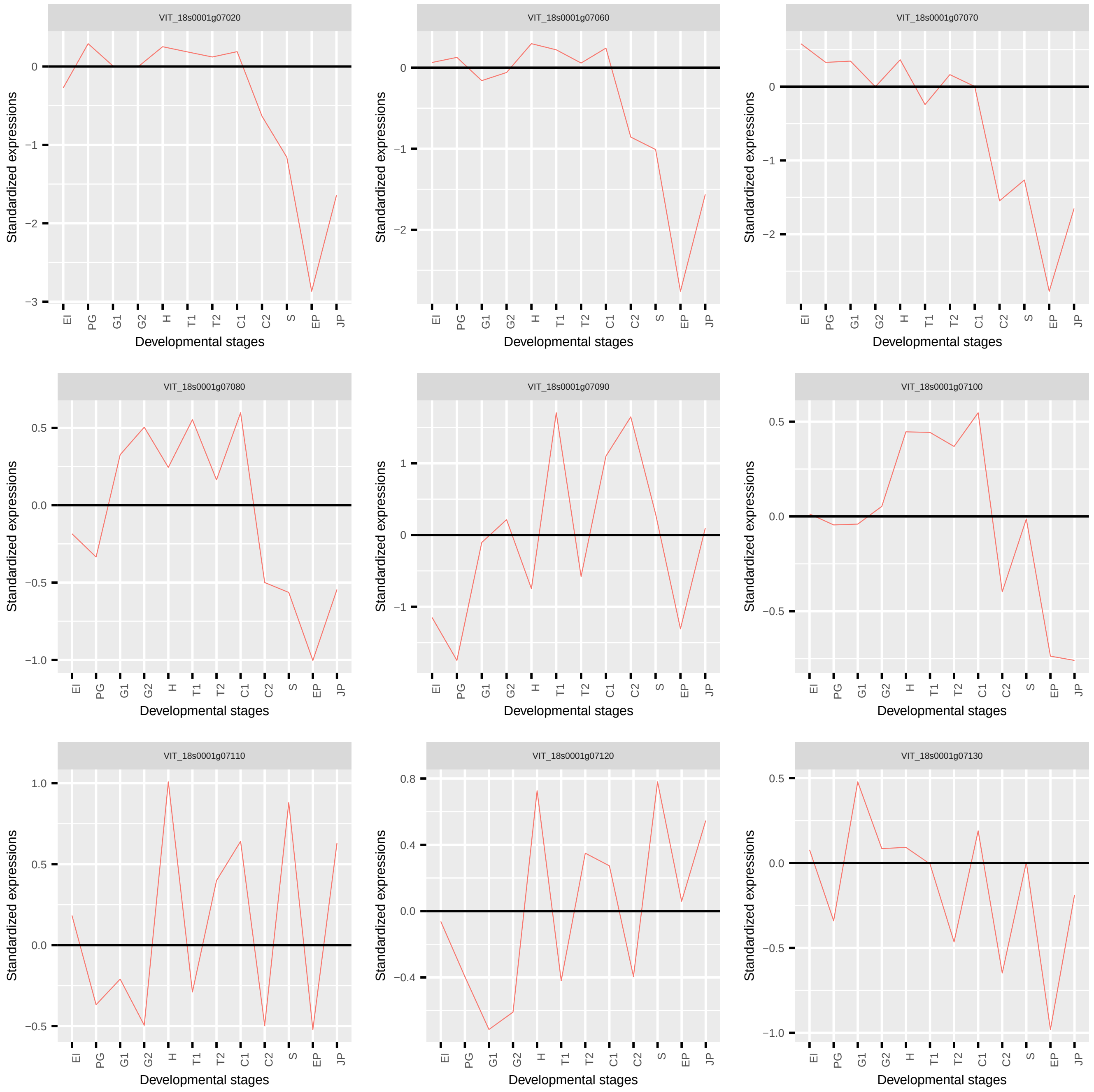

VIT_18s0001g07020
VIT_18s0001g07060
VIT_18s0001g07070
VIT_18s0001g07080
VIT_18s0001g07090
VIT_18s0001g07100
VIT_18s0001g07110
VIT_18s0001g07120
VIT_18s0001g07130
Standardized expressions
Developmental stages
EI PG G1 G2 H T1 T2 C1 C2 S EP JP

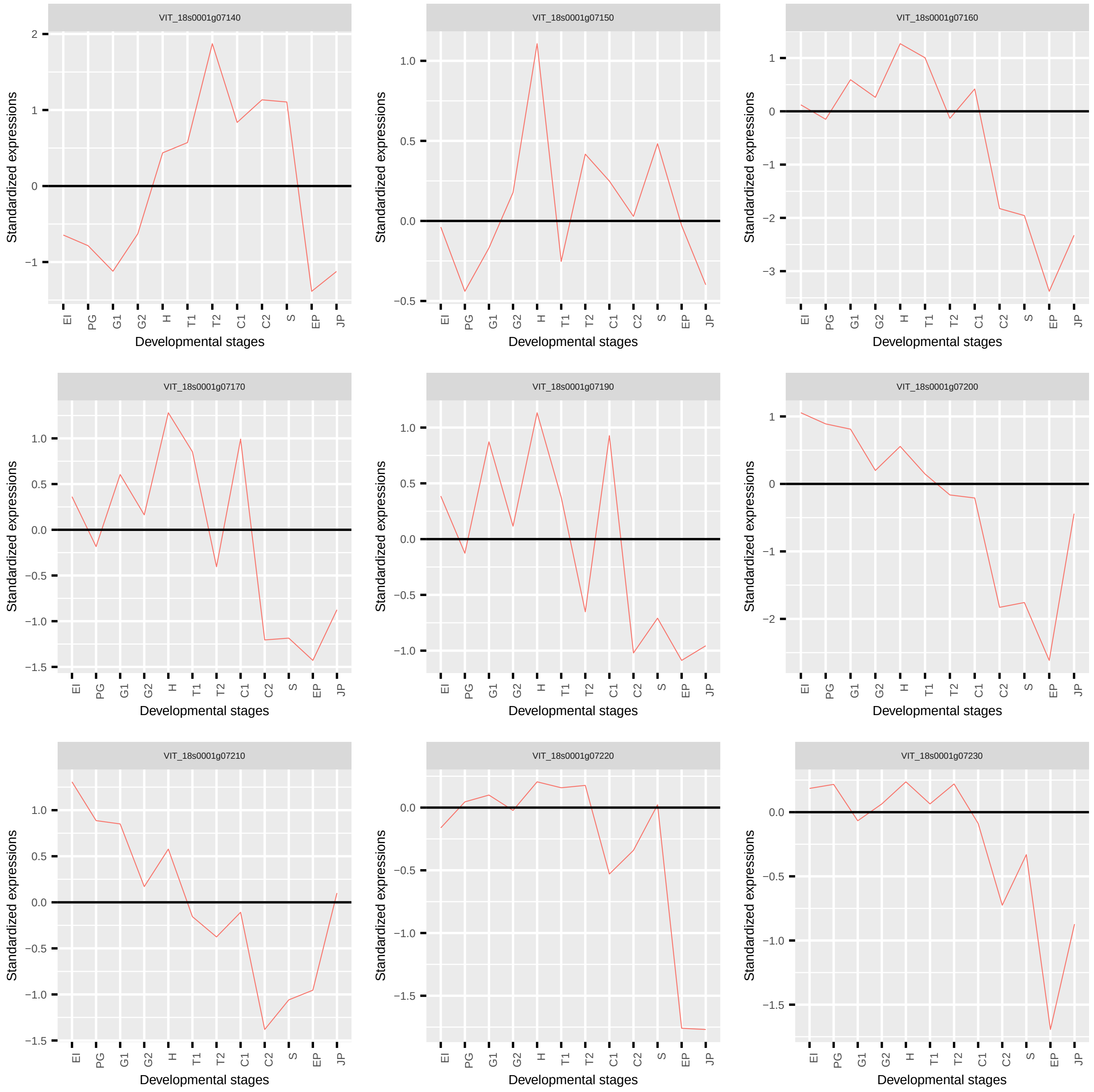

VIT_18s0001g07140
VIT_18s0001g07150
VIT_18s0001g07160
VIT_18s0001g07170
VIT_18s0001g07190
VIT_18s0001g07200
VIT_18s0001g07210
VIT_18s0001g07220
VIT_18s0001g07230
Standardized expressions
Developmental stages
EI PG G1 G2 H T1 T2 C1 C2 S EP JP

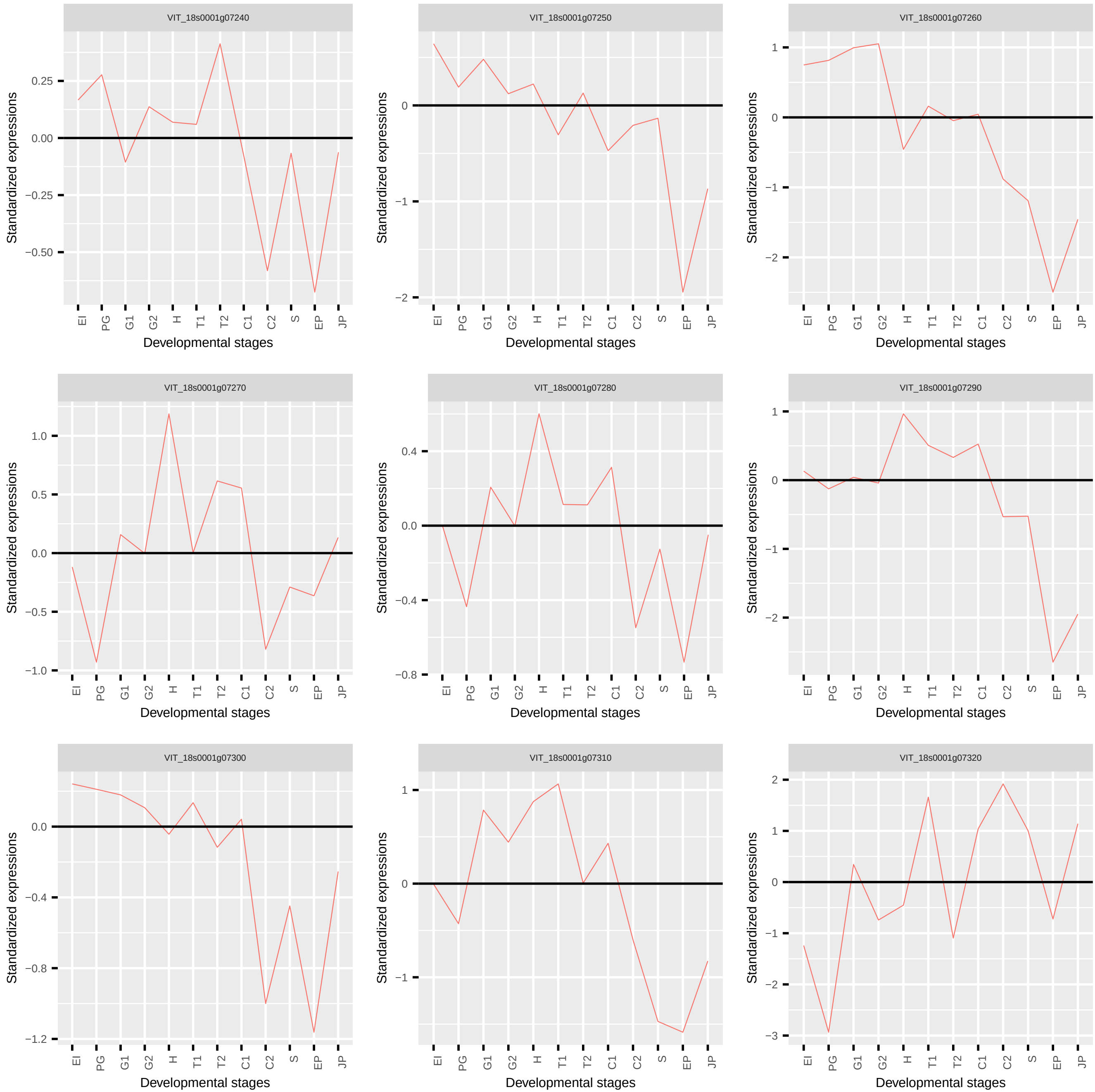
VIT_18s0001g07240
VIT_18s0001g07250
VIT_18s0001g07260
VIT_18s0001g07270
VIT_18s0001g07280
VIT_18s0001g07290
VIT_18s0001g07300
VIT_18s0001g07310
VIT_18s0001g07320
Standardized expressions
Developmental stages
EI PG G1 G2 H T1 T2 C1 C2 S EP JP

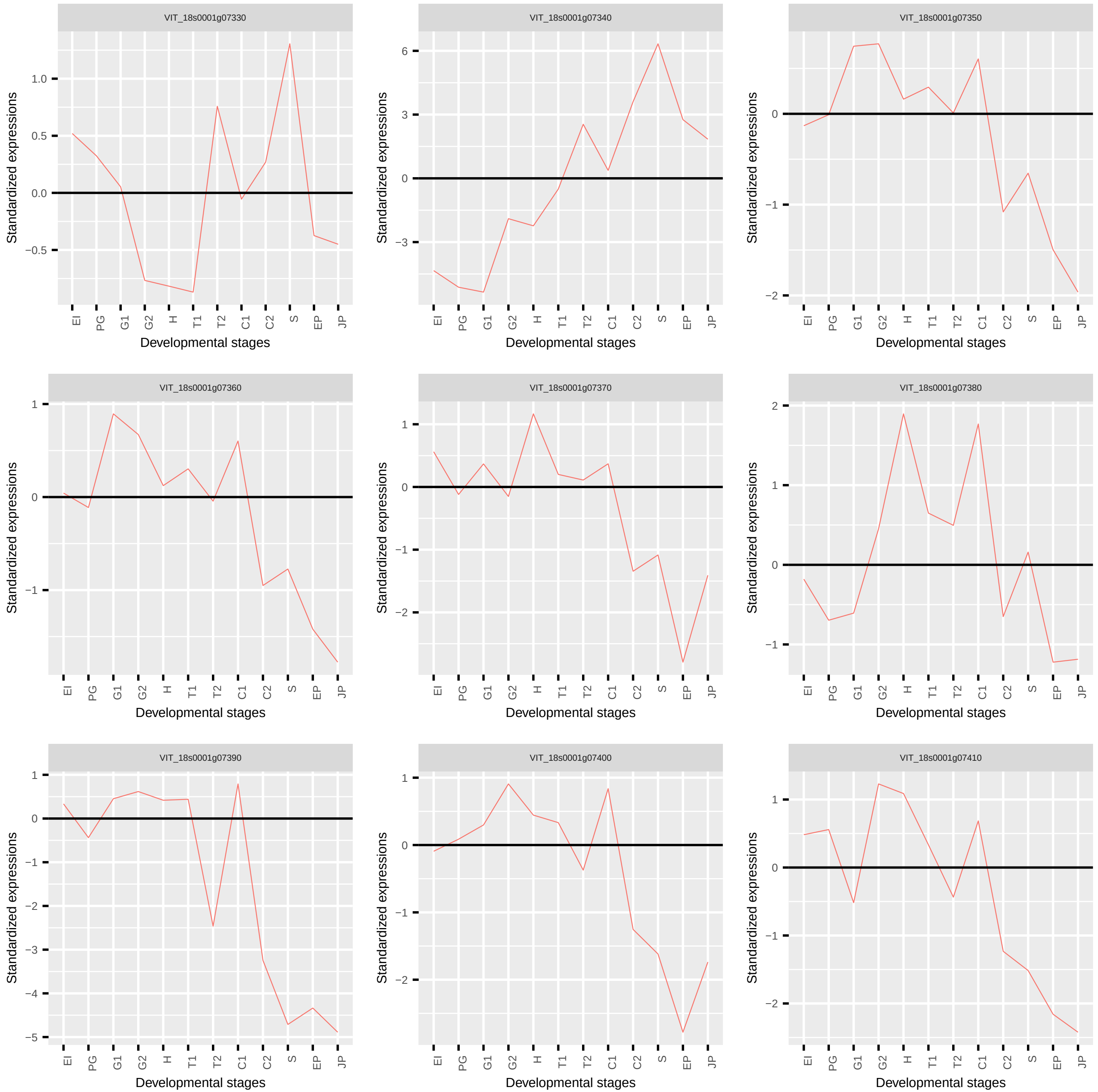
VIT_18s0001g07330
VIT_18s0001g07340
VIT_18s0001g07350
VIT_18s0001g07360
VIT_18s0001g07370
VIT_18s0001g07380
VIT_18s0001g07390
VIT_18s0001g07400
VIT_18s0001g07410
Standardized expressions
Developmental stages
EI PG G1 G2 H T1 T2 C1 C2 S EP JP

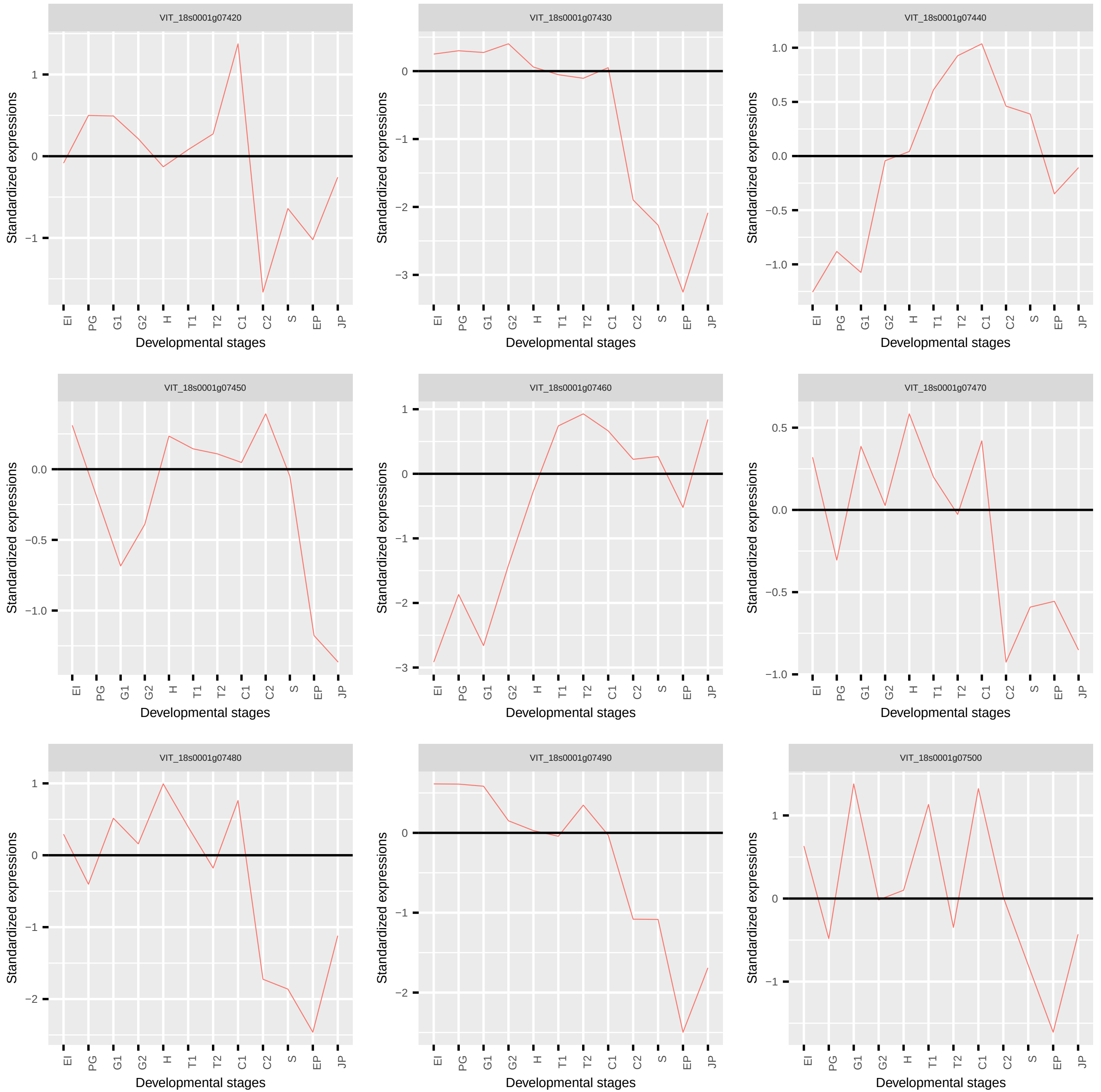

VIT_18s0001g07420
VIT_18s0001g07430
VIT_18s0001g07440
VIT_18s0001g07450
VIT_18s0001g07460
VIT_18s0001g07470
VIT_18s0001g07480
VIT_18s0001g07490
VIT_18s0001g07500
Standardized expressions
Developmental stages
EI PG G1 G2 H T1 T2 C1 C2 S EP JP

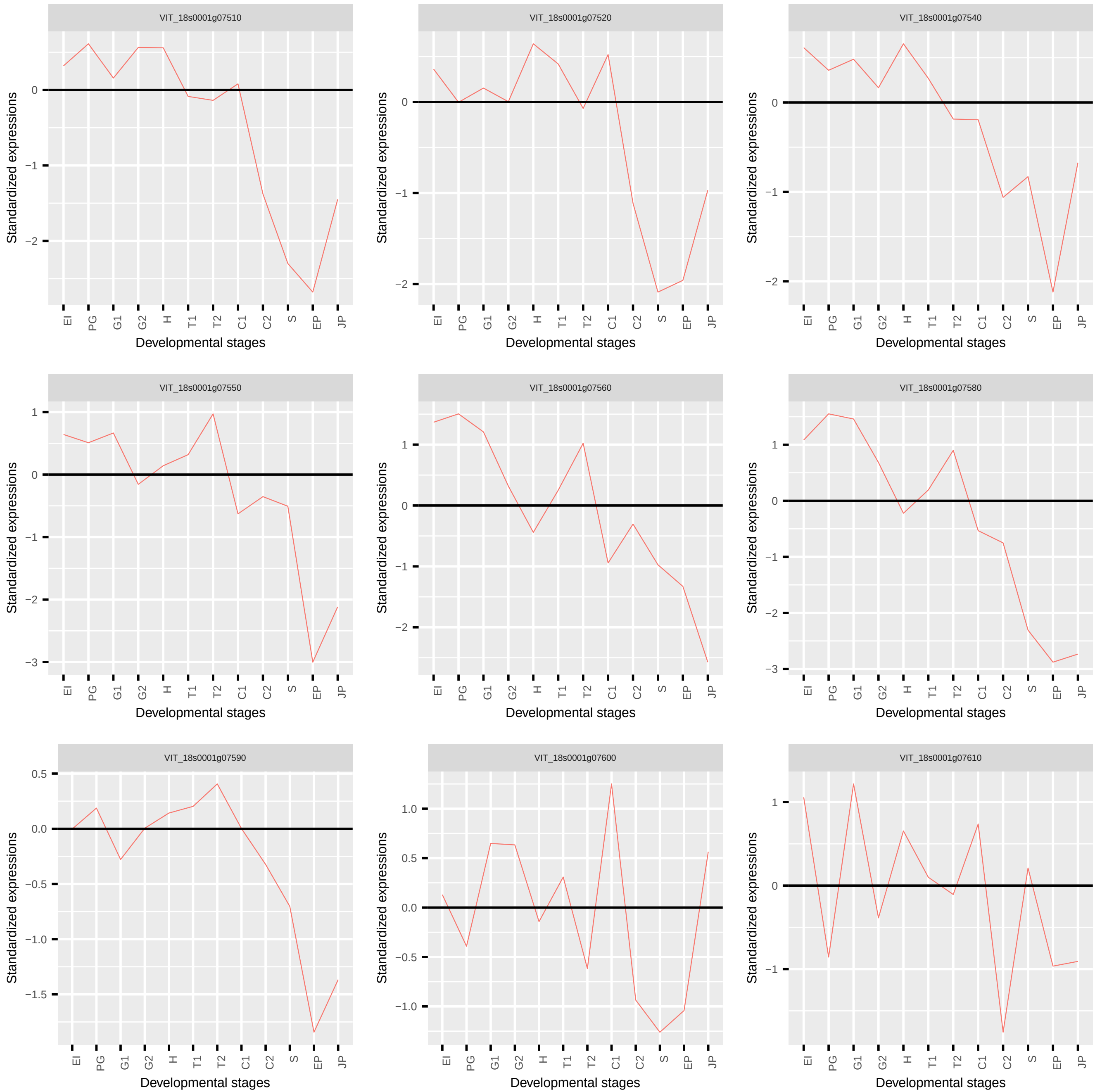

VIT_18s0001g07510
VIT_18s0001g07520
VIT_18s0001g07540
VIT_18s0001g07550
VIT_18s0001g07560
VIT_18s0001g07580
VIT_18s0001g07590
VIT_18s0001g07600
VIT_18s0001g07610
Standardized expressions
Developmental stages
EI PG G1 G2 H T1 T2 C1 C2 S EP JP

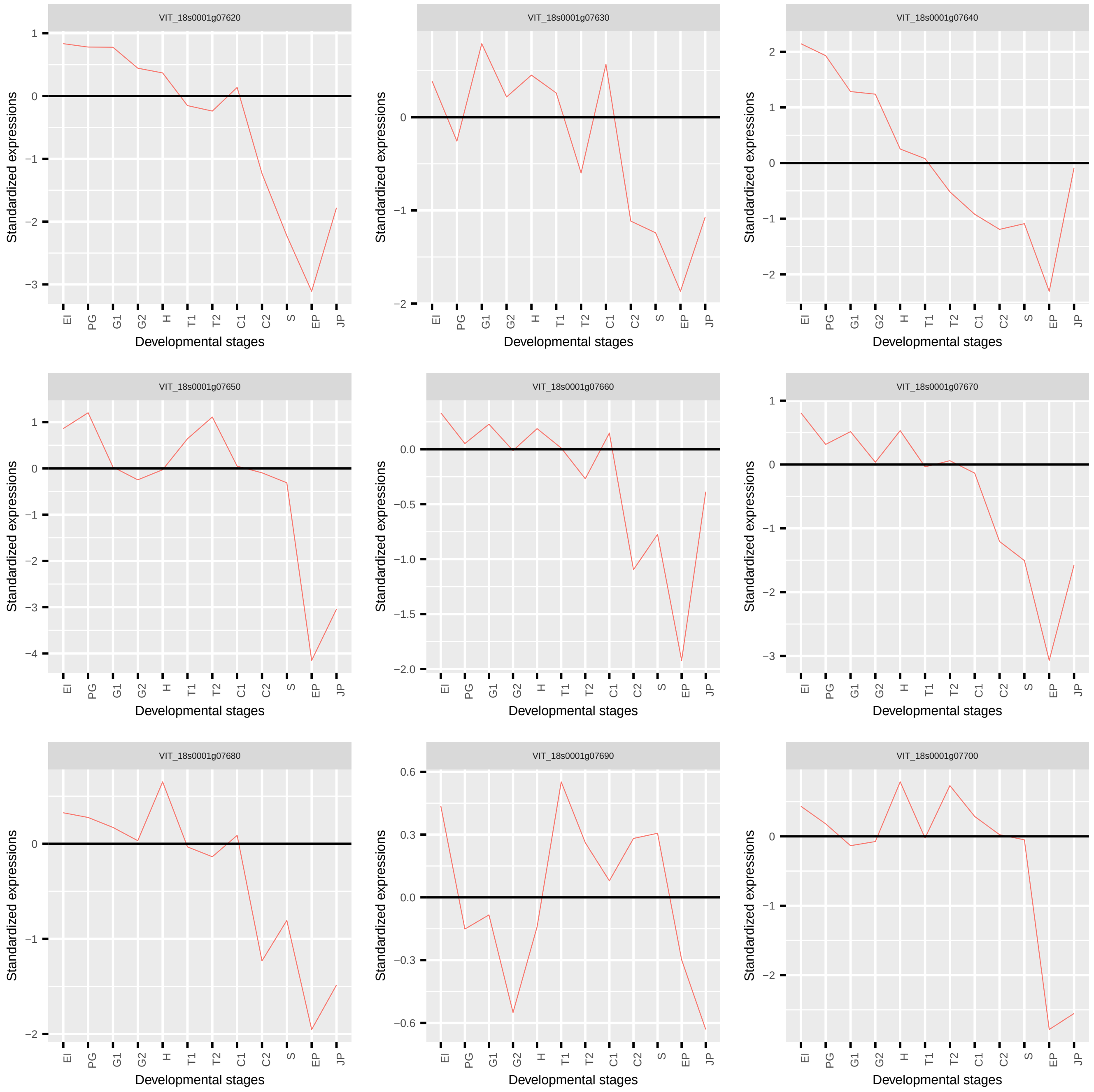
VIT_18s0001g07620
VIT_18s0001g07630
VIT_18s0001g07640
VIT_18s0001g07650
VIT_18s0001g07660
VIT_18s0001g07670
VIT_18s0001g07680
VIT_18s0001g07690
VIT_18s0001g07700
Standardized expressions
Developmental stages
EI PG G1 G2 H T1 T2 C1 C2 S EP JP

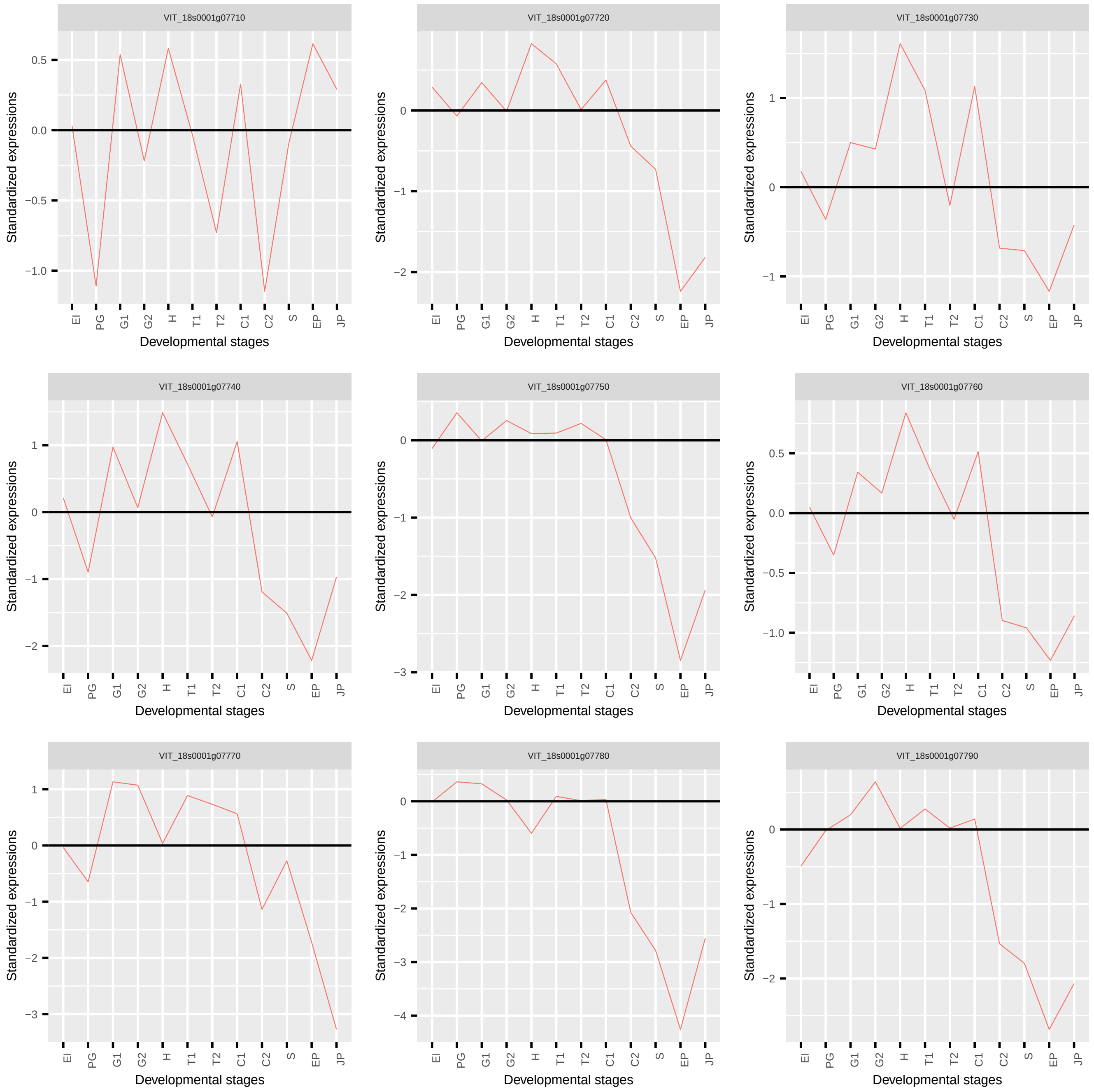

VIT_18s0001g07710
VIT_18s0001g07720
VIT_18s0001g07730
VIT_18s0001g07740
VIT_18s0001g07750
VIT_18s0001g07760
VIT_18s0001g07770
VIT_18s0001g07780
VIT_18s0001g07790
Standardized expressions
Developmental stages
EI
PG
G1
G2
H
T1
T2
C1
C2
S
EP
JP

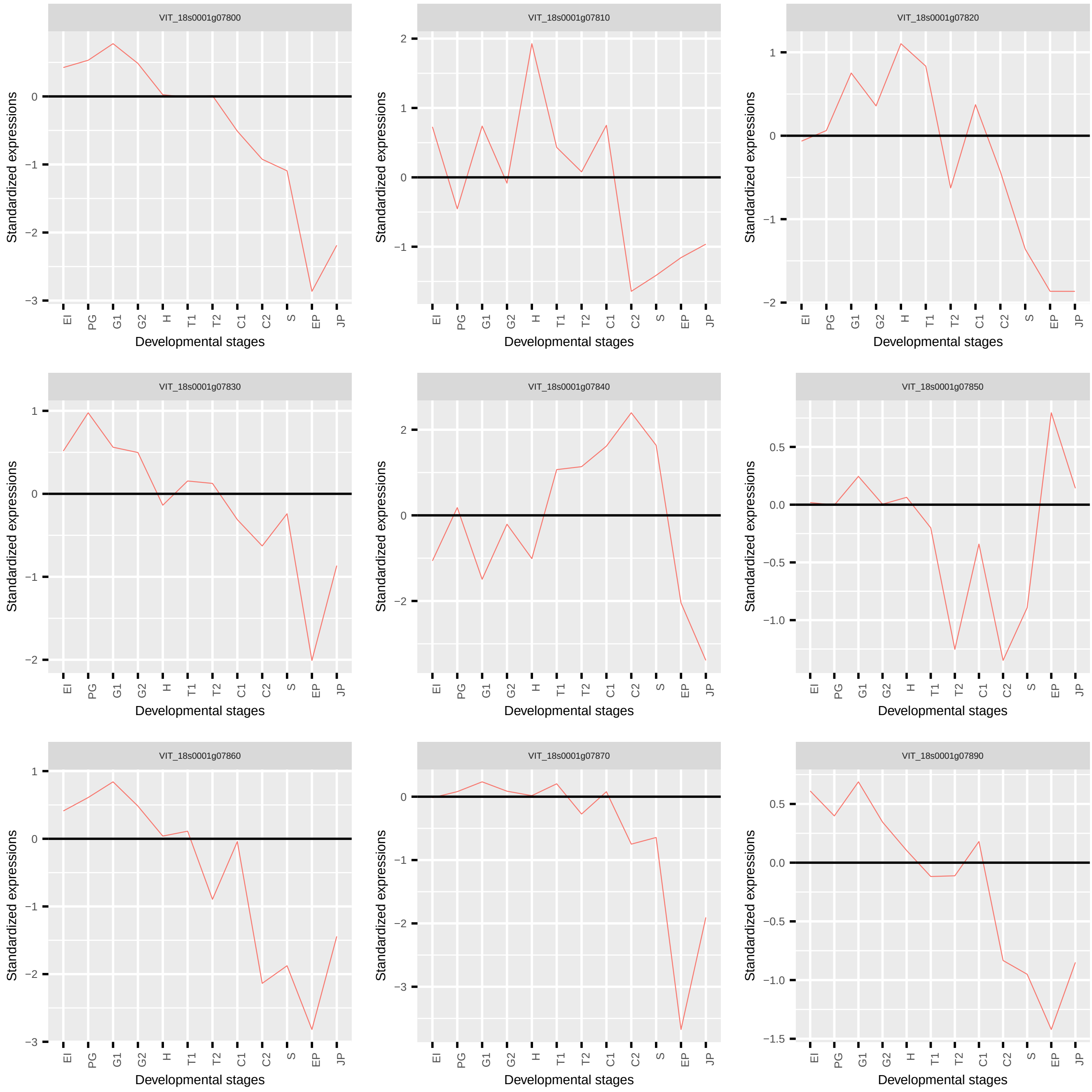
VIT_18s0001g07800
VIT_18s0001g07810
VIT_18s0001g07820
VIT_18s0001g07830
VIT_18s0001g07840
VIT_18s0001g07850
VIT_18s0001g07860
VIT_18s0001g07870
VIT_18s0001g07890
Standardized expressions
Developmental stages
EI PG G1 G2 H T1 T2 C1 C2 S EP JP

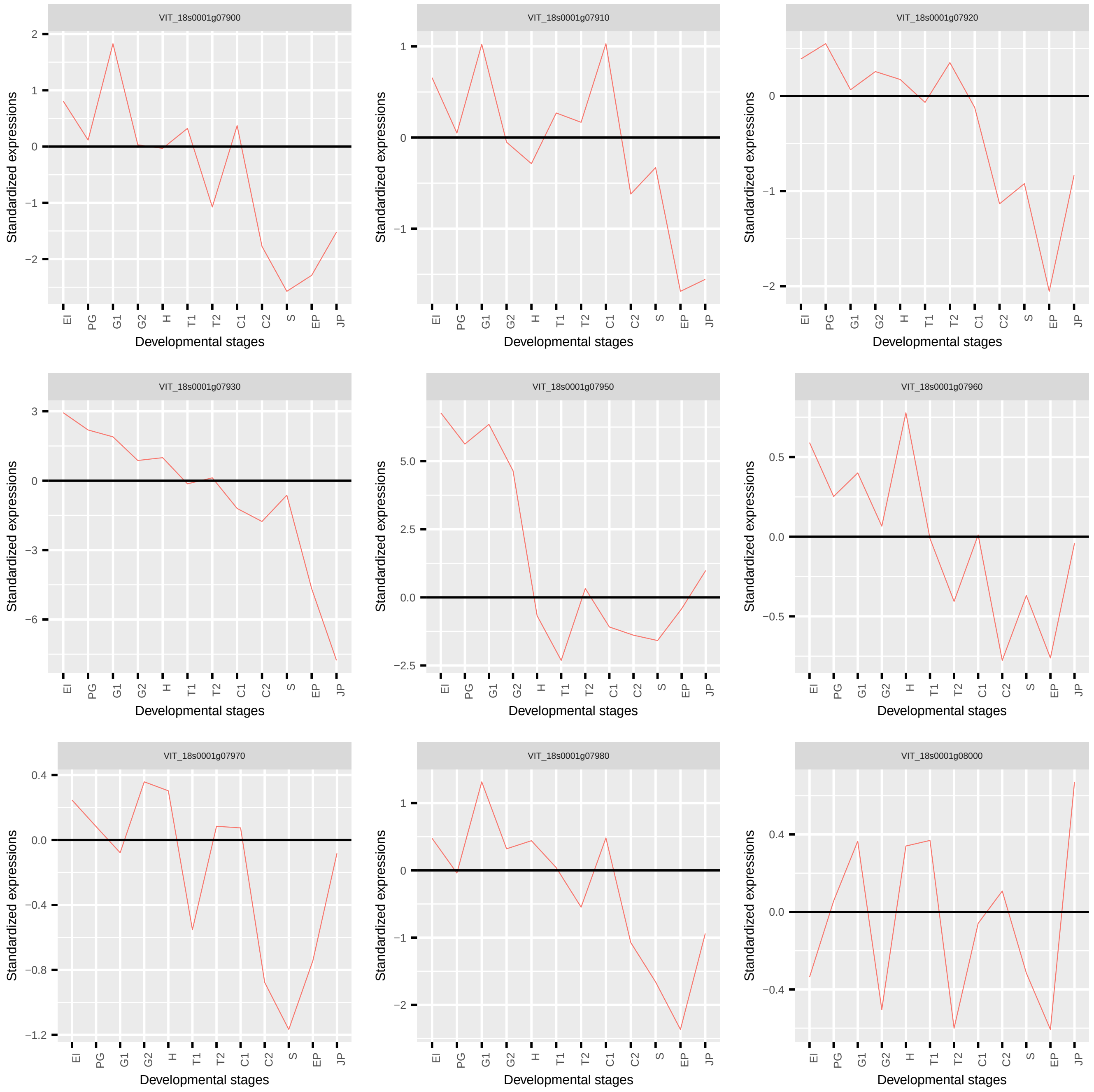
VIT_18s0001g07900
VIT_18s0001g07910
VIT_18s0001g07920
VIT_18s0001g07930
VIT_18s0001g07950
VIT_18s0001g07960
VIT_18s0001g07970
VIT_18s0001g07980
VIT_18s0001g08000
Standardized expressions
Developmental stages
EI PG G1 G2 H T1 T2 C1 C2 S EP JP

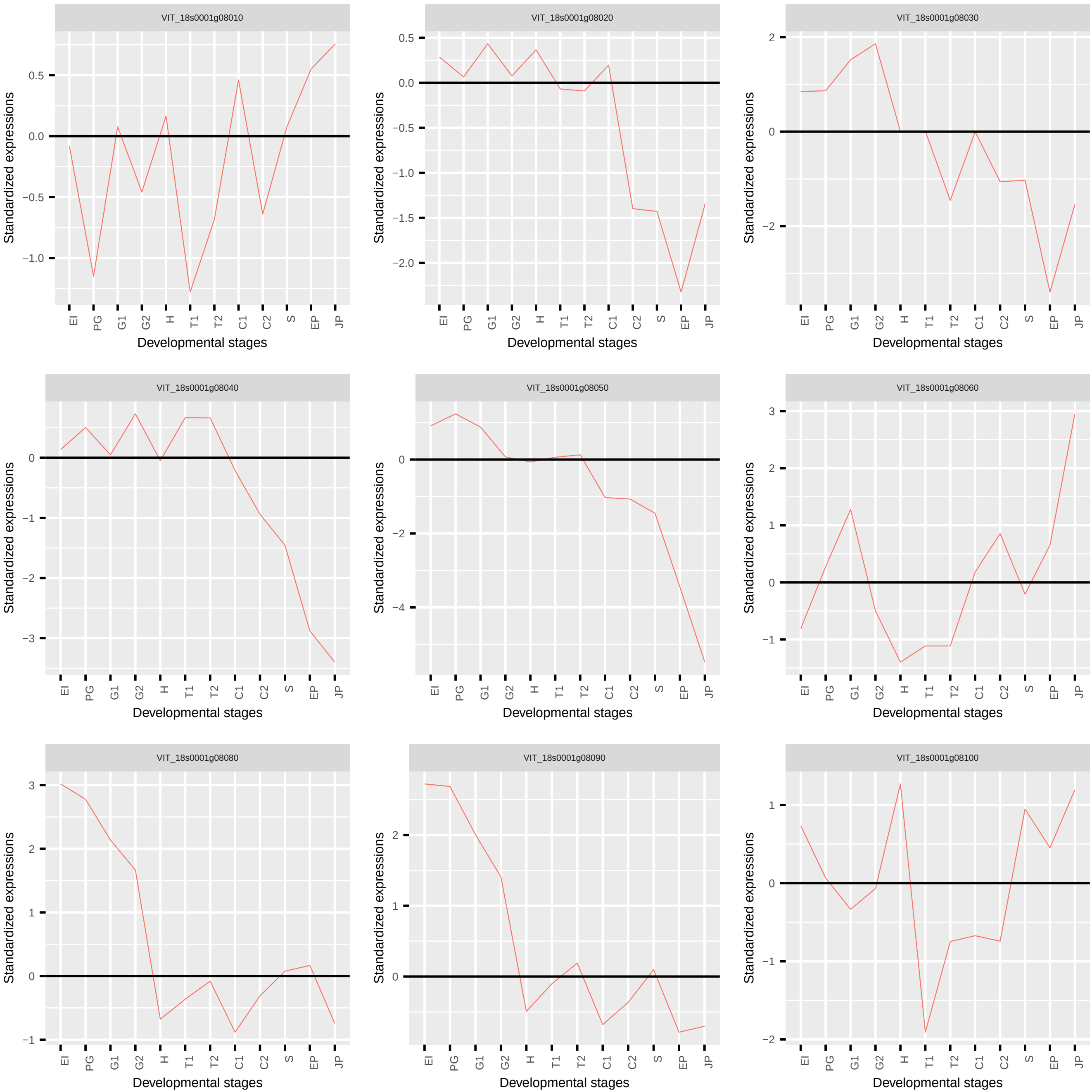
VIT_18s0001g08010
VIT_18s0001g08020
VIT_18s0001g08030
VIT_18s0001g08040
VIT_18s0001g08050
VIT_18s0001g08060
VIT_18s0001g08080
VIT_18s0001g08090
VIT_18s0001g08100
Standardized expressions
Developmental stages
EI PG G1 G2 H T1 T2 C1 C2 S EP JP

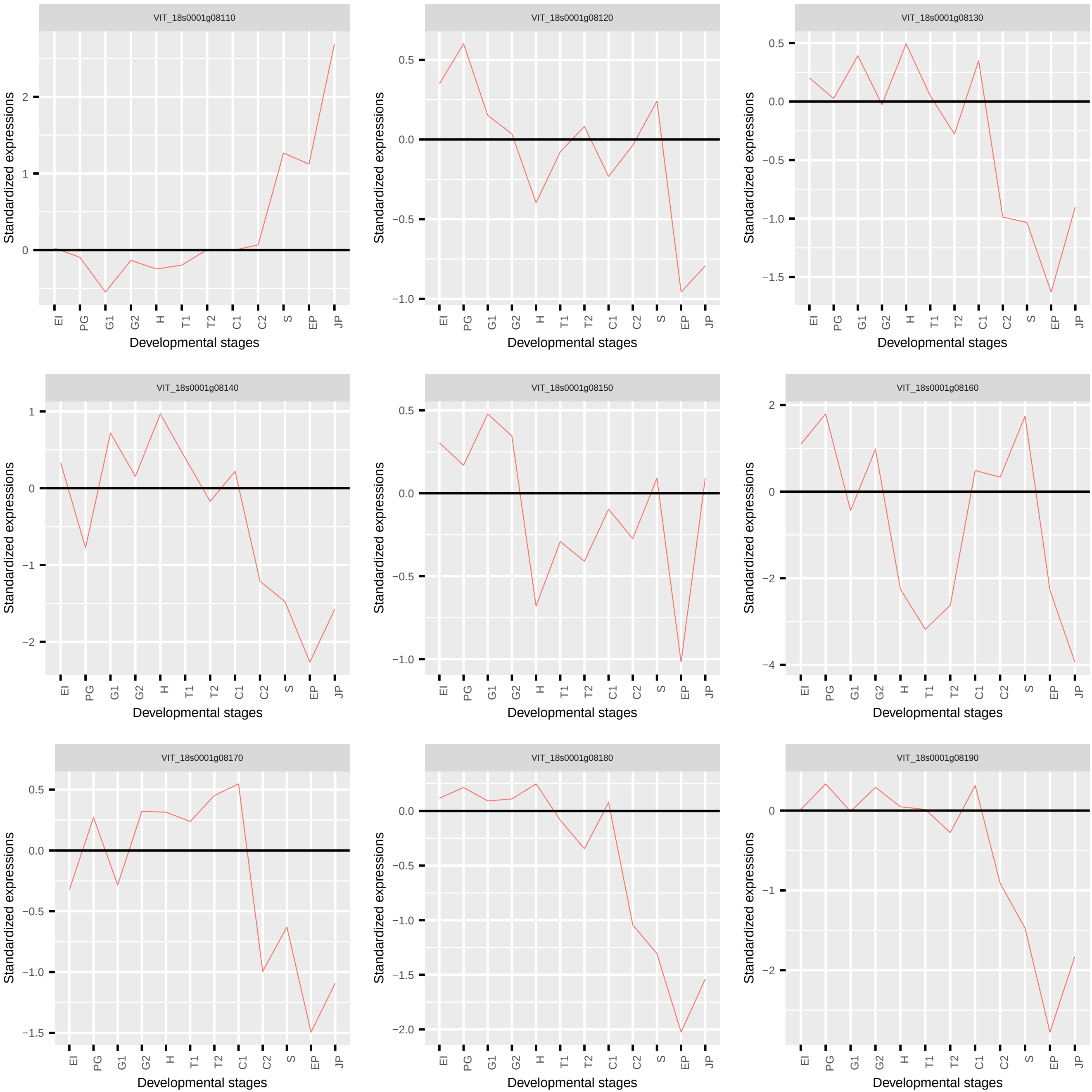

VIT_18s0001g08110
VIT_18s0001g08120
VIT_18s0001g08130
VIT_18s0001g08140
VIT_18s0001g08150
VIT_18s0001g08160
VIT_18s0001g08170
VIT_18s0001g08180
VIT_18s0001g08190
Standardized expressions
Developmental stages
EI
PG
G1
G2
H
T1
T2
C1
C2
S
EP
JP

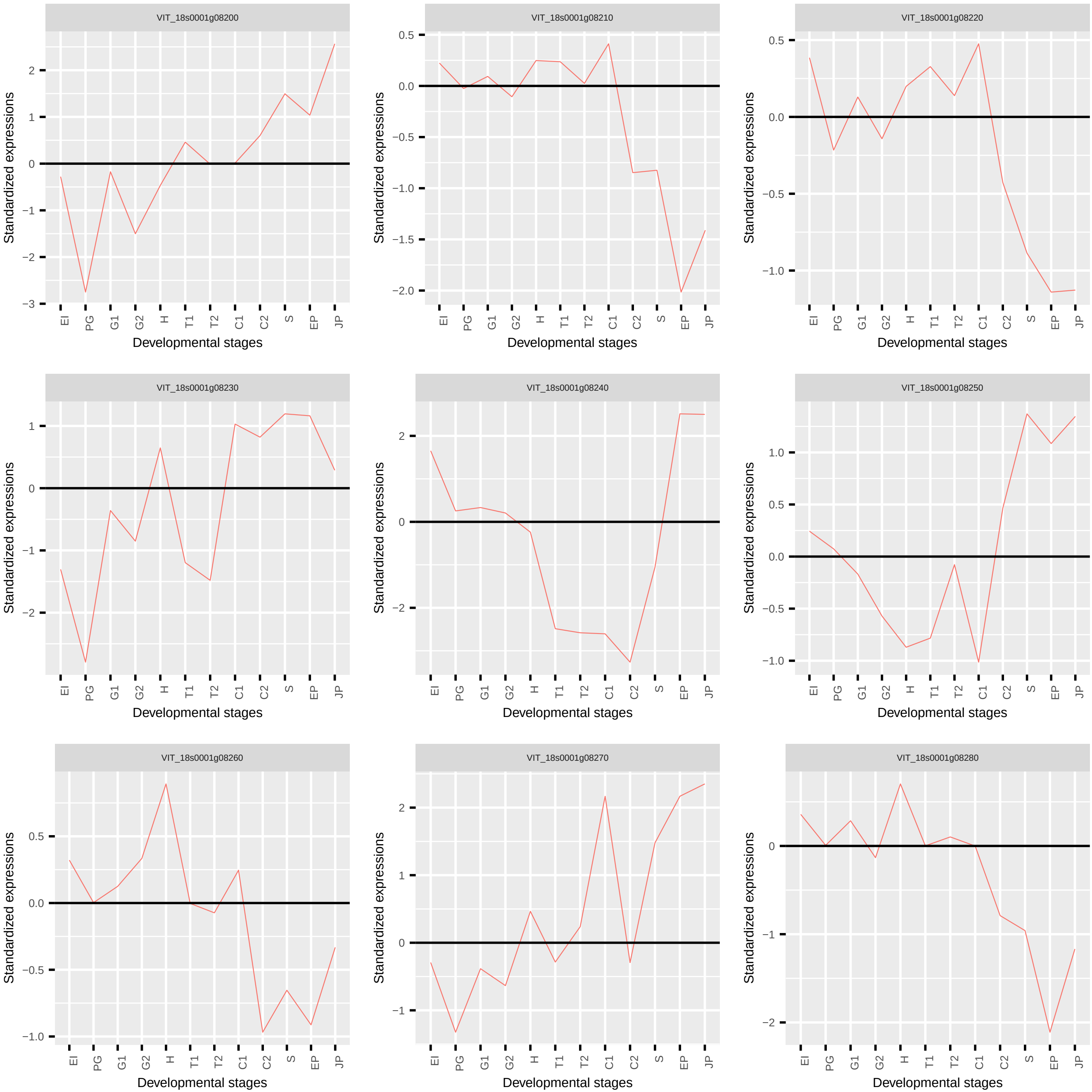
VIT_18s0001g08200
VIT_18s0001g08210
VIT_18s0001g08220
VIT_18s0001g08230
VIT_18s0001g08240
VIT_18s0001g08250
VIT_18s0001g08260
VIT_18s0001g08270
VIT_18s0001g08280
Standardized expressions
EI PG G1 G2 H T1 T2 C1 C2 S EP JP
Developmental stages

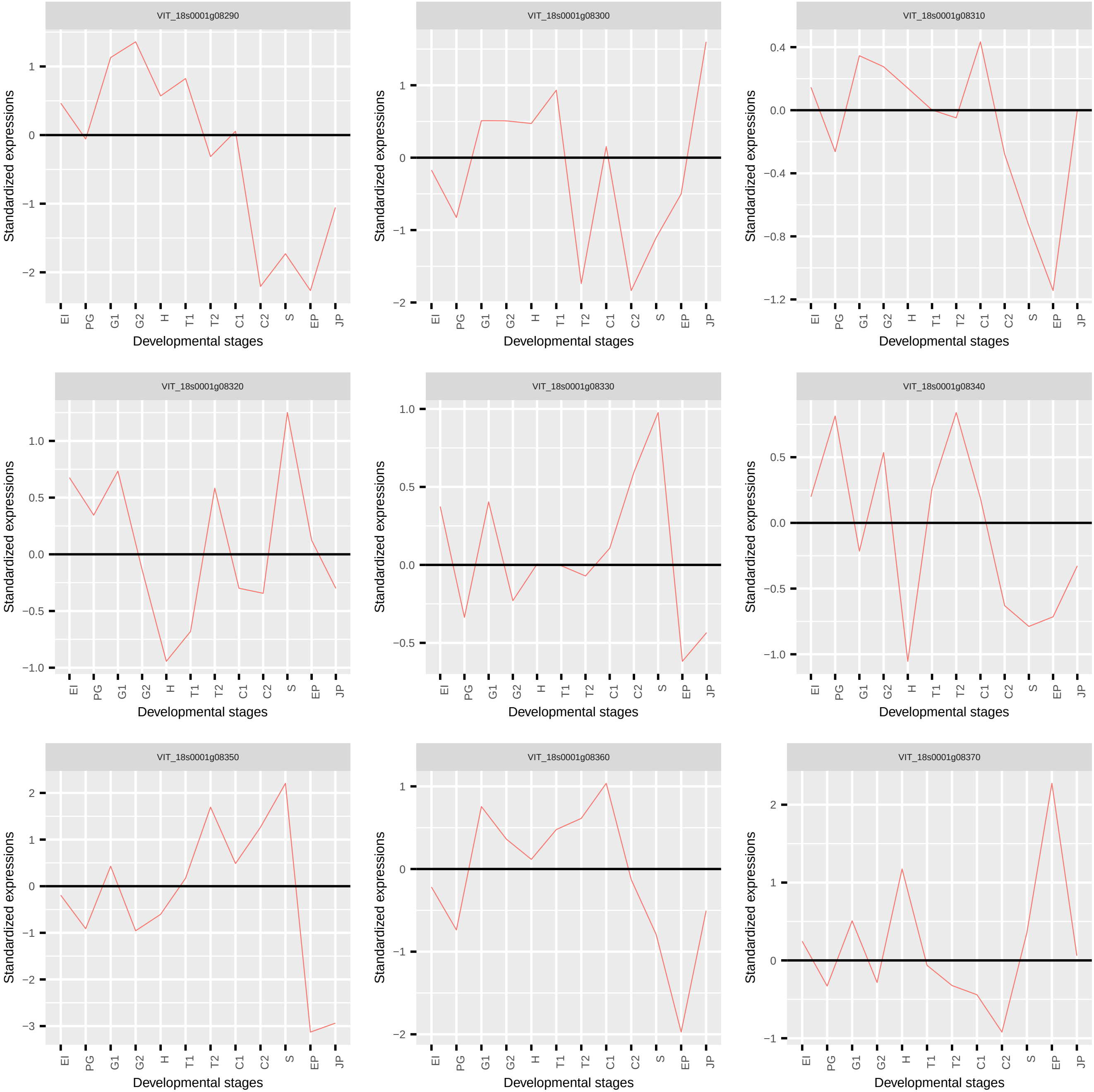
VIT_18s0001g08290
VIT_18s0001g08300
VIT_18s0001g08310
VIT_18s0001g08320
VIT_18s0001g08330
VIT_18s0001g08340
VIT_18s0001g08350
VIT_18s0001g08360
VIT_18s0001g08370
Standardized expressions
Developmental stages
EI
PG
G1
G2
H
T1
T2
C1
C2
S
EP
JP

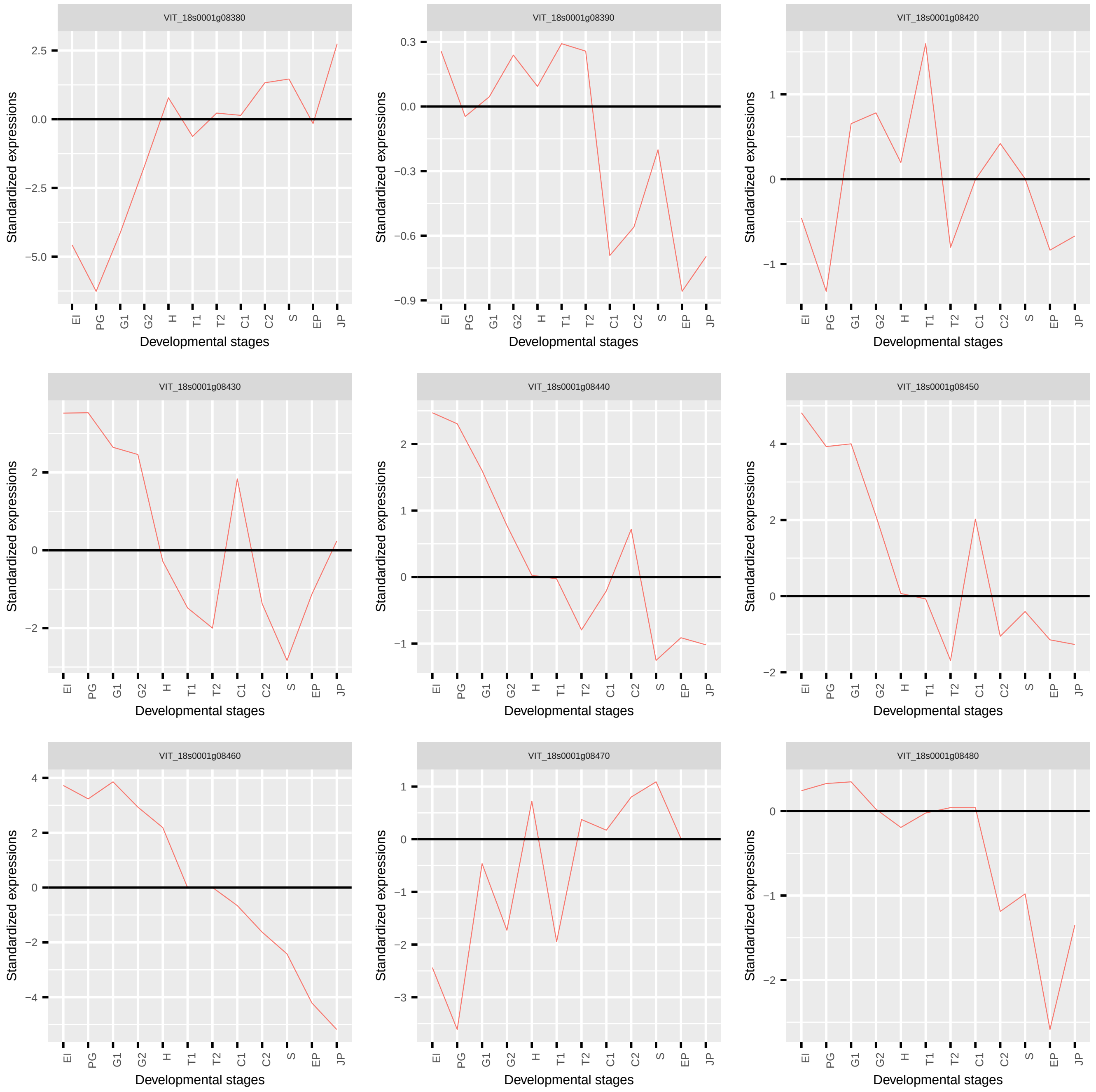
VIT_18s0001g08380
Standardized expressions
2.5
0.0
−2.5
−5.0
EI PG G1 G2 H T1 T2 C1 C2 S EP JP
Developmental stages
VIT_18s0001g08390
Standardized expressions
0.3
0.0
−0.3
−0.6
−0.9
EI PG G1 G2 H T1 T2 C1 C2 S EP JP
Developmental stages
VIT_18s0001g08420
Standardized expressions
1
0
−1
EI PG G1 G2 H T1 T2 C1 C2 S EP JP
Developmental stages
VIT_18s0001g08430
Standardized expressions
2
0
−2
EI PG G1 G2 H T1 T2 C1 C2 S EP JP
Developmental stages
VIT_18s0001g08440
Standardized expressions
2
1
0
−1
EI PG G1 G2 H T1 T2 C1 C2 S EP JP
Developmental stages
VIT_18s0001g08450
Standardized expressions
4
2
0
−2
EI PG G1 G2 H T1 T2 C1 C2 S EP JP
Developmental stages
VIT_18s0001g08460
Standardized expressions
4
2
0
−2
−4
EI PG G1 G2 H T1 T2 C1 C2 S EP JP
Developmental stages
VIT_18s0001g08470
Standardized expressions
1
0
−1
−2
−3
EI PG G1 G2 H T1 T2 C1 C2 S EP JP
Developmental stages
VIT_18s0001g08480
Standardized expressions
0
−1
−2
EI PG G1 G2 H T1 T2 C1 C2 S EP JP
Developmental stages

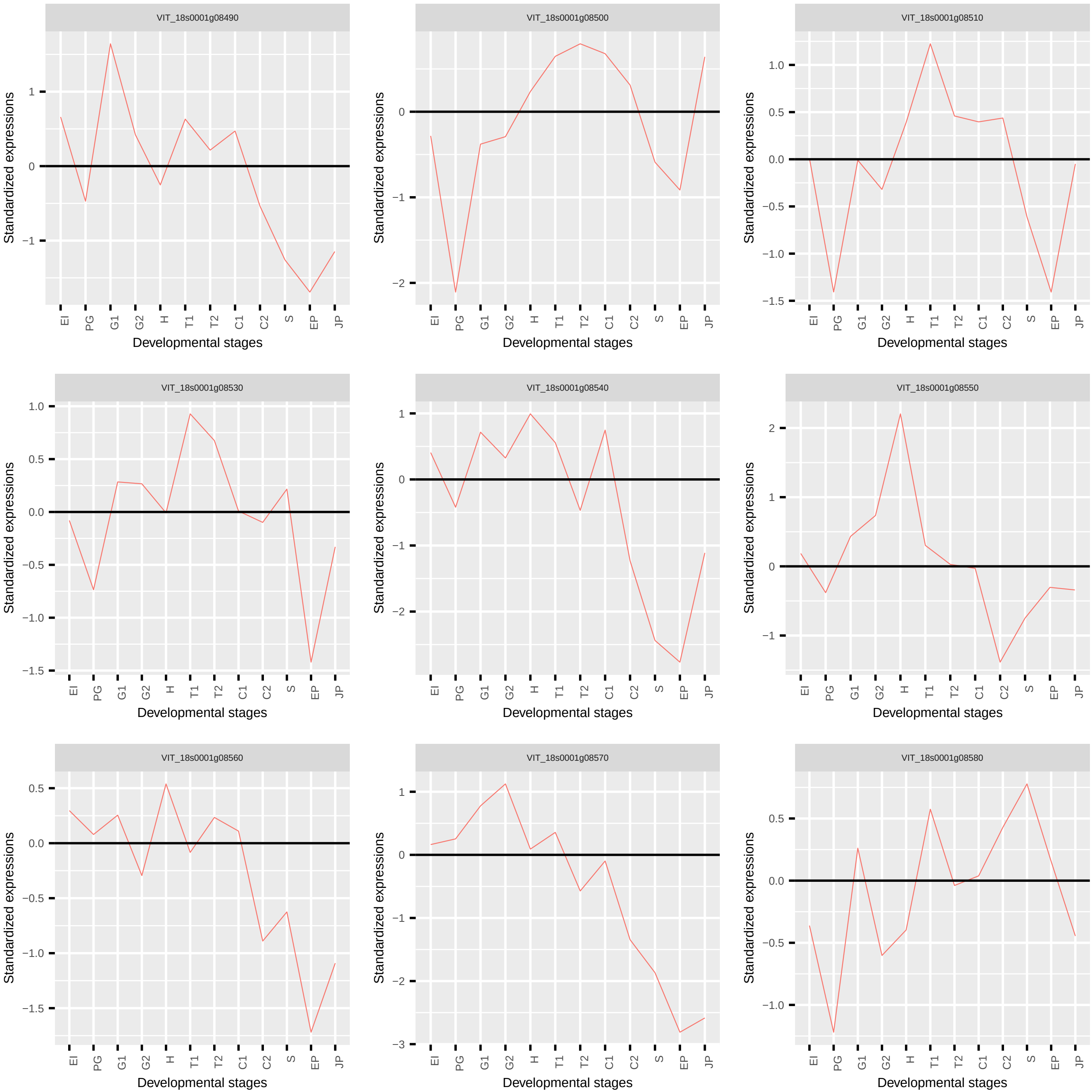
VIT_18s0001g08490
VIT_18s0001g08500
VIT_18s0001g08510
VIT_18s0001g08530
VIT_18s0001g08540
VIT_18s0001g08550
VIT_18s0001g08560
VIT_18s0001g08570
VIT_18s0001g08580
Standardized expressions
Developmental stages
EI PG G1 G2 H T1 T2 C1 C2 S EP JP

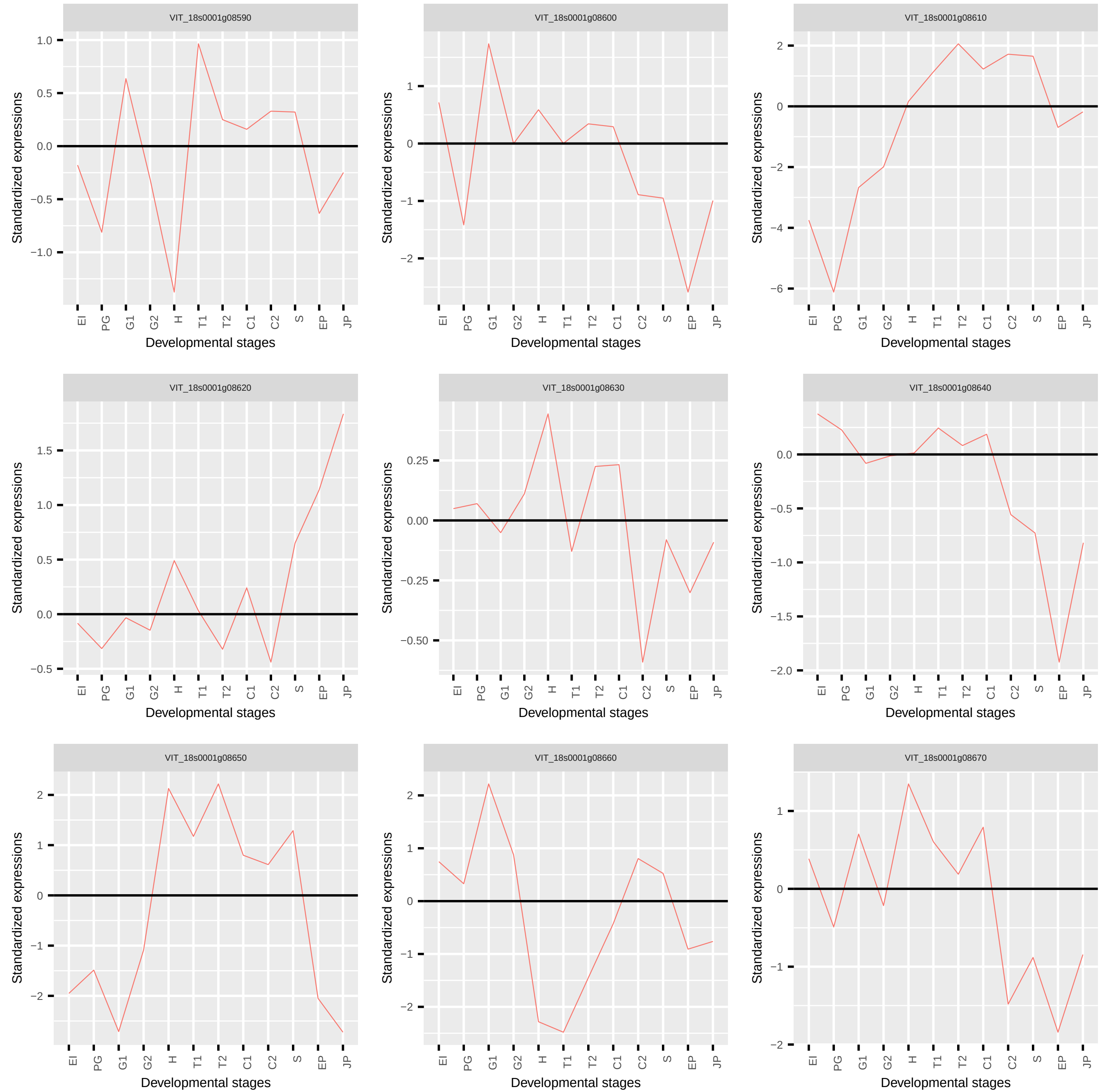

VIT_18s0001g08590
VIT_18s0001g08600
VIT_18s0001g08610
VIT_18s0001g08620
VIT_18s0001g08630
VIT_18s0001g08640
VIT_18s0001g08650
VIT_18s0001g08660
VIT_18s0001g08670
Standardized expressions
Developmental stages
EI PG G1 G2 H T1 T2 C1 C2 S EP JP
1.0 0.5 0.0 −0.5 −1.0
1 0 −1 −2
2 0 −2 −4 −6
1.5 1.0 0.5 0.0 −0.5
0.25 0.00 −0.25 −0.50
0.0 −0.5 −1.0 −1.5 −2.0
2 1 0 −1 −2
1 0 −1 −2

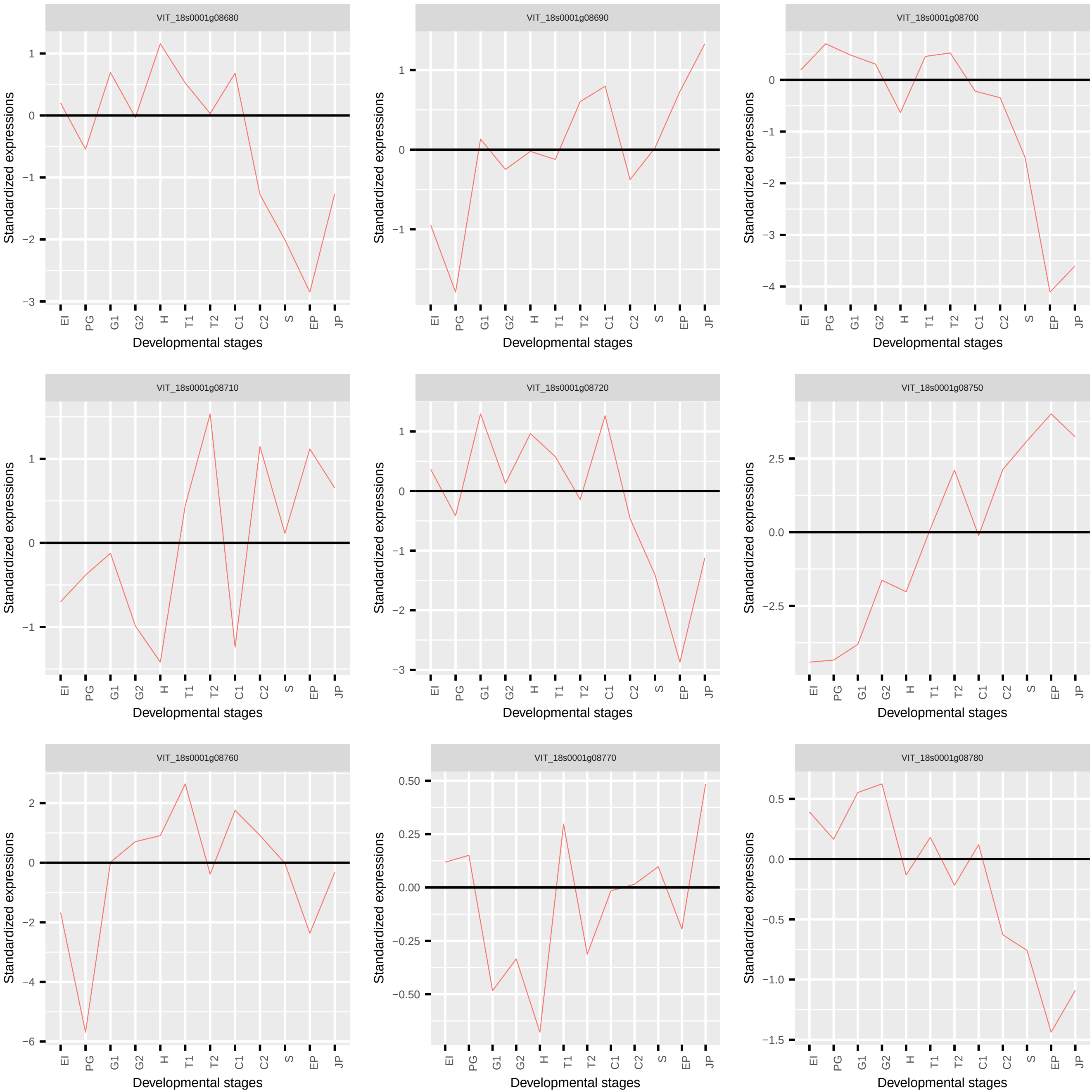

VIT_18s0001g08680
VIT_18s0001g08690
VIT_18s0001g08700
VIT_18s0001g08710
VIT_18s0001g08720
VIT_18s0001g08750
VIT_18s0001g08760
VIT_18s0001g08770
VIT_18s0001g08780
Standardized expressions
Developmental stages
EI
PG
G1
G2
H
T1
T2
C1
C2
S
EP
JP

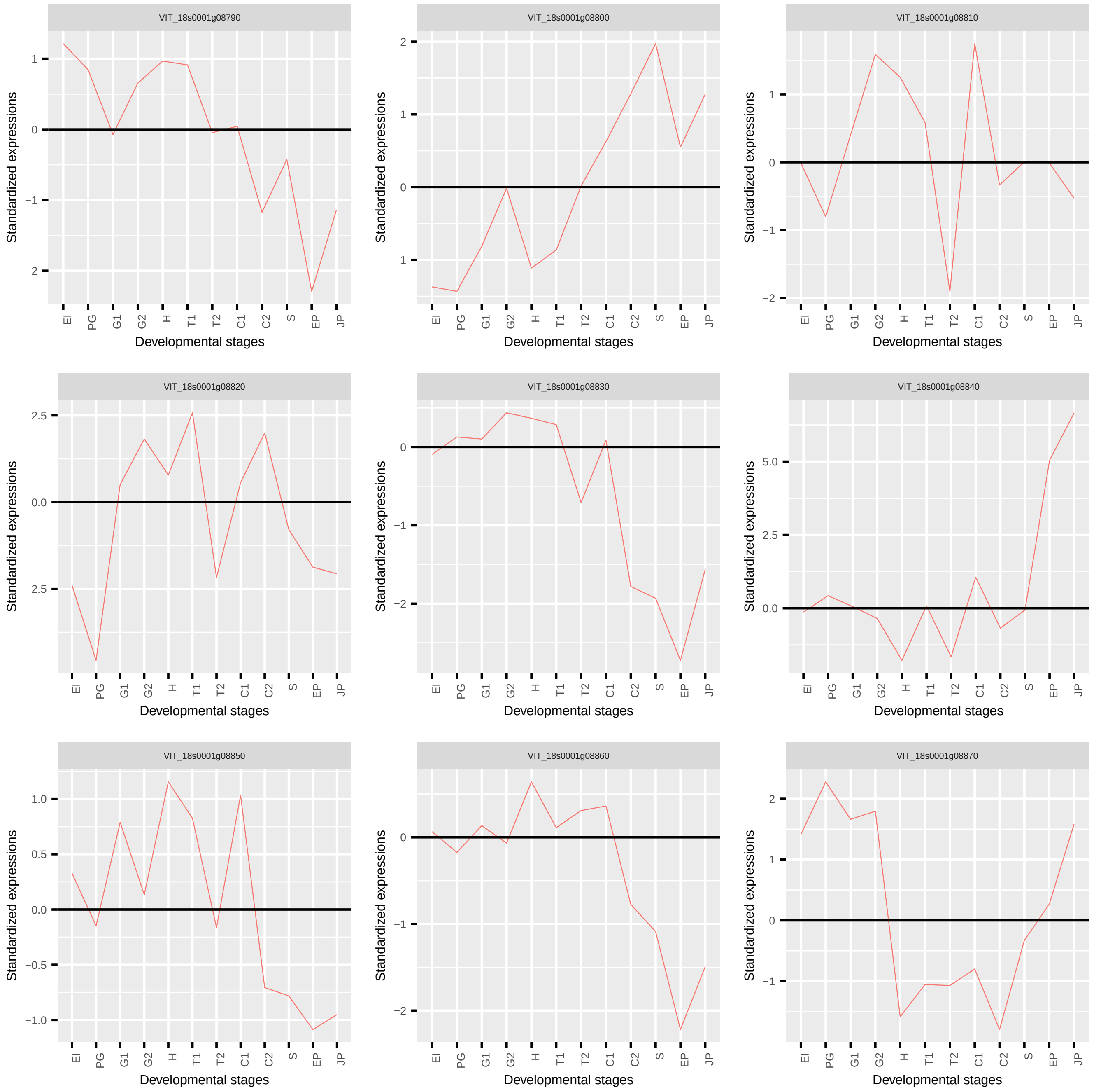
VIT_18s0001g08790
VIT_18s0001g08800
VIT_18s0001g08810
VIT_18s0001g08820
VIT_18s0001g08830
VIT_18s0001g08840
VIT_18s0001g08850
VIT_18s0001g08860
VIT_18s0001g08870
Standardized expressions
Developmental stages
EI PG G1 G2 H T1 T2 C1 C2 S EP JP

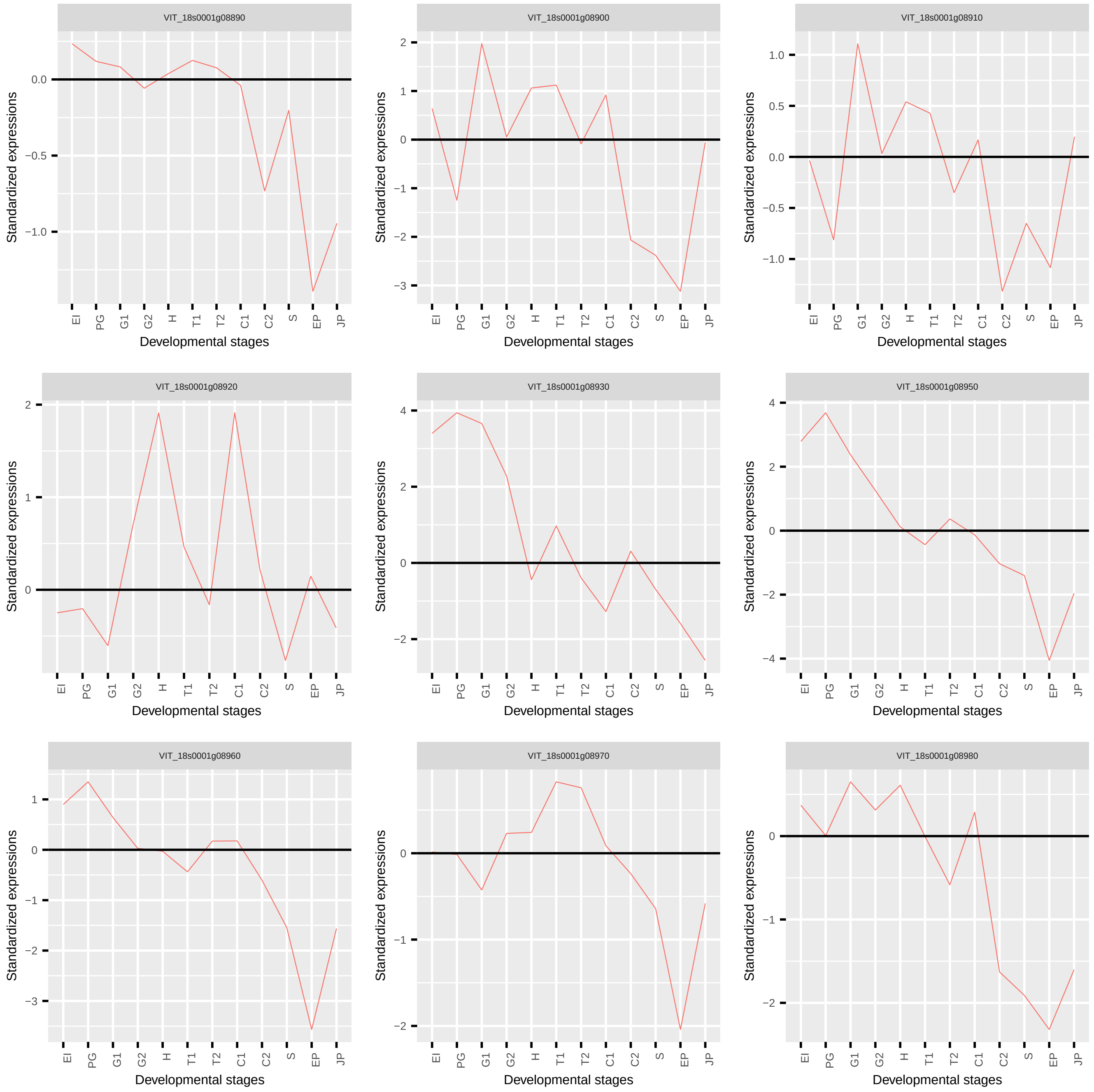

VIT_18s0001g08890
VIT_18s0001g08900
VIT_18s0001g08910
VIT_18s0001g08920
VIT_18s0001g08930
VIT_18s0001g08950
VIT_18s0001g08960
VIT_18s0001g08970
VIT_18s0001g08980
Standardized expressions
Developmental stages
EI PG G1 G2 H T1 T2 C1 C2 S EP JP

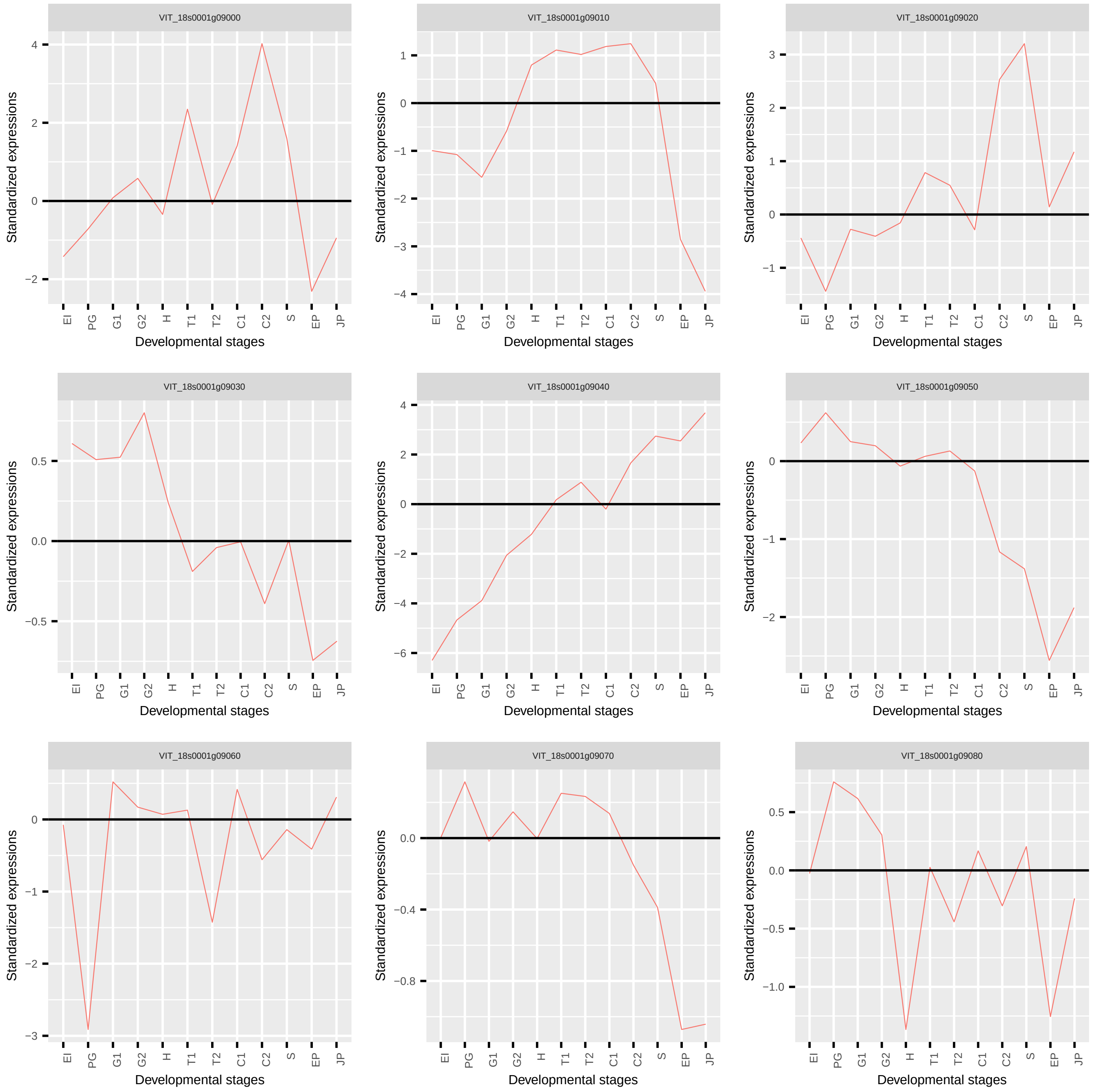
VIT_18s0001g09000
VIT_18s0001g09010
VIT_18s0001g09020
VIT_18s0001g09030
VIT_18s0001g09040
VIT_18s0001g09050
VIT_18s0001g09060
VIT_18s0001g09070
VIT_18s0001g09080
Standardized expressions
Developmental stages
EI PG G1 G2 H T1 T2 C1 C2 S EP JP

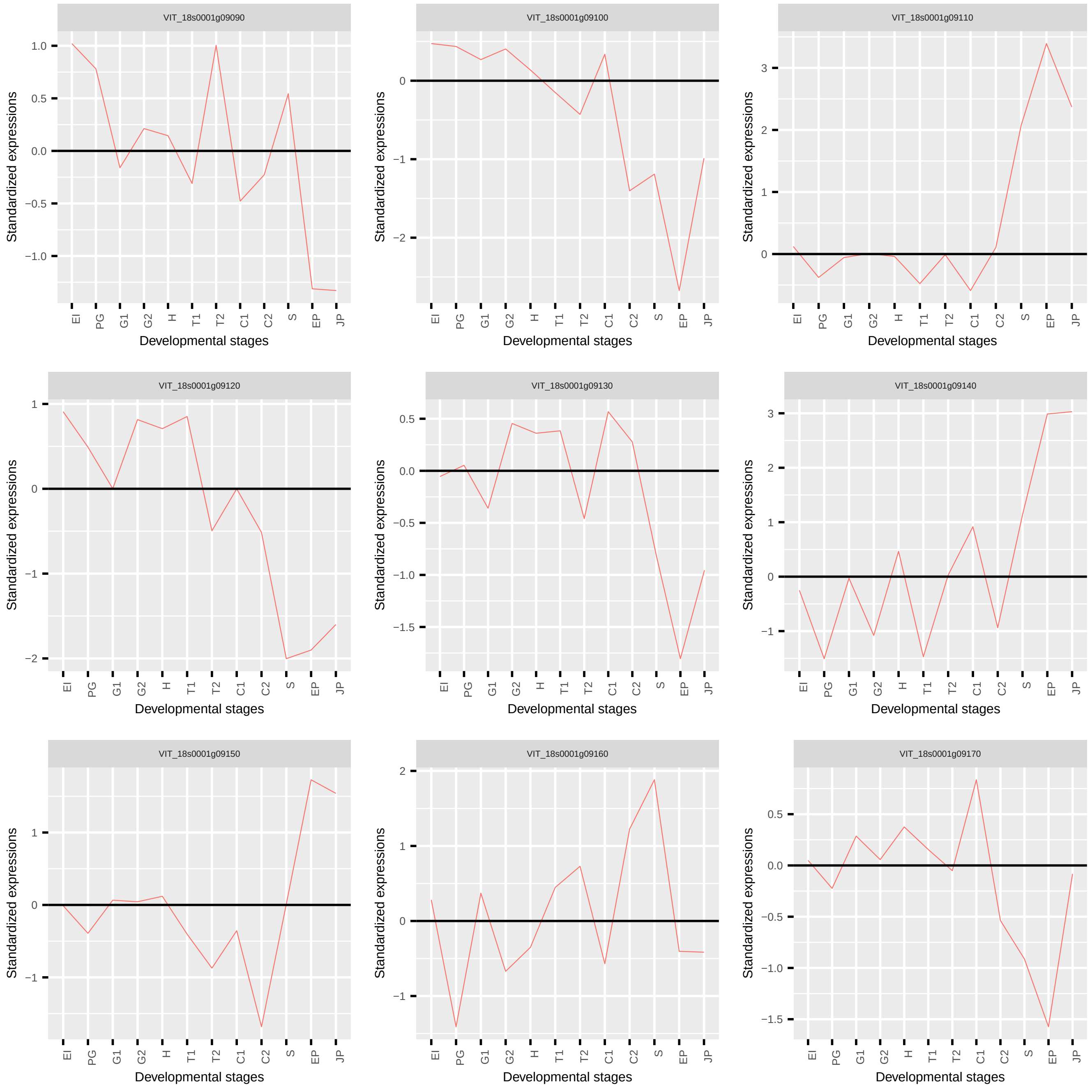
VIT_18s0001g09090
VIT_18s0001g09100
VIT_18s0001g09110
VIT_18s0001g09120
VIT_18s0001g09130
VIT_18s0001g09140
VIT_18s0001g09150
VIT_18s0001g09160
VIT_18s0001g09170
Standardized expressions
Developmental stages
EI PG G1 G2 H T1 T2 C1 C2 S EP JP

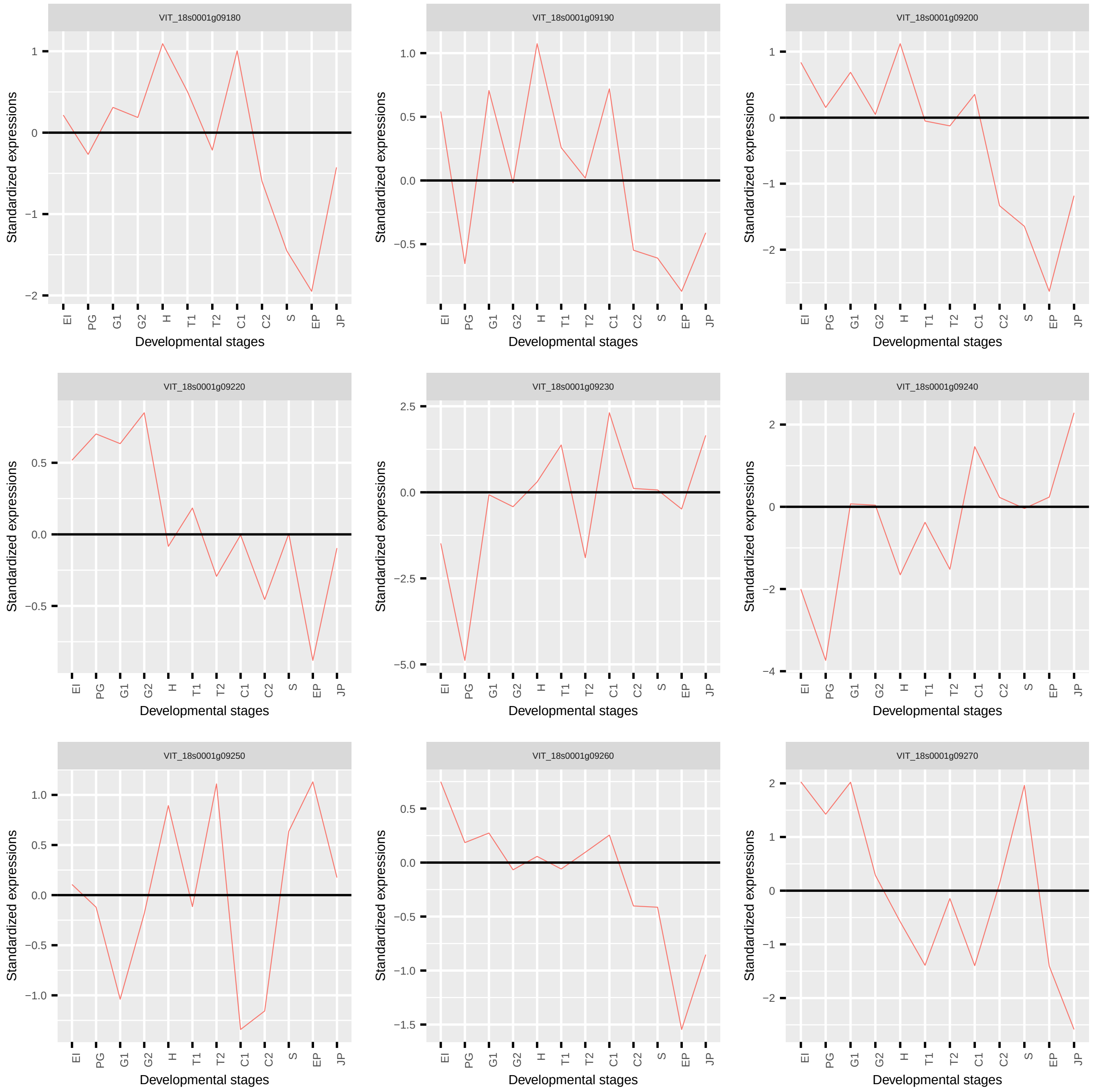
VIT_18s0001g09180
VIT_18s0001g09190
VIT_18s0001g09200
VIT_18s0001g09220
VIT_18s0001g09230
VIT_18s0001g09240
VIT_18s0001g09250
VIT_18s0001g09260
VIT_18s0001g09270
Standardized expressions
Developmental stages
EI PG G1 G2 H T1 T2 C1 C2 S EP JP
1 0 −1 −2
1.0 0.5 0.0 −0.5
1 0 −1 −2
0.5 0.0 −0.5
2.5 0.0 −2.5 −5.0
2 0 −2 −4
1.0 0.5 0.0 −0.5 −1.0
0.5 0.0 −0.5 −1.0 −1.5
2 1 0 −1 −2

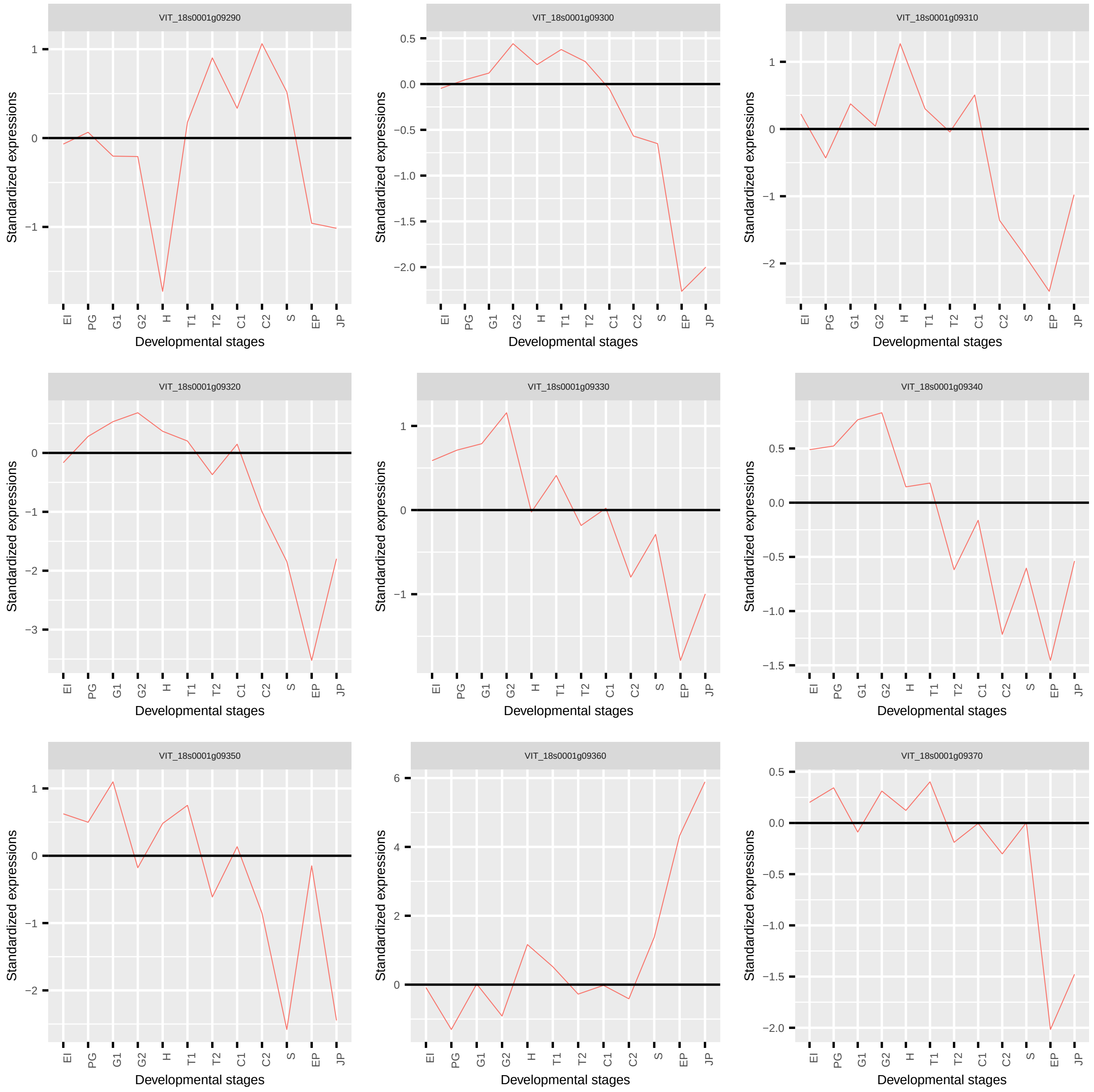
VIT_18s0001g09290
VIT_18s0001g09300
VIT_18s0001g09310
VIT_18s0001g09320
VIT_18s0001g09330
VIT_18s0001g09340
VIT_18s0001g09350
VIT_18s0001g09360
VIT_18s0001g09370
Standardized expressions
Developmental stages
EI PG G1 G2 H T1 T2 C1 C2 S EP JP

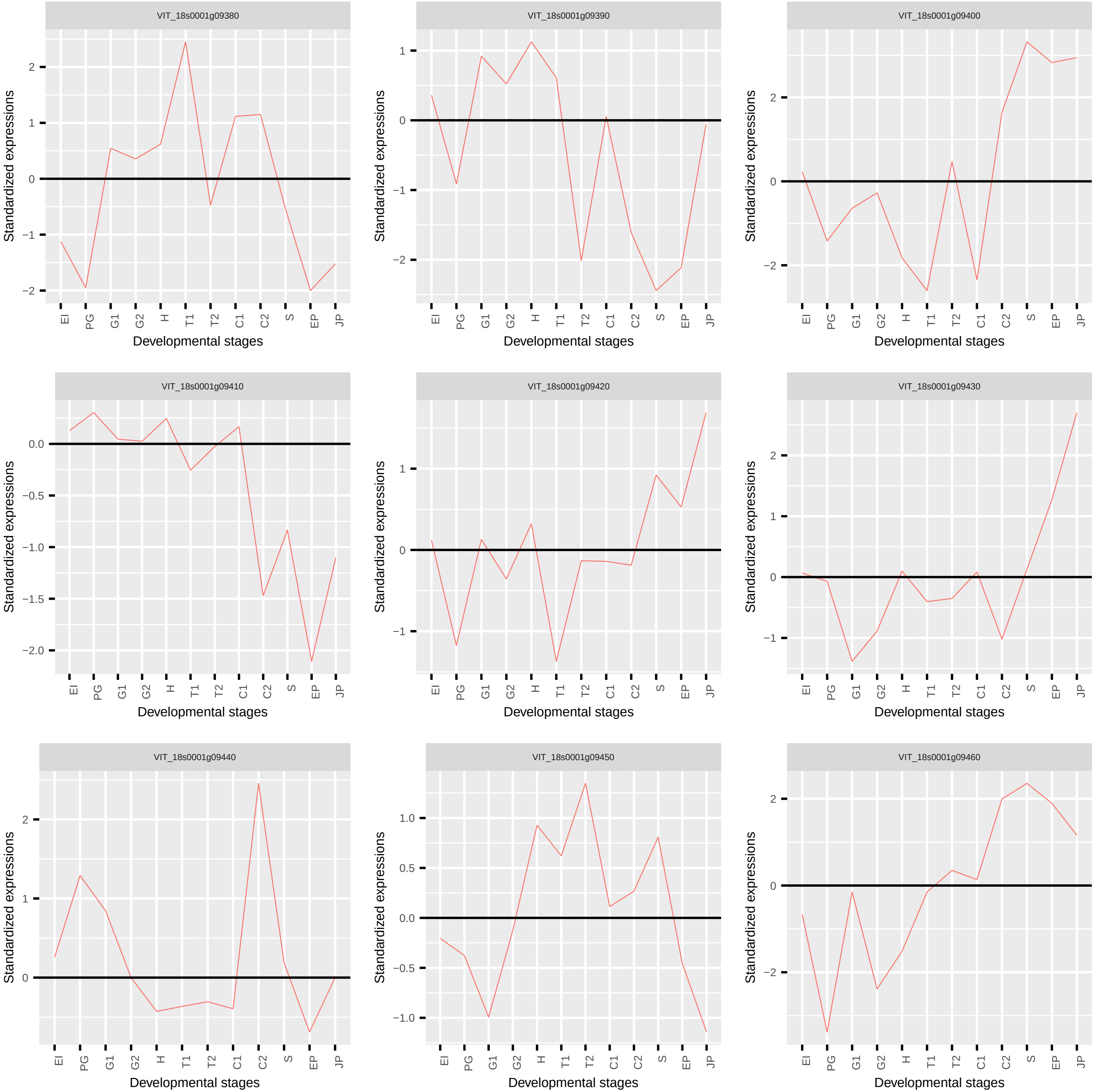
VIT_18s0001g09380
VIT_18s0001g09390
VIT_18s0001g09400
VIT_18s0001g09410
VIT_18s0001g09420
VIT_18s0001g09430
VIT_18s0001g09440
VIT_18s0001g09450
VIT_18s0001g09460
Standardized expressions
Developmental stages
EI PG G1 G2 H T1 T2 C1 C2 S EP JP

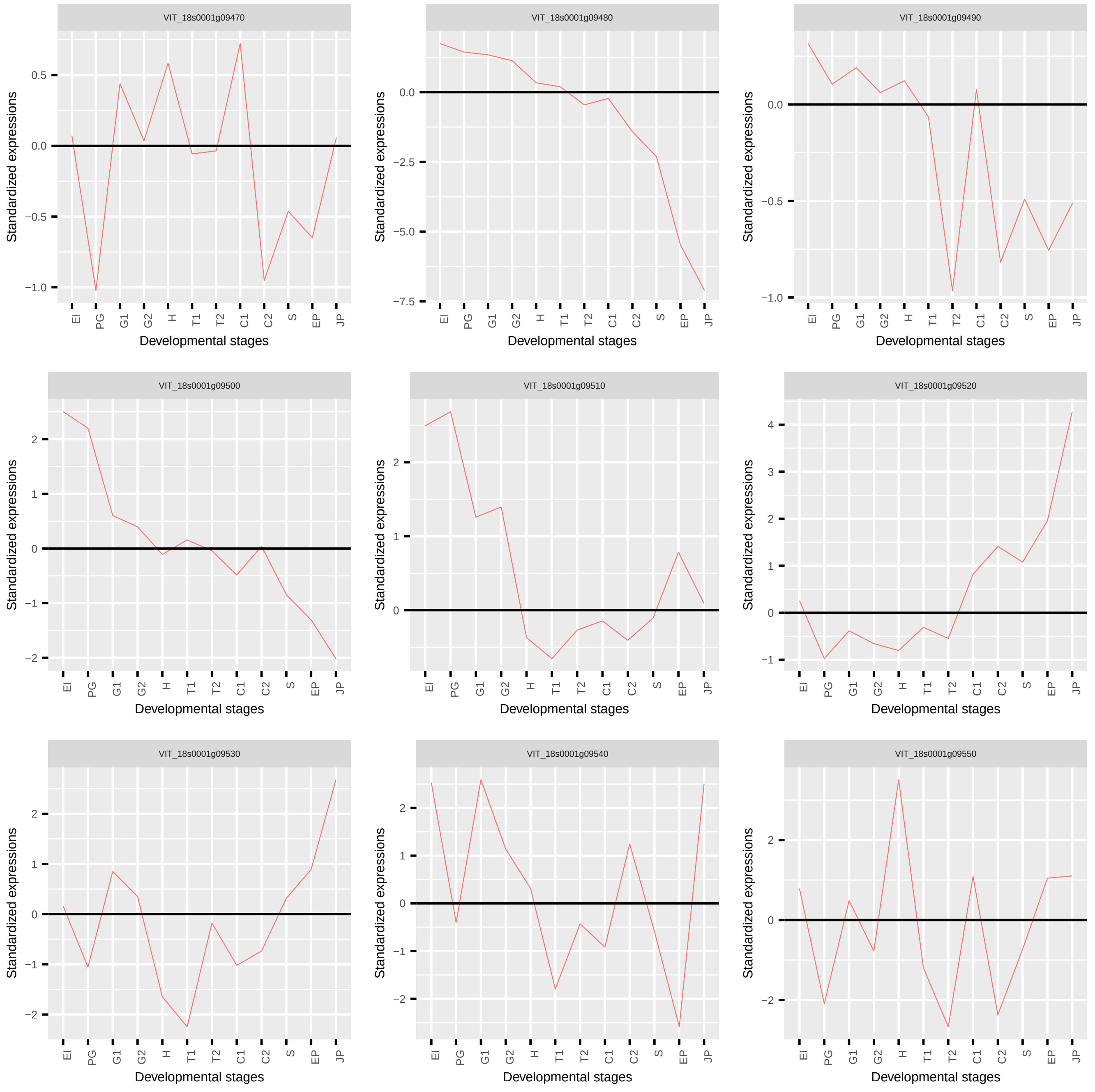
VIT_18s0001g09470
VIT_18s0001g09480
VIT_18s0001g09490
VIT_18s0001g09500
VIT_18s0001g09510
VIT_18s0001g09520
VIT_18s0001g09530
VIT_18s0001g09540
VIT_18s0001g09550
Standardized expressions
Developmental stages
EI PG G1 G2 H T1 T2 C1 C2 S EP JP
0.5
0.0
−0.5
−1.0
−2.5
−5.0
−7.5
4
3
2
1
0
−1
−2

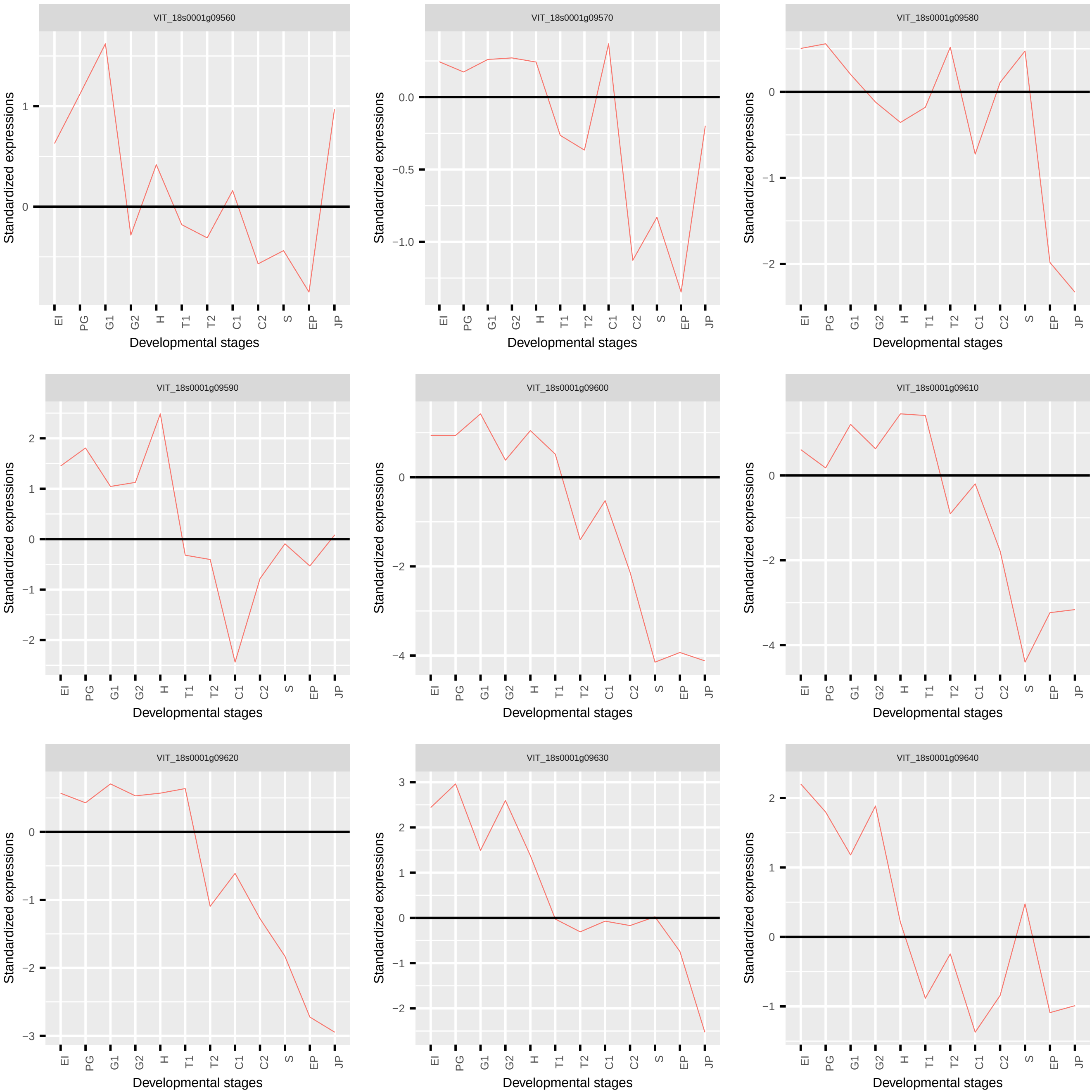

VIT_18s0001g09560
VIT_18s0001g09570
VIT_18s0001g09580
VIT_18s0001g09590
VIT_18s0001g09600
VIT_18s0001g09610
VIT_18s0001g09620
VIT_18s0001g09630
VIT_18s0001g09640
Standardized expressions
Developmental stages
EI
PG
G1
G2
H
T1
T2
C1
C2
S
EP
JP

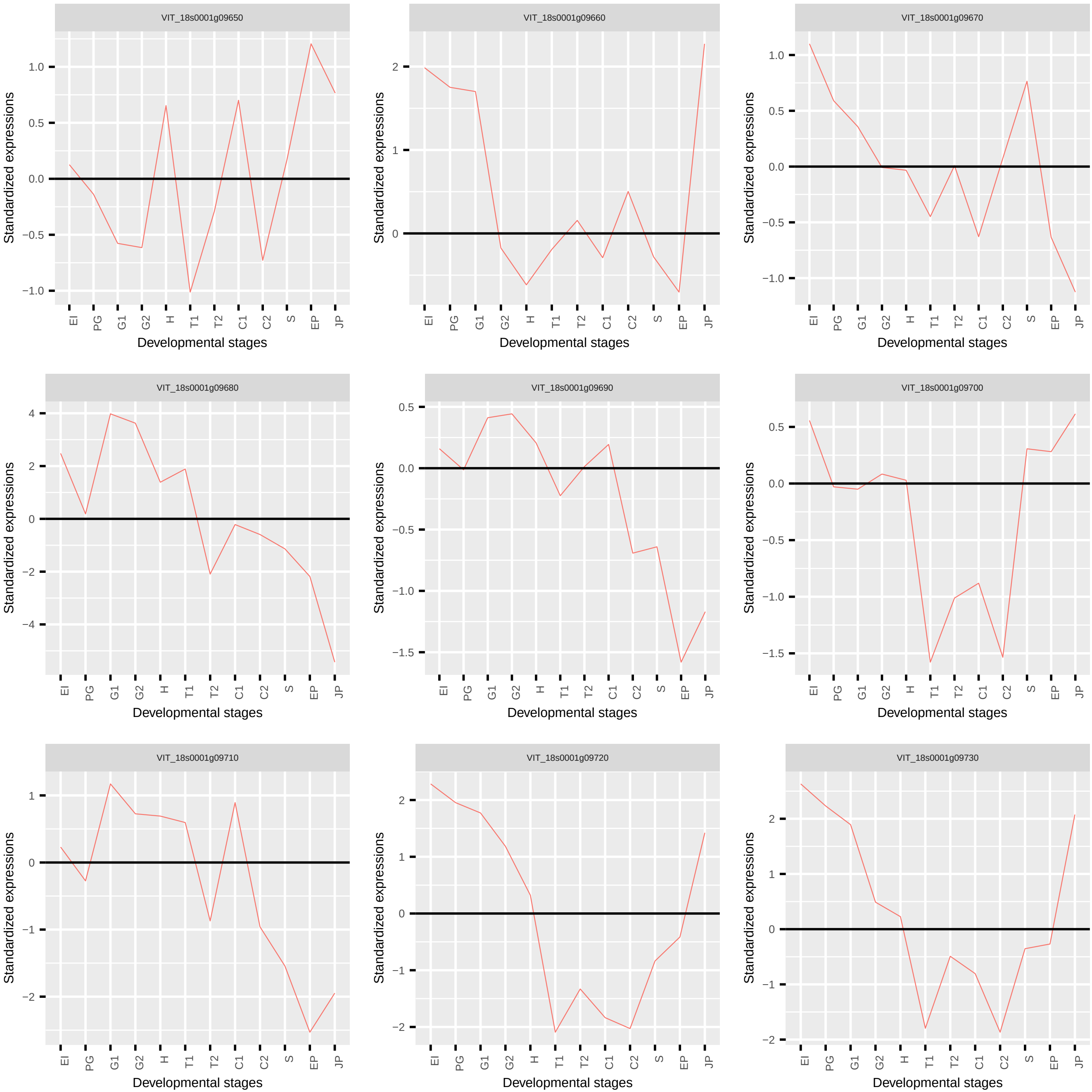
VIT_18s0001g09650
VIT_18s0001g09660
VIT_18s0001g09670
VIT_18s0001g09680
VIT_18s0001g09690
VIT_18s0001g09700
VIT_18s0001g09710
VIT_18s0001g09720
VIT_18s0001g09730
Standardized expressions
Developmental stages
EI PG G1 G2 H T1 T2 C1 C2 S EP JP

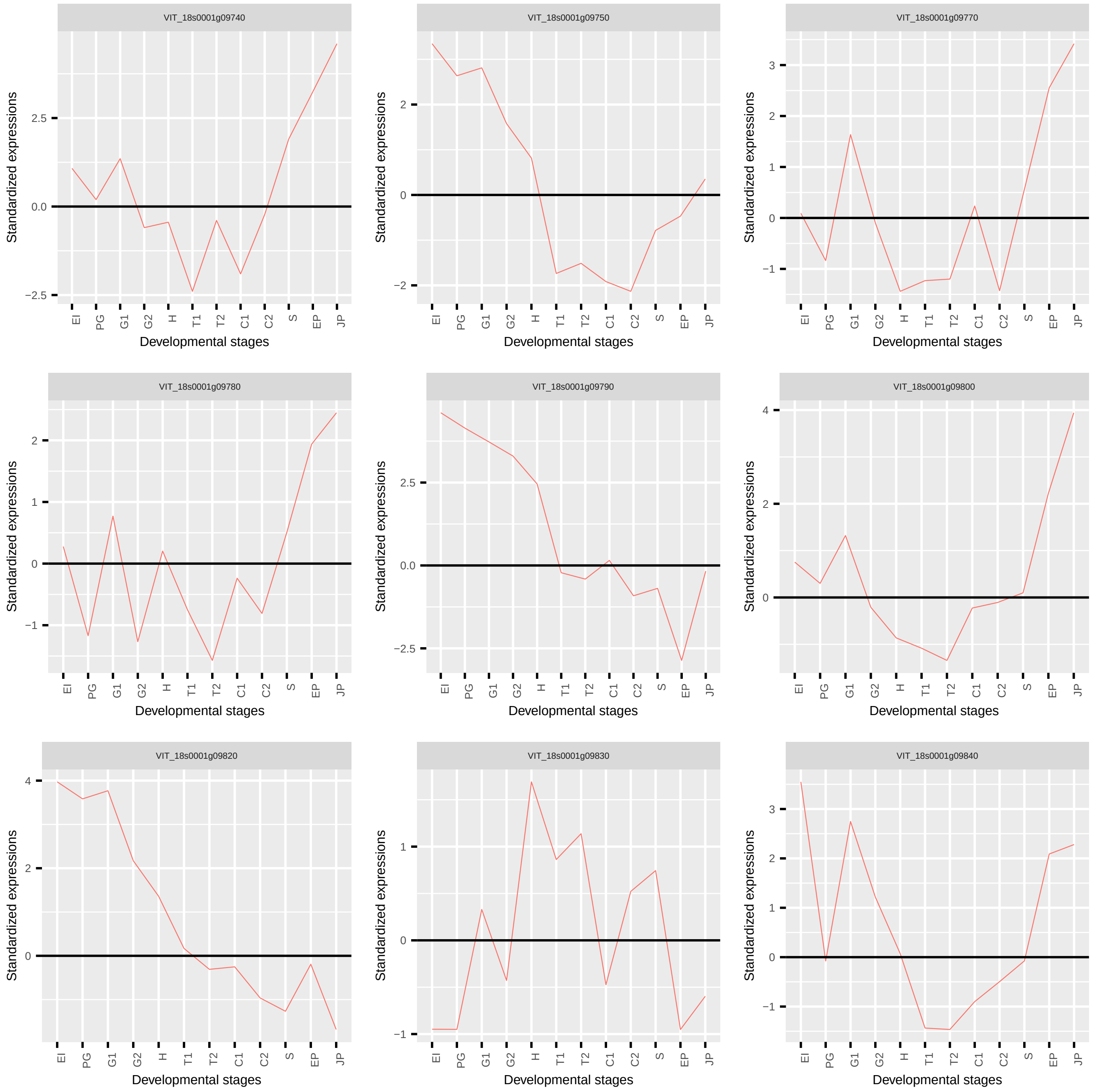
VIT_18s0001g09740
2.5
0.0
−2.5
Standardized expressions
EI PG G1 G2 H T1 T2 C1 C2 S EP JP
Developmental stages
VIT_18s0001g09750
2
0
−2
Standardized expressions
EI PG G1 G2 H T1 T2 C1 C2 S EP JP
Developmental stages
VIT_18s0001g09770
3
2
1
0
−1
Standardized expressions
EI PG G1 G2 H T1 T2 C1 C2 S EP JP
Developmental stages
VIT_18s0001g09780
2
1
0
−1
Standardized expressions
EI PG G1 G2 H T1 T2 C1 C2 S EP JP
Developmental stages
VIT_18s0001g09790
2.5
0.0
−2.5
Standardized expressions
EI PG G1 G2 H T1 T2 C1 C2 S EP JP
Developmental stages
VIT_18s0001g09800
4
2
0
Standardized expressions
EI PG G1 G2 H T1 T2 C1 C2 S EP JP
Developmental stages
VIT_18s0001g09820
4
2
0
Standardized expressions
EI PG G1 G2 H T1 T2 C1 C2 S EP JP
Developmental stages
VIT_18s0001g09830
1
0
−1
Standardized expressions
EI PG G1 G2 H T1 T2 C1 C2 S EP JP
Developmental stages
VIT_18s0001g09840
3
2
1
0
−1
Standardized expressions
EI PG G1 G2 H T1 T2 C1 C2 S EP JP
Developmental stages

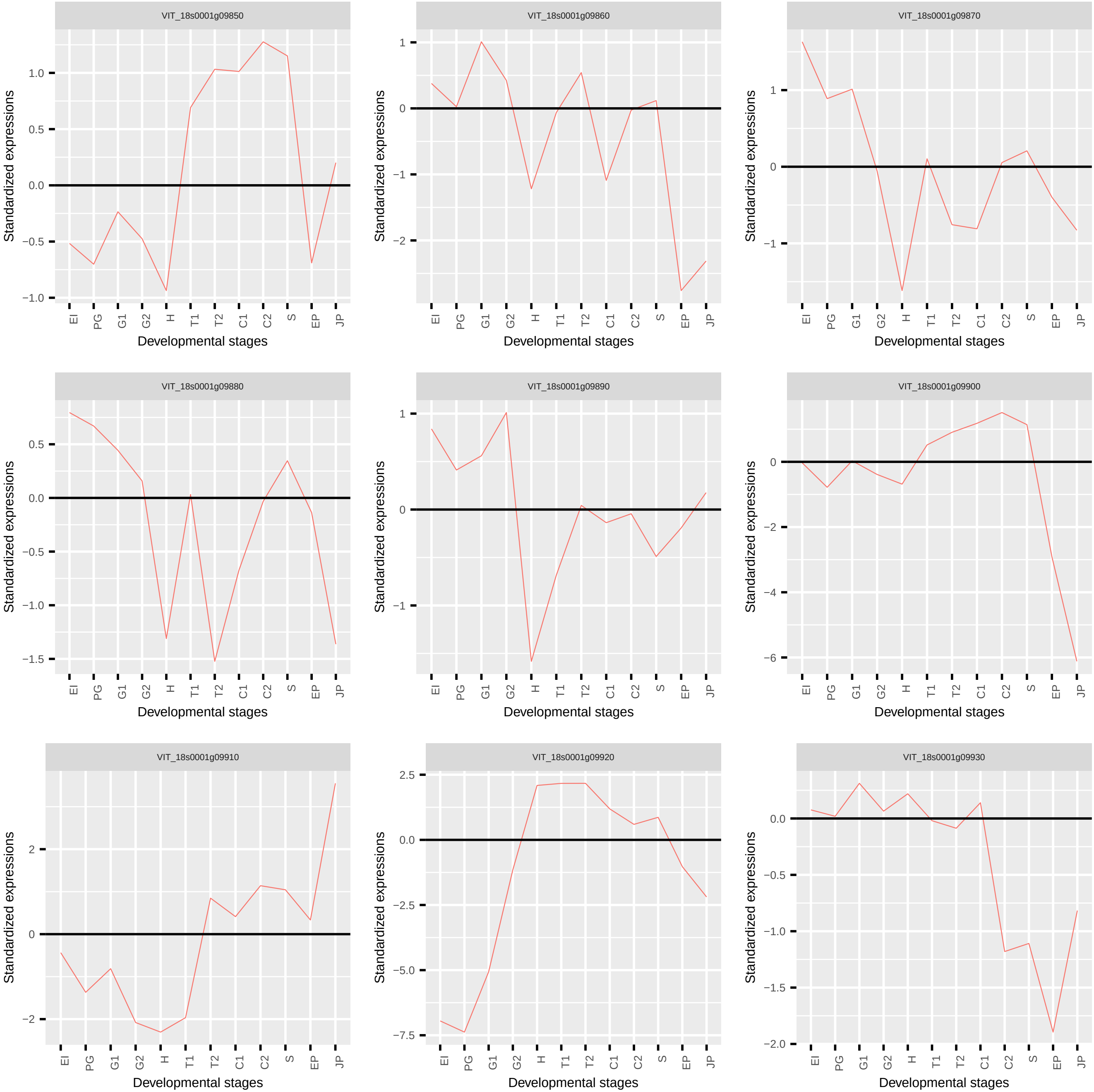
VIT_18s0001g09850
VIT_18s0001g09860
VIT_18s0001g09870
VIT_18s0001g09880
VIT_18s0001g09890
VIT_18s0001g09900
VIT_18s0001g09910
VIT_18s0001g09920
VIT_18s0001g09930
Standardized expressions
Developmental stages
EI PG G1 G2 H T1 T2 C1 C2 S EP JP

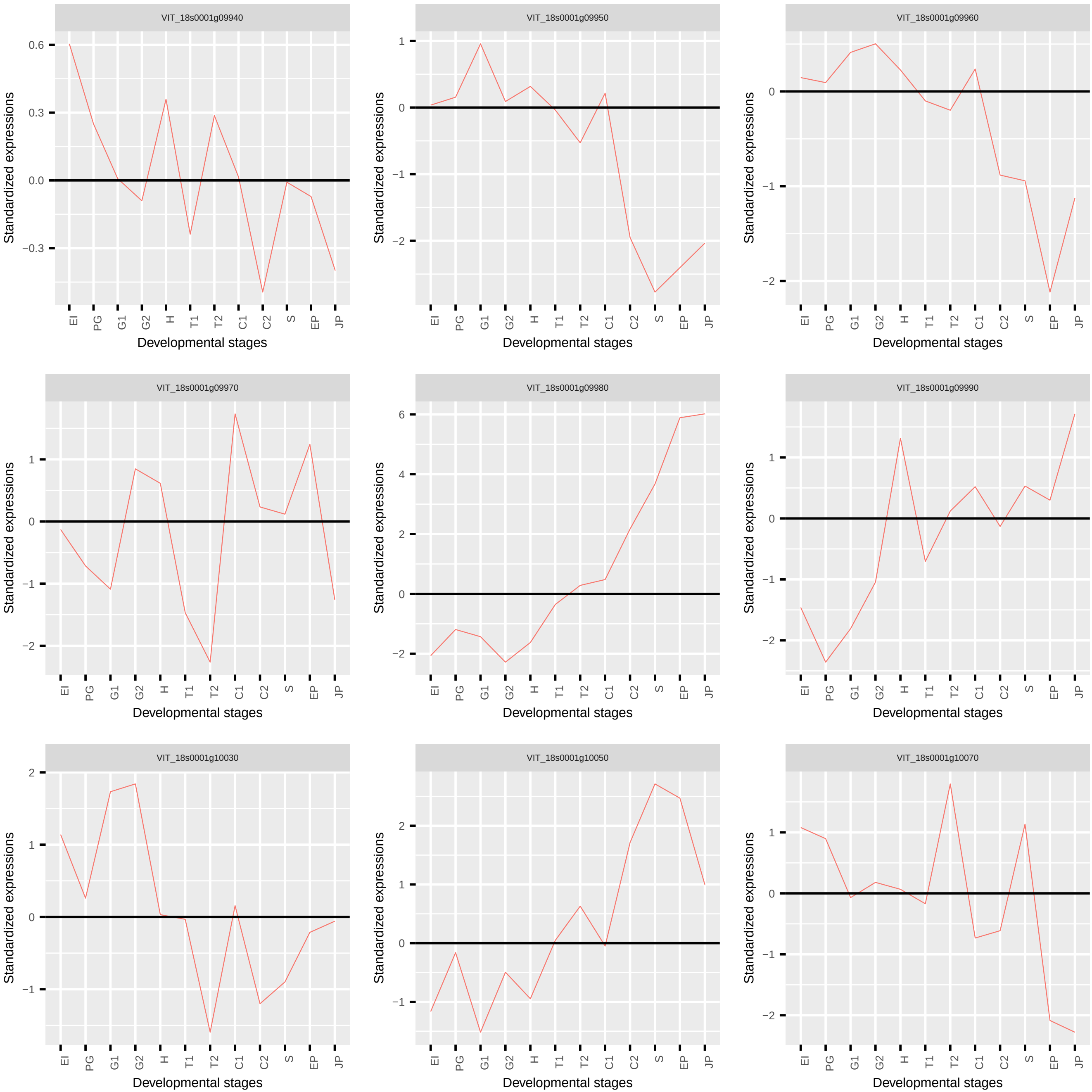

VIT_18s0001g09940
VIT_18s0001g09950
VIT_18s0001g09960
VIT_18s0001g09970
VIT_18s0001g09980
VIT_18s0001g09990
VIT_18s0001g10030
VIT_18s0001g10050
VIT_18s0001g10070
Standardized expressions
Developmental stages
EI PG G1 G2 H T1 T2 C1 C2 S EP JP
0.6
0.3
0.0
−0.3
1
0
−1
−2
6
4
2

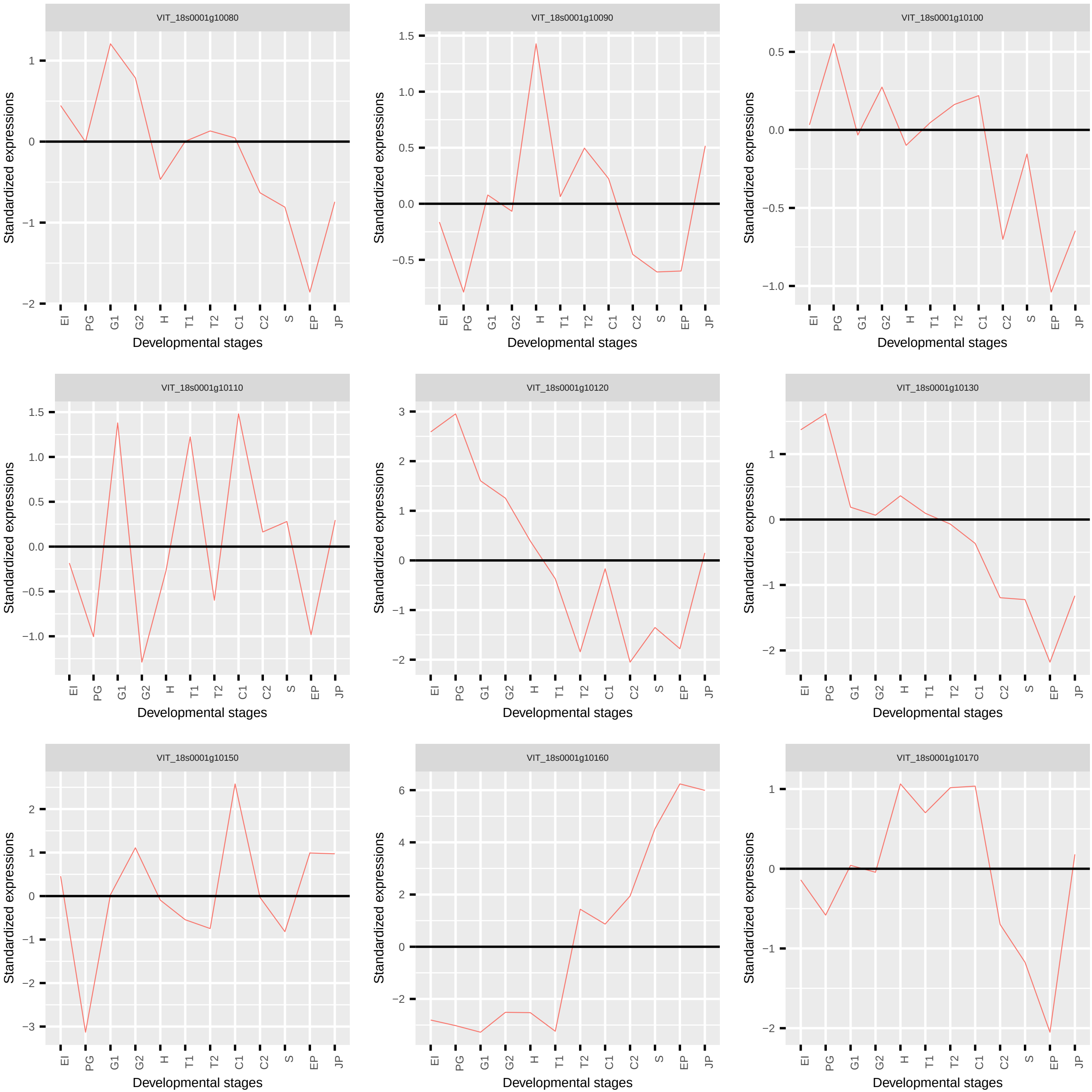
VIT_18s0001g10080
VIT_18s0001g10090
VIT_18s0001g10100
VIT_18s0001g10110
VIT_18s0001g10120
VIT_18s0001g10130
VIT_18s0001g10150
VIT_18s0001g10160
VIT_18s0001g10170
Standardized expressions
Developmental stages
EI PG G1 G2 H T1 T2 C1 C2 S EP JP

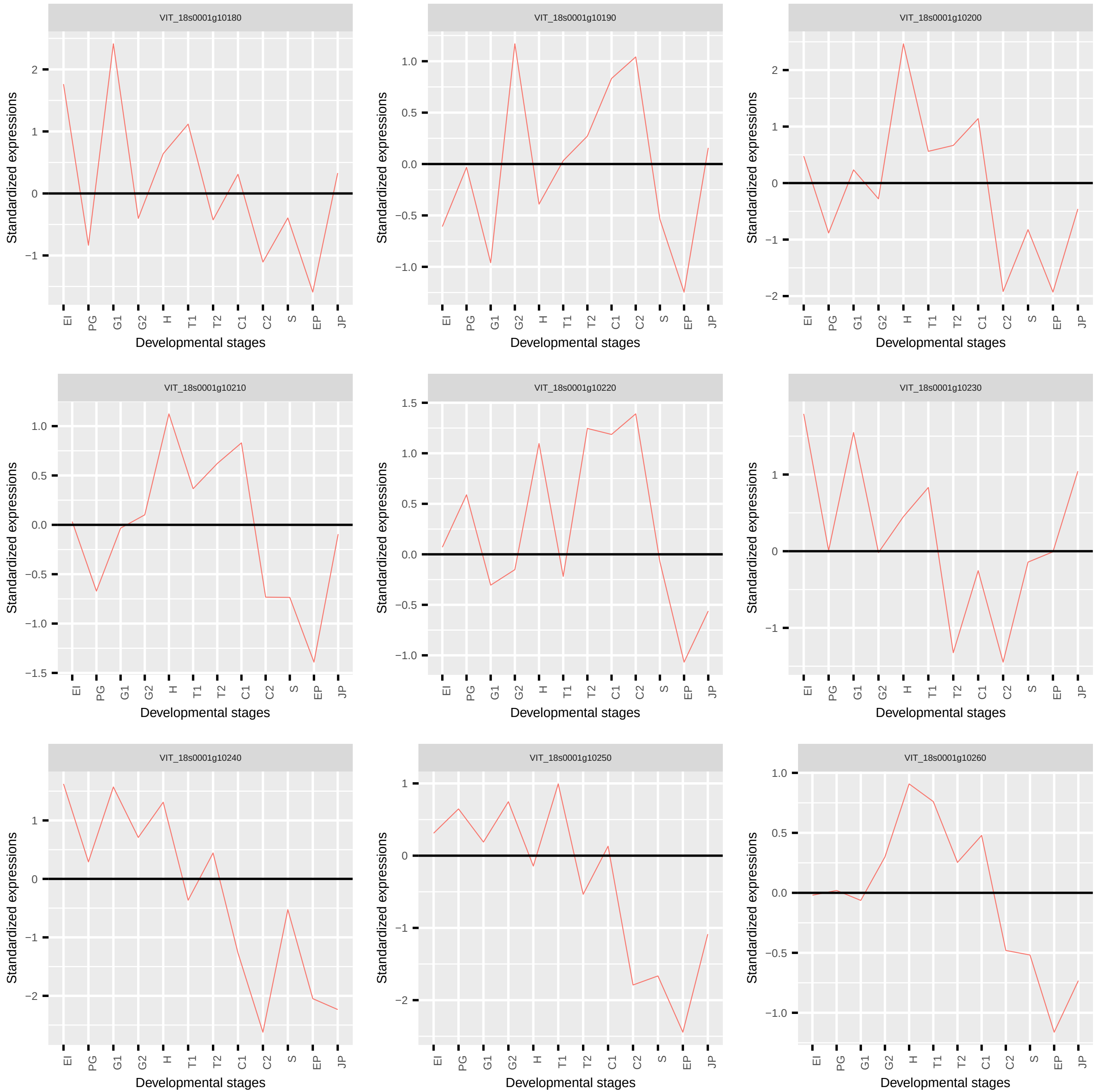
VIT_18s0001g10180
VIT_18s0001g10190
VIT_18s0001g10200
VIT_18s0001g10210
VIT_18s0001g10220
VIT_18s0001g10230
VIT_18s0001g10240
VIT_18s0001g10250
VIT_18s0001g10260
Standardized expressions
Developmental stages
EI PG G1 G2 H T1 T2 C1 C2 S EP JP

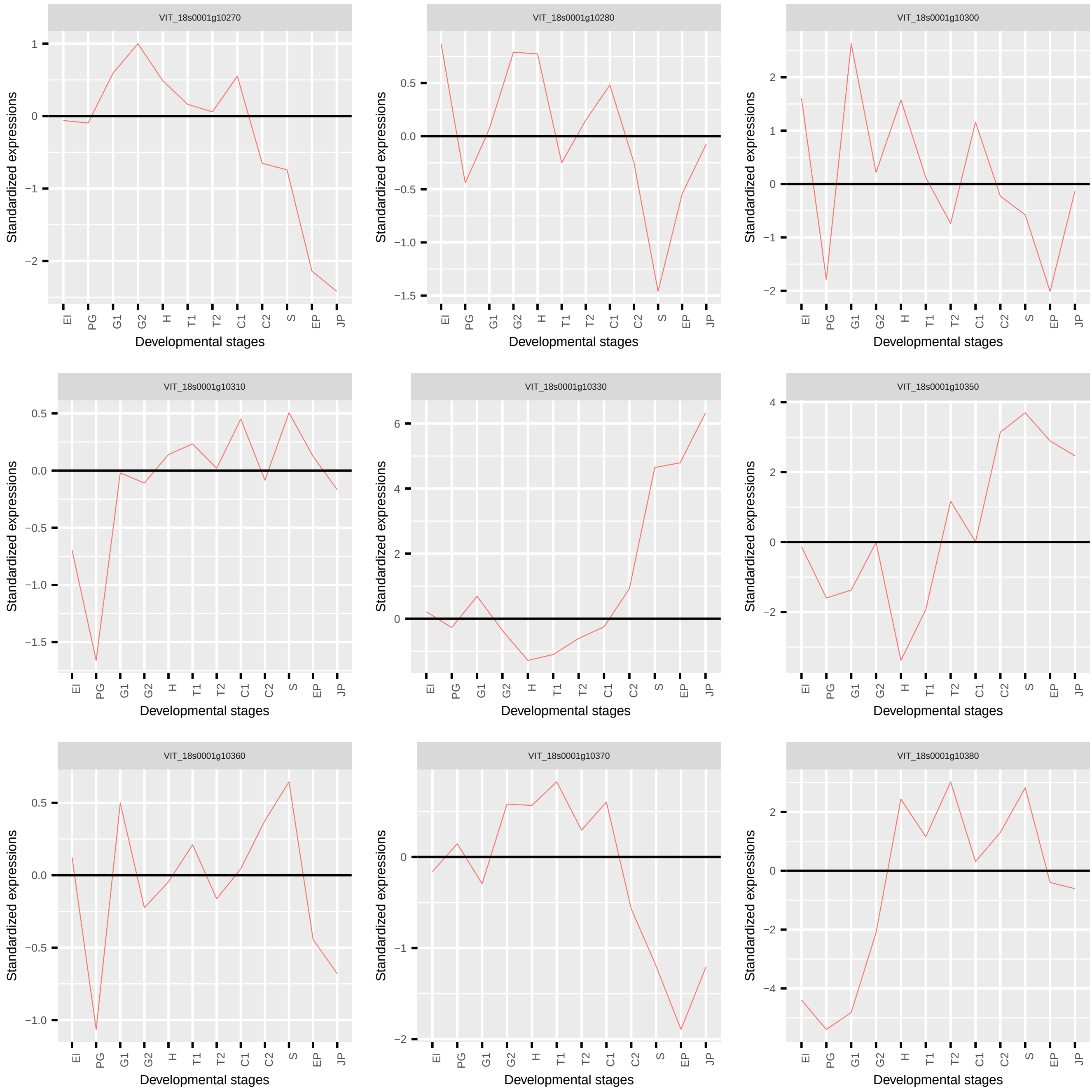
VIT_18s0001g10270
VIT_18s0001g10280
VIT_18s0001g10300
VIT_18s0001g10310
VIT_18s0001g10330
VIT_18s0001g10350
VIT_18s0001g10360
VIT_18s0001g10370
VIT_18s0001g10380
Standardized expressions
Developmental stages
EI PG G1 G2 H T1 T2 C1 C2 S EP JP

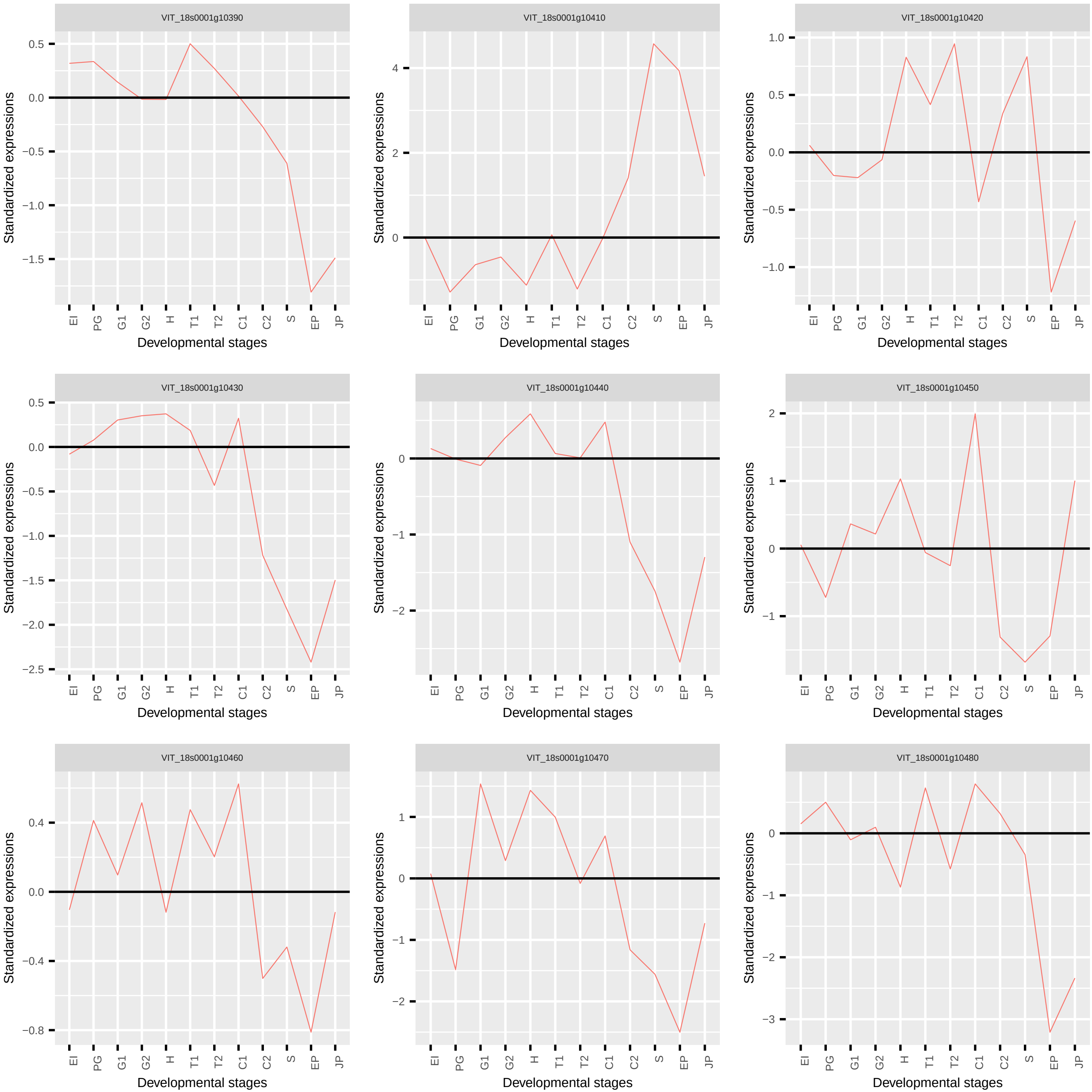

VIT_18s0001g10390
VIT_18s0001g10410
VIT_18s0001g10420
VIT_18s0001g10430
VIT_18s0001g10440
VIT_18s0001g10450
VIT_18s0001g10460
VIT_18s0001g10470
VIT_18s0001g10480
Standardized expressions
Developmental stages
EI PG G1 G2 H T1 T2 C1 C2 S EP JP

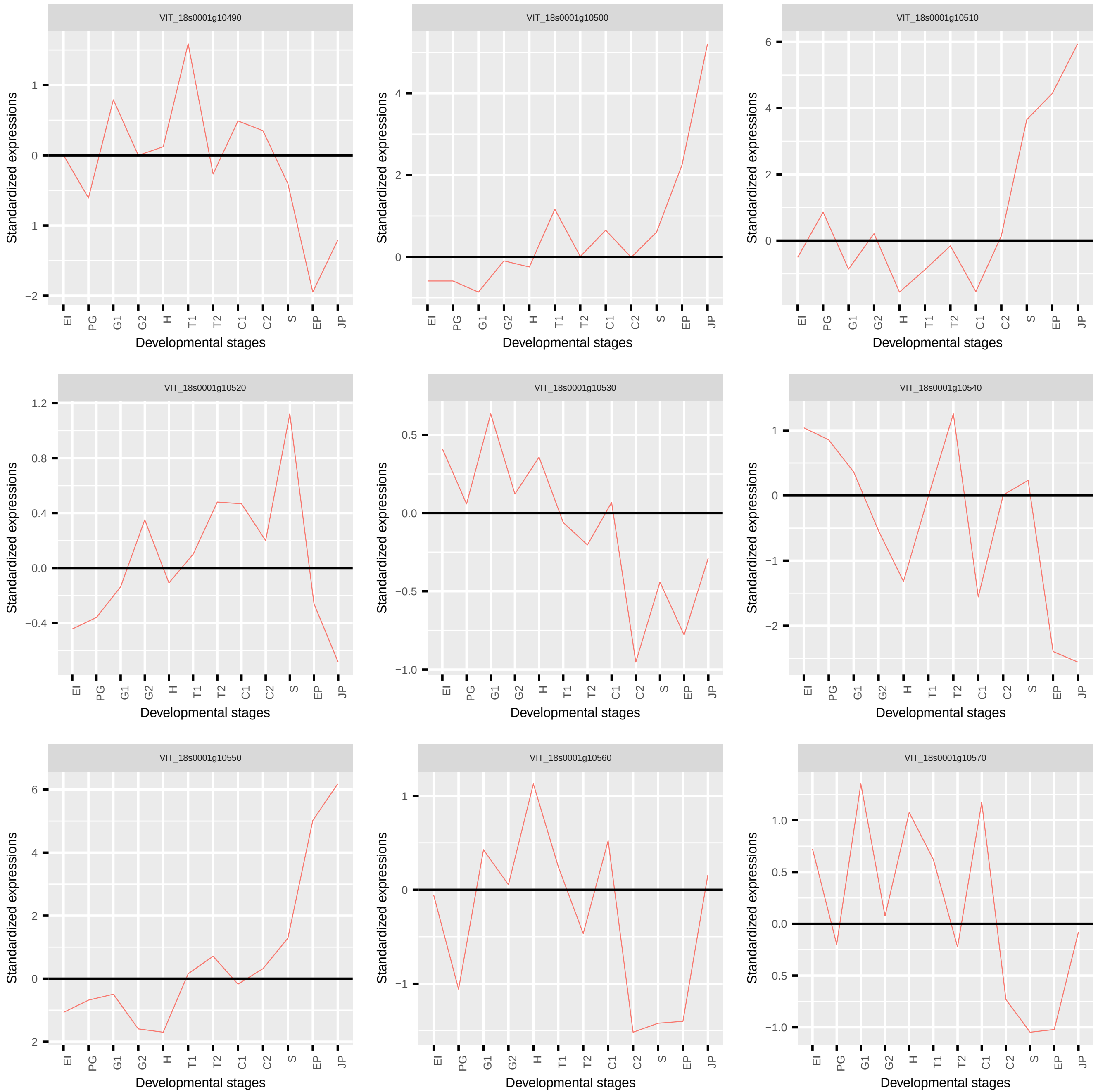

VIT_18s0001g10490
VIT_18s0001g10500
VIT_18s0001g10510
VIT_18s0001g10520
VIT_18s0001g10530
VIT_18s0001g10540
VIT_18s0001g10550
VIT_18s0001g10560
VIT_18s0001g10570
Standardized expressions
Developmental stages
EI PG G1 G2 H T1 T2 C1 C2 S EP JP

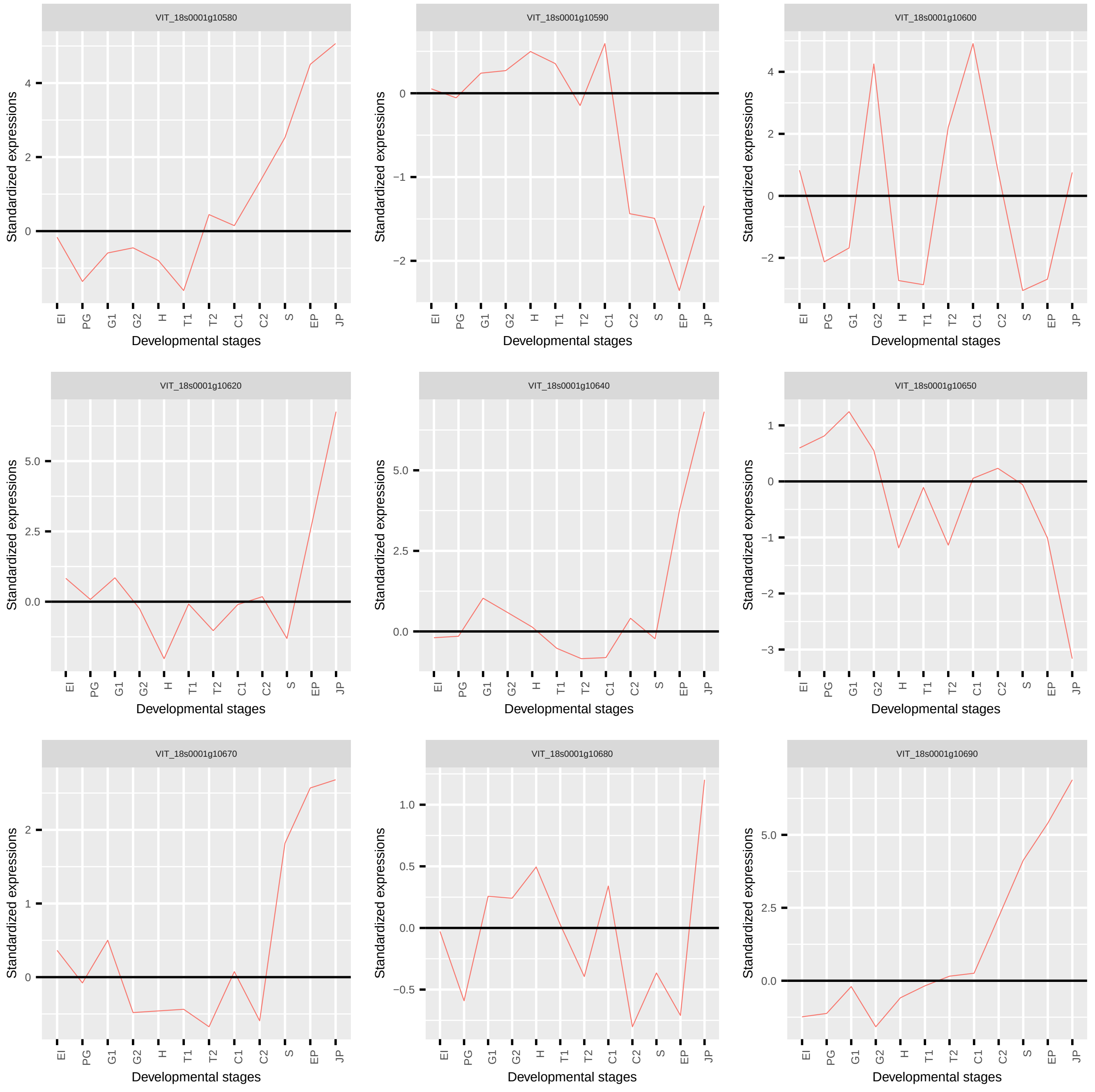

VIT_18s0001g10580
Standardized expressions
4
2
0
EI
PG
G1
G2
H
T1
T2
C1
C2
S
EP
JP
Developmental stages
VIT_18s0001g10590
Standardized expressions
0
−1
−2
EI
PG
G1
G2
H
T1
T2
C1
C2
S
EP
JP
Developmental stages
VIT_18s0001g10600
Standardized expressions
4
2
0
−2
EI
PG
G1
G2
H
T1
T2
C1
C2
S
EP
JP
Developmental stages
VIT_18s0001g10620
Standardized expressions
5.0
2.5
0.0
EI
PG
G1
G2
H
T1
T2
C1
C2
S
EP
JP
Developmental stages
VIT_18s0001g10640
Standardized expressions
5.0
2.5
0.0
EI
PG
G1
G2
H
T1
T2
C1
C2
S
EP
JP
Developmental stages
VIT_18s0001g10650
Standardized expressions
1
0
−1
−2
−3
EI
PG
G1
G2
H
T1
T2
C1
C2
S
EP
JP
Developmental stages
VIT_18s0001g10670
Standardized expressions
2
1
0
EI
PG
G1
G2
H
T1
T2
C1
C2
S
EP
JP
Developmental stages
VIT_18s0001g10680
Standardized expressions
1.0
0.5
0.0
−0.5
EI
PG
G1
G2
H
T1
T2
C1
C2
S
EP
JP
Developmental stages
VIT_18s0001g10690
Standardized expressions
5.0
2.5
0.0
EI
PG
G1
G2
H
T1
T2
C1
C2
S
EP
JP
Developmental stages

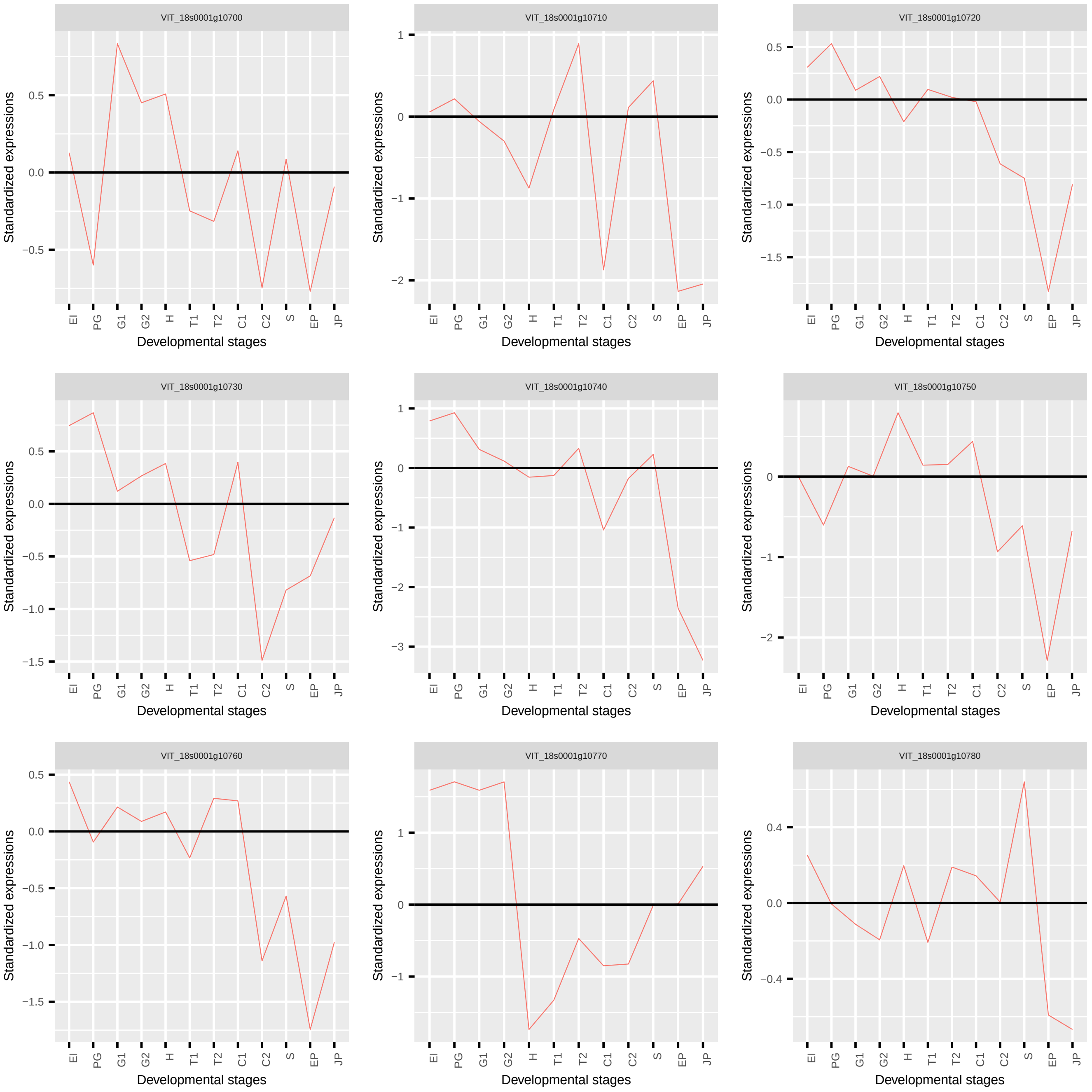

VIT_18s0001g10700
VIT_18s0001g10710
VIT_18s0001g10720
VIT_18s0001g10730
VIT_18s0001g10740
VIT_18s0001g10750
VIT_18s0001g10760
VIT_18s0001g10770
VIT_18s0001g10780
Standardized expressions
Developmental stages
EI PG G1 G2 H T1 T2 C1 C2 S EP JP
0.5
0.0
−0.5
−1.0
−1.5
1
0
−1
−2
−3
0.4
−0.4

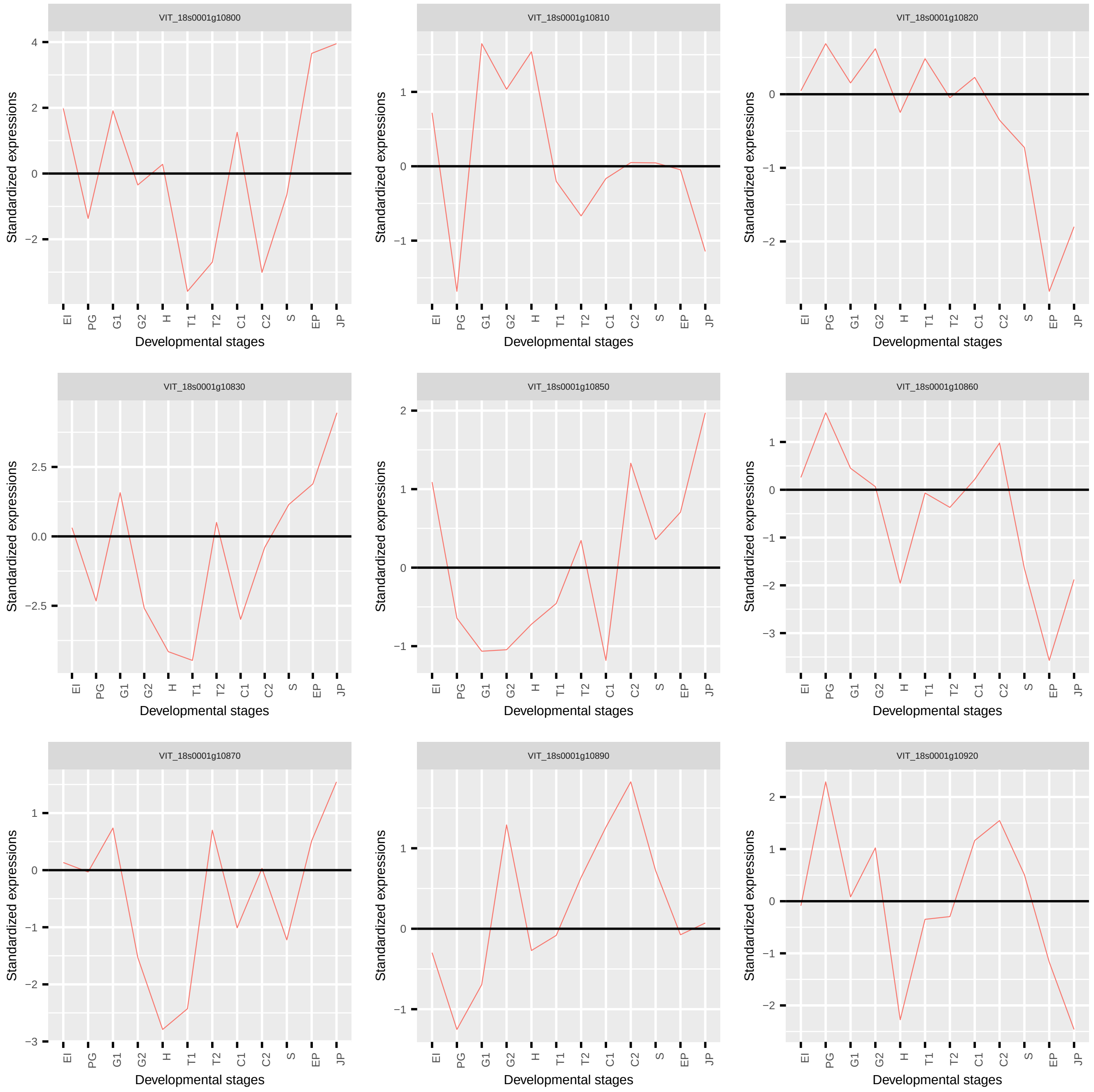

VIT_18s0001g10800
VIT_18s0001g10810
VIT_18s0001g10820
VIT_18s0001g10830
VIT_18s0001g10850
VIT_18s0001g10860
VIT_18s0001g10870
VIT_18s0001g10890
VIT_18s0001g10920
Standardized expressions
Developmental stages
EI PG G1 G2 H T1 T2 C1 C2 S EP JP

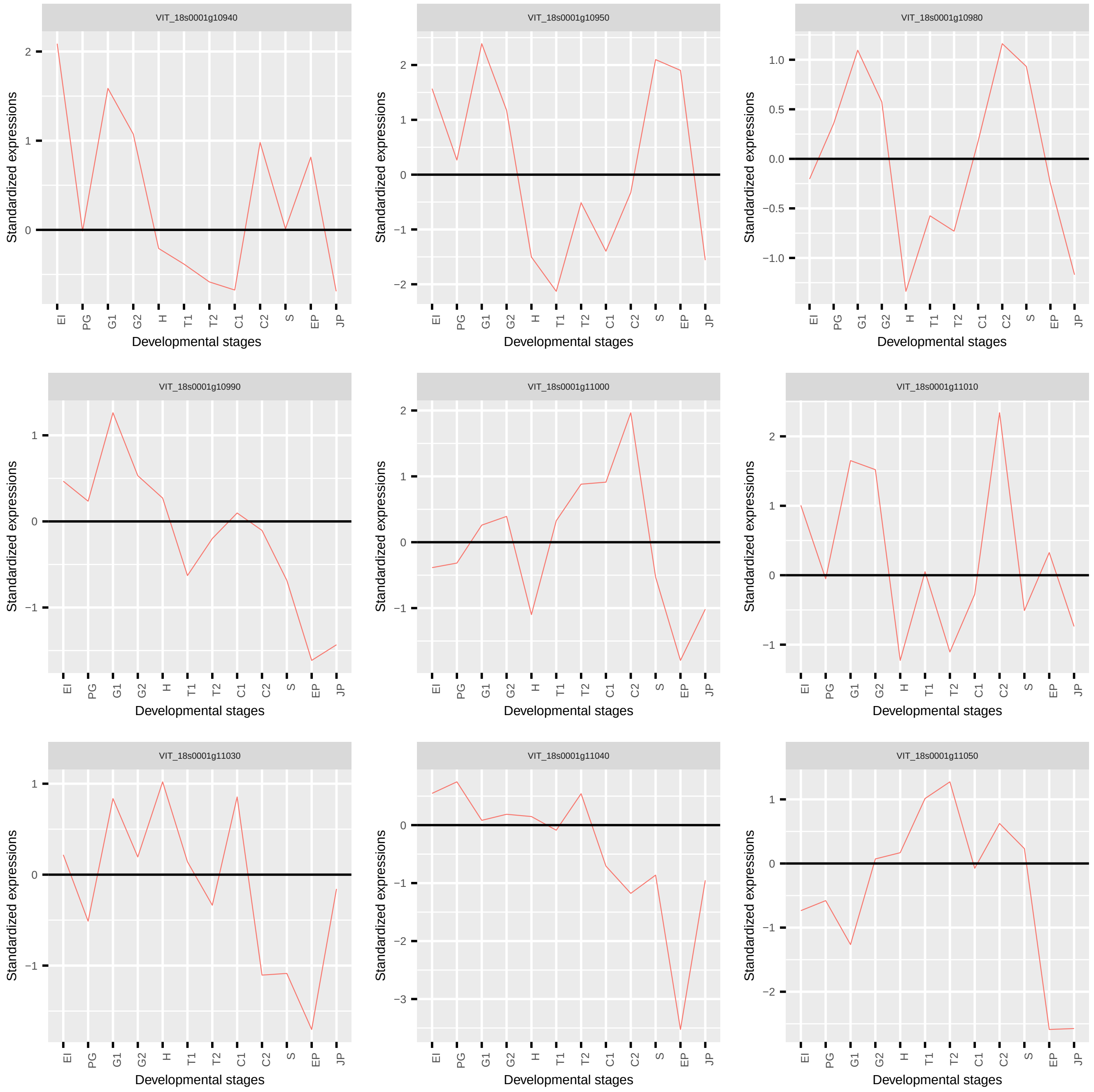
VIT_18s0001g10940
VIT_18s0001g10950
VIT_18s0001g10980
VIT_18s0001g10990
VIT_18s0001g11000
VIT_18s0001g11010
VIT_18s0001g11030
VIT_18s0001g11040
VIT_18s0001g11050
Standardized expressions
Developmental stages
EI
PG
G1
G2
H
T1
T2
C1
C2
S
EP
JP

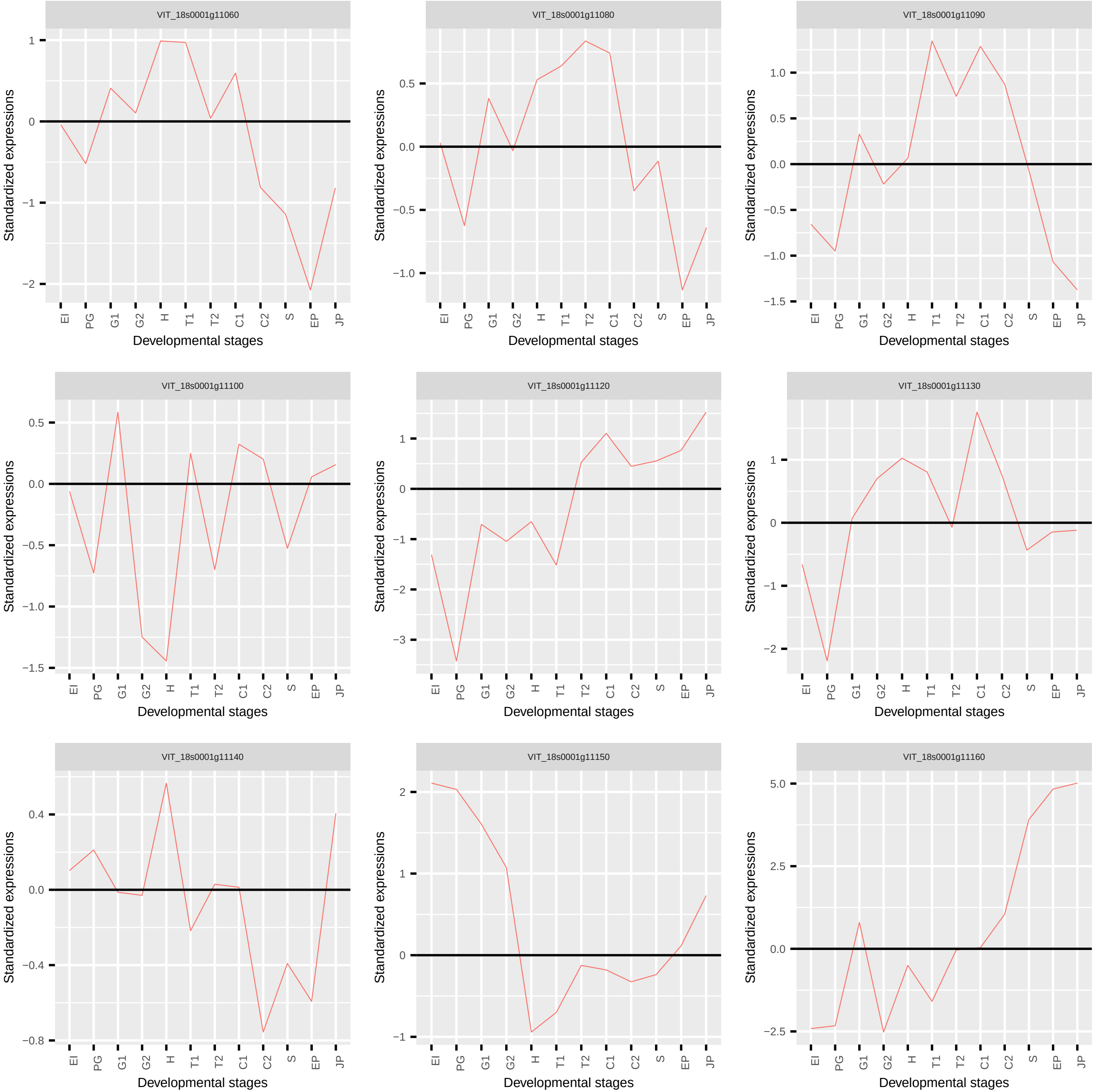
VIT_18s0001g11060
1
0
−1
−2
Standardized expressions
EI PG G1 G2 H T1 T2 C1 C2 S EP JP
Developmental stages
VIT_18s0001g11080
0.5
0.0
−0.5
−1.0
Standardized expressions
EI PG G1 G2 H T1 T2 C1 C2 S EP JP
Developmental stages
VIT_18s0001g11090
1.0
0.5
0.0
−0.5
−1.0
−1.5
Standardized expressions
EI PG G1 G2 H T1 T2 C1 C2 S EP JP
Developmental stages
VIT_18s0001g11100
0.5
0.0
−0.5
−1.0
−1.5
Standardized expressions
EI PG G1 G2 H T1 T2 C1 C2 S EP JP
Developmental stages
VIT_18s0001g11120
1
0
−1
−2
−3
Standardized expressions
EI PG G1 G2 H T1 T2 C1 C2 S EP JP
Developmental stages
VIT_18s0001g11130
1
0
−1
−2
Standardized expressions
EI PG G1 G2 H T1 T2 C1 C2 S EP JP
Developmental stages
VIT_18s0001g11140
0.4
0.0
−0.4
−0.8
Standardized expressions
EI PG G1 G2 H T1 T2 C1 C2 S EP JP
Developmental stages
VIT_18s0001g11150
2
1
0
−1
Standardized expressions
EI PG G1 G2 H T1 T2 C1 C2 S EP JP
Developmental stages
VIT_18s0001g11160
5.0
2.5
0.0
−2.5
Standardized expressions
EI PG G1 G2 H T1 T2 C1 C2 S EP JP
Developmental stages

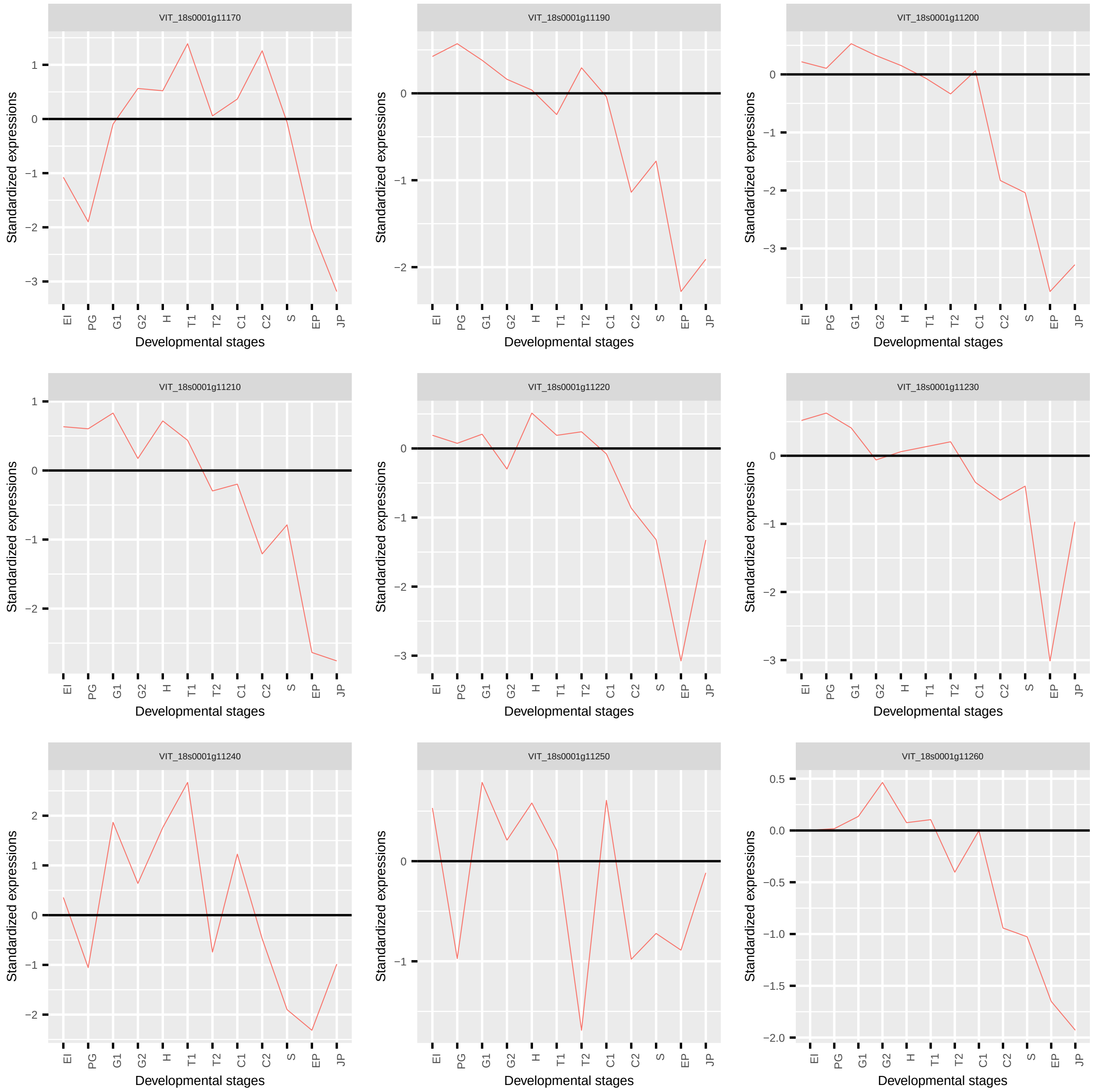
VIT_18s0001g11170
VIT_18s0001g11190
VIT_18s0001g11200
VIT_18s0001g11210
VIT_18s0001g11220
VIT_18s0001g11230
VIT_18s0001g11240
VIT_18s0001g11250
VIT_18s0001g11260
Standardized expressions
Developmental stages
EI PG G1 G2 H T1 T2 C1 C2 S EP JP

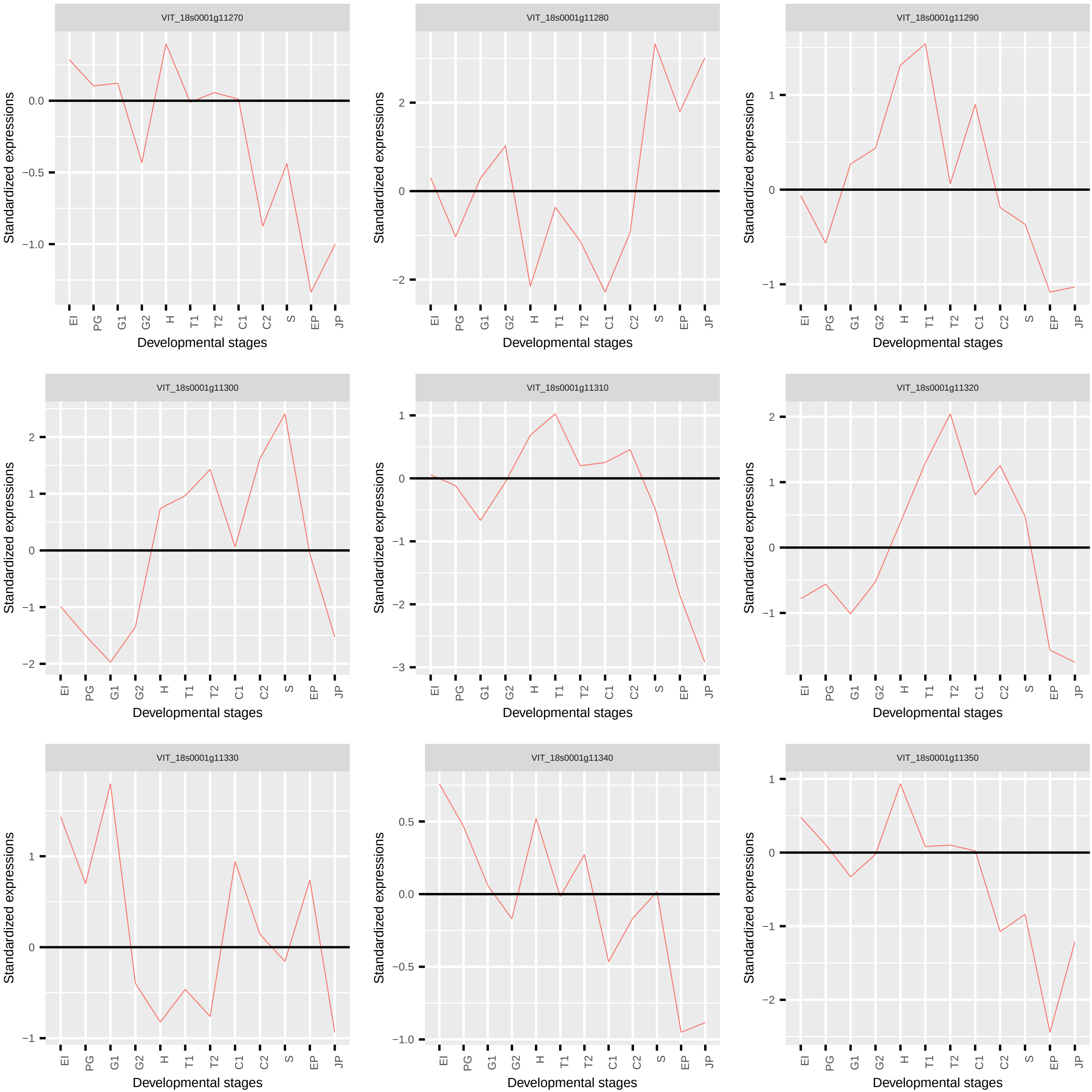
VIT_18s0001g11270
VIT_18s0001g11280
VIT_18s0001g11290
VIT_18s0001g11300
VIT_18s0001g11310
VIT_18s0001g11320
VIT_18s0001g11330
VIT_18s0001g11340
VIT_18s0001g11350
Standardized expressions
Developmental stages
EI PG G1 G2 H T1 T2 C1 C2 S EP JP
0.0 −0.5 −1.0
2 0 −2
1 0 −1
2 1 0 −1 −2
1 0 −1 −2 −3
2 1 0 −1
1 0 −1
0.5 0.0 −0.5 −1.0
1 0 −1 −2

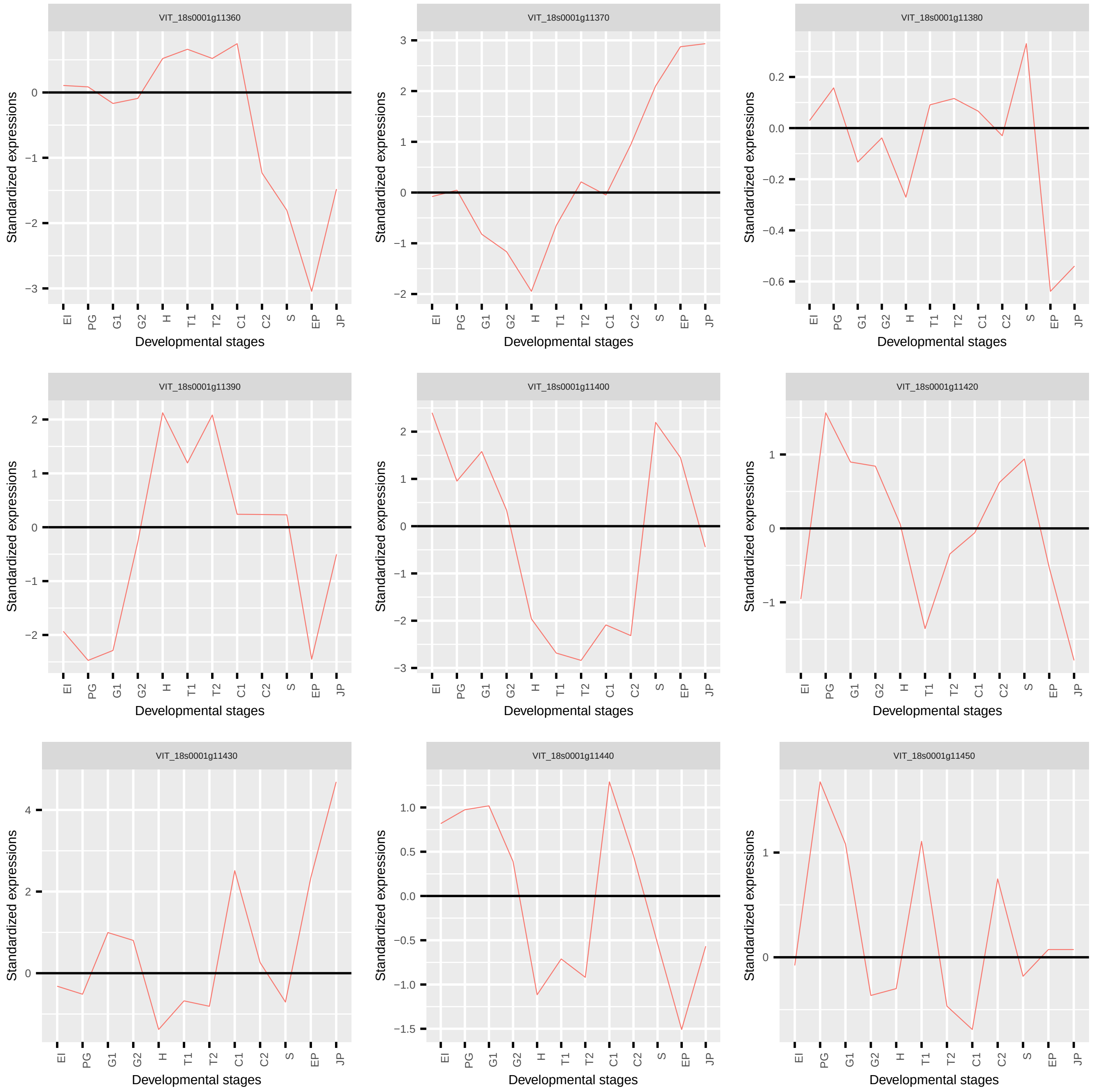

VIT_18s0001g11360
VIT_18s0001g11370
VIT_18s0001g11380
VIT_18s0001g11390
VIT_18s0001g11400
VIT_18s0001g11420
VIT_18s0001g11430
VIT_18s0001g11440
VIT_18s0001g11450
Standardized expressions
Developmental stages
EI PG G1 G2 H T1 T2 C1 C2 S EP JP

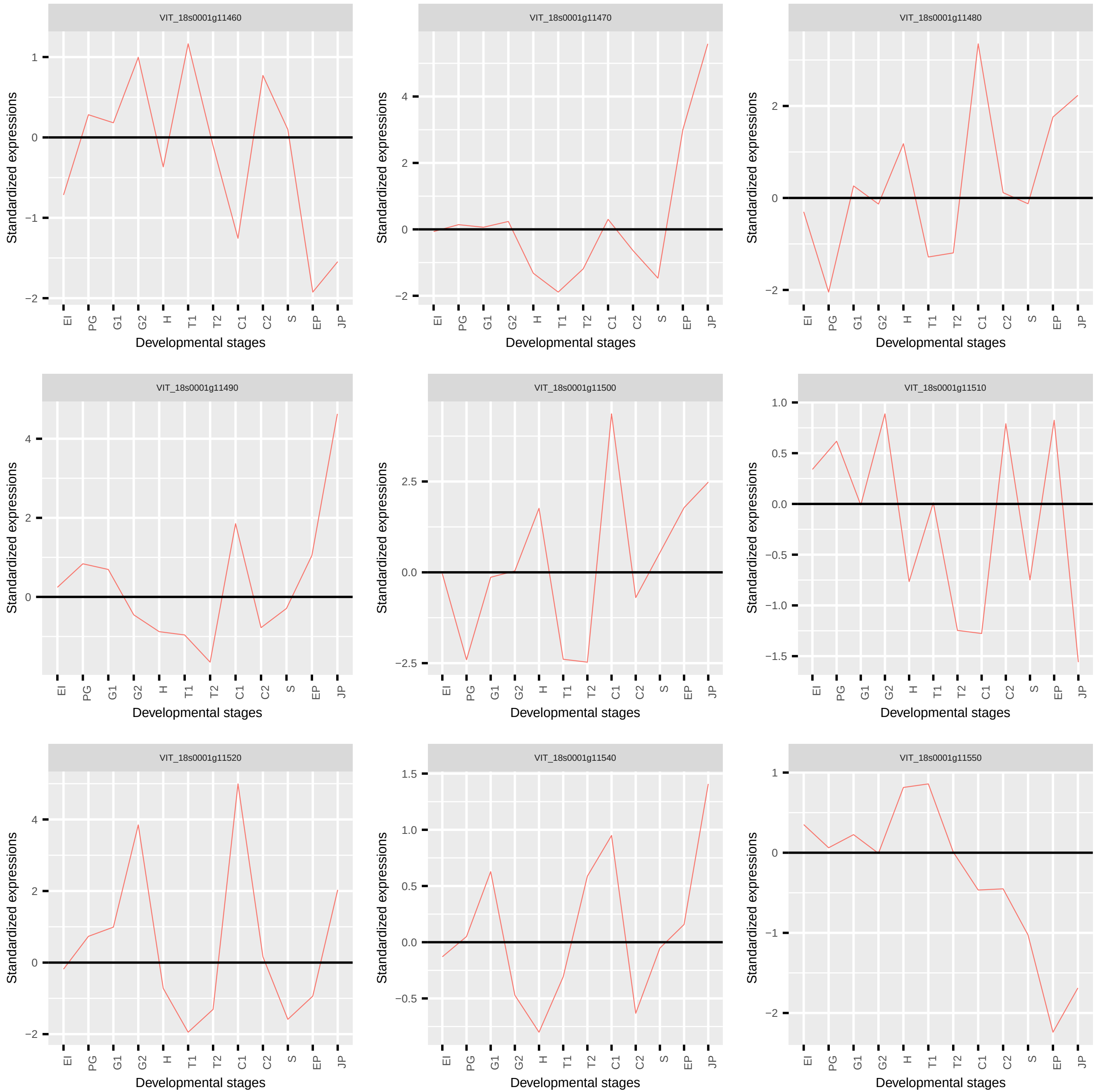
VIT_18s0001g11460
VIT_18s0001g11470
VIT_18s0001g11480
VIT_18s0001g11490
VIT_18s0001g11500
VIT_18s0001g11510
VIT_18s0001g11520
VIT_18s0001g11540
VIT_18s0001g11550
Standardized expressions
Developmental stages
EI PG G1 G2 H T1 T2 C1 C2 S EP JP

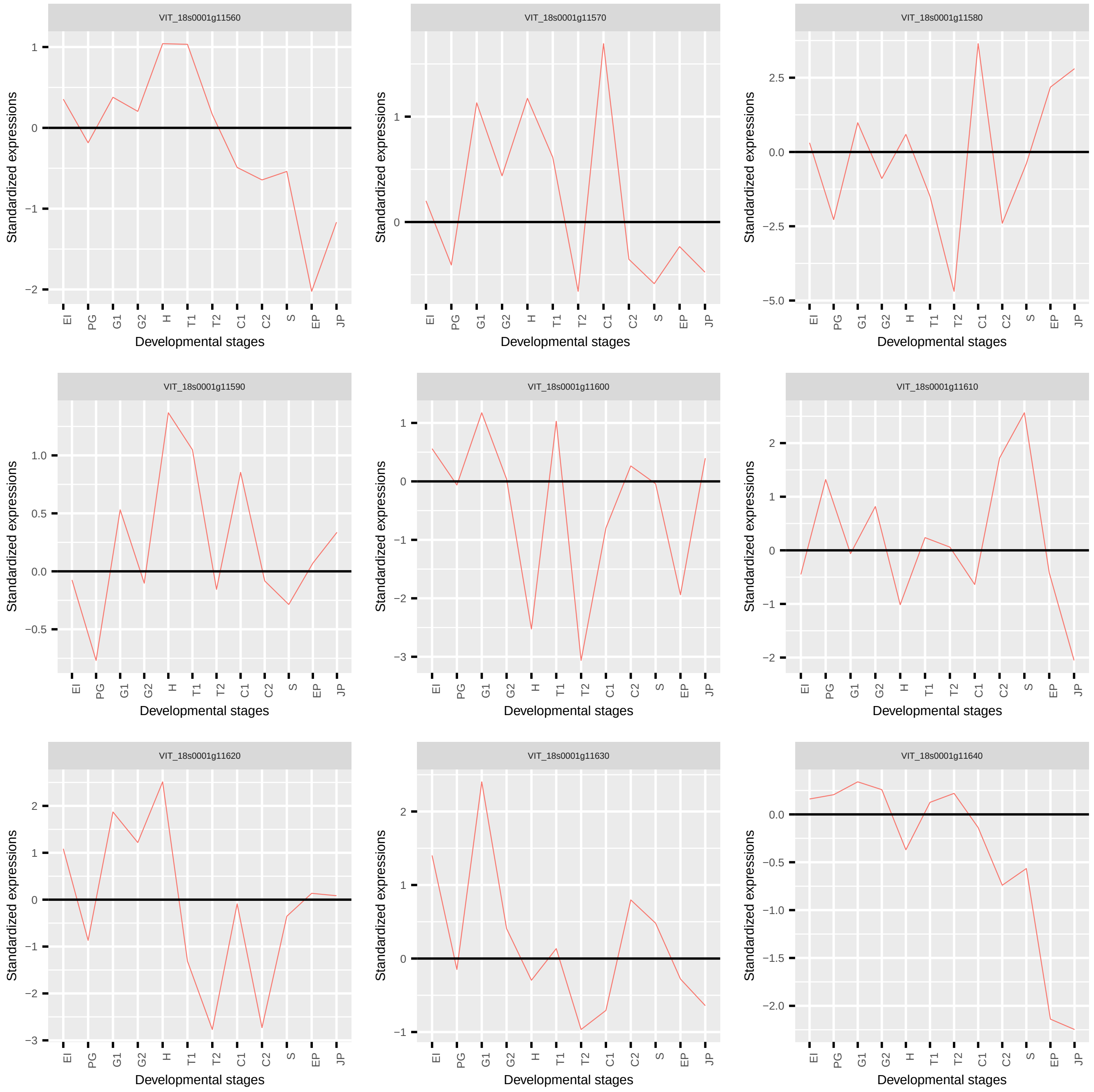

VIT_18s0001g11560
Standardized expressions
1
0
−1
−2
VIT_18s0001g11570
Standardized expressions
1
0
VIT_18s0001g11580
Standardized expressions
2.5
0.0
−2.5
−5.0
VIT_18s0001g11590
Standardized expressions
1.0
0.5
0.0
−0.5
VIT_18s0001g11600
Standardized expressions
1
0
−1
−2
−3
VIT_18s0001g11610
Standardized expressions
2
1
0
−1
−2
VIT_18s0001g11620
Standardized expressions
2
1
0
−1
−2
−3
VIT_18s0001g11630
Standardized expressions
2
1
0
−1
VIT_18s0001g11640
Standardized expressions
0.0
−0.5
−1.0
−1.5
−2.0
EI PG G1 G2 H T1 T2 C1 C2 S EP JP
Developmental stages

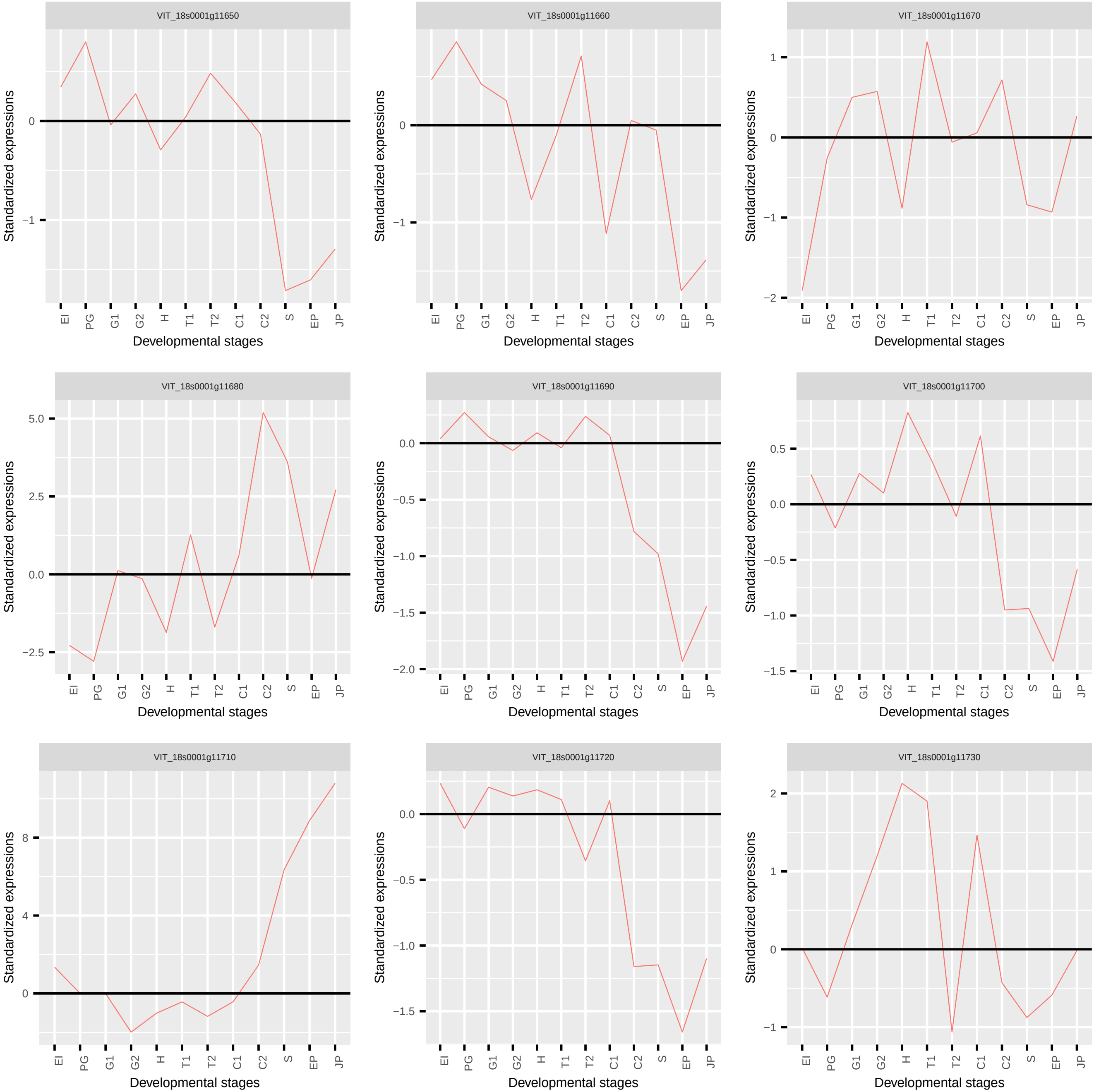

VIT_18s0001g11650
Standardized expressions
0
−1
EI
PG
G1
G2
H
T1
T2
C1
C2
S
EP
JP
Developmental stages
VIT_18s0001g11660
Standardized expressions
0
−1
EI
PG
G1
G2
H
T1
T2
C1
C2
S
EP
JP
Developmental stages
VIT_18s0001g11670
Standardized expressions
1
0
−1
−2
EI
PG
G1
G2
H
T1
T2
C1
C2
S
EP
JP
Developmental stages
VIT_18s0001g11680
Standardized expressions
5.0
2.5
0.0
−2.5
EI
PG
G1
G2
H
T1
T2
C1
C2
S
EP
JP
Developmental stages
VIT_18s0001g11690
Standardized expressions
0.0
−0.5
−1.0
−1.5
−2.0
EI
PG
G1
G2
H
T1
T2
C1
C2
S
EP
JP
Developmental stages
VIT_18s0001g11700
Standardized expressions
0.5
0.0
−0.5
−1.0
−1.5
EI
PG
G1
G2
H
T1
T2
C1
C2
S
EP
JP
Developmental stages
VIT_18s0001g11710
Standardized expressions
8
4
0
EI
PG
G1
G2
H
T1
T2
C1
C2
S
EP
JP
Developmental stages
VIT_18s0001g11720
Standardized expressions
0.0
−0.5
−1.0
−1.5
EI
PG
G1
G2
H
T1
T2
C1
C2
S
EP
JP
Developmental stages
VIT_18s0001g11730
Standardized expressions
2
1
0
−1
EI
PG
G1
G2
H
T1
T2
C1
C2
S
EP
JP
Developmental stages

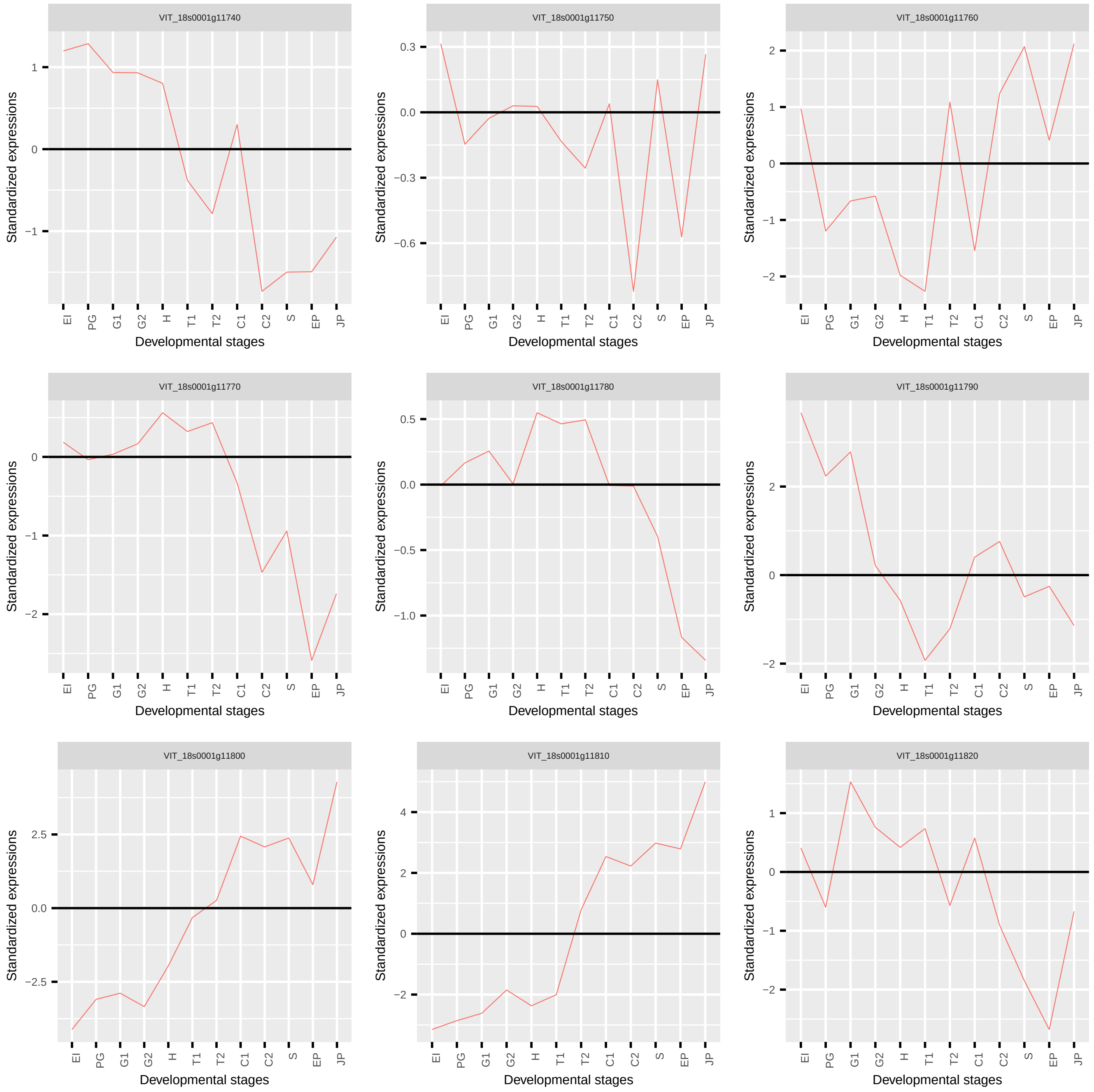
VIT_18s0001g11740
Standardized expressions
1
0
−1
EI PG G1 G2 H T1 T2 C1 C2 S EP JP
Developmental stages
VIT_18s0001g11750
Standardized expressions
0.3
0.0
−0.3
−0.6
EI PG G1 G2 H T1 T2 C1 C2 S EP JP
Developmental stages
VIT_18s0001g11760
Standardized expressions
2
1
0
−1
−2
EI PG G1 G2 H T1 T2 C1 C2 S EP JP
Developmental stages
VIT_18s0001g11770
Standardized expressions
0
−1
−2
EI PG G1 G2 H T1 T2 C1 C2 S EP JP
Developmental stages
VIT_18s0001g11780
Standardized expressions
0.5
0.0
−0.5
−1.0
EI PG G1 G2 H T1 T2 C1 C2 S EP JP
Developmental stages
VIT_18s0001g11790
Standardized expressions
2
0
−2
EI PG G1 G2 H T1 T2 C1 C2 S EP JP
Developmental stages
VIT_18s0001g11800
Standardized expressions
2.5
0.0
−2.5
EI PG G1 G2 H T1 T2 C1 C2 S EP JP
Developmental stages
VIT_18s0001g11810
Standardized expressions
4
2
0
−2
EI PG G1 G2 H T1 T2 C1 C2 S EP JP
Developmental stages
VIT_18s0001g11820
Standardized expressions
1
0
−1
−2
EI PG G1 G2 H T1 T2 C1 C2 S EP JP
Developmental stages

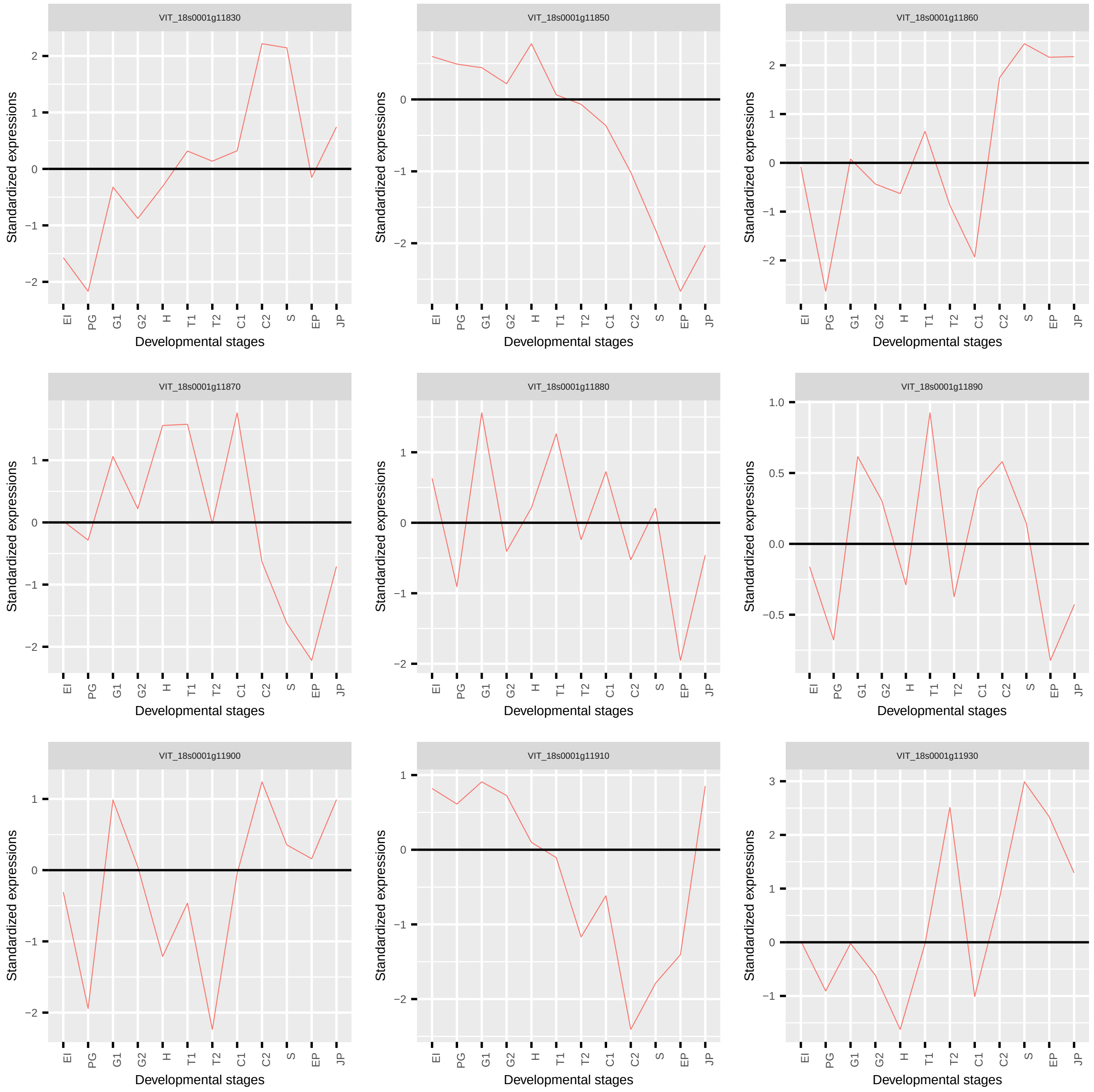
VIT_18s0001g11830
VIT_18s0001g11850
VIT_18s0001g11860
VIT_18s0001g11870
VIT_18s0001g11880
VIT_18s0001g11890
VIT_18s0001g11900
VIT_18s0001g11910
VIT_18s0001g11930
Standardized expressions
Developmental stages
EI PG G1 G2 H T1 T2 C1 C2 S EP JP

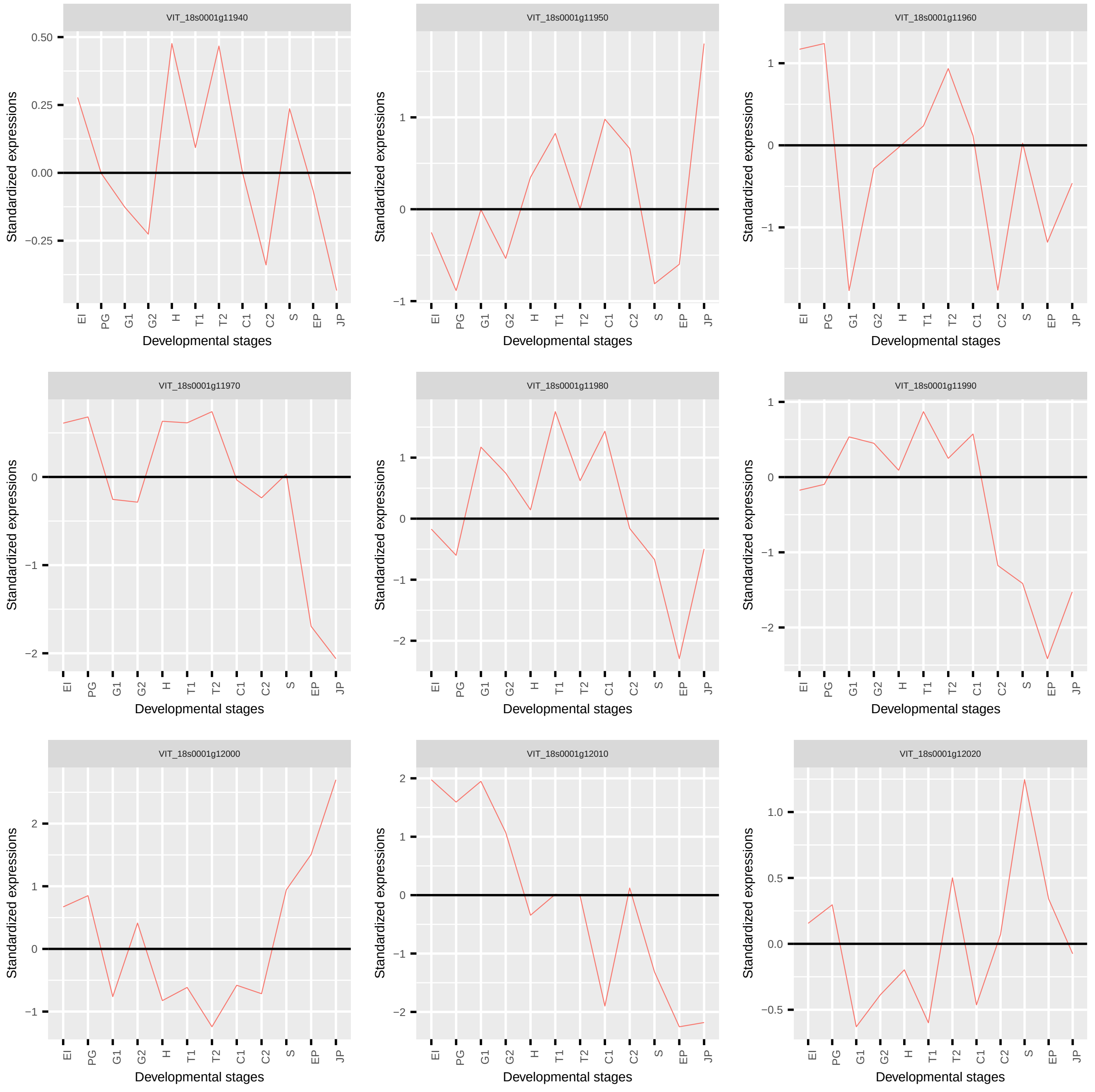
VIT_18s0001g11940
VIT_18s0001g11950
VIT_18s0001g11960
VIT_18s0001g11970
VIT_18s0001g11980
VIT_18s0001g11990
VIT_18s0001g12000
VIT_18s0001g12010
VIT_18s0001g12020
Standardized expressions
Developmental stages
EI PG G1 G2 H T1 T2 C1 C2 S EP JP

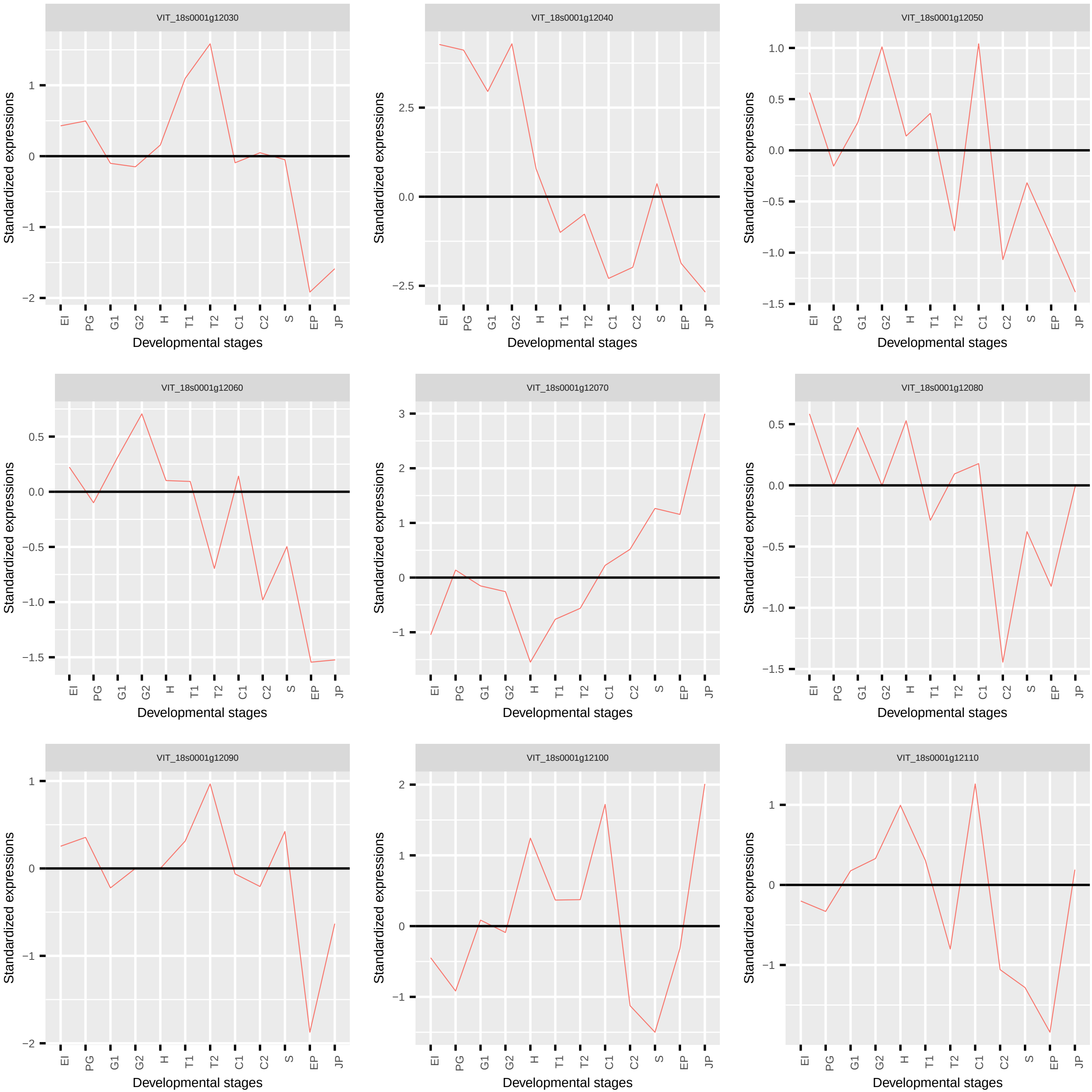
VIT_18s0001g12030
VIT_18s0001g12040
VIT_18s0001g12050
VIT_18s0001g12060
VIT_18s0001g12070
VIT_18s0001g12080
VIT_18s0001g12090
VIT_18s0001g12100
VIT_18s0001g12110
Standardized expressions
Developmental stages
EI PG G1 G2 H T1 T2 C1 C2 S EP JP

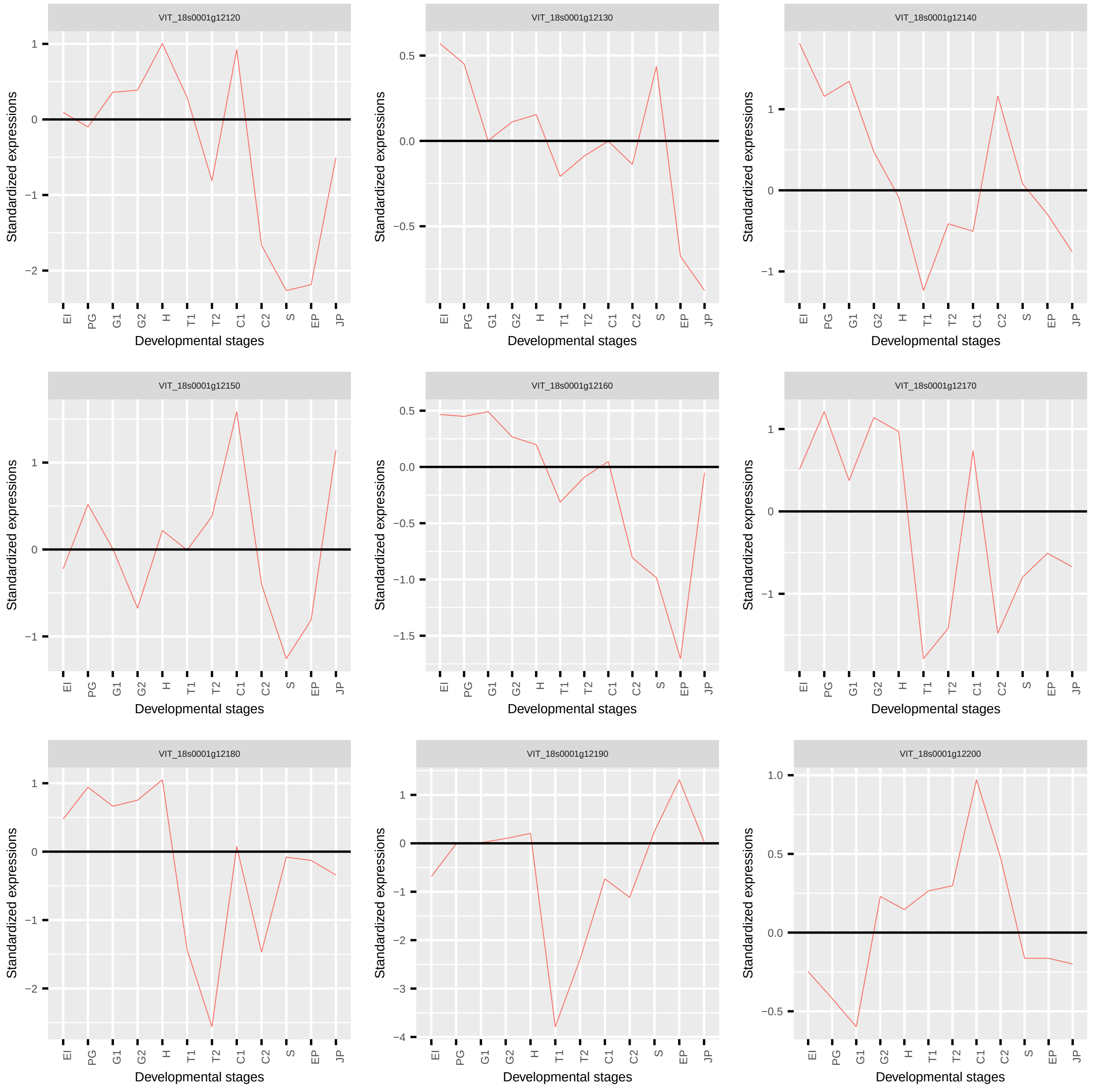
VIT_18s0001g12120
VIT_18s0001g12130
VIT_18s0001g12140
VIT_18s0001g12150
VIT_18s0001g12160
VIT_18s0001g12170
VIT_18s0001g12180
VIT_18s0001g12190
VIT_18s0001g12200
Standardized expressions
Developmental stages
EI PG G1 G2 H T1 T2 C1 C2 S EP JP

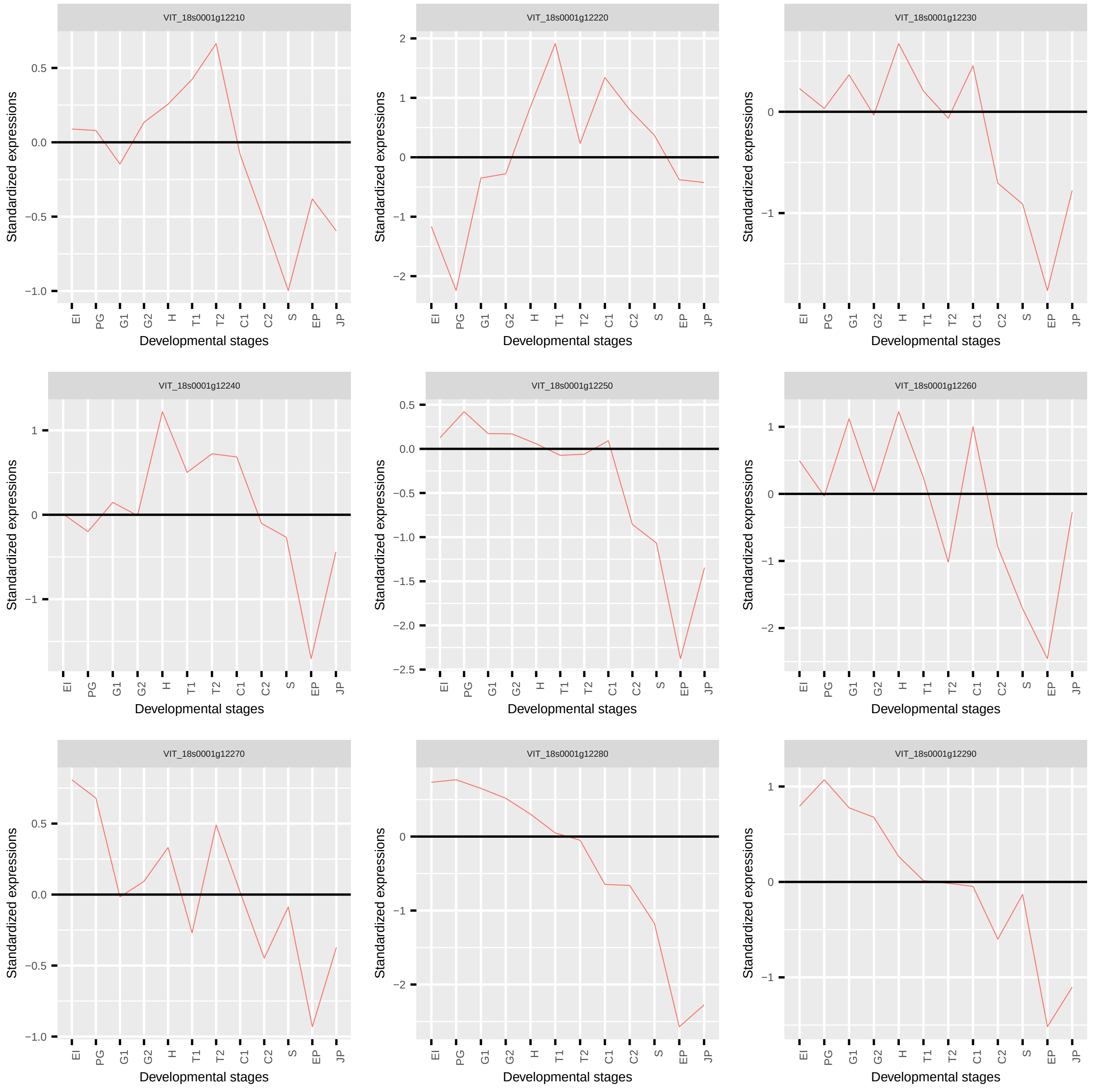
VIT_18s0001g12210
VIT_18s0001g12220
VIT_18s0001g12230
VIT_18s0001g12240
VIT_18s0001g12250
VIT_18s0001g12260
VIT_18s0001g12270
VIT_18s0001g12280
VIT_18s0001g12290
Standardized expressions
Developmental stages
EI PG G1 G2 H T1 T2 C1 C2 S EP JP

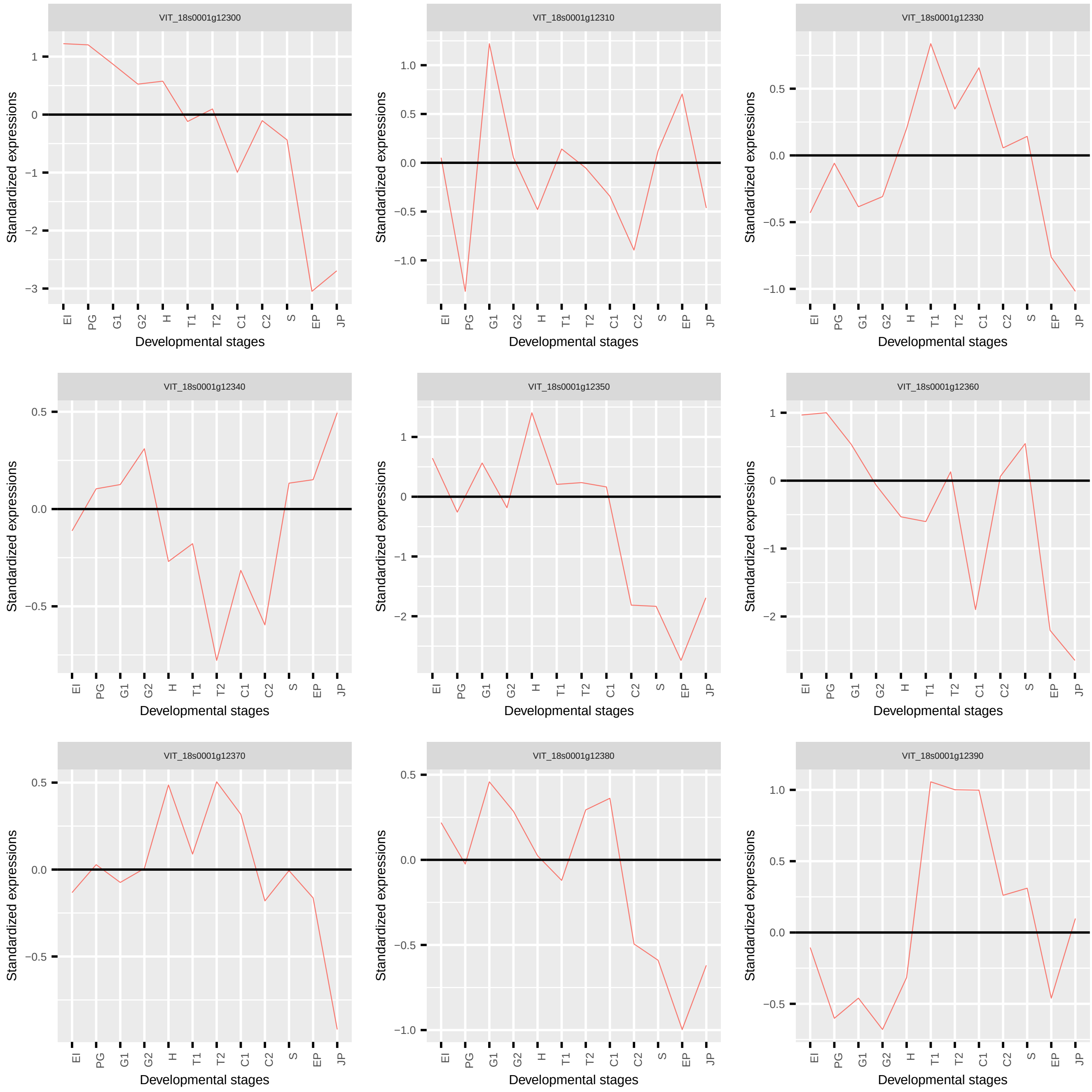
VIT_18s0001g12300
VIT_18s0001g12310
VIT_18s0001g12330
VIT_18s0001g12340
VIT_18s0001g12350
VIT_18s0001g12360
VIT_18s0001g12370
VIT_18s0001g12380
VIT_18s0001g12390
Standardized expressions
Developmental stages
EI PG G1 G2 H T1 T2 C1 C2 S EP JP

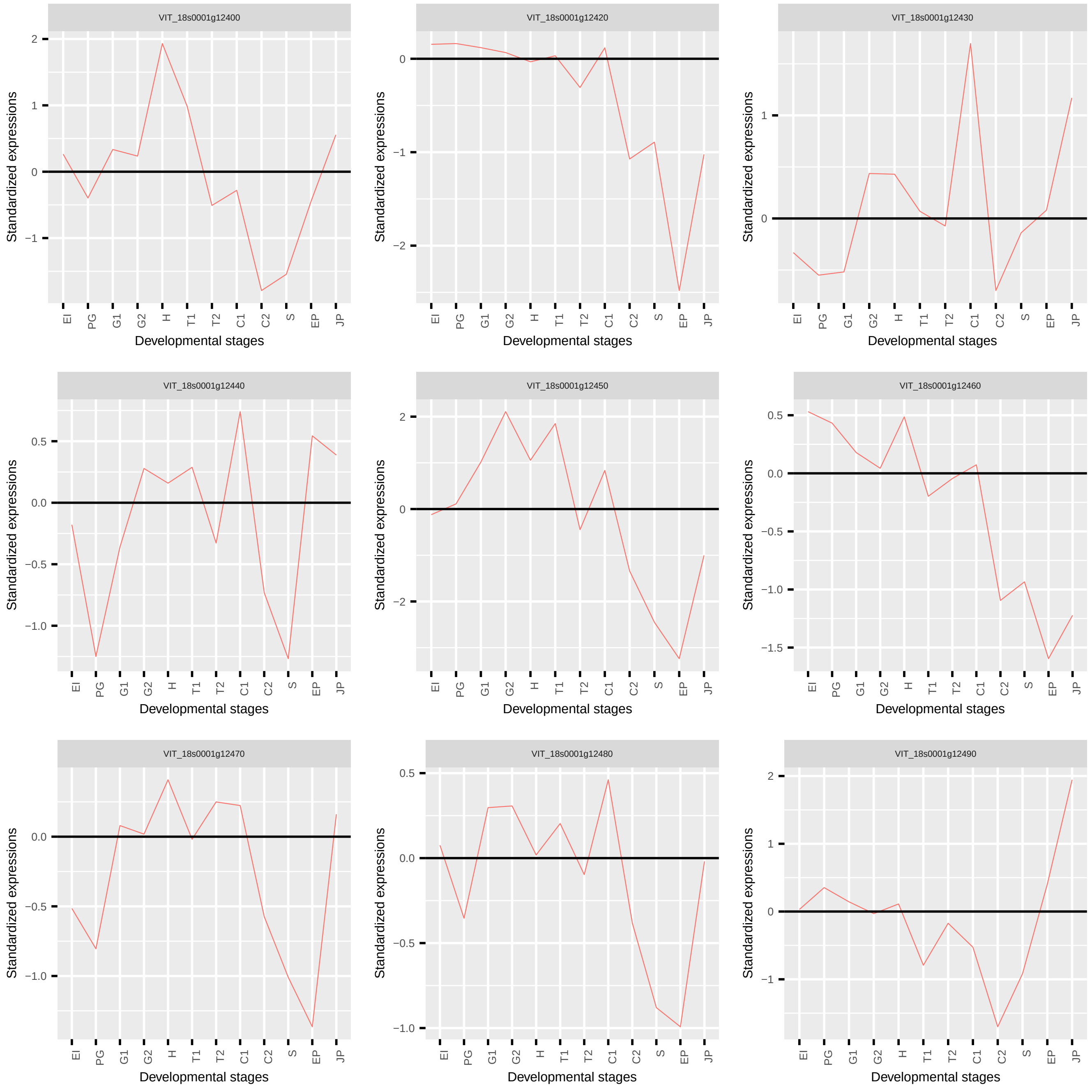
VIT_18s0001g12400
VIT_18s0001g12420
VIT_18s0001g12430
VIT_18s0001g12440
VIT_18s0001g12450
VIT_18s0001g12460
VIT_18s0001g12470
VIT_18s0001g12480
VIT_18s0001g12490
Standardized expressions
Developmental stages
EI PG G1 G2 H T1 T2 C1 C2 S EP JP

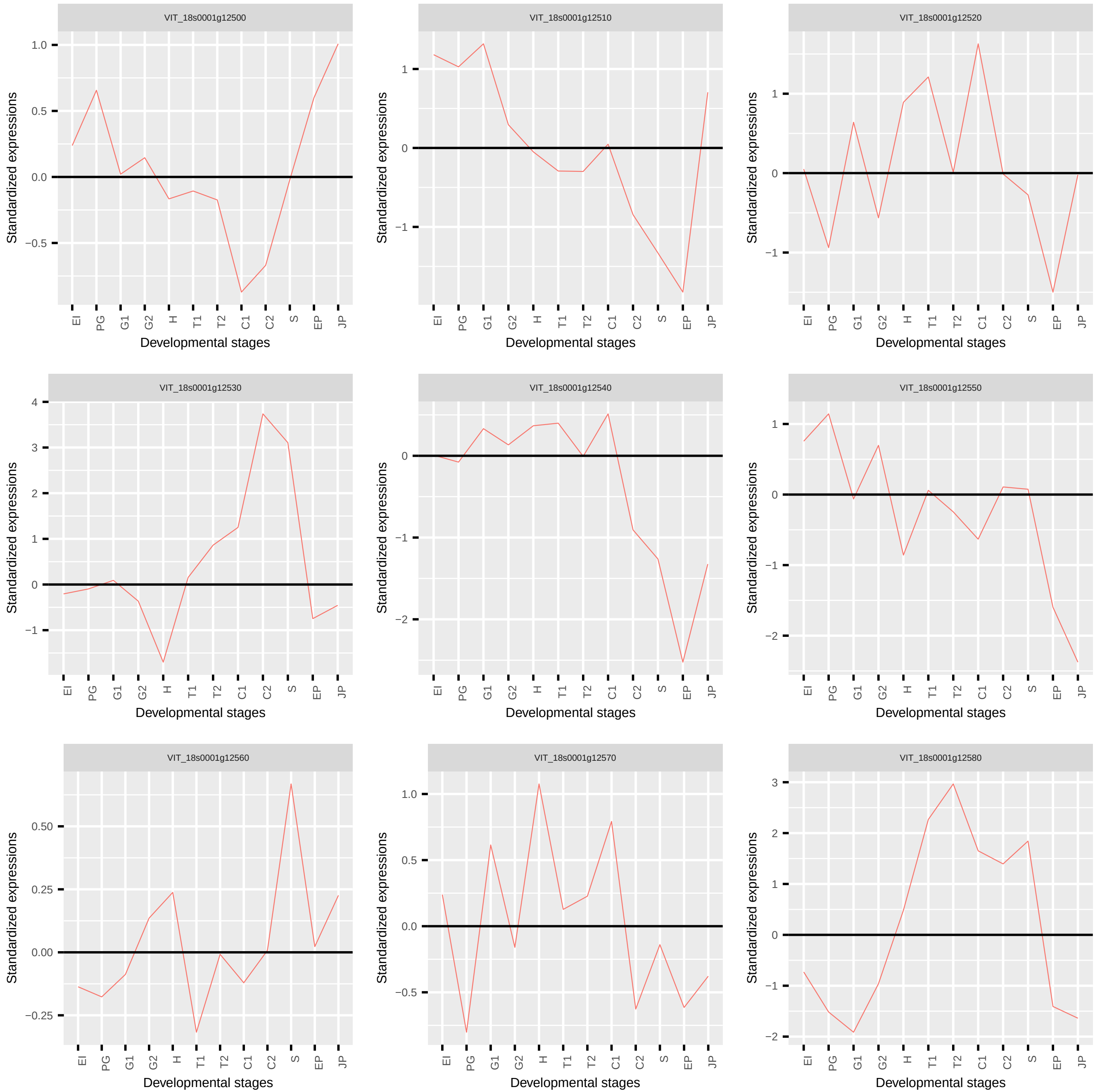
VIT_18s0001g12500
Standardized expressions
1.0
0.5
0.0
−0.5
EI PG G1 G2 H T1 T2 C1 C2 S EP JP
Developmental stages
VIT_18s0001g12510
Standardized expressions
1
0
−1
EI PG G1 G2 H T1 T2 C1 C2 S EP JP
Developmental stages
VIT_18s0001g12520
Standardized expressions
1
0
−1
EI PG G1 G2 H T1 T2 C1 C2 S EP JP
Developmental stages
VIT_18s0001g12530
Standardized expressions
4
3
2
1
0
−1
EI PG G1 G2 H T1 T2 C1 C2 S EP JP
Developmental stages
VIT_18s0001g12540
Standardized expressions
0
−1
−2
EI PG G1 G2 H T1 T2 C1 C2 S EP JP
Developmental stages
VIT_18s0001g12550
Standardized expressions
1
0
−1
−2
EI PG G1 G2 H T1 T2 C1 C2 S EP JP
Developmental stages
VIT_18s0001g12560
Standardized expressions
0.50
0.25
0.00
−0.25
EI PG G1 G2 H T1 T2 C1 C2 S EP JP
Developmental stages
VIT_18s0001g12570
Standardized expressions
1.0
0.5
0.0
−0.5
EI PG G1 G2 H T1 T2 C1 C2 S EP JP
Developmental stages
VIT_18s0001g12580
Standardized expressions
3
2
1
0
−1
−2
EI PG G1 G2 H T1 T2 C1 C2 S EP JP
Developmental stages

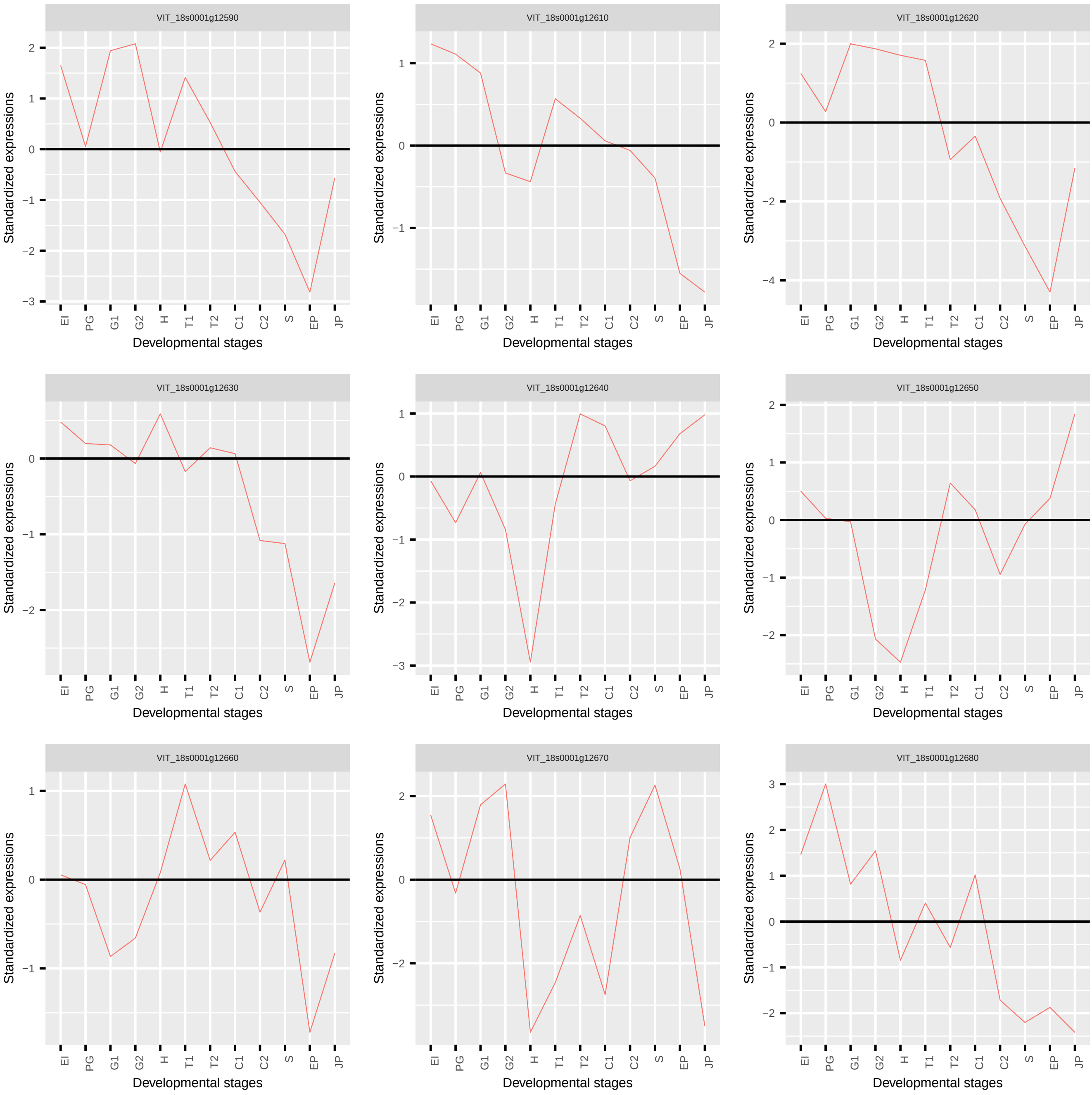
VIT_18s0001g12590
VIT_18s0001g12610
VIT_18s0001g12620
VIT_18s0001g12630
VIT_18s0001g12640
VIT_18s0001g12650
VIT_18s0001g12660
VIT_18s0001g12670
VIT_18s0001g12680
Standardized expressions
Developmental stages
EI
PG
G1
G2
H
T1
T2
C1
C2
S
EP
JP

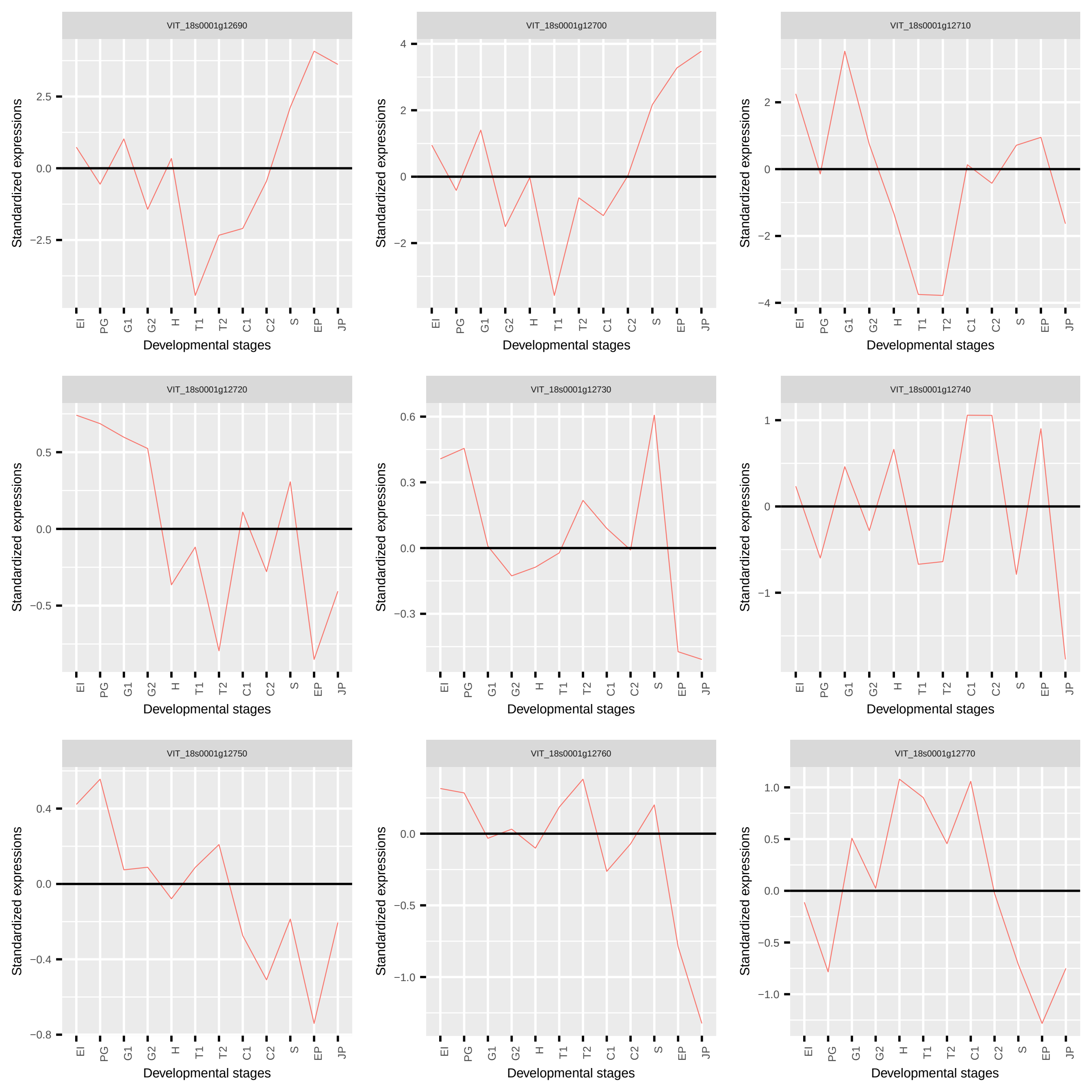

VIT_18s0001g12690
VIT_18s0001g12700
VIT_18s0001g12710
VIT_18s0001g12720
VIT_18s0001g12730
VIT_18s0001g12740
VIT_18s0001g12750
VIT_18s0001g12760
VIT_18s0001g12770
Standardized expressions
Developmental stages
EI PG G1 G2 H T1 T2 C1 C2 S EP JP

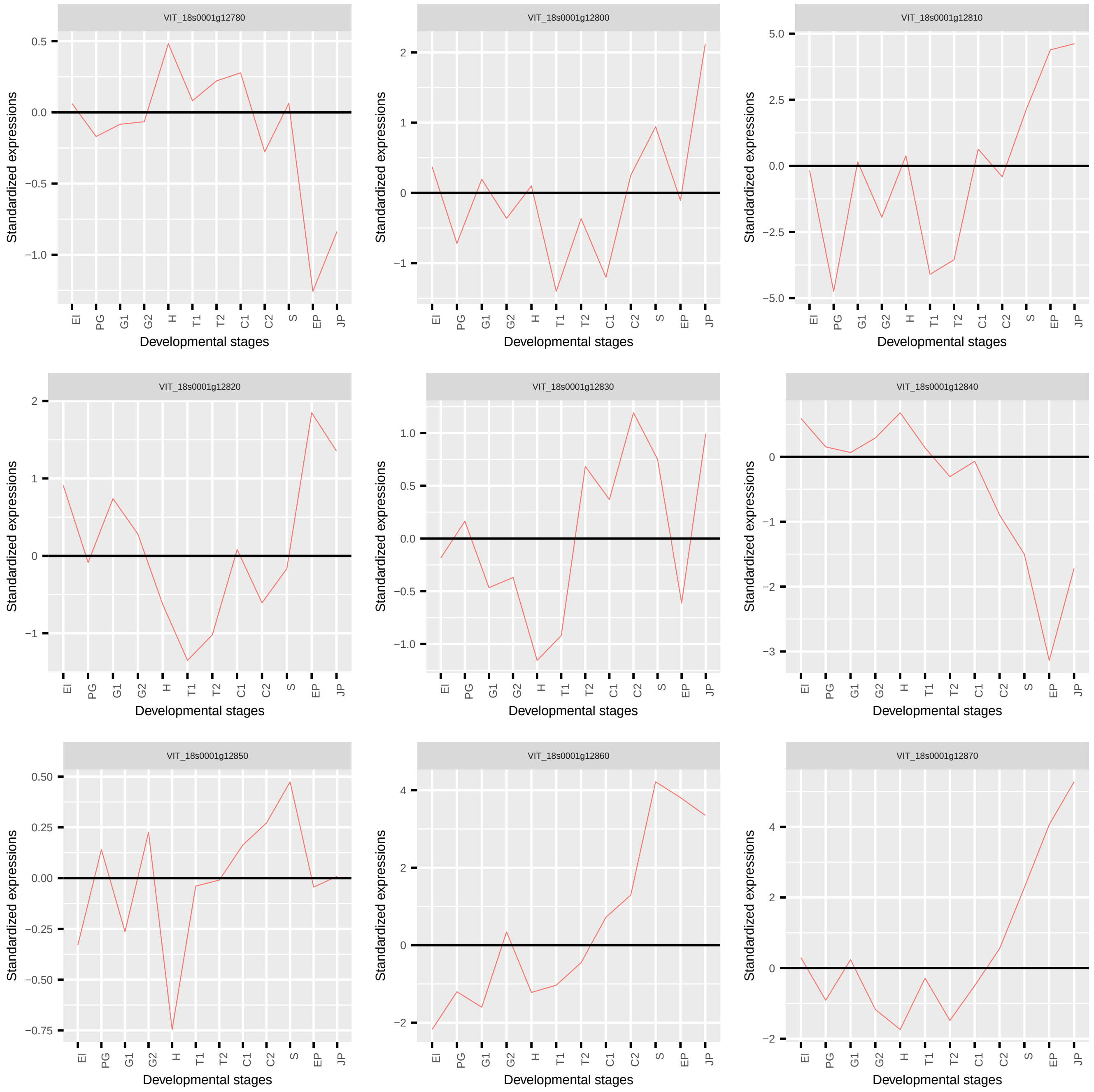
VIT_18s0001g12780
VIT_18s0001g12800
VIT_18s0001g12810
VIT_18s0001g12820
VIT_18s0001g12830
VIT_18s0001g12840
VIT_18s0001g12850
VIT_18s0001g12860
VIT_18s0001g12870
Standardized expressions
Developmental stages
EI
PG
G1
G2
H
T1
T2
C1
C2
S
EP
JP

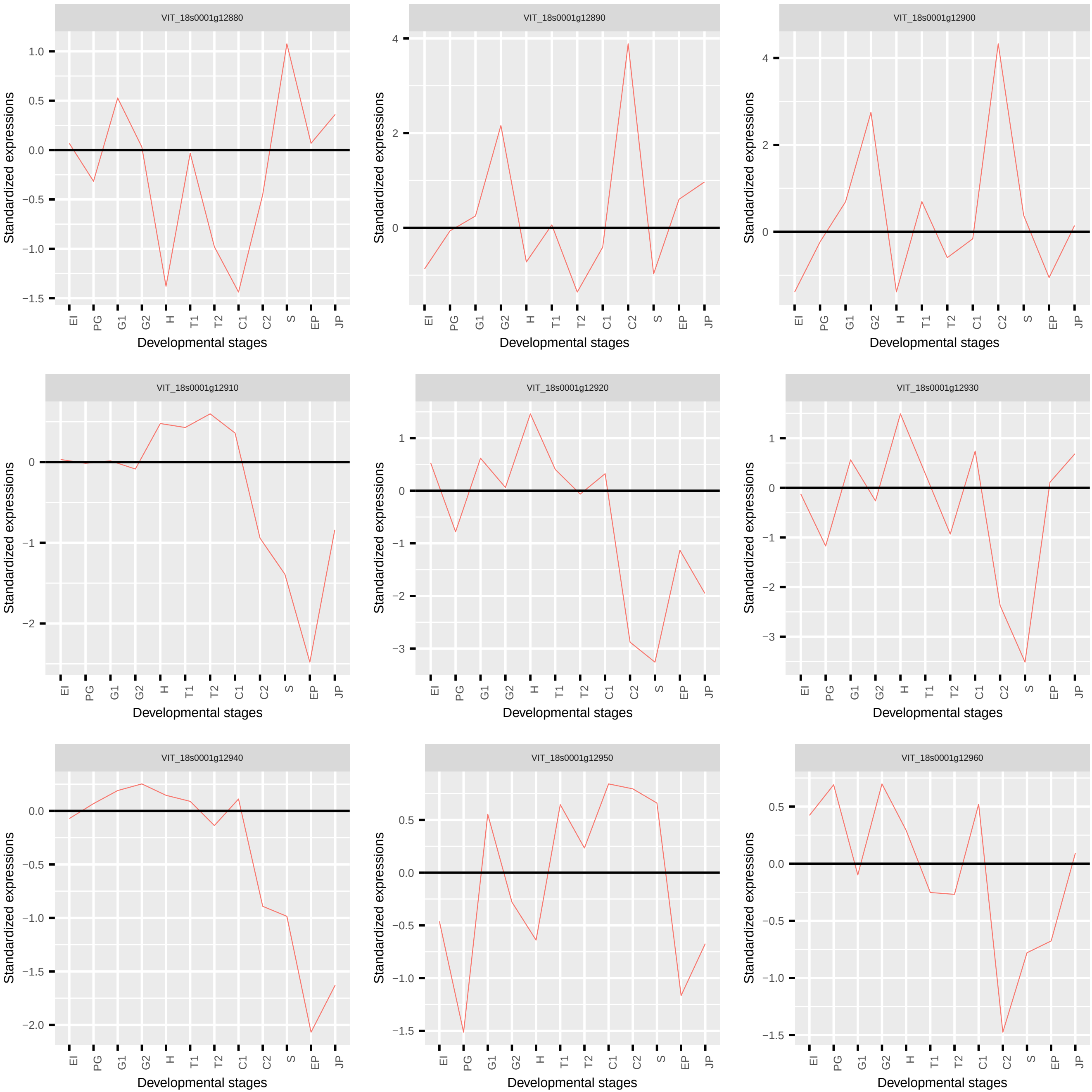
VIT_18s0001g12880
VIT_18s0001g12890
VIT_18s0001g12900
VIT_18s0001g12910
VIT_18s0001g12920
VIT_18s0001g12930
VIT_18s0001g12940
VIT_18s0001g12950
VIT_18s0001g12960
Standardized expressions
Developmental stages
EI
PG
G1
G2
H
T1
T2
C1
C2
S
EP
JP

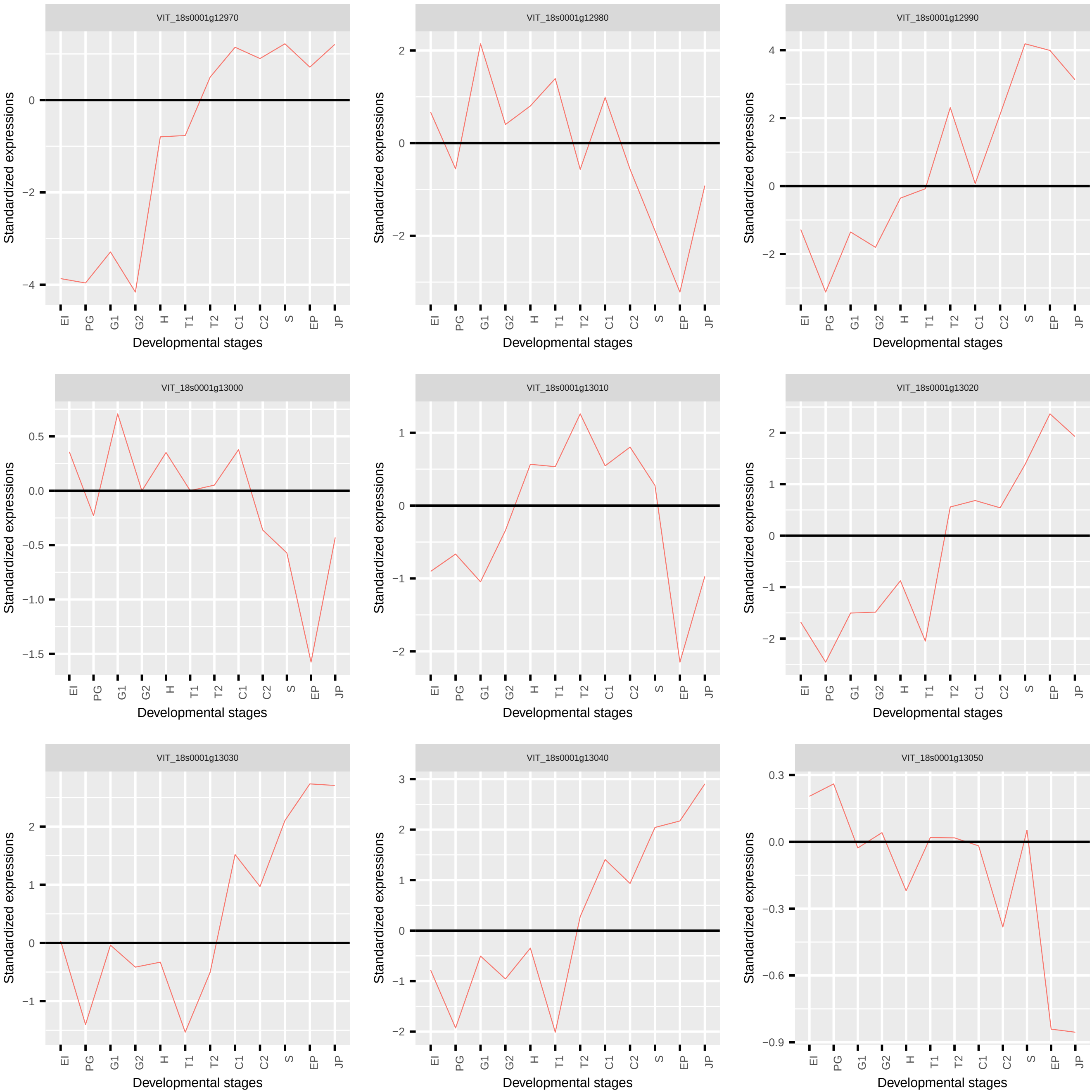
VIT_18s0001g12970
VIT_18s0001g12980
VIT_18s0001g12990
VIT_18s0001g13000
VIT_18s0001g13010
VIT_18s0001g13020
VIT_18s0001g13030
VIT_18s0001g13040
VIT_18s0001g13050
Standardized expressions
Developmental stages
EI PG G1 G2 H T1 T2 C1 C2 S EP JP

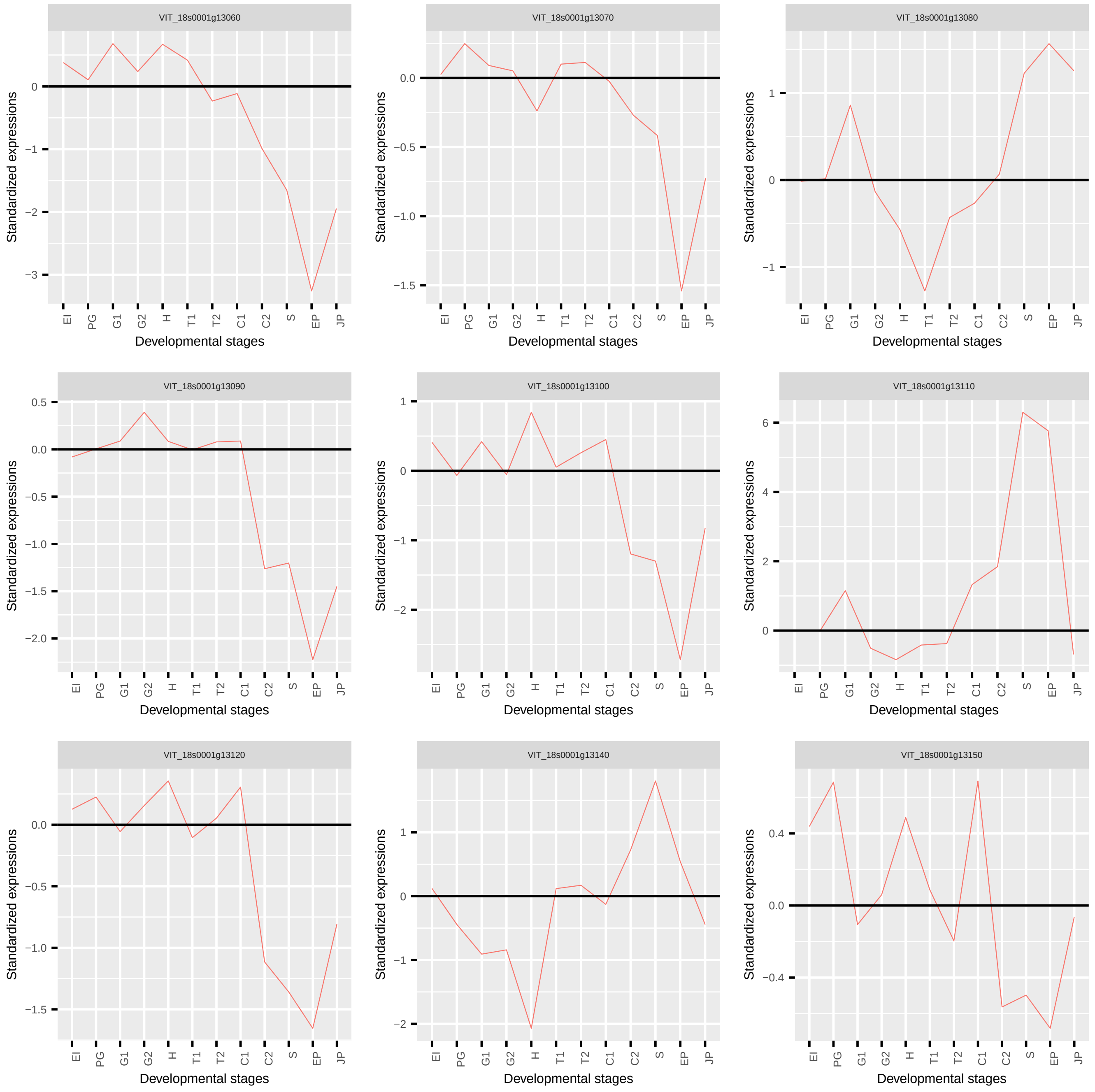
VIT_18s0001g13060
VIT_18s0001g13070
VIT_18s0001g13080
VIT_18s0001g13090
VIT_18s0001g13100
VIT_18s0001g13110
VIT_18s0001g13120
VIT_18s0001g13140
VIT_18s0001g13150
Standardized expressions
Developmental stages
EI PG G1 G2 H T1 T2 C1 C2 S EP JP

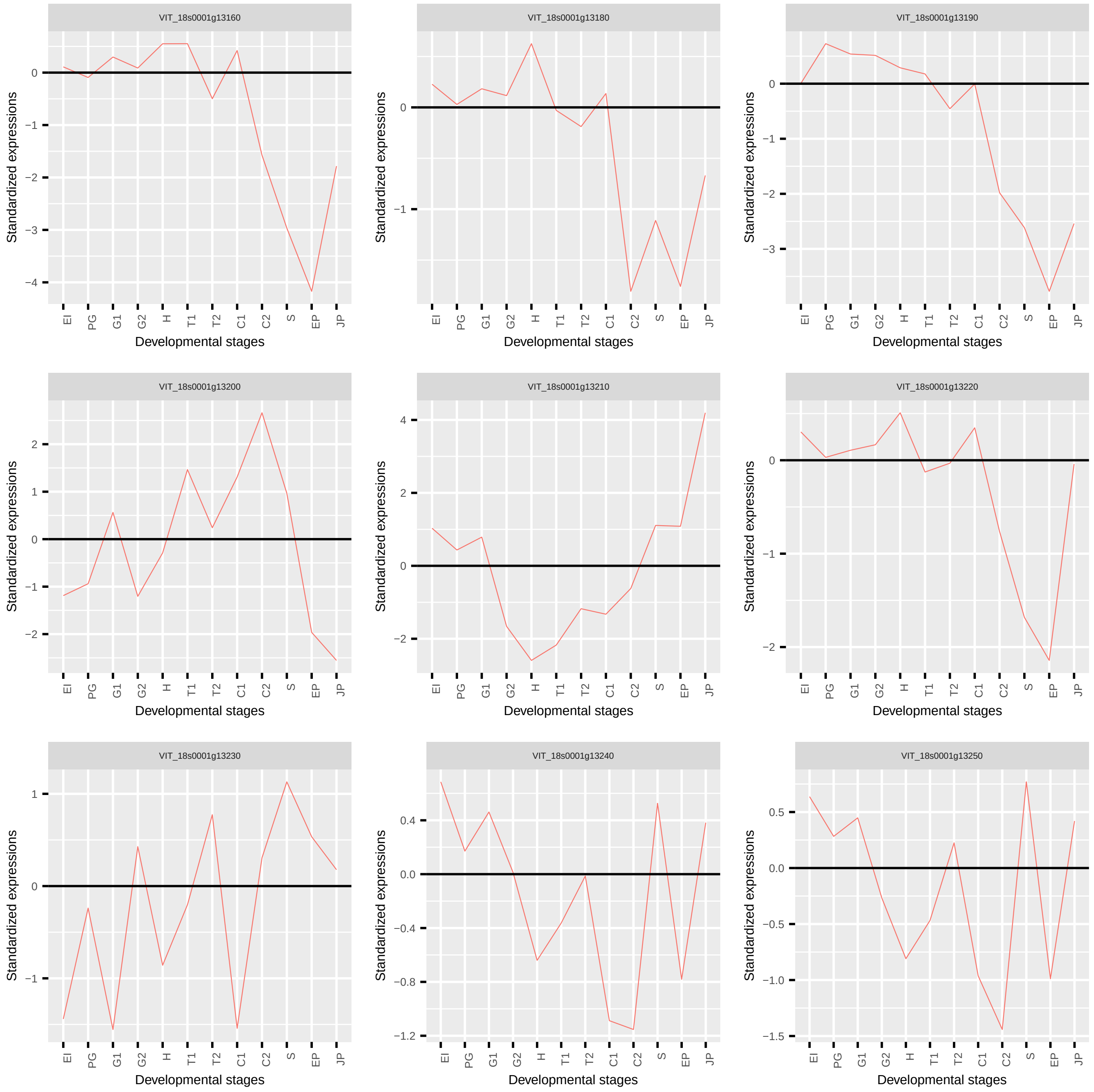
VIT_18s0001g13160
VIT_18s0001g13180
VIT_18s0001g13190
VIT_18s0001g13200
VIT_18s0001g13210
VIT_18s0001g13220
VIT_18s0001g13230
VIT_18s0001g13240
VIT_18s0001g13250
Standardized expressions
Developmental stages
EI
PG
G1
G2
H
T1
T2
C1
C2
S
EP
JP

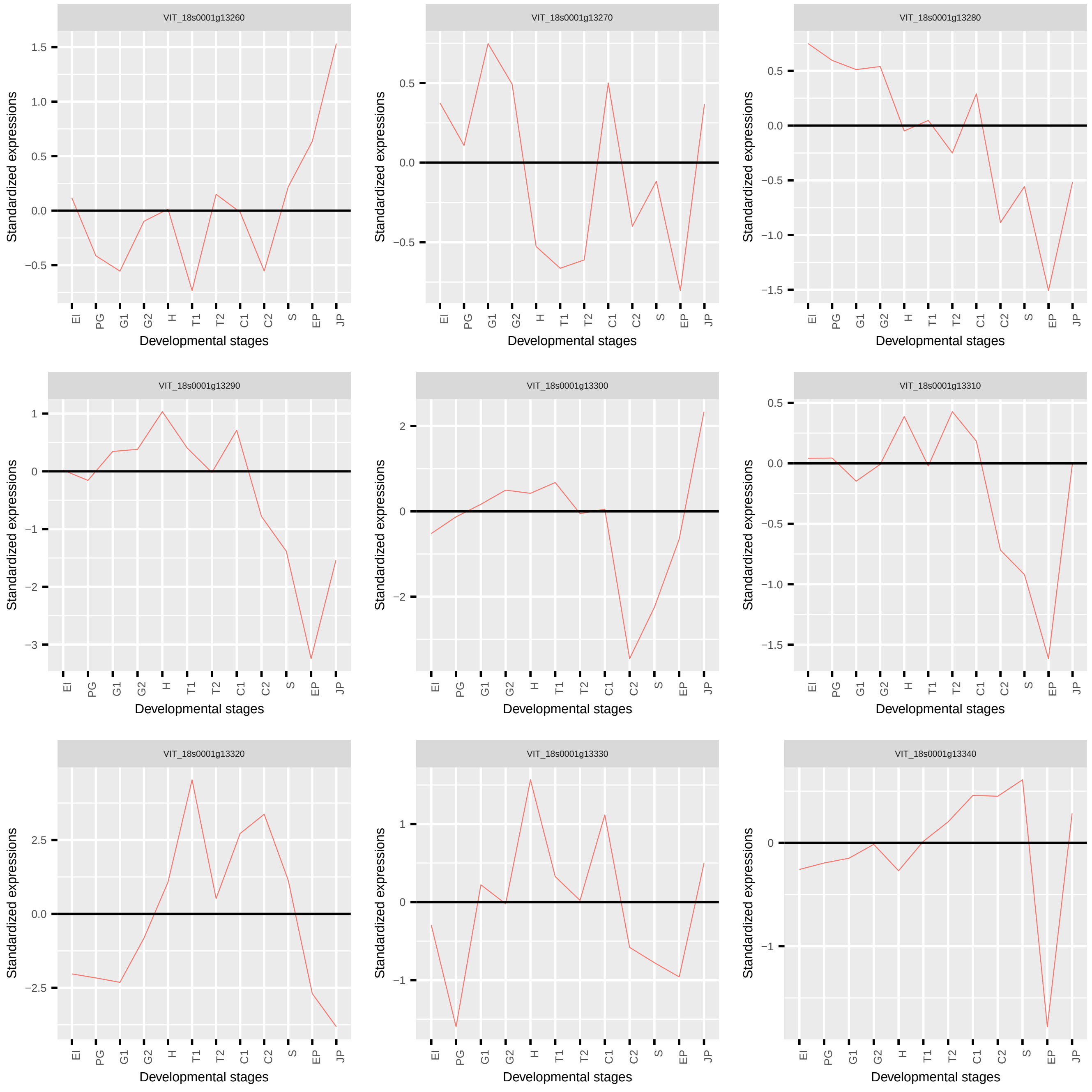
VIT_18s0001g13260
VIT_18s0001g13270
VIT_18s0001g13280
VIT_18s0001g13290
VIT_18s0001g13300
VIT_18s0001g13310
VIT_18s0001g13320
VIT_18s0001g13330
VIT_18s0001g13340
Standardized expressions
Developmental stages
EI PG G1 G2 H T1 T2 C1 C2 S EP JP

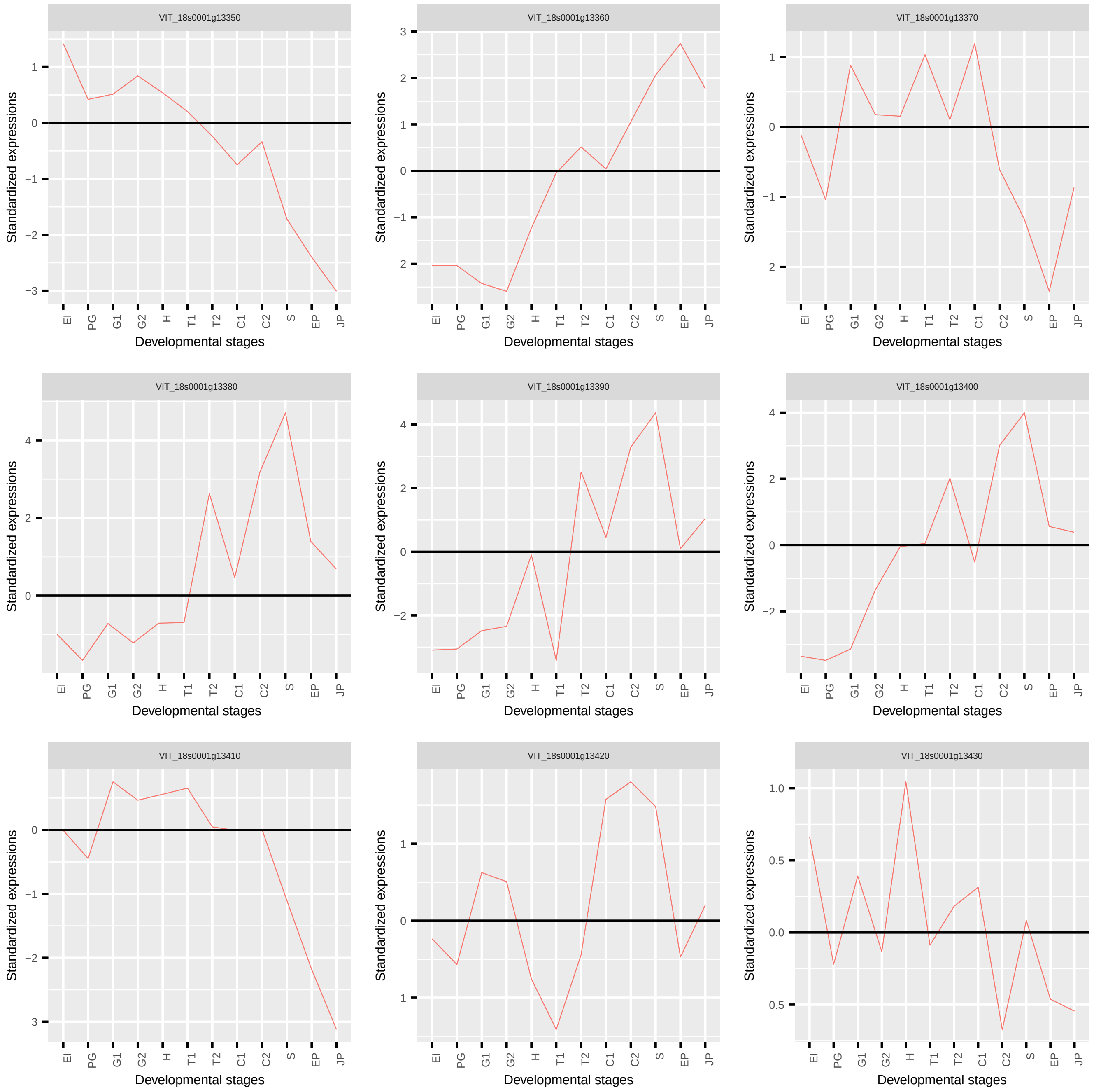

VIT_18s0001g13350
VIT_18s0001g13360
VIT_18s0001g13370
VIT_18s0001g13380
VIT_18s0001g13390
VIT_18s0001g13400
VIT_18s0001g13410
VIT_18s0001g13420
VIT_18s0001g13430
Standardized expressions
Developmental stages
EI PG G1 G2 H T1 T2 C1 C2 S EP JP

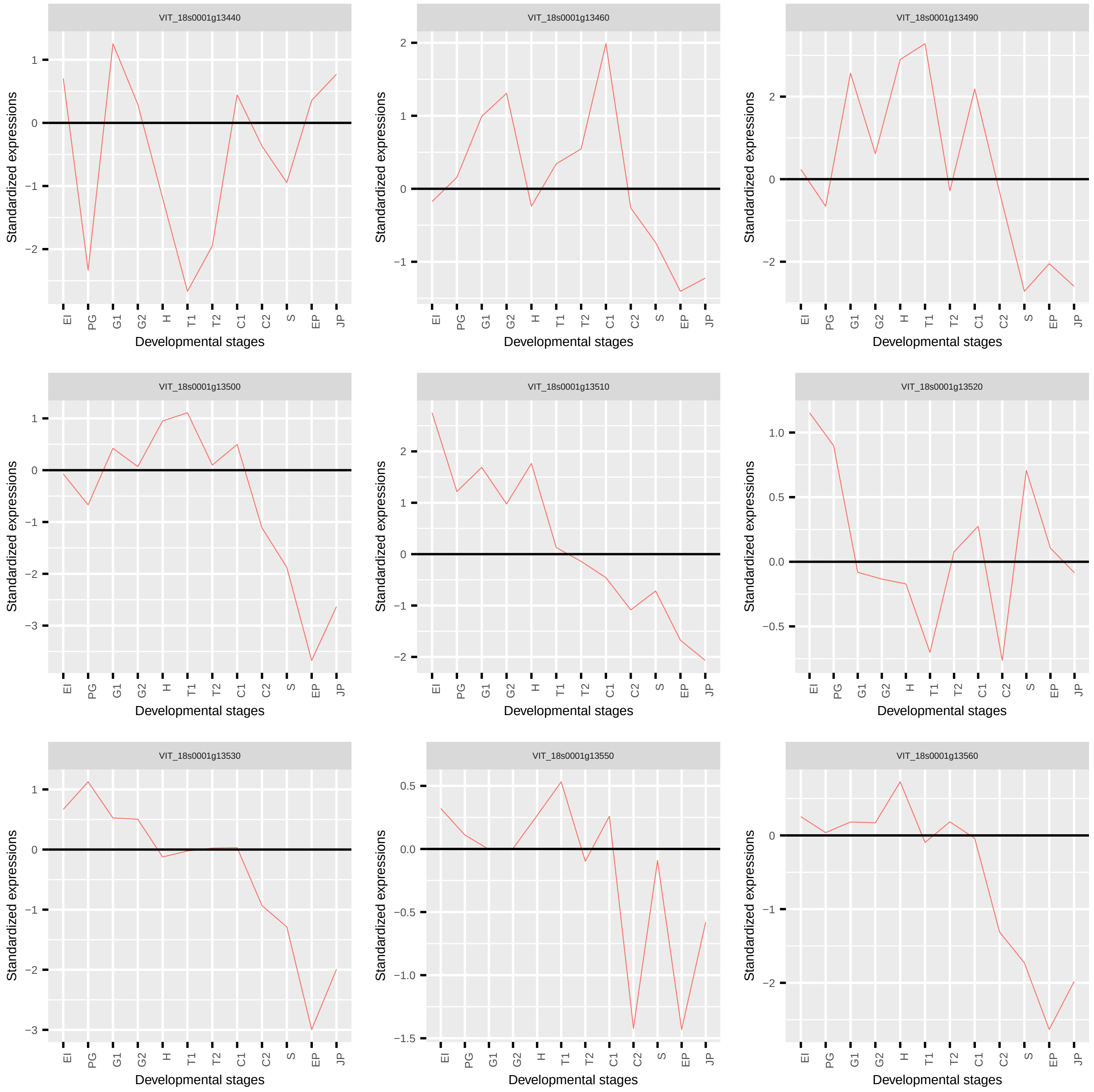
VIT_18s0001g13440
Standardized expressions
1
0
−1
−2
EI PG G1 G2 H T1 T2 C1 C2 S EP JP
Developmental stages
VIT_18s0001g13460
Standardized expressions
2
1
0
−1
EI PG G1 G2 H T1 T2 C1 C2 S EP JP
Developmental stages
VIT_18s0001g13490
Standardized expressions
2
0
−2
EI PG G1 G2 H T1 T2 C1 C2 S EP JP
Developmental stages
VIT_18s0001g13500
Standardized expressions
1
0
−1
−2
−3
EI PG G1 G2 H T1 T2 C1 C2 S EP JP
Developmental stages
VIT_18s0001g13510
Standardized expressions
2
1
0
−1
−2
EI PG G1 G2 H T1 T2 C1 C2 S EP JP
Developmental stages
VIT_18s0001g13520
Standardized expressions
1.0
0.5
0.0
−0.5
EI PG G1 G2 H T1 T2 C1 C2 S EP JP
Developmental stages
VIT_18s0001g13530
Standardized expressions
1
0
−1
−2
−3
EI PG G1 G2 H T1 T2 C1 C2 S EP JP
Developmental stages
VIT_18s0001g13550
Standardized expressions
0.5
0.0
−0.5
−1.0
−1.5
EI PG G1 G2 H T1 T2 C1 C2 S EP JP
Developmental stages
VIT_18s0001g13560
Standardized expressions
0
−1
−2
EI PG G1 G2 H T1 T2 C1 C2 S EP JP
Developmental stages

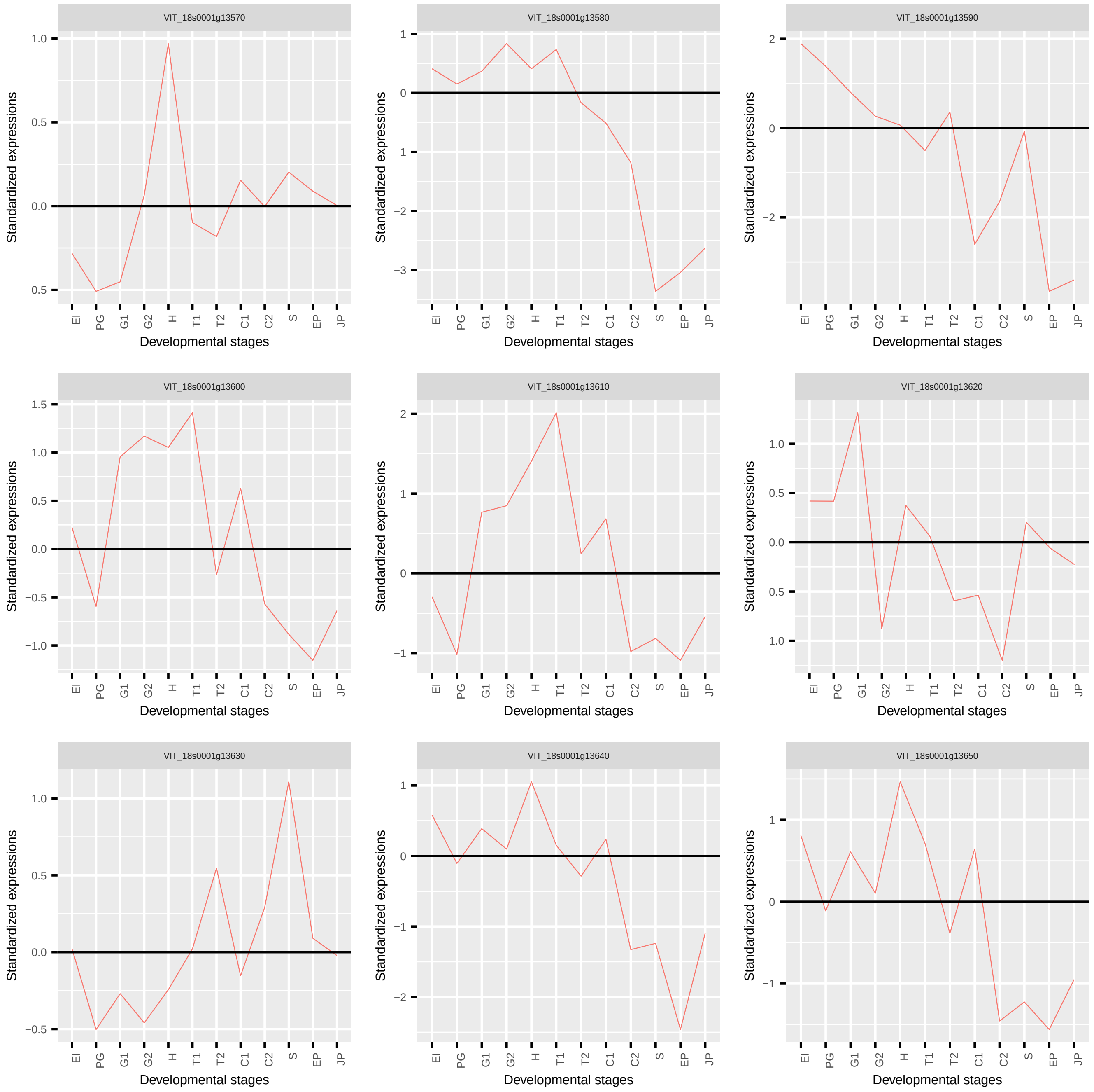

VIT_18s0001g13570
VIT_18s0001g13580
VIT_18s0001g13590
VIT_18s0001g13600
VIT_18s0001g13610
VIT_18s0001g13620
VIT_18s0001g13630
VIT_18s0001g13640
VIT_18s0001g13650
Standardized expressions
Developmental stages
EI PG G1 G2 H T1 T2 C1 C2 S EP JP

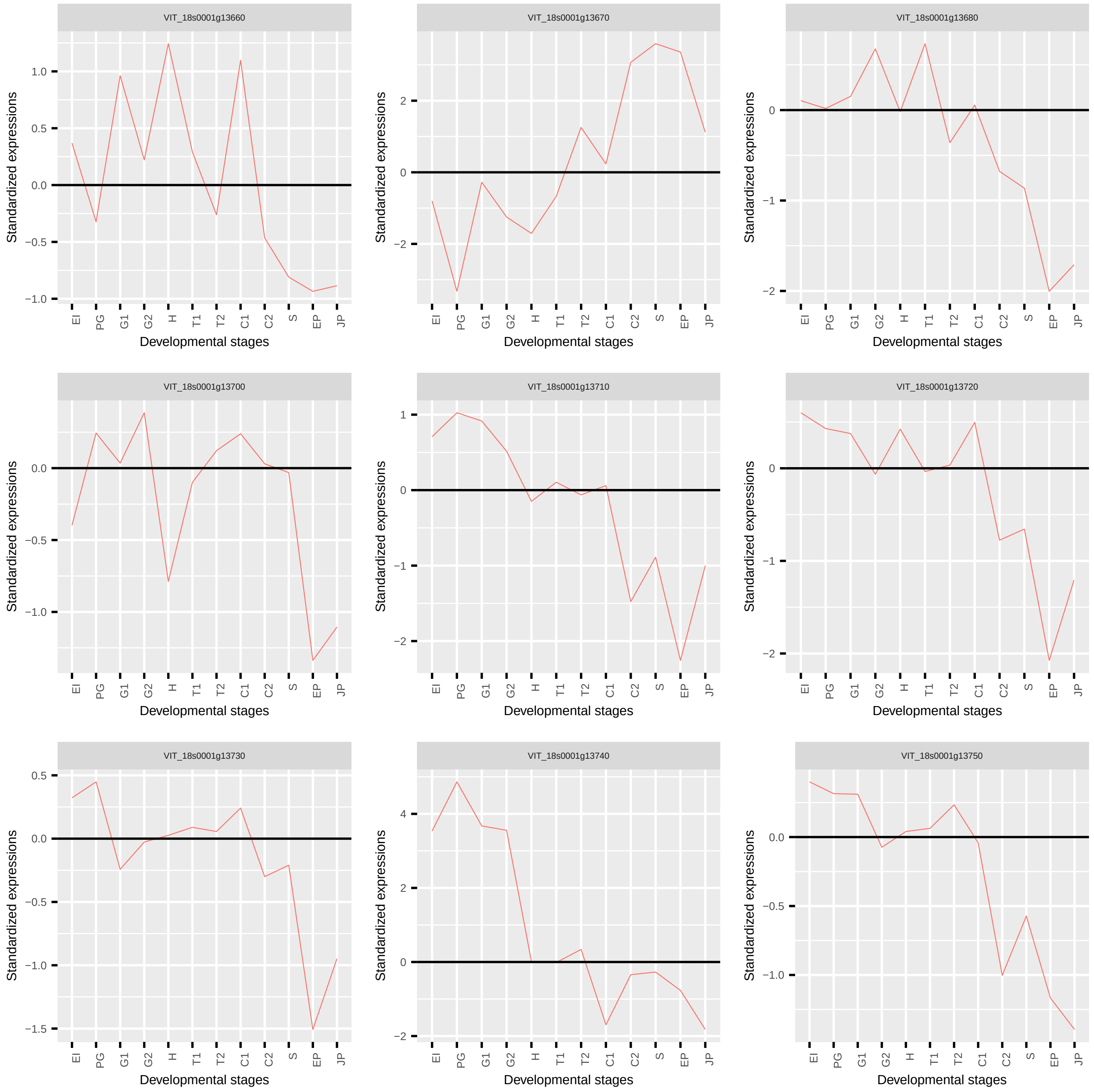
VIT_18s0001g13660
VIT_18s0001g13670
VIT_18s0001g13680
VIT_18s0001g13700
VIT_18s0001g13710
VIT_18s0001g13720
VIT_18s0001g13730
VIT_18s0001g13740
VIT_18s0001g13750
Standardized expressions
Developmental stages
EI PG G1 G2 H T1 T2 C1 C2 S EP JP

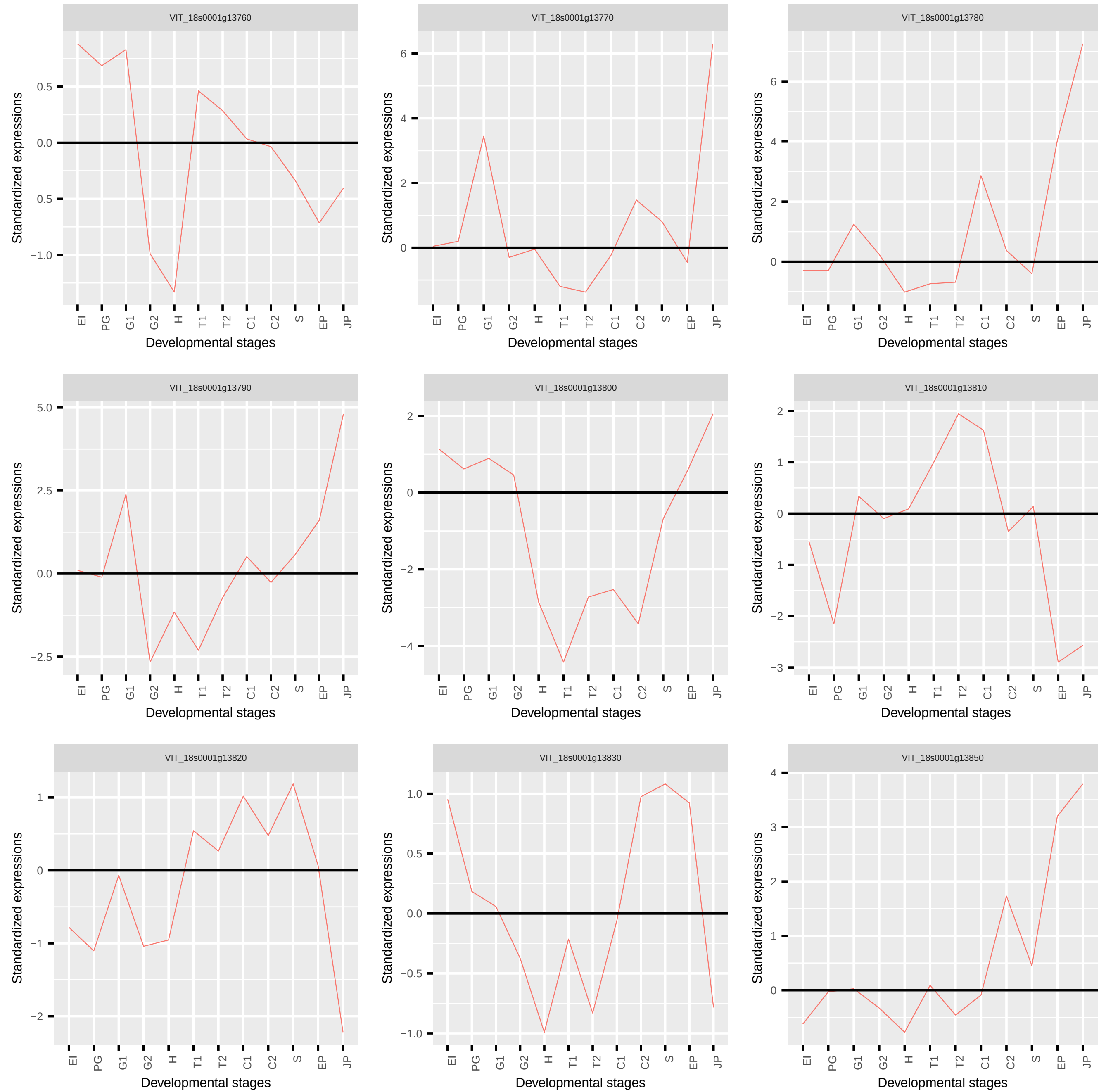
VIT_18s0001g13760
Standardized expressions
0.5
0.0
−0.5
−1.0
EI PG G1 G2 H T1 T2 C1 C2 S EP JP
Developmental stages
VIT_18s0001g13770
Standardized expressions
6
4
2
0
EI PG G1 G2 H T1 T2 C1 C2 S EP JP
Developmental stages
VIT_18s0001g13780
Standardized expressions
6
4
2
0
EI PG G1 G2 H T1 T2 C1 C2 S EP JP
Developmental stages
VIT_18s0001g13790
Standardized expressions
5.0
2.5
0.0
−2.5
EI PG G1 G2 H T1 T2 C1 C2 S EP JP
Developmental stages
VIT_18s0001g13800
Standardized expressions
2
0
−2
−4
EI PG G1 G2 H T1 T2 C1 C2 S EP JP
Developmental stages
VIT_18s0001g13810
Standardized expressions
2
1
0
−1
−2
−3
EI PG G1 G2 H T1 T2 C1 C2 S EP JP
Developmental stages
VIT_18s0001g13820
Standardized expressions
1
0
−1
−2
EI PG G1 G2 H T1 T2 C1 C2 S EP JP
Developmental stages
VIT_18s0001g13830
Standardized expressions
1.0
0.5
0.0
−0.5
−1.0
EI PG G1 G2 H T1 T2 C1 C2 S EP JP
Developmental stages
VIT_18s0001g13850
Standardized expressions
4
3
2
1
0
EI PG G1 G2 H T1 T2 C1 C2 S EP JP
Developmental stages

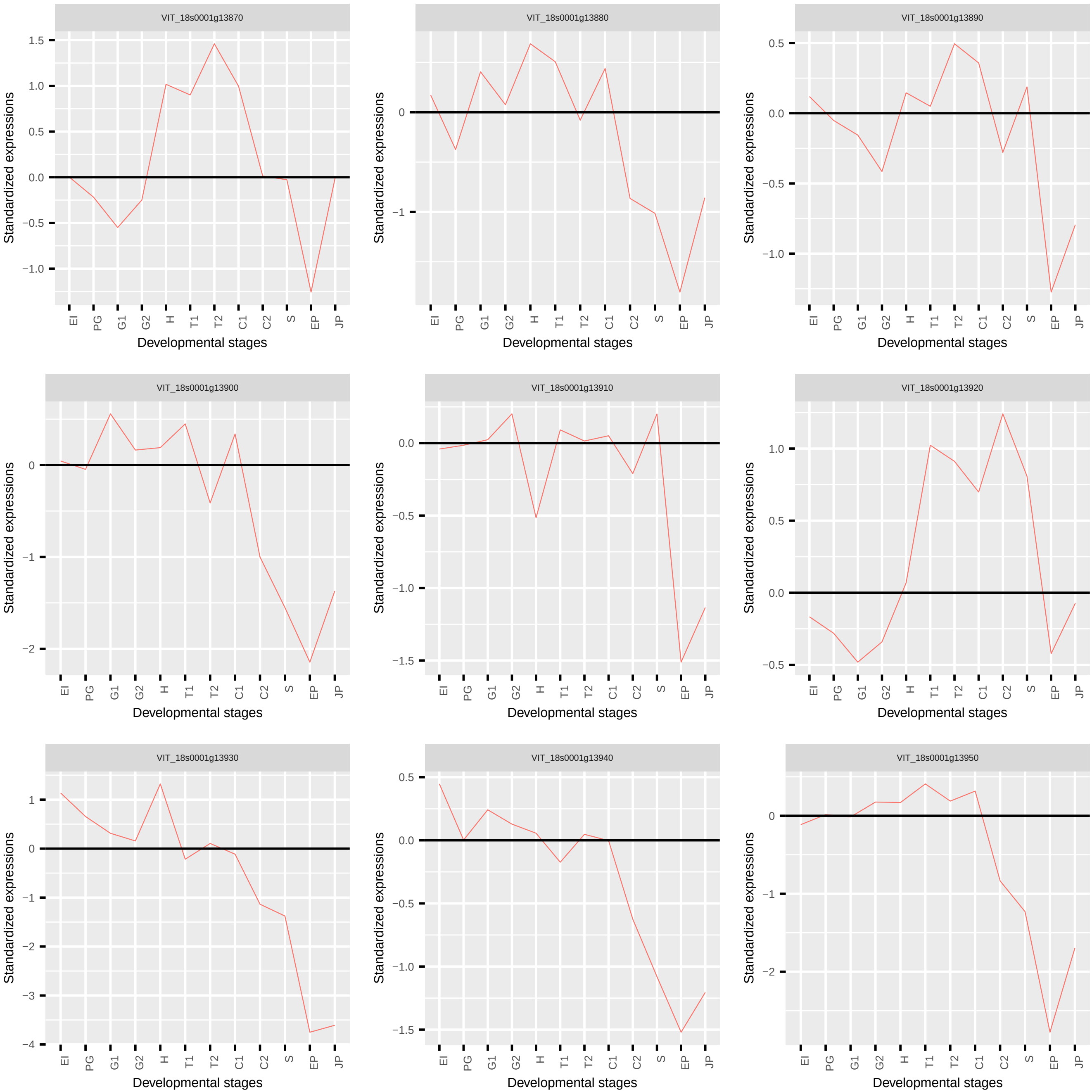
VIT_18s0001g13870
VIT_18s0001g13880
VIT_18s0001g13890
VIT_18s0001g13900
VIT_18s0001g13910
VIT_18s0001g13920
VIT_18s0001g13930
VIT_18s0001g13940
VIT_18s0001g13950
Standardized expressions
Developmental stages
EI
PG
G1
G2
H
T1
T2
C1
C2
S
EP
JP

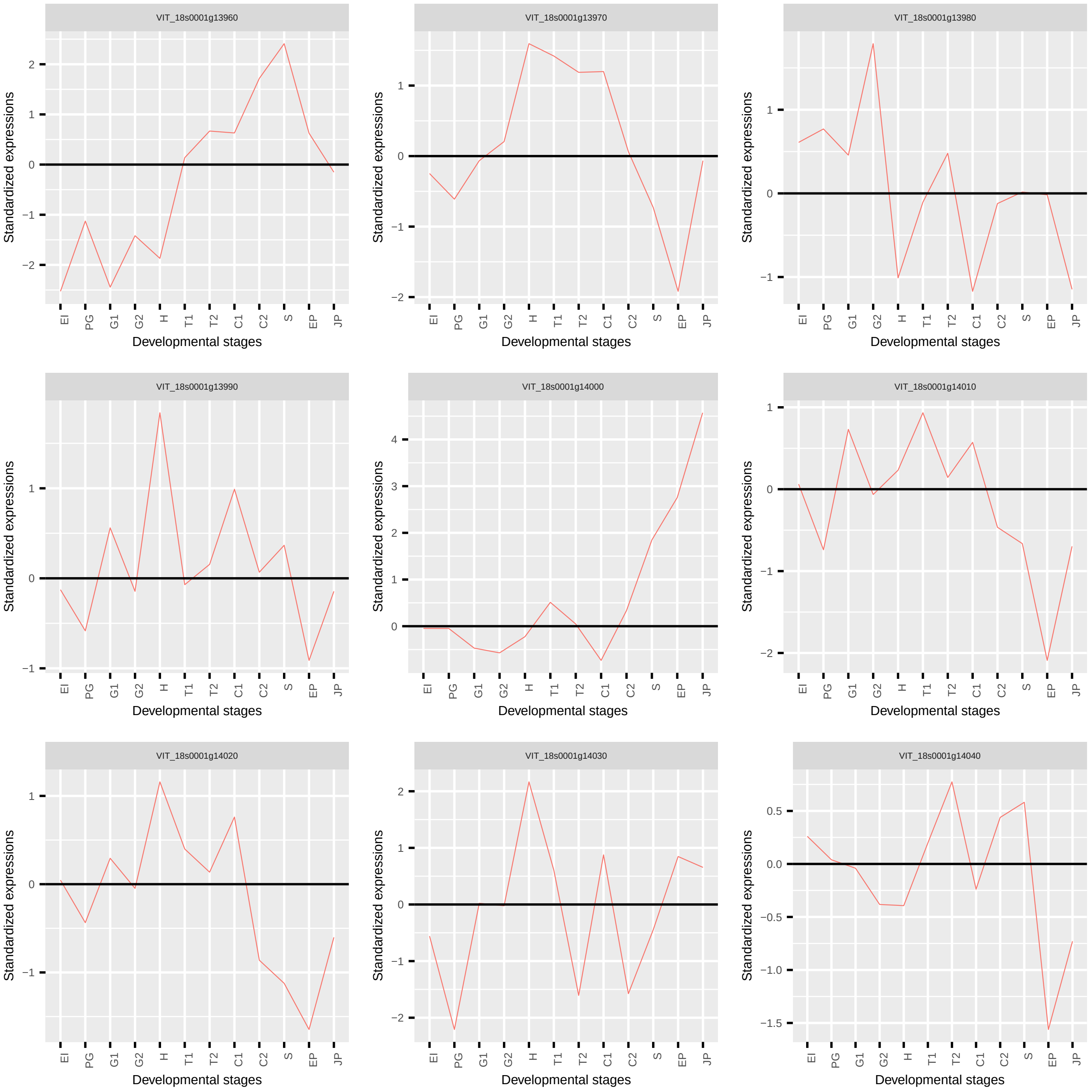
VIT_18s0001g13960
VIT_18s0001g13970
VIT_18s0001g13980
VIT_18s0001g13990
VIT_18s0001g14000
VIT_18s0001g14010
VIT_18s0001g14020
VIT_18s0001g14030
VIT_18s0001g14040
Standardized expressions
Developmental stages
EI
PG
G1
G2
H
T1
T2
C1
C2
S
EP
JP

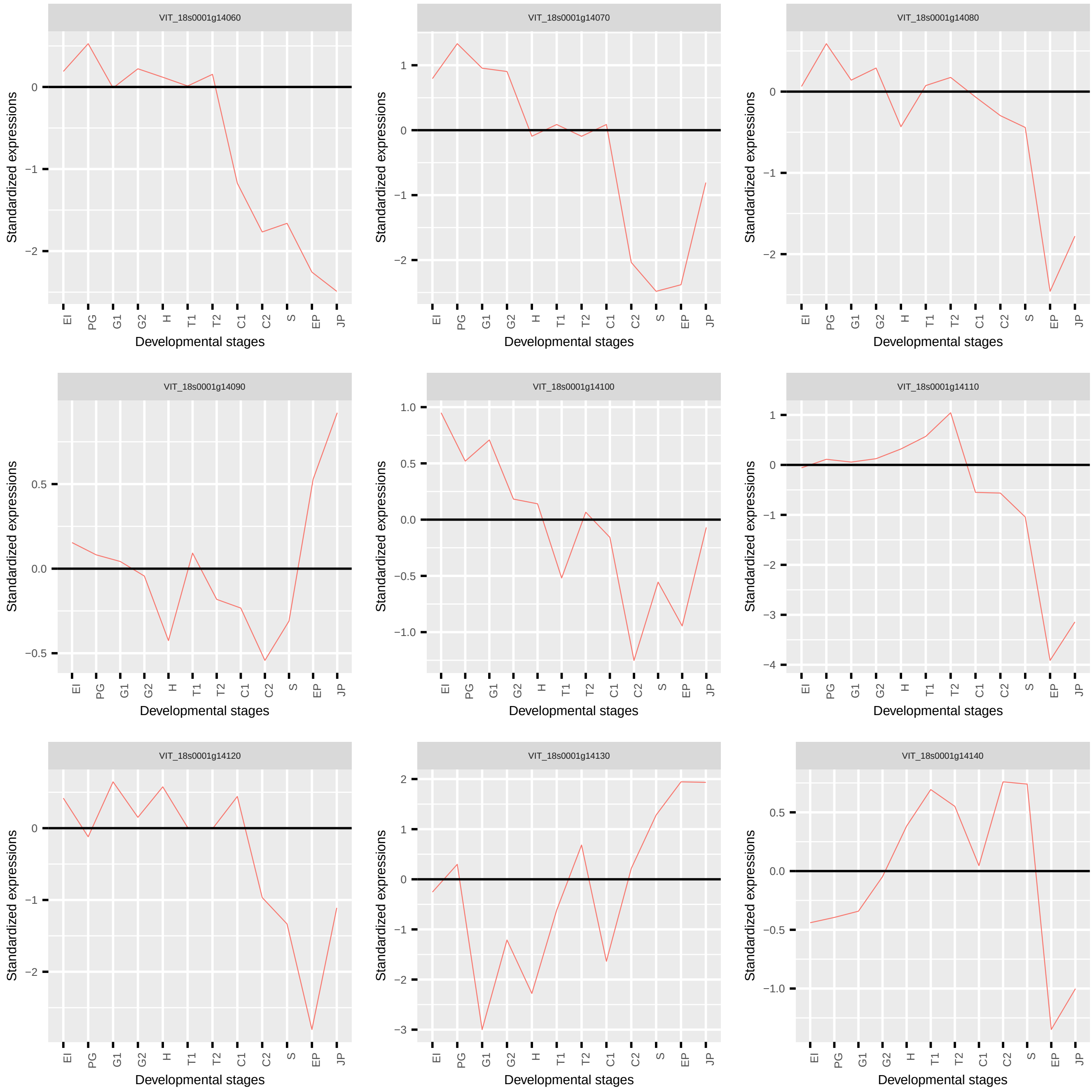

VIT_18s0001g14060
VIT_18s0001g14070
VIT_18s0001g14080
VIT_18s0001g14090
VIT_18s0001g14100
VIT_18s0001g14110
VIT_18s0001g14120
VIT_18s0001g14130
VIT_18s0001g14140
Standardized expressions
Developmental stages
EI
PG
G1
G2
H
T1
T2
C1
C2
S
EP
JP

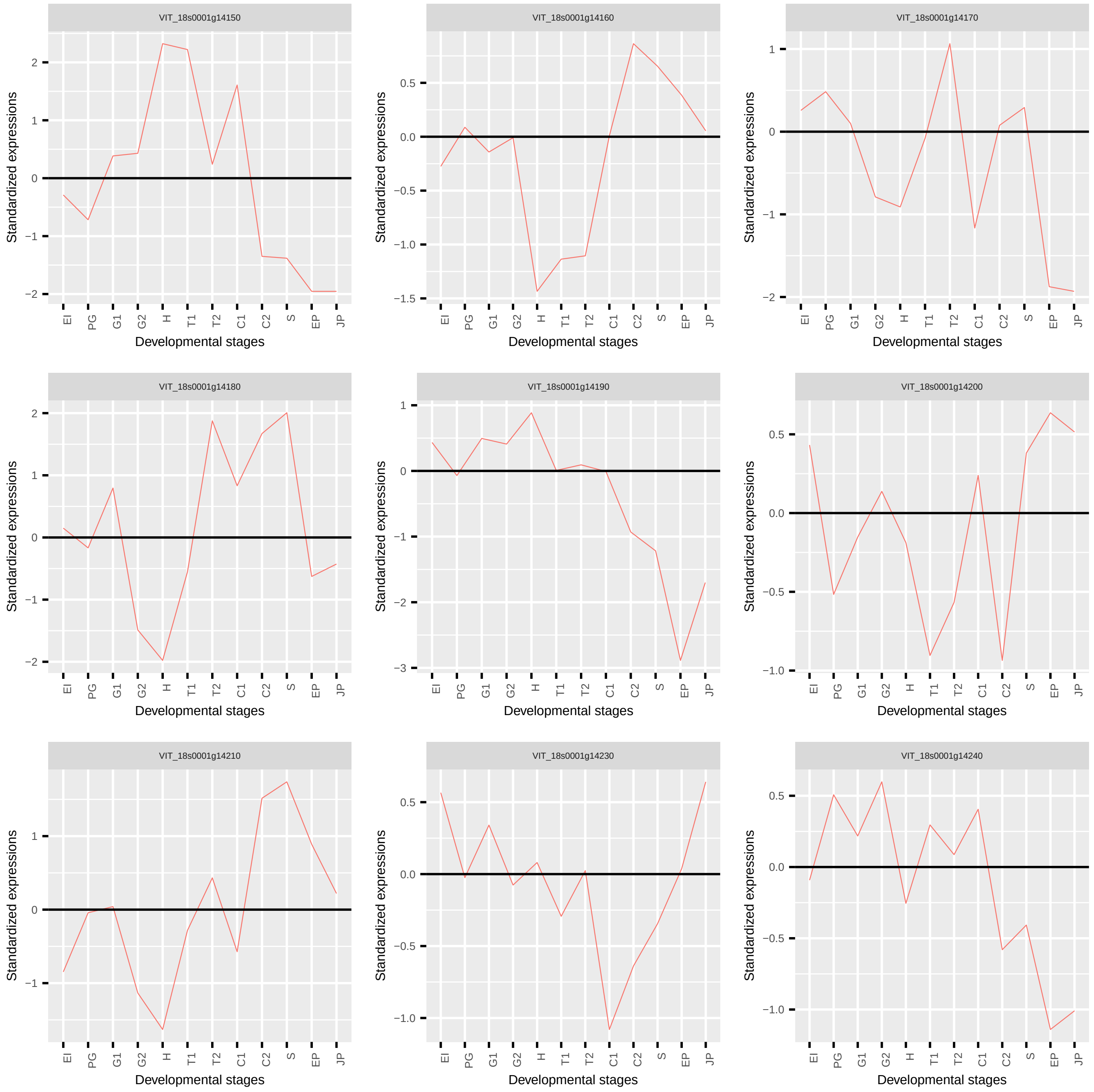
VIT_18s0001g14150
VIT_18s0001g14160
VIT_18s0001g14170
VIT_18s0001g14180
VIT_18s0001g14190
VIT_18s0001g14200
VIT_18s0001g14210
VIT_18s0001g14230
VIT_18s0001g14240
Standardized expressions
Developmental stages
EI PG G1 G2 H T1 T2 C1 C2 S EP JP

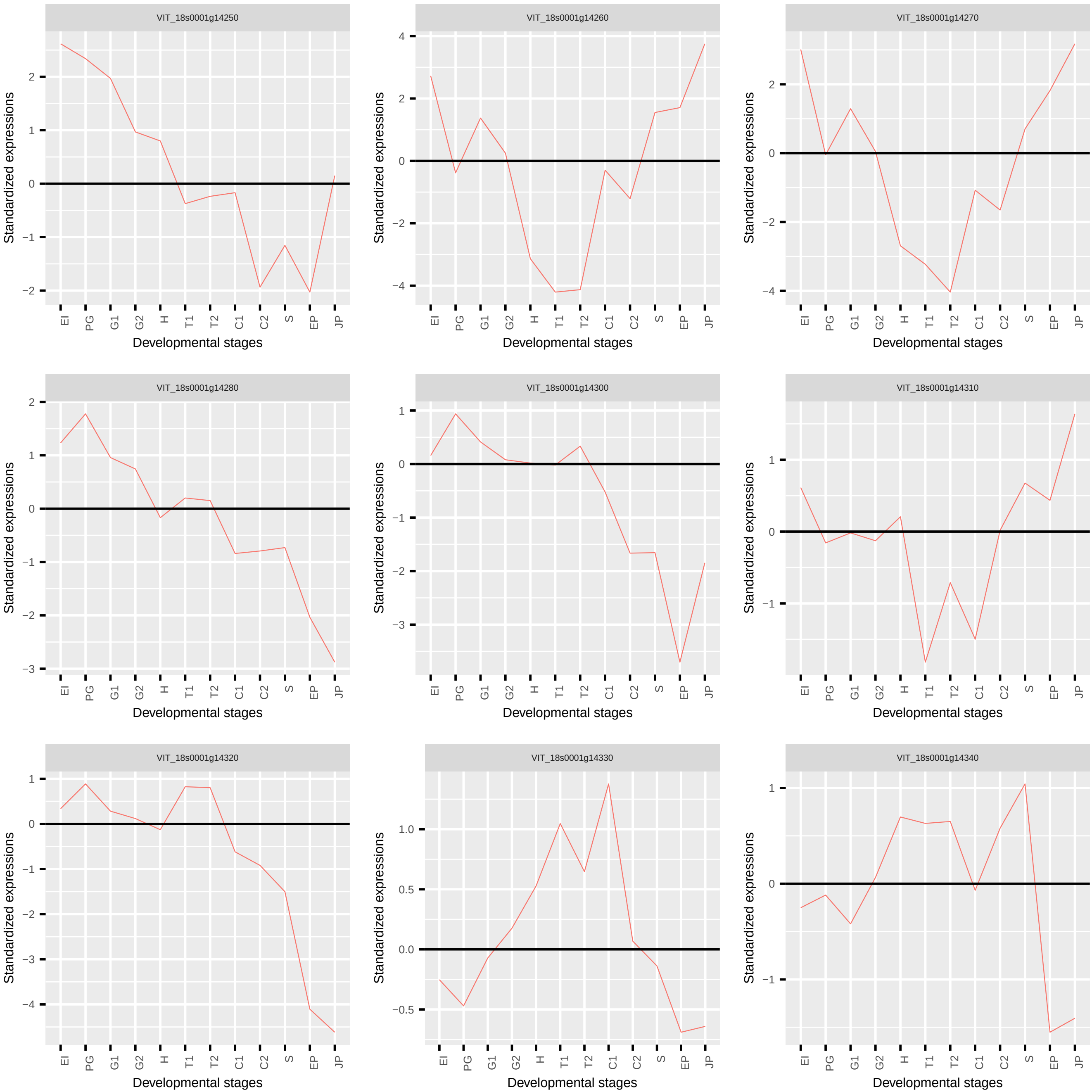
VIT_18s0001g14250
Standardized expressions
2
1
0
−1
−2
EI PG G1 G2 H T1 T2 C1 C2 S EP JP
Developmental stages
VIT_18s0001g14260
Standardized expressions
4
2
0
−2
−4
EI PG G1 G2 H T1 T2 C1 C2 S EP JP
Developmental stages
VIT_18s0001g14270
Standardized expressions
2
0
−2
−4
EI PG G1 G2 H T1 T2 C1 C2 S EP JP
Developmental stages
VIT_18s0001g14280
Standardized expressions
2
1
0
−1
−2
−3
EI PG G1 G2 H T1 T2 C1 C2 S EP JP
Developmental stages
VIT_18s0001g14300
Standardized expressions
1
0
−1
−2
−3
EI PG G1 G2 H T1 T2 C1 C2 S EP JP
Developmental stages
VIT_18s0001g14310
Standardized expressions
1
0
−1
EI PG G1 G2 H T1 T2 C1 C2 S EP JP
Developmental stages
VIT_18s0001g14320
Standardized expressions
1
0
−1
−2
−3
−4
EI PG G1 G2 H T1 T2 C1 C2 S EP JP
Developmental stages
VIT_18s0001g14330
Standardized expressions
1.0
0.5
0.0
−0.5
EI PG G1 G2 H T1 T2 C1 C2 S EP JP
Developmental stages
VIT_18s0001g14340
Standardized expressions
1
0
−1
EI PG G1 G2 H T1 T2 C1 C2 S EP JP
Developmental stages

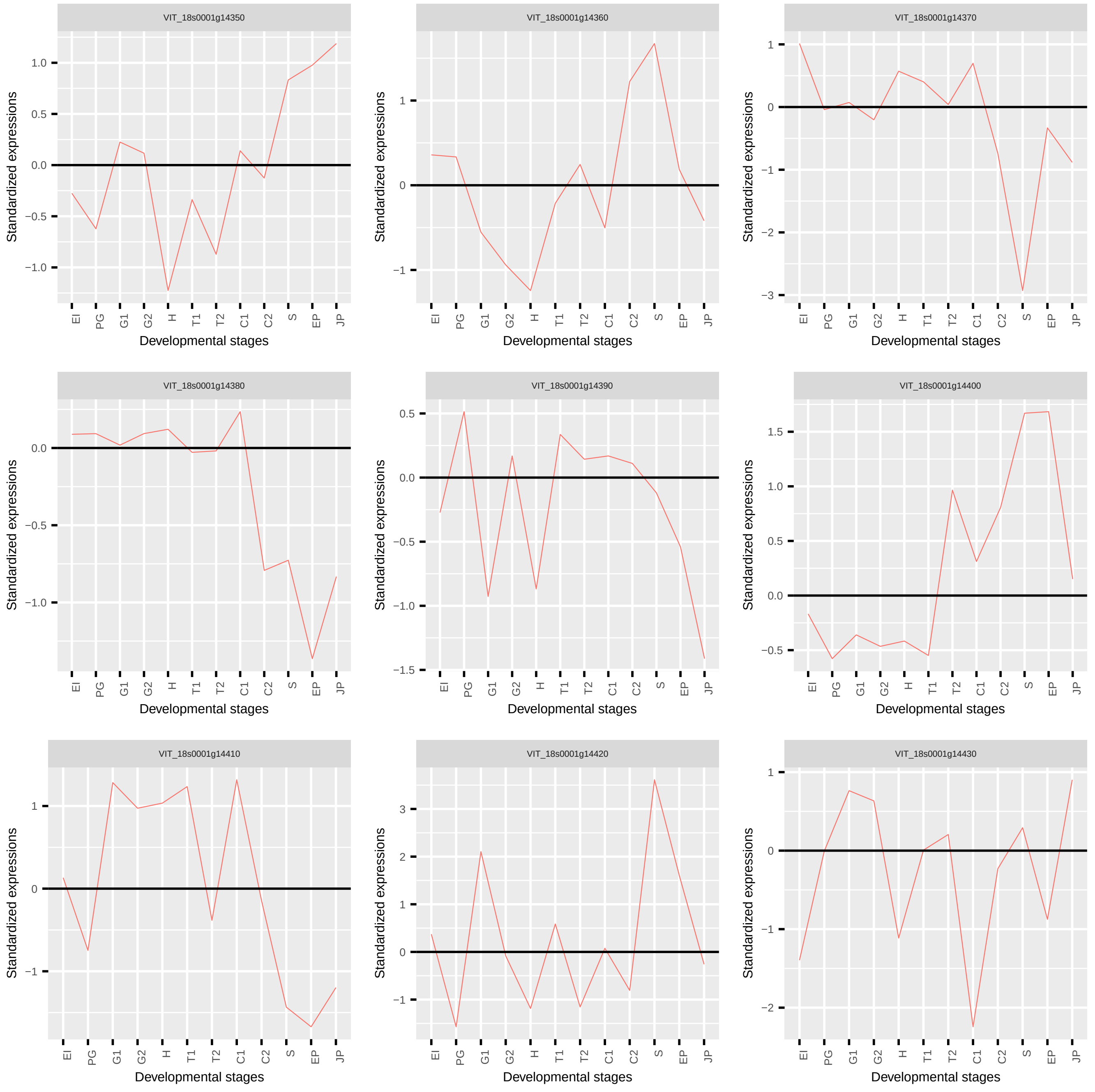
VIT_18s0001g14350
VIT_18s0001g14360
VIT_18s0001g14370
VIT_18s0001g14380
VIT_18s0001g14390
VIT_18s0001g14400
VIT_18s0001g14410
VIT_18s0001g14420
VIT_18s0001g14430
Standardized expressions
Developmental stages
EI PG G1 G2 H T1 T2 C1 C2 S EP JP

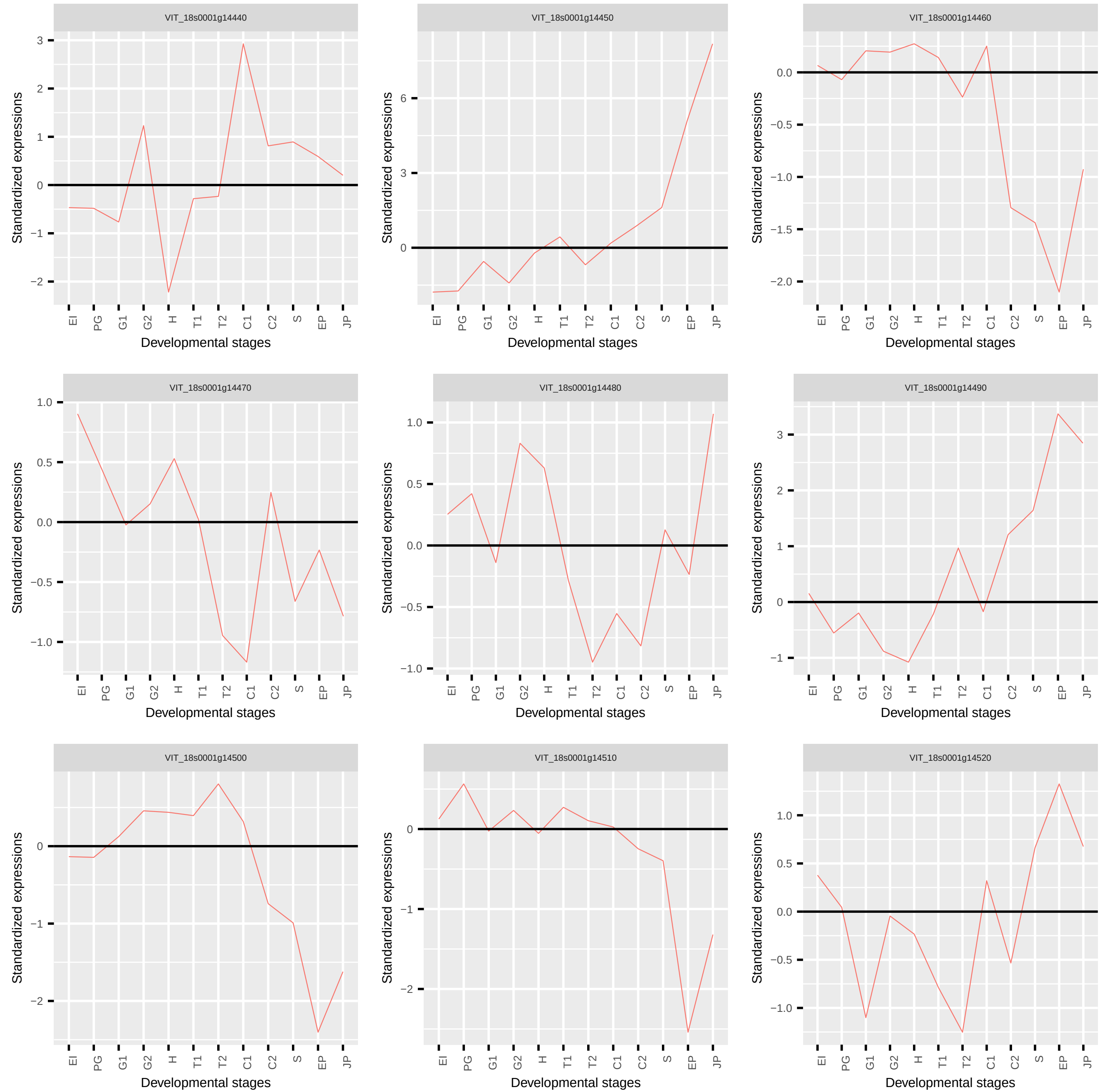

VIT_18s0001g14440
3
2
1
0
−1
−2
EI PG G1 G2 H T1 T2 C1 C2 S EP JP
Developmental stages
Standardized expressions
VIT_18s0001g14450
6
3
0
EI PG G1 G2 H T1 T2 C1 C2 S EP JP
Developmental stages
Standardized expressions
VIT_18s0001g14460
0.0
−0.5
−1.0
−1.5
−2.0
EI PG G1 G2 H T1 T2 C1 C2 S EP JP
Developmental stages
Standardized expressions
VIT_18s0001g14470
1.0
0.5
0.0
−0.5
−1.0
EI PG G1 G2 H T1 T2 C1 C2 S EP JP
Developmental stages
Standardized expressions
VIT_18s0001g14480
1.0
0.5
0.0
−0.5
−1.0
EI PG G1 G2 H T1 T2 C1 C2 S EP JP
Developmental stages
Standardized expressions
VIT_18s0001g14490
3
2
1
0
−1
EI PG G1 G2 H T1 T2 C1 C2 S EP JP
Developmental stages
Standardized expressions
VIT_18s0001g14500
0
−1
−2
EI PG G1 G2 H T1 T2 C1 C2 S EP JP
Developmental stages
Standardized expressions
VIT_18s0001g14510
0
−1
−2
EI PG G1 G2 H T1 T2 C1 C2 S EP JP
Developmental stages
Standardized expressions
VIT_18s0001g14520
1.0
0.5
0.0
−0.5
−1.0
EI PG G1 G2 H T1 T2 C1 C2 S EP JP
Developmental stages
Standardized expressions

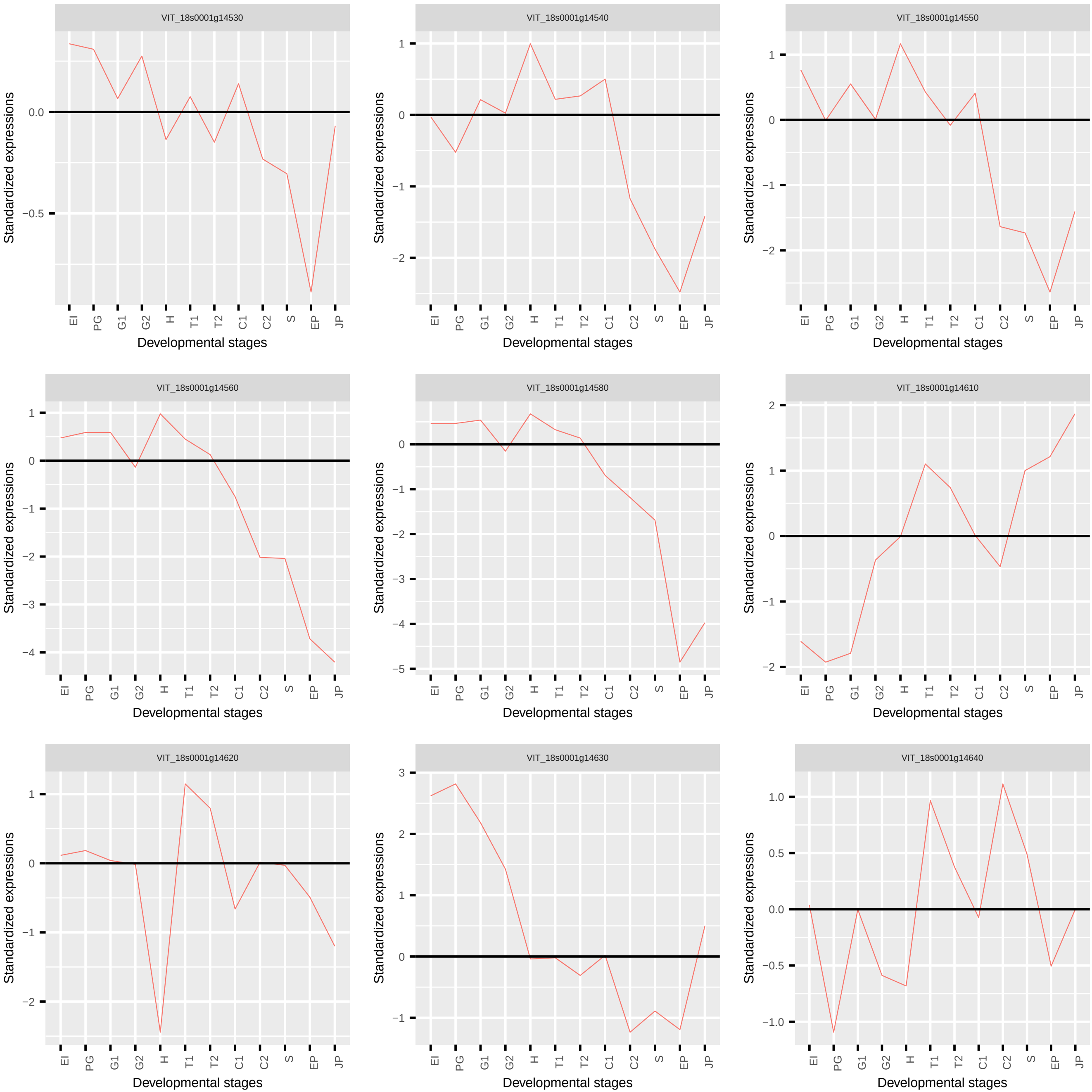
VIT_18s0001g14530
VIT_18s0001g14540
VIT_18s0001g14550
VIT_18s0001g14560
VIT_18s0001g14580
VIT_18s0001g14610
VIT_18s0001g14620
VIT_18s0001g14630
VIT_18s0001g14640
Standardized expressions
Developmental stages
EI PG G1 G2 H T1 T2 C1 C2 S EP JP

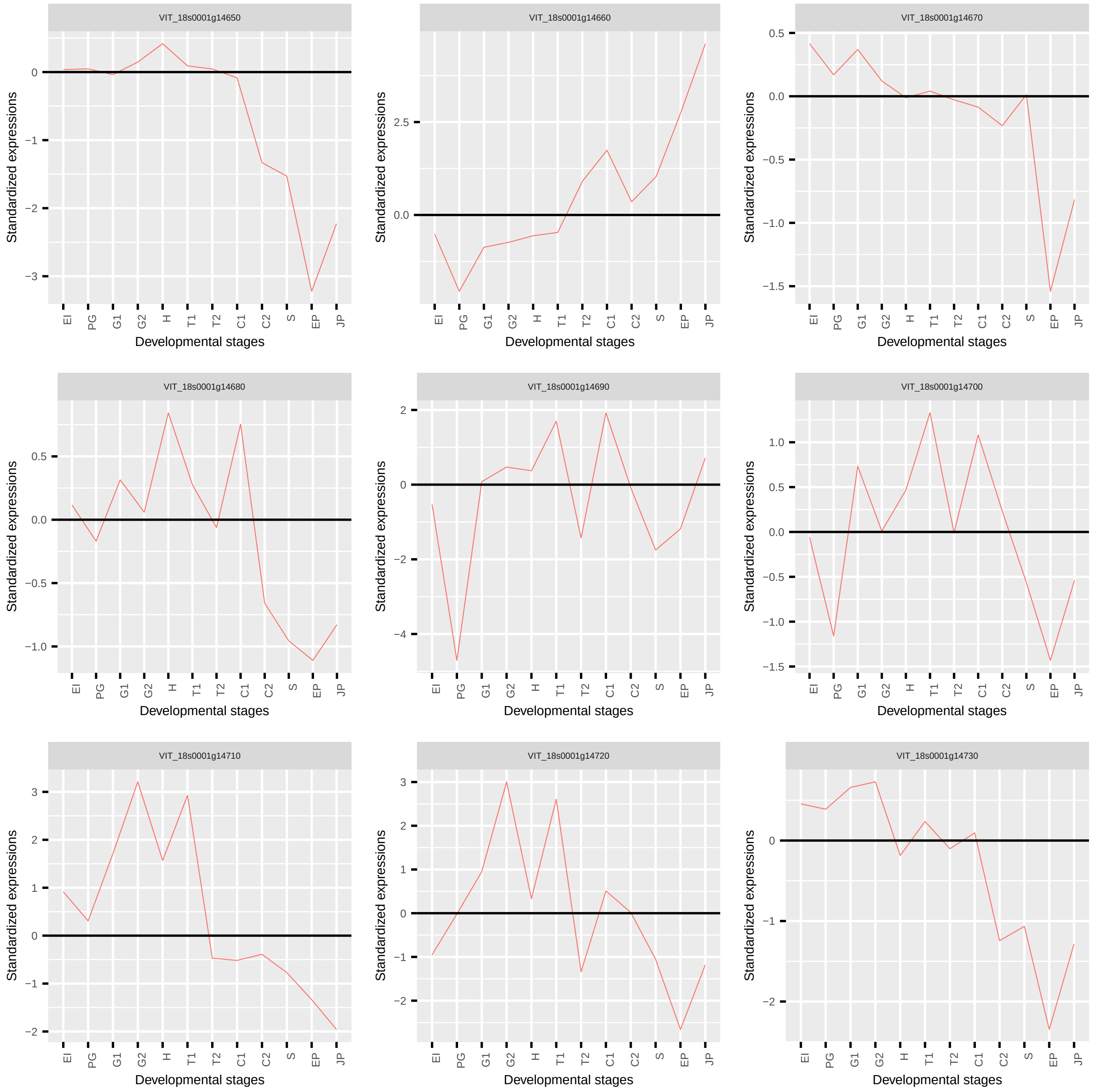
VIT_18s0001g14650
0
−1
−2
−3
Standardized expressions
EI PG G1 G2 H T1 T2 C1 C2 S EP JP
Developmental stages
VIT_18s0001g14660
2.5
0.0
Standardized expressions
EI PG G1 G2 H T1 T2 C1 C2 S EP JP
Developmental stages
VIT_18s0001g14670
0.5
0.0
−0.5
−1.0
−1.5
Standardized expressions
EI PG G1 G2 H T1 T2 C1 C2 S EP JP
Developmental stages
VIT_18s0001g14680
0.5
0.0
−0.5
−1.0
Standardized expressions
EI PG G1 G2 H T1 T2 C1 C2 S EP JP
Developmental stages
VIT_18s0001g14690
2
0
−2
−4
Standardized expressions
EI PG G1 G2 H T1 T2 C1 C2 S EP JP
Developmental stages
VIT_18s0001g14700
1.0
0.5
0.0
−0.5
−1.0
−1.5
Standardized expressions
EI PG G1 G2 H T1 T2 C1 C2 S EP JP
Developmental stages
VIT_18s0001g14710
3
2
1
0
−1
−2
Standardized expressions
EI PG G1 G2 H T1 T2 C1 C2 S EP JP
Developmental stages
VIT_18s0001g14720
3
2
1
0
−1
−2
Standardized expressions
EI PG G1 G2 H T1 T2 C1 C2 S EP JP
Developmental stages
VIT_18s0001g14730
0
−1
−2
Standardized expressions
EI PG G1 G2 H T1 T2 C1 C2 S EP JP
Developmental stages

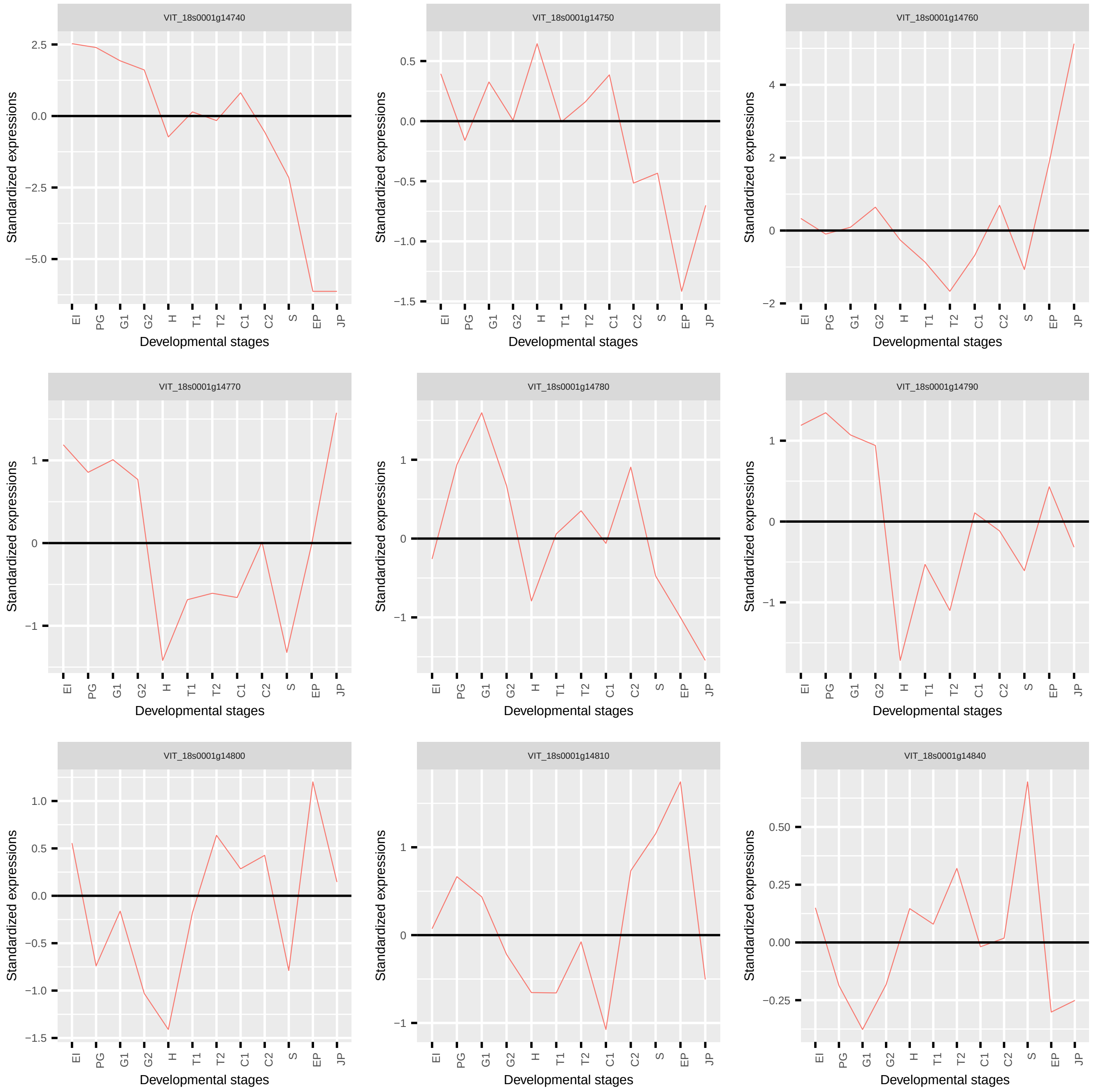
VIT_18s0001g14740
VIT_18s0001g14750
VIT_18s0001g14760
VIT_18s0001g14770
VIT_18s0001g14780
VIT_18s0001g14790
VIT_18s0001g14800
VIT_18s0001g14810
VIT_18s0001g14840
Standardized expressions
Developmental stages
EI PG G1 G2 H T1 T2 C1 C2 S EP JP

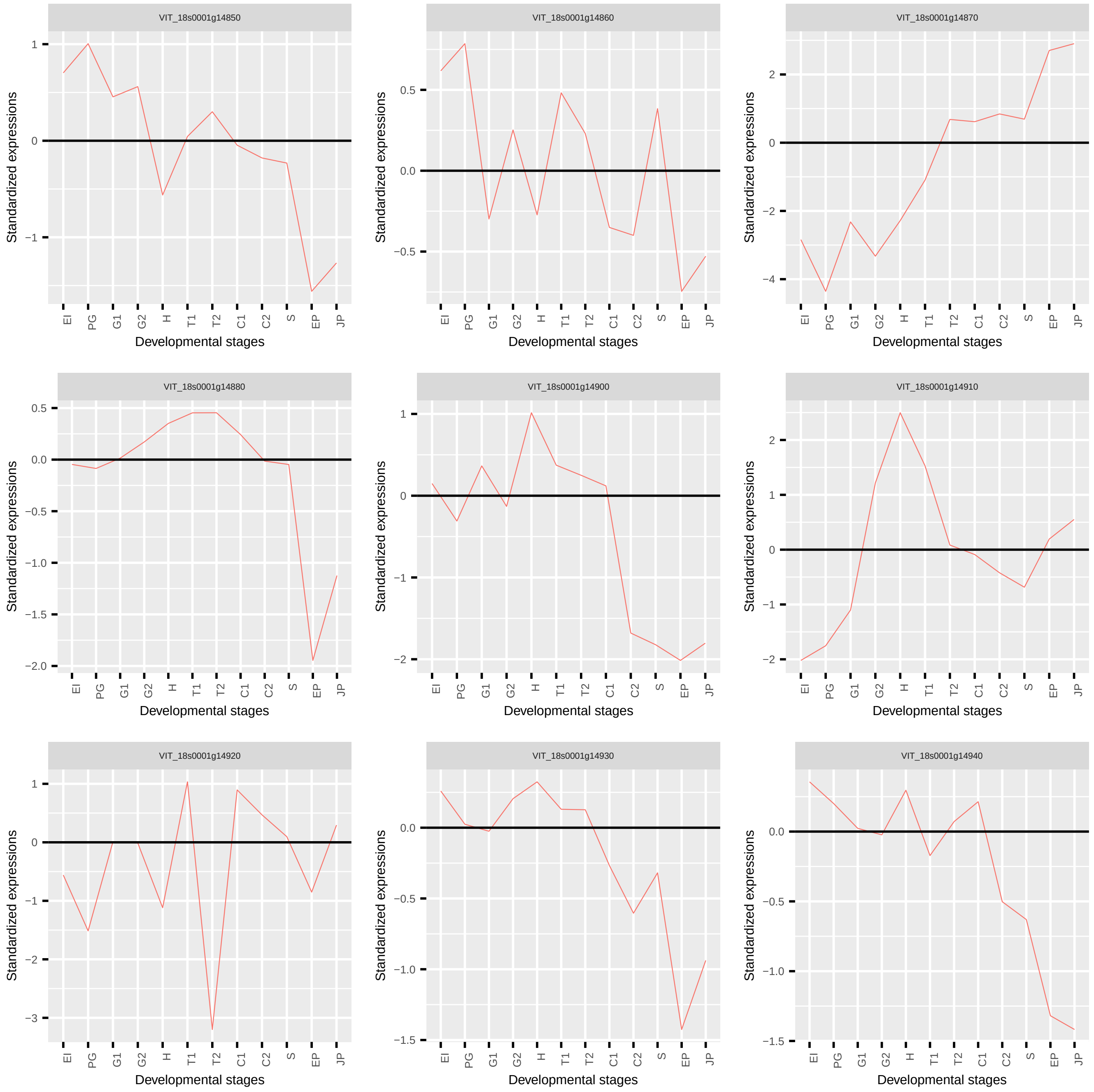
VIT_18s0001g14850
VIT_18s0001g14860
VIT_18s0001g14870
VIT_18s0001g14880
VIT_18s0001g14900
VIT_18s0001g14910
VIT_18s0001g14920
VIT_18s0001g14930
VIT_18s0001g14940
Standardized expressions
Developmental stages
EI PG G1 G2 H T1 T2 C1 C2 S EP JP

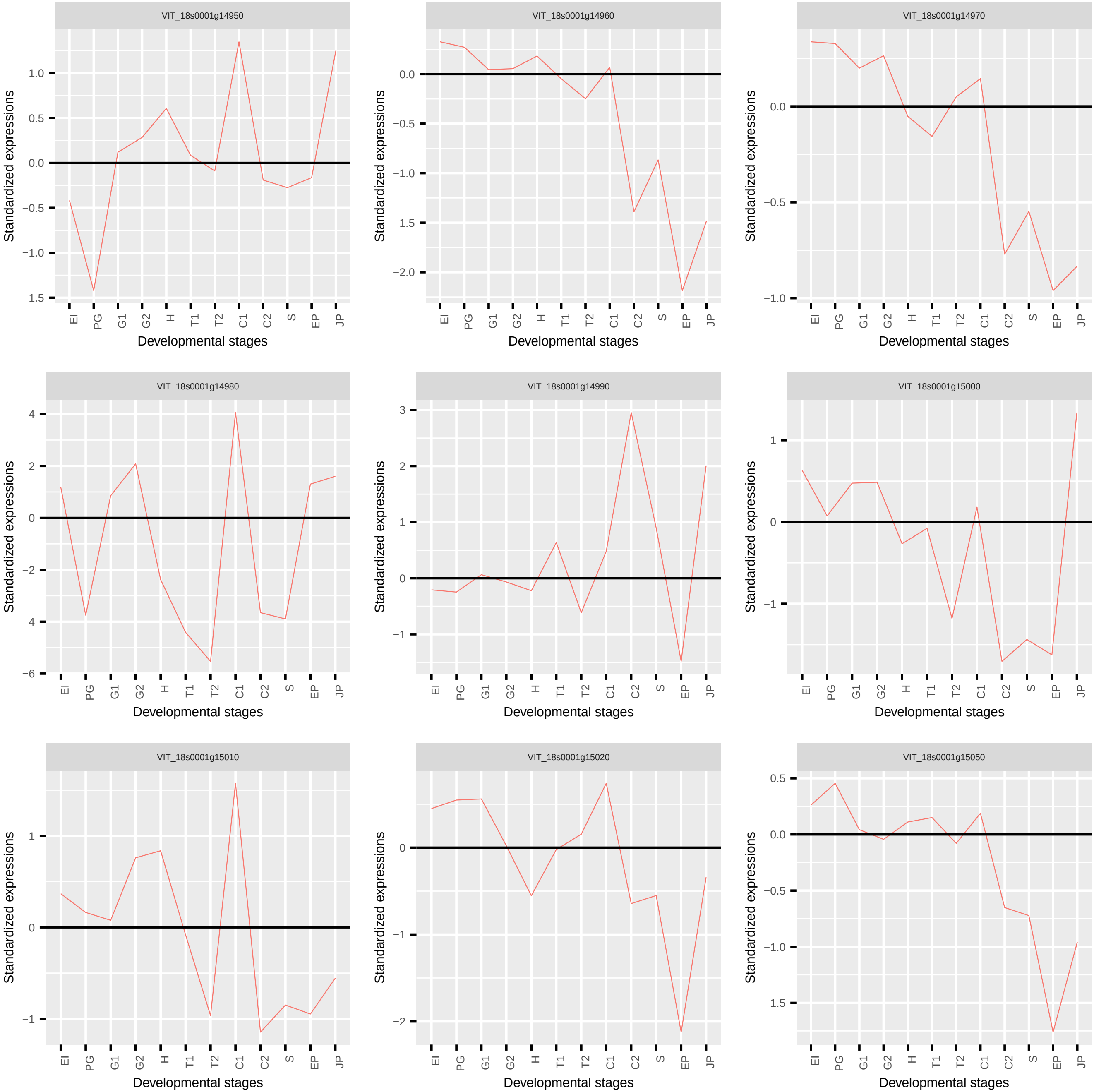
VIT_18s0001g14950
VIT_18s0001g14960
VIT_18s0001g14970
VIT_18s0001g14980
VIT_18s0001g14990
VIT_18s0001g15000
VIT_18s0001g15010
VIT_18s0001g15020
VIT_18s0001g15050
Standardized expressions
Developmental stages
EI PG G1 G2 H T1 T2 C1 C2 S EP JP

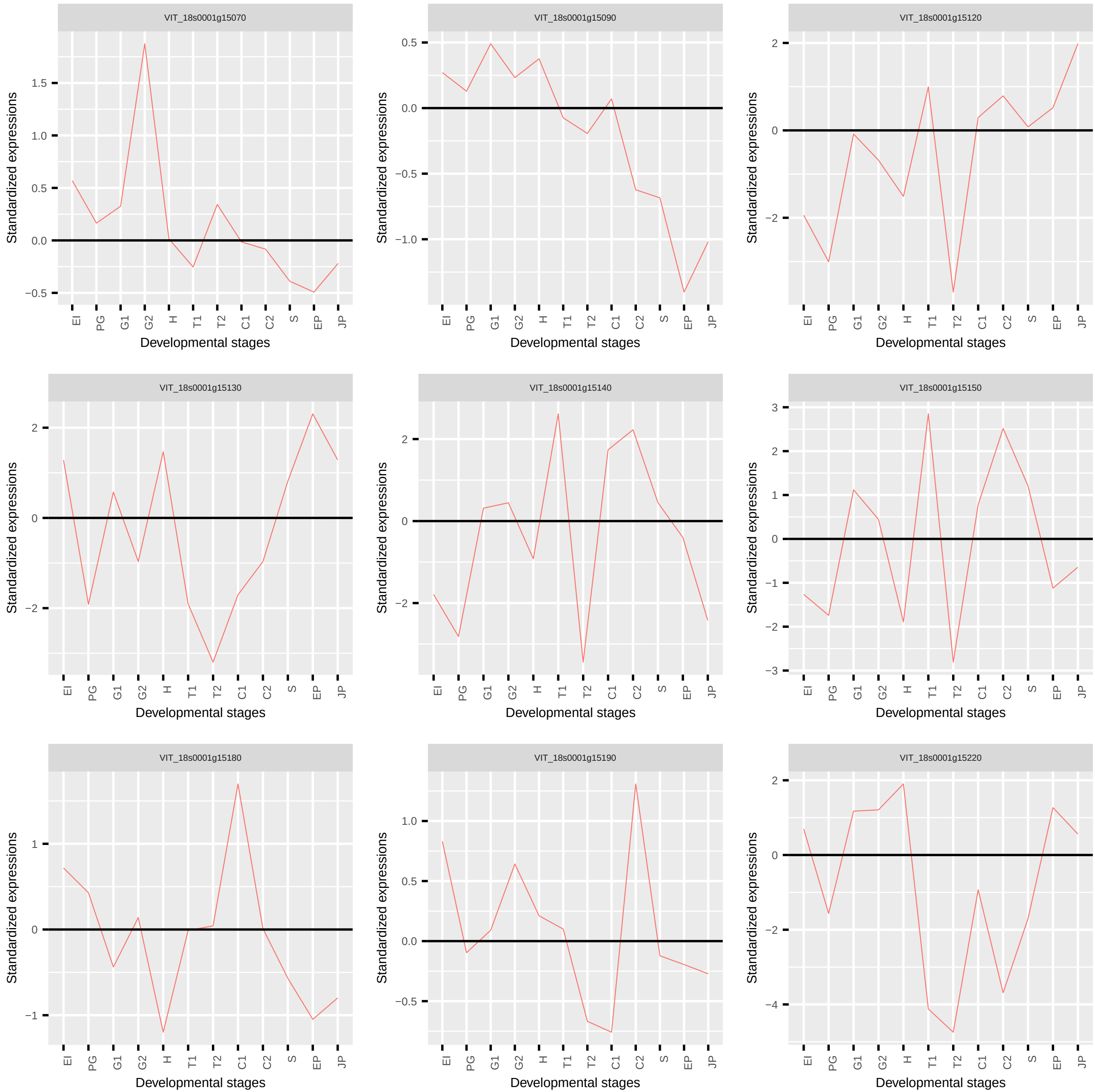
VIT_18s0001g15070
VIT_18s0001g15090
VIT_18s0001g15120
VIT_18s0001g15130
VIT_18s0001g15140
VIT_18s0001g15150
VIT_18s0001g15180
VIT_18s0001g15190
VIT_18s0001g15220
Standardized expressions
Developmental stages
EI PG G1 G2 H T1 T2 C1 C2 S EP JP

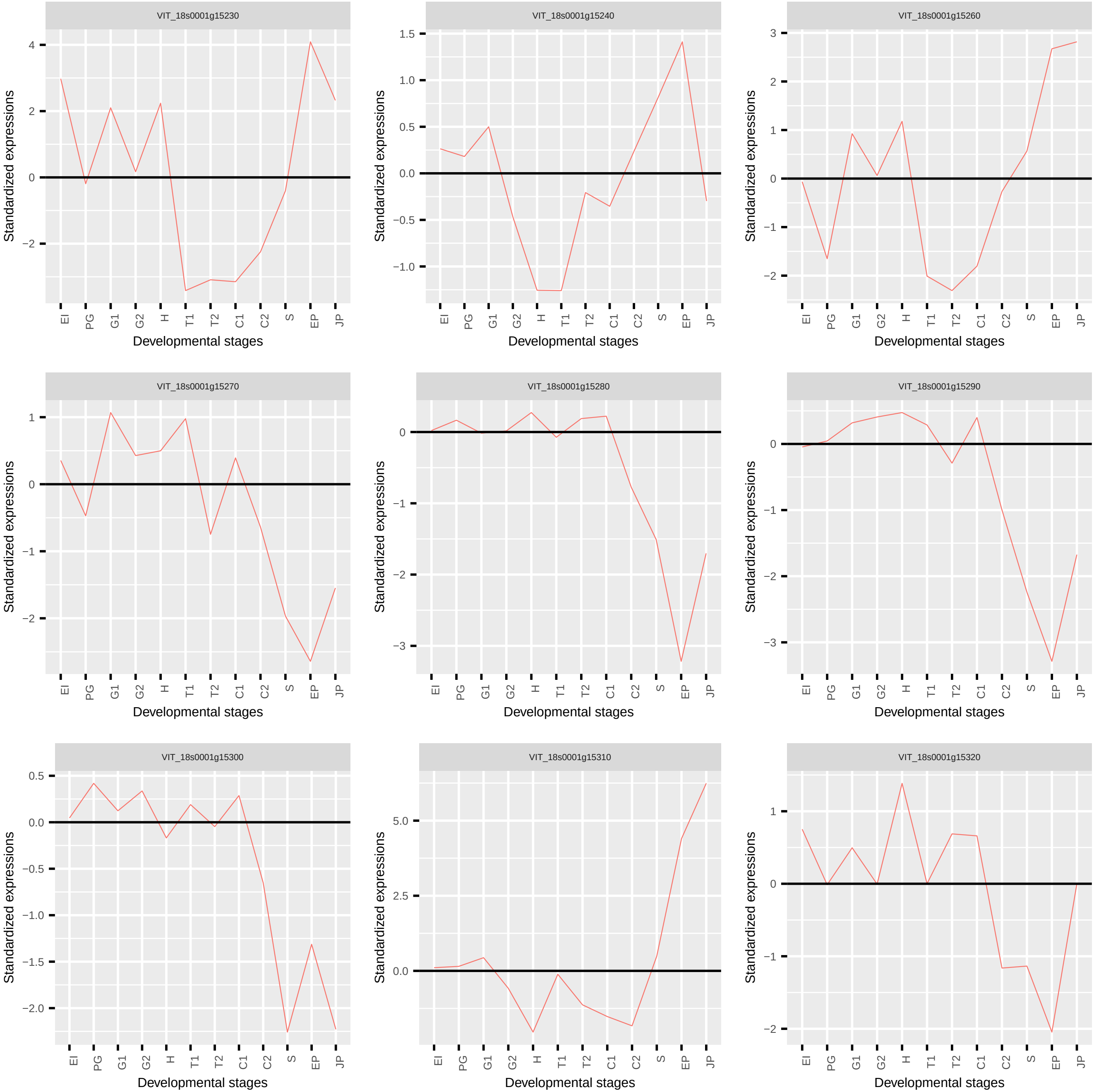
VIT_18s0001g15230
VIT_18s0001g15240
VIT_18s0001g15260
VIT_18s0001g15270
VIT_18s0001g15280
VIT_18s0001g15290
VIT_18s0001g15300
VIT_18s0001g15310
VIT_18s0001g15320
Standardized expressions
Developmental stages
EI PG G1 G2 H T1 T2 C1 C2 S EP JP

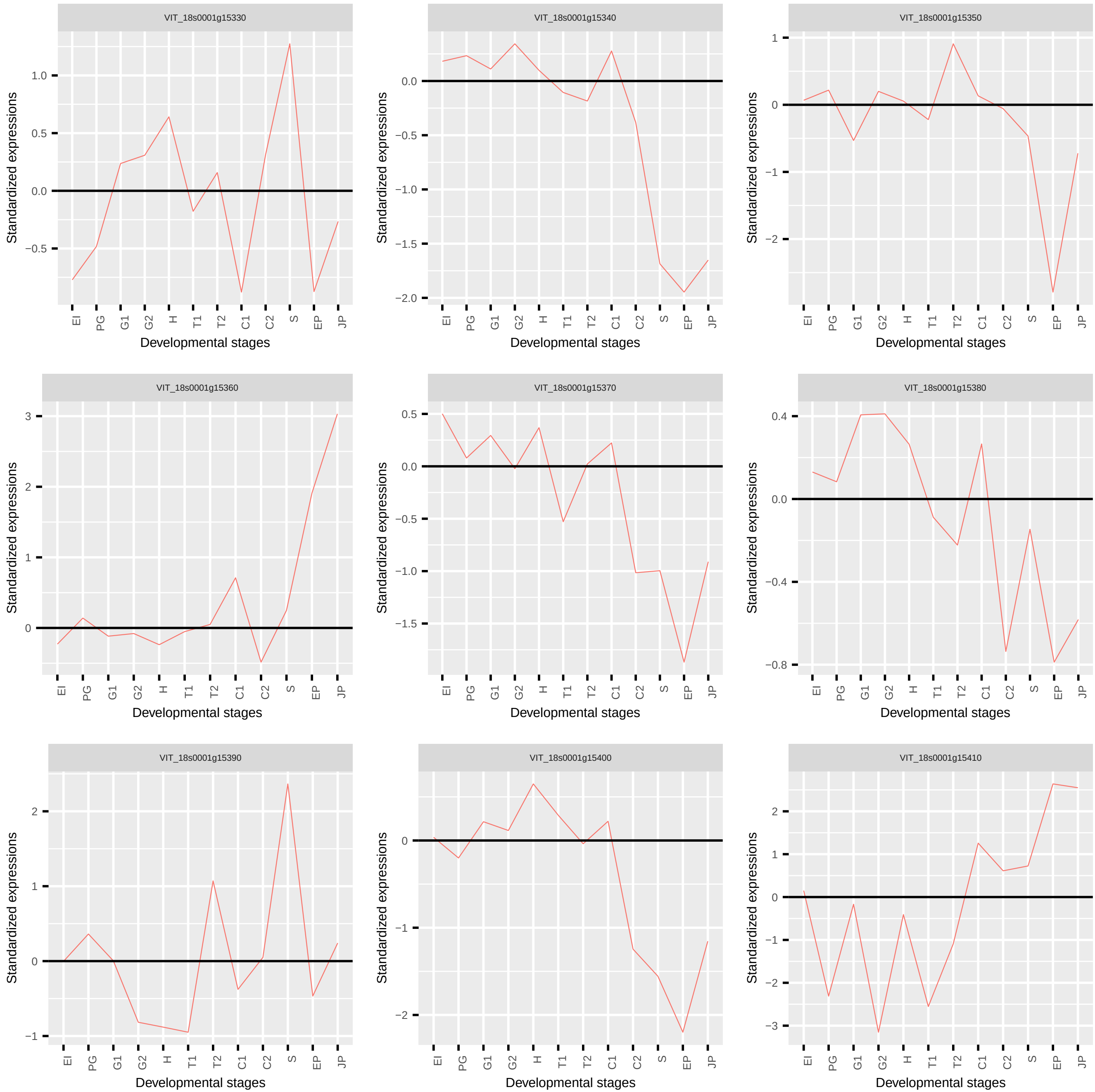

VIT_18s0001g15330
VIT_18s0001g15340
VIT_18s0001g15350
VIT_18s0001g15360
VIT_18s0001g15370
VIT_18s0001g15380
VIT_18s0001g15390
VIT_18s0001g15400
VIT_18s0001g15410
Standardized expressions
Developmental stages
EI PG G1 G2 H T1 T2 C1 C2 S EP JP

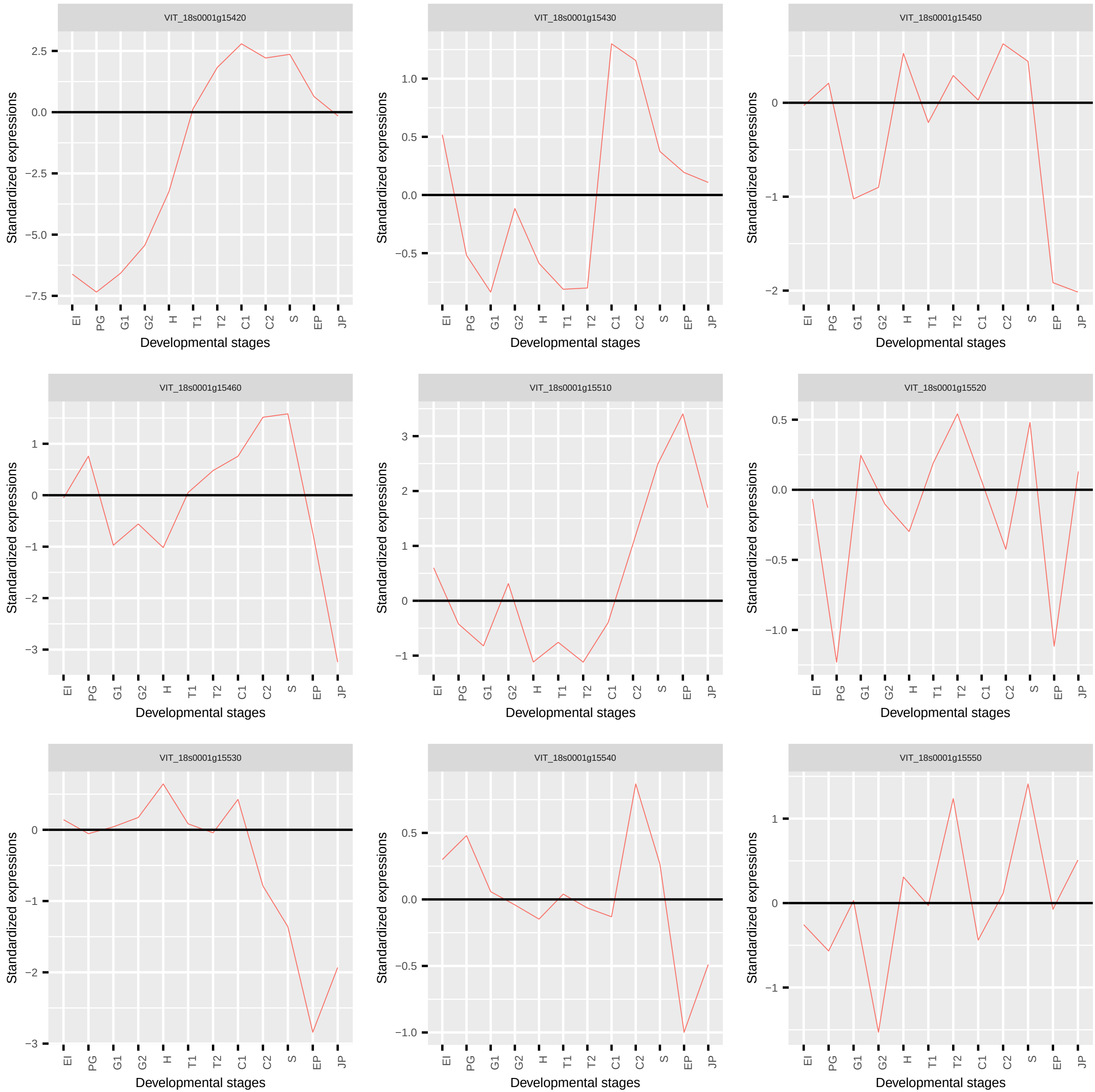

VIT_18s0001g15420
VIT_18s0001g15430
VIT_18s0001g15450
VIT_18s0001g15460
VIT_18s0001g15510
VIT_18s0001g15520
VIT_18s0001g15530
VIT_18s0001g15540
VIT_18s0001g15550
Standardized expressions
Developmental stages
EI PG G1 G2 H T1 T2 C1 C2 S EP JP

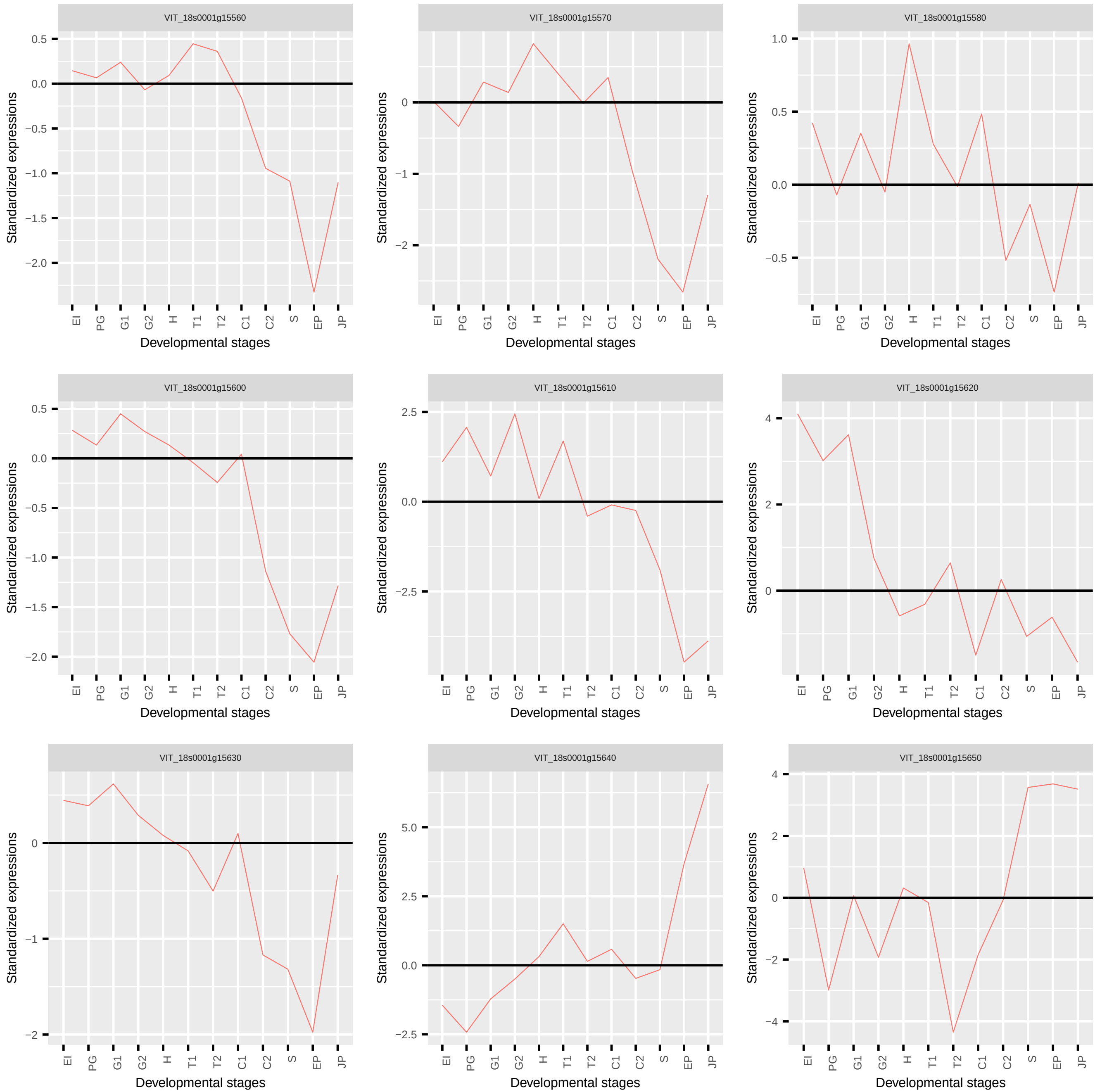

VIT_18s0001g15560
VIT_18s0001g15570
VIT_18s0001g15580
VIT_18s0001g15600
VIT_18s0001g15610
VIT_18s0001g15620
VIT_18s0001g15630
VIT_18s0001g15640
VIT_18s0001g15650
Standardized expressions
Developmental stages
EI PG G1 G2 H T1 T2 C1 C2 S EP JP

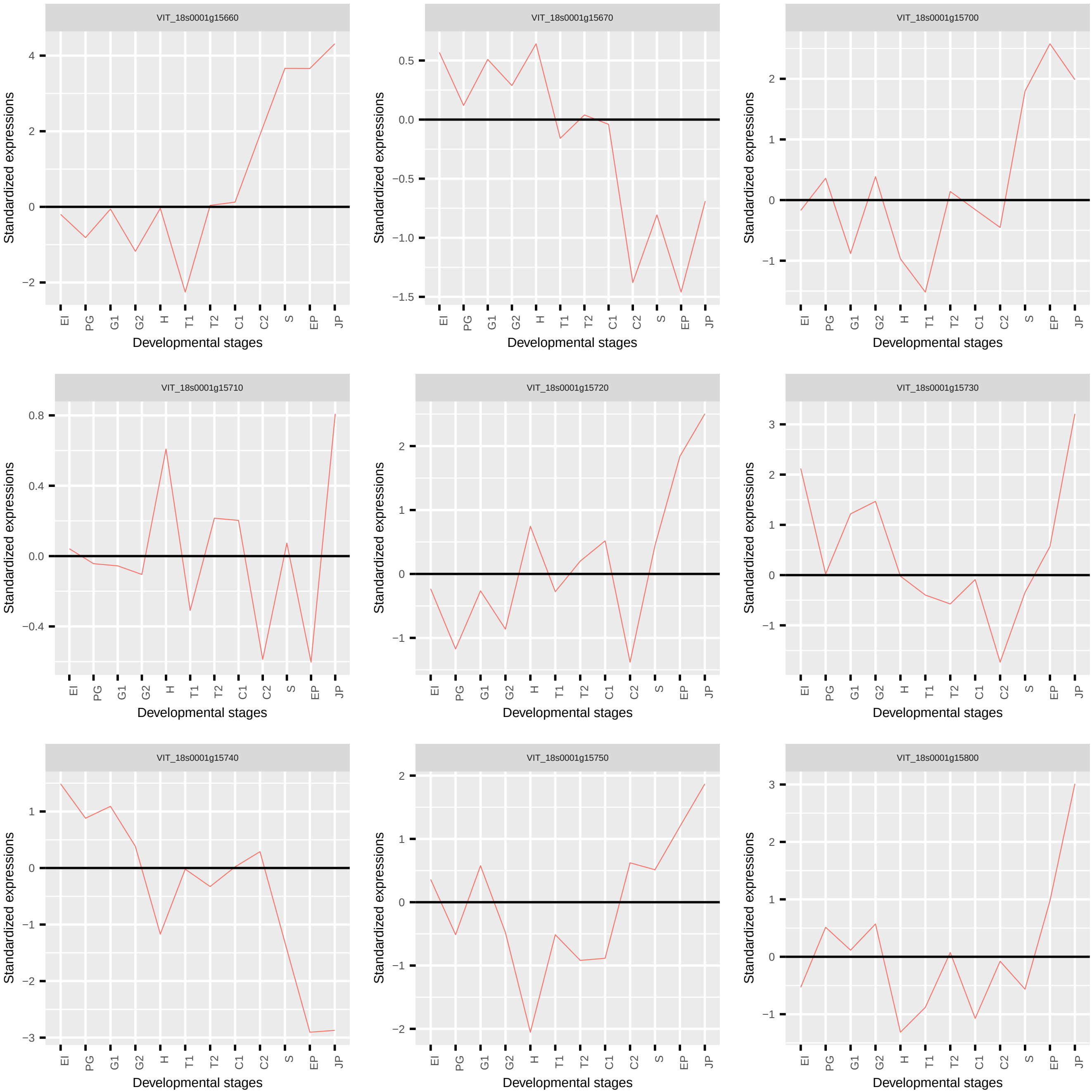

VIT_18s0001g15660
VIT_18s0001g15670
VIT_18s0001g15700
VIT_18s0001g15710
VIT_18s0001g15720
VIT_18s0001g15730
VIT_18s0001g15740
VIT_18s0001g15750
VIT_18s0001g15800
Standardized expressions
Developmental stages
EI PG G1 G2 H T1 T2 C1 C2 S EP JP

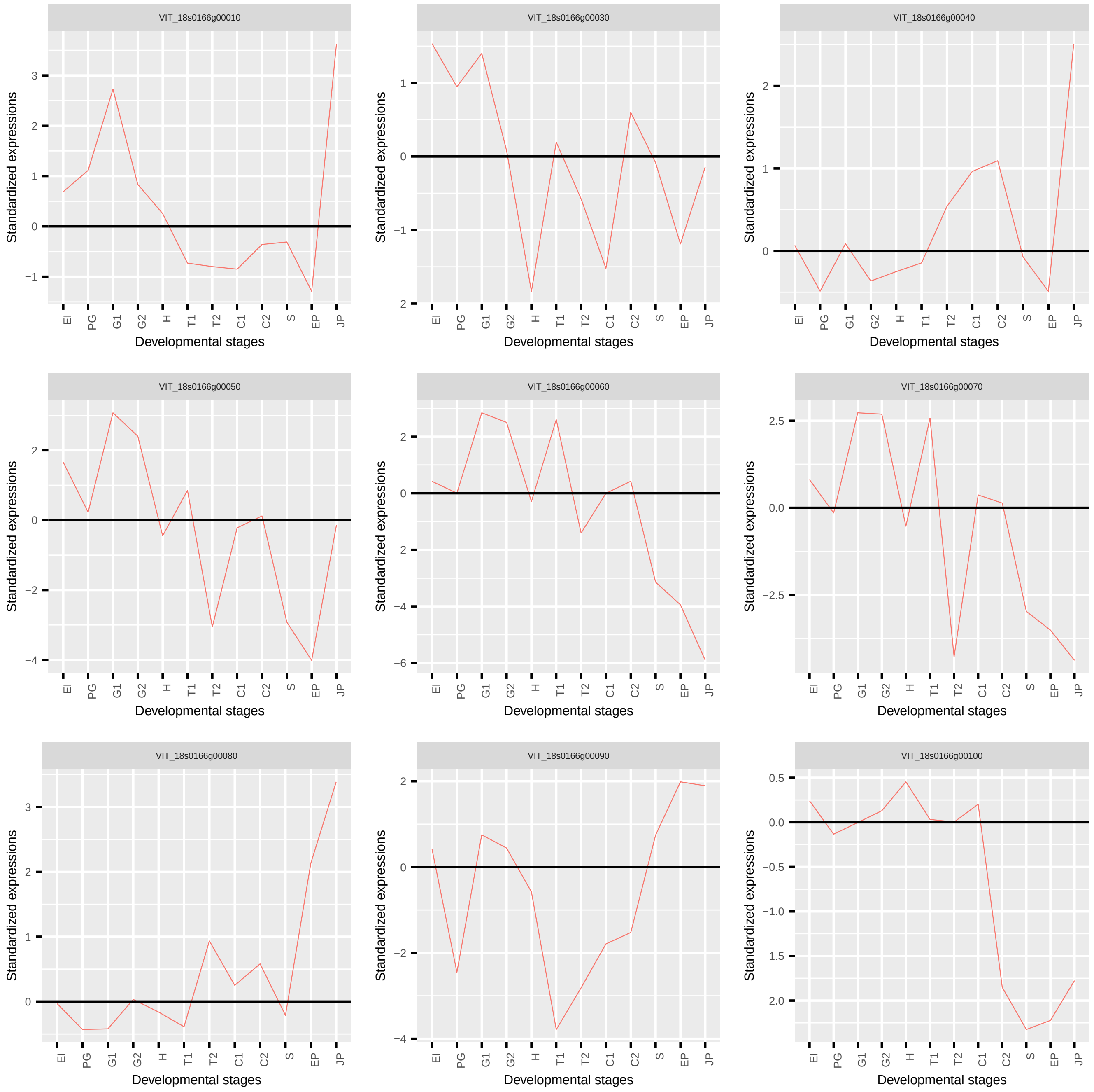
VIT_18s0166g00010
VIT_18s0166g00030
VIT_18s0166g00040
VIT_18s0166g00050
VIT_18s0166g00060
VIT_18s0166g00070
VIT_18s0166g00080
VIT_18s0166g00090
VIT_18s0166g00100
Standardized expressions
Developmental stages
EI PG G1 G2 H T1 T2 C1 C2 S EP JP

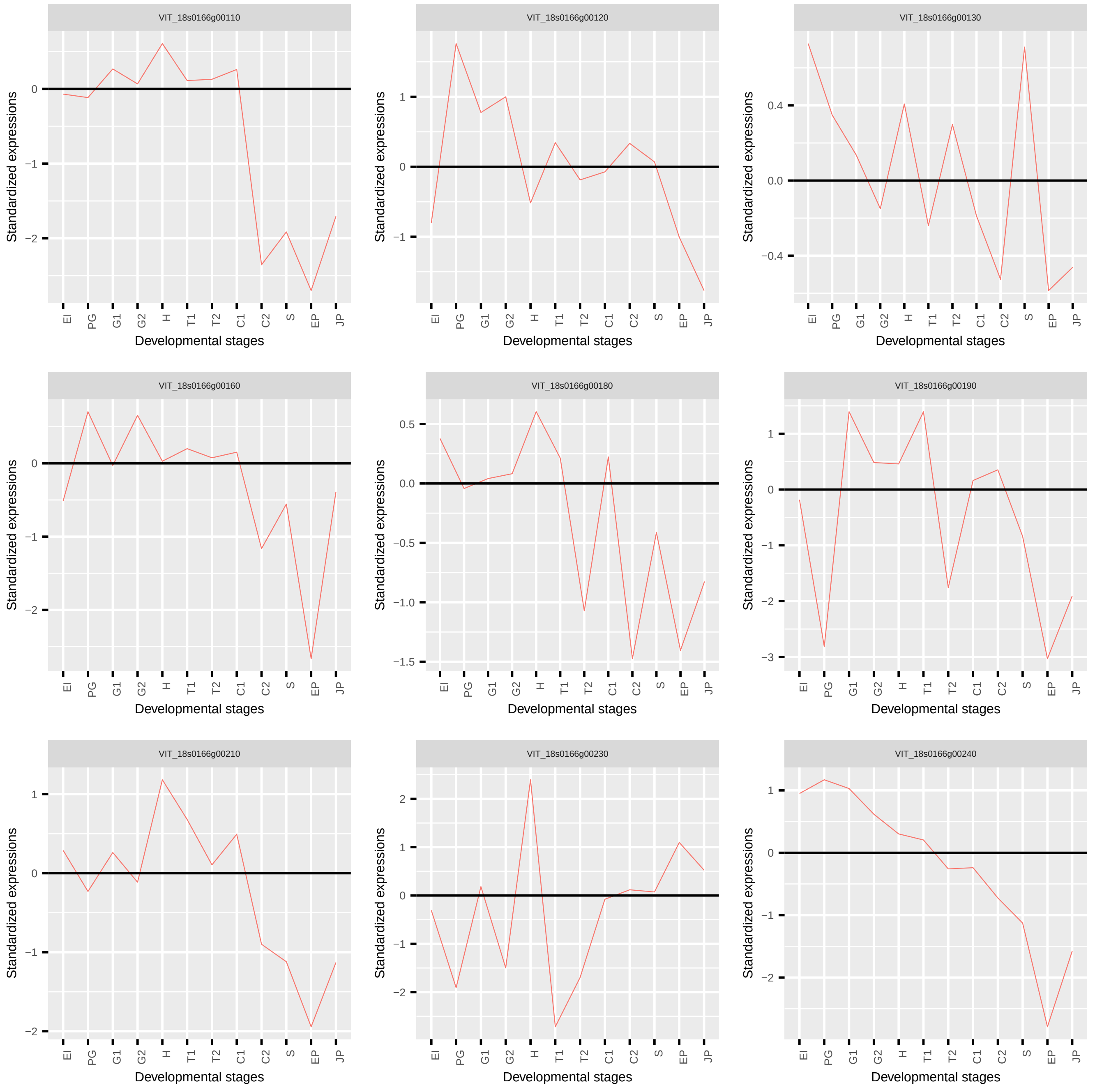
VIT_18s0166g00110
VIT_18s0166g00120
VIT_18s0166g00130
VIT_18s0166g00160
VIT_18s0166g00180
VIT_18s0166g00190
VIT_18s0166g00210
VIT_18s0166g00230
VIT_18s0166g00240
Standardized expressions
Developmental stages
EI
PG
G1
G2
H
T1
T2
C1
C2
S
EP
JP

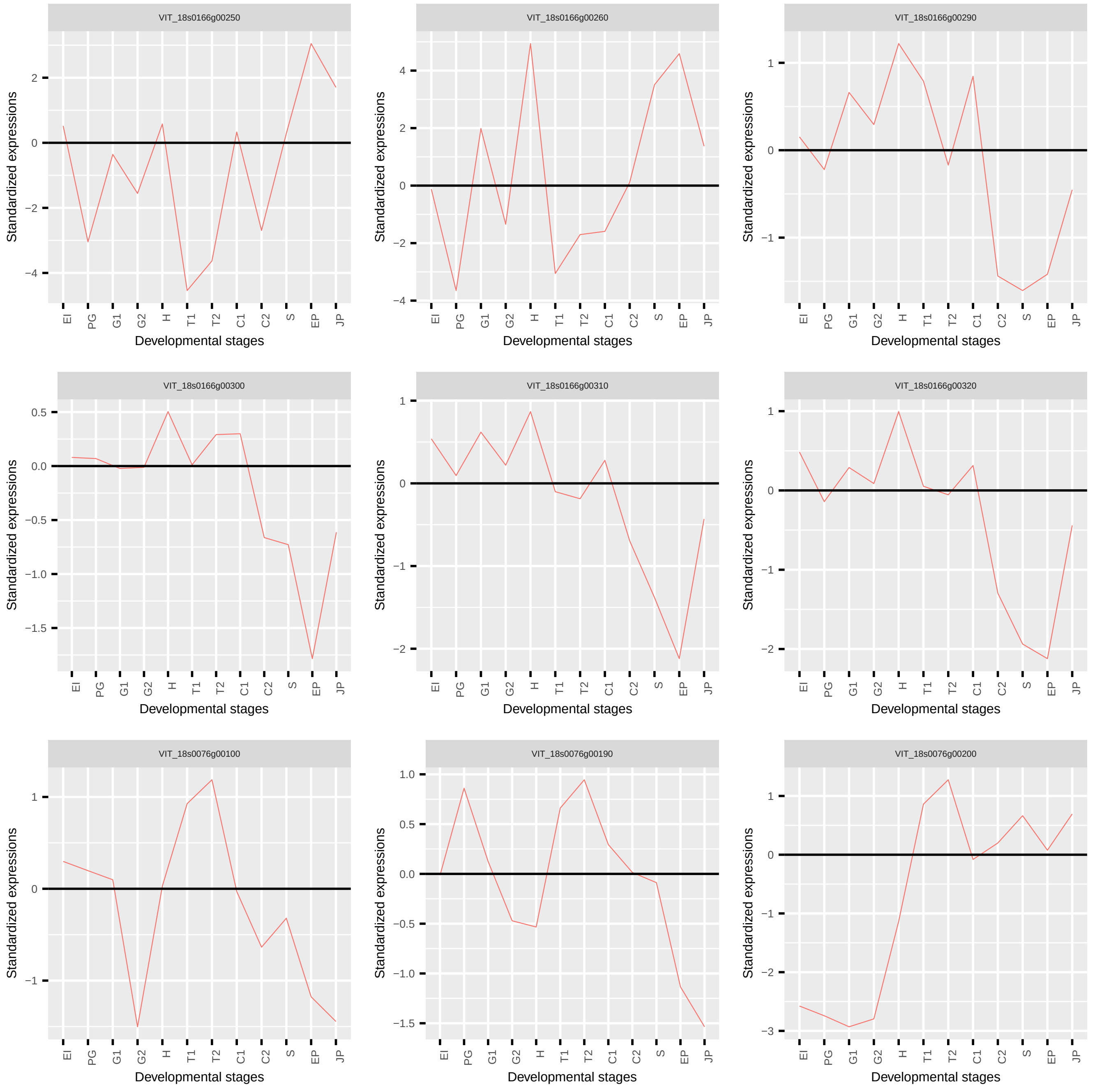
VIT_18s0166g00250
VIT_18s0166g00260
VIT_18s0166g00290
VIT_18s0166g00300
VIT_18s0166g00310
VIT_18s0166g00320
VIT_18s0076g00100
VIT_18s0076g00190
VIT_18s0076g00200
Standardized expressions
Developmental stages
EI PG G1 G2 H T1 T2 C1 C2 S EP JP

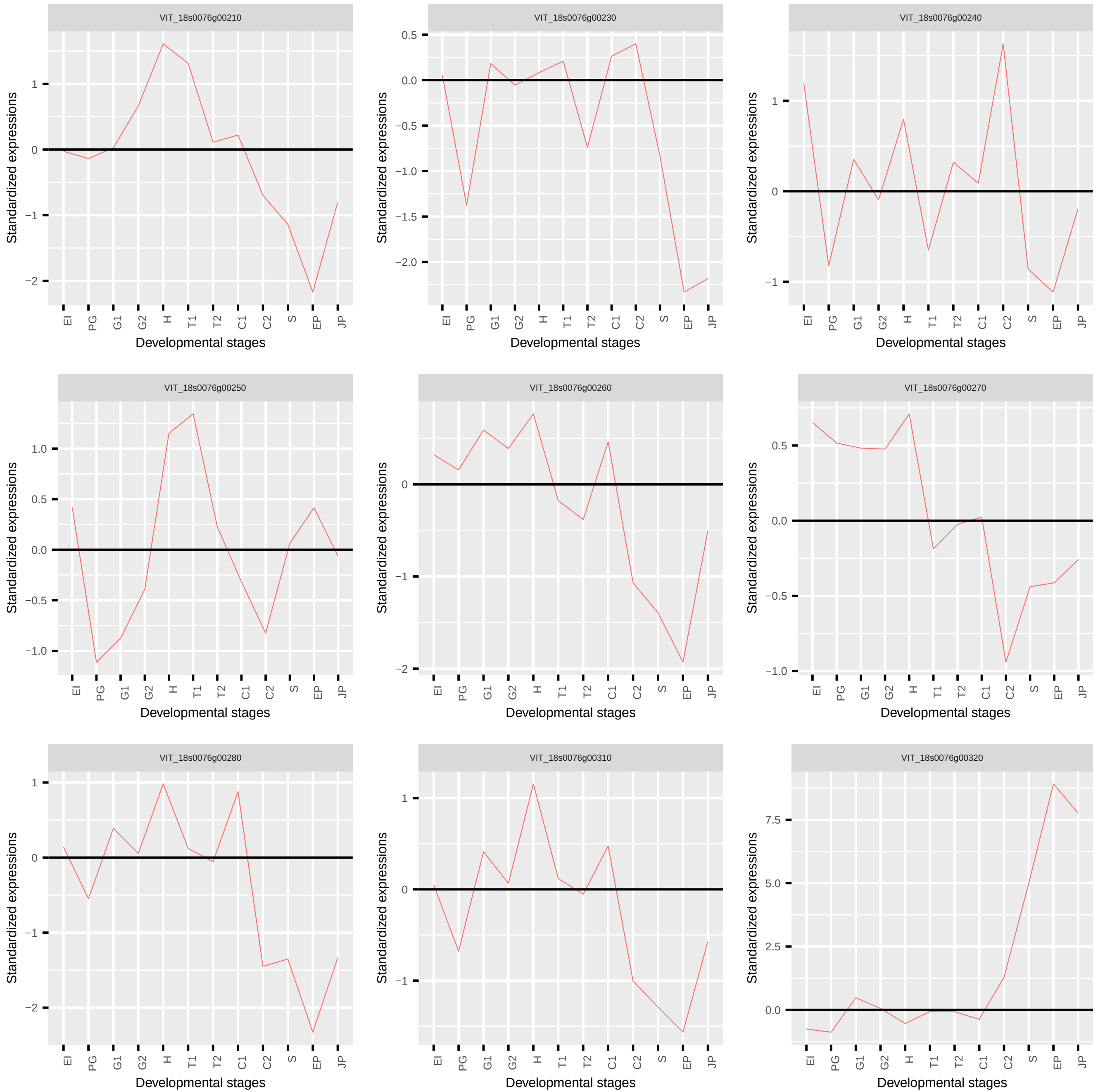

VIT_18s0076g00210
VIT_18s0076g00230
VIT_18s0076g00240
VIT_18s0076g00250
VIT_18s0076g00260
VIT_18s0076g00270
VIT_18s0076g00280
VIT_18s0076g00310
VIT_18s0076g00320
Standardized expressions
Developmental stages
EI PG G1 G2 H T1 T2 C1 C2 S EP JP

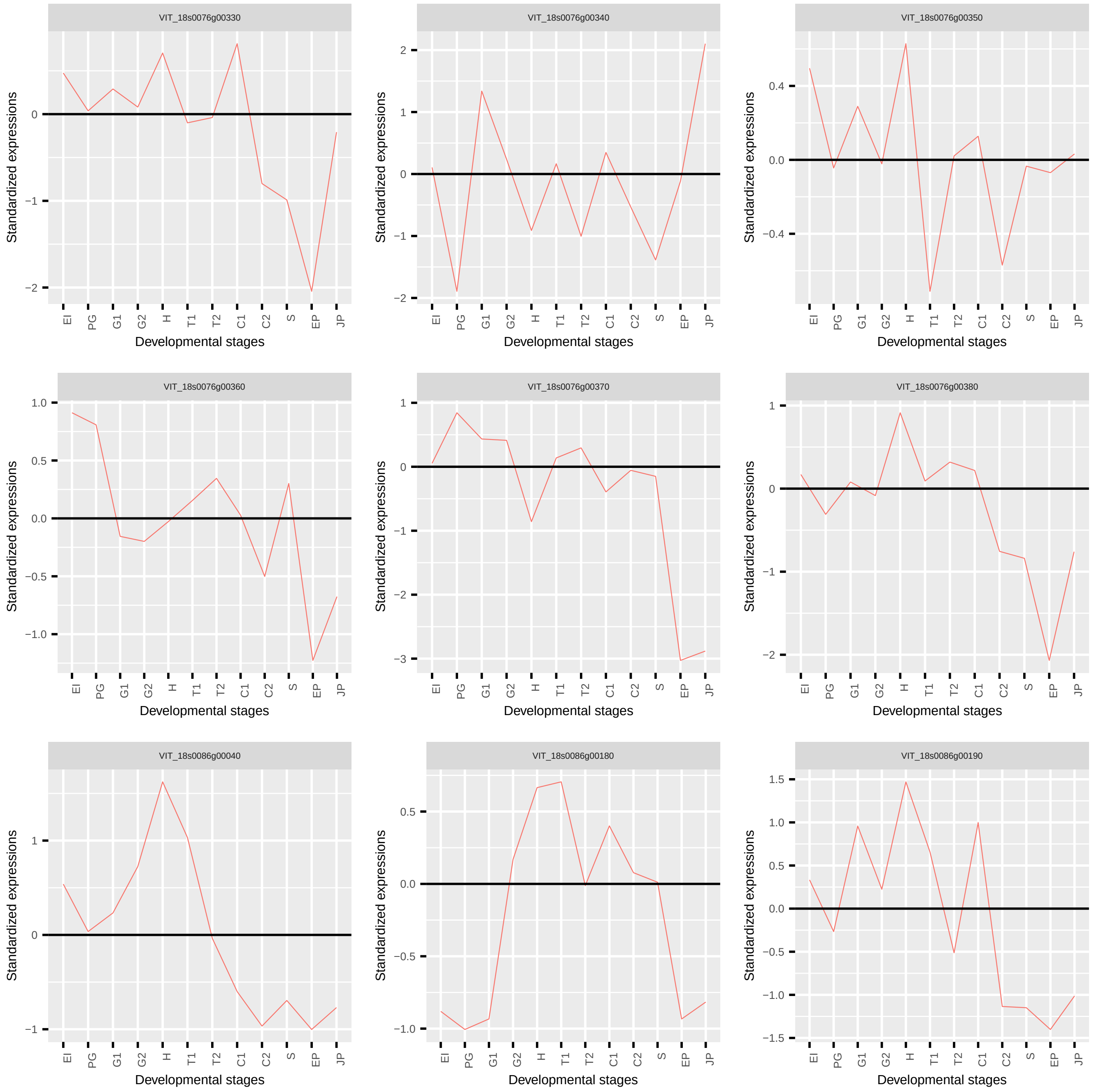
VIT_18s0076g00330
VIT_18s0076g00340
VIT_18s0076g00350
VIT_18s0076g00360
VIT_18s0076g00370
VIT_18s0076g00380
VIT_18s0086g00040
VIT_18s0086g00180
VIT_18s0086g00190
Standardized expressions
Developmental stages
EI PG G1 G2 H T1 T2 C1 C2 S EP JP

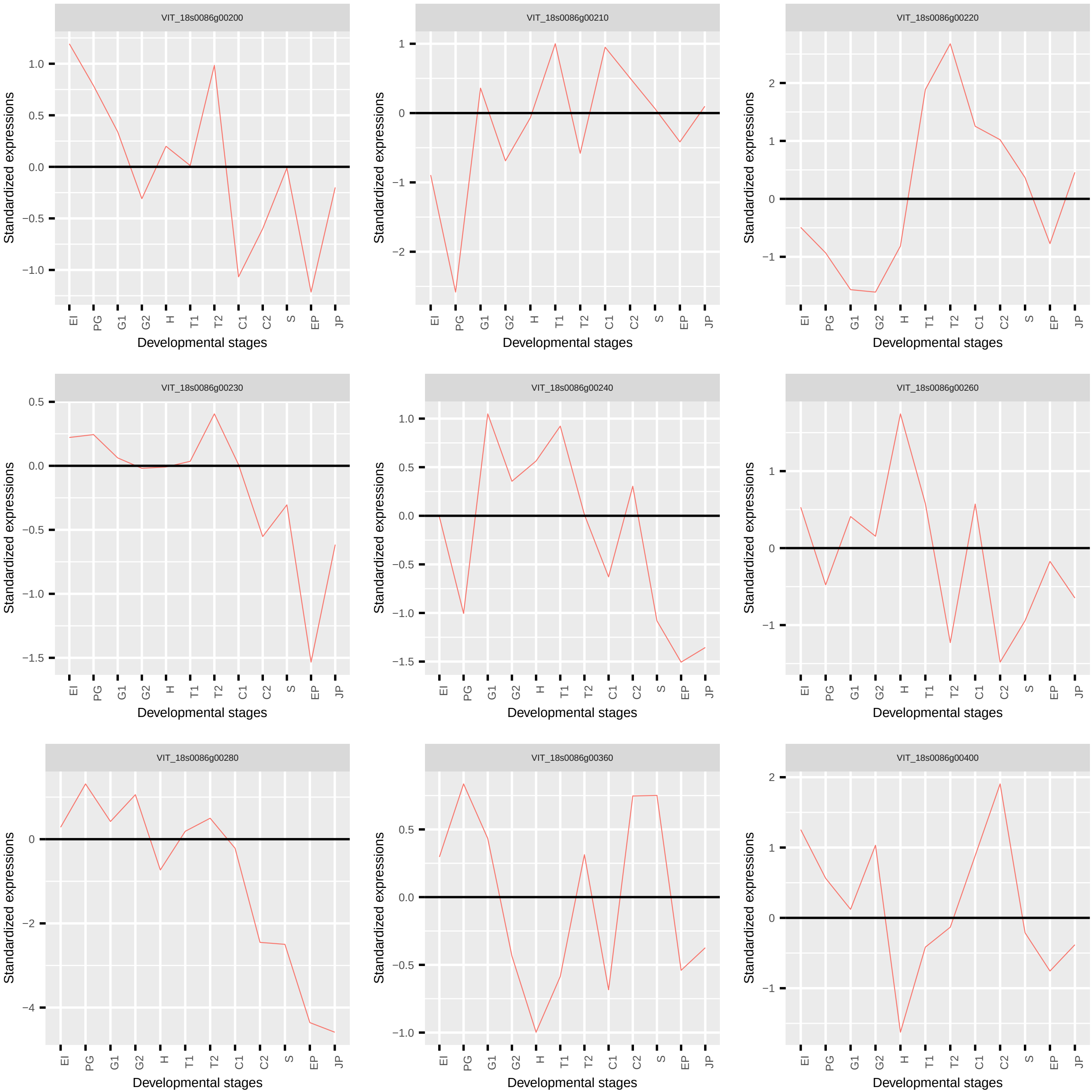
VIT_18s0086g00200
Standardized expressions
1.0
0.5
0.0
−0.5
−1.0
EI PG G1 G2 H T1 T2 C1 C2 S EP JP
Developmental stages
VIT_18s0086g00210
Standardized expressions
1
0
−1
−2
EI PG G1 G2 H T1 T2 C1 C2 S EP JP
Developmental stages
VIT_18s0086g00220
Standardized expressions
2
1
0
−1
EI PG G1 G2 H T1 T2 C1 C2 S EP JP
Developmental stages
VIT_18s0086g00230
Standardized expressions
0.5
0.0
−0.5
−1.0
−1.5
EI PG G1 G2 H T1 T2 C1 C2 S EP JP
Developmental stages
VIT_18s0086g00240
Standardized expressions
1.0
0.5
0.0
−0.5
−1.0
−1.5
EI PG G1 G2 H T1 T2 C1 C2 S EP JP
Developmental stages
VIT_18s0086g00260
Standardized expressions
1
0
−1
EI PG G1 G2 H T1 T2 C1 C2 S EP JP
Developmental stages
VIT_18s0086g00280
Standardized expressions
0
−2
−4
EI PG G1 G2 H T1 T2 C1 C2 S EP JP
Developmental stages
VIT_18s0086g00360
Standardized expressions
0.5
0.0
−0.5
−1.0
EI PG G1 G2 H T1 T2 C1 C2 S EP JP
Developmental stages
VIT_18s0086g00400
Standardized expressions
2
1
0
−1
EI PG G1 G2 H T1 T2 C1 C2 S EP JP
Developmental stages

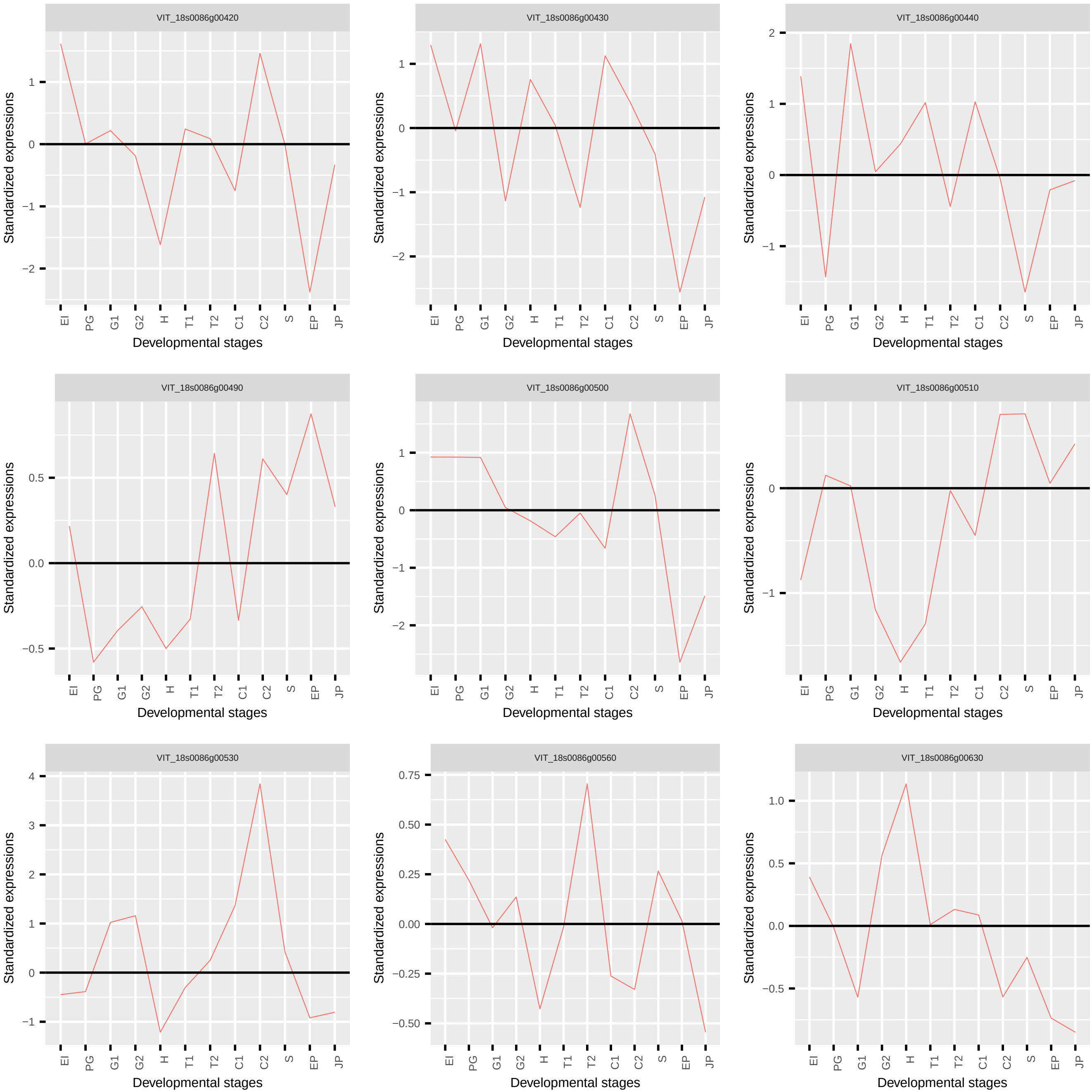
VIT_18s0086g00420
VIT_18s0086g00430
VIT_18s0086g00440
VIT_18s0086g00490
VIT_18s0086g00500
VIT_18s0086g00510
VIT_18s0086g00530
VIT_18s0086g00560
VIT_18s0086g00630
Standardized expressions
Developmental stages
EI PG G1 G2 H T1 T2 C1 C2 S EP JP

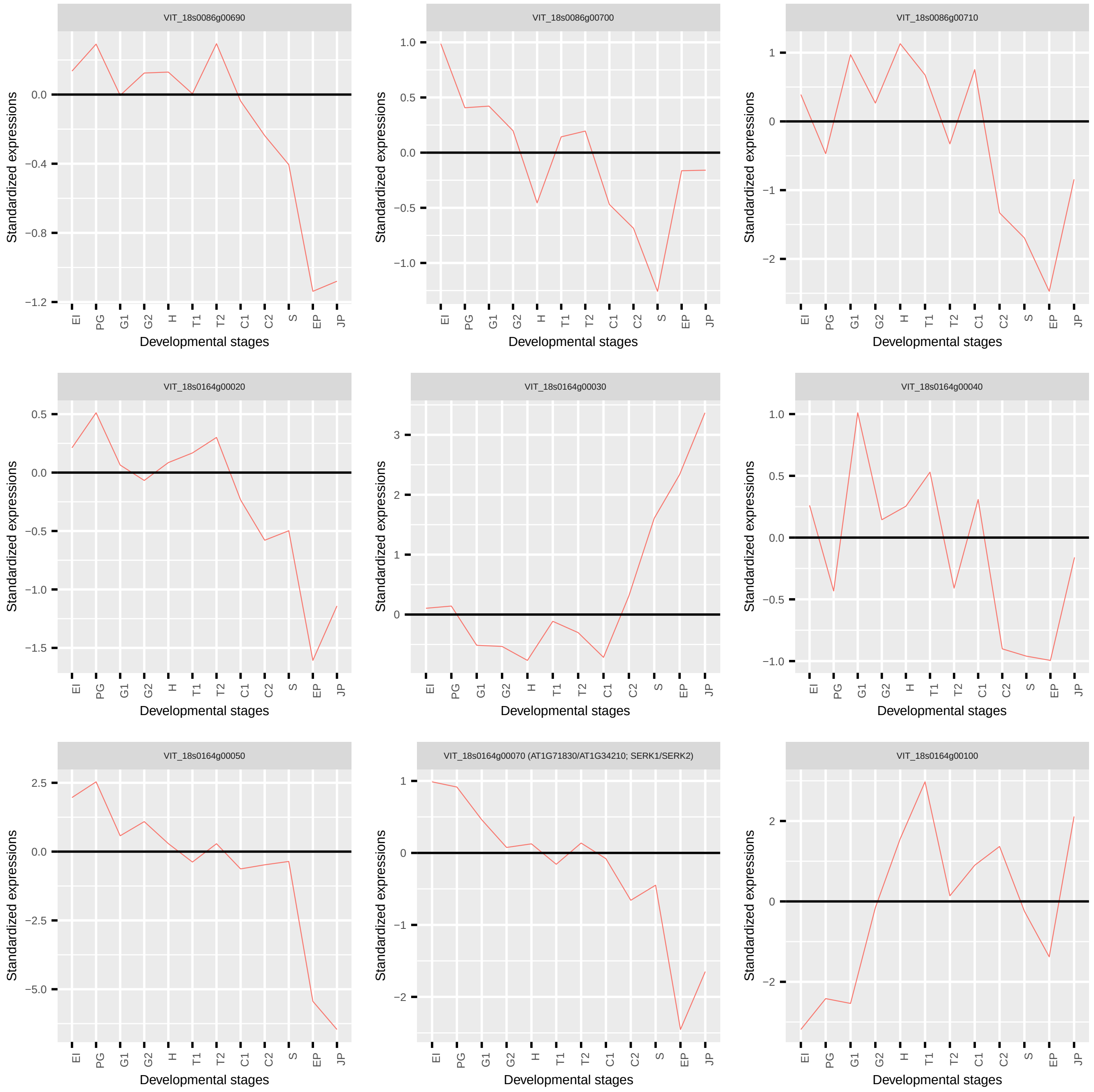
VIT_18s0086g00690
VIT_18s0086g00700
VIT_18s0086g00710
VIT_18s0164g00020
VIT_18s0164g00030
VIT_18s0164g00040
VIT_18s0164g00050
VIT_18s0164g00070 (AT1G71830/AT1G34210; SERK1/SERK2)
VIT_18s0164g00100
Standardized expressions
Developmental stages
EI PG G1 G2 H T1 T2 C1 C2 S EP JP

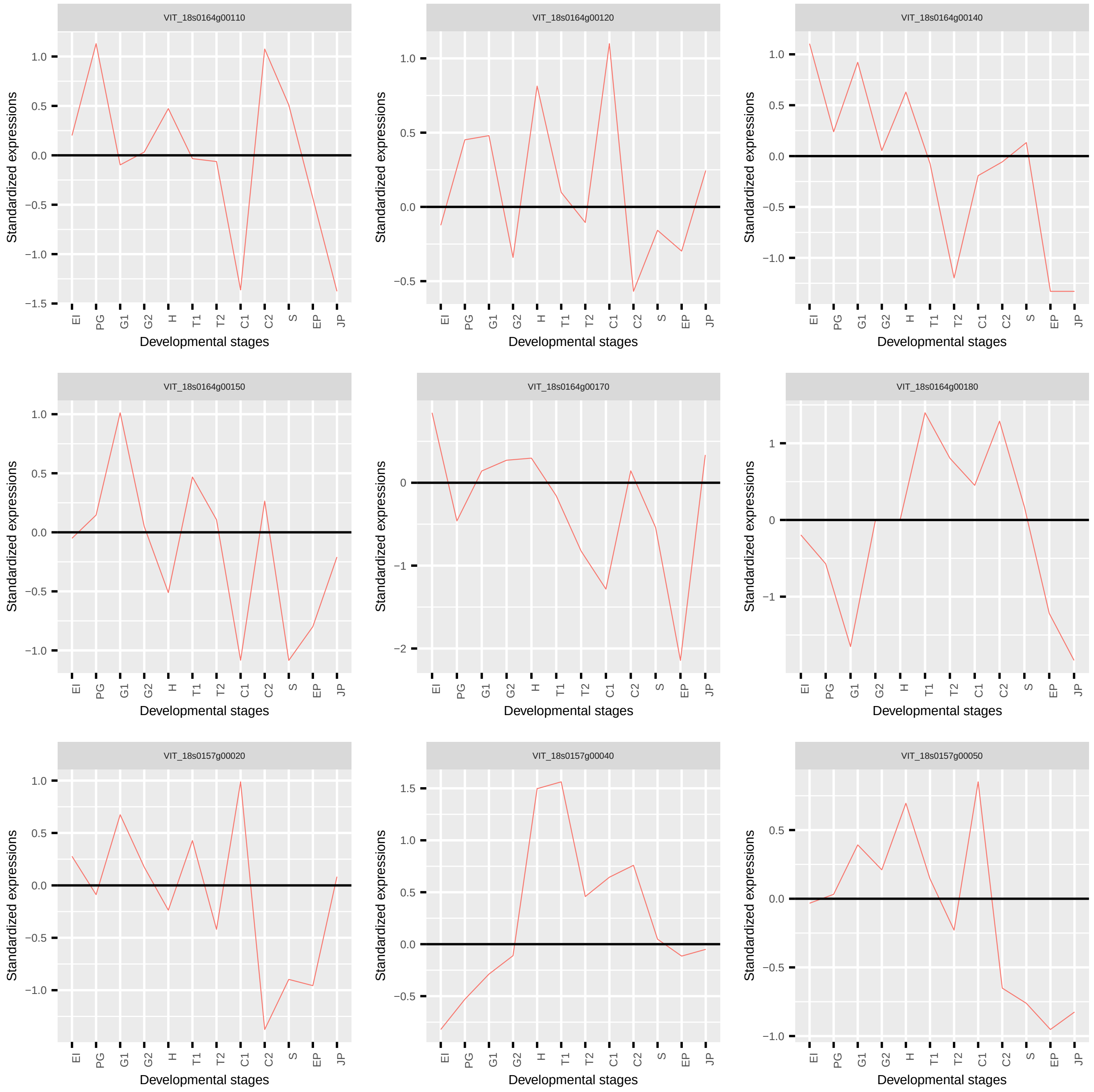

VIT_18s0164g00110
VIT_18s0164g00120
VIT_18s0164g00140
VIT_18s0164g00150
VIT_18s0164g00170
VIT_18s0164g00180
VIT_18s0157g00020
VIT_18s0157g00040
VIT_18s0157g00050
Standardized expressions
Developmental stages
EI PG G1 G2 H T1 T2 C1 C2 S EP JP

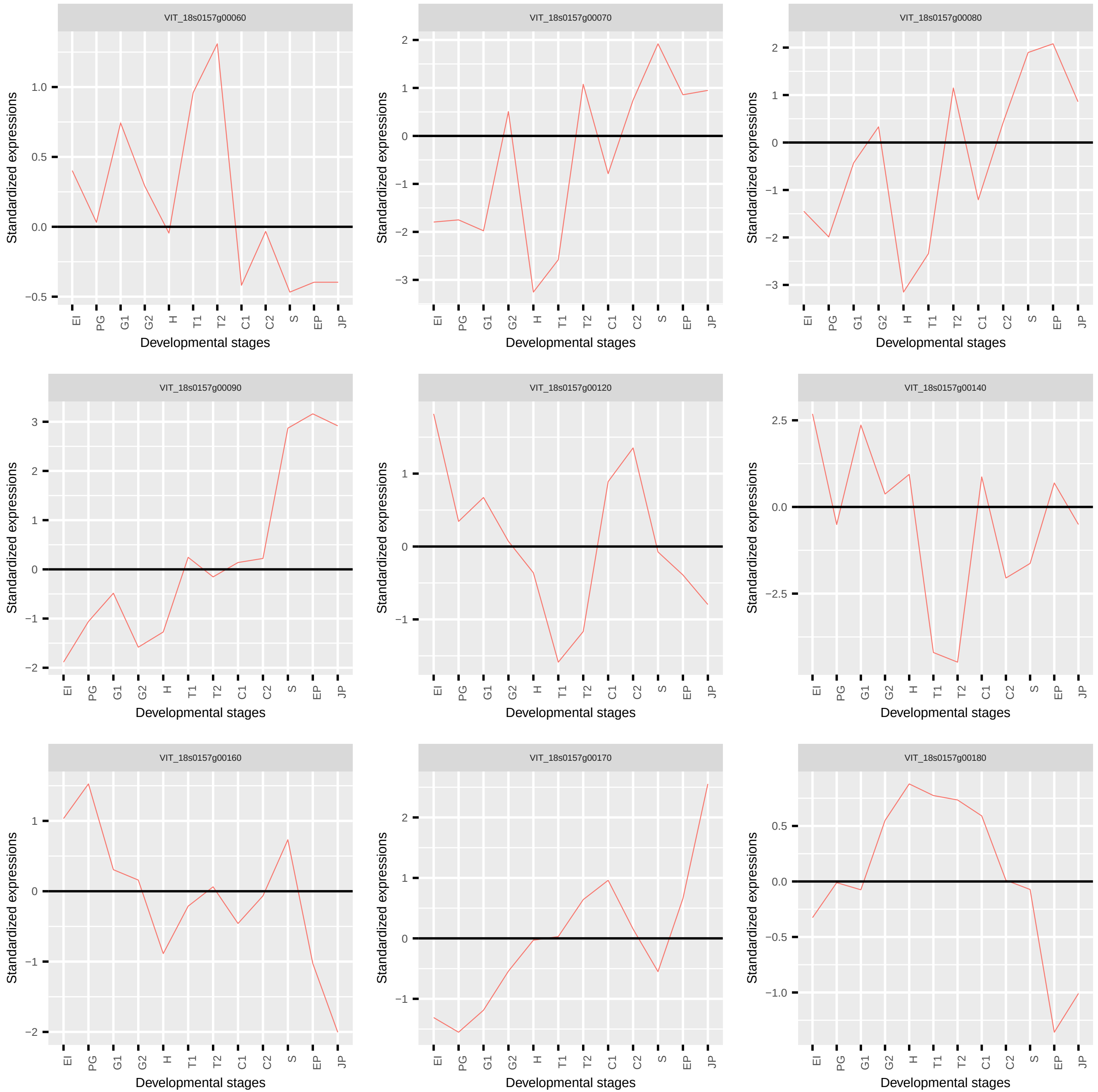
VIT_18s0157g00060
VIT_18s0157g00070
VIT_18s0157g00080
VIT_18s0157g00090
VIT_18s0157g00120
VIT_18s0157g00140
VIT_18s0157g00160
VIT_18s0157g00170
VIT_18s0157g00180
Standardized expressions
Developmental stages
EI PG G1 G2 H T1 T2 C1 C2 S EP JP

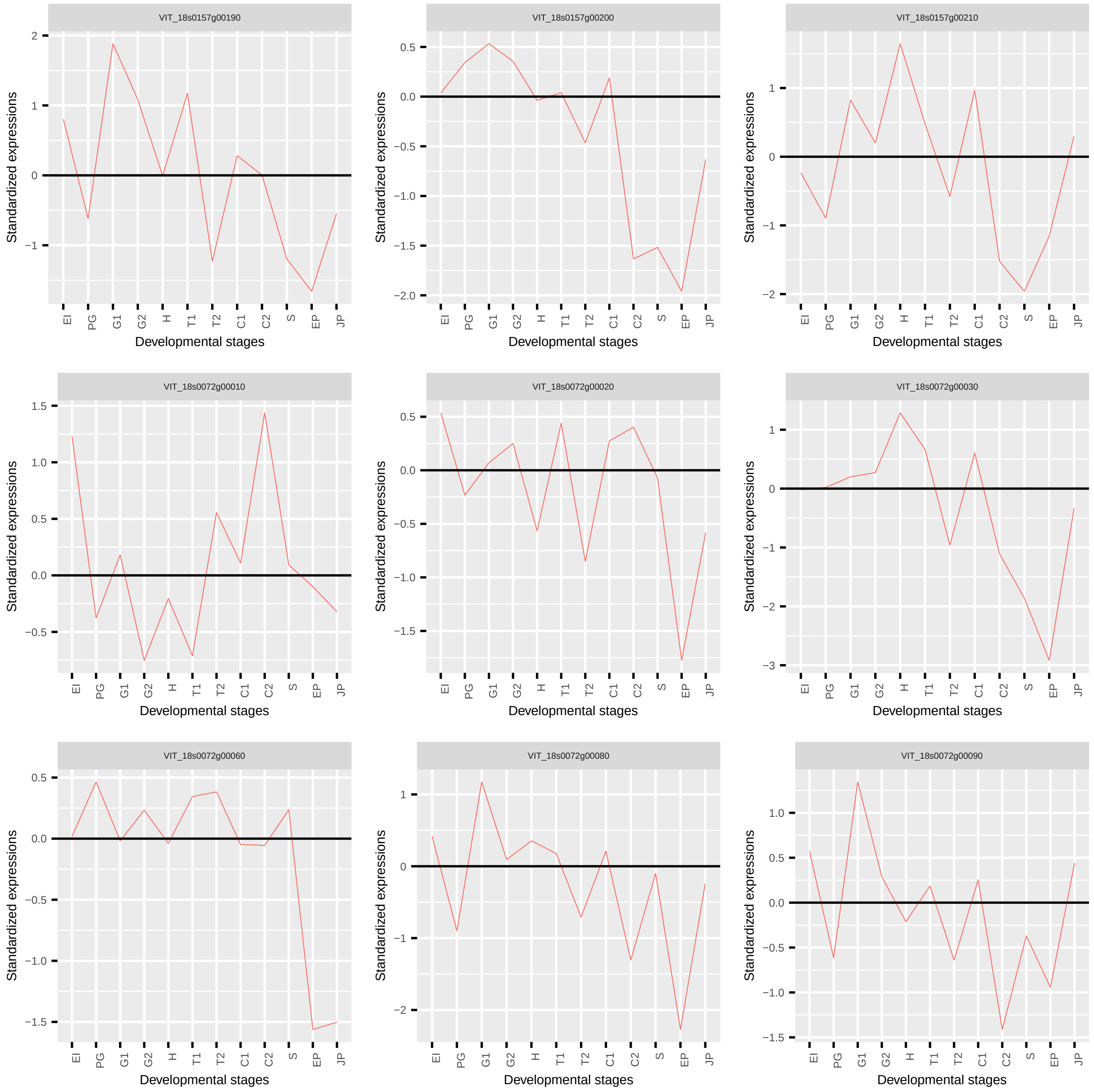
VIT_18s0157g00190
VIT_18s0157g00200
VIT_18s0157g00210
VIT_18s0072g00010
VIT_18s0072g00020
VIT_18s0072g00030
VIT_18s0072g00060
VIT_18s0072g00080
VIT_18s0072g00090
Standardized expressions
Developmental stages
EI
PG
G1
G2
H
T1
T2
C1
C2
S
EP
JP

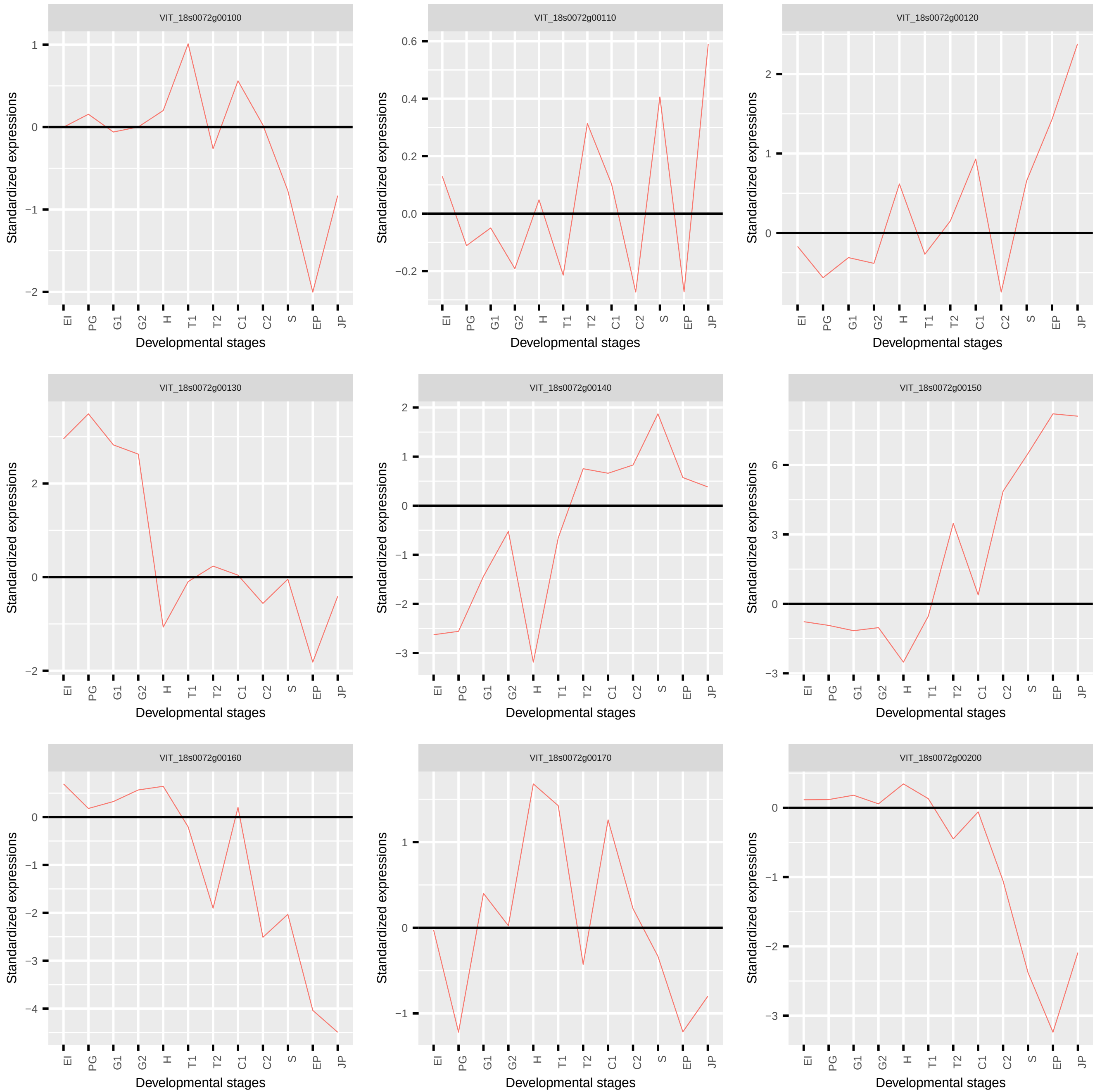
VIT_18s0072g00100
VIT_18s0072g00110
VIT_18s0072g00120
VIT_18s0072g00130
VIT_18s0072g00140
VIT_18s0072g00150
VIT_18s0072g00160
VIT_18s0072g00170
VIT_18s0072g00200
Standardized expressions
Developmental stages
EI PG G1 G2 H T1 T2 C1 C2 S EP JP

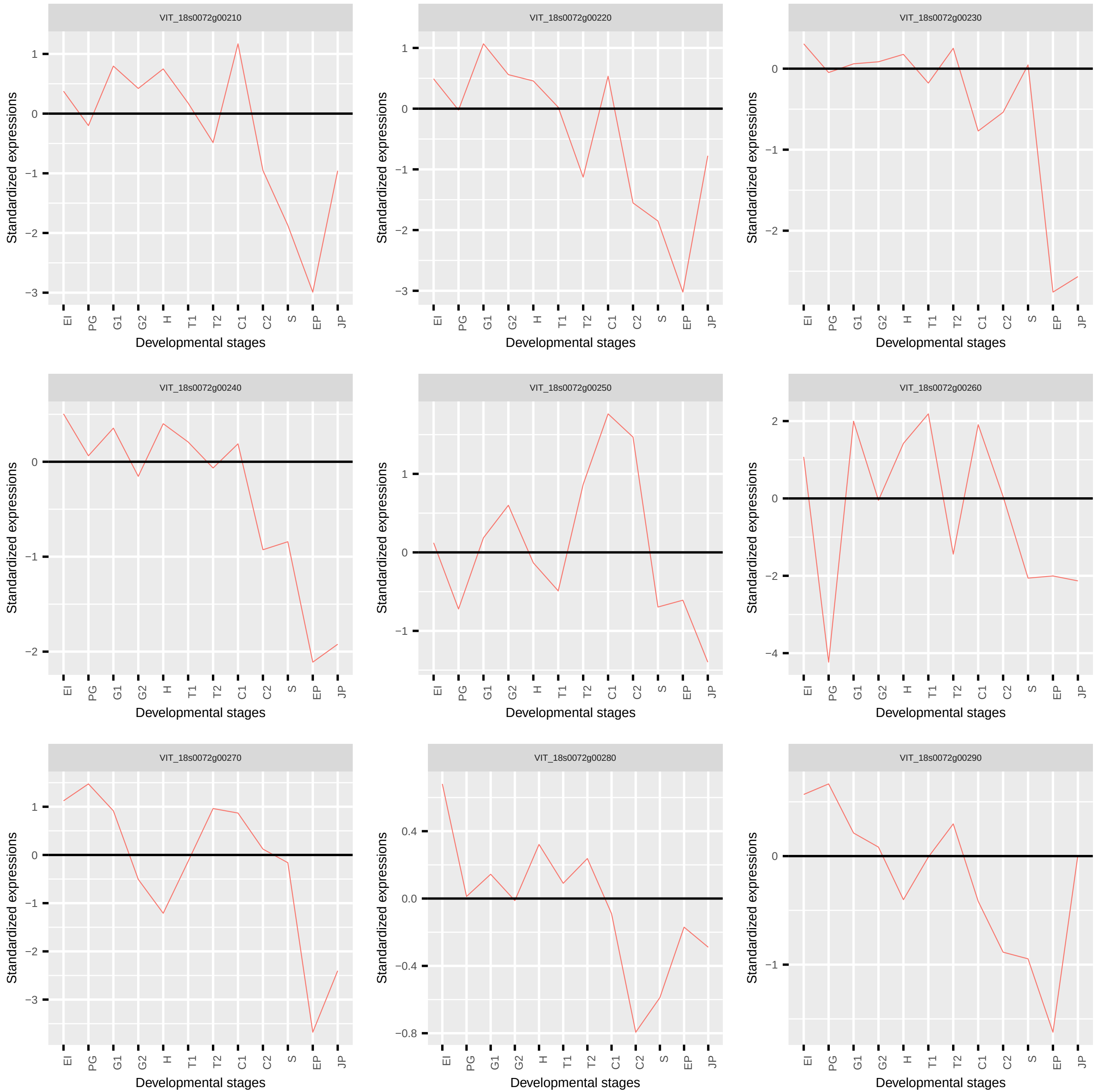

VIT_18s0072g00210
VIT_18s0072g00220
VIT_18s0072g00230
VIT_18s0072g00240
VIT_18s0072g00250
VIT_18s0072g00260
VIT_18s0072g00270
VIT_18s0072g00280
VIT_18s0072g00290
Standardized expressions
Developmental stages
EI PG G1 G2 H T1 T2 C1 C2 S EP JP

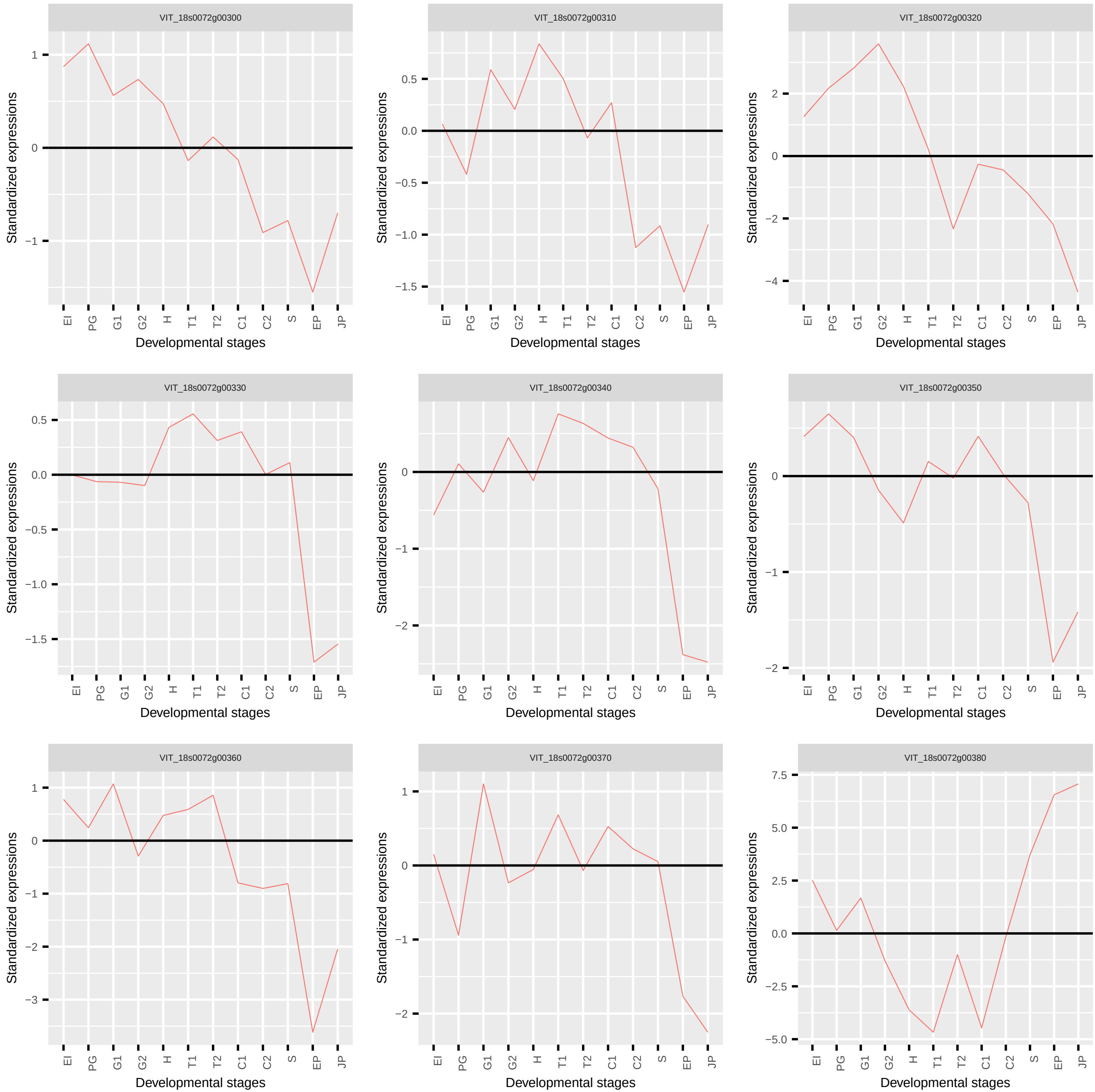
VIT_18s0072g00300
VIT_18s0072g00310
VIT_18s0072g00320
VIT_18s0072g00330
VIT_18s0072g00340
VIT_18s0072g00350
VIT_18s0072g00360
VIT_18s0072g00370
VIT_18s0072g00380
Standardized expressions
Developmental stages
EI PG G1 G2 H T1 T2 C1 C2 S EP JP

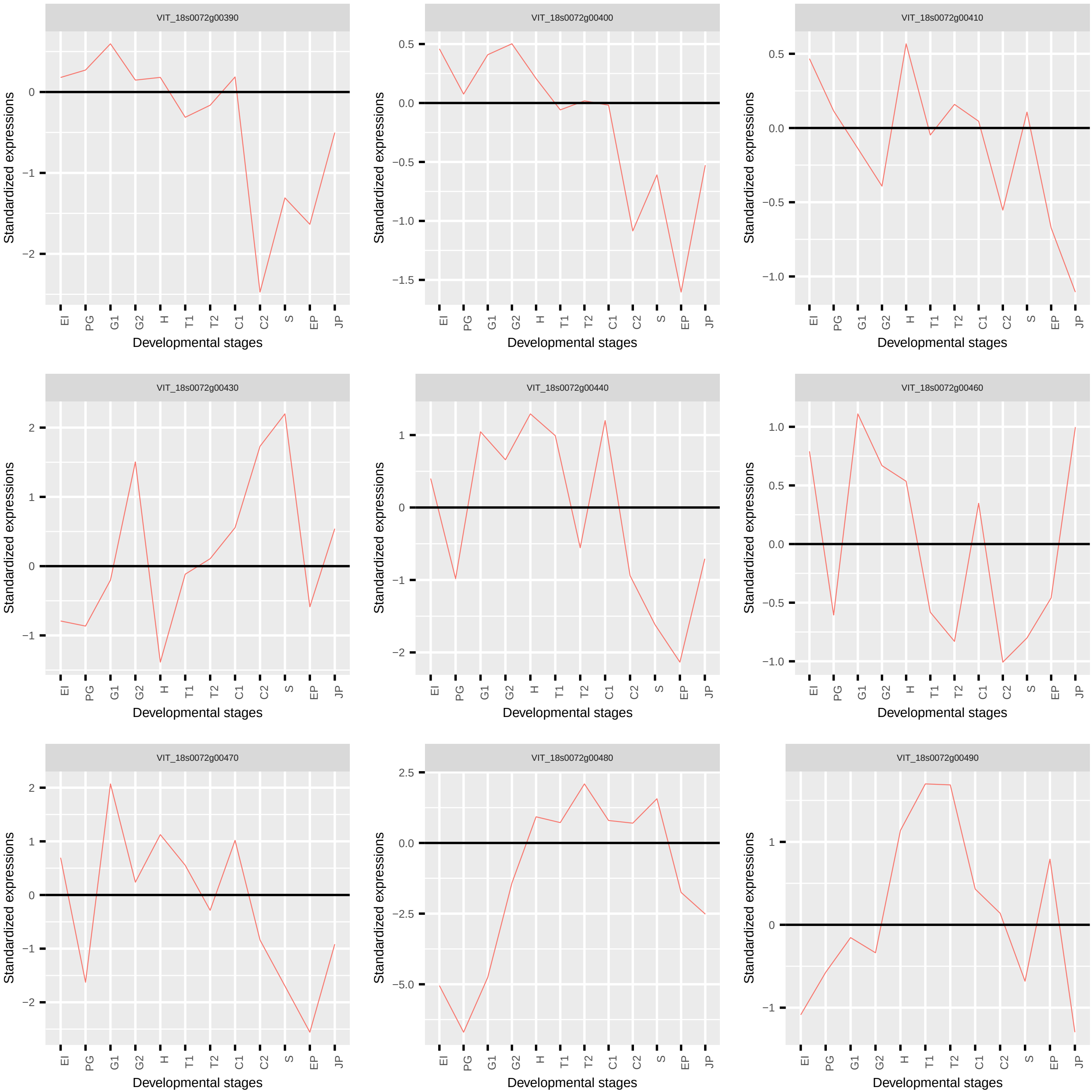
VIT_18s0072g00390
VIT_18s0072g00400
VIT_18s0072g00410
VIT_18s0072g00430
VIT_18s0072g00440
VIT_18s0072g00460
VIT_18s0072g00470
VIT_18s0072g00480
VIT_18s0072g00490
Standardized expressions
Developmental stages
EI PG G1 G2 H T1 T2 C1 C2 S EP JP

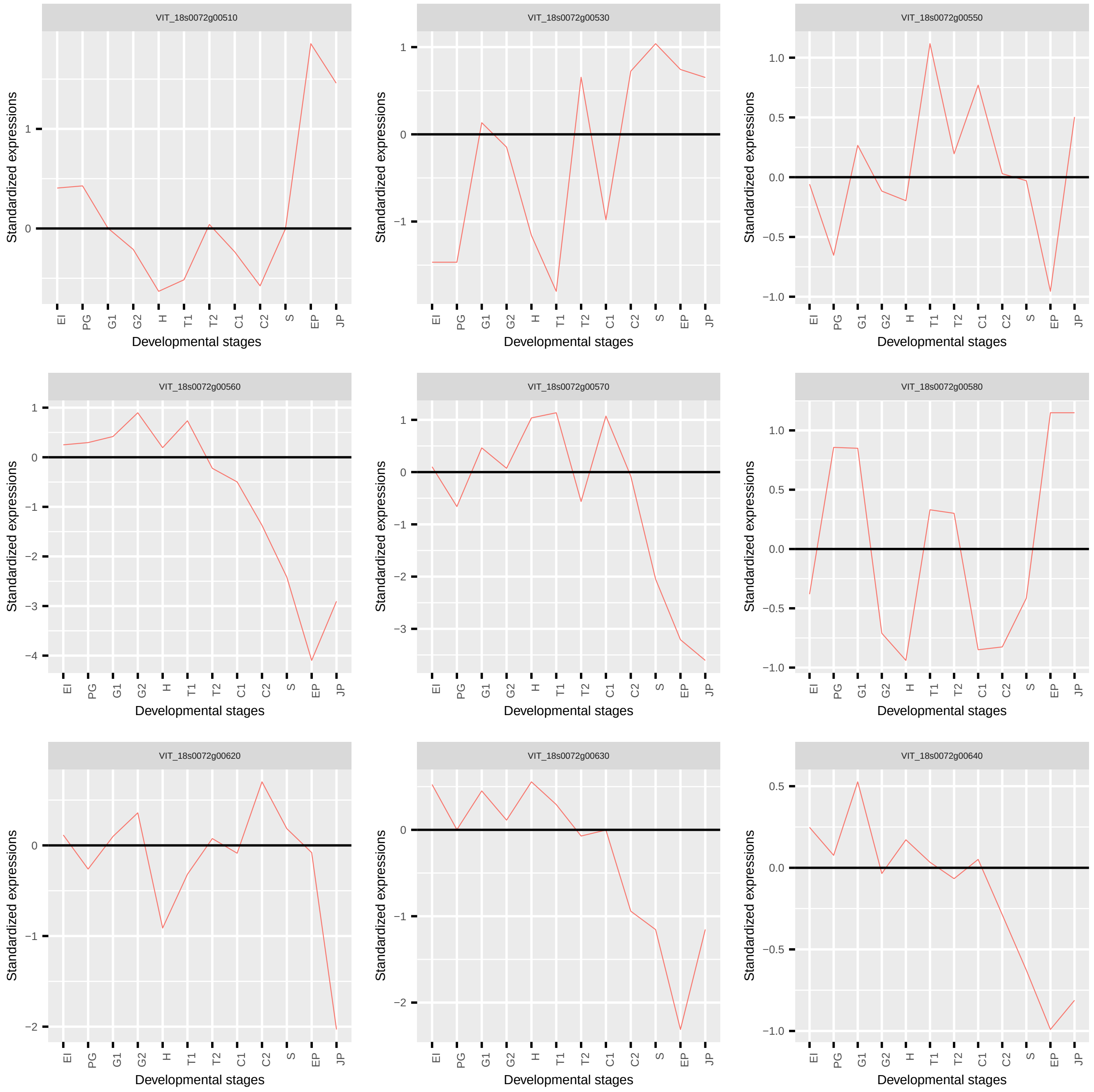

VIT_18s0072g00510
VIT_18s0072g00530
VIT_18s0072g00550
VIT_18s0072g00560
VIT_18s0072g00570
VIT_18s0072g00580
VIT_18s0072g00620
VIT_18s0072g00630
VIT_18s0072g00640
Standardized expressions
Developmental stages
EI
PG
G1
G2
H
T1
T2
C1
C2
S
EP
JP

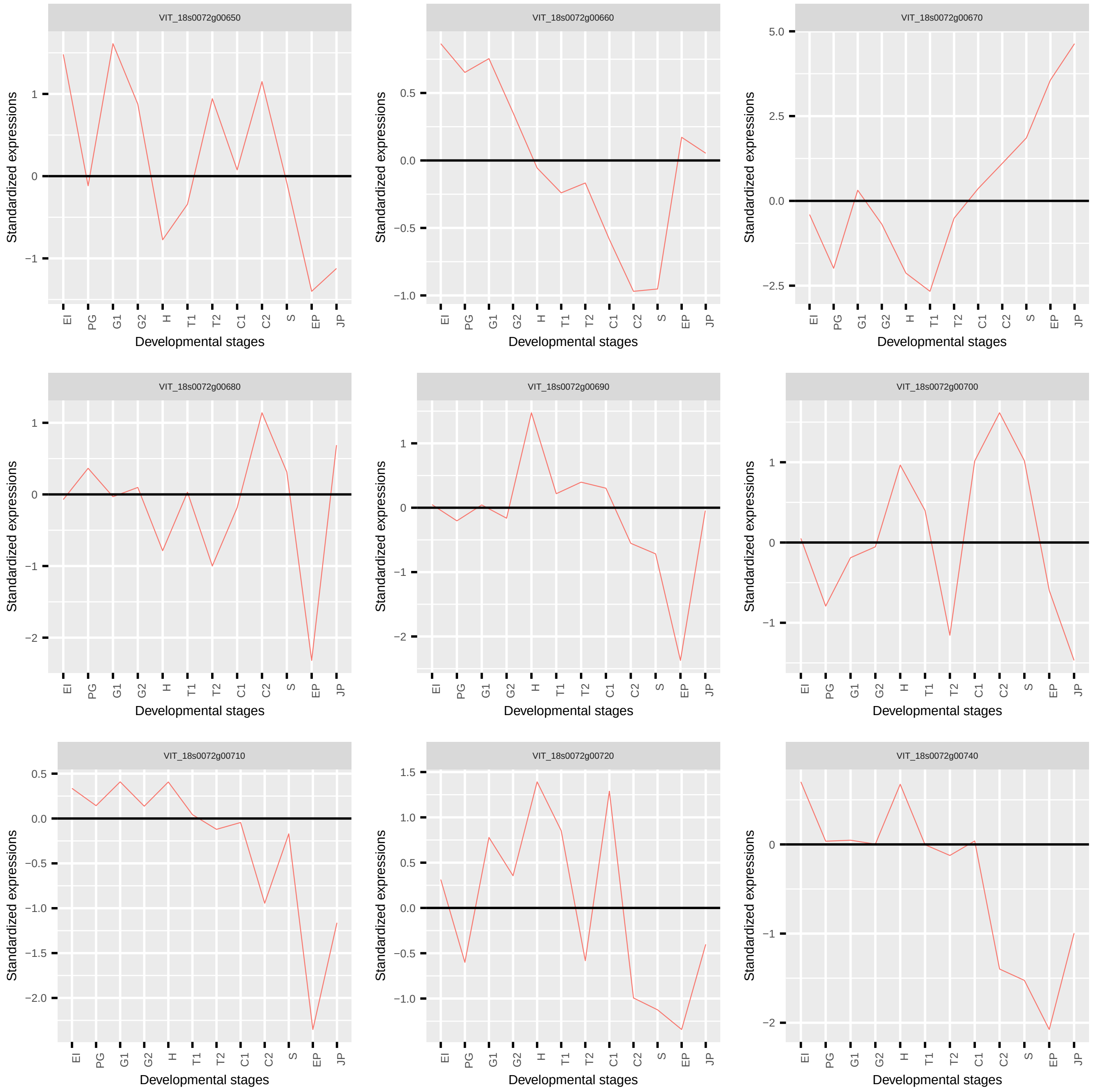
VIT_18s0072g00650
VIT_18s0072g00660
VIT_18s0072g00670
VIT_18s0072g00680
VIT_18s0072g00690
VIT_18s0072g00700
VIT_18s0072g00710
VIT_18s0072g00720
VIT_18s0072g00740
Standardized expressions
Developmental stages
EI PG G1 G2 H T1 T2 C1 C2 S EP JP

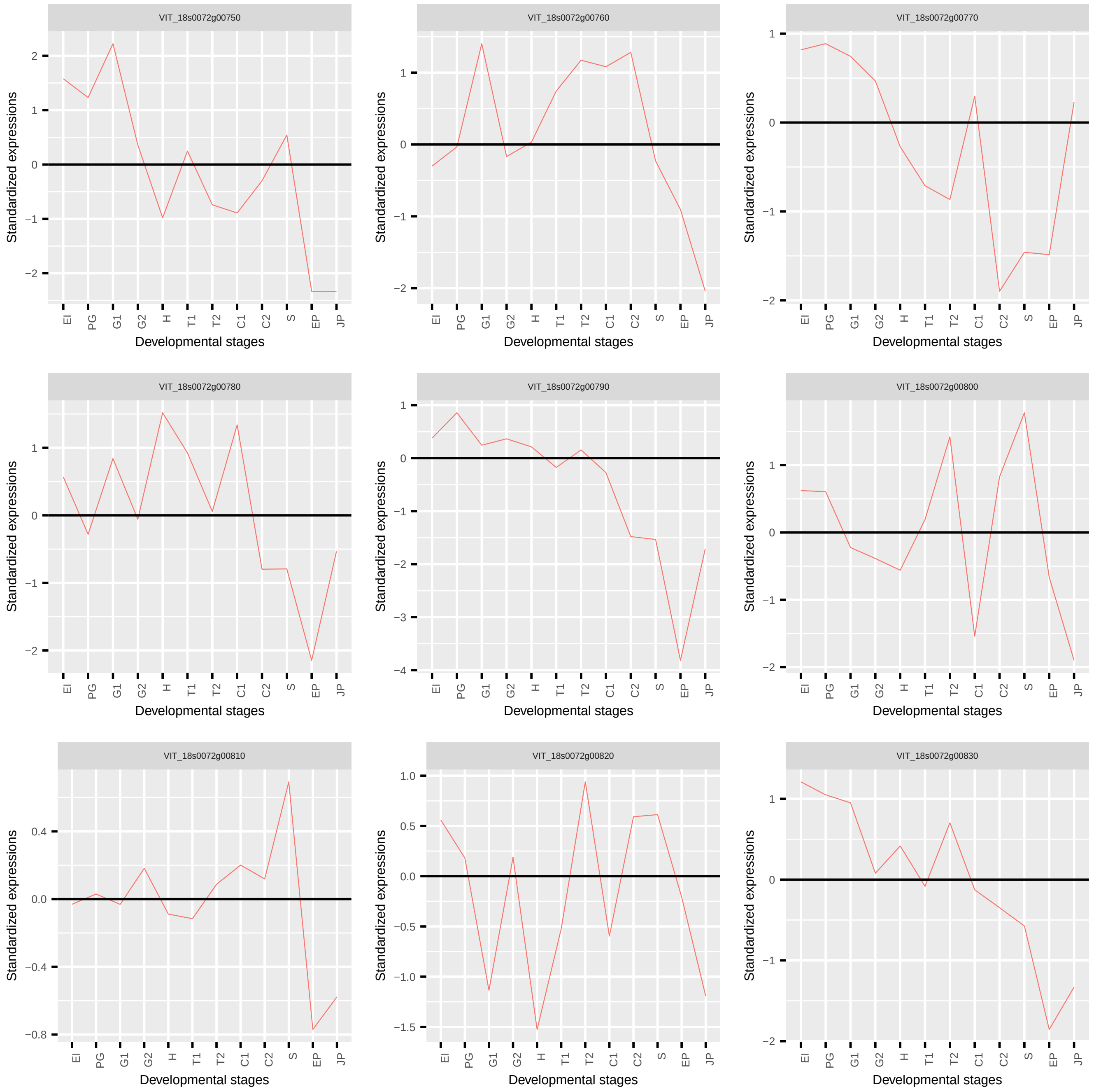
VIT_18s0072g00750
Standardized expressions
2
1
0
−1
−2
EI PG G1 G2 H T1 T2 C1 C2 S EP JP
Developmental stages
VIT_18s0072g00760
Standardized expressions
1
0
−1
−2
EI PG G1 G2 H T1 T2 C1 C2 S EP JP
Developmental stages
VIT_18s0072g00770
Standardized expressions
1
0
−1
−2
EI PG G1 G2 H T1 T2 C1 C2 S EP JP
Developmental stages
VIT_18s0072g00780
Standardized expressions
1
0
−1
−2
EI PG G1 G2 H T1 T2 C1 C2 S EP JP
Developmental stages
VIT_18s0072g00790
Standardized expressions
1
0
−1
−2
−3
−4
EI PG G1 G2 H T1 T2 C1 C2 S EP JP
Developmental stages
VIT_18s0072g00800
Standardized expressions
1
0
−1
−2
EI PG G1 G2 H T1 T2 C1 C2 S EP JP
Developmental stages
VIT_18s0072g00810
Standardized expressions
0.4
0.0
−0.4
−0.8
EI PG G1 G2 H T1 T2 C1 C2 S EP JP
Developmental stages
VIT_18s0072g00820
Standardized expressions
1.0
0.5
0.0
−0.5
−1.0
−1.5
EI PG G1 G2 H T1 T2 C1 C2 S EP JP
Developmental stages
VIT_18s0072g00830
Standardized expressions
1
0
−1
−2
EI PG G1 G2 H T1 T2 C1 C2 S EP JP
Developmental stages

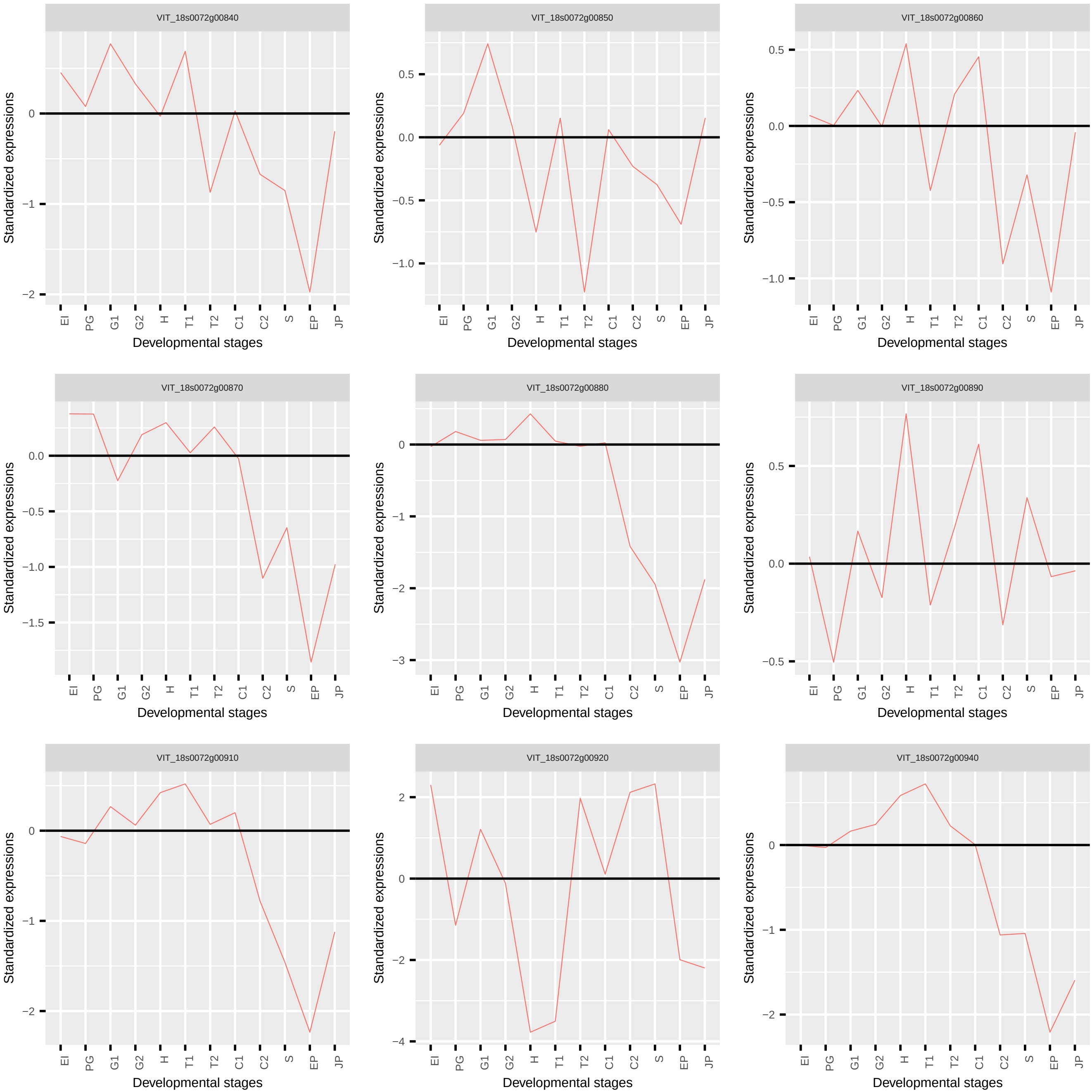
VIT_18s0072g00840
VIT_18s0072g00850
VIT_18s0072g00860
VIT_18s0072g00870
VIT_18s0072g00880
VIT_18s0072g00890
VIT_18s0072g00910
VIT_18s0072g00920
VIT_18s0072g00940
Standardized expressions
Developmental stages
EI PG G1 G2 H T1 T2 C1 C2 S EP JP
0
−1
−2
0.5
0.0
−0.5
−1.0
−1.5
−3
2
−4

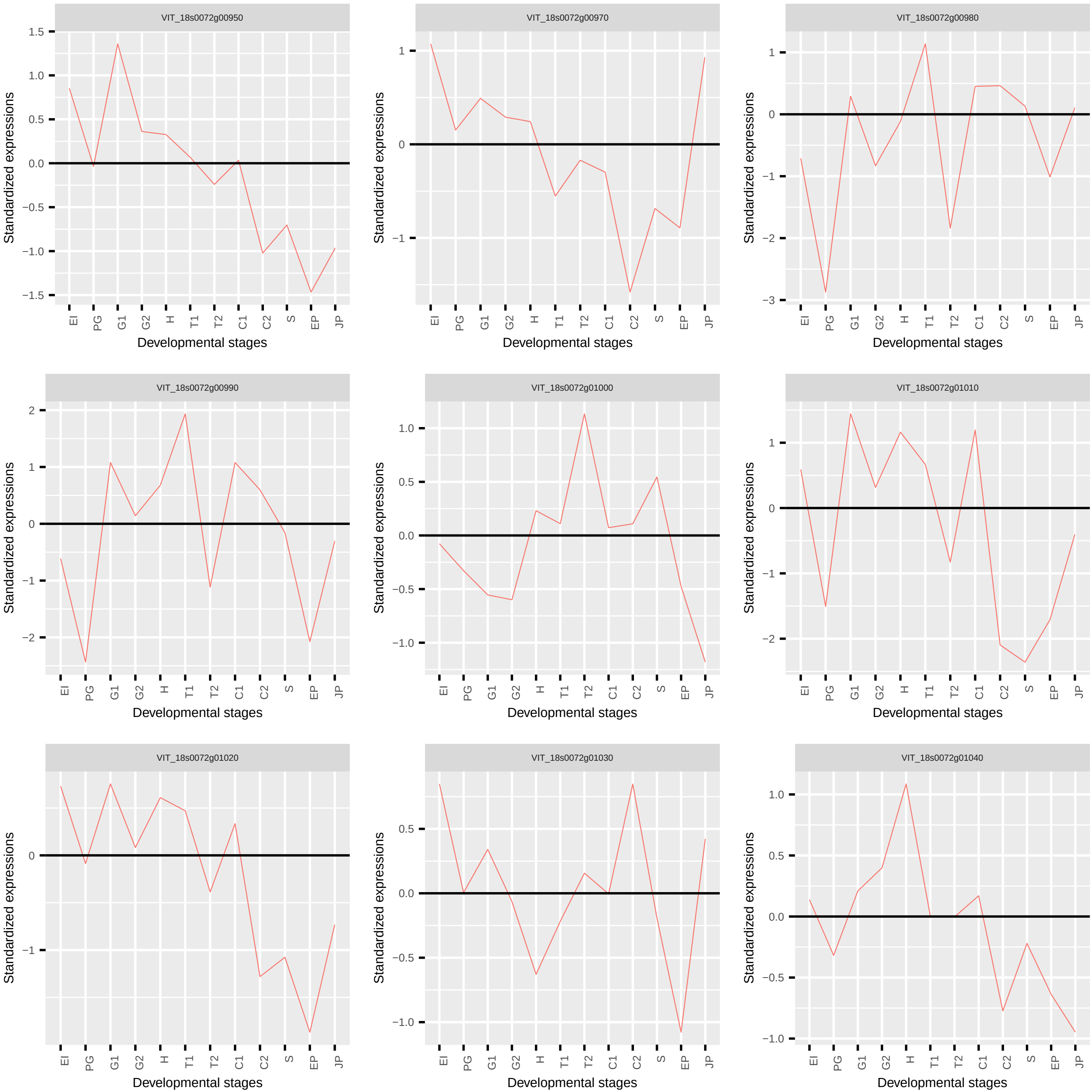
VIT_18s0072g00950
VIT_18s0072g00970
VIT_18s0072g00980
VIT_18s0072g00990
VIT_18s0072g01000
VIT_18s0072g01010
VIT_18s0072g01020
VIT_18s0072g01030
VIT_18s0072g01040
Standardized expressions
Developmental stages
EI PG G1 G2 H T1 T2 C1 C2 S EP JP

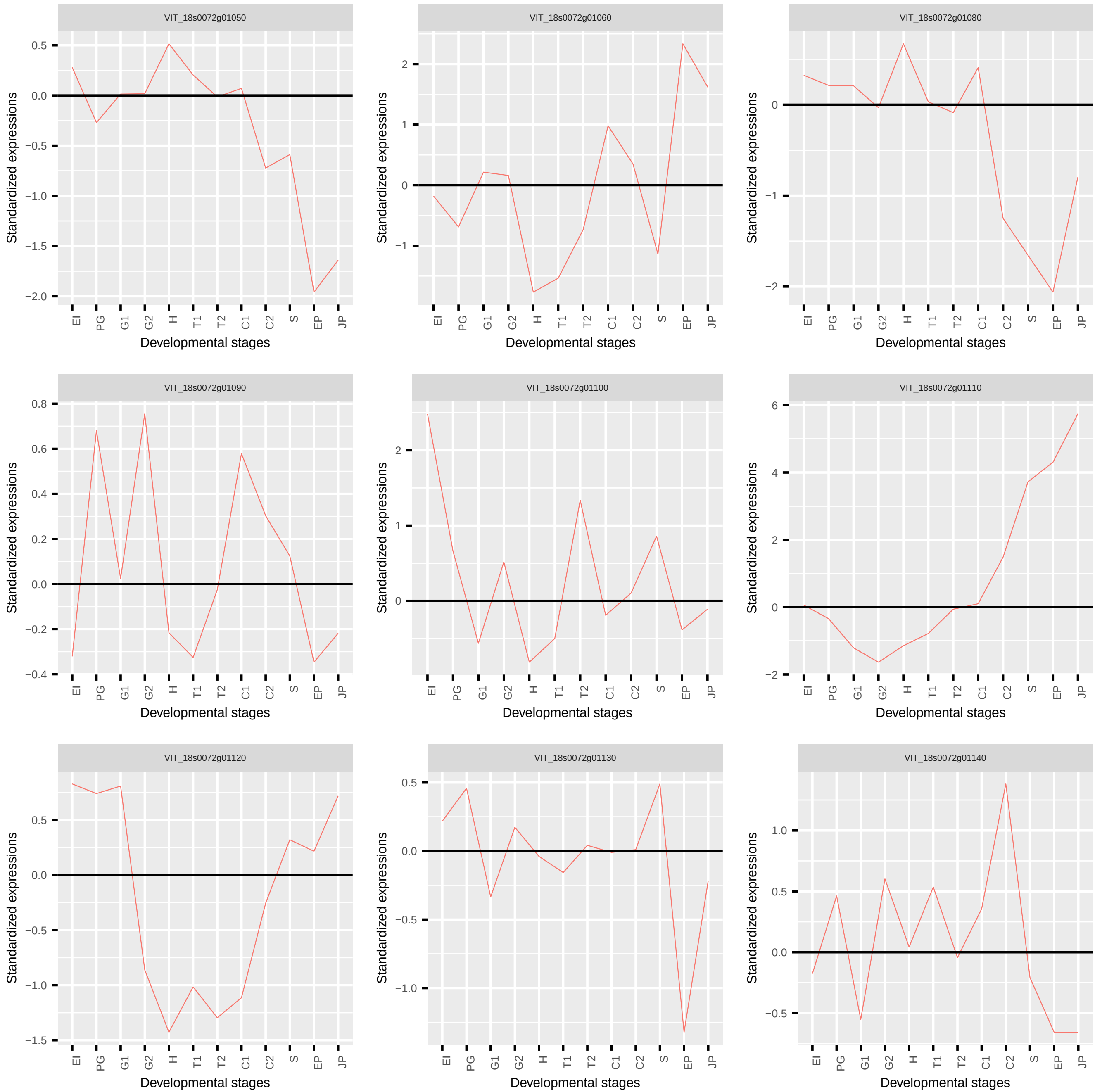
VIT_18s0072g01050
VIT_18s0072g01060
VIT_18s0072g01080
VIT_18s0072g01090
VIT_18s0072g01100
VIT_18s0072g01110
VIT_18s0072g01120
VIT_18s0072g01130
VIT_18s0072g01140
Standardized expressions
Developmental stages
EI PG G1 G2 H T1 T2 C1 C2 S EP JP

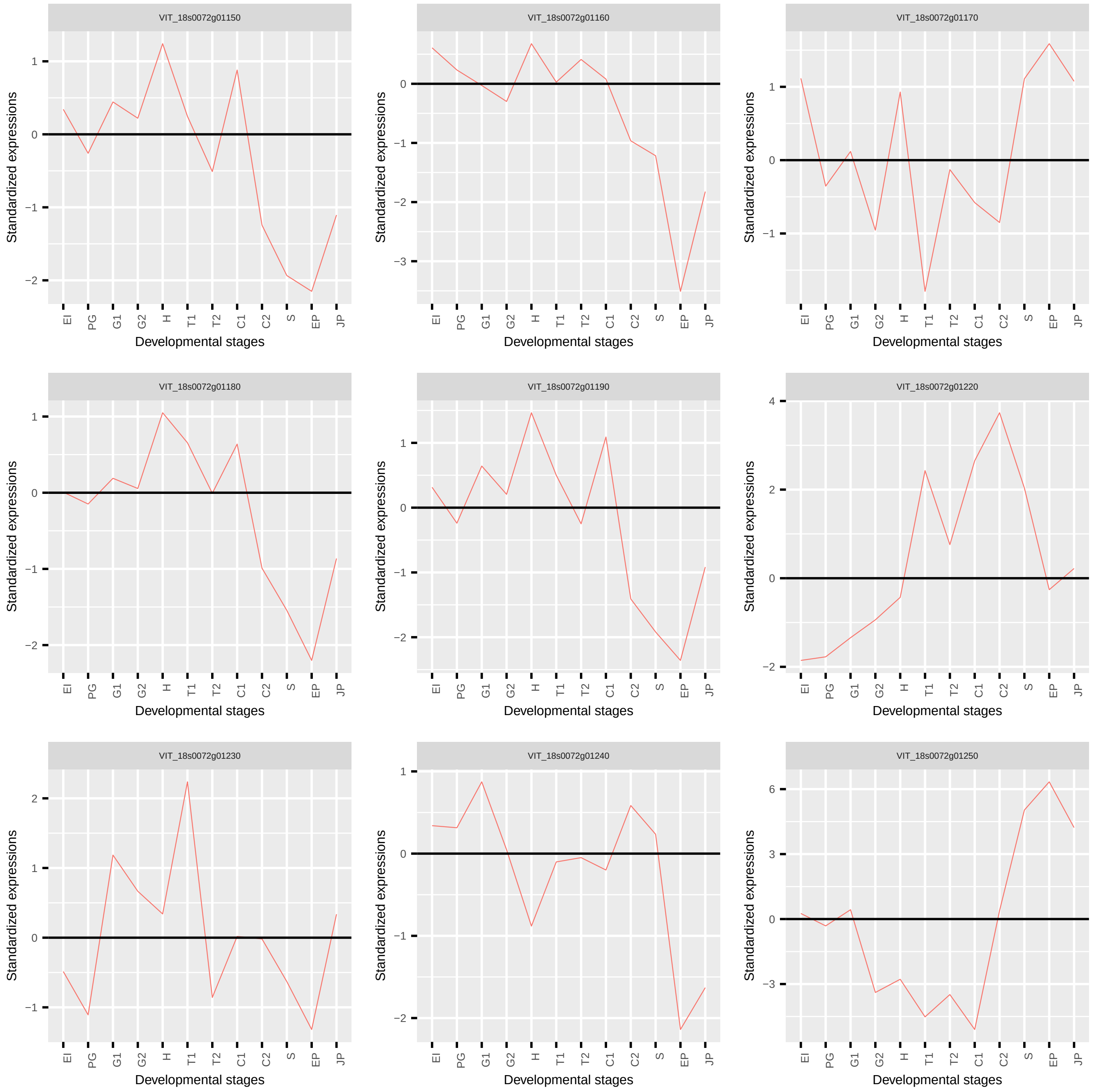
VIT_18s0072g01150
VIT_18s0072g01160
VIT_18s0072g01170
VIT_18s0072g01180
VIT_18s0072g01190
VIT_18s0072g01220
VIT_18s0072g01230
VIT_18s0072g01240
VIT_18s0072g01250
Standardized expressions
Developmental stages
EI
PG
G1
G2
H
T1
T2
C1
C2
S
EP
JP

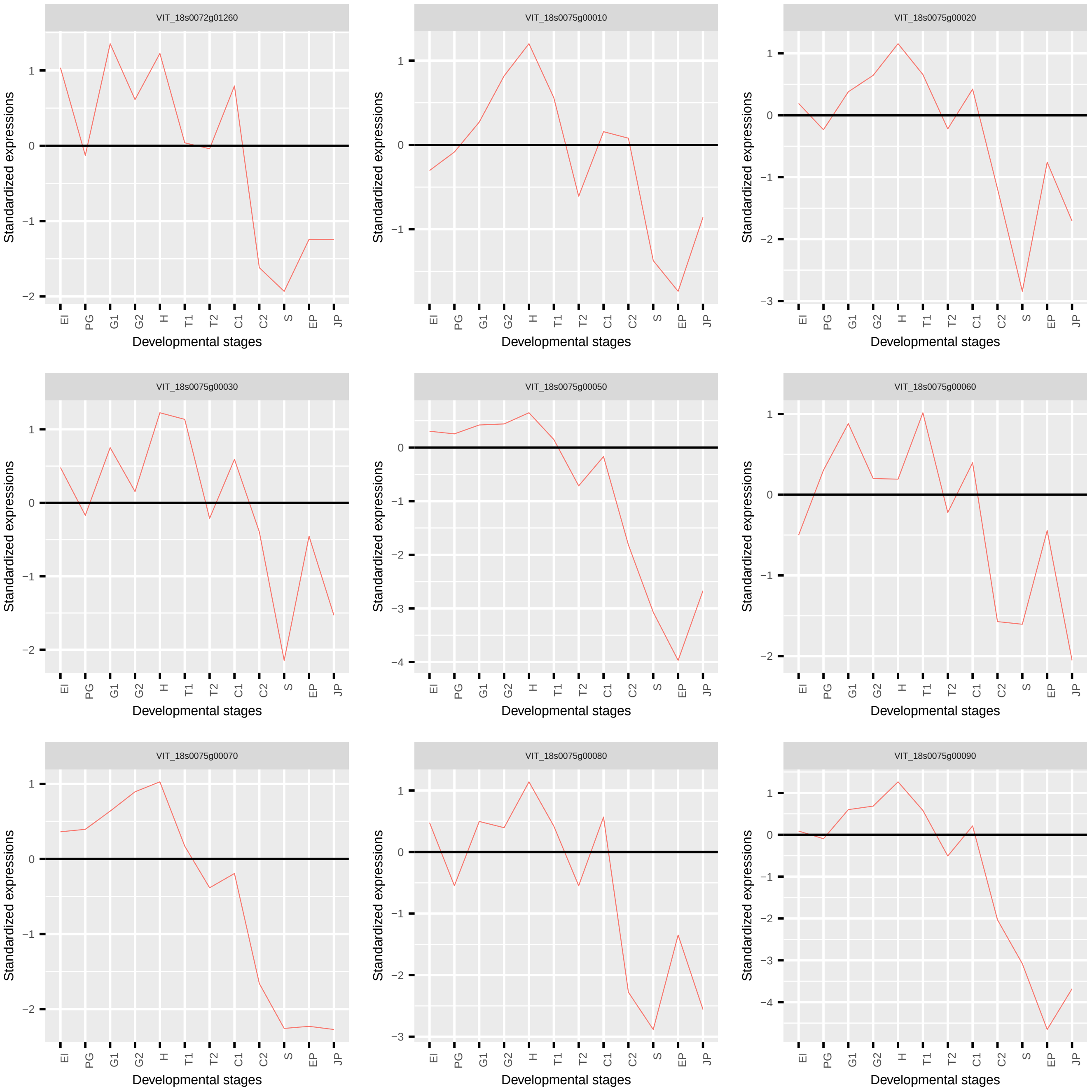
VIT_18s0072g01260
VIT_18s0075g00010
VIT_18s0075g00020
VIT_18s0075g00030
VIT_18s0075g00050
VIT_18s0075g00060
VIT_18s0075g00070
VIT_18s0075g00080
VIT_18s0075g00090
Standardized expressions
Developmental stages
EI
PG
G1
G2
H
T1
T2
C1
C2
S
EP
JP

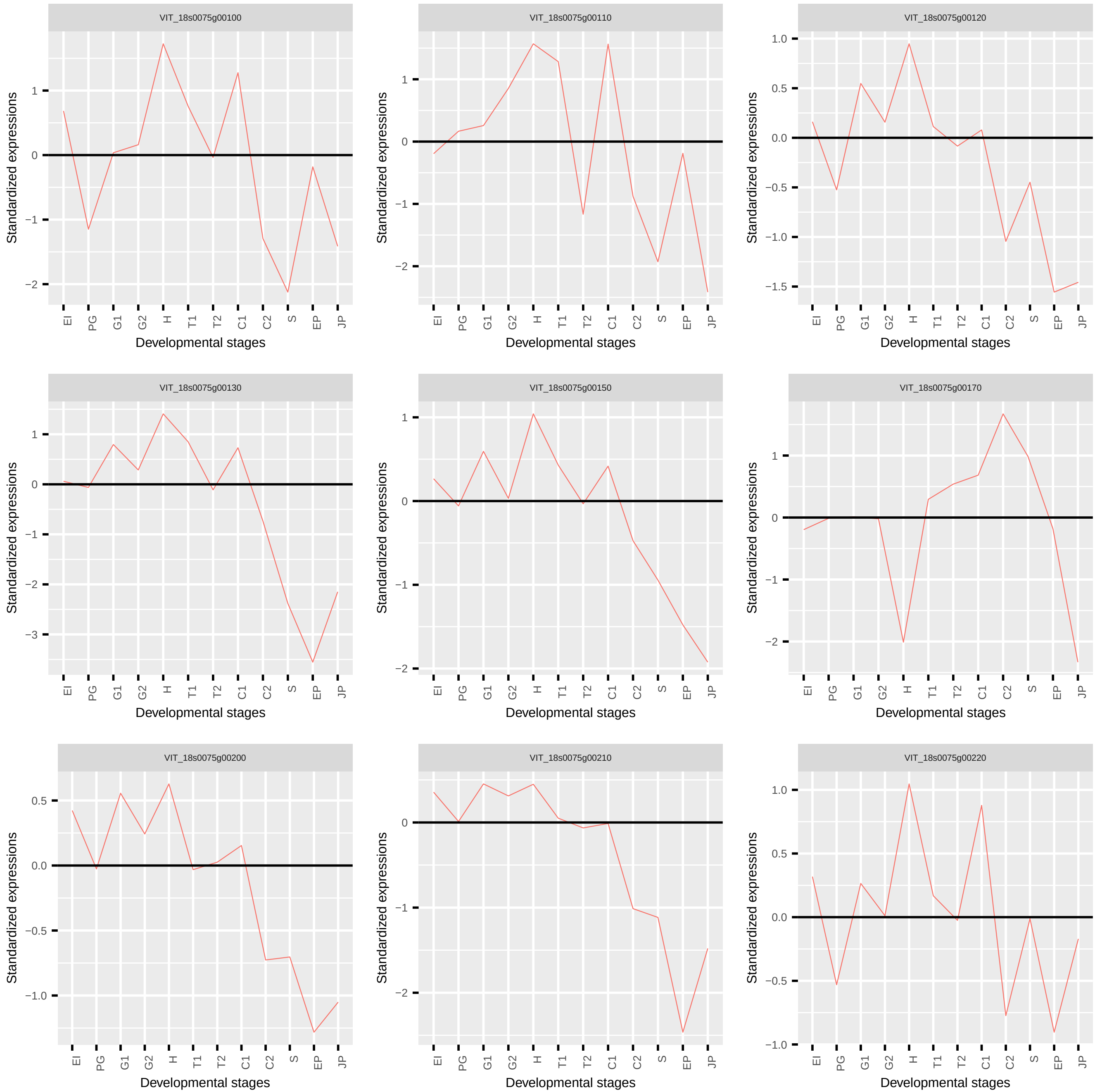
VIT_18s0075g00100
VIT_18s0075g00110
VIT_18s0075g00120
VIT_18s0075g00130
VIT_18s0075g00150
VIT_18s0075g00170
VIT_18s0075g00200
VIT_18s0075g00210
VIT_18s0075g00220
Standardized expressions
Developmental stages
EI
PG
G1
G2
H
T1
T2
C1
C2
S
EP
JP

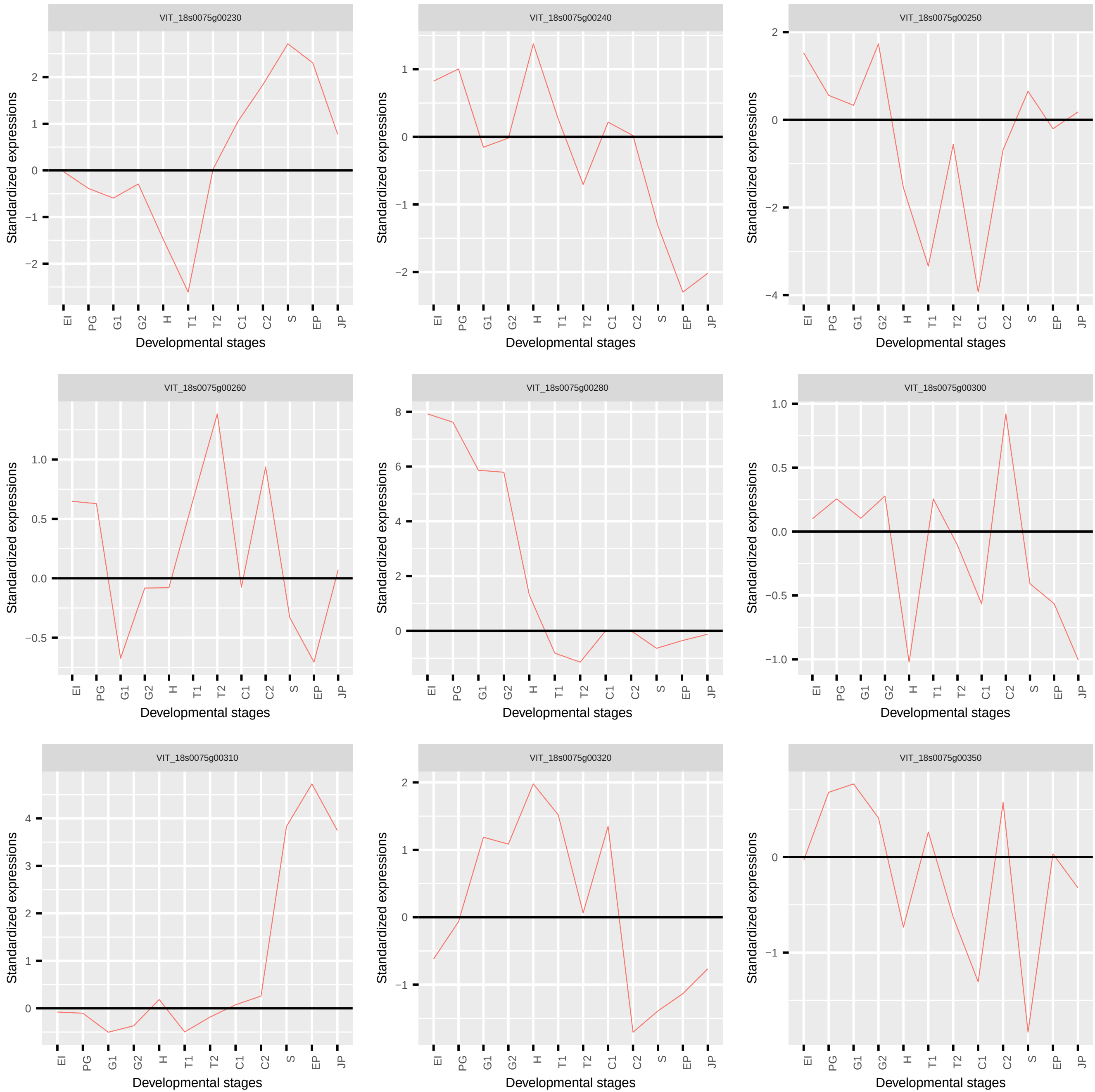
VIT_18s0075g00230
VIT_18s0075g00240
VIT_18s0075g00250
VIT_18s0075g00260
VIT_18s0075g00280
VIT_18s0075g00300
VIT_18s0075g00310
VIT_18s0075g00320
VIT_18s0075g00350
Standardized expressions
Developmental stages
EI
PG
G1
G2
H
T1
T2
C1
C2
S
EP
JP

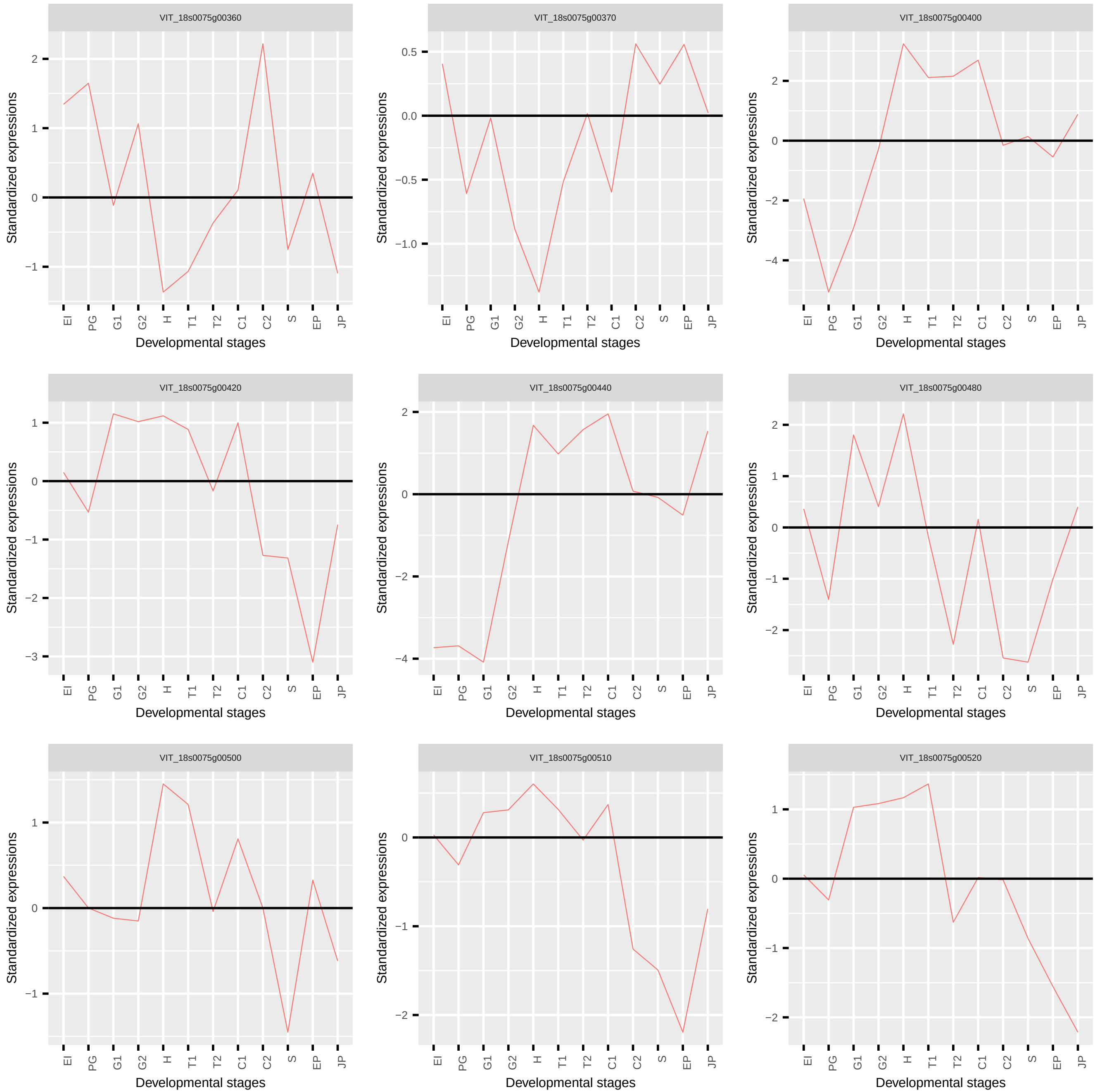

VIT_18s0075g00360
VIT_18s0075g00370
VIT_18s0075g00400
VIT_18s0075g00420
VIT_18s0075g00440
VIT_18s0075g00480
VIT_18s0075g00500
VIT_18s0075g00510
VIT_18s0075g00520
Standardized expressions
Developmental stages
EI PG G1 G2 H T1 T2 C1 C2 S EP JP

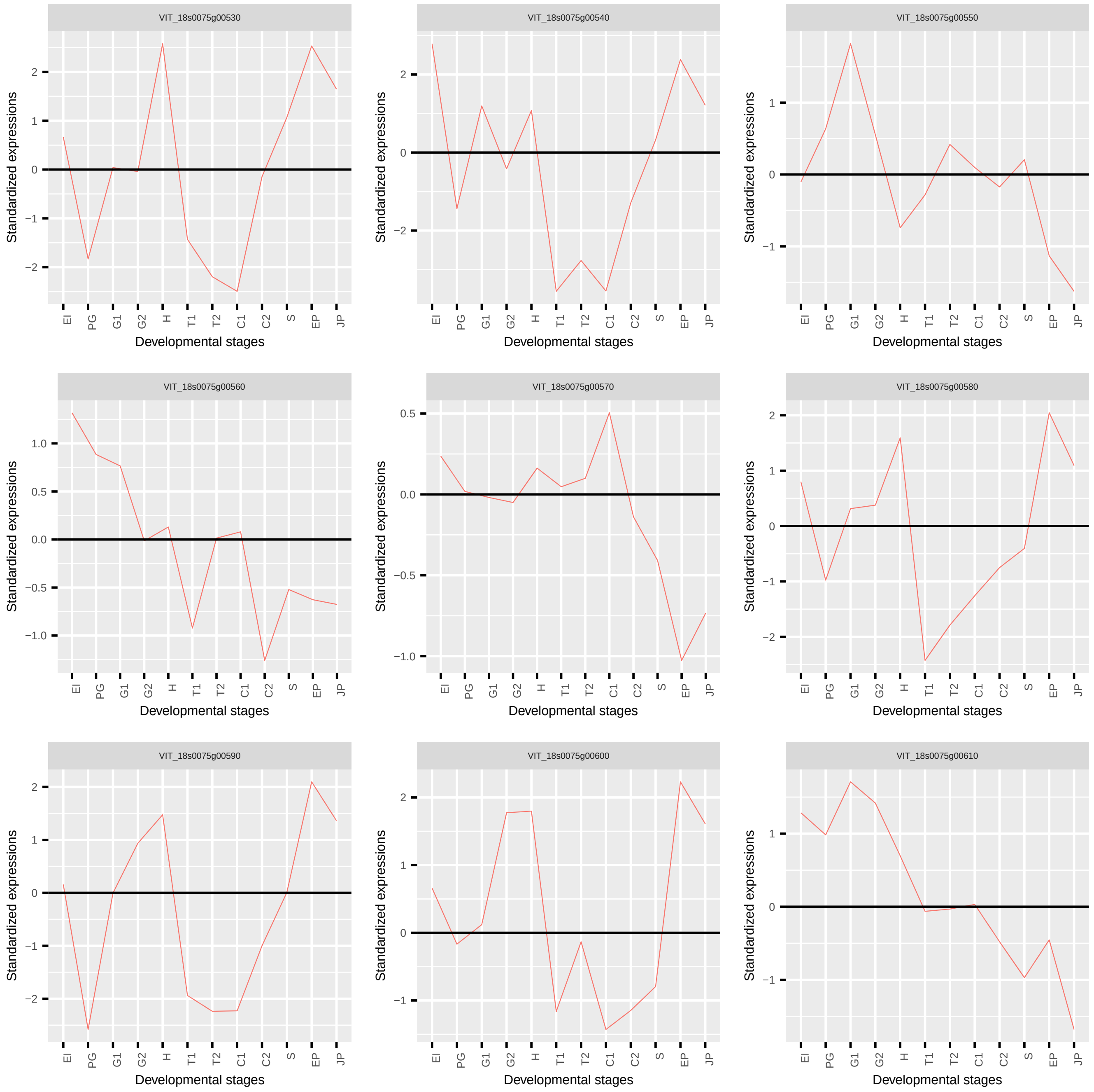
VIT_18s0075g00530
Standardized expressions
2
1
0
−1
−2
EI PG G1 G2 H T1 T2 C1 C2 S EP JP
Developmental stages
VIT_18s0075g00540
Standardized expressions
2
0
−2
EI PG G1 G2 H T1 T2 C1 C2 S EP JP
Developmental stages
VIT_18s0075g00550
Standardized expressions
1
0
−1
EI PG G1 G2 H T1 T2 C1 C2 S EP JP
Developmental stages
VIT_18s0075g00560
Standardized expressions
1.0
0.5
0.0
−0.5
−1.0
EI PG G1 G2 H T1 T2 C1 C2 S EP JP
Developmental stages
VIT_18s0075g00570
Standardized expressions
0.5
0.0
−0.5
−1.0
EI PG G1 G2 H T1 T2 C1 C2 S EP JP
Developmental stages
VIT_18s0075g00580
Standardized expressions
2
1
0
−1
−2
EI PG G1 G2 H T1 T2 C1 C2 S EP JP
Developmental stages
VIT_18s0075g00590
Standardized expressions
2
1
0
−1
−2
EI PG G1 G2 H T1 T2 C1 C2 S EP JP
Developmental stages
VIT_18s0075g00600
Standardized expressions
2
1
0
−1
EI PG G1 G2 H T1 T2 C1 C2 S EP JP
Developmental stages
VIT_18s0075g00610
Standardized expressions
1
0
−1
EI PG G1 G2 H T1 T2 C1 C2 S EP JP
Developmental stages

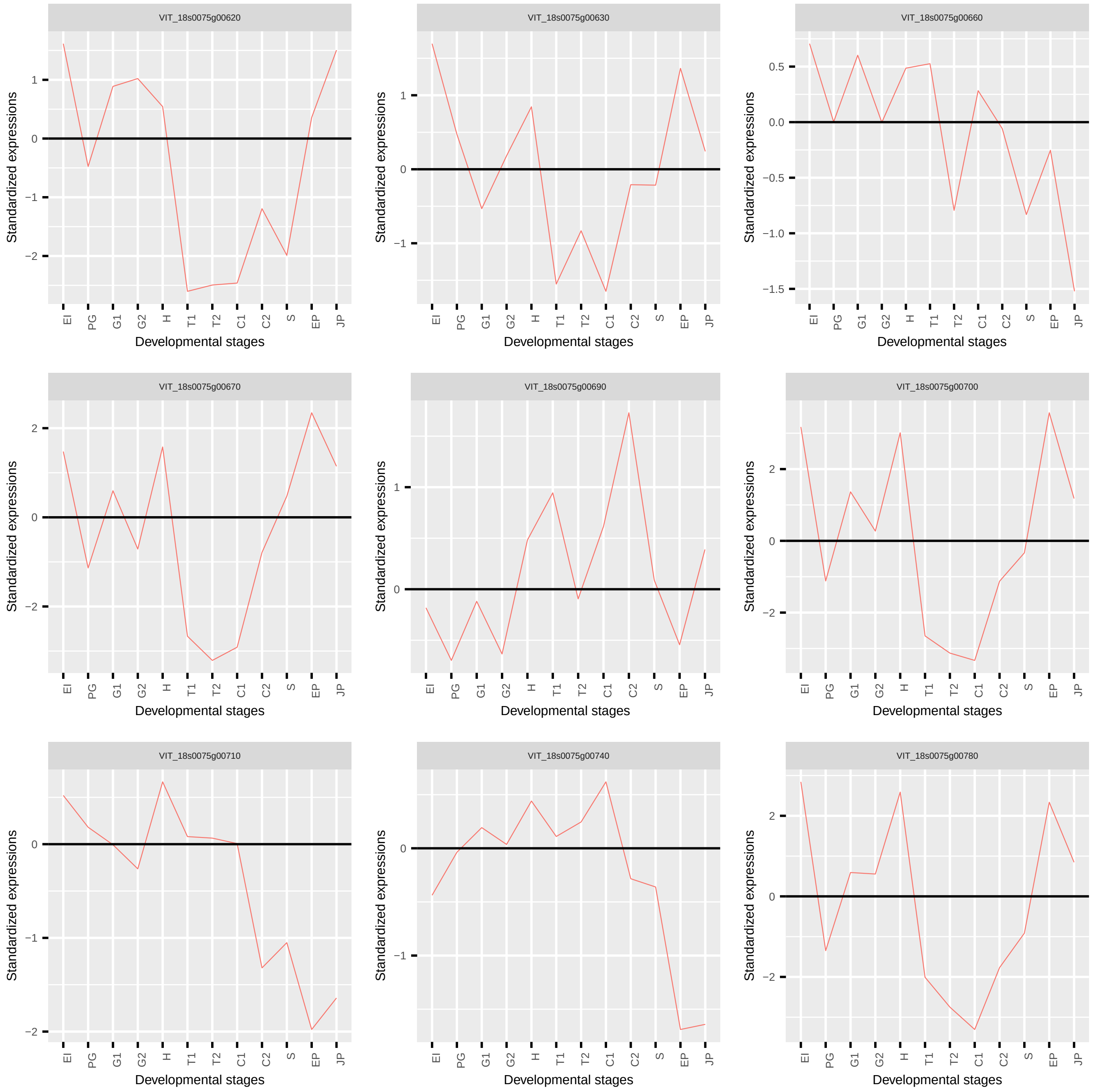

VIT_18s0075g00620
VIT_18s0075g00630
VIT_18s0075g00660
VIT_18s0075g00670
VIT_18s0075g00690
VIT_18s0075g00700
VIT_18s0075g00710
VIT_18s0075g00740
VIT_18s0075g00780
Standardized expressions
Developmental stages
EI PG G1 G2 H T1 T2 C1 C2 S EP JP

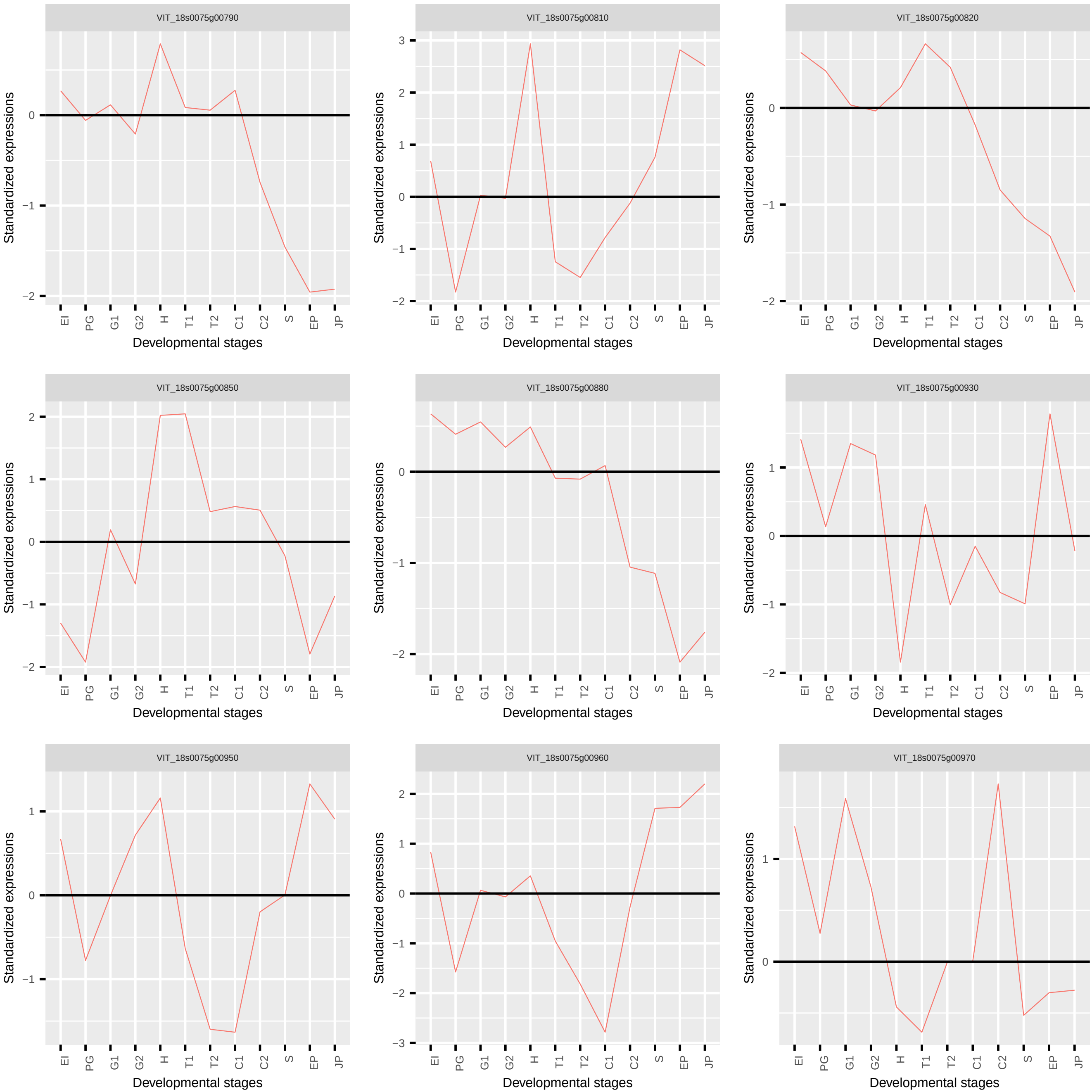
VIT_18s0075g00790
VIT_18s0075g00810
VIT_18s0075g00820
VIT_18s0075g00850
VIT_18s0075g00880
VIT_18s0075g00930
VIT_18s0075g00950
VIT_18s0075g00960
VIT_18s0075g00970
Standardized expressions
Developmental stages
EI
PG
G1
G2
H
T1
T2
C1
C2
S
EP
JP

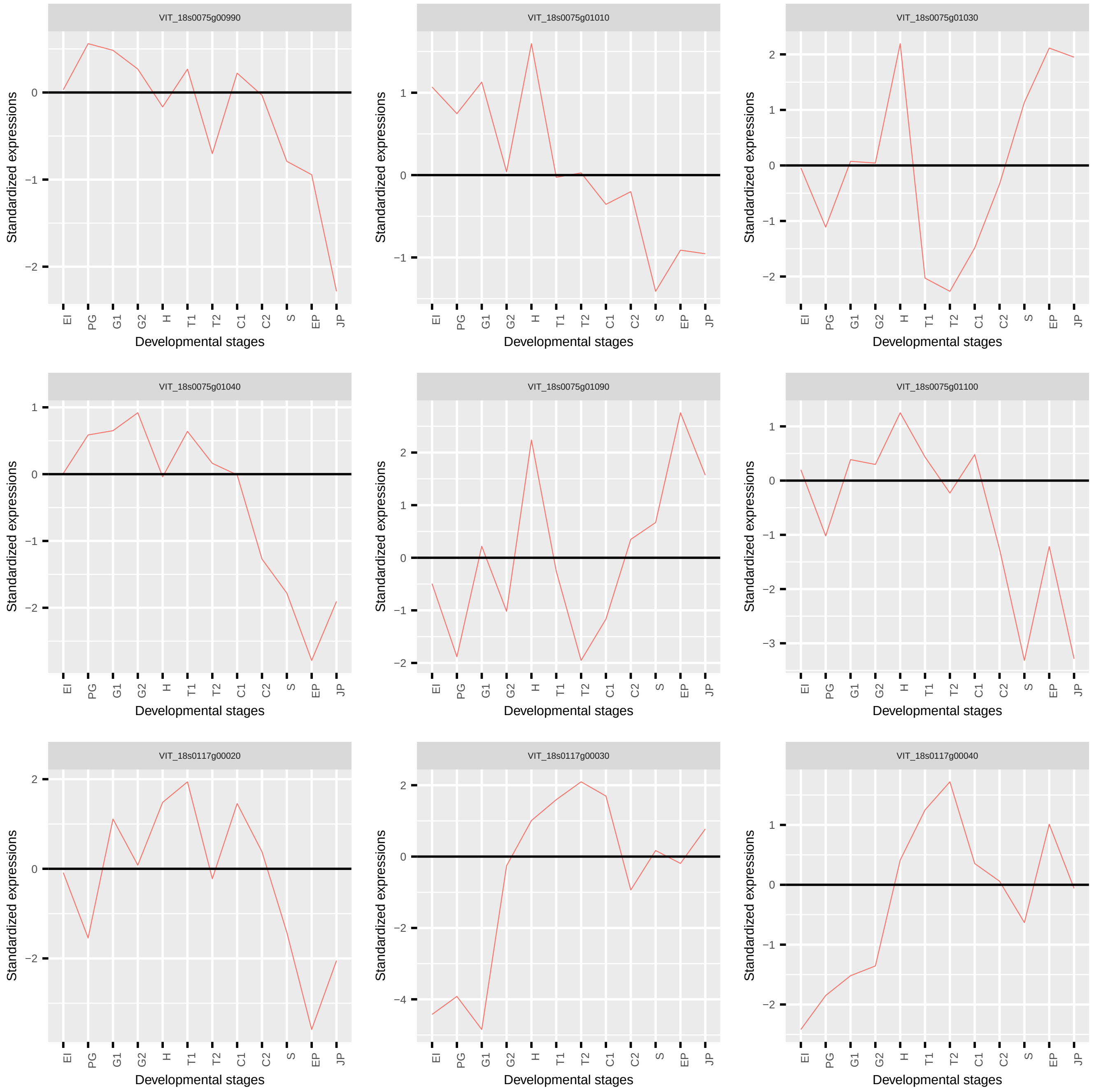
VIT_18s0075g00990
VIT_18s0075g01010
VIT_18s0075g01030
VIT_18s0075g01040
VIT_18s0075g01090
VIT_18s0075g01100
VIT_18s0117g00020
VIT_18s0117g00030
VIT_18s0117g00040
Standardized expressions
Developmental stages
EI PG G1 G2 H T1 T2 C1 C2 S EP JP

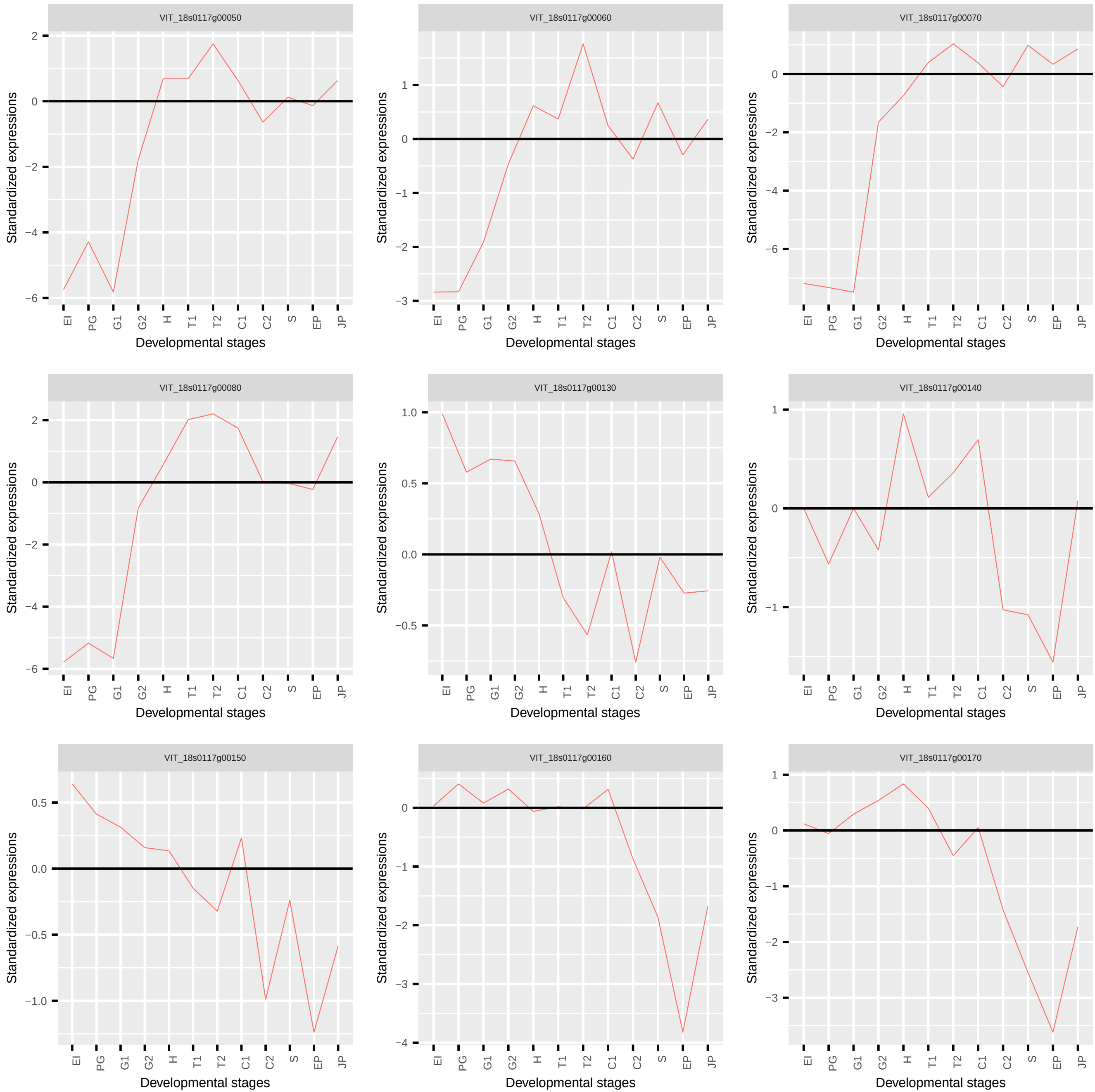
VIT_18s0117g00050
VIT_18s0117g00060
VIT_18s0117g00070
VIT_18s0117g00080
VIT_18s0117g00130
VIT_18s0117g00140
VIT_18s0117g00150
VIT_18s0117g00160
VIT_18s0117g00170
Standardized expressions
Developmental stages
EI PG G1 G2 H T1 T2 C1 C2 S EP JP
2 0 −2 −4 −6
1 0 −1 −2 −3
0 −2 −4 −6
1.0 0.5 0.0 −0.5
1 0 −1
0.5 0.0 −0.5 −1.0
0 −1 −2 −3 −4
1 0 −1 −2 −3

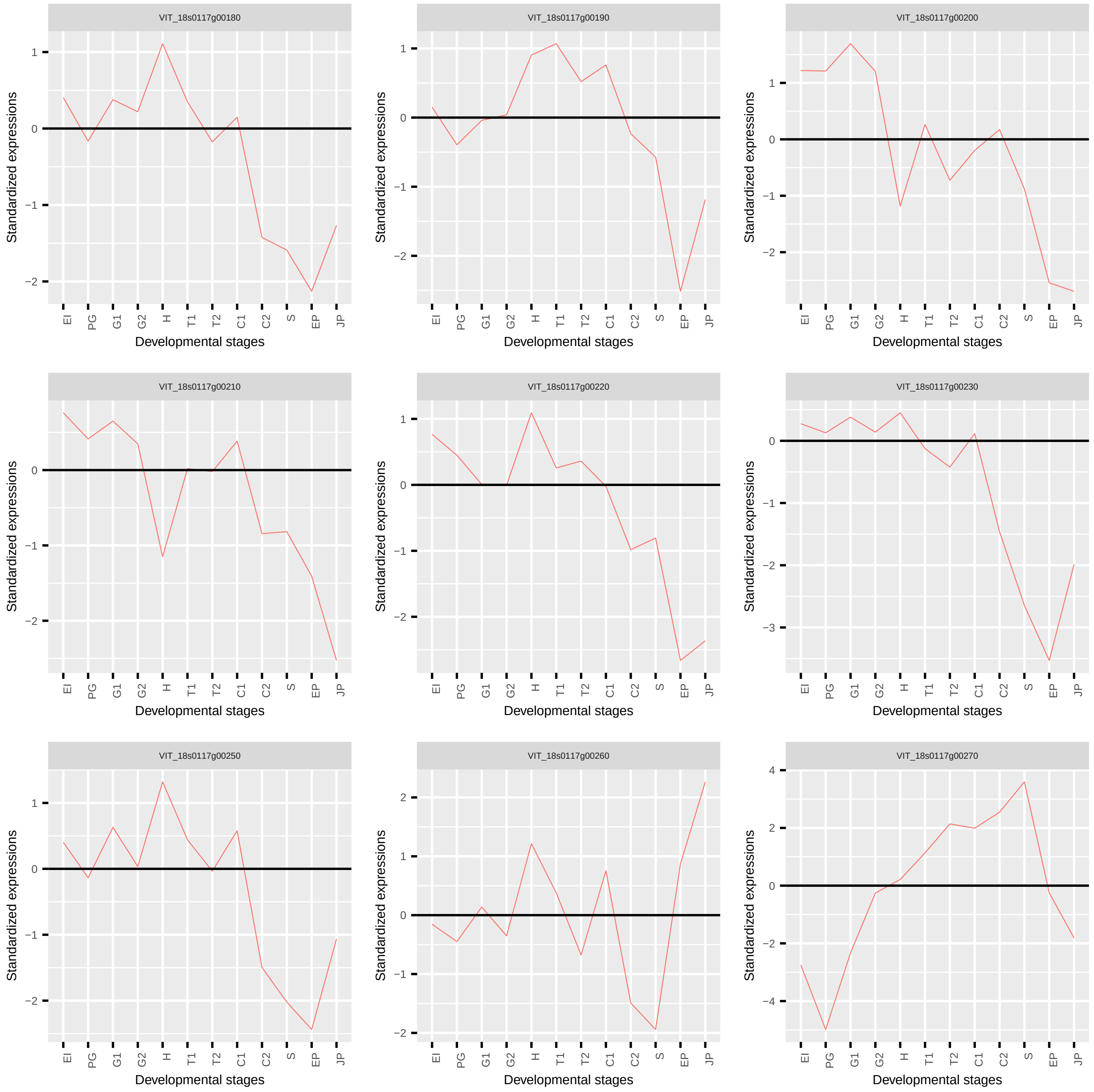
VIT_18s0117g00180
VIT_18s0117g00190
VIT_18s0117g00200
VIT_18s0117g00210
VIT_18s0117g00220
VIT_18s0117g00230
VIT_18s0117g00250
VIT_18s0117g00260
VIT_18s0117g00270
Standardized expressions
Developmental stages
EI PG G1 G2 H T1 T2 C1 C2 S EP JP

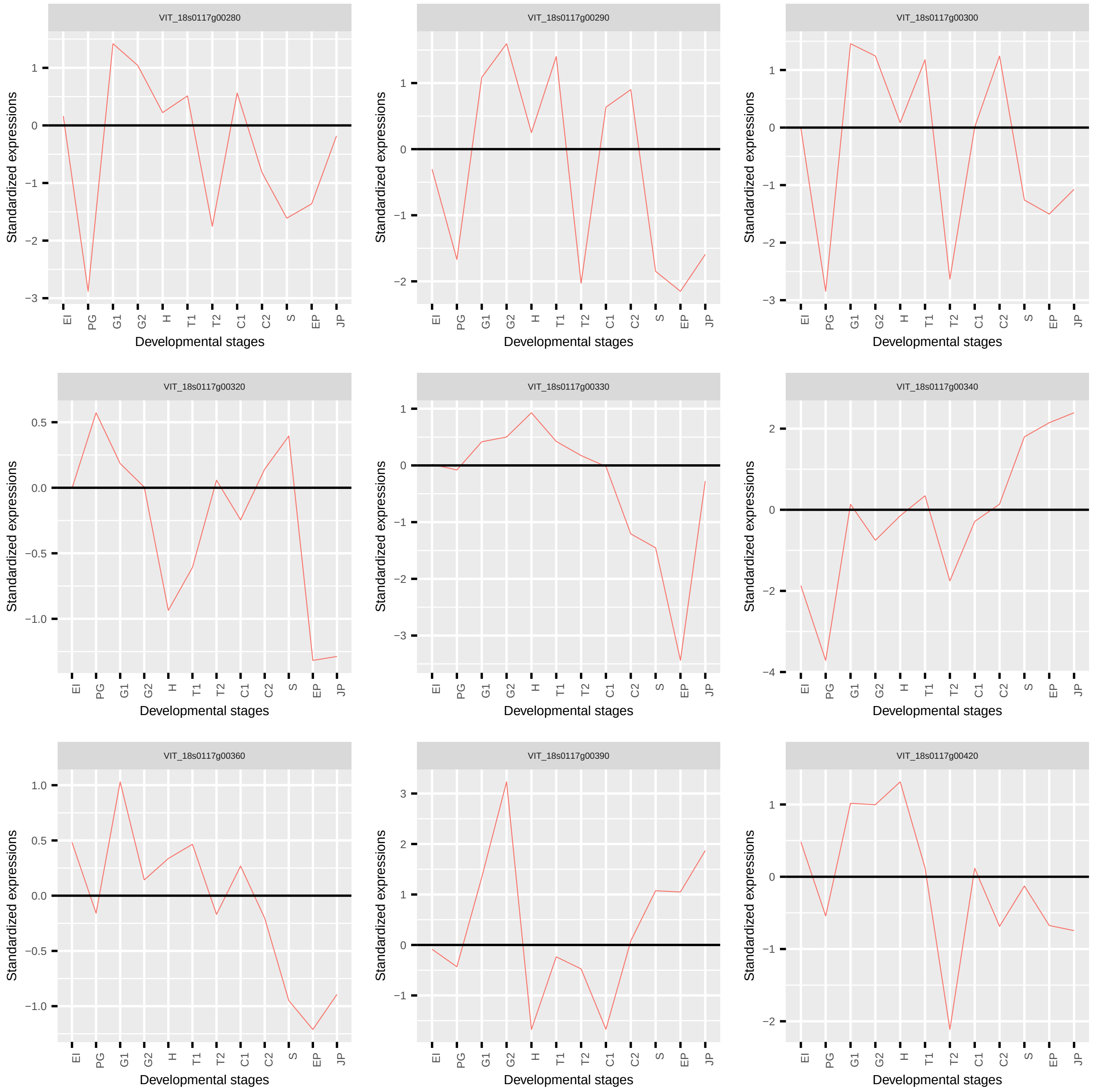
VIT_18s0117g00280
VIT_18s0117g00290
VIT_18s0117g00300
VIT_18s0117g00320
VIT_18s0117g00330
VIT_18s0117g00340
VIT_18s0117g00360
VIT_18s0117g00390
VIT_18s0117g00420
Standardized expressions
Developmental stages
EI PG G1 G2 H T1 T2 C1 C2 S EP JP

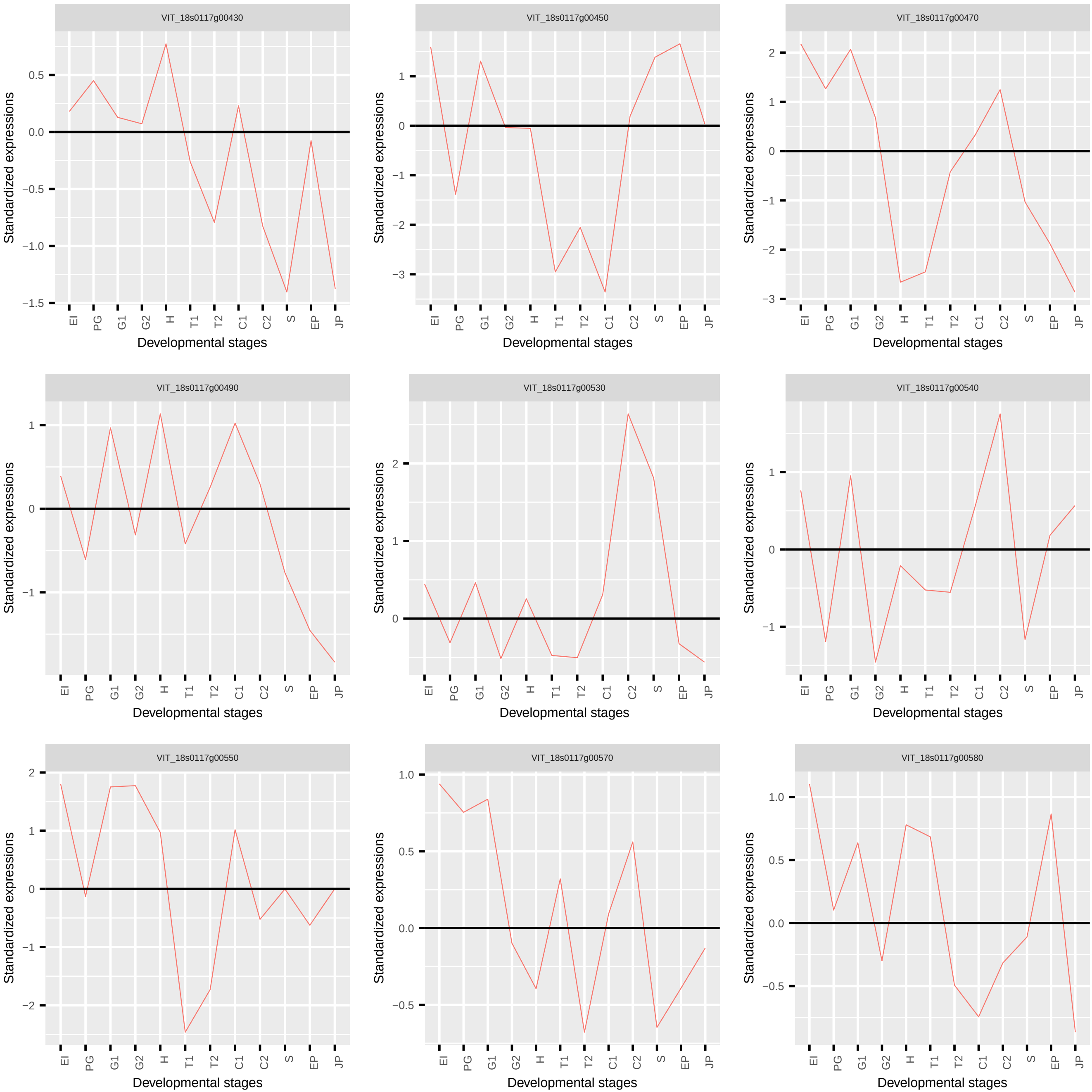
VIT_18s0117g00430
VIT_18s0117g00450
VIT_18s0117g00470
VIT_18s0117g00490
VIT_18s0117g00530
VIT_18s0117g00540
VIT_18s0117g00550
VIT_18s0117g00570
VIT_18s0117g00580
Standardized expressions
Developmental stages
EI
PG
G1
G2
H
T1
T2
C1
C2
S
EP
JP

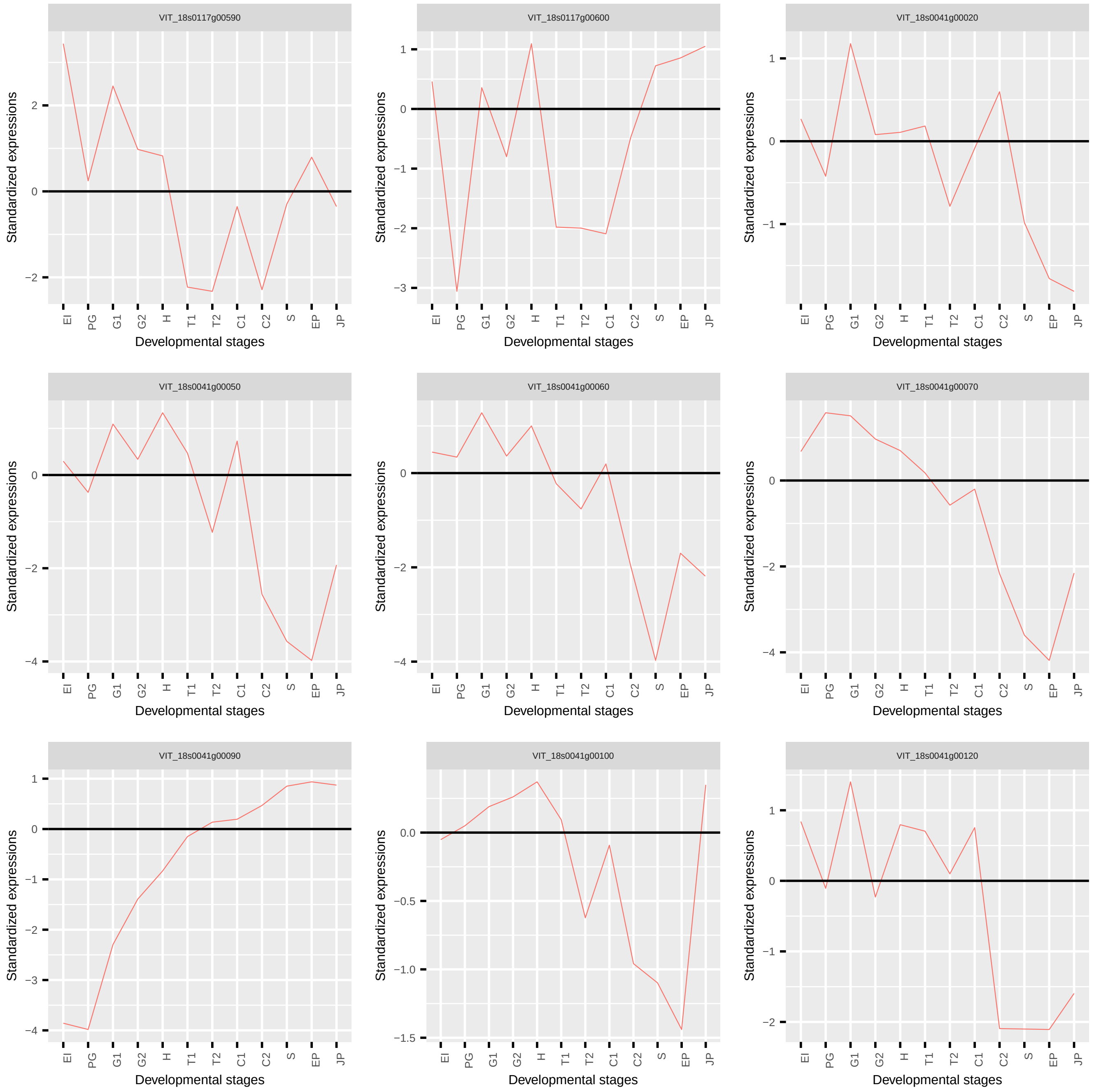
VIT_18s0117g00590
VIT_18s0117g00600
VIT_18s0041g00020
VIT_18s0041g00050
VIT_18s0041g00060
VIT_18s0041g00070
VIT_18s0041g00090
VIT_18s0041g00100
VIT_18s0041g00120
Standardized expressions
Developmental stages
EI PG G1 G2 H T1 T2 C1 C2 S EP JP

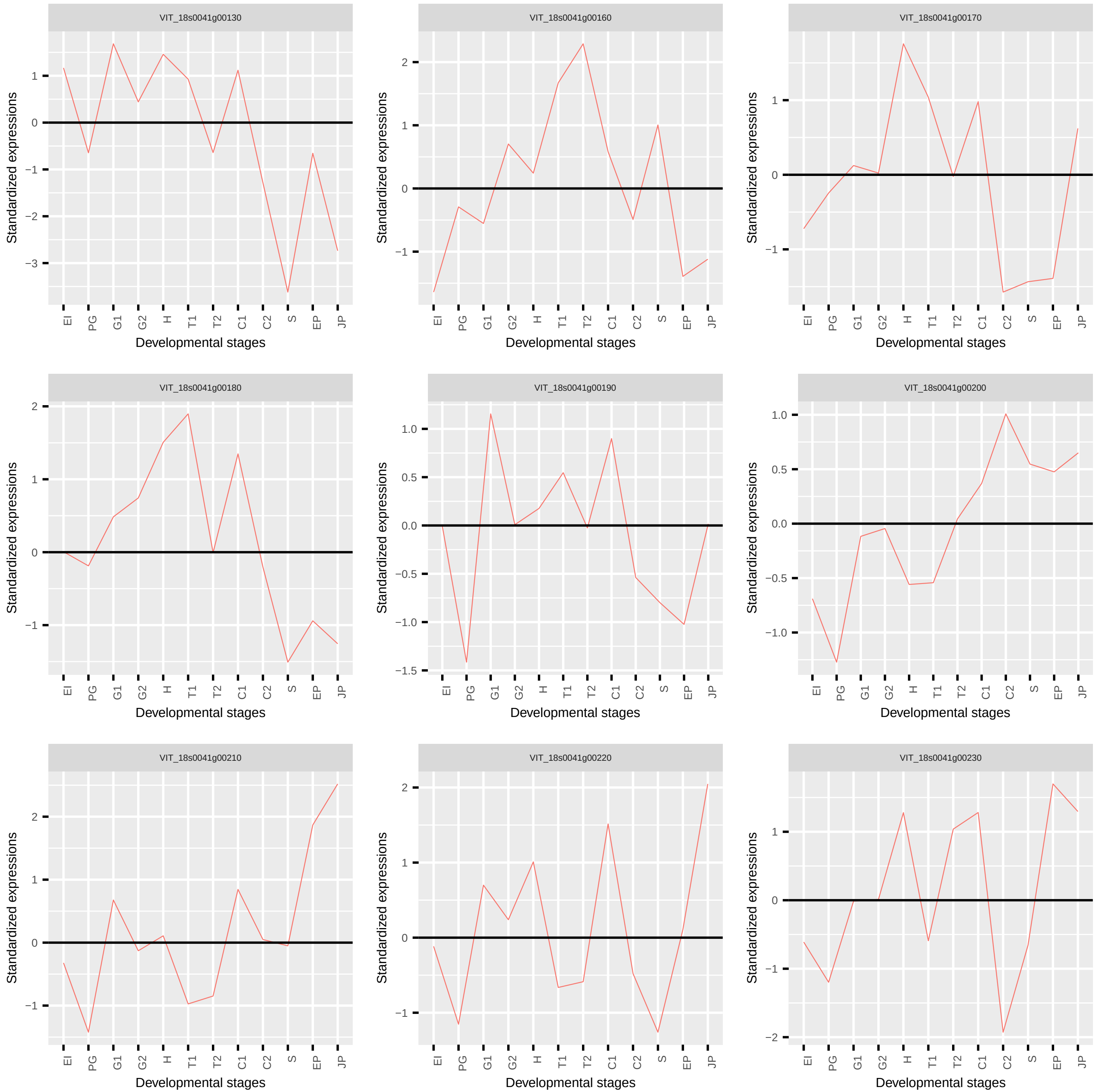
VIT_18s0041g00130
VIT_18s0041g00160
VIT_18s0041g00170
VIT_18s0041g00180
VIT_18s0041g00190
VIT_18s0041g00200
VIT_18s0041g00210
VIT_18s0041g00220
VIT_18s0041g00230
Standardized expressions
Developmental stages
EI PG G1 G2 H T1 T2 C1 C2 S EP JP

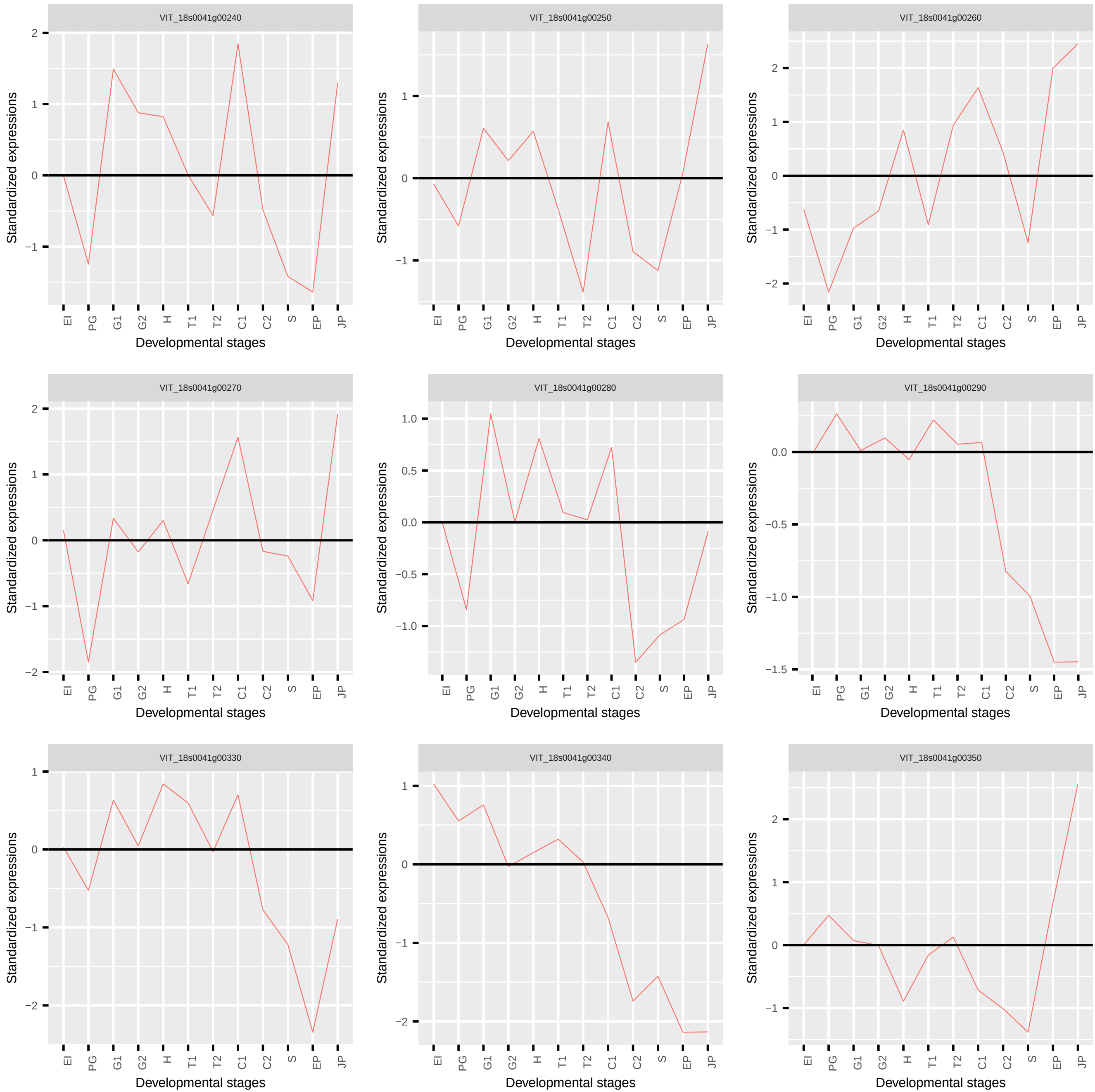
VIT_18s0041g00240
VIT_18s0041g00250
VIT_18s0041g00260
VIT_18s0041g00270
VIT_18s0041g00280
VIT_18s0041g00290
VIT_18s0041g00330
VIT_18s0041g00340
VIT_18s0041g00350
Standardized expressions
Developmental stages
EI PG G1 G2 H T1 T2 C1 C2 S EP JP

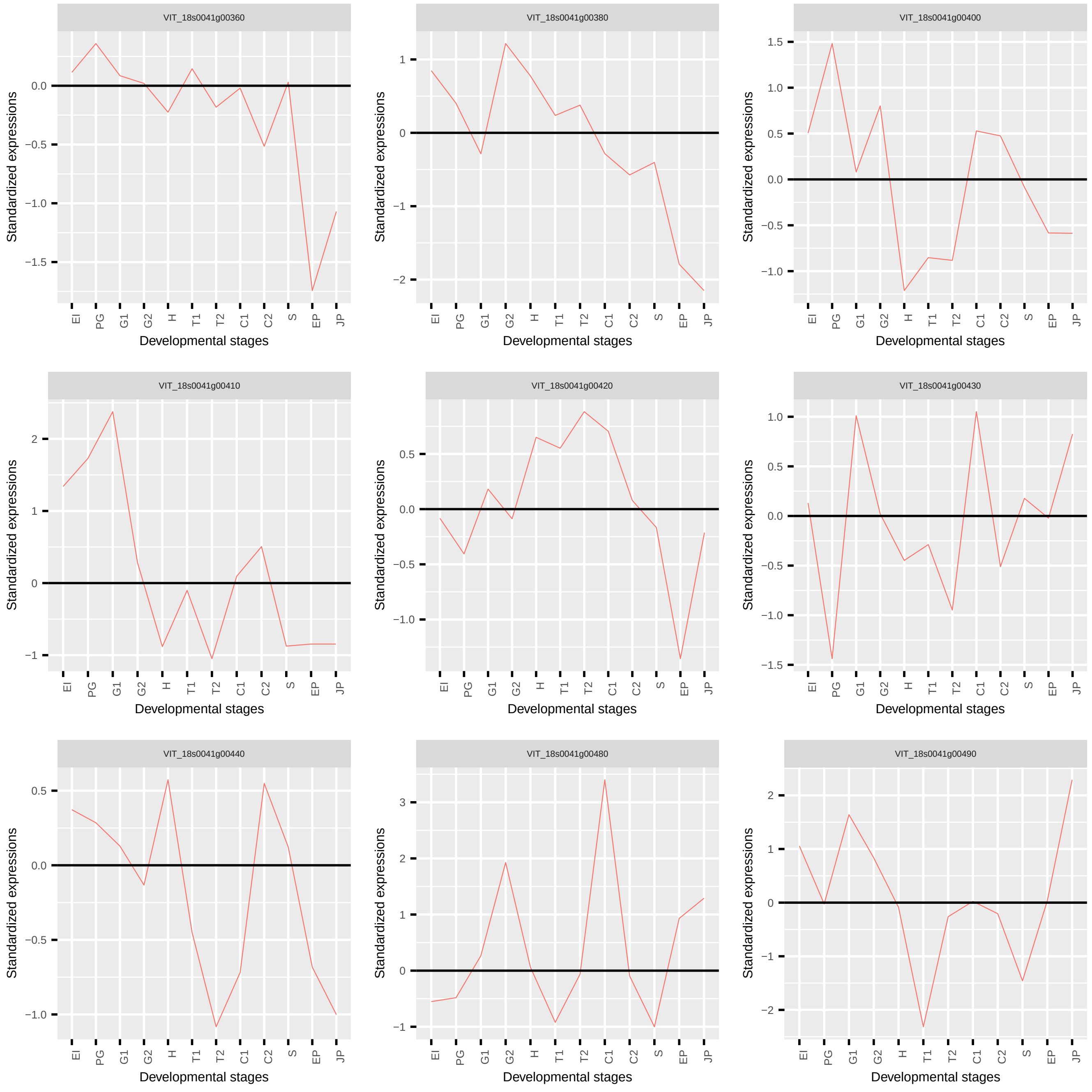

VIT_18s0041g00360
0.0
−0.5
−1.0
−1.5
Standardized expressions
EI PG G1 G2 H T1 T2 C1 C2 S EP JP
Developmental stages
VIT_18s0041g00380
1
0
−1
−2
Standardized expressions
EI PG G1 G2 H T1 T2 C1 C2 S EP JP
Developmental stages
VIT_18s0041g00400
1.5
1.0
0.5
0.0
−0.5
−1.0
Standardized expressions
EI PG G1 G2 H T1 T2 C1 C2 S EP JP
Developmental stages
VIT_18s0041g00410
2
1
0
−1
Standardized expressions
EI PG G1 G2 H T1 T2 C1 C2 S EP JP
Developmental stages
VIT_18s0041g00420
0.5
0.0
−0.5
−1.0
Standardized expressions
EI PG G1 G2 H T1 T2 C1 C2 S EP JP
Developmental stages
VIT_18s0041g00430
1.0
0.5
0.0
−0.5
−1.0
−1.5
Standardized expressions
EI PG G1 G2 H T1 T2 C1 C2 S EP JP
Developmental stages
VIT_18s0041g00440
0.5
0.0
−0.5
−1.0
Standardized expressions
EI PG G1 G2 H T1 T2 C1 C2 S EP JP
Developmental stages
VIT_18s0041g00480
3
2
1
0
−1
Standardized expressions
EI PG G1 G2 H T1 T2 C1 C2 S EP JP
Developmental stages
VIT_18s0041g00490
2
1
0
−1
−2
Standardized expressions
EI PG G1 G2 H T1 T2 C1 C2 S EP JP
Developmental stages

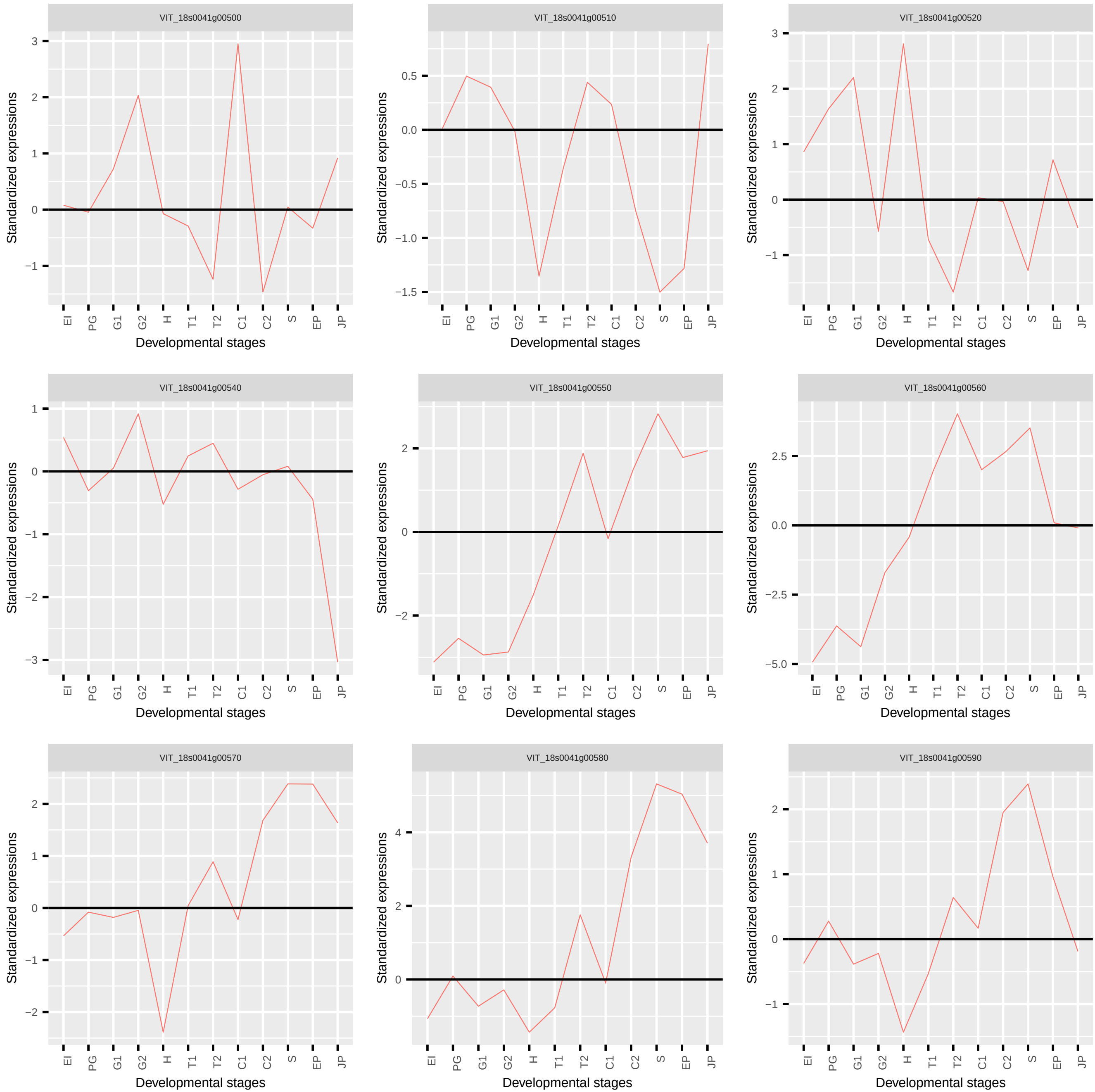
VIT_18s0041g00500
VIT_18s0041g00510
VIT_18s0041g00520
VIT_18s0041g00540
VIT_18s0041g00550
VIT_18s0041g00560
VIT_18s0041g00570
VIT_18s0041g00580
VIT_18s0041g00590
Standardized expressions
Developmental stages
EI PG G1 G2 H T1 T2 C1 C2 S EP JP

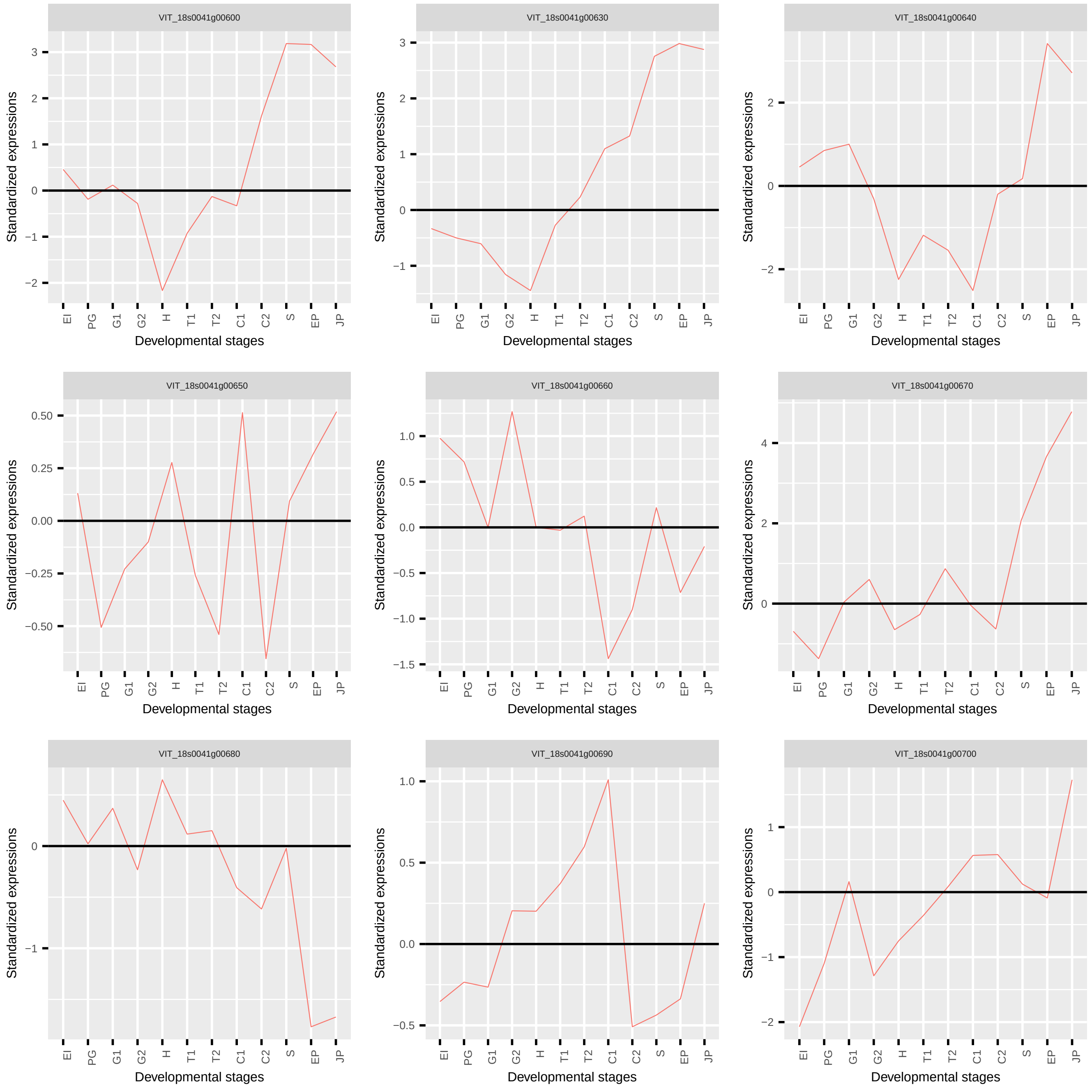
VIT_18s0041g00600
VIT_18s0041g00630
VIT_18s0041g00640
VIT_18s0041g00650
VIT_18s0041g00660
VIT_18s0041g00670
VIT_18s0041g00680
VIT_18s0041g00690
VIT_18s0041g00700
Standardized expressions
Developmental stages
EI PG G1 G2 H T1 T2 C1 C2 S EP JP

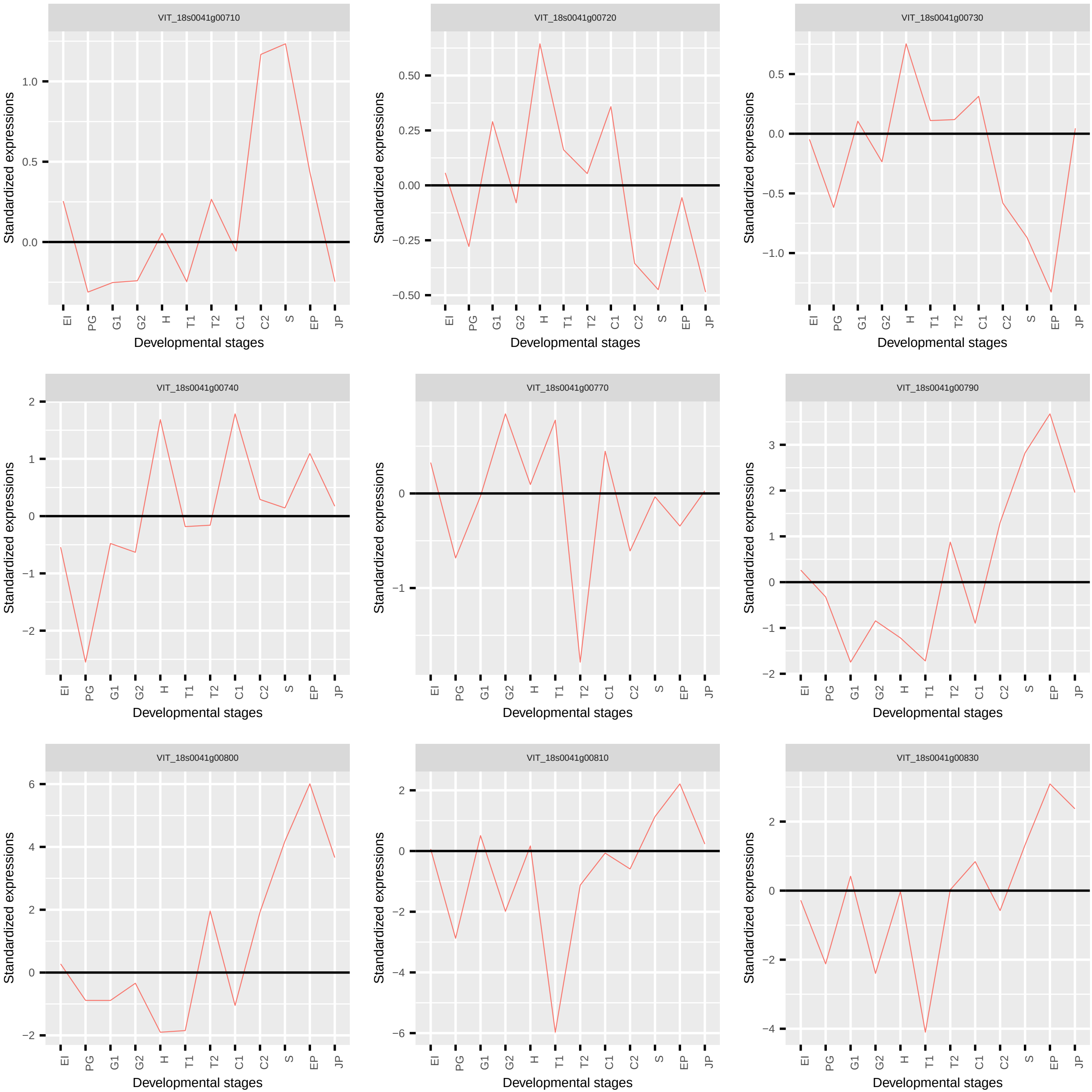
VIT_18s0041g00710
VIT_18s0041g00720
VIT_18s0041g00730
VIT_18s0041g00740
VIT_18s0041g00770
VIT_18s0041g00790
VIT_18s0041g00800
VIT_18s0041g00810
VIT_18s0041g00830
Standardized expressions
Developmental stages
EI PG G1 G2 H T1 T2 C1 C2 S EP JP

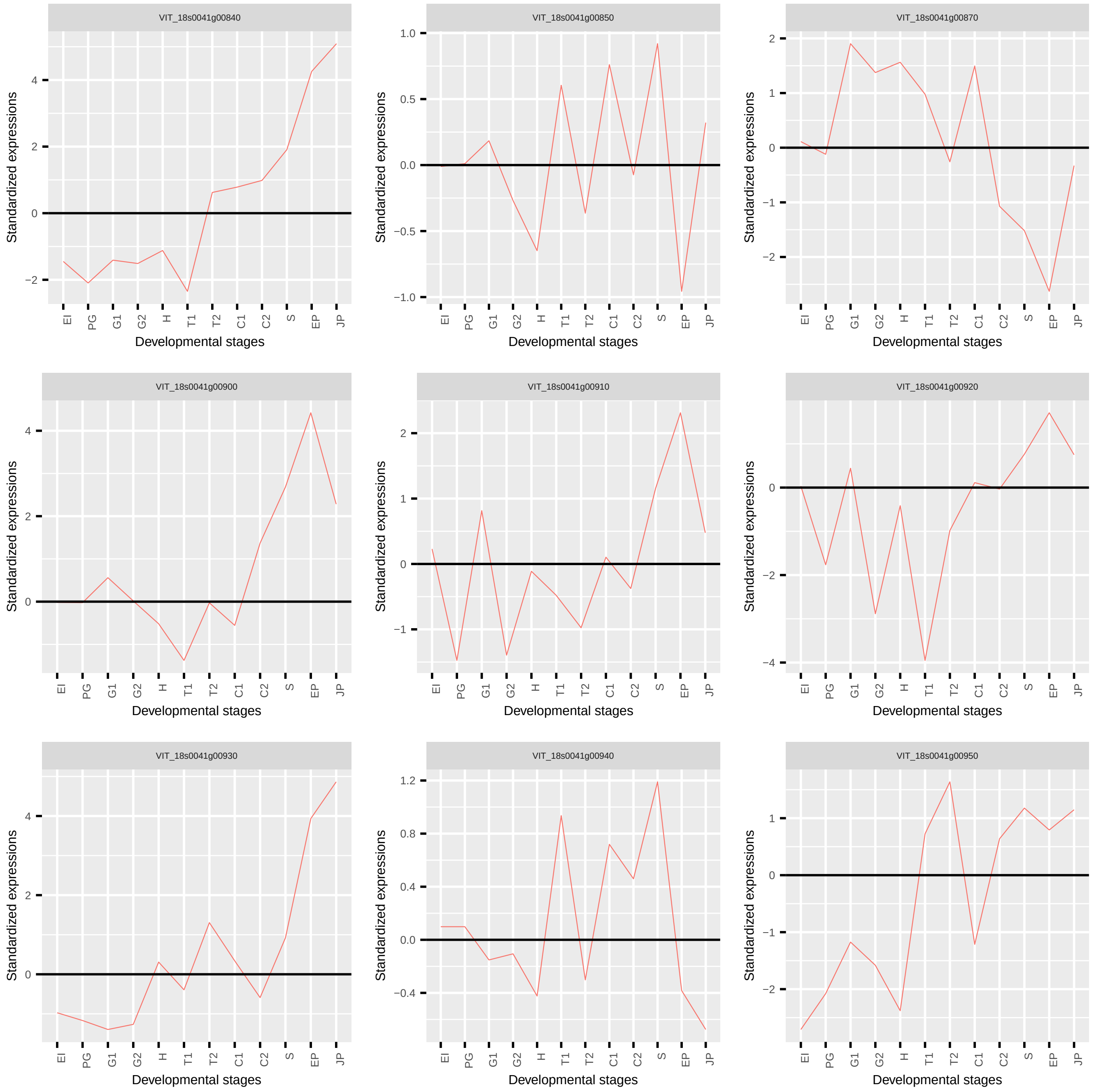
VIT_18s0041g00840
VIT_18s0041g00850
VIT_18s0041g00870
VIT_18s0041g00900
VIT_18s0041g00910
VIT_18s0041g00920
VIT_18s0041g00930
VIT_18s0041g00940
VIT_18s0041g00950
Standardized expressions
Developmental stages
EI PG G1 G2 H T1 T2 C1 C2 S EP JP

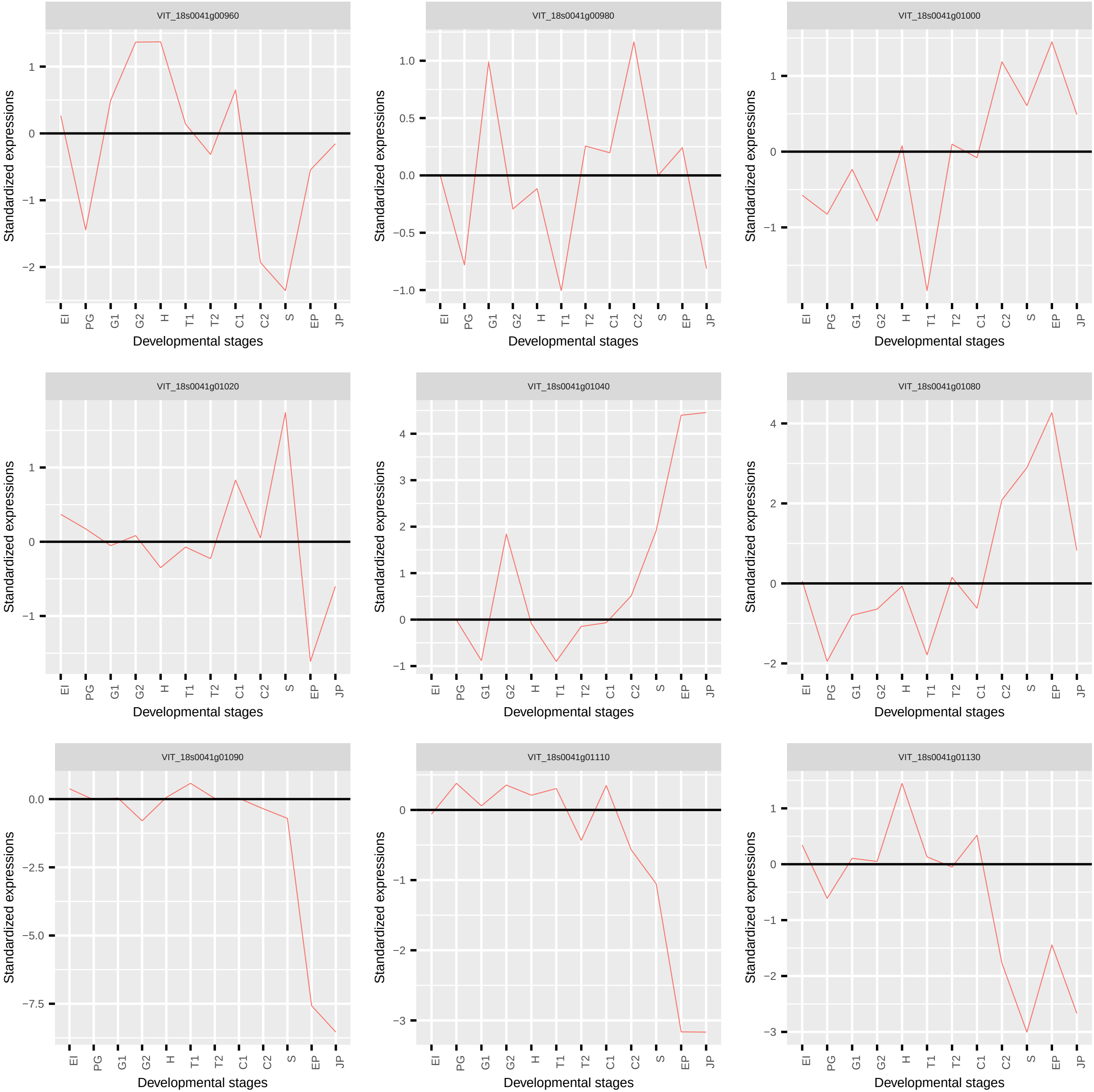
VIT_18s0041g00960
VIT_18s0041g00980
VIT_18s0041g01000
VIT_18s0041g01020
VIT_18s0041g01040
VIT_18s0041g01080
VIT_18s0041g01090
VIT_18s0041g01110
VIT_18s0041g01130
Standardized expressions
Developmental stages
EI PG G1 G2 H T1 T2 C1 C2 S EP JP

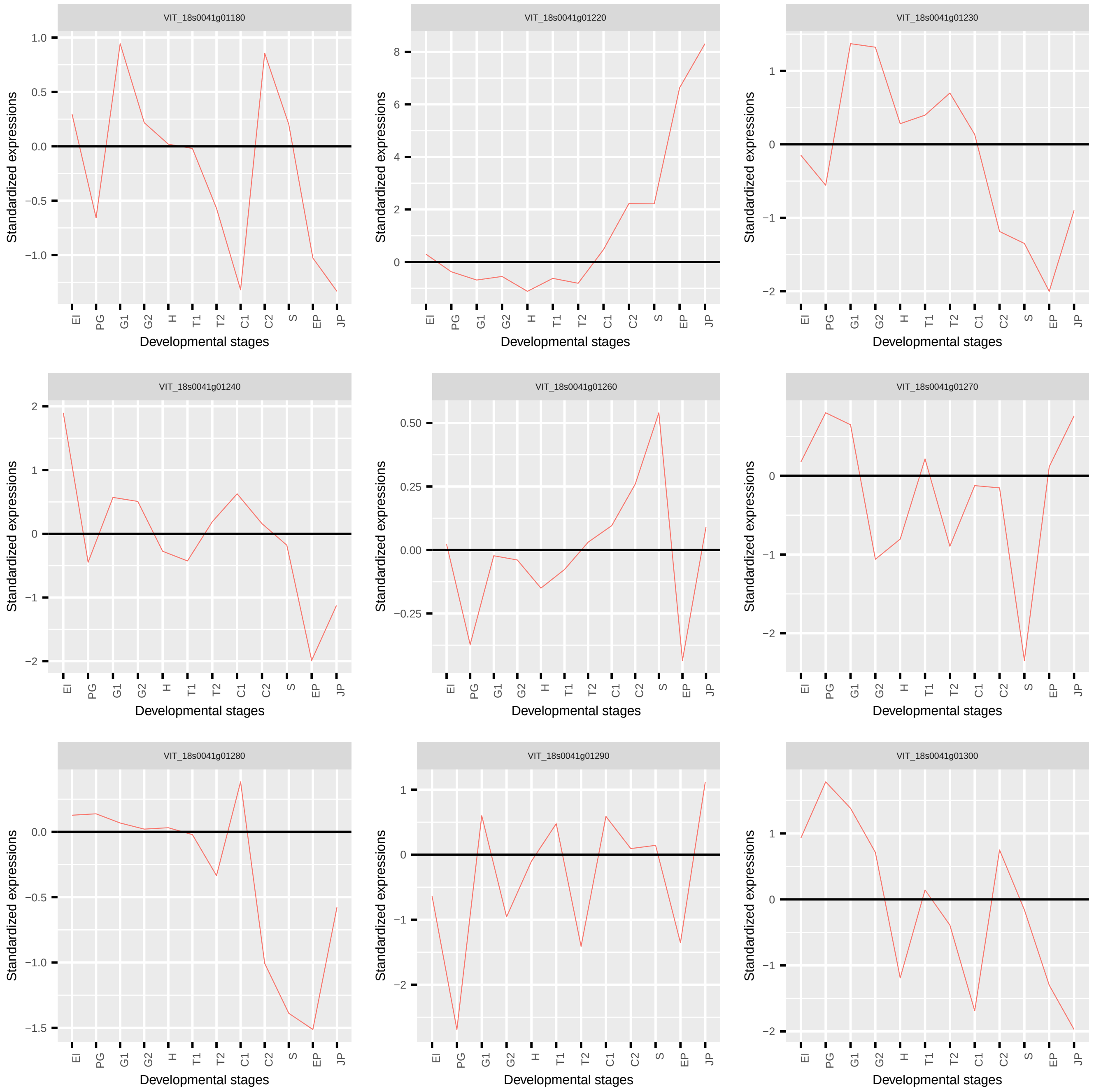
VIT_18s0041g01180
VIT_18s0041g01220
VIT_18s0041g01230
VIT_18s0041g01240
VIT_18s0041g01260
VIT_18s0041g01270
VIT_18s0041g01280
VIT_18s0041g01290
VIT_18s0041g01300
Standardized expressions
Developmental stages
EI PG G1 G2 H T1 T2 C1 C2 S EP JP
1.0 0.5 0.0 −0.5 −1.0
8 6 4 2 0
1 0 −1 −2
2 1 0 −1 −2
0.50 0.25 0.00 −0.25
0 −1 −2
0.0 −0.5 −1.0 −1.5
1 0 −1 −2
1 0 −1 −2

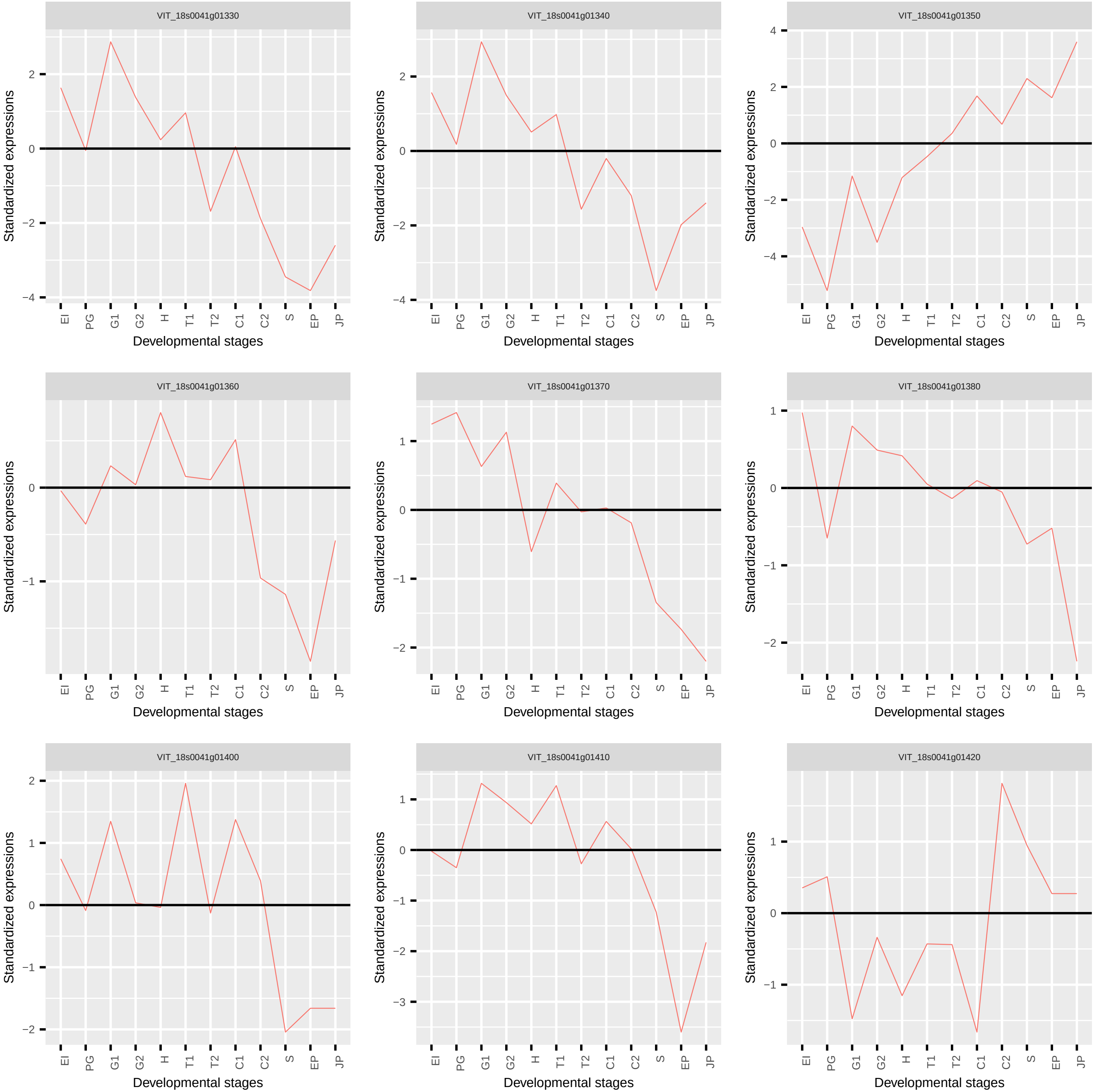
VIT_18s0041g01330
VIT_18s0041g01340
VIT_18s0041g01350
VIT_18s0041g01360
VIT_18s0041g01370
VIT_18s0041g01380
VIT_18s0041g01400
VIT_18s0041g01410
VIT_18s0041g01420
Standardized expressions
Developmental stages
EI PG G1 G2 H T1 T2 C1 C2 S EP JP

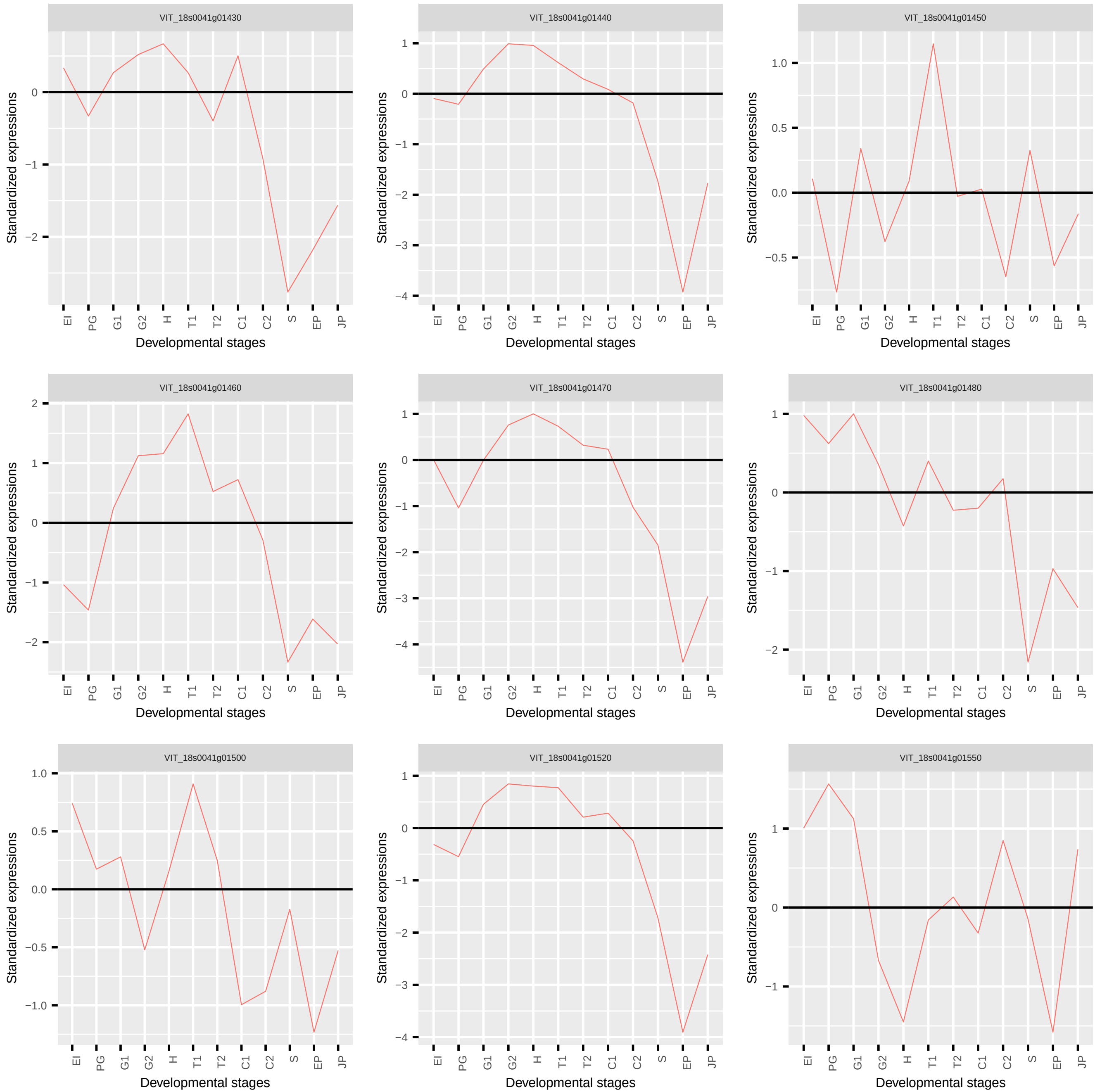

VIT_18s0041g01430
VIT_18s0041g01440
VIT_18s0041g01450
VIT_18s0041g01460
VIT_18s0041g01470
VIT_18s0041g01480
VIT_18s0041g01500
VIT_18s0041g01520
VIT_18s0041g01550
Standardized expressions
Developmental stages
EI
PG
G1
G2
H
T1
T2
C1
C2
S
EP
JP

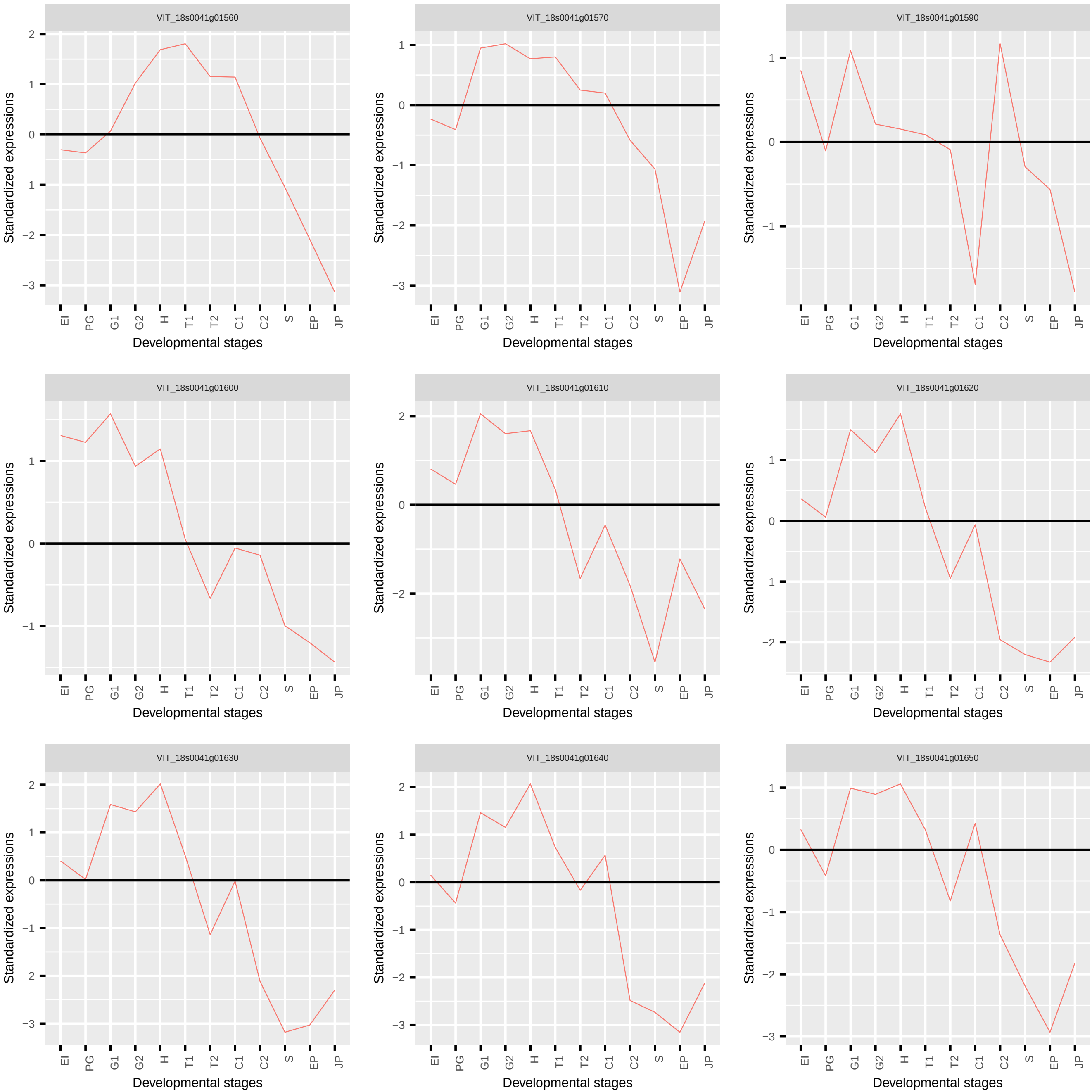
VIT_18s0041g01560
VIT_18s0041g01570
VIT_18s0041g01590
VIT_18s0041g01600
VIT_18s0041g01610
VIT_18s0041g01620
VIT_18s0041g01630
VIT_18s0041g01640
VIT_18s0041g01650
Standardized expressions
Developmental stages
EI PG G1 G2 H T1 T2 C1 C2 S EP JP

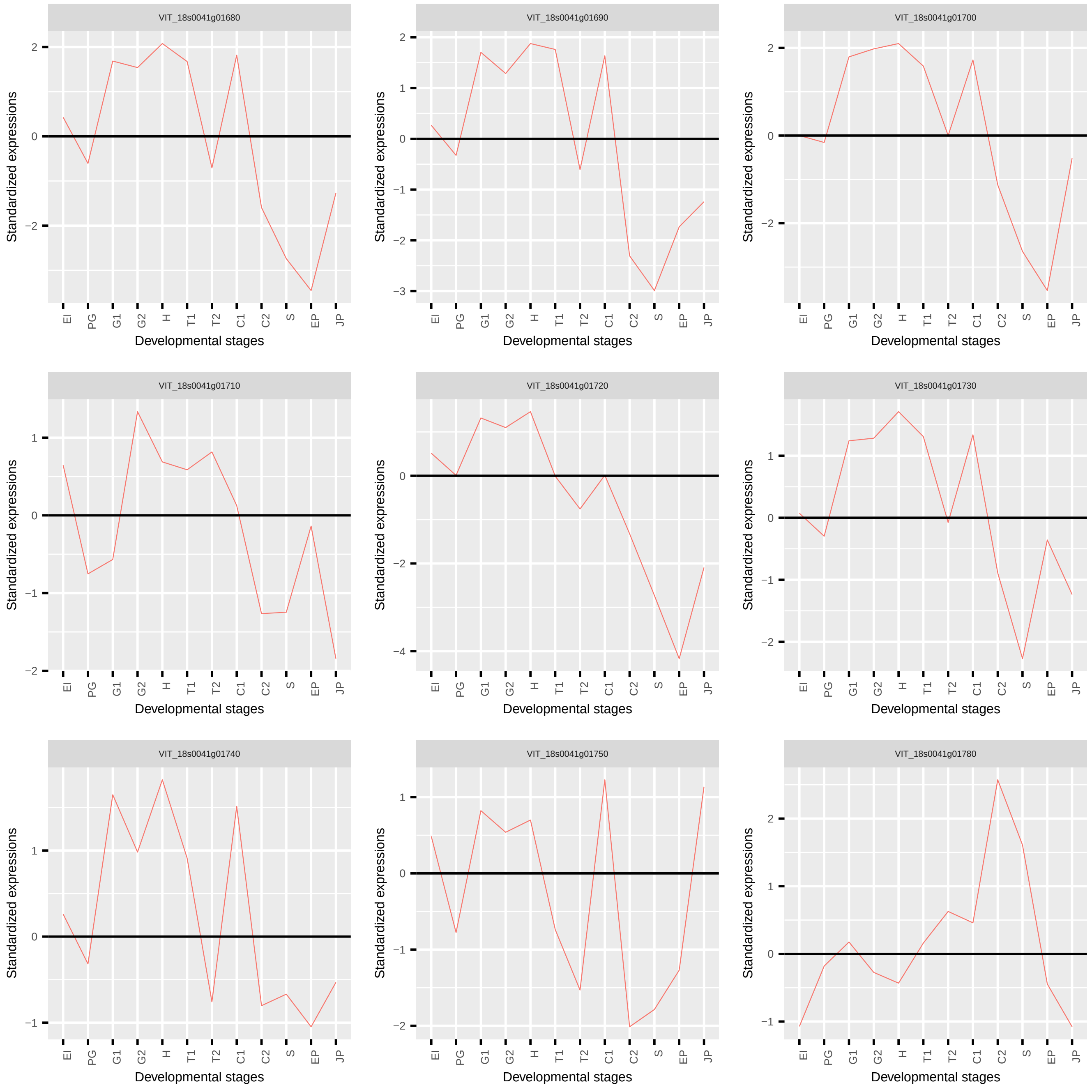

VIT_18s0041g01680
VIT_18s0041g01690
VIT_18s0041g01700
VIT_18s0041g01710
VIT_18s0041g01720
VIT_18s0041g01730
VIT_18s0041g01740
VIT_18s0041g01750
VIT_18s0041g01780
Standardized expressions
Developmental stages
EI
PG
G1
G2
H
T1
T2
C1
C2
S
EP
JP

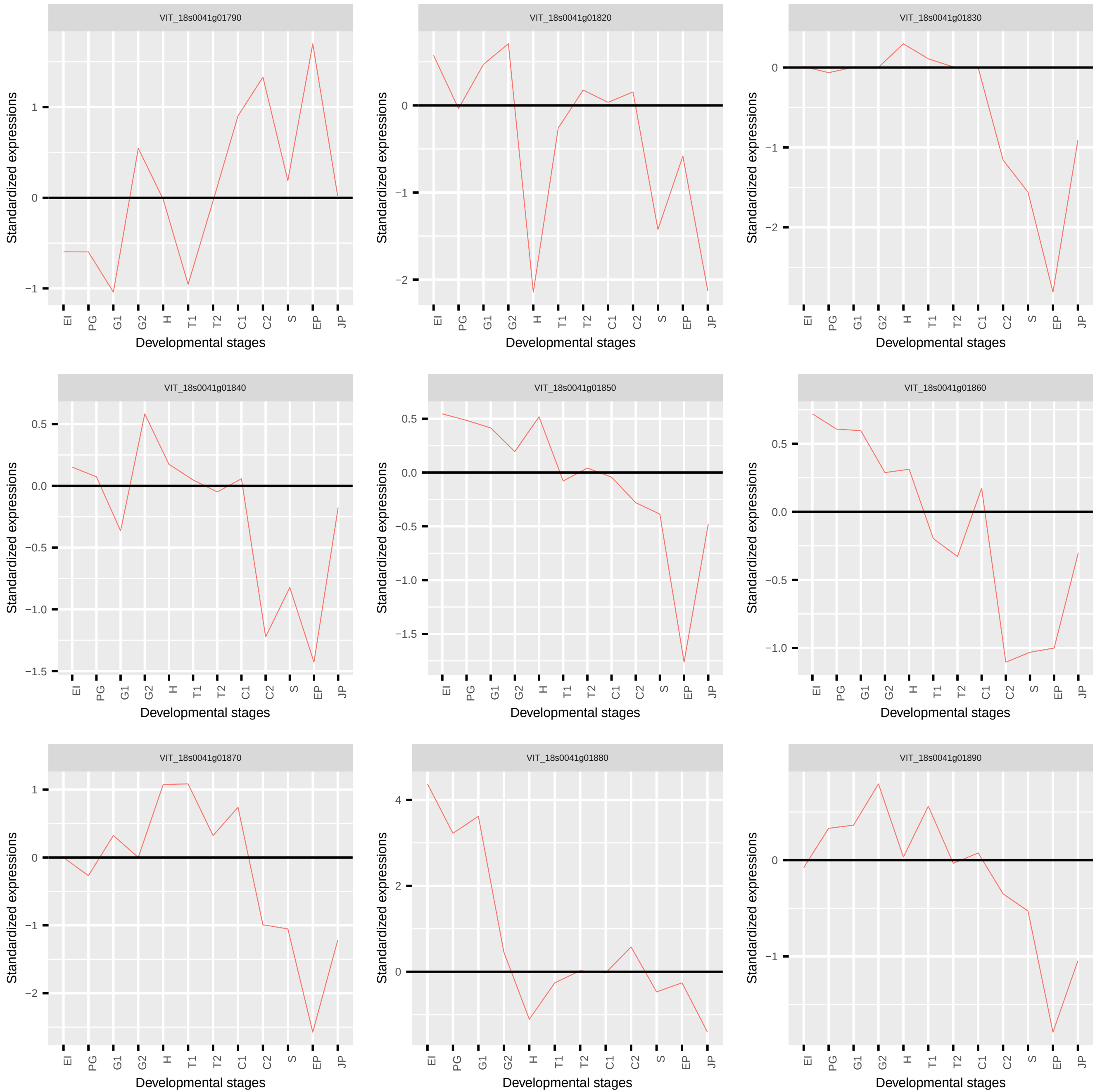
VIT_18s0041g01790
VIT_18s0041g01820
VIT_18s0041g01830
VIT_18s0041g01840
VIT_18s0041g01850
VIT_18s0041g01860
VIT_18s0041g01870
VIT_18s0041g01880
VIT_18s0041g01890
Standardized expressions
Developmental stages
EI PG G1 G2 H T1 T2 C1 C2 S EP JP

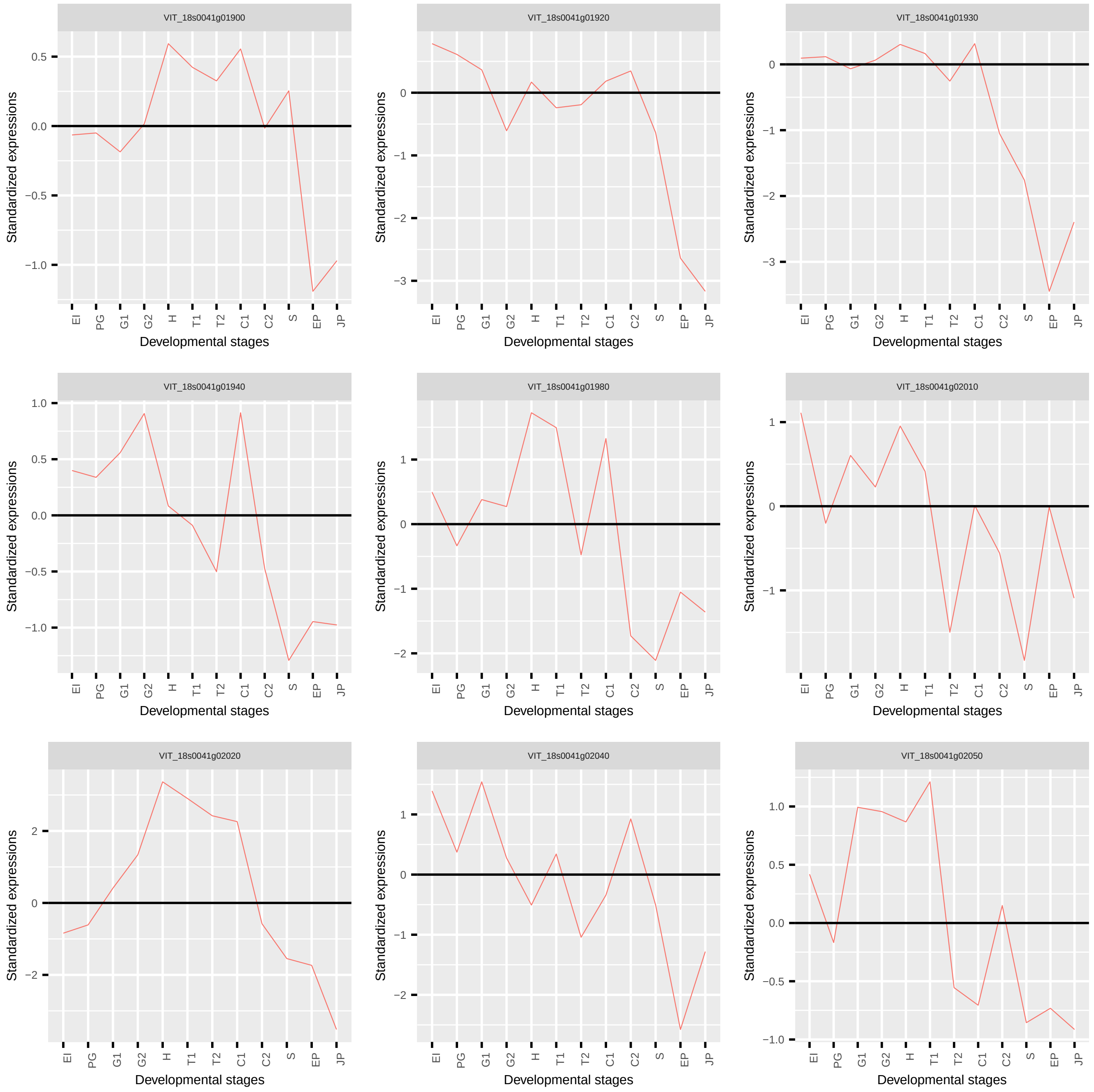
VIT_18s0041g01900
Standardized expressions
0.5
0.0
−0.5
−1.0
EI PG G1 G2 H T1 T2 C1 C2 S EP JP
Developmental stages
VIT_18s0041g01920
Standardized expressions
0
−1
−2
−3
EI PG G1 G2 H T1 T2 C1 C2 S EP JP
Developmental stages
VIT_18s0041g01930
Standardized expressions
0
−1
−2
−3
EI PG G1 G2 H T1 T2 C1 C2 S EP JP
Developmental stages
VIT_18s0041g01940
Standardized expressions
1.0
0.5
0.0
−0.5
−1.0
EI PG G1 G2 H T1 T2 C1 C2 S EP JP
Developmental stages
VIT_18s0041g01980
Standardized expressions
1
0
−1
−2
EI PG G1 G2 H T1 T2 C1 C2 S EP JP
Developmental stages
VIT_18s0041g02010
Standardized expressions
1
0
−1
EI PG G1 G2 H T1 T2 C1 C2 S EP JP
Developmental stages
VIT_18s0041g02020
Standardized expressions
2
0
−2
EI PG G1 G2 H T1 T2 C1 C2 S EP JP
Developmental stages
VIT_18s0041g02040
Standardized expressions
1
0
−1
−2
EI PG G1 G2 H T1 T2 C1 C2 S EP JP
Developmental stages
VIT_18s0041g02050
Standardized expressions
1.0
0.5
0.0
−0.5
−1.0
EI PG G1 G2 H T1 T2 C1 C2 S EP JP
Developmental stages

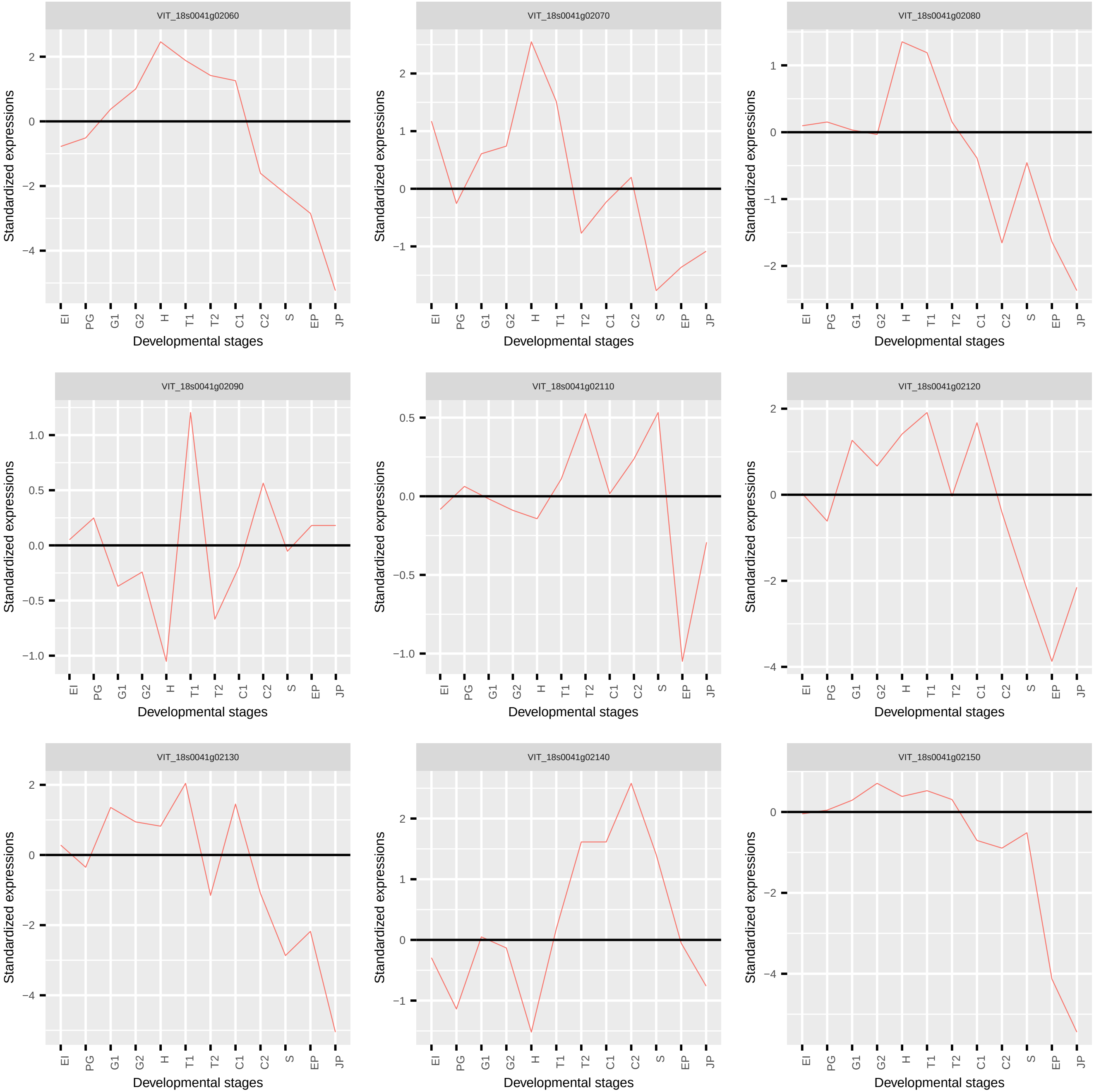
VIT_18s0041g02060
Standardized expressions
2
0
−2
−4
EI PG G1 G2 H T1 T2 C1 C2 S EP JP
Developmental stages
VIT_18s0041g02070
Standardized expressions
2
1
0
−1
EI PG G1 G2 H T1 T2 C1 C2 S EP JP
Developmental stages
VIT_18s0041g02080
Standardized expressions
1
0
−1
−2
EI PG G1 G2 H T1 T2 C1 C2 S EP JP
Developmental stages
VIT_18s0041g02090
Standardized expressions
1.0
0.5
0.0
−0.5
−1.0
EI PG G1 G2 H T1 T2 C1 C2 S EP JP
Developmental stages
VIT_18s0041g02110
Standardized expressions
0.5
0.0
−0.5
−1.0
EI PG G1 G2 H T1 T2 C1 C2 S EP JP
Developmental stages
VIT_18s0041g02120
Standardized expressions
2
0
−2
−4
EI PG G1 G2 H T1 T2 C1 C2 S EP JP
Developmental stages
VIT_18s0041g02130
Standardized expressions
2
0
−2
−4
EI PG G1 G2 H T1 T2 C1 C2 S EP JP
Developmental stages
VIT_18s0041g02140
Standardized expressions
2
1
0
−1
EI PG G1 G2 H T1 T2 C1 C2 S EP JP
Developmental stages
VIT_18s0041g02150
Standardized expressions
0
−2
−4
EI PG G1 G2 H T1 T2 C1 C2 S EP JP
Developmental stages

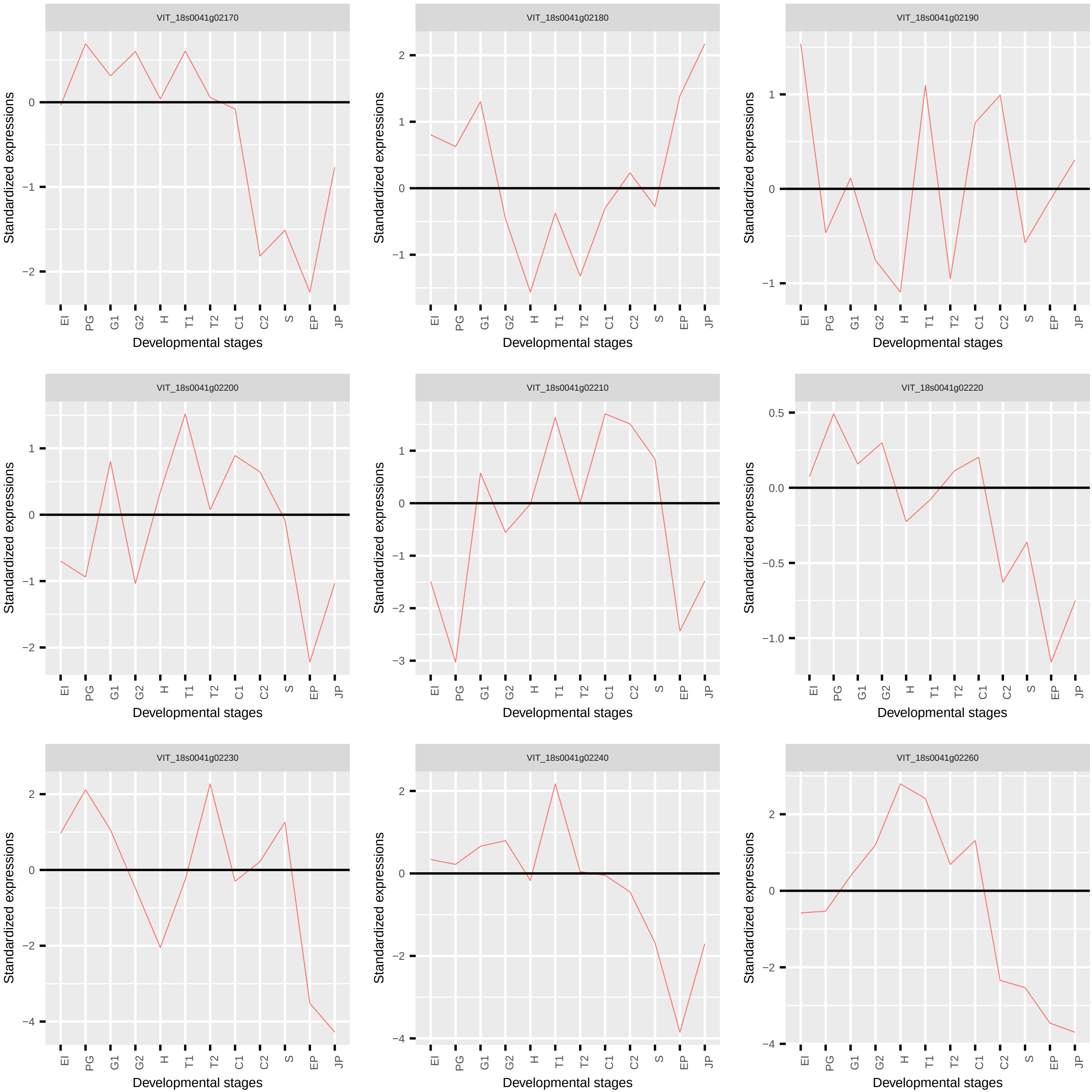

VIT_18s0041g02170
VIT_18s0041g02180
VIT_18s0041g02190
VIT_18s0041g02200
VIT_18s0041g02210
VIT_18s0041g02220
VIT_18s0041g02230
VIT_18s0041g02240
VIT_18s0041g02260
Standardized expressions
Developmental stages
EI PG G1 G2 H T1 T2 C1 C2 S EP JP

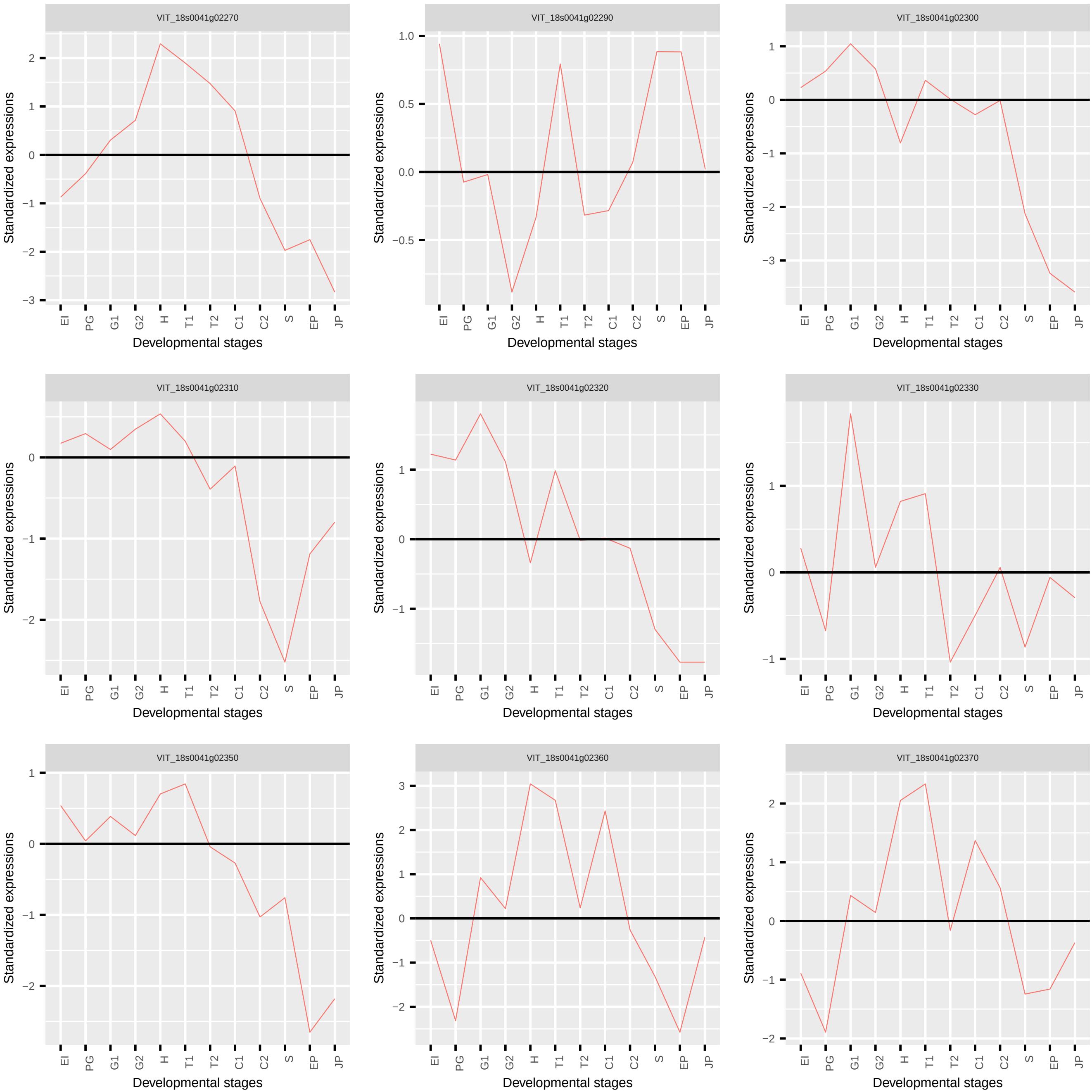
VIT_18s0041g02270
VIT_18s0041g02290
VIT_18s0041g02300
VIT_18s0041g02310
VIT_18s0041g02320
VIT_18s0041g02330
VIT_18s0041g02350
VIT_18s0041g02360
VIT_18s0041g02370
Standardized expressions
Developmental stages
EI PG G1 G2 H T1 T2 C1 C2 S EP JP

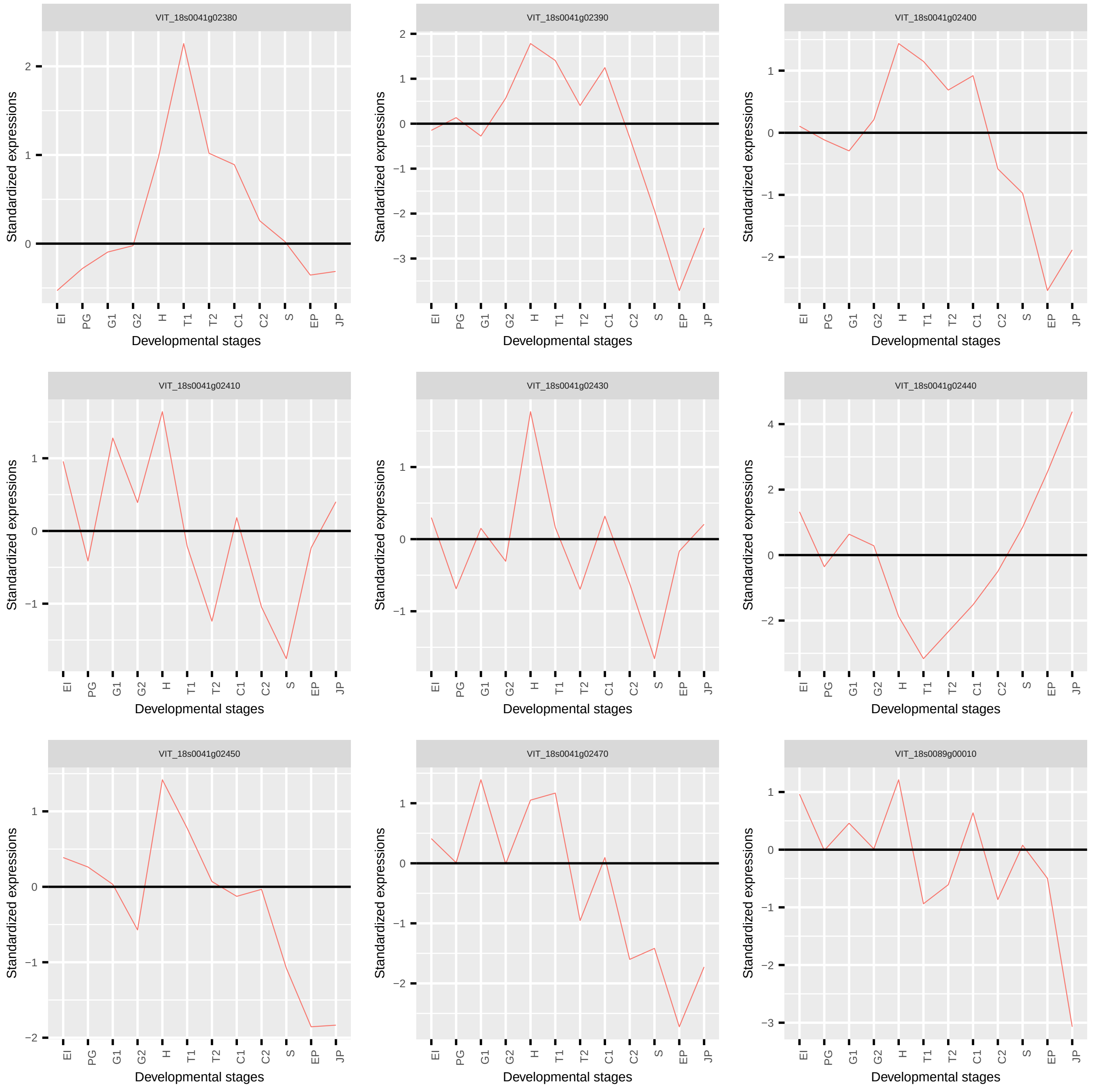

VIT_18s0041g02380
VIT_18s0041g02390
VIT_18s0041g02400
VIT_18s0041g02410
VIT_18s0041g02430
VIT_18s0041g02440
VIT_18s0041g02450
VIT_18s0041g02470
VIT_18s0089g00010
Standardized expressions
Developmental stages
EI PG G1 G2 H T1 T2 C1 C2 S EP JP

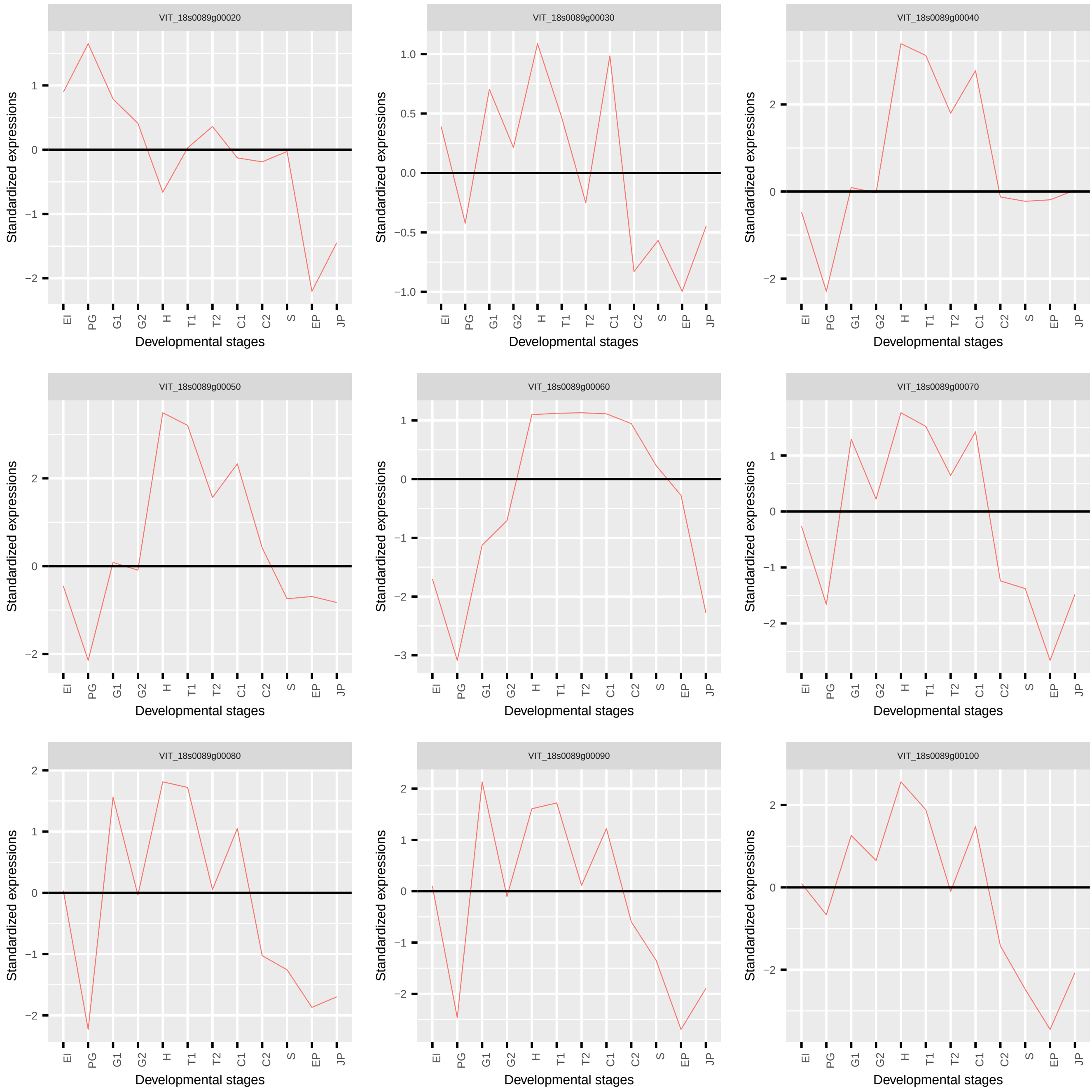
VIT_18s0089g00020
VIT_18s0089g00030
VIT_18s0089g00040
VIT_18s0089g00050
VIT_18s0089g00060
VIT_18s0089g00070
VIT_18s0089g00080
VIT_18s0089g00090
VIT_18s0089g00100
Standardized expressions
Developmental stages
EI
PG
G1
G2
H
T1
T2
C1
C2
S
EP
JP

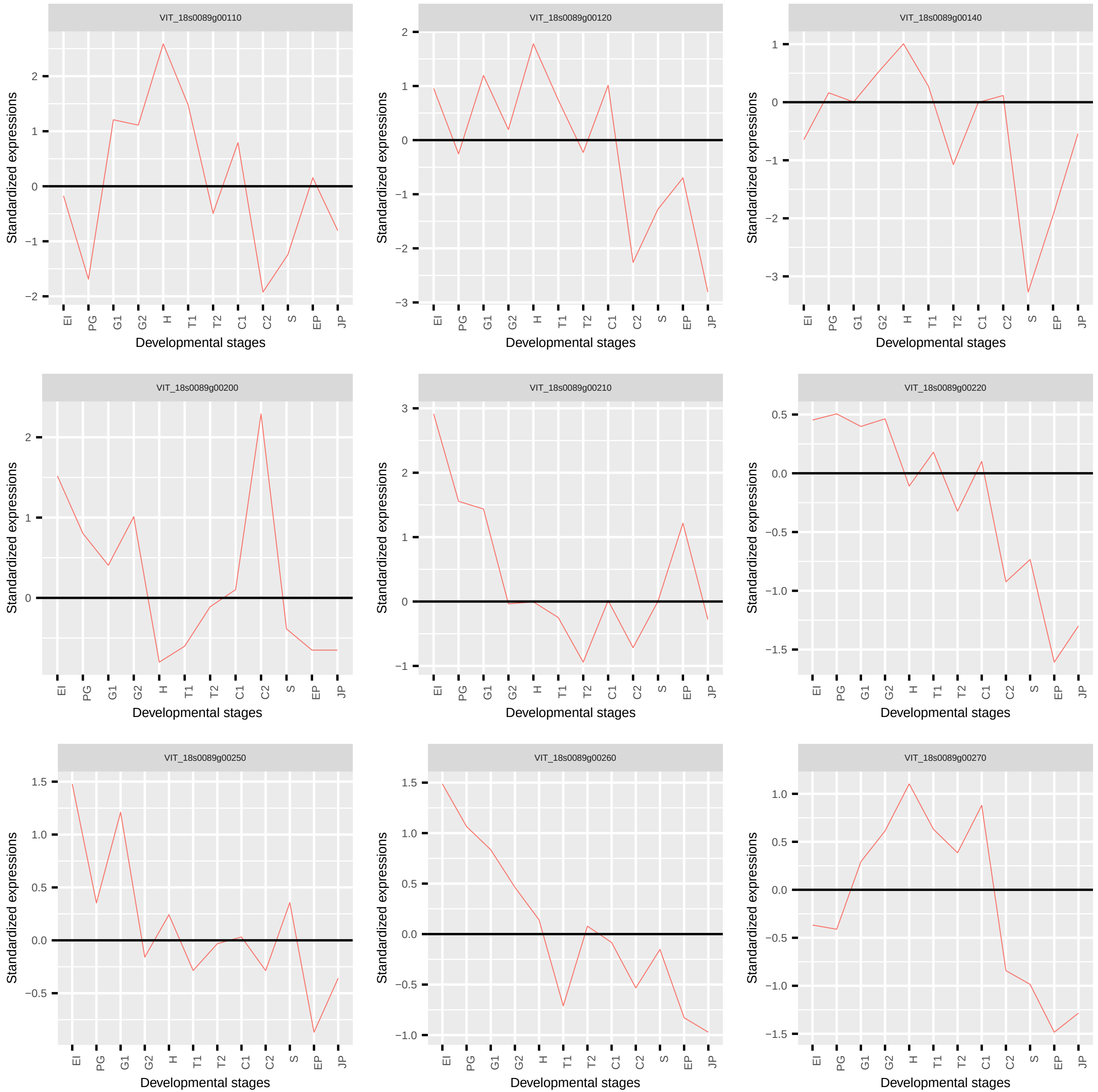

VIT_18s0089g00110
VIT_18s0089g00120
VIT_18s0089g00140
VIT_18s0089g00200
VIT_18s0089g00210
VIT_18s0089g00220
VIT_18s0089g00250
VIT_18s0089g00260
VIT_18s0089g00270
Standardized expressions
Developmental stages
EI PG G1 G2 H T1 T2 C1 C2 S EP JP

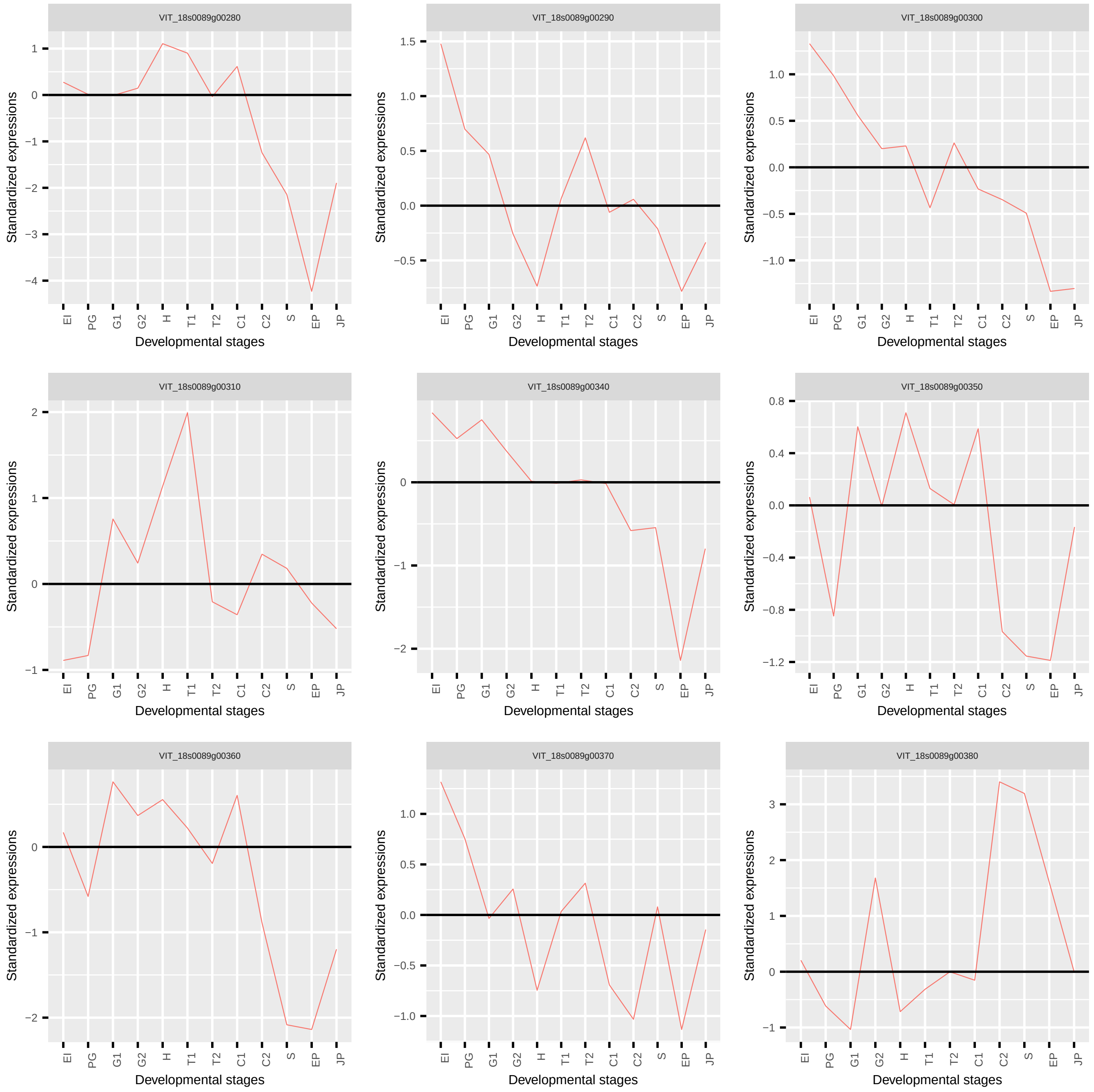

VIT_18s0089g00280
VIT_18s0089g00290
VIT_18s0089g00300
VIT_18s0089g00310
VIT_18s0089g00340
VIT_18s0089g00350
VIT_18s0089g00360
VIT_18s0089g00370
VIT_18s0089g00380
Standardized expressions
Developmental stages
EI PG G1 G2 H T1 T2 C1 C2 S EP JP

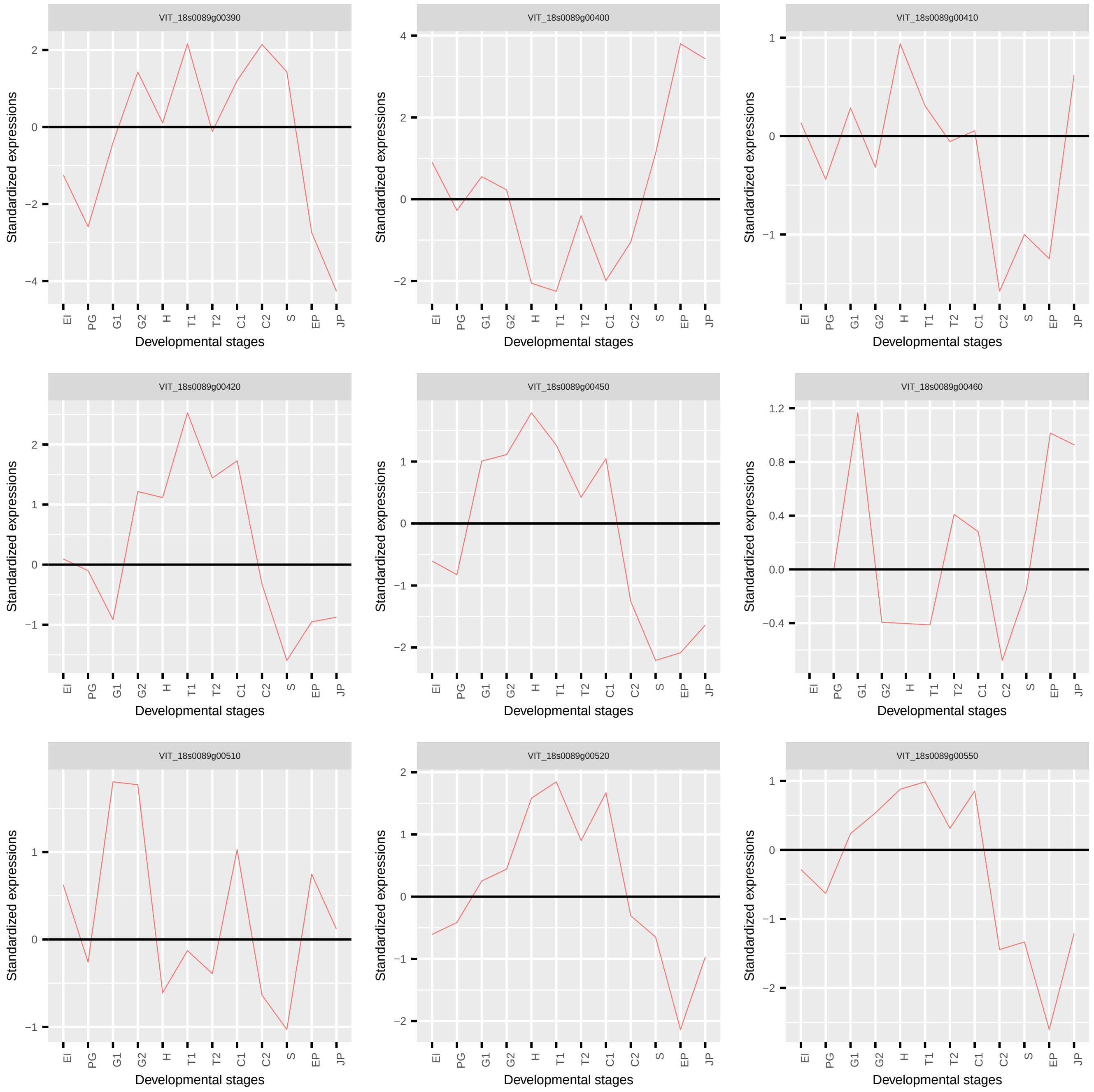
VIT_18s0089g00390
VIT_18s0089g00400
VIT_18s0089g00410
VIT_18s0089g00420
VIT_18s0089g00450
VIT_18s0089g00460
VIT_18s0089g00510
VIT_18s0089g00520
VIT_18s0089g00550
Standardized expressions
Developmental stages
EI PG G1 G2 H T1 T2 C1 C2 S EP JP

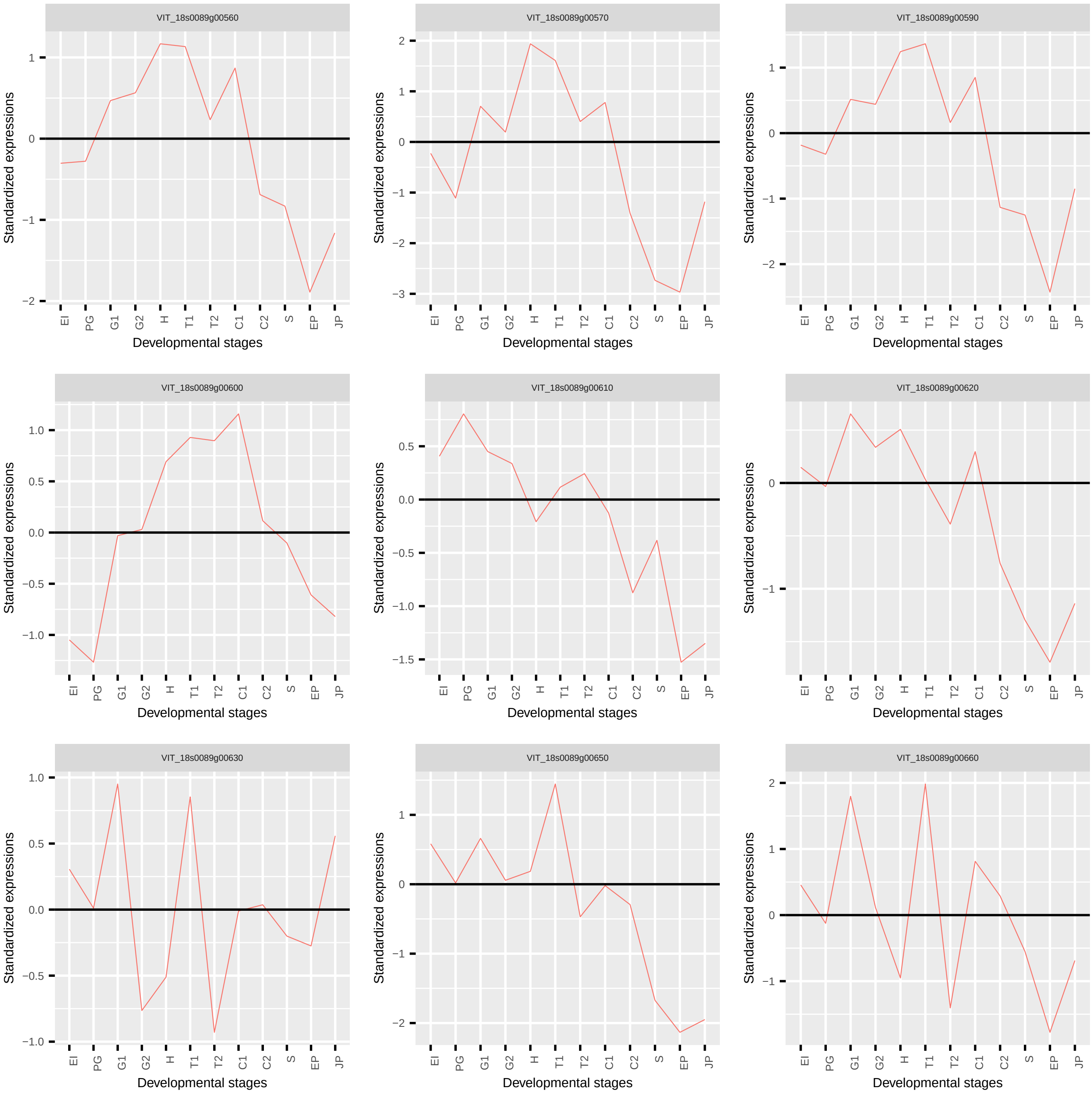
VIT_18s0089g00560
VIT_18s0089g00570
VIT_18s0089g00590
VIT_18s0089g00600
VIT_18s0089g00610
VIT_18s0089g00620
VIT_18s0089g00630
VIT_18s0089g00650
VIT_18s0089g00660
Standardized expressions
Developmental stages
EI
PG
G1
G2
H
T1
T2
C1
C2
S
EP
JP

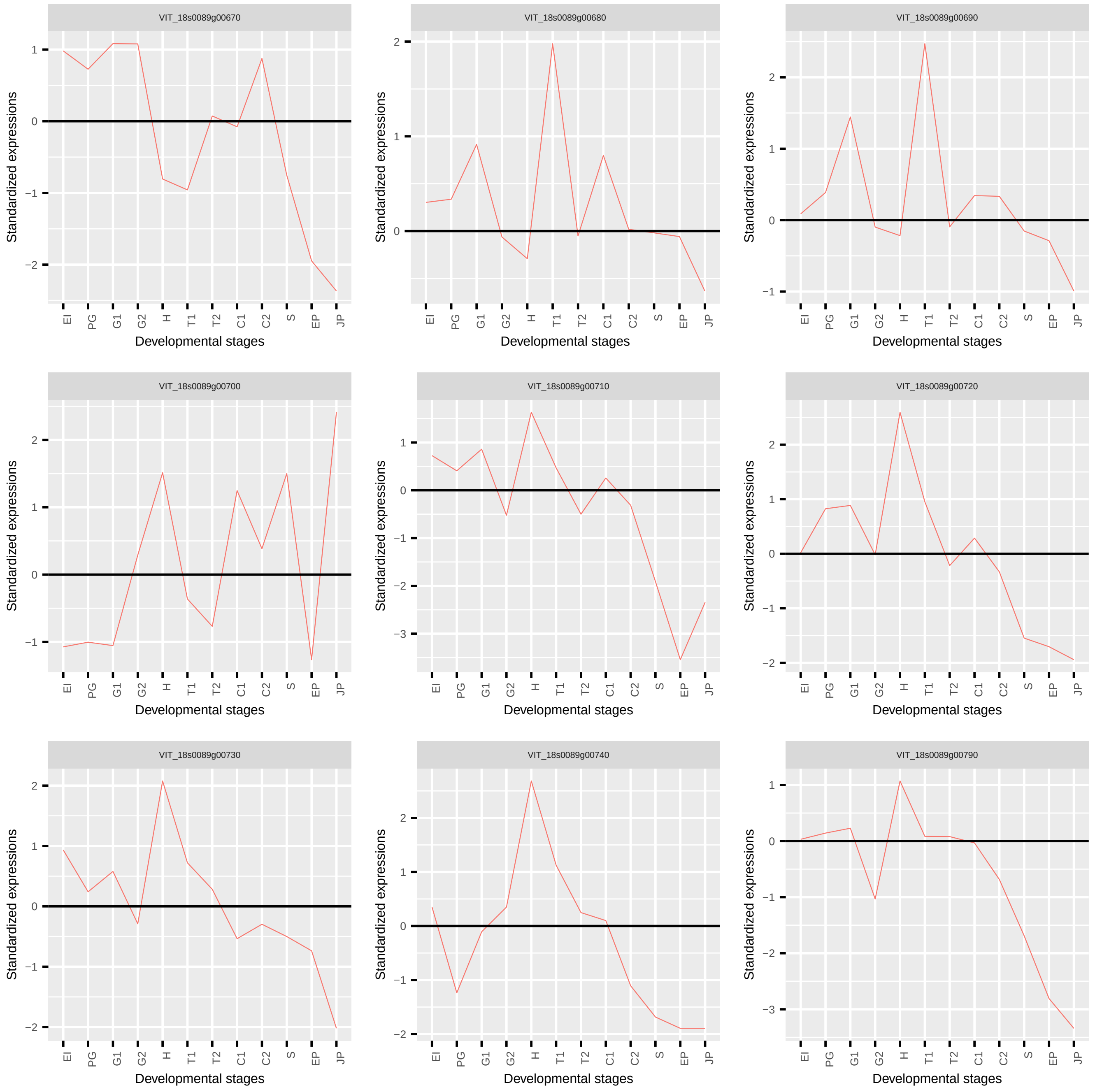

VIT_18s0089g00670
VIT_18s0089g00680
VIT_18s0089g00690
VIT_18s0089g00700
VIT_18s0089g00710
VIT_18s0089g00720
VIT_18s0089g00730
VIT_18s0089g00740
VIT_18s0089g00790
Standardized expressions
Developmental stages
EI PG G1 G2 H T1 T2 C1 C2 S EP JP

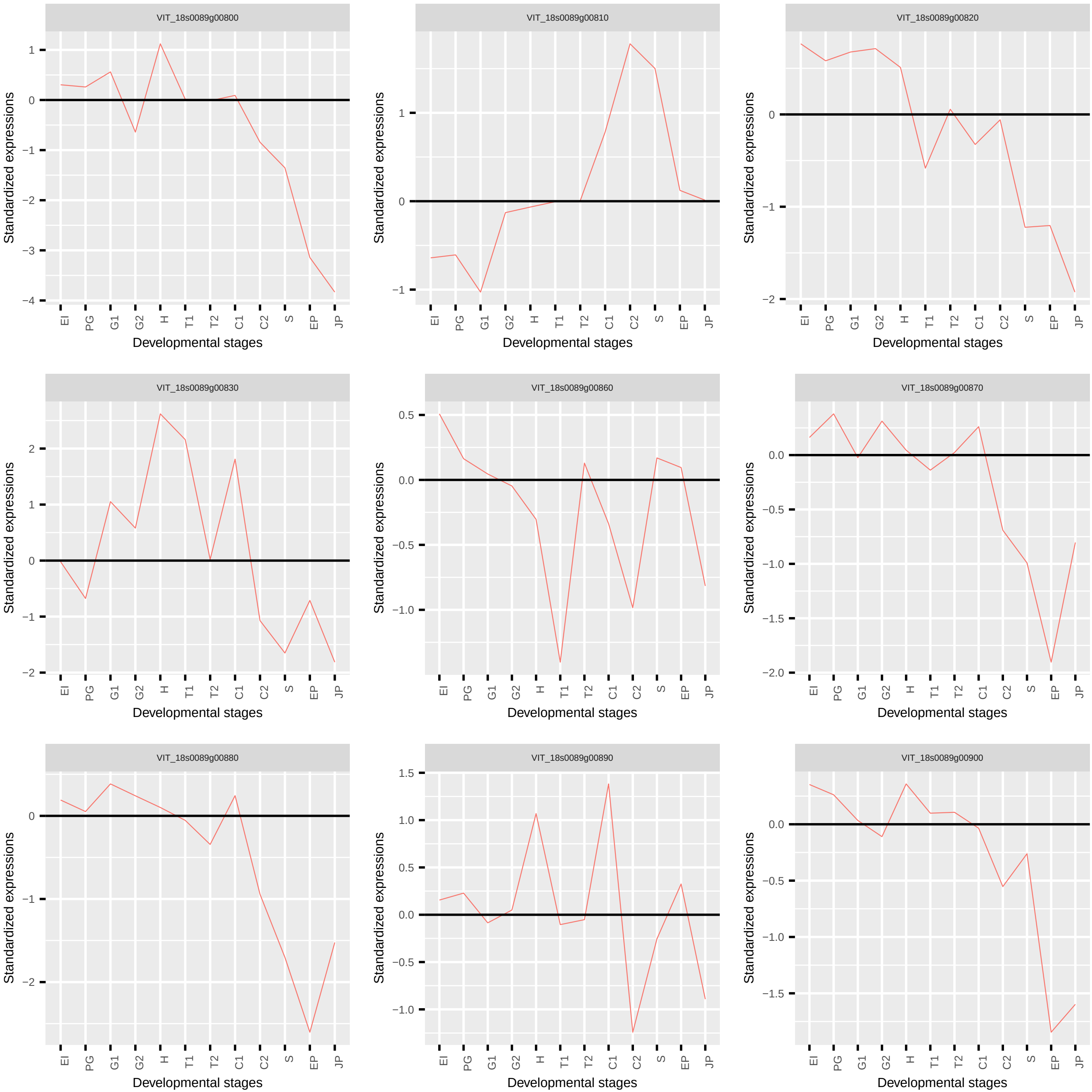
VIT_18s0089g00800
VIT_18s0089g00810
VIT_18s0089g00820
VIT_18s0089g00830
VIT_18s0089g00860
VIT_18s0089g00870
VIT_18s0089g00880
VIT_18s0089g00890
VIT_18s0089g00900
Standardized expressions
Developmental stages
EI PG G1 G2 H T1 T2 C1 C2 S EP JP

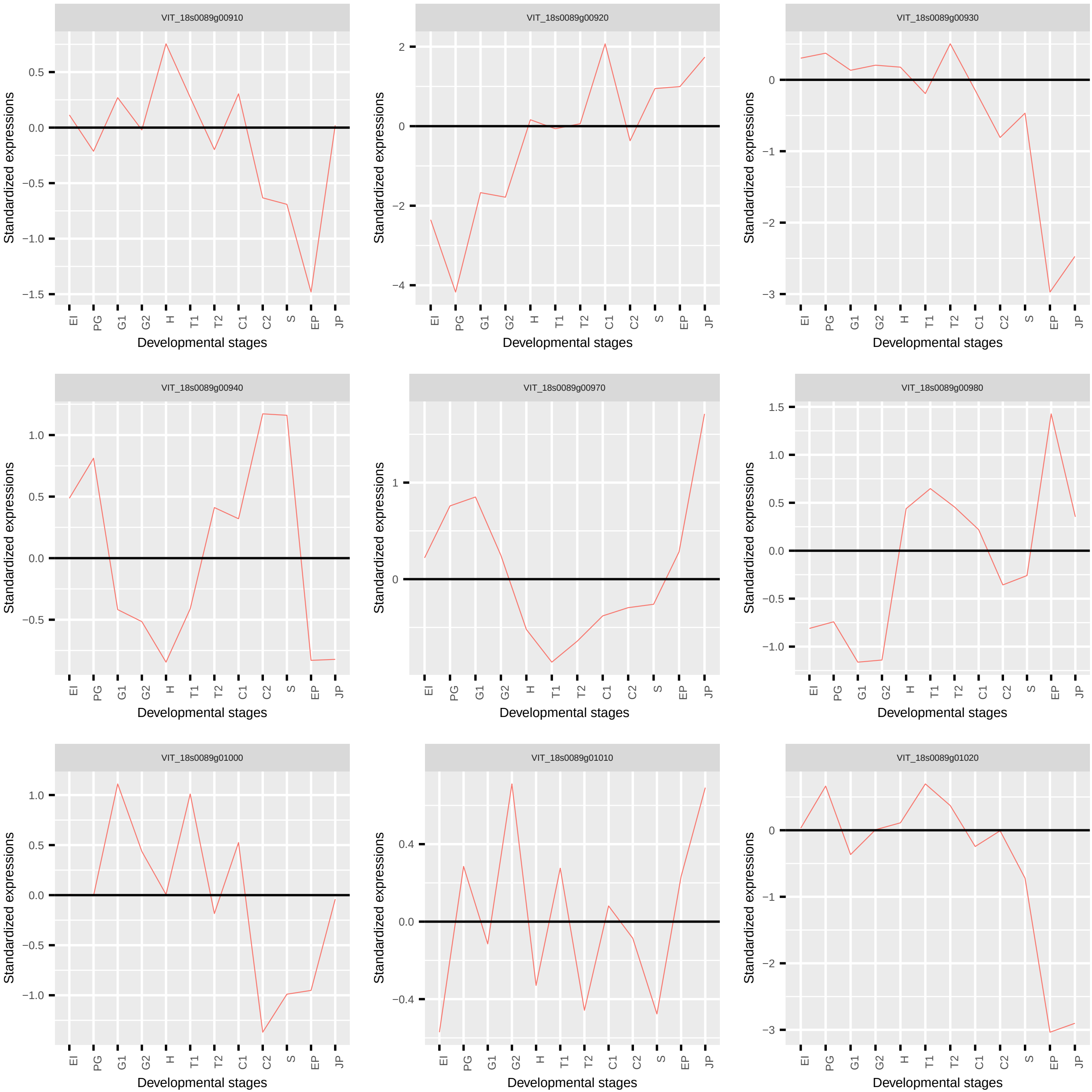
VIT_18s0089g00910
VIT_18s0089g00920
VIT_18s0089g00930
VIT_18s0089g00940
VIT_18s0089g00970
VIT_18s0089g00980
VIT_18s0089g01000
VIT_18s0089g01010
VIT_18s0089g01020
Standardized expressions
Developmental stages
EI PG G1 G2 H T1 T2 C1 C2 S EP JP

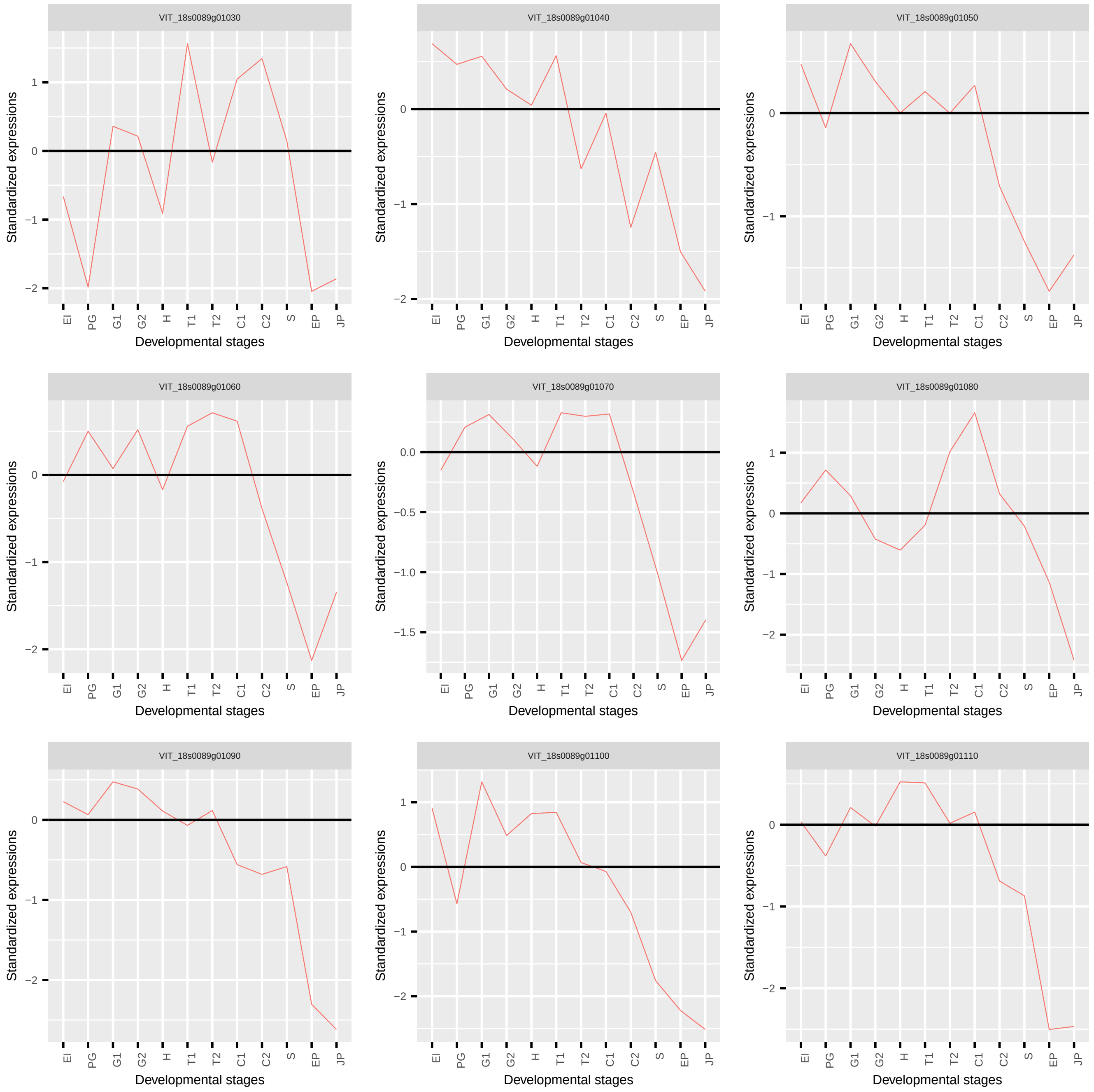
VIT_18s0089g01030
VIT_18s0089g01040
VIT_18s0089g01050
VIT_18s0089g01060
VIT_18s0089g01070
VIT_18s0089g01080
VIT_18s0089g01090
VIT_18s0089g01100
VIT_18s0089g01110
Standardized expressions
Developmental stages
EI PG G1 G2 H T1 T2 C1 C2 S EP JP

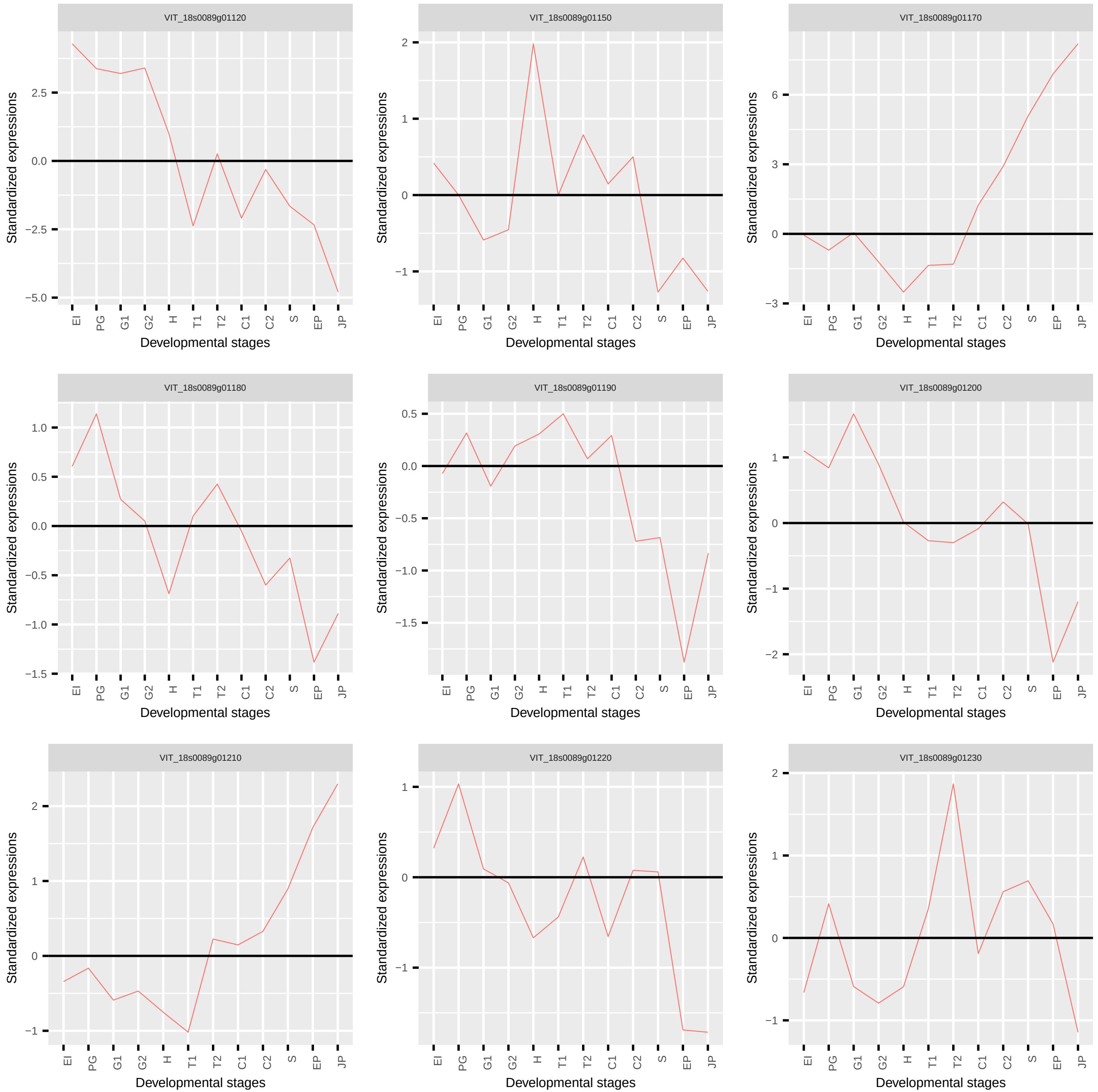
VIT_18s0089g01120
VIT_18s0089g01150
VIT_18s0089g01170
VIT_18s0089g01180
VIT_18s0089g01190
VIT_18s0089g01200
VIT_18s0089g01210
VIT_18s0089g01220
VIT_18s0089g01230
Standardized expressions
Developmental stages
EI
PG
G1
G2
H
T1
T2
C1
C2
S
EP
JP

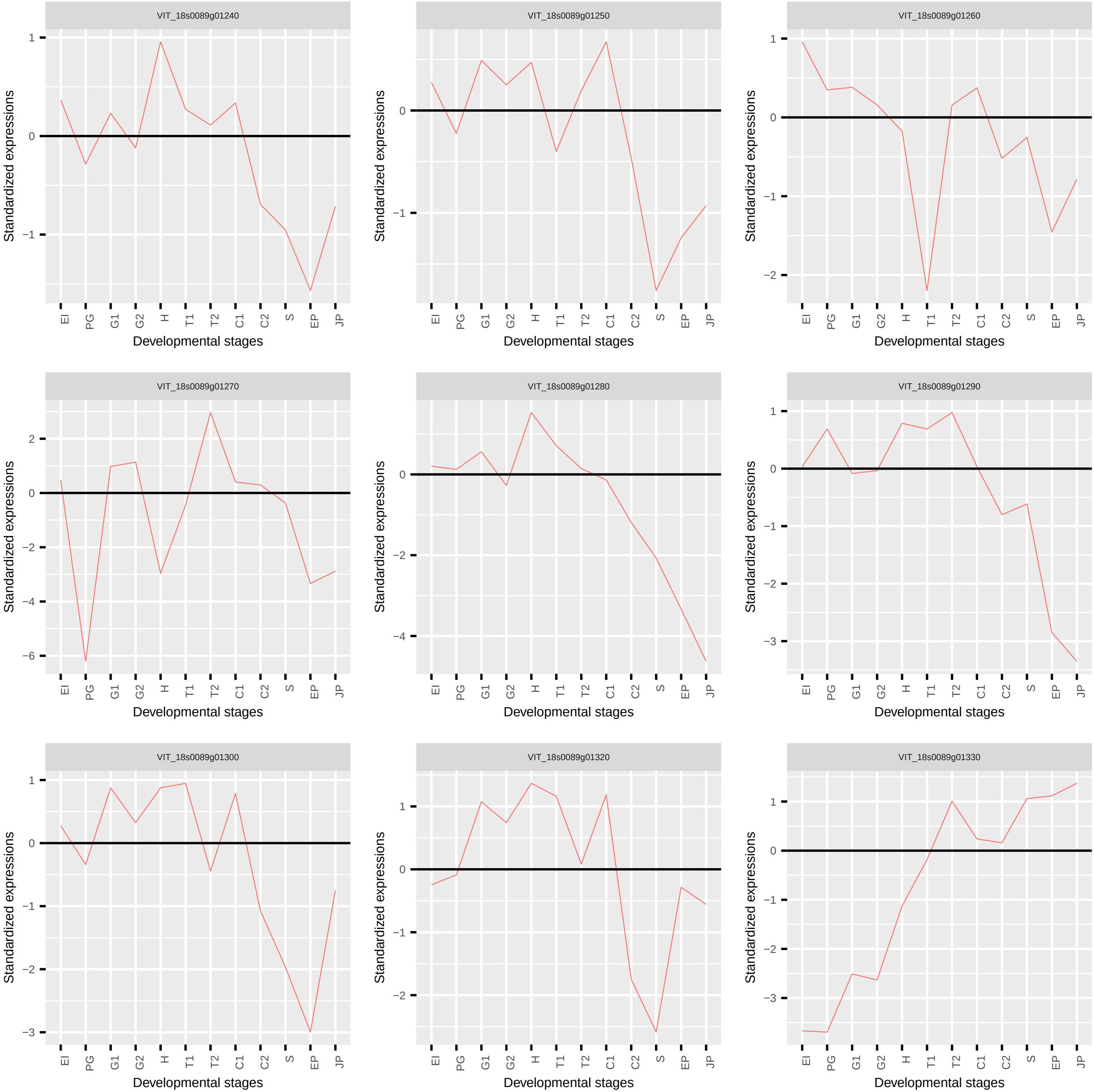

VIT_18s0089g01240
VIT_18s0089g01250
VIT_18s0089g01260
VIT_18s0089g01270
VIT_18s0089g01280
VIT_18s0089g01290
VIT_18s0089g01300
VIT_18s0089g01320
VIT_18s0089g01330
Standardized expressions
Developmental stages
EI PG G1 G2 H T1 T2 C1 C2 S EP JP

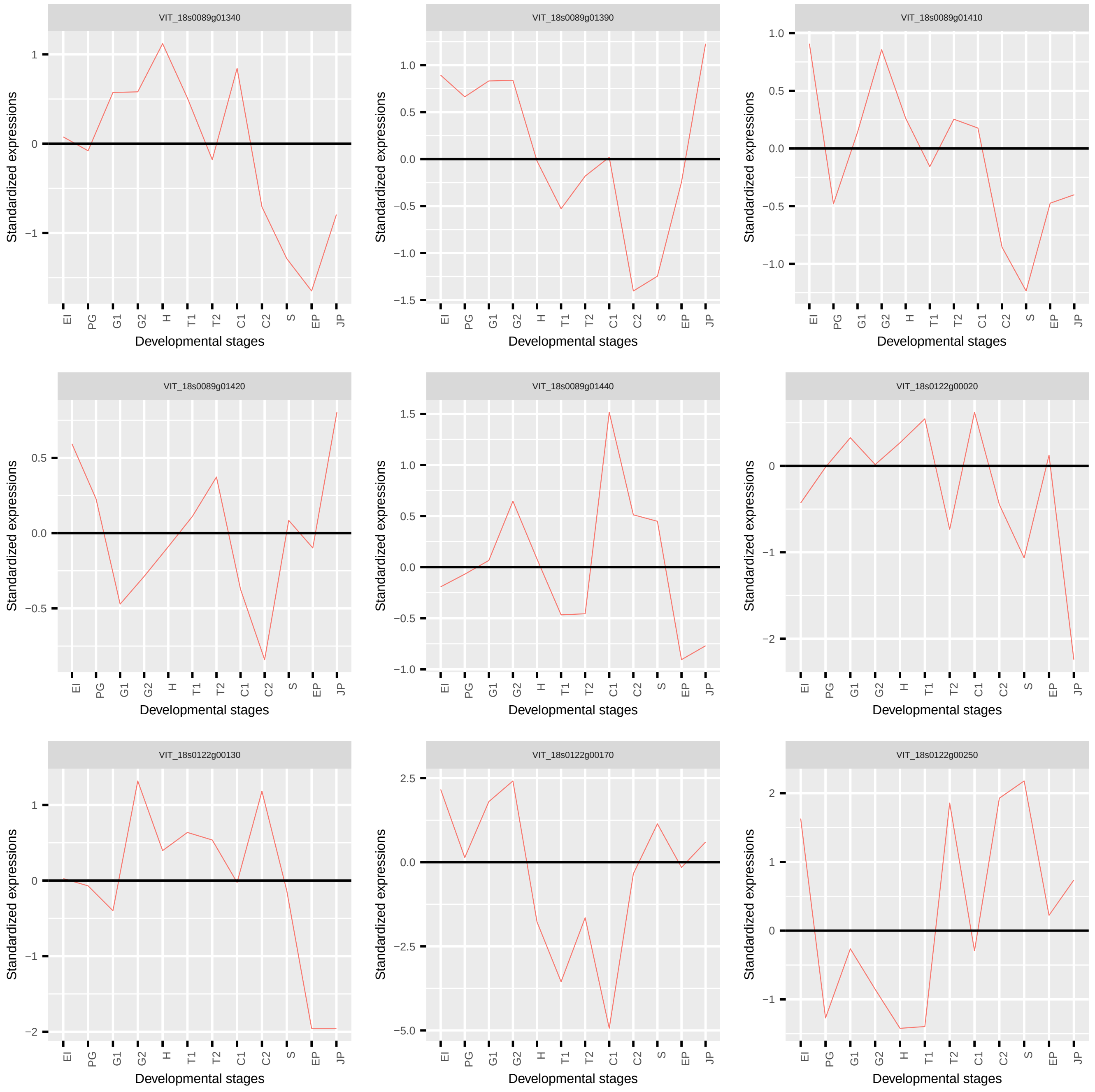
VIT_18s0089g01340
VIT_18s0089g01390
VIT_18s0089g01410
VIT_18s0089g01420
VIT_18s0089g01440
VIT_18s0122g00020
VIT_18s0122g00130
VIT_18s0122g00170
VIT_18s0122g00250
Standardized expressions
EI PG G1 G2 H T1 T2 C1 C2 S EP JP
Developmental stages

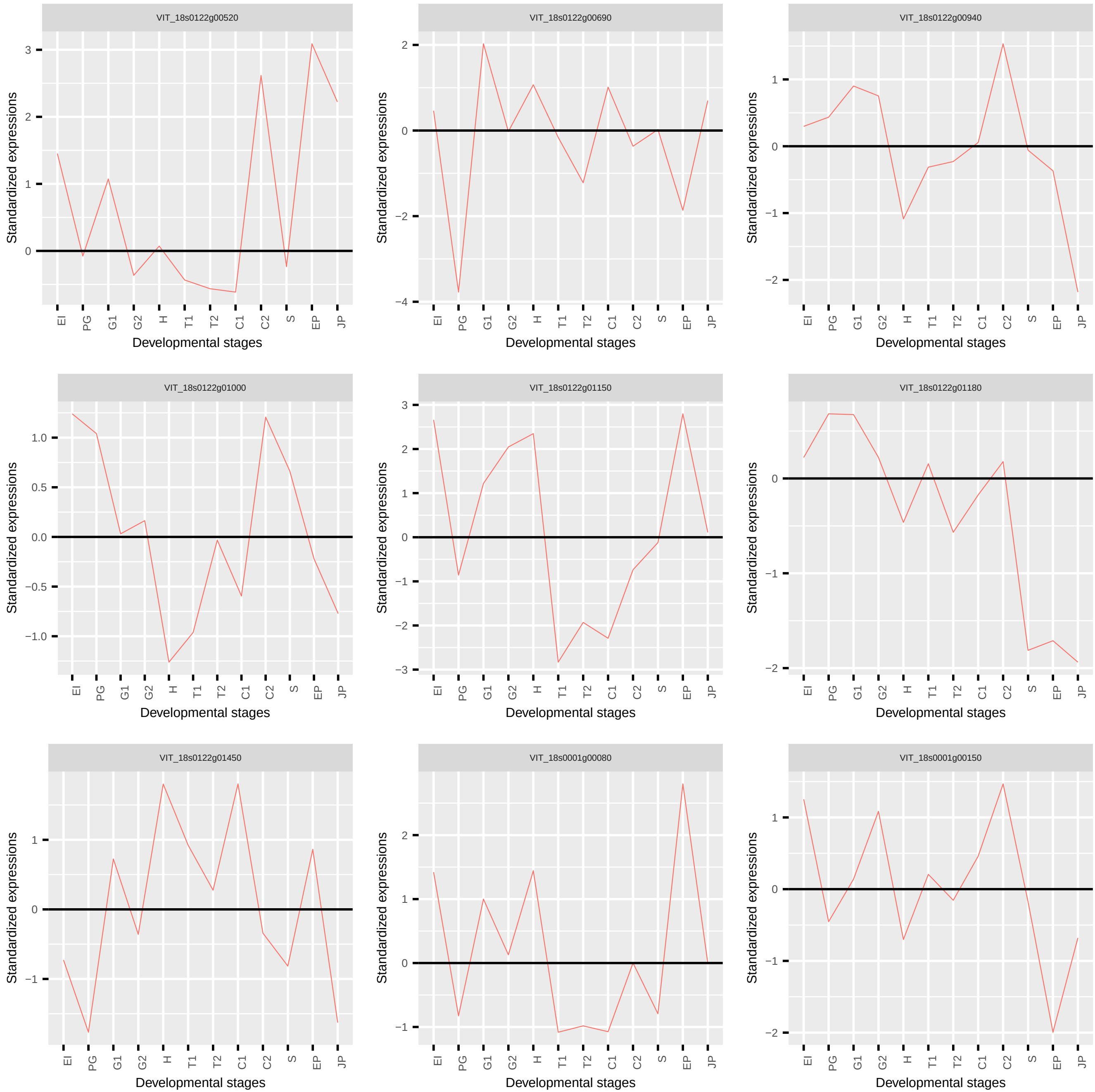
VIT_18s0122g00520
VIT_18s0122g00690
VIT_18s0122g00940
VIT_18s0122g01000
VIT_18s0122g01150
VIT_18s0122g01180
VIT_18s0122g01450
VIT_18s0001g00080
VIT_18s0001g00150
Standardized expressions
Developmental stages
EI
PG
G1
G2
H
T1
T2
C1
C2
S
EP
JP

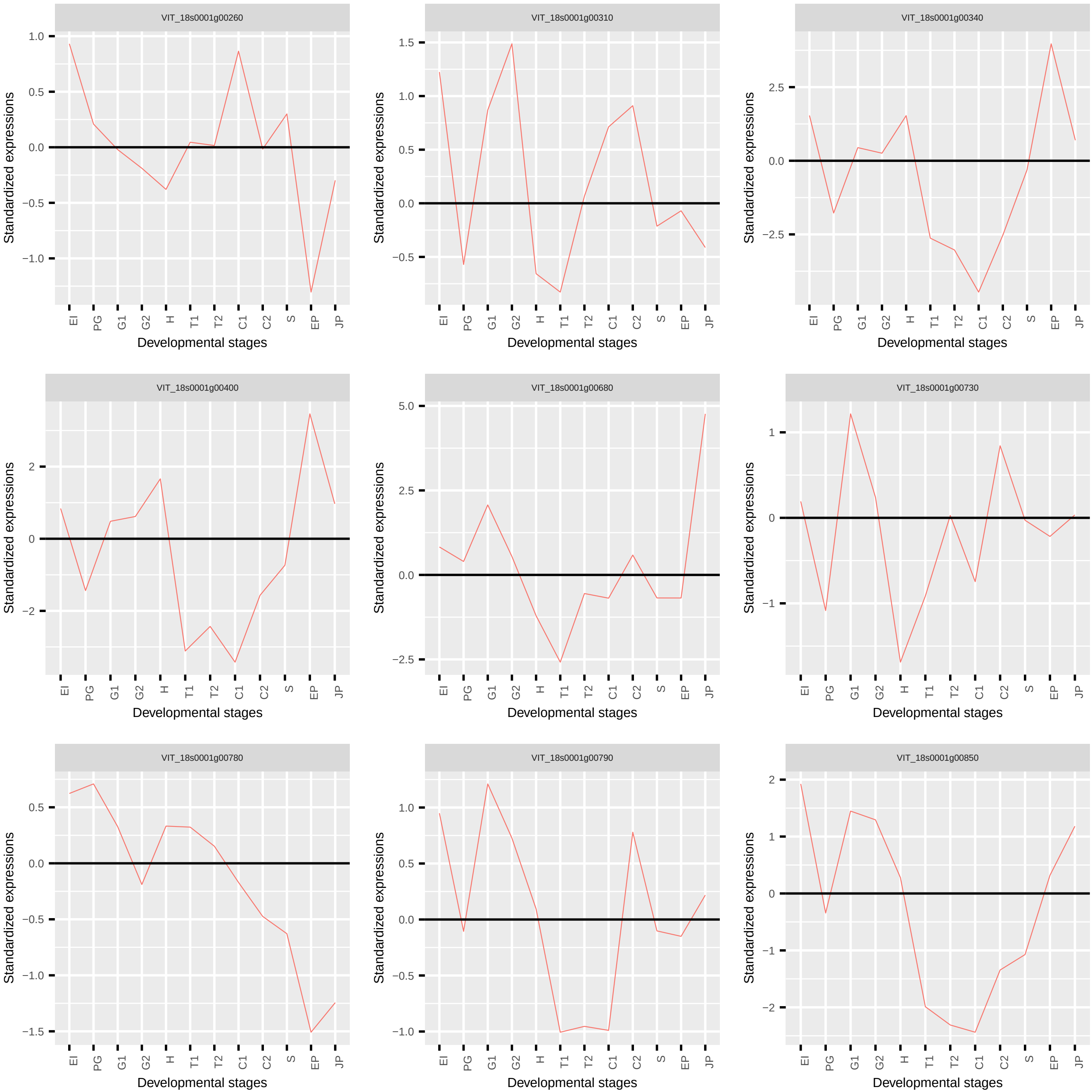
VIT_18s0001g00260
VIT_18s0001g00310
VIT_18s0001g00340
VIT_18s0001g00400
VIT_18s0001g00680
VIT_18s0001g00730
VIT_18s0001g00780
VIT_18s0001g00790
VIT_18s0001g00850
Standardized expressions
Developmental stages
EI PG G1 G2 H T1 T2 C1 C2 S EP JP

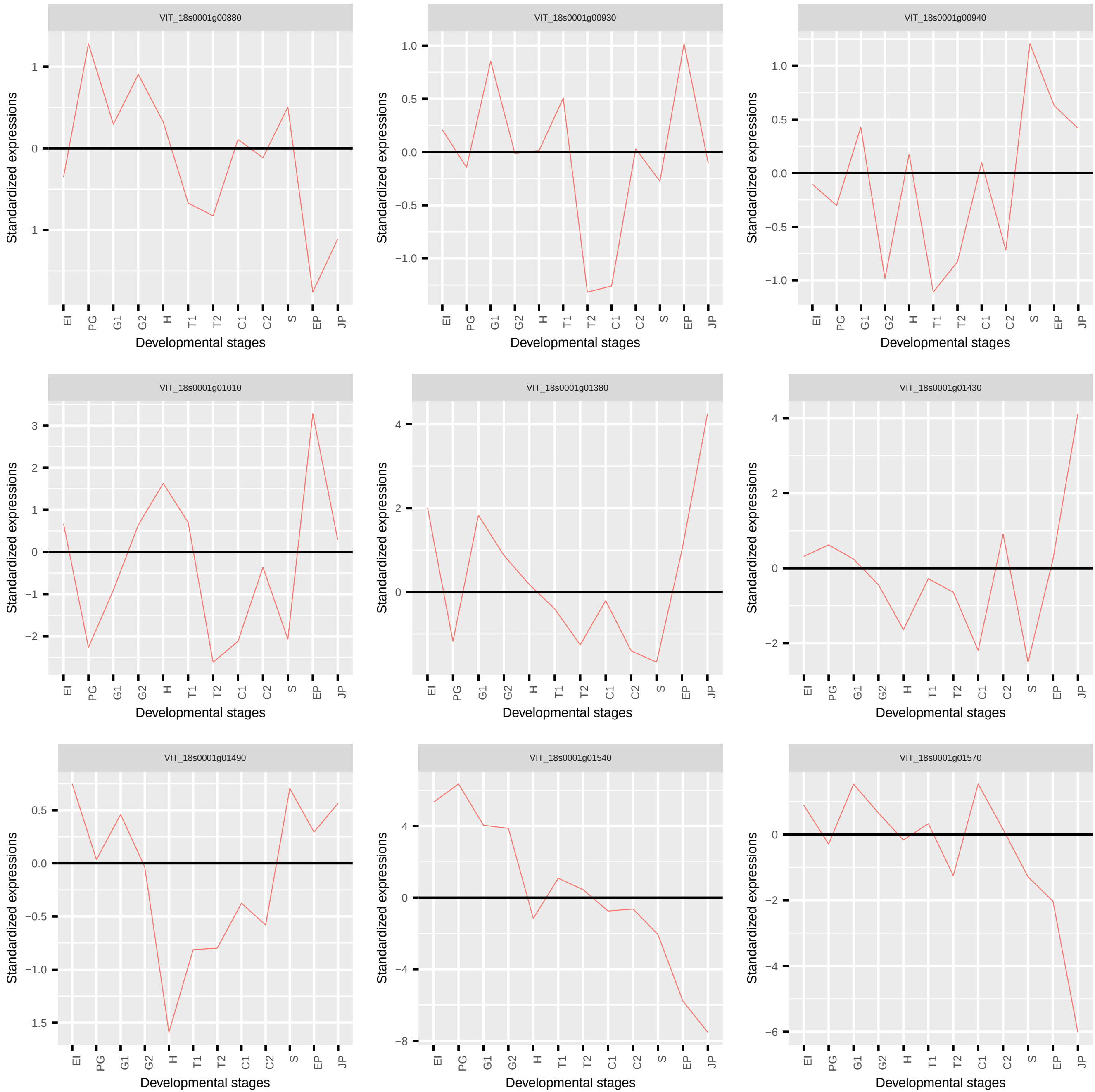
VIT_18s0001g00880
VIT_18s0001g00930
VIT_18s0001g00940
VIT_18s0001g01010
VIT_18s0001g01380
VIT_18s0001g01430
VIT_18s0001g01490
VIT_18s0001g01540
VIT_18s0001g01570
Standardized expressions
Developmental stages
EI
PG
G1
G2
H
T1
T2
C1
C2
S
EP
JP

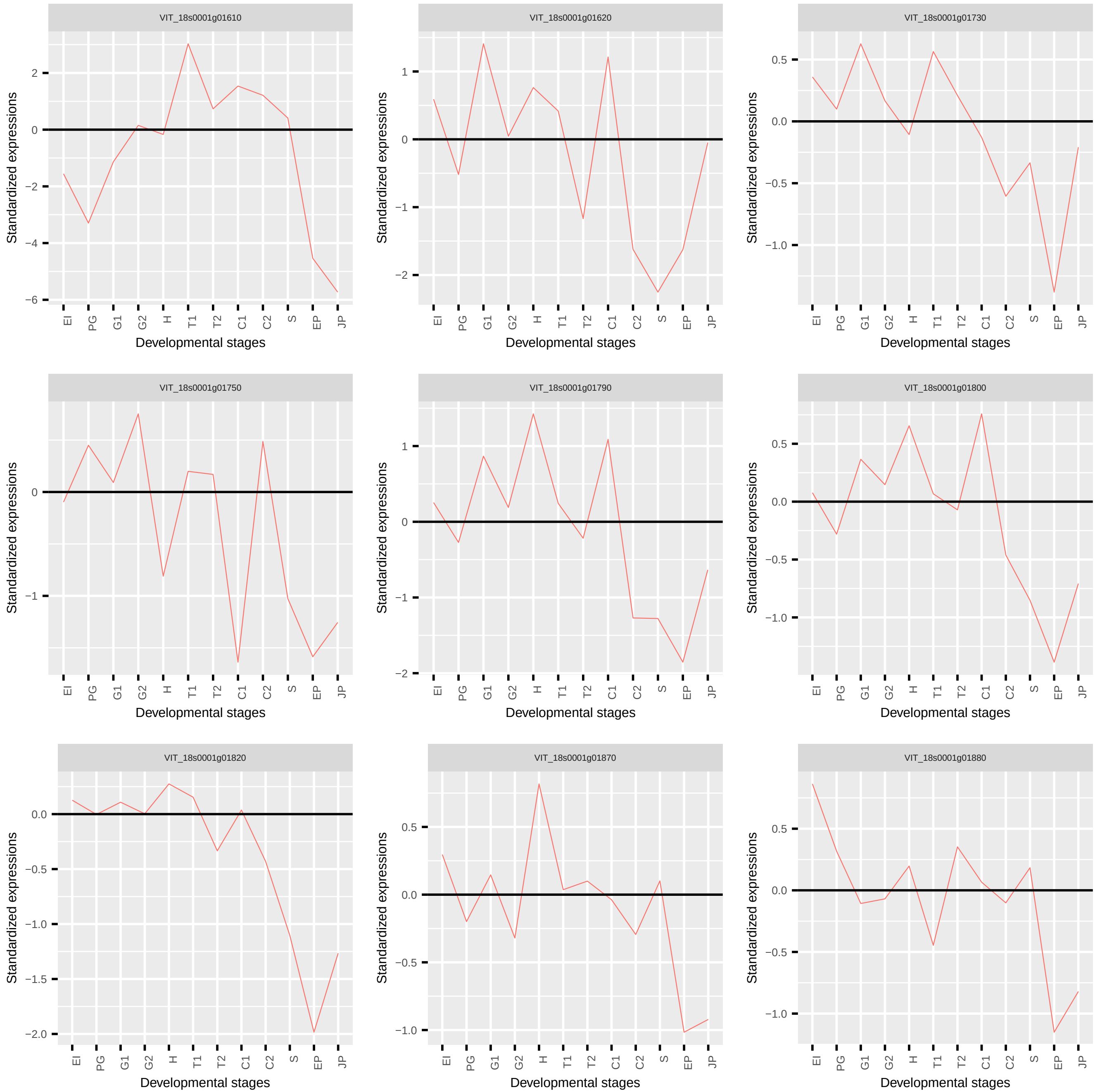

VIT_18s0001g01610
VIT_18s0001g01620
VIT_18s0001g01730
VIT_18s0001g01750
VIT_18s0001g01790
VIT_18s0001g01800
VIT_18s0001g01820
VIT_18s0001g01870
VIT_18s0001g01880
Standardized expressions
Developmental stages
EI
PG
G1
G2
H
T1
T2
C1
C2
S
EP
JP

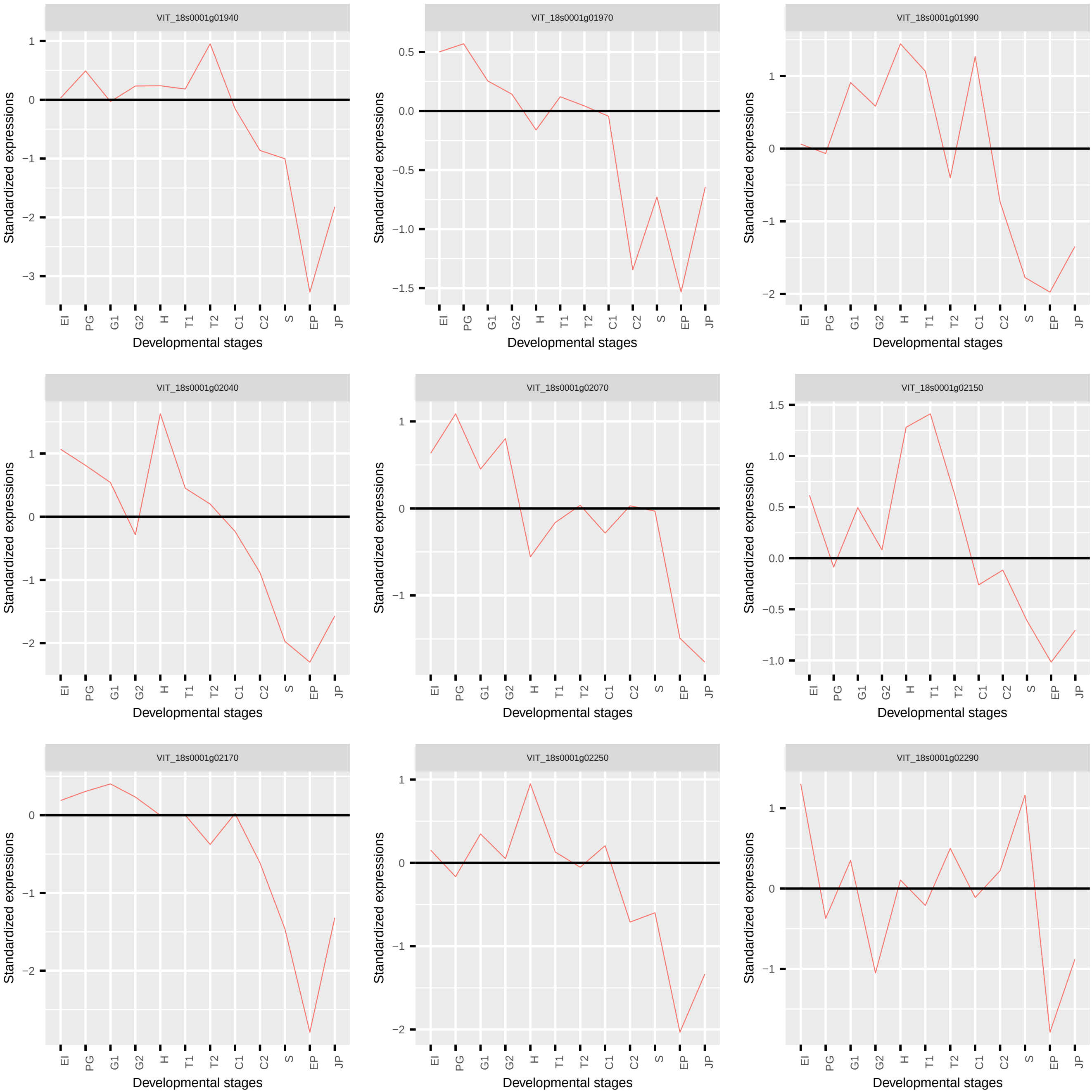
VIT_18s0001g01940
VIT_18s0001g01970
VIT_18s0001g01990
VIT_18s0001g02040
VIT_18s0001g02070
VIT_18s0001g02150
VIT_18s0001g02170
VIT_18s0001g02250
VIT_18s0001g02290
Standardized expressions
Developmental stages
EI PG G1 G2 H T1 T2 C1 C2 S EP JP

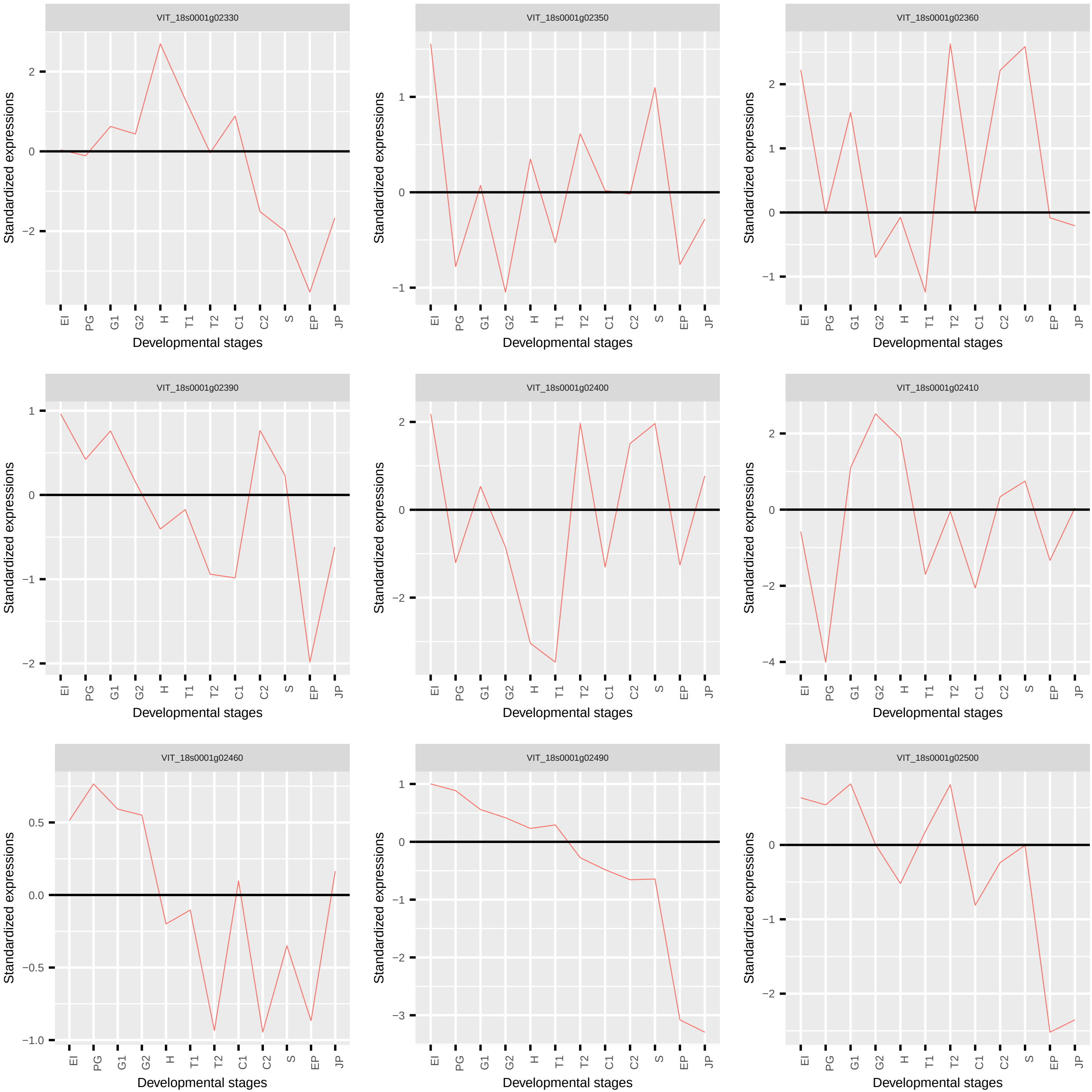
VIT_18s0001g02330
VIT_18s0001g02350
VIT_18s0001g02360
VIT_18s0001g02390
VIT_18s0001g02400
VIT_18s0001g02410
VIT_18s0001g02460
VIT_18s0001g02490
VIT_18s0001g02500
Standardized expressions
Developmental stages
EI PG G1 G2 H T1 T2 C1 C2 S EP JP

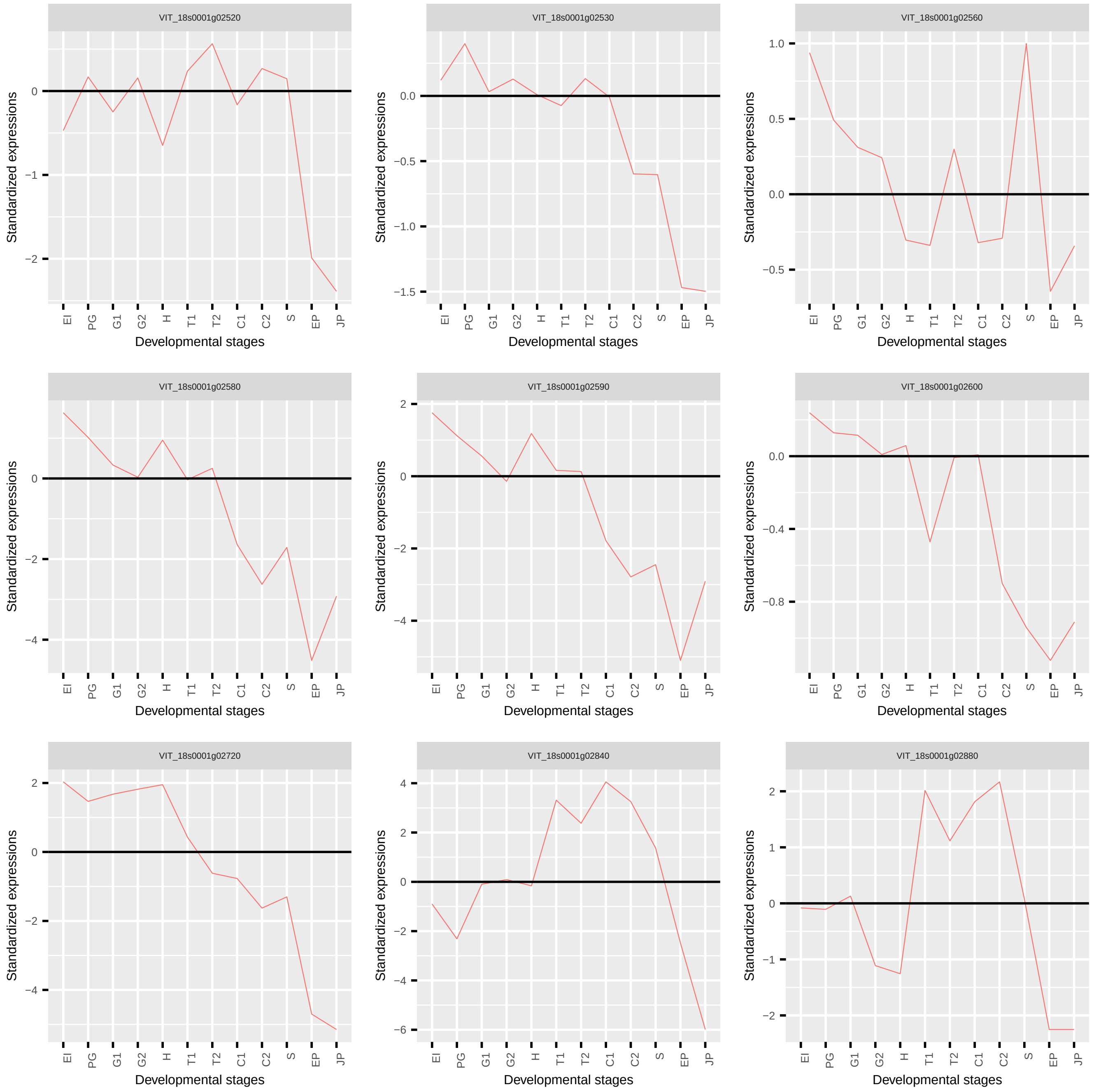
VIT_18s0001g02520
VIT_18s0001g02530
VIT_18s0001g02560
VIT_18s0001g02580
VIT_18s0001g02590
VIT_18s0001g02600
VIT_18s0001g02720
VIT_18s0001g02840
VIT_18s0001g02880
Standardized expressions
Developmental stages
EI PG G1 G2 H T1 T2 C1 C2 S EP JP

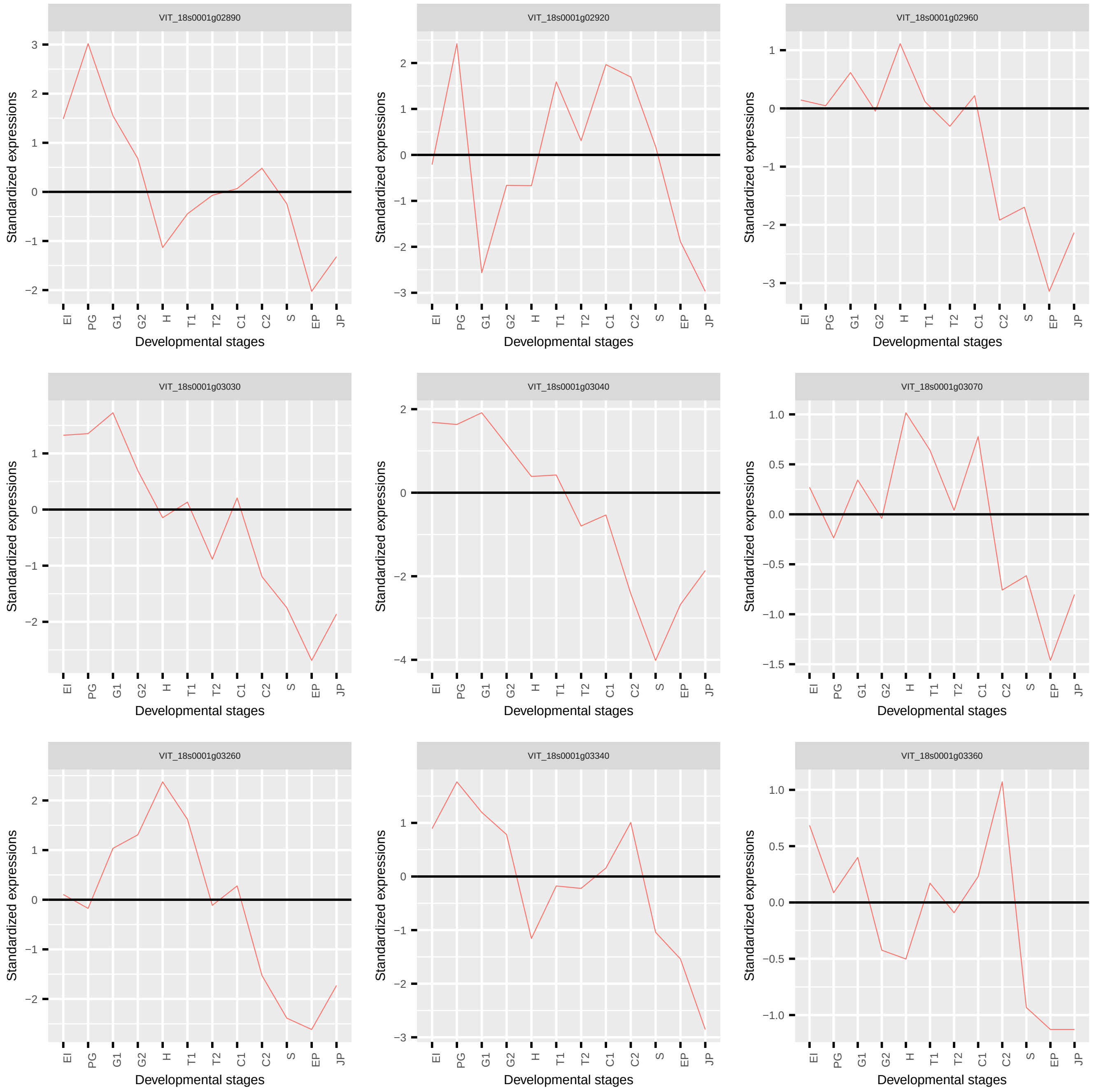
VIT_18s0001g02890
VIT_18s0001g02920
VIT_18s0001g02960
VIT_18s0001g03030
VIT_18s0001g03040
VIT_18s0001g03070
VIT_18s0001g03260
VIT_18s0001g03340
VIT_18s0001g03360
Standardized expressions
Developmental stages
EI PG G1 G2 H T1 T2 C1 C2 S EP JP

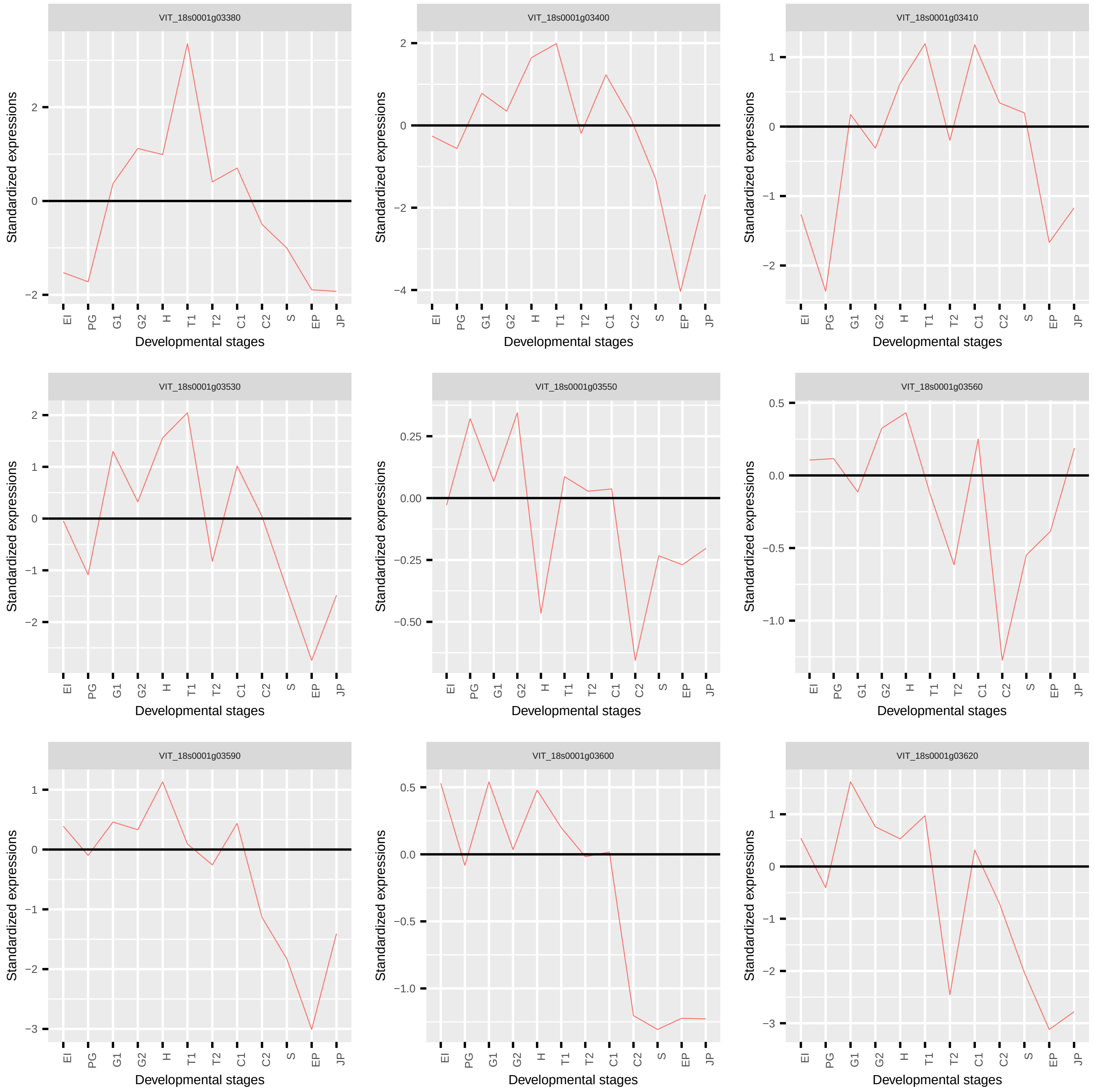

VIT_18s0001g03380
VIT_18s0001g03400
VIT_18s0001g03410
VIT_18s0001g03530
VIT_18s0001g03550
VIT_18s0001g03560
VIT_18s0001g03590
VIT_18s0001g03600
VIT_18s0001g03620
Standardized expressions
Developmental stages
EI PG G1 G2 H T1 T2 C1 C2 S EP JP

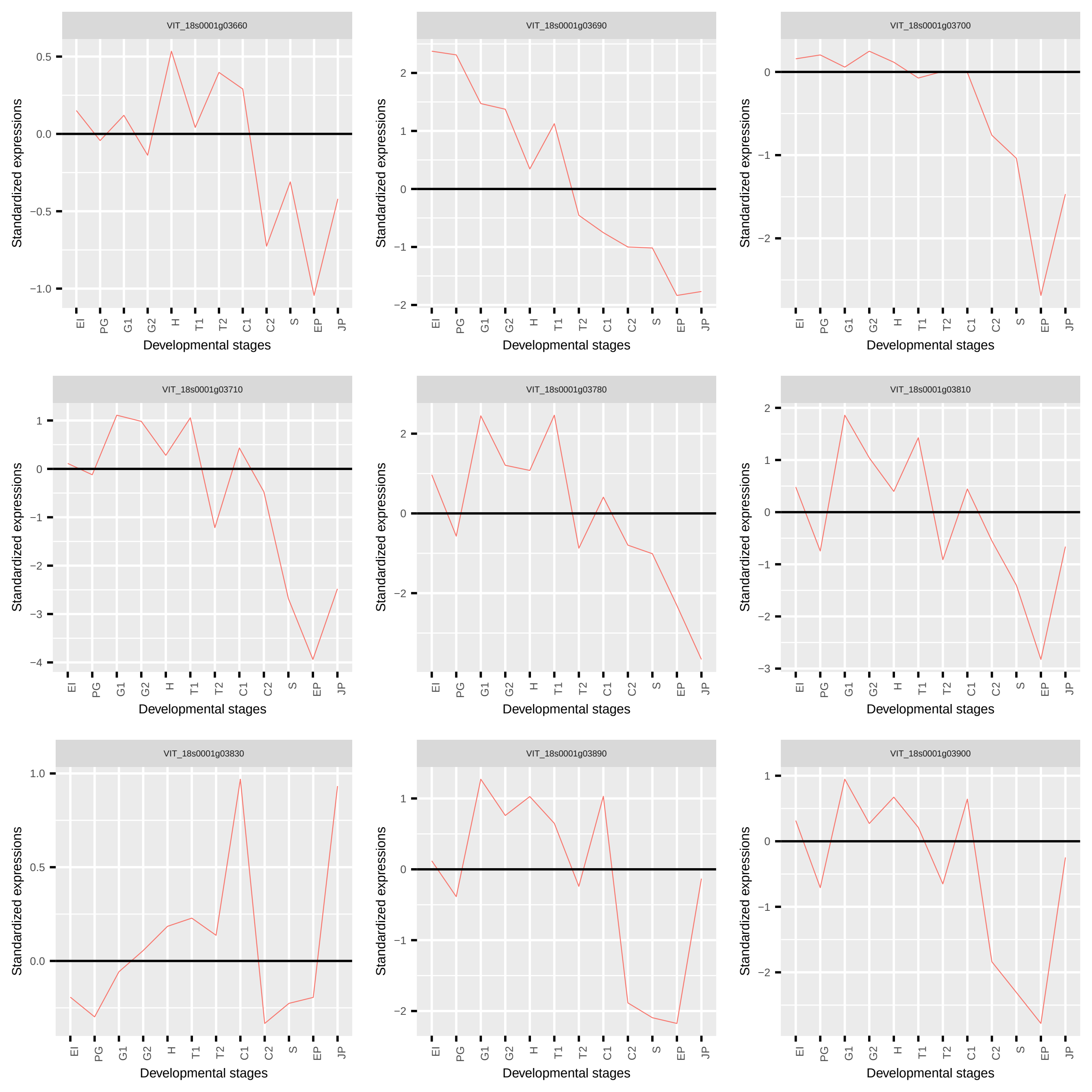
VIT_18s0001g03660
VIT_18s0001g03690
VIT_18s0001g03700
VIT_18s0001g03710
VIT_18s0001g03780
VIT_18s0001g03810
VIT_18s0001g03830
VIT_18s0001g03890
VIT_18s0001g03900
Standardized expressions
Developmental stages
EI PG G1 G2 H T1 T2 C1 C2 S EP JP

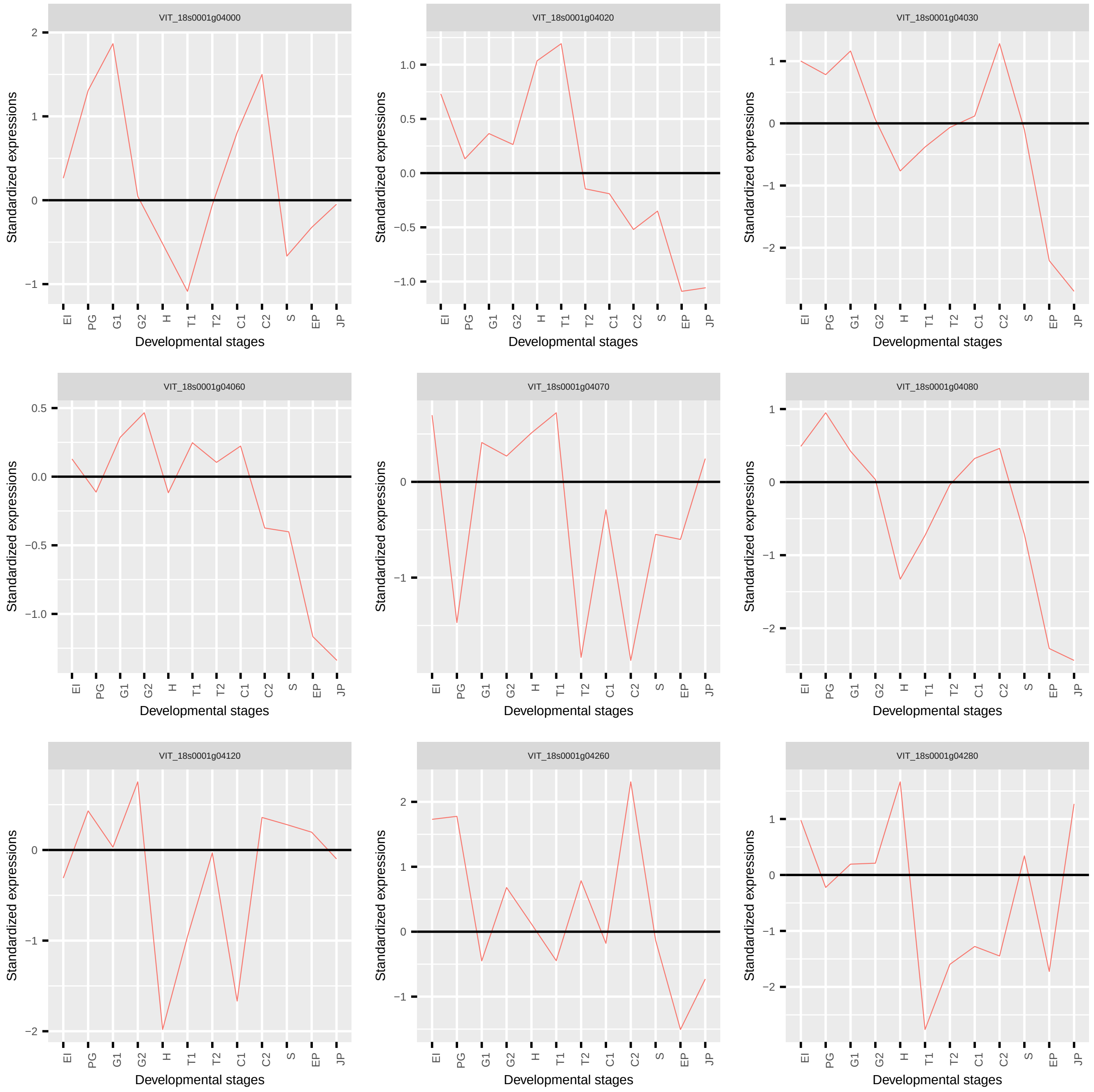
VIT_18s0001g04000
VIT_18s0001g04020
VIT_18s0001g04030
VIT_18s0001g04060
VIT_18s0001g04070
VIT_18s0001g04080
VIT_18s0001g04120
VIT_18s0001g04260
VIT_18s0001g04280
Standardized expressions
Developmental stages
EI
PG
G1
G2
H
T1
T2
C1
C2
S
EP
JP

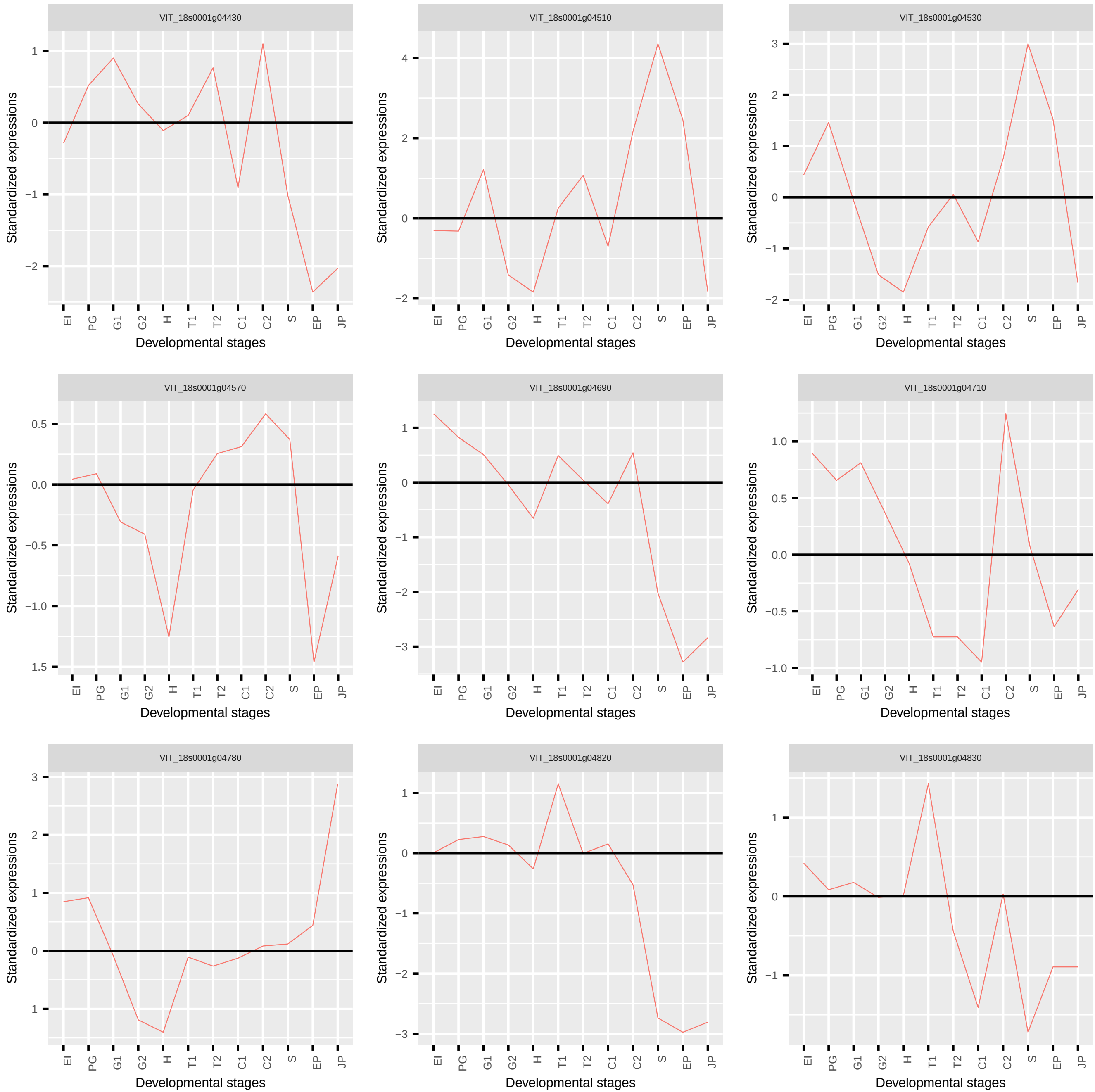
VIT_18s0001g04430
VIT_18s0001g04510
VIT_18s0001g04530
VIT_18s0001g04570
VIT_18s0001g04690
VIT_18s0001g04710
VIT_18s0001g04780
VIT_18s0001g04820
VIT_18s0001g04830
Standardized expressions
Developmental stages
EI PG G1 G2 H T1 T2 C1 C2 S EP JP

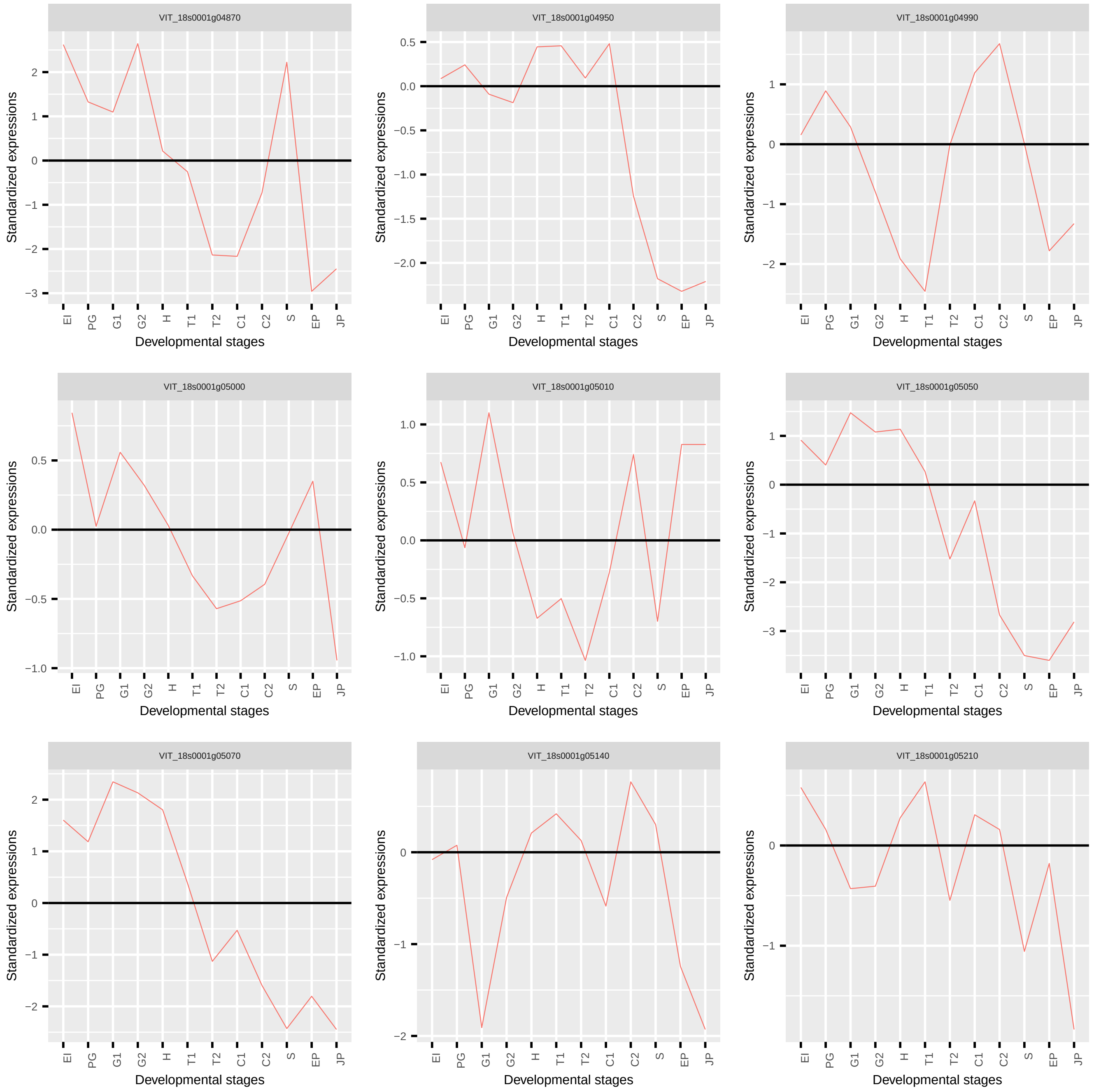
VIT_18s0001g04870
VIT_18s0001g04950
VIT_18s0001g04990
VIT_18s0001g05000
VIT_18s0001g05010
VIT_18s0001g05050
VIT_18s0001g05070
VIT_18s0001g05140
VIT_18s0001g05210
Standardized expressions
Developmental stages
EI PG G1 G2 H T1 T2 C1 C2 S EP JP

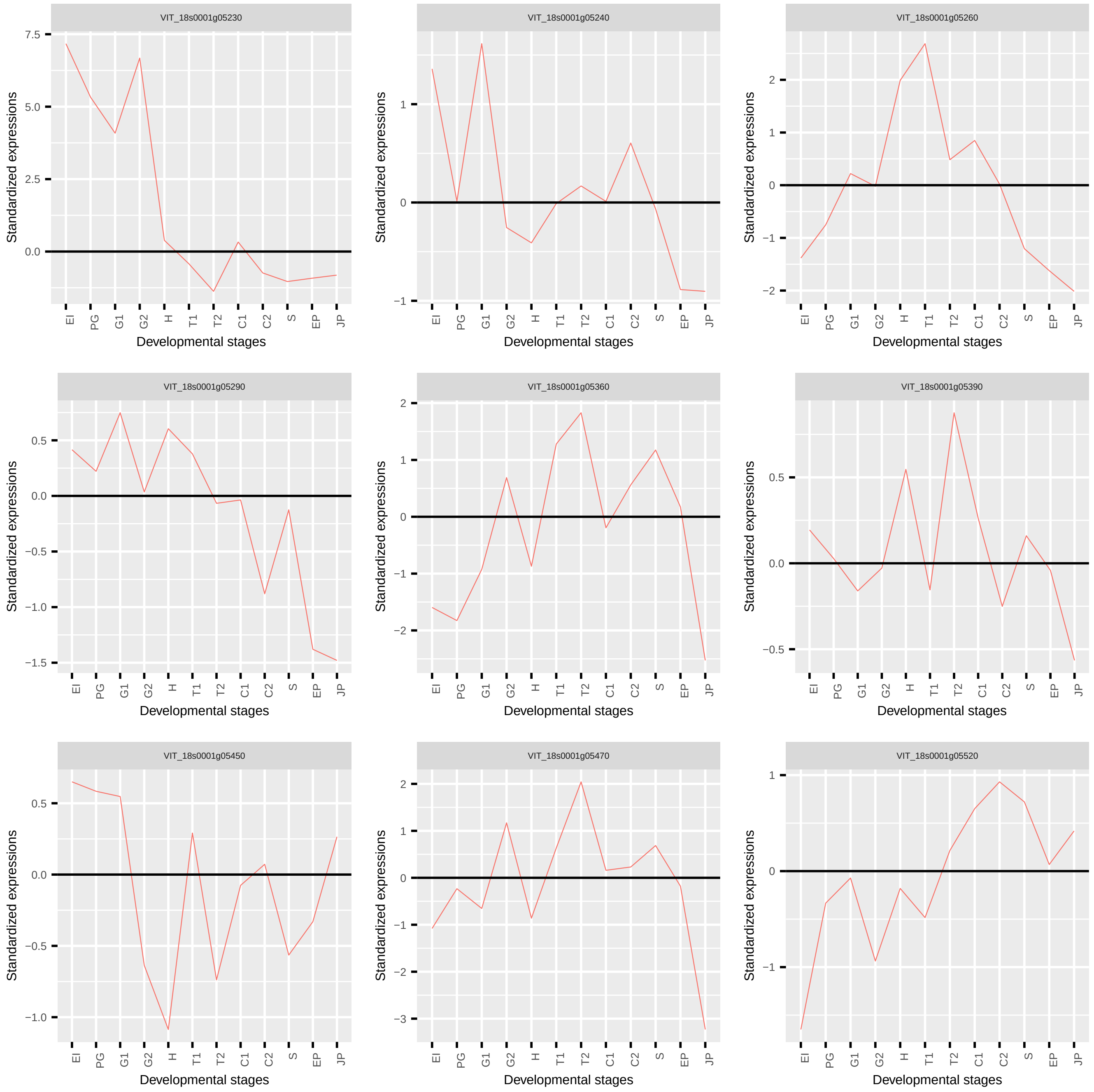
VIT_18s0001g05230
VIT_18s0001g05240
VIT_18s0001g05260
VIT_18s0001g05290
VIT_18s0001g05360
VIT_18s0001g05390
VIT_18s0001g05450
VIT_18s0001g05470
VIT_18s0001g05520
Standardized expressions
Developmental stages
EI PG G1 G2 H T1 T2 C1 C2 S EP JP

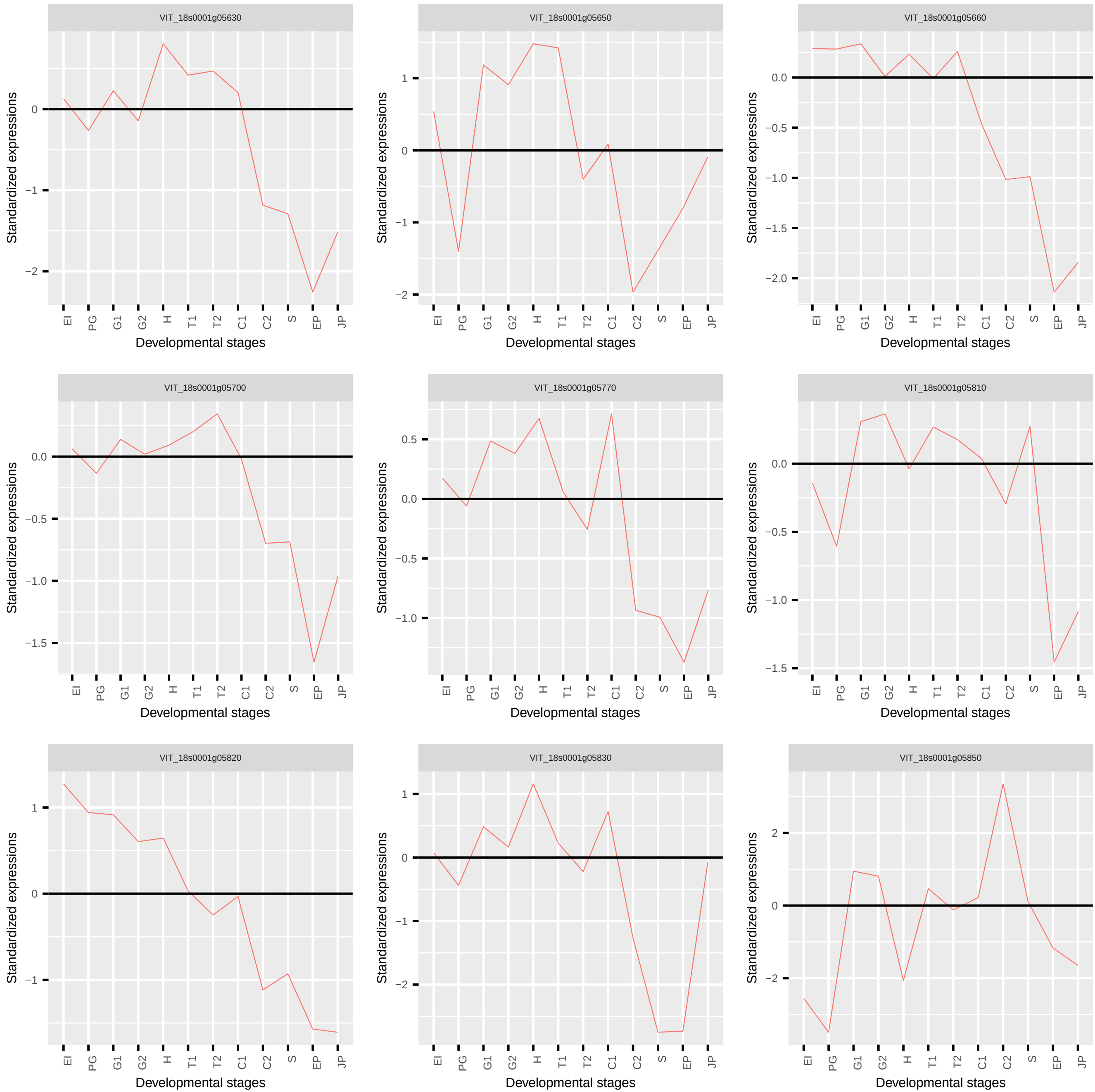
VIT_18s0001g05630
VIT_18s0001g05650
VIT_18s0001g05660
VIT_18s0001g05700
VIT_18s0001g05770
VIT_18s0001g05810
VIT_18s0001g05820
VIT_18s0001g05830
VIT_18s0001g05850
Standardized expressions
Developmental stages
EI
PG
G1
G2
H
T1
T2
C1
C2
S
EP
JP

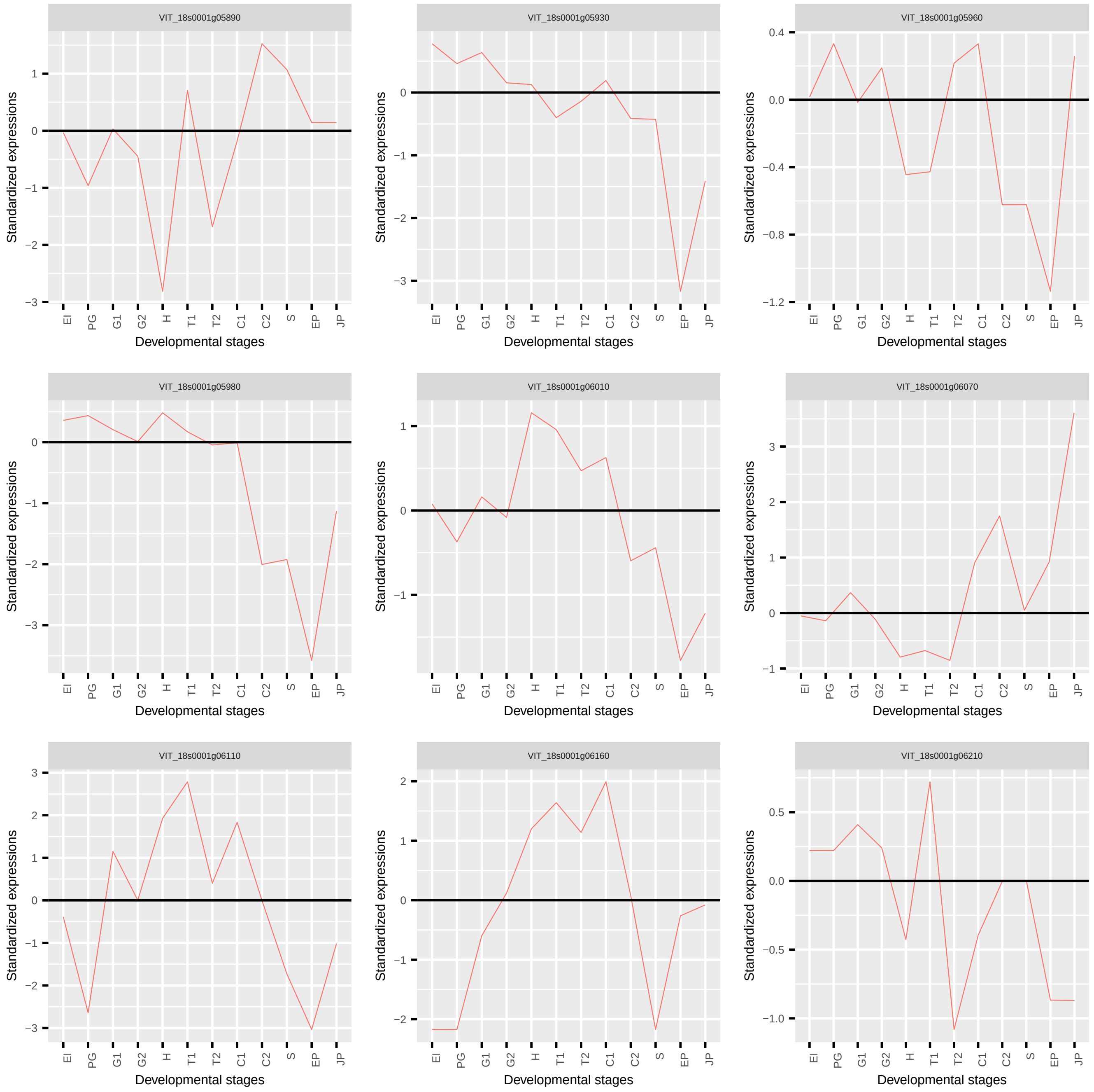
VIT_18s0001g05890
VIT_18s0001g05930
VIT_18s0001g05960
VIT_18s0001g05980
VIT_18s0001g06010
VIT_18s0001g06070
VIT_18s0001g06110
VIT_18s0001g06160
VIT_18s0001g06210
Standardized expressions
Developmental stages
EI PG G1 G2 H T1 T2 C1 C2 S EP JP

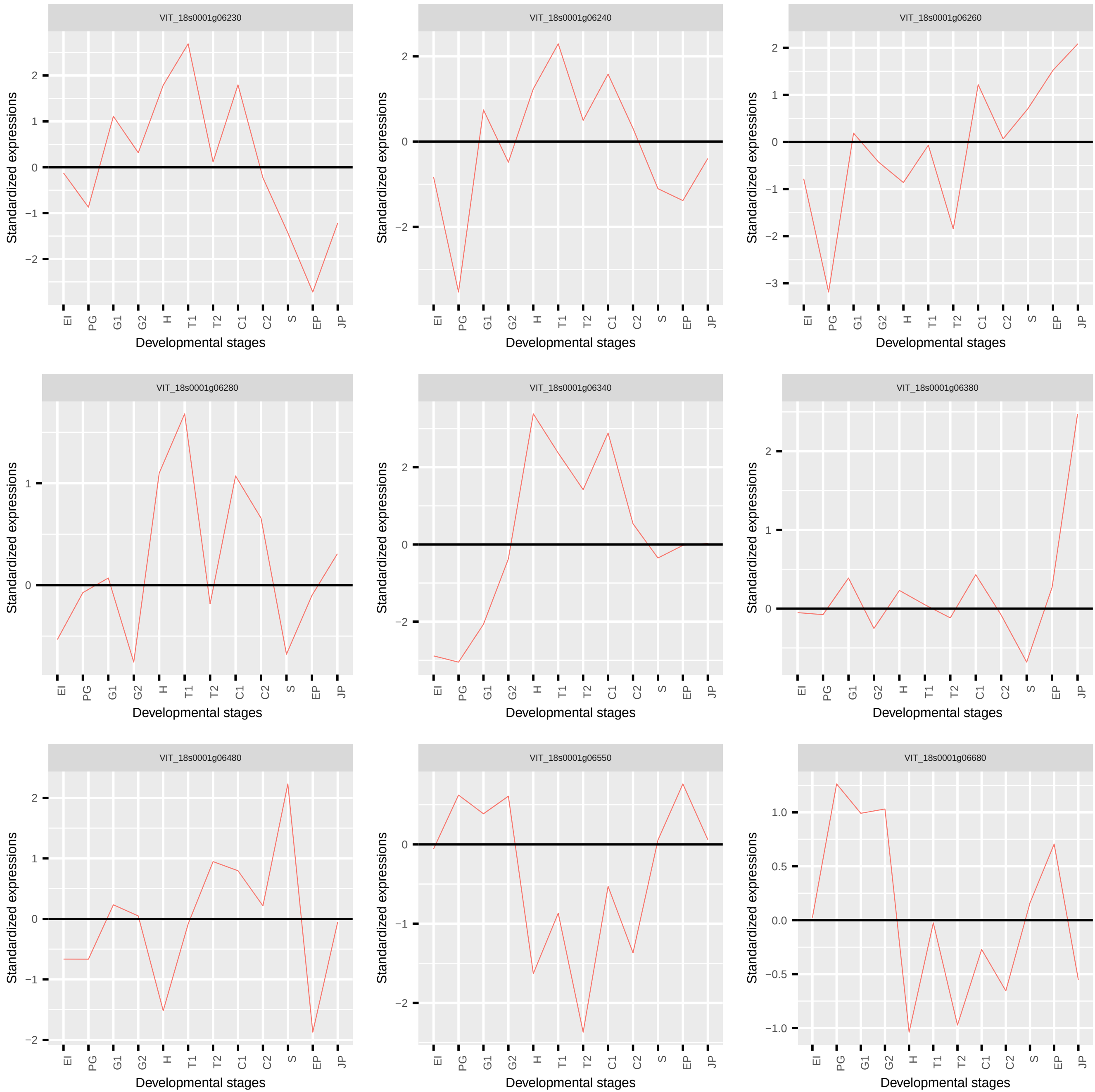
VIT_18s0001g06230
VIT_18s0001g06240
VIT_18s0001g06260
VIT_18s0001g06280
VIT_18s0001g06340
VIT_18s0001g06380
VIT_18s0001g06480
VIT_18s0001g06550
VIT_18s0001g06680
Standardized expressions
Developmental stages
EI PG G1 G2 H T1 T2 C1 C2 S EP JP

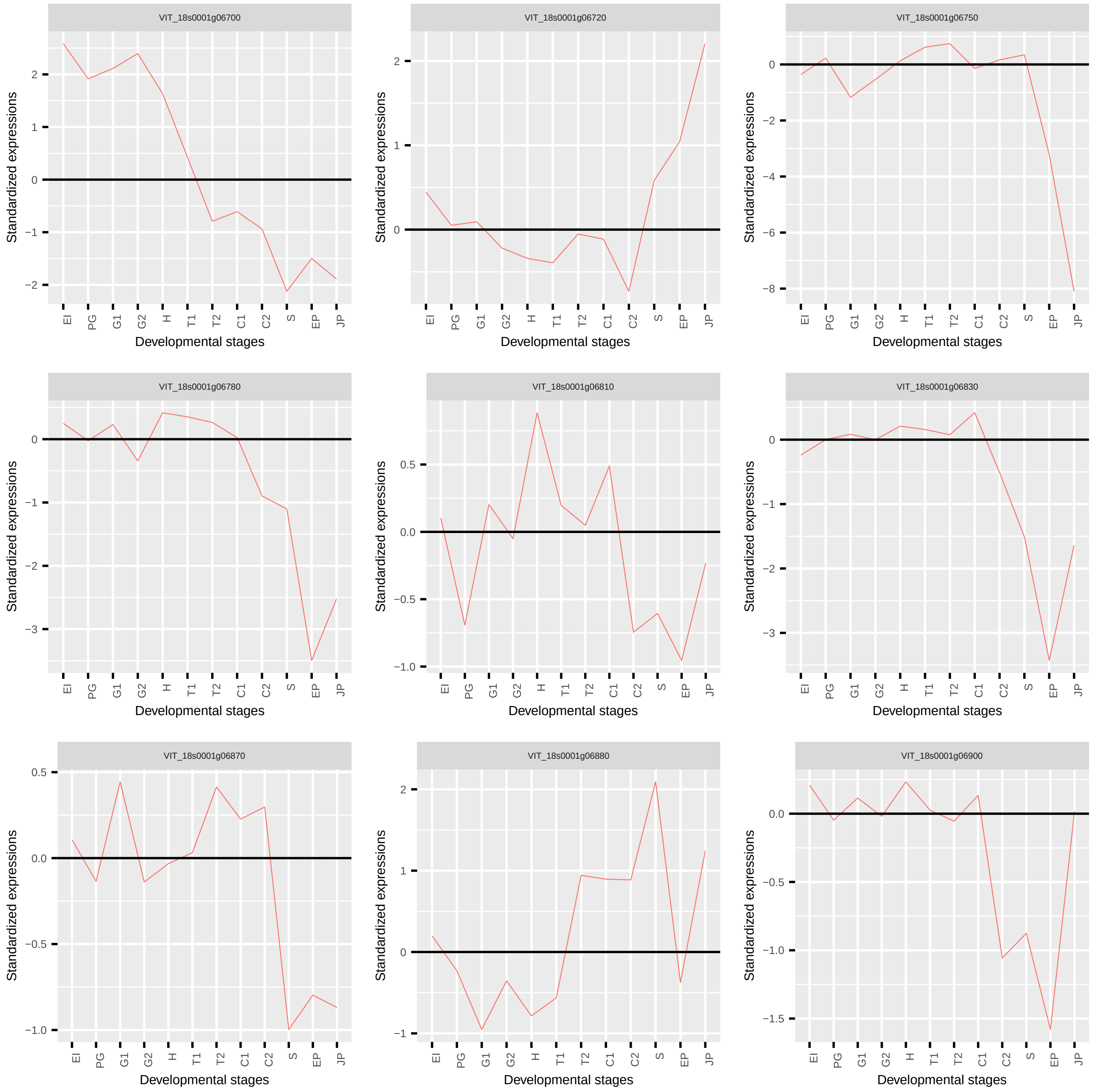

VIT_18s0001g06700
VIT_18s0001g06720
VIT_18s0001g06750
VIT_18s0001g06780
VIT_18s0001g06810
VIT_18s0001g06830
VIT_18s0001g06870
VIT_18s0001g06880
VIT_18s0001g06900
Standardized expressions
Developmental stages
EI PG G1 G2 H T1 T2 C1 C2 S EP JP

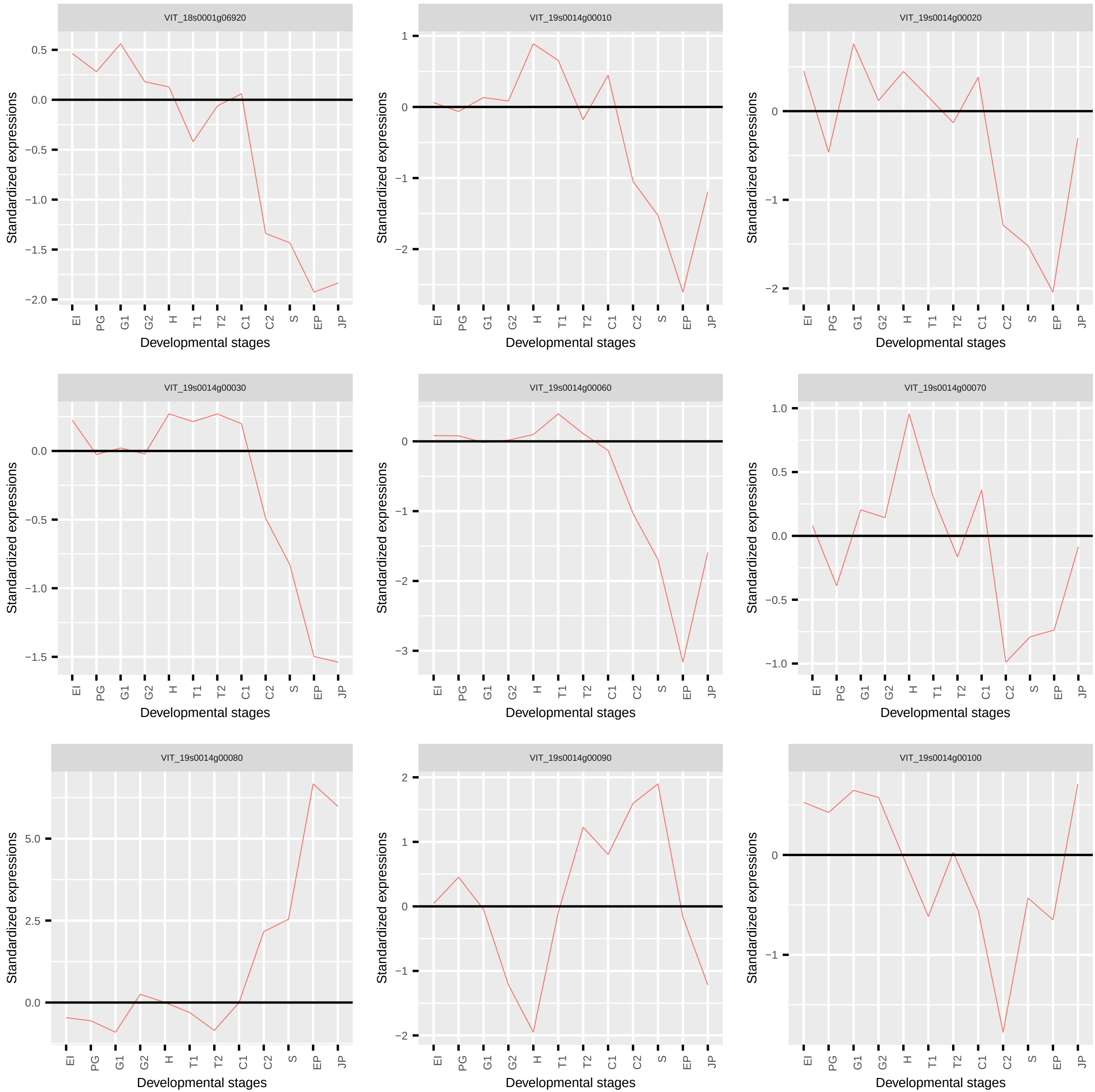

VIT_18s0001g06920
VIT_19s0014g00010
VIT_19s0014g00020
VIT_19s0014g00030
VIT_19s0014g00060
VIT_19s0014g00070
VIT_19s0014g00080
VIT_19s0014g00090
VIT_19s0014g00100
Standardized expressions
Developmental stages
EI PG G1 G2 H T1 T2 C1 C2 S EP JP

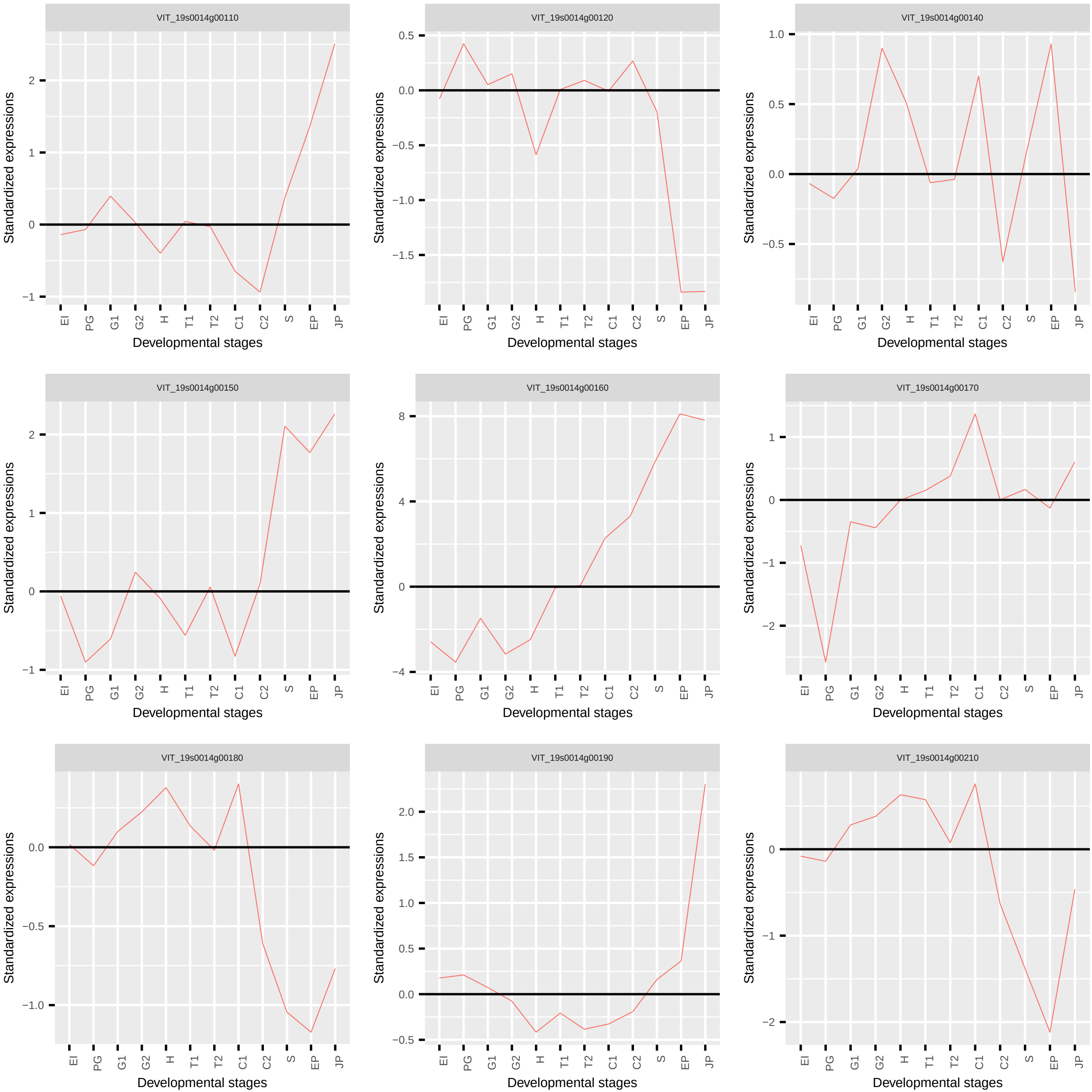
VIT_19s0014g00110
VIT_19s0014g00120
VIT_19s0014g00140
VIT_19s0014g00150
VIT_19s0014g00160
VIT_19s0014g00170
VIT_19s0014g00180
VIT_19s0014g00190
VIT_19s0014g00210
Standardized expressions
Developmental stages
EI PG G1 G2 H T1 T2 C1 C2 S EP JP

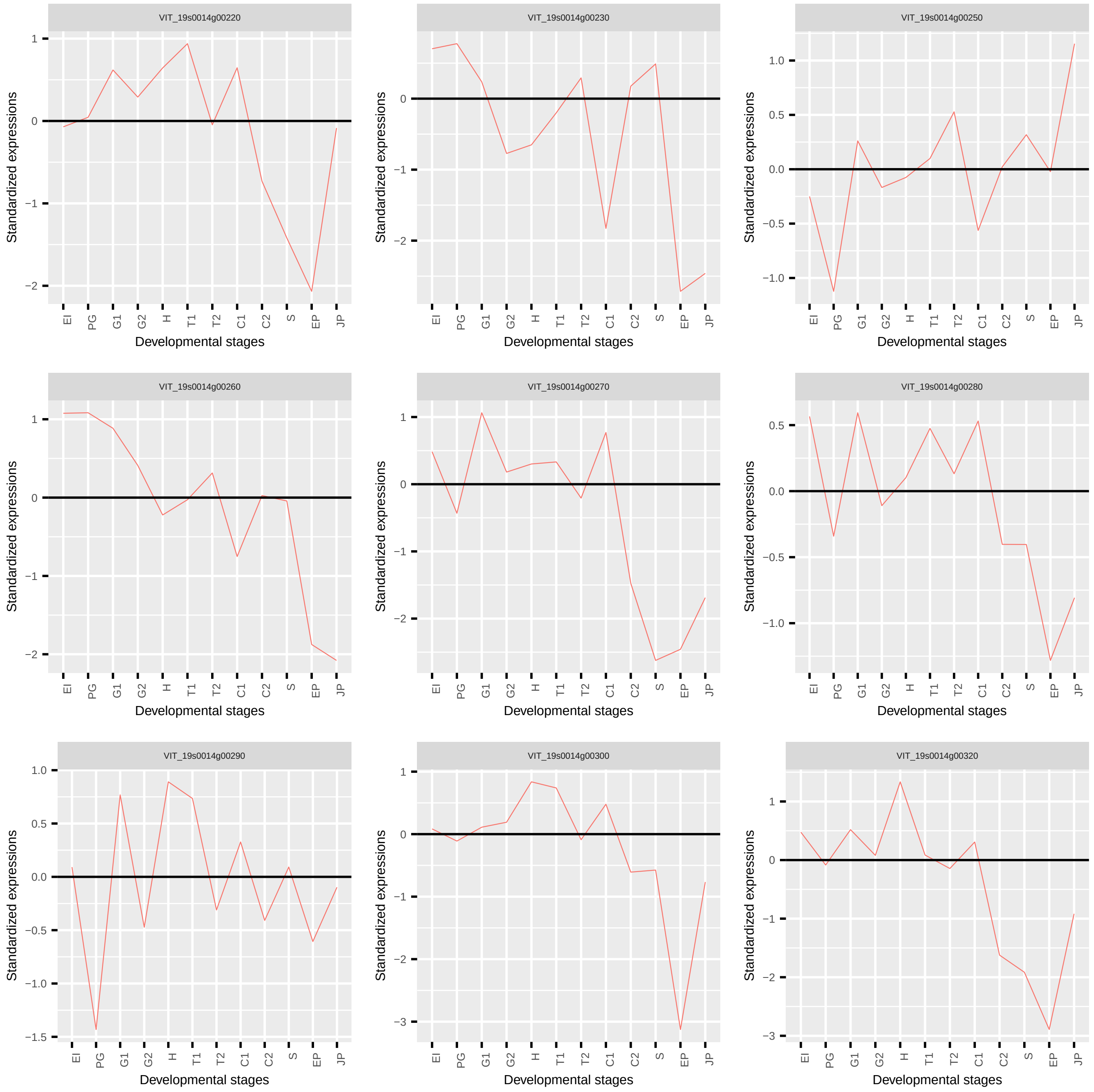
VIT_19s0014g00220
VIT_19s0014g00230
VIT_19s0014g00250
VIT_19s0014g00260
VIT_19s0014g00270
VIT_19s0014g00280
VIT_19s0014g00290
VIT_19s0014g00300
VIT_19s0014g00320
Standardized expressions
Developmental stages
EI PG G1 G2 H T1 T2 C1 C2 S EP JP

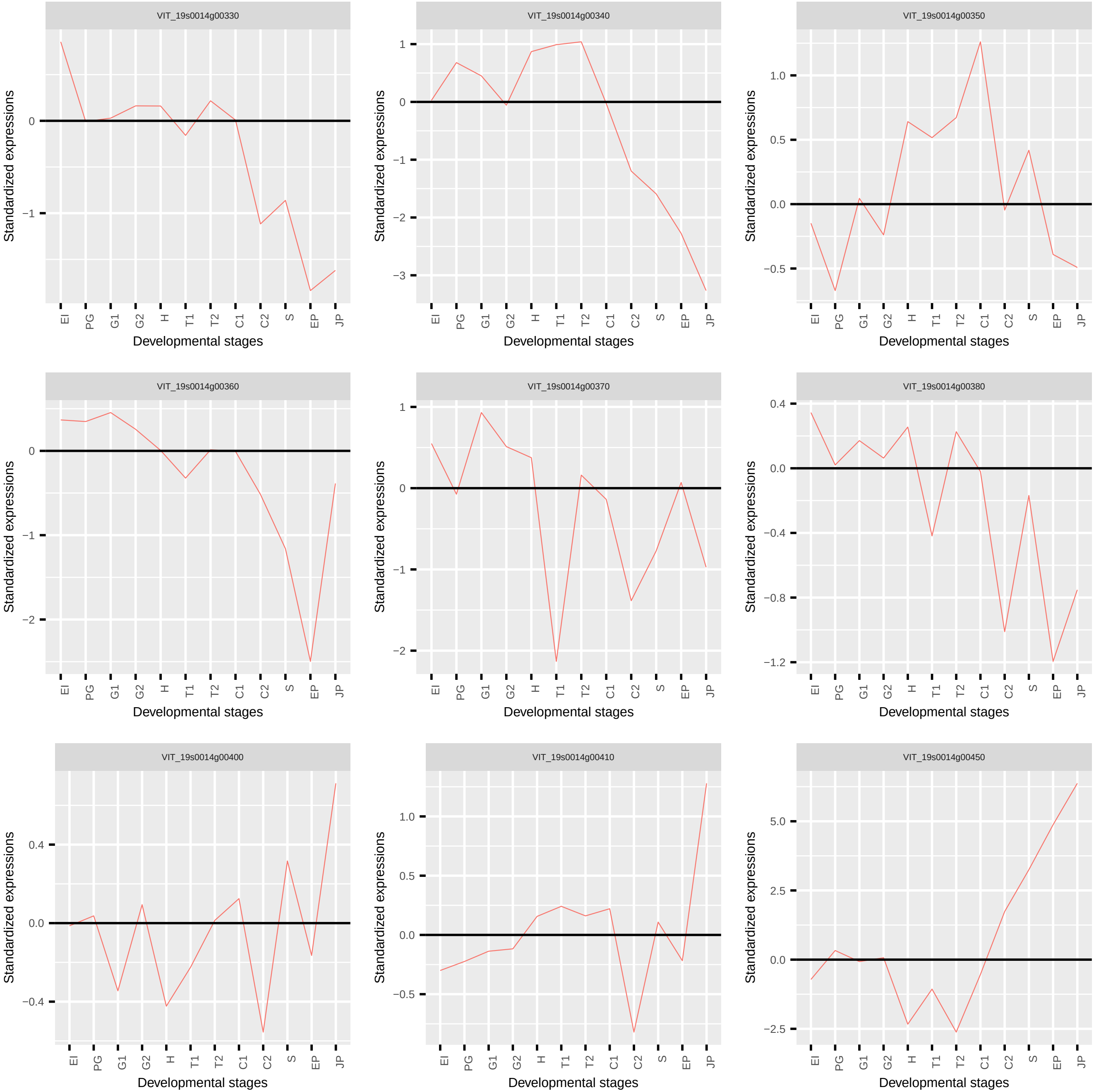
VIT_19s0014g00330
VIT_19s0014g00340
VIT_19s0014g00350
VIT_19s0014g00360
VIT_19s0014g00370
VIT_19s0014g00380
VIT_19s0014g00400
VIT_19s0014g00410
VIT_19s0014g00450
Standardized expressions
Developmental stages
EI PG G1 G2 H T1 T2 C1 C2 S EP JP

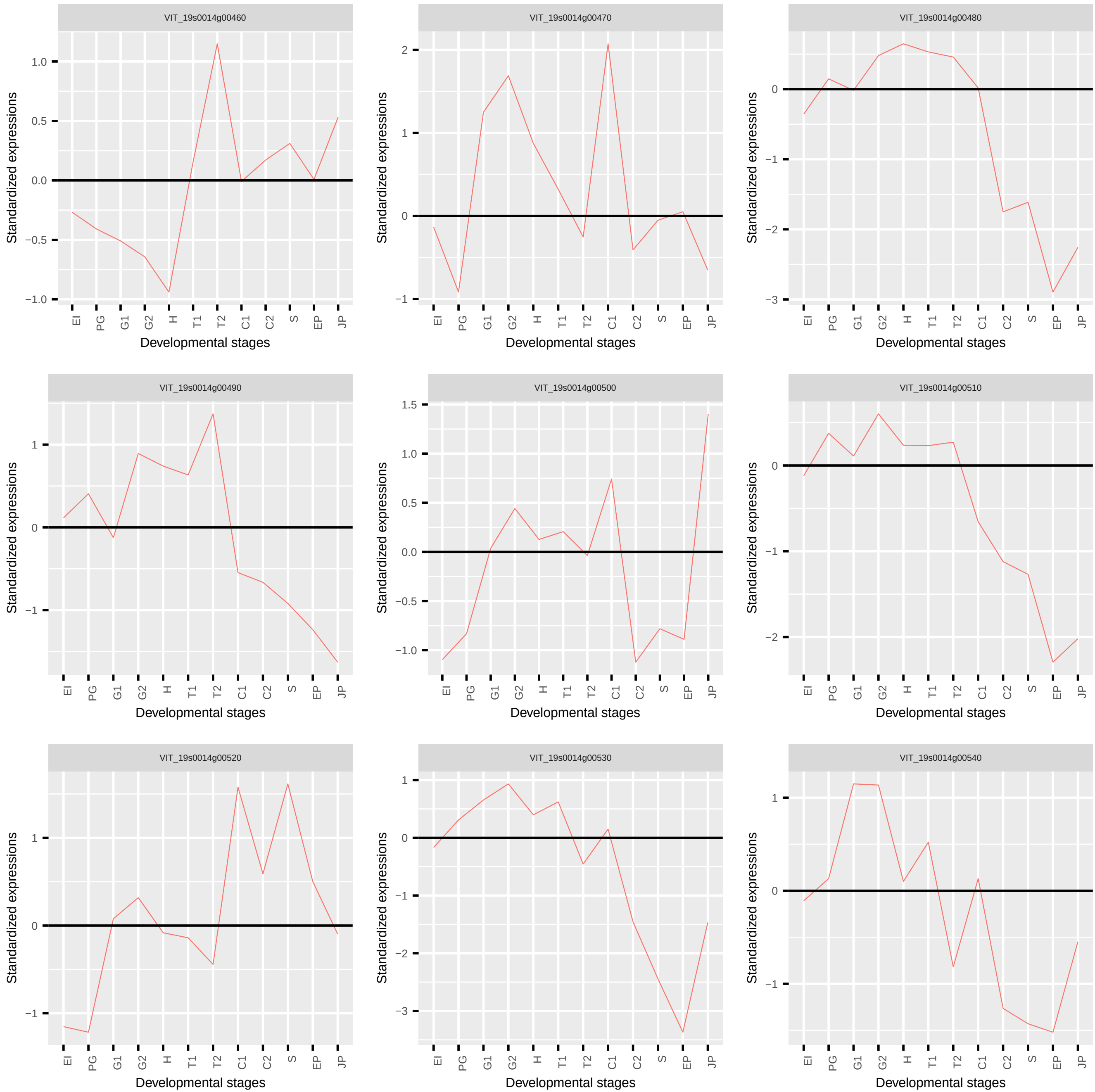

VIT_19s0014g00460
VIT_19s0014g00470
VIT_19s0014g00480
VIT_19s0014g00490
VIT_19s0014g00500
VIT_19s0014g00510
VIT_19s0014g00520
VIT_19s0014g00530
VIT_19s0014g00540
Standardized expressions
Developmental stages
EI PG G1 G2 H T1 T2 C1 C2 S EP JP

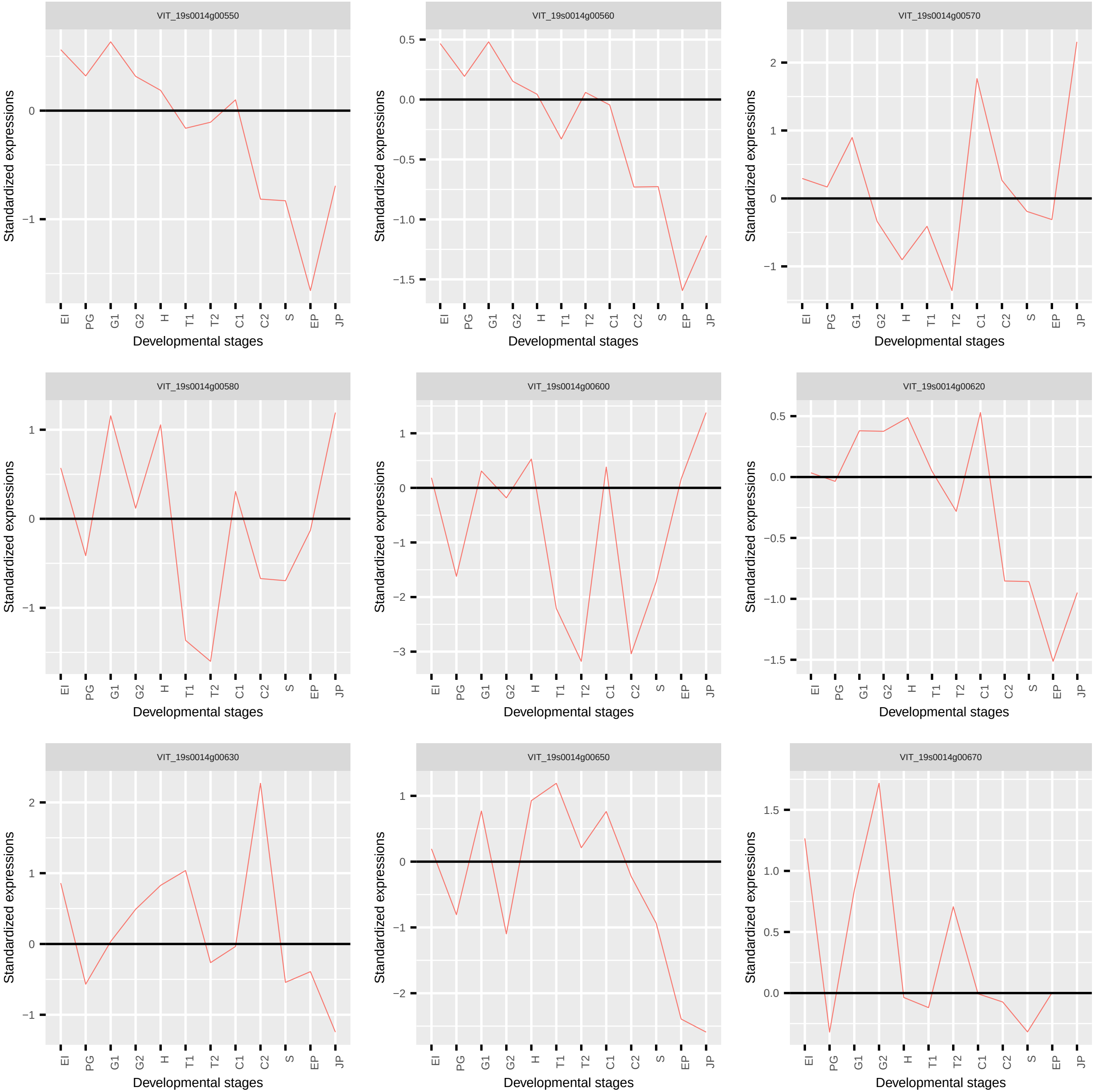
VIT_19s0014g00550
VIT_19s0014g00560
VIT_19s0014g00570
VIT_19s0014g00580
VIT_19s0014g00600
VIT_19s0014g00620
VIT_19s0014g00630
VIT_19s0014g00650
VIT_19s0014g00670
Standardized expressions
Developmental stages
EI PG G1 G2 H T1 T2 C1 C2 S EP JP

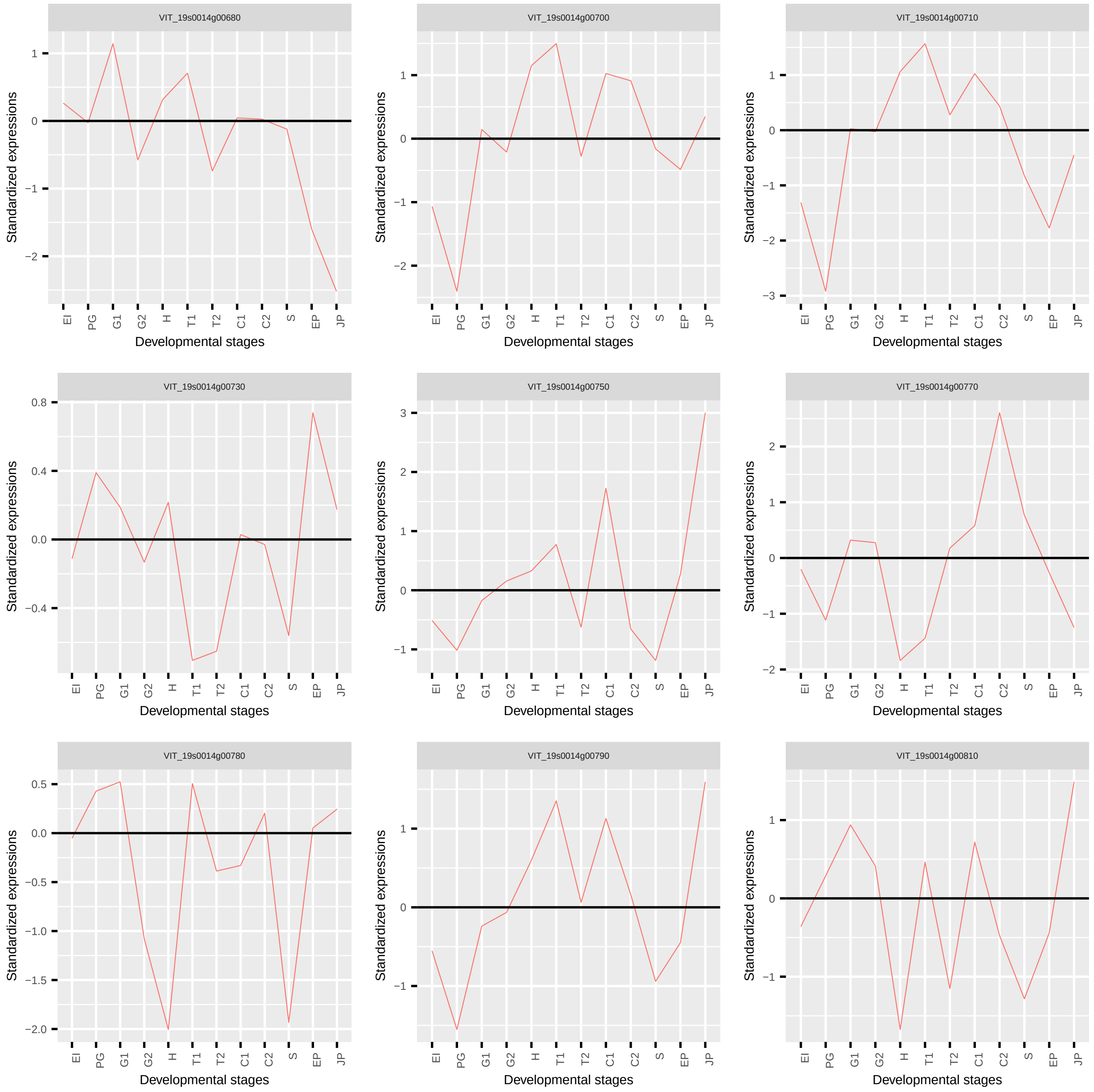
VIT_19s0014g00680
VIT_19s0014g00700
VIT_19s0014g00710
VIT_19s0014g00730
VIT_19s0014g00750
VIT_19s0014g00770
VIT_19s0014g00780
VIT_19s0014g00790
VIT_19s0014g00810
Standardized expressions
Developmental stages
EI PG G1 G2 H T1 T2 C1 C2 S EP JP

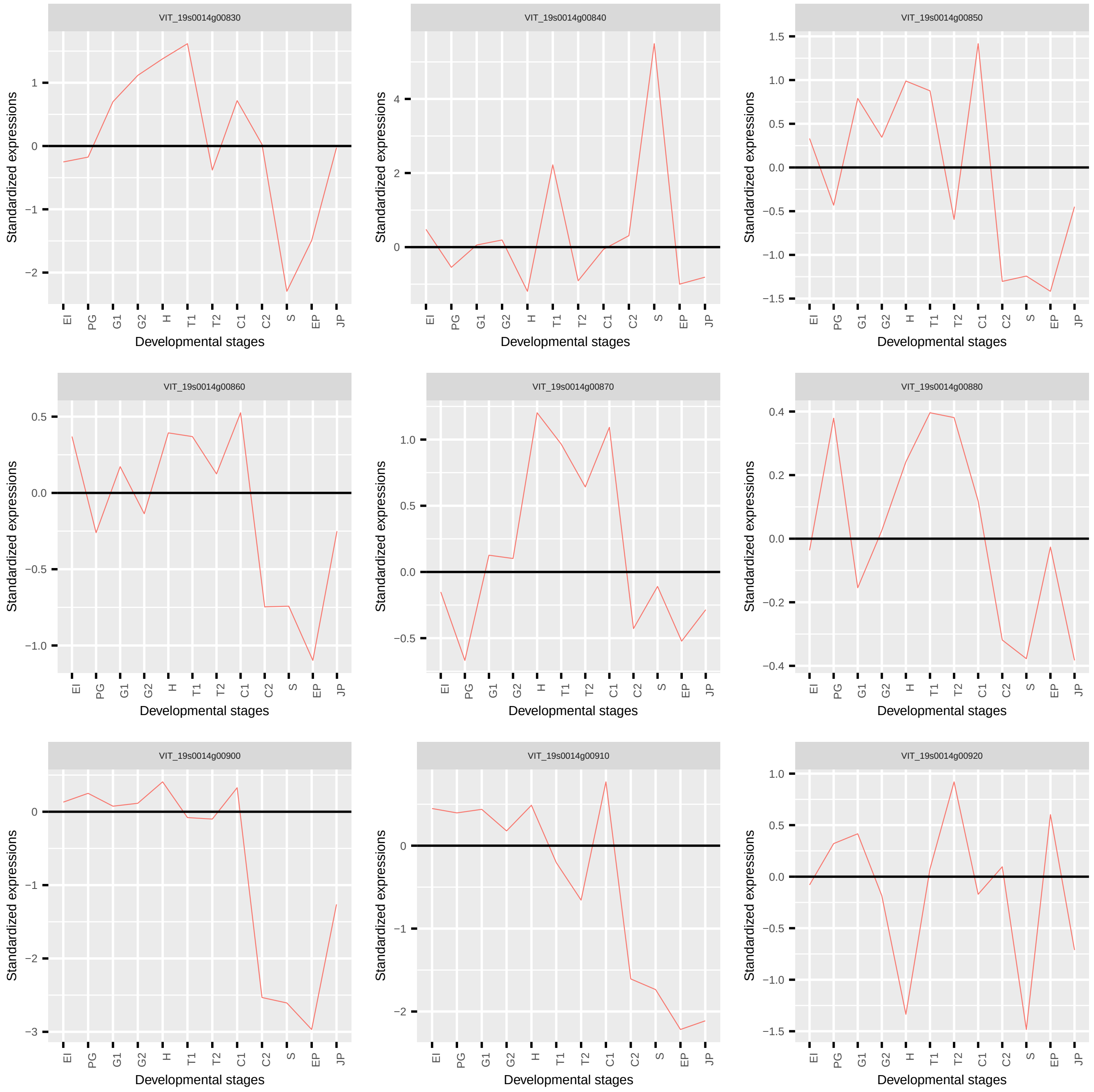

VIT_19s0014g00830
VIT_19s0014g00840
VIT_19s0014g00850
VIT_19s0014g00860
VIT_19s0014g00870
VIT_19s0014g00880
VIT_19s0014g00900
VIT_19s0014g00910
VIT_19s0014g00920
Standardized expressions
Developmental stages
EI PG G1 G2 H T1 T2 C1 C2 S EP JP

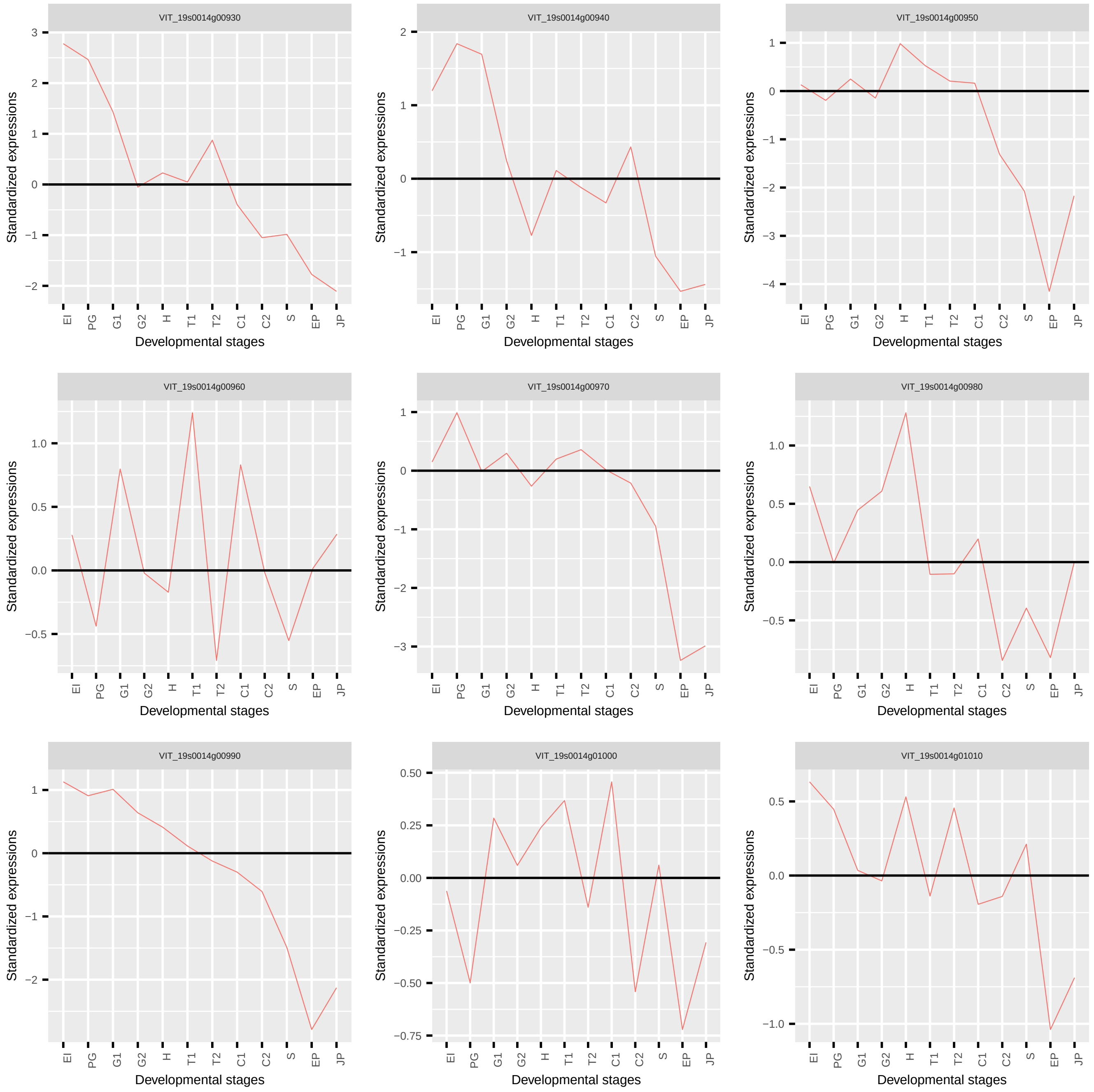
VIT_19s0014g00930
VIT_19s0014g00940
VIT_19s0014g00950
VIT_19s0014g00960
VIT_19s0014g00970
VIT_19s0014g00980
VIT_19s0014g00990
VIT_19s0014g01000
VIT_19s0014g01010
Standardized expressions
Developmental stages
EI PG G1 G2 H T1 T2 C1 C2 S EP JP

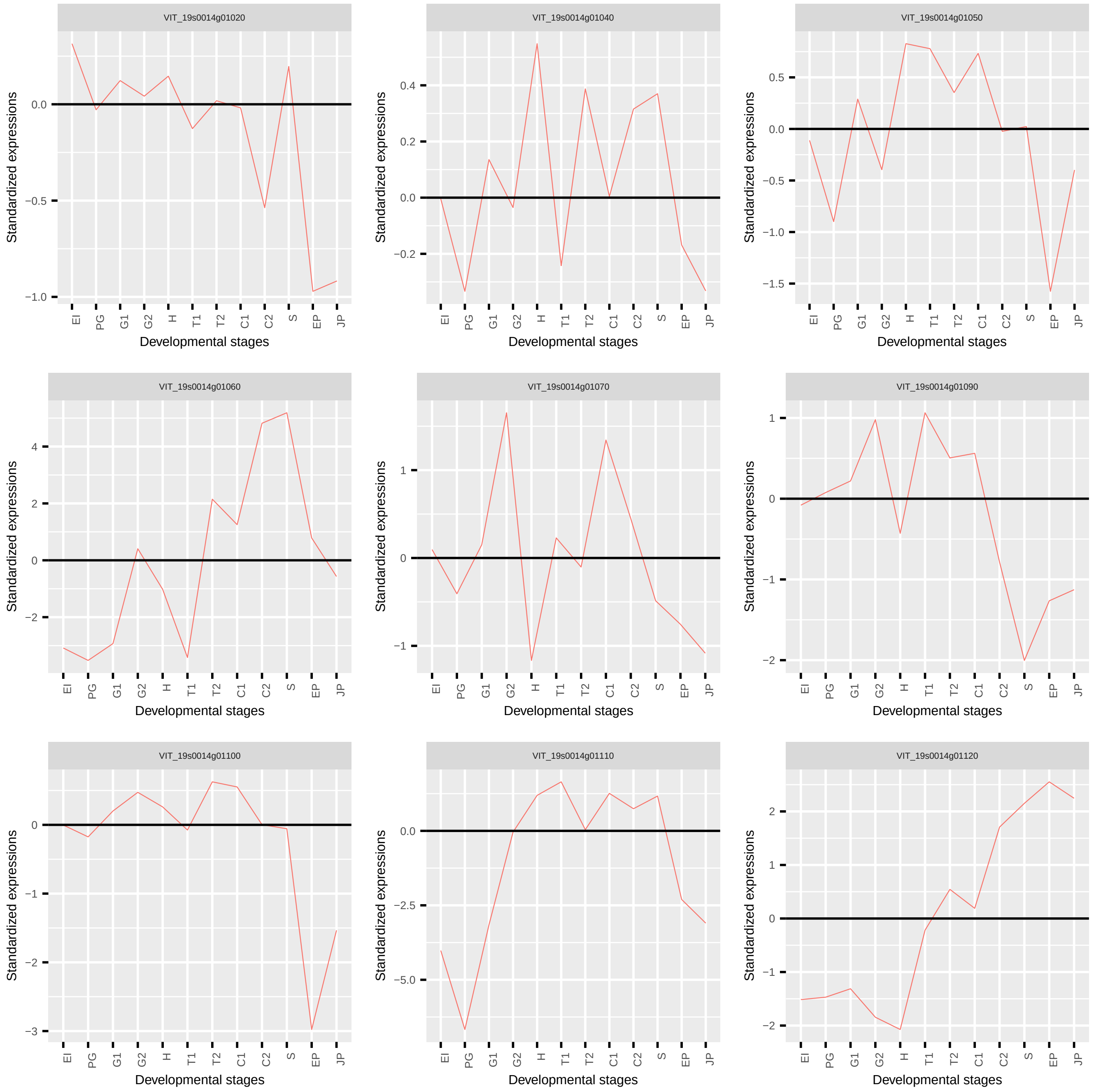

VIT_19s0014g01020
VIT_19s0014g01040
VIT_19s0014g01050
VIT_19s0014g01060
VIT_19s0014g01070
VIT_19s0014g01090
VIT_19s0014g01100
VIT_19s0014g01110
VIT_19s0014g01120
Standardized expressions
Developmental stages
EI PG G1 G2 H T1 T2 C1 C2 S EP JP

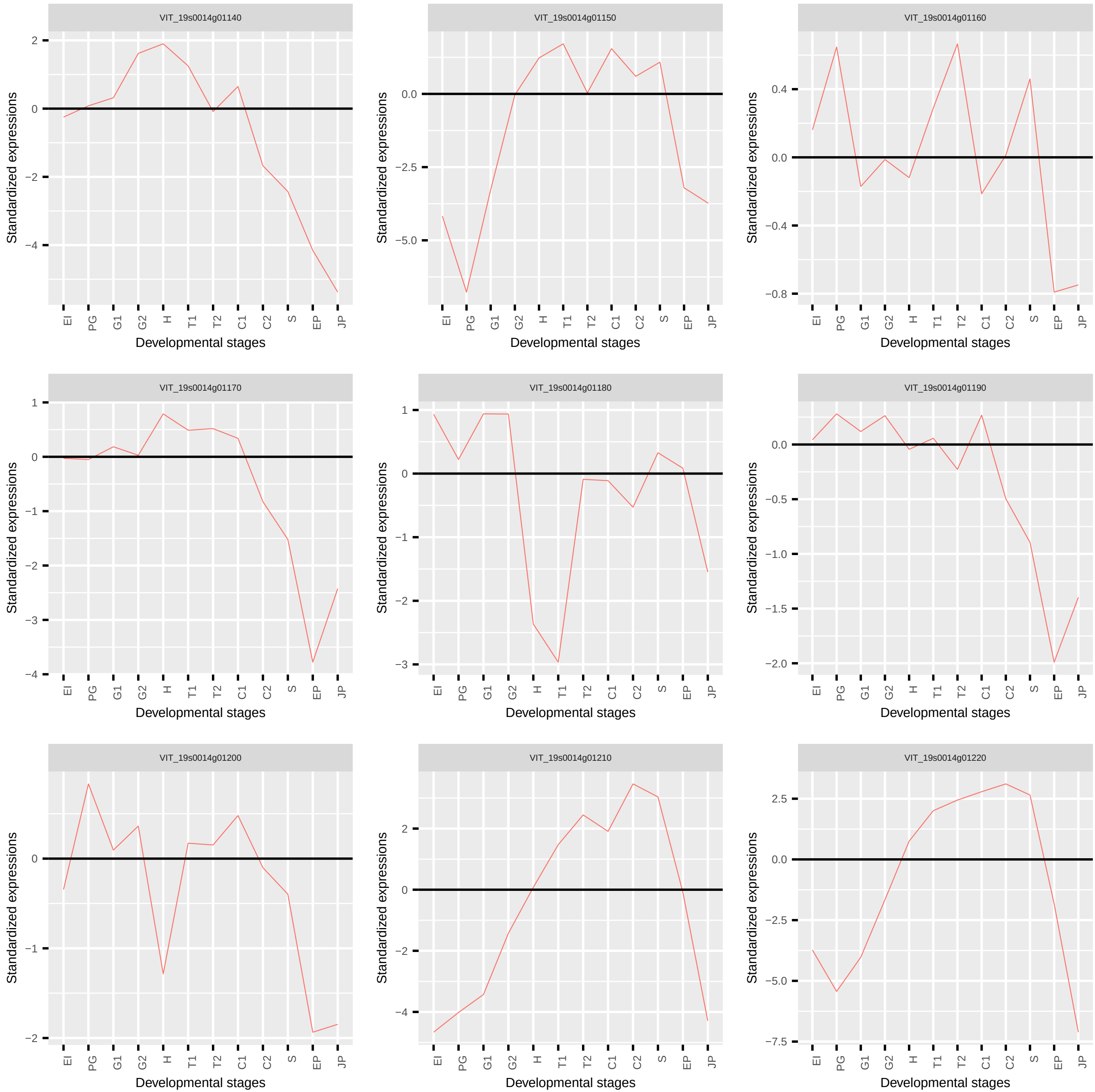

VIT_19s0014g01140
VIT_19s0014g01150
VIT_19s0014g01160
VIT_19s0014g01170
VIT_19s0014g01180
VIT_19s0014g01190
VIT_19s0014g01200
VIT_19s0014g01210
VIT_19s0014g01220
Standardized expressions
Developmental stages
EI PG G1 G2 H T1 T2 C1 C2 S EP JP

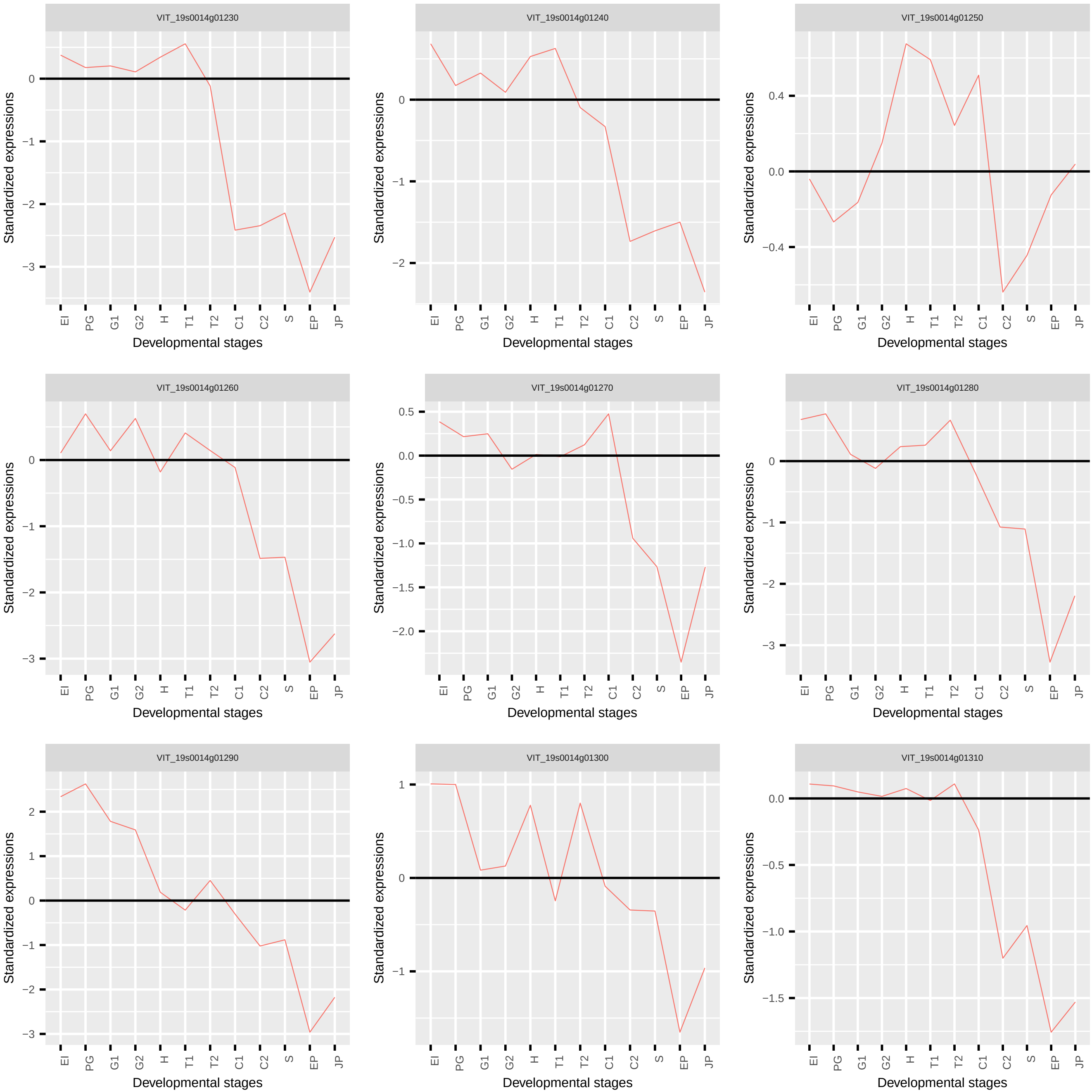
VIT_19s0014g01230
VIT_19s0014g01240
VIT_19s0014g01250
VIT_19s0014g01260
VIT_19s0014g01270
VIT_19s0014g01280
VIT_19s0014g01290
VIT_19s0014g01300
VIT_19s0014g01310
Standardized expressions
Developmental stages
EI PG G1 G2 H T1 T2 C1 C2 S EP JP
0 −1 −2 −3
0.4 0.0 −0.4
0.5 0.0 −0.5 −1.0 −1.5 −2.0
2 1 0 −1 −2 −3
1 0 −1
0.0 −0.5 −1.0 −1.5

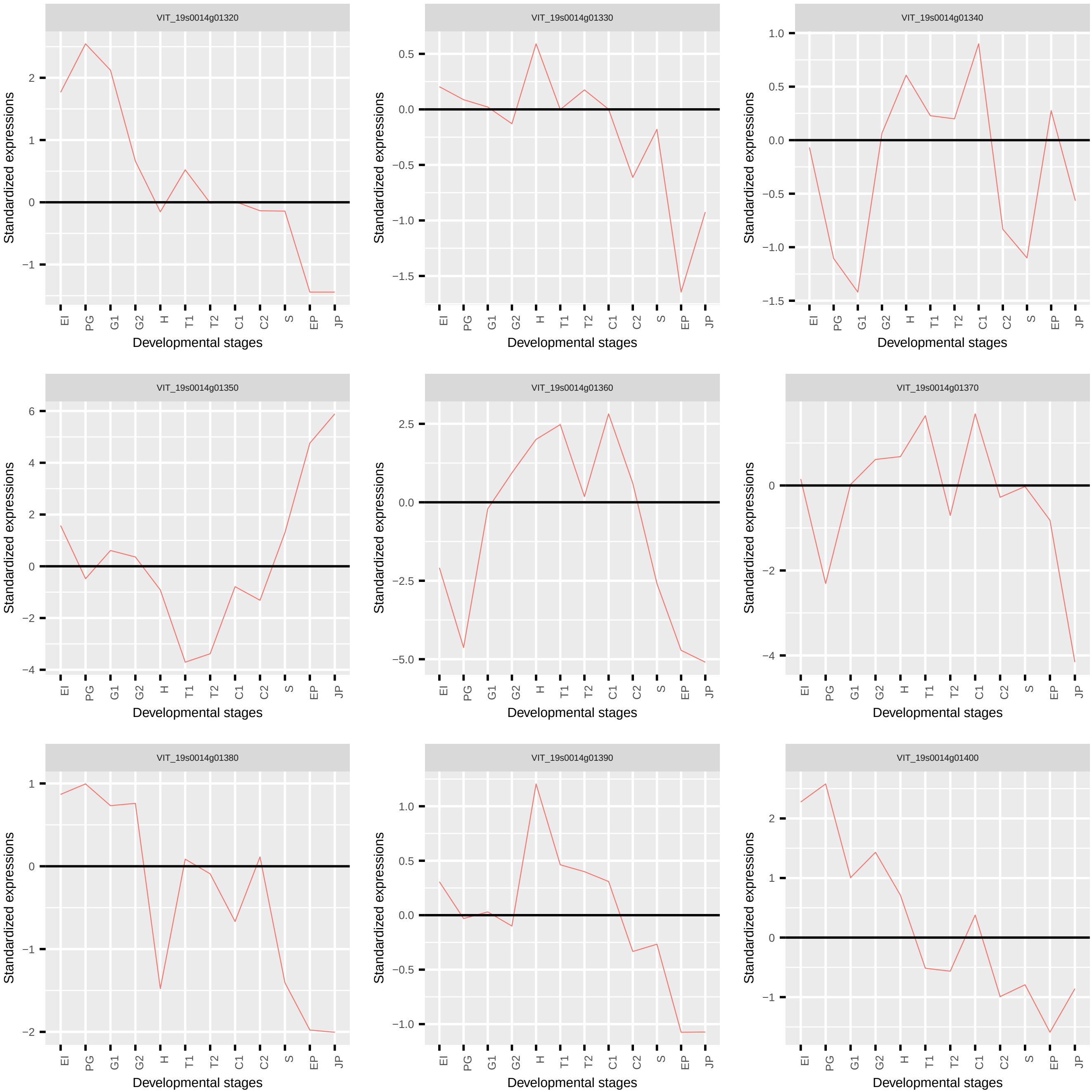
VIT_19s0014g01320
VIT_19s0014g01330
VIT_19s0014g01340
VIT_19s0014g01350
VIT_19s0014g01360
VIT_19s0014g01370
VIT_19s0014g01380
VIT_19s0014g01390
VIT_19s0014g01400
Standardized expressions
Developmental stages
EI PG G1 G2 H T1 T2 C1 C2 S EP JP

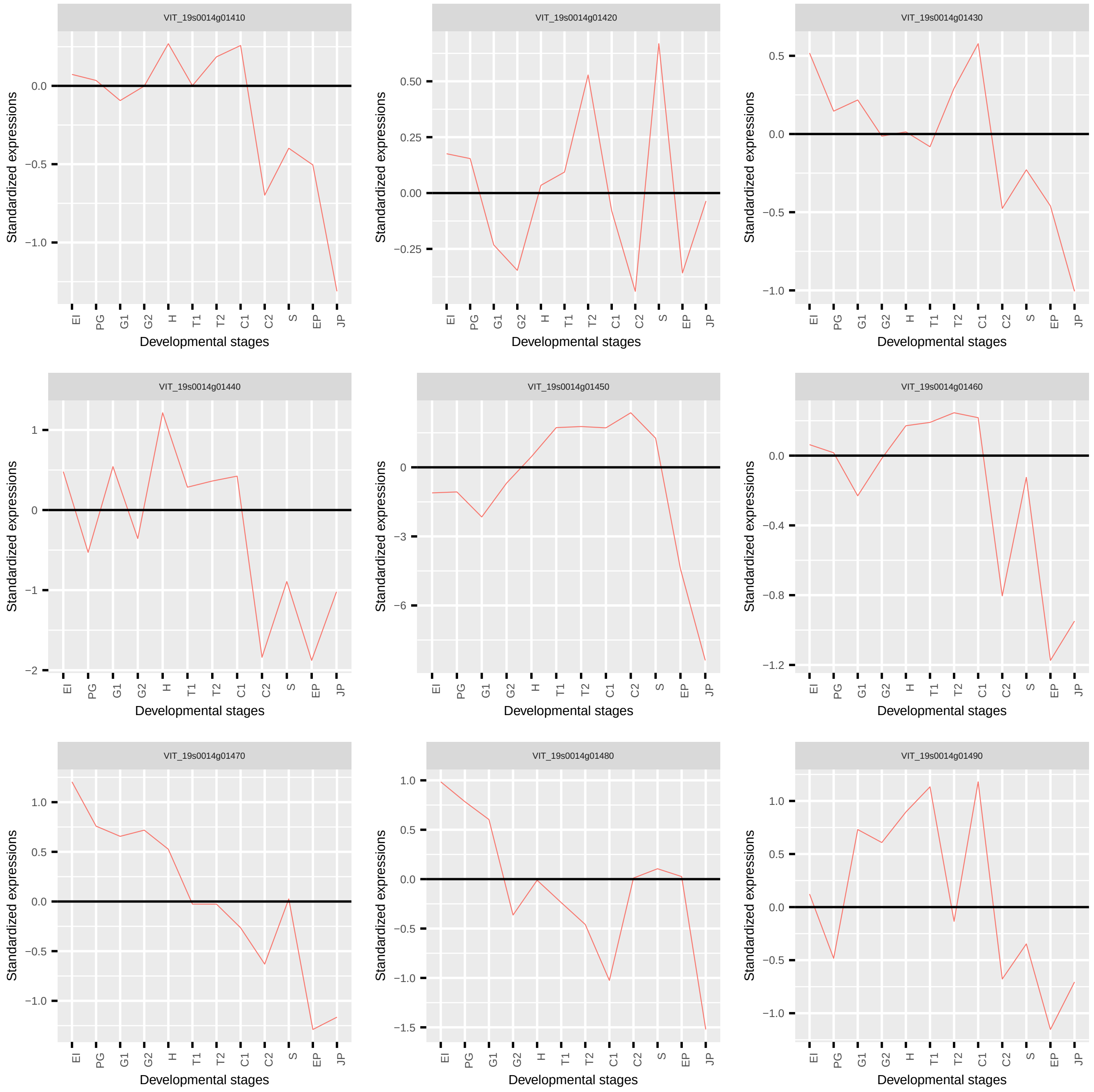
VIT_19s0014g01410
0.0
−0.5
−1.0
VIT_19s0014g01420
0.50
0.25
0.00
−0.25
VIT_19s0014g01430
0.5
0.0
−0.5
−1.0
VIT_19s0014g01440
1
0
−1
−2
VIT_19s0014g01450
0
−3
−6
VIT_19s0014g01460
0.0
−0.4
−0.8
−1.2
VIT_19s0014g01470
1.0
0.5
0.0
−0.5
−1.0
VIT_19s0014g01480
1.0
0.5
0.0
−0.5
−1.0
−1.5
VIT_19s0014g01490
1.0
0.5
0.0
−0.5
−1.0
Standardized expressions
EI
PG
G1
G2
H
T1
T2
C1
C2
S
EP
JP
Developmental stages

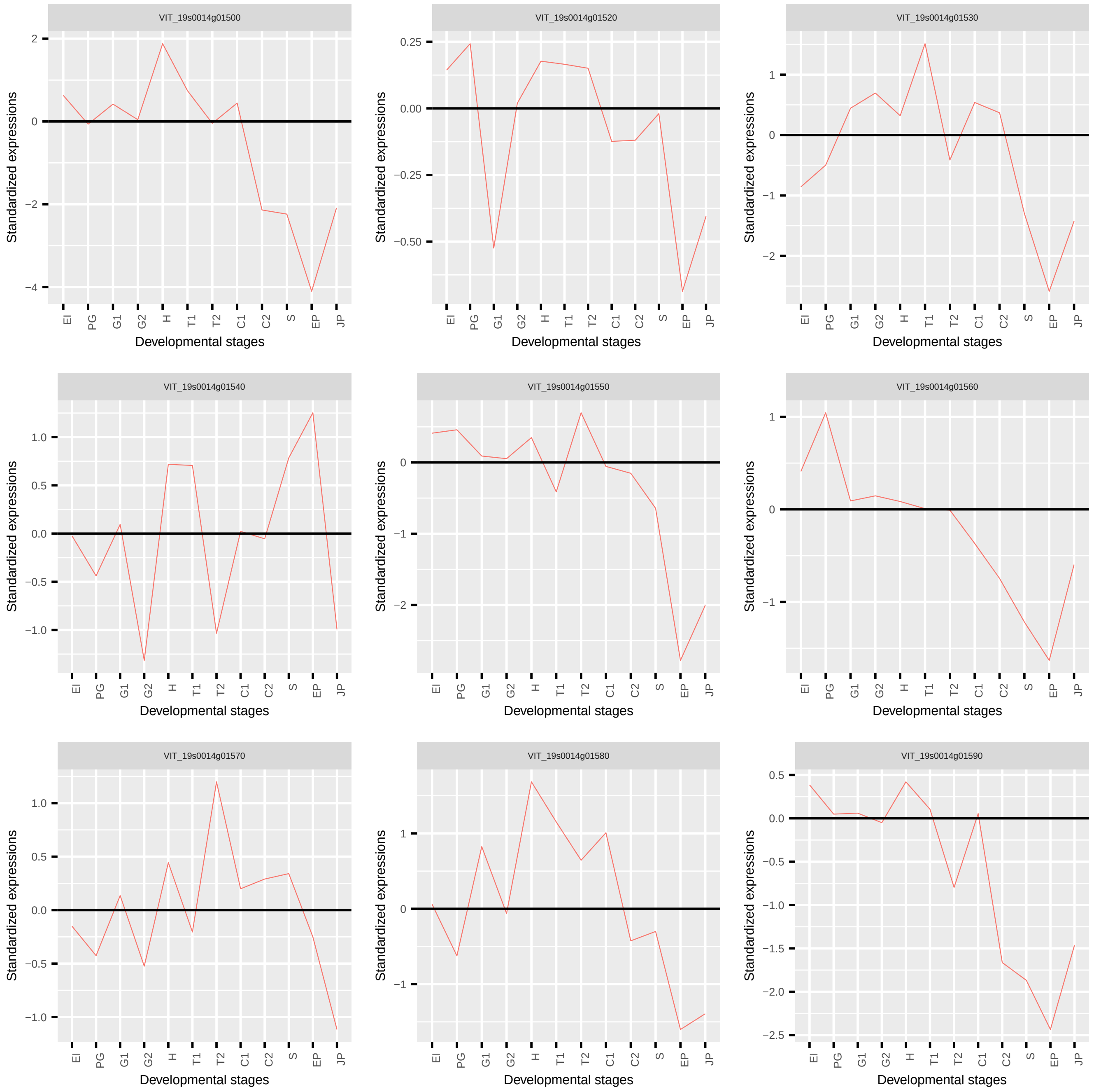
VIT_19s0014g01500
VIT_19s0014g01520
VIT_19s0014g01530
VIT_19s0014g01540
VIT_19s0014g01550
VIT_19s0014g01560
VIT_19s0014g01570
VIT_19s0014g01580
VIT_19s0014g01590
Standardized expressions
Developmental stages
EI
PG
G1
G2
H
T1
T2
C1
C2
S
EP
JP

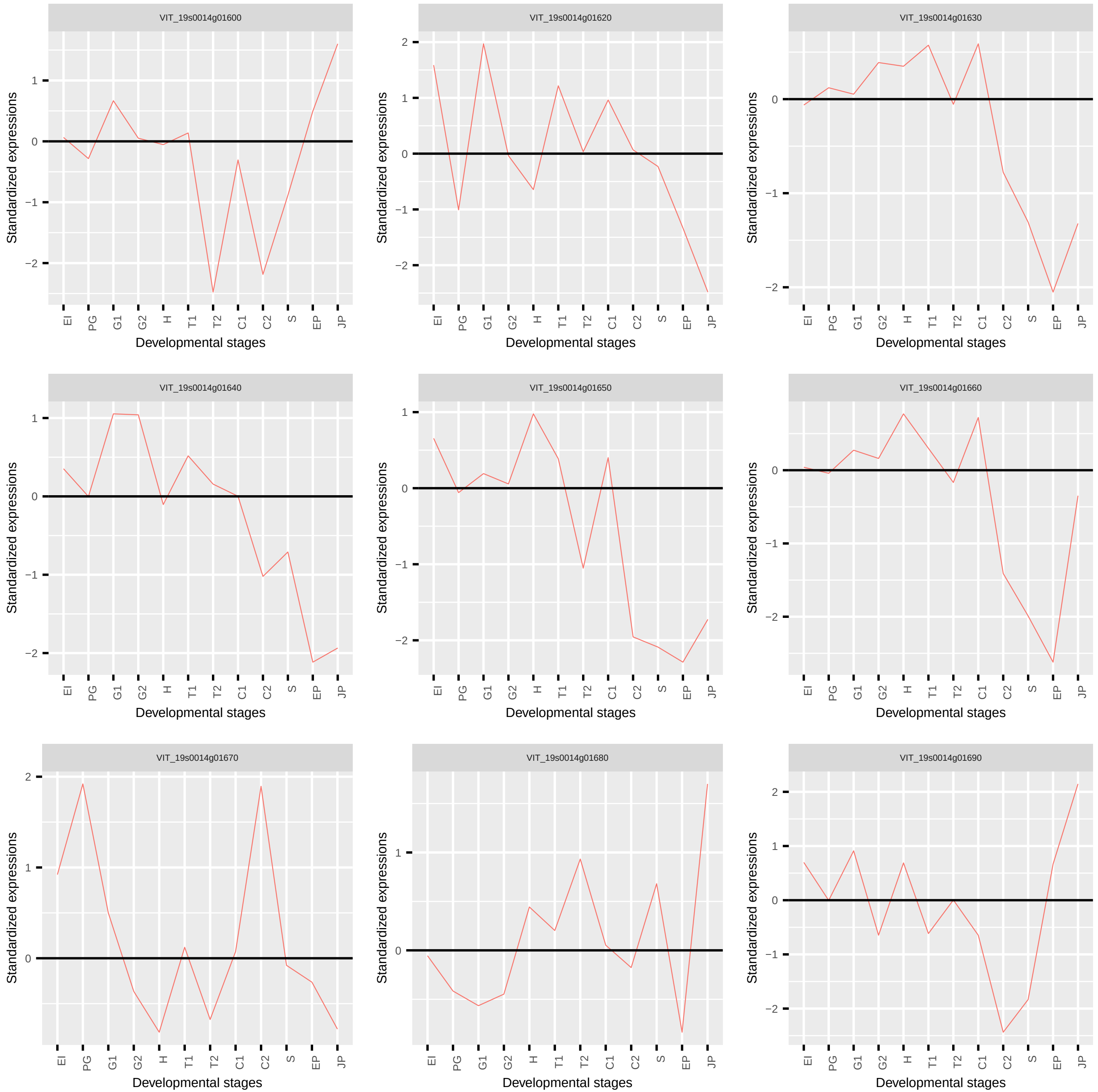

VIT_19s0014g01600
VIT_19s0014g01620
VIT_19s0014g01630
VIT_19s0014g01640
VIT_19s0014g01650
VIT_19s0014g01660
VIT_19s0014g01670
VIT_19s0014g01680
VIT_19s0014g01690
Standardized expressions
Developmental stages
EI PG G1 G2 H T1 T2 C1 C2 S EP JP

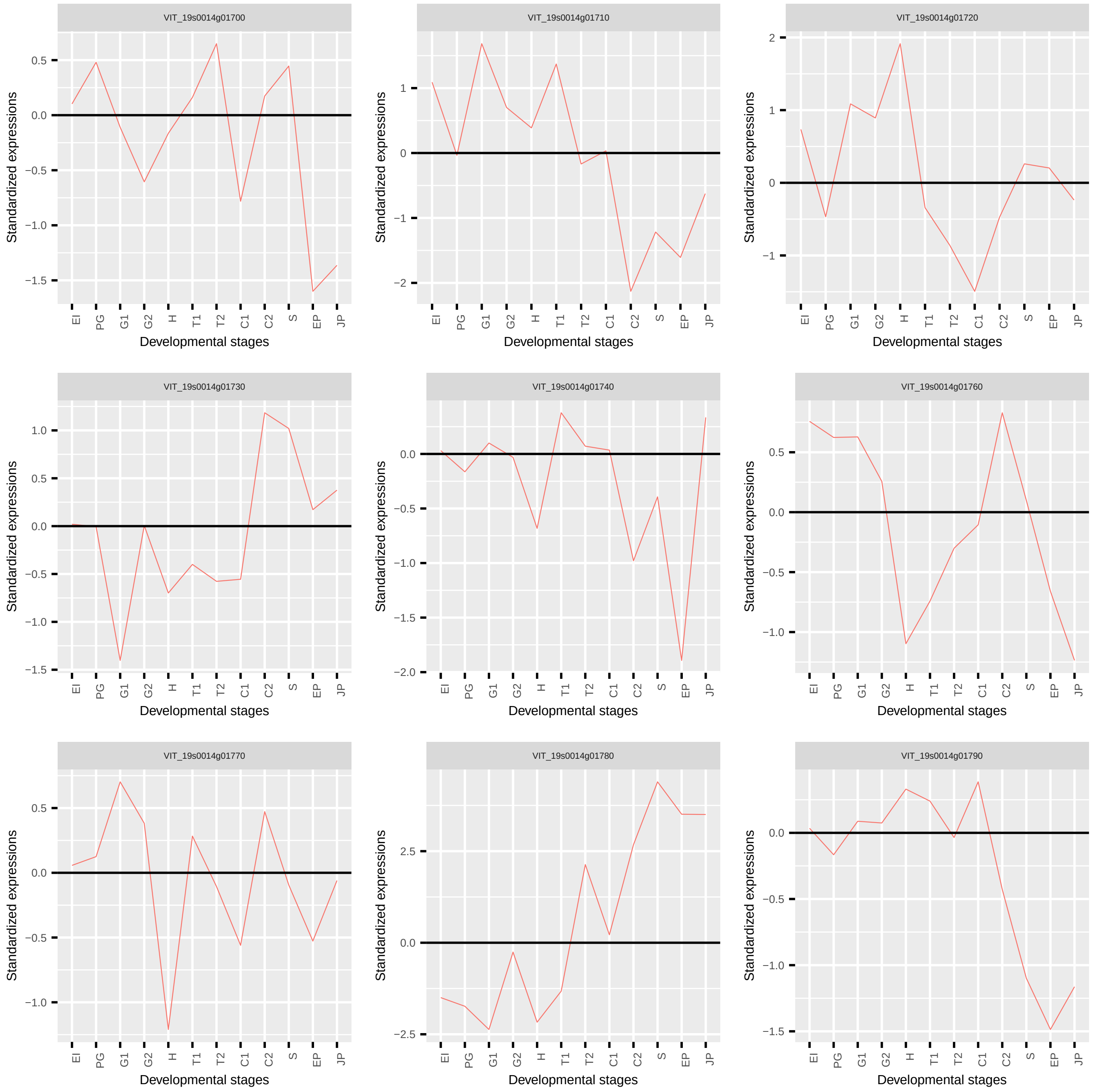

VIT_19s0014g01700
VIT_19s0014g01710
VIT_19s0014g01720
VIT_19s0014g01730
VIT_19s0014g01740
VIT_19s0014g01760
VIT_19s0014g01770
VIT_19s0014g01780
VIT_19s0014g01790
Standardized expressions
Developmental stages
EI PG G1 G2 H T1 T2 C1 C2 S EP JP

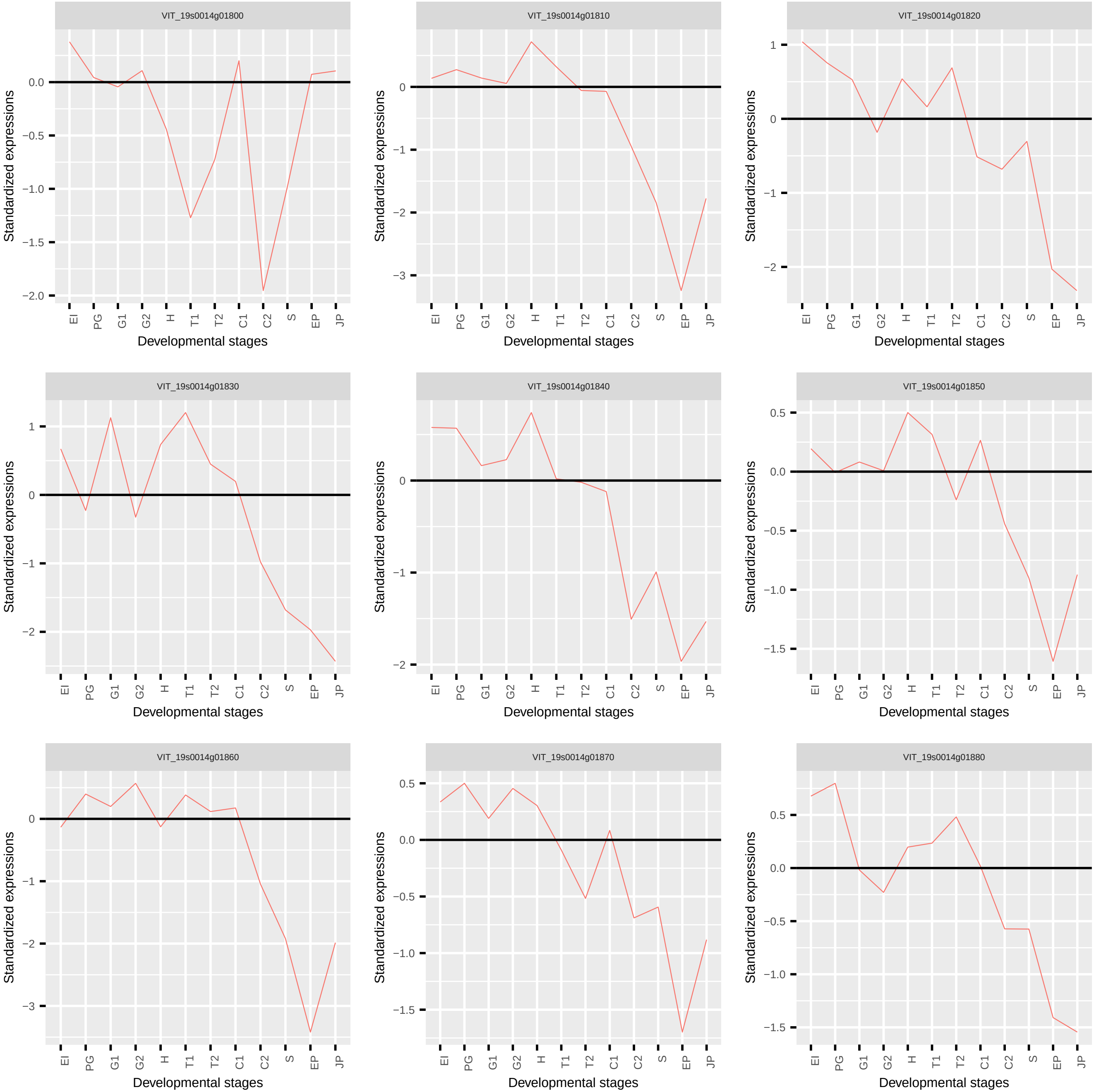
VIT_19s0014g01800
VIT_19s0014g01810
VIT_19s0014g01820
VIT_19s0014g01830
VIT_19s0014g01840
VIT_19s0014g01850
VIT_19s0014g01860
VIT_19s0014g01870
VIT_19s0014g01880
Standardized expressions
Developmental stages
EI PG G1 G2 H T1 T2 C1 C2 S EP JP

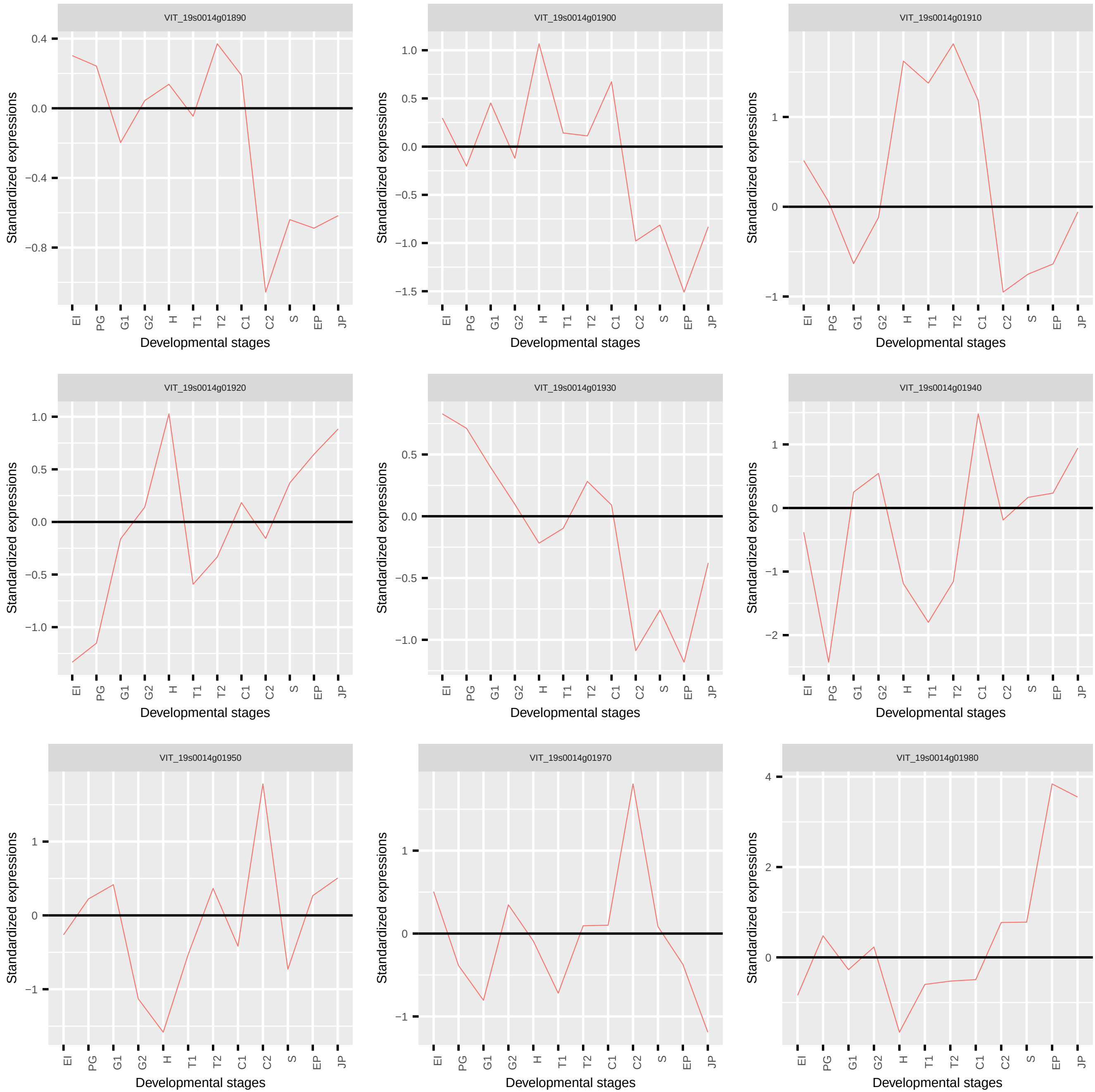
VIT_19s0014g01890
VIT_19s0014g01900
VIT_19s0014g01910
VIT_19s0014g01920
VIT_19s0014g01930
VIT_19s0014g01940
VIT_19s0014g01950
VIT_19s0014g01970
VIT_19s0014g01980
Standardized expressions
Developmental stages
EI PG G1 G2 H T1 T2 C1 C2 S EP JP

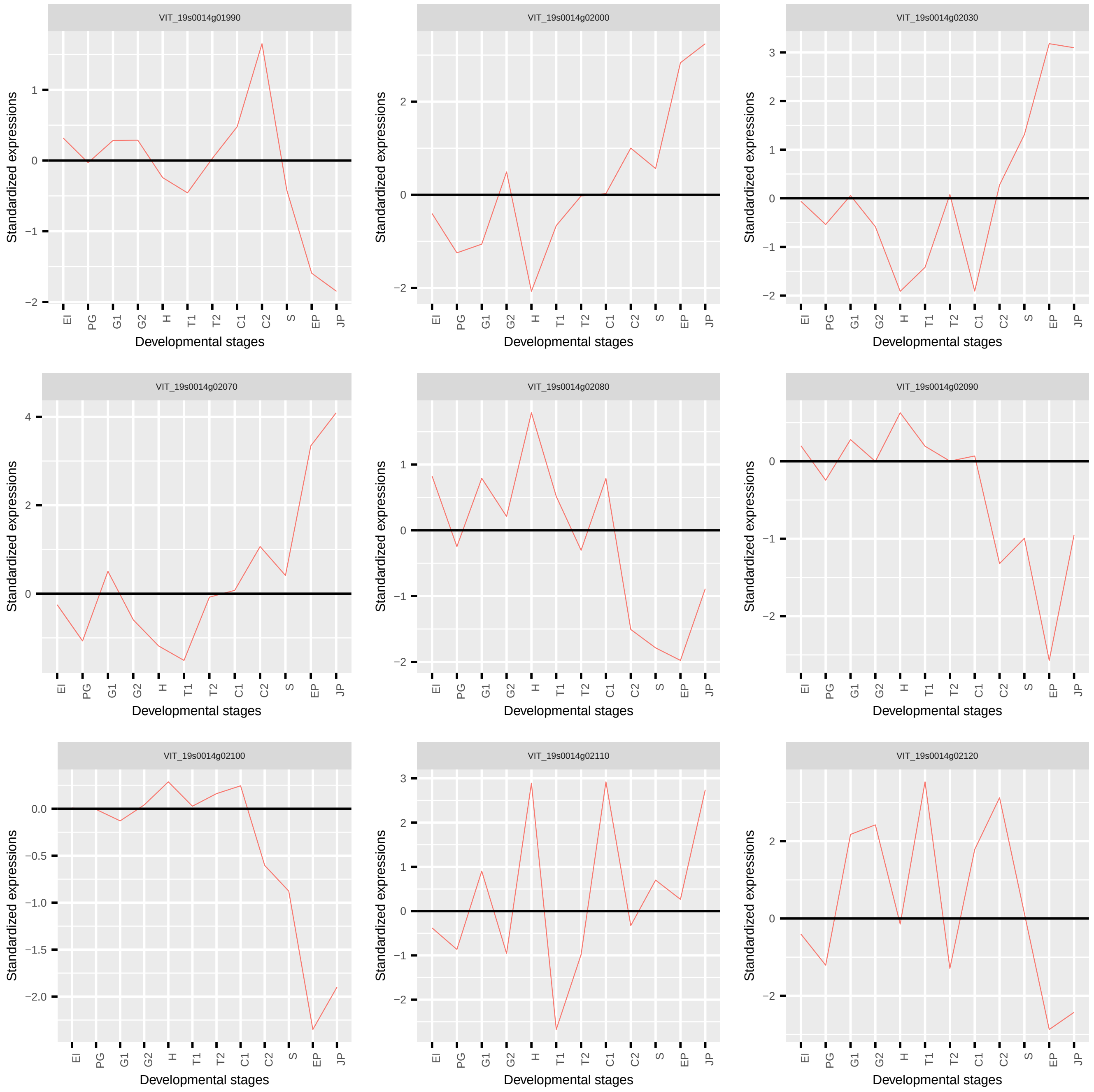

VIT_19s0014g01990
VIT_19s0014g02000
VIT_19s0014g02030
VIT_19s0014g02070
VIT_19s0014g02080
VIT_19s0014g02090
VIT_19s0014g02100
VIT_19s0014g02110
VIT_19s0014g02120
Standardized expressions
Developmental stages
EI PG G1 G2 H T1 T2 C1 C2 S EP JP

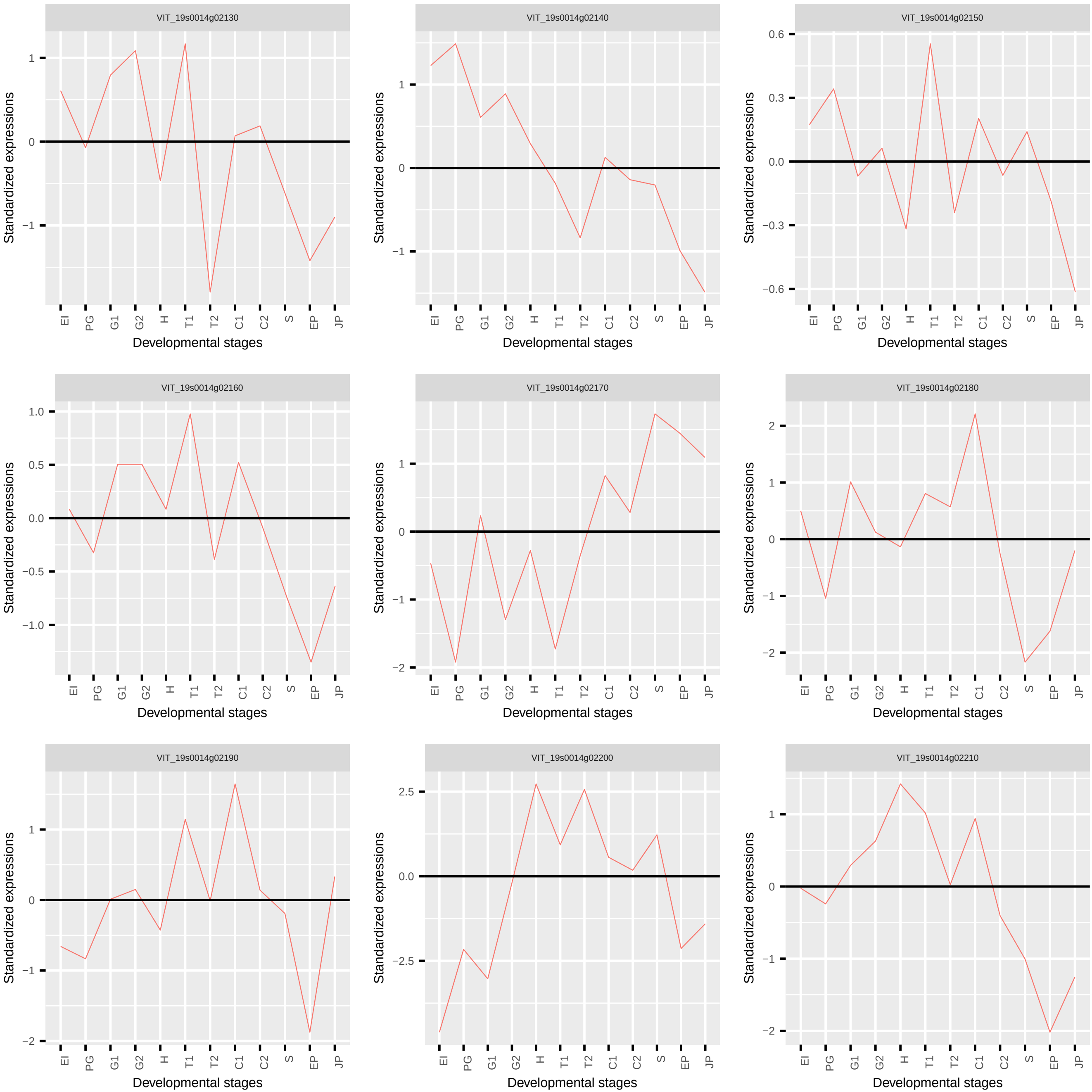
VIT_19s0014g02130
VIT_19s0014g02140
VIT_19s0014g02150
VIT_19s0014g02160
VIT_19s0014g02170
VIT_19s0014g02180
VIT_19s0014g02190
VIT_19s0014g02200
VIT_19s0014g02210
Standardized expressions
Developmental stages
EI PG G1 G2 H T1 T2 C1 C2 S EP JP

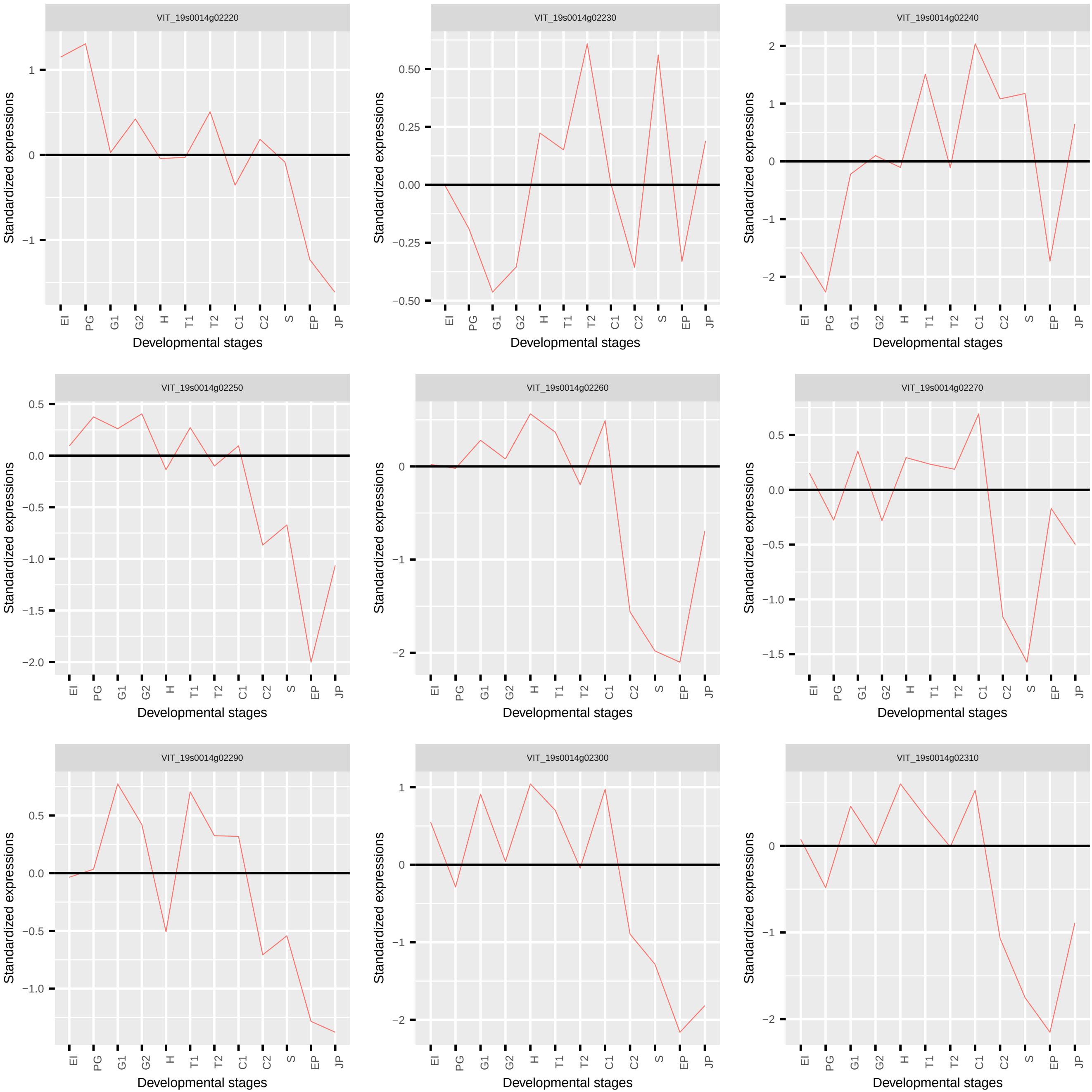
VIT_19s0014g02220
VIT_19s0014g02230
VIT_19s0014g02240
VIT_19s0014g02250
VIT_19s0014g02260
VIT_19s0014g02270
VIT_19s0014g02290
VIT_19s0014g02300
VIT_19s0014g02310
Standardized expressions
Developmental stages
EI
PG
G1
G2
H
T1
T2
C1
C2
S
EP
JP

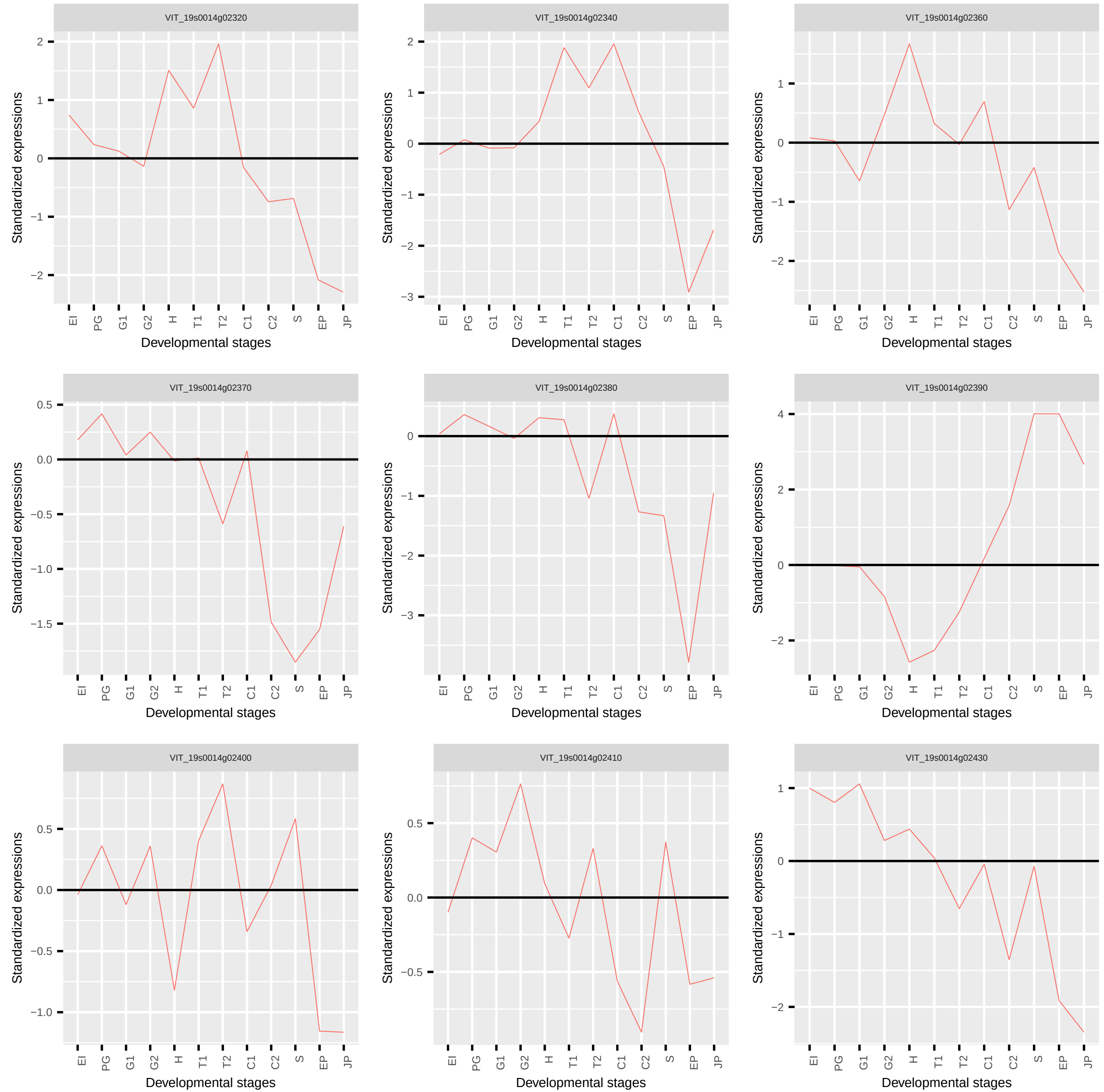

VIT_19s0014g02320
VIT_19s0014g02340
VIT_19s0014g02360
VIT_19s0014g02370
VIT_19s0014g02380
VIT_19s0014g02390
VIT_19s0014g02400
VIT_19s0014g02410
VIT_19s0014g02430
Standardized expressions
Developmental stages
EI PG G1 G2 H T1 T2 C1 C2 S EP JP

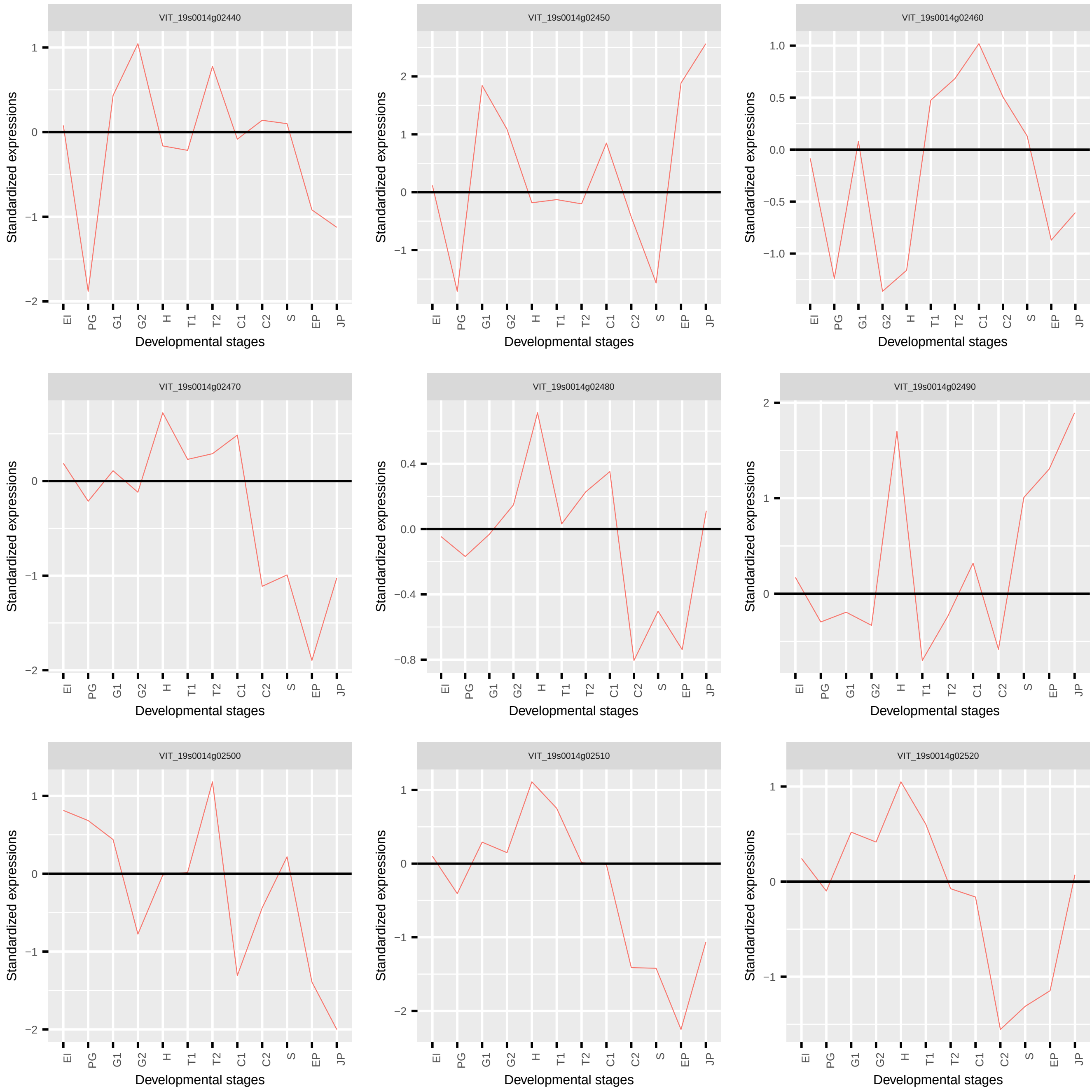
VIT_19s0014g02440
VIT_19s0014g02450
VIT_19s0014g02460
VIT_19s0014g02470
VIT_19s0014g02480
VIT_19s0014g02490
VIT_19s0014g02500
VIT_19s0014g02510
VIT_19s0014g02520
Standardized expressions
Developmental stages
EI PG G1 G2 H T1 T2 C1 C2 S EP JP

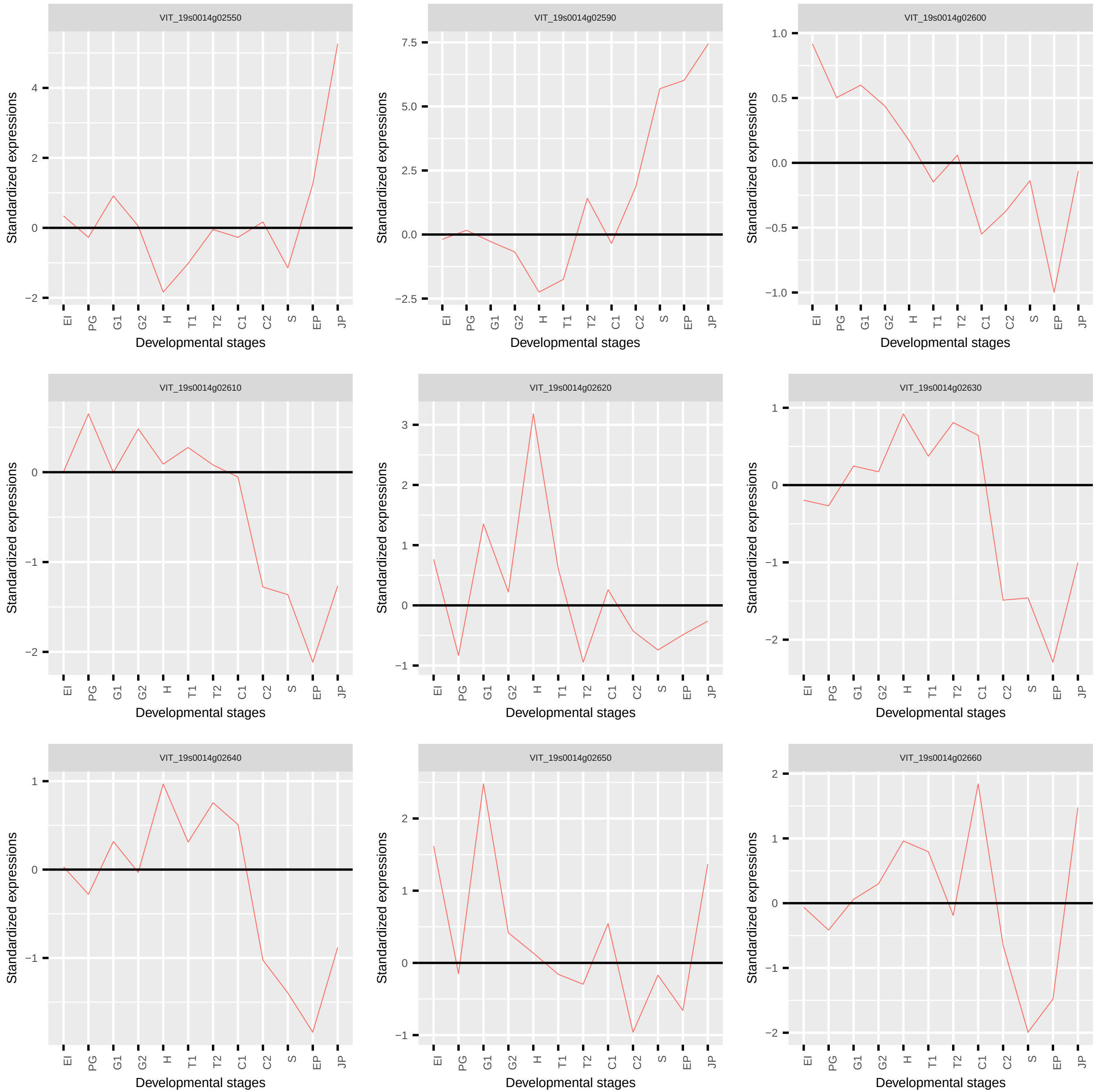
VIT_19s0014g02550
VIT_19s0014g02590
VIT_19s0014g02600
VIT_19s0014g02610
VIT_19s0014g02620
VIT_19s0014g02630
VIT_19s0014g02640
VIT_19s0014g02650
VIT_19s0014g02660
Standardized expressions
Developmental stages
EI
PG
G1
G2
H
T1
T2
C1
C2
S
EP
JP

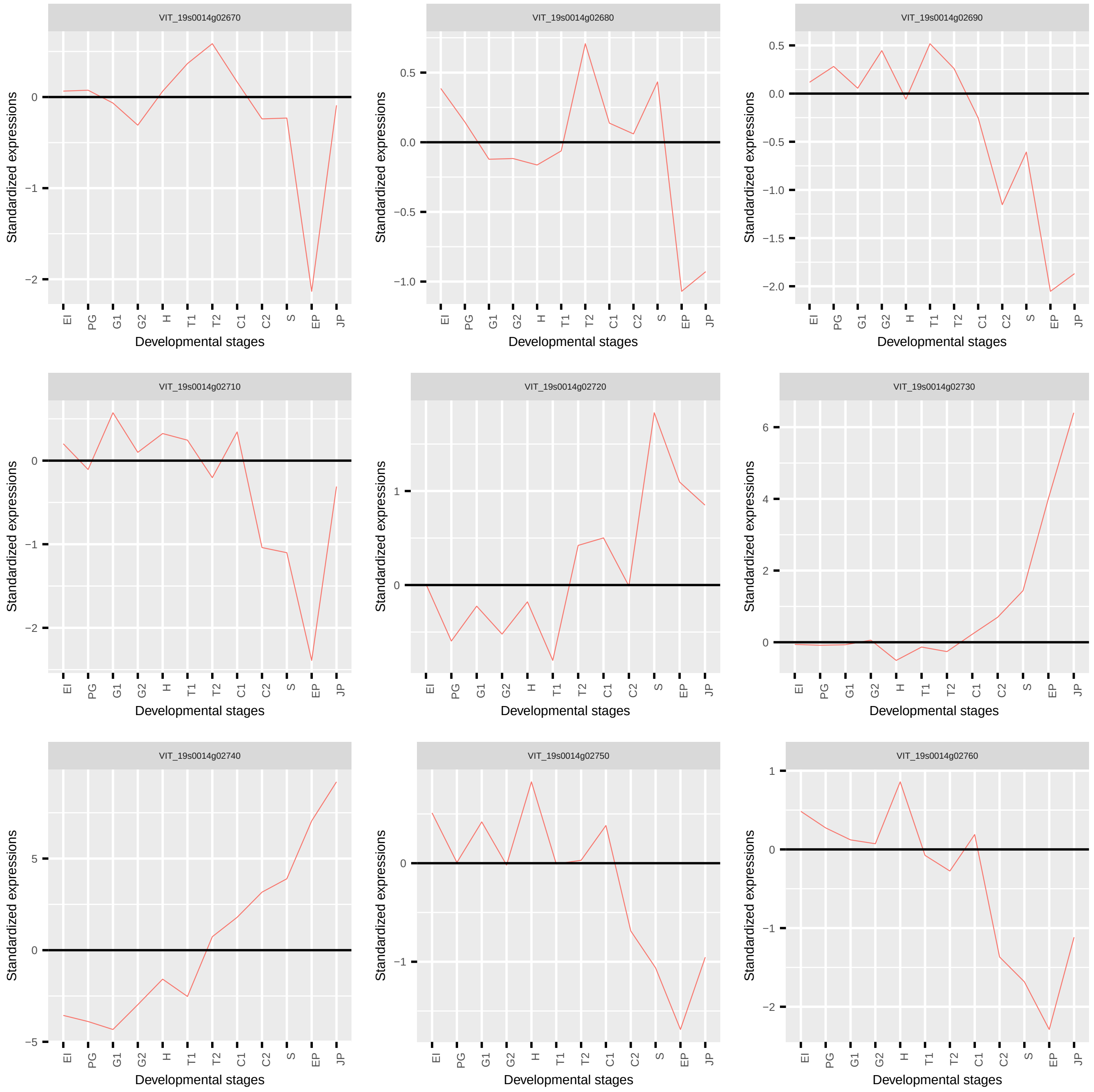

VIT_19s0014g02670
VIT_19s0014g02680
VIT_19s0014g02690
VIT_19s0014g02710
VIT_19s0014g02720
VIT_19s0014g02730
VIT_19s0014g02740
VIT_19s0014g02750
VIT_19s0014g02760
Standardized expressions
Developmental stages
EI PG G1 G2 H T1 T2 C1 C2 S EP JP

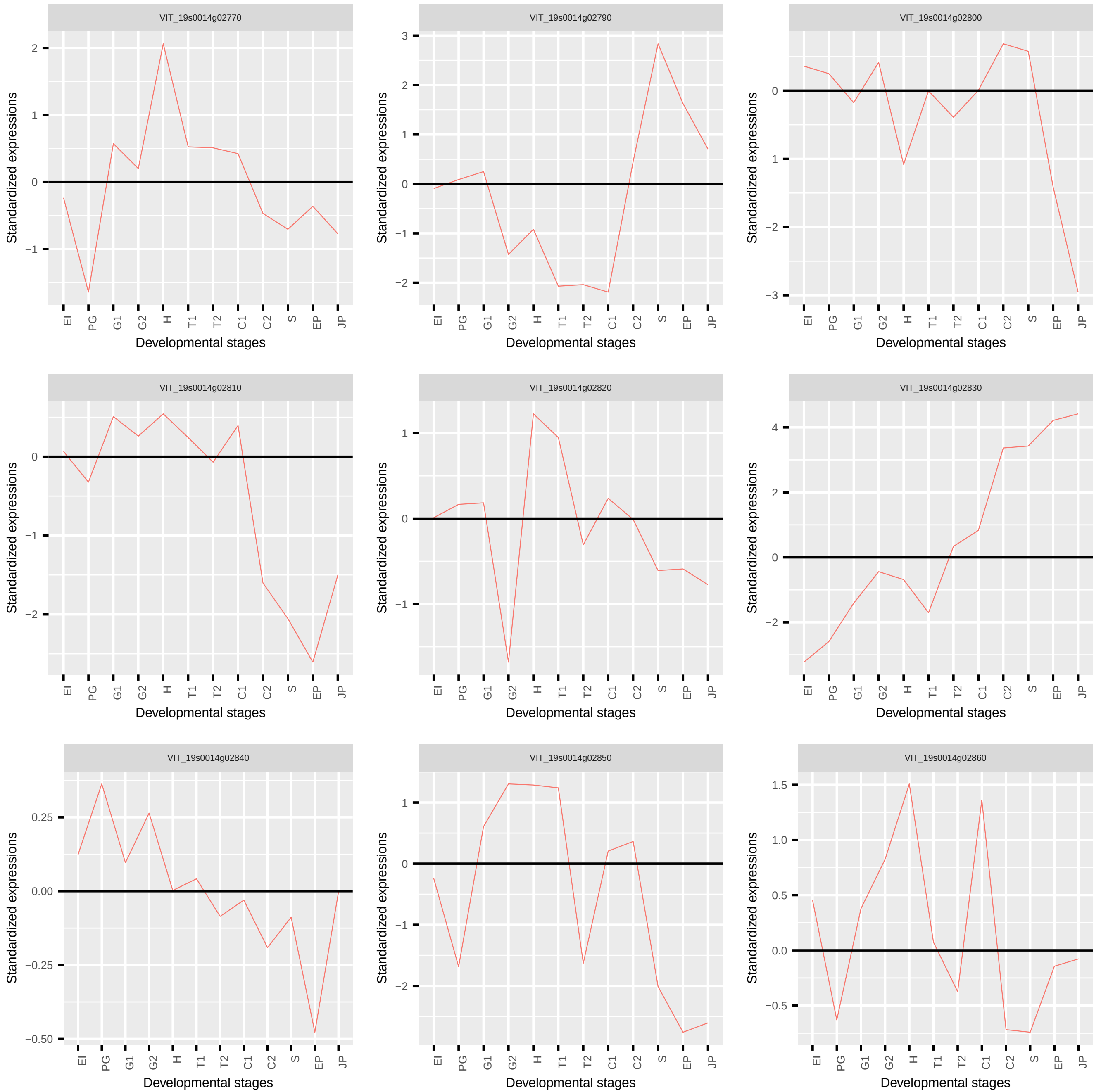
VIT_19s0014g02770
VIT_19s0014g02790
VIT_19s0014g02800
VIT_19s0014g02810
VIT_19s0014g02820
VIT_19s0014g02830
VIT_19s0014g02840
VIT_19s0014g02850
VIT_19s0014g02860
Standardized expressions
Developmental stages
EI PG G1 G2 H T1 T2 C1 C2 S EP JP

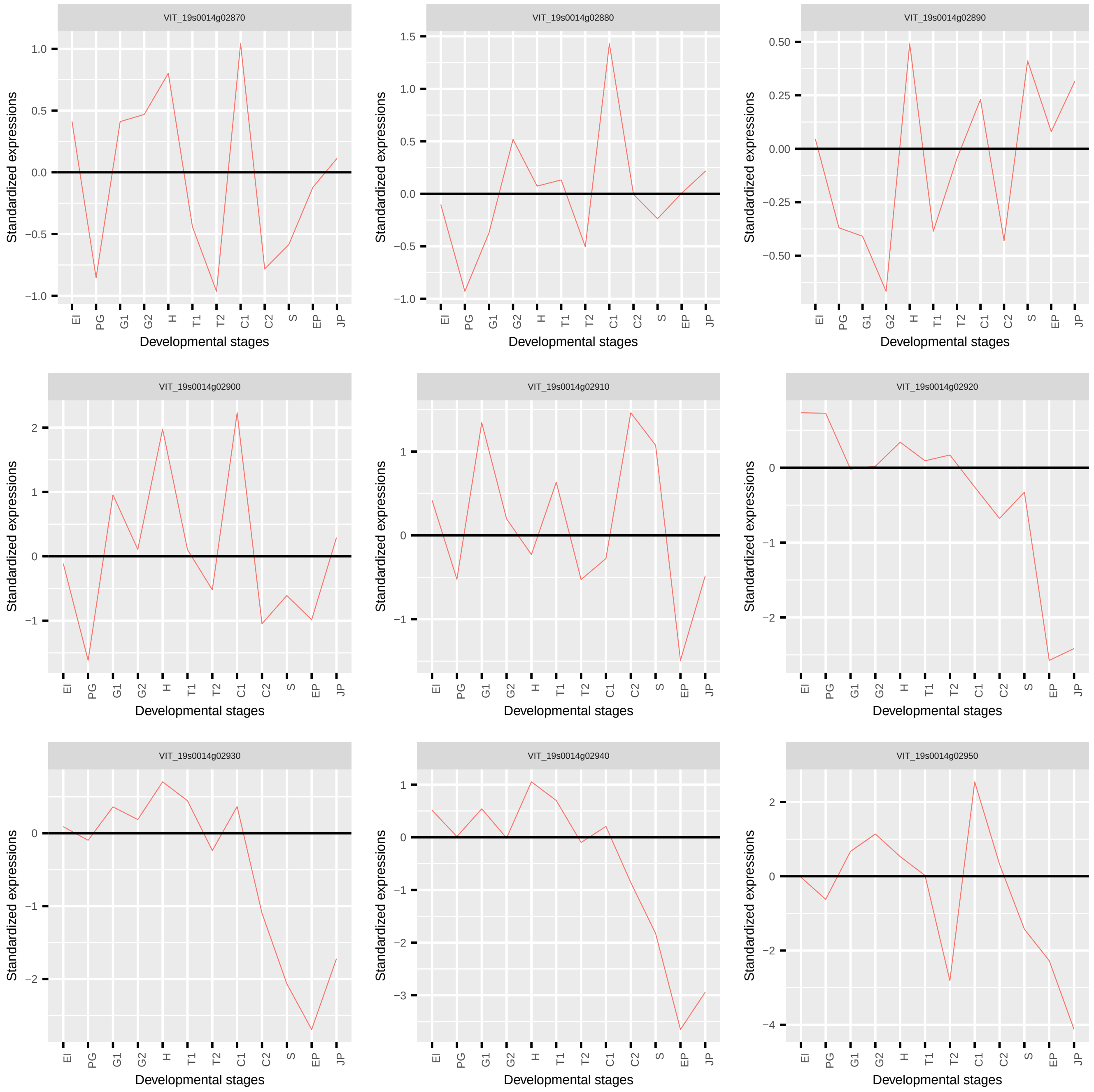
VIT_19s0014g02870
VIT_19s0014g02880
VIT_19s0014g02890
VIT_19s0014g02900
VIT_19s0014g02910
VIT_19s0014g02920
VIT_19s0014g02930
VIT_19s0014g02940
VIT_19s0014g02950
Standardized expressions
Developmental stages
EI PG G1 G2 H T1 T2 C1 C2 S EP JP

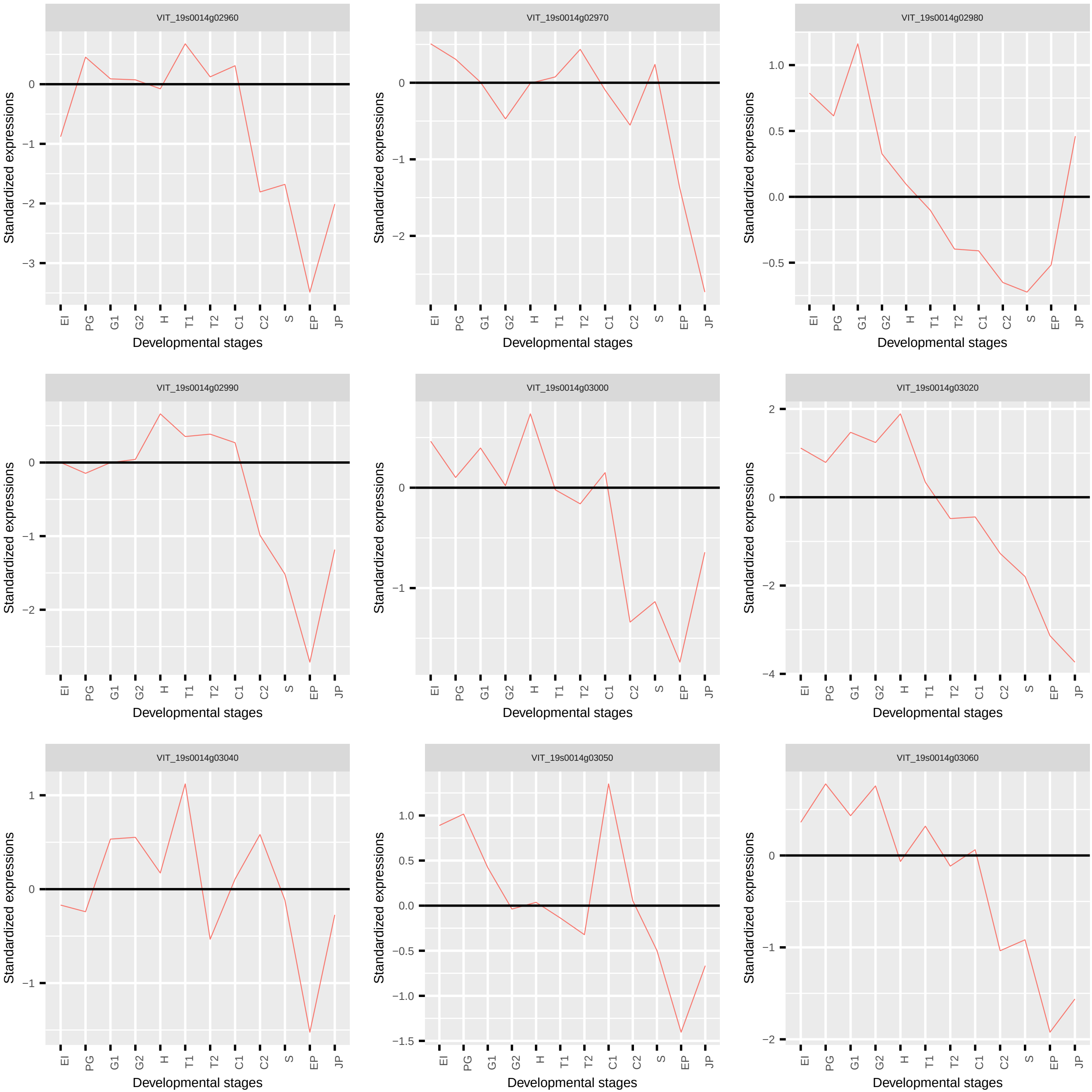
VIT_19s0014g02960
VIT_19s0014g02970
VIT_19s0014g02980
VIT_19s0014g02990
VIT_19s0014g03000
VIT_19s0014g03020
VIT_19s0014g03040
VIT_19s0014g03050
VIT_19s0014g03060
Standardized expressions
Developmental stages
EI PG G1 G2 H T1 T2 C1 C2 S EP JP

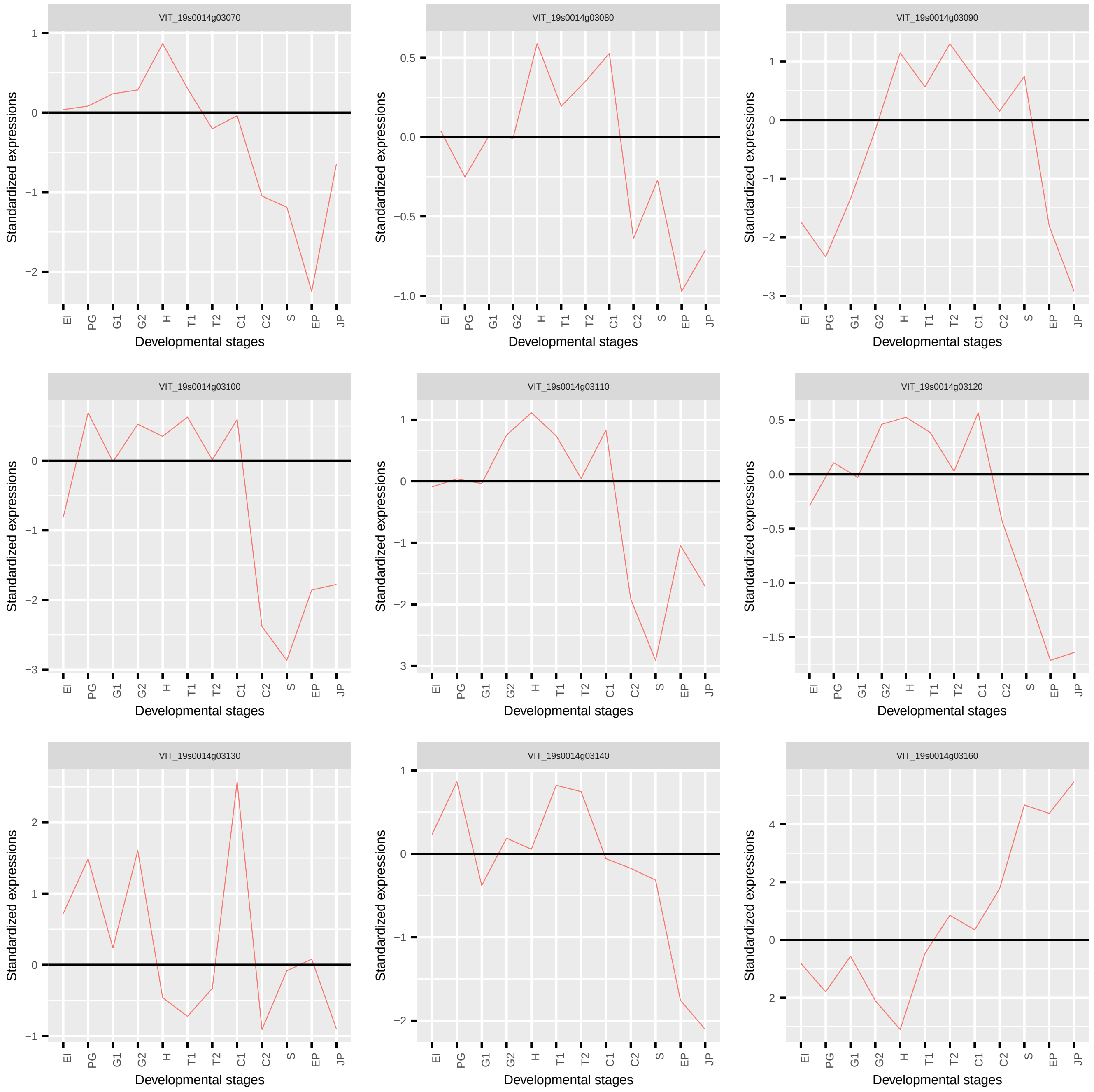
VIT_19s0014g03070
VIT_19s0014g03080
VIT_19s0014g03090
VIT_19s0014g03100
VIT_19s0014g03110
VIT_19s0014g03120
VIT_19s0014g03130
VIT_19s0014g03140
VIT_19s0014g03160
Standardized expressions
Developmental stages
EI
PG
G1
G2
H
T1
T2
C1
C2
S
EP
JP

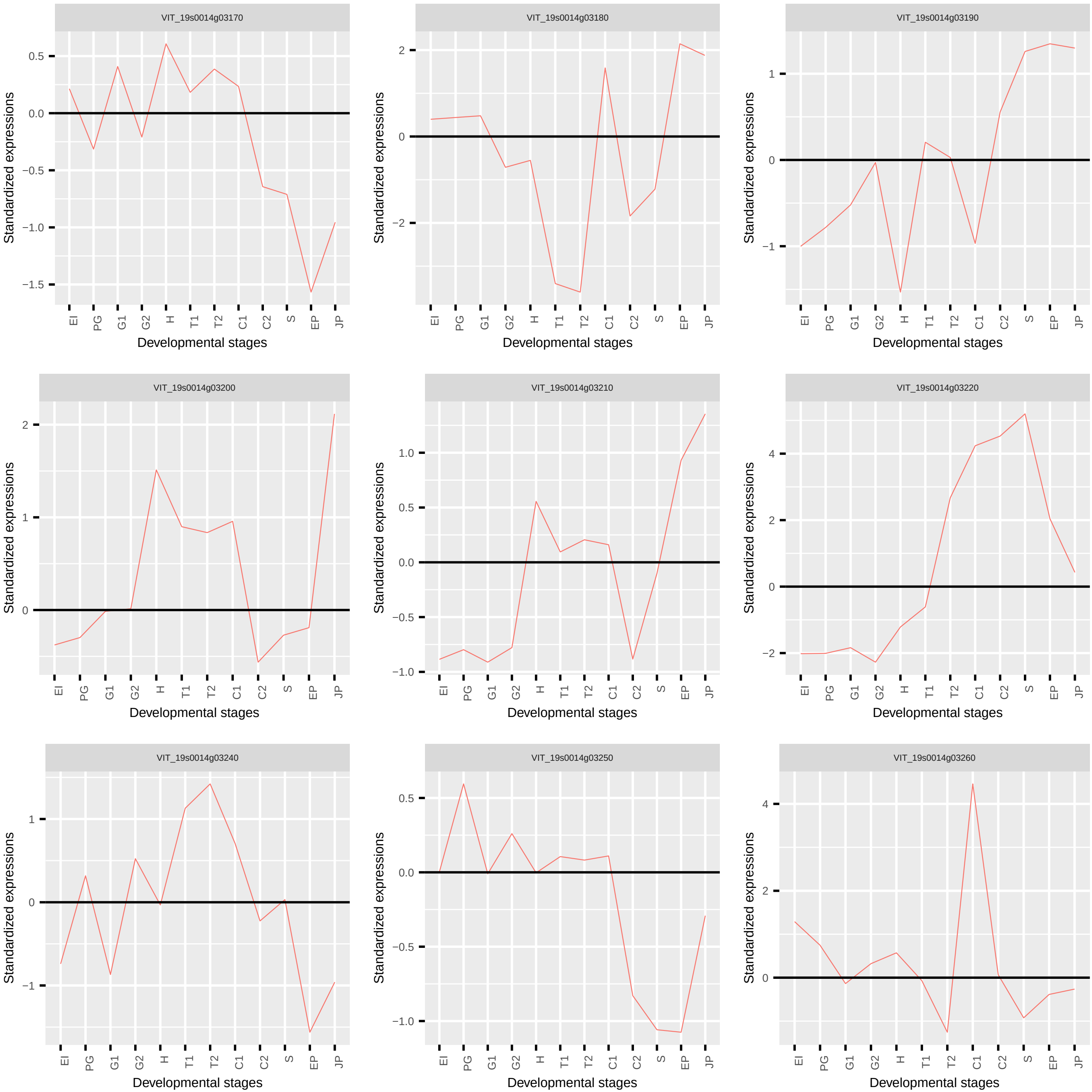
VIT_19s0014g03170
VIT_19s0014g03180
VIT_19s0014g03190
VIT_19s0014g03200
VIT_19s0014g03210
VIT_19s0014g03220
VIT_19s0014g03240
VIT_19s0014g03250
VIT_19s0014g03260
Standardized expressions
Developmental stages
EI PG G1 G2 H T1 T2 C1 C2 S EP JP
0.5 0.0 −0.5 −1.0 −1.5
2 0 −2
1 0 −1
2 1 0
1.0 0.5 0.0 −0.5 −1.0
4 2 0 −2
1 0 −1
0.5 0.0 −0.5 −1.0
4 2 0

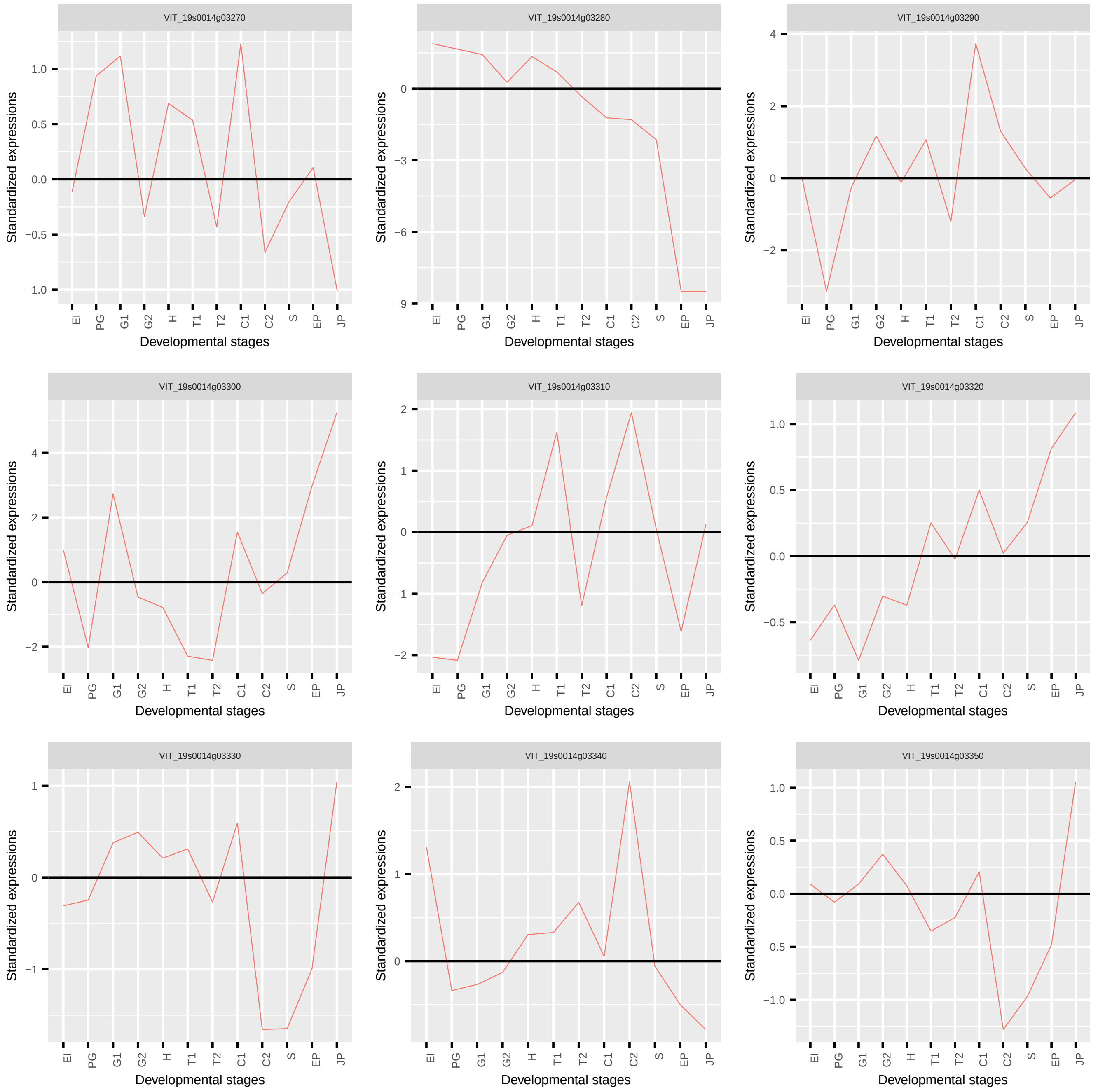
VIT_19s0014g03270
Standardized expressions
1.0
0.5
0.0
−0.5
−1.0
EI PG G1 G2 H T1 T2 C1 C2 S EP JP
Developmental stages
VIT_19s0014g03280
Standardized expressions
0
−3
−6
−9
EI PG G1 G2 H T1 T2 C1 C2 S EP JP
Developmental stages
VIT_19s0014g03290
Standardized expressions
4
2
0
−2
EI PG G1 G2 H T1 T2 C1 C2 S EP JP
Developmental stages
VIT_19s0014g03300
Standardized expressions
4
2
0
−2
EI PG G1 G2 H T1 T2 C1 C2 S EP JP
Developmental stages
VIT_19s0014g03310
Standardized expressions
2
1
0
−1
−2
EI PG G1 G2 H T1 T2 C1 C2 S EP JP
Developmental stages
VIT_19s0014g03320
Standardized expressions
1.0
0.5
0.0
−0.5
EI PG G1 G2 H T1 T2 C1 C2 S EP JP
Developmental stages
VIT_19s0014g03330
Standardized expressions
1
0
−1
EI PG G1 G2 H T1 T2 C1 C2 S EP JP
Developmental stages
VIT_19s0014g03340
Standardized expressions
2
1
0
EI PG G1 G2 H T1 T2 C1 C2 S EP JP
Developmental stages
VIT_19s0014g03350
Standardized expressions
1.0
0.5
0.0
−0.5
−1.0
EI PG G1 G2 H T1 T2 C1 C2 S EP JP
Developmental stages

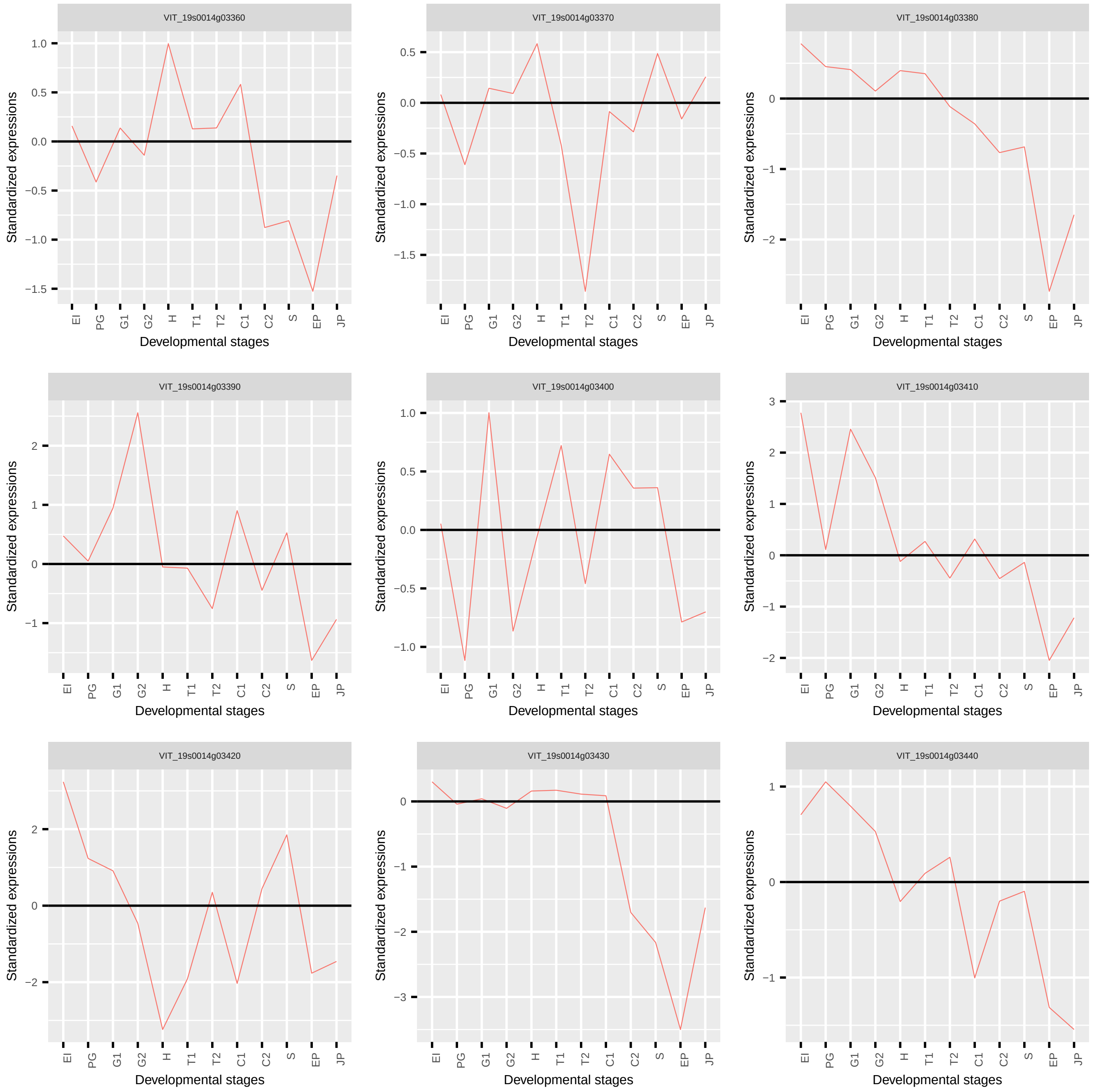
VIT_19s0014g03360
VIT_19s0014g03370
VIT_19s0014g03380
VIT_19s0014g03390
VIT_19s0014g03400
VIT_19s0014g03410
VIT_19s0014g03420
VIT_19s0014g03430
VIT_19s0014g03440
Standardized expressions
Developmental stages
EI PG G1 G2 H T1 T2 C1 C2 S EP JP

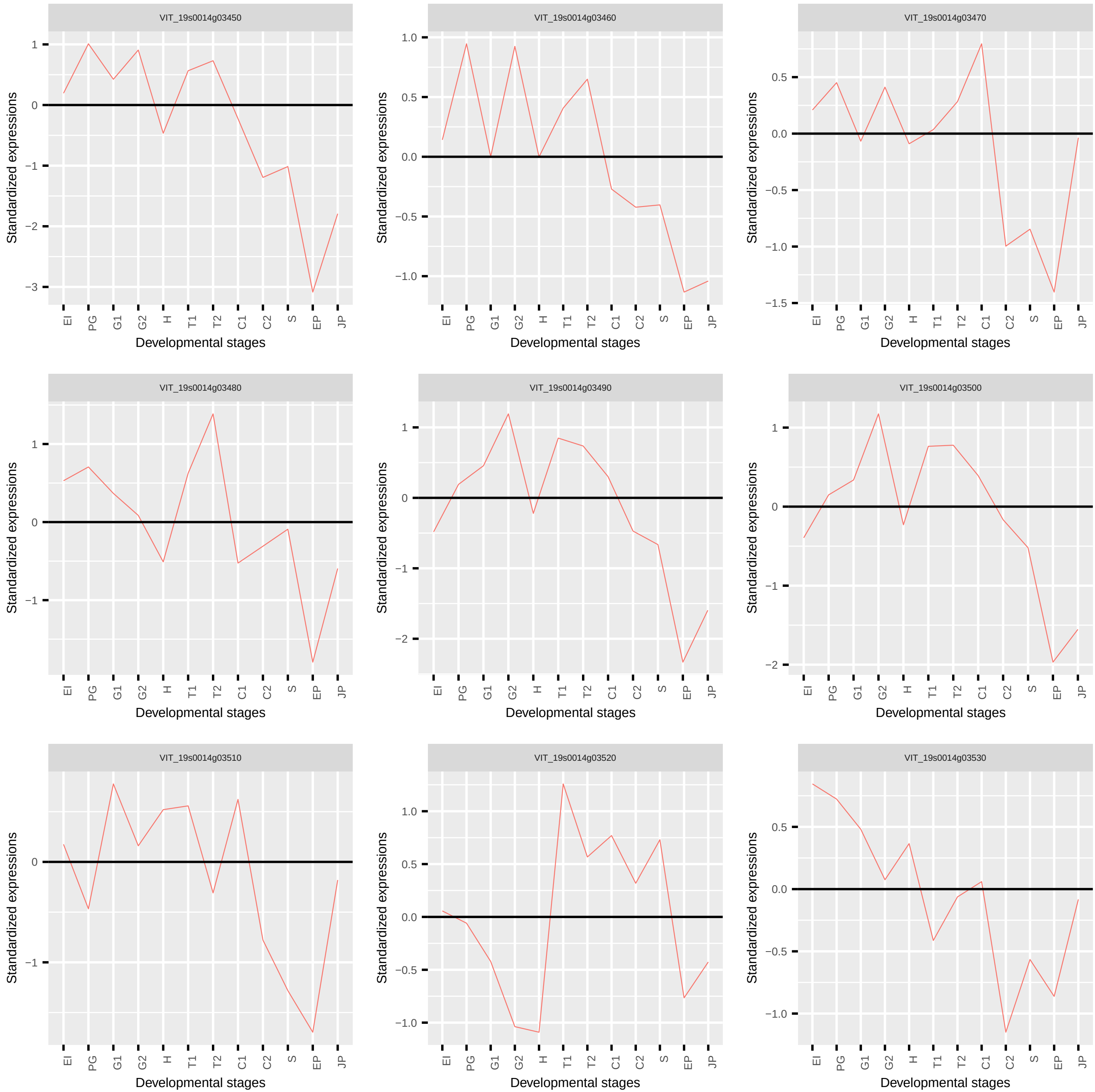

VIT_19s0014g03450
VIT_19s0014g03460
VIT_19s0014g03470
VIT_19s0014g03480
VIT_19s0014g03490
VIT_19s0014g03500
VIT_19s0014g03510
VIT_19s0014g03520
VIT_19s0014g03530
Standardized expressions
Developmental stages
EI PG G1 G2 H T1 T2 C1 C2 S EP JP

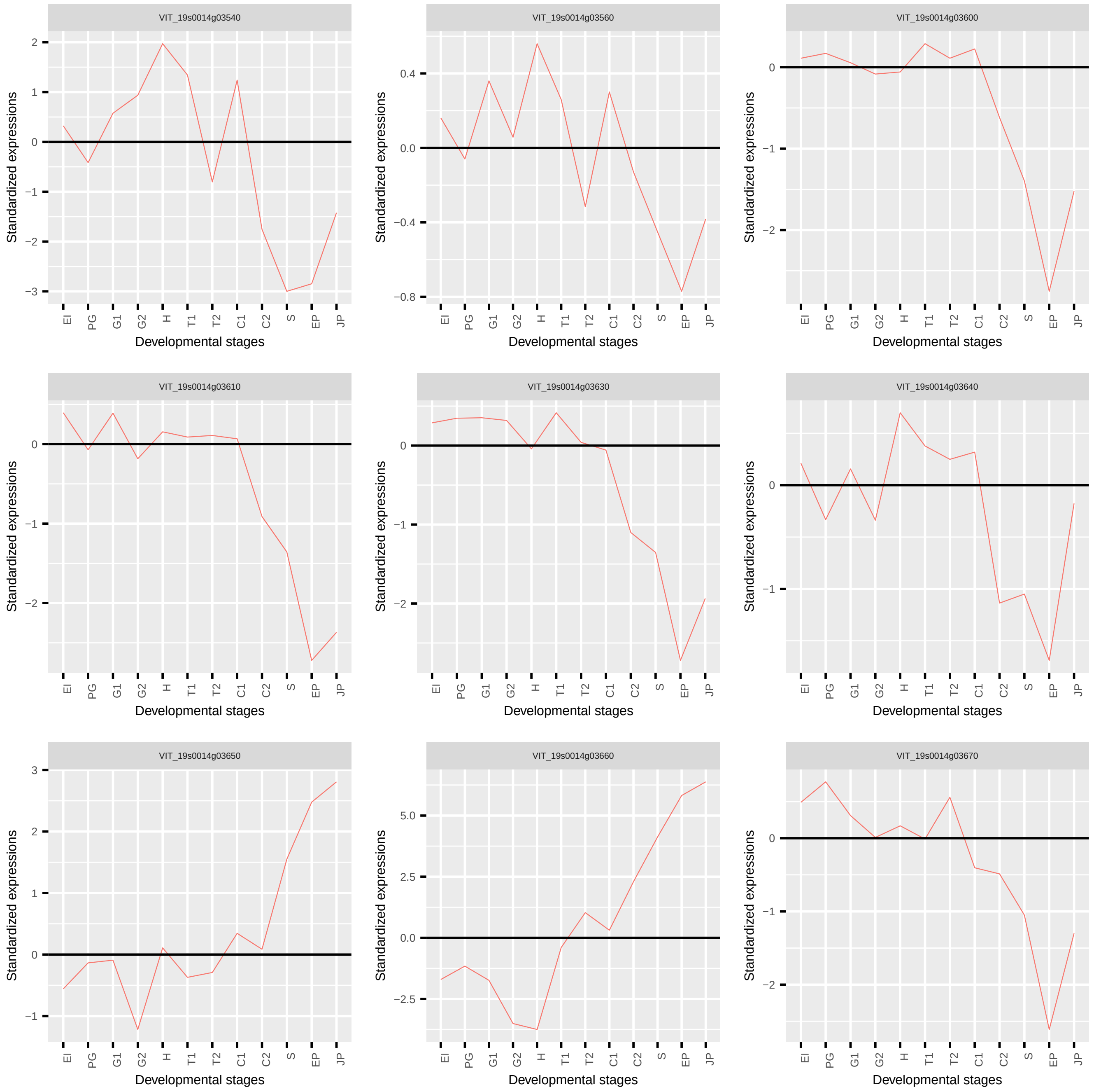

VIT_19s0014g03540
VIT_19s0014g03560
VIT_19s0014g03600
VIT_19s0014g03610
VIT_19s0014g03630
VIT_19s0014g03640
VIT_19s0014g03650
VIT_19s0014g03660
VIT_19s0014g03670
Standardized expressions
Developmental stages
EI PG G1 G2 H T1 T2 C1 C2 S EP JP

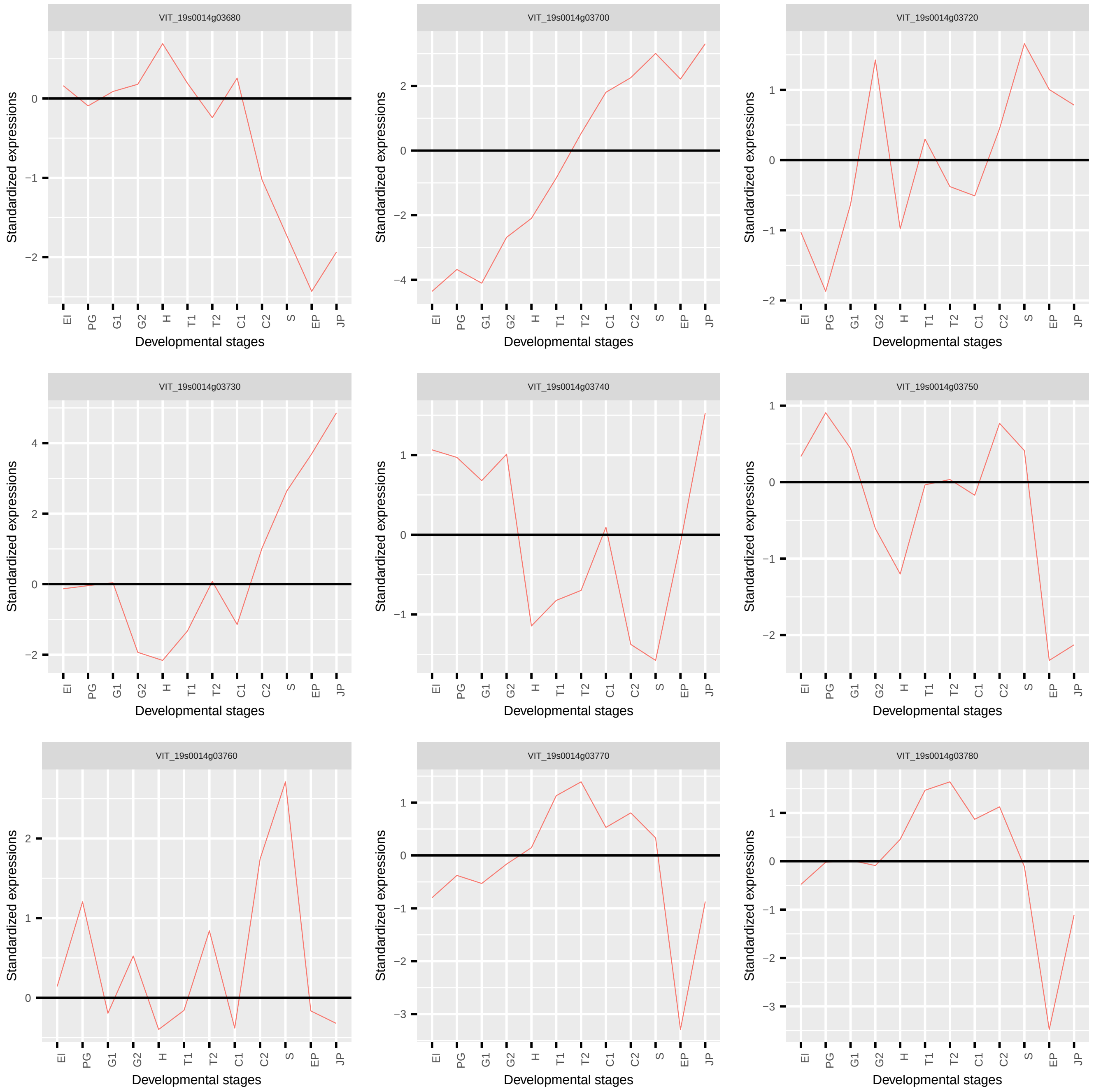

VIT_19s0014g03680
VIT_19s0014g03700
VIT_19s0014g03720
VIT_19s0014g03730
VIT_19s0014g03740
VIT_19s0014g03750
VIT_19s0014g03760
VIT_19s0014g03770
VIT_19s0014g03780
Standardized expressions
Developmental stages
EI
PG
G1
G2
H
T1
T2
C1
C2
S
EP
JP

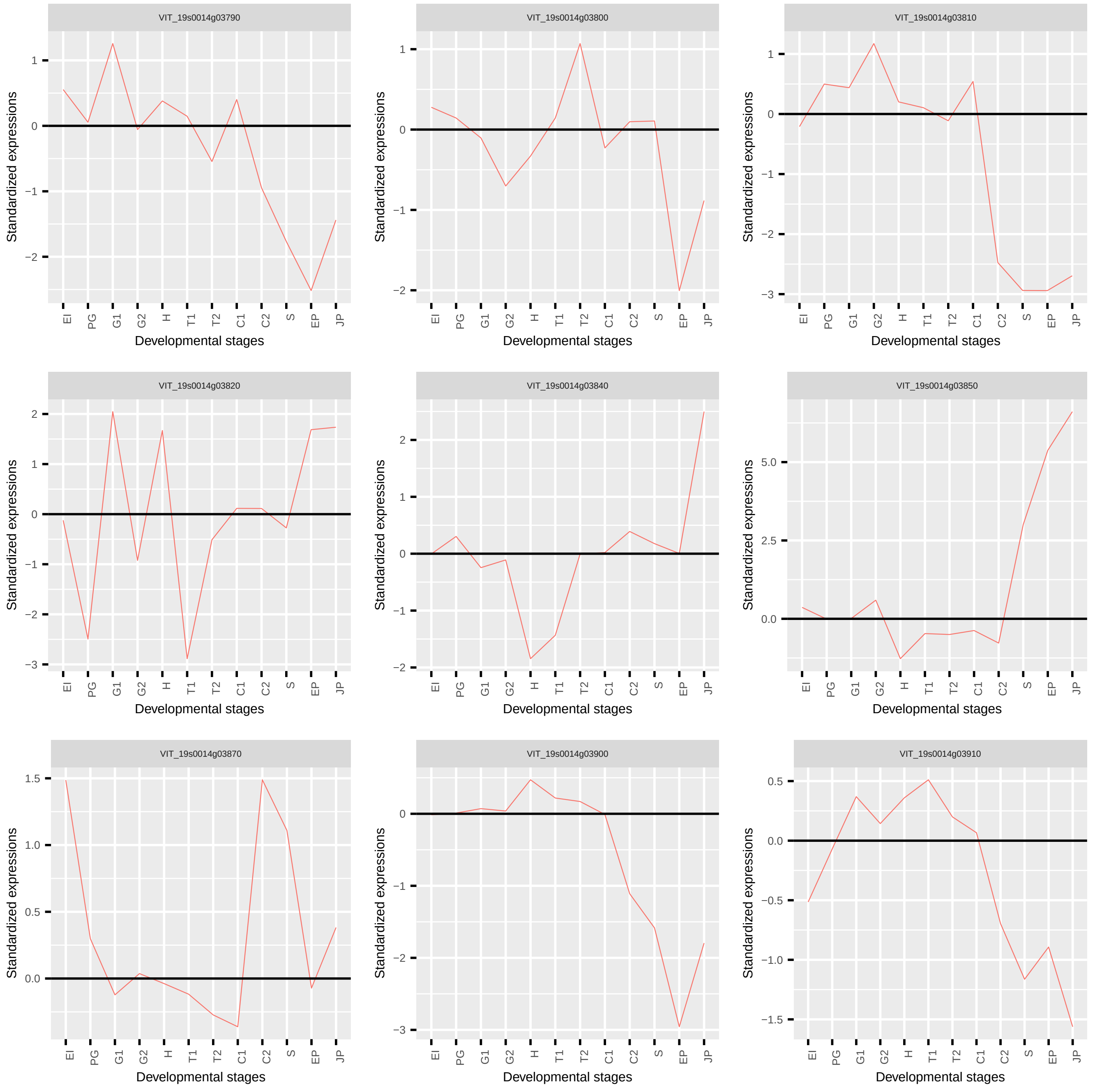

VIT_19s0014g03790
VIT_19s0014g03800
VIT_19s0014g03810
VIT_19s0014g03820
VIT_19s0014g03840
VIT_19s0014g03850
VIT_19s0014g03870
VIT_19s0014g03900
VIT_19s0014g03910
Standardized expressions
Developmental stages
EI
PG
G1
G2
H
T1
T2
C1
C2
S
EP
JP

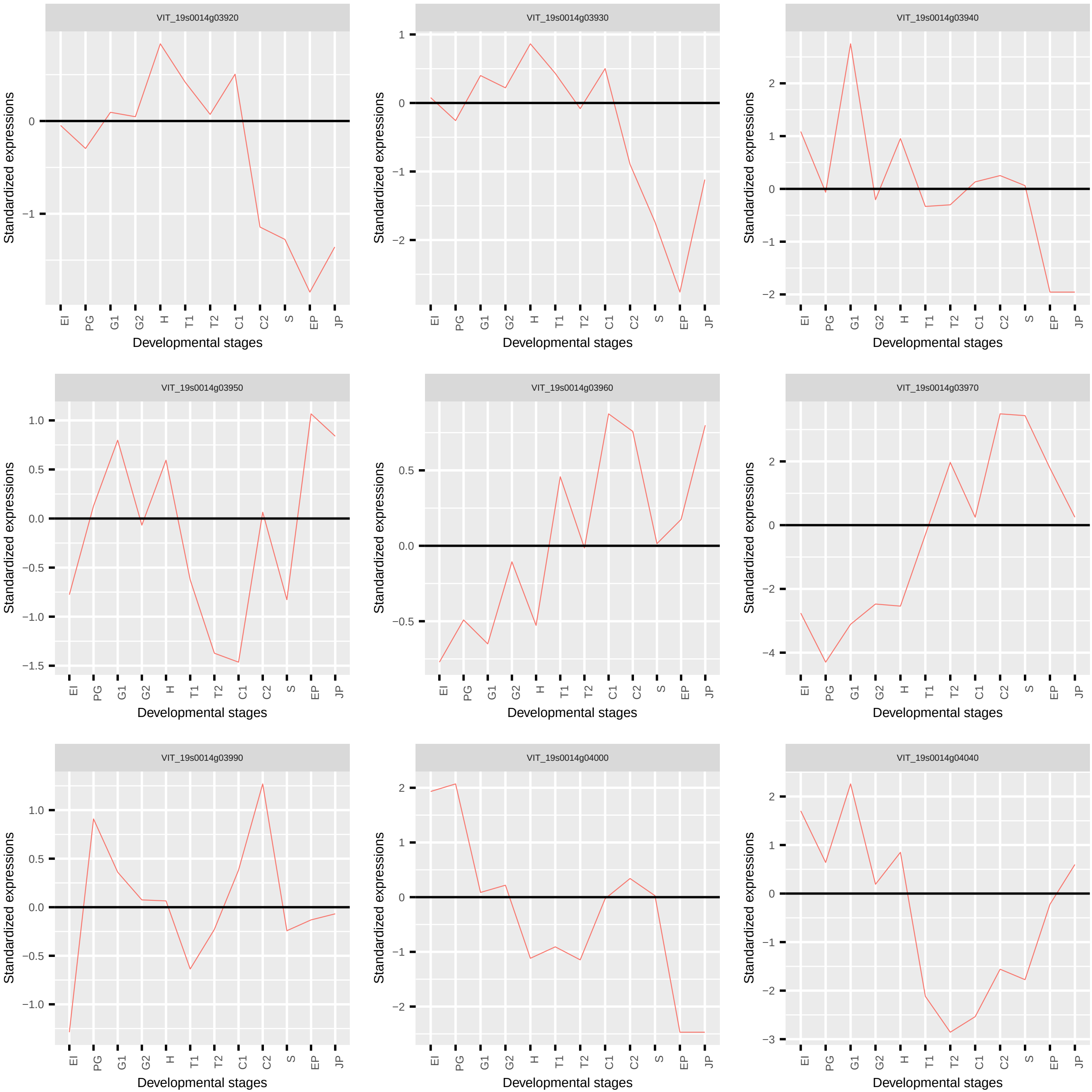
VIT_19s0014g03920
VIT_19s0014g03930
VIT_19s0014g03940
VIT_19s0014g03950
VIT_19s0014g03960
VIT_19s0014g03970
VIT_19s0014g03990
VIT_19s0014g04000
VIT_19s0014g04040
Standardized expressions
Developmental stages
EI PG G1 G2 H T1 T2 C1 C2 S EP JP

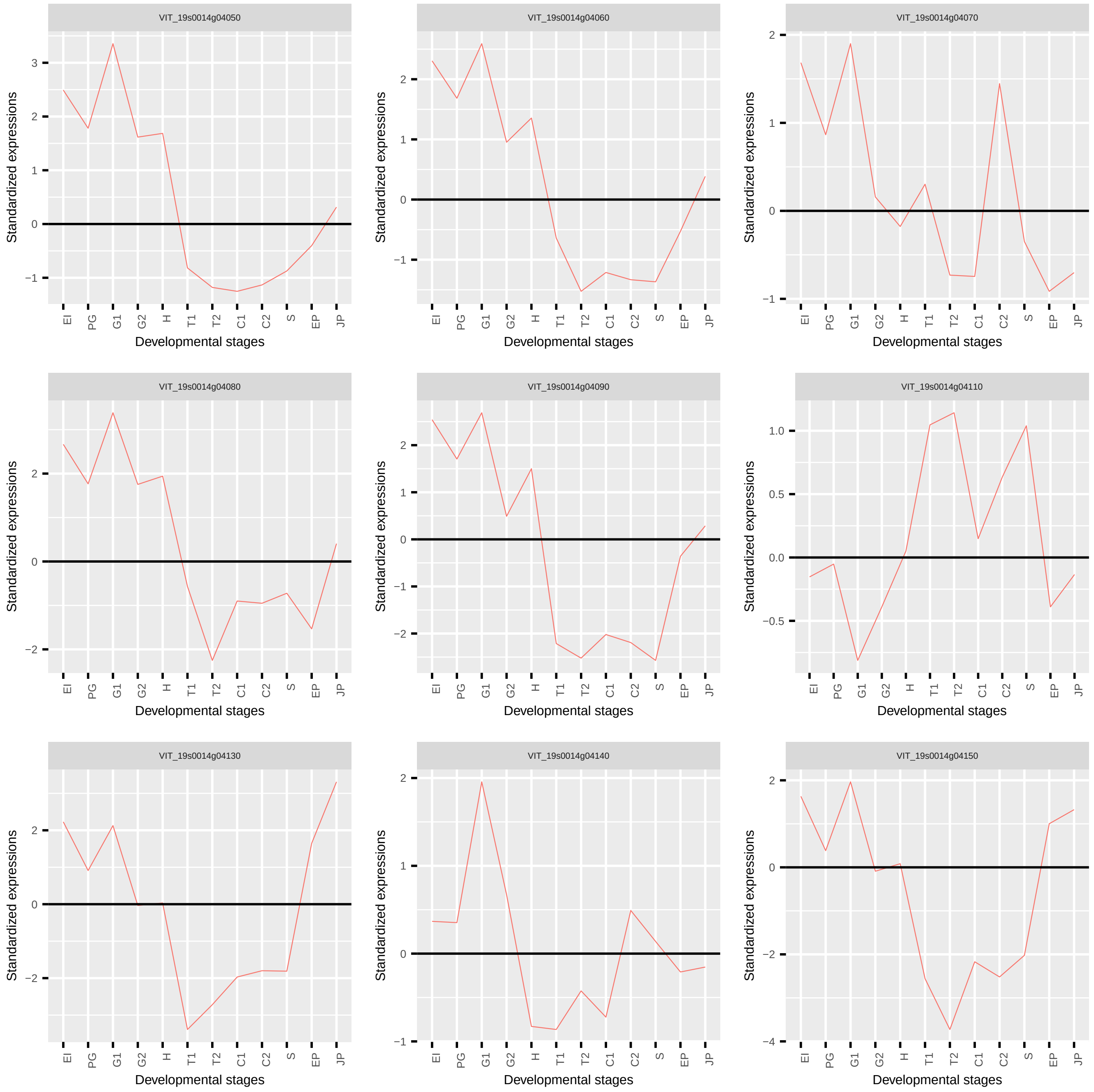
VIT_19s0014g04050
VIT_19s0014g04060
VIT_19s0014g04070
VIT_19s0014g04080
VIT_19s0014g04090
VIT_19s0014g04110
VIT_19s0014g04130
VIT_19s0014g04140
VIT_19s0014g04150
Standardized expressions
Developmental stages
EI
PG
G1
G2
H
T1
T2
C1
C2
S
EP
JP

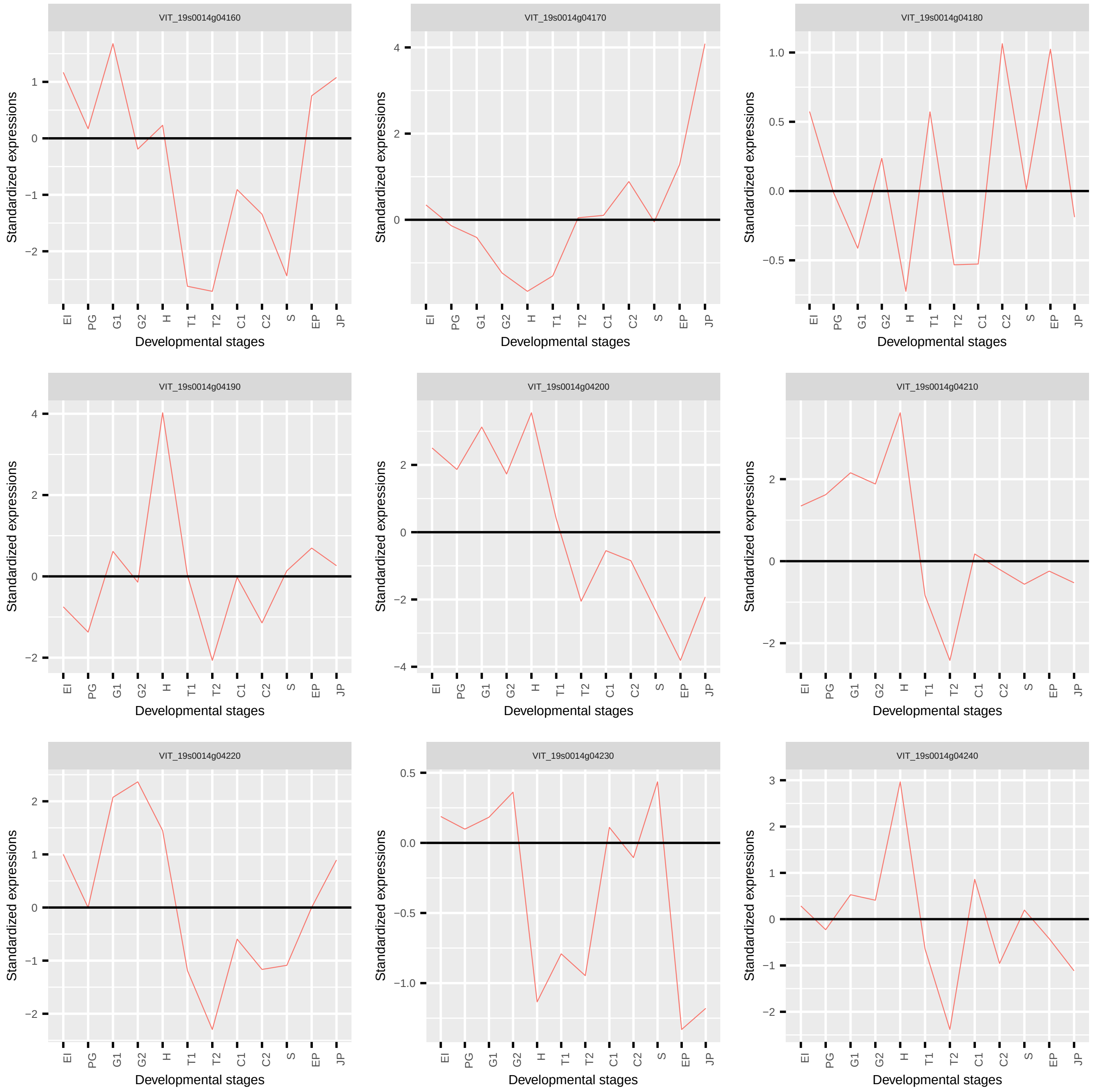

VIT_19s0014g04160
VIT_19s0014g04170
VIT_19s0014g04180
VIT_19s0014g04190
VIT_19s0014g04200
VIT_19s0014g04210
VIT_19s0014g04220
VIT_19s0014g04230
VIT_19s0014g04240
Standardized expressions
Developmental stages
EI PG G1 G2 H T1 T2 C1 C2 S EP JP

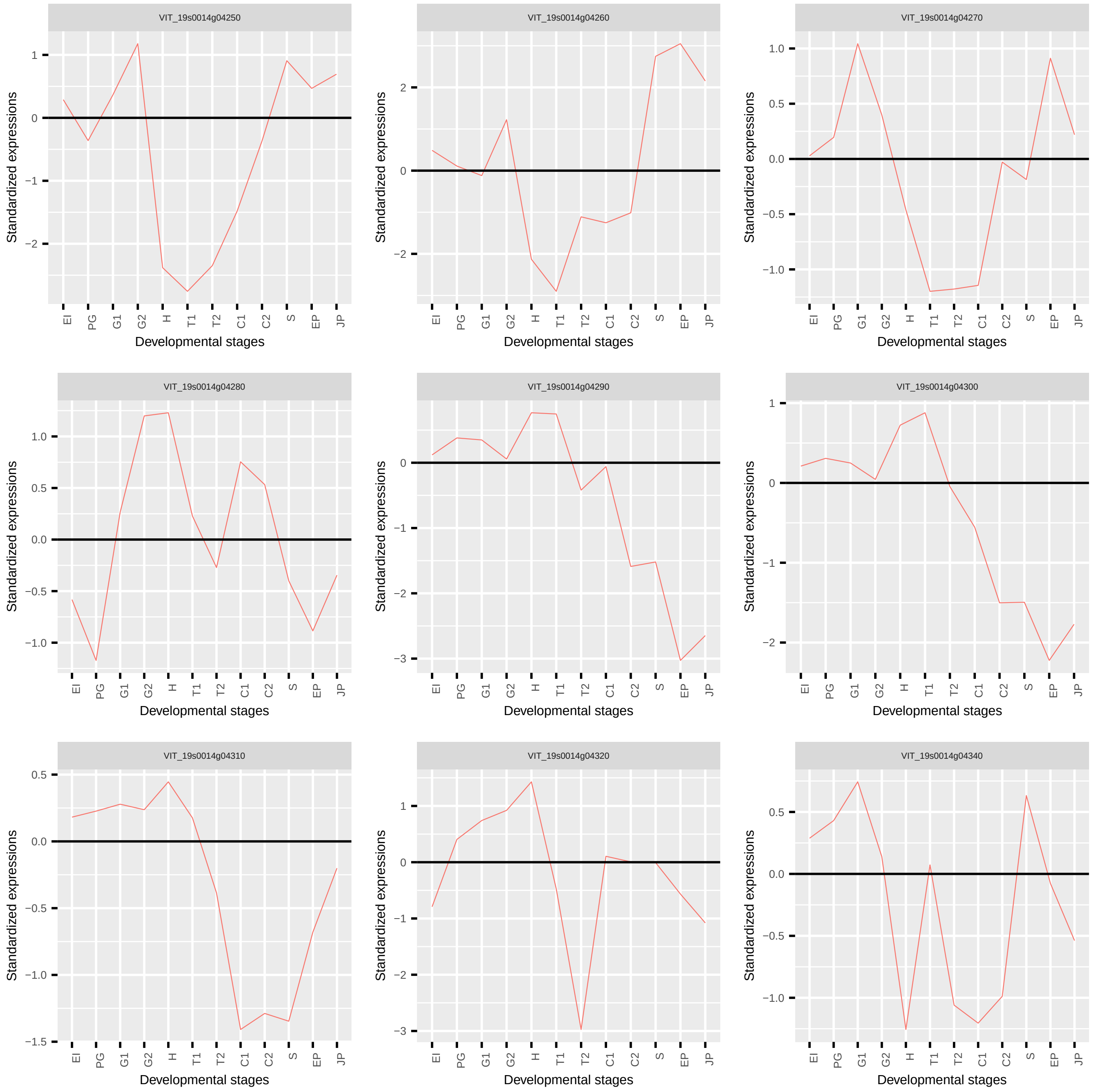

VIT_19s0014g04250
VIT_19s0014g04260
VIT_19s0014g04270
VIT_19s0014g04280
VIT_19s0014g04290
VIT_19s0014g04300
VIT_19s0014g04310
VIT_19s0014g04320
VIT_19s0014g04340
Standardized expressions
Developmental stages
EI PG G1 G2 H T1 T2 C1 C2 S EP JP

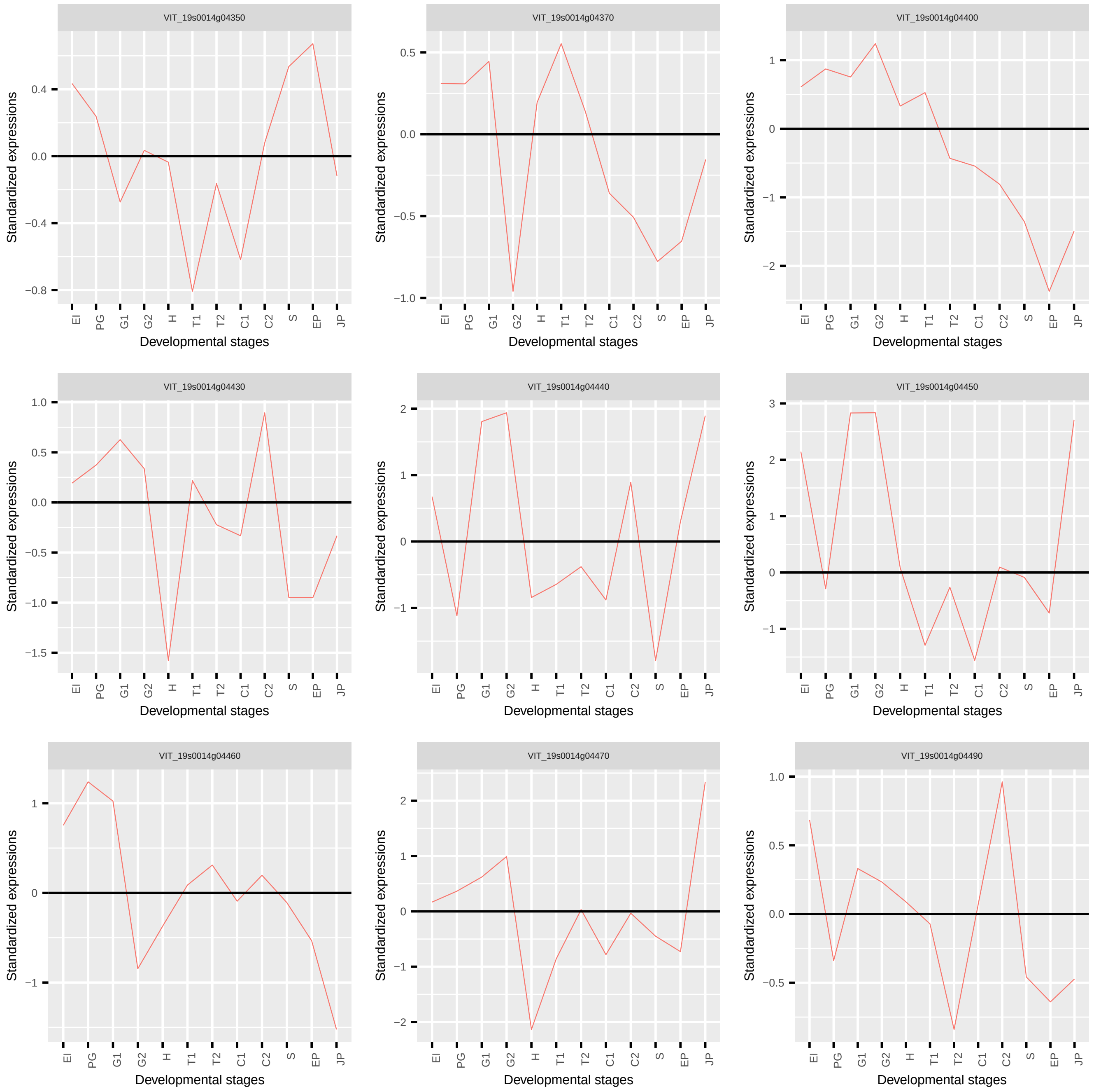
VIT_19s0014g04350
VIT_19s0014g04370
VIT_19s0014g04400
VIT_19s0014g04430
VIT_19s0014g04440
VIT_19s0014g04450
VIT_19s0014g04460
VIT_19s0014g04470
VIT_19s0014g04490
Standardized expressions
Developmental stages
EI PG G1 G2 H T1 T2 C1 C2 S EP JP

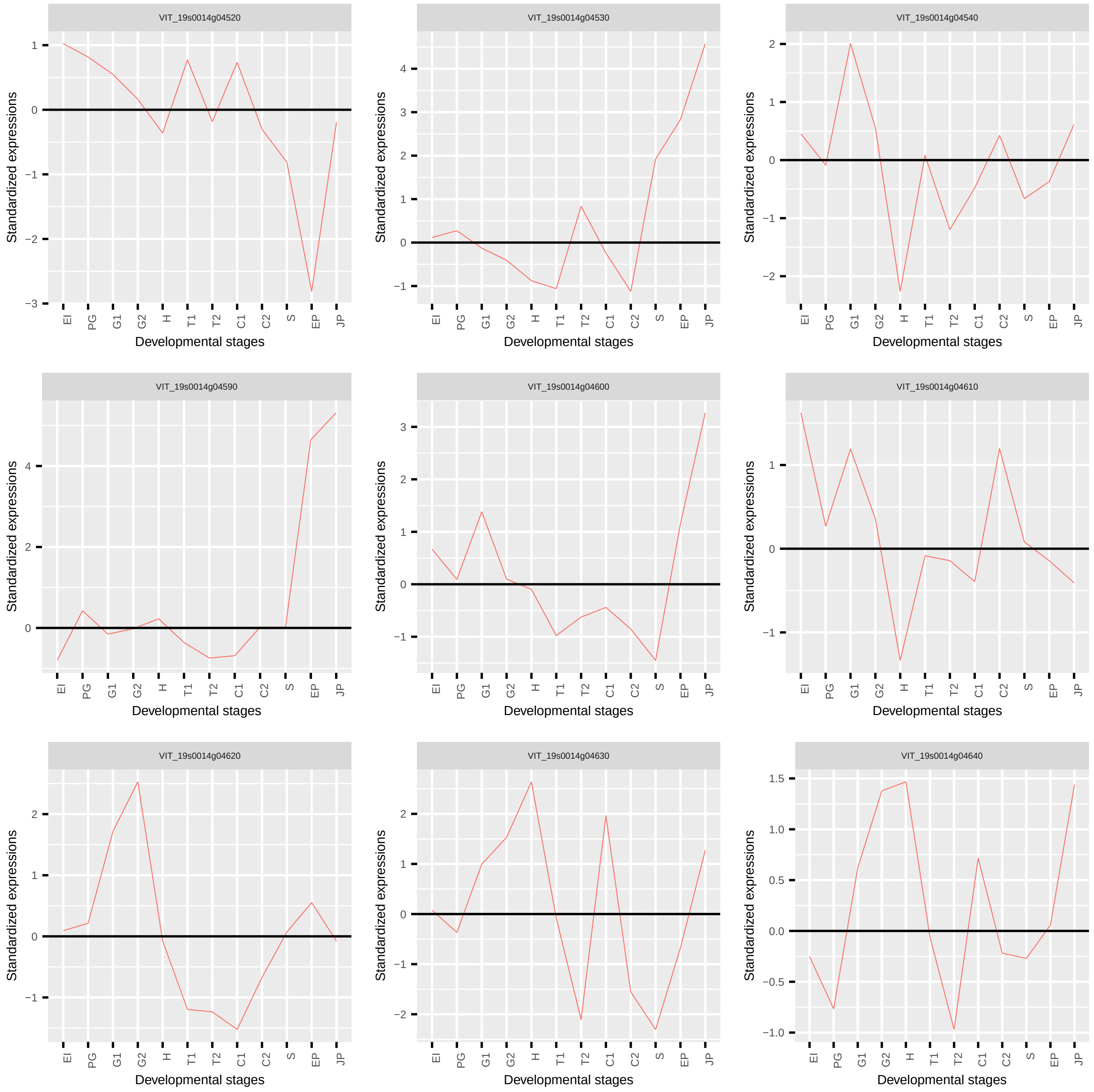
VIT_19s0014g04520
VIT_19s0014g04530
VIT_19s0014g04540
VIT_19s0014g04590
VIT_19s0014g04600
VIT_19s0014g04610
VIT_19s0014g04620
VIT_19s0014g04630
VIT_19s0014g04640
Standardized expressions
Developmental stages
EI PG G1 G2 H T1 T2 C1 C2 S EP JP

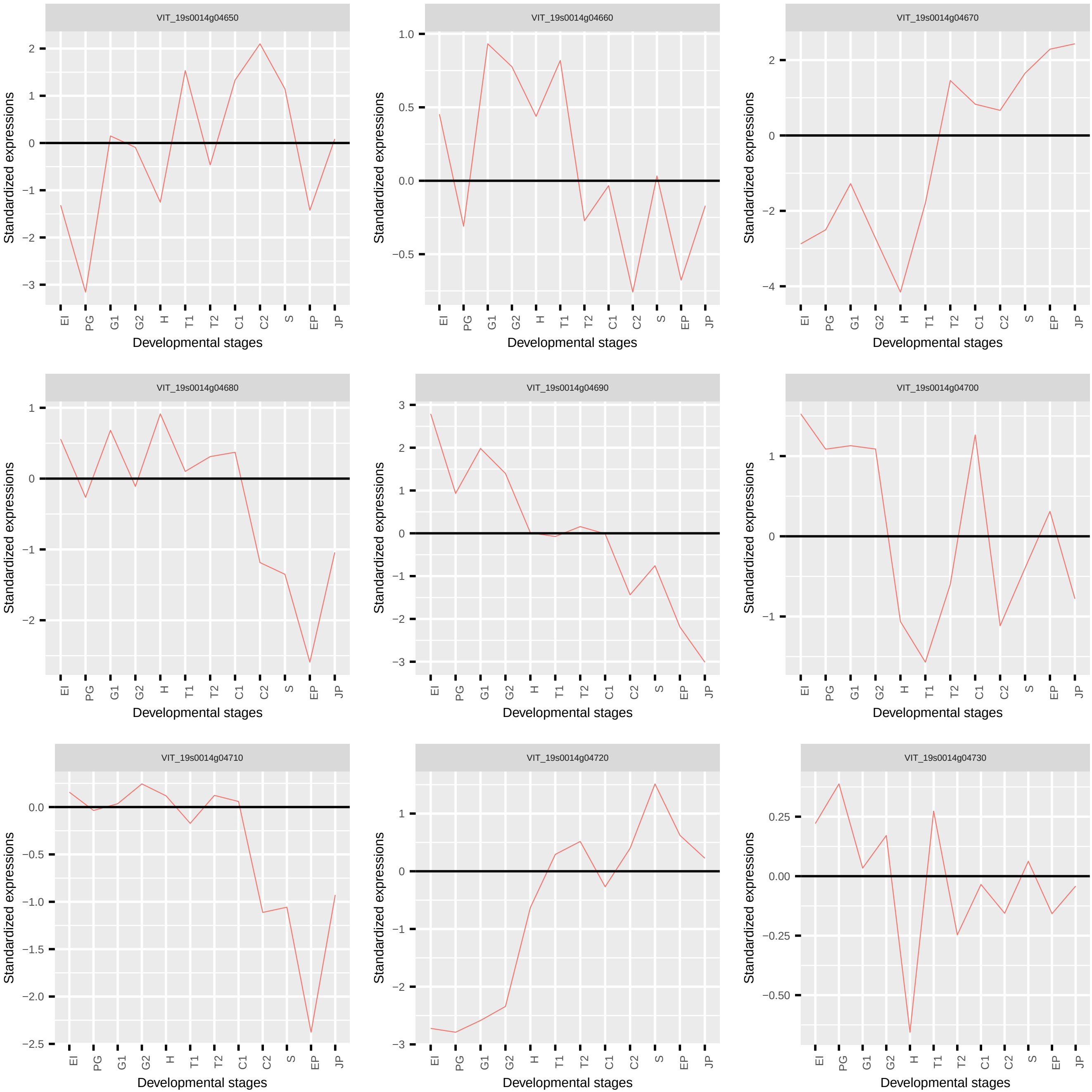
VIT_19s0014g04650
VIT_19s0014g04660
VIT_19s0014g04670
VIT_19s0014g04680
VIT_19s0014g04690
VIT_19s0014g04700
VIT_19s0014g04710
VIT_19s0014g04720
VIT_19s0014g04730
Standardized expressions
Developmental stages
EI PG G1 G2 H T1 T2 C1 C2 S EP JP

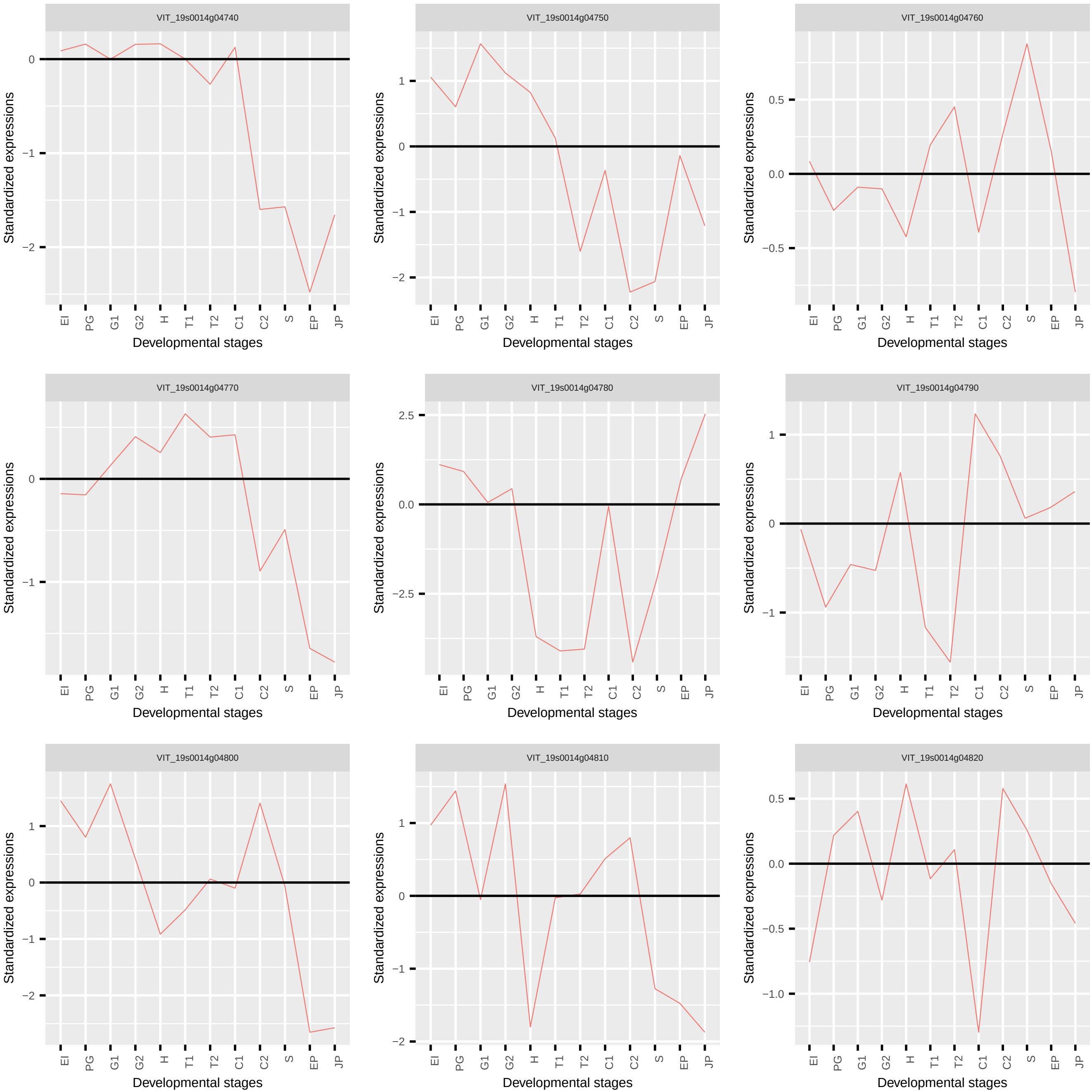
VIT_19s0014g04740
VIT_19s0014g04750
VIT_19s0014g04760
VIT_19s0014g04770
VIT_19s0014g04780
VIT_19s0014g04790
VIT_19s0014g04800
VIT_19s0014g04810
VIT_19s0014g04820
Standardized expressions
Developmental stages
EI PG G1 G2 H T1 T2 C1 C2 S EP JP

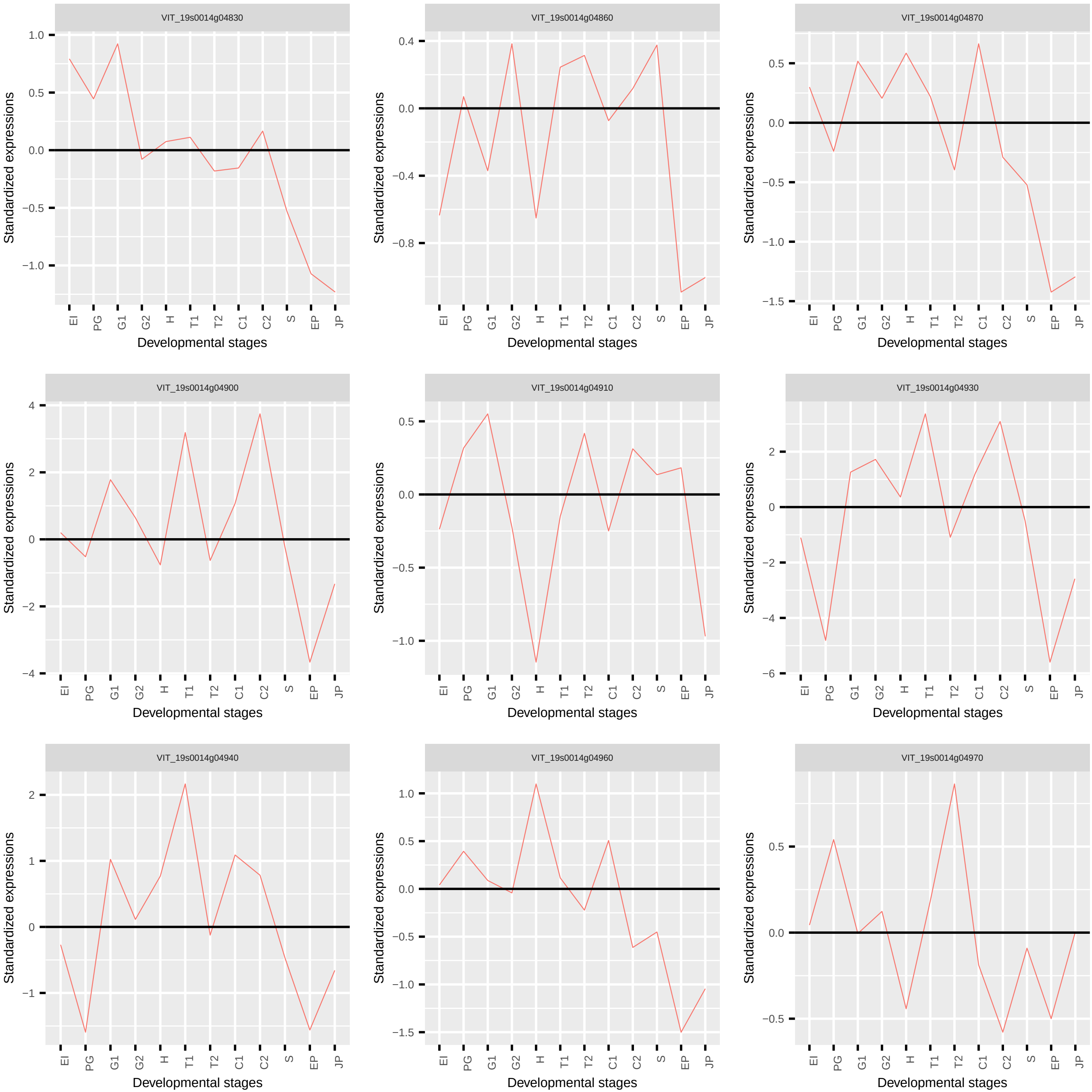
VIT_19s0014g04830
VIT_19s0014g04860
VIT_19s0014g04870
VIT_19s0014g04900
VIT_19s0014g04910
VIT_19s0014g04930
VIT_19s0014g04940
VIT_19s0014g04960
VIT_19s0014g04970
Standardized expressions
Developmental stages
EI PG G1 G2 H T1 T2 C1 C2 S EP JP

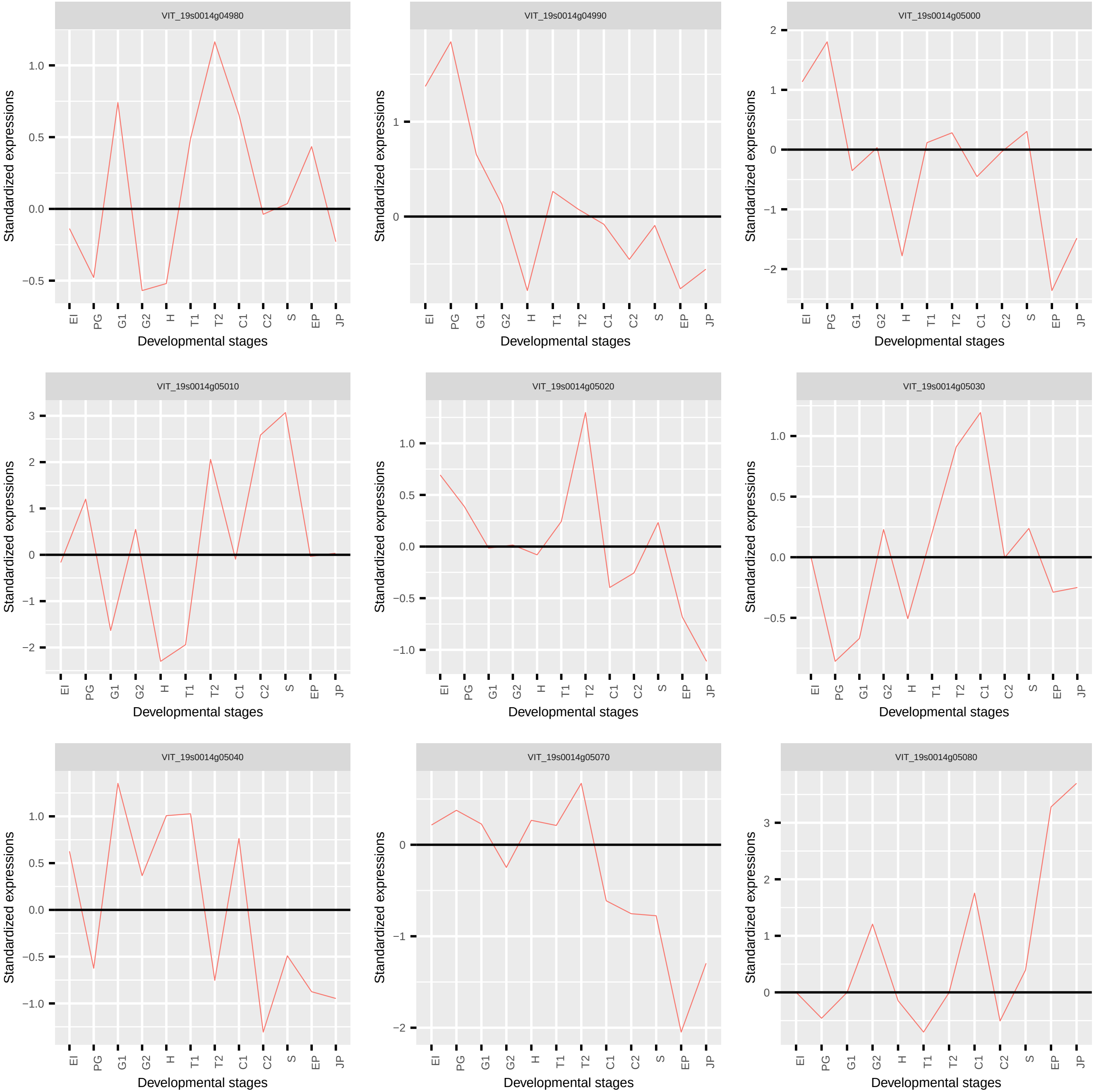
VIT_19s0014g04980
1.0
0.5
0.0
−0.5
Standardized expressions
EI PG G1 G2 H T1 T2 C1 C2 S EP JP
Developmental stages
VIT_19s0014g04990
1
0
Standardized expressions
EI PG G1 G2 H T1 T2 C1 C2 S EP JP
Developmental stages
VIT_19s0014g05000
2
1
0
−1
−2
Standardized expressions
EI PG G1 G2 H T1 T2 C1 C2 S EP JP
Developmental stages
VIT_19s0014g05010
3
2
1
0
−1
−2
Standardized expressions
EI PG G1 G2 H T1 T2 C1 C2 S EP JP
Developmental stages
VIT_19s0014g05020
1.0
0.5
0.0
−0.5
−1.0
Standardized expressions
EI PG G1 G2 H T1 T2 C1 C2 S EP JP
Developmental stages
VIT_19s0014g05030
1.0
0.5
0.0
−0.5
Standardized expressions
EI PG G1 G2 H T1 T2 C1 C2 S EP JP
Developmental stages
VIT_19s0014g05040
1.0
0.5
0.0
−0.5
−1.0
Standardized expressions
EI PG G1 G2 H T1 T2 C1 C2 S EP JP
Developmental stages
VIT_19s0014g05070
0
−1
−2
Standardized expressions
EI PG G1 G2 H T1 T2 C1 C2 S EP JP
Developmental stages
VIT_19s0014g05080
3
2
1
0
Standardized expressions
EI PG G1 G2 H T1 T2 C1 C2 S EP JP
Developmental stages

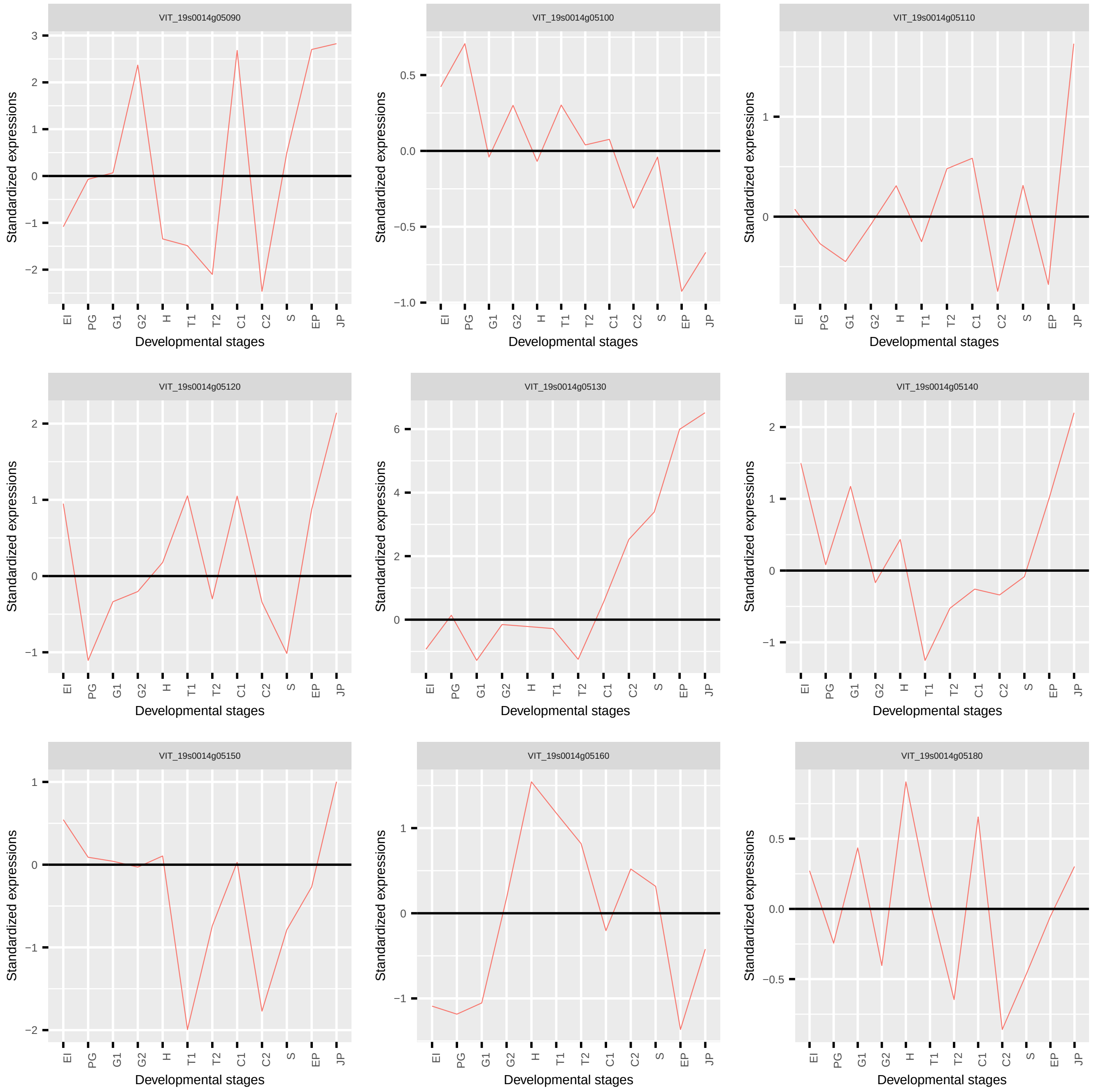

VIT_19s0014g05090
VIT_19s0014g05100
VIT_19s0014g05110
VIT_19s0014g05120
VIT_19s0014g05130
VIT_19s0014g05140
VIT_19s0014g05150
VIT_19s0014g05160
VIT_19s0014g05180
Standardized expressions
Developmental stages
EI PG G1 G2 H T1 T2 C1 C2 S EP JP

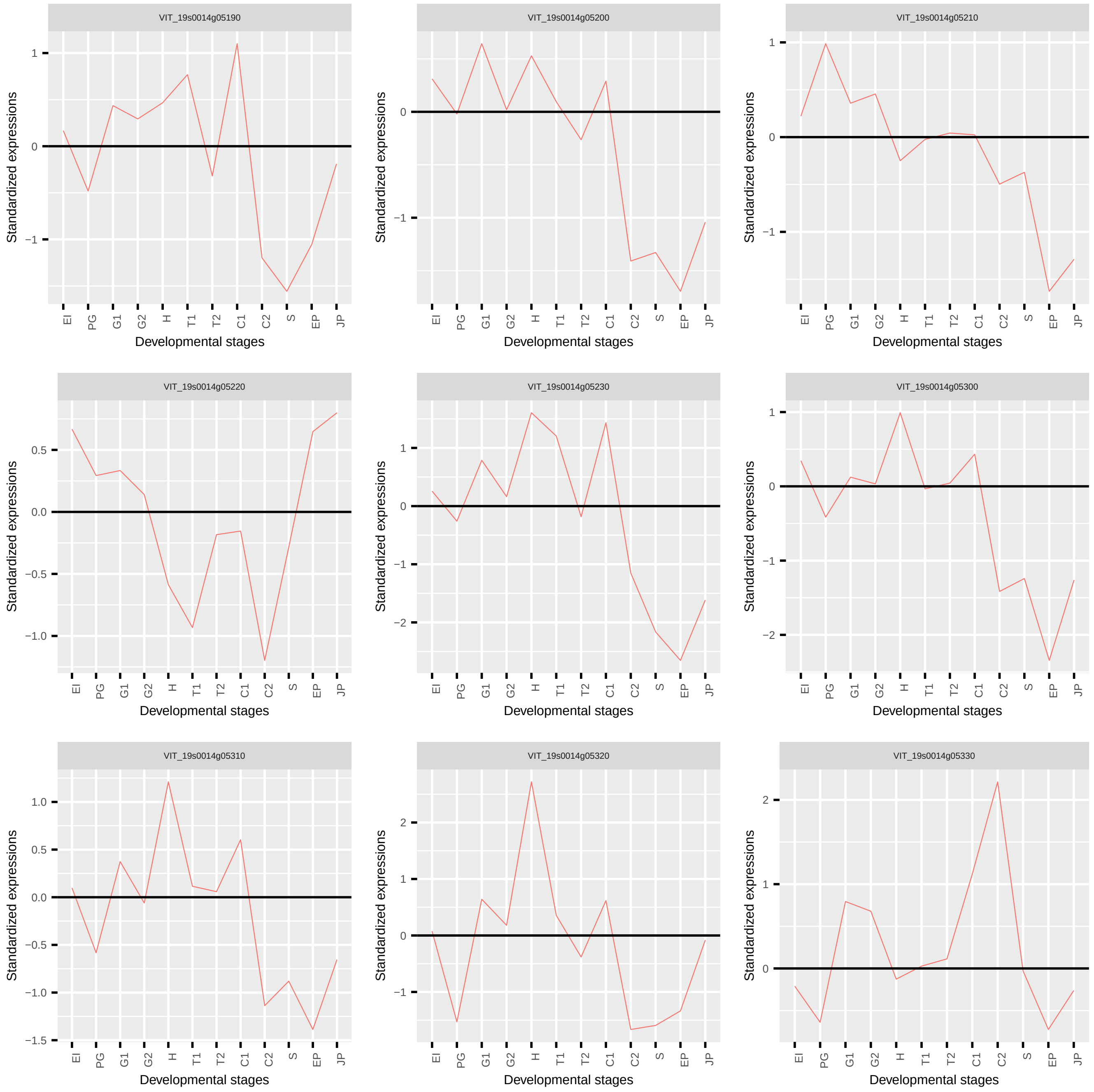
VIT_19s0014g05190
VIT_19s0014g05200
VIT_19s0014g05210
VIT_19s0014g05220
VIT_19s0014g05230
VIT_19s0014g05300
VIT_19s0014g05310
VIT_19s0014g05320
VIT_19s0014g05330
Standardized expressions
Developmental stages
EI PG G1 G2 H T1 T2 C1 C2 S EP JP

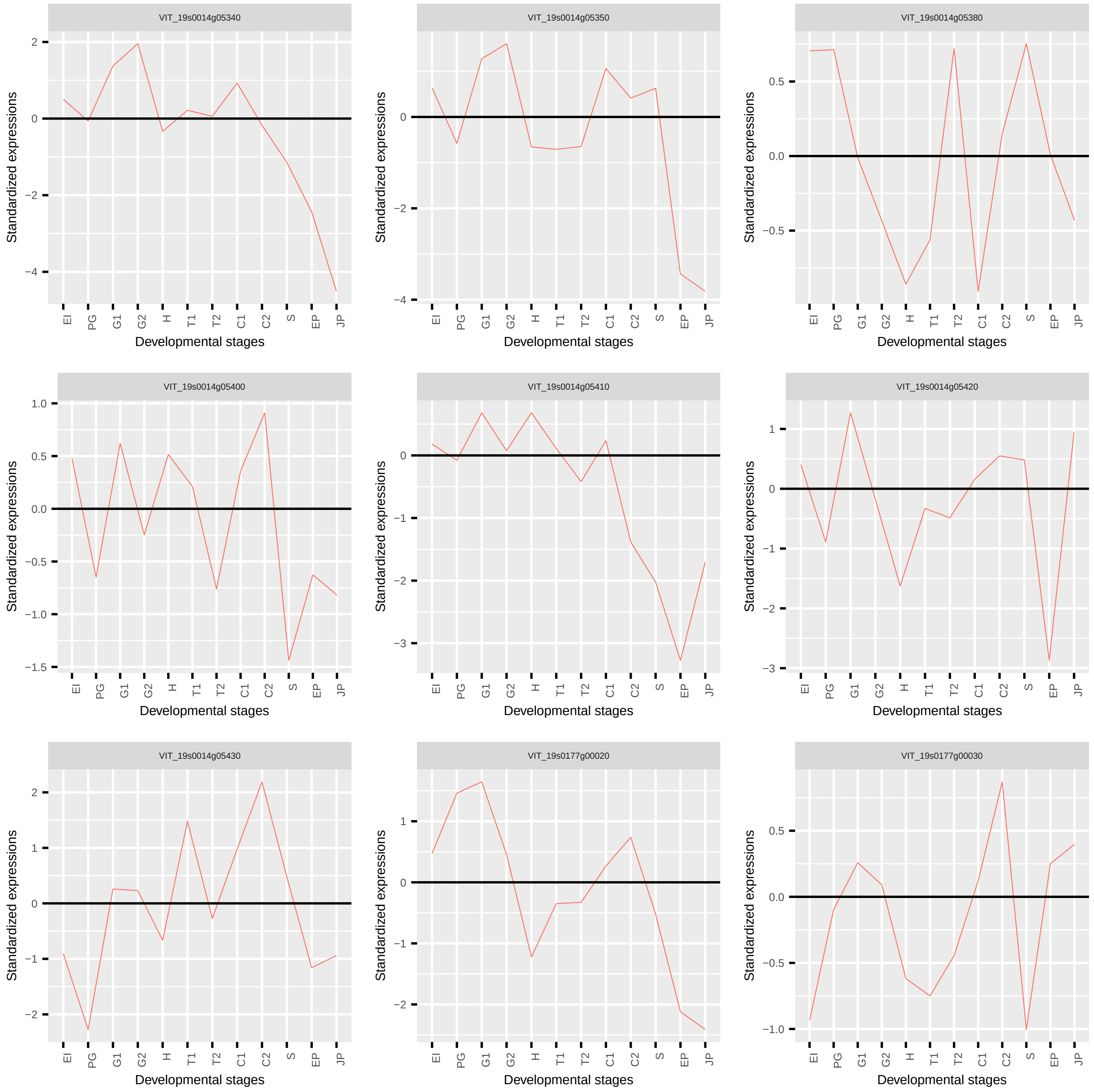
VIT_19s0014g05340
VIT_19s0014g05350
VIT_19s0014g05380
VIT_19s0014g05400
VIT_19s0014g05410
VIT_19s0014g05420
VIT_19s0014g05430
VIT_19s0177g00020
VIT_19s0177g00030
Standardized expressions
Developmental stages
EI PG G1 G2 H T1 T2 C1 C2 S EP JP

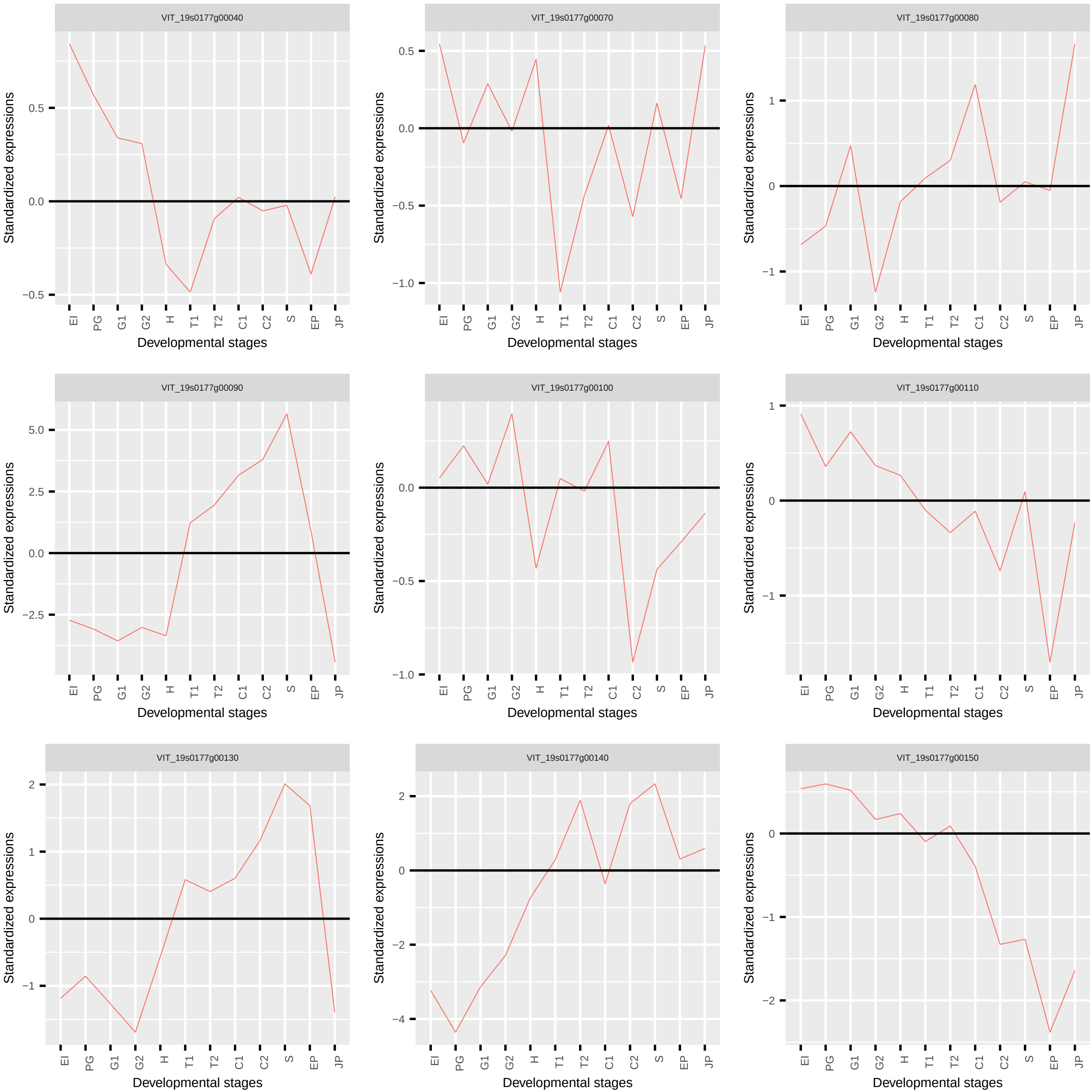
VIT_19s0177g00040
VIT_19s0177g00070
VIT_19s0177g00080
VIT_19s0177g00090
VIT_19s0177g00100
VIT_19s0177g00110
VIT_19s0177g00130
VIT_19s0177g00140
VIT_19s0177g00150
Standardized expressions
Developmental stages
EI PG G1 G2 H T1 T2 C1 C2 S EP JP

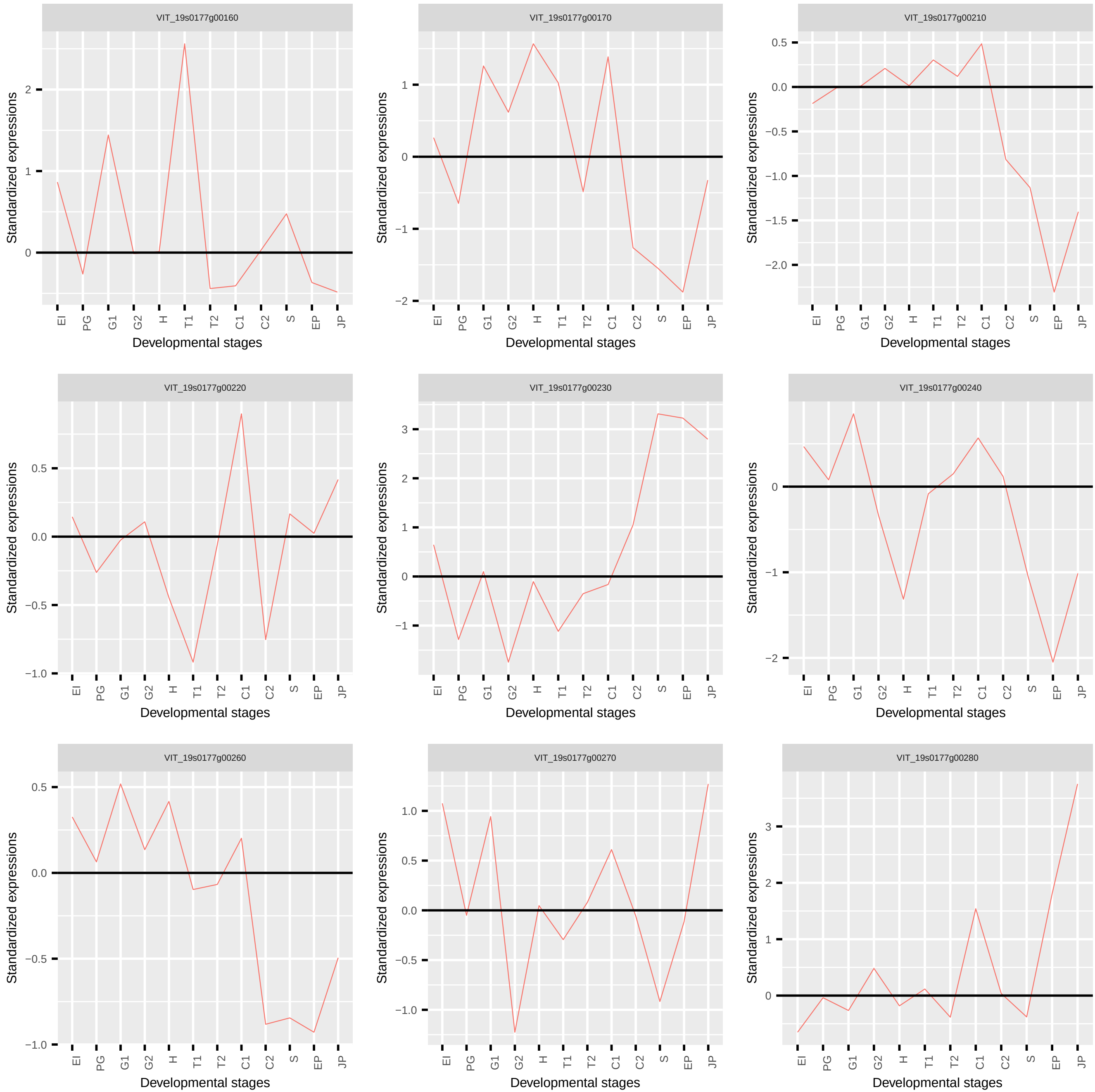
VIT_19s0177g00160
VIT_19s0177g00170
VIT_19s0177g00210
VIT_19s0177g00220
VIT_19s0177g00230
VIT_19s0177g00240
VIT_19s0177g00260
VIT_19s0177g00270
VIT_19s0177g00280
Standardized expressions
Developmental stages
EI PG G1 G2 H T1 T2 C1 C2 S EP JP

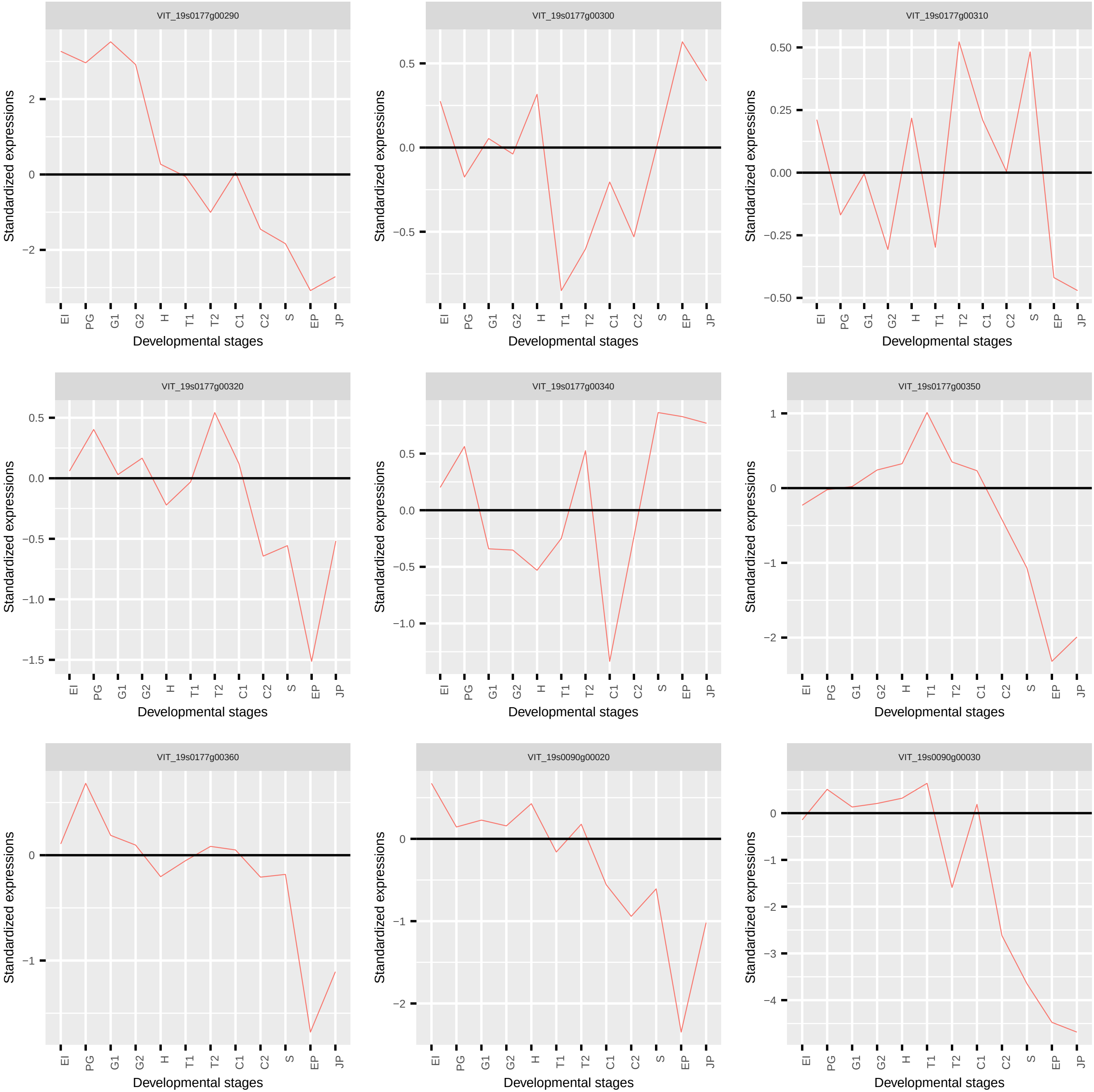
VIT_19s0177g00290
VIT_19s0177g00300
VIT_19s0177g00310
VIT_19s0177g00320
VIT_19s0177g00340
VIT_19s0177g00350
VIT_19s0177g00360
VIT_19s0090g00020
VIT_19s0090g00030
Standardized expressions
Developmental stages
EI
PG
G1
G2
H
T1
T2
C1
C2
S
EP
JP

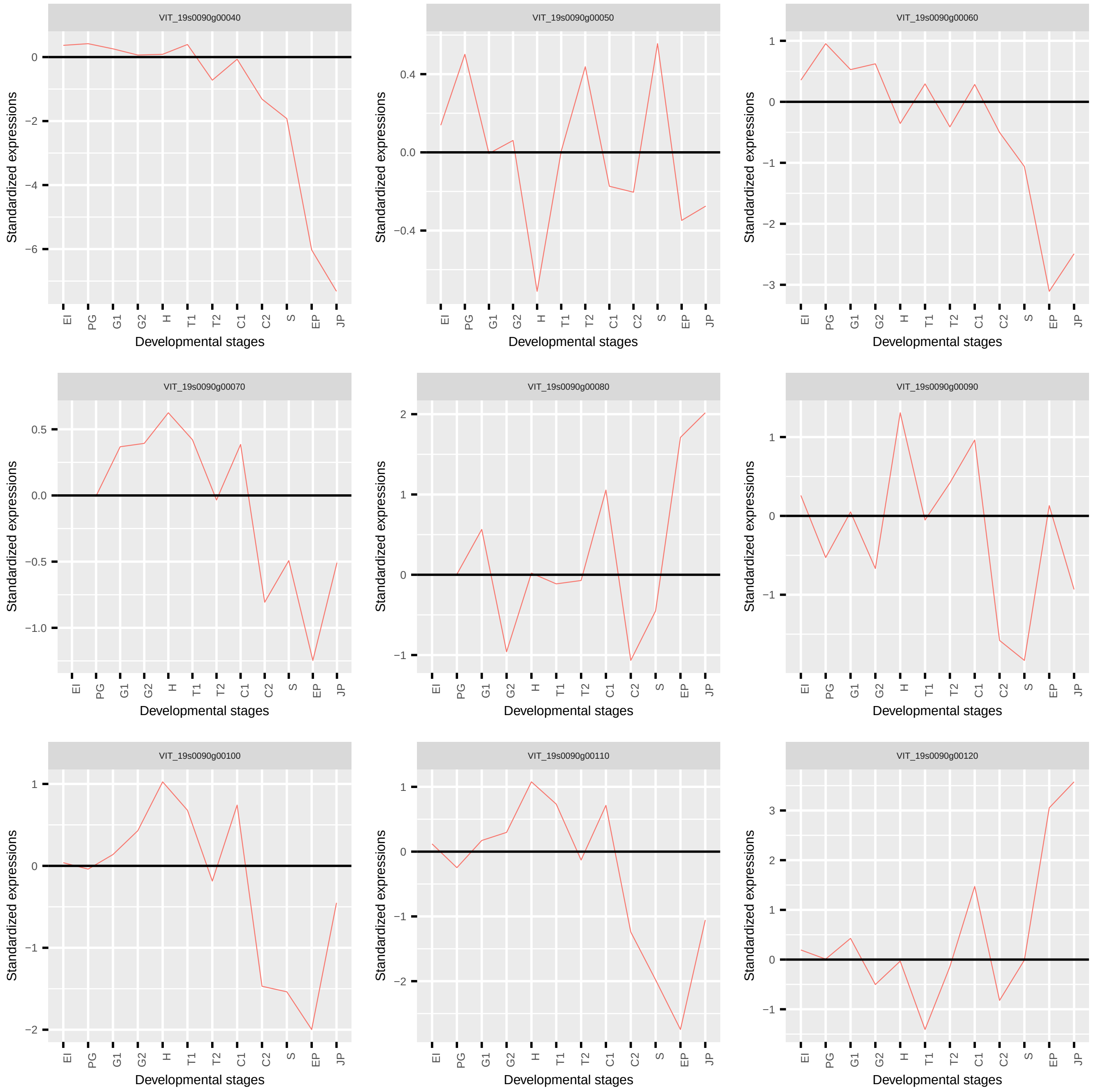
VIT_19s0090g00040
VIT_19s0090g00050
VIT_19s0090g00060
VIT_19s0090g00070
VIT_19s0090g00080
VIT_19s0090g00090
VIT_19s0090g00100
VIT_19s0090g00110
VIT_19s0090g00120
Standardized expressions
Developmental stages
EI
PG
G1
G2
H
T1
T2
C1
C2
S
EP
JP

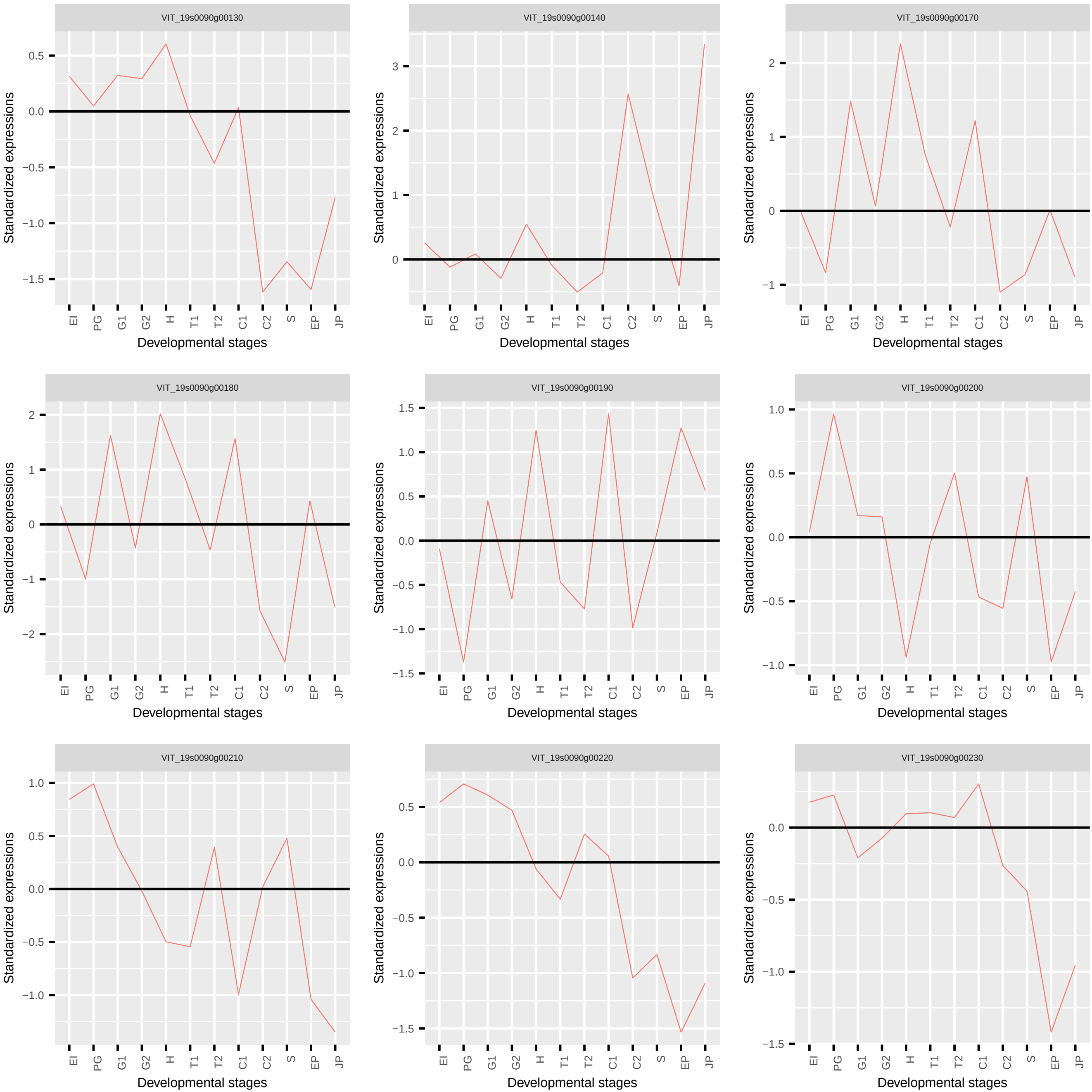
VIT_19s0090g00130
VIT_19s0090g00140
VIT_19s0090g00170
VIT_19s0090g00180
VIT_19s0090g00190
VIT_19s0090g00200
VIT_19s0090g00210
VIT_19s0090g00220
VIT_19s0090g00230
Standardized expressions
Developmental stages
EI PG G1 G2 H T1 T2 C1 C2 S EP JP

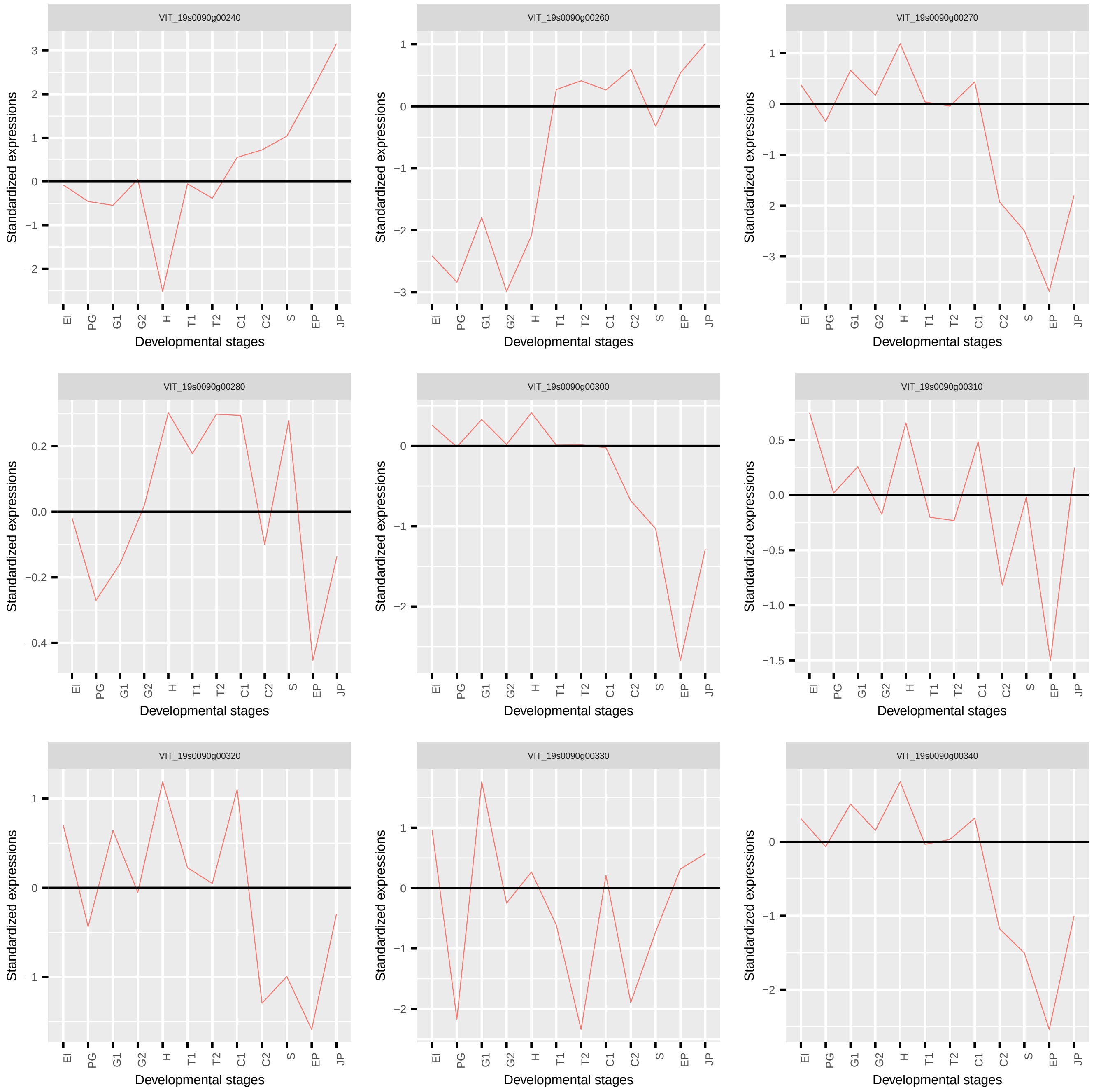
VIT_19s0090g00240
VIT_19s0090g00260
VIT_19s0090g00270
VIT_19s0090g00280
VIT_19s0090g00300
VIT_19s0090g00310
VIT_19s0090g00320
VIT_19s0090g00330
VIT_19s0090g00340
Standardized expressions
Developmental stages
EI PG G1 G2 H T1 T2 C1 C2 S EP JP

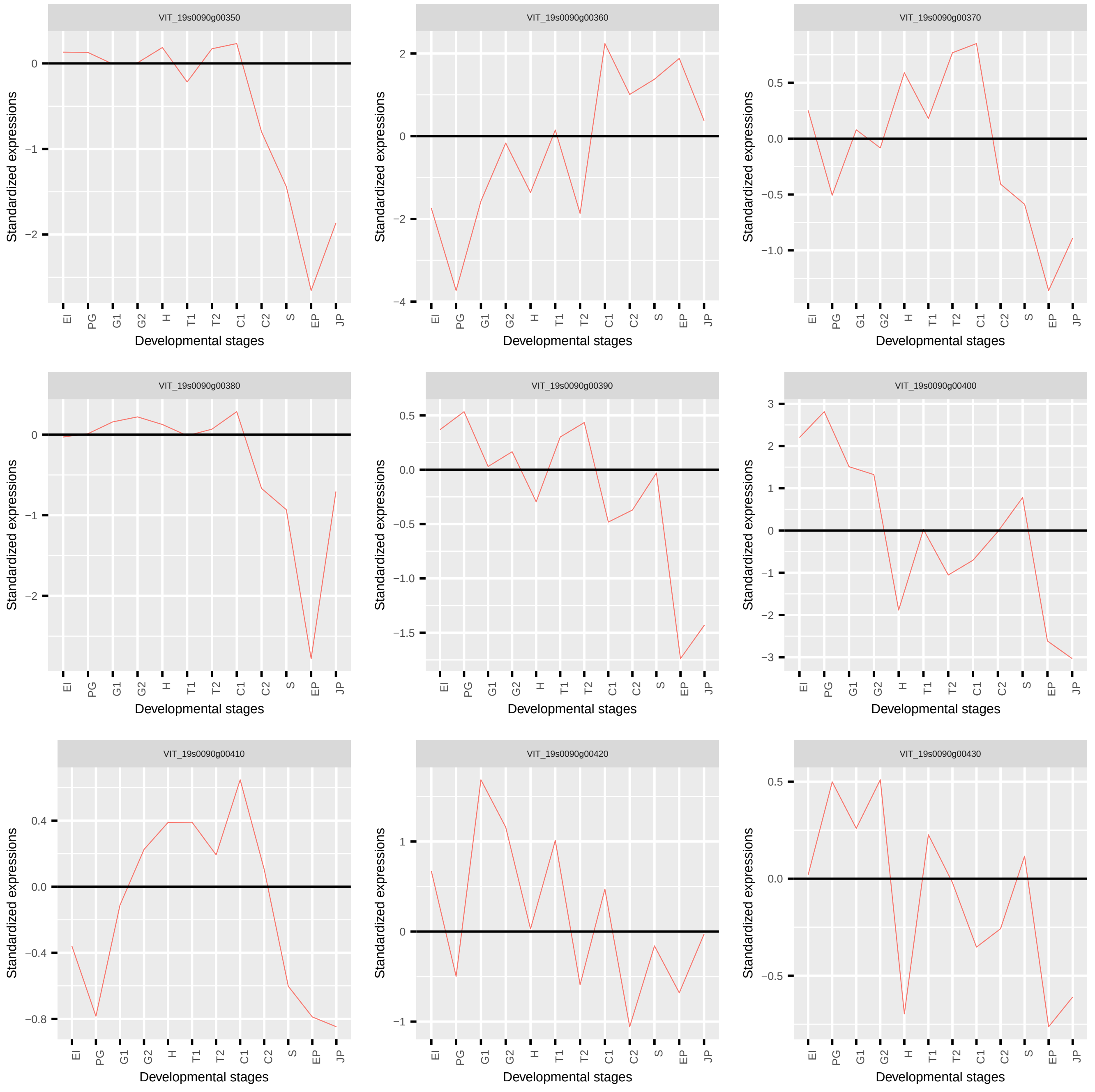
VIT_19s0090g00350
VIT_19s0090g00360
VIT_19s0090g00370
VIT_19s0090g00380
VIT_19s0090g00390
VIT_19s0090g00400
VIT_19s0090g00410
VIT_19s0090g00420
VIT_19s0090g00430
Standardized expressions
Developmental stages
EI PG G1 G2 H T1 T2 C1 C2 S EP JP

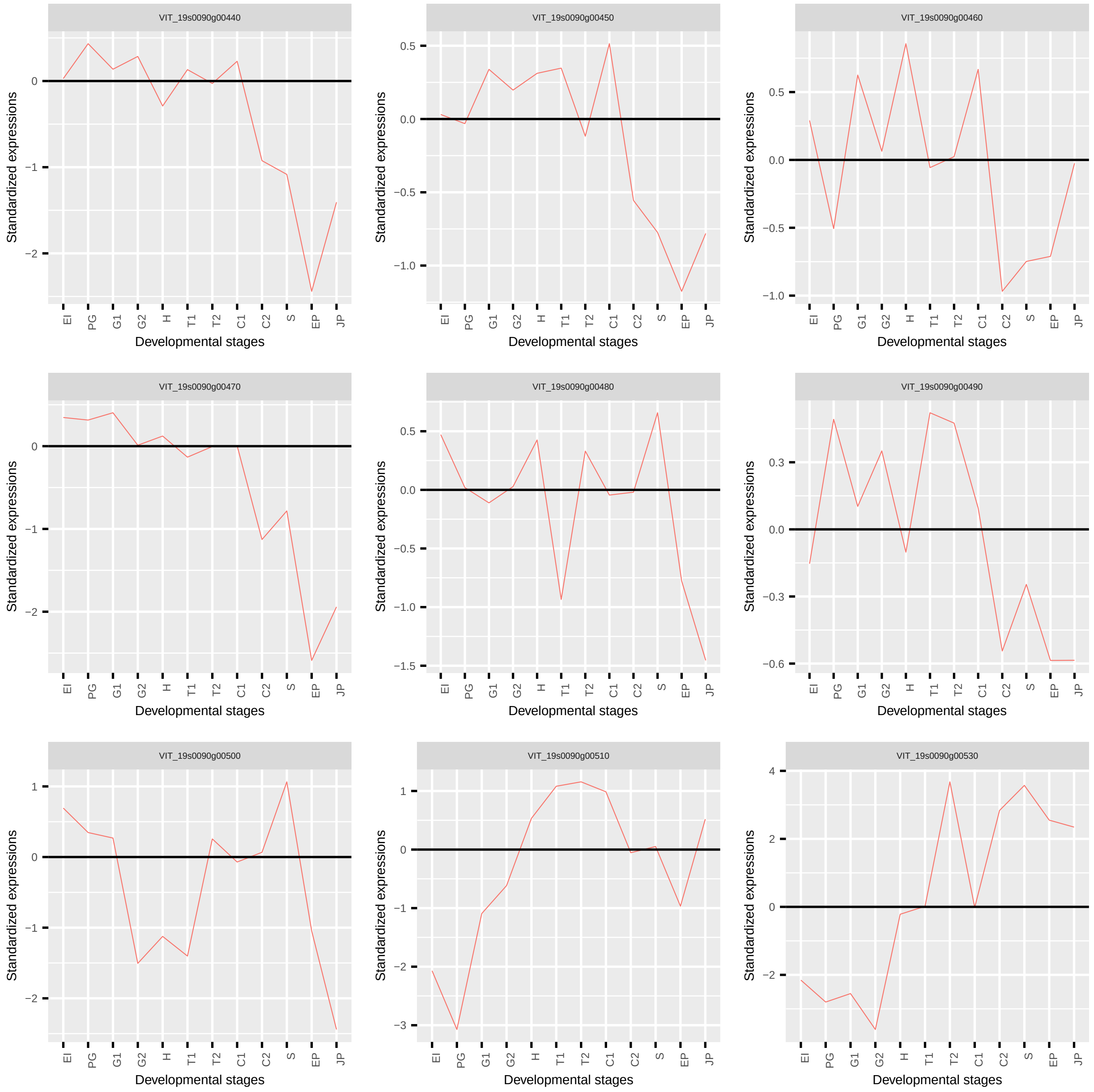
VIT_19s0090g00440
0
−1
−2
Standardized expressions
EI PG G1 G2 H T1 T2 C1 C2 S EP JP
Developmental stages
VIT_19s0090g00450
0.5
0.0
−0.5
−1.0
Standardized expressions
EI PG G1 G2 H T1 T2 C1 C2 S EP JP
Developmental stages
VIT_19s0090g00460
0.5
0.0
−0.5
−1.0
Standardized expressions
EI PG G1 G2 H T1 T2 C1 C2 S EP JP
Developmental stages
VIT_19s0090g00470
0
−1
−2
Standardized expressions
EI PG G1 G2 H T1 T2 C1 C2 S EP JP
Developmental stages
VIT_19s0090g00480
0.5
0.0
−0.5
−1.0
−1.5
Standardized expressions
EI PG G1 G2 H T1 T2 C1 C2 S EP JP
Developmental stages
VIT_19s0090g00490
0.3
0.0
−0.3
−0.6
Standardized expressions
EI PG G1 G2 H T1 T2 C1 C2 S EP JP
Developmental stages
VIT_19s0090g00500
1
0
−1
−2
Standardized expressions
EI PG G1 G2 H T1 T2 C1 C2 S EP JP
Developmental stages
VIT_19s0090g00510
1
0
−1
−2
−3
Standardized expressions
EI PG G1 G2 H T1 T2 C1 C2 S EP JP
Developmental stages
VIT_19s0090g00530
4
2
0
−2
Standardized expressions
EI PG G1 G2 H T1 T2 C1 C2 S EP JP
Developmental stages

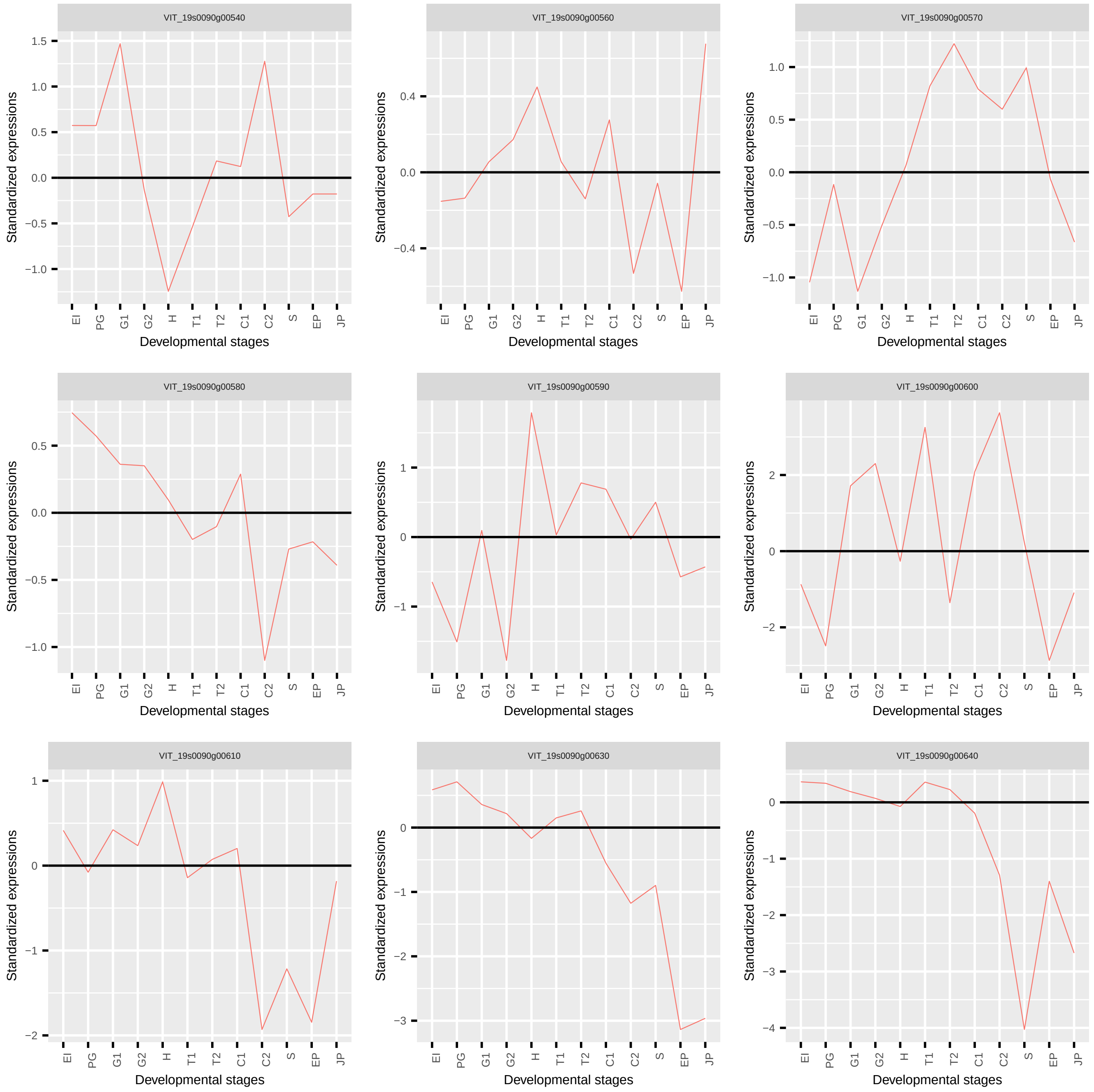
VIT_19s0090g00540
1.5
1.0
0.5
0.0
−0.5
−1.0
Standardized expressions
EI PG G1 G2 H T1 T2 C1 C2 S EP JP
Developmental stages
VIT_19s0090g00560
0.4
0.0
−0.4
Standardized expressions
EI PG G1 G2 H T1 T2 C1 C2 S EP JP
Developmental stages
VIT_19s0090g00570
1.0
0.5
0.0
−0.5
−1.0
Standardized expressions
EI PG G1 G2 H T1 T2 C1 C2 S EP JP
Developmental stages
VIT_19s0090g00580
0.5
0.0
−0.5
−1.0
Standardized expressions
EI PG G1 G2 H T1 T2 C1 C2 S EP JP
Developmental stages
VIT_19s0090g00590
1
0
−1
Standardized expressions
EI PG G1 G2 H T1 T2 C1 C2 S EP JP
Developmental stages
VIT_19s0090g00600
2
0
−2
Standardized expressions
EI PG G1 G2 H T1 T2 C1 C2 S EP JP
Developmental stages
VIT_19s0090g00610
1
0
−1
−2
Standardized expressions
EI PG G1 G2 H T1 T2 C1 C2 S EP JP
Developmental stages
VIT_19s0090g00630
0
−1
−2
−3
Standardized expressions
EI PG G1 G2 H T1 T2 C1 C2 S EP JP
Developmental stages
VIT_19s0090g00640
0
−1
−2
−3
−4
Standardized expressions
EI PG G1 G2 H T1 T2 C1 C2 S EP JP
Developmental stages

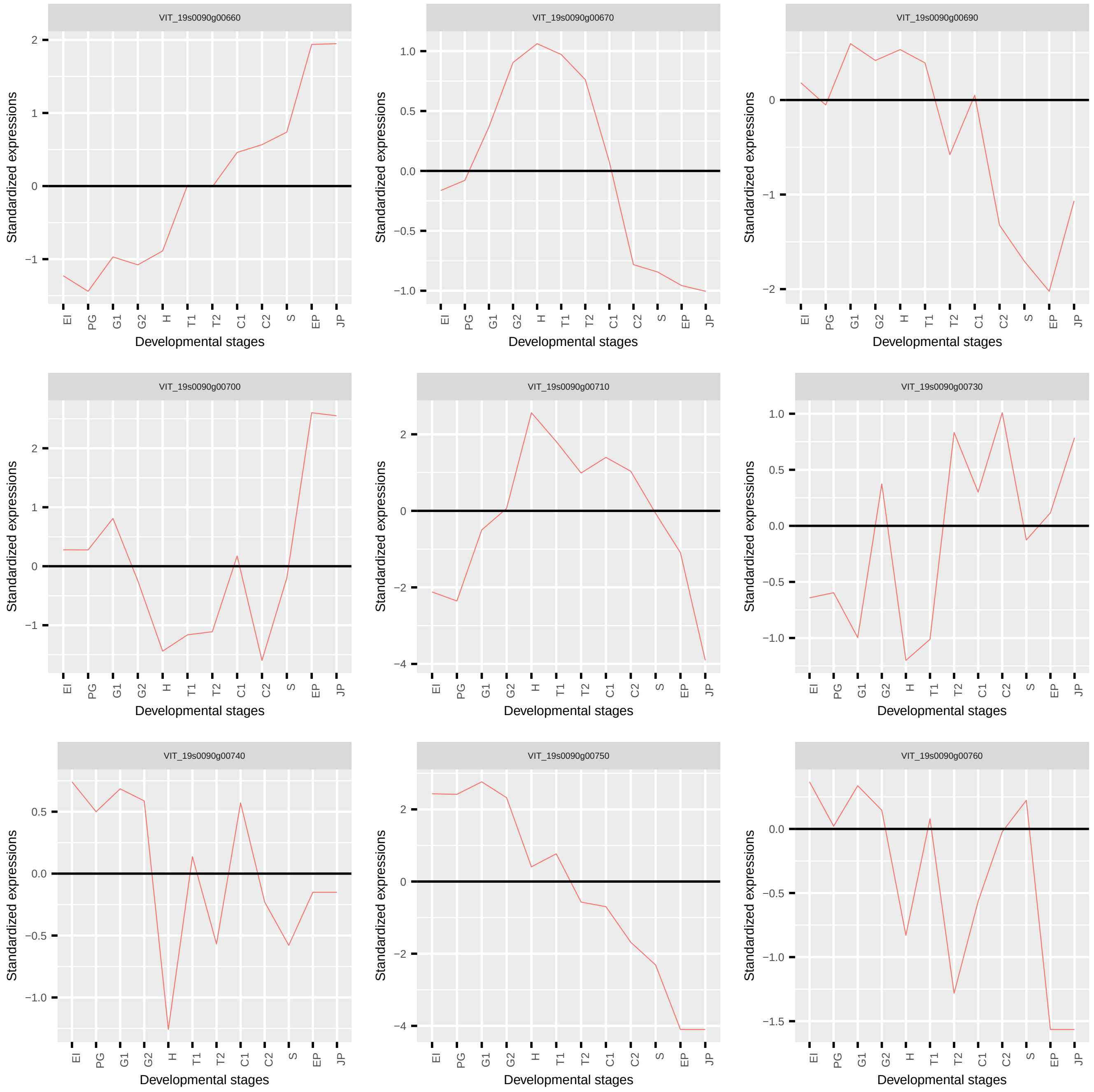
VIT_19s0090g00660
VIT_19s0090g00670
VIT_19s0090g00690
VIT_19s0090g00700
VIT_19s0090g00710
VIT_19s0090g00730
VIT_19s0090g00740
VIT_19s0090g00750
VIT_19s0090g00760
Standardized expressions
Developmental stages
EI PG G1 G2 H T1 T2 C1 C2 S EP JP

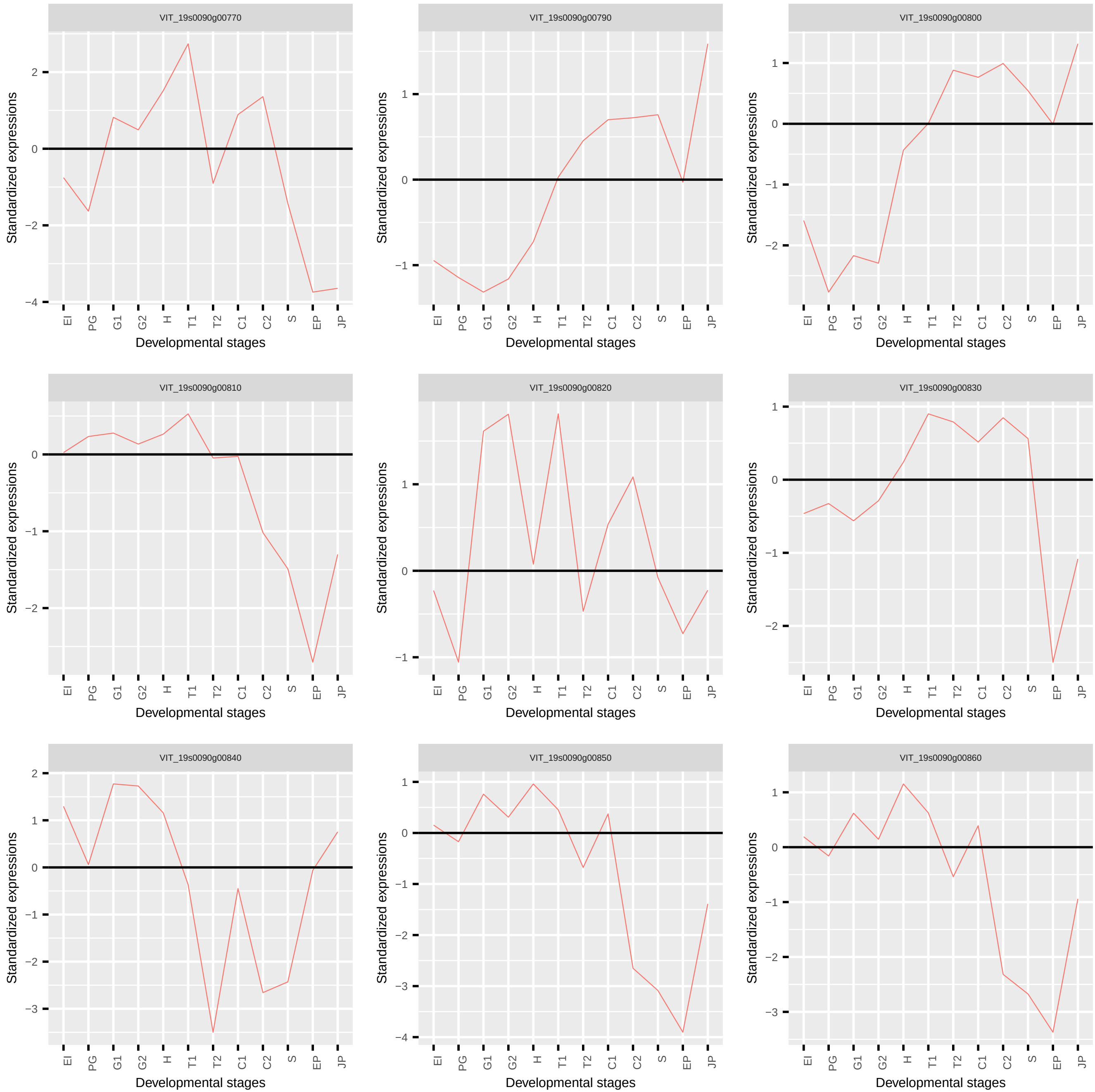

VIT_19s0090g00770
VIT_19s0090g00790
VIT_19s0090g00800
VIT_19s0090g00810
VIT_19s0090g00820
VIT_19s0090g00830
VIT_19s0090g00840
VIT_19s0090g00850
VIT_19s0090g00860
Standardized expressions
Developmental stages
EI
PG
G1
G2
H
T1
T2
C1
C2
S
EP
JP

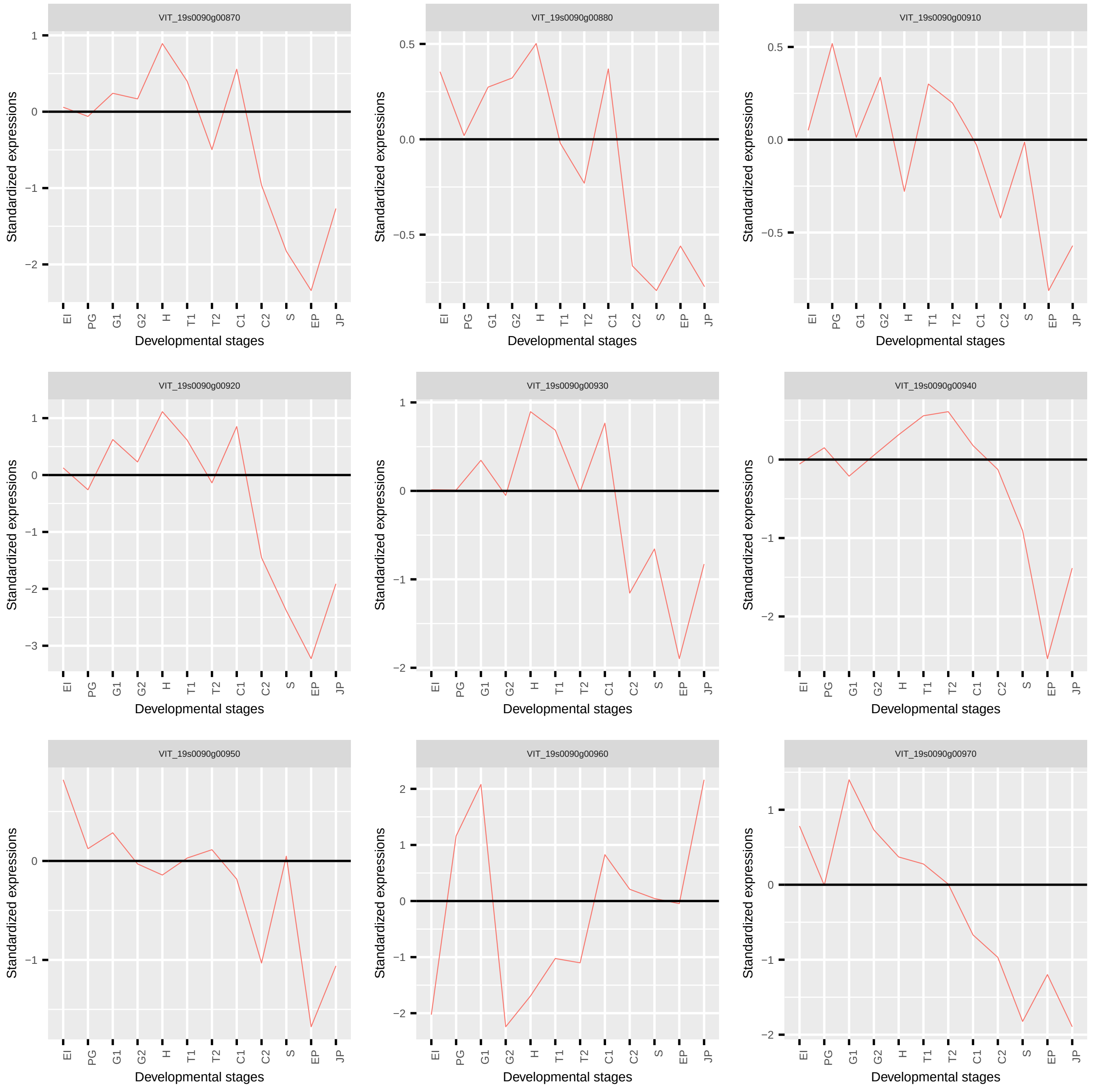
VIT_19s0090g00870
VIT_19s0090g00880
VIT_19s0090g00910
VIT_19s0090g00920
VIT_19s0090g00930
VIT_19s0090g00940
VIT_19s0090g00950
VIT_19s0090g00960
VIT_19s0090g00970
Standardized expressions
Developmental stages
EI PG G1 G2 H T1 T2 C1 C2 S EP JP

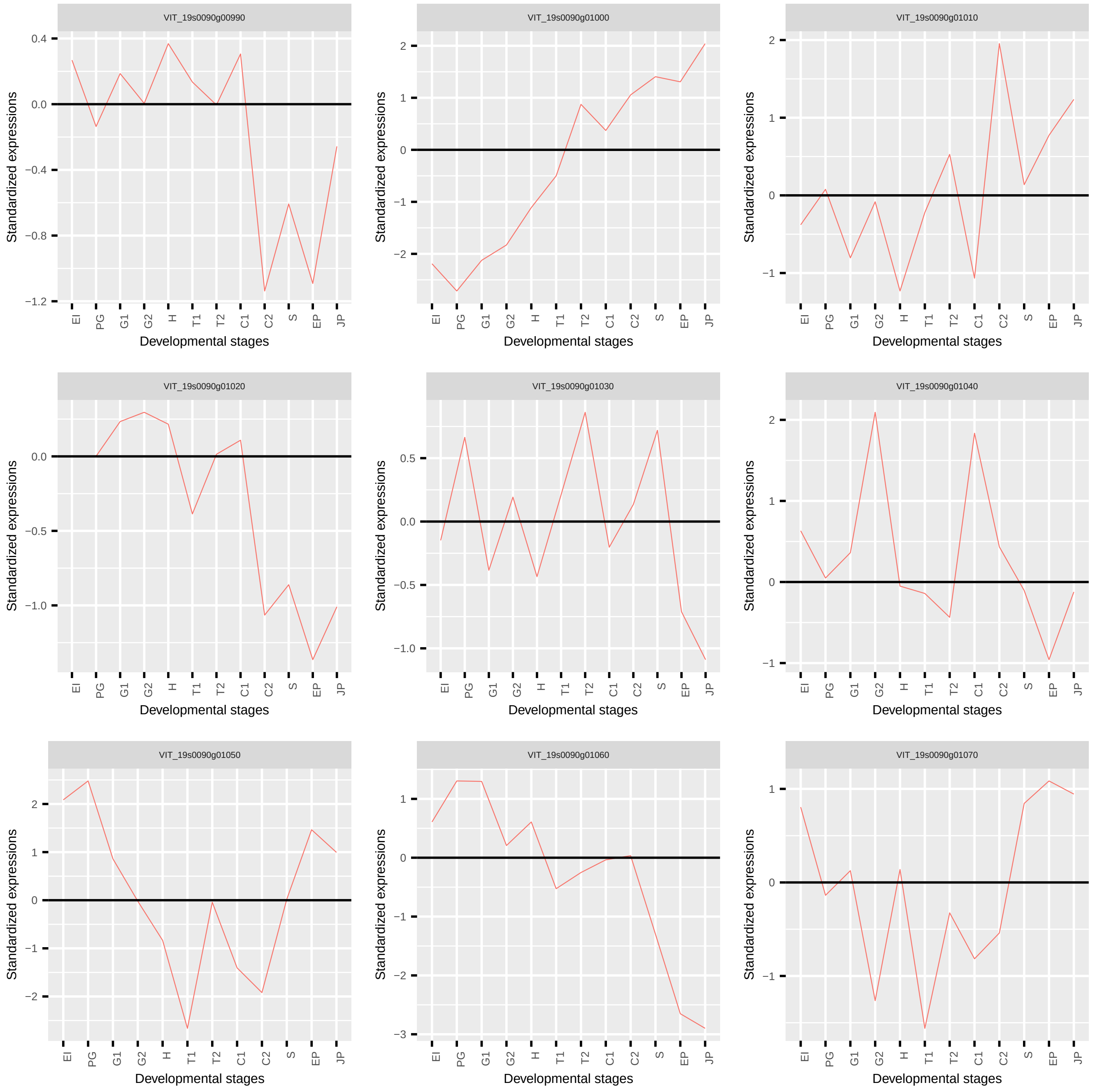

VIT_19s0090g00990
VIT_19s0090g01000
VIT_19s0090g01010
VIT_19s0090g01020
VIT_19s0090g01030
VIT_19s0090g01040
VIT_19s0090g01050
VIT_19s0090g01060
VIT_19s0090g01070
Standardized expressions
Developmental stages
EI PG G1 G2 H T1 T2 C1 C2 S EP JP

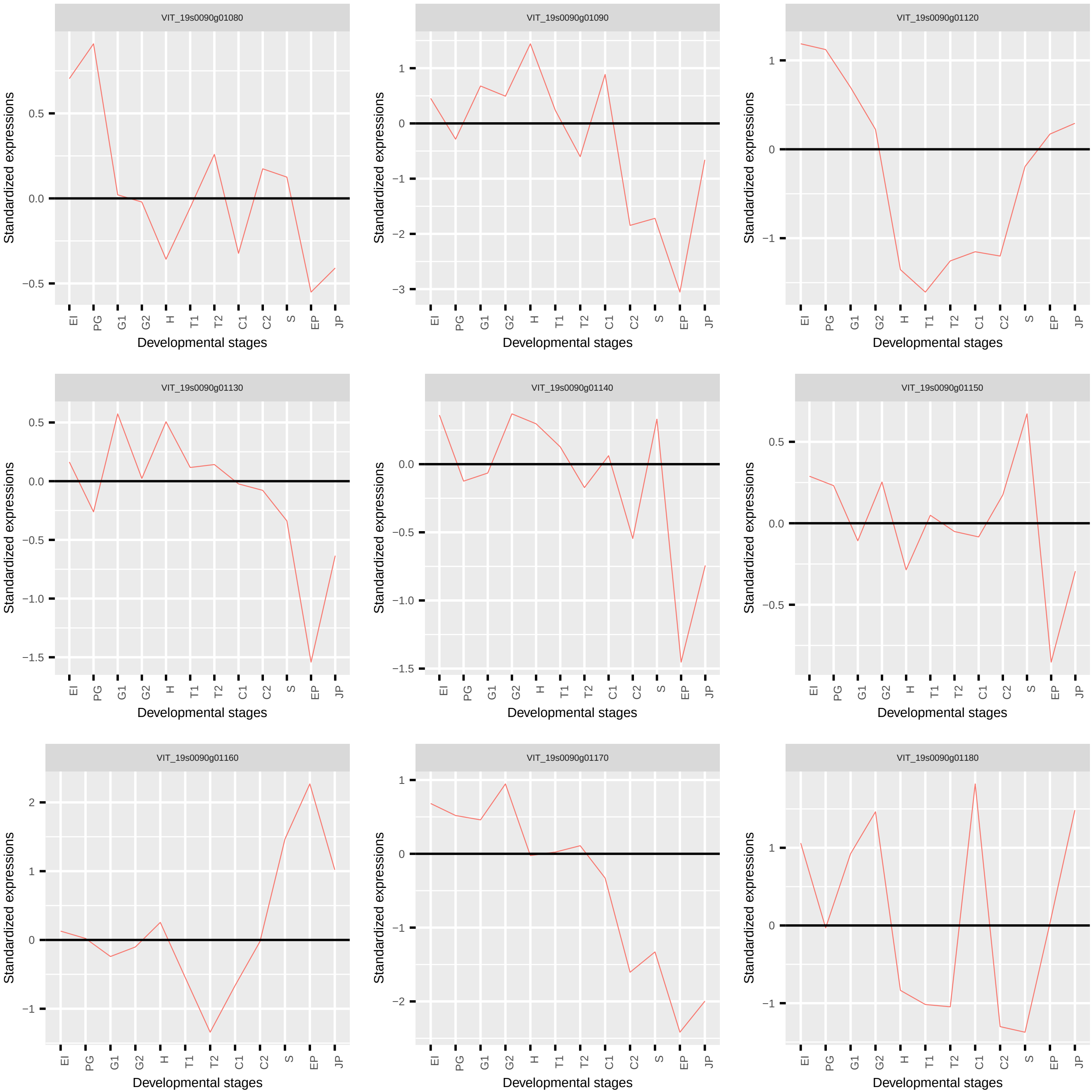

VIT_19s0090g01080
VIT_19s0090g01090
VIT_19s0090g01120
VIT_19s0090g01130
VIT_19s0090g01140
VIT_19s0090g01150
VIT_19s0090g01160
VIT_19s0090g01170
VIT_19s0090g01180
Standardized expressions
Developmental stages
EI
PG
G1
G2
H
T1
T2
C1
C2
S
EP
JP

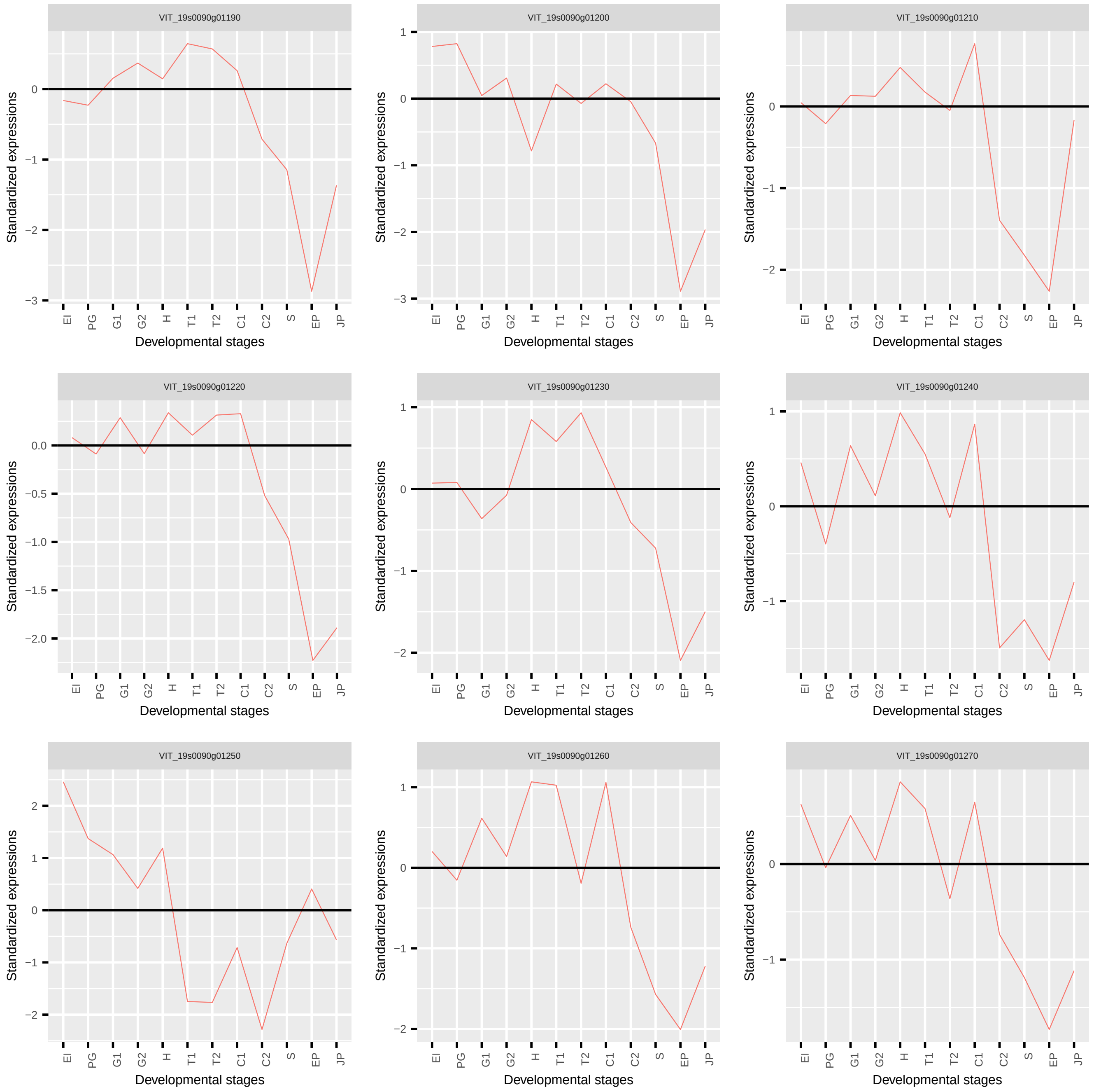
VIT_19s0090g01190
VIT_19s0090g01200
VIT_19s0090g01210
VIT_19s0090g01220
VIT_19s0090g01230
VIT_19s0090g01240
VIT_19s0090g01250
VIT_19s0090g01260
VIT_19s0090g01270
Standardized expressions
Developmental stages
EI PG G1 G2 H T1 T2 C1 C2 S EP JP

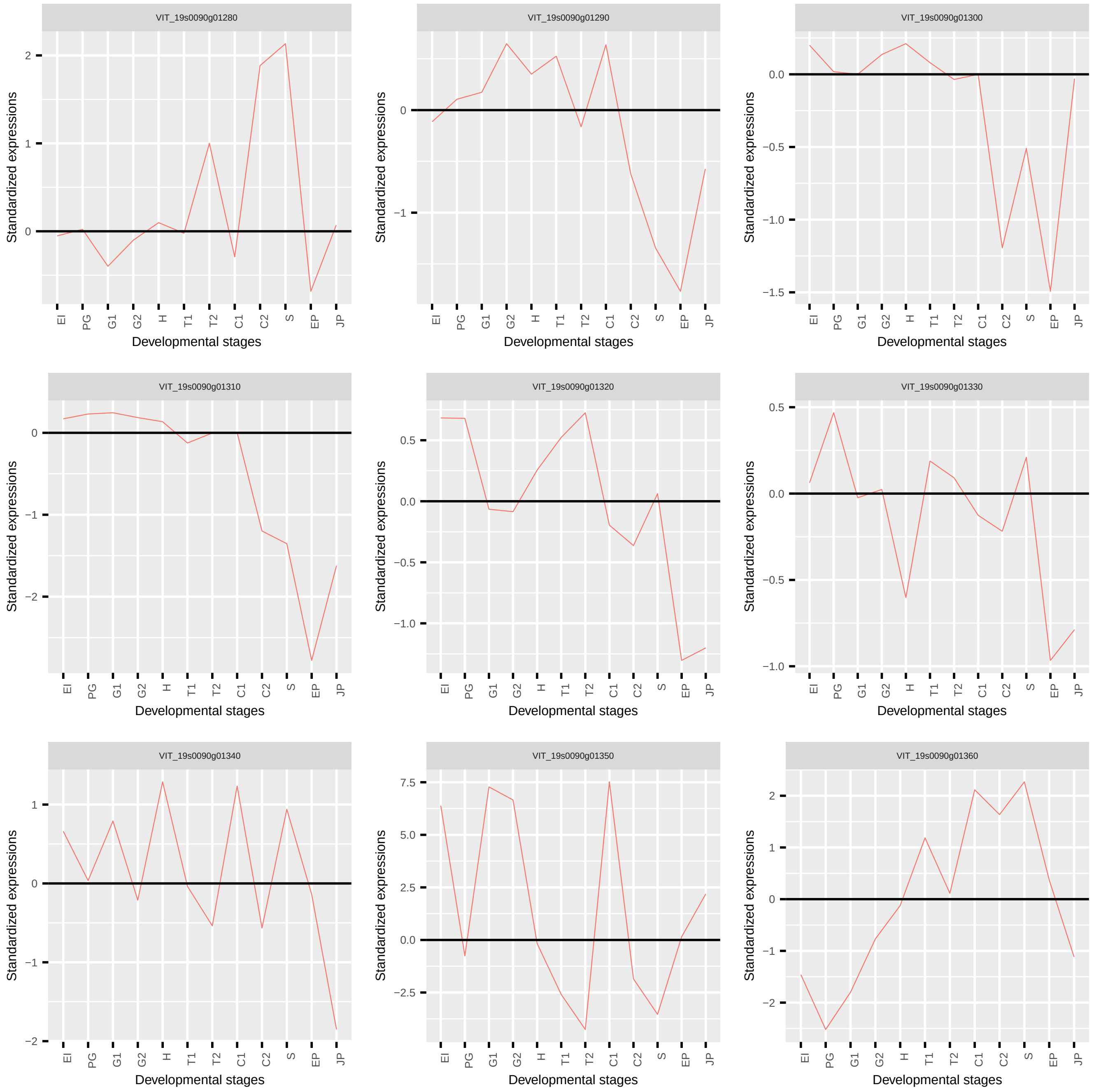

VIT_19s0090g01280
VIT_19s0090g01290
VIT_19s0090g01300
VIT_19s0090g01310
VIT_19s0090g01320
VIT_19s0090g01330
VIT_19s0090g01340
VIT_19s0090g01350
VIT_19s0090g01360
Standardized expressions
Developmental stages
EI
PG
G1
G2
H
T1
T2
C1
C2
S
EP
JP

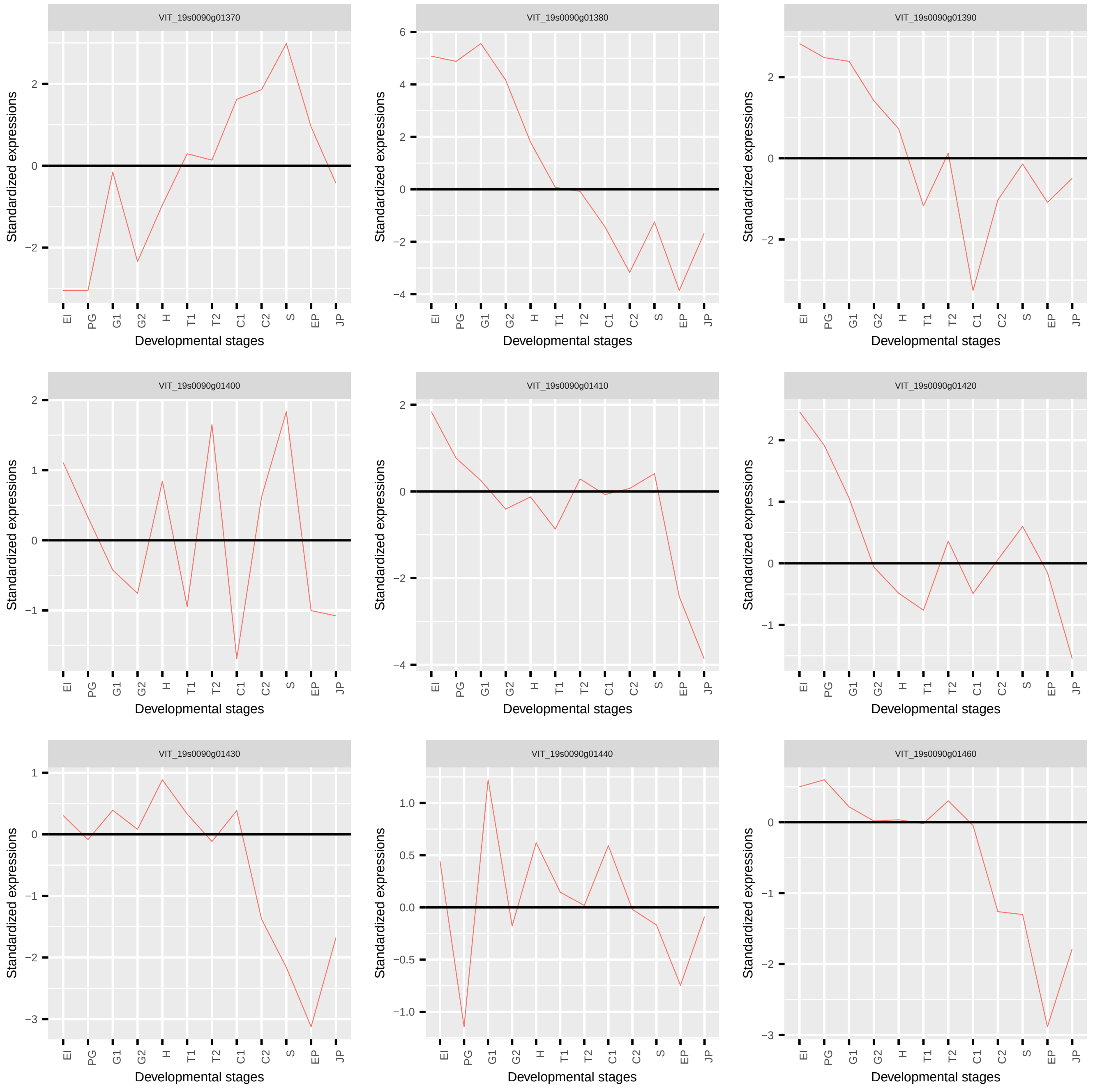
VIT_19s0090g01370
VIT_19s0090g01380
VIT_19s0090g01390
VIT_19s0090g01400
VIT_19s0090g01410
VIT_19s0090g01420
VIT_19s0090g01430
VIT_19s0090g01440
VIT_19s0090g01460
Standardized expressions
Developmental stages
EI PG G1 G2 H T1 T2 C1 C2 S EP JP
2 0 −2
6 4 2 0 −2 −4
2 1 0 −1
1 0 −1 −2 −3
1.0 0.5 0.0 −0.5 −1.0

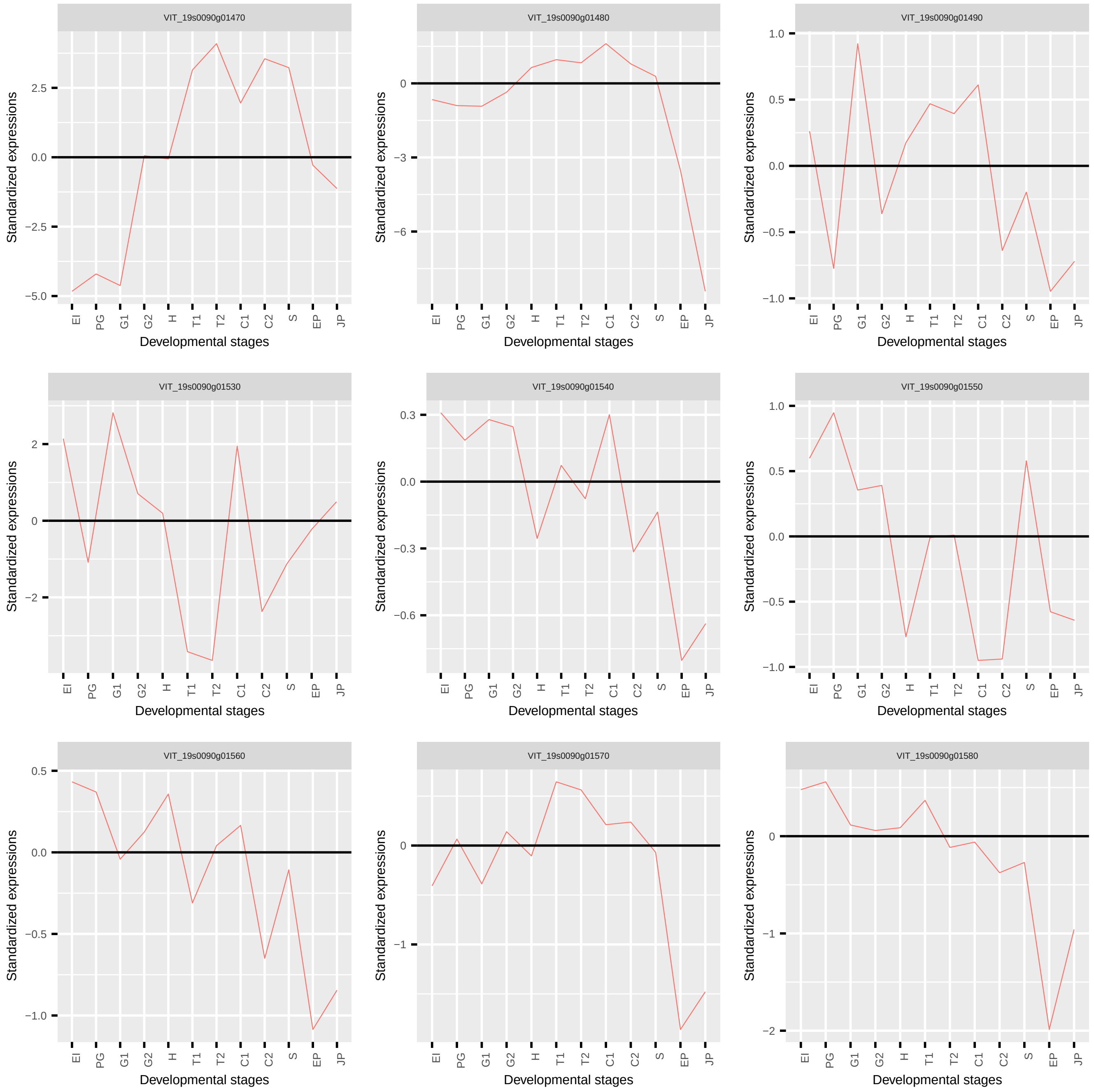
VIT_19s0090g01470
VIT_19s0090g01480
VIT_19s0090g01490
VIT_19s0090g01530
VIT_19s0090g01540
VIT_19s0090g01550
VIT_19s0090g01560
VIT_19s0090g01570
VIT_19s0090g01580
Standardized expressions
Developmental stages
EI PG G1 G2 H T1 T2 C1 C2 S EP JP

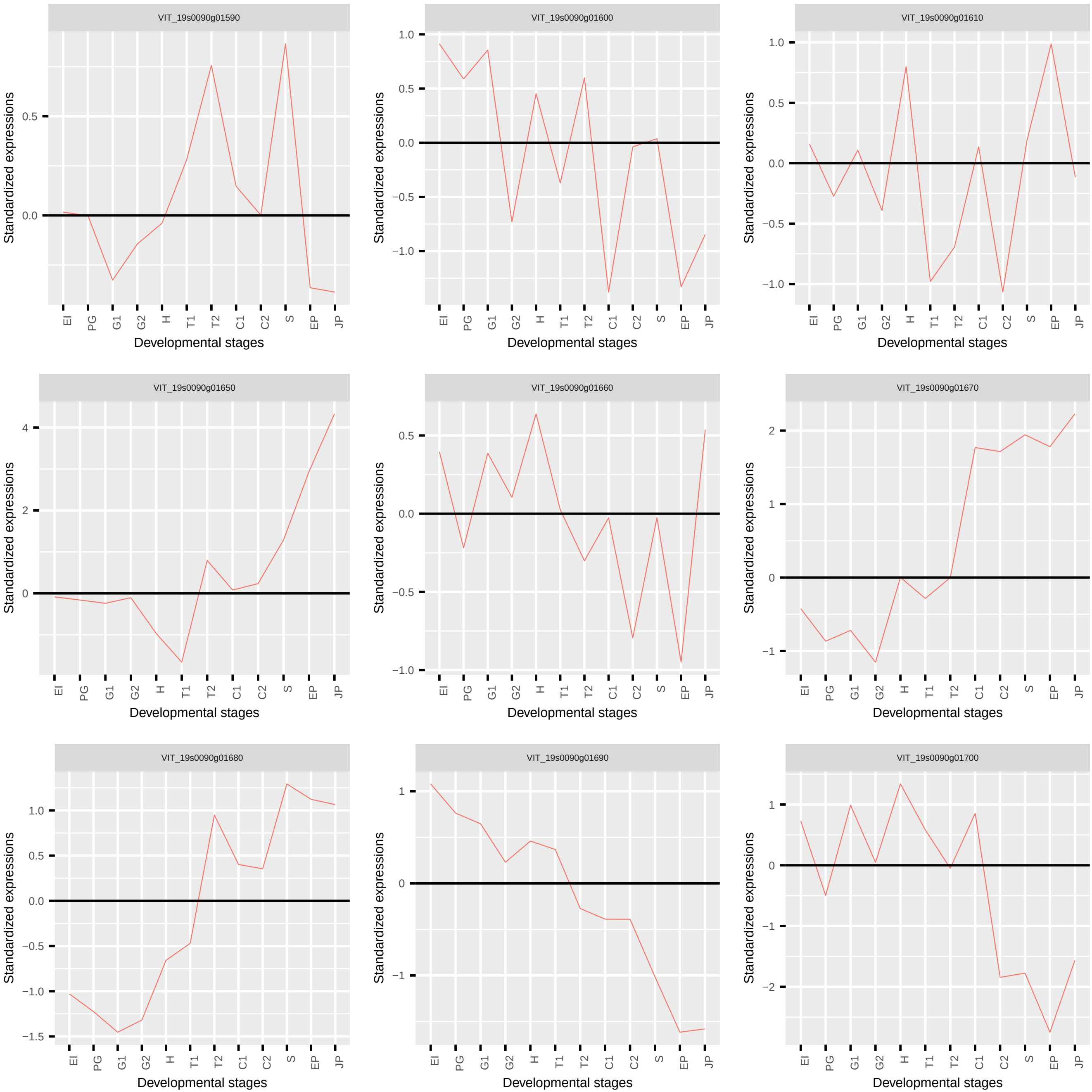
VIT_19s0090g01590
VIT_19s0090g01600
VIT_19s0090g01610
VIT_19s0090g01650
VIT_19s0090g01660
VIT_19s0090g01670
VIT_19s0090g01680
VIT_19s0090g01690
VIT_19s0090g01700
Standardized expressions
Developmental stages
EI PG G1 G2 H T1 T2 C1 C2 S EP JP

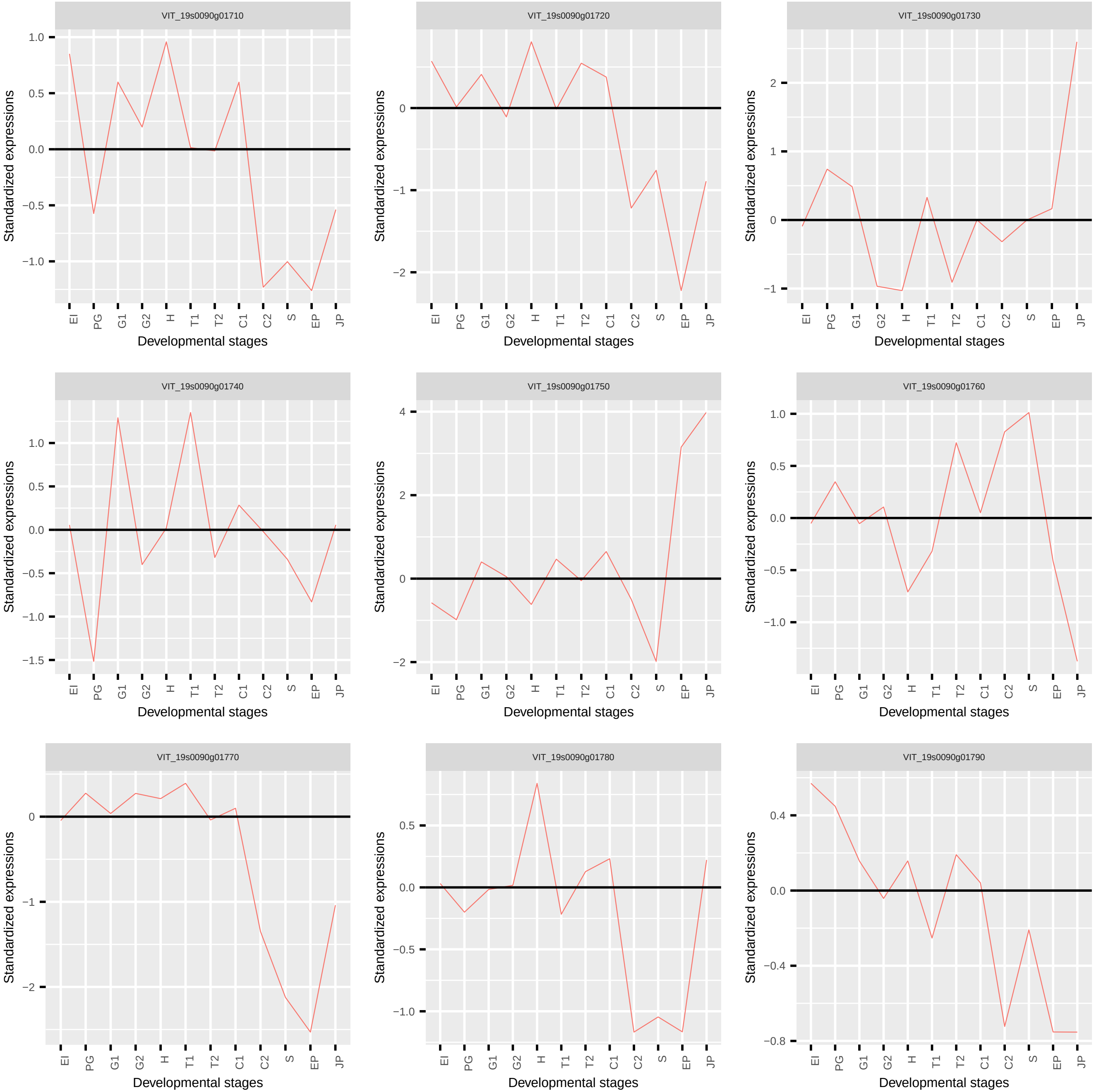
VIT_19s0090g01710
VIT_19s0090g01720
VIT_19s0090g01730
VIT_19s0090g01740
VIT_19s0090g01750
VIT_19s0090g01760
VIT_19s0090g01770
VIT_19s0090g01780
VIT_19s0090g01790
Standardized expressions
Developmental stages
EI PG G1 G2 H T1 T2 C1 C2 S EP JP

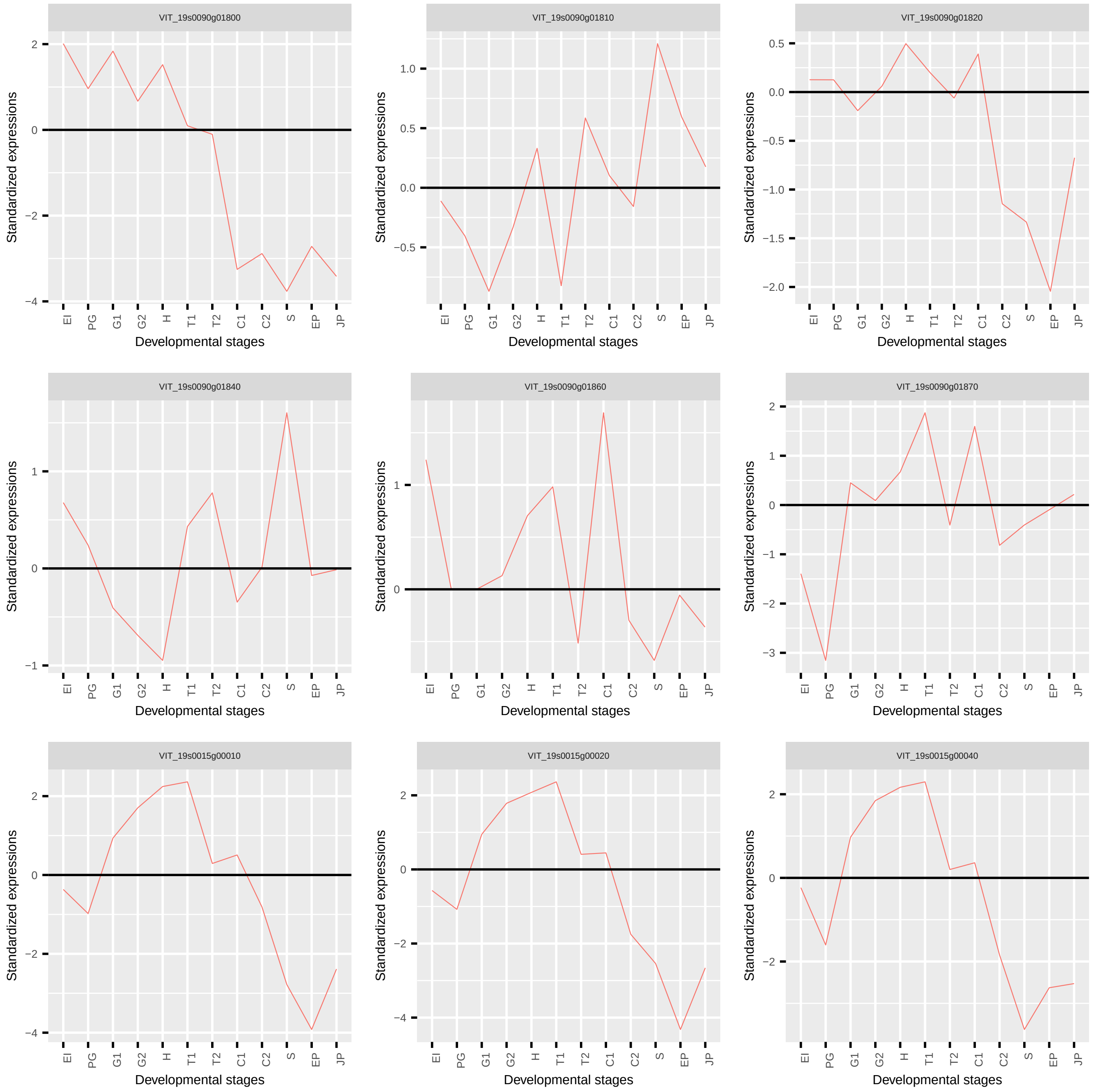
VIT_19s0090g01800
VIT_19s0090g01810
VIT_19s0090g01820
VIT_19s0090g01840
VIT_19s0090g01860
VIT_19s0090g01870
VIT_19s0015g00010
VIT_19s0015g00020
VIT_19s0015g00040
Standardized expressions
Developmental stages
EI PG G1 G2 H T1 T2 C1 C2 S EP JP

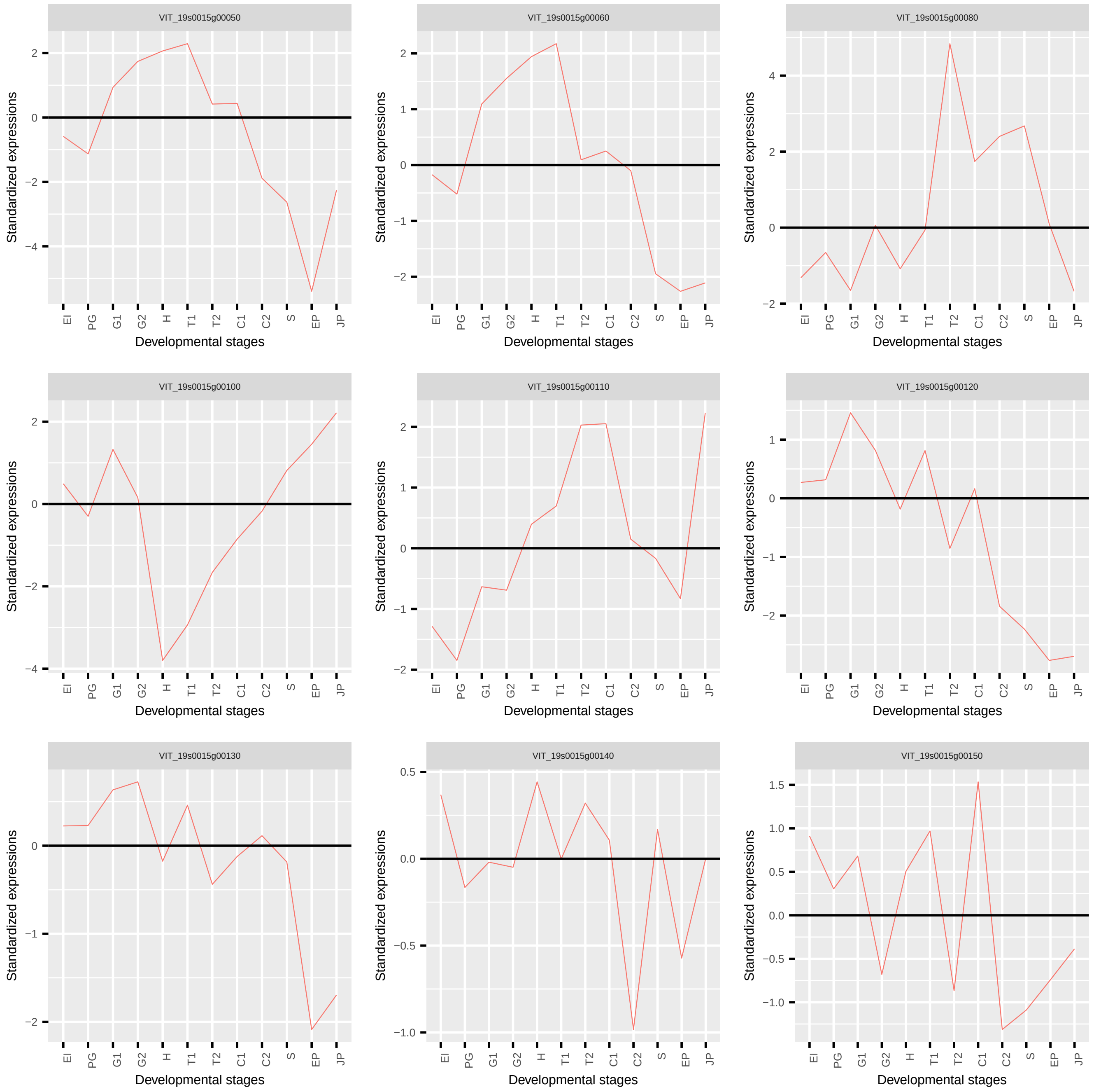

VIT_19s0015g00050
VIT_19s0015g00060
VIT_19s0015g00080
VIT_19s0015g00100
VIT_19s0015g00110
VIT_19s0015g00120
VIT_19s0015g00130
VIT_19s0015g00140
VIT_19s0015g00150
Standardized expressions
Developmental stages
EI PG G1 G2 H T1 T2 C1 C2 S EP JP

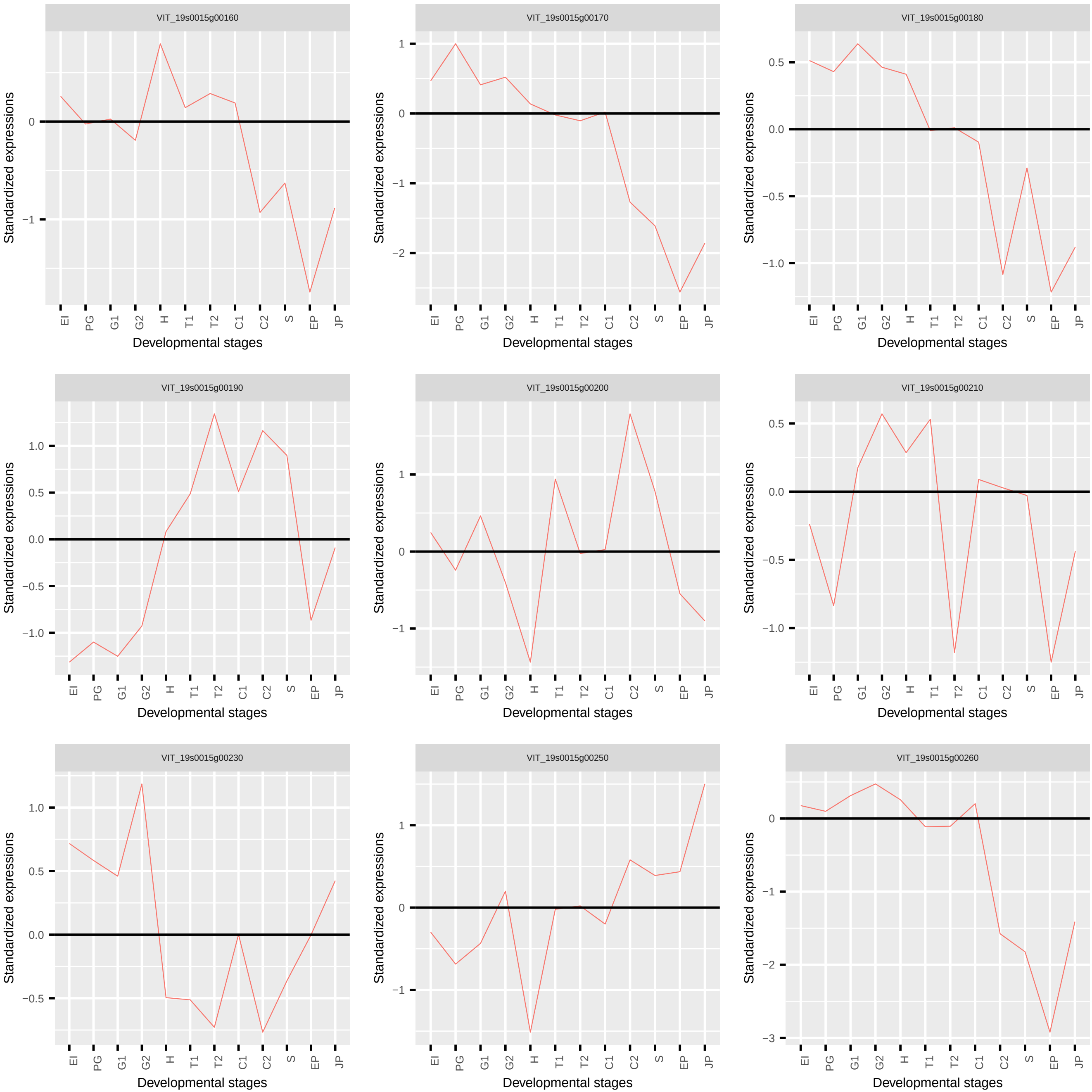
VIT_19s0015g00160
VIT_19s0015g00170
VIT_19s0015g00180
VIT_19s0015g00190
VIT_19s0015g00200
VIT_19s0015g00210
VIT_19s0015g00230
VIT_19s0015g00250
VIT_19s0015g00260
Standardized expressions
Developmental stages
EI PG G1 G2 H T1 T2 C1 C2 S EP JP

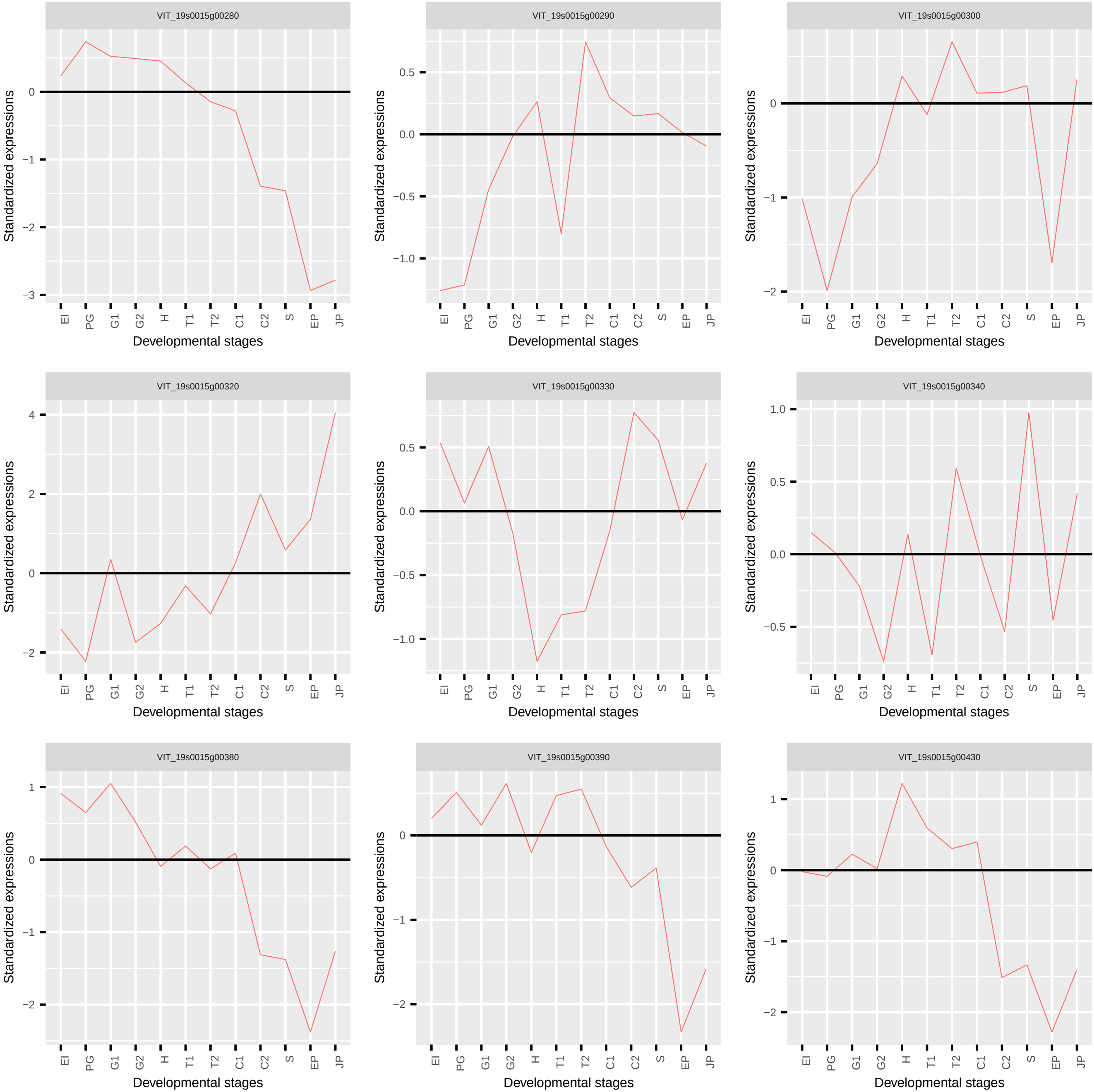
VIT_19s0015g00280
VIT_19s0015g00290
VIT_19s0015g00300
VIT_19s0015g00320
VIT_19s0015g00330
VIT_19s0015g00340
VIT_19s0015g00380
VIT_19s0015g00390
VIT_19s0015g00430
Standardized expressions
Developmental stages
EI PG G1 G2 H T1 T2 C1 C2 S EP JP

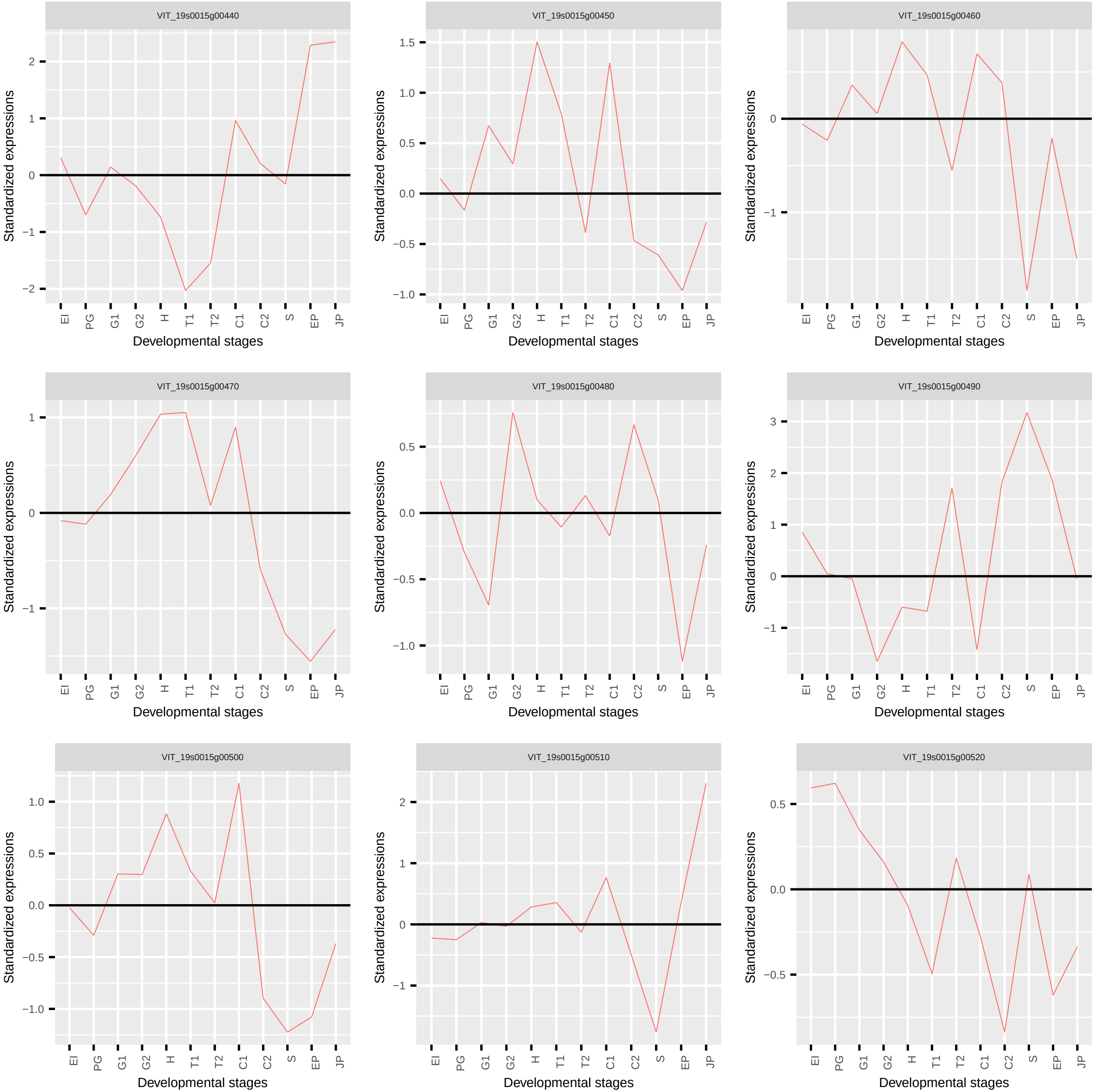
VIT_19s0015g00440
VIT_19s0015g00450
VIT_19s0015g00460
VIT_19s0015g00470
VIT_19s0015g00480
VIT_19s0015g00490
VIT_19s0015g00500
VIT_19s0015g00510
VIT_19s0015g00520
Standardized expressions
Developmental stages
EI PG G1 G2 H T1 T2 C1 C2 S EP JP

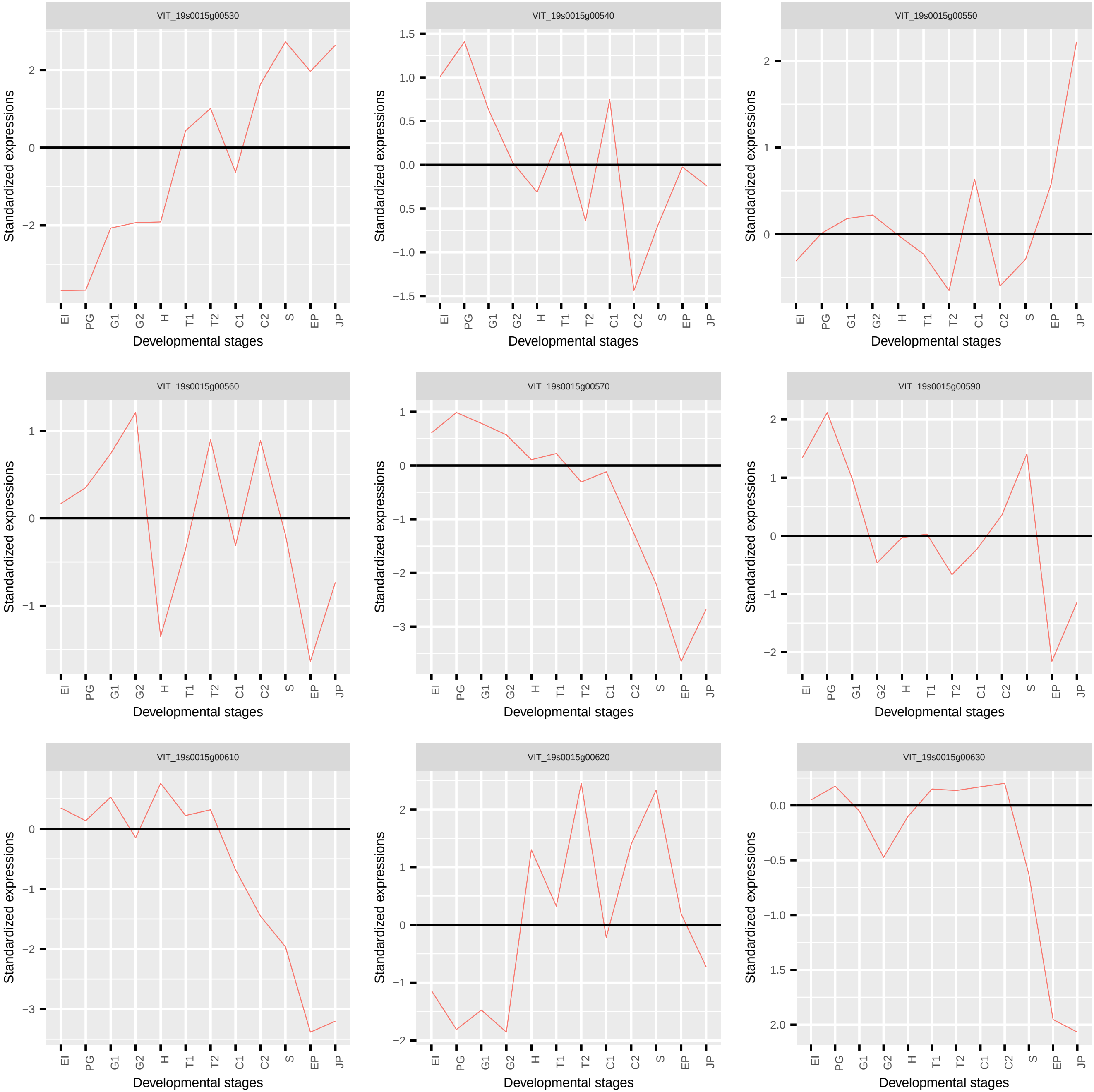

VIT_19s0015g00530
VIT_19s0015g00540
VIT_19s0015g00550
VIT_19s0015g00560
VIT_19s0015g00570
VIT_19s0015g00590
VIT_19s0015g00610
VIT_19s0015g00620
VIT_19s0015g00630
Standardized expressions
Developmental stages
EI PG G1 G2 H T1 T2 C1 C2 S EP JP

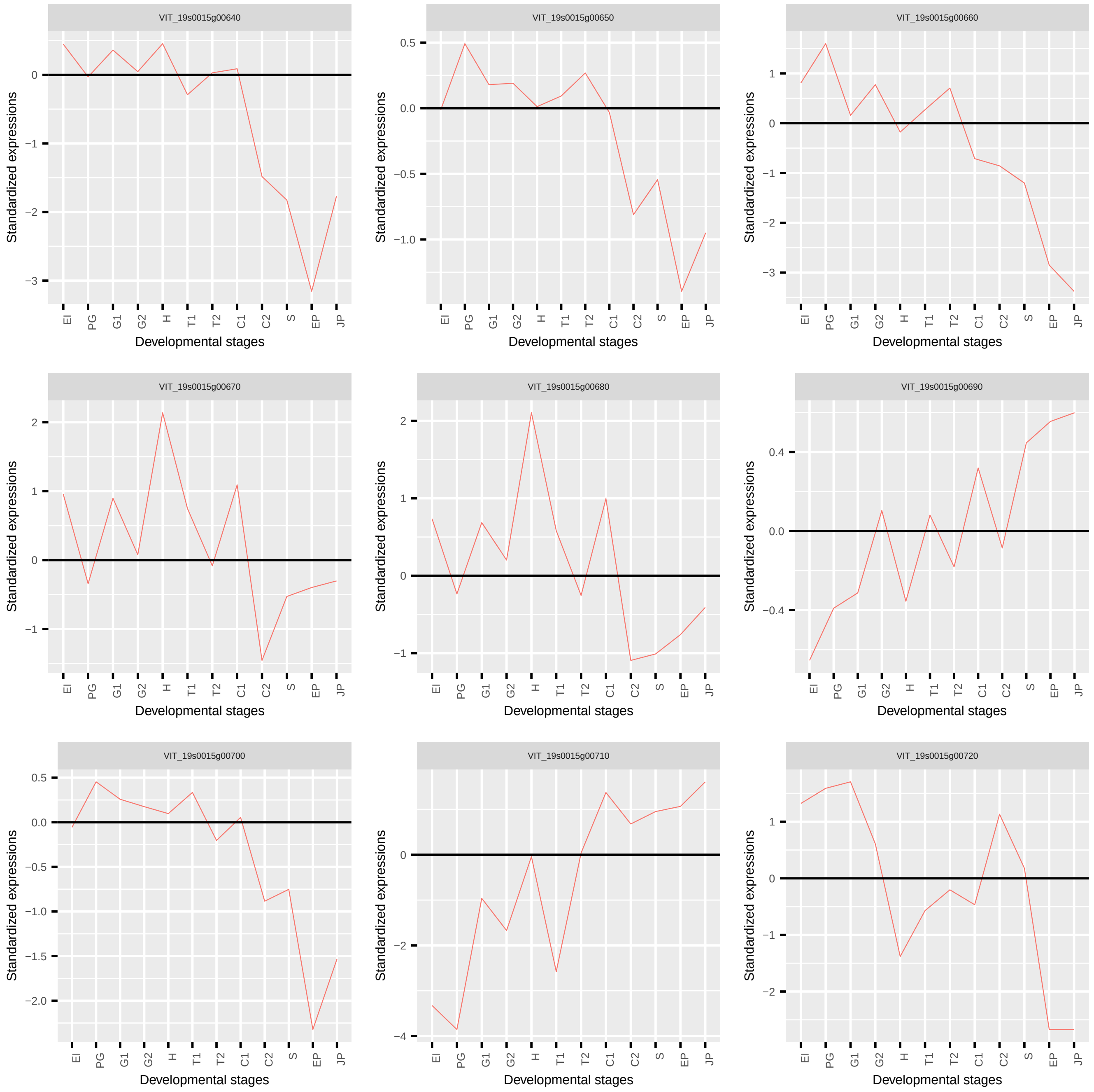

VIT_19s0015g00640
VIT_19s0015g00650
VIT_19s0015g00660
VIT_19s0015g00670
VIT_19s0015g00680
VIT_19s0015g00690
VIT_19s0015g00700
VIT_19s0015g00710
VIT_19s0015g00720
Standardized expressions
Developmental stages
EI
PG
G1
G2
H
T1
T2
C1
C2
S
EP
JP

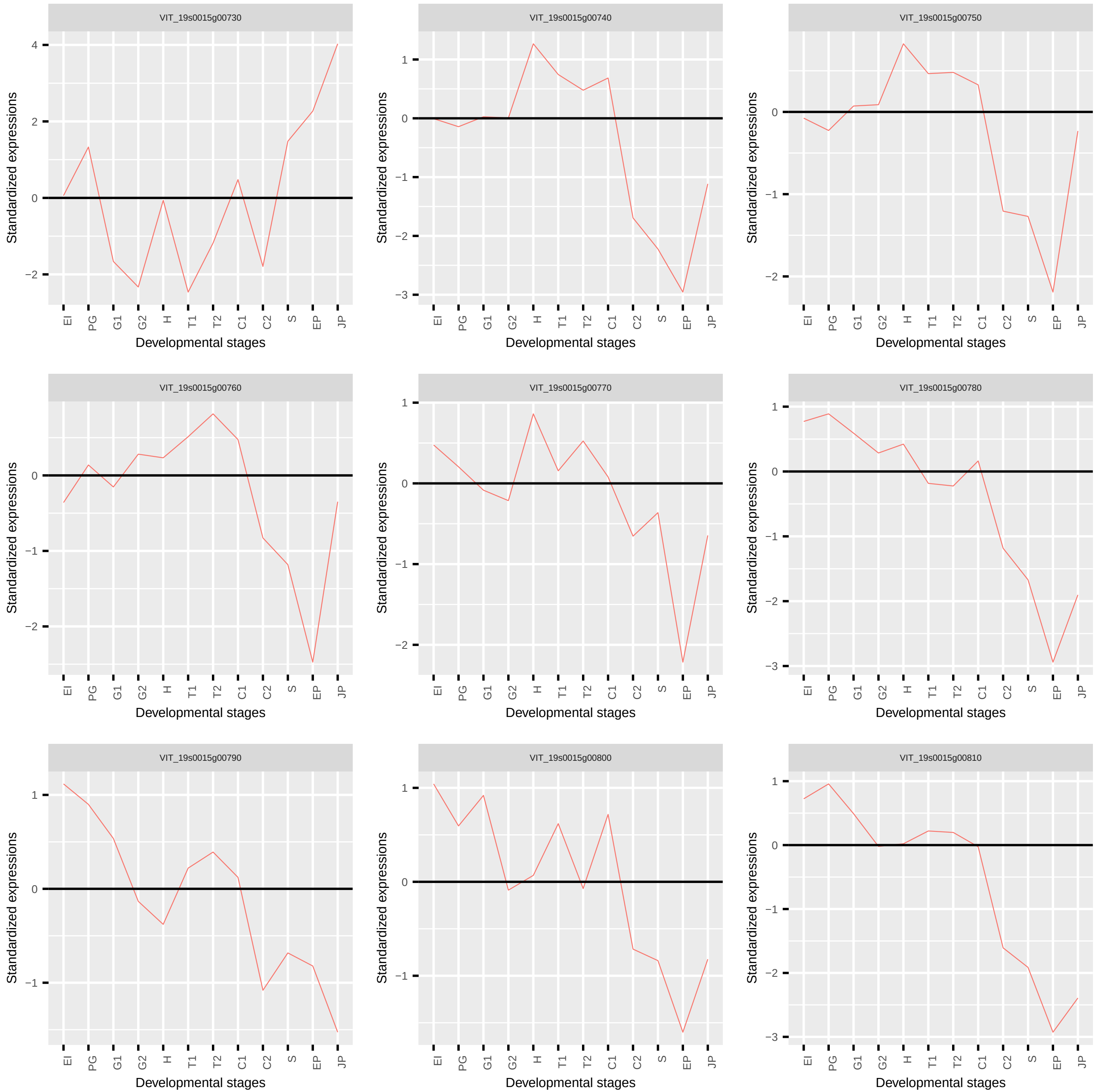

VIT_19s0015g00730
VIT_19s0015g00740
VIT_19s0015g00750
VIT_19s0015g00760
VIT_19s0015g00770
VIT_19s0015g00780
VIT_19s0015g00790
VIT_19s0015g00800
VIT_19s0015g00810
Standardized expressions
Developmental stages
EI PG G1 G2 H T1 T2 C1 C2 S EP JP

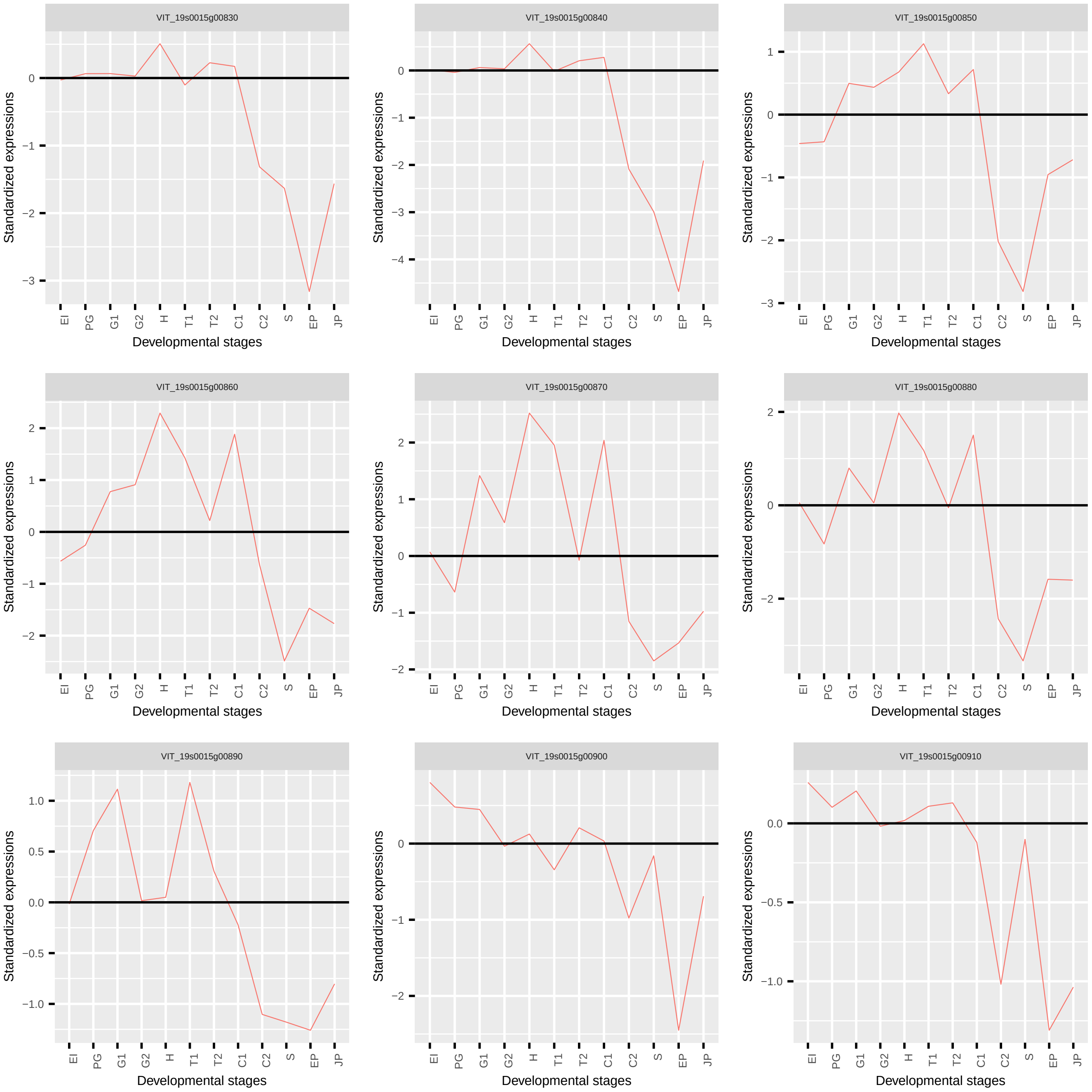
VIT_19s0015g00830
VIT_19s0015g00840
VIT_19s0015g00850
VIT_19s0015g00860
VIT_19s0015g00870
VIT_19s0015g00880
VIT_19s0015g00890
VIT_19s0015g00900
VIT_19s0015g00910
Standardized expressions
Developmental stages
EI PG G1 G2 H T1 T2 C1 C2 S EP JP

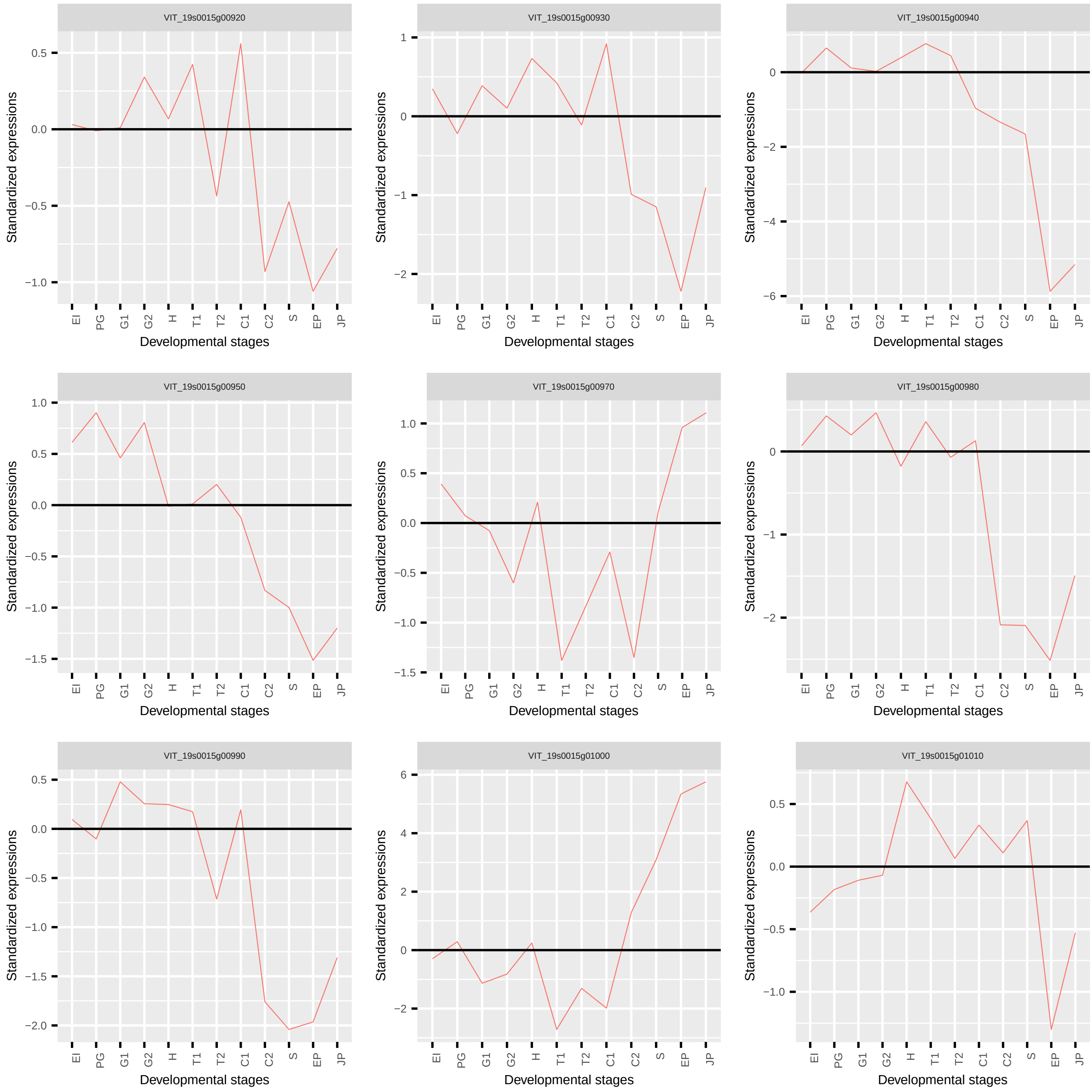
VIT_19s0015g00920
VIT_19s0015g00930
VIT_19s0015g00940
VIT_19s0015g00950
VIT_19s0015g00970
VIT_19s0015g00980
VIT_19s0015g00990
VIT_19s0015g01000
VIT_19s0015g01010
Standardized expressions
Developmental stages
EI PG G1 G2 H T1 T2 C1 C2 S EP JP

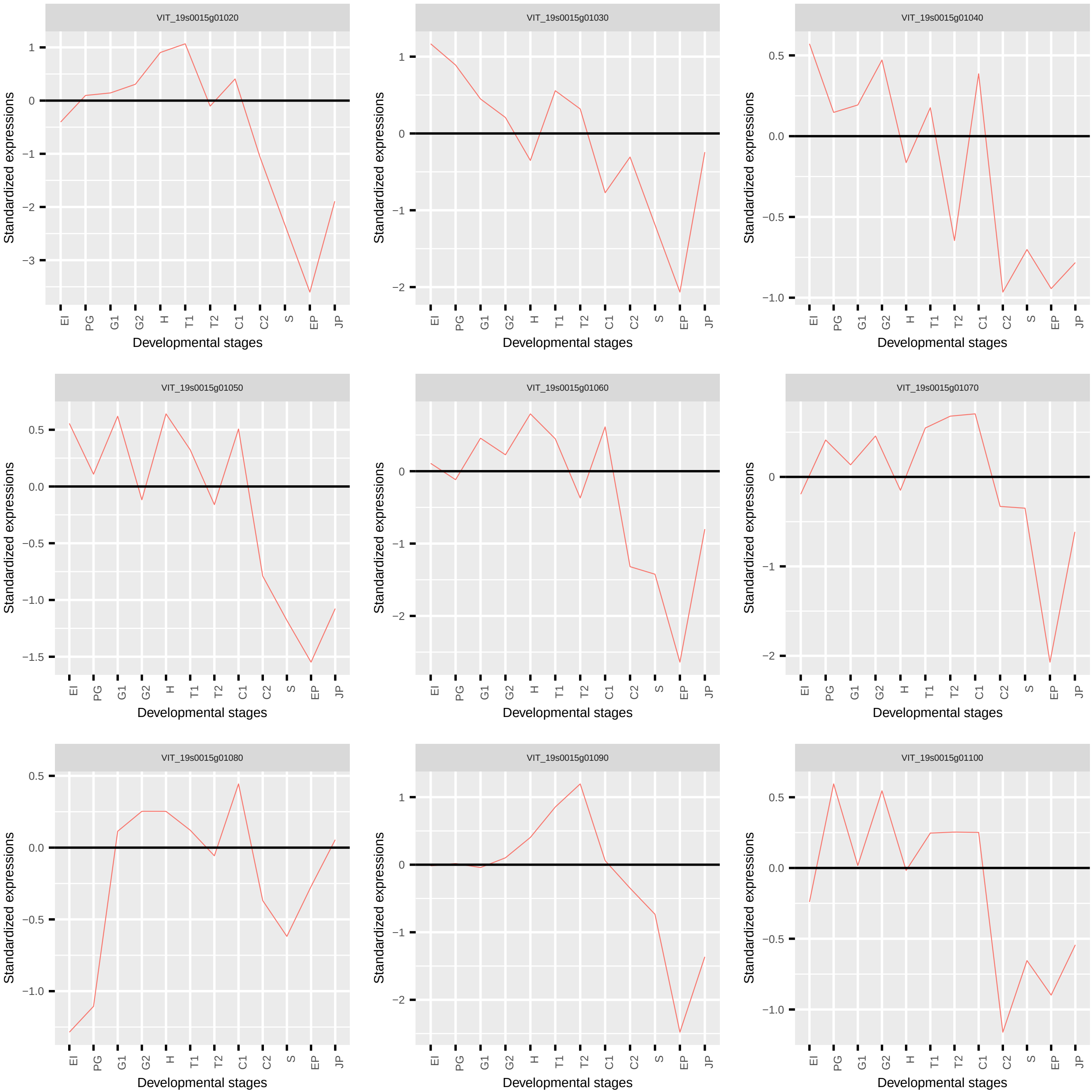

VIT_19s0015g01020
VIT_19s0015g01030
VIT_19s0015g01040
VIT_19s0015g01050
VIT_19s0015g01060
VIT_19s0015g01070
VIT_19s0015g01080
VIT_19s0015g01090
VIT_19s0015g01100
Standardized expressions
Developmental stages
EI PG G1 G2 H T1 T2 C1 C2 S EP JP

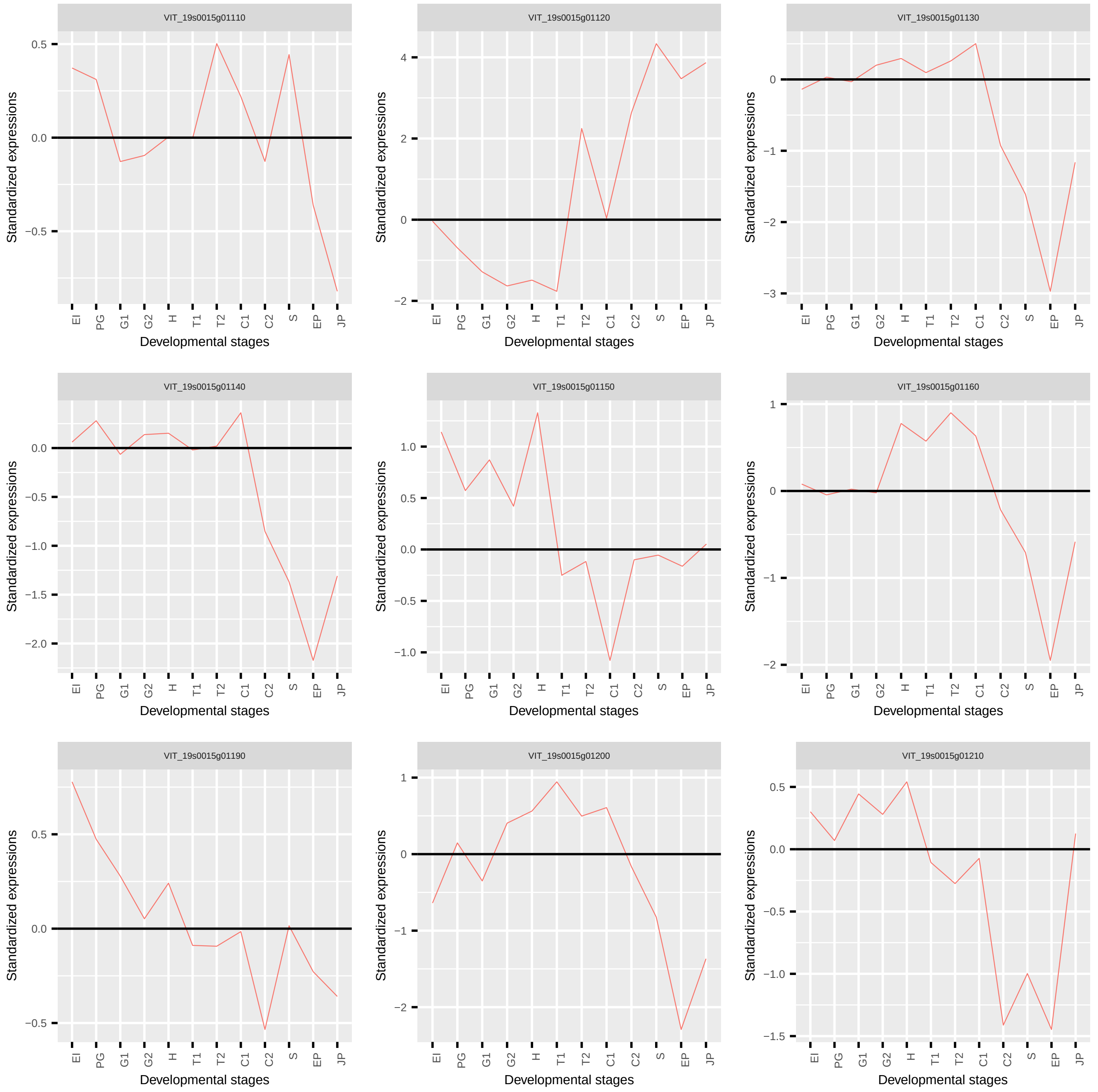

VIT_19s0015g01110
VIT_19s0015g01120
VIT_19s0015g01130
VIT_19s0015g01140
VIT_19s0015g01150
VIT_19s0015g01160
VIT_19s0015g01190
VIT_19s0015g01200
VIT_19s0015g01210
Standardized expressions
Developmental stages
EI PG G1 G2 H T1 T2 C1 C2 S EP JP

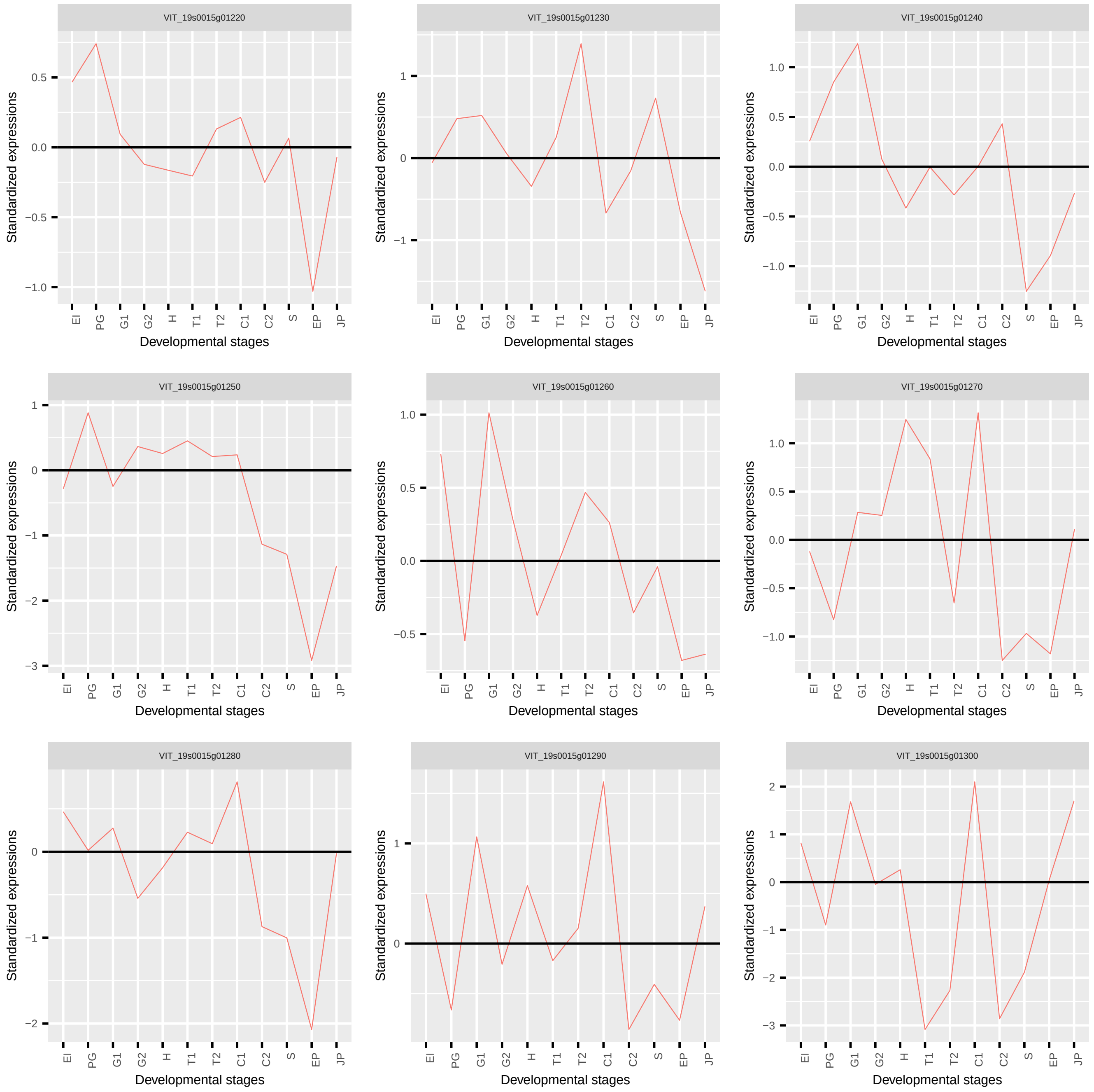
VIT_19s0015g01220
VIT_19s0015g01230
VIT_19s0015g01240
VIT_19s0015g01250
VIT_19s0015g01260
VIT_19s0015g01270
VIT_19s0015g01280
VIT_19s0015g01290
VIT_19s0015g01300
Standardized expressions
Developmental stages
EI
PG
G1
G2
H
T1
T2
C1
C2
S
EP
JP

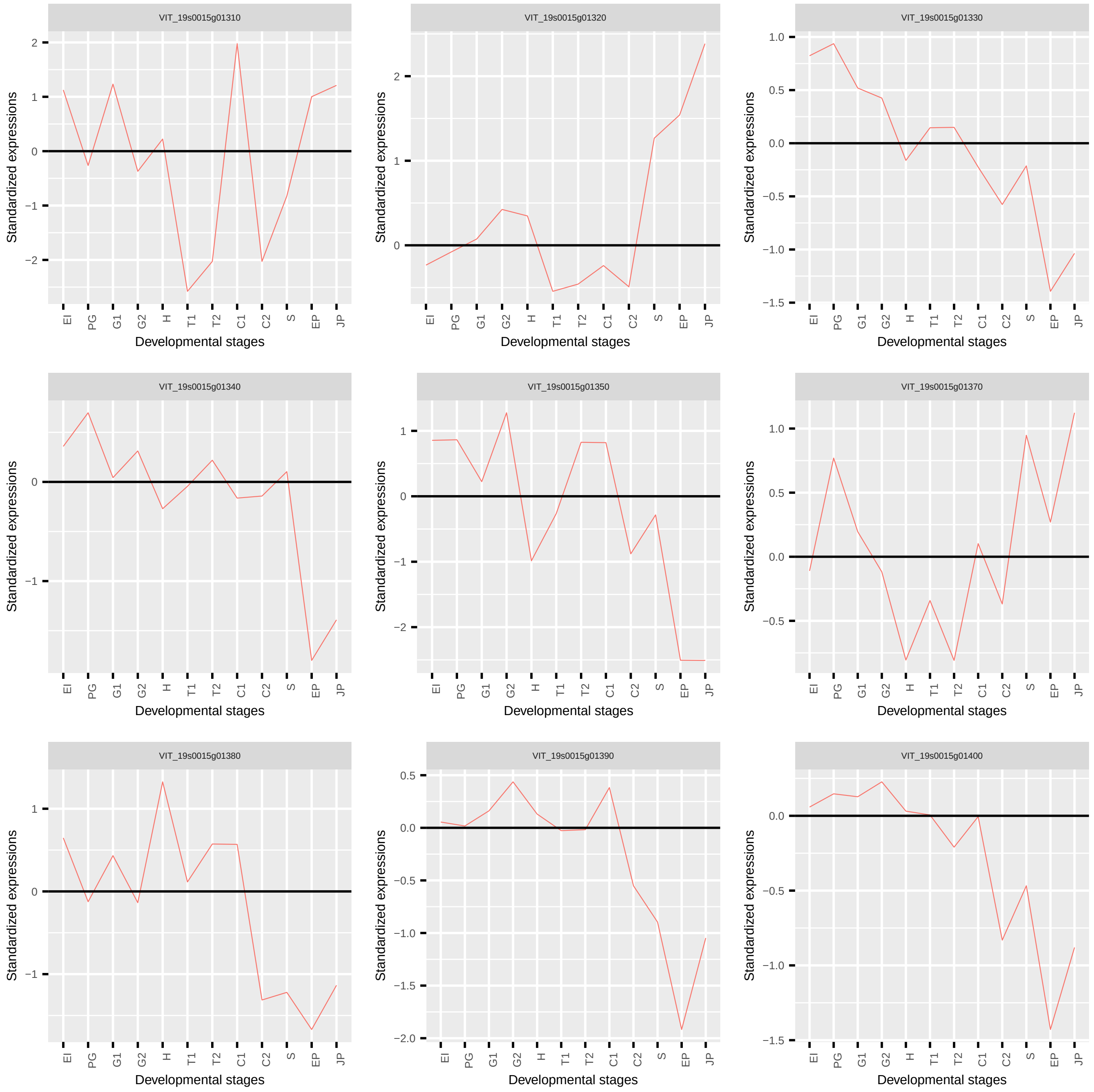
VIT_19s0015g01310
VIT_19s0015g01320
VIT_19s0015g01330
VIT_19s0015g01340
VIT_19s0015g01350
VIT_19s0015g01370
VIT_19s0015g01380
VIT_19s0015g01390
VIT_19s0015g01400
Standardized expressions
Developmental stages
EI PG G1 G2 H T1 T2 C1 C2 S EP JP

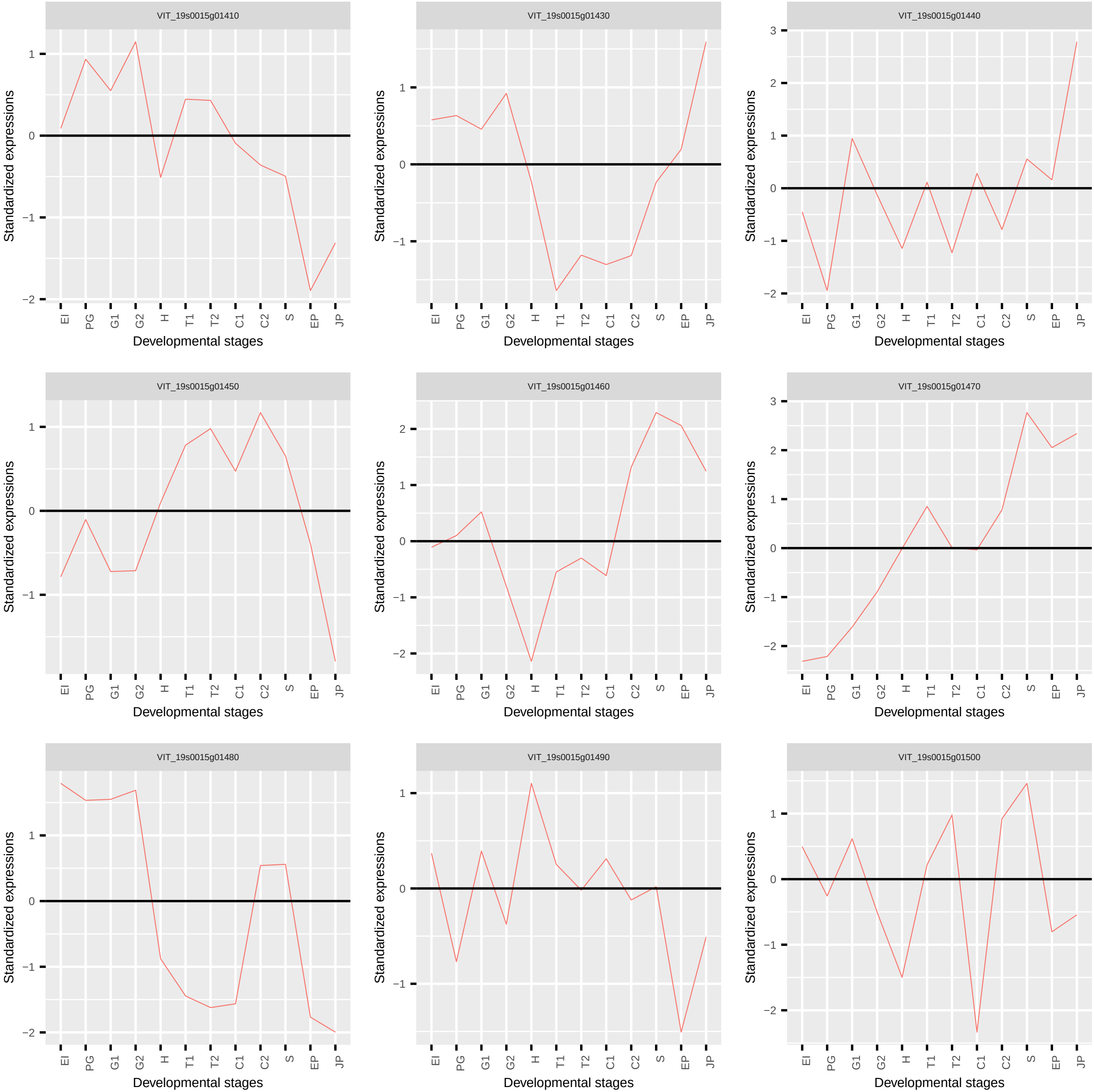
VIT_19s0015g01410
VIT_19s0015g01430
VIT_19s0015g01440
VIT_19s0015g01450
VIT_19s0015g01460
VIT_19s0015g01470
VIT_19s0015g01480
VIT_19s0015g01490
VIT_19s0015g01500
Standardized expressions
Developmental stages
EI PG G1 G2 H T1 T2 C1 C2 S EP JP

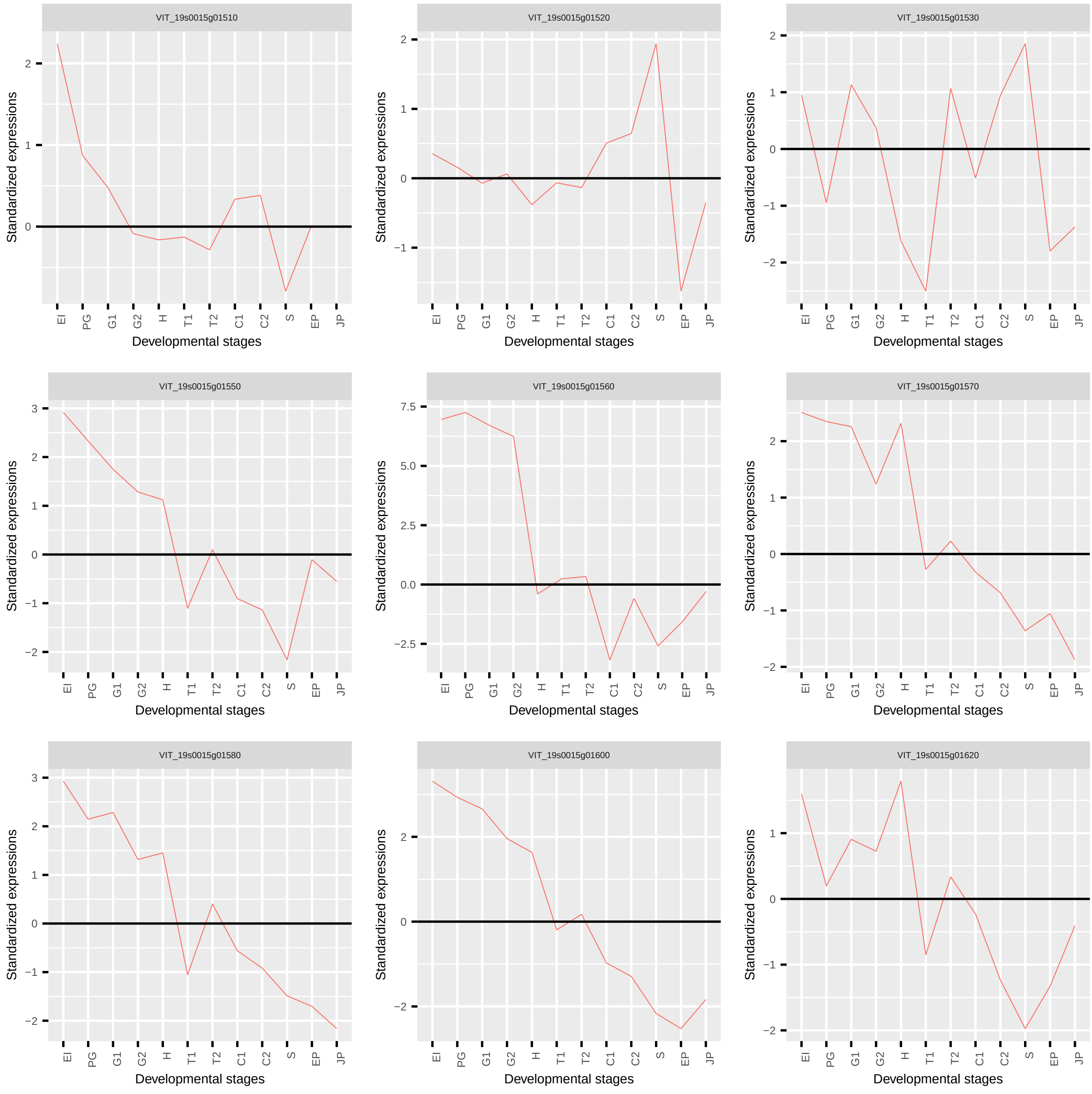
VIT_19s0015g01510
VIT_19s0015g01520
VIT_19s0015g01530
VIT_19s0015g01550
VIT_19s0015g01560
VIT_19s0015g01570
VIT_19s0015g01580
VIT_19s0015g01600
VIT_19s0015g01620
Standardized expressions
Developmental stages
EI PG G1 G2 H T1 T2 C1 C2 S EP JP

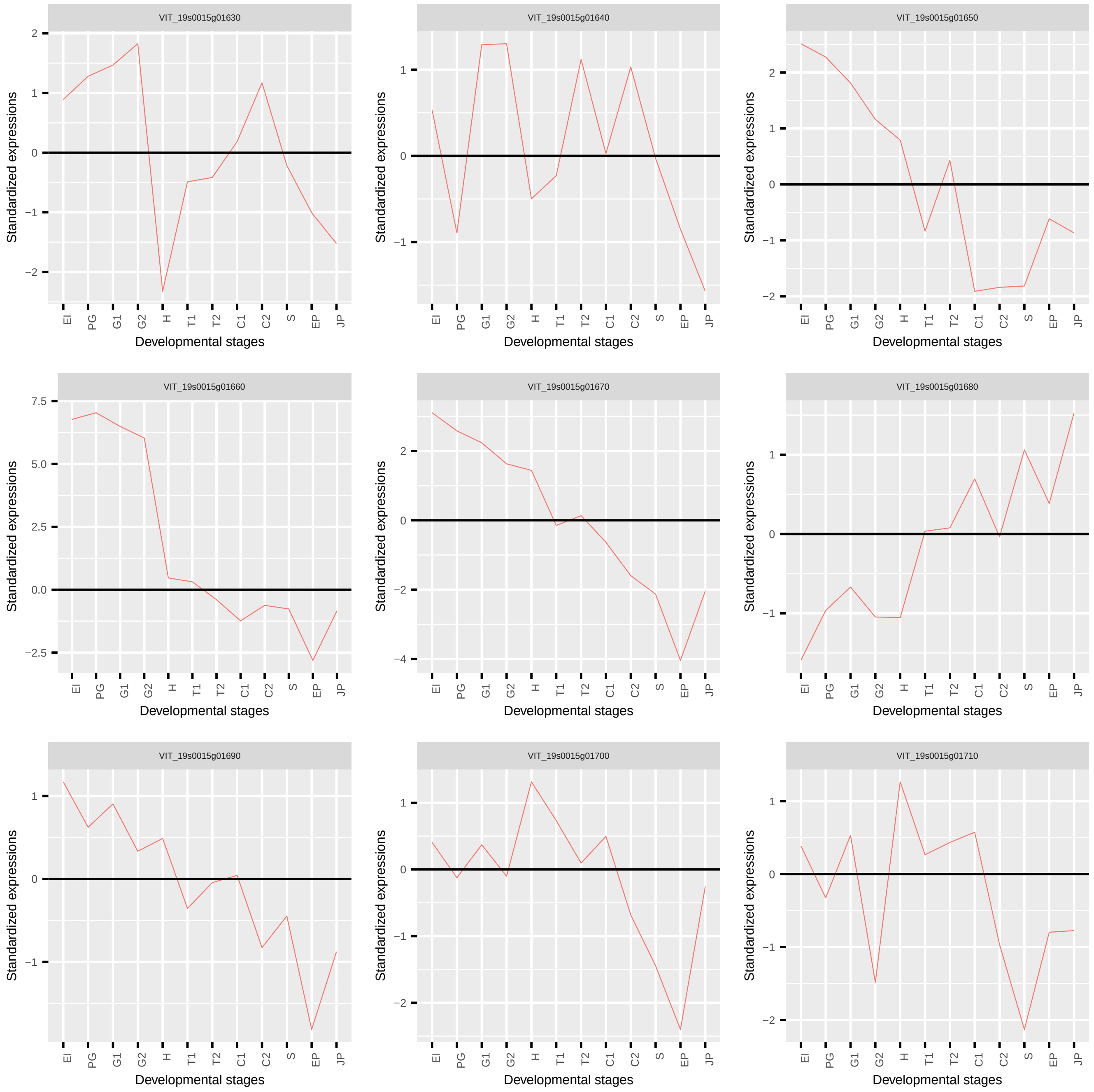
VIT_19s0015g01630
VIT_19s0015g01640
VIT_19s0015g01650
VIT_19s0015g01660
VIT_19s0015g01670
VIT_19s0015g01680
VIT_19s0015g01690
VIT_19s0015g01700
VIT_19s0015g01710
Standardized expressions
Developmental stages
EI PG G1 G2 H T1 T2 C1 C2 S EP JP

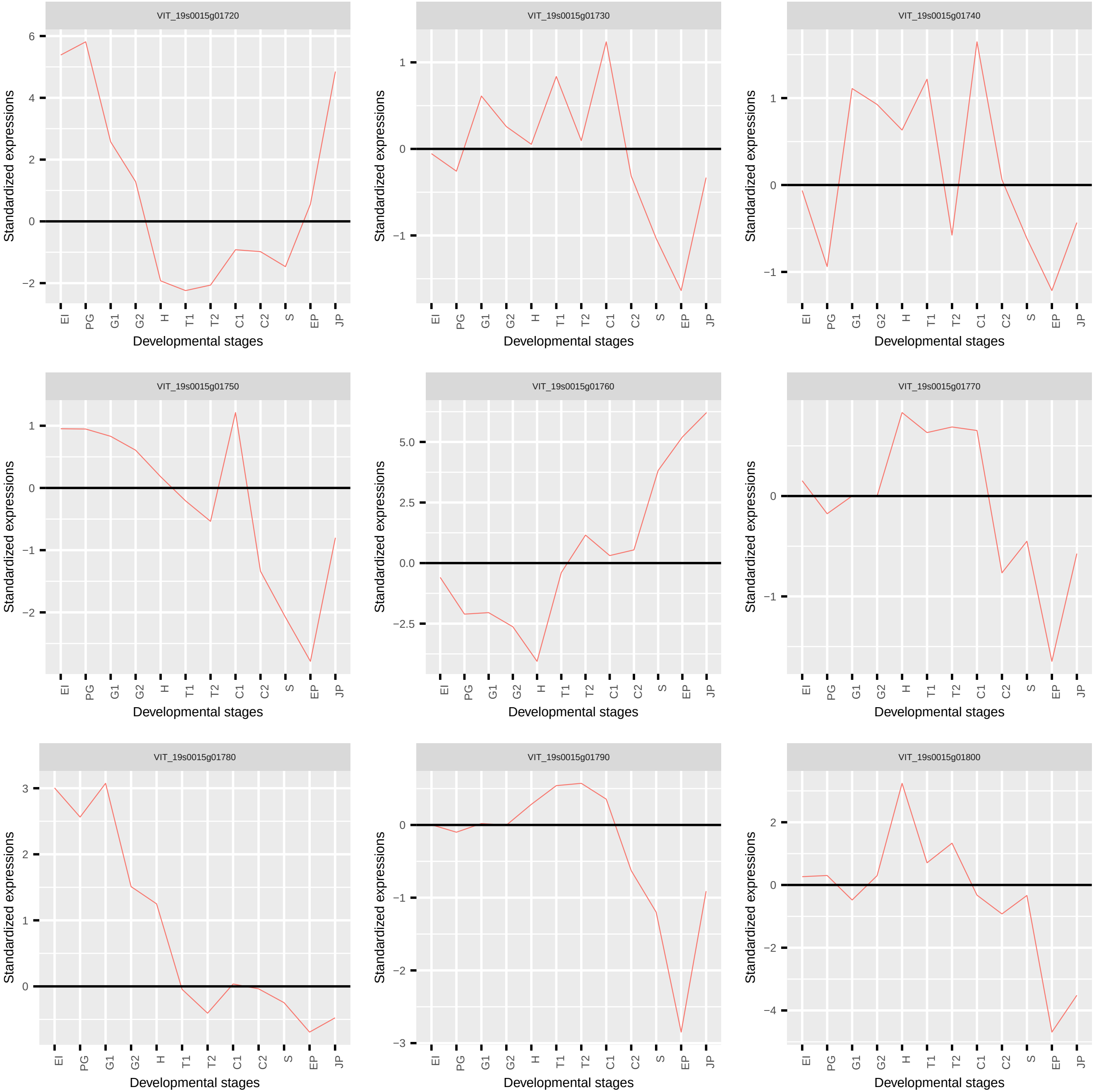
VIT_19s0015g01720
VIT_19s0015g01730
VIT_19s0015g01740
VIT_19s0015g01750
VIT_19s0015g01760
VIT_19s0015g01770
VIT_19s0015g01780
VIT_19s0015g01790
VIT_19s0015g01800
Standardized expressions
Developmental stages
EI PG G1 G2 H T1 T2 C1 C2 S EP JP

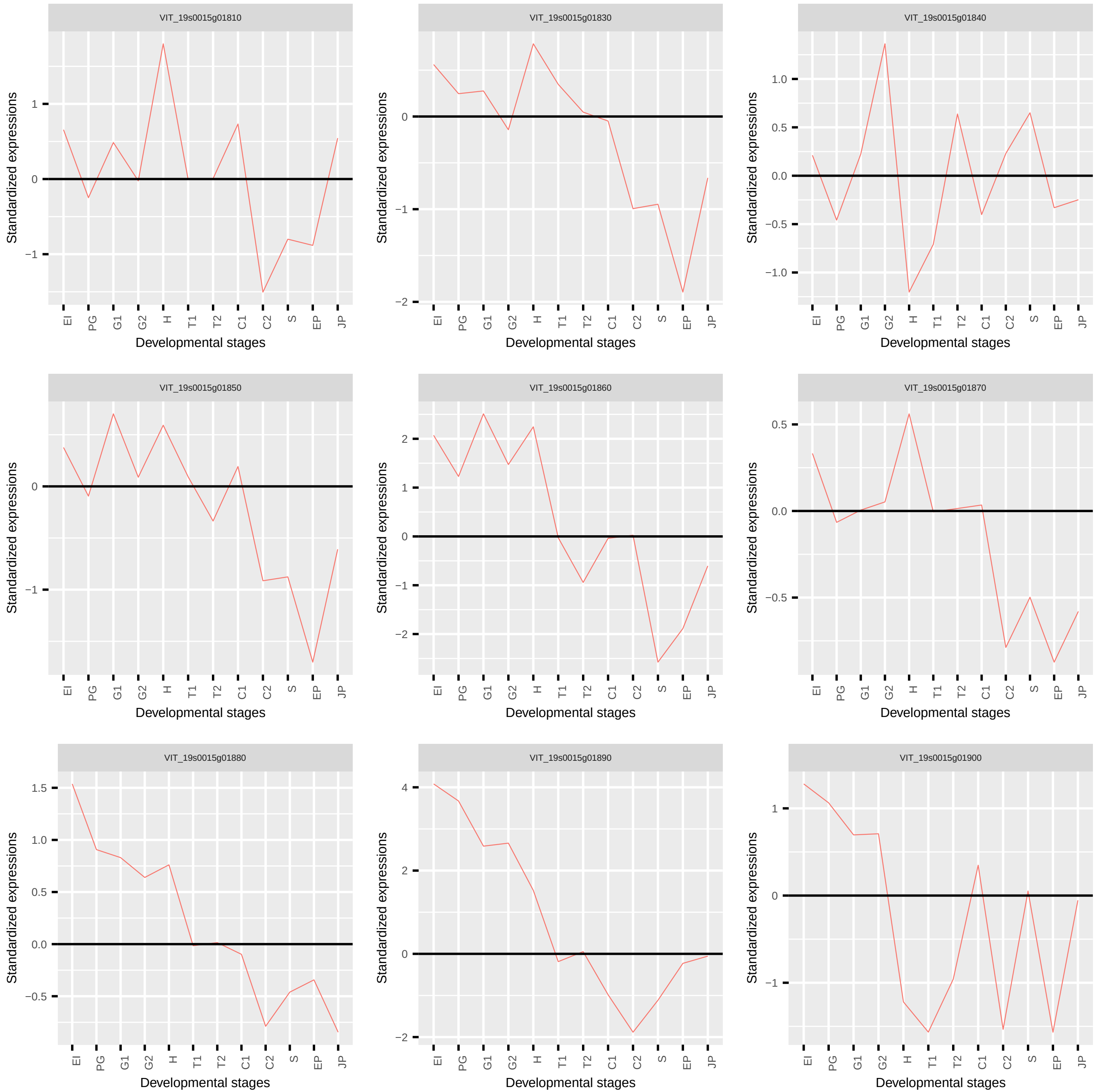
VIT_19s0015g01810
VIT_19s0015g01830
VIT_19s0015g01840
VIT_19s0015g01850
VIT_19s0015g01860
VIT_19s0015g01870
VIT_19s0015g01880
VIT_19s0015g01890
VIT_19s0015g01900
Standardized expressions
Developmental stages
EI
PG
G1
G2
H
T1
T2
C1
C2
S
EP
JP

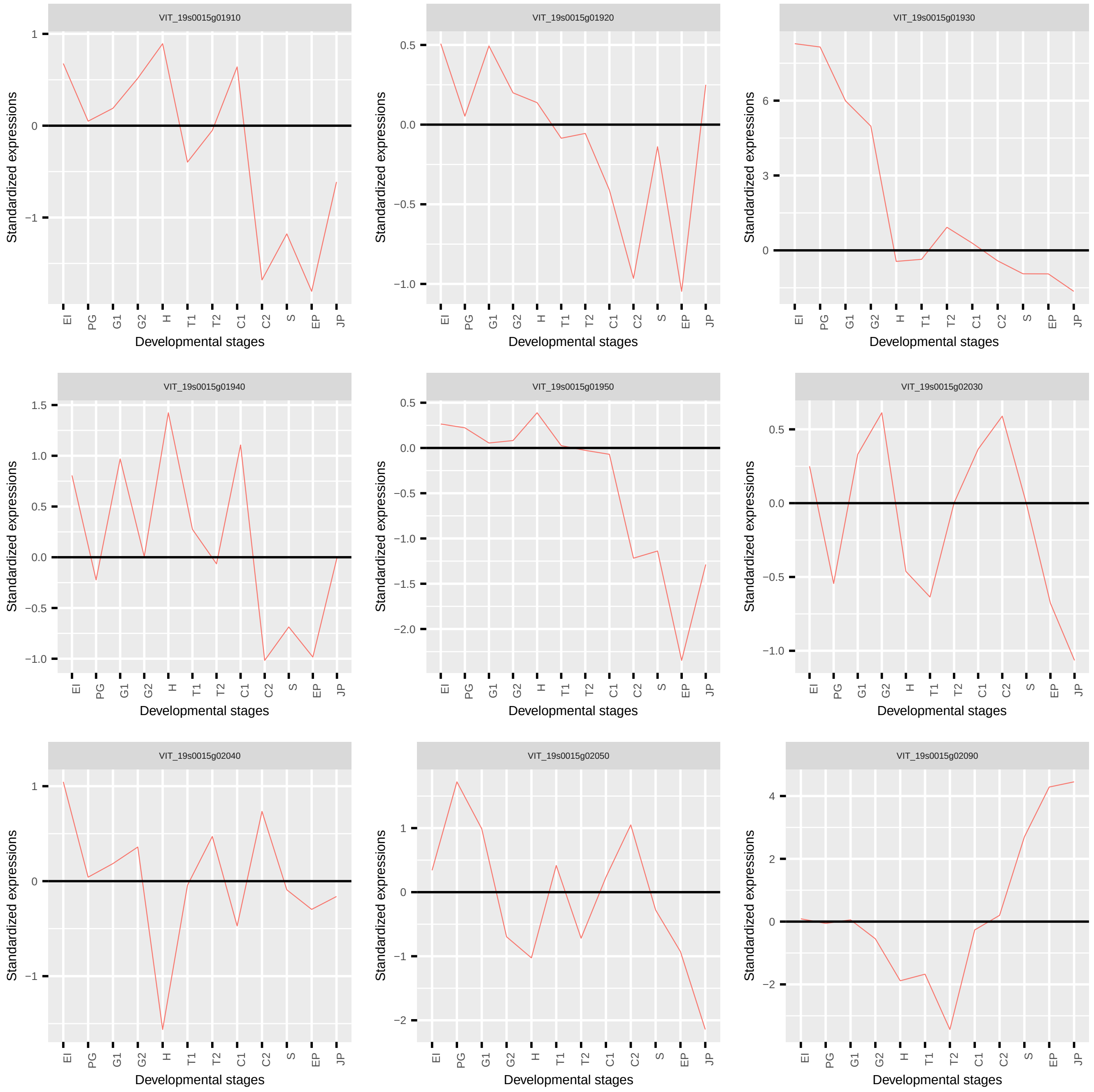
VIT_19s0015g01910
VIT_19s0015g01920
VIT_19s0015g01930
VIT_19s0015g01940
VIT_19s0015g01950
VIT_19s0015g02030
VIT_19s0015g02040
VIT_19s0015g02050
VIT_19s0015g02090
Standardized expressions
Developmental stages
EI PG G1 G2 H T1 T2 C1 C2 S EP JP

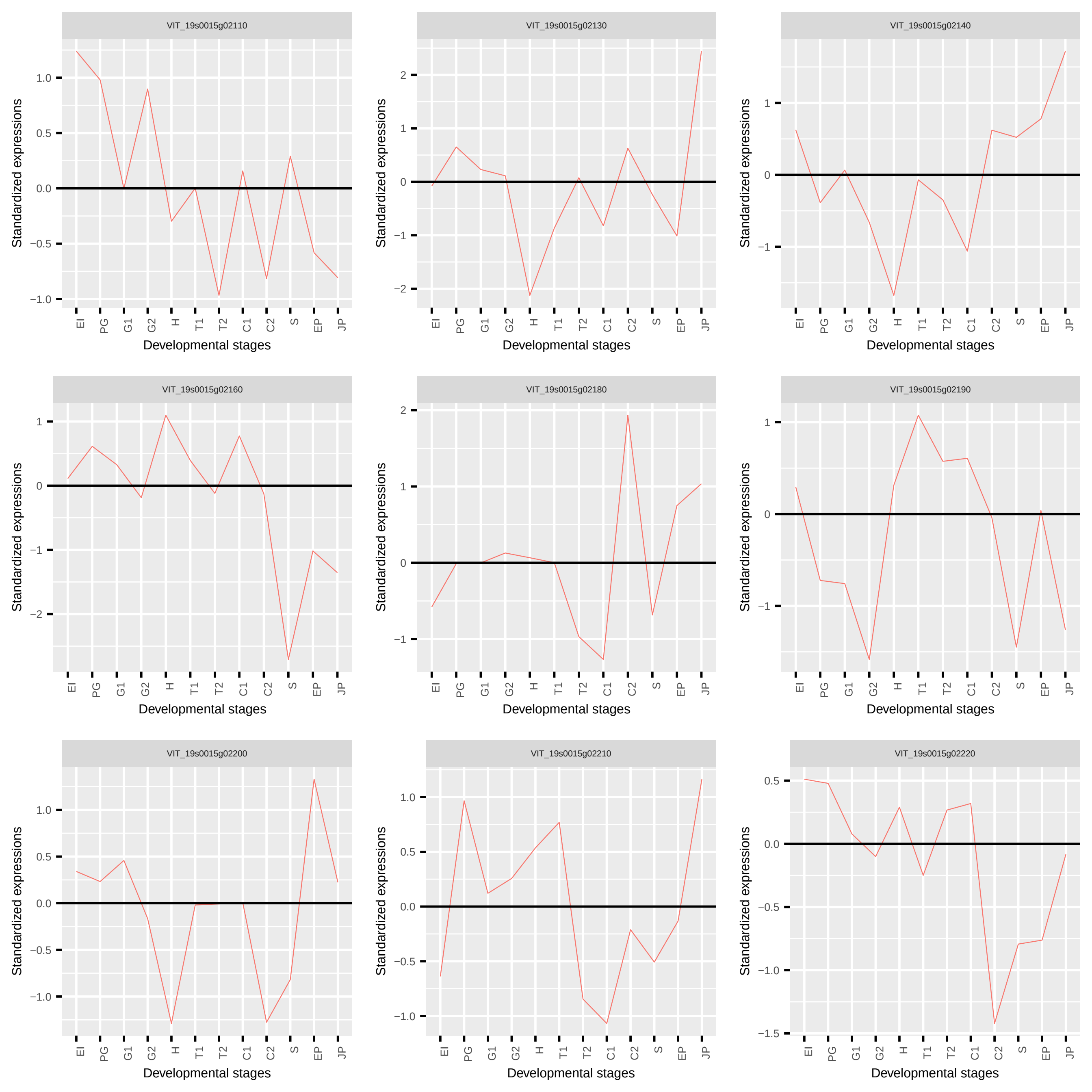
VIT_19s0015g02110
VIT_19s0015g02130
VIT_19s0015g02140
VIT_19s0015g02160
VIT_19s0015g02180
VIT_19s0015g02190
VIT_19s0015g02200
VIT_19s0015g02210
VIT_19s0015g02220
Standardized expressions
Developmental stages
EI PG G1 G2 H T1 T2 C1 C2 S EP JP

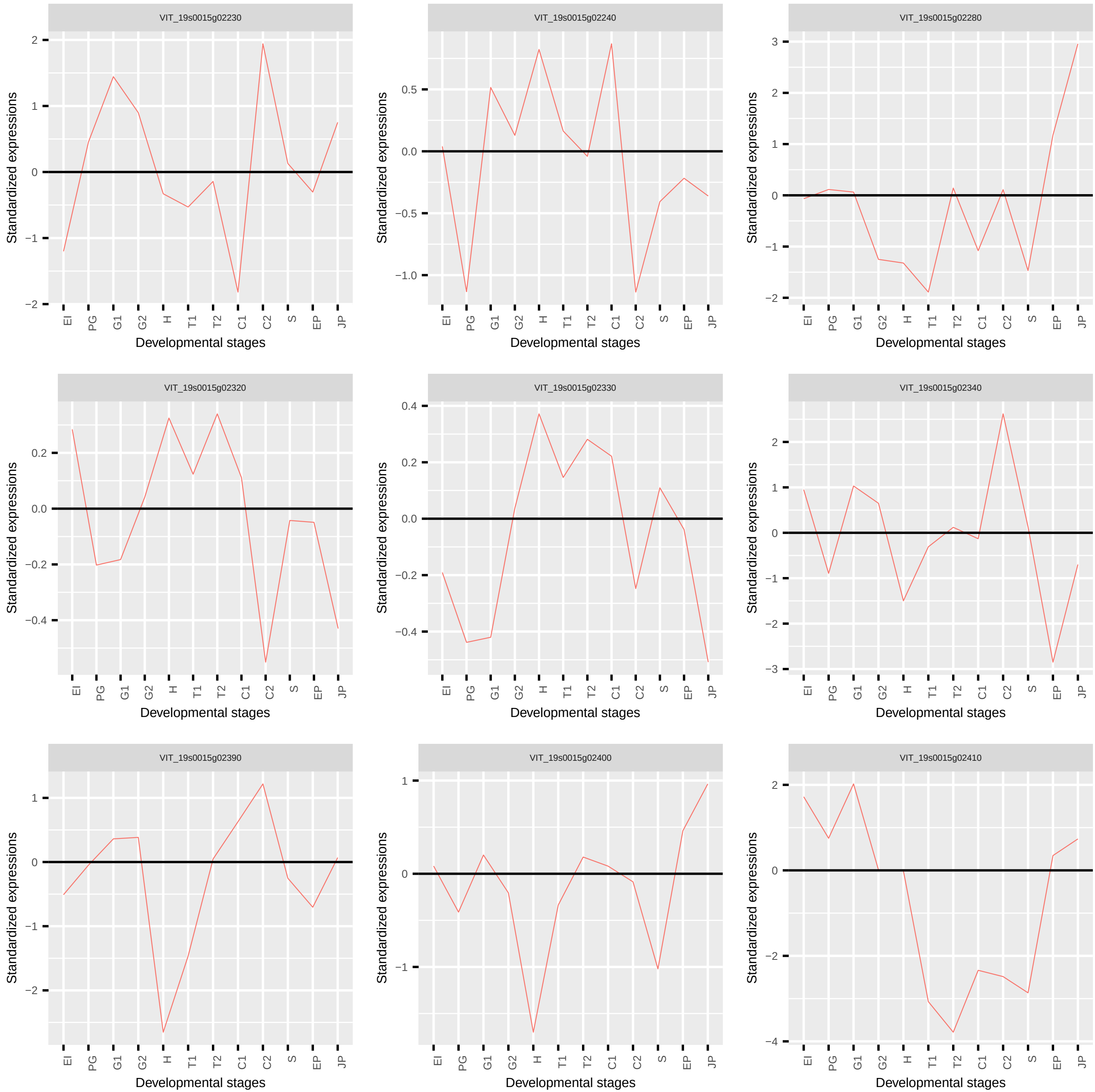
VIT_19s0015g02230
VIT_19s0015g02240
VIT_19s0015g02280
VIT_19s0015g02320
VIT_19s0015g02330
VIT_19s0015g02340
VIT_19s0015g02390
VIT_19s0015g02400
VIT_19s0015g02410
Standardized expressions
Developmental stages
EI PG G1 G2 H T1 T2 C1 C2 S EP JP

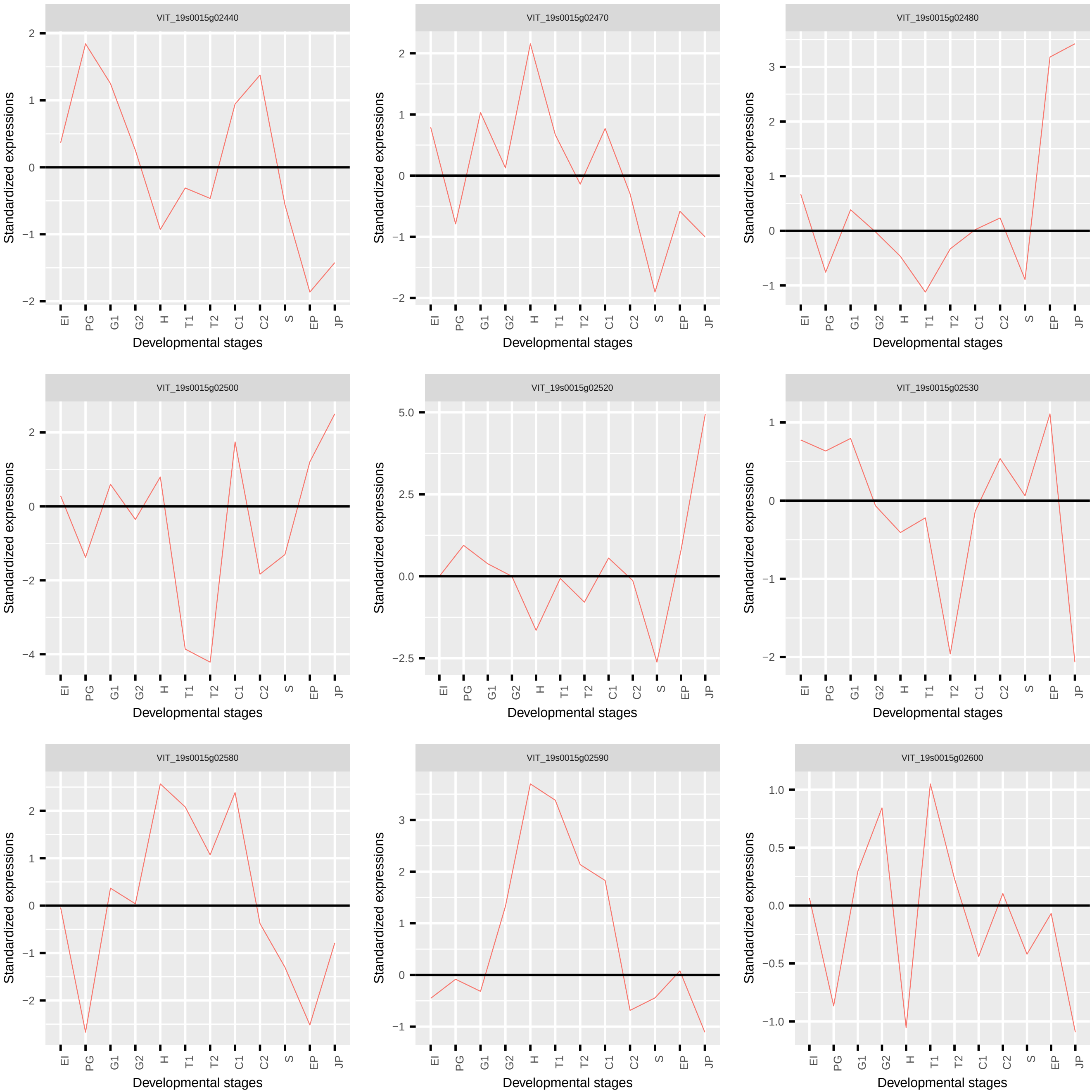
VIT_19s0015g02440
VIT_19s0015g02470
VIT_19s0015g02480
VIT_19s0015g02500
VIT_19s0015g02520
VIT_19s0015g02530
VIT_19s0015g02580
VIT_19s0015g02590
VIT_19s0015g02600
Standardized expressions
Developmental stages
EI PG G1 G2 H T1 T2 C1 C2 S EP JP

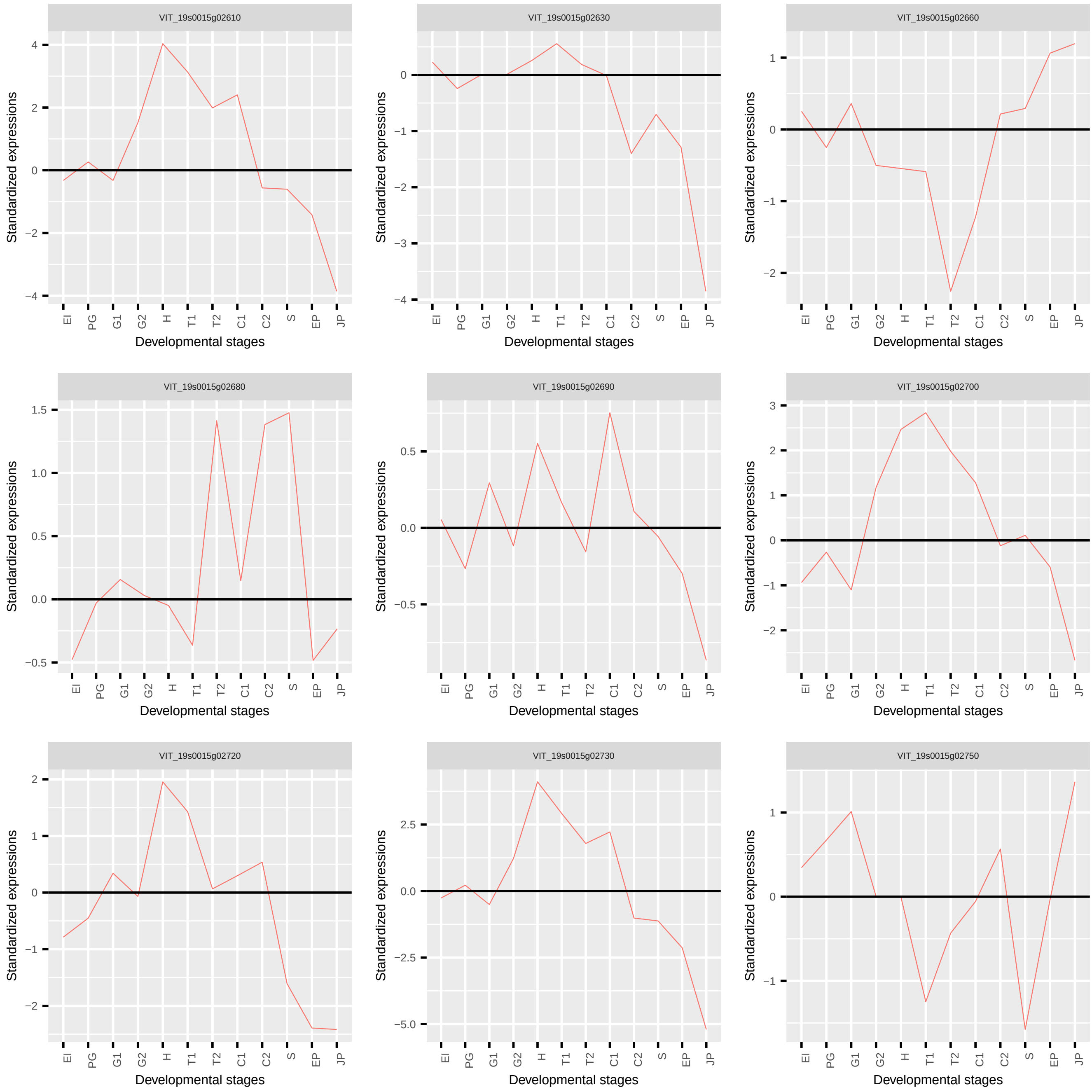
VIT_19s0015g02610
VIT_19s0015g02630
VIT_19s0015g02660
VIT_19s0015g02680
VIT_19s0015g02690
VIT_19s0015g02700
VIT_19s0015g02720
VIT_19s0015g02730
VIT_19s0015g02750
Standardized expressions
Developmental stages
EI
PG
G1
G2
H
T1
T2
C1
C2
S
EP
JP
4
2
0
−2
−4
−1
−3
1
1.5
1.0
0.5
0.0
−0.5
3
2.5
−2.5
−5.0

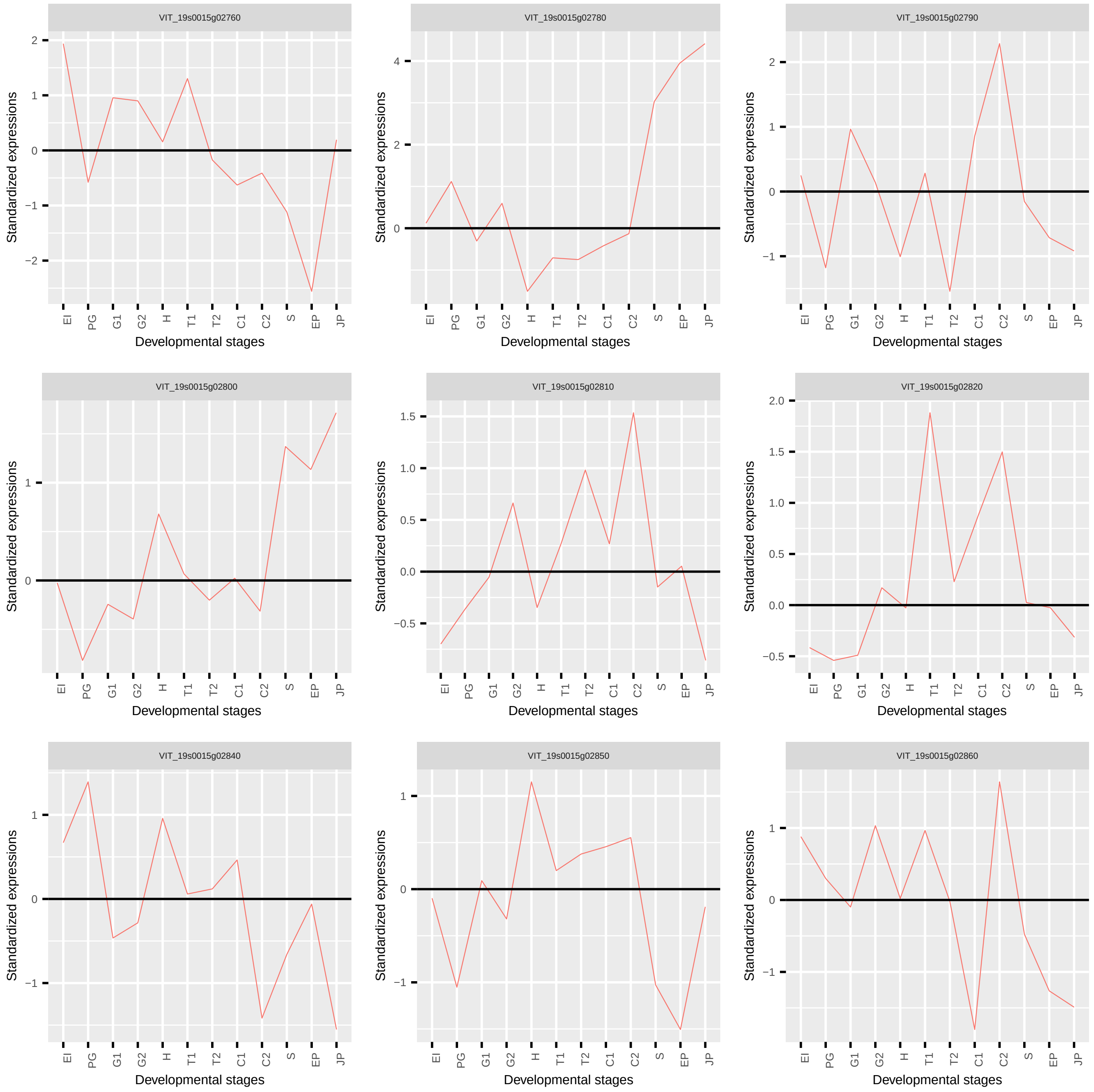
VIT_19s0015g02760
VIT_19s0015g02780
VIT_19s0015g02790
VIT_19s0015g02800
VIT_19s0015g02810
VIT_19s0015g02820
VIT_19s0015g02840
VIT_19s0015g02850
VIT_19s0015g02860
Standardized expressions
Developmental stages
EI PG G1 G2 H T1 T2 C1 C2 S EP JP

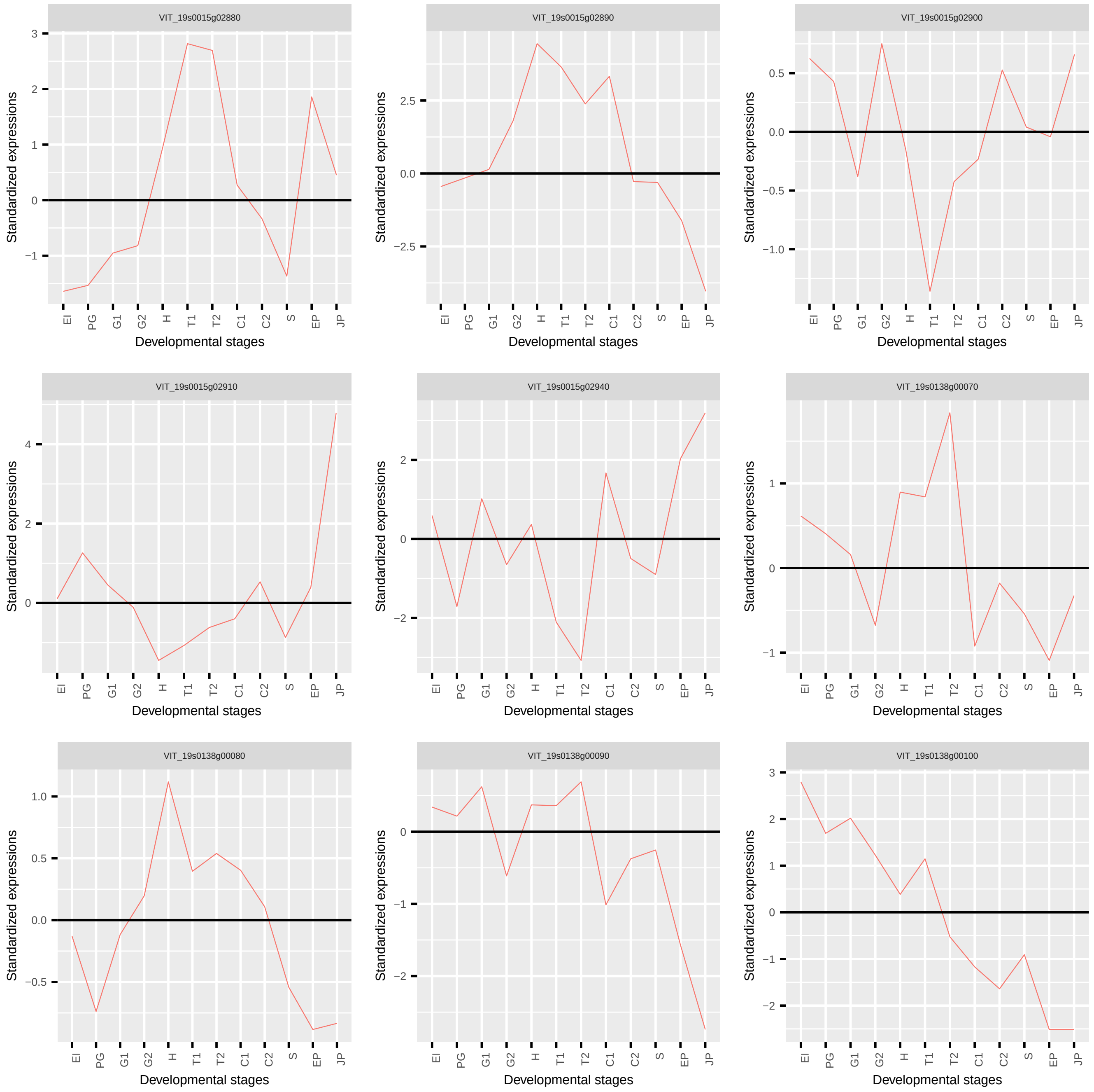
VIT_19s0015g02880
VIT_19s0015g02890
VIT_19s0015g02900
VIT_19s0015g02910
VIT_19s0015g02940
VIT_19s0138g00070
VIT_19s0138g00080
VIT_19s0138g00090
VIT_19s0138g00100
Standardized expressions
Developmental stages
EI PG G1 G2 H T1 T2 C1 C2 S EP JP

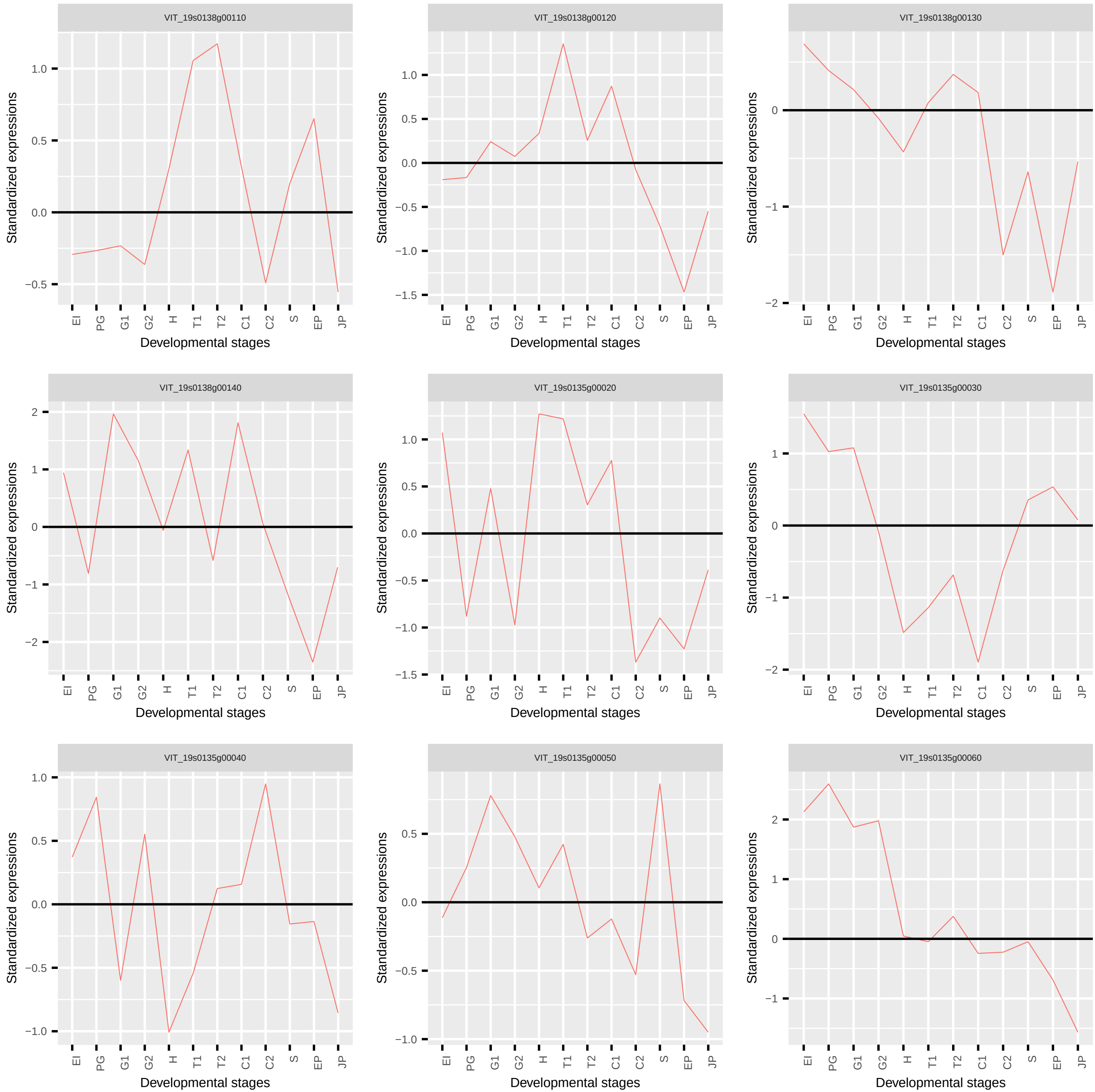

VIT_19s0138g00110
VIT_19s0138g00120
VIT_19s0138g00130
VIT_19s0138g00140
VIT_19s0135g00020
VIT_19s0135g00030
VIT_19s0135g00040
VIT_19s0135g00050
VIT_19s0135g00060
Standardized expressions
Developmental stages
EI PG G1 G2 H T1 T2 C1 C2 S EP JP

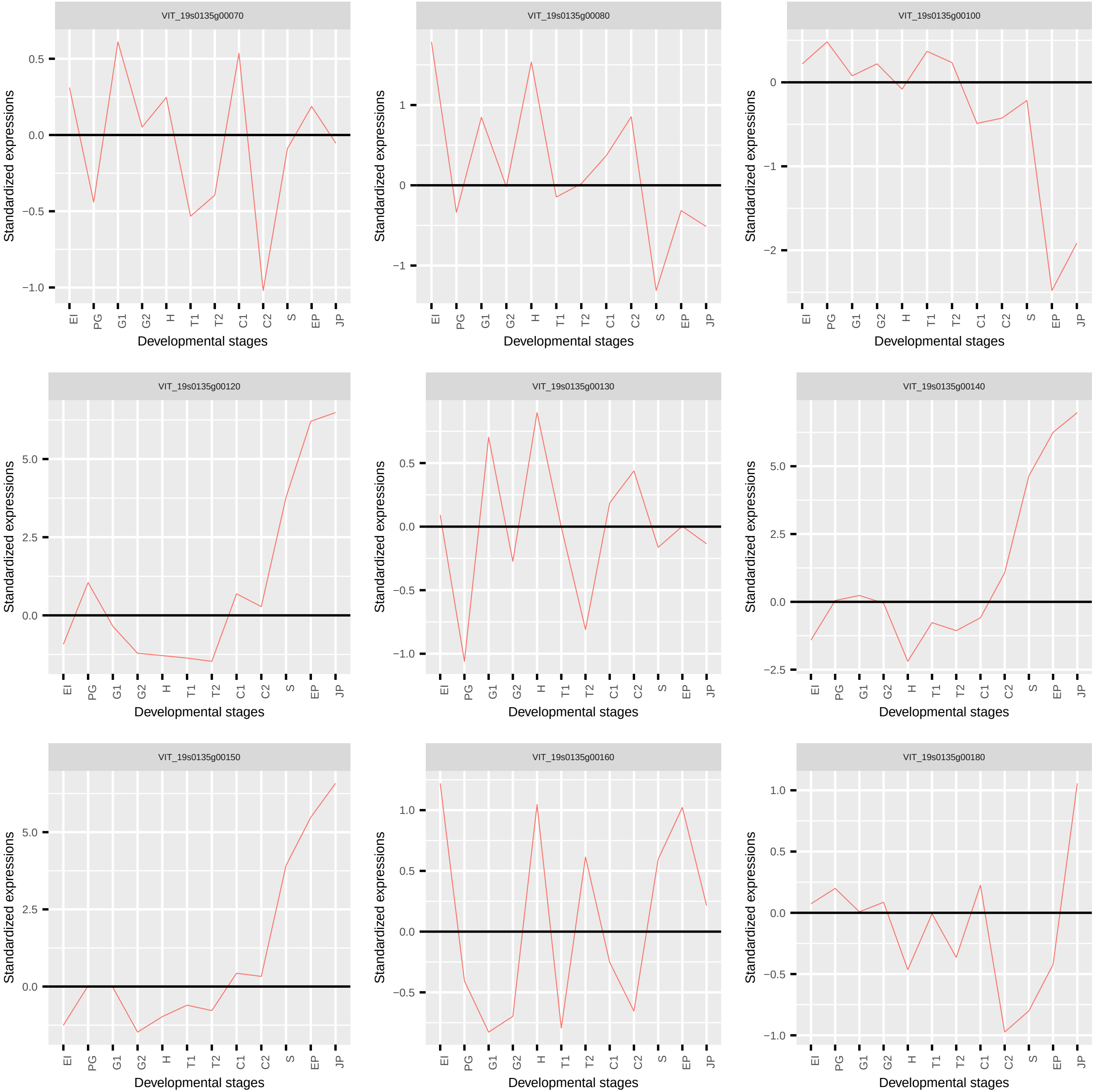
VIT_19s0135g00070
VIT_19s0135g00080
VIT_19s0135g00100
VIT_19s0135g00120
VIT_19s0135g00130
VIT_19s0135g00140
VIT_19s0135g00150
VIT_19s0135g00160
VIT_19s0135g00180
Standardized expressions
Developmental stages
EI PG G1 G2 H T1 T2 C1 C2 S EP JP

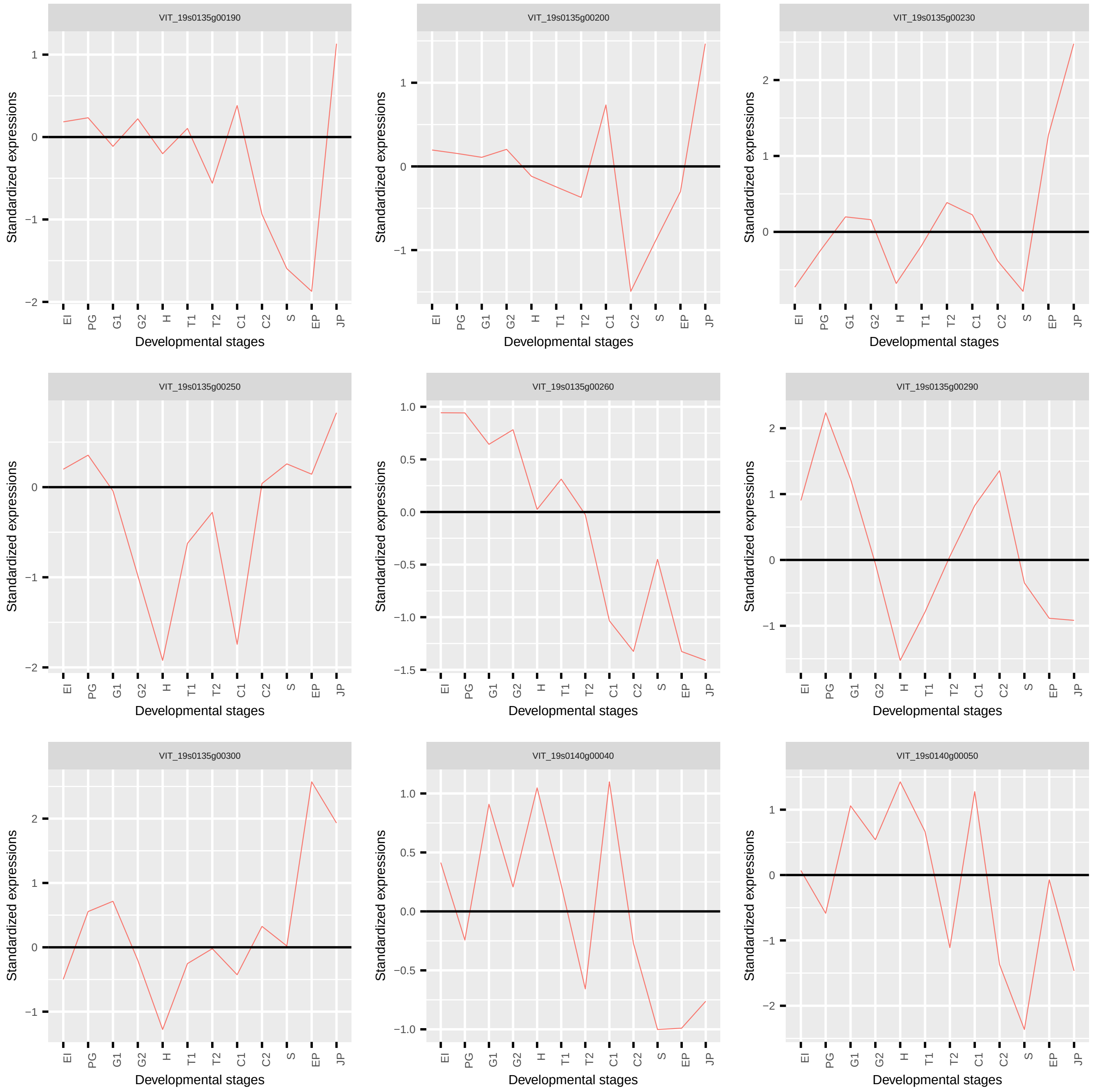
VIT_19s0135g00190
VIT_19s0135g00200
VIT_19s0135g00230
VIT_19s0135g00250
VIT_19s0135g00260
VIT_19s0135g00290
VIT_19s0135g00300
VIT_19s0140g00040
VIT_19s0140g00050
Standardized expressions
Developmental stages
EI PG G1 G2 H T1 T2 C1 C2 S EP JP

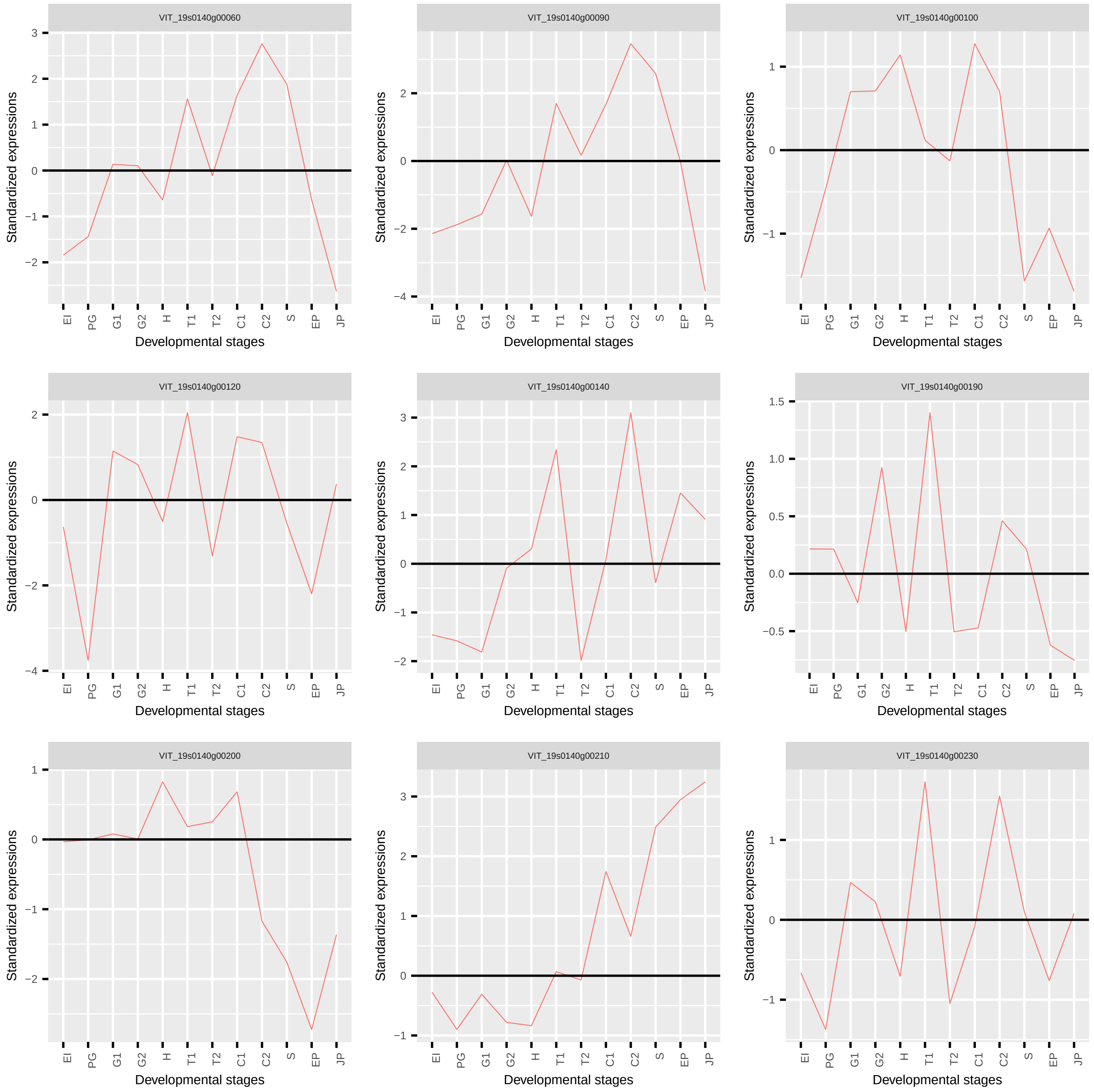

VIT_19s0140g00060
VIT_19s0140g00090
VIT_19s0140g00100
VIT_19s0140g00120
VIT_19s0140g00140
VIT_19s0140g00190
VIT_19s0140g00200
VIT_19s0140g00210
VIT_19s0140g00230
Standardized expressions
Developmental stages
EI
PG
G1
G2
H
T1
T2
C1
C2
S
EP
JP

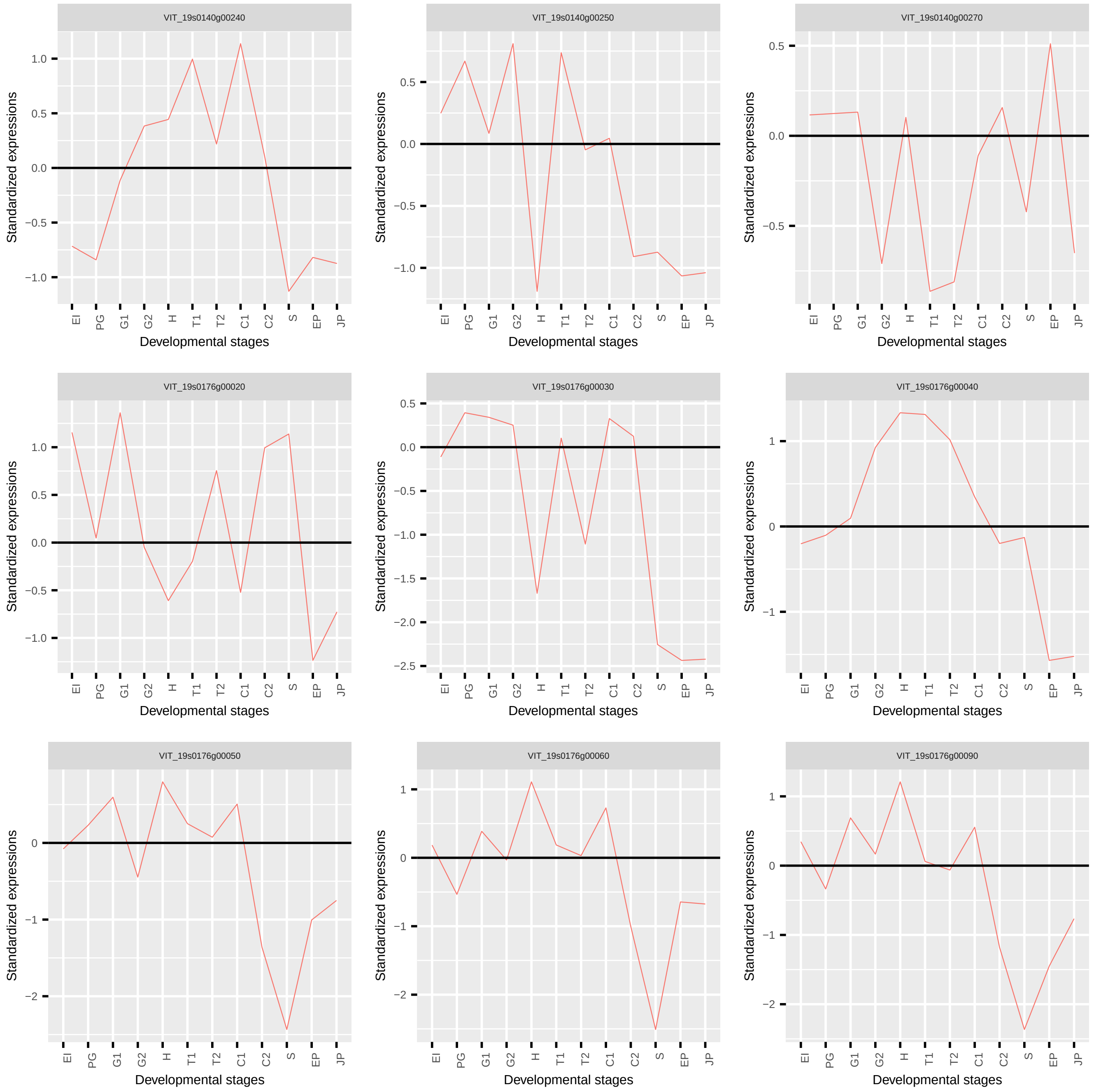

VIT_19s0140g00240
VIT_19s0140g00250
VIT_19s0140g00270
VIT_19s0176g00020
VIT_19s0176g00030
VIT_19s0176g00040
VIT_19s0176g00050
VIT_19s0176g00060
VIT_19s0176g00090
Standardized expressions
Developmental stages
EI
PG
G1
G2
H
T1
T2
C1
C2
S
EP
JP

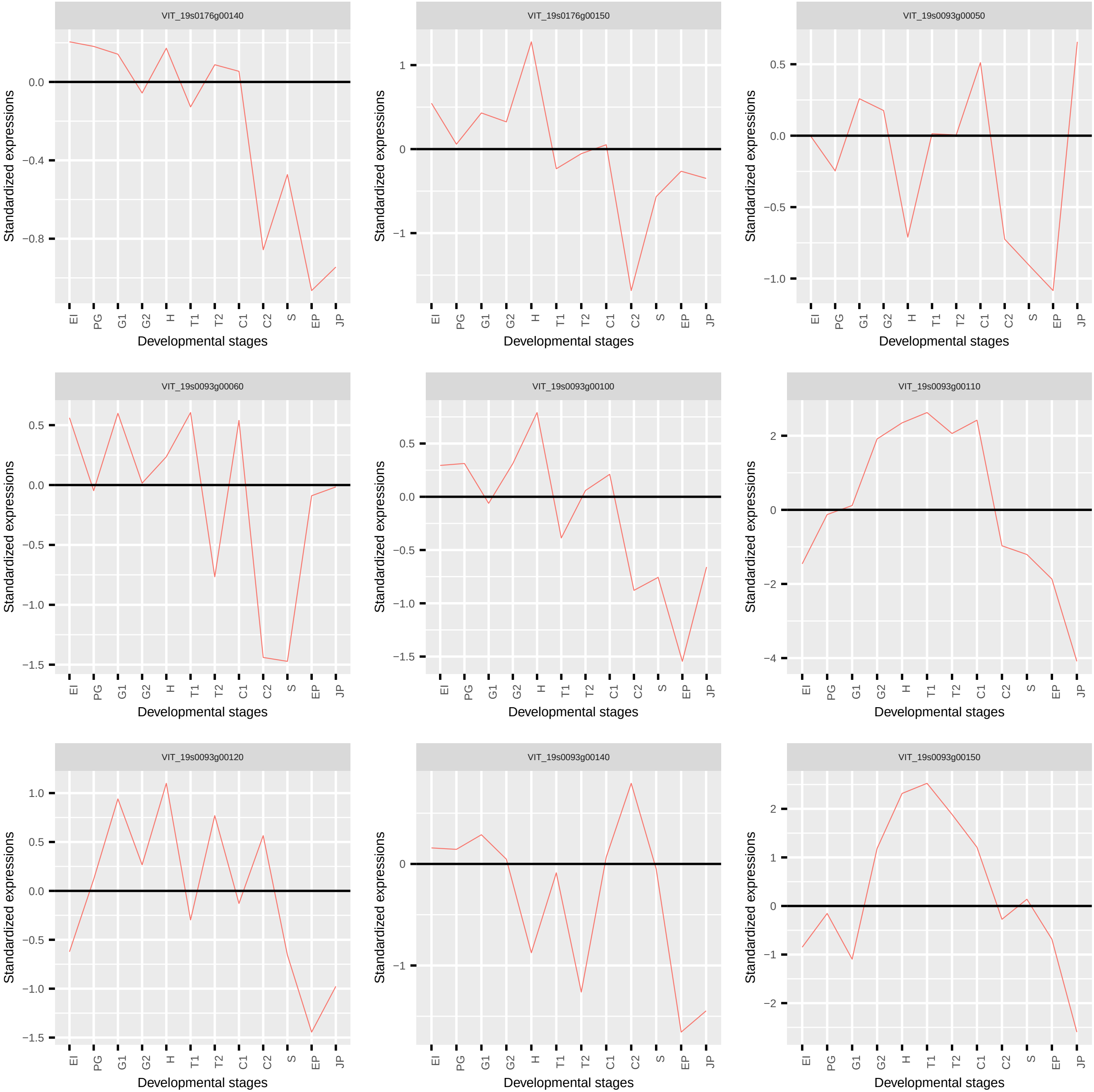
VIT_19s0176g00140
VIT_19s0176g00150
VIT_19s0093g00050
VIT_19s0093g00060
VIT_19s0093g00100
VIT_19s0093g00110
VIT_19s0093g00120
VIT_19s0093g00140
VIT_19s0093g00150
Standardized expressions
Developmental stages
EI PG G1 G2 H T1 T2 C1 C2 S EP JP
0.0 −0.4 −0.8
1 0 −1
0.5 0.0 −0.5 −1.0
0.5 0.0 −0.5 −1.0 −1.5
0.5 0.0 −0.5 −1.0 −1.5
2 0 −2 −4
1.0 0.5 0.0 −0.5 −1.0 −1.5
0 −1
2 1 0 −1 −2

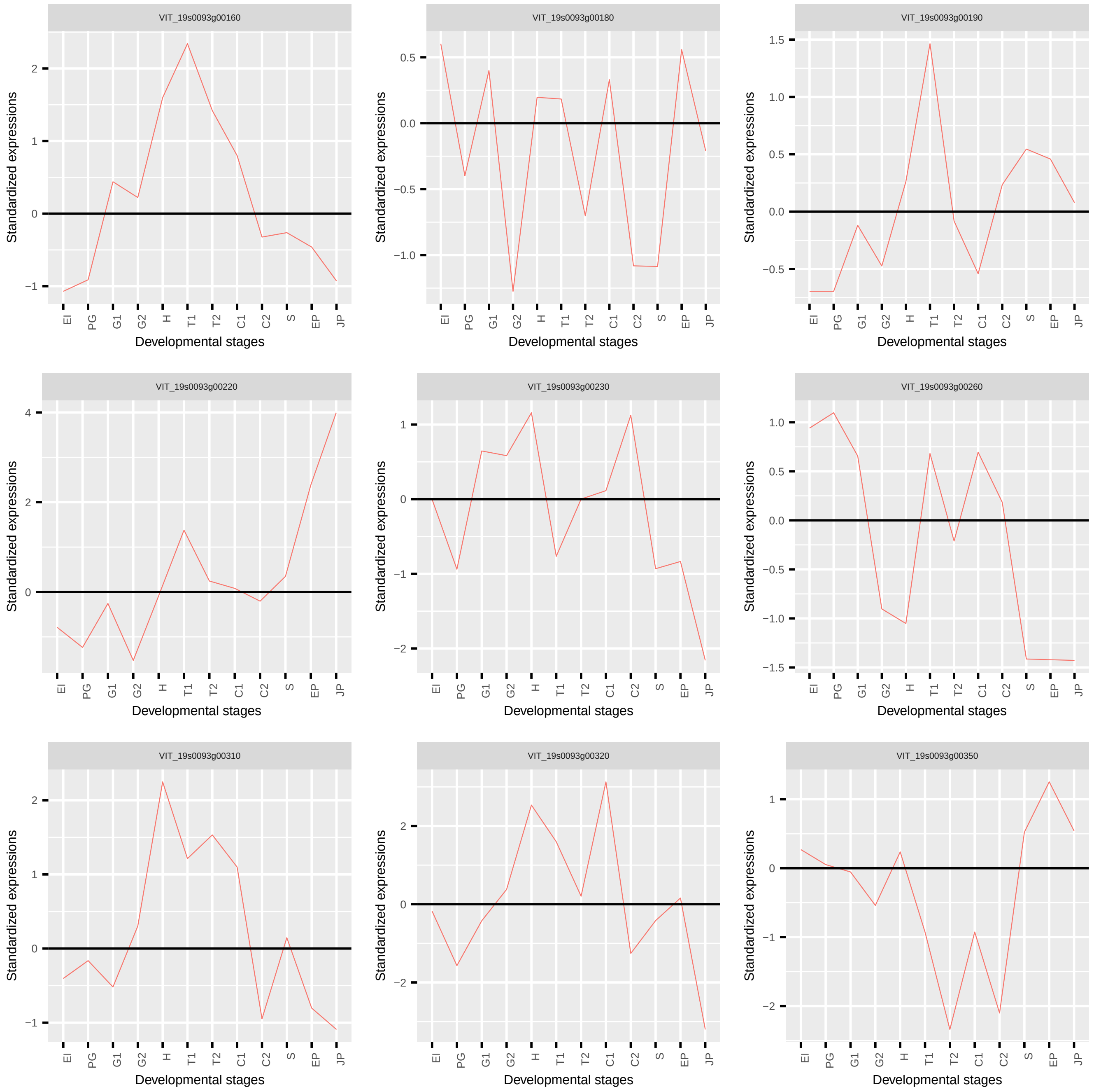
VIT_19s0093g00160
VIT_19s0093g00180
VIT_19s0093g00190
VIT_19s0093g00220
VIT_19s0093g00230
VIT_19s0093g00260
VIT_19s0093g00310
VIT_19s0093g00320
VIT_19s0093g00350
Standardized expressions
Developmental stages
EI
PG
G1
G2
H
T1
T2
C1
C2
S
EP
JP

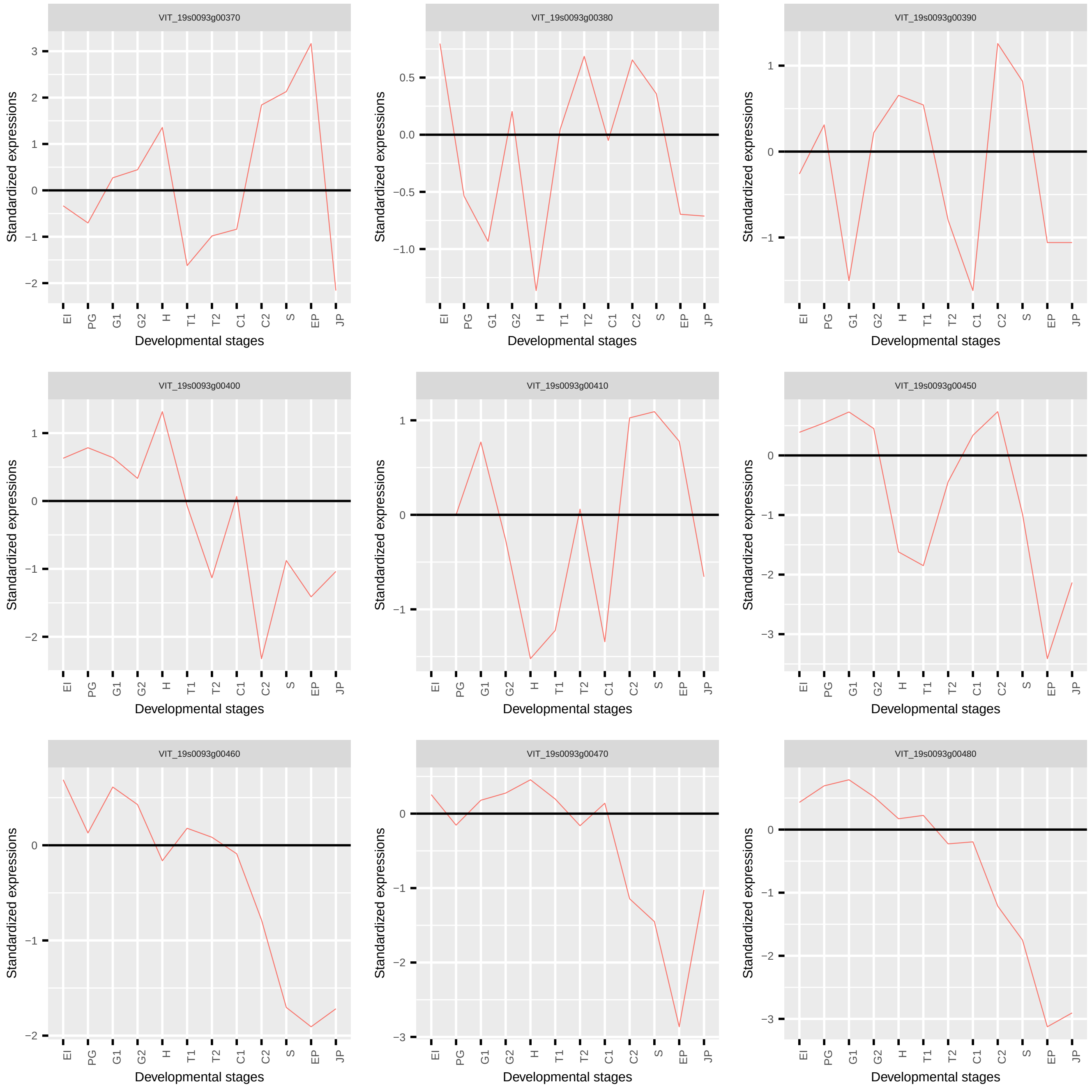

VIT_19s0093g00370
VIT_19s0093g00380
VIT_19s0093g00390
VIT_19s0093g00400
VIT_19s0093g00410
VIT_19s0093g00450
VIT_19s0093g00460
VIT_19s0093g00470
VIT_19s0093g00480
Standardized expressions
Developmental stages
EI PG G1 G2 H T1 T2 C1 C2 S EP JP

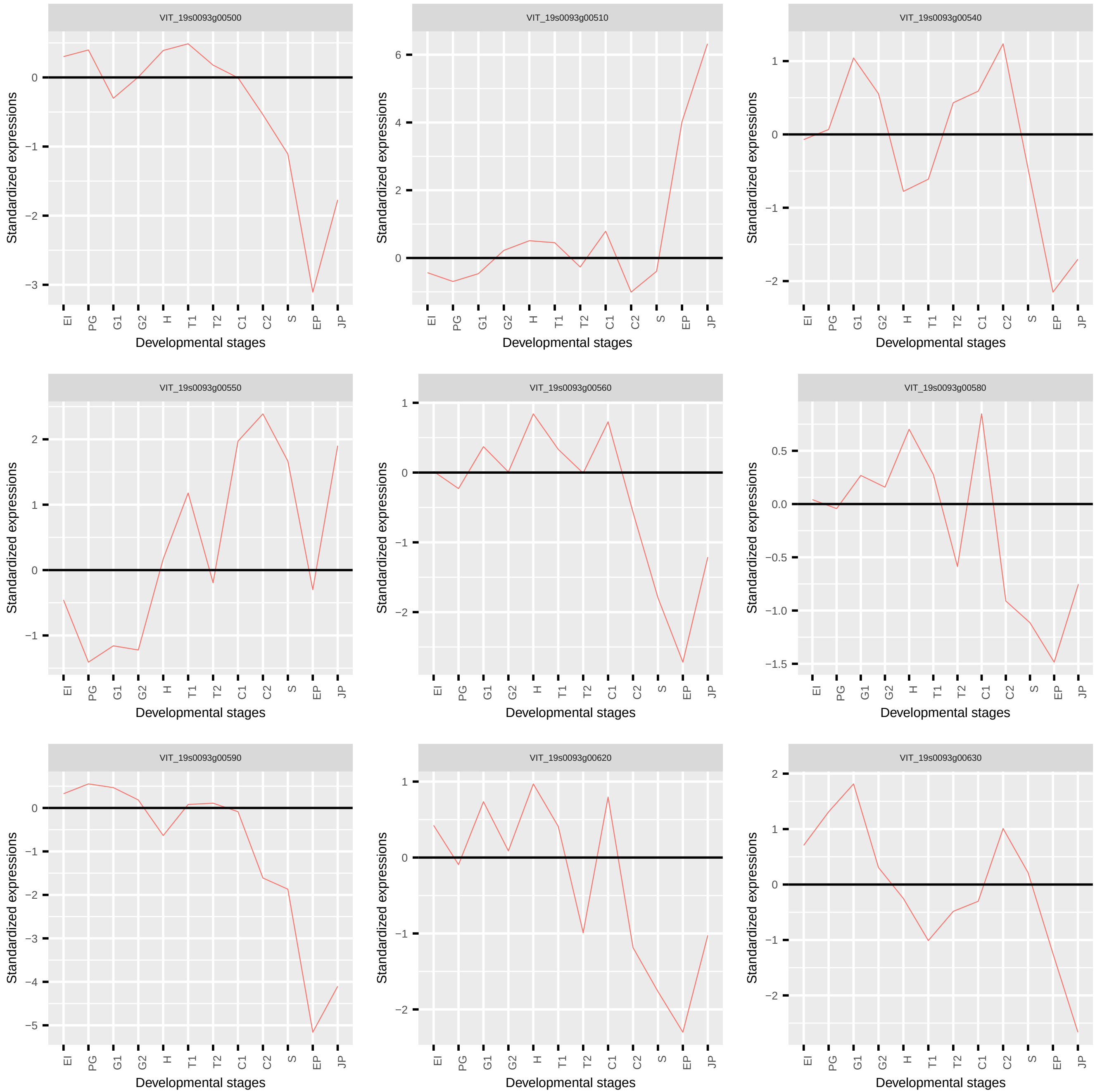
VIT_19s0093g00500
VIT_19s0093g00510
VIT_19s0093g00540
VIT_19s0093g00550
VIT_19s0093g00560
VIT_19s0093g00580
VIT_19s0093g00590
VIT_19s0093g00620
VIT_19s0093g00630
Standardized expressions
Developmental stages
EI PG G1 G2 H T1 T2 C1 C2 S EP JP

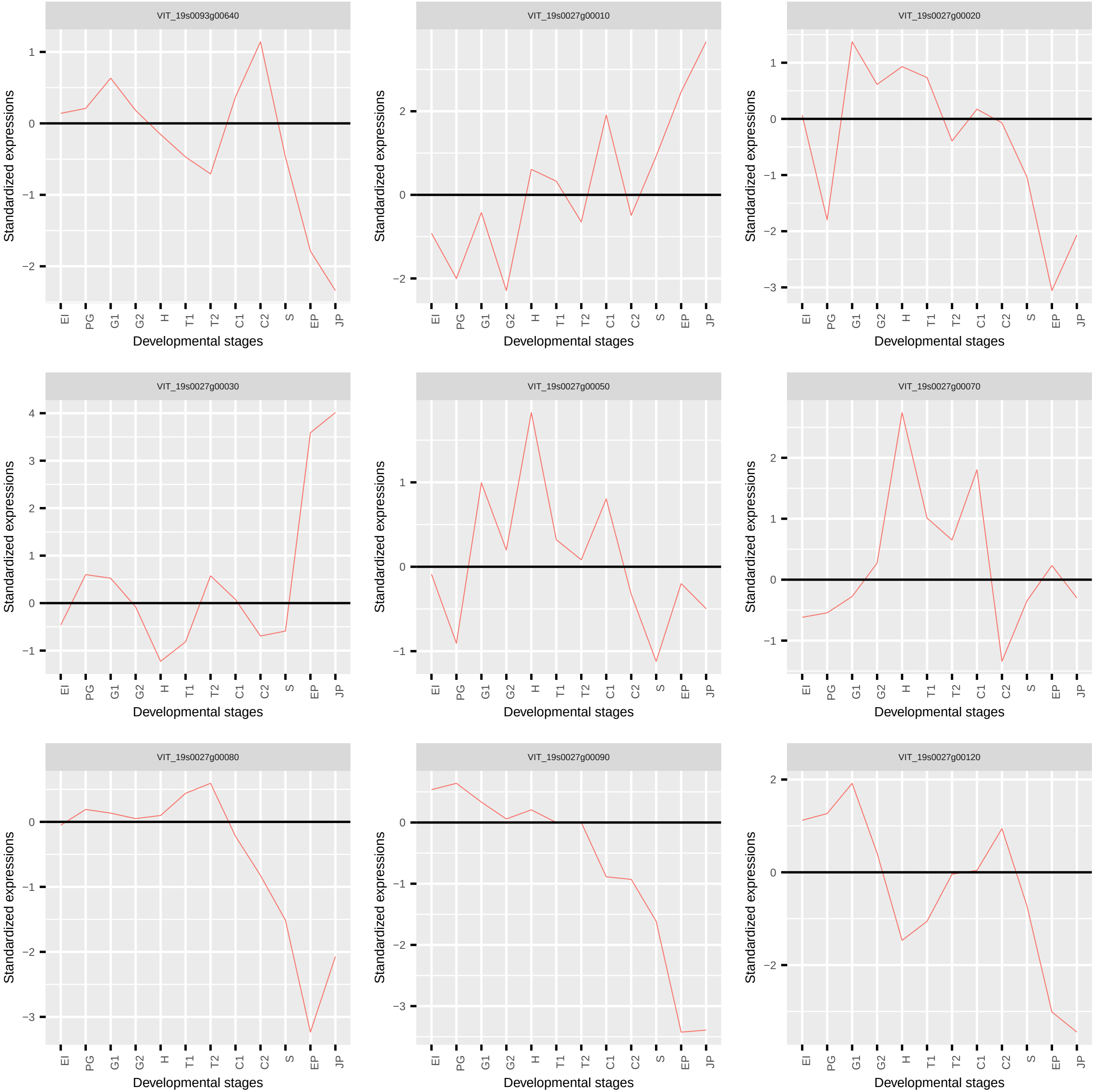
VIT_19s0093g00640
VIT_19s0027g00010
VIT_19s0027g00020
VIT_19s0027g00030
VIT_19s0027g00050
VIT_19s0027g00070
VIT_19s0027g00080
VIT_19s0027g00090
VIT_19s0027g00120
Standardized expressions
Developmental stages
EI PG G1 G2 H T1 T2 C1 C2 S EP JP

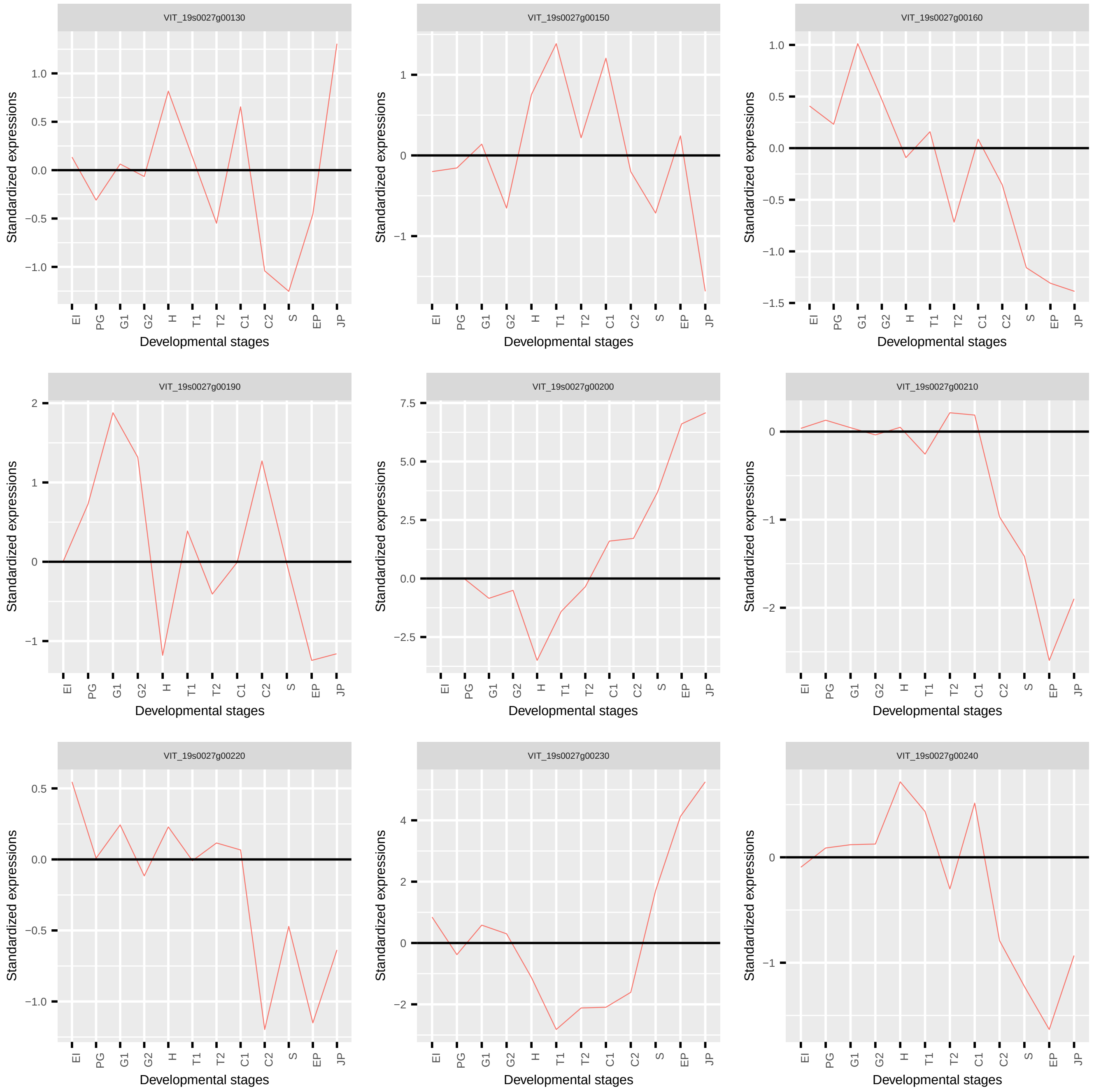

VIT_19s0027g00130
VIT_19s0027g00150
VIT_19s0027g00160
VIT_19s0027g00190
VIT_19s0027g00200
VIT_19s0027g00210
VIT_19s0027g00220
VIT_19s0027g00230
VIT_19s0027g00240
Standardized expressions
Developmental stages
EI PG G1 G2 H T1 T2 C1 C2 S EP JP

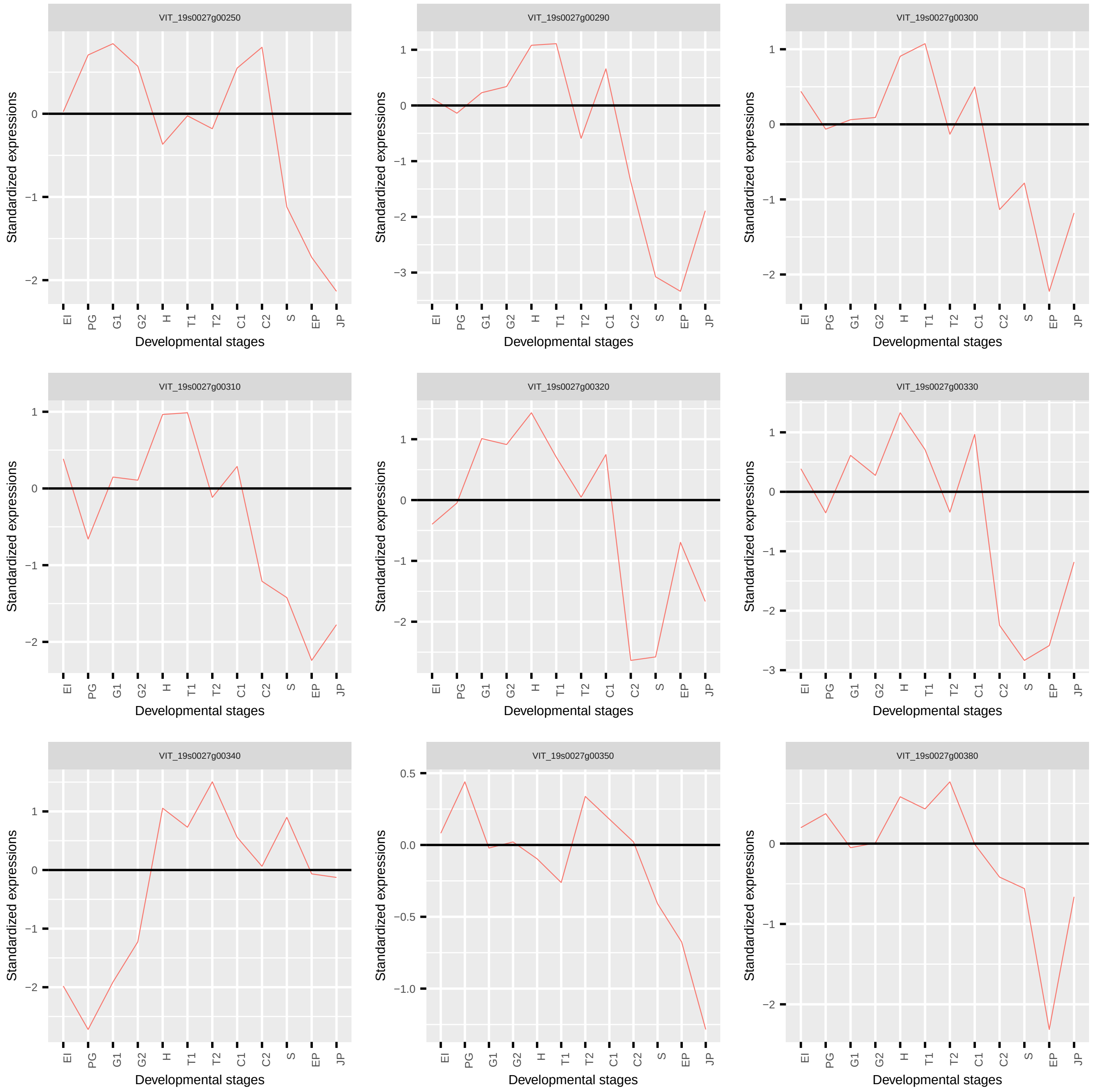
VIT_19s0027g00250
VIT_19s0027g00290
VIT_19s0027g00300
VIT_19s0027g00310
VIT_19s0027g00320
VIT_19s0027g00330
VIT_19s0027g00340
VIT_19s0027g00350
VIT_19s0027g00380
Standardized expressions
Developmental stages
EI PG G1 G2 H T1 T2 C1 C2 S EP JP

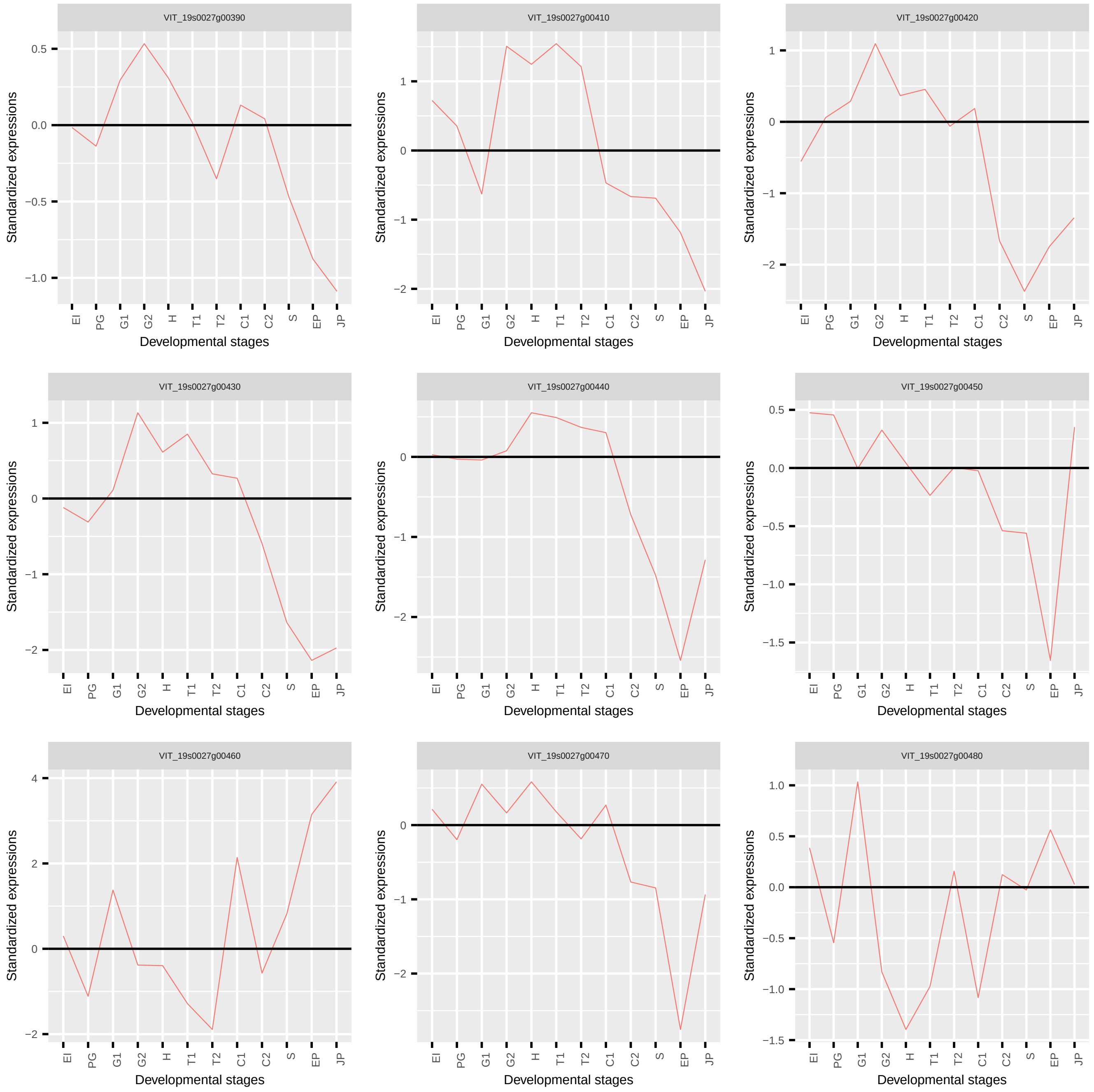
VIT_19s0027g00390
VIT_19s0027g00410
VIT_19s0027g00420
VIT_19s0027g00430
VIT_19s0027g00440
VIT_19s0027g00450
VIT_19s0027g00460
VIT_19s0027g00470
VIT_19s0027g00480
Standardized expressions
Developmental stages
EI PG G1 G2 H T1 T2 C1 C2 S EP JP

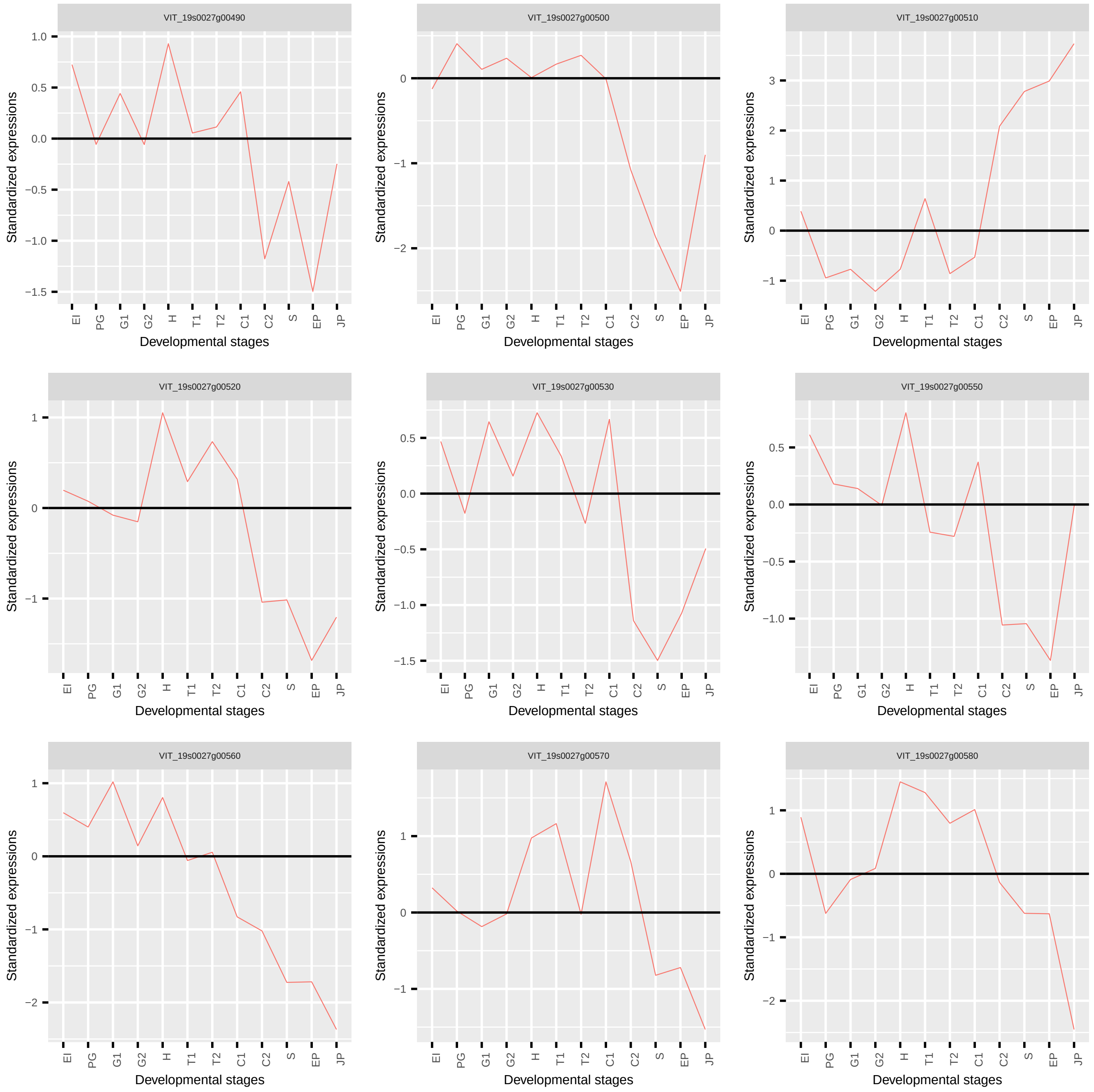
VIT_19s0027g00490
VIT_19s0027g00500
VIT_19s0027g00510
VIT_19s0027g00520
VIT_19s0027g00530
VIT_19s0027g00550
VIT_19s0027g00560
VIT_19s0027g00570
VIT_19s0027g00580
Standardized expressions
Developmental stages
EI PG G1 G2 H T1 T2 C1 C2 S EP JP

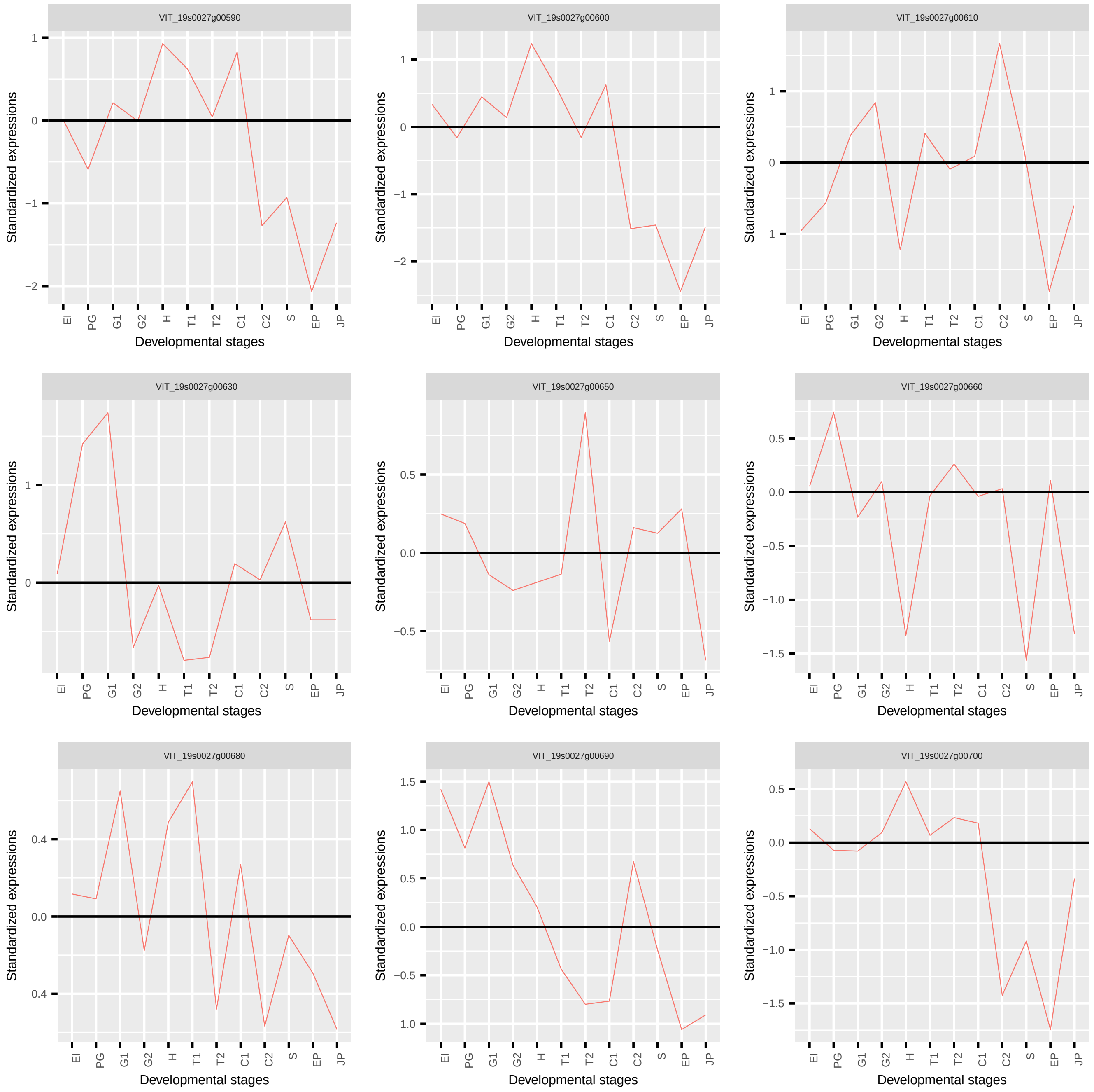
VIT_19s0027g00590
VIT_19s0027g00600
VIT_19s0027g00610
VIT_19s0027g00630
VIT_19s0027g00650
VIT_19s0027g00660
VIT_19s0027g00680
VIT_19s0027g00690
VIT_19s0027g00700
Standardized expressions
Developmental stages
EI PG G1 G2 H T1 T2 C1 C2 S EP JP

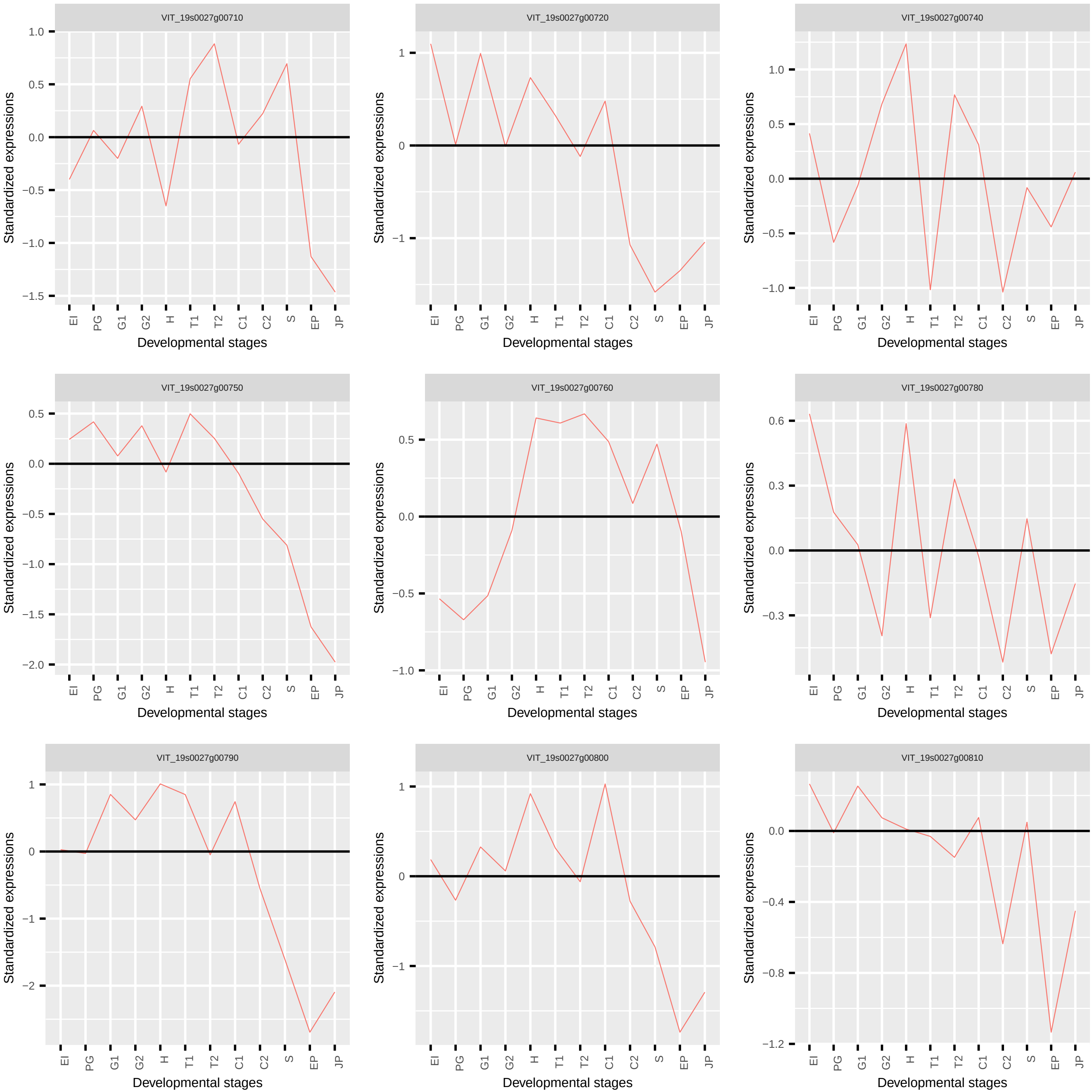

VIT_19s0027g00710
VIT_19s0027g00720
VIT_19s0027g00740
VIT_19s0027g00750
VIT_19s0027g00760
VIT_19s0027g00780
VIT_19s0027g00790
VIT_19s0027g00800
VIT_19s0027g00810
Standardized expressions
Developmental stages
EI PG G1 G2 H T1 T2 C1 C2 S EP JP

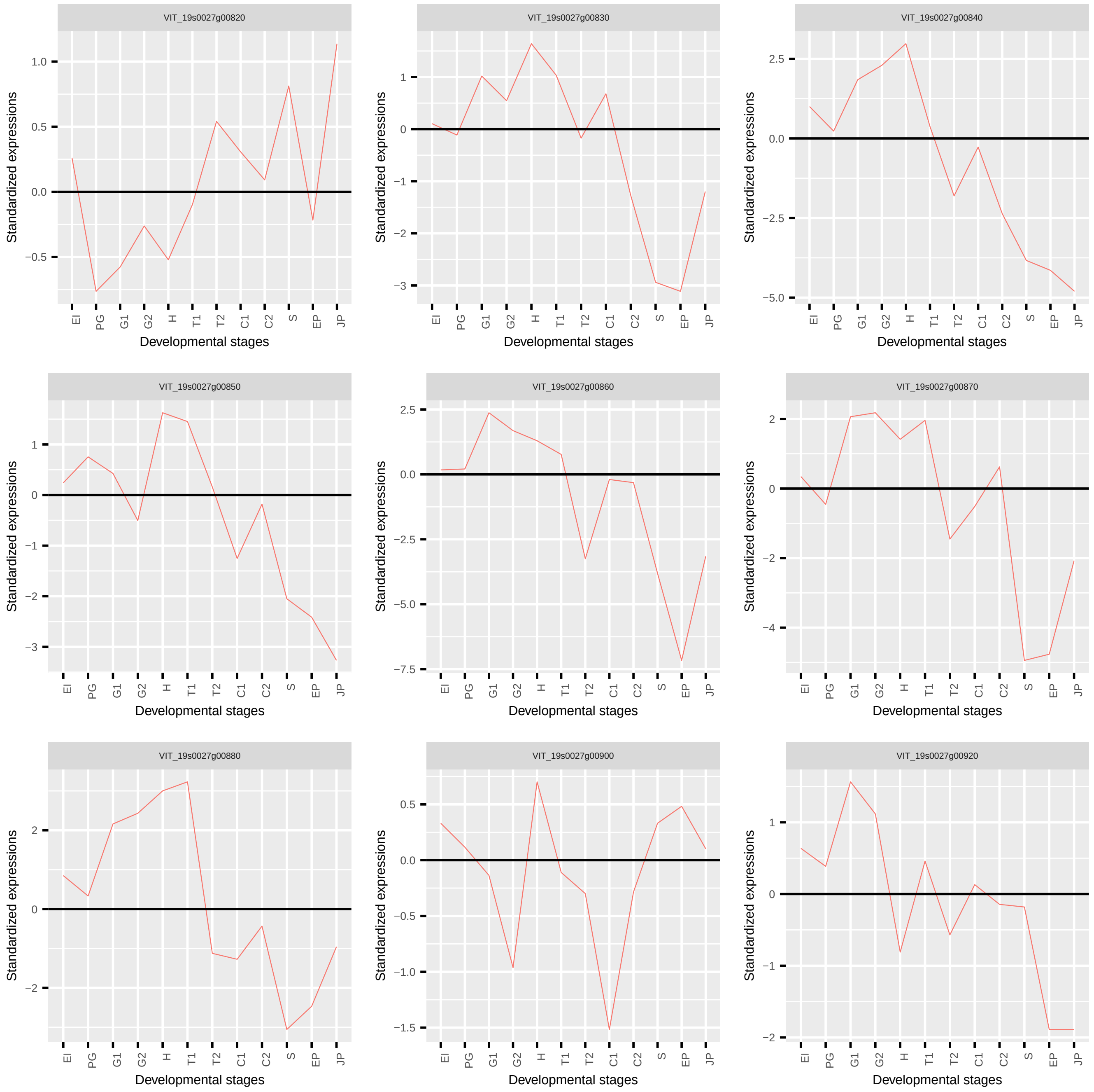
VIT_19s0027g00820
VIT_19s0027g00830
VIT_19s0027g00840
VIT_19s0027g00850
VIT_19s0027g00860
VIT_19s0027g00870
VIT_19s0027g00880
VIT_19s0027g00900
VIT_19s0027g00920
Standardized expressions
Developmental stages
EI PG G1 G2 H T1 T2 C1 C2 S EP JP

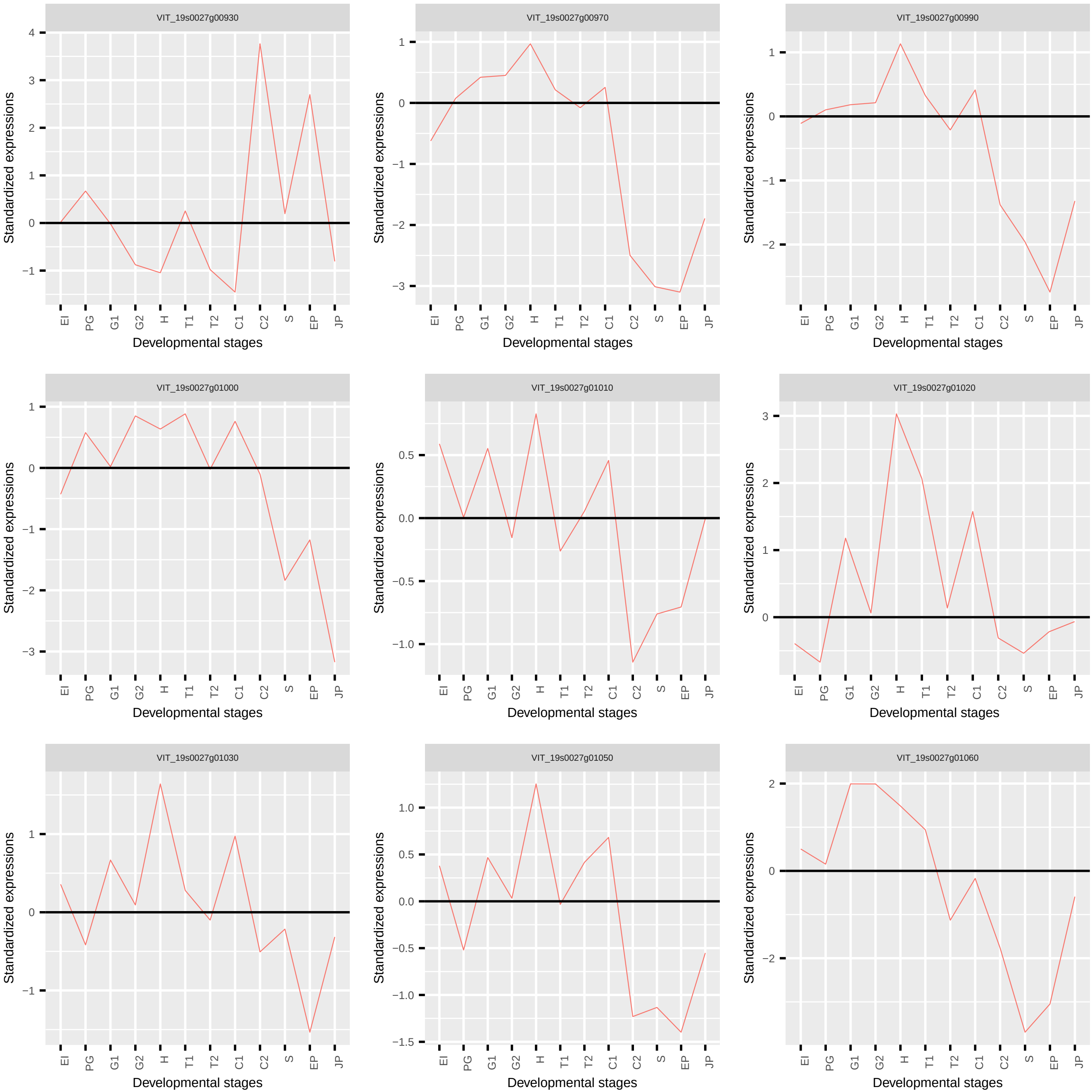

VIT_19s0027g00930
VIT_19s0027g00970
VIT_19s0027g00990
VIT_19s0027g01000
VIT_19s0027g01010
VIT_19s0027g01020
VIT_19s0027g01030
VIT_19s0027g01050
VIT_19s0027g01060
Standardized expressions
Developmental stages
EI PG G1 G2 H T1 T2 C1 C2 S EP JP

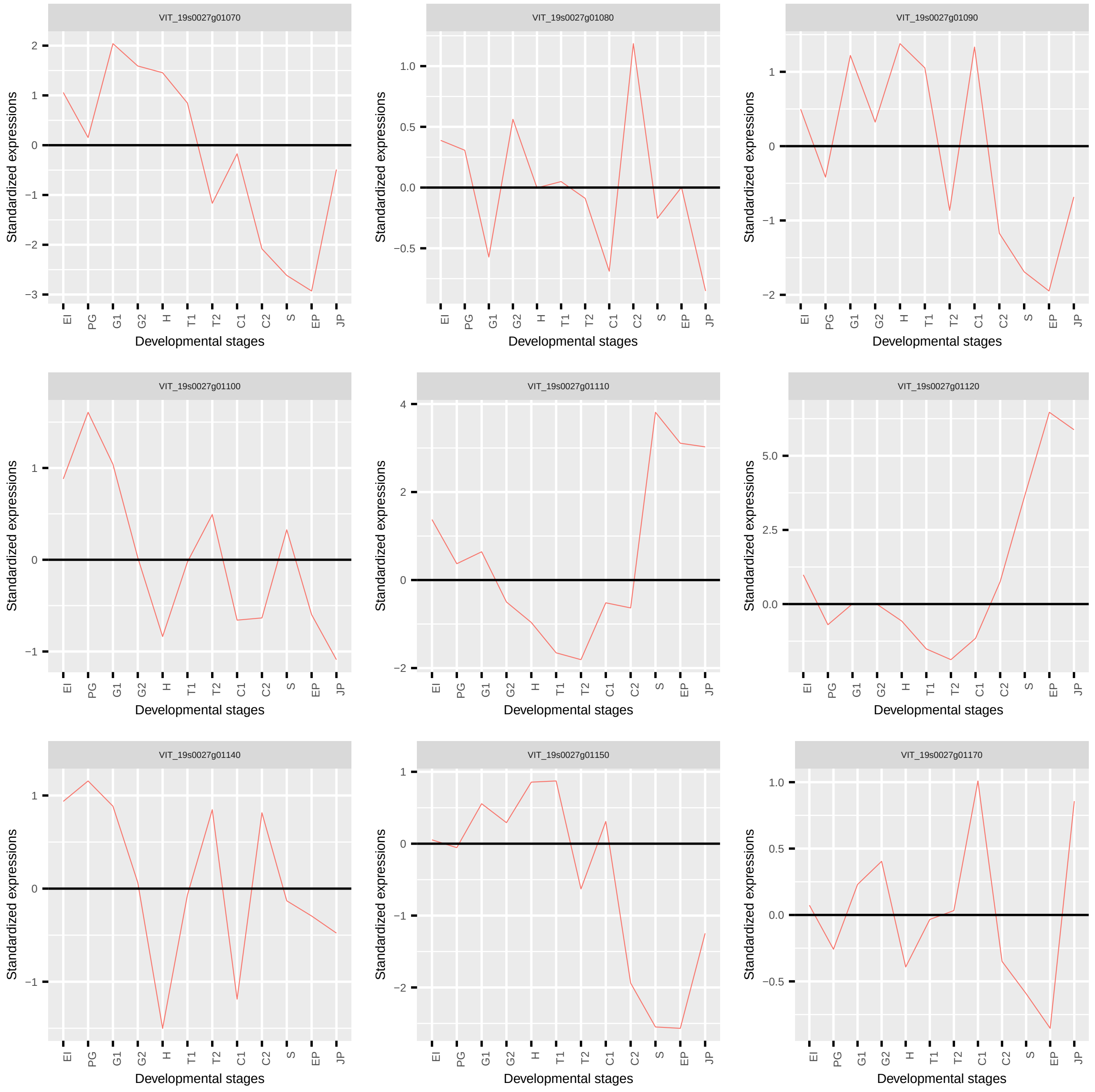

VIT_19s0027g01070
VIT_19s0027g01080
VIT_19s0027g01090
VIT_19s0027g01100
VIT_19s0027g01110
VIT_19s0027g01120
VIT_19s0027g01140
VIT_19s0027g01150
VIT_19s0027g01170
Standardized expressions
Developmental stages
EI
PG
G1
G2
H
T1
T2
C1
C2
S
EP
JP

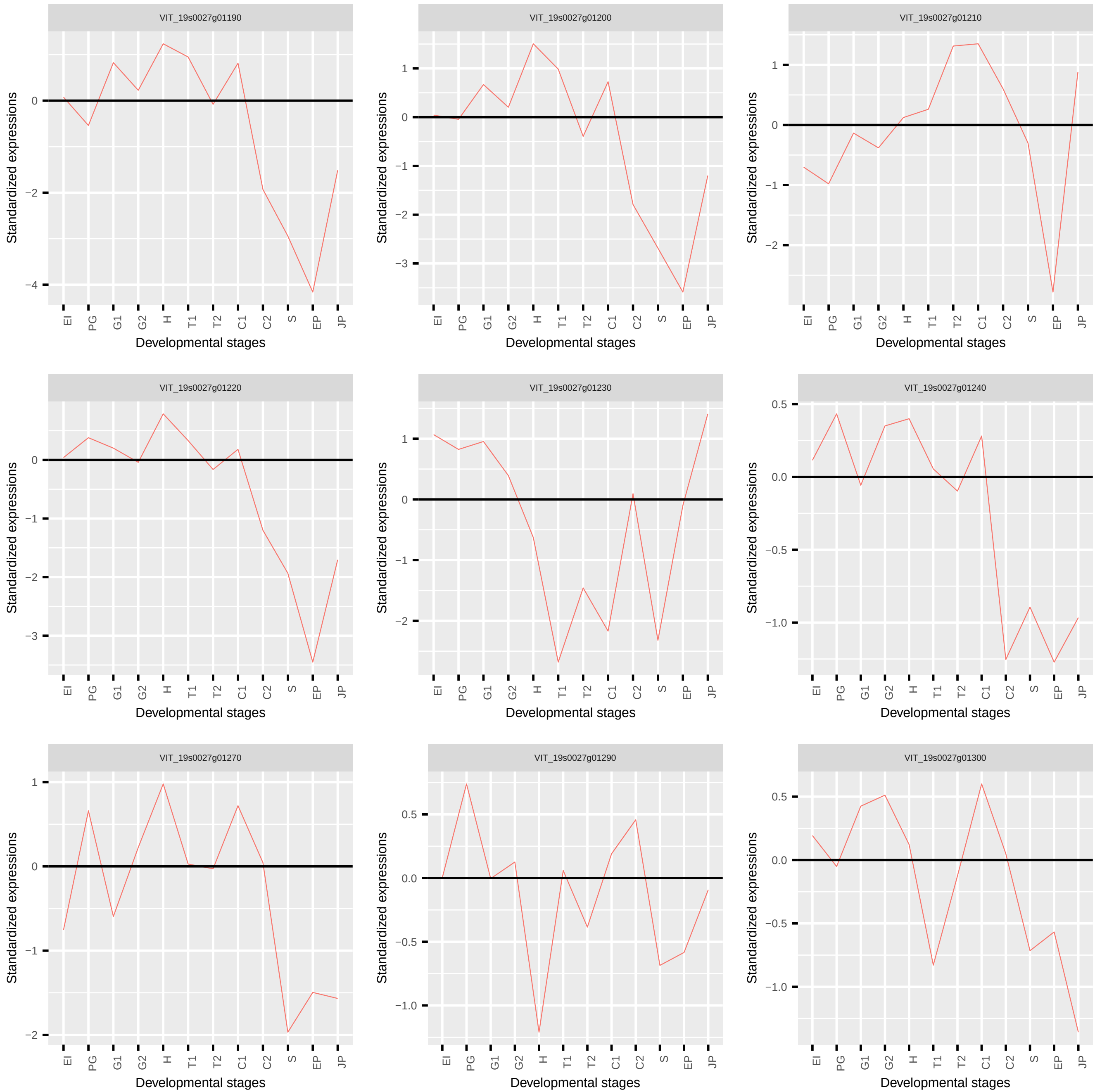
VIT_19s0027g01190
VIT_19s0027g01200
VIT_19s0027g01210
VIT_19s0027g01220
VIT_19s0027g01230
VIT_19s0027g01240
VIT_19s0027g01270
VIT_19s0027g01290
VIT_19s0027g01300
Standardized expressions
Developmental stages
EI
PG
G1
G2
H
T1
T2
C1
C2
S
EP
JP

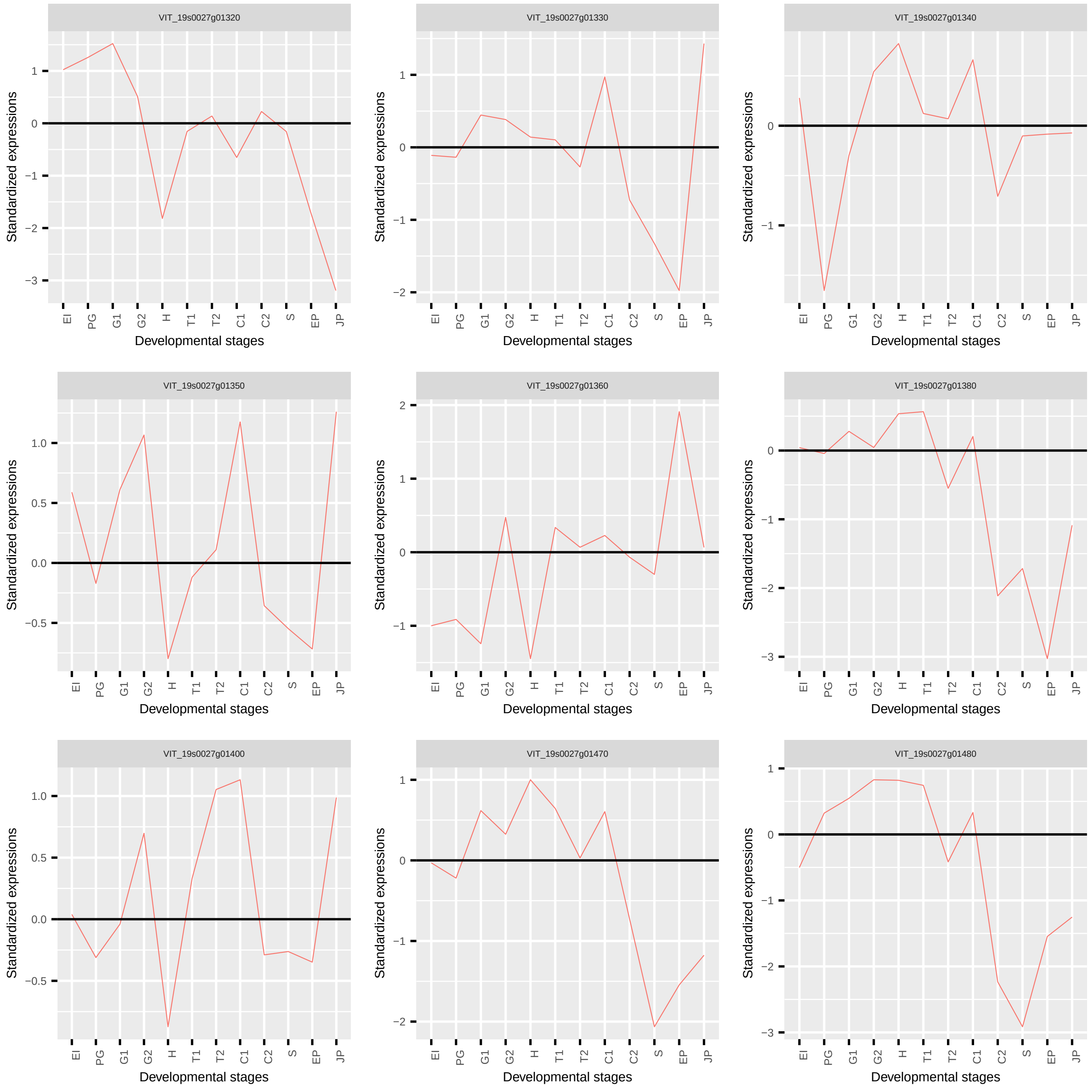
VIT_19s0027g01320
VIT_19s0027g01330
VIT_19s0027g01340
VIT_19s0027g01350
VIT_19s0027g01360
VIT_19s0027g01380
VIT_19s0027g01400
VIT_19s0027g01470
VIT_19s0027g01480
Standardized expressions
Developmental stages
EI PG G1 G2 H T1 T2 C1 C2 S EP JP

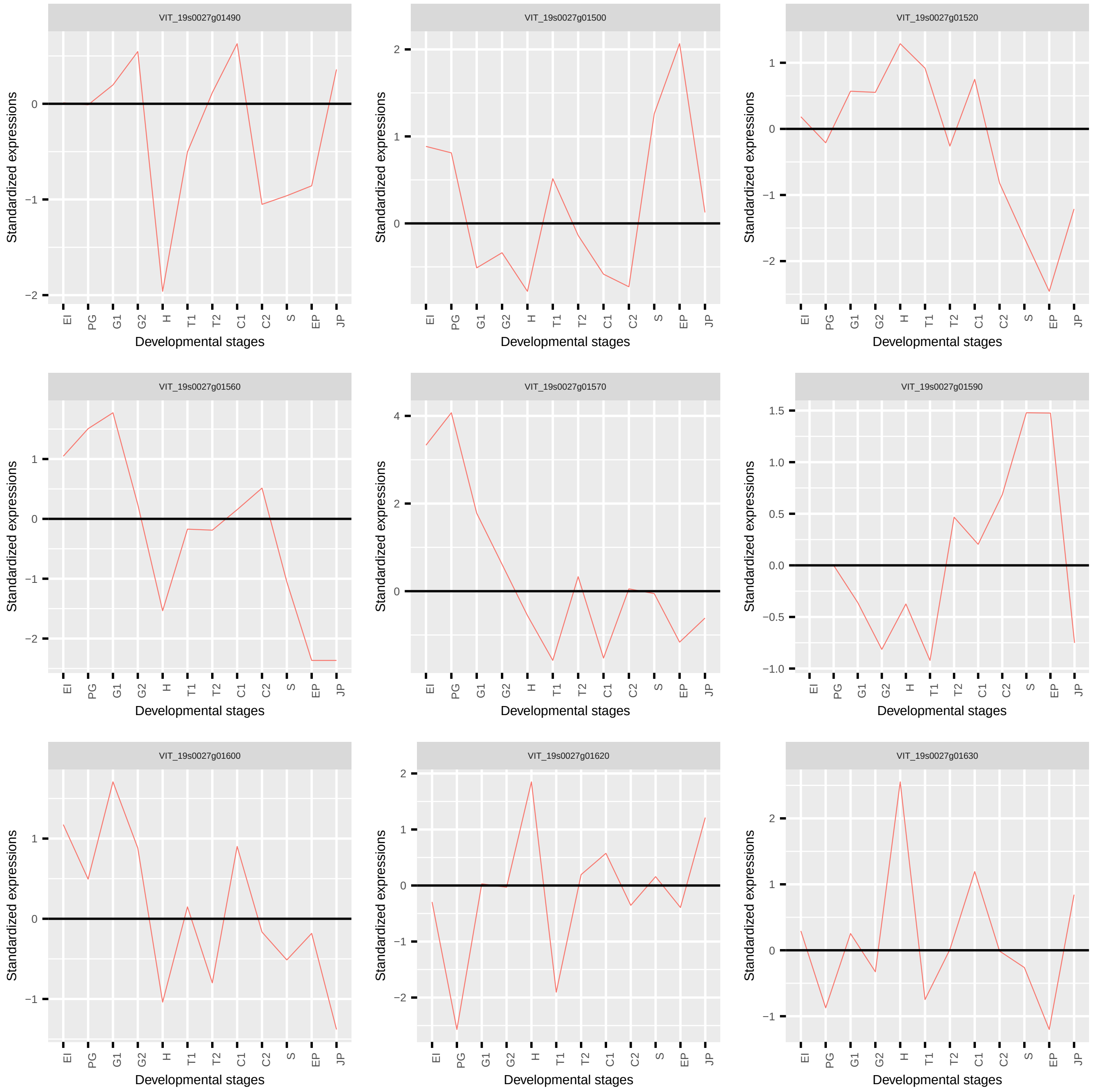
VIT_19s0027g01490
VIT_19s0027g01500
VIT_19s0027g01520
VIT_19s0027g01560
VIT_19s0027g01570
VIT_19s0027g01590
VIT_19s0027g01600
VIT_19s0027g01620
VIT_19s0027g01630
Standardized expressions
Developmental stages
EI PG G1 G2 H T1 T2 C1 C2 S EP JP

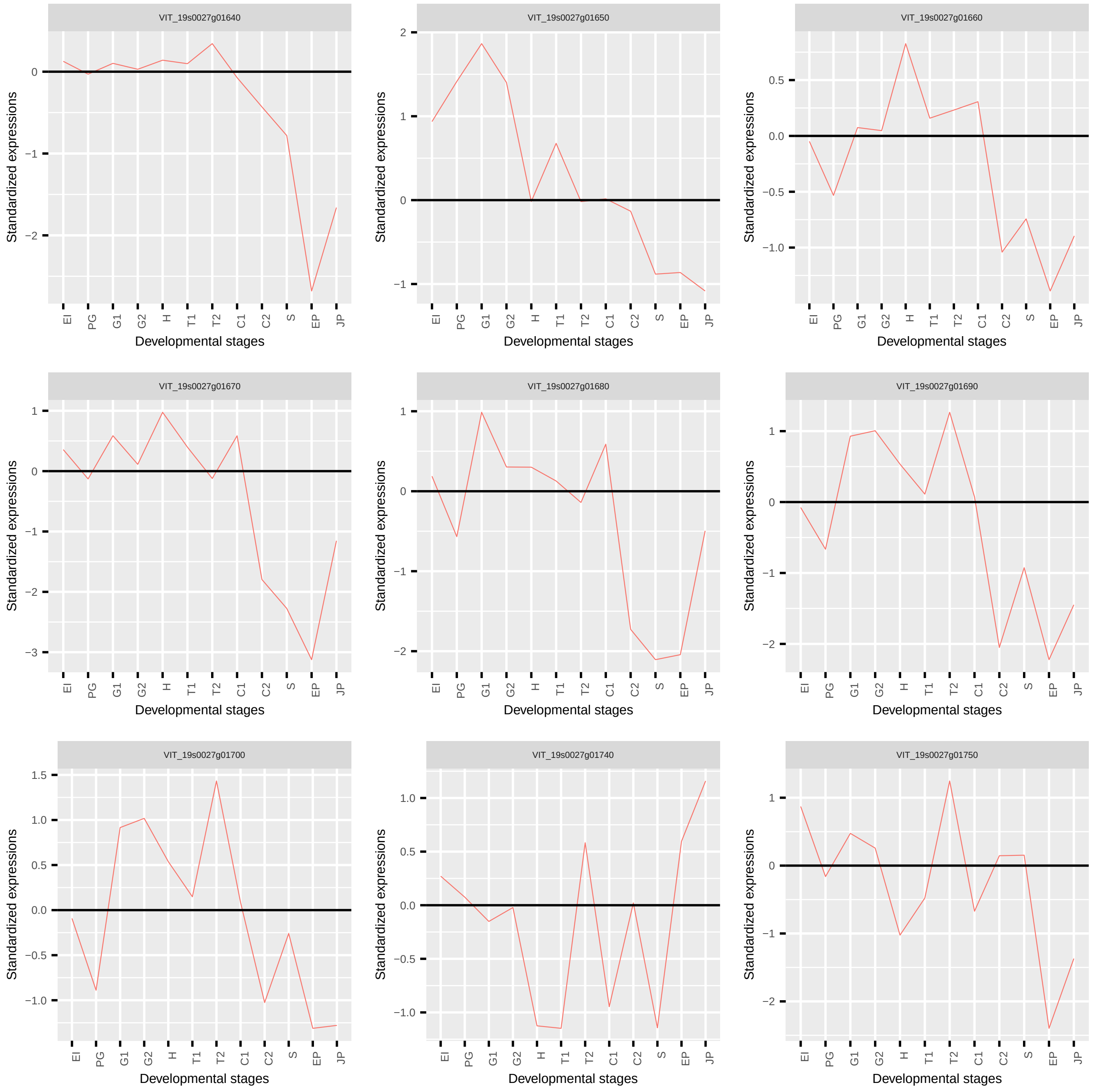

VIT_19s0027g01640
VIT_19s0027g01650
VIT_19s0027g01660
VIT_19s0027g01670
VIT_19s0027g01680
VIT_19s0027g01690
VIT_19s0027g01700
VIT_19s0027g01740
VIT_19s0027g01750
Standardized expressions
Developmental stages
EI
PG
G1
G2
H
T1
T2
C1
C2
S
EP
JP

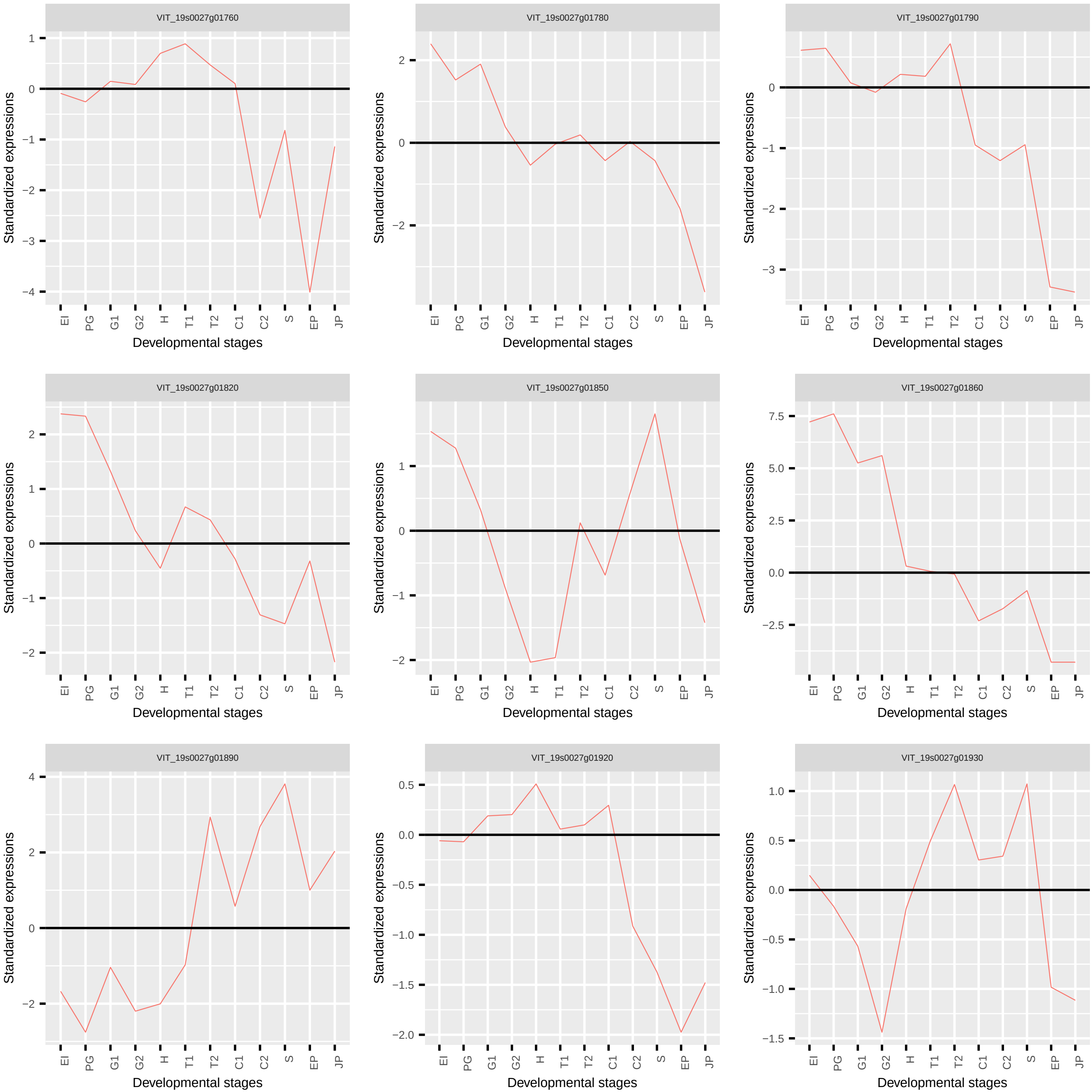
VIT_19s0027g01760
VIT_19s0027g01780
VIT_19s0027g01790
VIT_19s0027g01820
VIT_19s0027g01850
VIT_19s0027g01860
VIT_19s0027g01890
VIT_19s0027g01920
VIT_19s0027g01930
Standardized expressions
Developmental stages
EI PG G1 G2 H T1 T2 C1 C2 S EP JP

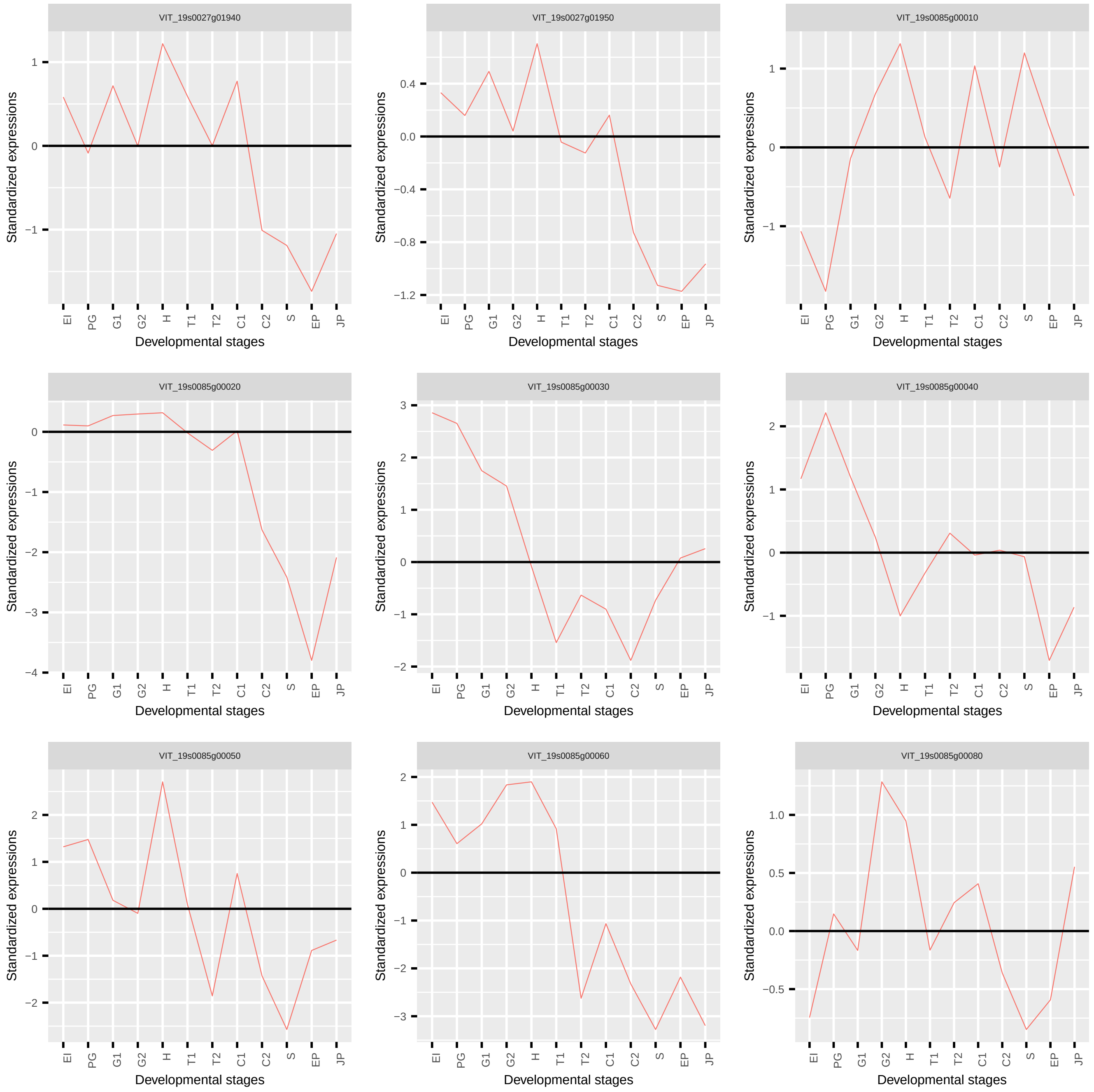
VIT_19s0027g01940
VIT_19s0027g01950
VIT_19s0085g00010
VIT_19s0085g00020
VIT_19s0085g00030
VIT_19s0085g00040
VIT_19s0085g00050
VIT_19s0085g00060
VIT_19s0085g00080
Standardized expressions
Developmental stages
EI
PG
G1
G2
H
T1
T2
C1
C2
S
EP
JP

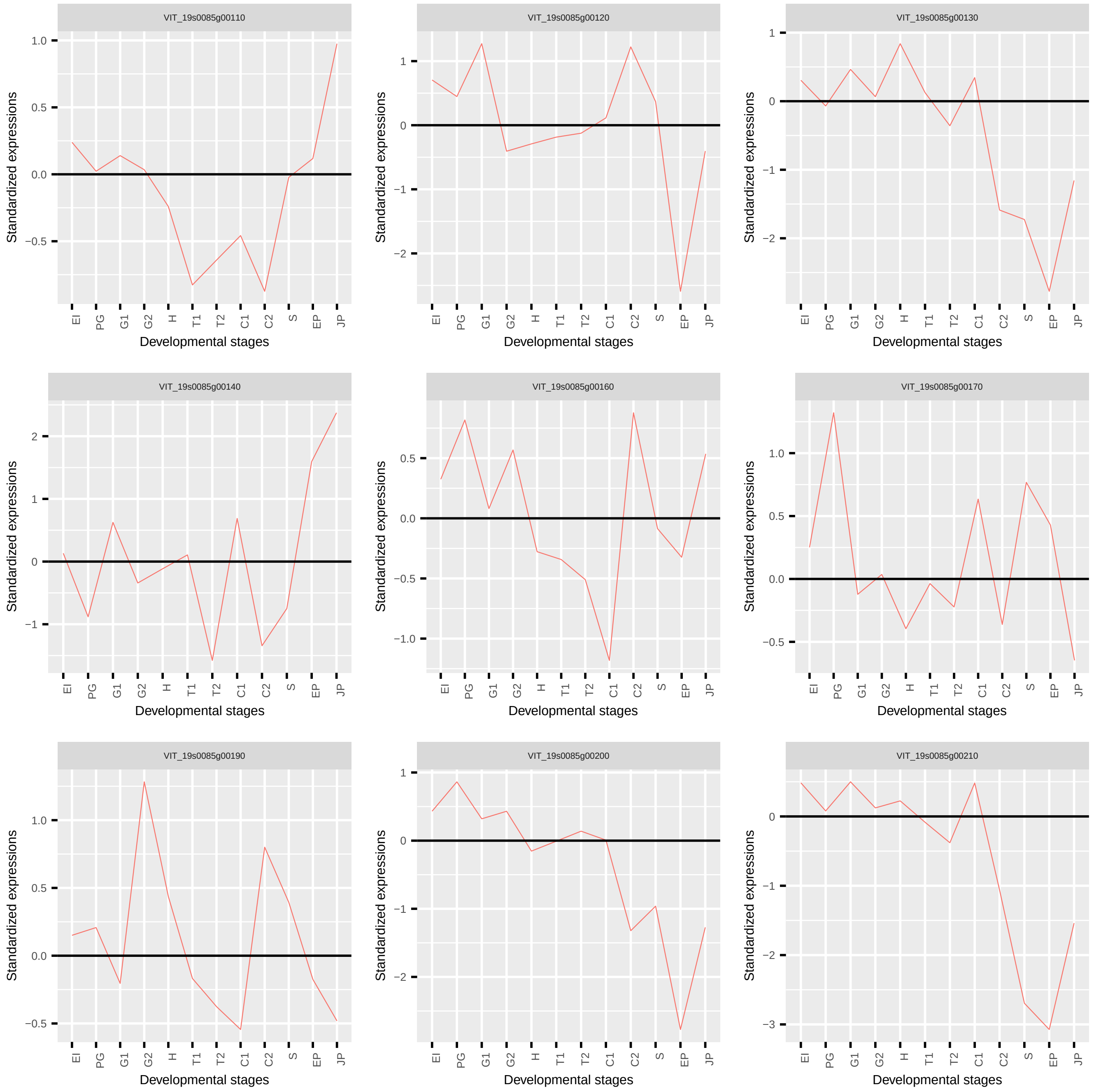

VIT_19s0085g00110
1.0
0.5
0.0
−0.5
Standardized expressions
EI PG G1 G2 H T1 T2 C1 C2 S EP JP
Developmental stages
VIT_19s0085g00120
1
0
−1
−2
Standardized expressions
EI PG G1 G2 H T1 T2 C1 C2 S EP JP
Developmental stages
VIT_19s0085g00130
1
0
−1
−2
Standardized expressions
EI PG G1 G2 H T1 T2 C1 C2 S EP JP
Developmental stages
VIT_19s0085g00140
2
1
0
−1
Standardized expressions
EI PG G1 G2 H T1 T2 C1 C2 S EP JP
Developmental stages
VIT_19s0085g00160
0.5
0.0
−0.5
−1.0
Standardized expressions
EI PG G1 G2 H T1 T2 C1 C2 S EP JP
Developmental stages
VIT_19s0085g00170
1.0
0.5
0.0
−0.5
Standardized expressions
EI PG G1 G2 H T1 T2 C1 C2 S EP JP
Developmental stages
VIT_19s0085g00190
1.0
0.5
0.0
−0.5
Standardized expressions
EI PG G1 G2 H T1 T2 C1 C2 S EP JP
Developmental stages
VIT_19s0085g00200
1
0
−1
−2
Standardized expressions
EI PG G1 G2 H T1 T2 C1 C2 S EP JP
Developmental stages
VIT_19s0085g00210
0
−1
−2
−3
Standardized expressions
EI PG G1 G2 H T1 T2 C1 C2 S EP JP
Developmental stages

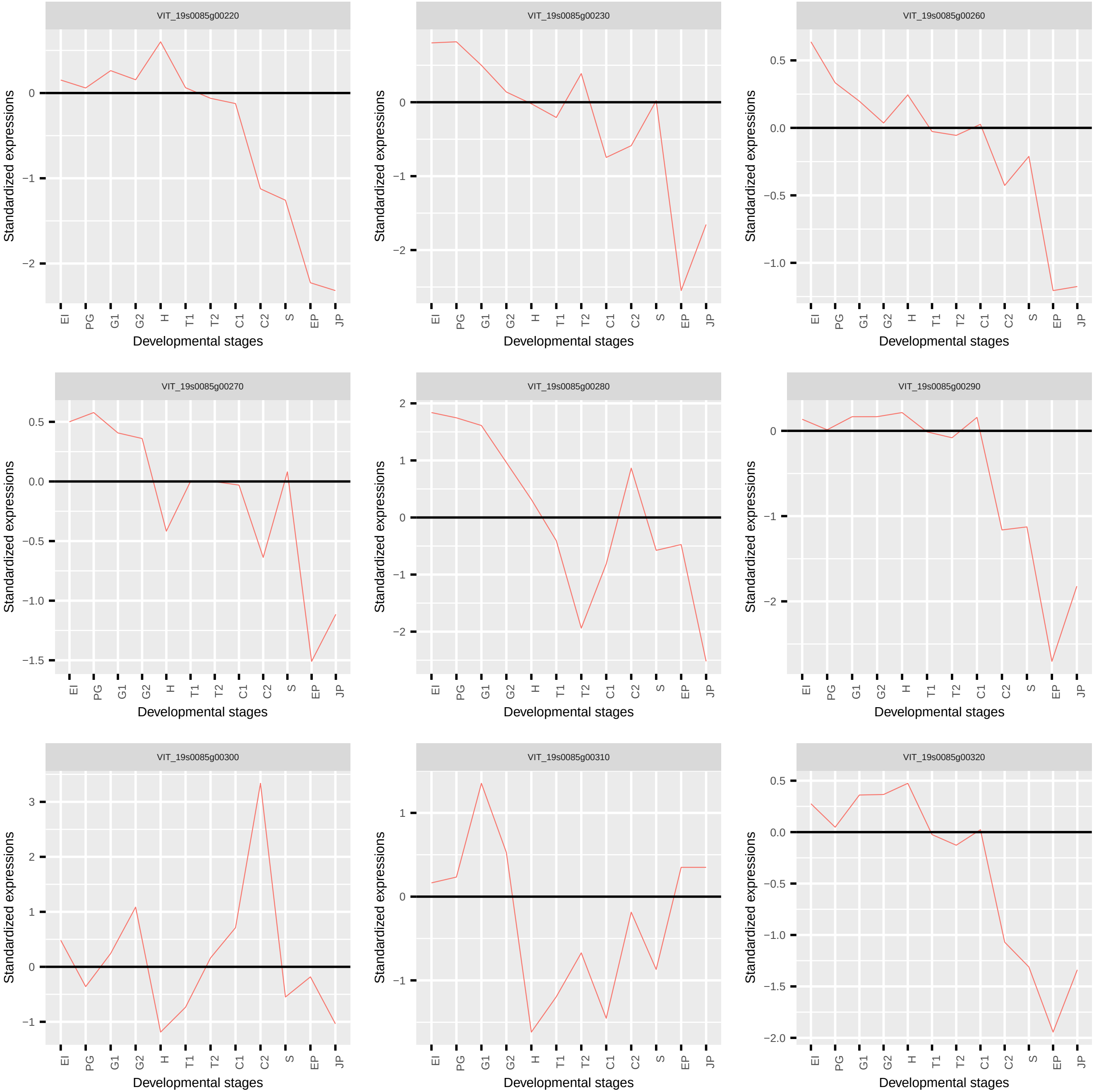
VIT_19s0085g00220
VIT_19s0085g00230
VIT_19s0085g00260
VIT_19s0085g00270
VIT_19s0085g00280
VIT_19s0085g00290
VIT_19s0085g00300
VIT_19s0085g00310
VIT_19s0085g00320
Standardized expressions
Developmental stages
EI PG G1 G2 H T1 T2 C1 C2 S EP JP

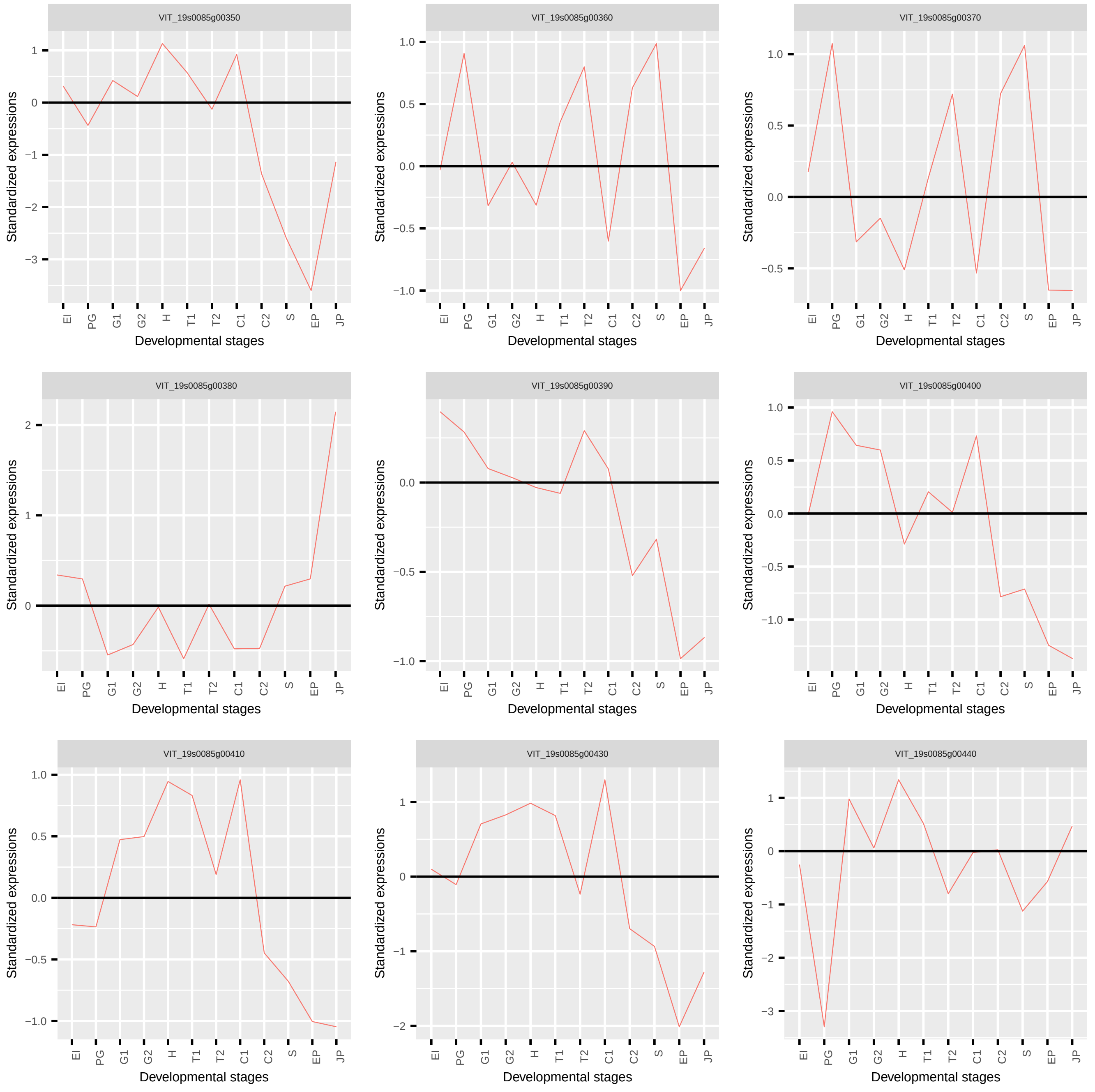
VIT_19s0085g00350
VIT_19s0085g00360
VIT_19s0085g00370
VIT_19s0085g00380
VIT_19s0085g00390
VIT_19s0085g00400
VIT_19s0085g00410
VIT_19s0085g00430
VIT_19s0085g00440
Standardized expressions
Developmental stages
EI PG G1 G2 H T1 T2 C1 C2 S EP JP

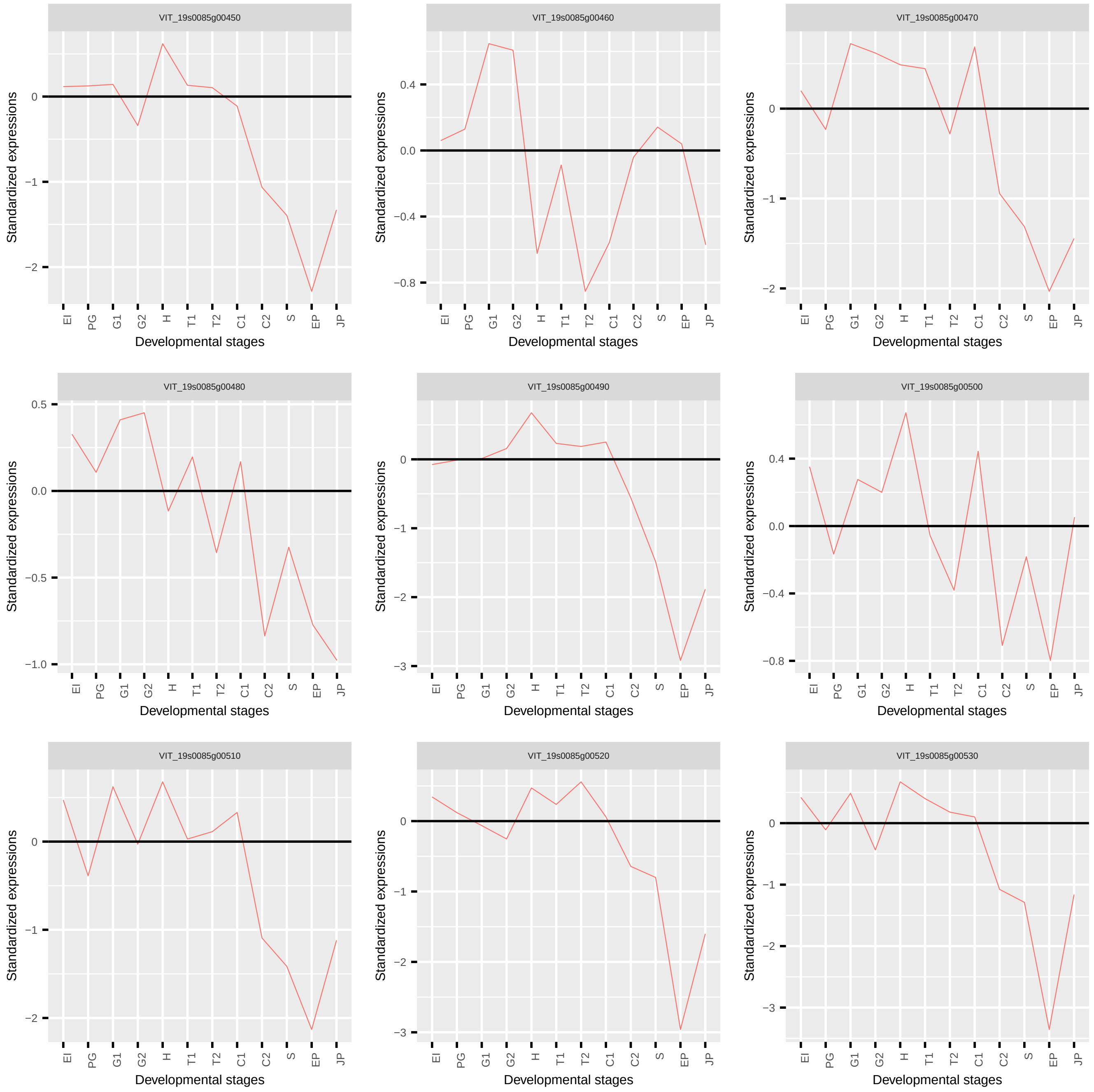
VIT_19s0085g00450
VIT_19s0085g00460
VIT_19s0085g00470
VIT_19s0085g00480
VIT_19s0085g00490
VIT_19s0085g00500
VIT_19s0085g00510
VIT_19s0085g00520
VIT_19s0085g00530
Standardized expressions
Developmental stages
EI PG G1 G2 H T1 T2 C1 C2 S EP JP

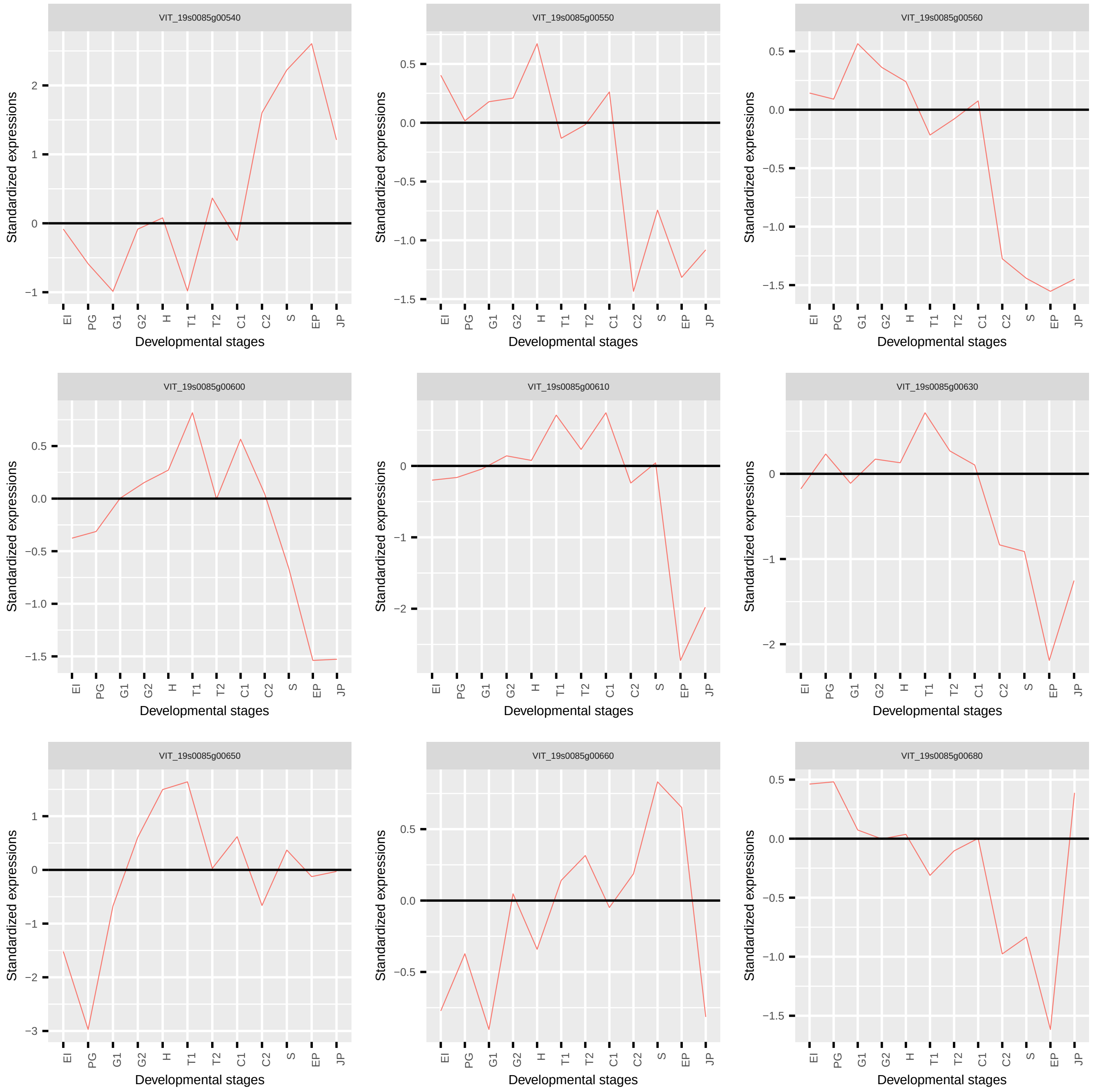
VIT_19s0085g00540
VIT_19s0085g00550
VIT_19s0085g00560
VIT_19s0085g00600
VIT_19s0085g00610
VIT_19s0085g00630
VIT_19s0085g00650
VIT_19s0085g00660
VIT_19s0085g00680
Standardized expressions
Developmental stages
EI PG G1 G2 H T1 T2 C1 C2 S EP JP

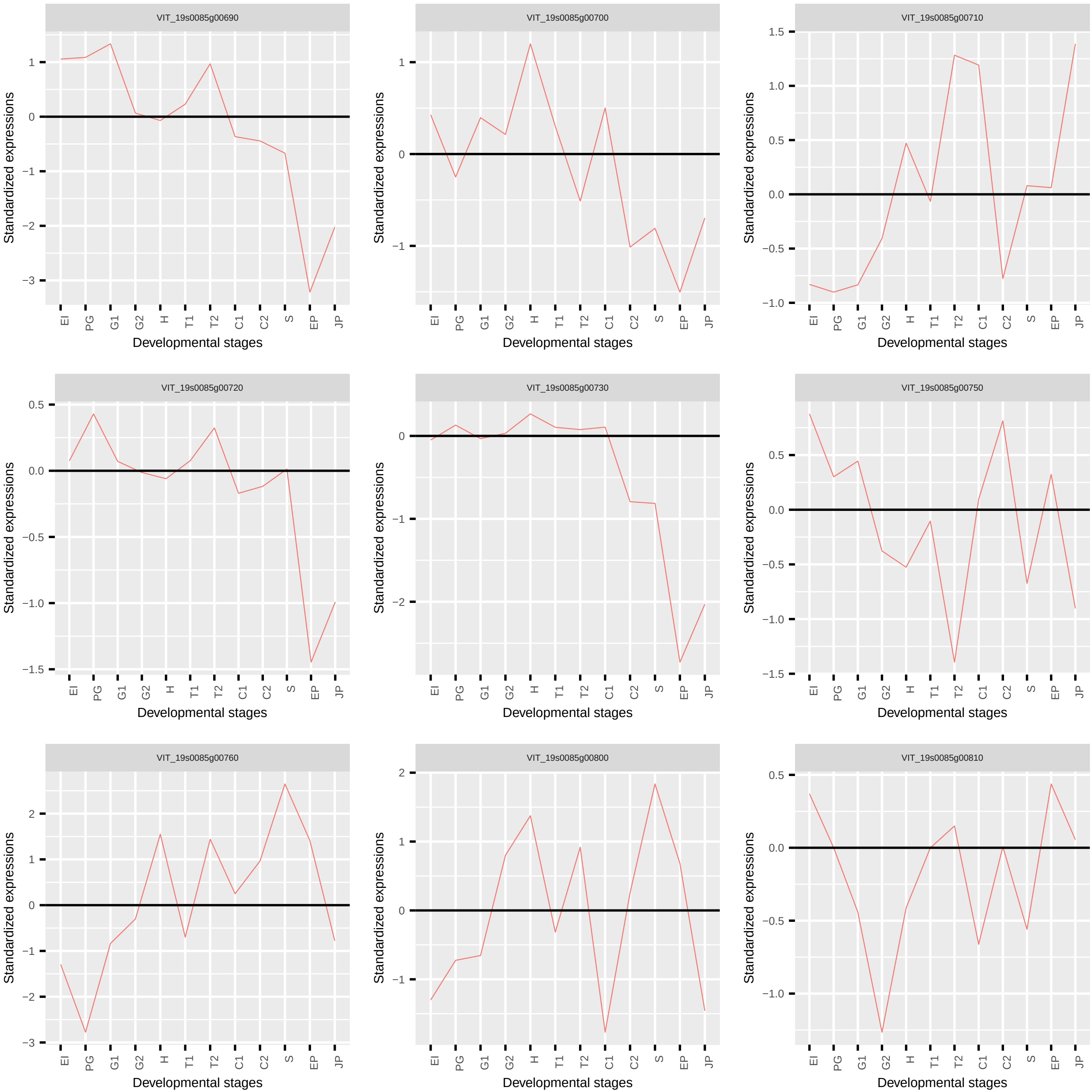
VIT_19s0085g00690
VIT_19s0085g00700
VIT_19s0085g00710
VIT_19s0085g00720
VIT_19s0085g00730
VIT_19s0085g00750
VIT_19s0085g00760
VIT_19s0085g00800
VIT_19s0085g00810
Standardized expressions
Developmental stages
EI PG G1 G2 H T1 T2 C1 C2 S EP JP

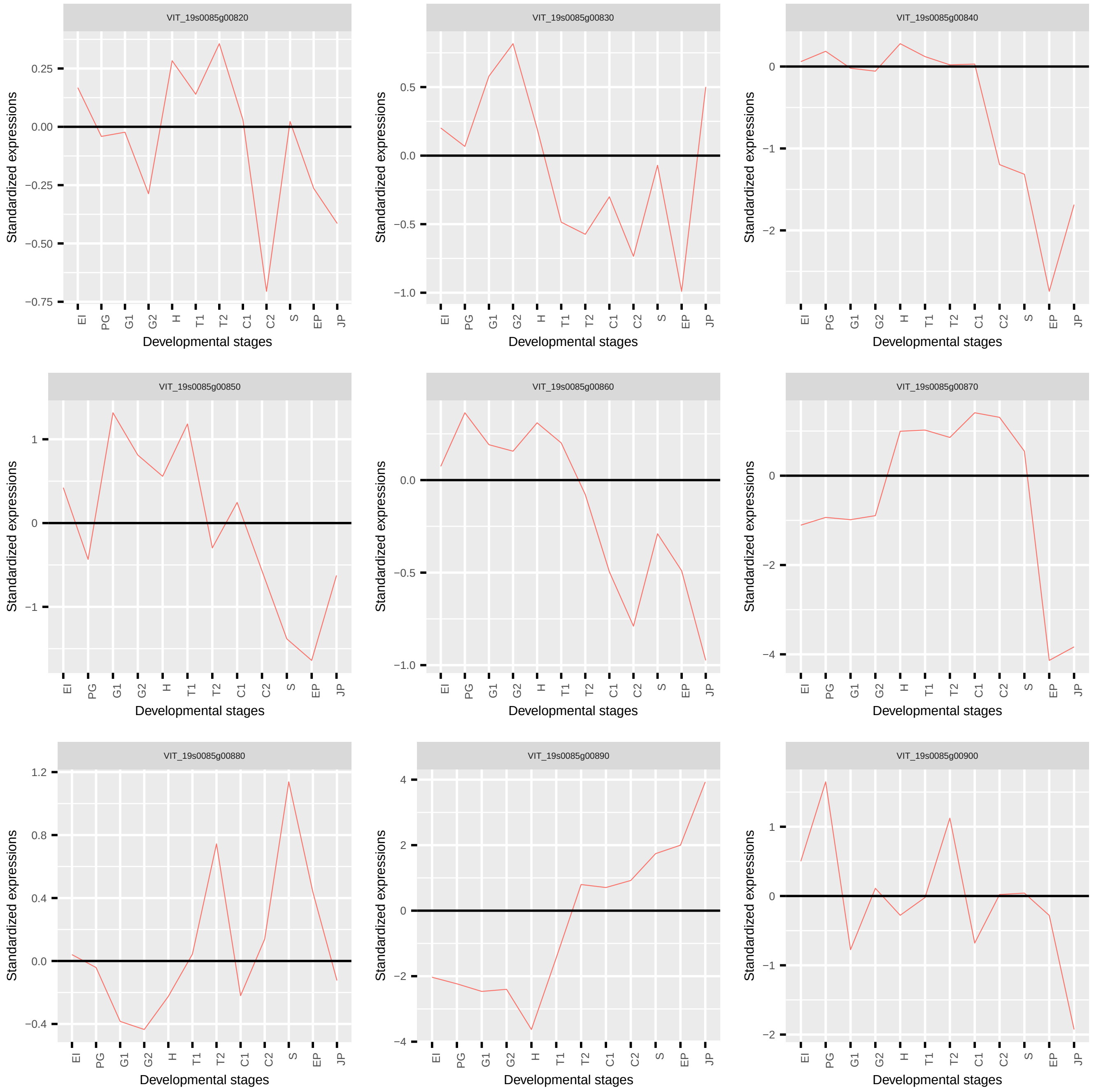
VIT_19s0085g00820
VIT_19s0085g00830
VIT_19s0085g00840
VIT_19s0085g00850
VIT_19s0085g00860
VIT_19s0085g00870
VIT_19s0085g00880
VIT_19s0085g00890
VIT_19s0085g00900
Standardized expressions
Developmental stages
EI PG G1 G2 H T1 T2 C1 C2 S EP JP
0.25 0.00 −0.25 −0.50 −0.75
0.5 0.0 −0.5 −1.0
0 −1 −2
1 0 −1
0.0 −0.5 −1.0
0 −2 −4
1.2 0.8 0.4 0.0 -0.4
4 2 0 -2 -4
1 0 −1 −2

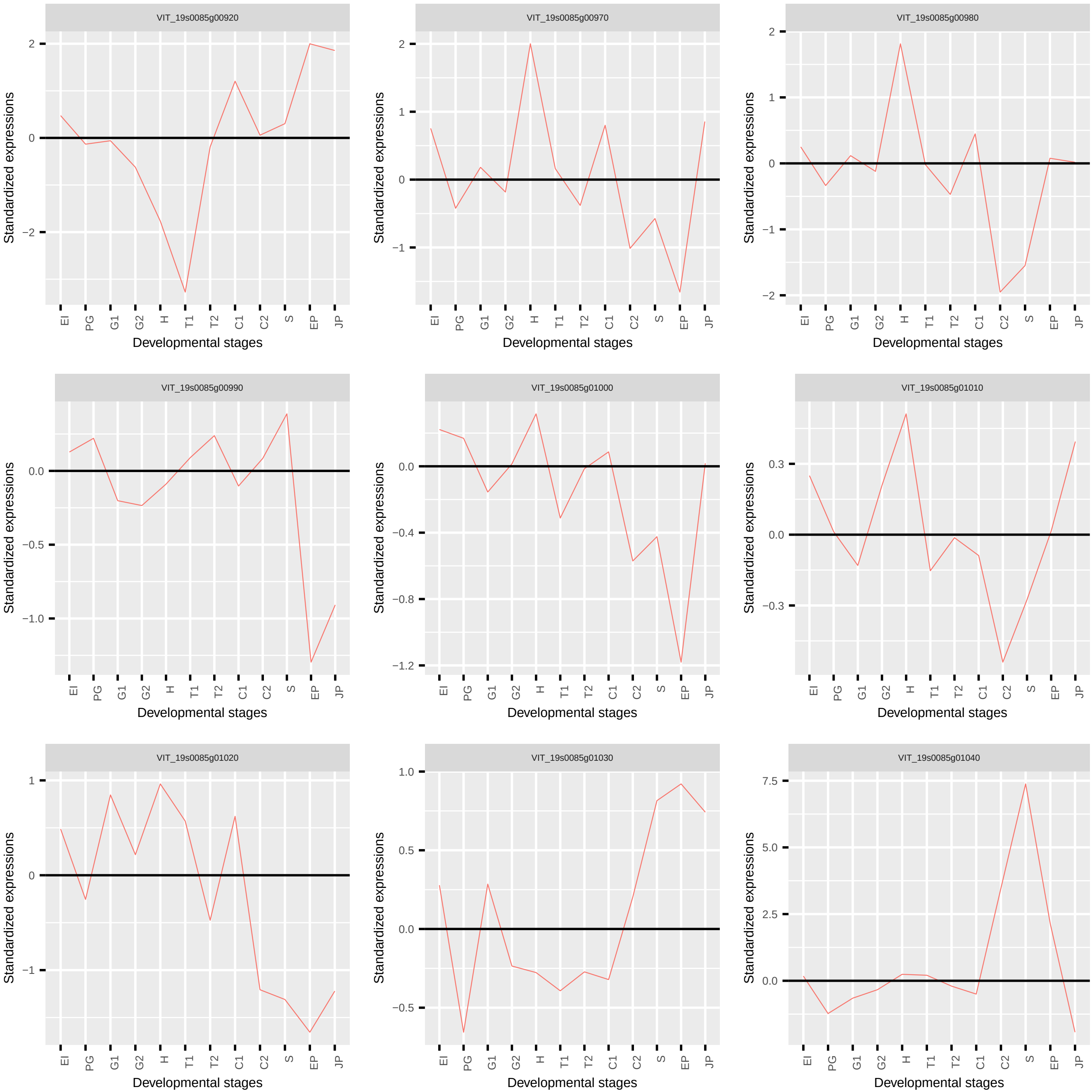
VIT_19s0085g00920
VIT_19s0085g00970
VIT_19s0085g00980
VIT_19s0085g00990
VIT_19s0085g01000
VIT_19s0085g01010
VIT_19s0085g01020
VIT_19s0085g01030
VIT_19s0085g01040
Standardized expressions
Developmental stages
EI PG G1 G2 H T1 T2 C1 C2 S EP JP

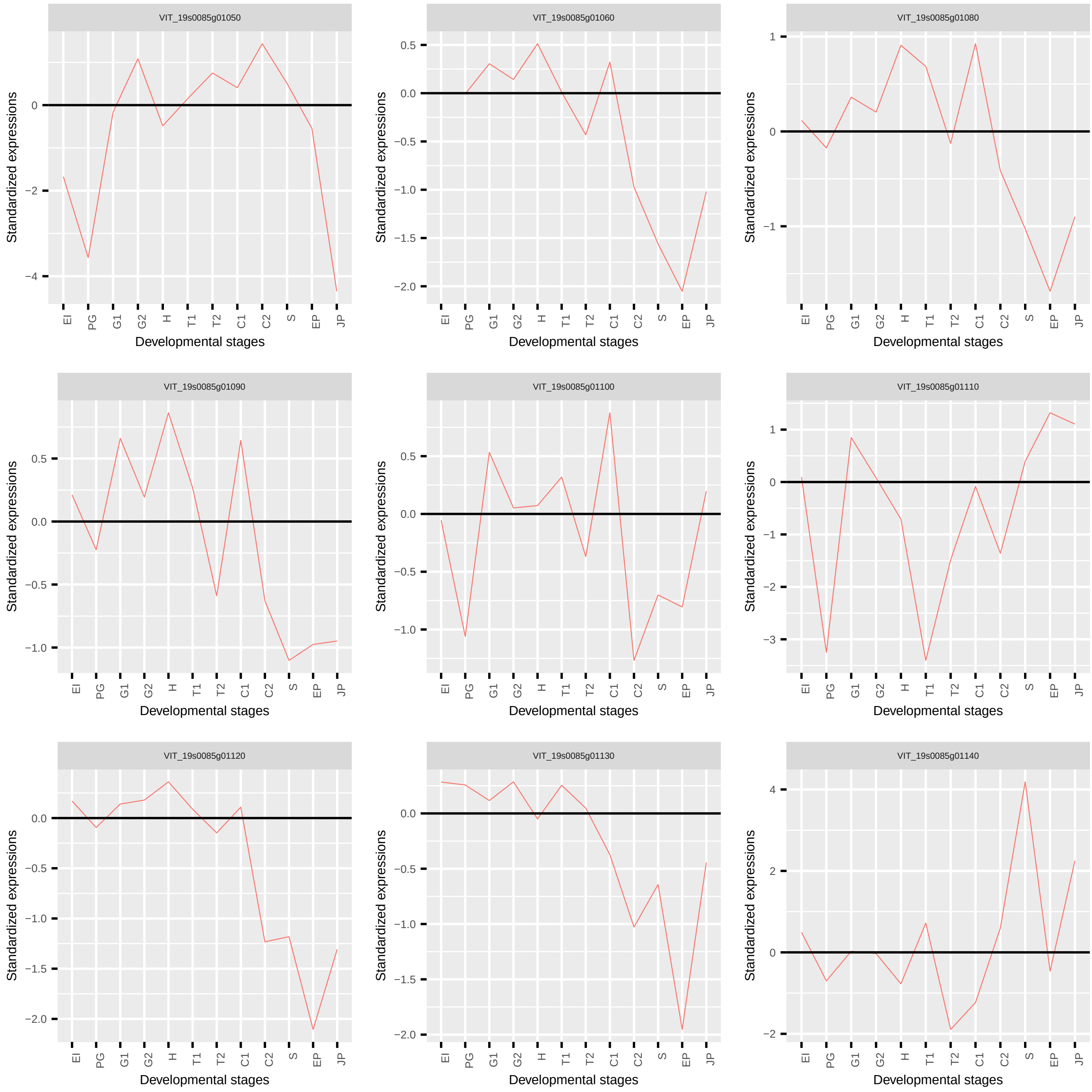

VIT_19s0085g01050
VIT_19s0085g01060
VIT_19s0085g01080
VIT_19s0085g01090
VIT_19s0085g01100
VIT_19s0085g01110
VIT_19s0085g01120
VIT_19s0085g01130
VIT_19s0085g01140
Standardized expressions
Developmental stages
EI
PG
G1
G2
H
T1
T2
C1
C2
S
EP
JP

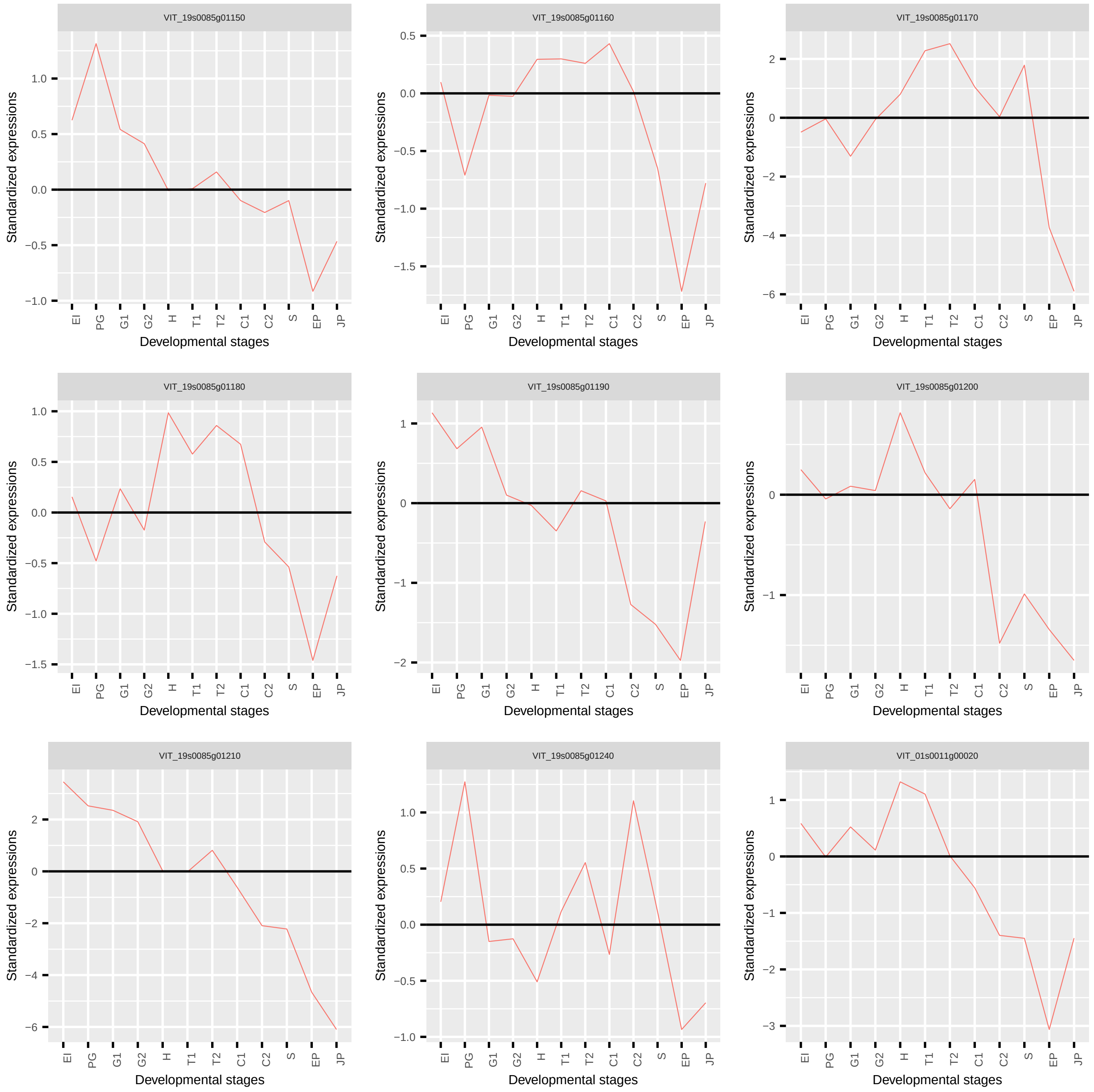
VIT_19s0085g01150
VIT_19s0085g01160
VIT_19s0085g01170
VIT_19s0085g01180
VIT_19s0085g01190
VIT_19s0085g01200
VIT_19s0085g01210
VIT_19s0085g01240
VIT_01s0011g00020
Standardized expressions
Developmental stages
EI PG G1 G2 H T1 T2 C1 C2 S EP JP

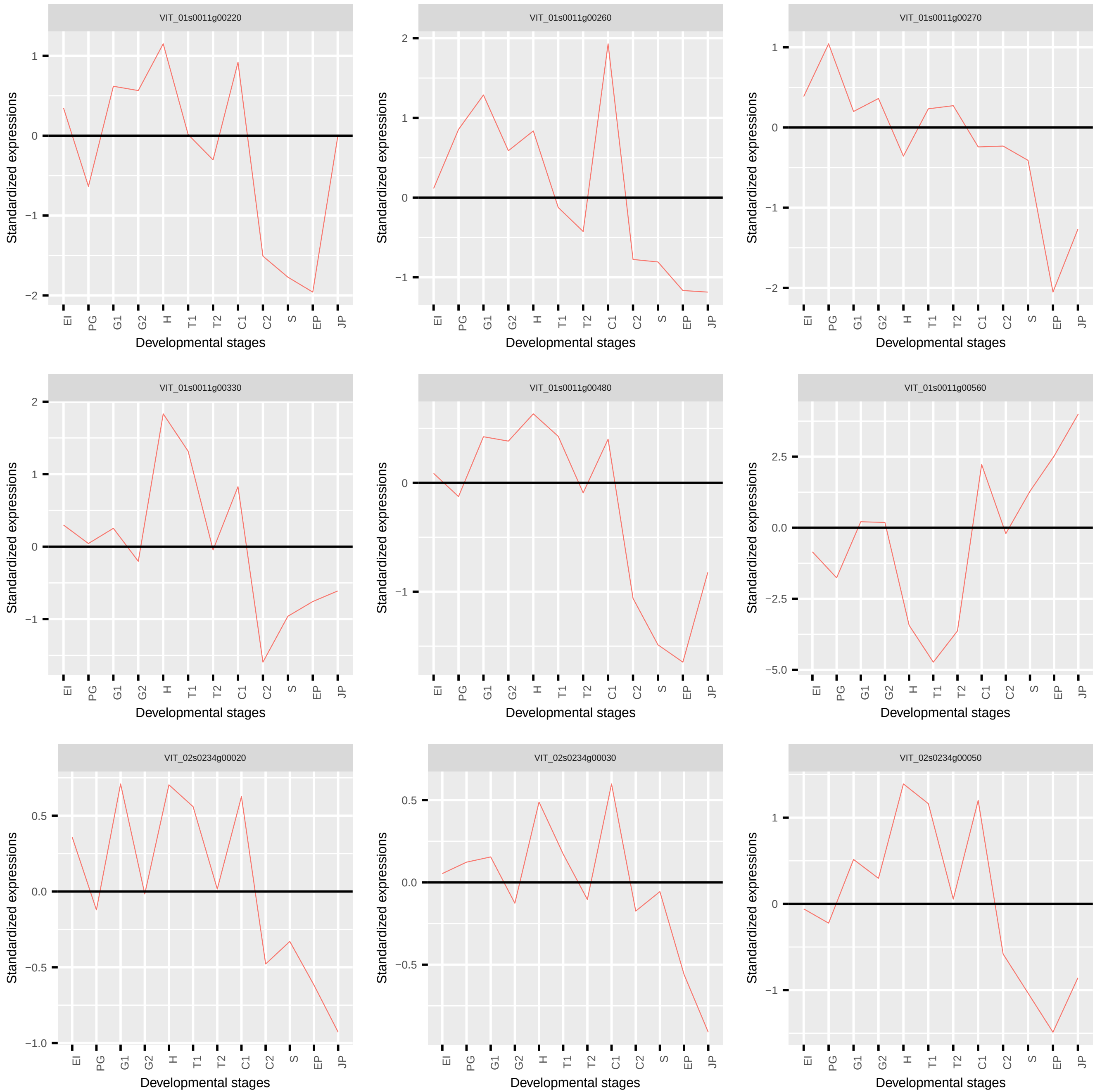
VIT_01s0011g00220
VIT_01s0011g00260
VIT_01s0011g00270
VIT_01s0011g00330
VIT_01s0011g00480
VIT_01s0011g00560
VIT_02s0234g00020
VIT_02s0234g00030
VIT_02s0234g00050
Standardized expressions
Developmental stages
EI PG G1 G2 H T1 T2 C1 C2 S EP JP

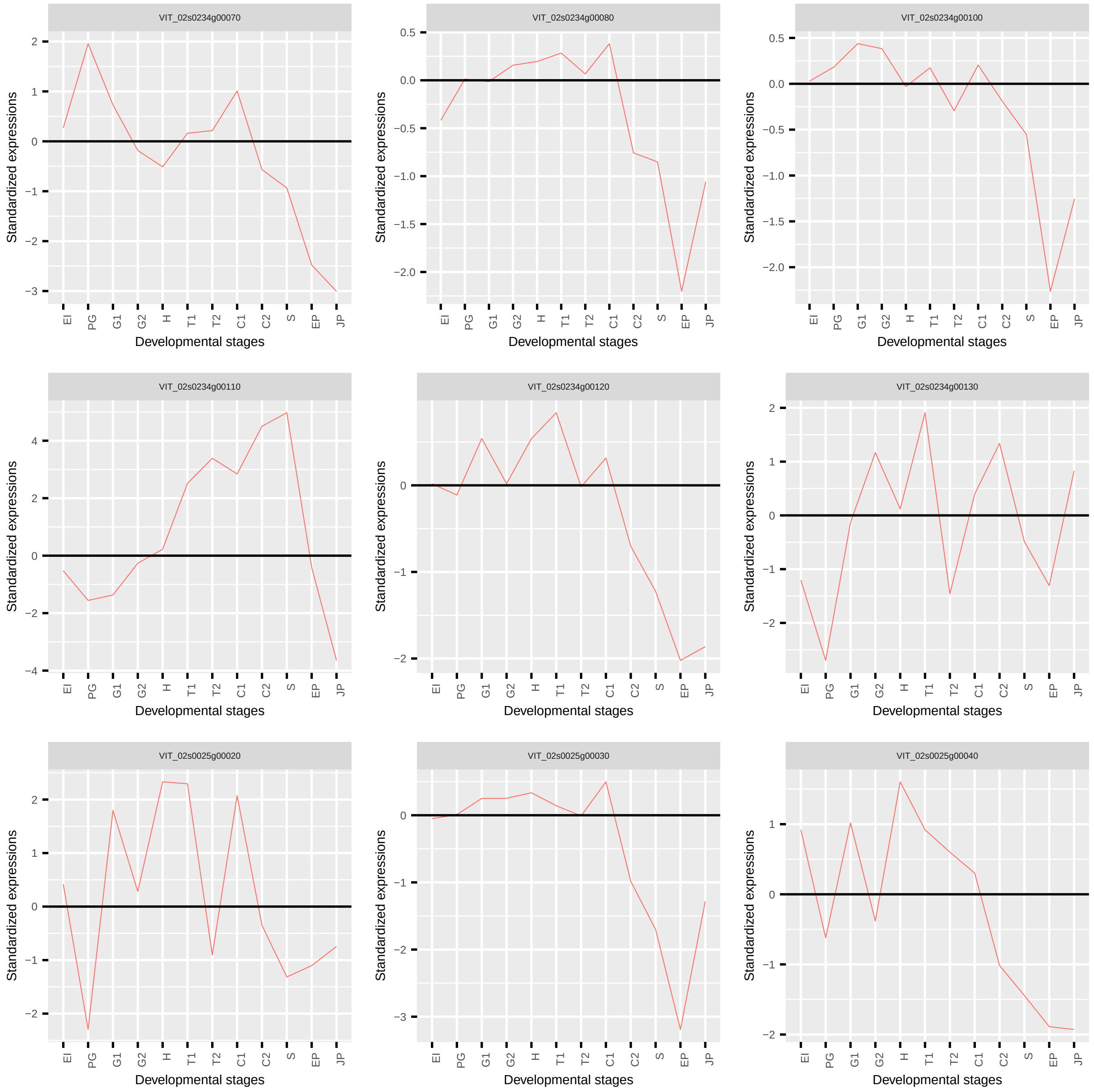

VIT_02s0234g00070
2
1
0
−1
−2
−3
Standardized expressions
EI PG G1 G2 H T1 T2 C1 C2 S EP JP
Developmental stages
VIT_02s0234g00080
0.5
0.0
−0.5
−1.0
−1.5
−2.0
Standardized expressions
EI PG G1 G2 H T1 T2 C1 C2 S EP JP
Developmental stages
VIT_02s0234g00100
0.5
0.0
−0.5
−1.0
−1.5
−2.0
Standardized expressions
EI PG G1 G2 H T1 T2 C1 C2 S EP JP
Developmental stages
VIT_02s0234g00110
4
2
0
−2
−4
Standardized expressions
EI PG G1 G2 H T1 T2 C1 C2 S EP JP
Developmental stages
VIT_02s0234g00120
0
−1
−2
Standardized expressions
EI PG G1 G2 H T1 T2 C1 C2 S EP JP
Developmental stages
VIT_02s0234g00130
2
1
0
−1
−2
Standardized expressions
EI PG G1 G2 H T1 T2 C1 C2 S EP JP
Developmental stages
VIT_02s0025g00020
2
1
0
−1
−2
Standardized expressions
EI PG G1 G2 H T1 T2 C1 C2 S EP JP
Developmental stages
VIT_02s0025g00030
0
−1
−2
−3
Standardized expressions
EI PG G1 G2 H T1 T2 C1 C2 S EP JP
Developmental stages
VIT_02s0025g00040
1
0
−1
−2
Standardized expressions
EI PG G1 G2 H T1 T2 C1 C2 S EP JP
Developmental stages

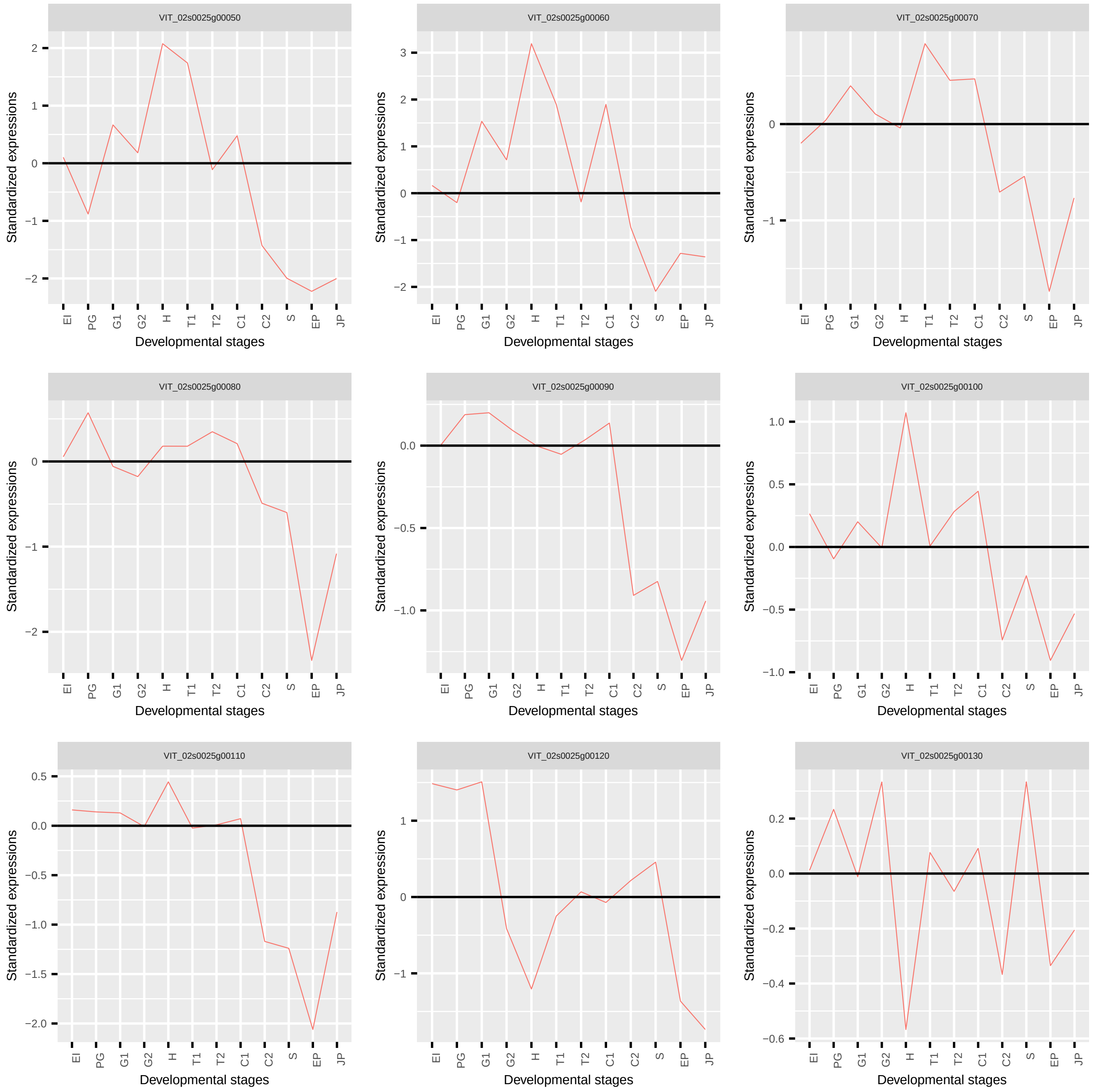
VIT_02s0025g00050
VIT_02s0025g00060
VIT_02s0025g00070
VIT_02s0025g00080
VIT_02s0025g00090
VIT_02s0025g00100
VIT_02s0025g00110
VIT_02s0025g00120
VIT_02s0025g00130
Standardized expressions
Developmental stages
EI
PG
G1
G2
H
T1
T2
C1
C2
S
EP
JP

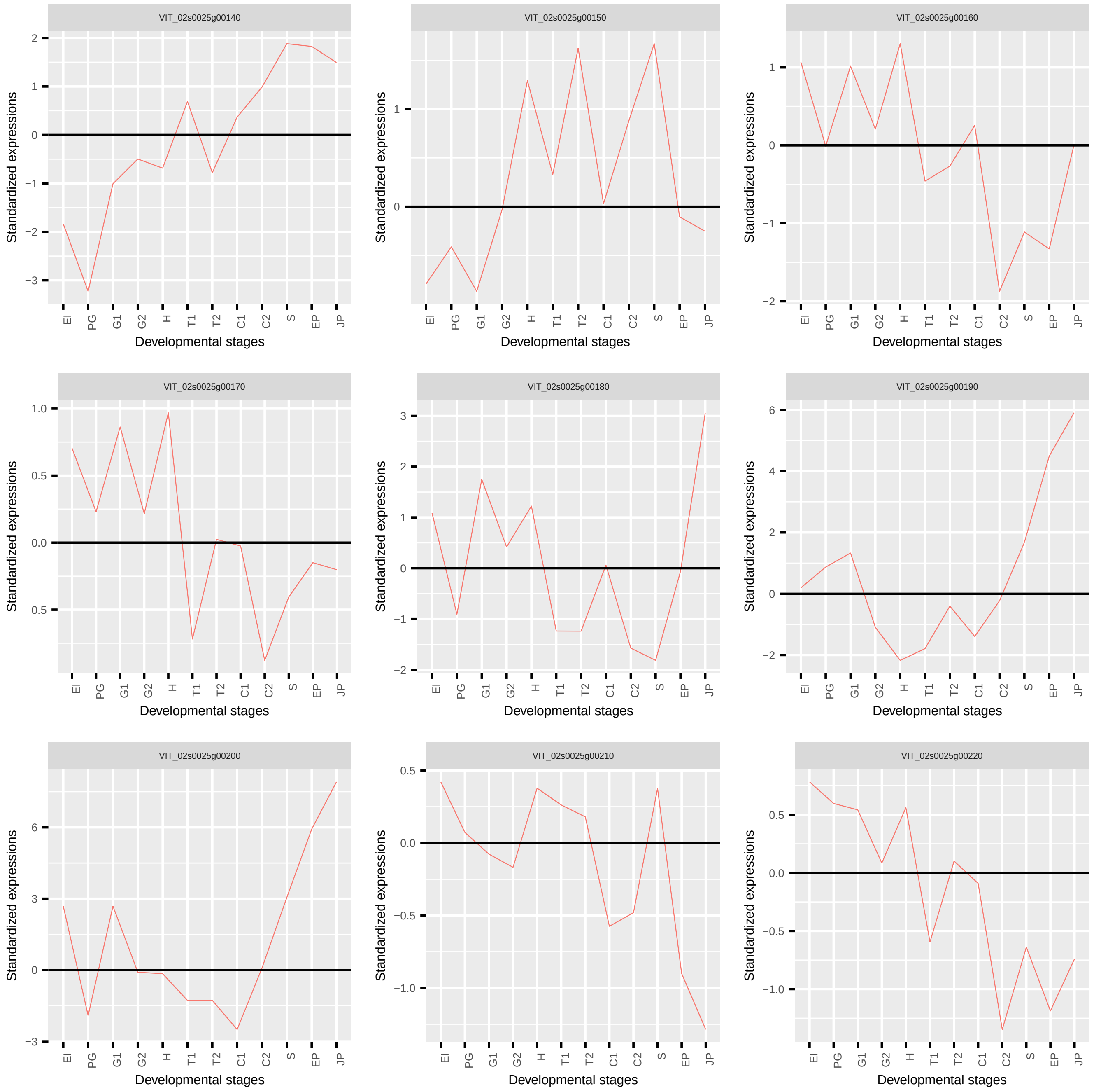

VIT_02s0025g00140
VIT_02s0025g00150
VIT_02s0025g00160
VIT_02s0025g00170
VIT_02s0025g00180
VIT_02s0025g00190
VIT_02s0025g00200
VIT_02s0025g00210
VIT_02s0025g00220
Standardized expressions
Developmental stages
EI PG G1 G2 H T1 T2 C1 C2 S EP JP

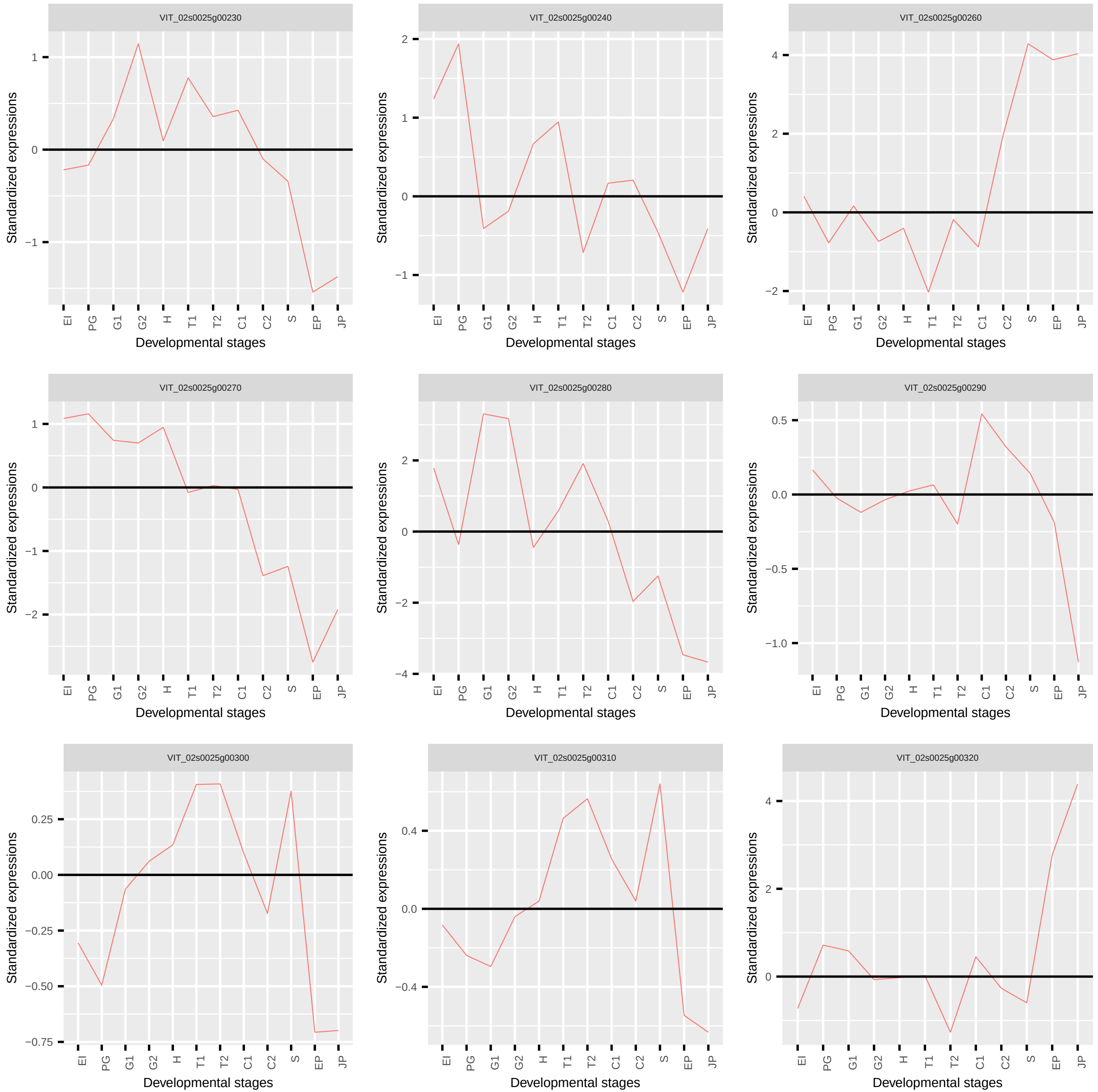
VIT_02s0025g00230
VIT_02s0025g00240
VIT_02s0025g00260
VIT_02s0025g00270
VIT_02s0025g00280
VIT_02s0025g00290
VIT_02s0025g00300
VIT_02s0025g00310
VIT_02s0025g00320
Standardized expressions
Developmental stages
EI PG G1 G2 H T1 T2 C1 C2 S EP JP

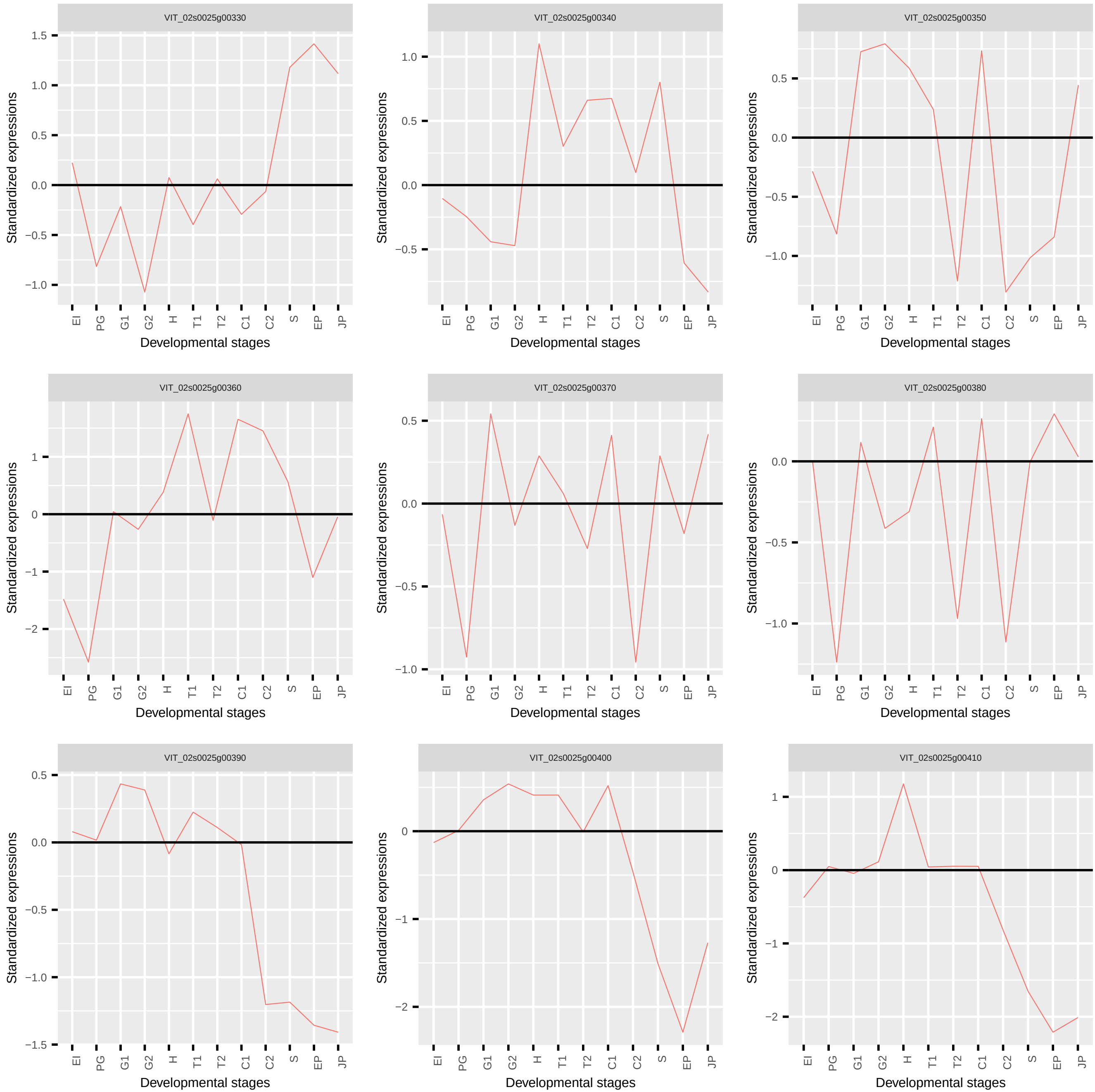
VIT_02s0025g00330
VIT_02s0025g00340
VIT_02s0025g00350
VIT_02s0025g00360
VIT_02s0025g00370
VIT_02s0025g00380
VIT_02s0025g00390
VIT_02s0025g00400
VIT_02s0025g00410
Standardized expressions
Developmental stages
EI
PG
G1
G2
H
T1
T2
C1
C2
S
EP
JP

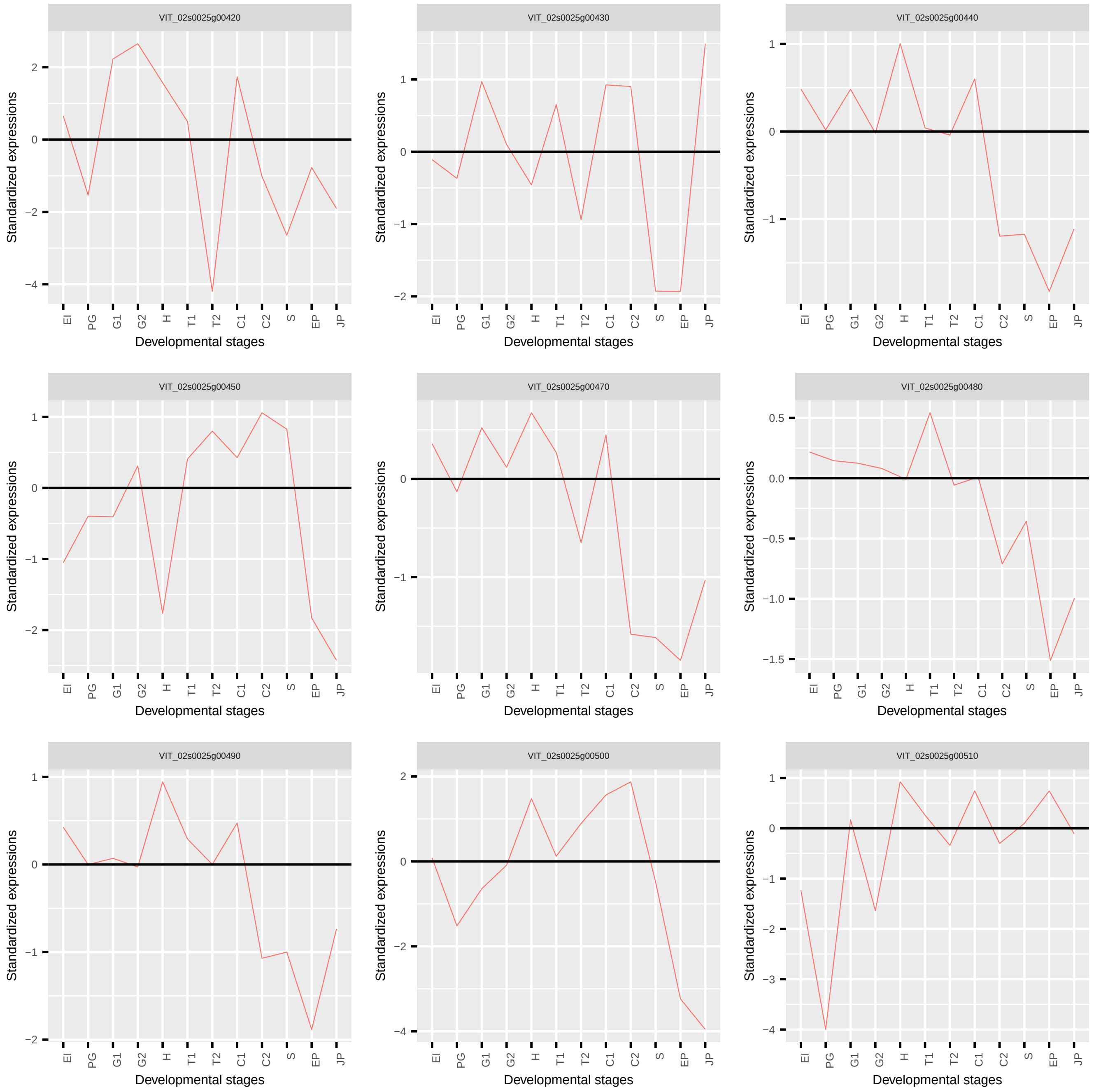
VIT_02s0025g00420
VIT_02s0025g00430
VIT_02s0025g00440
VIT_02s0025g00450
VIT_02s0025g00470
VIT_02s0025g00480
VIT_02s0025g00490
VIT_02s0025g00500
VIT_02s0025g00510
Standardized expressions
Developmental stages
EI
PG
G1
G2
H
T1
T2
C1
C2
S
EP
JP

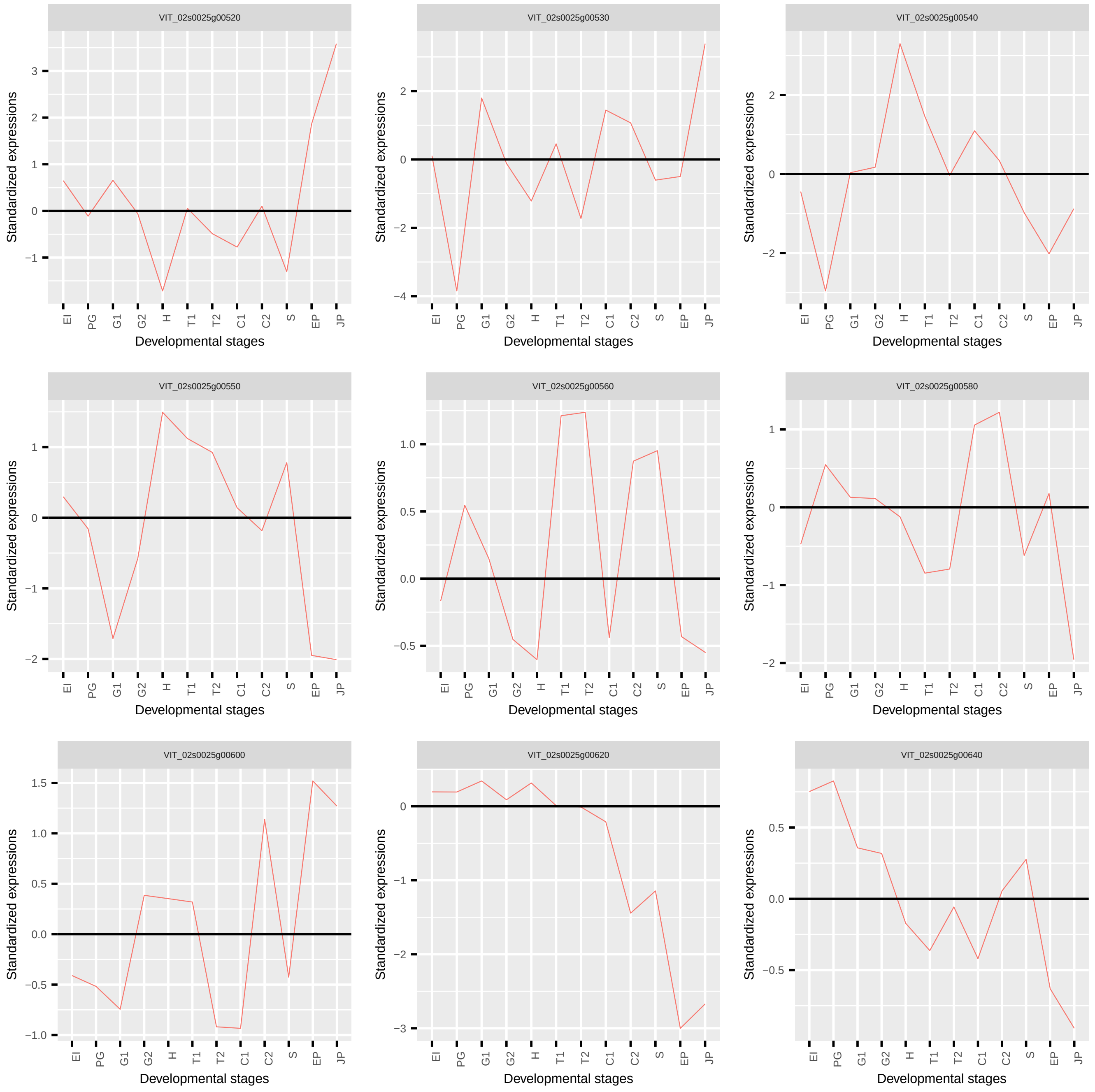

VIT_02s0025g00520
Standardized expressions
3
2
1
0
−1
EI PG G1 G2 H T1 T2 C1 C2 S EP JP
Developmental stages
VIT_02s0025g00530
Standardized expressions
2
0
−2
−4
EI PG G1 G2 H T1 T2 C1 C2 S EP JP
Developmental stages
VIT_02s0025g00540
Standardized expressions
2
0
−2
EI PG G1 G2 H T1 T2 C1 C2 S EP JP
Developmental stages
VIT_02s0025g00550
Standardized expressions
1
0
−1
−2
EI PG G1 G2 H T1 T2 C1 C2 S EP JP
Developmental stages
VIT_02s0025g00560
Standardized expressions
1.0
0.5
0.0
−0.5
EI PG G1 G2 H T1 T2 C1 C2 S EP JP
Developmental stages
VIT_02s0025g00580
Standardized expressions
1
0
−1
−2
EI PG G1 G2 H T1 T2 C1 C2 S EP JP
Developmental stages
VIT_02s0025g00600
Standardized expressions
1.5
1.0
0.5
0.0
−0.5
−1.0
EI PG G1 G2 H T1 T2 C1 C2 S EP JP
Developmental stages
VIT_02s0025g00620
Standardized expressions
0
−1
−2
−3
EI PG G1 G2 H T1 T2 C1 C2 S EP JP
Developmental stages
VIT_02s0025g00640
Standardized expressions
0.5
0.0
−0.5
EI PG G1 G2 H T1 T2 C1 C2 S EP JP
Developmental stages

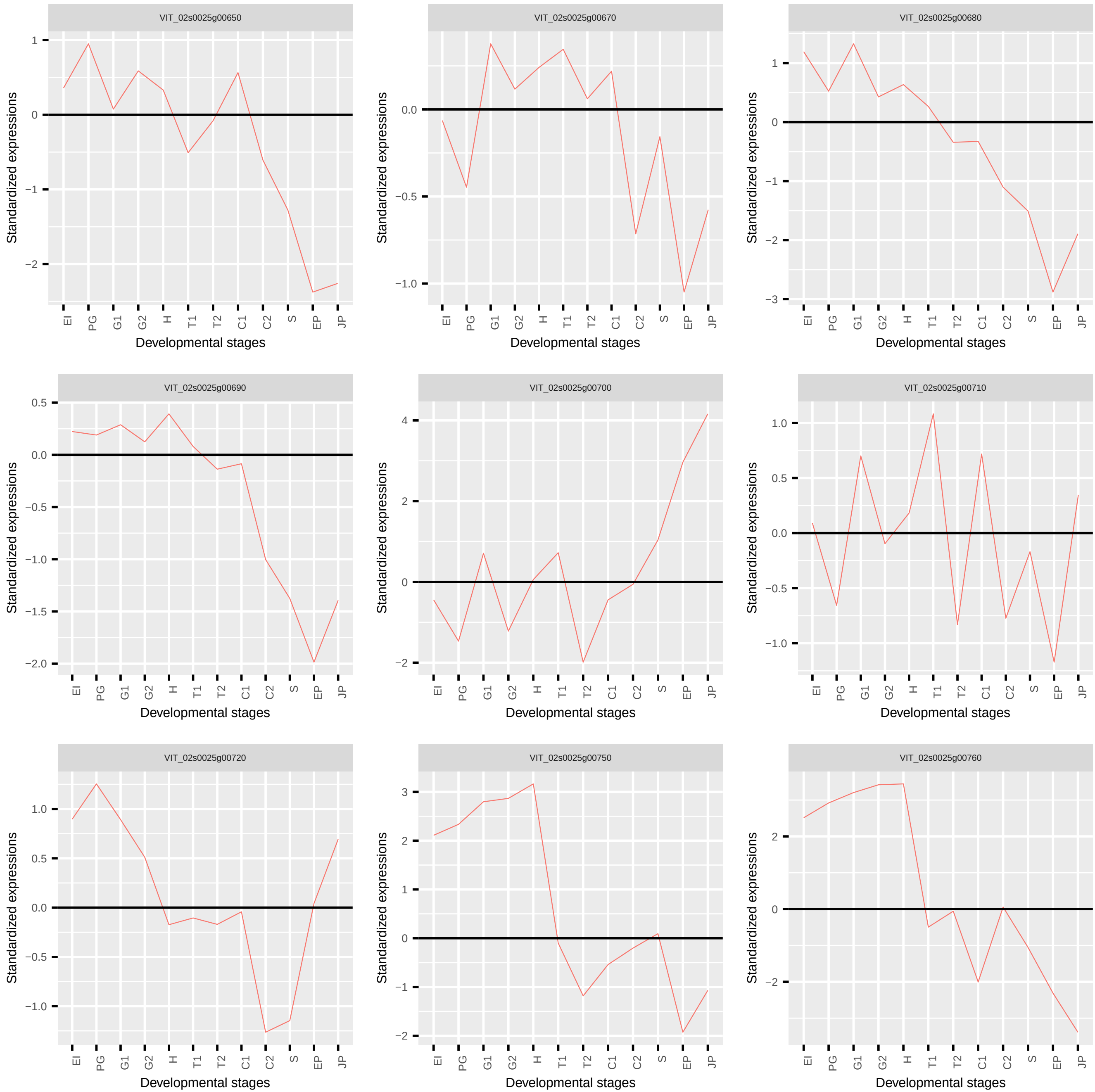
VIT_02s0025g00650
VIT_02s0025g00670
VIT_02s0025g00680
VIT_02s0025g00690
VIT_02s0025g00700
VIT_02s0025g00710
VIT_02s0025g00720
VIT_02s0025g00750
VIT_02s0025g00760
Standardized expressions
Developmental stages
EI PG G1 G2 H T1 T2 C1 C2 S EP JP

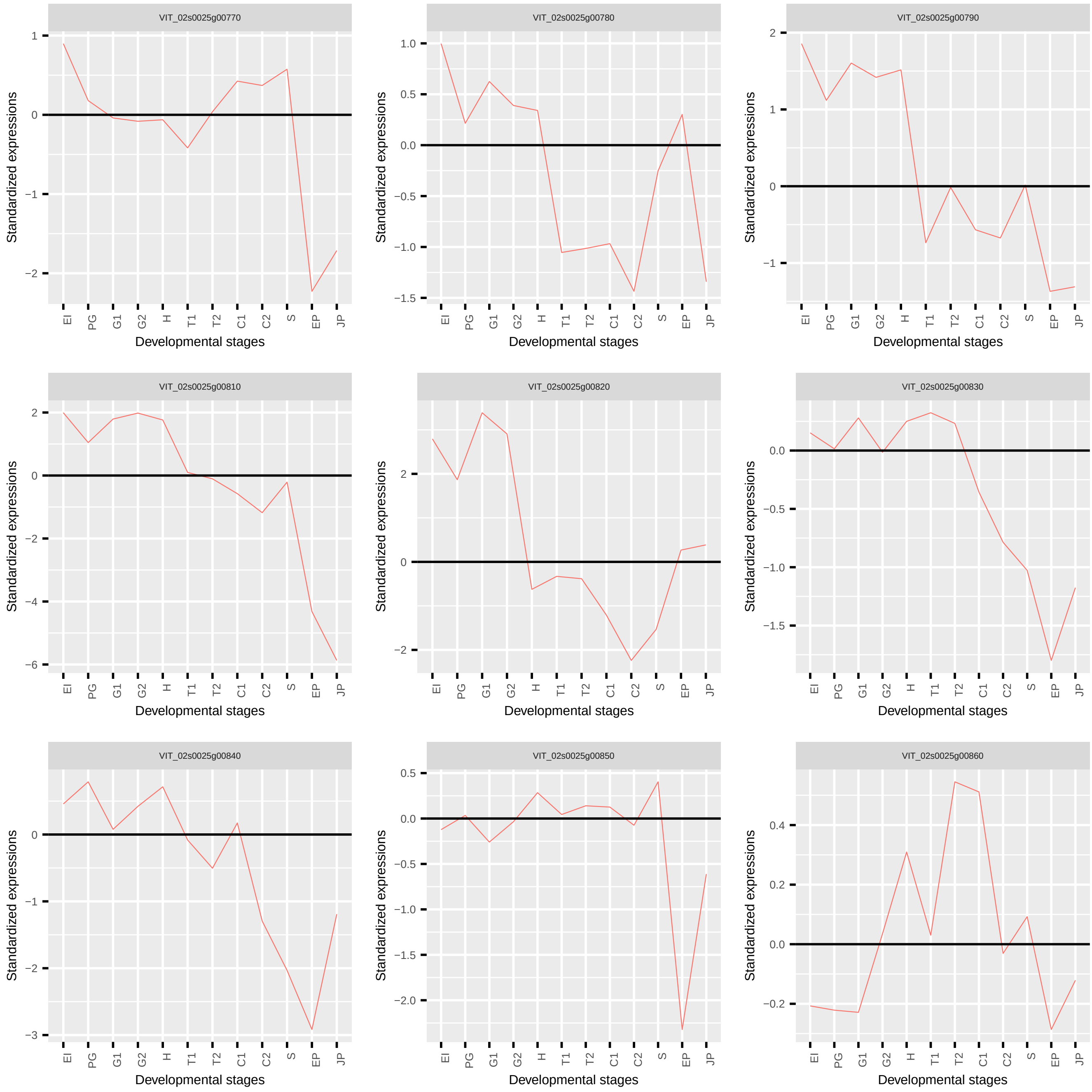
VIT_02s0025g00770
VIT_02s0025g00780
VIT_02s0025g00790
VIT_02s0025g00810
VIT_02s0025g00820
VIT_02s0025g00830
VIT_02s0025g00840
VIT_02s0025g00850
VIT_02s0025g00860
Standardized expressions
Developmental stages
EI PG G1 G2 H T1 T2 C1 C2 S EP JP

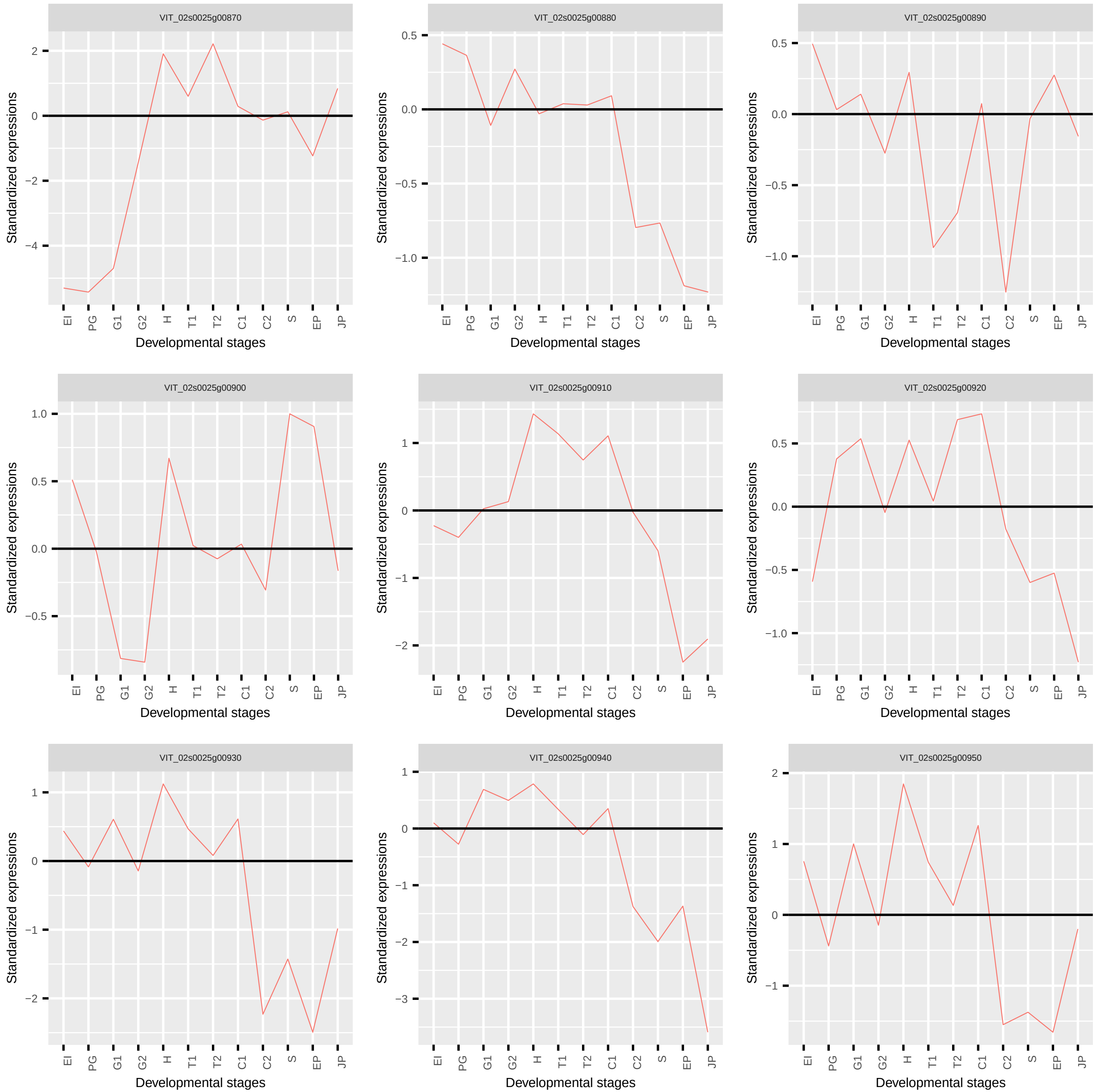
VIT_02s0025g00870
2
0
−2
−4
Standardized expressions
EI PG G1 G2 H T1 T2 C1 C2 S EP JP
Developmental stages
VIT_02s0025g00880
0.5
0.0
−0.5
−1.0
Standardized expressions
EI PG G1 G2 H T1 T2 C1 C2 S EP JP
Developmental stages
VIT_02s0025g00890
0.5
0.0
−0.5
−1.0
Standardized expressions
EI PG G1 G2 H T1 T2 C1 C2 S EP JP
Developmental stages
VIT_02s0025g00900
1.0
0.5
0.0
−0.5
Standardized expressions
EI PG G1 G2 H T1 T2 C1 C2 S EP JP
Developmental stages
VIT_02s0025g00910
1
0
−1
−2
Standardized expressions
EI PG G1 G2 H T1 T2 C1 C2 S EP JP
Developmental stages
VIT_02s0025g00920
0.5
0.0
−0.5
−1.0
Standardized expressions
EI PG G1 G2 H T1 T2 C1 C2 S EP JP
Developmental stages
VIT_02s0025g00930
1
0
−1
−2
Standardized expressions
EI PG G1 G2 H T1 T2 C1 C2 S EP JP
Developmental stages
VIT_02s0025g00940
1
0
−1
−2
−3
Standardized expressions
EI PG G1 G2 H T1 T2 C1 C2 S EP JP
Developmental stages
VIT_02s0025g00950
2
1
0
−1
Standardized expressions
EI PG G1 G2 H T1 T2 C1 C2 S EP JP
Developmental stages

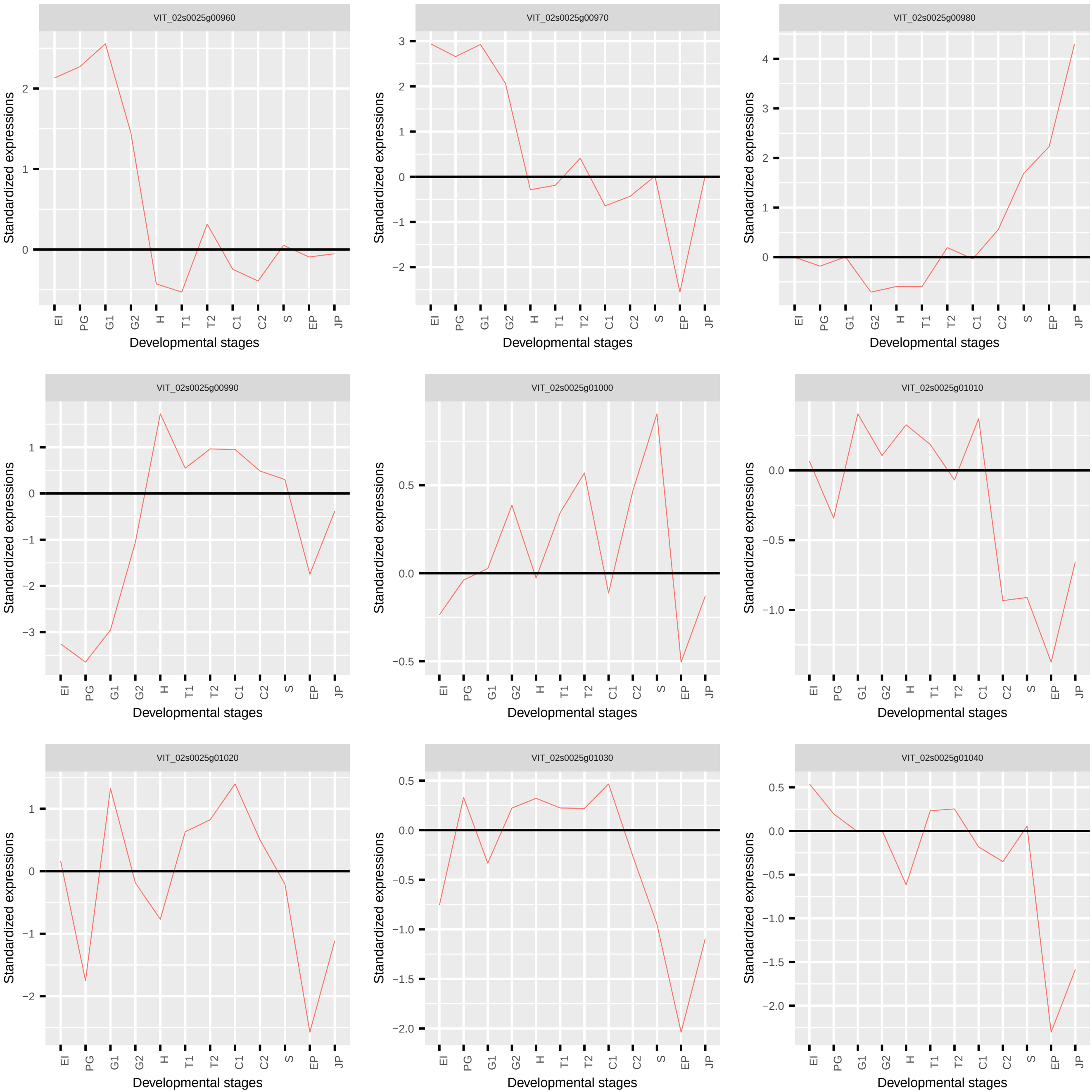
VIT_02s0025g00960
VIT_02s0025g00970
VIT_02s0025g00980
VIT_02s0025g00990
VIT_02s0025g01000
VIT_02s0025g01010
VIT_02s0025g01020
VIT_02s0025g01030
VIT_02s0025g01040
Standardized expressions
Developmental stages
EI PG G1 G2 H T1 T2 C1 C2 S EP JP

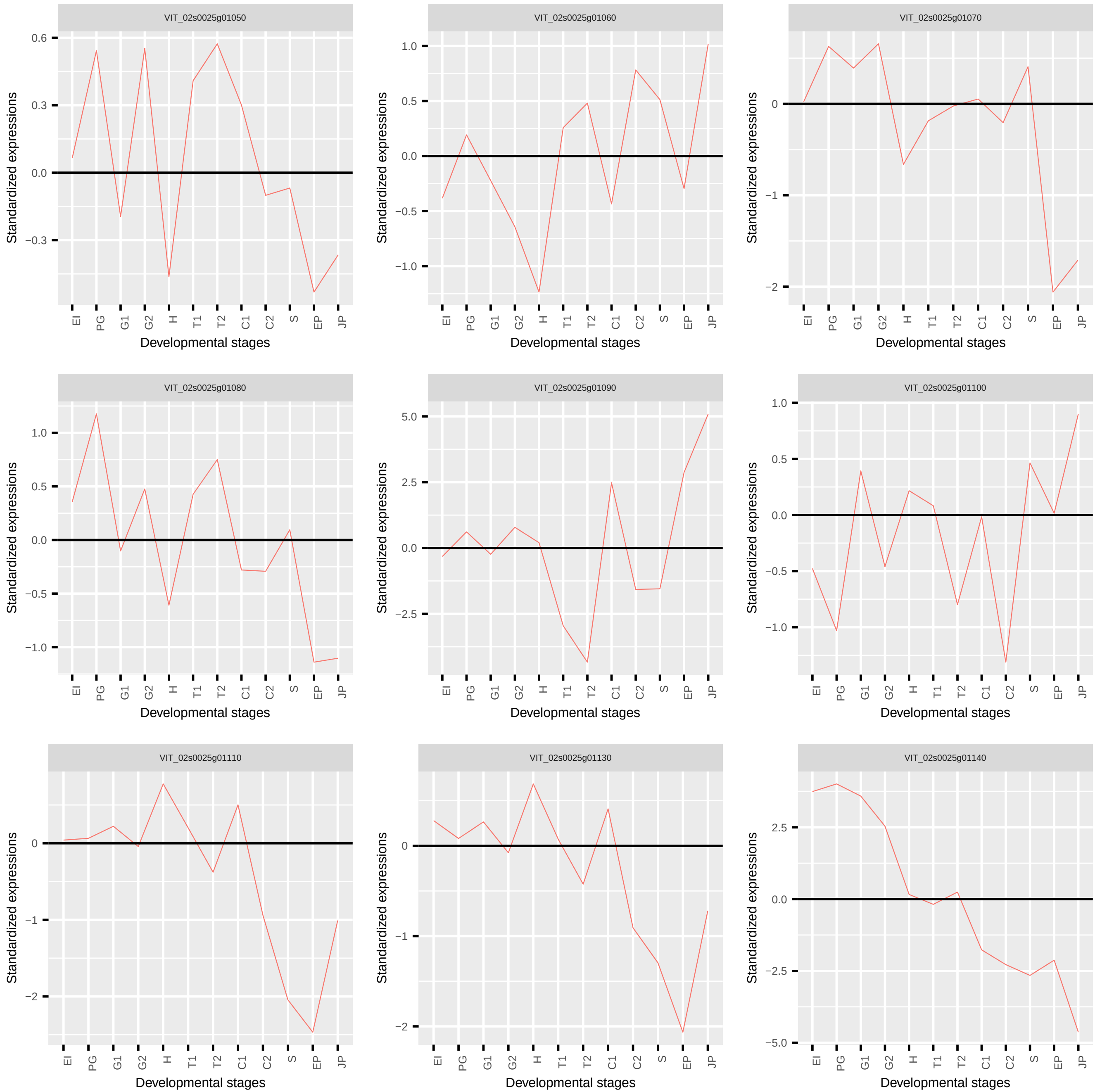
VIT_02s0025g01050
VIT_02s0025g01060
VIT_02s0025g01070
VIT_02s0025g01080
VIT_02s0025g01090
VIT_02s0025g01100
VIT_02s0025g01110
VIT_02s0025g01130
VIT_02s0025g01140
Standardized expressions
Developmental stages
EI
PG
G1
G2
H
T1
T2
C1
C2
S
EP
JP
0.6
0.3
0.0
−0.3
1.0
0.5
−0.5
−1.0
0
−1
−2
5.0
2.5
−2.5
−5.0

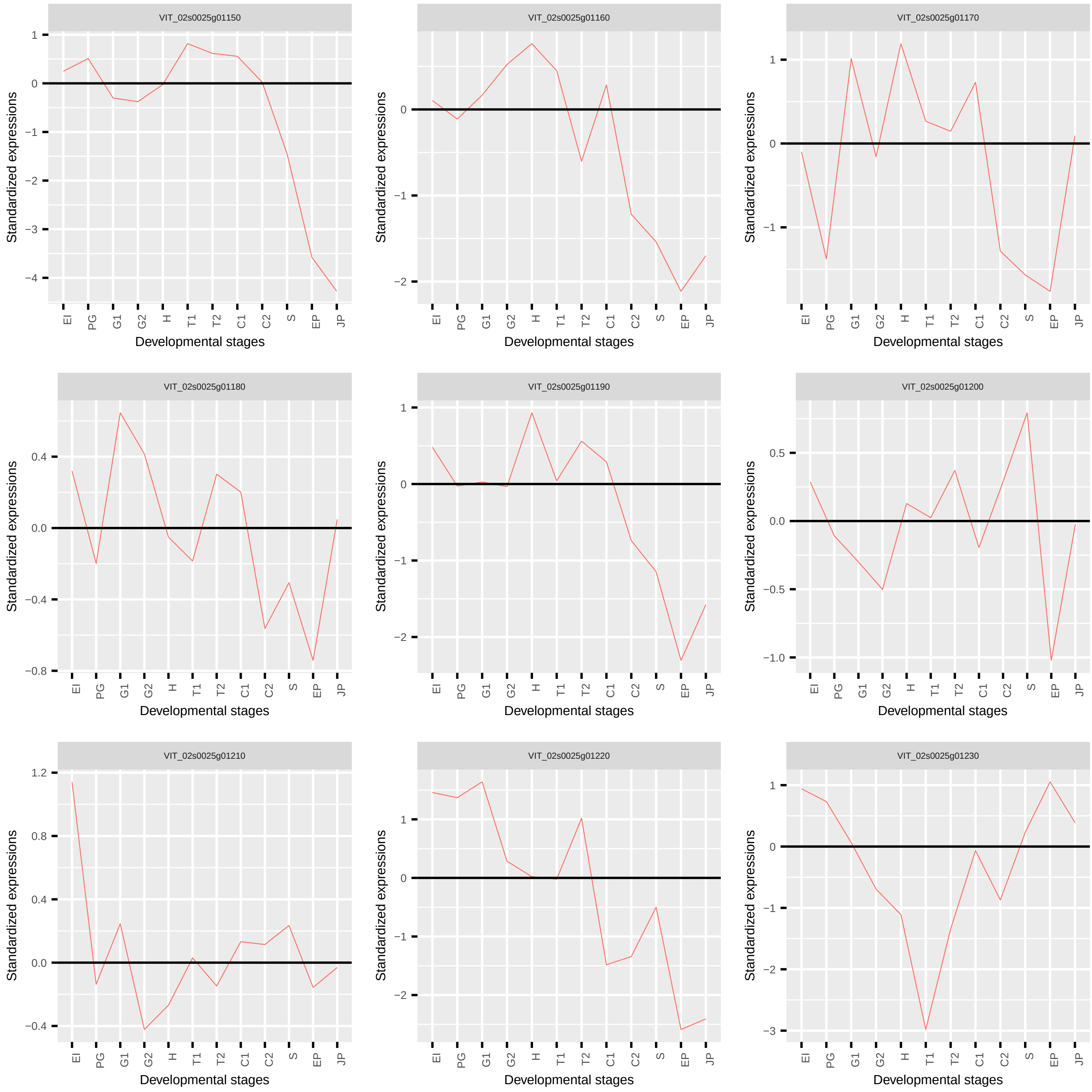

VIT_02s0025g01150
VIT_02s0025g01160
VIT_02s0025g01170
VIT_02s0025g01180
VIT_02s0025g01190
VIT_02s0025g01200
VIT_02s0025g01210
VIT_02s0025g01220
VIT_02s0025g01230
Standardized expressions
Developmental stages
EI PG G1 G2 H T1 T2 C1 C2 S EP JP

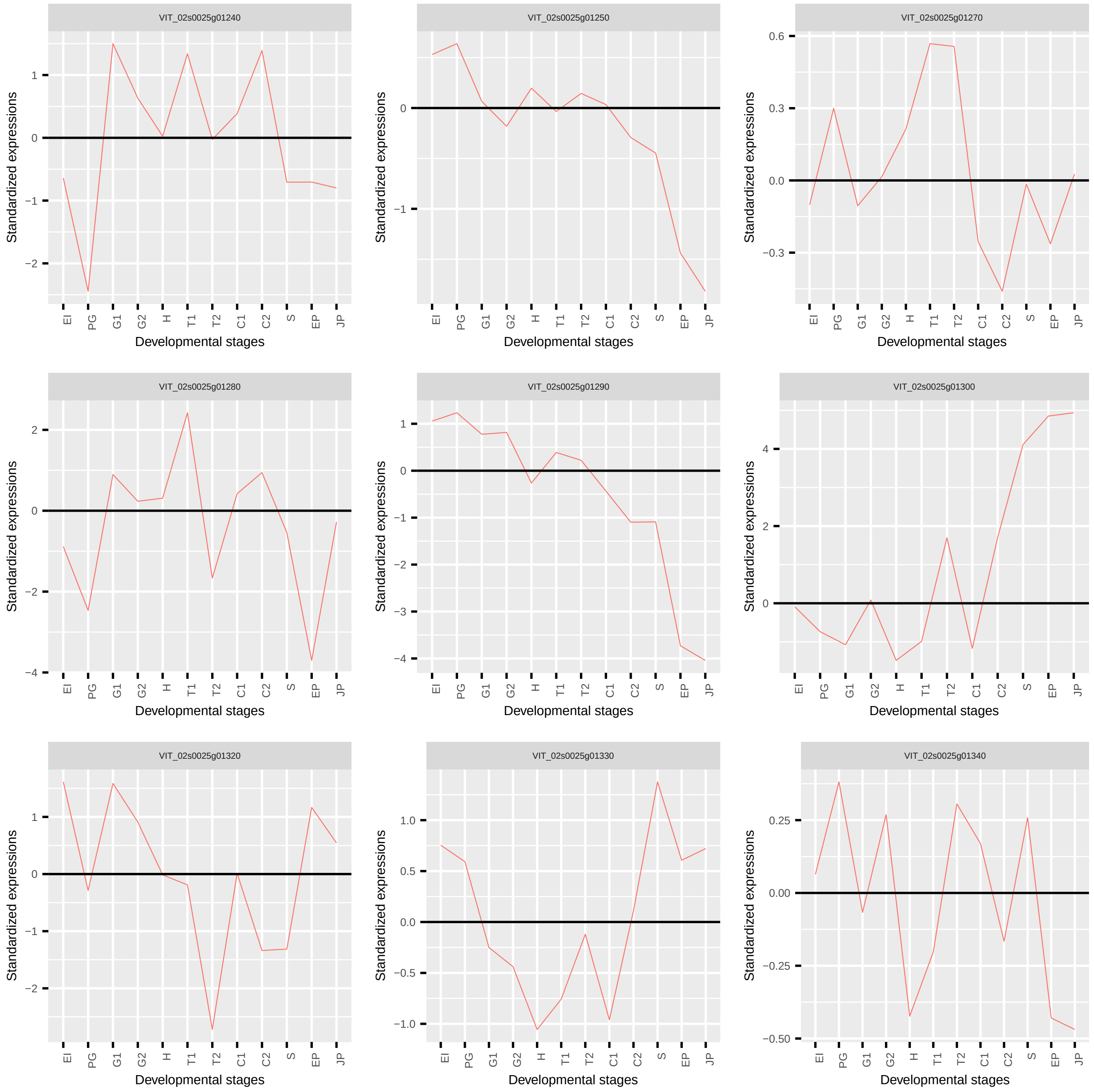

VIT_02s0025g01240
VIT_02s0025g01250
VIT_02s0025g01270
VIT_02s0025g01280
VIT_02s0025g01290
VIT_02s0025g01300
VIT_02s0025g01320
VIT_02s0025g01330
VIT_02s0025g01340
Standardized expressions
Developmental stages
EI
PG
G1
G2
H
T1
T2
C1
C2
S
EP
JP

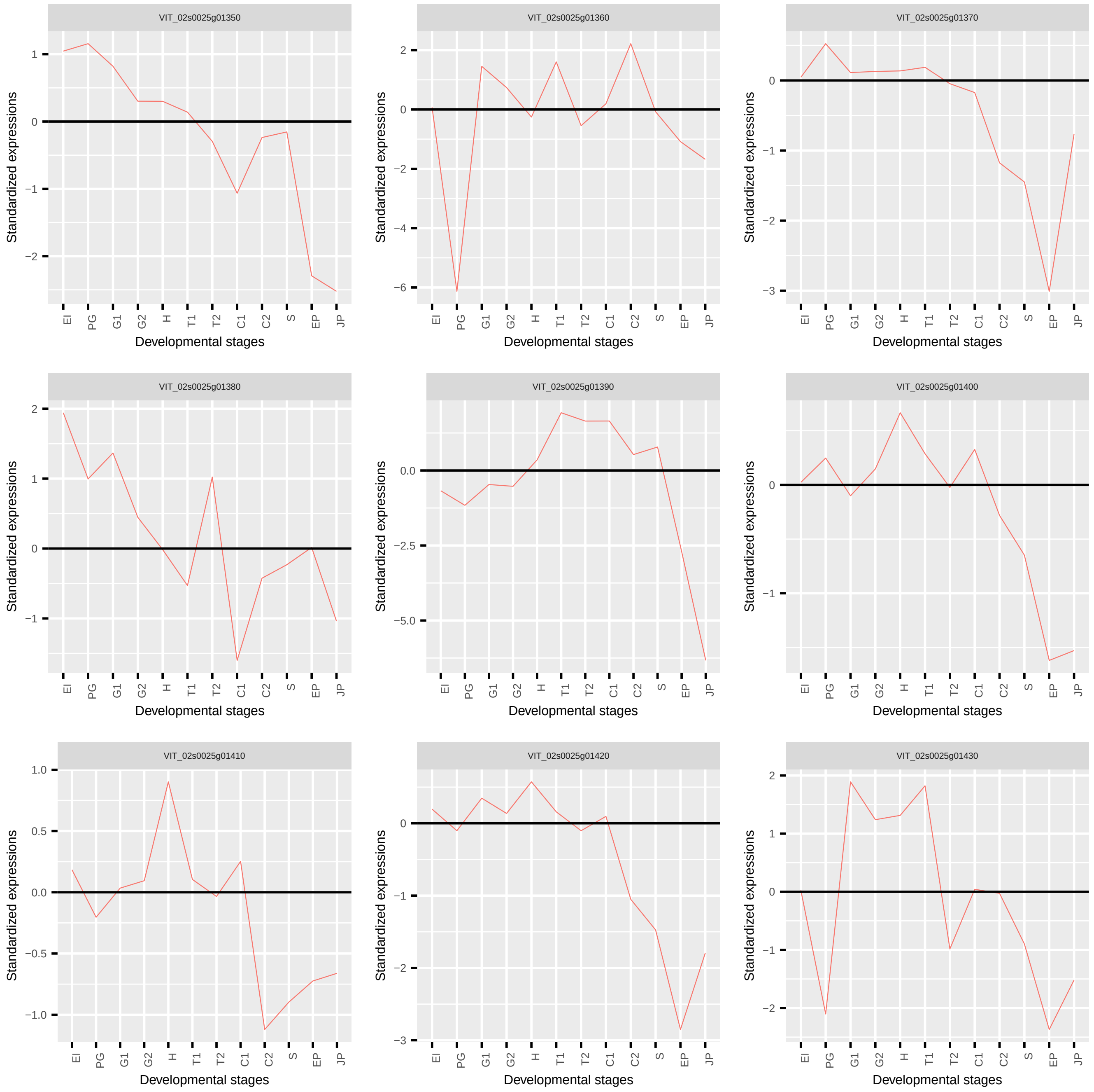
VIT_02s0025g01350
VIT_02s0025g01360
VIT_02s0025g01370
VIT_02s0025g01380
VIT_02s0025g01390
VIT_02s0025g01400
VIT_02s0025g01410
VIT_02s0025g01420
VIT_02s0025g01430
Standardized expressions
Developmental stages
EI
PG
G1
G2
H
T1
T2
C1
C2
S
EP
JP

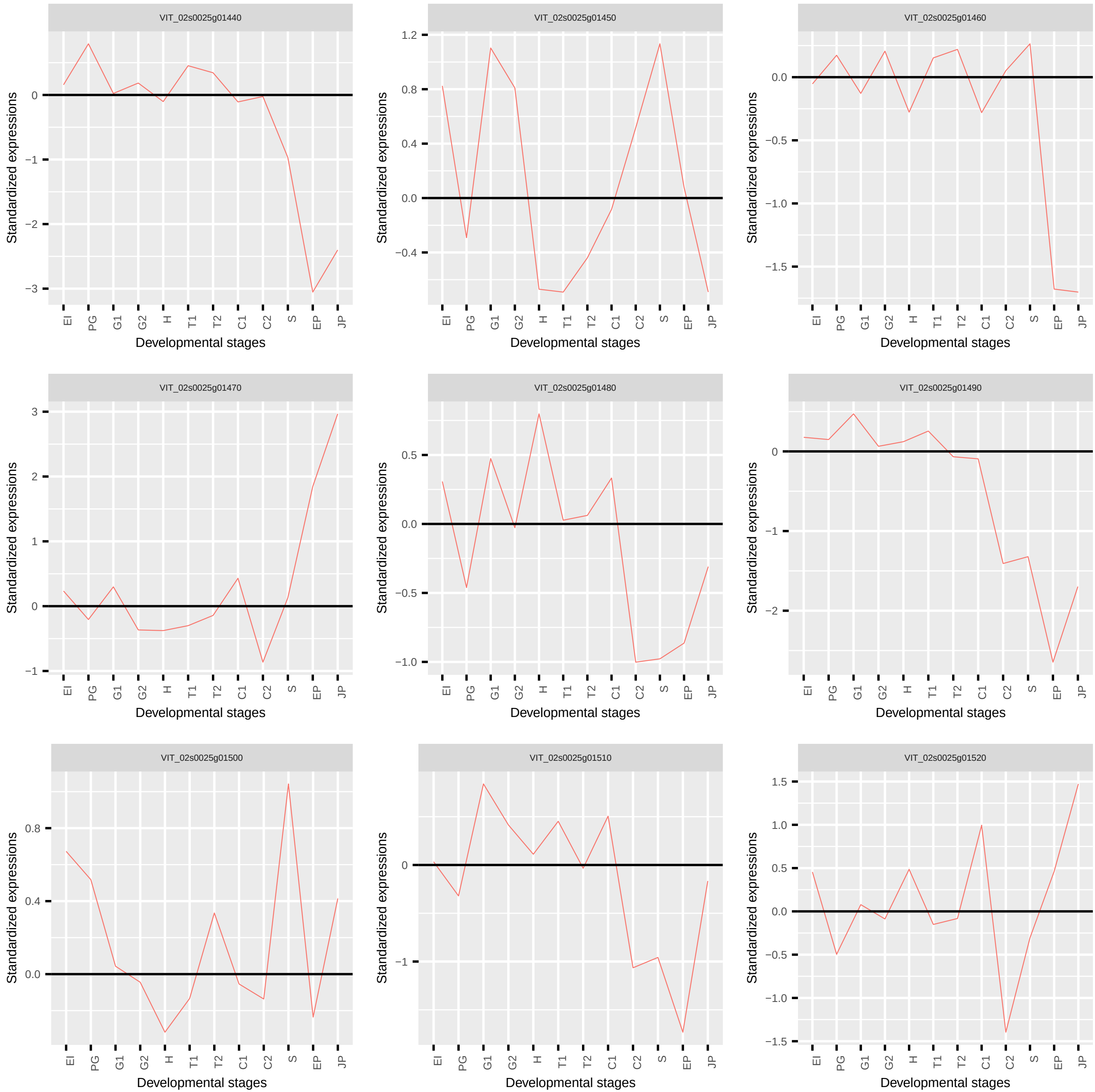
VIT_02s0025g01440
VIT_02s0025g01450
VIT_02s0025g01460
VIT_02s0025g01470
VIT_02s0025g01480
VIT_02s0025g01490
VIT_02s0025g01500
VIT_02s0025g01510
VIT_02s0025g01520
Standardized expressions
Developmental stages
EI PG G1 G2 H T1 T2 C1 C2 S EP JP

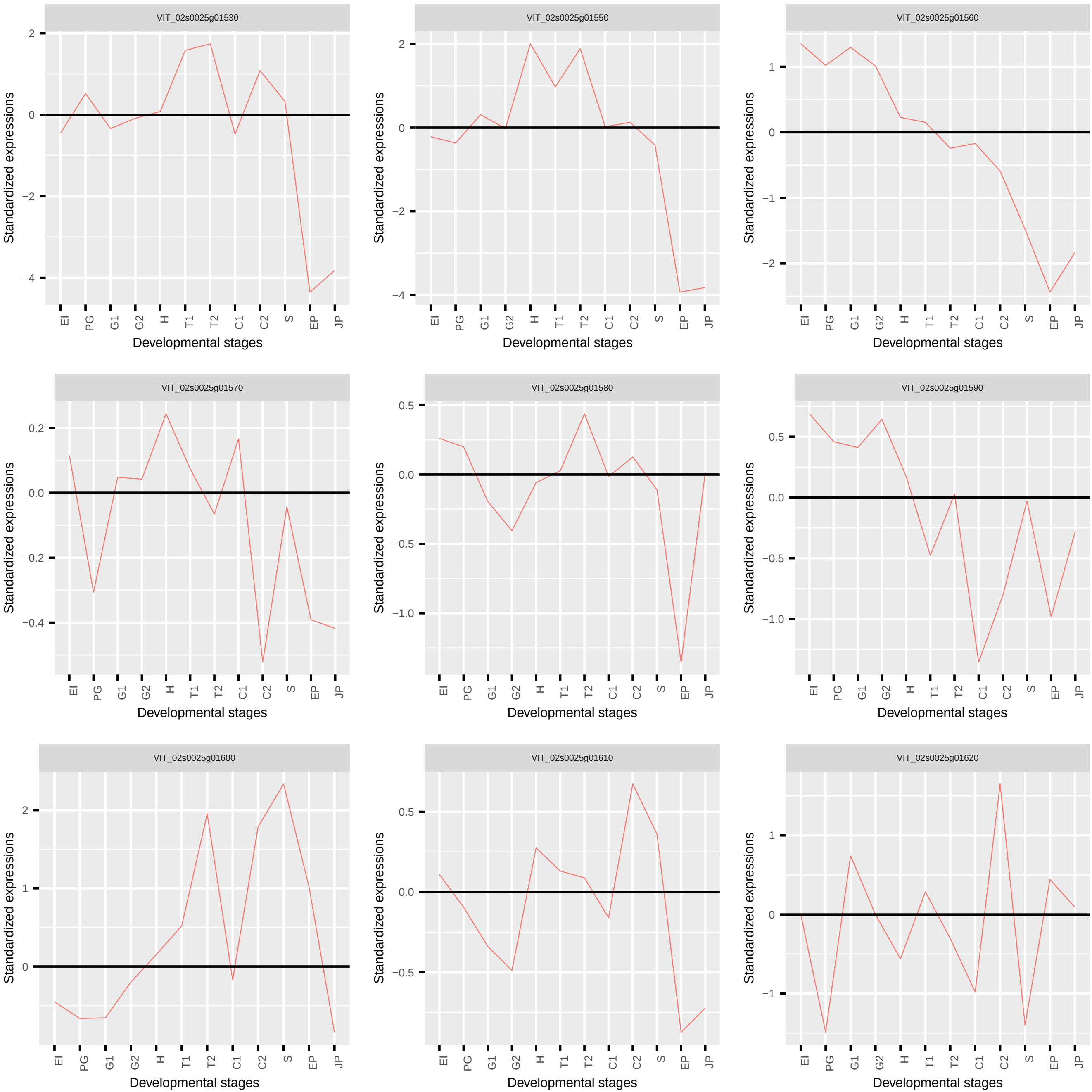
VIT_02s0025g01530
VIT_02s0025g01550
VIT_02s0025g01560
VIT_02s0025g01570
VIT_02s0025g01580
VIT_02s0025g01590
VIT_02s0025g01600
VIT_02s0025g01610
VIT_02s0025g01620
Standardized expressions
Developmental stages
EI
PG
G1
G2
H
T1
T2
C1
C2
S
EP
JP

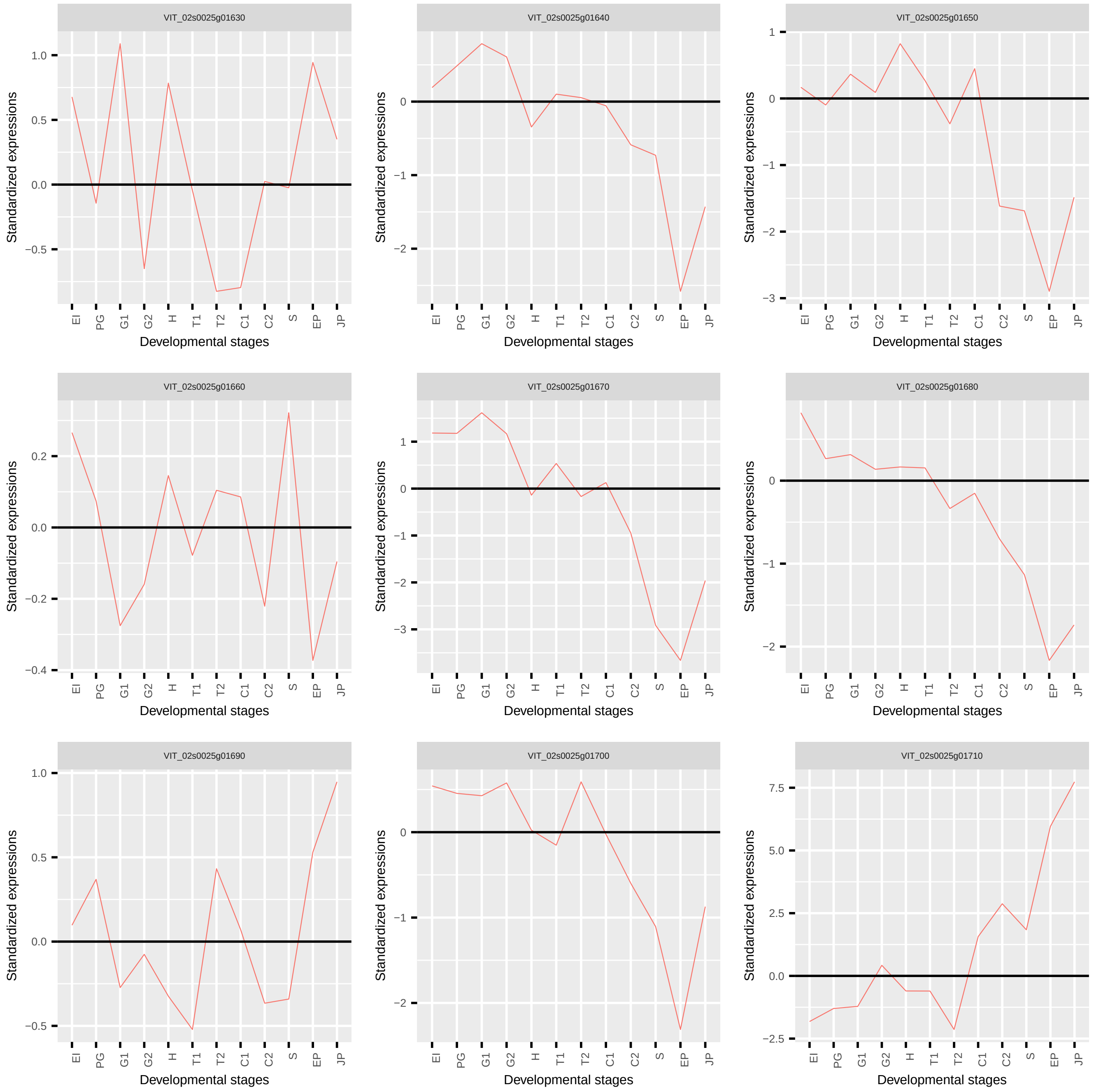

VIT_02s0025g01630
VIT_02s0025g01640
VIT_02s0025g01650
VIT_02s0025g01660
VIT_02s0025g01670
VIT_02s0025g01680
VIT_02s0025g01690
VIT_02s0025g01700
VIT_02s0025g01710
Standardized expressions
Developmental stages
EI
PG
G1
G2
H
T1
T2
C1
C2
S
EP
JP

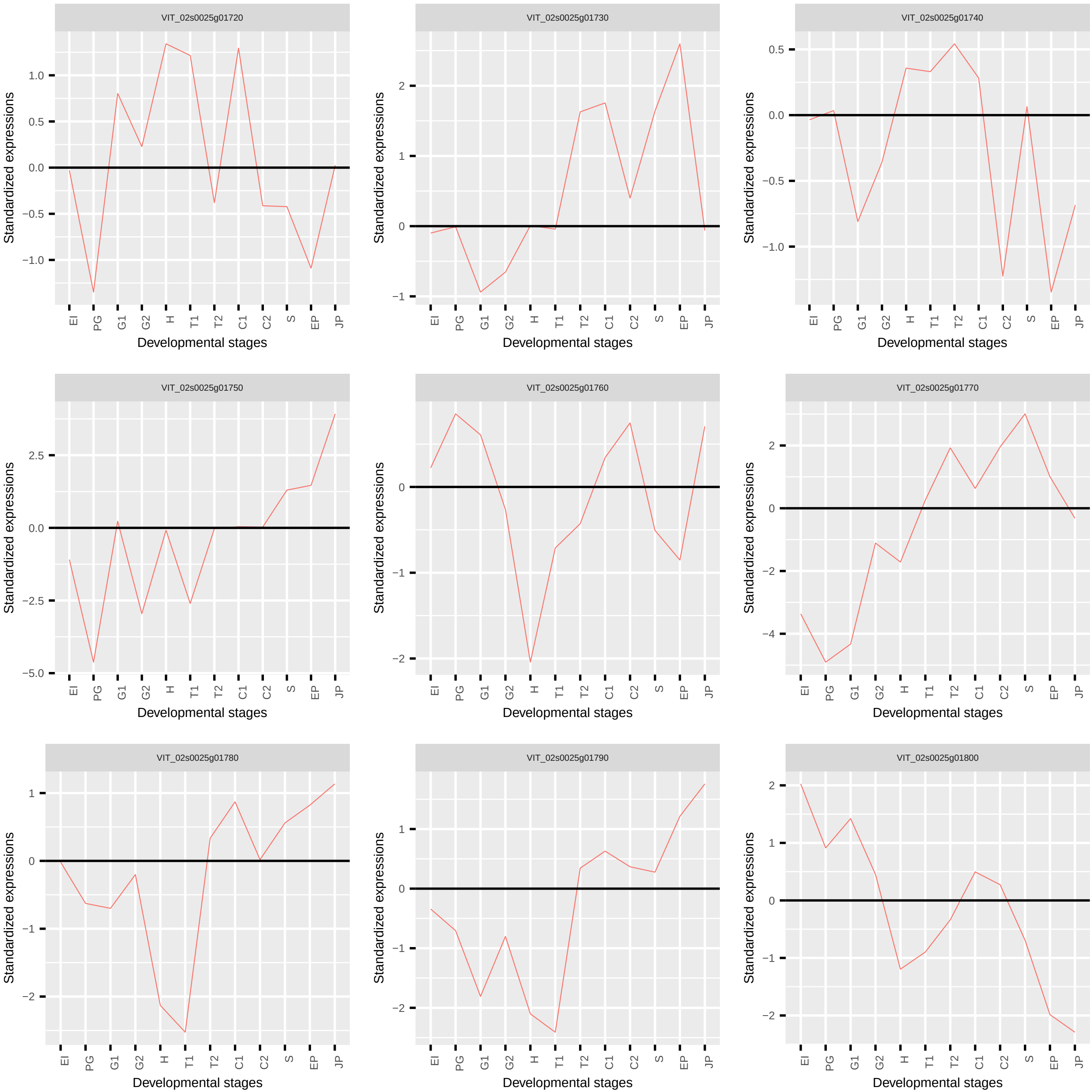

VIT_02s0025g01720
1.0
0.5
0.0
−0.5
−1.0
VIT_02s0025g01730
2
1
0
−1
VIT_02s0025g01740
0.5
0.0
−0.5
−1.0
VIT_02s0025g01750
2.5
0.0
−2.5
−5.0
VIT_02s0025g01760
0
−1
−2
VIT_02s0025g01770
2
0
−2
−4
VIT_02s0025g01780
1
0
−1
−2
VIT_02s0025g01790
1
0
−1
−2
VIT_02s0025g01800
2
1
0
−1
−2
Standardized expressions
EI
PG
G1
G2
H
T1
T2
C1
C2
S
EP
JP
Developmental stages

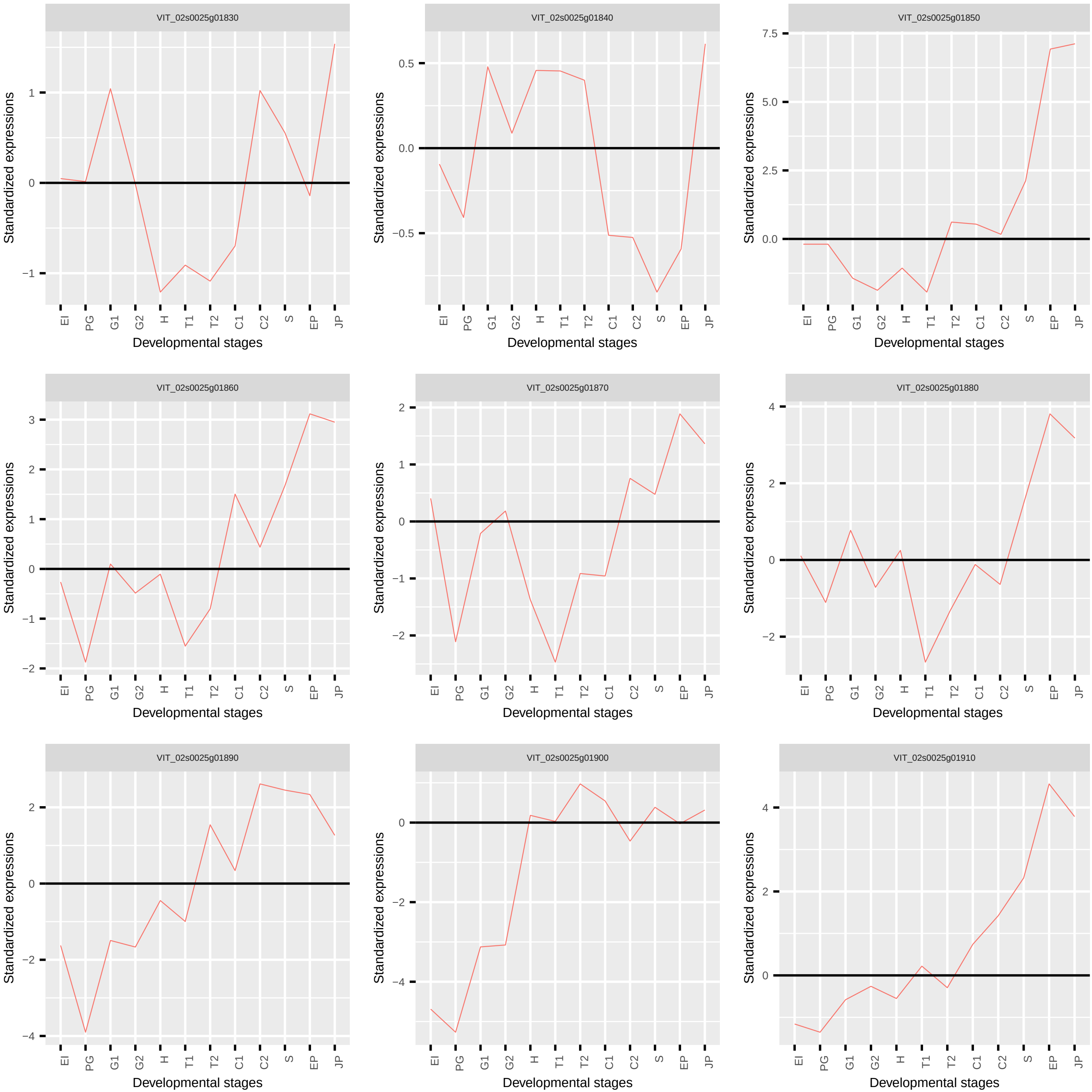
VIT_02s0025g01830
VIT_02s0025g01840
VIT_02s0025g01850
VIT_02s0025g01860
VIT_02s0025g01870
VIT_02s0025g01880
VIT_02s0025g01890
VIT_02s0025g01900
VIT_02s0025g01910
Standardized expressions
Developmental stages
EI PG G1 G2 H T1 T2 C1 C2 S EP JP

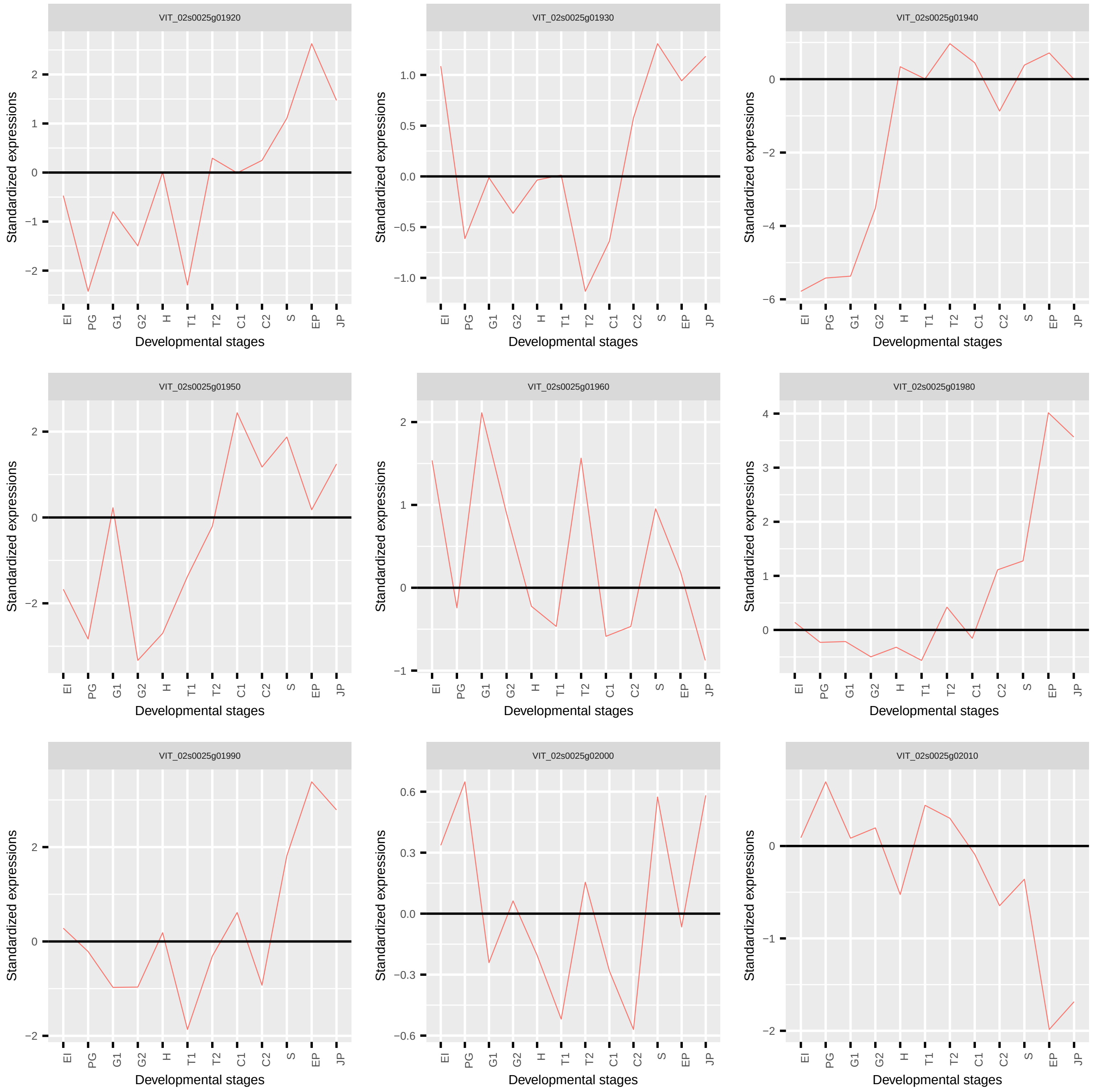
VIT_02s0025g01920
VIT_02s0025g01930
VIT_02s0025g01940
VIT_02s0025g01950
VIT_02s0025g01960
VIT_02s0025g01980
VIT_02s0025g01990
VIT_02s0025g02000
VIT_02s0025g02010
Standardized expressions
Developmental stages
EI PG G1 G2 H T1 T2 C1 C2 S EP JP

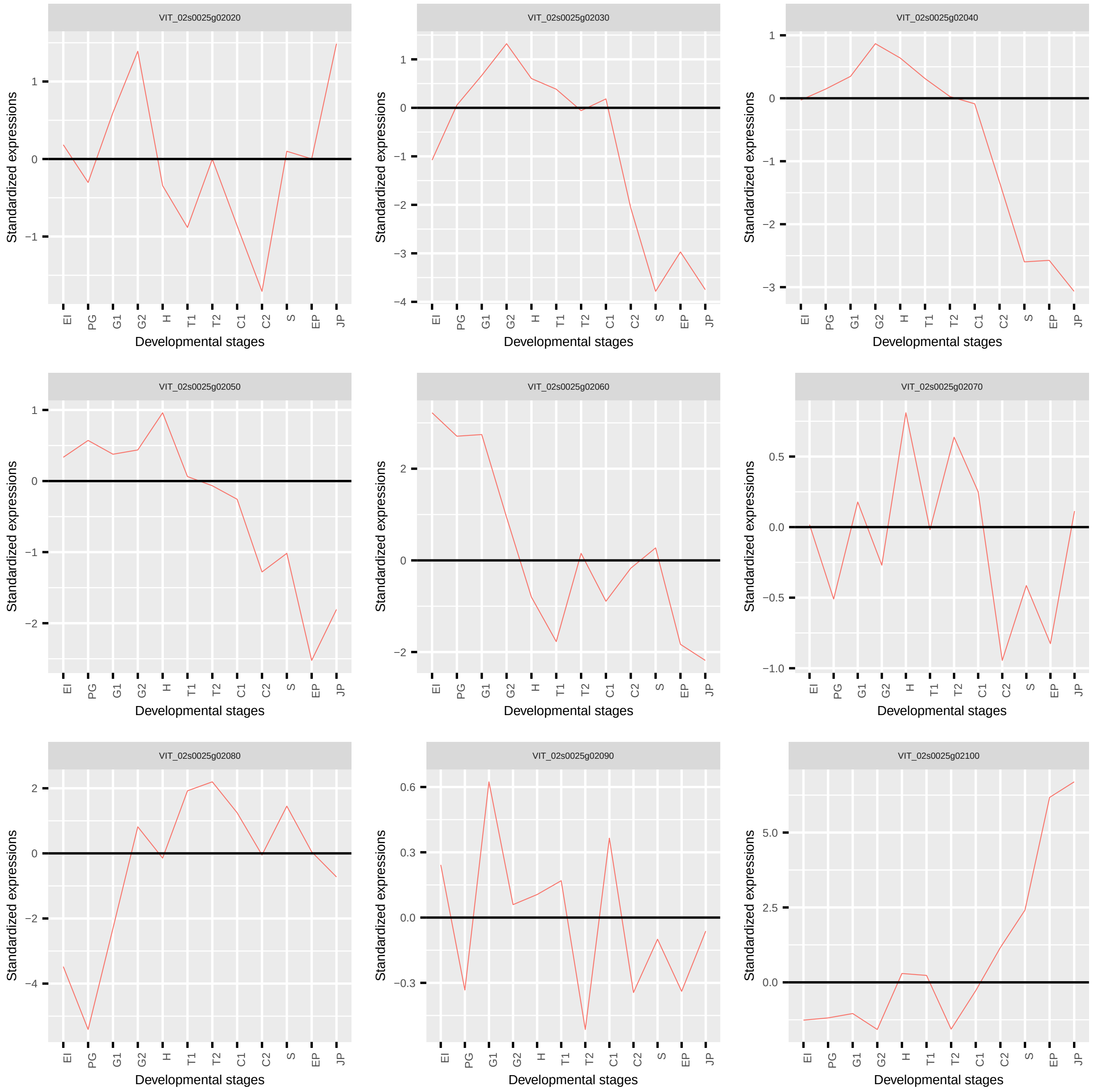
VIT_02s0025g02020
VIT_02s0025g02030
VIT_02s0025g02040
VIT_02s0025g02050
VIT_02s0025g02060
VIT_02s0025g02070
VIT_02s0025g02080
VIT_02s0025g02090
VIT_02s0025g02100
Standardized expressions
Developmental stages
EI PG G1 G2 H T1 T2 C1 C2 S EP JP

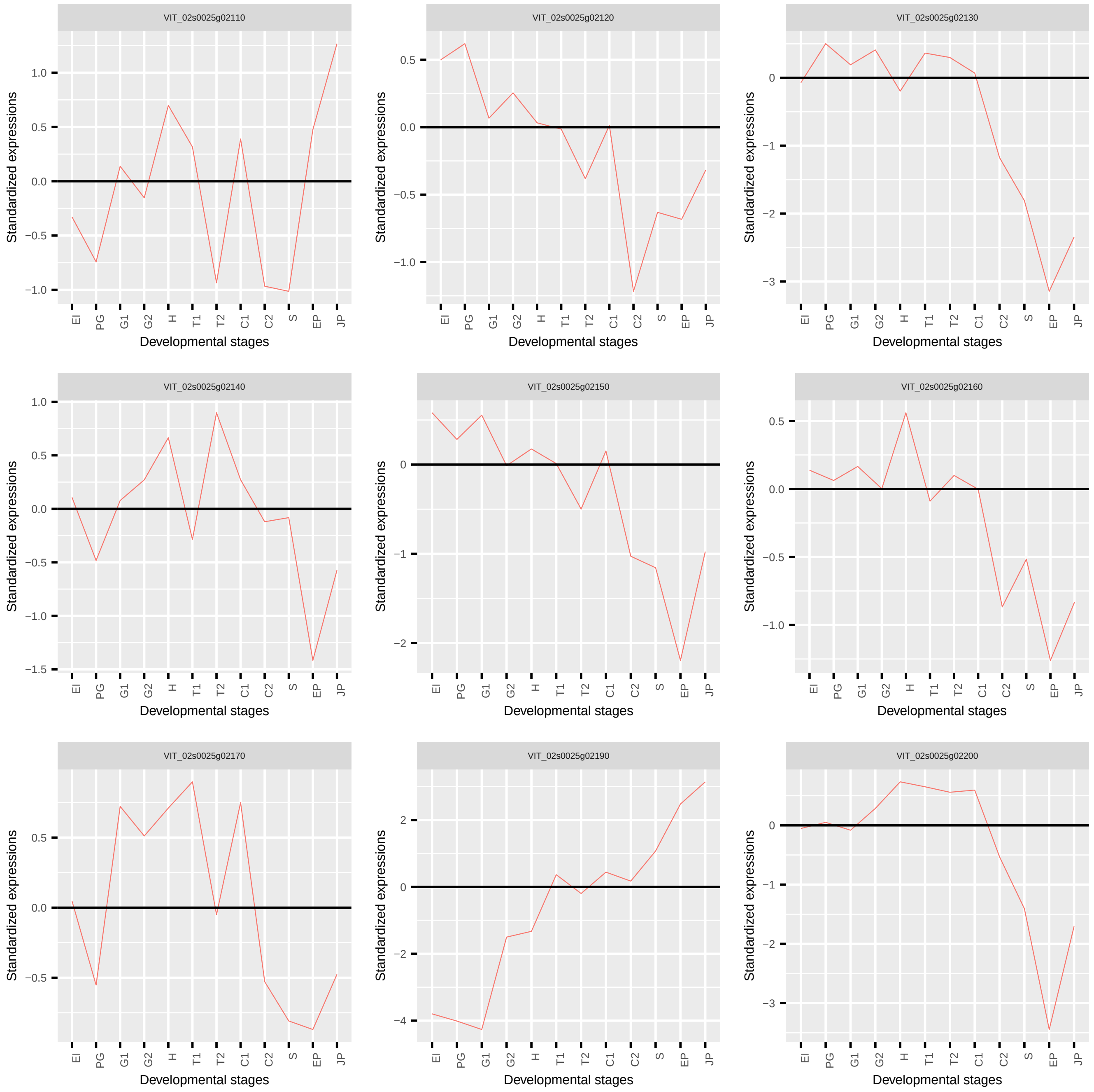

VIT_02s0025g02110
VIT_02s0025g02120
VIT_02s0025g02130
VIT_02s0025g02140
VIT_02s0025g02150
VIT_02s0025g02160
VIT_02s0025g02170
VIT_02s0025g02190
VIT_02s0025g02200
Standardized expressions
Developmental stages
EI PG G1 G2 H T1 T2 C1 C2 S EP JP

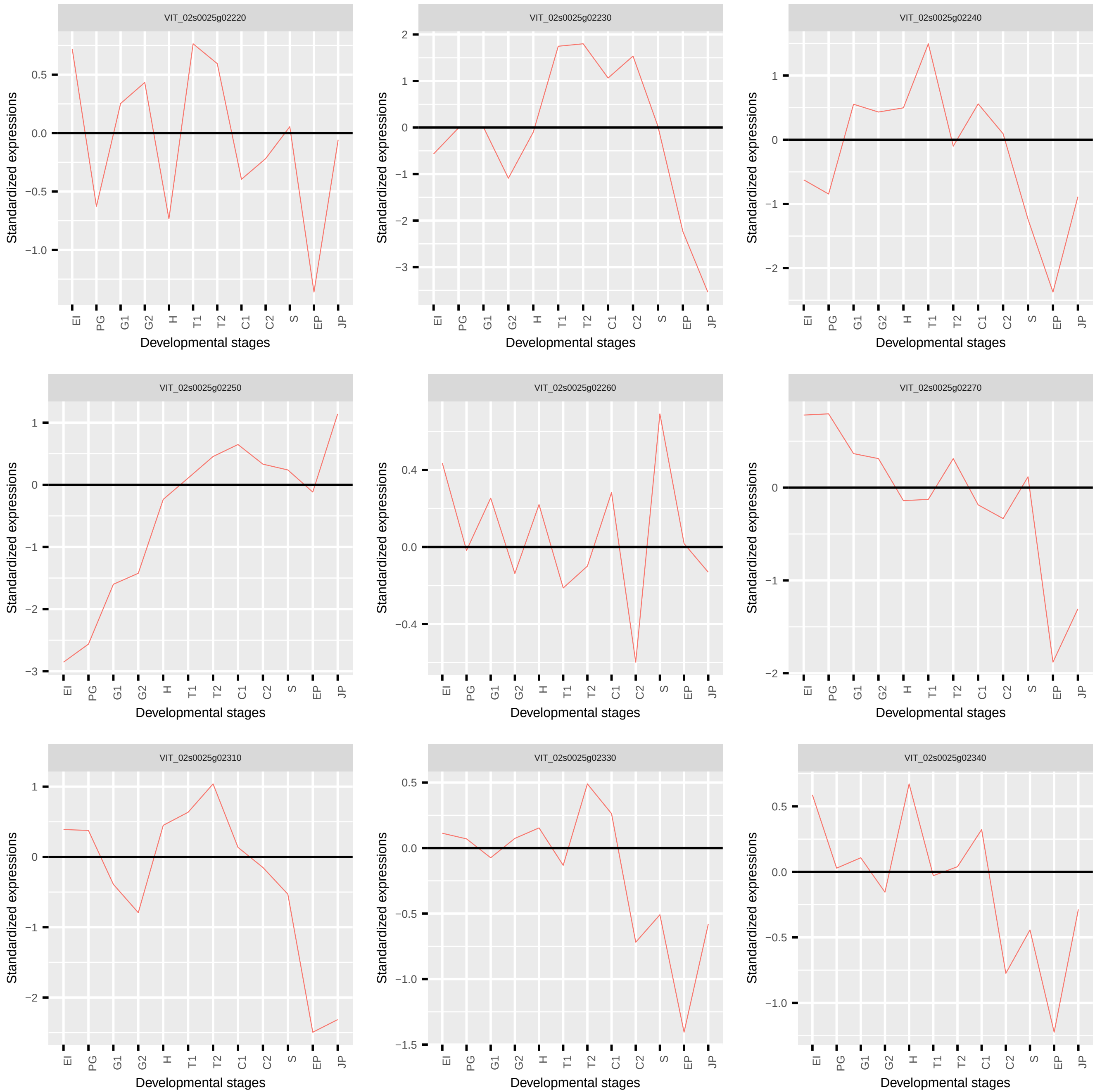
VIT_02s0025g02220
VIT_02s0025g02230
VIT_02s0025g02240
VIT_02s0025g02250
VIT_02s0025g02260
VIT_02s0025g02270
VIT_02s0025g02310
VIT_02s0025g02330
VIT_02s0025g02340
Standardized expressions
Developmental stages
EI PG G1 G2 H T1 T2 C1 C2 S EP JP

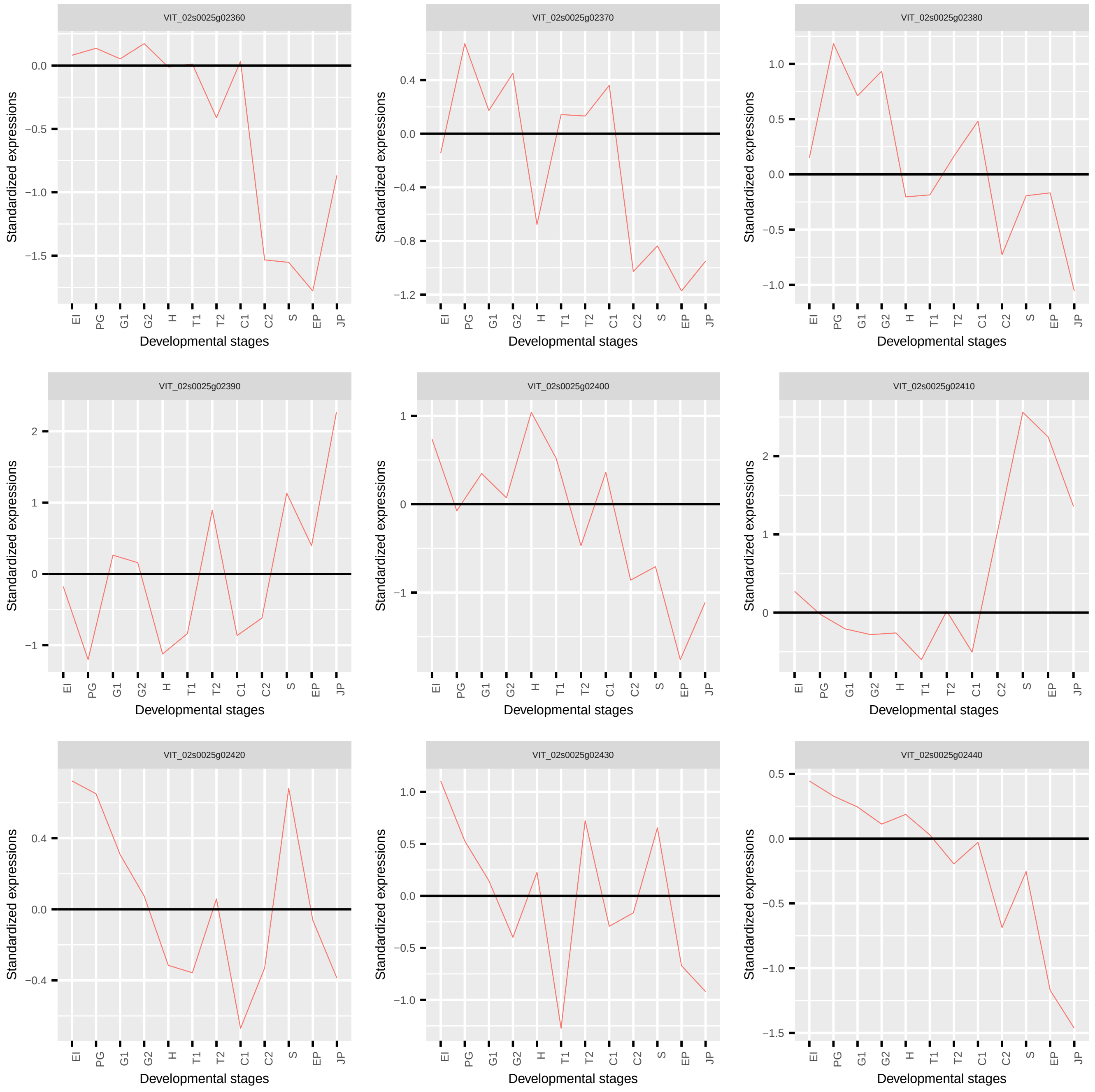

VIT_02s0025g02360
0.0
−0.5
−1.0
−1.5
VIT_02s0025g02370
0.4
0.0
−0.4
−0.8
−1.2
VIT_02s0025g02380
1.0
0.5
0.0
−0.5
−1.0
VIT_02s0025g02390
2
1
0
−1
VIT_02s0025g02400
1
0
−1
VIT_02s0025g02410
2
1
0
VIT_02s0025g02420
0.4
0.0
−0.4
VIT_02s0025g02430
1.0
0.5
0.0
−0.5
−1.0
VIT_02s0025g02440
0.5
0.0
−0.5
−1.0
−1.5
Standardized expressions
EI PG G1 G2 H T1 T2 C1 C2 S EP JP
Developmental stages

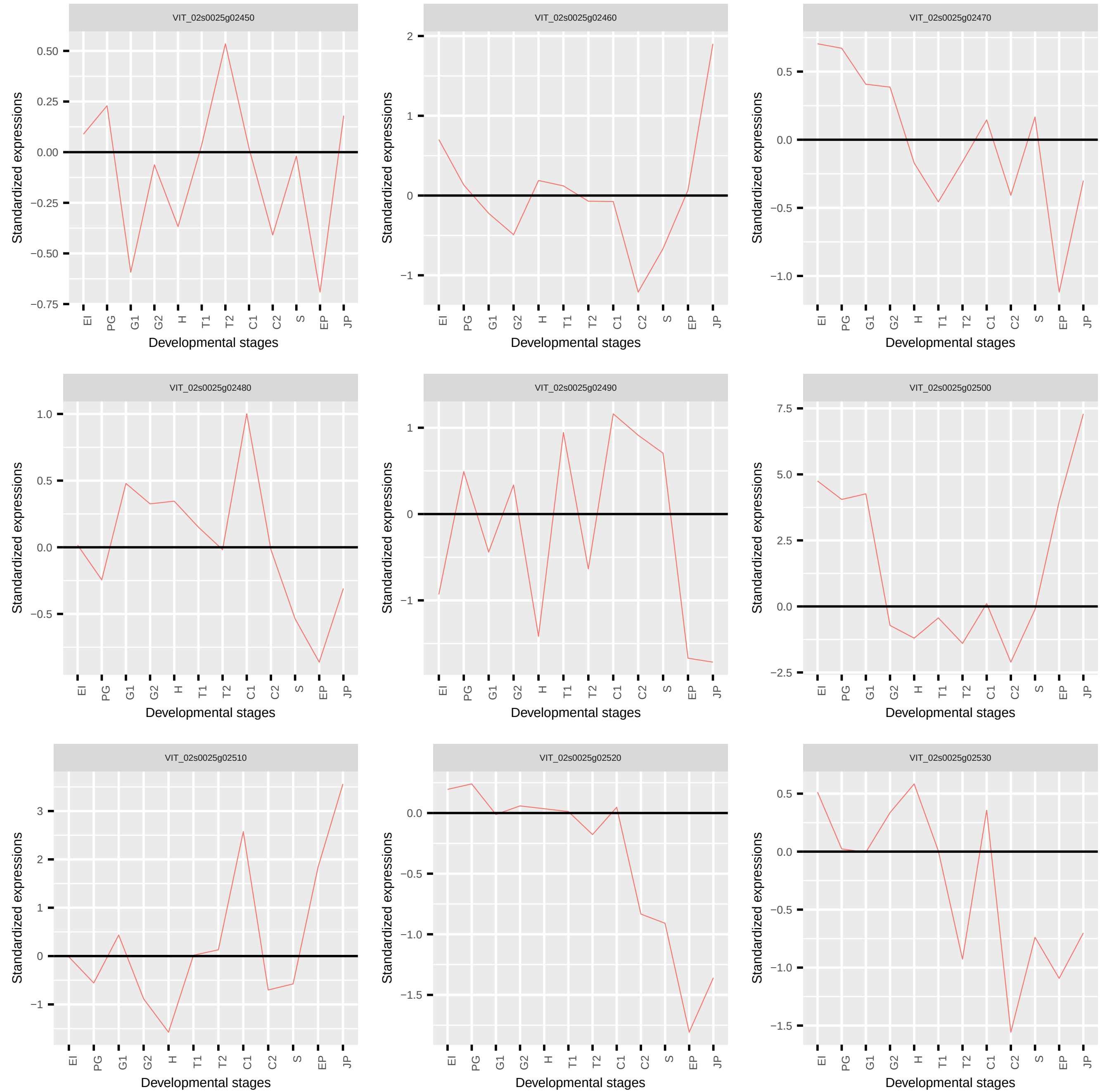

VIT_02s0025g02450
VIT_02s0025g02460
VIT_02s0025g02470
VIT_02s0025g02480
VIT_02s0025g02490
VIT_02s0025g02500
VIT_02s0025g02510
VIT_02s0025g02520
VIT_02s0025g02530
Standardized expressions
Developmental stages
EI PG G1 G2 H T1 T2 C1 C2 S EP JP
0.50 0.25 0.00 −0.25 −0.50 −0.75
2 1 0 −1
0.5 0.0 −0.5 −1.0
1.0 0.5 0.0 −0.5
1 0 −1
7.5 5.0 2.5 0.0 −2.5
3 2 1 0 −1
0.0 −0.5 −1.0 −1.5
0.5 0.0 −0.5 −1.0 −1.5

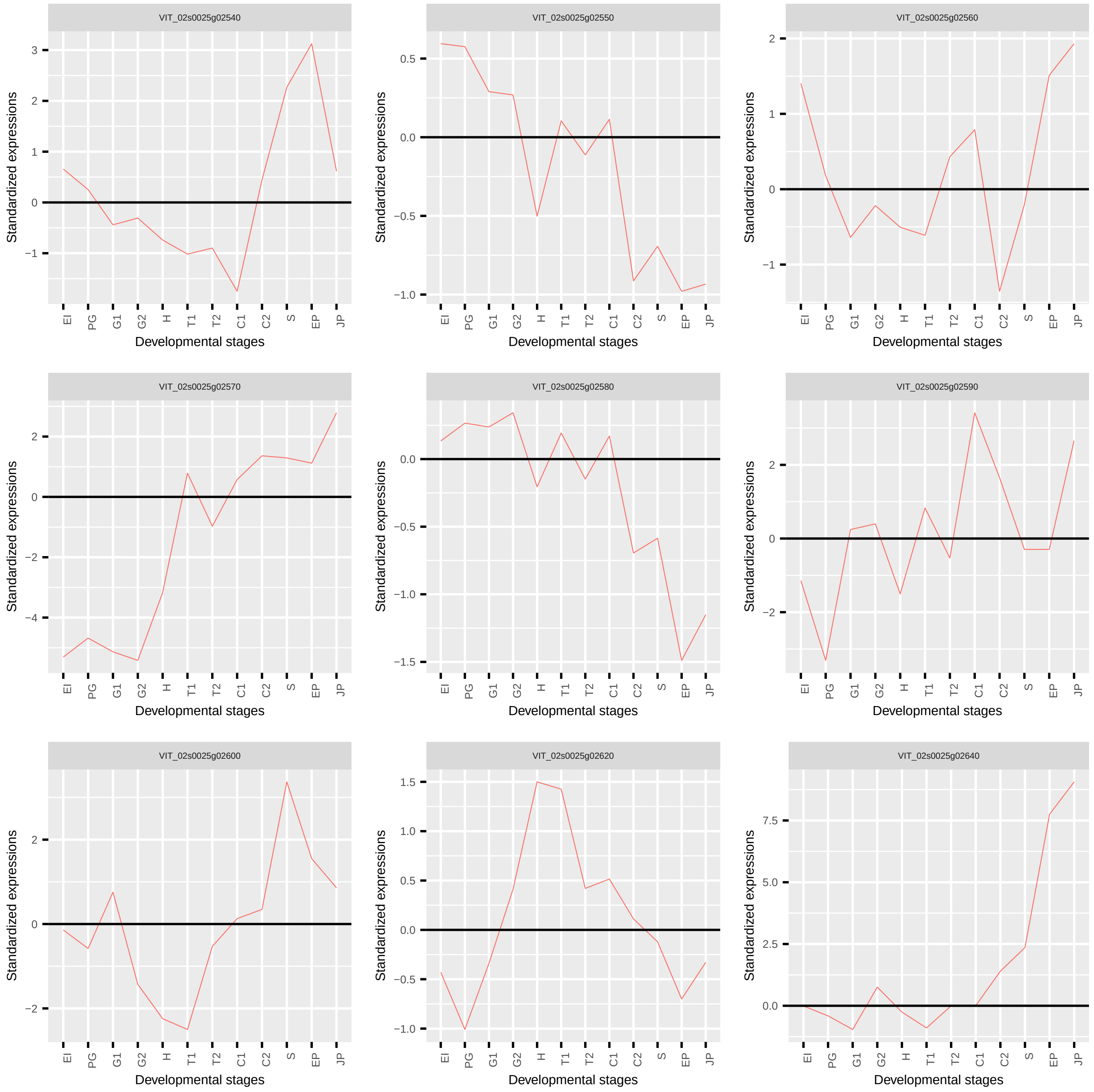
VIT_02s0025g02540
VIT_02s0025g02550
VIT_02s0025g02560
VIT_02s0025g02570
VIT_02s0025g02580
VIT_02s0025g02590
VIT_02s0025g02600
VIT_02s0025g02620
VIT_02s0025g02640
Standardized expressions
Developmental stages
EI PG G1 G2 H T1 T2 C1 C2 S EP JP

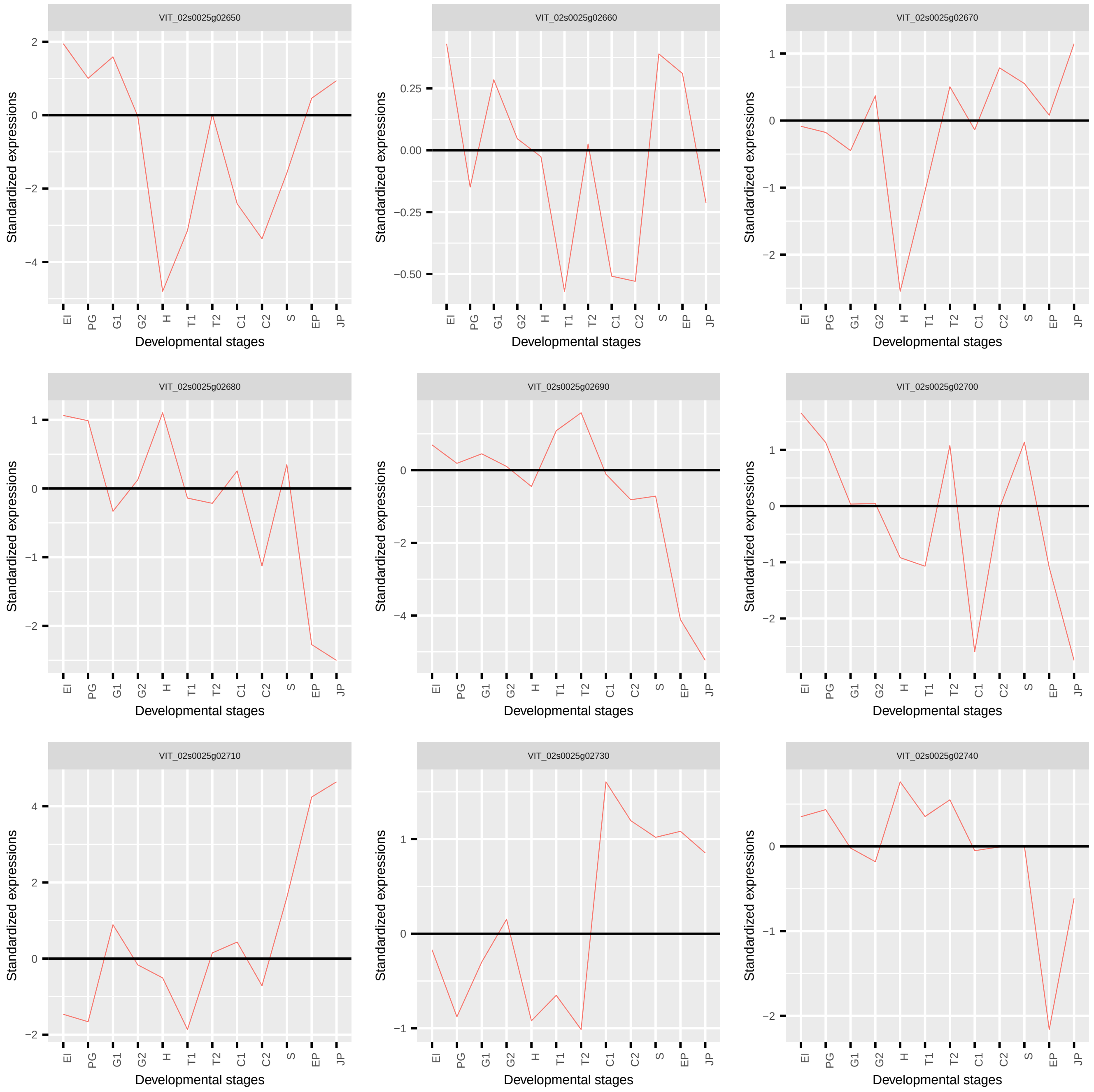
VIT_02s0025g02650
VIT_02s0025g02660
VIT_02s0025g02670
VIT_02s0025g02680
VIT_02s0025g02690
VIT_02s0025g02700
VIT_02s0025g02710
VIT_02s0025g02730
VIT_02s0025g02740
Standardized expressions
Developmental stages
EI PG G1 G2 H T1 T2 C1 C2 S EP JP

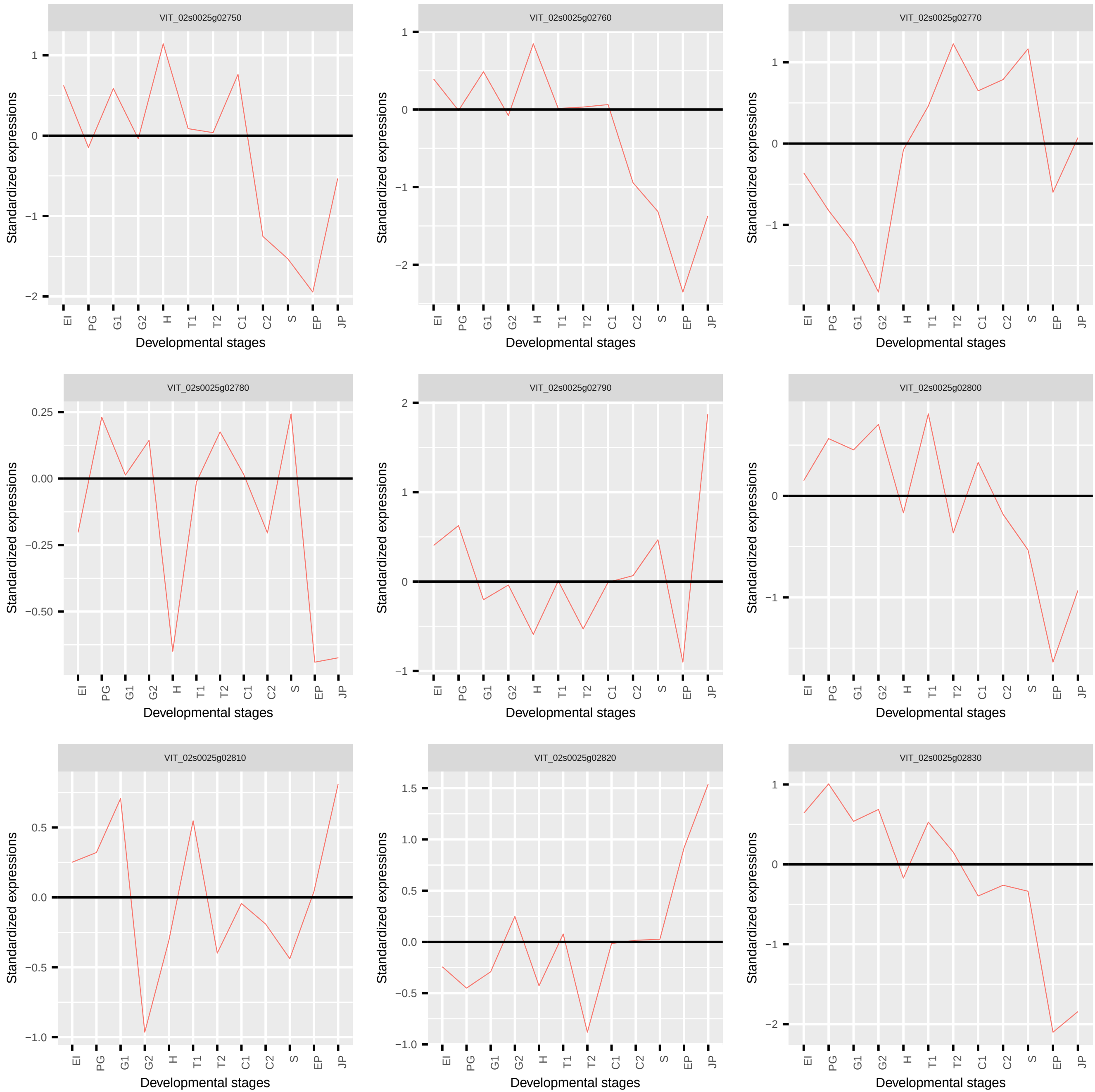

VIT_02s0025g02750
VIT_02s0025g02760
VIT_02s0025g02770
VIT_02s0025g02780
VIT_02s0025g02790
VIT_02s0025g02800
VIT_02s0025g02810
VIT_02s0025g02820
VIT_02s0025g02830
Standardized expressions
EI PG G1 G2 H T1 T2 C1 C2 S EP JP
Developmental stages

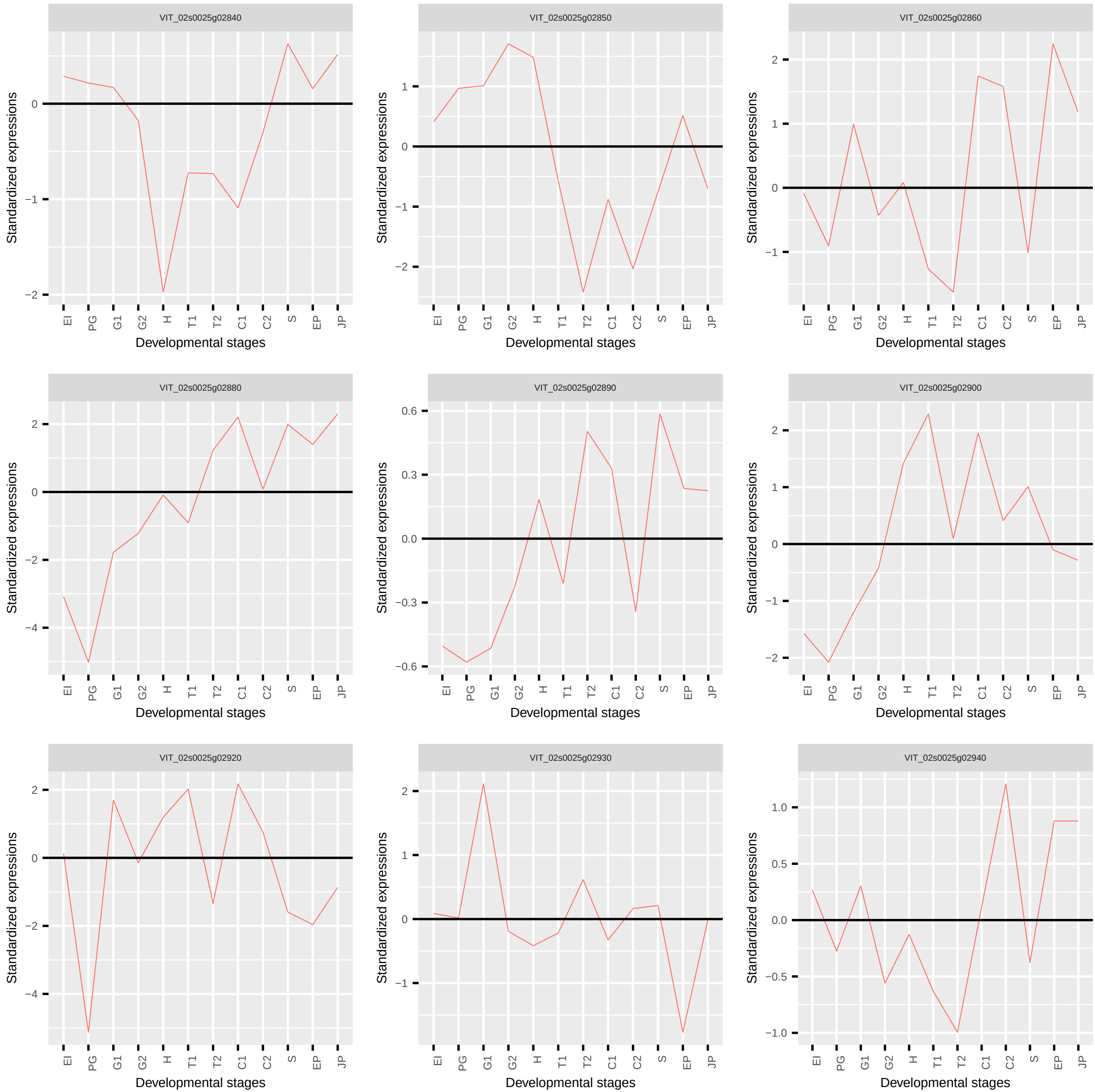
VIT_02s0025g02840
VIT_02s0025g02850
VIT_02s0025g02860
VIT_02s0025g02880
VIT_02s0025g02890
VIT_02s0025g02900
VIT_02s0025g02920
VIT_02s0025g02930
VIT_02s0025g02940
Standardized expressions
Developmental stages
EI PG G1 G2 H T1 T2 C1 C2 S EP JP

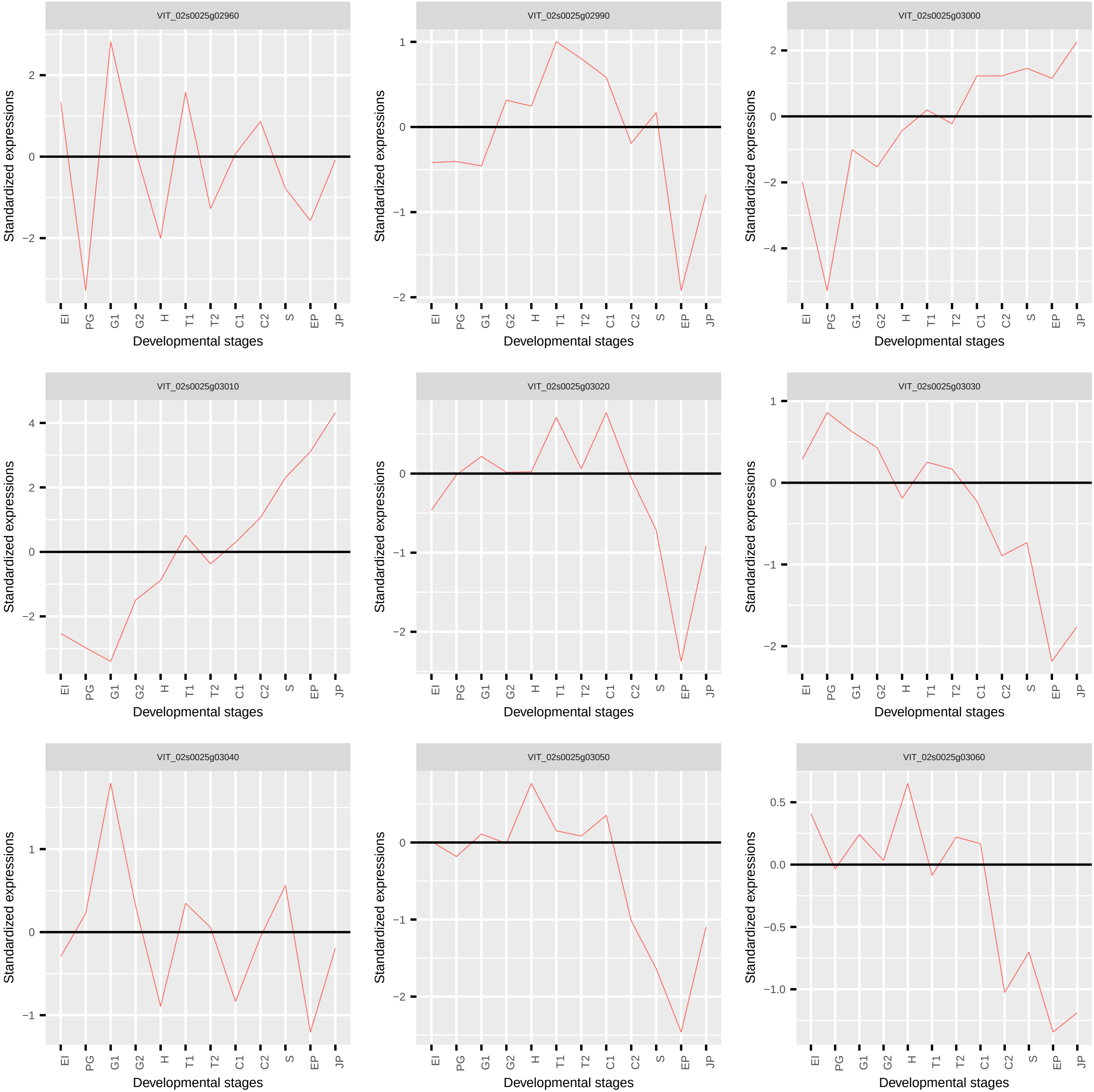
VIT_02s0025g02960
VIT_02s0025g02990
VIT_02s0025g03000
VIT_02s0025g03010
VIT_02s0025g03020
VIT_02s0025g03030
VIT_02s0025g03040
VIT_02s0025g03050
VIT_02s0025g03060
Standardized expressions
Developmental stages
EI PG G1 G2 H T1 T2 C1 C2 S EP JP
−2 0 2
−2 −1 0 1
−4 −2 0 2
−2 0 2 4
−2 −1 0
−2 −1 0 1
−1 0 1
−2 −1 0
−1.0 −0.5 0.0 0.5

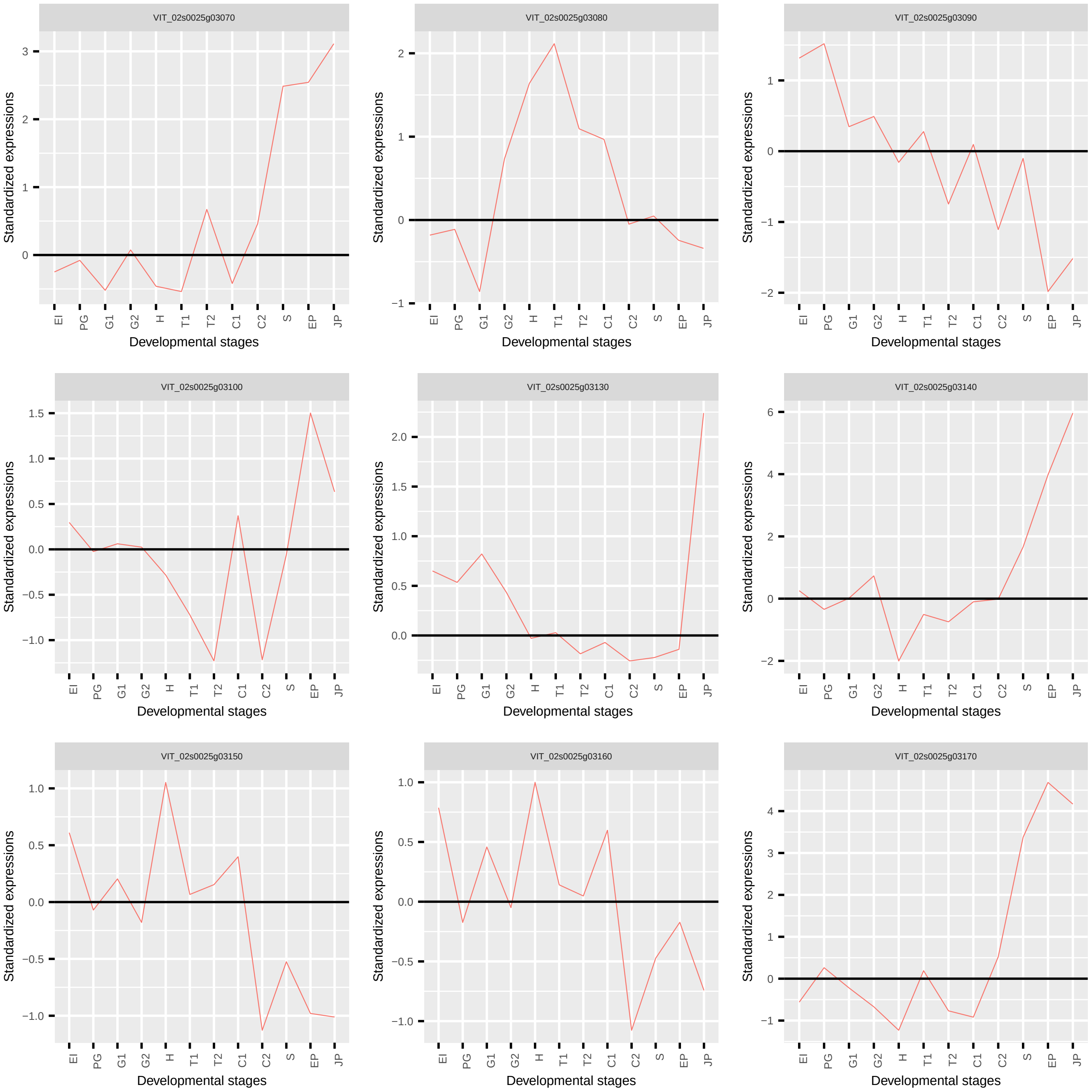
VIT_02s0025g03070
VIT_02s0025g03080
VIT_02s0025g03090
VIT_02s0025g03100
VIT_02s0025g03130
VIT_02s0025g03140
VIT_02s0025g03150
VIT_02s0025g03160
VIT_02s0025g03170
Standardized expressions
Developmental stages
EI PG G1 G2 H T1 T2 C1 C2 S EP JP

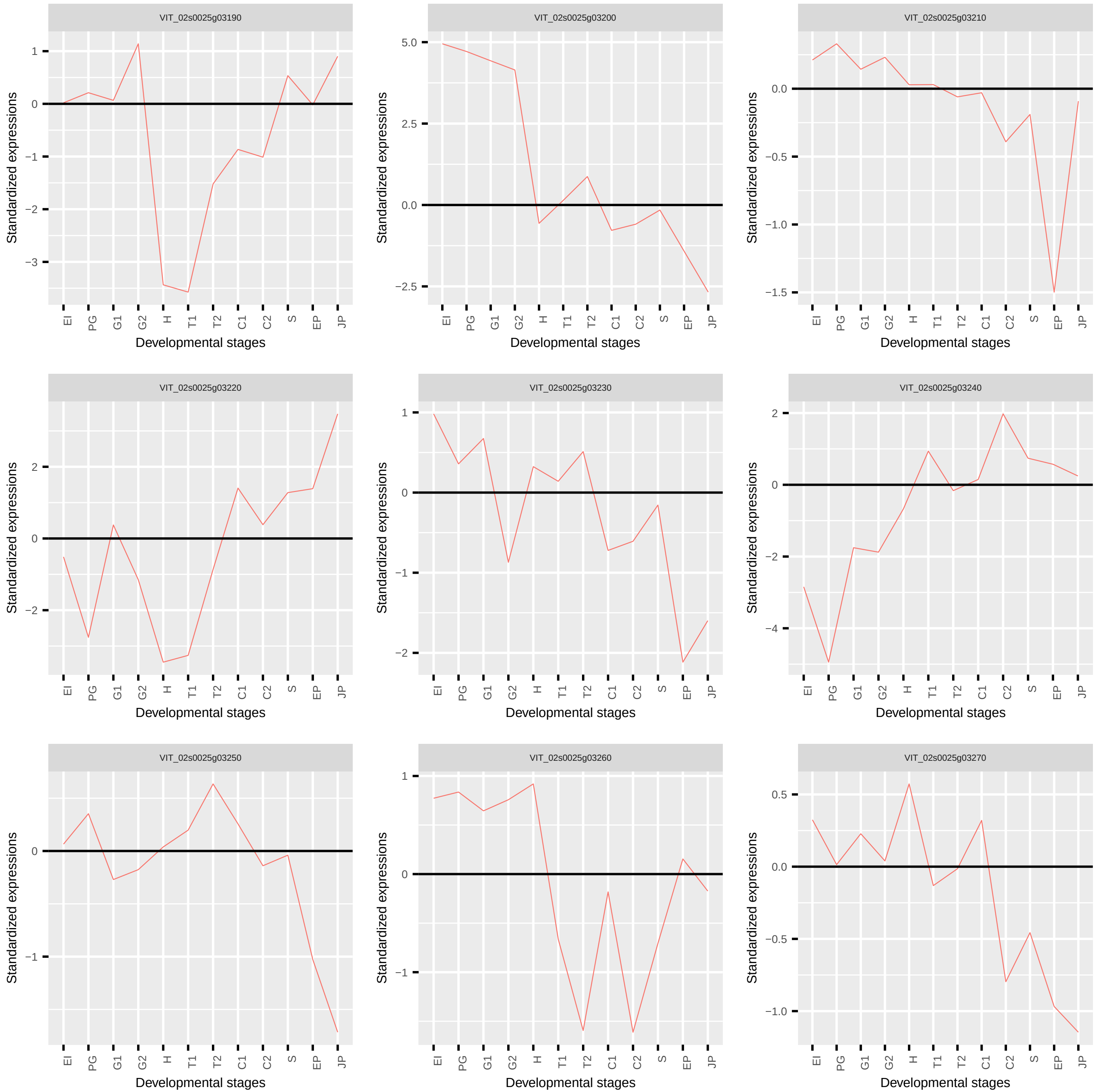
VIT_02s0025g03190
VIT_02s0025g03200
VIT_02s0025g03210
VIT_02s0025g03220
VIT_02s0025g03230
VIT_02s0025g03240
VIT_02s0025g03250
VIT_02s0025g03260
VIT_02s0025g03270
Standardized expressions
Developmental stages
EI PG G1 G2 H T1 T2 C1 C2 S EP JP

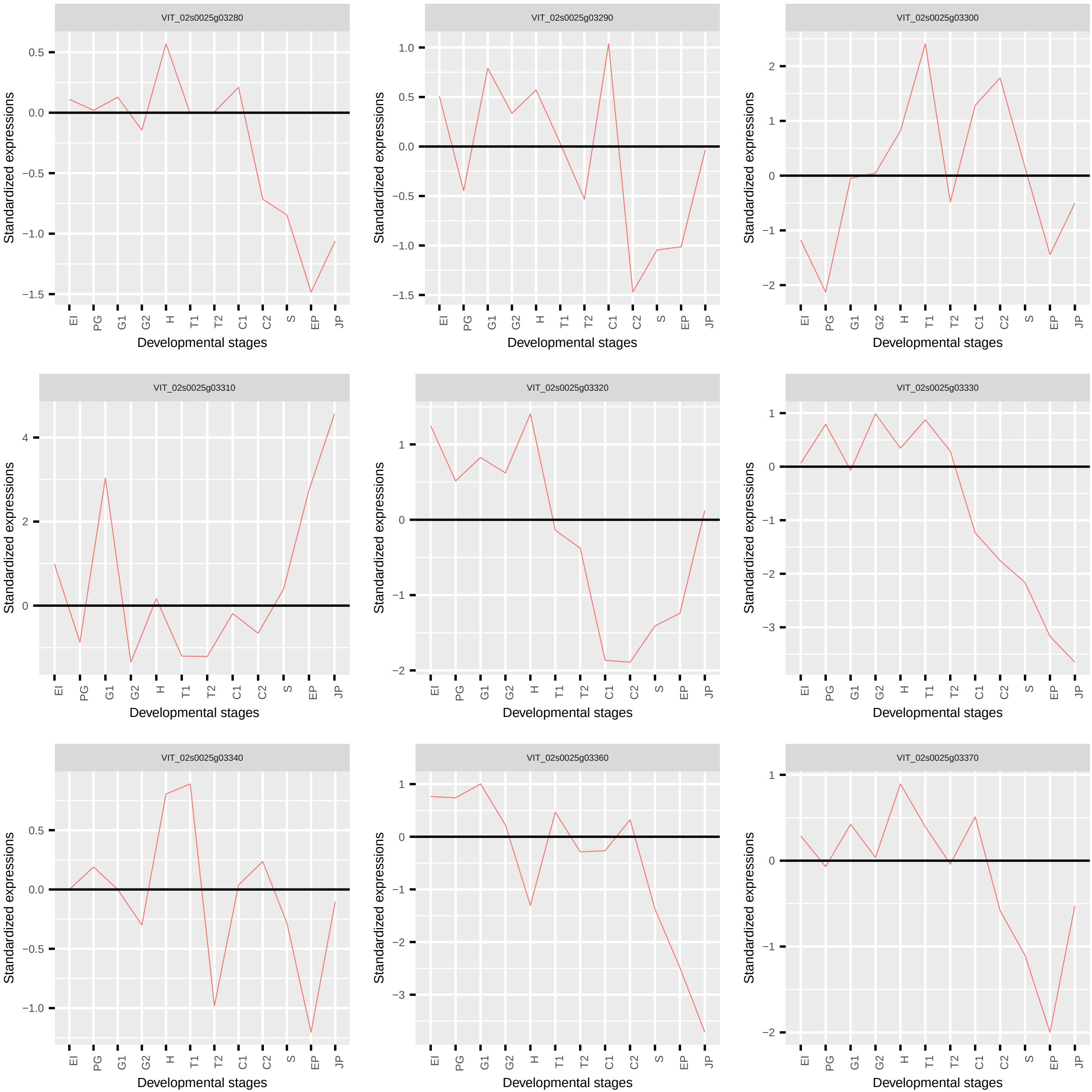

VIT_02s0025g03280
VIT_02s0025g03290
VIT_02s0025g03300
VIT_02s0025g03310
VIT_02s0025g03320
VIT_02s0025g03330
VIT_02s0025g03340
VIT_02s0025g03360
VIT_02s0025g03370
Standardized expressions
Developmental stages
EI
PG
G1
G2
H
T1
T2
C1
C2
S
EP
JP

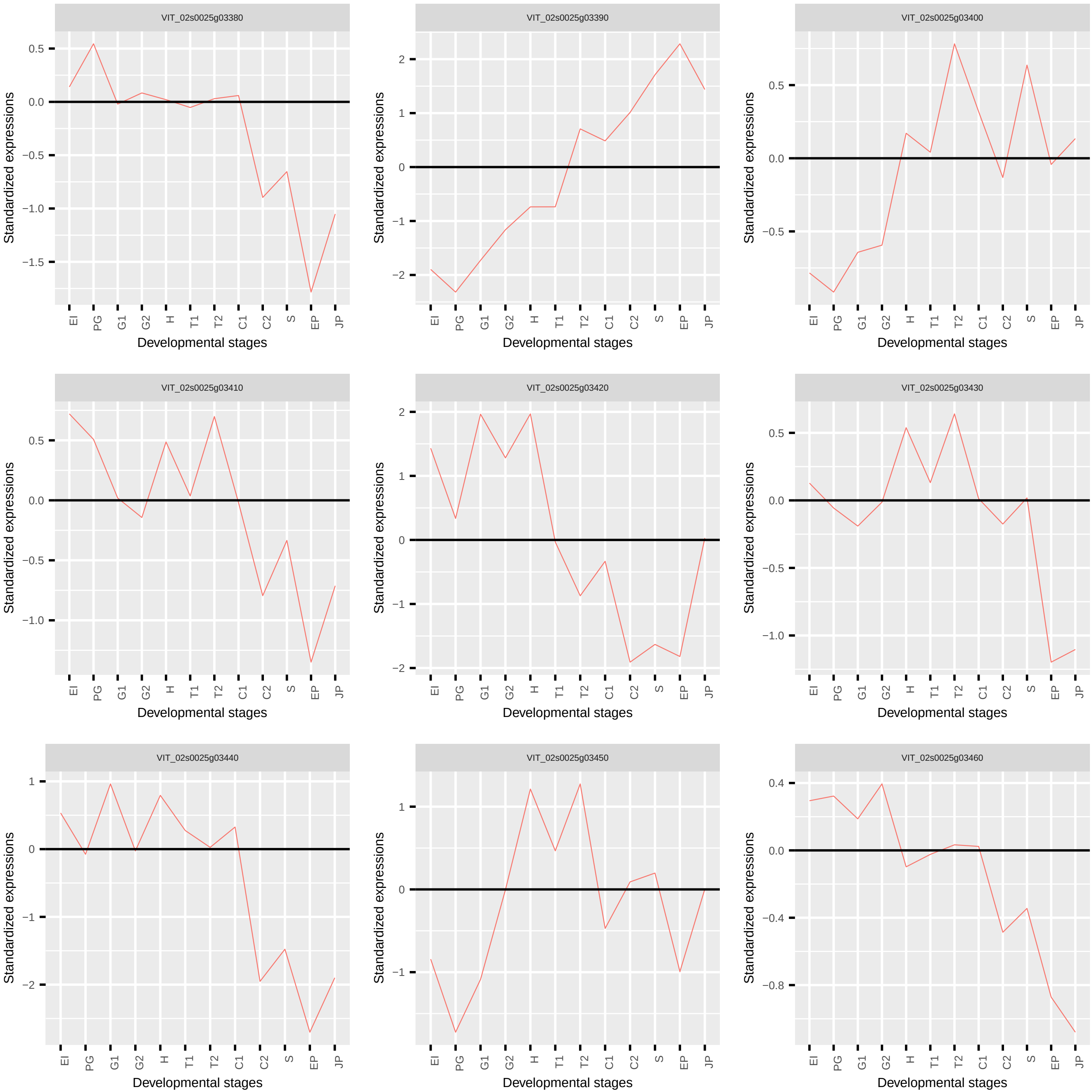

VIT_02s0025g03380
VIT_02s0025g03390
VIT_02s0025g03400
VIT_02s0025g03410
VIT_02s0025g03420
VIT_02s0025g03430
VIT_02s0025g03440
VIT_02s0025g03450
VIT_02s0025g03460
Standardized expressions
Developmental stages
EI PG G1 G2 H T1 T2 C1 C2 S EP JP

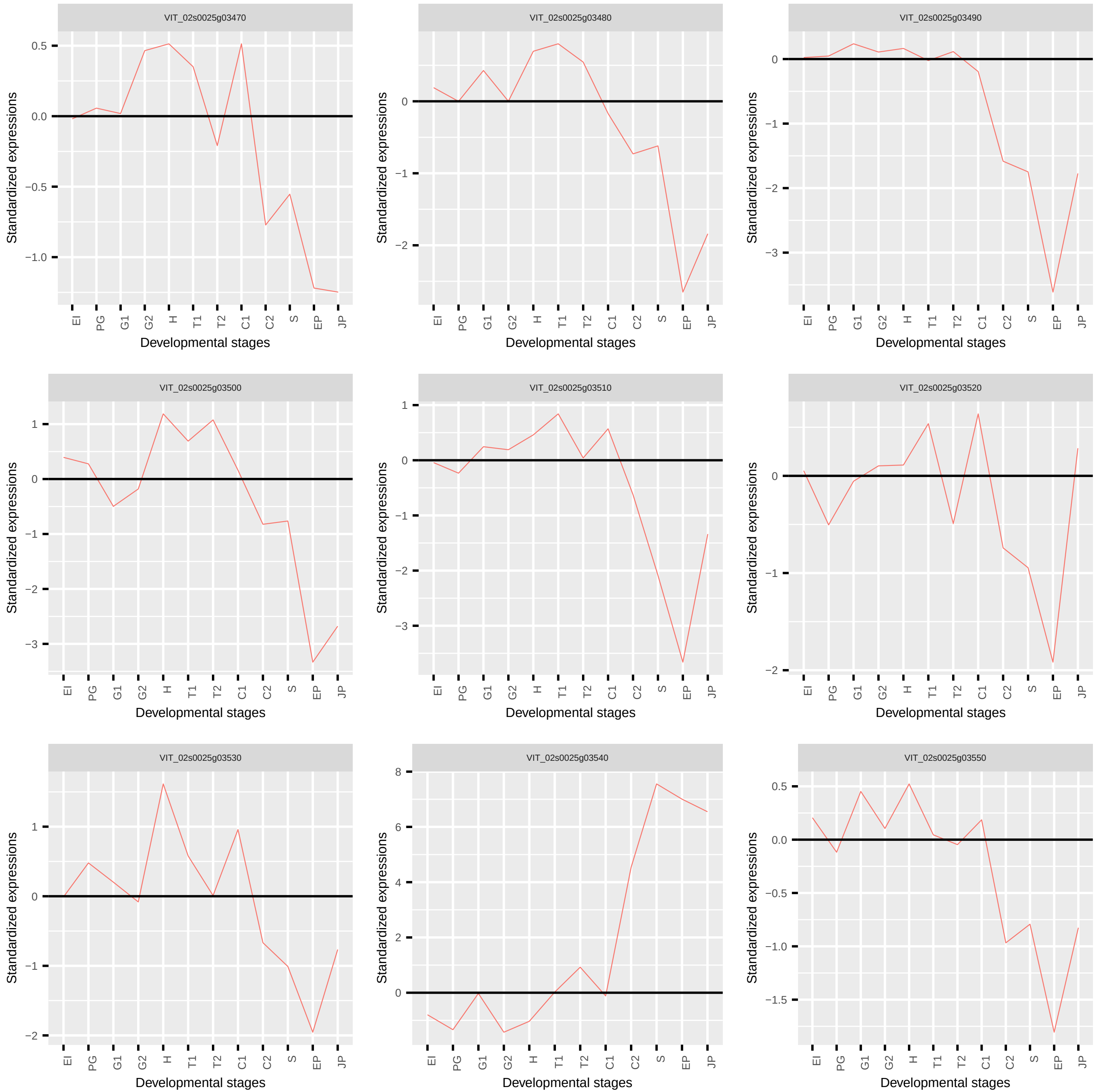

VIT_02s0025g03470
VIT_02s0025g03480
VIT_02s0025g03490
VIT_02s0025g03500
VIT_02s0025g03510
VIT_02s0025g03520
VIT_02s0025g03530
VIT_02s0025g03540
VIT_02s0025g03550
Standardized expressions
Developmental stages
EI PG G1 G2 H T1 T2 C1 C2 S EP JP

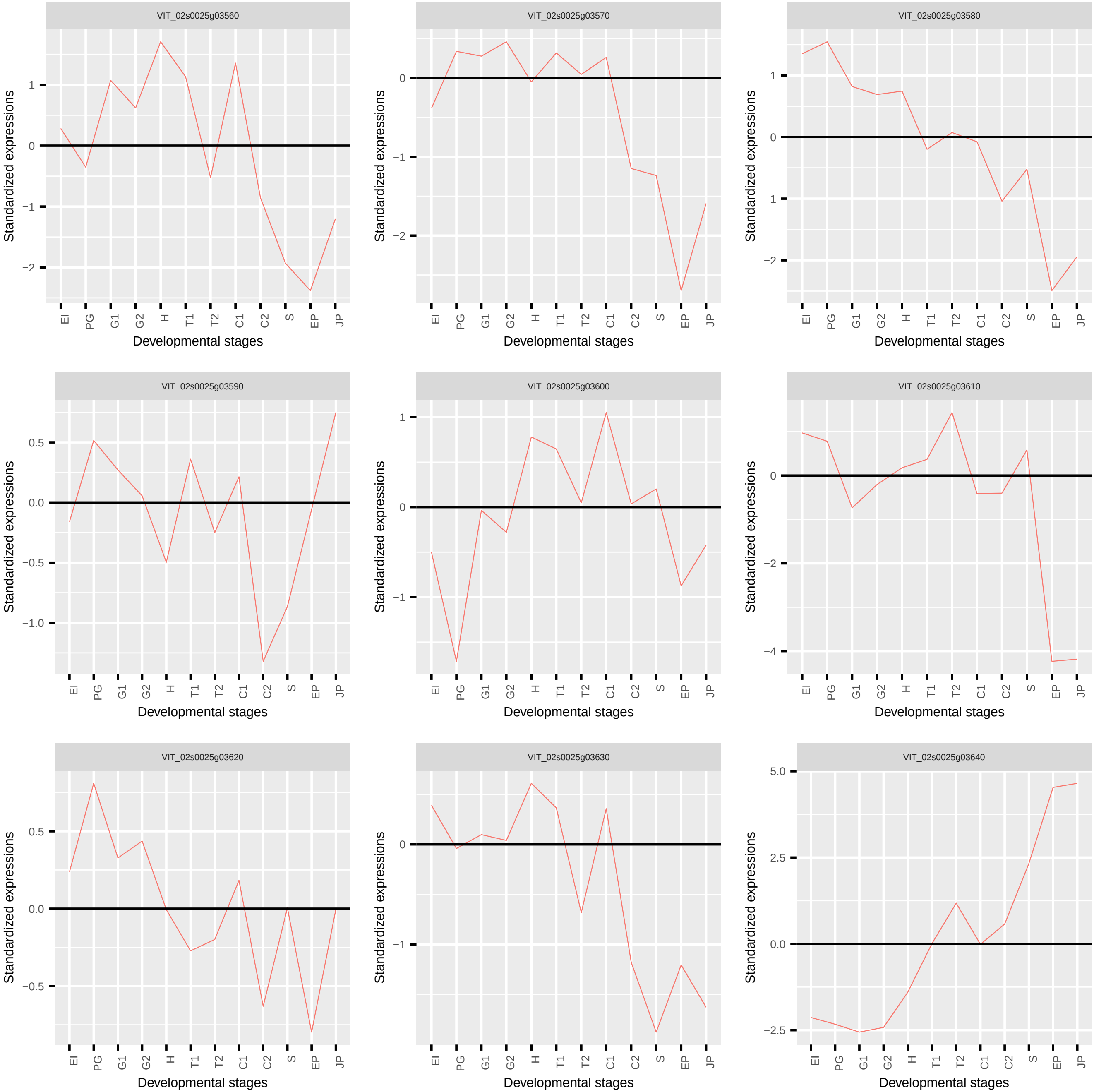
VIT_02s0025g03560
Standardized expressions
1
0
−1
−2
EI PG G1 G2 H T1 T2 C1 C2 S EP JP
Developmental stages
VIT_02s0025g03570
Standardized expressions
0
−1
−2
EI PG G1 G2 H T1 T2 C1 C2 S EP JP
Developmental stages
VIT_02s0025g03580
Standardized expressions
1
0
−1
−2
EI PG G1 G2 H T1 T2 C1 C2 S EP JP
Developmental stages
VIT_02s0025g03590
Standardized expressions
0.5
0.0
−0.5
−1.0
EI PG G1 G2 H T1 T2 C1 C2 S EP JP
Developmental stages
VIT_02s0025g03600
Standardized expressions
1
0
−1
EI PG G1 G2 H T1 T2 C1 C2 S EP JP
Developmental stages
VIT_02s0025g03610
Standardized expressions
0
−2
−4
EI PG G1 G2 H T1 T2 C1 C2 S EP JP
Developmental stages
VIT_02s0025g03620
Standardized expressions
0.5
0.0
−0.5
EI PG G1 G2 H T1 T2 C1 C2 S EP JP
Developmental stages
VIT_02s0025g03630
Standardized expressions
0
−1
EI PG G1 G2 H T1 T2 C1 C2 S EP JP
Developmental stages
VIT_02s0025g03640
Standardized expressions
5.0
2.5
0.0
−2.5
EI PG G1 G2 H T1 T2 C1 C2 S EP JP
Developmental stages

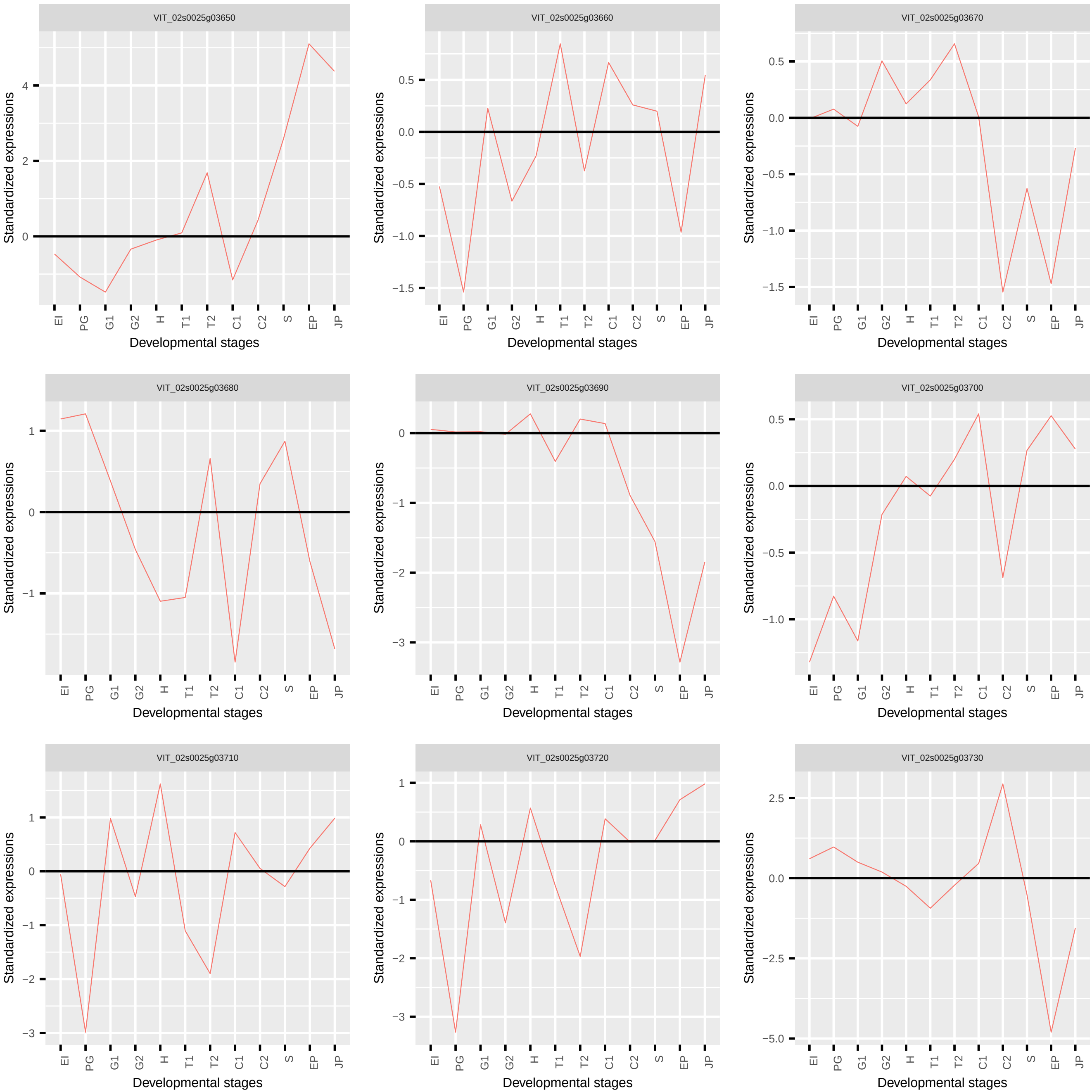
VIT_02s0025g03650
VIT_02s0025g03660
VIT_02s0025g03670
VIT_02s0025g03680
VIT_02s0025g03690
VIT_02s0025g03700
VIT_02s0025g03710
VIT_02s0025g03720
VIT_02s0025g03730
Standardized expressions
Developmental stages
EI PG G1 G2 H T1 T2 C1 C2 S EP JP

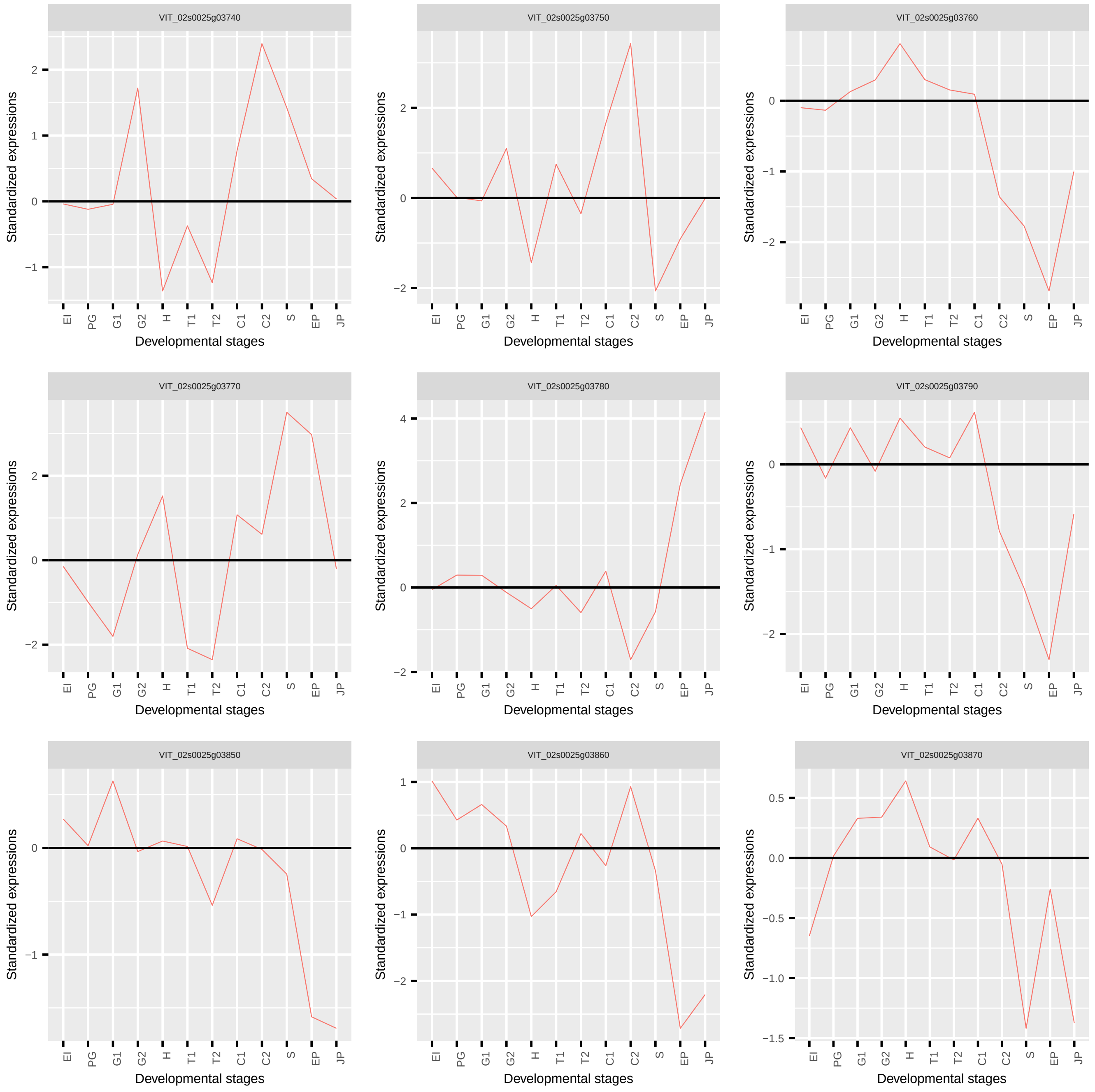

VIT_02s0025g03740
VIT_02s0025g03750
VIT_02s0025g03760
VIT_02s0025g03770
VIT_02s0025g03780
VIT_02s0025g03790
VIT_02s0025g03850
VIT_02s0025g03860
VIT_02s0025g03870
Standardized expressions
Developmental stages
EI PG G1 G2 H T1 T2 C1 C2 S EP JP

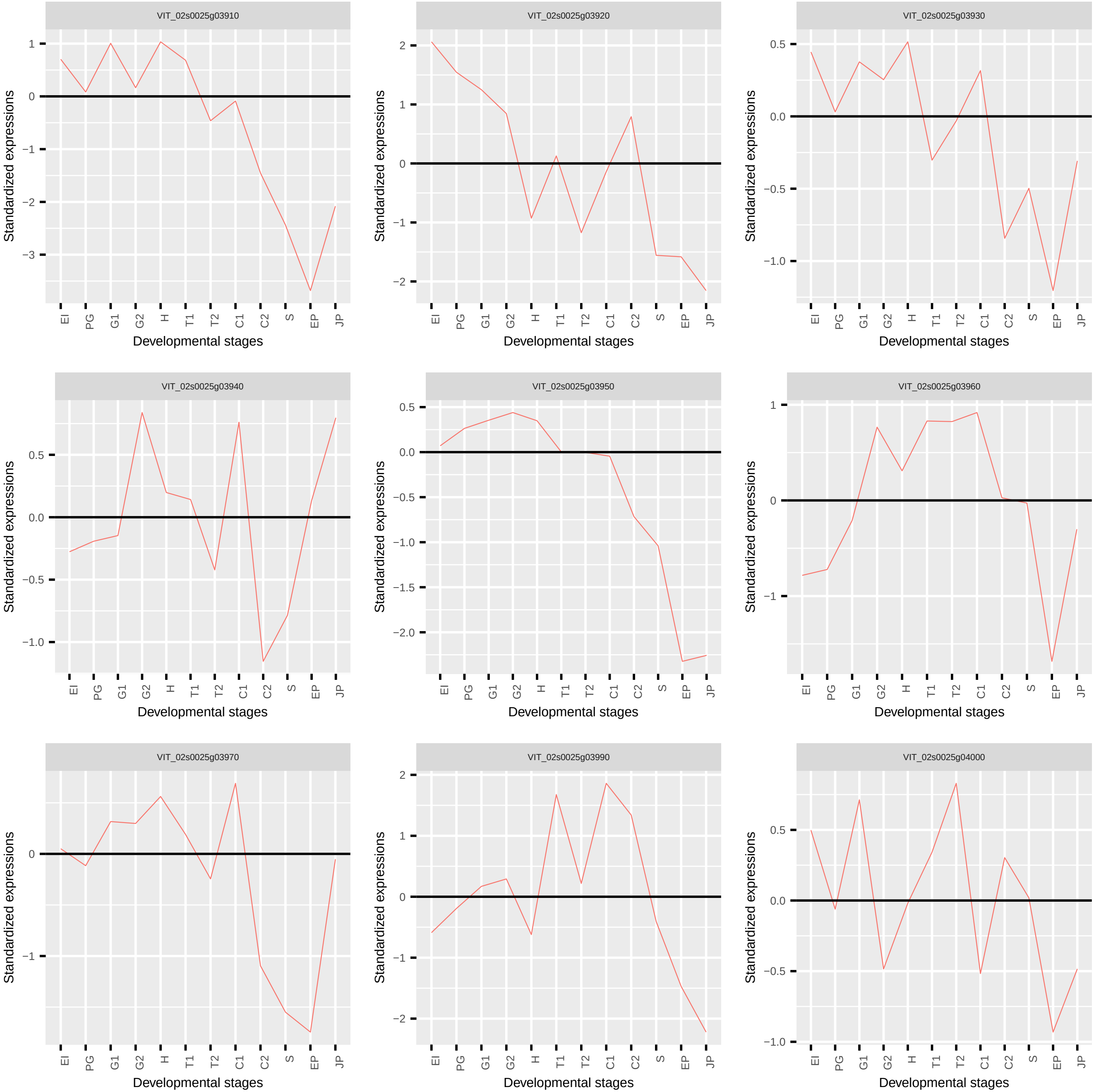
VIT_02s0025g03910
VIT_02s0025g03920
VIT_02s0025g03930
VIT_02s0025g03940
VIT_02s0025g03950
VIT_02s0025g03960
VIT_02s0025g03970
VIT_02s0025g03990
VIT_02s0025g04000
Standardized expressions
Developmental stages
EI PG G1 G2 H T1 T2 C1 C2 S EP JP

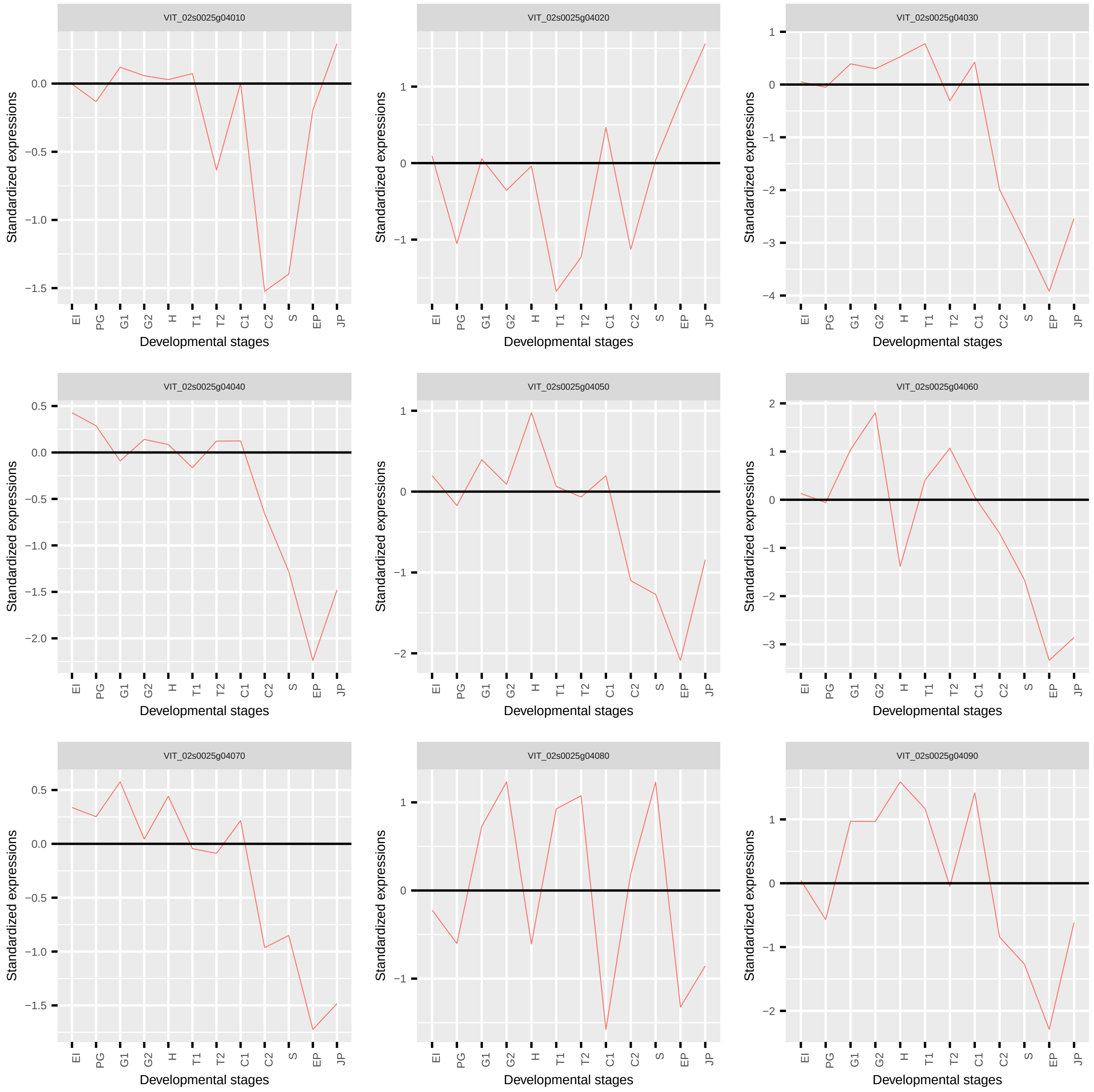
VIT_02s0025g04010
VIT_02s0025g04020
VIT_02s0025g04030
VIT_02s0025g04040
VIT_02s0025g04050
VIT_02s0025g04060
VIT_02s0025g04070
VIT_02s0025g04080
VIT_02s0025g04090
Standardized expressions
Developmental stages
EI PG G1 G2 H T1 T2 C1 C2 S EP JP

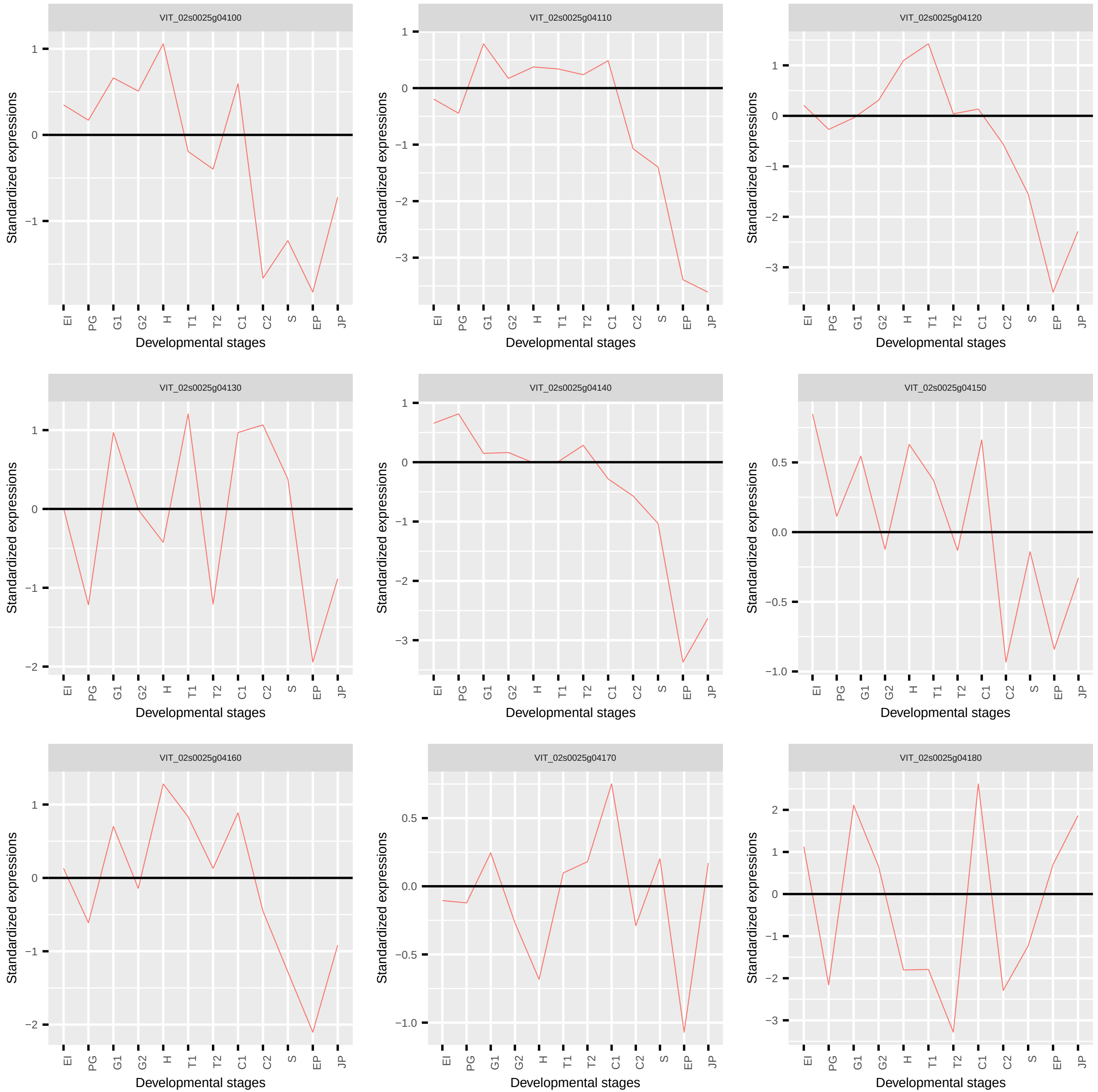
VIT_02s0025g04100
VIT_02s0025g04110
VIT_02s0025g04120
VIT_02s0025g04130
VIT_02s0025g04140
VIT_02s0025g04150
VIT_02s0025g04160
VIT_02s0025g04170
VIT_02s0025g04180
Standardized expressions
Developmental stages
EI PG G1 G2 H T1 T2 C1 C2 S EP JP

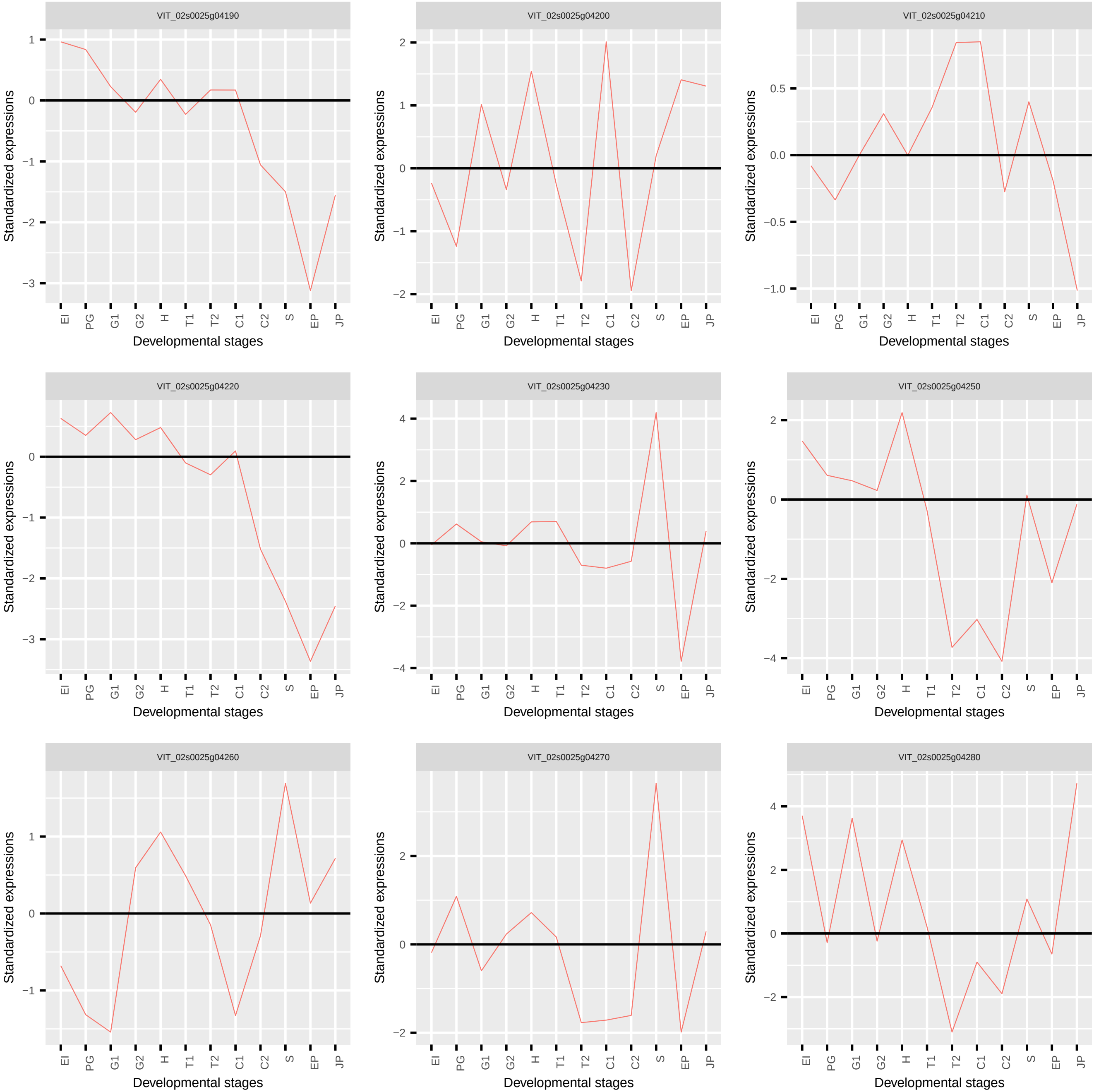
VIT_02s0025g04190
VIT_02s0025g04200
VIT_02s0025g04210
VIT_02s0025g04220
VIT_02s0025g04230
VIT_02s0025g04250
VIT_02s0025g04260
VIT_02s0025g04270
VIT_02s0025g04280
Standardized expressions
Developmental stages
EI PG G1 G2 H T1 T2 C1 C2 S EP JP

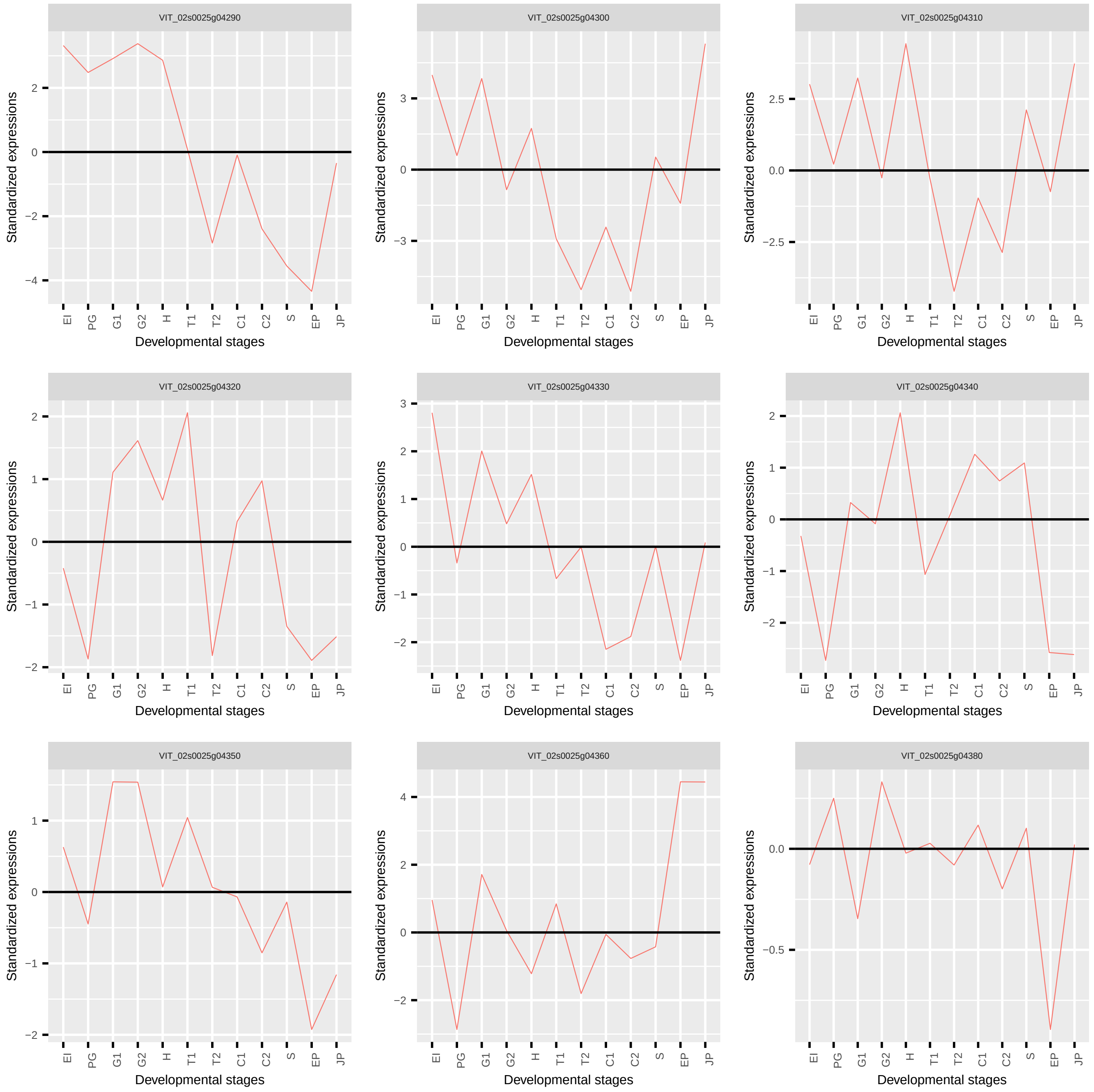
VIT_02s0025g04290
VIT_02s0025g04300
VIT_02s0025g04310
VIT_02s0025g04320
VIT_02s0025g04330
VIT_02s0025g04340
VIT_02s0025g04350
VIT_02s0025g04360
VIT_02s0025g04380
Standardized expressions
Developmental stages
EI PG G1 G2 H T1 T2 C1 C2 S EP JP

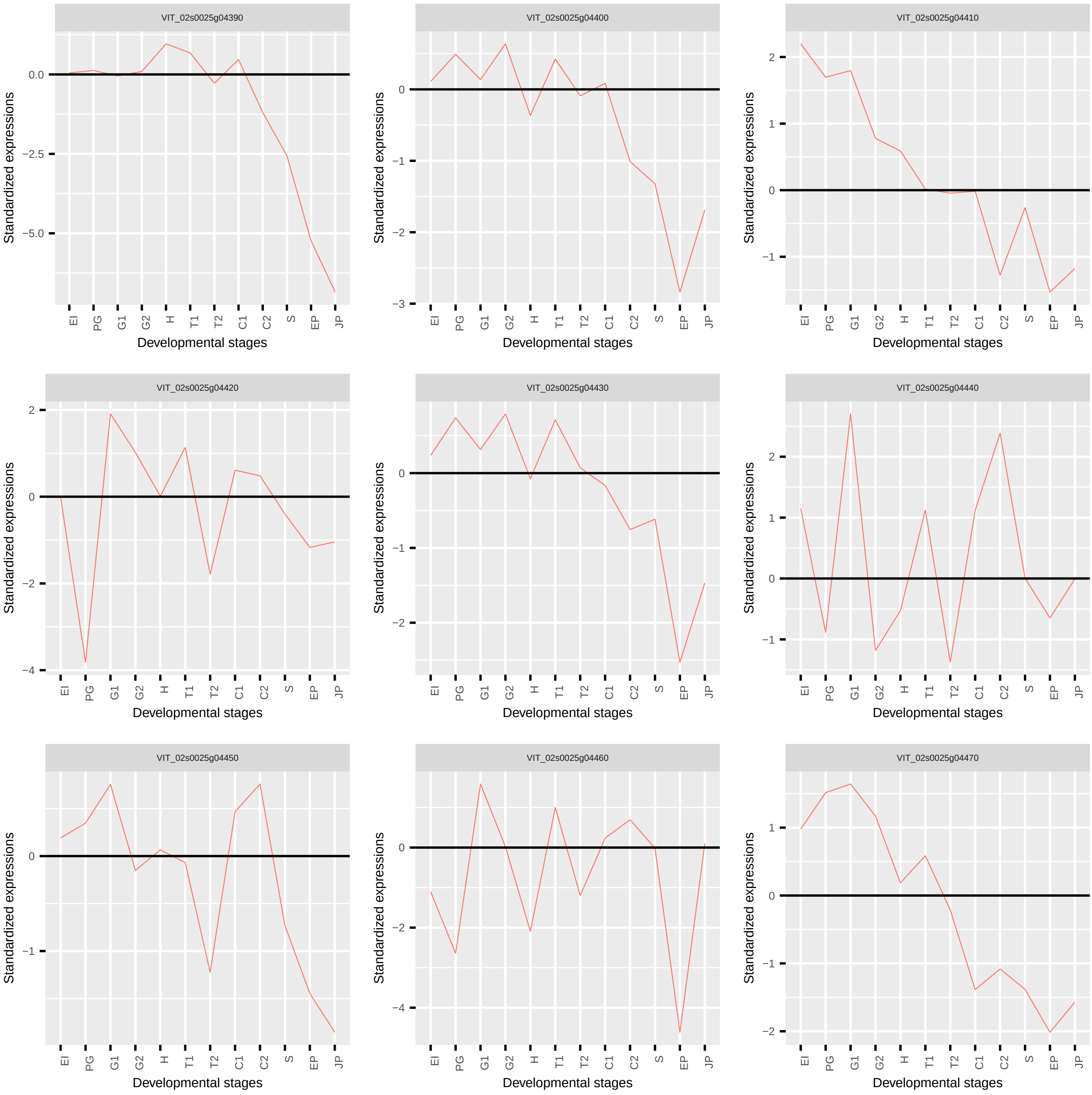
VIT_02s0025g04390
VIT_02s0025g04400
VIT_02s0025g04410
VIT_02s0025g04420
VIT_02s0025g04430
VIT_02s0025g04440
VIT_02s0025g04450
VIT_02s0025g04460
VIT_02s0025g04470
Standardized expressions
Developmental stages
EI PG G1 G2 H T1 T2 C1 C2 S EP JP

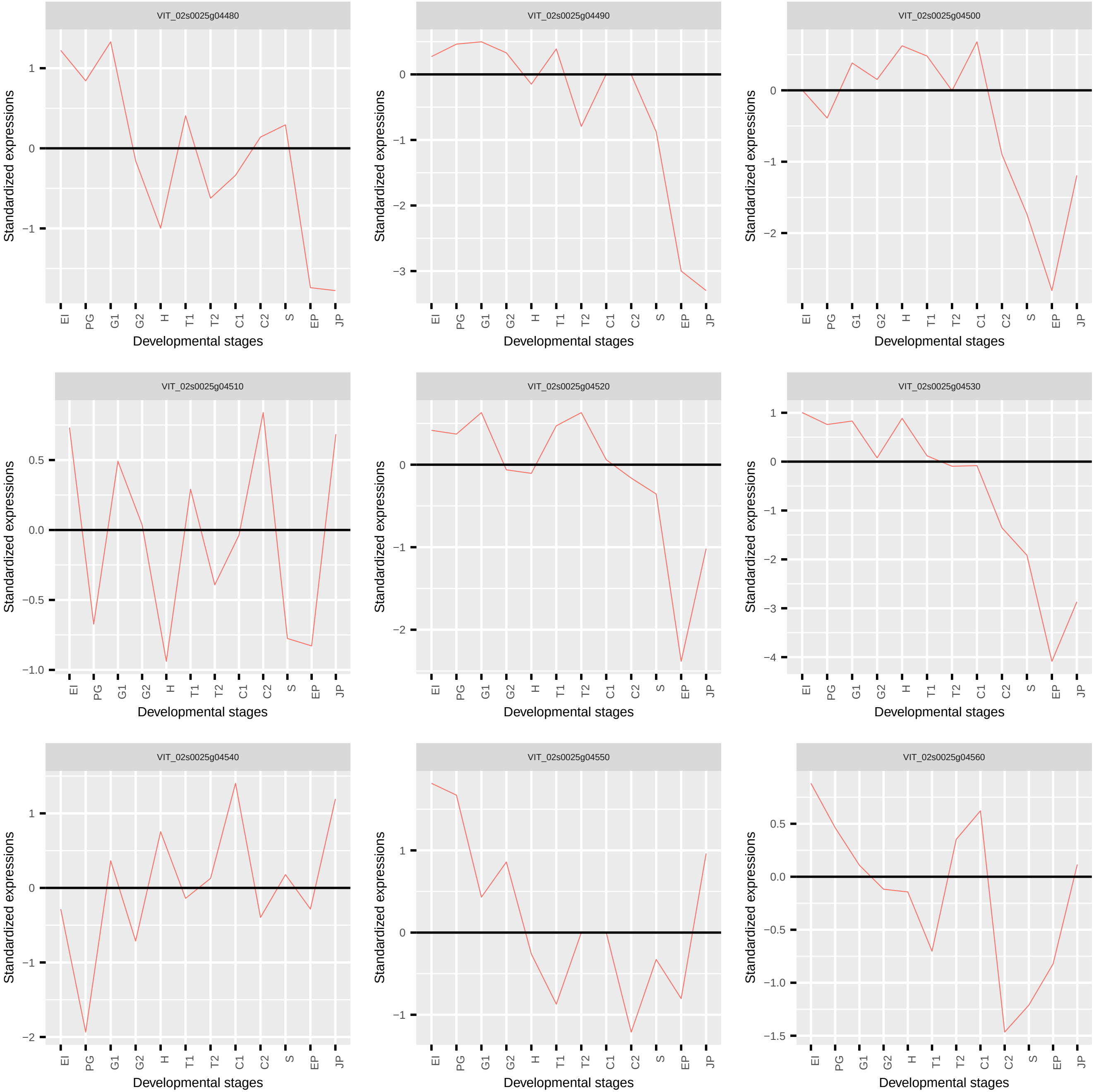
VIT_02s0025g04480
VIT_02s0025g04490
VIT_02s0025g04500
VIT_02s0025g04510
VIT_02s0025g04520
VIT_02s0025g04530
VIT_02s0025g04540
VIT_02s0025g04550
VIT_02s0025g04560
Standardized expressions
Developmental stages
EI PG G1 G2 H T1 T2 C1 C2 S EP JP

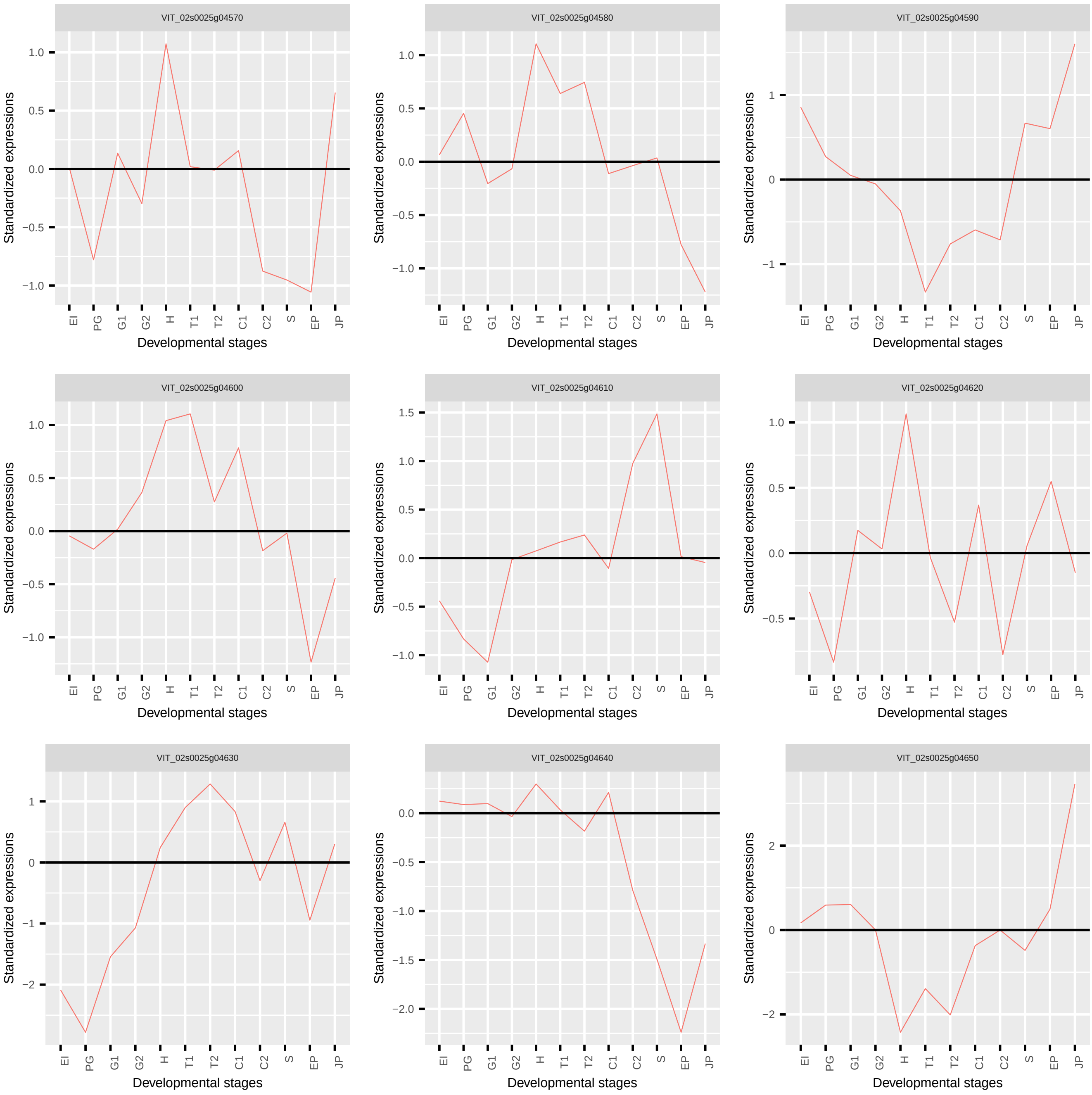
VIT_02s0025g04570
VIT_02s0025g04580
VIT_02s0025g04590
VIT_02s0025g04600
VIT_02s0025g04610
VIT_02s0025g04620
VIT_02s0025g04630
VIT_02s0025g04640
VIT_02s0025g04650
Standardized expressions
Developmental stages
EI PG G1 G2 H T1 T2 C1 C2 S EP JP

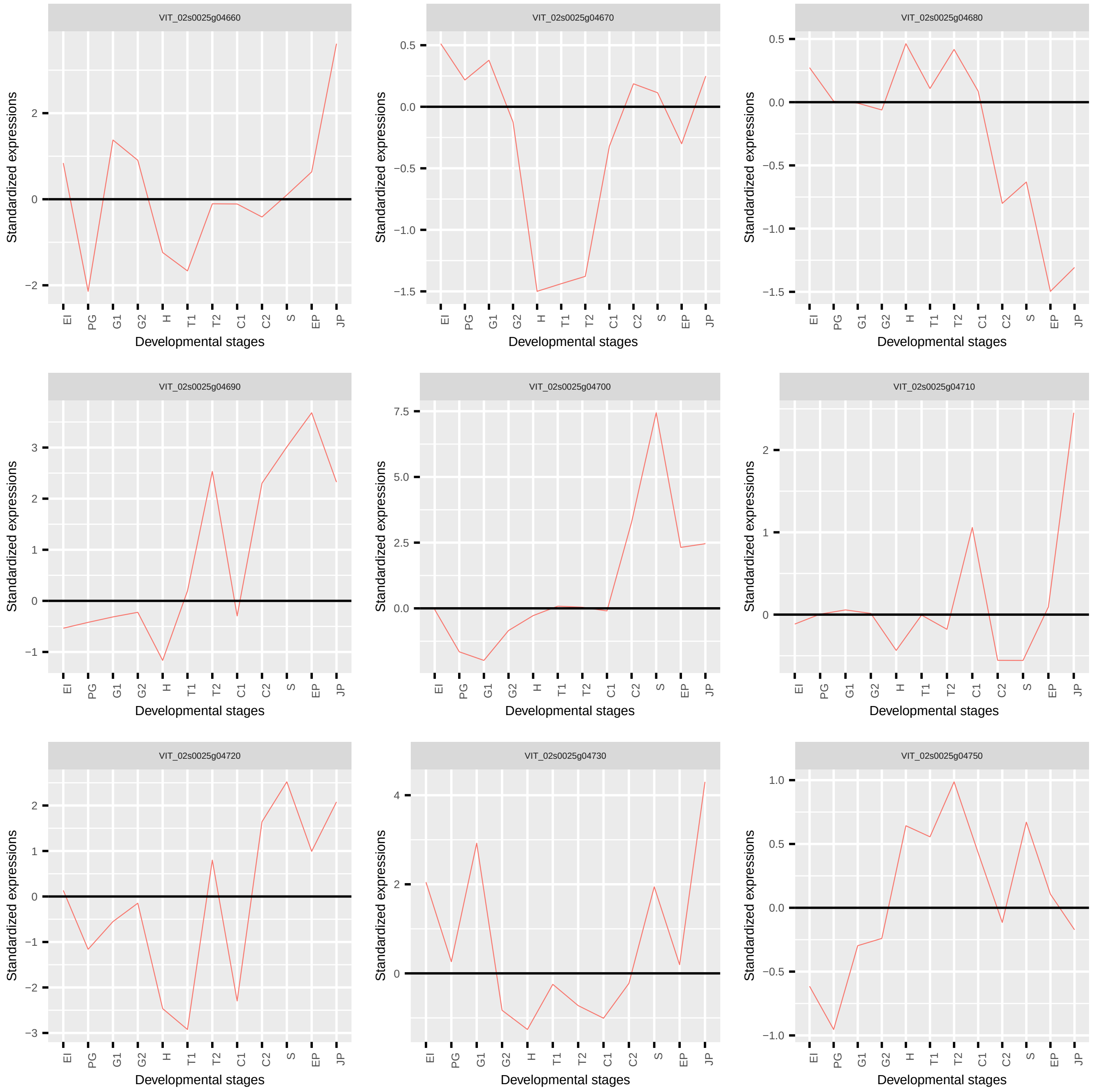
VIT_02s0025g04660
VIT_02s0025g04670
VIT_02s0025g04680
VIT_02s0025g04690
VIT_02s0025g04700
VIT_02s0025g04710
VIT_02s0025g04720
VIT_02s0025g04730
VIT_02s0025g04750
Standardized expressions
Developmental stages
EI PG G1 G2 H T1 T2 C1 C2 S EP JP

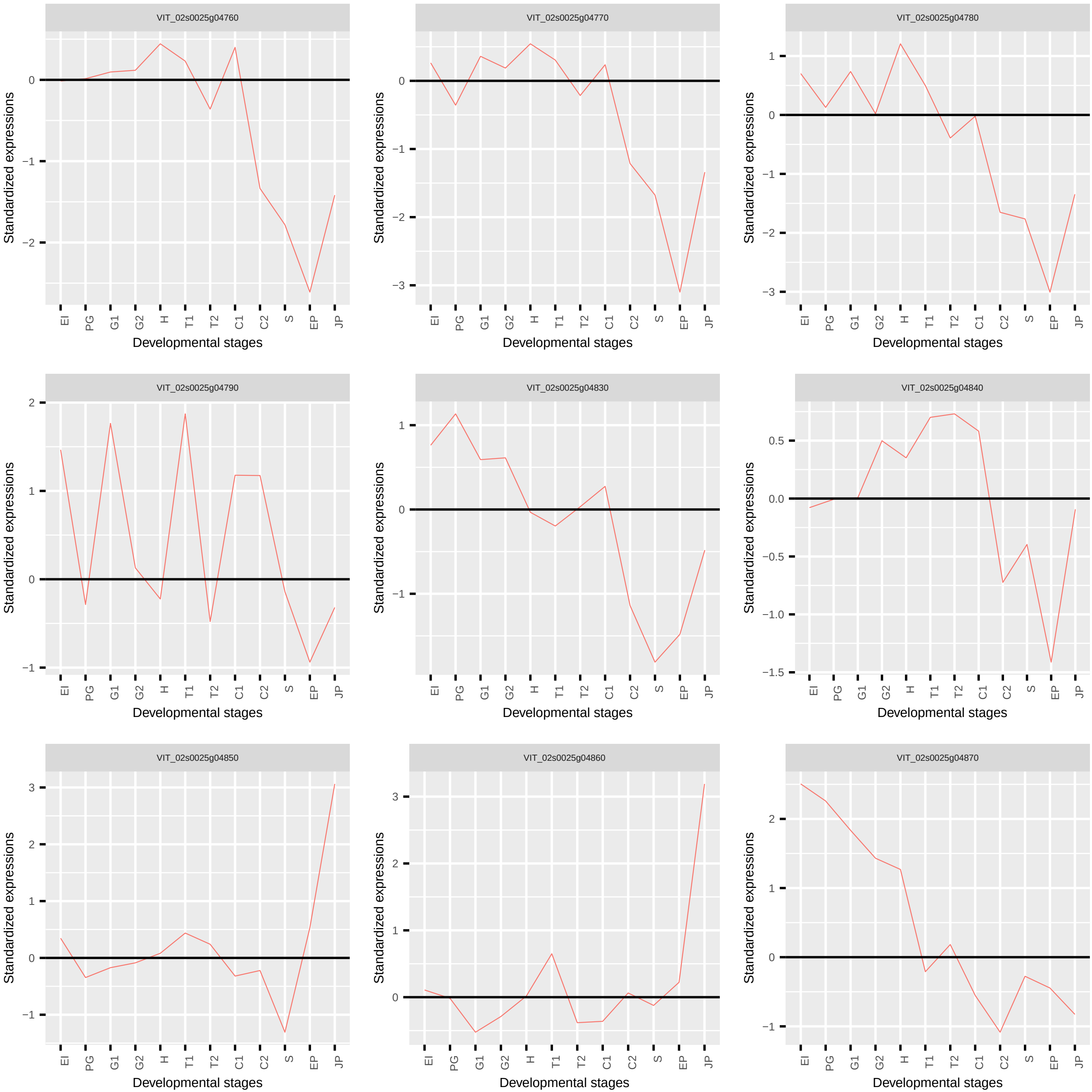

VIT_02s0025g04760
Standardized expressions
0
−1
−2
EI PG G1 G2 H T1 T2 C1 C2 S EP JP
Developmental stages
VIT_02s0025g04770
Standardized expressions
0
−1
−2
−3
EI PG G1 G2 H T1 T2 C1 C2 S EP JP
Developmental stages
VIT_02s0025g04780
Standardized expressions
1
0
−1
−2
−3
EI PG G1 G2 H T1 T2 C1 C2 S EP JP
Developmental stages
VIT_02s0025g04790
Standardized expressions
2
1
0
−1
EI PG G1 G2 H T1 T2 C1 C2 S EP JP
Developmental stages
VIT_02s0025g04830
Standardized expressions
1
0
−1
EI PG G1 G2 H T1 T2 C1 C2 S EP JP
Developmental stages
VIT_02s0025g04840
Standardized expressions
0.5
0.0
−0.5
−1.0
−1.5
EI PG G1 G2 H T1 T2 C1 C2 S EP JP
Developmental stages
VIT_02s0025g04850
Standardized expressions
3
2
1
0
−1
EI PG G1 G2 H T1 T2 C1 C2 S EP JP
Developmental stages
VIT_02s0025g04860
Standardized expressions
3
2
1
0
EI PG G1 G2 H T1 T2 C1 C2 S EP JP
Developmental stages
VIT_02s0025g04870
Standardized expressions
2
1
0
−1
EI PG G1 G2 H T1 T2 C1 C2 S EP JP
Developmental stages

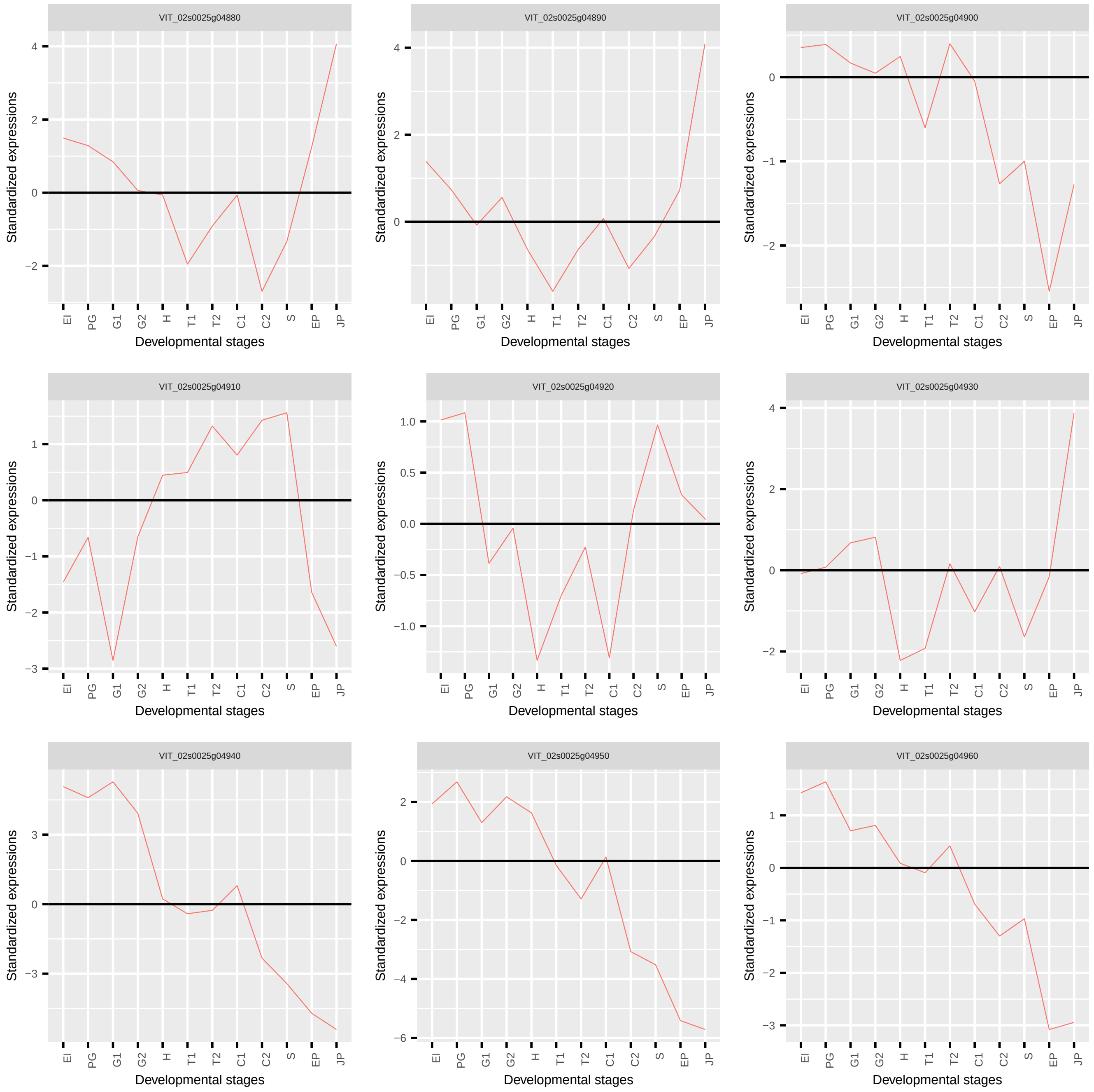
VIT_02s0025g04880
VIT_02s0025g04890
VIT_02s0025g04900
VIT_02s0025g04910
VIT_02s0025g04920
VIT_02s0025g04930
VIT_02s0025g04940
VIT_02s0025g04950
VIT_02s0025g04960
Standardized expressions
Developmental stages
EI PG G1 G2 H T1 T2 C1 C2 S EP JP

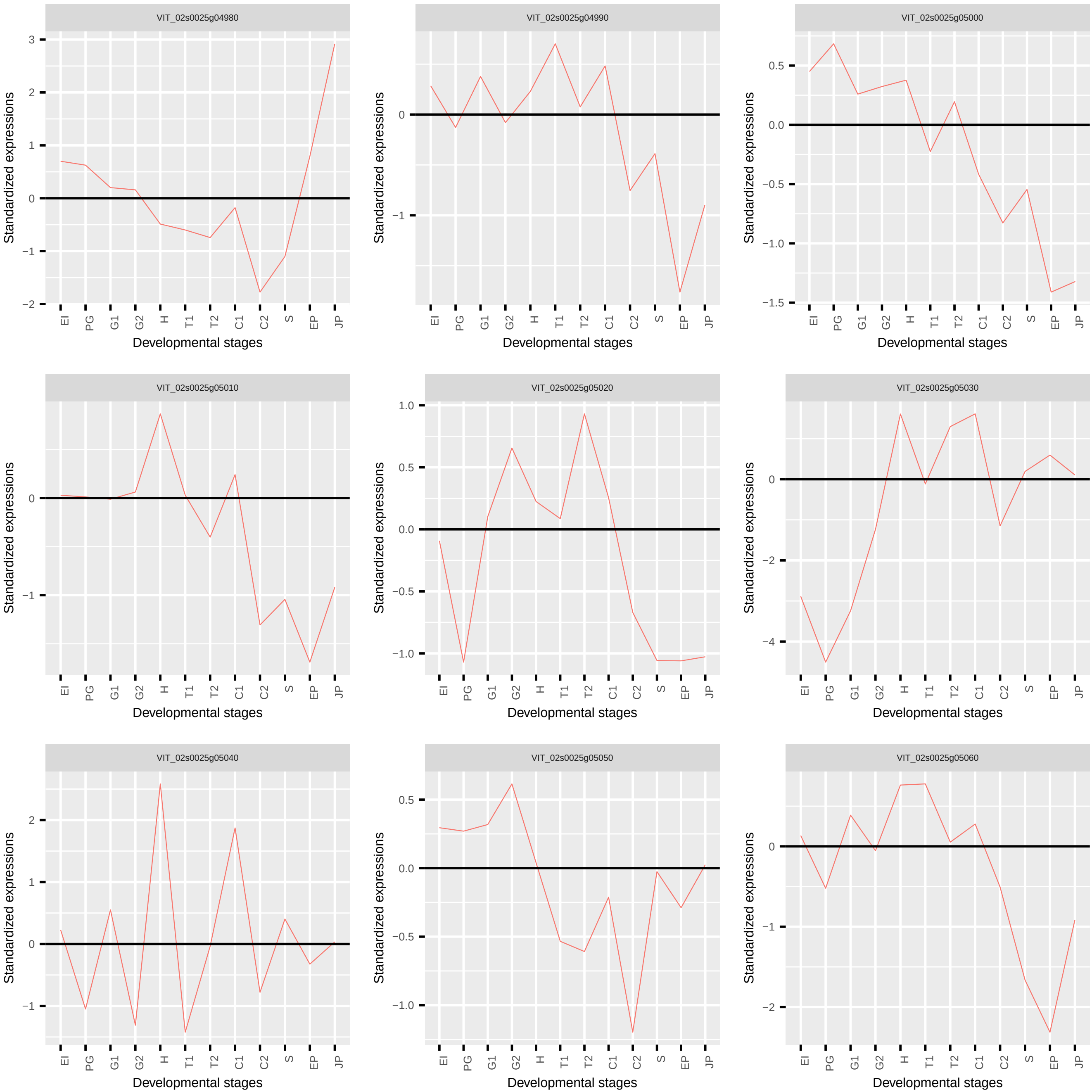
VIT_02s0025g04980
VIT_02s0025g04990
VIT_02s0025g05000
VIT_02s0025g05010
VIT_02s0025g05020
VIT_02s0025g05030
VIT_02s0025g05040
VIT_02s0025g05050
VIT_02s0025g05060
Standardized expressions
Developmental stages
EI PG G1 G2 H T1 T2 C1 C2 S EP JP

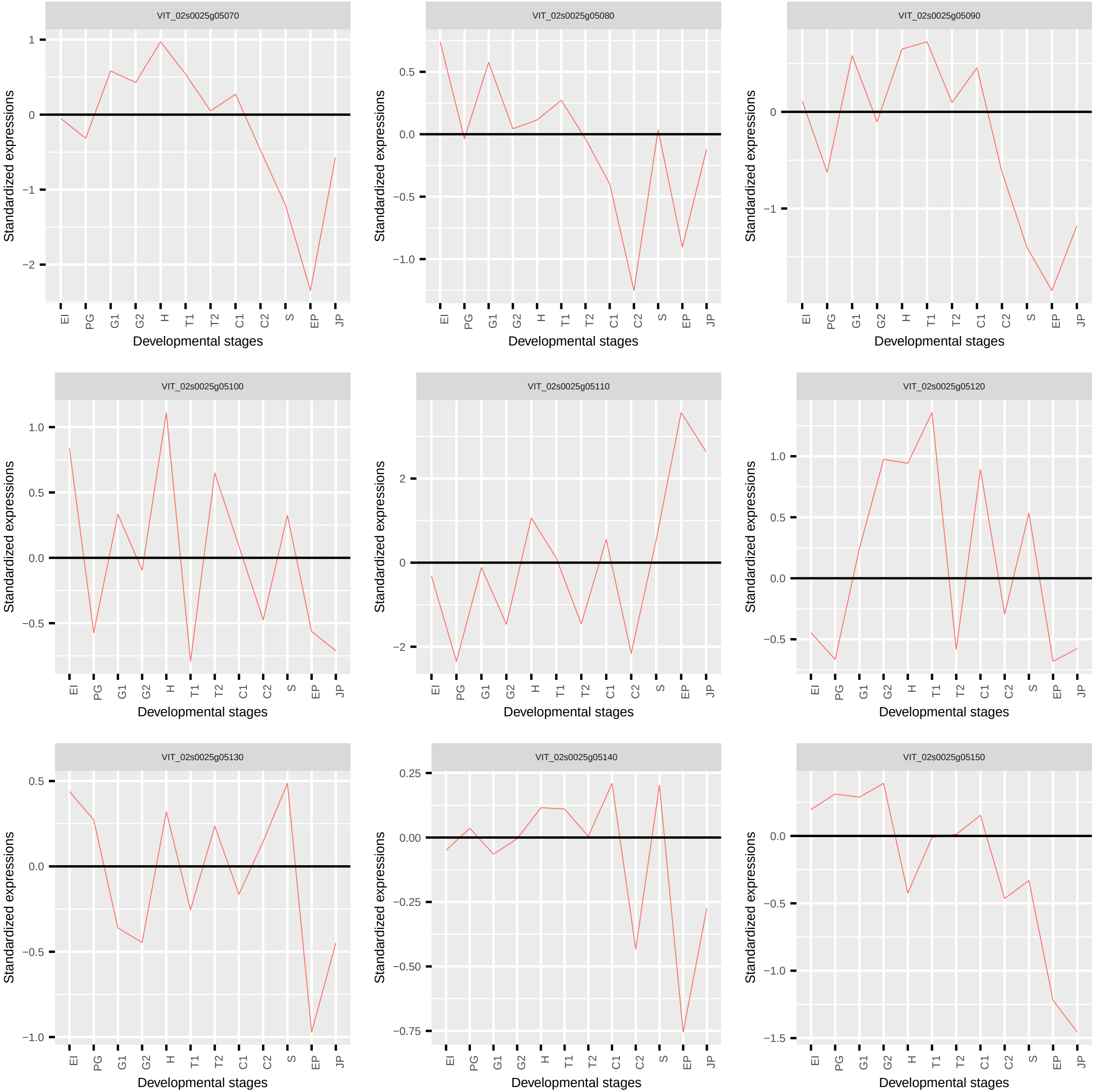

VIT_02s0025g05070
VIT_02s0025g05080
VIT_02s0025g05090
VIT_02s0025g05100
VIT_02s0025g05110
VIT_02s0025g05120
VIT_02s0025g05130
VIT_02s0025g05140
VIT_02s0025g05150
Standardized expressions
Developmental stages
EI PG G1 G2 H T1 T2 C1 C2 S EP JP
1 0 −1 −2
0.5 0.0 −0.5 −1.0
0 −1
1.0 0.5 0.0 −0.5
2 0 −2
1.0 0.5 0.0 −0.5
0.5 0.0 −0.5 −1.0
0.25 0.00 −0.25 −0.50 −0.75
0.0 −0.5 −1.0 −1.5

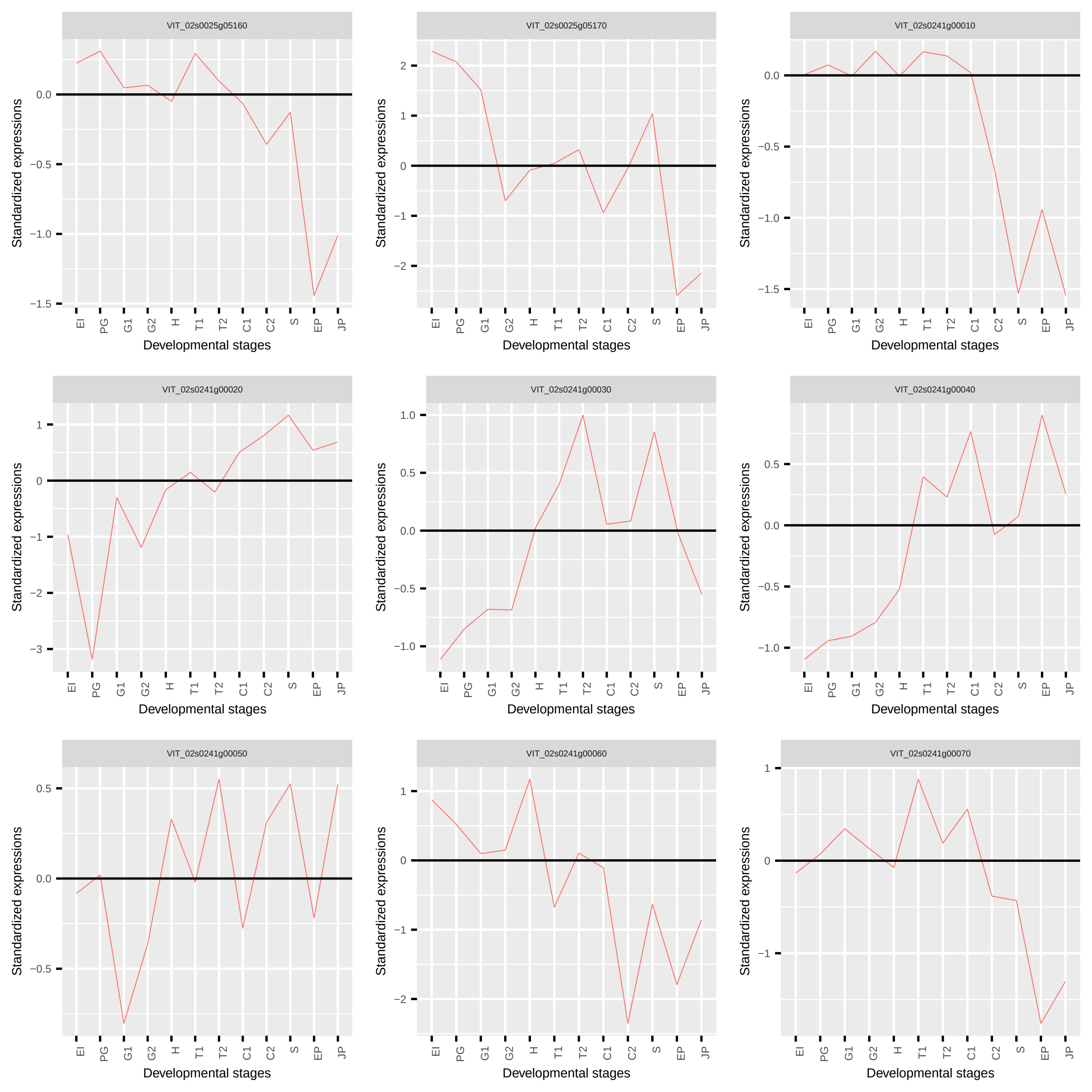
VIT_02s0025g05160
VIT_02s0025g05170
VIT_02s0241g00010
VIT_02s0241g00020
VIT_02s0241g00030
VIT_02s0241g00040
VIT_02s0241g00050
VIT_02s0241g00060
VIT_02s0241g00070
Standardized expressions
Developmental stages
EI PG G1 G2 H T1 T2 C1 C2 S EP JP

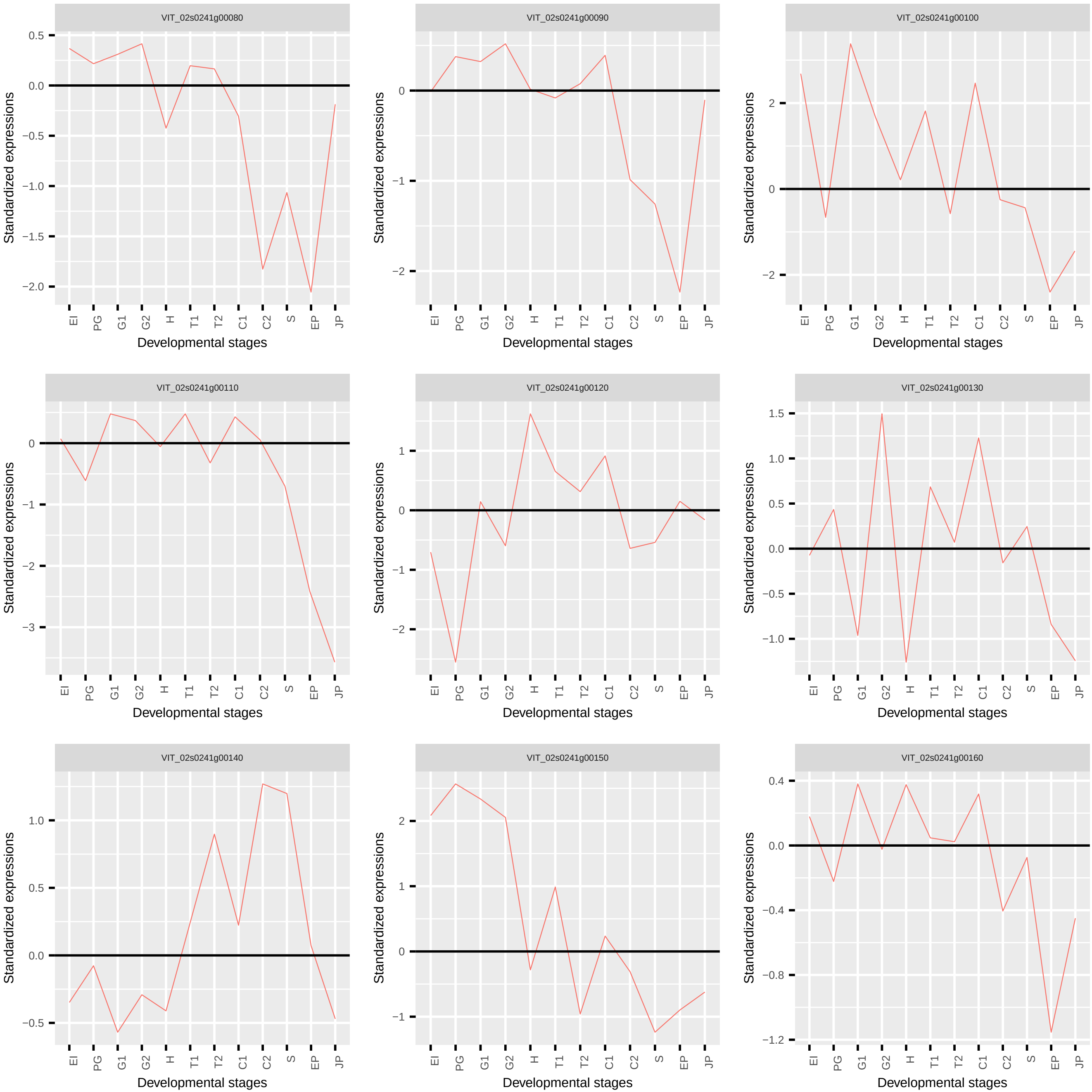
VIT_02s0241g00080
VIT_02s0241g00090
VIT_02s0241g00100
VIT_02s0241g00110
VIT_02s0241g00120
VIT_02s0241g00130
VIT_02s0241g00140
VIT_02s0241g00150
VIT_02s0241g00160
Standardized expressions
Developmental stages
EI PG G1 G2 H T1 T2 C1 C2 S EP JP

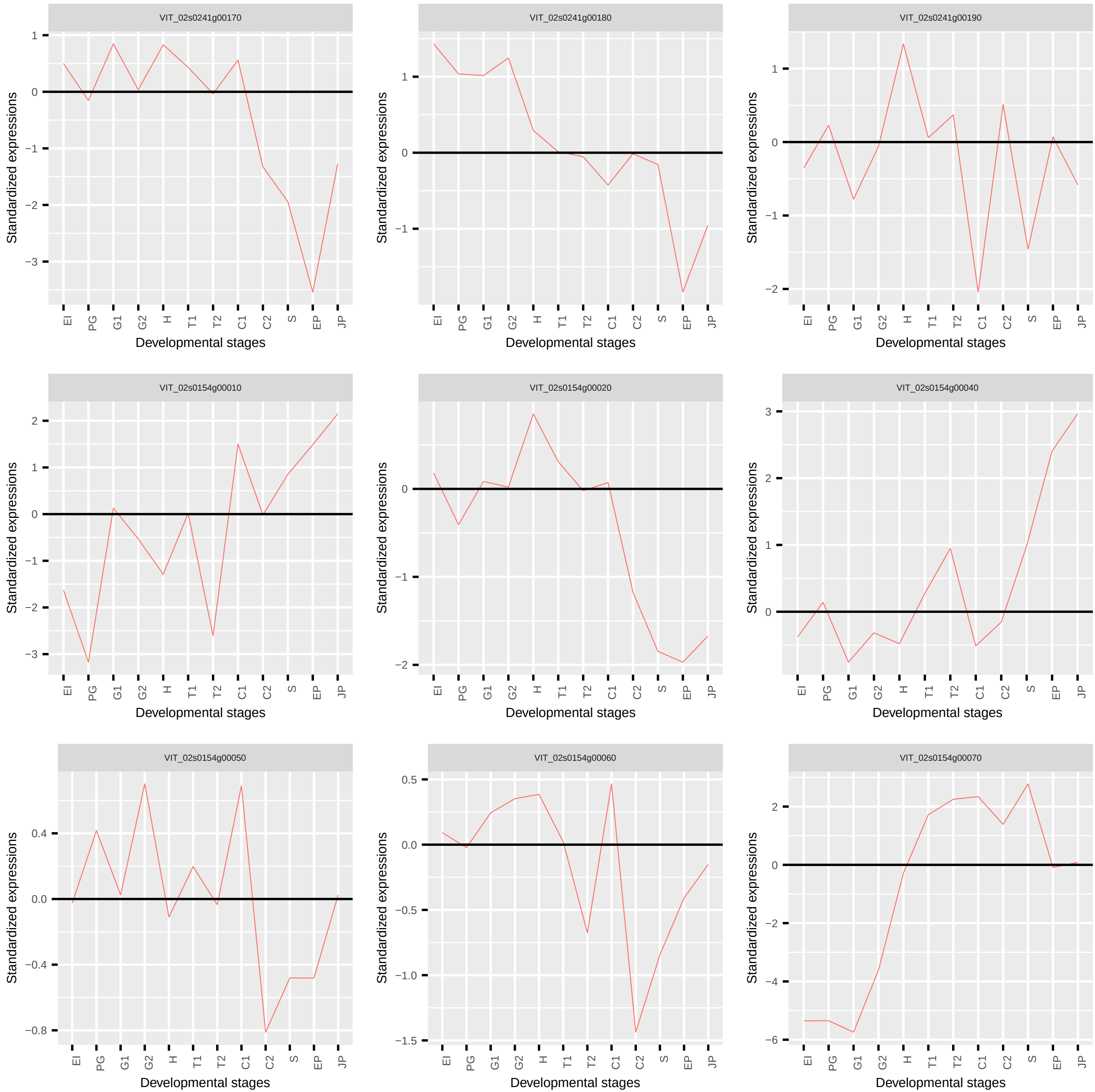
VIT_02s0241g00170
VIT_02s0241g00180
VIT_02s0241g00190
VIT_02s0154g00010
VIT_02s0154g00020
VIT_02s0154g00040
VIT_02s0154g00050
VIT_02s0154g00060
VIT_02s0154g00070
Standardized expressions
Developmental stages
EI PG G1 G2 H T1 T2 C1 C2 S EP JP

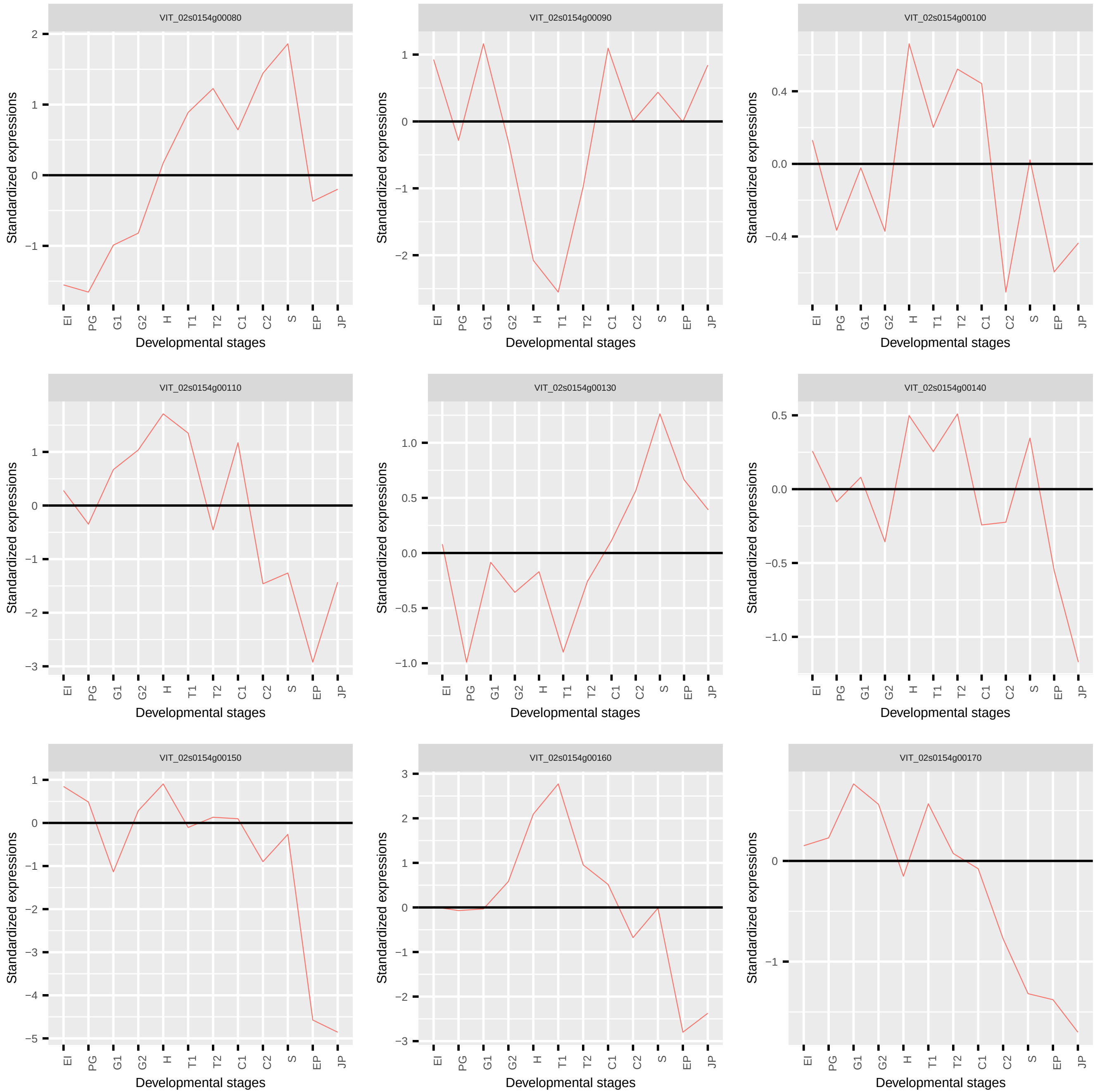
VIT_02s0154g00080
Standardized expressions
2
1
0
−1
EI
PG
G1
G2
H
T1
T2
C1
C2
S
EP
JP
Developmental stages
VIT_02s0154g00090
Standardized expressions
1
0
−1
−2
Developmental stages
VIT_02s0154g00100
Standardized expressions
0.4
0.0
−0.4
Developmental stages
VIT_02s0154g00110
Standardized expressions
1
0
−1
−2
−3
Developmental stages
VIT_02s0154g00130
Standardized expressions
1.0
0.5
0.0
−0.5
−1.0
Developmental stages
VIT_02s0154g00140
Standardized expressions
0.5
0.0
−0.5
−1.0
Developmental stages
VIT_02s0154g00150
Standardized expressions
1
0
−1
−2
−3
−4
−5
Developmental stages
VIT_02s0154g00160
Standardized expressions
3
2
1
0
−1
−2
−3
Developmental stages
VIT_02s0154g00170
Standardized expressions
0
−1
Developmental stages

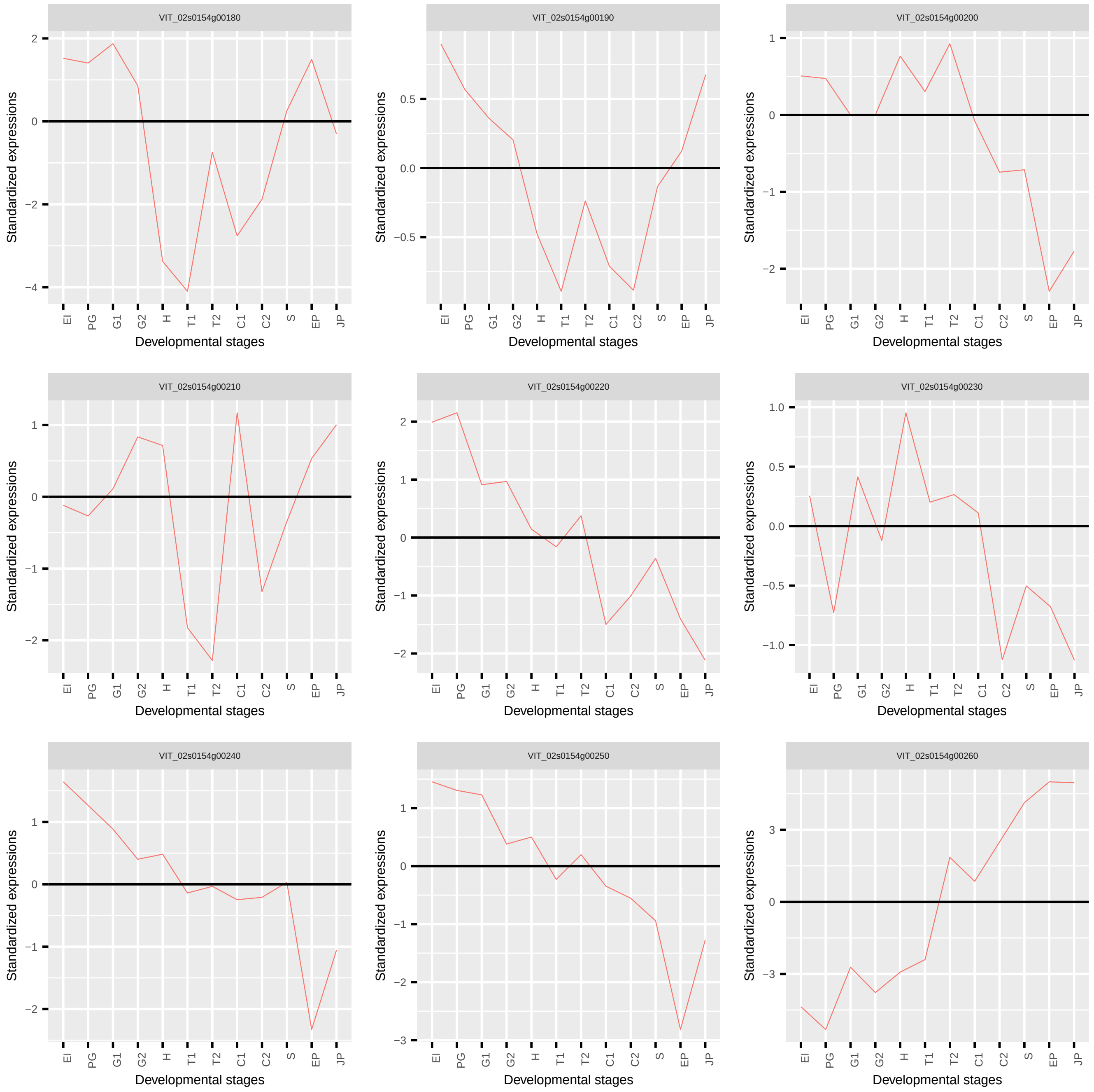
VIT_02s0154g00180
2
0
−2
−4
VIT_02s0154g00190
0.5
0.0
−0.5
VIT_02s0154g00200
1
0
−1
−2
VIT_02s0154g00210
1
0
−1
−2
VIT_02s0154g00220
2
1
0
−1
−2
VIT_02s0154g00230
1.0
0.5
0.0
−0.5
−1.0
VIT_02s0154g00240
1
0
−1
−2
VIT_02s0154g00250
1
0
−1
−2
−3
VIT_02s0154g00260
3
0
−3
Standardized expressions
EI PG G1 G2 H T1 T2 C1 C2 S EP JP
Developmental stages

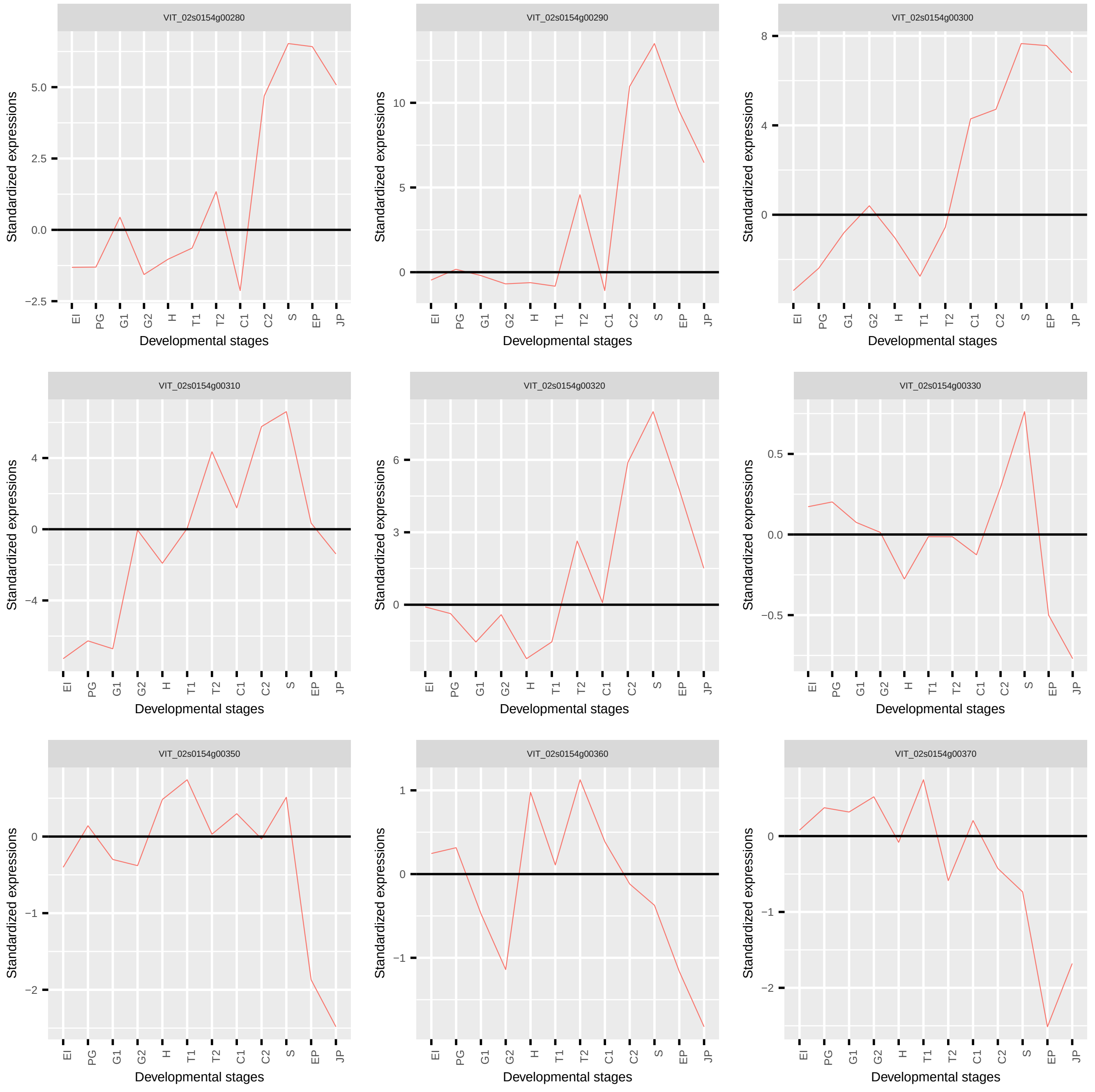

VIT_02s0154g00280
VIT_02s0154g00290
VIT_02s0154g00300
VIT_02s0154g00310
VIT_02s0154g00320
VIT_02s0154g00330
VIT_02s0154g00350
VIT_02s0154g00360
VIT_02s0154g00370
Standardized expressions
Developmental stages
EI PG G1 G2 H T1 T2 C1 C2 S EP JP

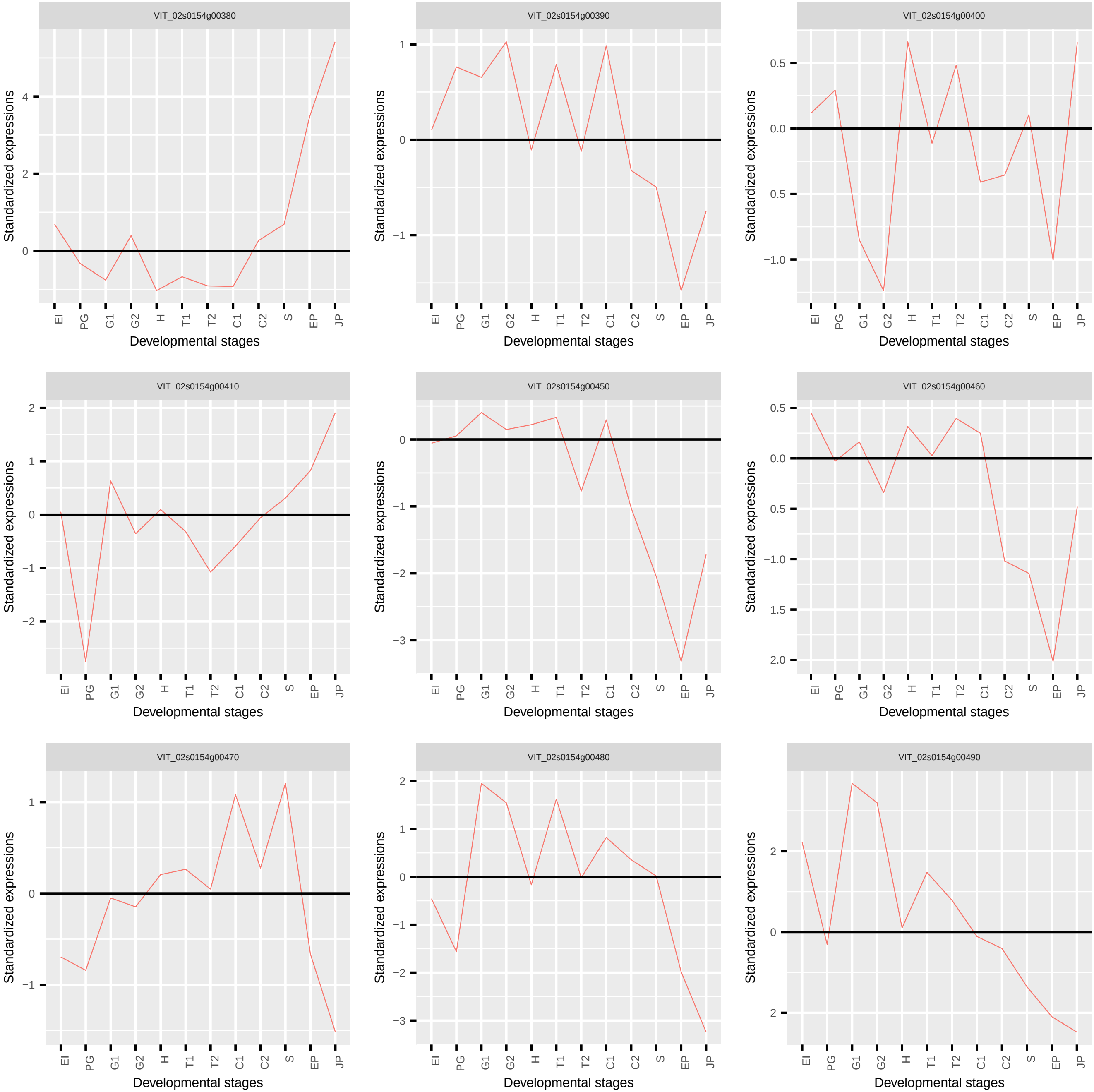
VIT_02s0154g00380
VIT_02s0154g00390
VIT_02s0154g00400
VIT_02s0154g00410
VIT_02s0154g00450
VIT_02s0154g00460
VIT_02s0154g00470
VIT_02s0154g00480
VIT_02s0154g00490
Standardized expressions
Developmental stages
EI PG G1 G2 H T1 T2 C1 C2 S EP JP

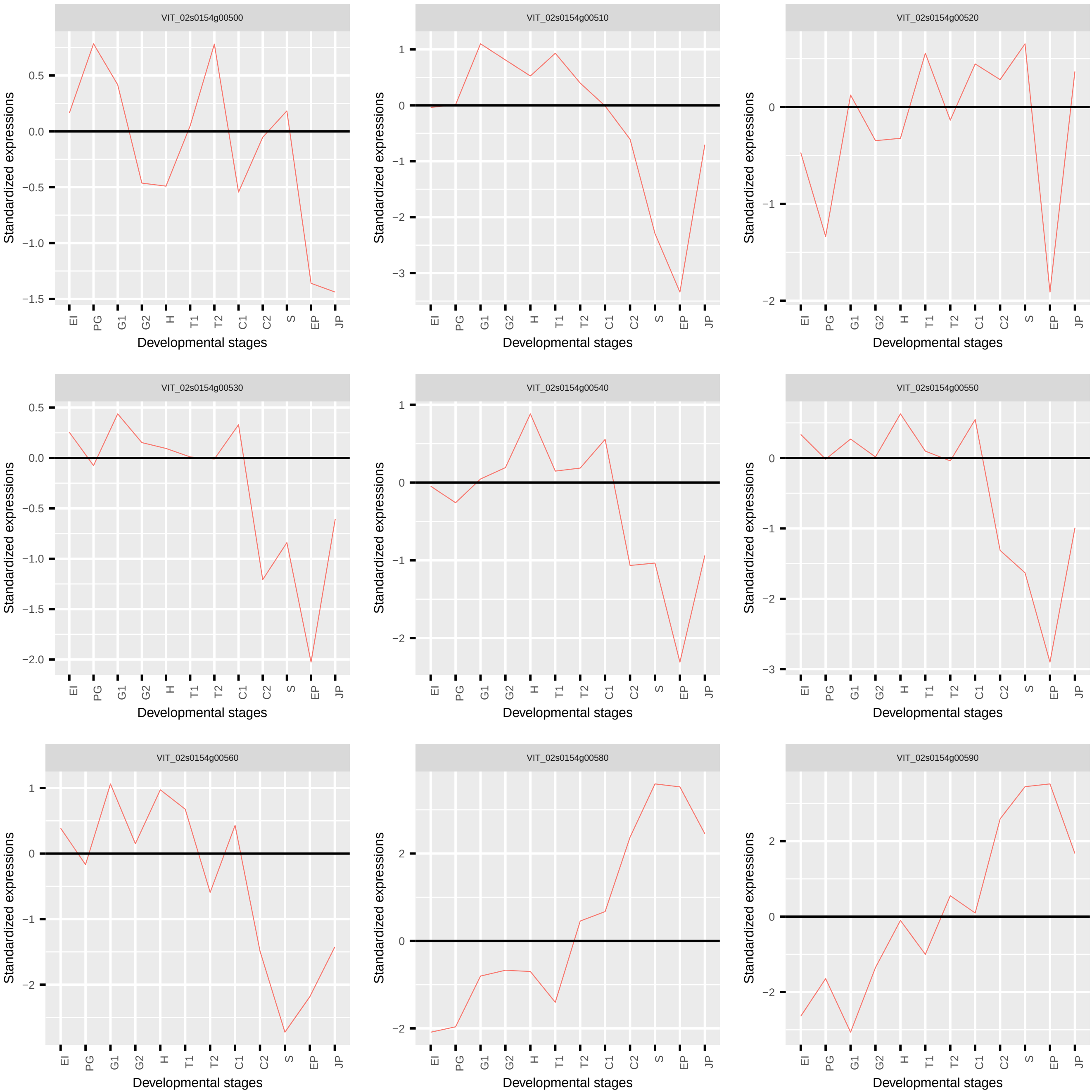
VIT_02s0154g00500
VIT_02s0154g00510
VIT_02s0154g00520
VIT_02s0154g00530
VIT_02s0154g00540
VIT_02s0154g00550
VIT_02s0154g00560
VIT_02s0154g00580
VIT_02s0154g00590
Standardized expressions
Developmental stages
EI PG G1 G2 H T1 T2 C1 C2 S EP JP

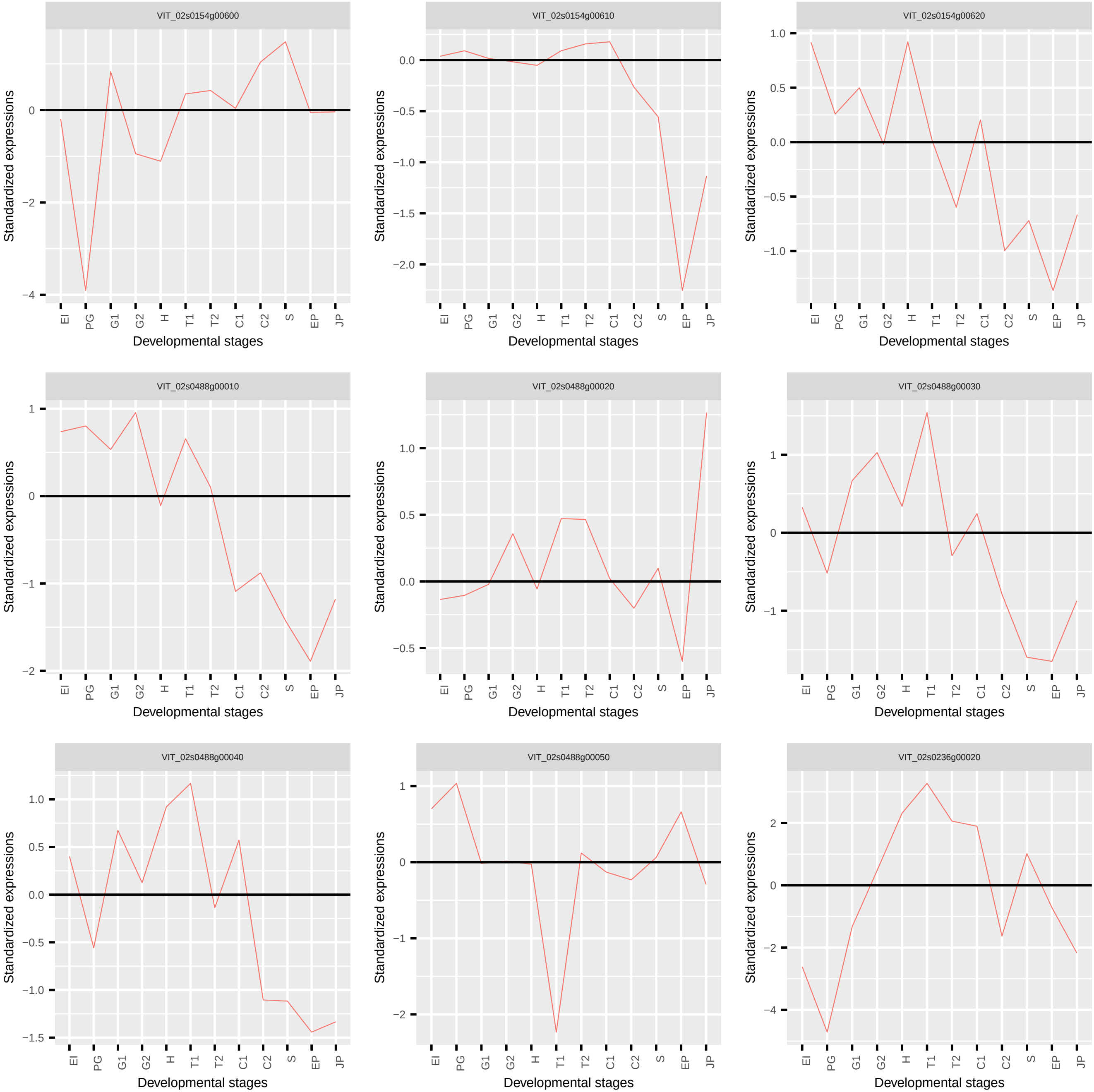

VIT_02s0154g00600
0
−2
−4
VIT_02s0154g00610
0.0
−0.5
−1.0
−1.5
−2.0
VIT_02s0154g00620
1.0
0.5
0.0
−0.5
−1.0
VIT_02s0488g00010
1
0
−1
−2
VIT_02s0488g00020
1.0
0.5
0.0
−0.5
VIT_02s0488g00030
1
0
−1
VIT_02s0488g00040
1.0
0.5
0.0
−0.5
−1.0
−1.5
VIT_02s0488g00050
1
0
−1
−2
VIT_02s0236g00020
2
0
−2
−4
Standardized expressions
EI PG G1 G2 H T1 T2 C1 C2 S EP JP
Developmental stages

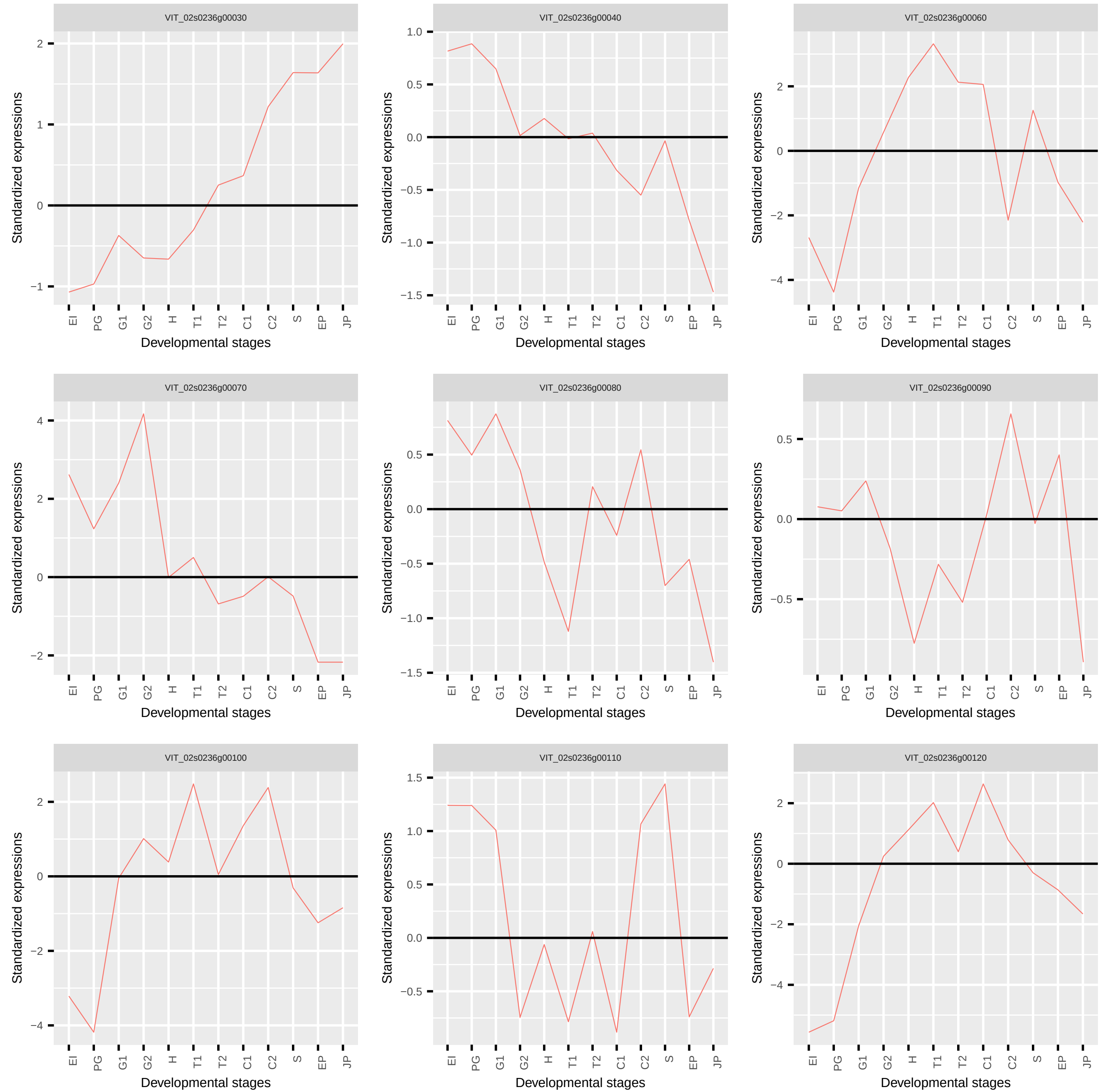
VIT_02s0236g00030
Standardized expressions
Developmental stages
EI PG G1 G2 H T1 T2 C1 C2 S EP JP
VIT_02s0236g00040
Standardized expressions
Developmental stages
EI PG G1 G2 H T1 T2 C1 C2 S EP JP
VIT_02s0236g00060
Standardized expressions
Developmental stages
EI PG G1 G2 H T1 T2 C1 C2 S EP JP
VIT_02s0236g00070
Standardized expressions
Developmental stages
EI PG G1 G2 H T1 T2 C1 C2 S EP JP
VIT_02s0236g00080
Standardized expressions
Developmental stages
EI PG G1 G2 H T1 T2 C1 C2 S EP JP
VIT_02s0236g00090
Standardized expressions
Developmental stages
EI PG G1 G2 H T1 T2 C1 C2 S EP JP
VIT_02s0236g00100
Standardized expressions
Developmental stages
EI PG G1 G2 H T1 T2 C1 C2 S EP JP
VIT_02s0236g00110
Standardized expressions
Developmental stages
EI PG G1 G2 H T1 T2 C1 C2 S EP JP
VIT_02s0236g00120
Standardized expressions
Developmental stages
EI PG G1 G2 H T1 T2 C1 C2 S EP JP

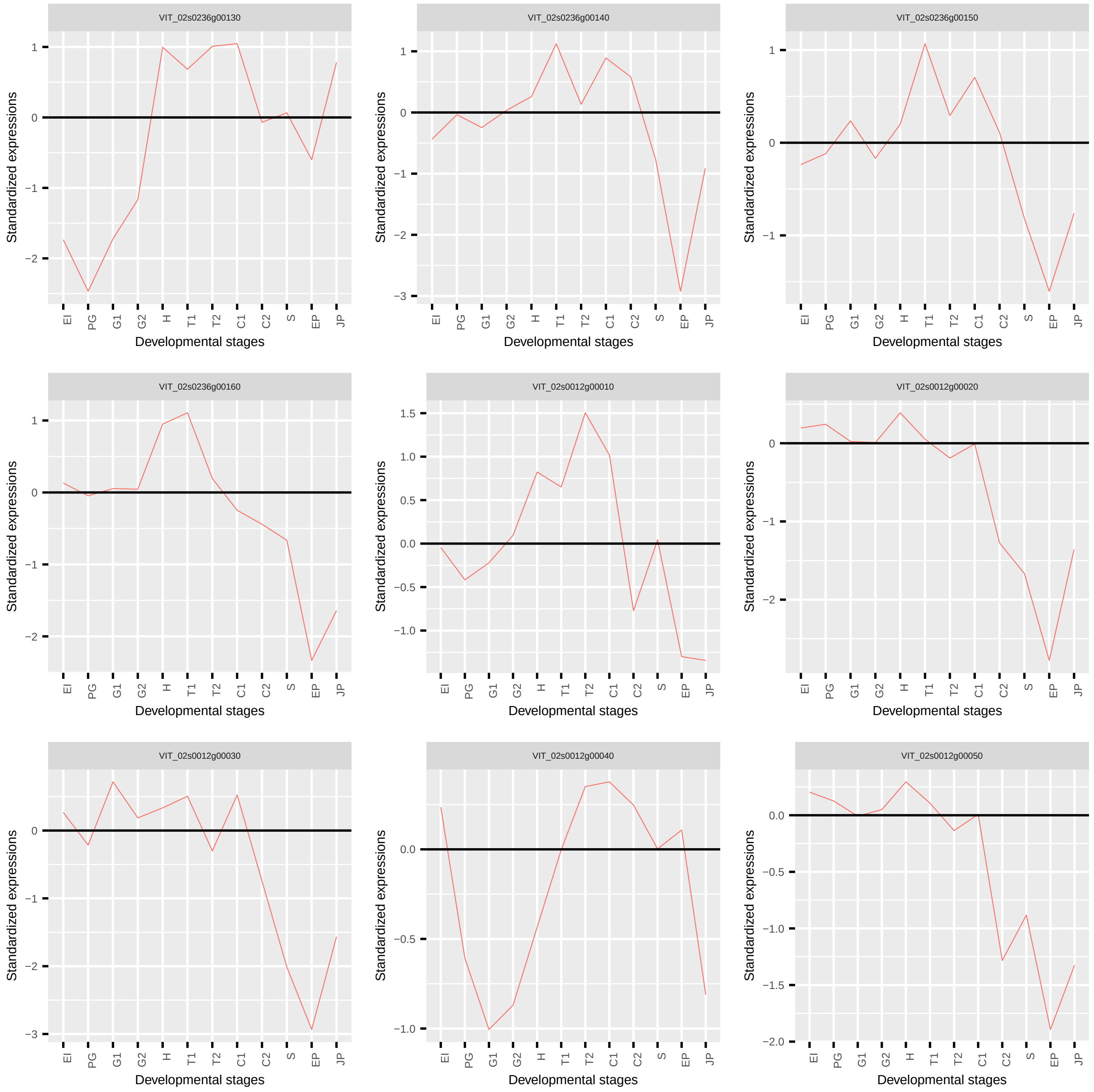
VIT_02s0236g00130
VIT_02s0236g00140
VIT_02s0236g00150
VIT_02s0236g00160
VIT_02s0012g00010
VIT_02s0012g00020
VIT_02s0012g00030
VIT_02s0012g00040
VIT_02s0012g00050
Standardized expressions
Developmental stages
EI PG G1 G2 H T1 T2 C1 C2 S EP JP

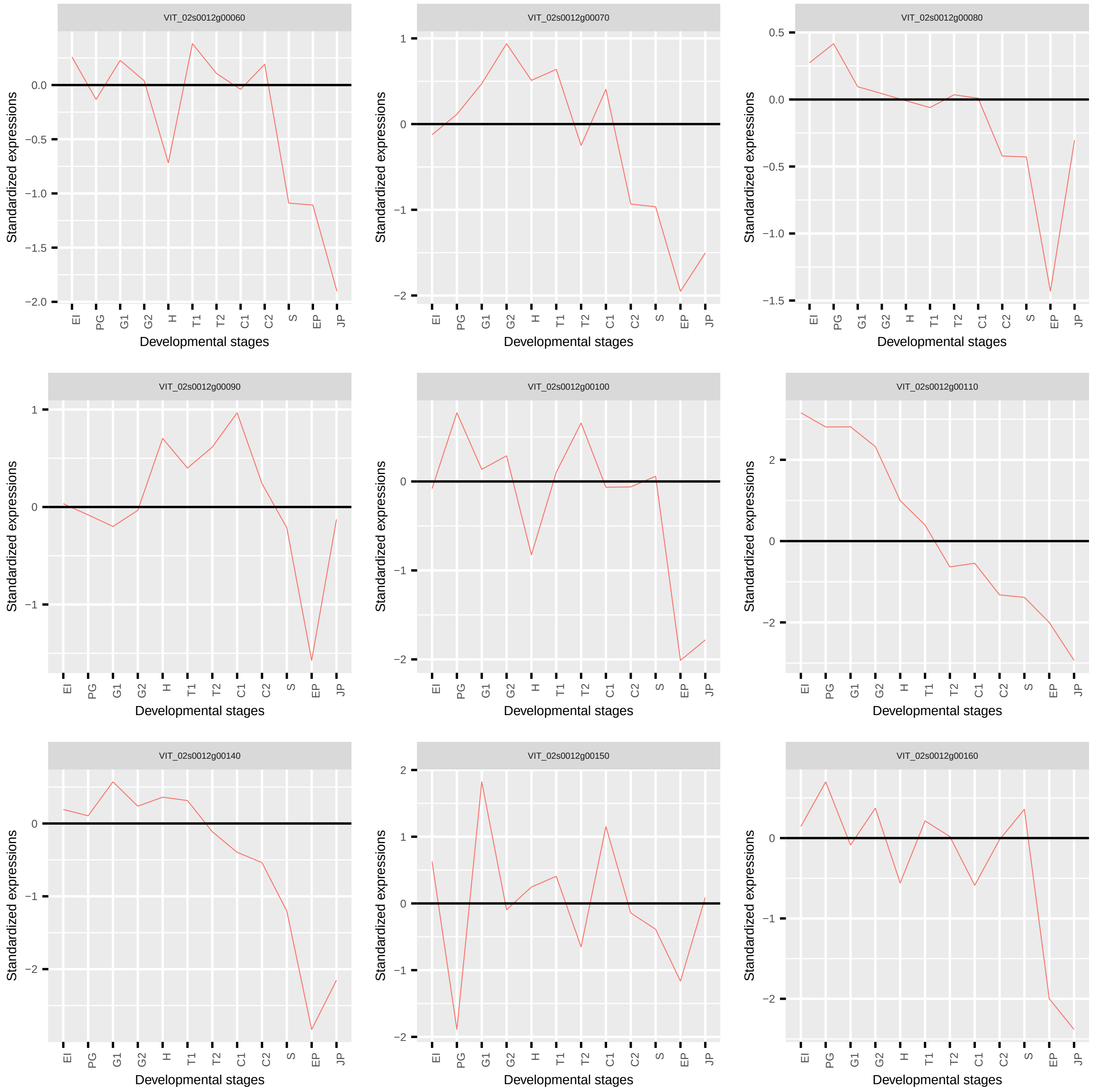
VIT_02s0012g00060
VIT_02s0012g00070
VIT_02s0012g00080
VIT_02s0012g00090
VIT_02s0012g00100
VIT_02s0012g00110
VIT_02s0012g00140
VIT_02s0012g00150
VIT_02s0012g00160
Standardized expressions
Developmental stages
EI PG G1 G2 H T1 T2 C1 C2 S EP JP

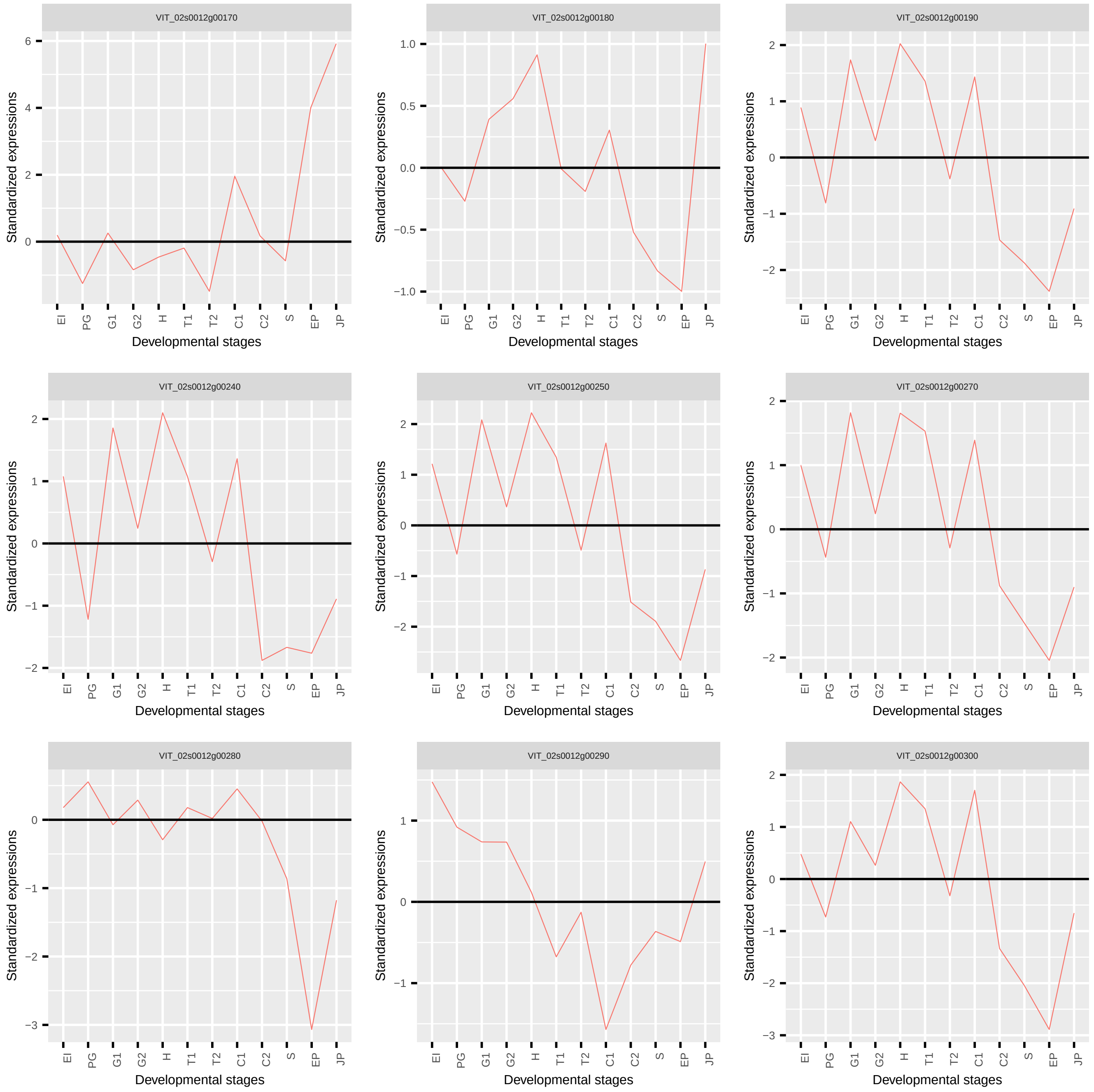
VIT_02s0012g00170
VIT_02s0012g00180
VIT_02s0012g00190
VIT_02s0012g00240
VIT_02s0012g00250
VIT_02s0012g00270
VIT_02s0012g00280
VIT_02s0012g00290
VIT_02s0012g00300
Standardized expressions
Developmental stages
EI
PG
G1
G2
H
T1
T2
C1
C2
S
EP
JP

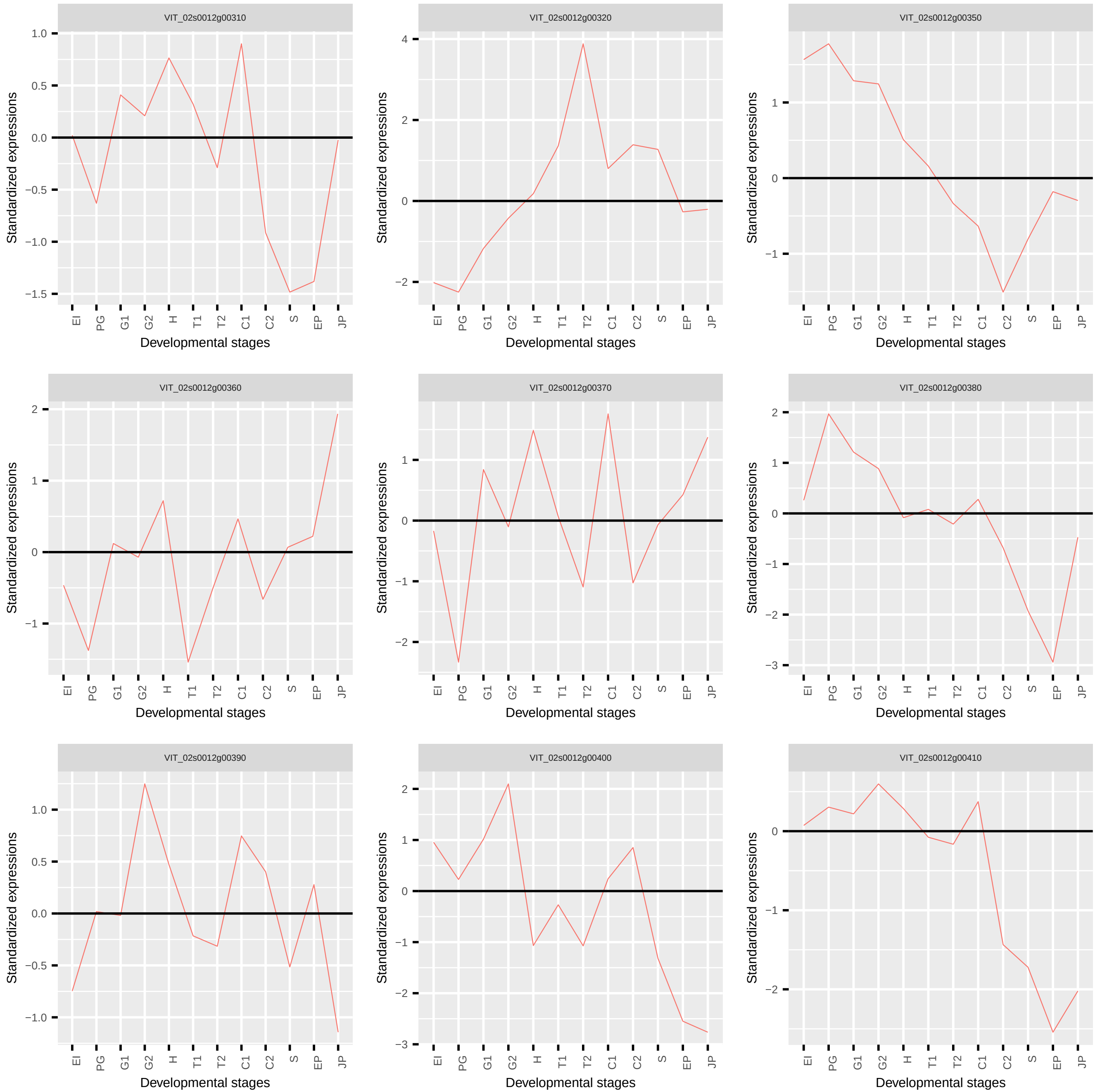
VIT_02s0012g00310
1.0 0.5 0.0 −0.5 −1.0 −1.5
VIT_02s0012g00320
4 2 0 −2
VIT_02s0012g00350
1 0 −1
VIT_02s0012g00360
2 1 0 −1
VIT_02s0012g00370
1 0 −1 −2
VIT_02s0012g00380
2 1 0 −1 −2 −3
VIT_02s0012g00390
1.0 0.5 0.0 −0.5 −1.0
VIT_02s0012g00400
2 1 0 −1 −2 −3
VIT_02s0012g00410
0 −1 −2
Standardized expressions
EI PG G1 G2 H T1 T2 C1 C2 S EP JP
Developmental stages

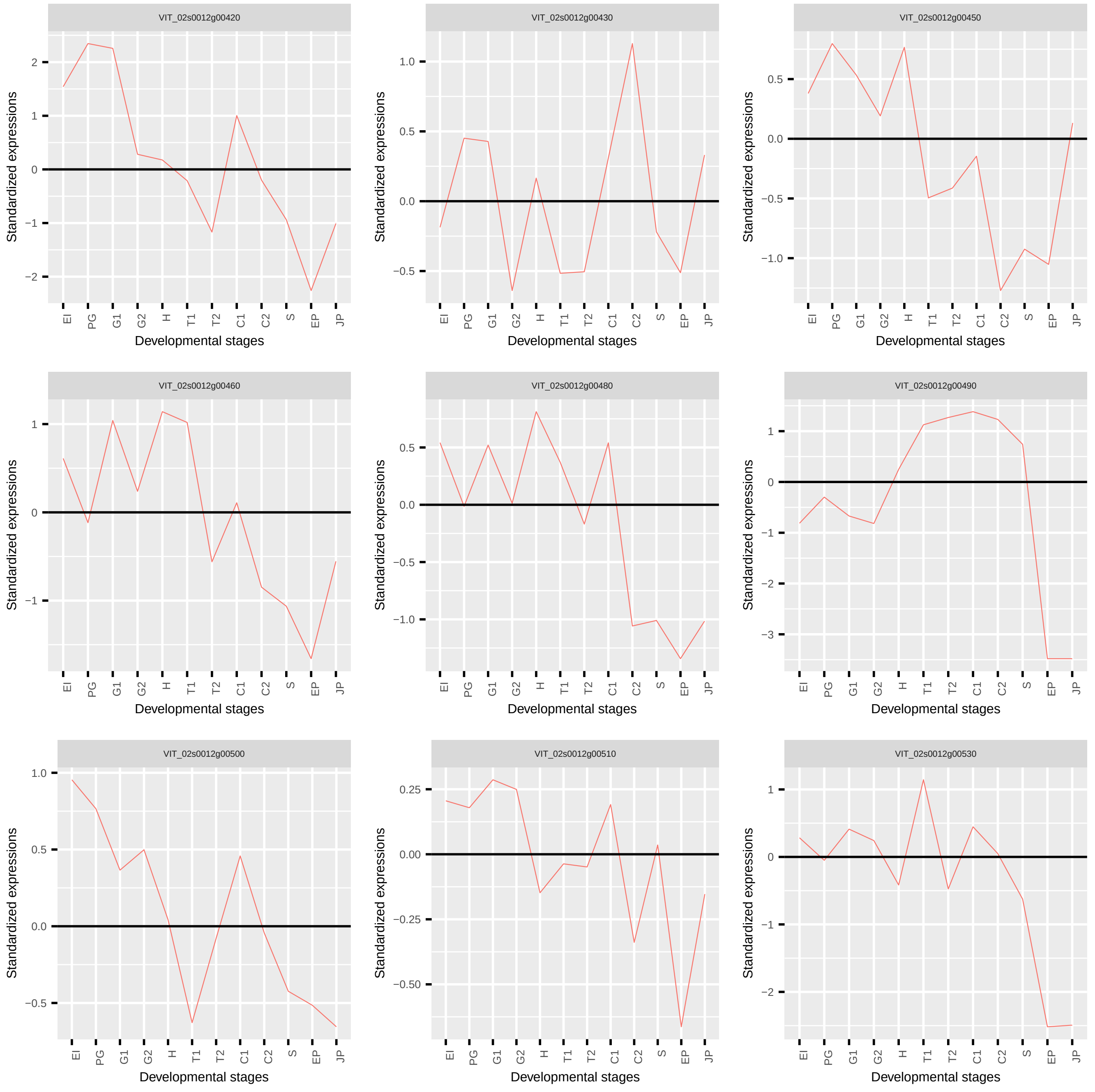
VIT_02s0012g00420
VIT_02s0012g00430
VIT_02s0012g00450
VIT_02s0012g00460
VIT_02s0012g00480
VIT_02s0012g00490
VIT_02s0012g00500
VIT_02s0012g00510
VIT_02s0012g00530
Standardized expressions
Developmental stages
EI PG G1 G2 H T1 T2 C1 C2 S EP JP

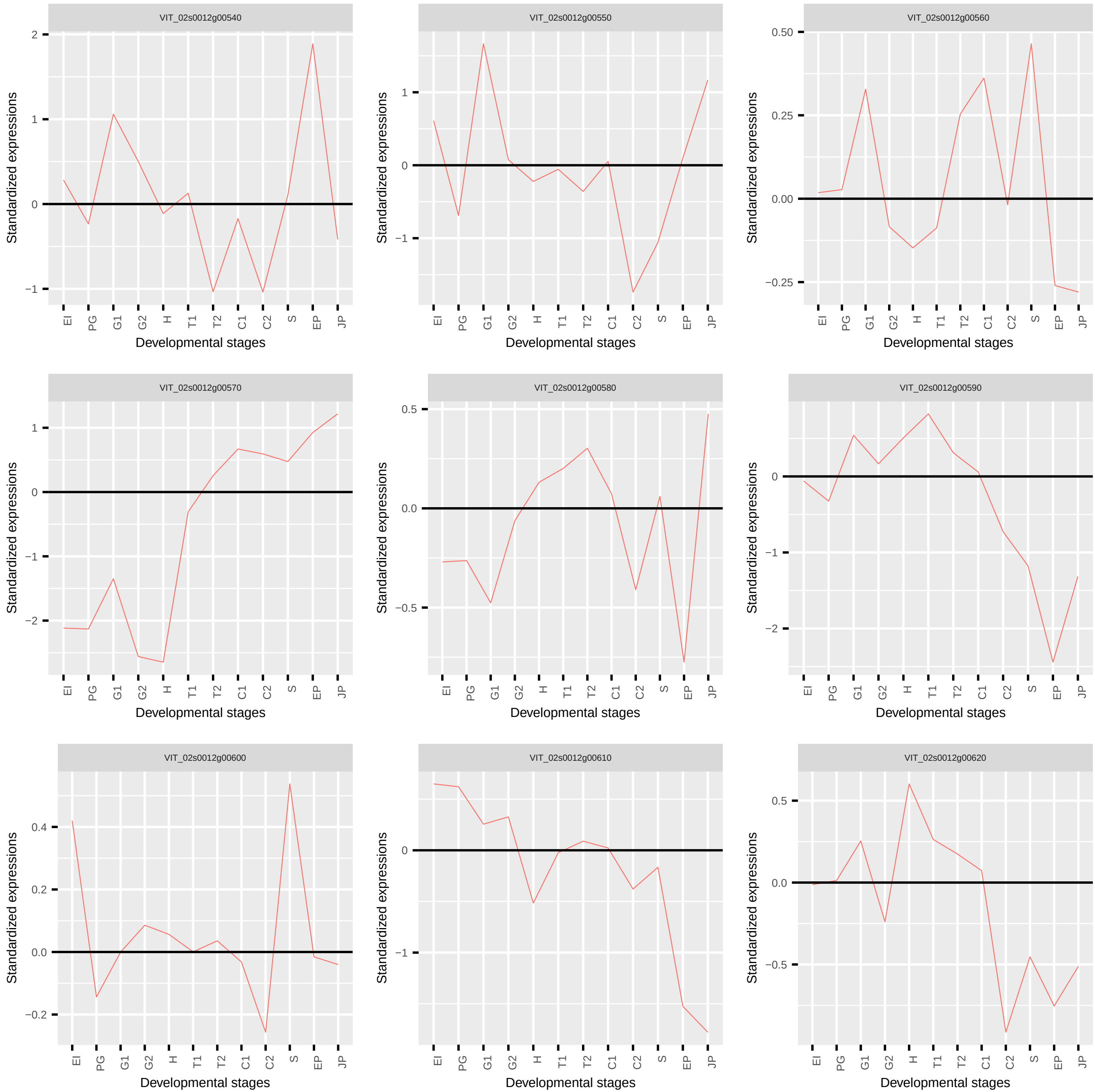

VIT_02s0012g00540
VIT_02s0012g00550
VIT_02s0012g00560
VIT_02s0012g00570
VIT_02s0012g00580
VIT_02s0012g00590
VIT_02s0012g00600
VIT_02s0012g00610
VIT_02s0012g00620
Standardized expressions
Developmental stages
EI PG G1 G2 H T1 T2 C1 C2 S EP JP

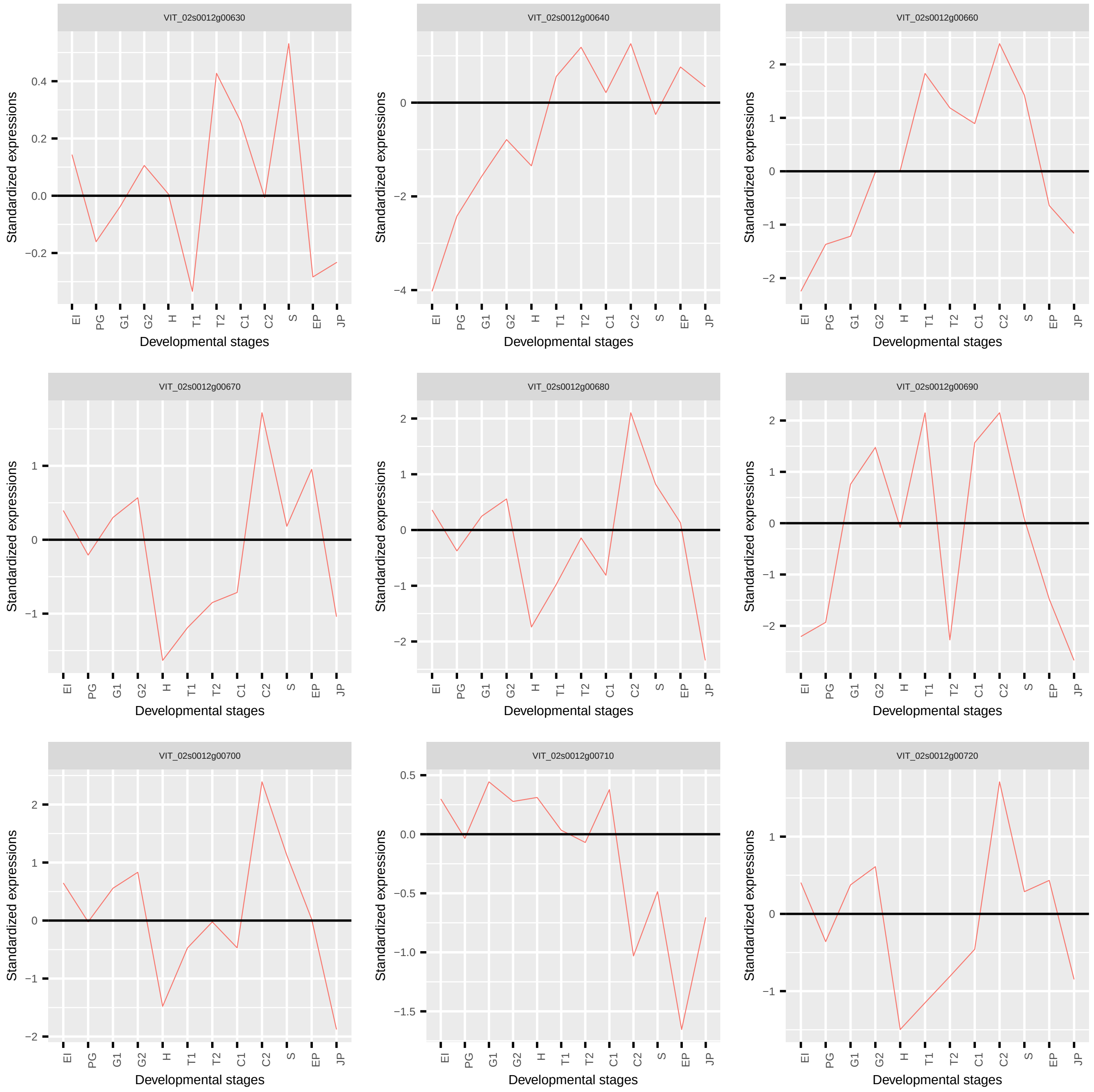
VIT_02s0012g00630
0.4
0.2
0.0
−0.2
Standardized expressions
EI PG G1 G2 H T1 T2 C1 C2 S EP JP
Developmental stages
VIT_02s0012g00640
0
−2
−4
Standardized expressions
EI PG G1 G2 H T1 T2 C1 C2 S EP JP
Developmental stages
VIT_02s0012g00660
2
1
0
−1
−2
Standardized expressions
EI PG G1 G2 H T1 T2 C1 C2 S EP JP
Developmental stages
VIT_02s0012g00670
1
0
−1
Standardized expressions
EI PG G1 G2 H T1 T2 C1 C2 S EP JP
Developmental stages
VIT_02s0012g00680
2
1
0
−1
−2
Standardized expressions
EI PG G1 G2 H T1 T2 C1 C2 S EP JP
Developmental stages
VIT_02s0012g00690
2
1
0
−1
−2
Standardized expressions
EI PG G1 G2 H T1 T2 C1 C2 S EP JP
Developmental stages
VIT_02s0012g00700
2
1
0
−1
−2
Standardized expressions
EI PG G1 G2 H T1 T2 C1 C2 S EP JP
Developmental stages
VIT_02s0012g00710
0.5
0.0
−0.5
−1.0
−1.5
Standardized expressions
EI PG G1 G2 H T1 T2 C1 C2 S EP JP
Developmental stages
VIT_02s0012g00720
1
0
−1
Standardized expressions
EI PG G1 G2 H T1 T2 C1 C2 S EP JP
Developmental stages

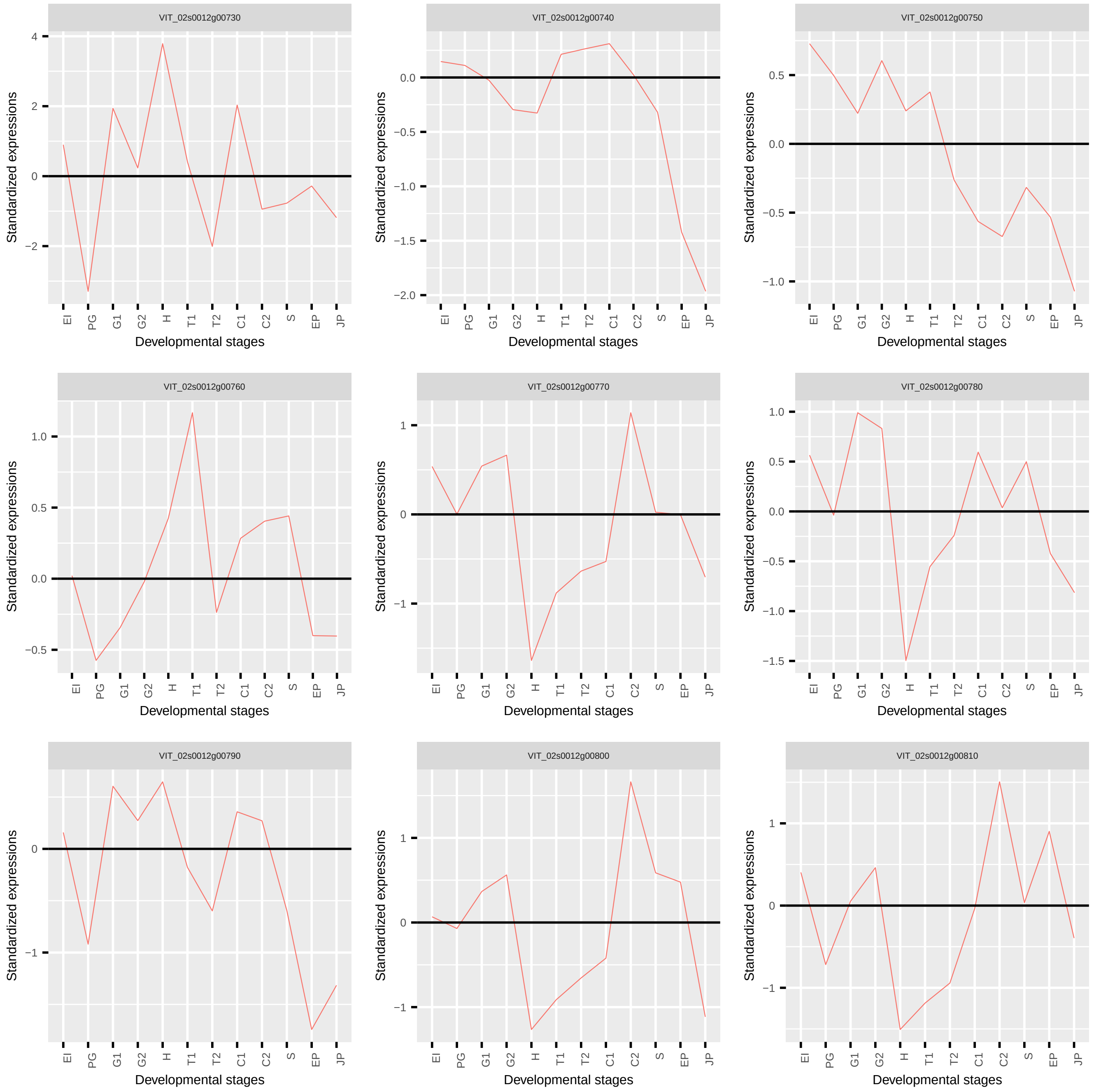
VIT_02s0012g00730
VIT_02s0012g00740
VIT_02s0012g00750
VIT_02s0012g00760
VIT_02s0012g00770
VIT_02s0012g00780
VIT_02s0012g00790
VIT_02s0012g00800
VIT_02s0012g00810
Standardized expressions
Developmental stages
EI PG G1 G2 H T1 T2 C1 C2 S EP JP

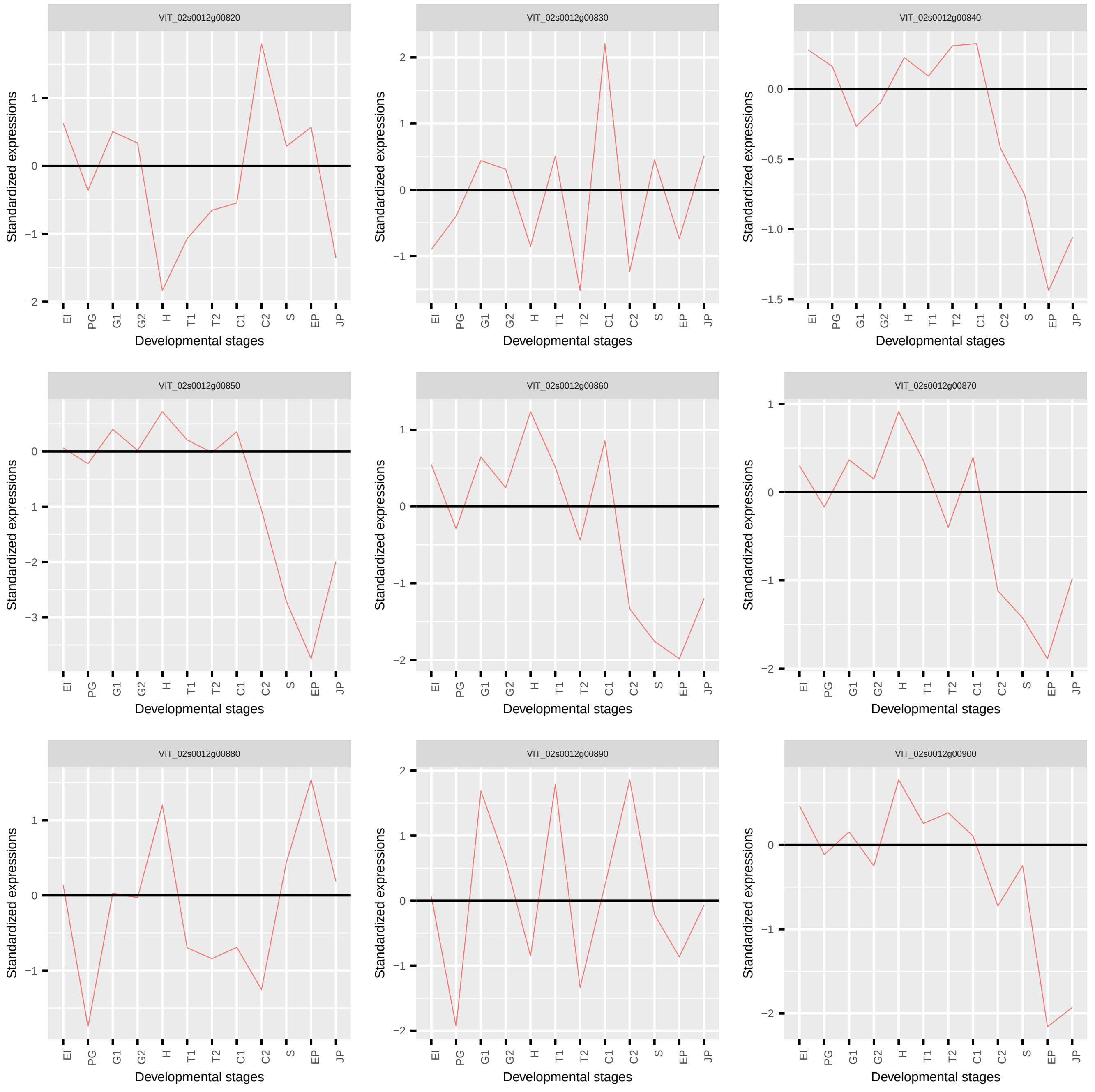
VIT_02s0012g00820
VIT_02s0012g00830
VIT_02s0012g00840
VIT_02s0012g00850
VIT_02s0012g00860
VIT_02s0012g00870
VIT_02s0012g00880
VIT_02s0012g00890
VIT_02s0012g00900
Standardized expressions
Developmental stages
EI
PG
G1
G2
H
T1
T2
C1
C2
S
EP
JP

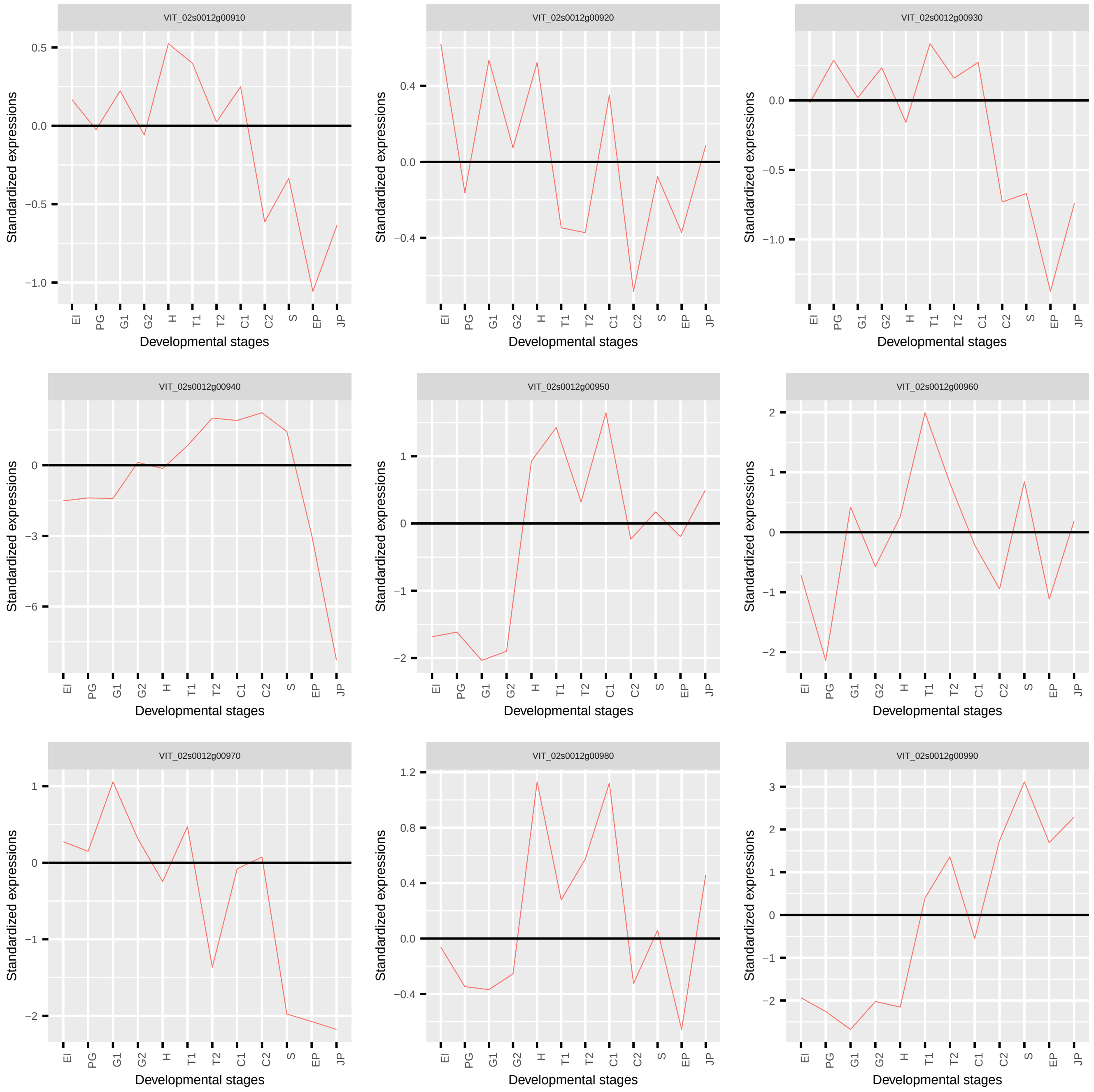
VIT_02s0012g00910
VIT_02s0012g00920
VIT_02s0012g00930
VIT_02s0012g00940
VIT_02s0012g00950
VIT_02s0012g00960
VIT_02s0012g00970
VIT_02s0012g00980
VIT_02s0012g00990
Standardized expressions
Developmental stages
EI
PG
G1
G2
H
T1
T2
C1
C2
S
EP
JP

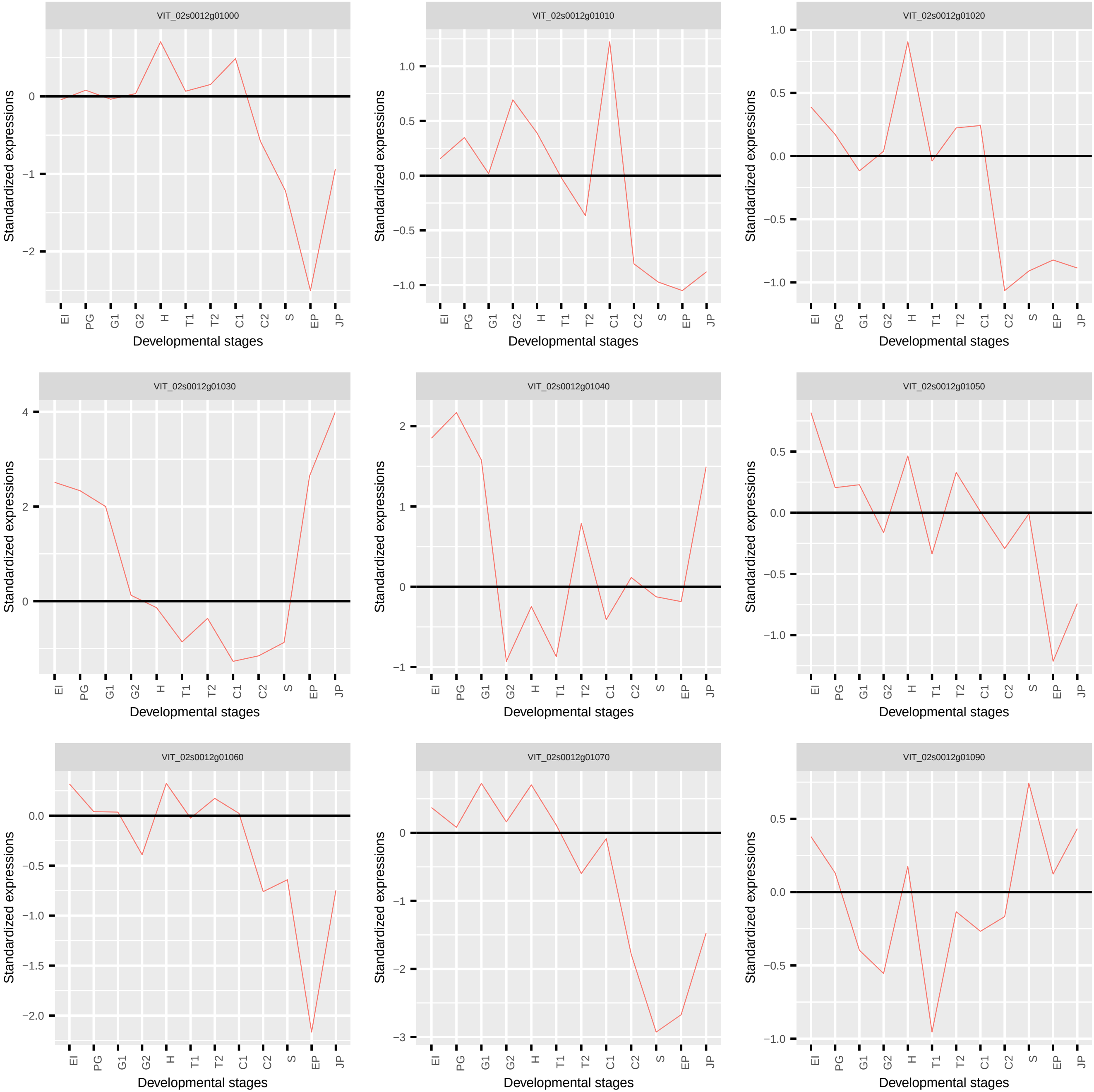
VIT_02s0012g01000
VIT_02s0012g01010
VIT_02s0012g01020
VIT_02s0012g01030
VIT_02s0012g01040
VIT_02s0012g01050
VIT_02s0012g01060
VIT_02s0012g01070
VIT_02s0012g01090
Standardized expressions
Developmental stages
EI PG G1 G2 H T1 T2 C1 C2 S EP JP

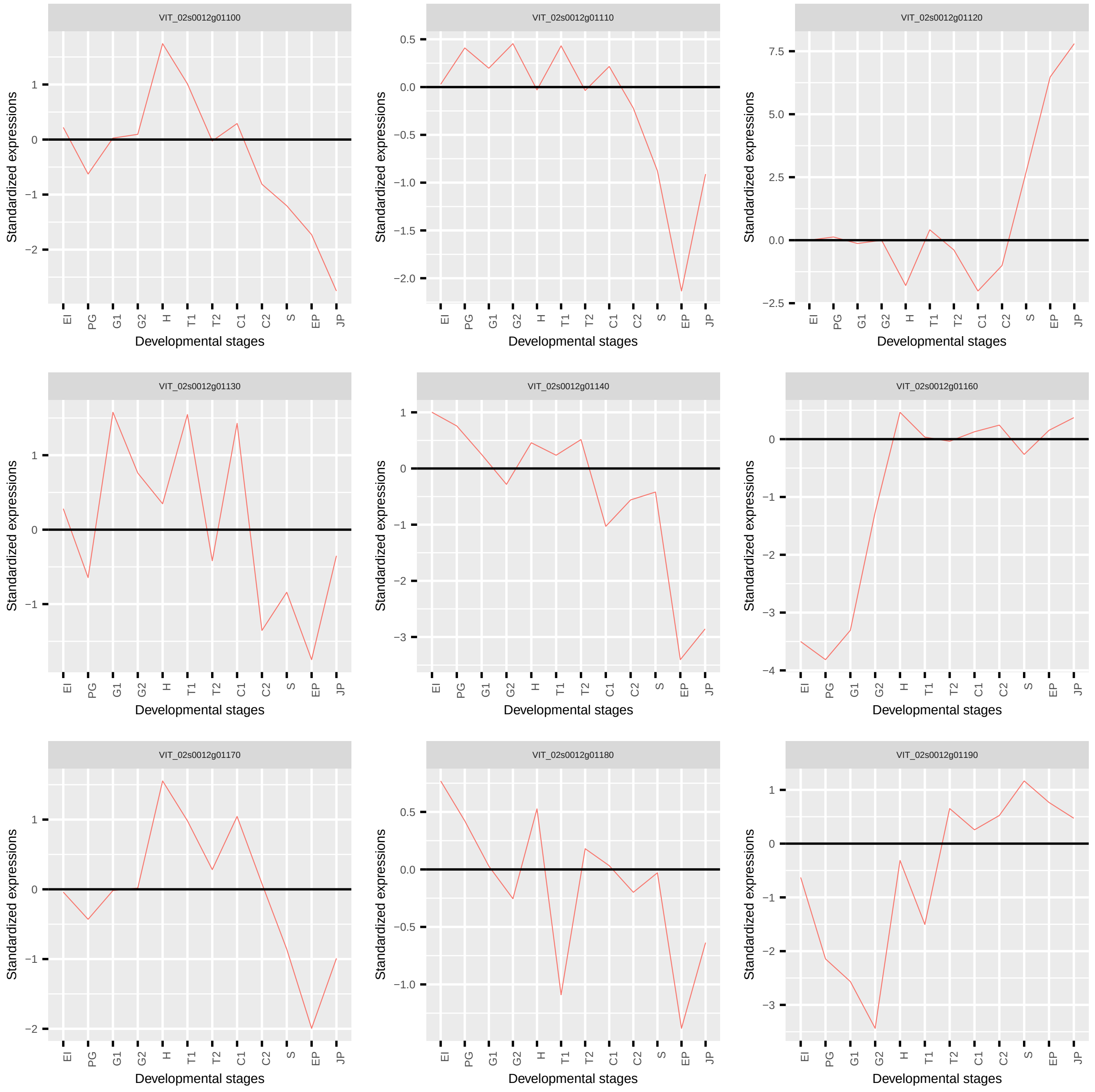
VIT_02s0012g01100
Standardized expressions
1
0
−1
−2
EI PG G1 G2 H T1 T2 C1 C2 S EP JP
Developmental stages
VIT_02s0012g01110
Standardized expressions
0.5
0.0
−0.5
−1.0
−1.5
−2.0
EI PG G1 G2 H T1 T2 C1 C2 S EP JP
Developmental stages
VIT_02s0012g01120
Standardized expressions
7.5
5.0
2.5
0.0
−2.5
EI PG G1 G2 H T1 T2 C1 C2 S EP JP
Developmental stages
VIT_02s0012g01130
Standardized expressions
1
0
−1
EI PG G1 G2 H T1 T2 C1 C2 S EP JP
Developmental stages
VIT_02s0012g01140
Standardized expressions
1
0
−1
−2
−3
EI PG G1 G2 H T1 T2 C1 C2 S EP JP
Developmental stages
VIT_02s0012g01160
Standardized expressions
0
−1
−2
−3
−4
EI PG G1 G2 H T1 T2 C1 C2 S EP JP
Developmental stages
VIT_02s0012g01170
Standardized expressions
1
0
−1
−2
EI PG G1 G2 H T1 T2 C1 C2 S EP JP
Developmental stages
VIT_02s0012g01180
Standardized expressions
0.5
0.0
−0.5
−1.0
EI PG G1 G2 H T1 T2 C1 C2 S EP JP
Developmental stages
VIT_02s0012g01190
Standardized expressions
1
0
−1
−2
−3
EI PG G1 G2 H T1 T2 C1 C2 S EP JP
Developmental stages

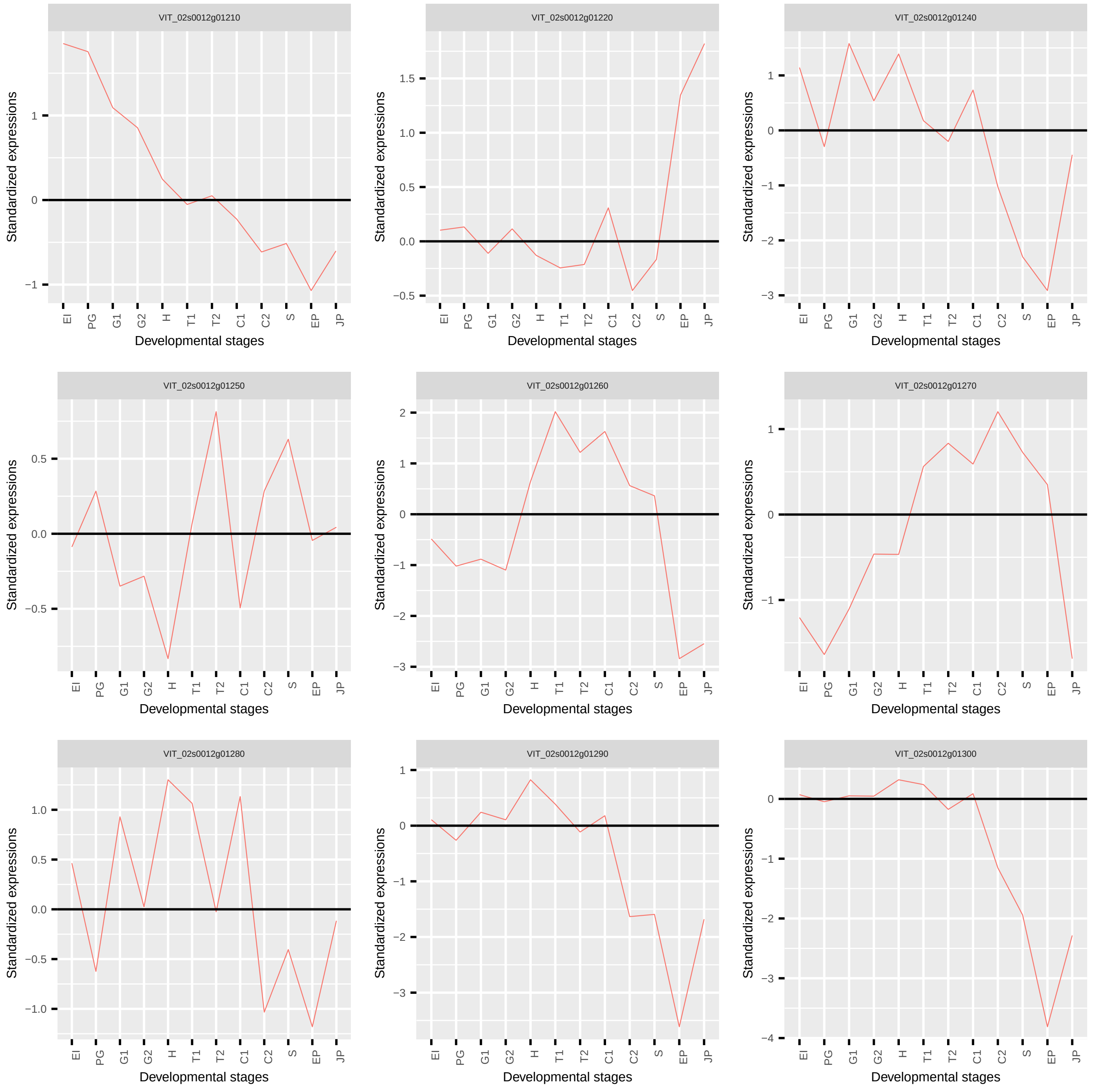
VIT_02s0012g01210
VIT_02s0012g01220
VIT_02s0012g01240
VIT_02s0012g01250
VIT_02s0012g01260
VIT_02s0012g01270
VIT_02s0012g01280
VIT_02s0012g01290
VIT_02s0012g01300
Standardized expressions
Developmental stages
EI PG G1 G2 H T1 T2 C1 C2 S EP JP

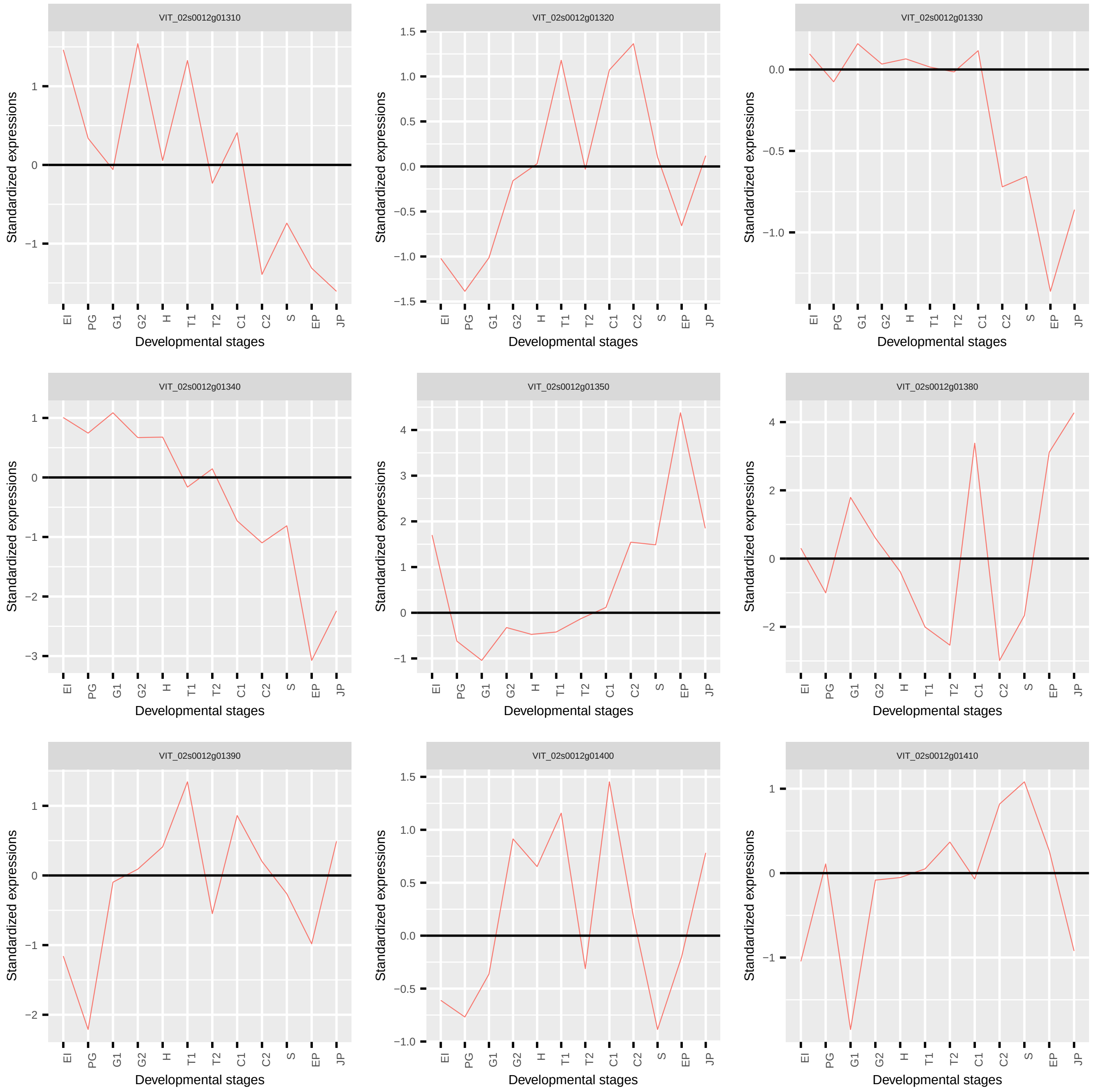
VIT_02s0012g01310
VIT_02s0012g01320
VIT_02s0012g01330
VIT_02s0012g01340
VIT_02s0012g01350
VIT_02s0012g01380
VIT_02s0012g01390
VIT_02s0012g01400
VIT_02s0012g01410
Standardized expressions
Developmental stages
EI PG G1 G2 H T1 T2 C1 C2 S EP JP

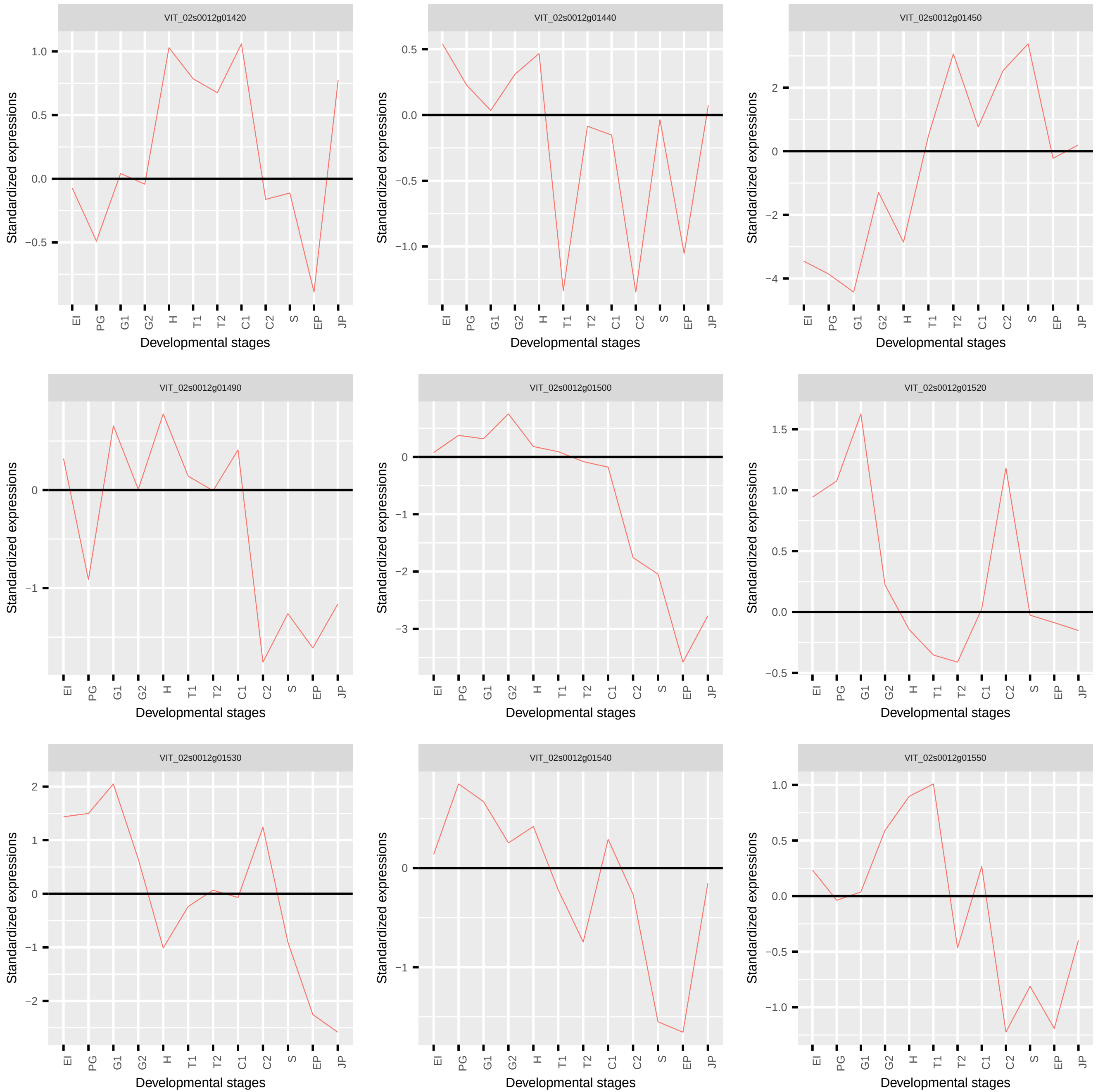
VIT_02s0012g01420
VIT_02s0012g01440
VIT_02s0012g01450
VIT_02s0012g01490
VIT_02s0012g01500
VIT_02s0012g01520
VIT_02s0012g01530
VIT_02s0012g01540
VIT_02s0012g01550
Standardized expressions
Developmental stages
EI PG G1 G2 H T1 T2 C1 C2 S EP JP

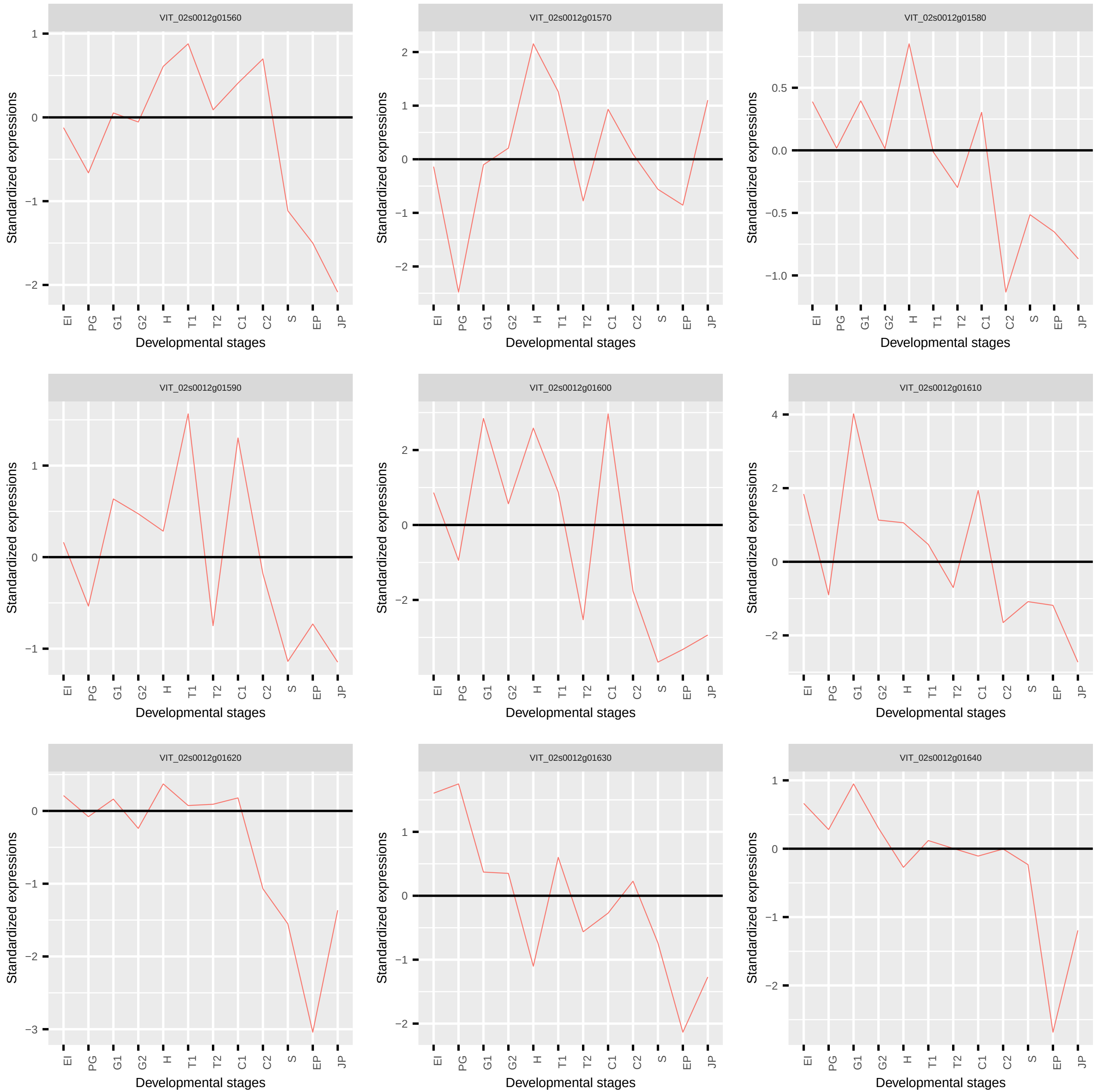
VIT_02s0012g01560
Standardized expressions
1
0
−1
−2
EI PG G1 G2 H T1 T2 C1 C2 S EP JP
Developmental stages
VIT_02s0012g01570
Standardized expressions
2
1
0
−1
−2
EI PG G1 G2 H T1 T2 C1 C2 S EP JP
Developmental stages
VIT_02s0012g01580
Standardized expressions
0.5
0.0
−0.5
−1.0
EI PG G1 G2 H T1 T2 C1 C2 S EP JP
Developmental stages
VIT_02s0012g01590
Standardized expressions
1
0
−1
EI PG G1 G2 H T1 T2 C1 C2 S EP JP
Developmental stages
VIT_02s0012g01600
Standardized expressions
2
0
−2
EI PG G1 G2 H T1 T2 C1 C2 S EP JP
Developmental stages
VIT_02s0012g01610
Standardized expressions
4
2
0
−2
EI PG G1 G2 H T1 T2 C1 C2 S EP JP
Developmental stages
VIT_02s0012g01620
Standardized expressions
0
−1
−2
−3
EI PG G1 G2 H T1 T2 C1 C2 S EP JP
Developmental stages
VIT_02s0012g01630
Standardized expressions
1
0
−1
−2
EI PG G1 G2 H T1 T2 C1 C2 S EP JP
Developmental stages
VIT_02s0012g01640
Standardized expressions
1
0
−1
−2
EI PG G1 G2 H T1 T2 C1 C2 S EP JP
Developmental stages

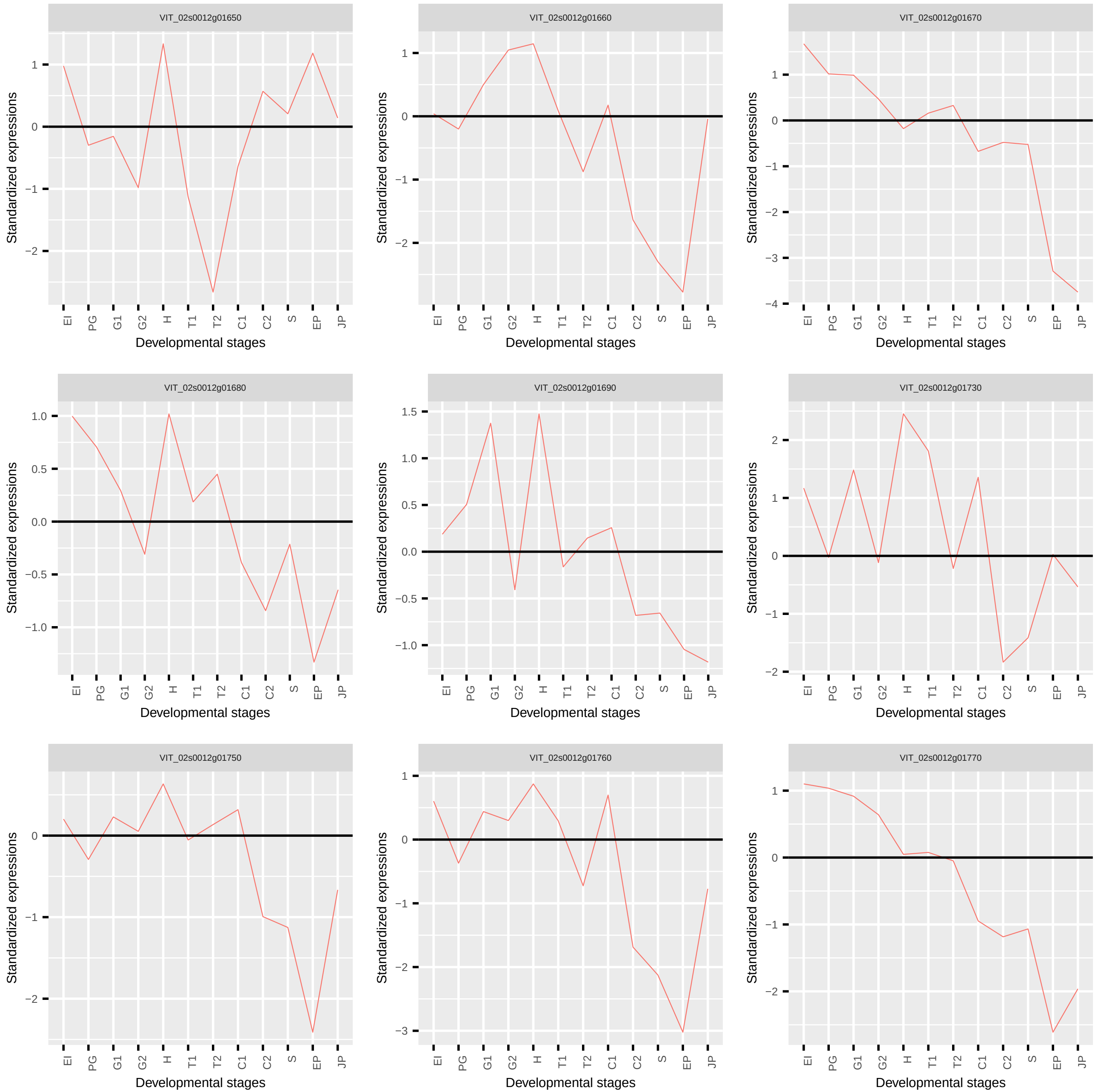

VIT_02s0012g01650
VIT_02s0012g01660
VIT_02s0012g01670
VIT_02s0012g01680
VIT_02s0012g01690
VIT_02s0012g01730
VIT_02s0012g01750
VIT_02s0012g01760
VIT_02s0012g01770
Standardized expressions
Developmental stages
EI PG G1 G2 H T1 T2 C1 C2 S EP JP

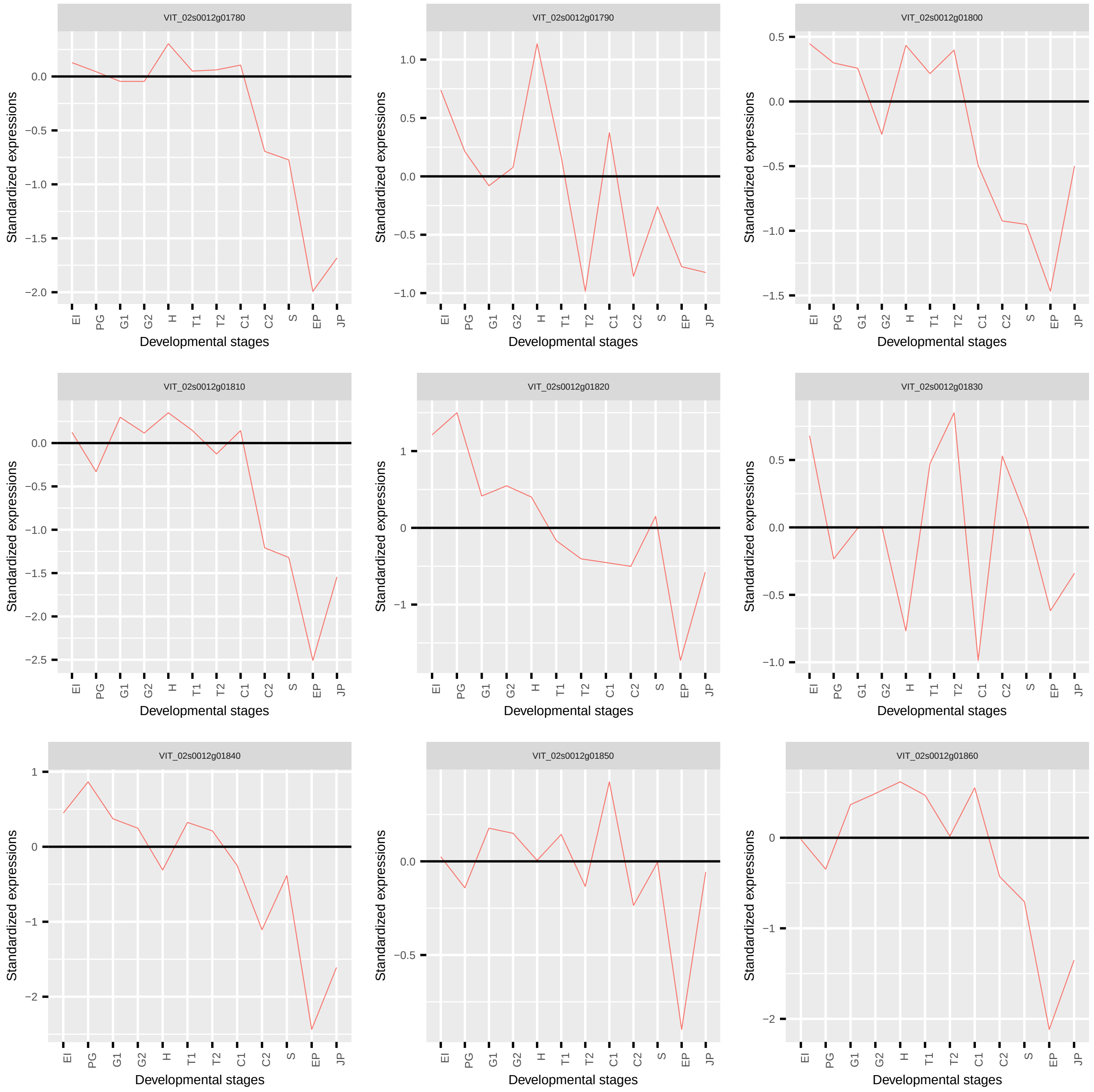
VIT_02s0012g01780
VIT_02s0012g01790
VIT_02s0012g01800
VIT_02s0012g01810
VIT_02s0012g01820
VIT_02s0012g01830
VIT_02s0012g01840
VIT_02s0012g01850
VIT_02s0012g01860
Standardized expressions
Developmental stages
EI PG G1 G2 H T1 T2 C1 C2 S EP JP

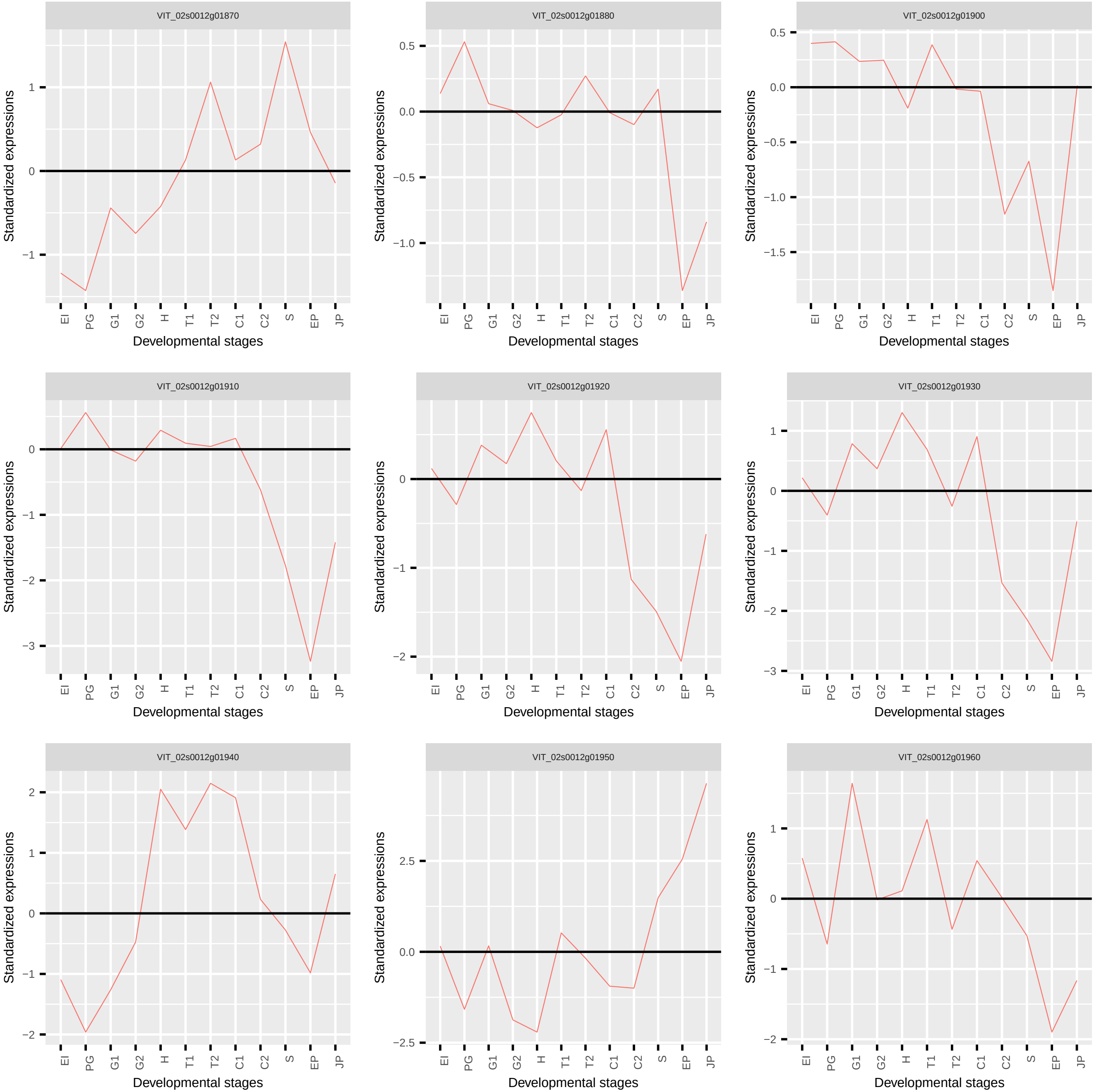
VIT_02s0012g01870
VIT_02s0012g01880
VIT_02s0012g01900
VIT_02s0012g01910
VIT_02s0012g01920
VIT_02s0012g01930
VIT_02s0012g01940
VIT_02s0012g01950
VIT_02s0012g01960
Standardized expressions
Developmental stages
EI PG G1 G2 H T1 T2 C1 C2 S EP JP

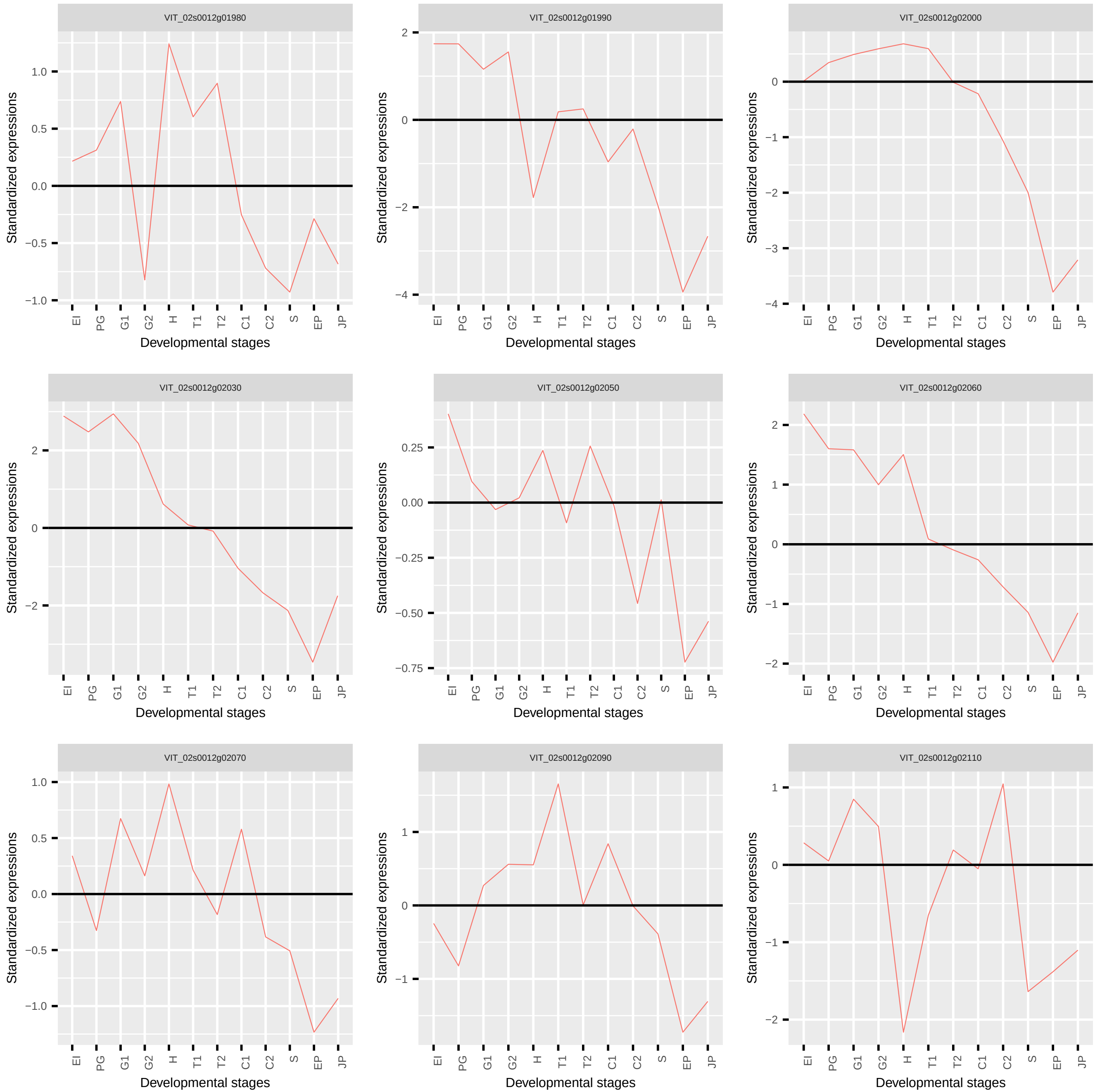
VIT_02s0012g01980
VIT_02s0012g01990
VIT_02s0012g02000
VIT_02s0012g02030
VIT_02s0012g02050
VIT_02s0012g02060
VIT_02s0012g02070
VIT_02s0012g02090
VIT_02s0012g02110
Standardized expressions
Developmental stages
EI PG G1 G2 H T1 T2 C1 C2 S EP JP

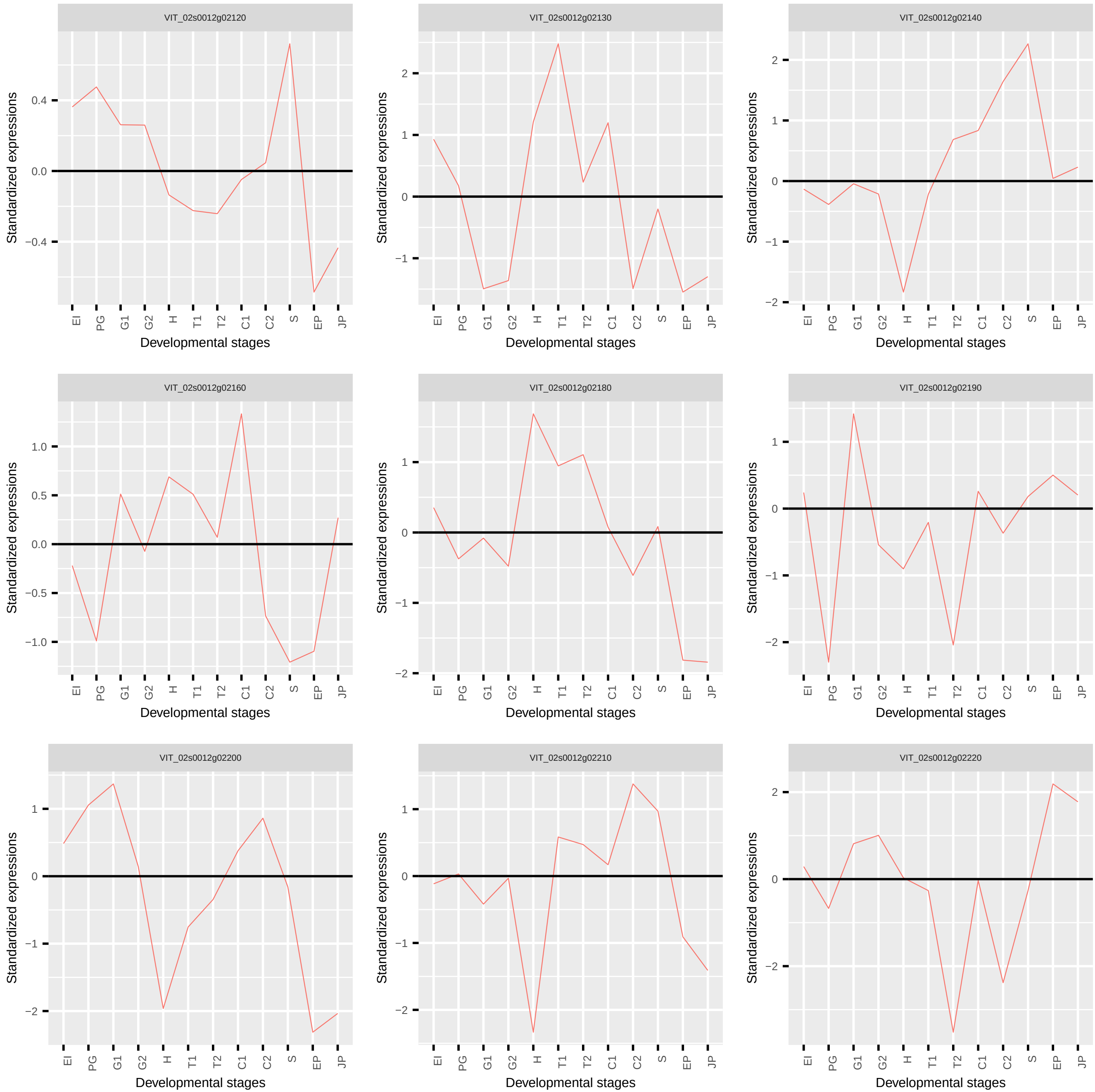
VIT_02s0012g02120
Standardized expressions
0.4
0.0
−0.4
EI PG G1 G2 H T1 T2 C1 C2 S EP JP
Developmental stages
VIT_02s0012g02130
Standardized expressions
2
1
0
−1
EI PG G1 G2 H T1 T2 C1 C2 S EP JP
Developmental stages
VIT_02s0012g02140
Standardized expressions
2
1
0
−1
−2
EI PG G1 G2 H T1 T2 C1 C2 S EP JP
Developmental stages
VIT_02s0012g02160
Standardized expressions
1.0
0.5
0.0
−0.5
−1.0
EI PG G1 G2 H T1 T2 C1 C2 S EP JP
Developmental stages
VIT_02s0012g02180
Standardized expressions
1
0
−1
−2
EI PG G1 G2 H T1 T2 C1 C2 S EP JP
Developmental stages
VIT_02s0012g02190
Standardized expressions
1
0
−1
−2
EI PG G1 G2 H T1 T2 C1 C2 S EP JP
Developmental stages
VIT_02s0012g02200
Standardized expressions
1
0
−1
−2
EI PG G1 G2 H T1 T2 C1 C2 S EP JP
Developmental stages
VIT_02s0012g02210
Standardized expressions
1
0
−1
−2
EI PG G1 G2 H T1 T2 C1 C2 S EP JP
Developmental stages
VIT_02s0012g02220
Standardized expressions
2
0
−2
EI PG G1 G2 H T1 T2 C1 C2 S EP JP
Developmental stages

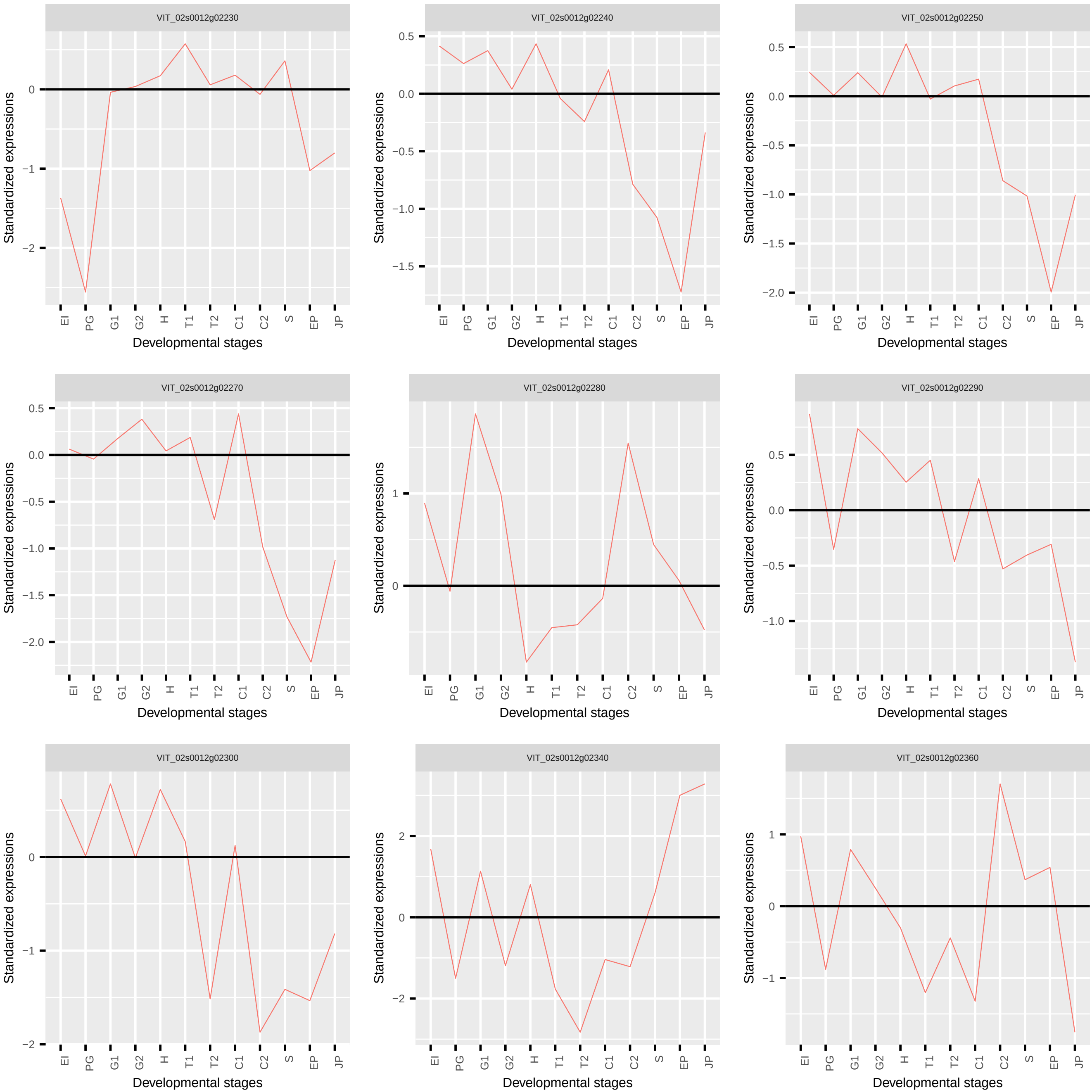
VIT_02s0012g02230
VIT_02s0012g02240
VIT_02s0012g02250
VIT_02s0012g02270
VIT_02s0012g02280
VIT_02s0012g02290
VIT_02s0012g02300
VIT_02s0012g02340
VIT_02s0012g02360
Standardized expressions
Developmental stages
EI PG G1 G2 H T1 T2 C1 C2 S EP JP

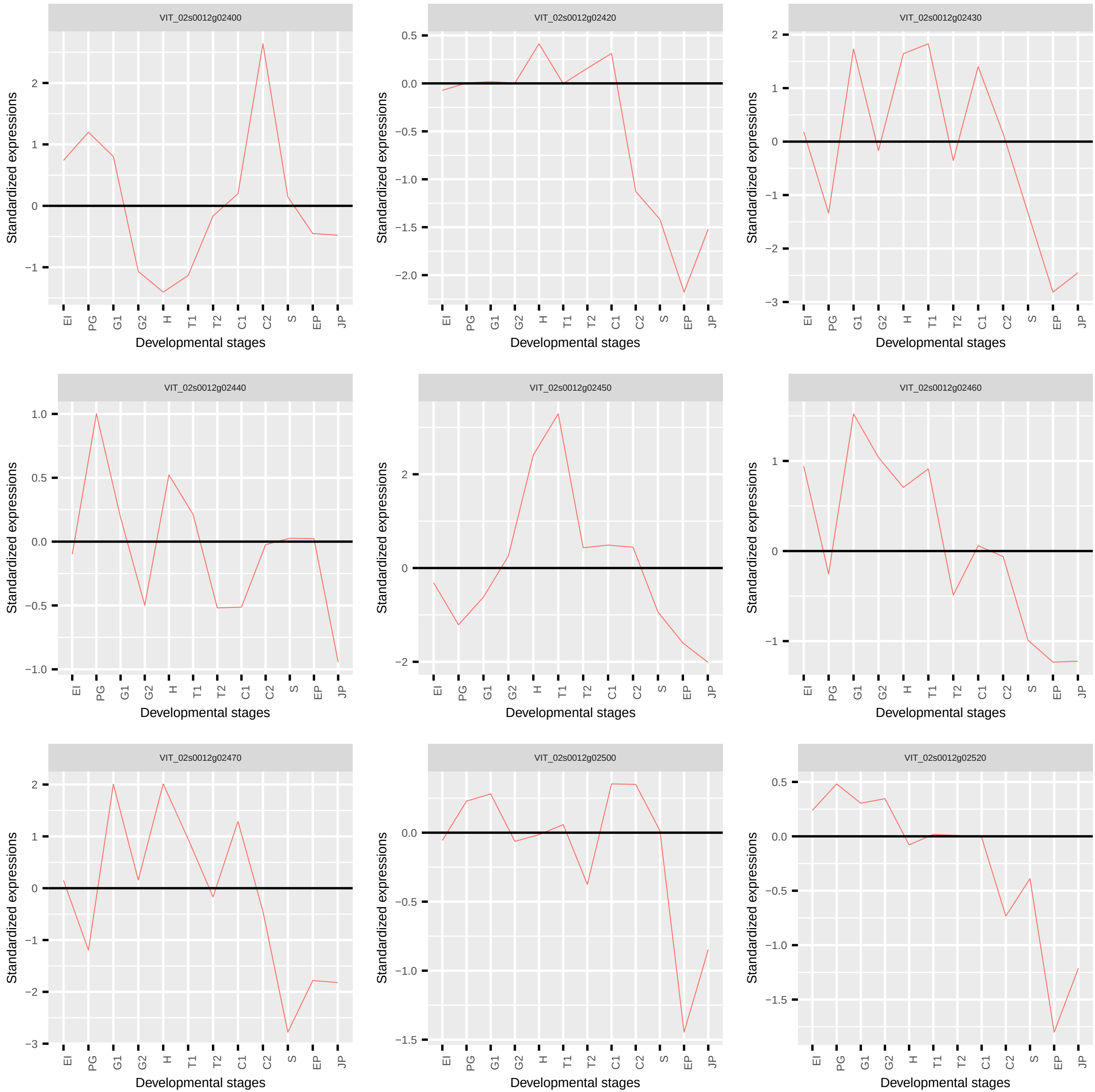
VIT_02s0012g02400
VIT_02s0012g02420
VIT_02s0012g02430
VIT_02s0012g02440
VIT_02s0012g02450
VIT_02s0012g02460
VIT_02s0012g02470
VIT_02s0012g02500
VIT_02s0012g02520
Standardized expressions
Developmental stages
EI
PG
G1
G2
H
T1
T2
C1
C2
S
EP
JP

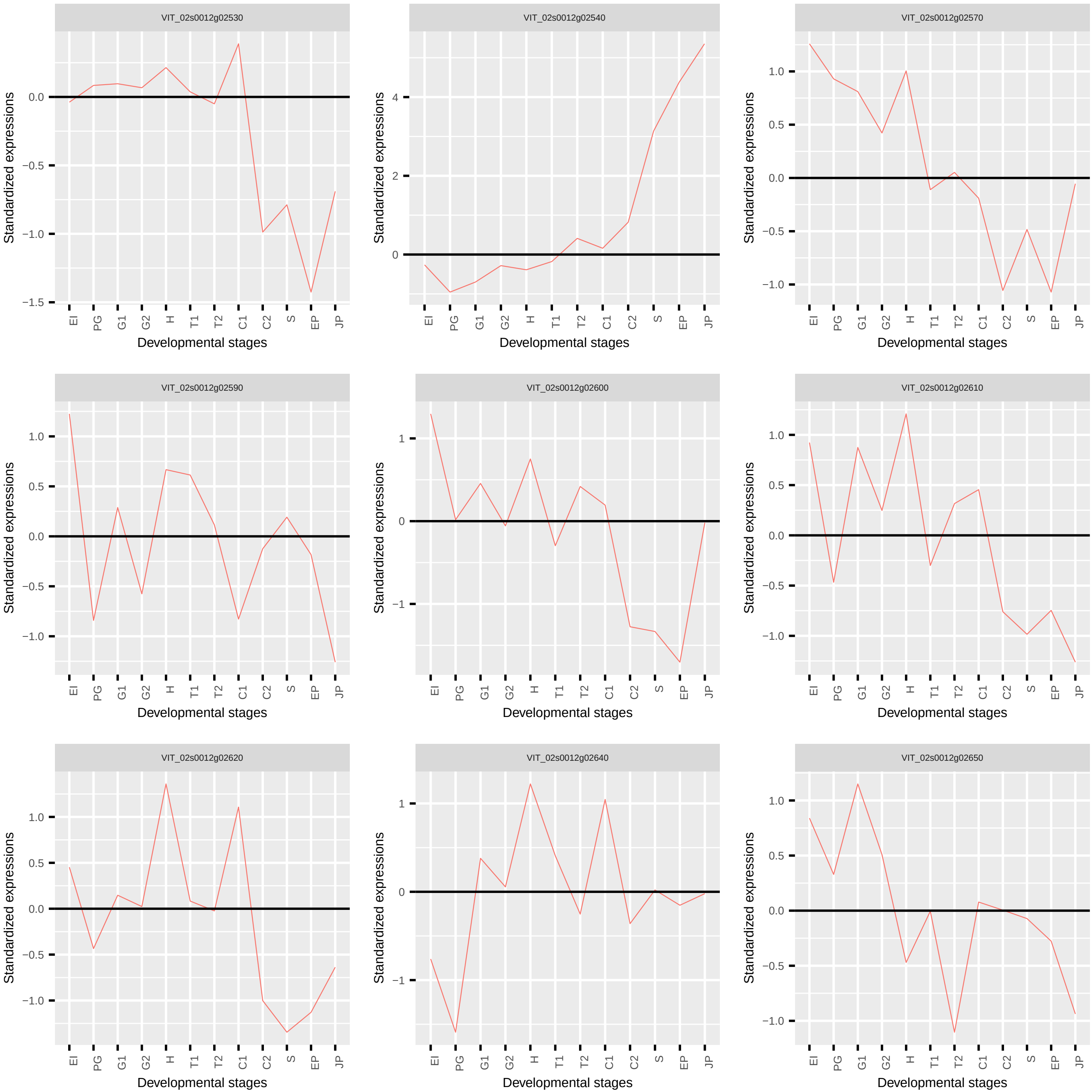
VIT_02s0012g02530
VIT_02s0012g02540
VIT_02s0012g02570
VIT_02s0012g02590
VIT_02s0012g02600
VIT_02s0012g02610
VIT_02s0012g02620
VIT_02s0012g02640
VIT_02s0012g02650
Standardized expressions
Developmental stages
EI PG G1 G2 H T1 T2 C1 C2 S EP JP

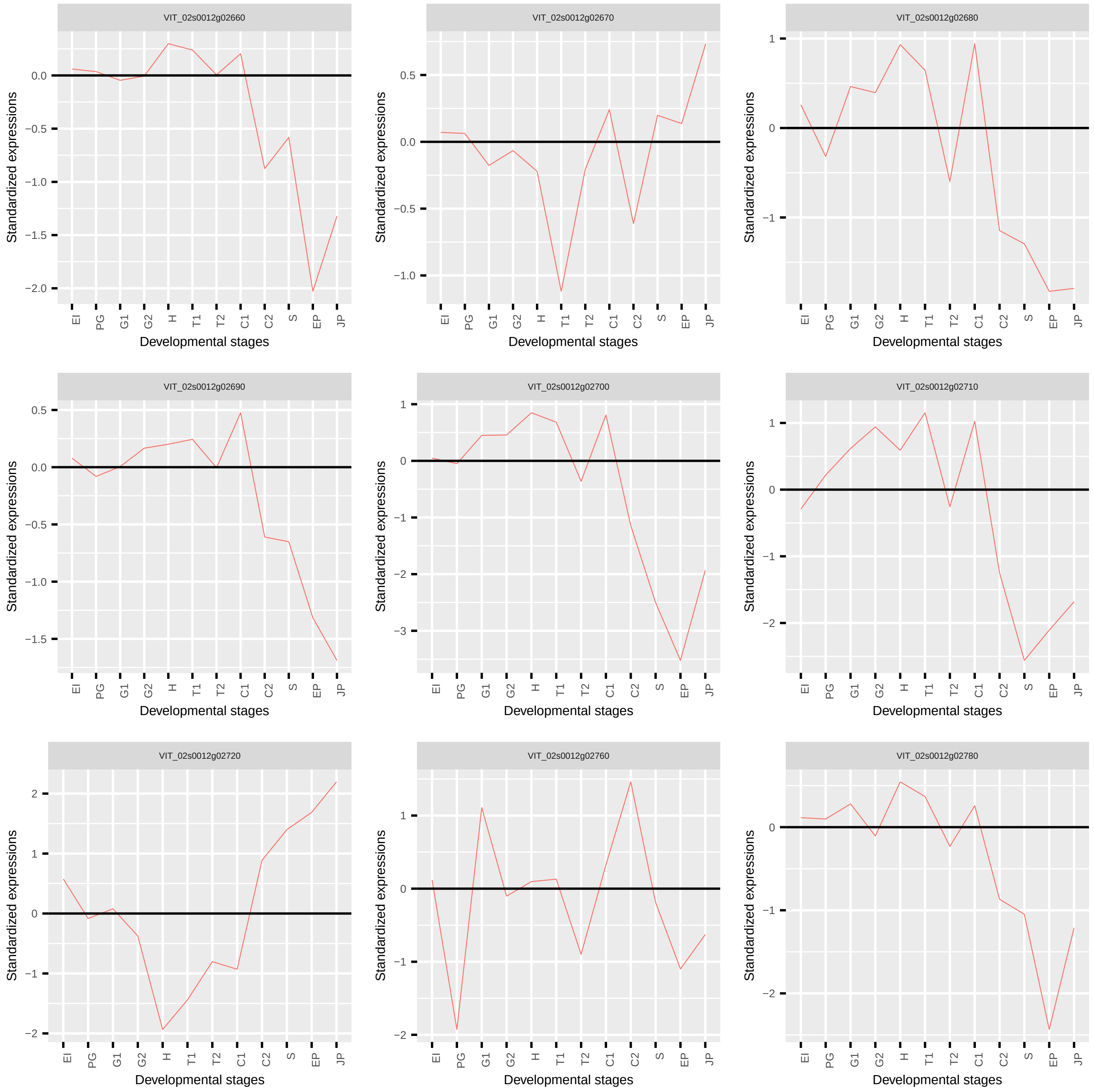
VIT_02s0012g02660
VIT_02s0012g02670
VIT_02s0012g02680
VIT_02s0012g02690
VIT_02s0012g02700
VIT_02s0012g02710
VIT_02s0012g02720
VIT_02s0012g02760
VIT_02s0012g02780
Standardized expressions
Developmental stages
EI PG G1 G2 H T1 T2 C1 C2 S EP JP

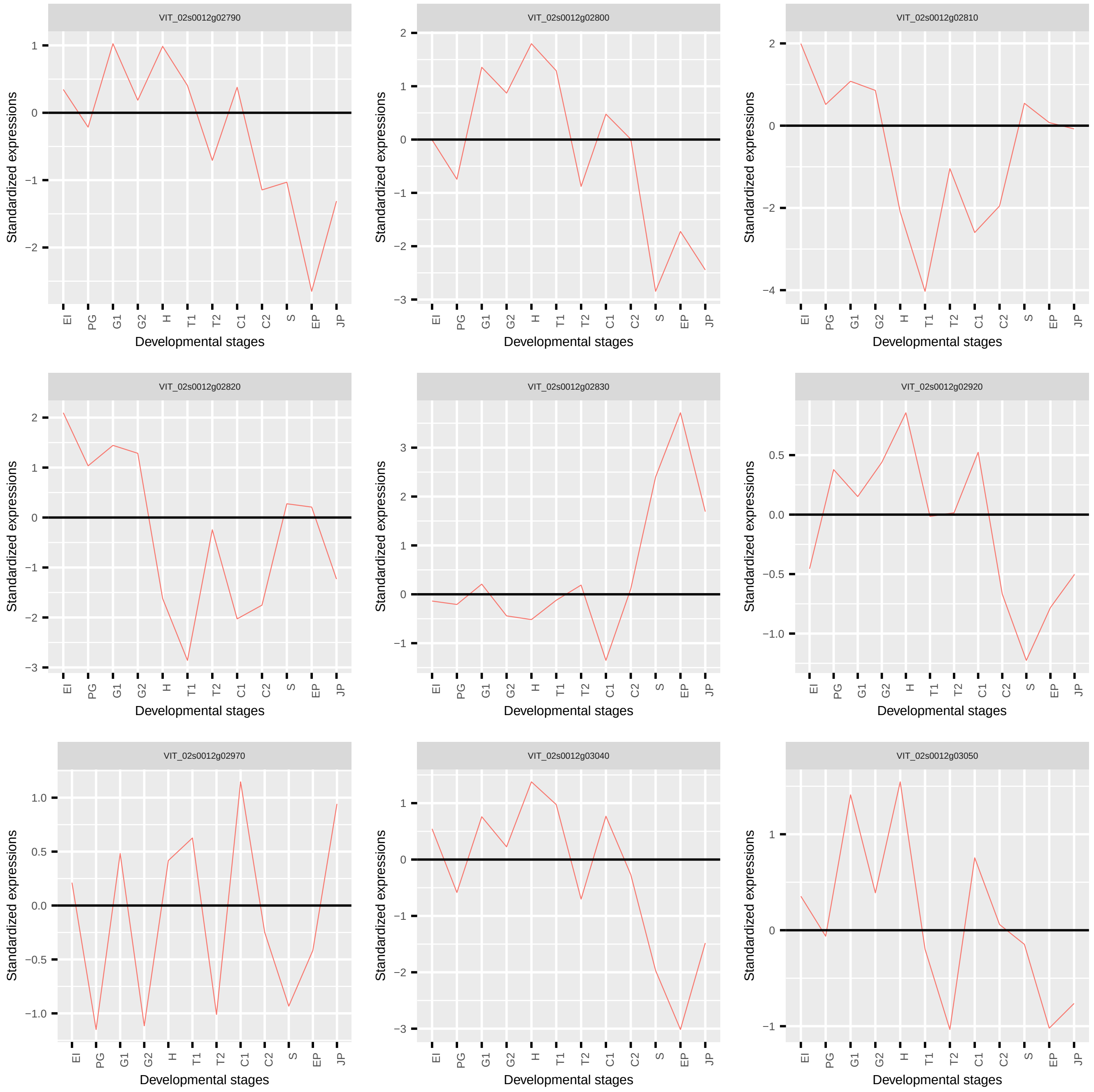
VIT_02s0012g02790
VIT_02s0012g02800
VIT_02s0012g02810
VIT_02s0012g02820
VIT_02s0012g02830
VIT_02s0012g02920
VIT_02s0012g02970
VIT_02s0012g03040
VIT_02s0012g03050
Standardized expressions
Developmental stages
EI PG G1 G2 H T1 T2 C1 C2 S EP JP

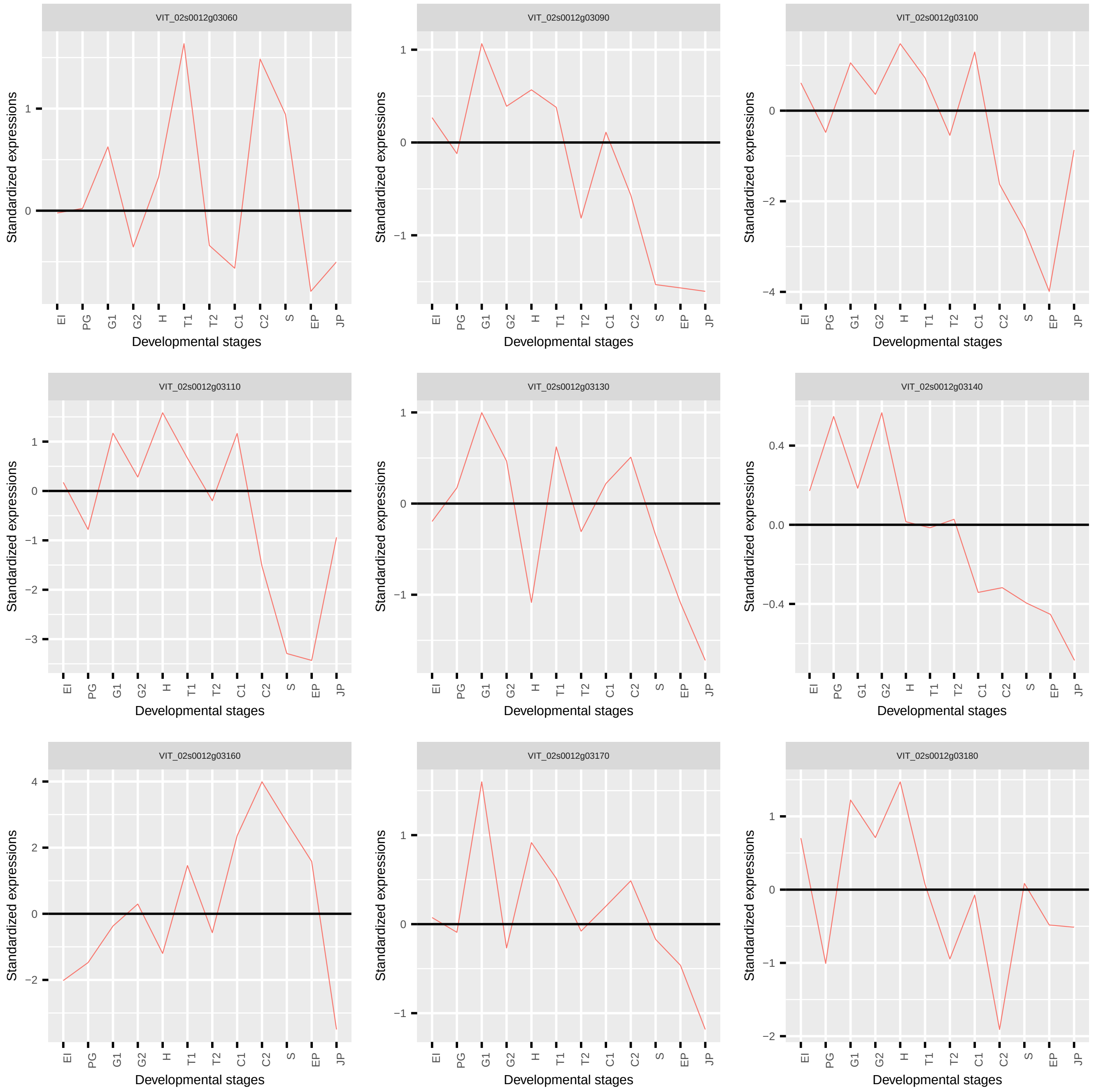
VIT_02s0012g03060
VIT_02s0012g03090
VIT_02s0012g03100
VIT_02s0012g03110
VIT_02s0012g03130
VIT_02s0012g03140
VIT_02s0012g03160
VIT_02s0012g03170
VIT_02s0012g03180
Standardized expressions
Developmental stages
EI PG G1 G2 H T1 T2 C1 C2 S EP JP

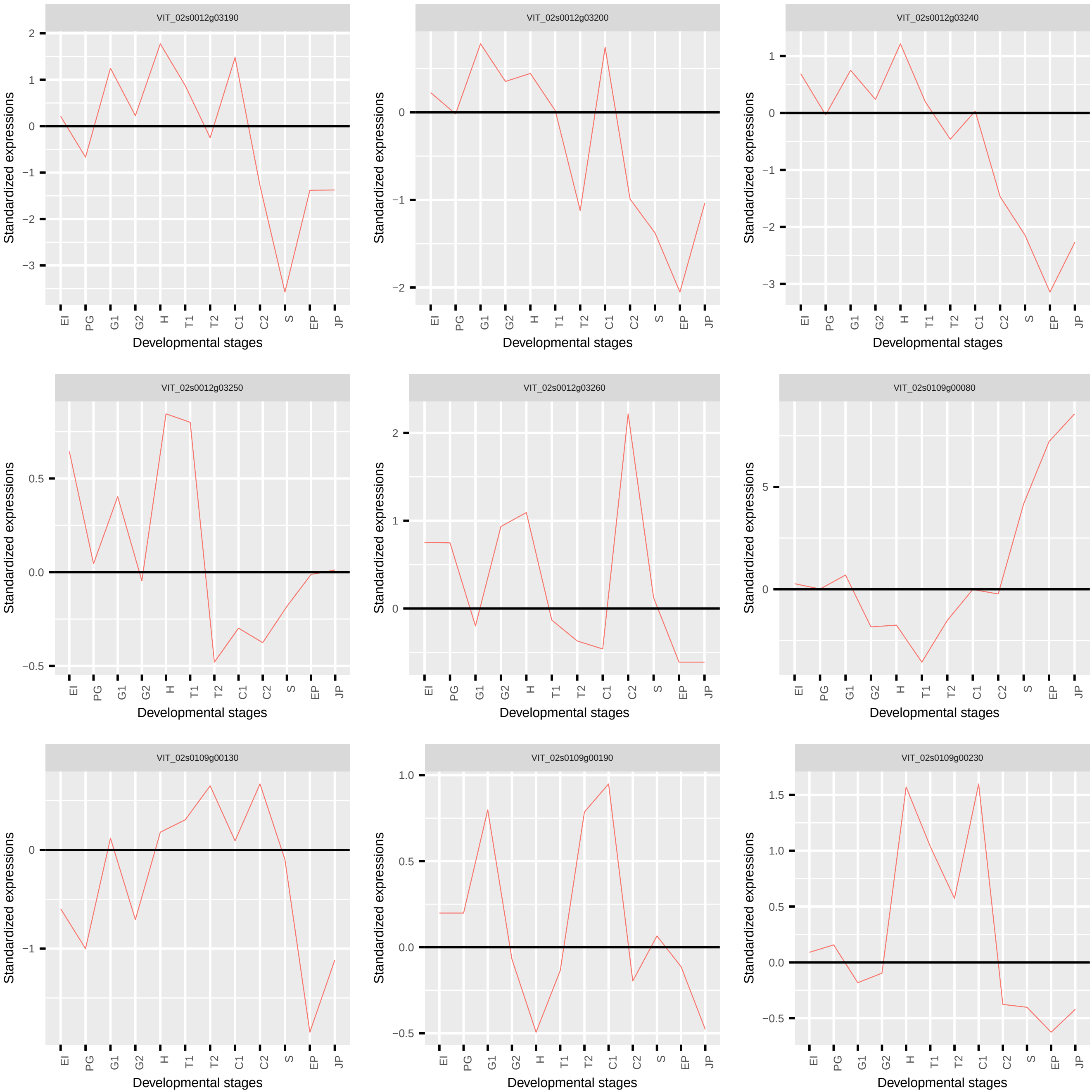
VIT_02s0012g03190
VIT_02s0012g03200
VIT_02s0012g03240
VIT_02s0012g03250
VIT_02s0012g03260
VIT_02s0109g00080
VIT_02s0109g00130
VIT_02s0109g00190
VIT_02s0109g00230
Standardized expressions
Developmental stages
EI PG G1 G2 H T1 T2 C1 C2 S EP JP

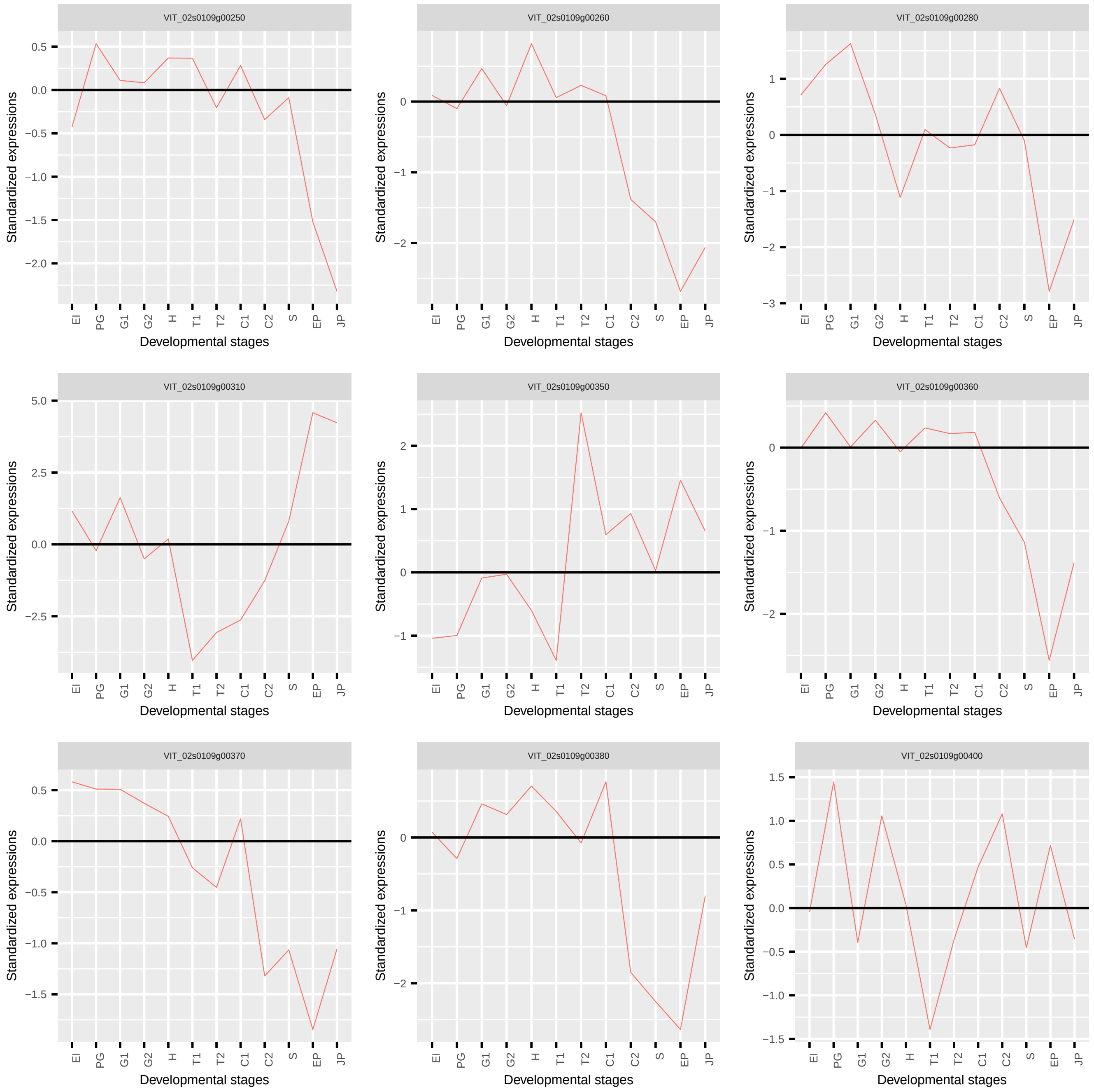
VIT_02s0109g00250
0.5
0.0
−0.5
−1.0
−1.5
−2.0
Standardized expressions
EI PG G1 G2 H T1 T2 C1 C2 S EP JP
Developmental stages
VIT_02s0109g00260
0
−1
−2
Standardized expressions
EI PG G1 G2 H T1 T2 C1 C2 S EP JP
Developmental stages
VIT_02s0109g00280
1
0
−1
−2
−3
Standardized expressions
EI PG G1 G2 H T1 T2 C1 C2 S EP JP
Developmental stages
VIT_02s0109g00310
5.0
2.5
0.0
−2.5
Standardized expressions
EI PG G1 G2 H T1 T2 C1 C2 S EP JP
Developmental stages
VIT_02s0109g00350
2
1
0
−1
Standardized expressions
EI PG G1 G2 H T1 T2 C1 C2 S EP JP
Developmental stages
VIT_02s0109g00360
0
−1
−2
Standardized expressions
EI PG G1 G2 H T1 T2 C1 C2 S EP JP
Developmental stages
VIT_02s0109g00370
0.5
0.0
−0.5
−1.0
−1.5
Standardized expressions
EI PG G1 G2 H T1 T2 C1 C2 S EP JP
Developmental stages
VIT_02s0109g00380
0
−1
−2
Standardized expressions
EI PG G1 G2 H T1 T2 C1 C2 S EP JP
Developmental stages
VIT_02s0109g00400
1.5
1.0
0.5
0.0
−0.5
−1.0
−1.5
Standardized expressions
EI PG G1 G2 H T1 T2 C1 C2 S EP JP
Developmental stages

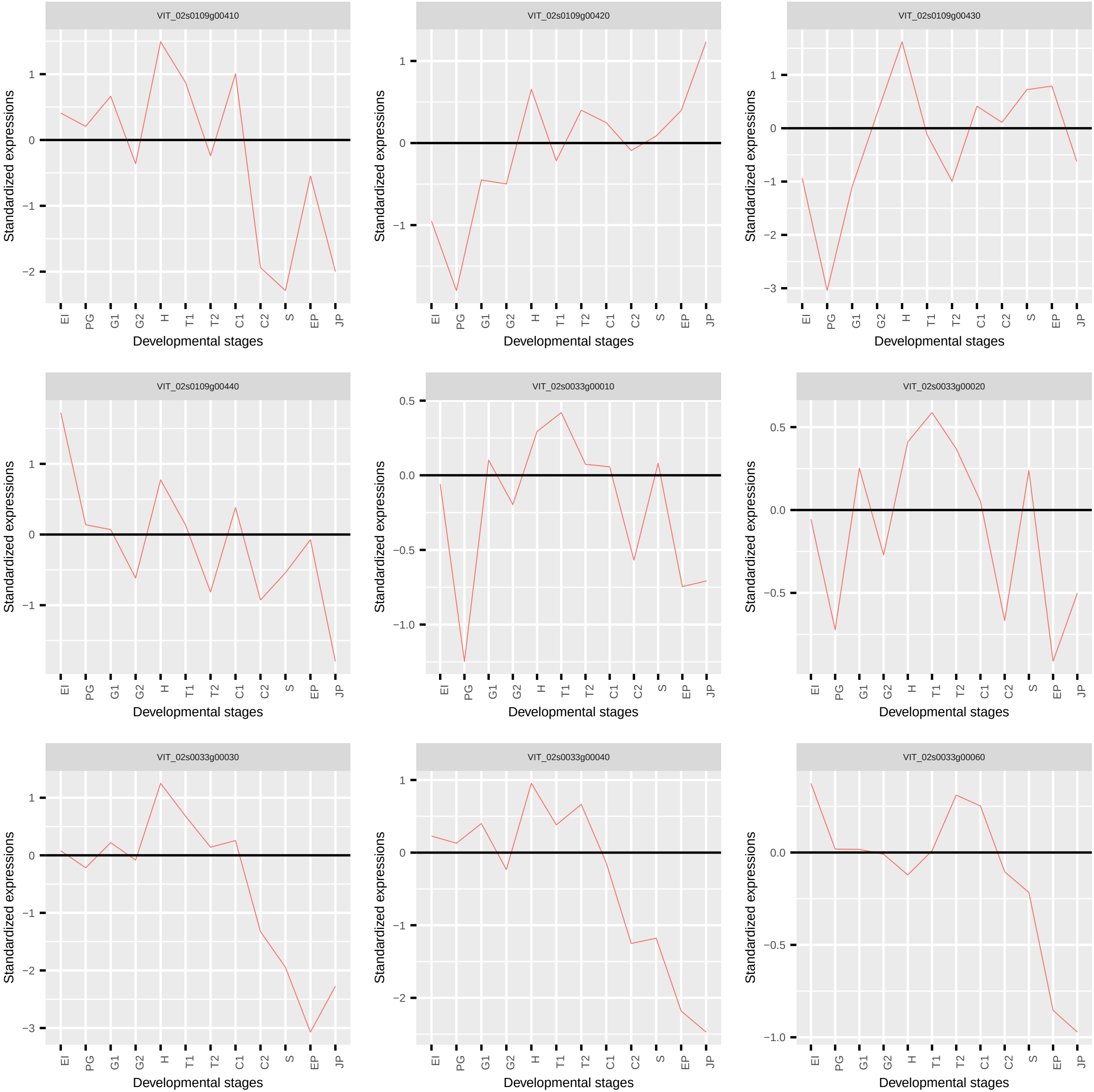

VIT_02s0109g00410
VIT_02s0109g00420
VIT_02s0109g00430
VIT_02s0109g00440
VIT_02s0033g00010
VIT_02s0033g00020
VIT_02s0033g00030
VIT_02s0033g00040
VIT_02s0033g00060
Standardized expressions
Developmental stages
EI
PG
G1
G2
H
T1
T2
C1
C2
S
EP
JP

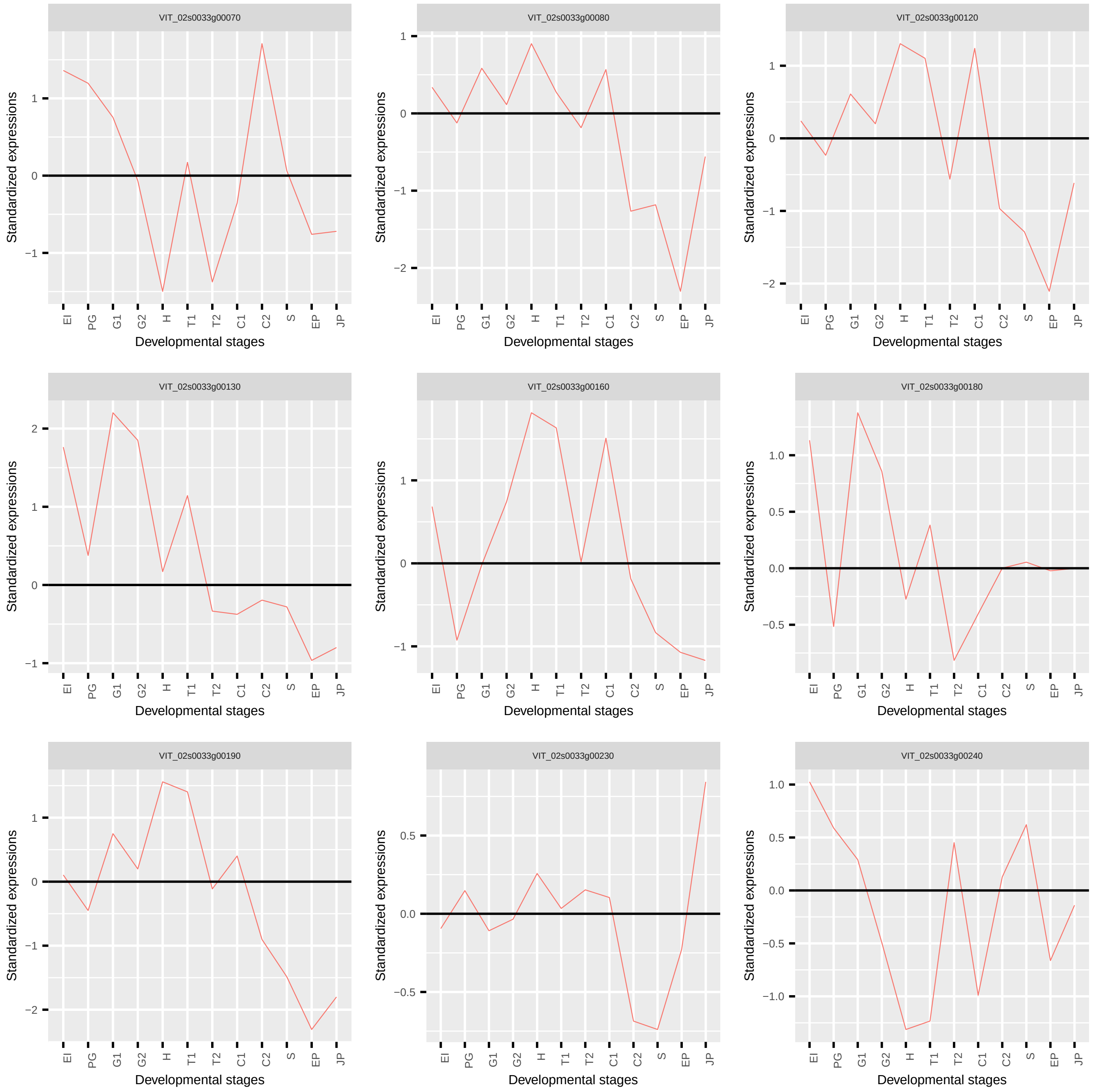
VIT_02s0033g00070
VIT_02s0033g00080
VIT_02s0033g00120
VIT_02s0033g00130
VIT_02s0033g00160
VIT_02s0033g00180
VIT_02s0033g00190
VIT_02s0033g00230
VIT_02s0033g00240
Standardized expressions
Developmental stages
EI PG G1 G2 H T1 T2 C1 C2 S EP JP

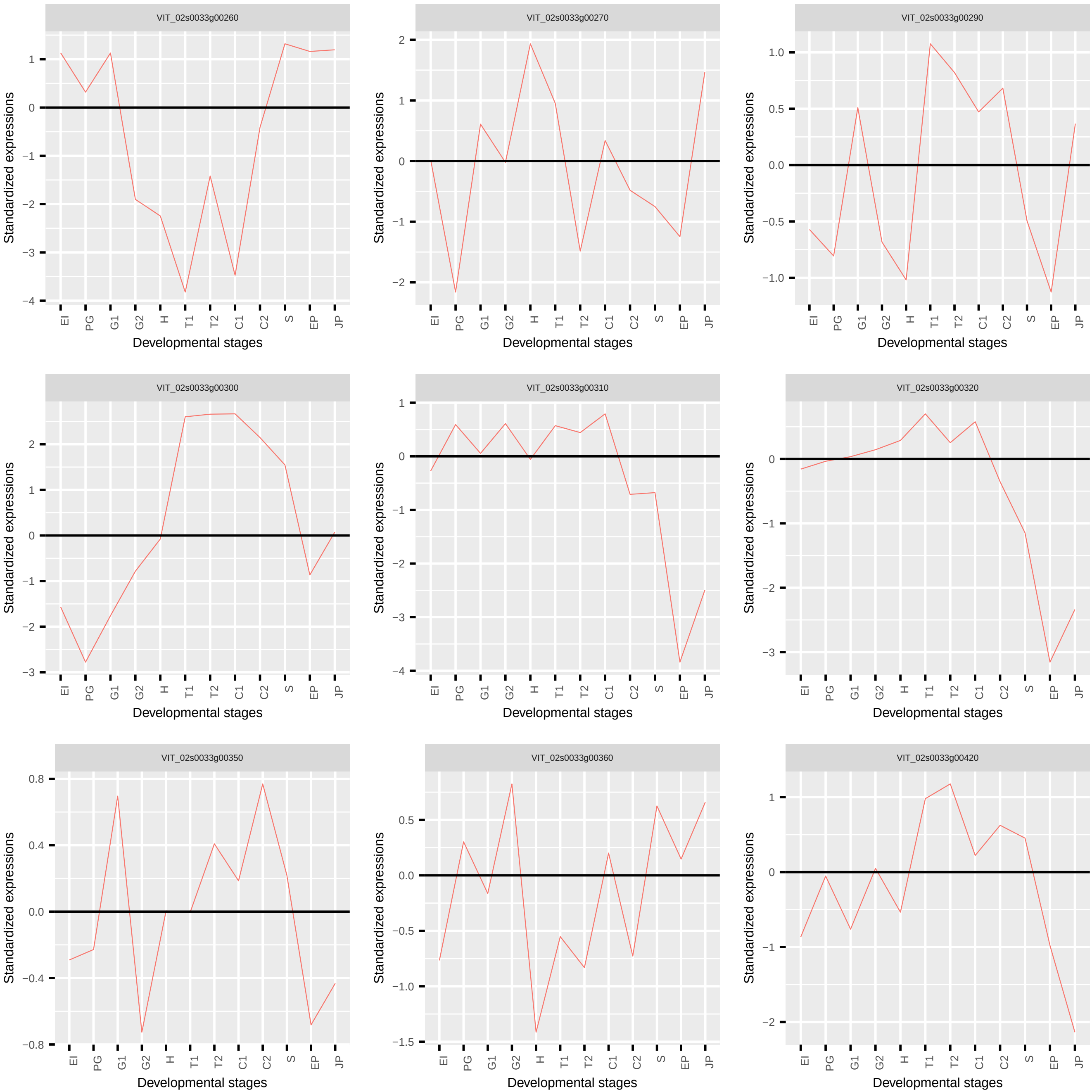
VIT_02s0033g00260
VIT_02s0033g00270
VIT_02s0033g00290
VIT_02s0033g00300
VIT_02s0033g00310
VIT_02s0033g00320
VIT_02s0033g00350
VIT_02s0033g00360
VIT_02s0033g00420
Standardized expressions
Developmental stages
EI PG G1 G2 H T1 T2 C1 C2 S EP JP

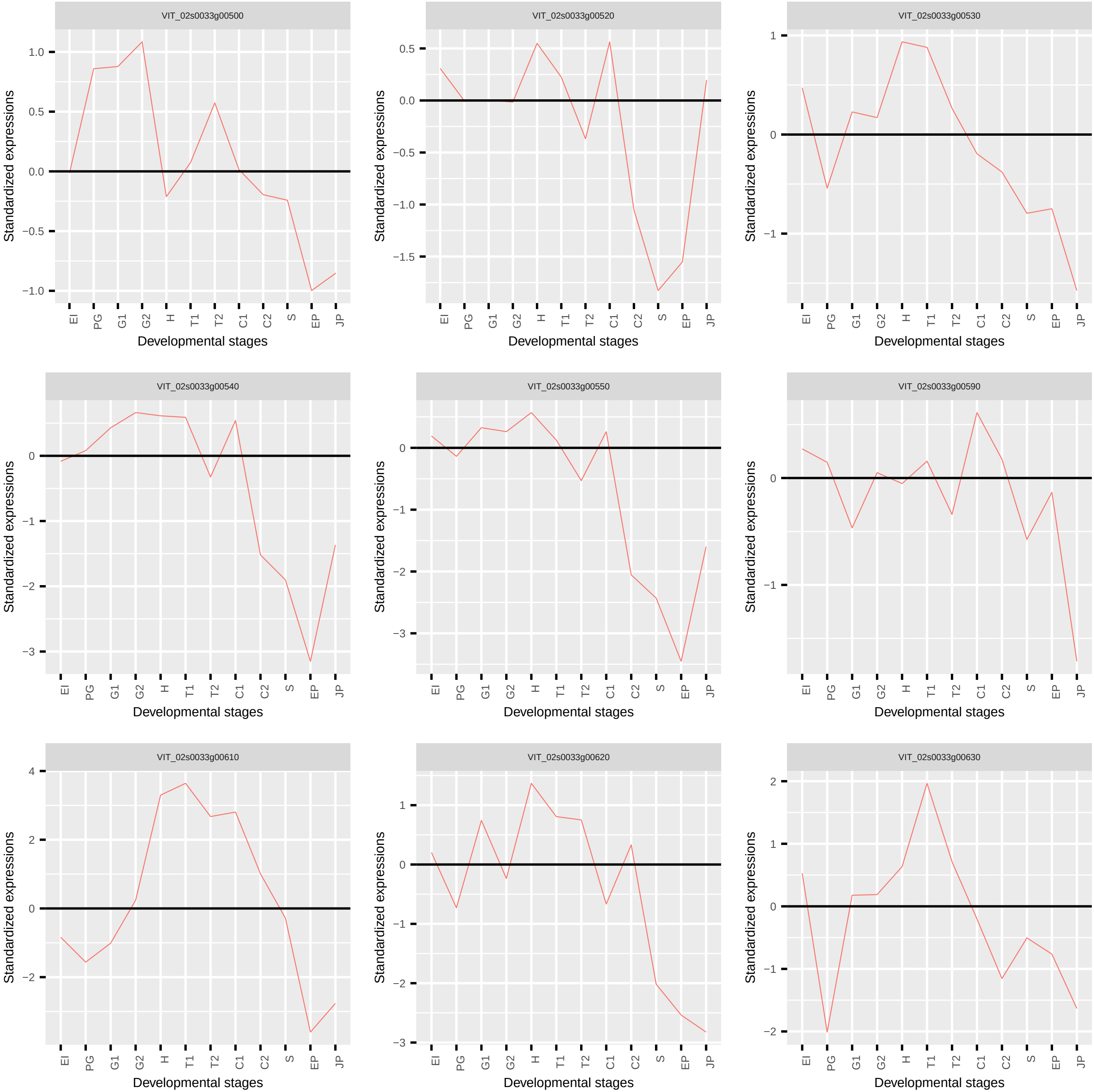
VIT_02s0033g00500
VIT_02s0033g00520
VIT_02s0033g00530
VIT_02s0033g00540
VIT_02s0033g00550
VIT_02s0033g00590
VIT_02s0033g00610
VIT_02s0033g00620
VIT_02s0033g00630
Standardized expressions
Developmental stages
EI PG G1 G2 H T1 T2 C1 C2 S EP JP

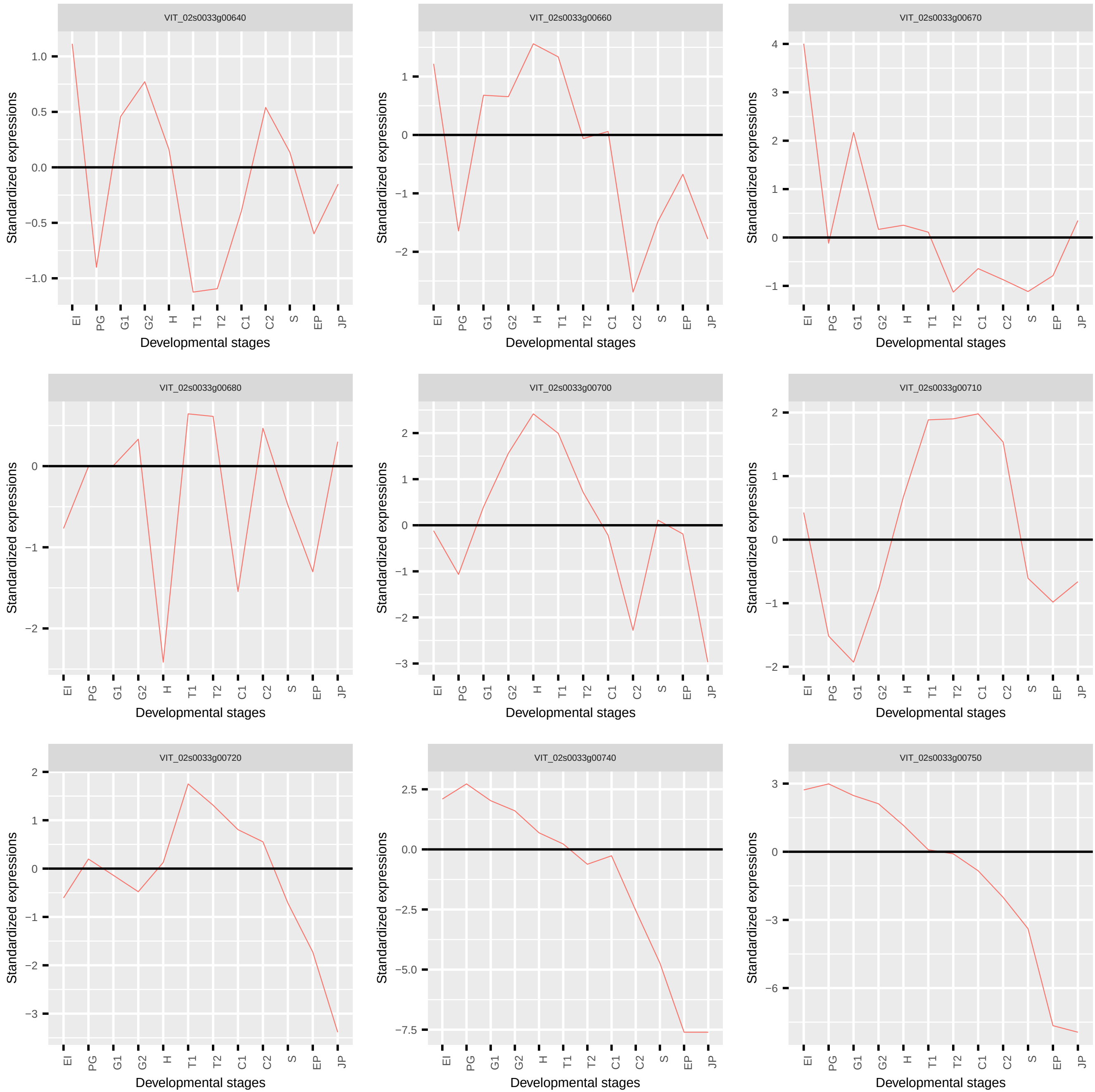
VIT_02s0033g00640
VIT_02s0033g00660
VIT_02s0033g00670
VIT_02s0033g00680
VIT_02s0033g00700
VIT_02s0033g00710
VIT_02s0033g00720
VIT_02s0033g00740
VIT_02s0033g00750
Standardized expressions
Developmental stages
EI
PG
G1
G2
H
T1
T2
C1
C2
S
EP
JP

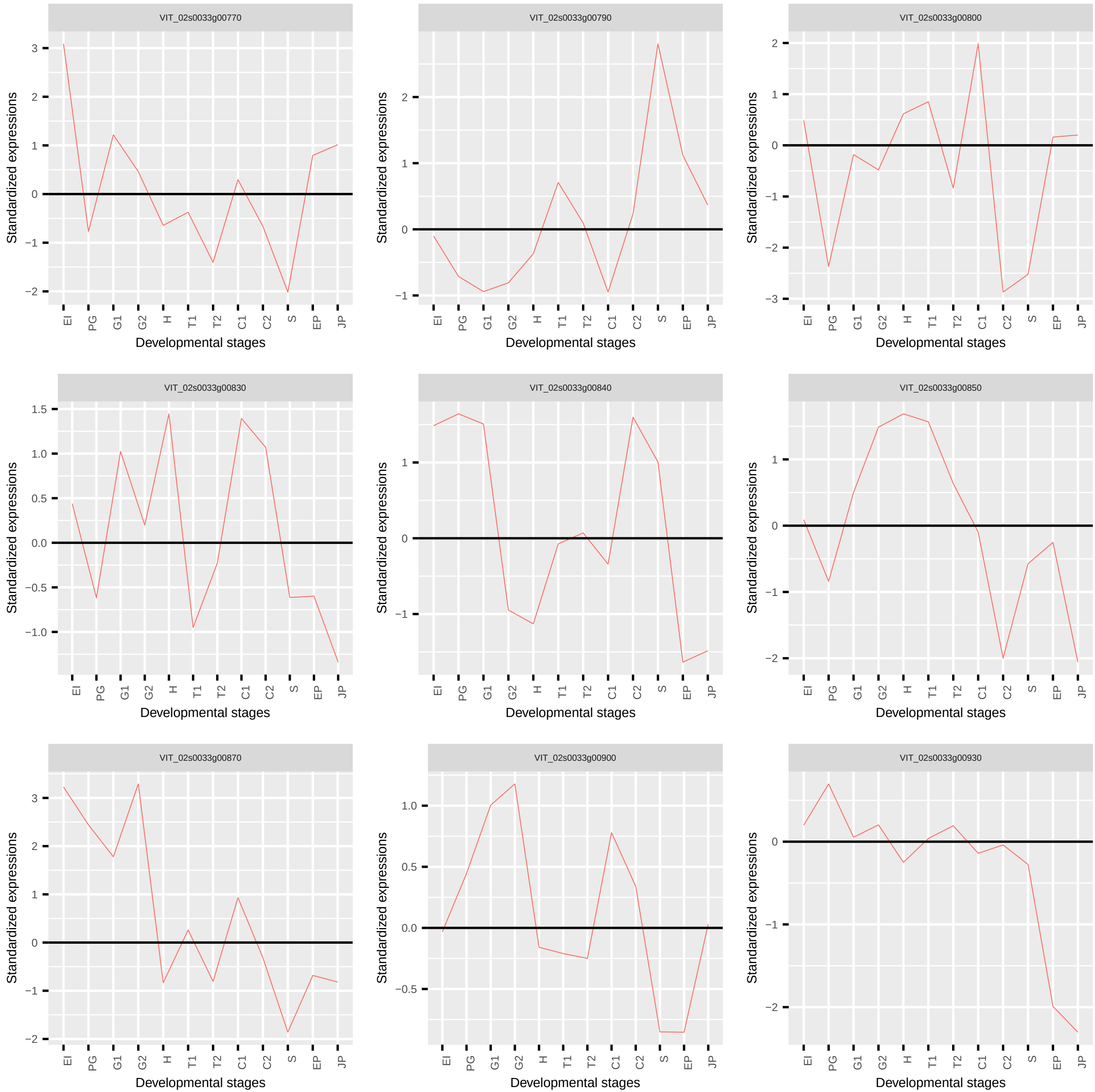

VIT_02s0033g00770
VIT_02s0033g00790
VIT_02s0033g00800
VIT_02s0033g00830
VIT_02s0033g00840
VIT_02s0033g00850
VIT_02s0033g00870
VIT_02s0033g00900
VIT_02s0033g00930
Standardized expressions
Developmental stages
EI PG G1 G2 H T1 T2 C1 C2 S EP JP

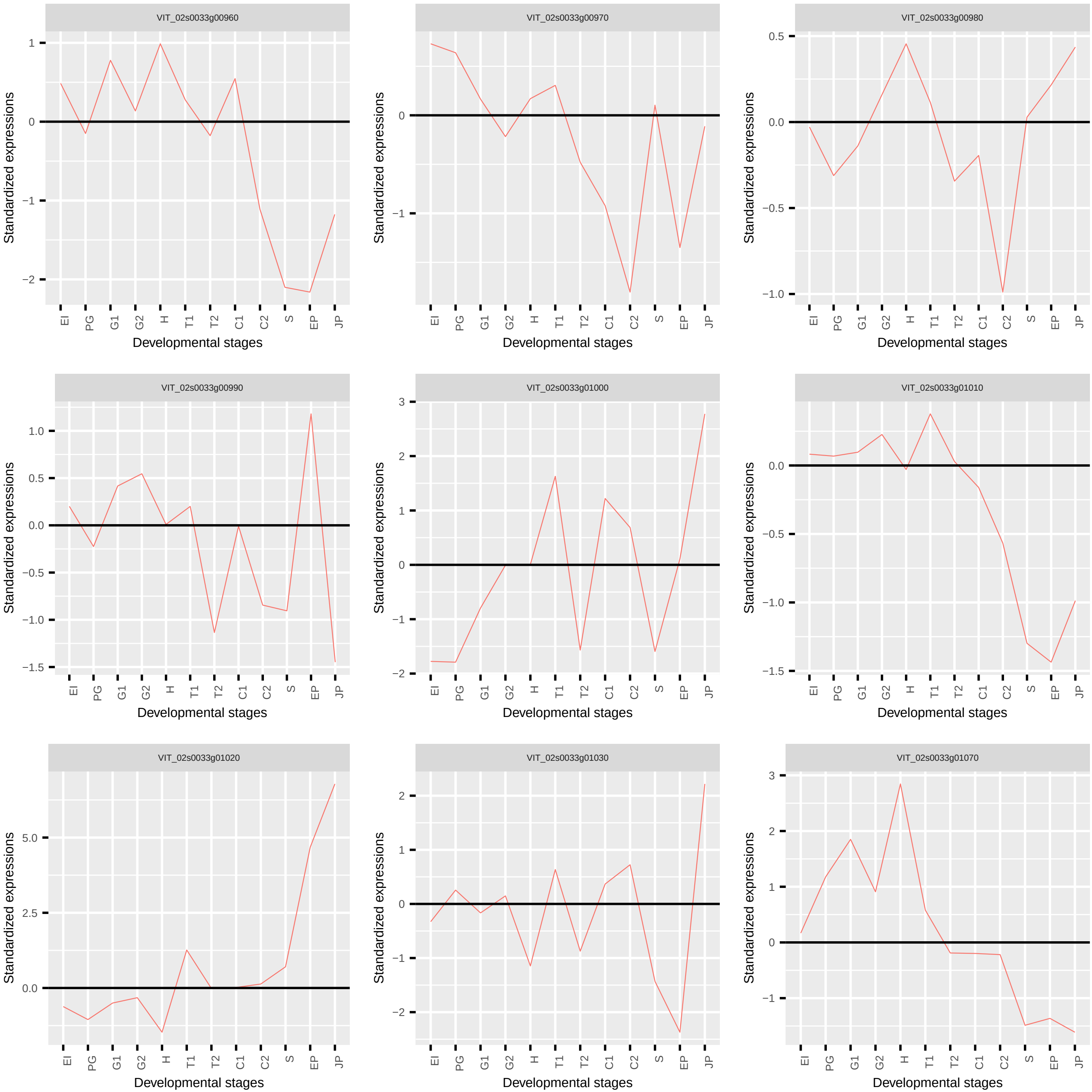
VIT_02s0033g00960
VIT_02s0033g00970
VIT_02s0033g00980
VIT_02s0033g00990
VIT_02s0033g01000
VIT_02s0033g01010
VIT_02s0033g01020
VIT_02s0033g01030
VIT_02s0033g01070
Standardized expressions
Developmental stages
EI PG G1 G2 H T1 T2 C1 C2 S EP JP

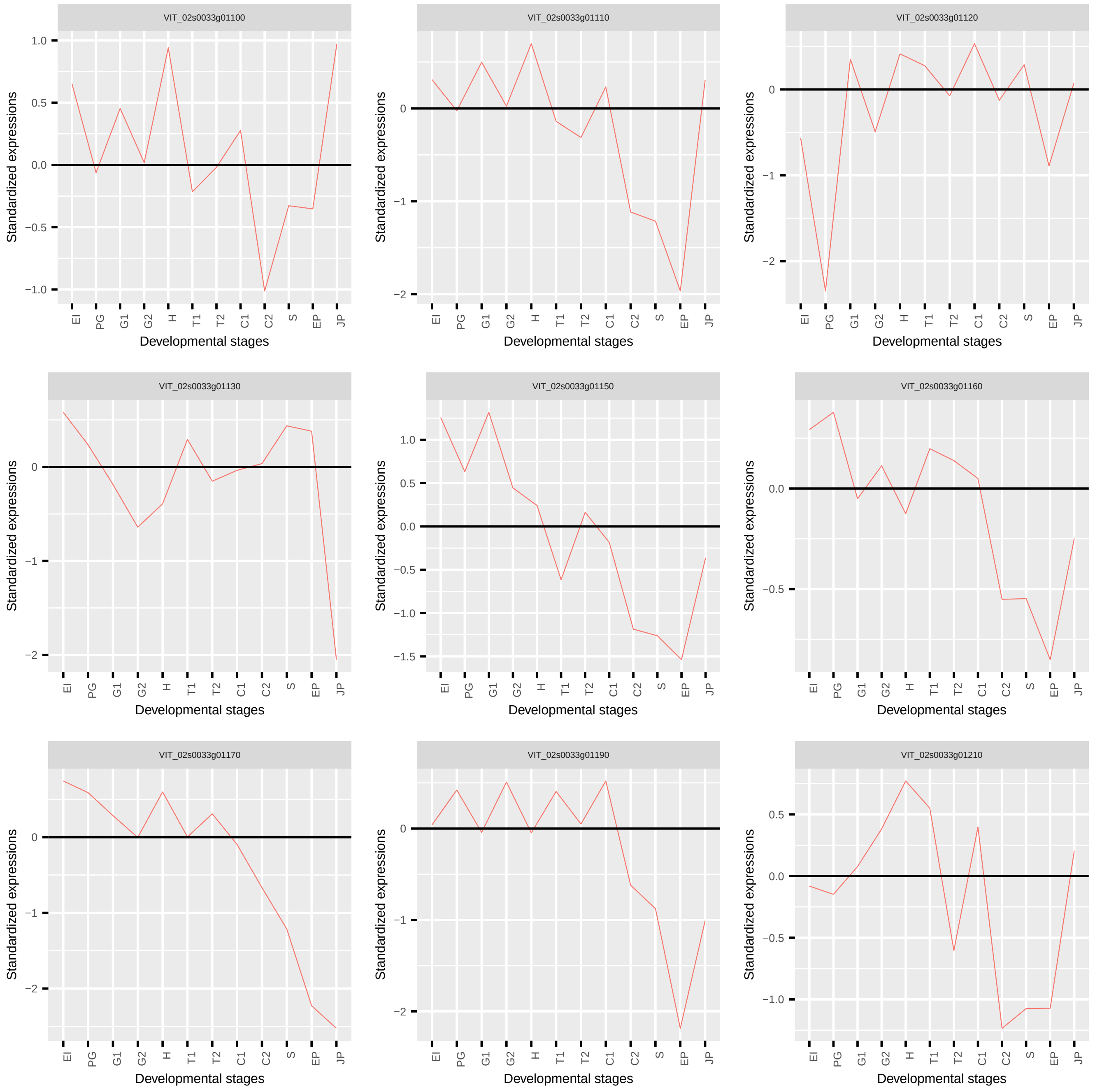
VIT_02s0033g01100
VIT_02s0033g01110
VIT_02s0033g01120
VIT_02s0033g01130
VIT_02s0033g01150
VIT_02s0033g01160
VIT_02s0033g01170
VIT_02s0033g01190
VIT_02s0033g01210
Standardized expressions
Developmental stages
EI PG G1 G2 H T1 T2 C1 C2 S EP JP

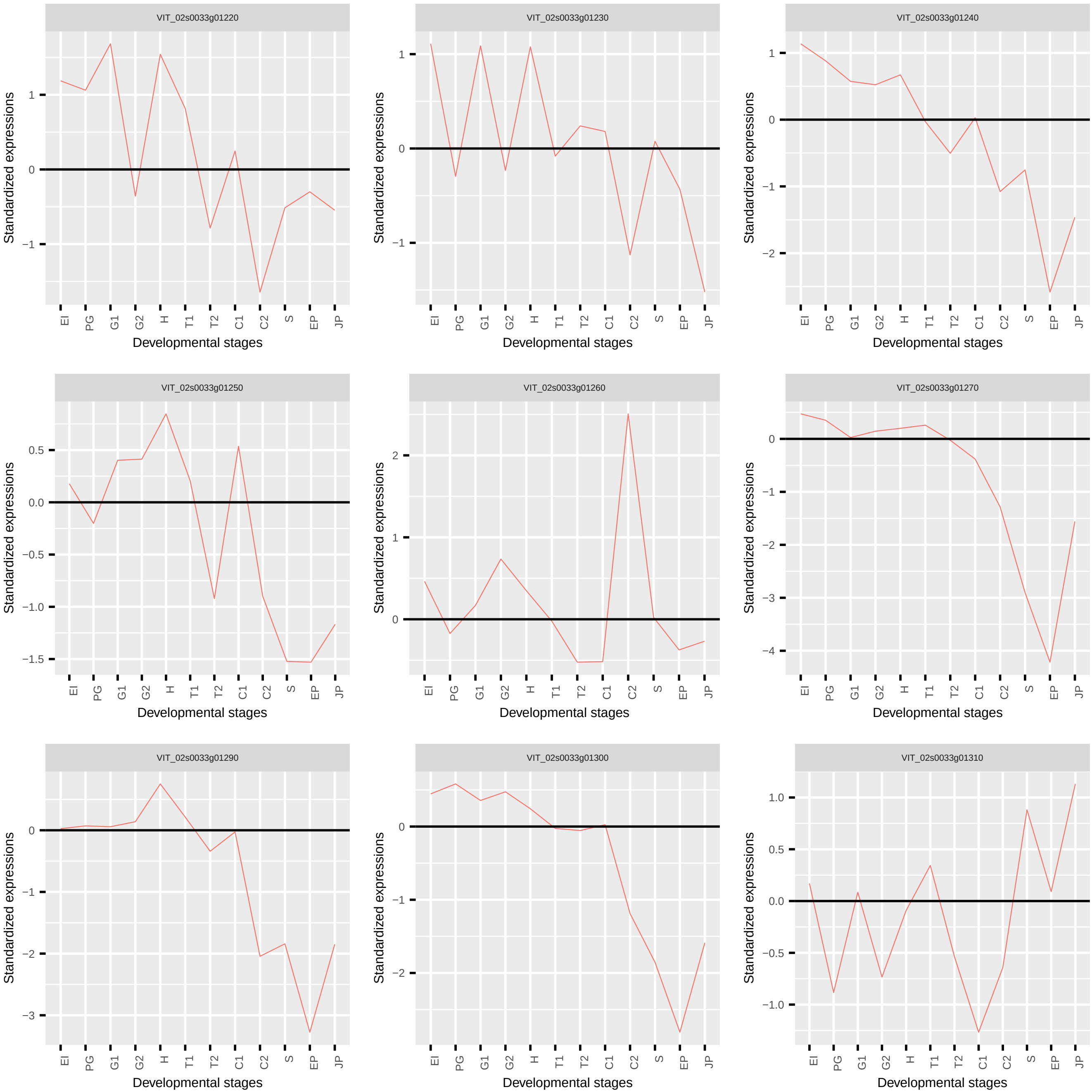
VIT_02s0033g01220
VIT_02s0033g01230
VIT_02s0033g01240
VIT_02s0033g01250
VIT_02s0033g01260
VIT_02s0033g01270
VIT_02s0033g01290
VIT_02s0033g01300
VIT_02s0033g01310
Standardized expressions
Developmental stages
EI PG G1 G2 H T1 T2 C1 C2 S EP JP

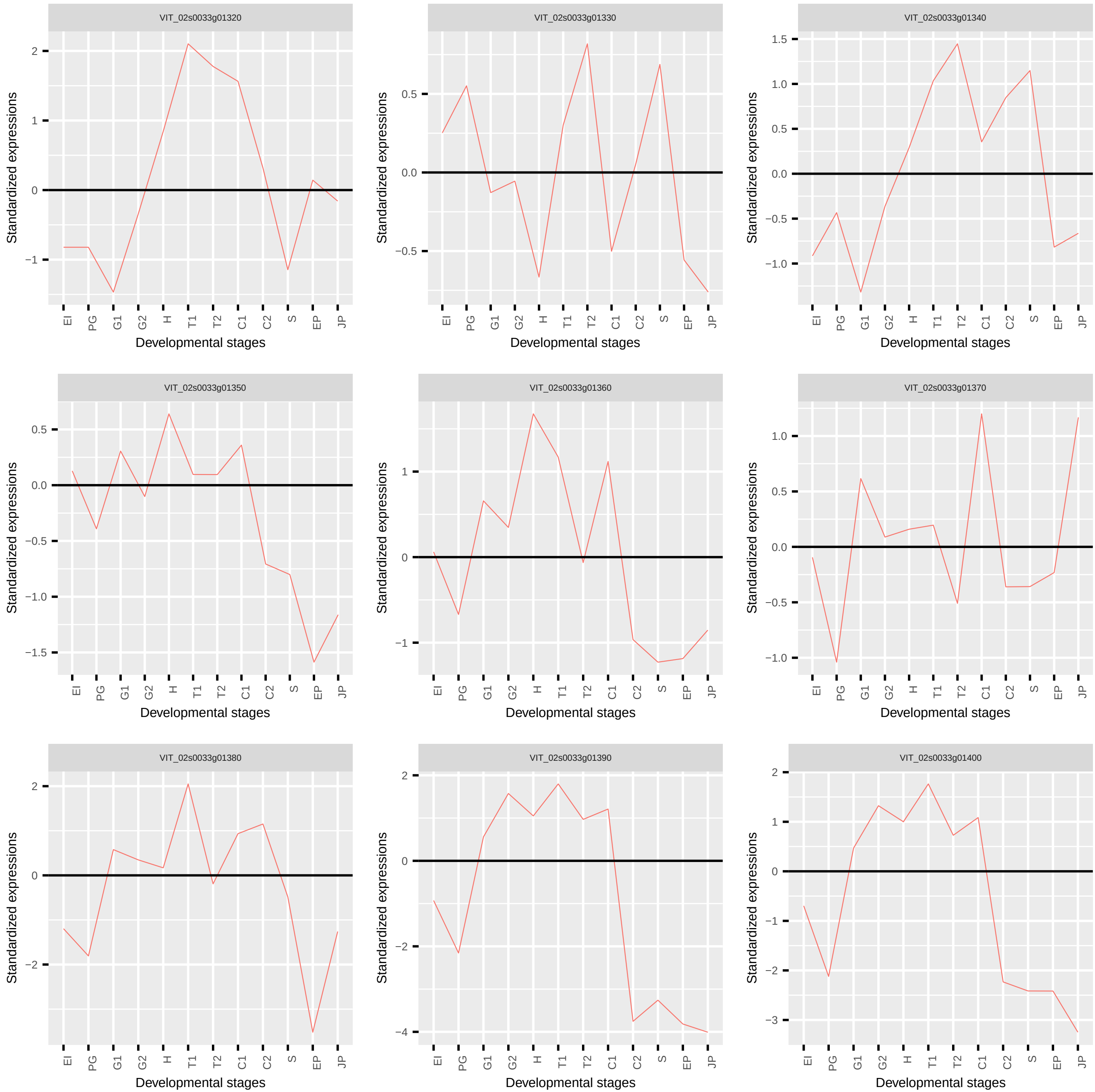

VIT_02s0033g01320
VIT_02s0033g01330
VIT_02s0033g01340
VIT_02s0033g01350
VIT_02s0033g01360
VIT_02s0033g01370
VIT_02s0033g01380
VIT_02s0033g01390
VIT_02s0033g01400
Standardized expressions
Developmental stages
EI
PG
G1
G2
H
T1
T2
C1
C2
S
EP
JP

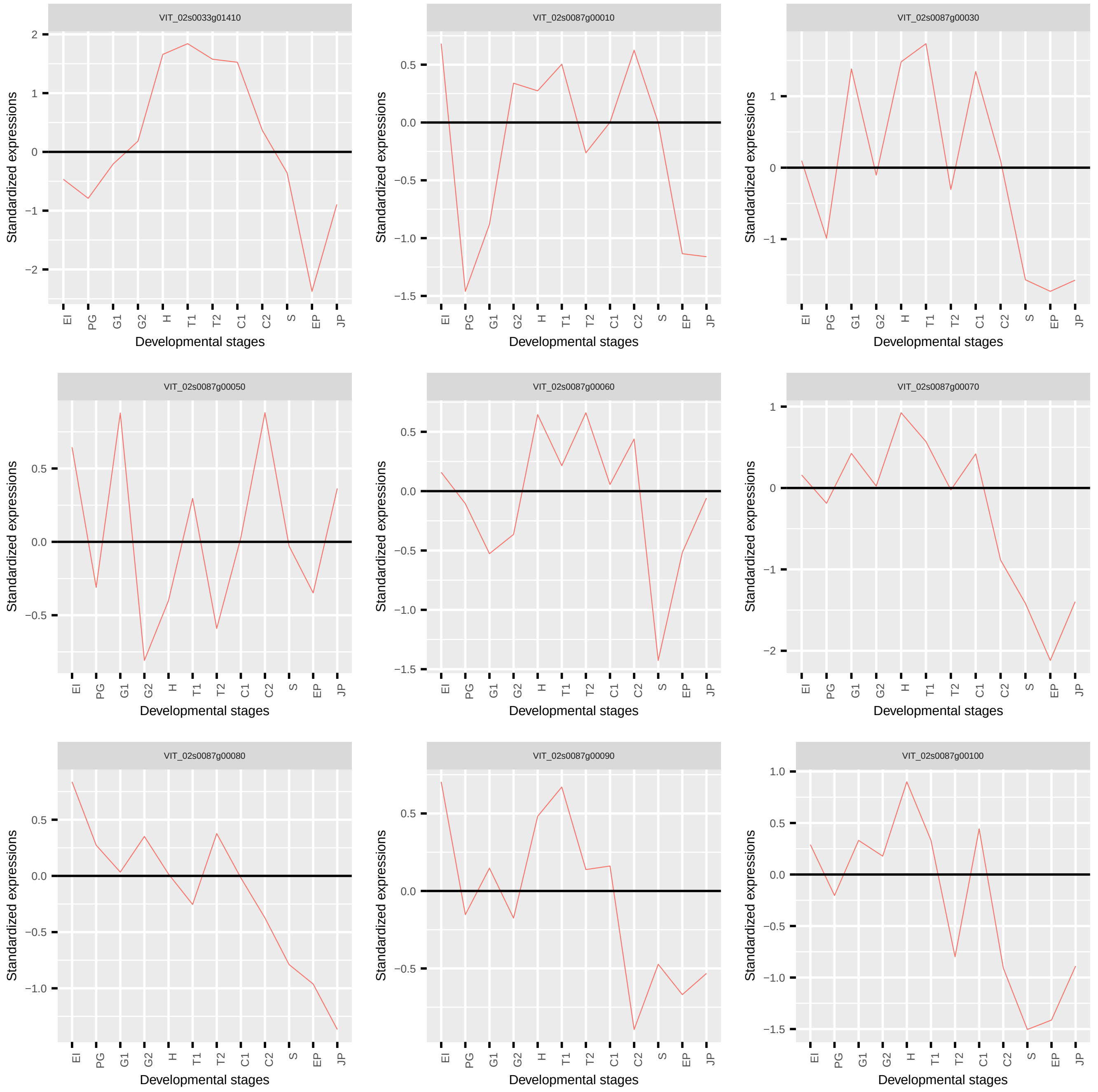
VIT_02s0033g01410
VIT_02s0087g00010
VIT_02s0087g00030
VIT_02s0087g00050
VIT_02s0087g00060
VIT_02s0087g00070
VIT_02s0087g00080
VIT_02s0087g00090
VIT_02s0087g00100
Standardized expressions
Developmental stages
EI PG G1 G2 H T1 T2 C1 C2 S EP JP
2
1
0
−1
−2
0.5
0.0
−0.5
−1.0
−1.5

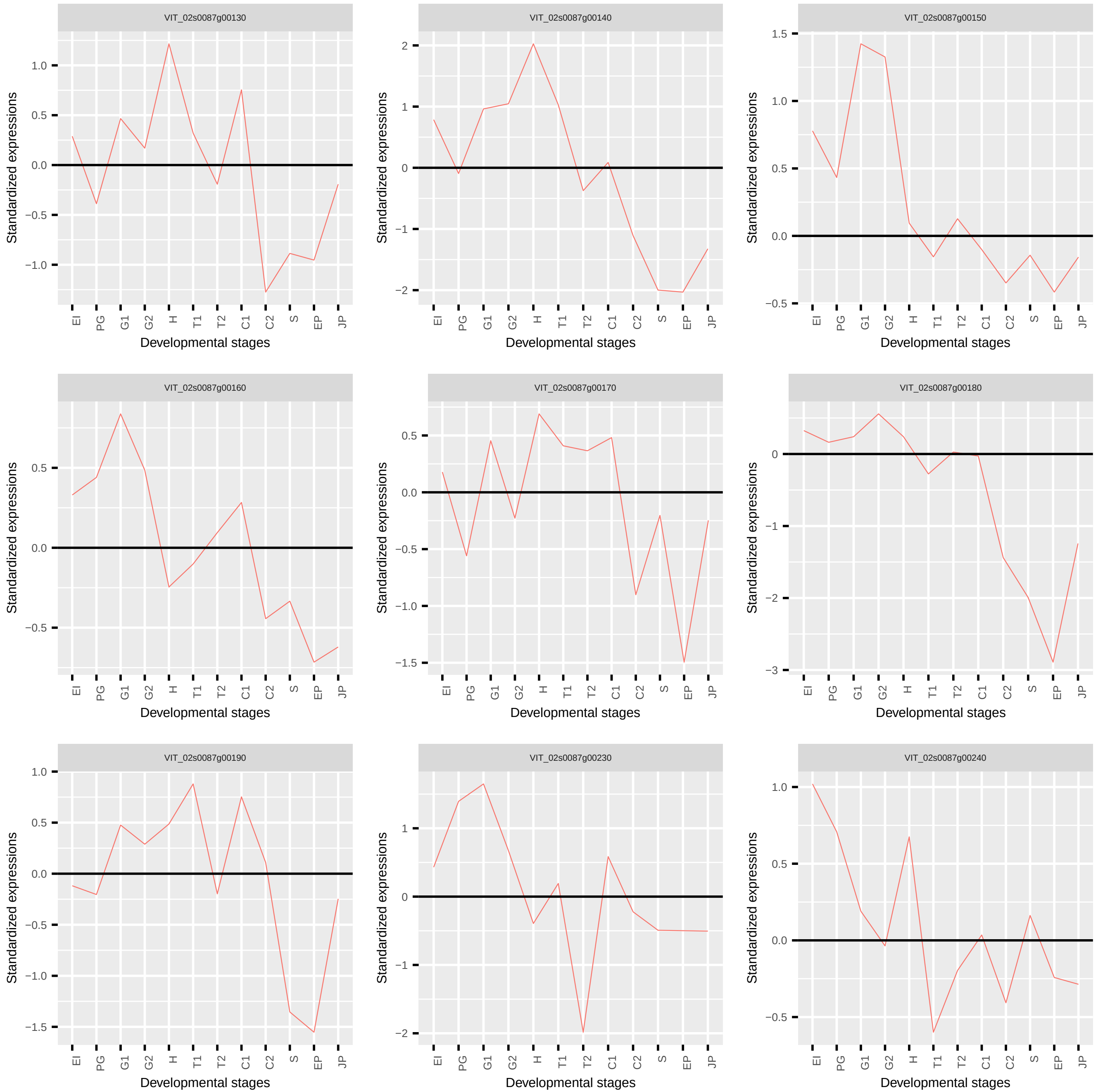

VIT_02s0087g00130
VIT_02s0087g00140
VIT_02s0087g00150
VIT_02s0087g00160
VIT_02s0087g00170
VIT_02s0087g00180
VIT_02s0087g00190
VIT_02s0087g00230
VIT_02s0087g00240
Standardized expressions
Developmental stages
EI PG G1 G2 H T1 T2 C1 C2 S EP JP

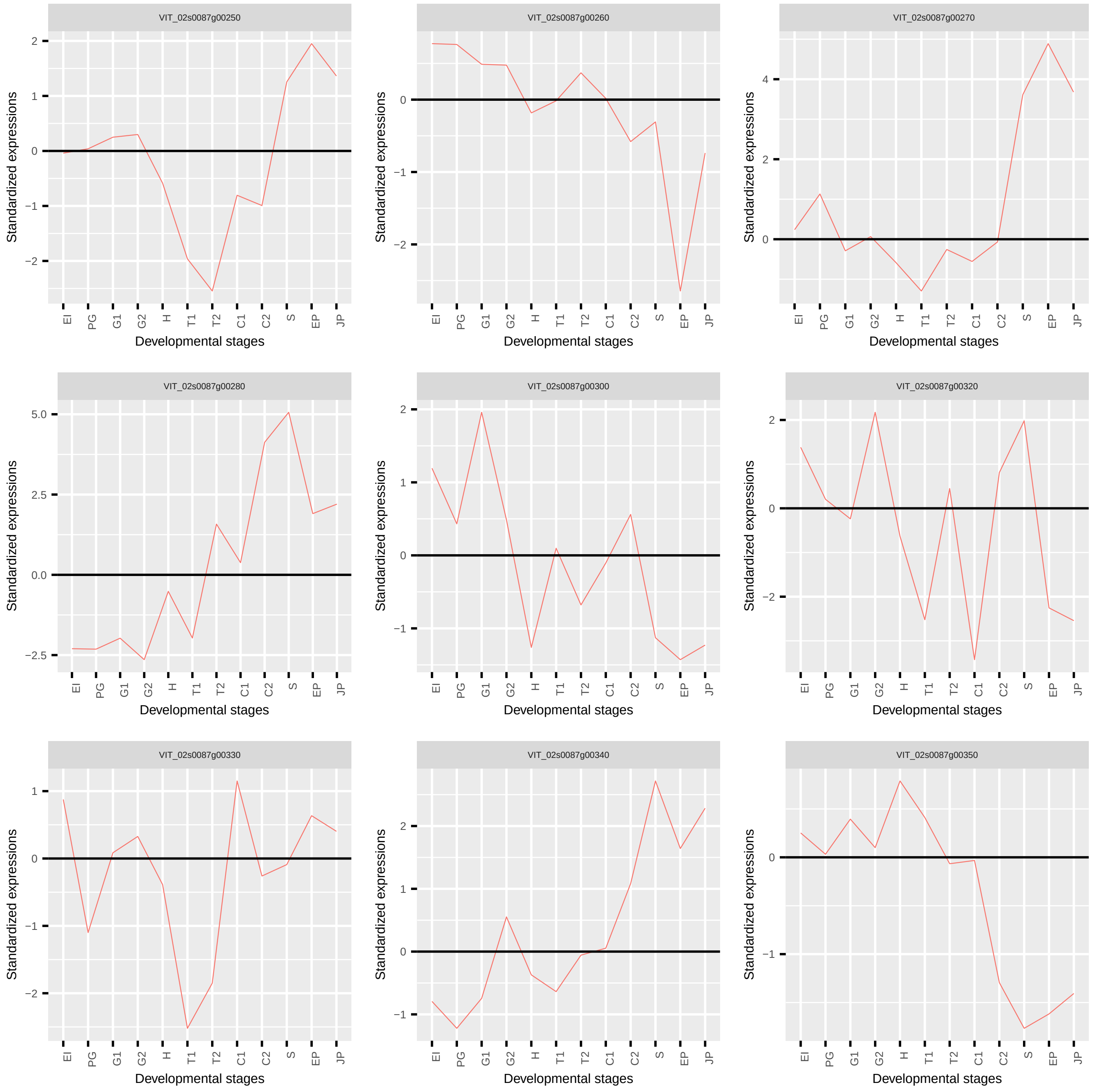
VIT_02s0087g00250
VIT_02s0087g00260
VIT_02s0087g00270
VIT_02s0087g00280
VIT_02s0087g00300
VIT_02s0087g00320
VIT_02s0087g00330
VIT_02s0087g00340
VIT_02s0087g00350
Standardized expressions
Developmental stages
EI PG G1 G2 H T1 T2 C1 C2 S EP JP

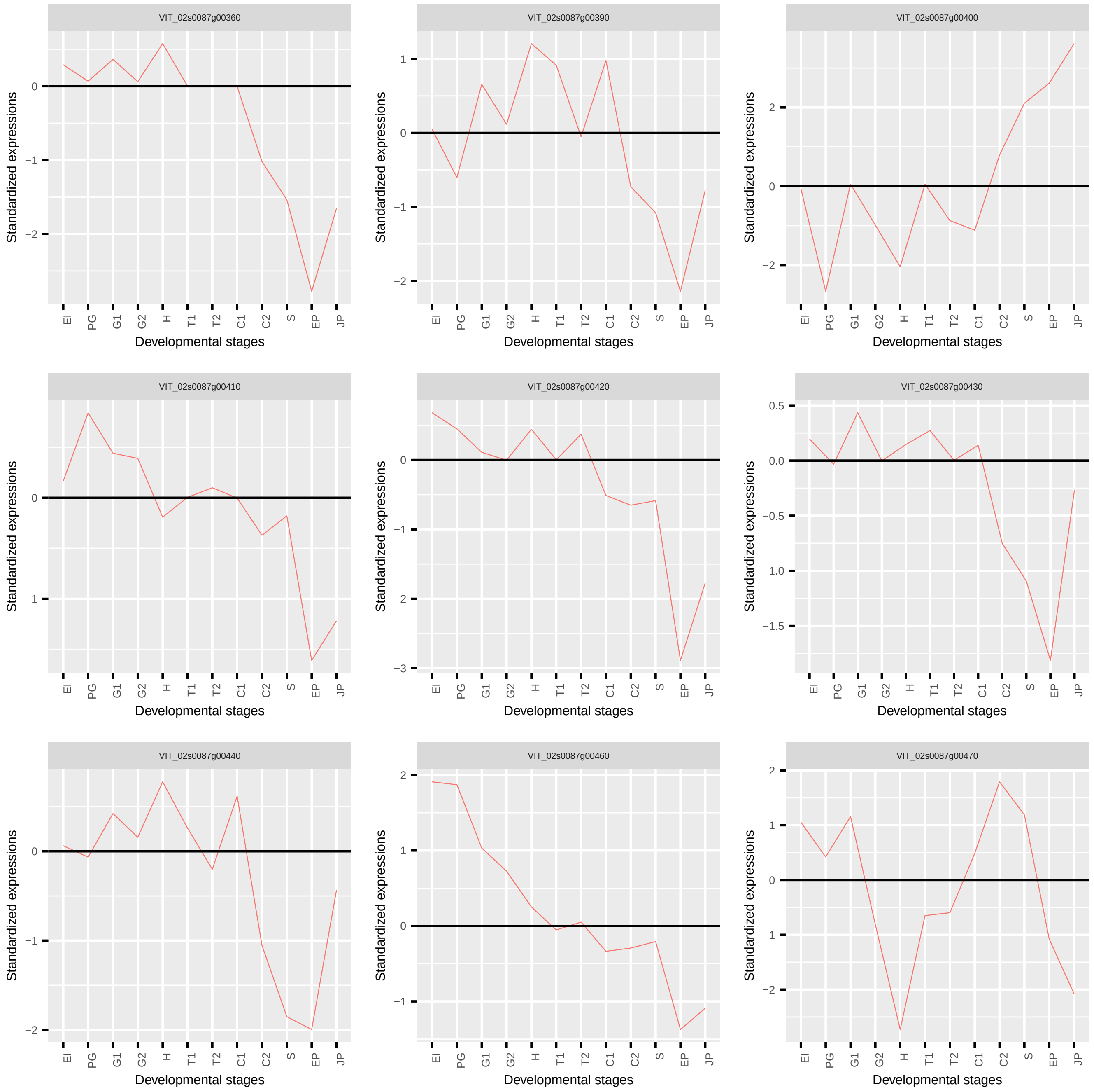
VIT_02s0087g00360
VIT_02s0087g00390
VIT_02s0087g00400
VIT_02s0087g00410
VIT_02s0087g00420
VIT_02s0087g00430
VIT_02s0087g00440
VIT_02s0087g00460
VIT_02s0087g00470
Standardized expressions
Developmental stages
EI
PG
G1
G2
H
T1
T2
C1
C2
S
EP
JP

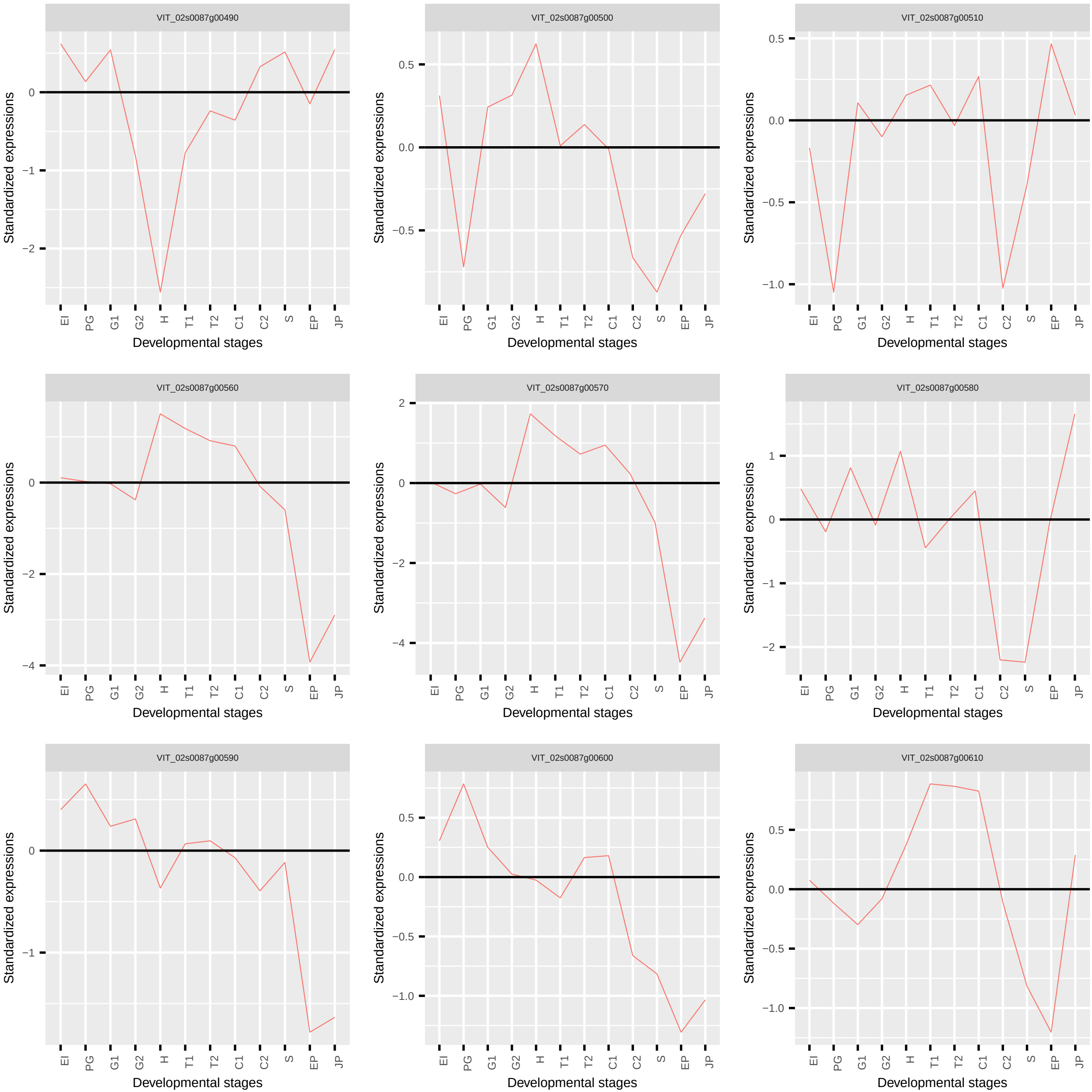
VIT_02s0087g00490
VIT_02s0087g00500
VIT_02s0087g00510
VIT_02s0087g00560
VIT_02s0087g00570
VIT_02s0087g00580
VIT_02s0087g00590
VIT_02s0087g00600
VIT_02s0087g00610
Standardized expressions
Developmental stages
EI
PG
G1
G2
H
T1
T2
C1
C2
S
EP
JP

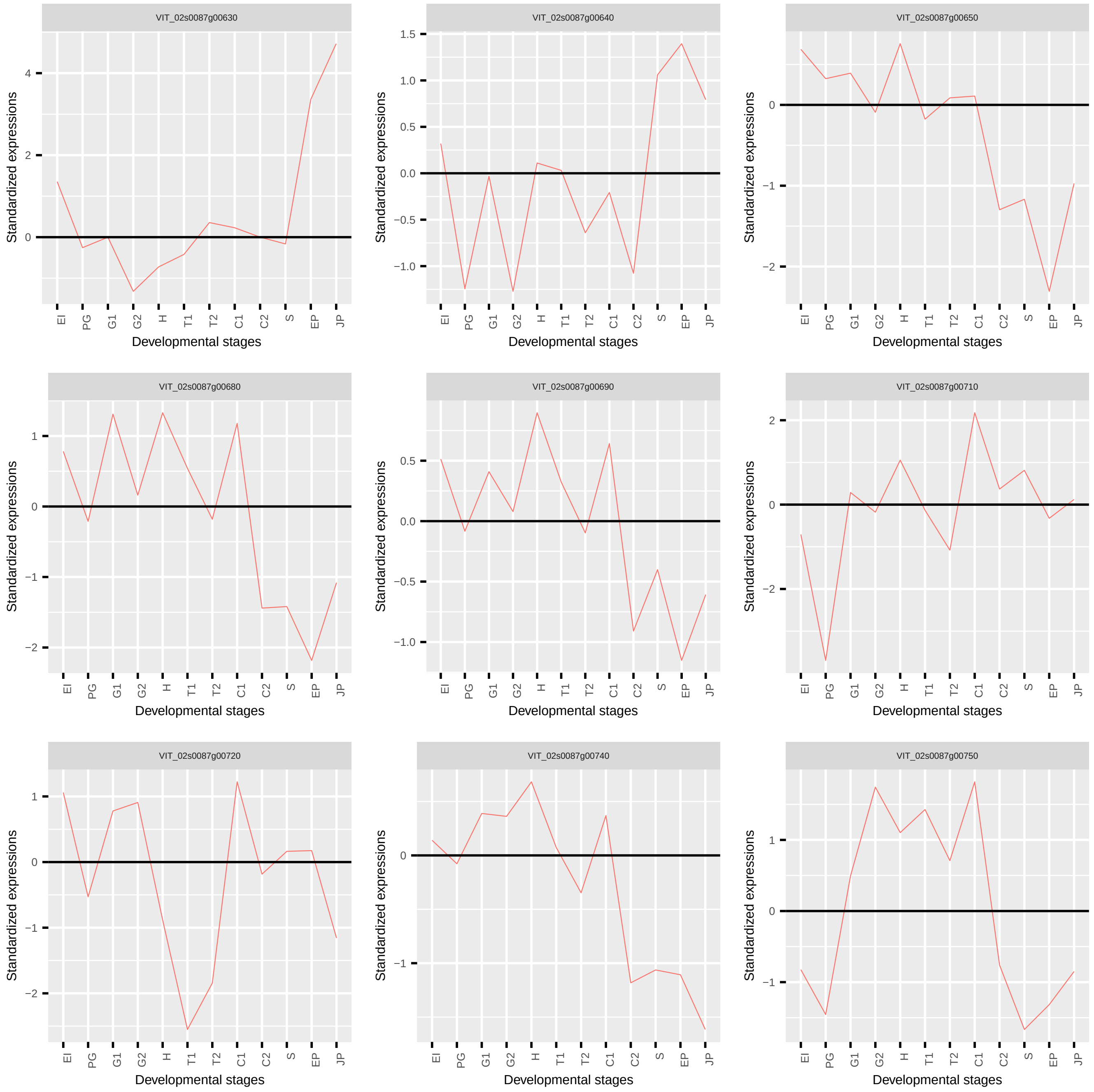

VIT_02s0087g00630
VIT_02s0087g00640
VIT_02s0087g00650
VIT_02s0087g00680
VIT_02s0087g00690
VIT_02s0087g00710
VIT_02s0087g00720
VIT_02s0087g00740
VIT_02s0087g00750
Standardized expressions
Developmental stages
EI PG G1 G2 H T1 T2 C1 C2 S EP JP

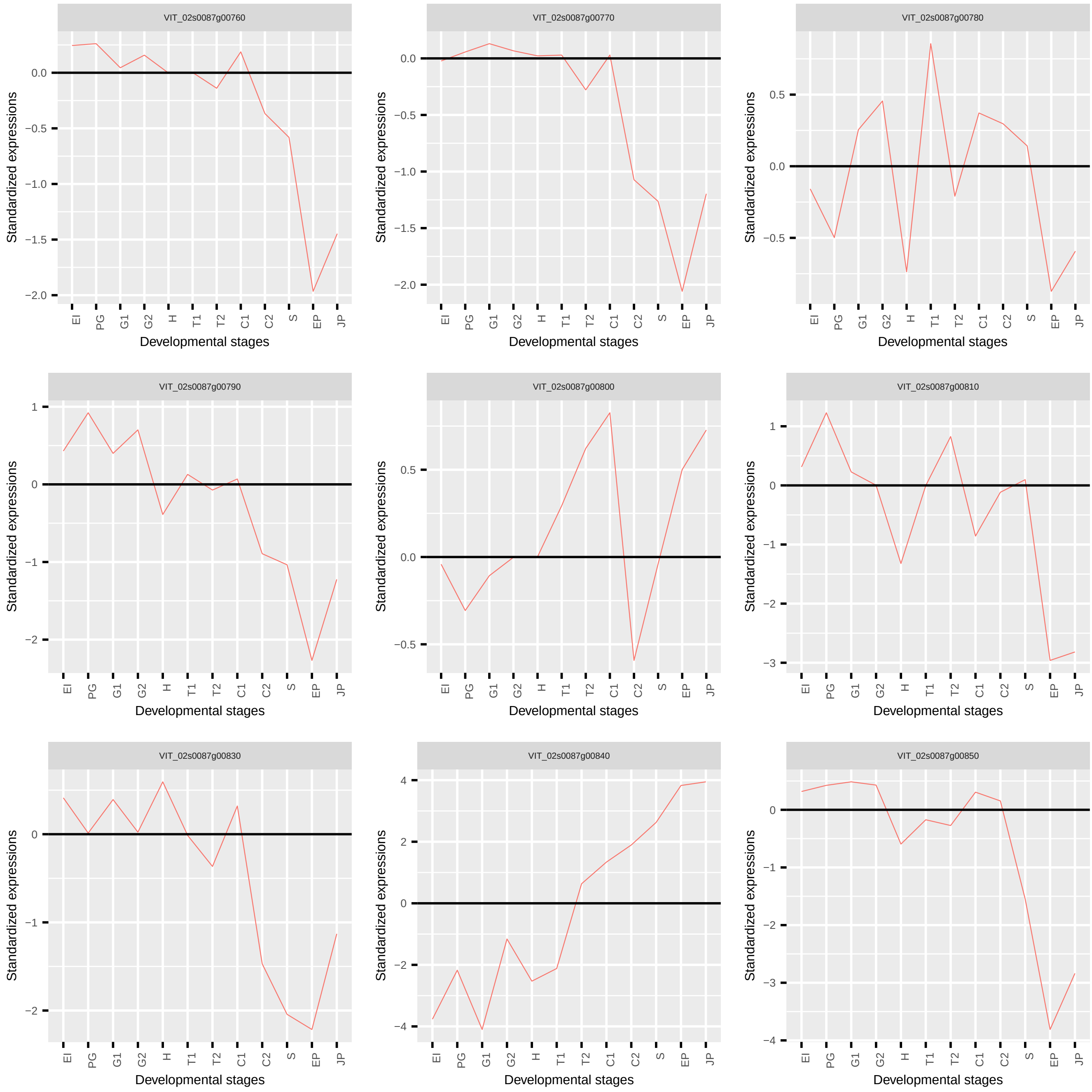
VIT_02s0087g00760
VIT_02s0087g00770
VIT_02s0087g00780
VIT_02s0087g00790
VIT_02s0087g00800
VIT_02s0087g00810
VIT_02s0087g00830
VIT_02s0087g00840
VIT_02s0087g00850
Standardized expressions
Developmental stages
EI
PG
G1
G2
H
T1
T2
C1
C2
S
EP
JP
0.0
−0.5
−1.0
−1.5
−2.0
0.5
1
0
−1
−2
−3
4
2
−4

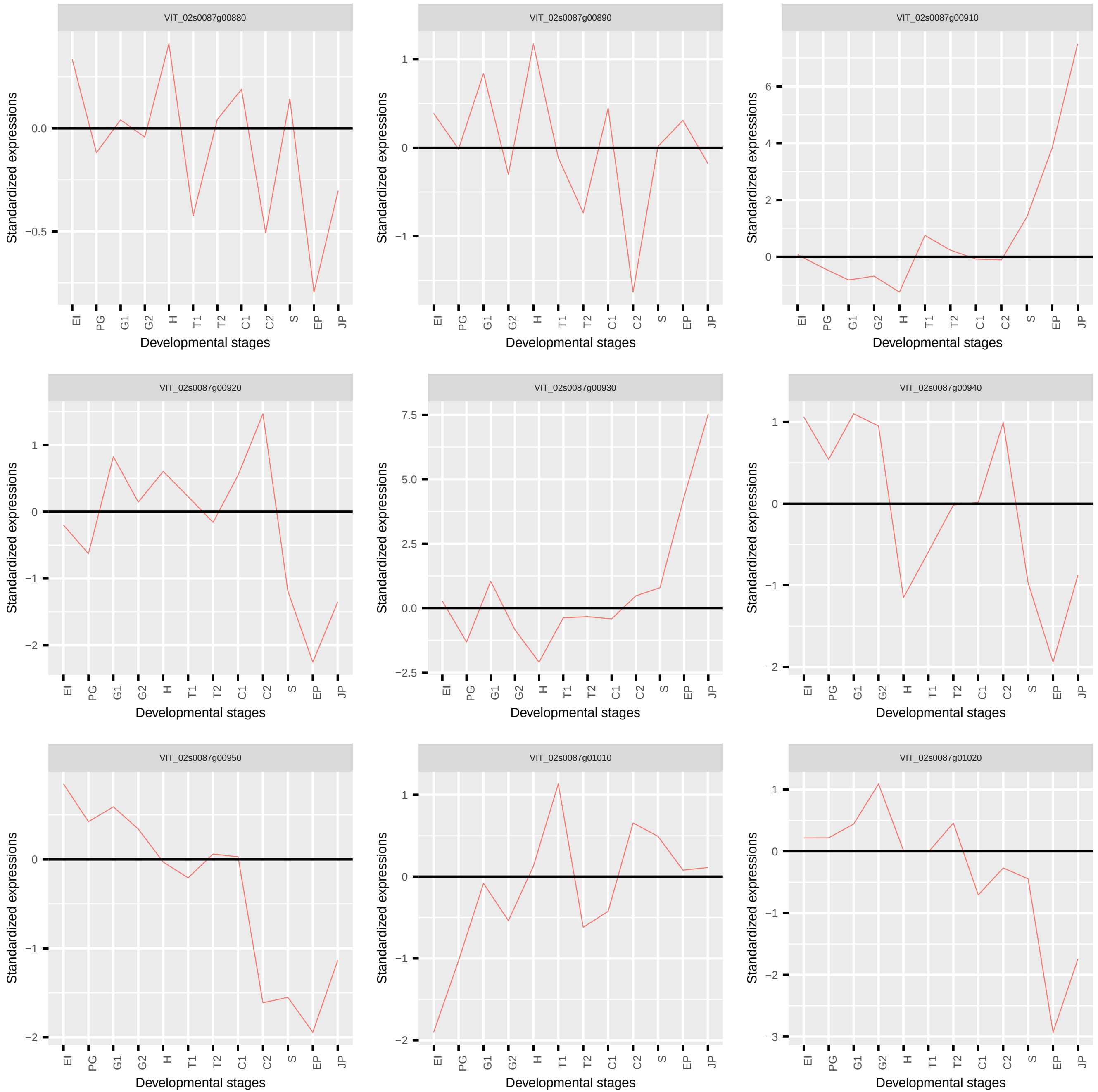
VIT_02s0087g00880
Standardized expressions
0.0
−0.5
EI
PG
G1
G2
H
T1
T2
C1
C2
S
EP
JP
Developmental stages
VIT_02s0087g00890
Standardized expressions
1
0
−1
EI
PG
G1
G2
H
T1
T2
C1
C2
S
EP
JP
Developmental stages
VIT_02s0087g00910
Standardized expressions
6
4
2
0
EI
PG
G1
G2
H
T1
T2
C1
C2
S
EP
JP
Developmental stages
VIT_02s0087g00920
Standardized expressions
1
0
−1
−2
EI
PG
G1
G2
H
T1
T2
C1
C2
S
EP
JP
Developmental stages
VIT_02s0087g00930
Standardized expressions
7.5
5.0
2.5
0.0
−2.5
EI
PG
G1
G2
H
T1
T2
C1
C2
S
EP
JP
Developmental stages
VIT_02s0087g00940
Standardized expressions
1
0
−1
−2
EI
PG
G1
G2
H
T1
T2
C1
C2
S
EP
JP
Developmental stages
VIT_02s0087g00950
Standardized expressions
0
−1
−2
EI
PG
G1
G2
H
T1
T2
C1
C2
S
EP
JP
Developmental stages
VIT_02s0087g01010
Standardized expressions
1
0
−1
−2
EI
PG
G1
G2
H
T1
T2
C1
C2
S
EP
JP
Developmental stages
VIT_02s0087g01020
Standardized expressions
1
0
−1
−2
−3
EI
PG
G1
G2
H
T1
T2
C1
C2
S
EP
JP
Developmental stages

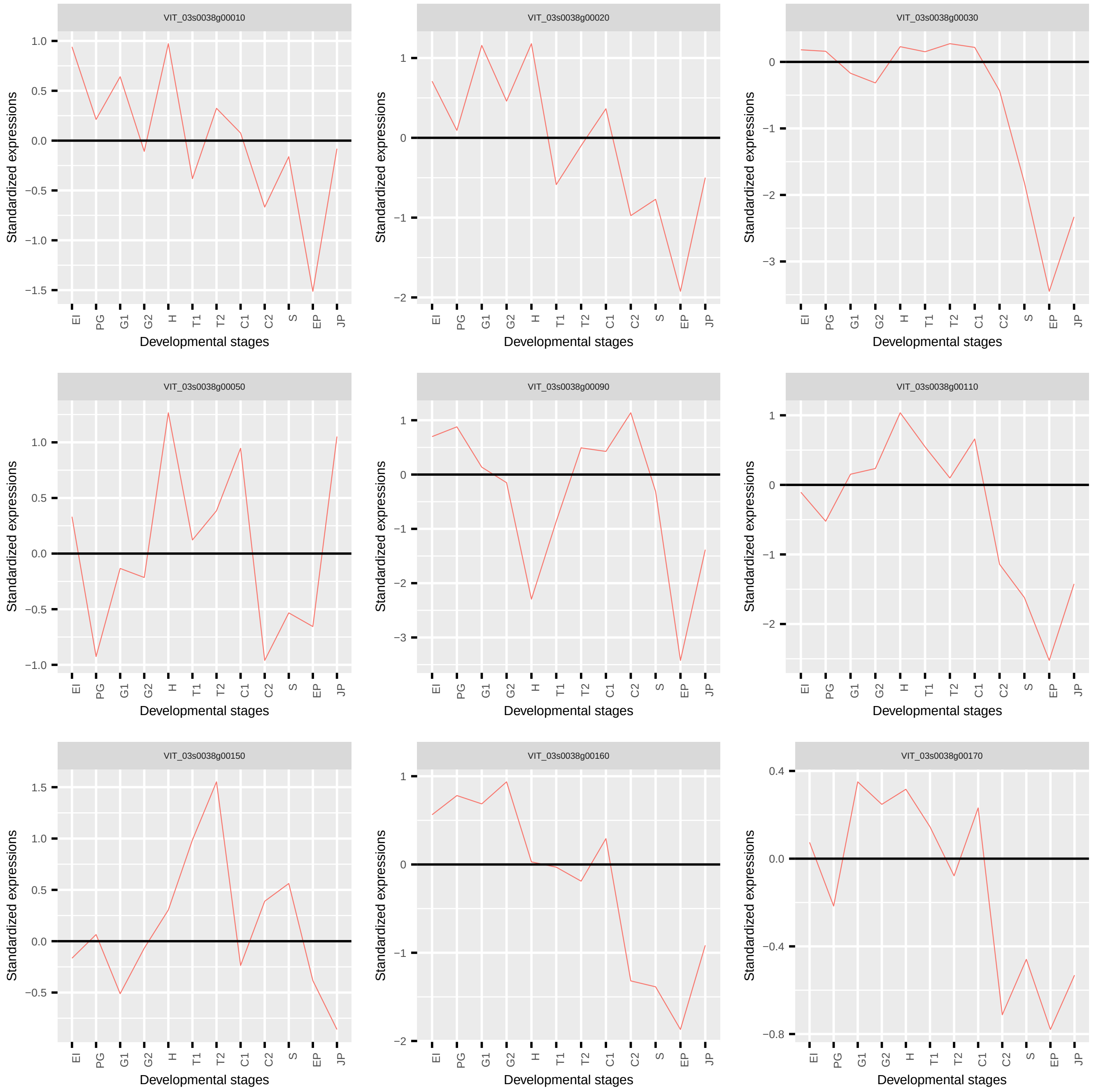

VIT_03s0038g00010
VIT_03s0038g00020
VIT_03s0038g00030
VIT_03s0038g00050
VIT_03s0038g00090
VIT_03s0038g00110
VIT_03s0038g00150
VIT_03s0038g00160
VIT_03s0038g00170
Standardized expressions
Developmental stages
EI PG G1 G2 H T1 T2 C1 C2 S EP JP

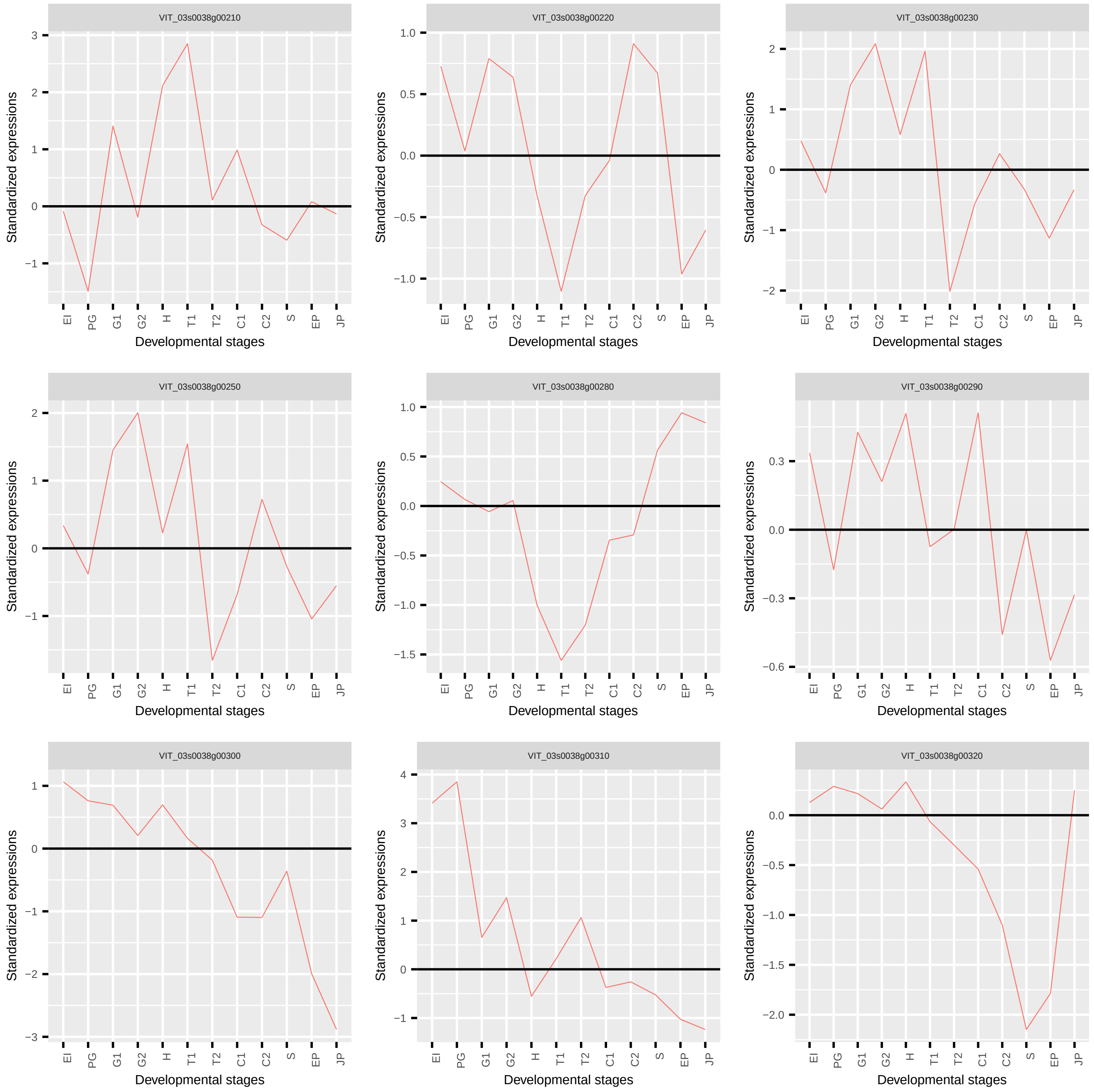
VIT_03s0038g00210
VIT_03s0038g00220
VIT_03s0038g00230
VIT_03s0038g00250
VIT_03s0038g00280
VIT_03s0038g00290
VIT_03s0038g00300
VIT_03s0038g00310
VIT_03s0038g00320
Standardized expressions
Developmental stages
EI PG G1 G2 H T1 T2 C1 C2 S EP JP

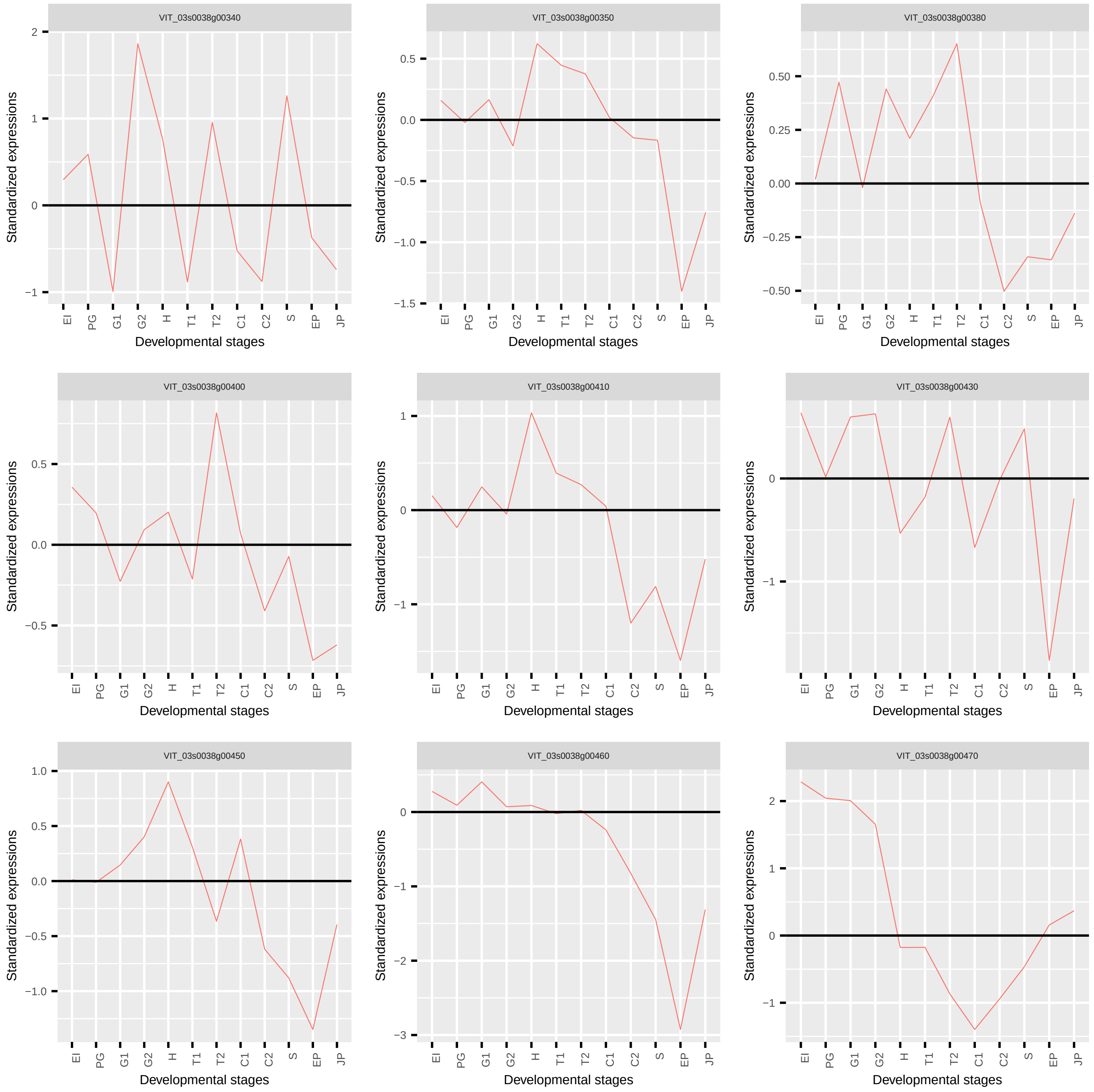
VIT_03s0038g00340
VIT_03s0038g00350
VIT_03s0038g00380
VIT_03s0038g00400
VIT_03s0038g00410
VIT_03s0038g00430
VIT_03s0038g00450
VIT_03s0038g00460
VIT_03s0038g00470
Standardized expressions
Developmental stages
EI PG G1 G2 H T1 T2 C1 C2 S EP JP

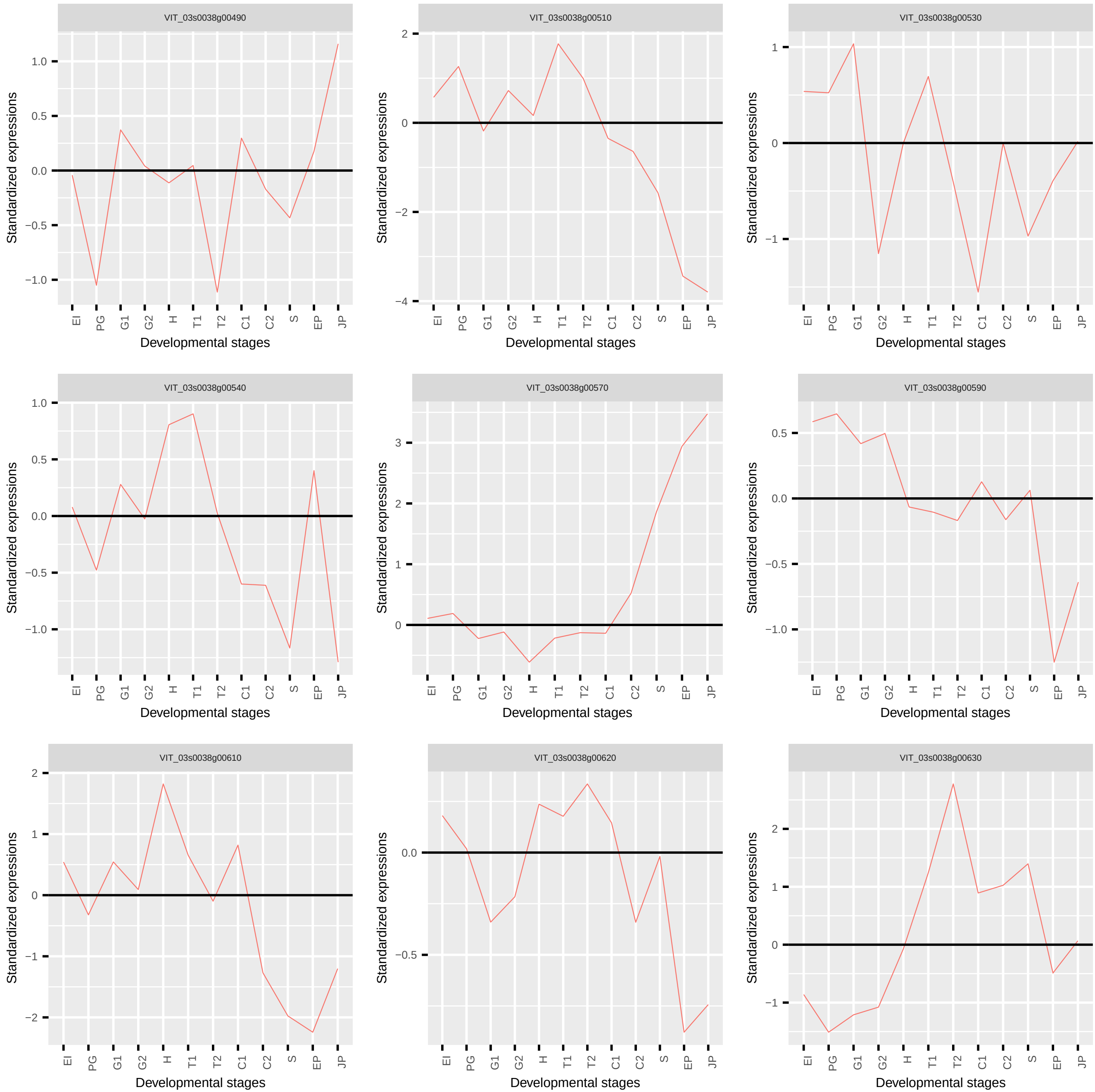
VIT_03s0038g00490
VIT_03s0038g00510
VIT_03s0038g00530
VIT_03s0038g00540
VIT_03s0038g00570
VIT_03s0038g00590
VIT_03s0038g00610
VIT_03s0038g00620
VIT_03s0038g00630
Standardized expressions
Developmental stages
EI PG G1 G2 H T1 T2 C1 C2 S EP JP

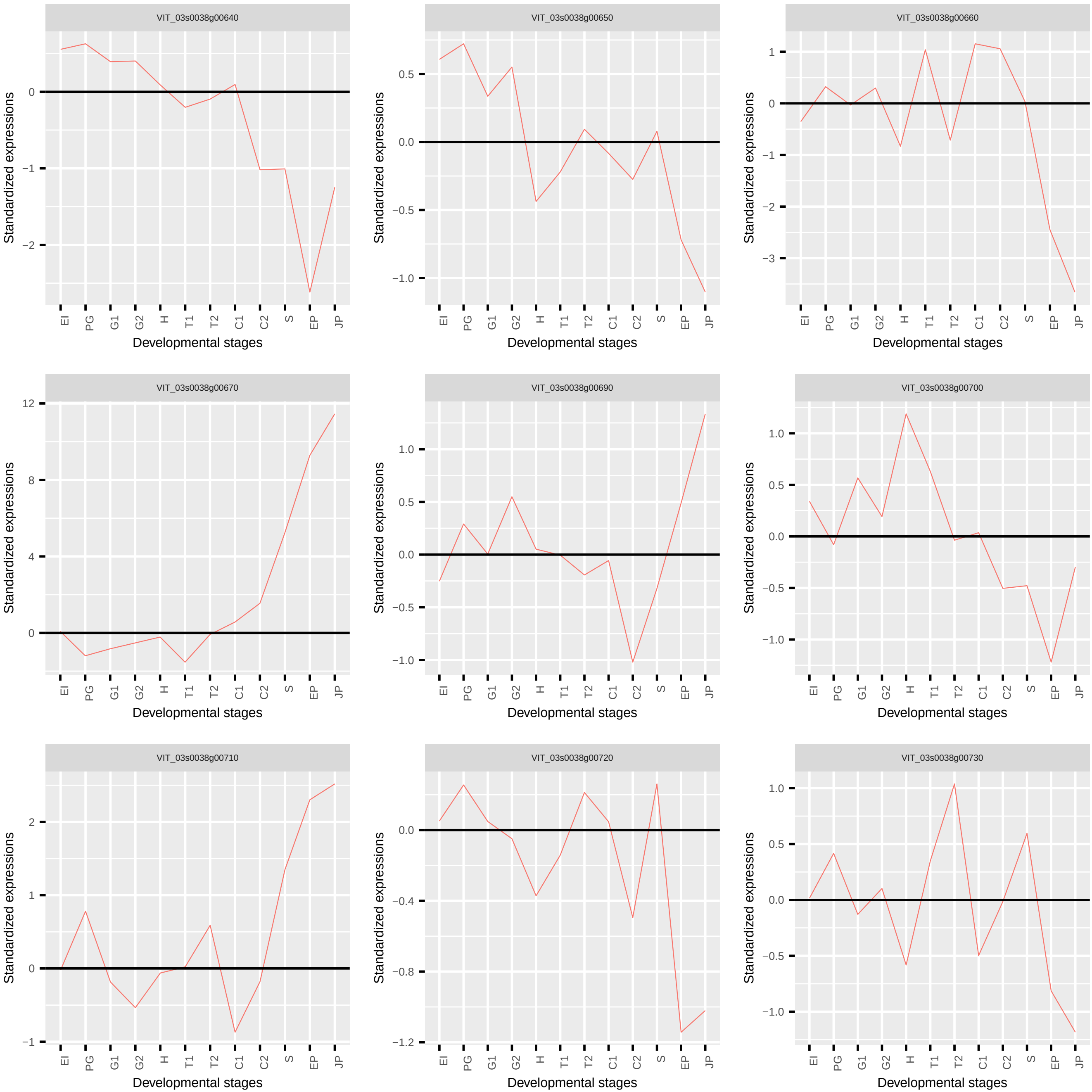
VIT_03s0038g00640
VIT_03s0038g00650
VIT_03s0038g00660
VIT_03s0038g00670
VIT_03s0038g00690
VIT_03s0038g00700
VIT_03s0038g00710
VIT_03s0038g00720
VIT_03s0038g00730
Standardized expressions
Developmental stages
EI PG G1 G2 H T1 T2 C1 C2 S EP JP

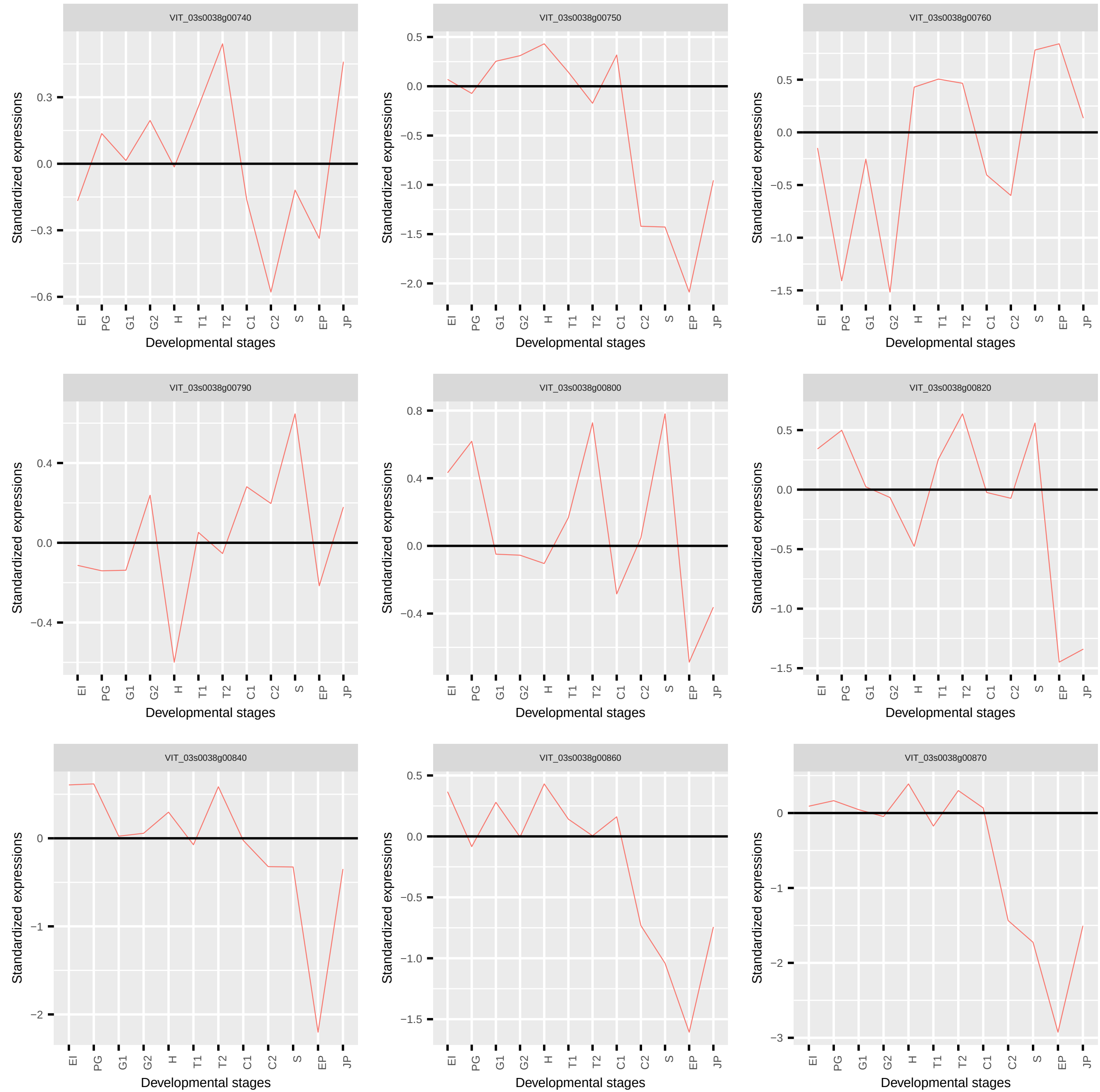
VIT_03s0038g00740
Standardized expressions
0.3
0.0
−0.3
−0.6
EI PG G1 G2 H T1 T2 C1 C2 S EP JP
Developmental stages
VIT_03s0038g00750
Standardized expressions
0.5
0.0
−0.5
−1.0
−1.5
−2.0
EI PG G1 G2 H T1 T2 C1 C2 S EP JP
Developmental stages
VIT_03s0038g00760
Standardized expressions
0.5
0.0
−0.5
−1.0
−1.5
EI PG G1 G2 H T1 T2 C1 C2 S EP JP
Developmental stages
VIT_03s0038g00790
Standardized expressions
0.4
0.0
−0.4
EI PG G1 G2 H T1 T2 C1 C2 S EP JP
Developmental stages
VIT_03s0038g00800
Standardized expressions
0.8
0.4
0.0
−0.4
EI PG G1 G2 H T1 T2 C1 C2 S EP JP
Developmental stages
VIT_03s0038g00820
Standardized expressions
0.5
0.0
−0.5
−1.0
−1.5
EI PG G1 G2 H T1 T2 C1 C2 S EP JP
Developmental stages
VIT_03s0038g00840
Standardized expressions
0
−1
−2
EI PG G1 G2 H T1 T2 C1 C2 S EP JP
Developmental stages
VIT_03s0038g00860
Standardized expressions
0.5
0.0
−0.5
−1.0
−1.5
EI PG G1 G2 H T1 T2 C1 C2 S EP JP
Developmental stages
VIT_03s0038g00870
Standardized expressions
0
−1
−2
−3
EI PG G1 G2 H T1 T2 C1 C2 S EP JP
Developmental stages

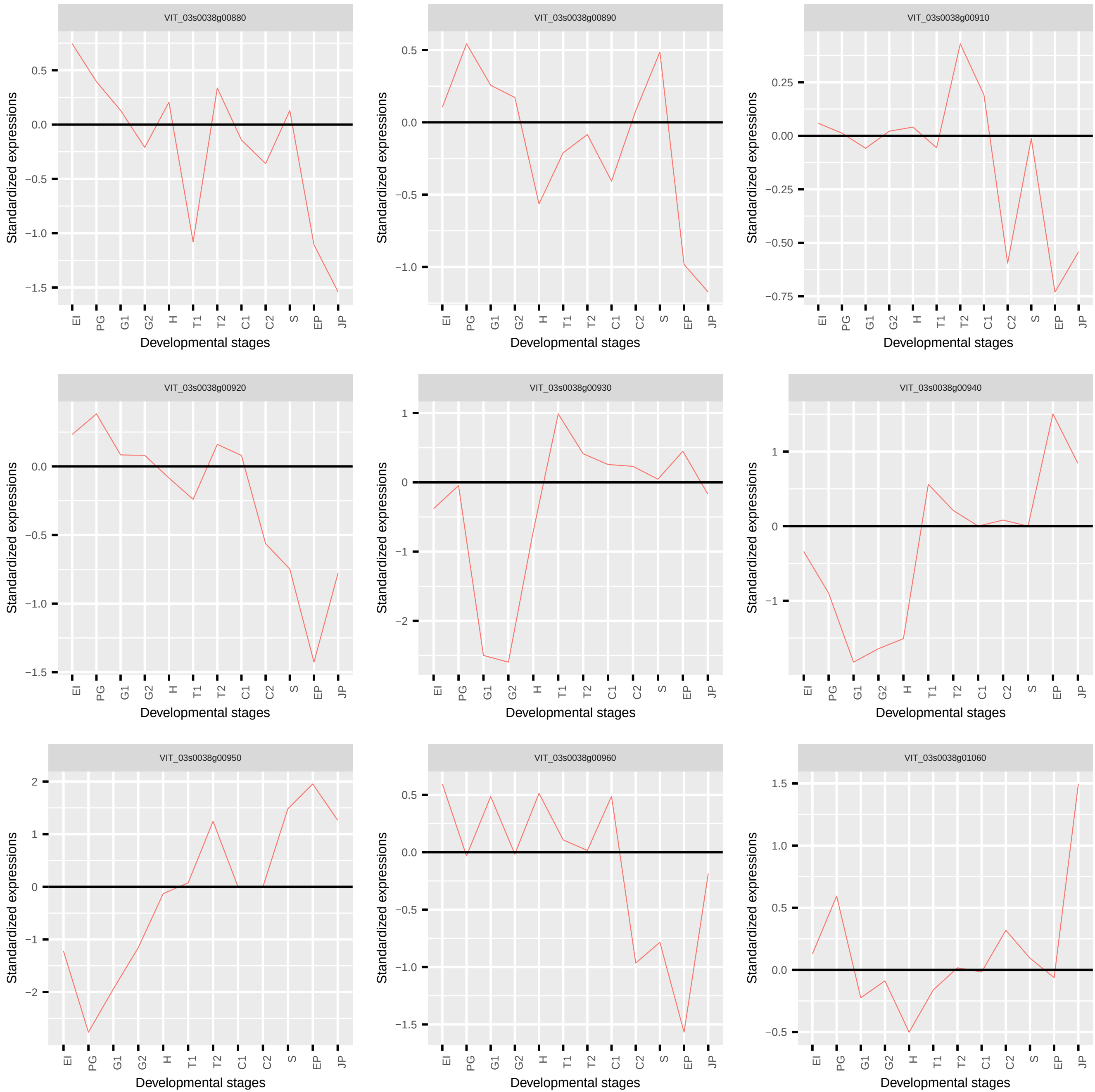

VIT_03s0038g00880
Standardized expressions
0.5
0.0
−0.5
−1.0
−1.5
EI PG G1 G2 H T1 T2 C1 C2 S EP JP
Developmental stages
VIT_03s0038g00890
Standardized expressions
0.5
0.0
−0.5
−1.0
EI PG G1 G2 H T1 T2 C1 C2 S EP JP
Developmental stages
VIT_03s0038g00910
Standardized expressions
0.25
0.00
−0.25
−0.50
−0.75
EI PG G1 G2 H T1 T2 C1 C2 S EP JP
Developmental stages
VIT_03s0038g00920
Standardized expressions
0.0
−0.5
−1.0
−1.5
EI PG G1 G2 H T1 T2 C1 C2 S EP JP
Developmental stages
VIT_03s0038g00930
Standardized expressions
1
0
−1
−2
EI PG G1 G2 H T1 T2 C1 C2 S EP JP
Developmental stages
VIT_03s0038g00940
Standardized expressions
1
0
−1
EI PG G1 G2 H T1 T2 C1 C2 S EP JP
Developmental stages
VIT_03s0038g00950
Standardized expressions
2
1
0
−1
−2
EI PG G1 G2 H T1 T2 C1 C2 S EP JP
Developmental stages
VIT_03s0038g00960
Standardized expressions
0.5
0.0
−0.5
−1.0
−1.5
EI PG G1 G2 H T1 T2 C1 C2 S EP JP
Developmental stages
VIT_03s0038g01060
Standardized expressions
1.5
1.0
0.5
0.0
−0.5
EI PG G1 G2 H T1 T2 C1 C2 S EP JP
Developmental stages

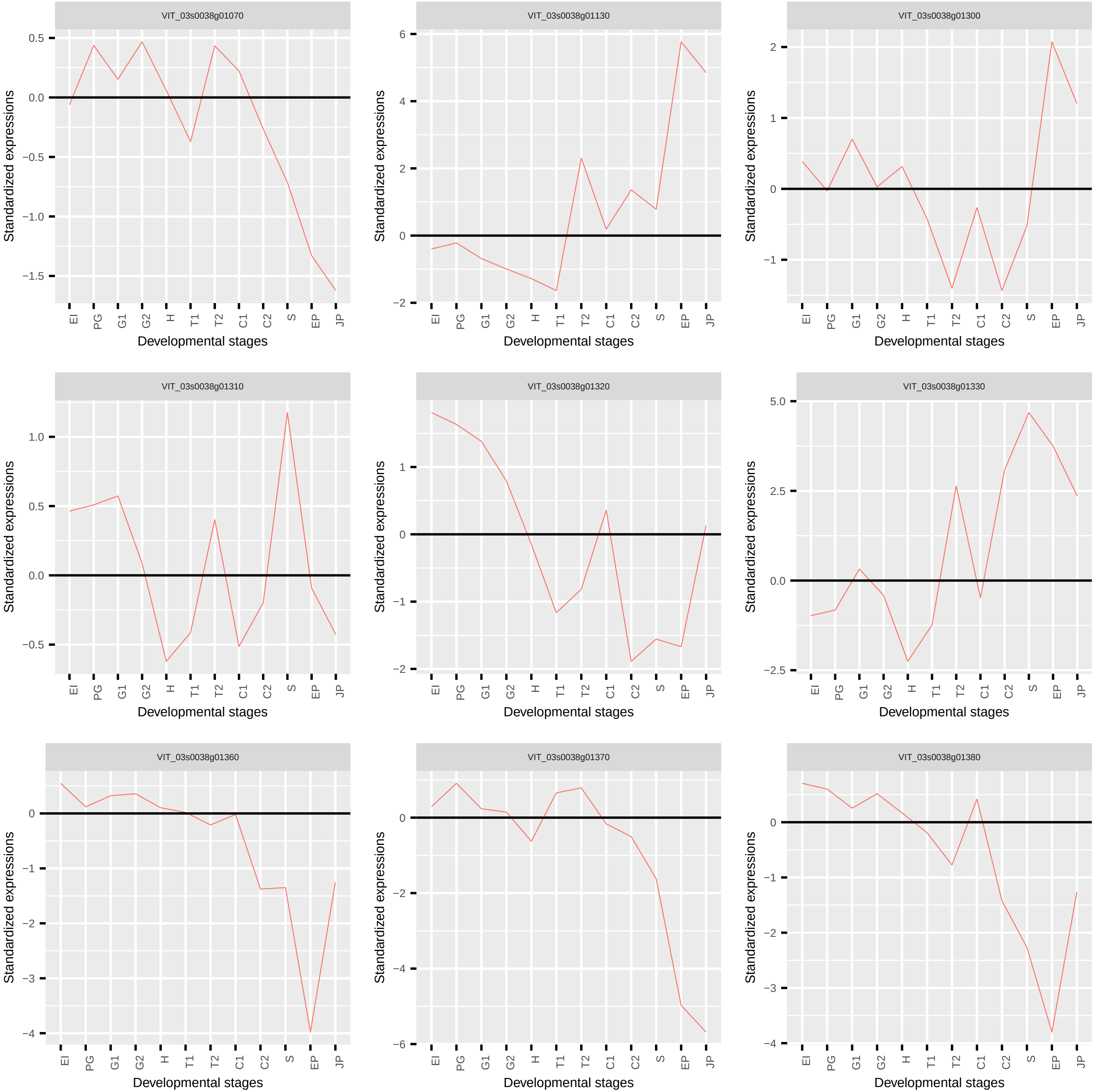
VIT_03s0038g01070
VIT_03s0038g01130
VIT_03s0038g01300
VIT_03s0038g01310
VIT_03s0038g01320
VIT_03s0038g01330
VIT_03s0038g01360
VIT_03s0038g01370
VIT_03s0038g01380
Standardized expressions
Developmental stages
EI PG G1 G2 H T1 T2 C1 C2 S EP JP

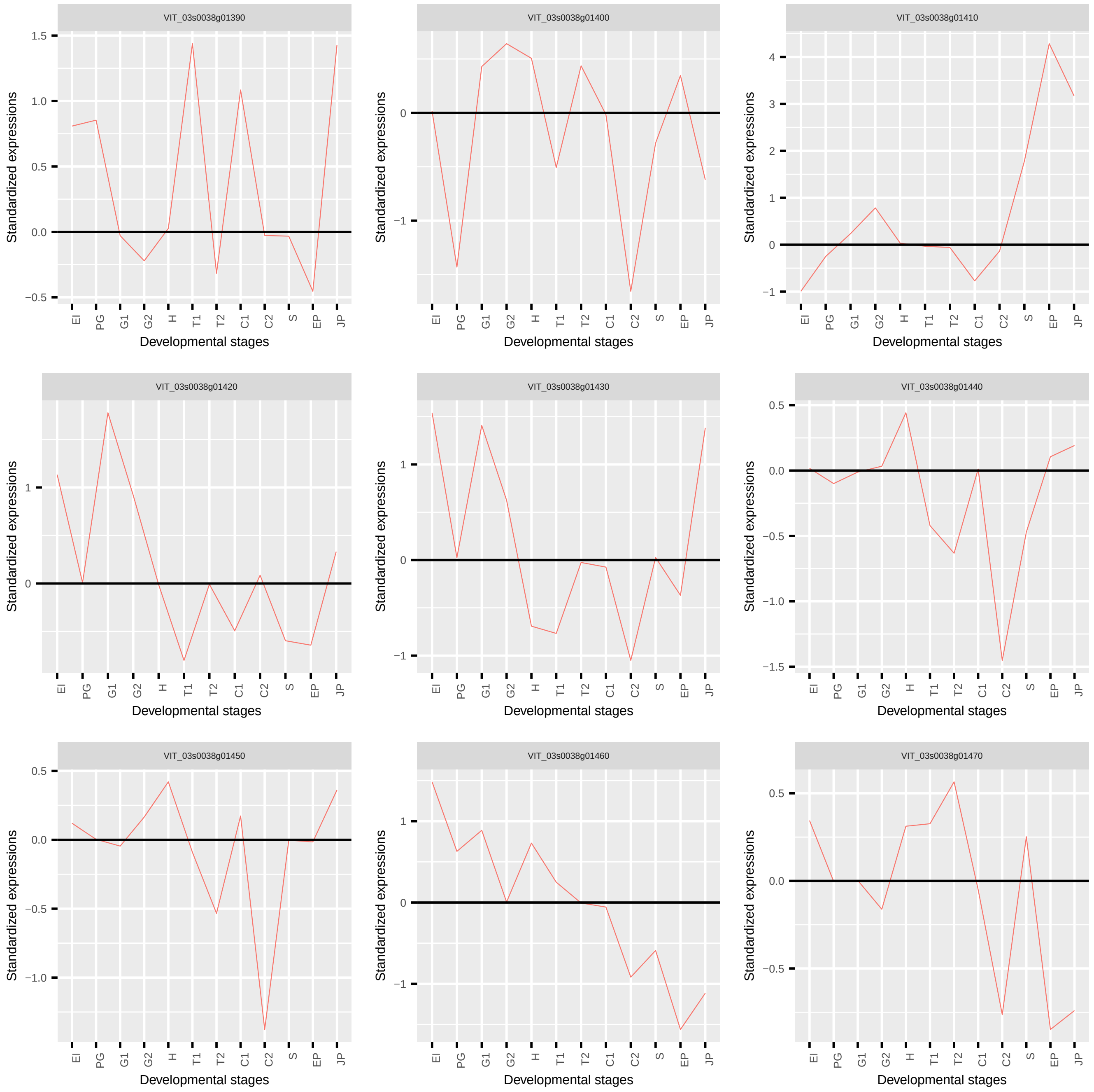

VIT_03s0038g01390
VIT_03s0038g01400
VIT_03s0038g01410
VIT_03s0038g01420
VIT_03s0038g01430
VIT_03s0038g01440
VIT_03s0038g01450
VIT_03s0038g01460
VIT_03s0038g01470
Standardized expressions
Developmental stages
EI PG G1 G2 H T1 T2 C1 C2 S EP JP

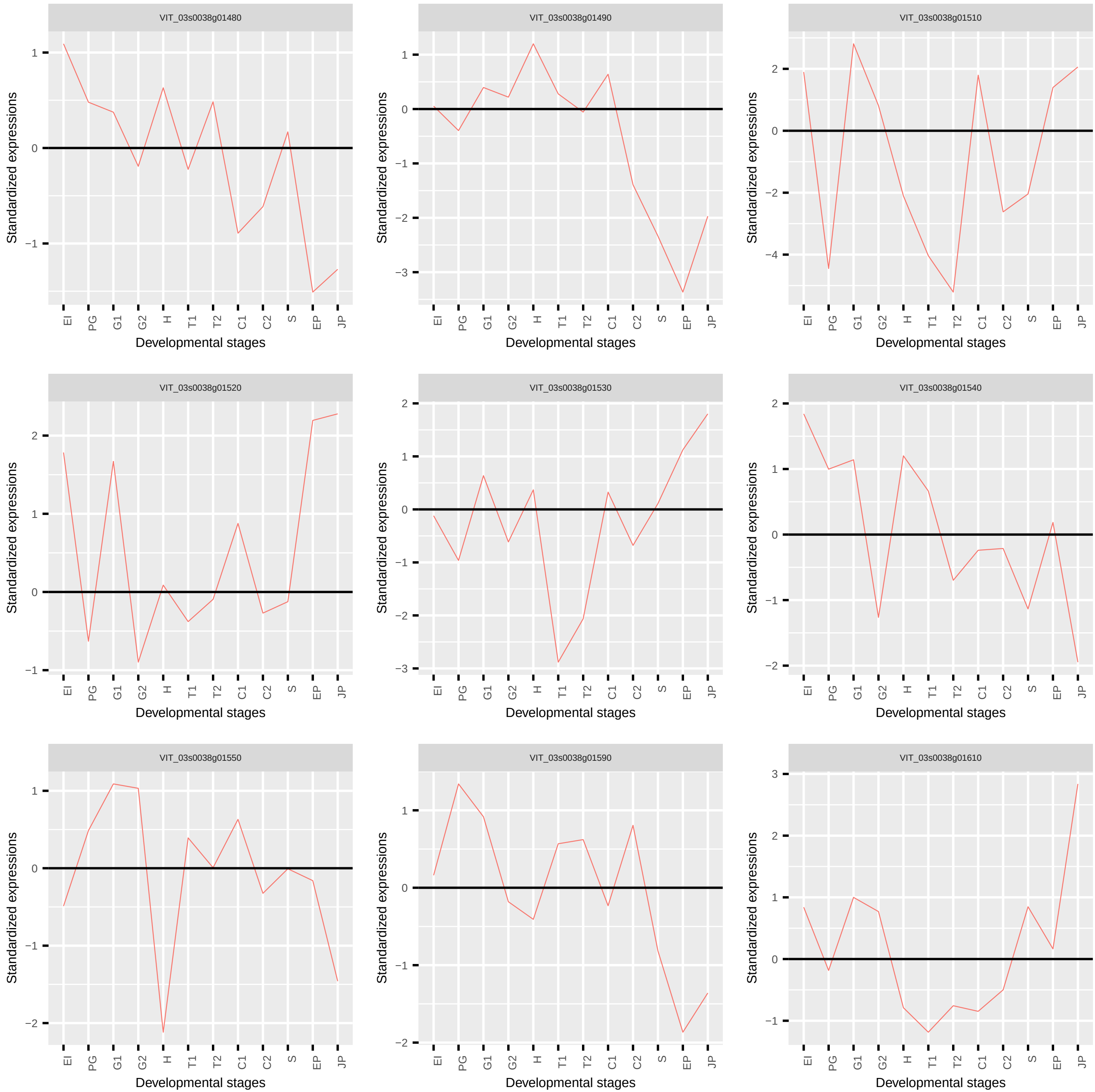

VIT_03s0038g01480
VIT_03s0038g01490
VIT_03s0038g01510
VIT_03s0038g01520
VIT_03s0038g01530
VIT_03s0038g01540
VIT_03s0038g01550
VIT_03s0038g01590
VIT_03s0038g01610
Standardized expressions
Developmental stages
EI PG G1 G2 H T1 T2 C1 C2 S EP JP

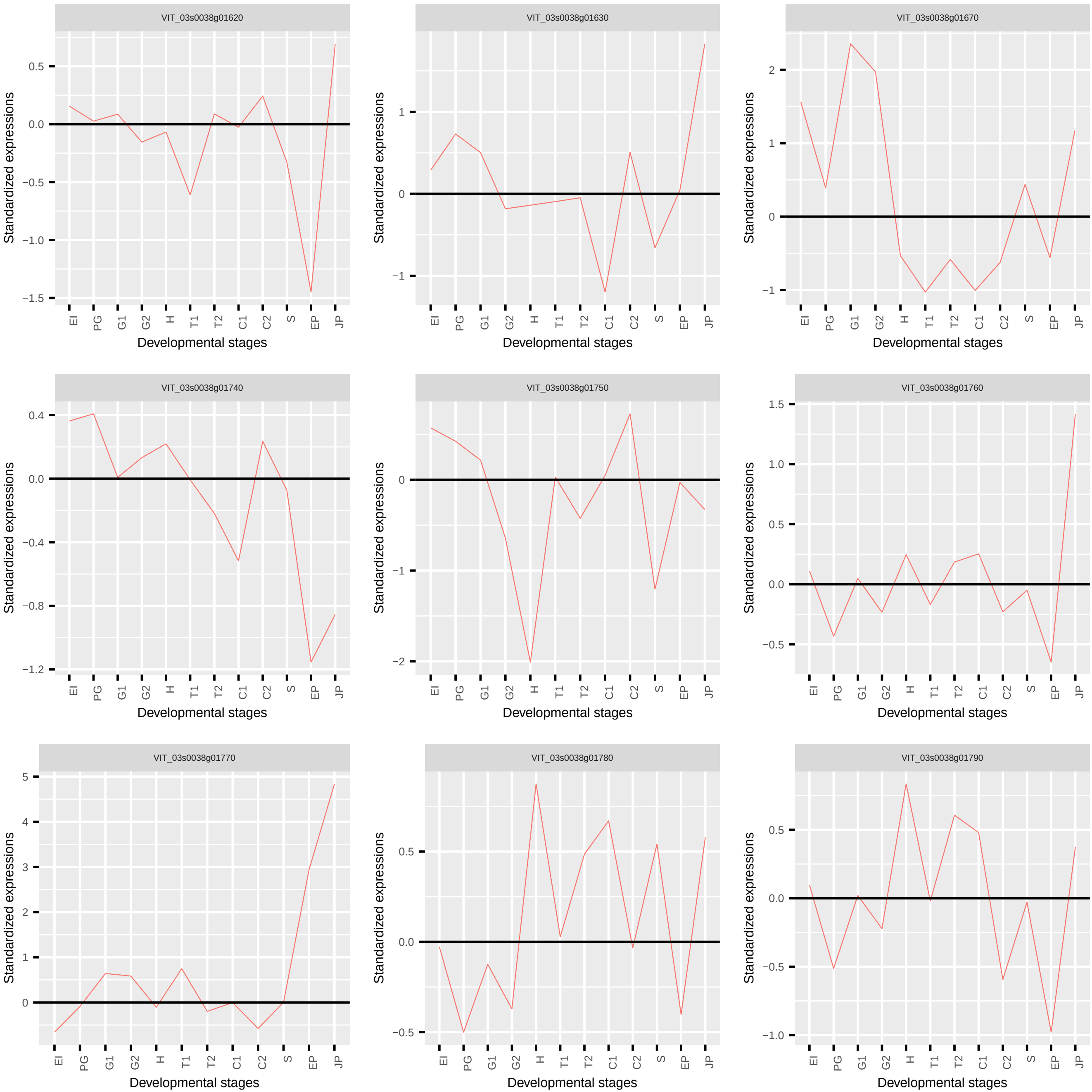
VIT_03s0038g01620
VIT_03s0038g01630
VIT_03s0038g01670
VIT_03s0038g01740
VIT_03s0038g01750
VIT_03s0038g01760
VIT_03s0038g01770
VIT_03s0038g01780
VIT_03s0038g01790
Standardized expressions
Developmental stages
EI PG G1 G2 H T1 T2 C1 C2 S EP JP

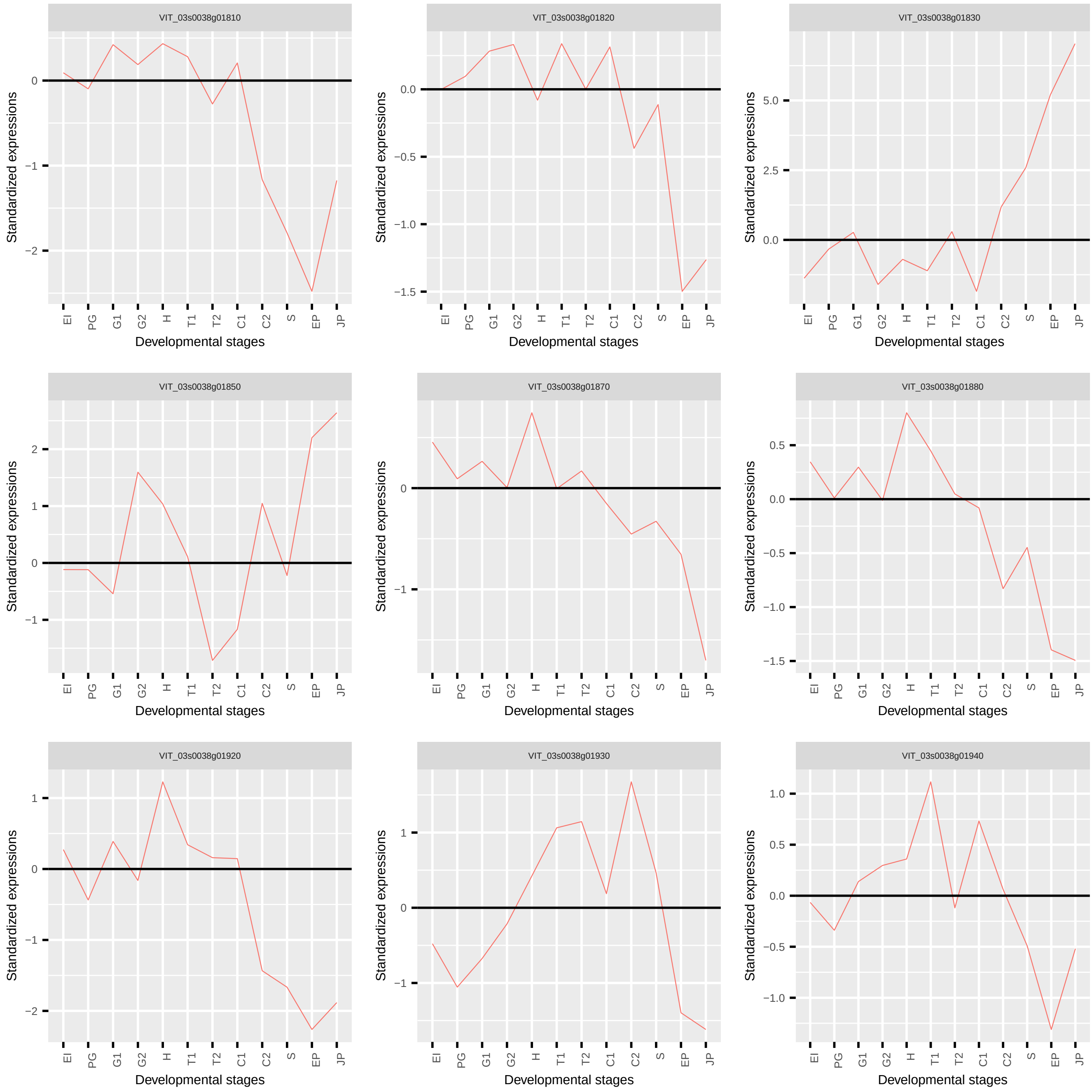
VIT_03s0038g01810
VIT_03s0038g01820
VIT_03s0038g01830
VIT_03s0038g01850
VIT_03s0038g01870
VIT_03s0038g01880
VIT_03s0038g01920
VIT_03s0038g01930
VIT_03s0038g01940
Standardized expressions
Developmental stages
EI PG G1 G2 H T1 T2 C1 C2 S EP JP

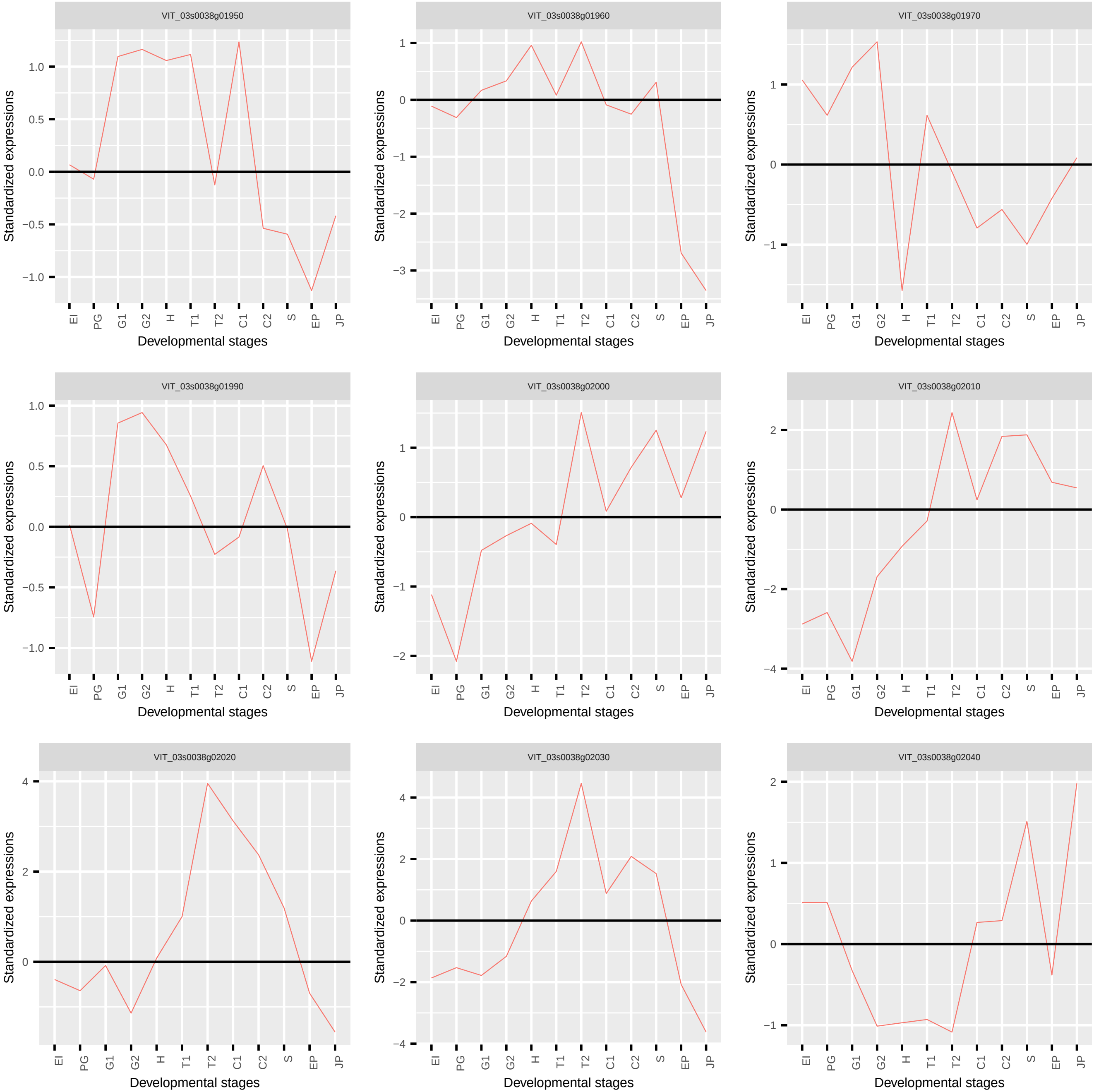

VIT_03s0038g01950
VIT_03s0038g01960
VIT_03s0038g01970
VIT_03s0038g01990
VIT_03s0038g02000
VIT_03s0038g02010
VIT_03s0038g02020
VIT_03s0038g02030
VIT_03s0038g02040
Standardized expressions
Developmental stages
EI PG G1 G2 H T1 T2 C1 C2 S EP JP

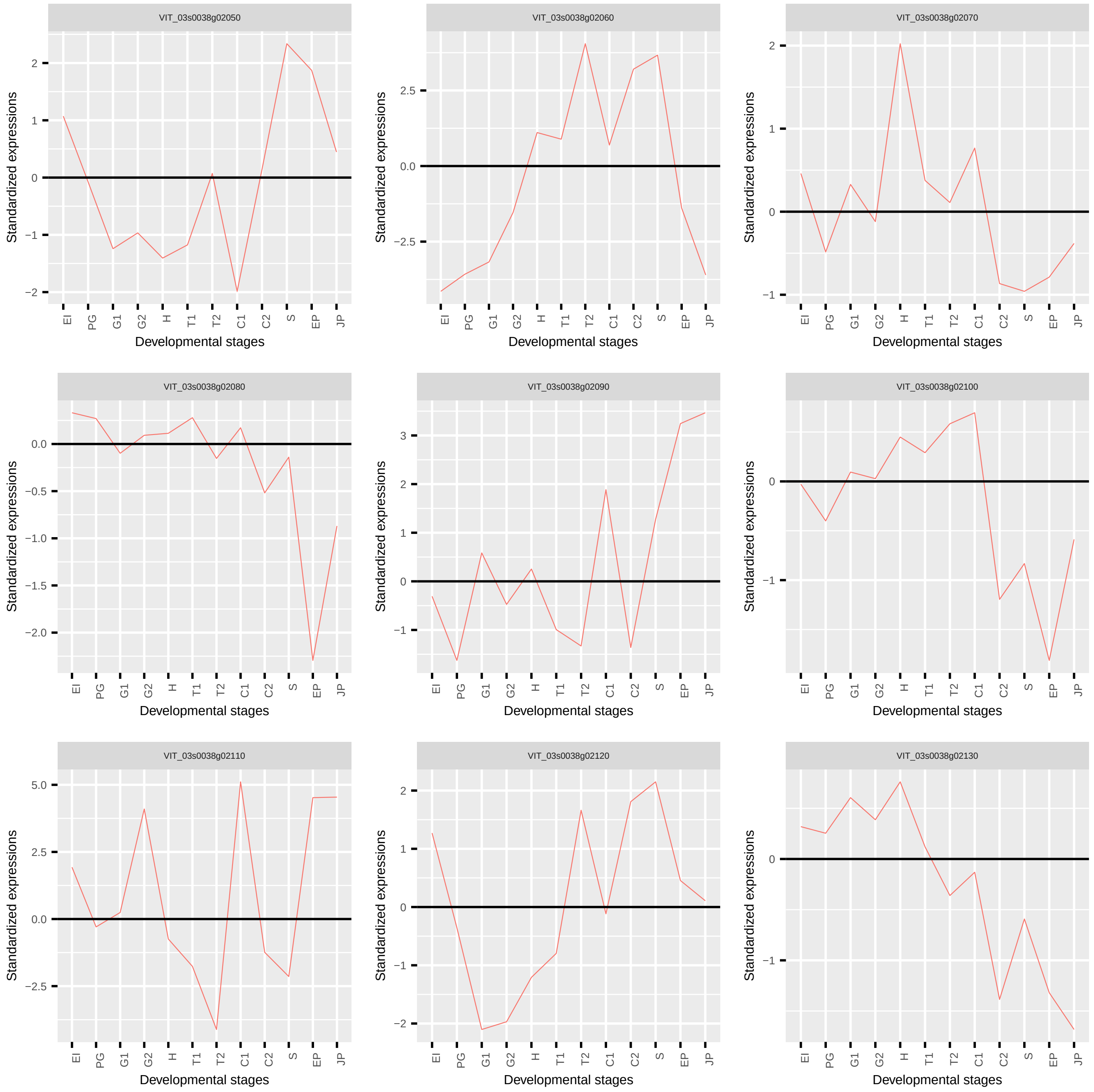
VIT_03s0038g02050
VIT_03s0038g02060
VIT_03s0038g02070
VIT_03s0038g02080
VIT_03s0038g02090
VIT_03s0038g02100
VIT_03s0038g02110
VIT_03s0038g02120
VIT_03s0038g02130
Standardized expressions
Developmental stages
EI PG G1 G2 H T1 T2 C1 C2 S EP JP

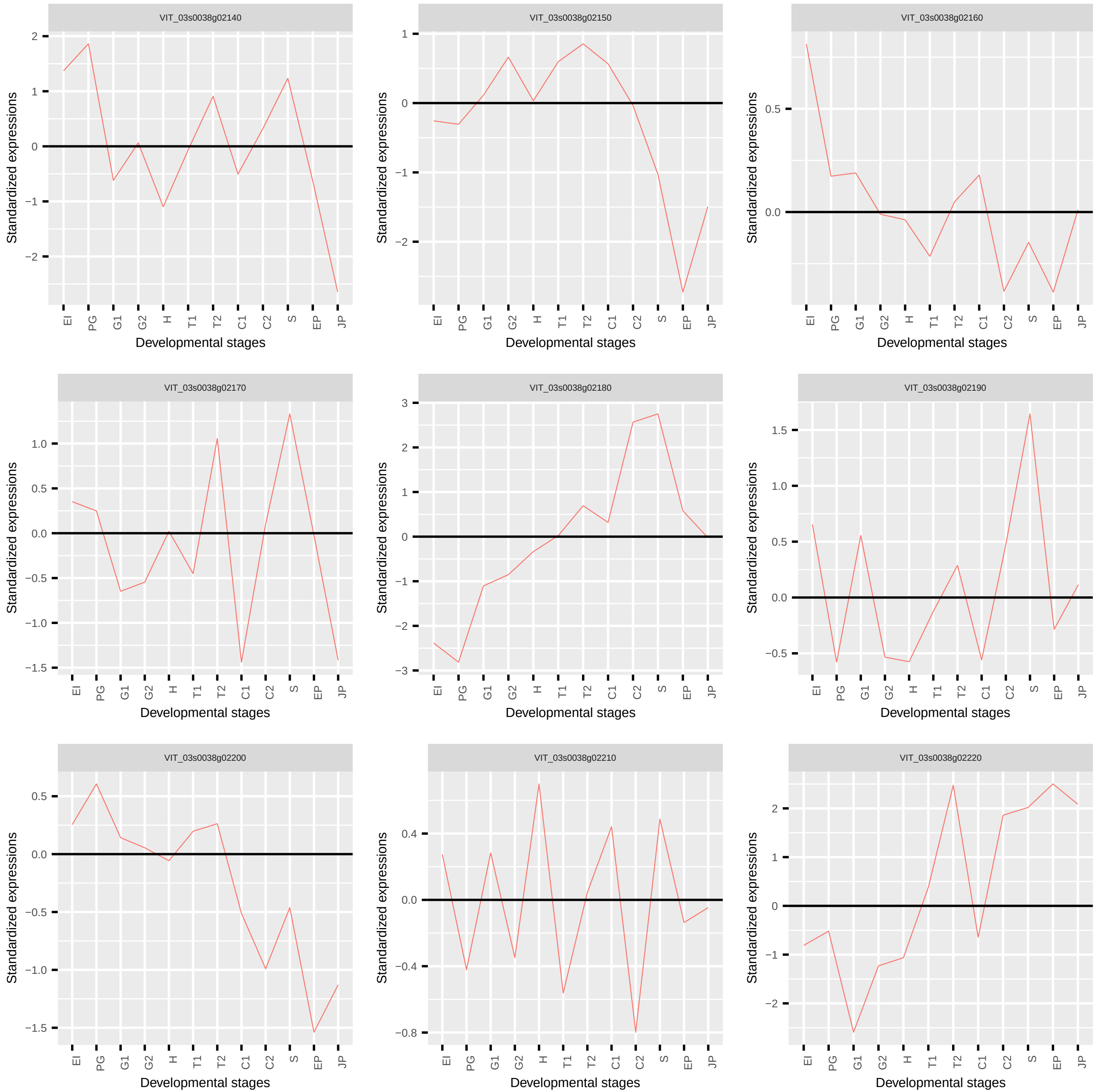
VIT_03s0038g02140
VIT_03s0038g02150
VIT_03s0038g02160
VIT_03s0038g02170
VIT_03s0038g02180
VIT_03s0038g02190
VIT_03s0038g02200
VIT_03s0038g02210
VIT_03s0038g02220
Standardized expressions
Developmental stages
EI PG G1 G2 H T1 T2 C1 C2 S EP JP

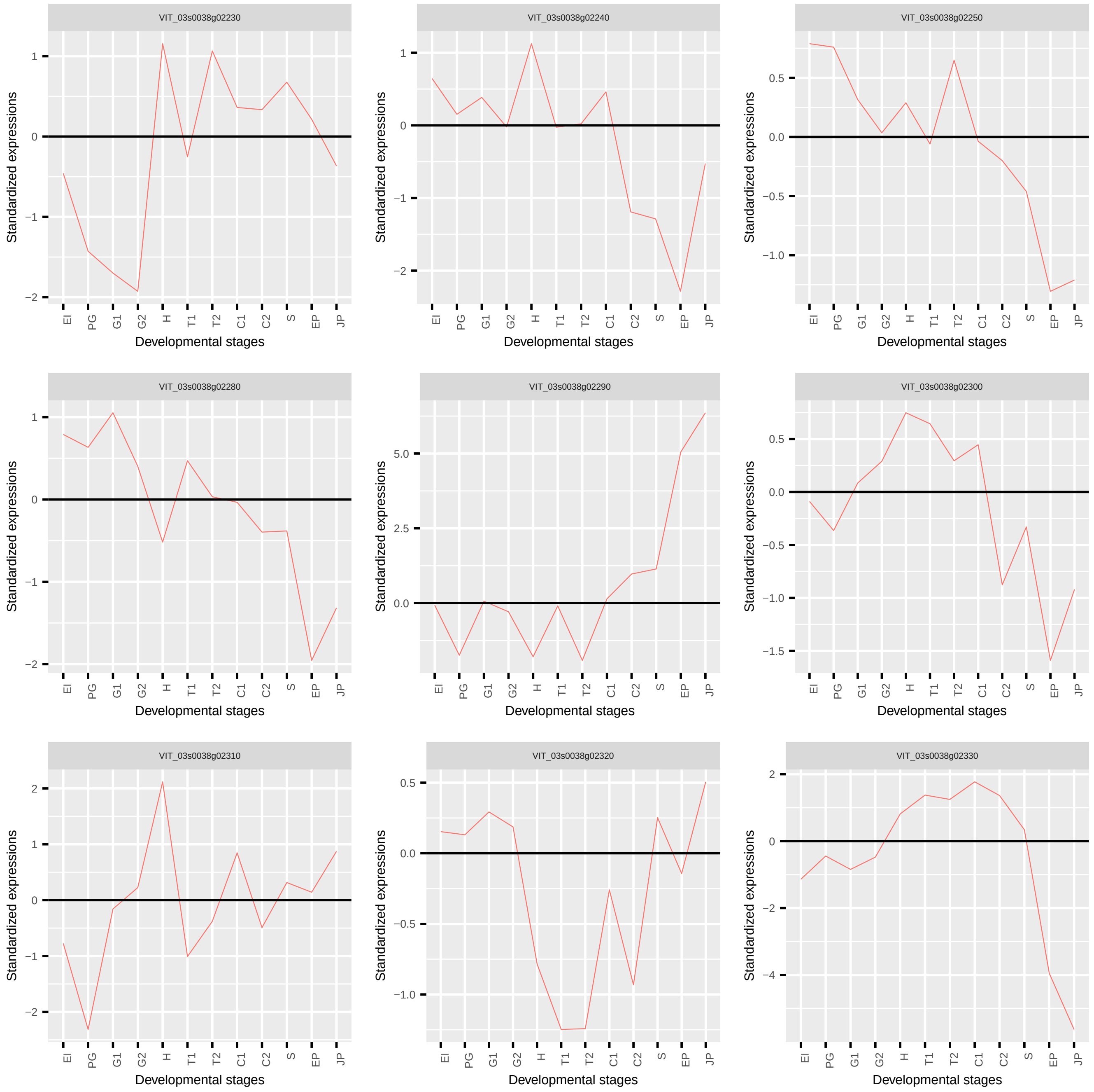
VIT_03s0038g02230
VIT_03s0038g02240
VIT_03s0038g02250
VIT_03s0038g02280
VIT_03s0038g02290
VIT_03s0038g02300
VIT_03s0038g02310
VIT_03s0038g02320
VIT_03s0038g02330
Standardized expressions
Developmental stages
EI PG G1 G2 H T1 T2 C1 C2 S EP JP

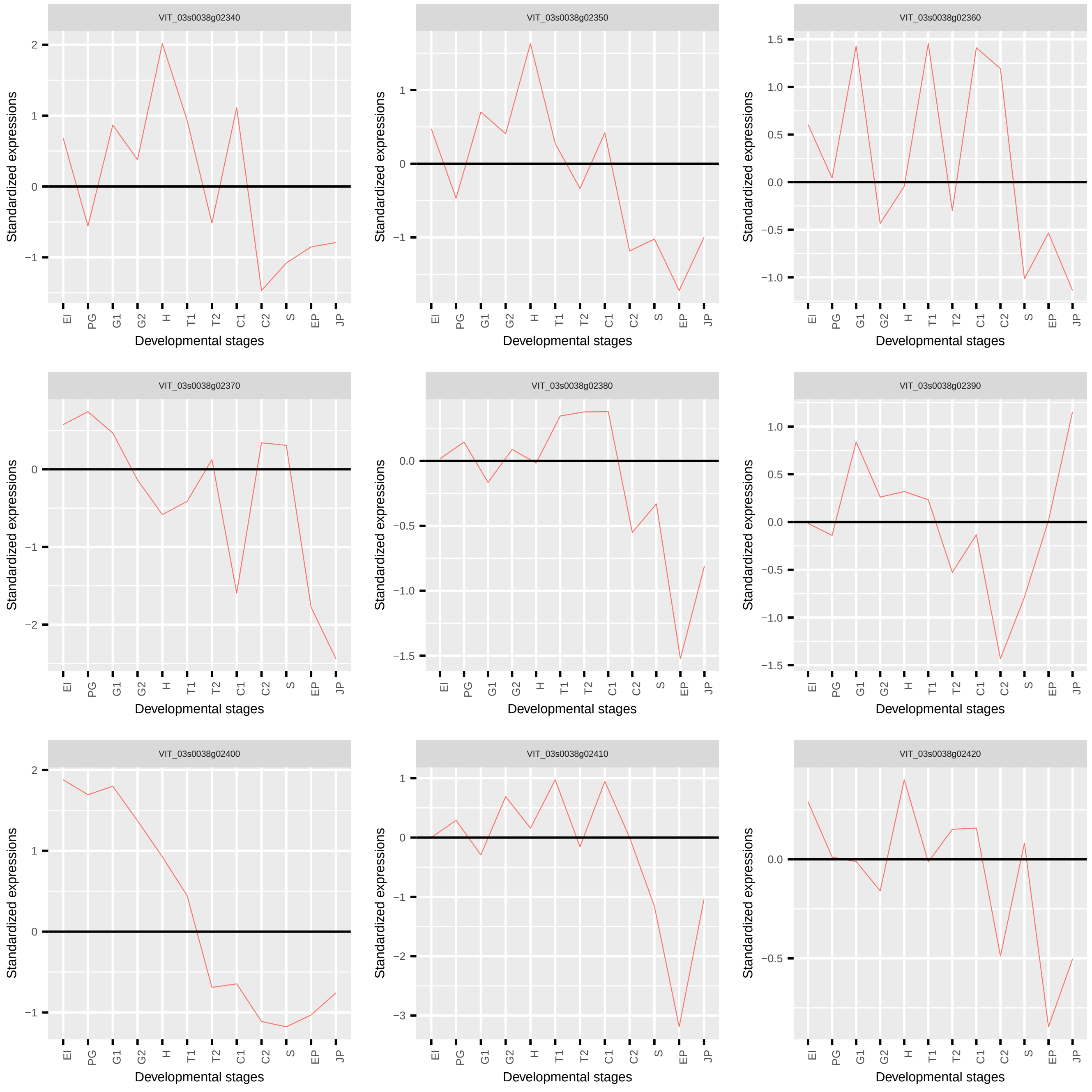
VIT_03s0038g02340
VIT_03s0038g02350
VIT_03s0038g02360
VIT_03s0038g02370
VIT_03s0038g02380
VIT_03s0038g02390
VIT_03s0038g02400
VIT_03s0038g02410
VIT_03s0038g02420
Standardized expressions
Developmental stages
EI PG G1 G2 H T1 T2 C1 C2 S EP JP

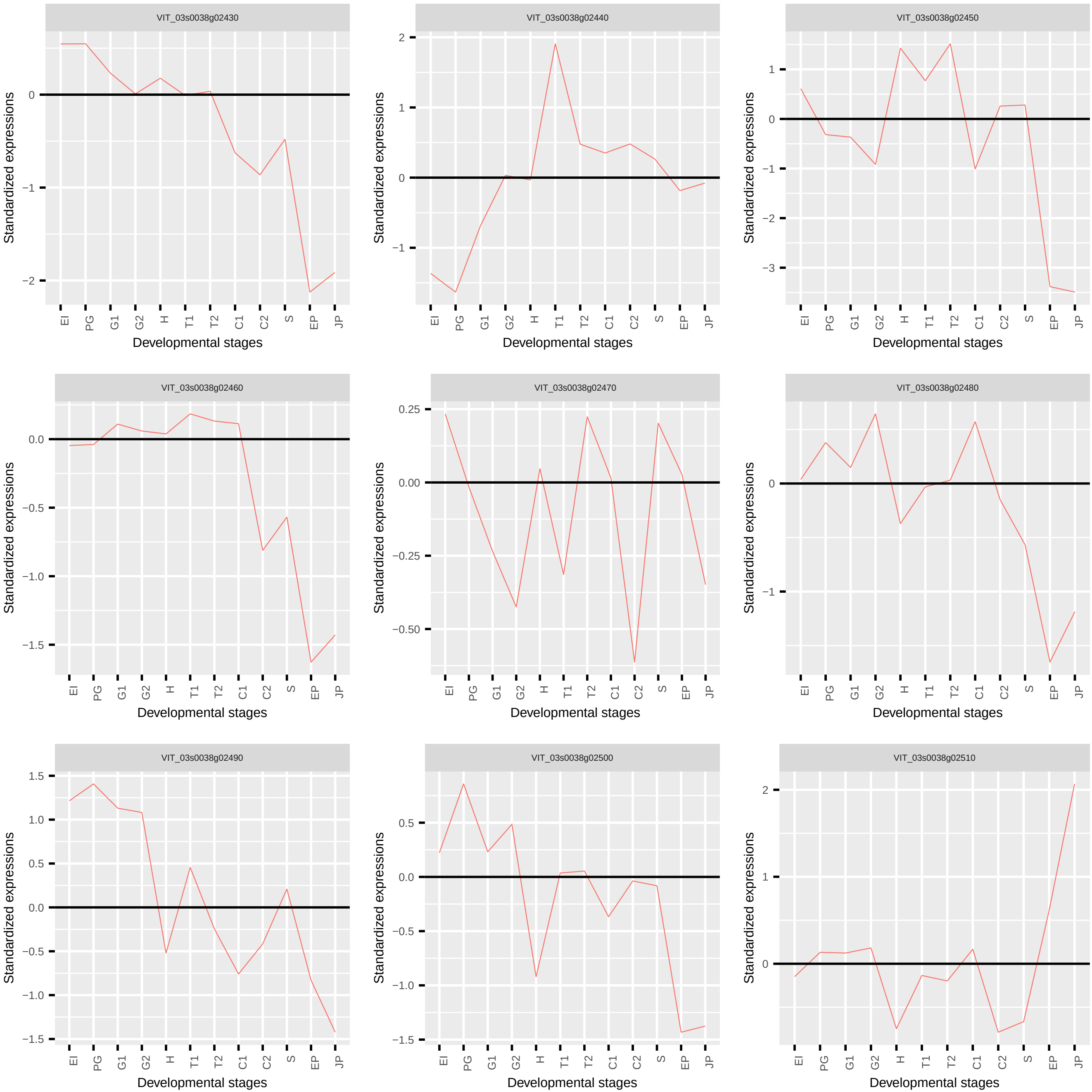
VIT_03s0038g02430
VIT_03s0038g02440
VIT_03s0038g02450
VIT_03s0038g02460
VIT_03s0038g02470
VIT_03s0038g02480
VIT_03s0038g02490
VIT_03s0038g02500
VIT_03s0038g02510
Standardized expressions
Developmental stages
EI PG G1 G2 H T1 T2 C1 C2 S EP JP

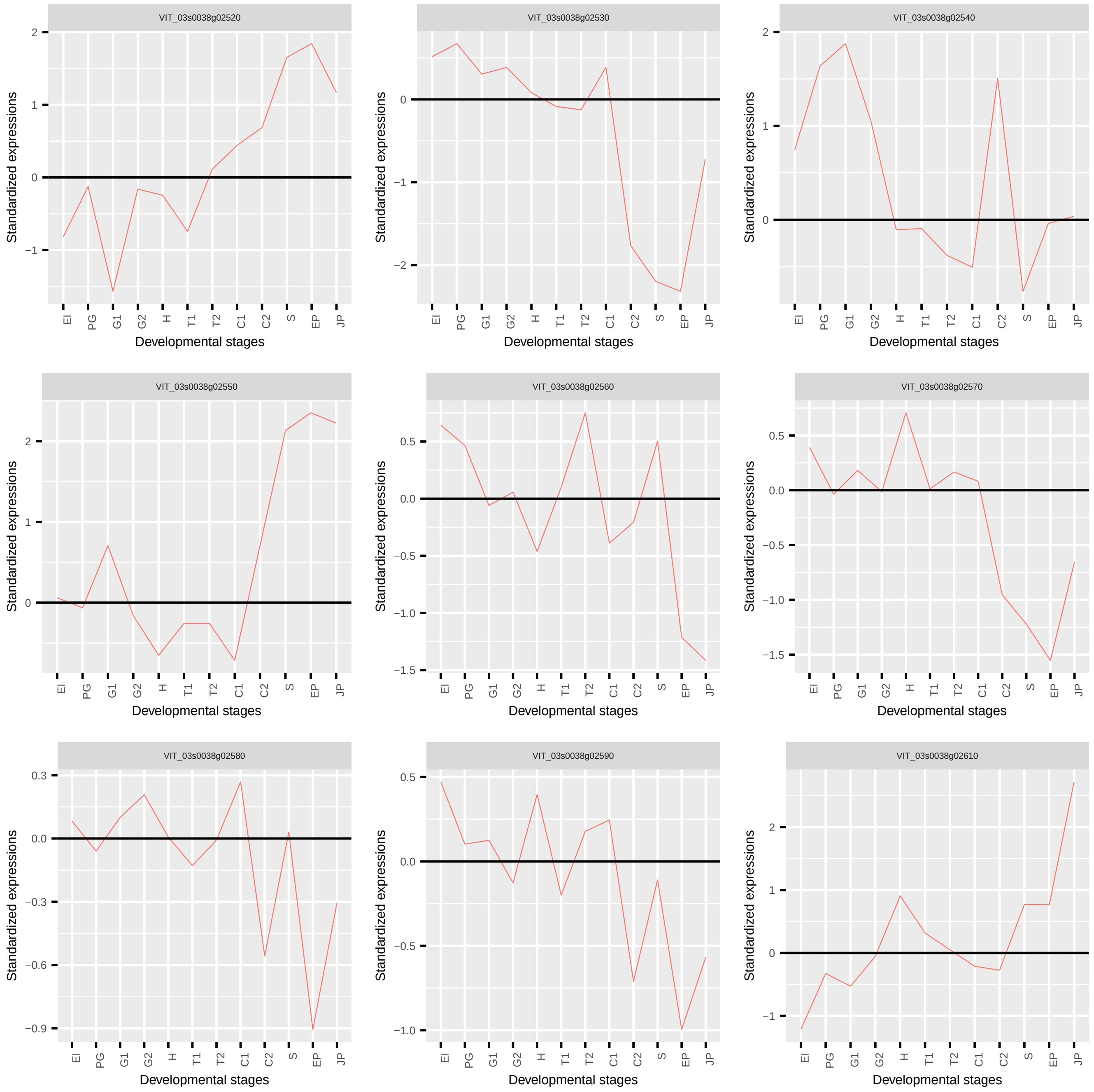
VIT_03s0038g02520
VIT_03s0038g02530
VIT_03s0038g02540
VIT_03s0038g02550
VIT_03s0038g02560
VIT_03s0038g02570
VIT_03s0038g02580
VIT_03s0038g02590
VIT_03s0038g02610
Standardized expressions
Developmental stages
EI PG G1 G2 H T1 T2 C1 C2 S EP JP

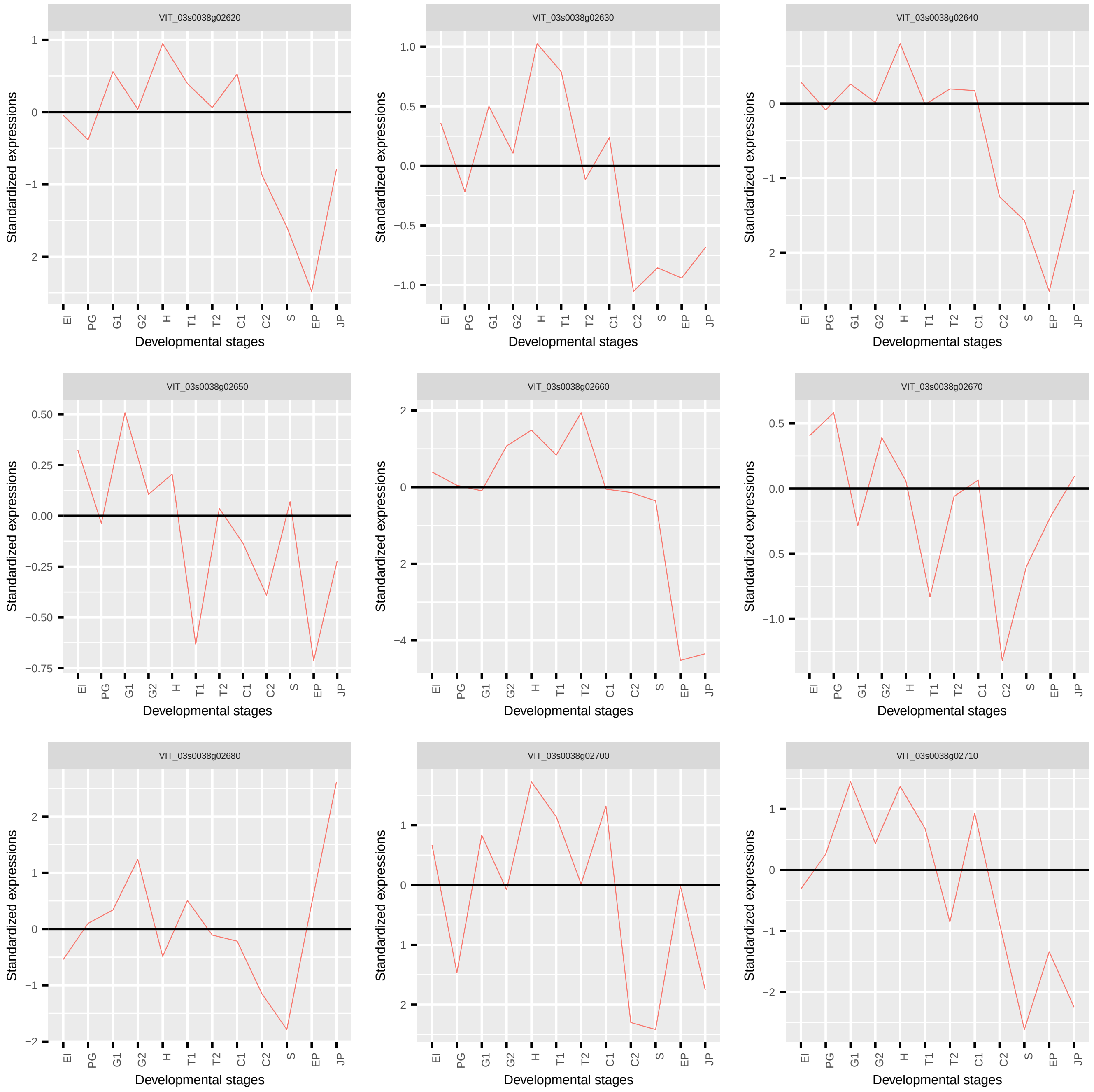
VIT_03s0038g02620
VIT_03s0038g02630
VIT_03s0038g02640
VIT_03s0038g02650
VIT_03s0038g02660
VIT_03s0038g02670
VIT_03s0038g02680
VIT_03s0038g02700
VIT_03s0038g02710
Standardized expressions
Developmental stages
EI PG G1 G2 H T1 T2 C1 C2 S EP JP

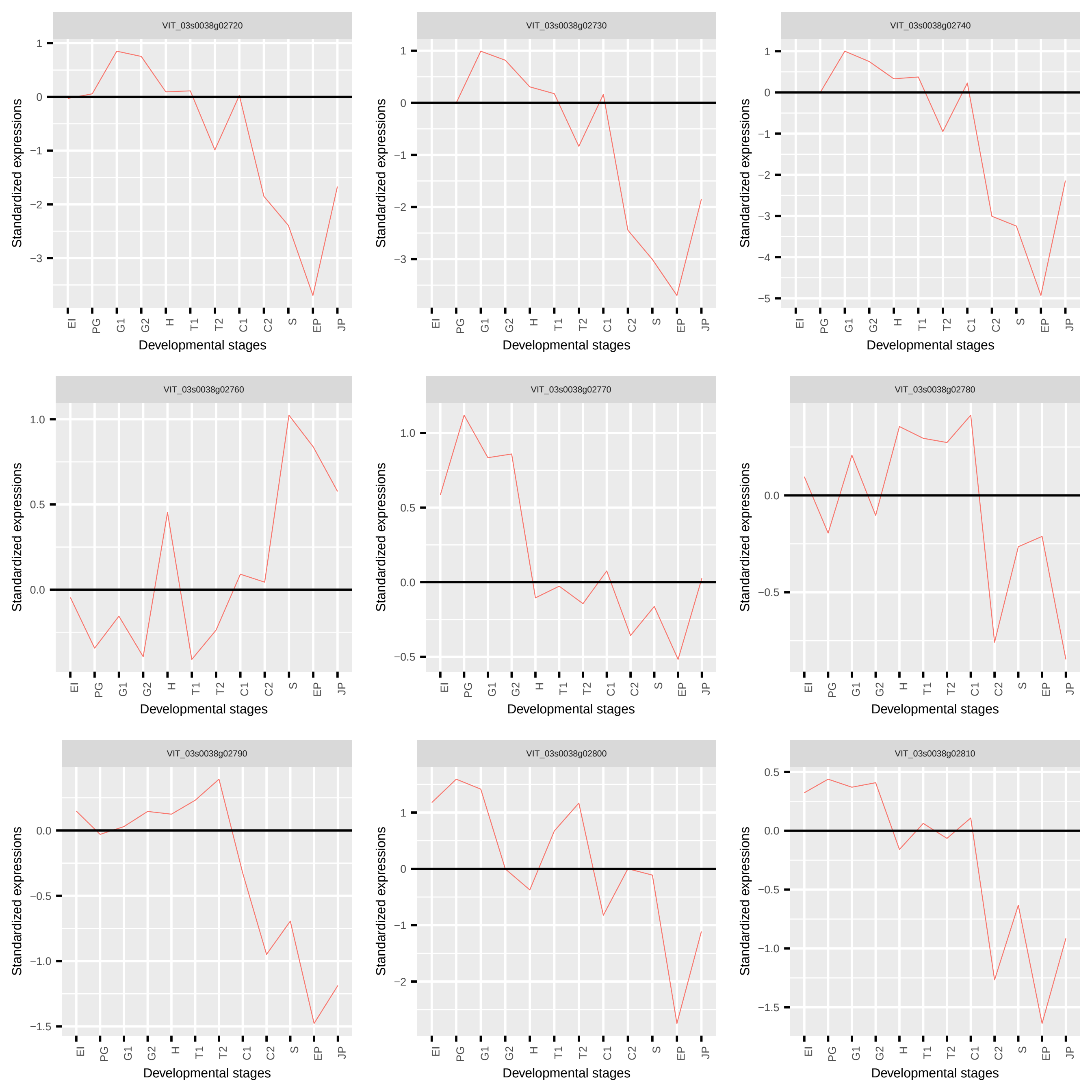

VIT_03s0038g02720
VIT_03s0038g02730
VIT_03s0038g02740
VIT_03s0038g02760
VIT_03s0038g02770
VIT_03s0038g02780
VIT_03s0038g02790
VIT_03s0038g02800
VIT_03s0038g02810
Standardized expressions
Developmental stages
EI PG G1 G2 H T1 T2 C1 C2 S EP JP

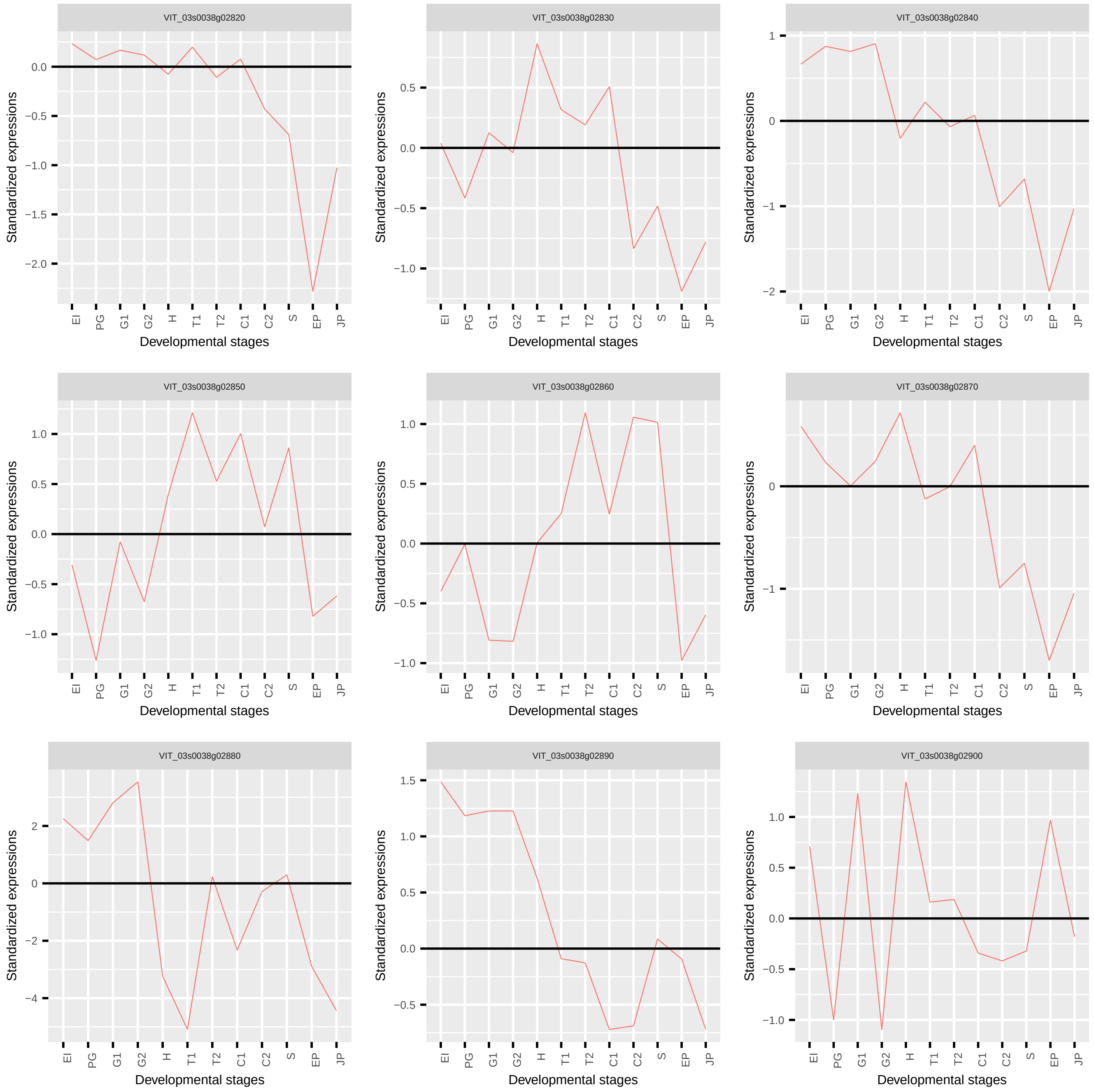
VIT_03s0038g02820
VIT_03s0038g02830
VIT_03s0038g02840
VIT_03s0038g02850
VIT_03s0038g02860
VIT_03s0038g02870
VIT_03s0038g02880
VIT_03s0038g02890
VIT_03s0038g02900
Standardized expressions
Developmental stages
EI PG G1 G2 H T1 T2 C1 C2 S EP JP
0.0 −0.5 −1.0 −1.5 −2.0
0.5 0.0 −0.5 −1.0
1 0 −1 −2
1.0 0.5 0.0 −0.5 −1.0
1.0 0.5 0.0 −0.5 −1.0
0 −1
2 0 −2 −4
1.5 1.0 0.5 0.0 −0.5
1.0 0.5 0.0 −0.5 −1.0

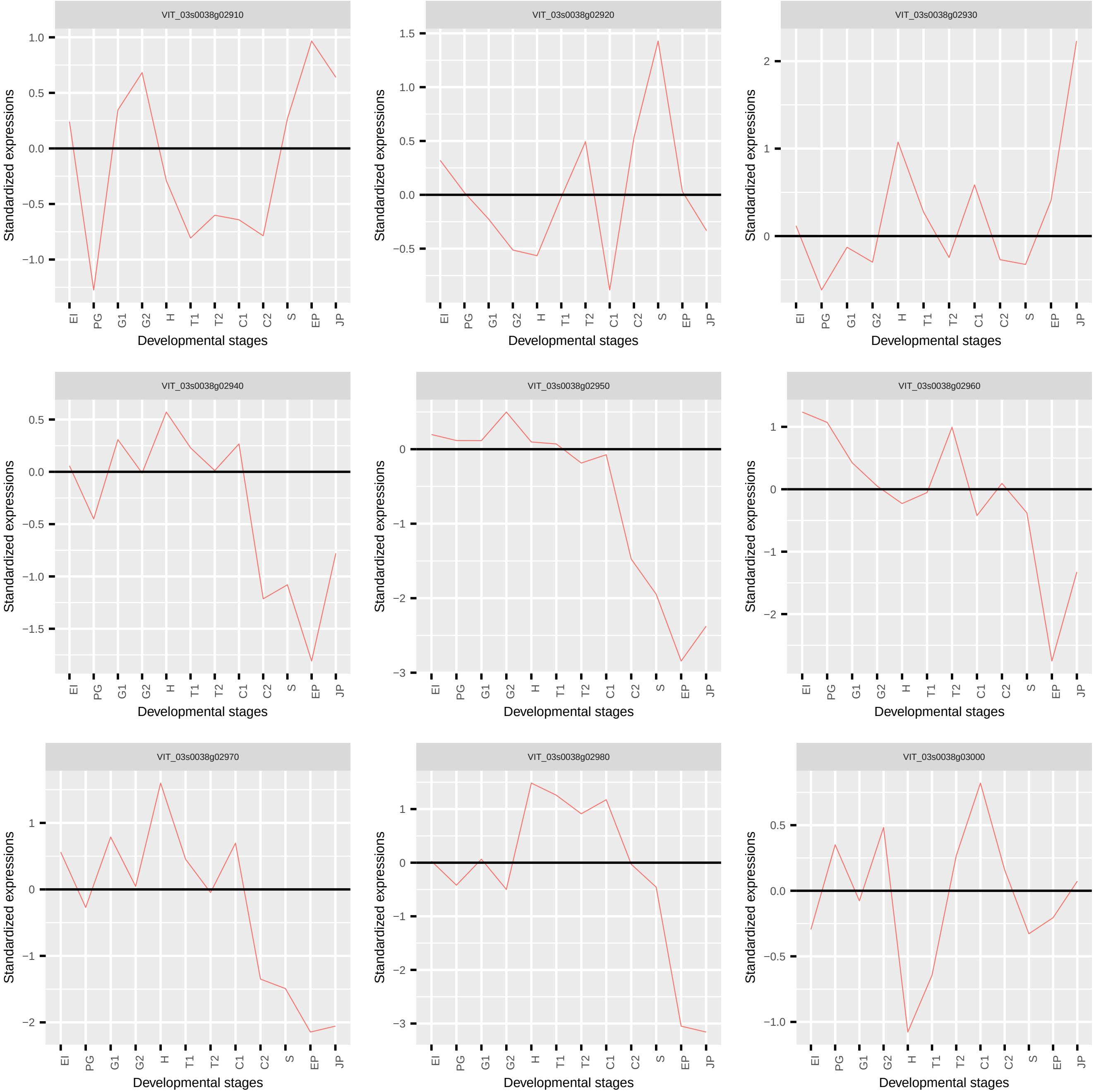
VIT_03s0038g02910
VIT_03s0038g02920
VIT_03s0038g02930
VIT_03s0038g02940
VIT_03s0038g02950
VIT_03s0038g02960
VIT_03s0038g02970
VIT_03s0038g02980
VIT_03s0038g03000
Standardized expressions
Developmental stages
EI PG G1 G2 H T1 T2 C1 C2 S EP JP

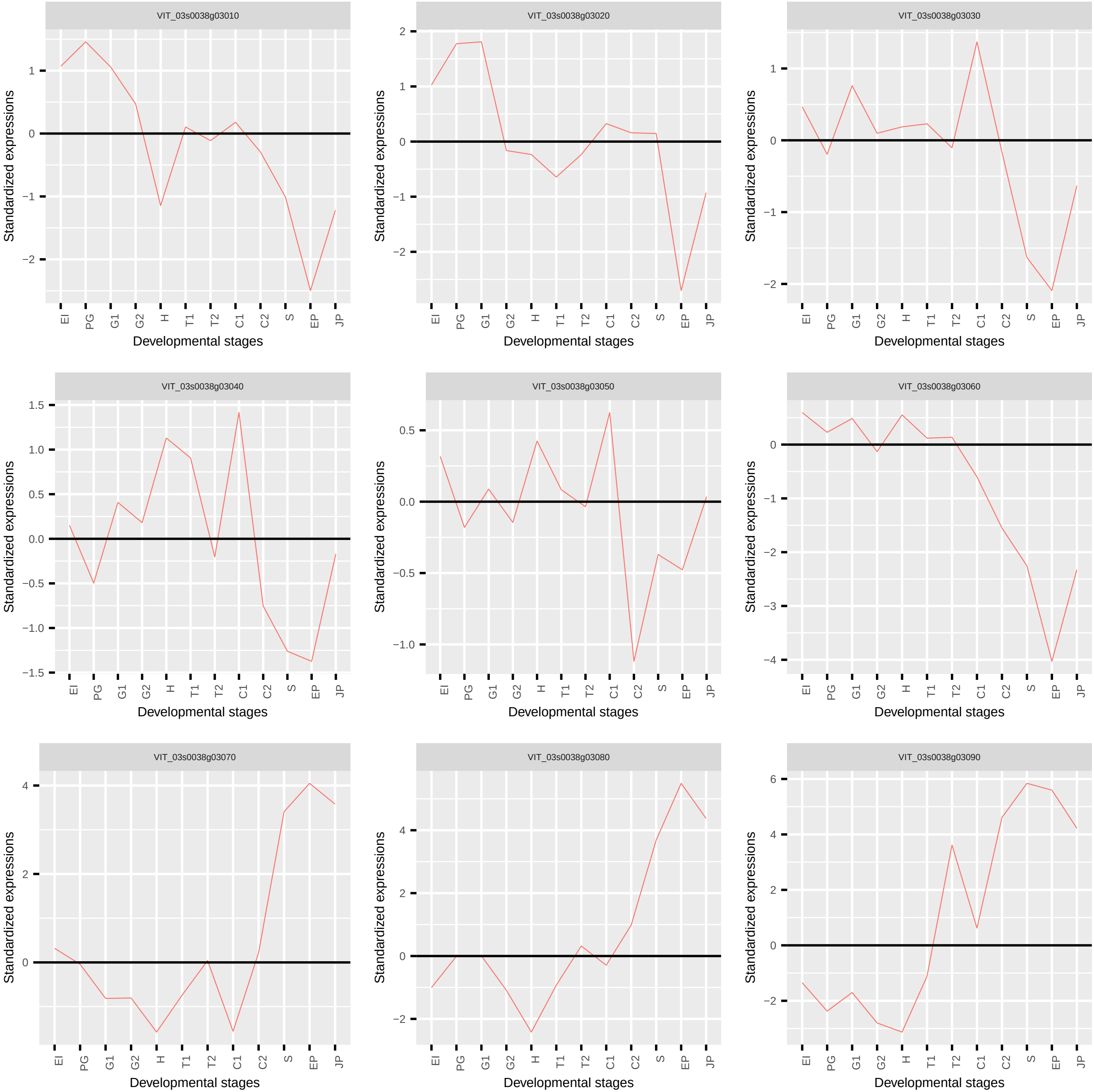

VIT_03s0038g03010
VIT_03s0038g03020
VIT_03s0038g03030
VIT_03s0038g03040
VIT_03s0038g03050
VIT_03s0038g03060
VIT_03s0038g03070
VIT_03s0038g03080
VIT_03s0038g03090
Standardized expressions
Developmental stages
EI PG G1 G2 H T1 T2 C1 C2 S EP JP

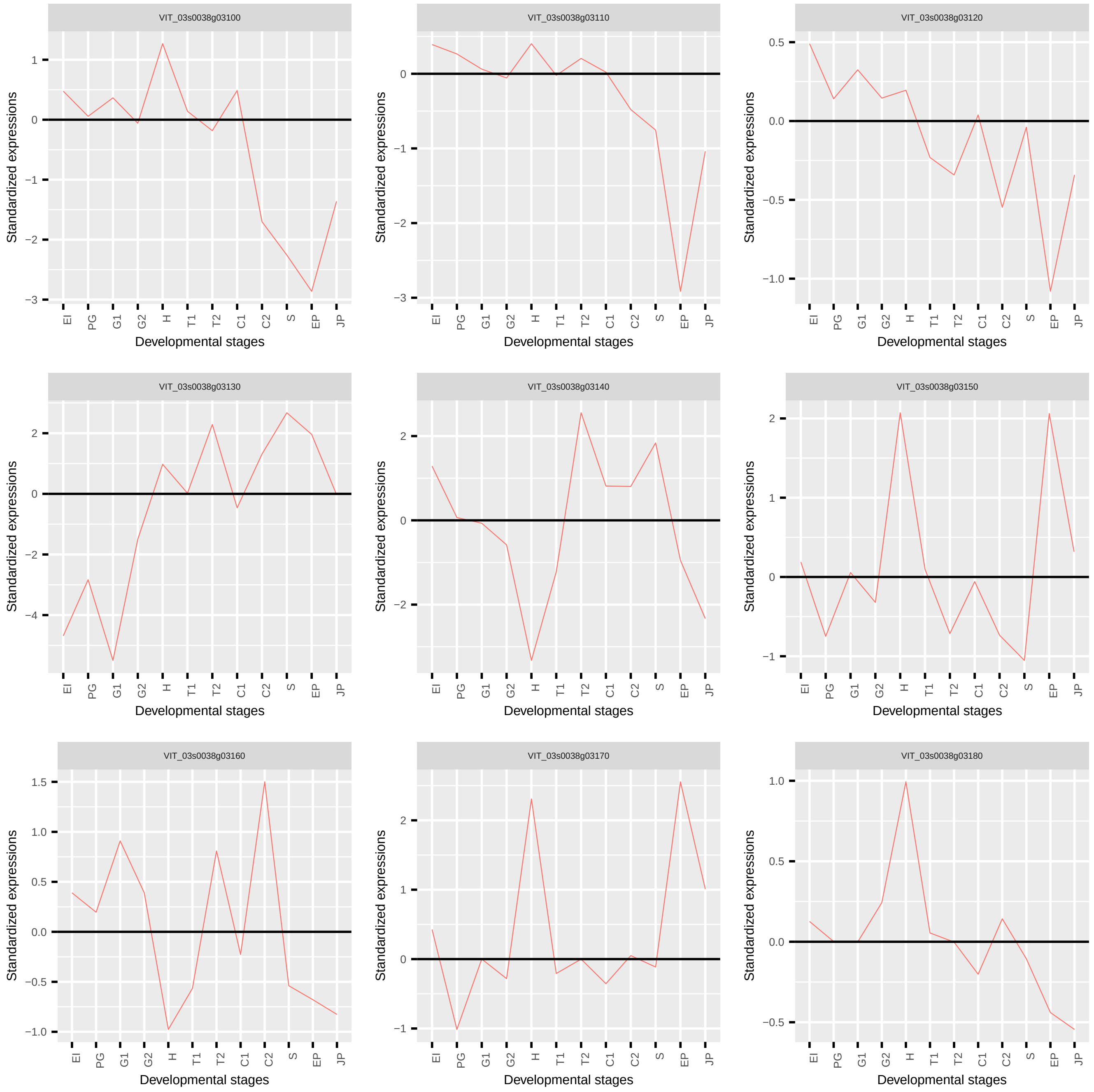
VIT_03s0038g03100
VIT_03s0038g03110
VIT_03s0038g03120
VIT_03s0038g03130
VIT_03s0038g03140
VIT_03s0038g03150
VIT_03s0038g03160
VIT_03s0038g03170
VIT_03s0038g03180
Standardized expressions
Developmental stages
EI PG G1 G2 H T1 T2 C1 C2 S EP JP

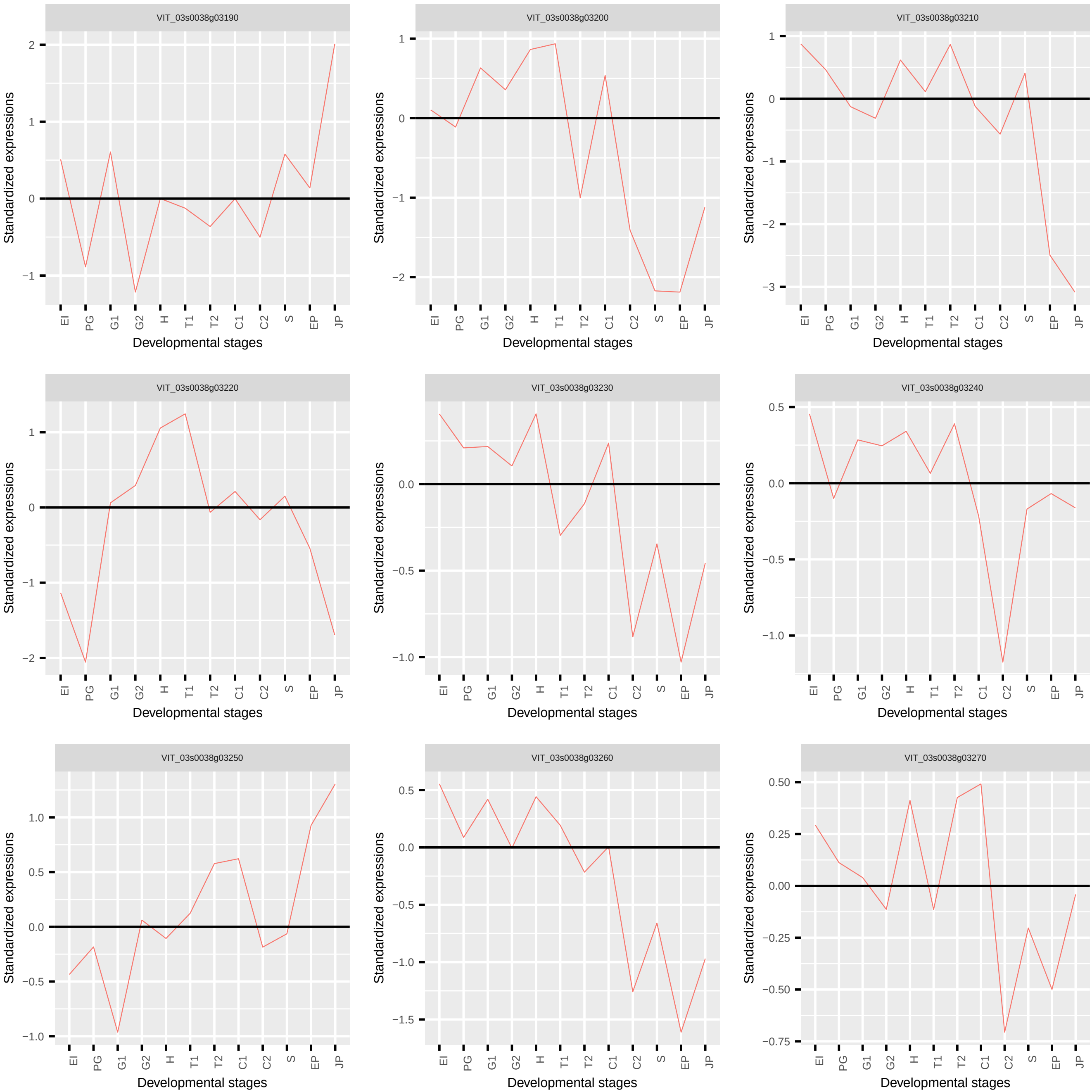

VIT_03s0038g03190
VIT_03s0038g03200
VIT_03s0038g03210
VIT_03s0038g03220
VIT_03s0038g03230
VIT_03s0038g03240
VIT_03s0038g03250
VIT_03s0038g03260
VIT_03s0038g03270
Standardized expressions
Developmental stages
EI
PG
G1
G2
H
T1
T2
C1
C2
S
EP
JP

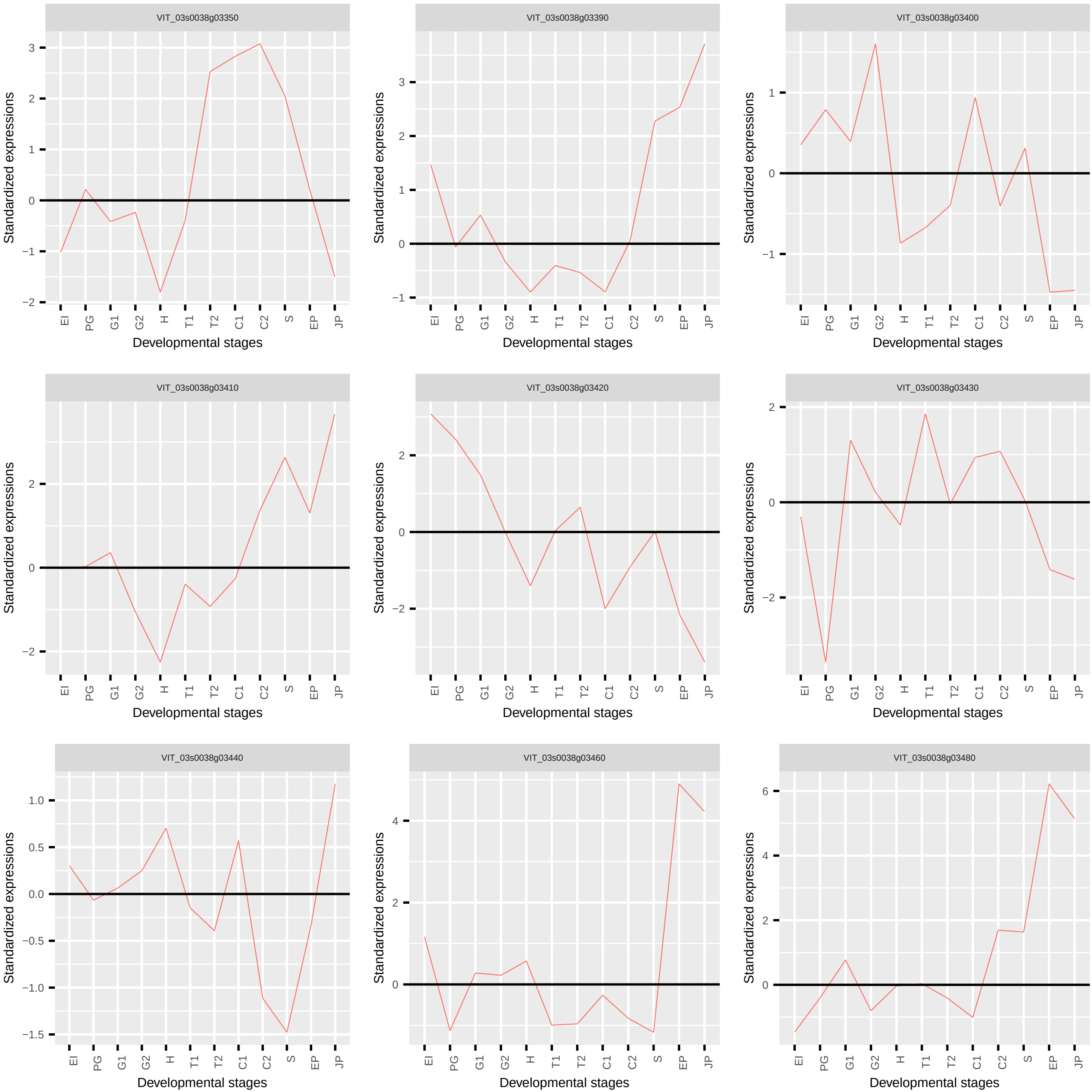
VIT_03s0038g03350
VIT_03s0038g03390
VIT_03s0038g03400
VIT_03s0038g03410
VIT_03s0038g03420
VIT_03s0038g03430
VIT_03s0038g03440
VIT_03s0038g03460
VIT_03s0038g03480
Standardized expressions
Developmental stages
EI
PG
G1
G2
H
T1
T2
C1
C2
S
EP
JP

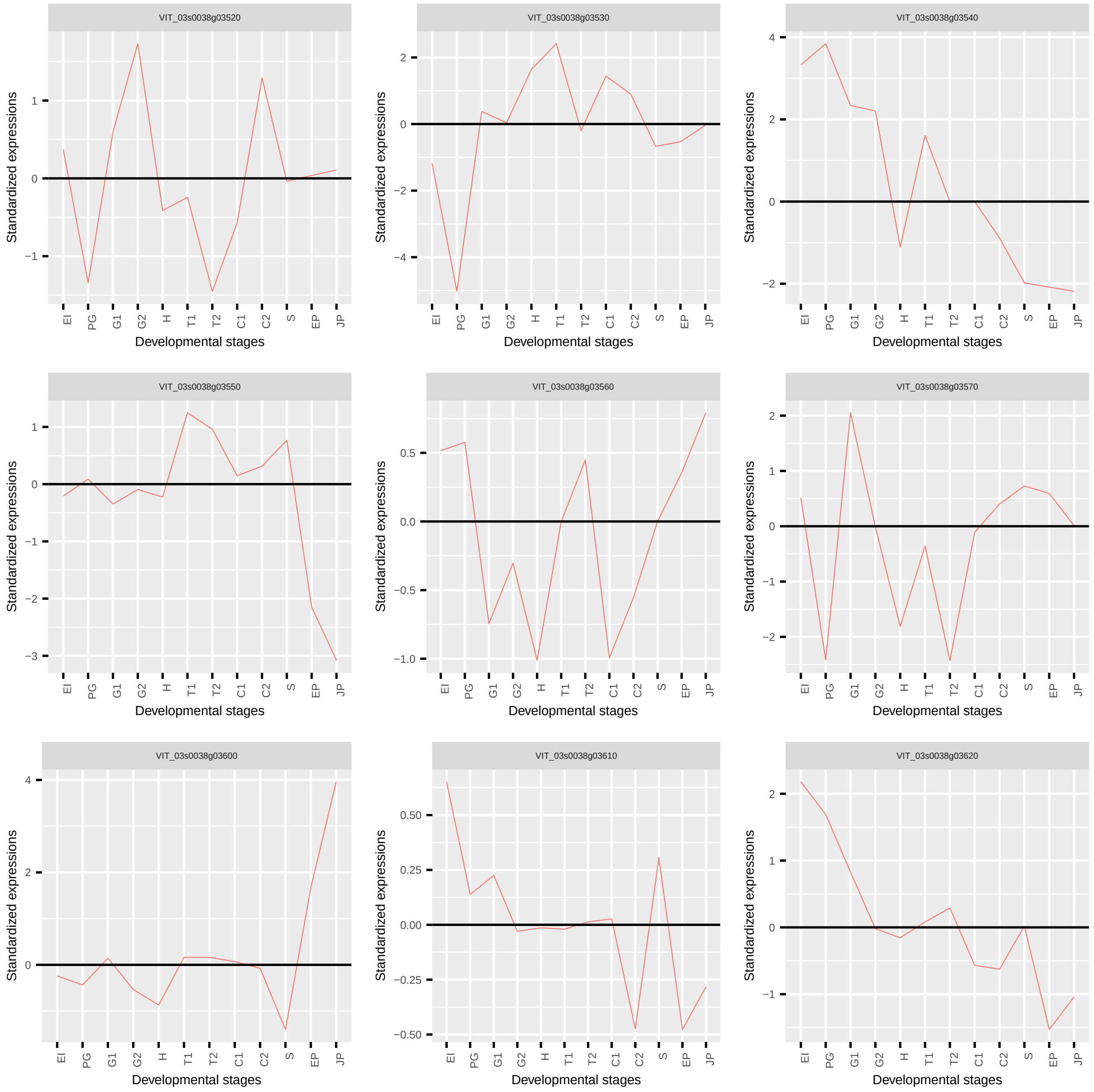
VIT_03s0038g03520
VIT_03s0038g03530
VIT_03s0038g03540
VIT_03s0038g03550
VIT_03s0038g03560
VIT_03s0038g03570
VIT_03s0038g03600
VIT_03s0038g03610
VIT_03s0038g03620
Standardized expressions
Developmental stages
EI PG G1 G2 H T1 T2 C1 C2 S EP JP

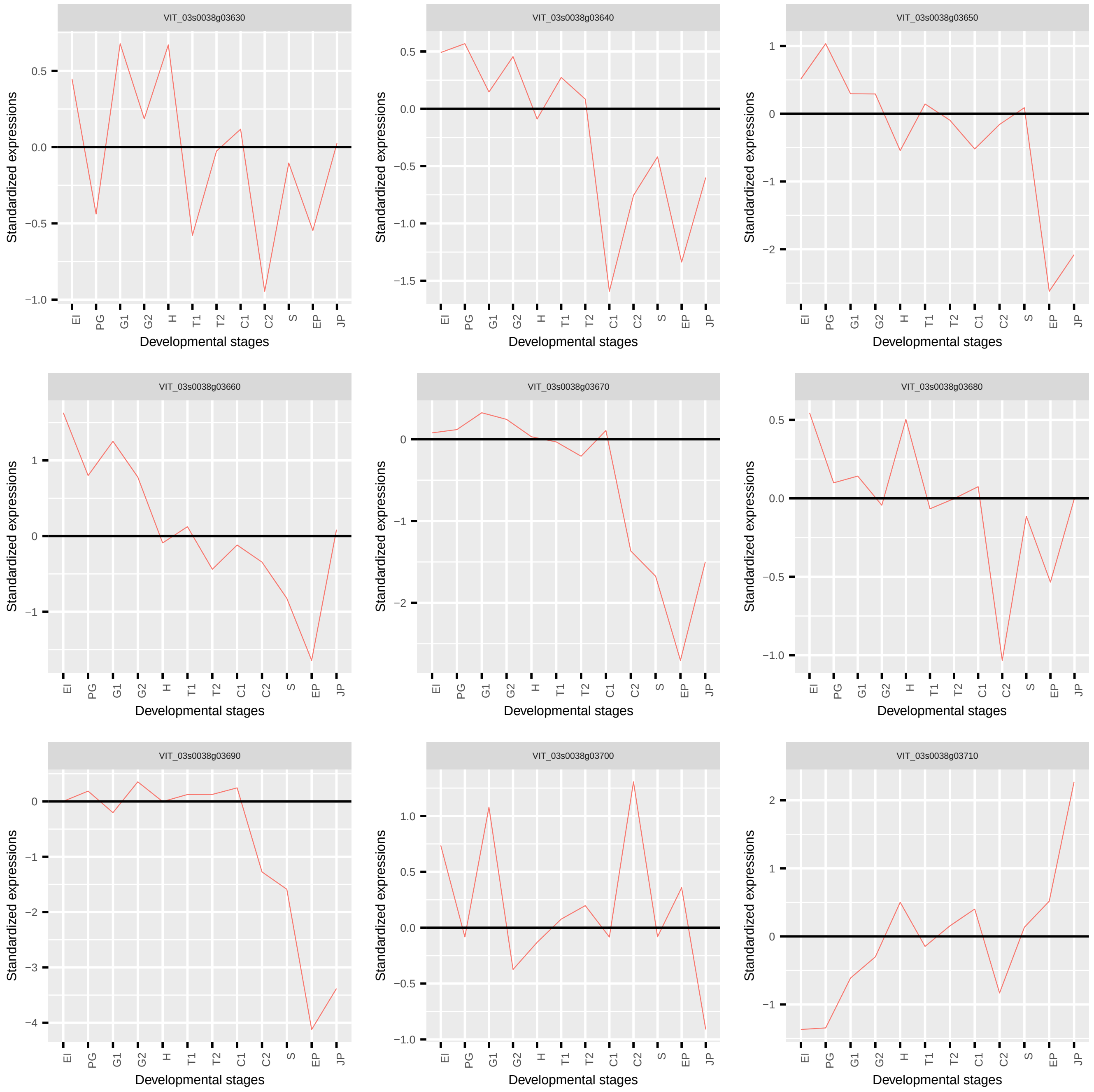
VIT_03s0038g03630
VIT_03s0038g03640
VIT_03s0038g03650
VIT_03s0038g03660
VIT_03s0038g03670
VIT_03s0038g03680
VIT_03s0038g03690
VIT_03s0038g03700
VIT_03s0038g03710
Standardized expressions
Developmental stages
EI PG G1 G2 H T1 T2 C1 C2 S EP JP

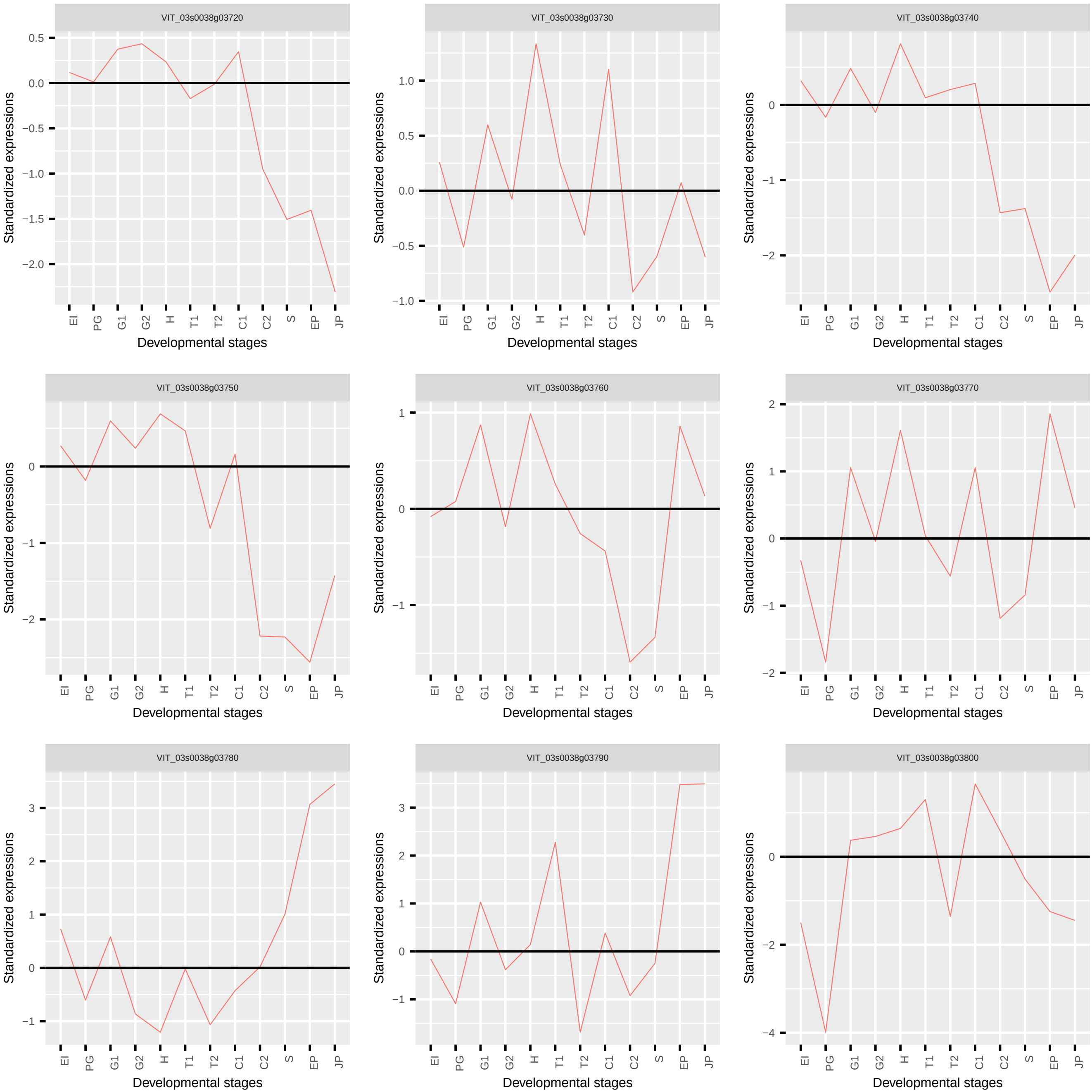
VIT_03s0038g03720
VIT_03s0038g03730
VIT_03s0038g03740
VIT_03s0038g03750
VIT_03s0038g03760
VIT_03s0038g03770
VIT_03s0038g03780
VIT_03s0038g03790
VIT_03s0038g03800
Standardized expressions
Developmental stages
EI PG G1 G2 H T1 T2 C1 C2 S EP JP
0.5 0.0 −0.5 −1.0 −1.5 −2.0
1.0 0.5 0.0 −0.5 −1.0
0 −1 −2
1 0 −1
2 1 0 −1 −2
3 2 1 0 −1
0 −2 −4

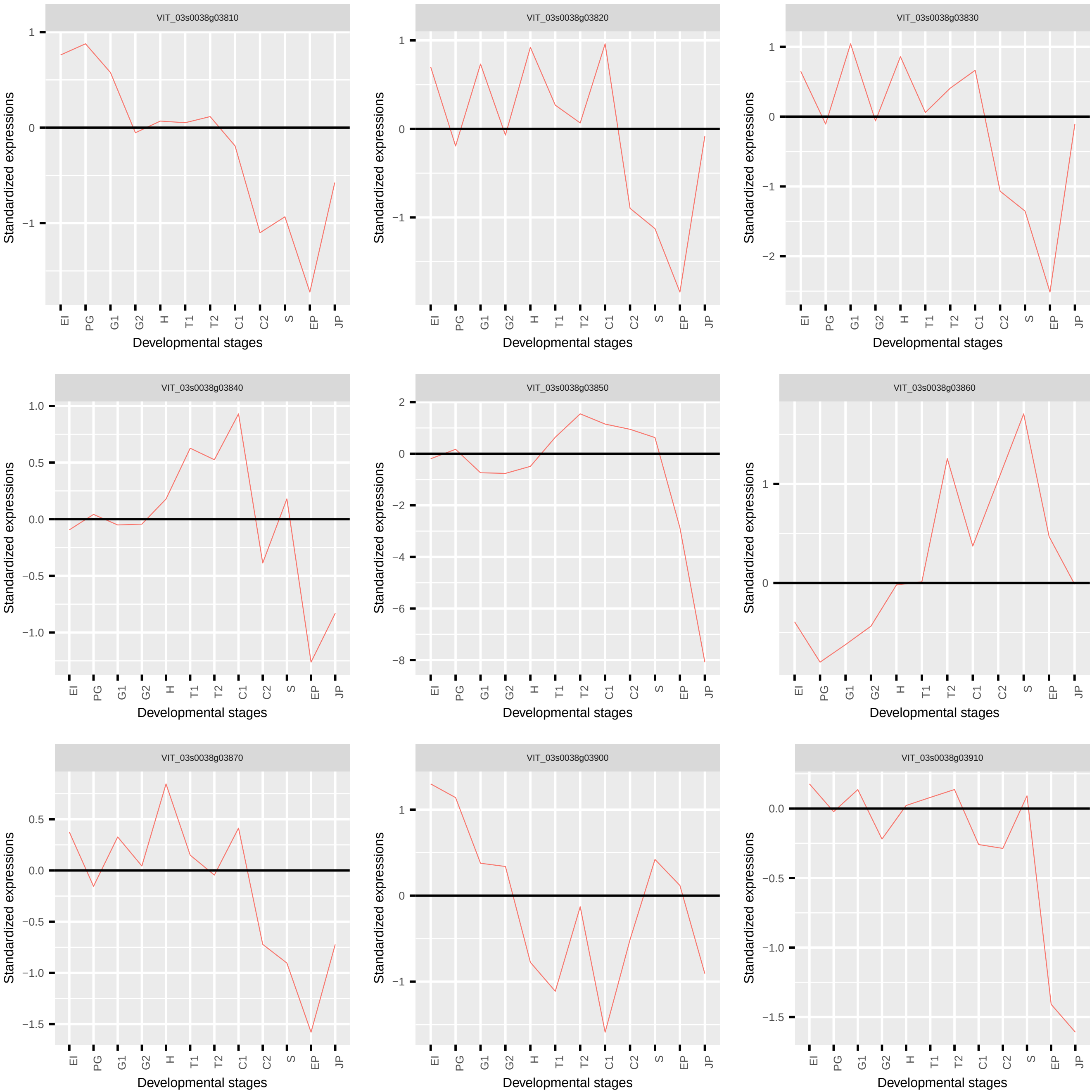
VIT_03s0038g03810
VIT_03s0038g03820
VIT_03s0038g03830
VIT_03s0038g03840
VIT_03s0038g03850
VIT_03s0038g03860
VIT_03s0038g03870
VIT_03s0038g03900
VIT_03s0038g03910
Standardized expressions
Developmental stages
EI PG G1 G2 H T1 T2 C1 C2 S EP JP

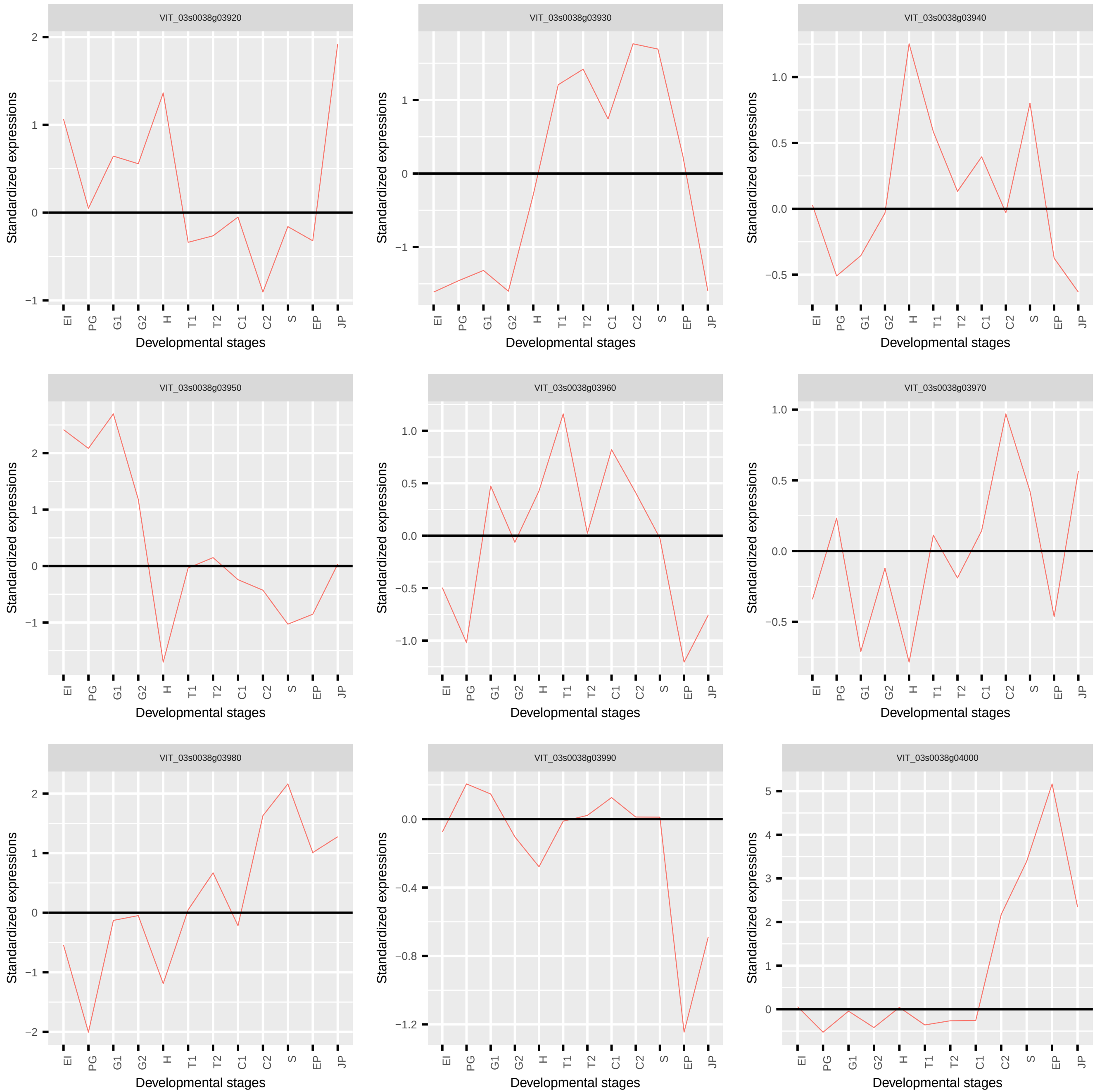
VIT_03s0038g03920
VIT_03s0038g03930
VIT_03s0038g03940
VIT_03s0038g03950
VIT_03s0038g03960
VIT_03s0038g03970
VIT_03s0038g03980
VIT_03s0038g03990
VIT_03s0038g04000
Standardized expressions
Developmental stages
EI PG G1 G2 H T1 T2 C1 C2 S EP JP

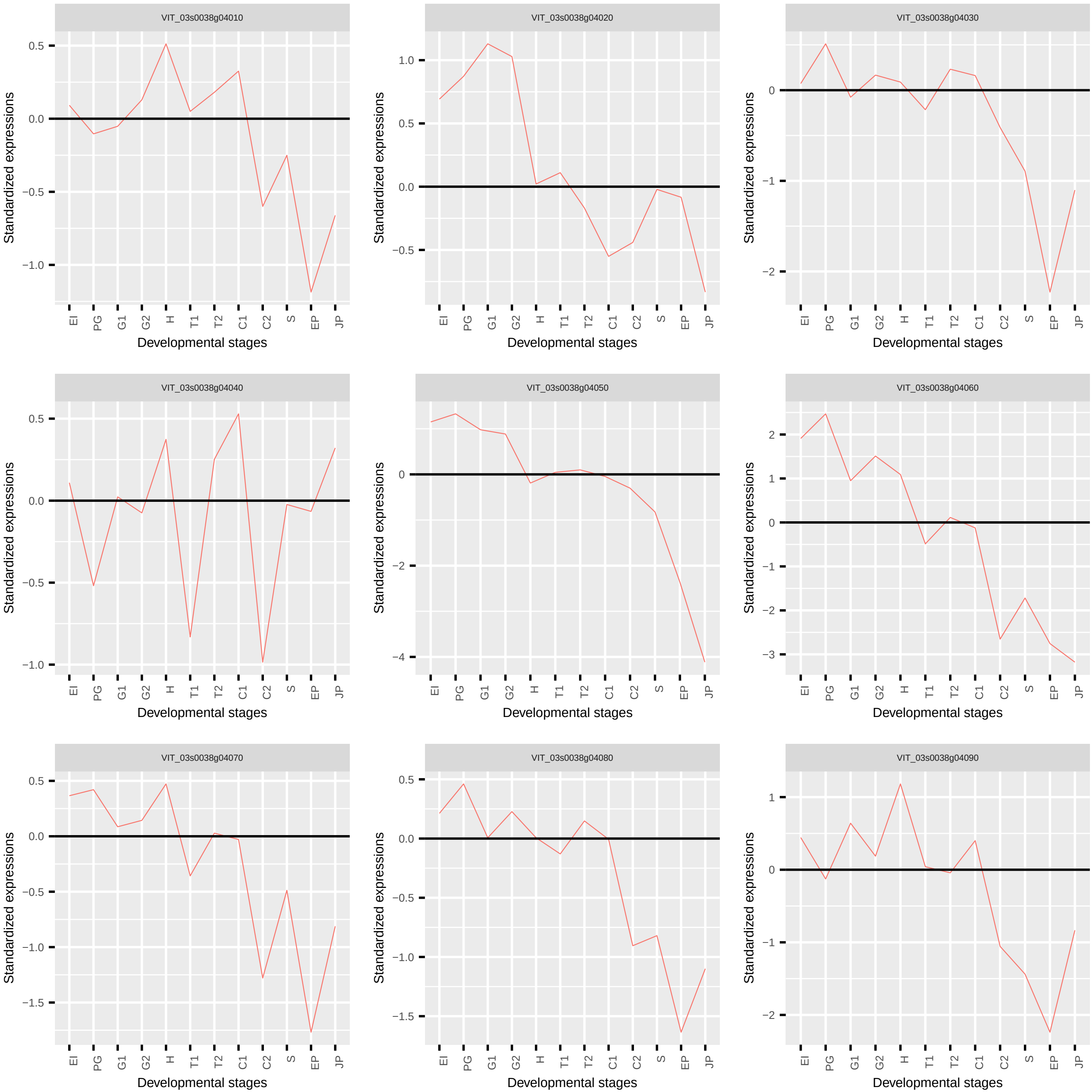
VIT_03s0038g04010
VIT_03s0038g04020
VIT_03s0038g04030
VIT_03s0038g04040
VIT_03s0038g04050
VIT_03s0038g04060
VIT_03s0038g04070
VIT_03s0038g04080
VIT_03s0038g04090
Standardized expressions
Developmental stages
EI PG G1 G2 H T1 T2 C1 C2 S EP JP

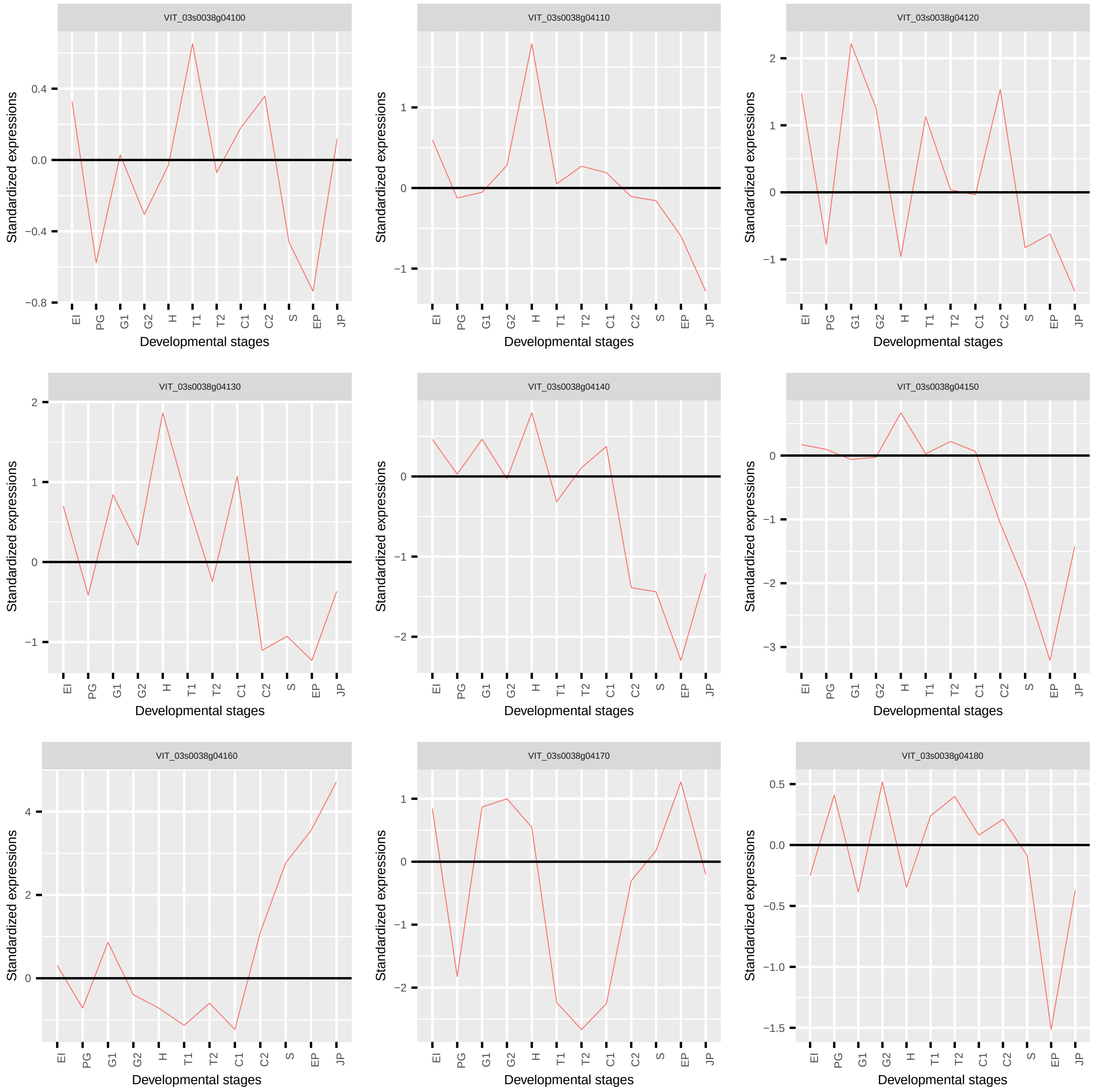
VIT_03s0038g04100
VIT_03s0038g04110
VIT_03s0038g04120
VIT_03s0038g04130
VIT_03s0038g04140
VIT_03s0038g04150
VIT_03s0038g04160
VIT_03s0038g04170
VIT_03s0038g04180
Standardized expressions
Developmental stages
EI PG G1 G2 H T1 T2 C1 C2 S EP JP

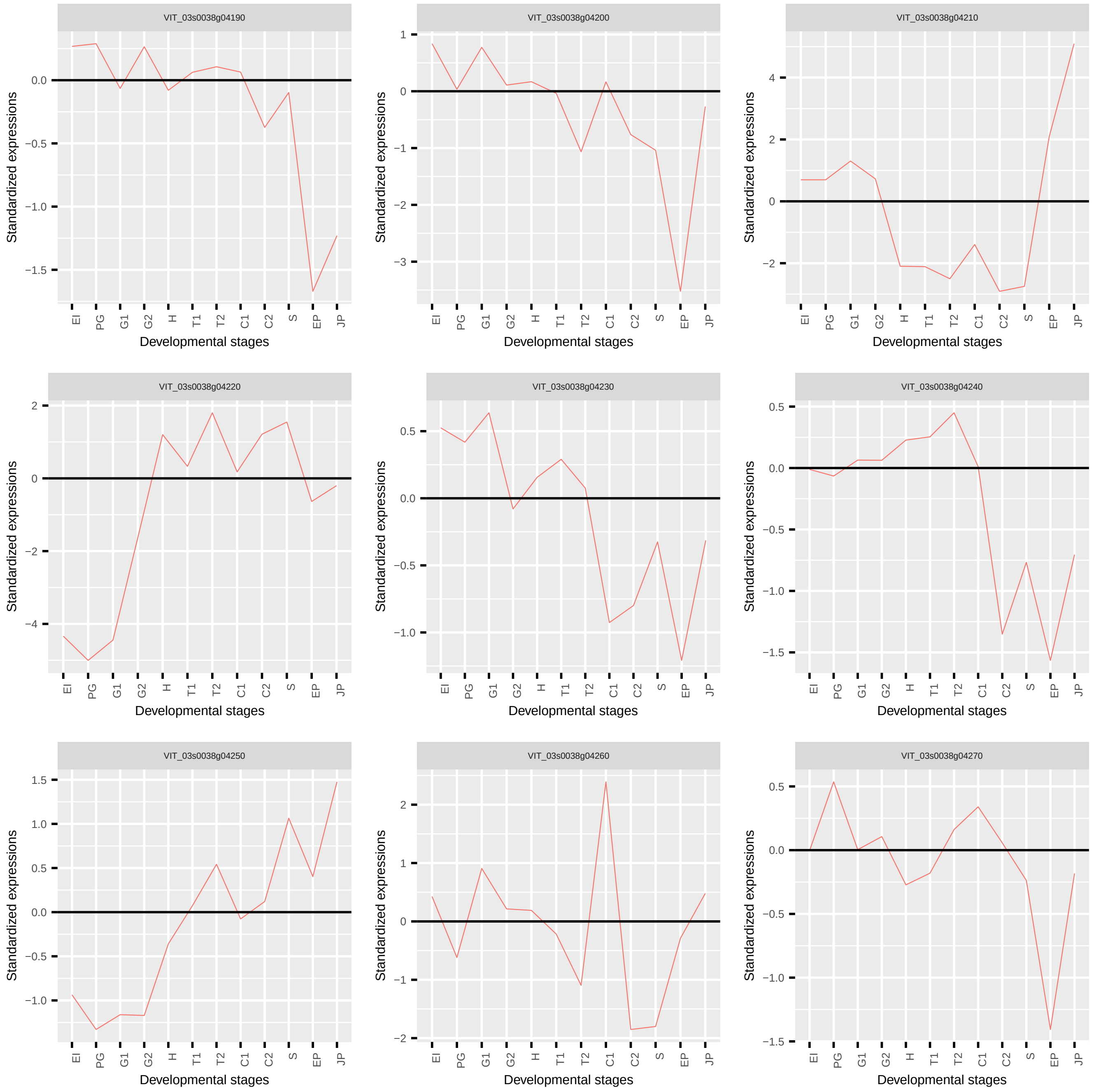
VIT_03s0038g04190
VIT_03s0038g04200
VIT_03s0038g04210
VIT_03s0038g04220
VIT_03s0038g04230
VIT_03s0038g04240
VIT_03s0038g04250
VIT_03s0038g04260
VIT_03s0038g04270
Standardized expressions
Developmental stages
EI PG G1 G2 H T1 T2 C1 C2 S EP JP
0.0 −0.5 −1.0 −1.5
1 0 −1 −2 −3
4 2 0 −2
2 0 −2 −4
0.5 0.0 −0.5 −1.0
0.5 0.0 −0.5 −1.0 −1.5
1.5 1.0 0.5 0.0 −0.5 −1.0
2 1 0 −1 −2
0.5 0.0 −0.5 −1.0 −1.5

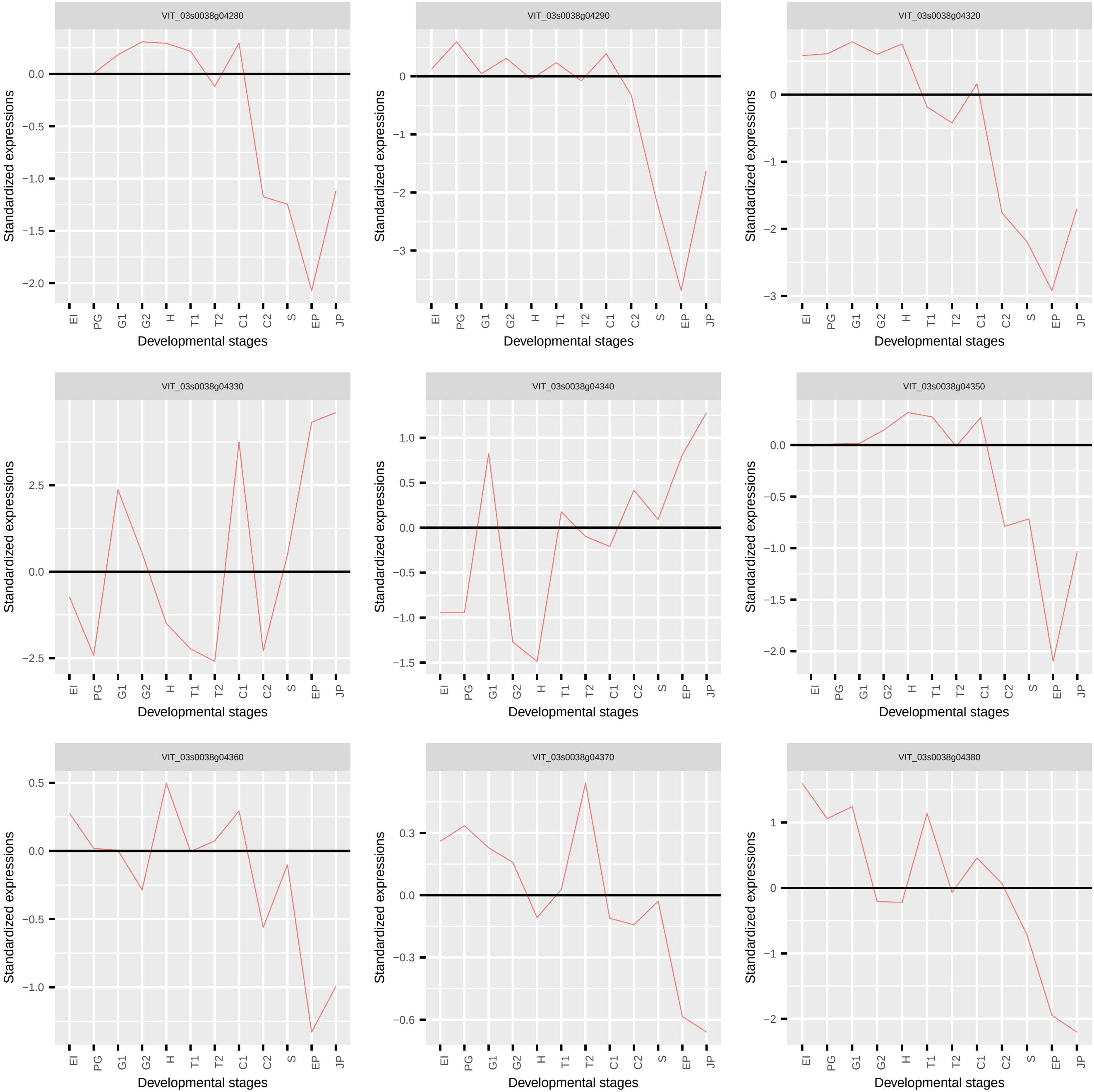

VIT_03s0038g04280
VIT_03s0038g04290
VIT_03s0038g04320
VIT_03s0038g04330
VIT_03s0038g04340
VIT_03s0038g04350
VIT_03s0038g04360
VIT_03s0038g04370
VIT_03s0038g04380
Standardized expressions
Developmental stages
EI PG G1 G2 H T1 T2 C1 C2 S EP JP
0.0 −0.5 −1.0 −1.5 −2.0
0 −1 −2 −3
2.5 0.0 −2.5
1.0 0.5 0.0 −0.5 −1.0 −1.5
0.5 0.0 −0.5 −1.0
0.3 0.0 −0.3 −0.6
1 0 −1 −2

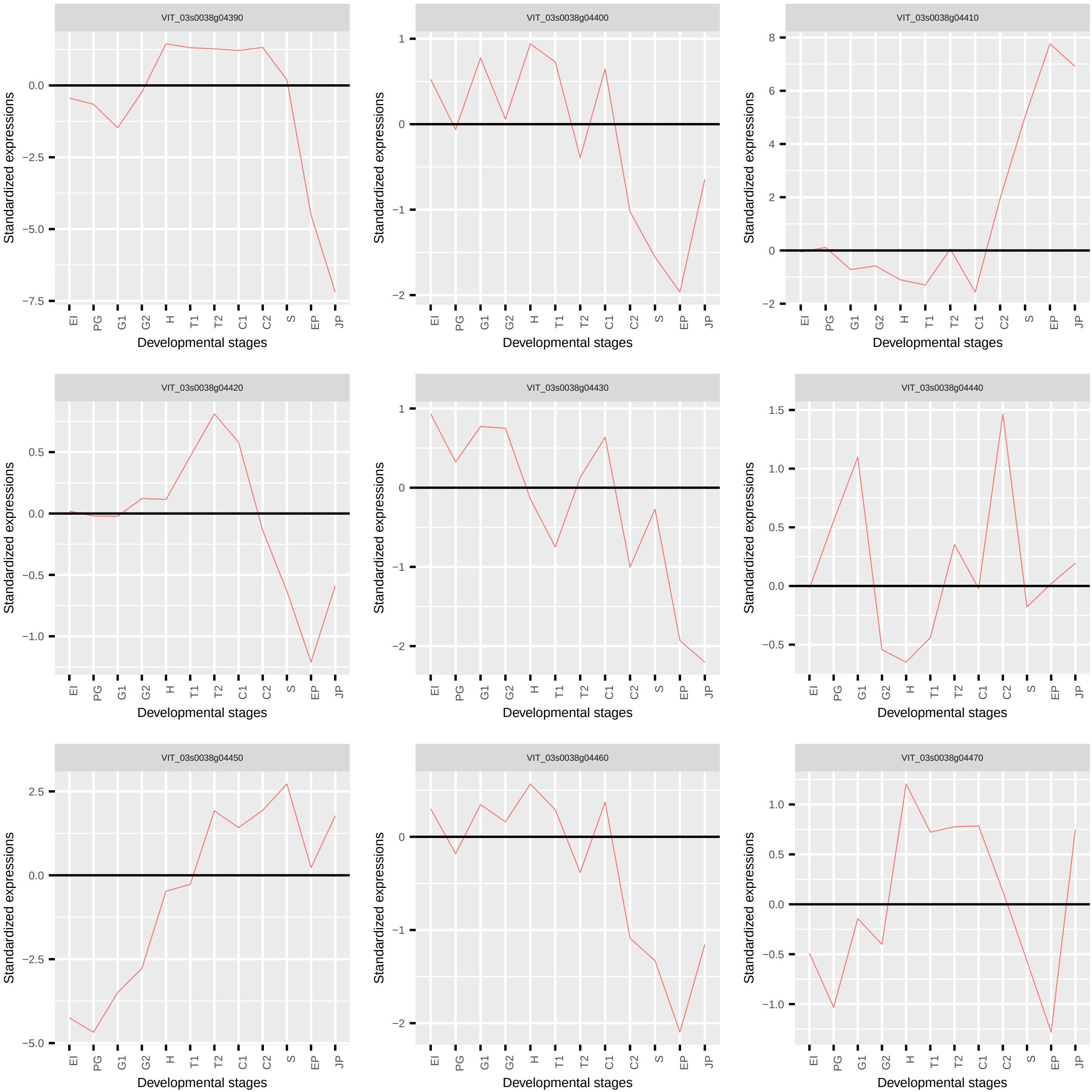
VIT_03s0038g04390
VIT_03s0038g04400
VIT_03s0038g04410
VIT_03s0038g04420
VIT_03s0038g04430
VIT_03s0038g04440
VIT_03s0038g04450
VIT_03s0038g04460
VIT_03s0038g04470
Standardized expressions
Developmental stages
EI PG G1 G2 H T1 T2 C1 C2 S EP JP

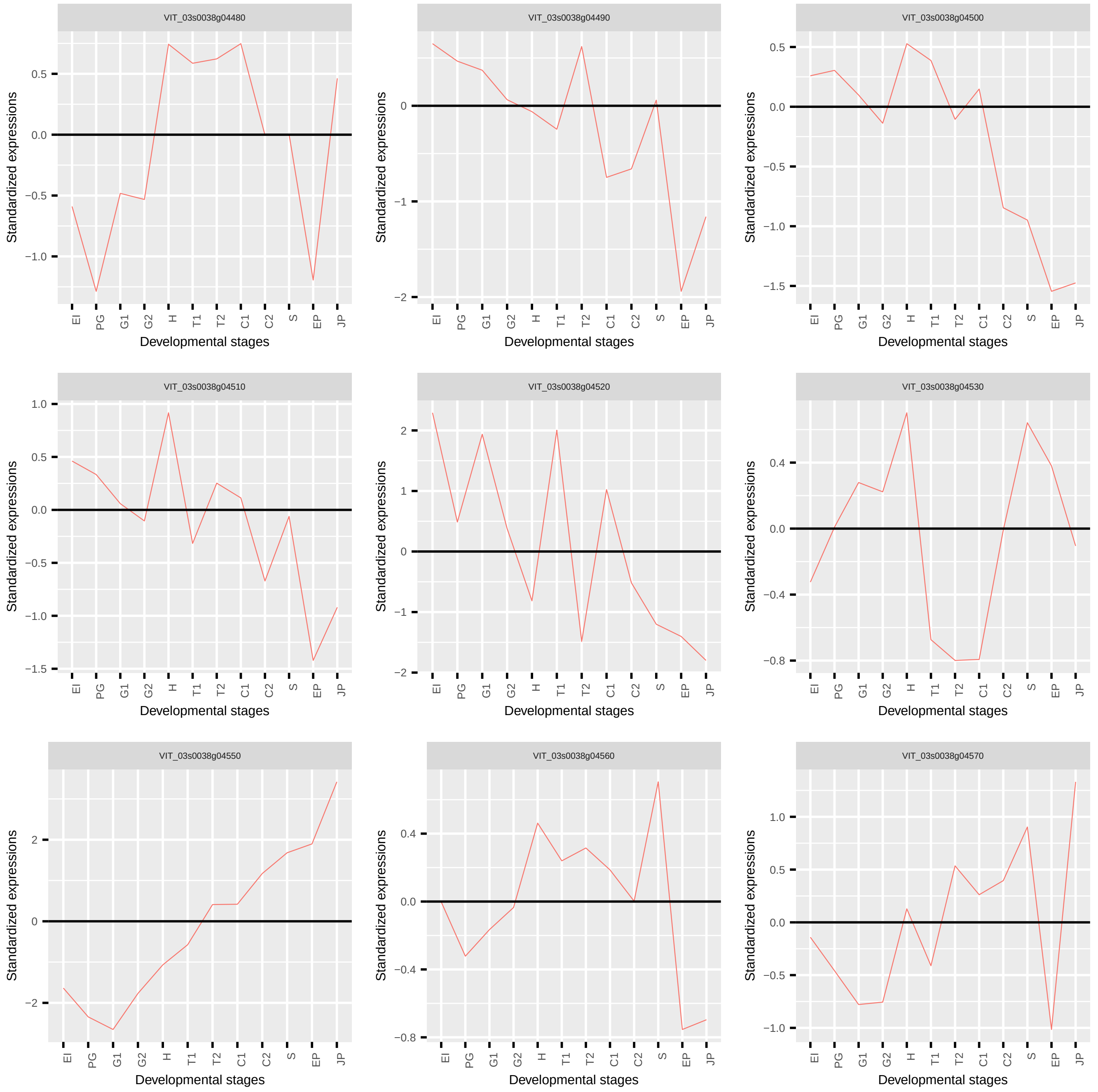

VIT_03s0038g04480
VIT_03s0038g04490
VIT_03s0038g04500
VIT_03s0038g04510
VIT_03s0038g04520
VIT_03s0038g04530
VIT_03s0038g04550
VIT_03s0038g04560
VIT_03s0038g04570
Standardized expressions
Developmental stages
EI PG G1 G2 H T1 T2 C1 C2 S EP JP

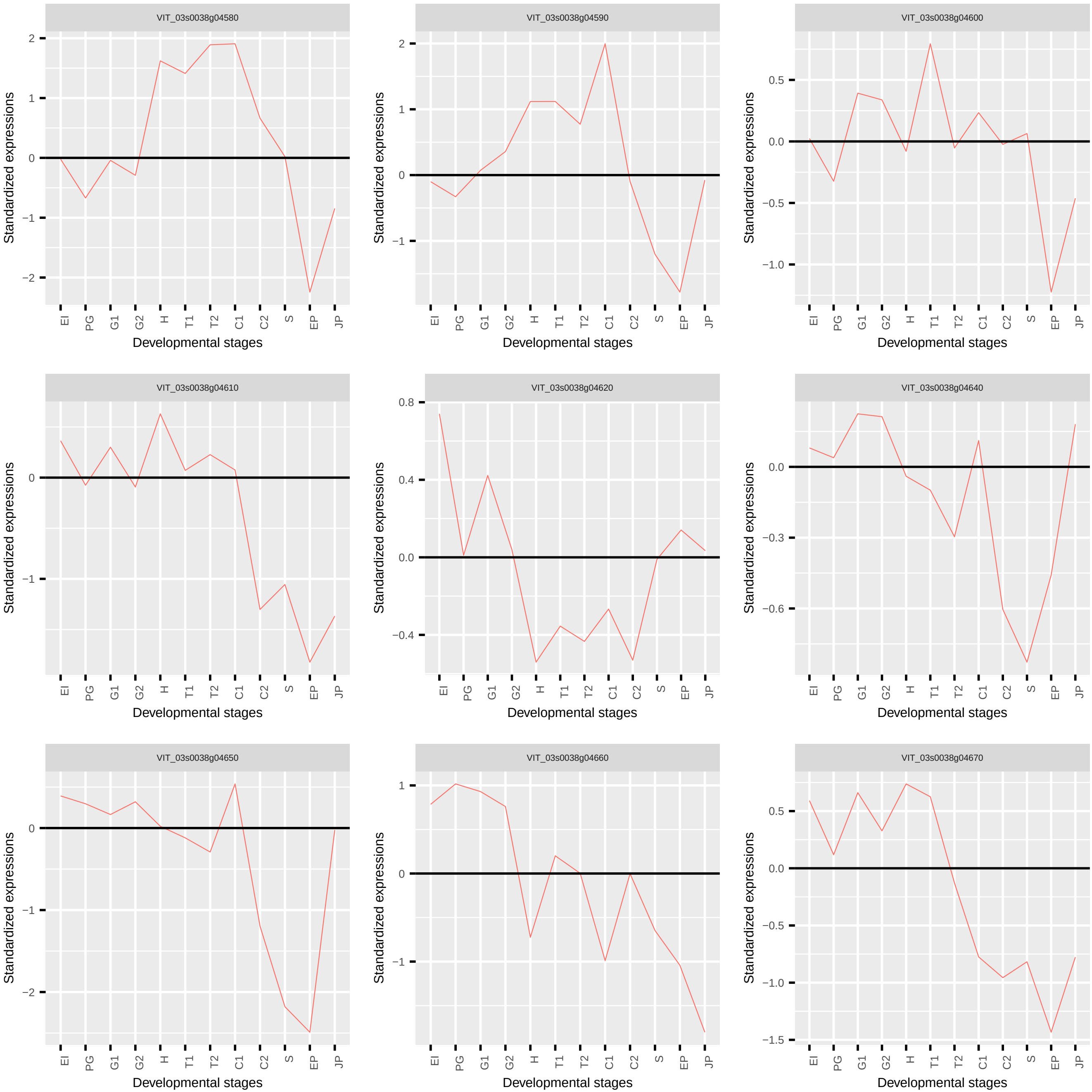
VIT_03s0038g04580
VIT_03s0038g04590
VIT_03s0038g04600
VIT_03s0038g04610
VIT_03s0038g04620
VIT_03s0038g04640
VIT_03s0038g04650
VIT_03s0038g04660
VIT_03s0038g04670
Standardized expressions
Developmental stages
EI
PG
G1
G2
H
T1
T2
C1
C2
S
EP
JP

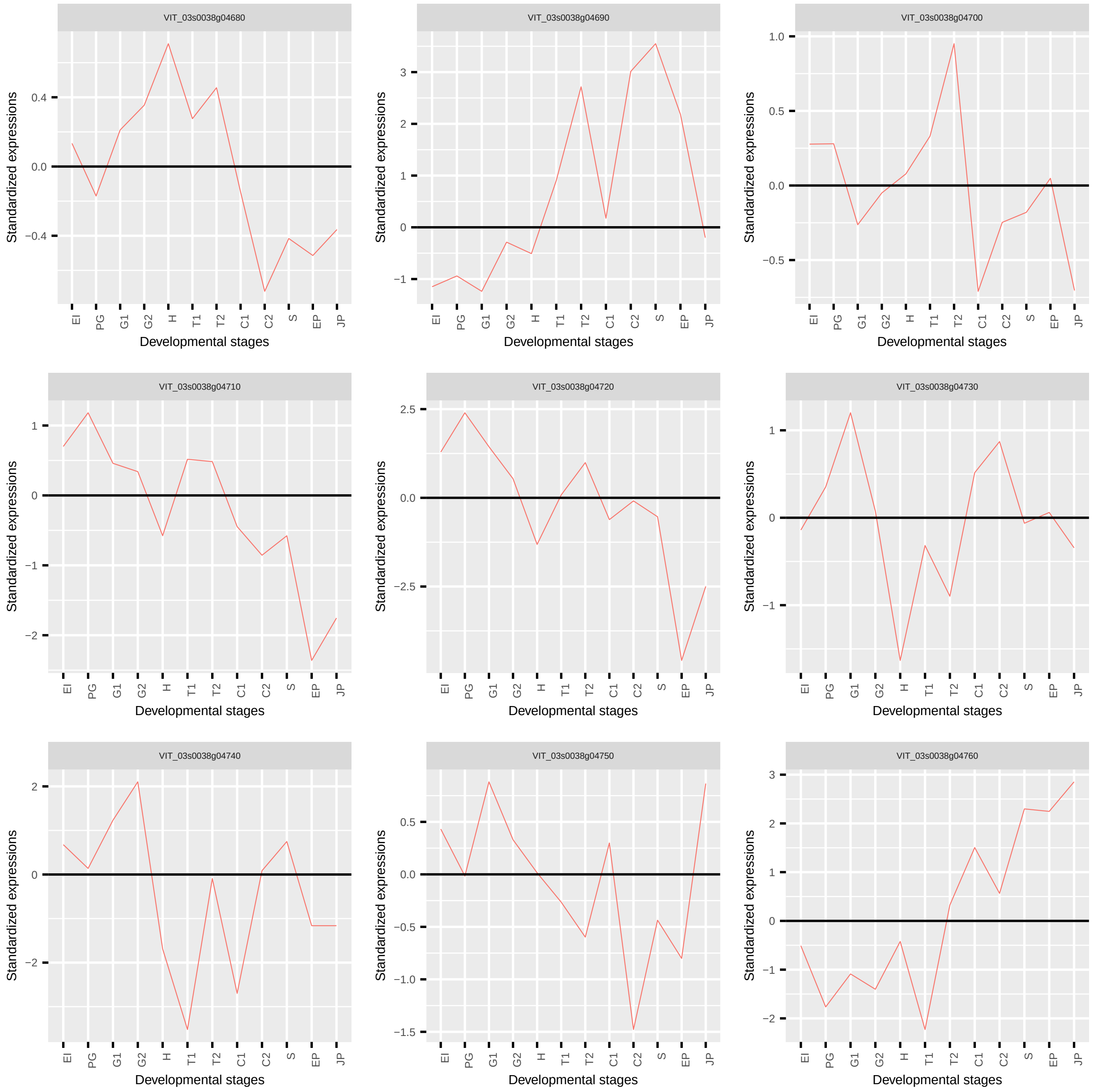
VIT_03s0038g04680
VIT_03s0038g04690
VIT_03s0038g04700
VIT_03s0038g04710
VIT_03s0038g04720
VIT_03s0038g04730
VIT_03s0038g04740
VIT_03s0038g04750
VIT_03s0038g04760
Standardized expressions
Developmental stages
EI PG G1 G2 H T1 T2 C1 C2 S EP JP

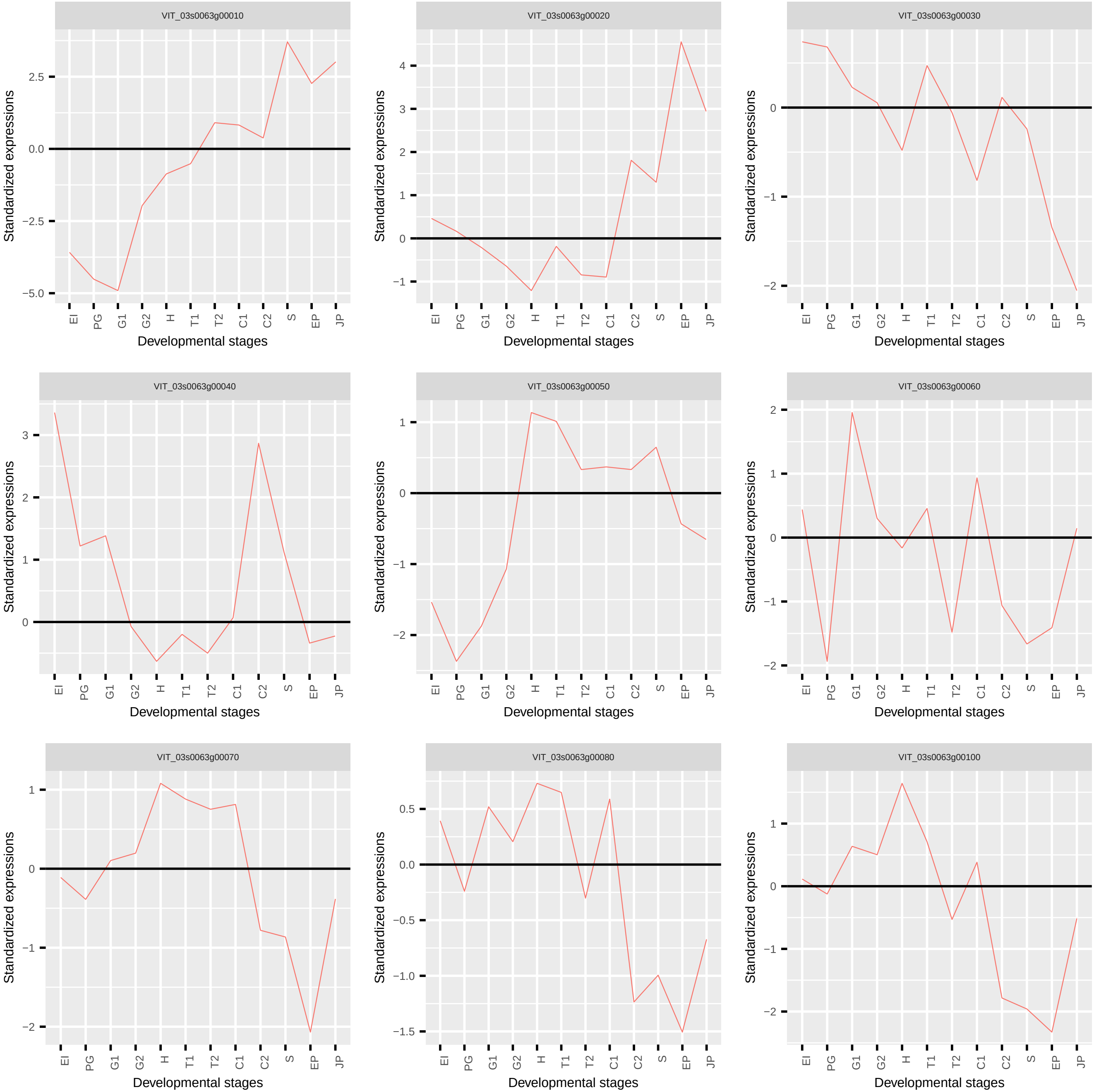
VIT_03s0063g00010
VIT_03s0063g00020
VIT_03s0063g00030
VIT_03s0063g00040
VIT_03s0063g00050
VIT_03s0063g00060
VIT_03s0063g00070
VIT_03s0063g00080
VIT_03s0063g00100
Standardized expressions
Developmental stages
EI PG G1 G2 H T1 T2 C1 C2 S EP JP
2.5
0.0
−2.5
−5.0
4
3
2
1
0
−1
−2
0.5
−0.5
−1.0
−1.5

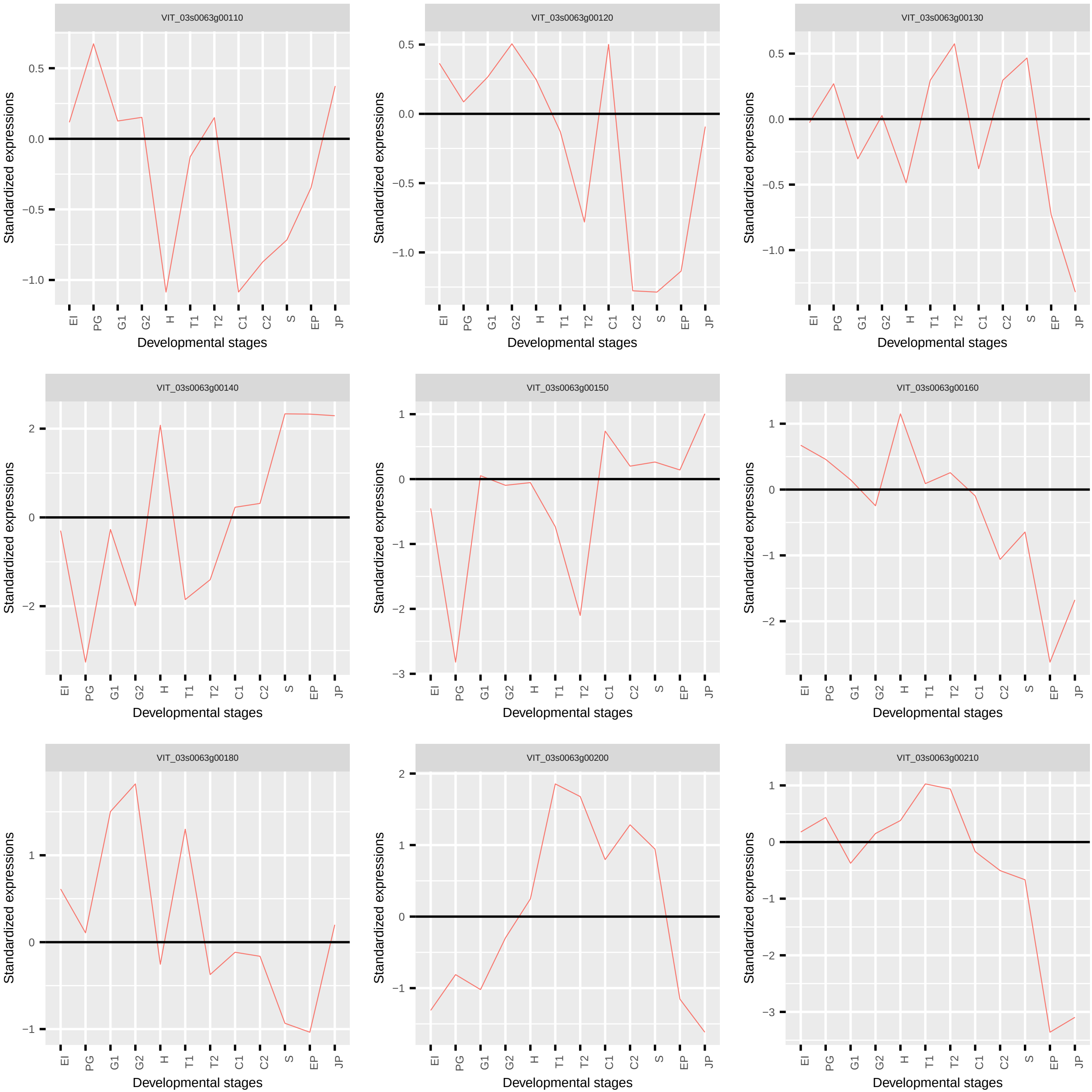
VIT_03s0063g00110
VIT_03s0063g00120
VIT_03s0063g00130
VIT_03s0063g00140
VIT_03s0063g00150
VIT_03s0063g00160
VIT_03s0063g00180
VIT_03s0063g00200
VIT_03s0063g00210
Standardized expressions
Developmental stages
EI
PG
G1
G2
H
T1
T2
C1
C2
S
EP
JP

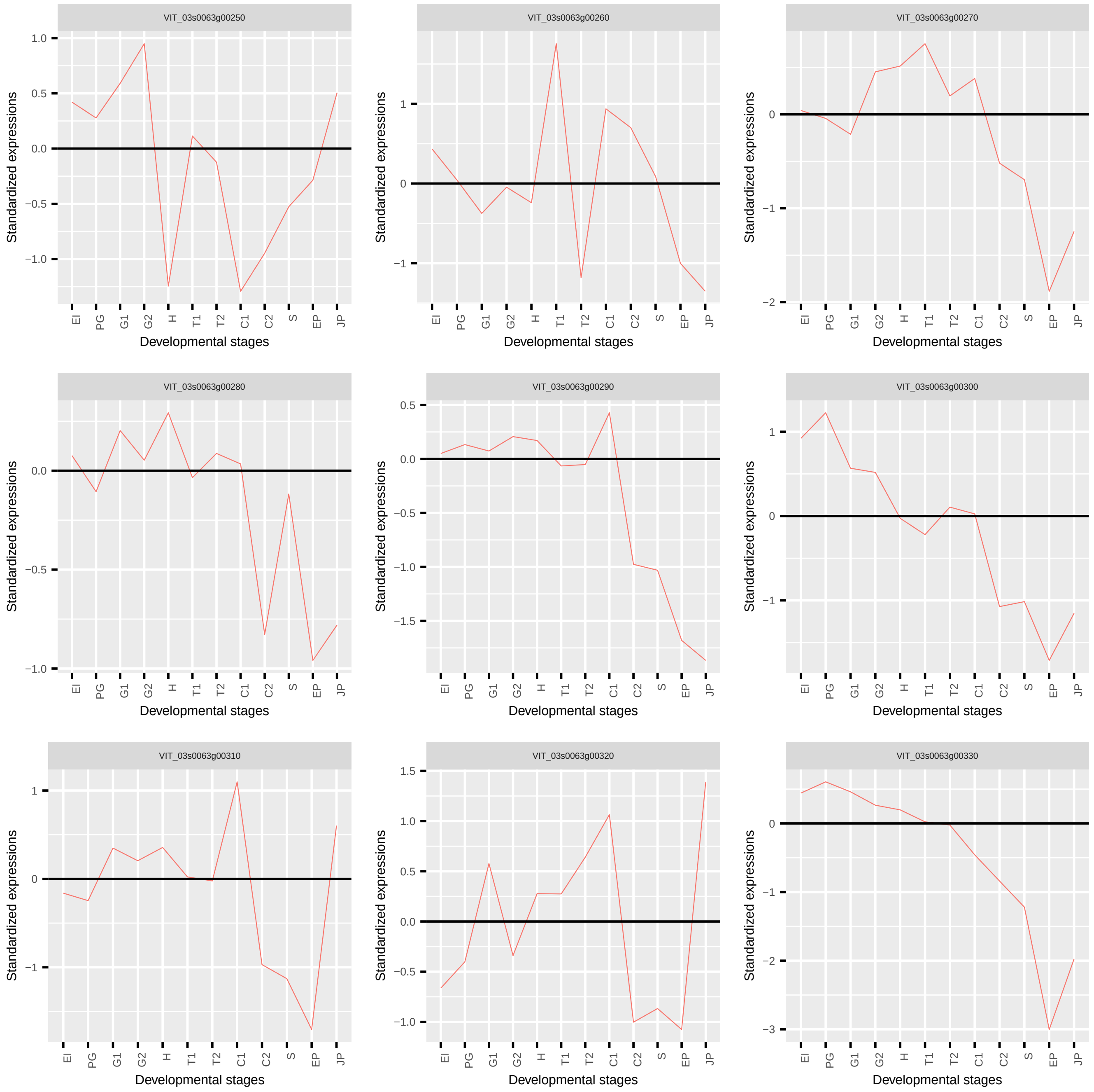
VIT_03s0063g00250
VIT_03s0063g00260
VIT_03s0063g00270
VIT_03s0063g00280
VIT_03s0063g00290
VIT_03s0063g00300
VIT_03s0063g00310
VIT_03s0063g00320
VIT_03s0063g00330
Standardized expressions
Developmental stages
EI PG G1 G2 H T1 T2 C1 C2 S EP JP

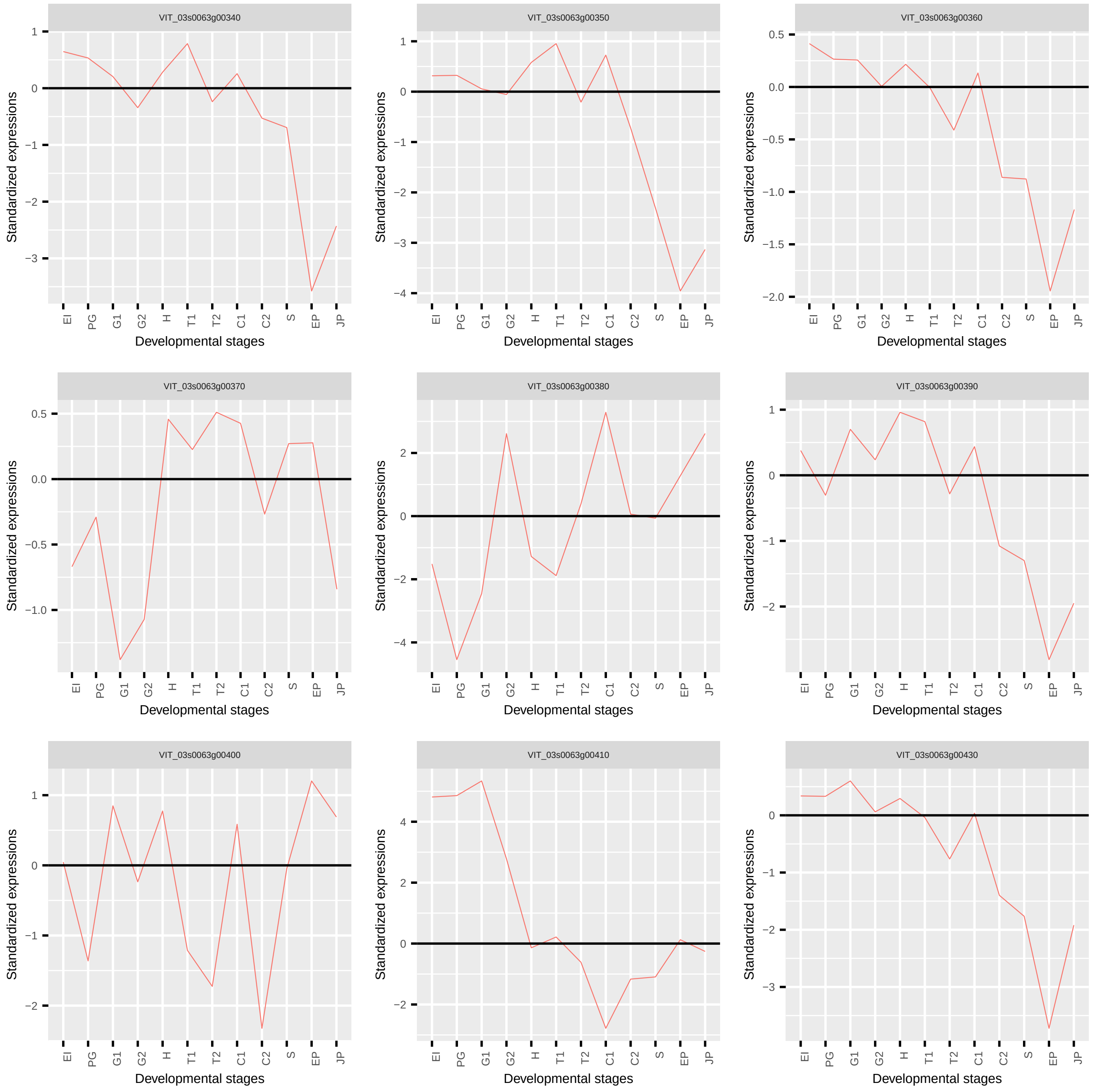
VIT_03s0063g00340
VIT_03s0063g00350
VIT_03s0063g00360
VIT_03s0063g00370
VIT_03s0063g00380
VIT_03s0063g00390
VIT_03s0063g00400
VIT_03s0063g00410
VIT_03s0063g00430
Standardized expressions
Developmental stages
EI PG G1 G2 H T1 T2 C1 C2 S EP JP

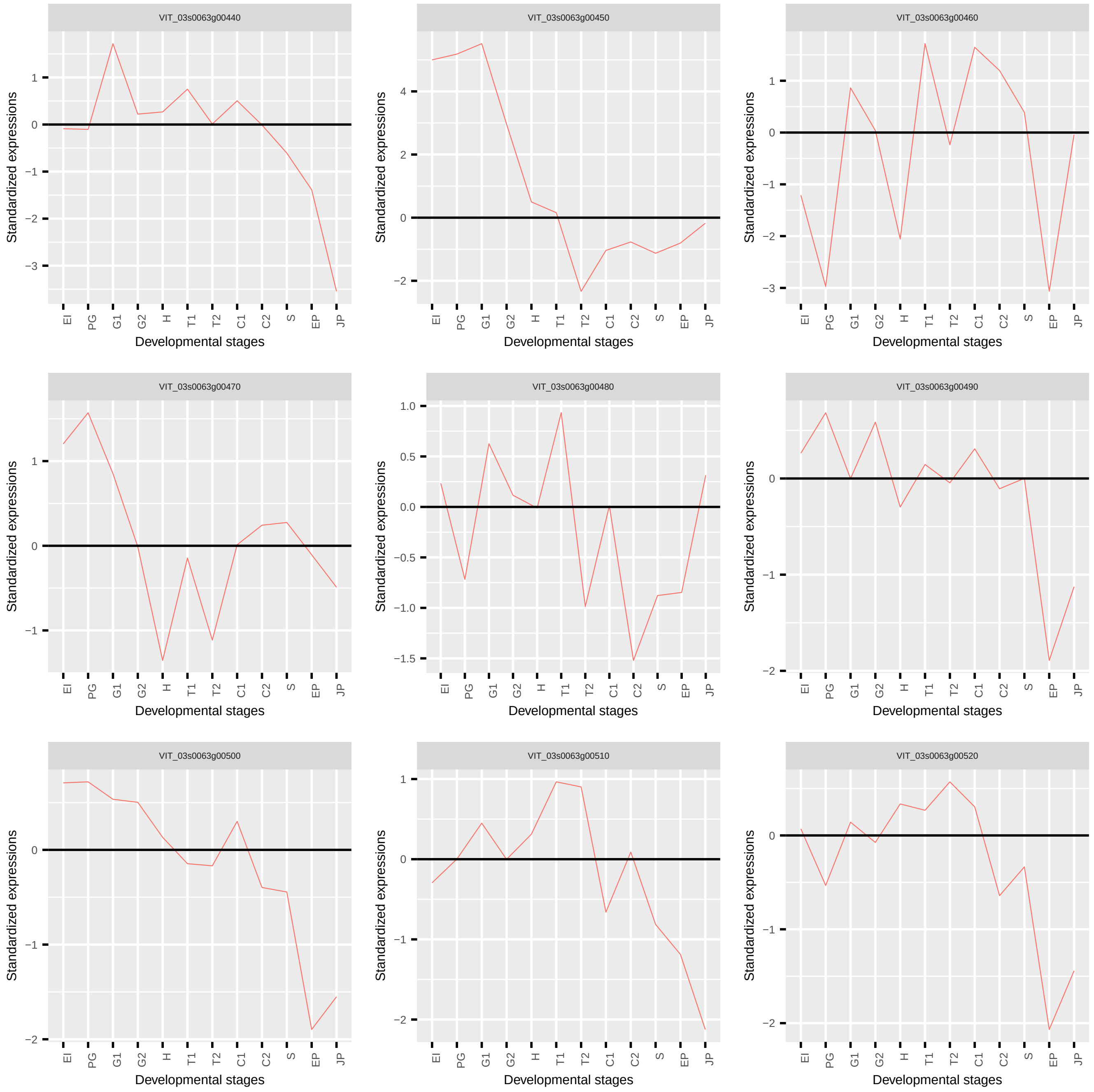
VIT_03s0063g00440
VIT_03s0063g00450
VIT_03s0063g00460
VIT_03s0063g00470
VIT_03s0063g00480
VIT_03s0063g00490
VIT_03s0063g00500
VIT_03s0063g00510
VIT_03s0063g00520
Standardized expressions
Developmental stages
EI PG G1 G2 H T1 T2 C1 C2 S EP JP

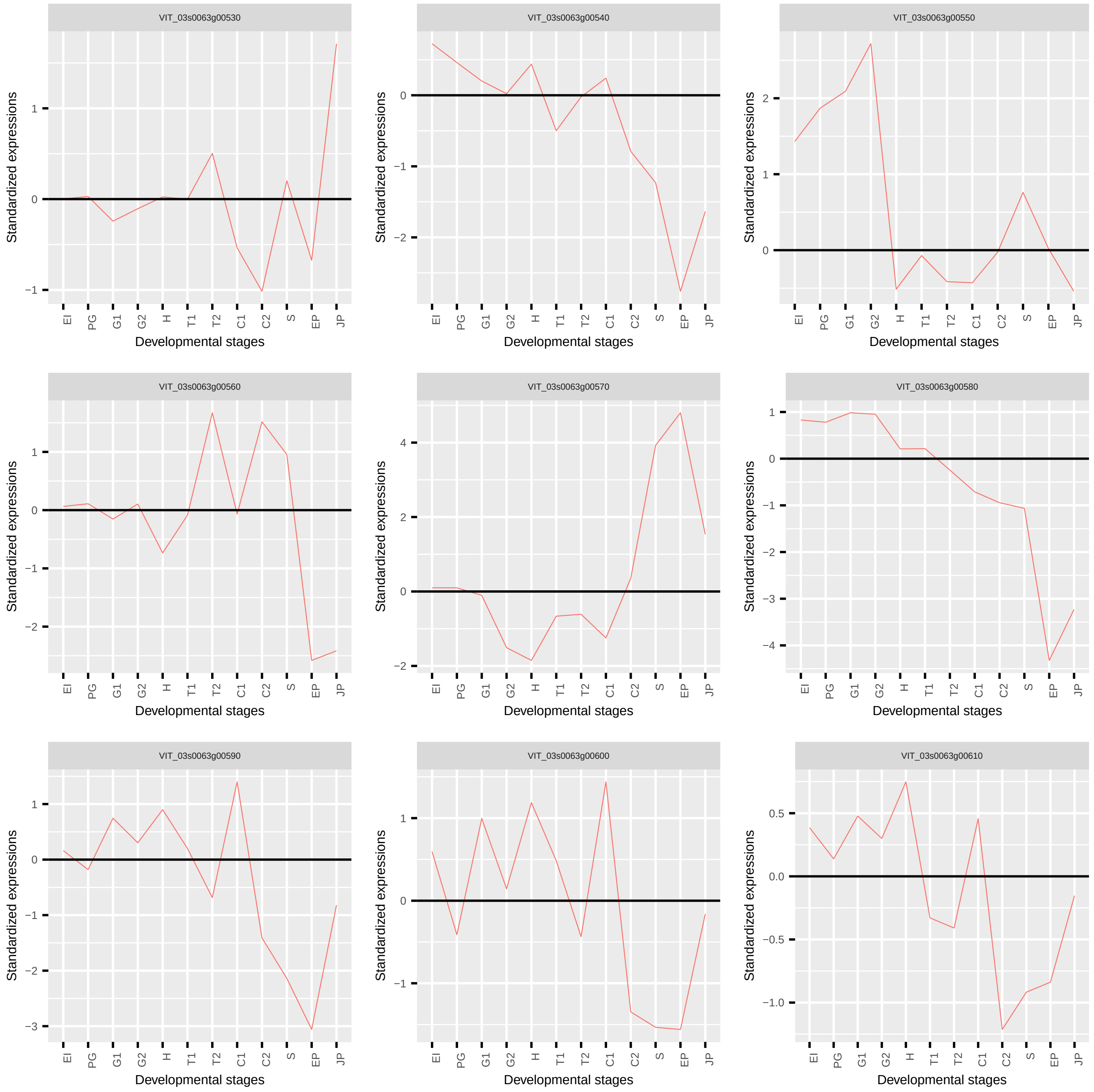

VIT_03s0063g00530
VIT_03s0063g00540
VIT_03s0063g00550
VIT_03s0063g00560
VIT_03s0063g00570
VIT_03s0063g00580
VIT_03s0063g00590
VIT_03s0063g00600
VIT_03s0063g00610
Standardized expressions
Developmental stages
EI PG G1 G2 H T1 T2 C1 C2 S EP JP

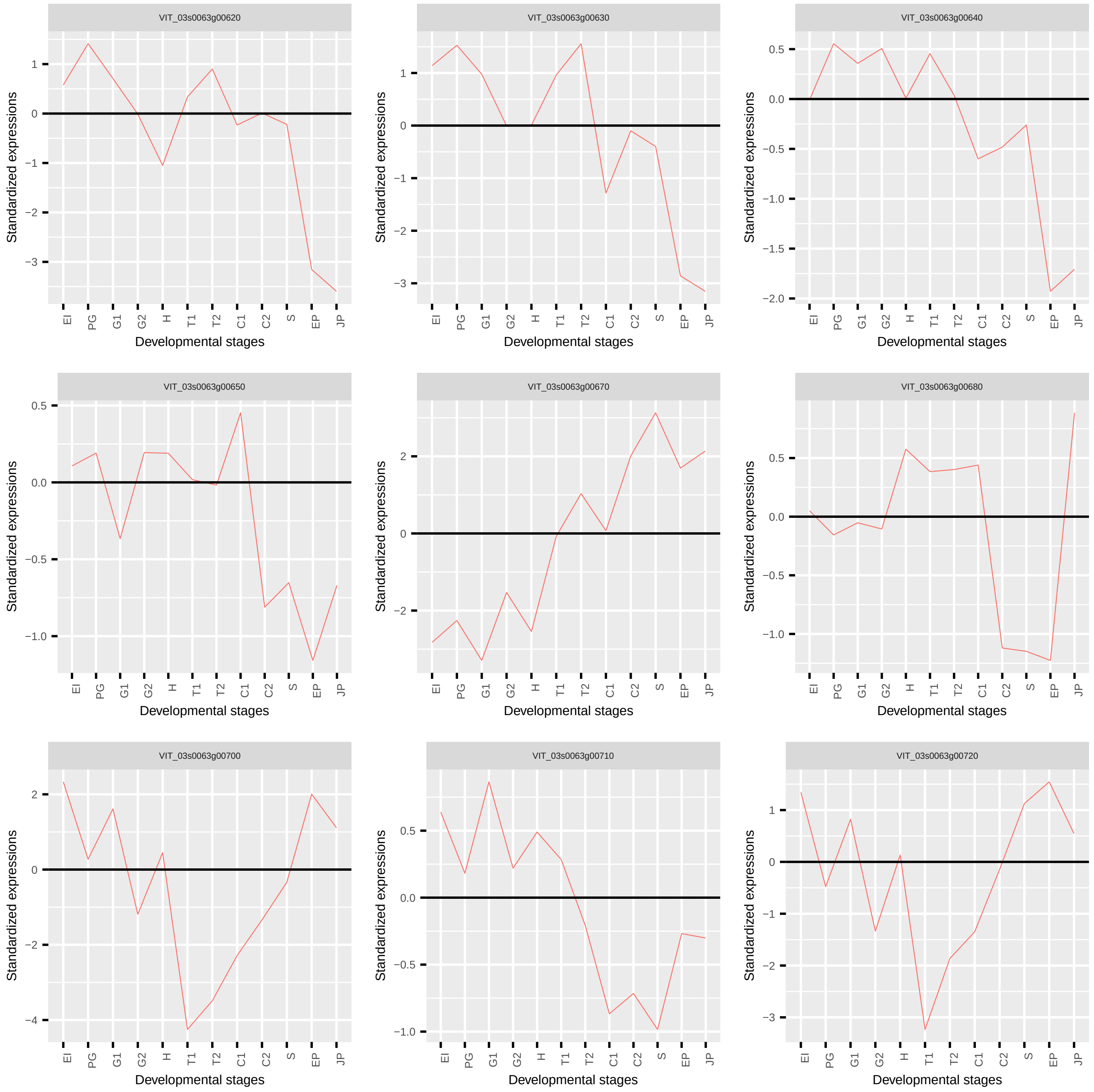

VIT_03s0063g00620
VIT_03s0063g00630
VIT_03s0063g00640
VIT_03s0063g00650
VIT_03s0063g00670
VIT_03s0063g00680
VIT_03s0063g00700
VIT_03s0063g00710
VIT_03s0063g00720
Standardized expressions
Developmental stages
EI PG G1 G2 H T1 T2 C1 C2 S EP JP

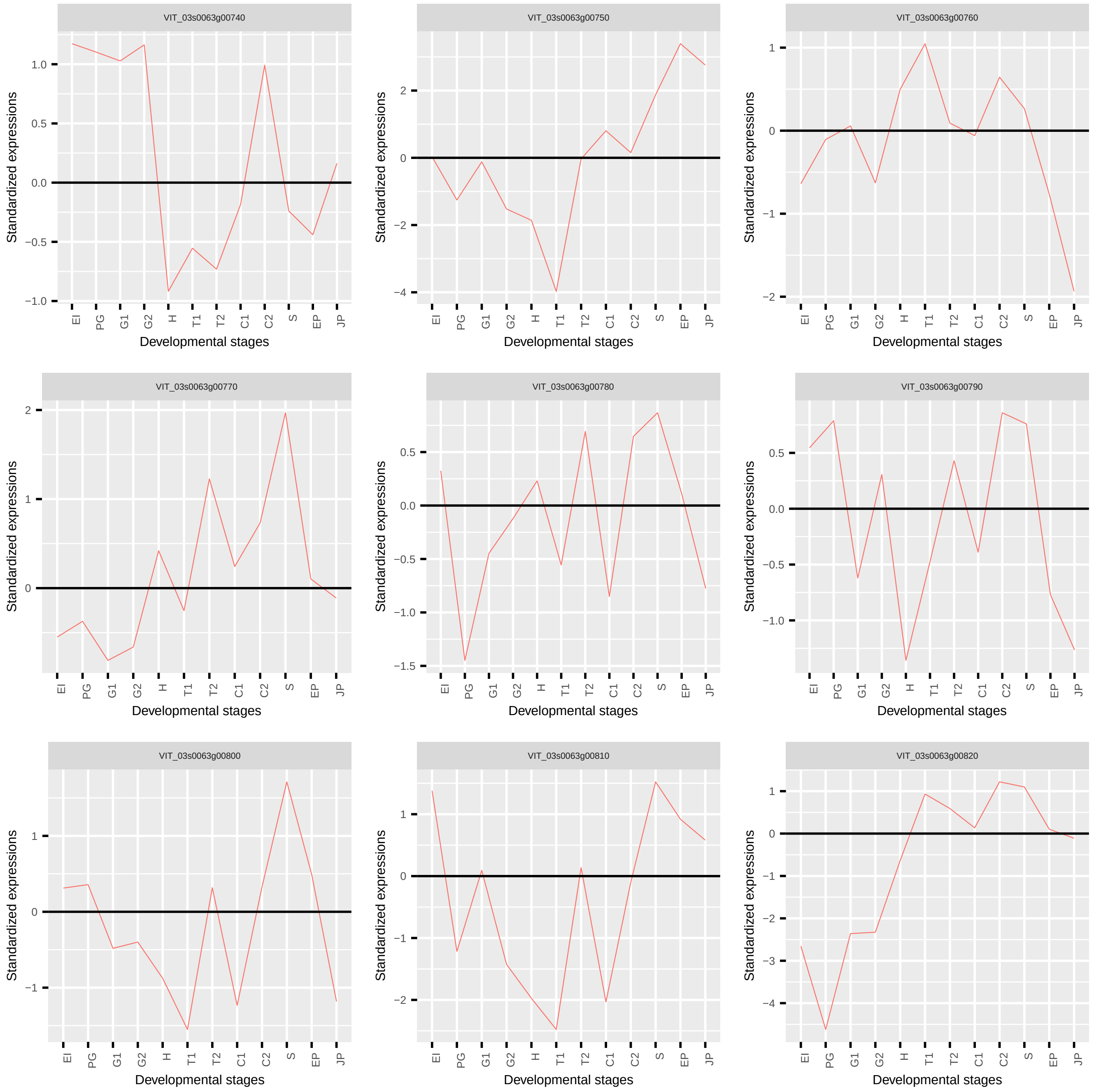
VIT_03s0063g00740
VIT_03s0063g00750
VIT_03s0063g00760
VIT_03s0063g00770
VIT_03s0063g00780
VIT_03s0063g00790
VIT_03s0063g00800
VIT_03s0063g00810
VIT_03s0063g00820
Standardized expressions
Developmental stages
EI PG G1 G2 H T1 T2 C1 C2 S EP JP

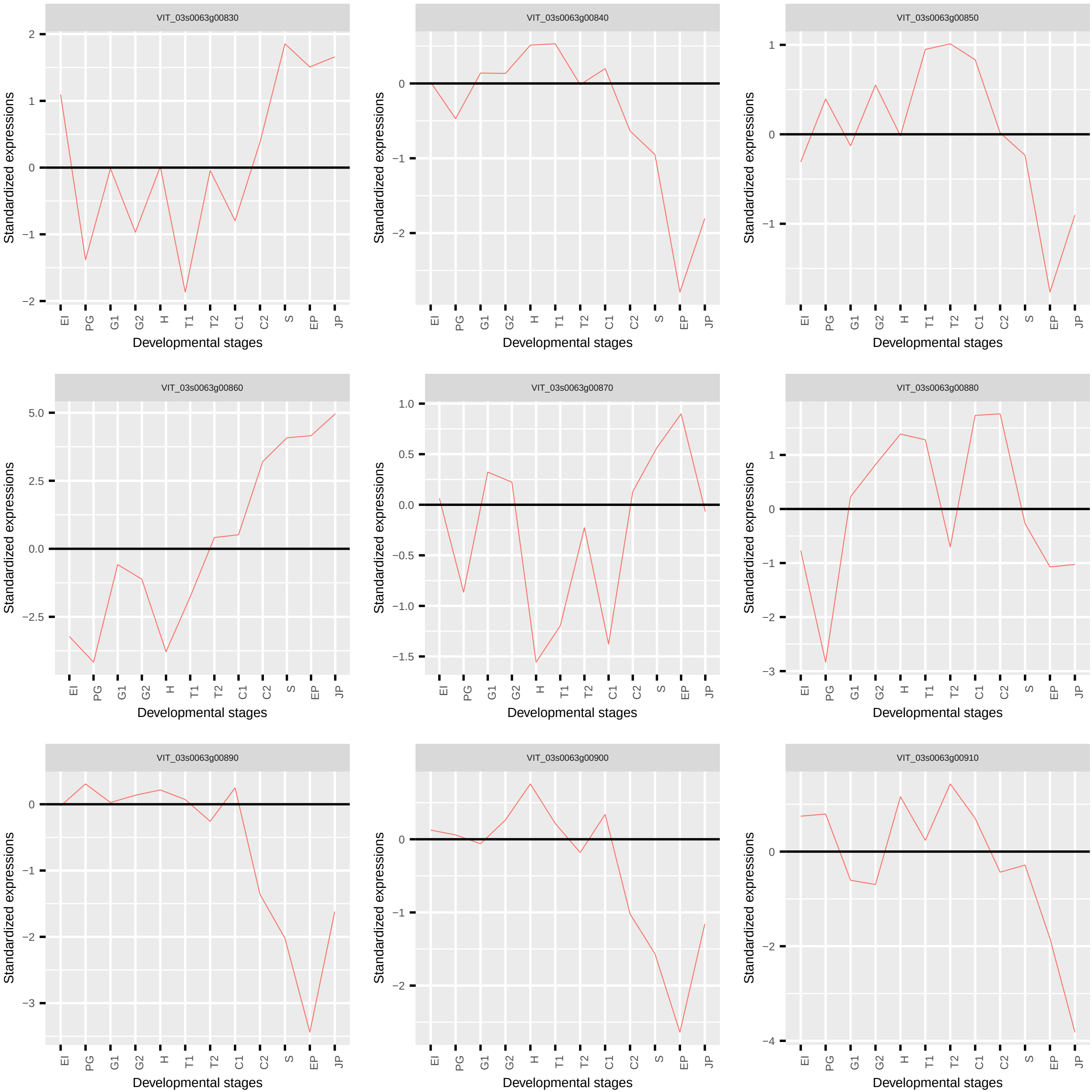
VIT_03s0063g00830
VIT_03s0063g00840
VIT_03s0063g00850
VIT_03s0063g00860
VIT_03s0063g00870
VIT_03s0063g00880
VIT_03s0063g00890
VIT_03s0063g00900
VIT_03s0063g00910
Standardized expressions
Developmental stages
EI PG G1 G2 H T1 T2 C1 C2 S EP JP
2 1 0 −1 −2
0 −1 −2
1 0 −1
5.0 2.5 0.0 −2.5
1.0 0.5 0.0 −0.5 −1.0 −1.5
1 0 −1 −2 −3
0 −1 −2 −3
0 −1 −2
0 −2 −4

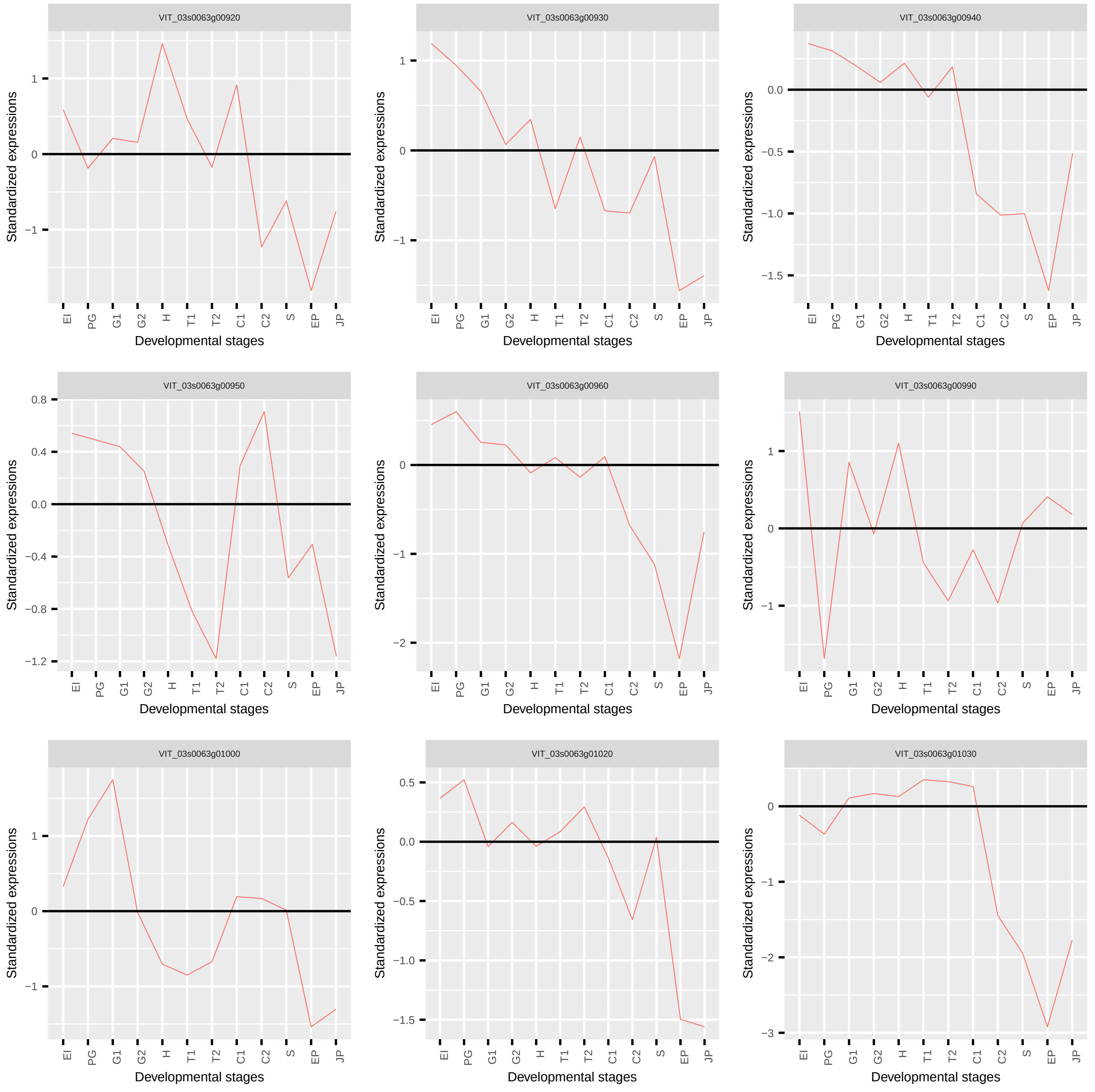
VIT_03s0063g00920
VIT_03s0063g00930
VIT_03s0063g00940
VIT_03s0063g00950
VIT_03s0063g00960
VIT_03s0063g00990
VIT_03s0063g01000
VIT_03s0063g01020
VIT_03s0063g01030
Standardized expressions
Developmental stages
EI PG G1 G2 H T1 T2 C1 C2 S EP JP

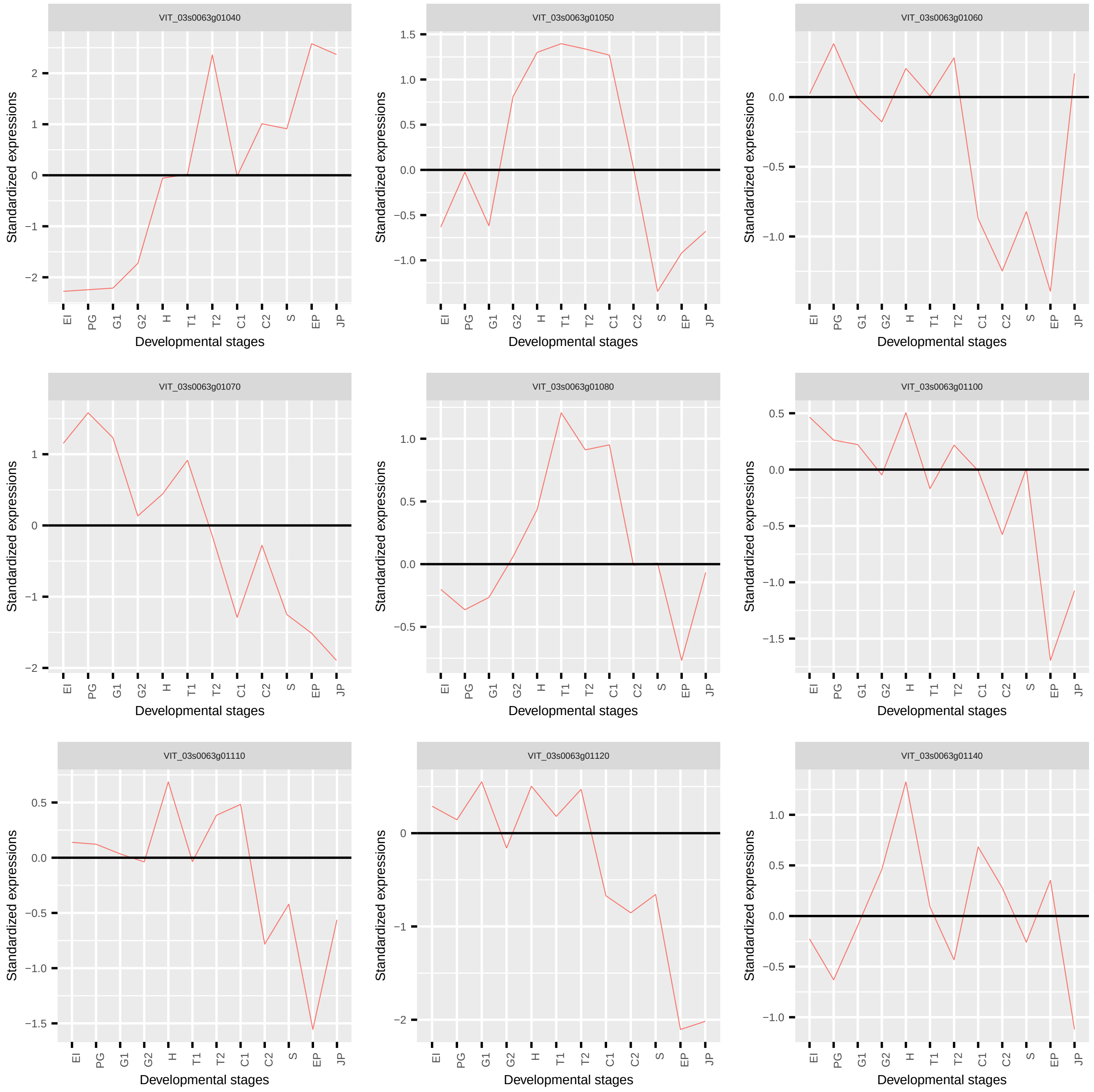

VIT_03s0063g01040
VIT_03s0063g01050
VIT_03s0063g01060
VIT_03s0063g01070
VIT_03s0063g01080
VIT_03s0063g01100
VIT_03s0063g01110
VIT_03s0063g01120
VIT_03s0063g01140
Standardized expressions
Developmental stages
EI PG G1 G2 H T1 T2 C1 C2 S EP JP

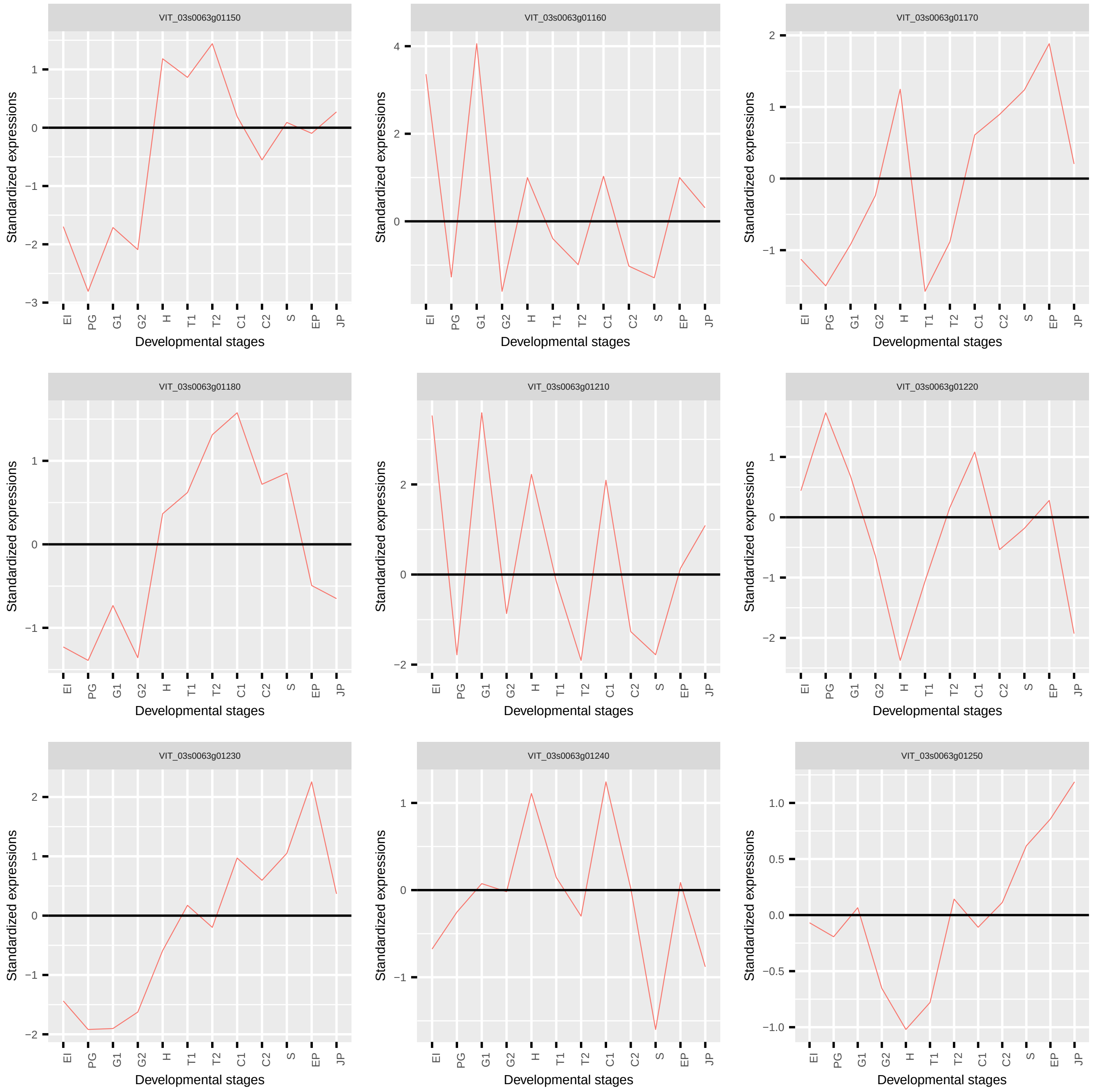
VIT_03s0063g01150
VIT_03s0063g01160
VIT_03s0063g01170
VIT_03s0063g01180
VIT_03s0063g01210
VIT_03s0063g01220
VIT_03s0063g01230
VIT_03s0063g01240
VIT_03s0063g01250
Standardized expressions
Developmental stages
EI
PG
G1
G2
H
T1
T2
C1
C2
S
EP
JP
1
0
−1
−2
−3
4
2
0
2
1
0
−1
1
0
−1
2
0
−2
1
0
−1
−2
2
1
0
−1
−2
1
0
−1
1.0
0.5
0.0
−0.5
−1.0

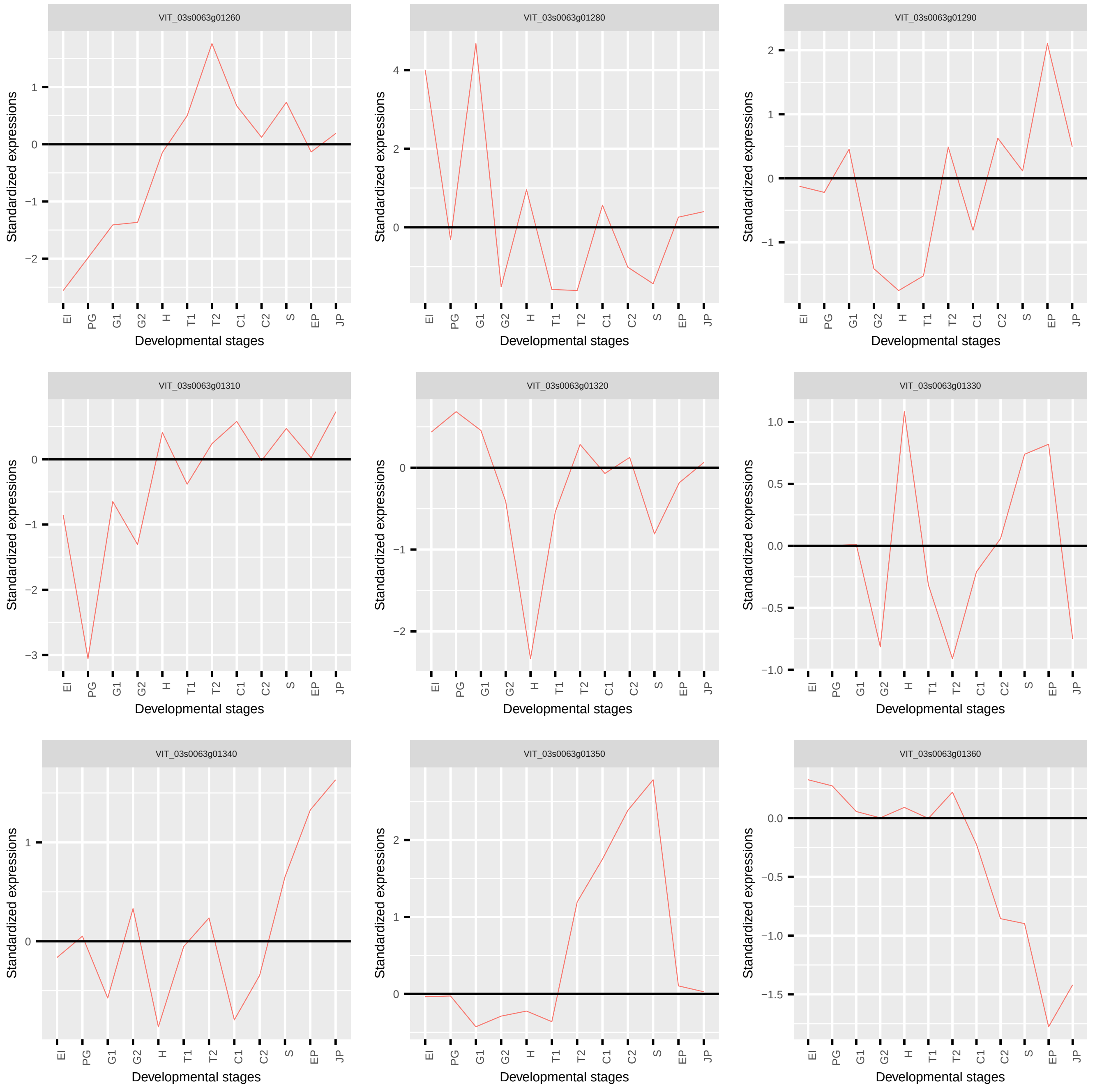
VIT_03s0063g01260
VIT_03s0063g01280
VIT_03s0063g01290
VIT_03s0063g01310
VIT_03s0063g01320
VIT_03s0063g01330
VIT_03s0063g01340
VIT_03s0063g01350
VIT_03s0063g01360
Standardized expressions
Developmental stages
EI PG G1 G2 H T1 T2 C1 C2 S EP JP

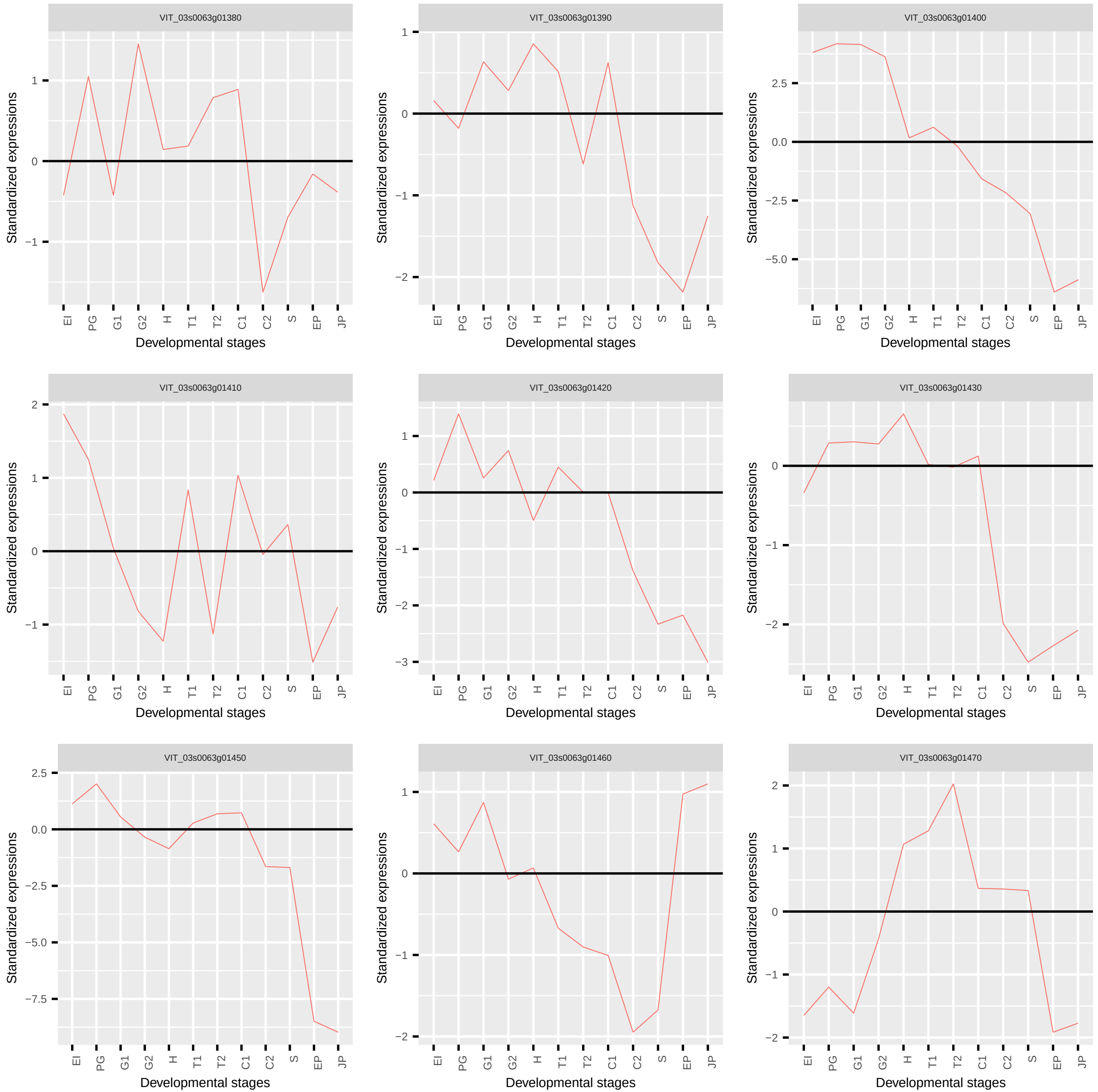
VIT_03s0063g01380
1
0
−1
Standardized expressions
EI PG G1 G2 H T1 T2 C1 C2 S EP JP
Developmental stages
VIT_03s0063g01390
1
0
−1
−2
Standardized expressions
EI PG G1 G2 H T1 T2 C1 C2 S EP JP
Developmental stages
VIT_03s0063g01400
2.5
0.0
−2.5
−5.0
Standardized expressions
EI PG G1 G2 H T1 T2 C1 C2 S EP JP
Developmental stages
VIT_03s0063g01410
2
1
0
−1
Standardized expressions
EI PG G1 G2 H T1 T2 C1 C2 S EP JP
Developmental stages
VIT_03s0063g01420
1
0
−1
−2
−3
Standardized expressions
EI PG G1 G2 H T1 T2 C1 C2 S EP JP
Developmental stages
VIT_03s0063g01430
0
−1
−2
Standardized expressions
EI PG G1 G2 H T1 T2 C1 C2 S EP JP
Developmental stages
VIT_03s0063g01450
2.5
0.0
−2.5
−5.0
−7.5
Standardized expressions
EI PG G1 G2 H T1 T2 C1 C2 S EP JP
Developmental stages
VIT_03s0063g01460
1
0
−1
−2
Standardized expressions
EI PG G1 G2 H T1 T2 C1 C2 S EP JP
Developmental stages
VIT_03s0063g01470
2
1
0
−1
−2
Standardized expressions
EI PG G1 G2 H T1 T2 C1 C2 S EP JP
Developmental stages

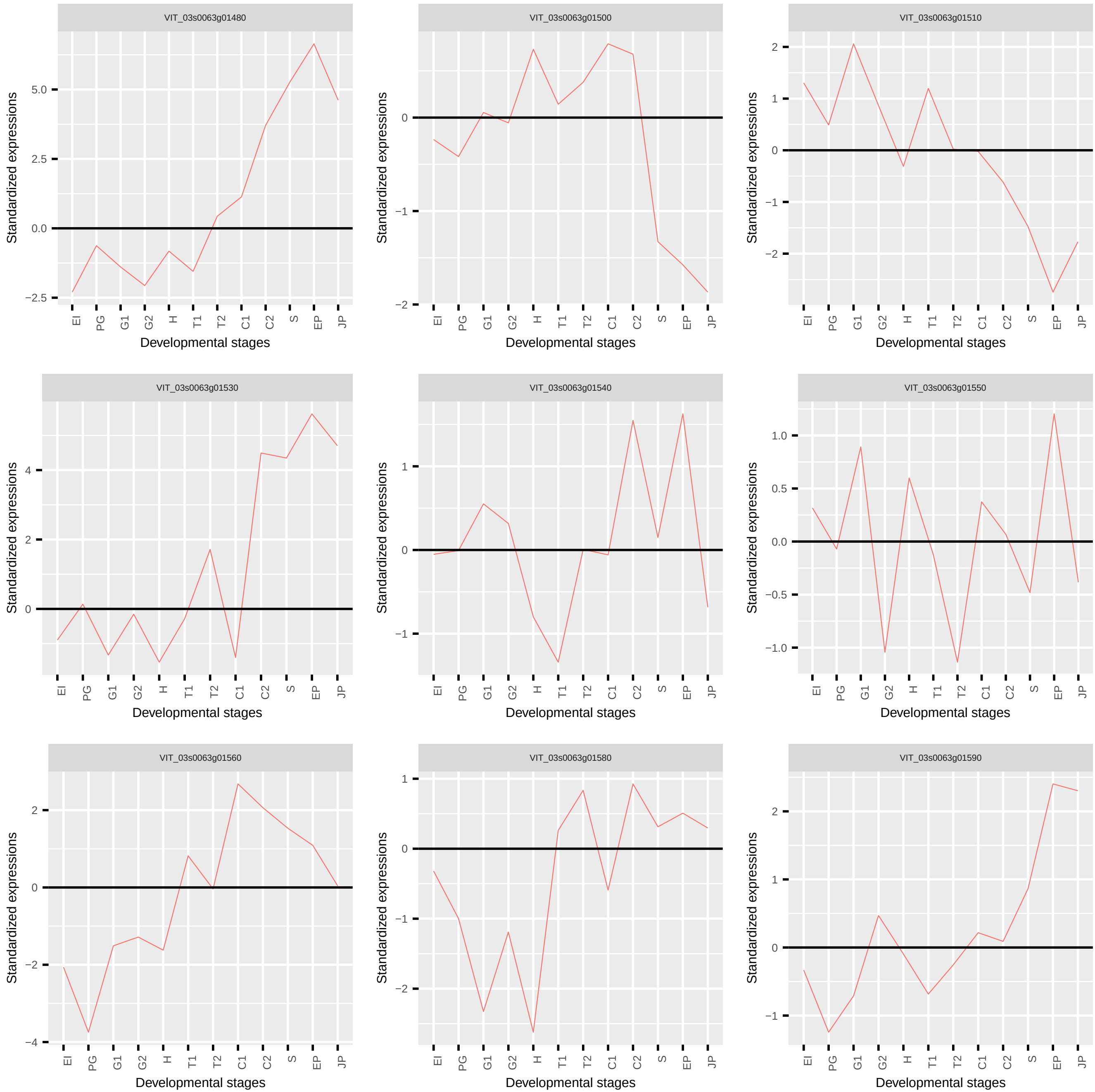

VIT_03s0063g01480
Standardized expressions
Developmental stages
5.0
2.5
0.0
−2.5
EI PG G1 G2 H T1 T2 C1 C2 S EP JP
VIT_03s0063g01500
Standardized expressions
Developmental stages
0
−1
−2
EI PG G1 G2 H T1 T2 C1 C2 S EP JP
VIT_03s0063g01510
Standardized expressions
Developmental stages
2
1
0
−1
−2
EI PG G1 G2 H T1 T2 C1 C2 S EP JP
VIT_03s0063g01530
Standardized expressions
Developmental stages
4
2
0
EI PG G1 G2 H T1 T2 C1 C2 S EP JP
VIT_03s0063g01540
Standardized expressions
Developmental stages
1
0
−1
EI PG G1 G2 H T1 T2 C1 C2 S EP JP
VIT_03s0063g01550
Standardized expressions
Developmental stages
1.0
0.5
0.0
−0.5
−1.0
EI PG G1 G2 H T1 T2 C1 C2 S EP JP
VIT_03s0063g01560
Standardized expressions
Developmental stages
2
0
−2
−4
EI PG G1 G2 H T1 T2 C1 C2 S EP JP
VIT_03s0063g01580
Standardized expressions
Developmental stages
1
0
−1
−2
EI PG G1 G2 H T1 T2 C1 C2 S EP JP
VIT_03s0063g01590
Standardized expressions
Developmental stages
2
1
0
−1
EI PG G1 G2 H T1 T2 C1 C2 S EP JP

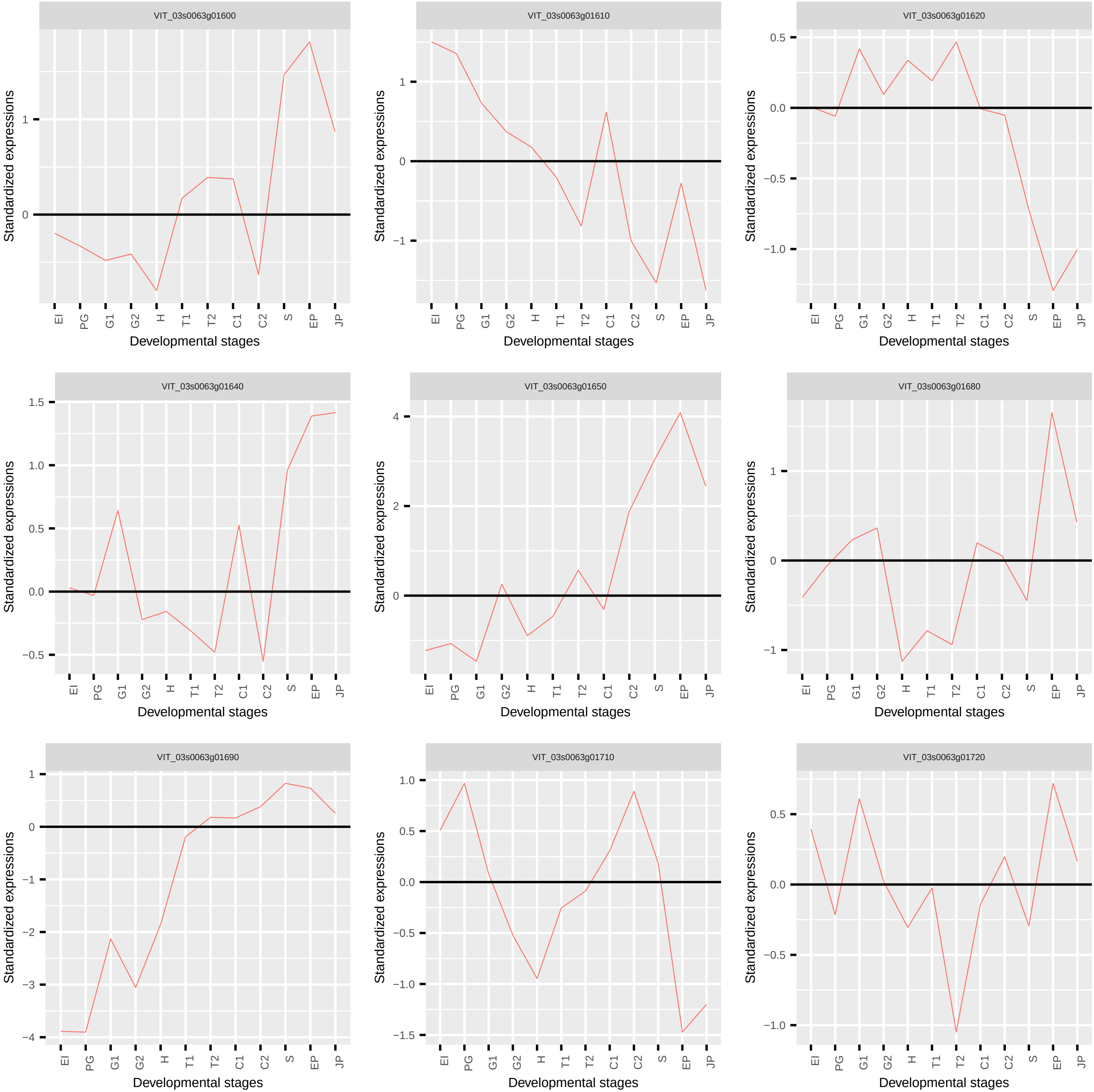
VIT_03s0063g01600
VIT_03s0063g01610
VIT_03s0063g01620
VIT_03s0063g01640
VIT_03s0063g01650
VIT_03s0063g01680
VIT_03s0063g01690
VIT_03s0063g01710
VIT_03s0063g01720
Standardized expressions
Developmental stages
EI PG G1 G2 H T1 T2 C1 C2 S EP JP

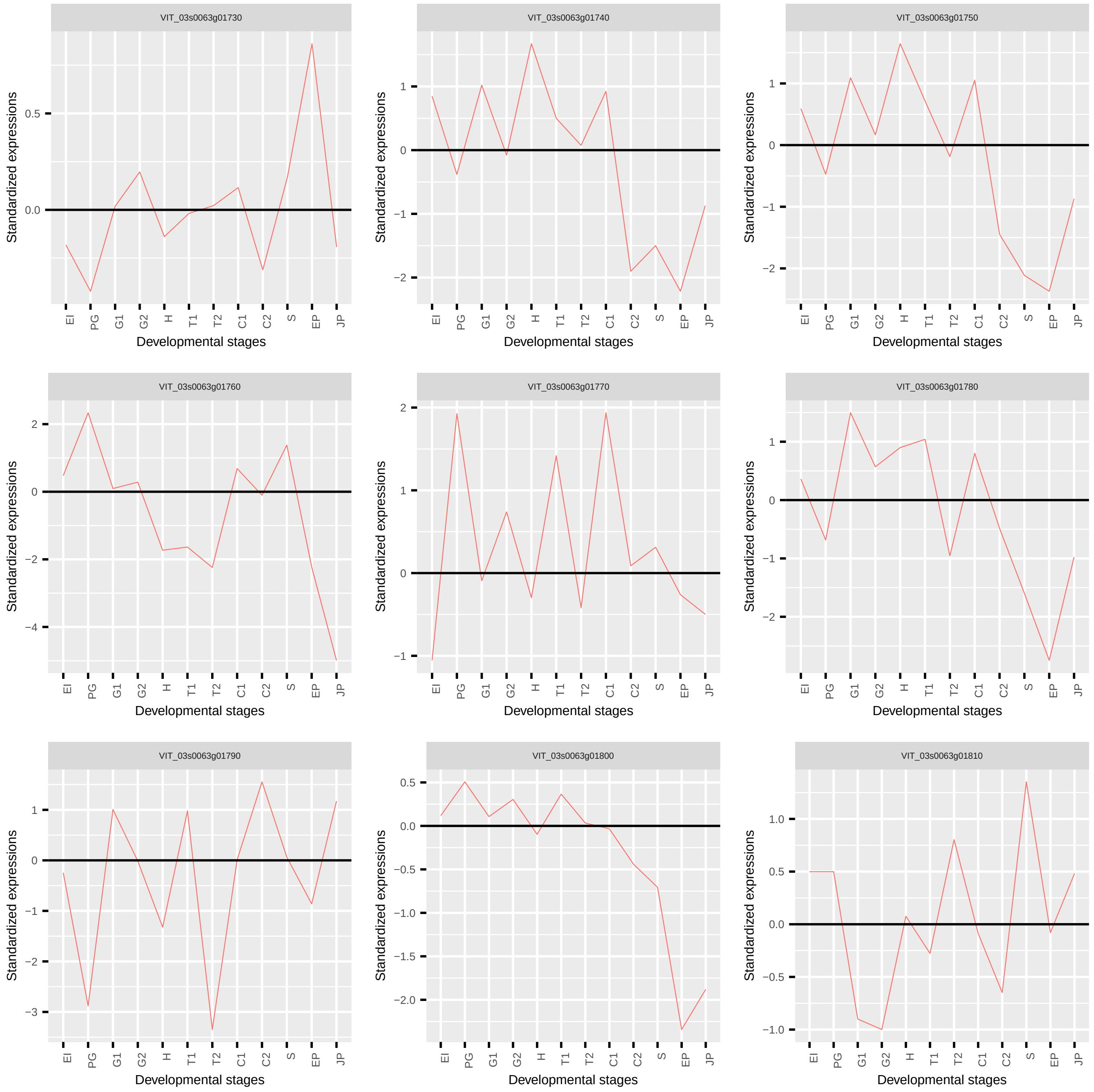
VIT_03s0063g01730
VIT_03s0063g01740
VIT_03s0063g01750
VIT_03s0063g01760
VIT_03s0063g01770
VIT_03s0063g01780
VIT_03s0063g01790
VIT_03s0063g01800
VIT_03s0063g01810
Standardized expressions
Developmental stages
EI PG G1 G2 H T1 T2 C1 C2 S EP JP

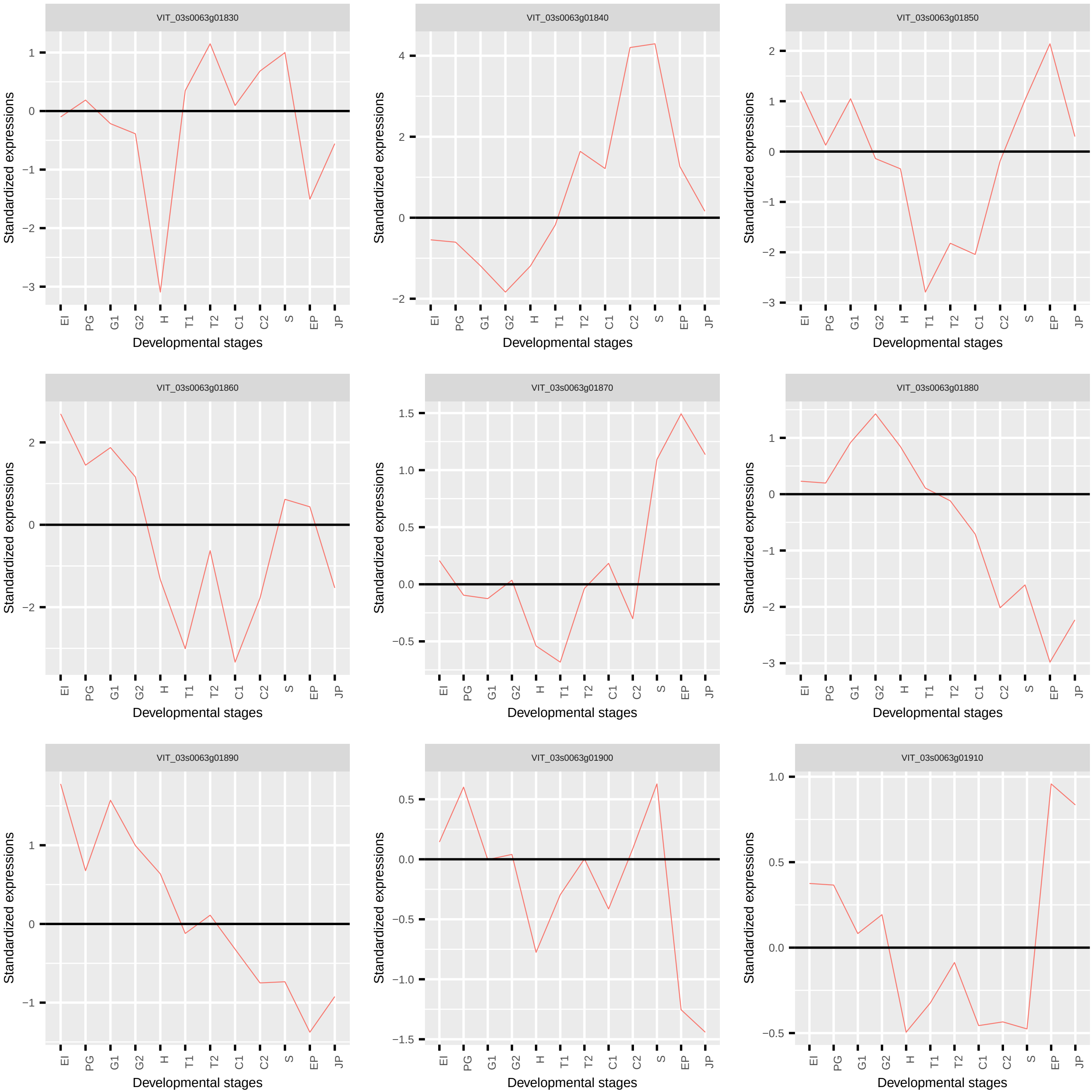

VIT_03s0063g01830
VIT_03s0063g01840
VIT_03s0063g01850
VIT_03s0063g01860
VIT_03s0063g01870
VIT_03s0063g01880
VIT_03s0063g01890
VIT_03s0063g01900
VIT_03s0063g01910
Standardized expressions
Developmental stages
EI PG G1 G2 H T1 T2 C1 C2 S EP JP

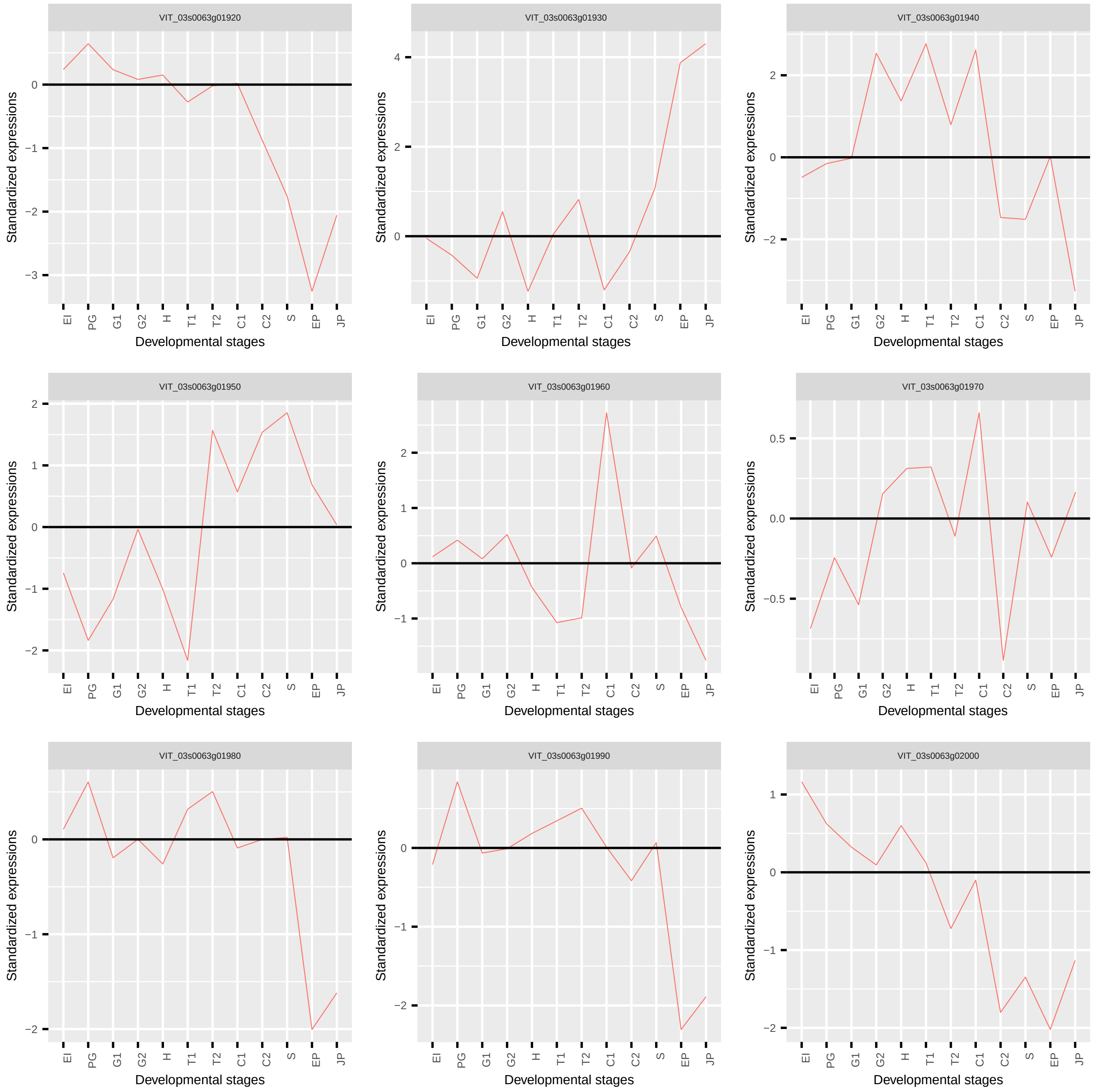
VIT_03s0063g01920
VIT_03s0063g01930
VIT_03s0063g01940
VIT_03s0063g01950
VIT_03s0063g01960
VIT_03s0063g01970
VIT_03s0063g01980
VIT_03s0063g01990
VIT_03s0063g02000
Standardized expressions
Developmental stages
EI PG G1 G2 H T1 T2 C1 C2 S EP JP

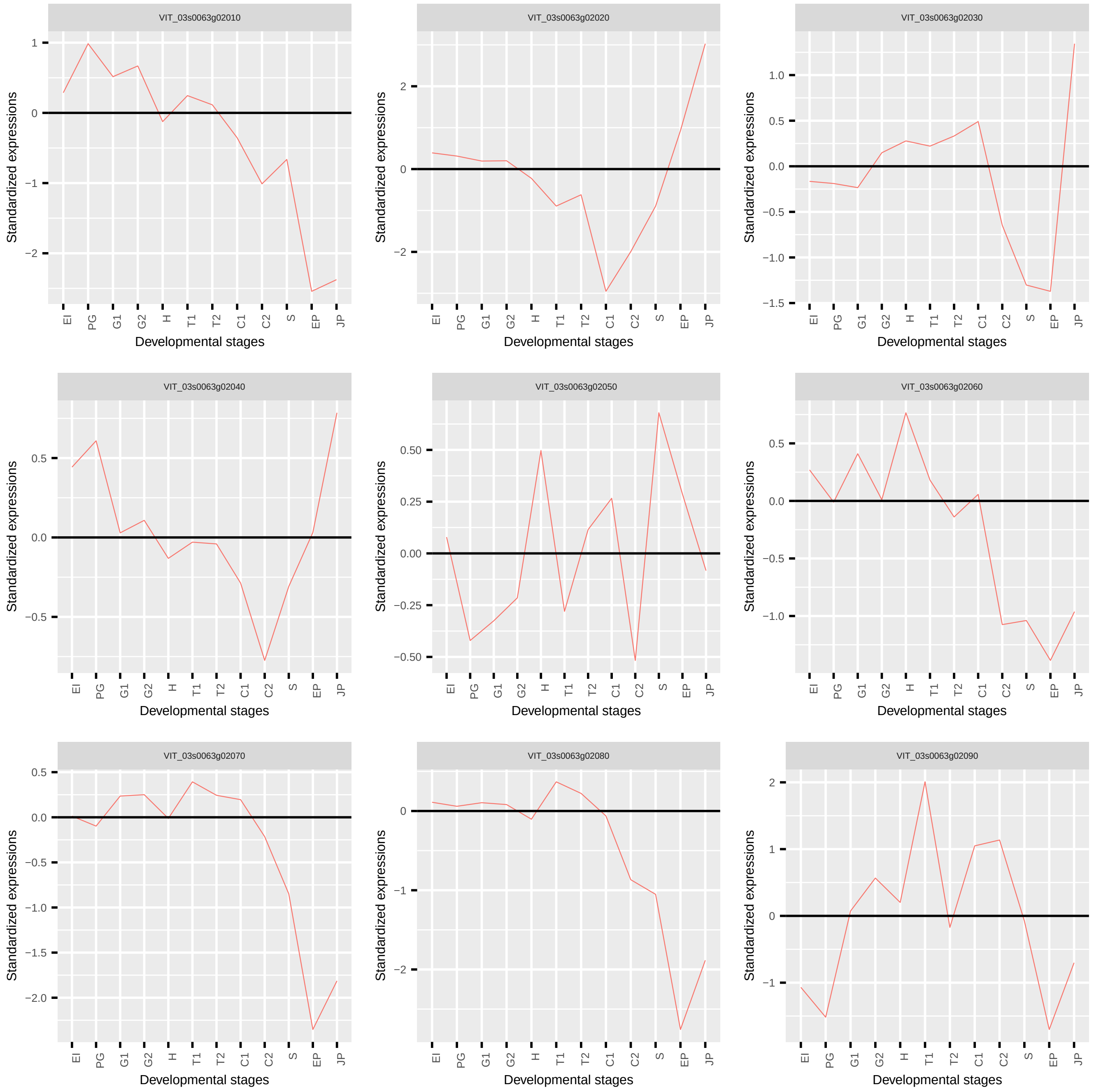
VIT_03s0063g02010
VIT_03s0063g02020
VIT_03s0063g02030
VIT_03s0063g02040
VIT_03s0063g02050
VIT_03s0063g02060
VIT_03s0063g02070
VIT_03s0063g02080
VIT_03s0063g02090
Standardized expressions
Developmental stages
EI
PG
G1
G2
H
T1
T2
C1
C2
S
EP
JP

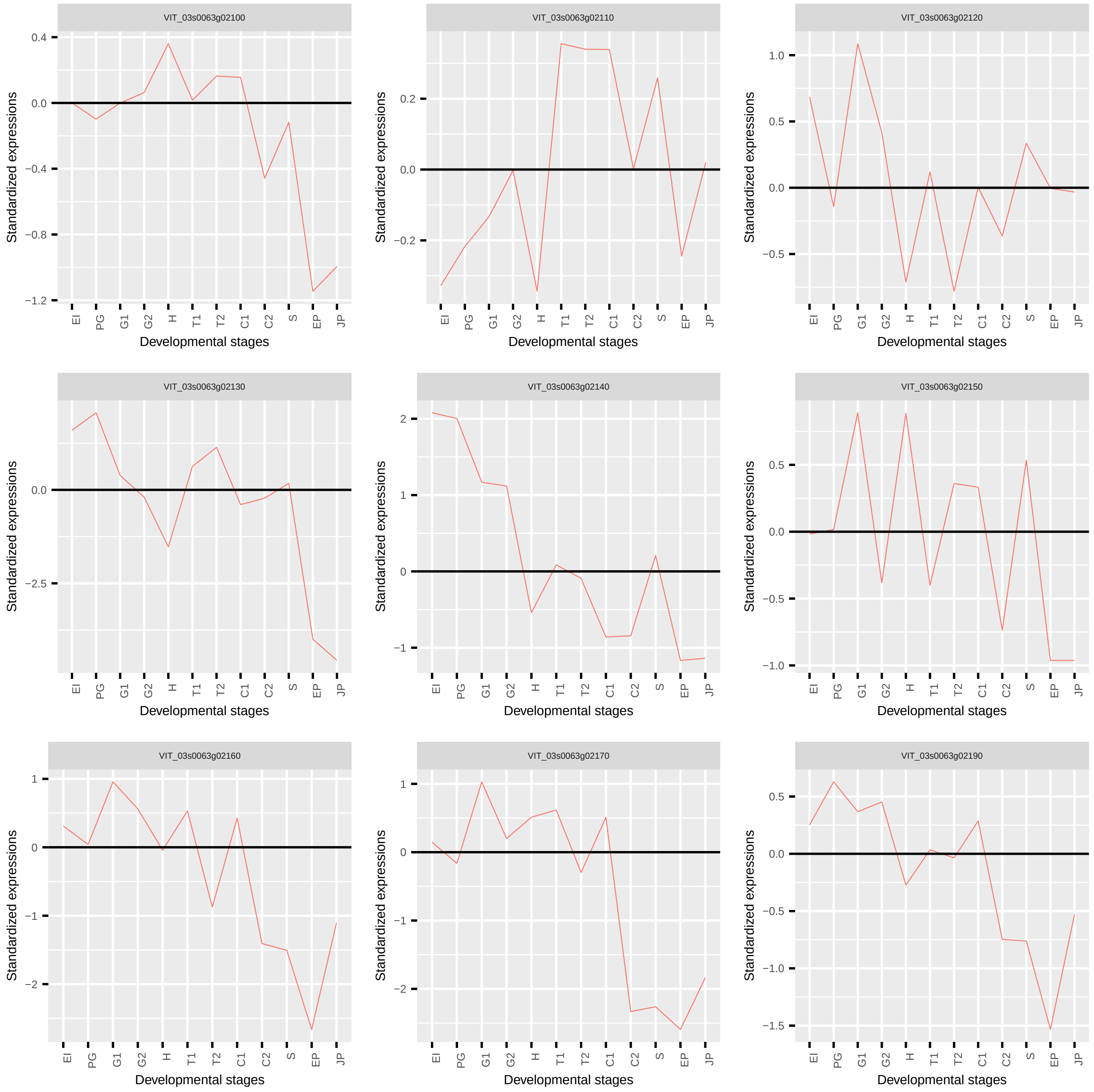

VIT_03s0063g02100
VIT_03s0063g02110
VIT_03s0063g02120
VIT_03s0063g02130
VIT_03s0063g02140
VIT_03s0063g02150
VIT_03s0063g02160
VIT_03s0063g02170
VIT_03s0063g02190
Standardized expressions
Developmental stages
EI PG G1 G2 H T1 T2 C1 C2 S EP JP

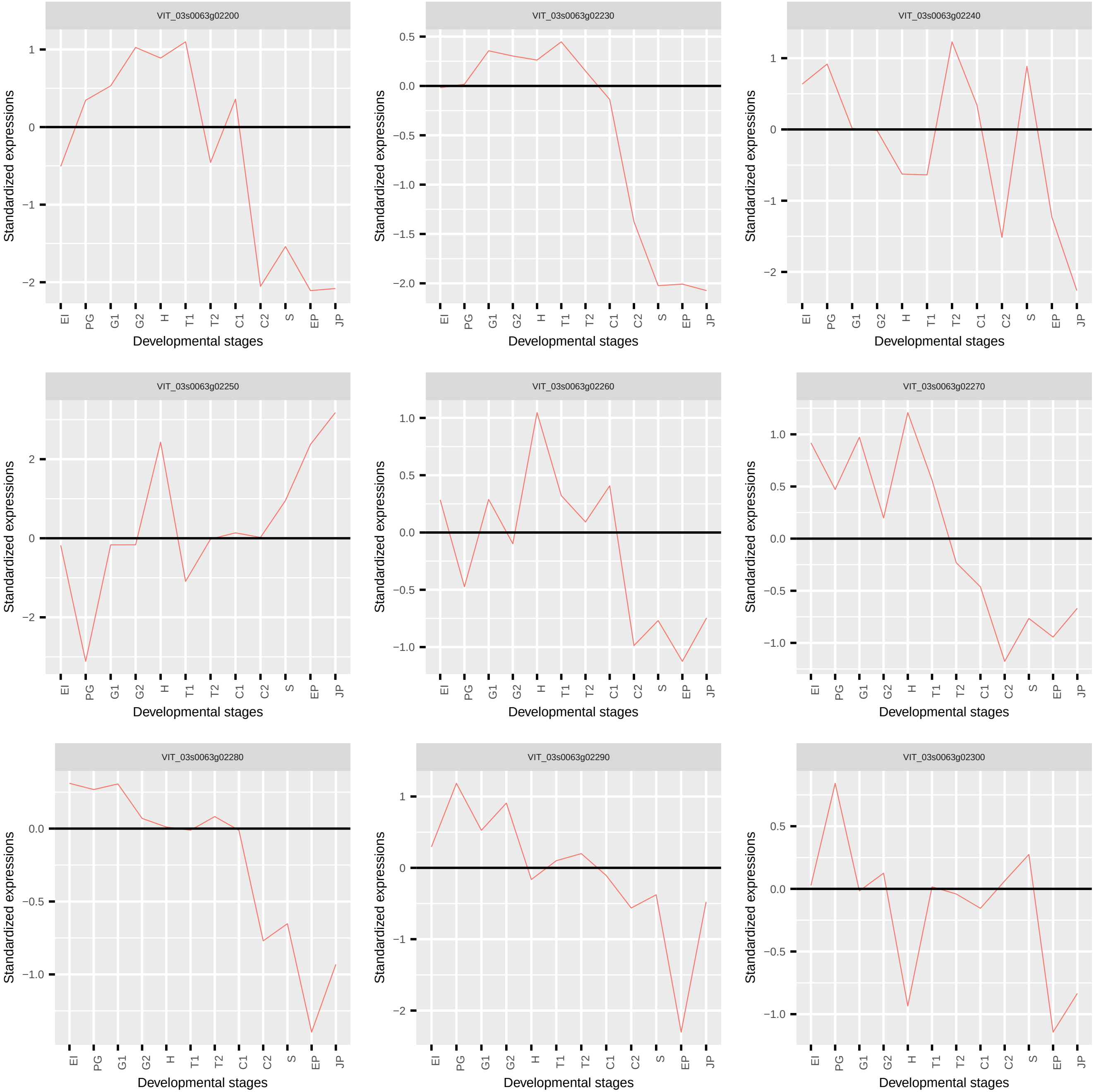
VIT_03s0063g02200
VIT_03s0063g02230
VIT_03s0063g02240
VIT_03s0063g02250
VIT_03s0063g02260
VIT_03s0063g02270
VIT_03s0063g02280
VIT_03s0063g02290
VIT_03s0063g02300
Standardized expressions
Developmental stages
EI
PG
G1
G2
H
T1
T2
C1
C2
S
EP
JP

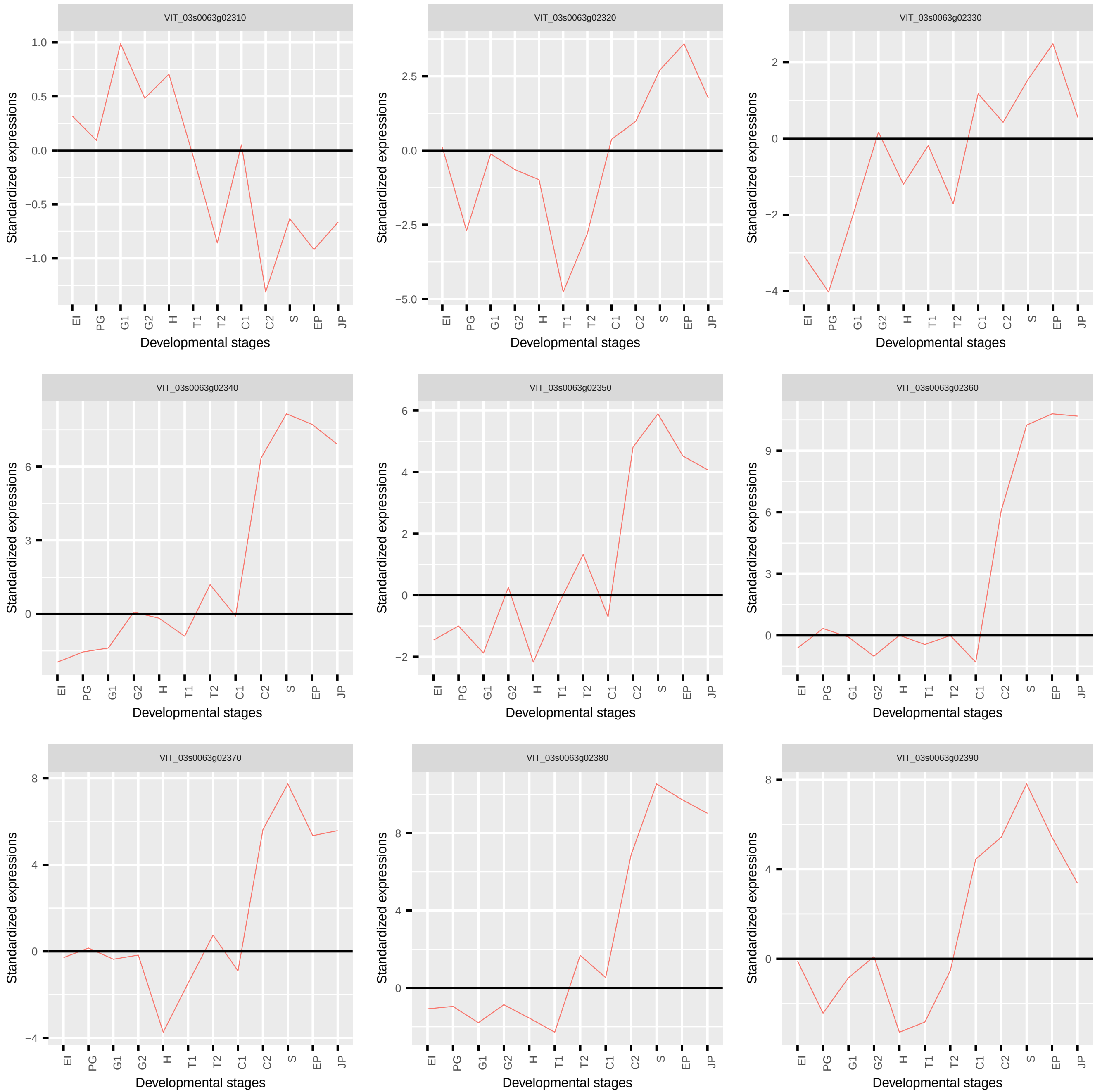
VIT_03s0063g02310
VIT_03s0063g02320
VIT_03s0063g02330
VIT_03s0063g02340
VIT_03s0063g02350
VIT_03s0063g02360
VIT_03s0063g02370
VIT_03s0063g02380
VIT_03s0063g02390
Standardized expressions
Developmental stages
EI PG G1 G2 H T1 T2 C1 C2 S EP JP
1.0 0.5 0.0 −0.5 −1.0
2.5 0.0 −2.5 −5.0
2 0 −2 −4
6 3 0
6 4 2 0 −2
9 6 3 0
8 4 0 −4
8 4 0
8 4 0

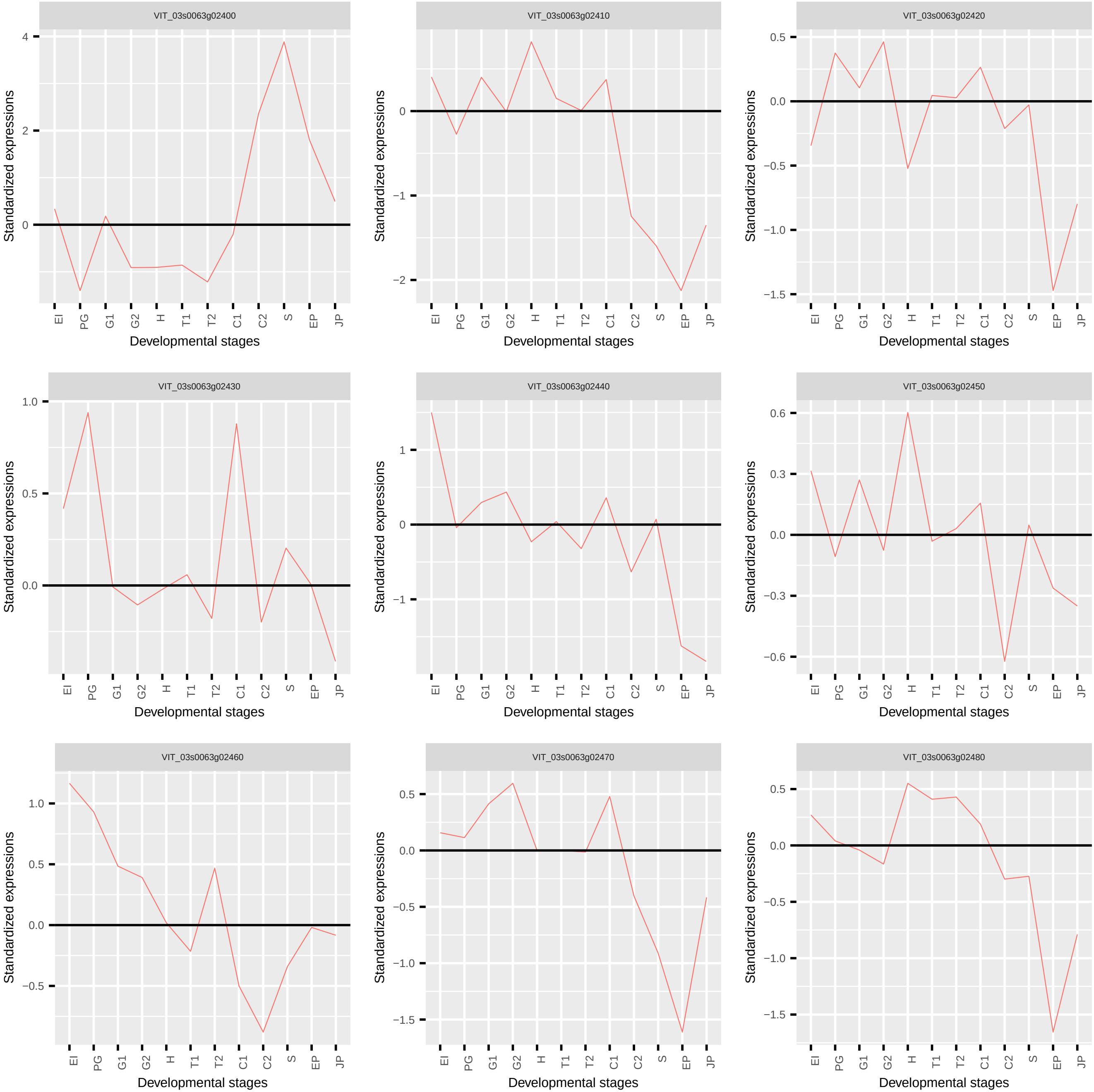
VIT_03s0063g02400
VIT_03s0063g02410
VIT_03s0063g02420
VIT_03s0063g02430
VIT_03s0063g02440
VIT_03s0063g02450
VIT_03s0063g02460
VIT_03s0063g02470
VIT_03s0063g02480
Standardized expressions
Developmental stages
EI PG G1 G2 H T1 T2 C1 C2 S EP JP

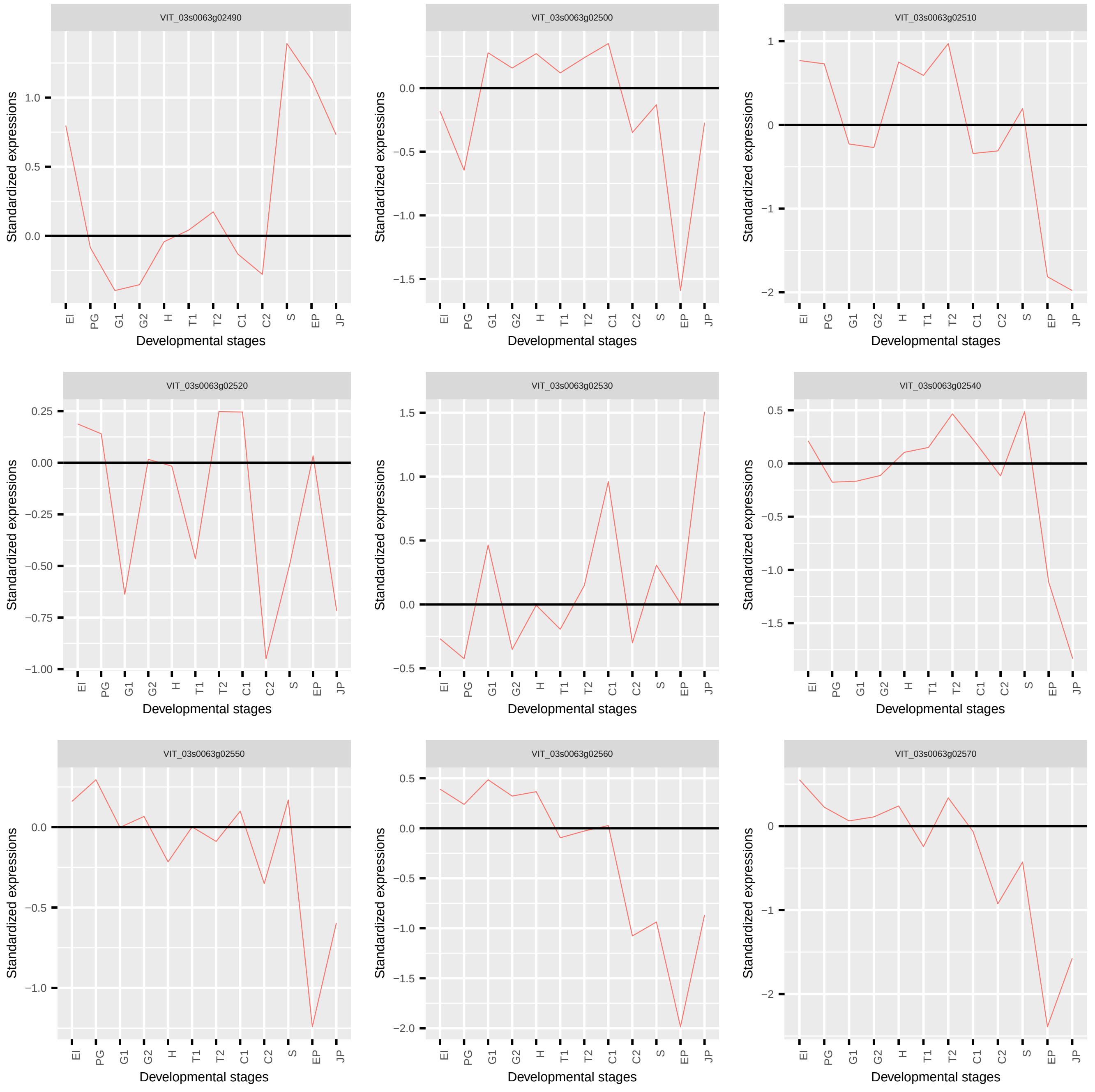
VIT_03s0063g02490
VIT_03s0063g02500
VIT_03s0063g02510
VIT_03s0063g02520
VIT_03s0063g02530
VIT_03s0063g02540
VIT_03s0063g02550
VIT_03s0063g02560
VIT_03s0063g02570
Standardized expressions
Developmental stages
EI PG G1 G2 H T1 T2 C1 C2 S EP JP

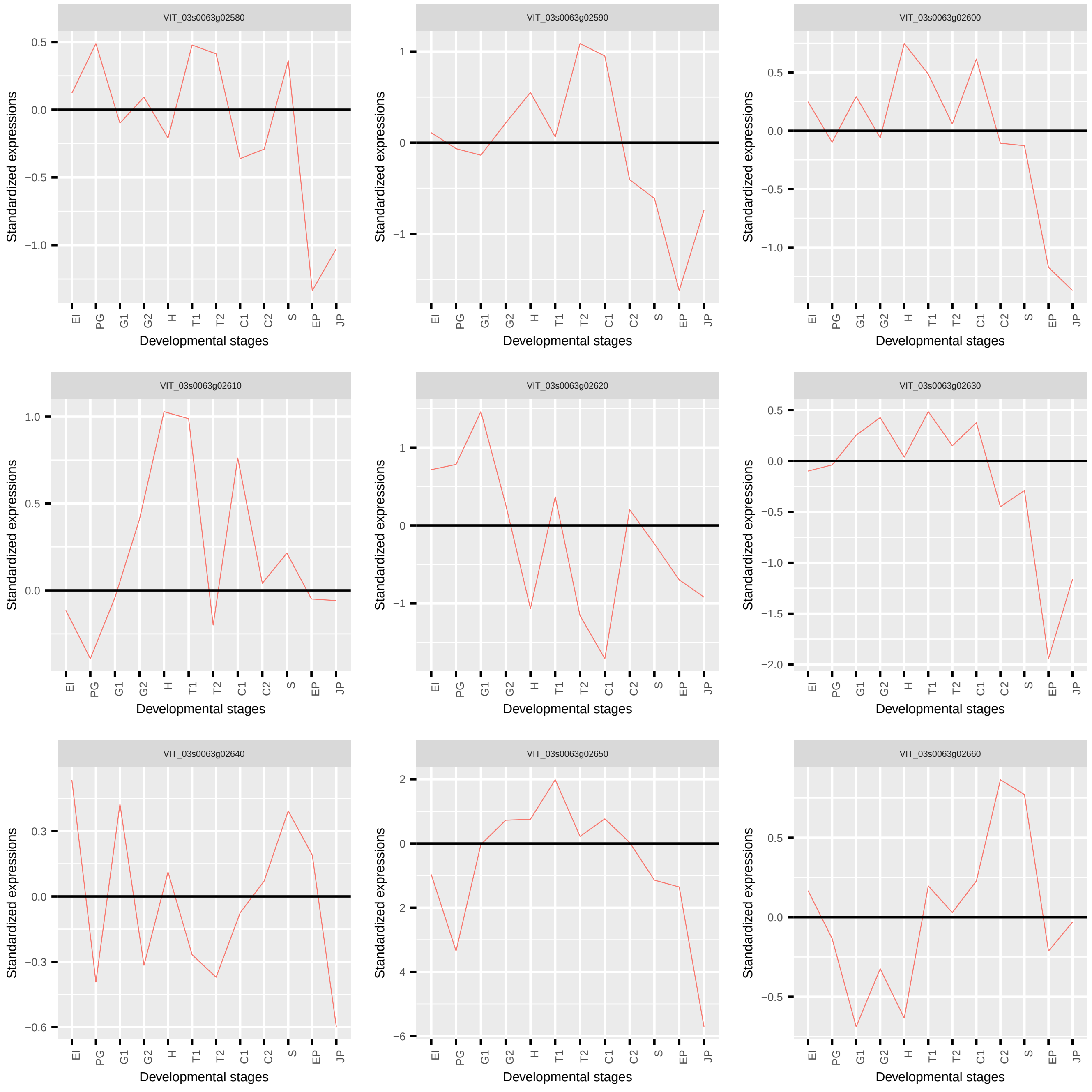
VIT_03s0063g02580
VIT_03s0063g02590
VIT_03s0063g02600
VIT_03s0063g02610
VIT_03s0063g02620
VIT_03s0063g02630
VIT_03s0063g02640
VIT_03s0063g02650
VIT_03s0063g02660
Standardized expressions
Developmental stages
EI PG G1 G2 H T1 T2 C1 C2 S EP JP
0.5
0.0
−0.5
−1.0
1.0
1
0
−1
−1.5
−2.0
0.3
−0.3
−0.6
2
−2
−4
−6

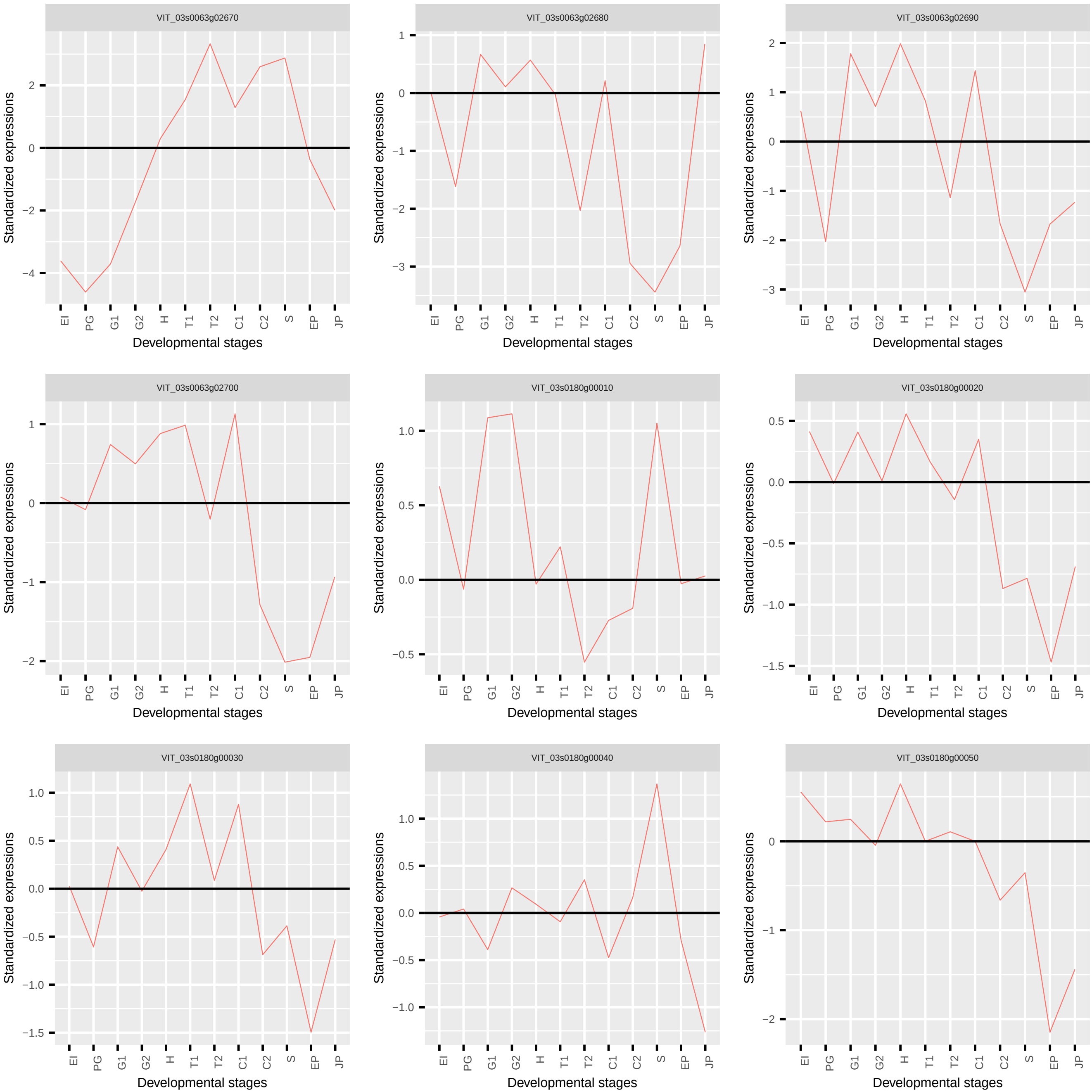
VIT_03s0063g02670
VIT_03s0063g02680
VIT_03s0063g02690
VIT_03s0063g02700
VIT_03s0180g00010
VIT_03s0180g00020
VIT_03s0180g00030
VIT_03s0180g00040
VIT_03s0180g00050
Standardized expressions
Developmental stages
EI PG G1 G2 H T1 T2 C1 C2 S EP JP

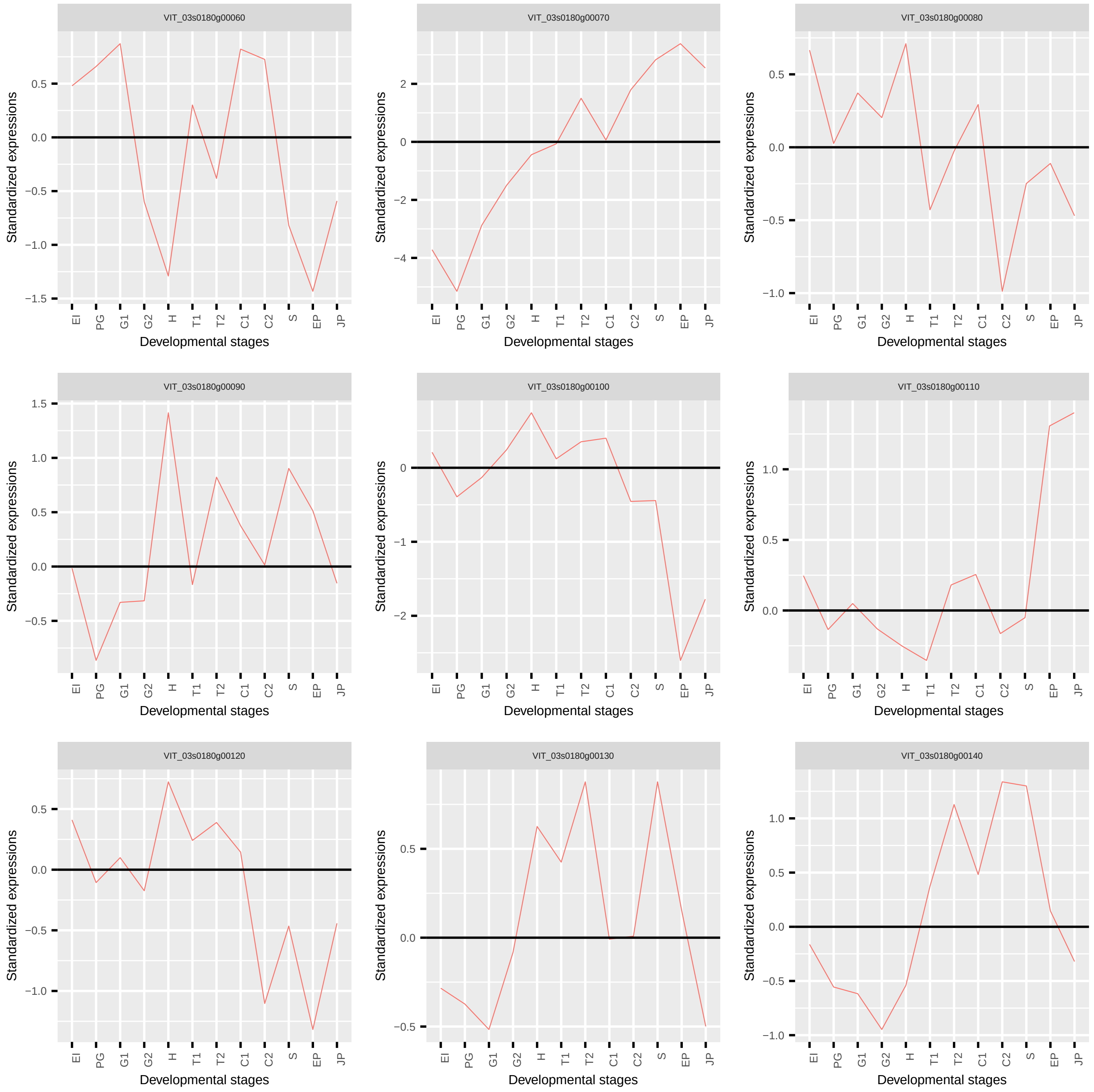
VIT_03s0180g00060
VIT_03s0180g00070
VIT_03s0180g00080
VIT_03s0180g00090
VIT_03s0180g00100
VIT_03s0180g00110
VIT_03s0180g00120
VIT_03s0180g00130
VIT_03s0180g00140
Standardized expressions
Developmental stages
EI PG G1 G2 H T1 T2 C1 C2 S EP JP

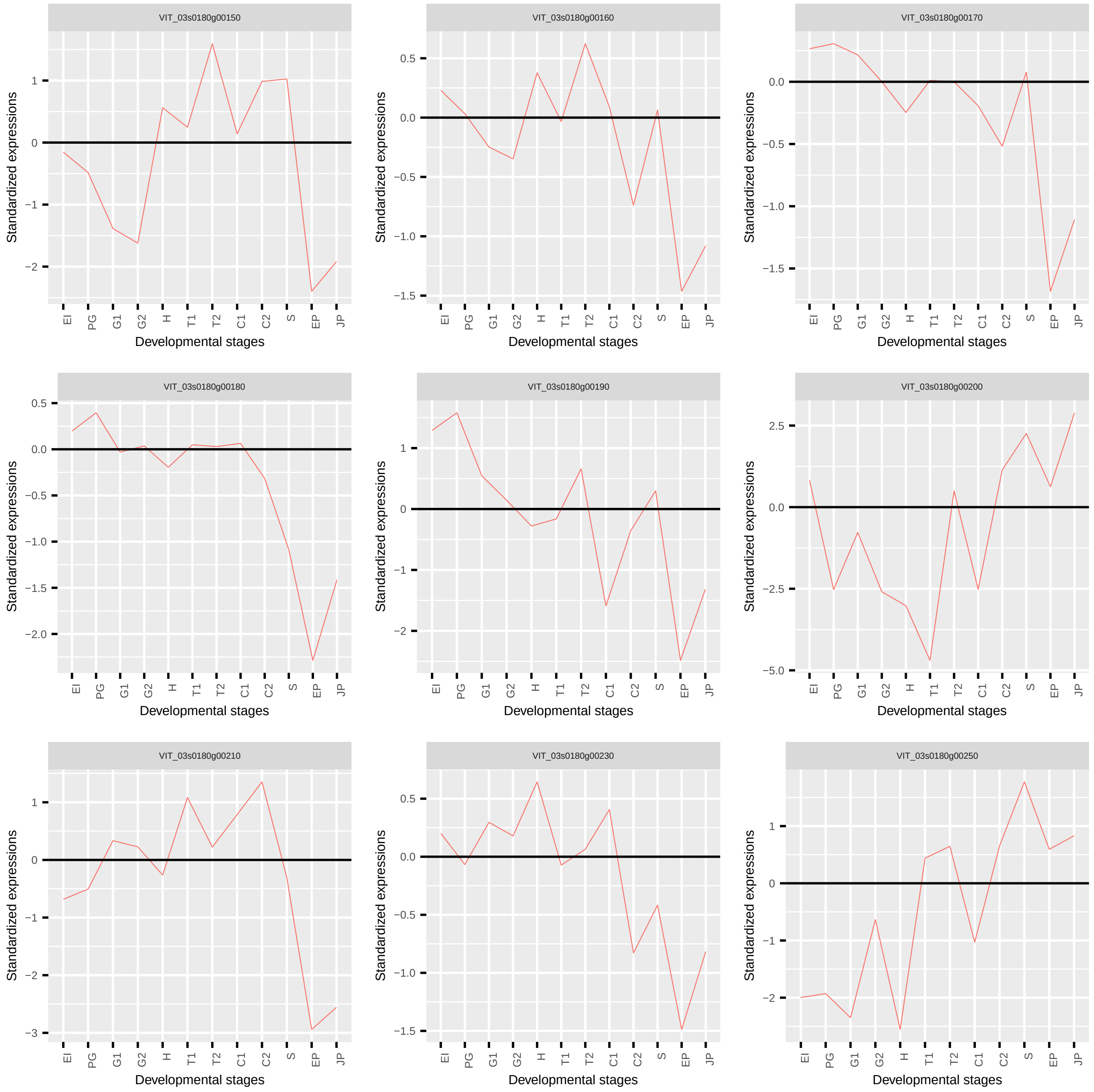
VIT_03s0180g00150
Standardized expressions
1
0
−1
−2
EI PG G1 G2 H T1 T2 C1 C2 S EP JP
Developmental stages
VIT_03s0180g00160
Standardized expressions
0.5
0.0
−0.5
−1.0
−1.5
EI PG G1 G2 H T1 T2 C1 C2 S EP JP
Developmental stages
VIT_03s0180g00170
Standardized expressions
0.0
−0.5
−1.0
−1.5
EI PG G1 G2 H T1 T2 C1 C2 S EP JP
Developmental stages
VIT_03s0180g00180
Standardized expressions
0.5
0.0
−0.5
−1.0
−1.5
−2.0
EI PG G1 G2 H T1 T2 C1 C2 S EP JP
Developmental stages
VIT_03s0180g00190
Standardized expressions
1
0
−1
−2
EI PG G1 G2 H T1 T2 C1 C2 S EP JP
Developmental stages
VIT_03s0180g00200
Standardized expressions
2.5
0.0
−2.5
−5.0
EI PG G1 G2 H T1 T2 C1 C2 S EP JP
Developmental stages
VIT_03s0180g00210
Standardized expressions
1
0
−1
−2
−3
EI PG G1 G2 H T1 T2 C1 C2 S EP JP
Developmental stages
VIT_03s0180g00230
Standardized expressions
0.5
0.0
−0.5
−1.0
−1.5
EI PG G1 G2 H T1 T2 C1 C2 S EP JP
Developmental stages
VIT_03s0180g00250
Standardized expressions
1
0
−1
−2
EI PG G1 G2 H T1 T2 C1 C2 S EP JP
Developmental stages

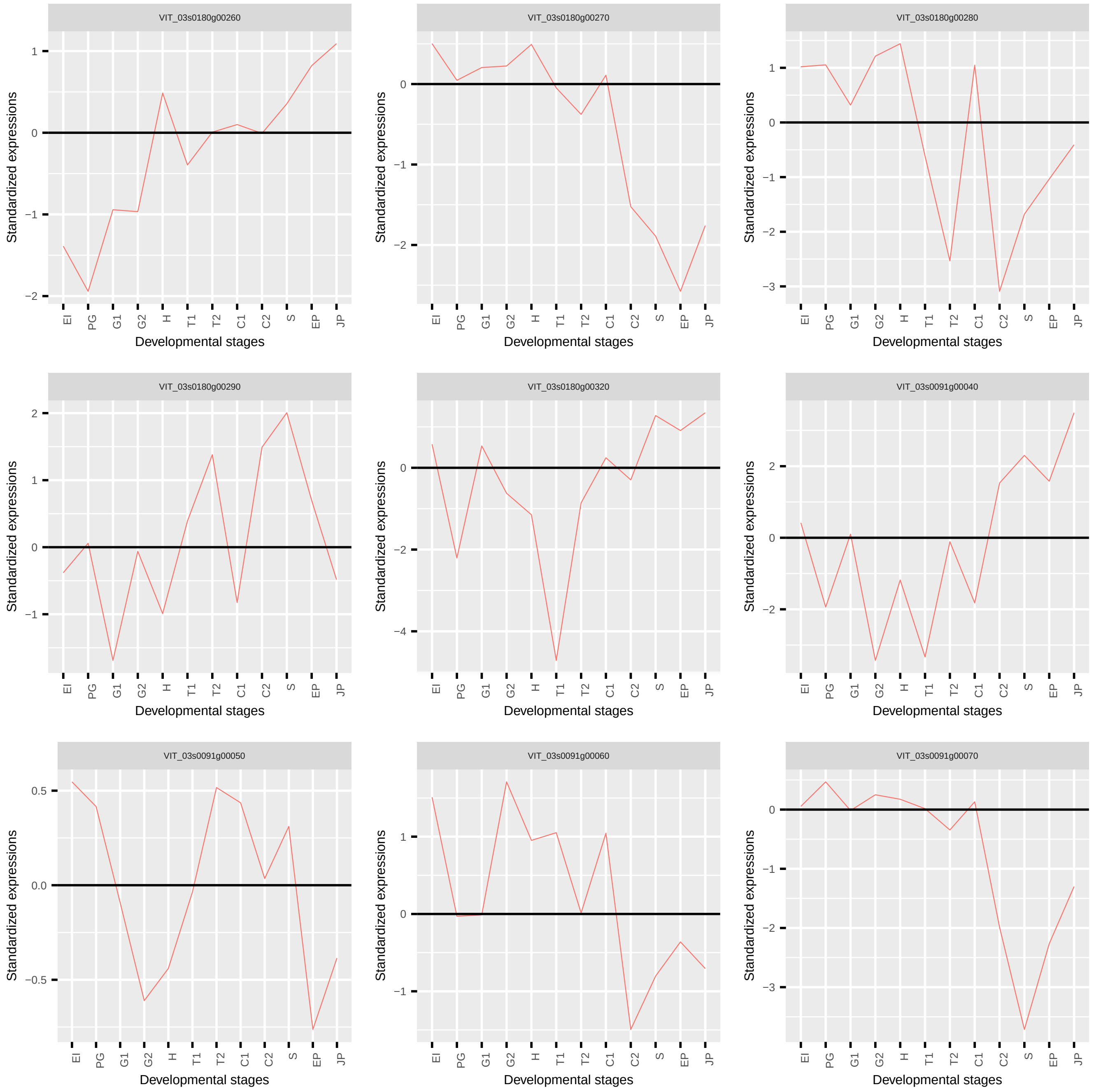
VIT_03s0180g00260
VIT_03s0180g00270
VIT_03s0180g00280
VIT_03s0180g00290
VIT_03s0180g00320
VIT_03s0091g00040
VIT_03s0091g00050
VIT_03s0091g00060
VIT_03s0091g00070
Standardized expressions
Developmental stages
EI PG G1 G2 H T1 T2 C1 C2 S EP JP

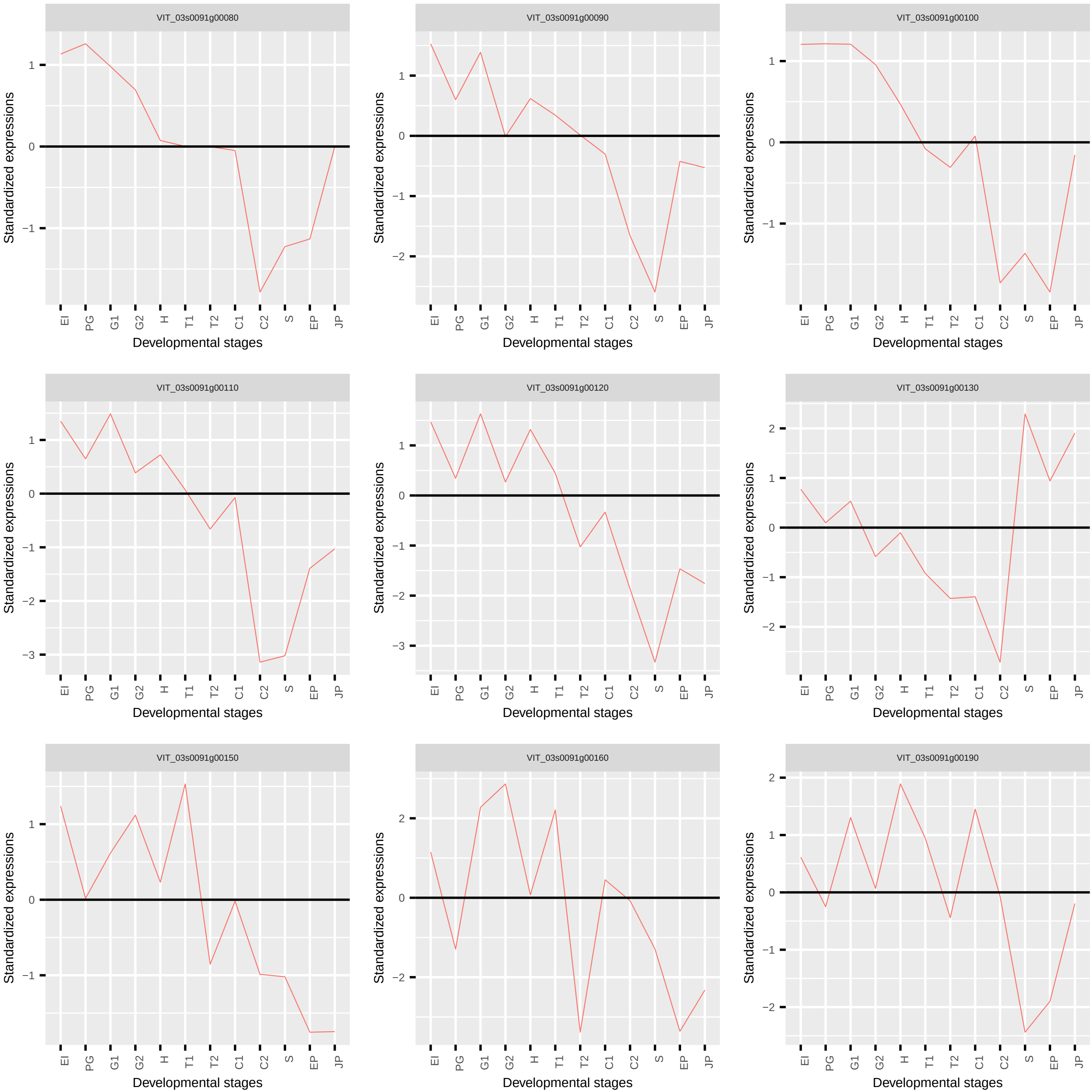
VIT_03s0091g00080
VIT_03s0091g00090
VIT_03s0091g00100
VIT_03s0091g00110
VIT_03s0091g00120
VIT_03s0091g00130
VIT_03s0091g00150
VIT_03s0091g00160
VIT_03s0091g00190
Standardized expressions
Developmental stages
EI PG G1 G2 H T1 T2 C1 C2 S EP JP

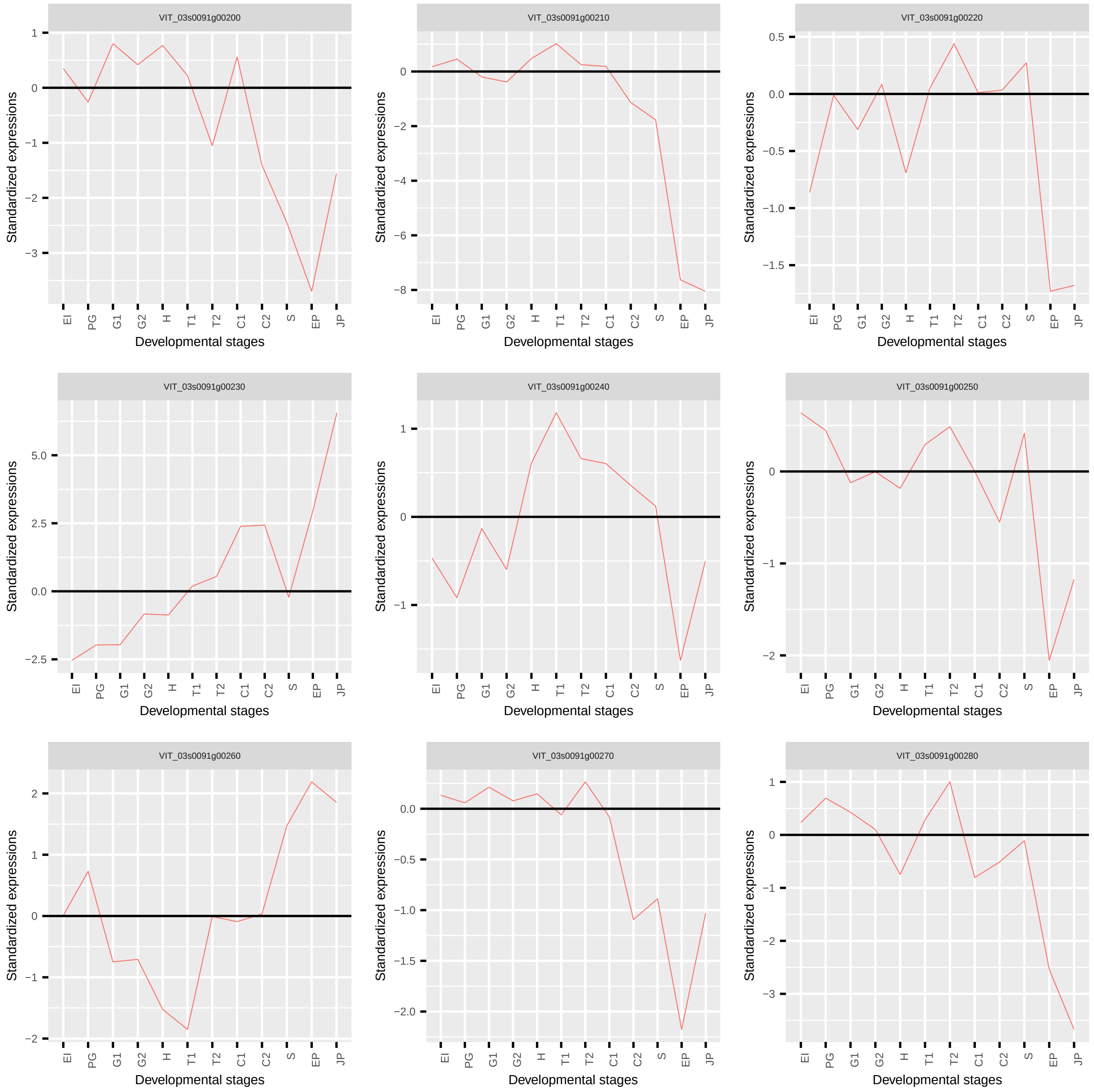

VIT_03s0091g00200
VIT_03s0091g00210
VIT_03s0091g00220
VIT_03s0091g00230
VIT_03s0091g00240
VIT_03s0091g00250
VIT_03s0091g00260
VIT_03s0091g00270
VIT_03s0091g00280
Standardized expressions
Developmental stages
EI PG G1 G2 H T1 T2 C1 C2 S EP JP

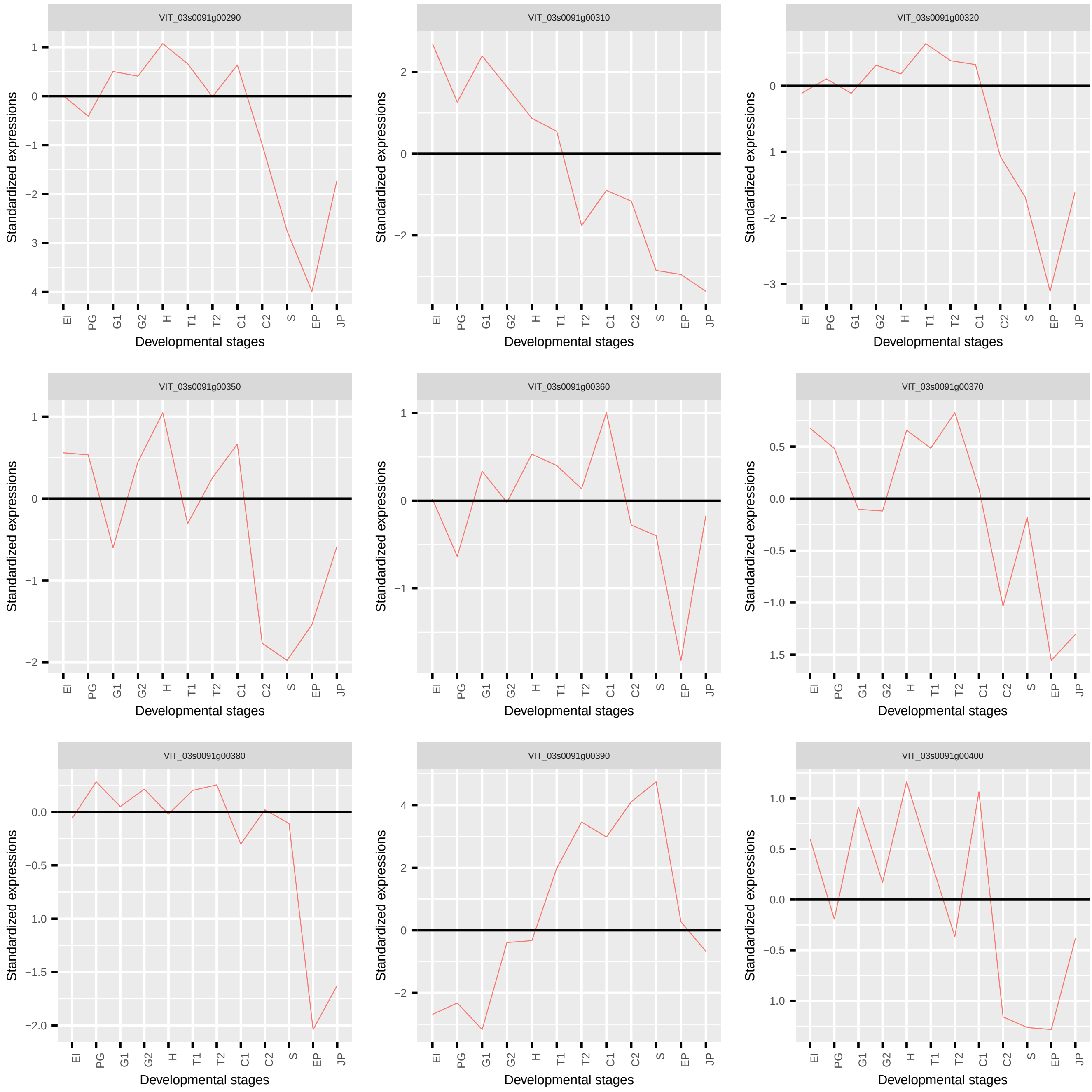

VIT_03s0091g00290
VIT_03s0091g00310
VIT_03s0091g00320
VIT_03s0091g00350
VIT_03s0091g00360
VIT_03s0091g00370
VIT_03s0091g00380
VIT_03s0091g00390
VIT_03s0091g00400
Standardized expressions
Developmental stages
EI PG G1 G2 H T1 T2 C1 C2 S EP JP

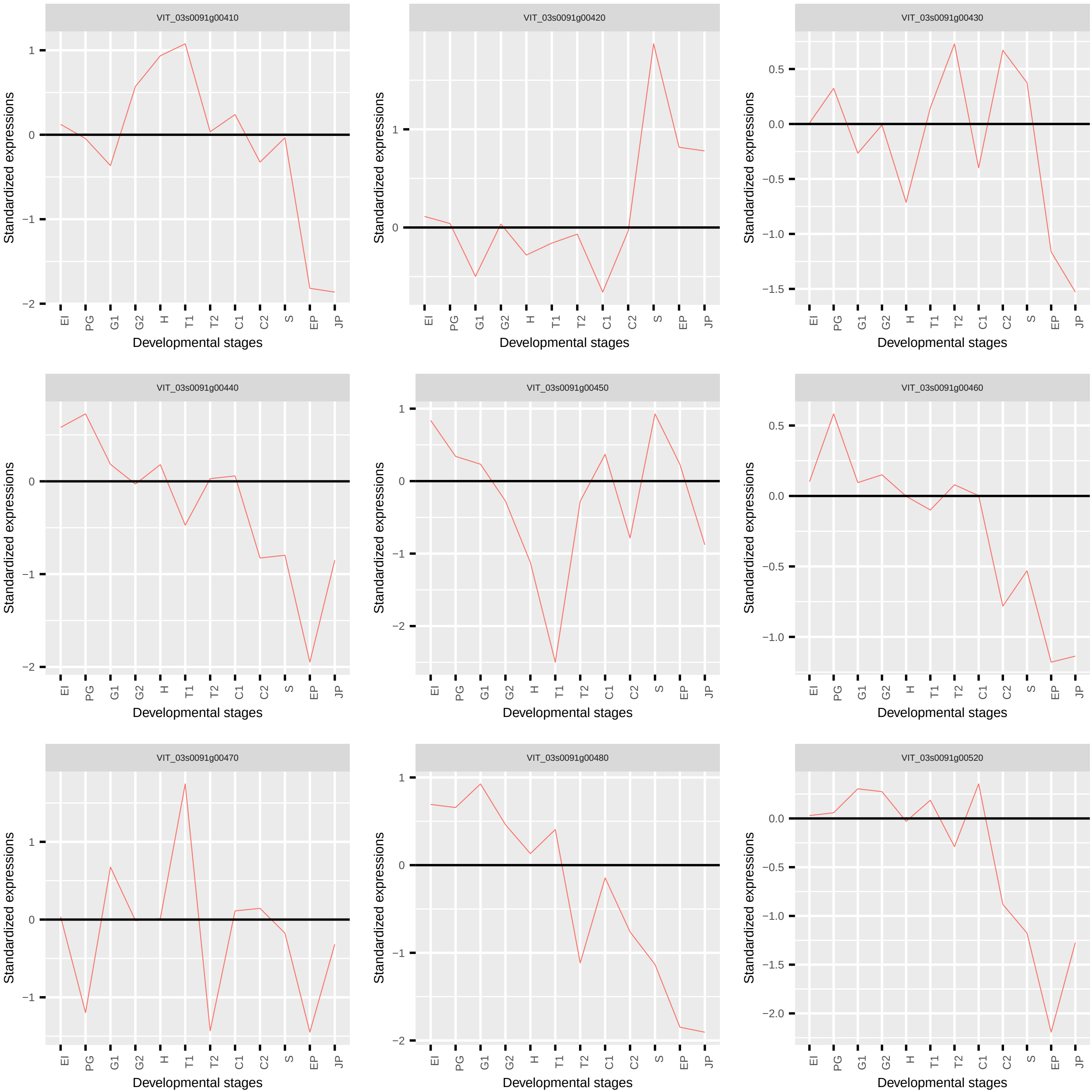
VIT_03s0091g00410
VIT_03s0091g00420
VIT_03s0091g00430
VIT_03s0091g00440
VIT_03s0091g00450
VIT_03s0091g00460
VIT_03s0091g00470
VIT_03s0091g00480
VIT_03s0091g00520
Standardized expressions
Developmental stages
EI PG G1 G2 H T1 T2 C1 C2 S EP JP

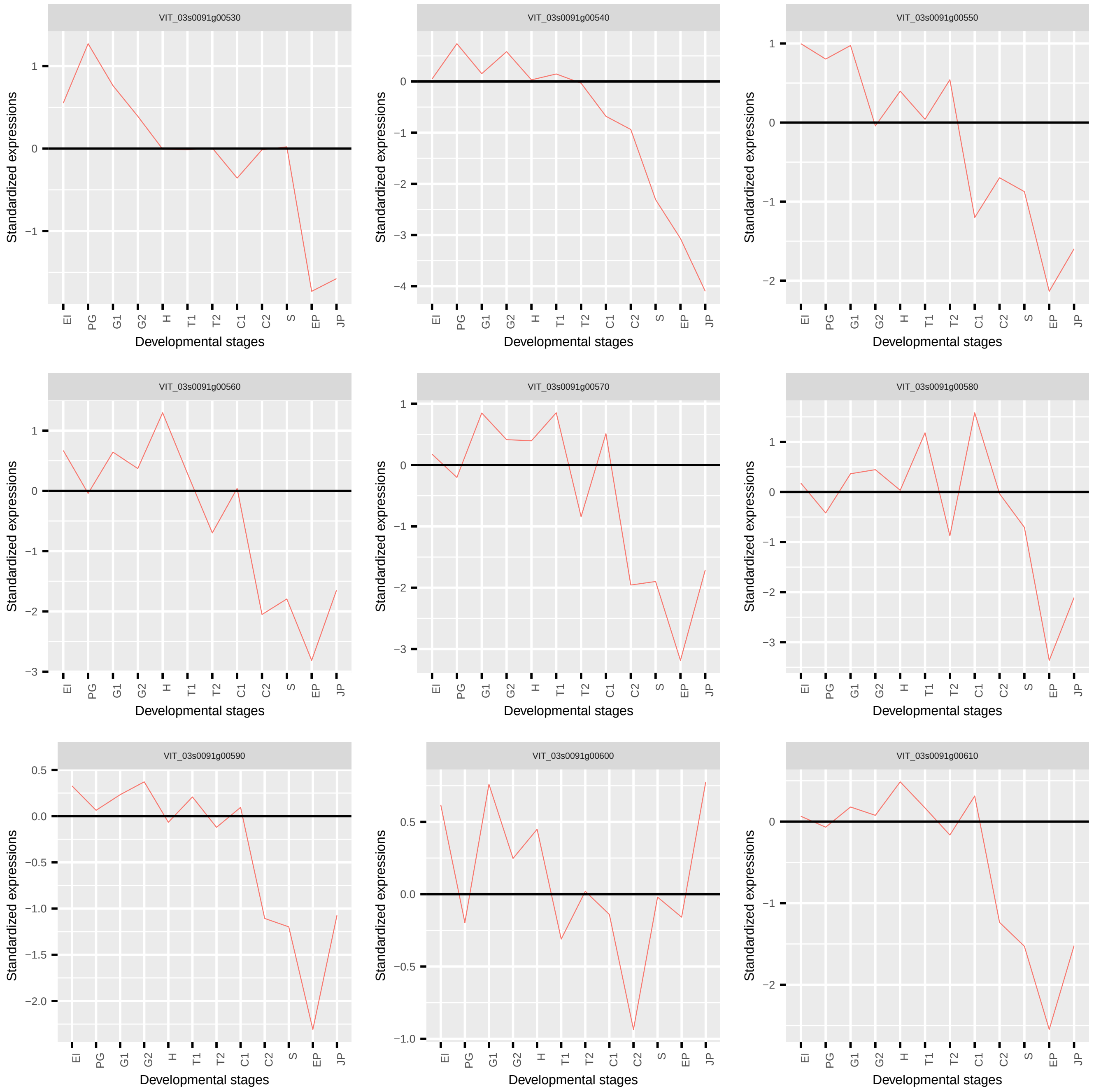
VIT_03s0091g00530
VIT_03s0091g00540
VIT_03s0091g00550
VIT_03s0091g00560
VIT_03s0091g00570
VIT_03s0091g00580
VIT_03s0091g00590
VIT_03s0091g00600
VIT_03s0091g00610
Standardized expressions
Developmental stages
EI PG G1 G2 H T1 T2 C1 C2 S EP JP

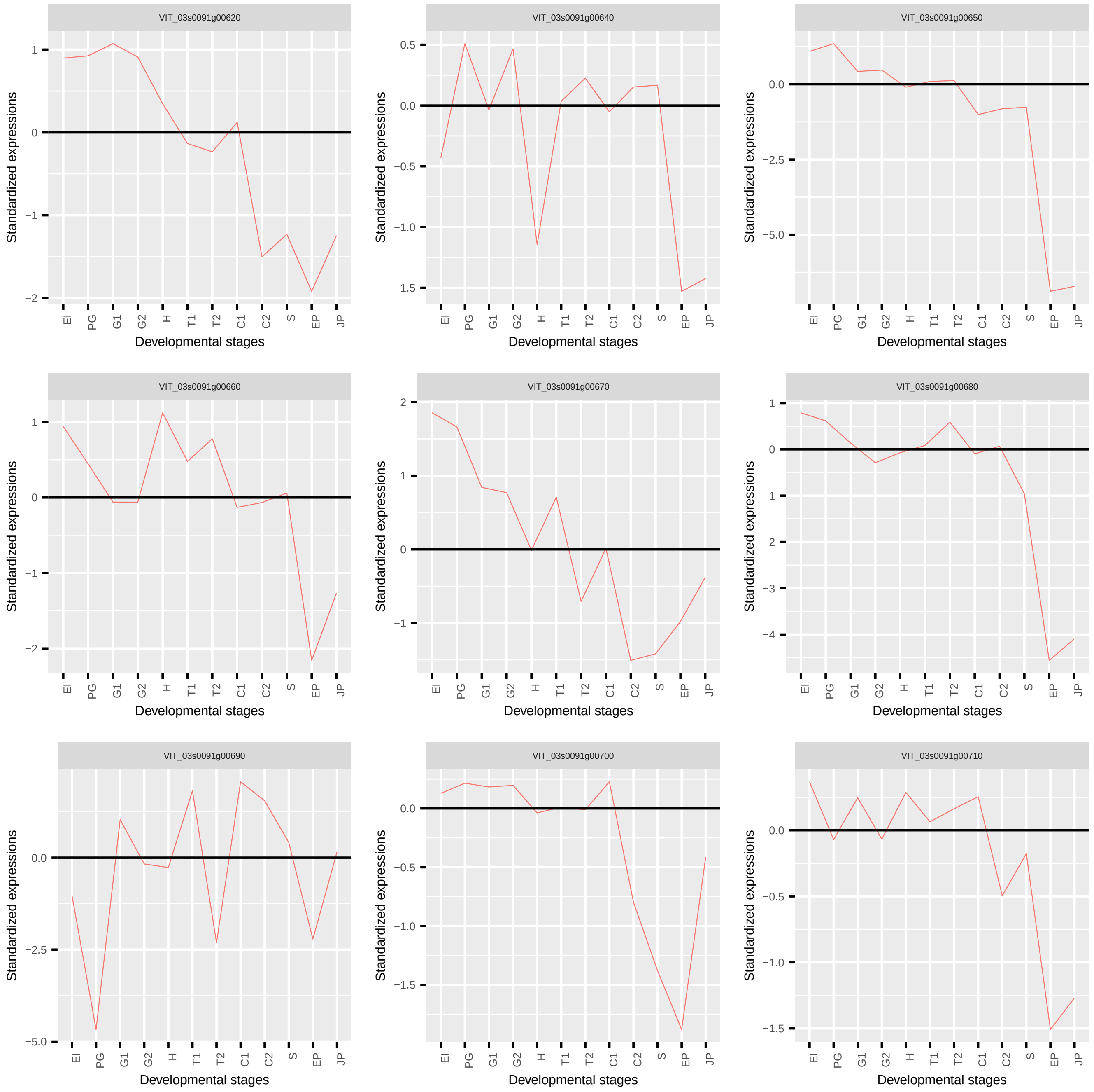

VIT_03s0091g00620
VIT_03s0091g00640
VIT_03s0091g00650
VIT_03s0091g00660
VIT_03s0091g00670
VIT_03s0091g00680
VIT_03s0091g00690
VIT_03s0091g00700
VIT_03s0091g00710
Standardized expressions
Developmental stages
EI PG G1 G2 H T1 T2 C1 C2 S EP JP

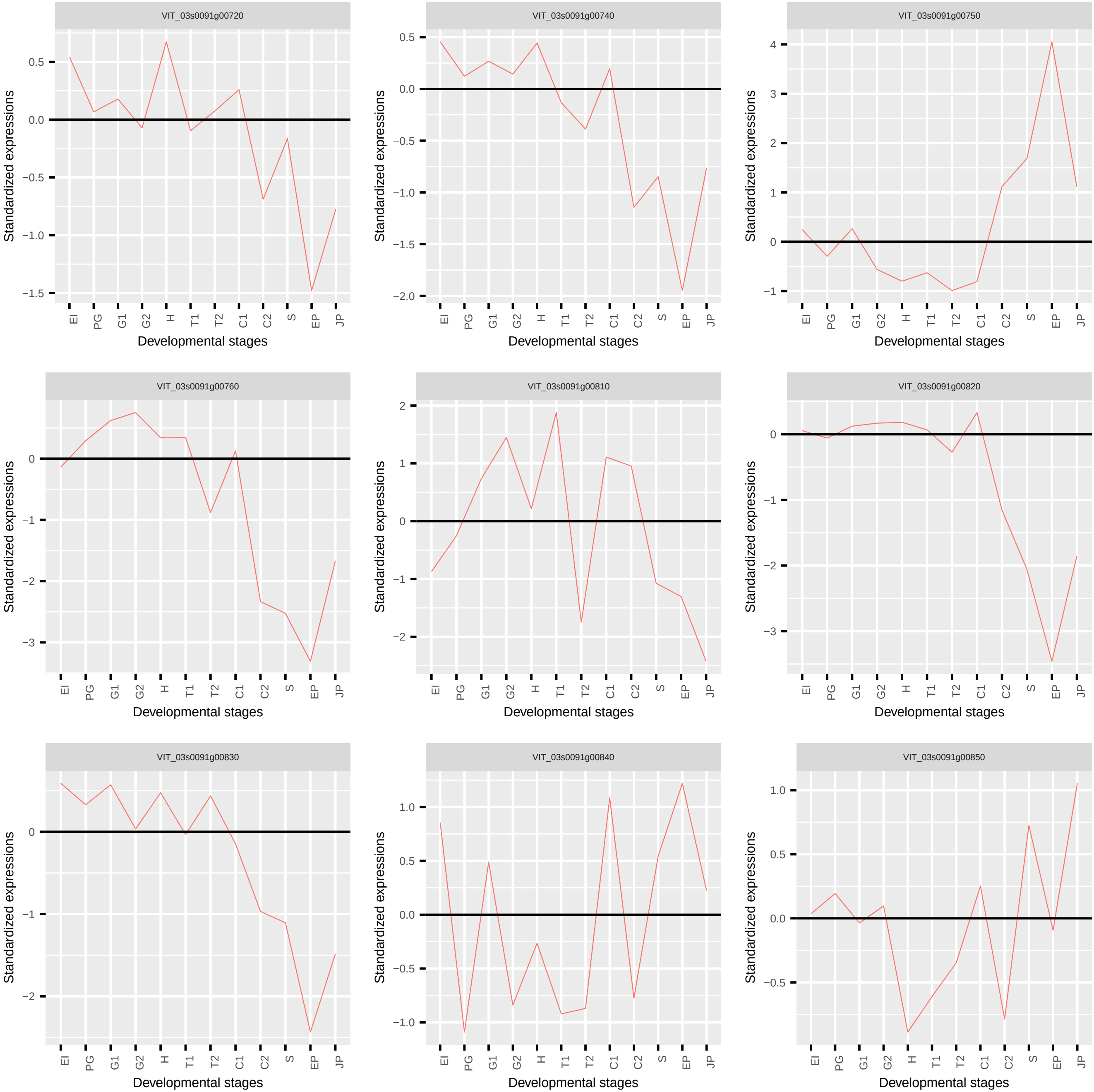
VIT_03s0091g00720
VIT_03s0091g00740
VIT_03s0091g00750
VIT_03s0091g00760
VIT_03s0091g00810
VIT_03s0091g00820
VIT_03s0091g00830
VIT_03s0091g00840
VIT_03s0091g00850
Standardized expressions
Developmental stages
EI PG G1 G2 H T1 T2 C1 C2 S EP JP

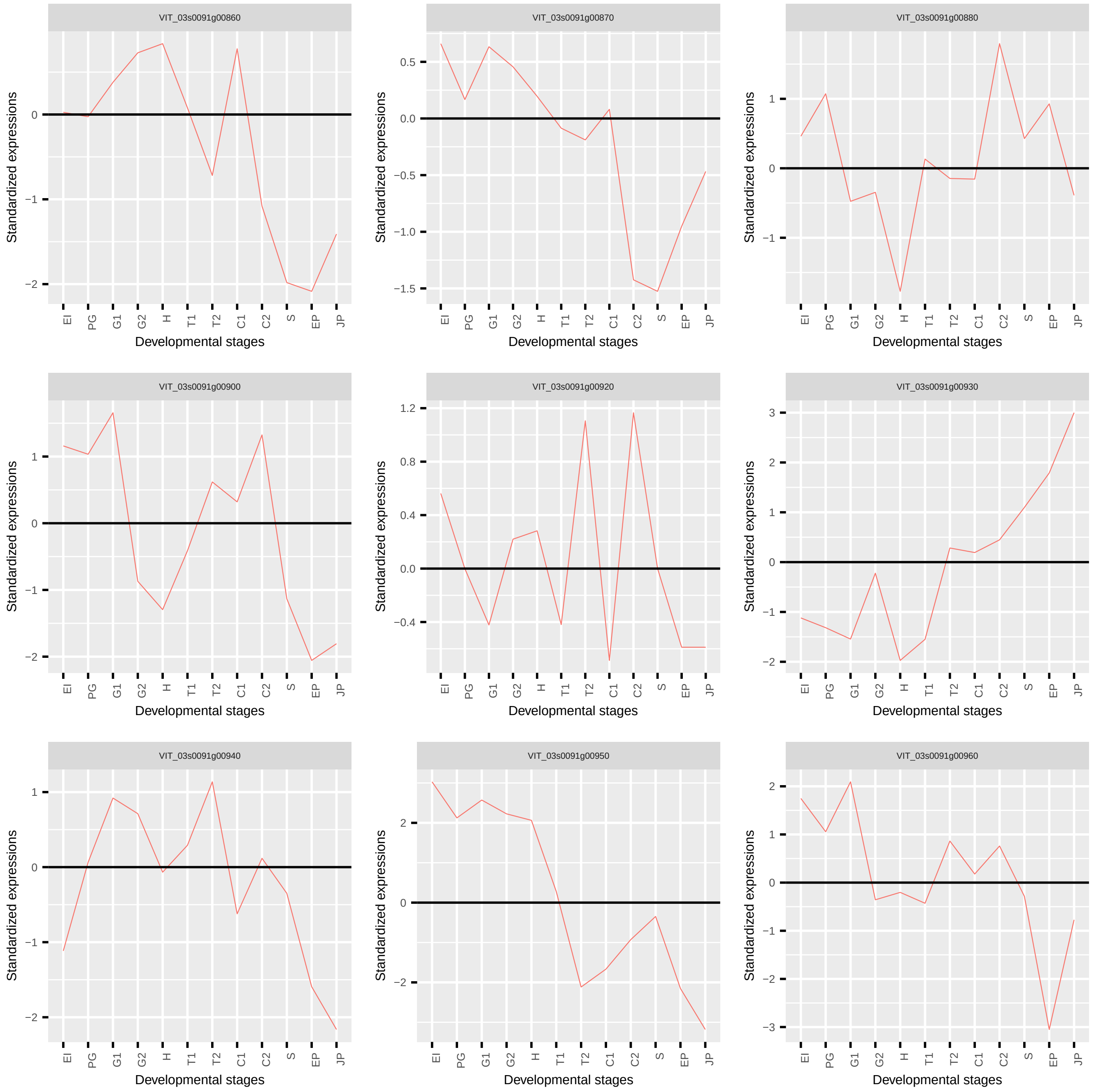

VIT_03s0091g00860
VIT_03s0091g00870
VIT_03s0091g00880
VIT_03s0091g00900
VIT_03s0091g00920
VIT_03s0091g00930
VIT_03s0091g00940
VIT_03s0091g00950
VIT_03s0091g00960
Standardized expressions
Developmental stages
EI PG G1 G2 H T1 T2 C1 C2 S EP JP

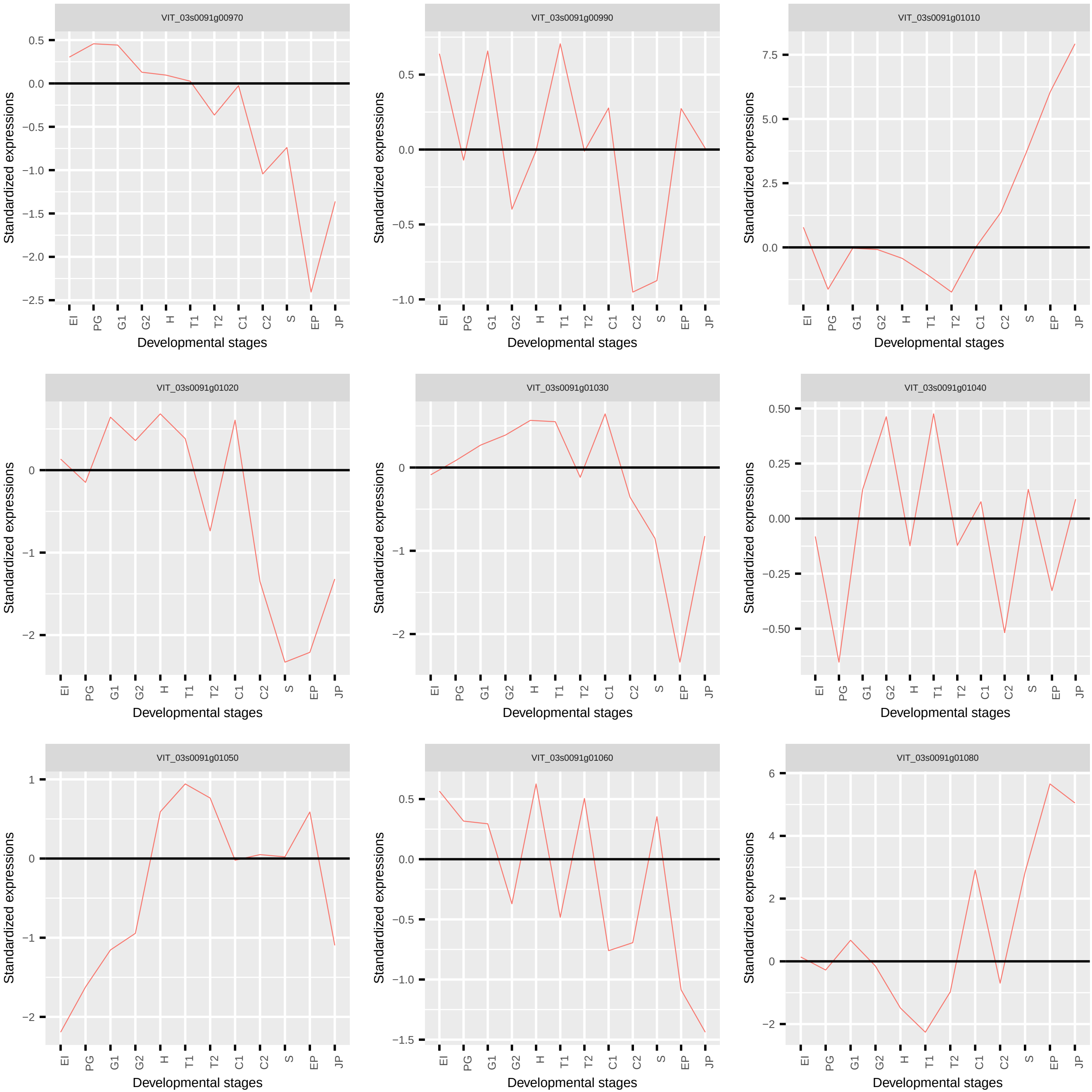
VIT_03s0091g00970
VIT_03s0091g00990
VIT_03s0091g01010
VIT_03s0091g01020
VIT_03s0091g01030
VIT_03s0091g01040
VIT_03s0091g01050
VIT_03s0091g01060
VIT_03s0091g01080
Standardized expressions
Developmental stages
EI PG G1 G2 H T1 T2 C1 C2 S EP JP
0.5 0.0 −0.5 −1.0 −1.5 −2.0 −2.5
7.5 5.0 2.5 0.0
0.50 0.25 0.00 −0.25 −0.50
1 0 −1 −2
6 4 2 0 −2

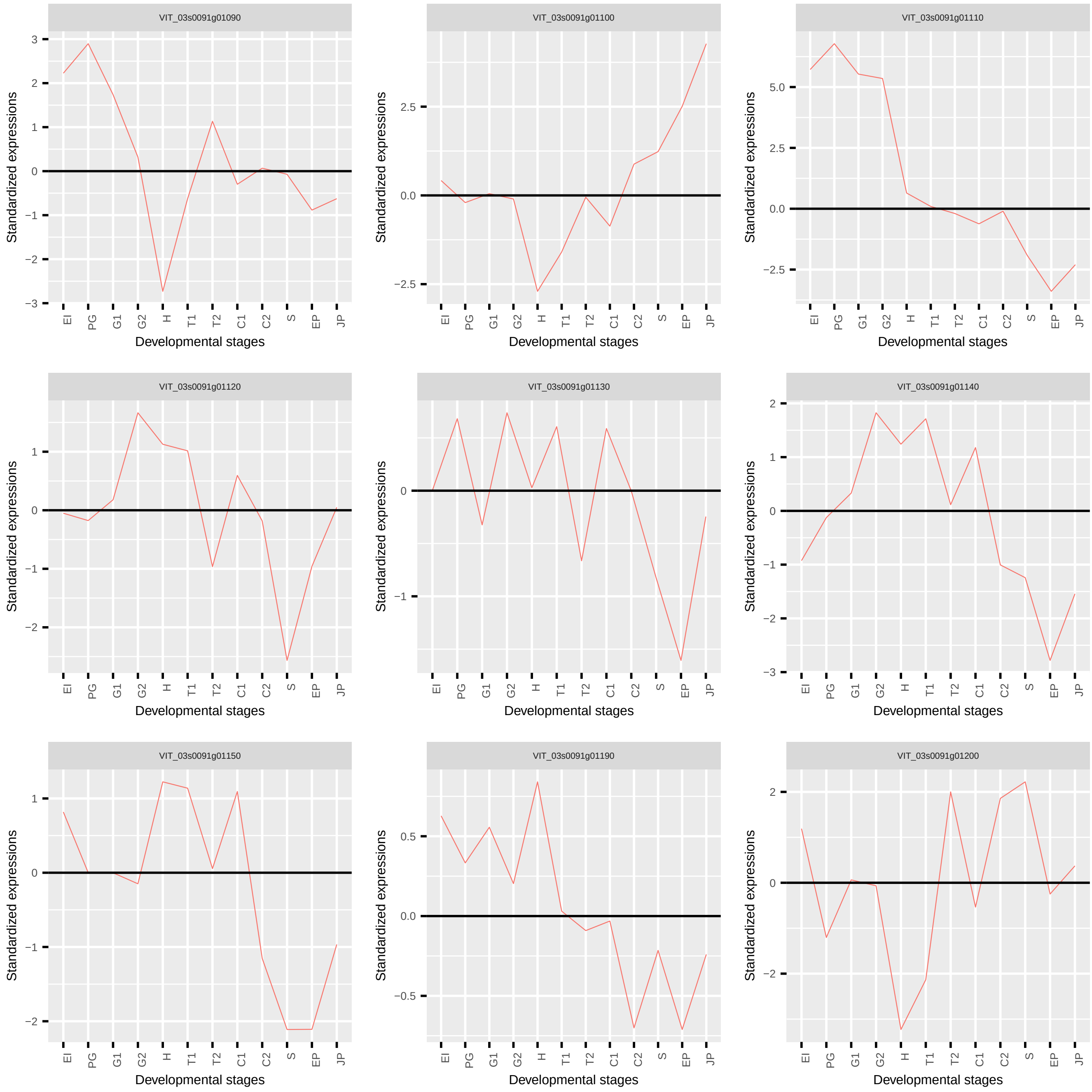
VIT_03s0091g01090
VIT_03s0091g01100
VIT_03s0091g01110
VIT_03s0091g01120
VIT_03s0091g01130
VIT_03s0091g01140
VIT_03s0091g01150
VIT_03s0091g01190
VIT_03s0091g01200
Standardized expressions
Developmental stages
EI PG G1 G2 H T1 T2 C1 C2 S EP JP
3 2 1 0 −1 −2 −3
2.5 0.0 −2.5
5.0 2.5 0.0 −2.5
1 0 −1 −2
0 −1
2 1 0 −1 −2 −3
1 0 −1 −2
0.5 0.0 −0.5
2 0 −2

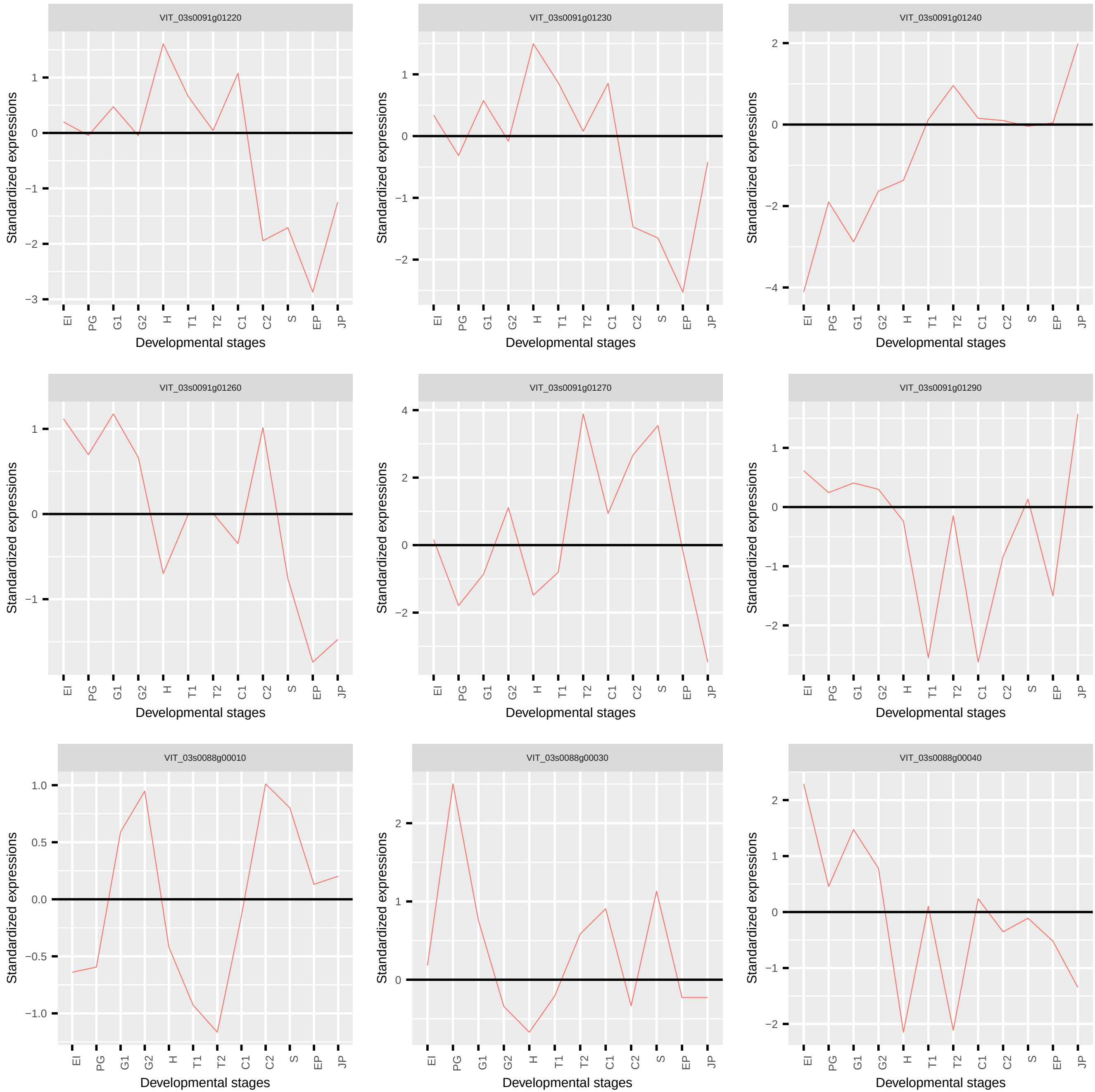

VIT_03s0091g01220
VIT_03s0091g01230
VIT_03s0091g01240
VIT_03s0091g01260
VIT_03s0091g01270
VIT_03s0091g01290
VIT_03s0088g00010
VIT_03s0088g00030
VIT_03s0088g00040
Standardized expressions
Developmental stages
EI
PG
G1
G2
H
T1
T2
C1
C2
S
EP
JP

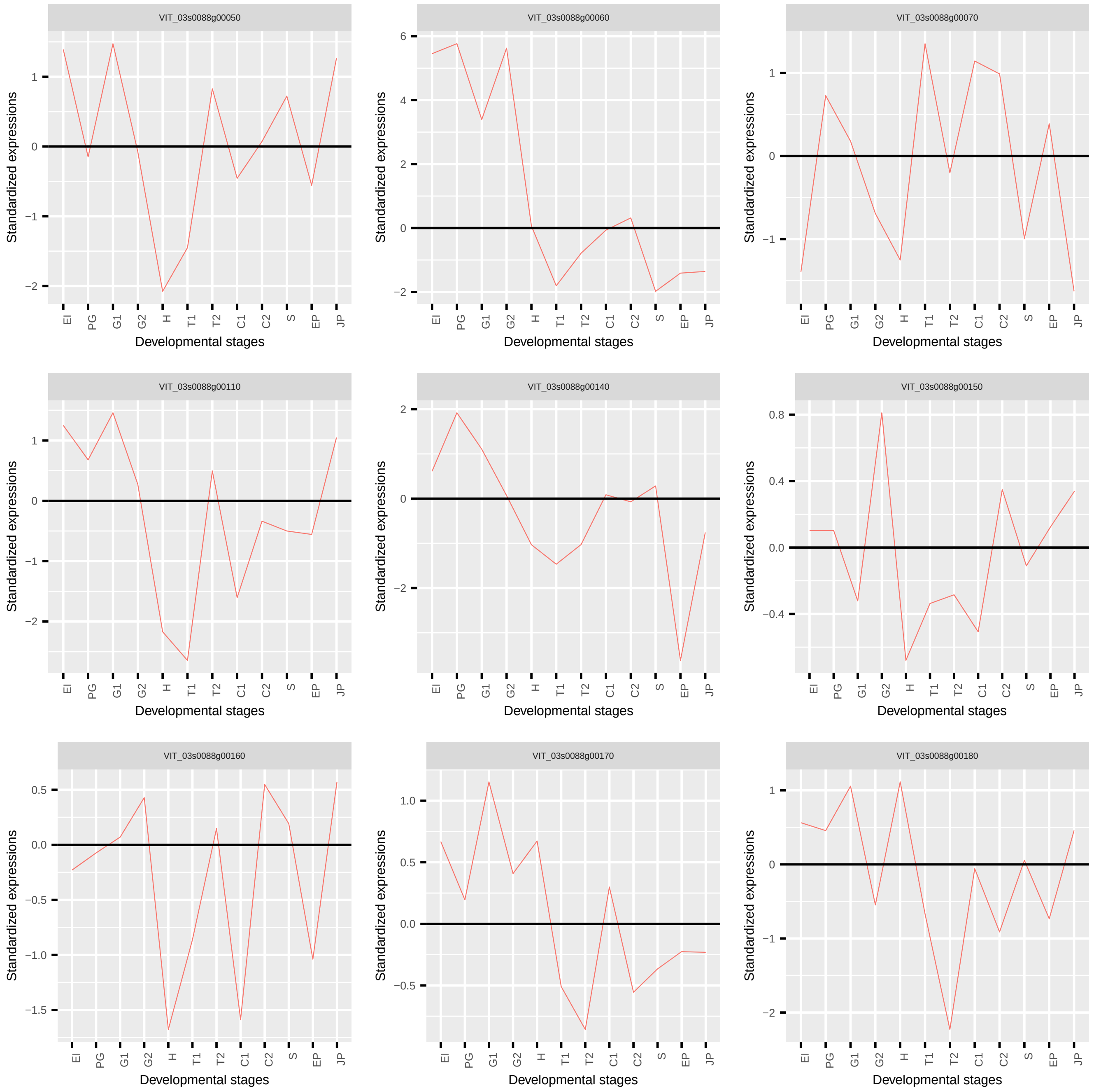
VIT_03s0088g00050
VIT_03s0088g00060
VIT_03s0088g00070
VIT_03s0088g00110
VIT_03s0088g00140
VIT_03s0088g00150
VIT_03s0088g00160
VIT_03s0088g00170
VIT_03s0088g00180
Standardized expressions
Developmental stages
EI PG G1 G2 H T1 T2 C1 C2 S EP JP

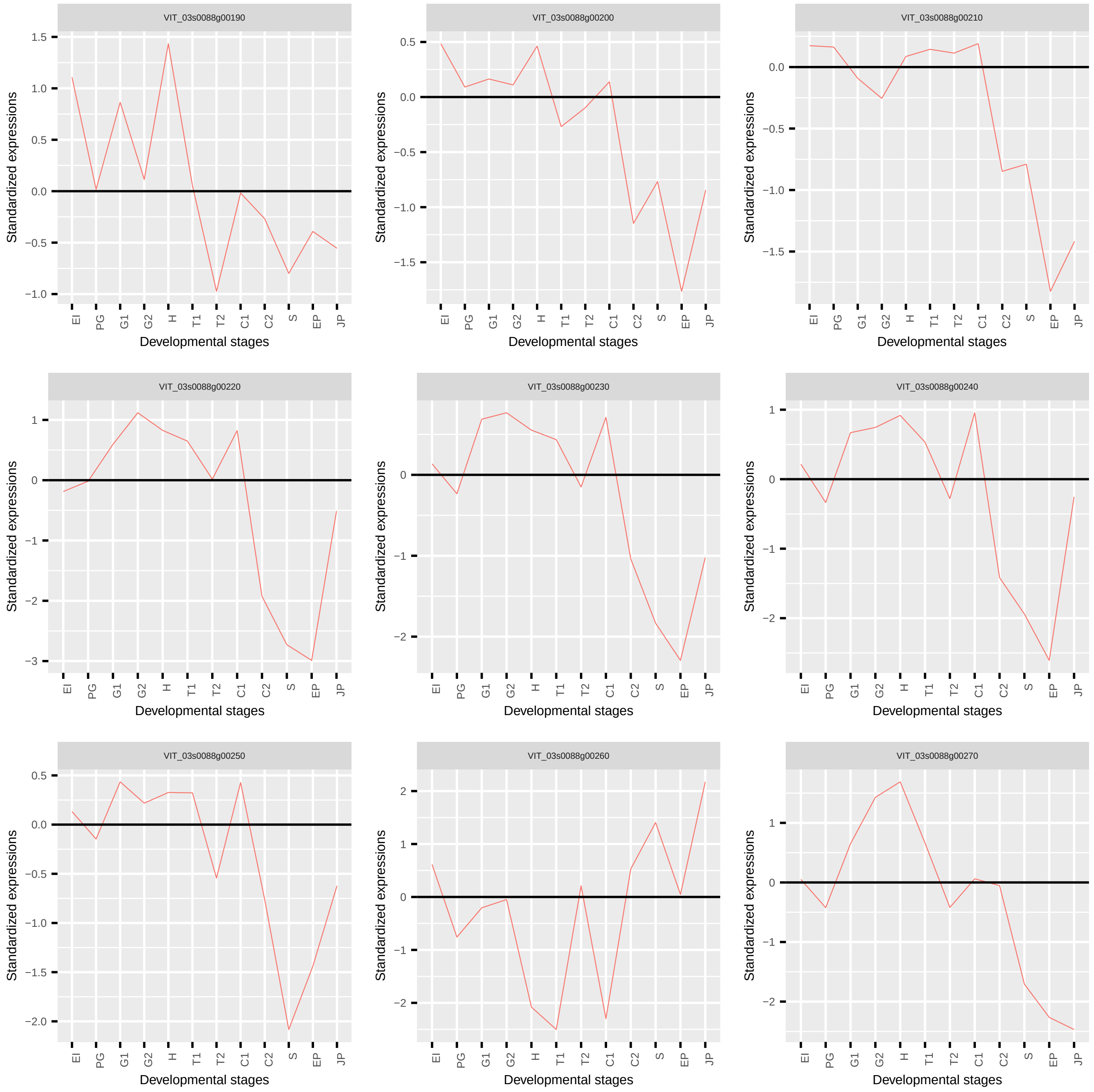
VIT_03s0088g00190
VIT_03s0088g00200
VIT_03s0088g00210
VIT_03s0088g00220
VIT_03s0088g00230
VIT_03s0088g00240
VIT_03s0088g00250
VIT_03s0088g00260
VIT_03s0088g00270
Standardized expressions
Developmental stages
EI PG G1 G2 H T1 T2 C1 C2 S EP JP

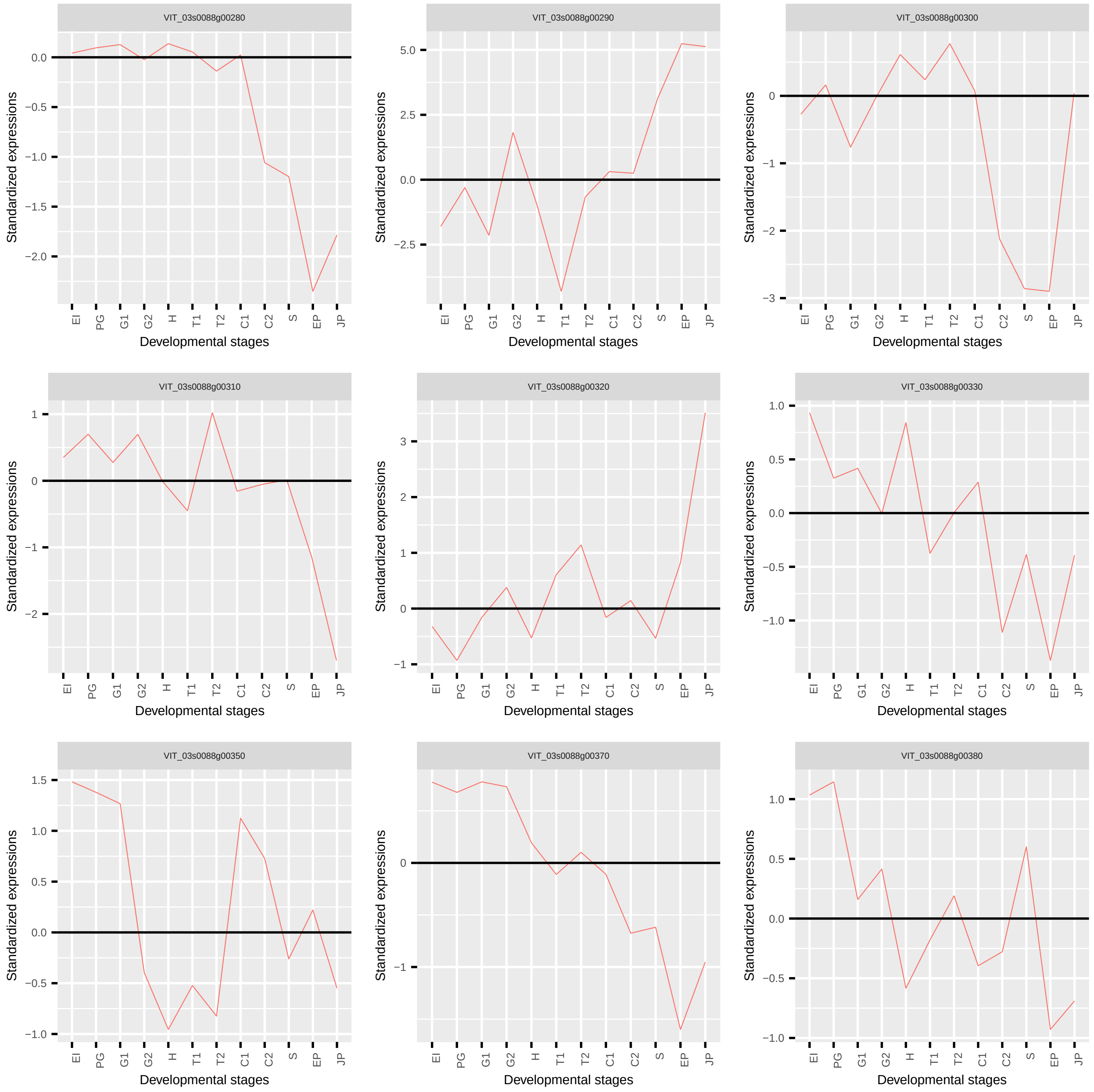
VIT_03s0088g00280
VIT_03s0088g00290
VIT_03s0088g00300
VIT_03s0088g00310
VIT_03s0088g00320
VIT_03s0088g00330
VIT_03s0088g00350
VIT_03s0088g00370
VIT_03s0088g00380
Standardized expressions
Developmental stages
EI PG G1 G2 H T1 T2 C1 C2 S EP JP

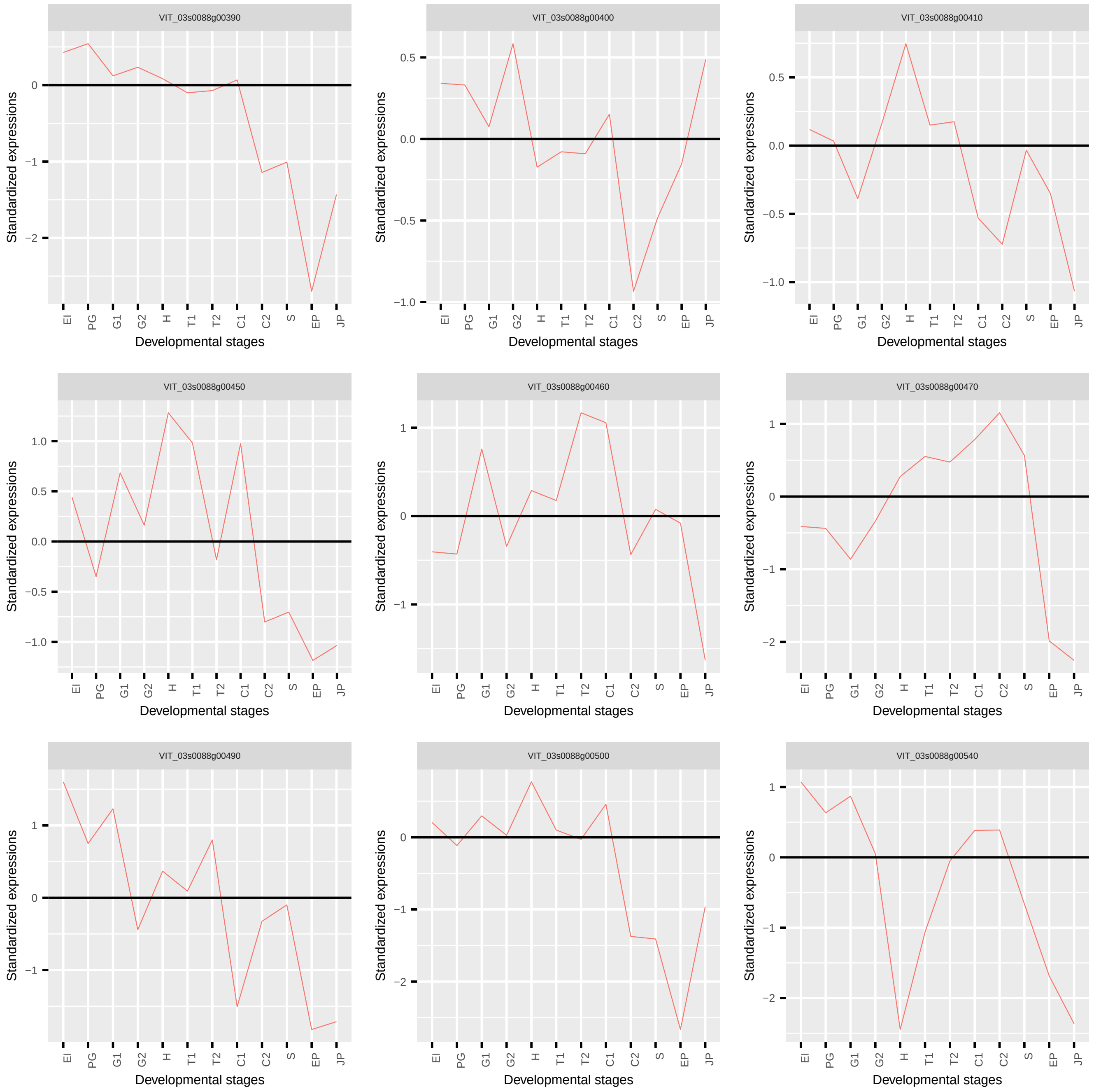

VIT_03s0088g00390
VIT_03s0088g00400
VIT_03s0088g00410
VIT_03s0088g00450
VIT_03s0088g00460
VIT_03s0088g00470
VIT_03s0088g00490
VIT_03s0088g00500
VIT_03s0088g00540
Standardized expressions
Developmental stages
EI PG G1 G2 H T1 T2 C1 C2 S EP JP

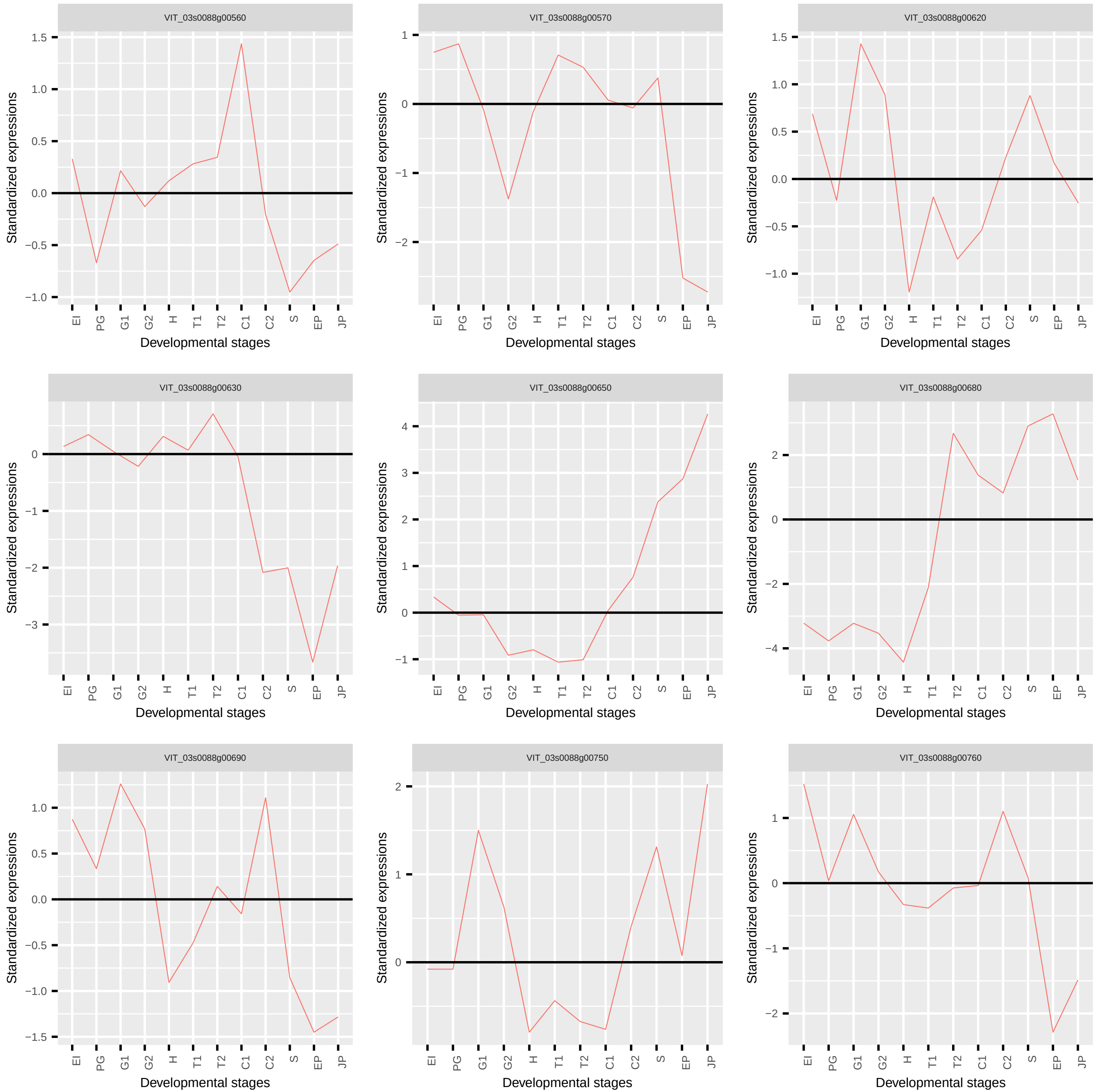
VIT_03s0088g00560
VIT_03s0088g00570
VIT_03s0088g00620
VIT_03s0088g00630
VIT_03s0088g00650
VIT_03s0088g00680
VIT_03s0088g00690
VIT_03s0088g00750
VIT_03s0088g00760
Standardized expressions
Developmental stages
EI PG G1 G2 H T1 T2 C1 C2 S EP JP

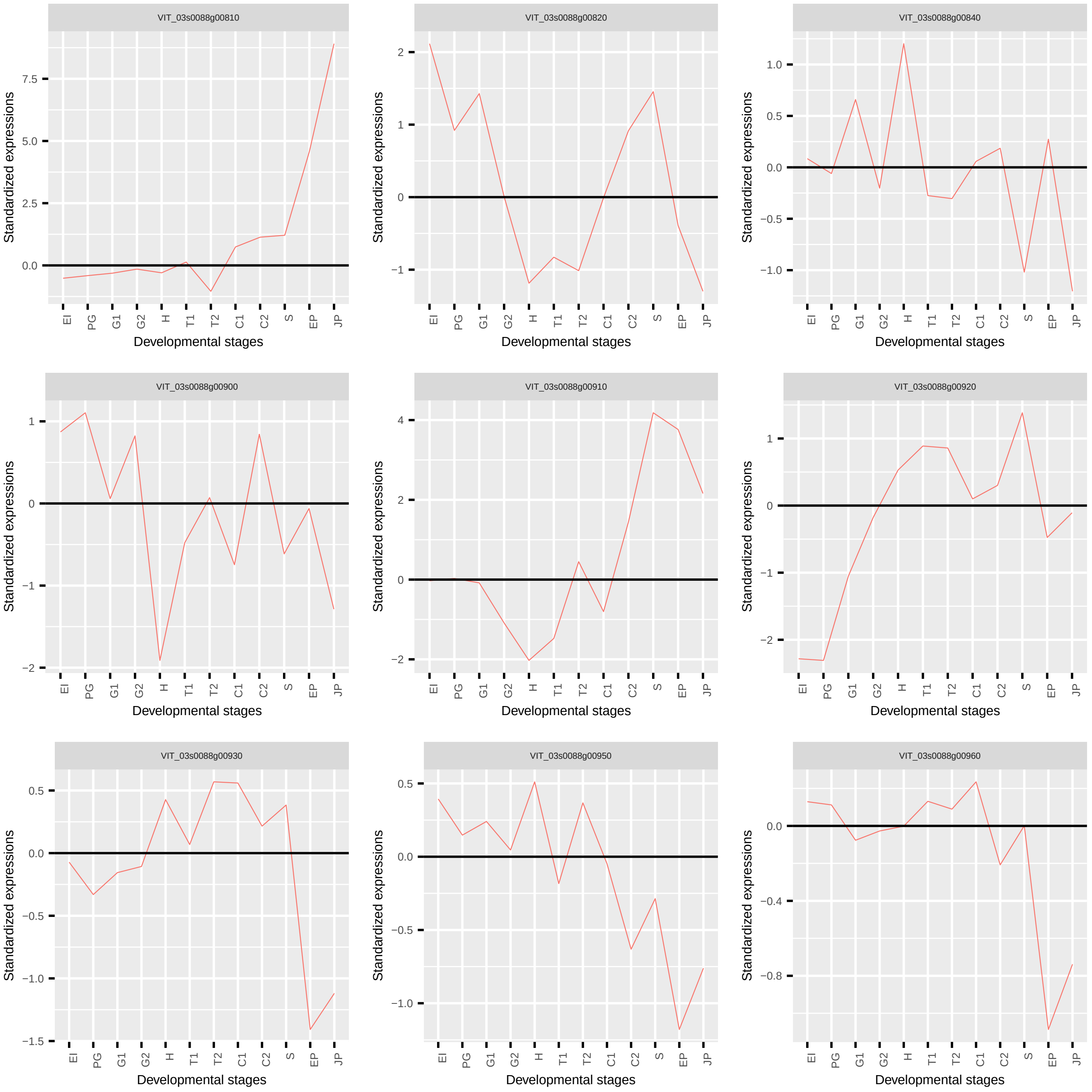

VIT_03s0088g00810
VIT_03s0088g00820
VIT_03s0088g00840
VIT_03s0088g00900
VIT_03s0088g00910
VIT_03s0088g00920
VIT_03s0088g00930
VIT_03s0088g00950
VIT_03s0088g00960
Standardized expressions
Developmental stages
EI PG G1 G2 H T1 T2 C1 C2 S EP JP

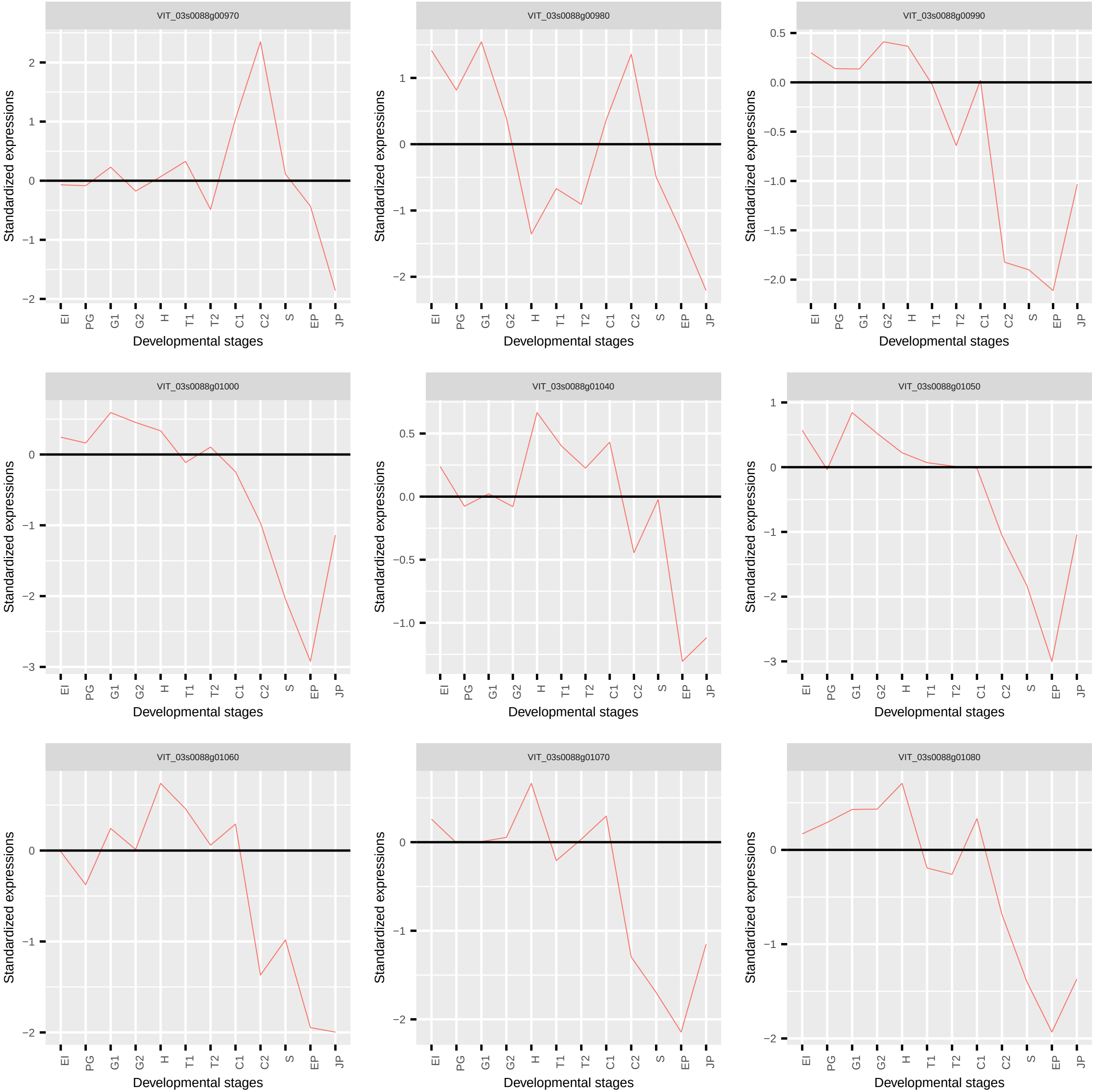
VIT_03s0088g00970
VIT_03s0088g00980
VIT_03s0088g00990
VIT_03s0088g01000
VIT_03s0088g01040
VIT_03s0088g01050
VIT_03s0088g01060
VIT_03s0088g01070
VIT_03s0088g01080
Standardized expressions
Developmental stages
EI PG G1 G2 H T1 T2 C1 C2 S EP JP

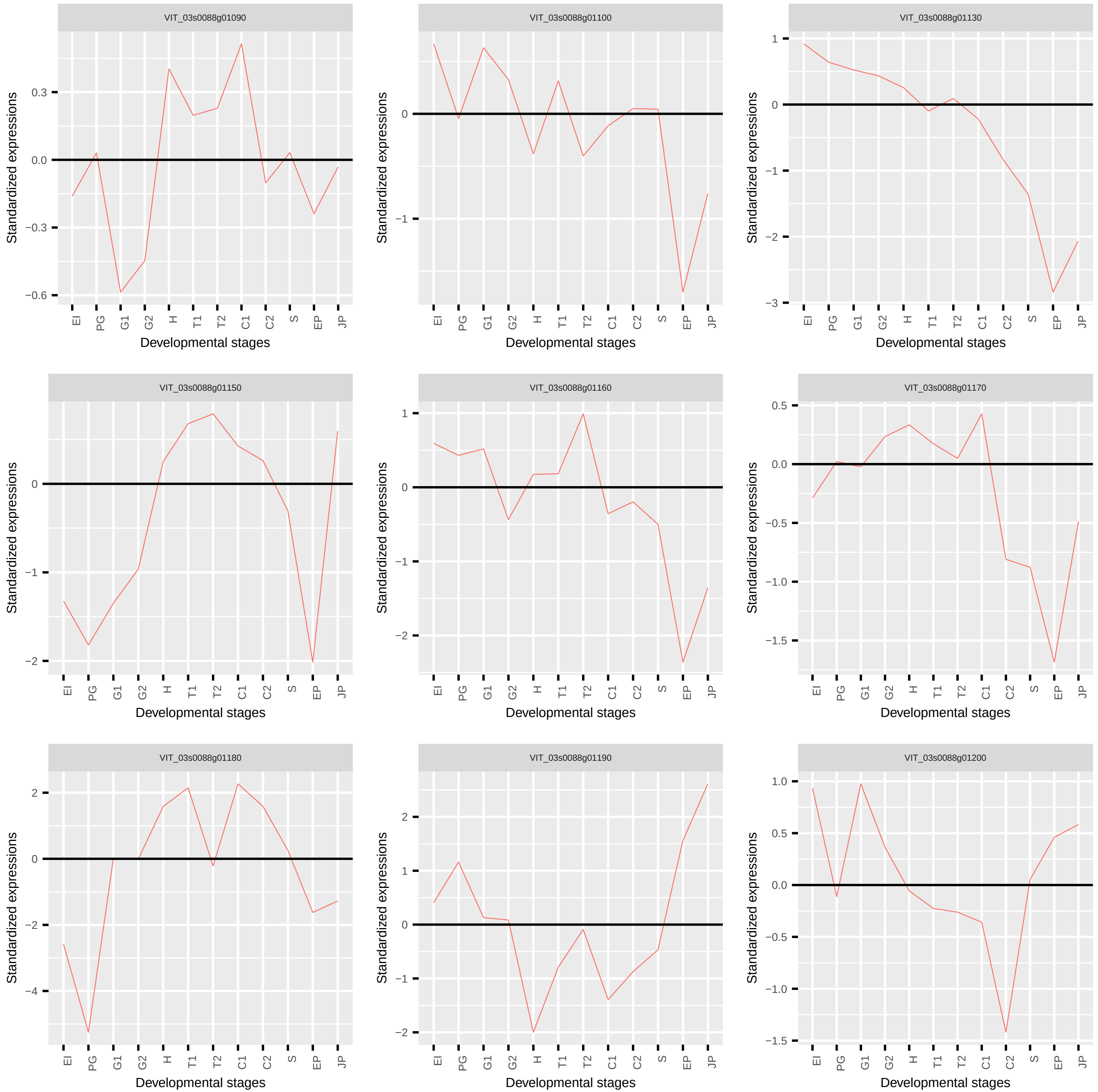
VIT_03s0088g01090
VIT_03s0088g01100
VIT_03s0088g01130
VIT_03s0088g01150
VIT_03s0088g01160
VIT_03s0088g01170
VIT_03s0088g01180
VIT_03s0088g01190
VIT_03s0088g01200
Standardized expressions
Developmental stages
EI PG G1 G2 H T1 T2 C1 C2 S EP JP

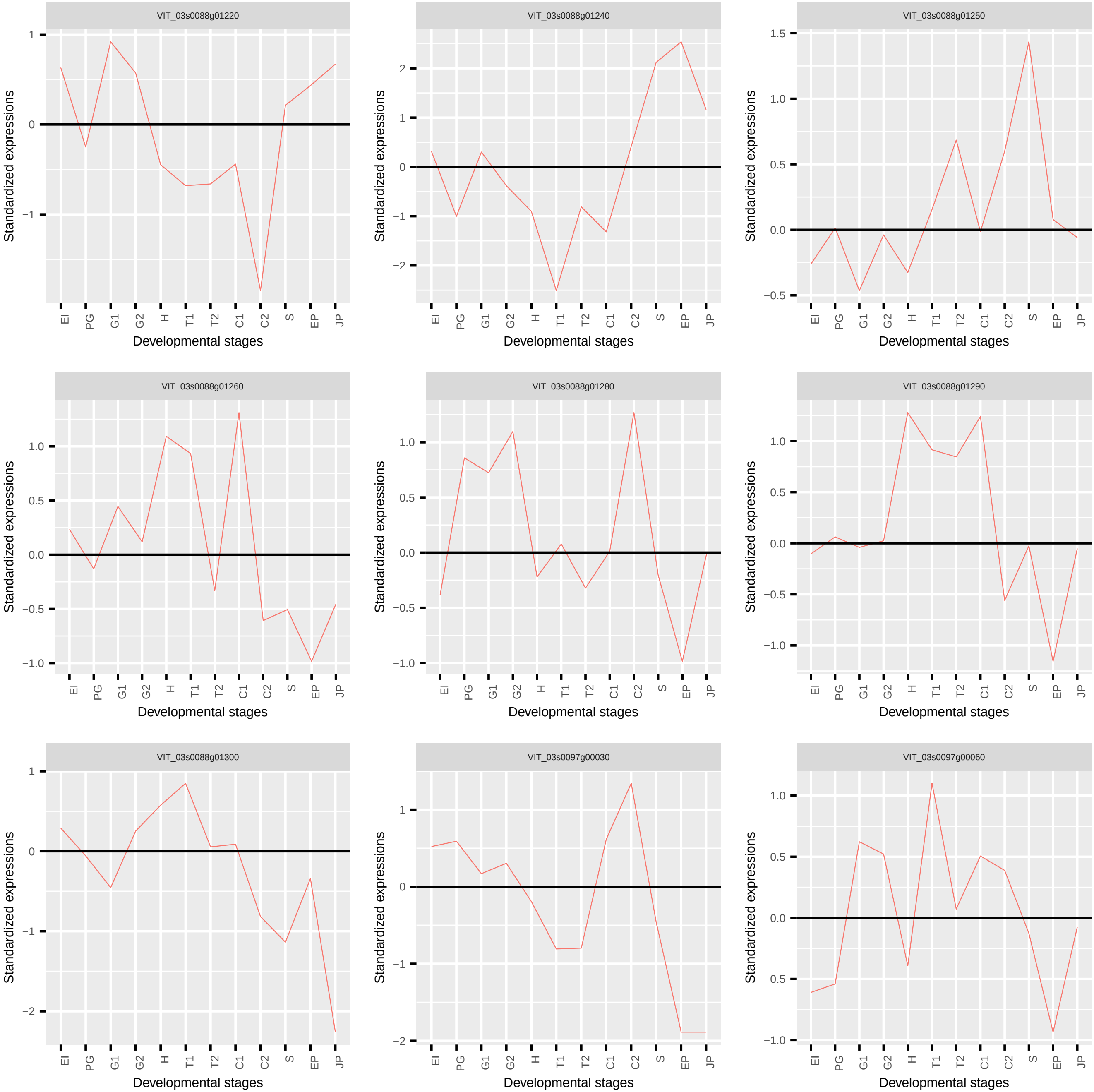
VIT_03s0088g01220
VIT_03s0088g01240
VIT_03s0088g01250
VIT_03s0088g01260
VIT_03s0088g01280
VIT_03s0088g01290
VIT_03s0088g01300
VIT_03s0097g00030
VIT_03s0097g00060
Standardized expressions
Developmental stages
EI PG G1 G2 H T1 T2 C1 C2 S EP JP

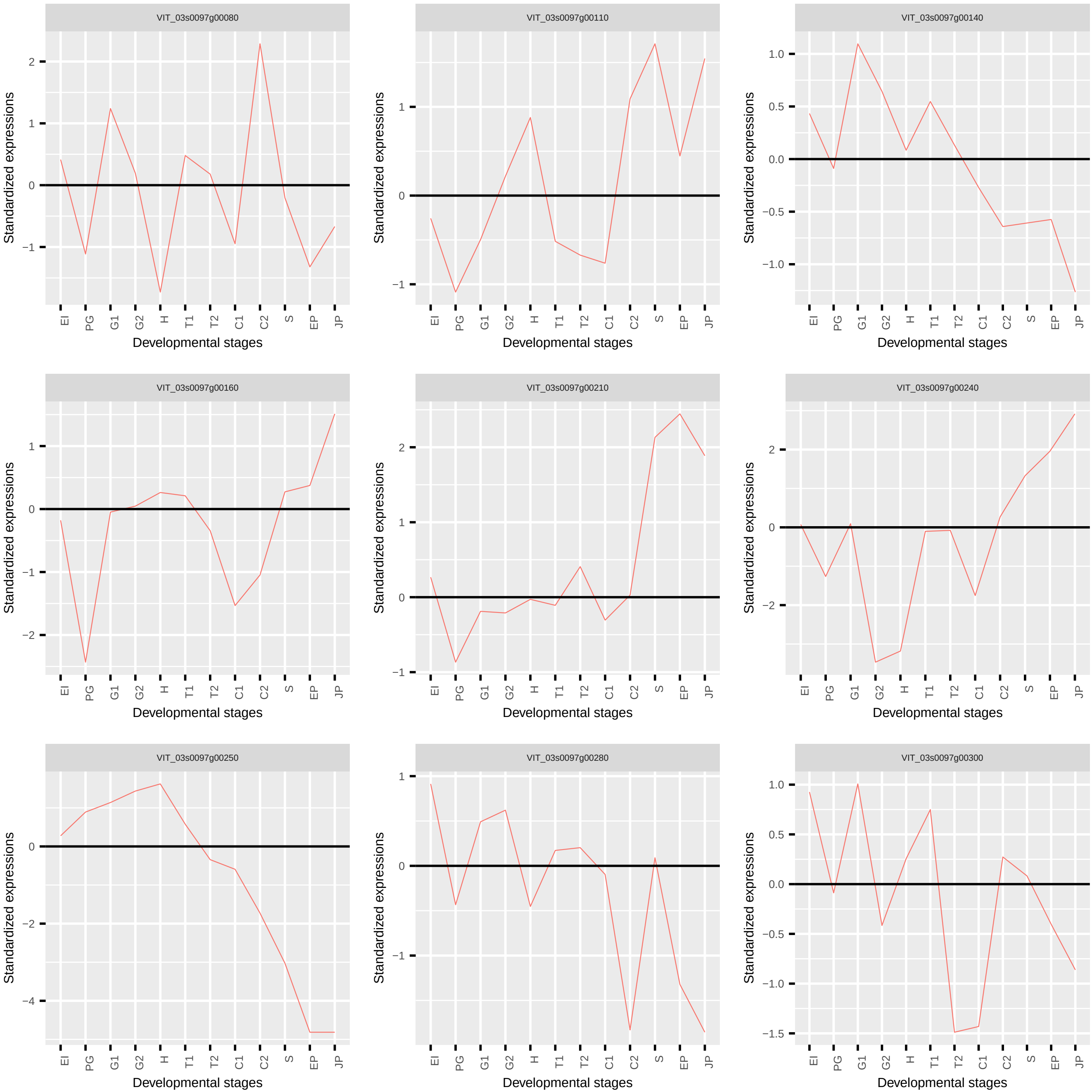
VIT_03s0097g00080
VIT_03s0097g00110
VIT_03s0097g00140
VIT_03s0097g00160
VIT_03s0097g00210
VIT_03s0097g00240
VIT_03s0097g00250
VIT_03s0097g00280
VIT_03s0097g00300
Standardized expressions
Developmental stages
EI PG G1 G2 H T1 T2 C1 C2 S EP JP

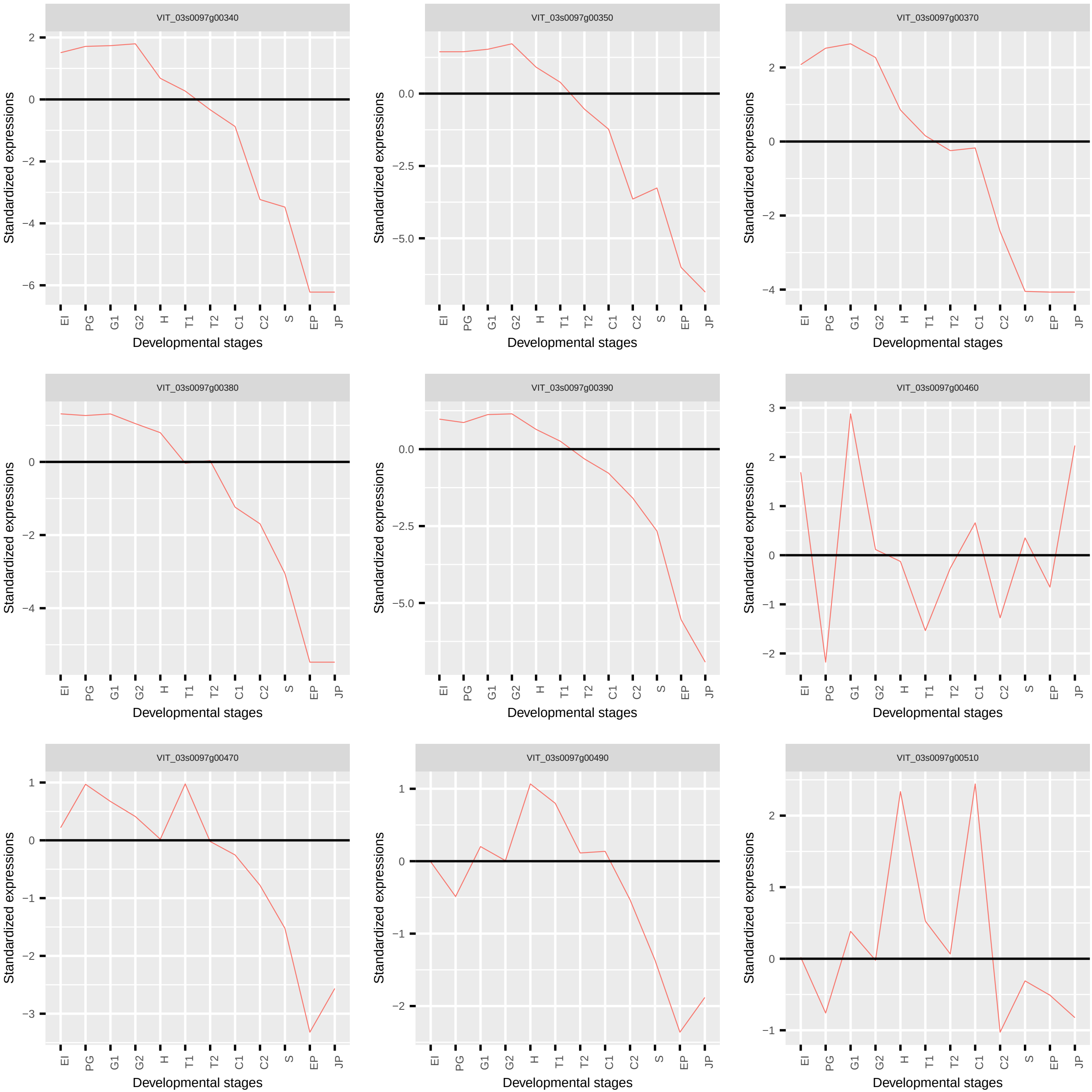
VIT_03s0097g00340
VIT_03s0097g00350
VIT_03s0097g00370
VIT_03s0097g00380
VIT_03s0097g00390
VIT_03s0097g00460
VIT_03s0097g00470
VIT_03s0097g00490
VIT_03s0097g00510
Standardized expressions
Developmental stages
EI PG G1 G2 H T1 T2 C1 C2 S EP JP

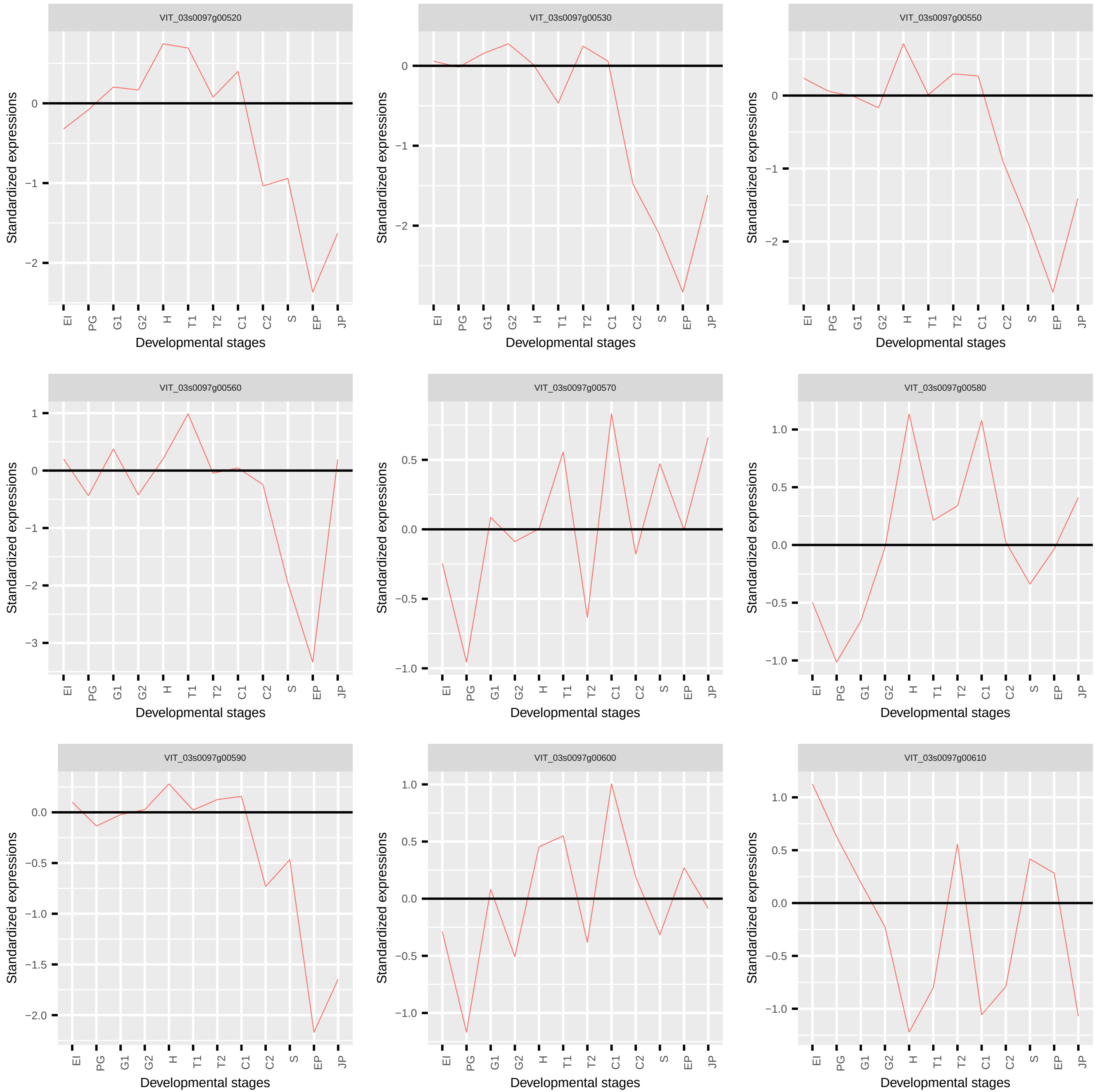
VIT_03s0097g00520
VIT_03s0097g00530
VIT_03s0097g00550
VIT_03s0097g00560
VIT_03s0097g00570
VIT_03s0097g00580
VIT_03s0097g00590
VIT_03s0097g00600
VIT_03s0097g00610
Standardized expressions
Developmental stages
EI PG G1 G2 H T1 T2 C1 C2 S EP JP
0
−1
−2
−3
1
0.5
0.0
−0.5
−1.0
1.0
−1.5
−2.0

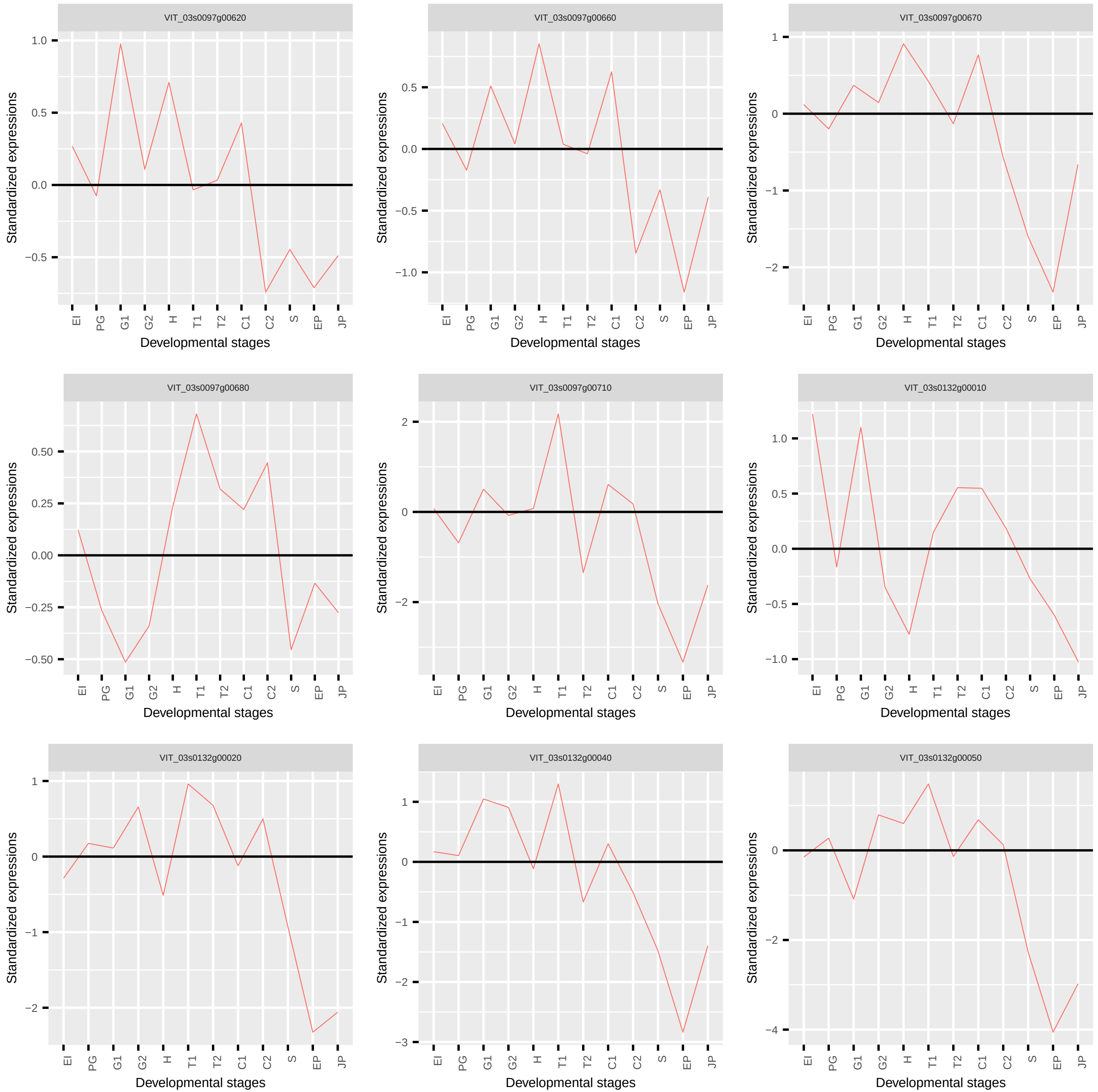

VIT_03s0097g00620
VIT_03s0097g00660
VIT_03s0097g00670
VIT_03s0097g00680
VIT_03s0097g00710
VIT_03s0132g00010
VIT_03s0132g00020
VIT_03s0132g00040
VIT_03s0132g00050
Standardized expressions
Developmental stages
EI
PG
G1
G2
H
T1
T2
C1
C2
S
EP
JP

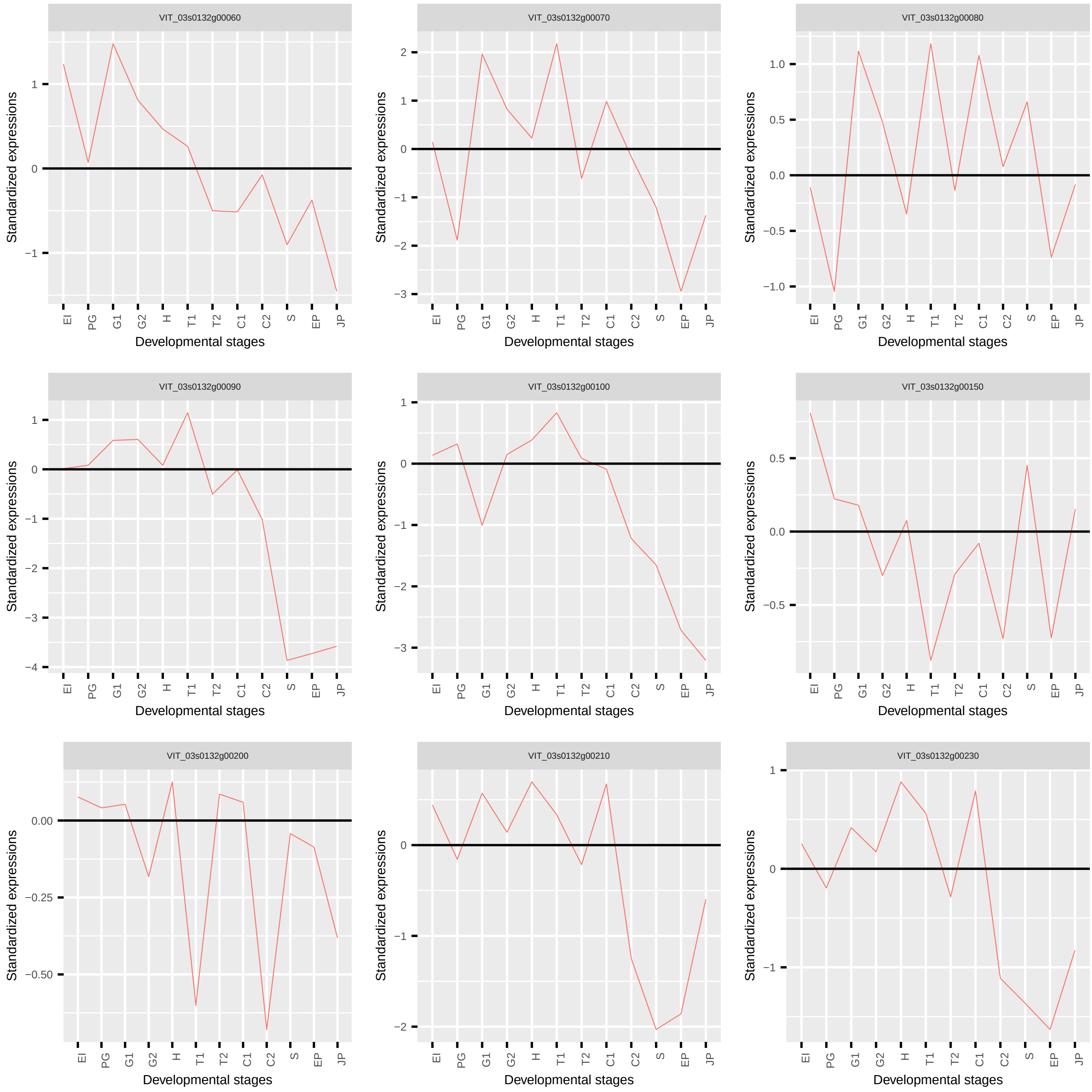
VIT_03s0132g00060
VIT_03s0132g00070
VIT_03s0132g00080
VIT_03s0132g00090
VIT_03s0132g00100
VIT_03s0132g00150
VIT_03s0132g00200
VIT_03s0132g00210
VIT_03s0132g00230
Standardized expressions
Developmental stages
EI PG G1 G2 H T1 T2 C1 C2 S EP JP

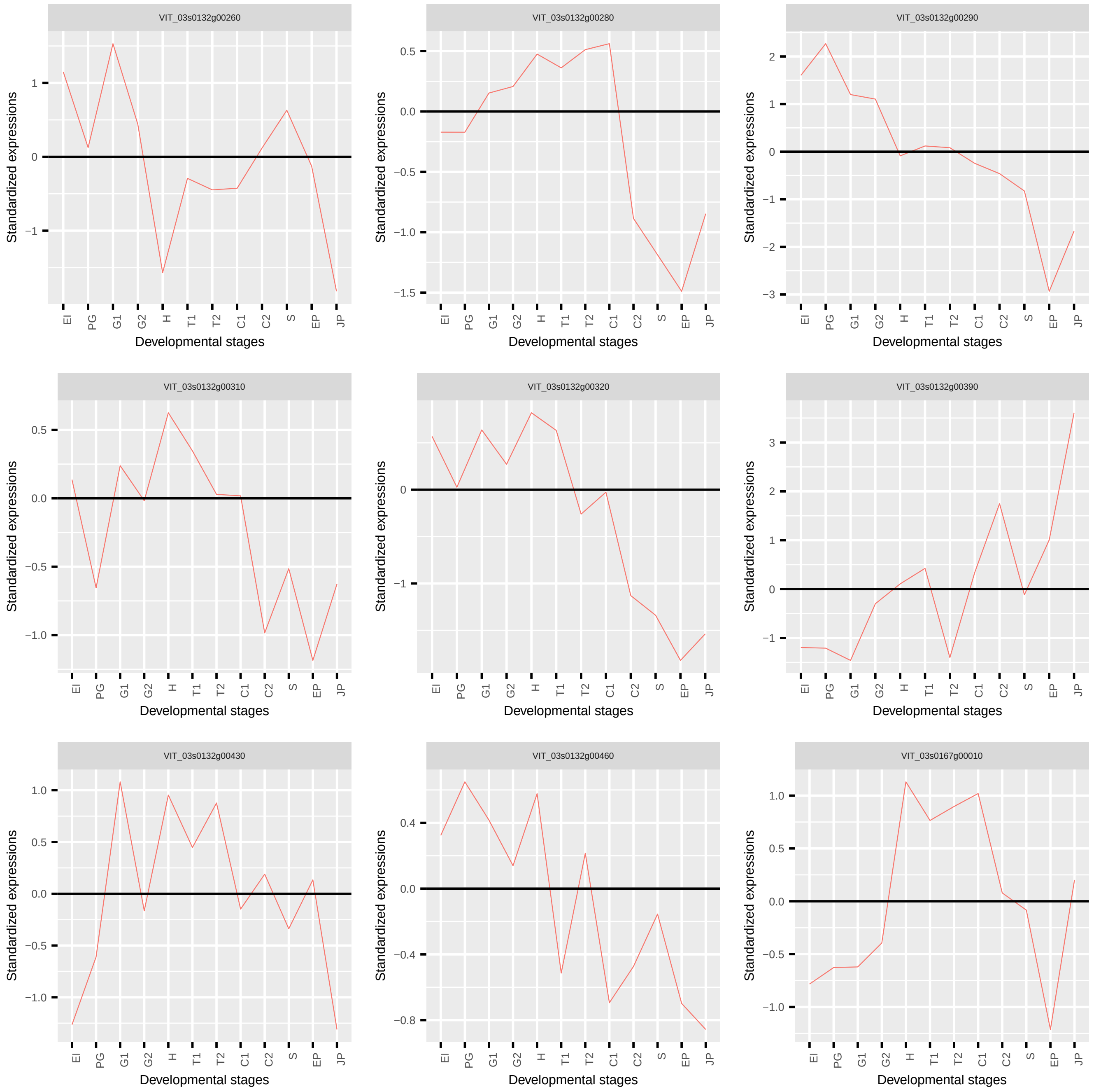
VIT_03s0132g00260
VIT_03s0132g00280
VIT_03s0132g00290
VIT_03s0132g00310
VIT_03s0132g00320
VIT_03s0132g00390
VIT_03s0132g00430
VIT_03s0132g00460
VIT_03s0167g00010
Standardized expressions
Developmental stages
EI PG G1 G2 H T1 T2 C1 C2 S EP JP

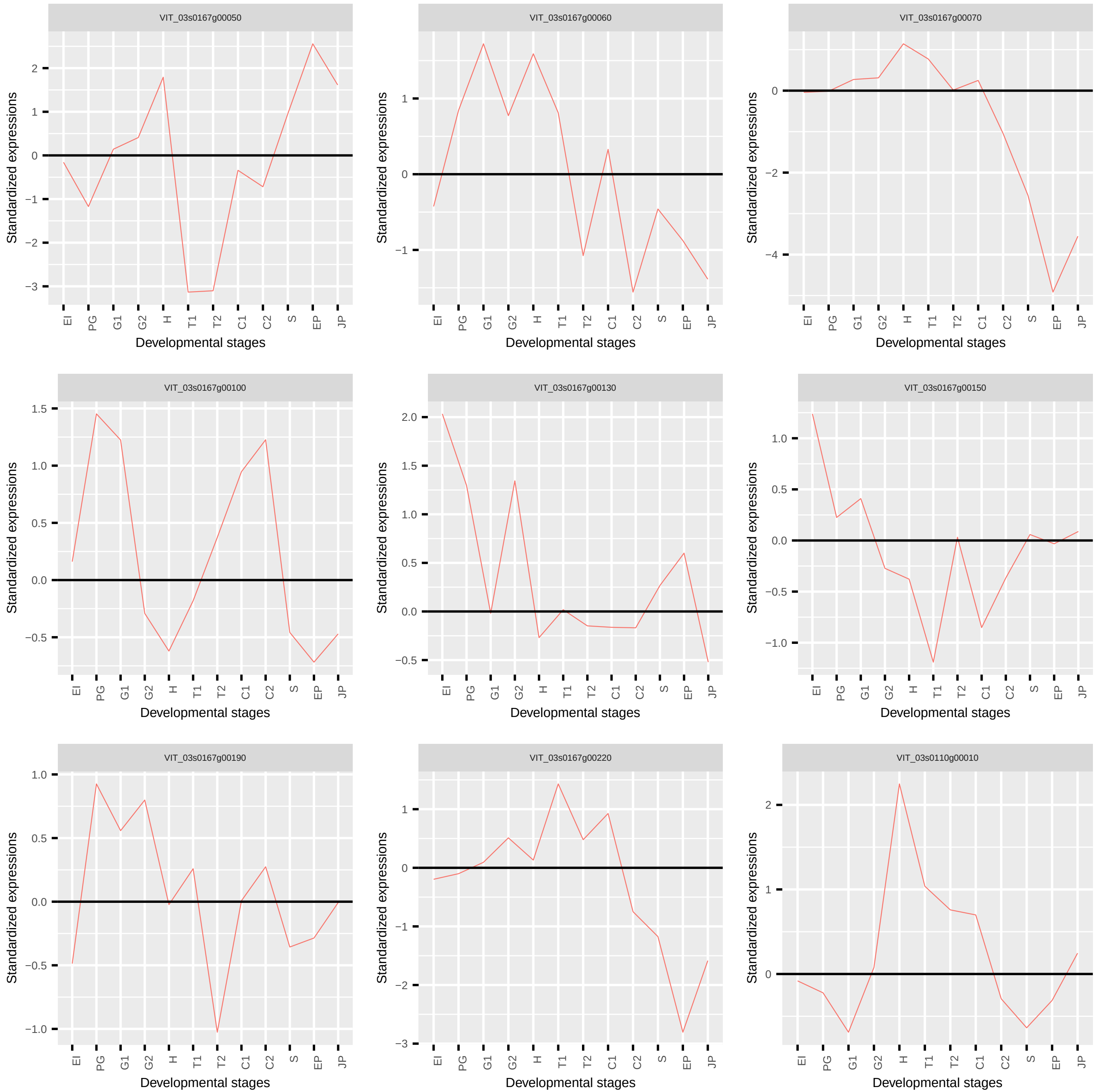

VIT_03s0167g00050
Standardized expressions
2
1
0
−1
−2
−3
EI PG G1 G2 H T1 T2 C1 C2 S EP JP
Developmental stages
VIT_03s0167g00060
Standardized expressions
1
0
−1
EI PG G1 G2 H T1 T2 C1 C2 S EP JP
Developmental stages
VIT_03s0167g00070
Standardized expressions
0
−2
−4
EI PG G1 G2 H T1 T2 C1 C2 S EP JP
Developmental stages
VIT_03s0167g00100
Standardized expressions
1.5
1.0
0.5
0.0
−0.5
EI PG G1 G2 H T1 T2 C1 C2 S EP JP
Developmental stages
VIT_03s0167g00130
Standardized expressions
2.0
1.5
1.0
0.5
0.0
−0.5
EI PG G1 G2 H T1 T2 C1 C2 S EP JP
Developmental stages
VIT_03s0167g00150
Standardized expressions
1.0
0.5
0.0
−0.5
−1.0
EI PG G1 G2 H T1 T2 C1 C2 S EP JP
Developmental stages
VIT_03s0167g00190
Standardized expressions
1.0
0.5
0.0
−0.5
−1.0
EI PG G1 G2 H T1 T2 C1 C2 S EP JP
Developmental stages
VIT_03s0167g00220
Standardized expressions
1
0
−1
−2
−3
EI PG G1 G2 H T1 T2 C1 C2 S EP JP
Developmental stages
VIT_03s0110g00010
Standardized expressions
2
1
0
EI PG G1 G2 H T1 T2 C1 C2 S EP JP
Developmental stages

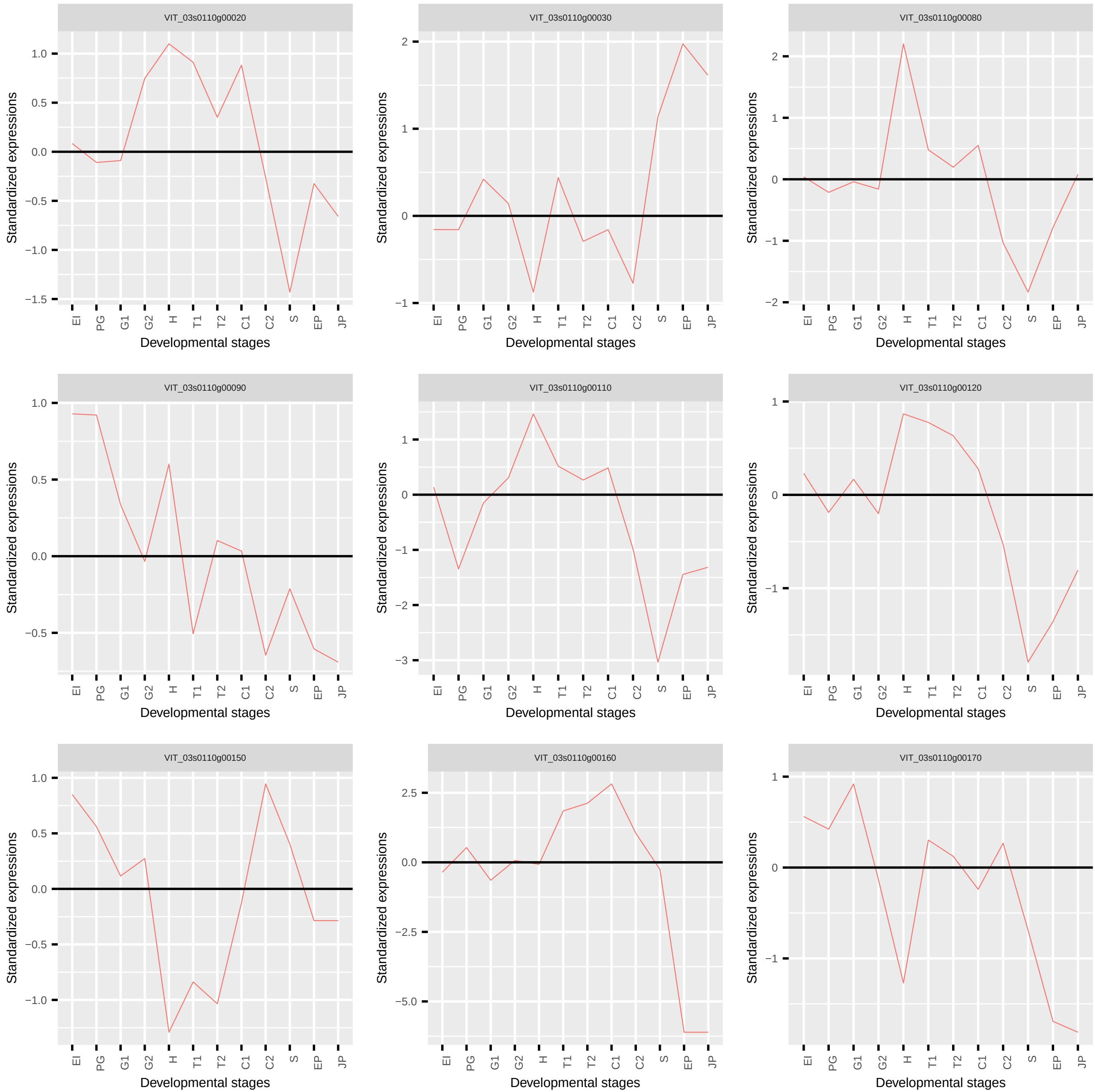

VIT_03s0110g00020
VIT_03s0110g00030
VIT_03s0110g00080
VIT_03s0110g00090
VIT_03s0110g00110
VIT_03s0110g00120
VIT_03s0110g00150
VIT_03s0110g00160
VIT_03s0110g00170
Standardized expressions
Developmental stages
EI PG G1 G2 H T1 T2 C1 C2 S EP JP

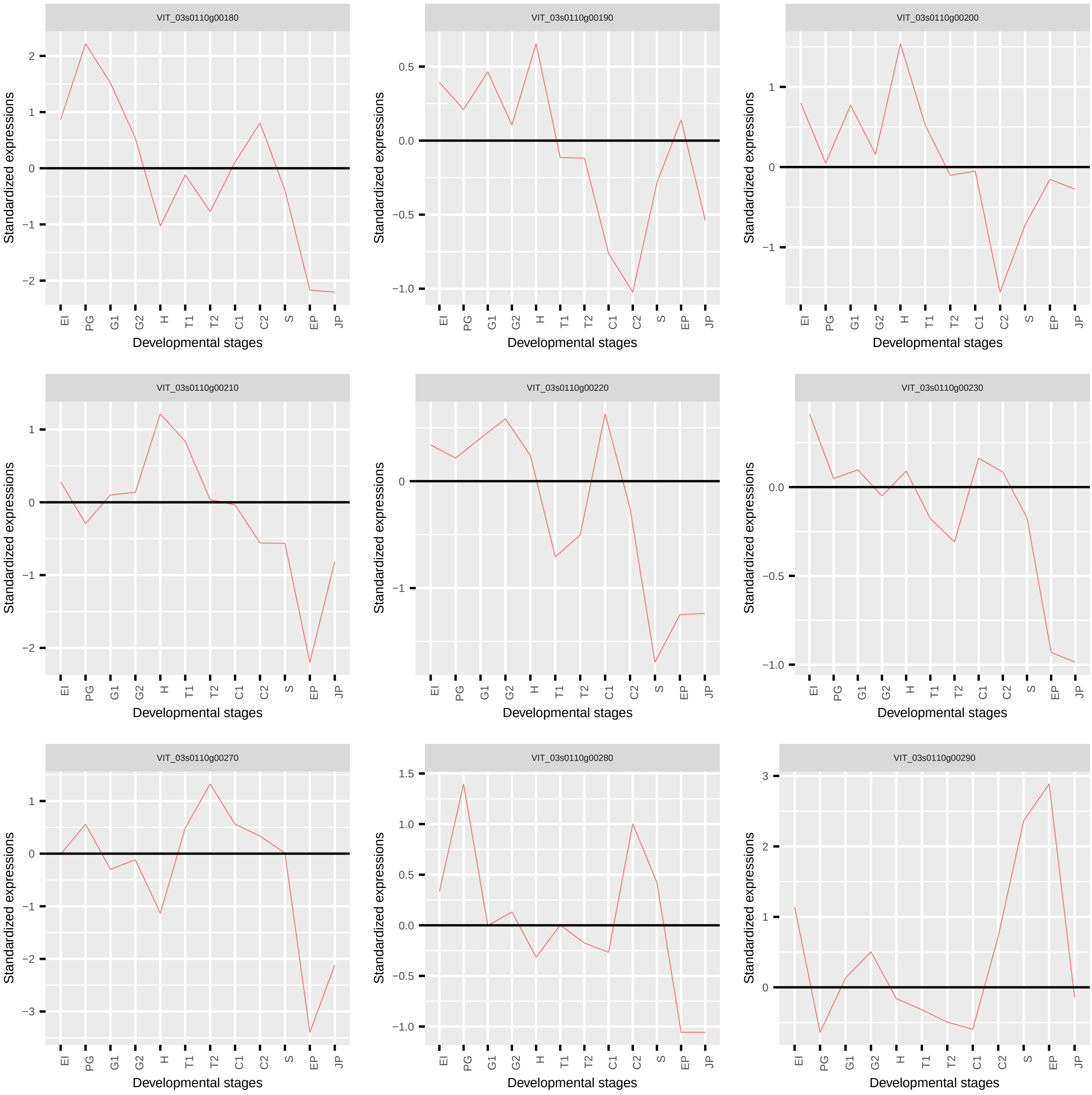
VIT_03s0110g00180
Standardized expressions
2
1
0
−1
−2
EI PG G1 G2 H T1 T2 C1 C2 S EP JP
Developmental stages
VIT_03s0110g00190
Standardized expressions
0.5
0.0
−0.5
−1.0
EI PG G1 G2 H T1 T2 C1 C2 S EP JP
Developmental stages
VIT_03s0110g00200
Standardized expressions
1
0
−1
EI PG G1 G2 H T1 T2 C1 C2 S EP JP
Developmental stages
VIT_03s0110g00210
Standardized expressions
1
0
−1
−2
EI PG G1 G2 H T1 T2 C1 C2 S EP JP
Developmental stages
VIT_03s0110g00220
Standardized expressions
0
−1
EI PG G1 G2 H T1 T2 C1 C2 S EP JP
Developmental stages
VIT_03s0110g00230
Standardized expressions
0.0
−0.5
−1.0
EI PG G1 G2 H T1 T2 C1 C2 S EP JP
Developmental stages
VIT_03s0110g00270
Standardized expressions
1
0
−1
−2
−3
EI PG G1 G2 H T1 T2 C1 C2 S EP JP
Developmental stages
VIT_03s0110g00280
Standardized expressions
1.5
1.0
0.5
0.0
−0.5
−1.0
EI PG G1 G2 H T1 T2 C1 C2 S EP JP
Developmental stages
VIT_03s0110g00290
Standardized expressions
3
2
1
0
EI PG G1 G2 H T1 T2 C1 C2 S EP JP
Developmental stages

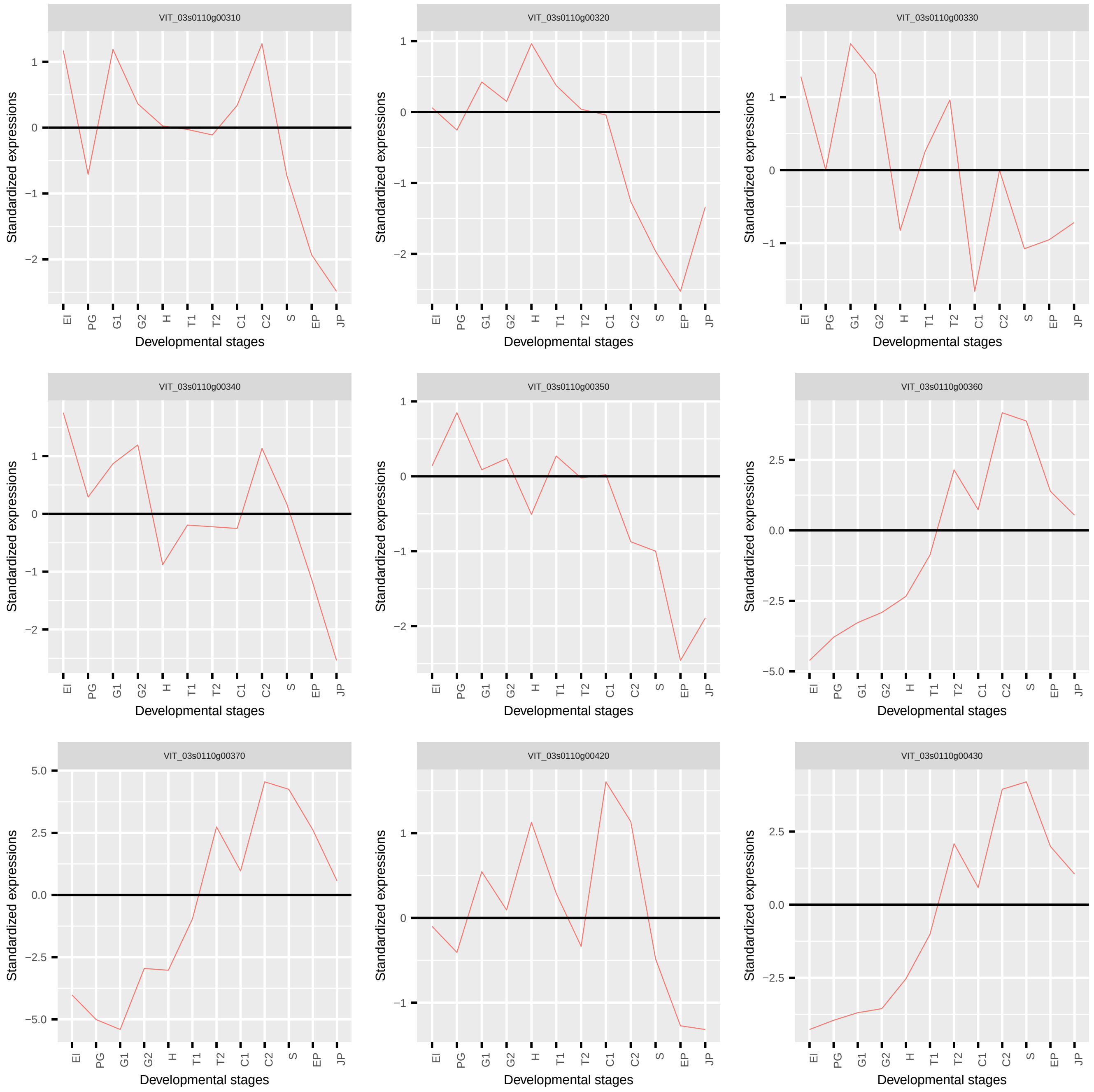
VIT_03s0110g00310
VIT_03s0110g00320
VIT_03s0110g00330
VIT_03s0110g00340
VIT_03s0110g00350
VIT_03s0110g00360
VIT_03s0110g00370
VIT_03s0110g00420
VIT_03s0110g00430
Standardized expressions
Developmental stages
EI PG G1 G2 H T1 T2 C1 C2 S EP JP
1 0 −1 −2
2.5 0.0 −2.5 −5.0
5.0

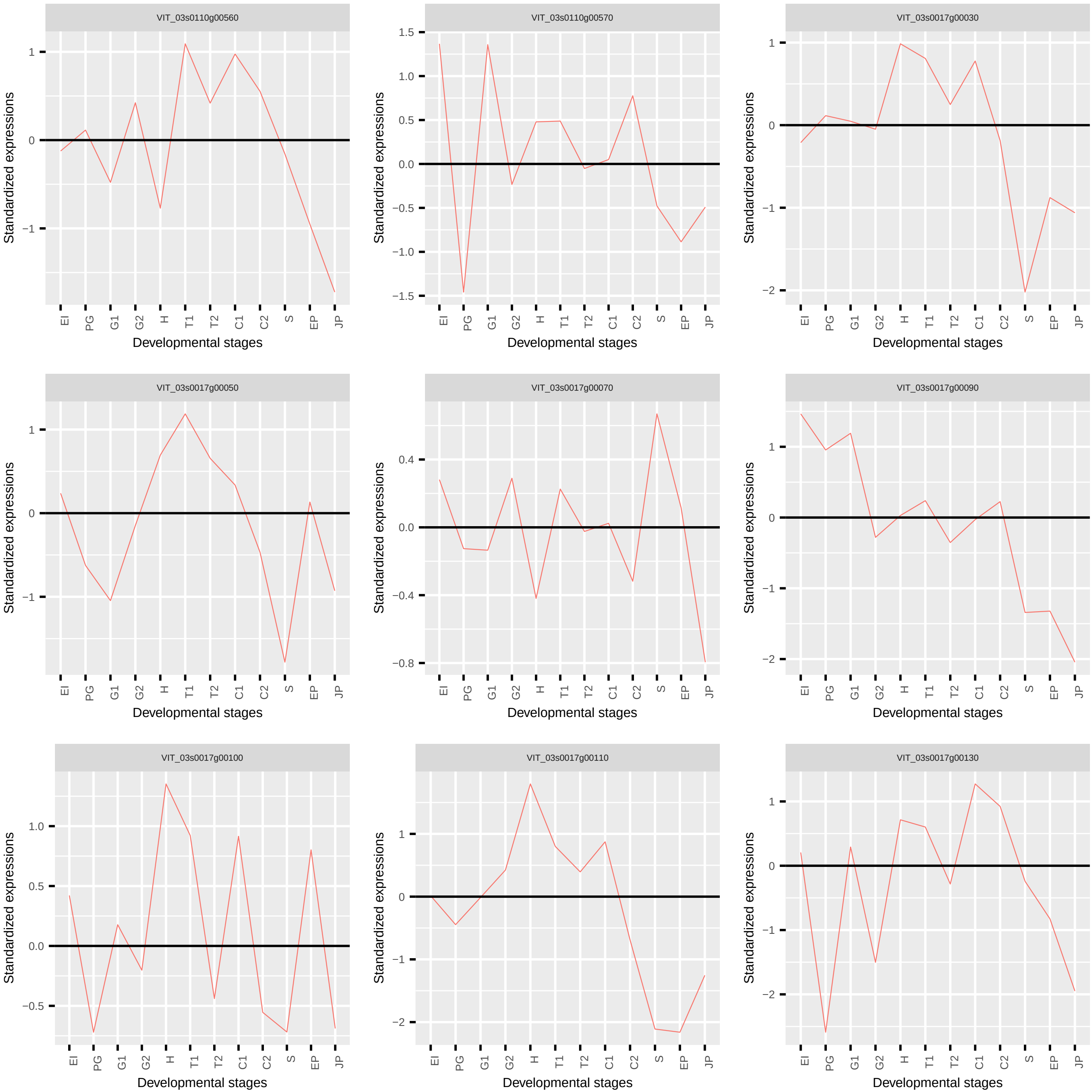
VIT_03s0110g00560
VIT_03s0110g00570
VIT_03s0017g00030
VIT_03s0017g00050
VIT_03s0017g00070
VIT_03s0017g00090
VIT_03s0017g00100
VIT_03s0017g00110
VIT_03s0017g00130
Standardized expressions
Developmental stages
EI PG G1 G2 H T1 T2 C1 C2 S EP JP

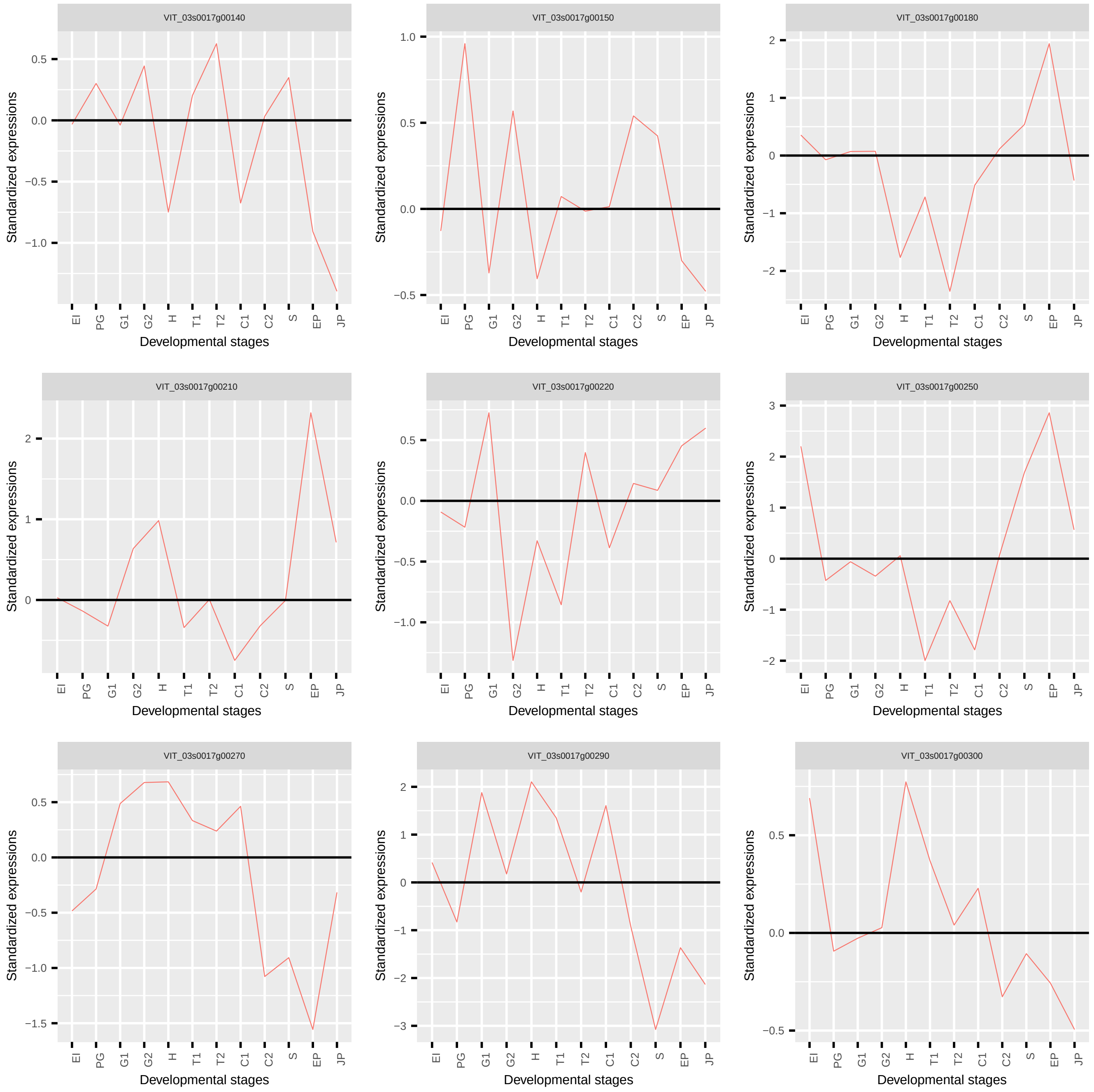
VIT_03s0017g00140
VIT_03s0017g00150
VIT_03s0017g00180
VIT_03s0017g00210
VIT_03s0017g00220
VIT_03s0017g00250
VIT_03s0017g00270
VIT_03s0017g00290
VIT_03s0017g00300
Standardized expressions
Developmental stages
EI
PG
G1
G2
H
T1
T2
C1
C2
S
EP
JP

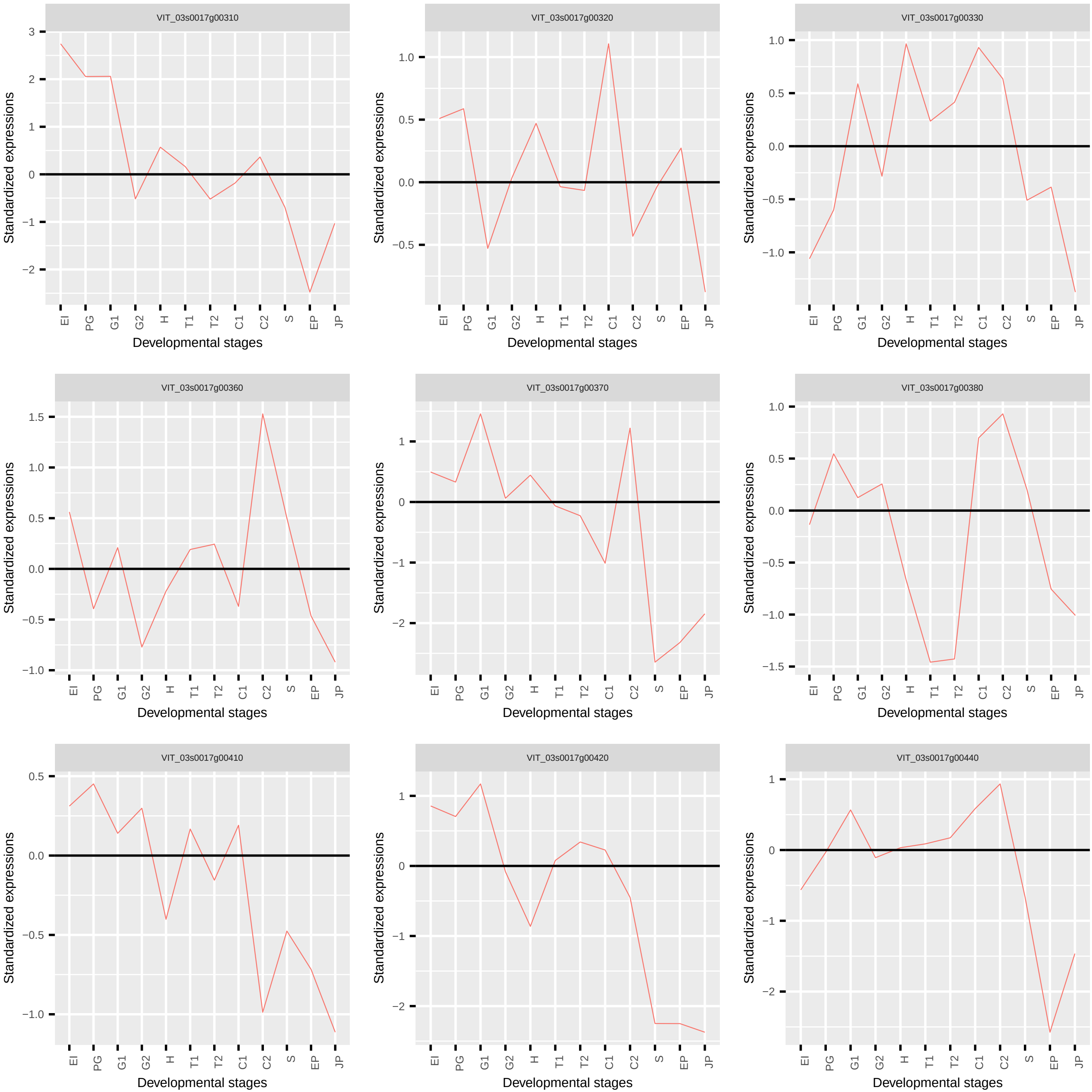
VIT_03s0017g00310
VIT_03s0017g00320
VIT_03s0017g00330
VIT_03s0017g00360
VIT_03s0017g00370
VIT_03s0017g00380
VIT_03s0017g00410
VIT_03s0017g00420
VIT_03s0017g00440
Standardized expressions
Developmental stages
EI
PG
G1
G2
H
T1
T2
C1
C2
S
EP
JP

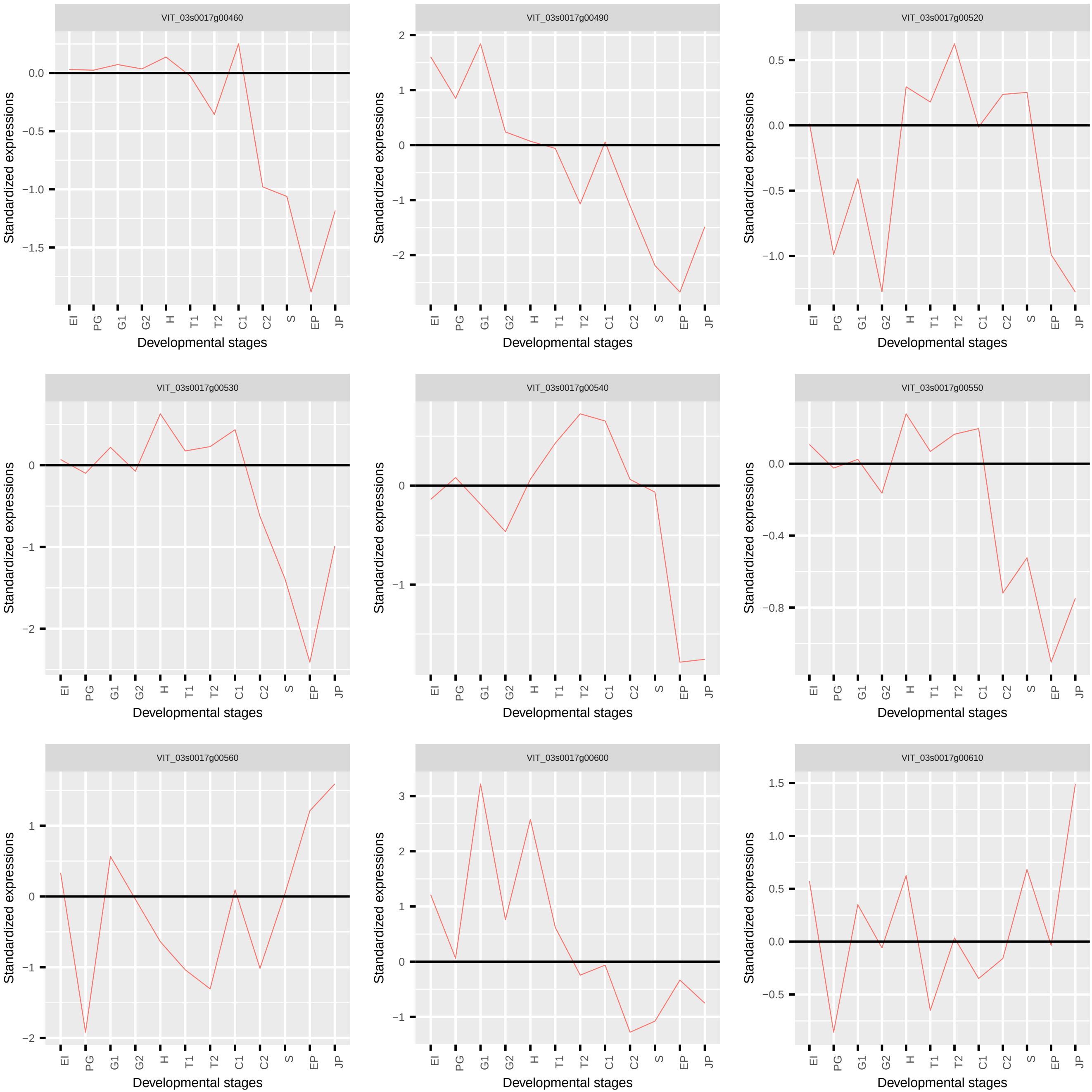
VIT_03s0017g00460
VIT_03s0017g00490
VIT_03s0017g00520
VIT_03s0017g00530
VIT_03s0017g00540
VIT_03s0017g00550
VIT_03s0017g00560
VIT_03s0017g00600
VIT_03s0017g00610
Standardized expressions
Developmental stages
EI PG G1 G2 H T1 T2 C1 C2 S EP JP

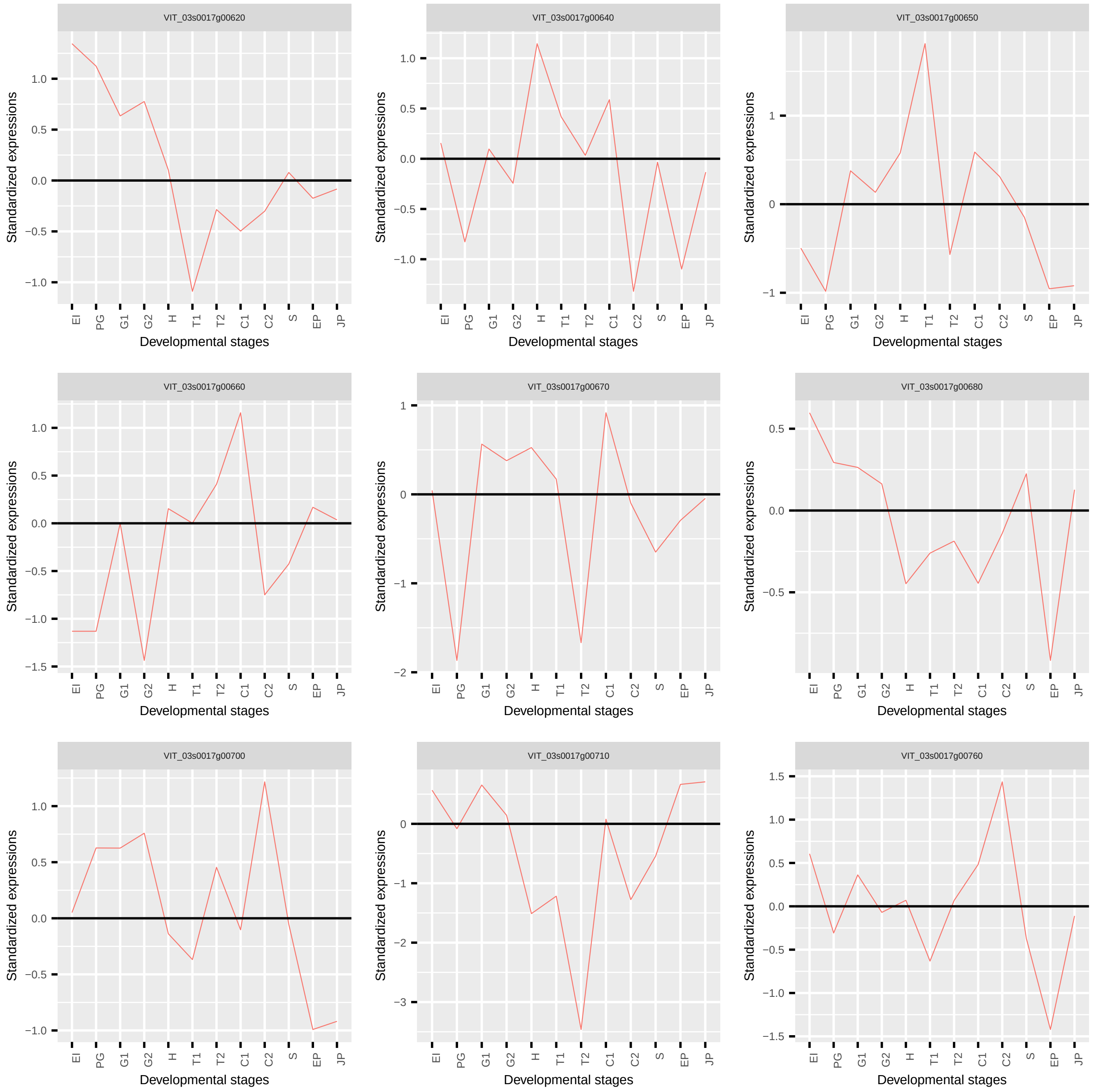
VIT_03s0017g00620
VIT_03s0017g00640
VIT_03s0017g00650
VIT_03s0017g00660
VIT_03s0017g00670
VIT_03s0017g00680
VIT_03s0017g00700
VIT_03s0017g00710
VIT_03s0017g00760
Standardized expressions
Developmental stages
EI PG G1 G2 H T1 T2 C1 C2 S EP JP

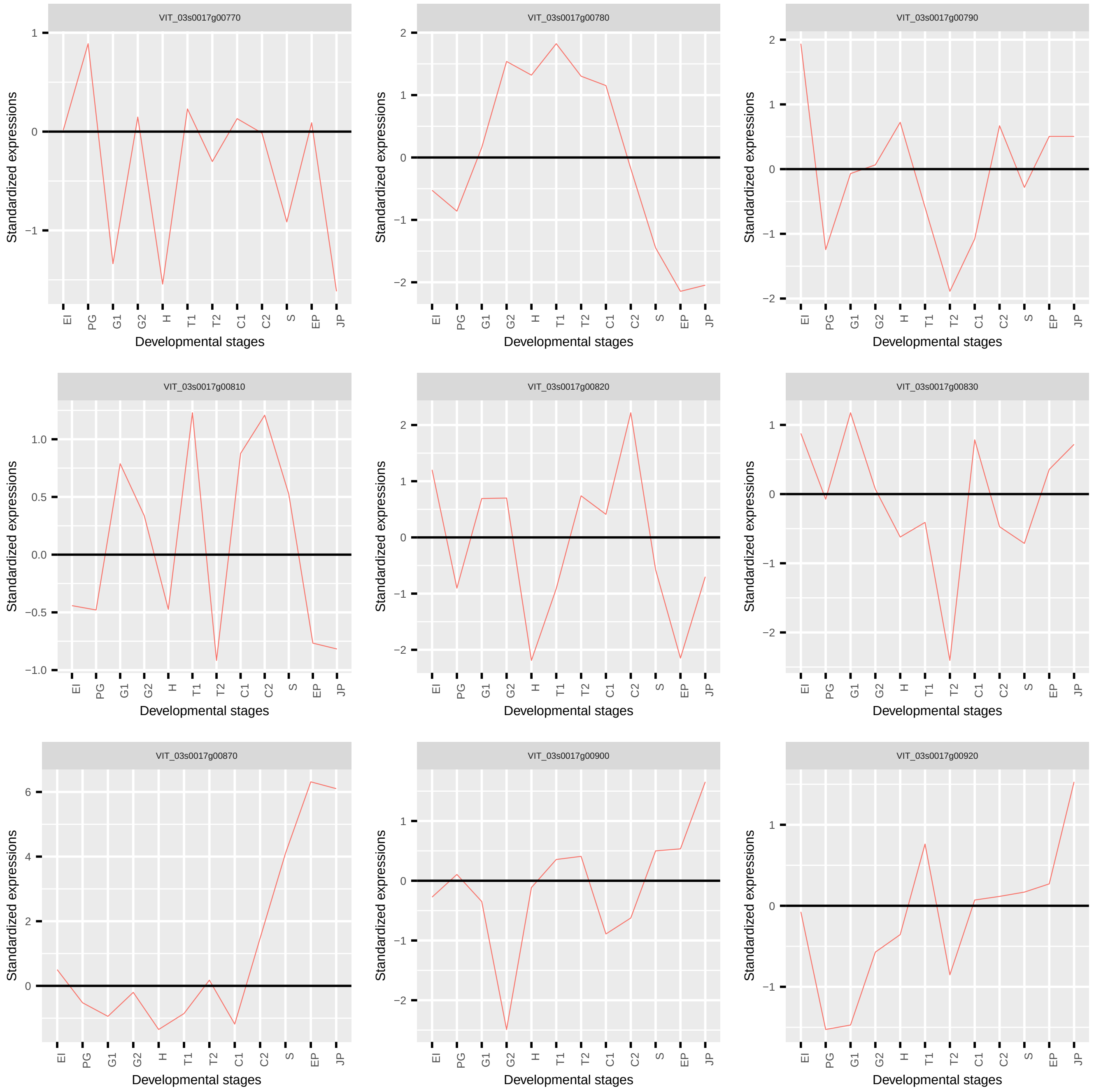
VIT_03s0017g00770
VIT_03s0017g00780
VIT_03s0017g00790
VIT_03s0017g00810
VIT_03s0017g00820
VIT_03s0017g00830
VIT_03s0017g00870
VIT_03s0017g00900
VIT_03s0017g00920
Standardized expressions
Developmental stages
EI
PG
G1
G2
H
T1
T2
C1
C2
S
EP
JP

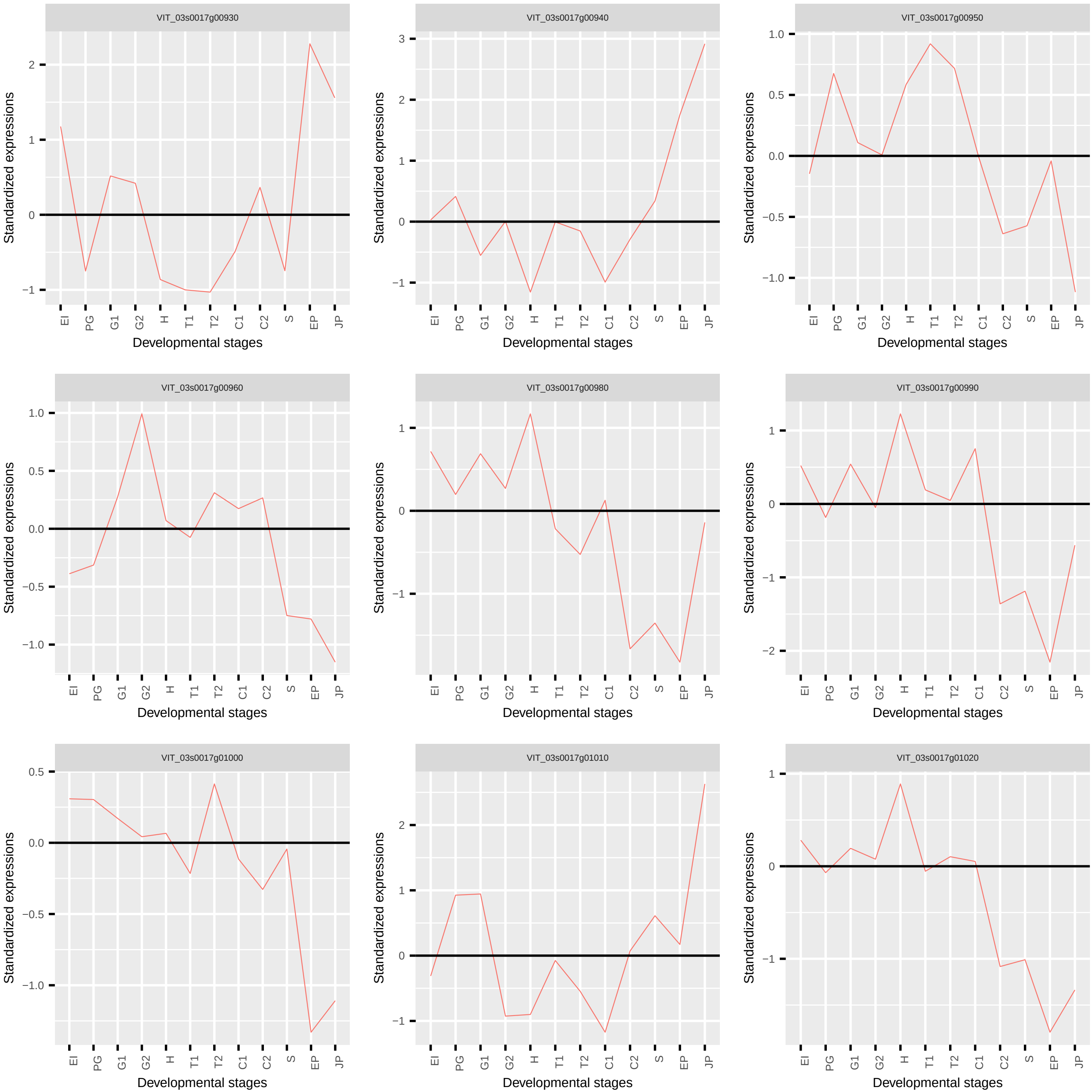
VIT_03s0017g00930
VIT_03s0017g00940
VIT_03s0017g00950
VIT_03s0017g00960
VIT_03s0017g00980
VIT_03s0017g00990
VIT_03s0017g01000
VIT_03s0017g01010
VIT_03s0017g01020
Standardized expressions
Developmental stages
EI
PG
G1
G2
H
T1
T2
C1
C2
S
EP
JP

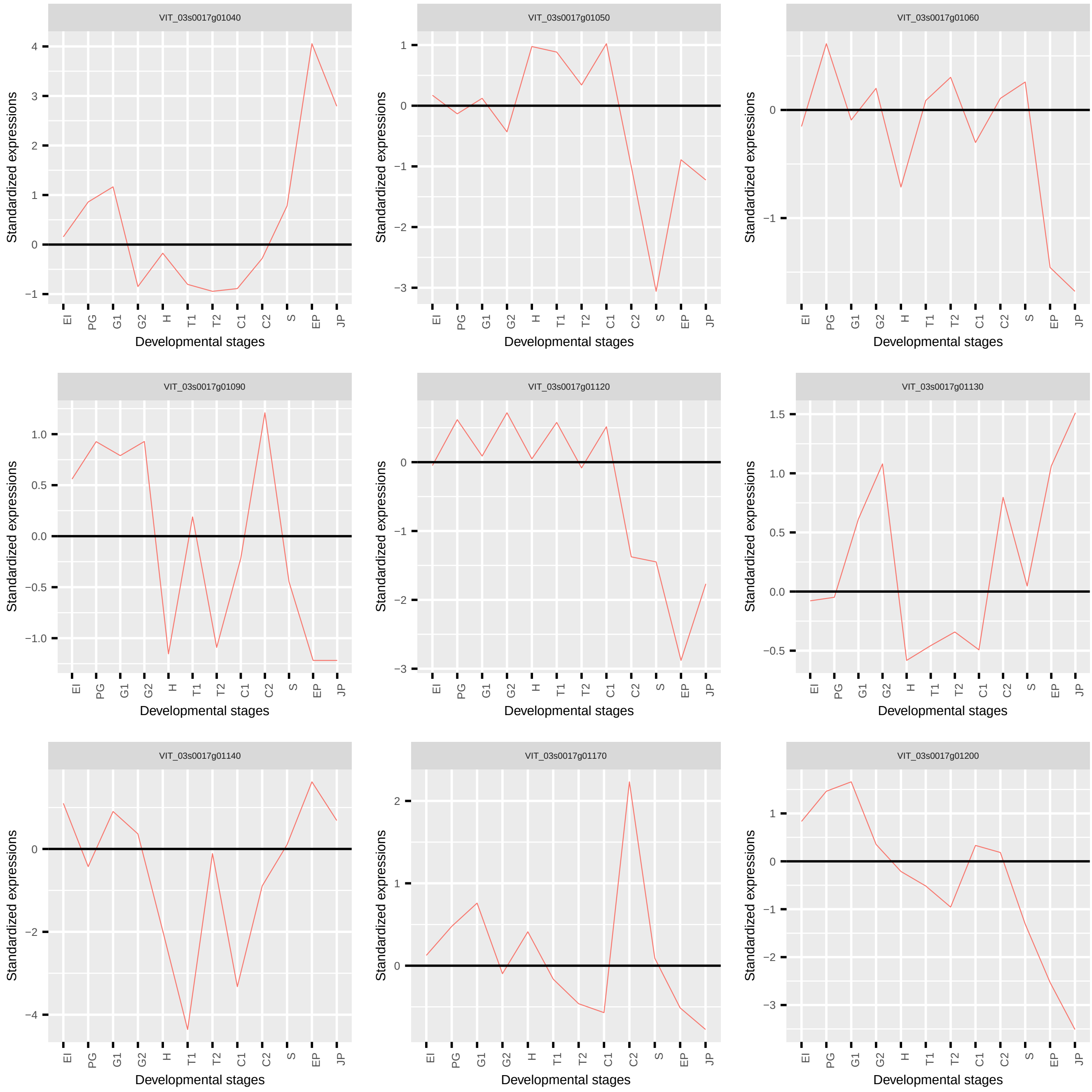

VIT_03s0017g01040
VIT_03s0017g01050
VIT_03s0017g01060
VIT_03s0017g01090
VIT_03s0017g01120
VIT_03s0017g01130
VIT_03s0017g01140
VIT_03s0017g01170
VIT_03s0017g01200
Standardized expressions
Developmental stages
EI PG G1 G2 H T1 T2 C1 C2 S EP JP

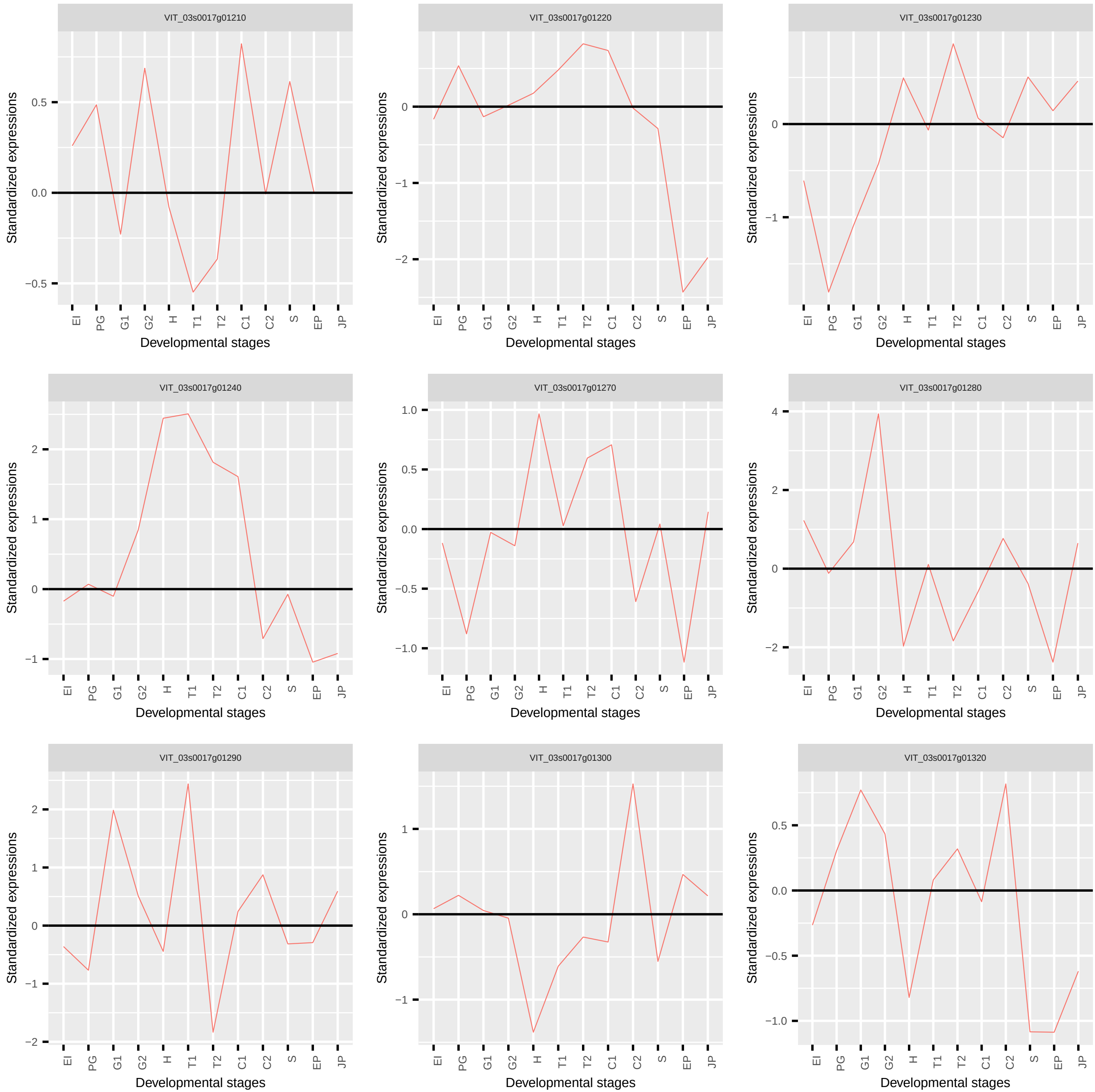
VIT_03s0017g01210
VIT_03s0017g01220
VIT_03s0017g01230
VIT_03s0017g01240
VIT_03s0017g01270
VIT_03s0017g01280
VIT_03s0017g01290
VIT_03s0017g01300
VIT_03s0017g01320
Standardized expressions
Developmental stages
EI PG G1 G2 H T1 T2 C1 C2 S EP JP

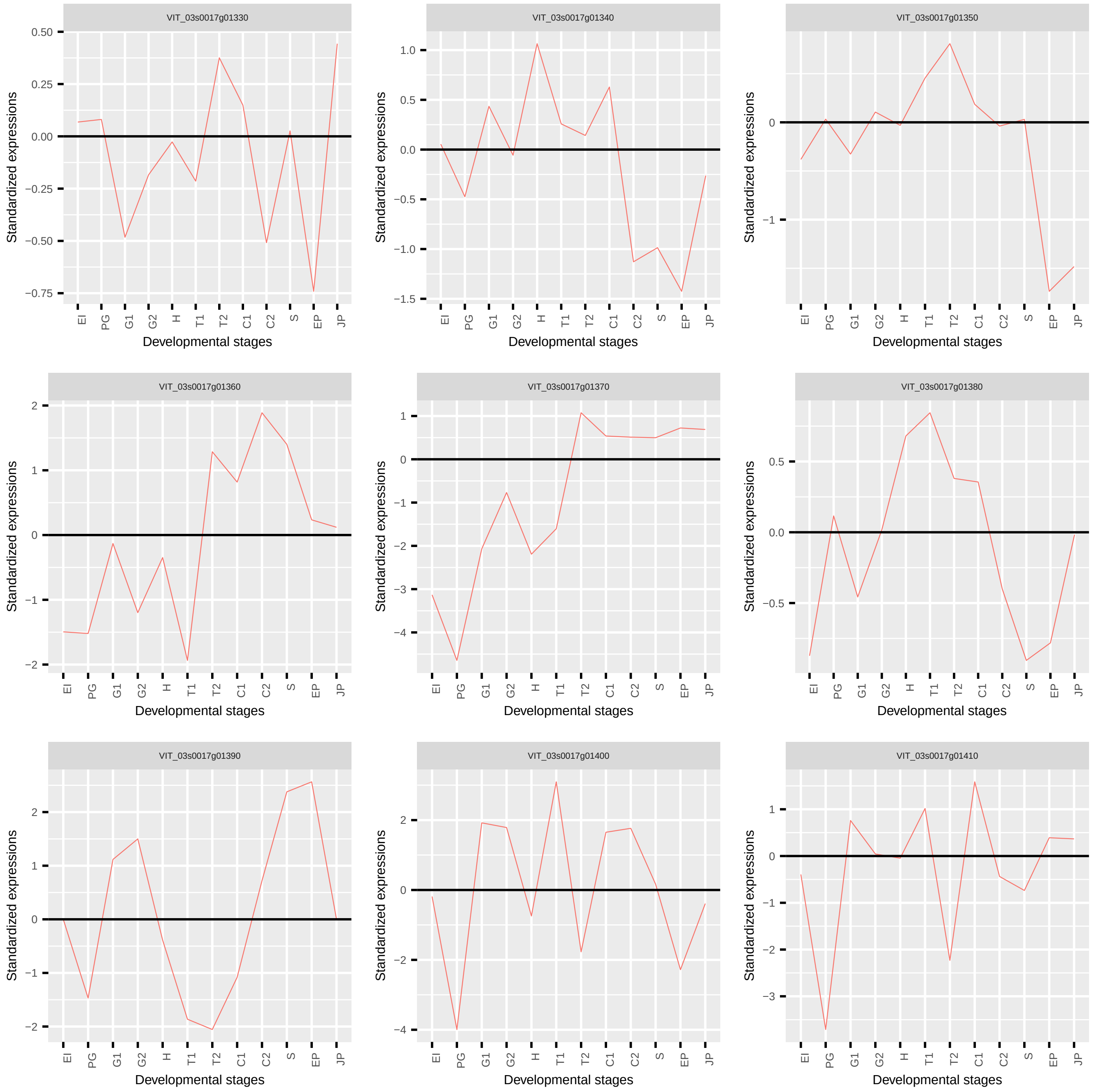

VIT_03s0017g01330
VIT_03s0017g01340
VIT_03s0017g01350
VIT_03s0017g01360
VIT_03s0017g01370
VIT_03s0017g01380
VIT_03s0017g01390
VIT_03s0017g01400
VIT_03s0017g01410
Standardized expressions
Developmental stages
EI
PG
G1
G2
H
T1
T2
C1
C2
S
EP
JP

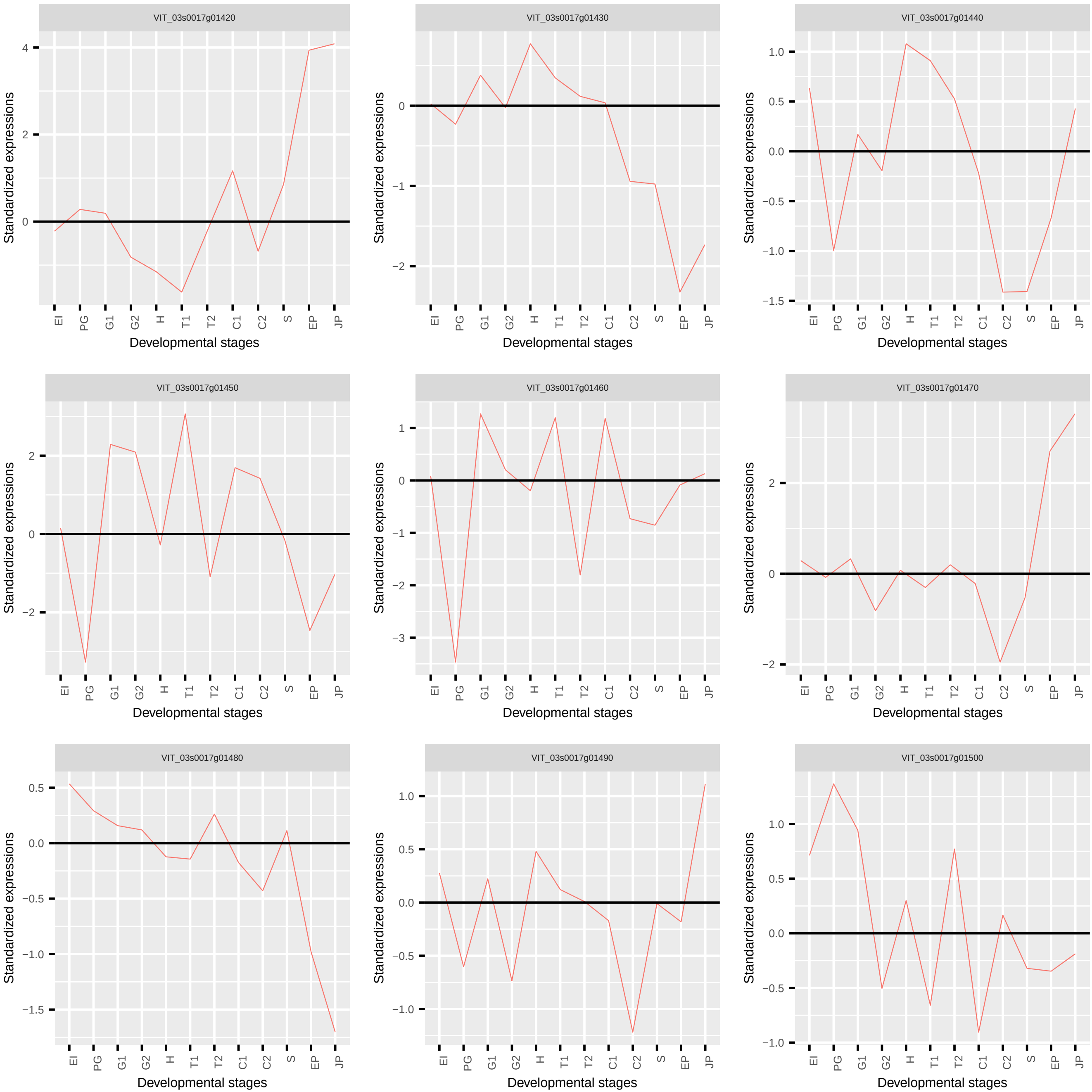
VIT_03s0017g01420
VIT_03s0017g01430
VIT_03s0017g01440
VIT_03s0017g01450
VIT_03s0017g01460
VIT_03s0017g01470
VIT_03s0017g01480
VIT_03s0017g01490
VIT_03s0017g01500
Standardized expressions
Developmental stages
EI PG G1 G2 H T1 T2 C1 C2 S EP JP

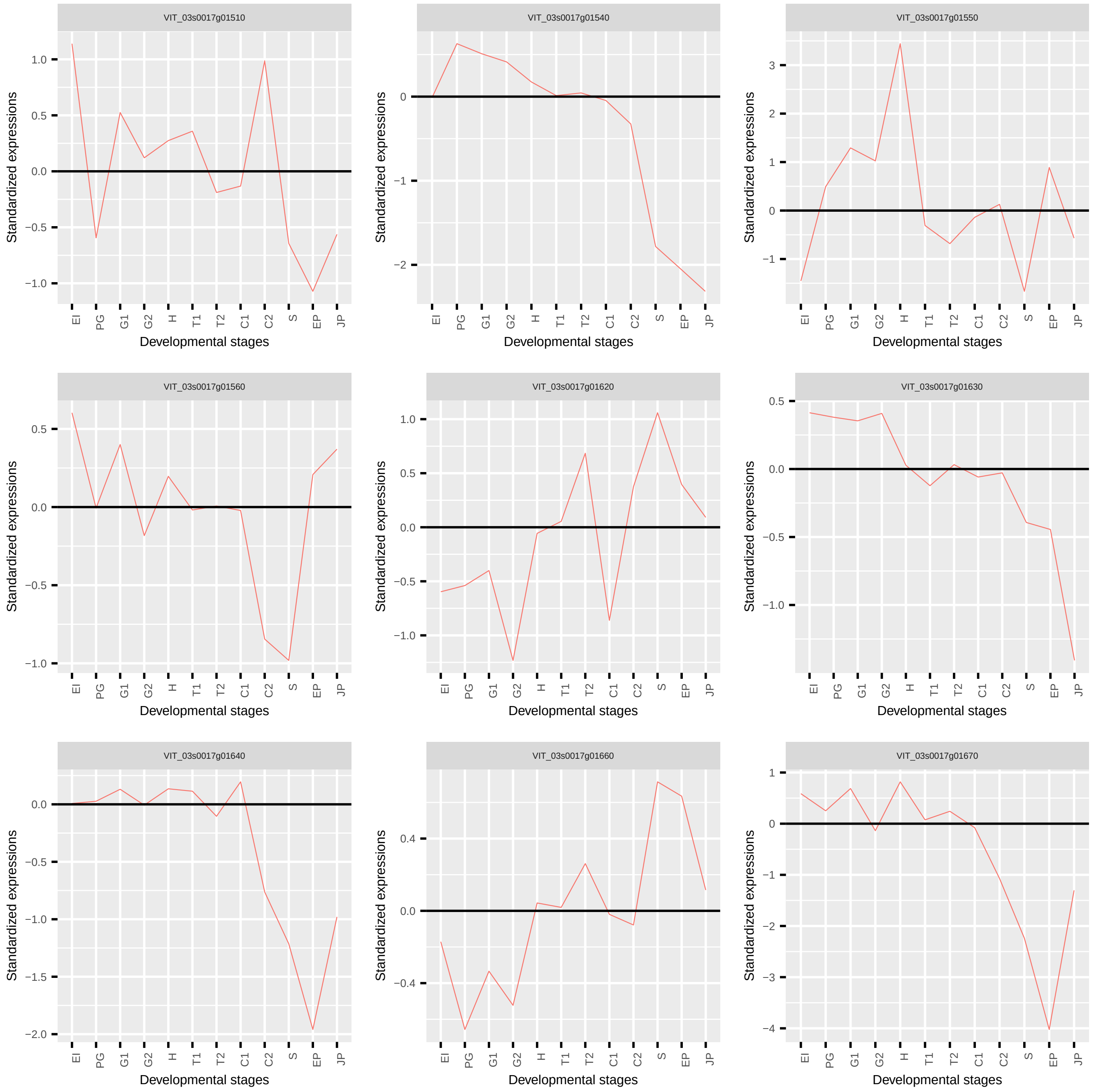
VIT_03s0017g01510
VIT_03s0017g01540
VIT_03s0017g01550
VIT_03s0017g01560
VIT_03s0017g01620
VIT_03s0017g01630
VIT_03s0017g01640
VIT_03s0017g01660
VIT_03s0017g01670
Standardized expressions
Developmental stages
EI PG G1 G2 H T1 T2 C1 C2 S EP JP

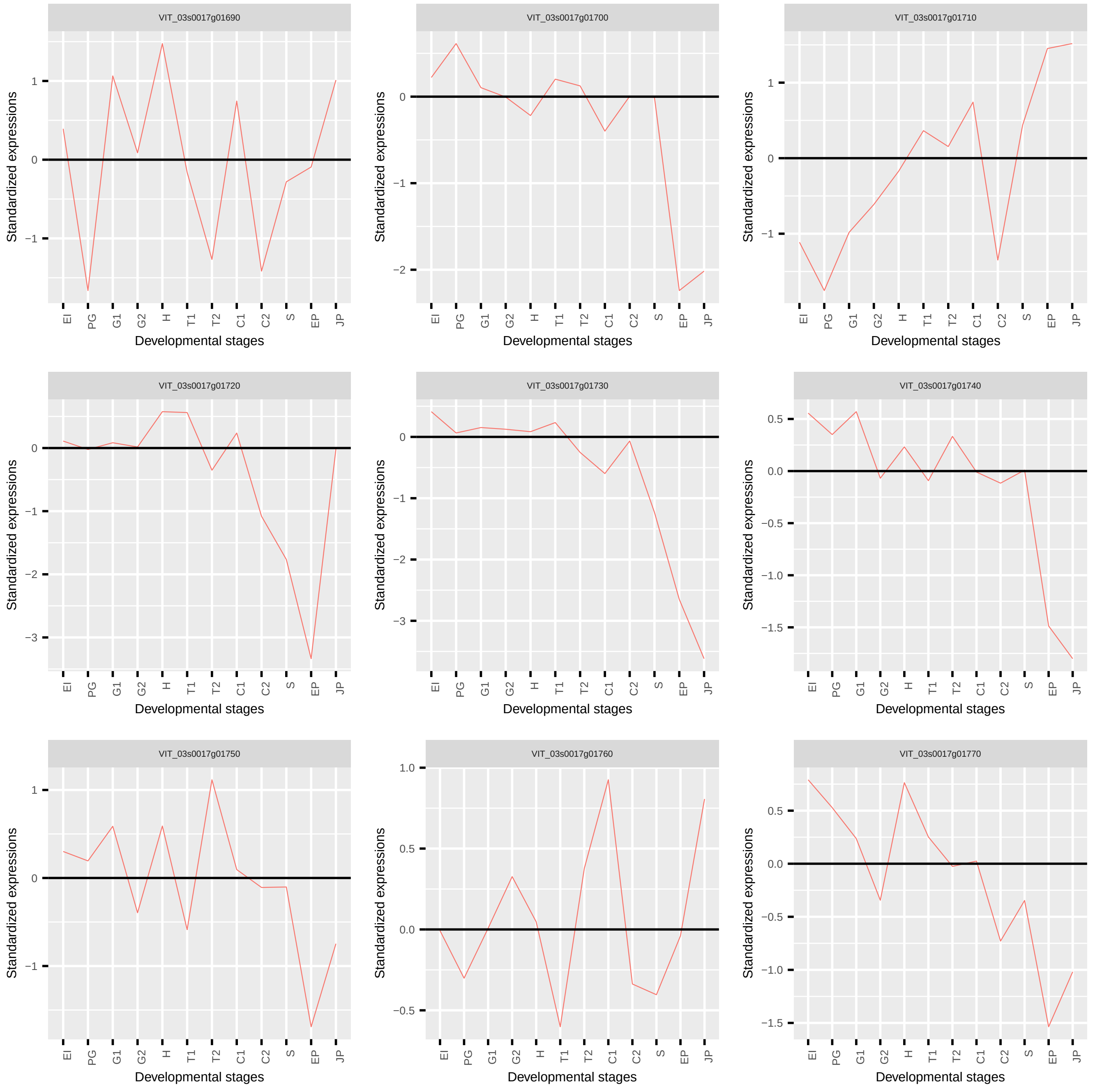

VIT_03s0017g01690
VIT_03s0017g01700
VIT_03s0017g01710
VIT_03s0017g01720
VIT_03s0017g01730
VIT_03s0017g01740
VIT_03s0017g01750
VIT_03s0017g01760
VIT_03s0017g01770
Standardized expressions
Developmental stages
EI PG G1 G2 H T1 T2 C1 C2 S EP JP

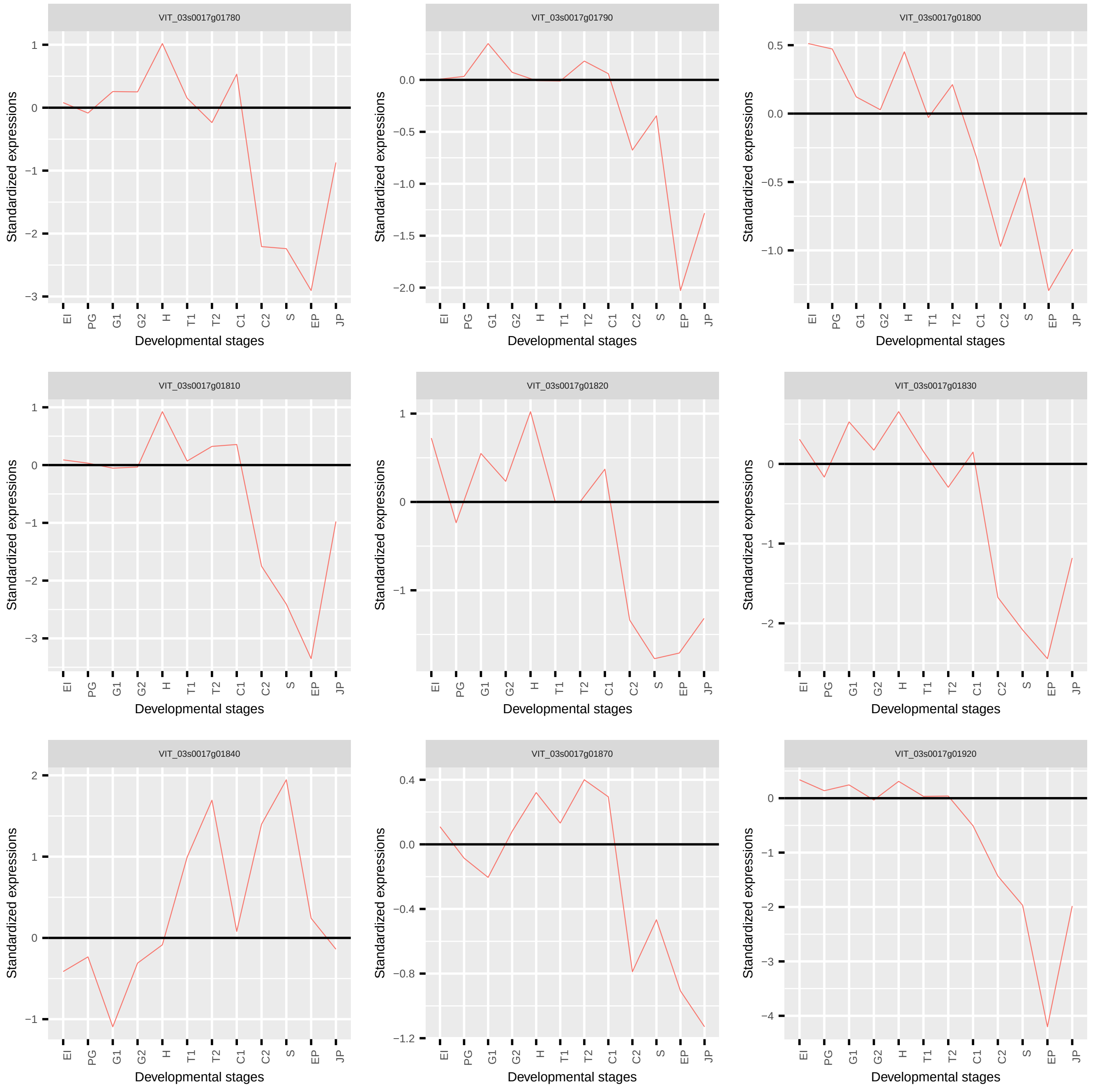
VIT_03s0017g01780
VIT_03s0017g01790
VIT_03s0017g01800
VIT_03s0017g01810
VIT_03s0017g01820
VIT_03s0017g01830
VIT_03s0017g01840
VIT_03s0017g01870
VIT_03s0017g01920
Standardized expressions
Developmental stages
EI PG G1 G2 H T1 T2 C1 C2 S EP JP

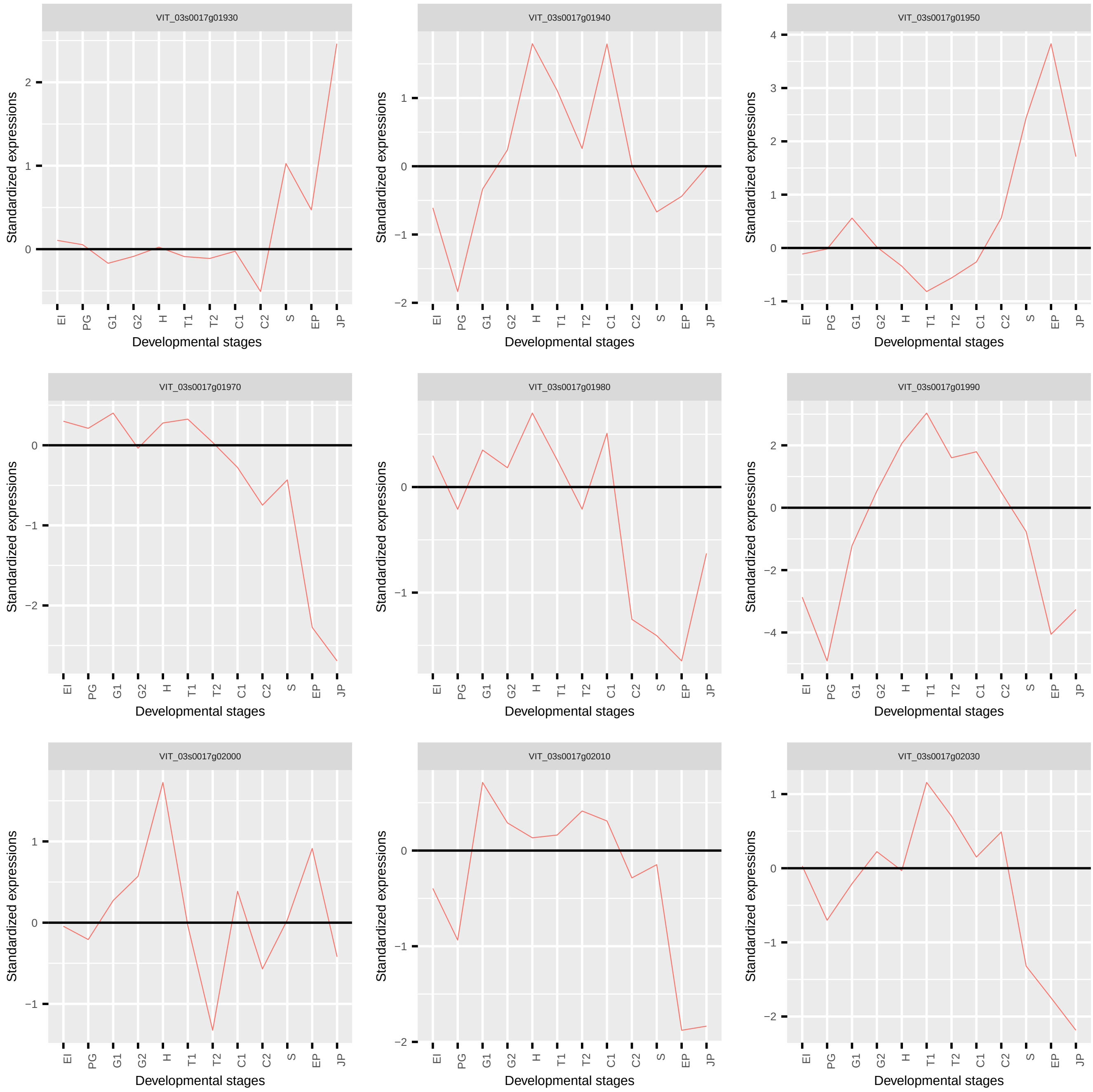
VIT_03s0017g01930
VIT_03s0017g01940
VIT_03s0017g01950
VIT_03s0017g01970
VIT_03s0017g01980
VIT_03s0017g01990
VIT_03s0017g02000
VIT_03s0017g02010
VIT_03s0017g02030
Standardized expressions
Developmental stages
EI
PG
G1
G2
H
T1
T2
C1
C2
S
EP
JP

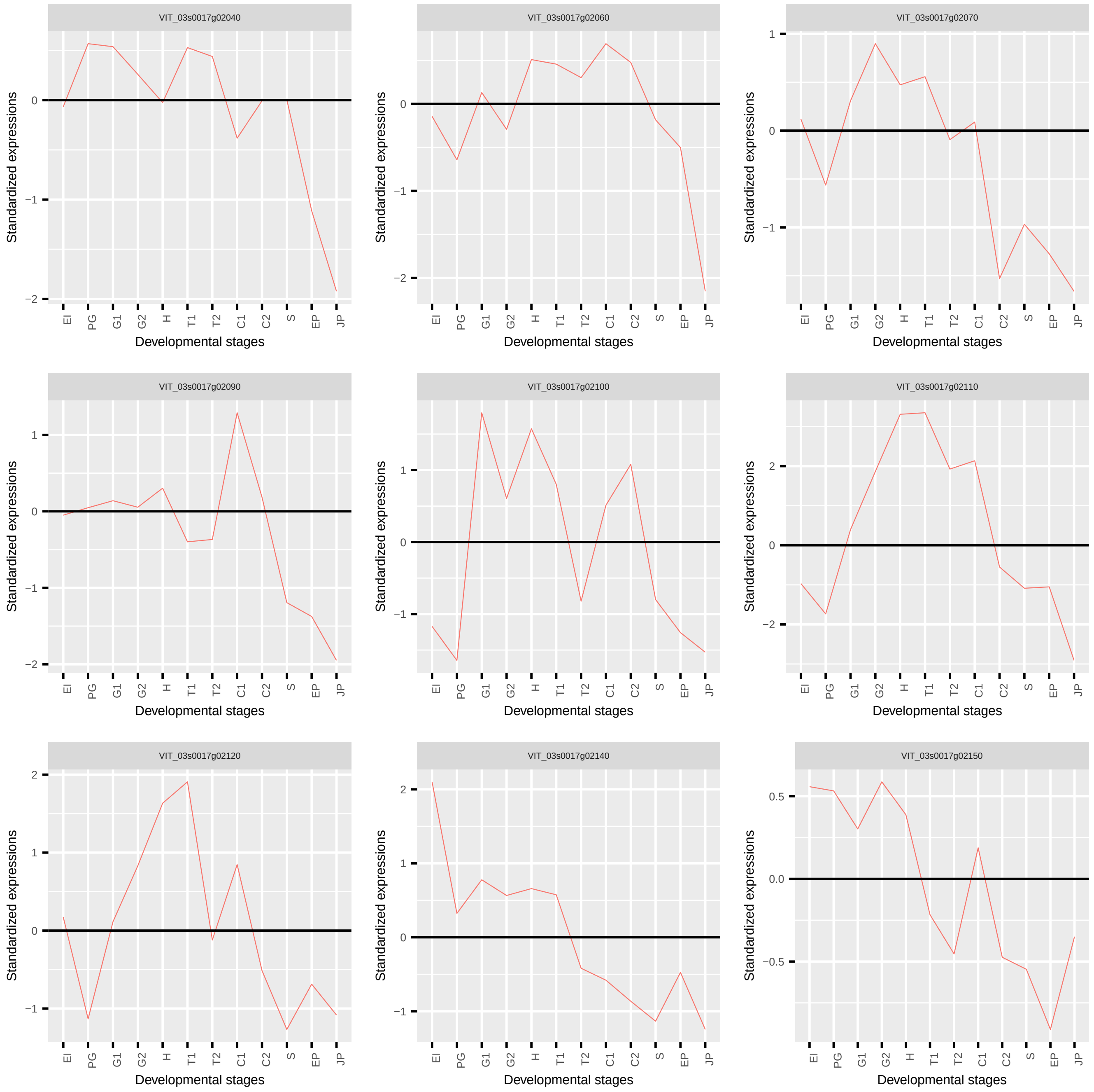

VIT_03s0017g02040
VIT_03s0017g02060
VIT_03s0017g02070
VIT_03s0017g02090
VIT_03s0017g02100
VIT_03s0017g02110
VIT_03s0017g02120
VIT_03s0017g02140
VIT_03s0017g02150
Standardized expressions
Developmental stages
EI PG G1 G2 H T1 T2 C1 C2 S EP JP

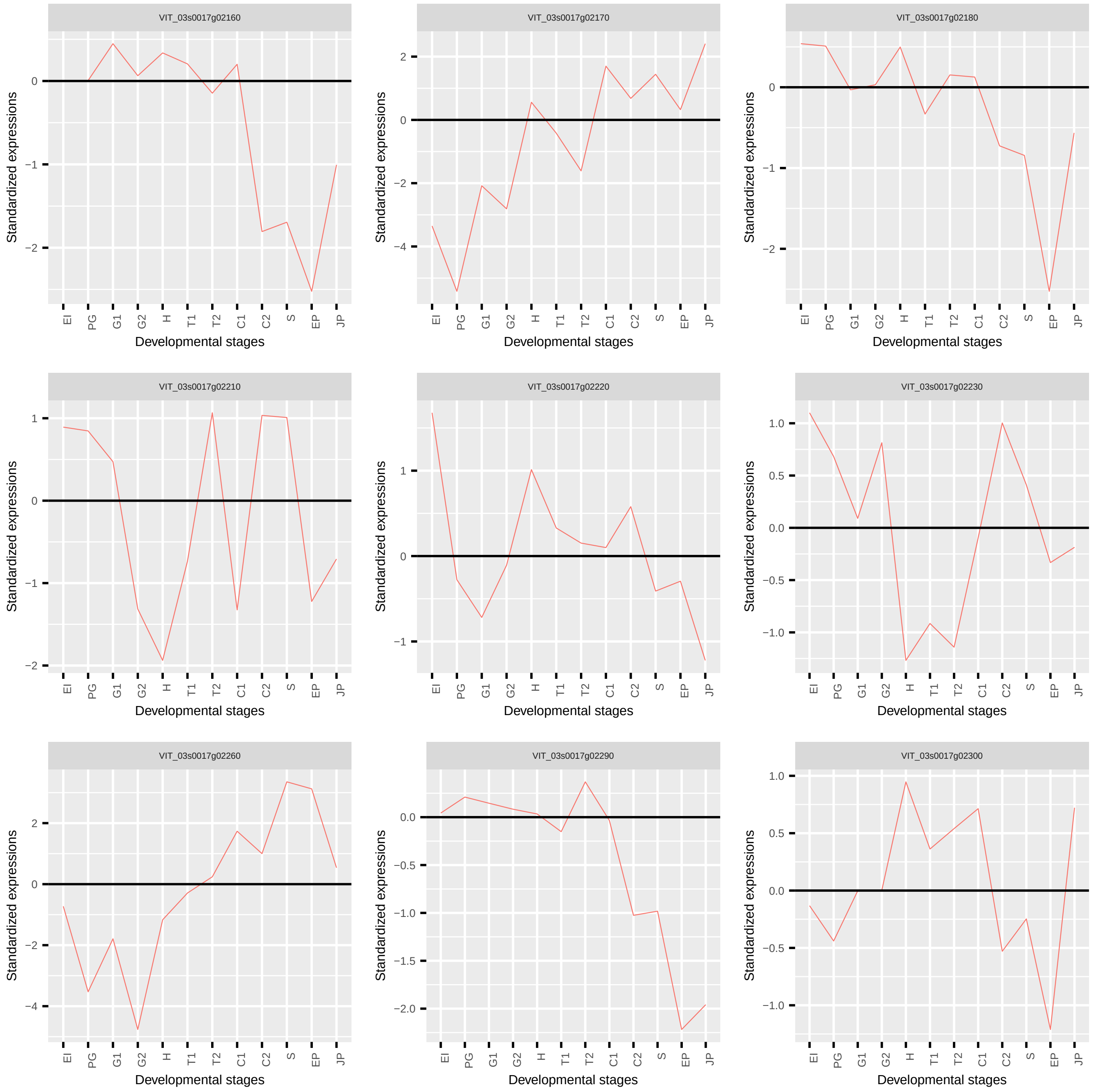
VIT_03s0017g02160
VIT_03s0017g02170
VIT_03s0017g02180
VIT_03s0017g02210
VIT_03s0017g02220
VIT_03s0017g02230
VIT_03s0017g02260
VIT_03s0017g02290
VIT_03s0017g02300
Standardized expressions
Developmental stages
EI
PG
G1
G2
H
T1
T2
C1
C2
S
EP
JP

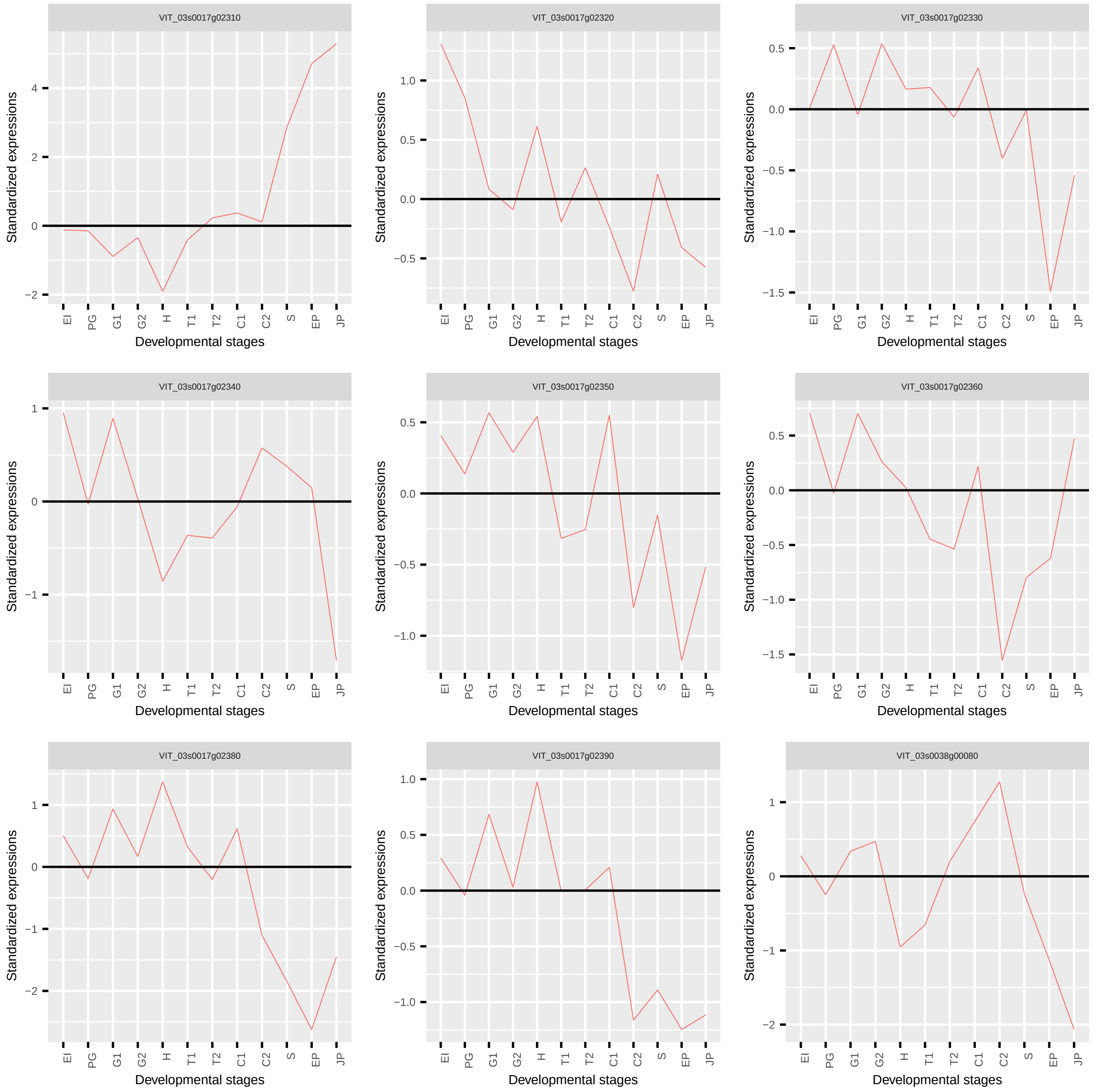
VIT_03s0017g02310
VIT_03s0017g02320
VIT_03s0017g02330
VIT_03s0017g02340
VIT_03s0017g02350
VIT_03s0017g02360
VIT_03s0017g02380
VIT_03s0017g02390
VIT_03s0038g00080
Standardized expressions
Developmental stages
EI PG G1 G2 H T1 T2 C1 C2 S EP JP

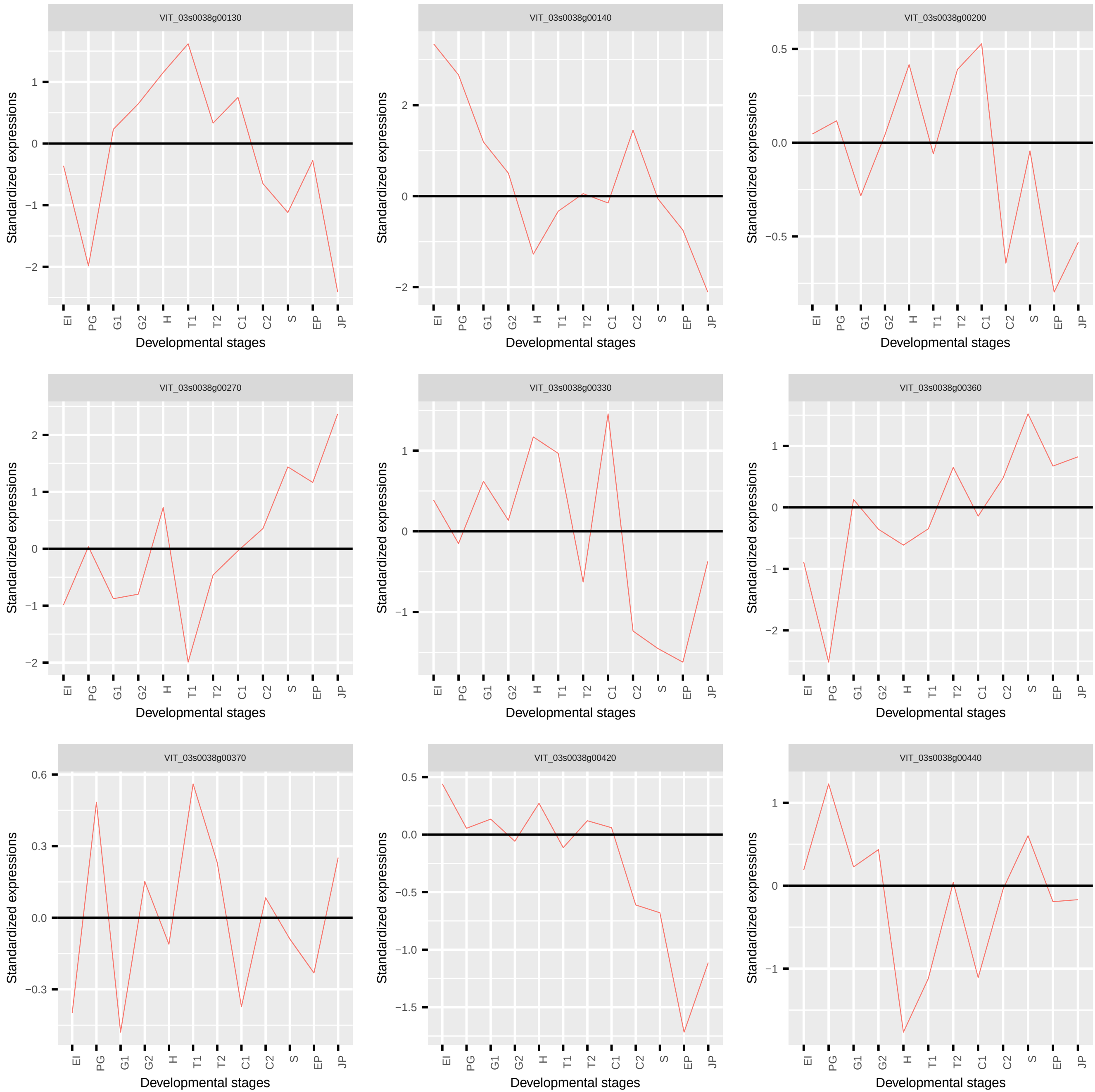
VIT_03s0038g00130
VIT_03s0038g00140
VIT_03s0038g00200
VIT_03s0038g00270
VIT_03s0038g00330
VIT_03s0038g00360
VIT_03s0038g00370
VIT_03s0038g00420
VIT_03s0038g00440
Standardized expressions
Developmental stages
EI PG G1 G2 H T1 T2 C1 C2 S EP JP

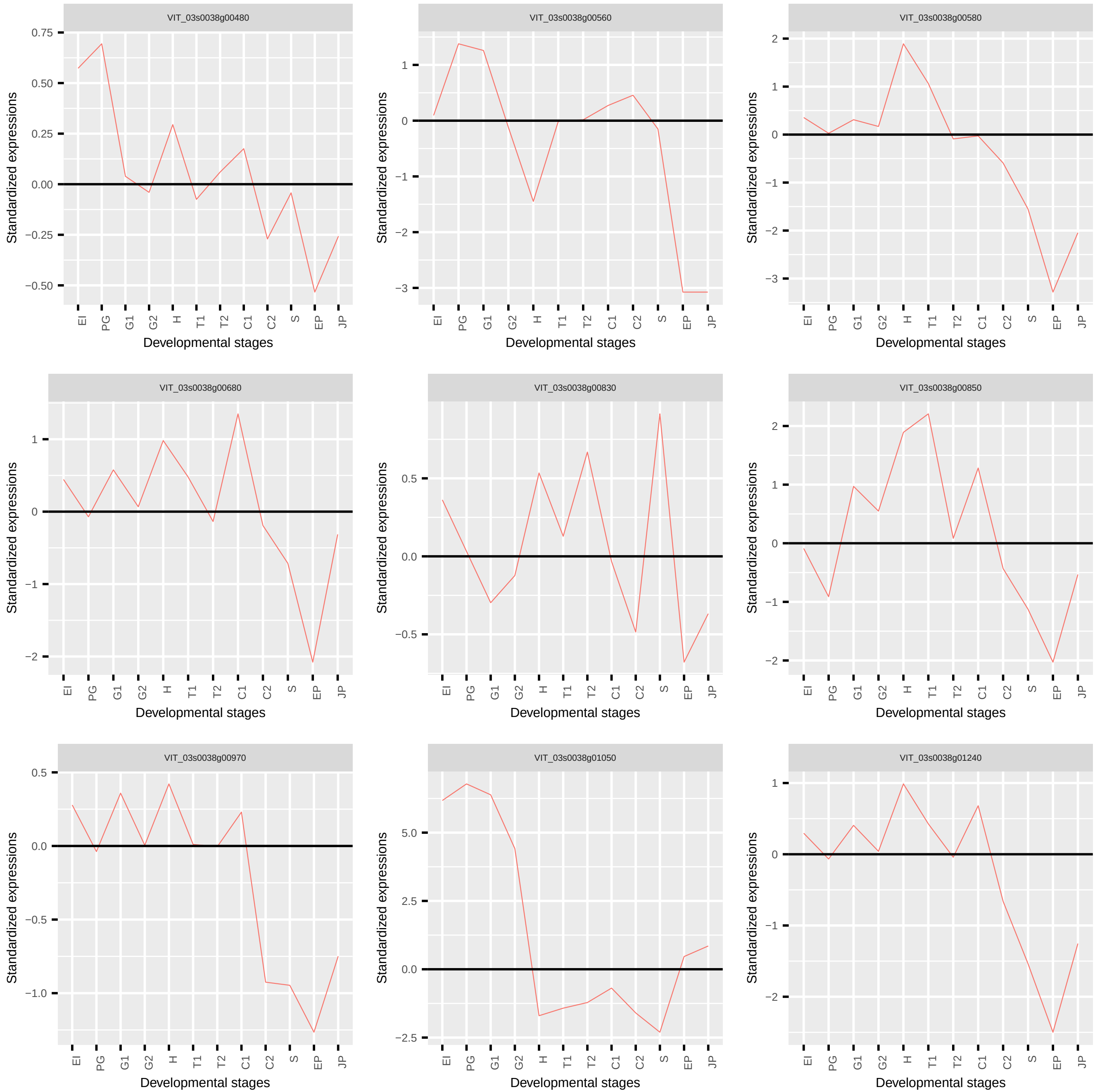

VIT_03s0038g00480
VIT_03s0038g00560
VIT_03s0038g00580
VIT_03s0038g00680
VIT_03s0038g00830
VIT_03s0038g00850
VIT_03s0038g00970
VIT_03s0038g01050
VIT_03s0038g01240
Standardized expressions
Developmental stages
EI PG G1 G2 H T1 T2 C1 C2 S EP JP

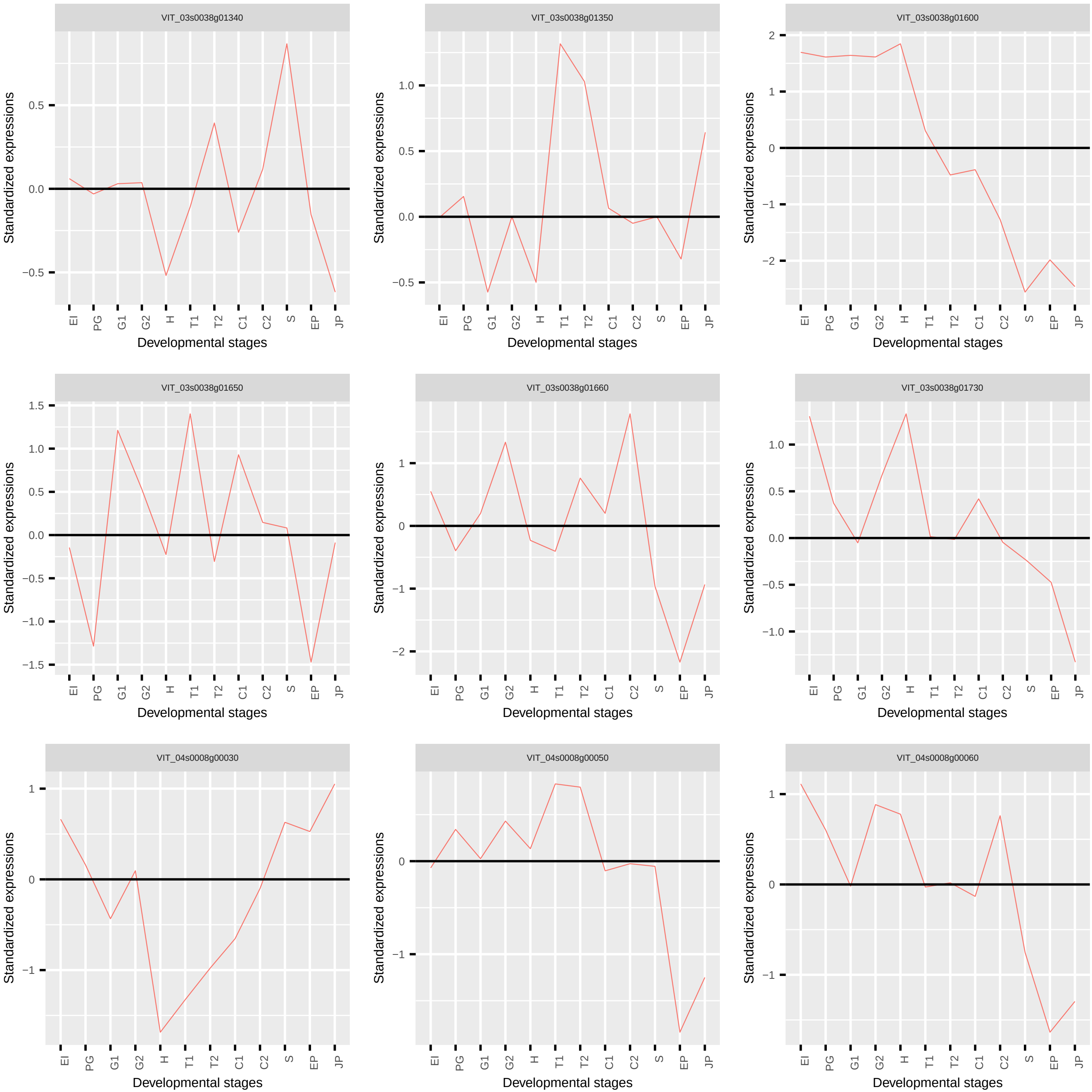
VIT_03s0038g01340
VIT_03s0038g01350
VIT_03s0038g01600
VIT_03s0038g01650
VIT_03s0038g01660
VIT_03s0038g01730
VIT_04s0008g00030
VIT_04s0008g00050
VIT_04s0008g00060
Standardized expressions
Developmental stages
EI PG G1 G2 H T1 T2 C1 C2 S EP JP

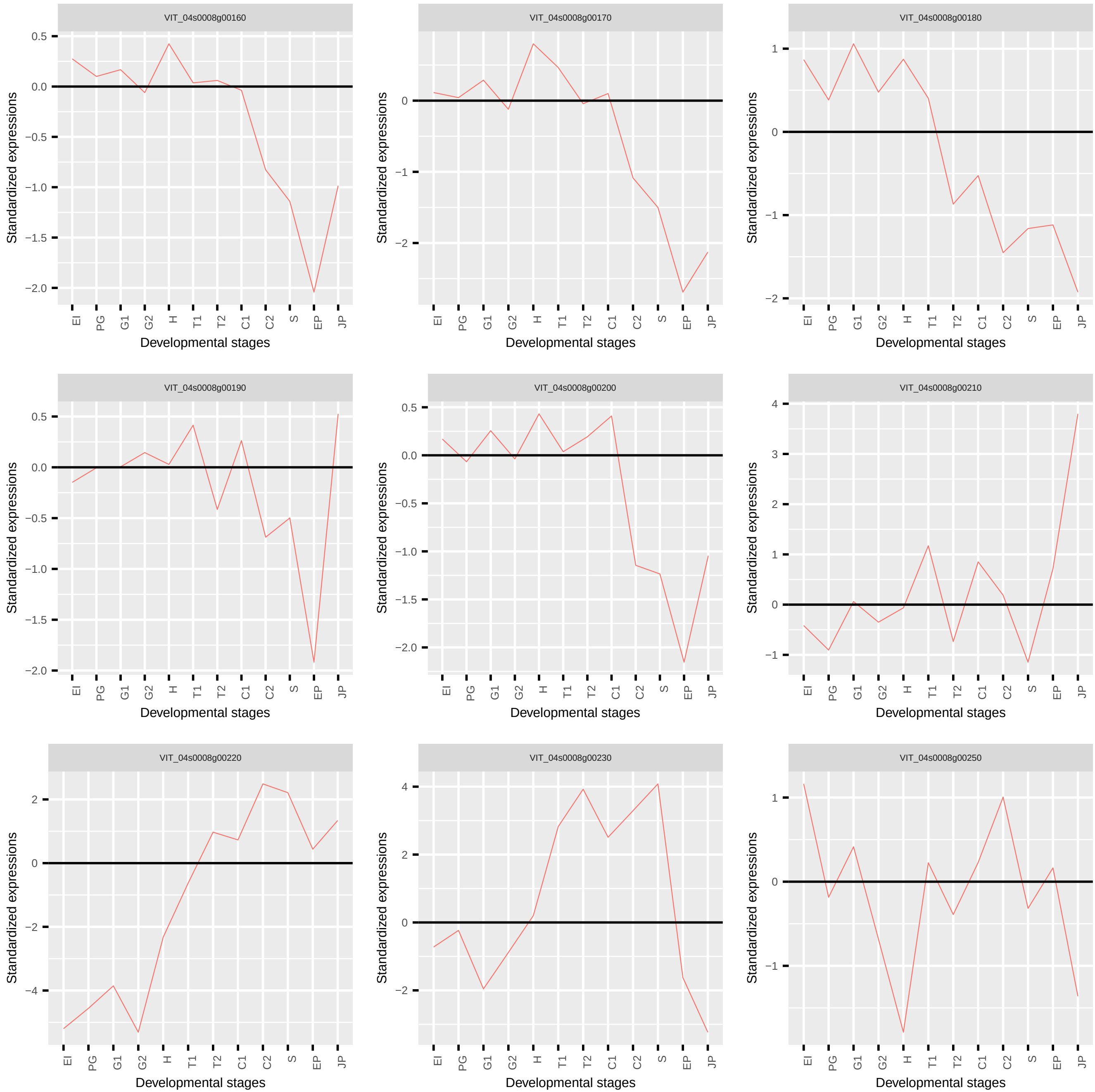
VIT_04s0008g00160
VIT_04s0008g00170
VIT_04s0008g00180
VIT_04s0008g00190
VIT_04s0008g00200
VIT_04s0008g00210
VIT_04s0008g00220
VIT_04s0008g00230
VIT_04s0008g00250
Standardized expressions
Developmental stages
EI
PG
G1
G2
H
T1
T2
C1
C2
S
EP
JP

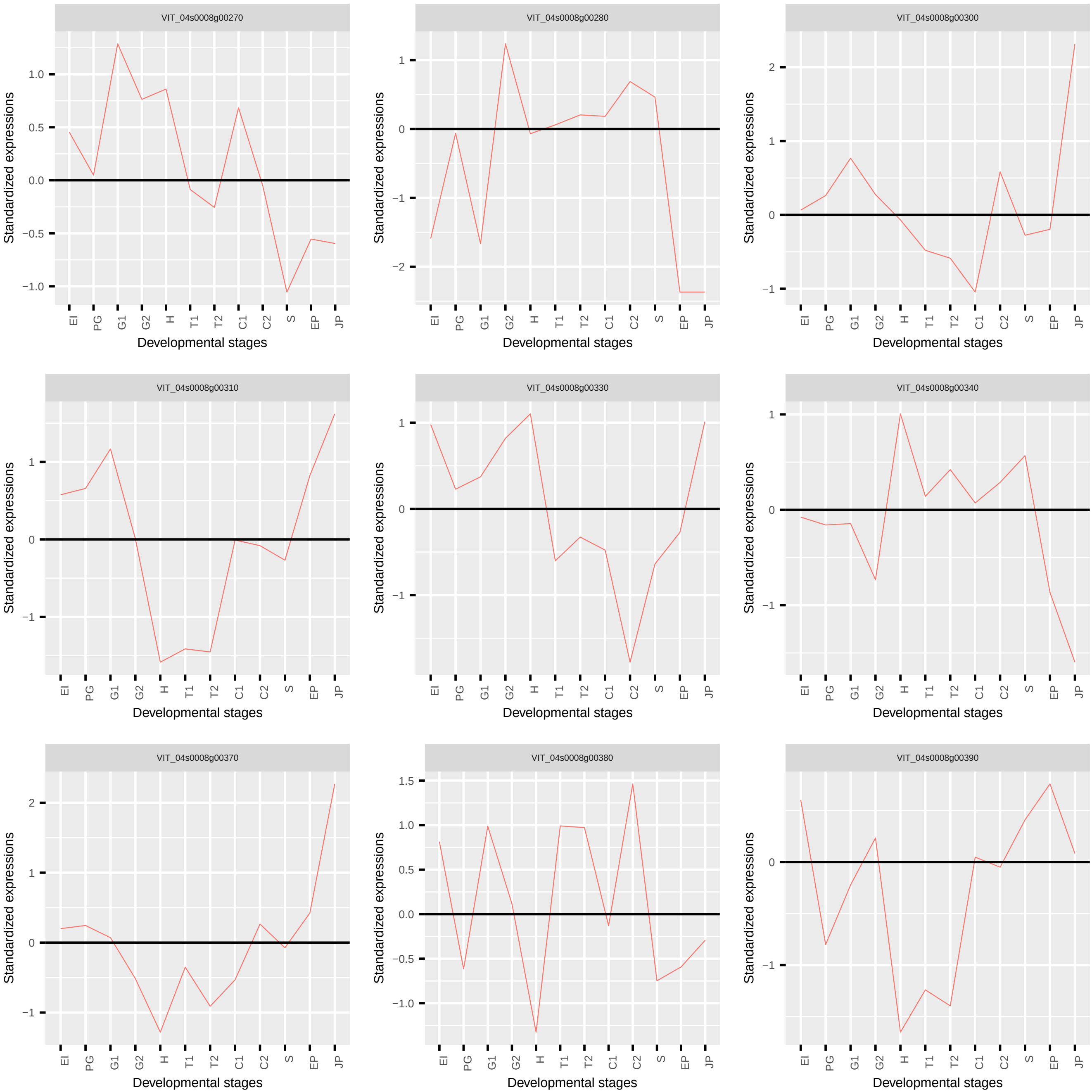
VIT_04s0008g00270
VIT_04s0008g00280
VIT_04s0008g00300
VIT_04s0008g00310
VIT_04s0008g00330
VIT_04s0008g00340
VIT_04s0008g00370
VIT_04s0008g00380
VIT_04s0008g00390
Standardized expressions
Developmental stages
EI PG G1 G2 H T1 T2 C1 C2 S EP JP
1.5 1.0 0.5 0.0 −0.5 −1.0
2 1 0 −1 −2

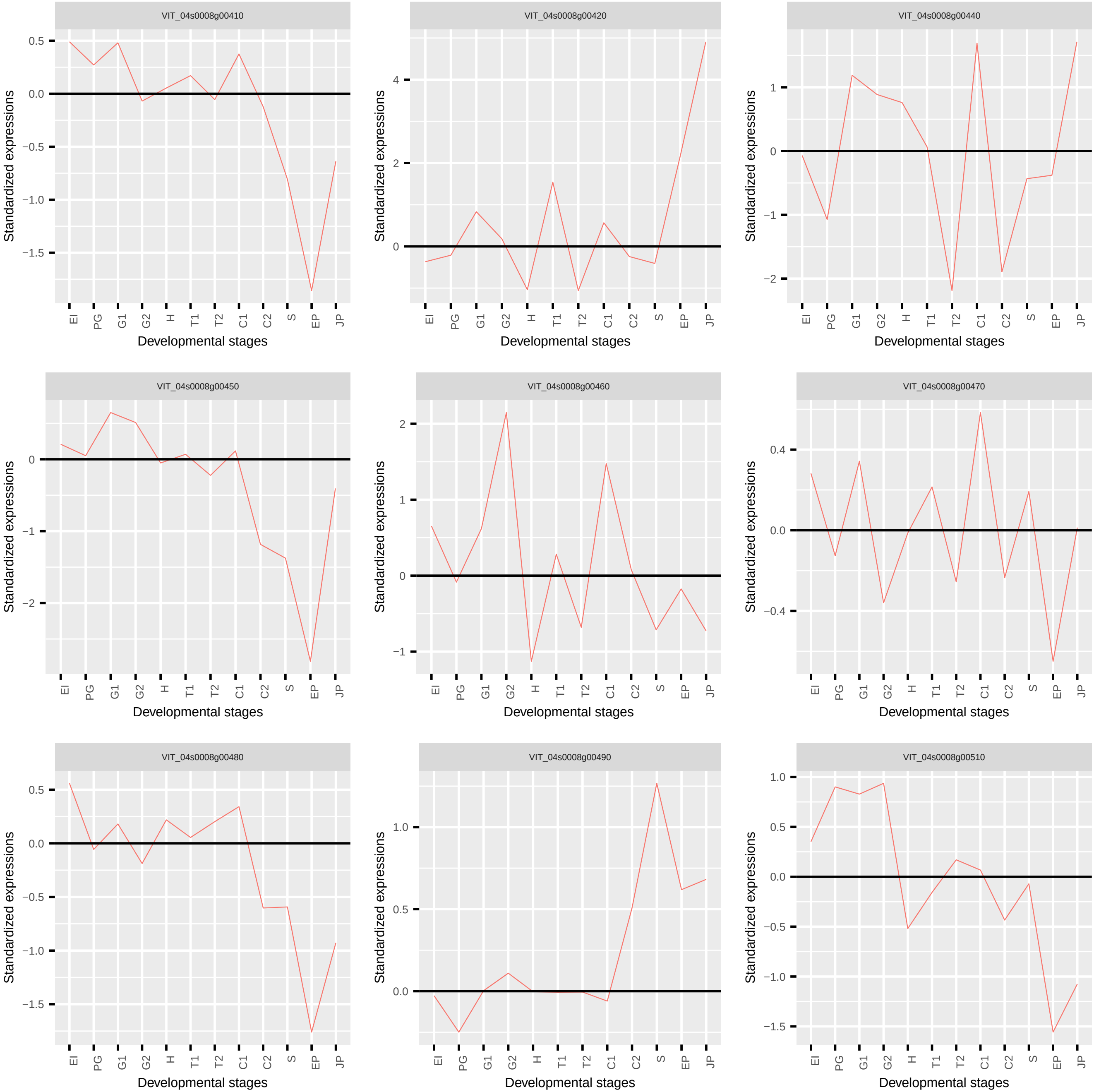
VIT_04s0008g00410
VIT_04s0008g00420
VIT_04s0008g00440
VIT_04s0008g00450
VIT_04s0008g00460
VIT_04s0008g00470
VIT_04s0008g00480
VIT_04s0008g00490
VIT_04s0008g00510
Standardized expressions
Developmental stages
EI PG G1 G2 H T1 T2 C1 C2 S EP JP

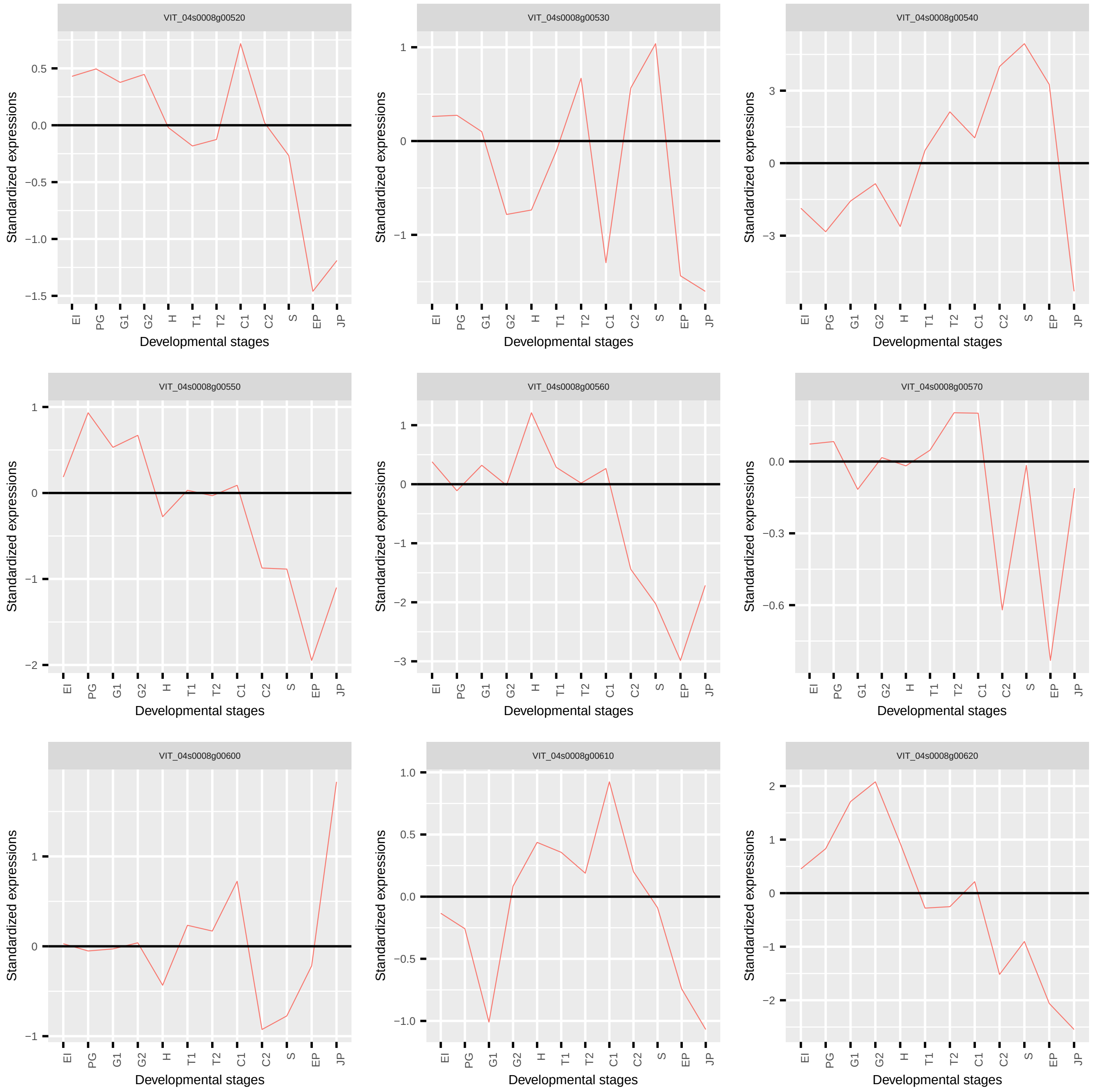
VIT_04s0008g00520
VIT_04s0008g00530
VIT_04s0008g00540
VIT_04s0008g00550
VIT_04s0008g00560
VIT_04s0008g00570
VIT_04s0008g00600
VIT_04s0008g00610
VIT_04s0008g00620
Standardized expressions
Developmental stages
EI PG G1 G2 H T1 T2 C1 C2 S EP JP

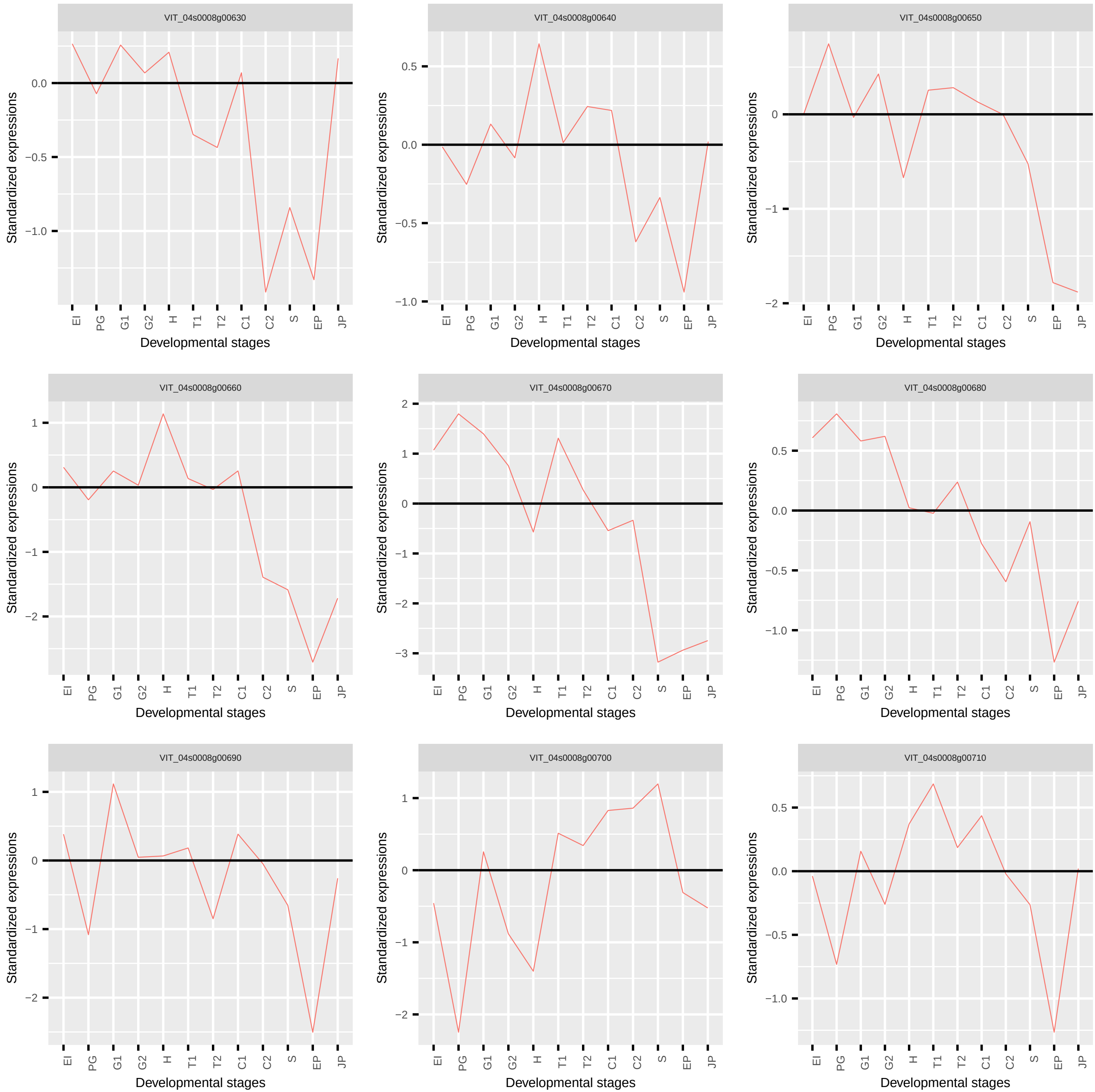
VIT_04s0008g00630
VIT_04s0008g00640
VIT_04s0008g00650
VIT_04s0008g00660
VIT_04s0008g00670
VIT_04s0008g00680
VIT_04s0008g00690
VIT_04s0008g00700
VIT_04s0008g00710
Standardized expressions
Developmental stages
EI PG G1 G2 H T1 T2 C1 C2 S EP JP

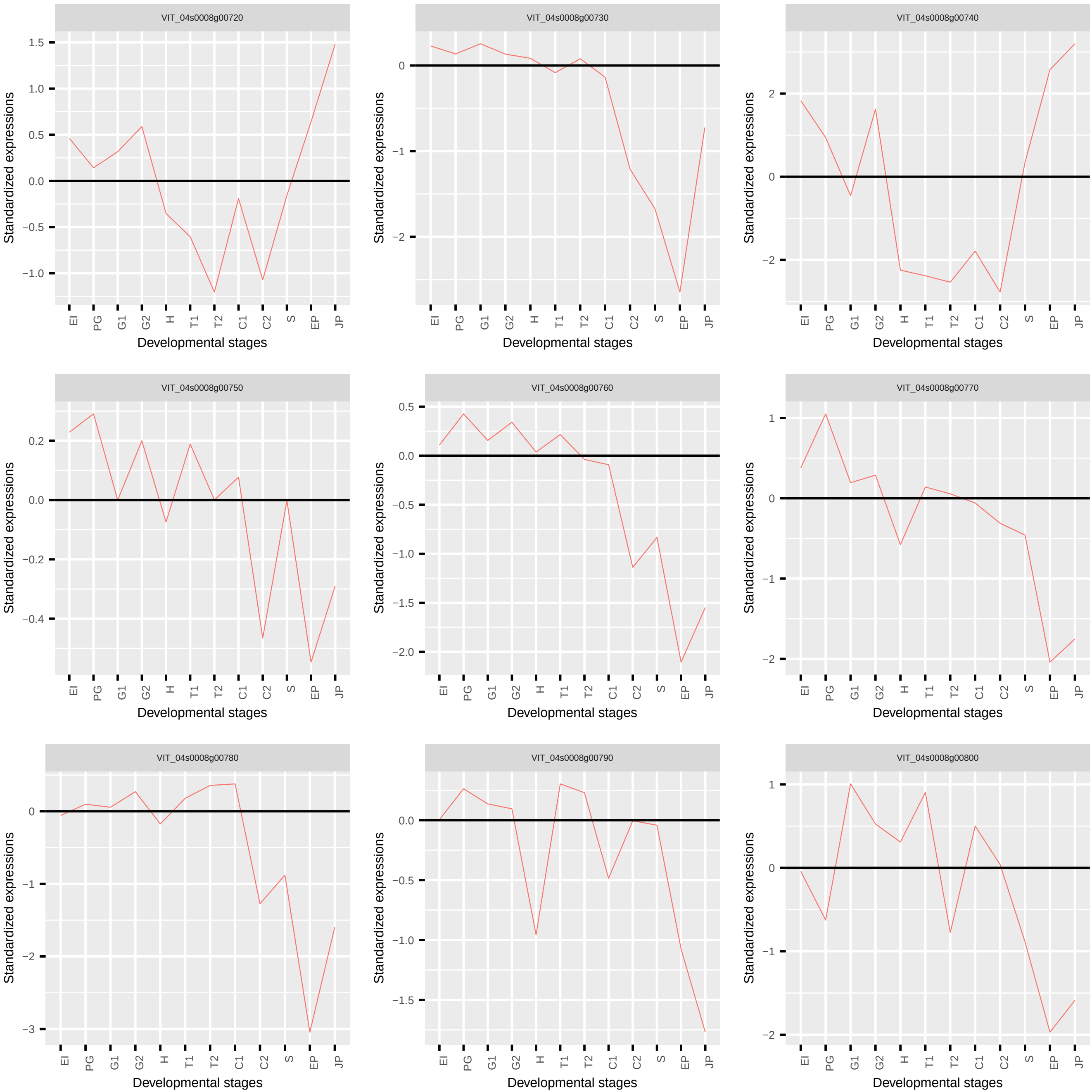
VIT_04s0008g00720
VIT_04s0008g00730
VIT_04s0008g00740
VIT_04s0008g00750
VIT_04s0008g00760
VIT_04s0008g00770
VIT_04s0008g00780
VIT_04s0008g00790
VIT_04s0008g00800
Standardized expressions
Developmental stages
EI
PG
G1
G2
H
T1
T2
C1
C2
S
EP
JP

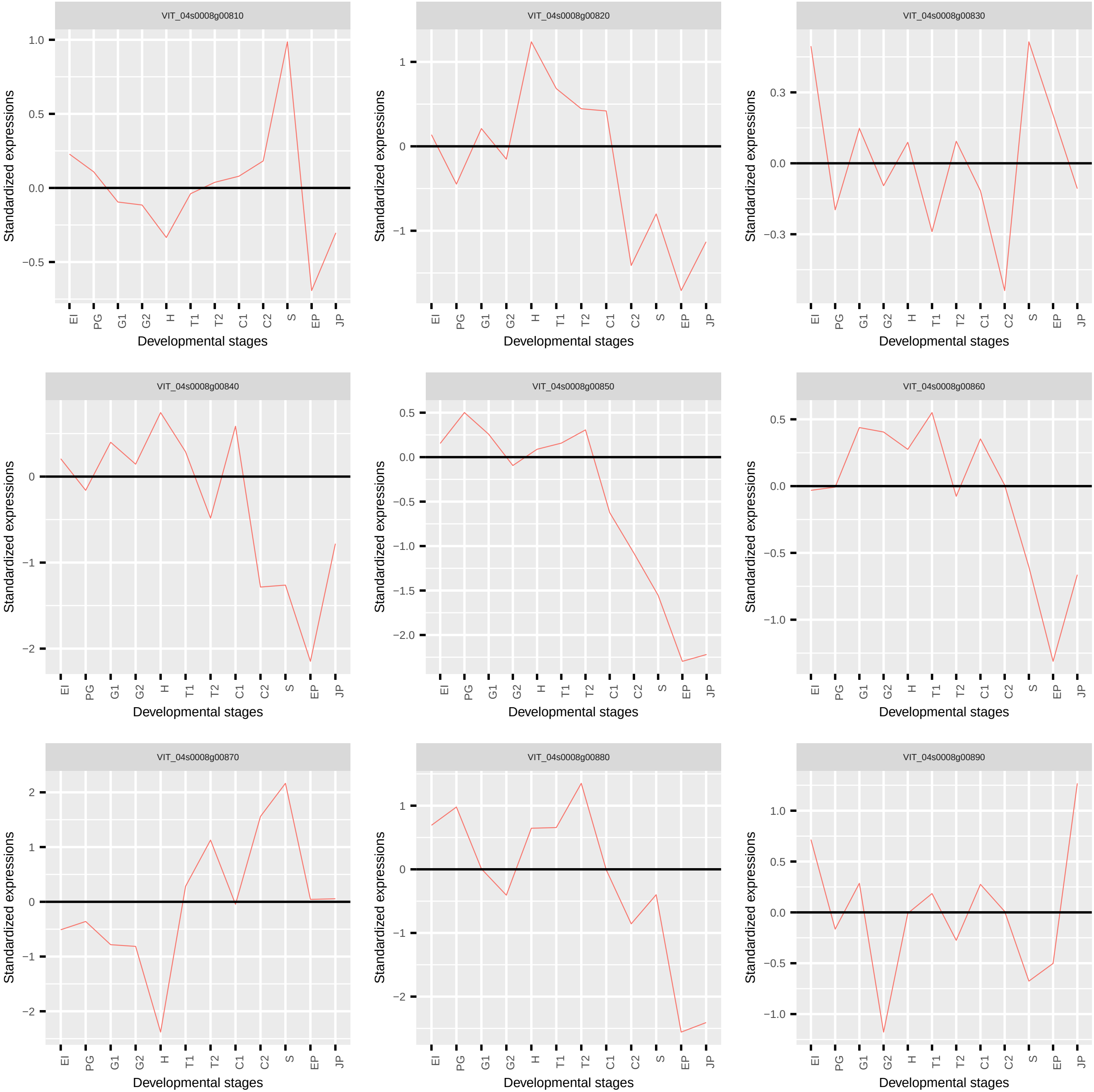

VIT_04s0008g00810
VIT_04s0008g00820
VIT_04s0008g00830
VIT_04s0008g00840
VIT_04s0008g00850
VIT_04s0008g00860
VIT_04s0008g00870
VIT_04s0008g00880
VIT_04s0008g00890
Standardized expressions
Developmental stages
EI PG G1 G2 H T1 T2 C1 C2 S EP JP
1.0 0.5 0.0 −0.5
1 0 −1
0.3 0.0 −0.3
0 −1 −2
0.5 0.0 −0.5 −1.0 −1.5 −2.0
0.5 0.0 −0.5 −1.0
2 1 0 −1 −2
1 0 −1 −2
1.0 0.5 0.0 −0.5 −1.0

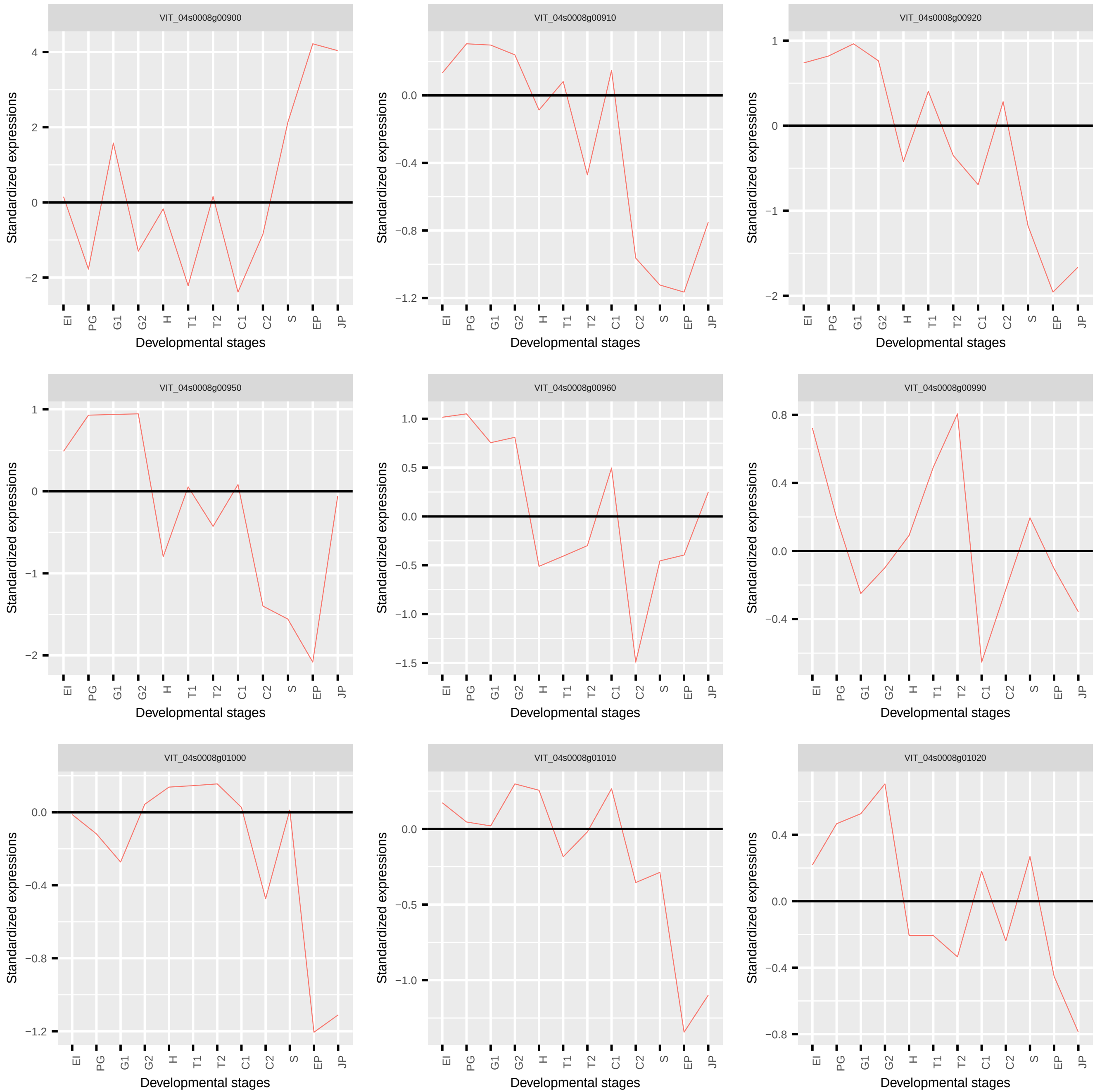

VIT_04s0008g00900
VIT_04s0008g00910
VIT_04s0008g00920
VIT_04s0008g00950
VIT_04s0008g00960
VIT_04s0008g00990
VIT_04s0008g01000
VIT_04s0008g01010
VIT_04s0008g01020
Standardized expressions
Developmental stages
EI
PG
G1
G2
H
T1
T2
C1
C2
S
EP
JP

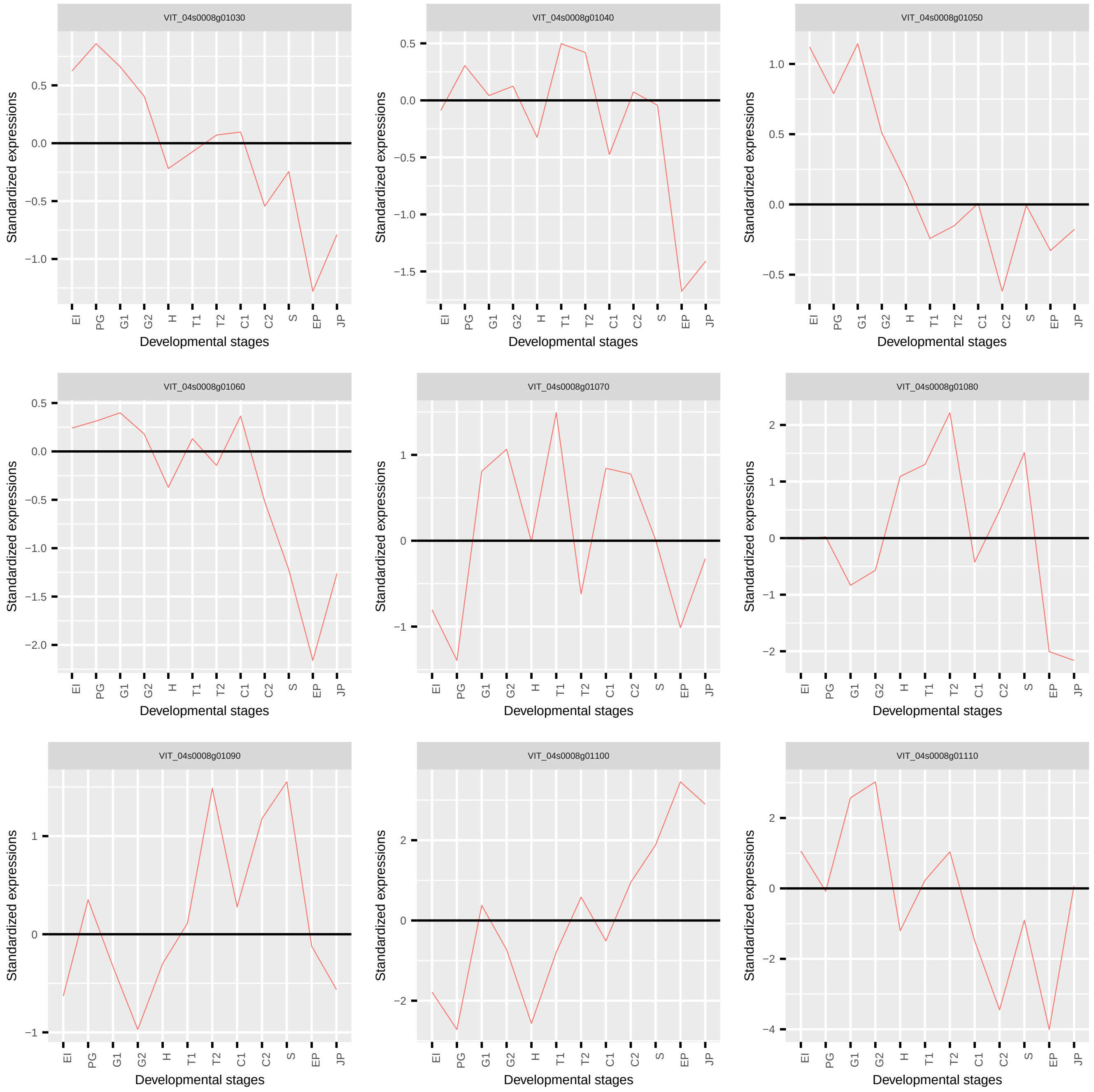

VIT_04s0008g01030
VIT_04s0008g01040
VIT_04s0008g01050
VIT_04s0008g01060
VIT_04s0008g01070
VIT_04s0008g01080
VIT_04s0008g01090
VIT_04s0008g01100
VIT_04s0008g01110
Standardized expressions
Developmental stages
EI PG G1 G2 H T1 T2 C1 C2 S EP JP

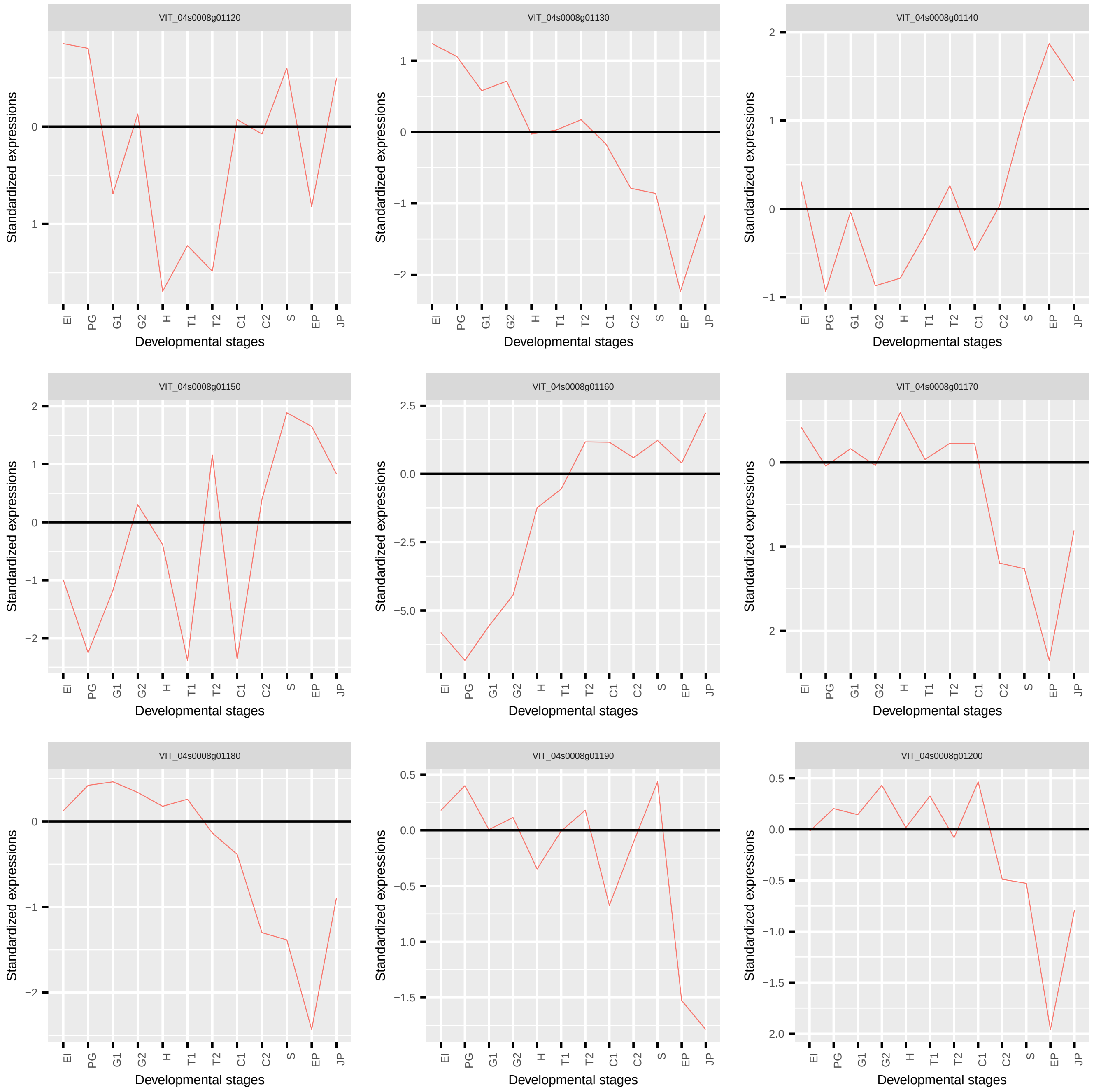
VIT_04s0008g01120
VIT_04s0008g01130
VIT_04s0008g01140
VIT_04s0008g01150
VIT_04s0008g01160
VIT_04s0008g01170
VIT_04s0008g01180
VIT_04s0008g01190
VIT_04s0008g01200
Standardized expressions
Developmental stages
EI PG G1 G2 H T1 T2 C1 C2 S EP JP

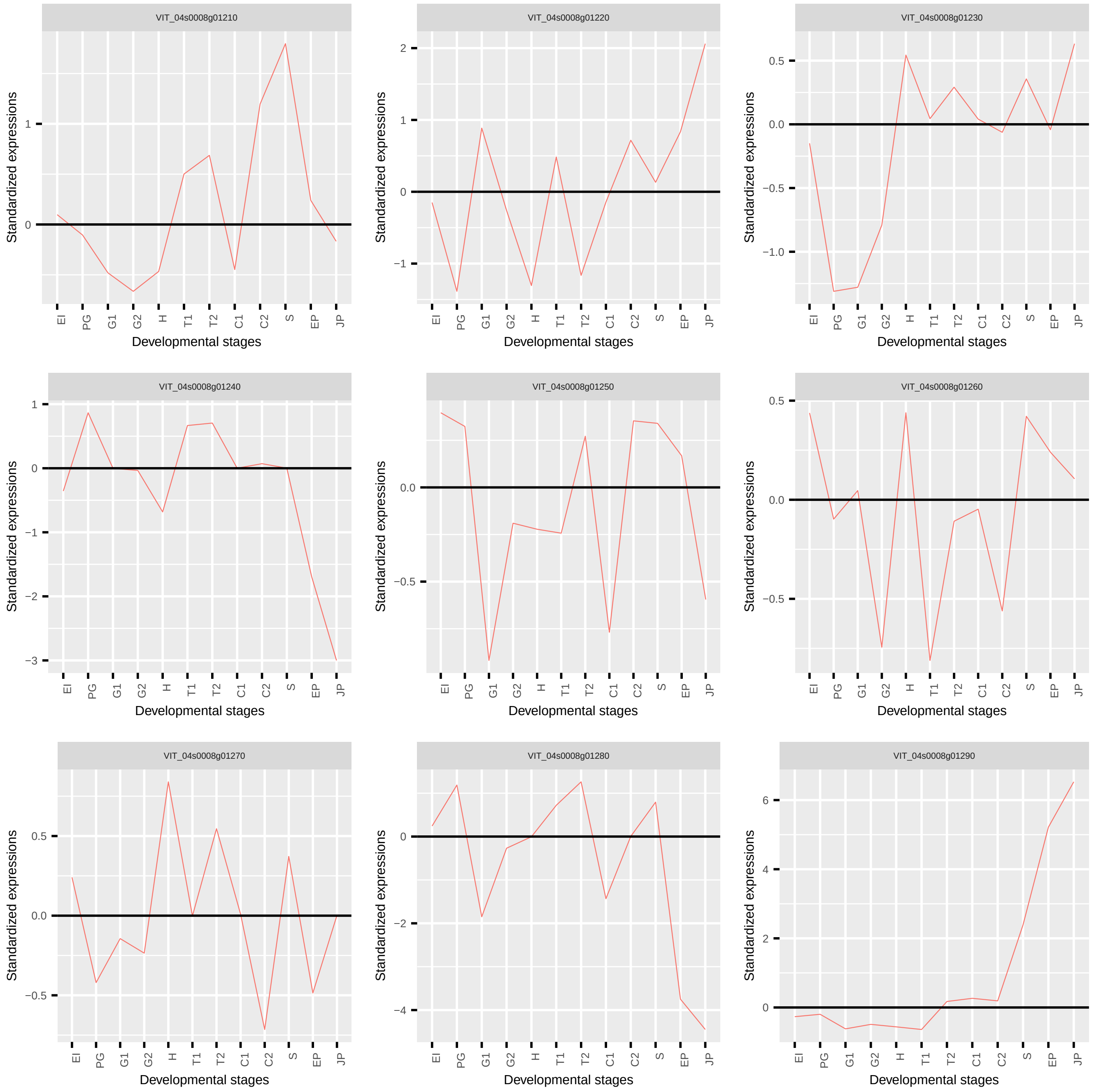
VIT_04s0008g01210
VIT_04s0008g01220
VIT_04s0008g01230
VIT_04s0008g01240
VIT_04s0008g01250
VIT_04s0008g01260
VIT_04s0008g01270
VIT_04s0008g01280
VIT_04s0008g01290
Standardized expressions
Developmental stages
EI PG G1 G2 H T1 T2 C1 C2 S EP JP

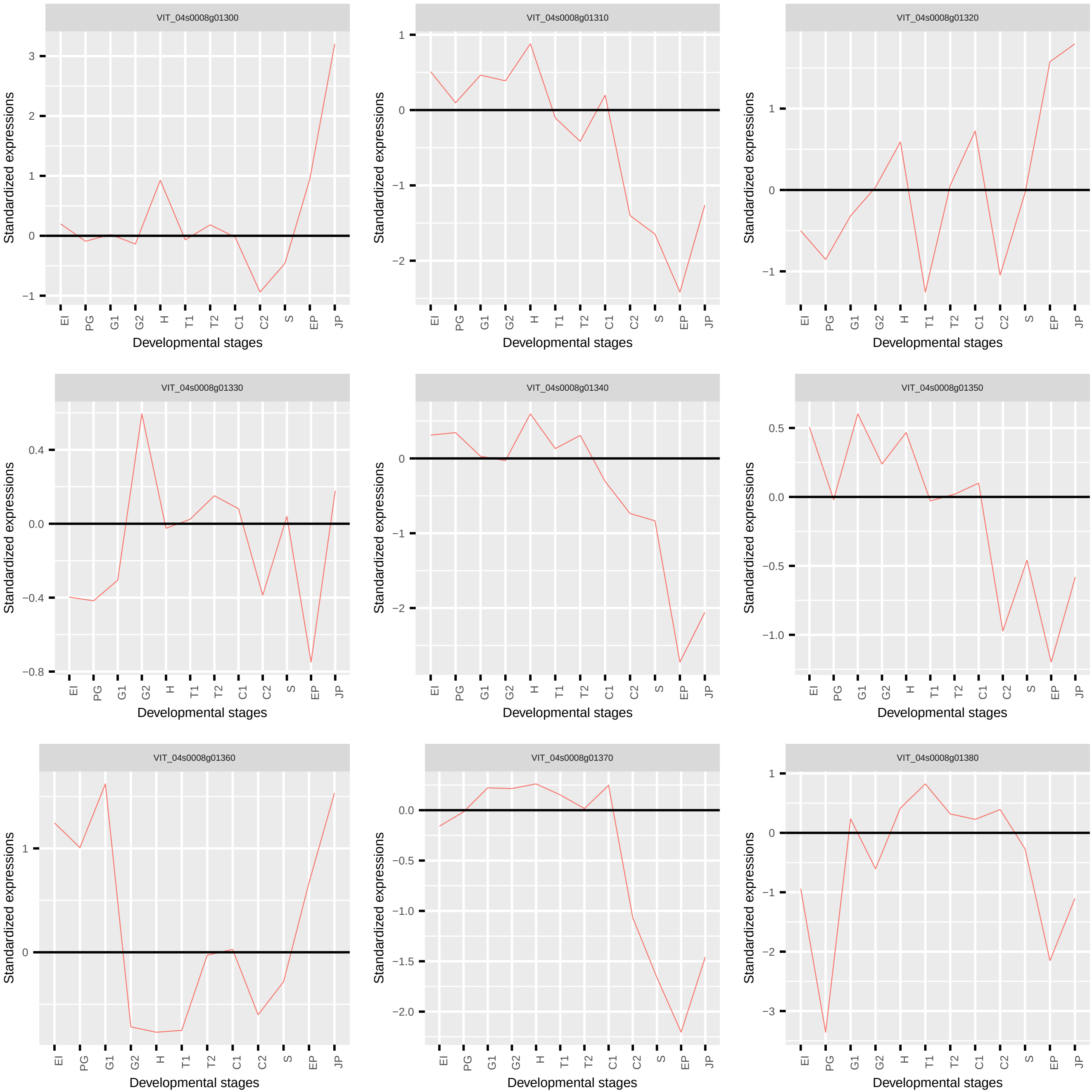
VIT_04s0008g01300
VIT_04s0008g01310
VIT_04s0008g01320
VIT_04s0008g01330
VIT_04s0008g01340
VIT_04s0008g01350
VIT_04s0008g01360
VIT_04s0008g01370
VIT_04s0008g01380
Standardized expressions
Developmental stages
EI
PG
G1
G2
H
T1
T2
C1
C2
S
EP
JP

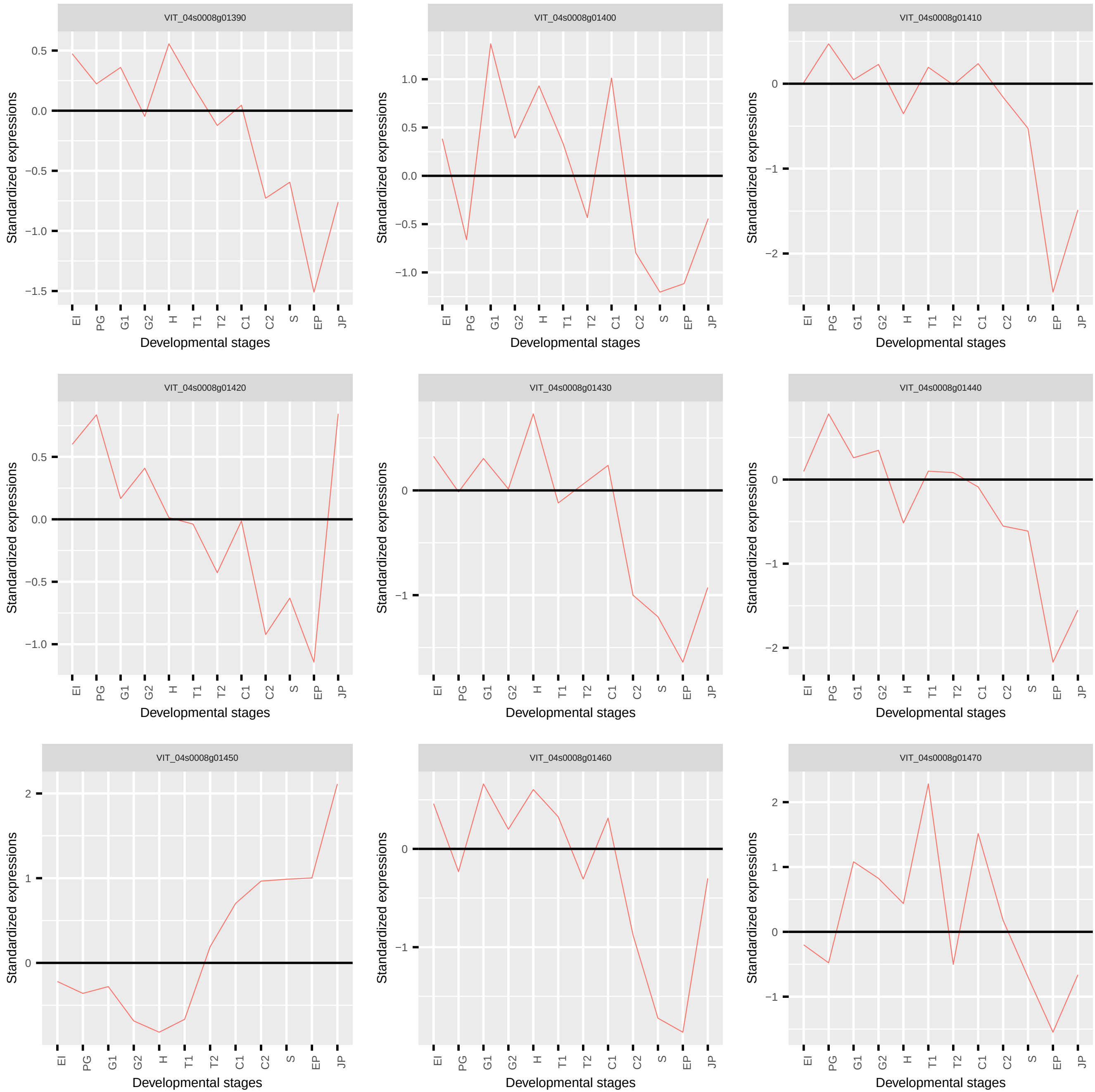
VIT_04s0008g01390
0.5
0.0
−0.5
−1.0
−1.5
Standardized expressions
EI PG G1 G2 H T1 T2 C1 C2 S EP JP
Developmental stages
VIT_04s0008g01400
1.0
0.5
0.0
−0.5
−1.0
Standardized expressions
EI PG G1 G2 H T1 T2 C1 C2 S EP JP
Developmental stages
VIT_04s0008g01410
0
−1
−2
Standardized expressions
EI PG G1 G2 H T1 T2 C1 C2 S EP JP
Developmental stages
VIT_04s0008g01420
0.5
0.0
−0.5
−1.0
Standardized expressions
EI PG G1 G2 H T1 T2 C1 C2 S EP JP
Developmental stages
VIT_04s0008g01430
0
−1
Standardized expressions
EI PG G1 G2 H T1 T2 C1 C2 S EP JP
Developmental stages
VIT_04s0008g01440
0
−1
−2
Standardized expressions
EI PG G1 G2 H T1 T2 C1 C2 S EP JP
Developmental stages
VIT_04s0008g01450
2
1
0
Standardized expressions
EI PG G1 G2 H T1 T2 C1 C2 S EP JP
Developmental stages
VIT_04s0008g01460
0
−1
Standardized expressions
EI PG G1 G2 H T1 T2 C1 C2 S EP JP
Developmental stages
VIT_04s0008g01470
2
1
0
−1
Standardized expressions
EI PG G1 G2 H T1 T2 C1 C2 S EP JP
Developmental stages

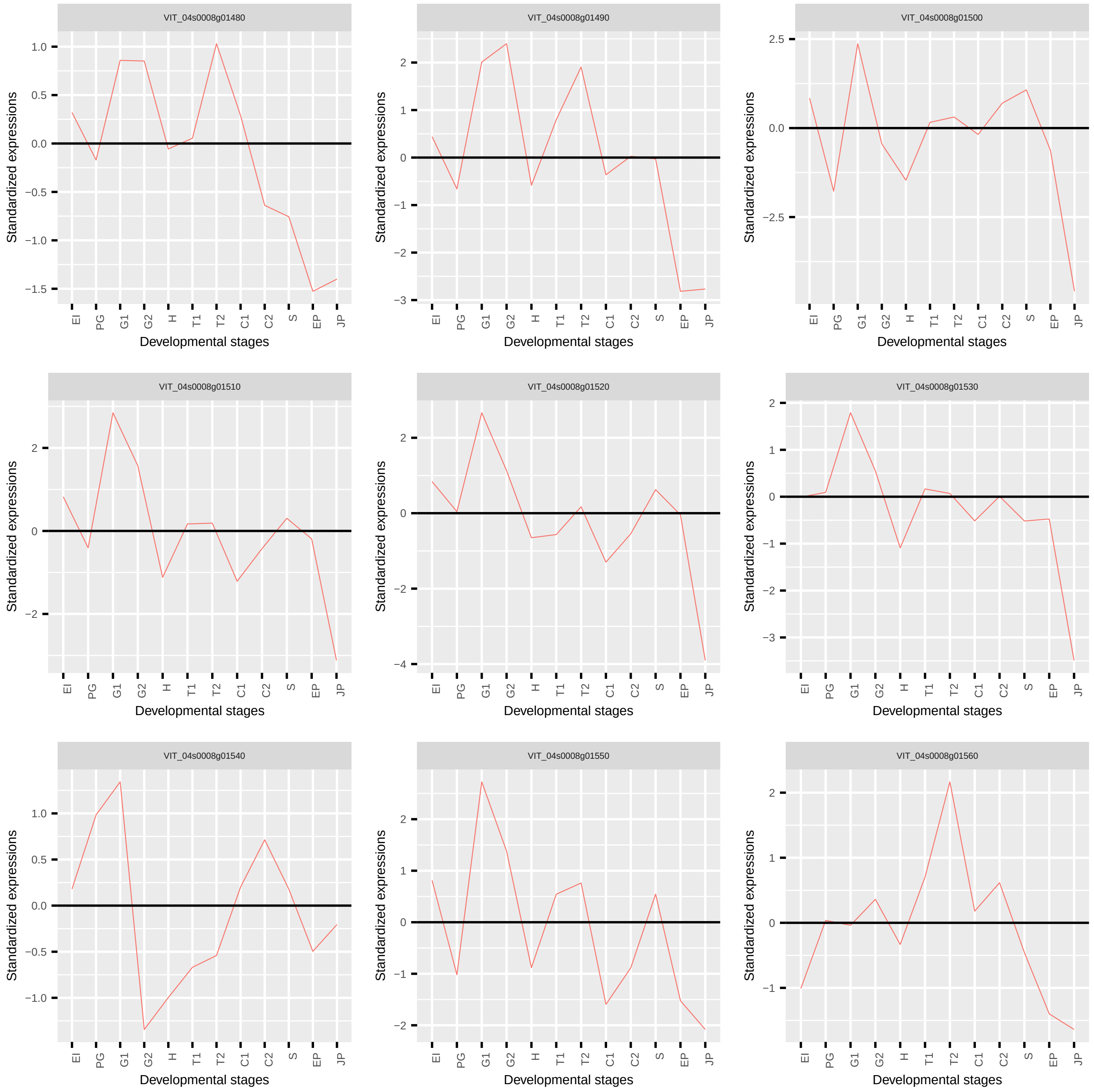
VIT_04s0008g01480
1.0
0.5
0.0
−0.5
−1.0
−1.5
Standardized expressions
EI PG G1 G2 H T1 T2 C1 C2 S EP JP
Developmental stages
VIT_04s0008g01490
2
1
0
−1
−2
−3
Standardized expressions
EI PG G1 G2 H T1 T2 C1 C2 S EP JP
Developmental stages
VIT_04s0008g01500
2.5
0.0
−2.5
Standardized expressions
EI PG G1 G2 H T1 T2 C1 C2 S EP JP
Developmental stages
VIT_04s0008g01510
2
0
−2
Standardized expressions
EI PG G1 G2 H T1 T2 C1 C2 S EP JP
Developmental stages
VIT_04s0008g01520
2
0
−2
−4
Standardized expressions
EI PG G1 G2 H T1 T2 C1 C2 S EP JP
Developmental stages
VIT_04s0008g01530
2
1
0
−1
−2
−3
Standardized expressions
EI PG G1 G2 H T1 T2 C1 C2 S EP JP
Developmental stages
VIT_04s0008g01540
1.0
0.5
0.0
−0.5
−1.0
Standardized expressions
EI PG G1 G2 H T1 T2 C1 C2 S EP JP
Developmental stages
VIT_04s0008g01550
2
1
0
−1
−2
Standardized expressions
EI PG G1 G2 H T1 T2 C1 C2 S EP JP
Developmental stages
VIT_04s0008g01560
2
1
0
−1
Standardized expressions
EI PG G1 G2 H T1 T2 C1 C2 S EP JP
Developmental stages

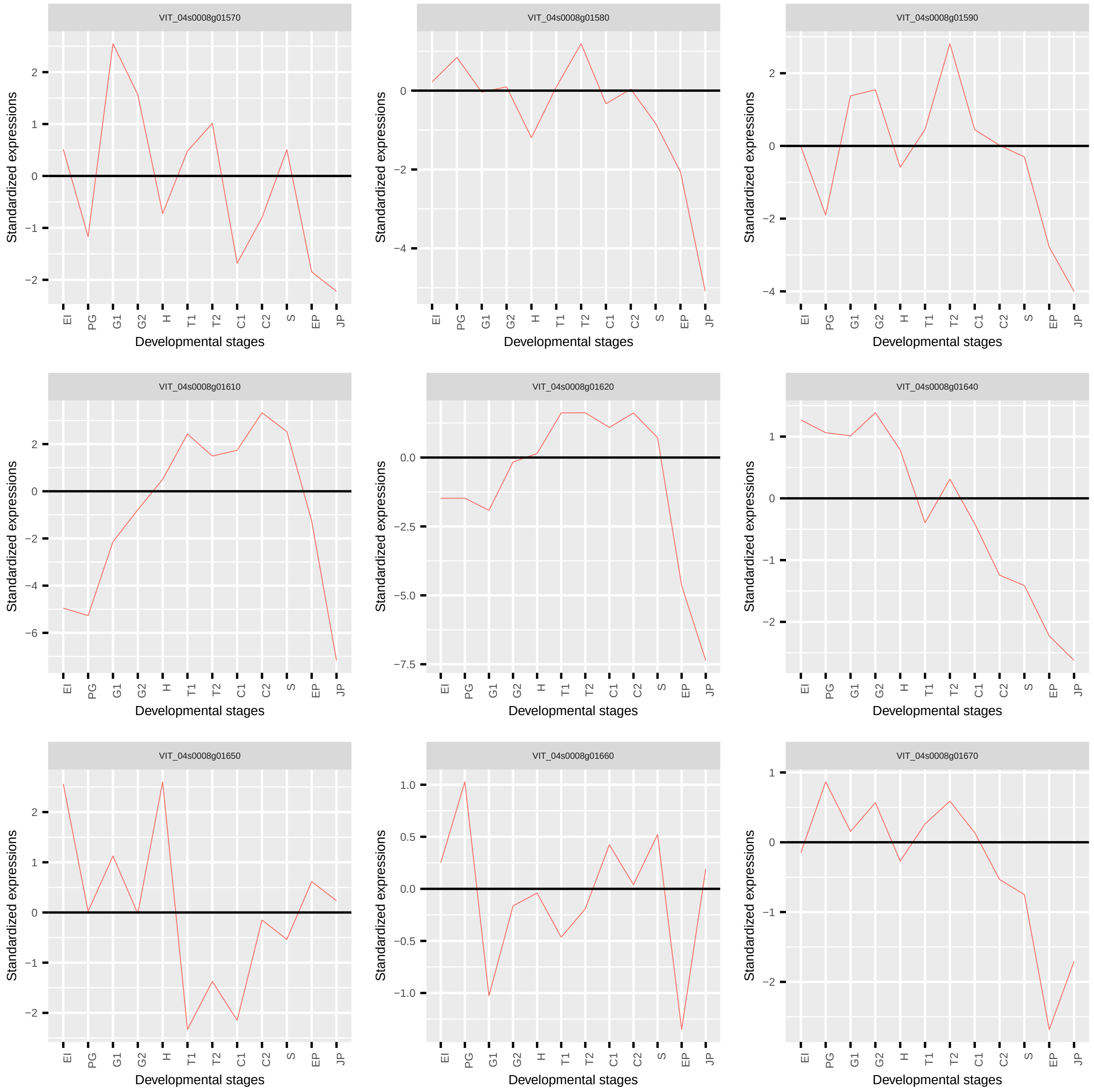
VIT_04s0008g01570
VIT_04s0008g01580
VIT_04s0008g01590
VIT_04s0008g01610
VIT_04s0008g01620
VIT_04s0008g01640
VIT_04s0008g01650
VIT_04s0008g01660
VIT_04s0008g01670
Standardized expressions
Developmental stages
EI PG G1 G2 H T1 T2 C1 C2 S EP JP

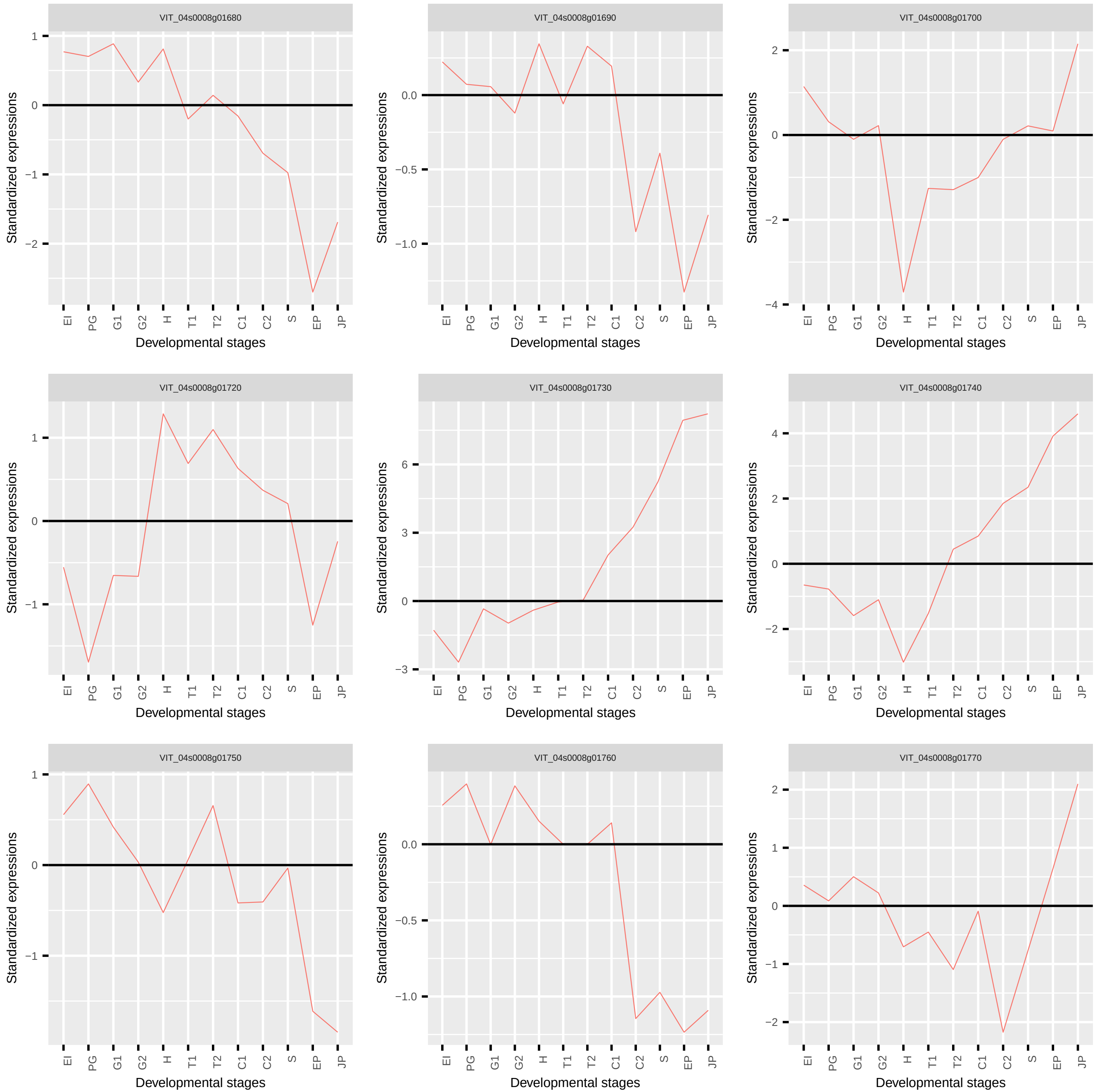
VIT_04s0008g01680
VIT_04s0008g01690
VIT_04s0008g01700
VIT_04s0008g01720
VIT_04s0008g01730
VIT_04s0008g01740
VIT_04s0008g01750
VIT_04s0008g01760
VIT_04s0008g01770
Standardized expressions
Developmental stages
EI PG G1 G2 H T1 T2 C1 C2 S EP JP
1 0 −1 −2
0.0 −0.5 −1.0
2 0 −2 −4
6 3 0 −3
4 2 0 −2

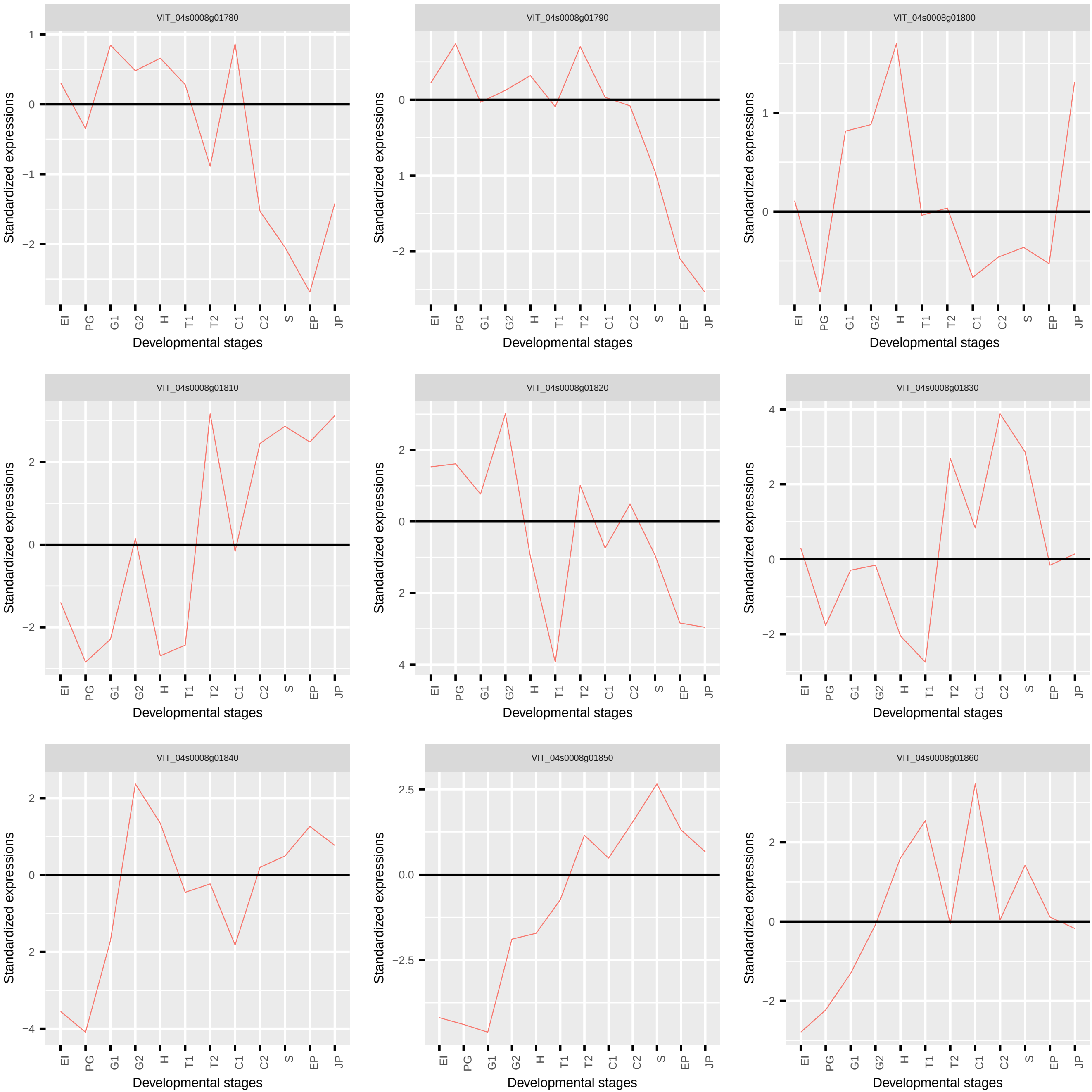

VIT_04s0008g01780
VIT_04s0008g01790
VIT_04s0008g01800
VIT_04s0008g01810
VIT_04s0008g01820
VIT_04s0008g01830
VIT_04s0008g01840
VIT_04s0008g01850
VIT_04s0008g01860
Standardized expressions
Developmental stages
EI PG G1 G2 H T1 T2 C1 C2 S EP JP

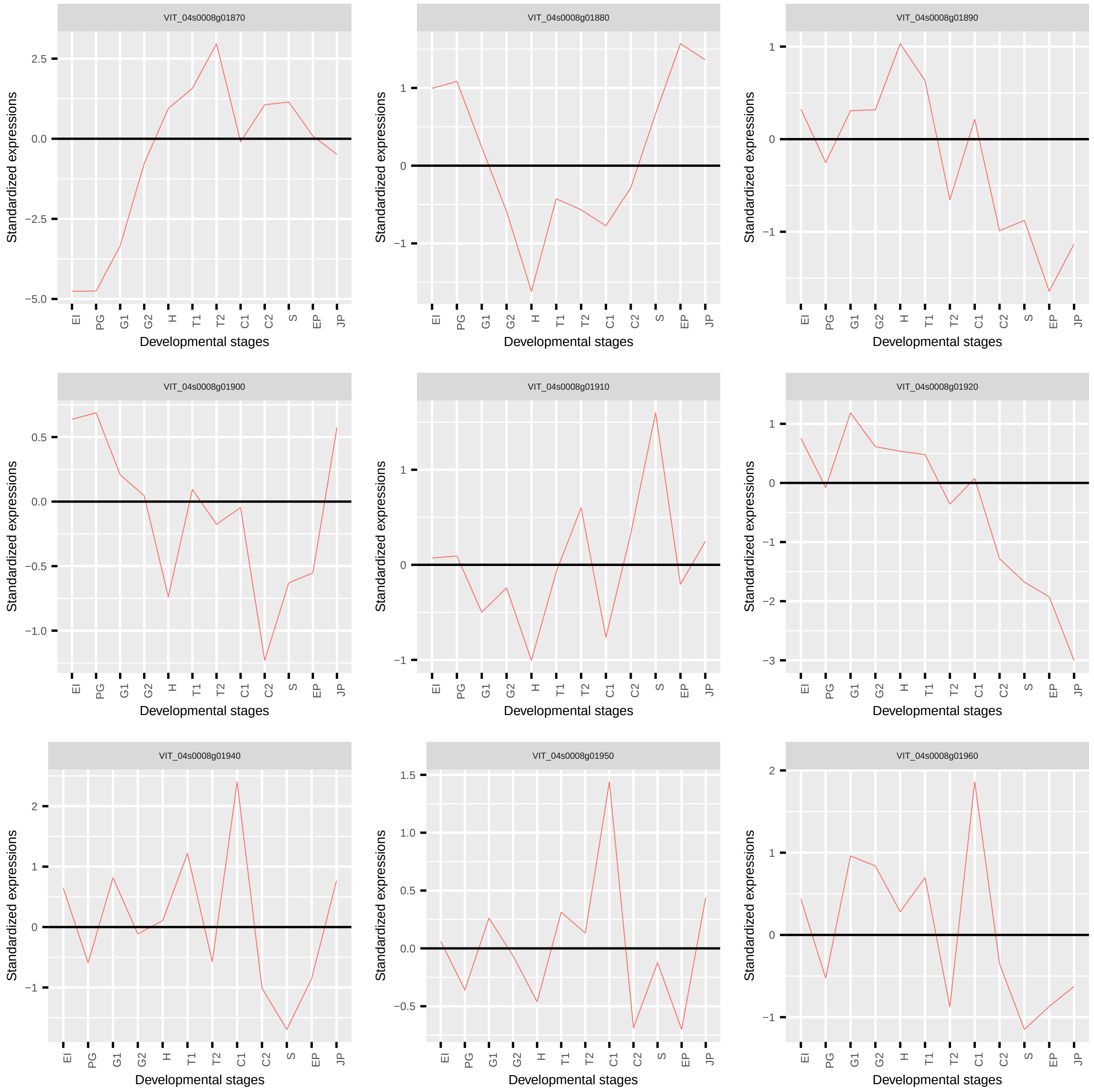
VIT_04s0008g01870
VIT_04s0008g01880
VIT_04s0008g01890
VIT_04s0008g01900
VIT_04s0008g01910
VIT_04s0008g01920
VIT_04s0008g01940
VIT_04s0008g01950
VIT_04s0008g01960
Standardized expressions
Developmental stages
EI PG G1 G2 H T1 T2 C1 C2 S EP JP

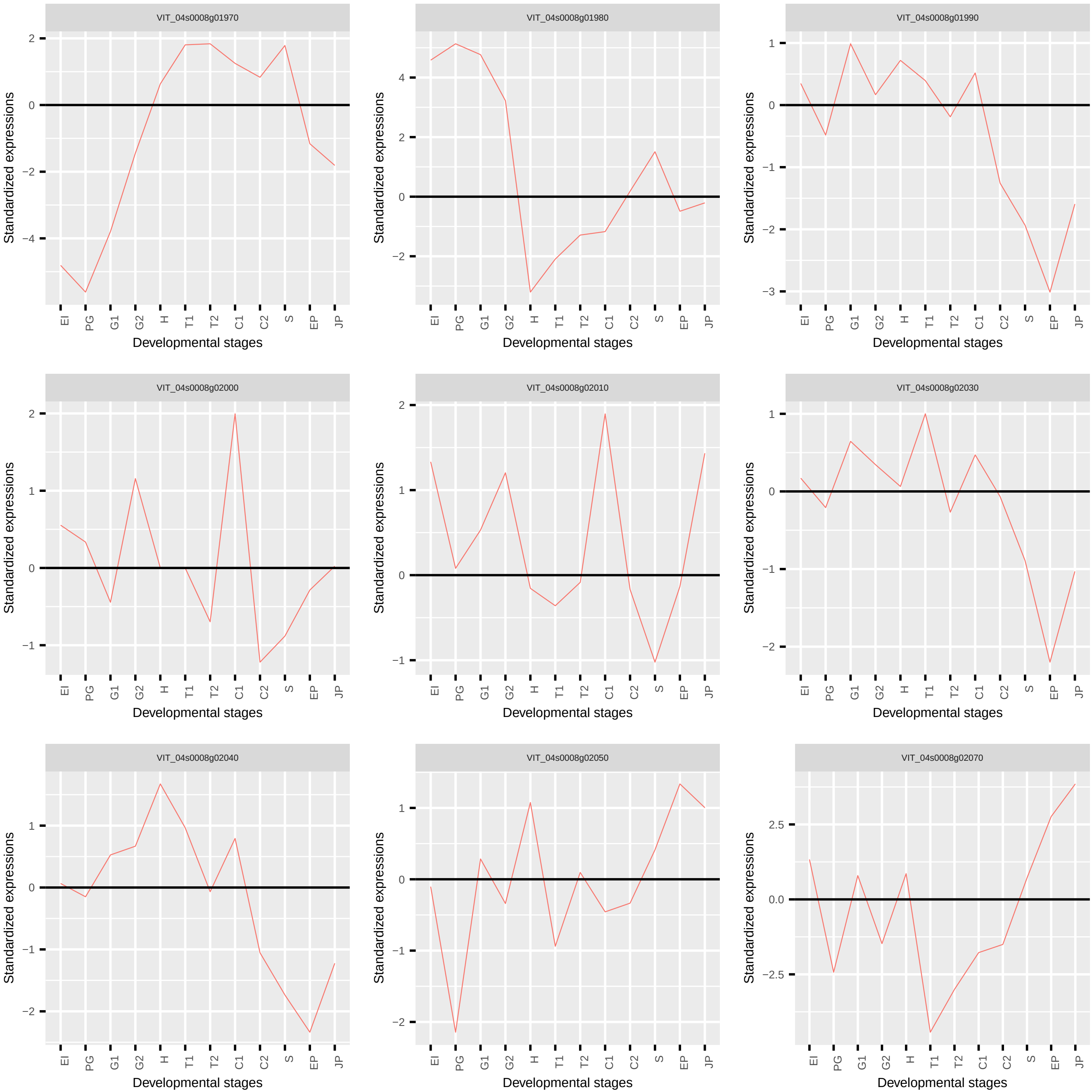
VIT_04s0008g01970
VIT_04s0008g01980
VIT_04s0008g01990
VIT_04s0008g02000
VIT_04s0008g02010
VIT_04s0008g02030
VIT_04s0008g02040
VIT_04s0008g02050
VIT_04s0008g02070
Standardized expressions
Developmental stages
EI PG G1 G2 H T1 T2 C1 C2 S EP JP

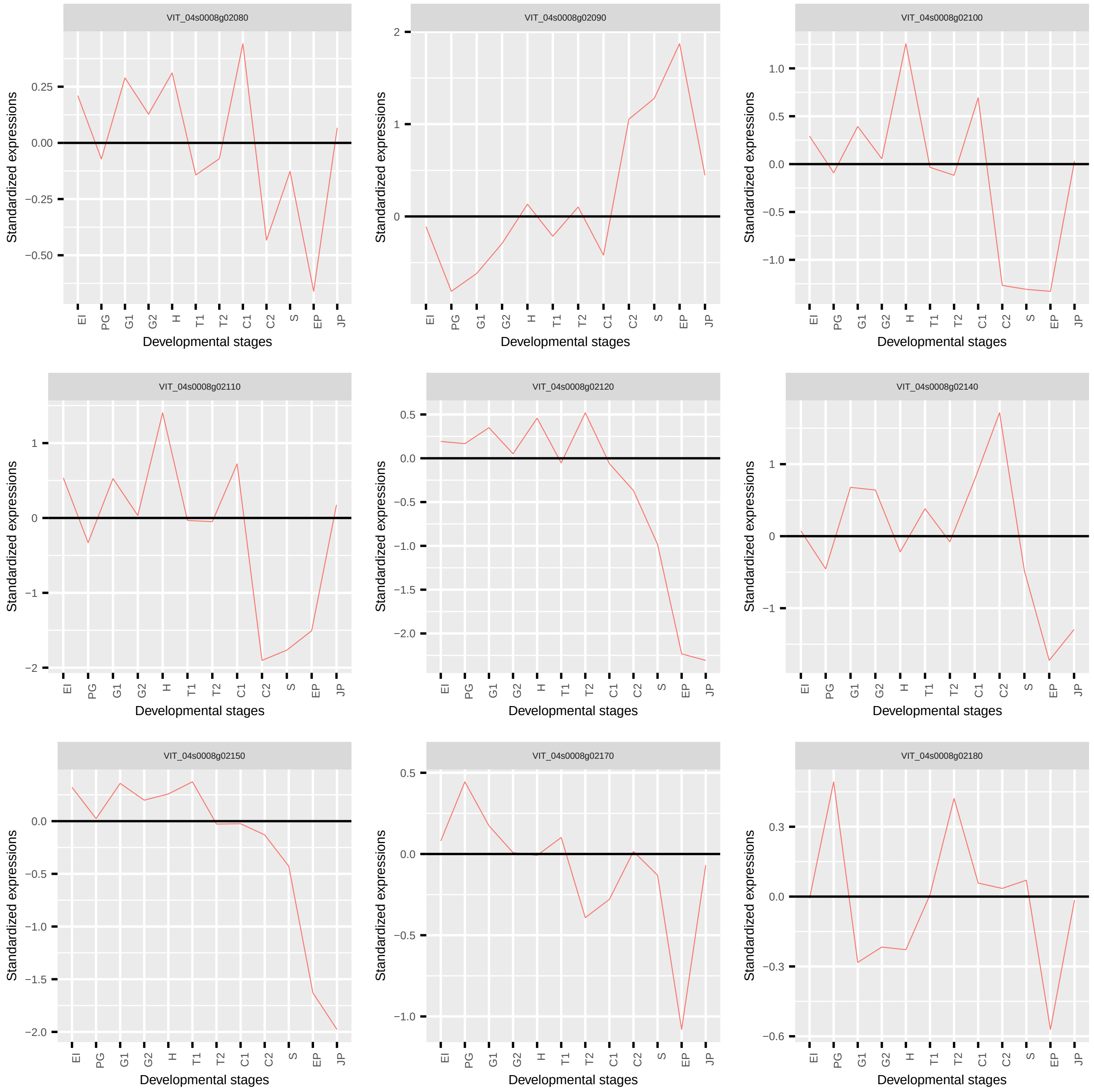
VIT_04s0008g02080
VIT_04s0008g02090
VIT_04s0008g02100
VIT_04s0008g02110
VIT_04s0008g02120
VIT_04s0008g02140
VIT_04s0008g02150
VIT_04s0008g02170
VIT_04s0008g02180
Standardized expressions
Developmental stages
EI
PG
G1
G2
H
T1
T2
C1
C2
S
EP
JP
0.25
0.00
−0.25
−0.50
2
1
0
1.0
0.5
0.0
−0.5
−1.0
−1
−2
−1.5
−2.0
0.3
−0.3
−0.6

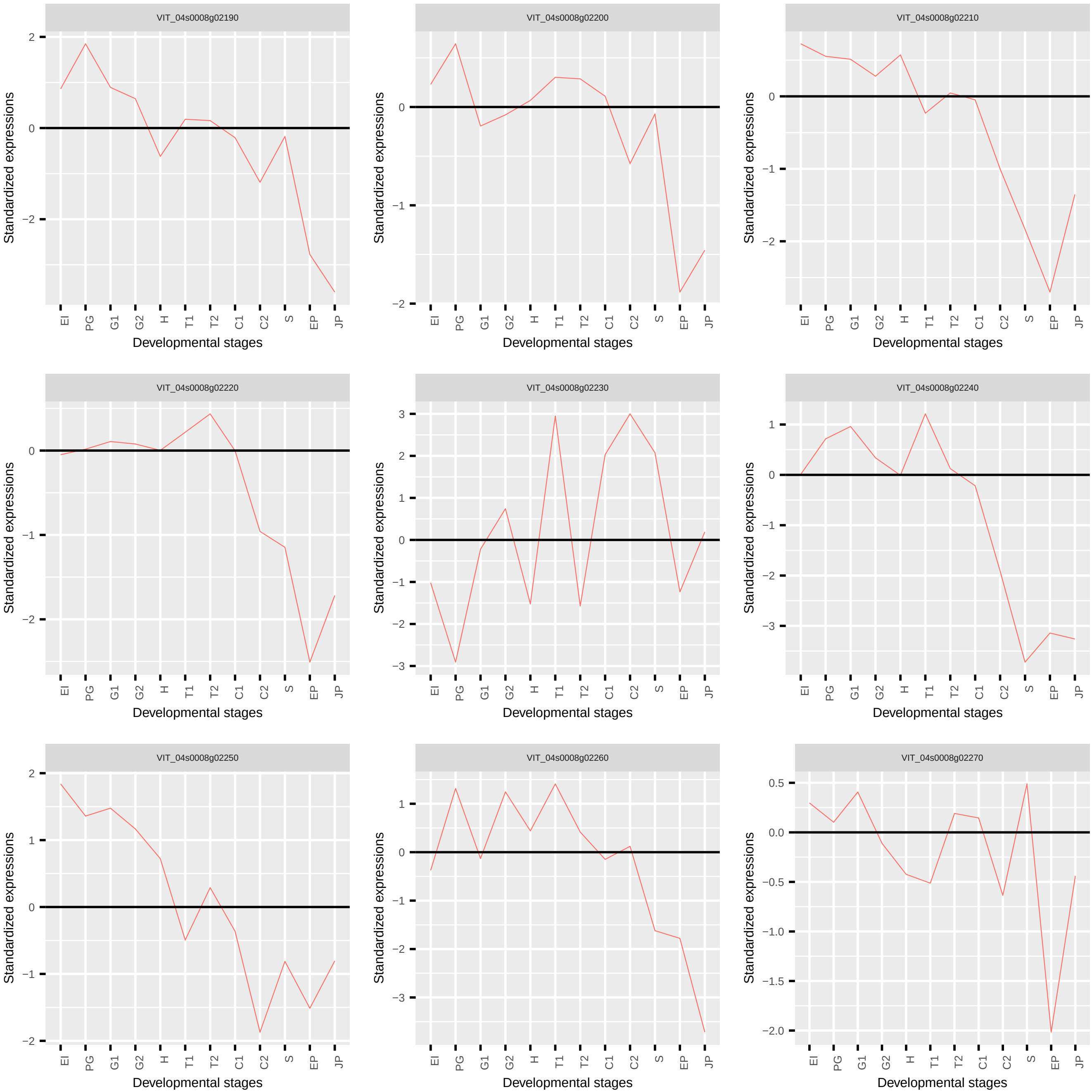
VIT_04s0008g02190
VIT_04s0008g02200
VIT_04s0008g02210
VIT_04s0008g02220
VIT_04s0008g02230
VIT_04s0008g02240
VIT_04s0008g02250
VIT_04s0008g02260
VIT_04s0008g02270
Standardized expressions
Developmental stages
EI
PG
G1
G2
H
T1
T2
C1
C2
S
EP
JP
2
1
0
−1
−2
−3
3
0.5
0.0
−0.5
−1.0
−1.5
−2.0

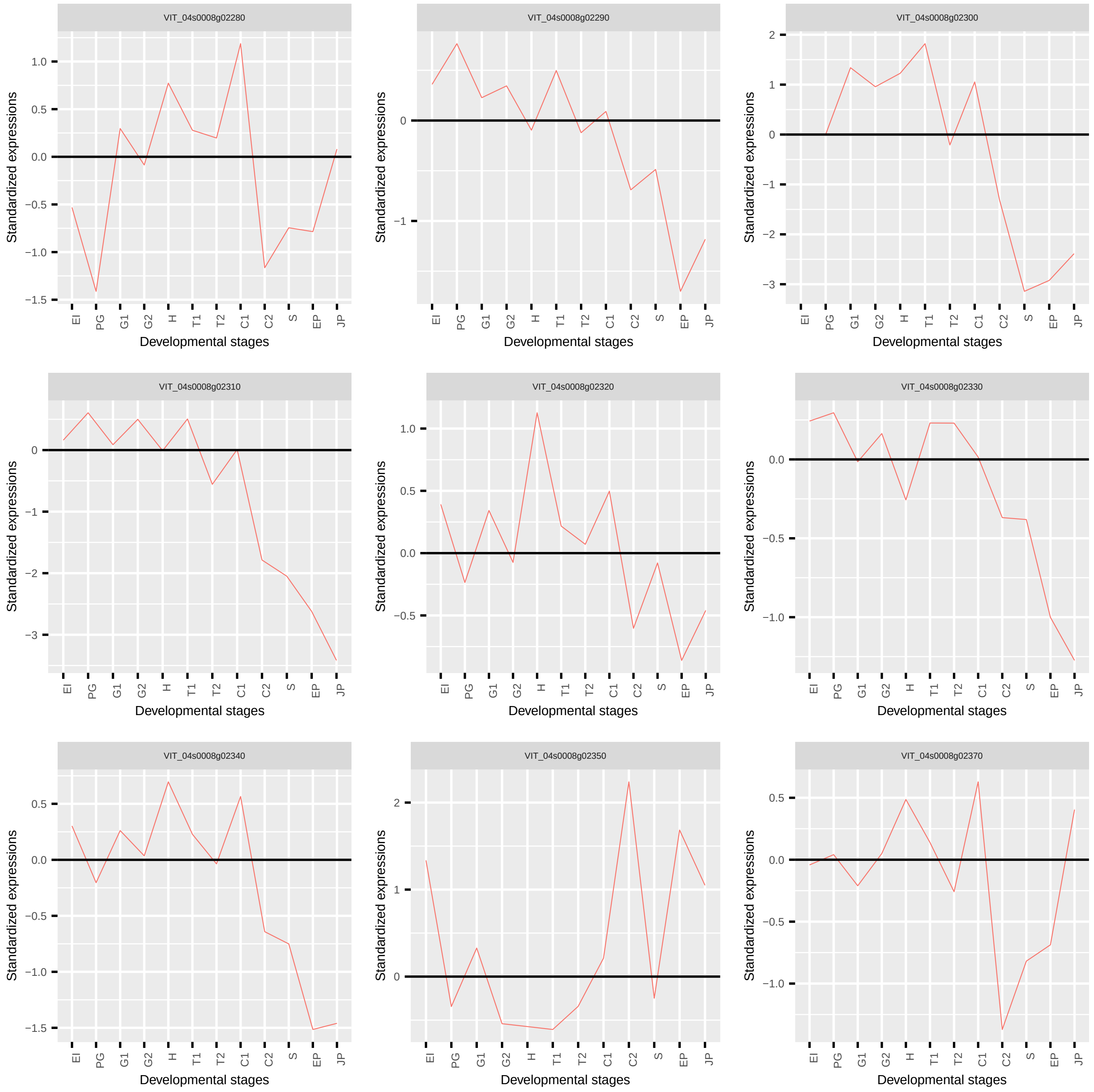
VIT_04s0008g02280
Standardized expressions
1.0
0.5
0.0
−0.5
−1.0
−1.5
EI PG G1 G2 H T1 T2 C1 C2 S EP JP
Developmental stages
VIT_04s0008g02290
Standardized expressions
0
−1
EI PG G1 G2 H T1 T2 C1 C2 S EP JP
Developmental stages
VIT_04s0008g02300
Standardized expressions
2
1
0
−1
−2
−3
EI PG G1 G2 H T1 T2 C1 C2 S EP JP
Developmental stages
VIT_04s0008g02310
Standardized expressions
0
−1
−2
−3
EI PG G1 G2 H T1 T2 C1 C2 S EP JP
Developmental stages
VIT_04s0008g02320
Standardized expressions
1.0
0.5
0.0
−0.5
EI PG G1 G2 H T1 T2 C1 C2 S EP JP
Developmental stages
VIT_04s0008g02330
Standardized expressions
0.0
−0.5
−1.0
EI PG G1 G2 H T1 T2 C1 C2 S EP JP
Developmental stages
VIT_04s0008g02340
Standardized expressions
0.5
0.0
−0.5
−1.0
−1.5
EI PG G1 G2 H T1 T2 C1 C2 S EP JP
Developmental stages
VIT_04s0008g02350
Standardized expressions
2
1
0
EI PG G1 G2 H T1 T2 C1 C2 S EP JP
Developmental stages
VIT_04s0008g02370
Standardized expressions
0.5
0.0
−0.5
−1.0
EI PG G1 G2 H T1 T2 C1 C2 S EP JP
Developmental stages

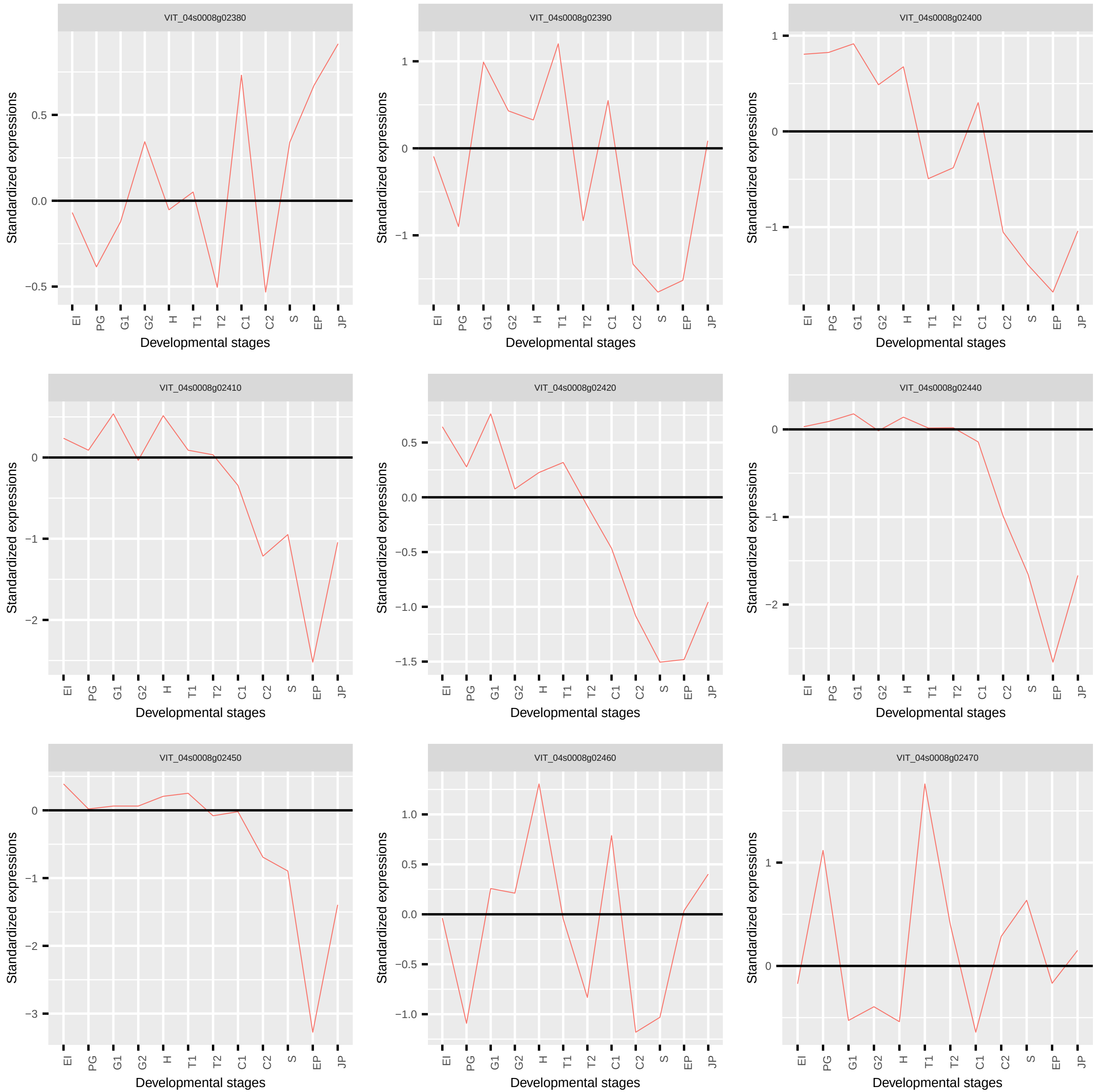

VIT_04s0008g02380
VIT_04s0008g02390
VIT_04s0008g02400
VIT_04s0008g02410
VIT_04s0008g02420
VIT_04s0008g02440
VIT_04s0008g02450
VIT_04s0008g02460
VIT_04s0008g02470
Standardized expressions
Developmental stages
EI PG G1 G2 H T1 T2 C1 C2 S EP JP

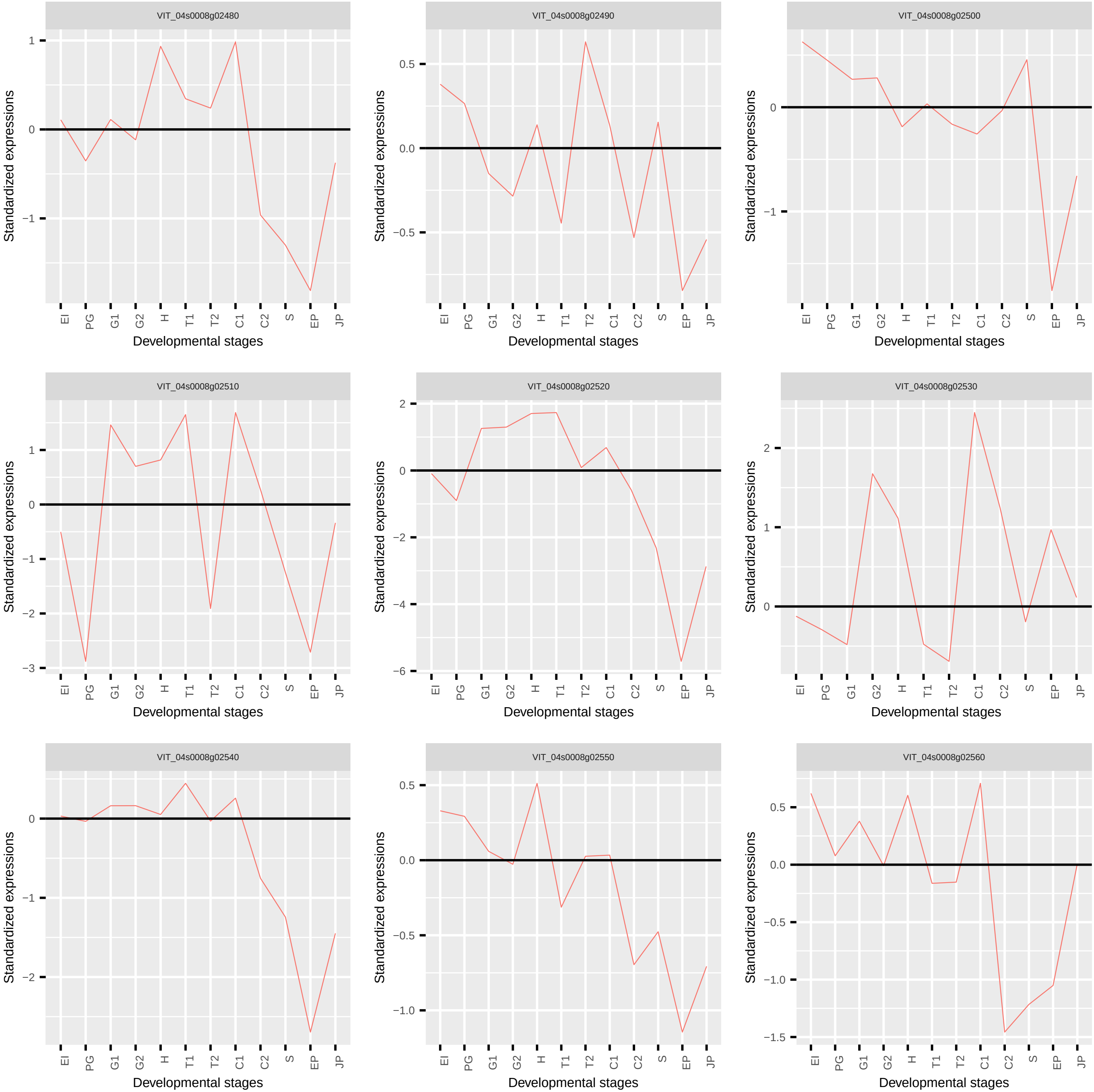
VIT_04s0008g02480
VIT_04s0008g02490
VIT_04s0008g02500
VIT_04s0008g02510
VIT_04s0008g02520
VIT_04s0008g02530
VIT_04s0008g02540
VIT_04s0008g02550
VIT_04s0008g02560
Standardized expressions
Developmental stages
EI
PG
G1
G2
H
T1
T2
C1
C2
S
EP
JP

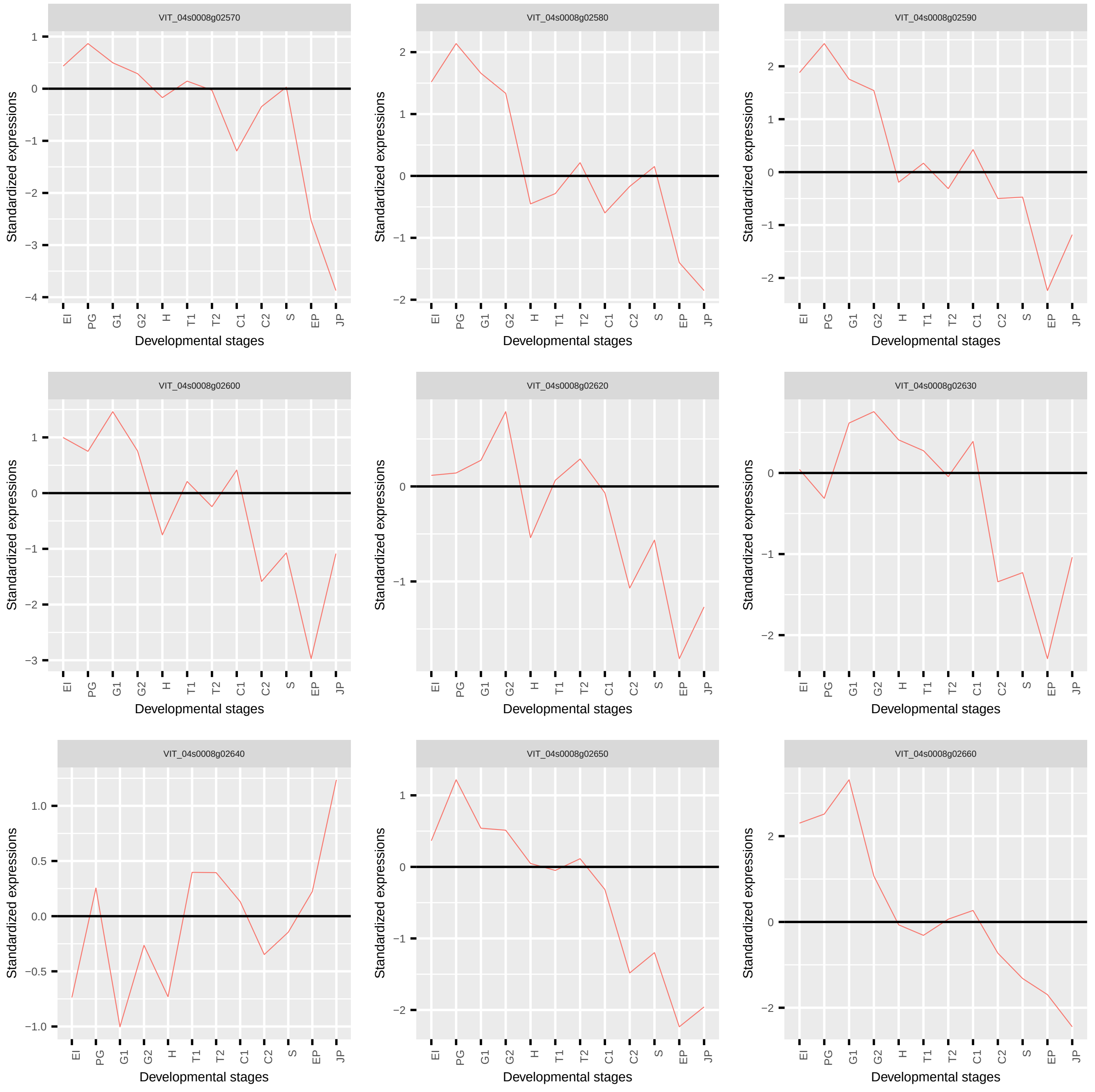

VIT_04s0008g02570
VIT_04s0008g02580
VIT_04s0008g02590
VIT_04s0008g02600
VIT_04s0008g02620
VIT_04s0008g02630
VIT_04s0008g02640
VIT_04s0008g02650
VIT_04s0008g02660
Standardized expressions
Developmental stages
EI PG G1 G2 H T1 T2 C1 C2 S EP JP

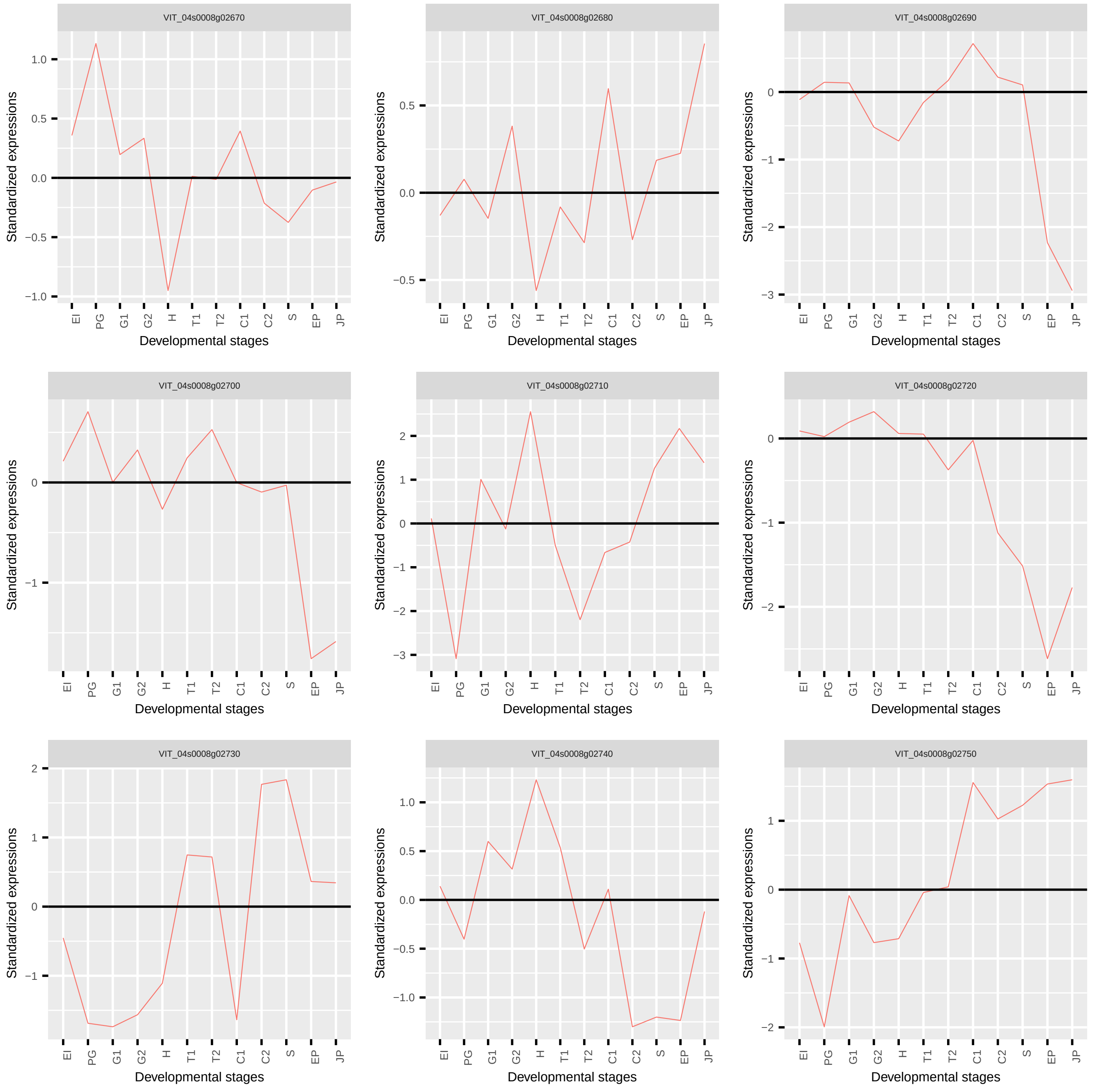
VIT_04s0008g02670
VIT_04s0008g02680
VIT_04s0008g02690
VIT_04s0008g02700
VIT_04s0008g02710
VIT_04s0008g02720
VIT_04s0008g02730
VIT_04s0008g02740
VIT_04s0008g02750
Standardized expressions
Developmental stages
EI PG G1 G2 H T1 T2 C1 C2 S EP JP
1.0 0.5 0.0 −0.5 −1.0
0.5 0.0 −0.5
0 −1 −2 −3
0 −1
2 1 0 −1 −2 −3
0 −1 −2
2 1 0 −1
1.0 0.5 0.0 −0.5 −1.0
1 0 −1 −2

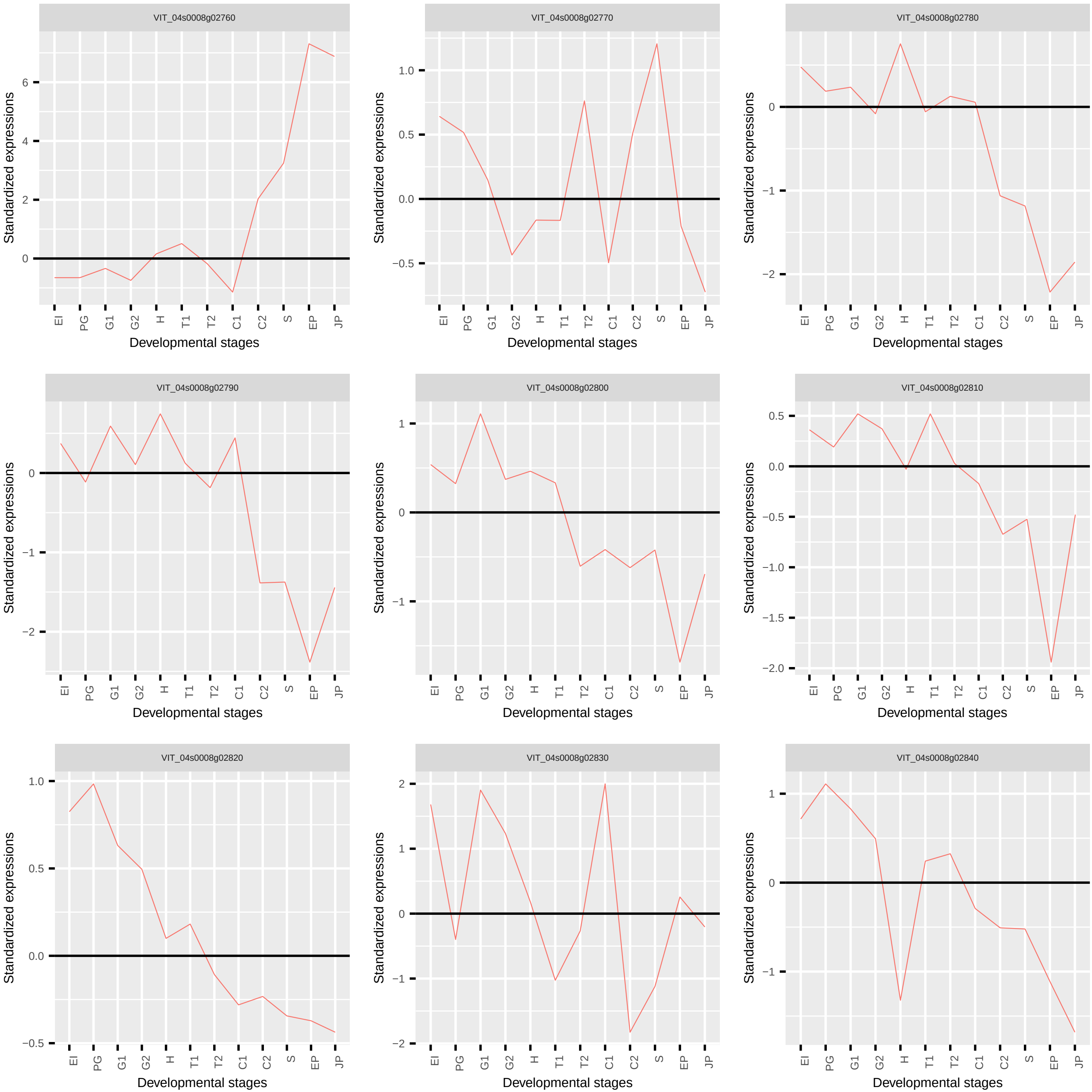
VIT_04s0008g02760
VIT_04s0008g02770
VIT_04s0008g02780
VIT_04s0008g02790
VIT_04s0008g02800
VIT_04s0008g02810
VIT_04s0008g02820
VIT_04s0008g02830
VIT_04s0008g02840
Standardized expressions
Developmental stages
EI PG G1 G2 H T1 T2 C1 C2 S EP JP

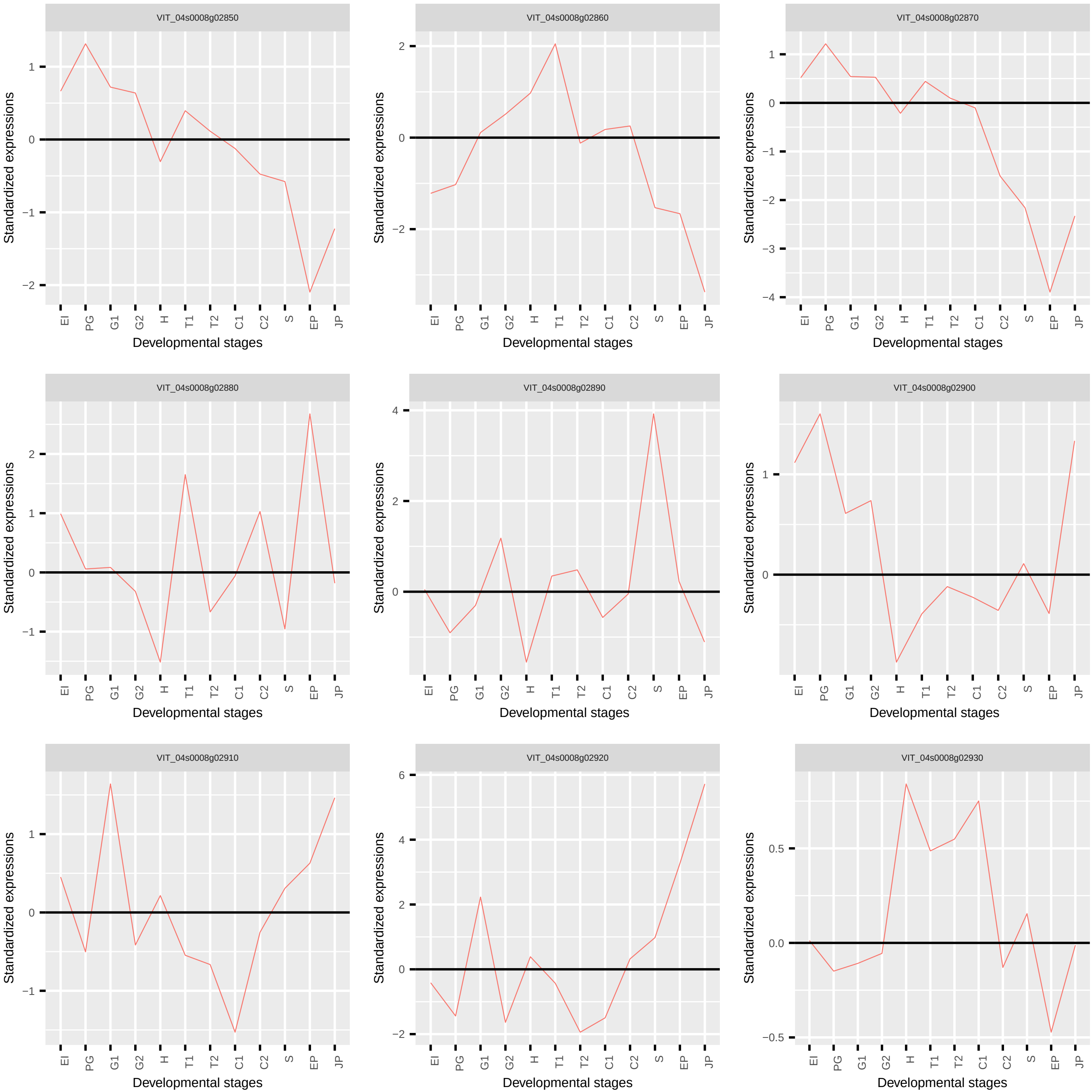
VIT_04s0008g02850
VIT_04s0008g02860
VIT_04s0008g02870
VIT_04s0008g02880
VIT_04s0008g02890
VIT_04s0008g02900
VIT_04s0008g02910
VIT_04s0008g02920
VIT_04s0008g02930
Standardized expressions
Developmental stages
EI PG G1 G2 H T1 T2 C1 C2 S EP JP

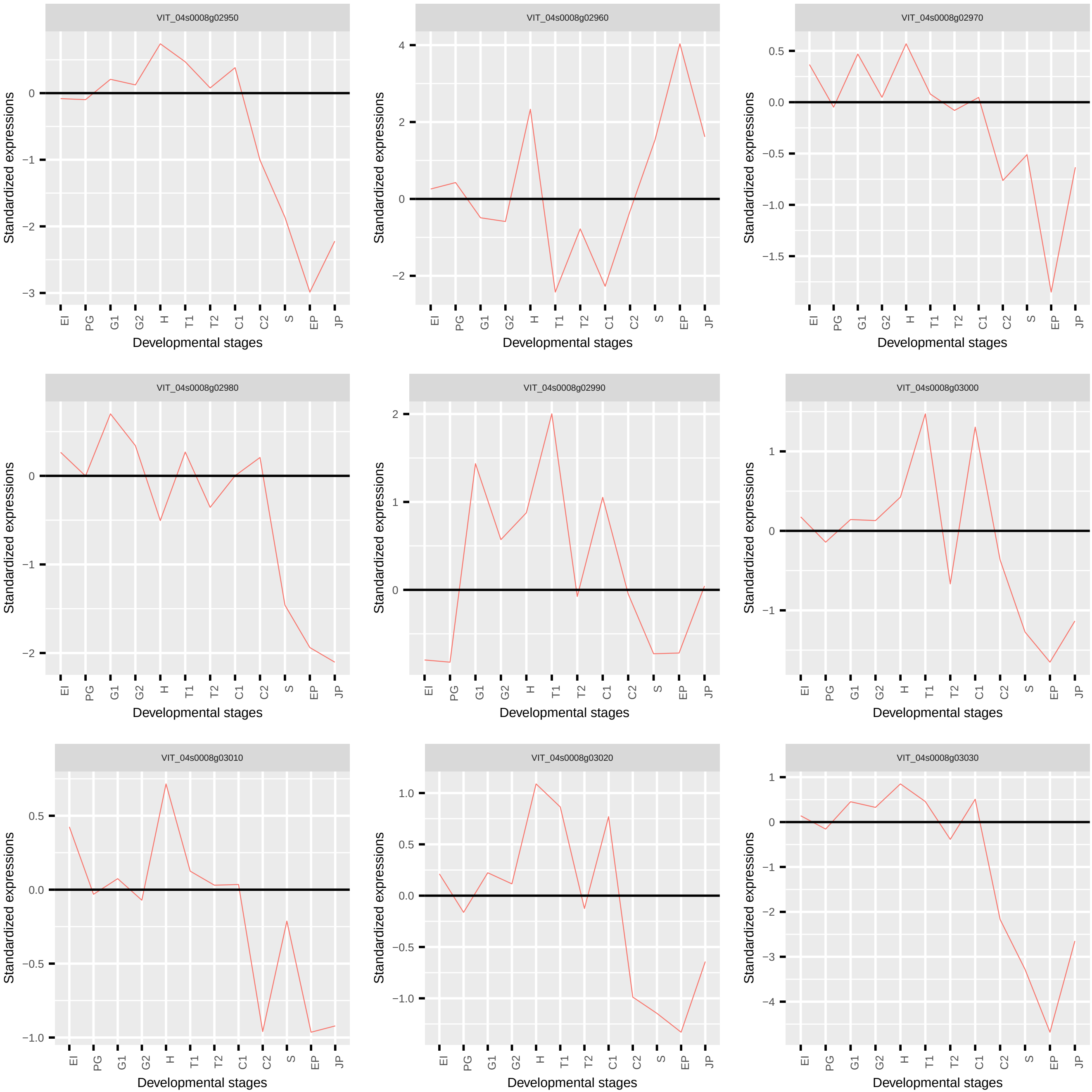
VIT_04s0008g02950
VIT_04s0008g02960
VIT_04s0008g02970
VIT_04s0008g02980
VIT_04s0008g02990
VIT_04s0008g03000
VIT_04s0008g03010
VIT_04s0008g03020
VIT_04s0008g03030
Standardized expressions
Developmental stages
EI PG G1 G2 H T1 T2 C1 C2 S EP JP

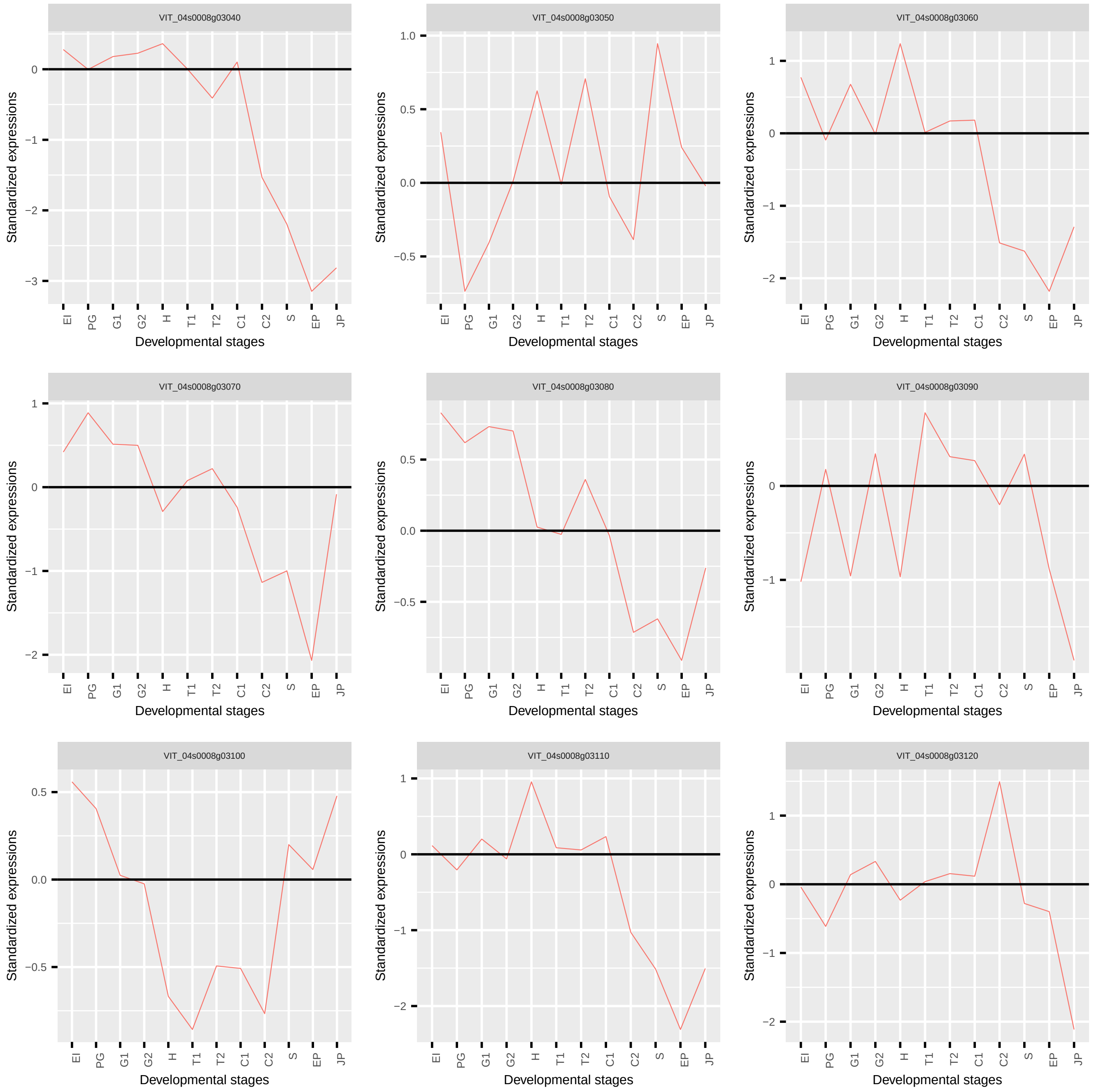

VIT_04s0008g03040
0
−1
−2
−3
Standardized expressions
EI PG G1 G2 H T1 T2 C1 C2 S EP JP
Developmental stages
VIT_04s0008g03050
1.0
0.5
0.0
−0.5
Standardized expressions
EI PG G1 G2 H T1 T2 C1 C2 S EP JP
Developmental stages
VIT_04s0008g03060
1
0
−1
−2
Standardized expressions
EI PG G1 G2 H T1 T2 C1 C2 S EP JP
Developmental stages
VIT_04s0008g03070
1
0
−1
−2
Standardized expressions
EI PG G1 G2 H T1 T2 C1 C2 S EP JP
Developmental stages
VIT_04s0008g03080
0.5
0.0
−0.5
Standardized expressions
EI PG G1 G2 H T1 T2 C1 C2 S EP JP
Developmental stages
VIT_04s0008g03090
0
−1
Standardized expressions
EI PG G1 G2 H T1 T2 C1 C2 S EP JP
Developmental stages
VIT_04s0008g03100
0.5
0.0
−0.5
Standardized expressions
EI PG G1 G2 H T1 T2 C1 C2 S EP JP
Developmental stages
VIT_04s0008g03110
1
0
−1
−2
Standardized expressions
EI PG G1 G2 H T1 T2 C1 C2 S EP JP
Developmental stages
VIT_04s0008g03120
1
0
−1
−2
Standardized expressions
EI PG G1 G2 H T1 T2 C1 C2 S EP JP
Developmental stages

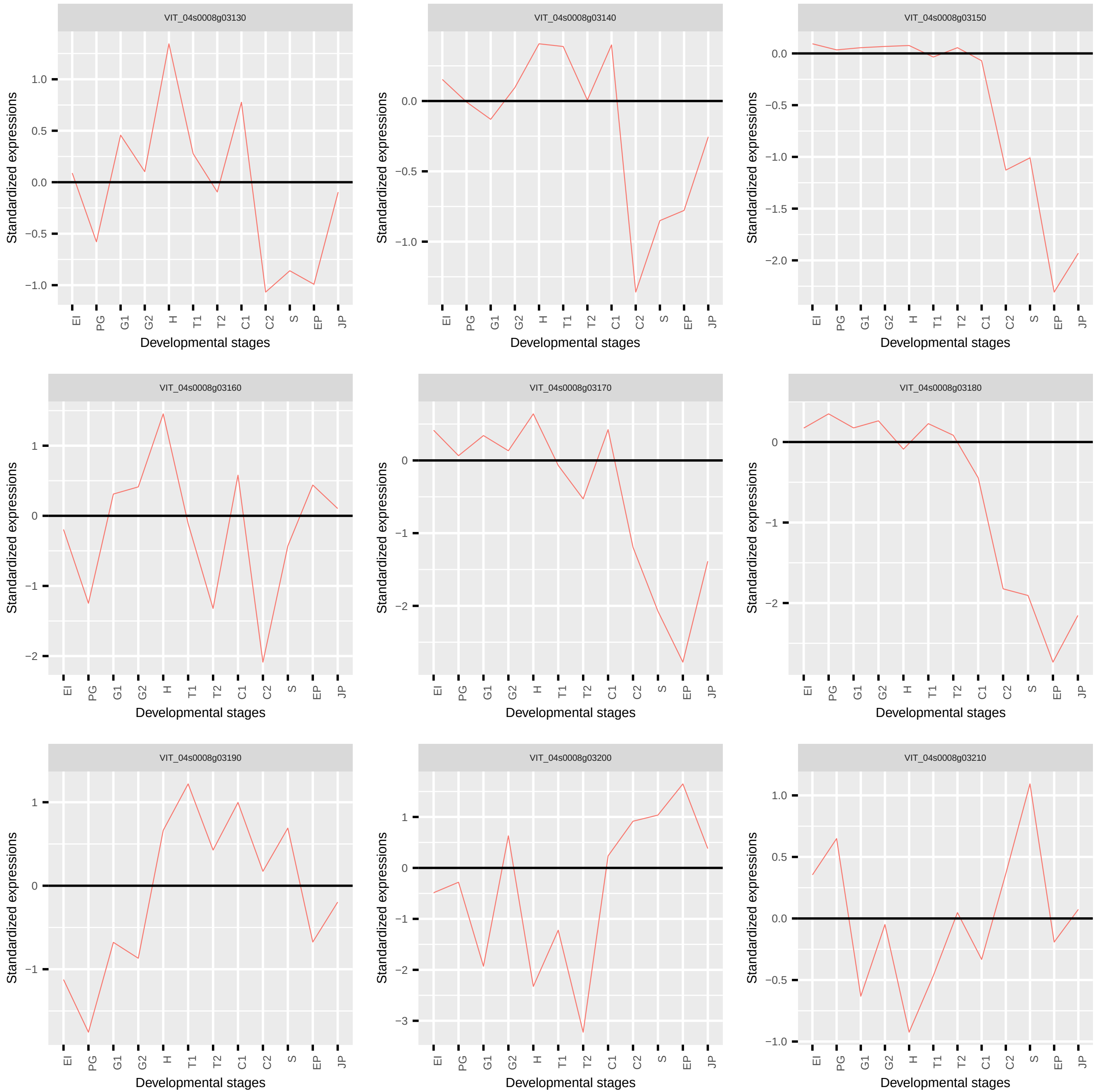
VIT_04s0008g03130
1.0
0.5
0.0
−0.5
−1.0
Standardized expressions
EI PG G1 G2 H T1 T2 C1 C2 S EP JP
Developmental stages
VIT_04s0008g03140
0.0
−0.5
−1.0
Standardized expressions
EI PG G1 G2 H T1 T2 C1 C2 S EP JP
Developmental stages
VIT_04s0008g03150
0.0
−0.5
−1.0
−1.5
−2.0
Standardized expressions
EI PG G1 G2 H T1 T2 C1 C2 S EP JP
Developmental stages
VIT_04s0008g03160
1
0
−1
−2
Standardized expressions
EI PG G1 G2 H T1 T2 C1 C2 S EP JP
Developmental stages
VIT_04s0008g03170
0
−1
−2
Standardized expressions
EI PG G1 G2 H T1 T2 C1 C2 S EP JP
Developmental stages
VIT_04s0008g03180
0
−1
−2
Standardized expressions
EI PG G1 G2 H T1 T2 C1 C2 S EP JP
Developmental stages
VIT_04s0008g03190
1
0
−1
Standardized expressions
EI PG G1 G2 H T1 T2 C1 C2 S EP JP
Developmental stages
VIT_04s0008g03200
1
0
−1
−2
−3
Standardized expressions
EI PG G1 G2 H T1 T2 C1 C2 S EP JP
Developmental stages
VIT_04s0008g03210
1.0
0.5
0.0
−0.5
−1.0
Standardized expressions
EI PG G1 G2 H T1 T2 C1 C2 S EP JP
Developmental stages

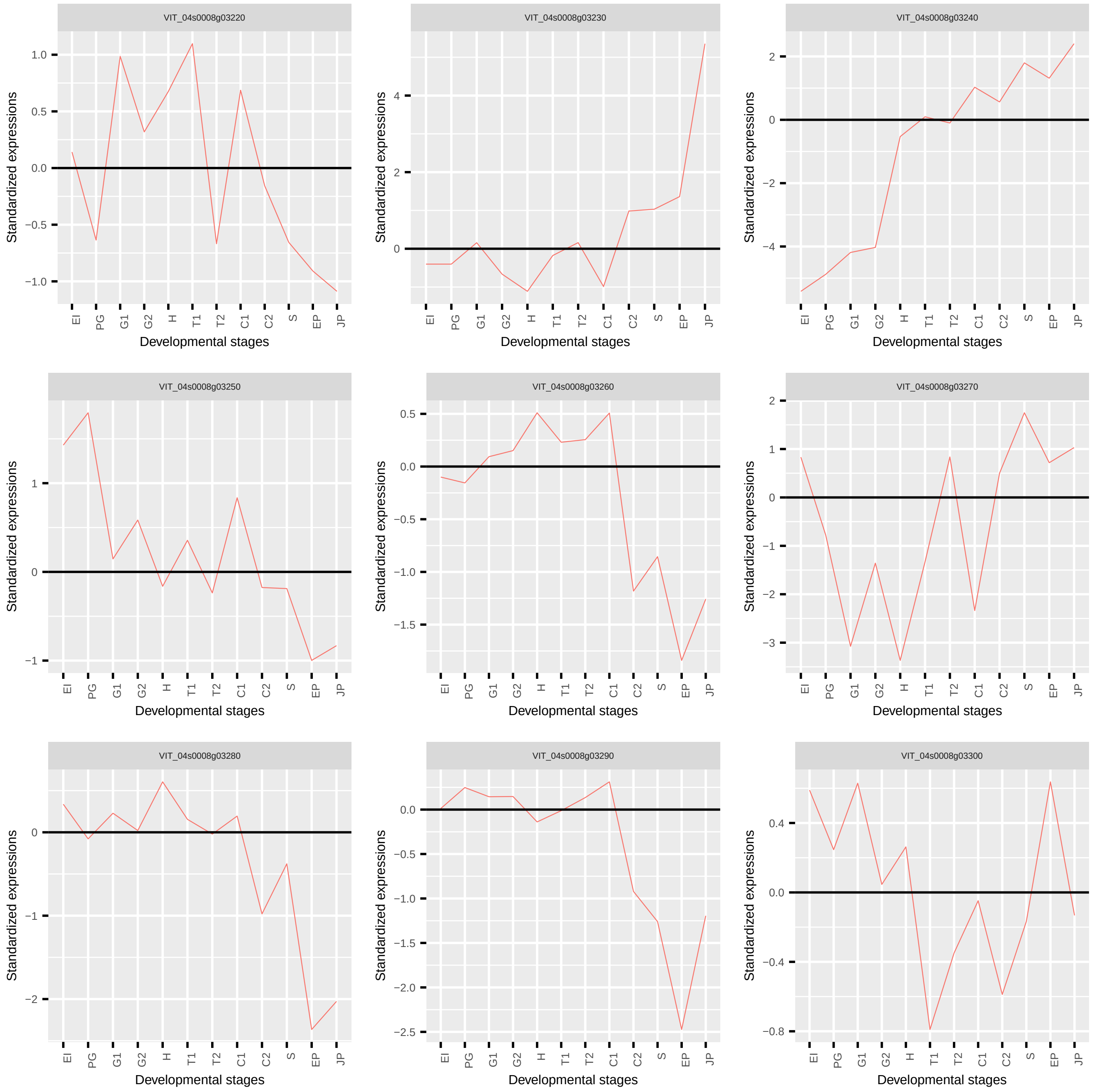
VIT_04s0008g03220
VIT_04s0008g03230
VIT_04s0008g03240
VIT_04s0008g03250
VIT_04s0008g03260
VIT_04s0008g03270
VIT_04s0008g03280
VIT_04s0008g03290
VIT_04s0008g03300
Standardized expressions
Developmental stages
EI PG G1 G2 H T1 T2 C1 C2 S EP JP

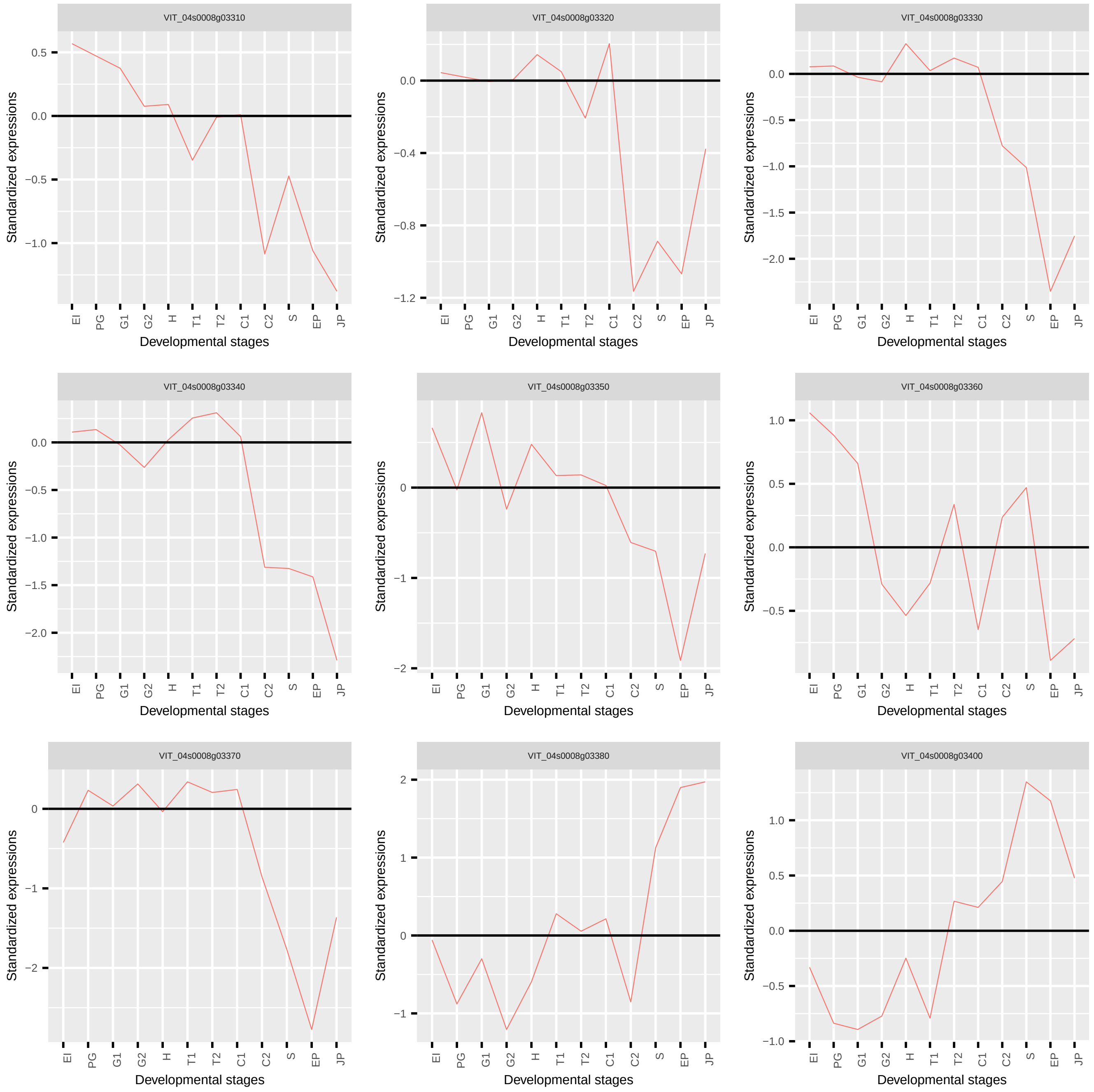
VIT_04s0008g03310
VIT_04s0008g03320
VIT_04s0008g03330
VIT_04s0008g03340
VIT_04s0008g03350
VIT_04s0008g03360
VIT_04s0008g03370
VIT_04s0008g03380
VIT_04s0008g03400
Standardized expressions
Developmental stages
EI PG G1 G2 H T1 T2 C1 C2 S EP JP

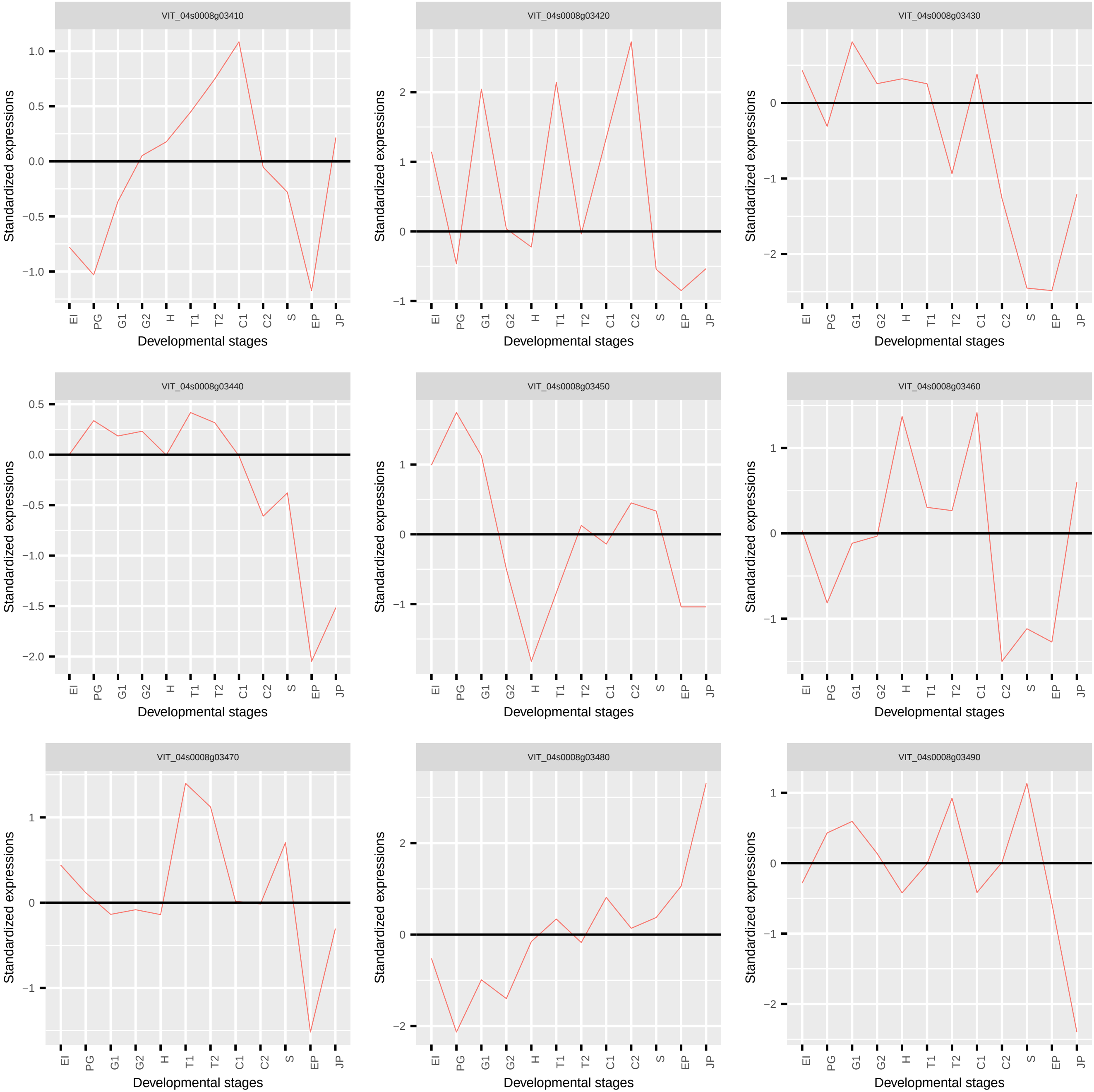
VIT_04s0008g03410
VIT_04s0008g03420
VIT_04s0008g03430
VIT_04s0008g03440
VIT_04s0008g03450
VIT_04s0008g03460
VIT_04s0008g03470
VIT_04s0008g03480
VIT_04s0008g03490
Standardized expressions
Developmental stages
EI PG G1 G2 H T1 T2 C1 C2 S EP JP

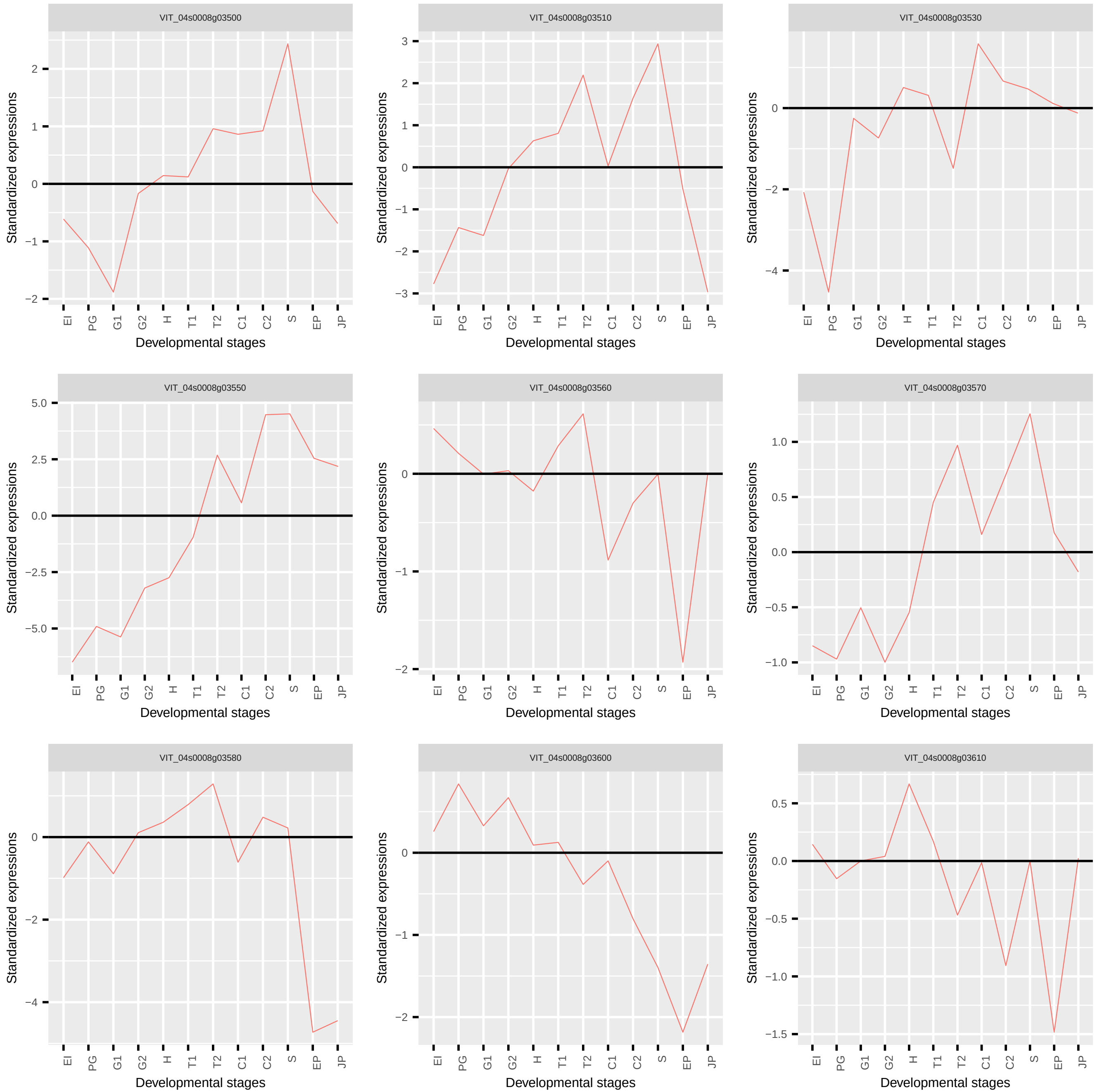
VIT_04s0008g03500
VIT_04s0008g03510
VIT_04s0008g03530
VIT_04s0008g03550
VIT_04s0008g03560
VIT_04s0008g03570
VIT_04s0008g03580
VIT_04s0008g03600
VIT_04s0008g03610
Standardized expressions
Developmental stages
EI PG G1 G2 H T1 T2 C1 C2 S EP JP

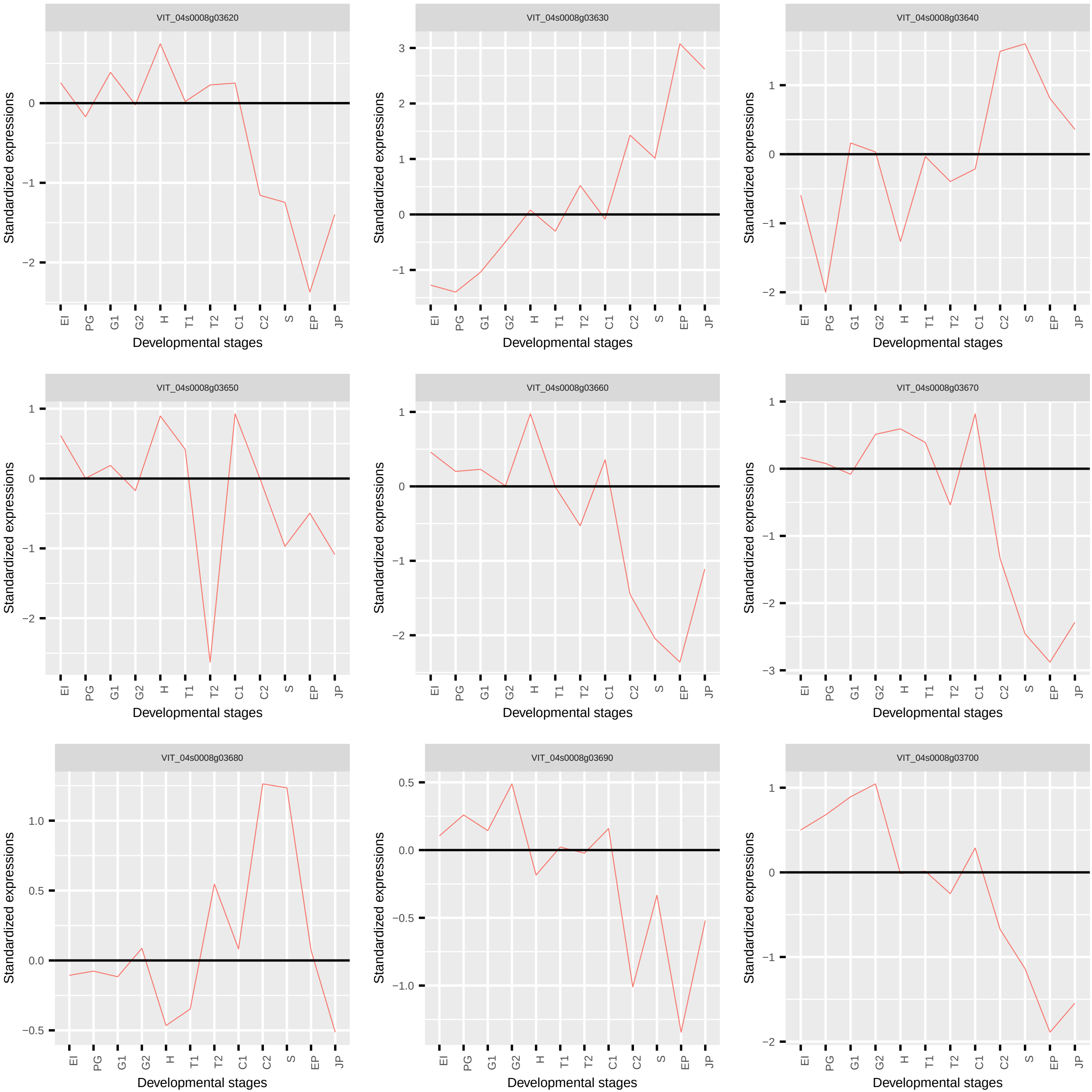
VIT_04s0008g03620
Standardized expressions
0
−1
−2
EI PG G1 G2 H T1 T2 C1 C2 S EP JP
Developmental stages
VIT_04s0008g03630
Standardized expressions
3
2
1
0
−1
EI PG G1 G2 H T1 T2 C1 C2 S EP JP
Developmental stages
VIT_04s0008g03640
Standardized expressions
1
0
−1
−2
EI PG G1 G2 H T1 T2 C1 C2 S EP JP
Developmental stages
VIT_04s0008g03650
Standardized expressions
1
0
−1
−2
EI PG G1 G2 H T1 T2 C1 C2 S EP JP
Developmental stages
VIT_04s0008g03660
Standardized expressions
1
0
−1
−2
EI PG G1 G2 H T1 T2 C1 C2 S EP JP
Developmental stages
VIT_04s0008g03670
Standardized expressions
1
0
−1
−2
−3
EI PG G1 G2 H T1 T2 C1 C2 S EP JP
Developmental stages
VIT_04s0008g03680
Standardized expressions
1.0
0.5
0.0
−0.5
EI PG G1 G2 H T1 T2 C1 C2 S EP JP
Developmental stages
VIT_04s0008g03690
Standardized expressions
0.5
0.0
−0.5
−1.0
EI PG G1 G2 H T1 T2 C1 C2 S EP JP
Developmental stages
VIT_04s0008g03700
Standardized expressions
1
0
−1
−2
EI PG G1 G2 H T1 T2 C1 C2 S EP JP
Developmental stages

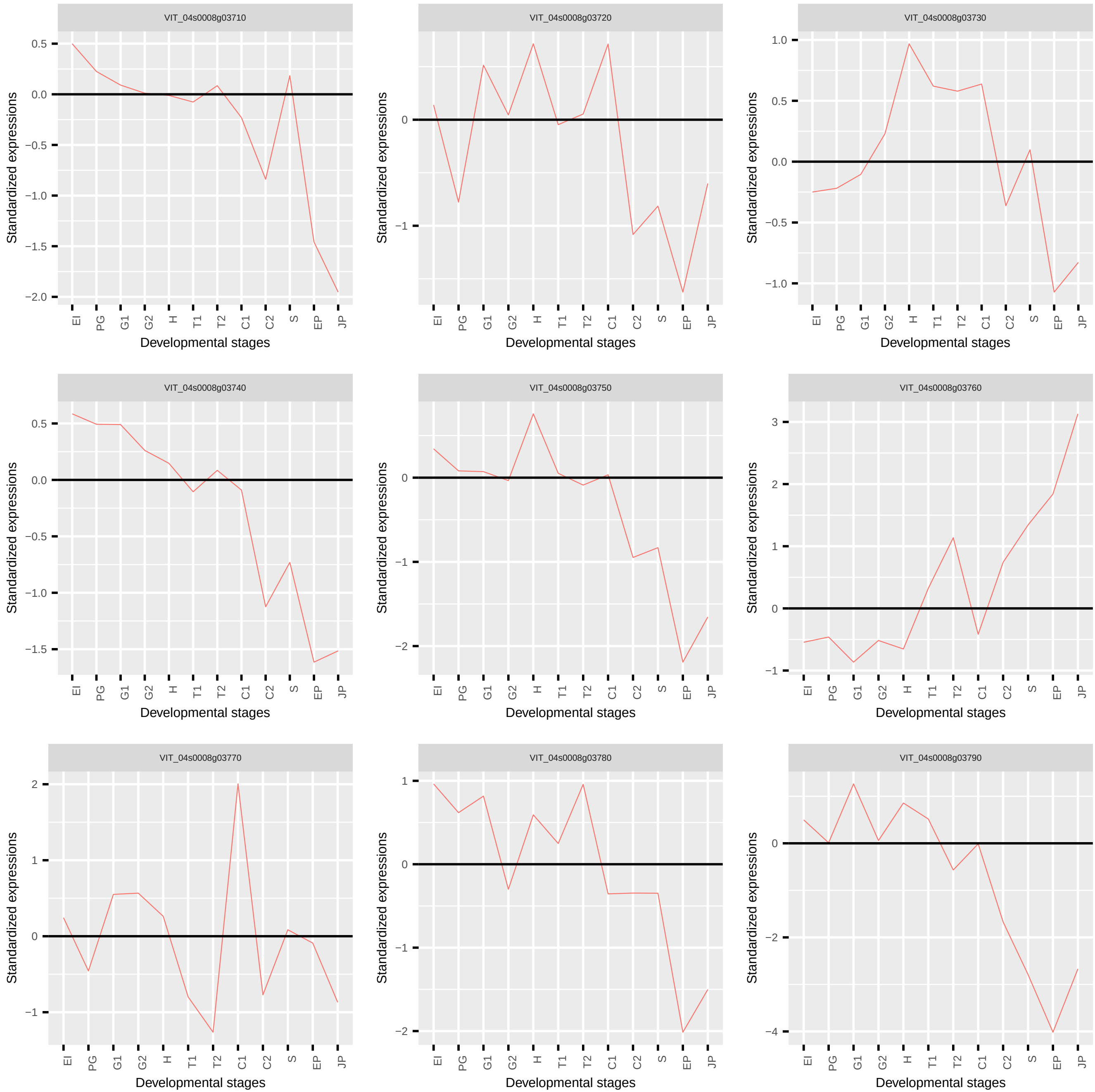
VIT_04s0008g03710
VIT_04s0008g03720
VIT_04s0008g03730
VIT_04s0008g03740
VIT_04s0008g03750
VIT_04s0008g03760
VIT_04s0008g03770
VIT_04s0008g03780
VIT_04s0008g03790
Standardized expressions
Developmental stages
EI PG G1 G2 H T1 T2 C1 C2 S EP JP

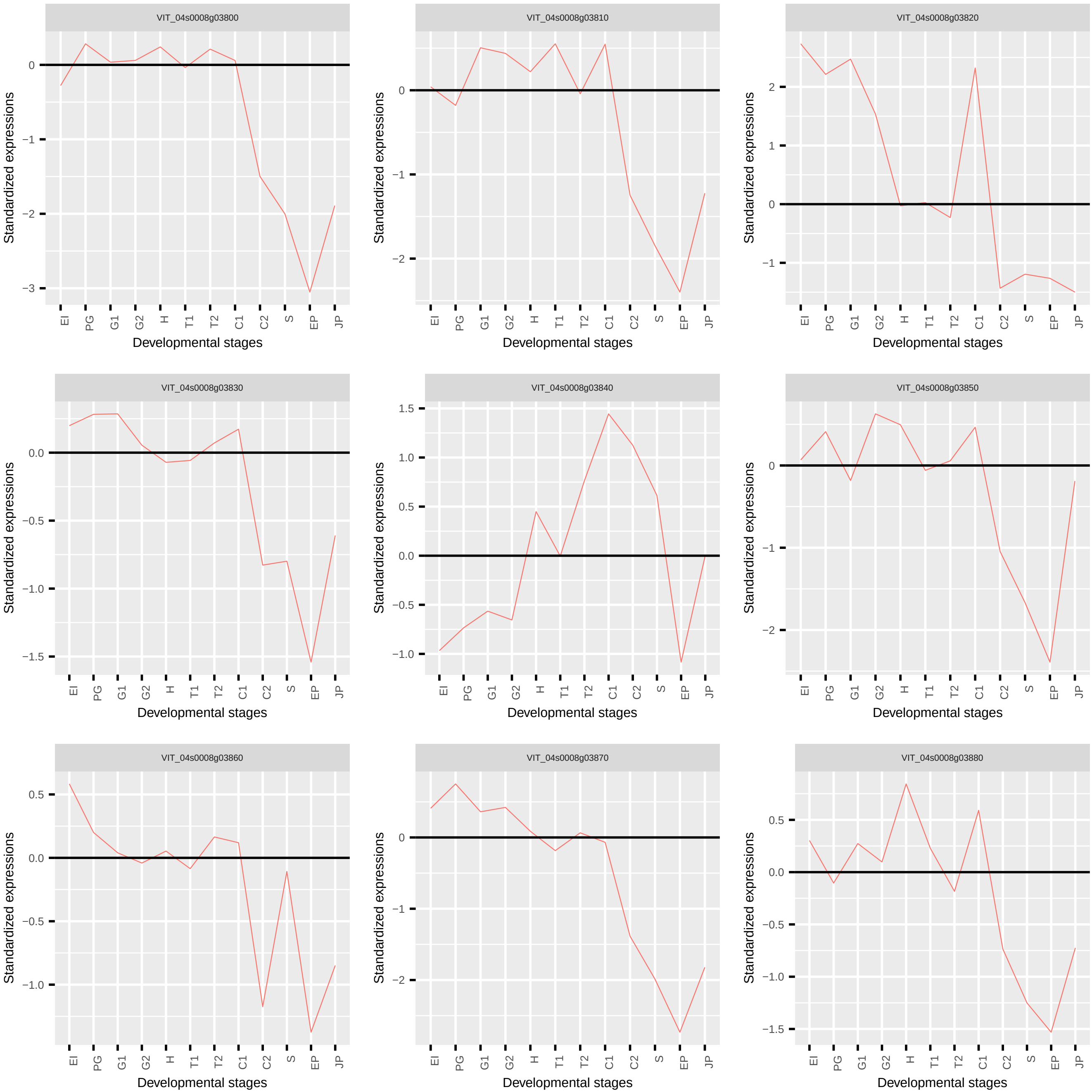
VIT_04s0008g03800
VIT_04s0008g03810
VIT_04s0008g03820
VIT_04s0008g03830
VIT_04s0008g03840
VIT_04s0008g03850
VIT_04s0008g03860
VIT_04s0008g03870
VIT_04s0008g03880
Standardized expressions
Developmental stages
EI PG G1 G2 H T1 T2 C1 C2 S EP JP

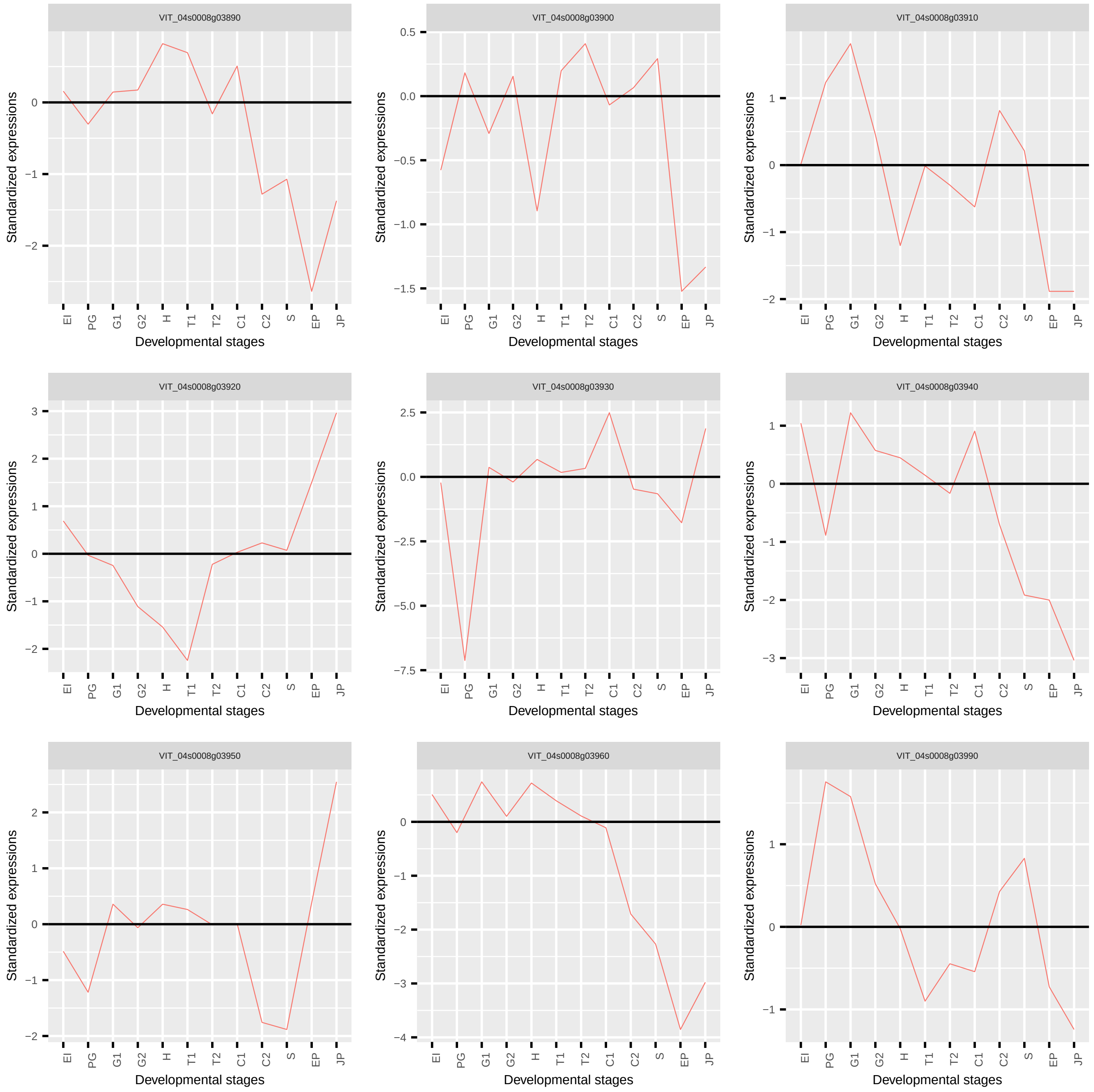
VIT_04s0008g03890
VIT_04s0008g03900
VIT_04s0008g03910
VIT_04s0008g03920
VIT_04s0008g03930
VIT_04s0008g03940
VIT_04s0008g03950
VIT_04s0008g03960
VIT_04s0008g03990
Standardized expressions
Developmental stages
EI PG G1 G2 H T1 T2 C1 C2 S EP JP

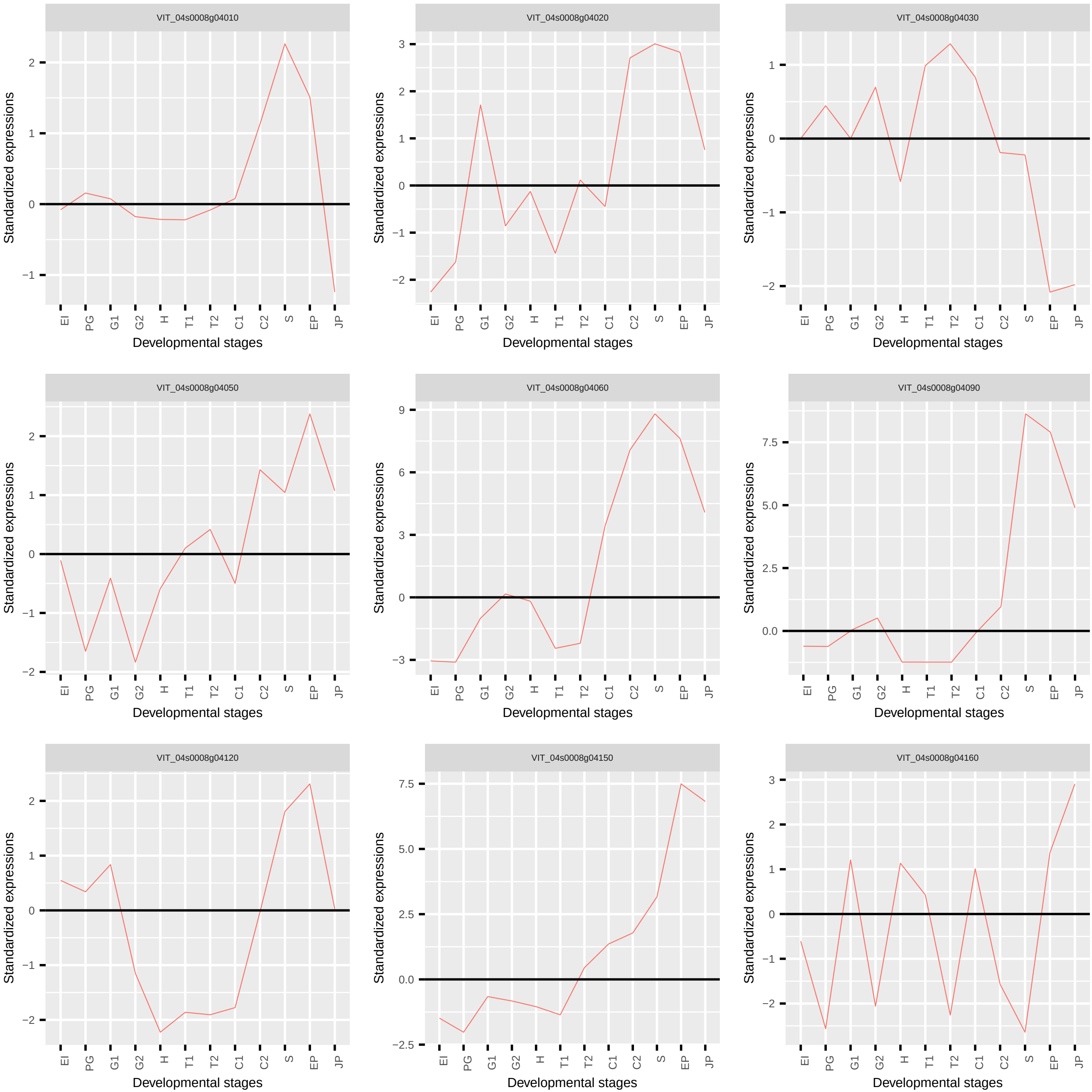
VIT_04s0008g04010
VIT_04s0008g04020
VIT_04s0008g04030
VIT_04s0008g04050
VIT_04s0008g04060
VIT_04s0008g04090
VIT_04s0008g04120
VIT_04s0008g04150
VIT_04s0008g04160
Standardized expressions
Developmental stages
EI PG G1 G2 H T1 T2 C1 C2 S EP JP

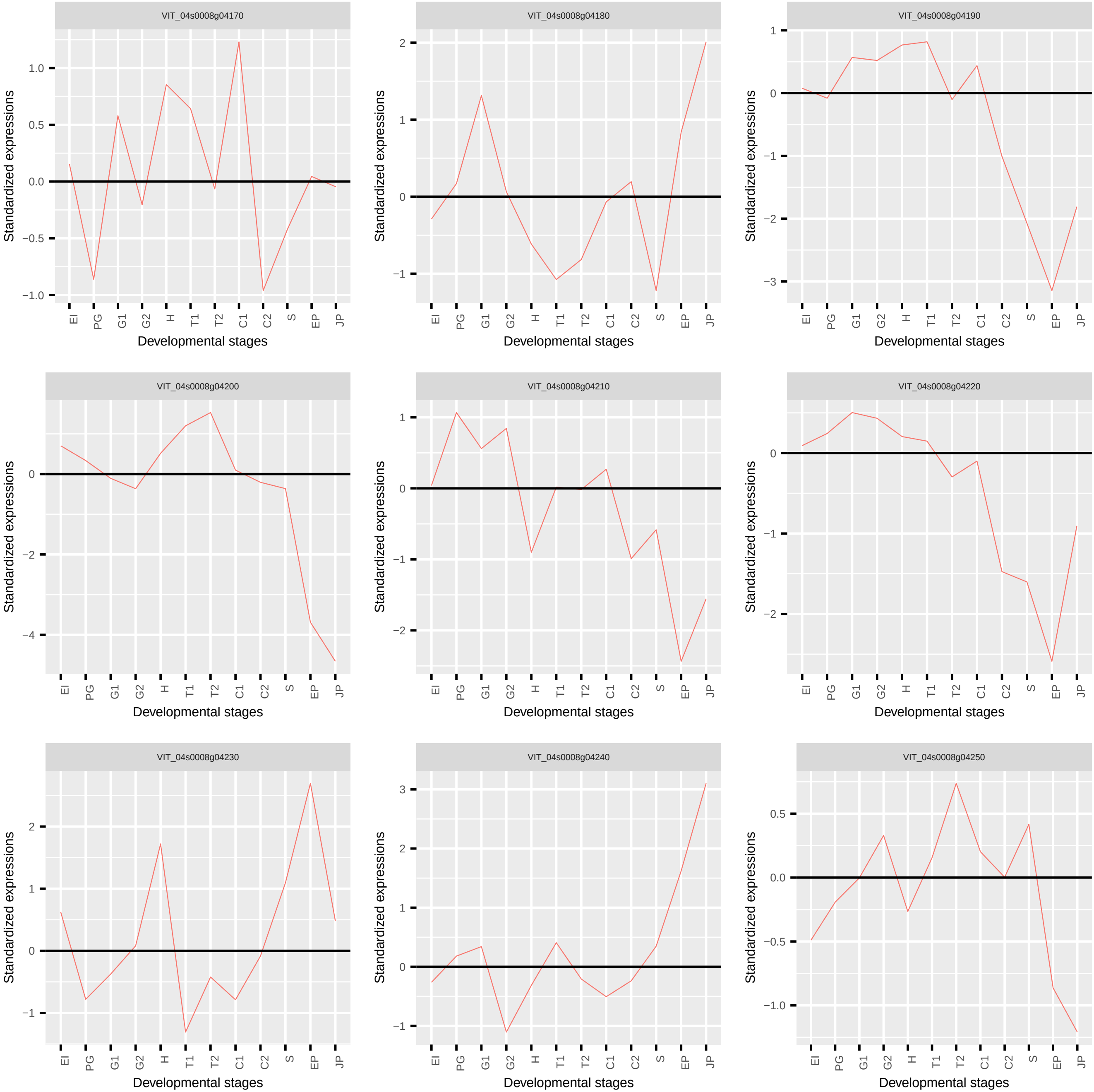
VIT_04s0008g04170
VIT_04s0008g04180
VIT_04s0008g04190
VIT_04s0008g04200
VIT_04s0008g04210
VIT_04s0008g04220
VIT_04s0008g04230
VIT_04s0008g04240
VIT_04s0008g04250
Standardized expressions
Developmental stages
EI PG G1 G2 H T1 T2 C1 C2 S EP JP

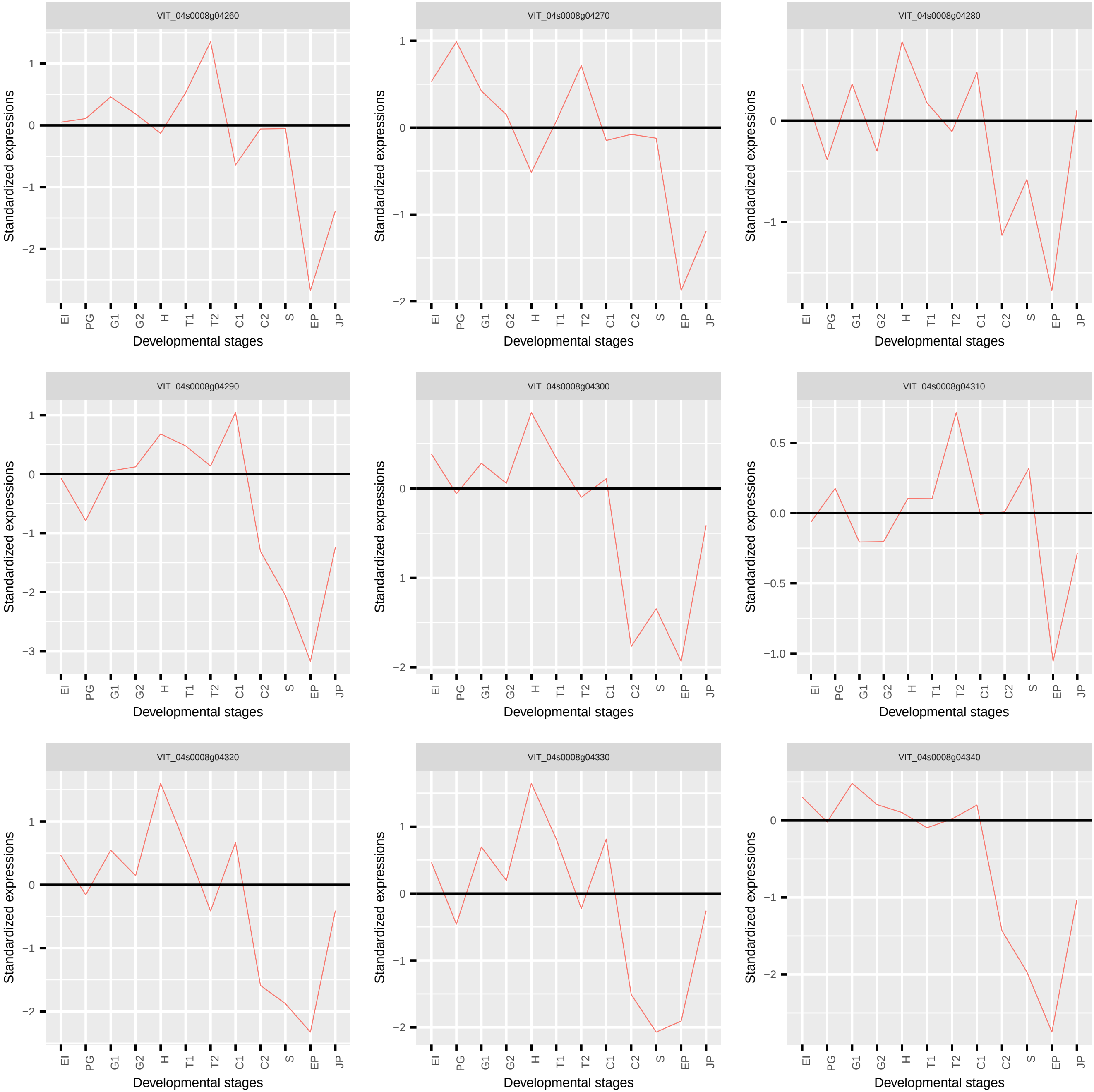
VIT_04s0008g04260
VIT_04s0008g04270
VIT_04s0008g04280
VIT_04s0008g04290
VIT_04s0008g04300
VIT_04s0008g04310
VIT_04s0008g04320
VIT_04s0008g04330
VIT_04s0008g04340
Standardized expressions
Developmental stages
EI PG G1 G2 H T1 T2 C1 C2 S EP JP

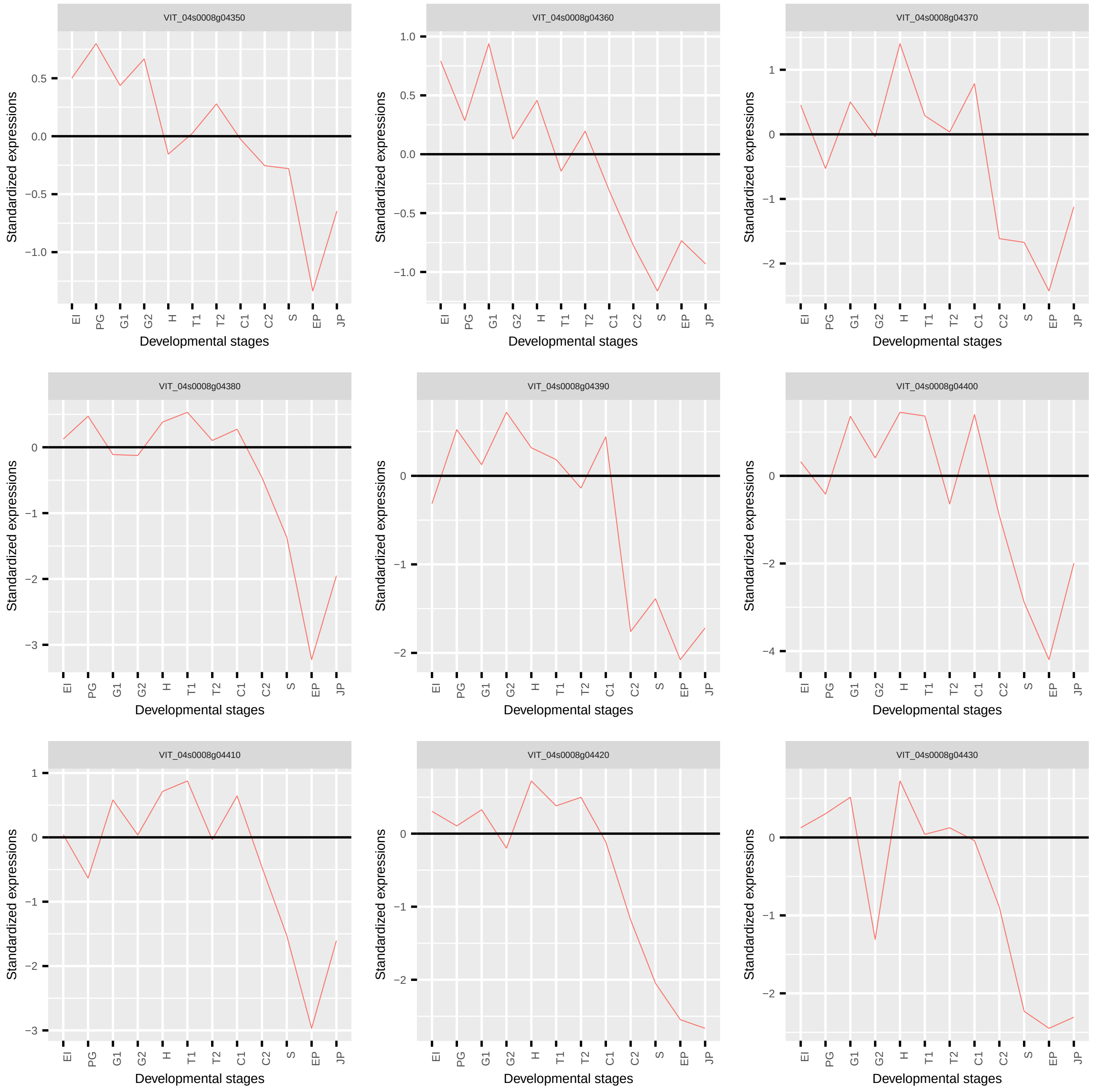
VIT_04s0008g04350
VIT_04s0008g04360
VIT_04s0008g04370
VIT_04s0008g04380
VIT_04s0008g04390
VIT_04s0008g04400
VIT_04s0008g04410
VIT_04s0008g04420
VIT_04s0008g04430
Standardized expressions
Developmental stages
EI PG G1 G2 H T1 T2 C1 C2 S EP JP

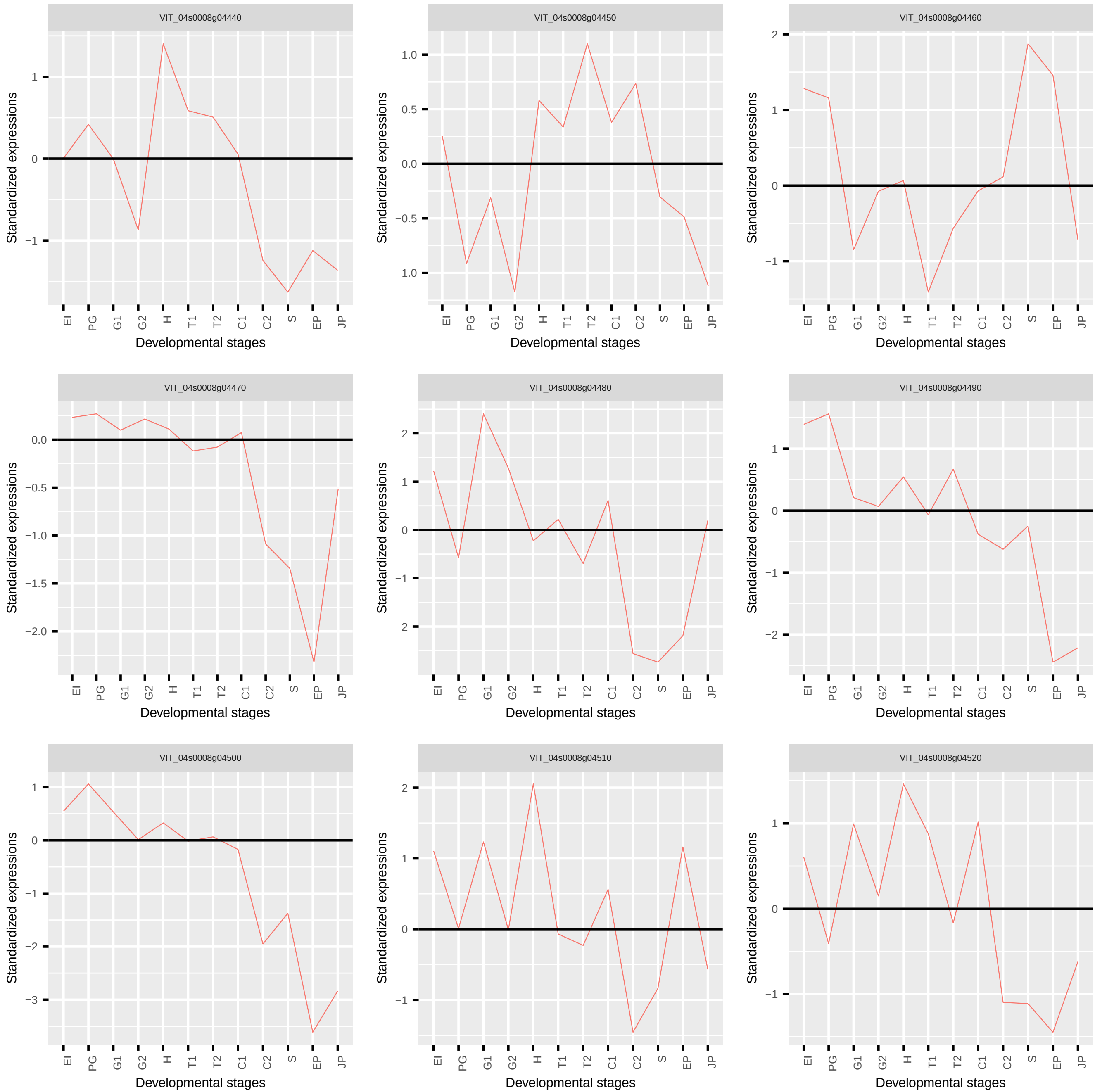
VIT_04s0008g04440
Standardized expressions
1
0
−1
EI PG G1 G2 H T1 T2 C1 C2 S EP JP
Developmental stages
VIT_04s0008g04450
Standardized expressions
1.0
0.5
0.0
−0.5
−1.0
EI PG G1 G2 H T1 T2 C1 C2 S EP JP
Developmental stages
VIT_04s0008g04460
Standardized expressions
2
1
0
−1
EI PG G1 G2 H T1 T2 C1 C2 S EP JP
Developmental stages
VIT_04s0008g04470
Standardized expressions
0.0
−0.5
−1.0
−1.5
−2.0
EI PG G1 G2 H T1 T2 C1 C2 S EP JP
Developmental stages
VIT_04s0008g04480
Standardized expressions
2
1
0
−1
−2
EI PG G1 G2 H T1 T2 C1 C2 S EP JP
Developmental stages
VIT_04s0008g04490
Standardized expressions
1
0
−1
−2
EI PG G1 G2 H T1 T2 C1 C2 S EP JP
Developmental stages
VIT_04s0008g04500
Standardized expressions
1
0
−1
−2
−3
EI PG G1 G2 H T1 T2 C1 C2 S EP JP
Developmental stages
VIT_04s0008g04510
Standardized expressions
2
1
0
−1
EI PG G1 G2 H T1 T2 C1 C2 S EP JP
Developmental stages
VIT_04s0008g04520
Standardized expressions
1
0
−1
EI PG G1 G2 H T1 T2 C1 C2 S EP JP
Developmental stages

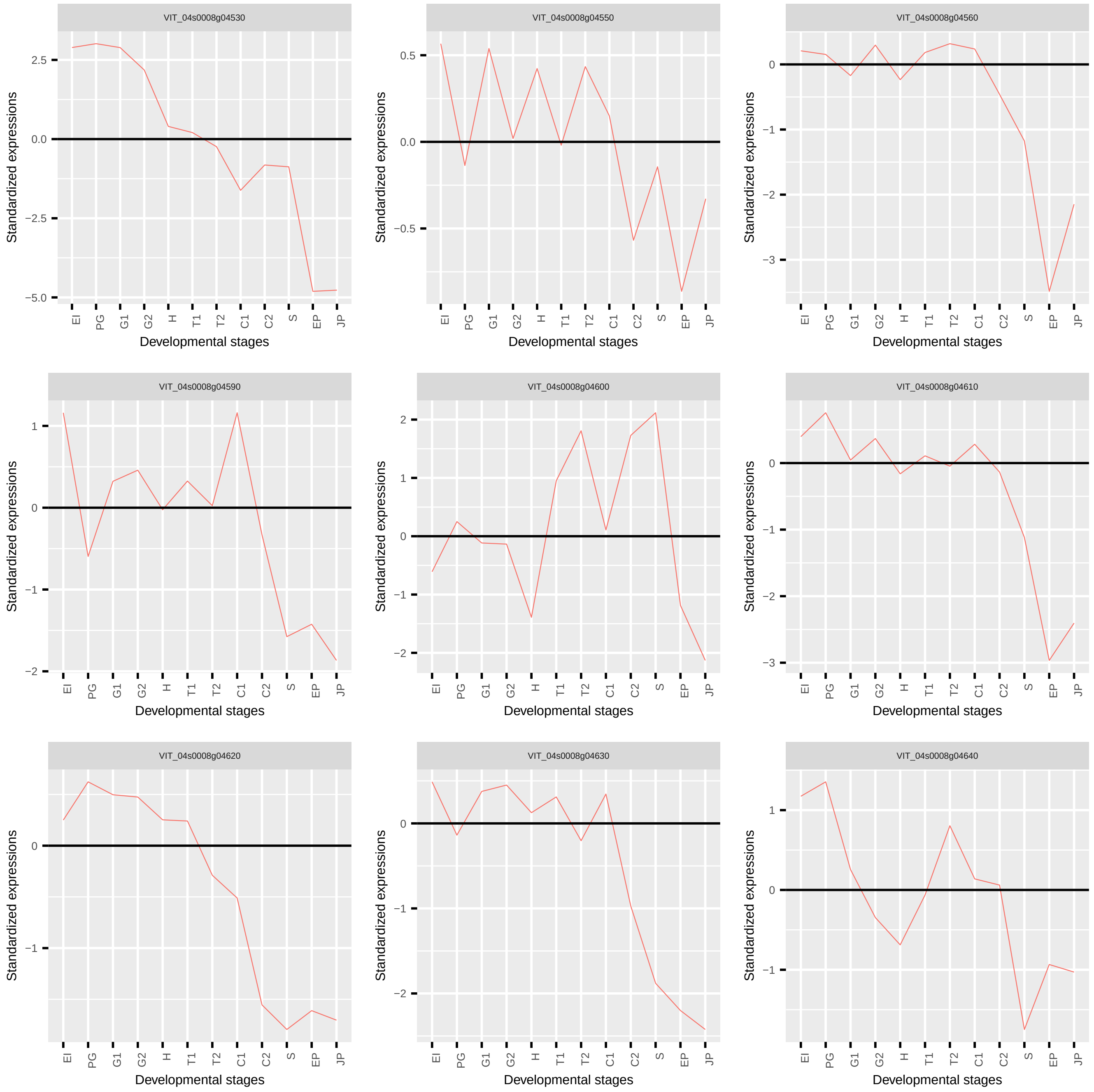
VIT_04s0008g04530
VIT_04s0008g04550
VIT_04s0008g04560
VIT_04s0008g04590
VIT_04s0008g04600
VIT_04s0008g04610
VIT_04s0008g04620
VIT_04s0008g04630
VIT_04s0008g04640
Standardized expressions
Developmental stages
EI PG G1 G2 H T1 T2 C1 C2 S EP JP
2.5 0.0 −2.5 −5.0
0.5 0.0 −0.5
0 −1 −2 −3
1 0 −1 −2
2 1 0 −1 −2
0 −1 −2 −3
0 −1
0 −1 −2
1 0 −1

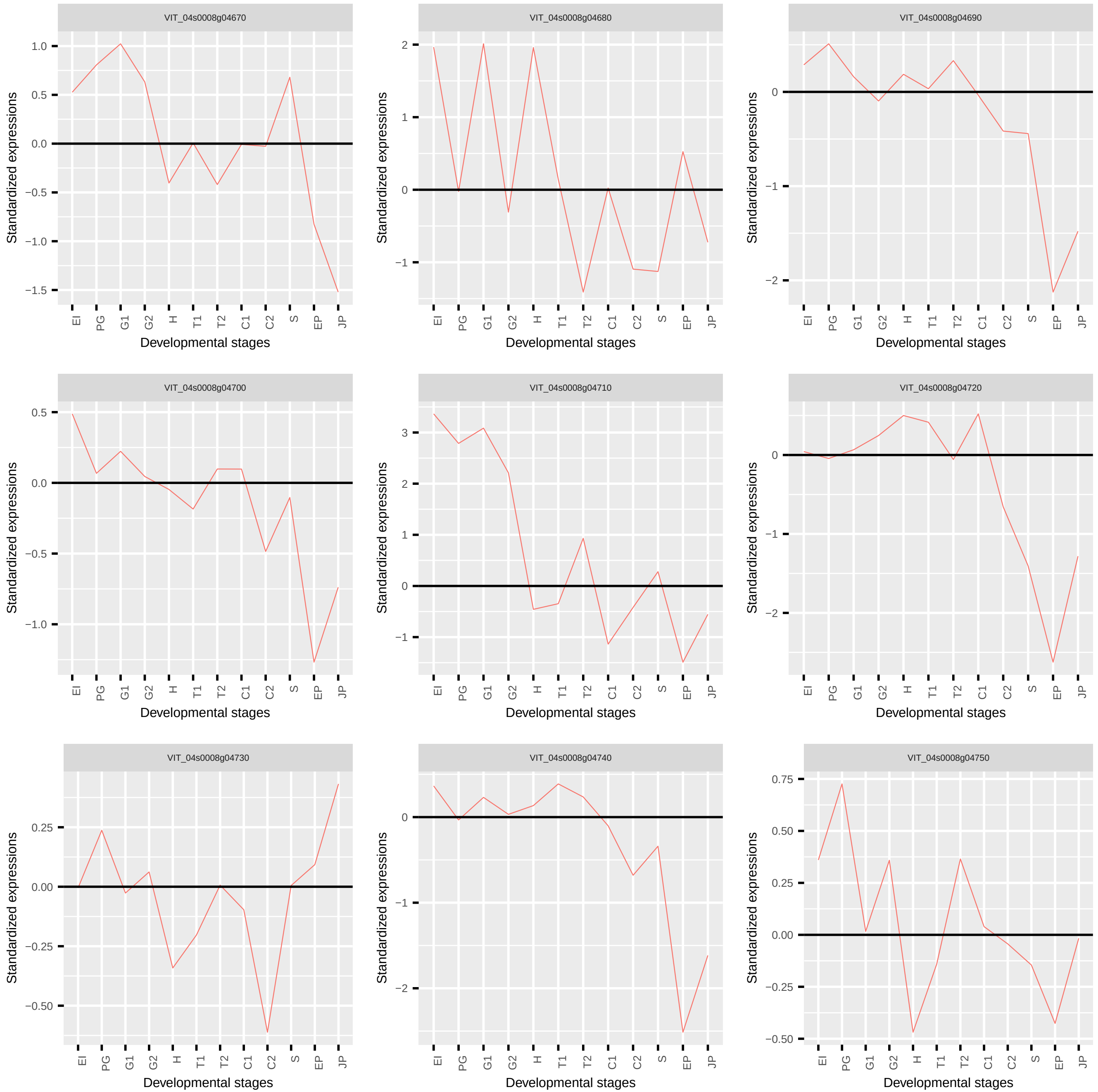

VIT_04s0008g04670
VIT_04s0008g04680
VIT_04s0008g04690
VIT_04s0008g04700
VIT_04s0008g04710
VIT_04s0008g04720
VIT_04s0008g04730
VIT_04s0008g04740
VIT_04s0008g04750
Standardized expressions
Developmental stages
EI PG G1 G2 H T1 T2 C1 C2 S EP JP

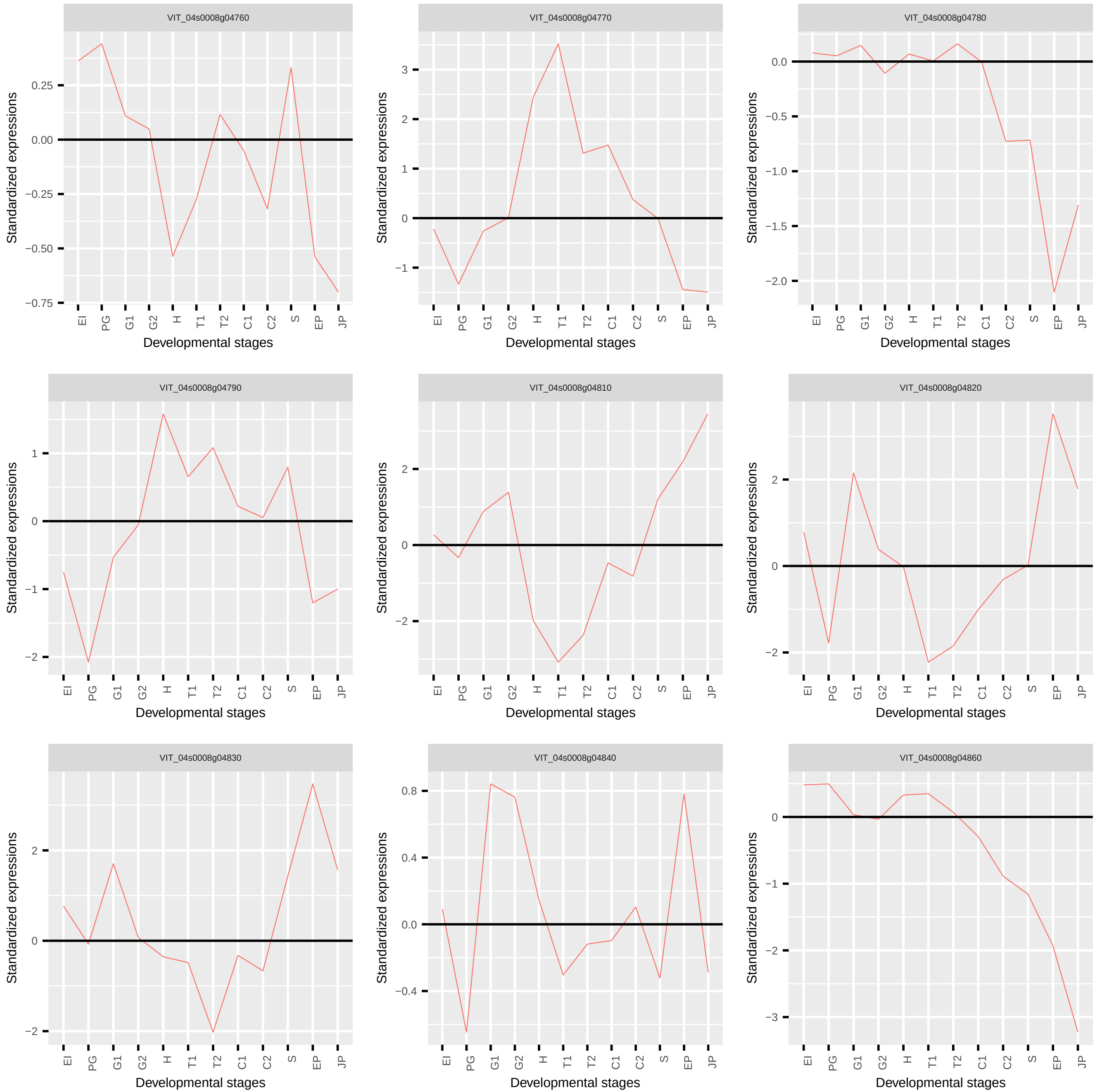
VIT_04s0008g04760
VIT_04s0008g04770
VIT_04s0008g04780
VIT_04s0008g04790
VIT_04s0008g04810
VIT_04s0008g04820
VIT_04s0008g04830
VIT_04s0008g04840
VIT_04s0008g04860
Standardized expressions
Developmental stages
EI PG G1 G2 H T1 T2 C1 C2 S EP JP
0.25
0.00
−0.25
−0.50
−0.75
3
2
1
0
−1
0.0
−0.5
−1.0
−1.5
−2.0
−2
−3
0.8
0.4
−0.4

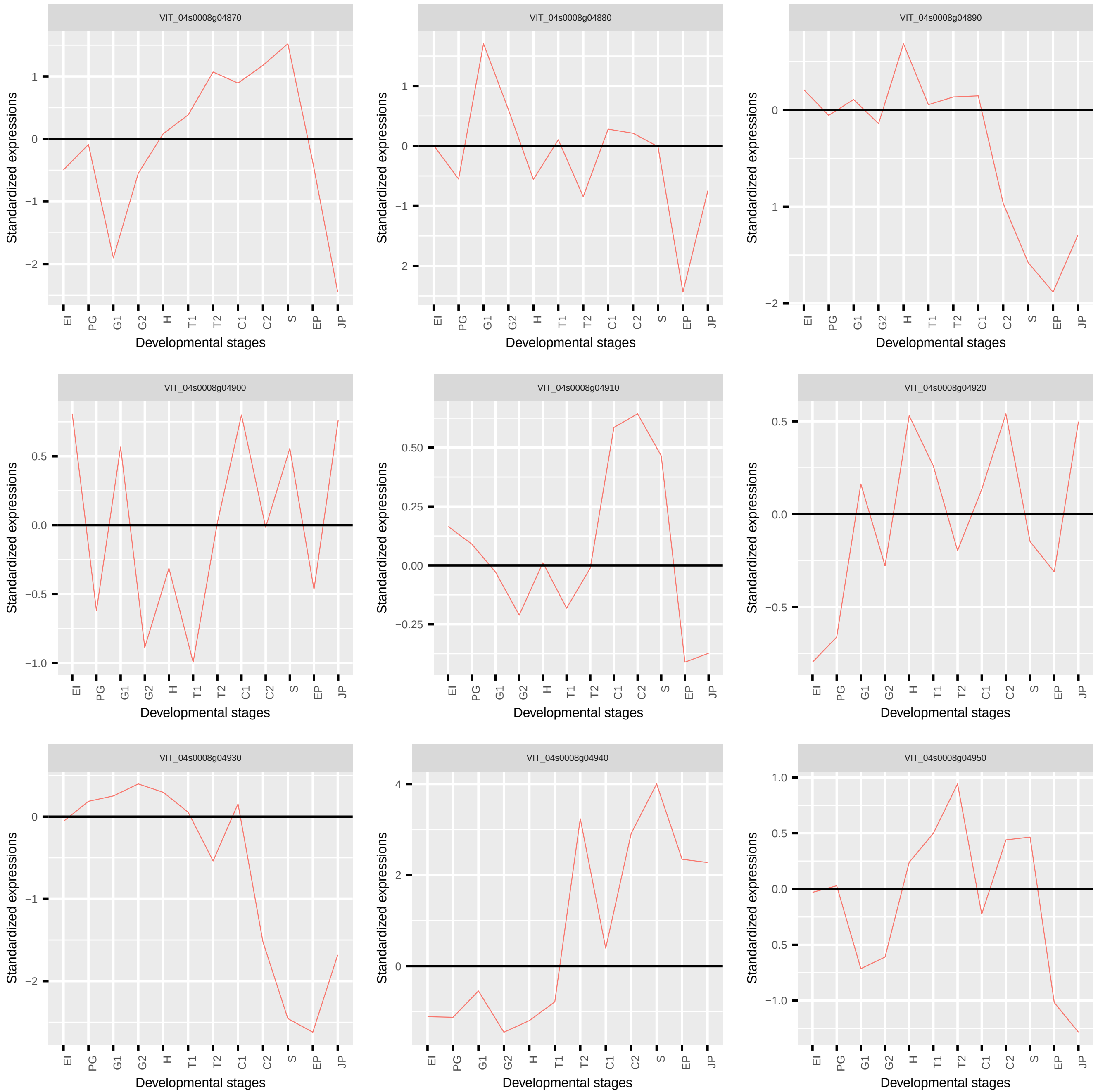

VIT_04s0008g04870
VIT_04s0008g04880
VIT_04s0008g04890
VIT_04s0008g04900
VIT_04s0008g04910
VIT_04s0008g04920
VIT_04s0008g04930
VIT_04s0008g04940
VIT_04s0008g04950
Standardized expressions
Developmental stages
EI
PG
G1
G2
H
T1
T2
C1
C2
S
EP
JP

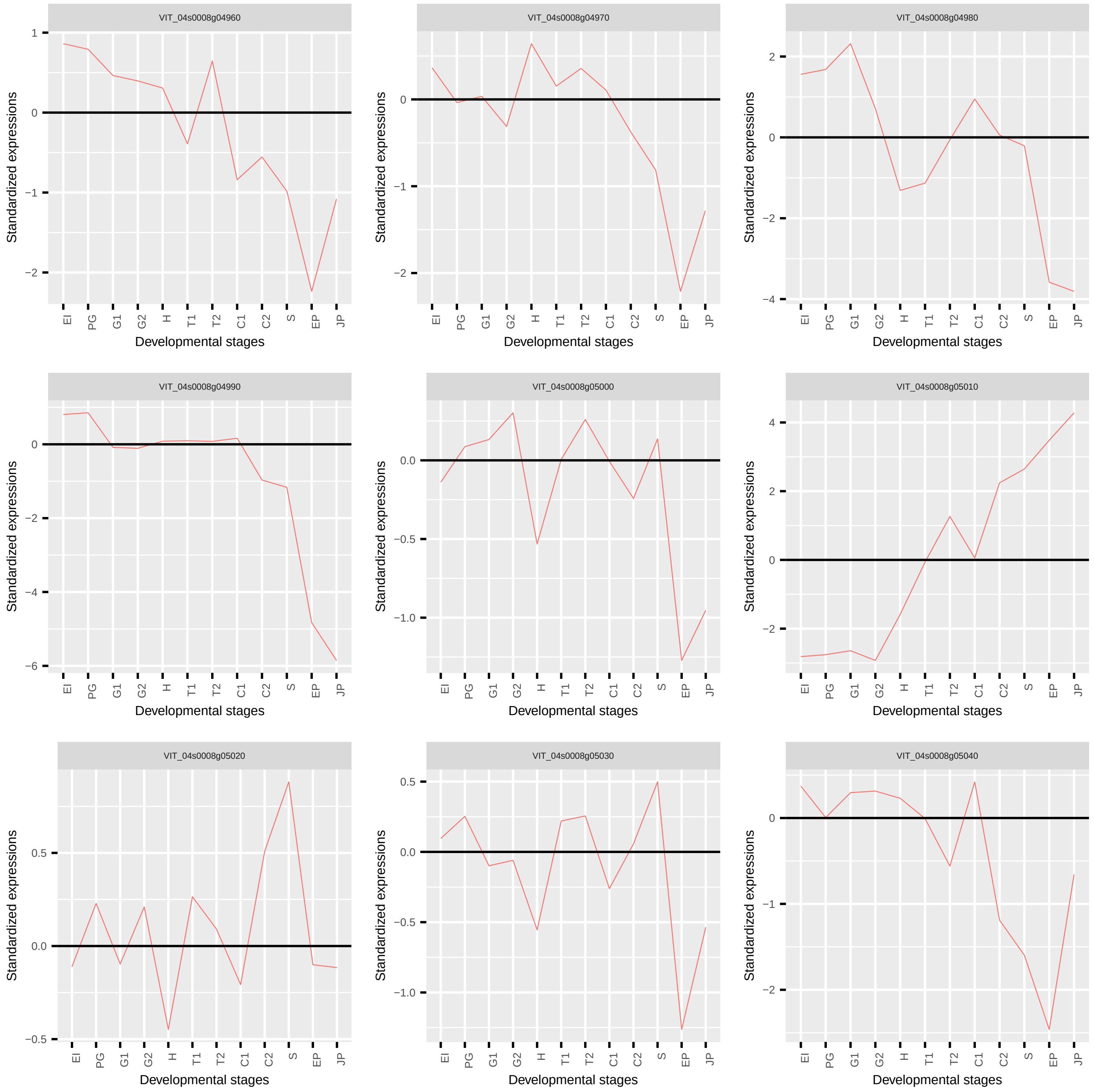

VIT_04s0008g04960
VIT_04s0008g04970
VIT_04s0008g04980
VIT_04s0008g04990
VIT_04s0008g05000
VIT_04s0008g05010
VIT_04s0008g05020
VIT_04s0008g05030
VIT_04s0008g05040
Standardized expressions
Developmental stages
EI PG G1 G2 H T1 T2 C1 C2 S EP JP

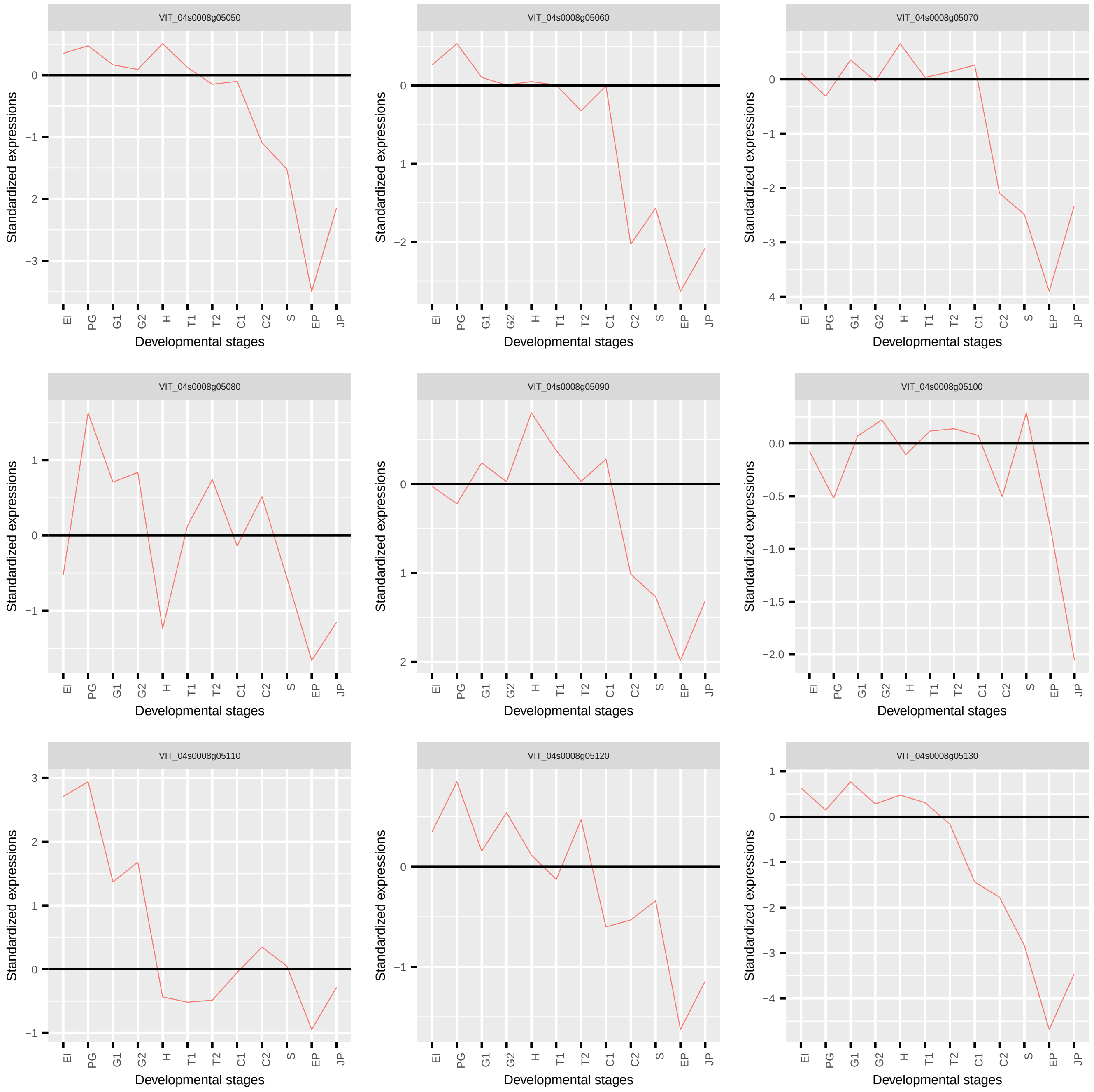

VIT_04s0008g05050
VIT_04s0008g05060
VIT_04s0008g05070
VIT_04s0008g05080
VIT_04s0008g05090
VIT_04s0008g05100
VIT_04s0008g05110
VIT_04s0008g05120
VIT_04s0008g05130
Standardized expressions
Developmental stages
EI PG G1 G2 H T1 T2 C1 C2 S EP JP
0 −1 −2 −3 −4
0.0 −0.5 −1.0 −1.5 −2.0
3 2 1 0 −1

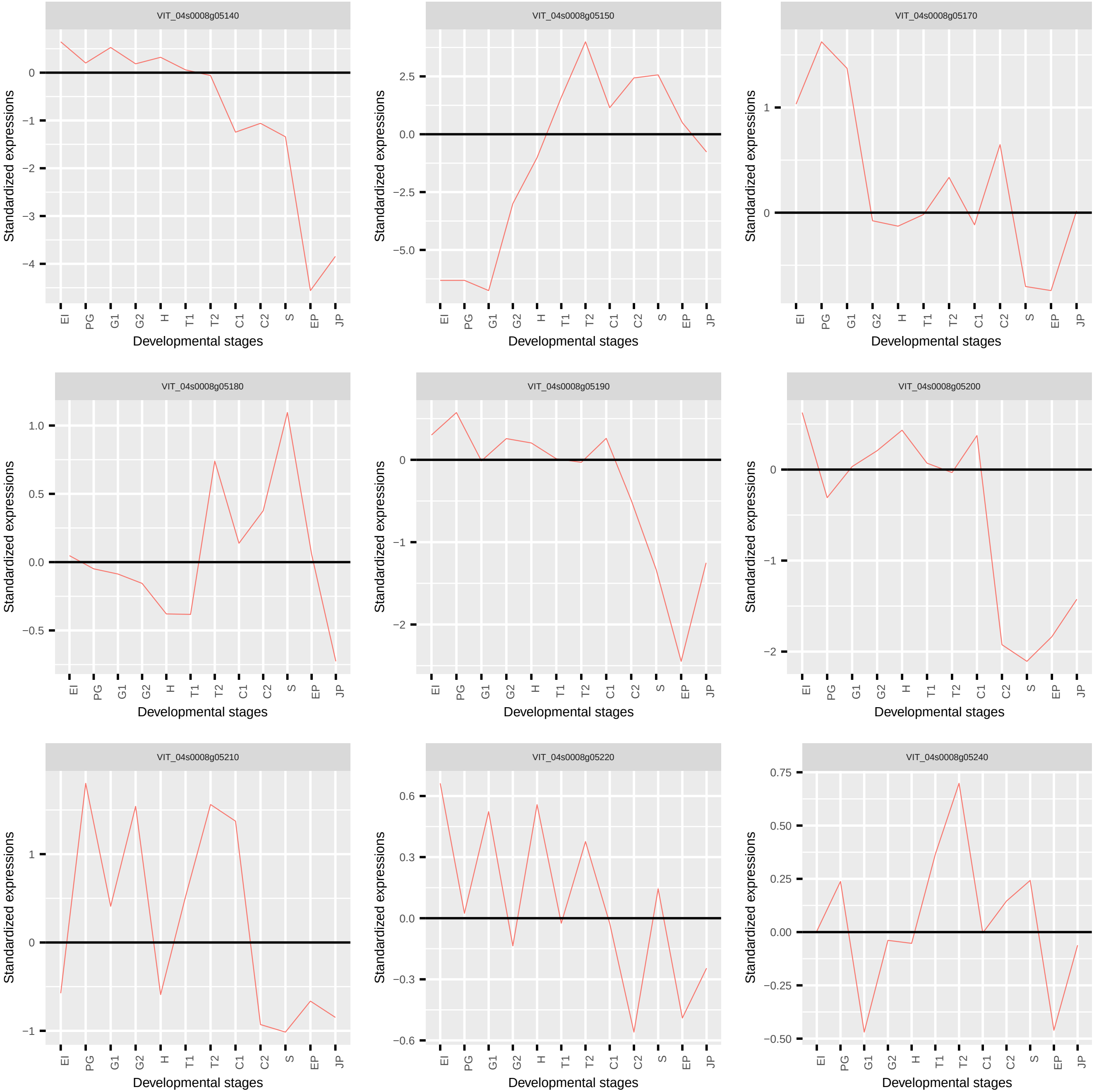
VIT_04s0008g05140
VIT_04s0008g05150
VIT_04s0008g05170
VIT_04s0008g05180
VIT_04s0008g05190
VIT_04s0008g05200
VIT_04s0008g05210
VIT_04s0008g05220
VIT_04s0008g05240
Standardized expressions
Developmental stages
EI PG G1 G2 H T1 T2 C1 C2 S EP JP
0
−1
−2
−3
−4
2.5
0.0
−2.5
−5.0
1
1.0
0.5
−0.5
0.75
0.50
0.25
0.00
−0.25
−0.50
0.6
0.3
−0.3
−0.6

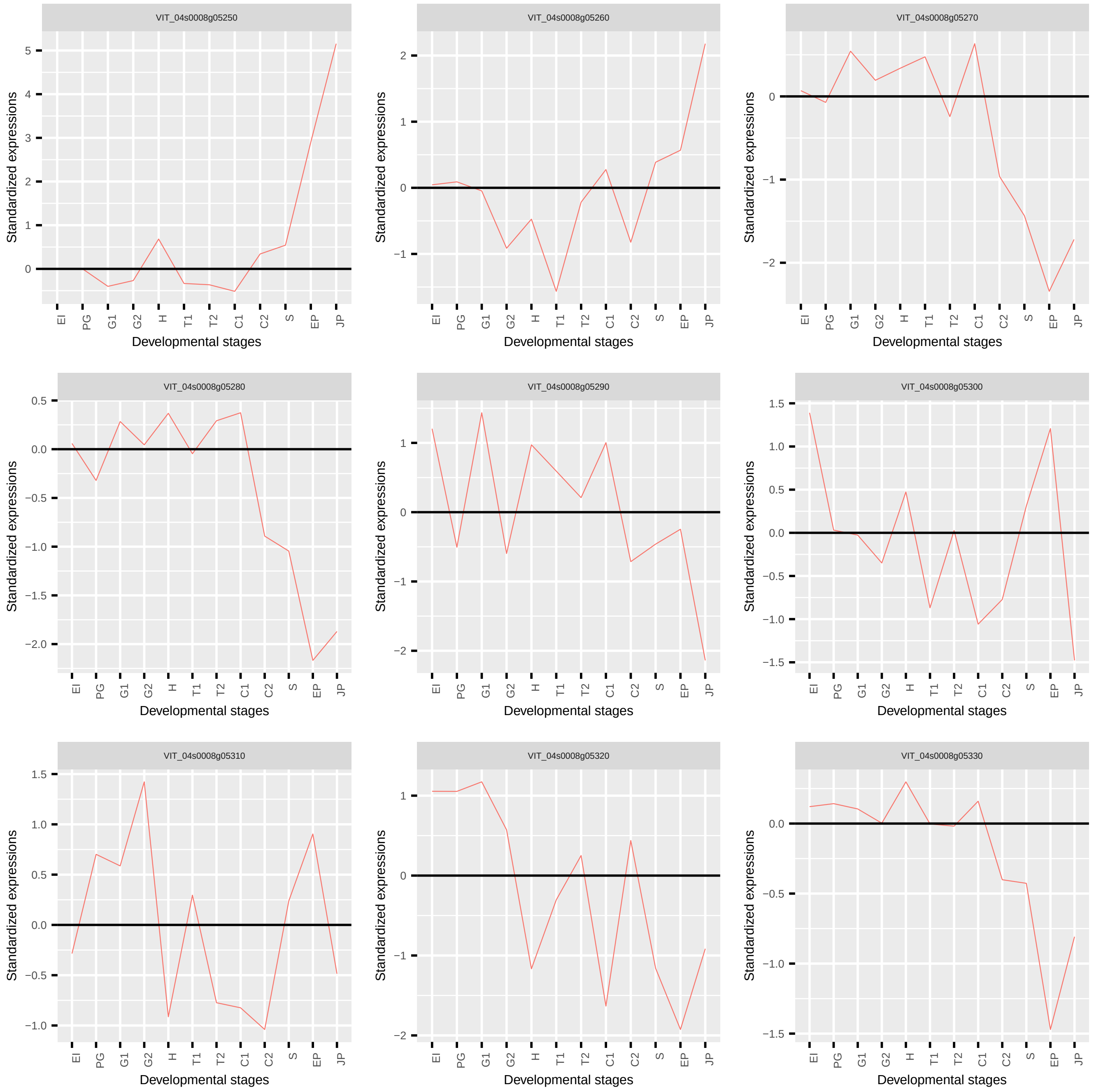

VIT_04s0008g05250
VIT_04s0008g05260
VIT_04s0008g05270
VIT_04s0008g05280
VIT_04s0008g05290
VIT_04s0008g05300
VIT_04s0008g05310
VIT_04s0008g05320
VIT_04s0008g05330
Standardized expressions
Developmental stages
EI PG G1 G2 H T1 T2 C1 C2 S EP JP

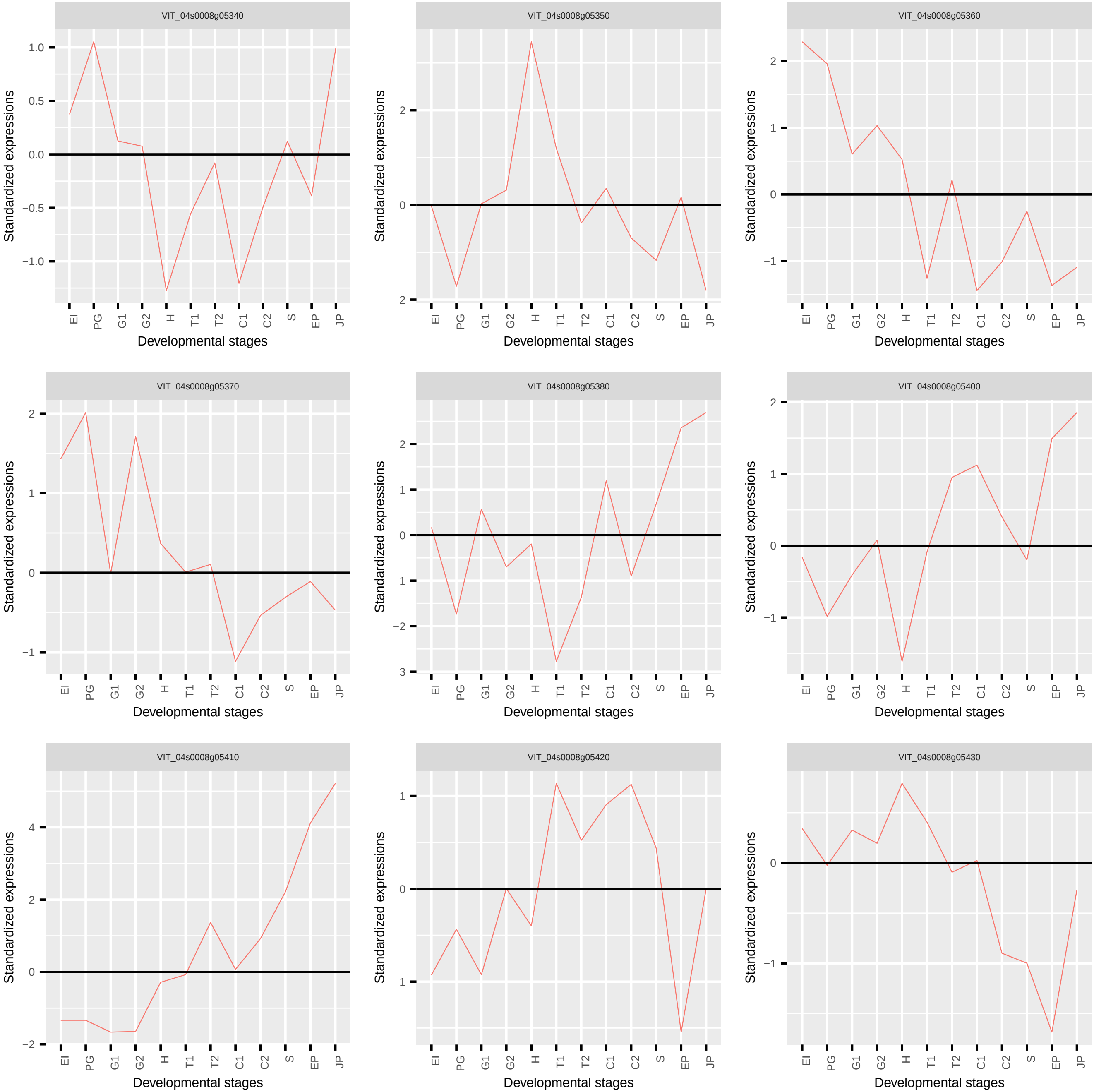
VIT_04s0008g05340
Standardized expressions
1.0
0.5
0.0
−0.5
−1.0
EI
PG
G1
G2
H
T1
T2
C1
C2
S
EP
JP
Developmental stages
VIT_04s0008g05350
Standardized expressions
2
0
−2
EI
PG
G1
G2
H
T1
T2
C1
C2
S
EP
JP
Developmental stages
VIT_04s0008g05360
Standardized expressions
2
1
0
−1
EI
PG
G1
G2
H
T1
T2
C1
C2
S
EP
JP
Developmental stages
VIT_04s0008g05370
Standardized expressions
2
1
0
−1
EI
PG
G1
G2
H
T1
T2
C1
C2
S
EP
JP
Developmental stages
VIT_04s0008g05380
Standardized expressions
2
1
0
−1
−2
−3
EI
PG
G1
G2
H
T1
T2
C1
C2
S
EP
JP
Developmental stages
VIT_04s0008g05400
Standardized expressions
2
1
0
−1
EI
PG
G1
G2
H
T1
T2
C1
C2
S
EP
JP
Developmental stages
VIT_04s0008g05410
Standardized expressions
4
2
0
−2
EI
PG
G1
G2
H
T1
T2
C1
C2
S
EP
JP
Developmental stages
VIT_04s0008g05420
Standardized expressions
1
0
−1
EI
PG
G1
G2
H
T1
T2
C1
C2
S
EP
JP
Developmental stages
VIT_04s0008g05430
Standardized expressions
0
−1
EI
PG
G1
G2
H
T1
T2
C1
C2
S
EP
JP
Developmental stages

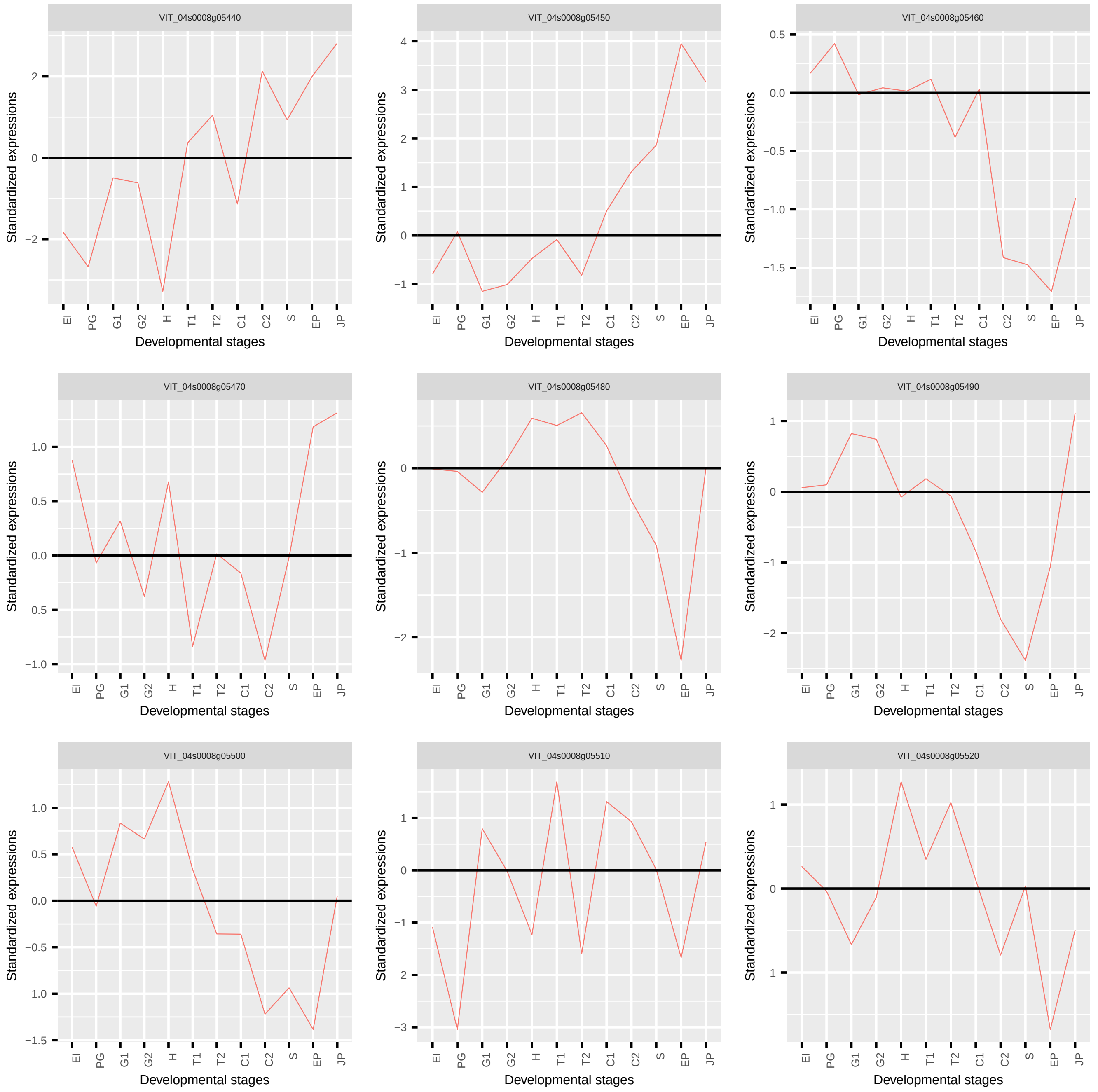
VIT_04s0008g05440
Standardized expressions
2
0
−2
EI PG G1 G2 H T1 T2 C1 C2 S EP JP
Developmental stages
VIT_04s0008g05450
Standardized expressions
4
3
2
1
0
−1
EI PG G1 G2 H T1 T2 C1 C2 S EP JP
Developmental stages
VIT_04s0008g05460
Standardized expressions
0.5
0.0
−0.5
−1.0
−1.5
EI PG G1 G2 H T1 T2 C1 C2 S EP JP
Developmental stages
VIT_04s0008g05470
Standardized expressions
1.0
0.5
0.0
−0.5
−1.0
EI PG G1 G2 H T1 T2 C1 C2 S EP JP
Developmental stages
VIT_04s0008g05480
Standardized expressions
0
−1
−2
EI PG G1 G2 H T1 T2 C1 C2 S EP JP
Developmental stages
VIT_04s0008g05490
Standardized expressions
1
0
−1
−2
EI PG G1 G2 H T1 T2 C1 C2 S EP JP
Developmental stages
VIT_04s0008g05500
Standardized expressions
1.0
0.5
0.0
−0.5
−1.0
−1.5
EI PG G1 G2 H T1 T2 C1 C2 S EP JP
Developmental stages
VIT_04s0008g05510
Standardized expressions
1
0
−1
−2
−3
EI PG G1 G2 H T1 T2 C1 C2 S EP JP
Developmental stages
VIT_04s0008g05520
Standardized expressions
1
0
−1
EI PG G1 G2 H T1 T2 C1 C2 S EP JP
Developmental stages

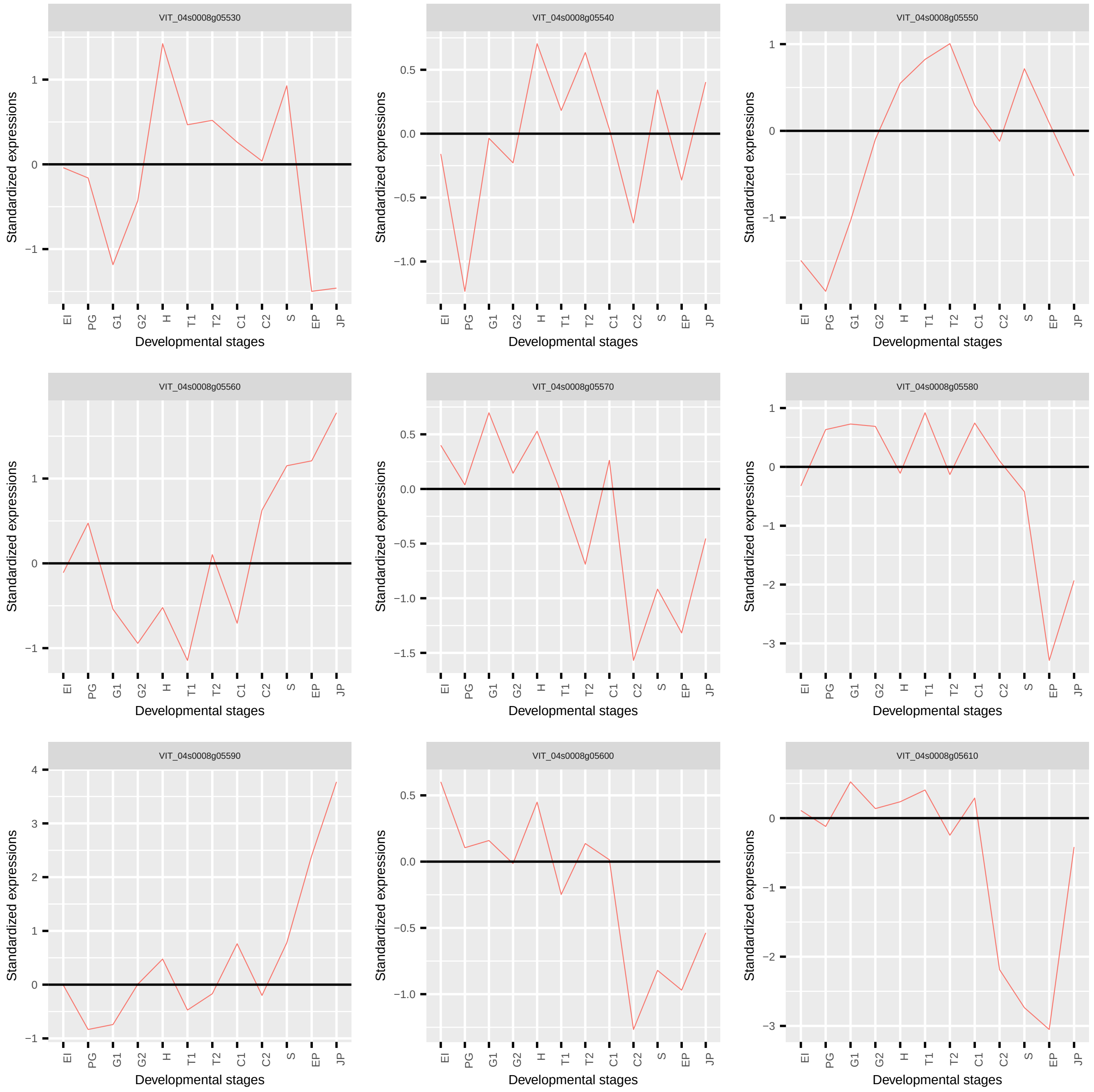
VIT_04s0008g05530
VIT_04s0008g05540
VIT_04s0008g05550
VIT_04s0008g05560
VIT_04s0008g05570
VIT_04s0008g05580
VIT_04s0008g05590
VIT_04s0008g05600
VIT_04s0008g05610
Standardized expressions
Developmental stages
EI PG G1 G2 H T1 T2 C1 C2 S EP JP

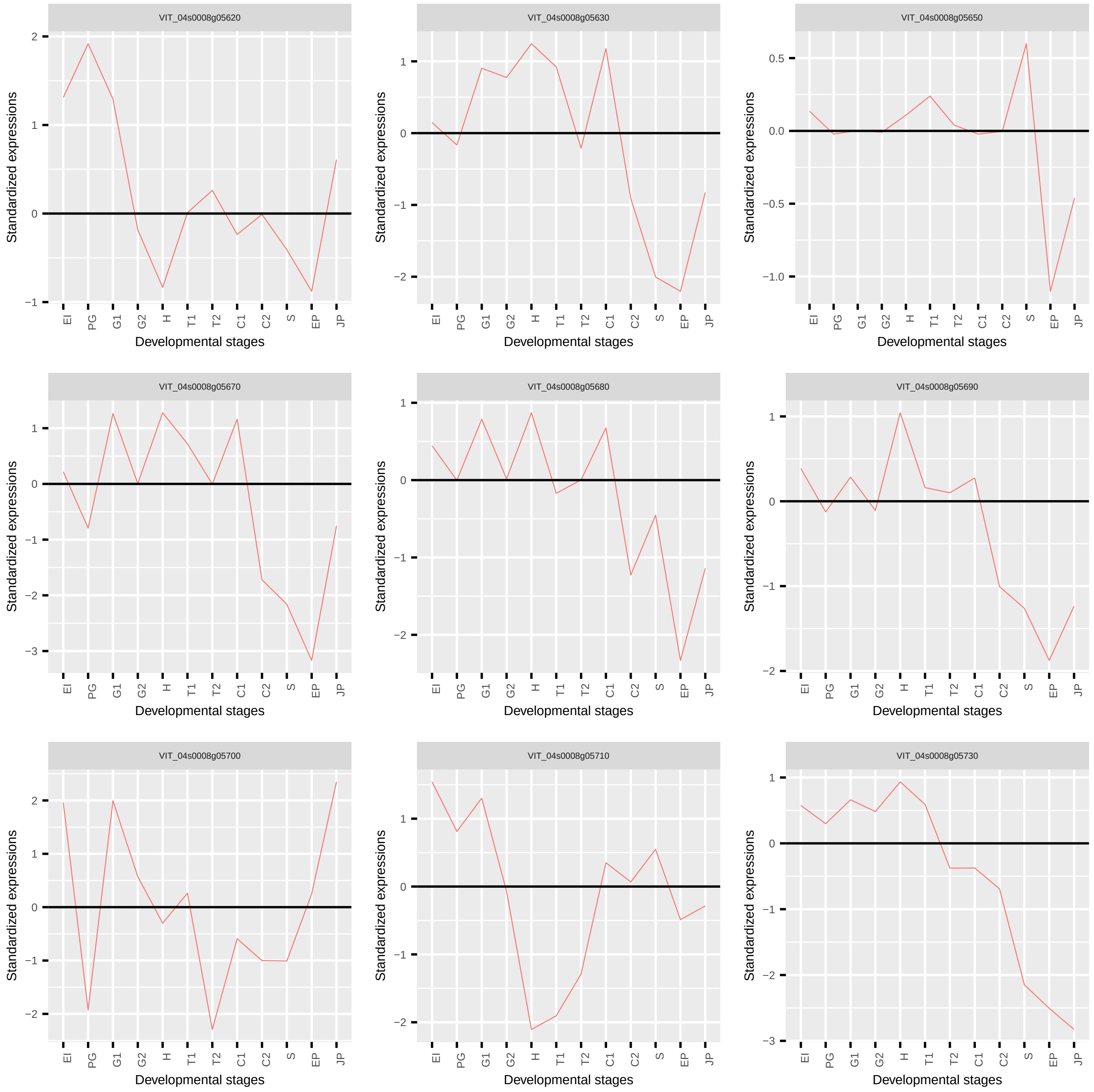

VIT_04s0008g05620
VIT_04s0008g05630
VIT_04s0008g05650
VIT_04s0008g05670
VIT_04s0008g05680
VIT_04s0008g05690
VIT_04s0008g05700
VIT_04s0008g05710
VIT_04s0008g05730
Standardized expressions
Developmental stages
EI
PG
G1
G2
H
T1
T2
C1
C2
S
EP
JP

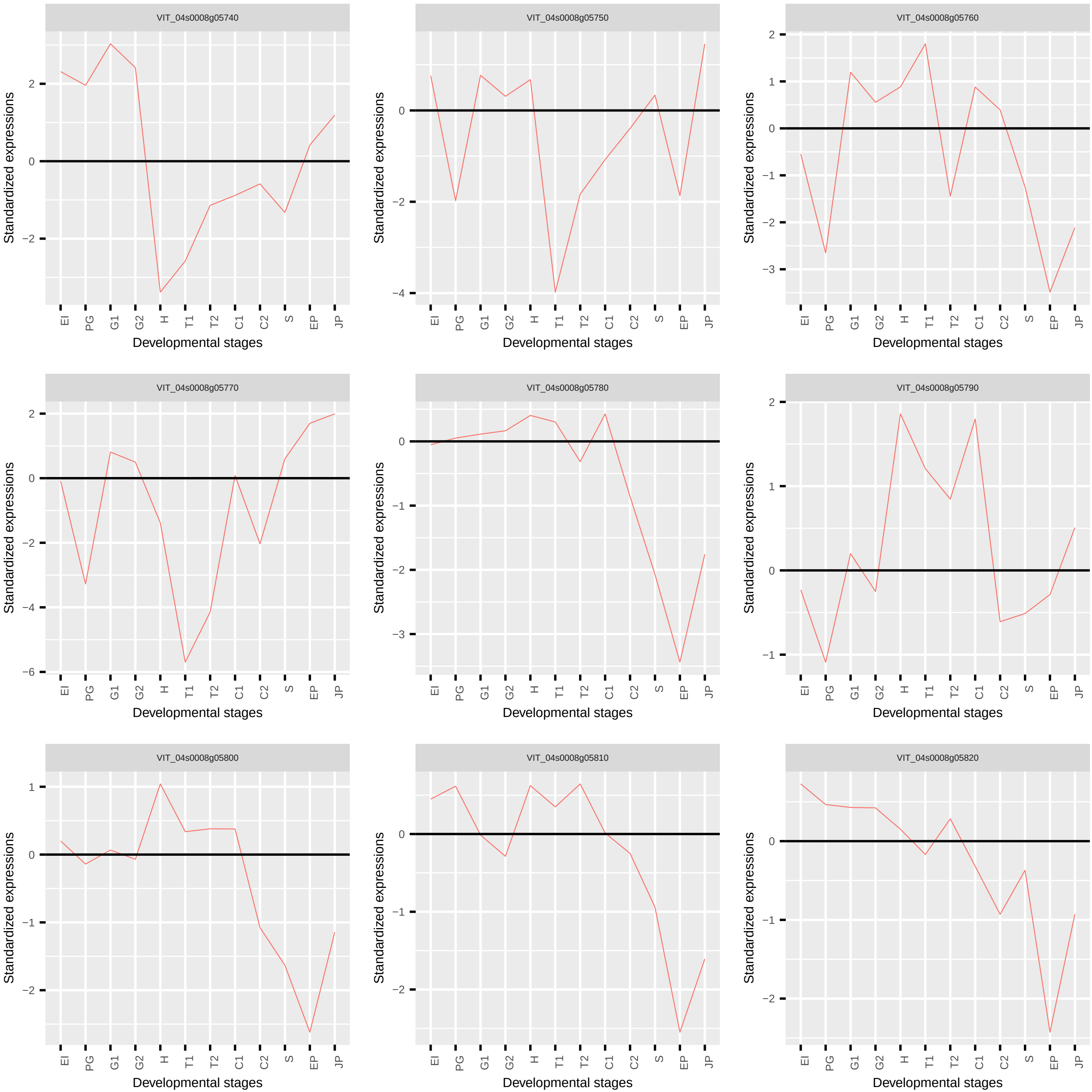
VIT_04s0008g05740
VIT_04s0008g05750
VIT_04s0008g05760
VIT_04s0008g05770
VIT_04s0008g05780
VIT_04s0008g05790
VIT_04s0008g05800
VIT_04s0008g05810
VIT_04s0008g05820
Standardized expressions
Developmental stages
EI PG G1 G2 H T1 T2 C1 C2 S EP JP

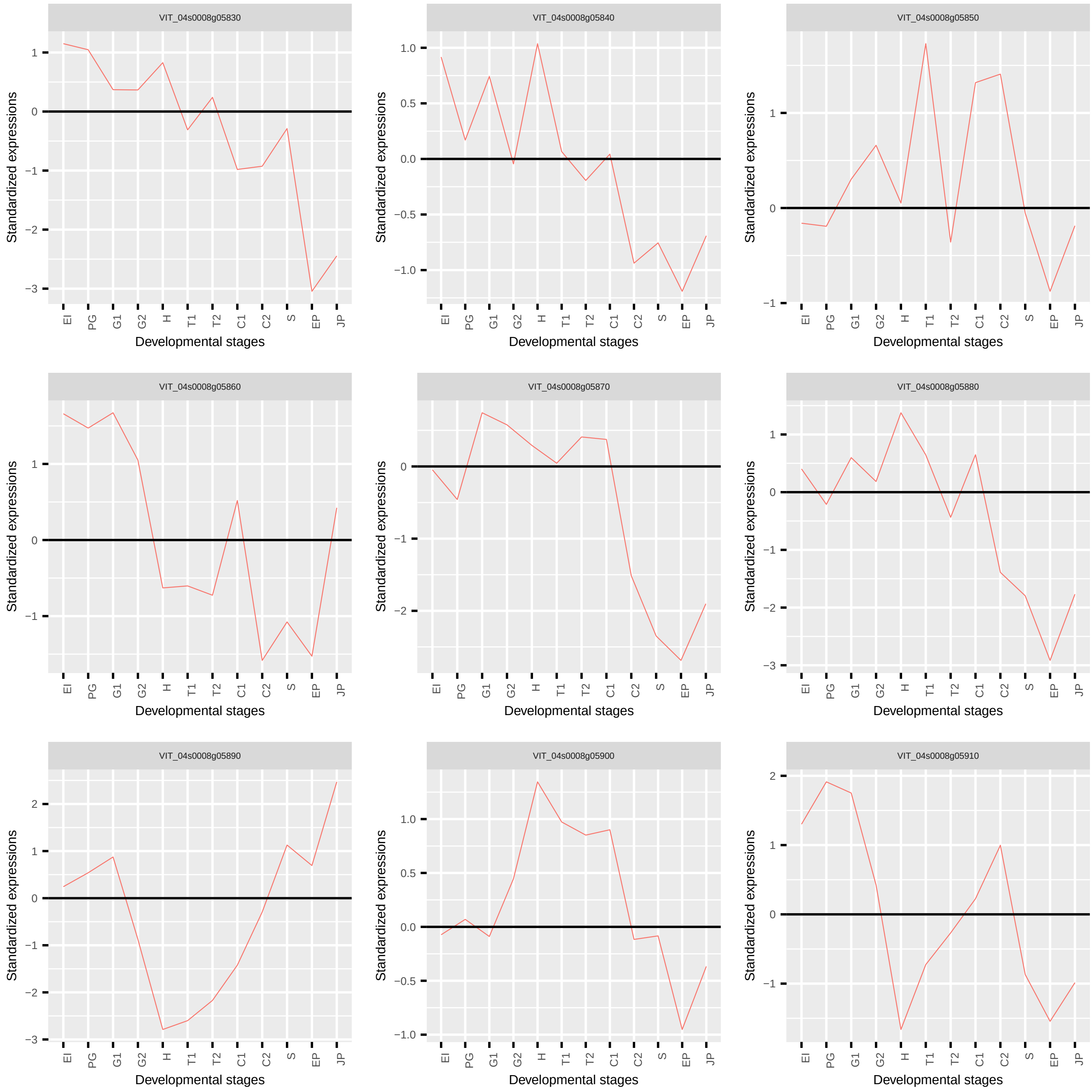
VIT_04s0008g05830
VIT_04s0008g05840
VIT_04s0008g05850
VIT_04s0008g05860
VIT_04s0008g05870
VIT_04s0008g05880
VIT_04s0008g05890
VIT_04s0008g05900
VIT_04s0008g05910
Standardized expressions
Developmental stages
EI PG G1 G2 H T1 T2 C1 C2 S EP JP

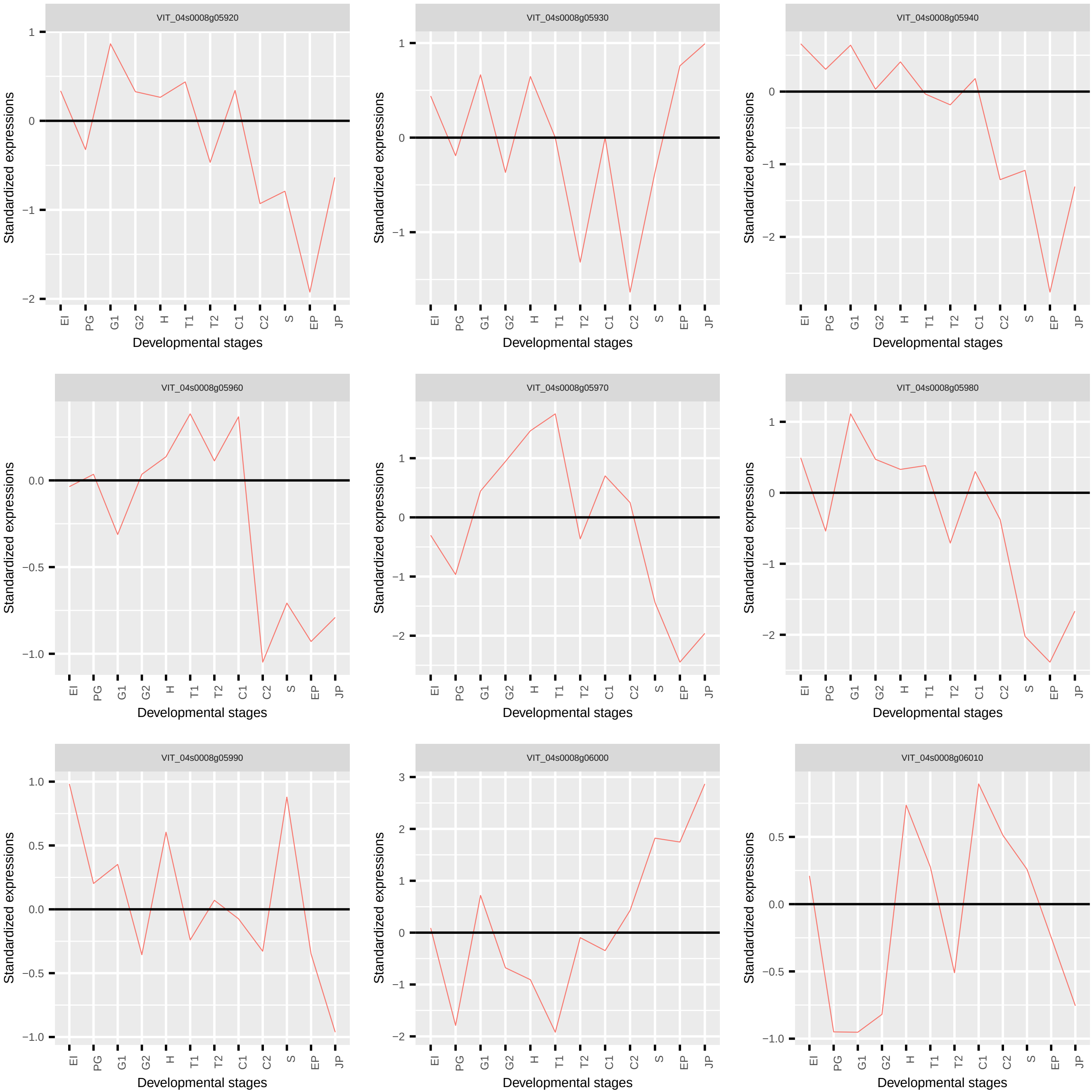
VIT_04s0008g05920
VIT_04s0008g05930
VIT_04s0008g05940
VIT_04s0008g05960
VIT_04s0008g05970
VIT_04s0008g05980
VIT_04s0008g05990
VIT_04s0008g06000
VIT_04s0008g06010
Standardized expressions
EI PG G1 G2 H T1 T2 C1 C2 S EP JP
Developmental stages

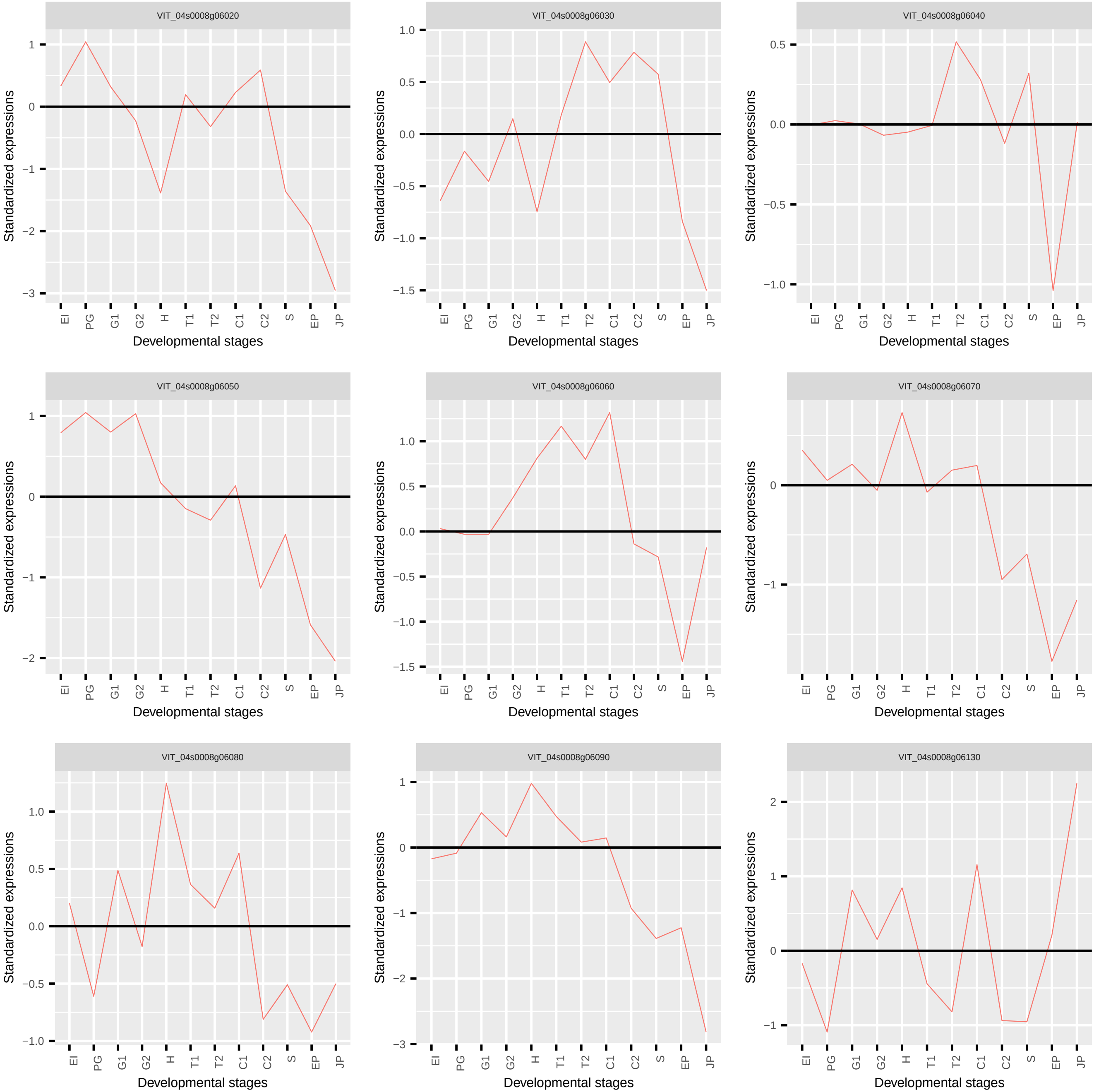
VIT_04s0008g06020
VIT_04s0008g06030
VIT_04s0008g06040
VIT_04s0008g06050
VIT_04s0008g06060
VIT_04s0008g06070
VIT_04s0008g06080
VIT_04s0008g06090
VIT_04s0008g06130
Standardized expressions
Developmental stages
EI PG G1 G2 H T1 T2 C1 C2 S EP JP

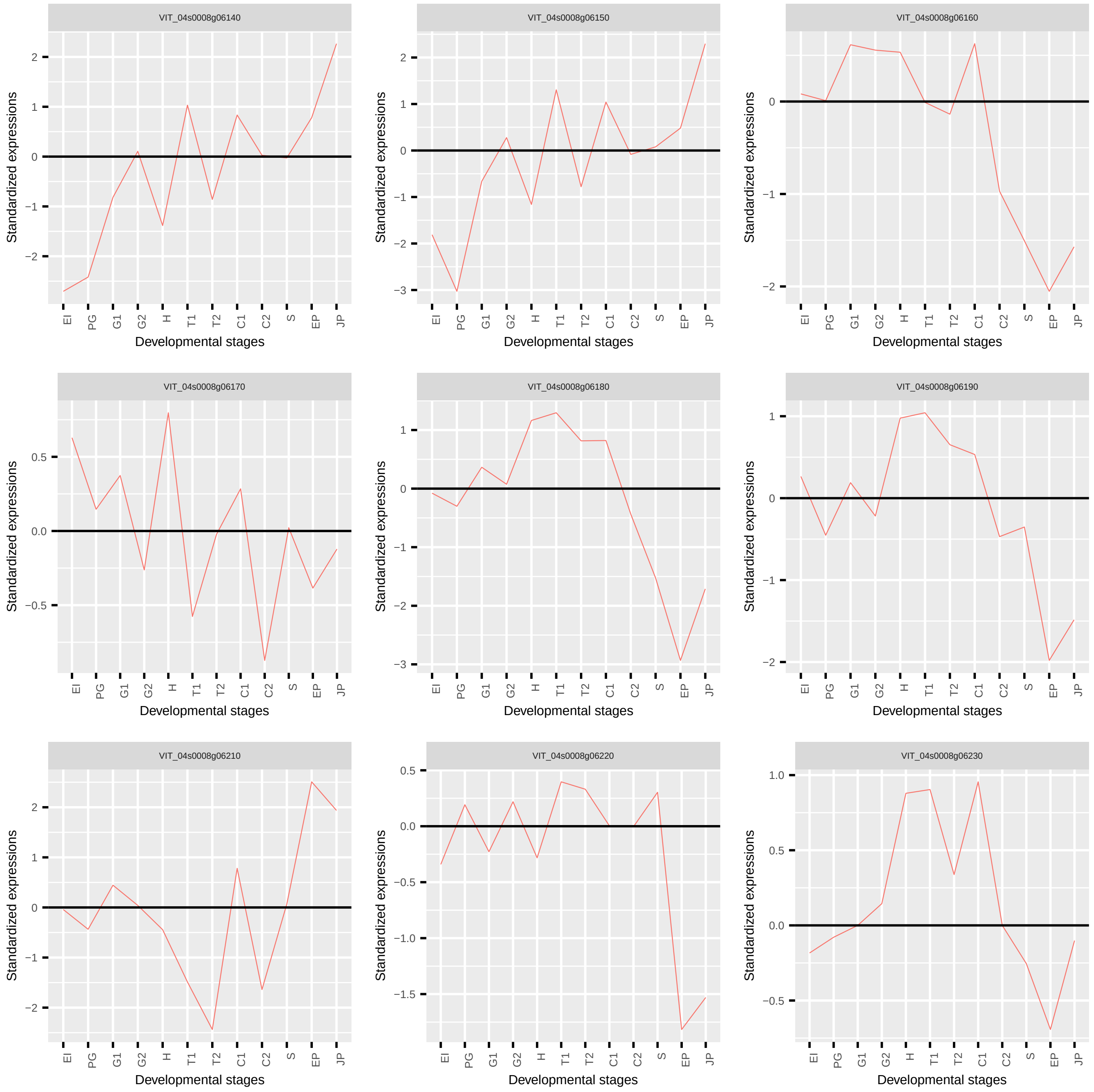

VIT_04s0008g06140
VIT_04s0008g06150
VIT_04s0008g06160
VIT_04s0008g06170
VIT_04s0008g06180
VIT_04s0008g06190
VIT_04s0008g06210
VIT_04s0008g06220
VIT_04s0008g06230
Standardized expressions
Developmental stages
EI PG G1 G2 H T1 T2 C1 C2 S EP JP

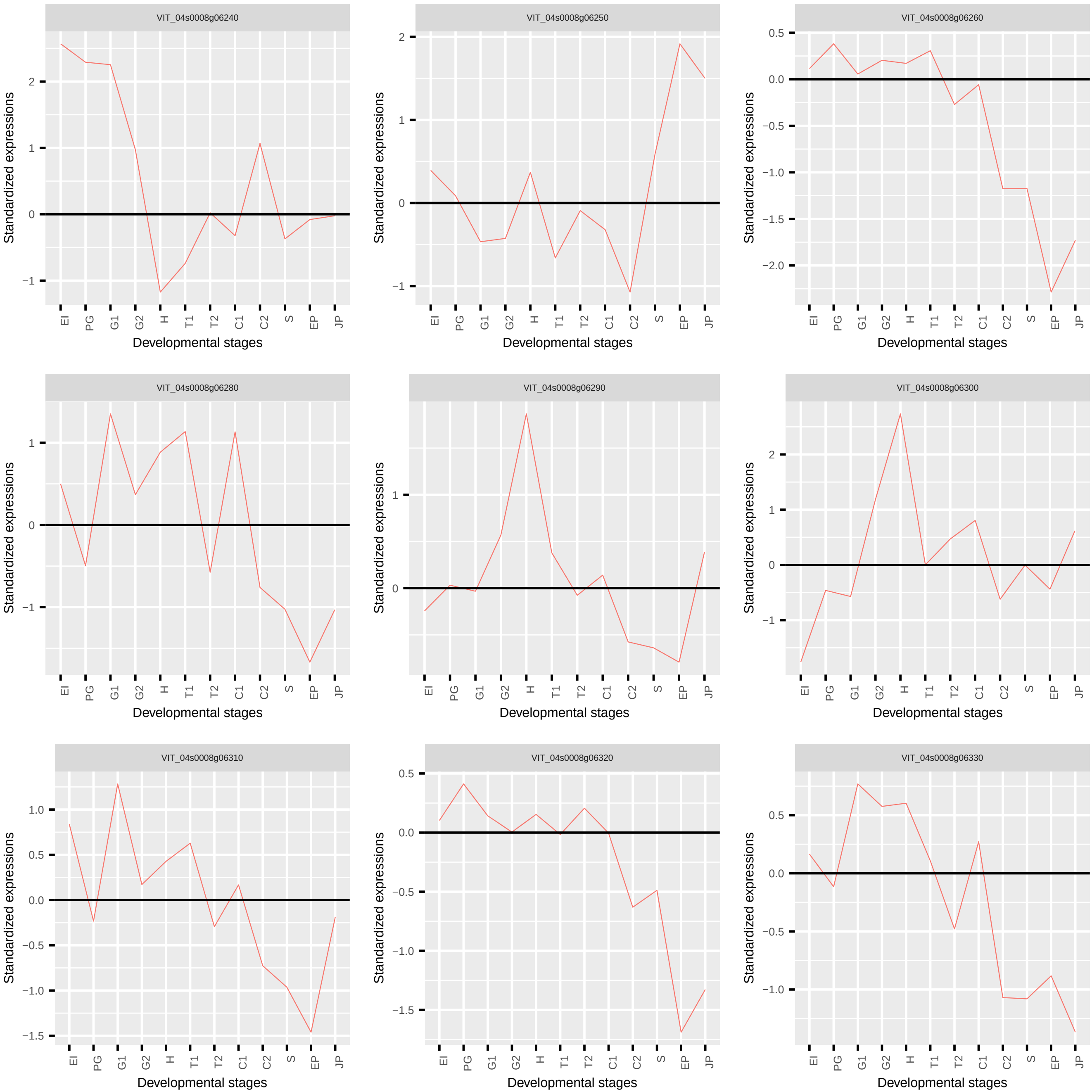
VIT_04s0008g06240
Standardized expressions
2
1
0
−1
EI PG G1 G2 H T1 T2 C1 C2 S EP JP
Developmental stages
VIT_04s0008g06250
Standardized expressions
2
1
0
−1
EI PG G1 G2 H T1 T2 C1 C2 S EP JP
Developmental stages
VIT_04s0008g06260
Standardized expressions
0.5
0.0
−0.5
−1.0
−1.5
−2.0
EI PG G1 G2 H T1 T2 C1 C2 S EP JP
Developmental stages
VIT_04s0008g06280
Standardized expressions
1
0
−1
EI PG G1 G2 H T1 T2 C1 C2 S EP JP
Developmental stages
VIT_04s0008g06290
Standardized expressions
1
0
EI PG G1 G2 H T1 T2 C1 C2 S EP JP
Developmental stages
VIT_04s0008g06300
Standardized expressions
2
1
0
−1
EI PG G1 G2 H T1 T2 C1 C2 S EP JP
Developmental stages
VIT_04s0008g06310
Standardized expressions
1.0
0.5
0.0
−0.5
−1.0
−1.5
EI PG G1 G2 H T1 T2 C1 C2 S EP JP
Developmental stages
VIT_04s0008g06320
Standardized expressions
0.5
0.0
−0.5
−1.0
−1.5
EI PG G1 G2 H T1 T2 C1 C2 S EP JP
Developmental stages
VIT_04s0008g06330
Standardized expressions
0.5
0.0
−0.5
−1.0
EI PG G1 G2 H T1 T2 C1 C2 S EP JP
Developmental stages

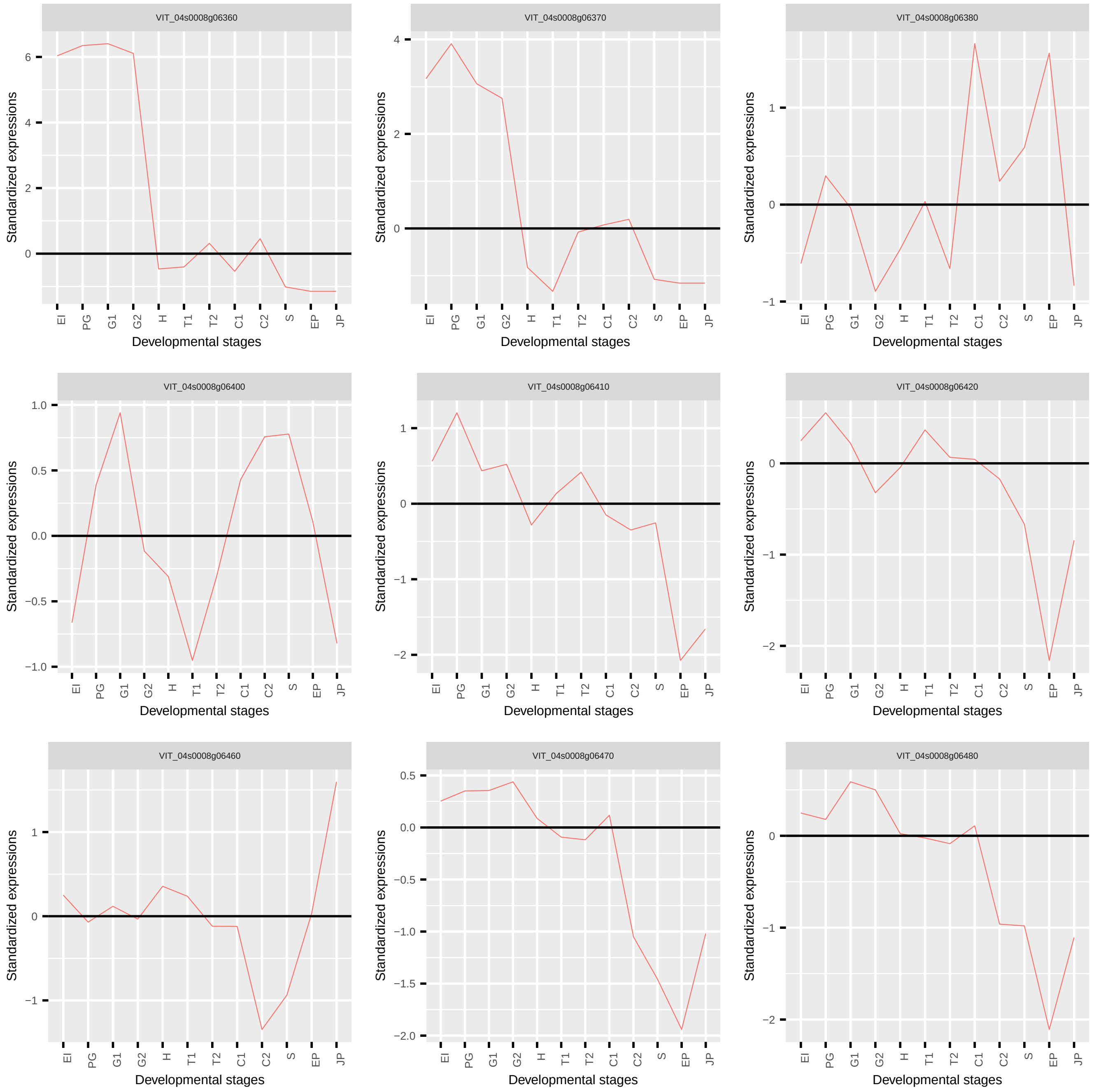
VIT_04s0008g06360
VIT_04s0008g06370
VIT_04s0008g06380
VIT_04s0008g06400
VIT_04s0008g06410
VIT_04s0008g06420
VIT_04s0008g06460
VIT_04s0008g06470
VIT_04s0008g06480
Standardized expressions
Developmental stages
EI PG G1 G2 H T1 T2 C1 C2 S EP JP

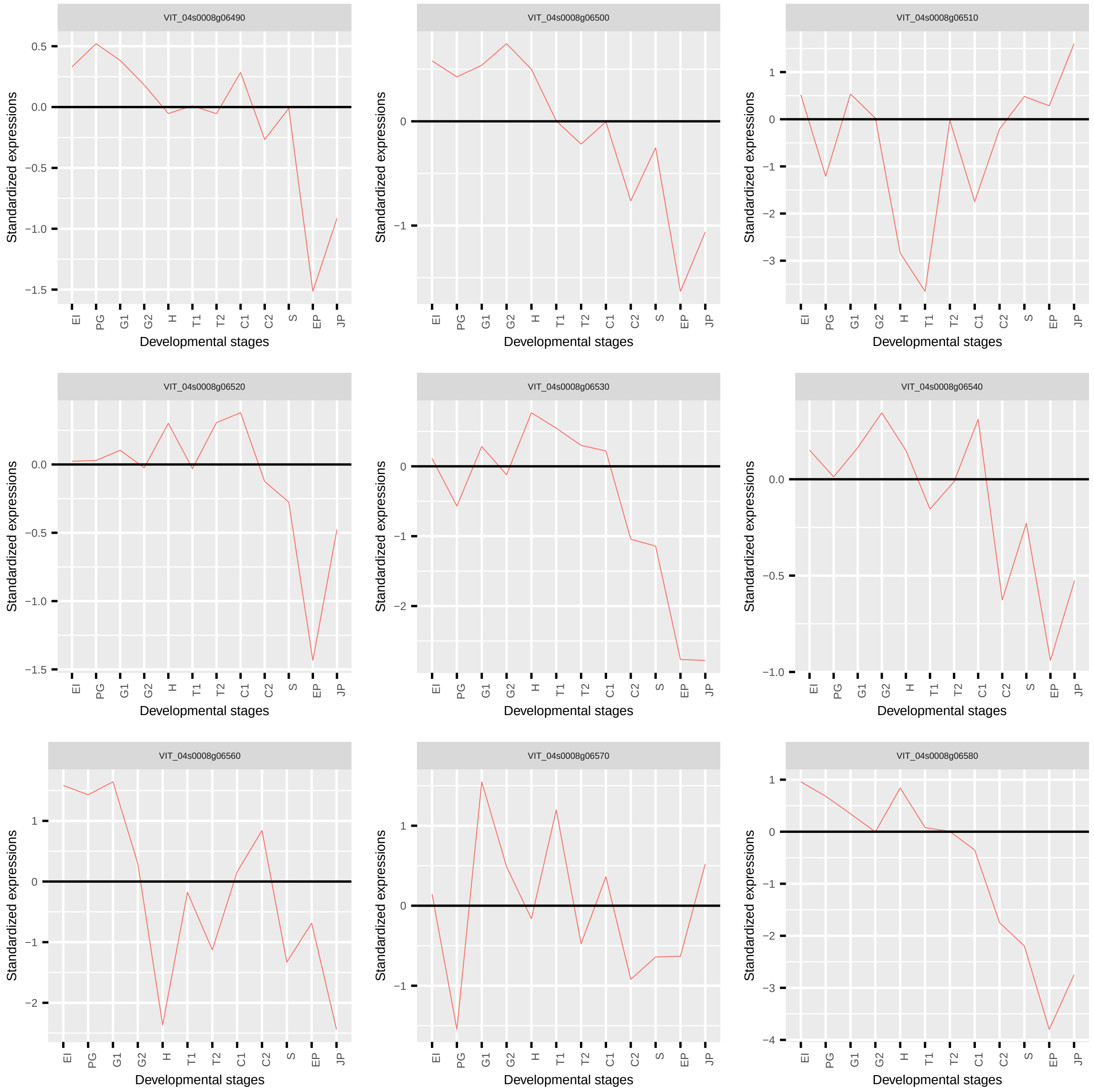

VIT_04s0008g06490
VIT_04s0008g06500
VIT_04s0008g06510
VIT_04s0008g06520
VIT_04s0008g06530
VIT_04s0008g06540
VIT_04s0008g06560
VIT_04s0008g06570
VIT_04s0008g06580
Standardized expressions
Developmental stages
EI PG G1 G2 H T1 T2 C1 C2 S EP JP

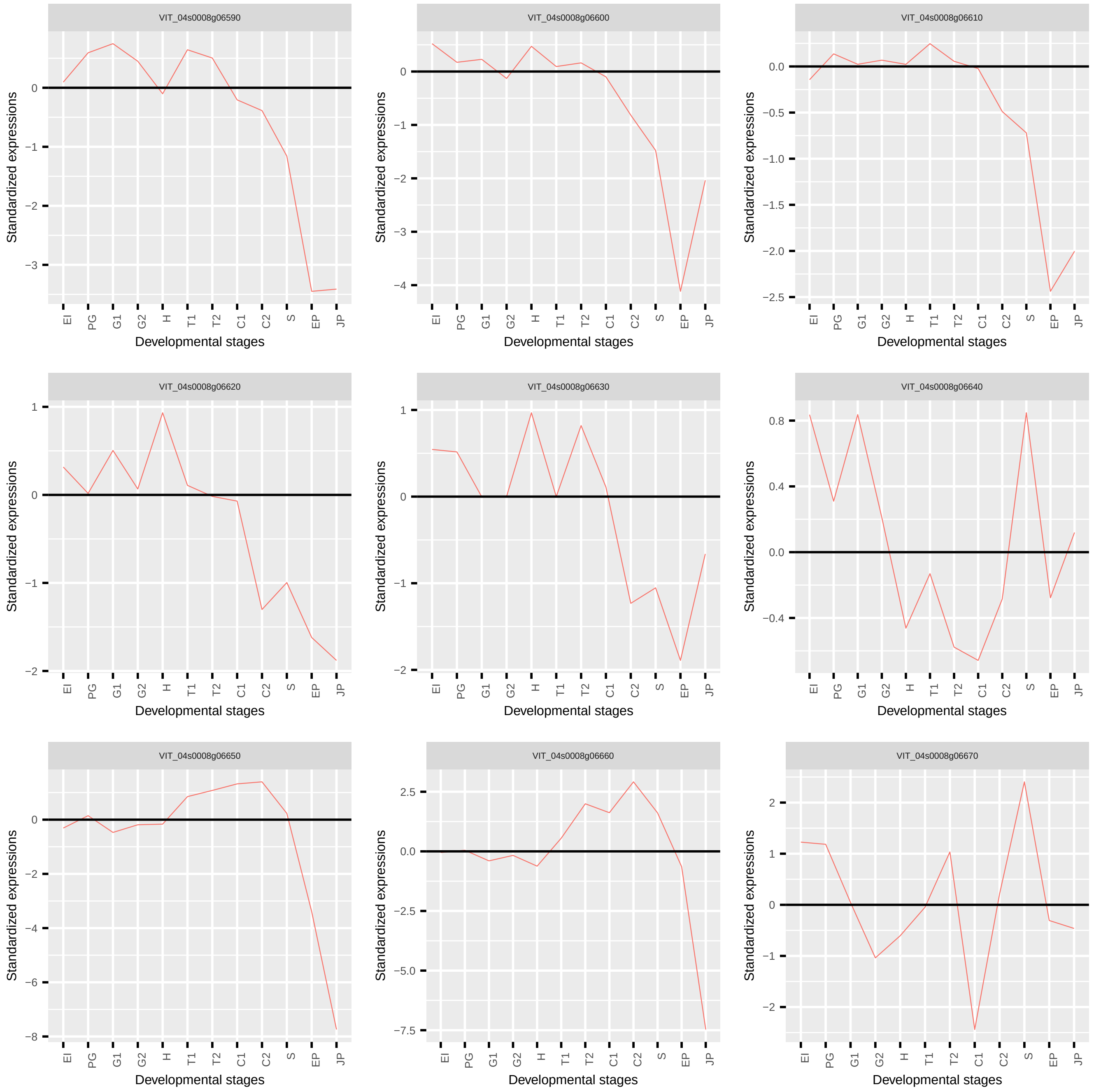
VIT_04s0008g06590
VIT_04s0008g06600
VIT_04s0008g06610
VIT_04s0008g06620
VIT_04s0008g06630
VIT_04s0008g06640
VIT_04s0008g06650
VIT_04s0008g06660
VIT_04s0008g06670
Standardized expressions
Developmental stages
EI
PG
G1
G2
H
T1
T2
C1
C2
S
EP
JP

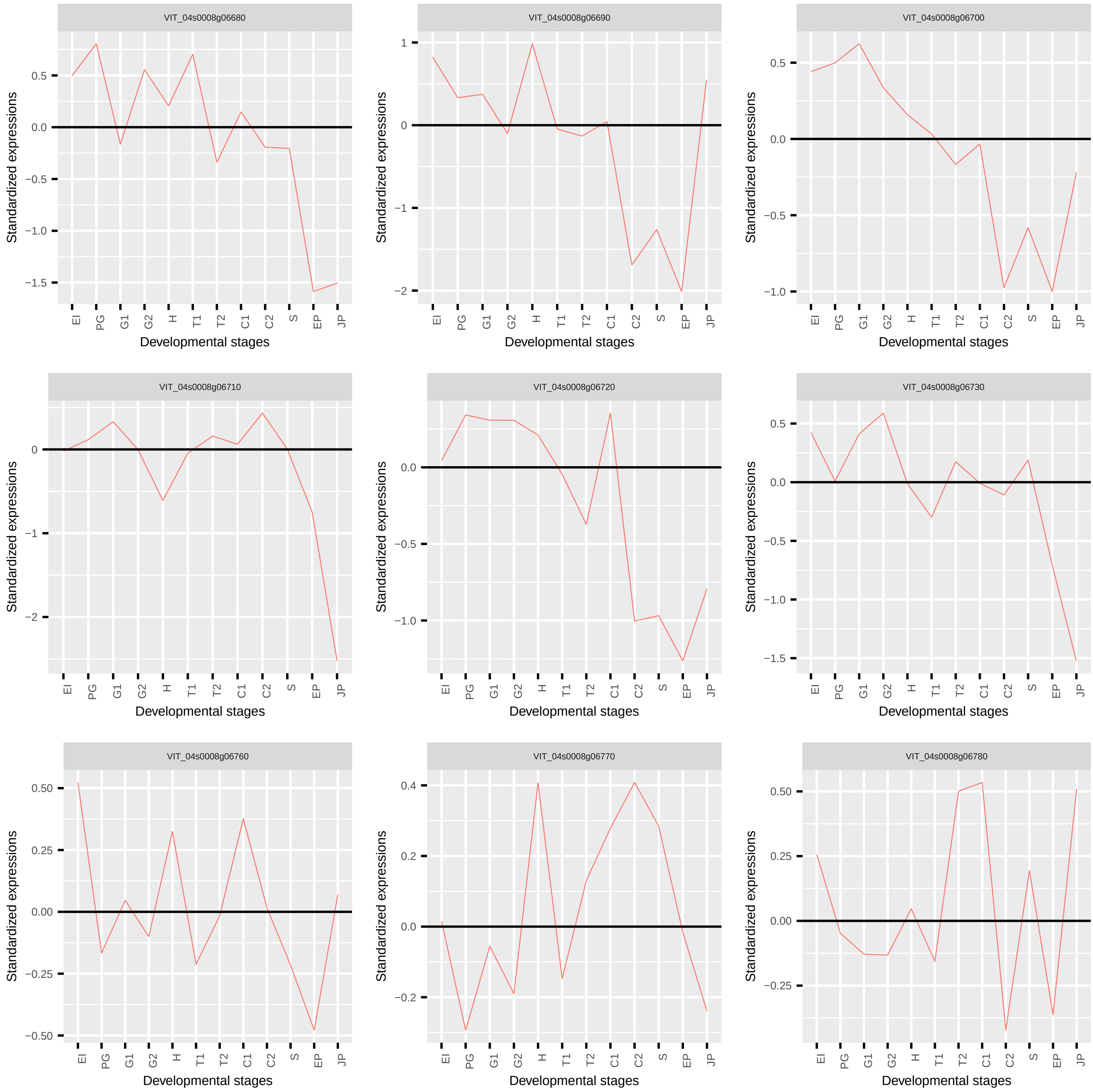
VIT_04s0008g06680
0.5
0.0
−0.5
−1.0
−1.5
Standardized expressions
EI PG G1 G2 H T1 T2 C1 C2 S EP JP
Developmental stages
VIT_04s0008g06690
1
0
−1
−2
Standardized expressions
EI PG G1 G2 H T1 T2 C1 C2 S EP JP
Developmental stages
VIT_04s0008g06700
0.5
0.0
−0.5
−1.0
Standardized expressions
EI PG G1 G2 H T1 T2 C1 C2 S EP JP
Developmental stages
VIT_04s0008g06710
0
−1
−2
Standardized expressions
EI PG G1 G2 H T1 T2 C1 C2 S EP JP
Developmental stages
VIT_04s0008g06720
0.0
−0.5
−1.0
Standardized expressions
EI PG G1 G2 H T1 T2 C1 C2 S EP JP
Developmental stages
VIT_04s0008g06730
0.5
0.0
−0.5
−1.0
−1.5
Standardized expressions
EI PG G1 G2 H T1 T2 C1 C2 S EP JP
Developmental stages
VIT_04s0008g06760
0.50
0.25
0.00
−0.25
−0.50
Standardized expressions
EI PG G1 G2 H T1 T2 C1 C2 S EP JP
Developmental stages
VIT_04s0008g06770
0.4
0.2
0.0
−0.2
Standardized expressions
EI PG G1 G2 H T1 T2 C1 C2 S EP JP
Developmental stages
VIT_04s0008g06780
0.50
0.25
0.00
−0.25
Standardized expressions
EI PG G1 G2 H T1 T2 C1 C2 S EP JP
Developmental stages

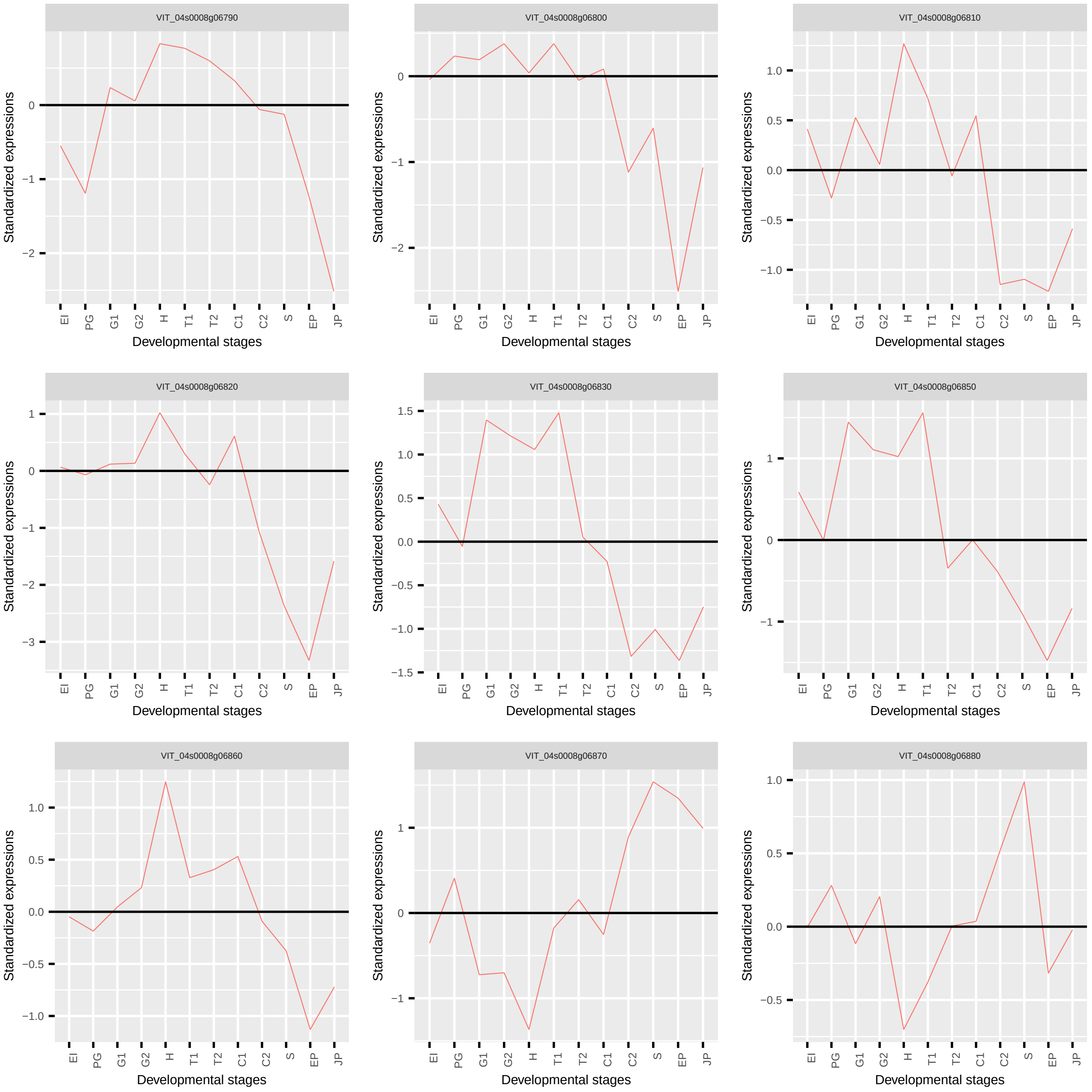
VIT_04s0008g06790
VIT_04s0008g06800
VIT_04s0008g06810
VIT_04s0008g06820
VIT_04s0008g06830
VIT_04s0008g06850
VIT_04s0008g06860
VIT_04s0008g06870
VIT_04s0008g06880
Standardized expressions
Developmental stages
EI
PG
G1
G2
H
T1
T2
C1
C2
S
EP
JP

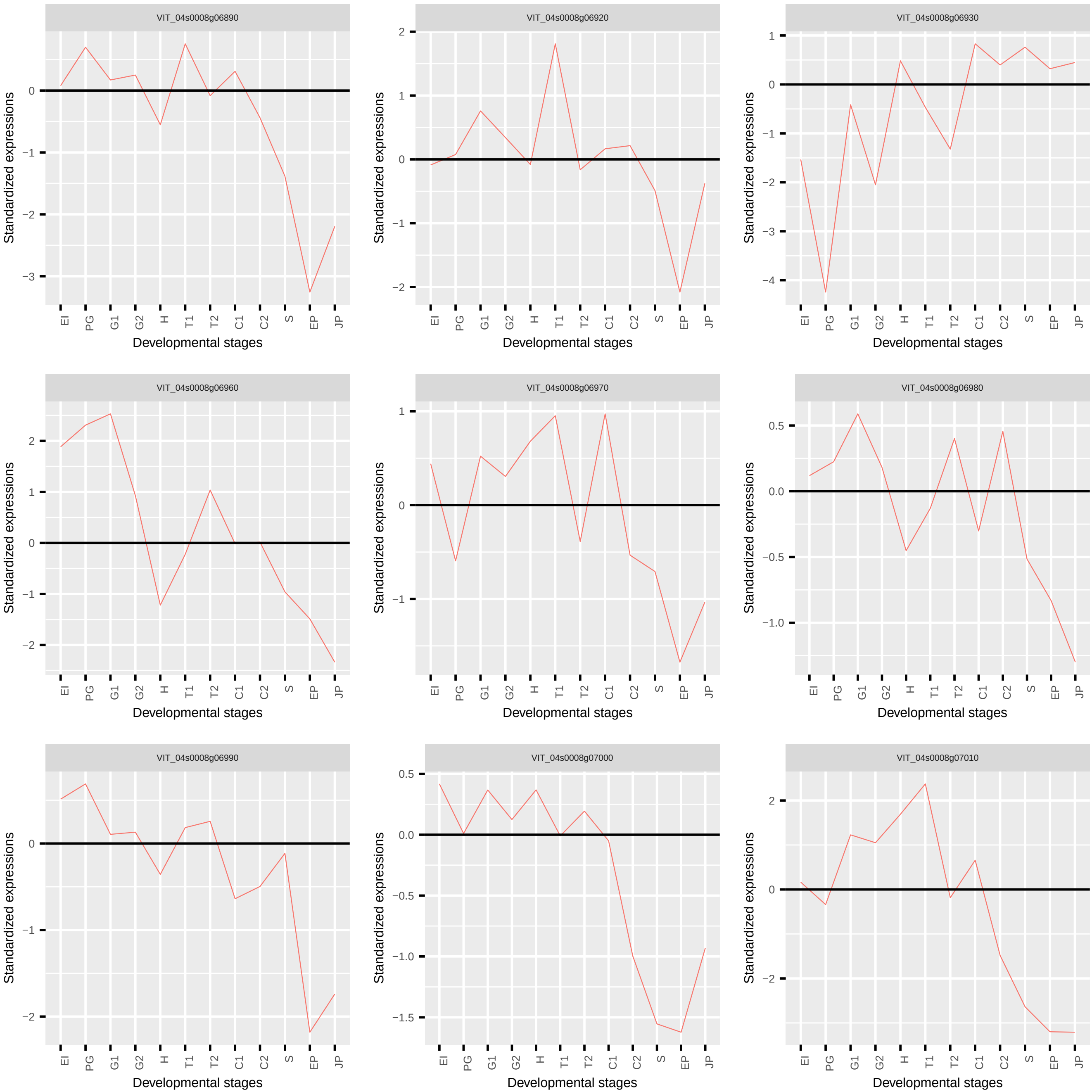

VIT_04s0008g06890
VIT_04s0008g06920
VIT_04s0008g06930
VIT_04s0008g06960
VIT_04s0008g06970
VIT_04s0008g06980
VIT_04s0008g06990
VIT_04s0008g07000
VIT_04s0008g07010
Standardized expressions
Developmental stages
EI PG G1 G2 H T1 T2 C1 C2 S EP JP

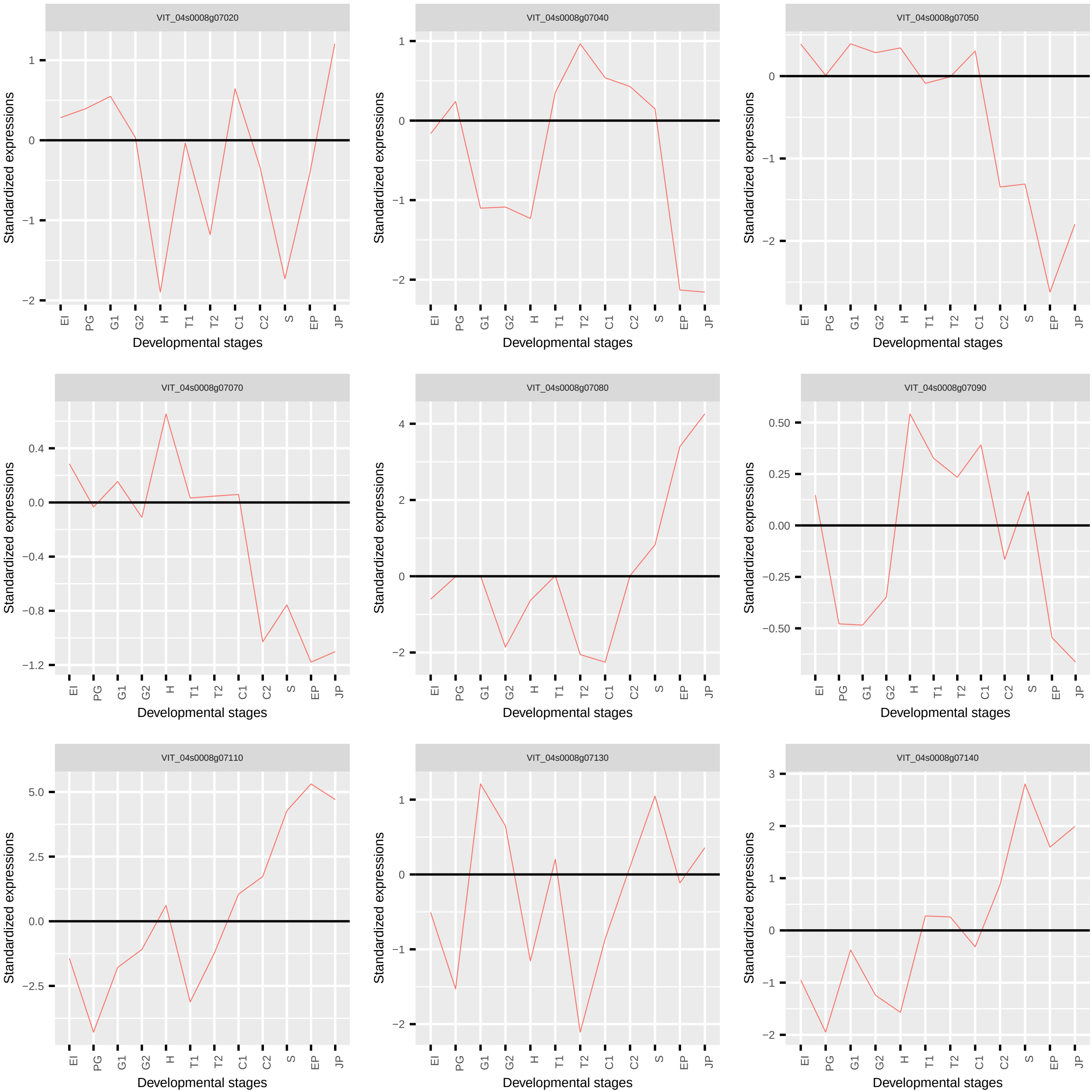
VIT_04s0008g07020
VIT_04s0008g07040
VIT_04s0008g07050
VIT_04s0008g07070
VIT_04s0008g07080
VIT_04s0008g07090
VIT_04s0008g07110
VIT_04s0008g07130
VIT_04s0008g07140
Standardized expressions
Developmental stages
EI
PG
G1
G2
H
T1
T2
C1
C2
S
EP
JP

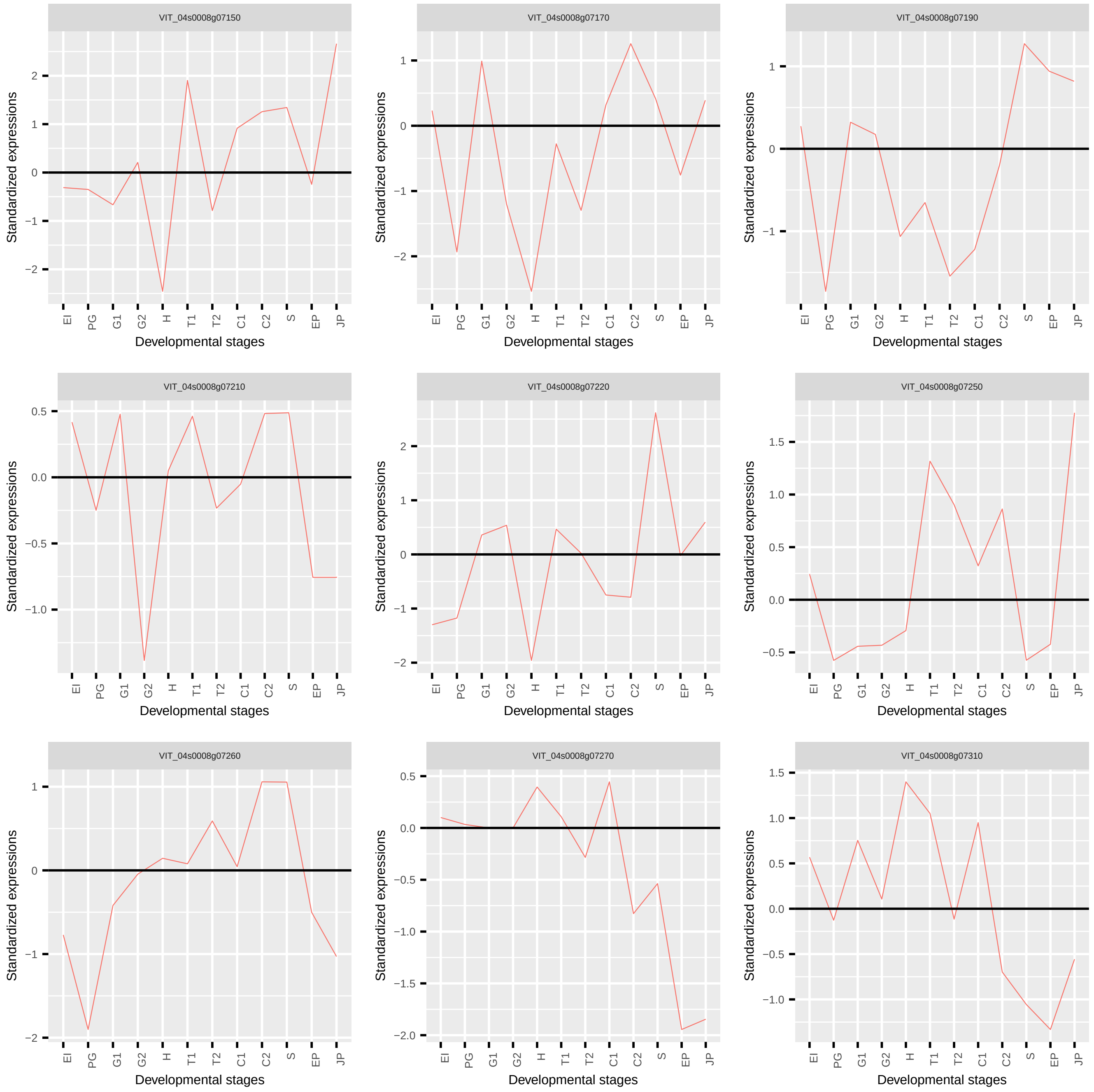

VIT_04s0008g07150
VIT_04s0008g07170
VIT_04s0008g07190
VIT_04s0008g07210
VIT_04s0008g07220
VIT_04s0008g07250
VIT_04s0008g07260
VIT_04s0008g07270
VIT_04s0008g07310
Standardized expressions
Developmental stages
EI
PG
G1
G2
H
T1
T2
C1
C2
S
EP
JP

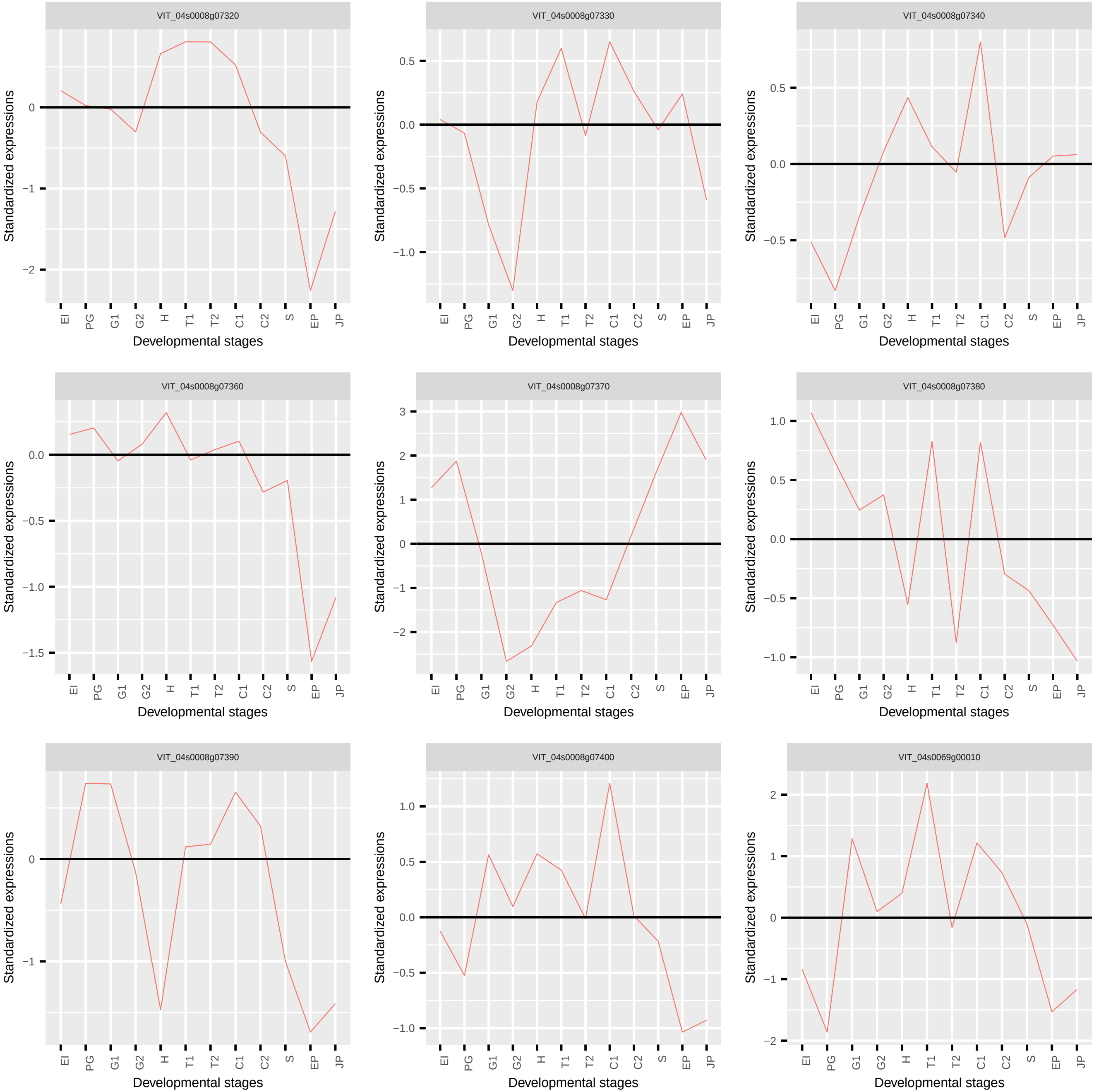
VIT_04s0008g07320
VIT_04s0008g07330
VIT_04s0008g07340
VIT_04s0008g07360
VIT_04s0008g07370
VIT_04s0008g07380
VIT_04s0008g07390
VIT_04s0008g07400
VIT_04s0069g00010
Standardized expressions
Developmental stages
EI PG G1 G2 H T1 T2 C1 C2 S EP JP

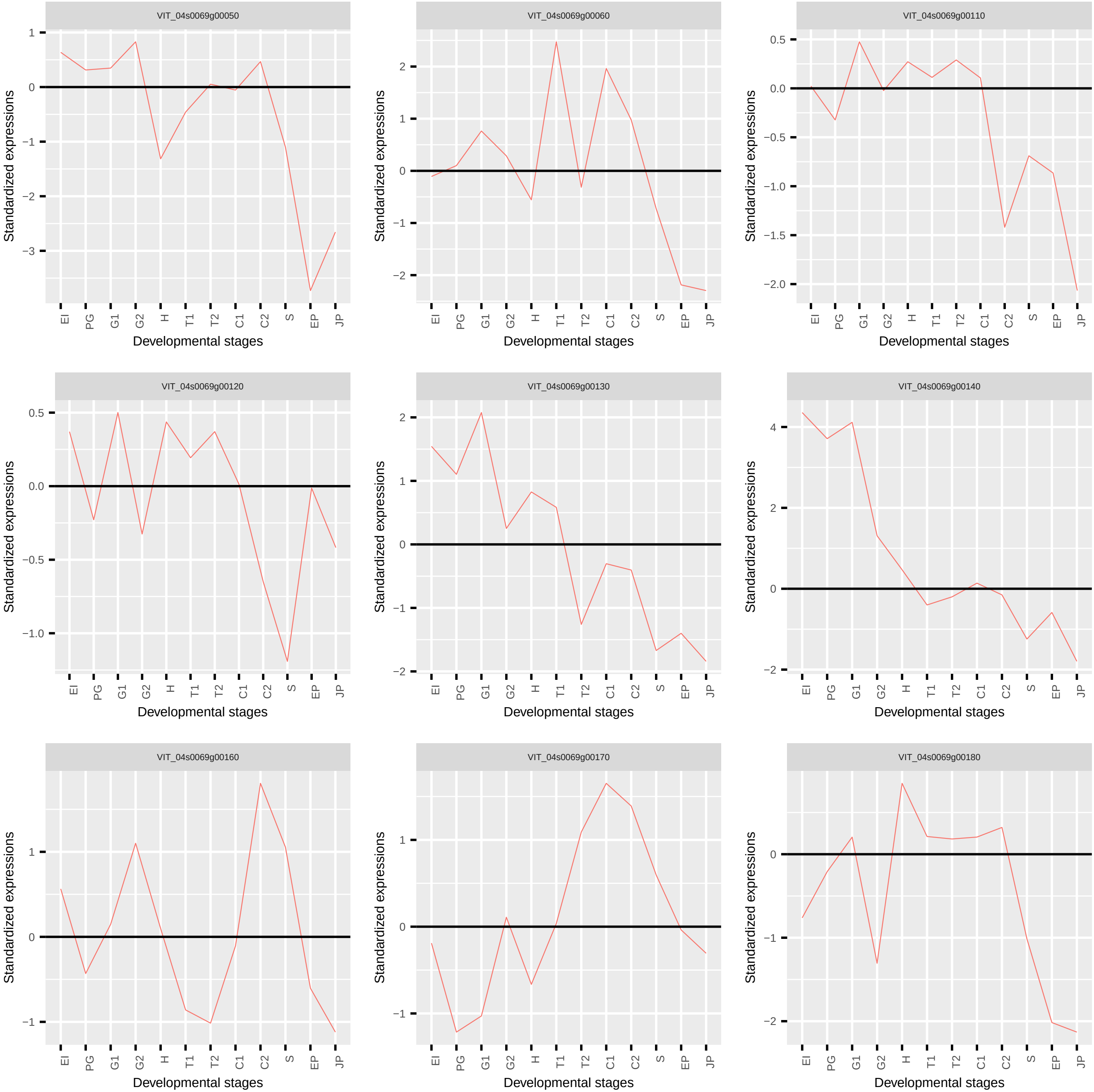

VIT_04s0069g00050
VIT_04s0069g00060
VIT_04s0069g00110
VIT_04s0069g00120
VIT_04s0069g00130
VIT_04s0069g00140
VIT_04s0069g00160
VIT_04s0069g00170
VIT_04s0069g00180
Standardized expressions
Developmental stages
EI PG G1 G2 H T1 T2 C1 C2 S EP JP

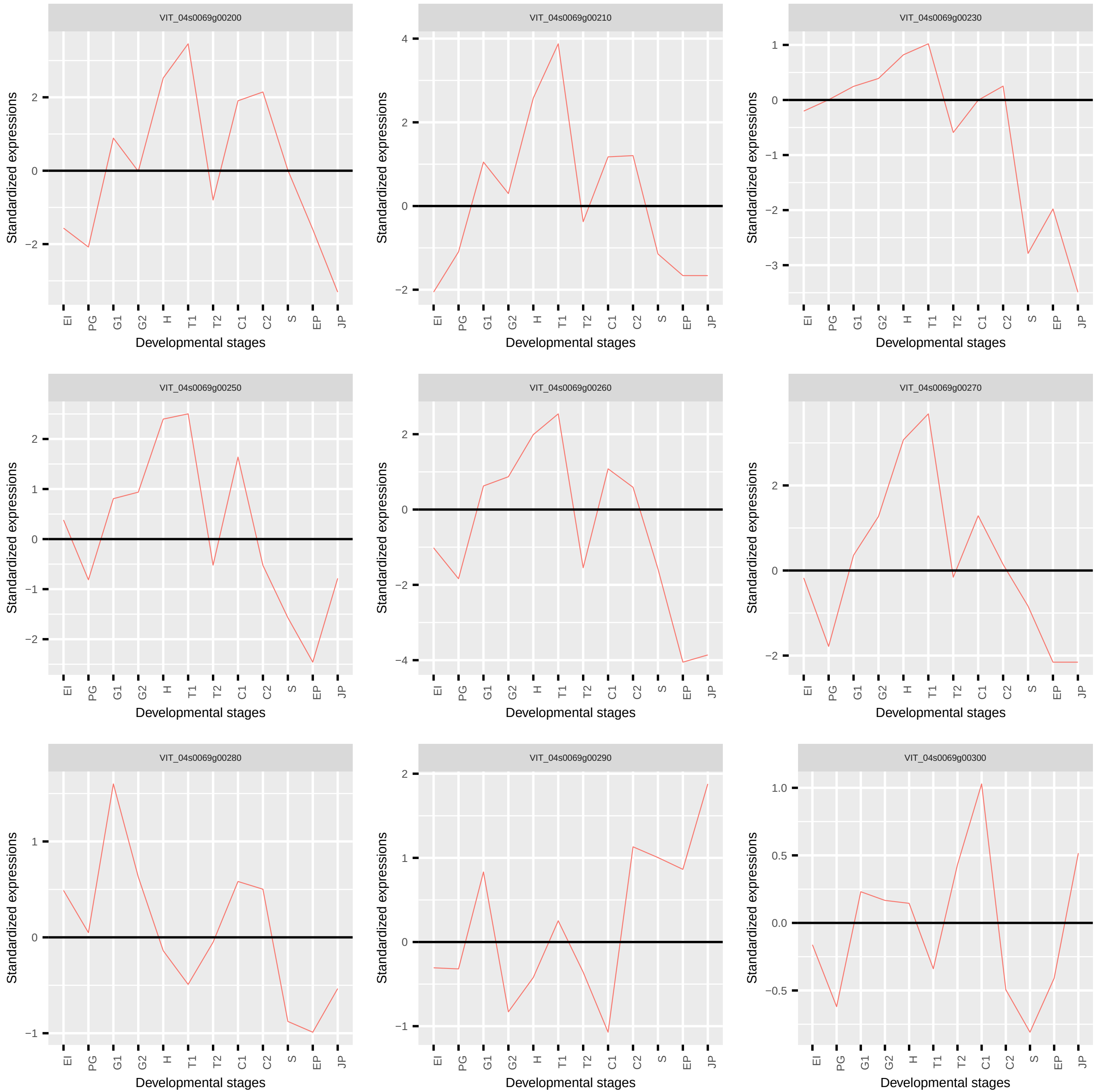
VIT_04s0069g00200
VIT_04s0069g00210
VIT_04s0069g00230
VIT_04s0069g00250
VIT_04s0069g00260
VIT_04s0069g00270
VIT_04s0069g00280
VIT_04s0069g00290
VIT_04s0069g00300
Standardized expressions
Developmental stages
EI PG G1 G2 H T1 T2 C1 C2 S EP JP

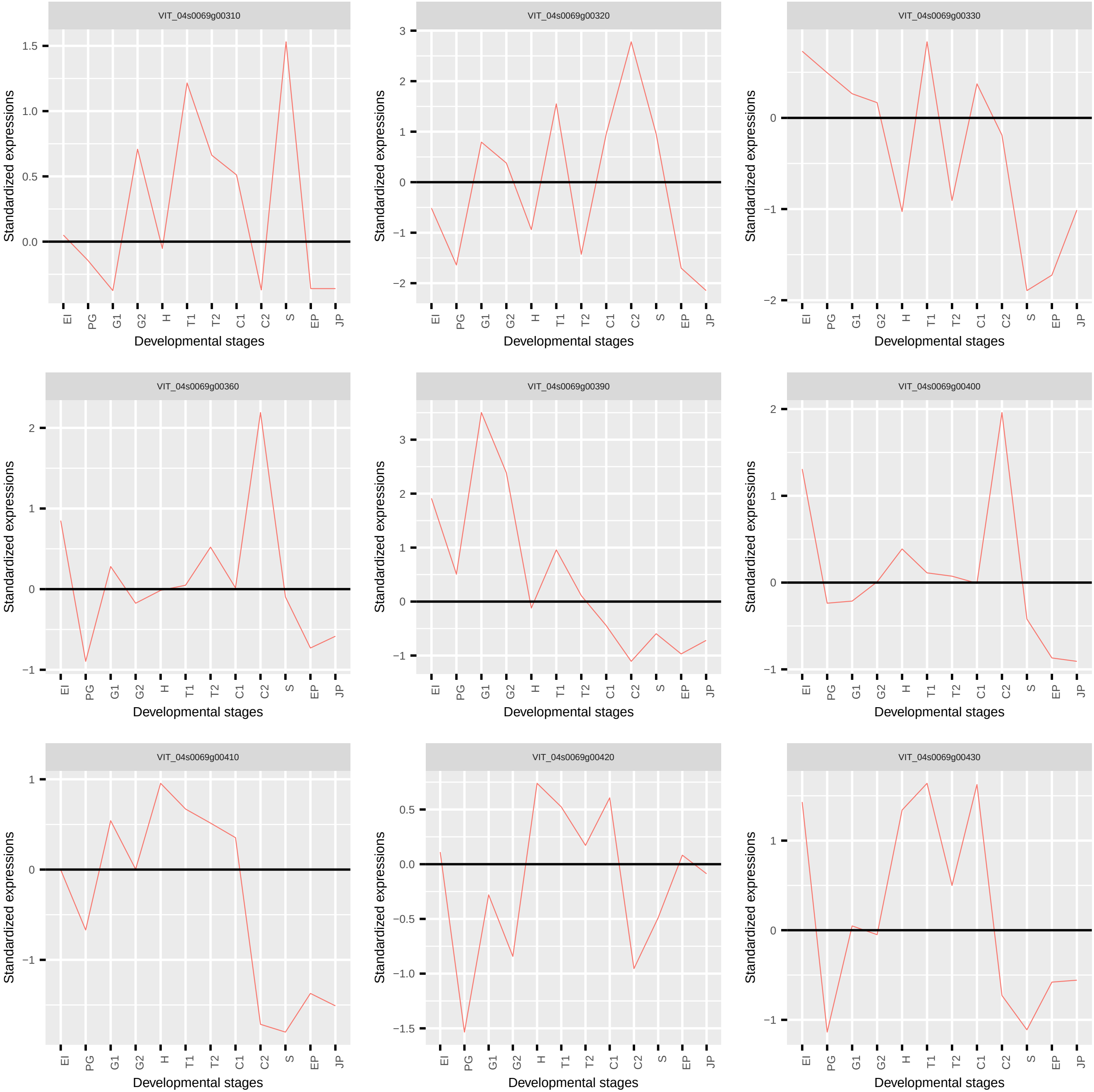

VIT_04s0069g00310
VIT_04s0069g00320
VIT_04s0069g00330
VIT_04s0069g00360
VIT_04s0069g00390
VIT_04s0069g00400
VIT_04s0069g00410
VIT_04s0069g00420
VIT_04s0069g00430
Standardized expressions
Developmental stages
EI PG G1 G2 H T1 T2 C1 C2 S EP JP

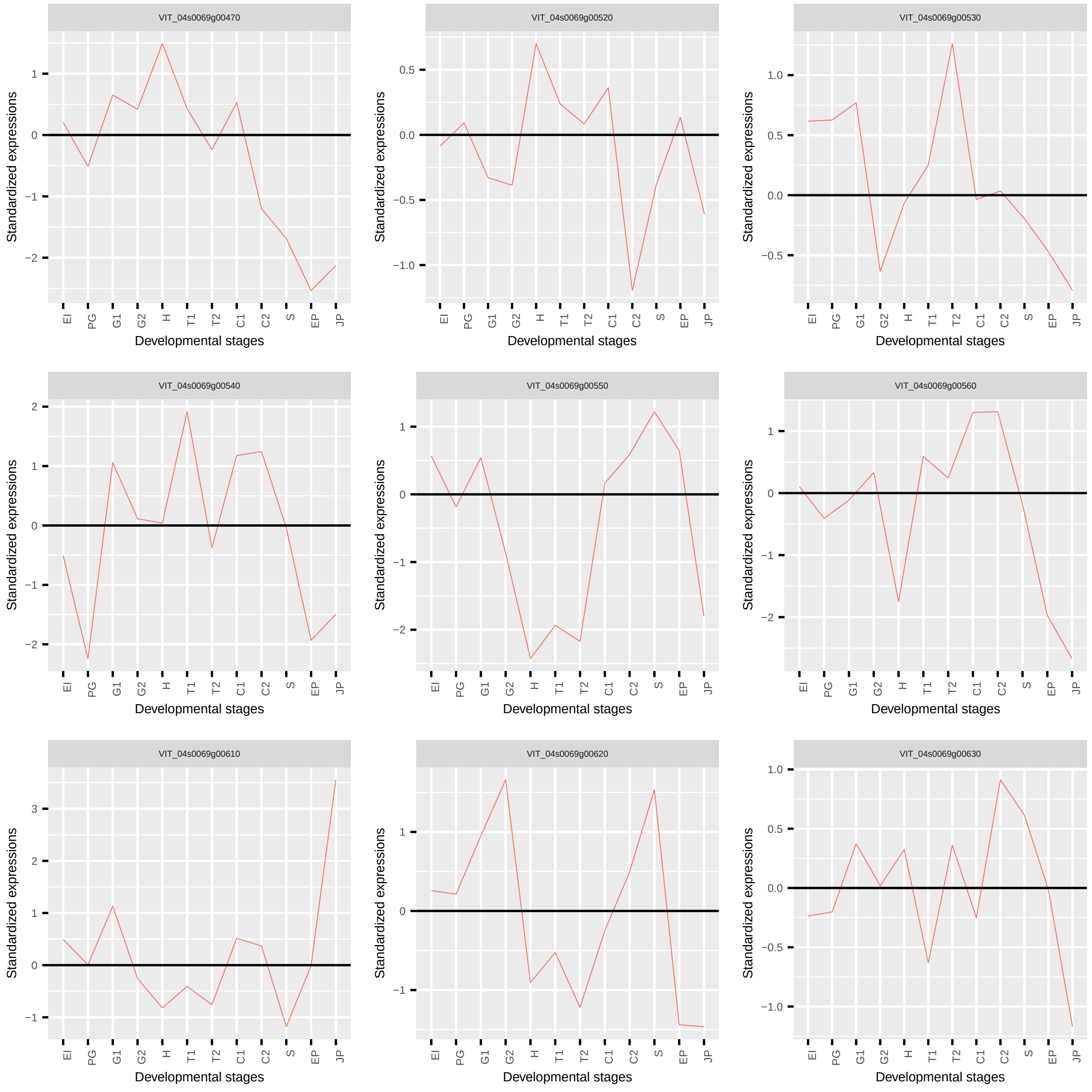
VIT_04s0069g00470
VIT_04s0069g00520
VIT_04s0069g00530
VIT_04s0069g00540
VIT_04s0069g00550
VIT_04s0069g00560
VIT_04s0069g00610
VIT_04s0069g00620
VIT_04s0069g00630
Standardized expressions
Developmental stages
EI PG G1 G2 H T1 T2 C1 C2 S EP JP

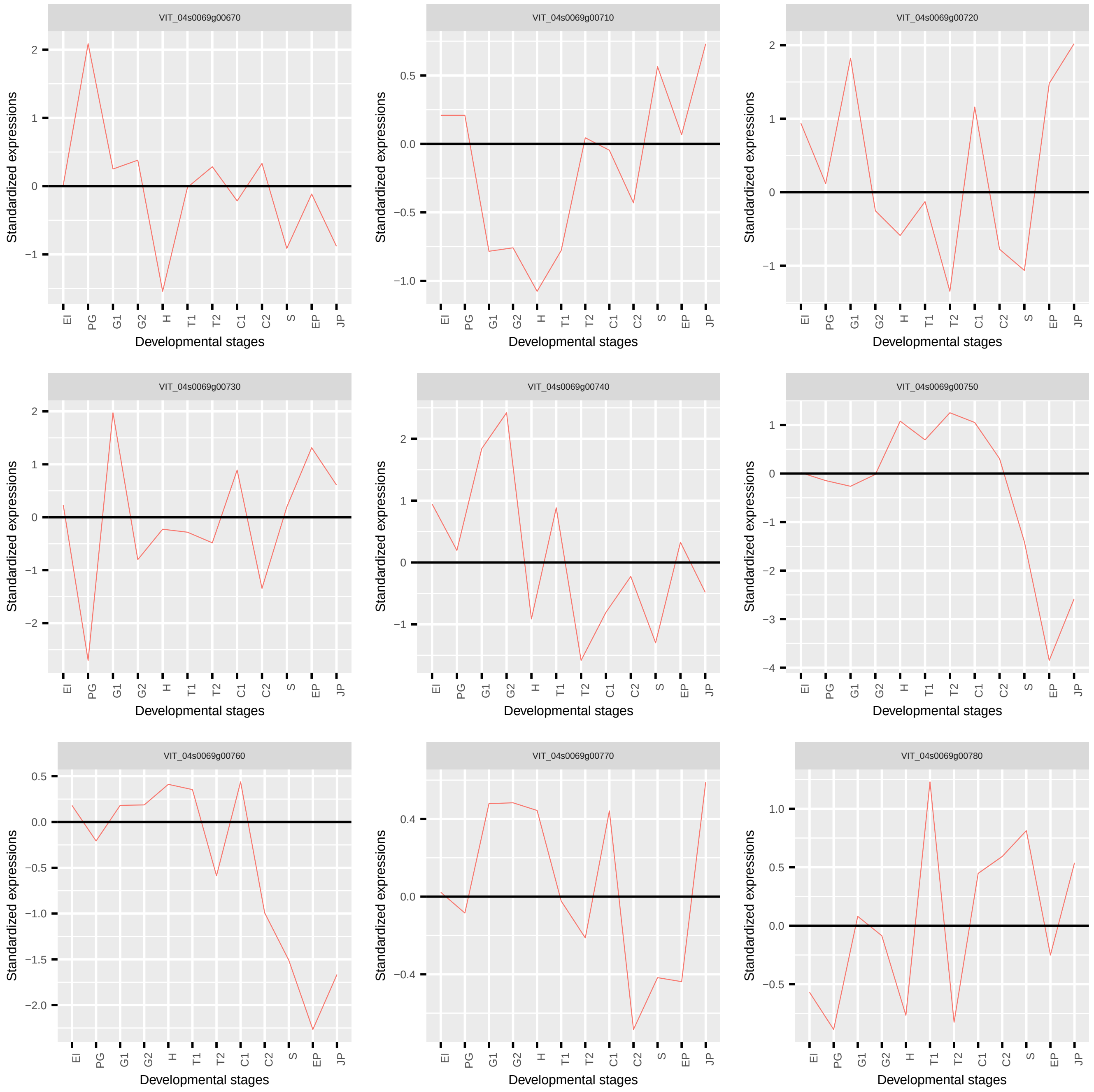
VIT_04s0069g00670
VIT_04s0069g00710
VIT_04s0069g00720
VIT_04s0069g00730
VIT_04s0069g00740
VIT_04s0069g00750
VIT_04s0069g00760
VIT_04s0069g00770
VIT_04s0069g00780
Standardized expressions
Developmental stages
EI PG G1 G2 H T1 T2 C1 C2 S EP JP

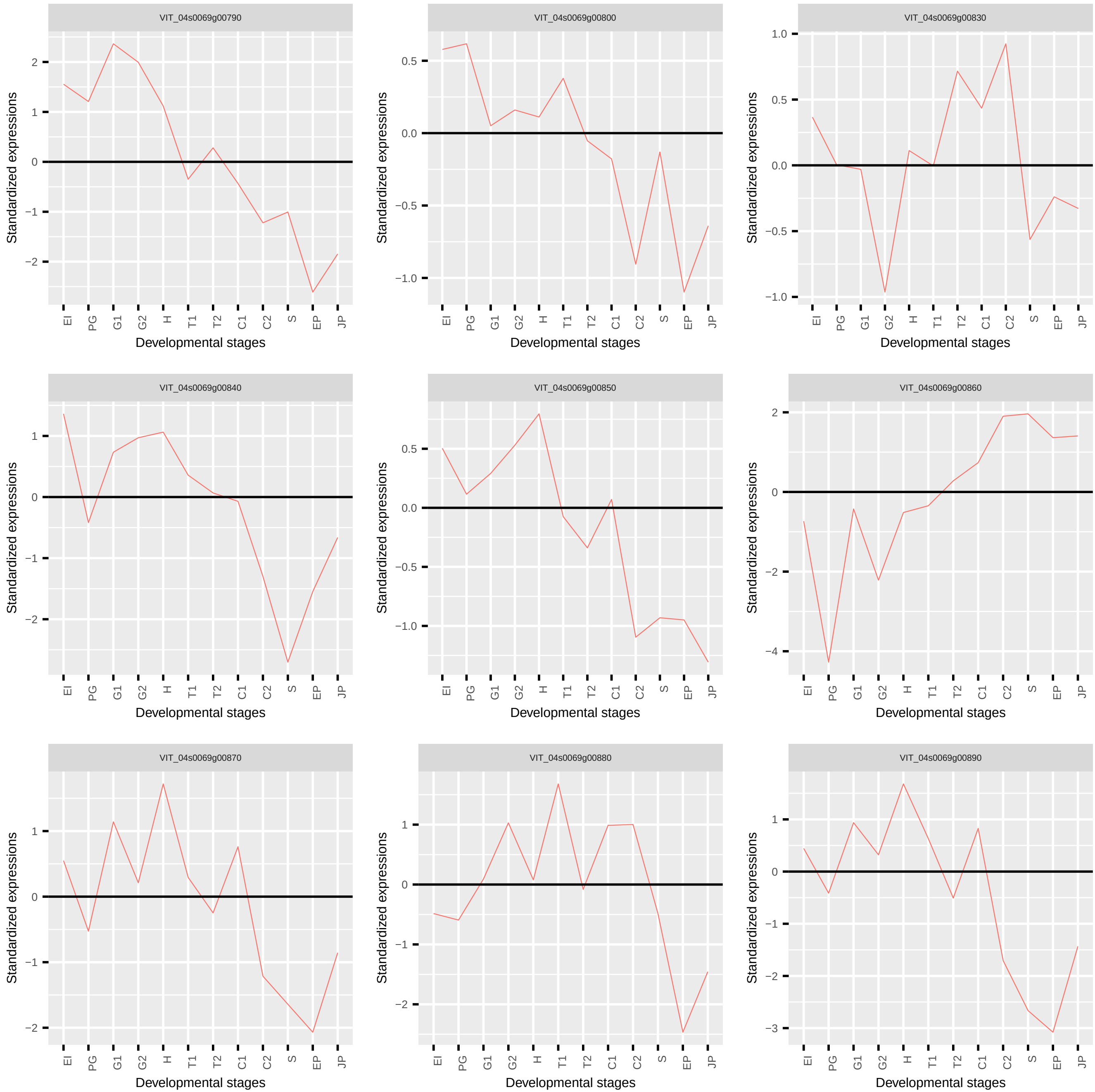
VIT_04s0069g00790
VIT_04s0069g00800
VIT_04s0069g00830
VIT_04s0069g00840
VIT_04s0069g00850
VIT_04s0069g00860
VIT_04s0069g00870
VIT_04s0069g00880
VIT_04s0069g00890
Standardized expressions
Developmental stages
EI PG G1 G2 H T1 T2 C1 C2 S EP JP

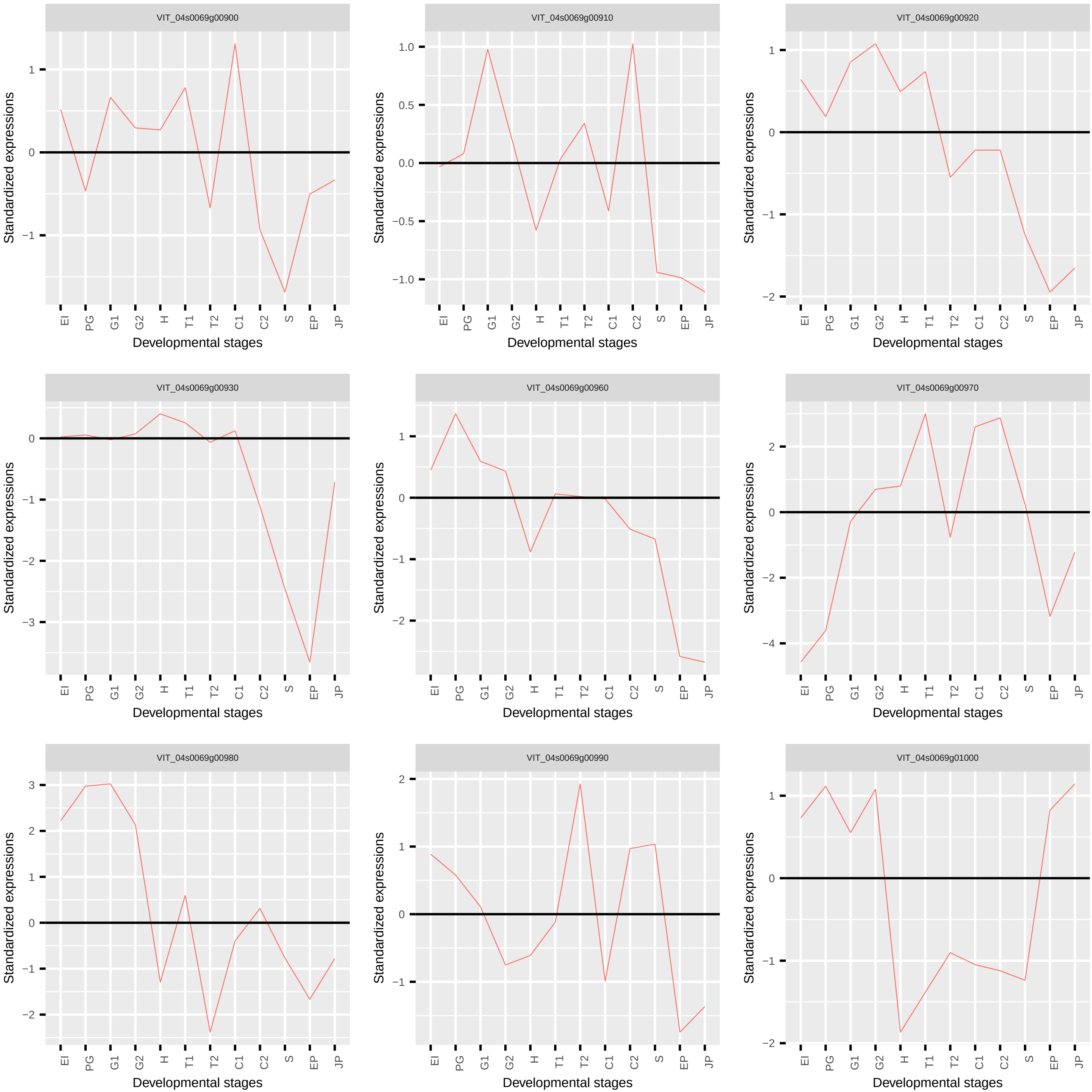
VIT_04s0069g00900
VIT_04s0069g00910
VIT_04s0069g00920
VIT_04s0069g00930
VIT_04s0069g00960
VIT_04s0069g00970
VIT_04s0069g00980
VIT_04s0069g00990
VIT_04s0069g01000
Standardized expressions
Developmental stages
EI
PG
G1
G2
H
T1
T2
C1
C2
S
EP
JP

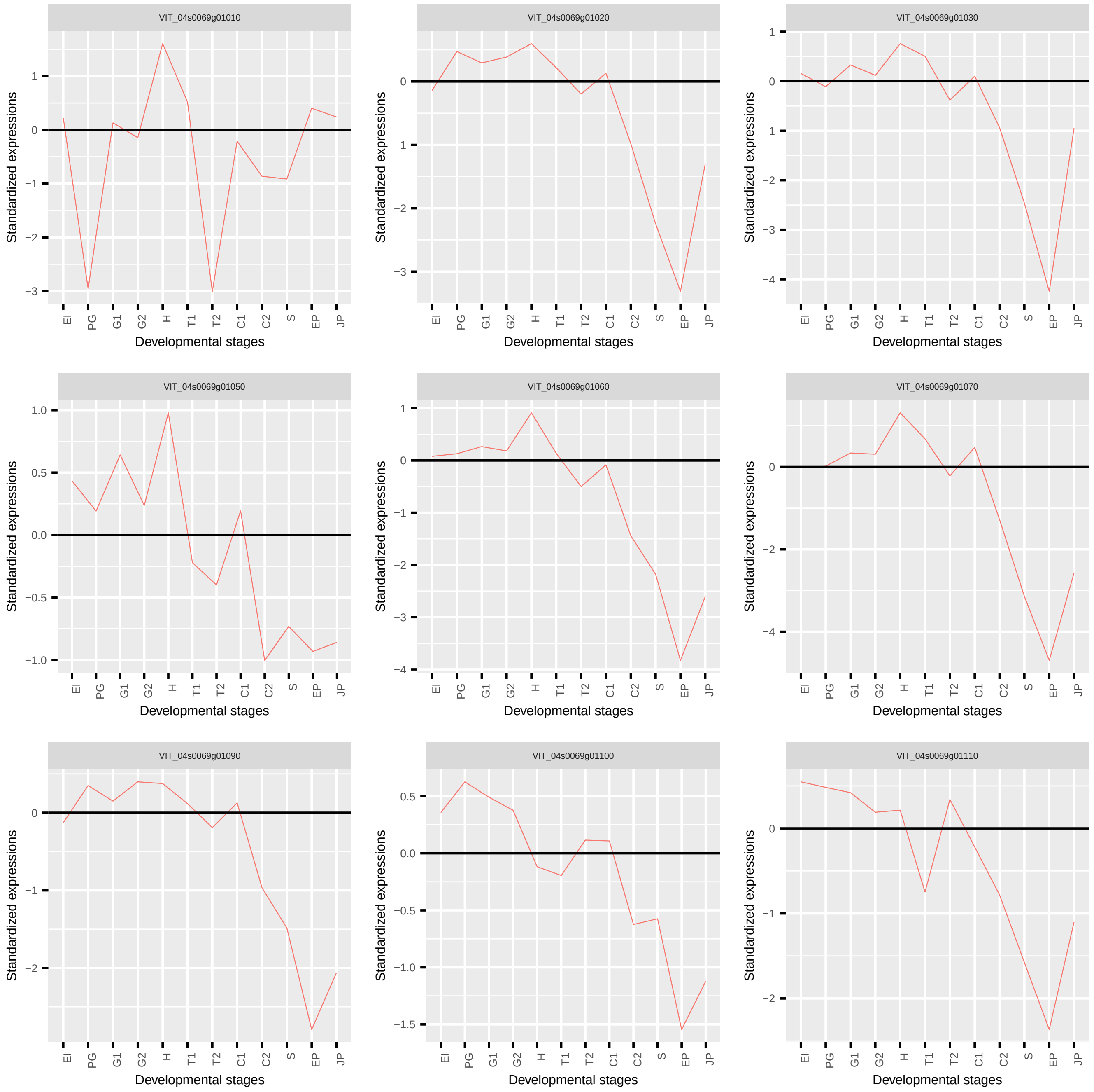
VIT_04s0069g01010
VIT_04s0069g01020
VIT_04s0069g01030
VIT_04s0069g01050
VIT_04s0069g01060
VIT_04s0069g01070
VIT_04s0069g01090
VIT_04s0069g01100
VIT_04s0069g01110
Standardized expressions
Developmental stages
EI
PG
G1
G2
H
T1
T2
C1
C2
S
EP
JP

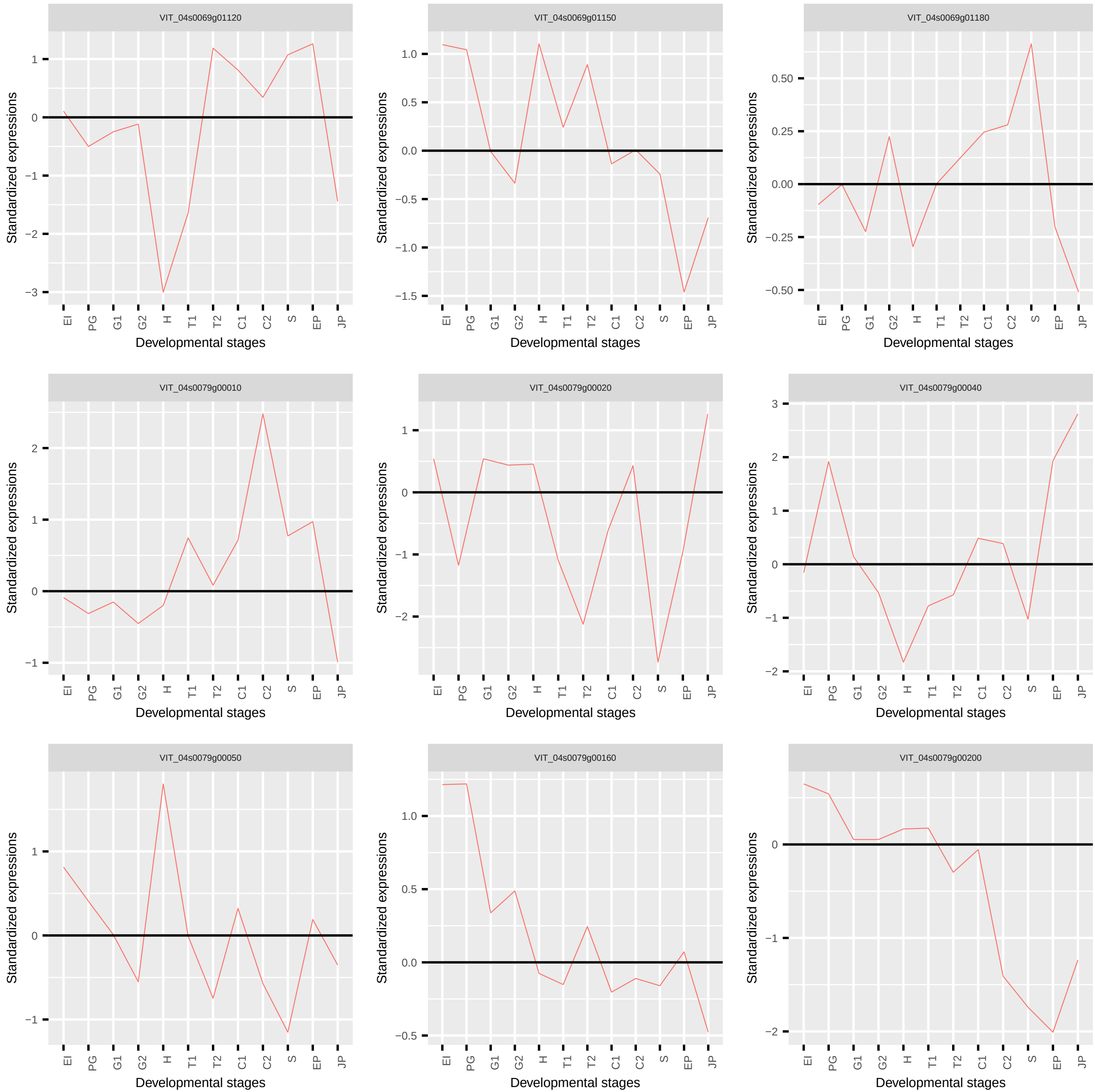
VIT_04s0069g01120
VIT_04s0069g01150
VIT_04s0069g01180
VIT_04s0079g00010
VIT_04s0079g00020
VIT_04s0079g00040
VIT_04s0079g00050
VIT_04s0079g00160
VIT_04s0079g00200
Standardized expressions
Developmental stages
EI PG G1 G2 H T1 T2 C1 C2 S EP JP

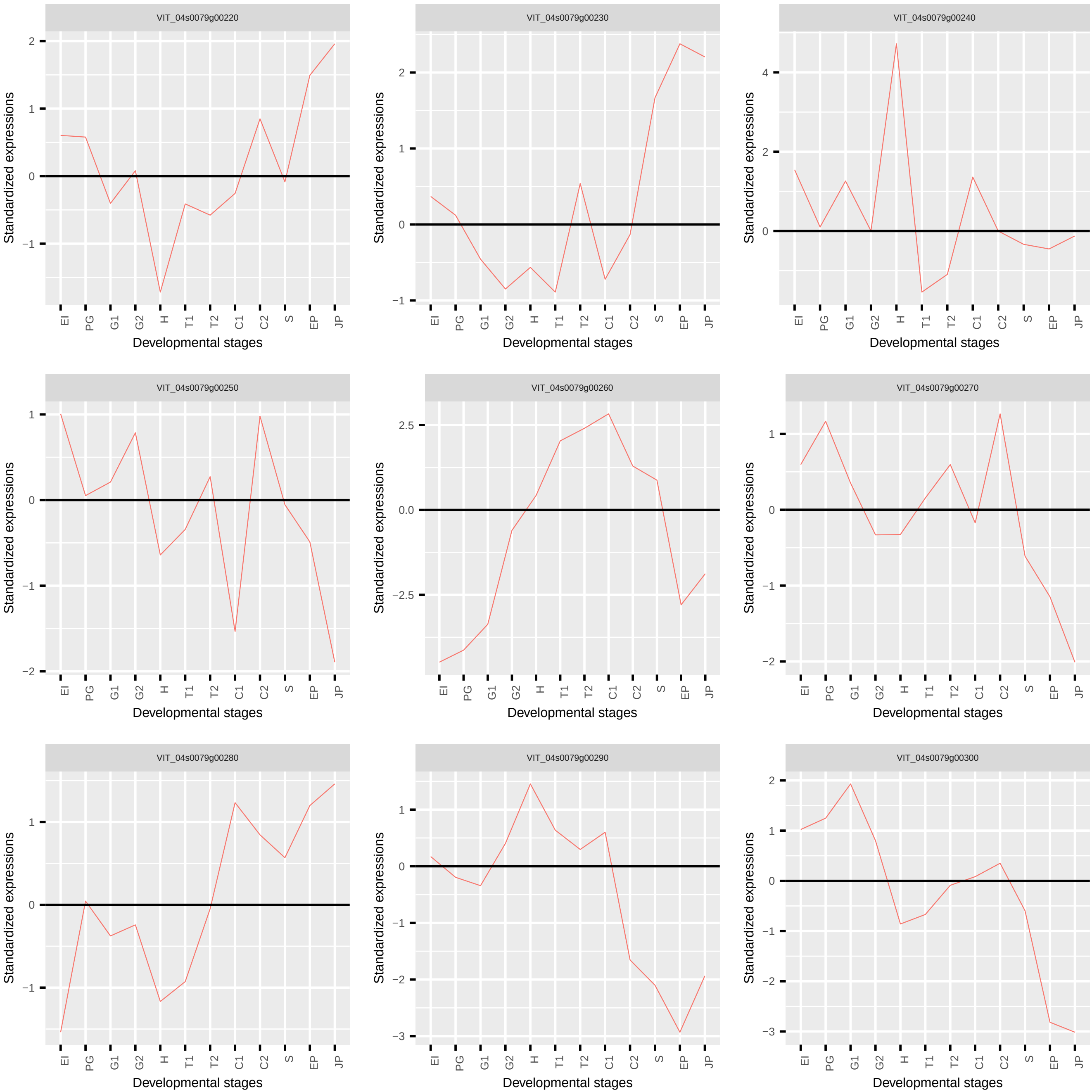
VIT_04s0079g00220
VIT_04s0079g00230
VIT_04s0079g00240
VIT_04s0079g00250
VIT_04s0079g00260
VIT_04s0079g00270
VIT_04s0079g00280
VIT_04s0079g00290
VIT_04s0079g00300
Standardized expressions
Developmental stages
EI PG G1 G2 H T1 T2 C1 C2 S EP JP

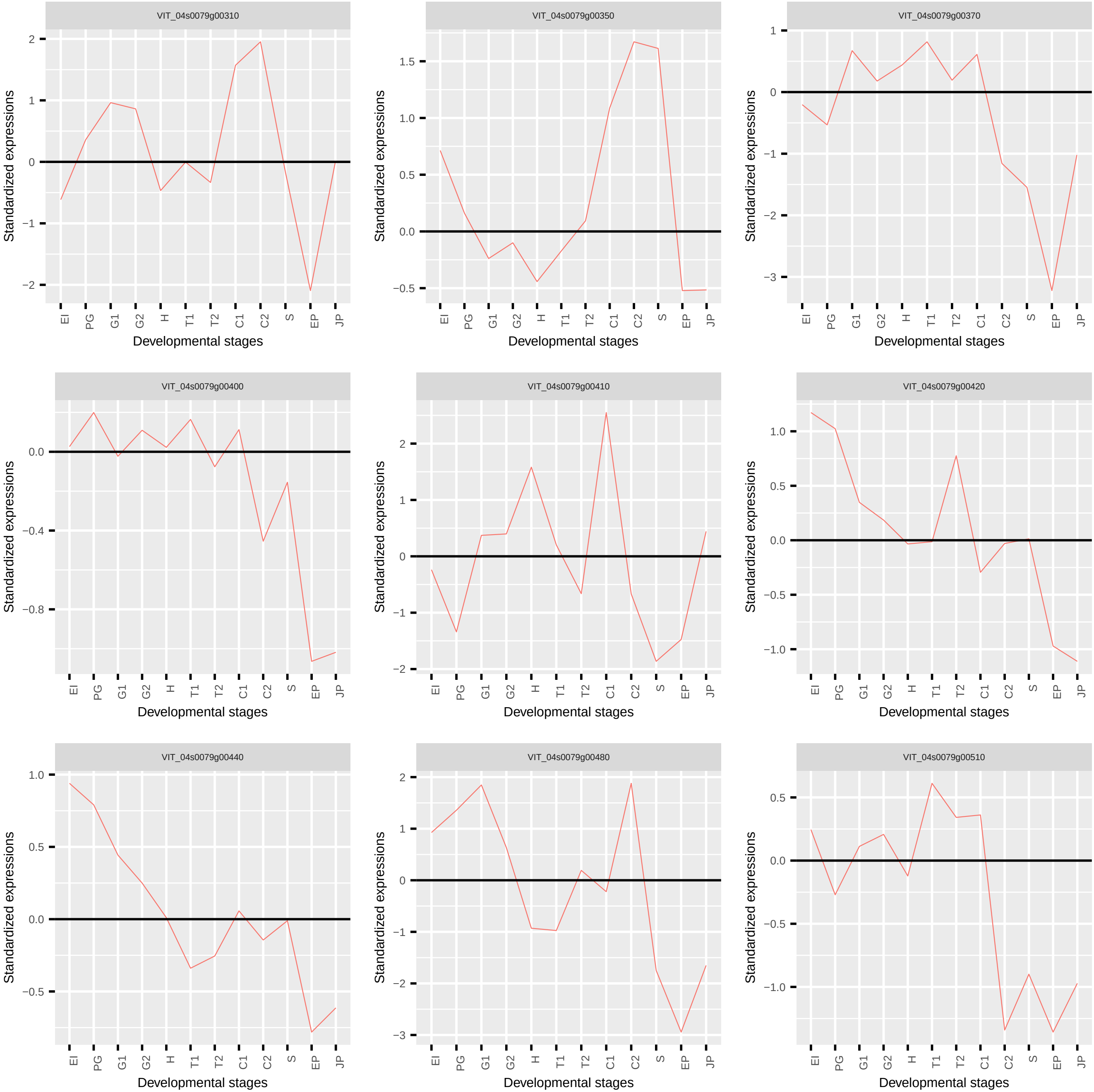
VIT_04s0079g00310
VIT_04s0079g00350
VIT_04s0079g00370
VIT_04s0079g00400
VIT_04s0079g00410
VIT_04s0079g00420
VIT_04s0079g00440
VIT_04s0079g00480
VIT_04s0079g00510
Standardized expressions
Developmental stages
EI PG G1 G2 H T1 T2 C1 C2 S EP JP

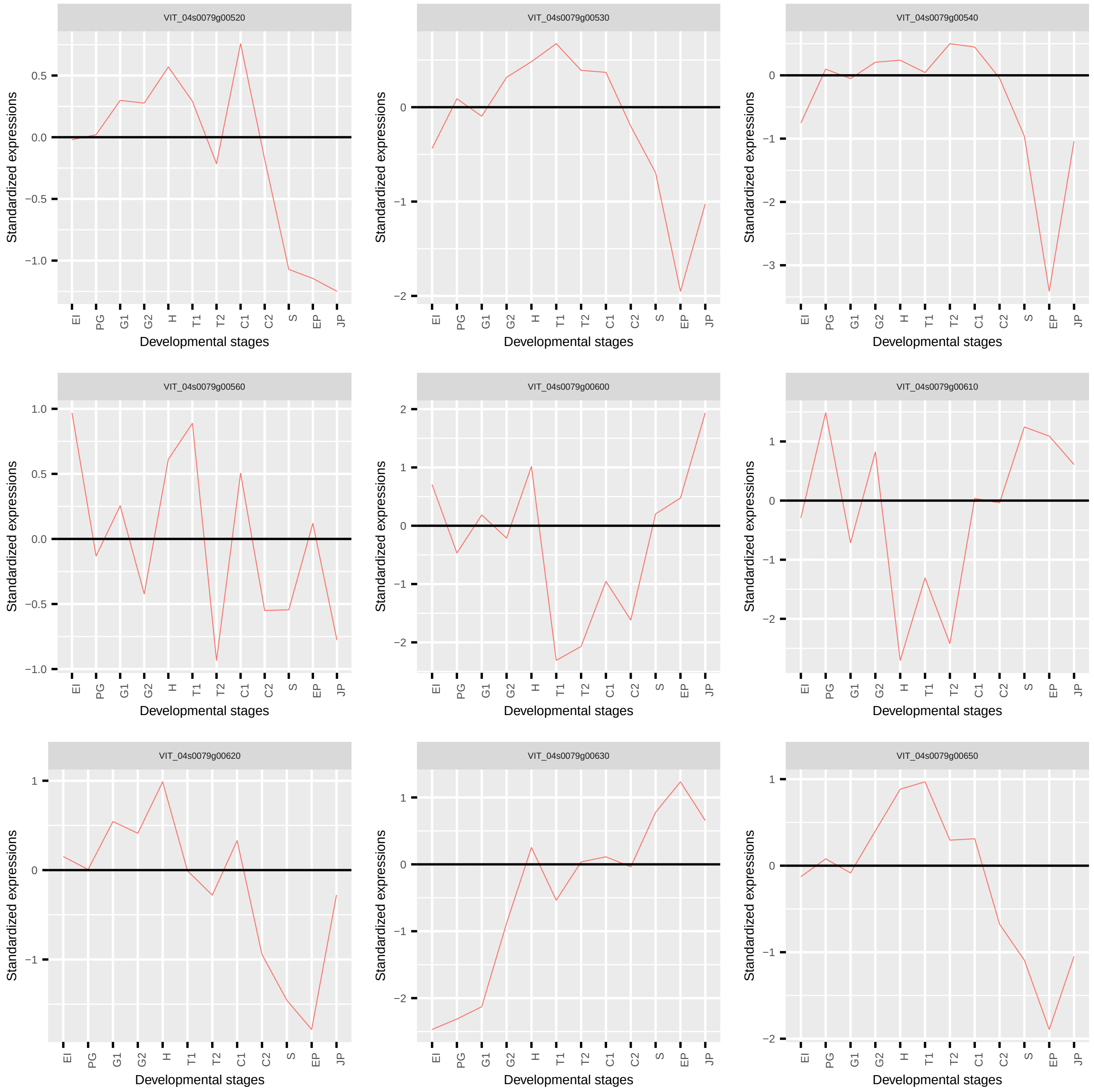

VIT_04s0079g00520
VIT_04s0079g00530
VIT_04s0079g00540
VIT_04s0079g00560
VIT_04s0079g00600
VIT_04s0079g00610
VIT_04s0079g00620
VIT_04s0079g00630
VIT_04s0079g00650
Standardized expressions
Developmental stages
EI PG G1 G2 H T1 T2 C1 C2 S EP JP

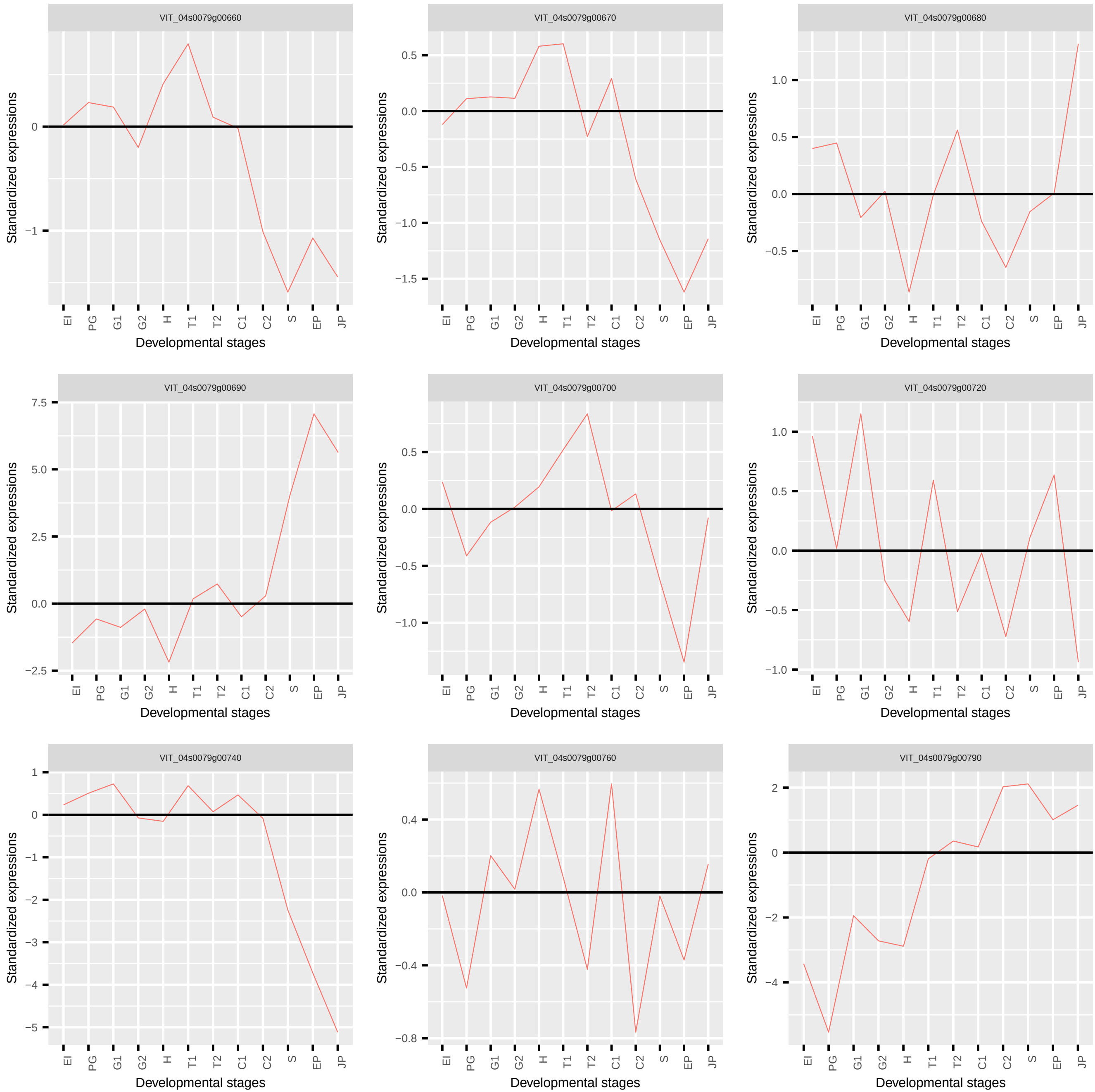
VIT_04s0079g00660
VIT_04s0079g00670
VIT_04s0079g00680
VIT_04s0079g00690
VIT_04s0079g00700
VIT_04s0079g00720
VIT_04s0079g00740
VIT_04s0079g00760
VIT_04s0079g00790
Standardized expressions
Developmental stages
EI PG G1 G2 H T1 T2 C1 C2 S EP JP

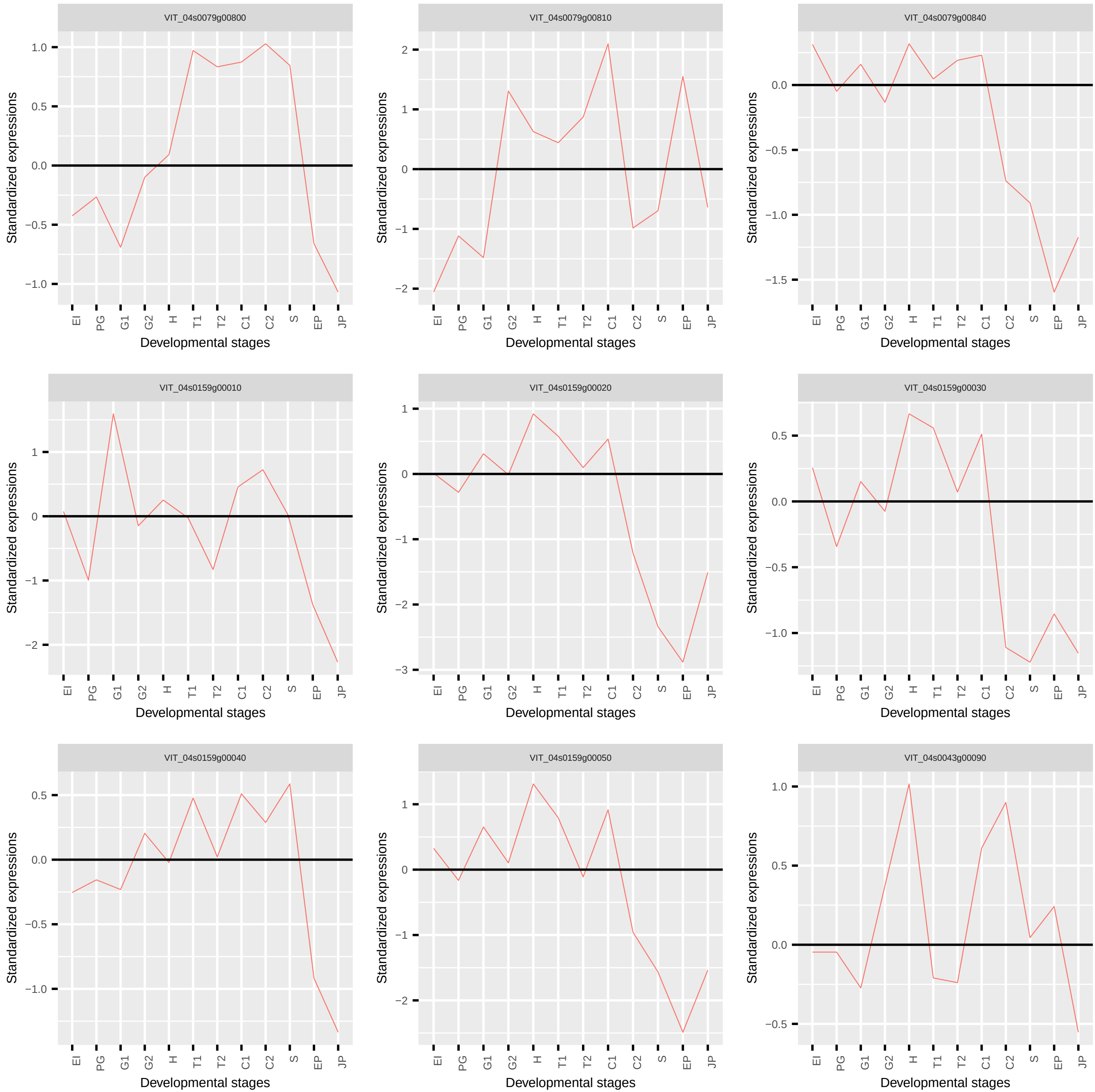
VIT_04s0079g00800
VIT_04s0079g00810
VIT_04s0079g00840
VIT_04s0159g00010
VIT_04s0159g00020
VIT_04s0159g00030
VIT_04s0159g00040
VIT_04s0159g00050
VIT_04s0043g00090
Standardized expressions
Developmental stages
EI PG G1 G2 H T1 T2 C1 C2 S EP JP

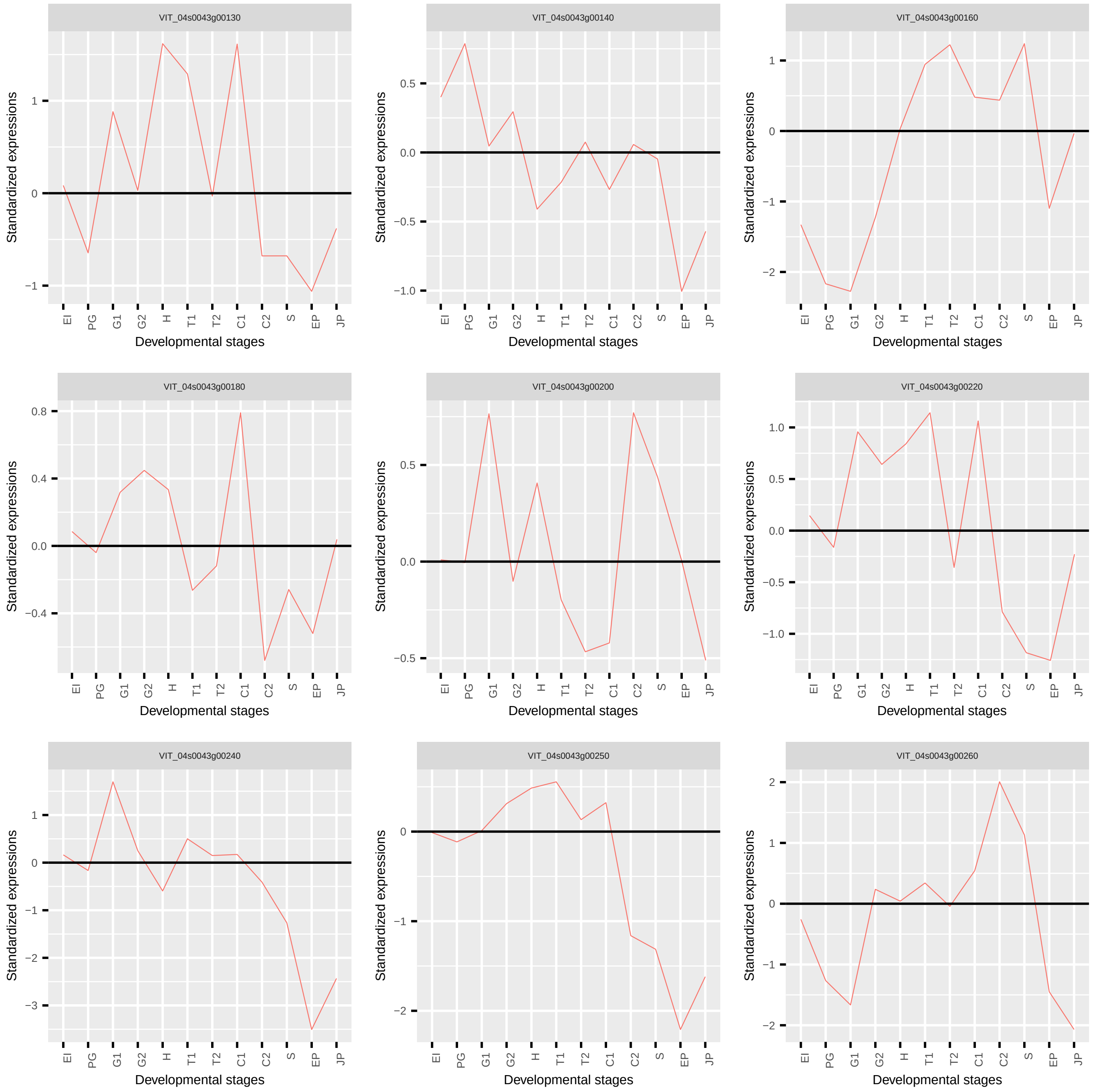

VIT_04s0043g00130
VIT_04s0043g00140
VIT_04s0043g00160
VIT_04s0043g00180
VIT_04s0043g00200
VIT_04s0043g00220
VIT_04s0043g00240
VIT_04s0043g00250
VIT_04s0043g00260
Standardized expressions
Developmental stages
EI
PG
G1
G2
H
T1
T2
C1
C2
S
EP
JP

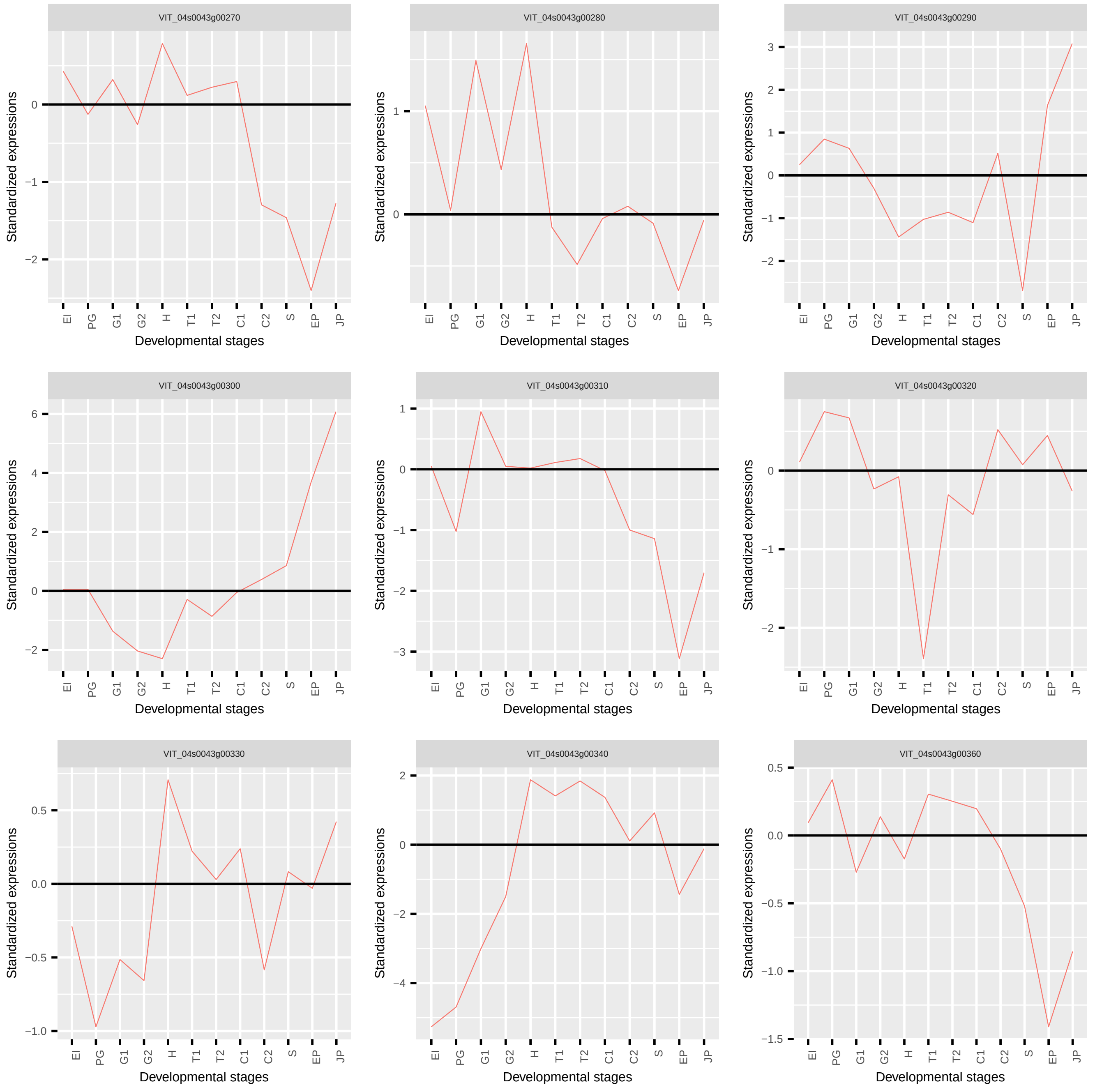
VIT_04s0043g00270
VIT_04s0043g00280
VIT_04s0043g00290
VIT_04s0043g00300
VIT_04s0043g00310
VIT_04s0043g00320
VIT_04s0043g00330
VIT_04s0043g00340
VIT_04s0043g00360
Standardized expressions
Developmental stages
EI
PG
G1
G2
H
T1
T2
C1
C2
S
EP
JP

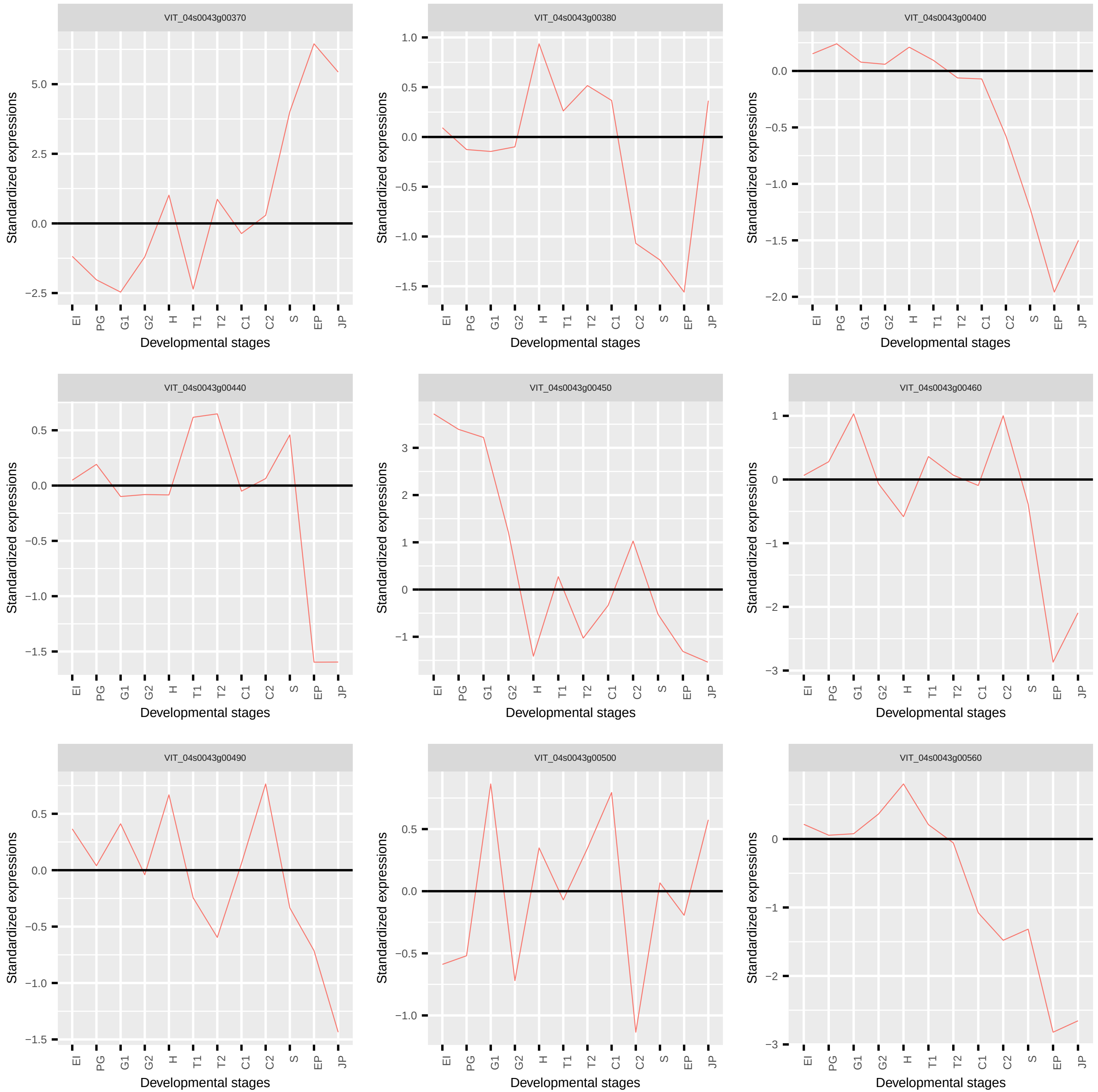
VIT_04s0043g00370
5.0
2.5
0.0
−2.5
Standardized expressions
EI
PG
G1
G2
H
T1
T2
C1
C2
S
EP
JP
Developmental stages
VIT_04s0043g00380
1.0
0.5
0.0
−0.5
−1.0
−1.5
Standardized expressions
Developmental stages
VIT_04s0043g00400
0.0
−0.5
−1.0
−1.5
−2.0
Standardized expressions
Developmental stages
VIT_04s0043g00440
0.5
0.0
−0.5
−1.0
−1.5
Standardized expressions
Developmental stages
VIT_04s0043g00450
3
2
1
0
−1
Standardized expressions
Developmental stages
VIT_04s0043g00460
1
0
−1
−2
−3
Standardized expressions
Developmental stages
VIT_04s0043g00490
0.5
0.0
−0.5
−1.0
−1.5
Standardized expressions
Developmental stages
VIT_04s0043g00500
0.5
0.0
−0.5
−1.0
Standardized expressions
Developmental stages
VIT_04s0043g00560
0
−1
−2
−3
Standardized expressions
Developmental stages

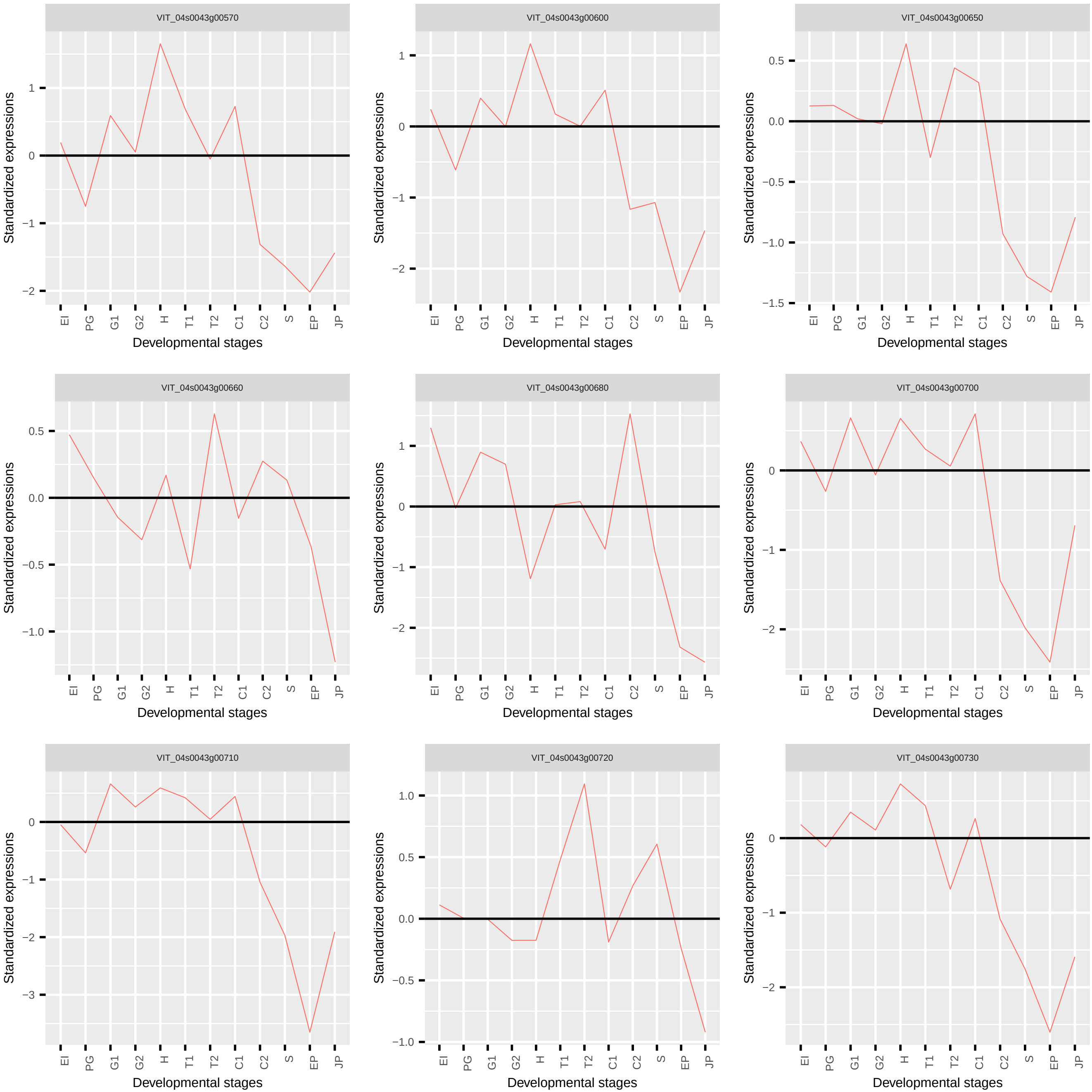
VIT_04s0043g00570
VIT_04s0043g00600
VIT_04s0043g00650
VIT_04s0043g00660
VIT_04s0043g00680
VIT_04s0043g00700
VIT_04s0043g00710
VIT_04s0043g00720
VIT_04s0043g00730
Standardized expressions
Developmental stages
EI PG G1 G2 H T1 T2 C1 C2 S EP JP
1
0
−1
−2
−3
1.0
0.5
0.0
−0.5
−1.0
−1.5

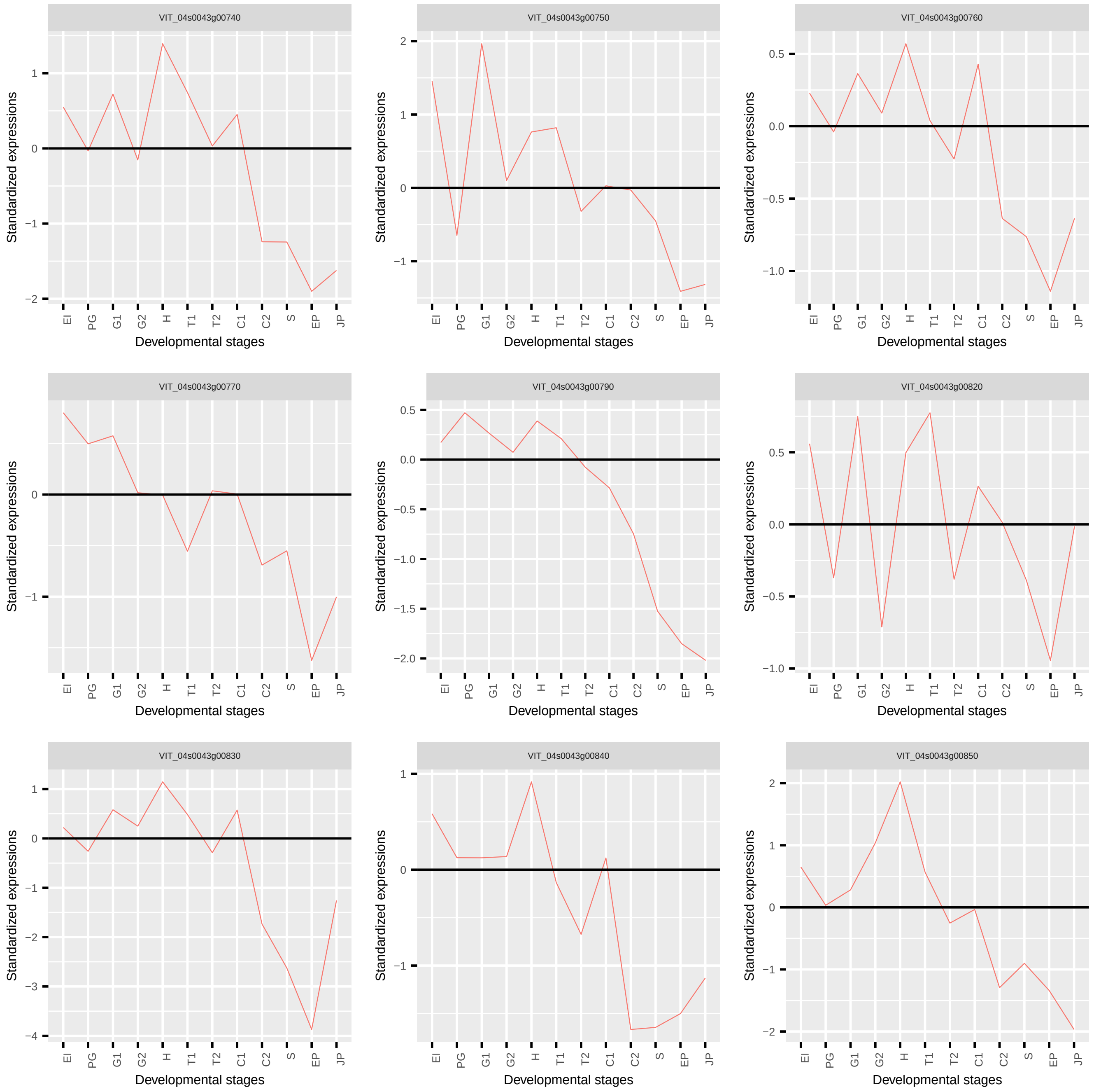
VIT_04s0043g00740
VIT_04s0043g00750
VIT_04s0043g00760
VIT_04s0043g00770
VIT_04s0043g00790
VIT_04s0043g00820
VIT_04s0043g00830
VIT_04s0043g00840
VIT_04s0043g00850
Standardized expressions
Developmental stages
EI PG G1 G2 H T1 T2 C1 C2 S EP JP

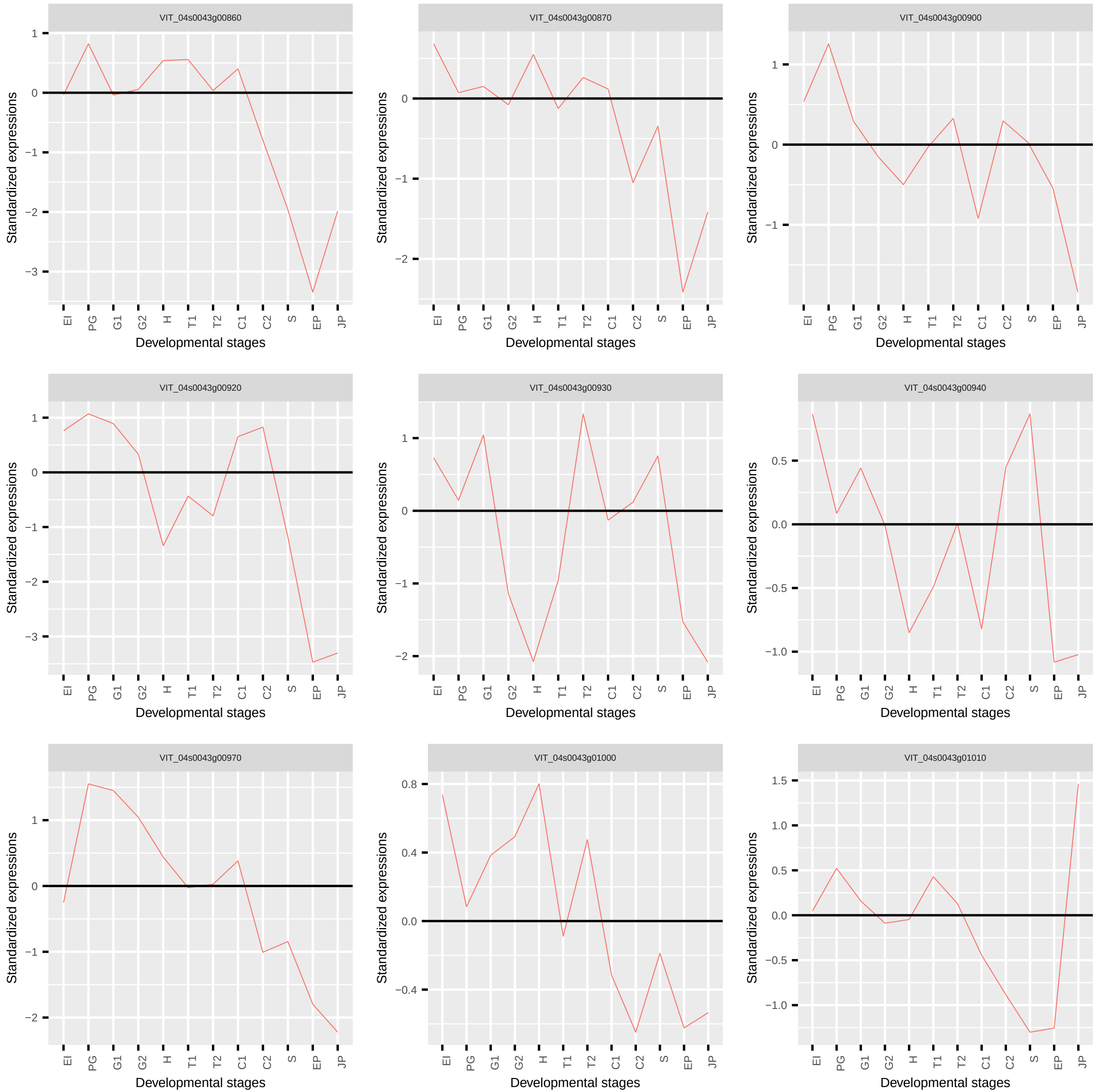

VIT_04s0043g00860
VIT_04s0043g00870
VIT_04s0043g00900
VIT_04s0043g00920
VIT_04s0043g00930
VIT_04s0043g00940
VIT_04s0043g00970
VIT_04s0043g01000
VIT_04s0043g01010
Standardized expressions
Developmental stages
EI PG G1 G2 H T1 T2 C1 C2 S EP JP

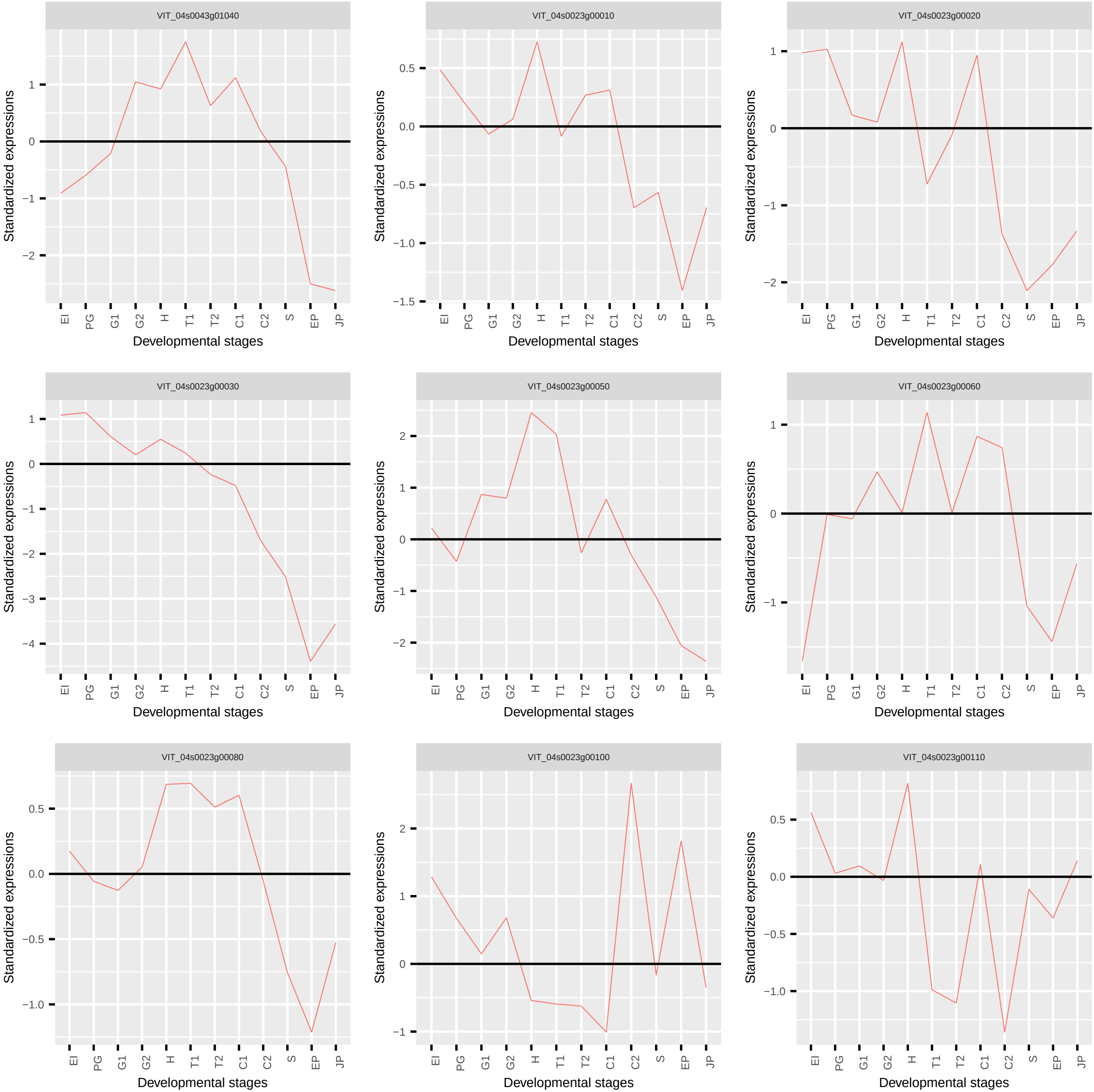
VIT_04s0043g01040
1
0
−1
−2
Standardized expressions
EI PG G1 G2 H T1 T2 C1 C2 S EP JP
Developmental stages
VIT_04s0023g00010
0.5
0.0
−0.5
−1.0
−1.5
Standardized expressions
EI PG G1 G2 H T1 T2 C1 C2 S EP JP
Developmental stages
VIT_04s0023g00020
1
0
−1
−2
Standardized expressions
EI PG G1 G2 H T1 T2 C1 C2 S EP JP
Developmental stages
VIT_04s0023g00030
1
0
−1
−2
−3
−4
Standardized expressions
EI PG G1 G2 H T1 T2 C1 C2 S EP JP
Developmental stages
VIT_04s0023g00050
2
1
0
−1
−2
Standardized expressions
EI PG G1 G2 H T1 T2 C1 C2 S EP JP
Developmental stages
VIT_04s0023g00060
1
0
−1
Standardized expressions
EI PG G1 G2 H T1 T2 C1 C2 S EP JP
Developmental stages
VIT_04s0023g00080
0.5
0.0
−0.5
−1.0
Standardized expressions
EI PG G1 G2 H T1 T2 C1 C2 S EP JP
Developmental stages
VIT_04s0023g00100
2
1
0
−1
Standardized expressions
EI PG G1 G2 H T1 T2 C1 C2 S EP JP
Developmental stages
VIT_04s0023g00110
0.5
0.0
−0.5
−1.0
Standardized expressions
EI PG G1 G2 H T1 T2 C1 C2 S EP JP
Developmental stages

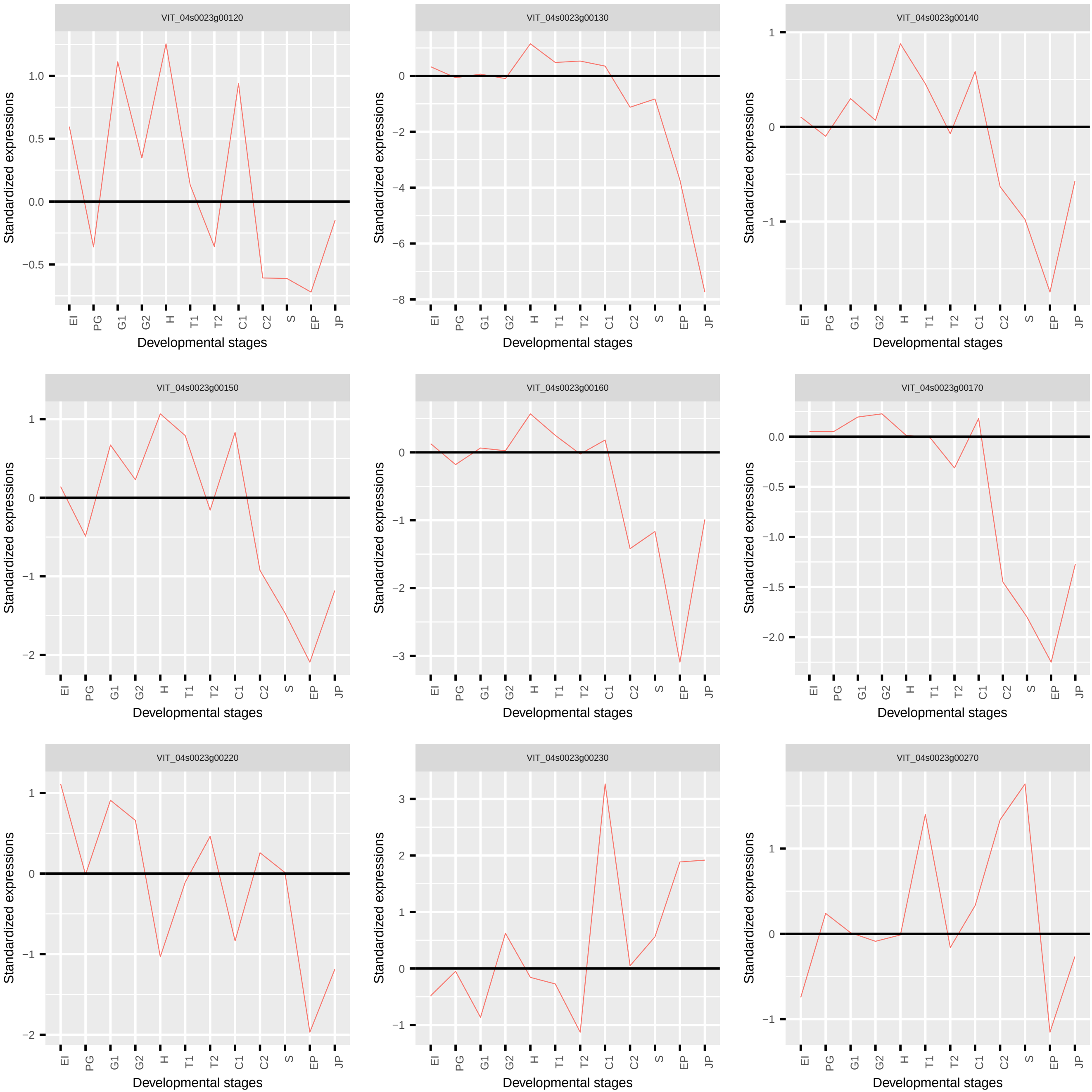
VIT_04s0023g00120
VIT_04s0023g00130
VIT_04s0023g00140
VIT_04s0023g00150
VIT_04s0023g00160
VIT_04s0023g00170
VIT_04s0023g00220
VIT_04s0023g00230
VIT_04s0023g00270
Standardized expressions
Developmental stages
EI PG G1 G2 H T1 T2 C1 C2 S EP JP

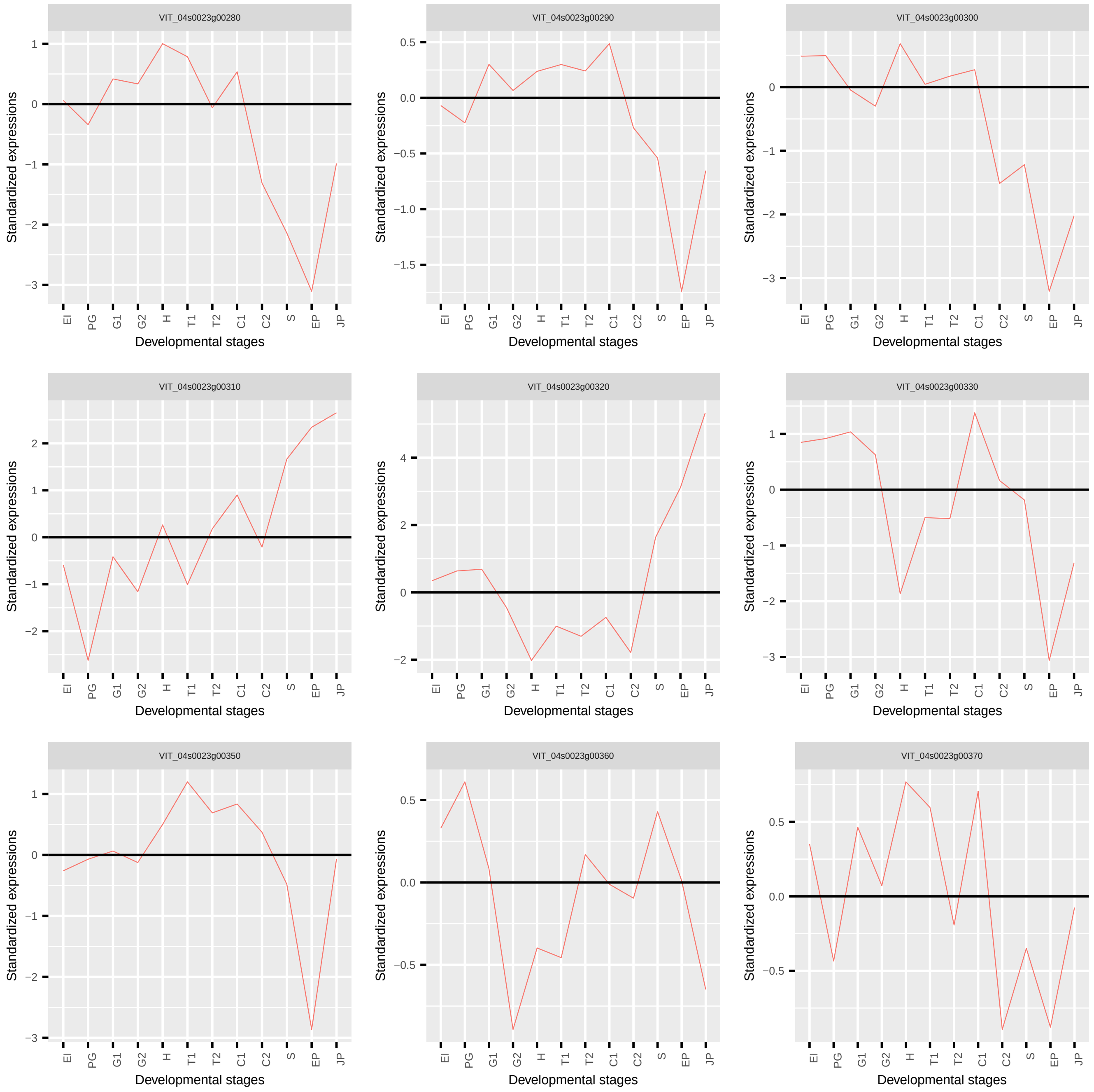
VIT_04s0023g00280
VIT_04s0023g00290
VIT_04s0023g00300
VIT_04s0023g00310
VIT_04s0023g00320
VIT_04s0023g00330
VIT_04s0023g00350
VIT_04s0023g00360
VIT_04s0023g00370
Standardized expressions
Developmental stages
EI
PG
G1
G2
H
T1
T2
C1
C2
S
EP
JP

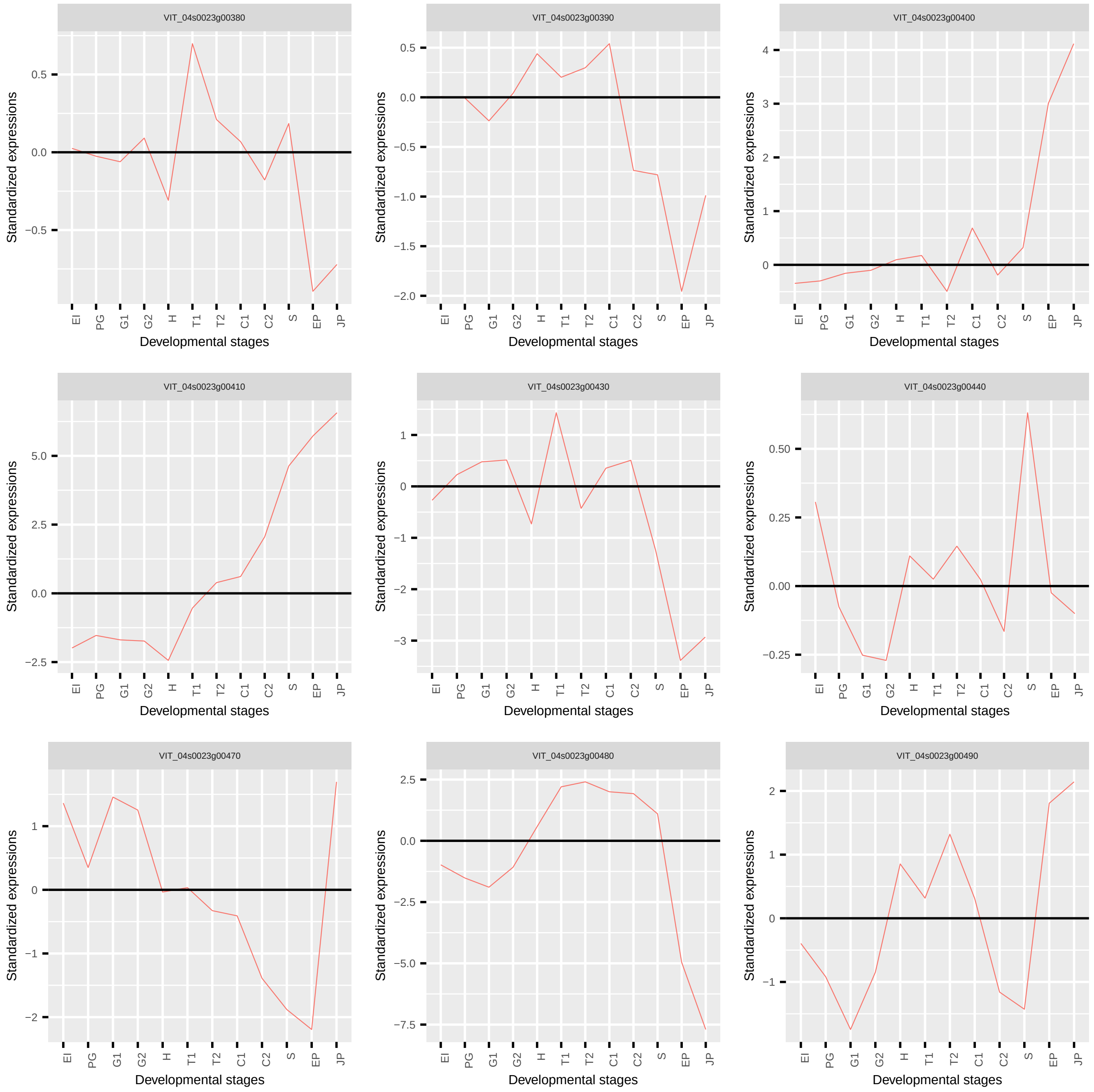
VIT_04s0023g00380
VIT_04s0023g00390
VIT_04s0023g00400
VIT_04s0023g00410
VIT_04s0023g00430
VIT_04s0023g00440
VIT_04s0023g00470
VIT_04s0023g00480
VIT_04s0023g00490
Standardized expressions
Developmental stages
EI
PG
G1
G2
H
T1
T2
C1
C2
S
EP
JP

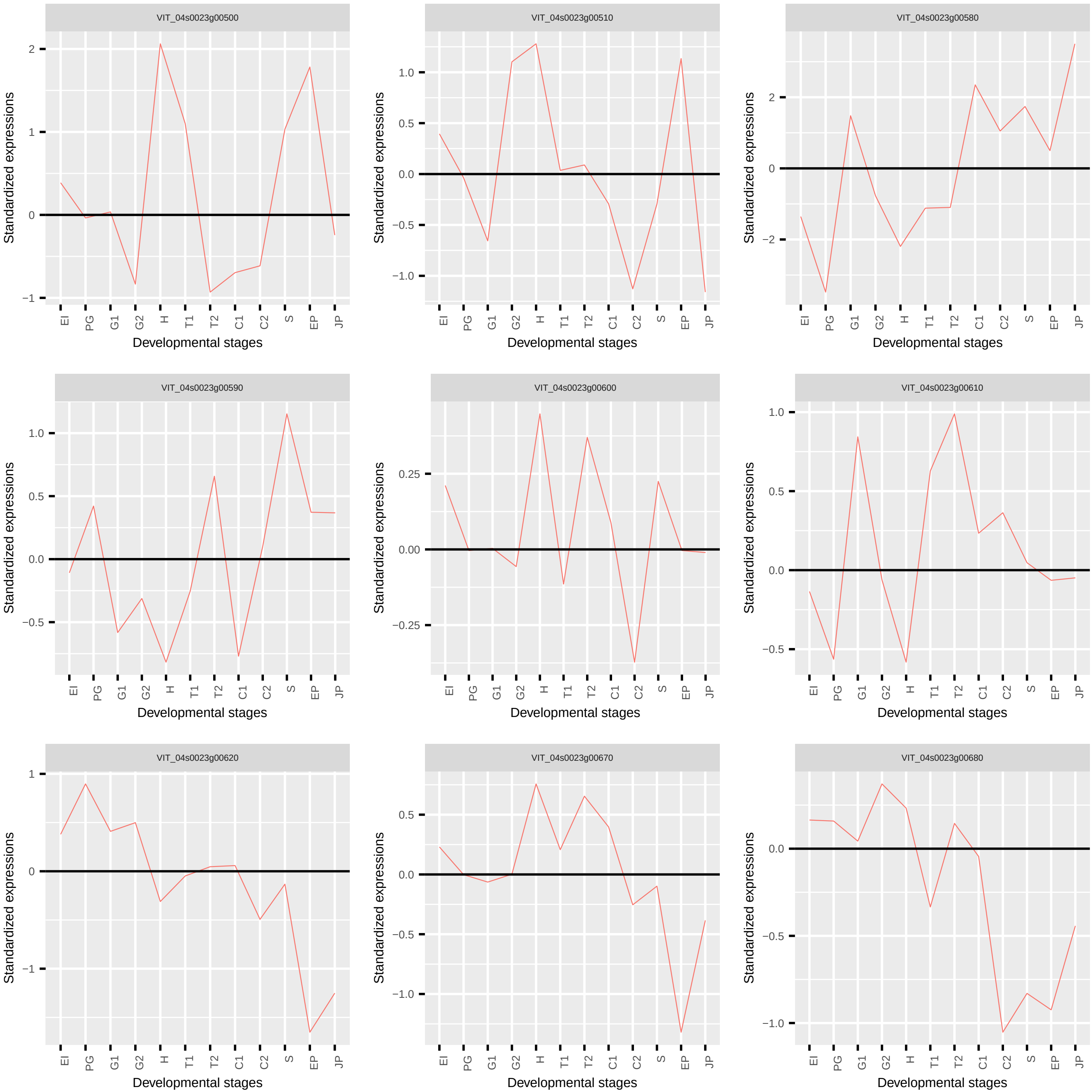

VIT_04s0023g00500
VIT_04s0023g00510
VIT_04s0023g00580
VIT_04s0023g00590
VIT_04s0023g00600
VIT_04s0023g00610
VIT_04s0023g00620
VIT_04s0023g00670
VIT_04s0023g00680
Standardized expressions
Developmental stages
EI
PG
G1
G2
H
T1
T2
C1
C2
S
EP
JP

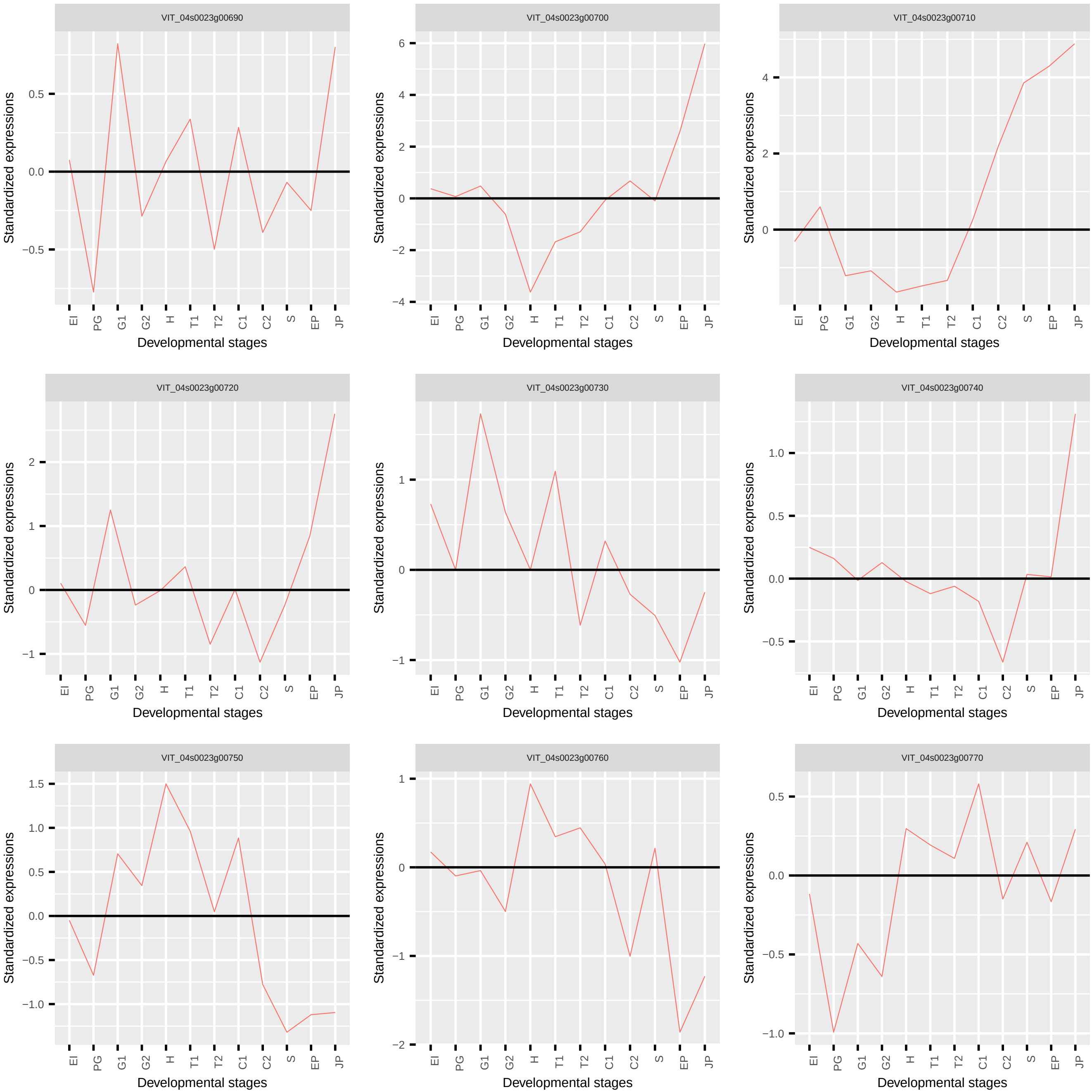
VIT_04s0023g00690
VIT_04s0023g00700
VIT_04s0023g00710
VIT_04s0023g00720
VIT_04s0023g00730
VIT_04s0023g00740
VIT_04s0023g00750
VIT_04s0023g00760
VIT_04s0023g00770
Standardized expressions
Developmental stages
EI PG G1 G2 H T1 T2 C1 C2 S EP JP

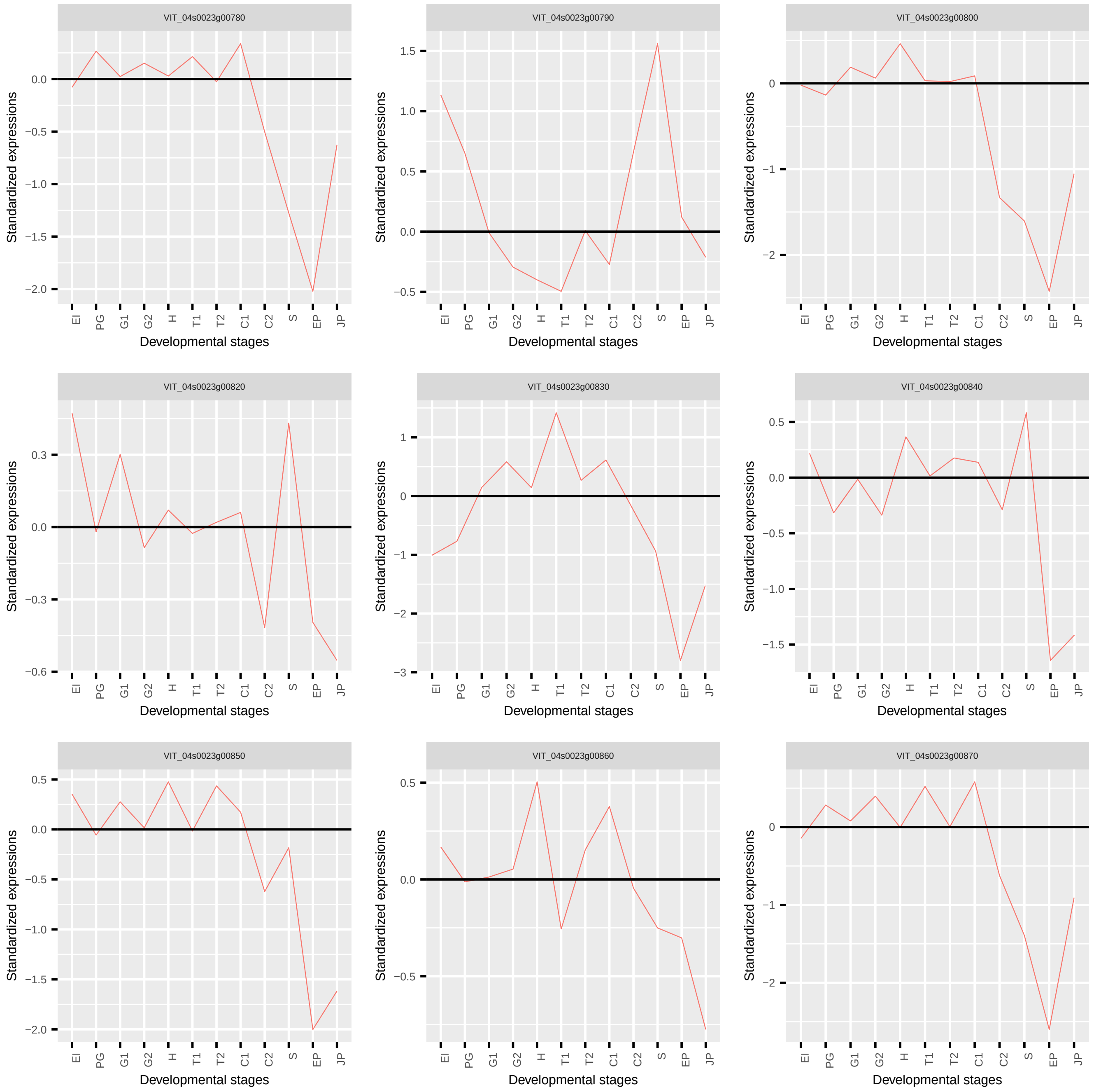
VIT_04s0023g00780
VIT_04s0023g00790
VIT_04s0023g00800
VIT_04s0023g00820
VIT_04s0023g00830
VIT_04s0023g00840
VIT_04s0023g00850
VIT_04s0023g00860
VIT_04s0023g00870
Standardized expressions
Developmental stages
EI PG G1 G2 H T1 T2 C1 C2 S EP JP

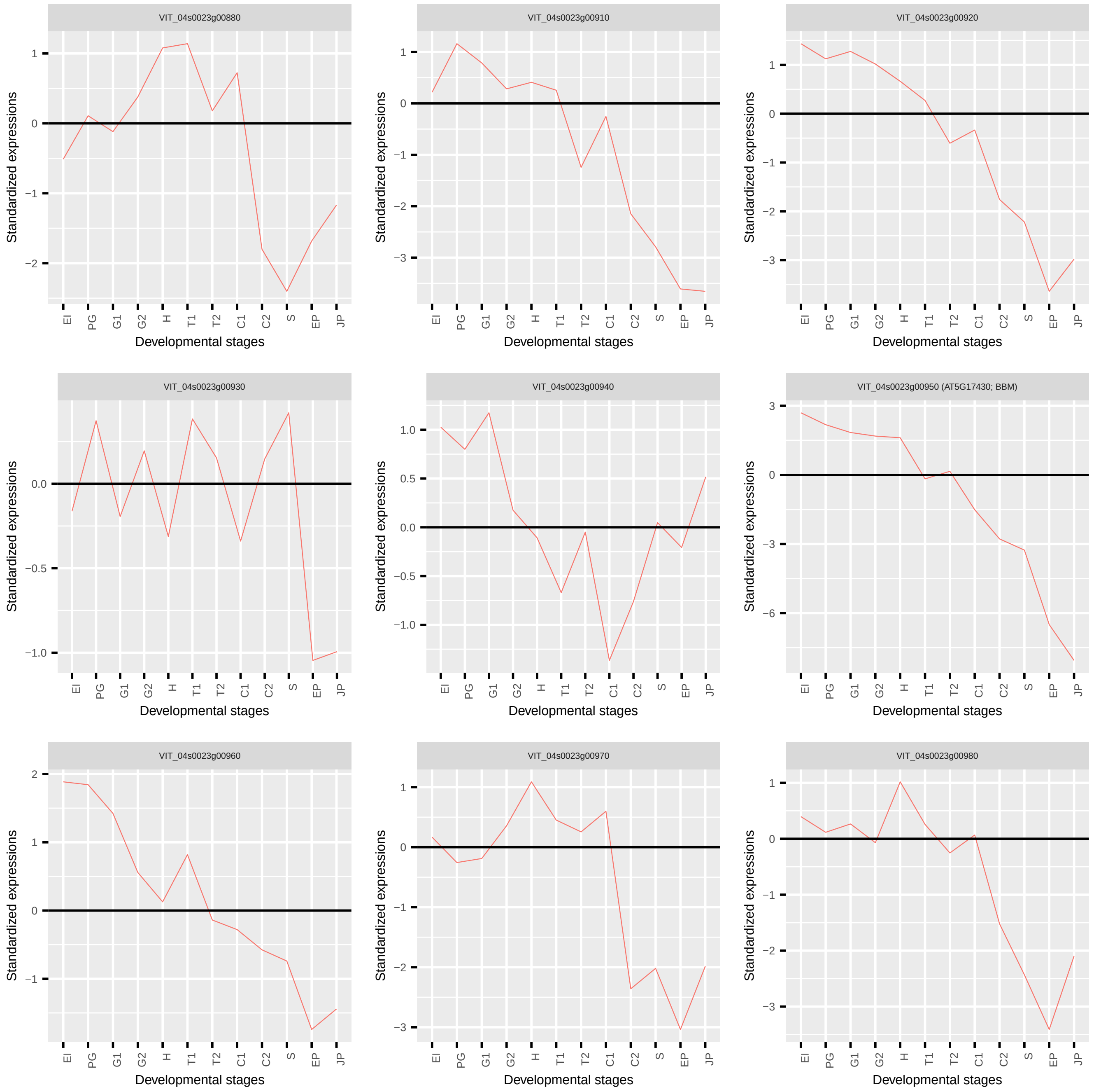
VIT_04s0023g00880
VIT_04s0023g00910
VIT_04s0023g00920
VIT_04s0023g00930
VIT_04s0023g00940
VIT_04s0023g00950 (AT5G17430; BBM)
VIT_04s0023g00960
VIT_04s0023g00970
VIT_04s0023g00980
Standardized expressions
Developmental stages
EI PG G1 G2 H T1 T2 C1 C2 S EP JP

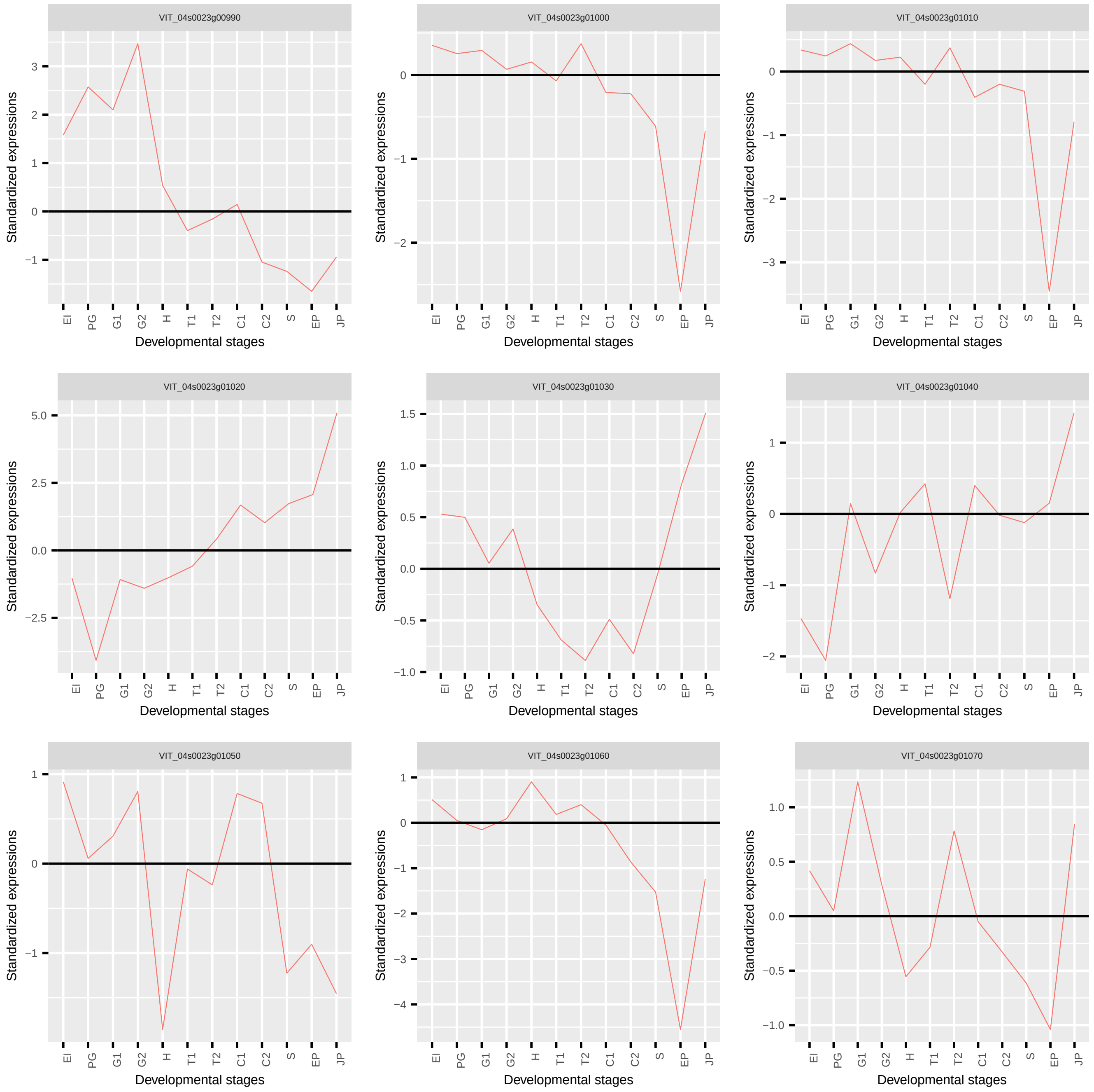
VIT_04s0023g00990
VIT_04s0023g01000
VIT_04s0023g01010
VIT_04s0023g01020
VIT_04s0023g01030
VIT_04s0023g01040
VIT_04s0023g01050
VIT_04s0023g01060
VIT_04s0023g01070
Standardized expressions
Developmental stages
EI PG G1 G2 H T1 T2 C1 C2 S EP JP

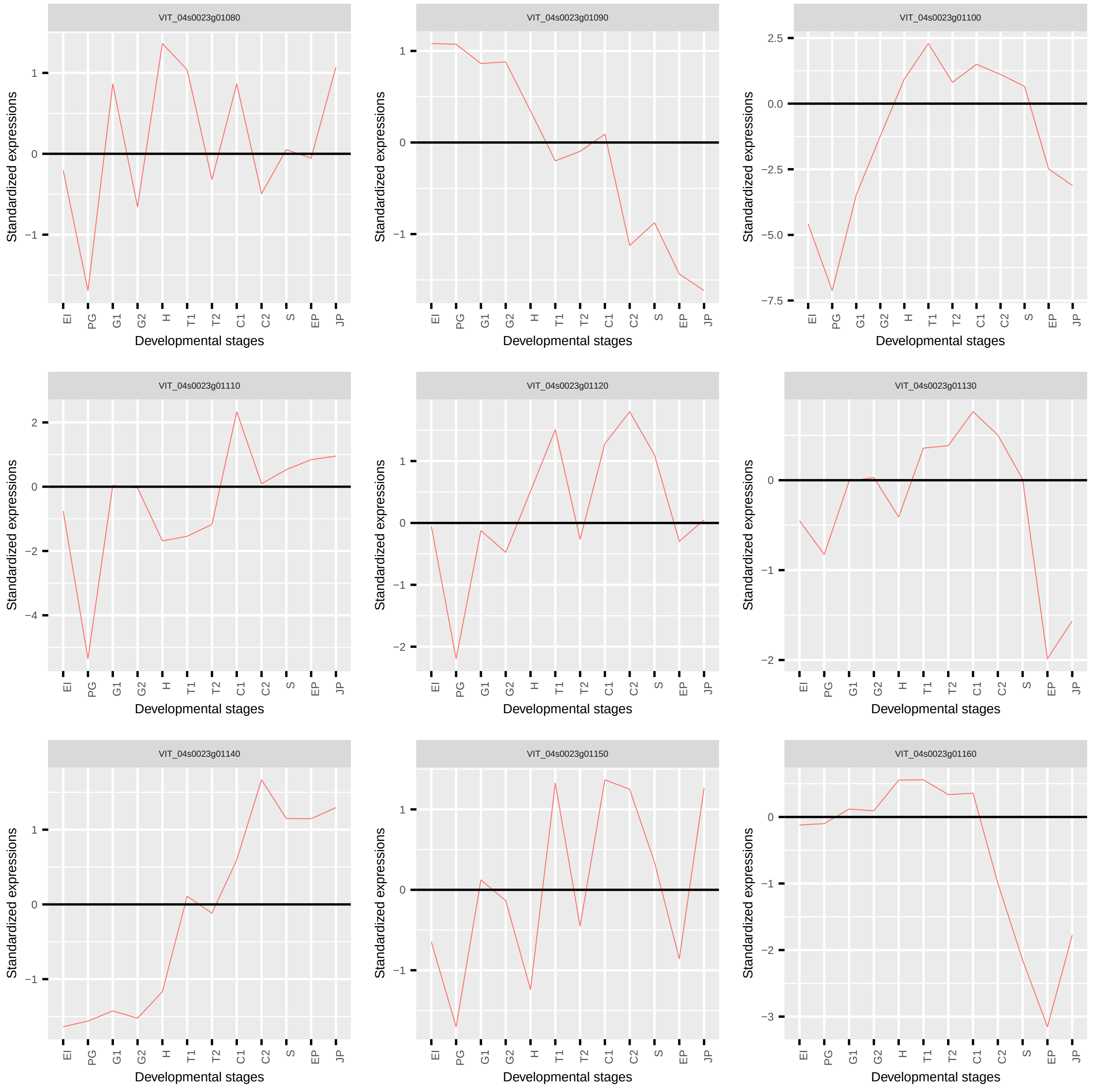

VIT_04s0023g01080
VIT_04s0023g01090
VIT_04s0023g01100
VIT_04s0023g01110
VIT_04s0023g01120
VIT_04s0023g01130
VIT_04s0023g01140
VIT_04s0023g01150
VIT_04s0023g01160
Standardized expressions
Developmental stages
EI PG G1 G2 H T1 T2 C1 C2 S EP JP

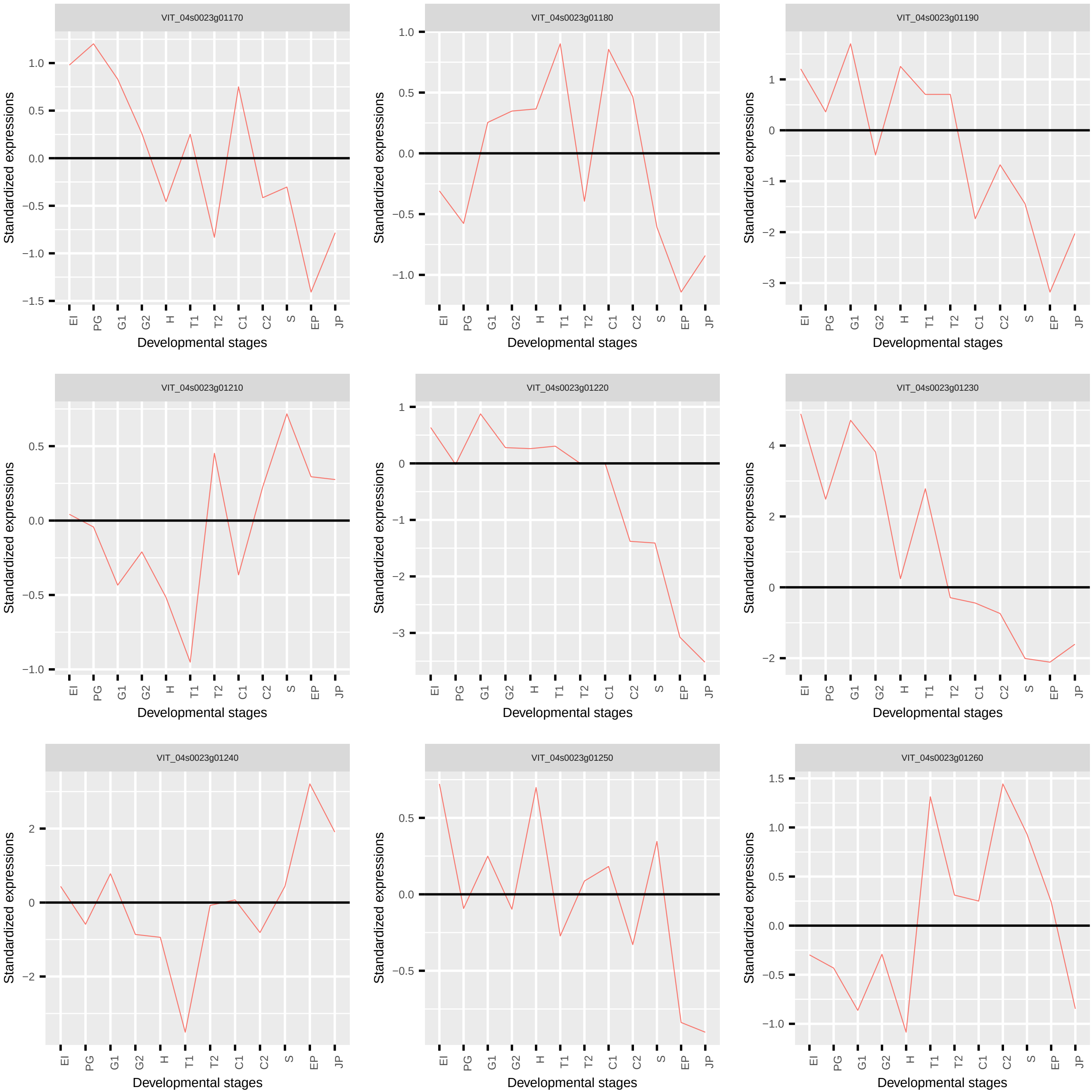

VIT_04s0023g01170
VIT_04s0023g01180
VIT_04s0023g01190
VIT_04s0023g01210
VIT_04s0023g01220
VIT_04s0023g01230
VIT_04s0023g01240
VIT_04s0023g01250
VIT_04s0023g01260
Standardized expressions
Developmental stages
EI PG G1 G2 H T1 T2 C1 C2 S EP JP

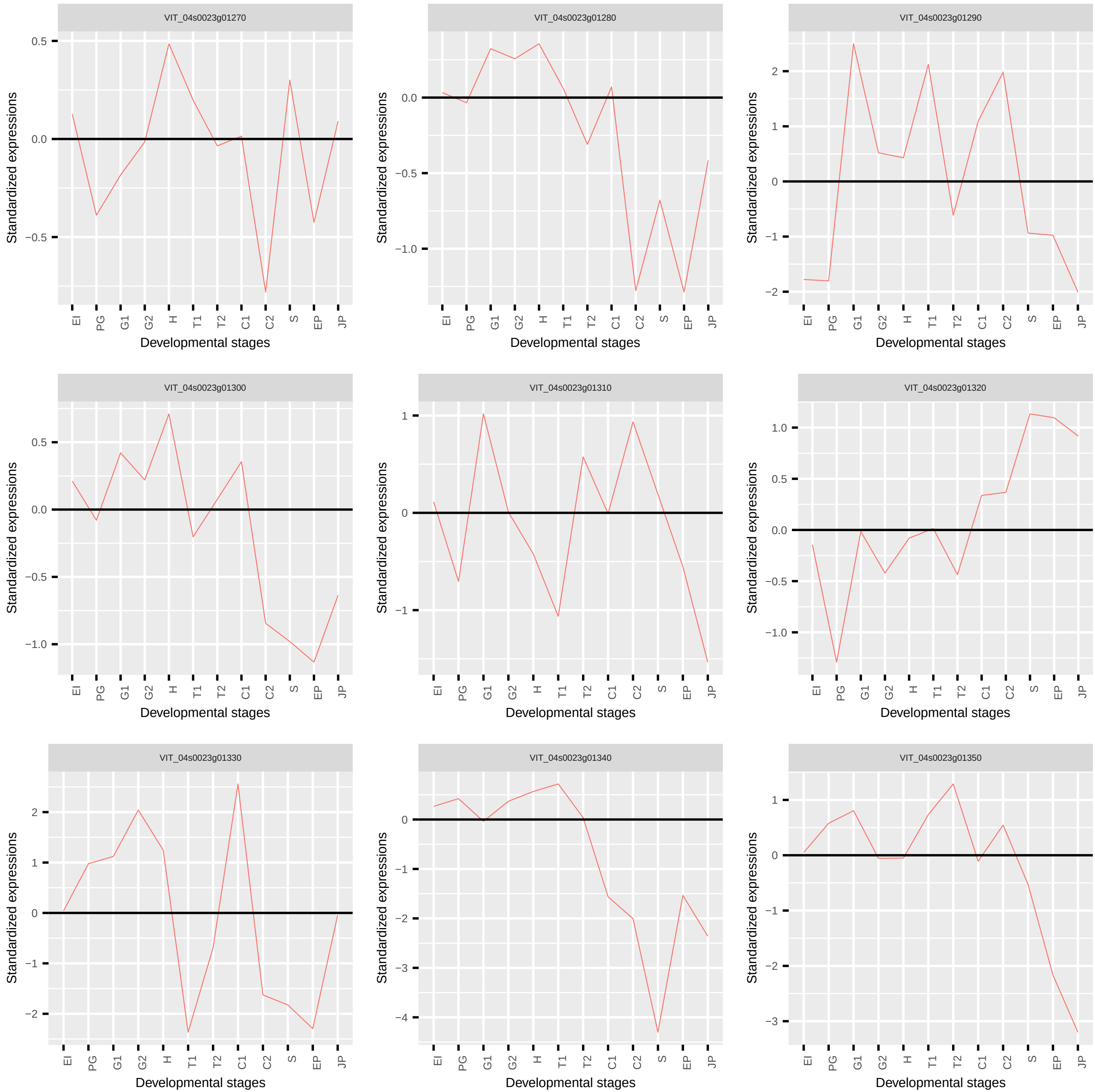

VIT_04s0023g01270
VIT_04s0023g01280
VIT_04s0023g01290
VIT_04s0023g01300
VIT_04s0023g01310
VIT_04s0023g01320
VIT_04s0023g01330
VIT_04s0023g01340
VIT_04s0023g01350
Standardized expressions
Developmental stages
EI PG G1 G2 H T1 T2 C1 C2 S EP JP

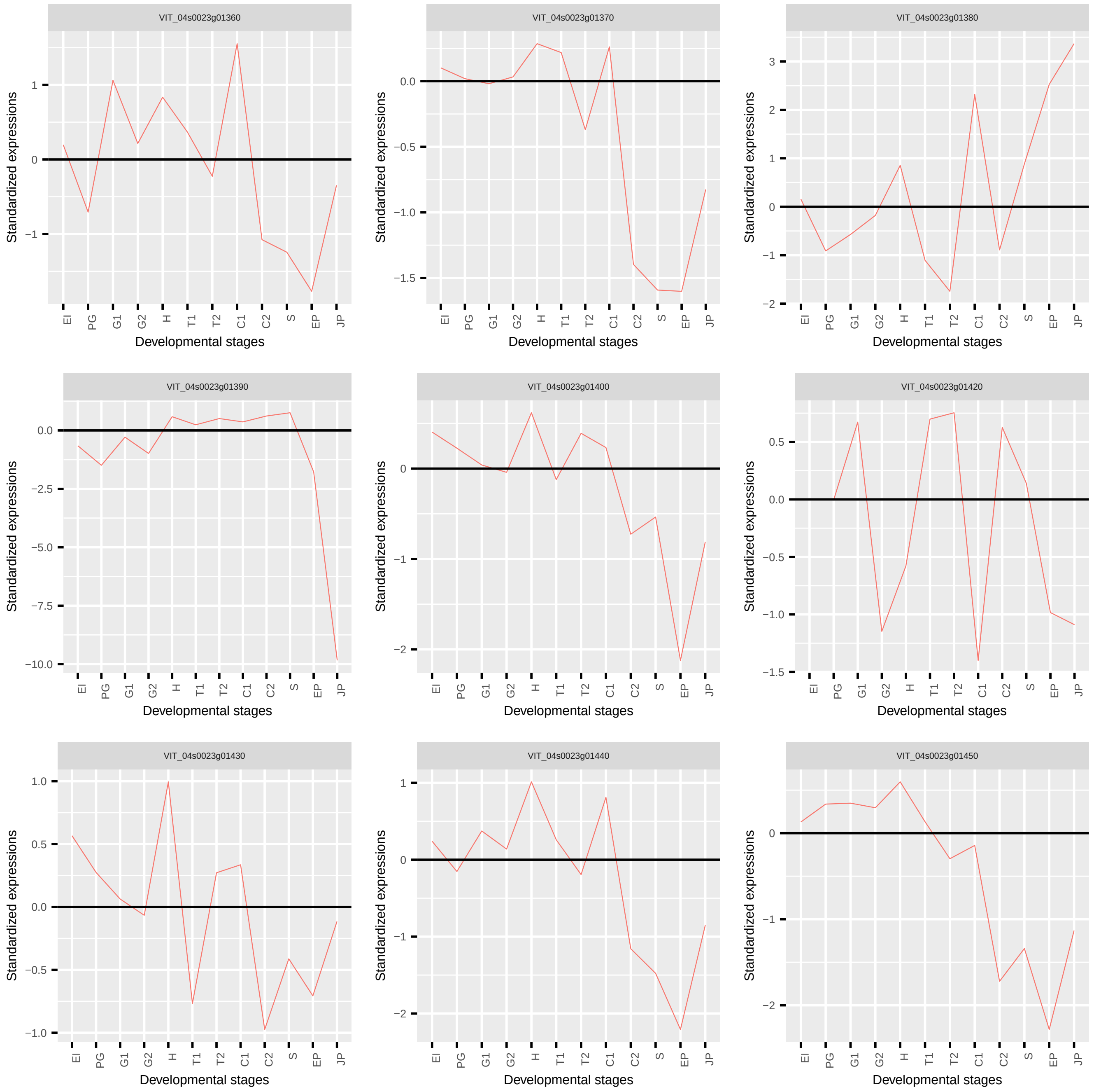
VIT_04s0023g01360
Standardized expressions
1
0
−1
EI PG G1 G2 H T1 T2 C1 C2 S EP JP
Developmental stages
VIT_04s0023g01370
Standardized expressions
0.0
−0.5
−1.0
−1.5
EI PG G1 G2 H T1 T2 C1 C2 S EP JP
Developmental stages
VIT_04s0023g01380
Standardized expressions
3
2
1
0
−1
−2
EI PG G1 G2 H T1 T2 C1 C2 S EP JP
Developmental stages
VIT_04s0023g01390
Standardized expressions
0.0
−2.5
−5.0
−7.5
−10.0
EI PG G1 G2 H T1 T2 C1 C2 S EP JP
Developmental stages
VIT_04s0023g01400
Standardized expressions
0
−1
−2
EI PG G1 G2 H T1 T2 C1 C2 S EP JP
Developmental stages
VIT_04s0023g01420
Standardized expressions
0.5
0.0
−0.5
−1.0
−1.5
EI PG G1 G2 H T1 T2 C1 C2 S EP JP
Developmental stages
VIT_04s0023g01430
Standardized expressions
1.0
0.5
0.0
−0.5
−1.0
EI PG G1 G2 H T1 T2 C1 C2 S EP JP
Developmental stages
VIT_04s0023g01440
Standardized expressions
1
0
−1
−2
EI PG G1 G2 H T1 T2 C1 C2 S EP JP
Developmental stages
VIT_04s0023g01450
Standardized expressions
0
−1
−2
EI PG G1 G2 H T1 T2 C1 C2 S EP JP
Developmental stages

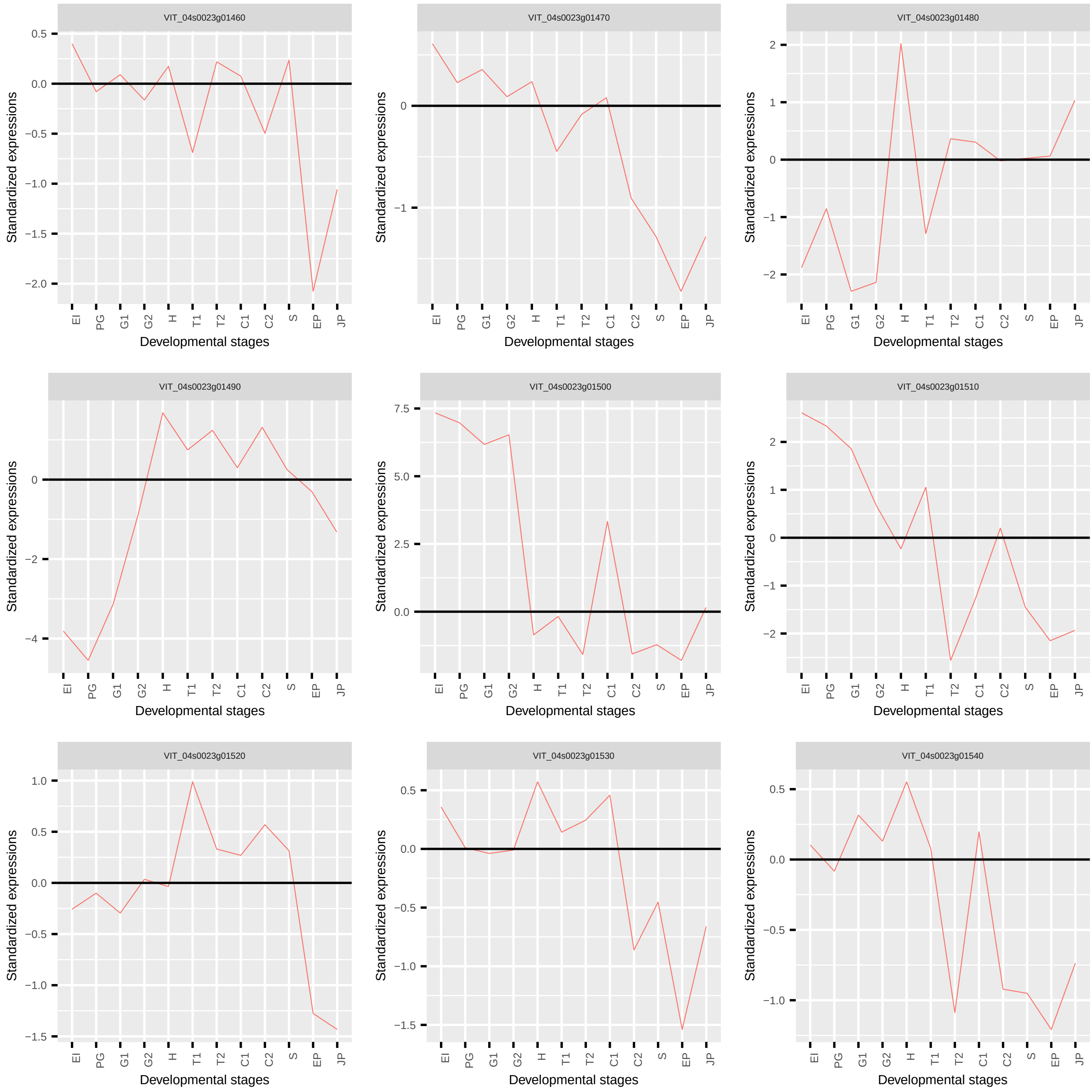
VIT_04s0023g01460
VIT_04s0023g01470
VIT_04s0023g01480
VIT_04s0023g01490
VIT_04s0023g01500
VIT_04s0023g01510
VIT_04s0023g01520
VIT_04s0023g01530
VIT_04s0023g01540
Standardized expressions
Developmental stages
EI PG G1 G2 H T1 T2 C1 C2 S EP JP
0.5 0.0 −0.5 −1.0 −1.5 −2.0
0 −1
2 1 0 −1 −2
0 −2 −4
7.5 5.0 2.5 0.0
2 1 0 −1 −2
1.0 0.5 0.0 −0.5 −1.0 −1.5
0.5 0.0 −0.5 −1.0 −1.5
0.5 0.0 −0.5 −1.0

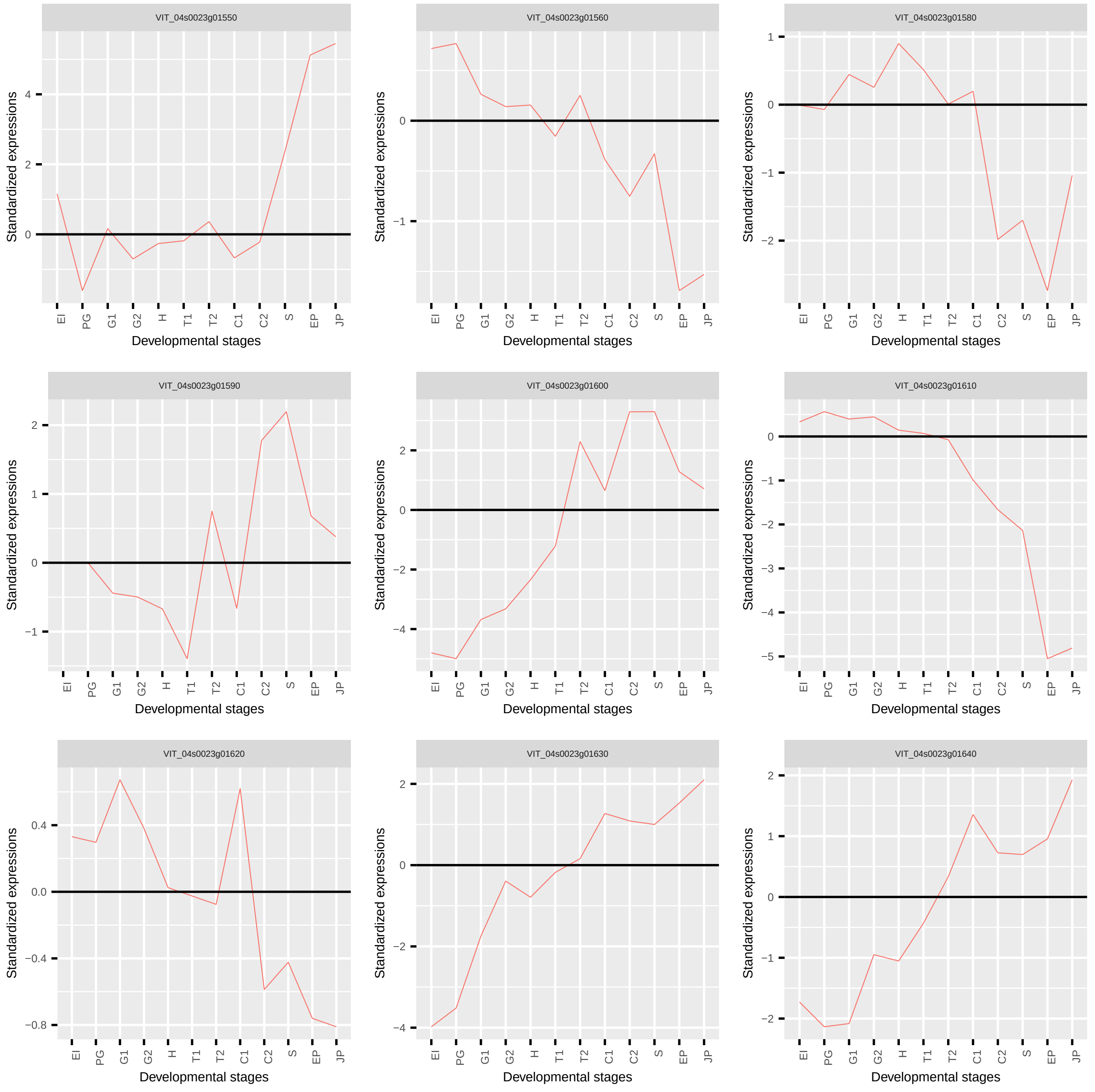
VIT_04s0023g01550
Standardized expressions
4
2
0
EI PG G1 G2 H T1 T2 C1 C2 S EP JP
Developmental stages
VIT_04s0023g01560
Standardized expressions
0
−1
EI PG G1 G2 H T1 T2 C1 C2 S EP JP
Developmental stages
VIT_04s0023g01580
Standardized expressions
1
0
−1
−2
EI PG G1 G2 H T1 T2 C1 C2 S EP JP
Developmental stages
VIT_04s0023g01590
Standardized expressions
2
1
0
−1
EI PG G1 G2 H T1 T2 C1 C2 S EP JP
Developmental stages
VIT_04s0023g01600
Standardized expressions
2
0
−2
−4
EI PG G1 G2 H T1 T2 C1 C2 S EP JP
Developmental stages
VIT_04s0023g01610
Standardized expressions
0
−1
−2
−3
−4
−5
EI PG G1 G2 H T1 T2 C1 C2 S EP JP
Developmental stages
VIT_04s0023g01620
Standardized expressions
0.4
0.0
−0.4
−0.8
EI PG G1 G2 H T1 T2 C1 C2 S EP JP
Developmental stages
VIT_04s0023g01630
Standardized expressions
2
0
−2
−4
EI PG G1 G2 H T1 T2 C1 C2 S EP JP
Developmental stages
VIT_04s0023g01640
Standardized expressions
2
1
0
−1
−2
EI PG G1 G2 H T1 T2 C1 C2 S EP JP
Developmental stages

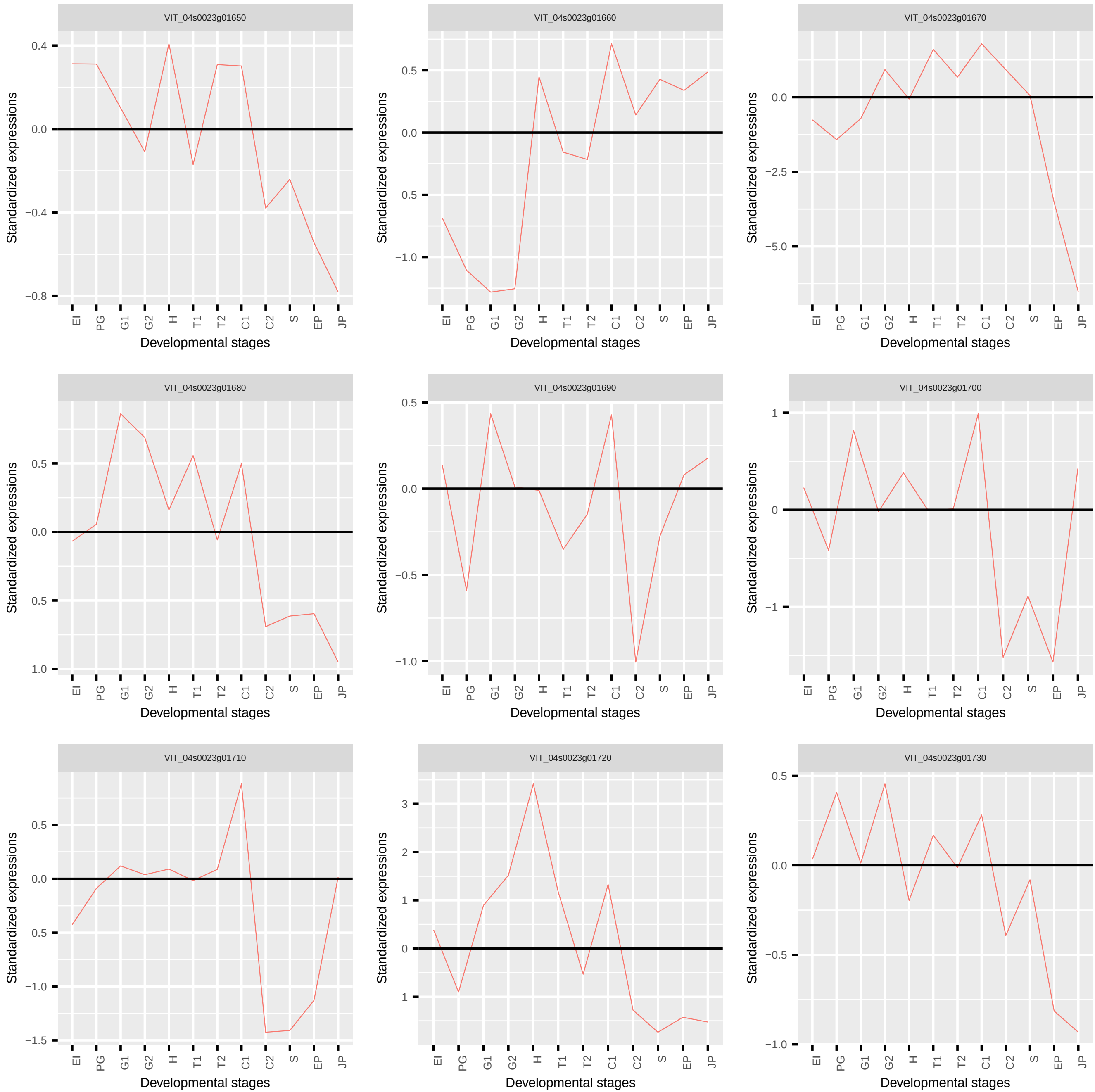
VIT_04s0023g01650
VIT_04s0023g01660
VIT_04s0023g01670
VIT_04s0023g01680
VIT_04s0023g01690
VIT_04s0023g01700
VIT_04s0023g01710
VIT_04s0023g01720
VIT_04s0023g01730
Standardized expressions
Developmental stages
EI
PG
G1
G2
H
T1
T2
C1
C2
S
EP
JP

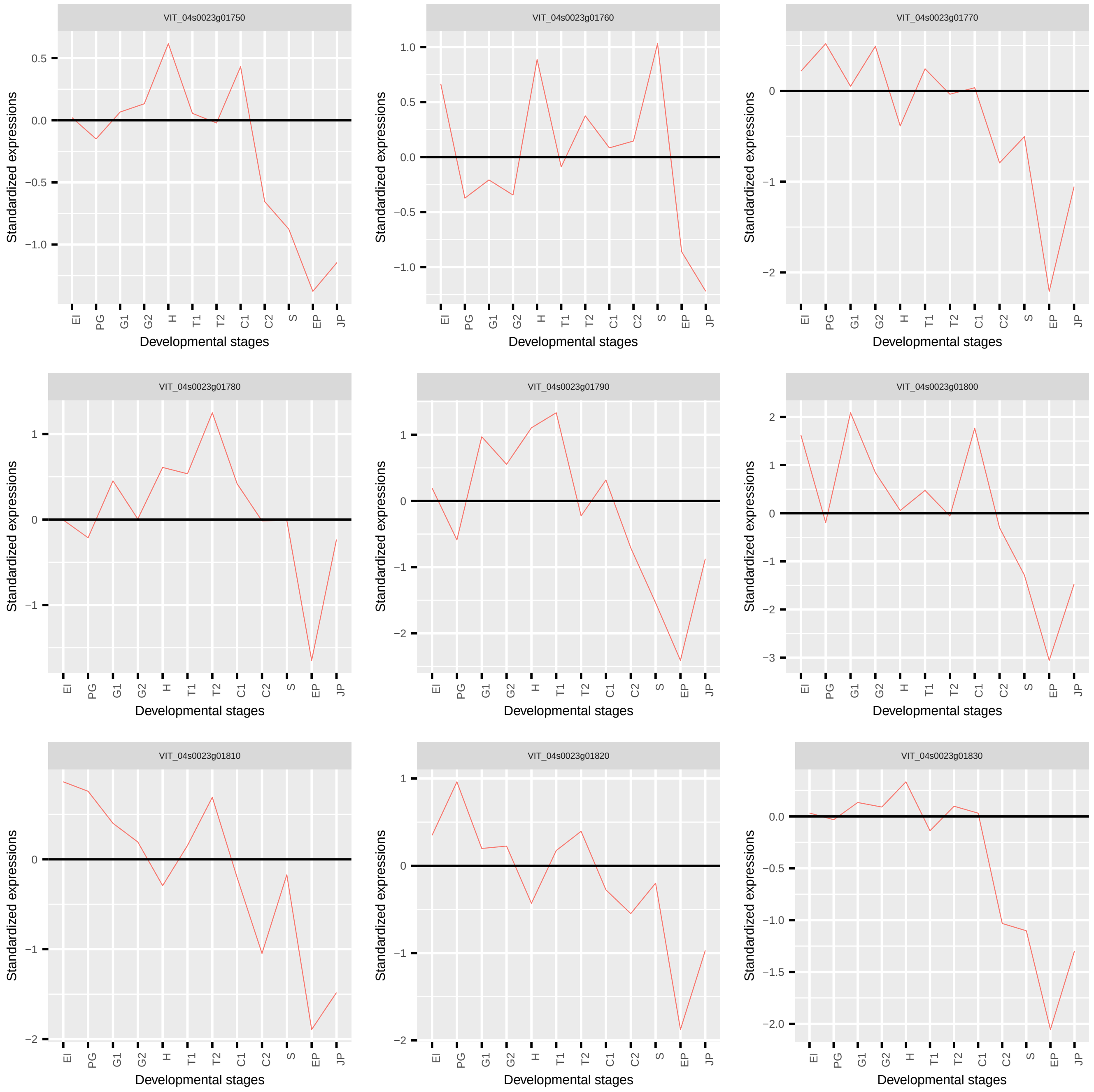
VIT_04s0023g01750
VIT_04s0023g01760
VIT_04s0023g01770
VIT_04s0023g01780
VIT_04s0023g01790
VIT_04s0023g01800
VIT_04s0023g01810
VIT_04s0023g01820
VIT_04s0023g01830
Standardized expressions
Developmental stages
EI PG G1 G2 H T1 T2 C1 C2 S EP JP

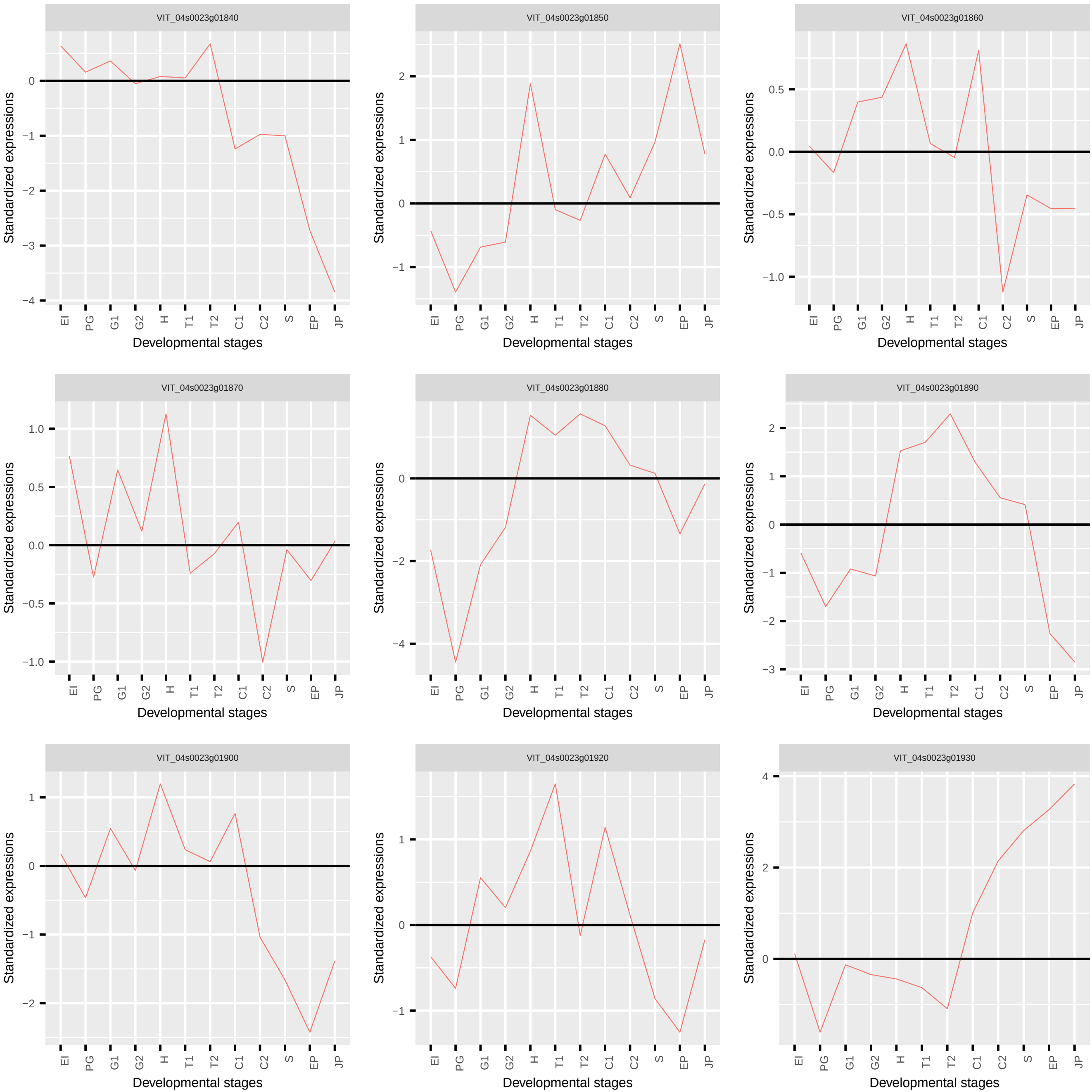
VIT_04s0023g01840
VIT_04s0023g01850
VIT_04s0023g01860
VIT_04s0023g01870
VIT_04s0023g01880
VIT_04s0023g01890
VIT_04s0023g01900
VIT_04s0023g01920
VIT_04s0023g01930
Standardized expressions
Developmental stages
EI PG G1 G2 H T1 T2 C1 C2 S EP JP

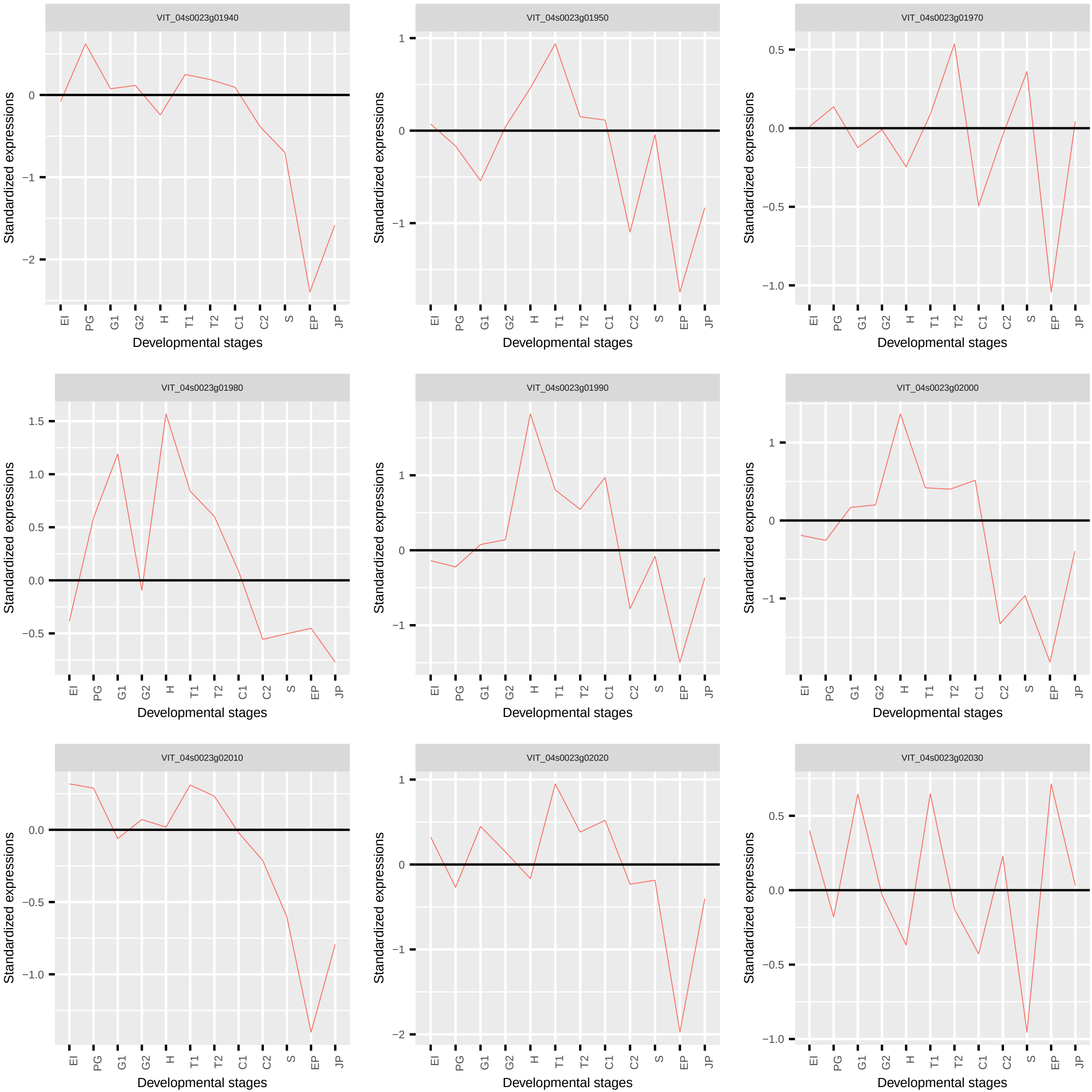
VIT_04s0023g01940
VIT_04s0023g01950
VIT_04s0023g01970
VIT_04s0023g01980
VIT_04s0023g01990
VIT_04s0023g02000
VIT_04s0023g02010
VIT_04s0023g02020
VIT_04s0023g02030
Standardized expressions
Developmental stages
EI
PG
G1
G2
H
T1
T2
C1
C2
S
EP
JP

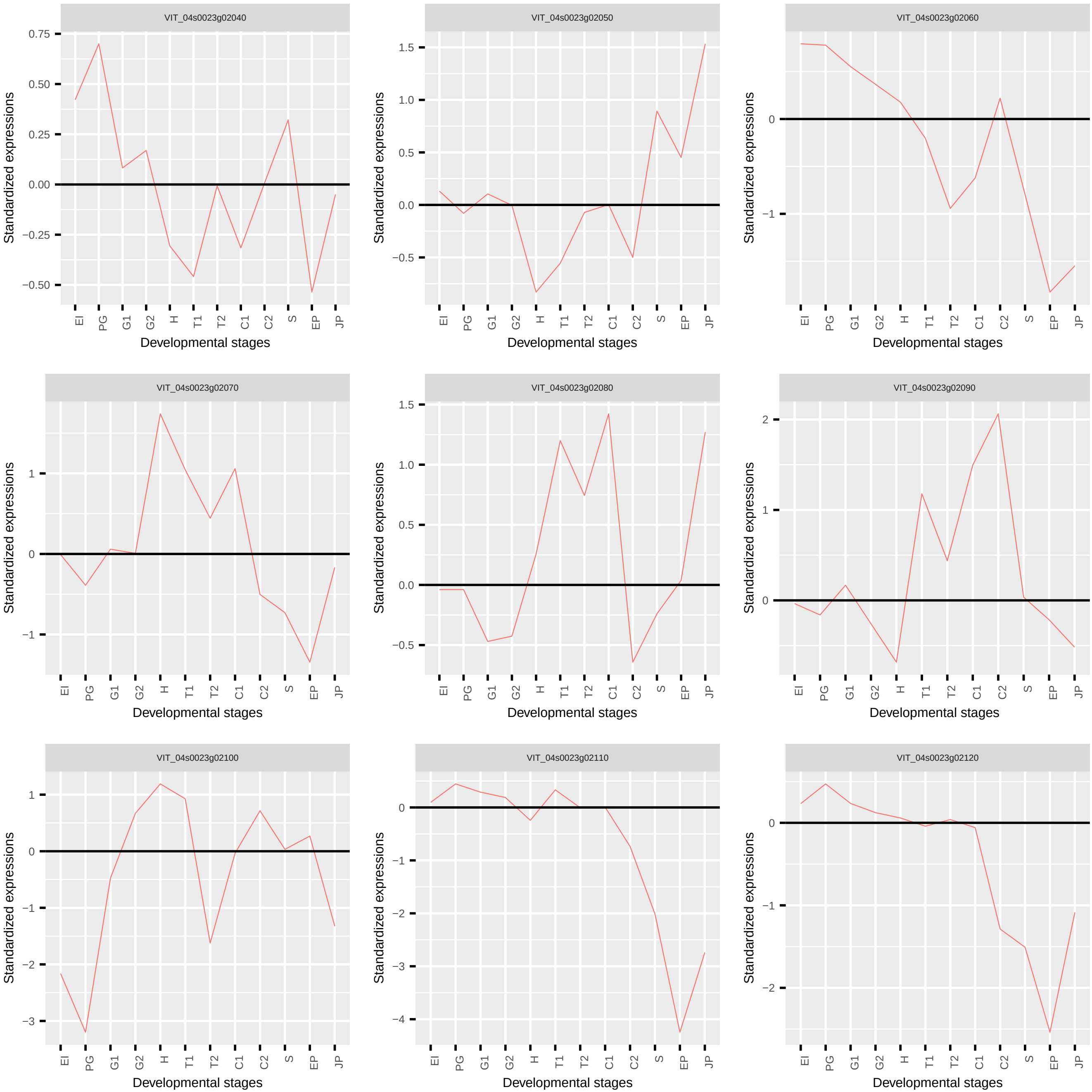

VIT_04s0023g02040
VIT_04s0023g02050
VIT_04s0023g02060
VIT_04s0023g02070
VIT_04s0023g02080
VIT_04s0023g02090
VIT_04s0023g02100
VIT_04s0023g02110
VIT_04s0023g02120
Standardized expressions
Developmental stages
EI PG G1 G2 H T1 T2 C1 C2 S EP JP

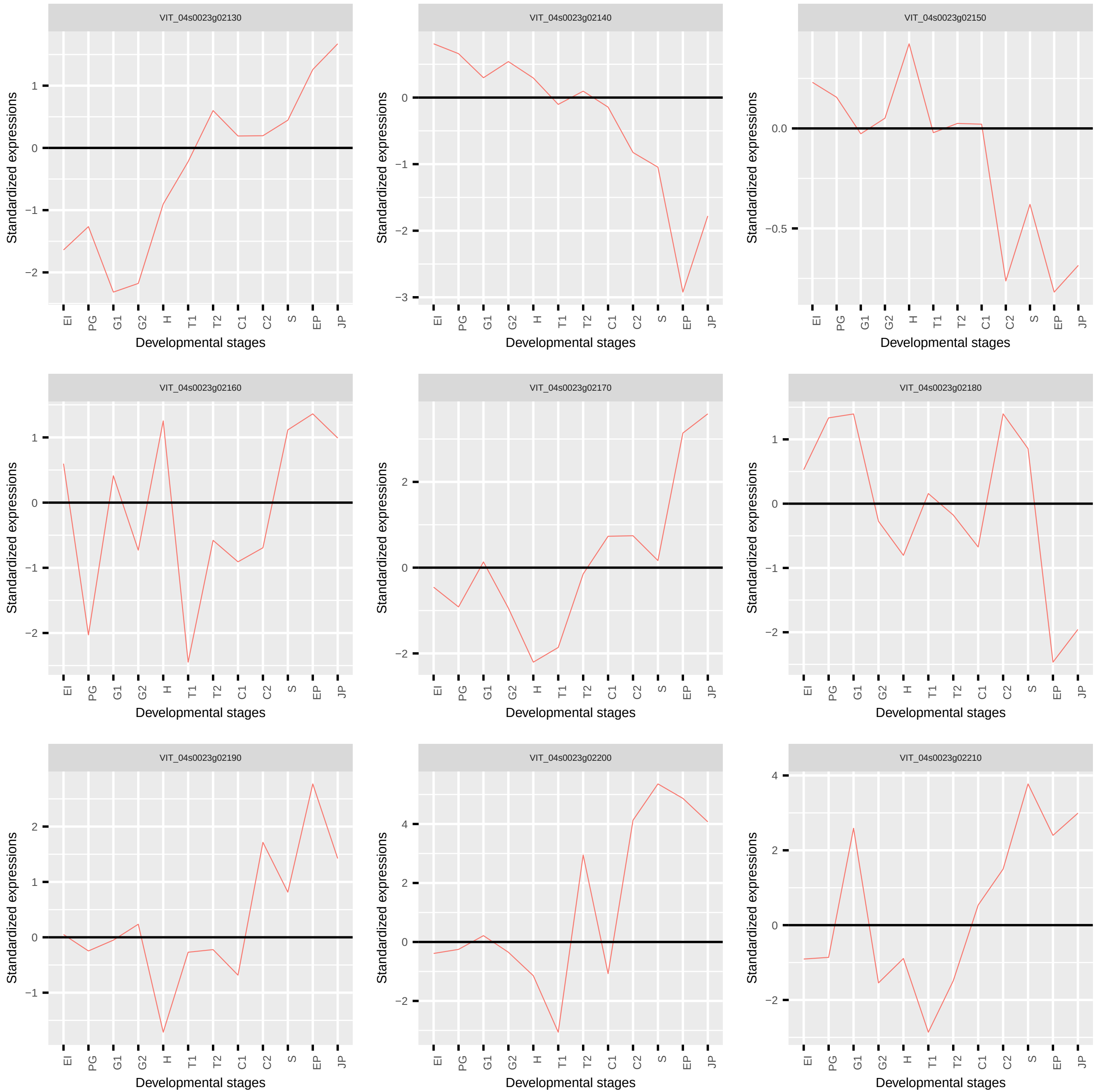
VIT_04s0023g02130
VIT_04s0023g02140
VIT_04s0023g02150
VIT_04s0023g02160
VIT_04s0023g02170
VIT_04s0023g02180
VIT_04s0023g02190
VIT_04s0023g02200
VIT_04s0023g02210
Standardized expressions
Developmental stages
EI PG G1 G2 H T1 T2 C1 C2 S EP JP

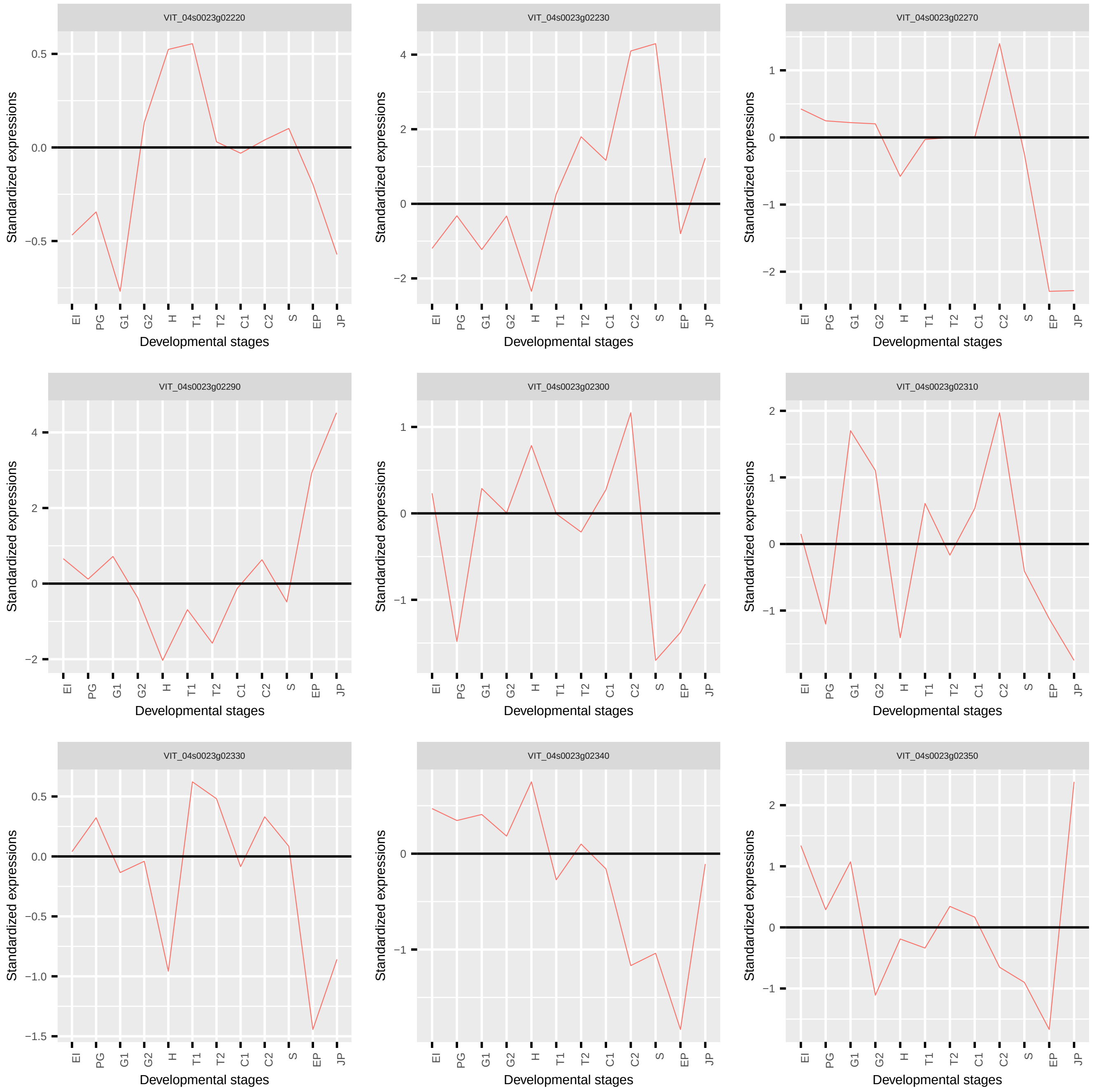
VIT_04s0023g02220
Standardized expressions
0.5
0.0
−0.5
EI
PG
G1
G2
H
T1
T2
C1
C2
S
EP
JP
Developmental stages
VIT_04s0023g02230
Standardized expressions
4
2
0
−2
EI
PG
G1
G2
H
T1
T2
C1
C2
S
EP
JP
Developmental stages
VIT_04s0023g02270
Standardized expressions
1
0
−1
−2
EI
PG
G1
G2
H
T1
T2
C1
C2
S
EP
JP
Developmental stages
VIT_04s0023g02290
Standardized expressions
4
2
0
−2
EI
PG
G1
G2
H
T1
T2
C1
C2
S
EP
JP
Developmental stages
VIT_04s0023g02300
Standardized expressions
1
0
−1
EI
PG
G1
G2
H
T1
T2
C1
C2
S
EP
JP
Developmental stages
VIT_04s0023g02310
Standardized expressions
2
1
0
−1
EI
PG
G1
G2
H
T1
T2
C1
C2
S
EP
JP
Developmental stages
VIT_04s0023g02330
Standardized expressions
0.5
0.0
−0.5
−1.0
−1.5
EI
PG
G1
G2
H
T1
T2
C1
C2
S
EP
JP
Developmental stages
VIT_04s0023g02340
Standardized expressions
0
−1
EI
PG
G1
G2
H
T1
T2
C1
C2
S
EP
JP
Developmental stages
VIT_04s0023g02350
Standardized expressions
2
1
0
−1
EI
PG
G1
G2
H
T1
T2
C1
C2
S
EP
JP
Developmental stages

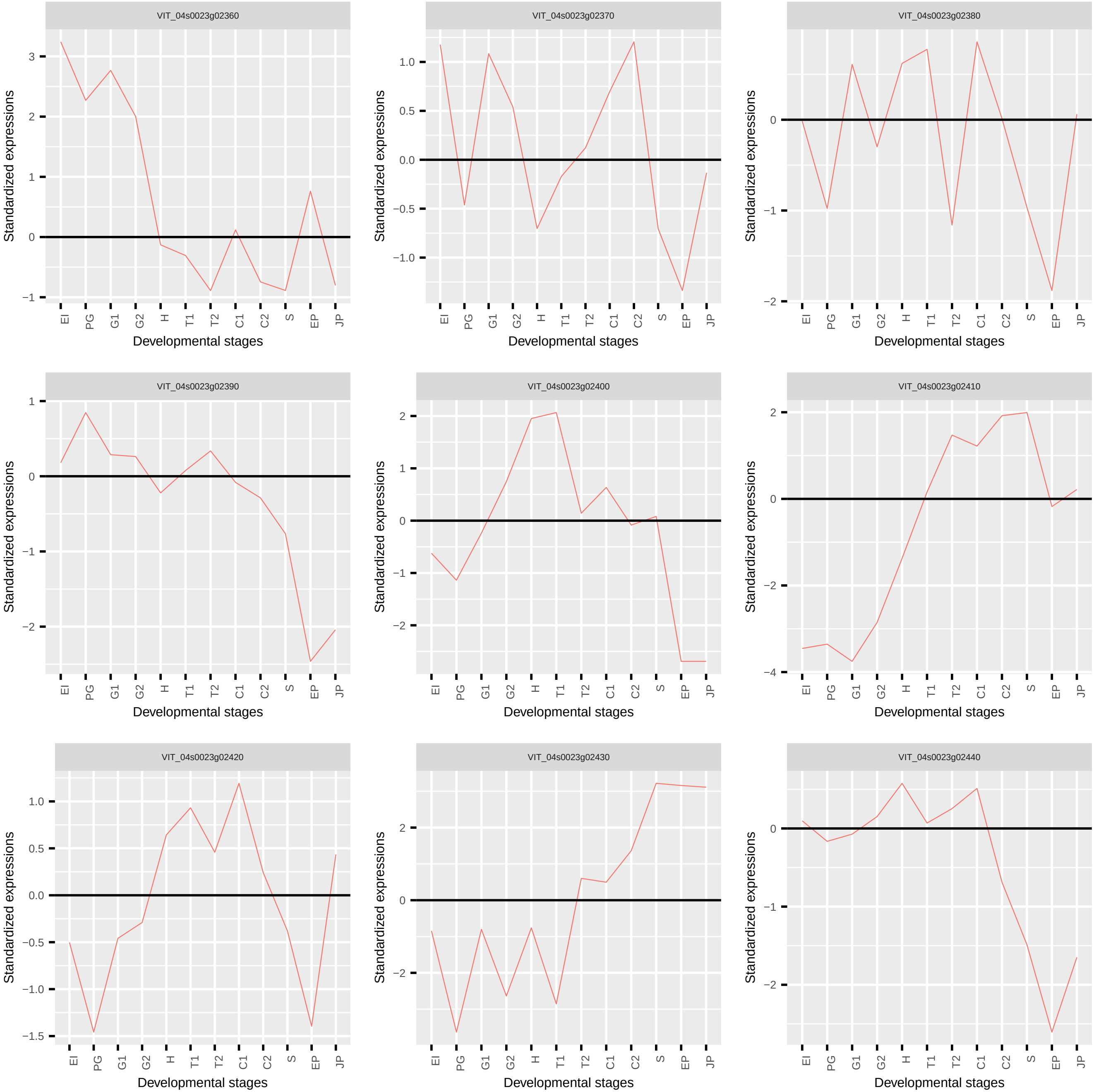

VIT_04s0023g02360
VIT_04s0023g02370
VIT_04s0023g02380
VIT_04s0023g02390
VIT_04s0023g02400
VIT_04s0023g02410
VIT_04s0023g02420
VIT_04s0023g02430
VIT_04s0023g02440
Standardized expressions
Developmental stages
EI PG G1 G2 H T1 T2 C1 C2 S EP JP

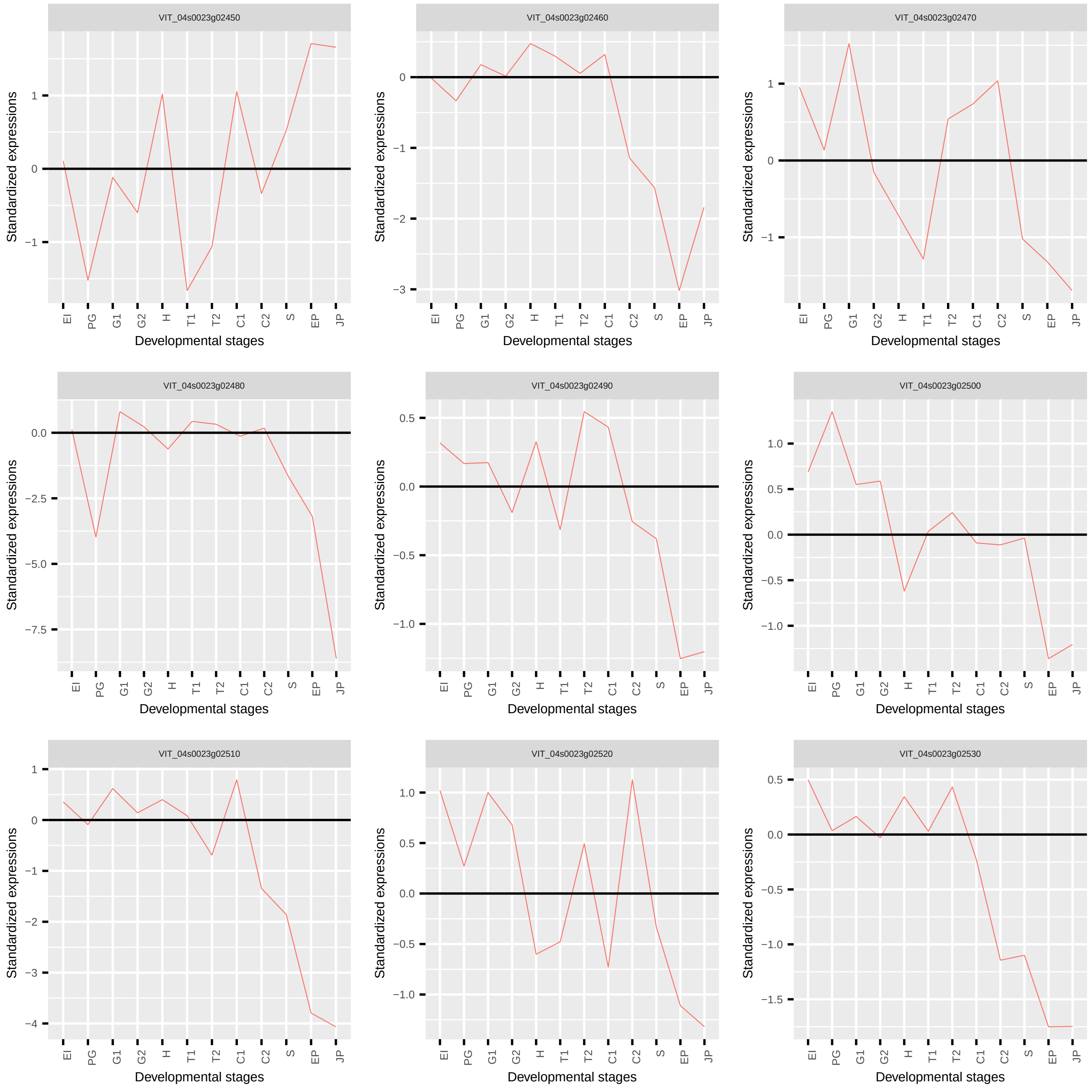
VIT_04s0023g02450
VIT_04s0023g02460
VIT_04s0023g02470
VIT_04s0023g02480
VIT_04s0023g02490
VIT_04s0023g02500
VIT_04s0023g02510
VIT_04s0023g02520
VIT_04s0023g02530
Standardized expressions
Developmental stages
EI PG G1 G2 H T1 T2 C1 C2 S EP JP

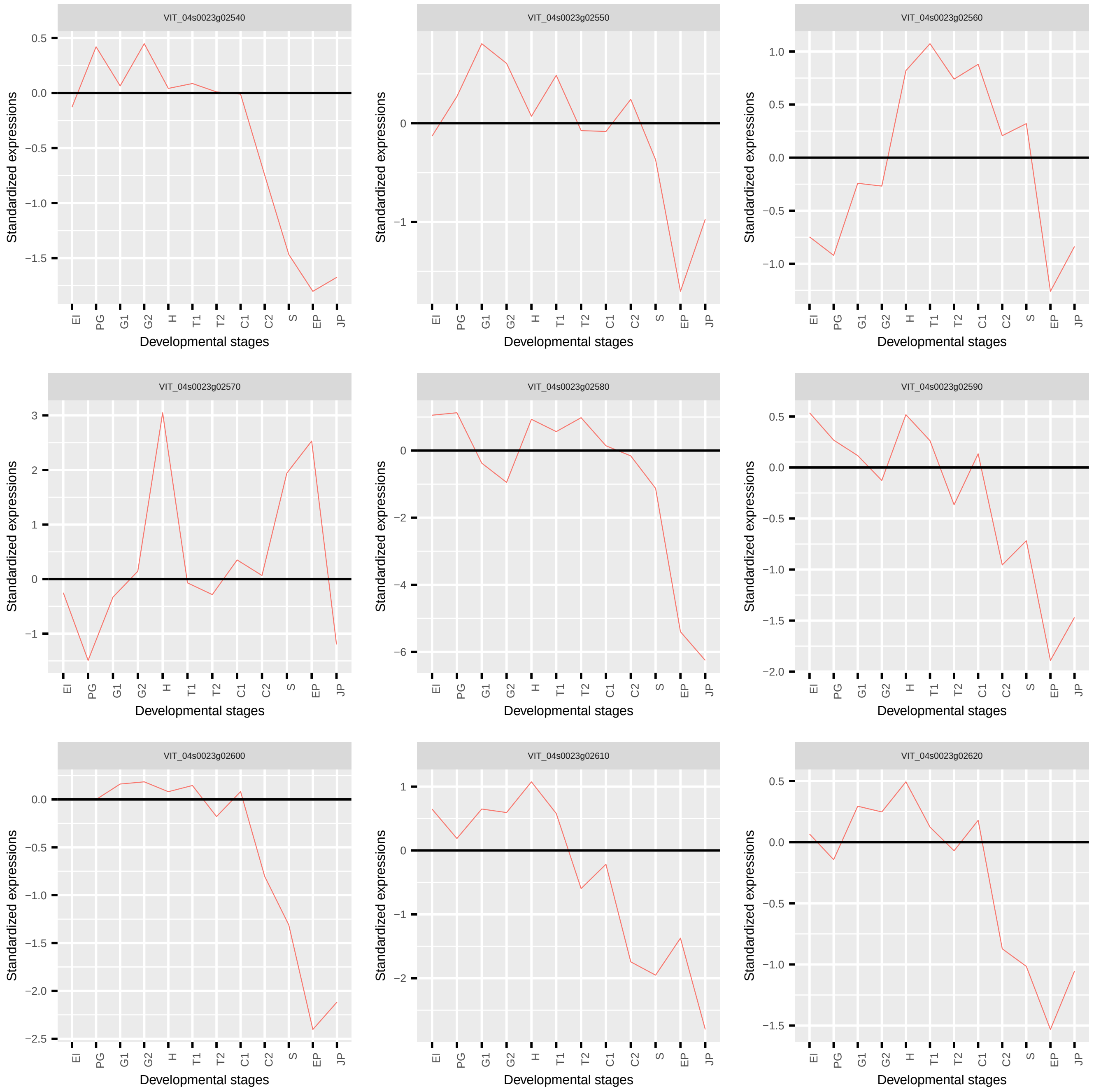
VIT_04s0023g02540
VIT_04s0023g02550
VIT_04s0023g02560
VIT_04s0023g02570
VIT_04s0023g02580
VIT_04s0023g02590
VIT_04s0023g02600
VIT_04s0023g02610
VIT_04s0023g02620
Standardized expressions
Developmental stages
EI PG G1 G2 H T1 T2 C1 C2 S EP JP

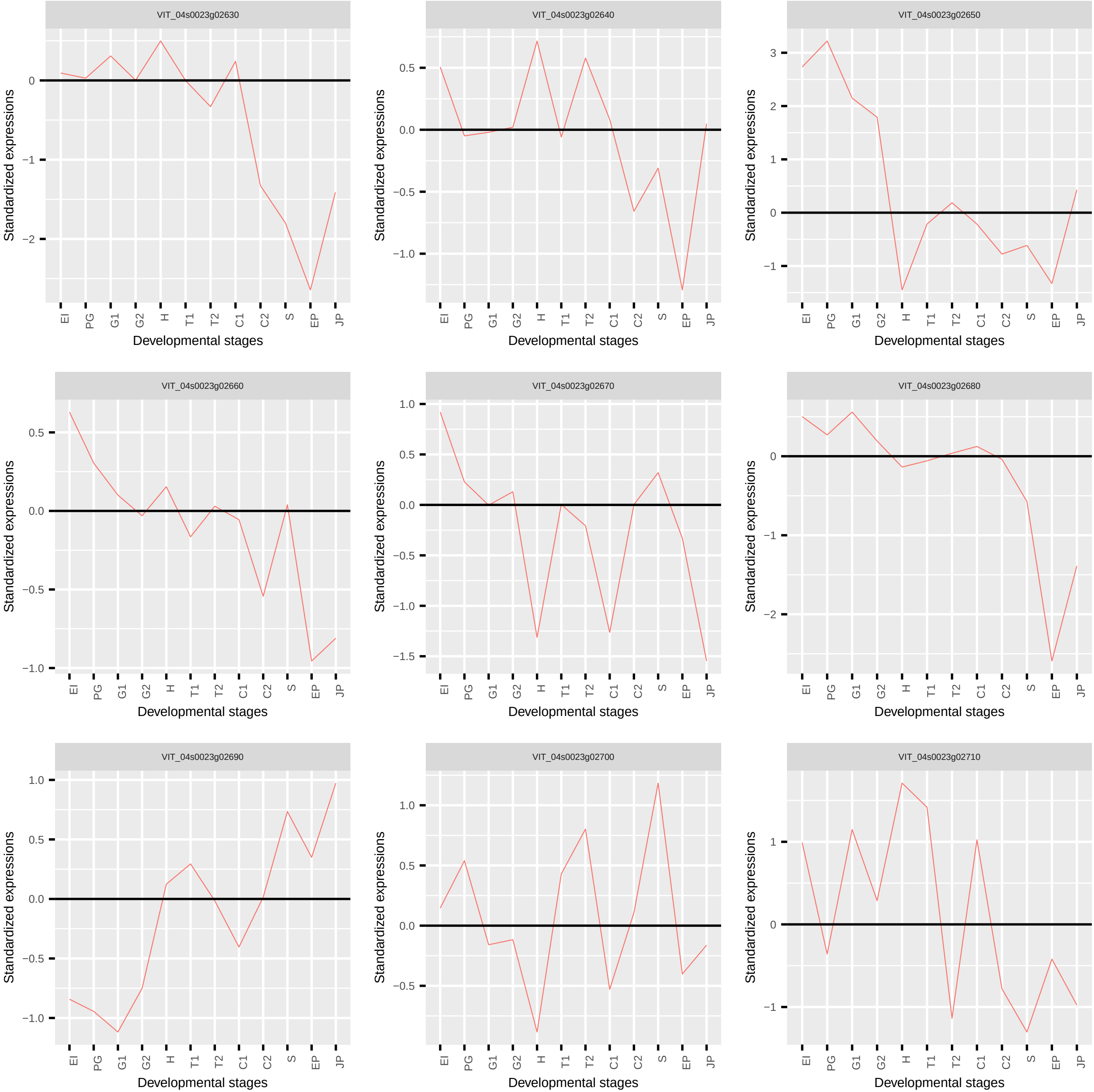
VIT_04s0023g02630
VIT_04s0023g02640
VIT_04s0023g02650
VIT_04s0023g02660
VIT_04s0023g02670
VIT_04s0023g02680
VIT_04s0023g02690
VIT_04s0023g02700
VIT_04s0023g02710
Standardized expressions
Developmental stages
EI PG G1 G2 H T1 T2 C1 C2 S EP JP

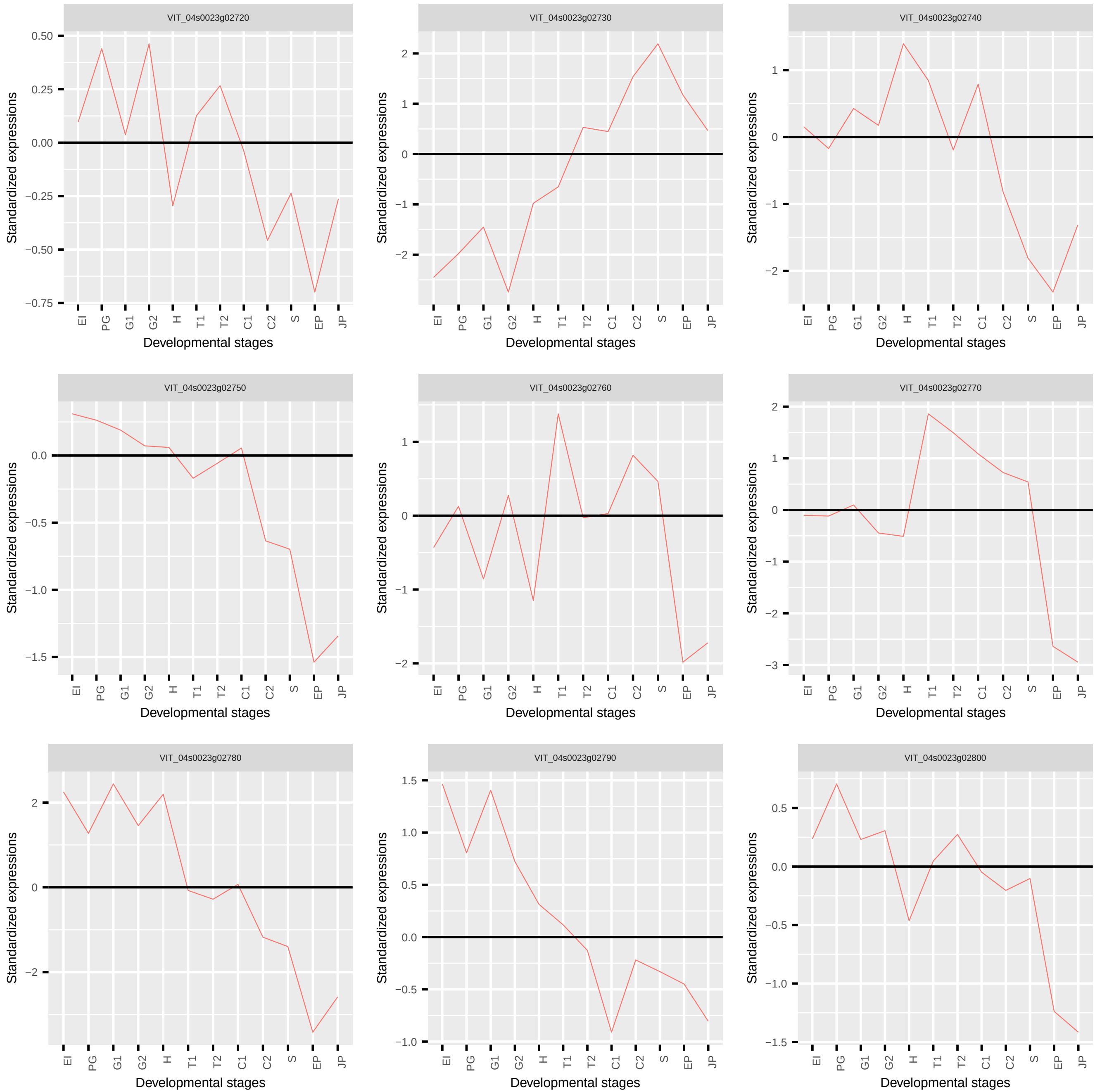
VIT_04s0023g02720
VIT_04s0023g02730
VIT_04s0023g02740
VIT_04s0023g02750
VIT_04s0023g02760
VIT_04s0023g02770
VIT_04s0023g02780
VIT_04s0023g02790
VIT_04s0023g02800
Standardized expressions
Developmental stages
EI PG G1 G2 H T1 T2 C1 C2 S EP JP

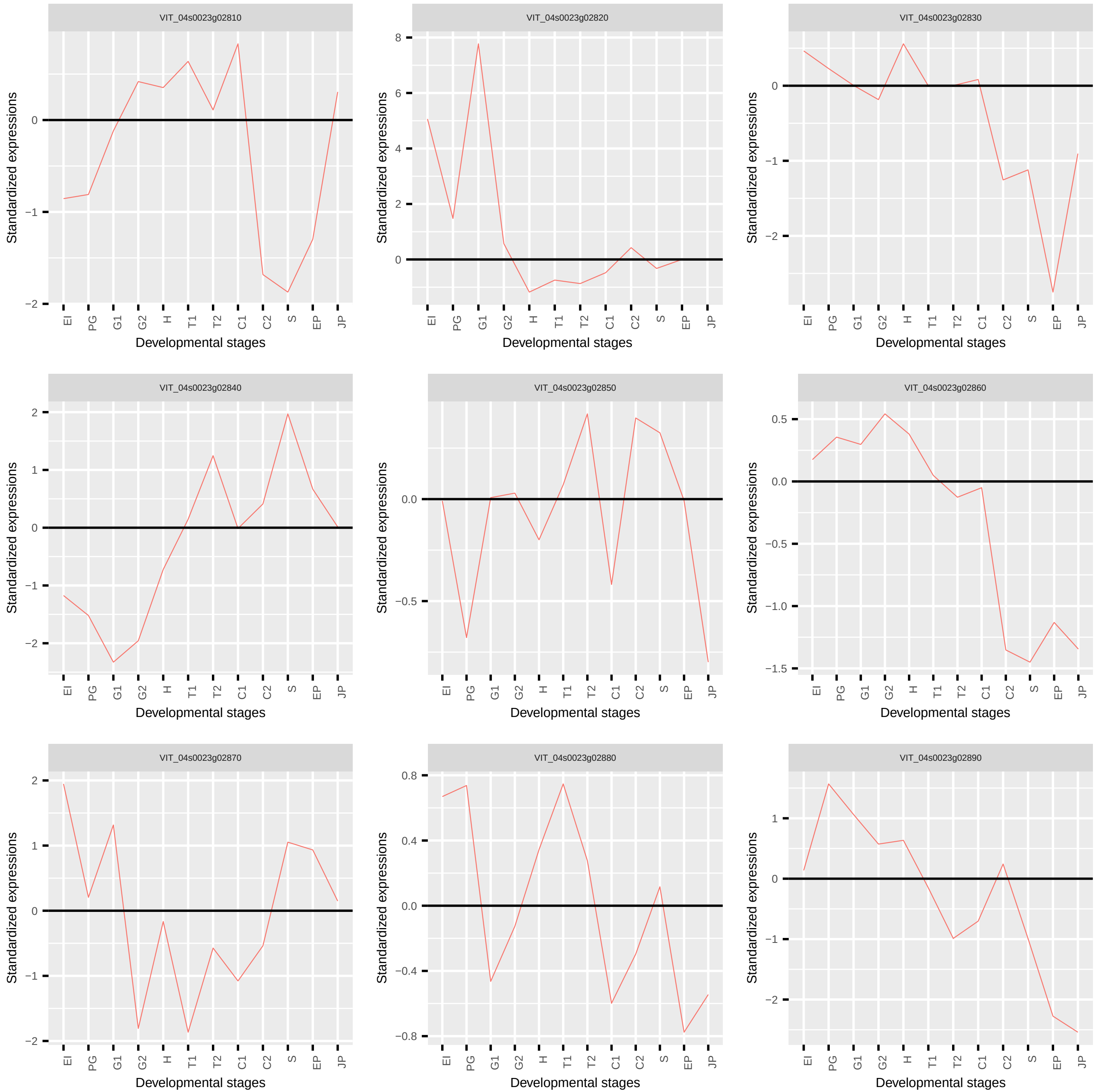
VIT_04s0023g02810
VIT_04s0023g02820
VIT_04s0023g02830
VIT_04s0023g02840
VIT_04s0023g02850
VIT_04s0023g02860
VIT_04s0023g02870
VIT_04s0023g02880
VIT_04s0023g02890
Standardized expressions
Developmental stages
EI PG G1 G2 H T1 T2 C1 C2 S EP JP

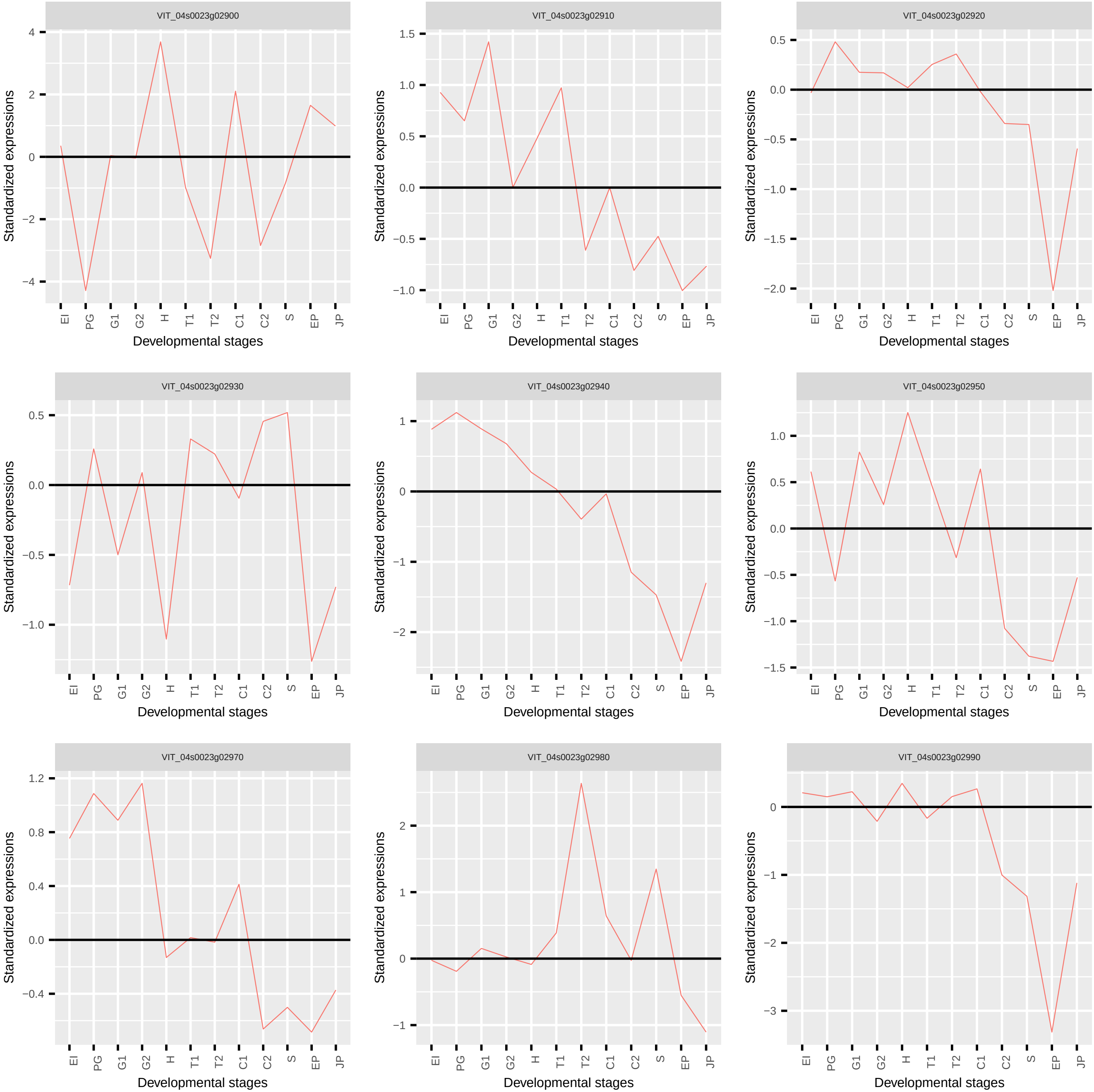
VIT_04s0023g02900
VIT_04s0023g02910
VIT_04s0023g02920
VIT_04s0023g02930
VIT_04s0023g02940
VIT_04s0023g02950
VIT_04s0023g02970
VIT_04s0023g02980
VIT_04s0023g02990
Standardized expressions
Developmental stages
EI PG G1 G2 H T1 T2 C1 C2 S EP JP

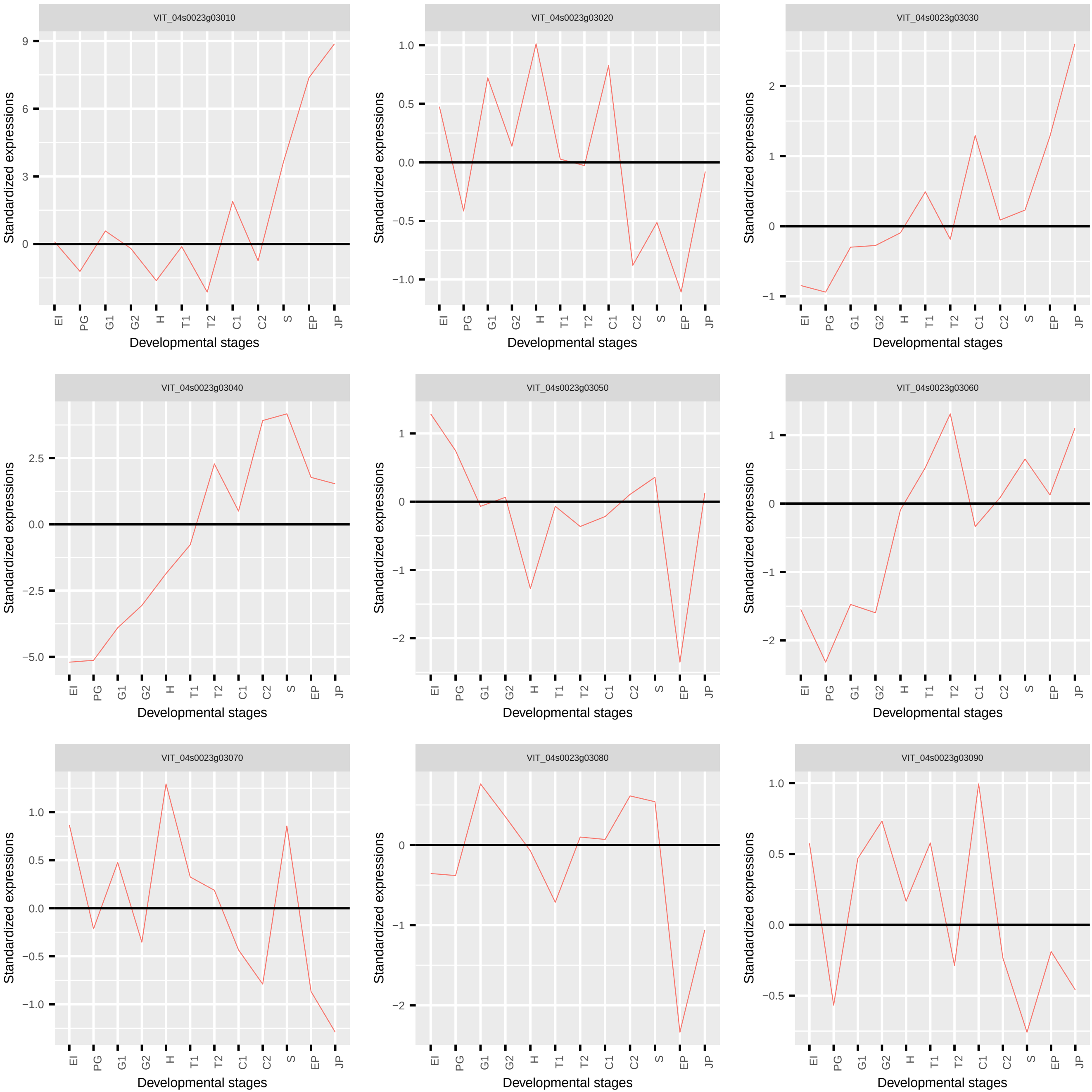

VIT_04s0023g03010
VIT_04s0023g03020
VIT_04s0023g03030
VIT_04s0023g03040
VIT_04s0023g03050
VIT_04s0023g03060
VIT_04s0023g03070
VIT_04s0023g03080
VIT_04s0023g03090
Standardized expressions
Developmental stages
EI PG G1 G2 H T1 T2 C1 C2 S EP JP

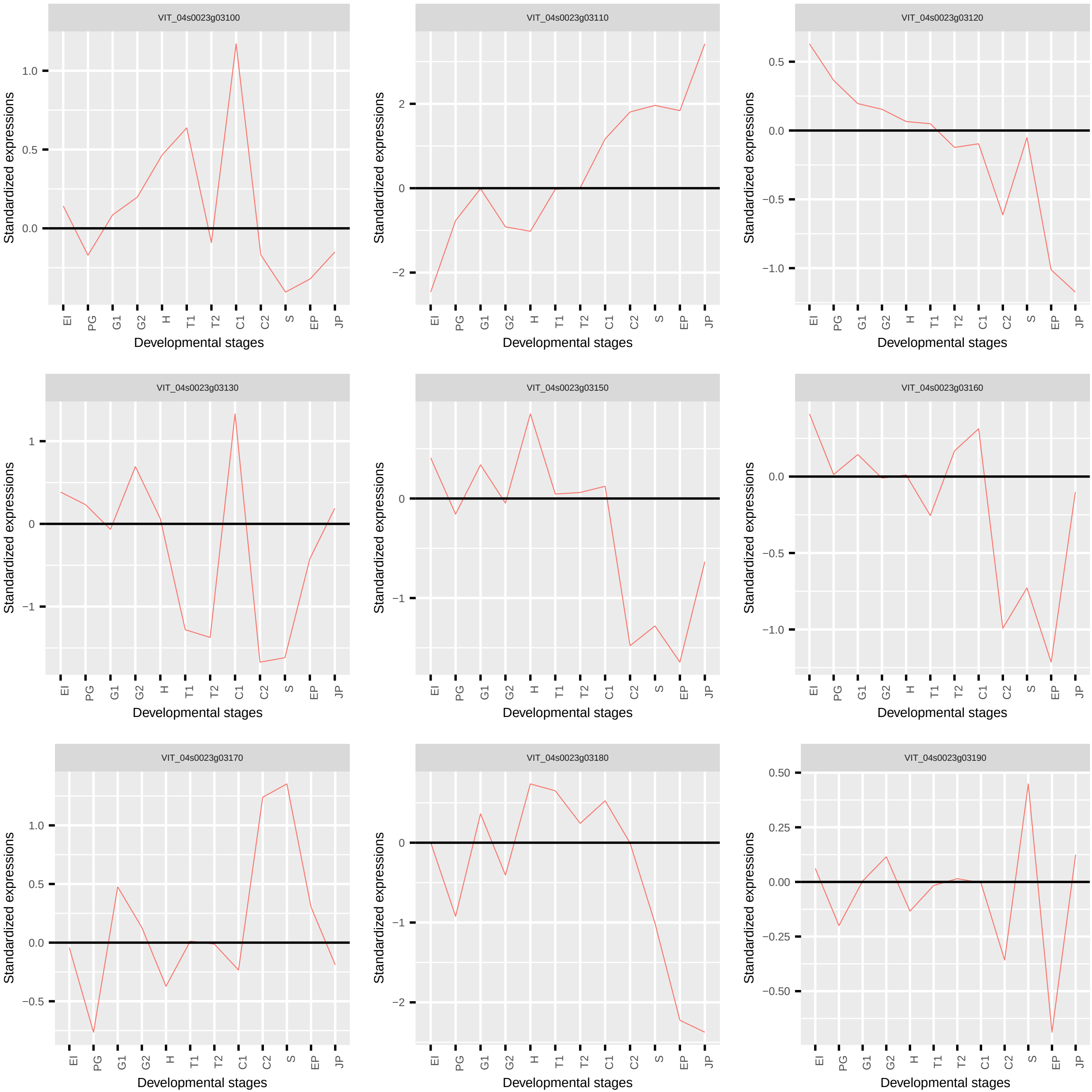
VIT_04s0023g03100
VIT_04s0023g03110
VIT_04s0023g03120
VIT_04s0023g03130
VIT_04s0023g03150
VIT_04s0023g03160
VIT_04s0023g03170
VIT_04s0023g03180
VIT_04s0023g03190
Standardized expressions
Developmental stages
EI PG G1 G2 H T1 T2 C1 C2 S EP JP

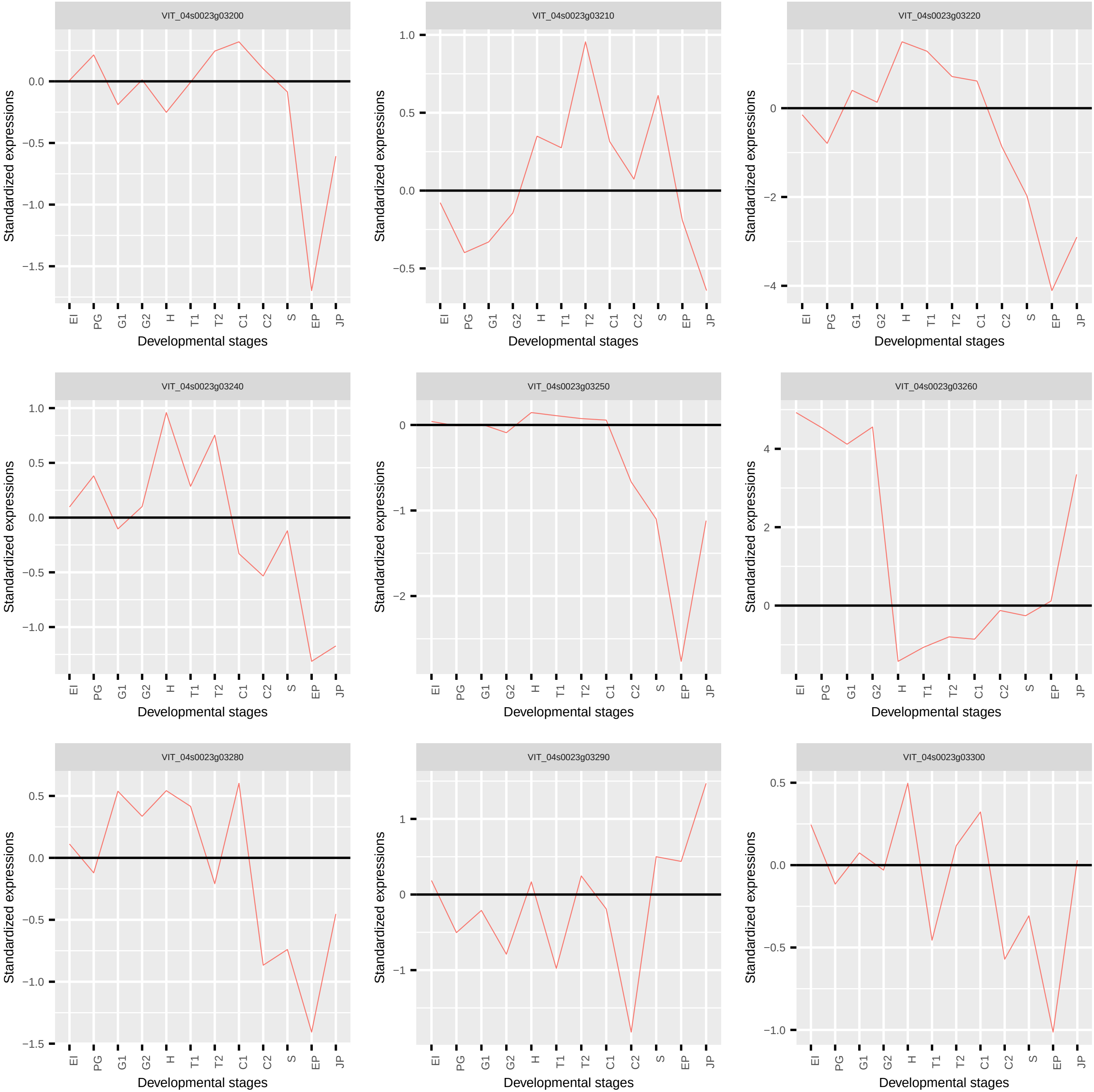
VIT_04s0023g03200
VIT_04s0023g03210
VIT_04s0023g03220
VIT_04s0023g03240
VIT_04s0023g03250
VIT_04s0023g03260
VIT_04s0023g03280
VIT_04s0023g03290
VIT_04s0023g03300
Standardized expressions
Developmental stages
EI PG G1 G2 H T1 T2 C1 C2 S EP JP

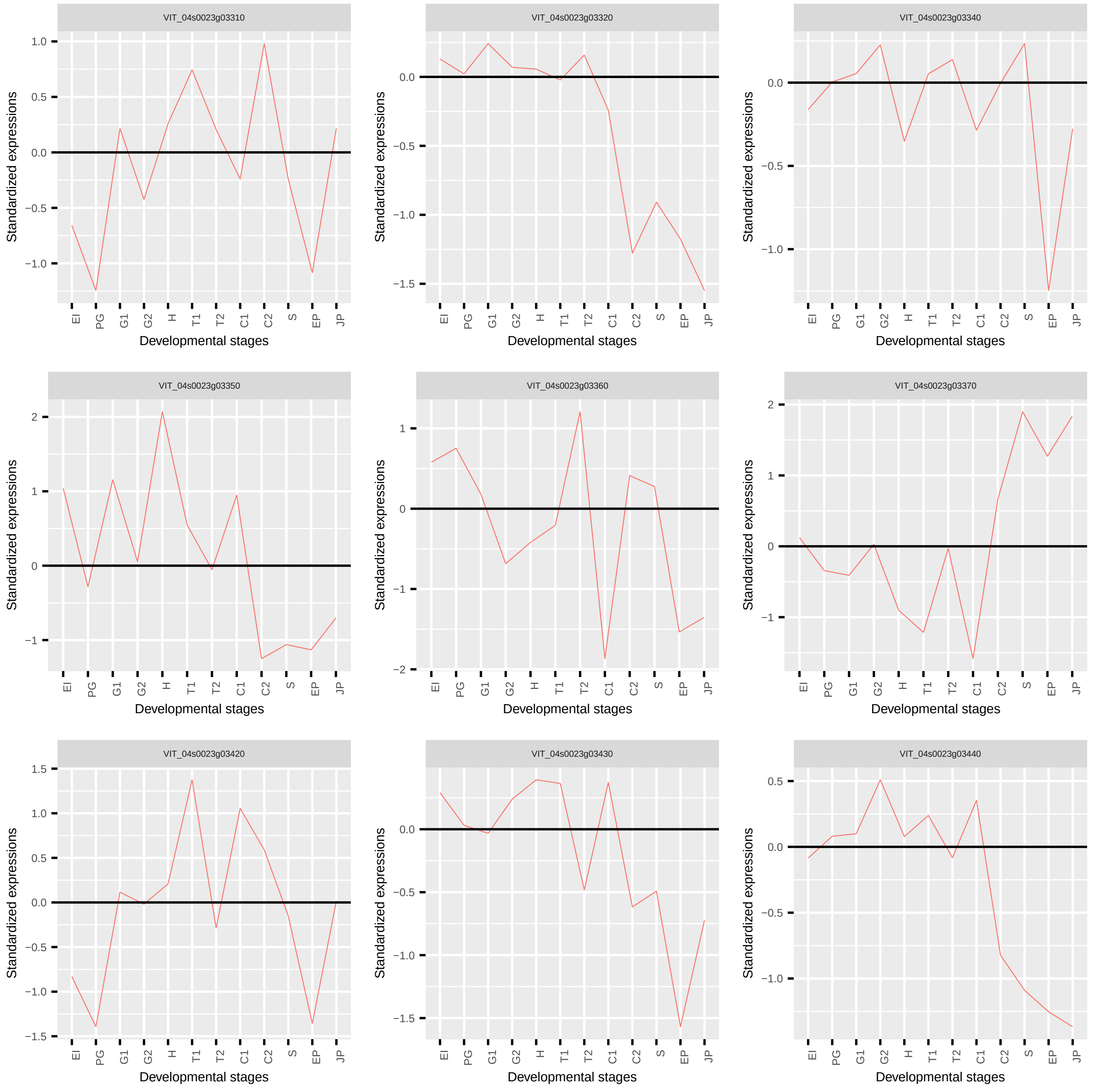
VIT_04s0023g03310
VIT_04s0023g03320
VIT_04s0023g03340
VIT_04s0023g03350
VIT_04s0023g03360
VIT_04s0023g03370
VIT_04s0023g03420
VIT_04s0023g03430
VIT_04s0023g03440
Standardized expressions
Developmental stages
EI PG G1 G2 H T1 T2 C1 C2 S EP JP

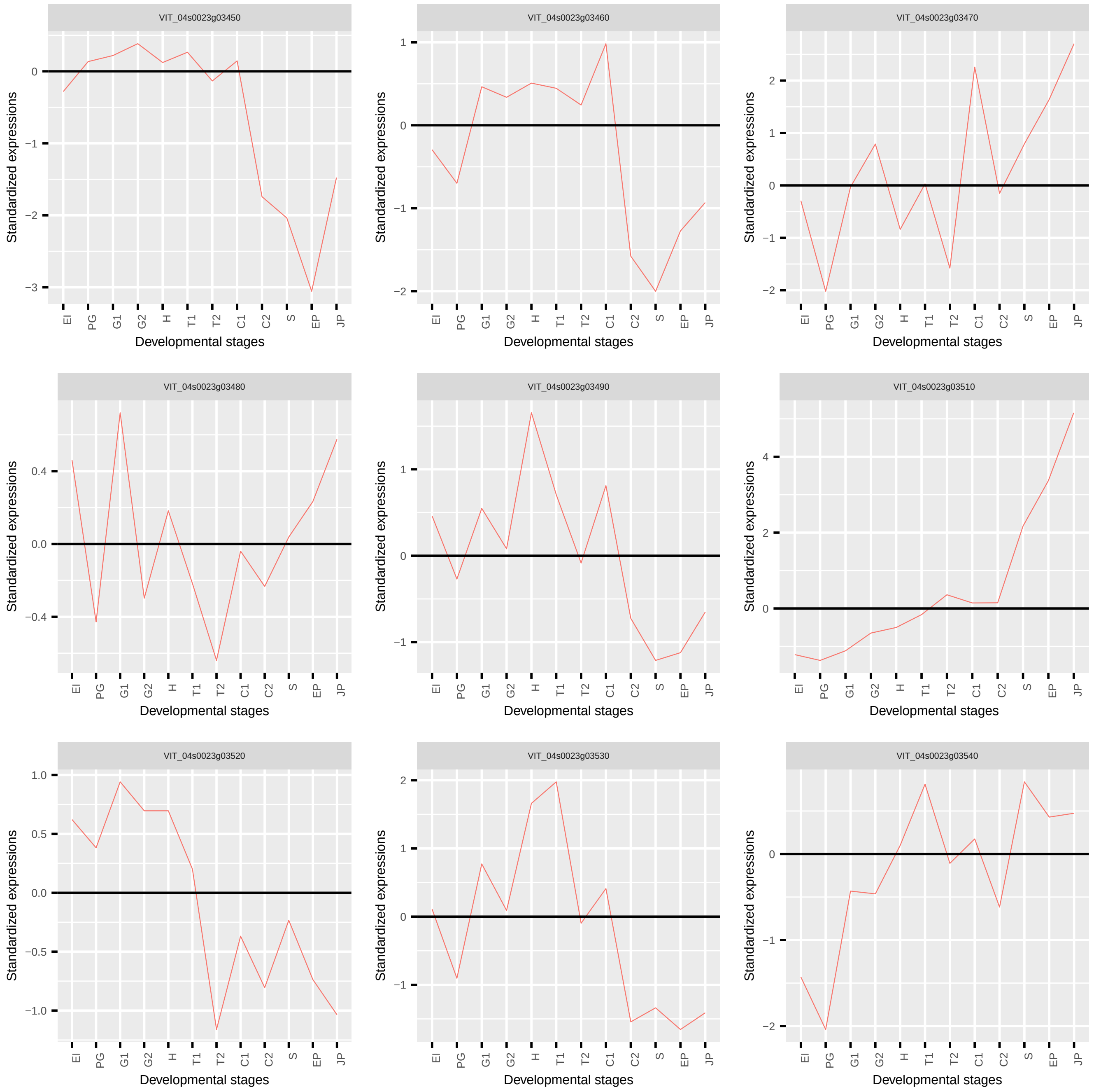
VIT_04s0023g03450
VIT_04s0023g03460
VIT_04s0023g03470
VIT_04s0023g03480
VIT_04s0023g03490
VIT_04s0023g03510
VIT_04s0023g03520
VIT_04s0023g03530
VIT_04s0023g03540
Standardized expressions
Developmental stages
EI PG G1 G2 H T1 T2 C1 C2 S EP JP

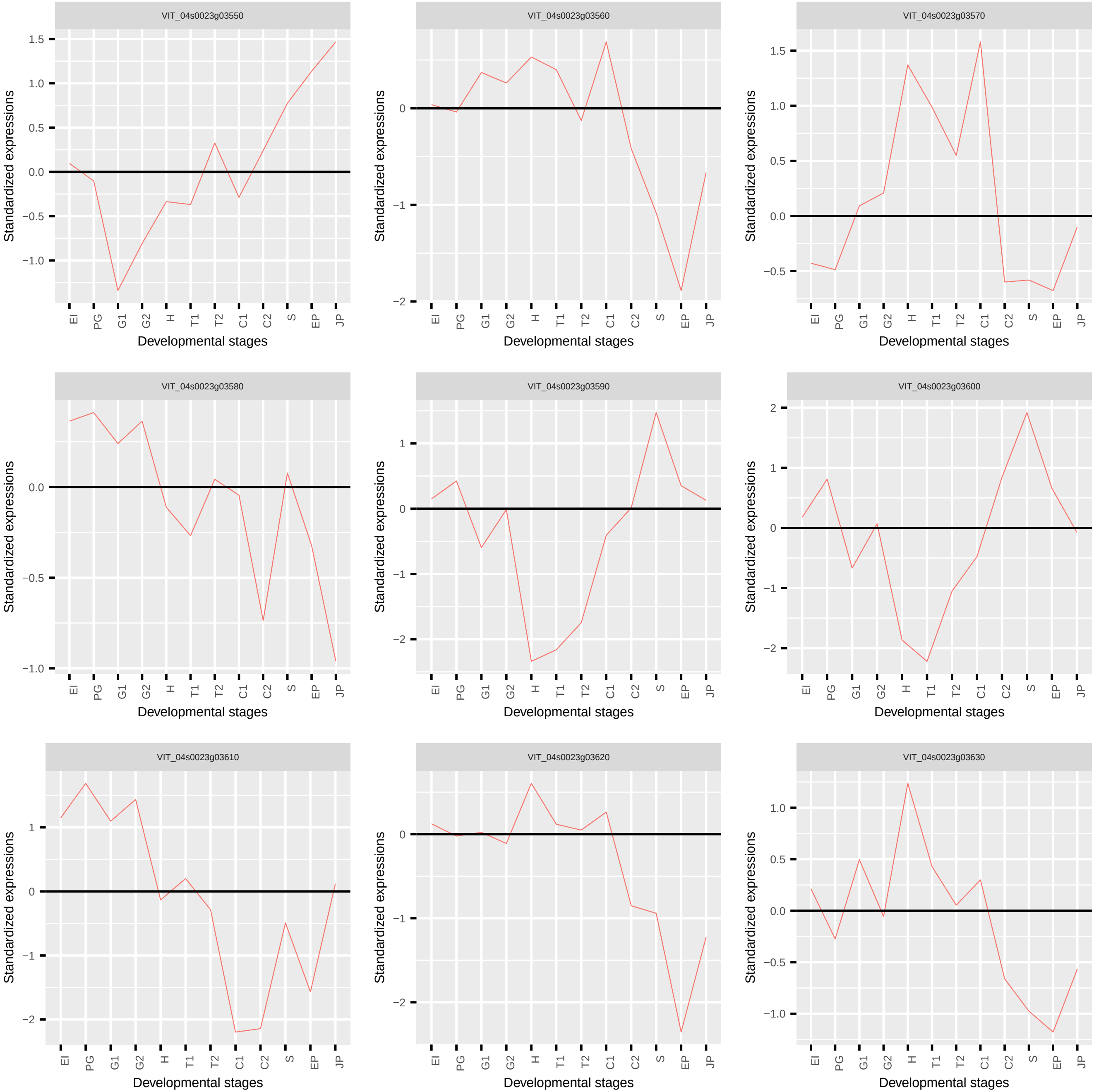
VIT_04s0023g03550
VIT_04s0023g03560
VIT_04s0023g03570
VIT_04s0023g03580
VIT_04s0023g03590
VIT_04s0023g03600
VIT_04s0023g03610
VIT_04s0023g03620
VIT_04s0023g03630
Standardized expressions
Developmental stages
EI PG G1 G2 H T1 T2 C1 C2 S EP JP

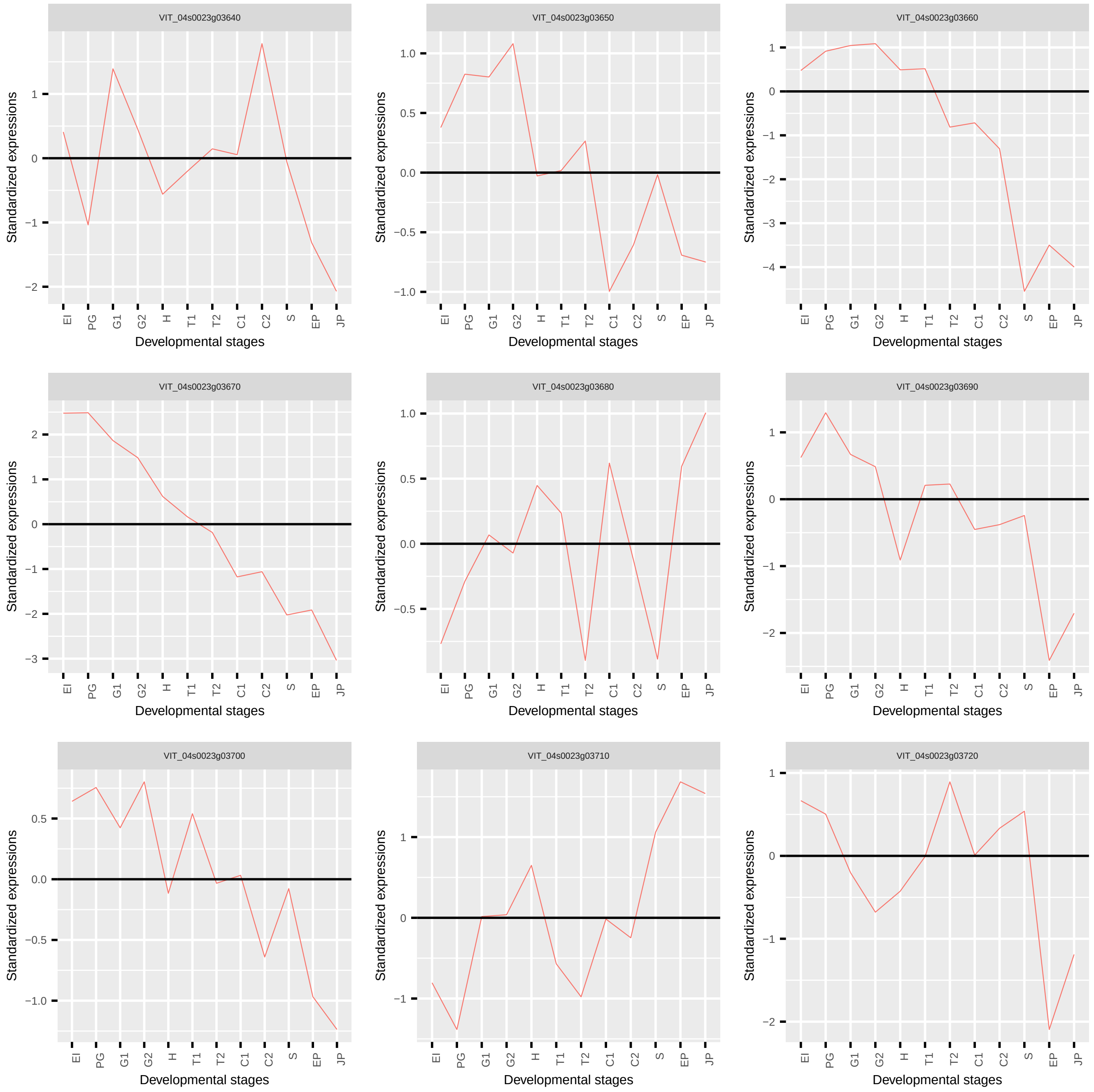
VIT_04s0023g03640
VIT_04s0023g03650
VIT_04s0023g03660
VIT_04s0023g03670
VIT_04s0023g03680
VIT_04s0023g03690
VIT_04s0023g03700
VIT_04s0023g03710
VIT_04s0023g03720
Standardized expressions
Developmental stages
EI PG G1 G2 H T1 T2 C1 C2 S EP JP

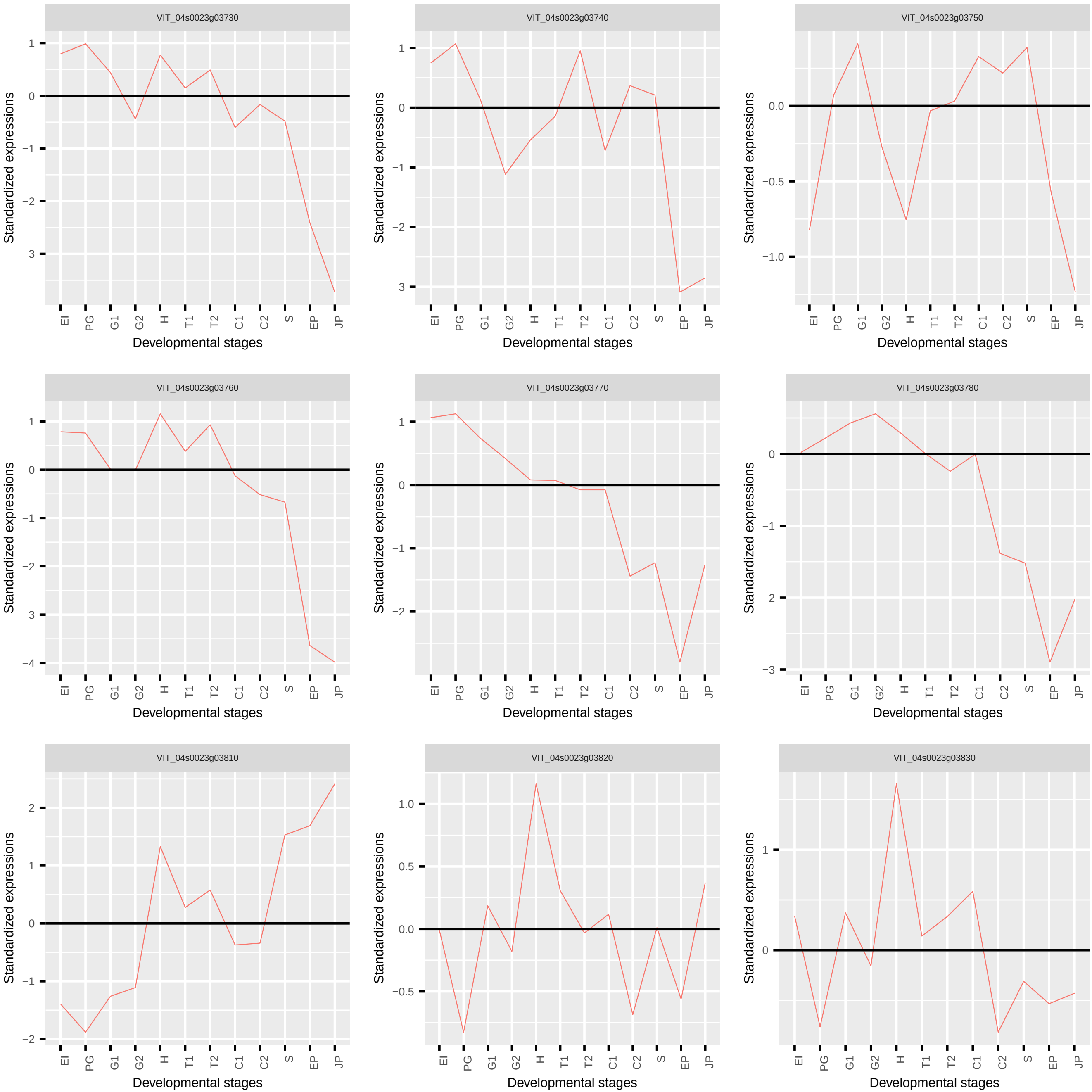

VIT_04s0023g03730
VIT_04s0023g03740
VIT_04s0023g03750
VIT_04s0023g03760
VIT_04s0023g03770
VIT_04s0023g03780
VIT_04s0023g03810
VIT_04s0023g03820
VIT_04s0023g03830
Standardized expressions
Developmental stages
EI
PG
G1
G2
H
T1
T2
C1
C2
S
EP
JP

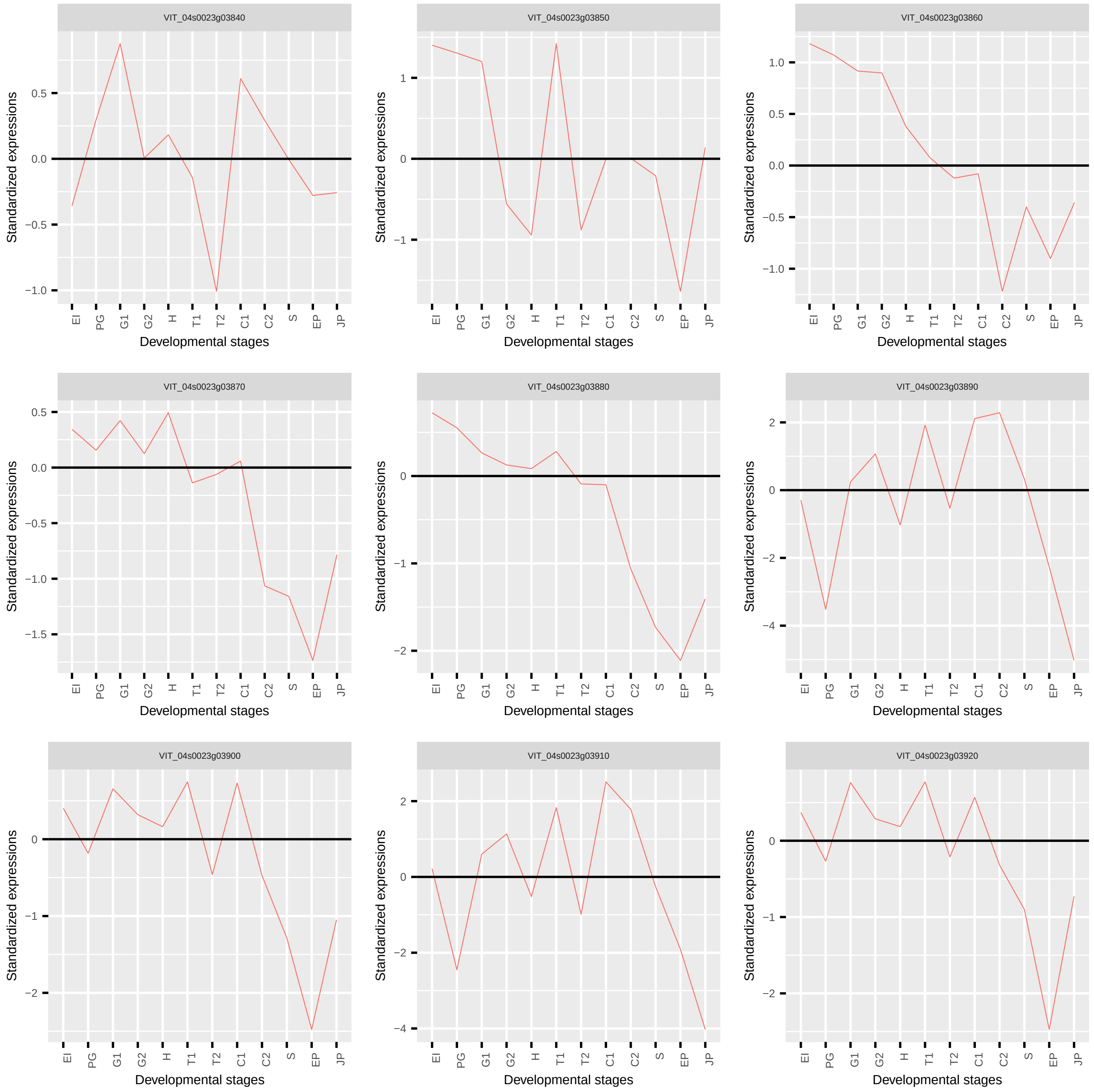
VIT_04s0023g03840
VIT_04s0023g03850
VIT_04s0023g03860
VIT_04s0023g03870
VIT_04s0023g03880
VIT_04s0023g03890
VIT_04s0023g03900
VIT_04s0023g03910
VIT_04s0023g03920
Standardized expressions
Developmental stages
EI
PG
G1
G2
H
T1
T2
C1
C2
S
EP
JP

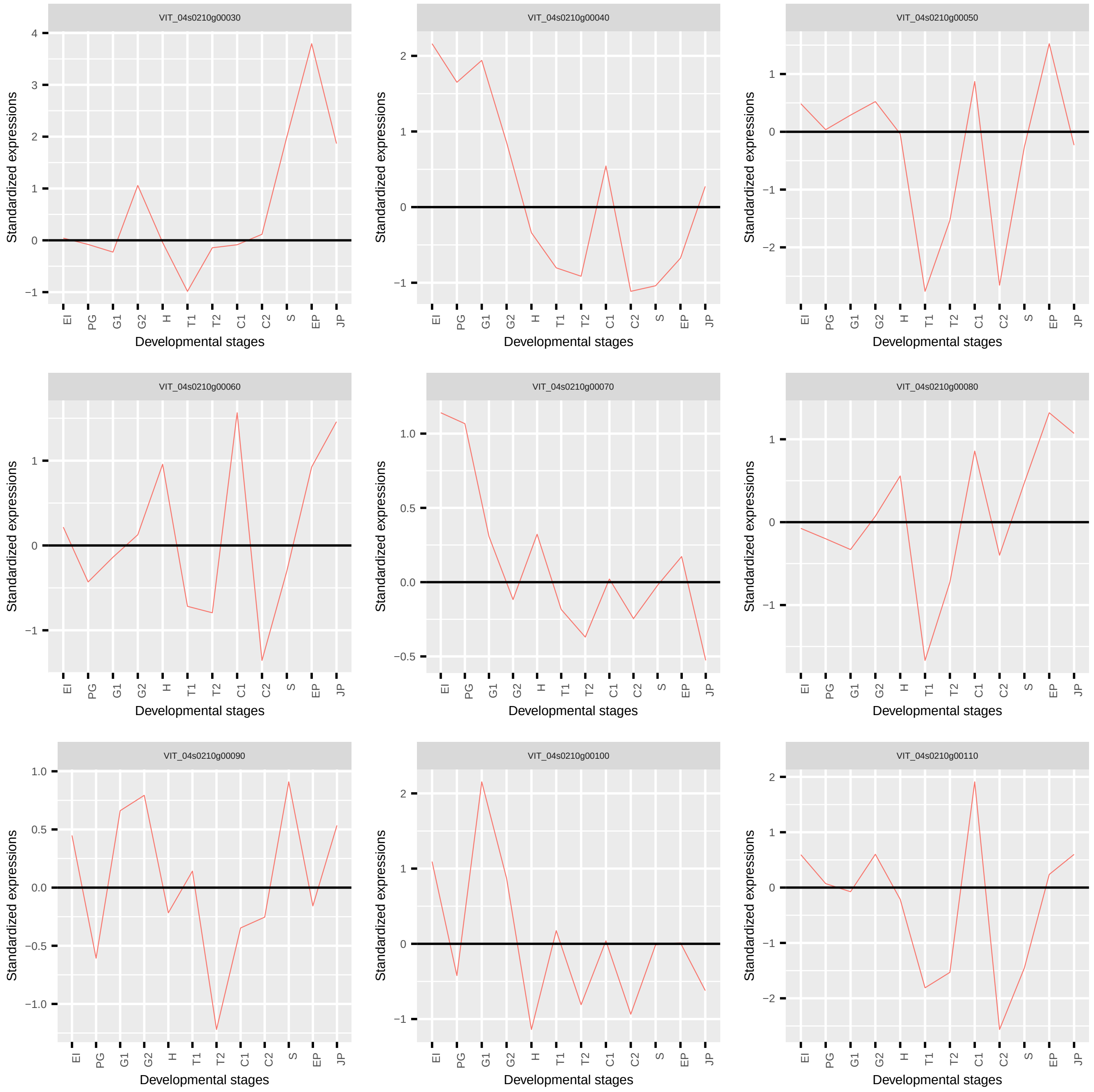
VIT_04s0210g00030
VIT_04s0210g00040
VIT_04s0210g00050
VIT_04s0210g00060
VIT_04s0210g00070
VIT_04s0210g00080
VIT_04s0210g00090
VIT_04s0210g00100
VIT_04s0210g00110
Standardized expressions
Developmental stages
EI PG G1 G2 H T1 T2 C1 C2 S EP JP

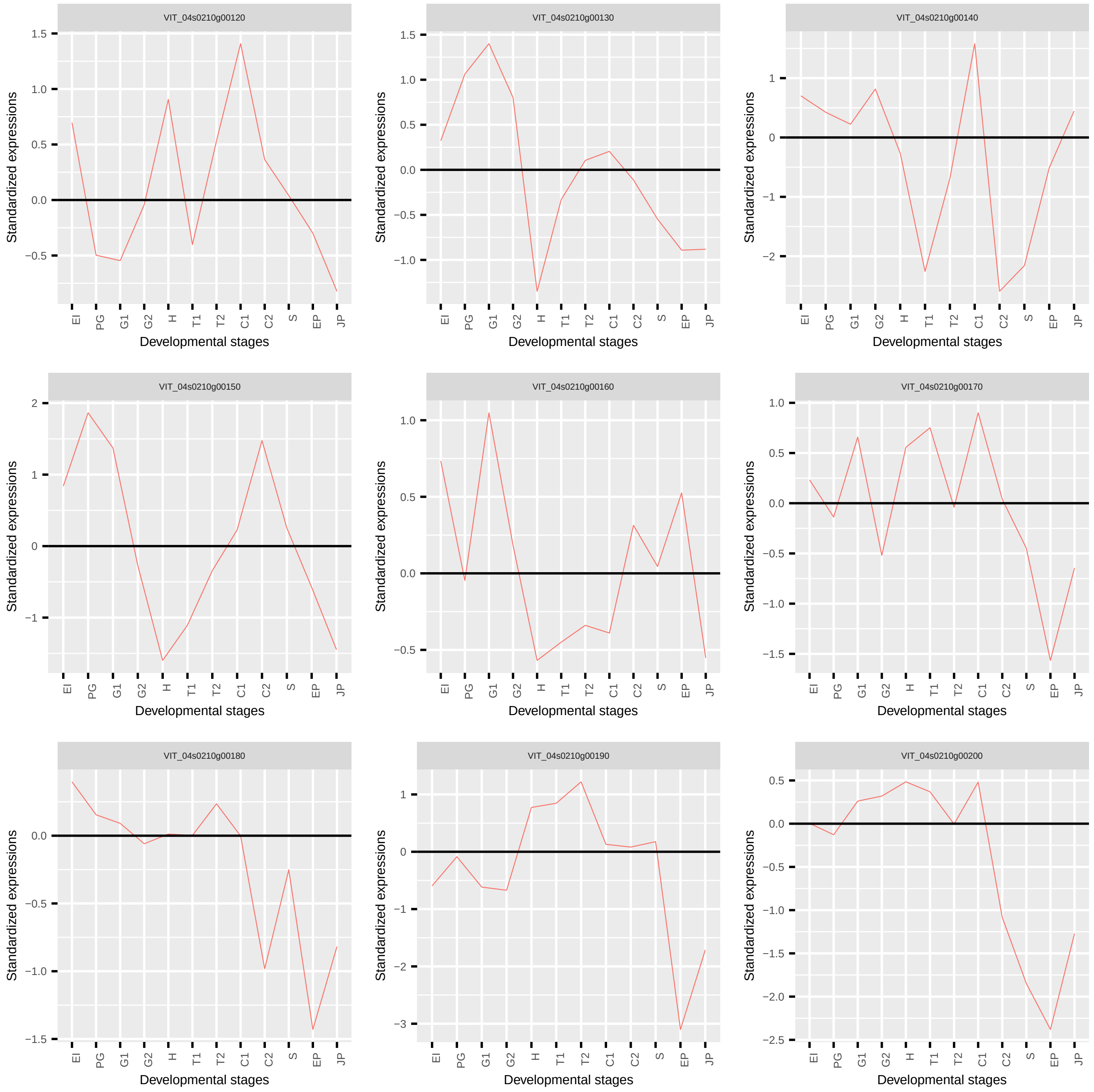
VIT_04s0210g00120
VIT_04s0210g00130
VIT_04s0210g00140
VIT_04s0210g00150
VIT_04s0210g00160
VIT_04s0210g00170
VIT_04s0210g00180
VIT_04s0210g00190
VIT_04s0210g00200
Standardized expressions
Developmental stages
EI PG G1 G2 H T1 T2 C1 C2 S EP JP

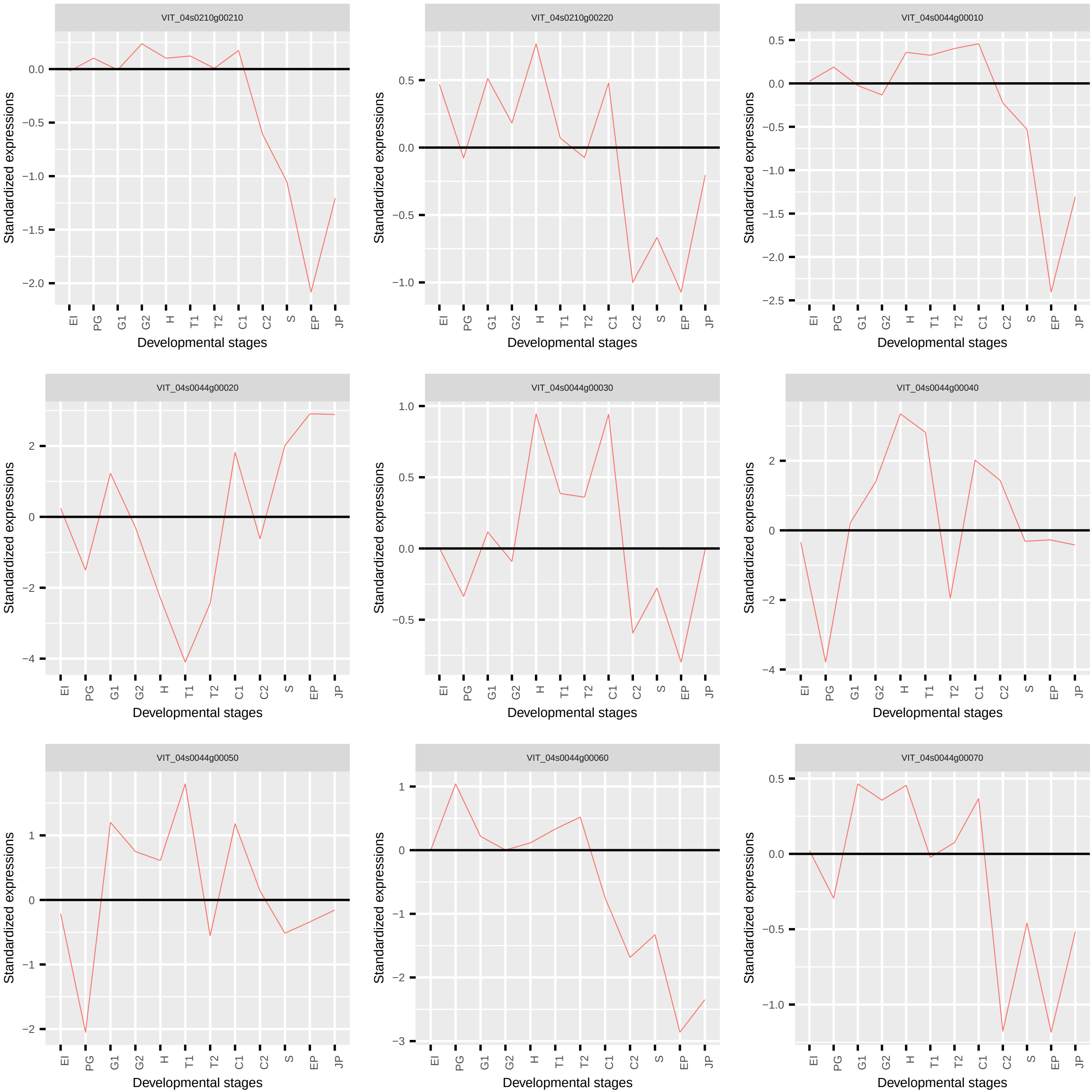
VIT_04s0210g00210
VIT_04s0210g00220
VIT_04s0044g00010
VIT_04s0044g00020
VIT_04s0044g00030
VIT_04s0044g00040
VIT_04s0044g00050
VIT_04s0044g00060
VIT_04s0044g00070
Standardized expressions
Developmental stages
EI PG G1 G2 H T1 T2 C1 C2 S EP JP

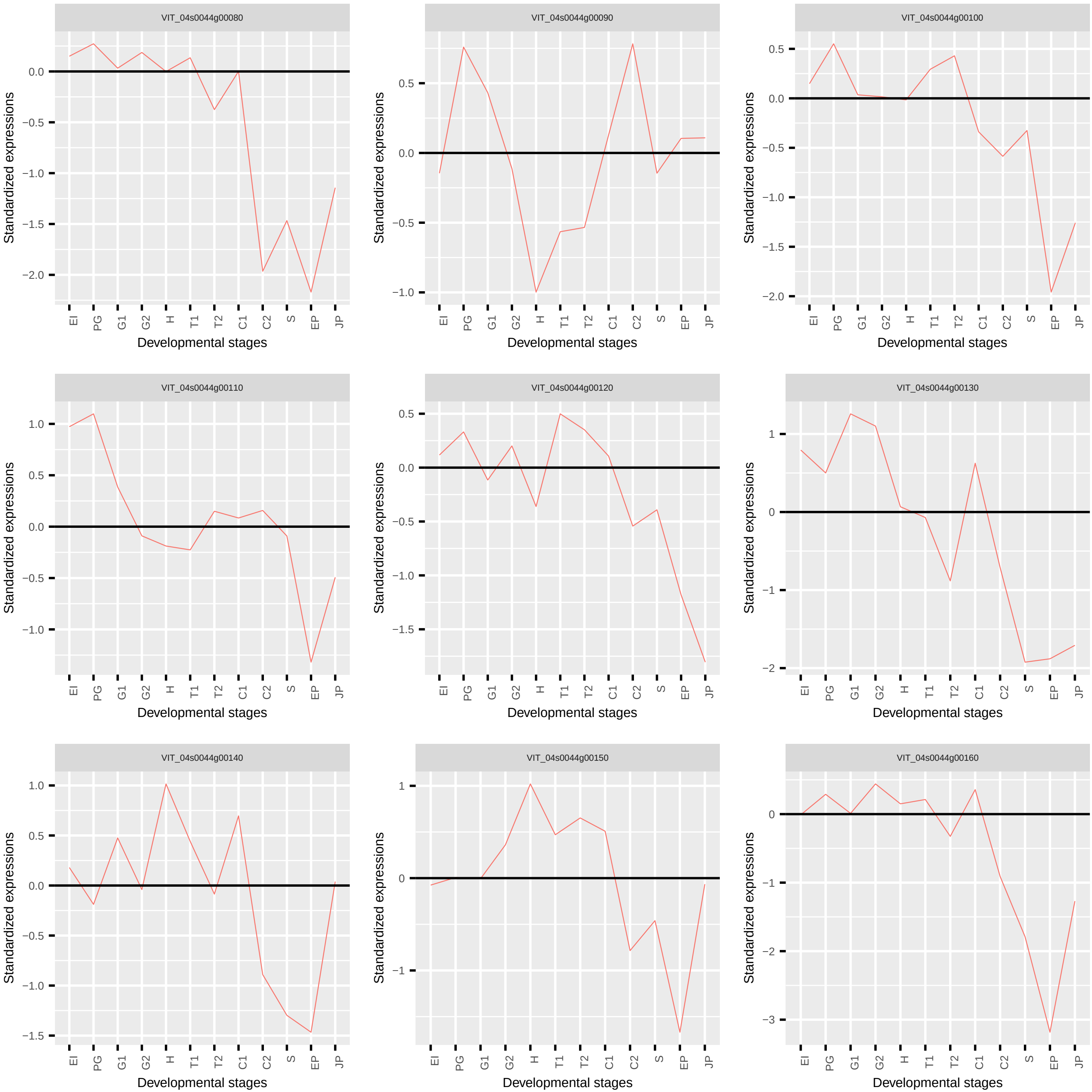
VIT_04s0044g00080
VIT_04s0044g00090
VIT_04s0044g00100
VIT_04s0044g00110
VIT_04s0044g00120
VIT_04s0044g00130
VIT_04s0044g00140
VIT_04s0044g00150
VIT_04s0044g00160
Standardized expressions
Developmental stages
EI PG G1 G2 H T1 T2 C1 C2 S EP JP

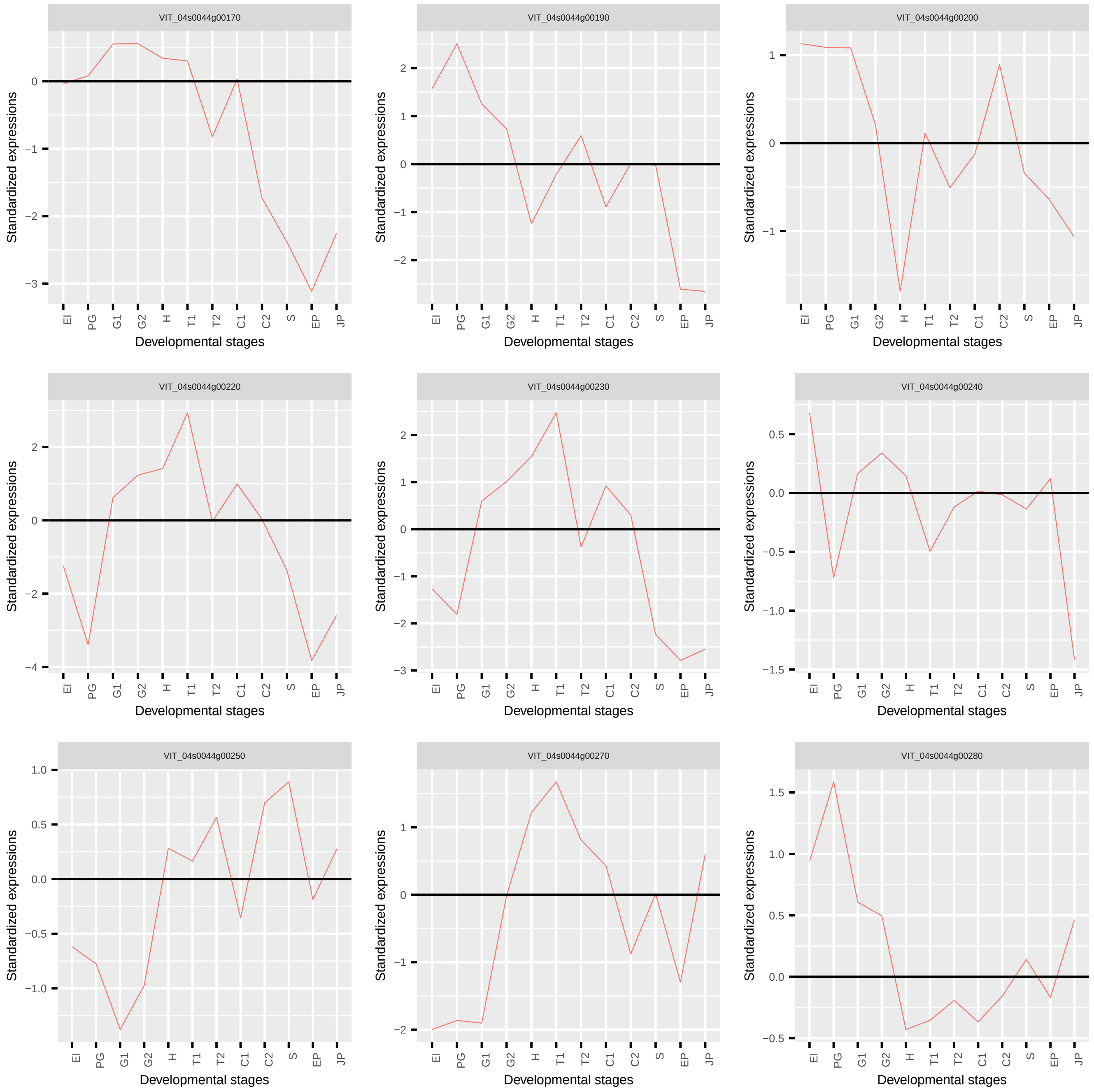
VIT_04s0044g00170
VIT_04s0044g00190
VIT_04s0044g00200
VIT_04s0044g00220
VIT_04s0044g00230
VIT_04s0044g00240
VIT_04s0044g00250
VIT_04s0044g00270
VIT_04s0044g00280
Standardized expressions
Developmental stages
EI
PG
G1
G2
H
T1
T2
C1
C2
S
EP
JP

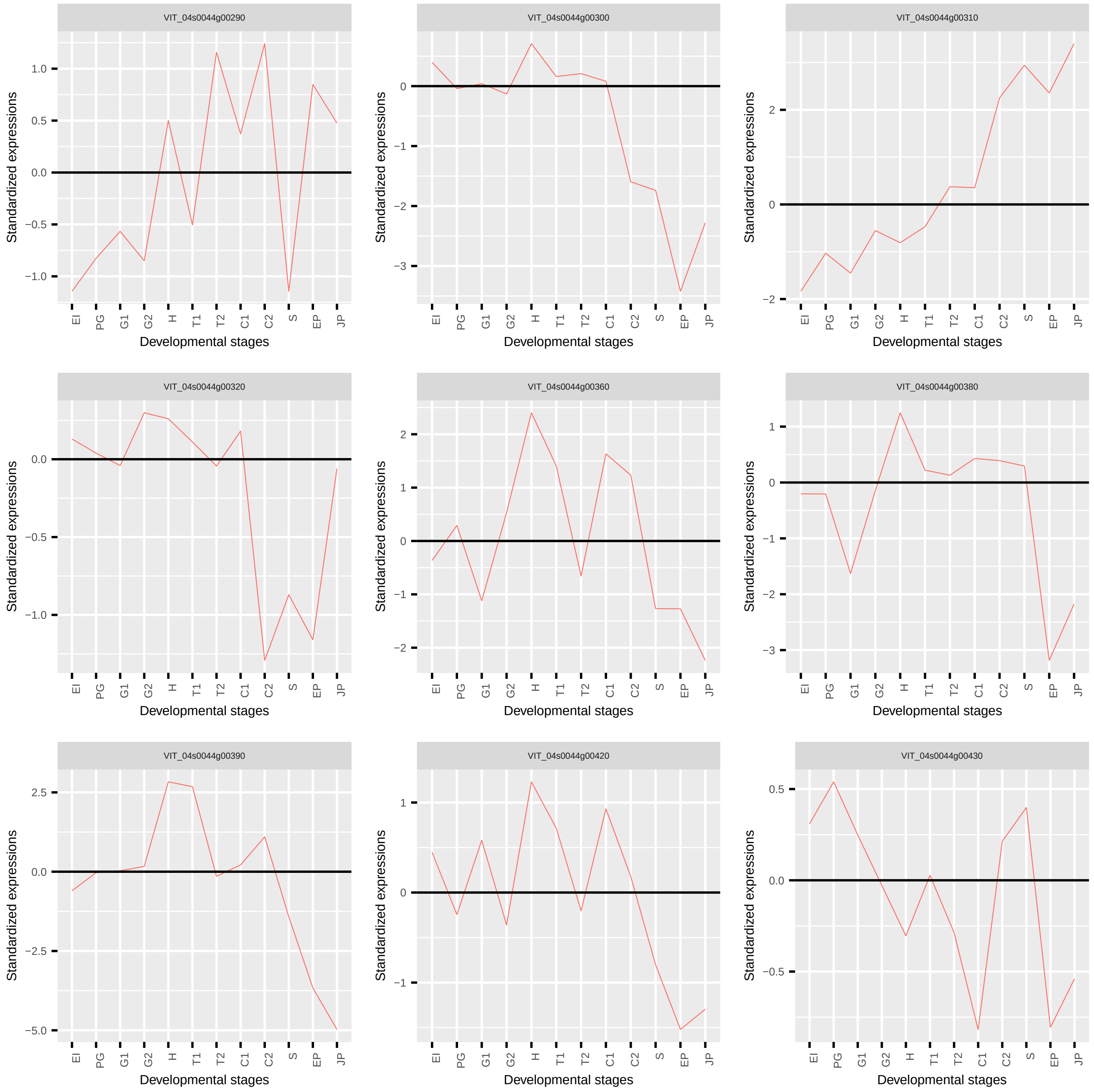
VIT_04s0044g00290
VIT_04s0044g00300
VIT_04s0044g00310
VIT_04s0044g00320
VIT_04s0044g00360
VIT_04s0044g00380
VIT_04s0044g00390
VIT_04s0044g00420
VIT_04s0044g00430
Standardized expressions
Developmental stages
EI PG G1 G2 H T1 T2 C1 C2 S EP JP

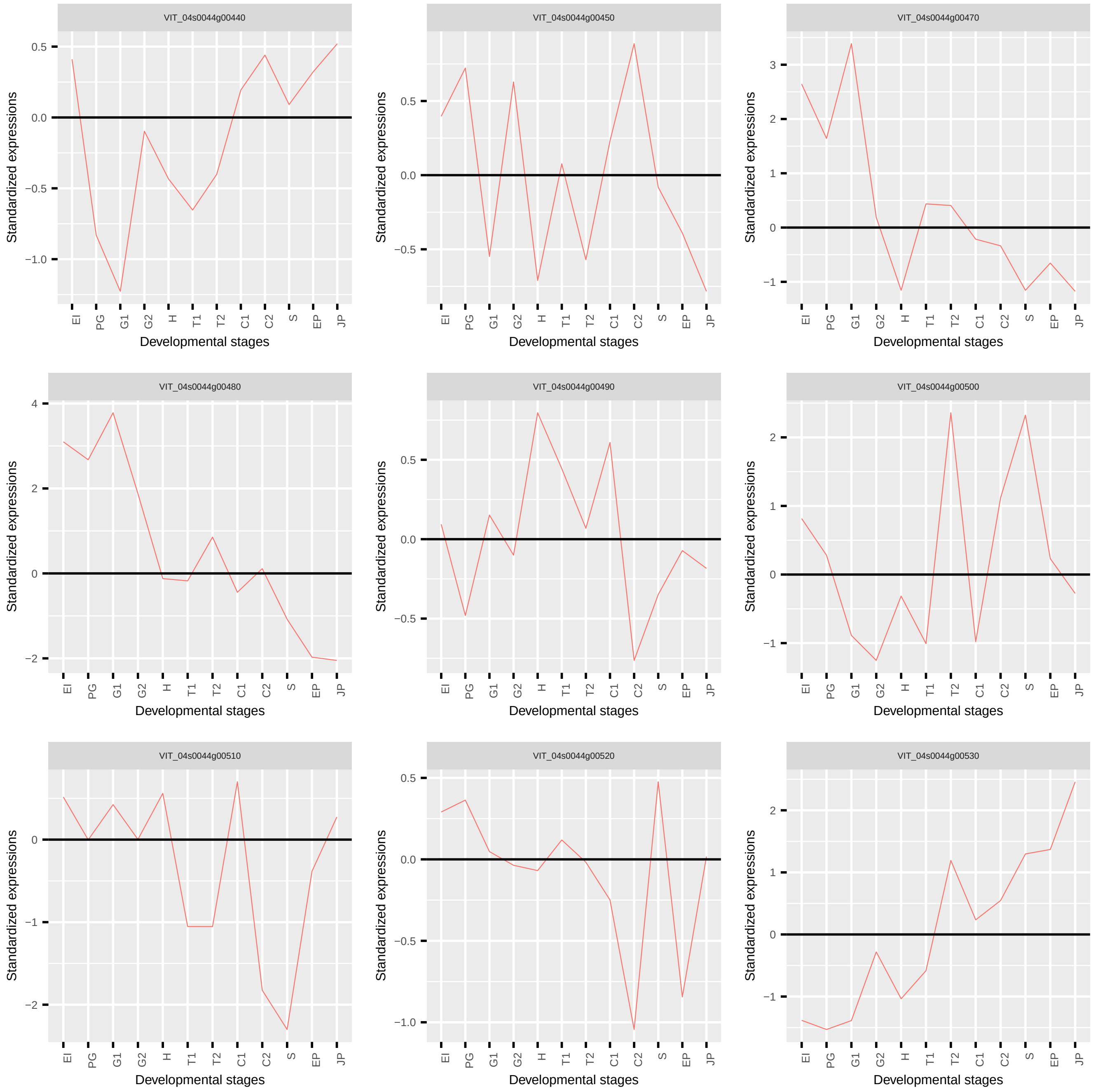
VIT_04s0044g00440
VIT_04s0044g00450
VIT_04s0044g00470
VIT_04s0044g00480
VIT_04s0044g00490
VIT_04s0044g00500
VIT_04s0044g00510
VIT_04s0044g00520
VIT_04s0044g00530
Standardized expressions
Developmental stages
EI
PG
G1
G2
H
T1
T2
C1
C2
S
EP
JP

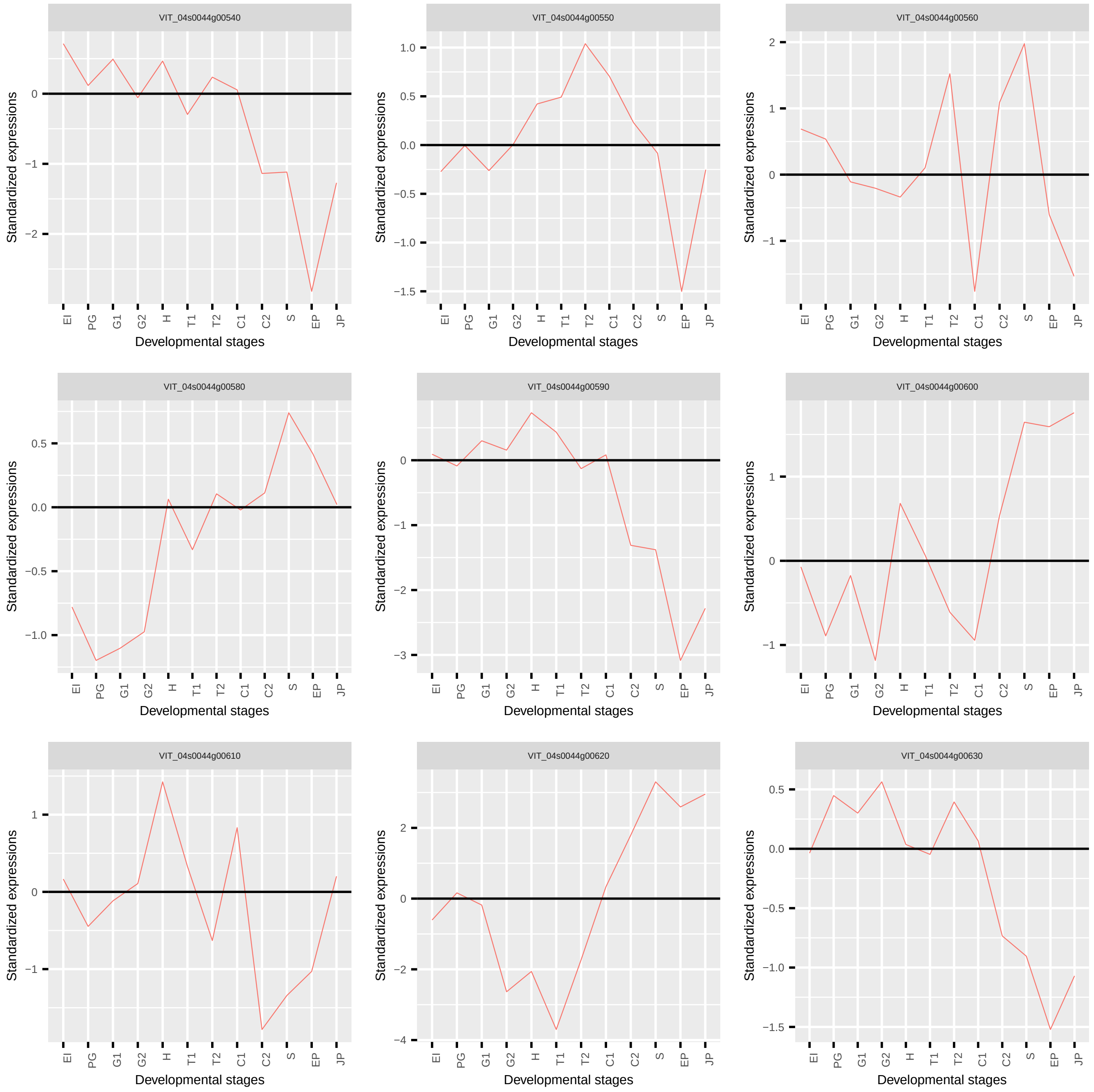

VIT_04s0044g00540
VIT_04s0044g00550
VIT_04s0044g00560
VIT_04s0044g00580
VIT_04s0044g00590
VIT_04s0044g00600
VIT_04s0044g00610
VIT_04s0044g00620
VIT_04s0044g00630
Standardized expressions
Developmental stages
EI
PG
G1
G2
H
T1
T2
C1
C2
S
EP
JP

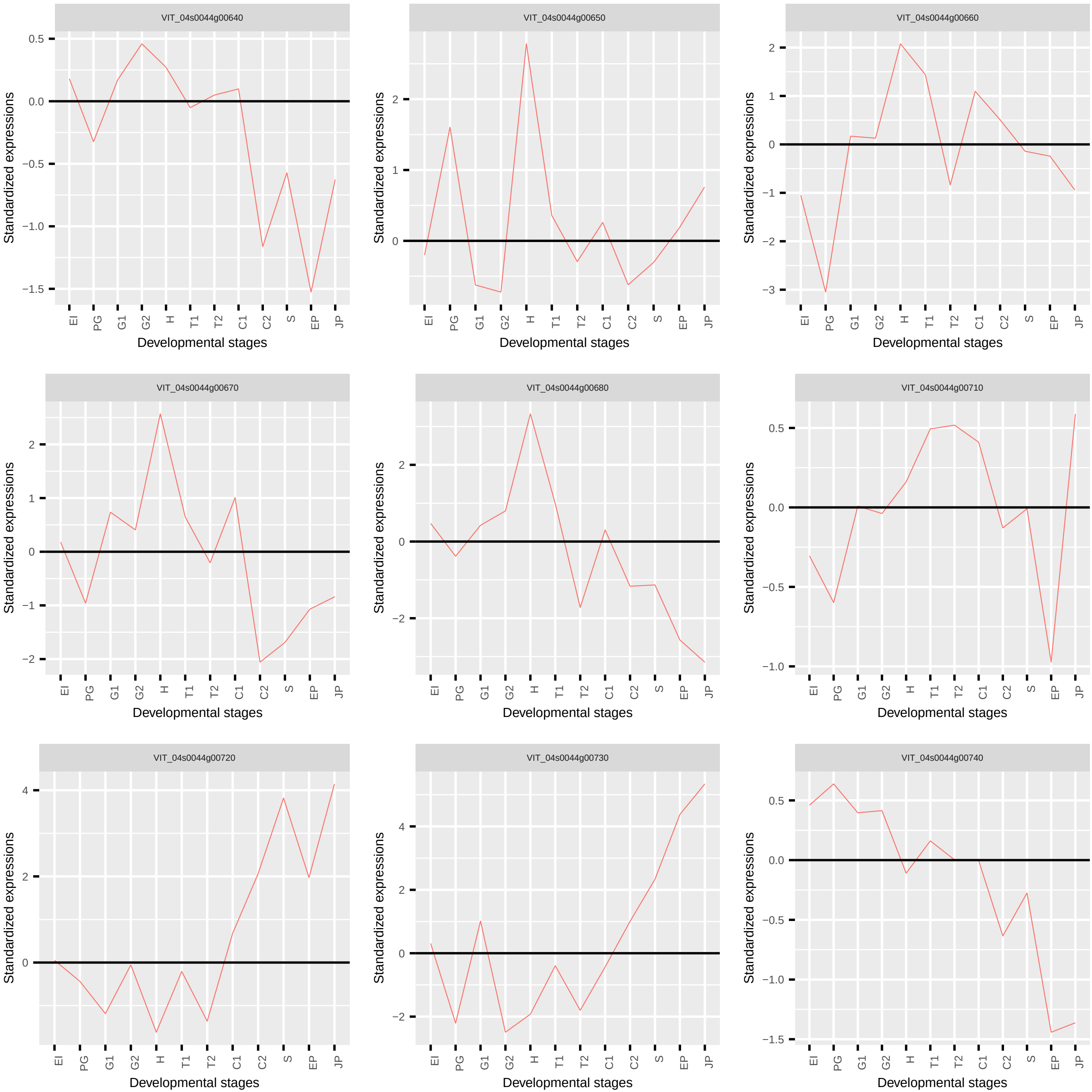

VIT_04s0044g00640
VIT_04s0044g00650
VIT_04s0044g00660
VIT_04s0044g00670
VIT_04s0044g00680
VIT_04s0044g00710
VIT_04s0044g00720
VIT_04s0044g00730
VIT_04s0044g00740
Standardized expressions
Developmental stages
EI PG G1 G2 H T1 T2 C1 C2 S EP JP

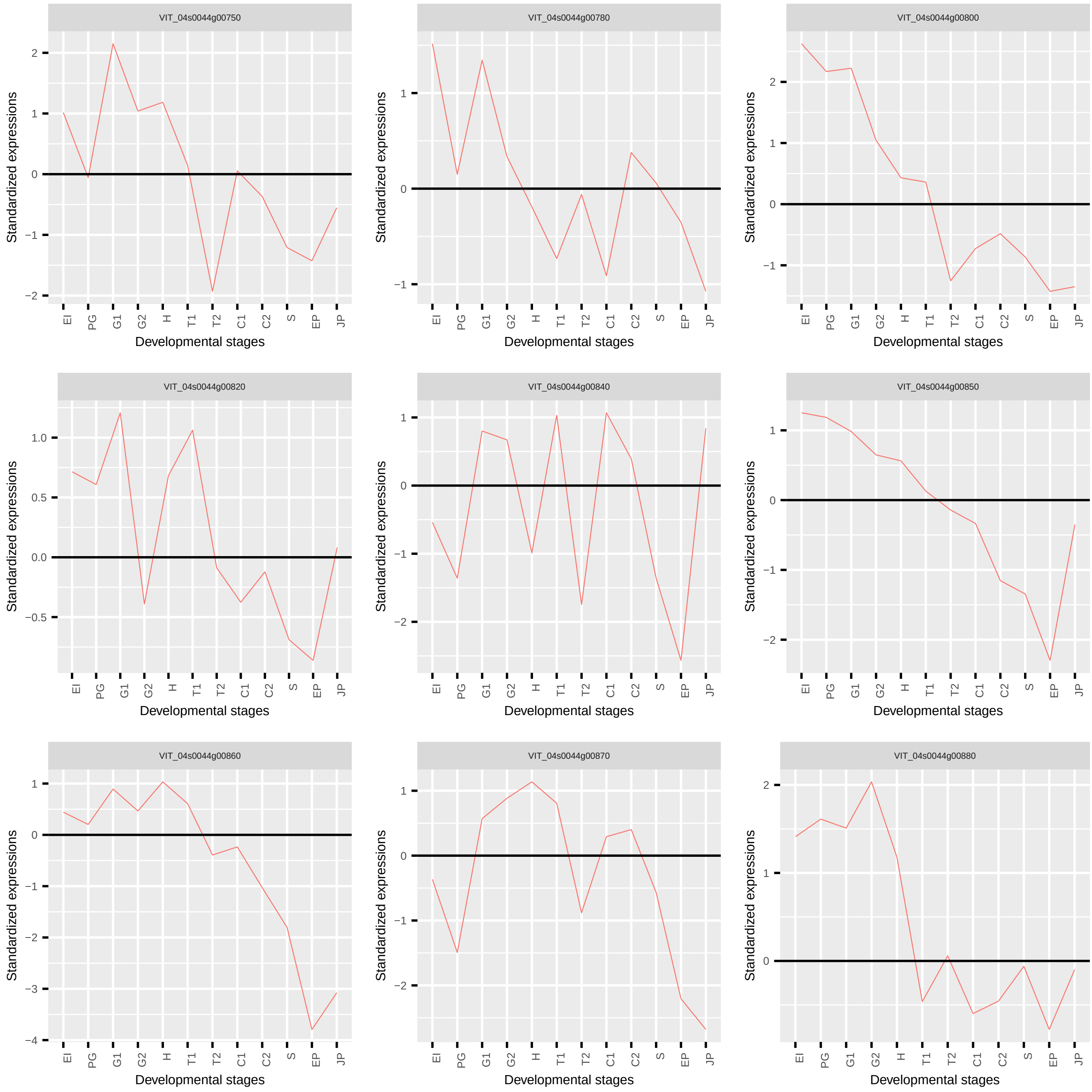
VIT_04s0044g00750
VIT_04s0044g00780
VIT_04s0044g00800
VIT_04s0044g00820
VIT_04s0044g00840
VIT_04s0044g00850
VIT_04s0044g00860
VIT_04s0044g00870
VIT_04s0044g00880
Standardized expressions
Developmental stages
EI
PG
G1
G2
H
T1
T2
C1
C2
S
EP
JP

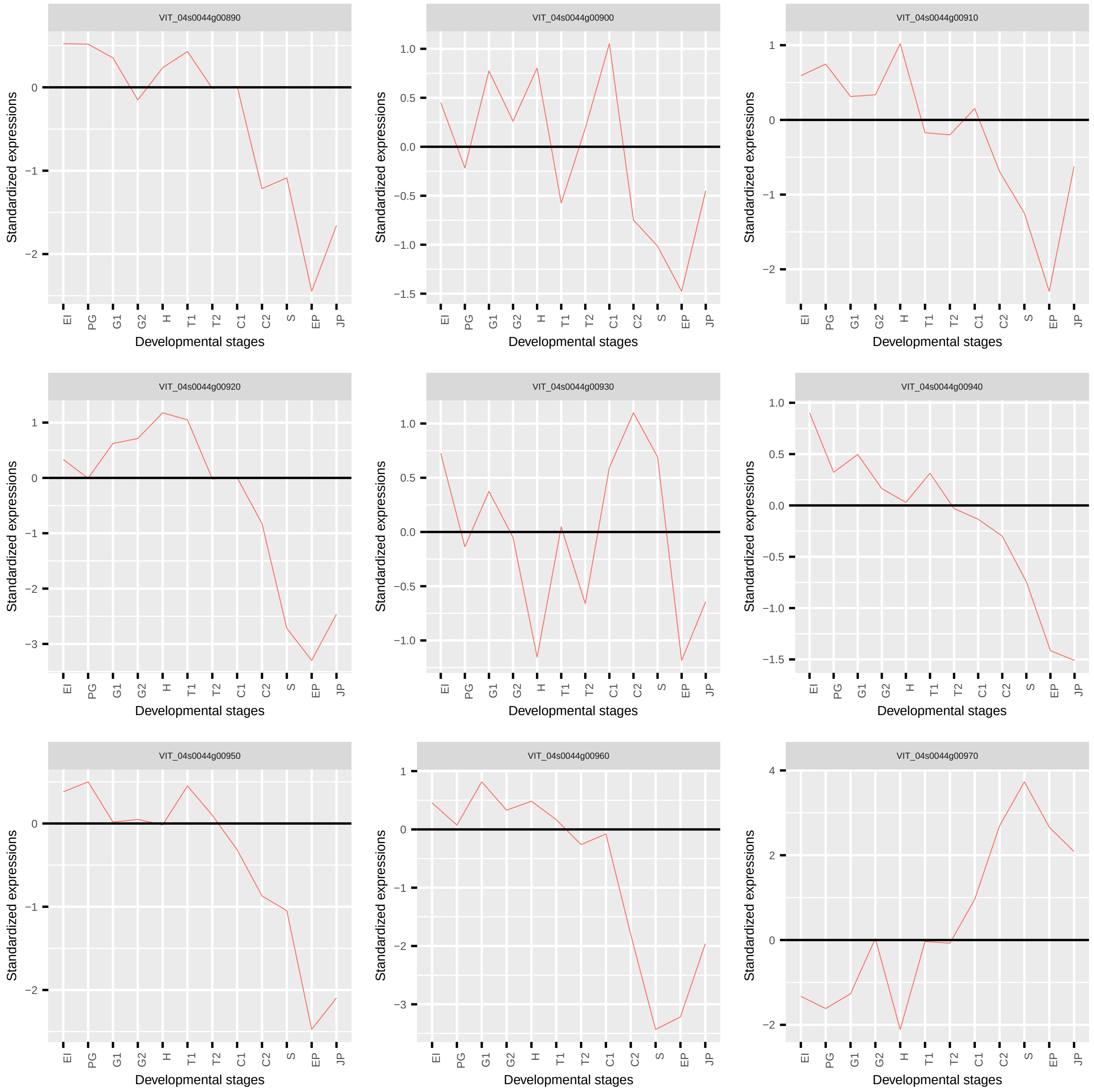
VIT_04s0044g00890
VIT_04s0044g00900
VIT_04s0044g00910
VIT_04s0044g00920
VIT_04s0044g00930
VIT_04s0044g00940
VIT_04s0044g00950
VIT_04s0044g00960
VIT_04s0044g00970
Standardized expressions
Developmental stages
EI PG G1 G2 H T1 T2 C1 C2 S EP JP

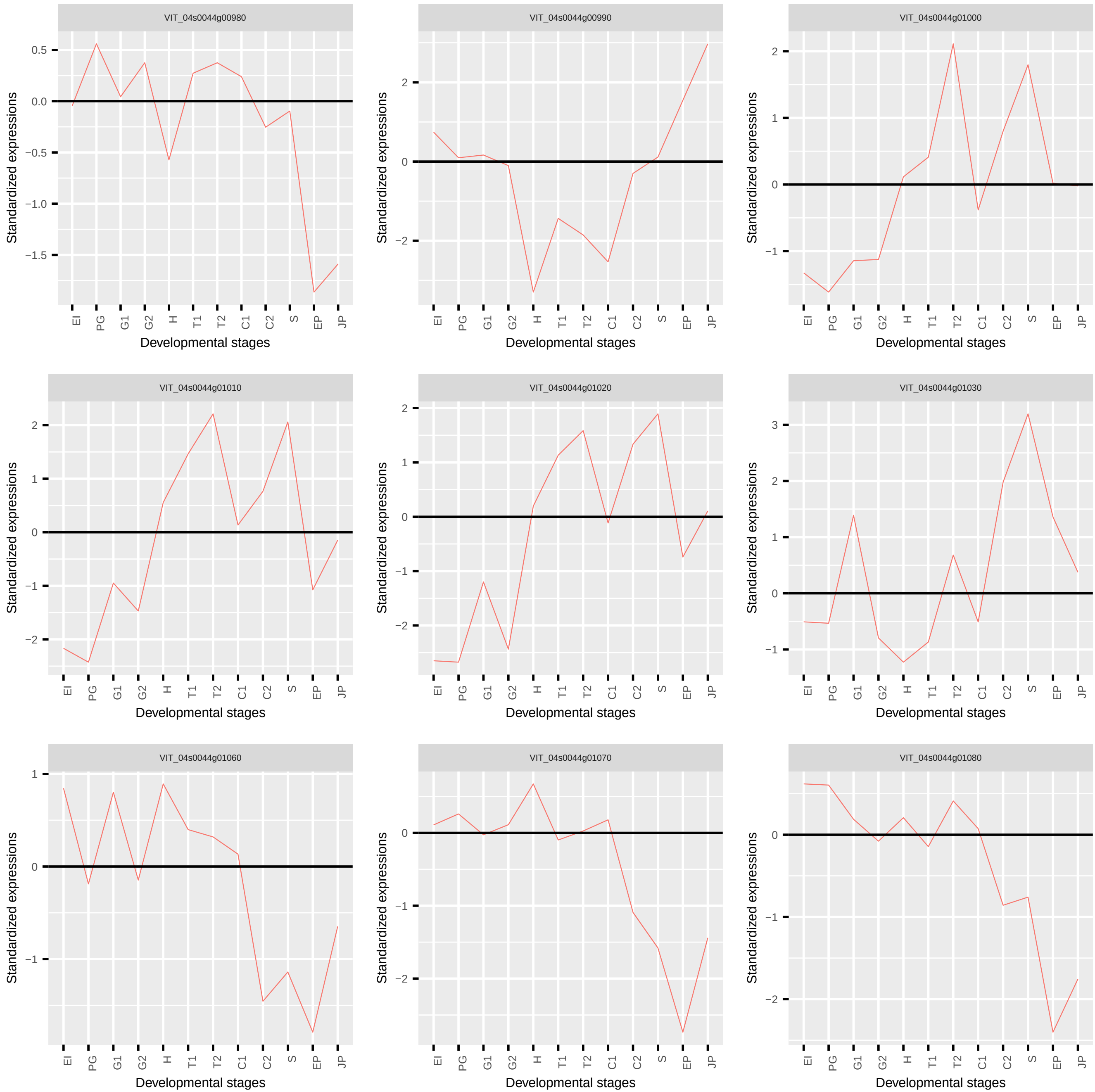

VIT_04s0044g00980
VIT_04s0044g00990
VIT_04s0044g01000
VIT_04s0044g01010
VIT_04s0044g01020
VIT_04s0044g01030
VIT_04s0044g01060
VIT_04s0044g01070
VIT_04s0044g01080
Standardized expressions
Developmental stages
EI
PG
G1
G2
H
T1
T2
C1
C2
S
EP
JP

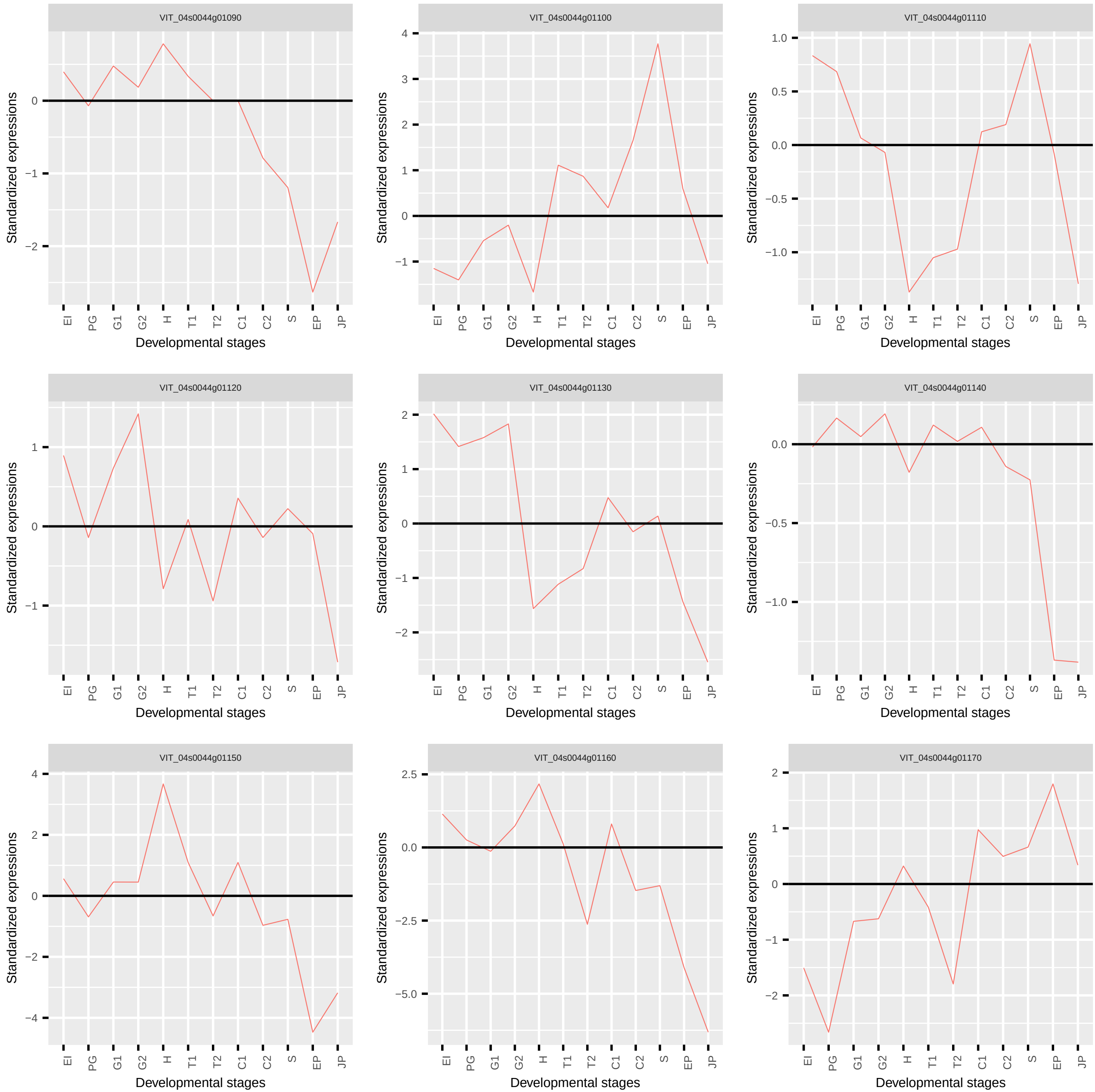
VIT_04s0044g01090
VIT_04s0044g01100
VIT_04s0044g01110
VIT_04s0044g01120
VIT_04s0044g01130
VIT_04s0044g01140
VIT_04s0044g01150
VIT_04s0044g01160
VIT_04s0044g01170
Standardized expressions
Developmental stages
EI PG G1 G2 H T1 T2 C1 C2 S EP JP

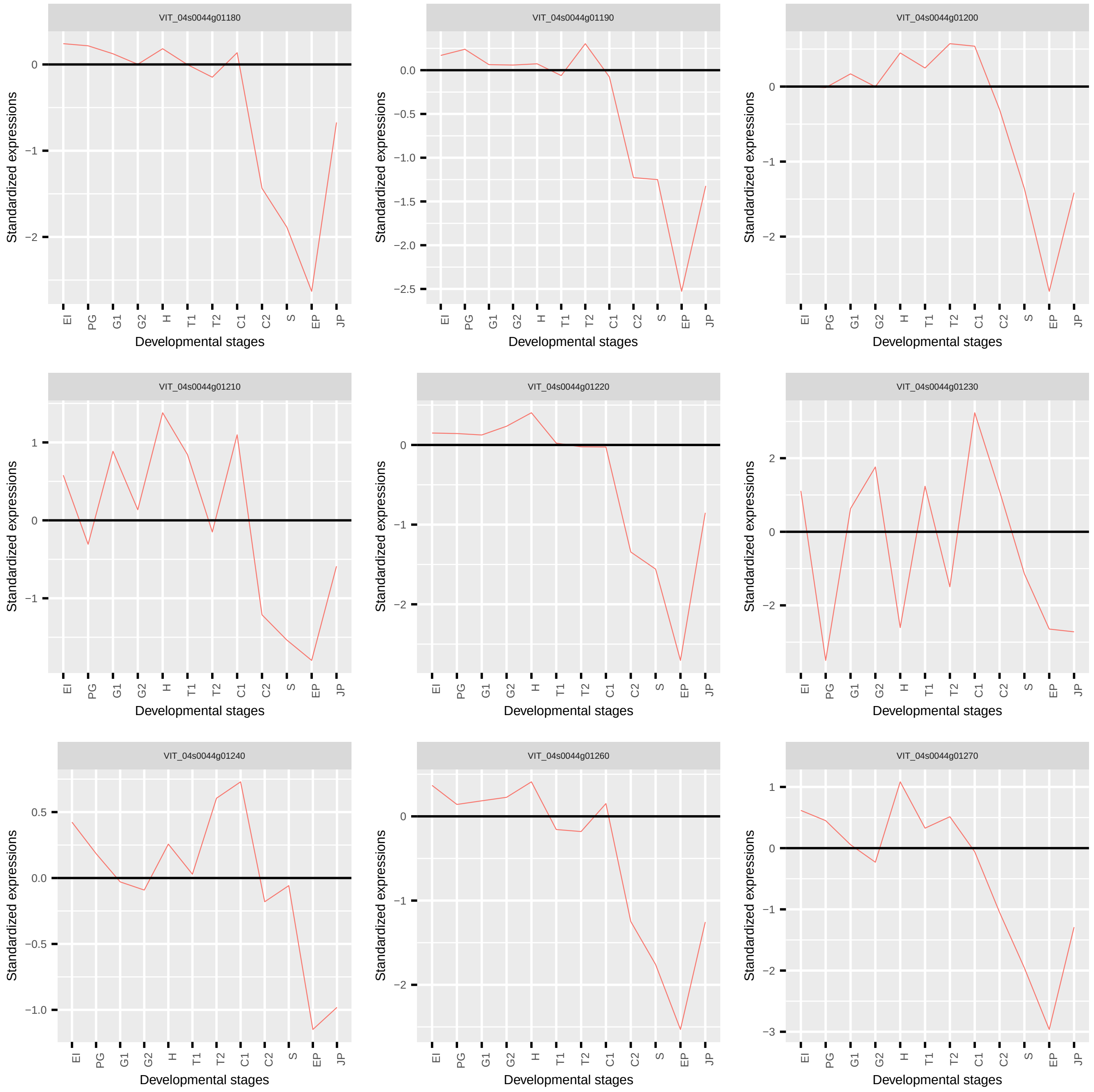

VIT_04s0044g01180
VIT_04s0044g01190
VIT_04s0044g01200
VIT_04s0044g01210
VIT_04s0044g01220
VIT_04s0044g01230
VIT_04s0044g01240
VIT_04s0044g01260
VIT_04s0044g01270
Standardized expressions
Developmental stages
EI
PG
G1
G2
H
T1
T2
C1
C2
S
EP
JP

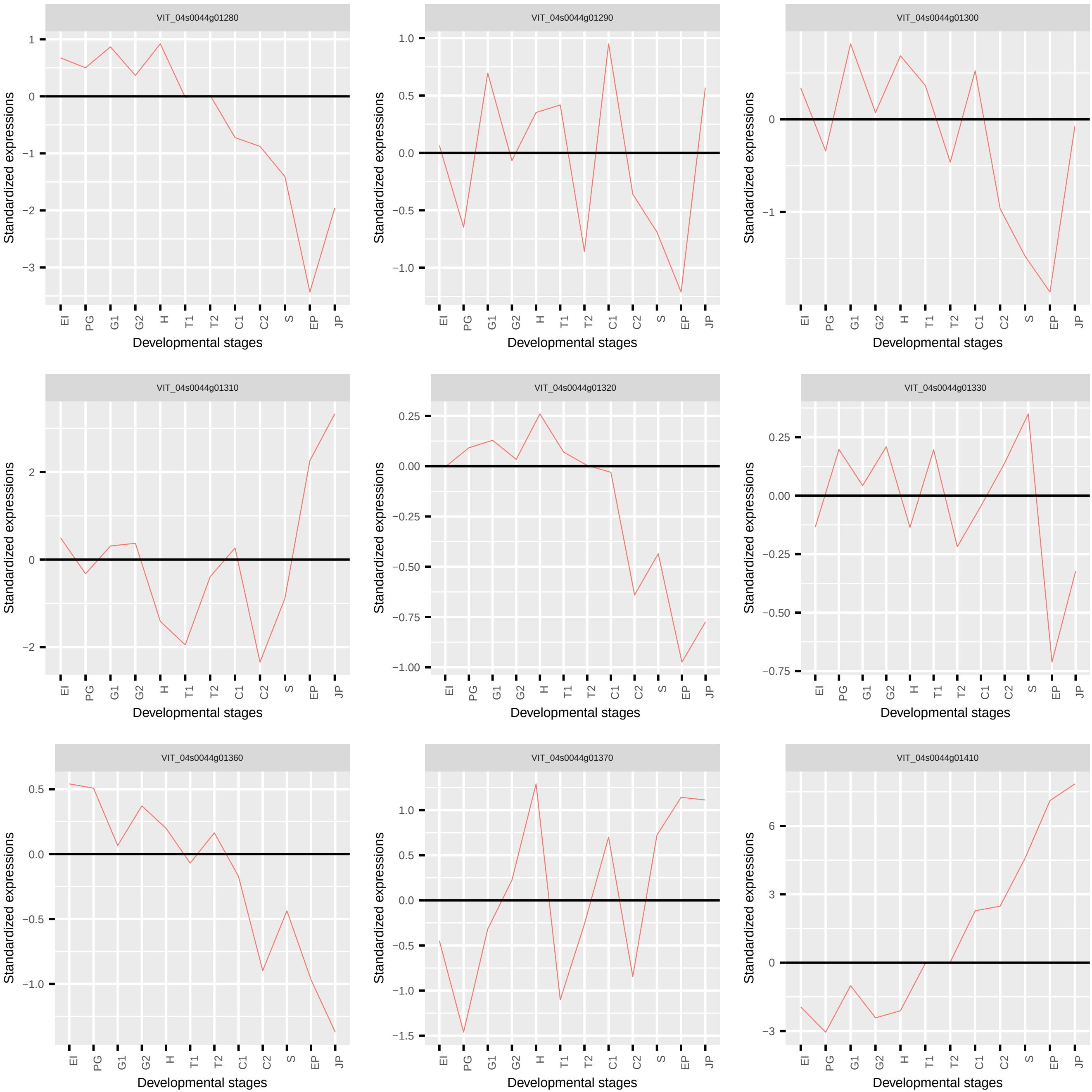
VIT_04s0044g01280
Standardized expressions
1
0
−1
−2
−3
EI PG G1 G2 H T1 T2 C1 C2 S EP JP
Developmental stages
VIT_04s0044g01290
Standardized expressions
1.0
0.5
0.0
−0.5
−1.0
EI PG G1 G2 H T1 T2 C1 C2 S EP JP
Developmental stages
VIT_04s0044g01300
Standardized expressions
0
−1
EI PG G1 G2 H T1 T2 C1 C2 S EP JP
Developmental stages
VIT_04s0044g01310
Standardized expressions
2
0
−2
EI PG G1 G2 H T1 T2 C1 C2 S EP JP
Developmental stages
VIT_04s0044g01320
Standardized expressions
0.25
0.00
−0.25
−0.50
−0.75
−1.00
EI PG G1 G2 H T1 T2 C1 C2 S EP JP
Developmental stages
VIT_04s0044g01330
Standardized expressions
0.25
0.00
−0.25
−0.50
−0.75
EI PG G1 G2 H T1 T2 C1 C2 S EP JP
Developmental stages
VIT_04s0044g01360
Standardized expressions
0.5
0.0
−0.5
−1.0
EI PG G1 G2 H T1 T2 C1 C2 S EP JP
Developmental stages
VIT_04s0044g01370
Standardized expressions
1.0
0.5
0.0
−0.5
−1.0
−1.5
EI PG G1 G2 H T1 T2 C1 C2 S EP JP
Developmental stages
VIT_04s0044g01410
Standardized expressions
6
3
0
−3
EI PG G1 G2 H T1 T2 C1 C2 S EP JP
Developmental stages

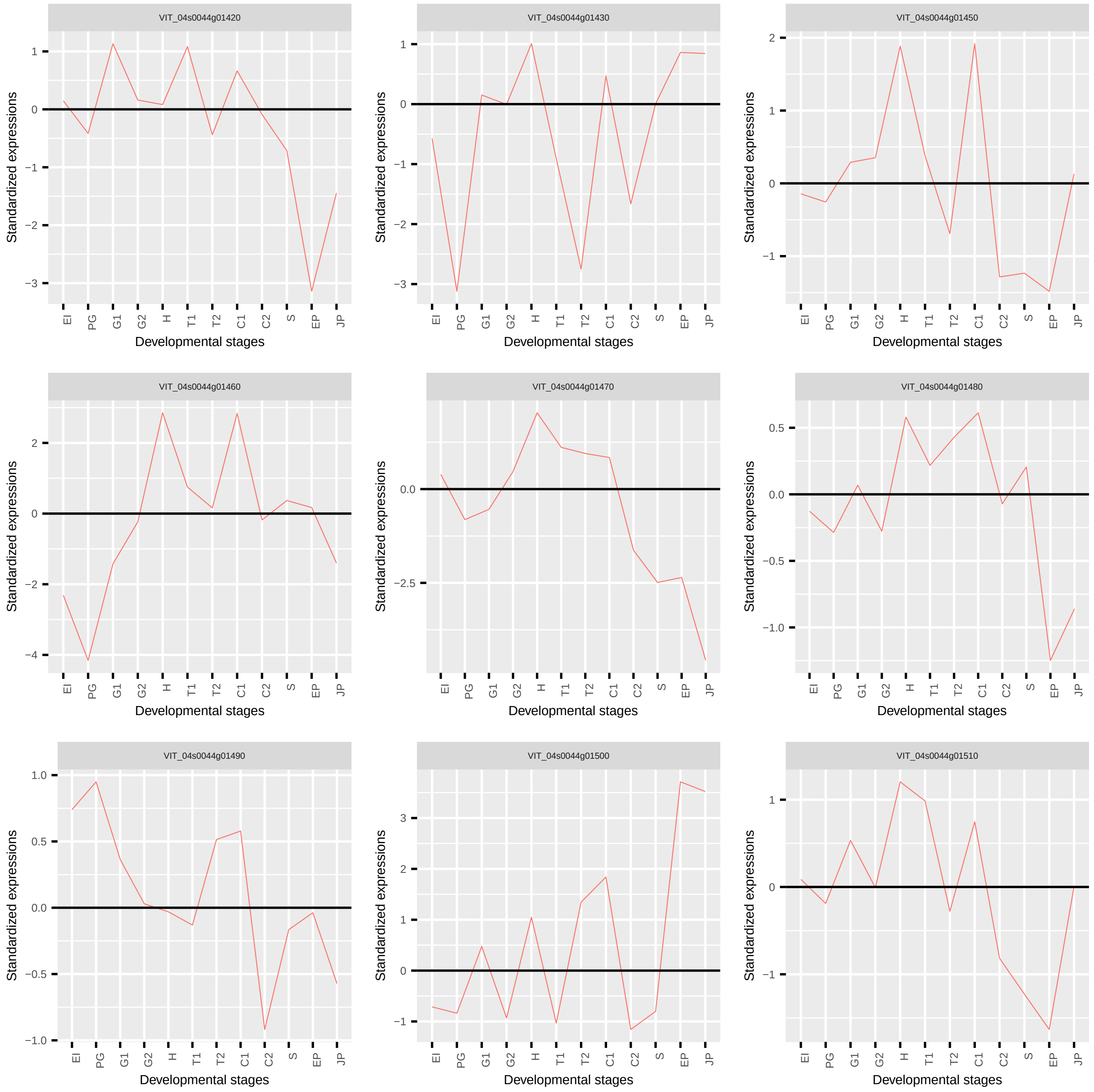

VIT_04s0044g01420
VIT_04s0044g01430
VIT_04s0044g01450
VIT_04s0044g01460
VIT_04s0044g01470
VIT_04s0044g01480
VIT_04s0044g01490
VIT_04s0044g01500
VIT_04s0044g01510
Standardized expressions
Developmental stages
EI PG G1 G2 H T1 T2 C1 C2 S EP JP

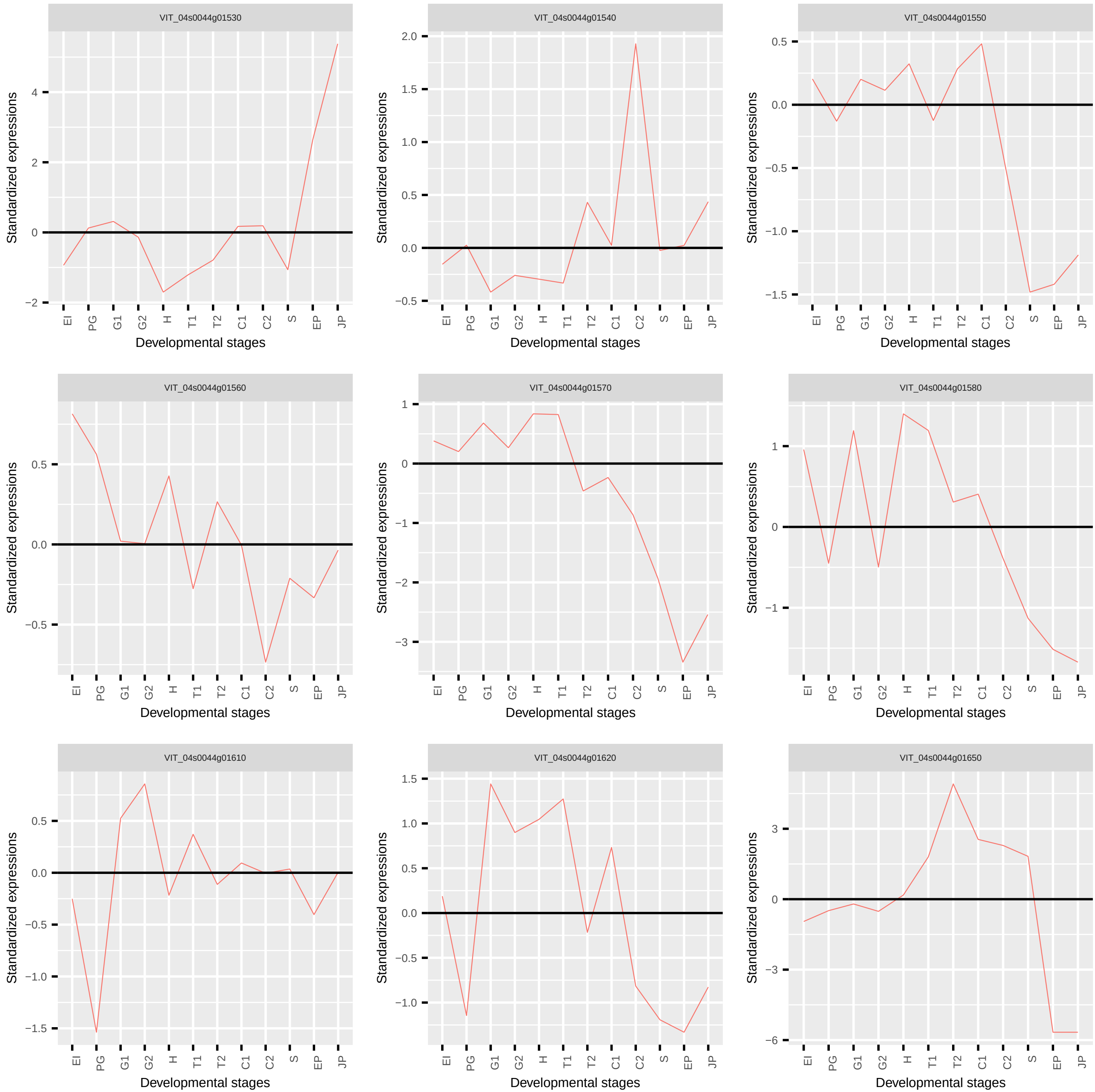
VIT_04s0044g01530
VIT_04s0044g01540
VIT_04s0044g01550
VIT_04s0044g01560
VIT_04s0044g01570
VIT_04s0044g01580
VIT_04s0044g01610
VIT_04s0044g01620
VIT_04s0044g01650
Standardized expressions
Developmental stages
EI PG G1 G2 H T1 T2 C1 C2 S EP JP

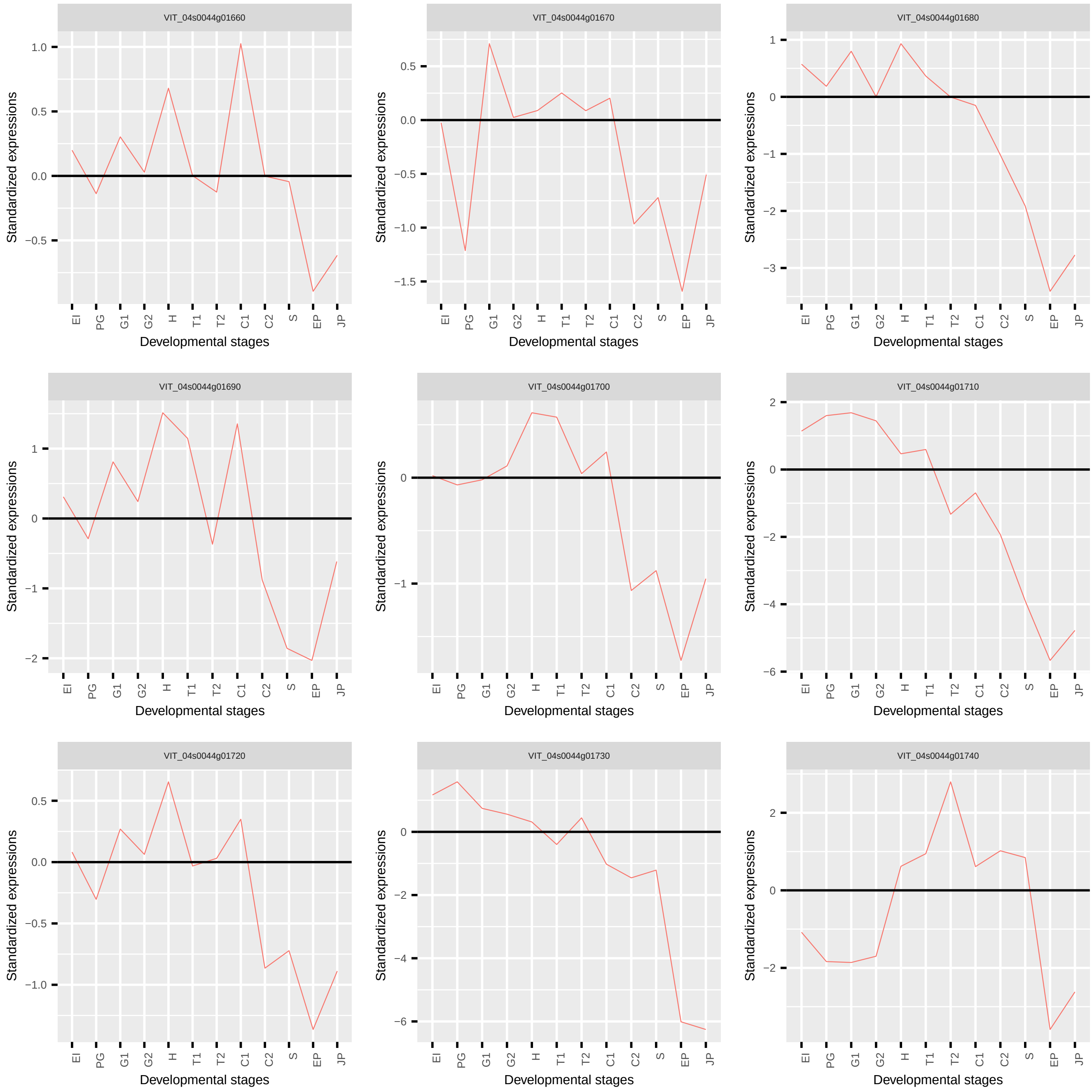
VIT_04s0044g01660
VIT_04s0044g01670
VIT_04s0044g01680
VIT_04s0044g01690
VIT_04s0044g01700
VIT_04s0044g01710
VIT_04s0044g01720
VIT_04s0044g01730
VIT_04s0044g01740
Standardized expressions
Developmental stages
EI PG G1 G2 H T1 T2 C1 C2 S EP JP

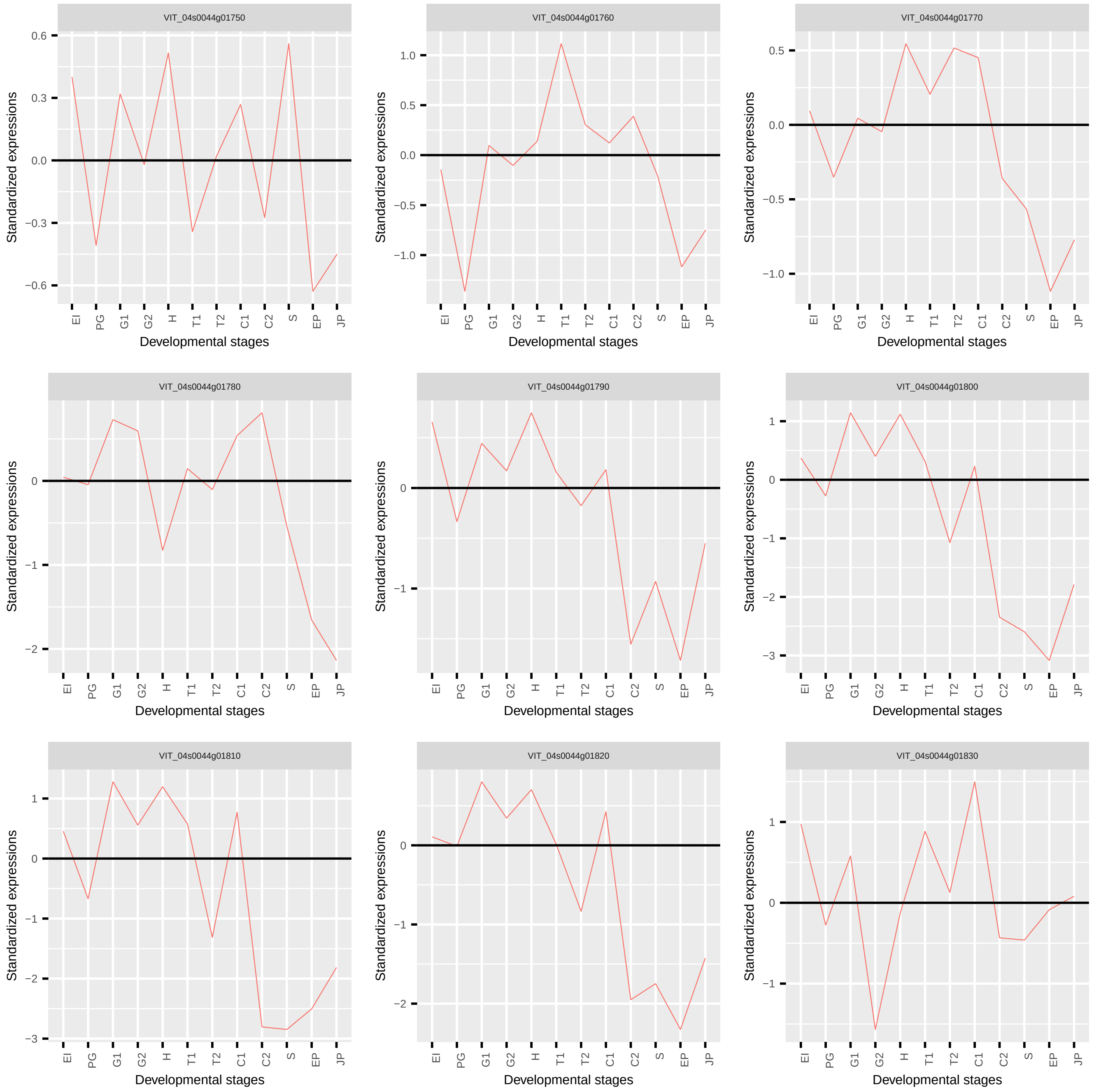
VIT_04s0044g01750
VIT_04s0044g01760
VIT_04s0044g01770
VIT_04s0044g01780
VIT_04s0044g01790
VIT_04s0044g01800
VIT_04s0044g01810
VIT_04s0044g01820
VIT_04s0044g01830
Standardized expressions
Developmental stages
EI PG G1 G2 H T1 T2 C1 C2 S EP JP
0.6 0.3 0.0 −0.3 −0.6
1.0 0.5 0.0 −0.5 −1.0
0.5 0.0 −0.5 −1.0
0 −1 −2
0 −1
1 0 −1 −2 −3
1 0 −1 −2 −3
0 −1 −2
1 0 −1

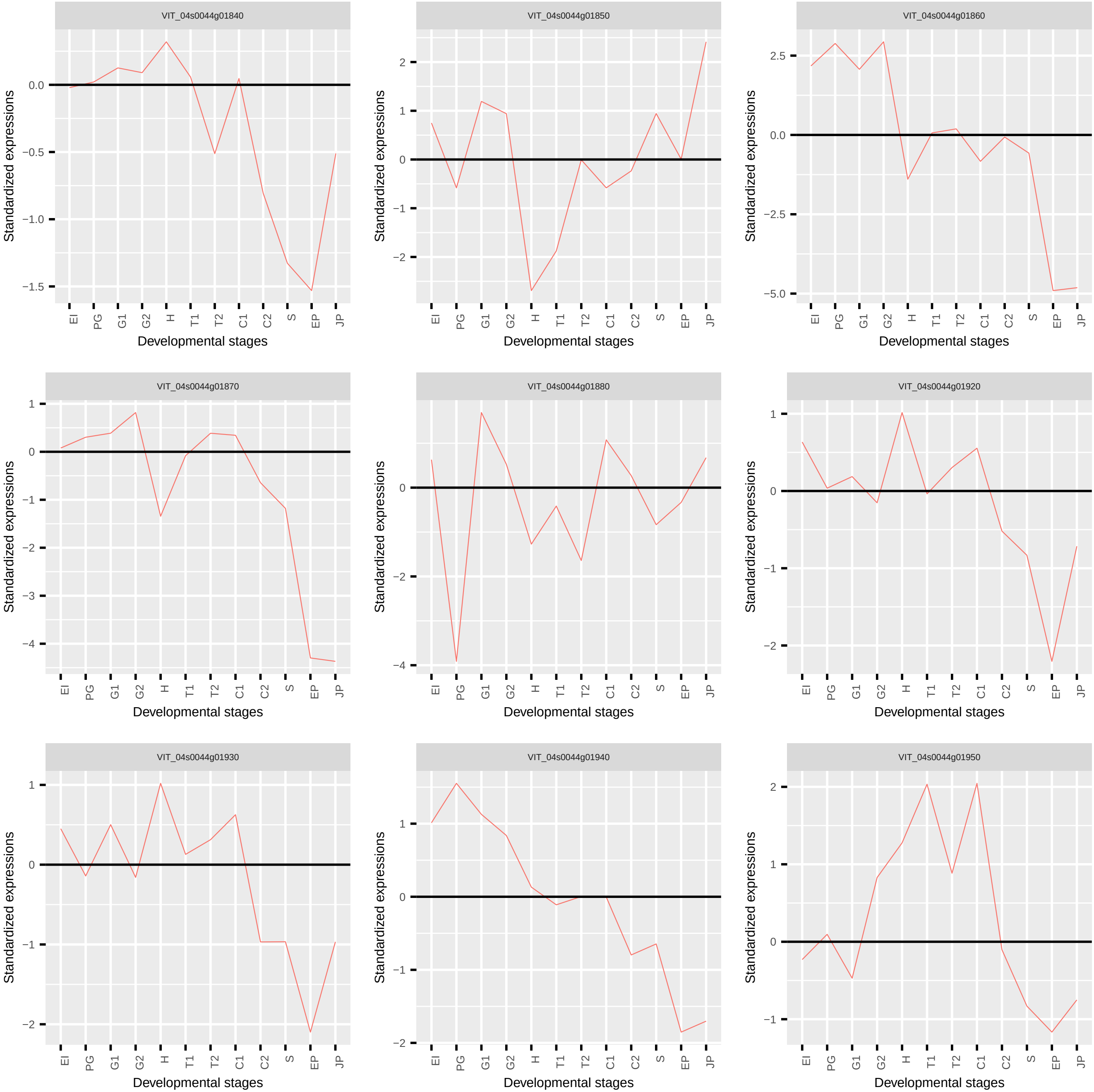
VIT_04s0044g01840
VIT_04s0044g01850
VIT_04s0044g01860
VIT_04s0044g01870
VIT_04s0044g01880
VIT_04s0044g01920
VIT_04s0044g01930
VIT_04s0044g01940
VIT_04s0044g01950
Standardized expressions
Developmental stages
EI PG G1 G2 H T1 T2 C1 C2 S EP JP

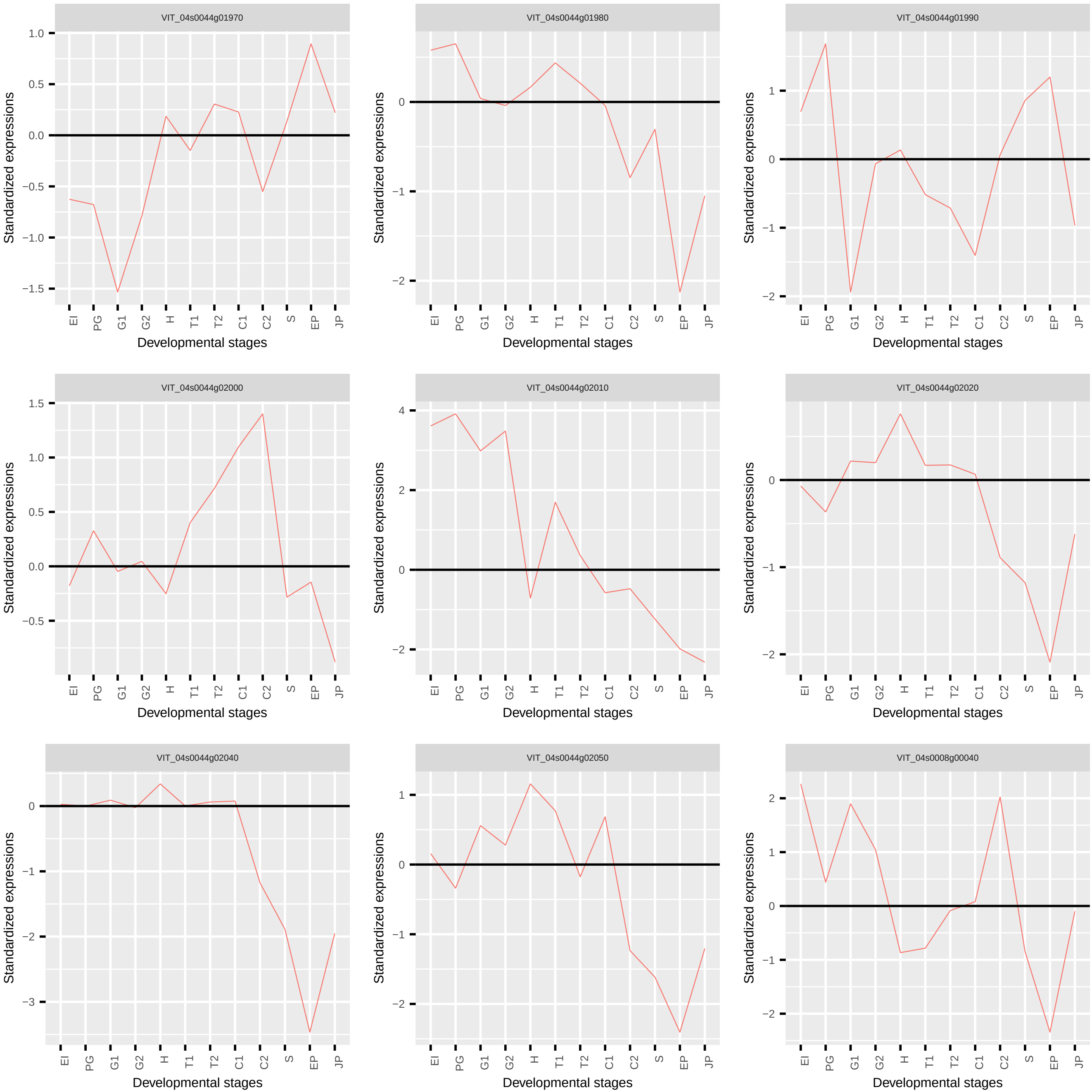
VIT_04s0044g01970
VIT_04s0044g01980
VIT_04s0044g01990
VIT_04s0044g02000
VIT_04s0044g02010
VIT_04s0044g02020
VIT_04s0044g02040
VIT_04s0044g02050
VIT_04s0008g00040
Standardized expressions
Developmental stages
EI PG G1 G2 H T1 T2 C1 C2 S EP JP

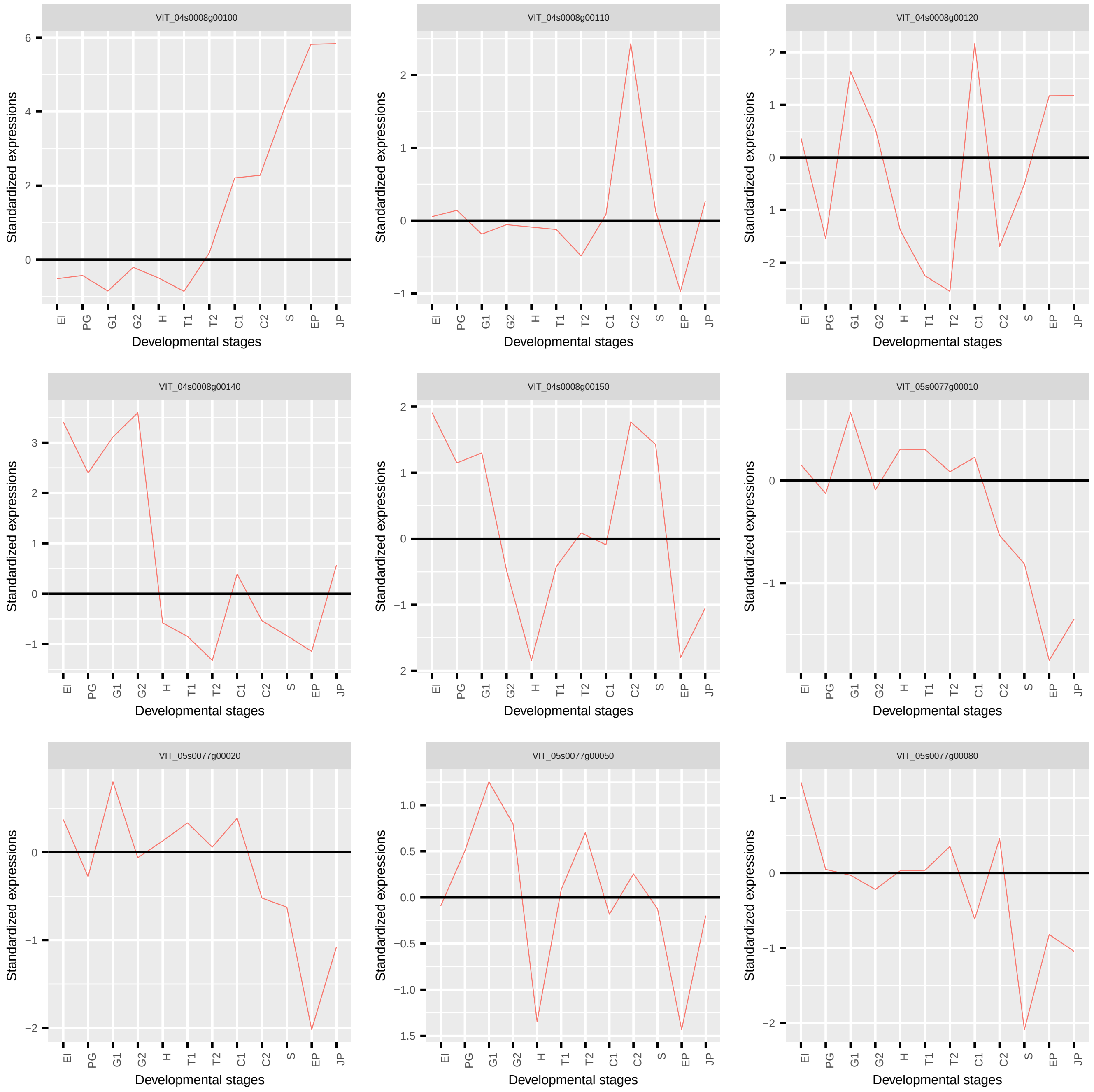

VIT_04s0008g00100
VIT_04s0008g00110
VIT_04s0008g00120
VIT_04s0008g00140
VIT_04s0008g00150
VIT_05s0077g00010
VIT_05s0077g00020
VIT_05s0077g00050
VIT_05s0077g00080
Standardized expressions
Developmental stages
EI PG G1 G2 H T1 T2 C1 C2 S EP JP

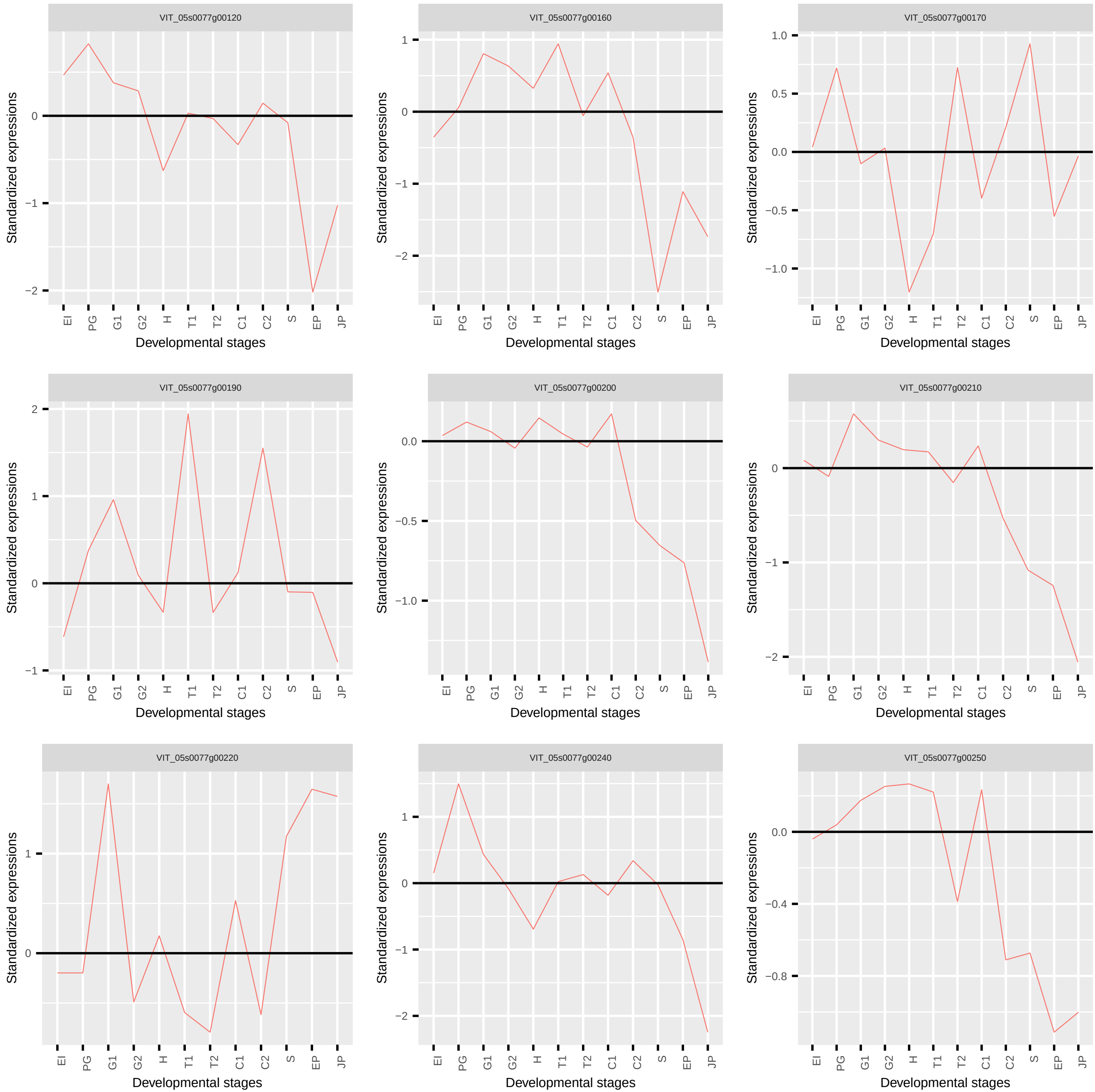

VIT_05s0077g00120
VIT_05s0077g00160
VIT_05s0077g00170
VIT_05s0077g00190
VIT_05s0077g00200
VIT_05s0077g00210
VIT_05s0077g00220
VIT_05s0077g00240
VIT_05s0077g00250
Standardized expressions
Developmental stages
EI PG G1 G2 H T1 T2 C1 C2 S EP JP

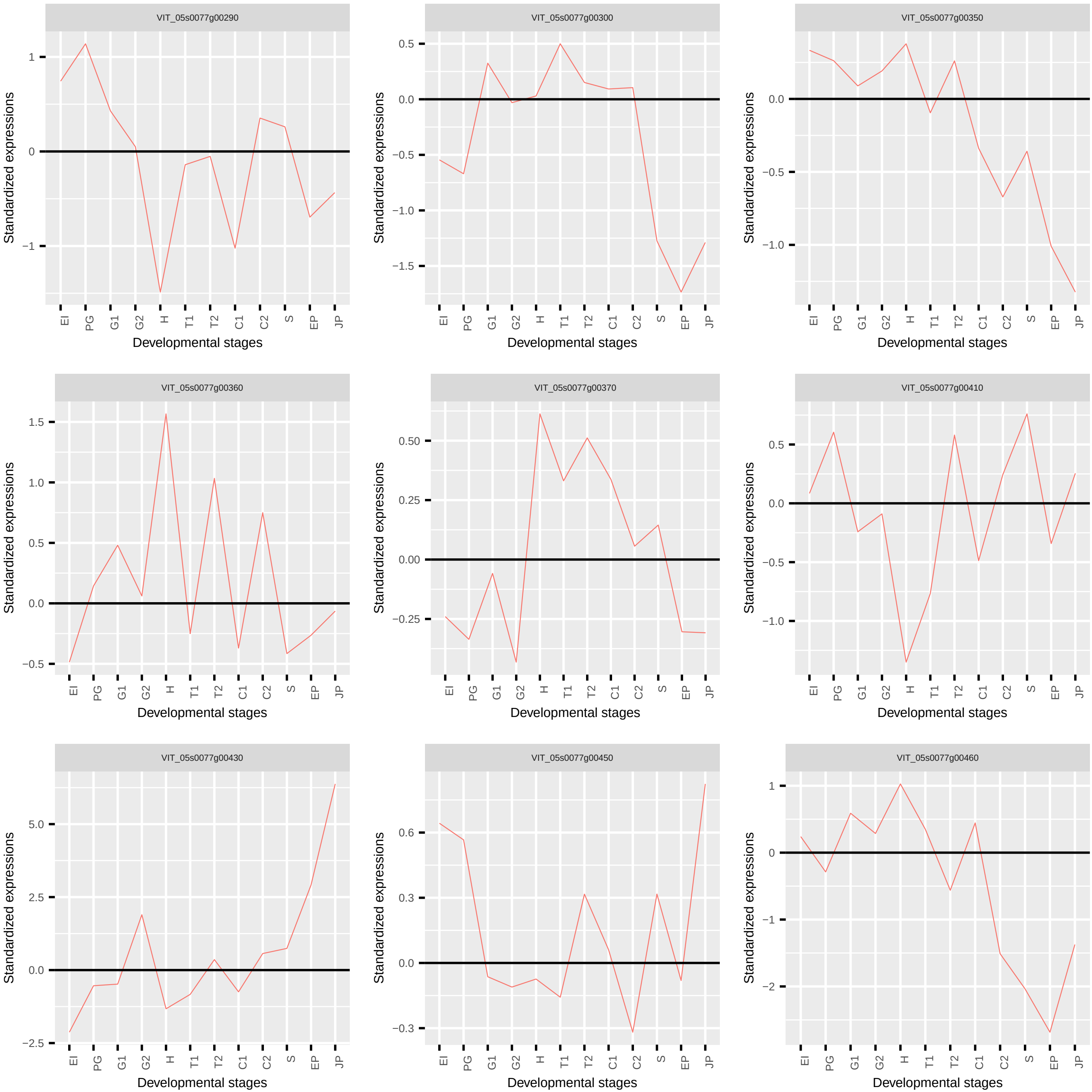

VIT_05s0077g00290
VIT_05s0077g00300
VIT_05s0077g00350
VIT_05s0077g00360
VIT_05s0077g00370
VIT_05s0077g00410
VIT_05s0077g00430
VIT_05s0077g00450
VIT_05s0077g00460
Standardized expressions
Developmental stages
EI PG G1 G2 H T1 T2 C1 C2 S EP JP

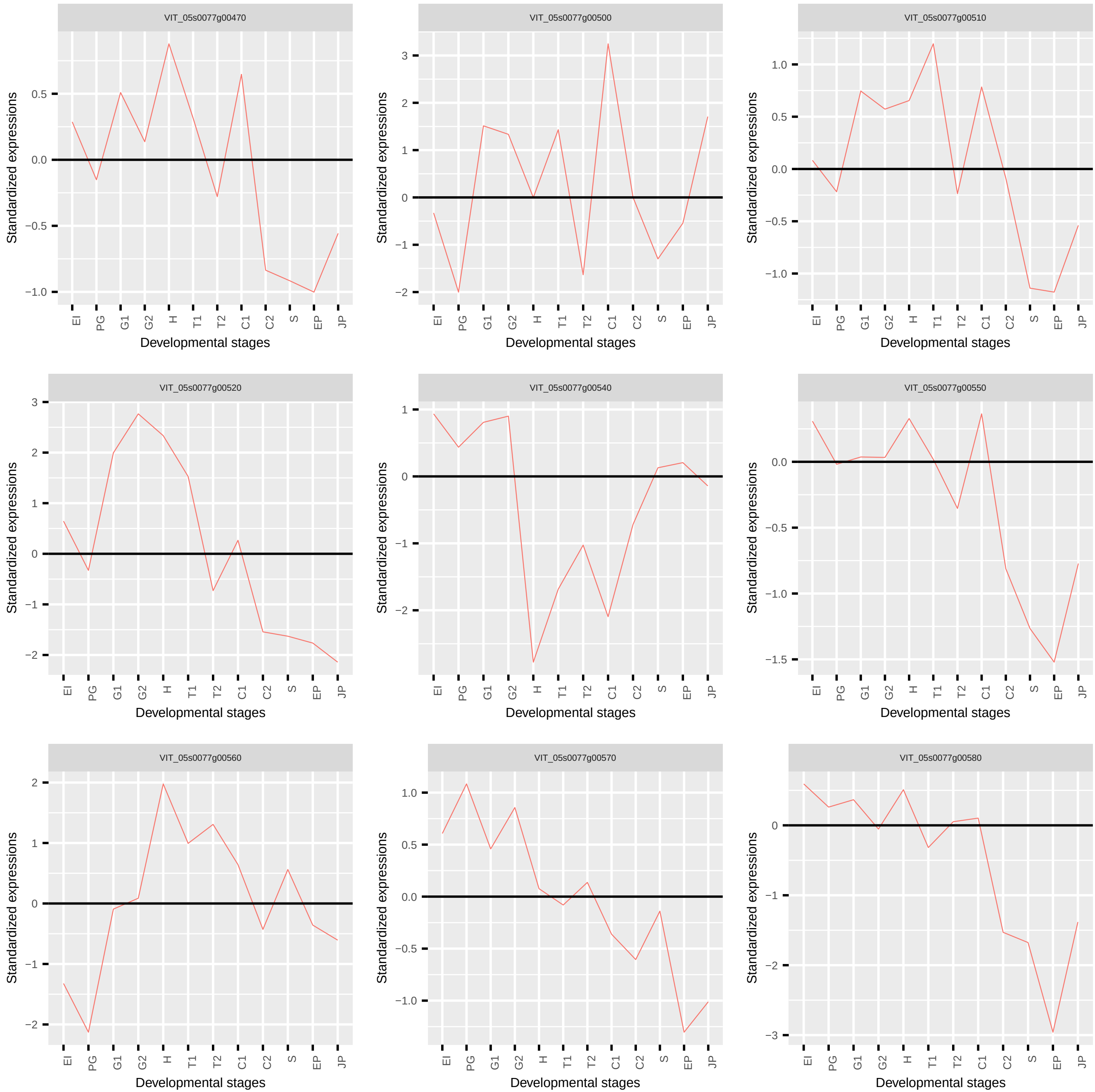
VIT_05s0077g00470
VIT_05s0077g00500
VIT_05s0077g00510
VIT_05s0077g00520
VIT_05s0077g00540
VIT_05s0077g00550
VIT_05s0077g00560
VIT_05s0077g00570
VIT_05s0077g00580
Standardized expressions
Developmental stages
EI PG G1 G2 H T1 T2 C1 C2 S EP JP

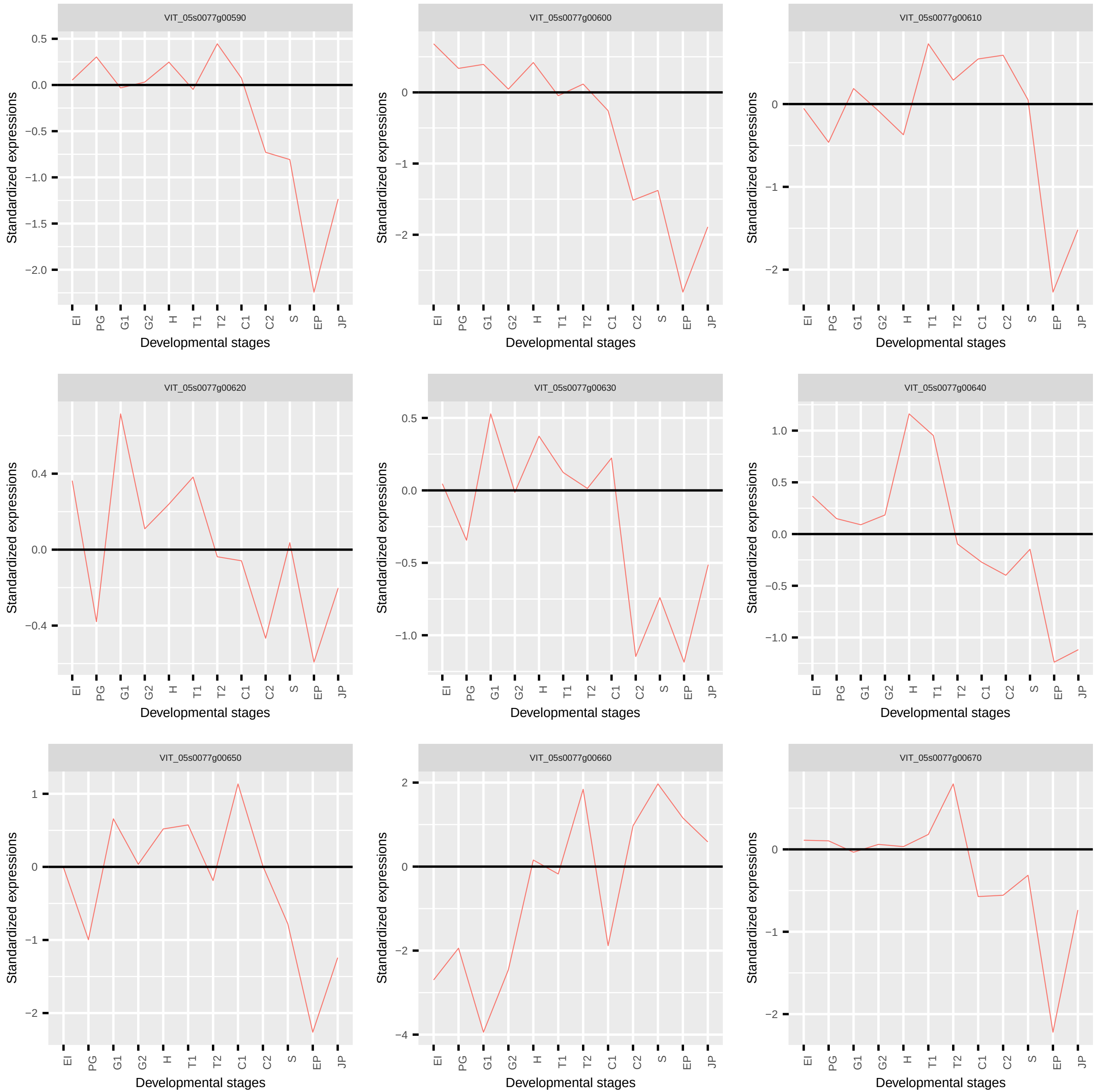
VIT_05s0077g00590
VIT_05s0077g00600
VIT_05s0077g00610
VIT_05s0077g00620
VIT_05s0077g00630
VIT_05s0077g00640
VIT_05s0077g00650
VIT_05s0077g00660
VIT_05s0077g00670
Standardized expressions
Developmental stages
EI PG G1 G2 H T1 T2 C1 C2 S EP JP

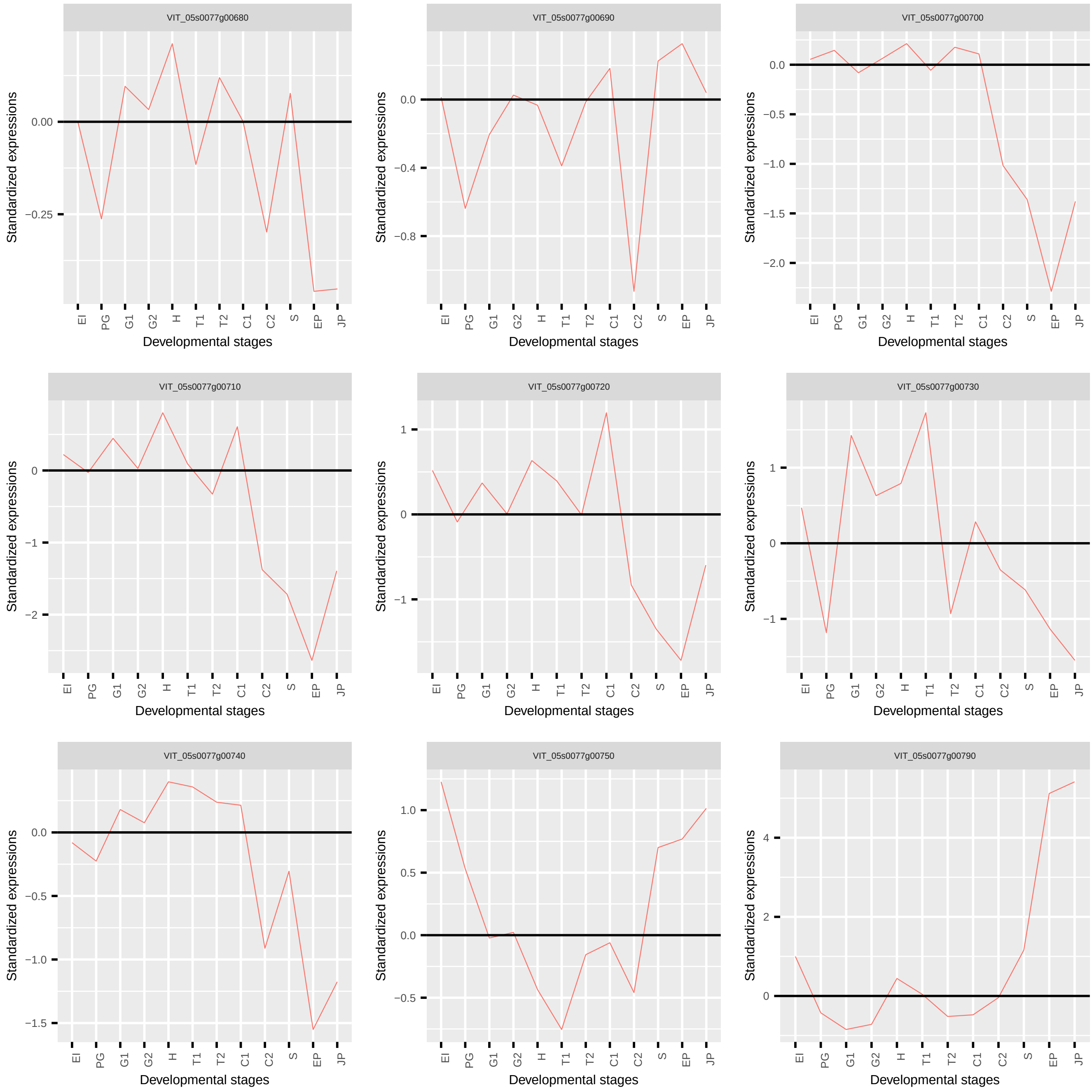
VIT_05s0077g00680
VIT_05s0077g00690
VIT_05s0077g00700
VIT_05s0077g00710
VIT_05s0077g00720
VIT_05s0077g00730
VIT_05s0077g00740
VIT_05s0077g00750
VIT_05s0077g00790
Standardized expressions
Developmental stages
EI PG G1 G2 H T1 T2 C1 C2 S EP JP

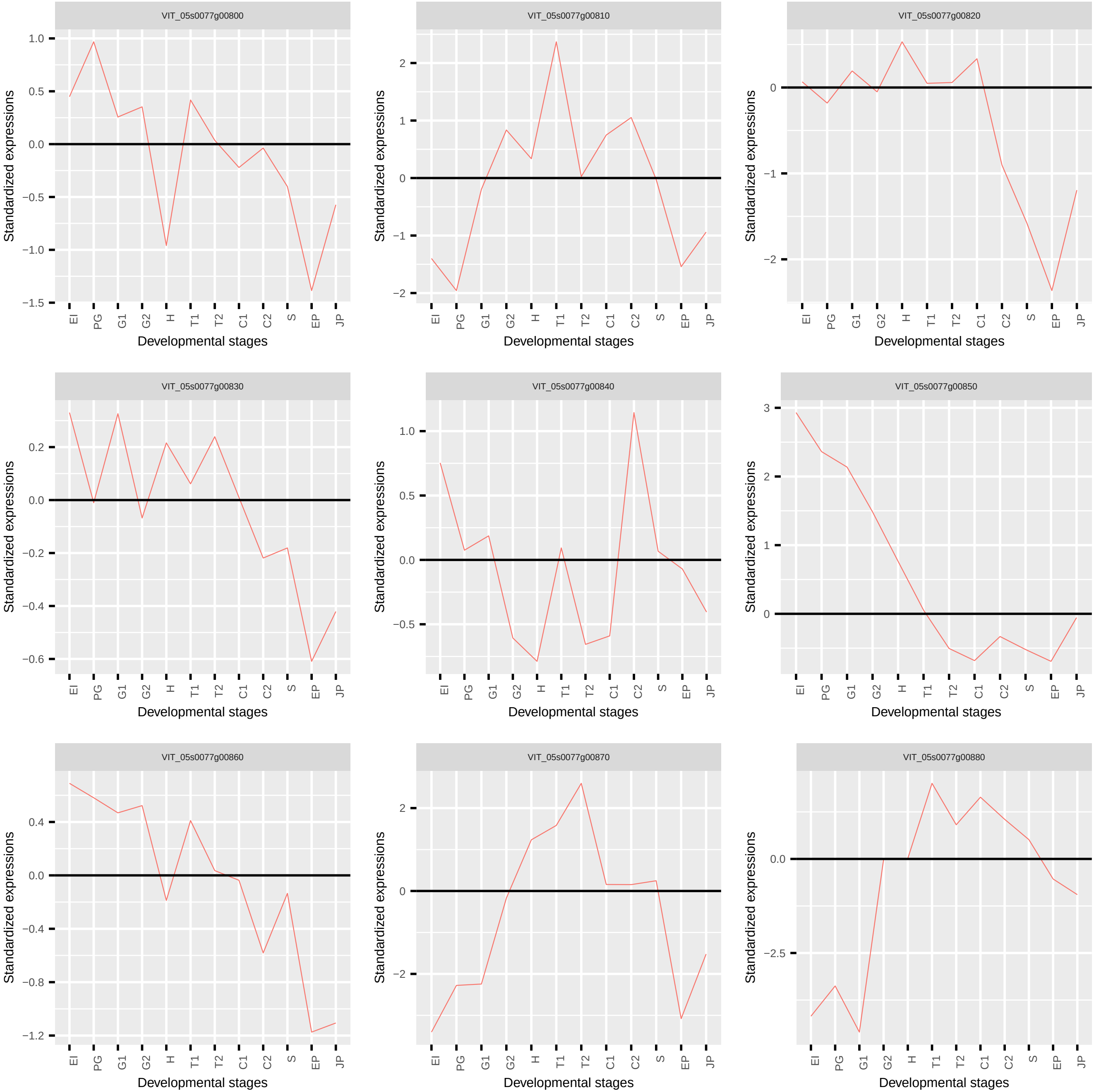
VIT_05s0077g00800
VIT_05s0077g00810
VIT_05s0077g00820
VIT_05s0077g00830
VIT_05s0077g00840
VIT_05s0077g00850
VIT_05s0077g00860
VIT_05s0077g00870
VIT_05s0077g00880
Standardized expressions
Developmental stages
EI PG G1 G2 H T1 T2 C1 C2 S EP JP

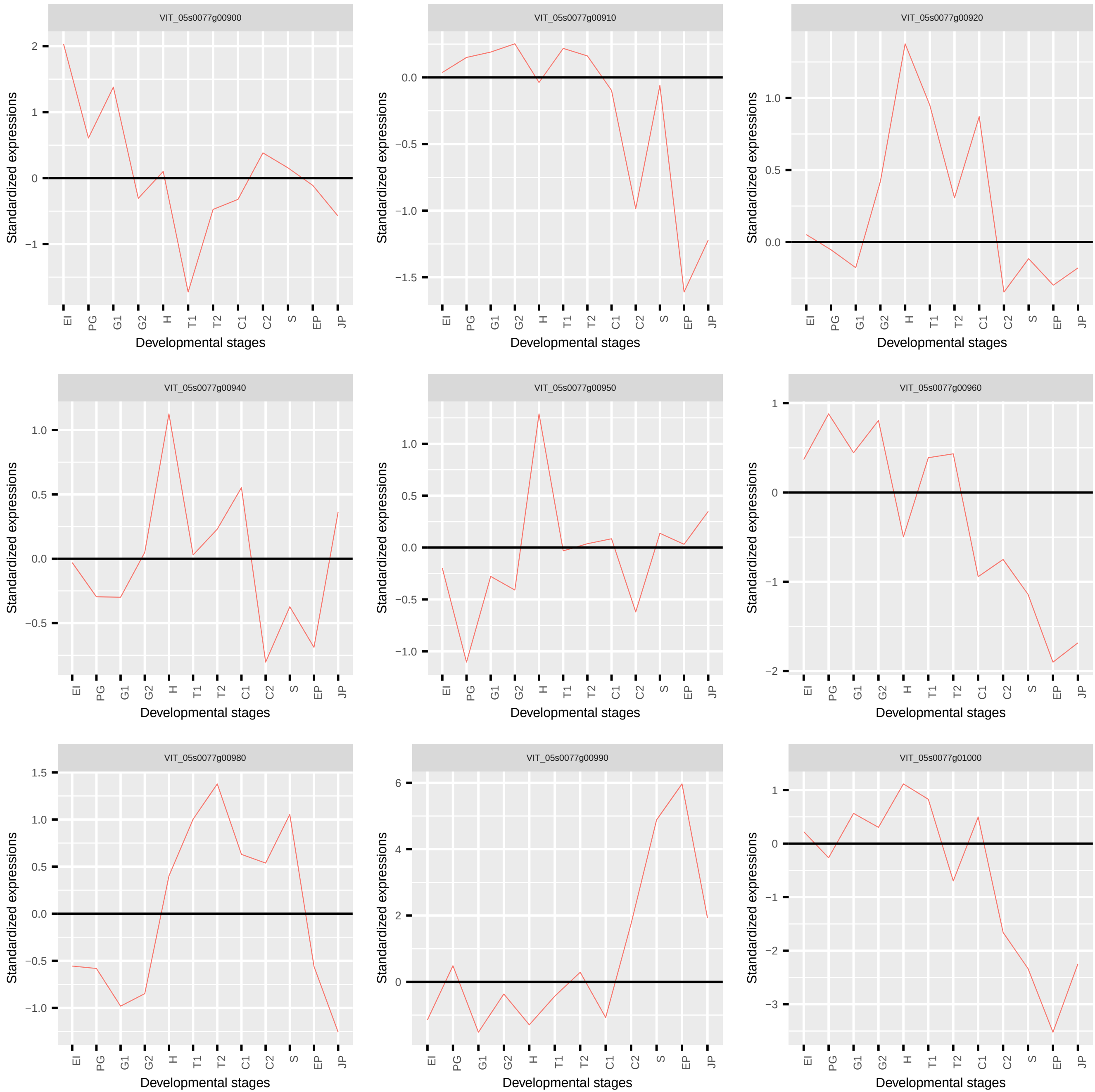

VIT_05s0077g00900
VIT_05s0077g00910
VIT_05s0077g00920
VIT_05s0077g00940
VIT_05s0077g00950
VIT_05s0077g00960
VIT_05s0077g00980
VIT_05s0077g00990
VIT_05s0077g01000
Standardized expressions
Developmental stages
EI PG G1 G2 H T1 T2 C1 C2 S EP JP

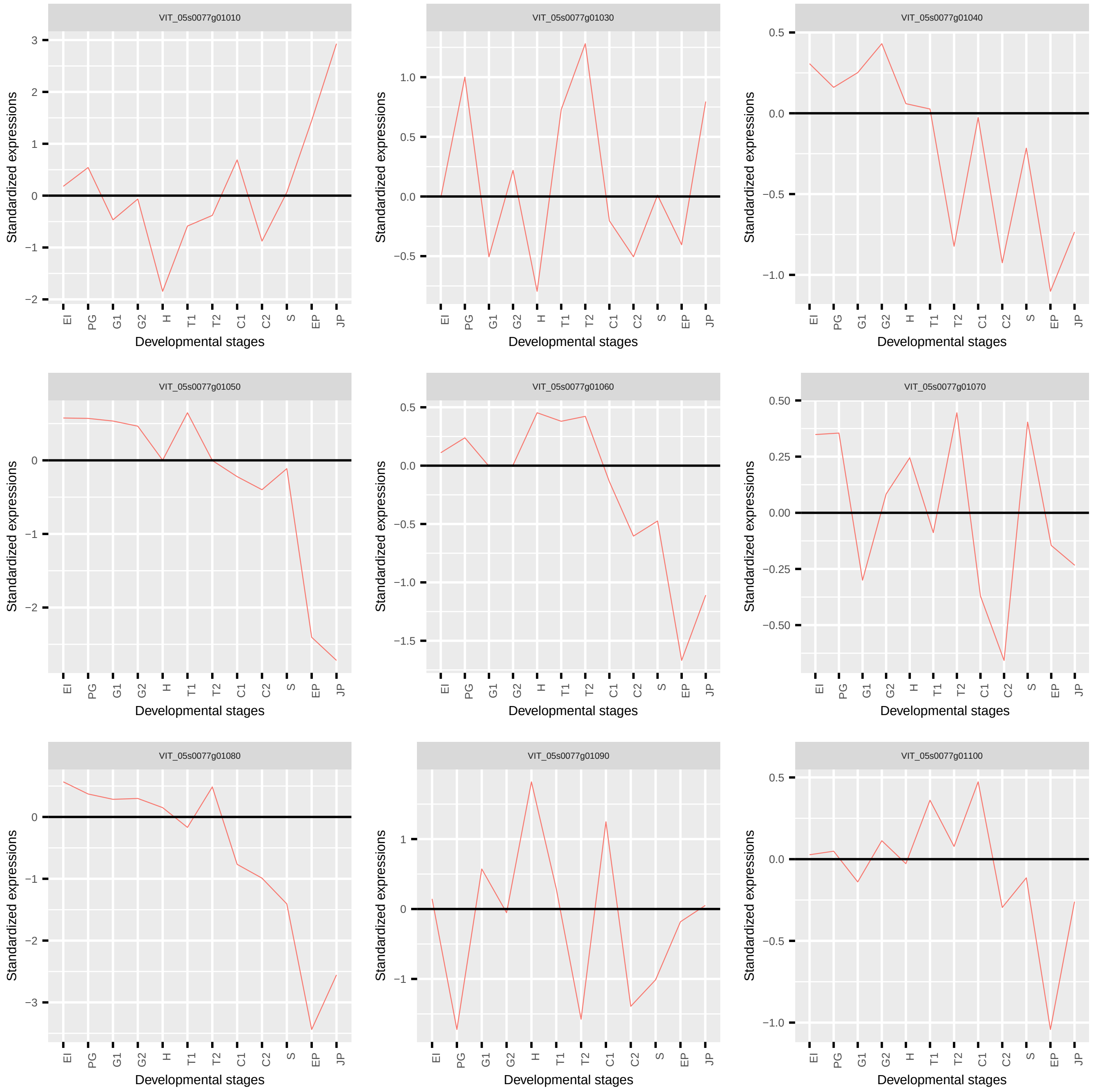
VIT_05s0077g01010
VIT_05s0077g01030
VIT_05s0077g01040
VIT_05s0077g01050
VIT_05s0077g01060
VIT_05s0077g01070
VIT_05s0077g01080
VIT_05s0077g01090
VIT_05s0077g01100
Standardized expressions
Developmental stages
EI PG G1 G2 H T1 T2 C1 C2 S EP JP

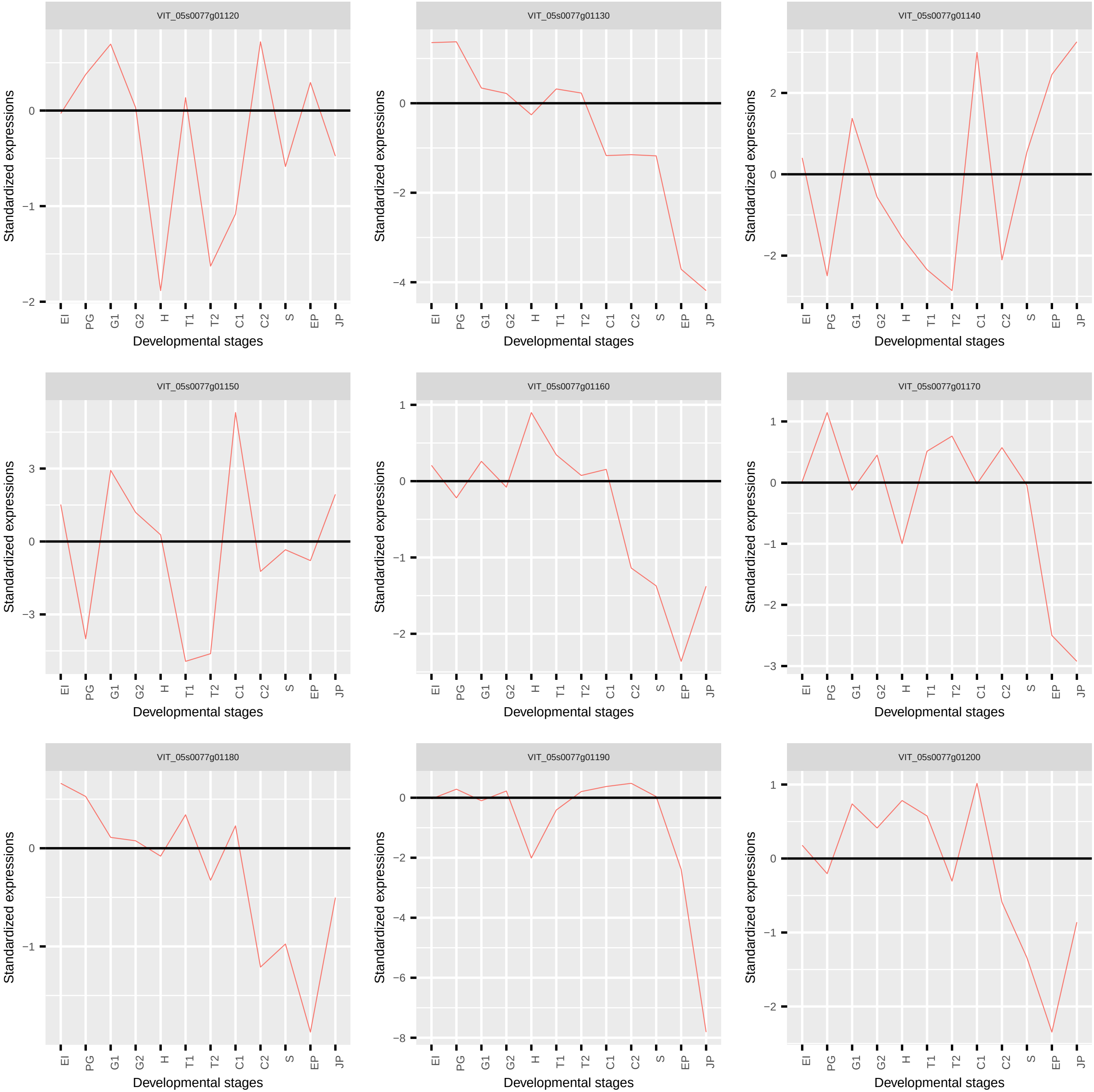
VIT_05s0077g01120
VIT_05s0077g01130
VIT_05s0077g01140
VIT_05s0077g01150
VIT_05s0077g01160
VIT_05s0077g01170
VIT_05s0077g01180
VIT_05s0077g01190
VIT_05s0077g01200
Standardized expressions
Developmental stages
EI
PG
G1
G2
H
T1
T2
C1
C2
S
EP
JP

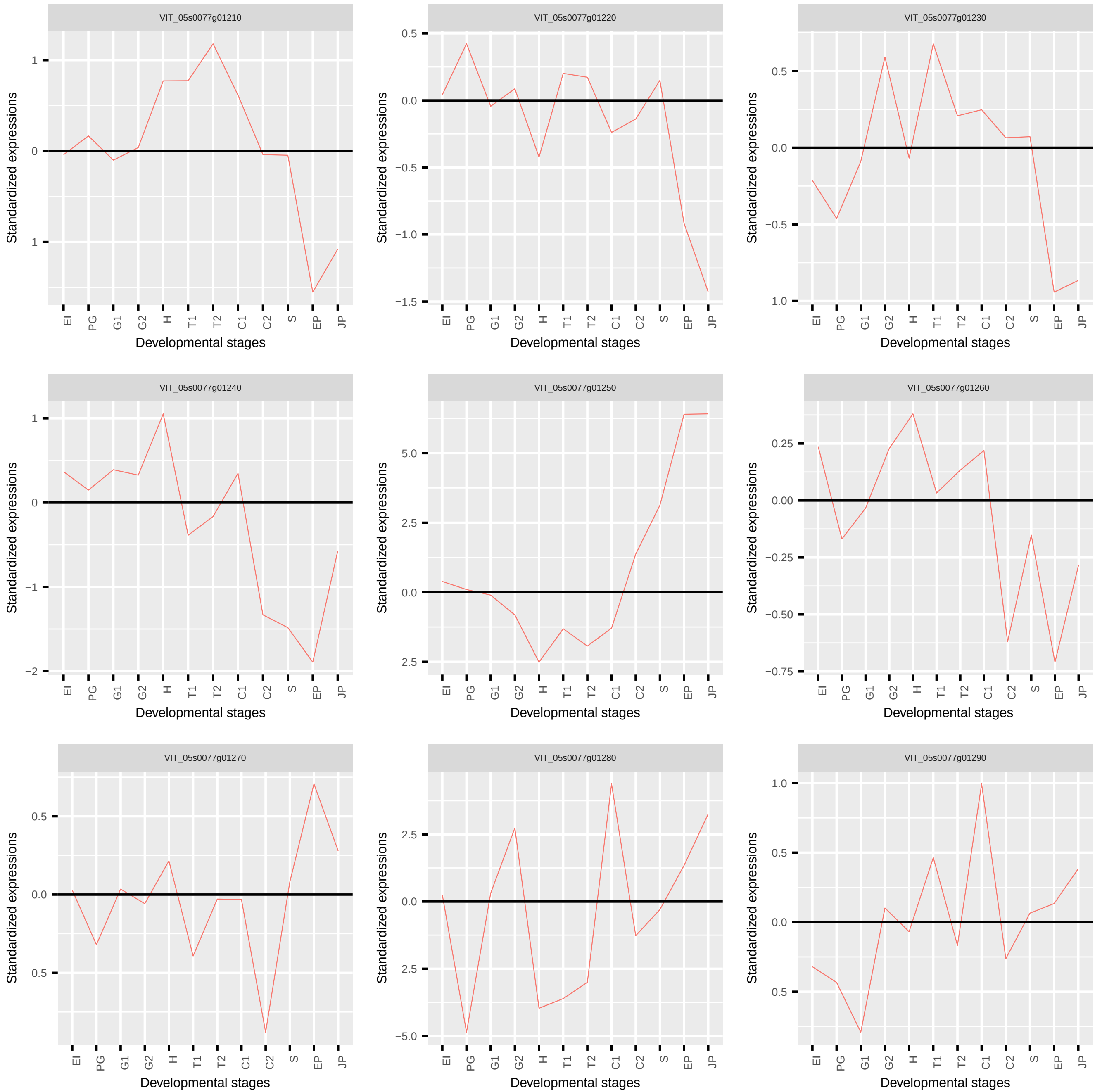
VIT_05s0077g01210
1
0
−1
Standardized expressions
EI PG G1 G2 H T1 T2 C1 C2 S EP JP
Developmental stages
VIT_05s0077g01220
0.5
0.0
−0.5
−1.0
−1.5
Standardized expressions
EI PG G1 G2 H T1 T2 C1 C2 S EP JP
Developmental stages
VIT_05s0077g01230
0.5
0.0
−0.5
−1.0
Standardized expressions
EI PG G1 G2 H T1 T2 C1 C2 S EP JP
Developmental stages
VIT_05s0077g01240
1
0
−1
−2
Standardized expressions
EI PG G1 G2 H T1 T2 C1 C2 S EP JP
Developmental stages
VIT_05s0077g01250
5.0
2.5
0.0
−2.5
Standardized expressions
EI PG G1 G2 H T1 T2 C1 C2 S EP JP
Developmental stages
VIT_05s0077g01260
0.25
0.00
−0.25
−0.50
−0.75
Standardized expressions
EI PG G1 G2 H T1 T2 C1 C2 S EP JP
Developmental stages
VIT_05s0077g01270
0.5
0.0
−0.5
Standardized expressions
EI PG G1 G2 H T1 T2 C1 C2 S EP JP
Developmental stages
VIT_05s0077g01280
2.5
0.0
−2.5
−5.0
Standardized expressions
EI PG G1 G2 H T1 T2 C1 C2 S EP JP
Developmental stages
VIT_05s0077g01290
1.0
0.5
0.0
−0.5
Standardized expressions
EI PG G1 G2 H T1 T2 C1 C2 S EP JP
Developmental stages

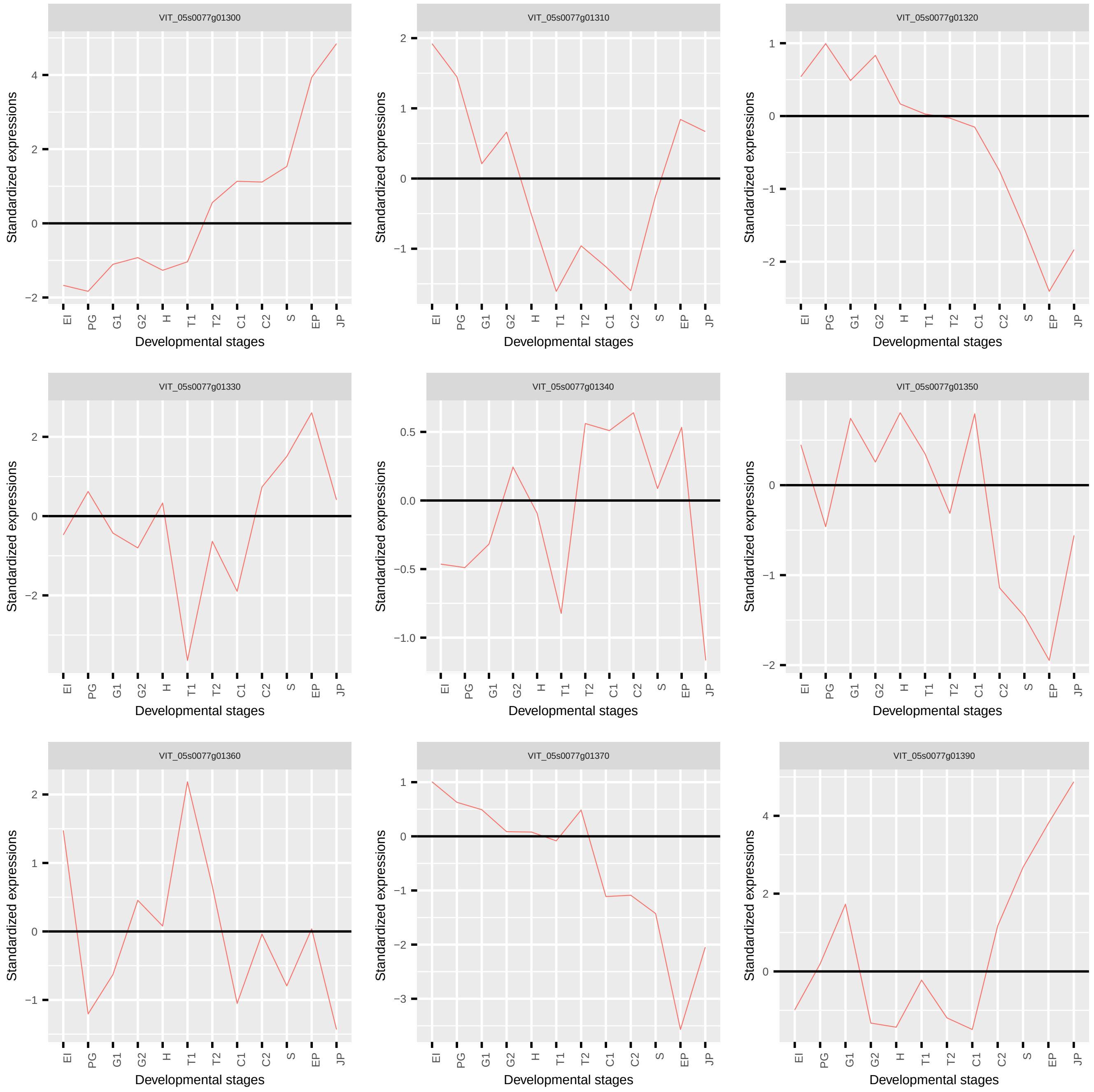
VIT_05s0077g01300
VIT_05s0077g01310
VIT_05s0077g01320
VIT_05s0077g01330
VIT_05s0077g01340
VIT_05s0077g01350
VIT_05s0077g01360
VIT_05s0077g01370
VIT_05s0077g01390
Standardized expressions
Developmental stages
EI PG G1 G2 H T1 T2 C1 C2 S EP JP

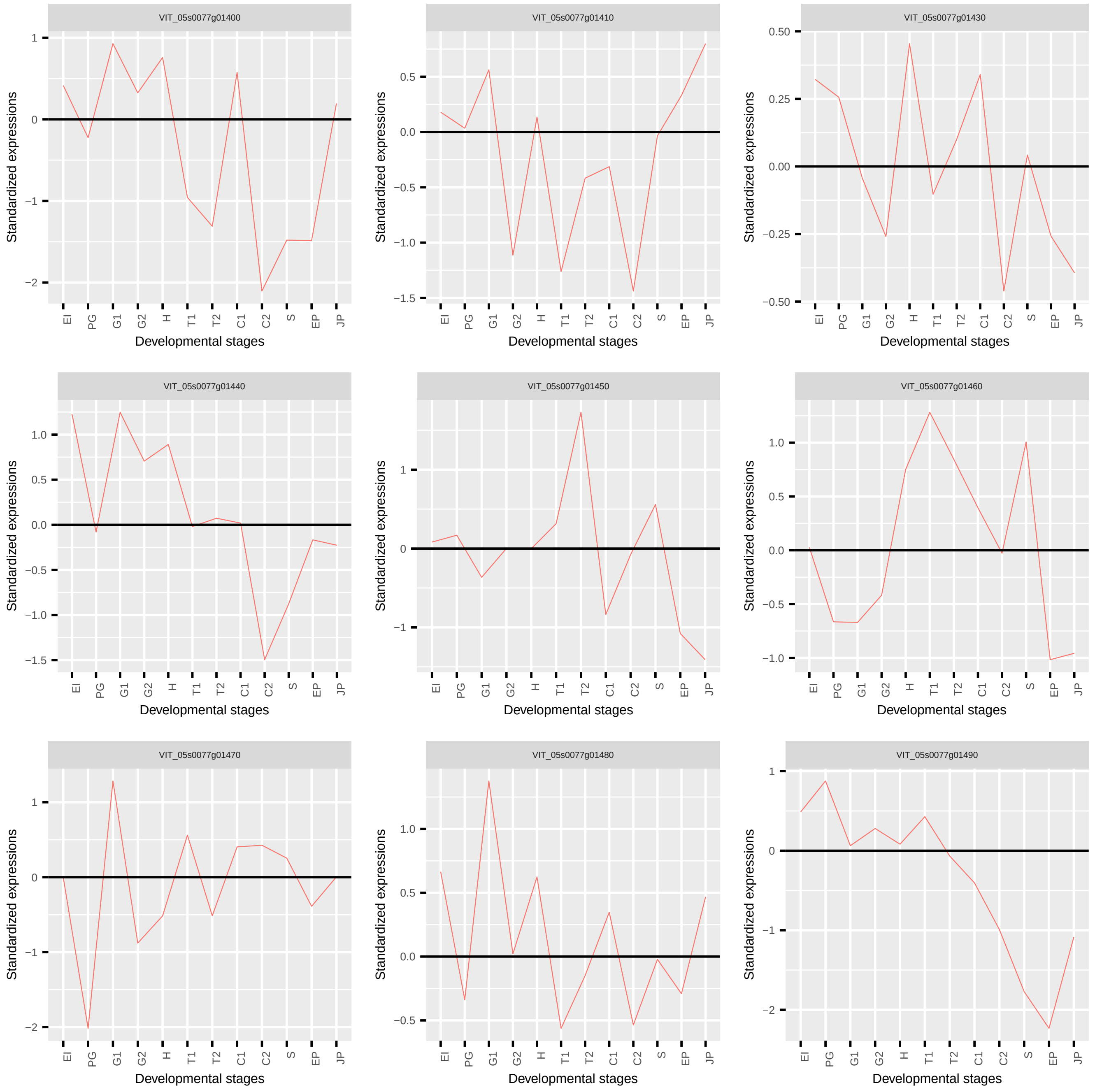
VIT_05s0077g01400
VIT_05s0077g01410
VIT_05s0077g01430
VIT_05s0077g01440
VIT_05s0077g01450
VIT_05s0077g01460
VIT_05s0077g01470
VIT_05s0077g01480
VIT_05s0077g01490
Standardized expressions
Developmental stages
EI PG G1 G2 H T1 T2 C1 C2 S EP JP

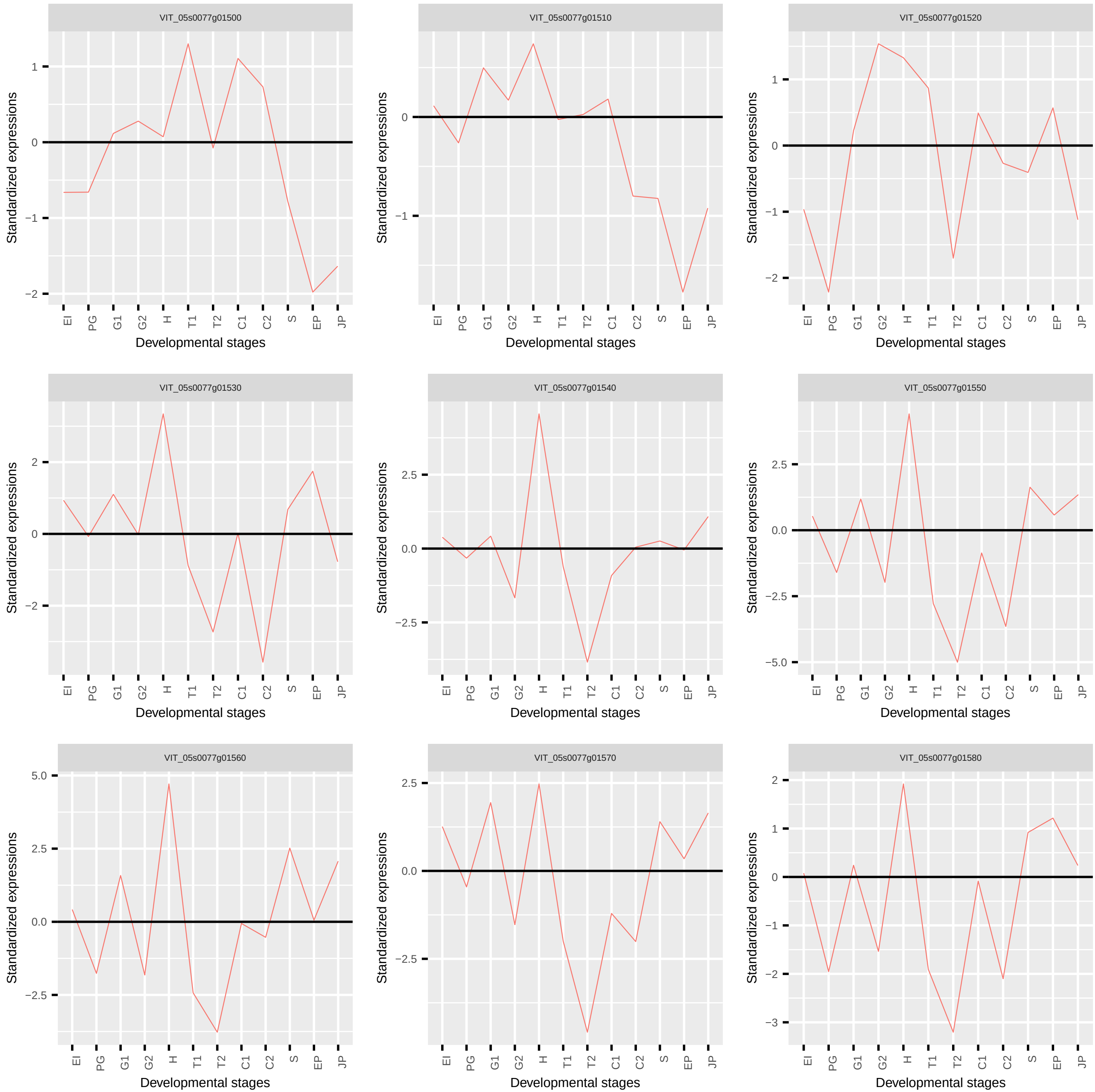

VIT_05s0077g01500
VIT_05s0077g01510
VIT_05s0077g01520
VIT_05s0077g01530
VIT_05s0077g01540
VIT_05s0077g01550
VIT_05s0077g01560
VIT_05s0077g01570
VIT_05s0077g01580
Standardized expressions
Developmental stages
EI PG G1 G2 H T1 T2 C1 C2 S EP JP

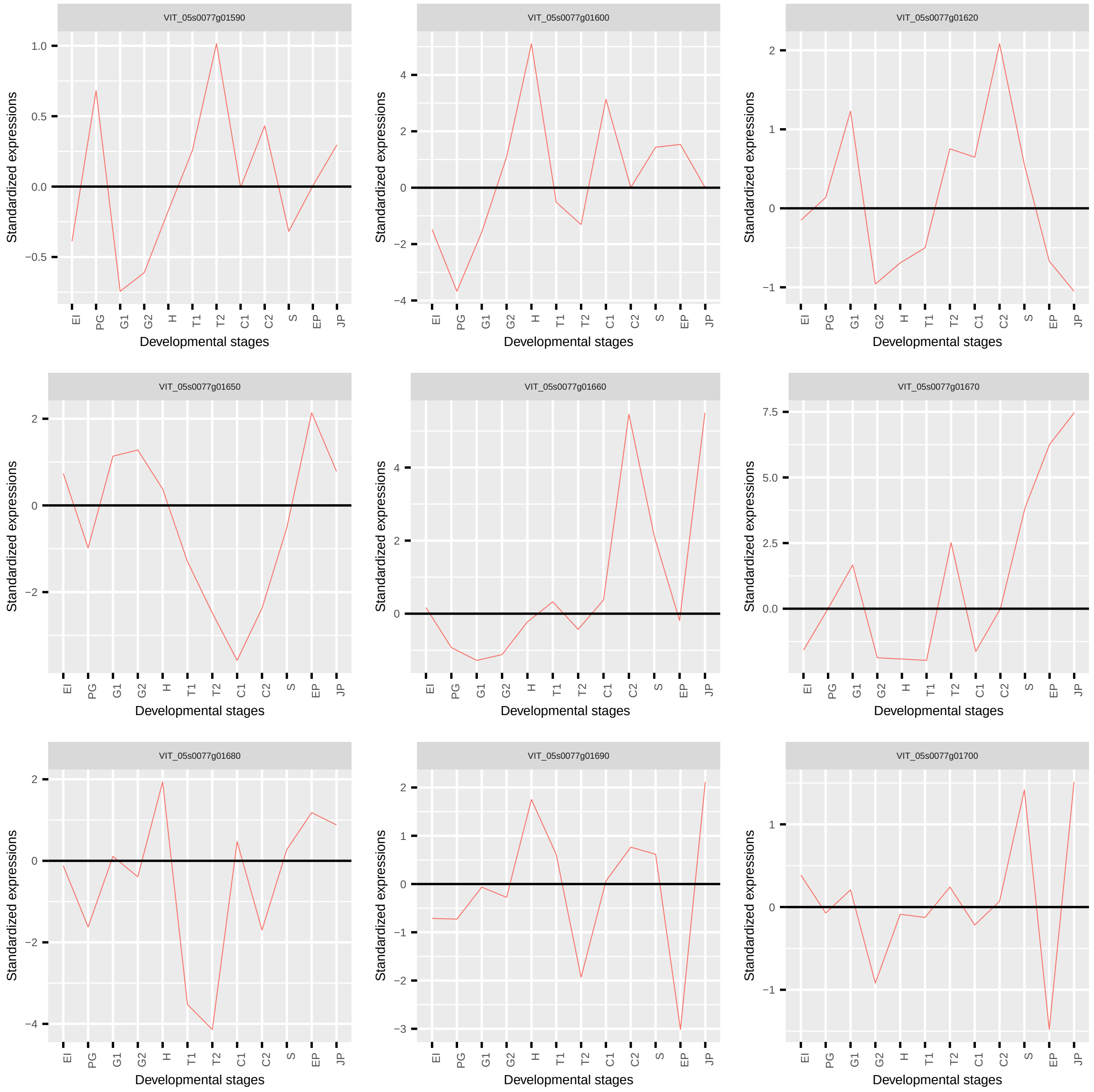
VIT_05s0077g01590
VIT_05s0077g01600
VIT_05s0077g01620
VIT_05s0077g01650
VIT_05s0077g01660
VIT_05s0077g01670
VIT_05s0077g01680
VIT_05s0077g01690
VIT_05s0077g01700
Standardized expressions
Developmental stages
EI PG G1 G2 H T1 T2 C1 C2 S EP JP

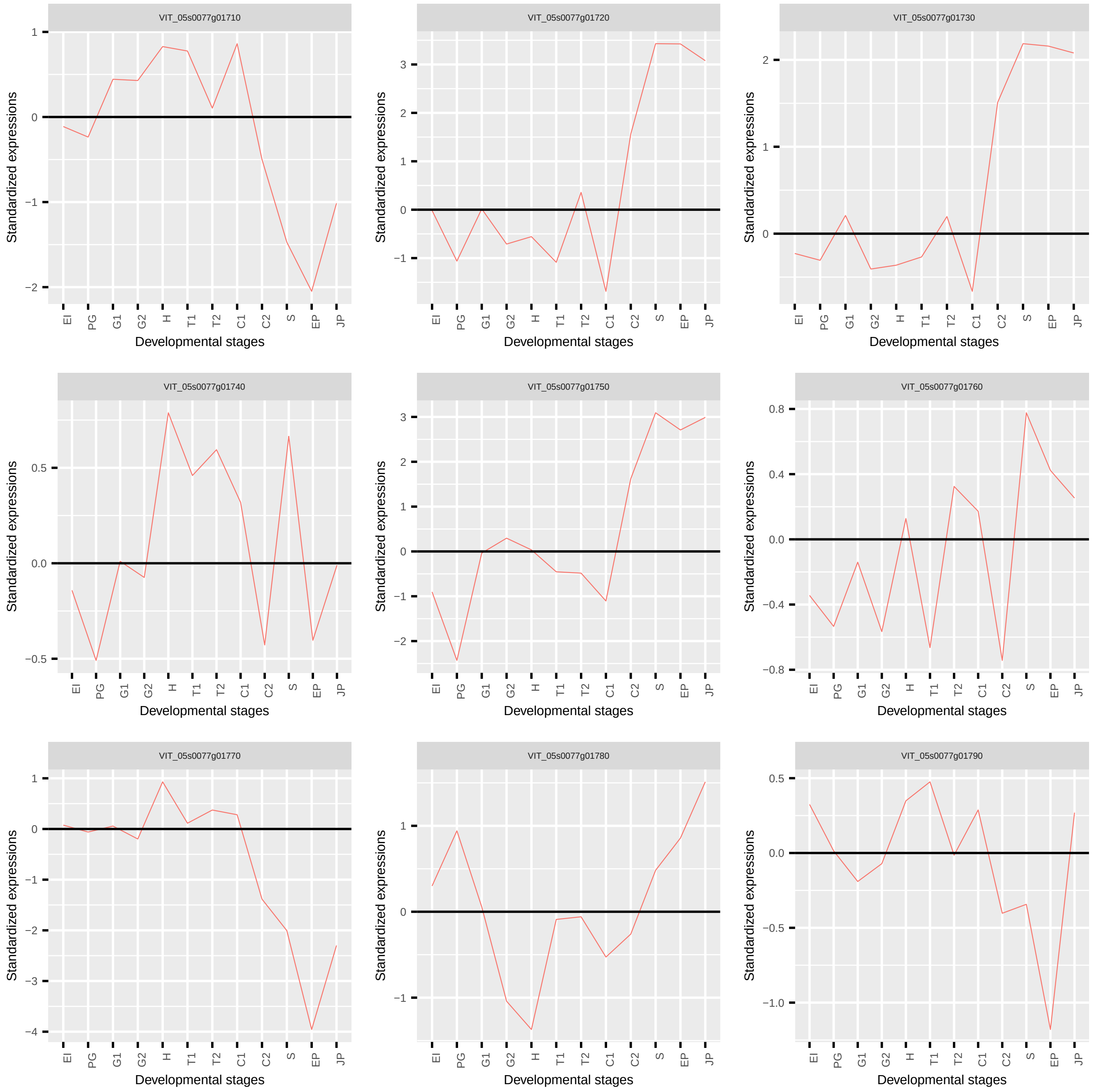
VIT_05s0077g01710
VIT_05s0077g01720
VIT_05s0077g01730
VIT_05s0077g01740
VIT_05s0077g01750
VIT_05s0077g01760
VIT_05s0077g01770
VIT_05s0077g01780
VIT_05s0077g01790
Standardized expressions
Developmental stages
EI PG G1 G2 H T1 T2 C1 C2 S EP JP

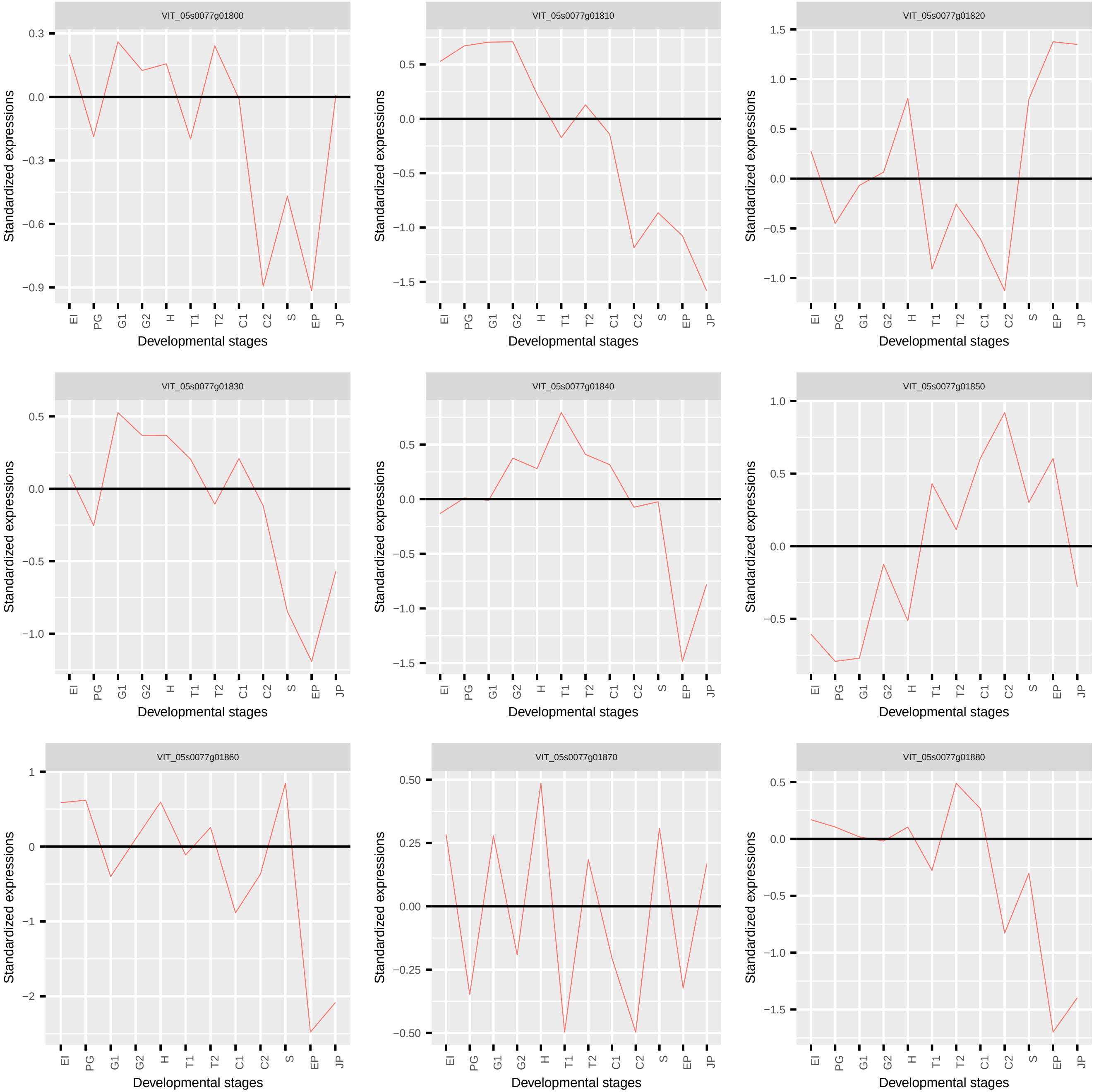
VIT_05s0077g01800
VIT_05s0077g01810
VIT_05s0077g01820
VIT_05s0077g01830
VIT_05s0077g01840
VIT_05s0077g01850
VIT_05s0077g01860
VIT_05s0077g01870
VIT_05s0077g01880
Standardized expressions
Developmental stages
EI PG G1 G2 H T1 T2 C1 C2 S EP JP

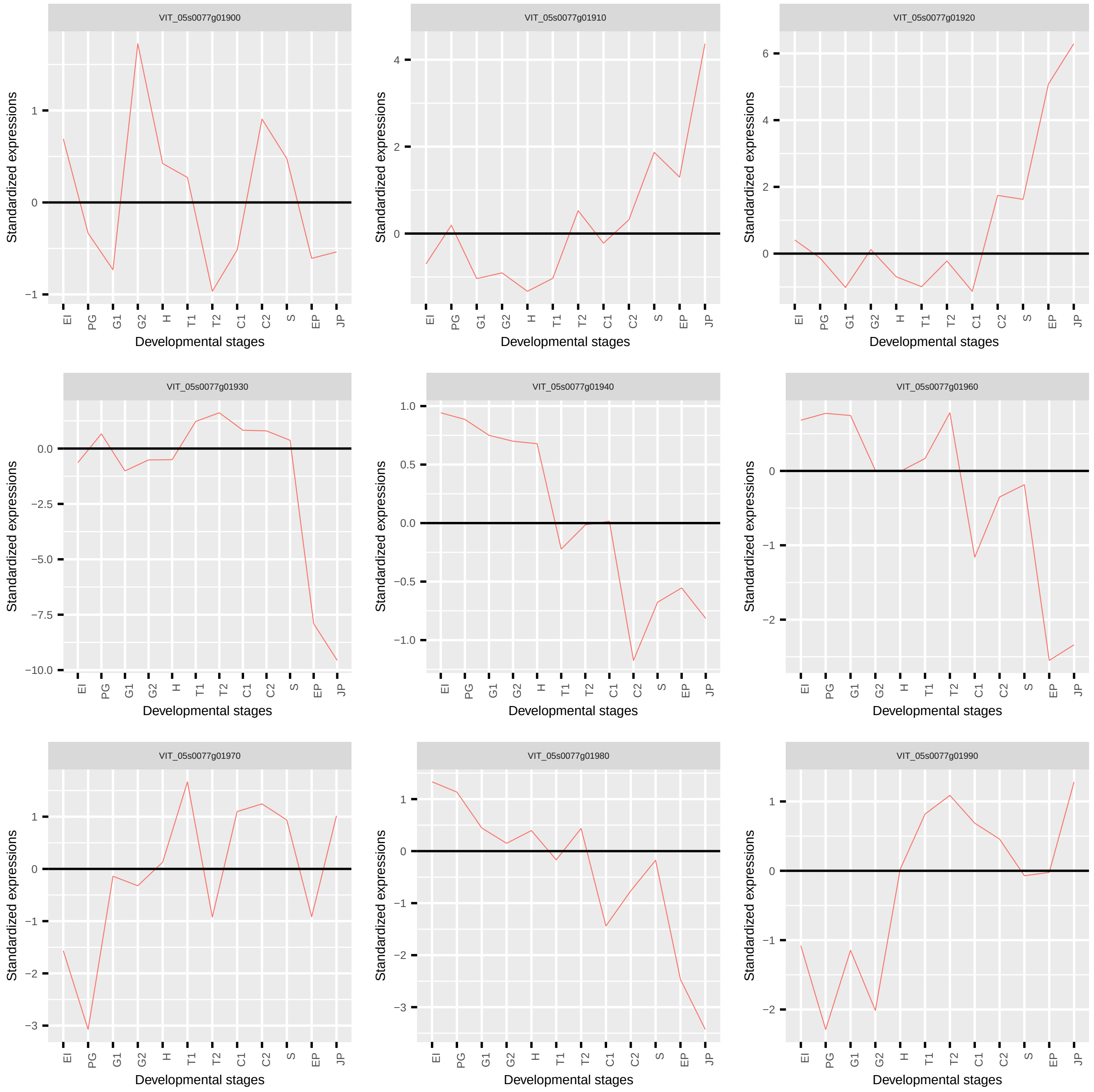
VIT_05s0077g01900
VIT_05s0077g01910
VIT_05s0077g01920
VIT_05s0077g01930
VIT_05s0077g01940
VIT_05s0077g01960
VIT_05s0077g01970
VIT_05s0077g01980
VIT_05s0077g01990
Standardized expressions
Developmental stages
EI PG G1 G2 H T1 T2 C1 C2 S EP JP

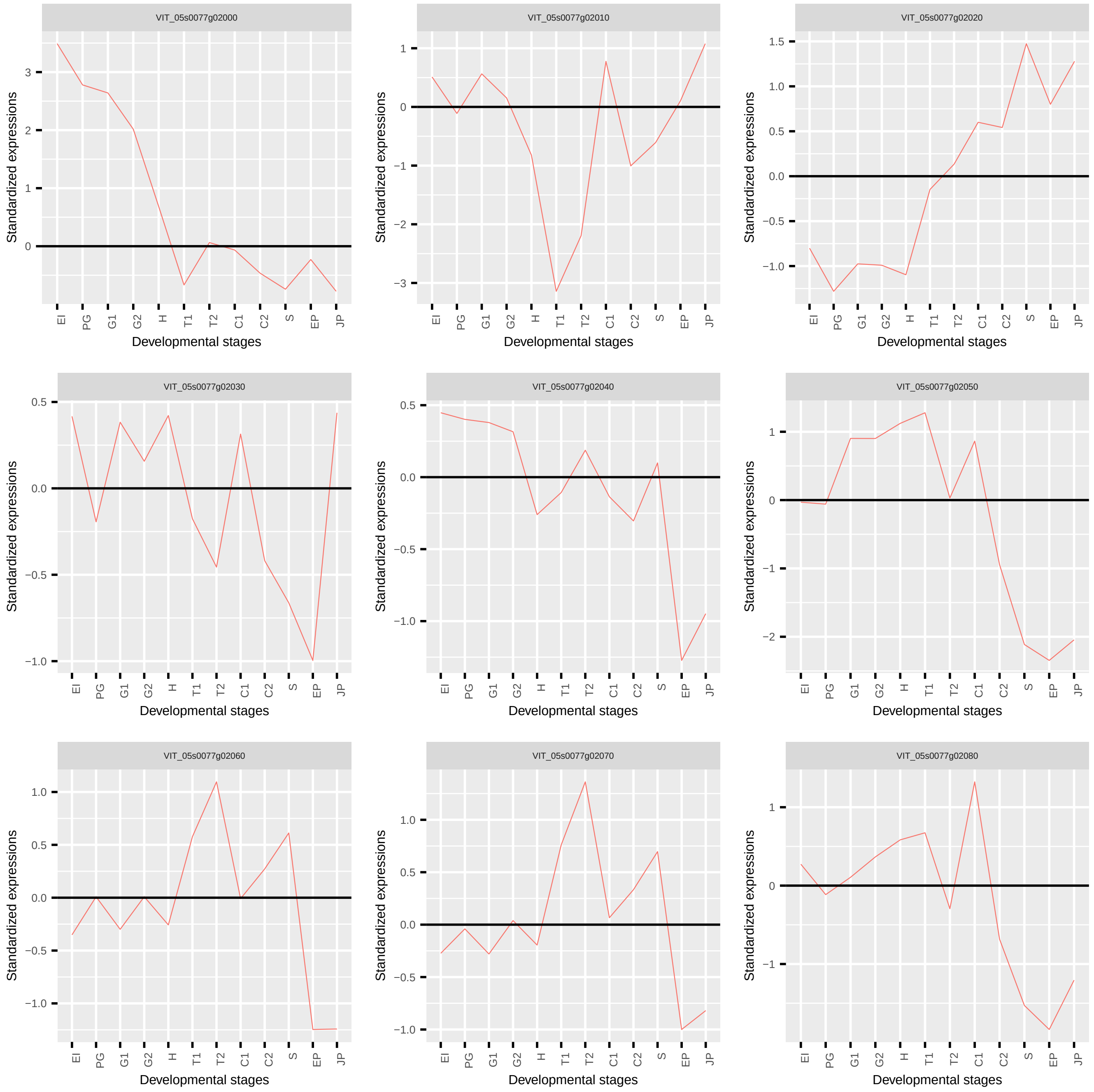
VIT_05s0077g02000
VIT_05s0077g02010
VIT_05s0077g02020
VIT_05s0077g02030
VIT_05s0077g02040
VIT_05s0077g02050
VIT_05s0077g02060
VIT_05s0077g02070
VIT_05s0077g02080
Standardized expressions
Developmental stages
EI PG G1 G2 H T1 T2 C1 C2 S EP JP

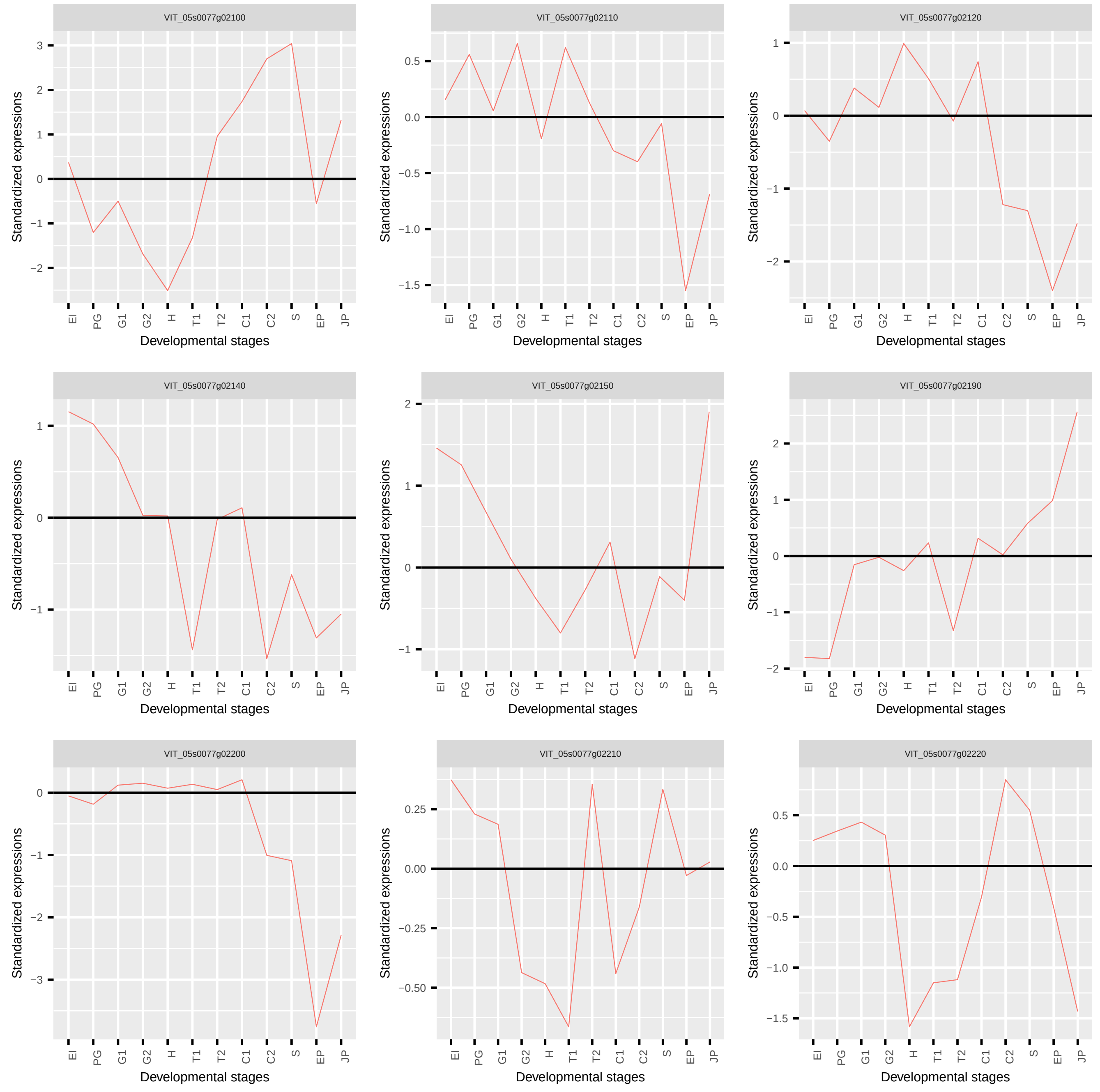
VIT_05s0077g02100
VIT_05s0077g02110
VIT_05s0077g02120
VIT_05s0077g02140
VIT_05s0077g02150
VIT_05s0077g02190
VIT_05s0077g02200
VIT_05s0077g02210
VIT_05s0077g02220
Standardized expressions
Developmental stages
EI PG G1 G2 H T1 T2 C1 C2 S EP JP

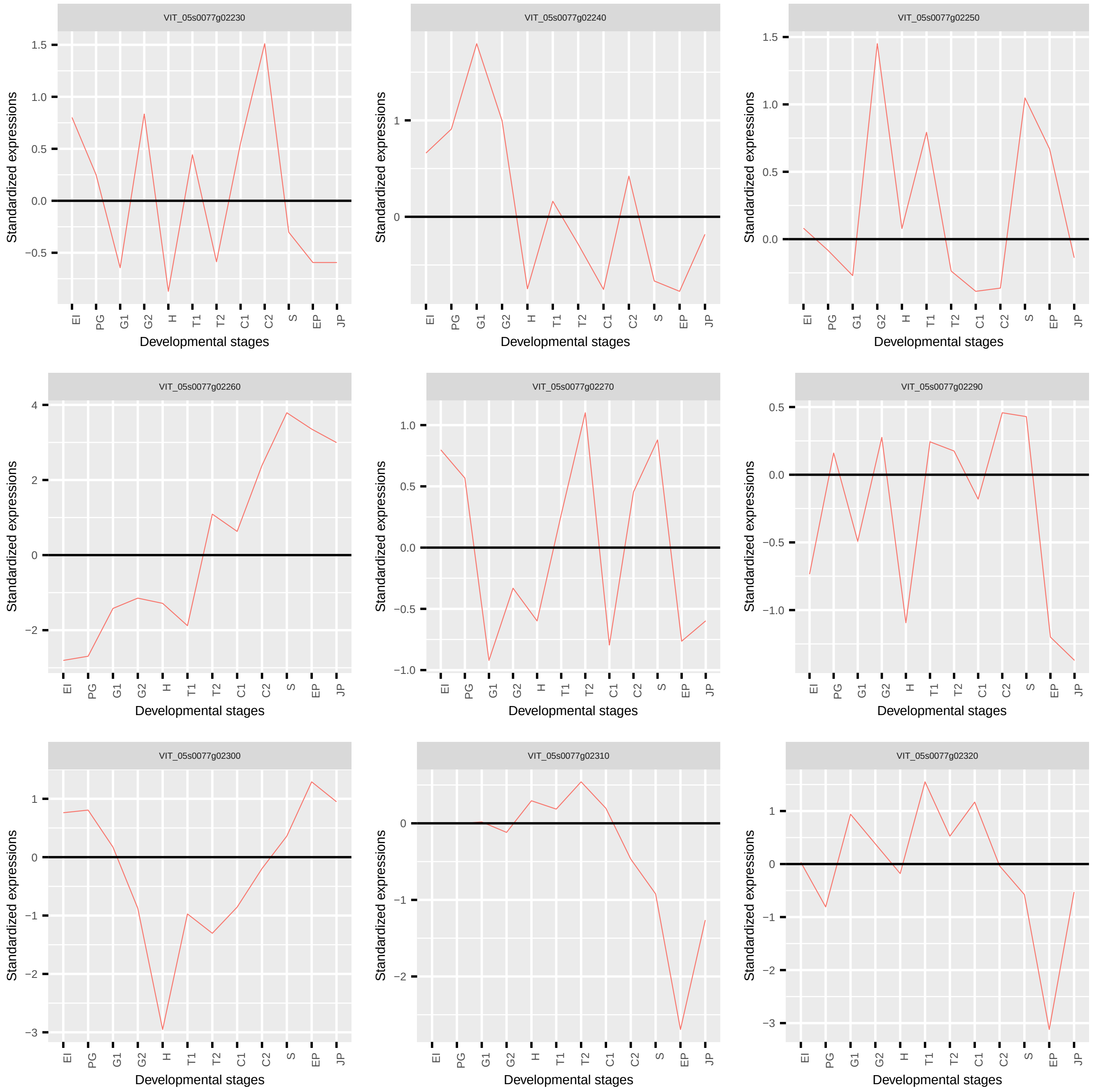

VIT_05s0077g02230
VIT_05s0077g02240
VIT_05s0077g02250
VIT_05s0077g02260
VIT_05s0077g02270
VIT_05s0077g02290
VIT_05s0077g02300
VIT_05s0077g02310
VIT_05s0077g02320
Standardized expressions
Developmental stages
EI PG G1 G2 H T1 T2 C1 C2 S EP JP

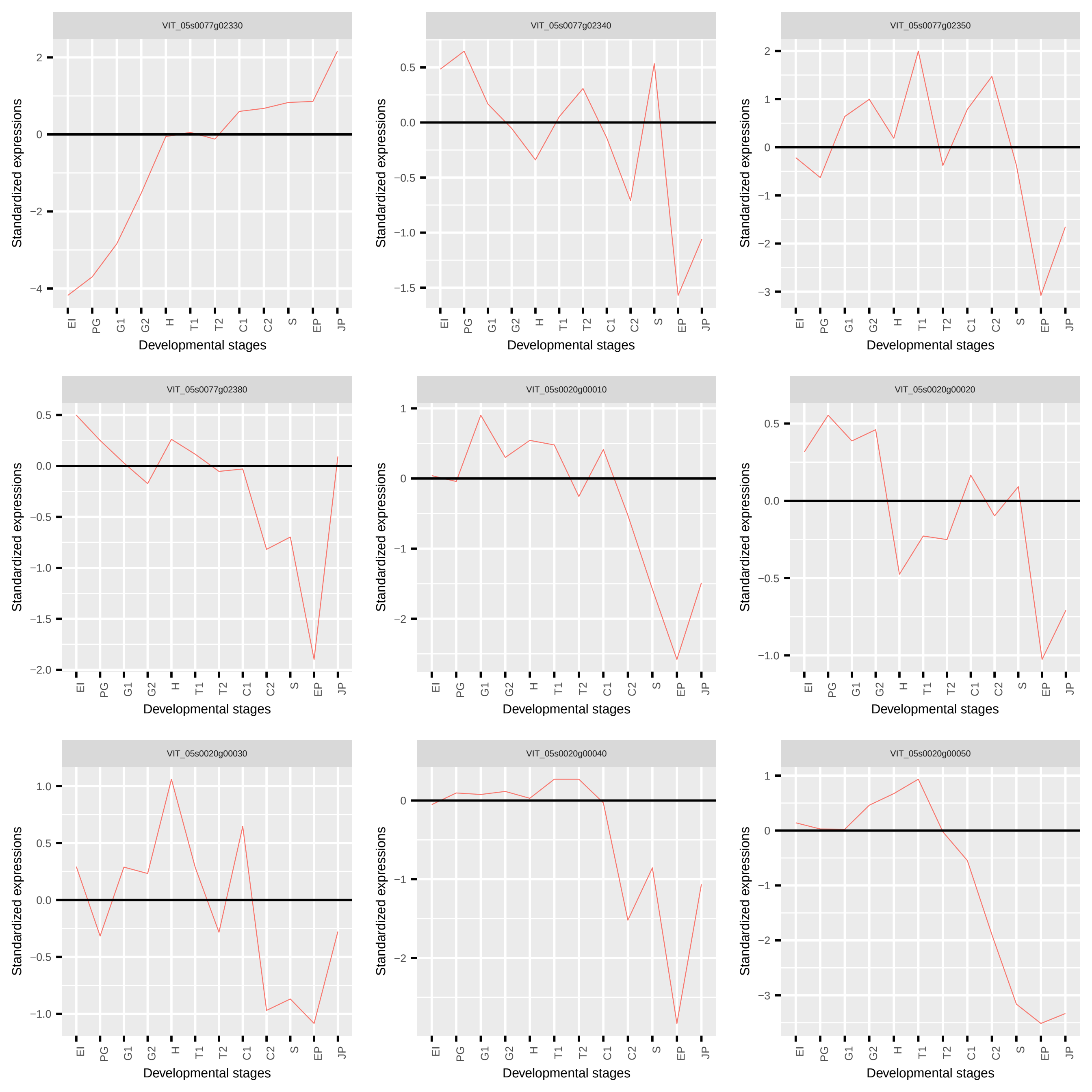
VIT_05s0077g02330
VIT_05s0077g02340
VIT_05s0077g02350
VIT_05s0077g02380
VIT_05s0020g00010
VIT_05s0020g00020
VIT_05s0020g00030
VIT_05s0020g00040
VIT_05s0020g00050
Standardized expressions
Developmental stages
EI PG G1 G2 H T1 T2 C1 C2 S EP JP

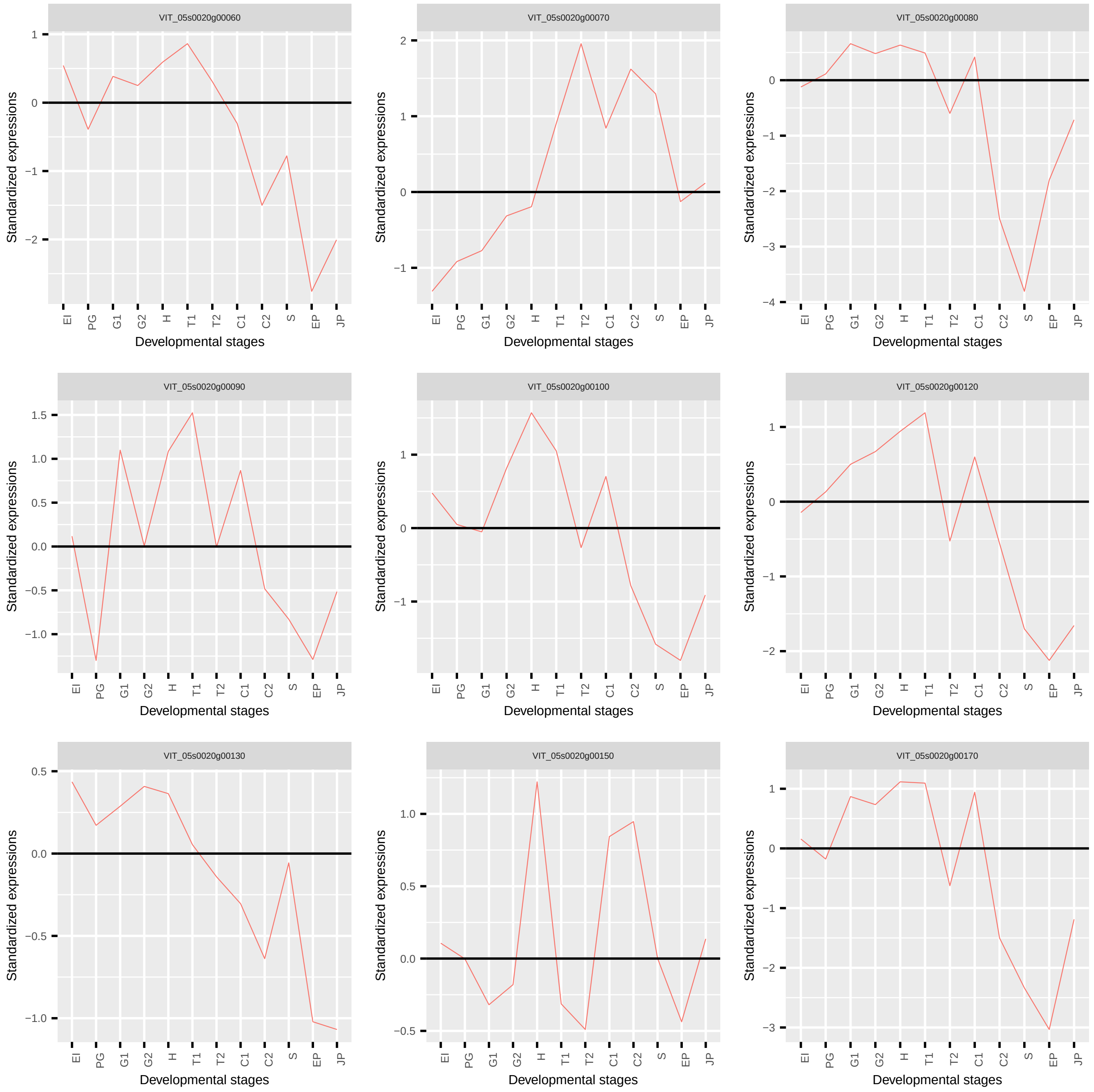
VIT_05s0020g00060
VIT_05s0020g00070
VIT_05s0020g00080
VIT_05s0020g00090
VIT_05s0020g00100
VIT_05s0020g00120
VIT_05s0020g00130
VIT_05s0020g00150
VIT_05s0020g00170
Standardized expressions
Developmental stages
EI PG G1 G2 H T1 T2 C1 C2 S EP JP

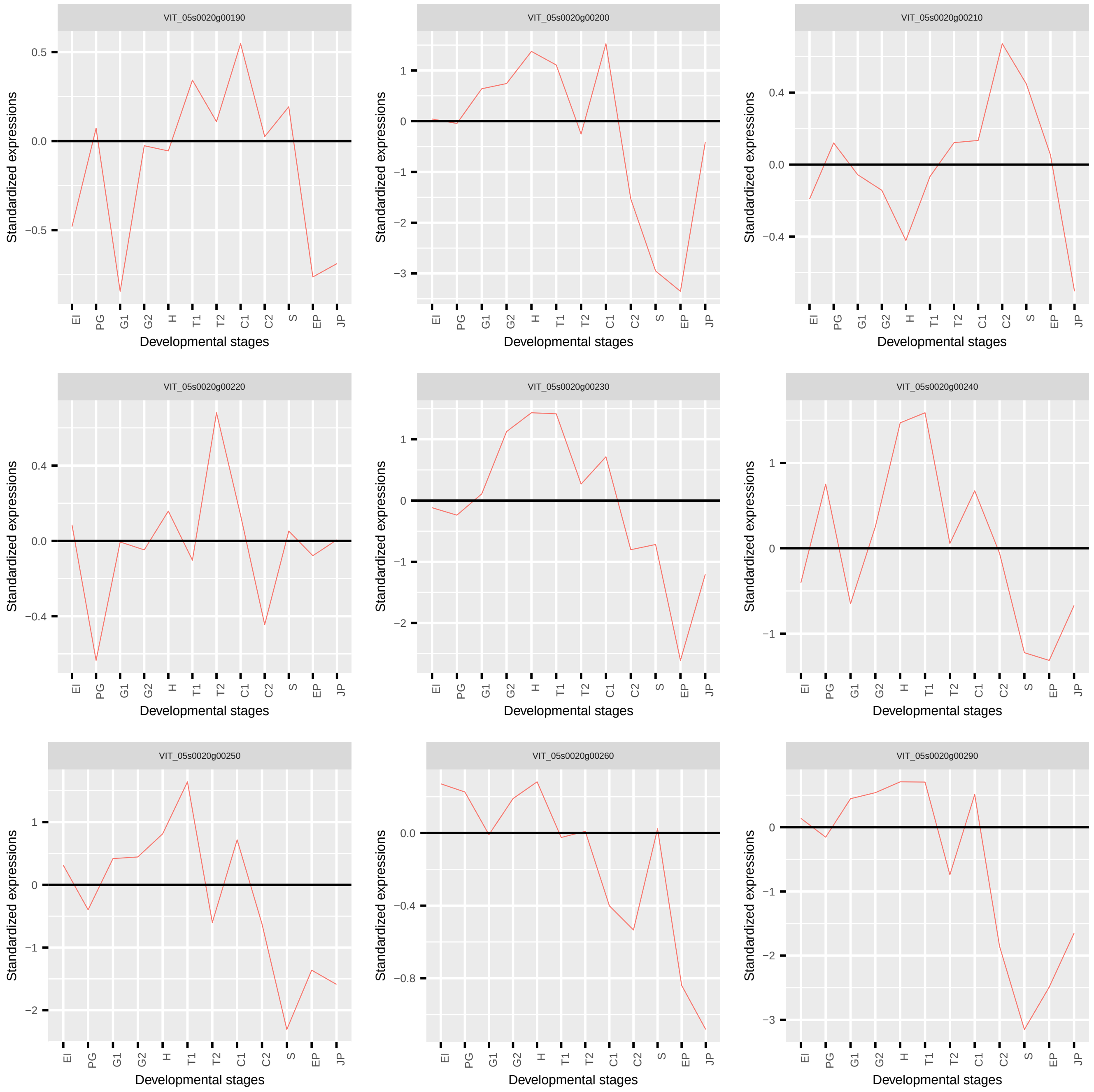
VIT_05s0020g00190
VIT_05s0020g00200
VIT_05s0020g00210
VIT_05s0020g00220
VIT_05s0020g00230
VIT_05s0020g00240
VIT_05s0020g00250
VIT_05s0020g00260
VIT_05s0020g00290
Standardized expressions
Developmental stages
EI PG G1 G2 H T1 T2 C1 C2 S EP JP

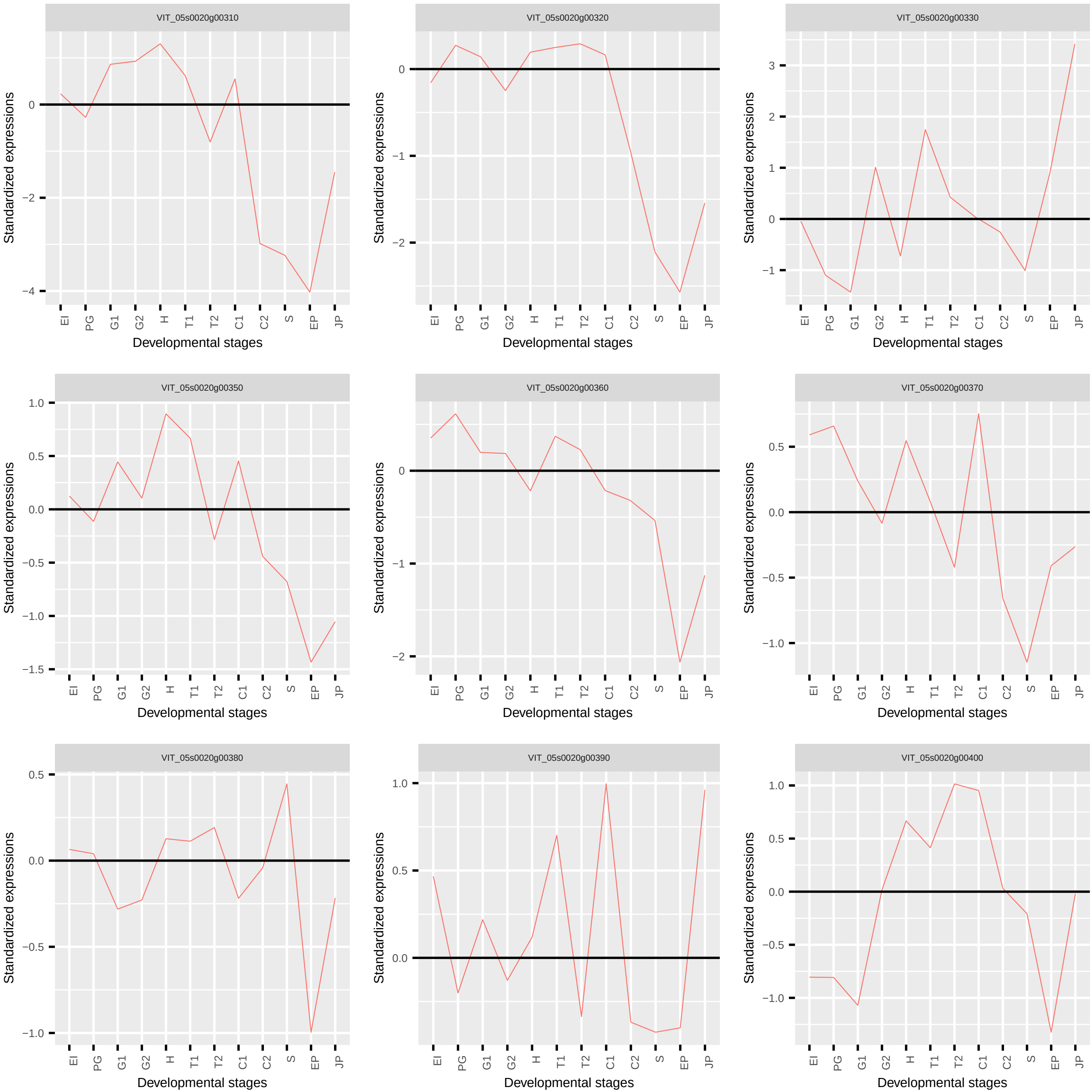
VIT_05s0020g00310
Standardized expressions
0
−2
−4
EI PG G1 G2 H T1 T2 C1 C2 S EP JP
Developmental stages
VIT_05s0020g00320
Standardized expressions
0
−1
−2
EI PG G1 G2 H T1 T2 C1 C2 S EP JP
Developmental stages
VIT_05s0020g00330
Standardized expressions
3
2
1
0
−1
EI PG G1 G2 H T1 T2 C1 C2 S EP JP
Developmental stages
VIT_05s0020g00350
Standardized expressions
1.0
0.5
0.0
−0.5
−1.0
−1.5
EI PG G1 G2 H T1 T2 C1 C2 S EP JP
Developmental stages
VIT_05s0020g00360
Standardized expressions
0
−1
−2
EI PG G1 G2 H T1 T2 C1 C2 S EP JP
Developmental stages
VIT_05s0020g00370
Standardized expressions
0.5
0.0
−0.5
−1.0
EI PG G1 G2 H T1 T2 C1 C2 S EP JP
Developmental stages
VIT_05s0020g00380
Standardized expressions
0.5
0.0
−0.5
−1.0
EI PG G1 G2 H T1 T2 C1 C2 S EP JP
Developmental stages
VIT_05s0020g00390
Standardized expressions
1.0
0.5
0.0
EI PG G1 G2 H T1 T2 C1 C2 S EP JP
Developmental stages
VIT_05s0020g00400
Standardized expressions
1.0
0.5
0.0
−0.5
−1.0
EI PG G1 G2 H T1 T2 C1 C2 S EP JP
Developmental stages

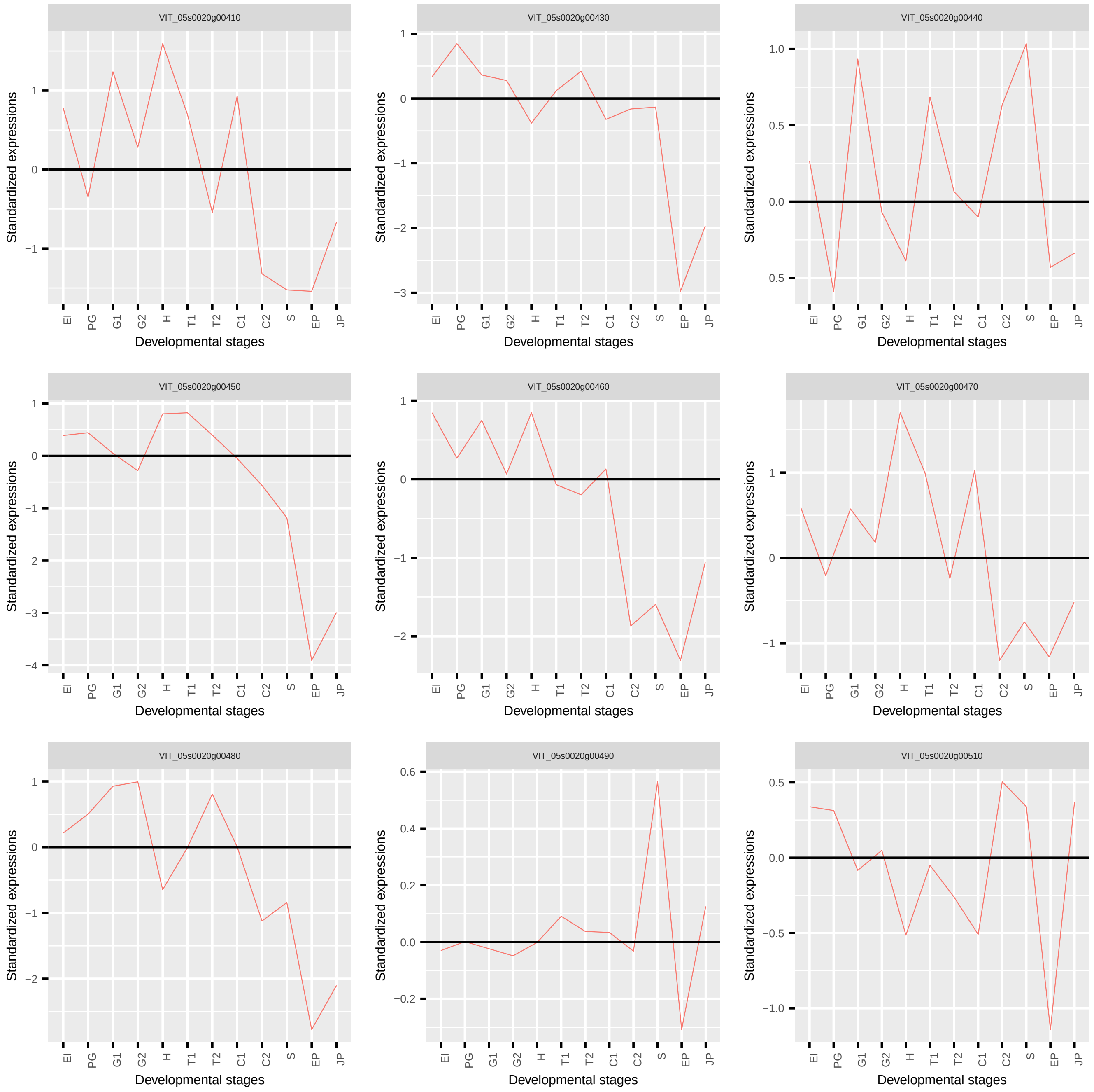
VIT_05s0020g00410
VIT_05s0020g00430
VIT_05s0020g00440
VIT_05s0020g00450
VIT_05s0020g00460
VIT_05s0020g00470
VIT_05s0020g00480
VIT_05s0020g00490
VIT_05s0020g00510
Standardized expressions
Developmental stages
EI
PG
G1
G2
H
T1
T2
C1
C2
S
EP
JP

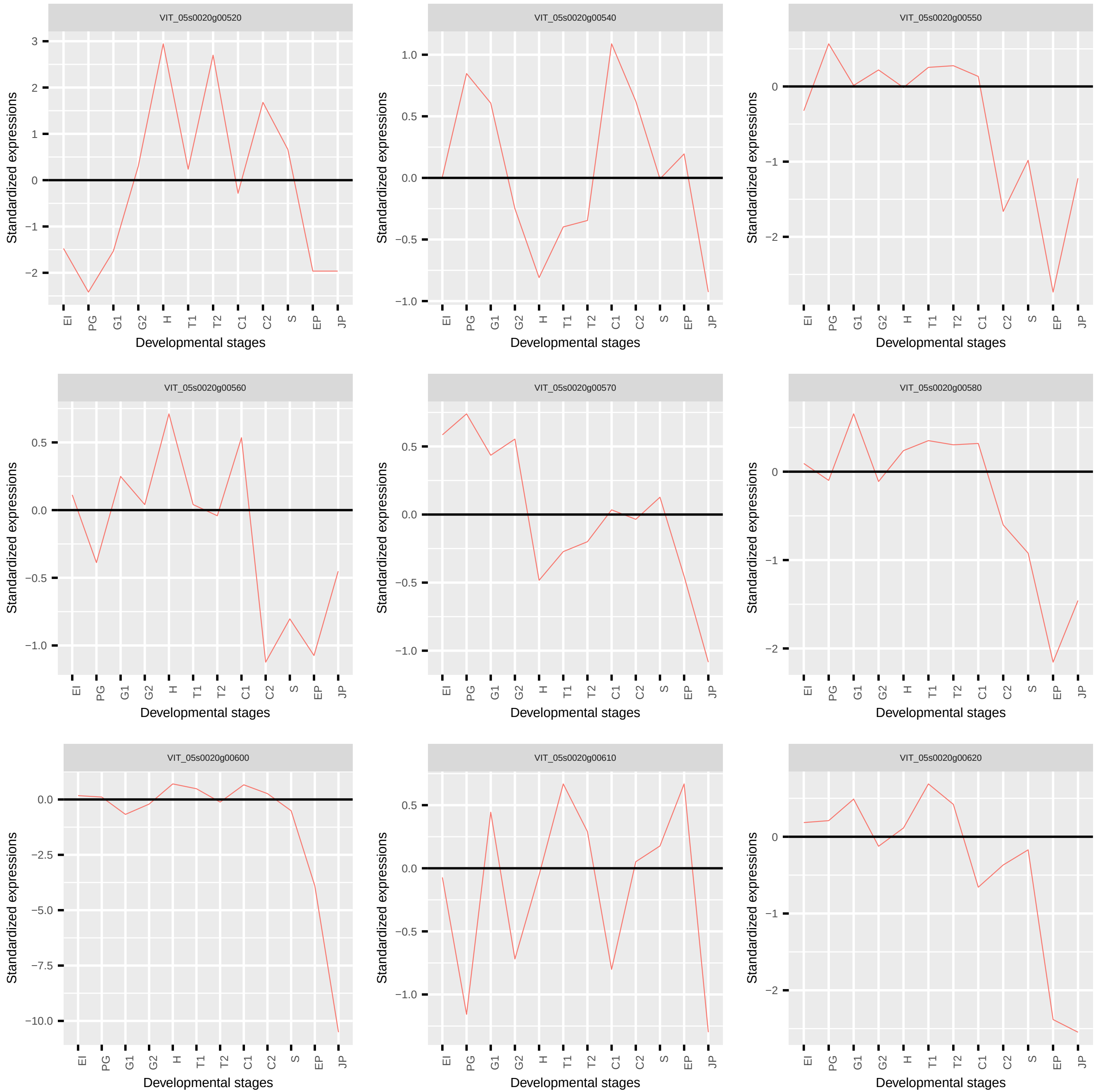
VIT_05s0020g00520
VIT_05s0020g00540
VIT_05s0020g00550
VIT_05s0020g00560
VIT_05s0020g00570
VIT_05s0020g00580
VIT_05s0020g00600
VIT_05s0020g00610
VIT_05s0020g00620
Standardized expressions
Developmental stages
EI
PG
G1
G2
H
T1
T2
C1
C2
S
EP
JP

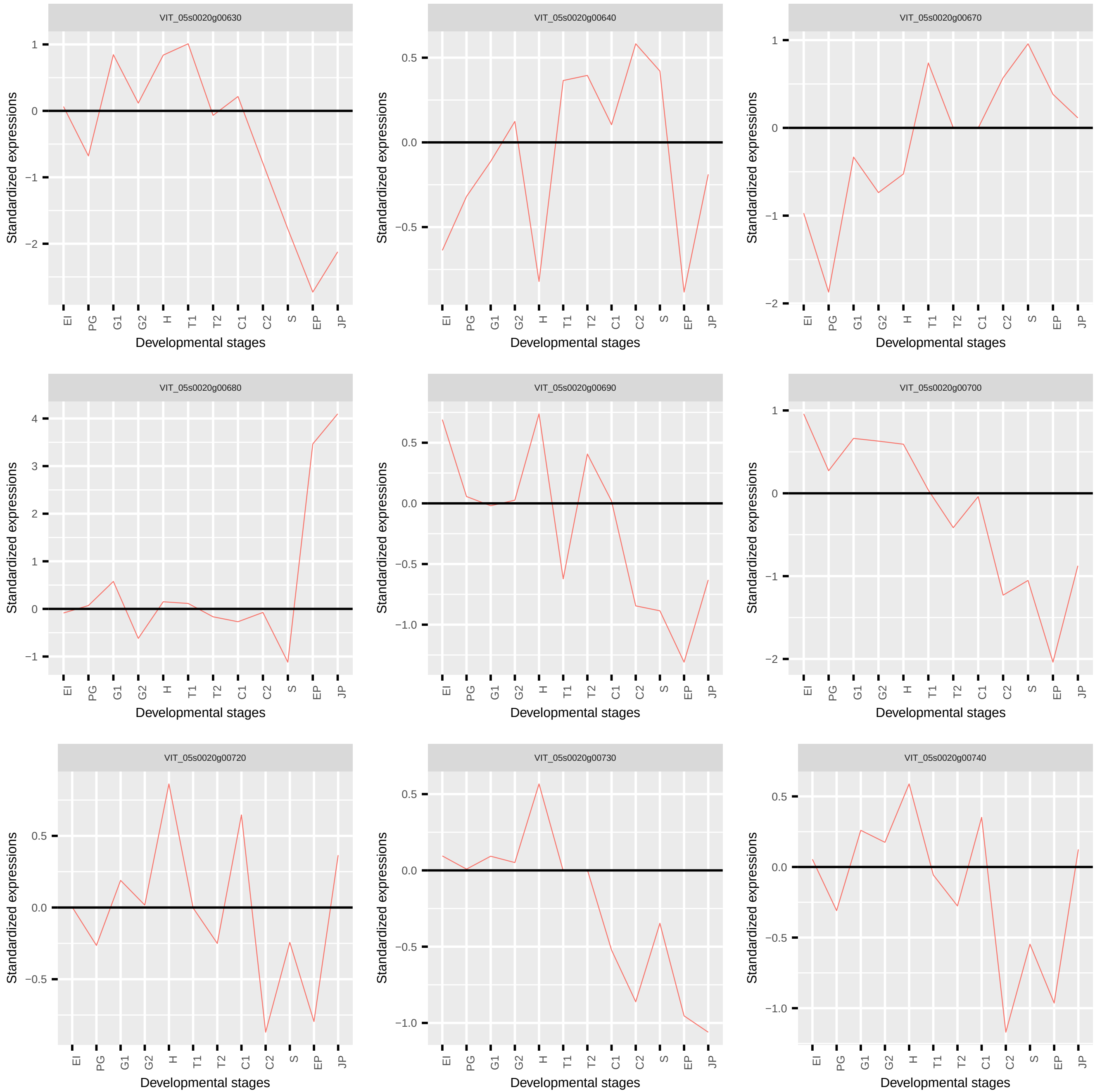
VIT_05s0020g00630
VIT_05s0020g00640
VIT_05s0020g00670
VIT_05s0020g00680
VIT_05s0020g00690
VIT_05s0020g00700
VIT_05s0020g00720
VIT_05s0020g00730
VIT_05s0020g00740
Standardized expressions
Developmental stages
EI PG G1 G2 H T1 T2 C1 C2 S EP JP

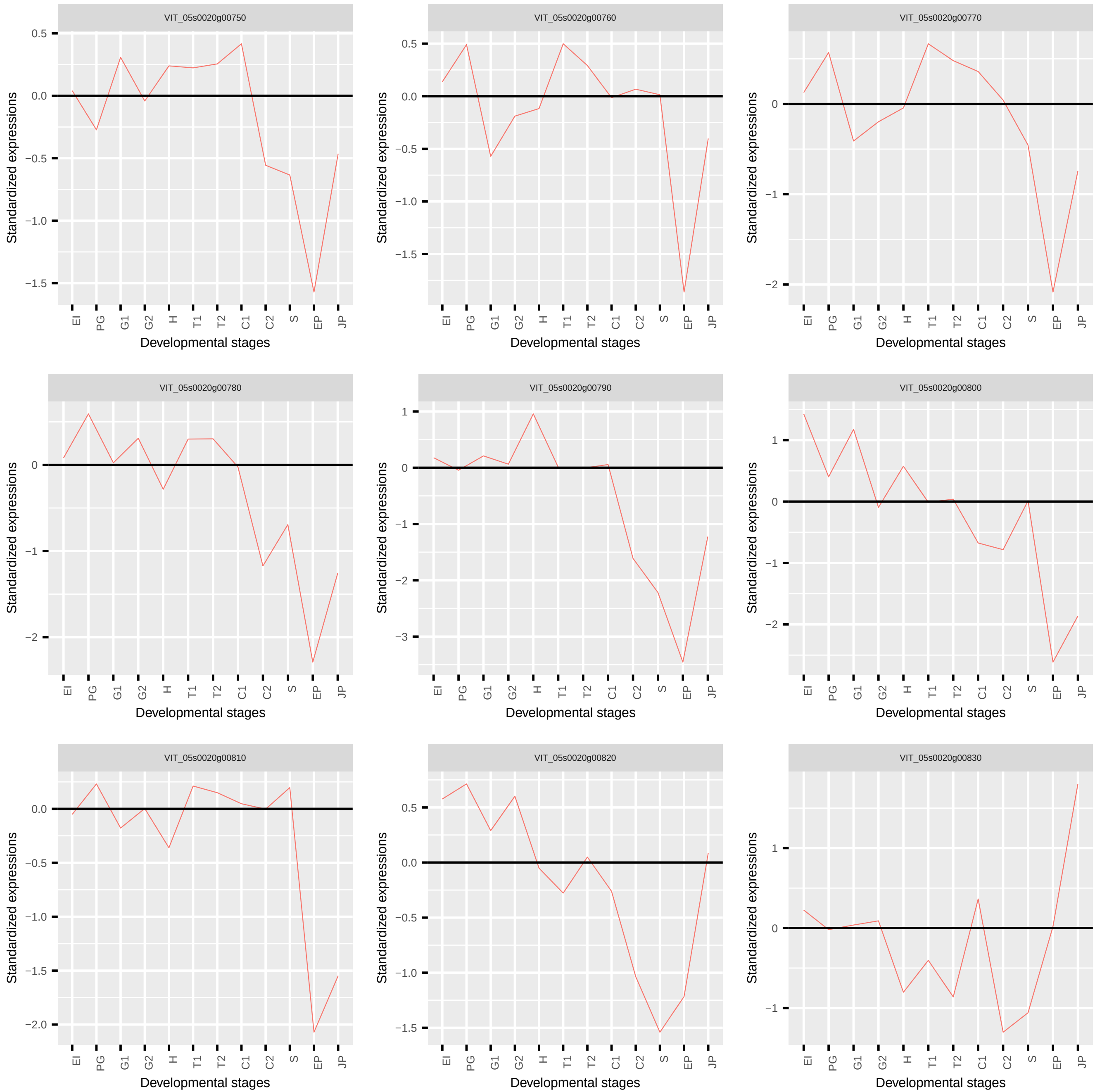
VIT_05s0020g00750
VIT_05s0020g00760
VIT_05s0020g00770
VIT_05s0020g00780
VIT_05s0020g00790
VIT_05s0020g00800
VIT_05s0020g00810
VIT_05s0020g00820
VIT_05s0020g00830
Standardized expressions
Developmental stages
EI PG G1 G2 H T1 T2 C1 C2 S EP JP

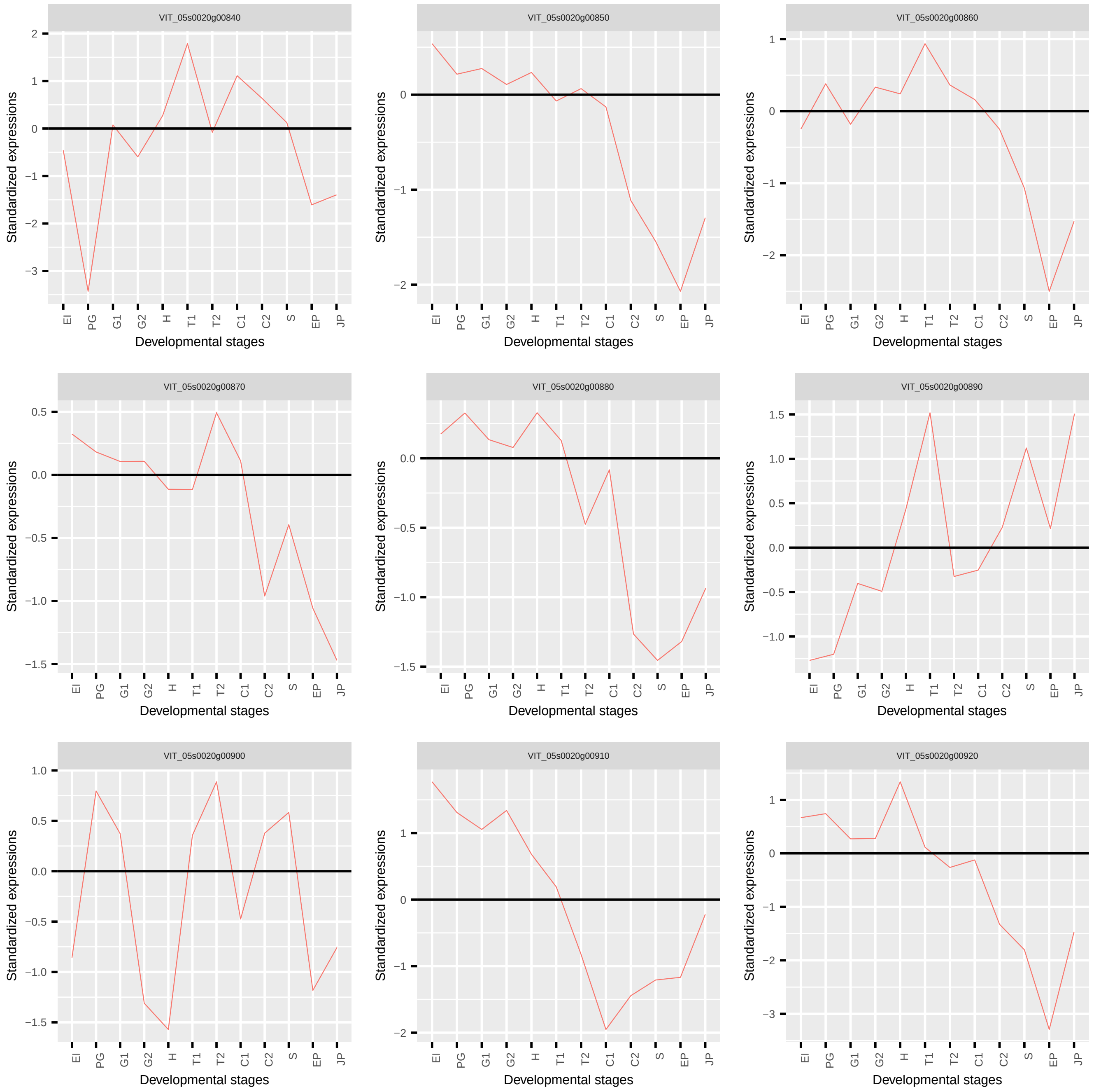
VIT_05s0020g00840
VIT_05s0020g00850
VIT_05s0020g00860
VIT_05s0020g00870
VIT_05s0020g00880
VIT_05s0020g00890
VIT_05s0020g00900
VIT_05s0020g00910
VIT_05s0020g00920
Standardized expressions
Developmental stages
EI PG G1 G2 H T1 T2 C1 C2 S EP JP

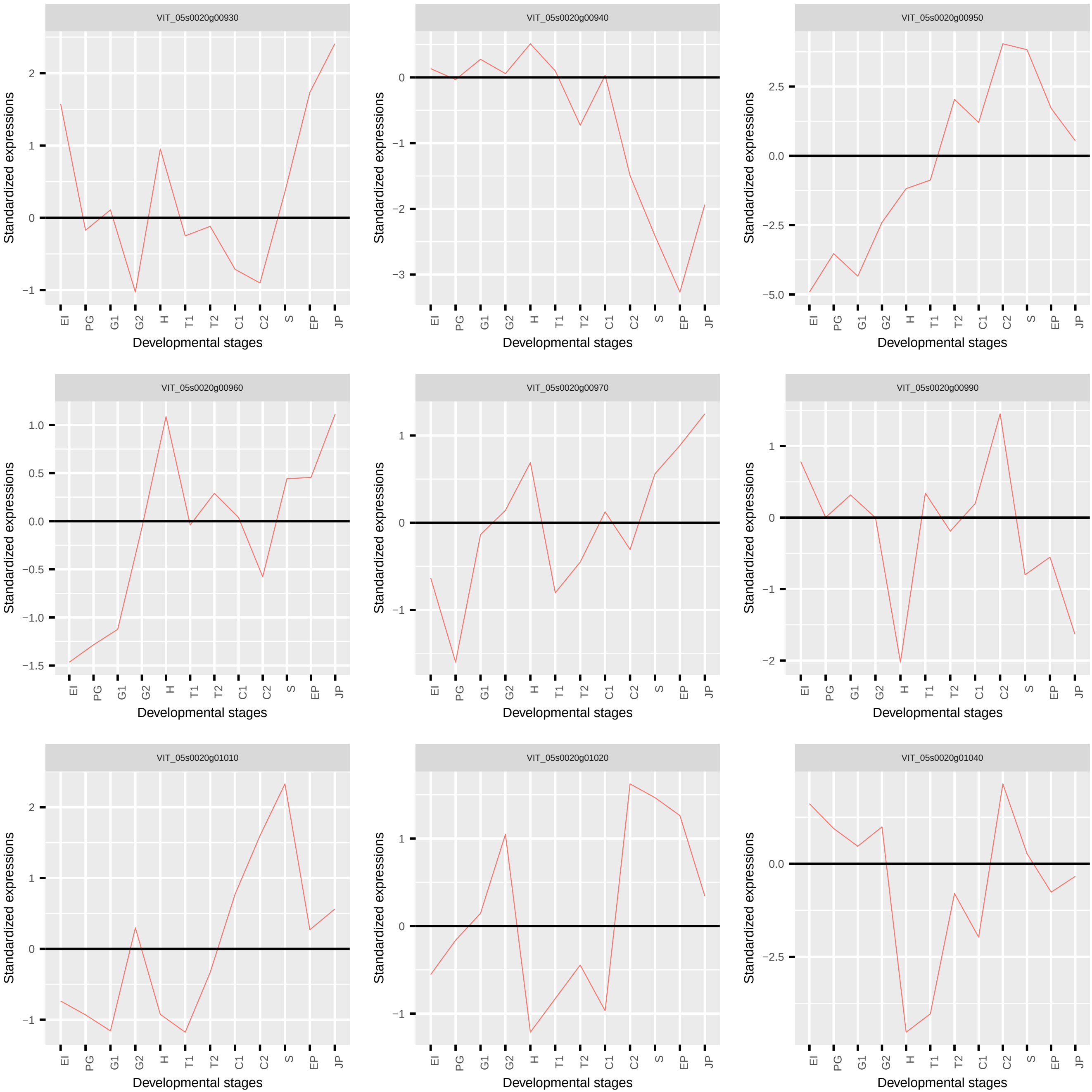
VIT_05s0020g00930
VIT_05s0020g00940
VIT_05s0020g00950
VIT_05s0020g00960
VIT_05s0020g00970
VIT_05s0020g00990
VIT_05s0020g01010
VIT_05s0020g01020
VIT_05s0020g01040
Standardized expressions
Developmental stages
EI PG G1 G2 H T1 T2 C1 C2 S EP JP

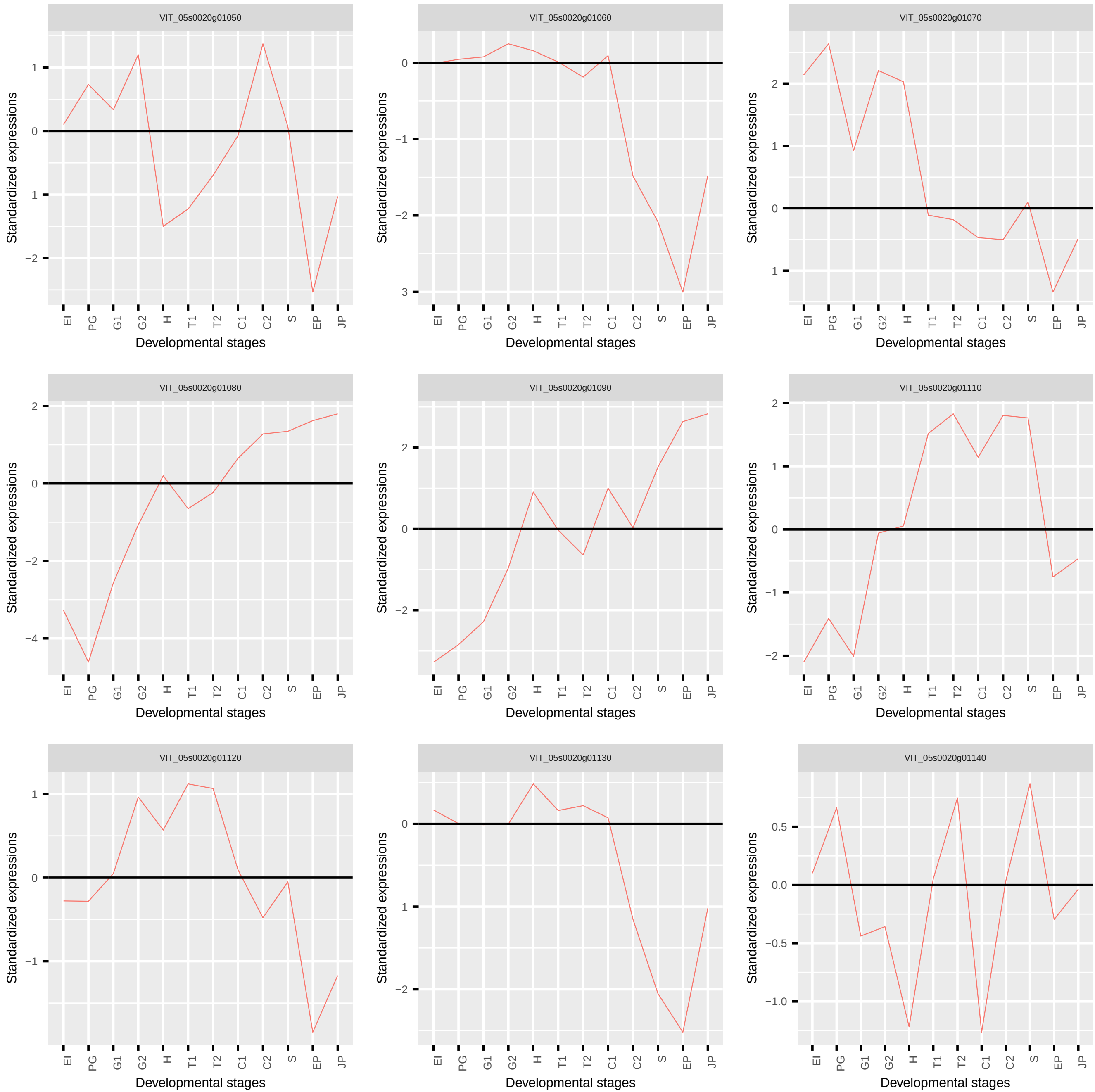
VIT_05s0020g01050
VIT_05s0020g01060
VIT_05s0020g01070
VIT_05s0020g01080
VIT_05s0020g01090
VIT_05s0020g01110
VIT_05s0020g01120
VIT_05s0020g01130
VIT_05s0020g01140
Standardized expressions
Developmental stages
EI PG G1 G2 H T1 T2 C1 C2 S EP JP

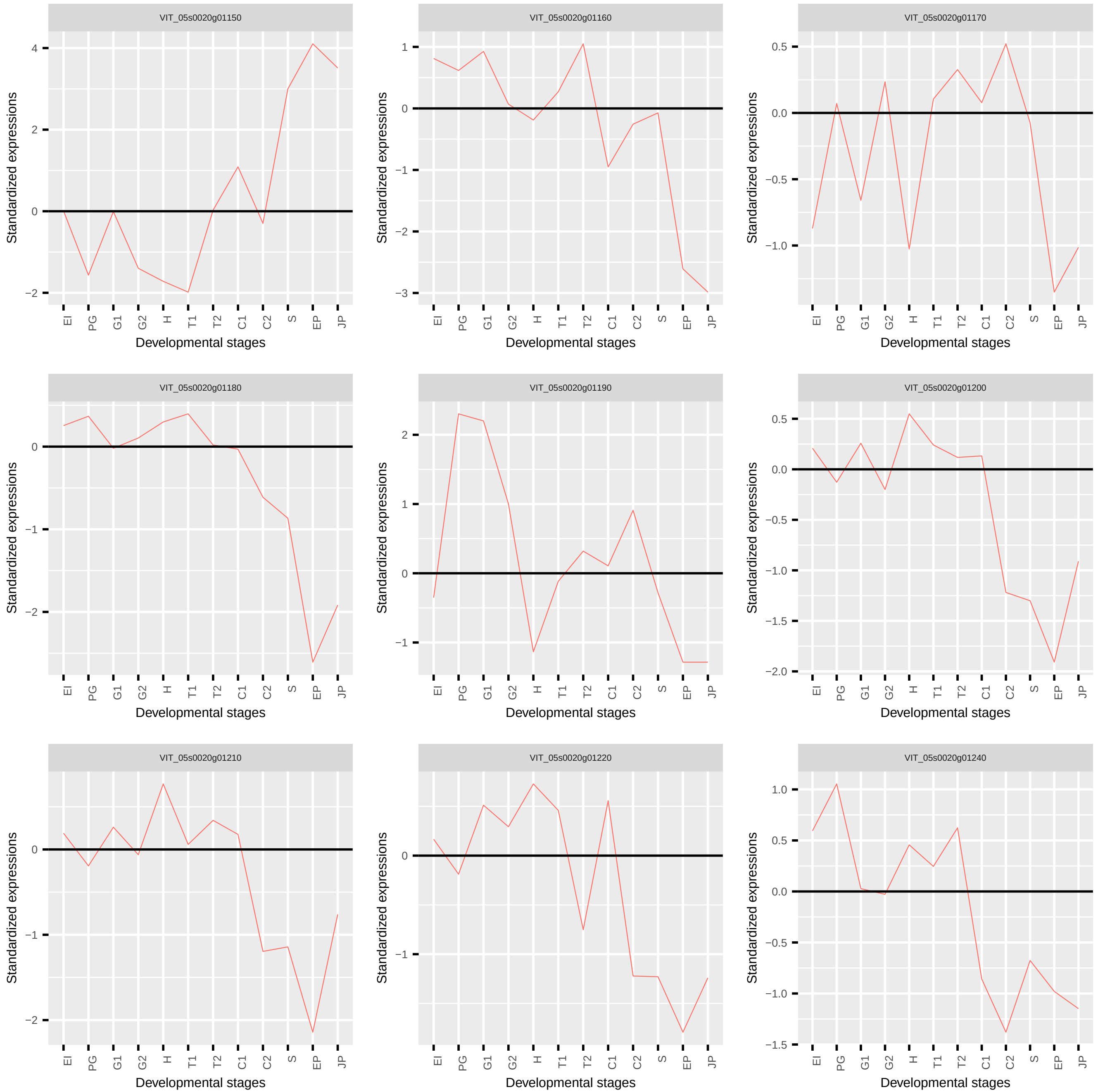
VIT_05s0020g01150
VIT_05s0020g01160
VIT_05s0020g01170
VIT_05s0020g01180
VIT_05s0020g01190
VIT_05s0020g01200
VIT_05s0020g01210
VIT_05s0020g01220
VIT_05s0020g01240
Standardized expressions
Developmental stages
EI PG G1 G2 H T1 T2 C1 C2 S EP JP

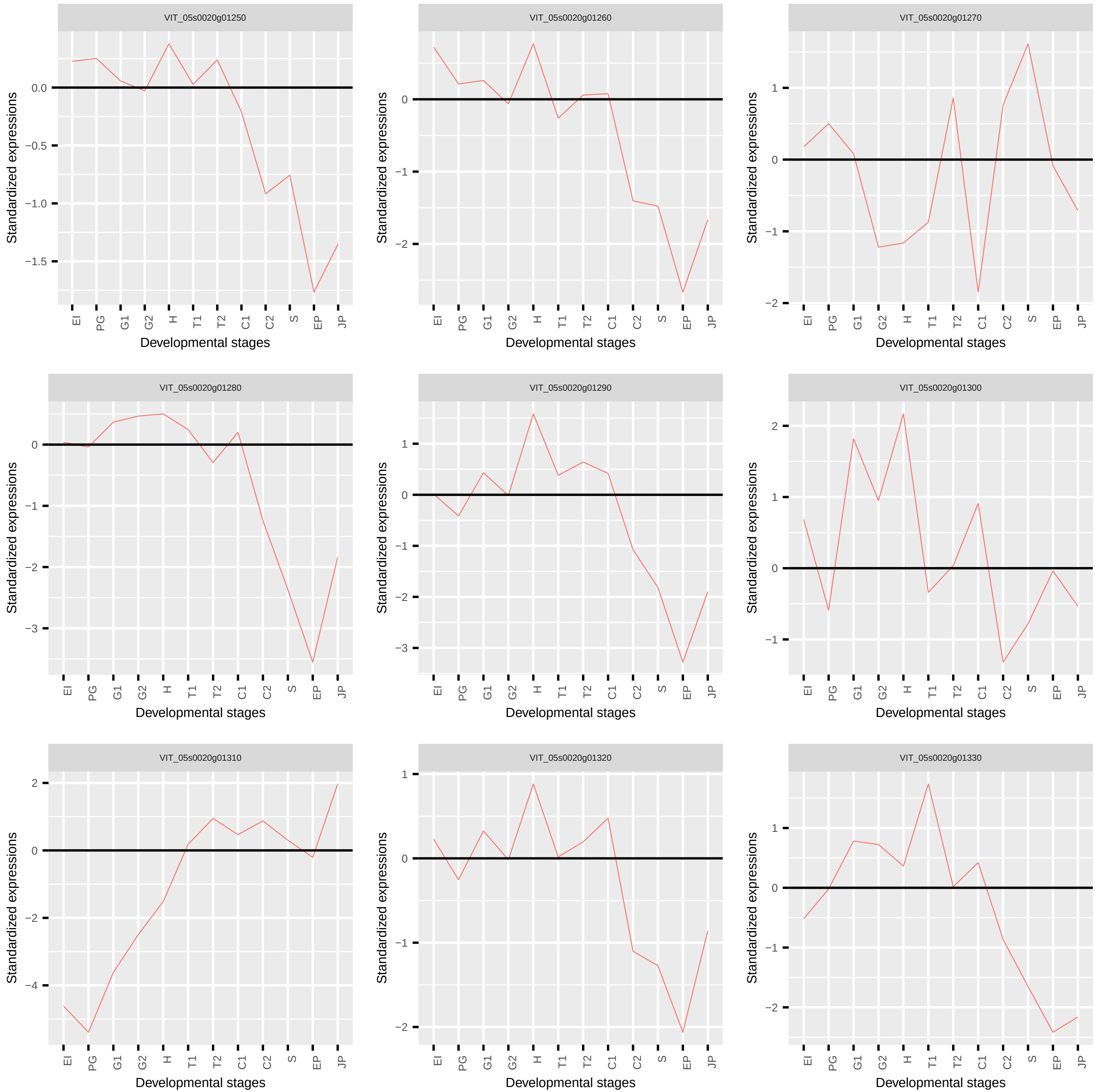

VIT_05s0020g01250
0.0
−0.5
−1.0
−1.5
VIT_05s0020g01260
0
−1
−2
VIT_05s0020g01270
1
0
−1
−2
VIT_05s0020g01280
0
−1
−2
−3
VIT_05s0020g01290
1
0
−1
−2
−3
VIT_05s0020g01300
2
1
0
−1
VIT_05s0020g01310
2
0
−2
−4
VIT_05s0020g01320
1
0
−1
−2
VIT_05s0020g01330
1
0
−1
−2
Standardized expressions
EI
PG
G1
G2
H
T1
T2
C1
C2
S
EP
JP
Developmental stages

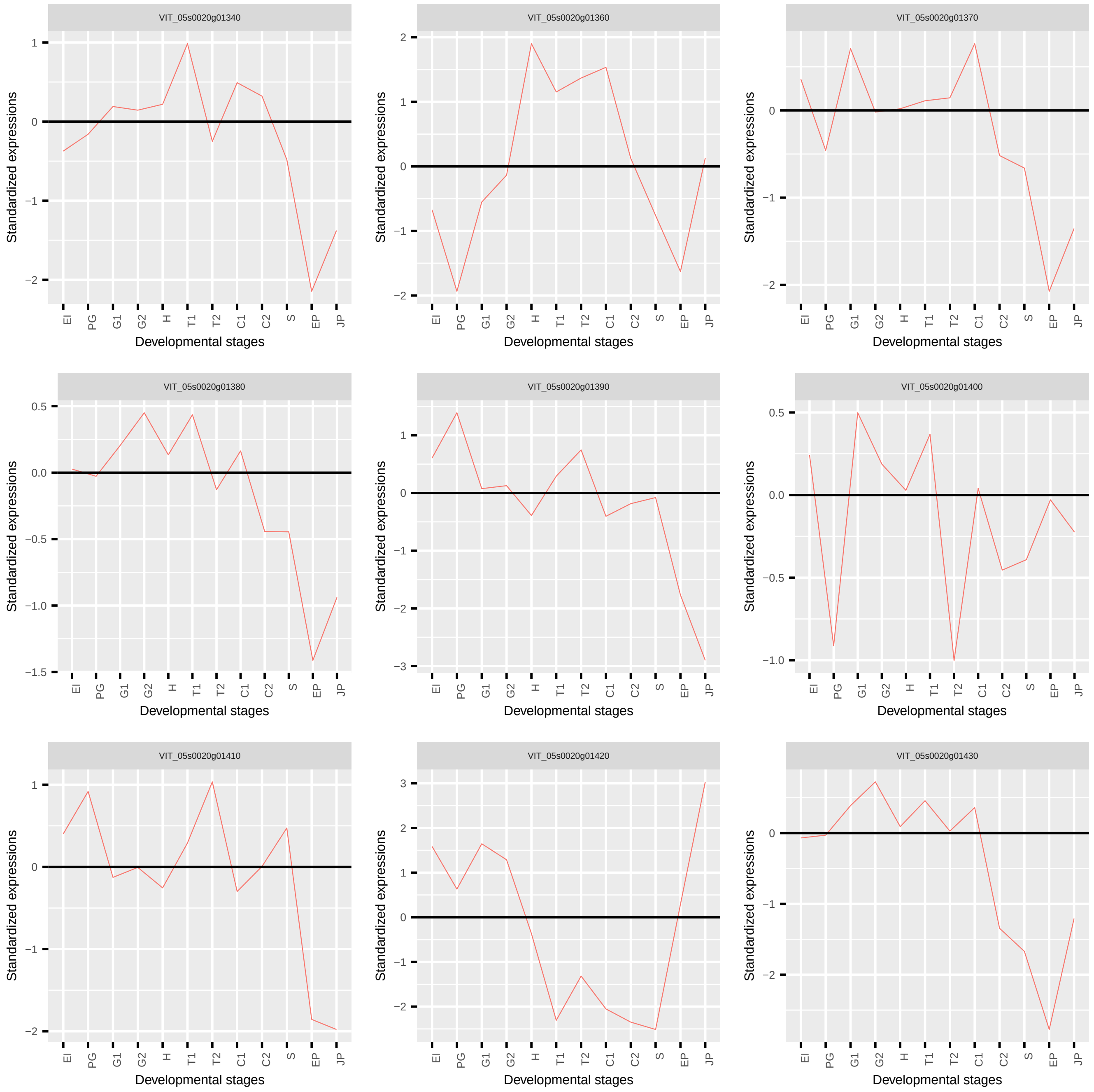

VIT_05s0020g01340
VIT_05s0020g01360
VIT_05s0020g01370
VIT_05s0020g01380
VIT_05s0020g01390
VIT_05s0020g01400
VIT_05s0020g01410
VIT_05s0020g01420
VIT_05s0020g01430
Standardized expressions
Developmental stages
EI
PG
G1
G2
H
T1
T2
C1
C2
S
EP
JP

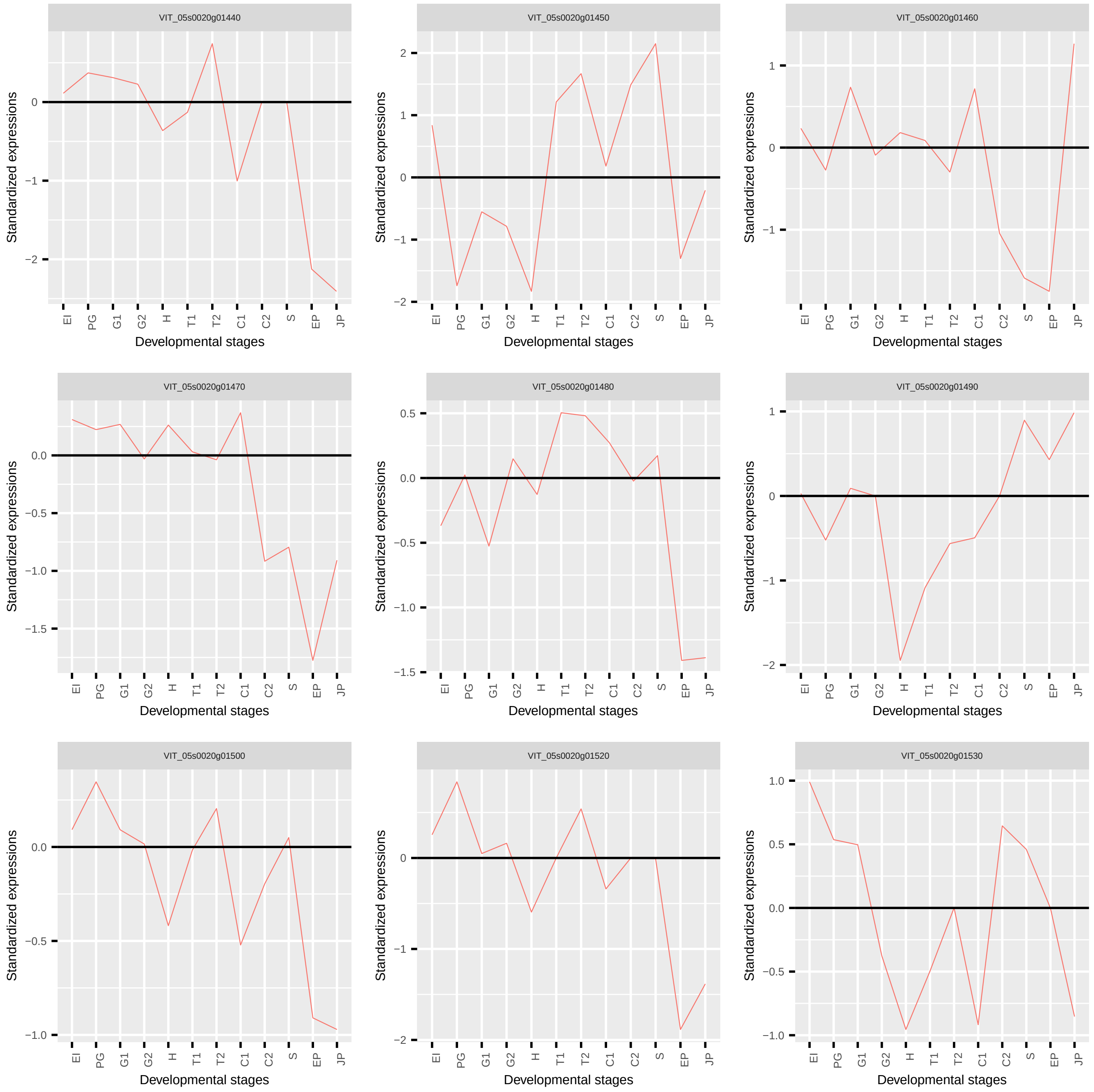
VIT_05s0020g01440
VIT_05s0020g01450
VIT_05s0020g01460
VIT_05s0020g01470
VIT_05s0020g01480
VIT_05s0020g01490
VIT_05s0020g01500
VIT_05s0020g01520
VIT_05s0020g01530
Standardized expressions
Developmental stages
EI PG G1 G2 H T1 T2 C1 C2 S EP JP

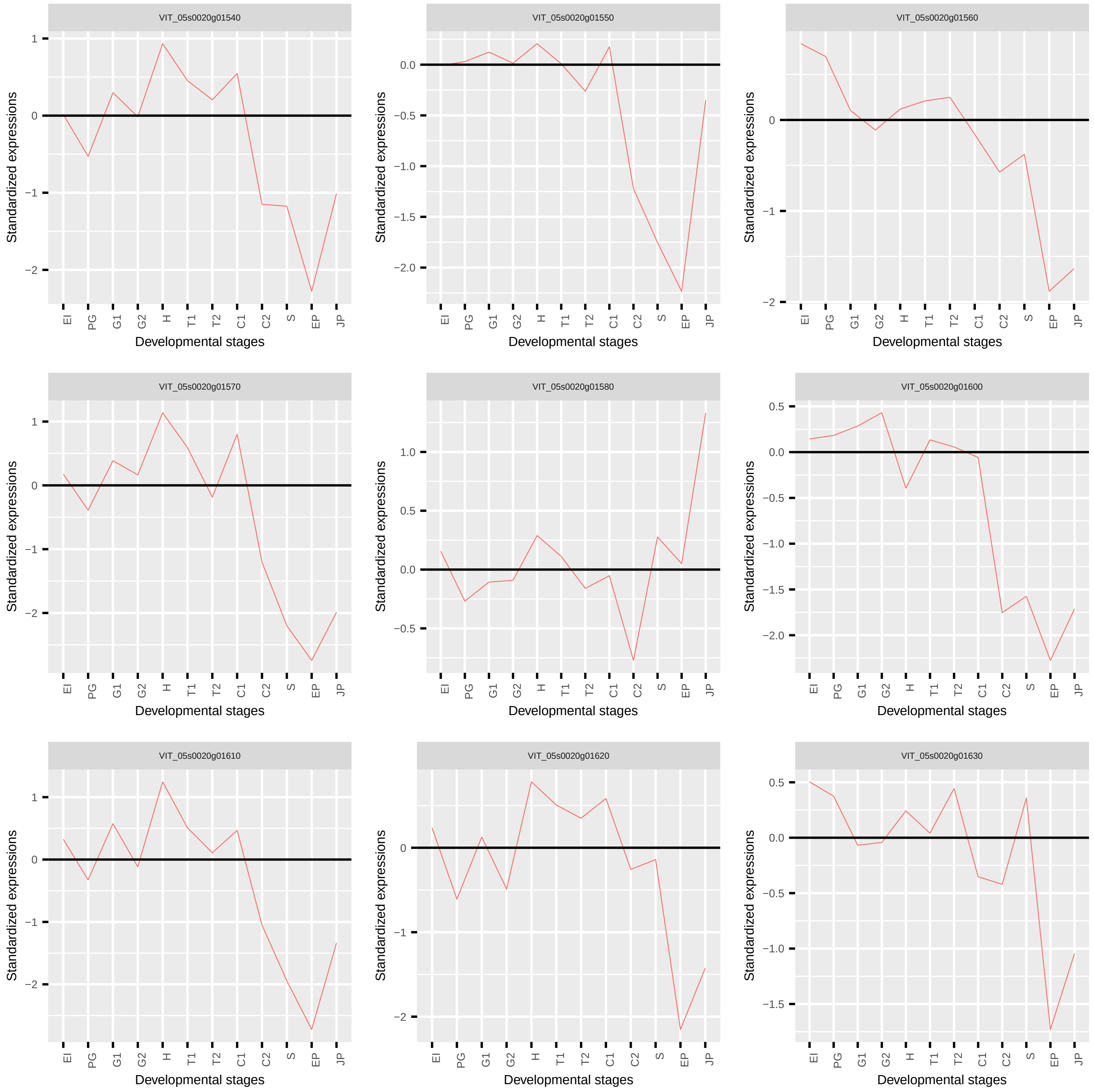
VIT_05s0020g01540
VIT_05s0020g01550
VIT_05s0020g01560
VIT_05s0020g01570
VIT_05s0020g01580
VIT_05s0020g01600
VIT_05s0020g01610
VIT_05s0020g01620
VIT_05s0020g01630
Standardized expressions
Developmental stages
EI PG G1 G2 H T1 T2 C1 C2 S EP JP

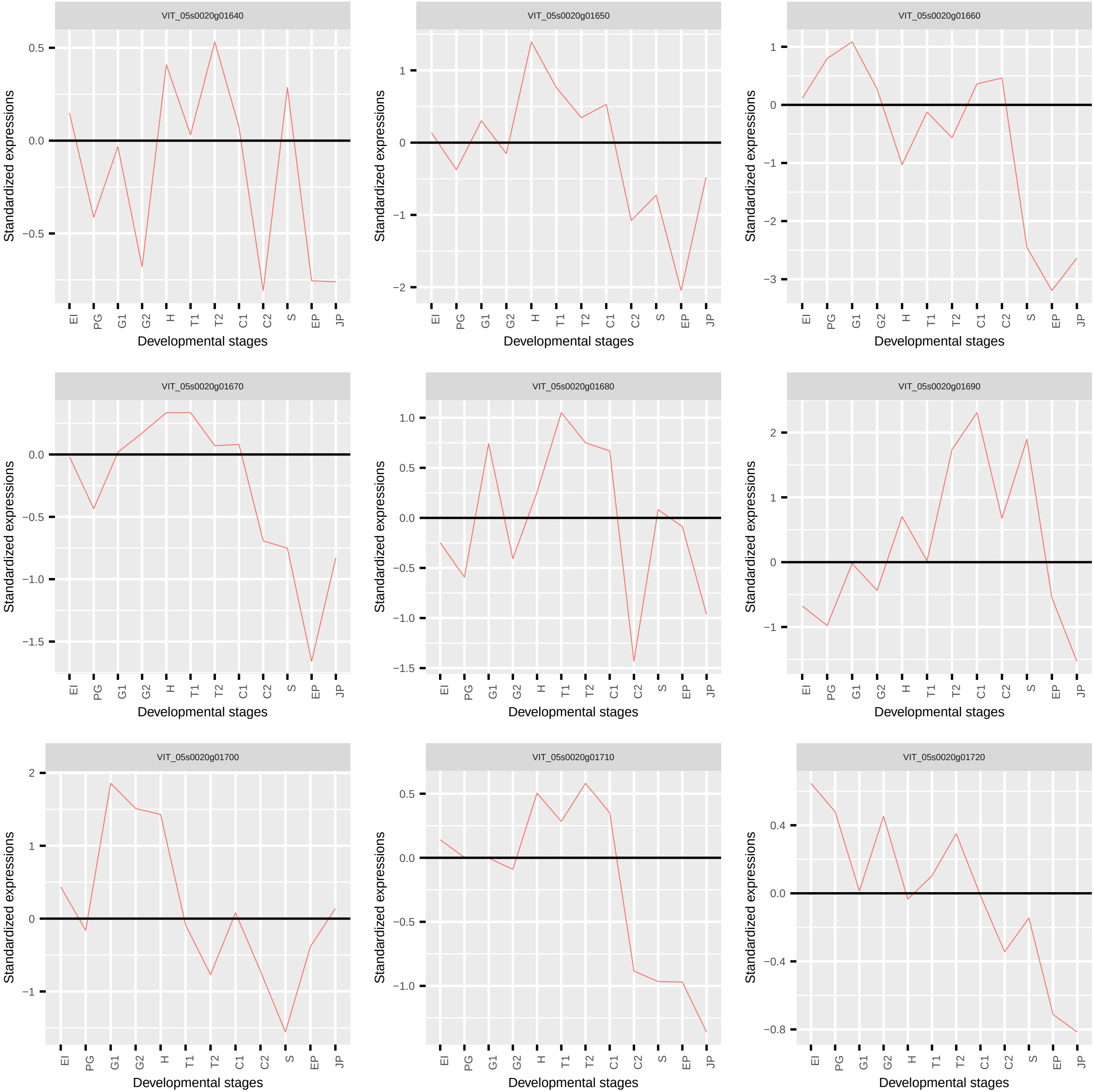
VIT_05s0020g01640
VIT_05s0020g01650
VIT_05s0020g01660
VIT_05s0020g01670
VIT_05s0020g01680
VIT_05s0020g01690
VIT_05s0020g01700
VIT_05s0020g01710
VIT_05s0020g01720
Standardized expressions
Developmental stages
EI PG G1 G2 H T1 T2 C1 C2 S EP JP
0.5
0.0
−0.5
1
0
−1
−2
−3
−1.0
−1.5
1.0
2
0.4
−0.4
−0.8

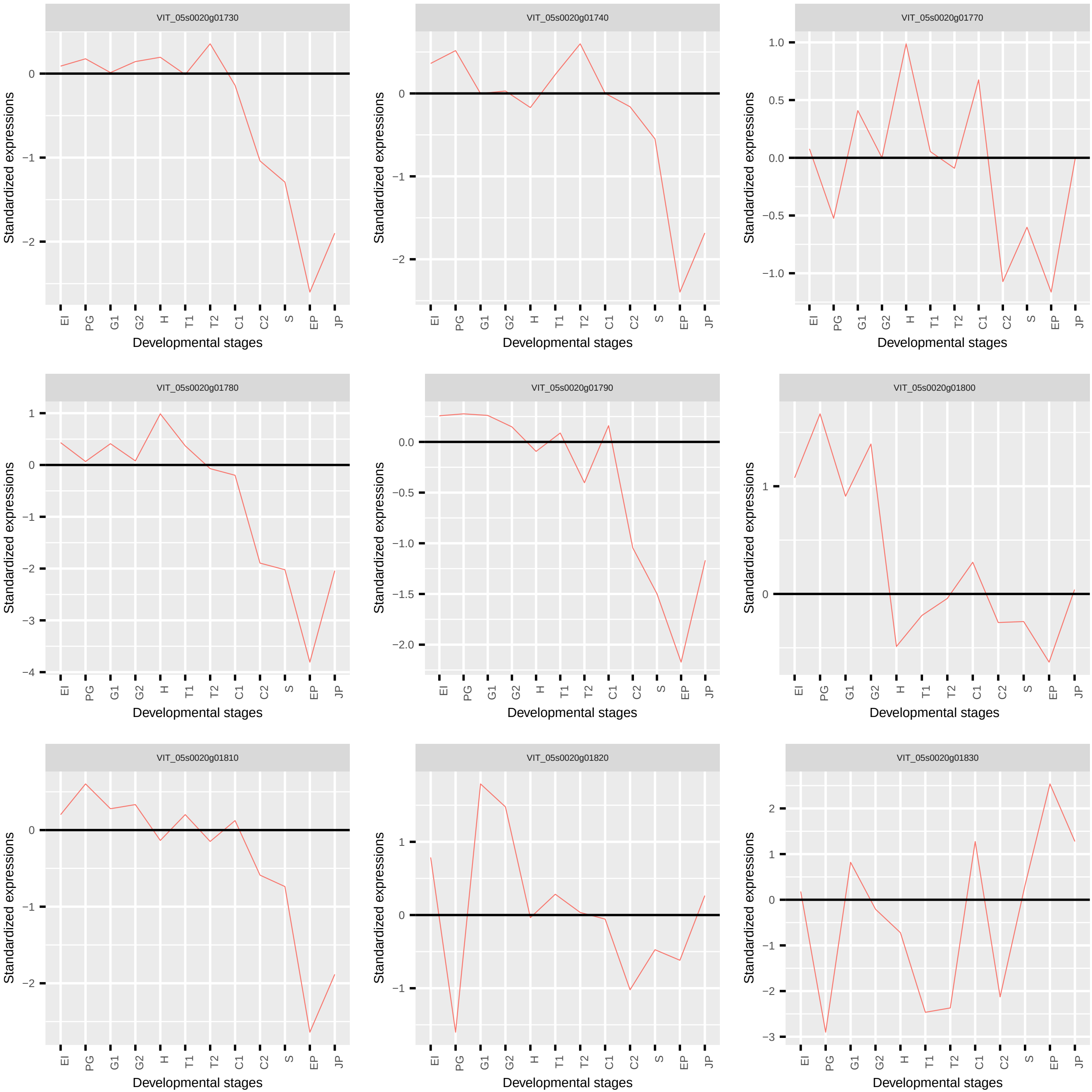
VIT_05s0020g01730
VIT_05s0020g01740
VIT_05s0020g01770
VIT_05s0020g01780
VIT_05s0020g01790
VIT_05s0020g01800
VIT_05s0020g01810
VIT_05s0020g01820
VIT_05s0020g01830
Standardized expressions
Developmental stages
EI PG G1 G2 H T1 T2 C1 C2 S EP JP

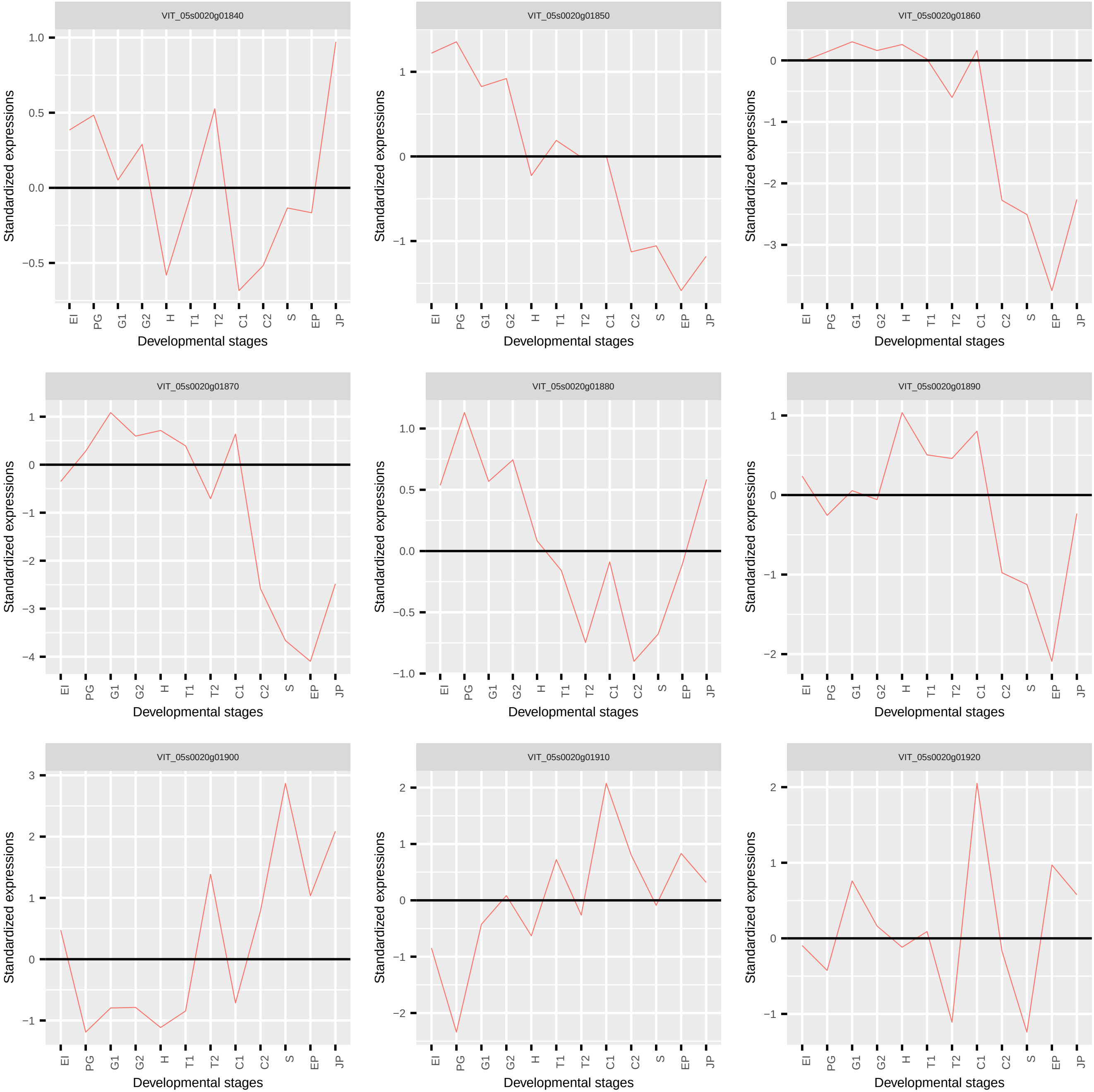
VIT_05s0020g01840
VIT_05s0020g01850
VIT_05s0020g01860
VIT_05s0020g01870
VIT_05s0020g01880
VIT_05s0020g01890
VIT_05s0020g01900
VIT_05s0020g01910
VIT_05s0020g01920
Standardized expressions
Developmental stages
EI PG G1 G2 H T1 T2 C1 C2 S EP JP

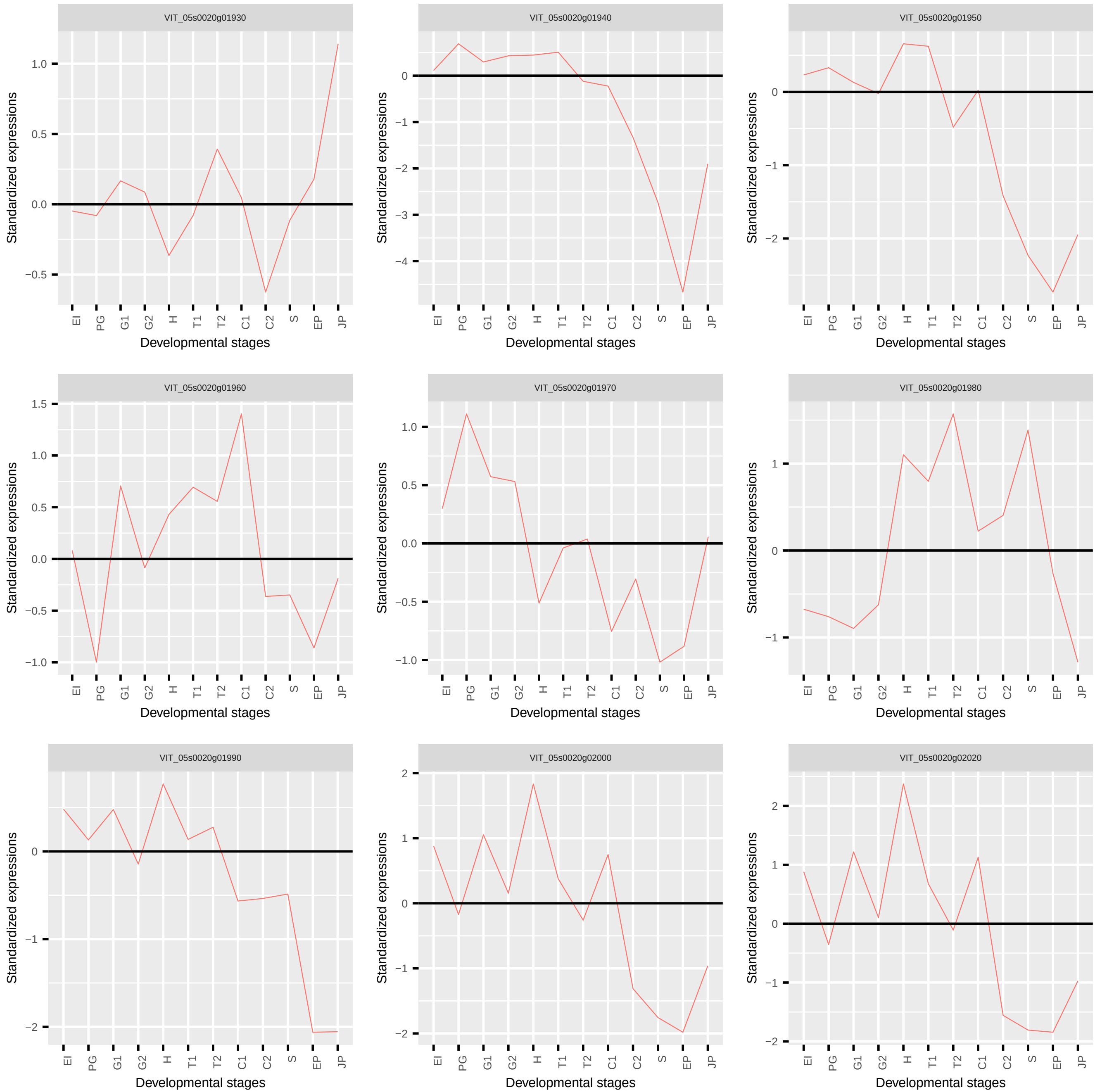
VIT_05s0020g01930
VIT_05s0020g01940
VIT_05s0020g01950
VIT_05s0020g01960
VIT_05s0020g01970
VIT_05s0020g01980
VIT_05s0020g01990
VIT_05s0020g02000
VIT_05s0020g02020
Standardized expressions
Developmental stages
EI PG G1 G2 H T1 T2 C1 C2 S EP JP

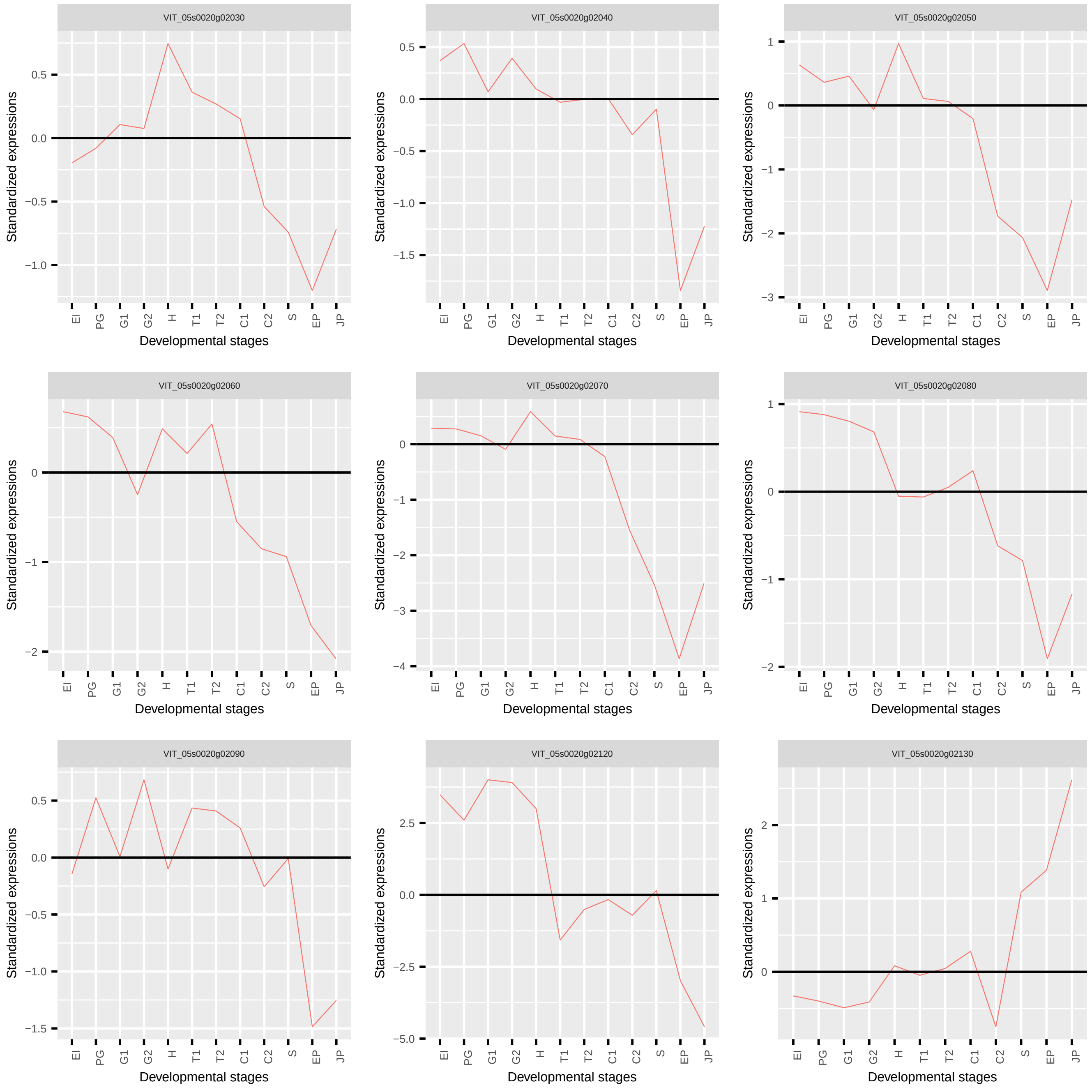

VIT_05s0020g02030
VIT_05s0020g02040
VIT_05s0020g02050
VIT_05s0020g02060
VIT_05s0020g02070
VIT_05s0020g02080
VIT_05s0020g02090
VIT_05s0020g02120
VIT_05s0020g02130
Standardized expressions
Developmental stages
EI PG G1 G2 H T1 T2 C1 C2 S EP JP

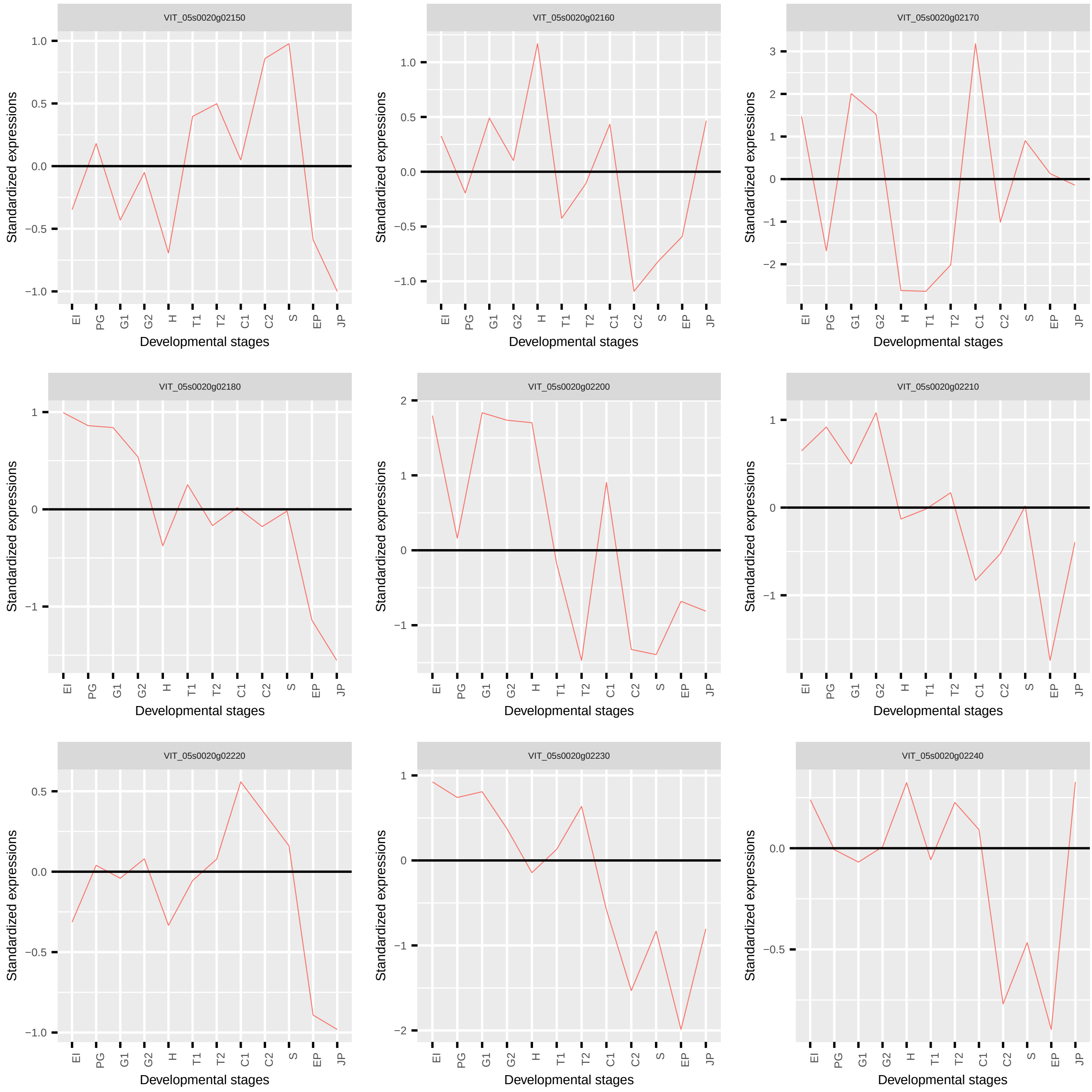
VIT_05s0020g02150
VIT_05s0020g02160
VIT_05s0020g02170
VIT_05s0020g02180
VIT_05s0020g02200
VIT_05s0020g02210
VIT_05s0020g02220
VIT_05s0020g02230
VIT_05s0020g02240
Standardized expressions
Developmental stages
EI PG G1 G2 H T1 T2 C1 C2 S EP JP

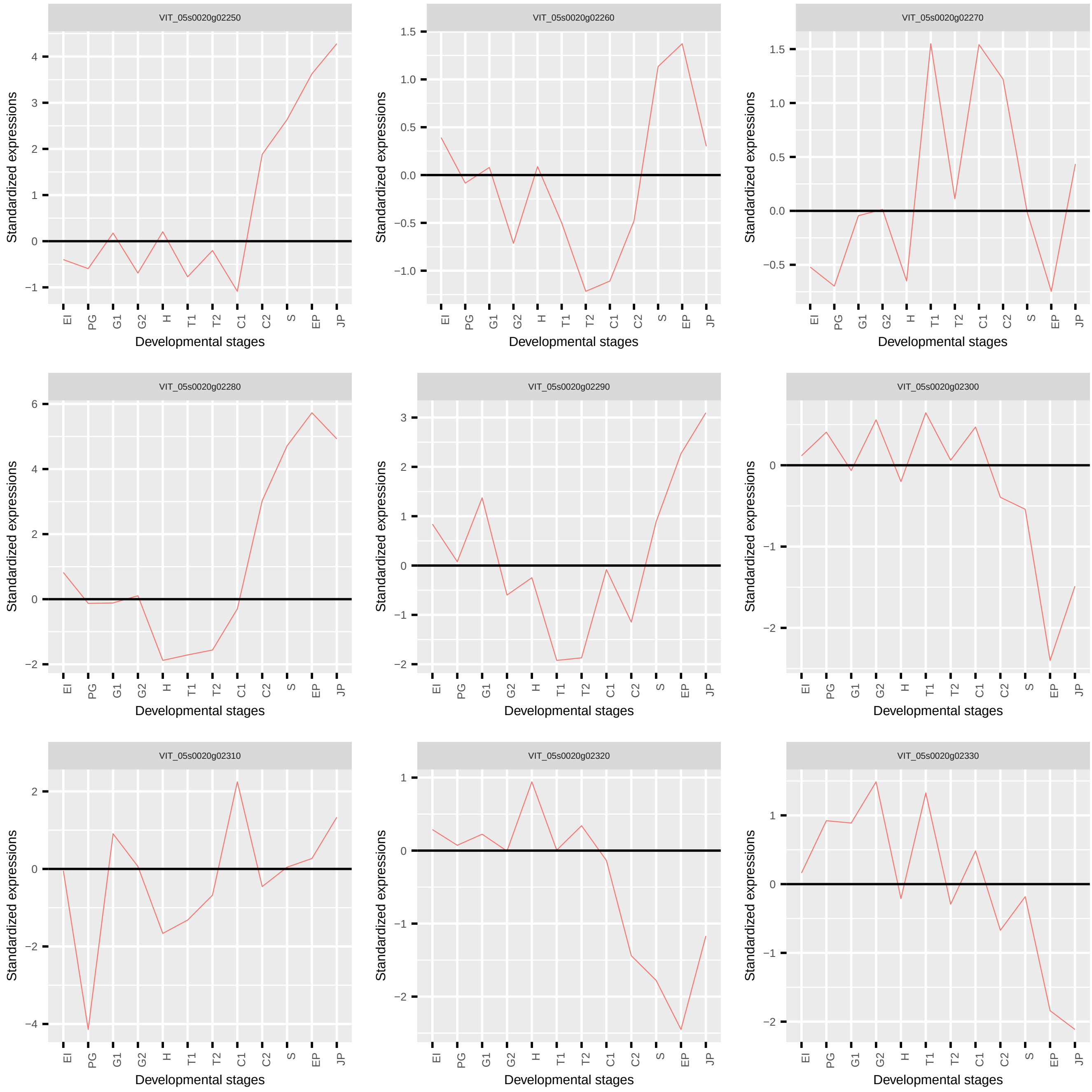
VIT_05s0020g02250
Standardized expressions
4
3
2
1
0
−1
EI
PG
G1
G2
H
T1
T2
C1
C2
S
EP
JP
Developmental stages
VIT_05s0020g02260
Standardized expressions
1.5
1.0
0.5
0.0
−0.5
−1.0
EI
PG
G1
G2
H
T1
T2
C1
C2
S
EP
JP
Developmental stages
VIT_05s0020g02270
Standardized expressions
1.5
1.0
0.5
0.0
−0.5
EI
PG
G1
G2
H
T1
T2
C1
C2
S
EP
JP
Developmental stages
VIT_05s0020g02280
Standardized expressions
6
4
2
0
−2
EI
PG
G1
G2
H
T1
T2
C1
C2
S
EP
JP
Developmental stages
VIT_05s0020g02290
Standardized expressions
3
2
1
0
−1
−2
EI
PG
G1
G2
H
T1
T2
C1
C2
S
EP
JP
Developmental stages
VIT_05s0020g02300
Standardized expressions
0
−1
−2
EI
PG
G1
G2
H
T1
T2
C1
C2
S
EP
JP
Developmental stages
VIT_05s0020g02310
Standardized expressions
2
0
−2
−4
EI
PG
G1
G2
H
T1
T2
C1
C2
S
EP
JP
Developmental stages
VIT_05s0020g02320
Standardized expressions
1
0
−1
−2
EI
PG
G1
G2
H
T1
T2
C1
C2
S
EP
JP
Developmental stages
VIT_05s0020g02330
Standardized expressions
1
0
−1
−2
EI
PG
G1
G2
H
T1
T2
C1
C2
S
EP
JP
Developmental stages

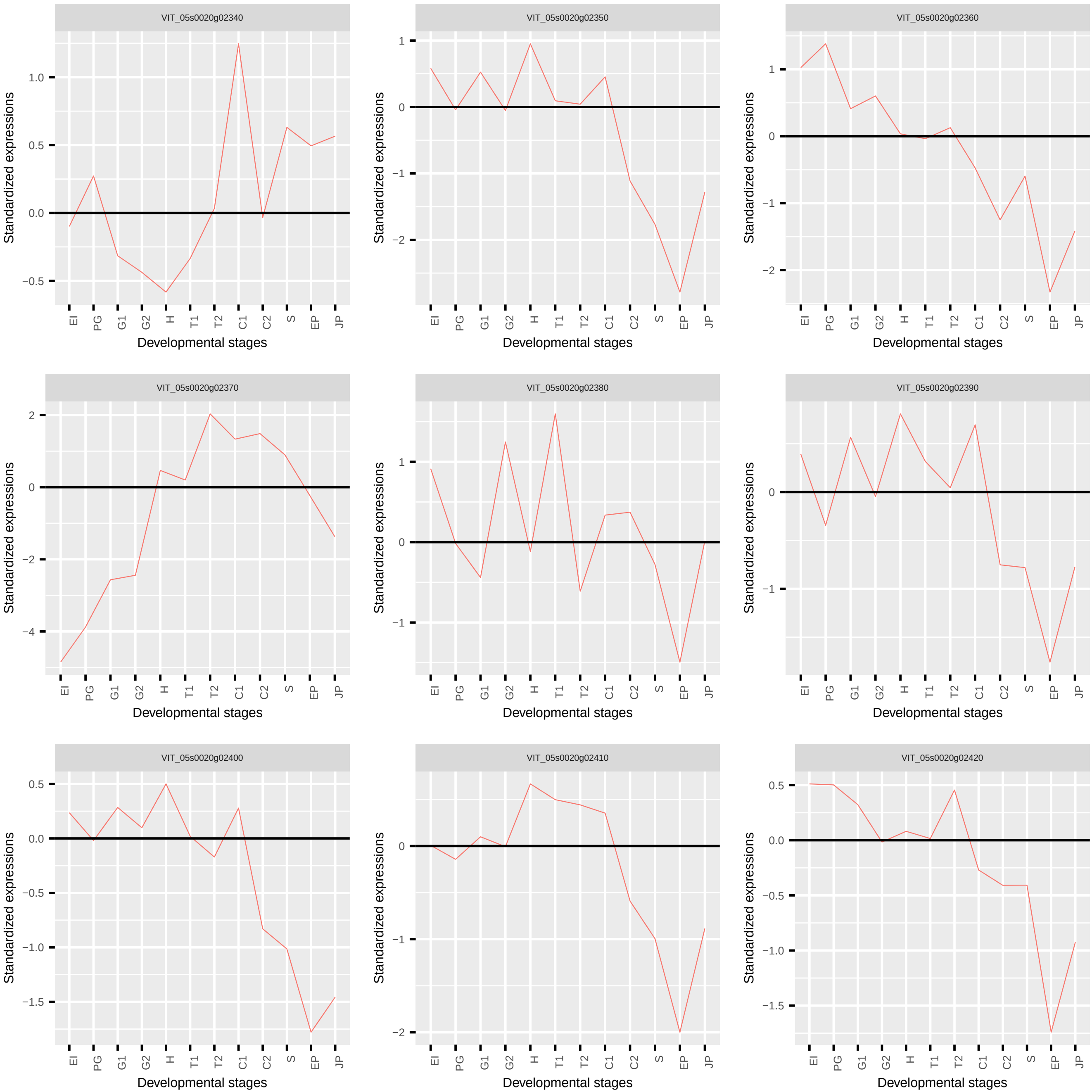
VIT_05s0020g02340
VIT_05s0020g02350
VIT_05s0020g02360
VIT_05s0020g02370
VIT_05s0020g02380
VIT_05s0020g02390
VIT_05s0020g02400
VIT_05s0020g02410
VIT_05s0020g02420
Standardized expressions
Developmental stages
EI PG G1 G2 H T1 T2 C1 C2 S EP JP

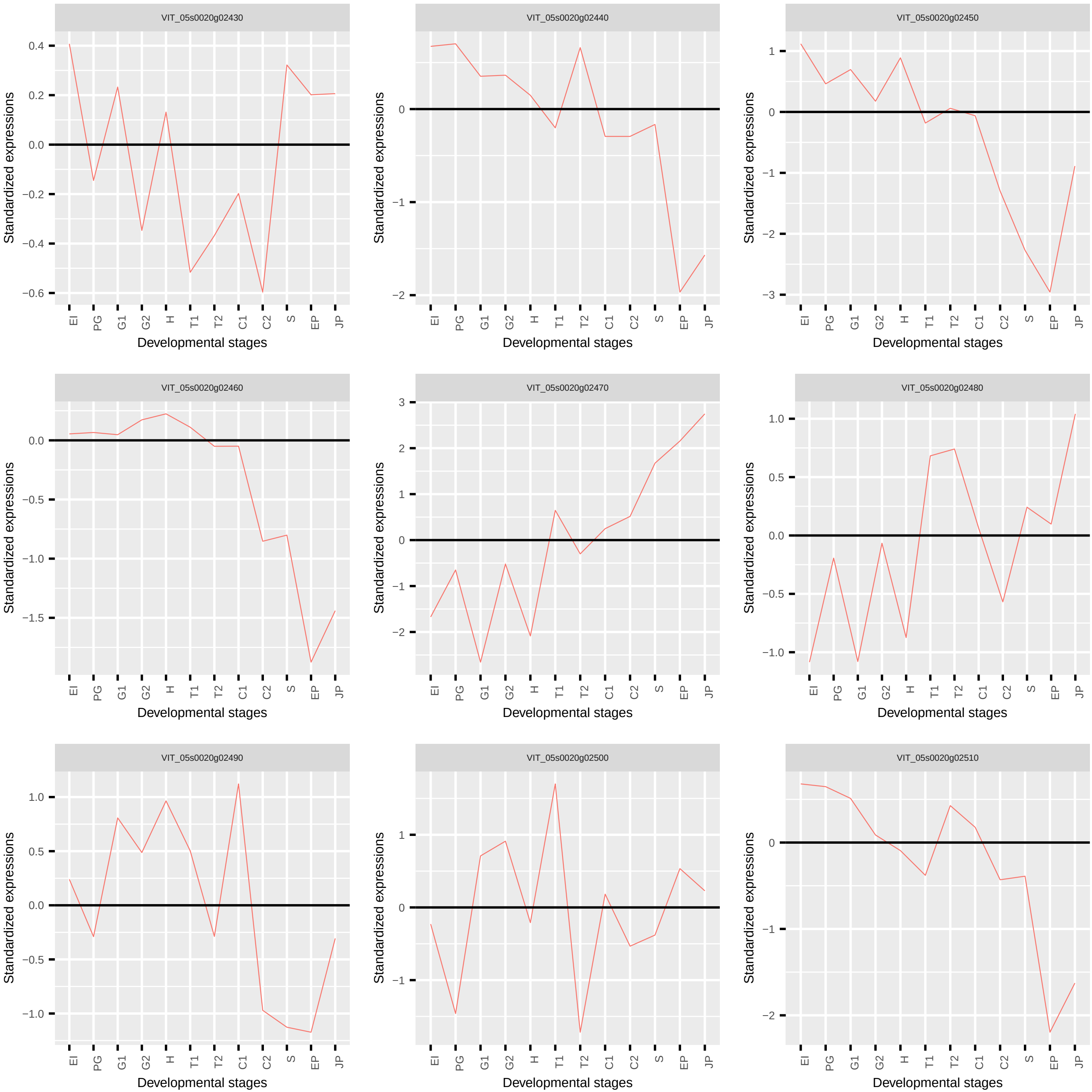

VIT_05s0020g02430
VIT_05s0020g02440
VIT_05s0020g02450
VIT_05s0020g02460
VIT_05s0020g02470
VIT_05s0020g02480
VIT_05s0020g02490
VIT_05s0020g02500
VIT_05s0020g02510
Standardized expressions
Developmental stages
EI PG G1 G2 H T1 T2 C1 C2 S EP JP

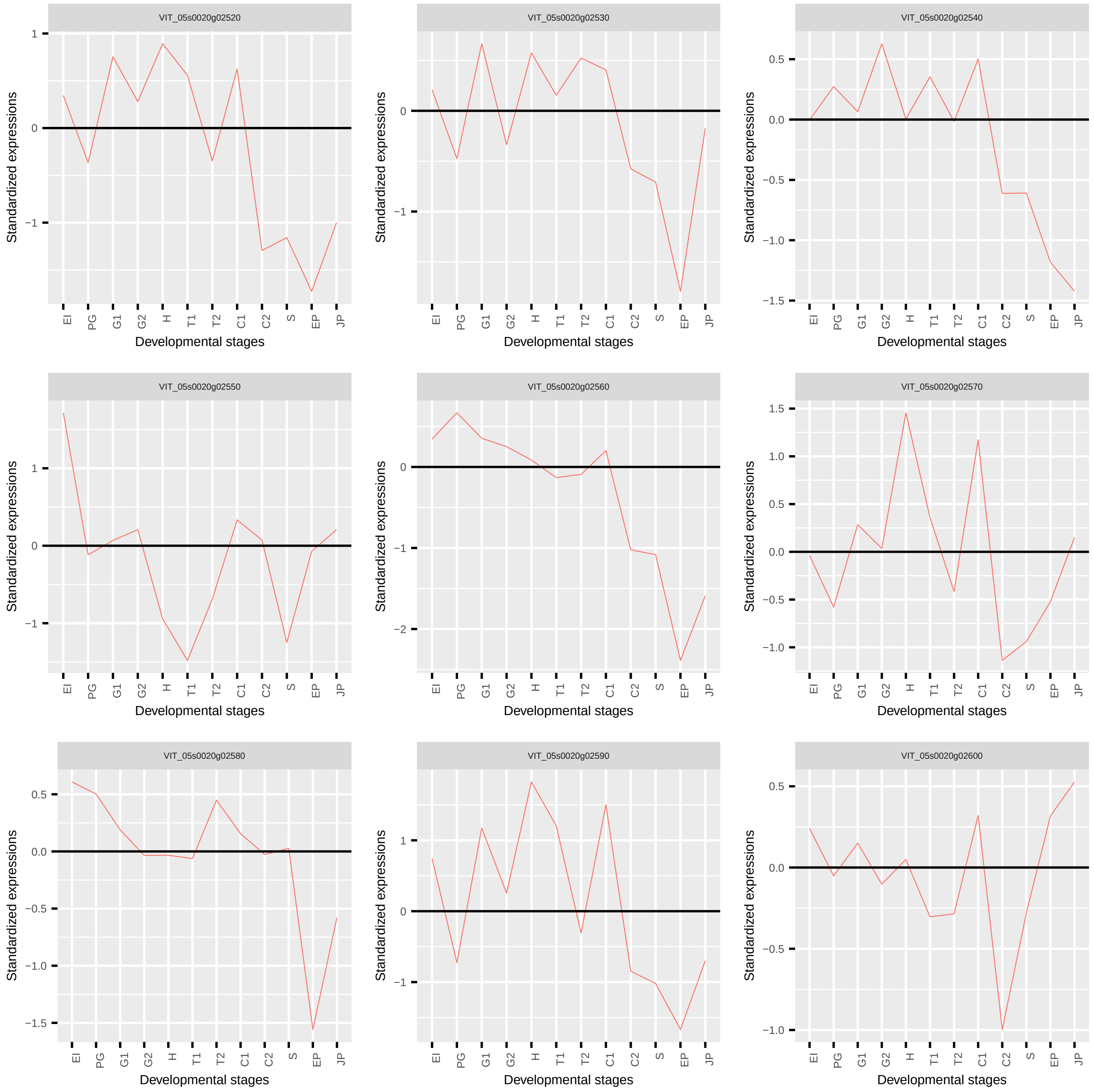
VIT_05s0020g02520
VIT_05s0020g02530
VIT_05s0020g02540
VIT_05s0020g02550
VIT_05s0020g02560
VIT_05s0020g02570
VIT_05s0020g02580
VIT_05s0020g02590
VIT_05s0020g02600
Standardized expressions
Developmental stages
EI
PG
G1
G2
H
T1
T2
C1
C2
S
EP
JP

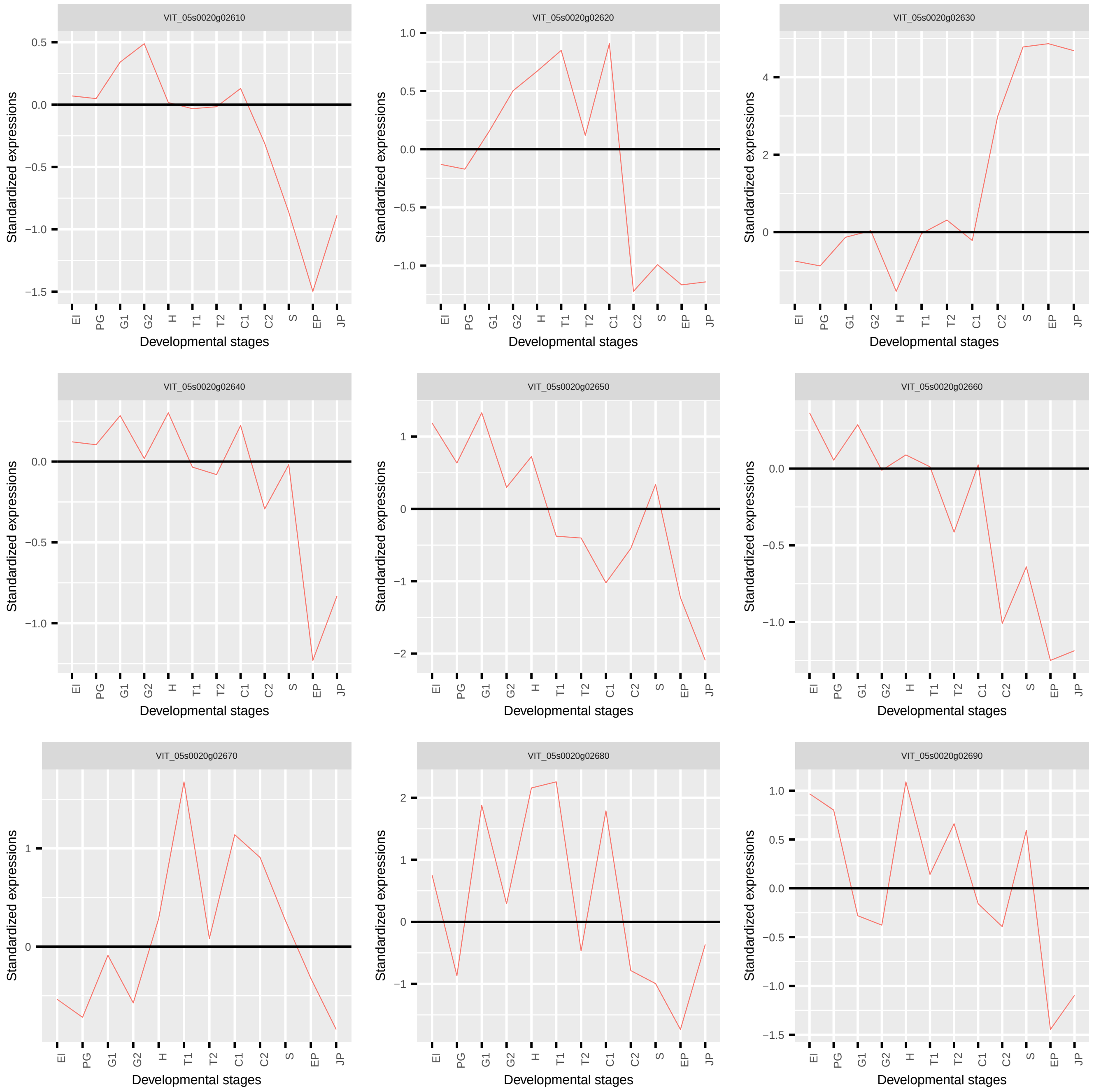
VIT_05s0020g02610
VIT_05s0020g02620
VIT_05s0020g02630
VIT_05s0020g02640
VIT_05s0020g02650
VIT_05s0020g02660
VIT_05s0020g02670
VIT_05s0020g02680
VIT_05s0020g02690
Standardized expressions
Developmental stages
EI PG G1 G2 H T1 T2 C1 C2 S EP JP

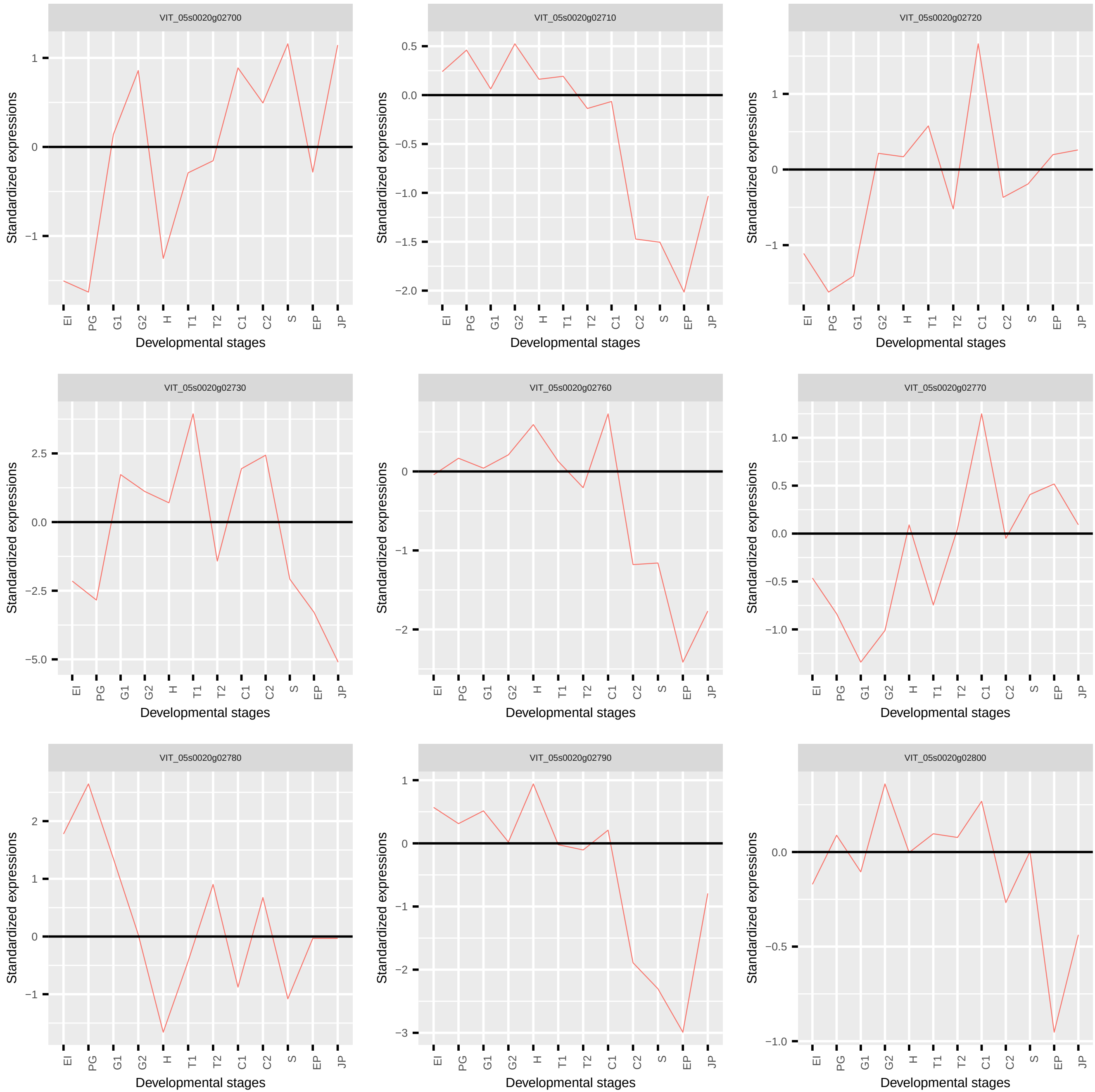
VIT_05s0020g02700
VIT_05s0020g02710
VIT_05s0020g02720
VIT_05s0020g02730
VIT_05s0020g02760
VIT_05s0020g02770
VIT_05s0020g02780
VIT_05s0020g02790
VIT_05s0020g02800
Standardized expressions
Developmental stages
EI
PG
G1
G2
H
T1
T2
C1
C2
S
EP
JP

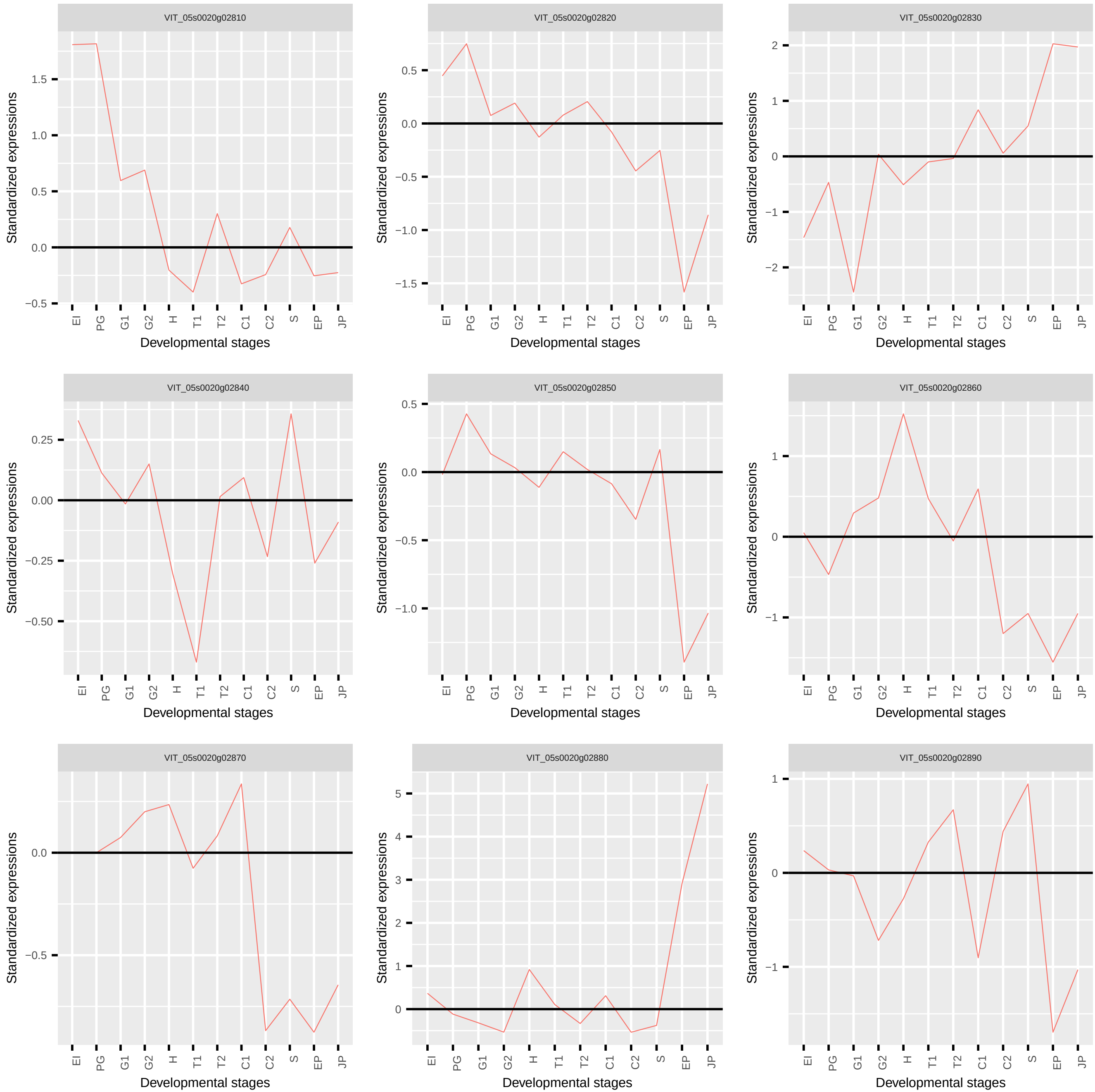

VIT_05s0020g02810
VIT_05s0020g02820
VIT_05s0020g02830
VIT_05s0020g02840
VIT_05s0020g02850
VIT_05s0020g02860
VIT_05s0020g02870
VIT_05s0020g02880
VIT_05s0020g02890
Standardized expressions
Developmental stages
EI PG G1 G2 H T1 T2 C1 C2 S EP JP

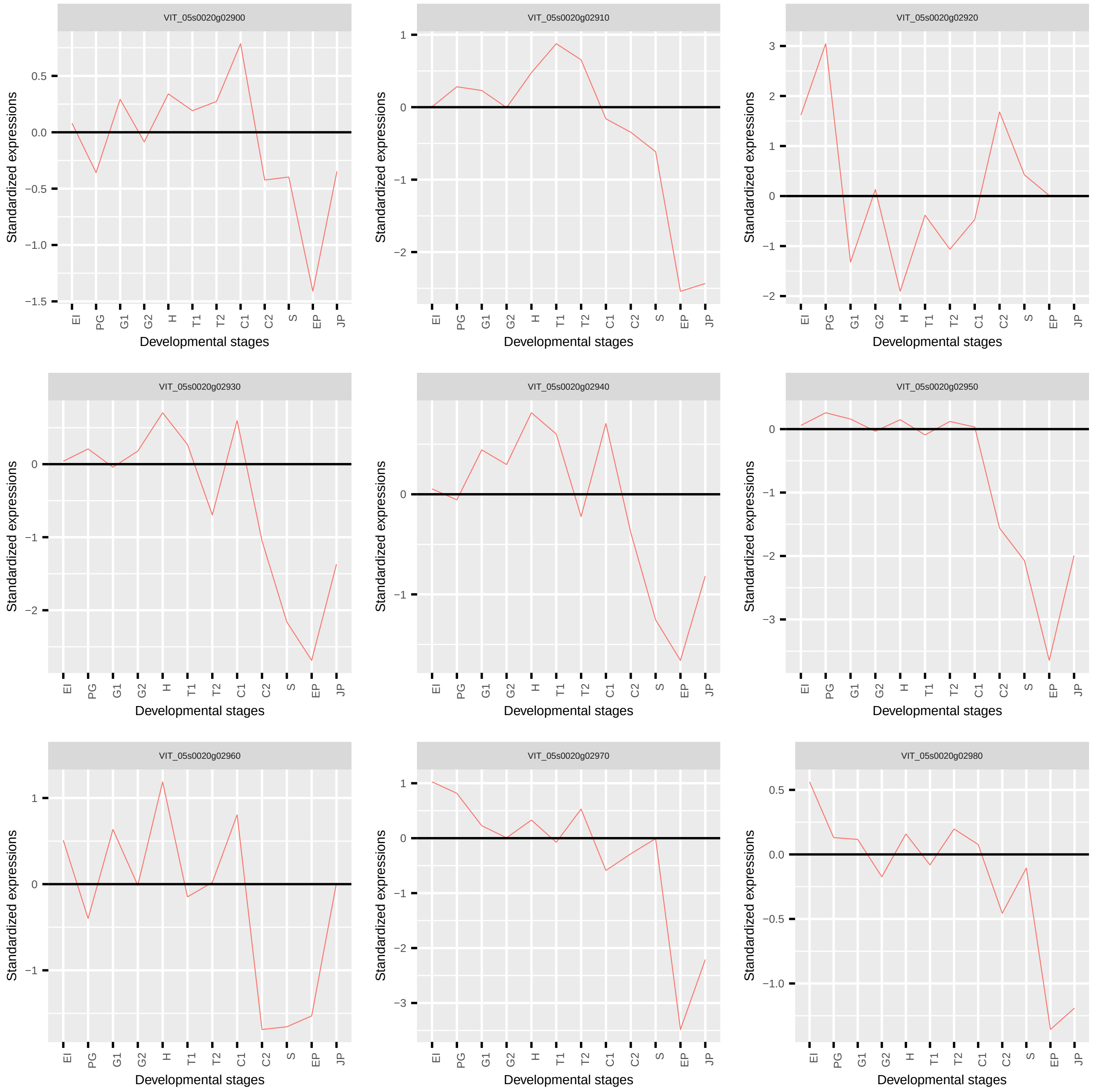

VIT_05s0020g02900
VIT_05s0020g02910
VIT_05s0020g02920
VIT_05s0020g02930
VIT_05s0020g02940
VIT_05s0020g02950
VIT_05s0020g02960
VIT_05s0020g02970
VIT_05s0020g02980
Standardized expressions
Developmental stages
EI
PG
G1
G2
H
T1
T2
C1
C2
S
EP
JP

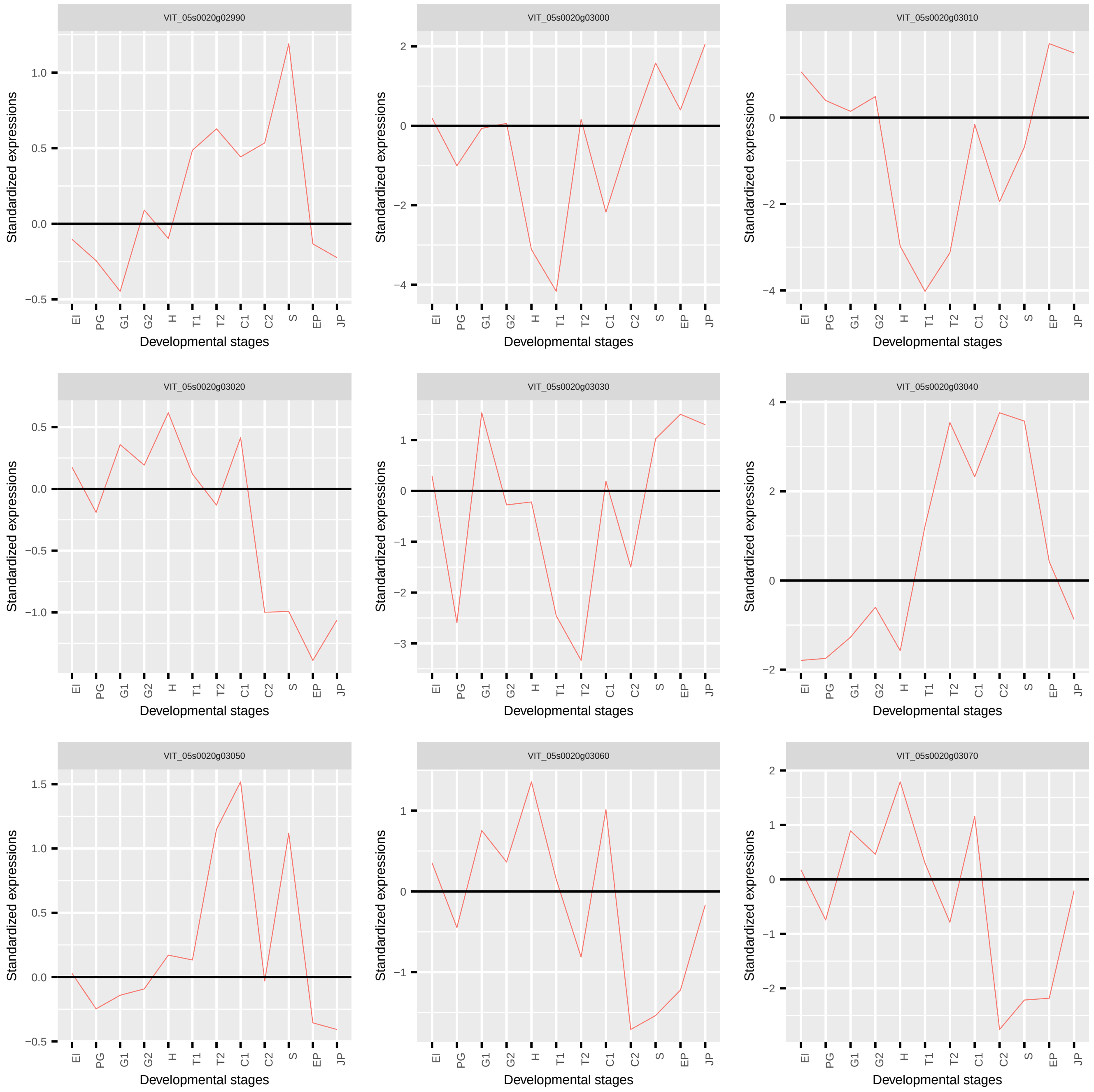
VIT_05s0020g02990
VIT_05s0020g03000
VIT_05s0020g03010
VIT_05s0020g03020
VIT_05s0020g03030
VIT_05s0020g03040
VIT_05s0020g03050
VIT_05s0020g03060
VIT_05s0020g03070
Standardized expressions
Developmental stages
EI PG G1 G2 H T1 T2 C1 C2 S EP JP

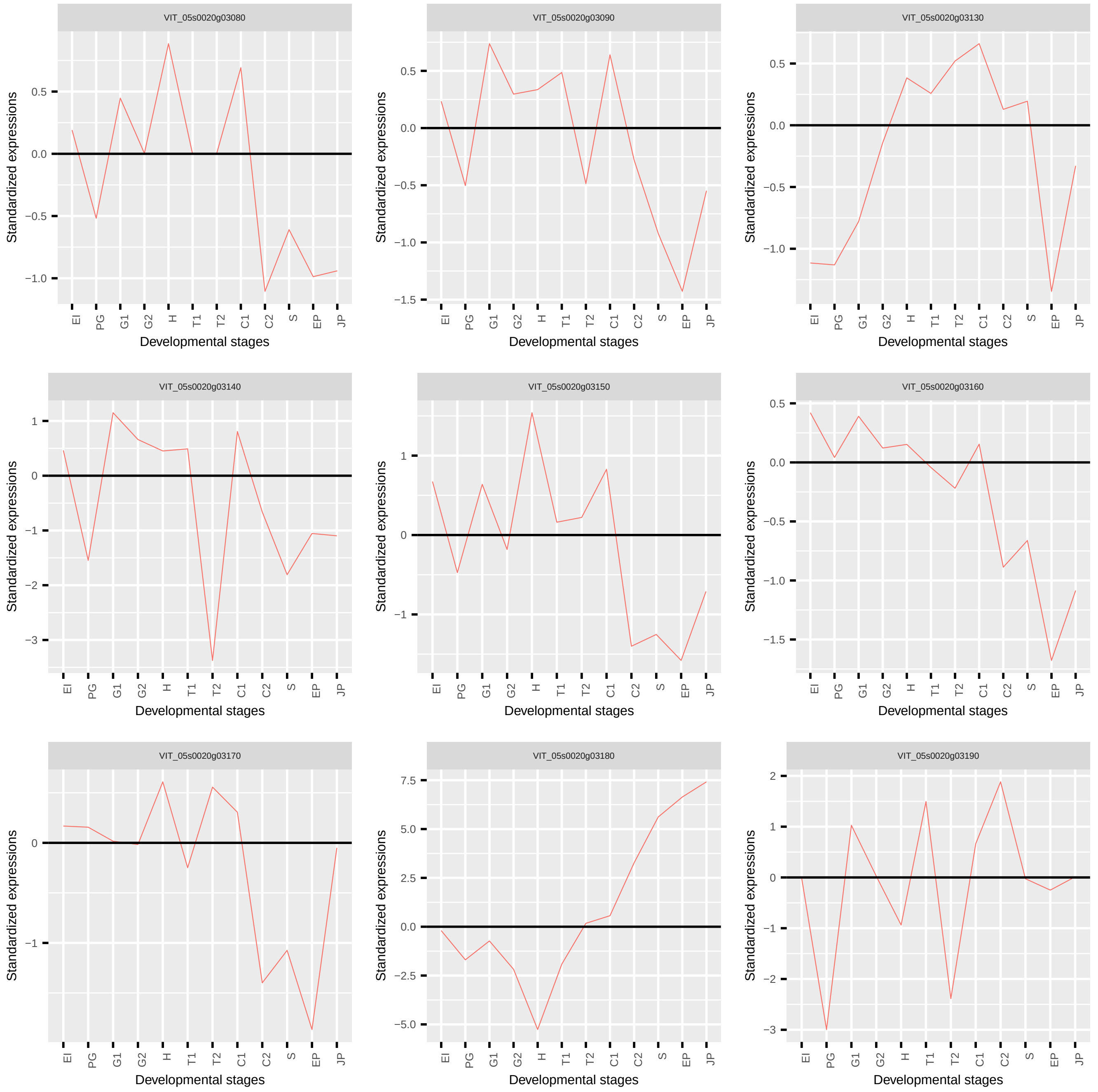
VIT_05s0020g03080
VIT_05s0020g03090
VIT_05s0020g03130
VIT_05s0020g03140
VIT_05s0020g03150
VIT_05s0020g03160
VIT_05s0020g03170
VIT_05s0020g03180
VIT_05s0020g03190
Standardized expressions
Developmental stages
EI PG G1 G2 H T1 T2 C1 C2 S EP JP

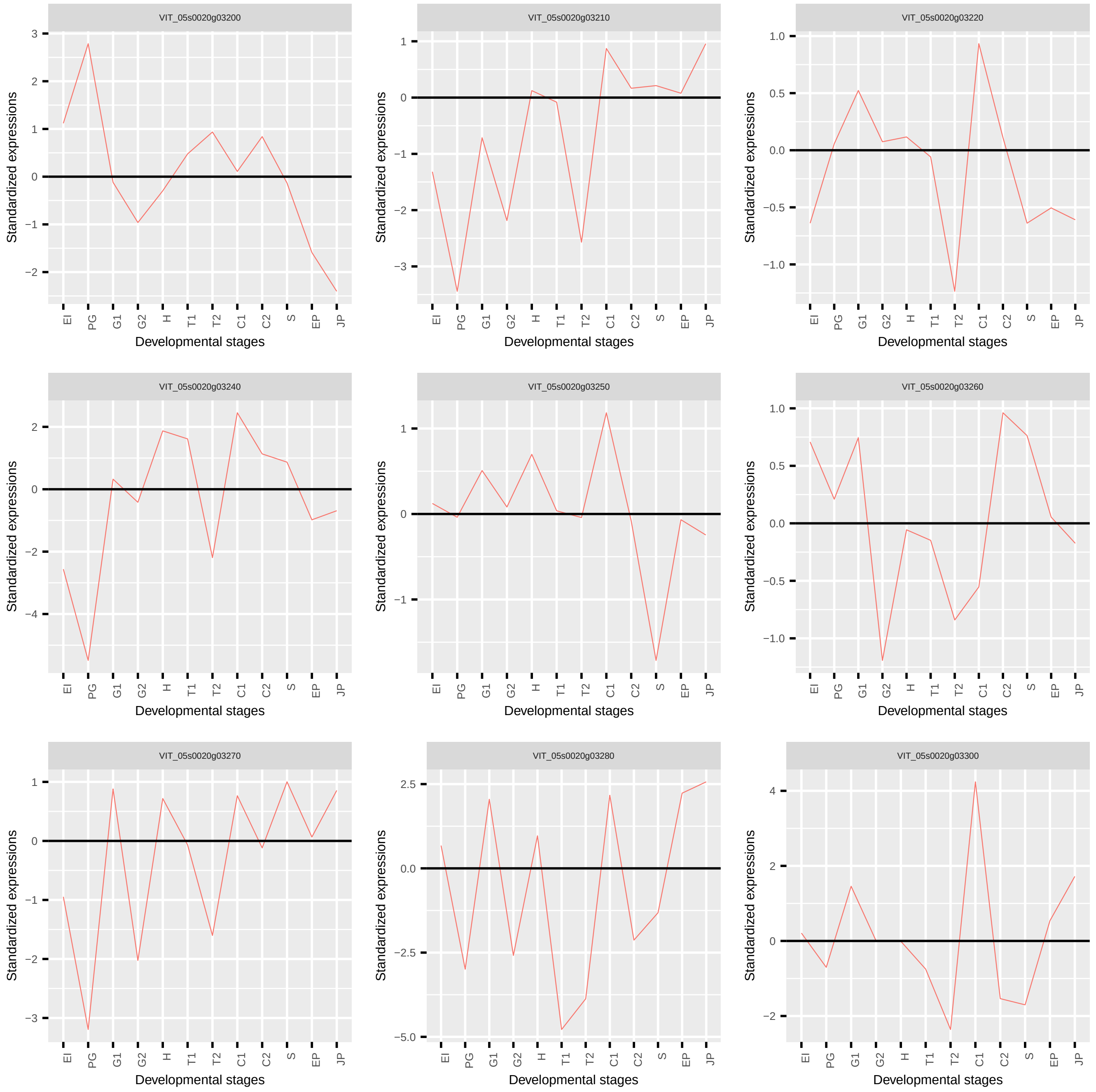
VIT_05s0020g03200
VIT_05s0020g03210
VIT_05s0020g03220
VIT_05s0020g03240
VIT_05s0020g03250
VIT_05s0020g03260
VIT_05s0020g03270
VIT_05s0020g03280
VIT_05s0020g03300
Standardized expressions
Developmental stages
EI PG G1 G2 H T1 T2 C1 C2 S EP JP

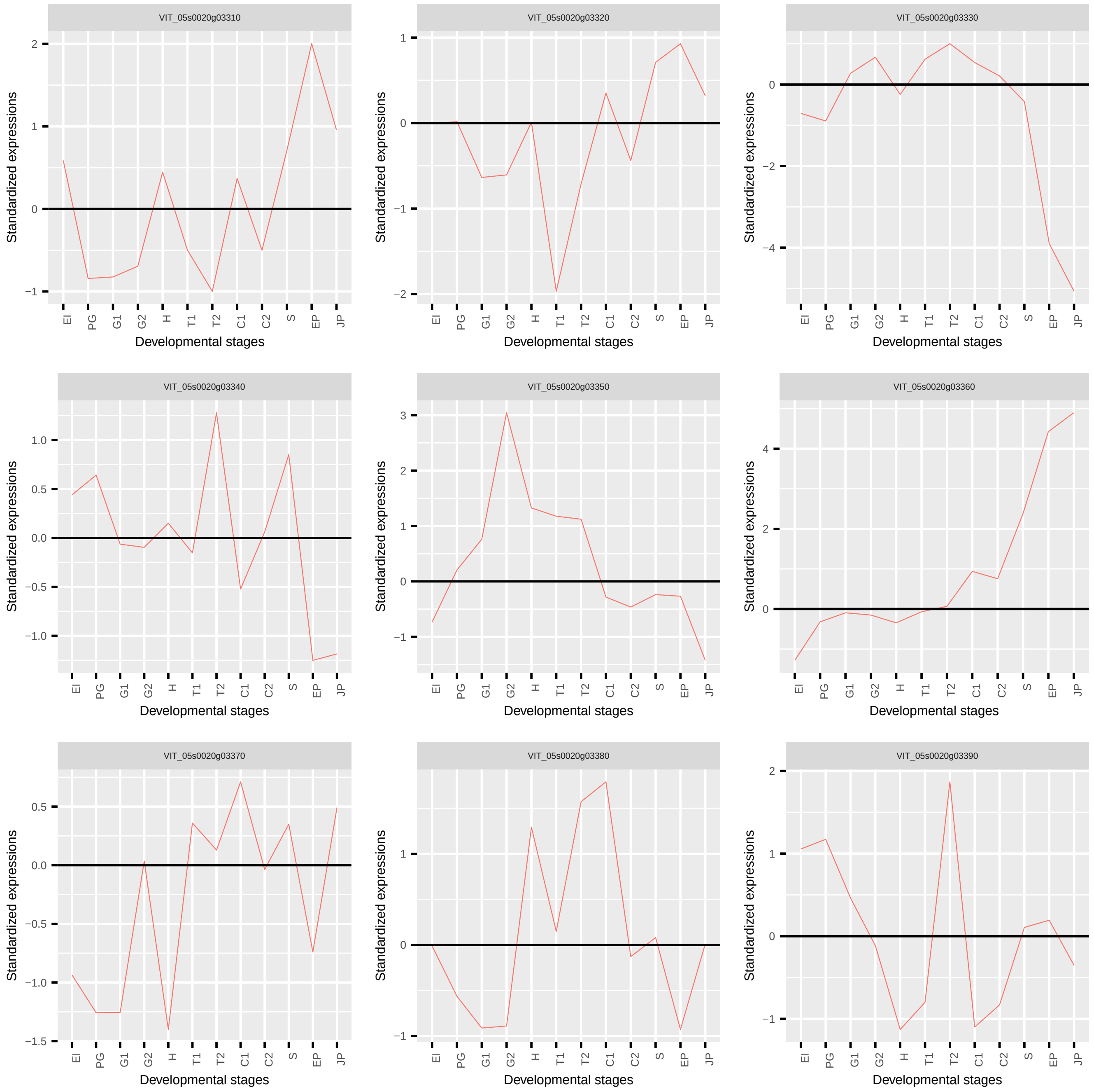
VIT_05s0020g03310
VIT_05s0020g03320
VIT_05s0020g03330
VIT_05s0020g03340
VIT_05s0020g03350
VIT_05s0020g03360
VIT_05s0020g03370
VIT_05s0020g03380
VIT_05s0020g03390
Standardized expressions
Developmental stages
EI PG G1 G2 H T1 T2 C1 C2 S EP JP

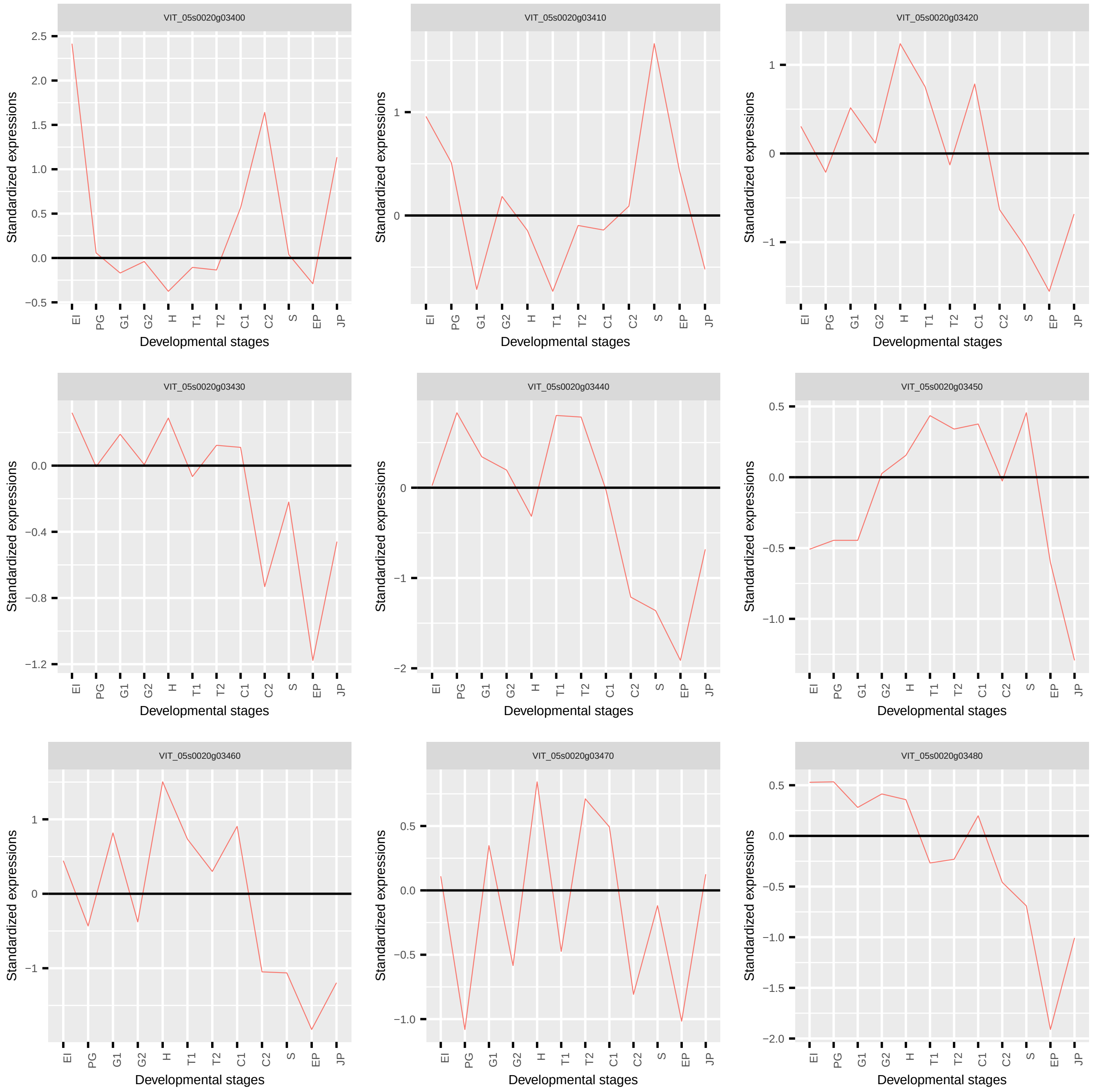
VIT_05s0020g03400
VIT_05s0020g03410
VIT_05s0020g03420
VIT_05s0020g03430
VIT_05s0020g03440
VIT_05s0020g03450
VIT_05s0020g03460
VIT_05s0020g03470
VIT_05s0020g03480
Standardized expressions
Developmental stages
EI PG G1 G2 H T1 T2 C1 C2 S EP JP
2.5 2.0 1.5 1.0 0.5 0.0 −0.5
1 0
1 0 −1
0.0 −0.4 −0.8 −1.2
0 −1 −2
0.5 0.0 −0.5 −1.0
1 0 −1
0.5 0.0 −0.5 −1.0
0.5 0.0 −0.5 −1.0 −1.5 −2.0

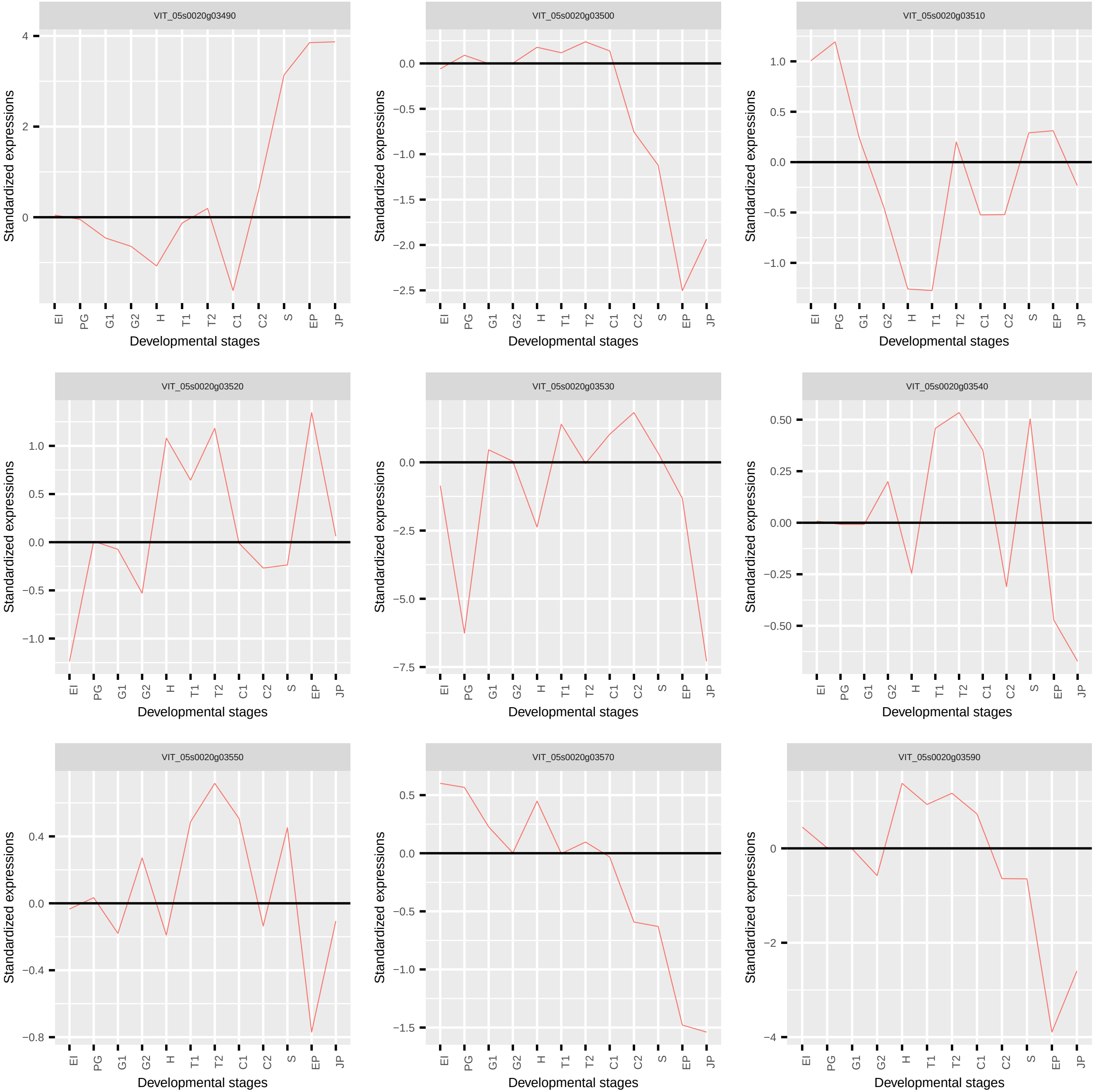

VIT_05s0020g03490
VIT_05s0020g03500
VIT_05s0020g03510
VIT_05s0020g03520
VIT_05s0020g03530
VIT_05s0020g03540
VIT_05s0020g03550
VIT_05s0020g03570
VIT_05s0020g03590
Standardized expressions
Developmental stages
EI PG G1 G2 H T1 T2 C1 C2 S EP JP

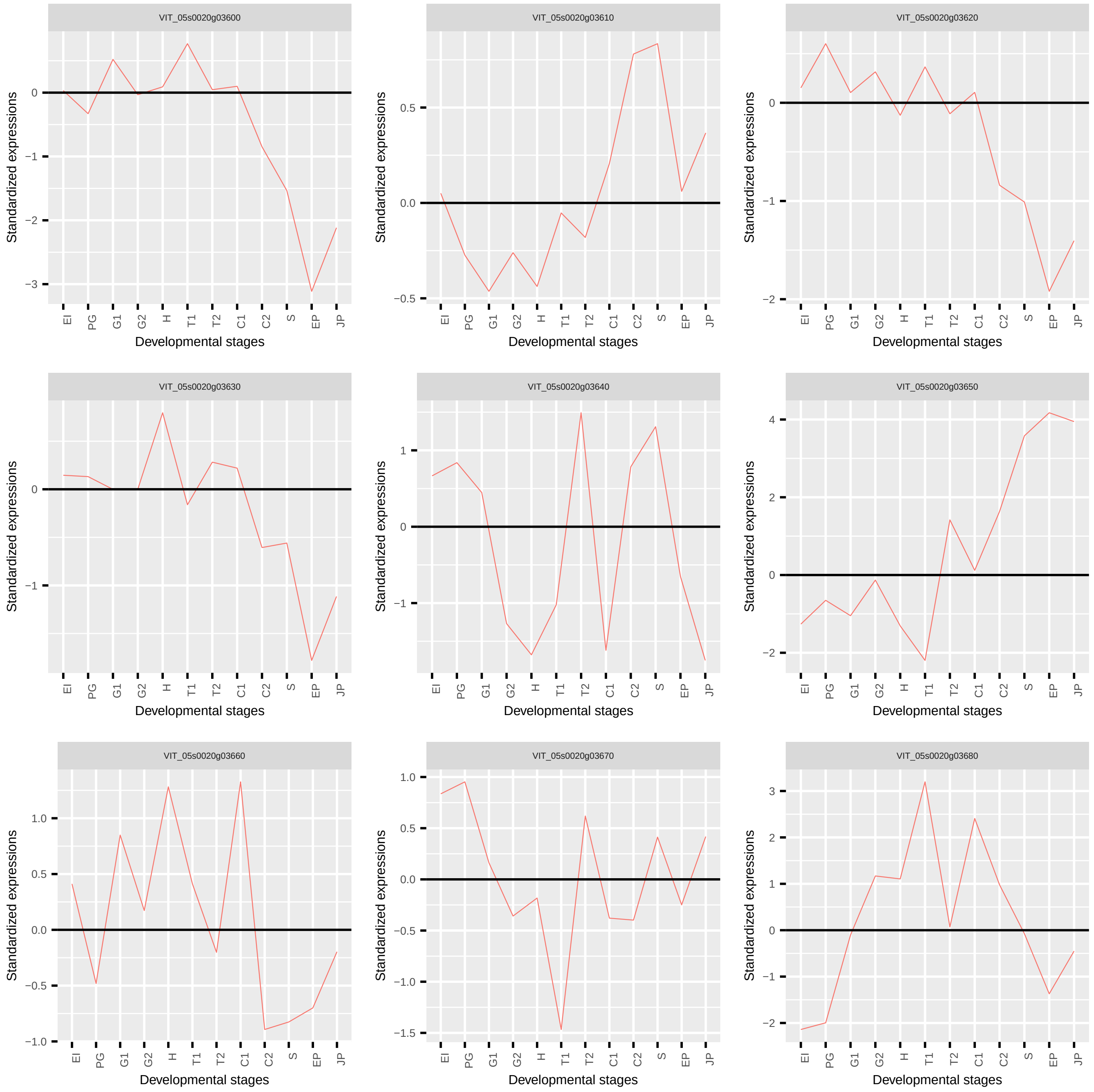
VIT_05s0020g03600
VIT_05s0020g03610
VIT_05s0020g03620
VIT_05s0020g03630
VIT_05s0020g03640
VIT_05s0020g03650
VIT_05s0020g03660
VIT_05s0020g03670
VIT_05s0020g03680
Standardized expressions
Developmental stages
EI PG G1 G2 H T1 T2 C1 C2 S EP JP

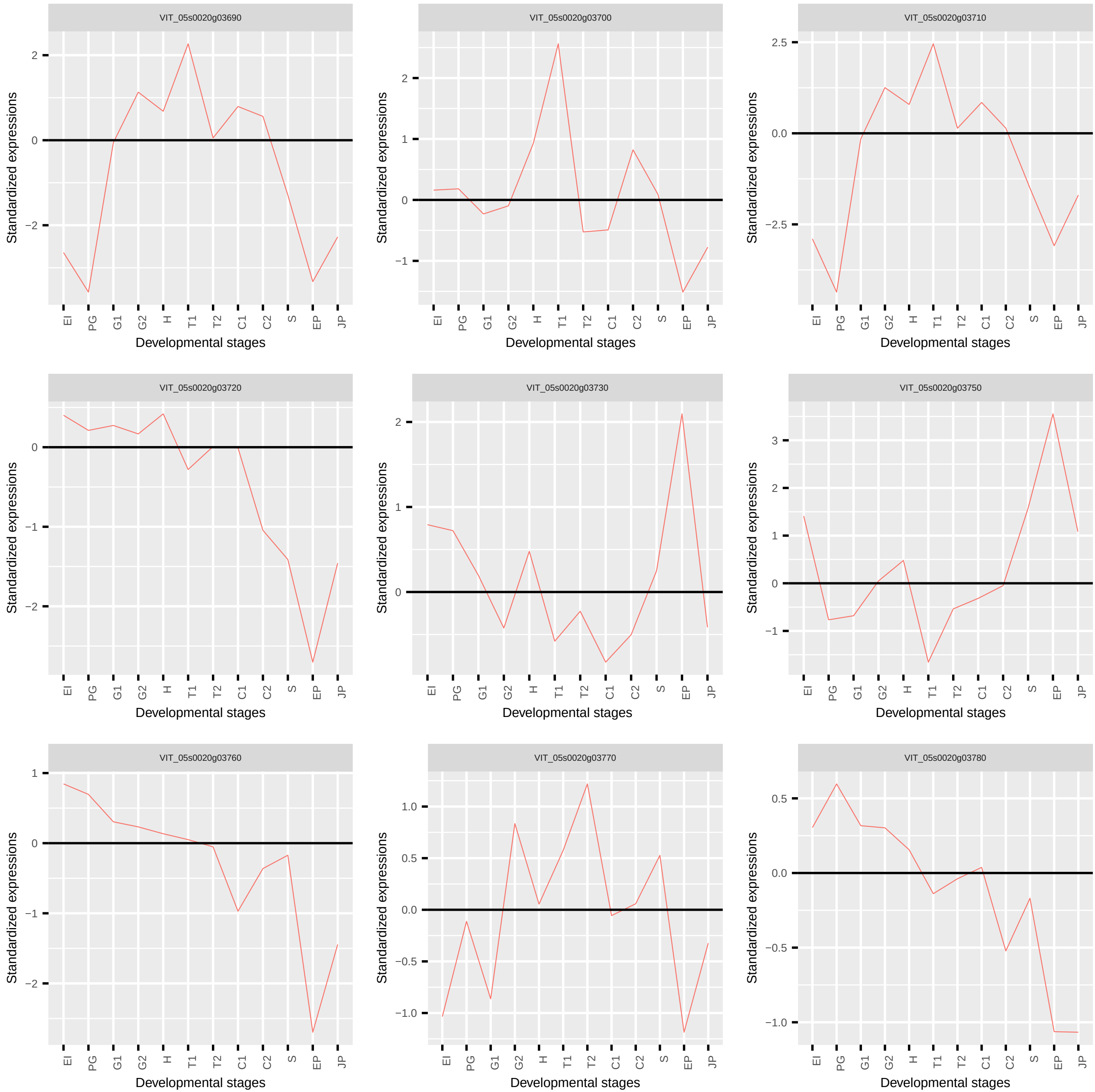
VIT_05s0020g03690
VIT_05s0020g03700
VIT_05s0020g03710
VIT_05s0020g03720
VIT_05s0020g03730
VIT_05s0020g03750
VIT_05s0020g03760
VIT_05s0020g03770
VIT_05s0020g03780
Standardized expressions
Developmental stages
EI
PG
G1
G2
H
T1
T2
C1
C2
S
EP
JP
2
0
−2
1
−1
2.5
0.0
−2.5
3
1.0
0.5
−0.5
−1.0

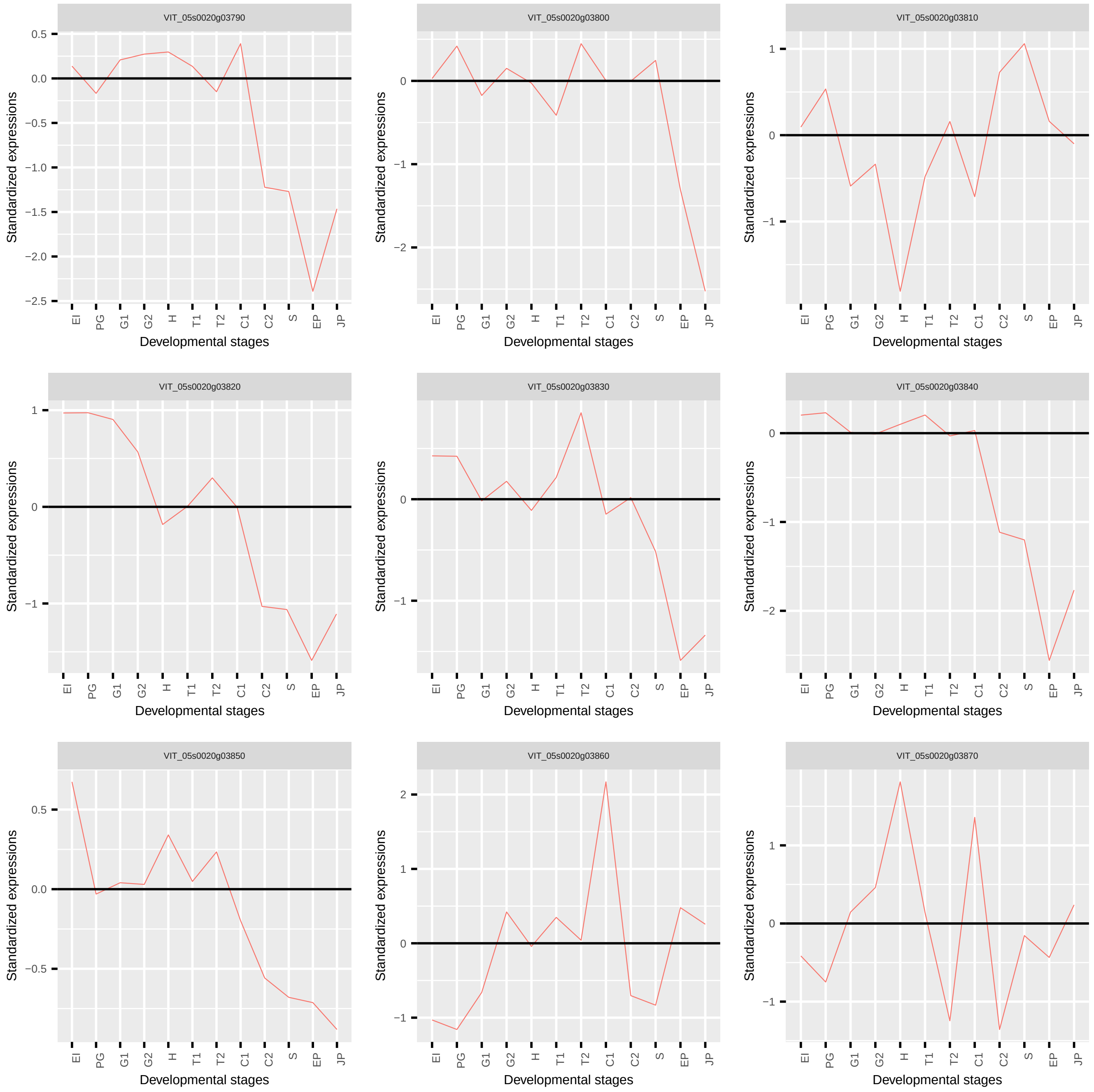
VIT_05s0020g03790
VIT_05s0020g03800
VIT_05s0020g03810
VIT_05s0020g03820
VIT_05s0020g03830
VIT_05s0020g03840
VIT_05s0020g03850
VIT_05s0020g03860
VIT_05s0020g03870
Standardized expressions
Developmental stages
EI PG G1 G2 H T1 T2 C1 C2 S EP JP
0.5
0.0
−0.5
−1.0
−1.5
−2.0
−2.5
2
1
0
−1
−2

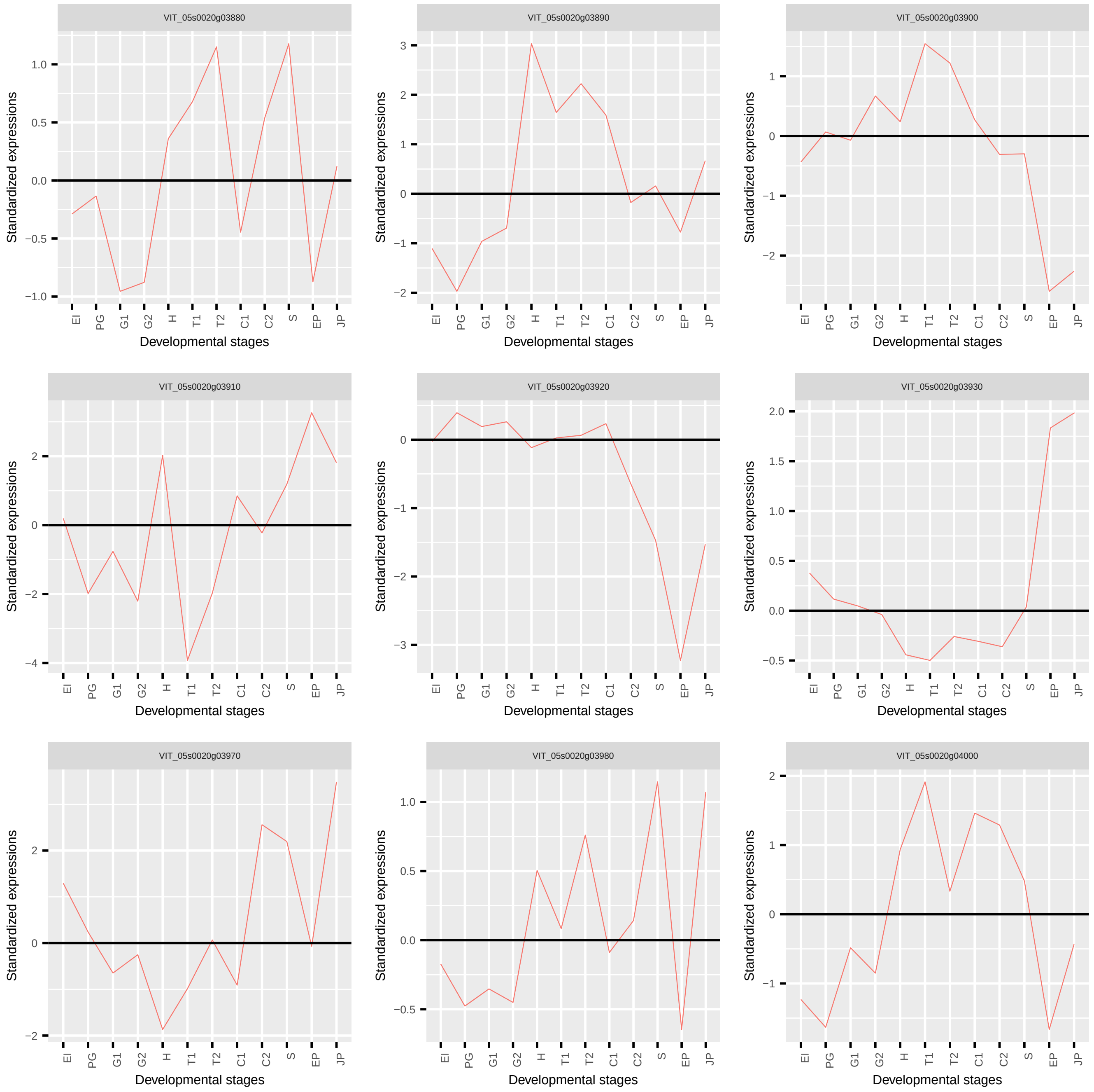
VIT_05s0020g03880
VIT_05s0020g03890
VIT_05s0020g03900
VIT_05s0020g03910
VIT_05s0020g03920
VIT_05s0020g03930
VIT_05s0020g03970
VIT_05s0020g03980
VIT_05s0020g04000
Standardized expressions
Developmental stages
EI
PG
G1
G2
H
T1
T2
C1
C2
S
EP
JP

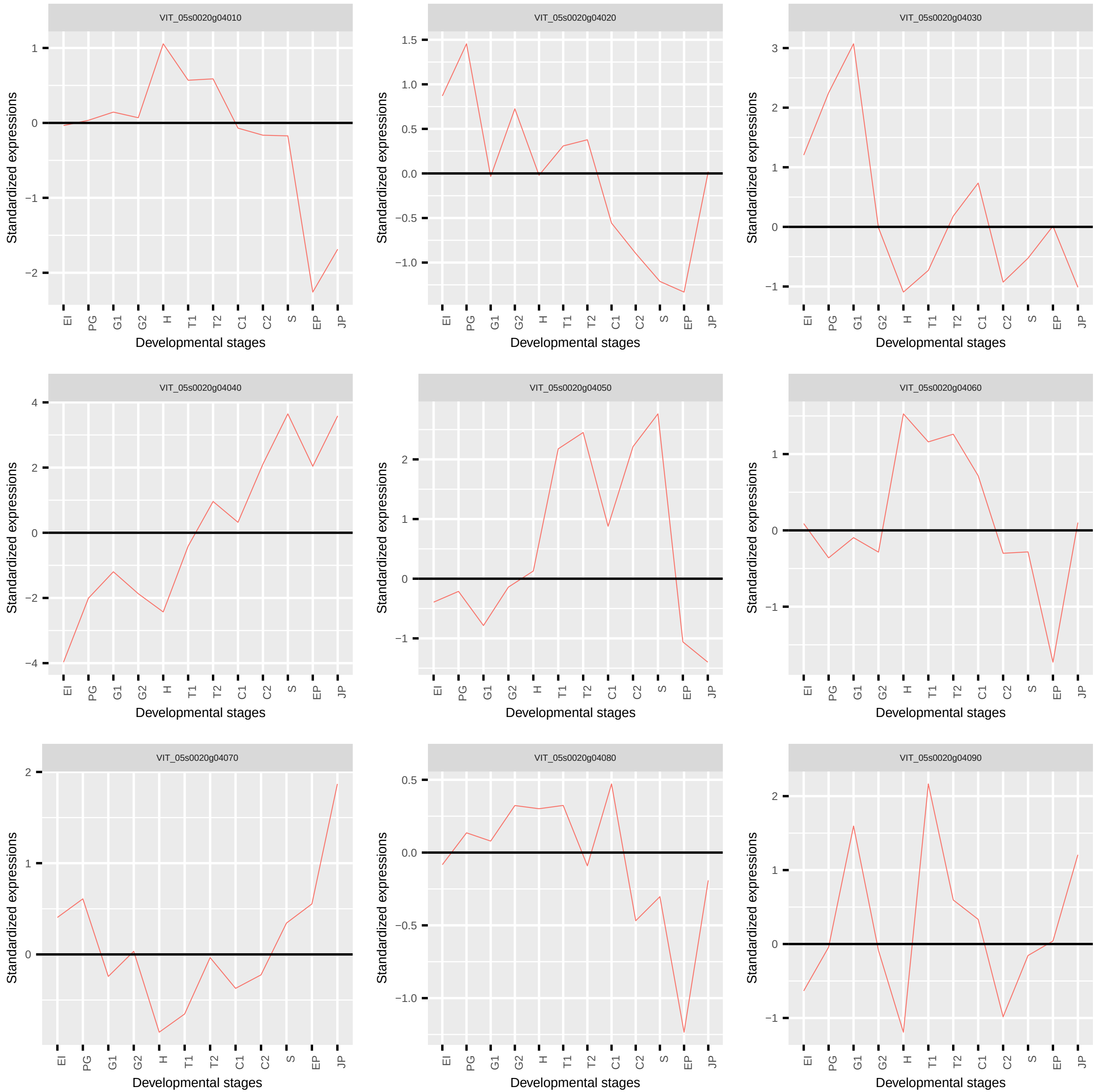

VIT_05s0020g04010
VIT_05s0020g04020
VIT_05s0020g04030
VIT_05s0020g04040
VIT_05s0020g04050
VIT_05s0020g04060
VIT_05s0020g04070
VIT_05s0020g04080
VIT_05s0020g04090
Standardized expressions
Developmental stages
EI PG G1 G2 H T1 T2 C1 C2 S EP JP

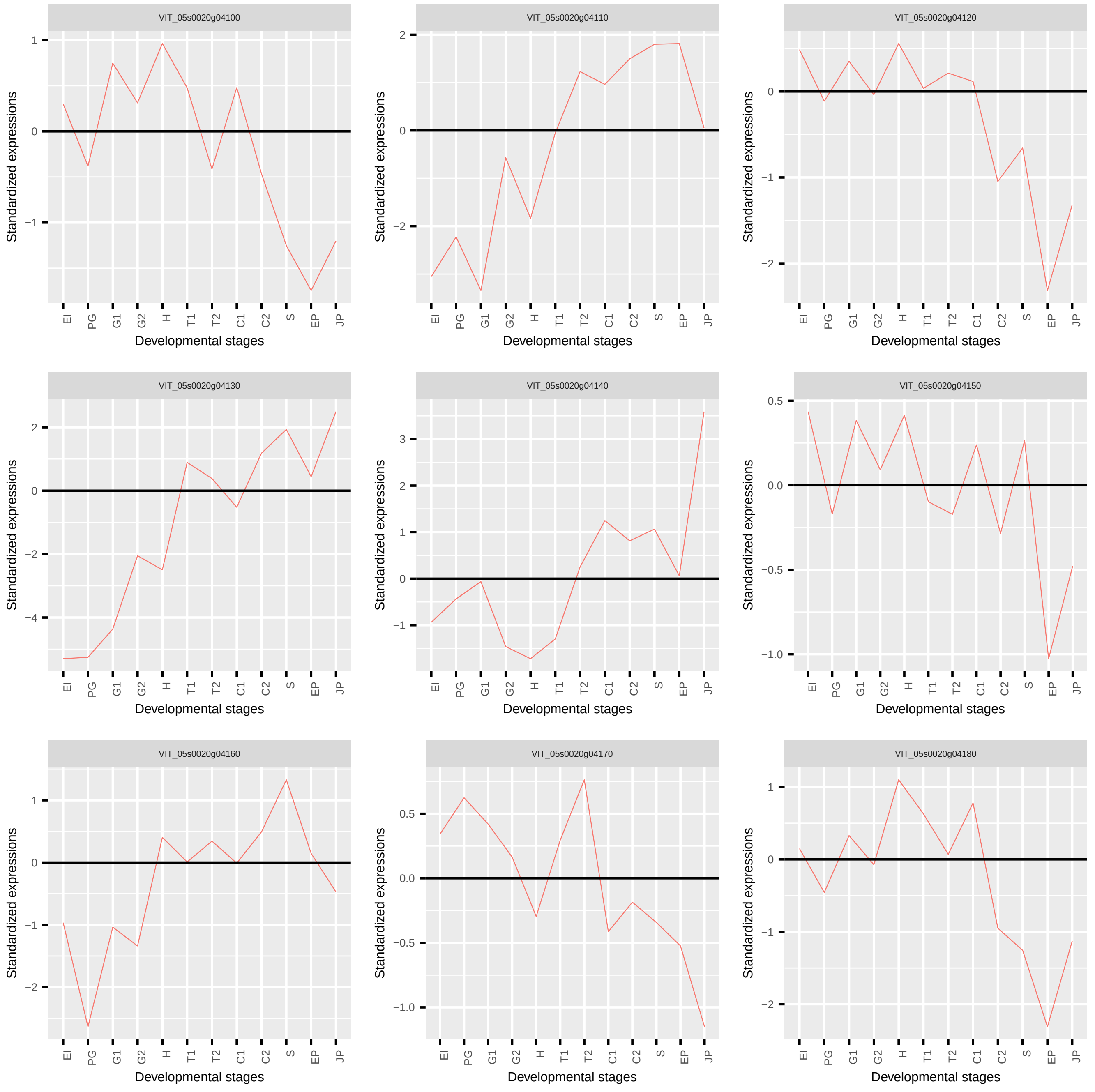
VIT_05s0020g04100
VIT_05s0020g04110
VIT_05s0020g04120
VIT_05s0020g04130
VIT_05s0020g04140
VIT_05s0020g04150
VIT_05s0020g04160
VIT_05s0020g04170
VIT_05s0020g04180
Standardized expressions
Developmental stages
EI PG G1 G2 H T1 T2 C1 C2 S EP JP

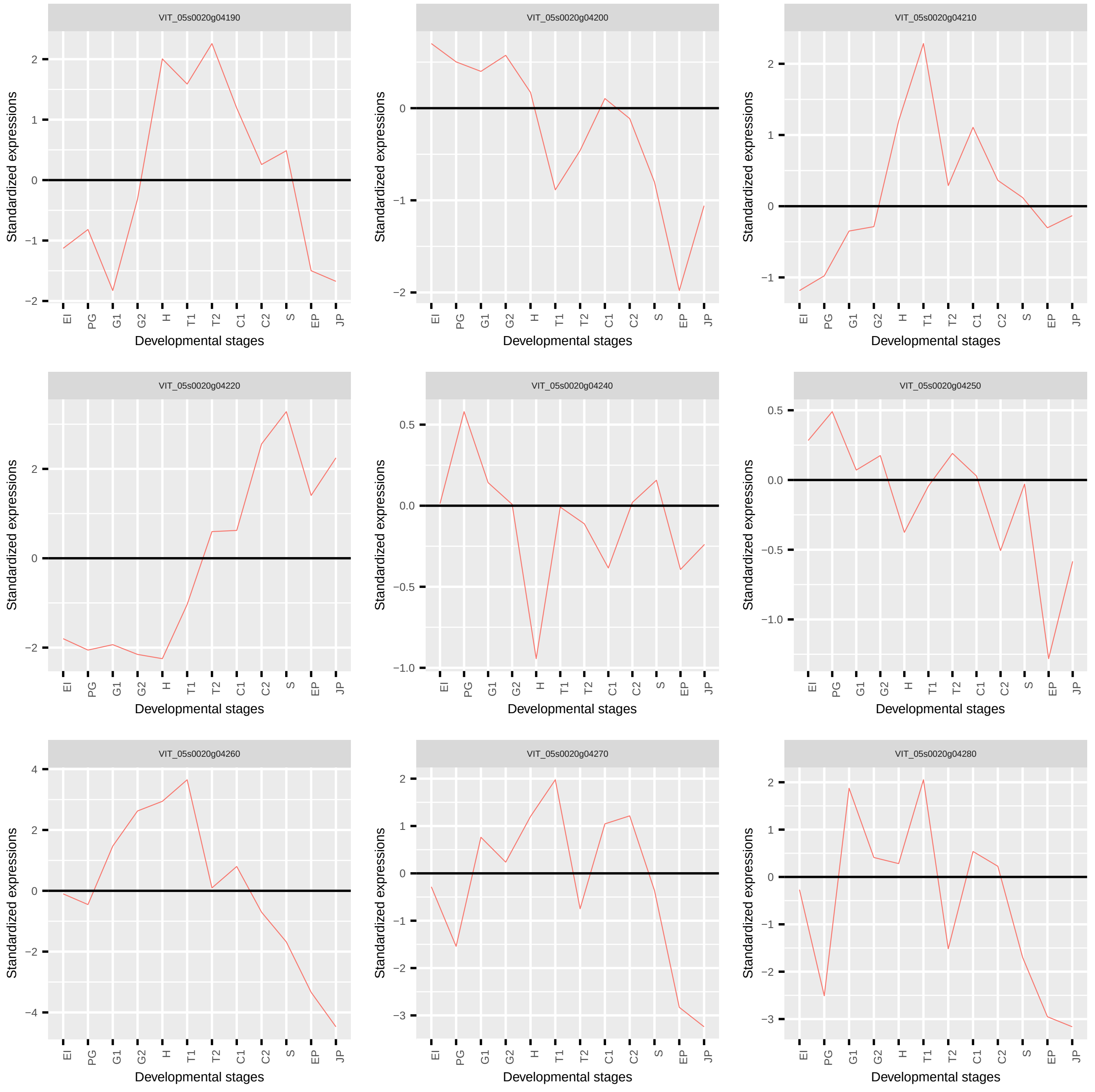
VIT_05s0020g04190
VIT_05s0020g04200
VIT_05s0020g04210
VIT_05s0020g04220
VIT_05s0020g04240
VIT_05s0020g04250
VIT_05s0020g04260
VIT_05s0020g04270
VIT_05s0020g04280
Standardized expressions
Developmental stages
EI PG G1 G2 H T1 T2 C1 C2 S EP JP

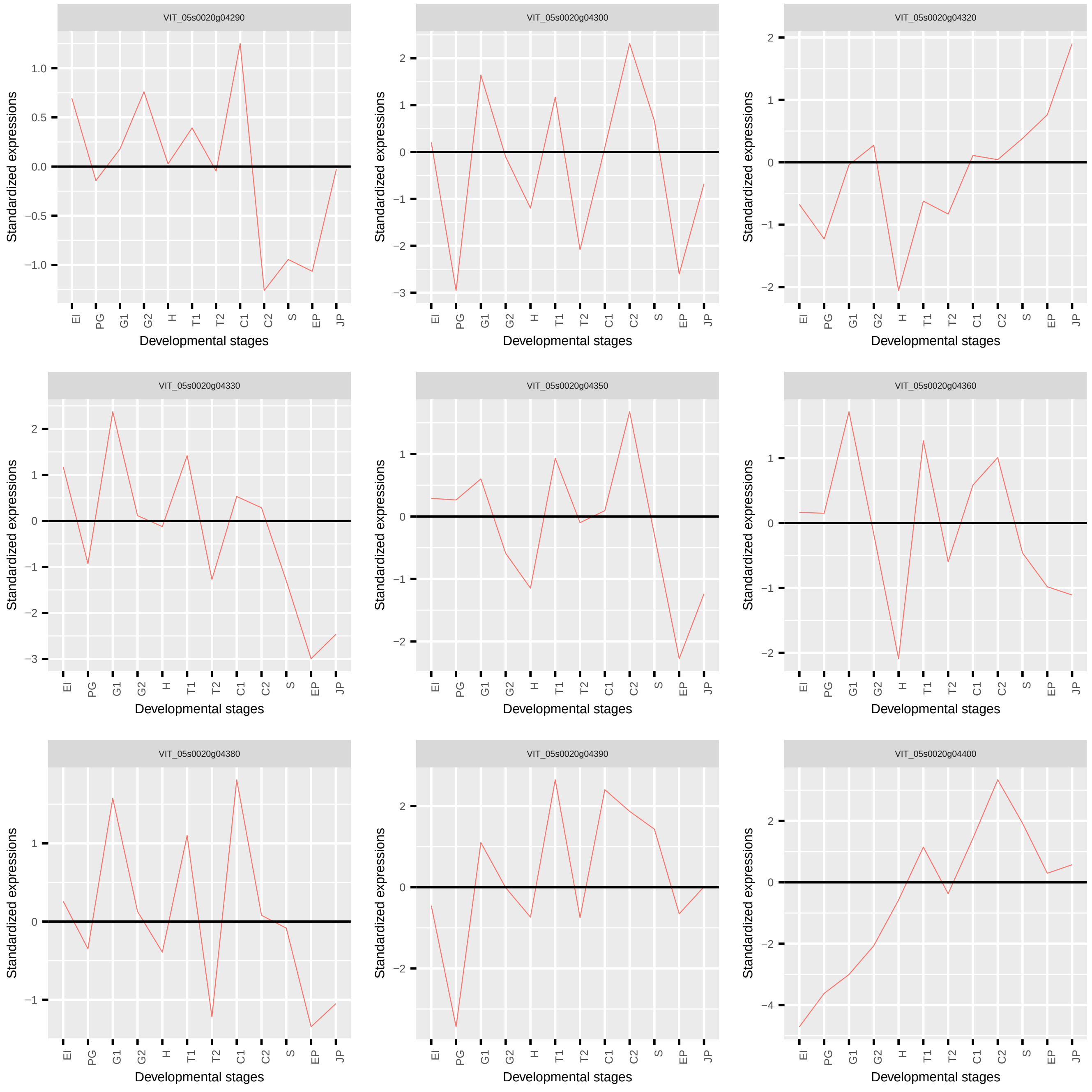
VIT_05s0020g04290
VIT_05s0020g04300
VIT_05s0020g04320
VIT_05s0020g04330
VIT_05s0020g04350
VIT_05s0020g04360
VIT_05s0020g04380
VIT_05s0020g04390
VIT_05s0020g04400
Standardized expressions
Developmental stages
EI PG G1 G2 H T1 T2 C1 C2 S EP JP

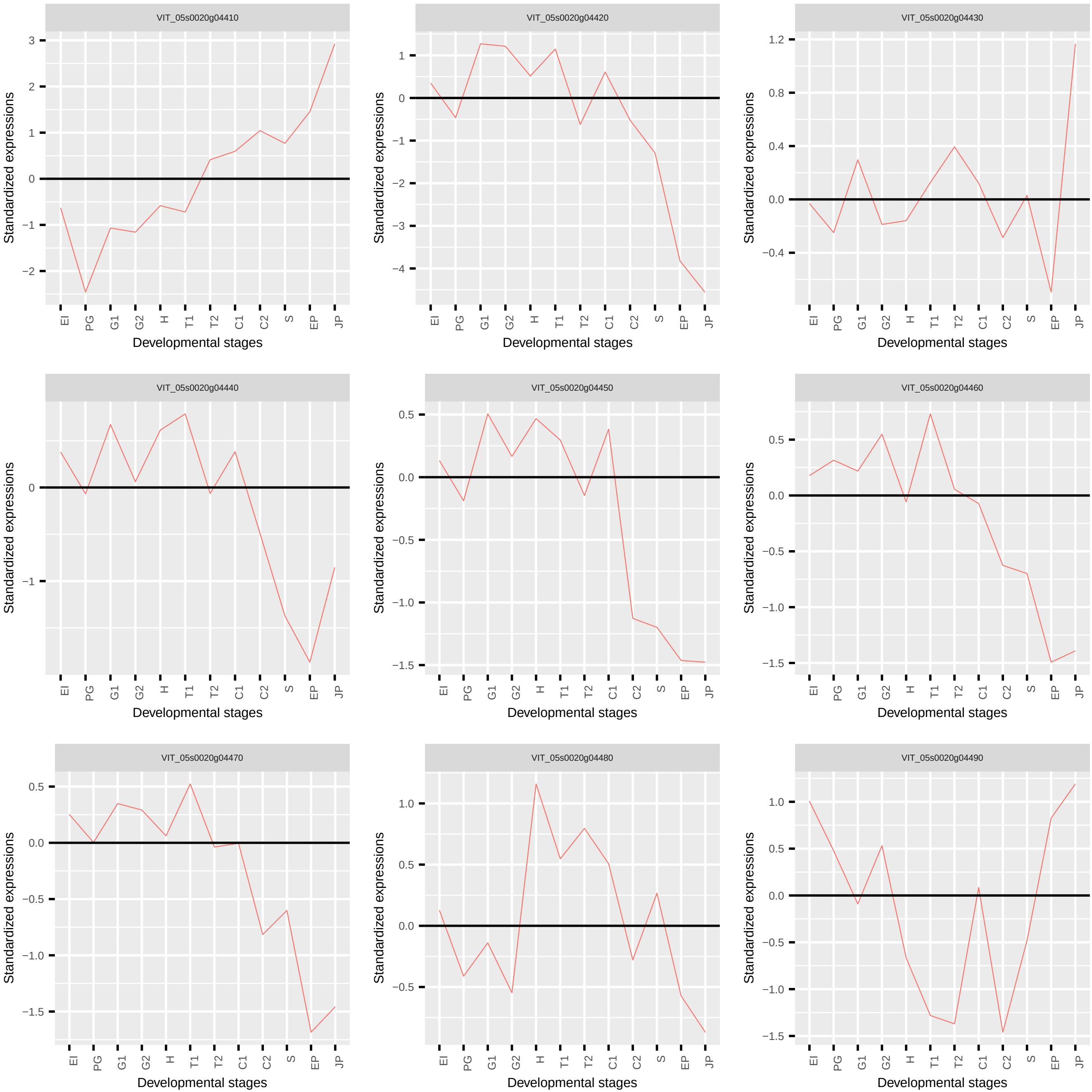
VIT_05s0020g04410
VIT_05s0020g04420
VIT_05s0020g04430
VIT_05s0020g04440
VIT_05s0020g04450
VIT_05s0020g04460
VIT_05s0020g04470
VIT_05s0020g04480
VIT_05s0020g04490
Standardized expressions
Developmental stages
EI PG G1 G2 H T1 T2 C1 C2 S EP JP

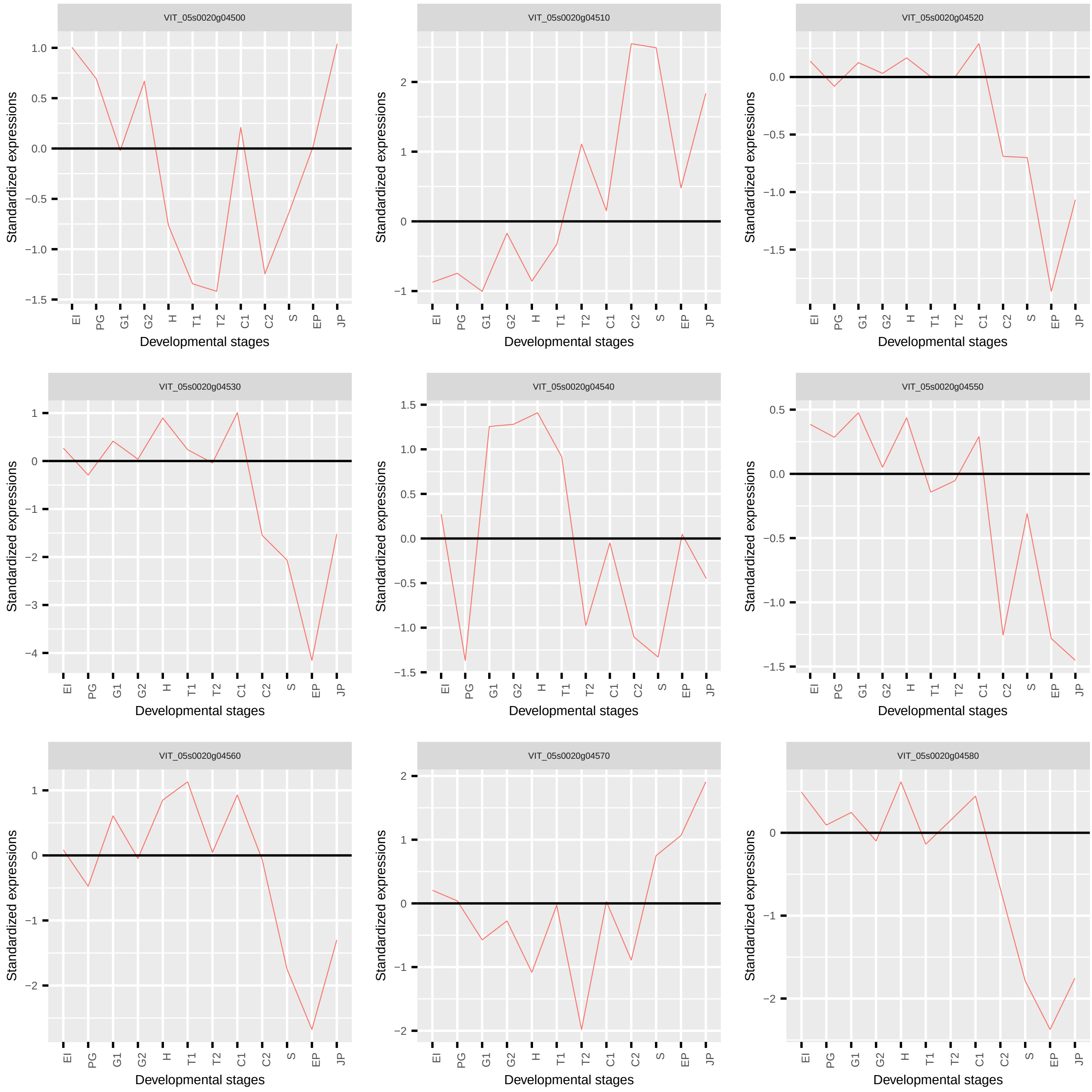
VIT_05s0020g04500
VIT_05s0020g04510
VIT_05s0020g04520
VIT_05s0020g04530
VIT_05s0020g04540
VIT_05s0020g04550
VIT_05s0020g04560
VIT_05s0020g04570
VIT_05s0020g04580
Standardized expressions
Developmental stages
EI PG G1 G2 H T1 T2 C1 C2 S EP JP

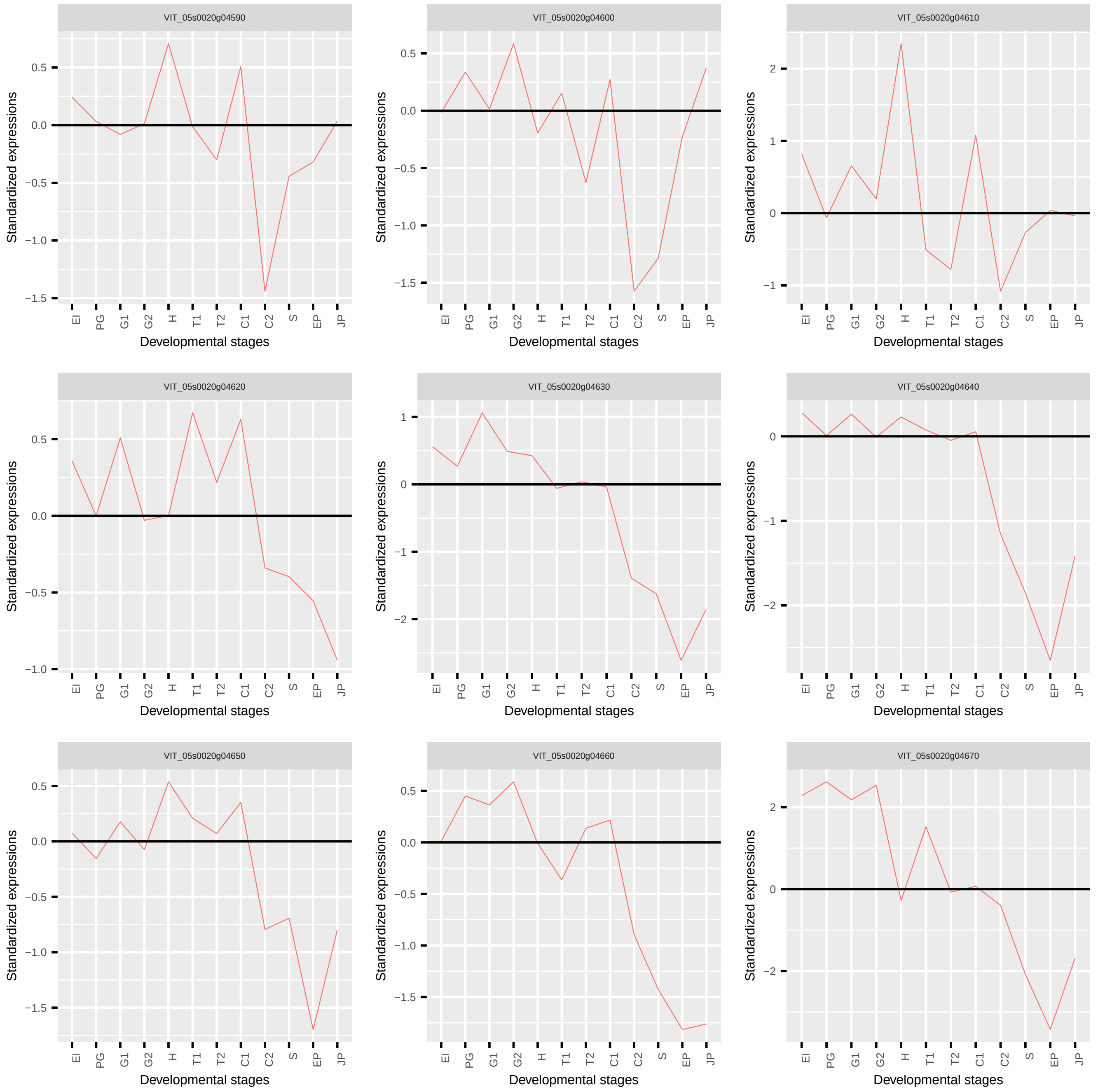
VIT_05s0020g04590
VIT_05s0020g04600
VIT_05s0020g04610
VIT_05s0020g04620
VIT_05s0020g04630
VIT_05s0020g04640
VIT_05s0020g04650
VIT_05s0020g04660
VIT_05s0020g04670
Standardized expressions
Developmental stages
EI PG G1 G2 H T1 T2 C1 C2 S EP JP

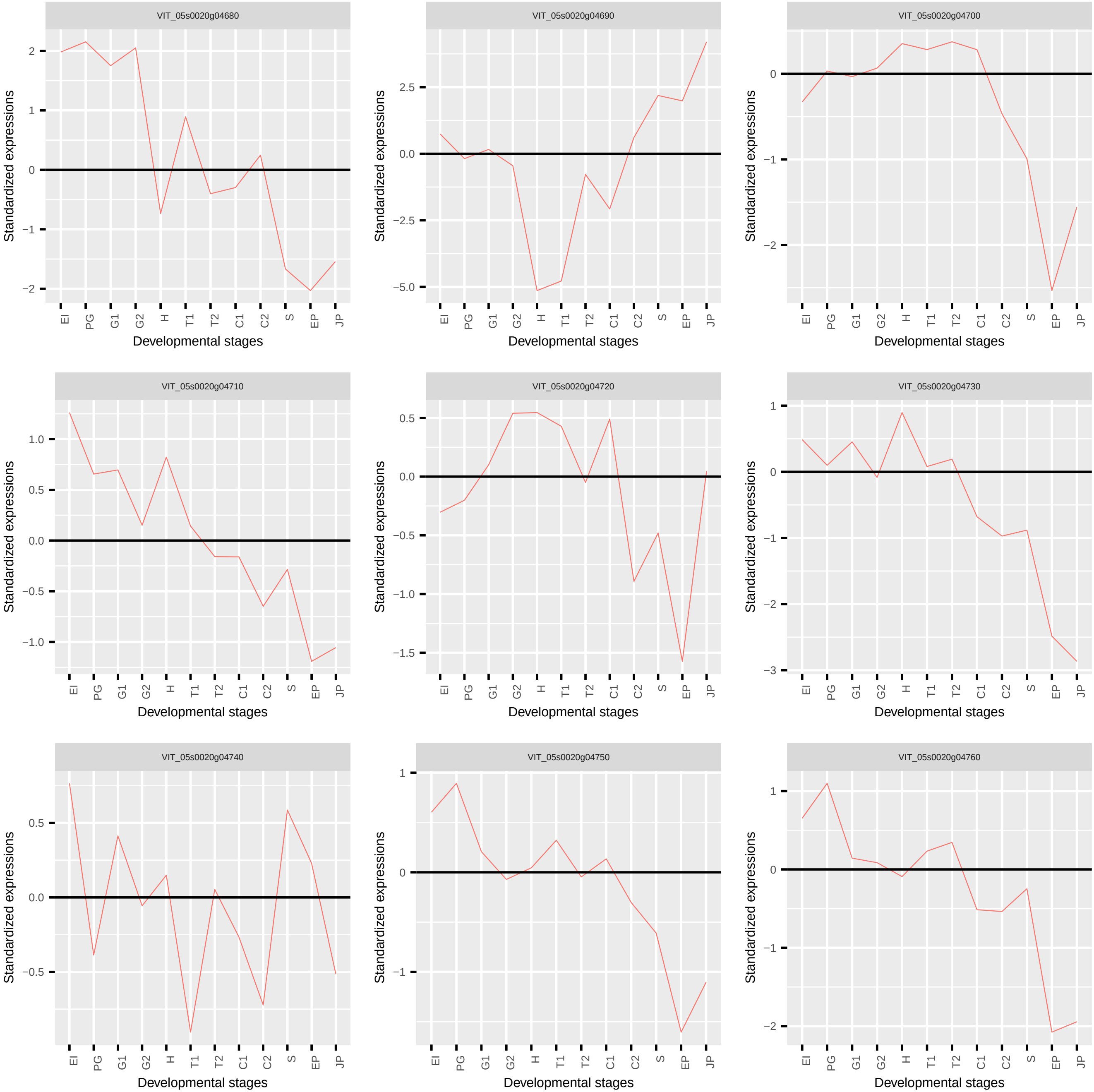
VIT_05s0020g04680
VIT_05s0020g04690
VIT_05s0020g04700
VIT_05s0020g04710
VIT_05s0020g04720
VIT_05s0020g04730
VIT_05s0020g04740
VIT_05s0020g04750
VIT_05s0020g04760
Standardized expressions
Developmental stages
EI PG G1 G2 H T1 T2 C1 C2 S EP JP
2 1 0 −1 −2
2.5 0.0 −2.5 −5.0
0 −1 −2
1.0 0.5 0.0 −0.5 −1.0
0.5 0.0 −0.5 −1.0 −1.5
1 0 −1 −2 −3
0.5 0.0 −0.5
1 0 −1
1 0 −1 −2

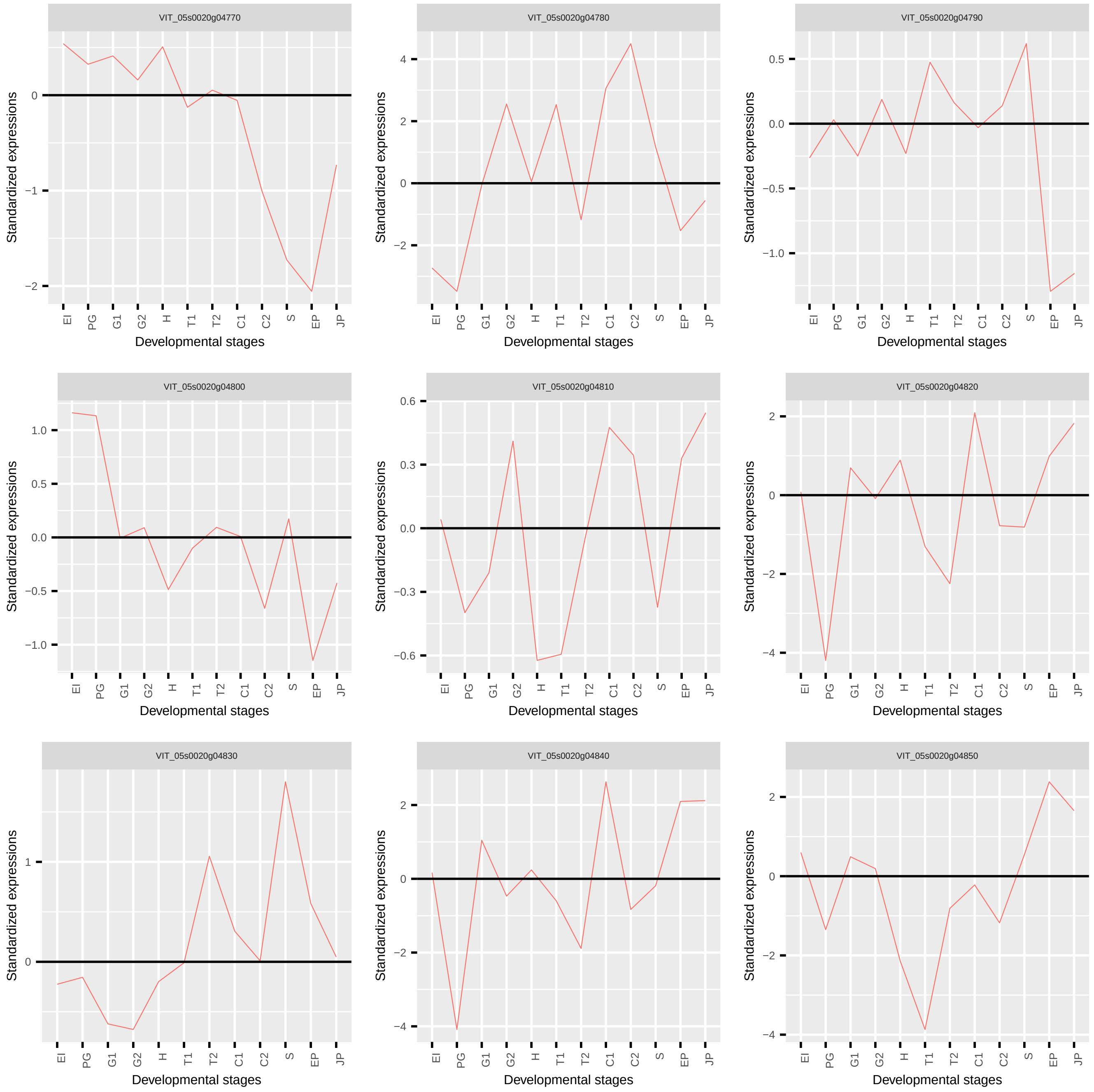
VIT_05s0020g04770
VIT_05s0020g04780
VIT_05s0020g04790
VIT_05s0020g04800
VIT_05s0020g04810
VIT_05s0020g04820
VIT_05s0020g04830
VIT_05s0020g04840
VIT_05s0020g04850
Standardized expressions
Developmental stages
EI PG G1 G2 H T1 T2 C1 C2 S EP JP

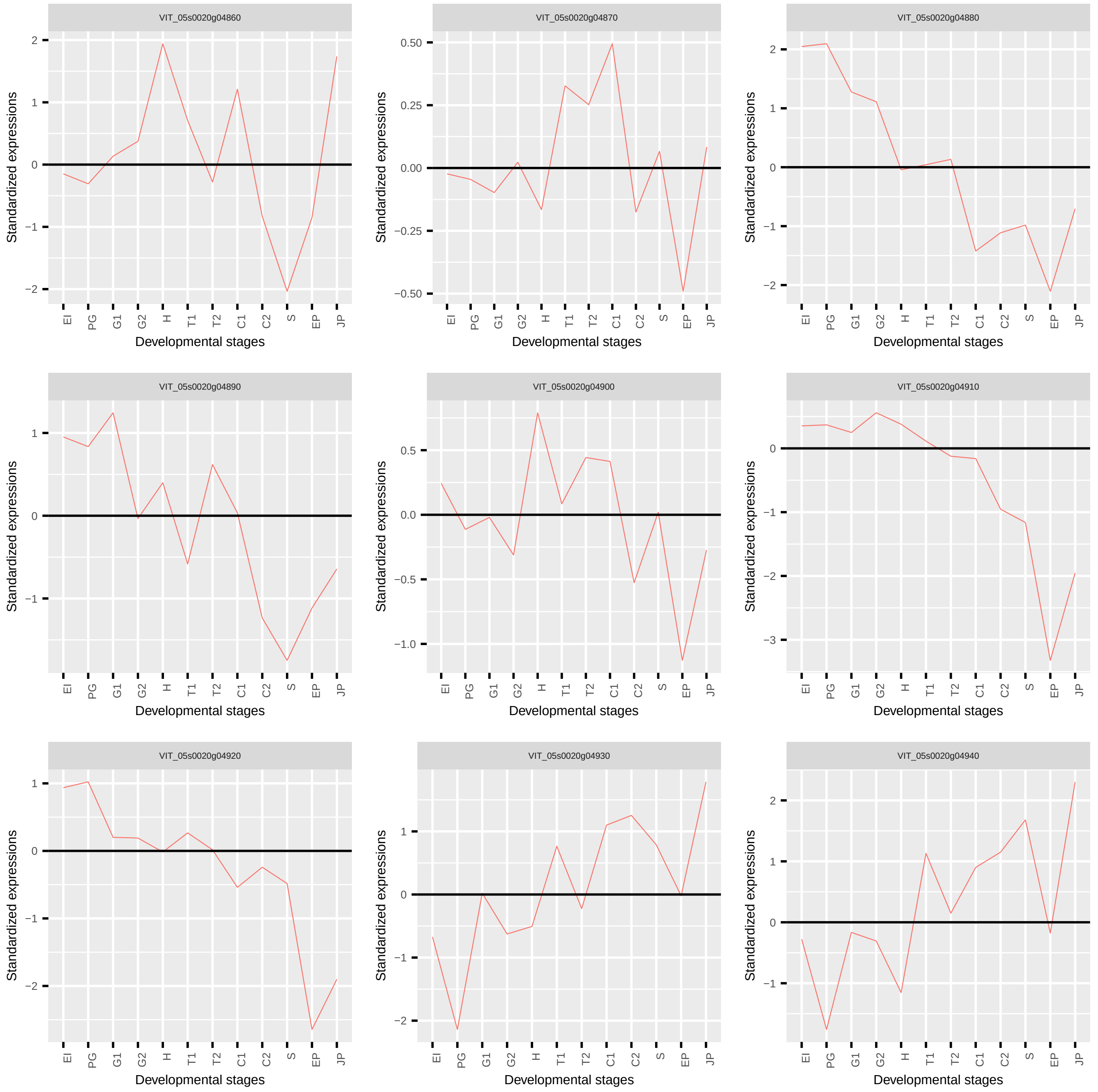

VIT_05s0020g04860
VIT_05s0020g04870
VIT_05s0020g04880
VIT_05s0020g04890
VIT_05s0020g04900
VIT_05s0020g04910
VIT_05s0020g04920
VIT_05s0020g04930
VIT_05s0020g04940
Standardized expressions
Developmental stages
EI
PG
G1
G2
H
T1
T2
C1
C2
S
EP
JP

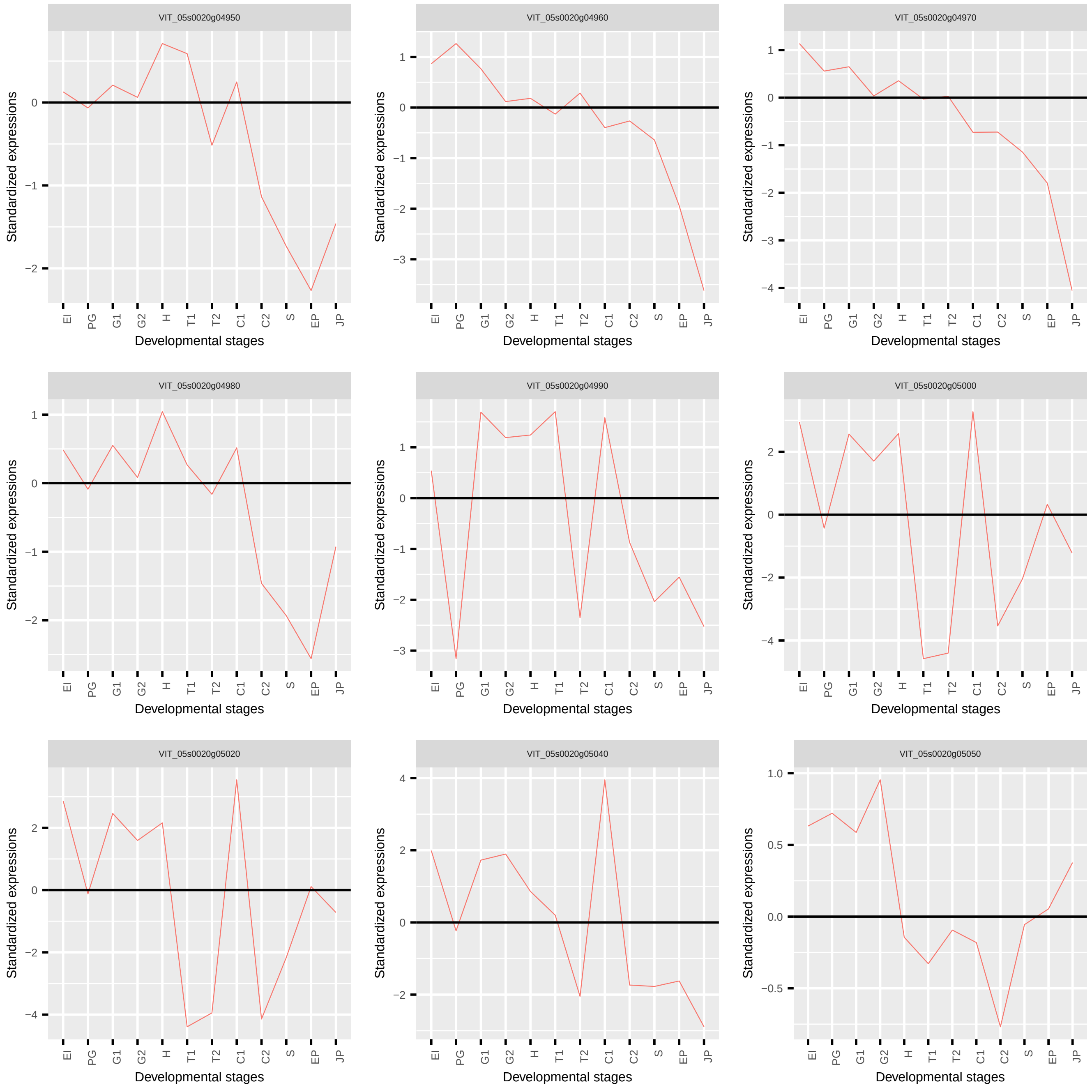
VIT_05s0020g04950
VIT_05s0020g04960
VIT_05s0020g04970
VIT_05s0020g04980
VIT_05s0020g04990
VIT_05s0020g05000
VIT_05s0020g05020
VIT_05s0020g05040
VIT_05s0020g05050
Standardized expressions
Developmental stages
EI
PG
G1
G2
H
T1
T2
C1
C2
S
EP
JP
0
−1
−2
1
−3
−4
2
4
1.0
0.5
0.0
−0.5

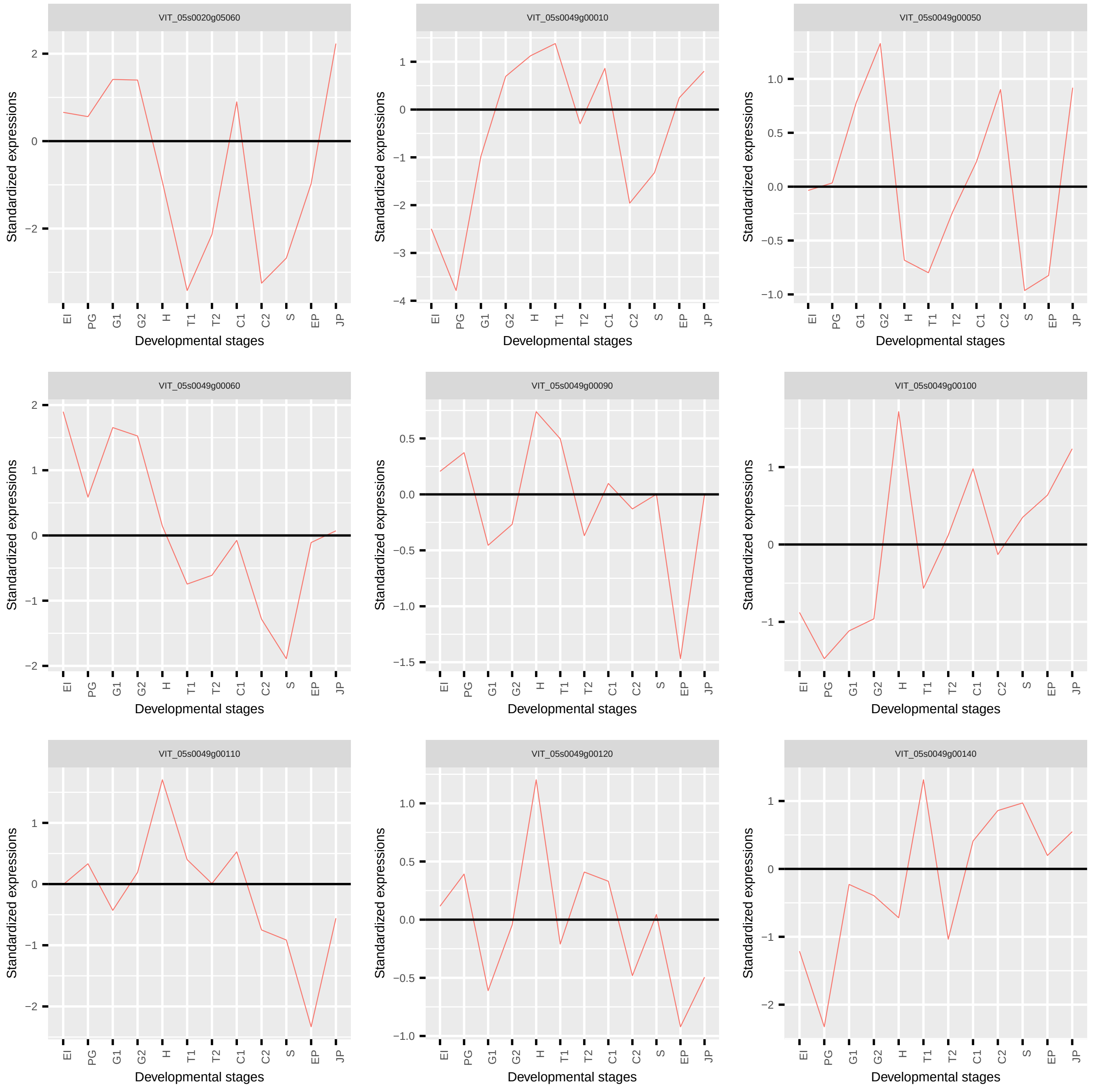
VIT_05s0020g05060
VIT_05s0049g00010
VIT_05s0049g00050
VIT_05s0049g00060
VIT_05s0049g00090
VIT_05s0049g00100
VIT_05s0049g00110
VIT_05s0049g00120
VIT_05s0049g00140
Standardized expressions
Developmental stages
EI PG G1 G2 H T1 T2 C1 C2 S EP JP

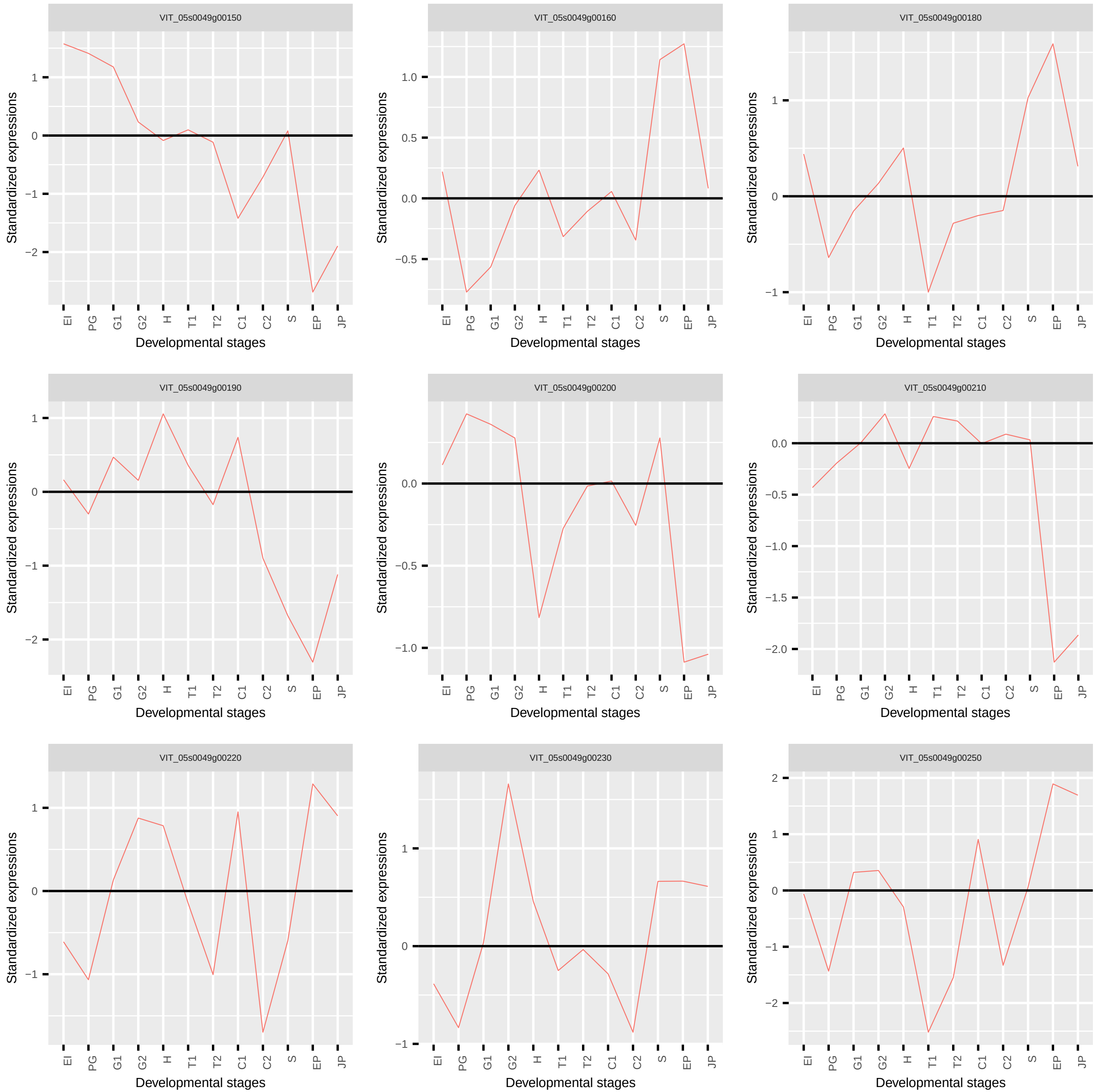

VIT_05s0049g00150
VIT_05s0049g00160
VIT_05s0049g00180
VIT_05s0049g00190
VIT_05s0049g00200
VIT_05s0049g00210
VIT_05s0049g00220
VIT_05s0049g00230
VIT_05s0049g00250
Standardized expressions
Developmental stages
EI PG G1 G2 H T1 T2 C1 C2 S EP JP

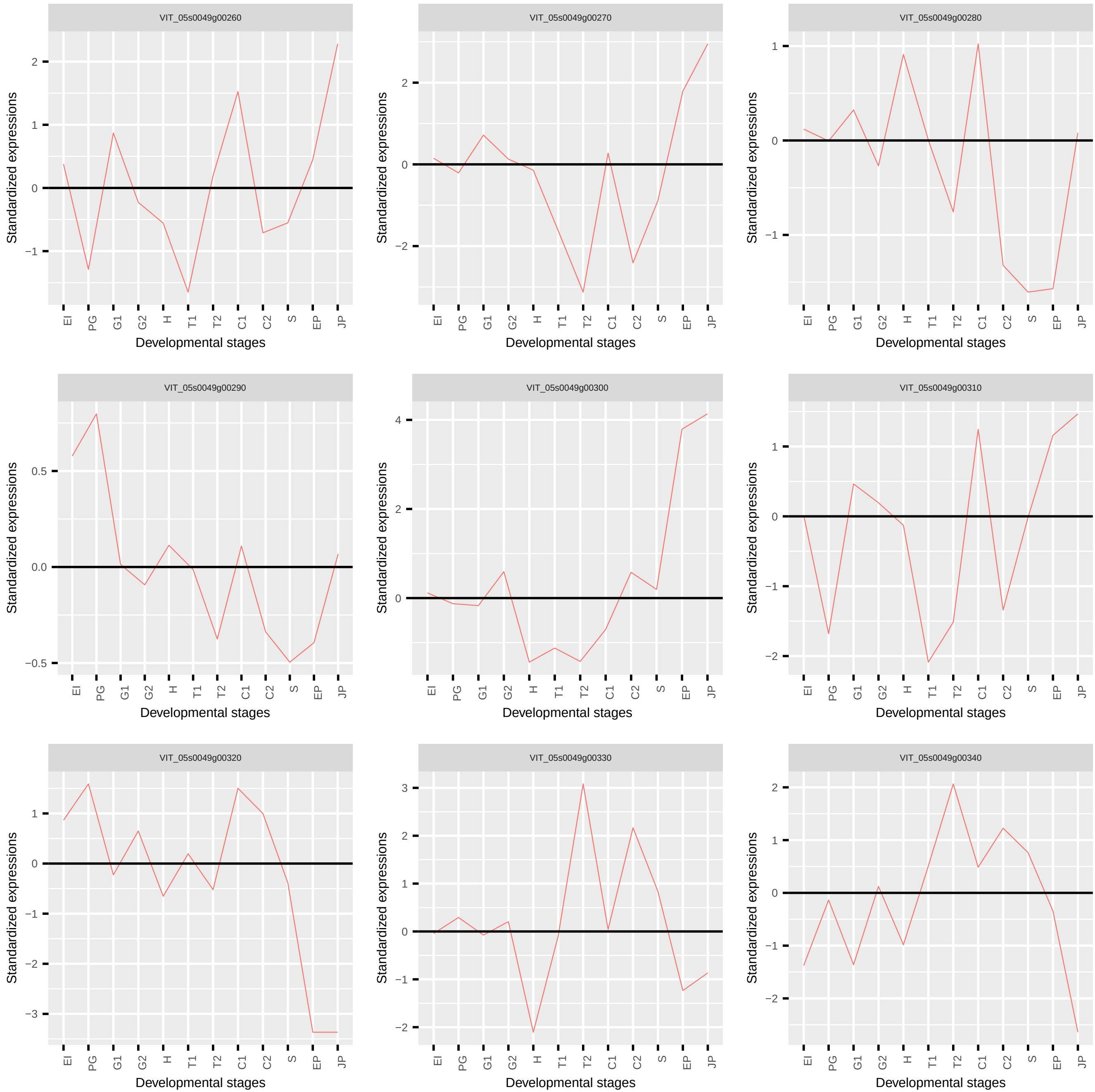
VIT_05s0049g00260
VIT_05s0049g00270
VIT_05s0049g00280
VIT_05s0049g00290
VIT_05s0049g00300
VIT_05s0049g00310
VIT_05s0049g00320
VIT_05s0049g00330
VIT_05s0049g00340
Standardized expressions
Developmental stages
EI
PG
G1
G2
H
T1
T2
C1
C2
S
EP
JP

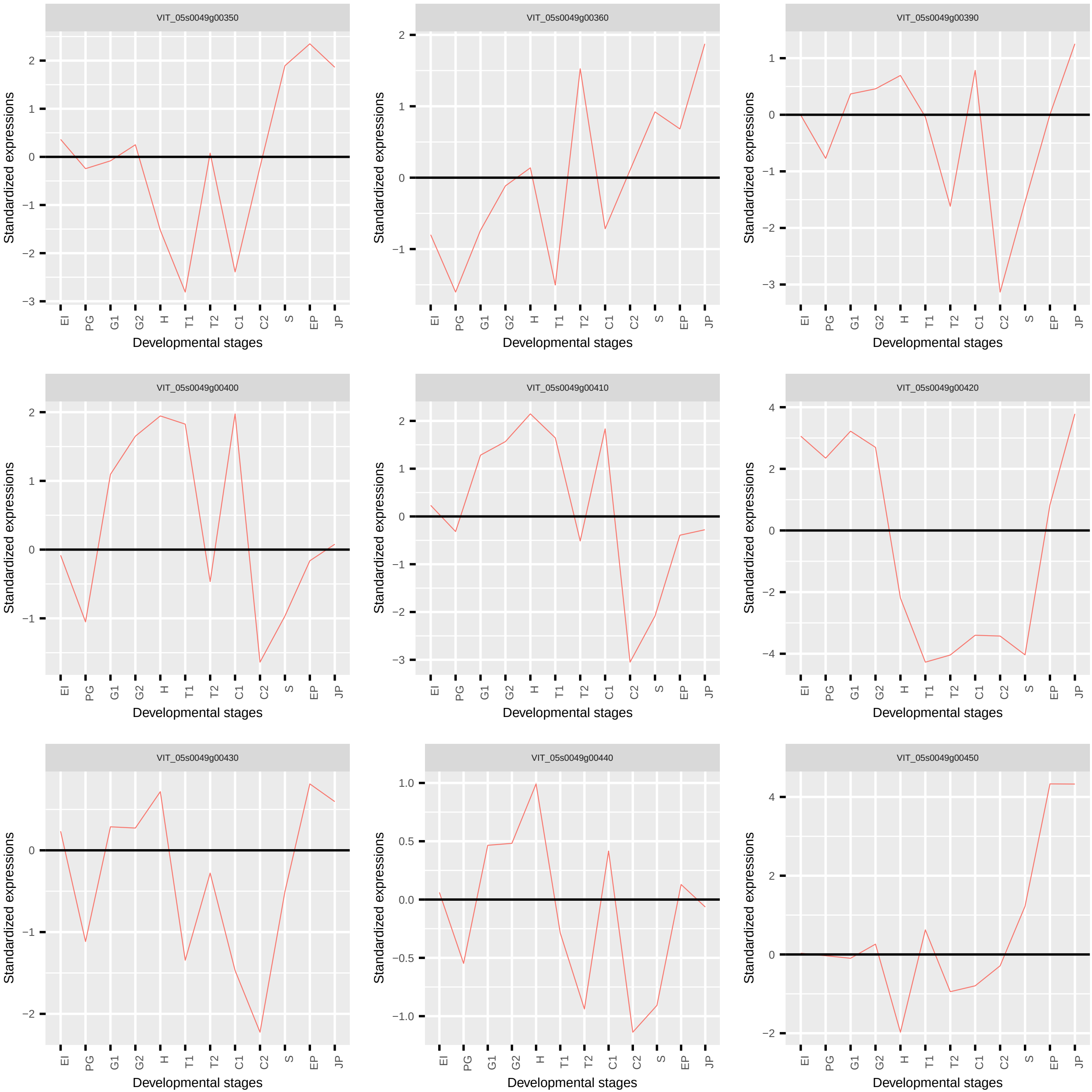

VIT_05s0049g00350
VIT_05s0049g00360
VIT_05s0049g00390
VIT_05s0049g00400
VIT_05s0049g00410
VIT_05s0049g00420
VIT_05s0049g00430
VIT_05s0049g00440
VIT_05s0049g00450
Standardized expressions
Developmental stages
EI PG G1 G2 H T1 T2 C1 C2 S EP JP

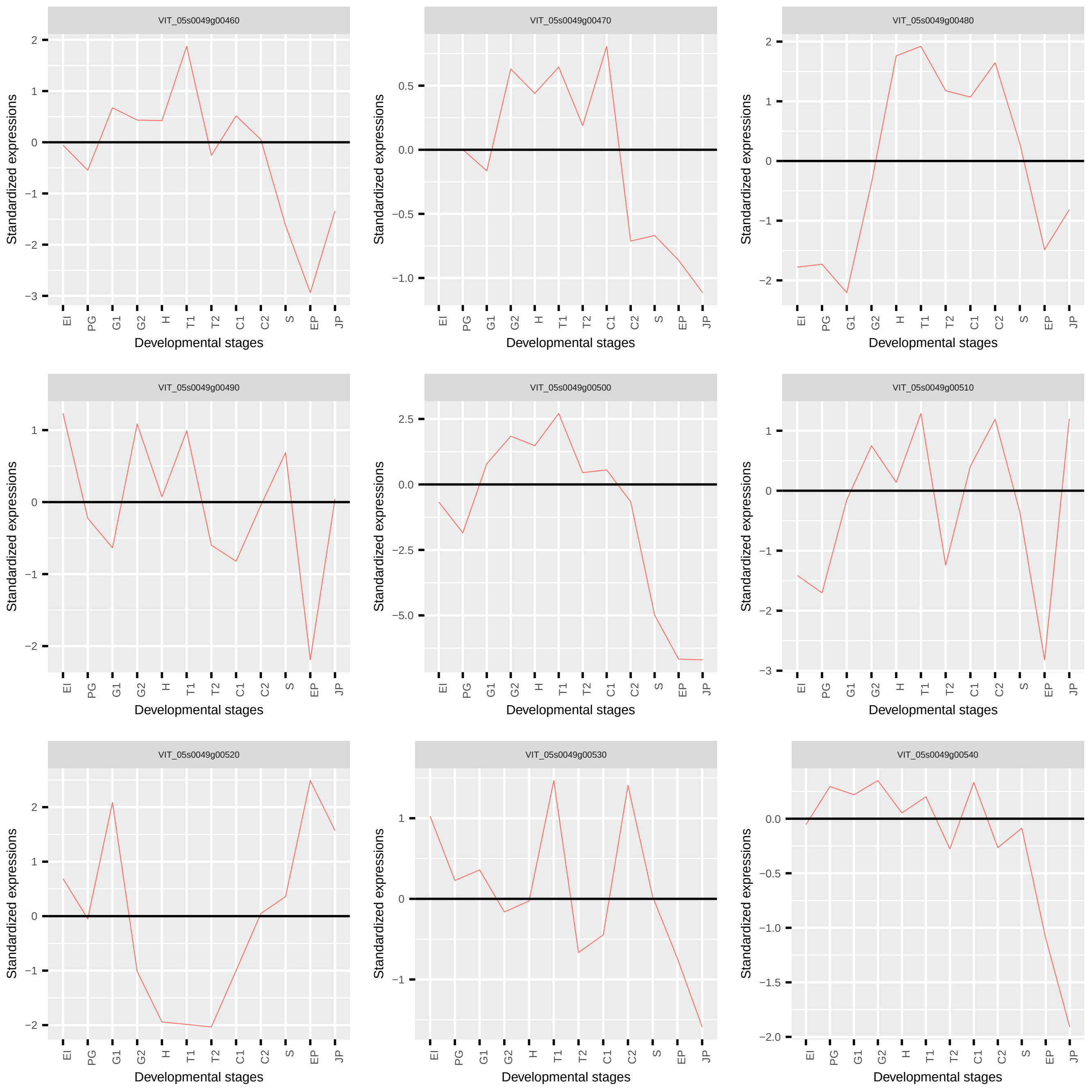

VIT_05s0049g00460
2
1
0
−1
−2
−3
Standardized expressions
EI PG G1 G2 H T1 T2 C1 C2 S EP JP
Developmental stages
VIT_05s0049g00470
0.5
0.0
−0.5
−1.0
Standardized expressions
EI PG G1 G2 H T1 T2 C1 C2 S EP JP
Developmental stages
VIT_05s0049g00480
2
1
0
−1
−2
Standardized expressions
EI PG G1 G2 H T1 T2 C1 C2 S EP JP
Developmental stages
VIT_05s0049g00490
1
0
−1
−2
Standardized expressions
EI PG G1 G2 H T1 T2 C1 C2 S EP JP
Developmental stages
VIT_05s0049g00500
2.5
0.0
−2.5
−5.0
Standardized expressions
EI PG G1 G2 H T1 T2 C1 C2 S EP JP
Developmental stages
VIT_05s0049g00510
1
0
−1
−2
−3
Standardized expressions
EI PG G1 G2 H T1 T2 C1 C2 S EP JP
Developmental stages
VIT_05s0049g00520
2
1
0
−1
−2
Standardized expressions
EI PG G1 G2 H T1 T2 C1 C2 S EP JP
Developmental stages
VIT_05s0049g00530
1
0
−1
Standardized expressions
EI PG G1 G2 H T1 T2 C1 C2 S EP JP
Developmental stages
VIT_05s0049g00540
0.0
−0.5
−1.0
−1.5
−2.0
Standardized expressions
EI PG G1 G2 H T1 T2 C1 C2 S EP JP
Developmental stages

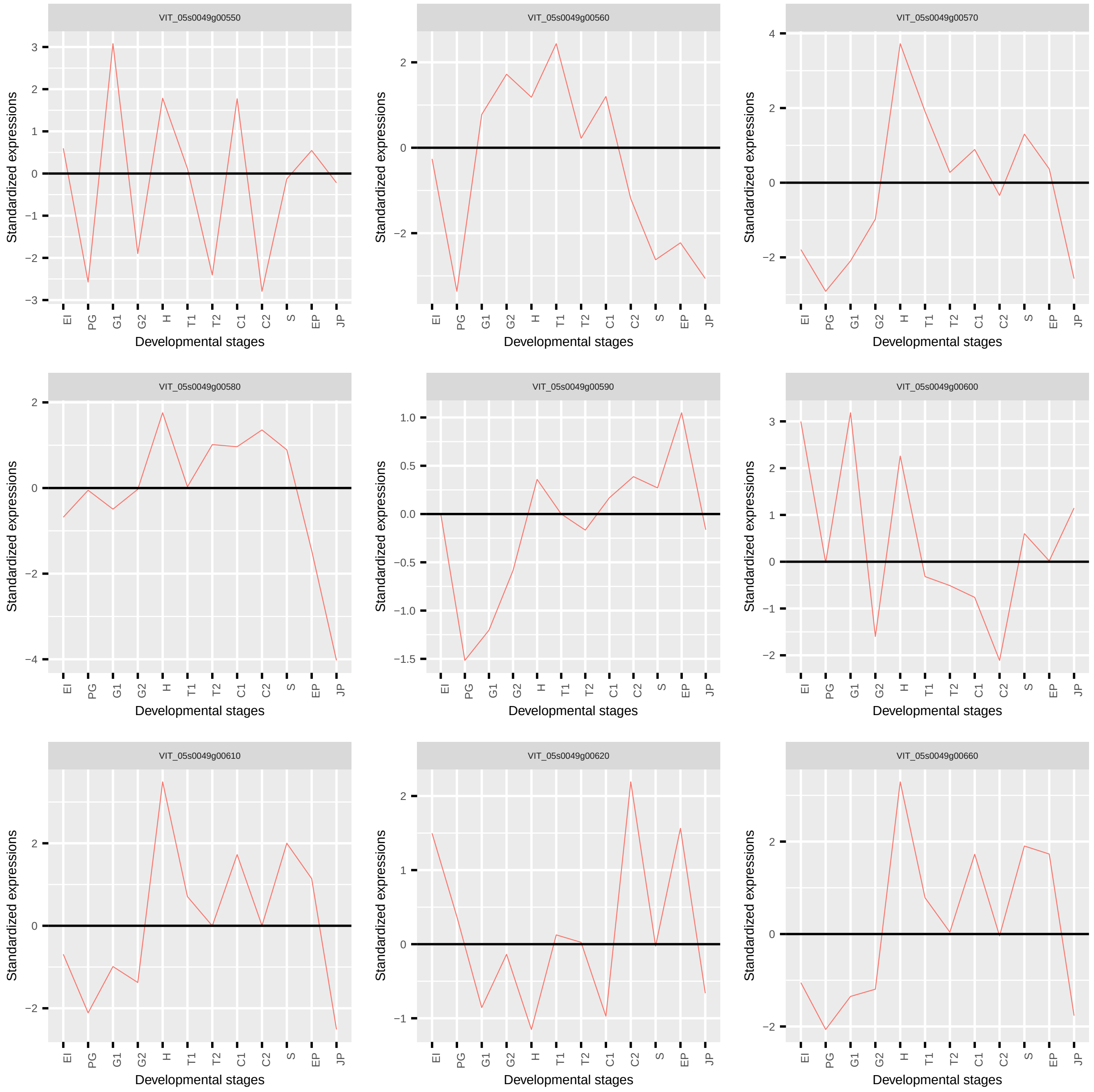
VIT_05s0049g00550
VIT_05s0049g00560
VIT_05s0049g00570
VIT_05s0049g00580
VIT_05s0049g00590
VIT_05s0049g00600
VIT_05s0049g00610
VIT_05s0049g00620
VIT_05s0049g00660
Standardized expressions
Developmental stages
EI
PG
G1
G2
H
T1
T2
C1
C2
S
EP
JP

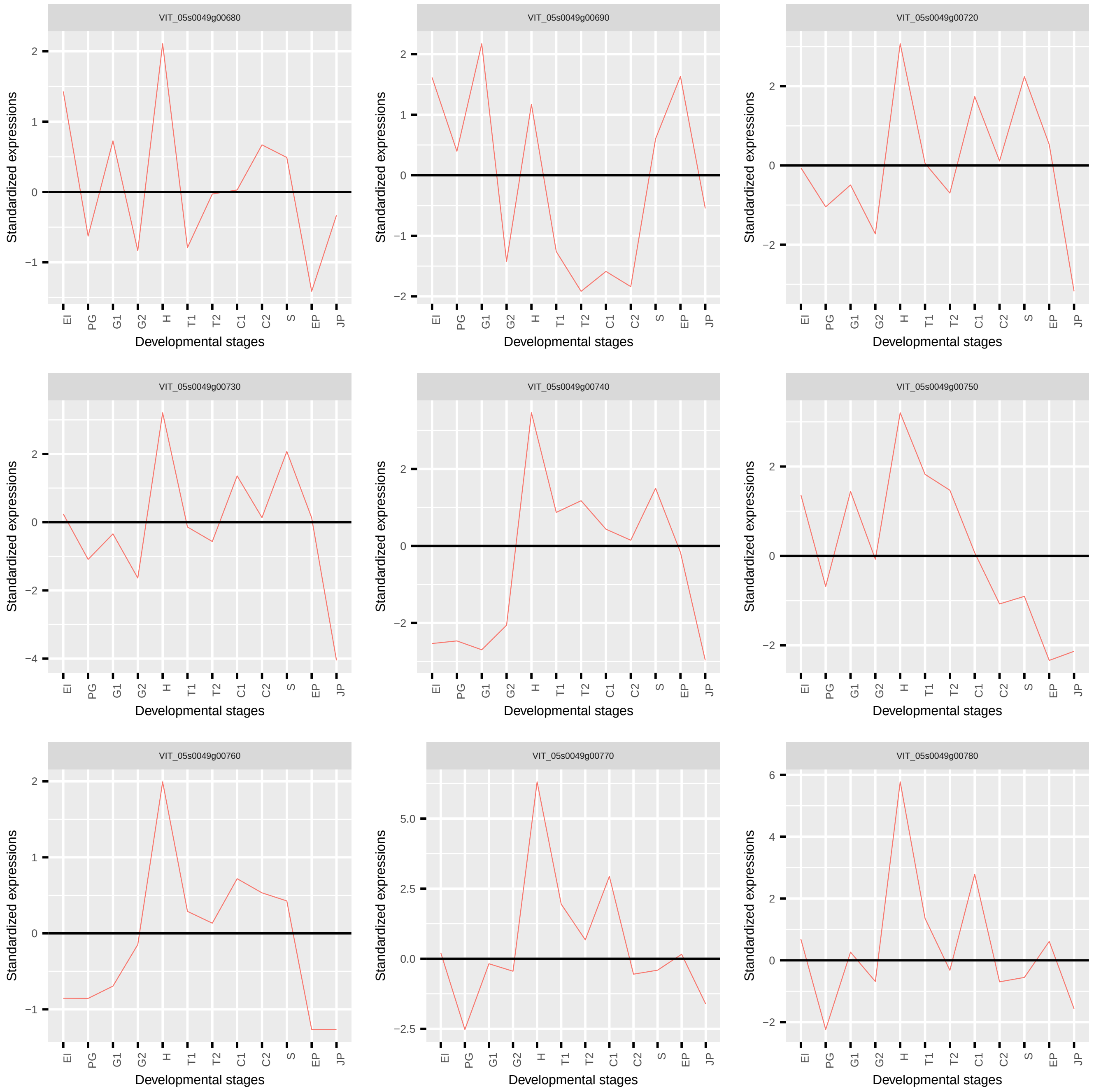
VIT_05s0049g00680
VIT_05s0049g00690
VIT_05s0049g00720
VIT_05s0049g00730
VIT_05s0049g00740
VIT_05s0049g00750
VIT_05s0049g00760
VIT_05s0049g00770
VIT_05s0049g00780
Standardized expressions
Developmental stages
EI
PG
G1
G2
H
T1
T2
C1
C2
S
EP
JP

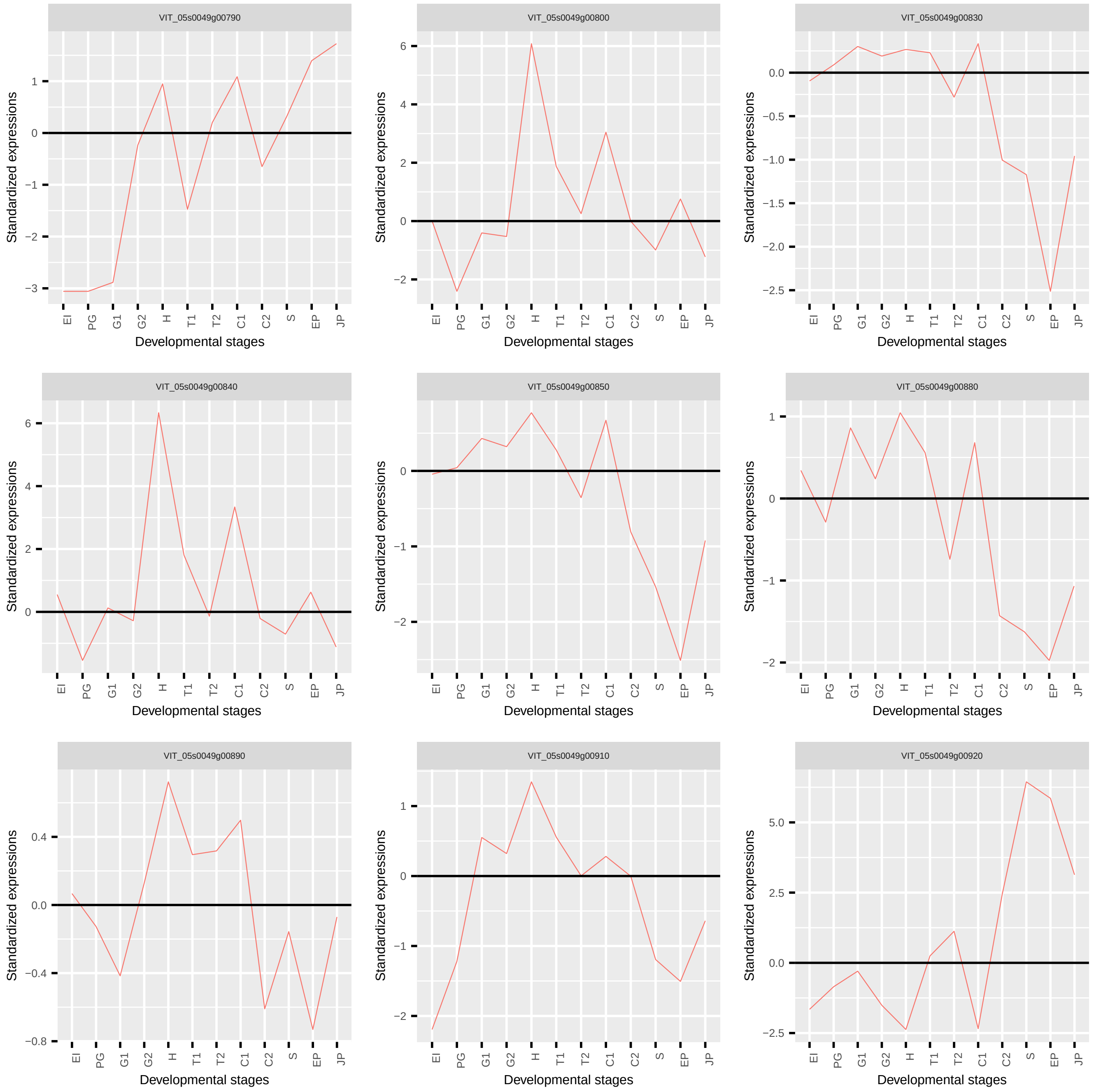
VIT_05s0049g00790
VIT_05s0049g00800
VIT_05s0049g00830
VIT_05s0049g00840
VIT_05s0049g00850
VIT_05s0049g00880
VIT_05s0049g00890
VIT_05s0049g00910
VIT_05s0049g00920
Standardized expressions
Developmental stages
EI
PG
G1
G2
H
T1
T2
C1
C2
S
EP
JP

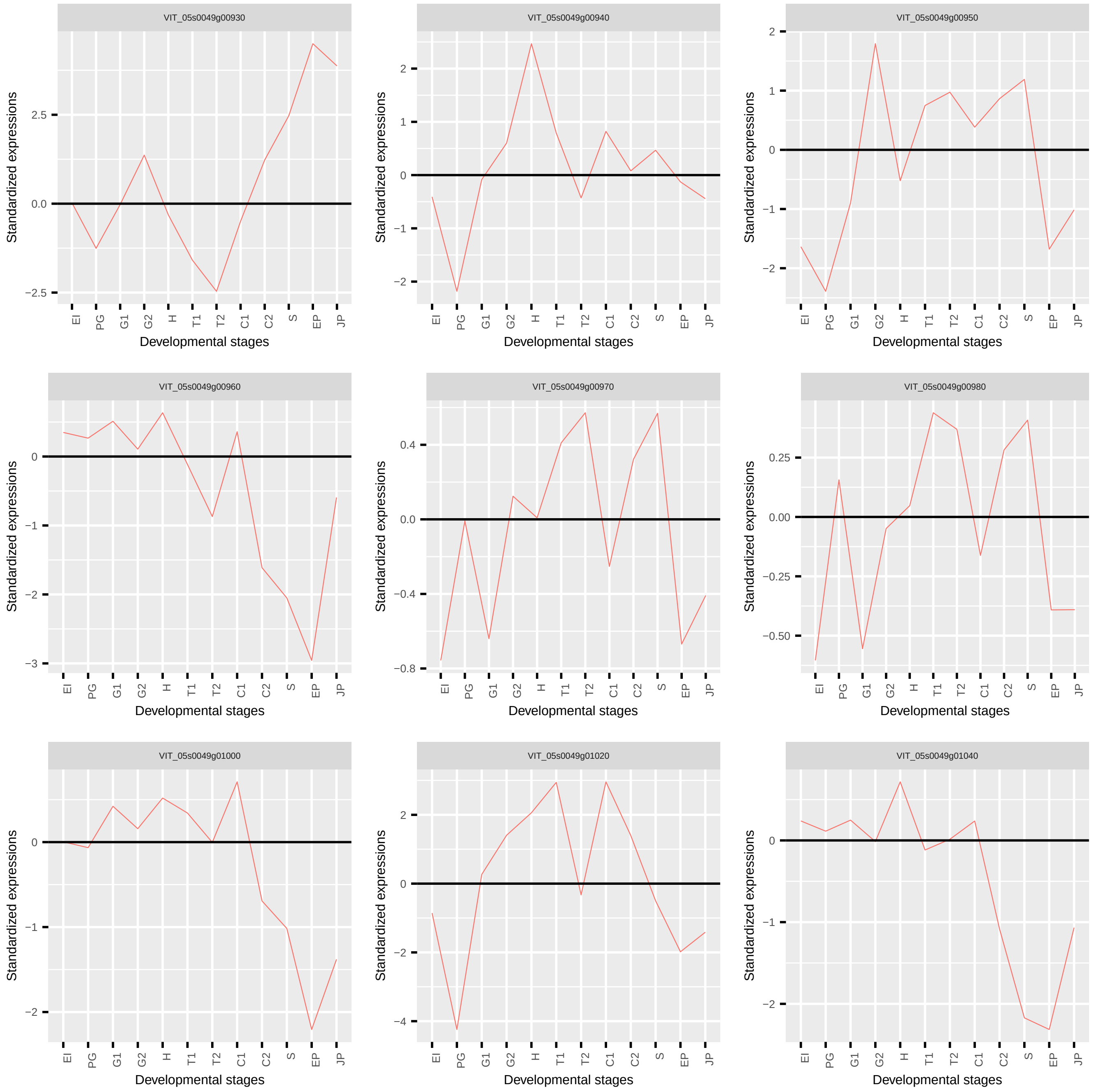
VIT_05s0049g00930
VIT_05s0049g00940
VIT_05s0049g00950
VIT_05s0049g00960
VIT_05s0049g00970
VIT_05s0049g00980
VIT_05s0049g01000
VIT_05s0049g01020
VIT_05s0049g01040
Standardized expressions
Developmental stages
EI PG G1 G2 H T1 T2 C1 C2 S EP JP

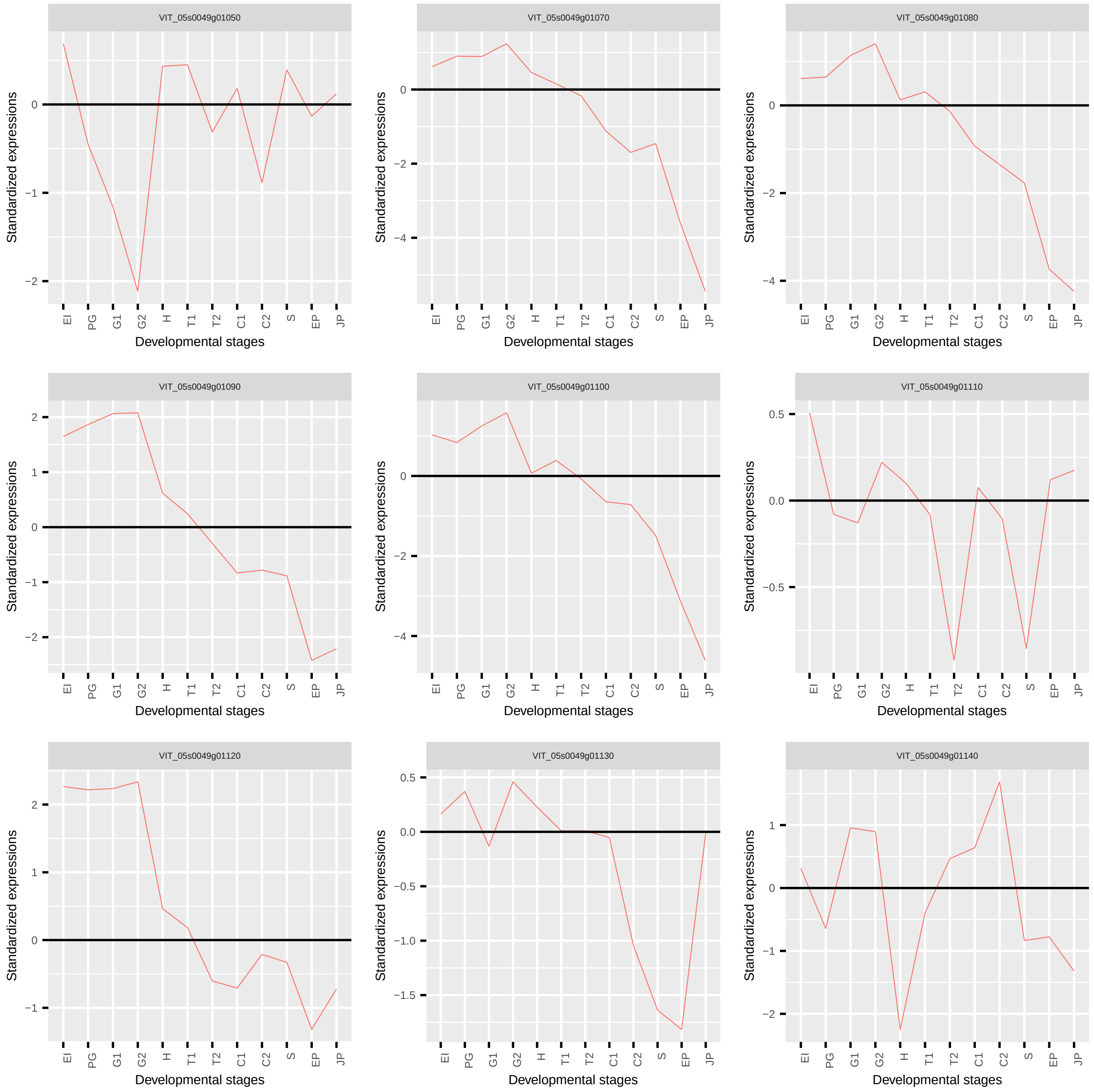

VIT_05s0049g01050
VIT_05s0049g01070
VIT_05s0049g01080
VIT_05s0049g01090
VIT_05s0049g01100
VIT_05s0049g01110
VIT_05s0049g01120
VIT_05s0049g01130
VIT_05s0049g01140
Standardized expressions
Developmental stages
EI
PG
G1
G2
H
T1
T2
C1
C2
S
EP
JP

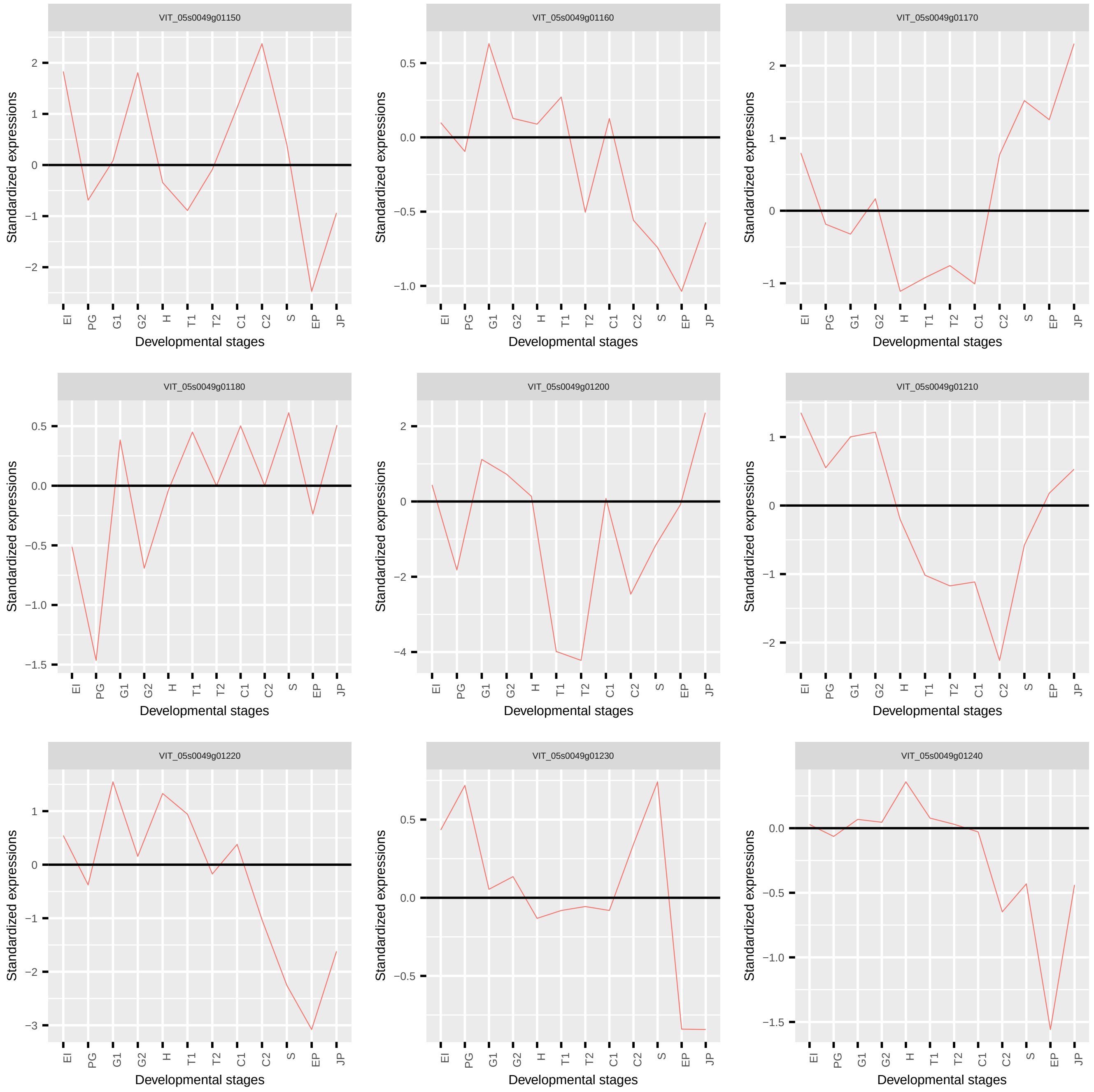
VIT_05s0049g01150
VIT_05s0049g01160
VIT_05s0049g01170
VIT_05s0049g01180
VIT_05s0049g01200
VIT_05s0049g01210
VIT_05s0049g01220
VIT_05s0049g01230
VIT_05s0049g01240
Standardized expressions
Developmental stages
EI PG G1 G2 H T1 T2 C1 C2 S EP JP

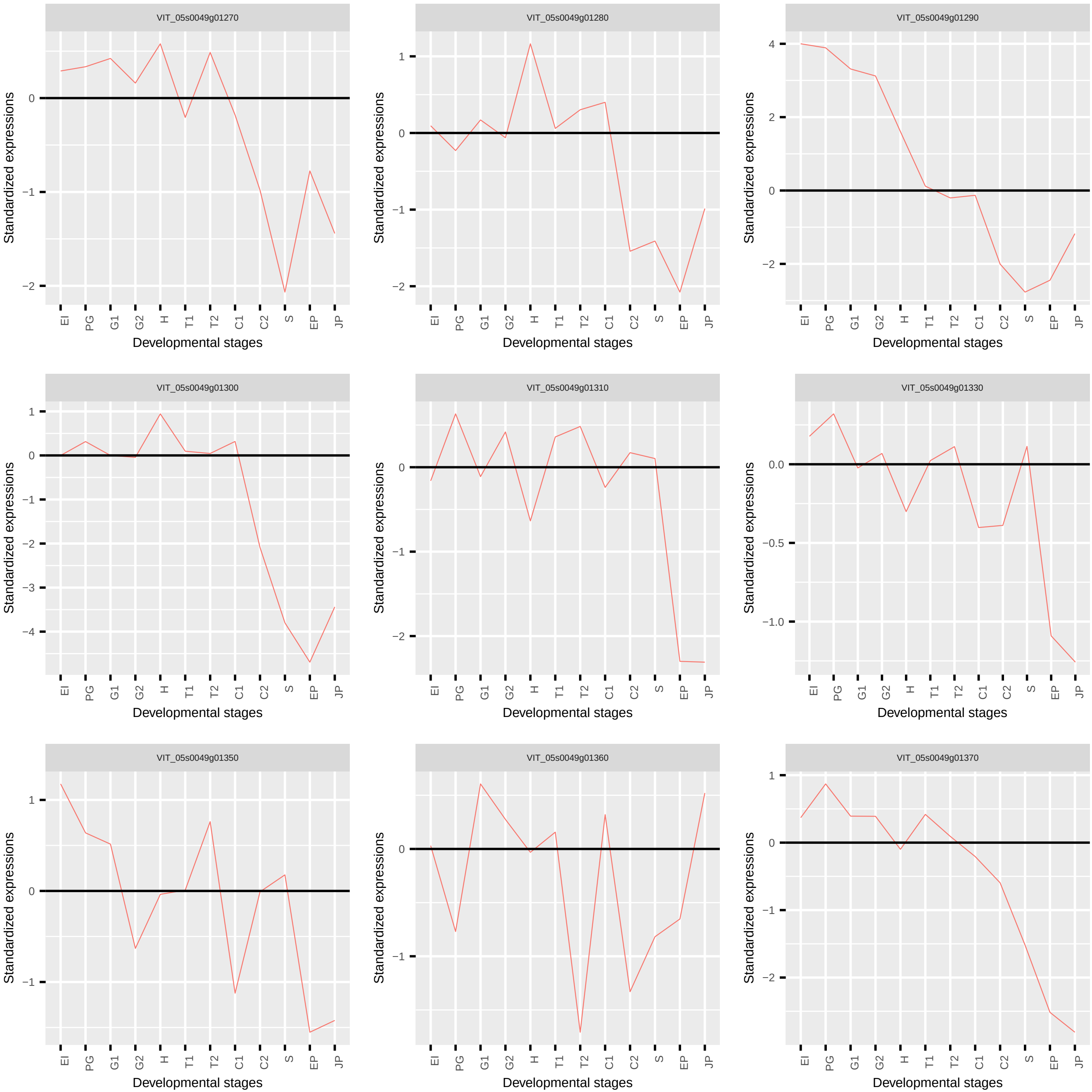
VIT_05s0049g01270
VIT_05s0049g01280
VIT_05s0049g01290
VIT_05s0049g01300
VIT_05s0049g01310
VIT_05s0049g01330
VIT_05s0049g01350
VIT_05s0049g01360
VIT_05s0049g01370
Standardized expressions
Developmental stages
EI PG G1 G2 H T1 T2 C1 C2 S EP JP

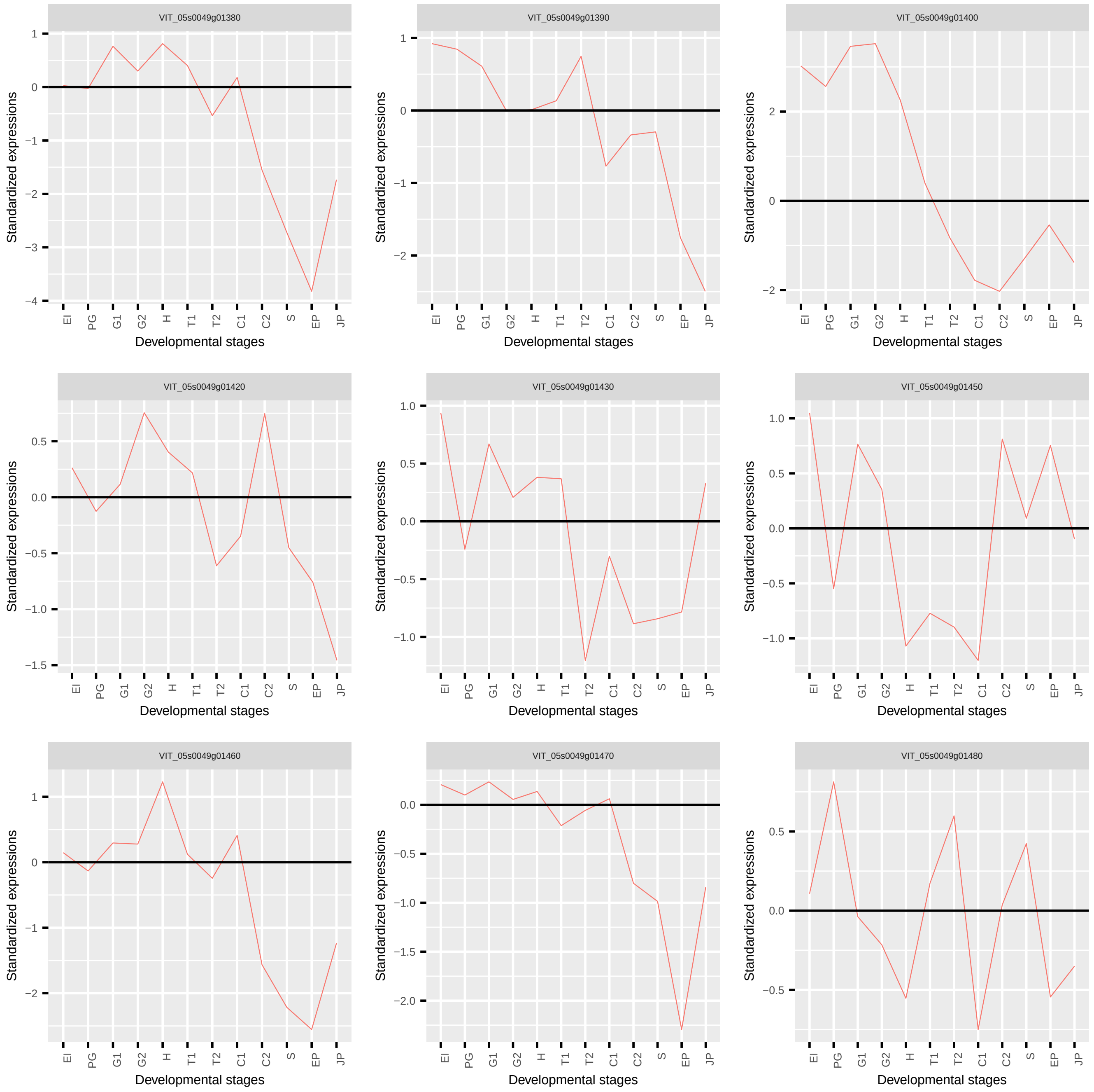
VIT_05s0049g01380
VIT_05s0049g01390
VIT_05s0049g01400
VIT_05s0049g01420
VIT_05s0049g01430
VIT_05s0049g01450
VIT_05s0049g01460
VIT_05s0049g01470
VIT_05s0049g01480
Standardized expressions
Developmental stages
EI PG G1 G2 H T1 T2 C1 C2 S EP JP

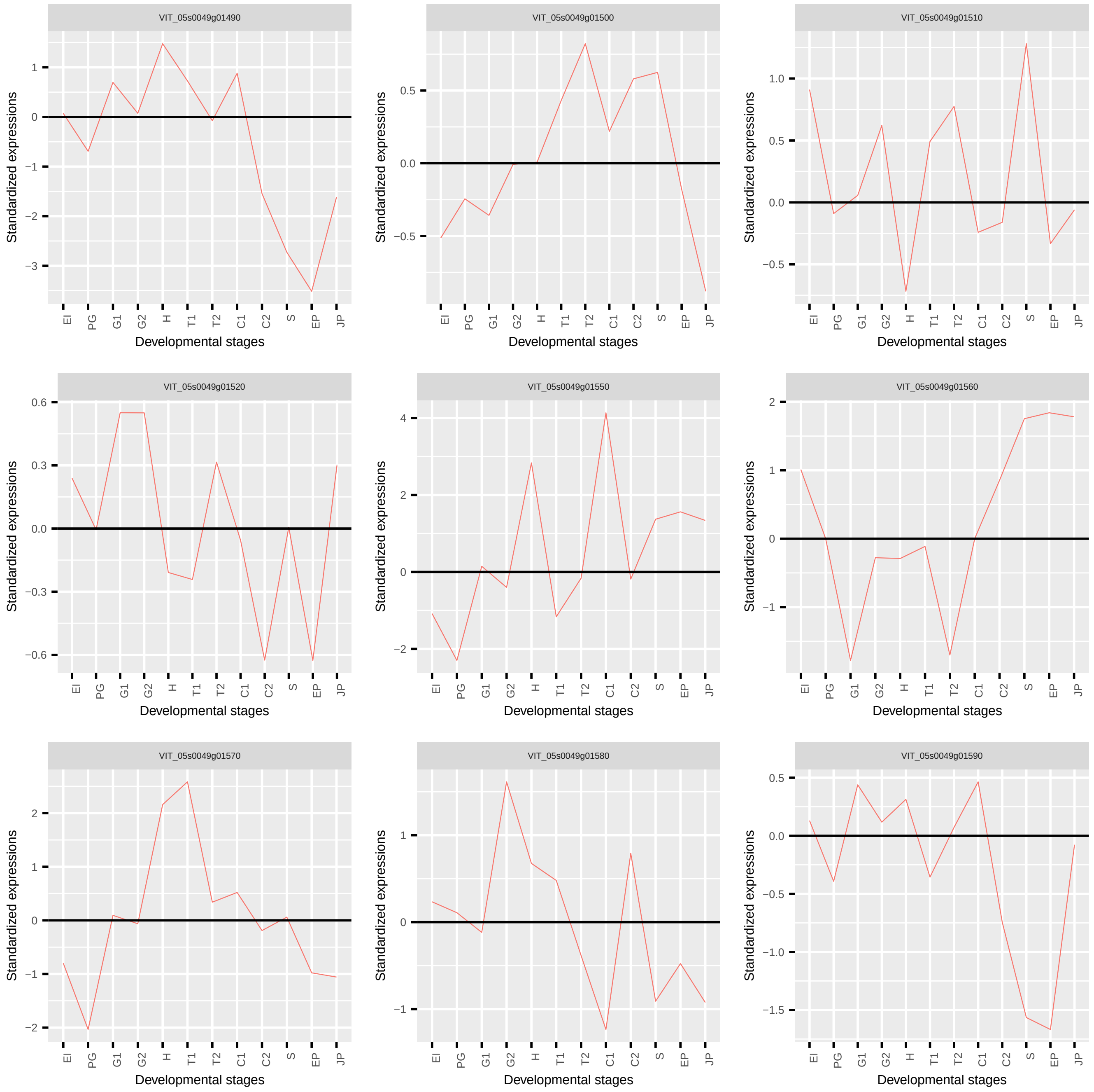
VIT_05s0049g01490
Standardized expressions
1
0
−1
−2
−3
EI PG G1 G2 H T1 T2 C1 C2 S EP JP
Developmental stages
VIT_05s0049g01500
Standardized expressions
0.5
0.0
−0.5
EI PG G1 G2 H T1 T2 C1 C2 S EP JP
Developmental stages
VIT_05s0049g01510
Standardized expressions
1.0
0.5
0.0
−0.5
EI PG G1 G2 H T1 T2 C1 C2 S EP JP
Developmental stages
VIT_05s0049g01520
Standardized expressions
0.6
0.3
0.0
−0.3
−0.6
EI PG G1 G2 H T1 T2 C1 C2 S EP JP
Developmental stages
VIT_05s0049g01550
Standardized expressions
4
2
0
−2
EI PG G1 G2 H T1 T2 C1 C2 S EP JP
Developmental stages
VIT_05s0049g01560
Standardized expressions
2
1
0
−1
EI PG G1 G2 H T1 T2 C1 C2 S EP JP
Developmental stages
VIT_05s0049g01570
Standardized expressions
2
1
0
−1
−2
EI PG G1 G2 H T1 T2 C1 C2 S EP JP
Developmental stages
VIT_05s0049g01580
Standardized expressions
1
0
−1
EI PG G1 G2 H T1 T2 C1 C2 S EP JP
Developmental stages
VIT_05s0049g01590
Standardized expressions
0.5
0.0
−0.5
−1.0
−1.5
EI PG G1 G2 H T1 T2 C1 C2 S EP JP
Developmental stages

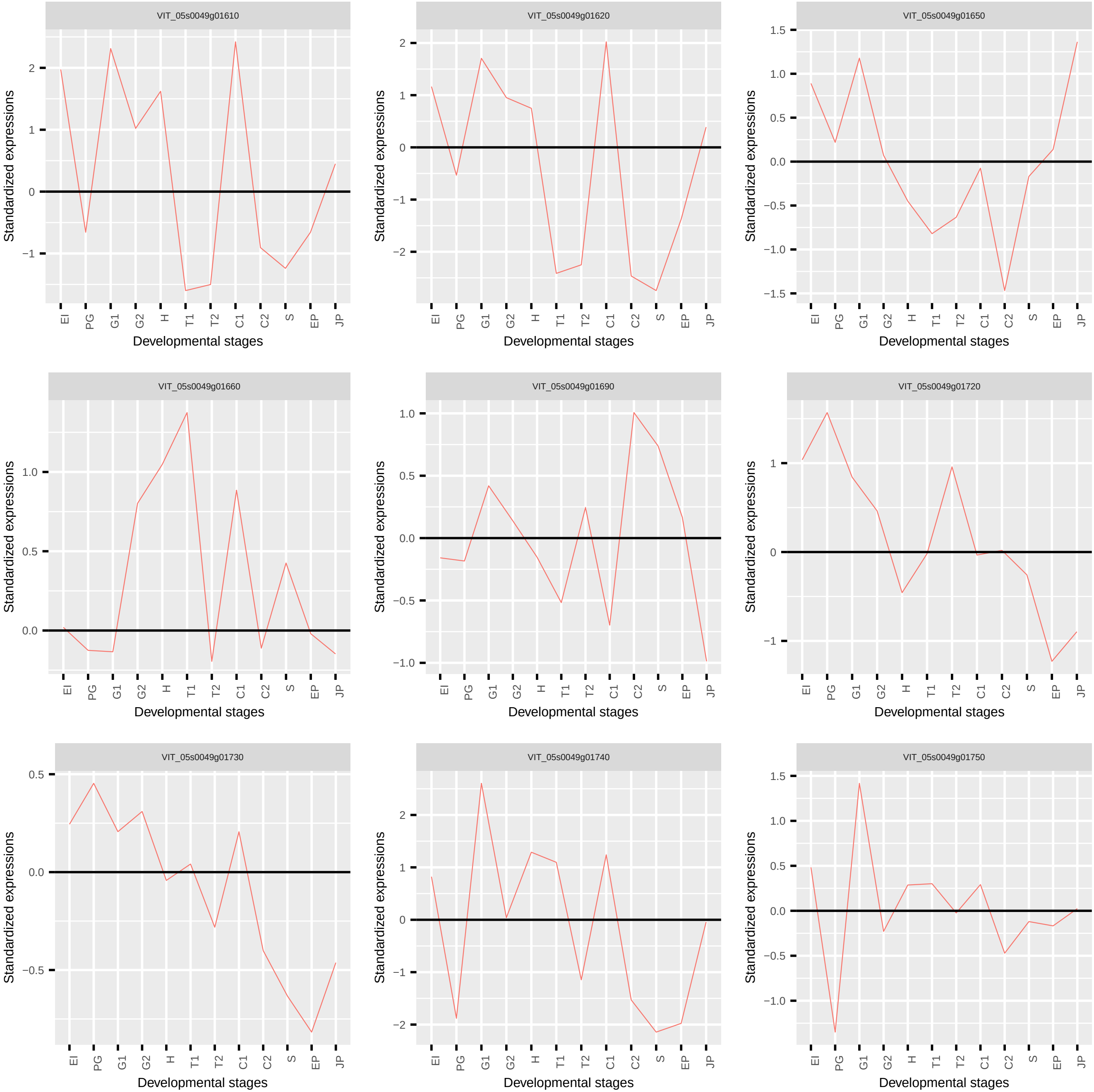
VIT_05s0049g01610
VIT_05s0049g01620
VIT_05s0049g01650
VIT_05s0049g01660
VIT_05s0049g01690
VIT_05s0049g01720
VIT_05s0049g01730
VIT_05s0049g01740
VIT_05s0049g01750
Standardized expressions
Developmental stages
EI
PG
G1
G2
H
T1
T2
C1
C2
S
EP
JP

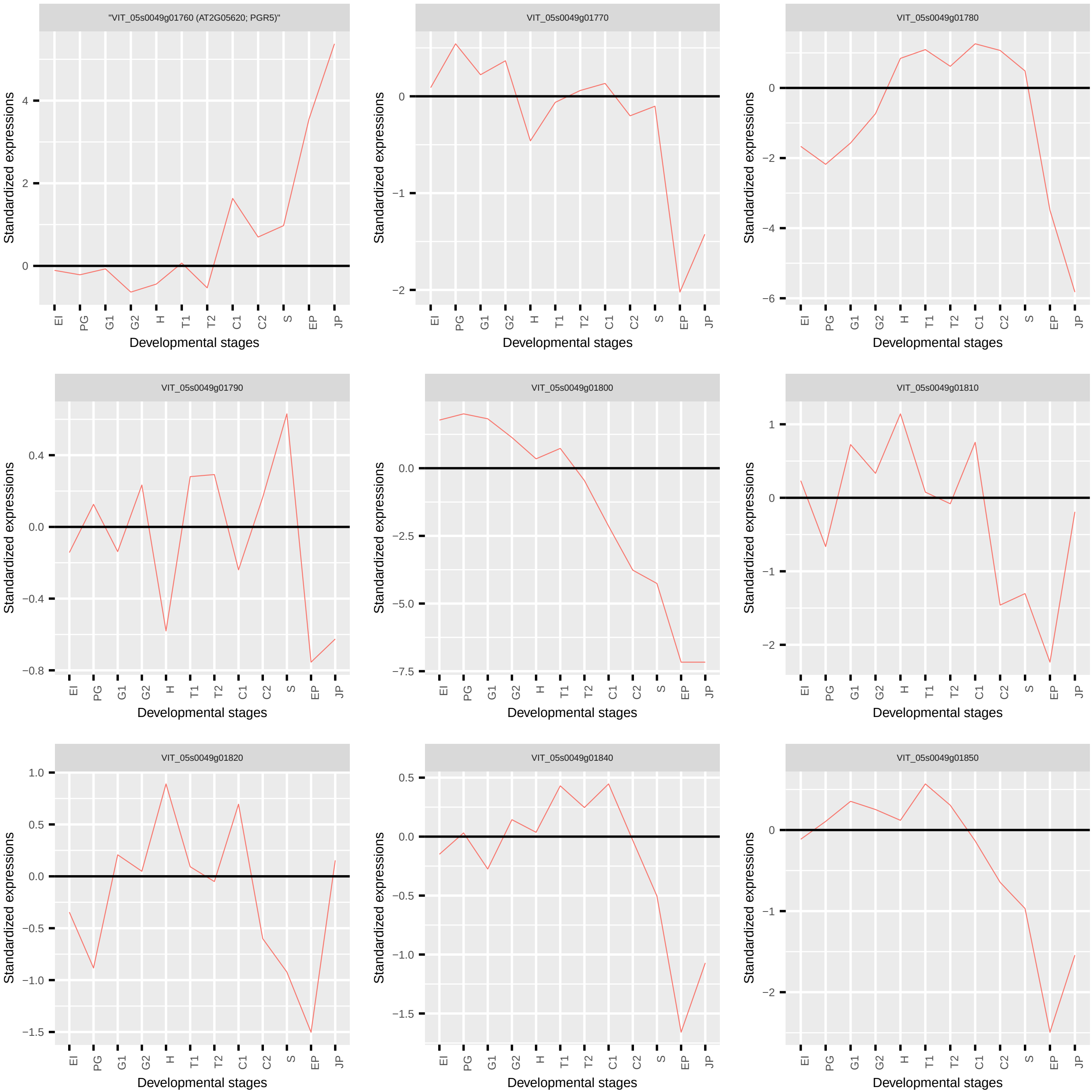

"VIT_05s0049g01760 (AT2G05620; PGR5)"
VIT_05s0049g01770
VIT_05s0049g01780
VIT_05s0049g01790
VIT_05s0049g01800
VIT_05s0049g01810
VIT_05s0049g01820
VIT_05s0049g01840
VIT_05s0049g01850
Standardized expressions
Developmental stages
EI PG G1 G2 H T1 T2 C1 C2 S EP JP

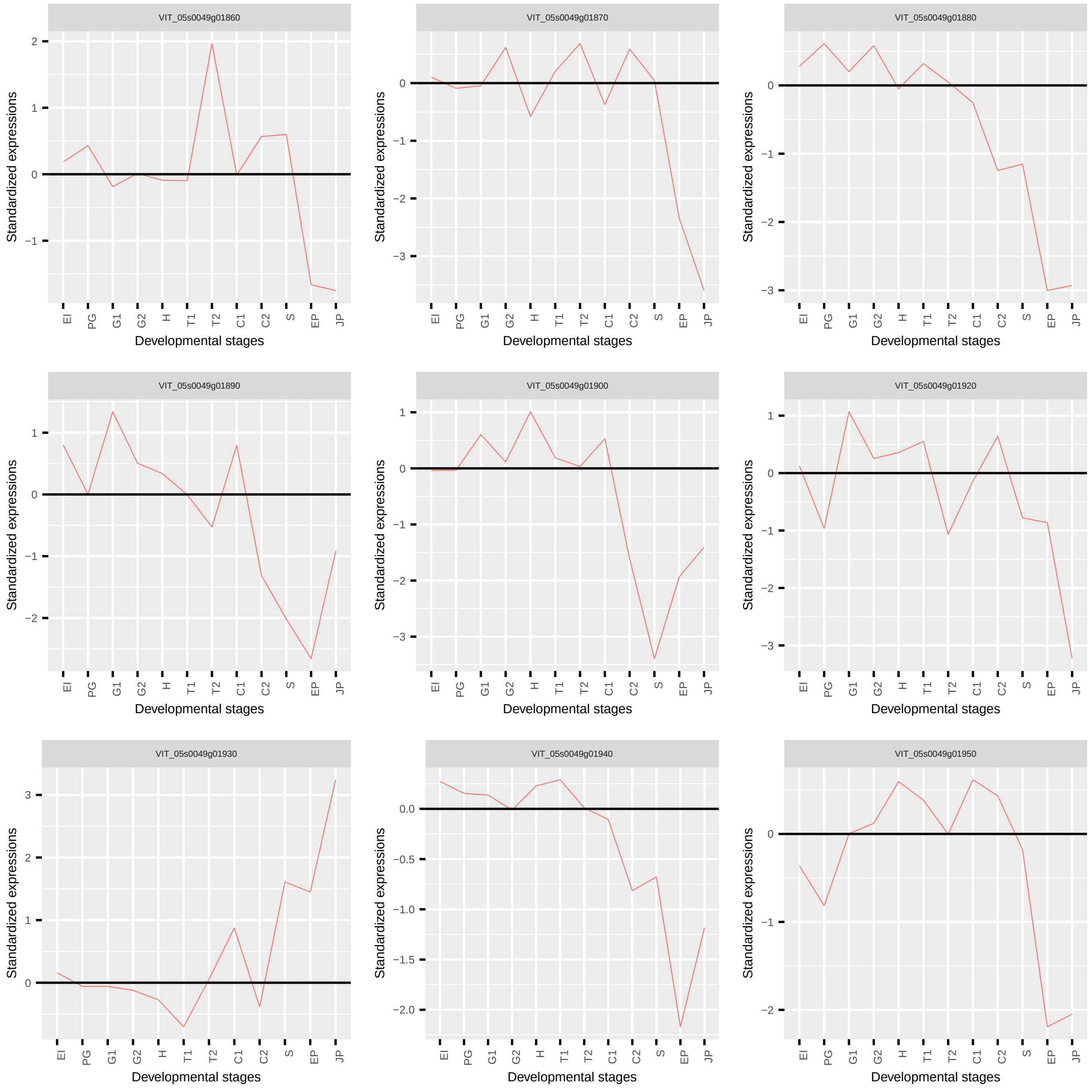

VIT_05s0049g01860
VIT_05s0049g01870
VIT_05s0049g01880
VIT_05s0049g01890
VIT_05s0049g01900
VIT_05s0049g01920
VIT_05s0049g01930
VIT_05s0049g01940
VIT_05s0049g01950
Standardized expressions
Developmental stages
EI PG G1 G2 H T1 T2 C1 C2 S EP JP

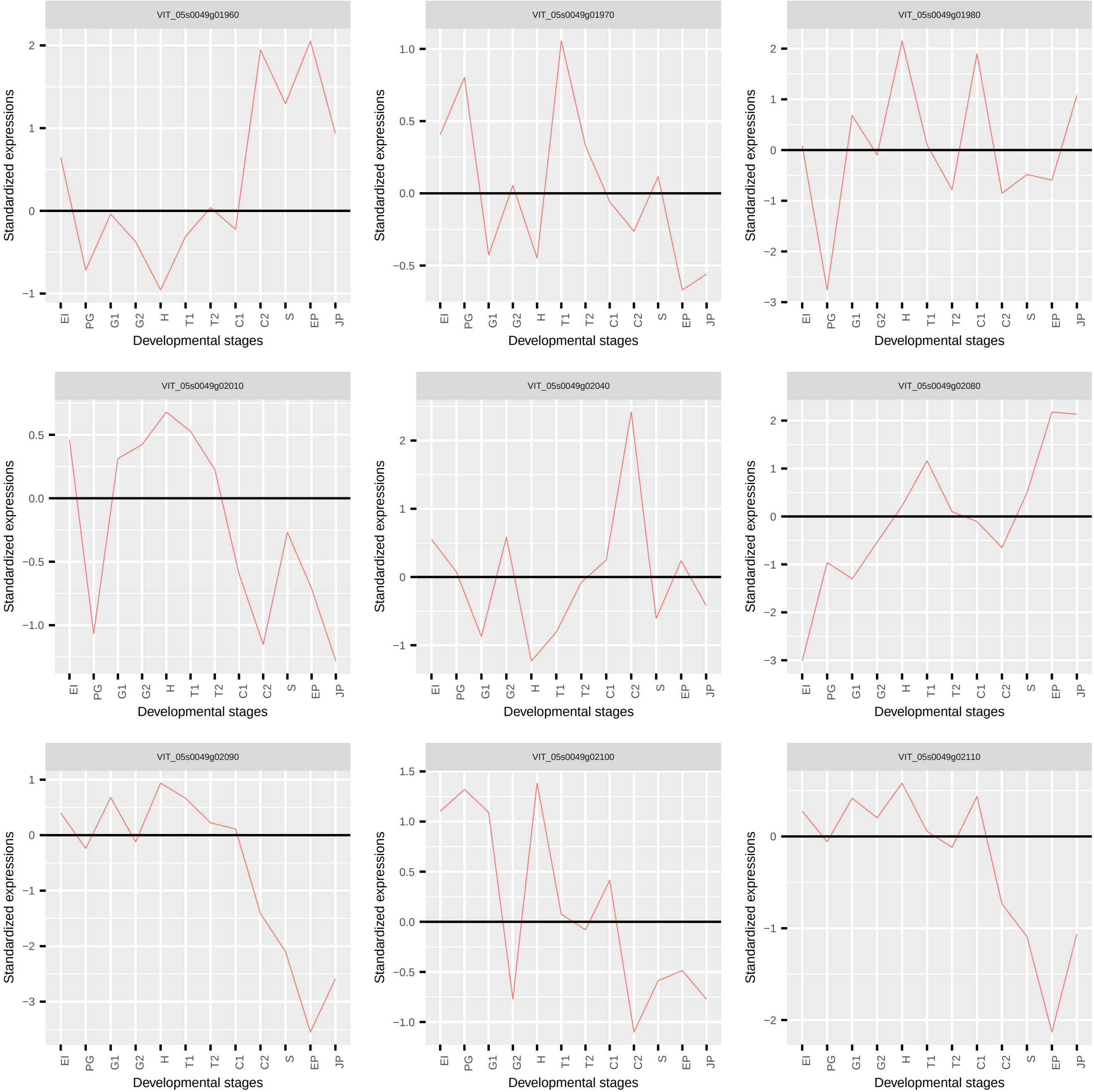
VIT_05s0049g01960
Standardized expressions
2
1
0
−1
EI PG G1 G2 H T1 T2 C1 C2 S EP JP
Developmental stages
VIT_05s0049g01970
Standardized expressions
1.0
0.5
0.0
−0.5
EI PG G1 G2 H T1 T2 C1 C2 S EP JP
Developmental stages
VIT_05s0049g01980
Standardized expressions
2
1
0
−1
−2
−3
EI PG G1 G2 H T1 T2 C1 C2 S EP JP
Developmental stages
VIT_05s0049g02010
Standardized expressions
0.5
0.0
−0.5
−1.0
EI PG G1 G2 H T1 T2 C1 C2 S EP JP
Developmental stages
VIT_05s0049g02040
Standardized expressions
2
1
0
−1
EI PG G1 G2 H T1 T2 C1 C2 S EP JP
Developmental stages
VIT_05s0049g02080
Standardized expressions
2
1
0
−1
−2
−3
EI PG G1 G2 H T1 T2 C1 C2 S EP JP
Developmental stages
VIT_05s0049g02090
Standardized expressions
1
0
−1
−2
−3
EI PG G1 G2 H T1 T2 C1 C2 S EP JP
Developmental stages
VIT_05s0049g02100
Standardized expressions
1.5
1.0
0.5
0.0
−0.5
−1.0
EI PG G1 G2 H T1 T2 C1 C2 S EP JP
Developmental stages
VIT_05s0049g02110
Standardized expressions
0
−1
−2
EI PG G1 G2 H T1 T2 C1 C2 S EP JP
Developmental stages

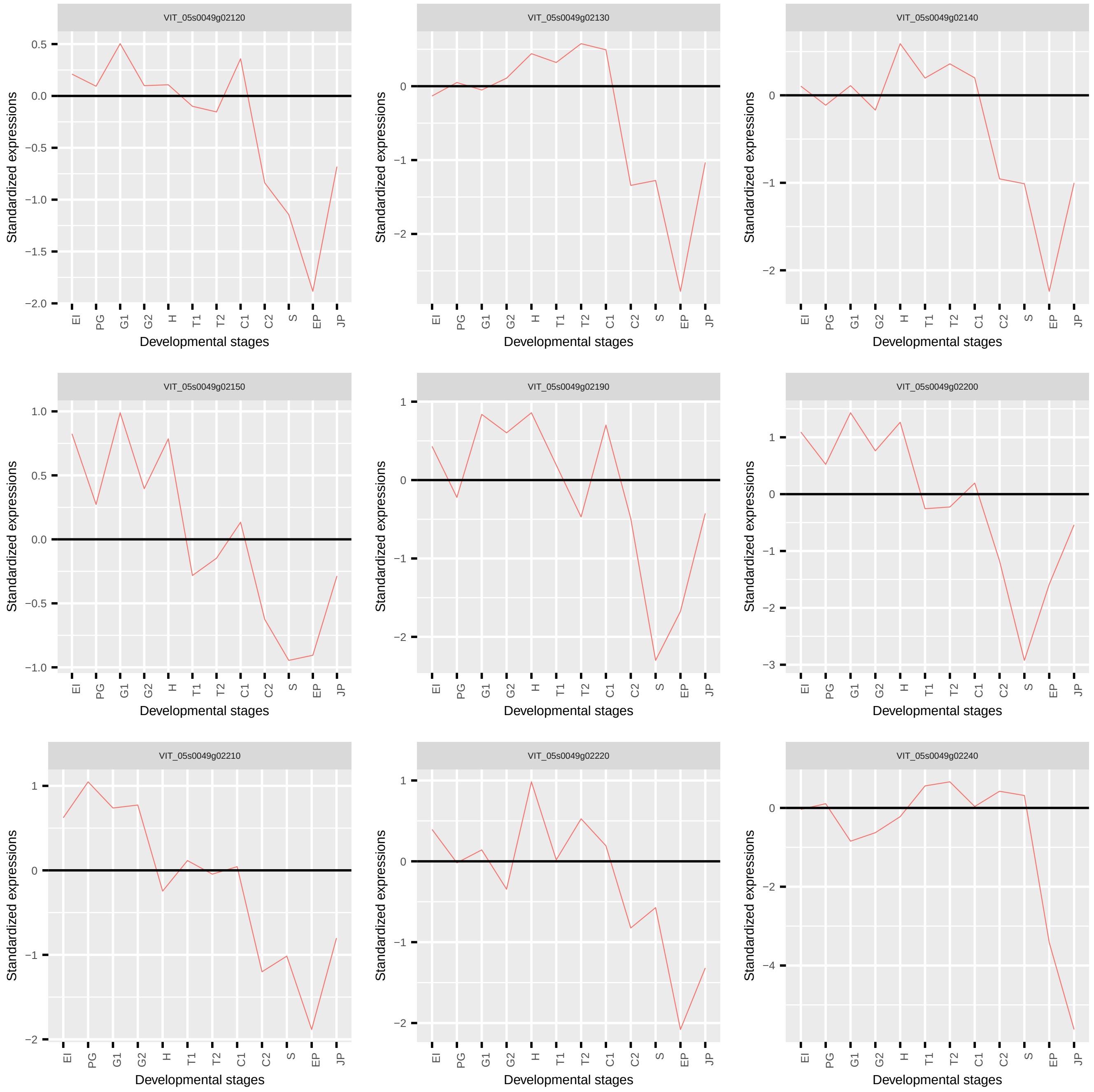
VIT_05s0049g02120
VIT_05s0049g02130
VIT_05s0049g02140
VIT_05s0049g02150
VIT_05s0049g02190
VIT_05s0049g02200
VIT_05s0049g02210
VIT_05s0049g02220
VIT_05s0049g02240
Standardized expressions
Developmental stages
EI PG G1 G2 H T1 T2 C1 C2 S EP JP

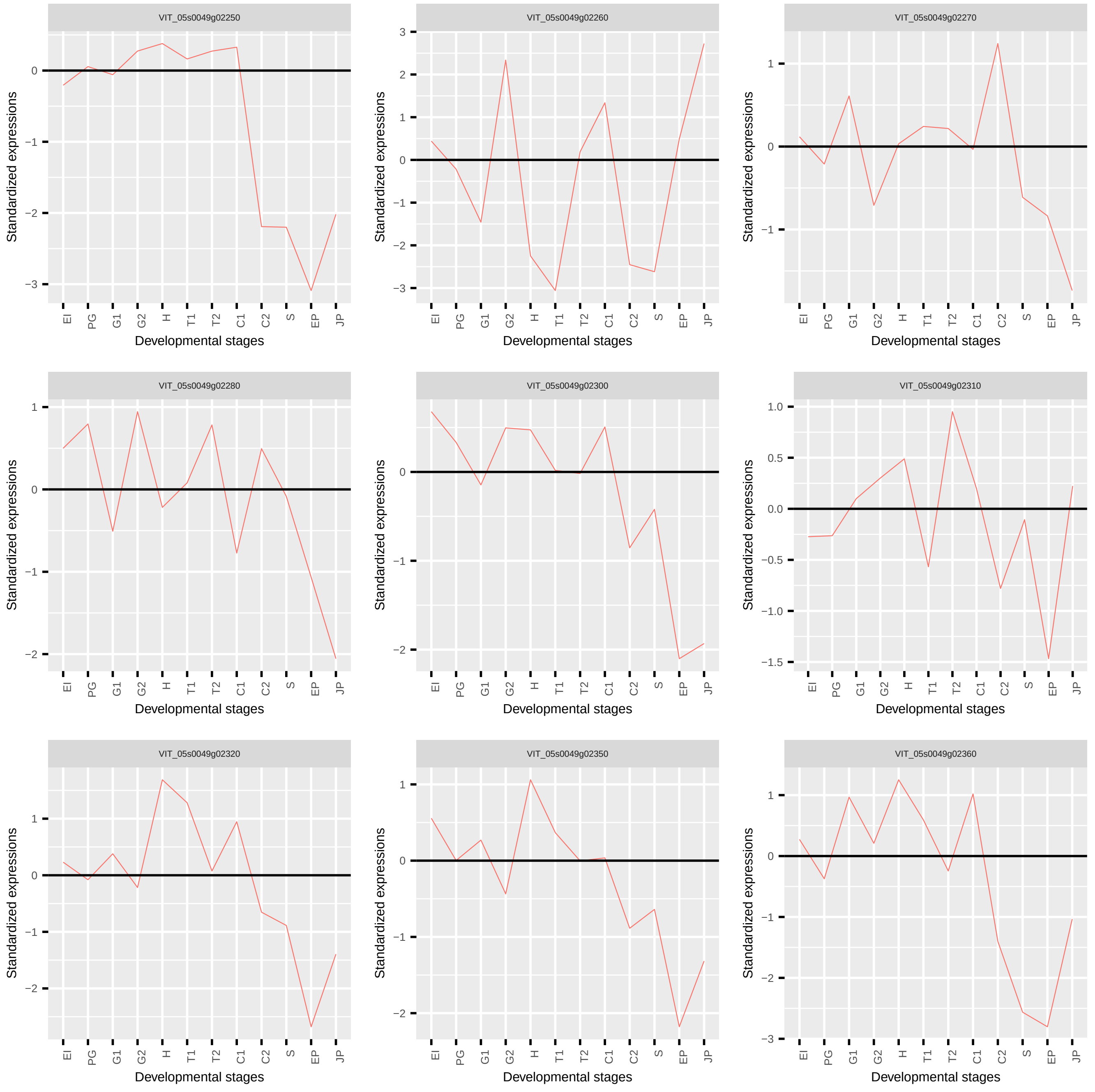
VIT_05s0049g02250
VIT_05s0049g02260
VIT_05s0049g02270
VIT_05s0049g02280
VIT_05s0049g02300
VIT_05s0049g02310
VIT_05s0049g02320
VIT_05s0049g02350
VIT_05s0049g02360
Standardized expressions
Developmental stages
EI PG G1 G2 H T1 T2 C1 C2 S EP JP

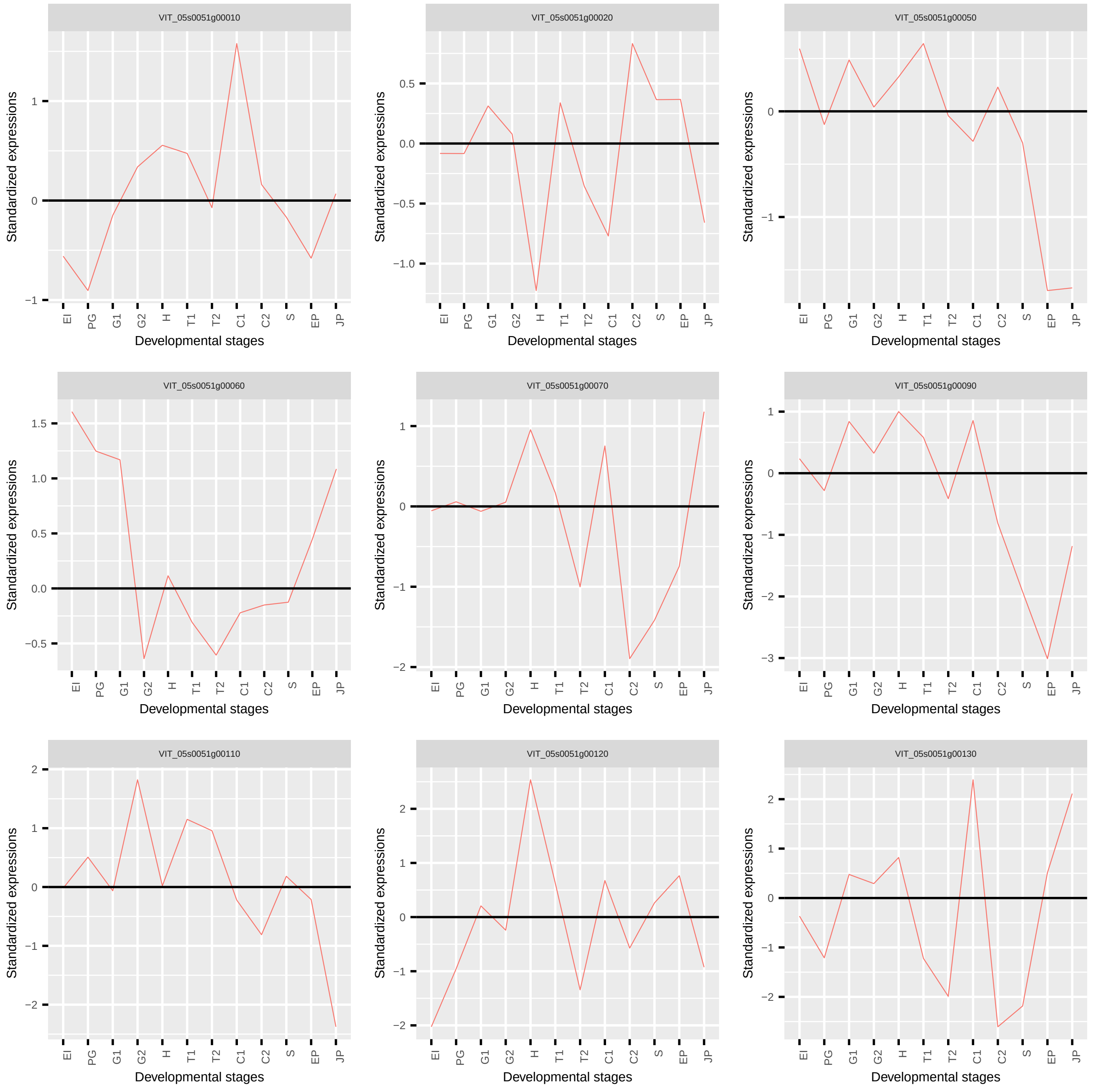
VIT_05s0051g00010
VIT_05s0051g00020
VIT_05s0051g00050
VIT_05s0051g00060
VIT_05s0051g00070
VIT_05s0051g00090
VIT_05s0051g00110
VIT_05s0051g00120
VIT_05s0051g00130
Standardized expressions
Developmental stages
EI PG G1 G2 H T1 T2 C1 C2 S EP JP

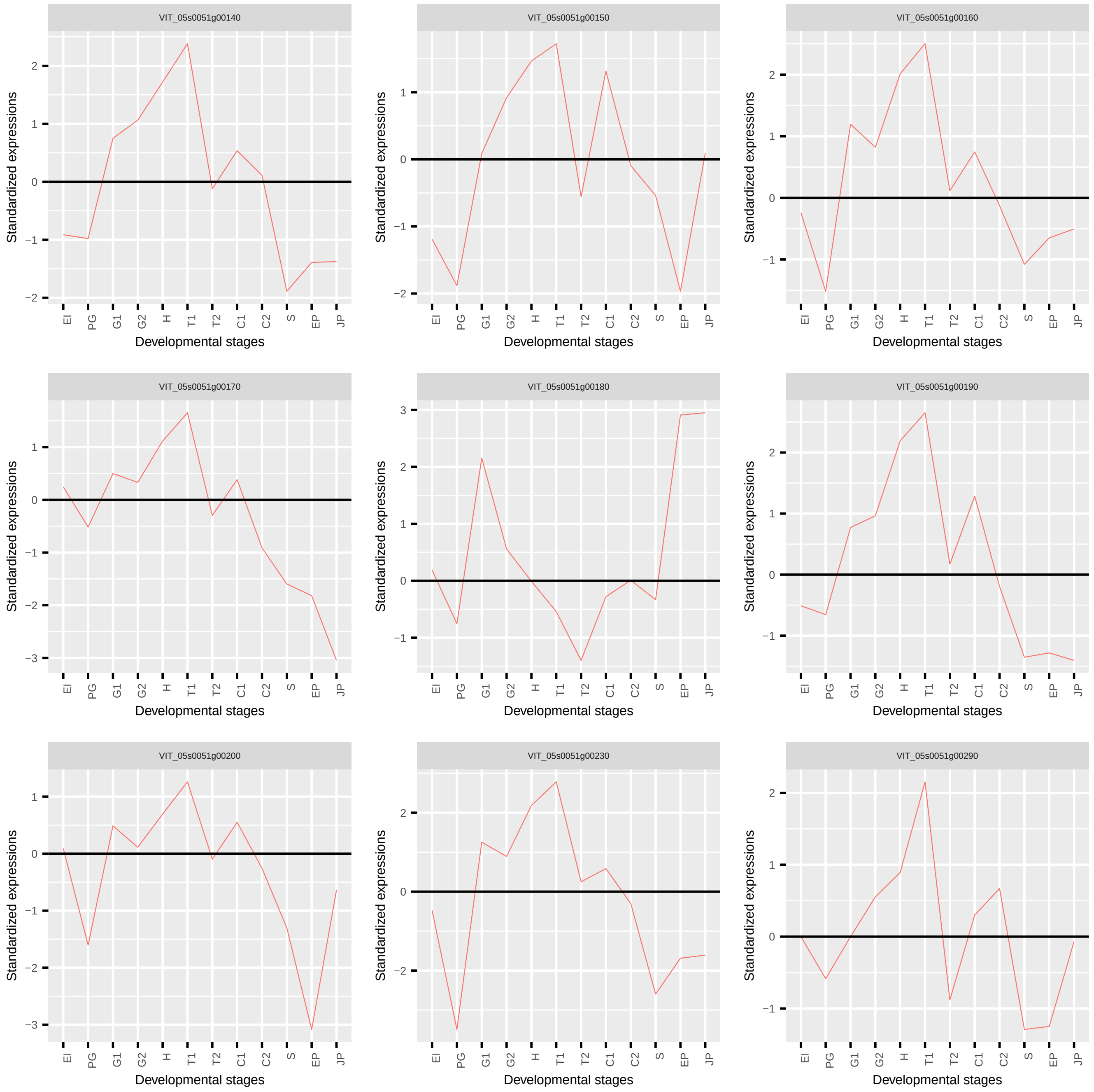
VIT_05s0051g00140
VIT_05s0051g00150
VIT_05s0051g00160
VIT_05s0051g00170
VIT_05s0051g00180
VIT_05s0051g00190
VIT_05s0051g00200
VIT_05s0051g00230
VIT_05s0051g00290
Standardized expressions
EI PG G1 G2 H T1 T2 C1 C2 S EP JP
Developmental stages

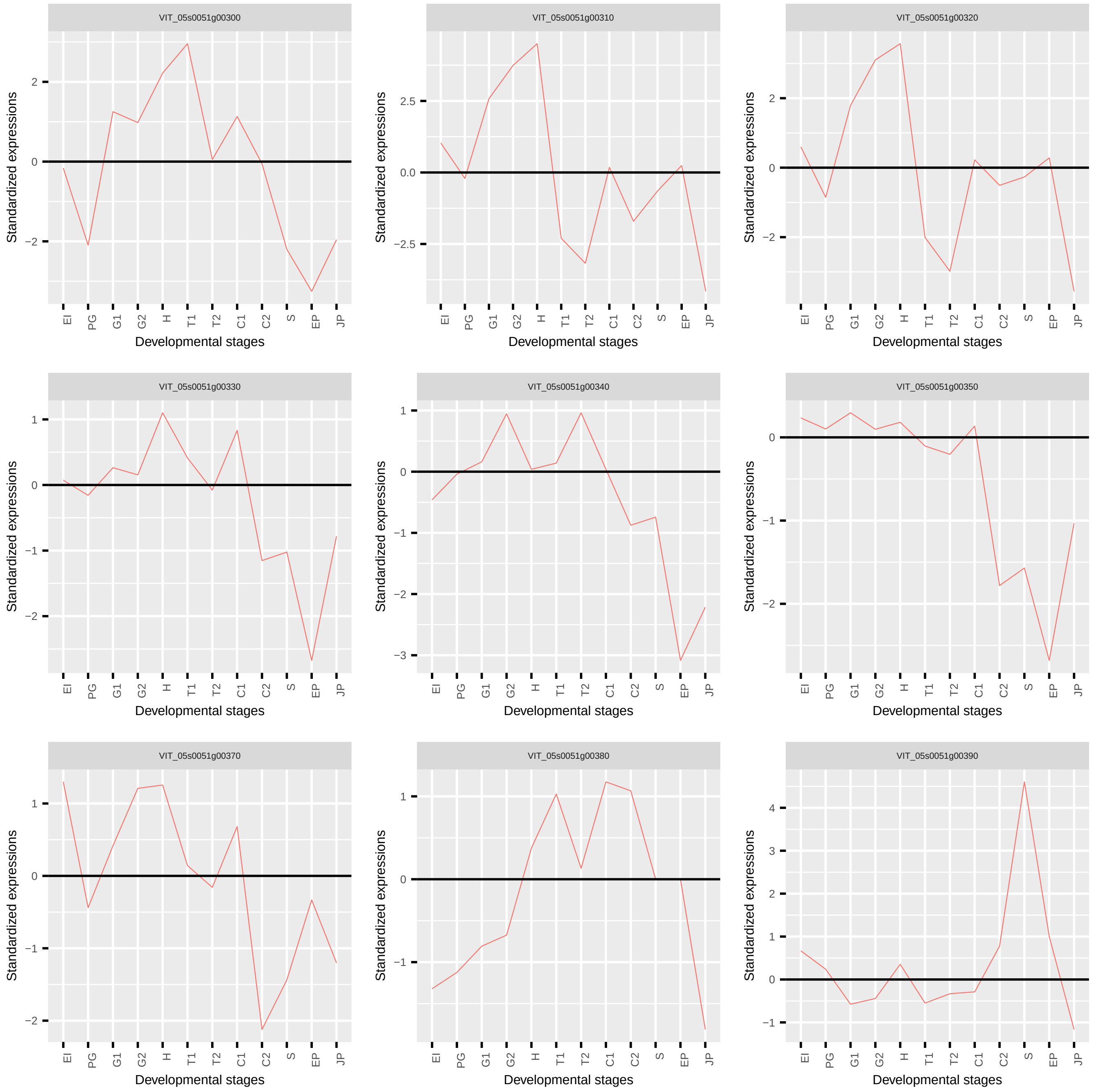
VIT_05s0051g00300
VIT_05s0051g00310
VIT_05s0051g00320
VIT_05s0051g00330
VIT_05s0051g00340
VIT_05s0051g00350
VIT_05s0051g00370
VIT_05s0051g00380
VIT_05s0051g00390
Standardized expressions
Developmental stages
EI PG G1 G2 H T1 T2 C1 C2 S EP JP

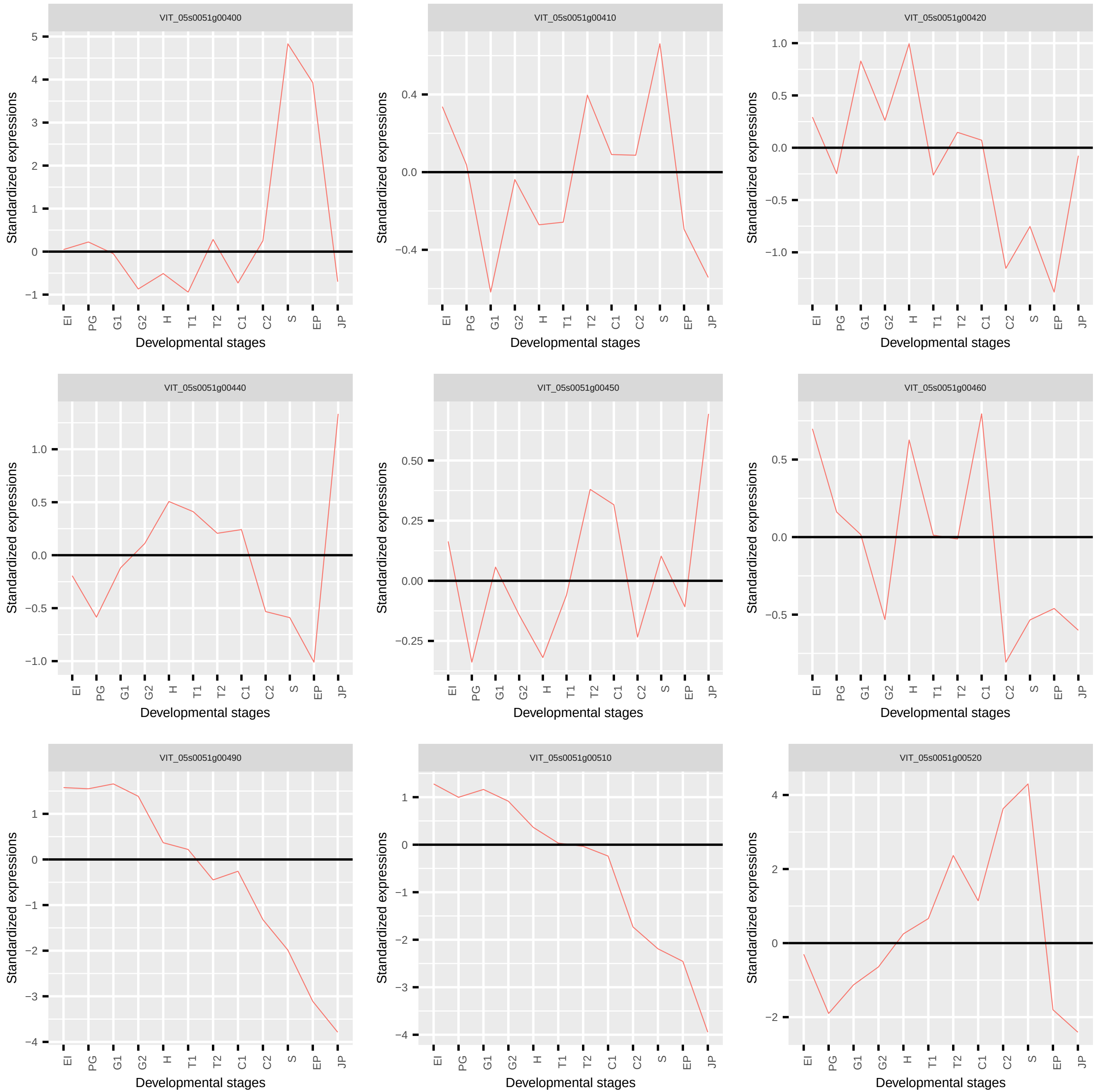
VIT_05s0051g00400
Standardized expressions
5
4
3
2
1
0
−1
EI
PG
G1
G2
H
T1
T2
C1
C2
S
EP
JP
Developmental stages
VIT_05s0051g00410
Standardized expressions
0.4
0.0
−0.4
EI
PG
G1
G2
H
T1
T2
C1
C2
S
EP
JP
Developmental stages
VIT_05s0051g00420
Standardized expressions
1.0
0.5
0.0
−0.5
−1.0
EI
PG
G1
G2
H
T1
T2
C1
C2
S
EP
JP
Developmental stages
VIT_05s0051g00440
Standardized expressions
1.0
0.5
0.0
−0.5
−1.0
EI
PG
G1
G2
H
T1
T2
C1
C2
S
EP
JP
Developmental stages
VIT_05s0051g00450
Standardized expressions
0.50
0.25
0.00
−0.25
EI
PG
G1
G2
H
T1
T2
C1
C2
S
EP
JP
Developmental stages
VIT_05s0051g00460
Standardized expressions
0.5
0.0
−0.5
EI
PG
G1
G2
H
T1
T2
C1
C2
S
EP
JP
Developmental stages
VIT_05s0051g00490
Standardized expressions
1
0
−1
−2
−3
−4
EI
PG
G1
G2
H
T1
T2
C1
C2
S
EP
JP
Developmental stages
VIT_05s0051g00510
Standardized expressions
1
0
−1
−2
−3
−4
EI
PG
G1
G2
H
T1
T2
C1
C2
S
EP
JP
Developmental stages
VIT_05s0051g00520
Standardized expressions
4
2
0
−2
EI
PG
G1
G2
H
T1
T2
C1
C2
S
EP
JP
Developmental stages

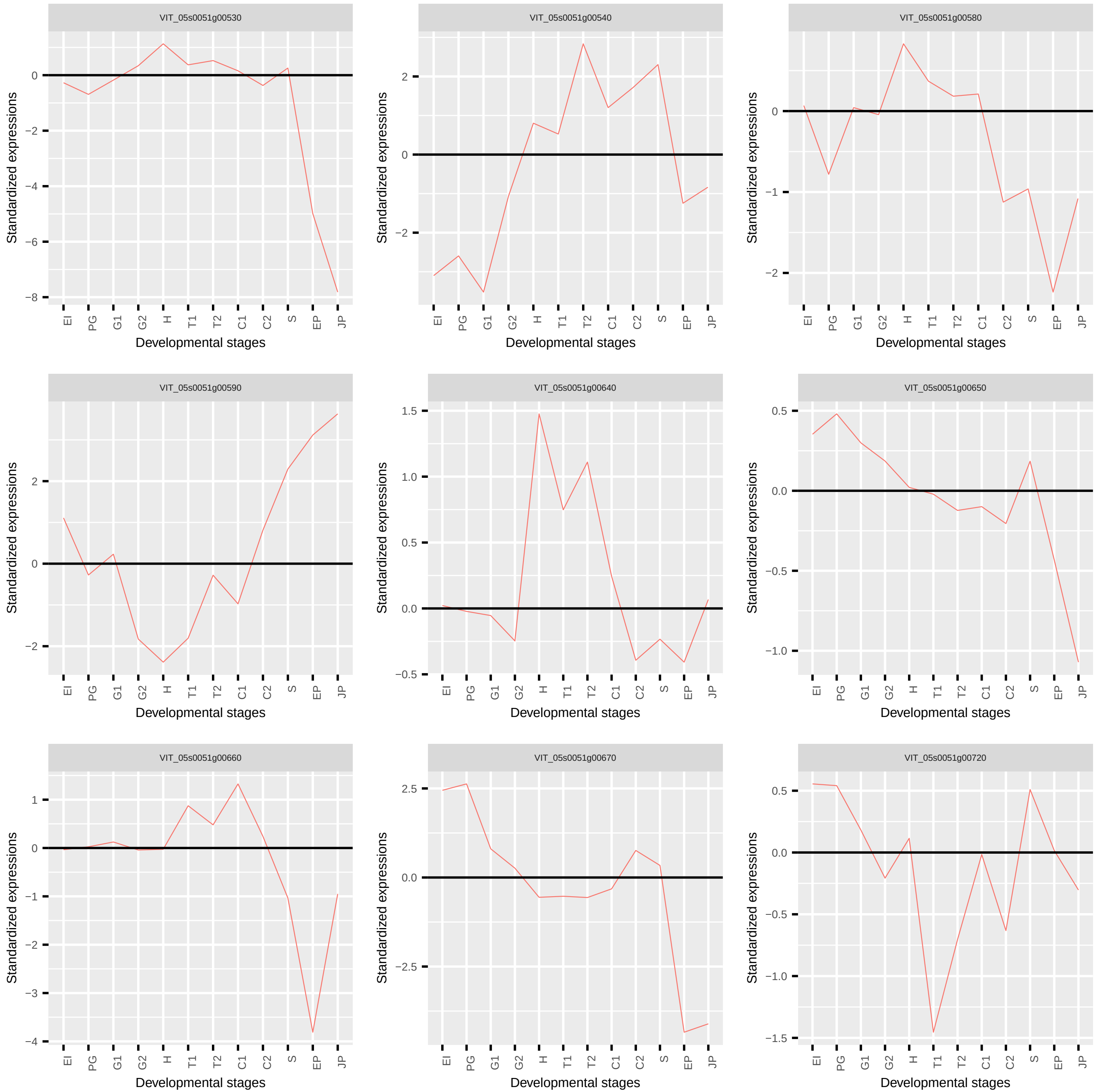

VIT_05s0051g00530
VIT_05s0051g00540
VIT_05s0051g00580
VIT_05s0051g00590
VIT_05s0051g00640
VIT_05s0051g00650
VIT_05s0051g00660
VIT_05s0051g00670
VIT_05s0051g00720
Standardized expressions
Developmental stages
EI
PG
G1
G2
H
T1
T2
C1
C2
S
EP
JP
0
−2
−4
−6
−8
2
−1
1
−3
1.5
1.0
0.5
0.0
−0.5
−1.0
−1.5
2.5
−2.5

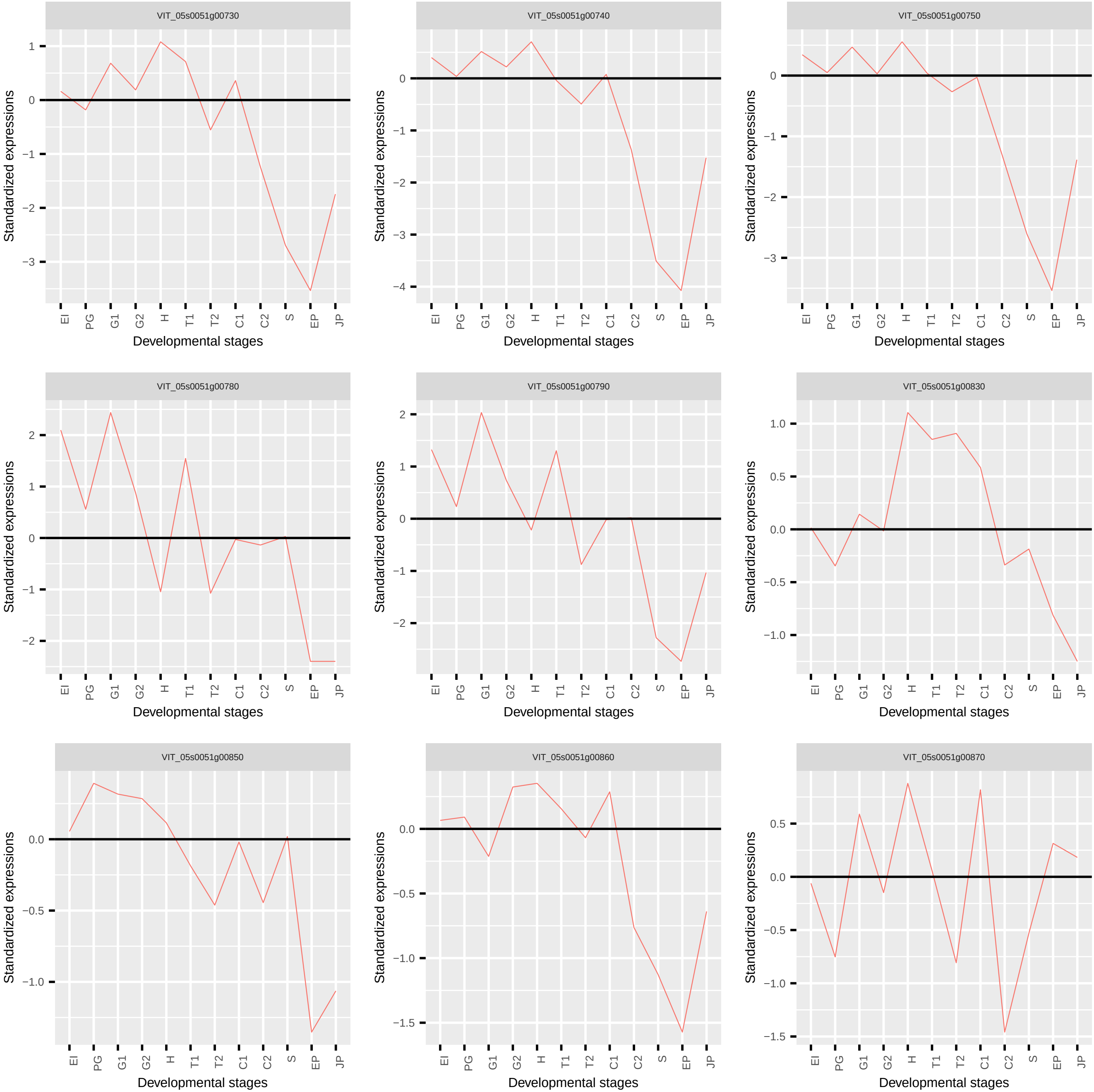

VIT_05s0051g00730
VIT_05s0051g00740
VIT_05s0051g00750
VIT_05s0051g00780
VIT_05s0051g00790
VIT_05s0051g00830
VIT_05s0051g00850
VIT_05s0051g00860
VIT_05s0051g00870
Standardized expressions
Developmental stages
EI
PG
G1
G2
H
T1
T2
C1
C2
S
EP
JP

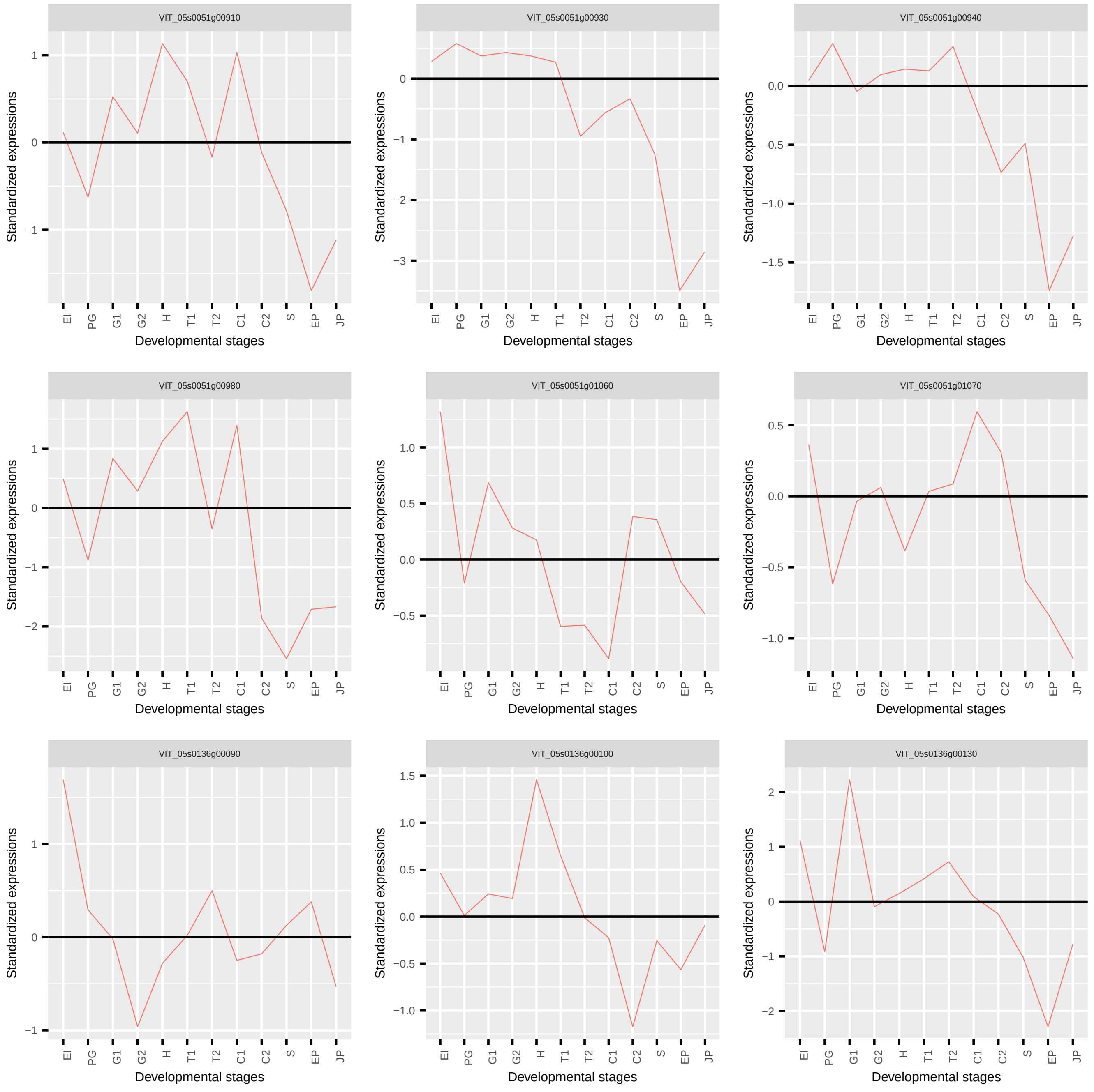
VIT_05s0051g00910
VIT_05s0051g00930
VIT_05s0051g00940
VIT_05s0051g00980
VIT_05s0051g01060
VIT_05s0051g01070
VIT_05s0136g00090
VIT_05s0136g00100
VIT_05s0136g00130
Standardized expressions
Developmental stages
EI PG G1 G2 H T1 T2 C1 C2 S EP JP

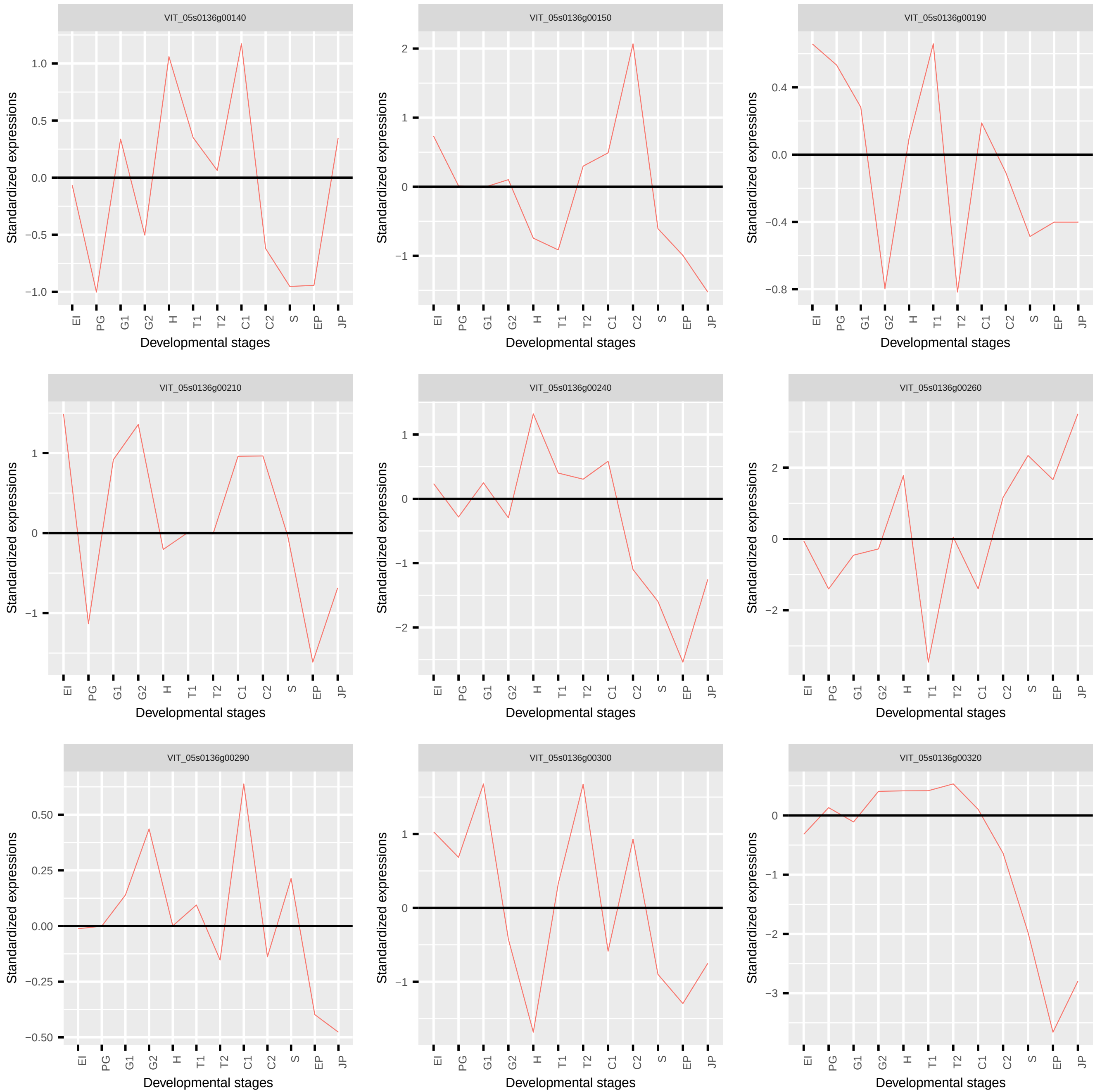
VIT_05s0136g00140
VIT_05s0136g00150
VIT_05s0136g00190
VIT_05s0136g00210
VIT_05s0136g00240
VIT_05s0136g00260
VIT_05s0136g00290
VIT_05s0136g00300
VIT_05s0136g00320
Standardized expressions
Developmental stages
EI PG G1 G2 H T1 T2 C1 C2 S EP JP

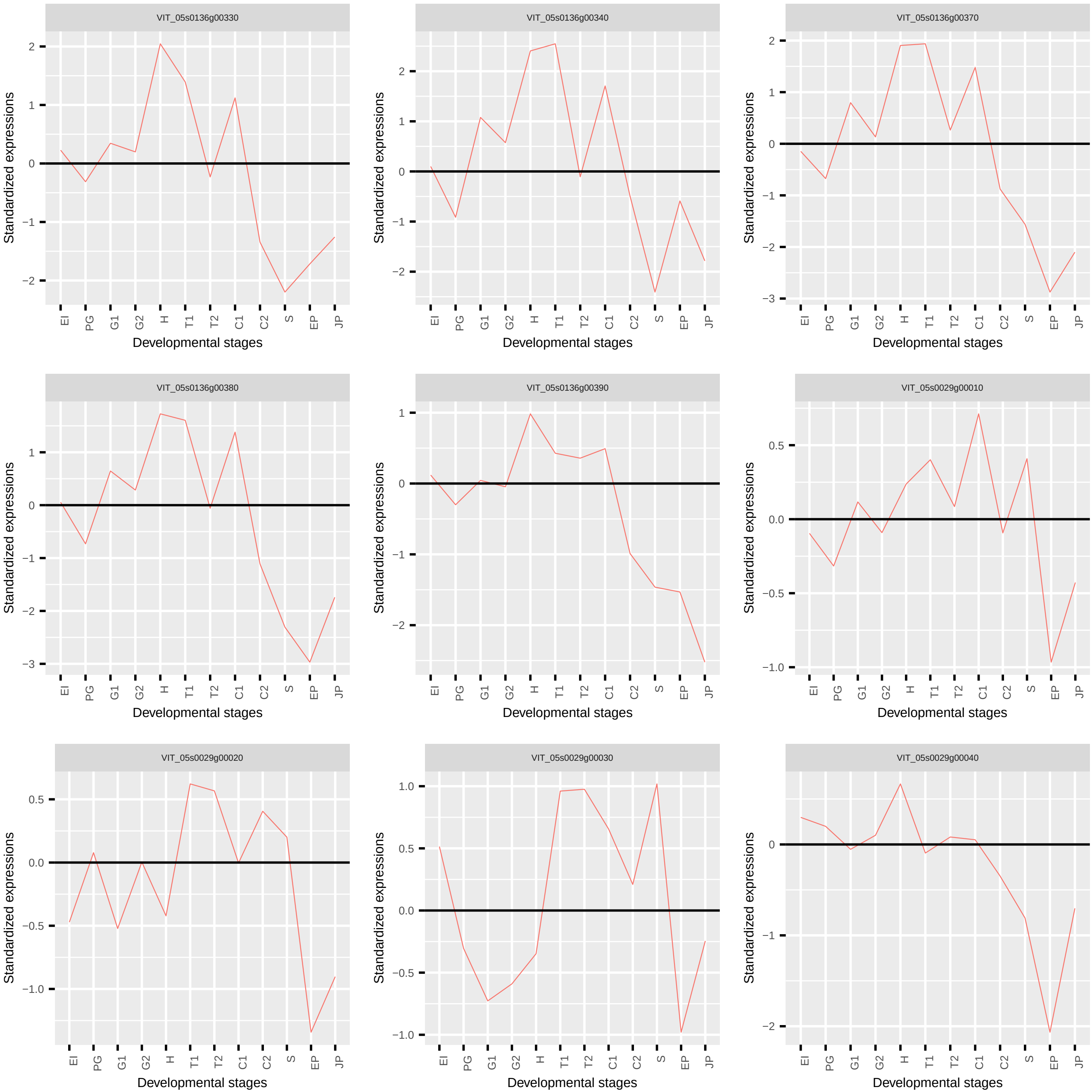
VIT_05s0136g00330
VIT_05s0136g00340
VIT_05s0136g00370
VIT_05s0136g00380
VIT_05s0136g00390
VIT_05s0029g00010
VIT_05s0029g00020
VIT_05s0029g00030
VIT_05s0029g00040
Standardized expressions
Developmental stages
EI
PG
G1
G2
H
T1
T2
C1
C2
S
EP
JP

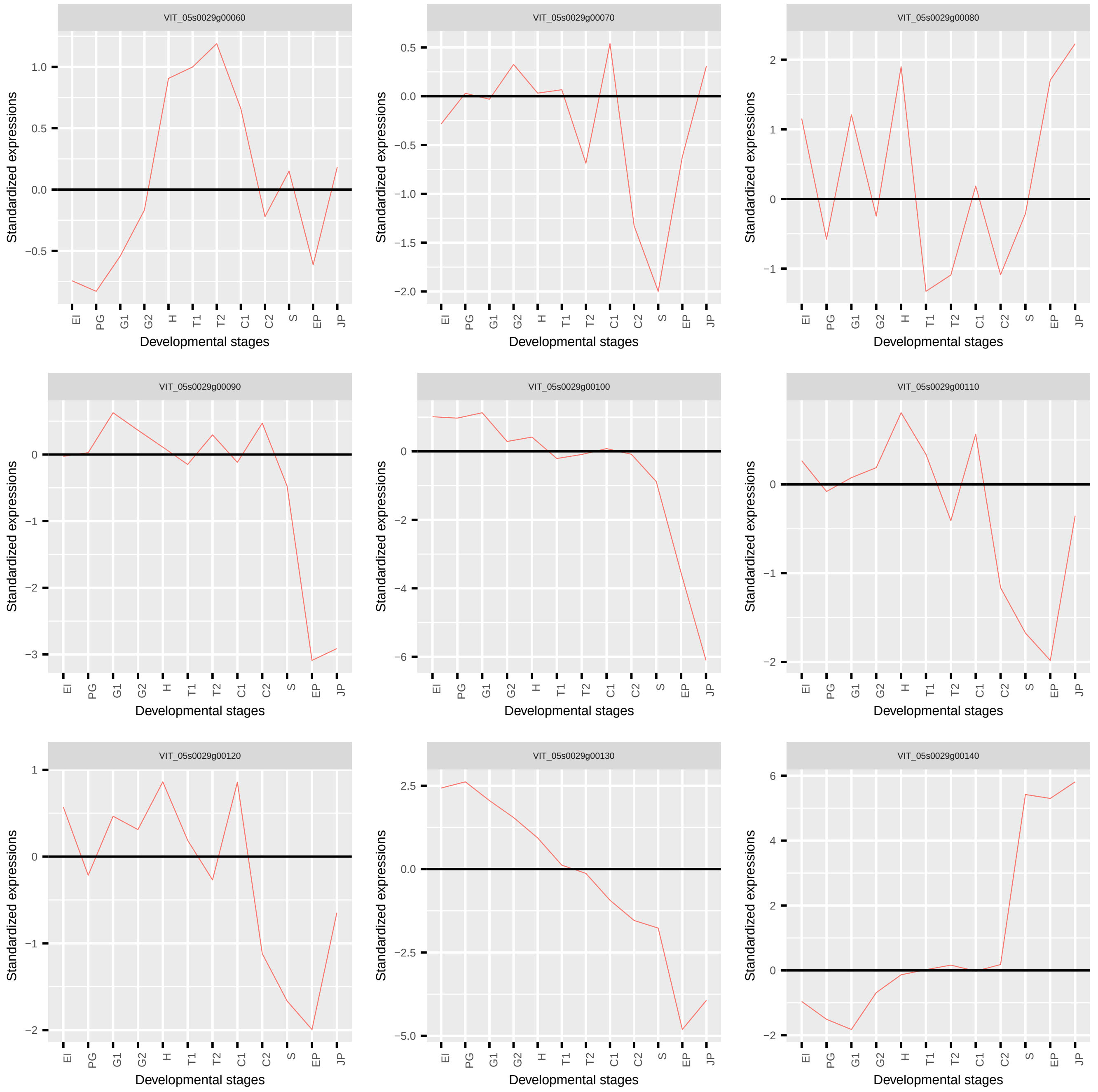

VIT_05s0029g00060
VIT_05s0029g00070
VIT_05s0029g00080
VIT_05s0029g00090
VIT_05s0029g00100
VIT_05s0029g00110
VIT_05s0029g00120
VIT_05s0029g00130
VIT_05s0029g00140
Standardized expressions
Developmental stages
EI PG G1 G2 H T1 T2 C1 C2 S EP JP
1.0 0.5 0.0 −0.5
0.5 0.0 −0.5 −1.0 −1.5 −2.0
2 1 0 −1
0 −1 −2 −3
0 −2 −4 −6
0 −1 −2
1 0 −1 −2
2.5 0.0 −2.5 −5.0
6 4 2 0 −2

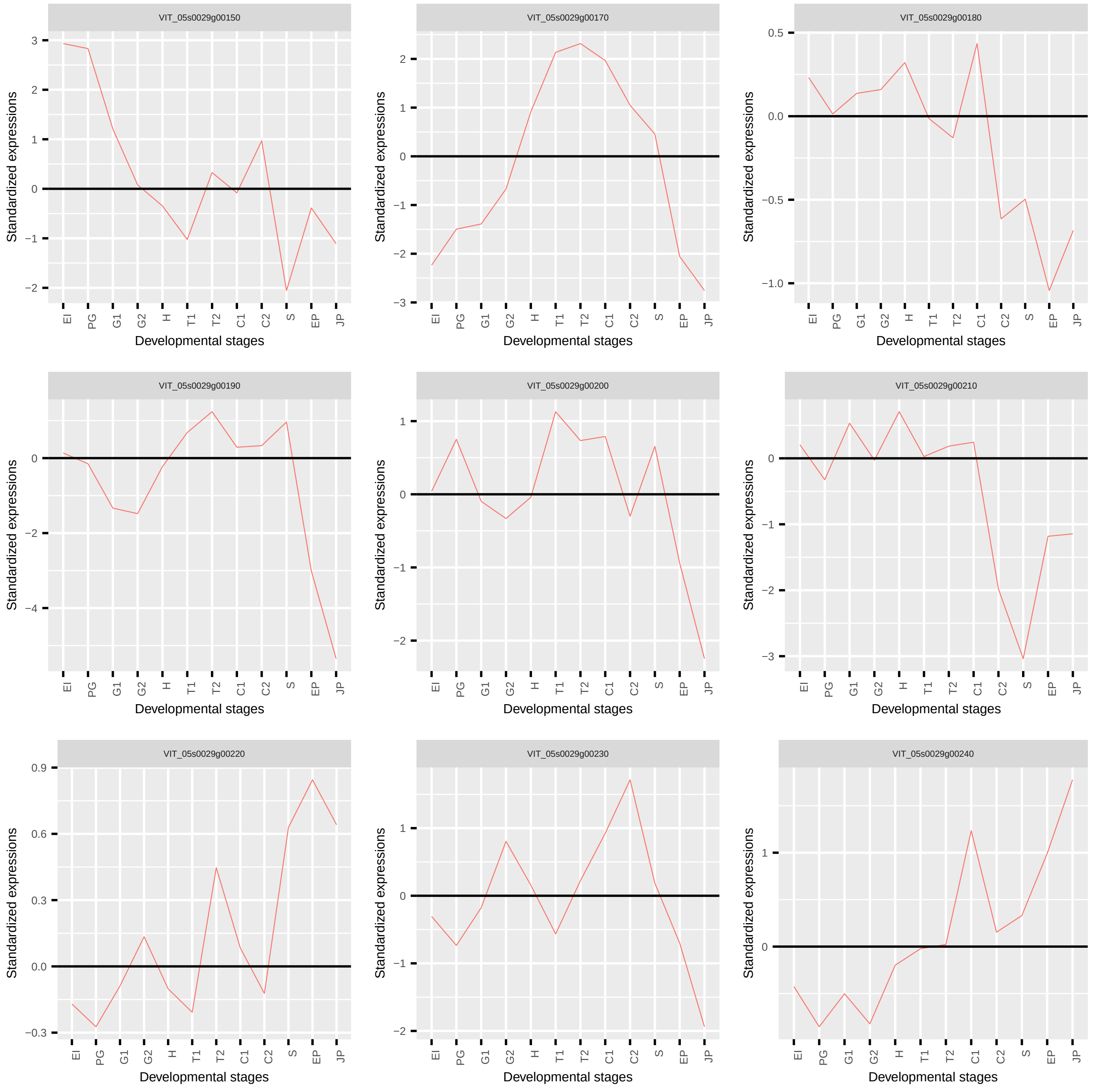
VIT_05s0029g00150
VIT_05s0029g00170
VIT_05s0029g00180
VIT_05s0029g00190
VIT_05s0029g00200
VIT_05s0029g00210
VIT_05s0029g00220
VIT_05s0029g00230
VIT_05s0029g00240
Standardized expressions
Developmental stages
EI PG G1 G2 H T1 T2 C1 C2 S EP JP

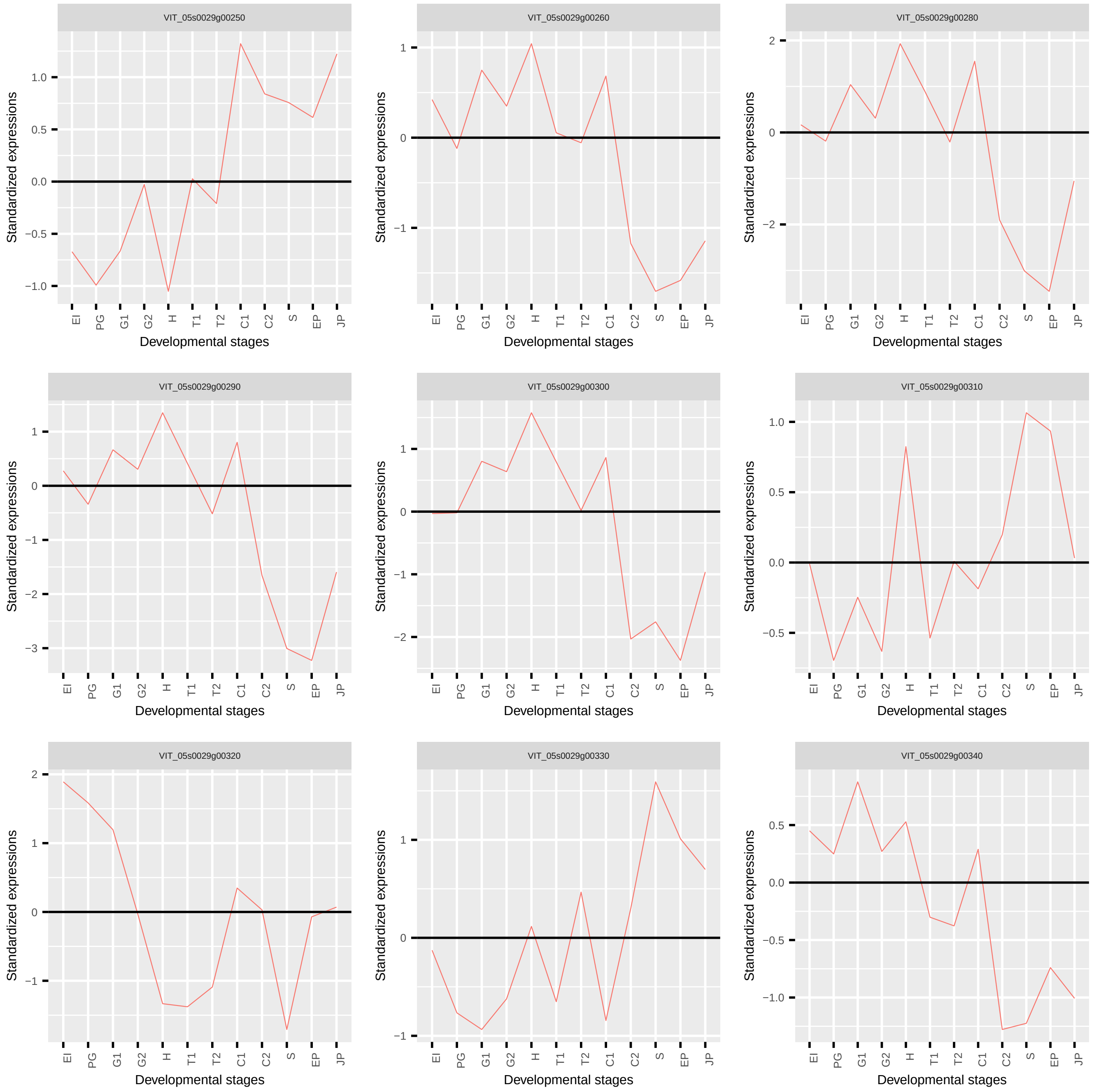
VIT_05s0029g00250
VIT_05s0029g00260
VIT_05s0029g00280
VIT_05s0029g00290
VIT_05s0029g00300
VIT_05s0029g00310
VIT_05s0029g00320
VIT_05s0029g00330
VIT_05s0029g00340
Standardized expressions
Developmental stages
EI PG G1 G2 H T1 T2 C1 C2 S EP JP

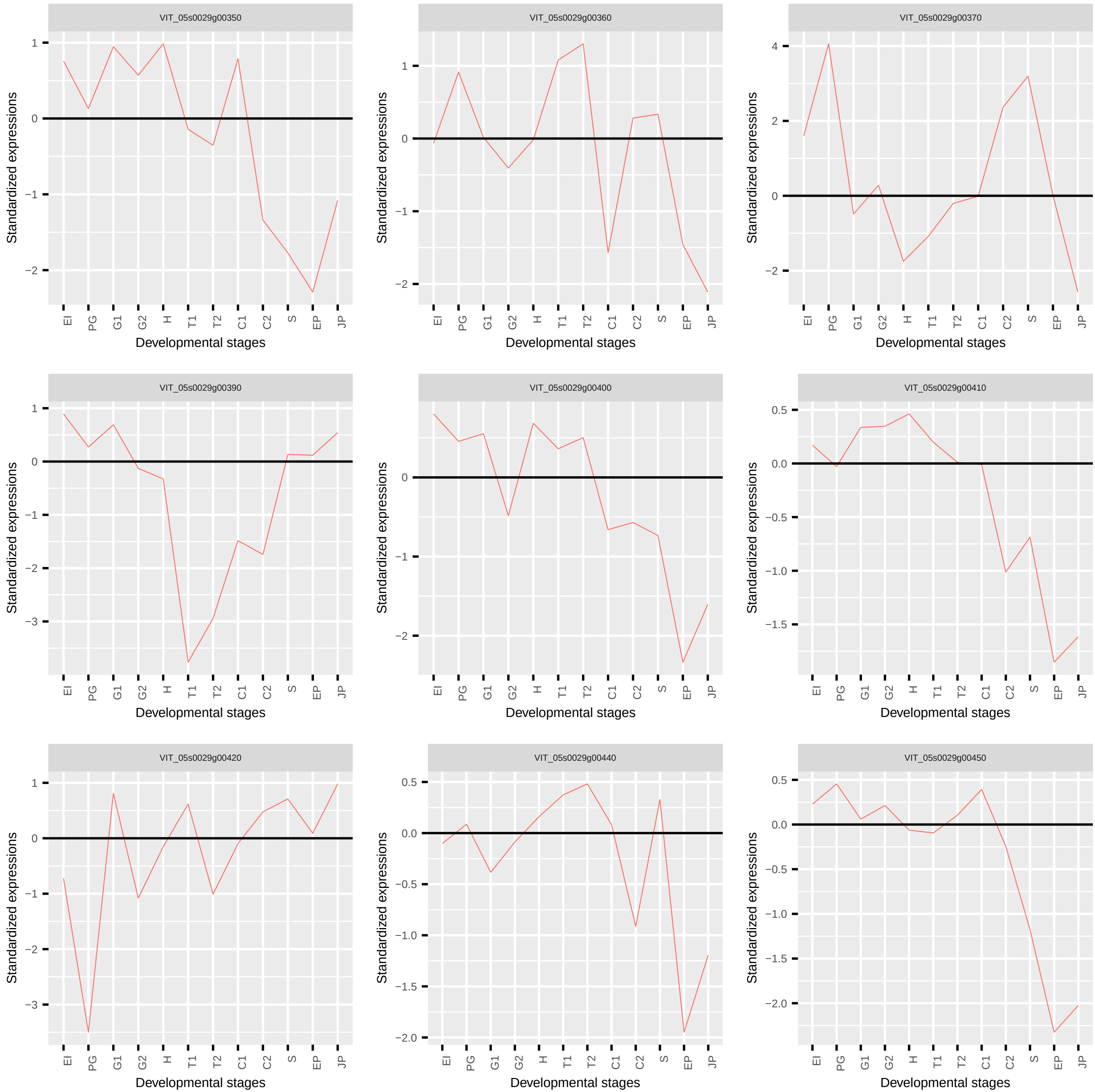

VIT_05s0029g00350
VIT_05s0029g00360
VIT_05s0029g00370
VIT_05s0029g00390
VIT_05s0029g00400
VIT_05s0029g00410
VIT_05s0029g00420
VIT_05s0029g00440
VIT_05s0029g00450
Standardized expressions
Developmental stages
EI PG G1 G2 H T1 T2 C1 C2 S EP JP

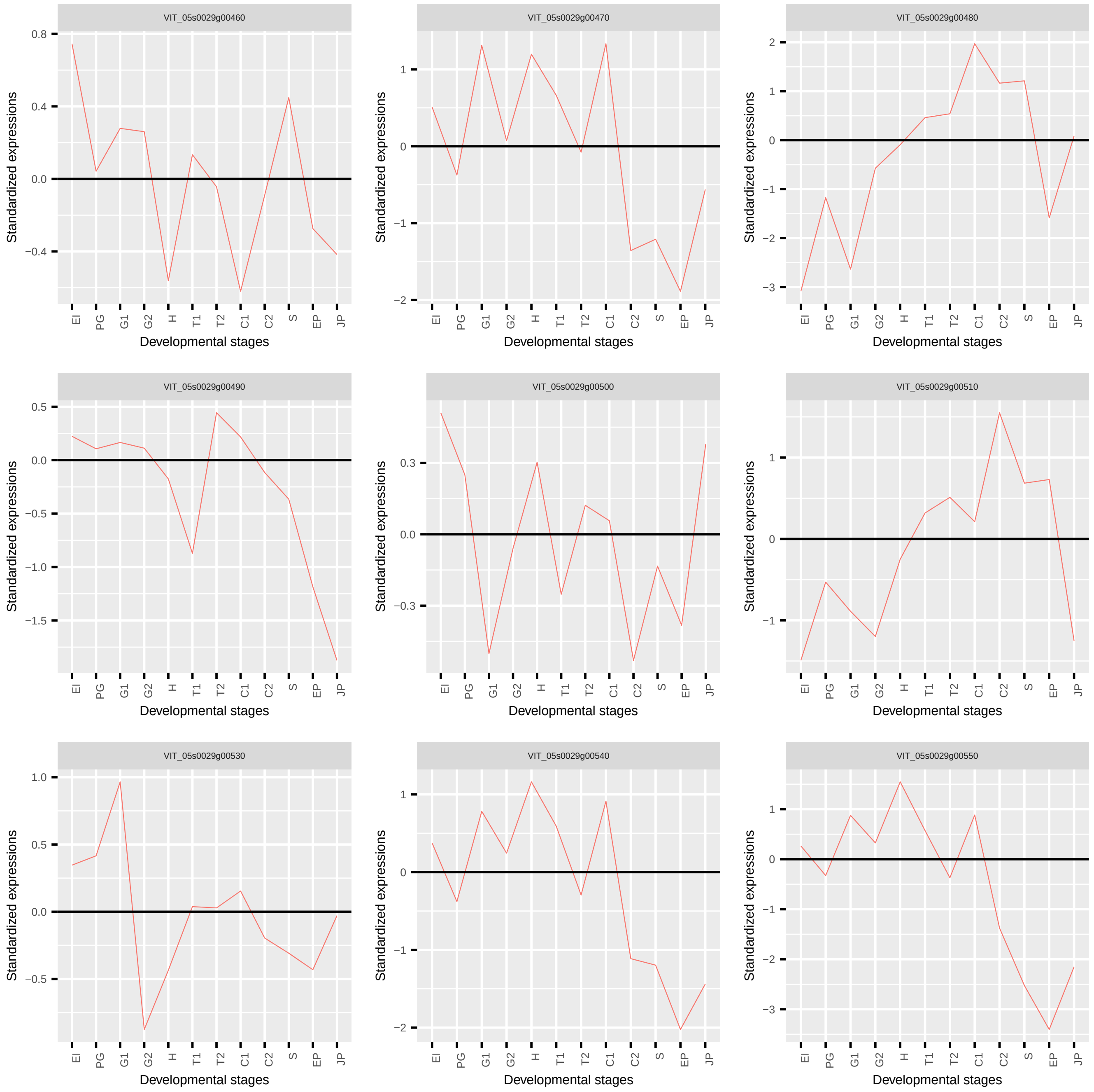
VIT_05s0029g00460
VIT_05s0029g00470
VIT_05s0029g00480
VIT_05s0029g00490
VIT_05s0029g00500
VIT_05s0029g00510
VIT_05s0029g00530
VIT_05s0029g00540
VIT_05s0029g00550
Standardized expressions
Developmental stages
EI PG G1 G2 H T1 T2 C1 C2 S EP JP

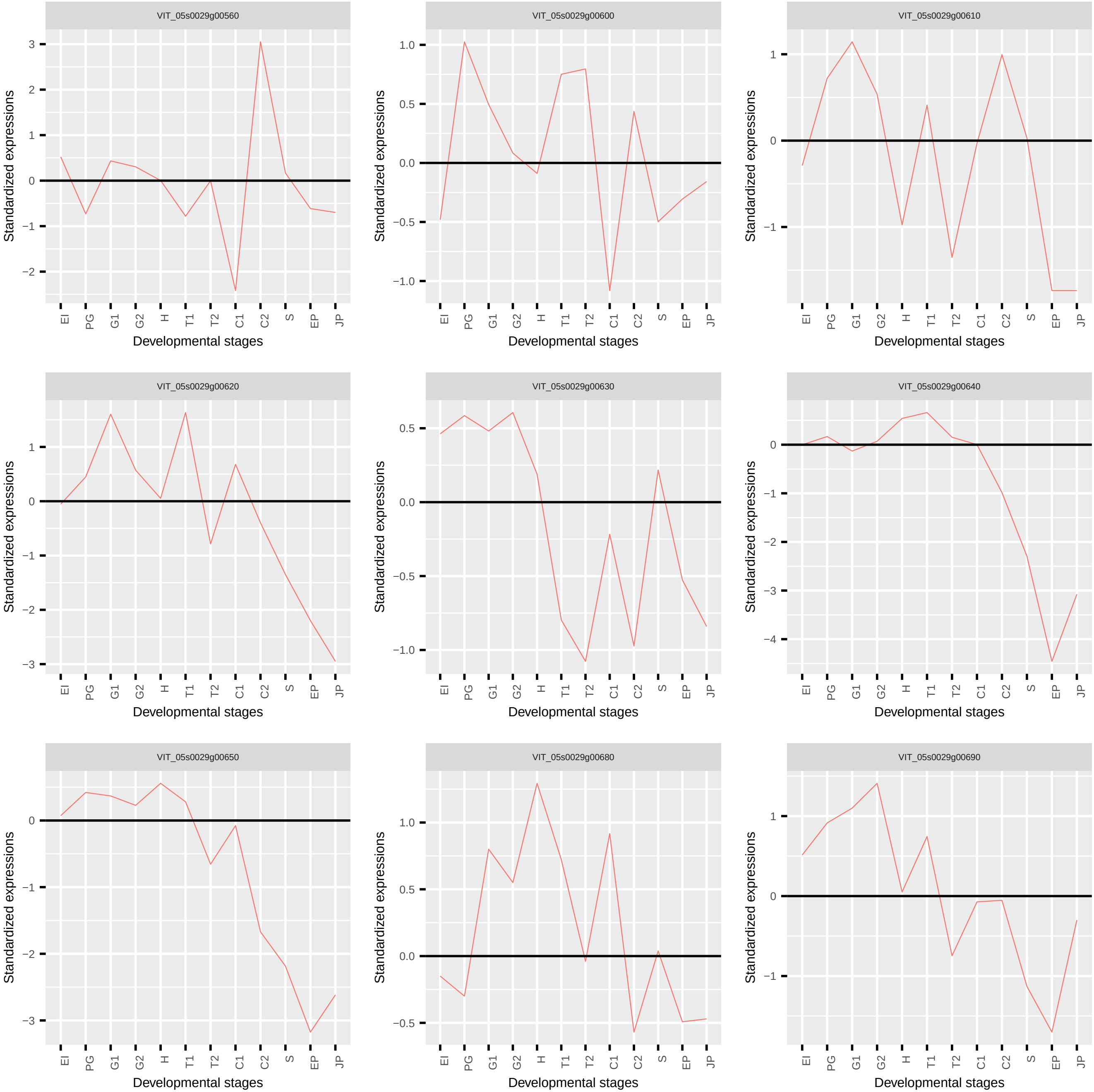
VIT_05s0029g00560
VIT_05s0029g00600
VIT_05s0029g00610
VIT_05s0029g00620
VIT_05s0029g00630
VIT_05s0029g00640
VIT_05s0029g00650
VIT_05s0029g00680
VIT_05s0029g00690
Standardized expressions
Developmental stages
EI PG G1 G2 H T1 T2 C1 C2 S EP JP

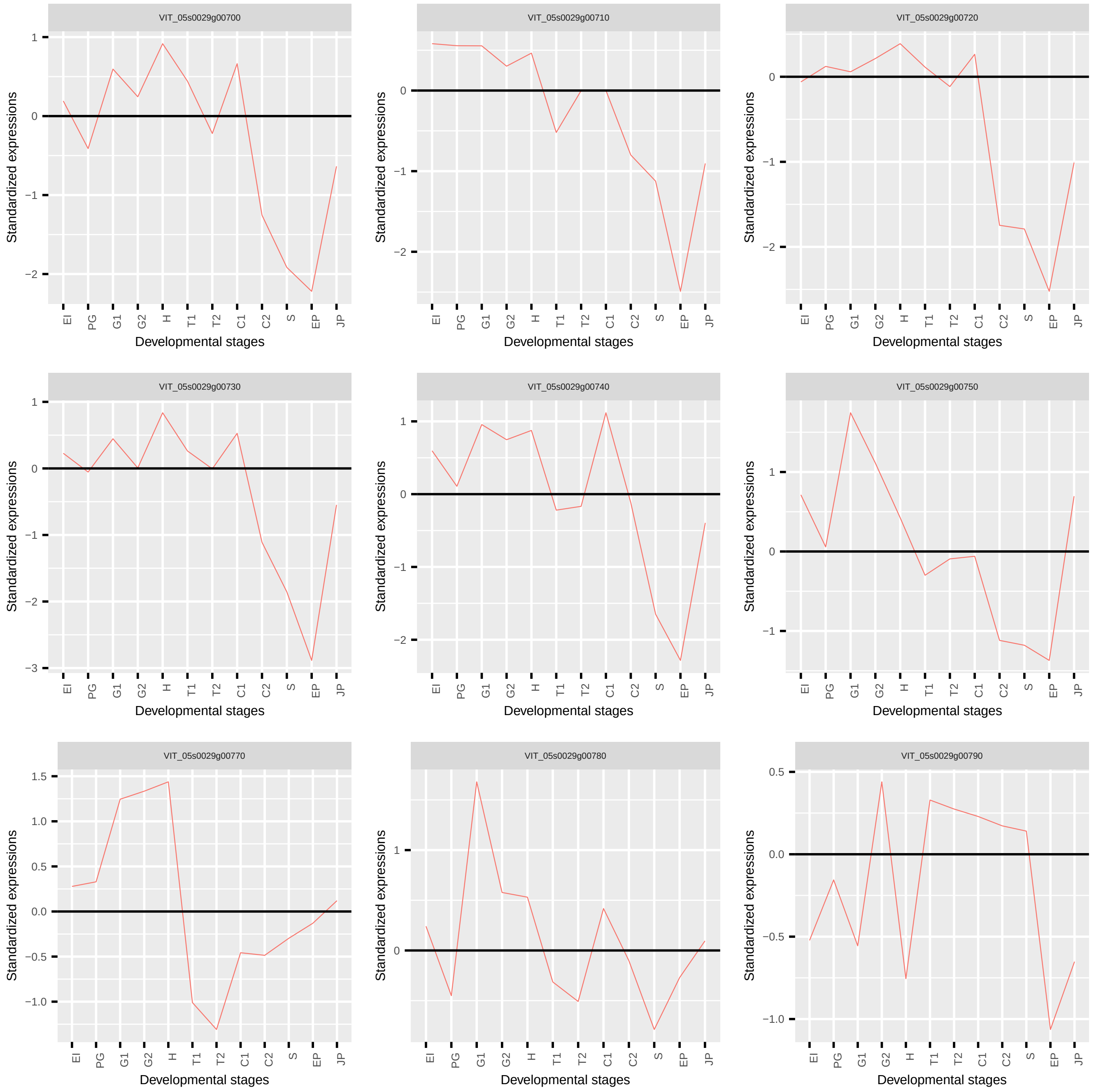

VIT_05s0029g00700
VIT_05s0029g00710
VIT_05s0029g00720
VIT_05s0029g00730
VIT_05s0029g00740
VIT_05s0029g00750
VIT_05s0029g00770
VIT_05s0029g00780
VIT_05s0029g00790
Standardized expressions
Developmental stages
EI
PG
G1
G2
H
T1
T2
C1
C2
S
EP
JP

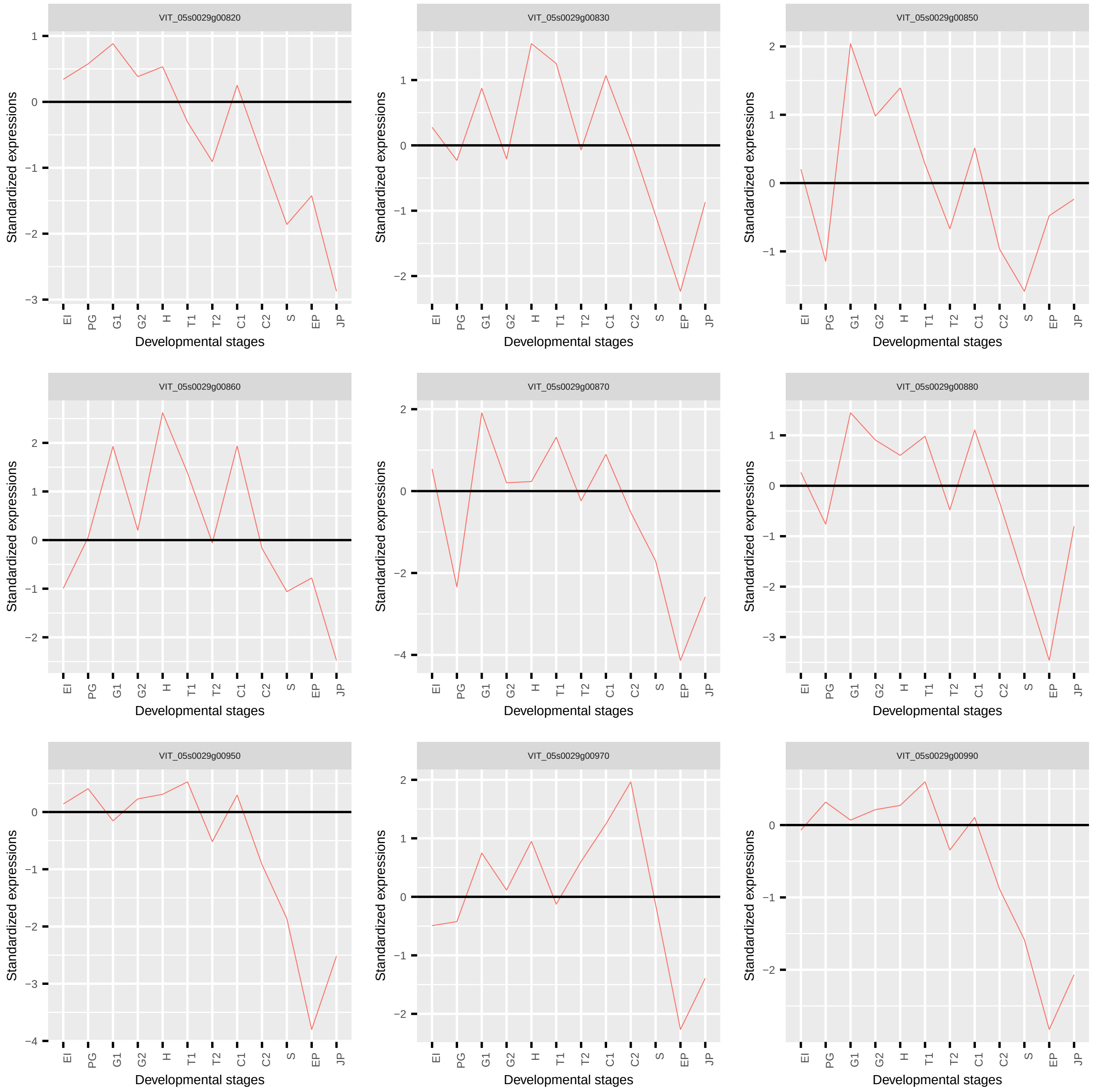
VIT_05s0029g00820
VIT_05s0029g00830
VIT_05s0029g00850
VIT_05s0029g00860
VIT_05s0029g00870
VIT_05s0029g00880
VIT_05s0029g00950
VIT_05s0029g00970
VIT_05s0029g00990
Standardized expressions
Developmental stages
EI PG G1 G2 H T1 T2 C1 C2 S EP JP

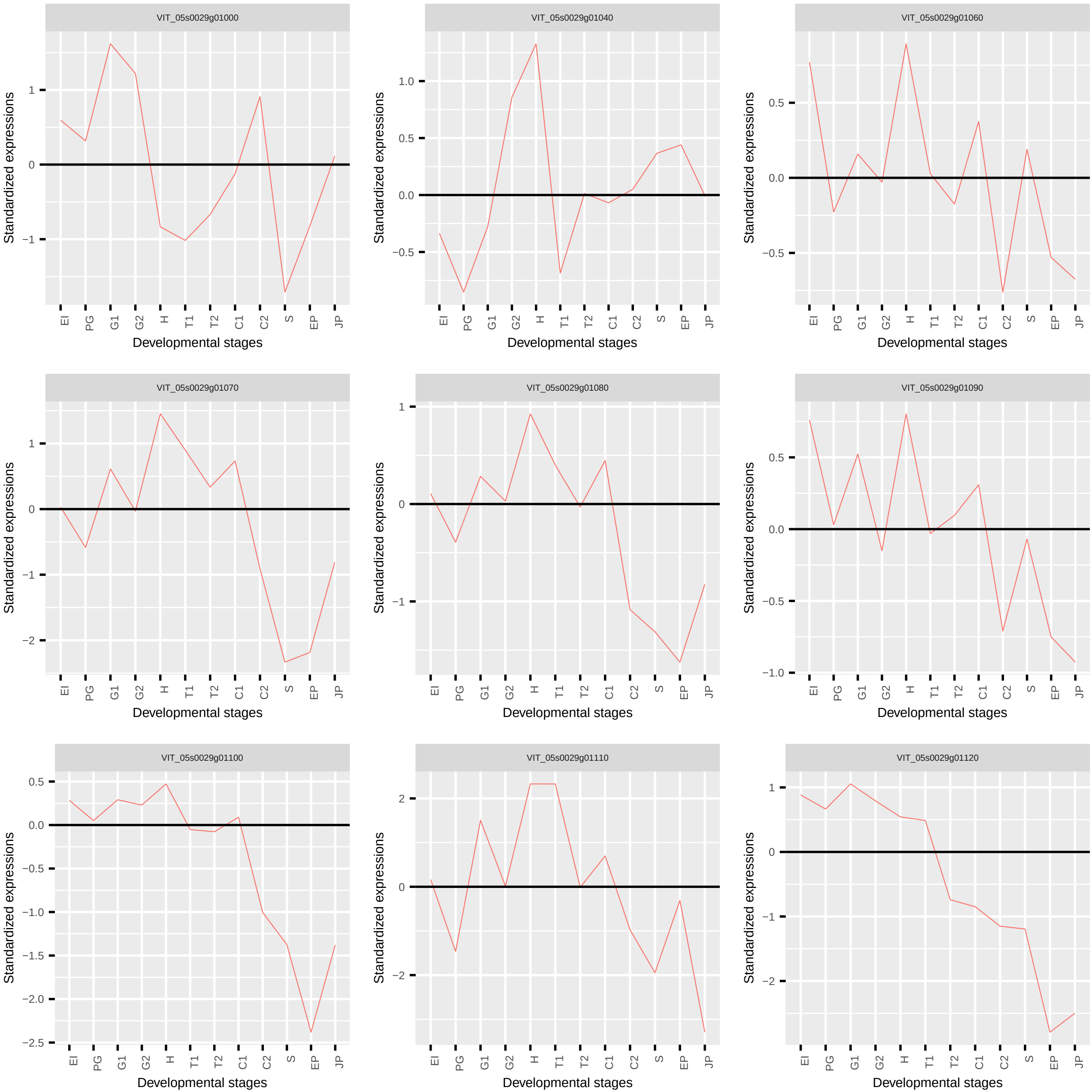

VIT_05s0029g01000
Standardized expressions
1
0
−1
EI PG G1 G2 H T1 T2 C1 C2 S EP JP
Developmental stages
VIT_05s0029g01040
Standardized expressions
1.0
0.5
0.0
−0.5
EI PG G1 G2 H T1 T2 C1 C2 S EP JP
Developmental stages
VIT_05s0029g01060
Standardized expressions
0.5
0.0
−0.5
EI PG G1 G2 H T1 T2 C1 C2 S EP JP
Developmental stages
VIT_05s0029g01070
Standardized expressions
1
0
−1
−2
EI PG G1 G2 H T1 T2 C1 C2 S EP JP
Developmental stages
VIT_05s0029g01080
Standardized expressions
1
0
−1
EI PG G1 G2 H T1 T2 C1 C2 S EP JP
Developmental stages
VIT_05s0029g01090
Standardized expressions
0.5
0.0
−0.5
−1.0
EI PG G1 G2 H T1 T2 C1 C2 S EP JP
Developmental stages
VIT_05s0029g01100
Standardized expressions
0.5
0.0
−0.5
−1.0
−1.5
−2.0
−2.5
EI PG G1 G2 H T1 T2 C1 C2 S EP JP
Developmental stages
VIT_05s0029g01110
Standardized expressions
2
0
−2
EI PG G1 G2 H T1 T2 C1 C2 S EP JP
Developmental stages
VIT_05s0029g01120
Standardized expressions
1
0
−1
−2
EI PG G1 G2 H T1 T2 C1 C2 S EP JP
Developmental stages

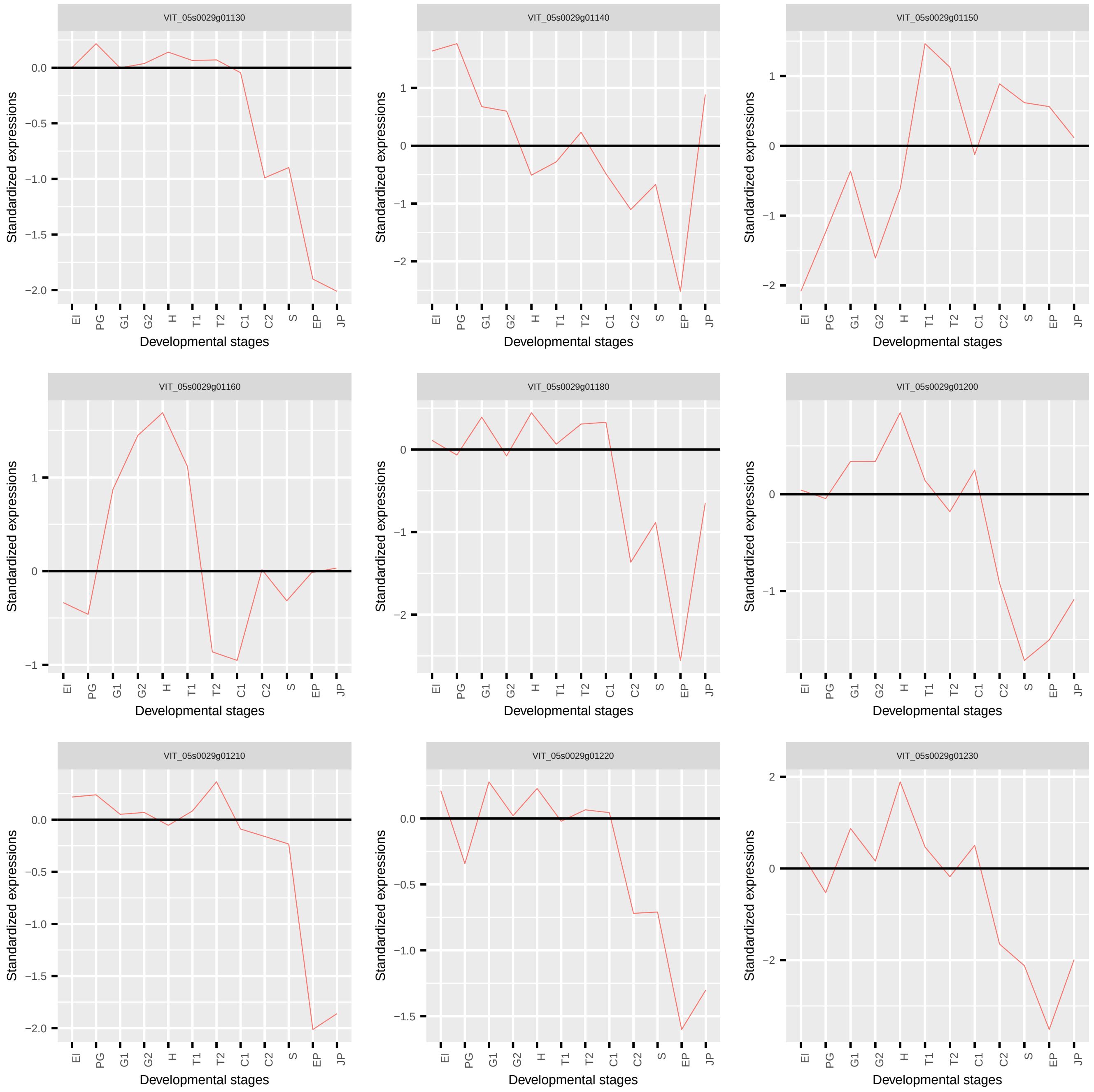
VIT_05s0029g01130
VIT_05s0029g01140
VIT_05s0029g01150
VIT_05s0029g01160
VIT_05s0029g01180
VIT_05s0029g01200
VIT_05s0029g01210
VIT_05s0029g01220
VIT_05s0029g01230
Standardized expressions
Developmental stages
EI PG G1 G2 H T1 T2 C1 C2 S EP JP

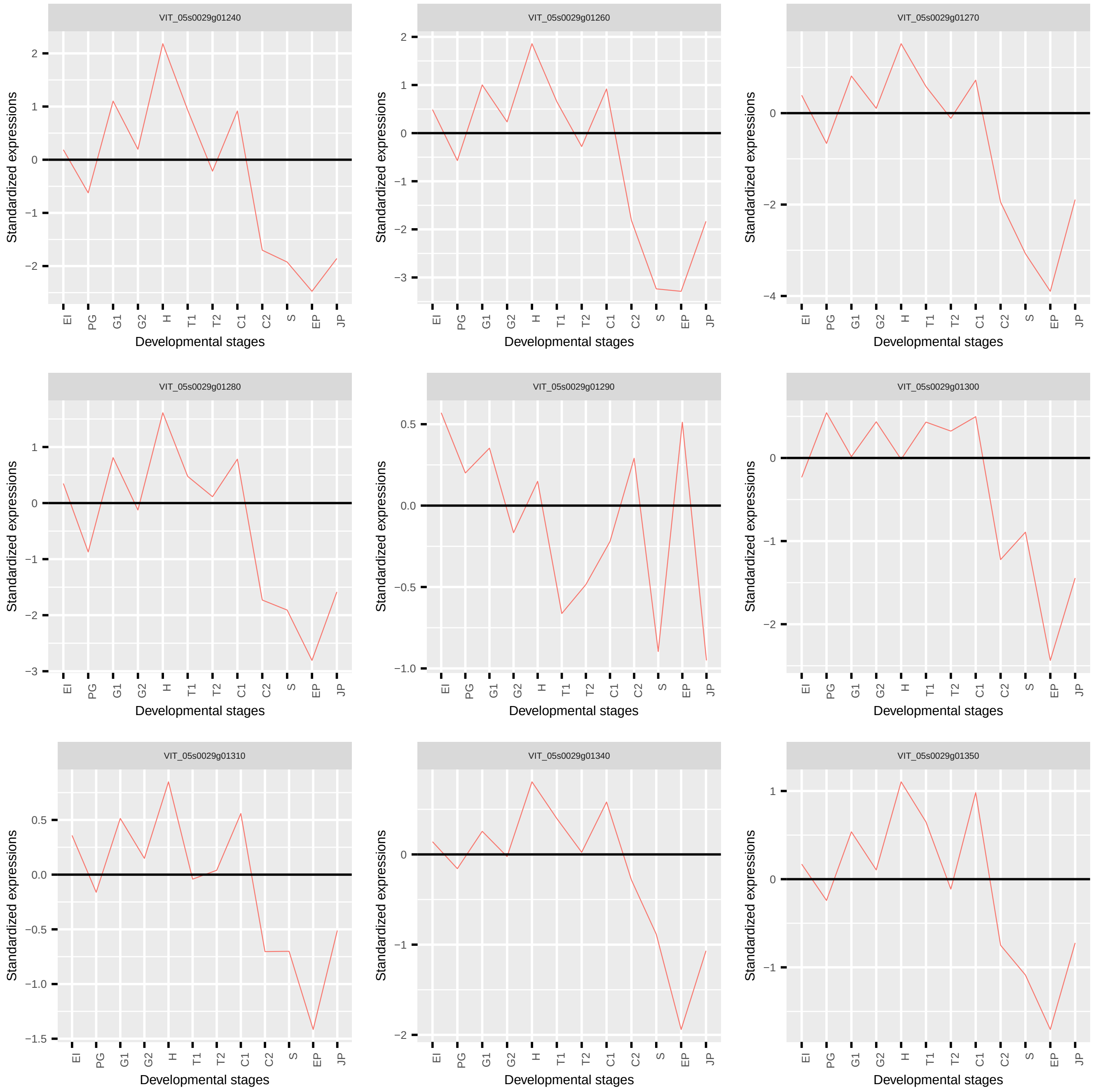
VIT_05s0029g01240
VIT_05s0029g01260
VIT_05s0029g01270
VIT_05s0029g01280
VIT_05s0029g01290
VIT_05s0029g01300
VIT_05s0029g01310
VIT_05s0029g01340
VIT_05s0029g01350
Standardized expressions
Developmental stages
EI PG G1 G2 H T1 T2 C1 C2 S EP JP

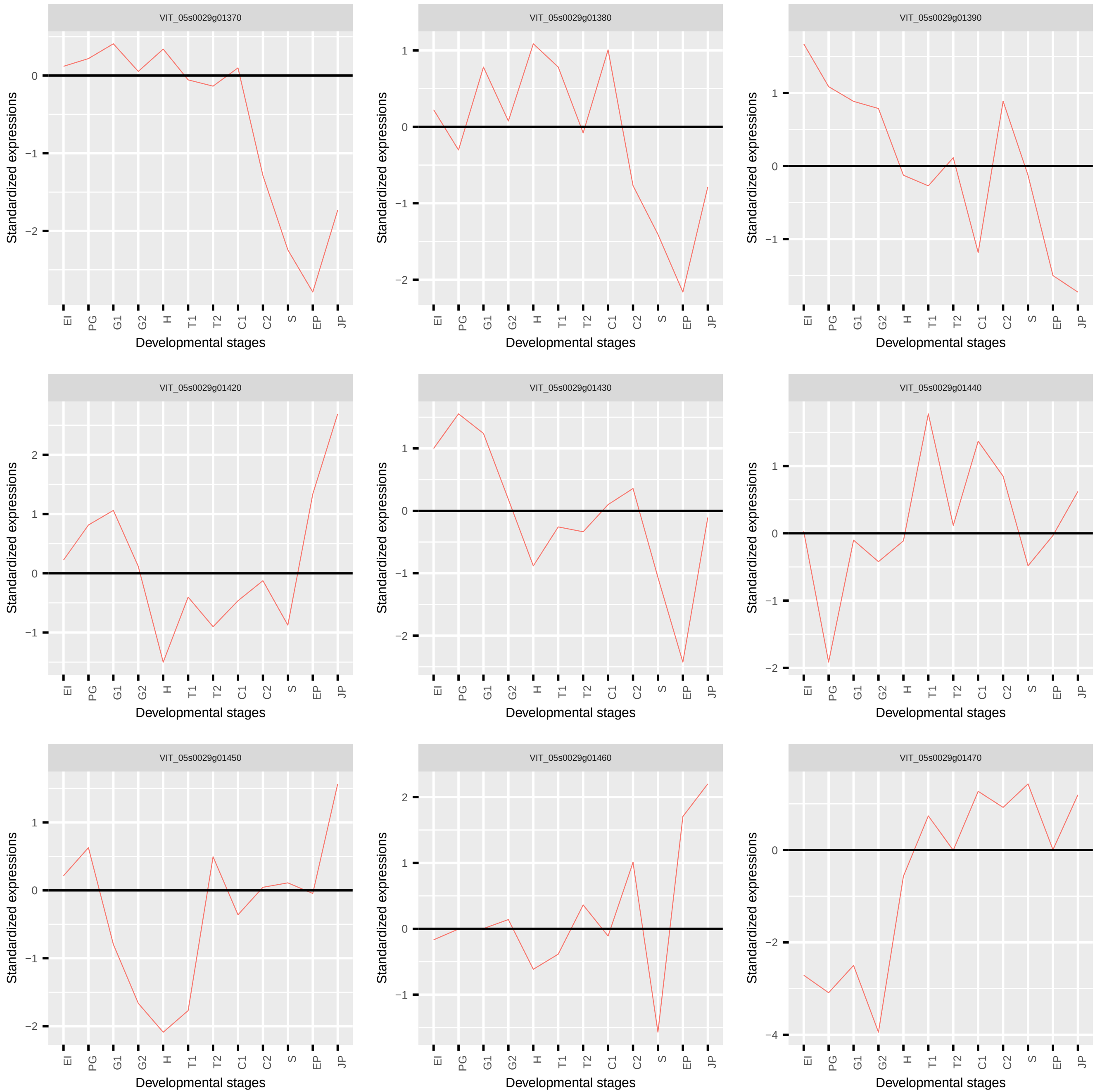
VIT_05s0029g01370
VIT_05s0029g01380
VIT_05s0029g01390
VIT_05s0029g01420
VIT_05s0029g01430
VIT_05s0029g01440
VIT_05s0029g01450
VIT_05s0029g01460
VIT_05s0029g01470
Standardized expressions
Developmental stages
EI
PG
G1
G2
H
T1
T2
C1
C2
S
EP
JP

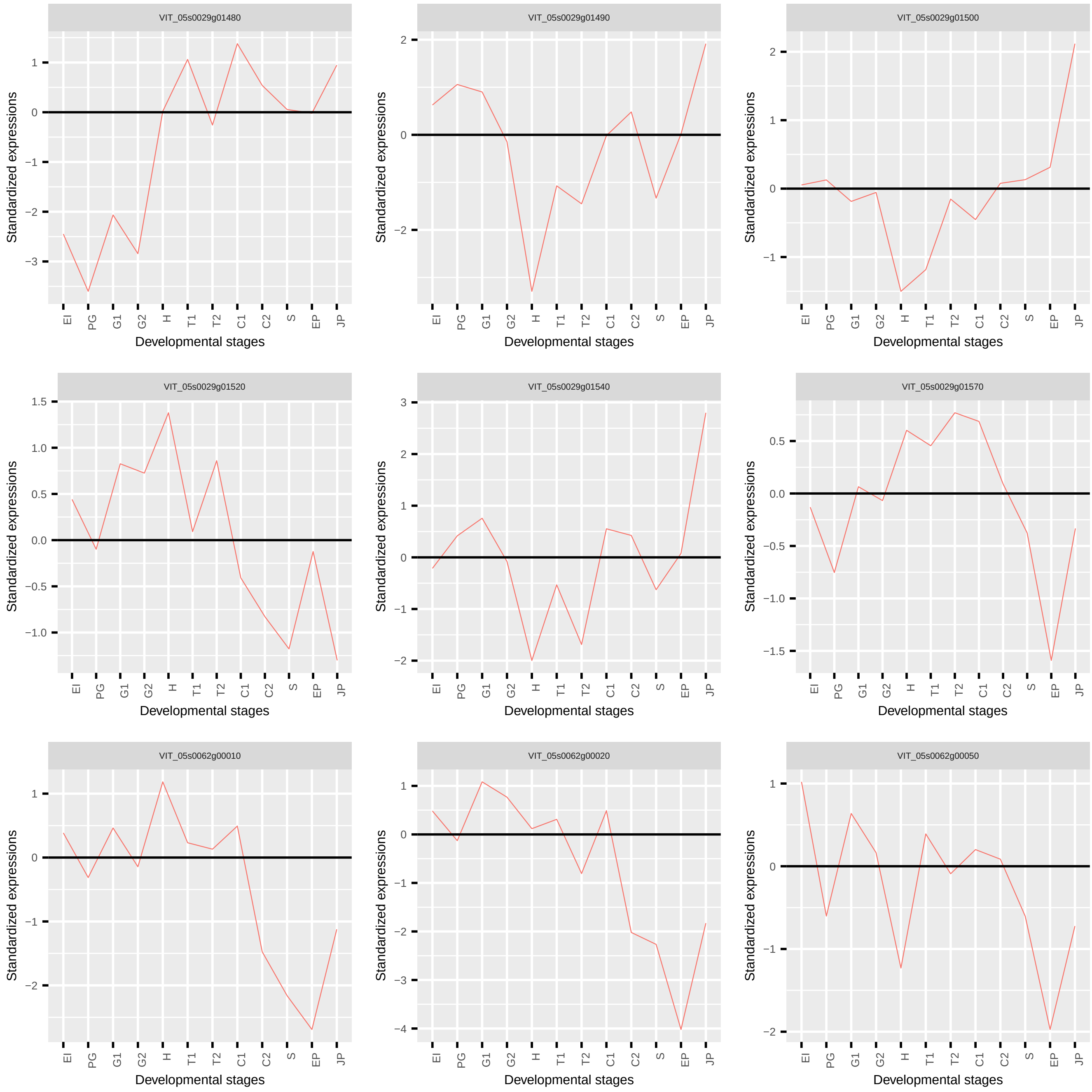
VIT_05s0029g01480
VIT_05s0029g01490
VIT_05s0029g01500
VIT_05s0029g01520
VIT_05s0029g01540
VIT_05s0029g01570
VIT_05s0062g00010
VIT_05s0062g00020
VIT_05s0062g00050
Standardized expressions
Developmental stages
EI PG G1 G2 H T1 T2 C1 C2 S EP JP

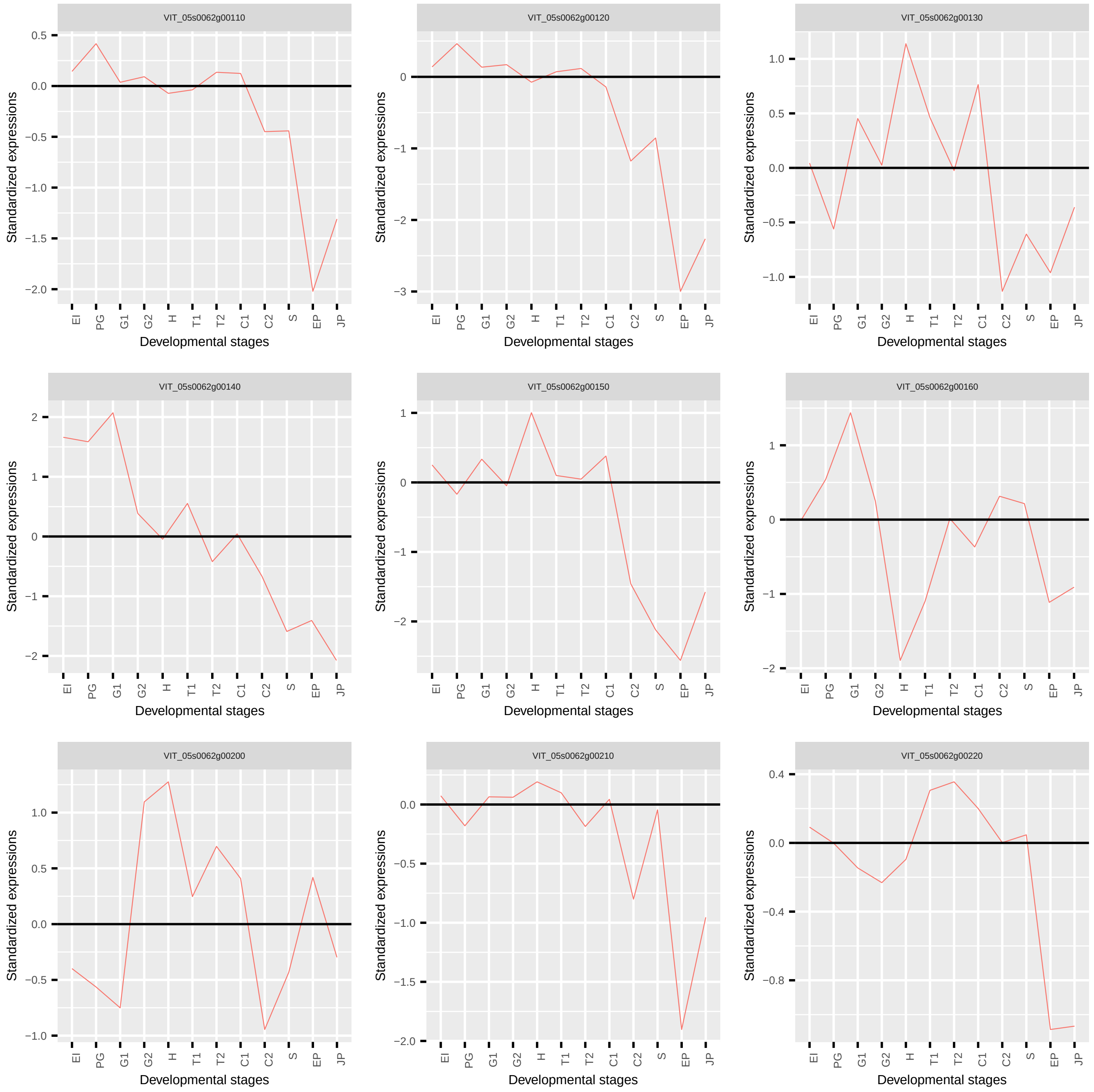

VIT_05s0062g00110
0.5
0.0
−0.5
−1.0
−1.5
−2.0
Standardized expressions
EI PG G1 G2 H T1 T2 C1 C2 S EP JP
Developmental stages
VIT_05s0062g00120
0
−1
−2
−3
Standardized expressions
EI PG G1 G2 H T1 T2 C1 C2 S EP JP
Developmental stages
VIT_05s0062g00130
1.0
0.5
0.0
−0.5
−1.0
Standardized expressions
EI PG G1 G2 H T1 T2 C1 C2 S EP JP
Developmental stages
VIT_05s0062g00140
2
1
0
−1
−2
Standardized expressions
EI PG G1 G2 H T1 T2 C1 C2 S EP JP
Developmental stages
VIT_05s0062g00150
1
0
−1
−2
Standardized expressions
EI PG G1 G2 H T1 T2 C1 C2 S EP JP
Developmental stages
VIT_05s0062g00160
1
0
−1
−2
Standardized expressions
EI PG G1 G2 H T1 T2 C1 C2 S EP JP
Developmental stages
VIT_05s0062g00200
1.0
0.5
0.0
−0.5
−1.0
Standardized expressions
EI PG G1 G2 H T1 T2 C1 C2 S EP JP
Developmental stages
VIT_05s0062g00210
0.0
−0.5
−1.0
−1.5
−2.0
Standardized expressions
EI PG G1 G2 H T1 T2 C1 C2 S EP JP
Developmental stages
VIT_05s0062g00220
0.4
0.0
−0.4
−0.8
Standardized expressions
EI PG G1 G2 H T1 T2 C1 C2 S EP JP
Developmental stages

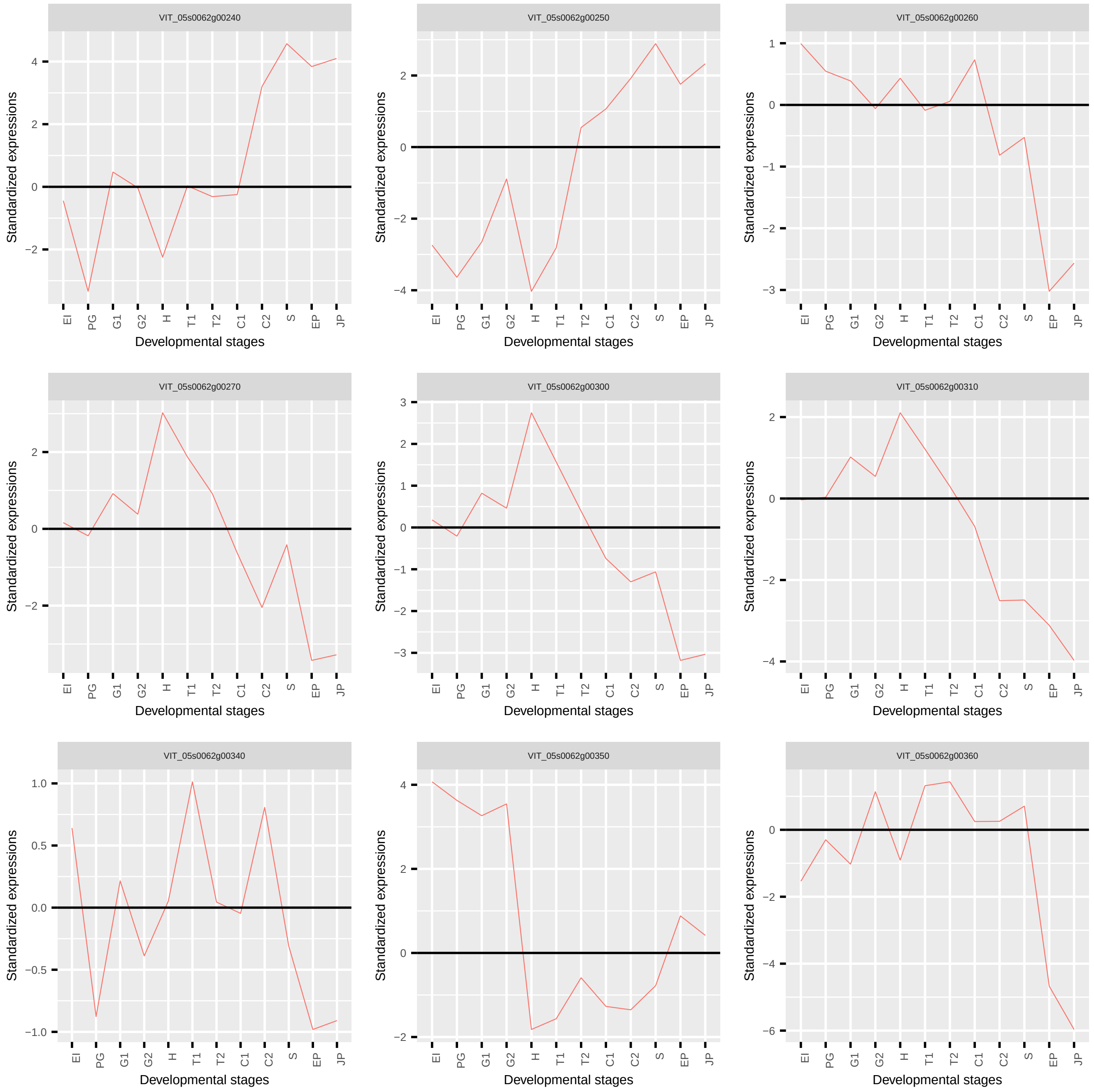
VIT_05s0062g00240
VIT_05s0062g00250
VIT_05s0062g00260
VIT_05s0062g00270
VIT_05s0062g00300
VIT_05s0062g00310
VIT_05s0062g00340
VIT_05s0062g00350
VIT_05s0062g00360
Standardized expressions
Developmental stages
EI PG G1 G2 H T1 T2 C1 C2 S EP JP

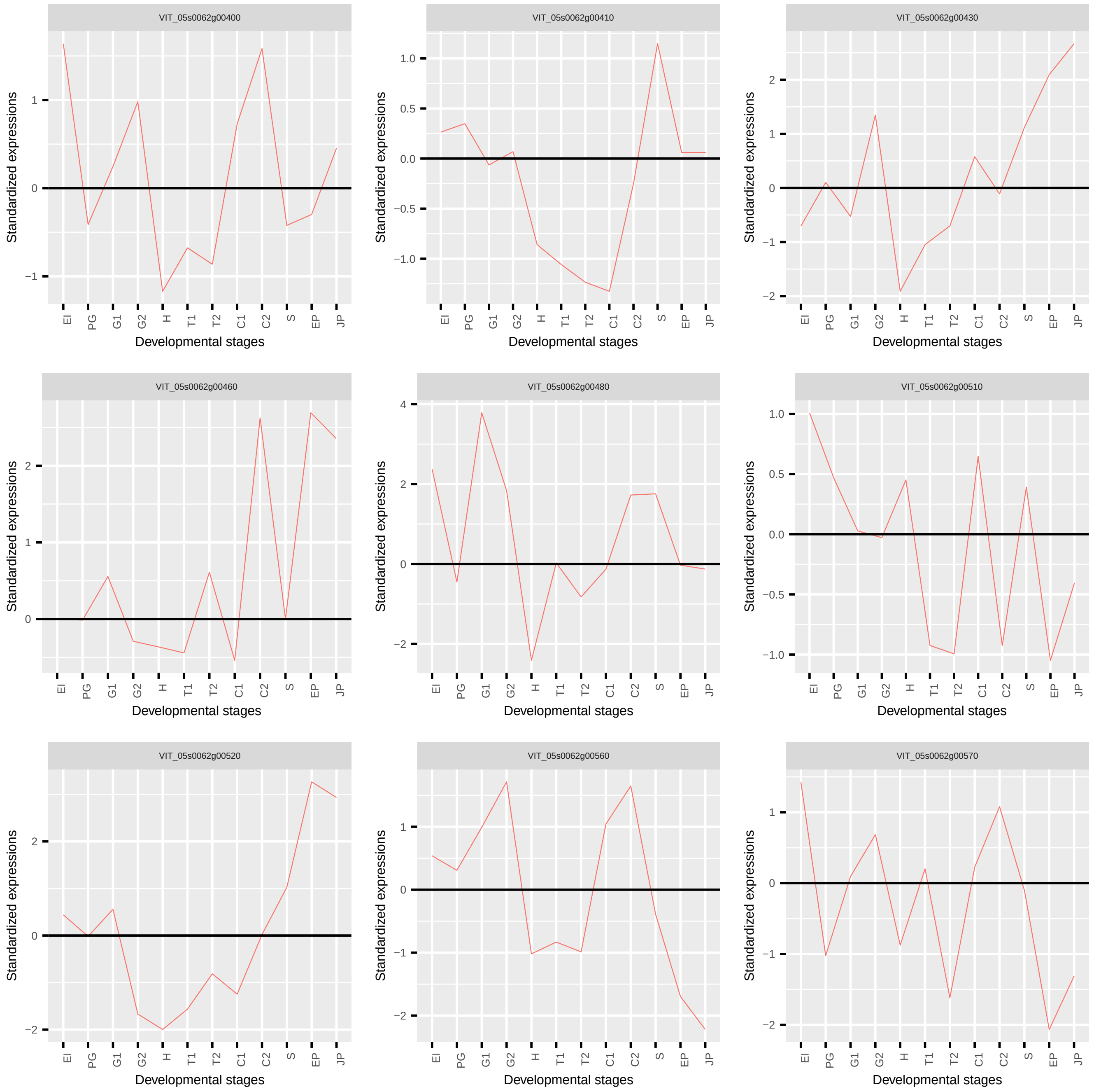
VIT_05s0062g00400
VIT_05s0062g00410
VIT_05s0062g00430
VIT_05s0062g00460
VIT_05s0062g00480
VIT_05s0062g00510
VIT_05s0062g00520
VIT_05s0062g00560
VIT_05s0062g00570
Standardized expressions
Developmental stages
EI
PG
G1
G2
H
T1
T2
C1
C2
S
EP
JP

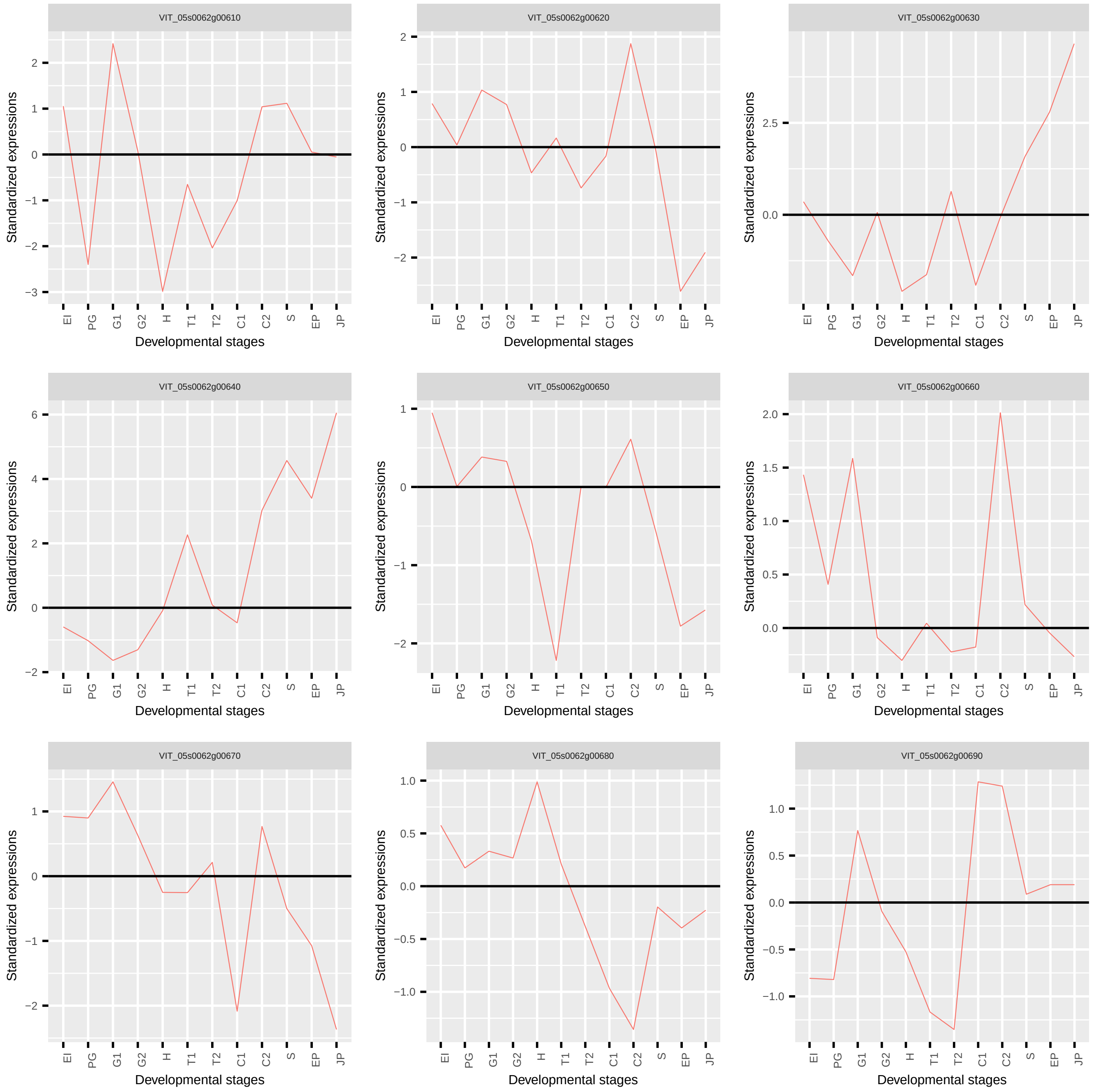

VIT_05s0062g00610
VIT_05s0062g00620
VIT_05s0062g00630
VIT_05s0062g00640
VIT_05s0062g00650
VIT_05s0062g00660
VIT_05s0062g00670
VIT_05s0062g00680
VIT_05s0062g00690
Standardized expressions
Developmental stages
EI PG G1 G2 H T1 T2 C1 C2 S EP JP

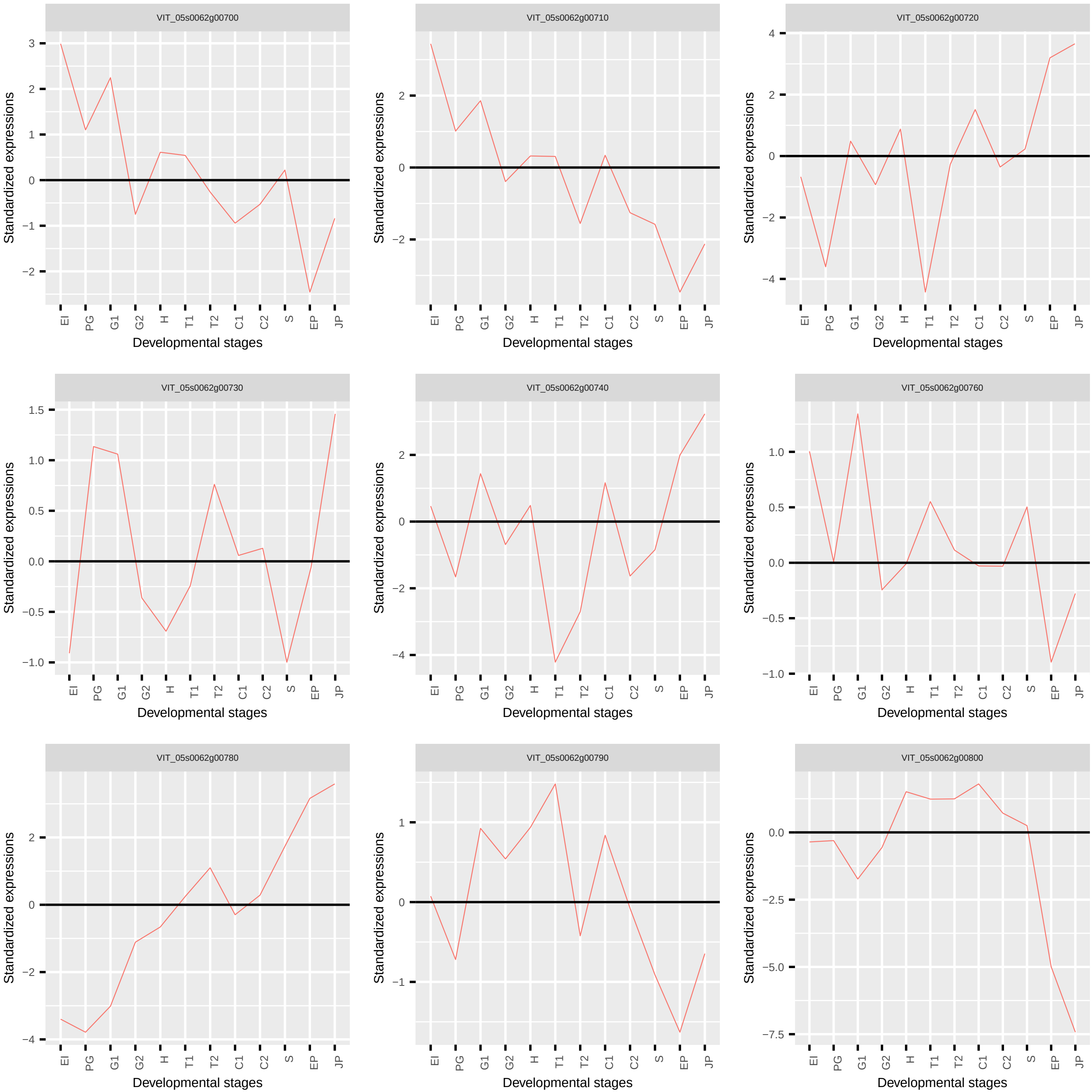
VIT_05s0062g00700
VIT_05s0062g00710
VIT_05s0062g00720
VIT_05s0062g00730
VIT_05s0062g00740
VIT_05s0062g00760
VIT_05s0062g00780
VIT_05s0062g00790
VIT_05s0062g00800
Standardized expressions
Developmental stages
EI
PG
G1
G2
H
T1
T2
C1
C2
S
EP
JP

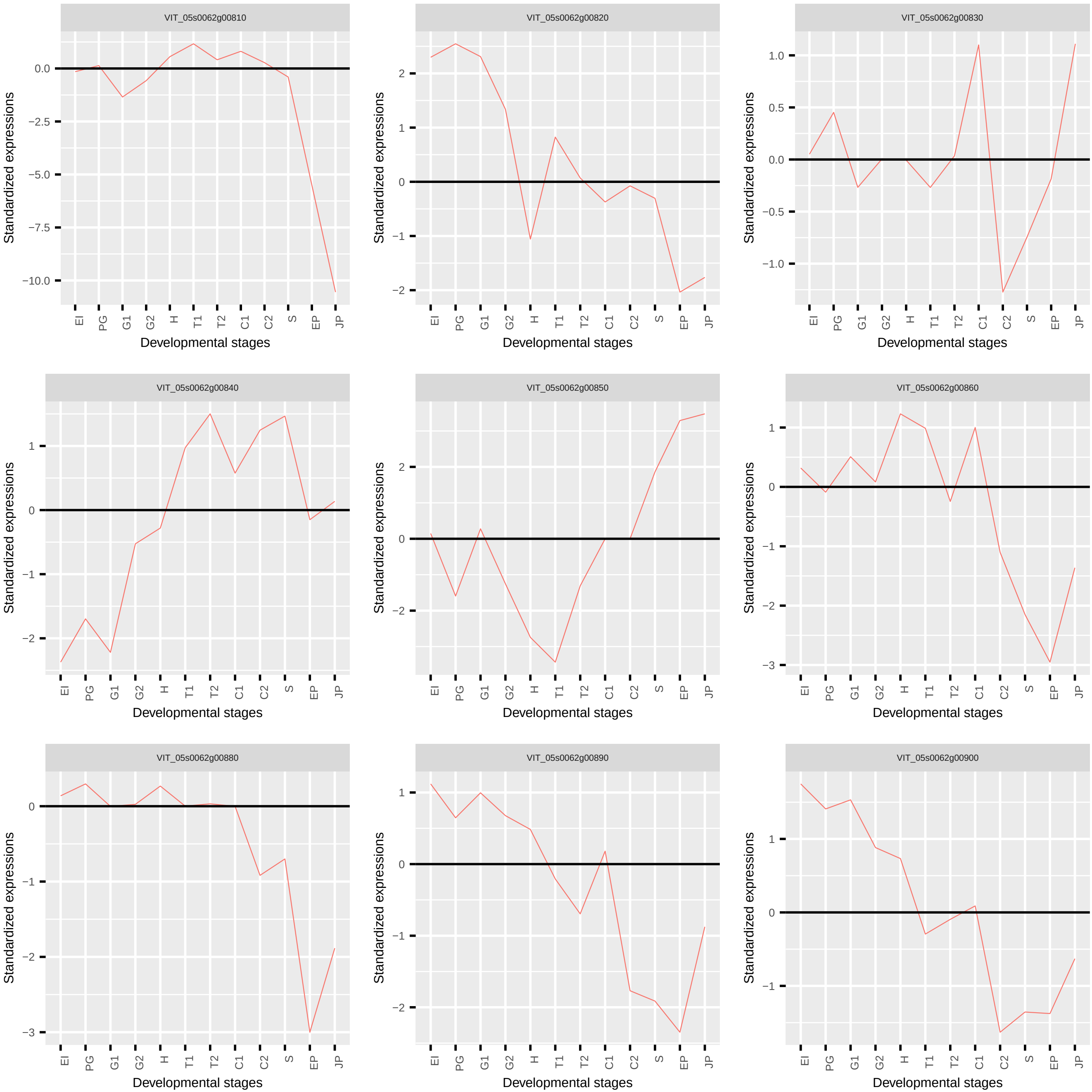
VIT_05s0062g00810
VIT_05s0062g00820
VIT_05s0062g00830
VIT_05s0062g00840
VIT_05s0062g00850
VIT_05s0062g00860
VIT_05s0062g00880
VIT_05s0062g00890
VIT_05s0062g00900
Standardized expressions
Developmental stages
EI
PG
G1
G2
H
T1
T2
C1
C2
S
EP
JP

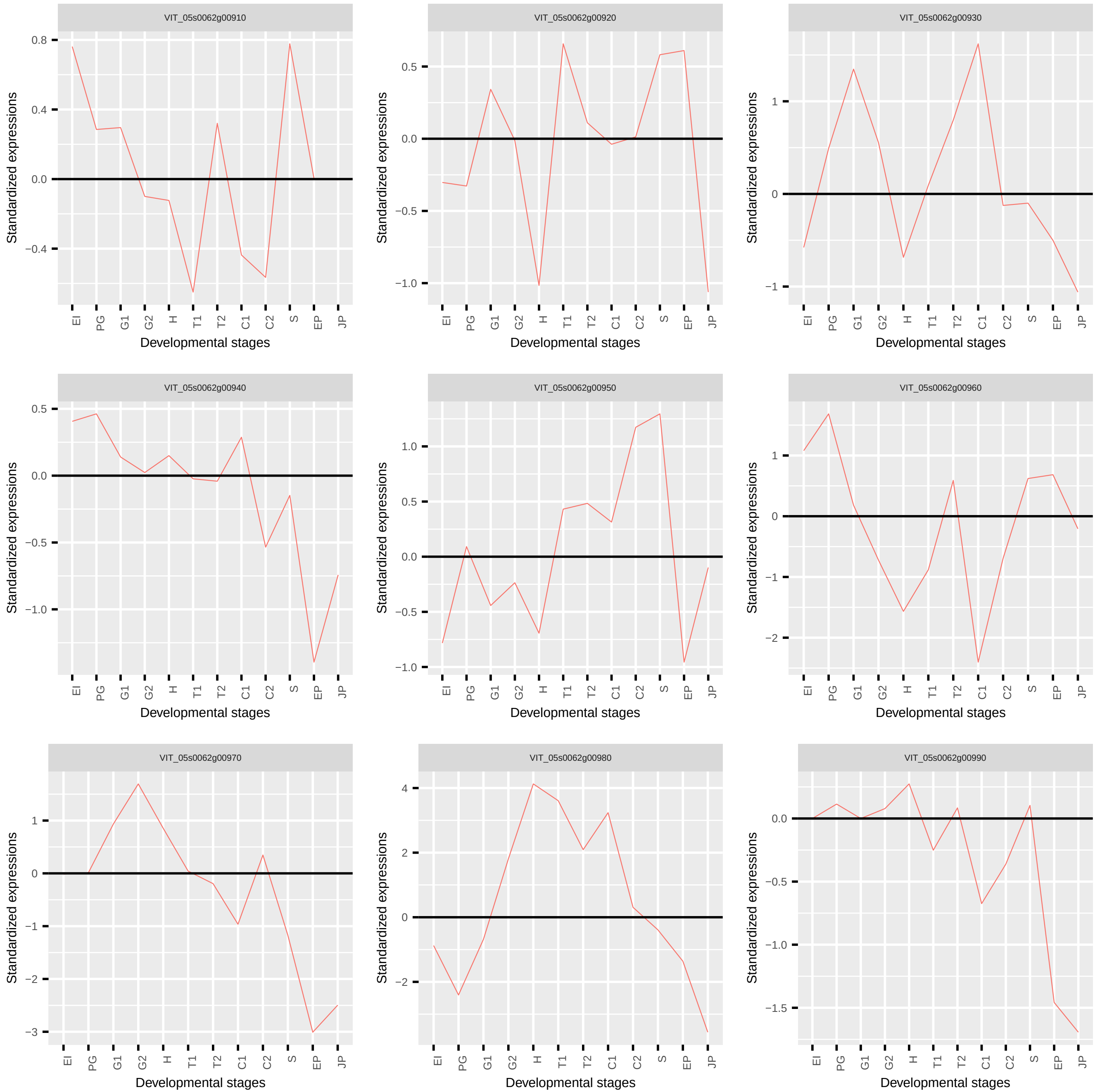

VIT_05s0062g00910
VIT_05s0062g00920
VIT_05s0062g00930
VIT_05s0062g00940
VIT_05s0062g00950
VIT_05s0062g00960
VIT_05s0062g00970
VIT_05s0062g00980
VIT_05s0062g00990
Standardized expressions
Developmental stages
EI PG G1 G2 H T1 T2 C1 C2 S EP JP
0.8 0.4 0.0 −0.4
0.5 0.0 −0.5 −1.0
1 0 −1
1.0 0.5 0.0 −0.5 −1.0
−2
−3
4 2 0 −2
−1.5

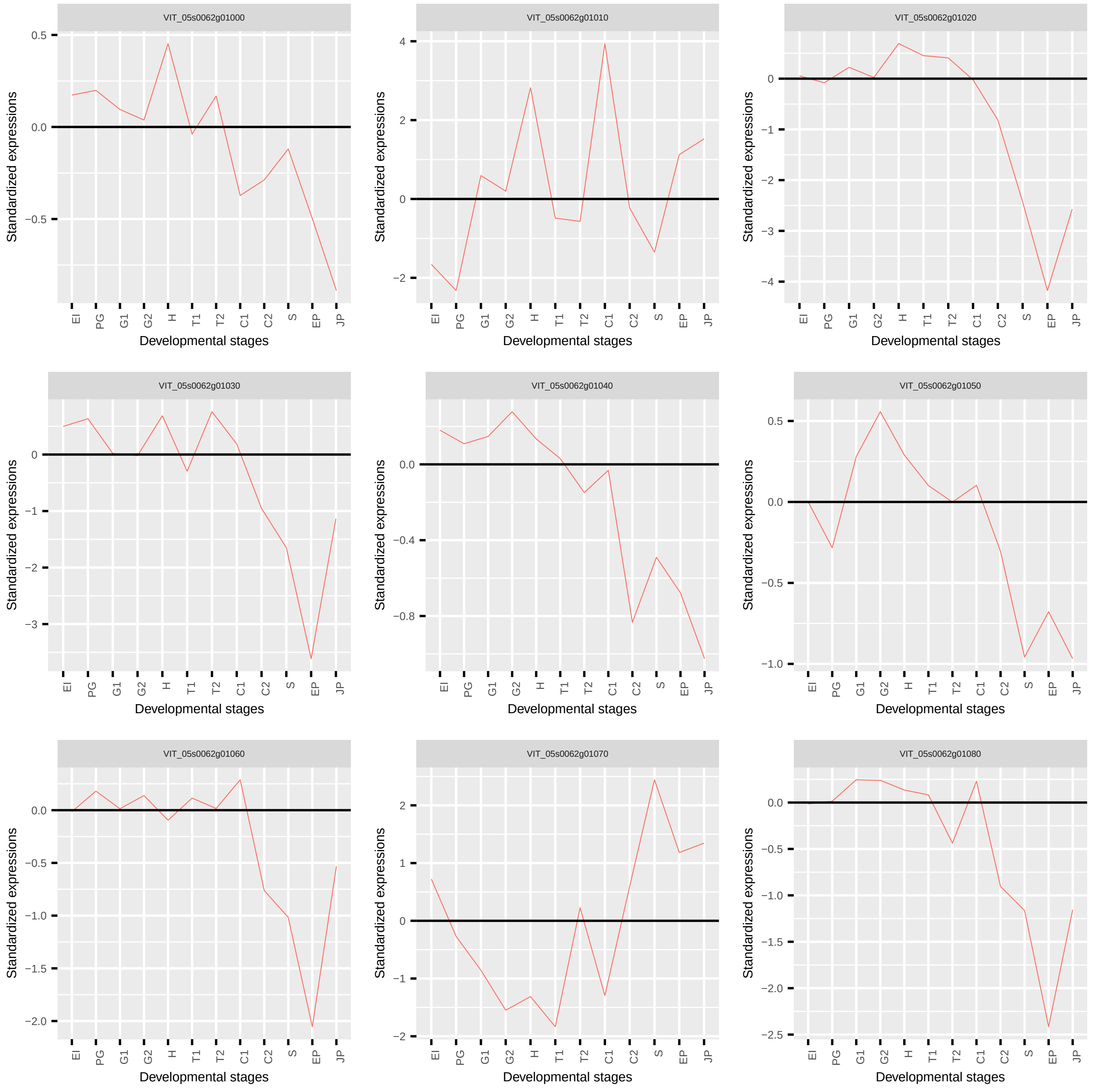
VIT_05s0062g01000
VIT_05s0062g01010
VIT_05s0062g01020
VIT_05s0062g01030
VIT_05s0062g01040
VIT_05s0062g01050
VIT_05s0062g01060
VIT_05s0062g01070
VIT_05s0062g01080
Standardized expressions
Developmental stages
EI
PG
G1
G2
H
T1
T2
C1
C2
S
EP
JP

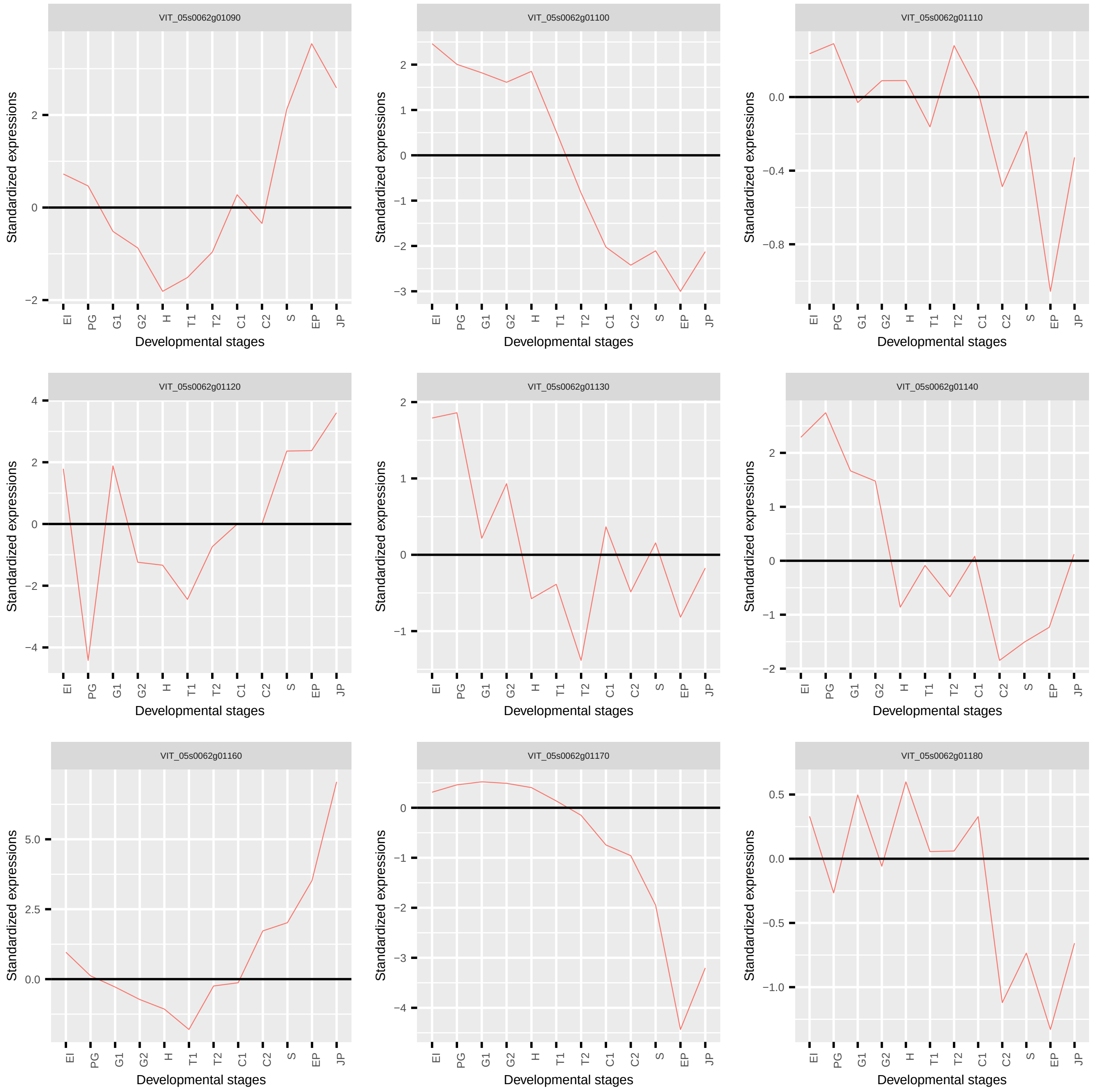
VIT_05s0062g01090
VIT_05s0062g01100
VIT_05s0062g01110
VIT_05s0062g01120
VIT_05s0062g01130
VIT_05s0062g01140
VIT_05s0062g01160
VIT_05s0062g01170
VIT_05s0062g01180
Standardized expressions
Developmental stages
EI
PG
G1
G2
H
T1
T2
C1
C2
S
EP
JP

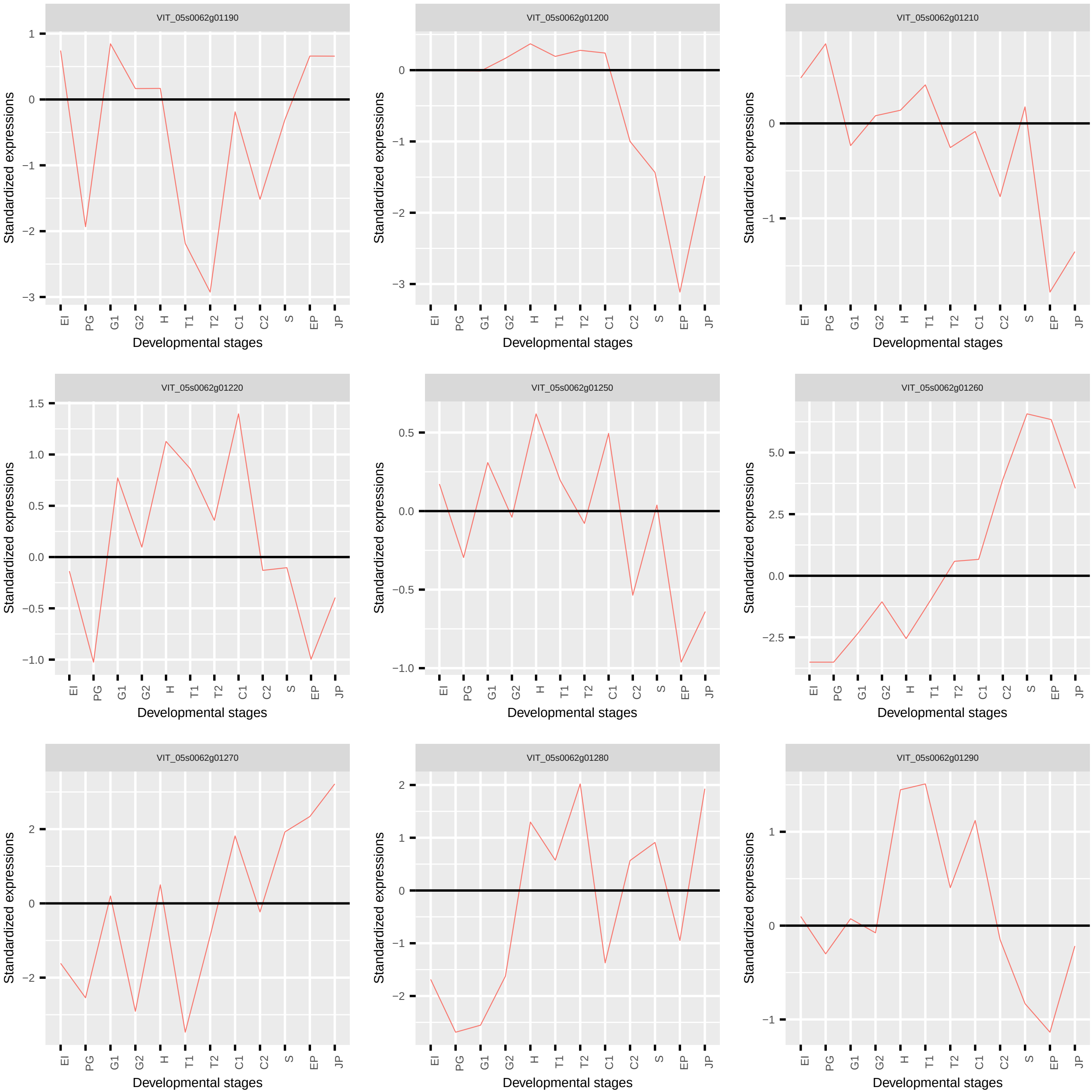
VIT_05s0062g01190
VIT_05s0062g01200
VIT_05s0062g01210
VIT_05s0062g01220
VIT_05s0062g01250
VIT_05s0062g01260
VIT_05s0062g01270
VIT_05s0062g01280
VIT_05s0062g01290
Standardized expressions
Developmental stages
EI PG G1 G2 H T1 T2 C1 C2 S EP JP

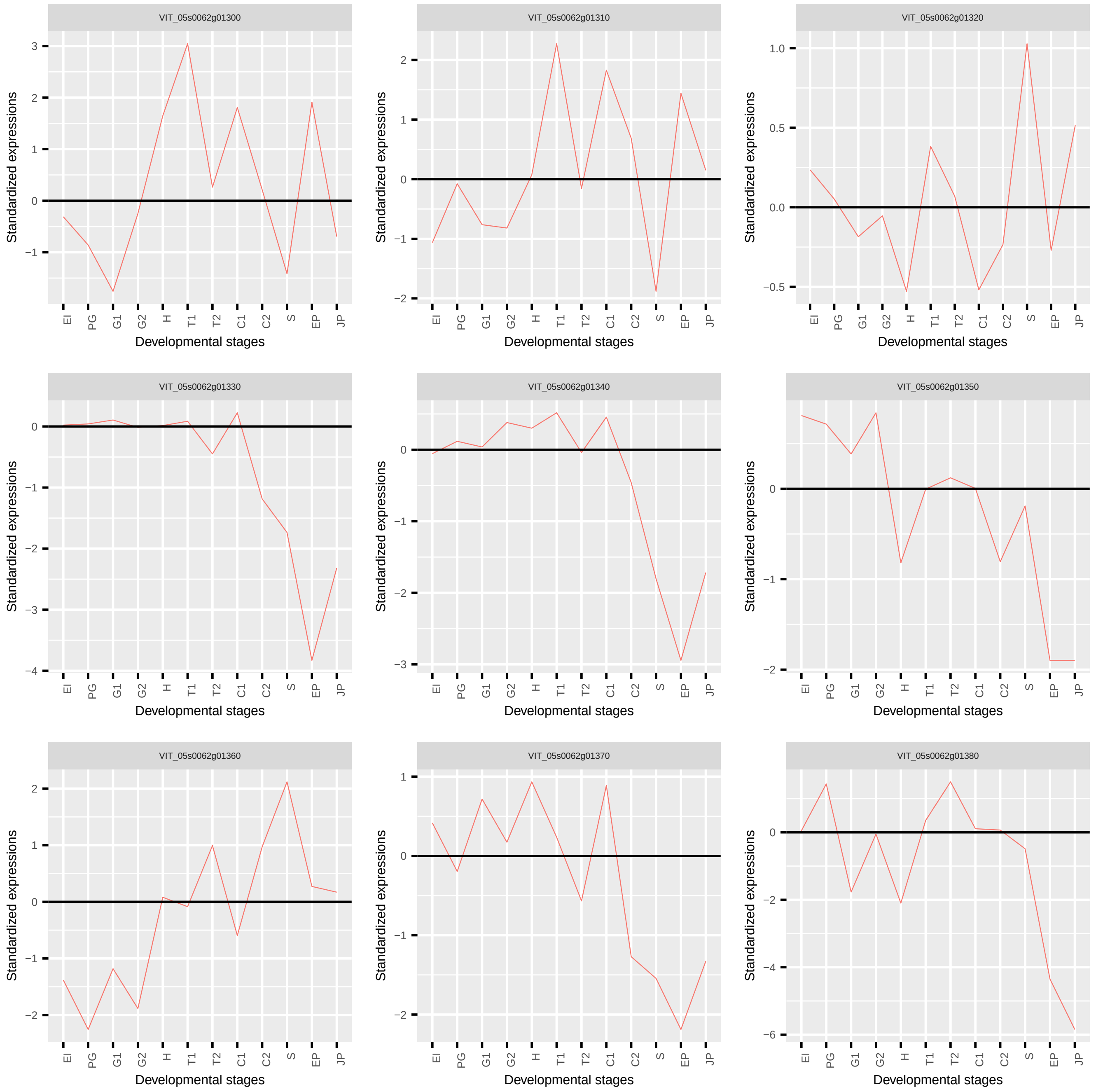
VIT_05s0062g01300
VIT_05s0062g01310
VIT_05s0062g01320
VIT_05s0062g01330
VIT_05s0062g01340
VIT_05s0062g01350
VIT_05s0062g01360
VIT_05s0062g01370
VIT_05s0062g01380
Standardized expressions
Developmental stages
EI PG G1 G2 H T1 T2 C1 C2 S EP JP

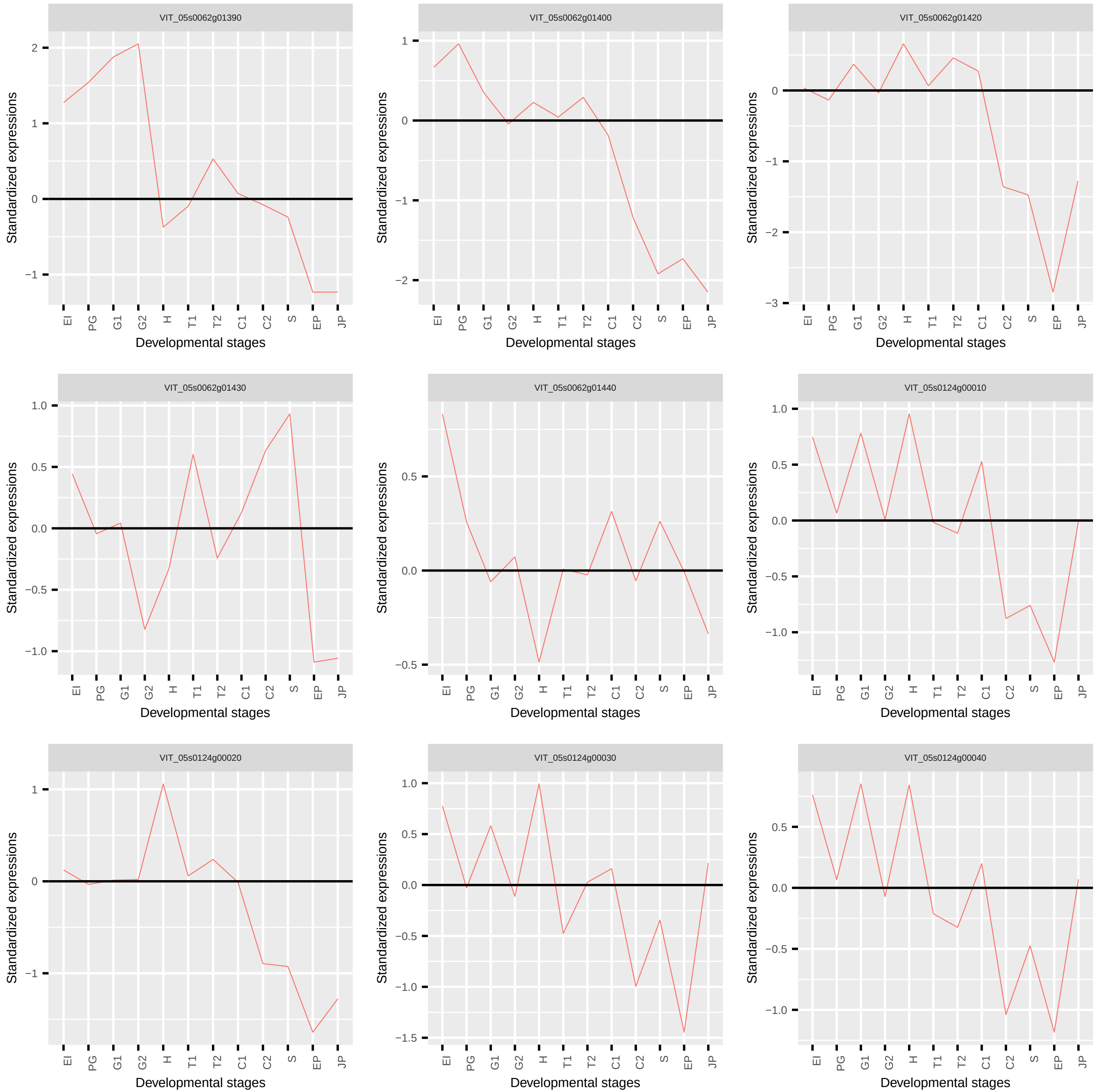
VIT_05s0062g01390
VIT_05s0062g01400
VIT_05s0062g01420
VIT_05s0062g01430
VIT_05s0062g01440
VIT_05s0124g00010
VIT_05s0124g00020
VIT_05s0124g00030
VIT_05s0124g00040
Standardized expressions
Developmental stages
EI PG G1 G2 H T1 T2 C1 C2 S EP JP

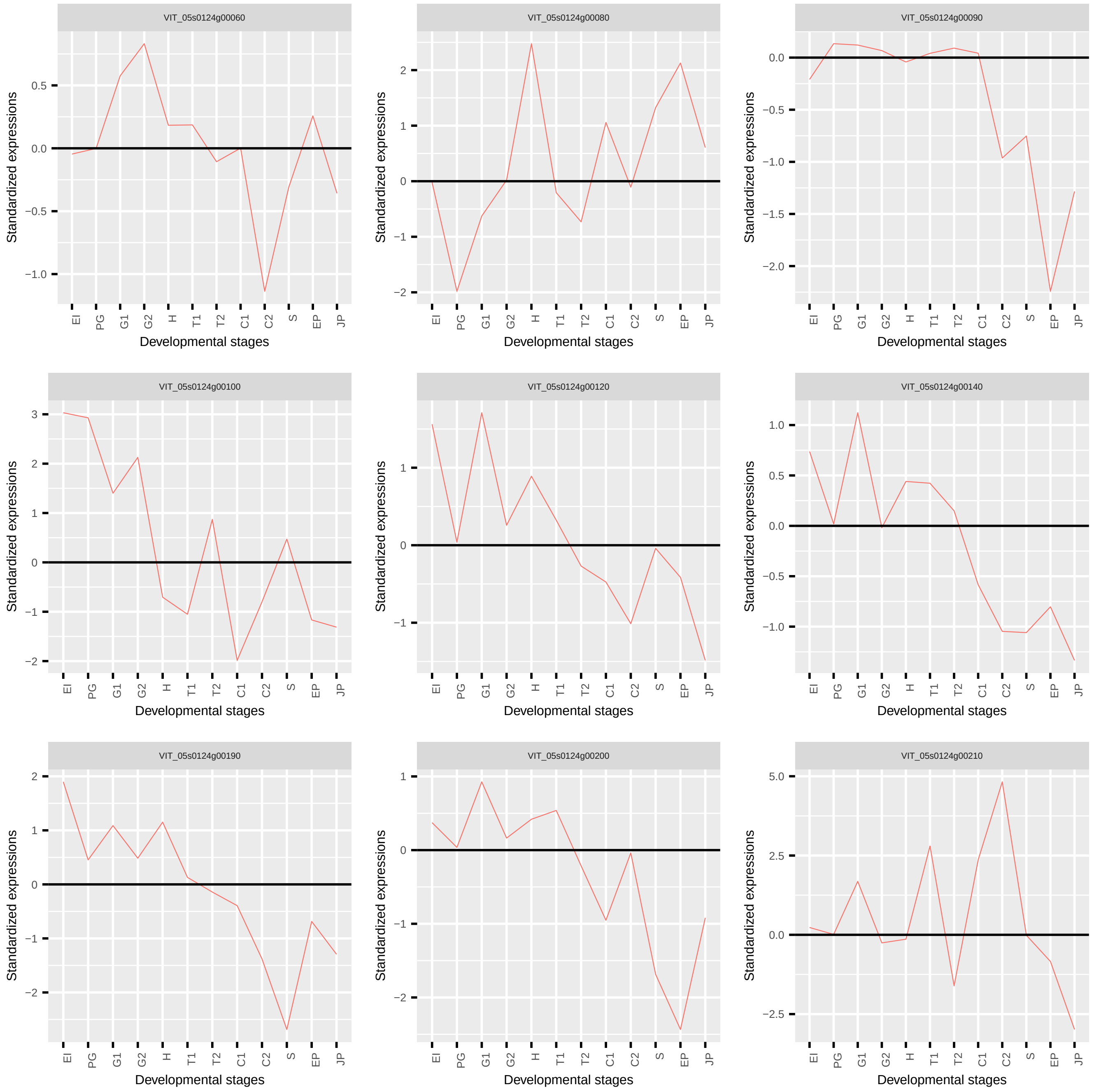
VIT_05s0124g00060
0.5
0.0
−0.5
−1.0
Standardized expressions
EI PG G1 G2 H T1 T2 C1 C2 S EP JP
Developmental stages
VIT_05s0124g00080
2
1
0
−1
−2
Standardized expressions
EI PG G1 G2 H T1 T2 C1 C2 S EP JP
Developmental stages
VIT_05s0124g00090
0.0
−0.5
−1.0
−1.5
−2.0
Standardized expressions
EI PG G1 G2 H T1 T2 C1 C2 S EP JP
Developmental stages
VIT_05s0124g00100
3
2
1
0
−1
−2
Standardized expressions
EI PG G1 G2 H T1 T2 C1 C2 S EP JP
Developmental stages
VIT_05s0124g00120
1
0
−1
Standardized expressions
EI PG G1 G2 H T1 T2 C1 C2 S EP JP
Developmental stages
VIT_05s0124g00140
1.0
0.5
0.0
−0.5
−1.0
Standardized expressions
EI PG G1 G2 H T1 T2 C1 C2 S EP JP
Developmental stages
VIT_05s0124g00190
2
1
0
−1
−2
Standardized expressions
EI PG G1 G2 H T1 T2 C1 C2 S EP JP
Developmental stages
VIT_05s0124g00200
1
0
−1
−2
Standardized expressions
EI PG G1 G2 H T1 T2 C1 C2 S EP JP
Developmental stages
VIT_05s0124g00210
5.0
2.5
0.0
−2.5
Standardized expressions
EI PG G1 G2 H T1 T2 C1 C2 S EP JP
Developmental stages

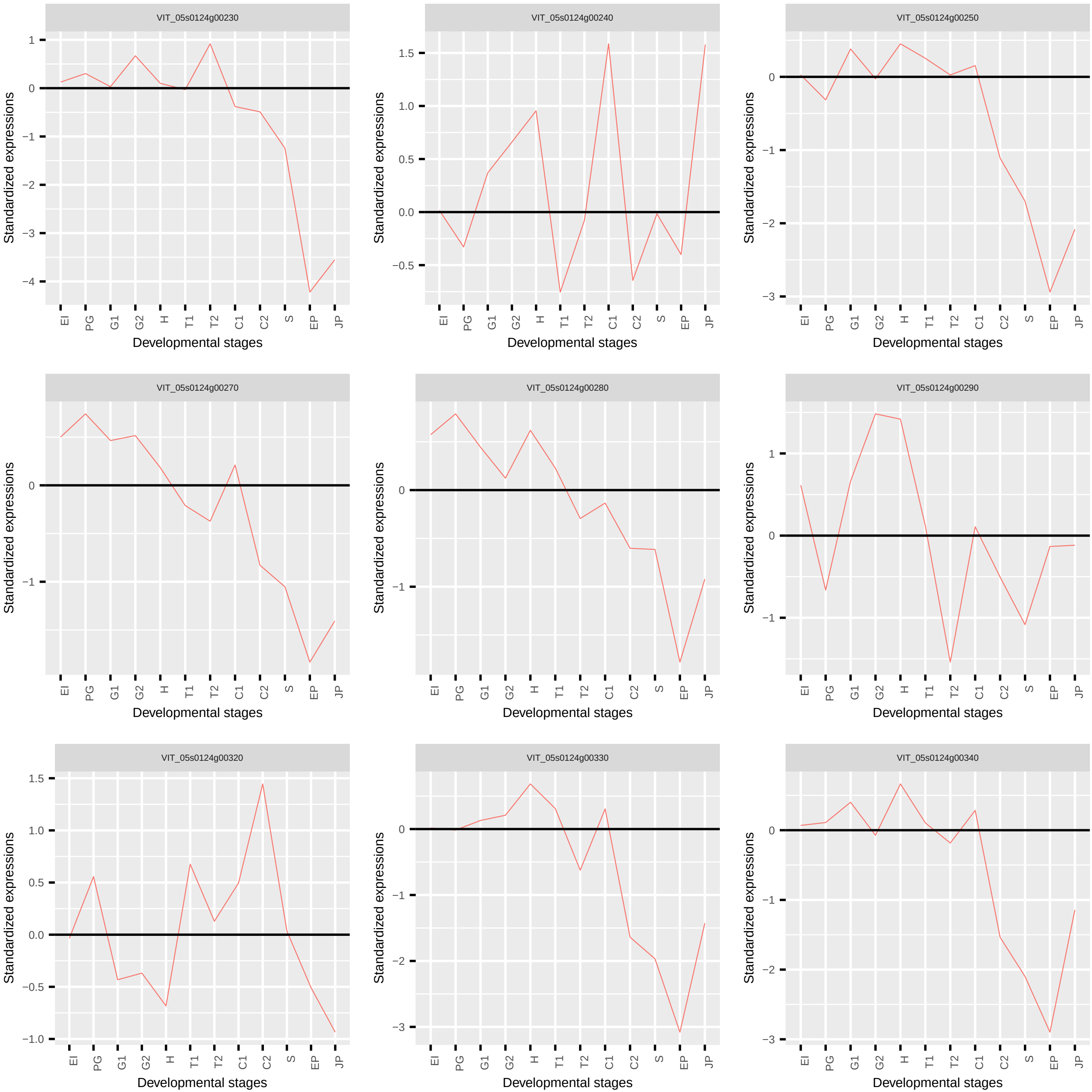
VIT_05s0124g00230
VIT_05s0124g00240
VIT_05s0124g00250
VIT_05s0124g00270
VIT_05s0124g00280
VIT_05s0124g00290
VIT_05s0124g00320
VIT_05s0124g00330
VIT_05s0124g00340
Standardized expressions
Developmental stages
EI
PG
G1
G2
H
T1
T2
C1
C2
S
EP
JP

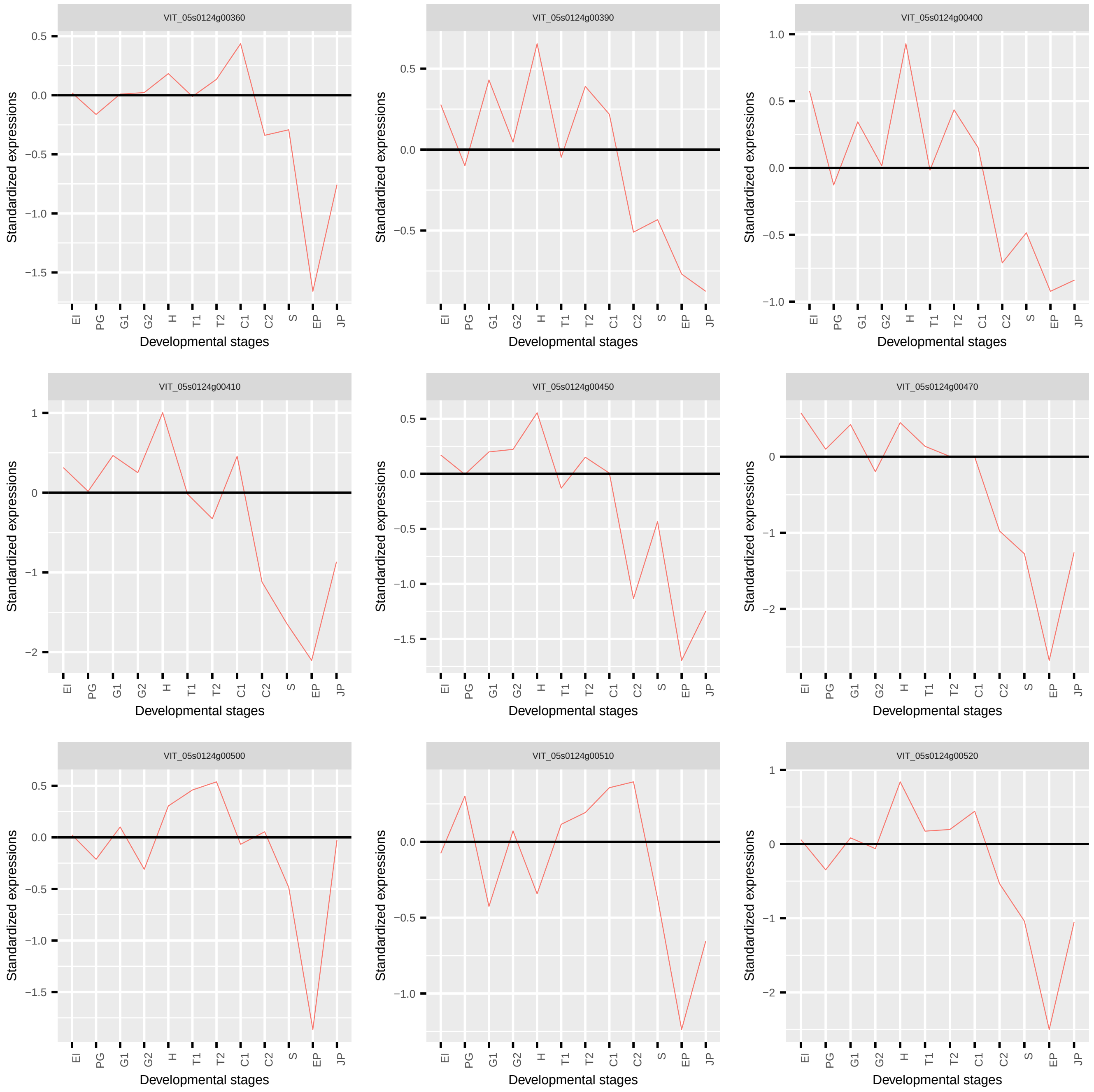
VIT_05s0124g00360
VIT_05s0124g00390
VIT_05s0124g00400
VIT_05s0124g00410
VIT_05s0124g00450
VIT_05s0124g00470
VIT_05s0124g00500
VIT_05s0124g00510
VIT_05s0124g00520
Standardized expressions
Developmental stages
EI PG G1 G2 H T1 T2 C1 C2 S EP JP

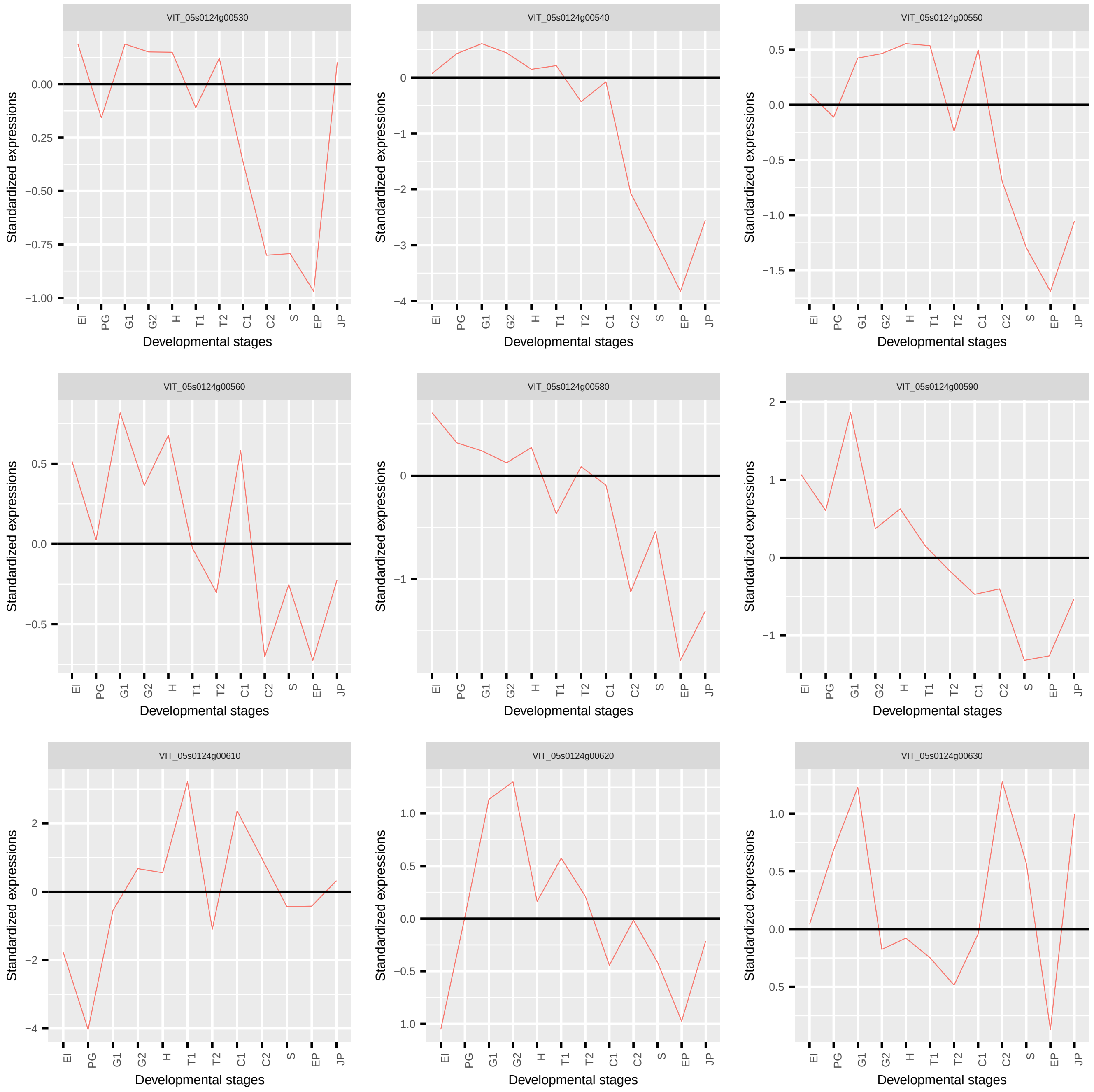
VIT_05s0124g00530
VIT_05s0124g00540
VIT_05s0124g00550
VIT_05s0124g00560
VIT_05s0124g00580
VIT_05s0124g00590
VIT_05s0124g00610
VIT_05s0124g00620
VIT_05s0124g00630
Standardized expressions
Developmental stages
EI PG G1 G2 H T1 T2 C1 C2 S EP JP

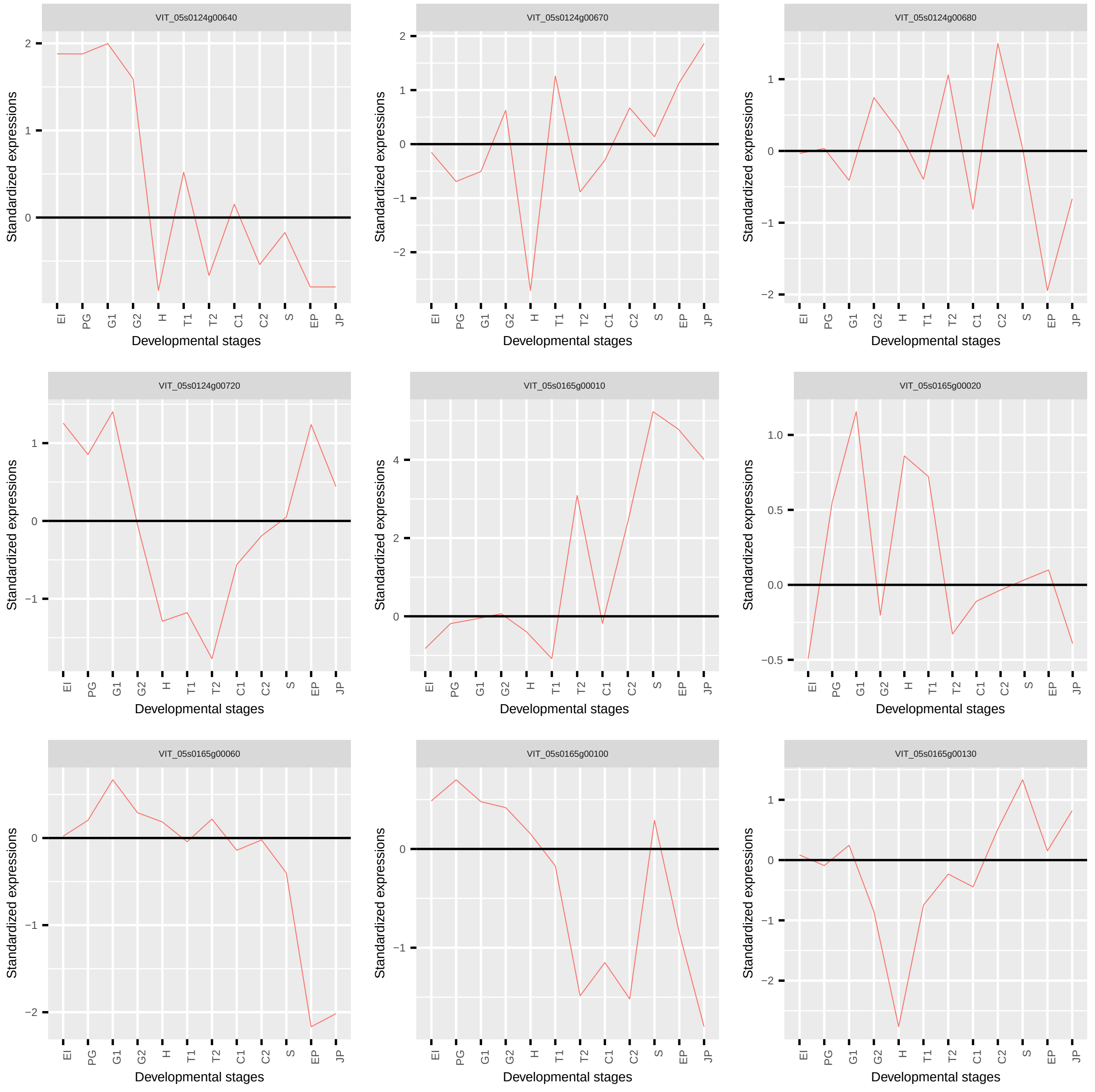
VIT_05s0124g00640
VIT_05s0124g00670
VIT_05s0124g00680
VIT_05s0124g00720
VIT_05s0165g00010
VIT_05s0165g00020
VIT_05s0165g00060
VIT_05s0165g00100
VIT_05s0165g00130
Standardized expressions
Developmental stages
EI PG G1 G2 H T1 T2 C1 C2 S EP JP

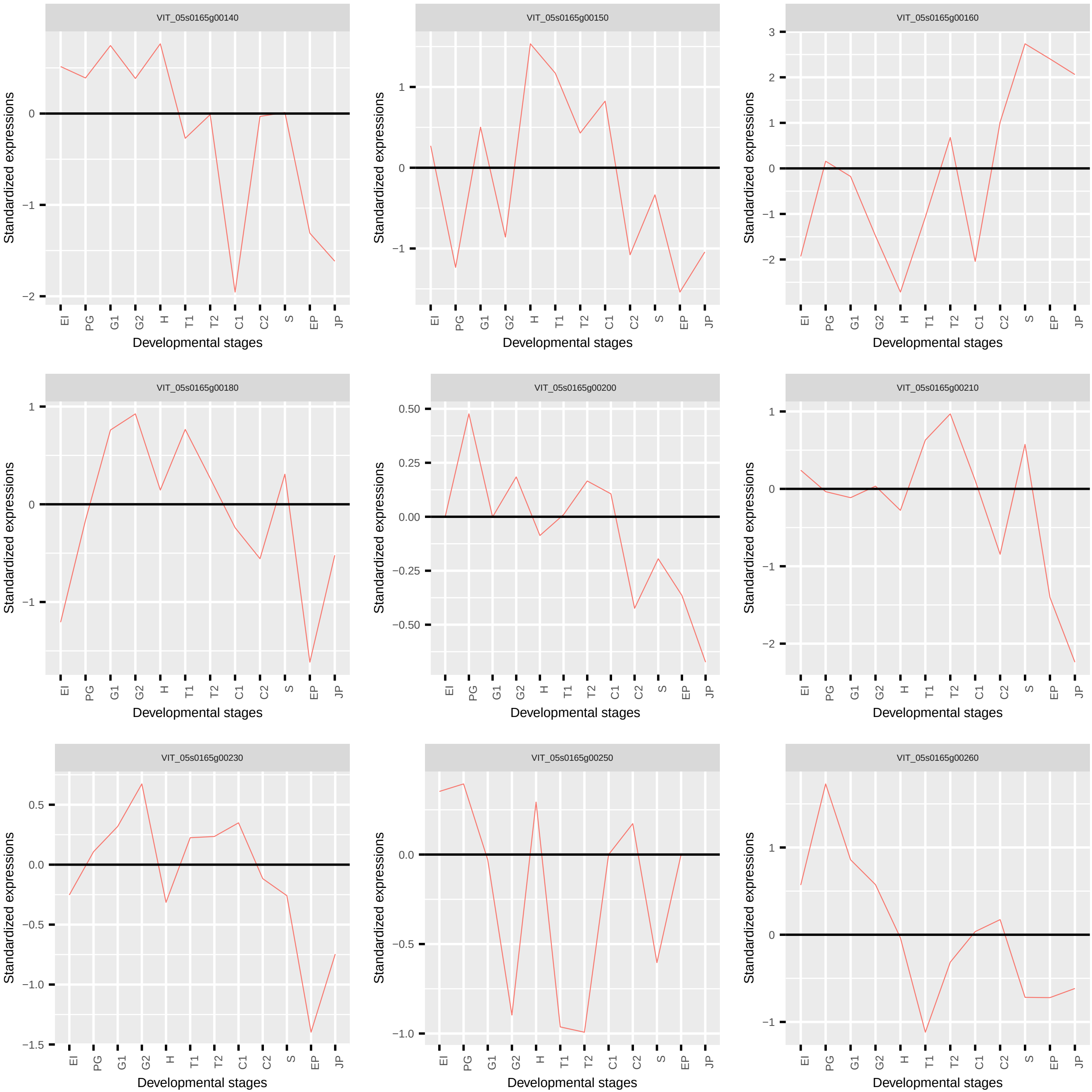
VIT_05s0165g00140
VIT_05s0165g00150
VIT_05s0165g00160
VIT_05s0165g00180
VIT_05s0165g00200
VIT_05s0165g00210
VIT_05s0165g00230
VIT_05s0165g00250
VIT_05s0165g00260
Standardized expressions
Developmental stages
EI PG G1 G2 H T1 T2 C1 C2 S EP JP
3 2 1 0 −1 −2
0.50 0.25 0.00 −0.25 −0.50
0.5 0.0 −0.5 −1.0 −1.5

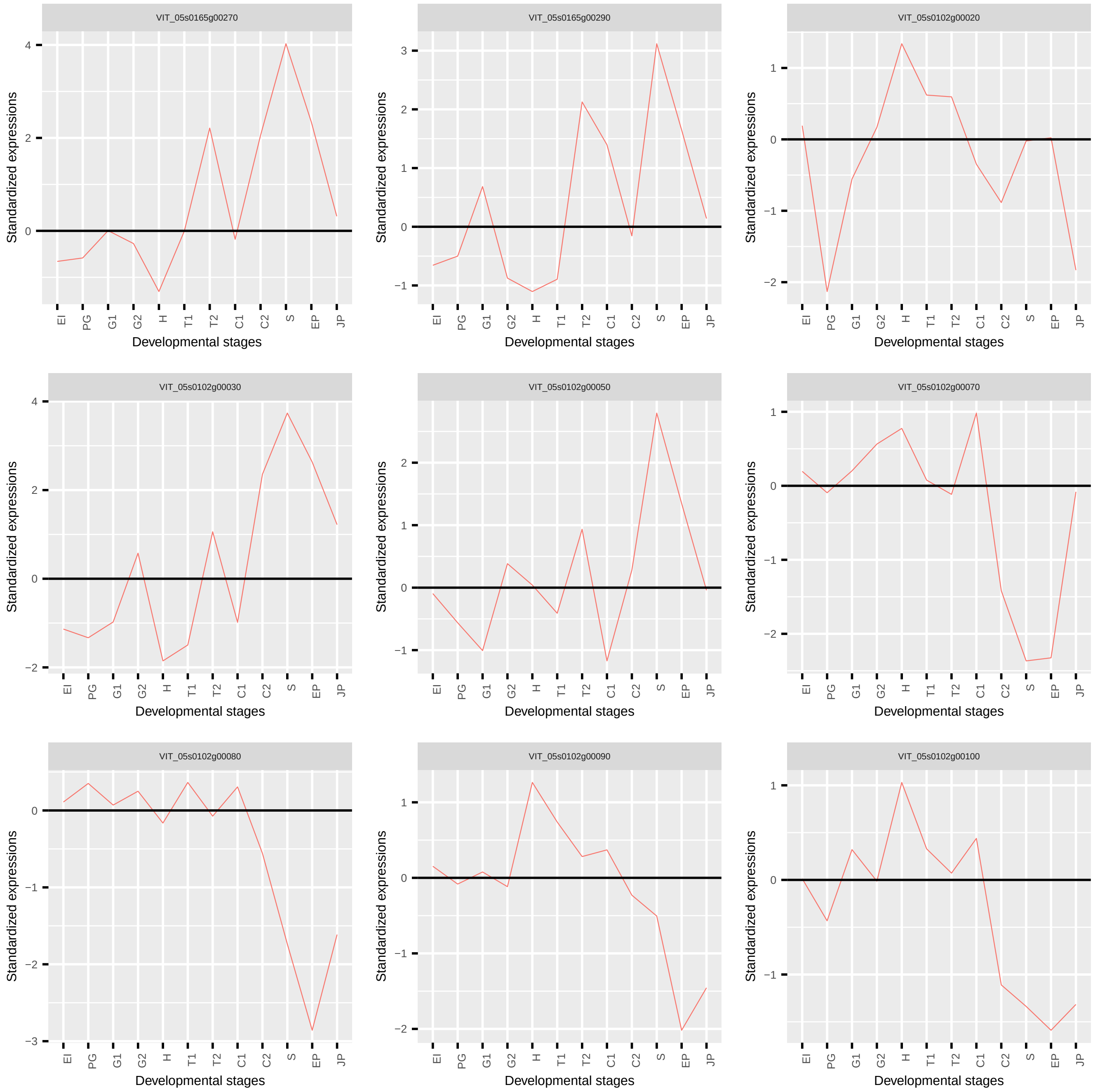
VIT_05s0165g00270
VIT_05s0165g00290
VIT_05s0102g00020
VIT_05s0102g00030
VIT_05s0102g00050
VIT_05s0102g00070
VIT_05s0102g00080
VIT_05s0102g00090
VIT_05s0102g00100
Standardized expressions
Developmental stages
EI PG G1 G2 H T1 T2 C1 C2 S EP JP

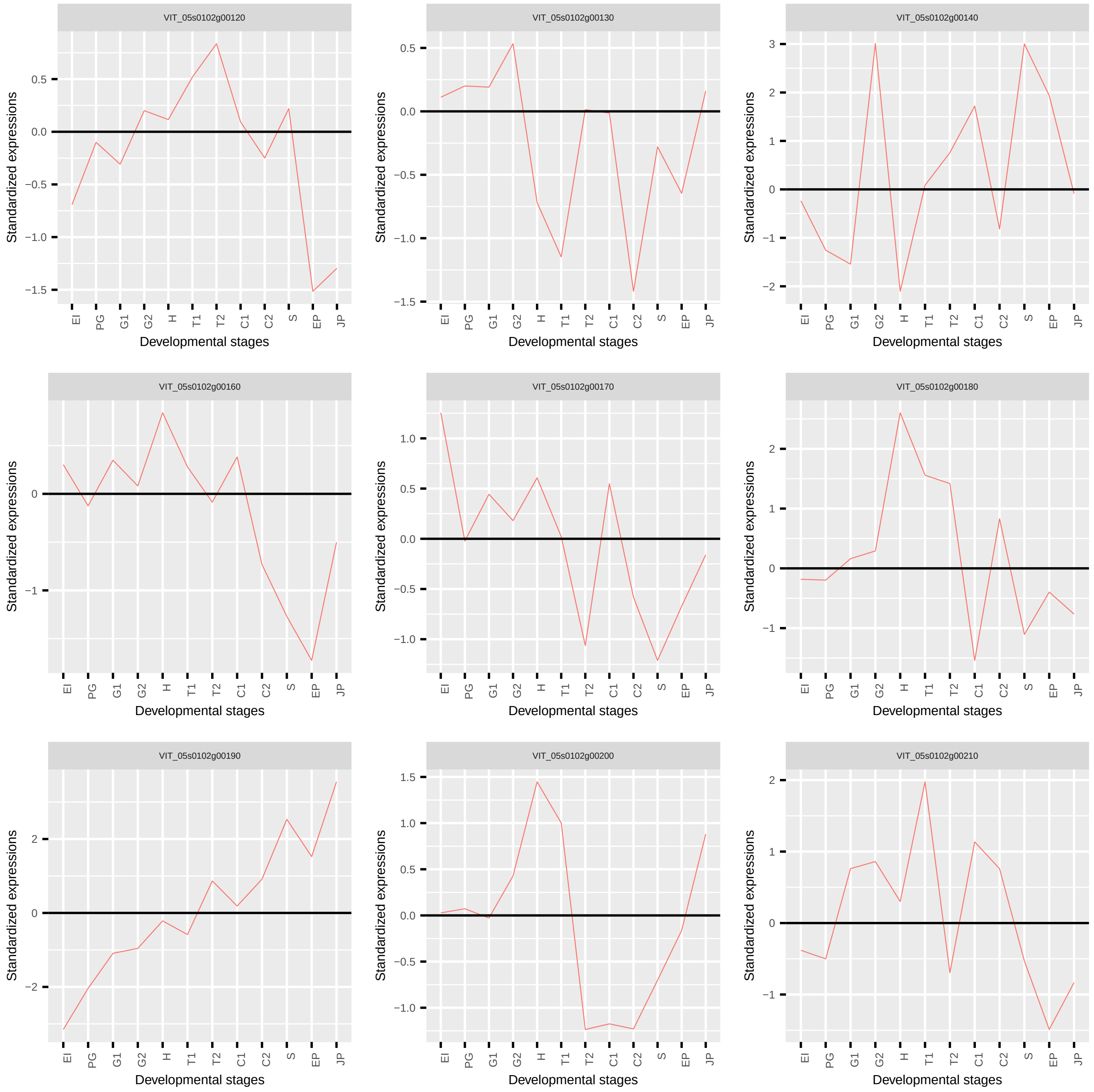
VIT_05s0102g00120
VIT_05s0102g00130
VIT_05s0102g00140
VIT_05s0102g00160
VIT_05s0102g00170
VIT_05s0102g00180
VIT_05s0102g00190
VIT_05s0102g00200
VIT_05s0102g00210
Standardized expressions
Developmental stages
EI PG G1 G2 H T1 T2 C1 C2 S EP JP

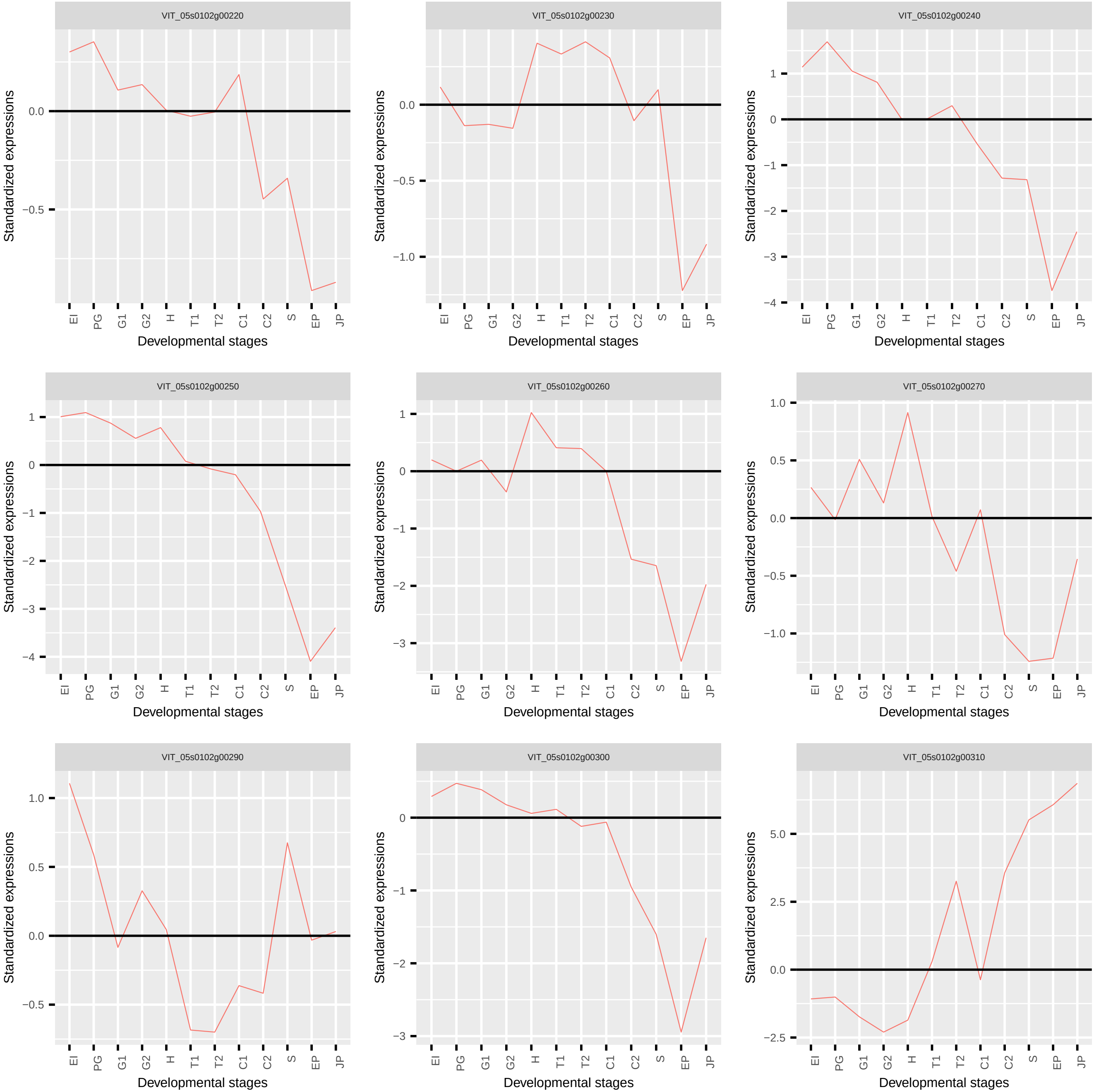
VIT_05s0102g00220
VIT_05s0102g00230
VIT_05s0102g00240
VIT_05s0102g00250
VIT_05s0102g00260
VIT_05s0102g00270
VIT_05s0102g00290
VIT_05s0102g00300
VIT_05s0102g00310
Standardized expressions
Developmental stages
EI PG G1 G2 H T1 T2 C1 C2 S EP JP

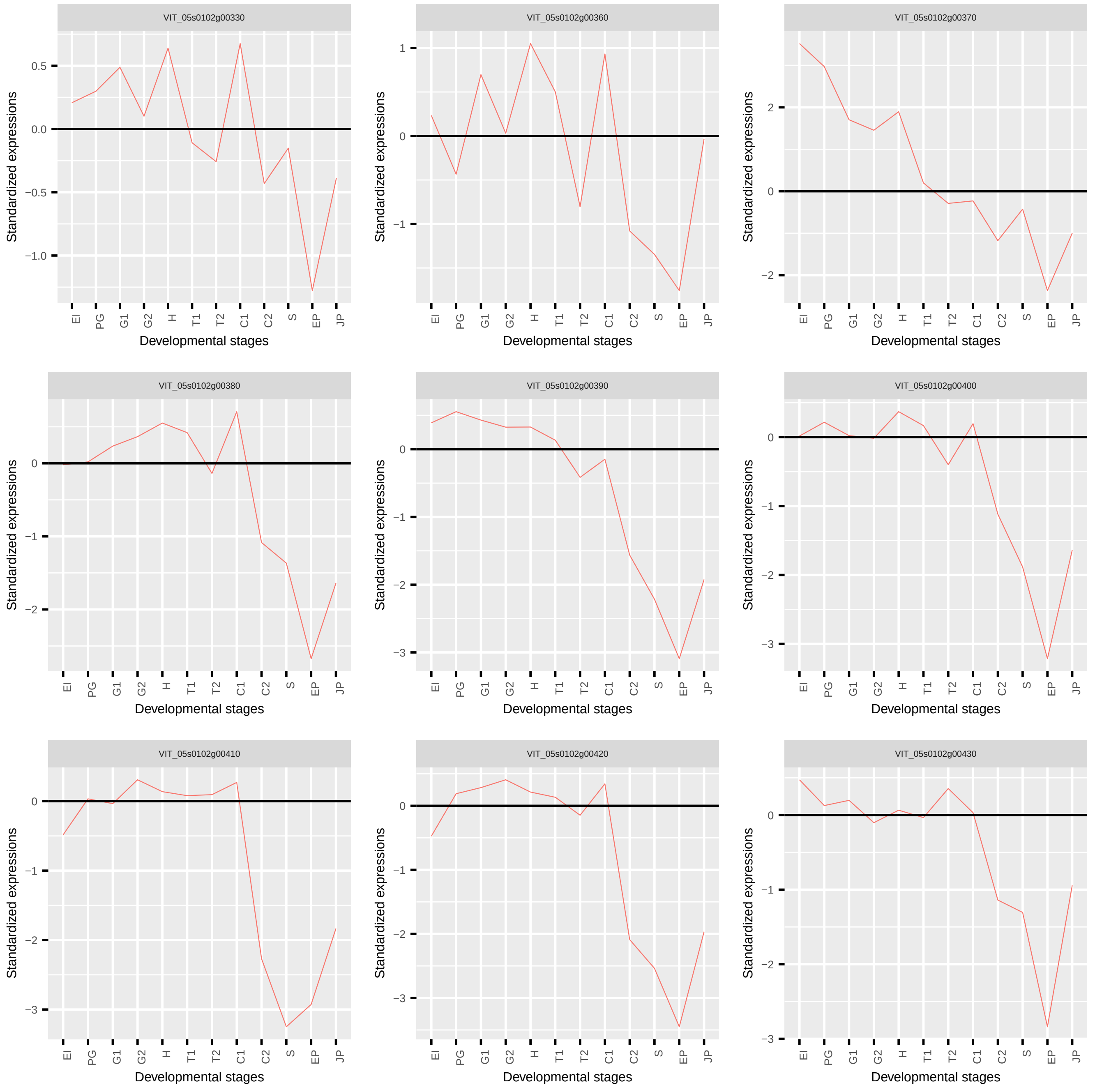
VIT_05s0102g00330
VIT_05s0102g00360
VIT_05s0102g00370
VIT_05s0102g00380
VIT_05s0102g00390
VIT_05s0102g00400
VIT_05s0102g00410
VIT_05s0102g00420
VIT_05s0102g00430
Standardized expressions
Developmental stages
EI PG G1 G2 H T1 T2 C1 C2 S EP JP

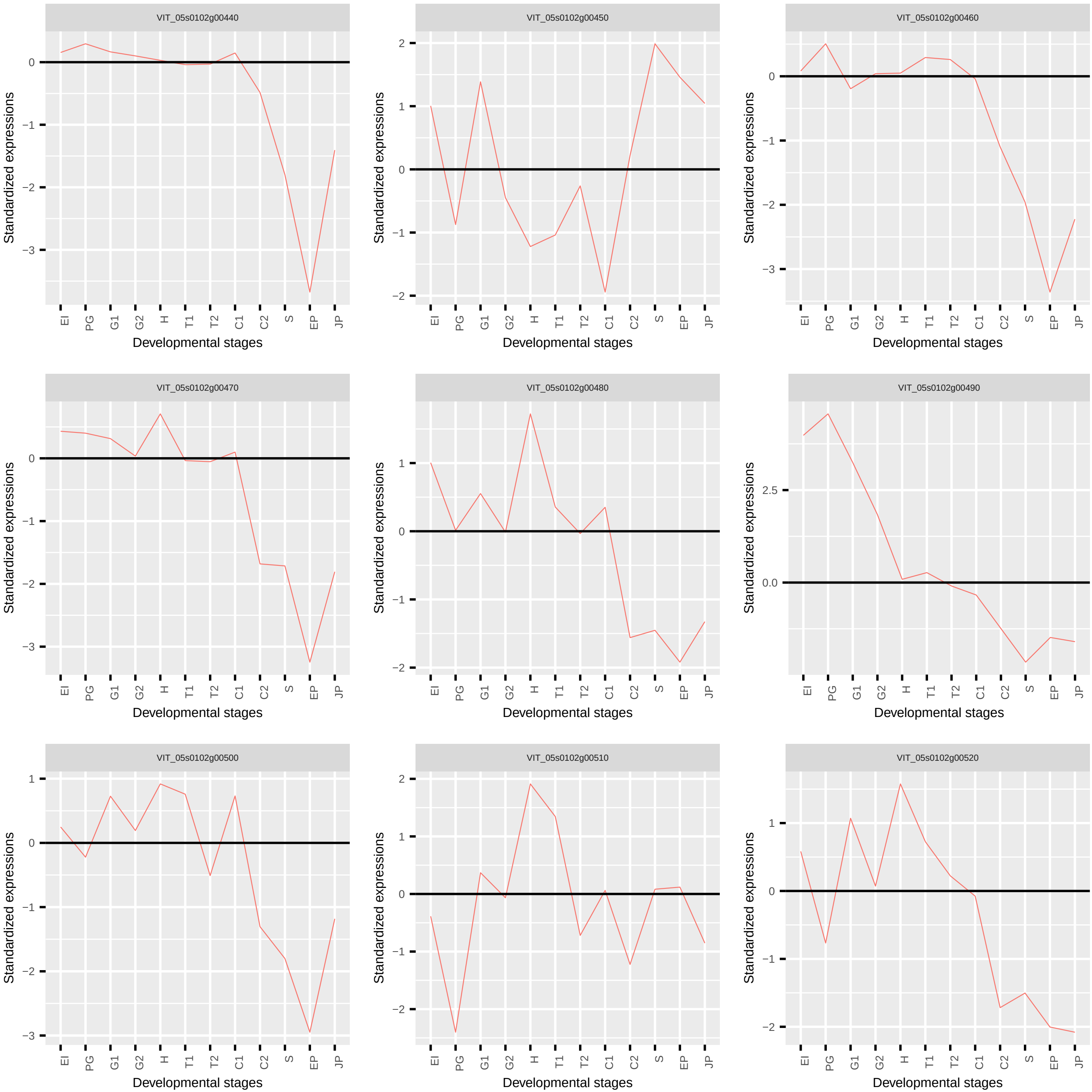
VIT_05s0102g00440
VIT_05s0102g00450
VIT_05s0102g00460
VIT_05s0102g00470
VIT_05s0102g00480
VIT_05s0102g00490
VIT_05s0102g00500
VIT_05s0102g00510
VIT_05s0102g00520
Standardized expressions
Developmental stages
EI PG G1 G2 H T1 T2 C1 C2 S EP JP
0 −1 −2 −3
2 1 0 −1 −2
0 −1 −2 −3
0 −1 −2 −3
1 0 −1 −2
2.5 0.0
1 0 −1 −2 −3
2 1 0 −1 −2
1 0 −1 −2

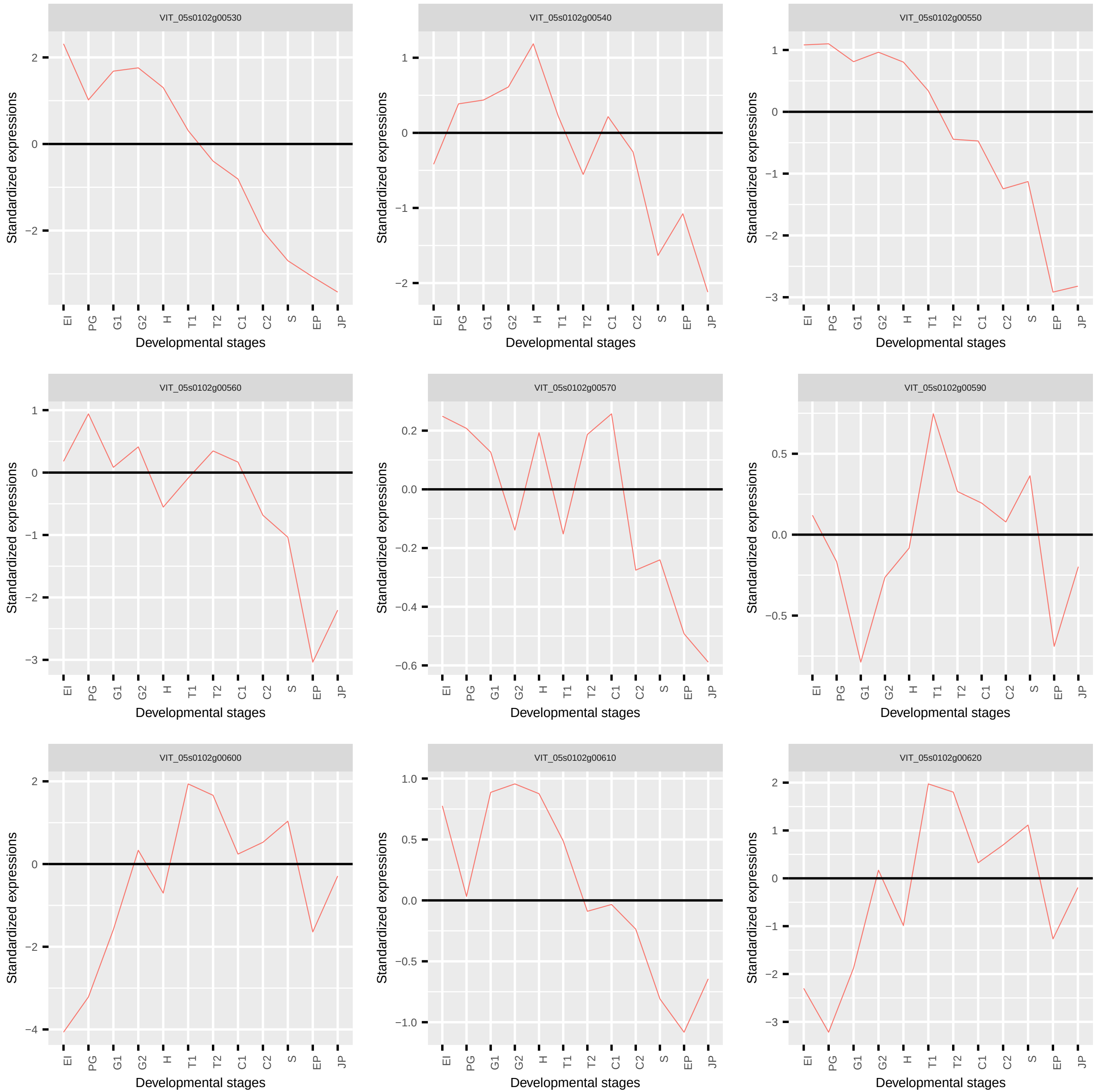
VIT_05s0102g00530
2
0
−2
Standardized expressions
EI PG G1 G2 H T1 T2 C1 C2 S EP JP
Developmental stages
VIT_05s0102g00540
1
0
−1
−2
Standardized expressions
EI PG G1 G2 H T1 T2 C1 C2 S EP JP
Developmental stages
VIT_05s0102g00550
1
0
−1
−2
−3
Standardized expressions
EI PG G1 G2 H T1 T2 C1 C2 S EP JP
Developmental stages
VIT_05s0102g00560
1
0
−1
−2
−3
Standardized expressions
EI PG G1 G2 H T1 T2 C1 C2 S EP JP
Developmental stages
VIT_05s0102g00570
0.2
0.0
−0.2
−0.4
−0.6
Standardized expressions
EI PG G1 G2 H T1 T2 C1 C2 S EP JP
Developmental stages
VIT_05s0102g00590
0.5
0.0
−0.5
Standardized expressions
EI PG G1 G2 H T1 T2 C1 C2 S EP JP
Developmental stages
VIT_05s0102g00600
2
0
−2
−4
Standardized expressions
EI PG G1 G2 H T1 T2 C1 C2 S EP JP
Developmental stages
VIT_05s0102g00610
1.0
0.5
0.0
−0.5
−1.0
Standardized expressions
EI PG G1 G2 H T1 T2 C1 C2 S EP JP
Developmental stages
VIT_05s0102g00620
2
1
0
−1
−2
−3
Standardized expressions
EI PG G1 G2 H T1 T2 C1 C2 S EP JP
Developmental stages

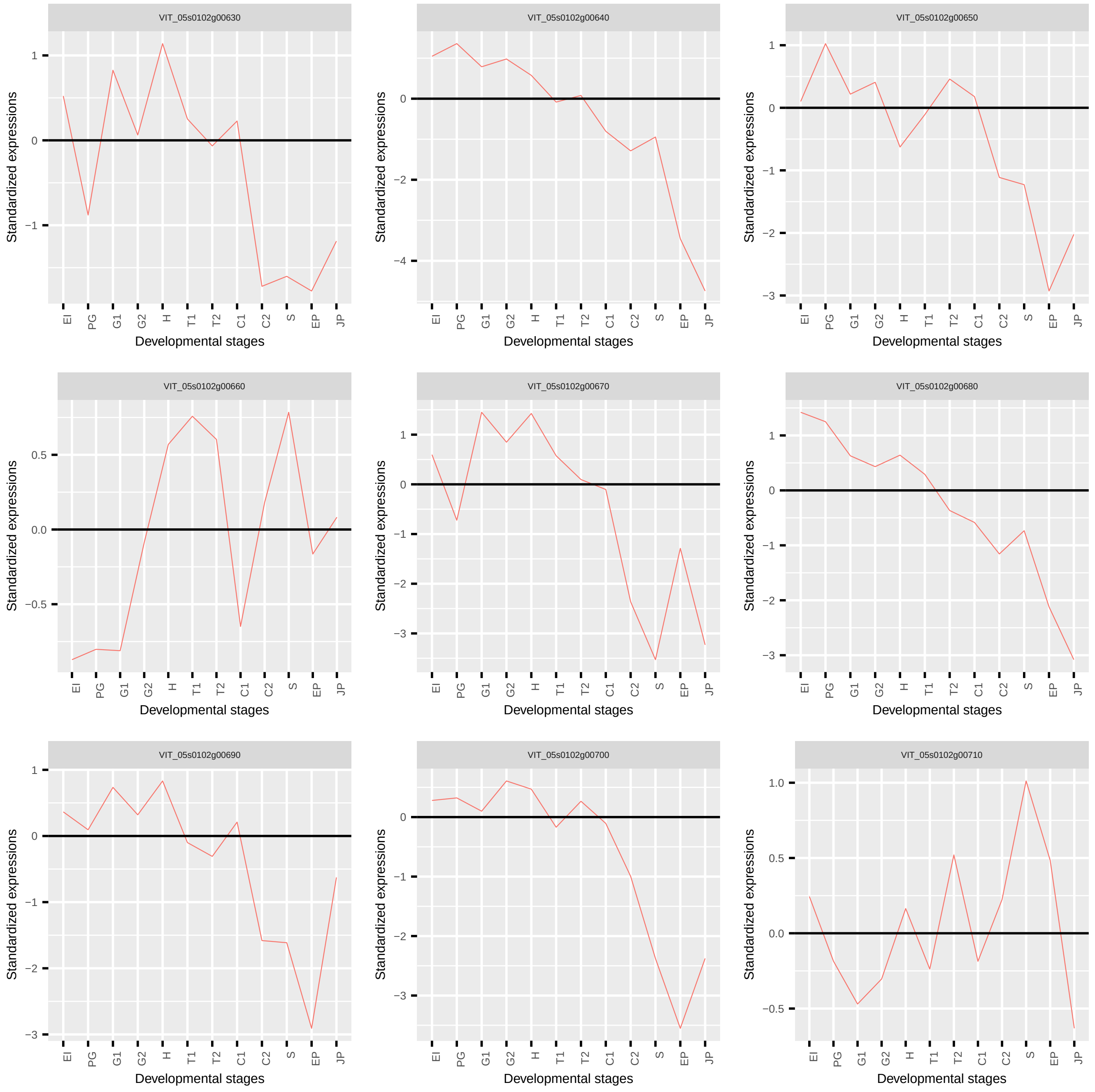
VIT_05s0102g00630
VIT_05s0102g00640
VIT_05s0102g00650
VIT_05s0102g00660
VIT_05s0102g00670
VIT_05s0102g00680
VIT_05s0102g00690
VIT_05s0102g00700
VIT_05s0102g00710
Standardized expressions
Developmental stages
EI PG G1 G2 H T1 T2 C1 C2 S EP JP

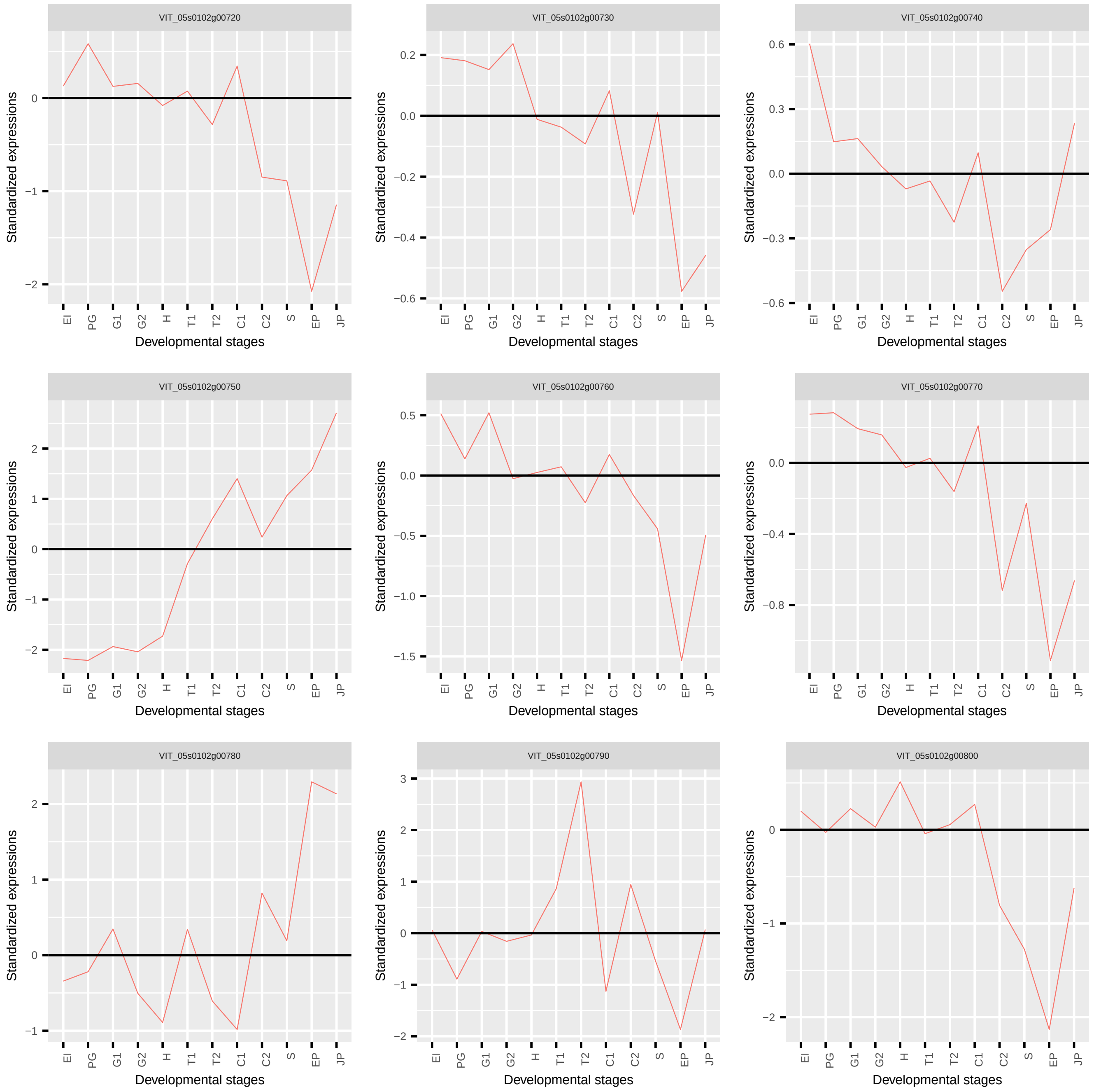
VIT_05s0102g00720
VIT_05s0102g00730
VIT_05s0102g00740
VIT_05s0102g00750
VIT_05s0102g00760
VIT_05s0102g00770
VIT_05s0102g00780
VIT_05s0102g00790
VIT_05s0102g00800
Standardized expressions
Developmental stages
EI PG G1 G2 H T1 T2 C1 C2 S EP JP

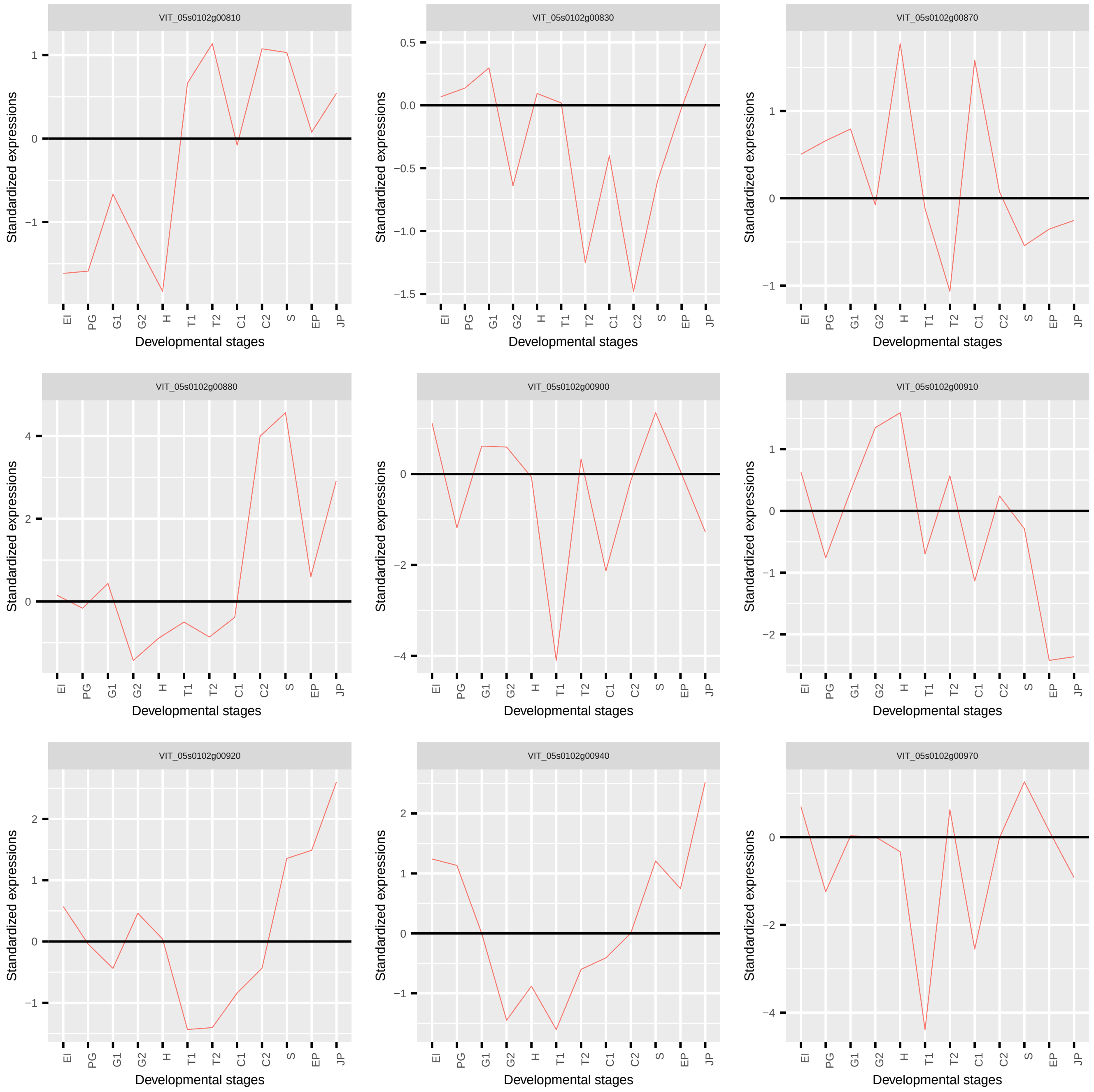
VIT_05s0102g00810
1
0
−1
Standardized expressions
EI PG G1 G2 H T1 T2 C1 C2 S EP JP
Developmental stages
VIT_05s0102g00830
0.5
0.0
−0.5
−1.0
−1.5
Standardized expressions
EI PG G1 G2 H T1 T2 C1 C2 S EP JP
Developmental stages
VIT_05s0102g00870
1
0
−1
Standardized expressions
EI PG G1 G2 H T1 T2 C1 C2 S EP JP
Developmental stages
VIT_05s0102g00880
4
2
0
Standardized expressions
EI PG G1 G2 H T1 T2 C1 C2 S EP JP
Developmental stages
VIT_05s0102g00900
0
−2
−4
Standardized expressions
EI PG G1 G2 H T1 T2 C1 C2 S EP JP
Developmental stages
VIT_05s0102g00910
1
0
−1
−2
Standardized expressions
EI PG G1 G2 H T1 T2 C1 C2 S EP JP
Developmental stages
VIT_05s0102g00920
2
1
0
−1
Standardized expressions
EI PG G1 G2 H T1 T2 C1 C2 S EP JP
Developmental stages
VIT_05s0102g00940
2
1
0
−1
Standardized expressions
EI PG G1 G2 H T1 T2 C1 C2 S EP JP
Developmental stages
VIT_05s0102g00970
0
−2
−4
Standardized expressions
EI PG G1 G2 H T1 T2 C1 C2 S EP JP
Developmental stages

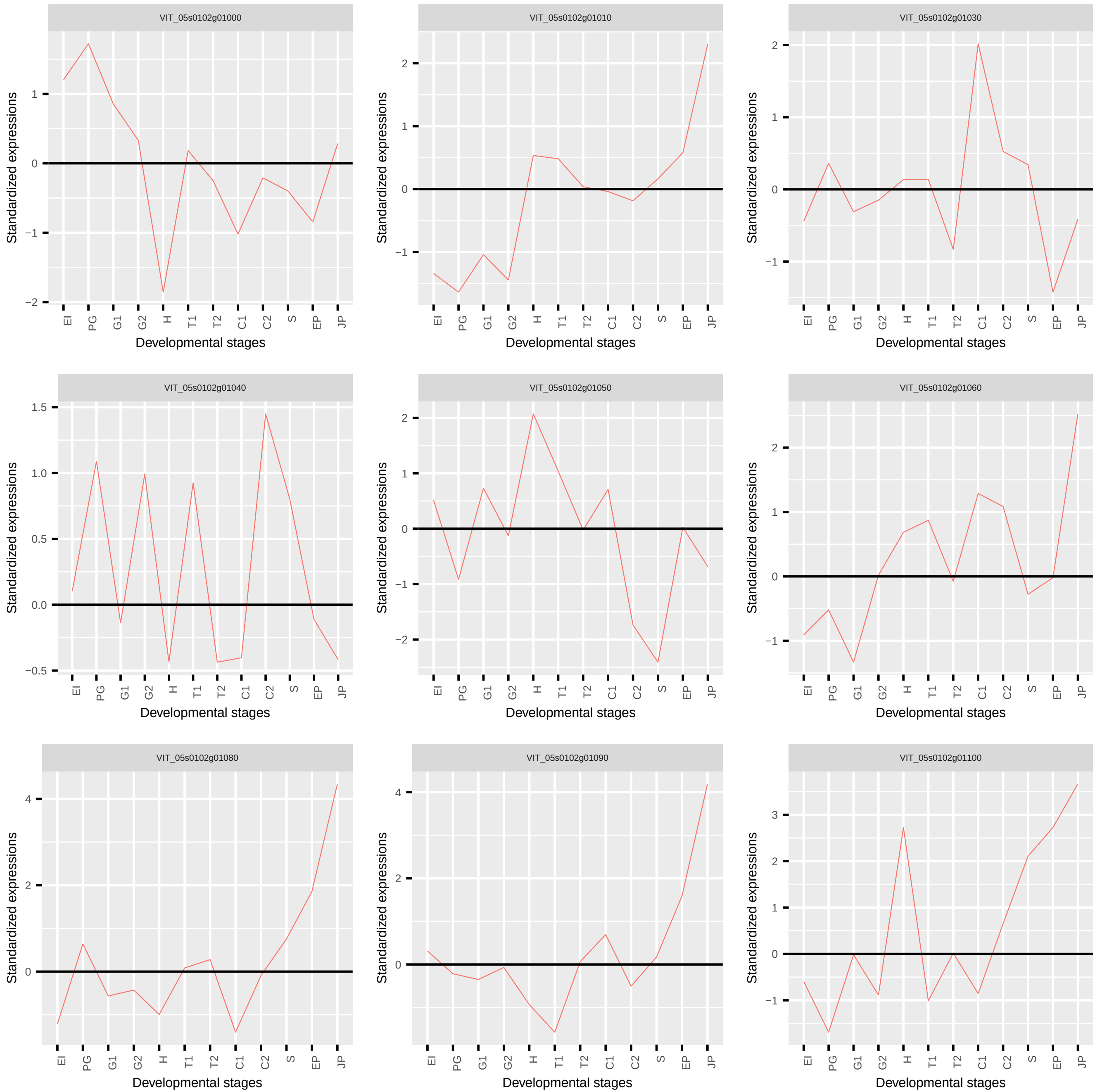
VIT_05s0102g01000
VIT_05s0102g01010
VIT_05s0102g01030
VIT_05s0102g01040
VIT_05s0102g01050
VIT_05s0102g01060
VIT_05s0102g01080
VIT_05s0102g01090
VIT_05s0102g01100
Standardized expressions
Developmental stages
EI
PG
G1
G2
H
T1
T2
C1
C2
S
EP
JP
1
0
−1
−2
2
3
4
1.5
1.0
0.5
0.0
−0.5

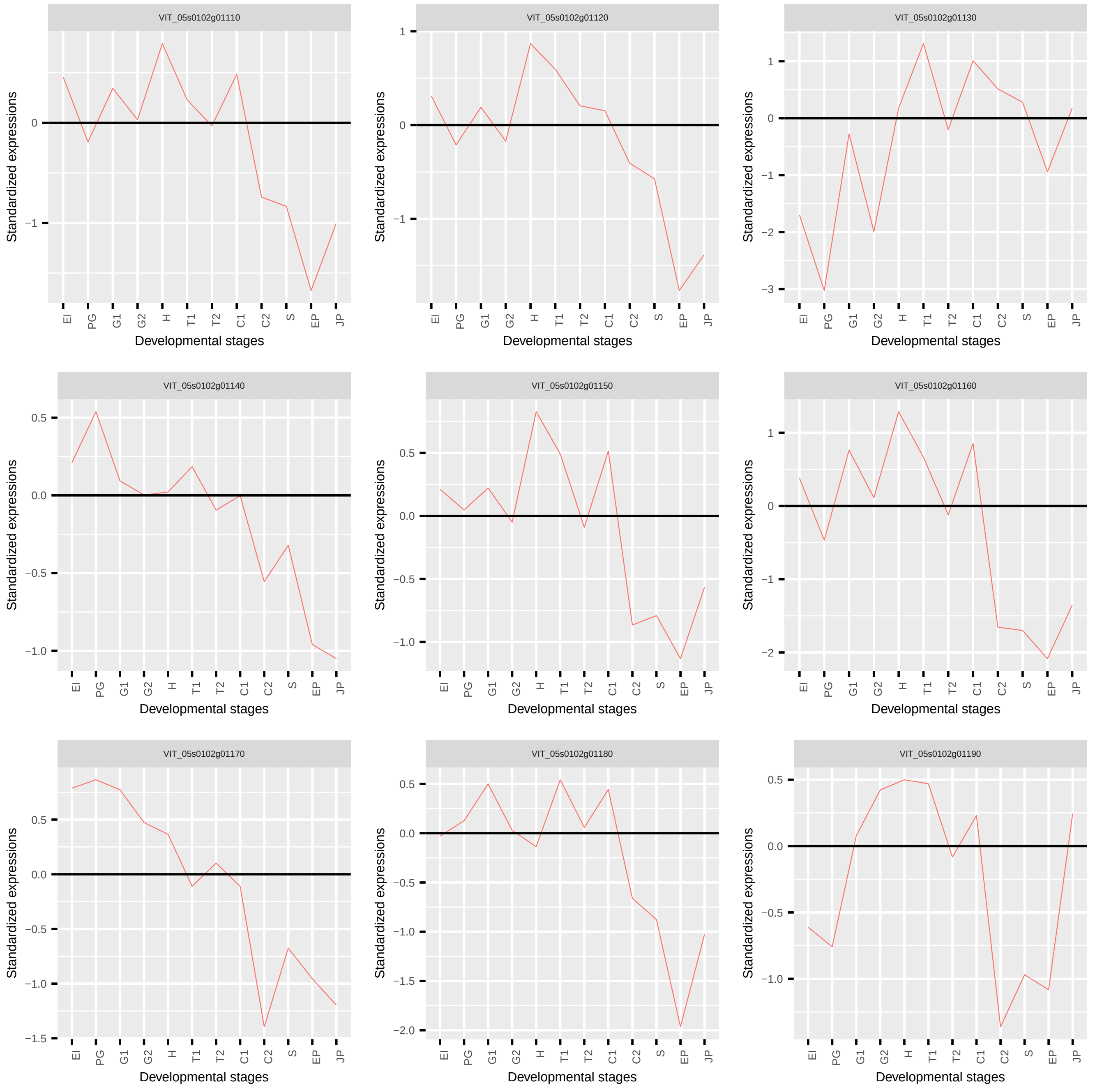
VIT_05s0102g01110
VIT_05s0102g01120
VIT_05s0102g01130
VIT_05s0102g01140
VIT_05s0102g01150
VIT_05s0102g01160
VIT_05s0102g01170
VIT_05s0102g01180
VIT_05s0102g01190
Standardized expressions
Developmental stages
EI PG G1 G2 H T1 T2 C1 C2 S EP JP

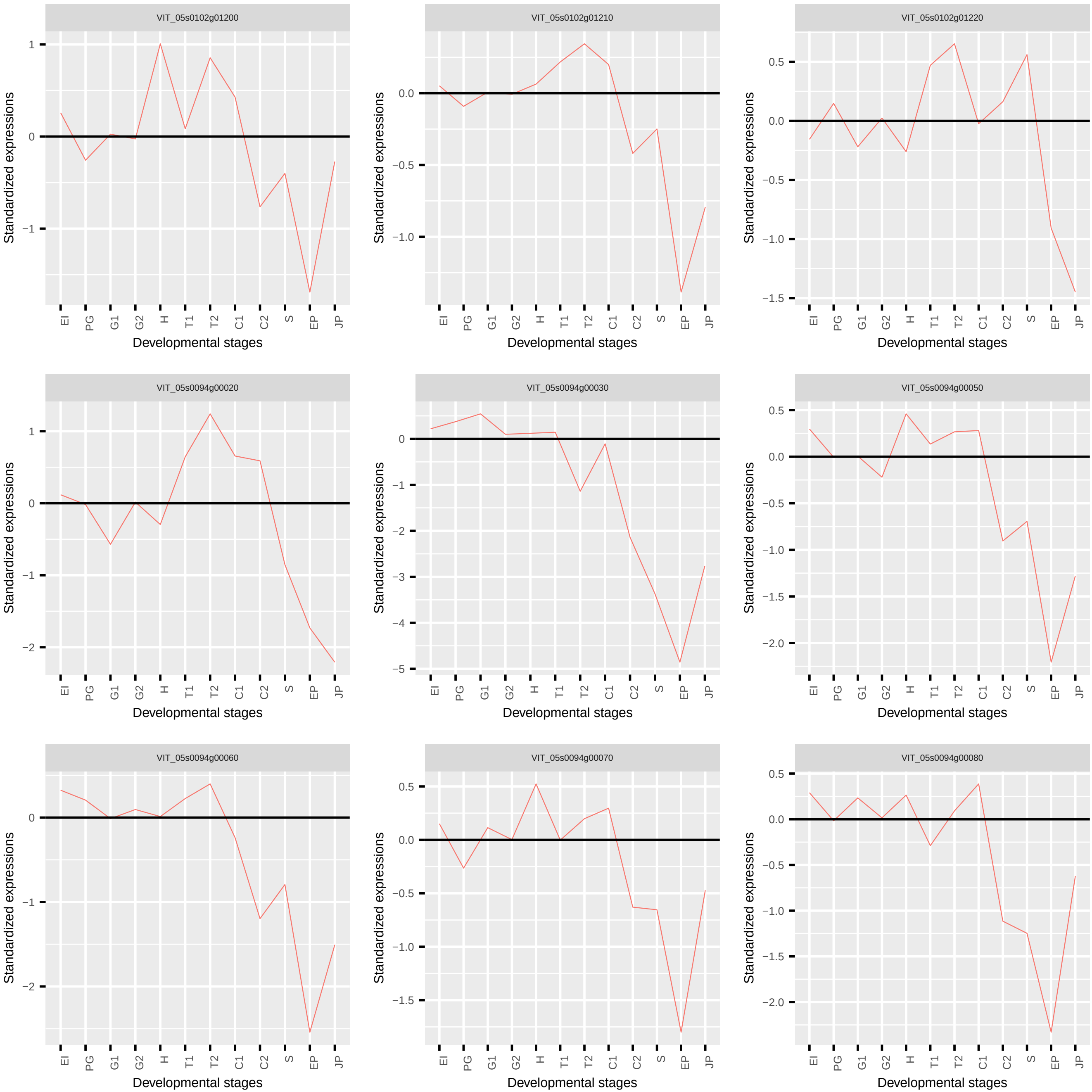
VIT_05s0102g01200
VIT_05s0102g01210
VIT_05s0102g01220
VIT_05s0094g00020
VIT_05s0094g00030
VIT_05s0094g00050
VIT_05s0094g00060
VIT_05s0094g00070
VIT_05s0094g00080
Standardized expressions
Developmental stages
EI
PG
G1
G2
H
T1
T2
C1
C2
S
EP
JP

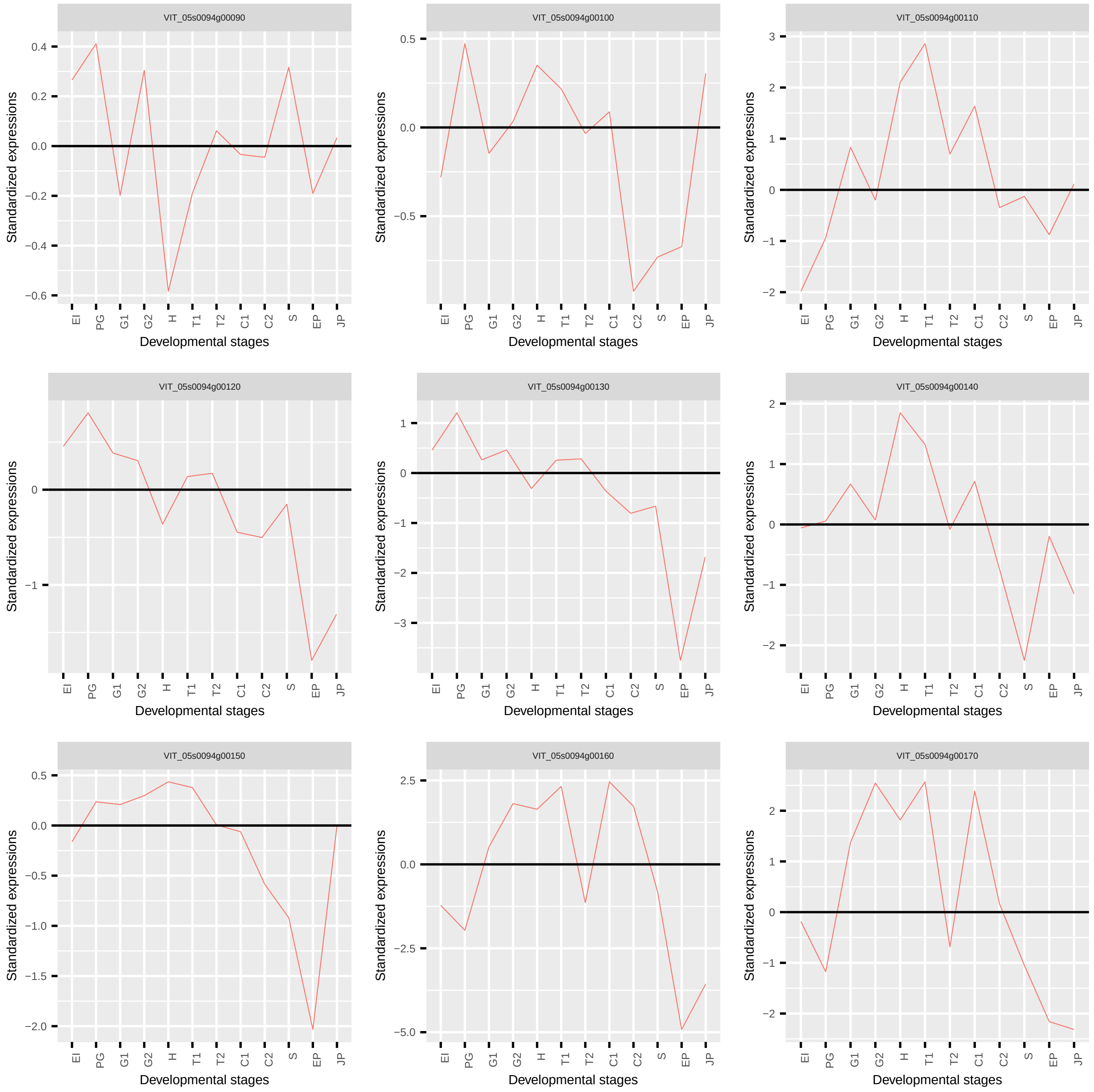
VIT_05s0094g00090
VIT_05s0094g00100
VIT_05s0094g00110
VIT_05s0094g00120
VIT_05s0094g00130
VIT_05s0094g00140
VIT_05s0094g00150
VIT_05s0094g00160
VIT_05s0094g00170
Standardized expressions
Developmental stages
EI PG G1 G2 H T1 T2 C1 C2 S EP JP

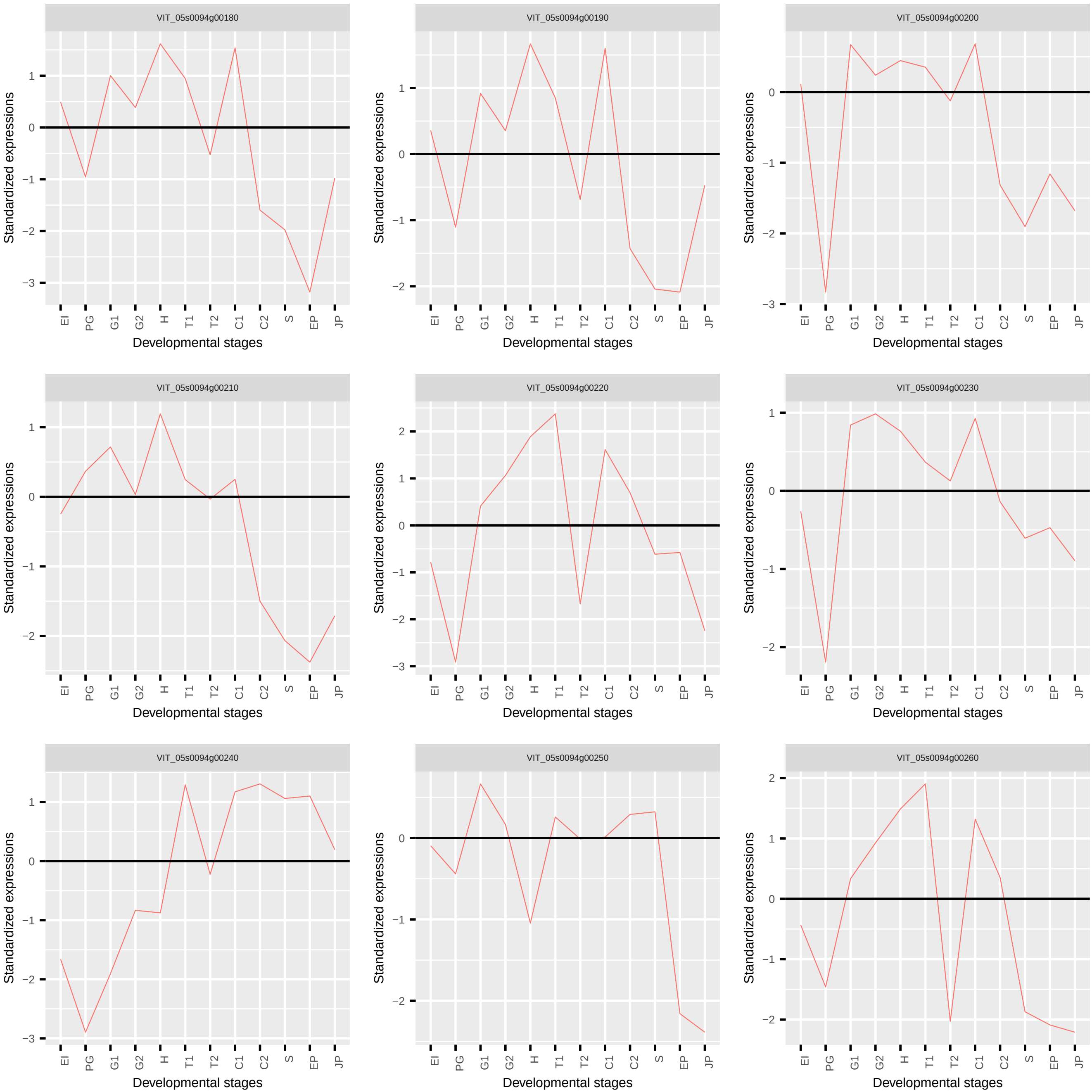
VIT_05s0094g00180
VIT_05s0094g00190
VIT_05s0094g00200
VIT_05s0094g00210
VIT_05s0094g00220
VIT_05s0094g00230
VIT_05s0094g00240
VIT_05s0094g00250
VIT_05s0094g00260
Standardized expressions
Developmental stages
EI PG G1 G2 H T1 T2 C1 C2 S EP JP

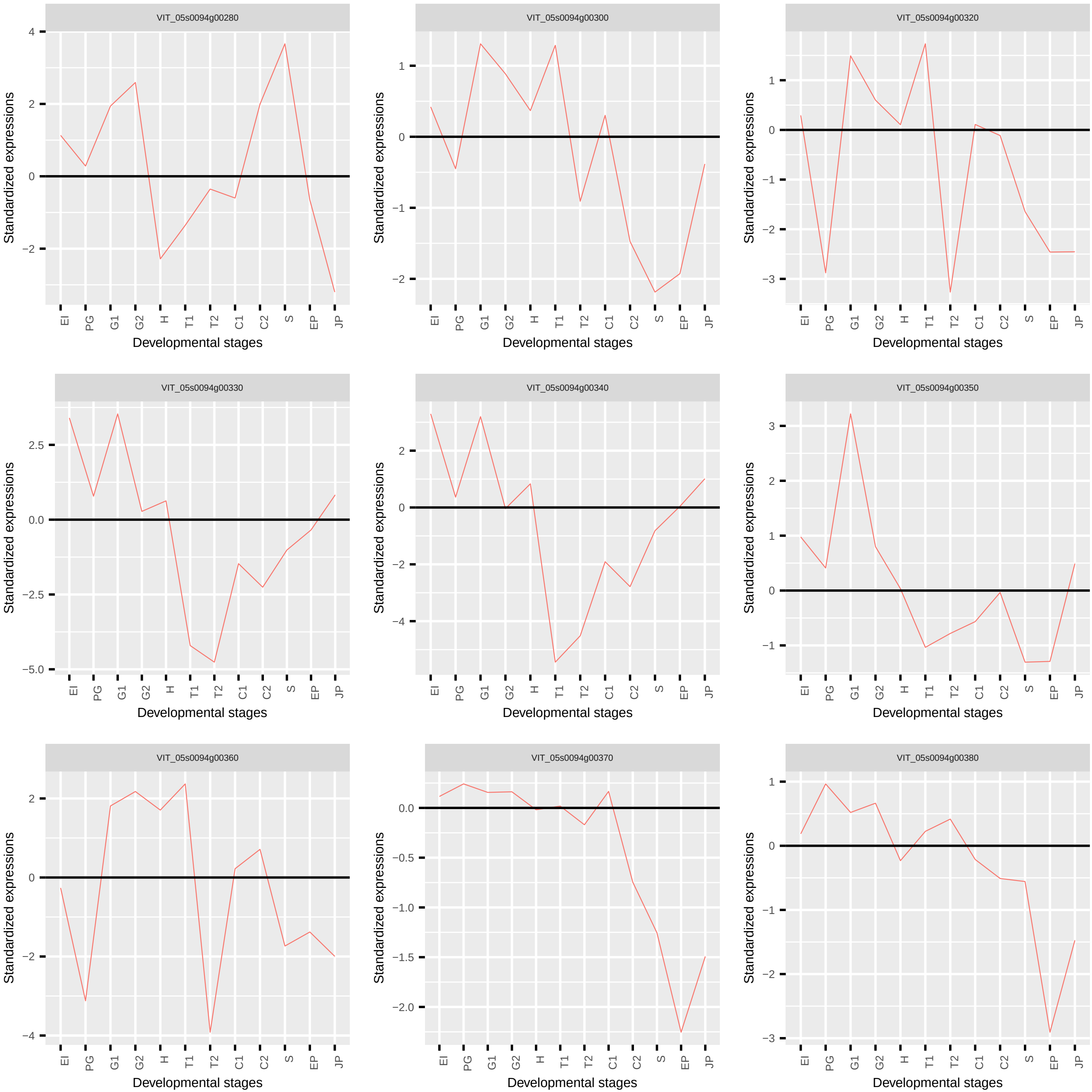
VIT_05s0094g00280
VIT_05s0094g00300
VIT_05s0094g00320
VIT_05s0094g00330
VIT_05s0094g00340
VIT_05s0094g00350
VIT_05s0094g00360
VIT_05s0094g00370
VIT_05s0094g00380
Standardized expressions
Developmental stages
EI PG G1 G2 H T1 T2 C1 C2 S EP JP
4 2 0 −2
1 0 −1 −2
1 0 −1 −2 −3
2.5 0.0 −2.5 −5.0
2 0 −2 −4
3 2 1 0 −1
2 0 −2 −4
0.0 −0.5 −1.0 −1.5 −2.0
1 0 −1 −2 −3

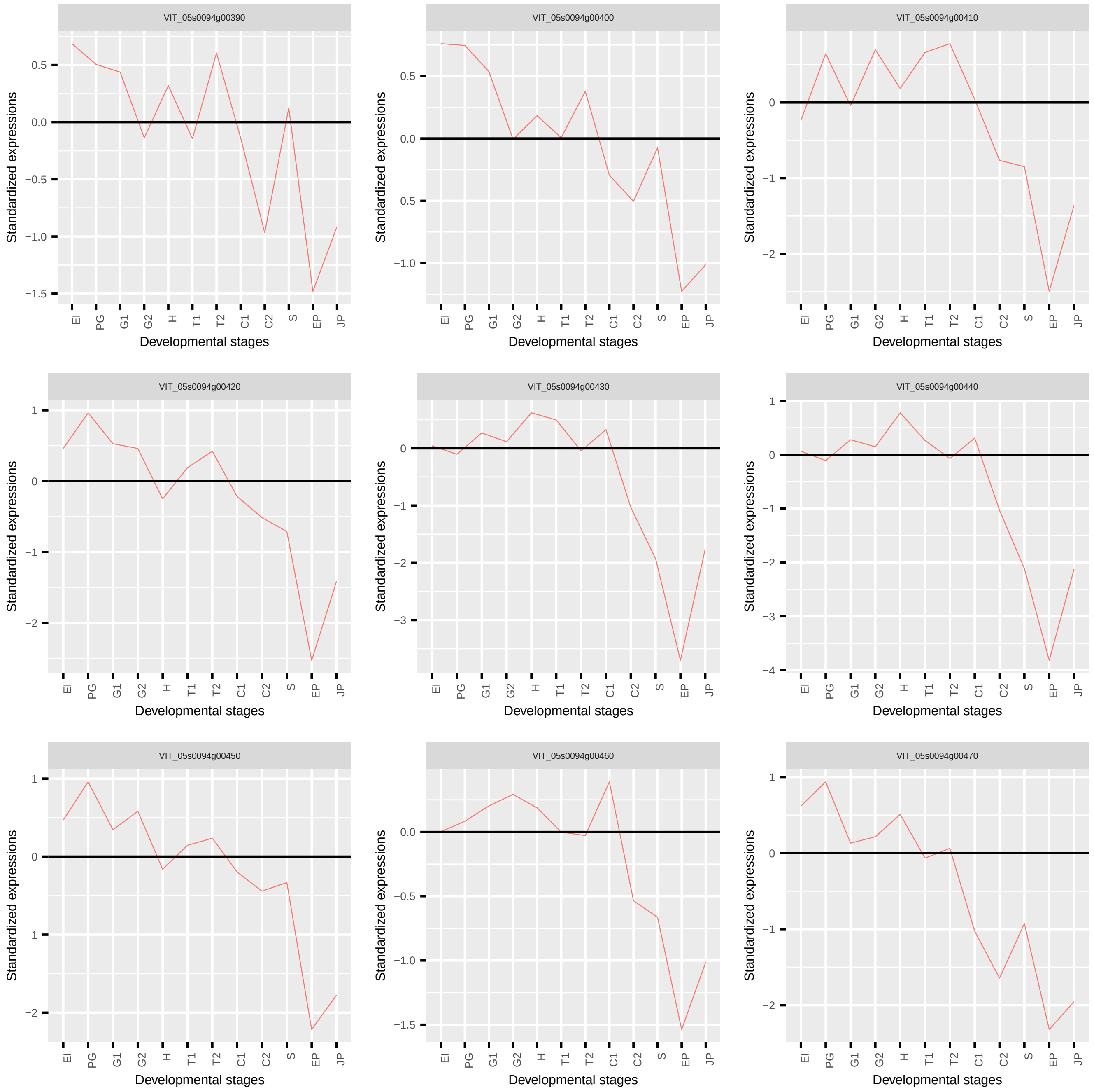
VIT_05s0094g00390
VIT_05s0094g00400
VIT_05s0094g00410
VIT_05s0094g00420
VIT_05s0094g00430
VIT_05s0094g00440
VIT_05s0094g00450
VIT_05s0094g00460
VIT_05s0094g00470
Standardized expressions
Developmental stages
EI
PG
G1
G2
H
T1
T2
C1
C2
S
EP
JP

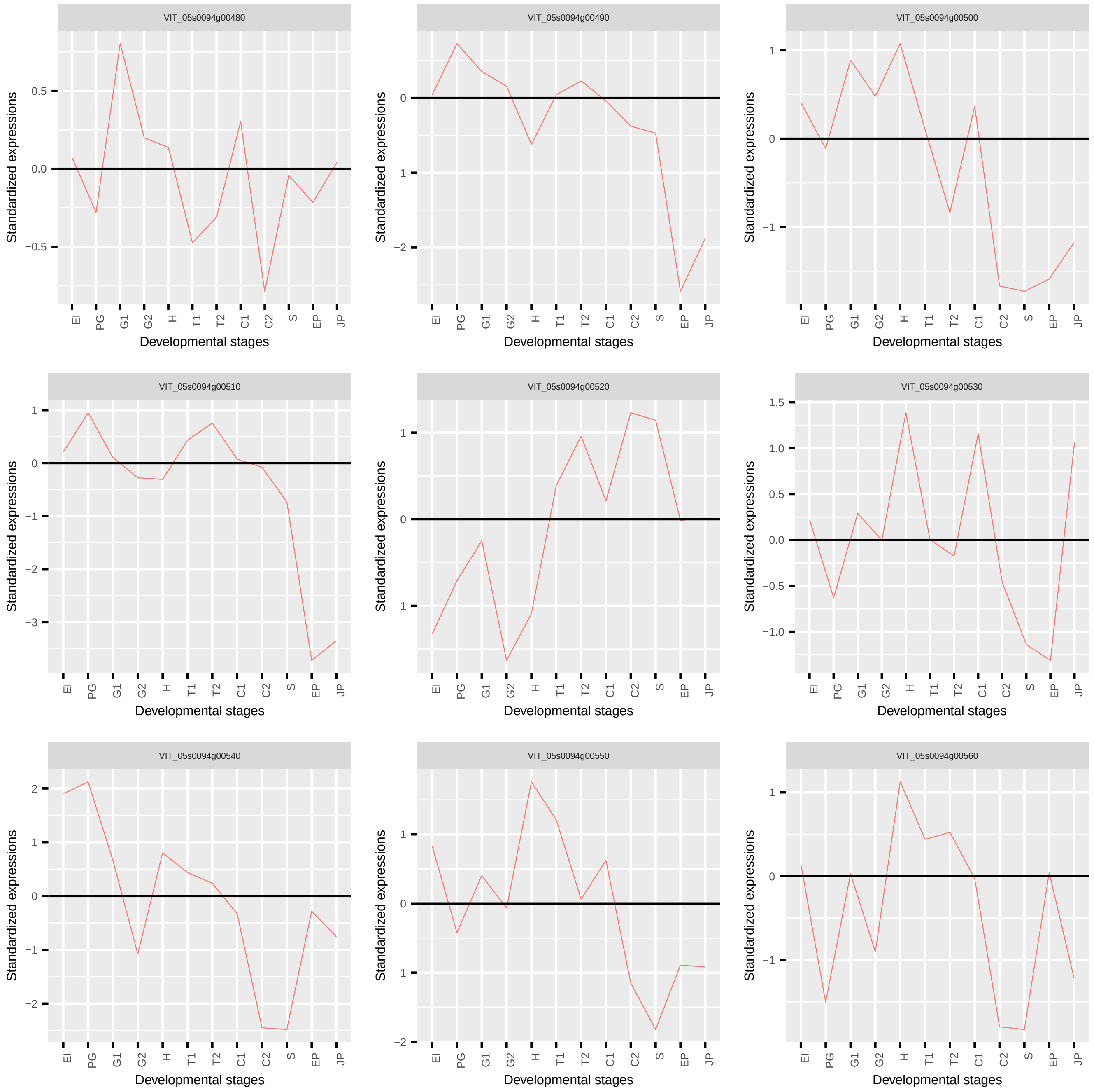
VIT_05s0094g00480
VIT_05s0094g00490
VIT_05s0094g00500
VIT_05s0094g00510
VIT_05s0094g00520
VIT_05s0094g00530
VIT_05s0094g00540
VIT_05s0094g00550
VIT_05s0094g00560
Standardized expressions
Developmental stages
EI PG G1 G2 H T1 T2 C1 C2 S EP JP

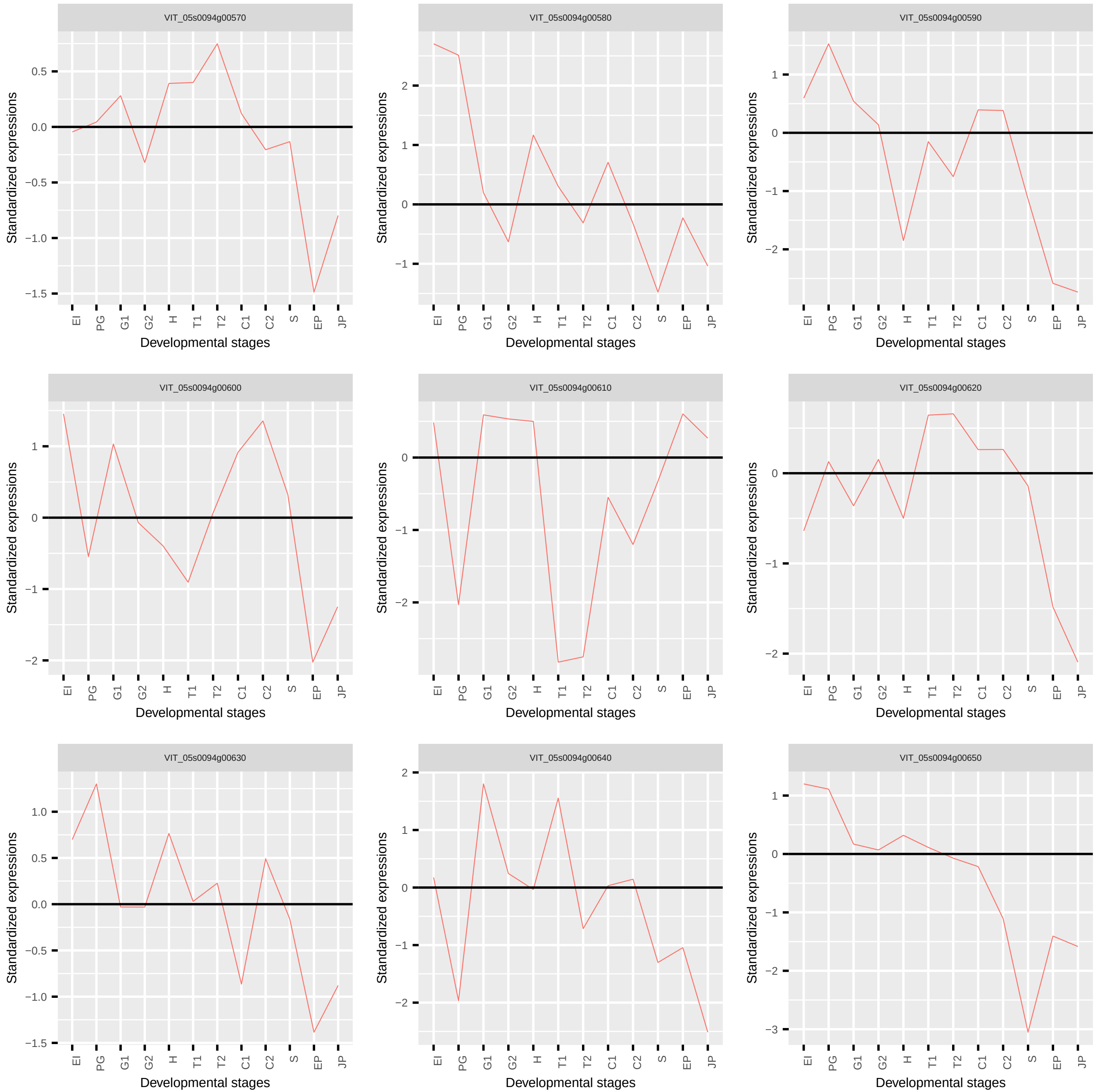
VIT_05s0094g00570
Standardized expressions
0.5
0.0
−0.5
−1.0
−1.5
EI PG G1 G2 H T1 T2 C1 C2 S EP JP
Developmental stages
VIT_05s0094g00580
Standardized expressions
2
1
0
−1
EI PG G1 G2 H T1 T2 C1 C2 S EP JP
Developmental stages
VIT_05s0094g00590
Standardized expressions
1
0
−1
−2
EI PG G1 G2 H T1 T2 C1 C2 S EP JP
Developmental stages
VIT_05s0094g00600
Standardized expressions
1
0
−1
−2
EI PG G1 G2 H T1 T2 C1 C2 S EP JP
Developmental stages
VIT_05s0094g00610
Standardized expressions
0
−1
−2
EI PG G1 G2 H T1 T2 C1 C2 S EP JP
Developmental stages
VIT_05s0094g00620
Standardized expressions
0
−1
−2
EI PG G1 G2 H T1 T2 C1 C2 S EP JP
Developmental stages
VIT_05s0094g00630
Standardized expressions
1.0
0.5
0.0
−0.5
−1.0
−1.5
EI PG G1 G2 H T1 T2 C1 C2 S EP JP
Developmental stages
VIT_05s0094g00640
Standardized expressions
2
1
0
−1
−2
EI PG G1 G2 H T1 T2 C1 C2 S EP JP
Developmental stages
VIT_05s0094g00650
Standardized expressions
1
0
−1
−2
−3
EI PG G1 G2 H T1 T2 C1 C2 S EP JP
Developmental stages

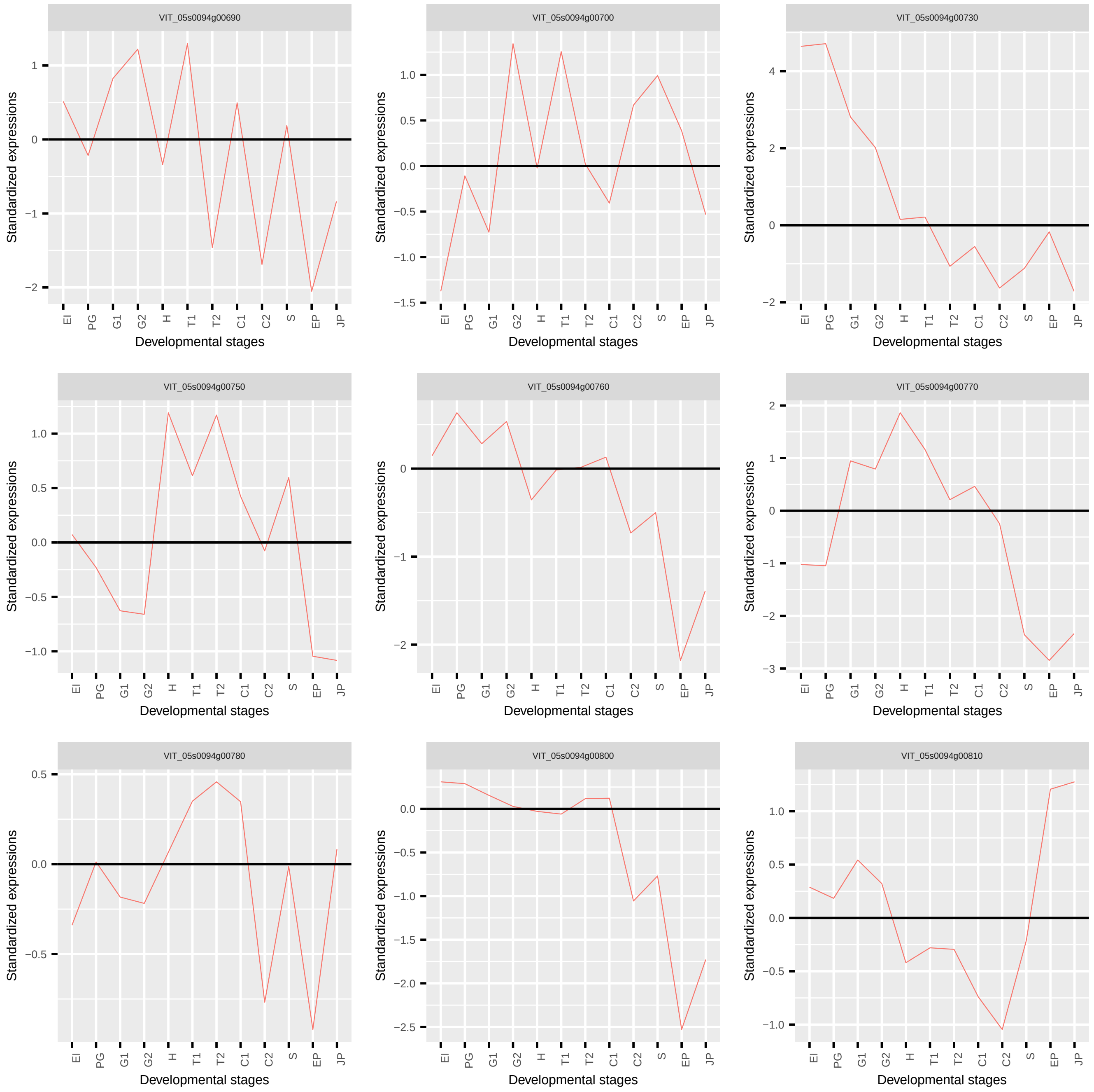

VIT_05s0094g00690
VIT_05s0094g00700
VIT_05s0094g00730
VIT_05s0094g00750
VIT_05s0094g00760
VIT_05s0094g00770
VIT_05s0094g00780
VIT_05s0094g00800
VIT_05s0094g00810
Standardized expressions
Developmental stages
EI
PG
G1
G2
H
T1
T2
C1
C2
S
EP
JP

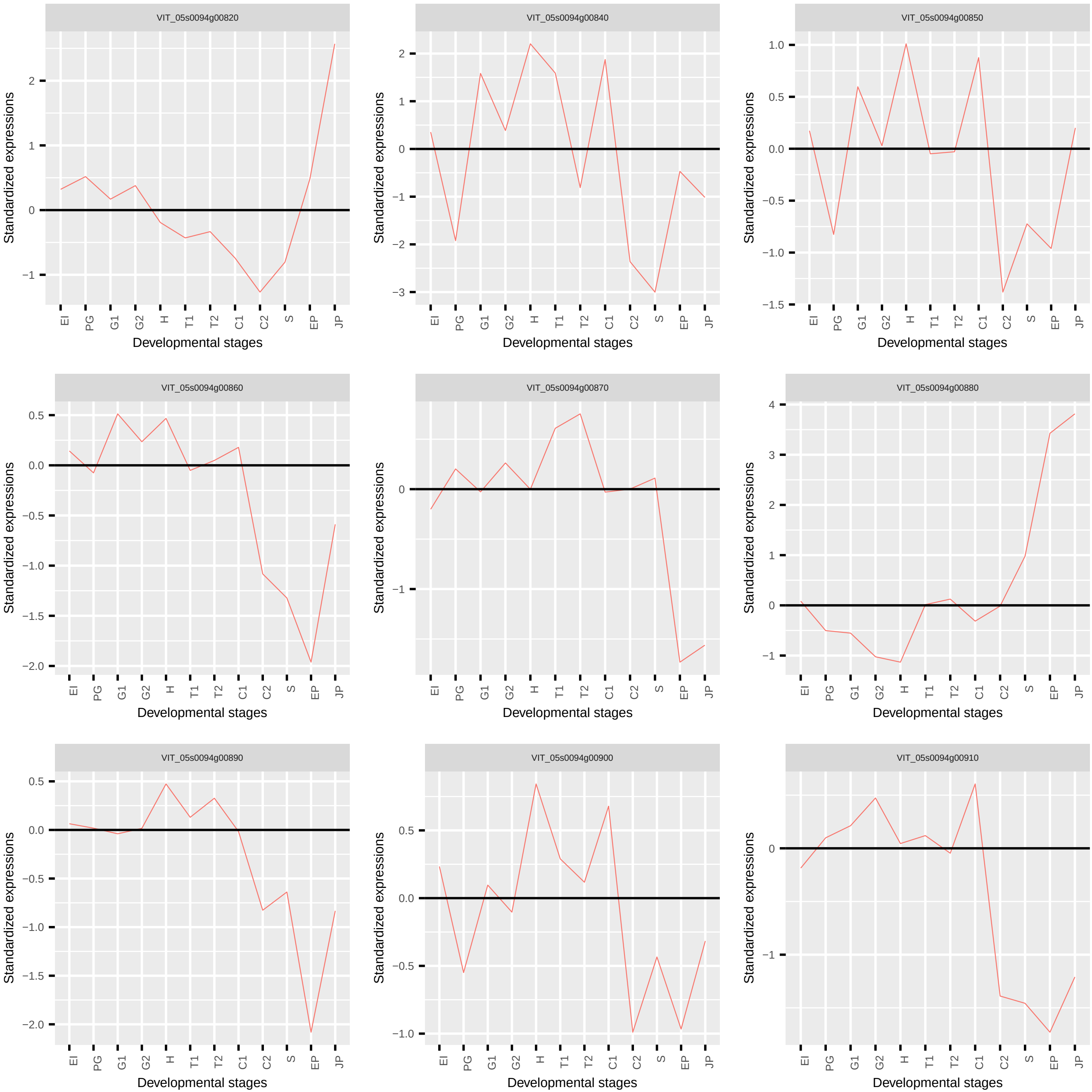
VIT_05s0094g00820
VIT_05s0094g00840
VIT_05s0094g00850
VIT_05s0094g00860
VIT_05s0094g00870
VIT_05s0094g00880
VIT_05s0094g00890
VIT_05s0094g00900
VIT_05s0094g00910
Standardized expressions
Developmental stages
EI PG G1 G2 H T1 T2 C1 C2 S EP JP

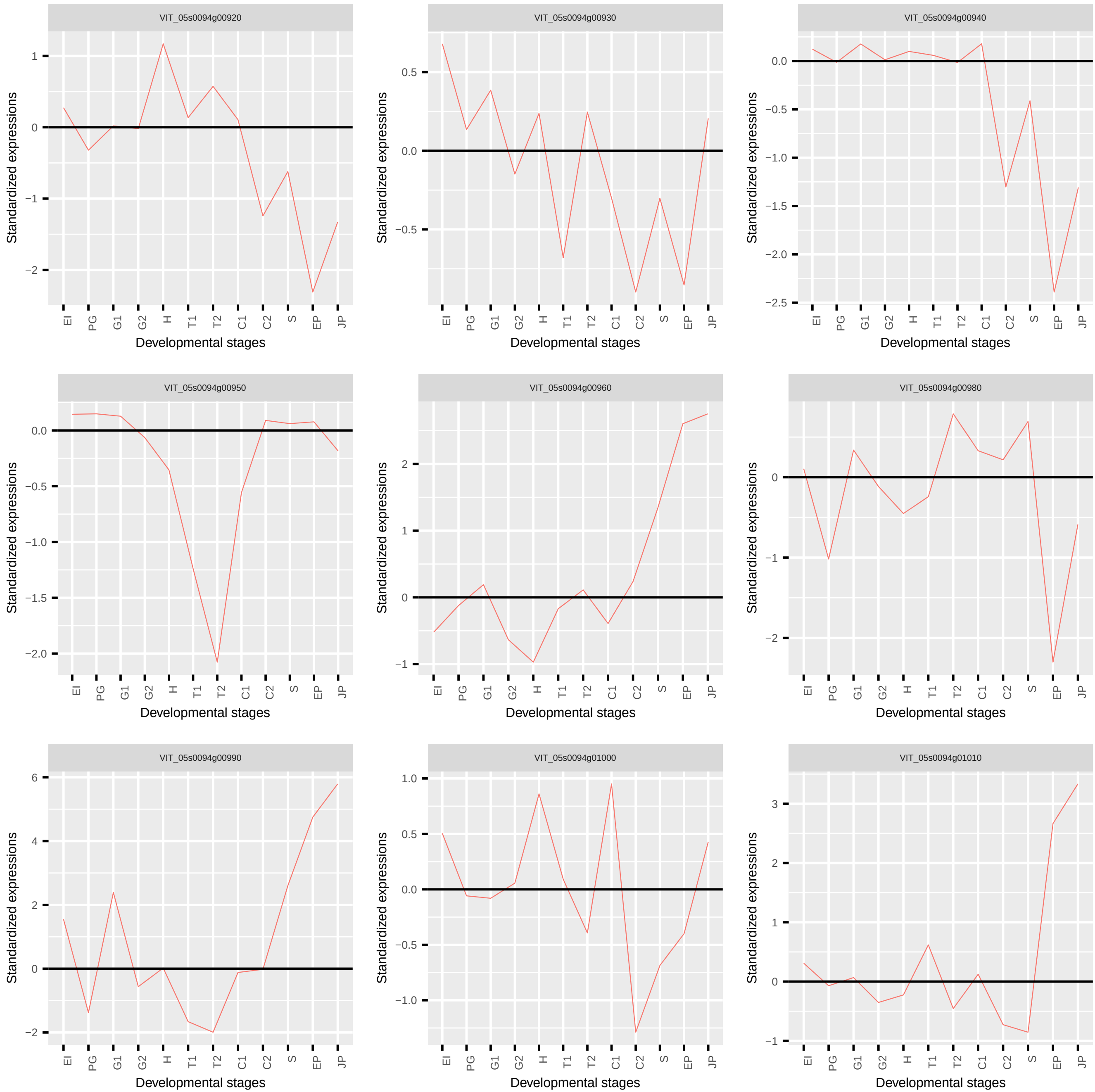
VIT_05s0094g00920
1
0
−1
−2
Standardized expressions
EI PG G1 G2 H T1 T2 C1 C2 S EP JP
Developmental stages
VIT_05s0094g00930
0.5
0.0
−0.5
Standardized expressions
EI PG G1 G2 H T1 T2 C1 C2 S EP JP
Developmental stages
VIT_05s0094g00940
0.0
−0.5
−1.0
−1.5
−2.0
−2.5
Standardized expressions
EI PG G1 G2 H T1 T2 C1 C2 S EP JP
Developmental stages
VIT_05s0094g00950
0.0
−0.5
−1.0
−1.5
−2.0
Standardized expressions
EI PG G1 G2 H T1 T2 C1 C2 S EP JP
Developmental stages
VIT_05s0094g00960
2
1
0
−1
Standardized expressions
EI PG G1 G2 H T1 T2 C1 C2 S EP JP
Developmental stages
VIT_05s0094g00980
0
−1
−2
Standardized expressions
EI PG G1 G2 H T1 T2 C1 C2 S EP JP
Developmental stages
VIT_05s0094g00990
6
4
2
0
−2
Standardized expressions
EI PG G1 G2 H T1 T2 C1 C2 S EP JP
Developmental stages
VIT_05s0094g01000
1.0
0.5
0.0
−0.5
−1.0
Standardized expressions
EI PG G1 G2 H T1 T2 C1 C2 S EP JP
Developmental stages
VIT_05s0094g01010
3
2
1
0
−1
Standardized expressions
EI PG G1 G2 H T1 T2 C1 C2 S EP JP
Developmental stages

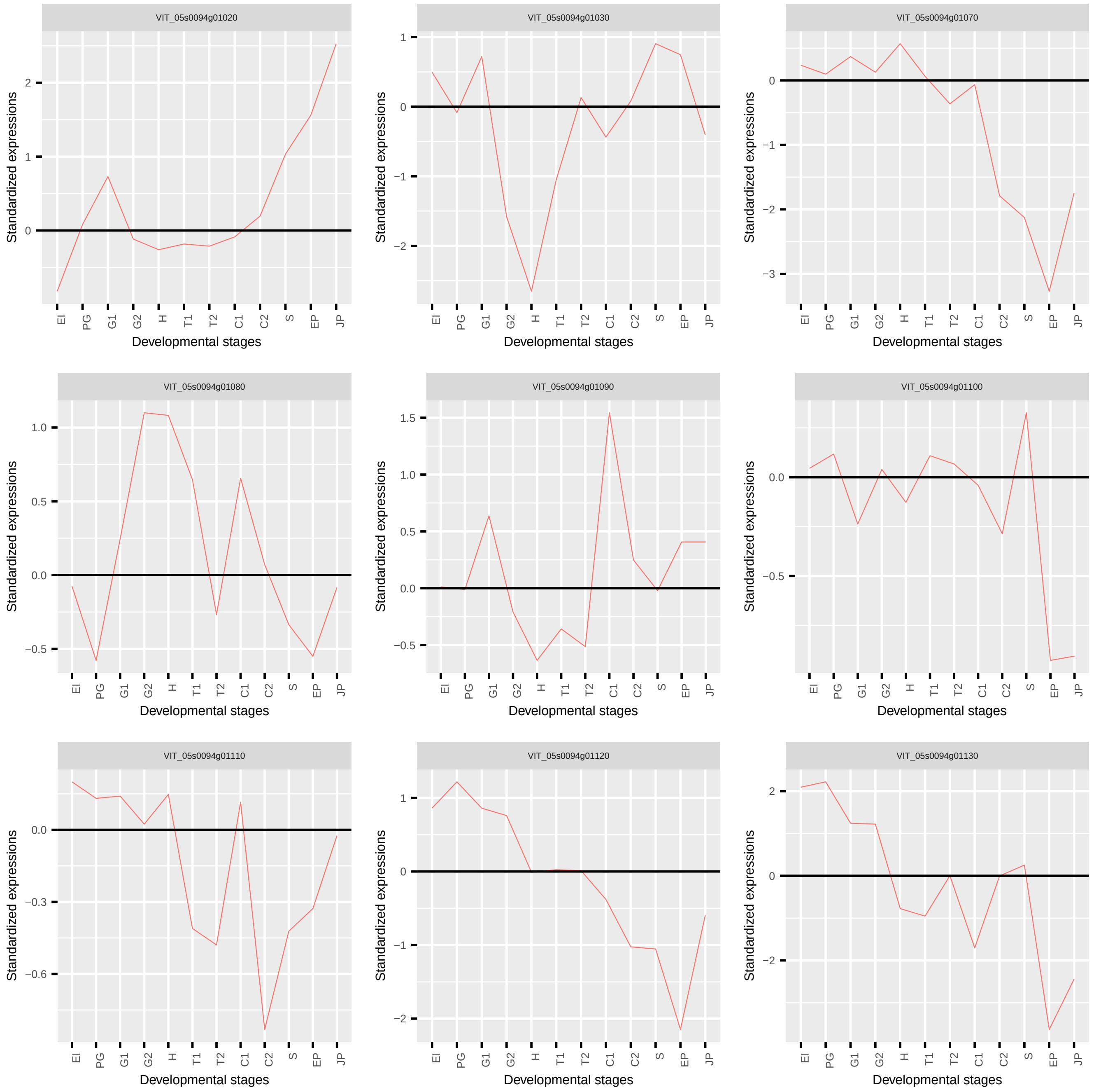
VIT_05s0094g01020
Standardized expressions
2
1
0
EI
PG
G1
G2
H
T1
T2
C1
C2
S
EP
JP
Developmental stages
VIT_05s0094g01030
Standardized expressions
1
0
−1
−2
EI
PG
G1
G2
H
T1
T2
C1
C2
S
EP
JP
Developmental stages
VIT_05s0094g01070
Standardized expressions
0
−1
−2
−3
EI
PG
G1
G2
H
T1
T2
C1
C2
S
EP
JP
Developmental stages
VIT_05s0094g01080
Standardized expressions
1.0
0.5
0.0
−0.5
EI
PG
G1
G2
H
T1
T2
C1
C2
S
EP
JP
Developmental stages
VIT_05s0094g01090
Standardized expressions
1.5
1.0
0.5
0.0
−0.5
EI
PG
G1
G2
H
T1
T2
C1
C2
S
EP
JP
Developmental stages
VIT_05s0094g01100
Standardized expressions
0.0
−0.5
EI
PG
G1
G2
H
T1
T2
C1
C2
S
EP
JP
Developmental stages
VIT_05s0094g01110
Standardized expressions
0.0
−0.3
−0.6
EI
PG
G1
G2
H
T1
T2
C1
C2
S
EP
JP
Developmental stages
VIT_05s0094g01120
Standardized expressions
1
0
−1
−2
EI
PG
G1
G2
H
T1
T2
C1
C2
S
EP
JP
Developmental stages
VIT_05s0094g01130
Standardized expressions
2
0
−2
EI
PG
G1
G2
H
T1
T2
C1
C2
S
EP
JP
Developmental stages

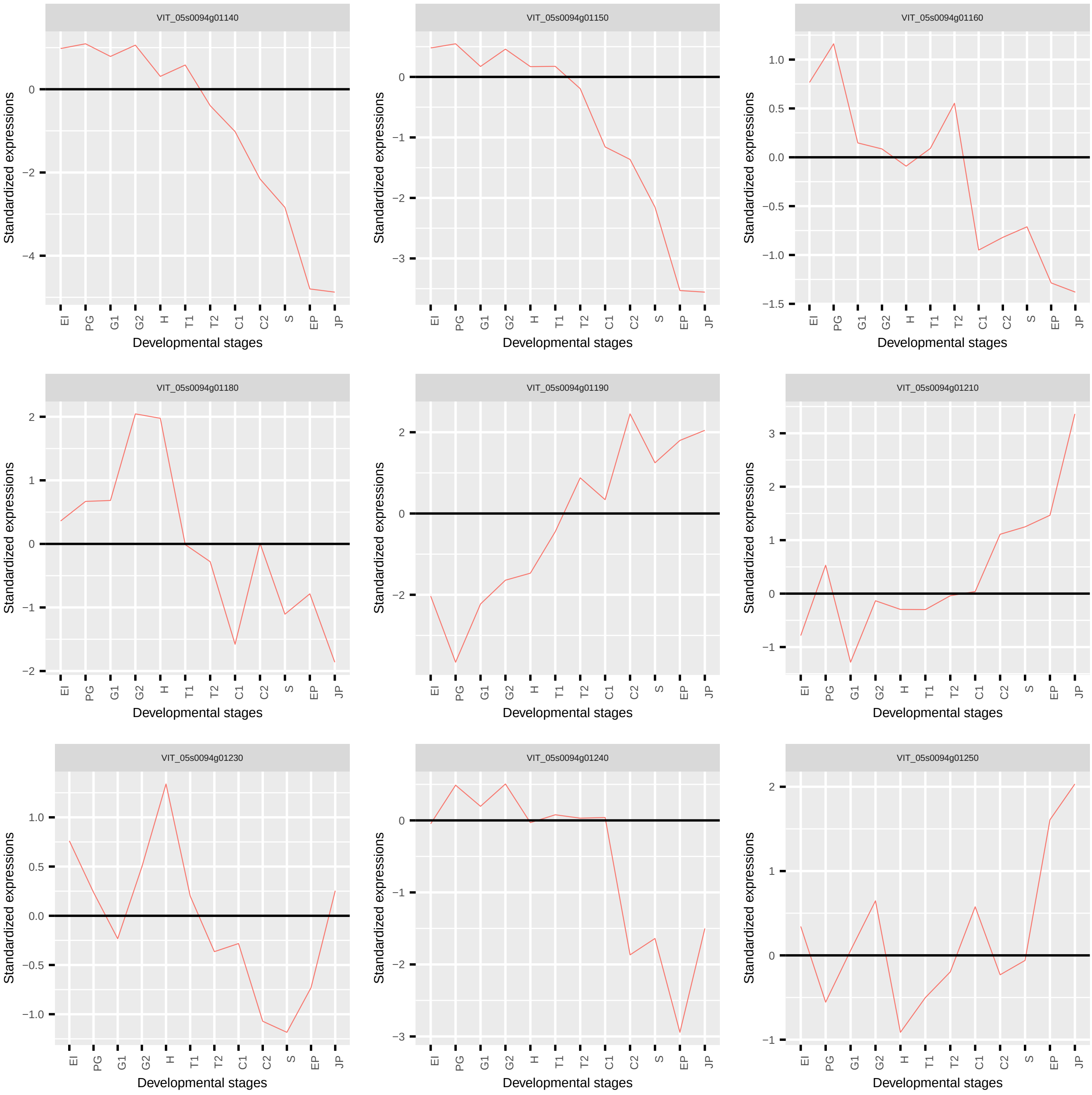

VIT_05s0094g01140
VIT_05s0094g01150
VIT_05s0094g01160
VIT_05s0094g01180
VIT_05s0094g01190
VIT_05s0094g01210
VIT_05s0094g01230
VIT_05s0094g01240
VIT_05s0094g01250
Standardized expressions
Developmental stages
EI PG G1 G2 H T1 T2 C1 C2 S EP JP
0 −2 −4
0 −1 −2 −3
1.0 0.5 0.0 −0.5 −1.0 −1.5
2 1 0 −1 −2
2 0 −2
3 2 1 0 −1
1.0 0.5 0.0 −0.5 −1.0
0 −1 −2 −3
2 1 0 −1

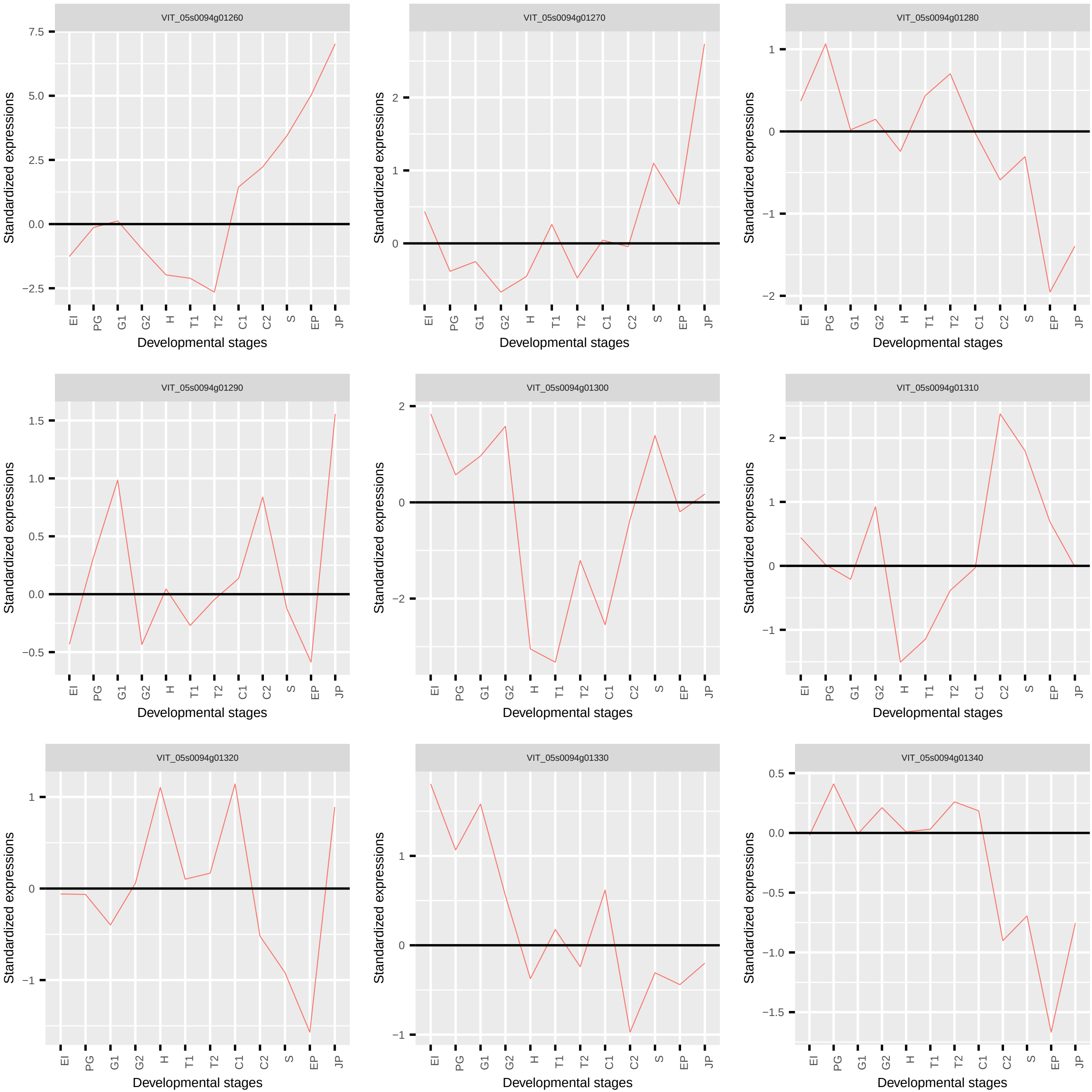

VIT_05s0094g01260
VIT_05s0094g01270
VIT_05s0094g01280
VIT_05s0094g01290
VIT_05s0094g01300
VIT_05s0094g01310
VIT_05s0094g01320
VIT_05s0094g01330
VIT_05s0094g01340
Standardized expressions
Developmental stages
EI PG G1 G2 H T1 T2 C1 C2 S EP JP

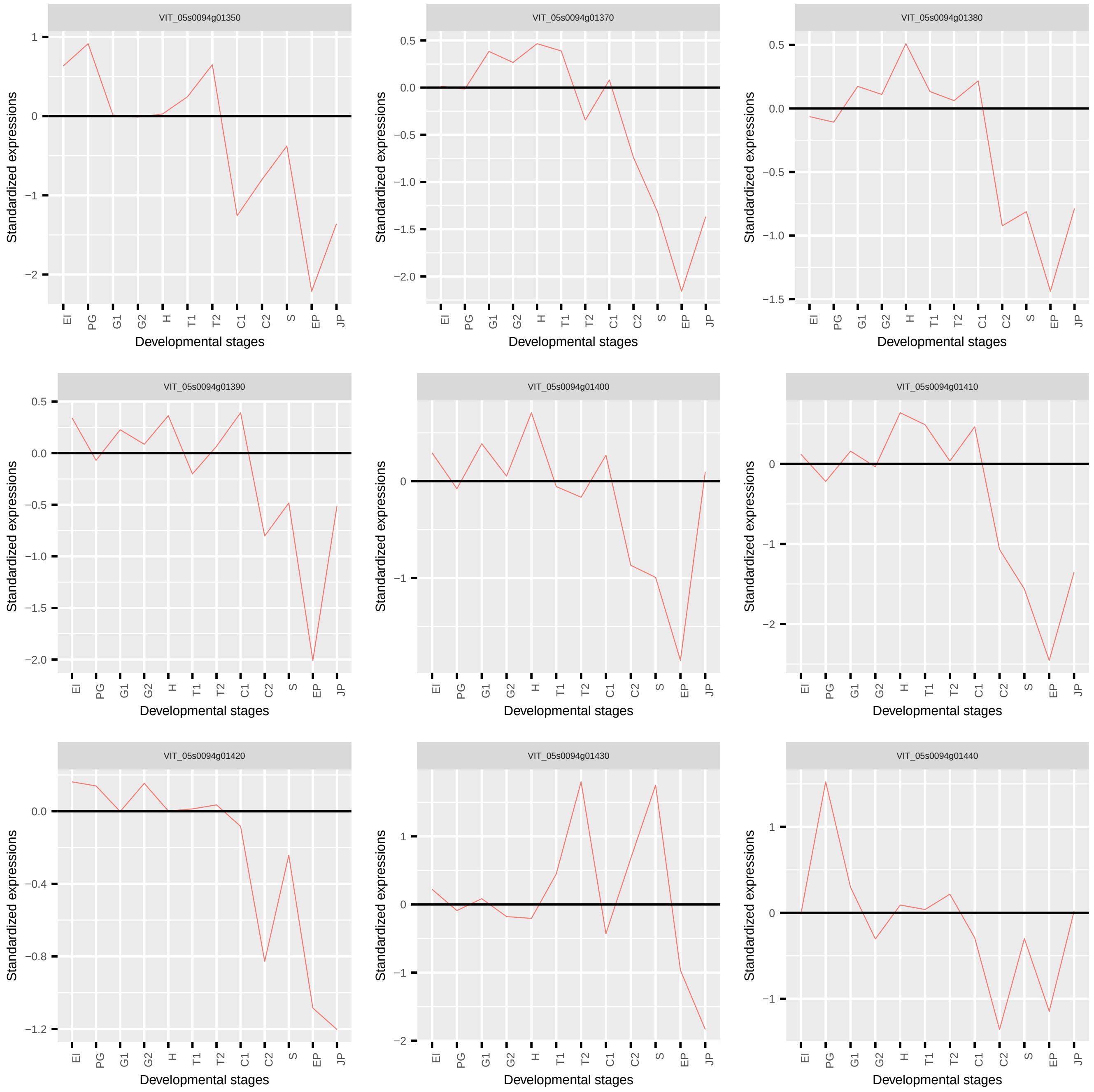
VIT_05s0094g01350
VIT_05s0094g01370
VIT_05s0094g01380
VIT_05s0094g01390
VIT_05s0094g01400
VIT_05s0094g01410
VIT_05s0094g01420
VIT_05s0094g01430
VIT_05s0094g01440
Standardized expressions
Developmental stages
EI PG G1 G2 H T1 T2 C1 C2 S EP JP

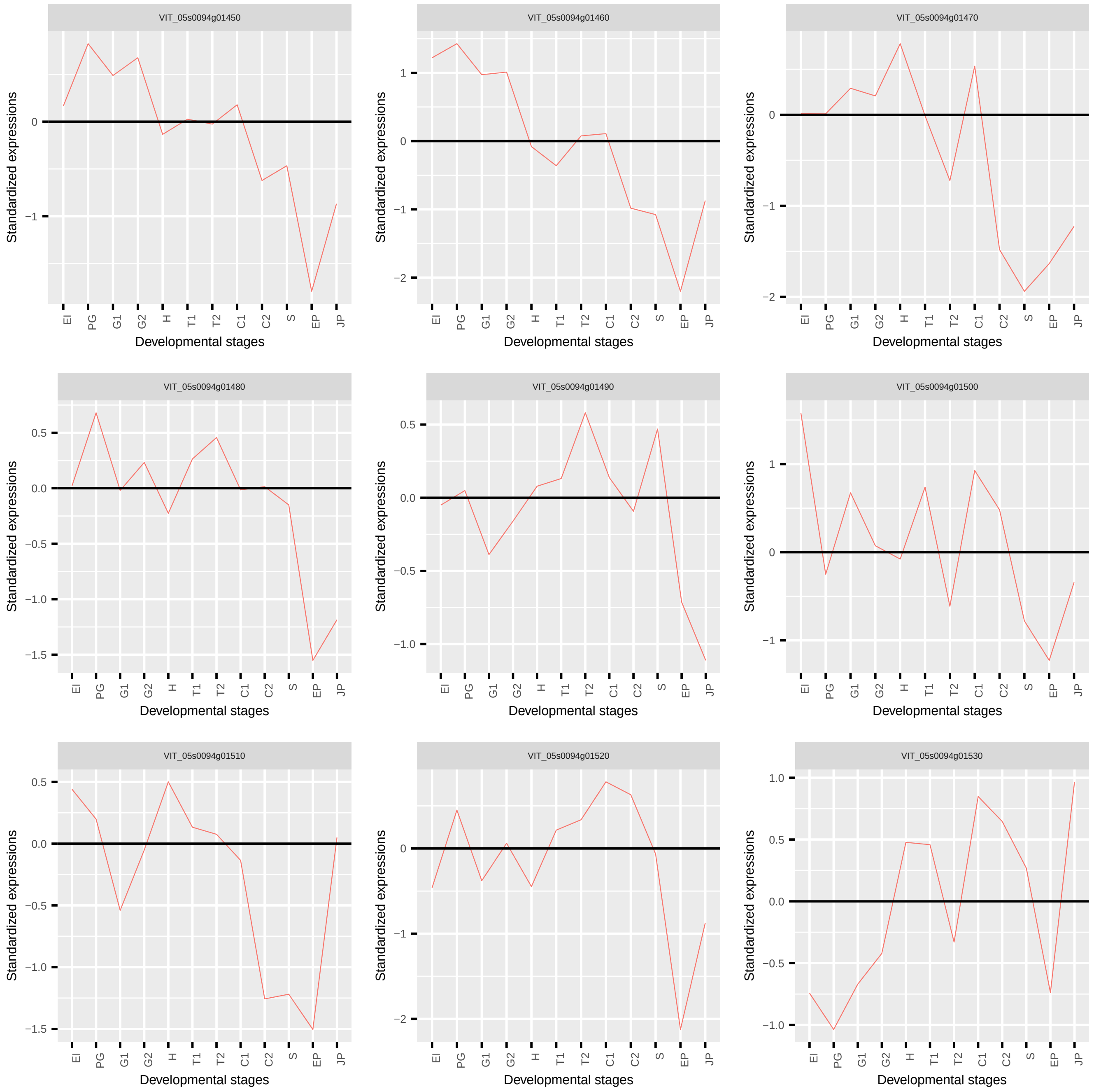
VIT_05s0094g01450
VIT_05s0094g01460
VIT_05s0094g01470
VIT_05s0094g01480
VIT_05s0094g01490
VIT_05s0094g01500
VIT_05s0094g01510
VIT_05s0094g01520
VIT_05s0094g01530
Standardized expressions
Developmental stages
EI PG G1 G2 H T1 T2 C1 C2 S EP JP

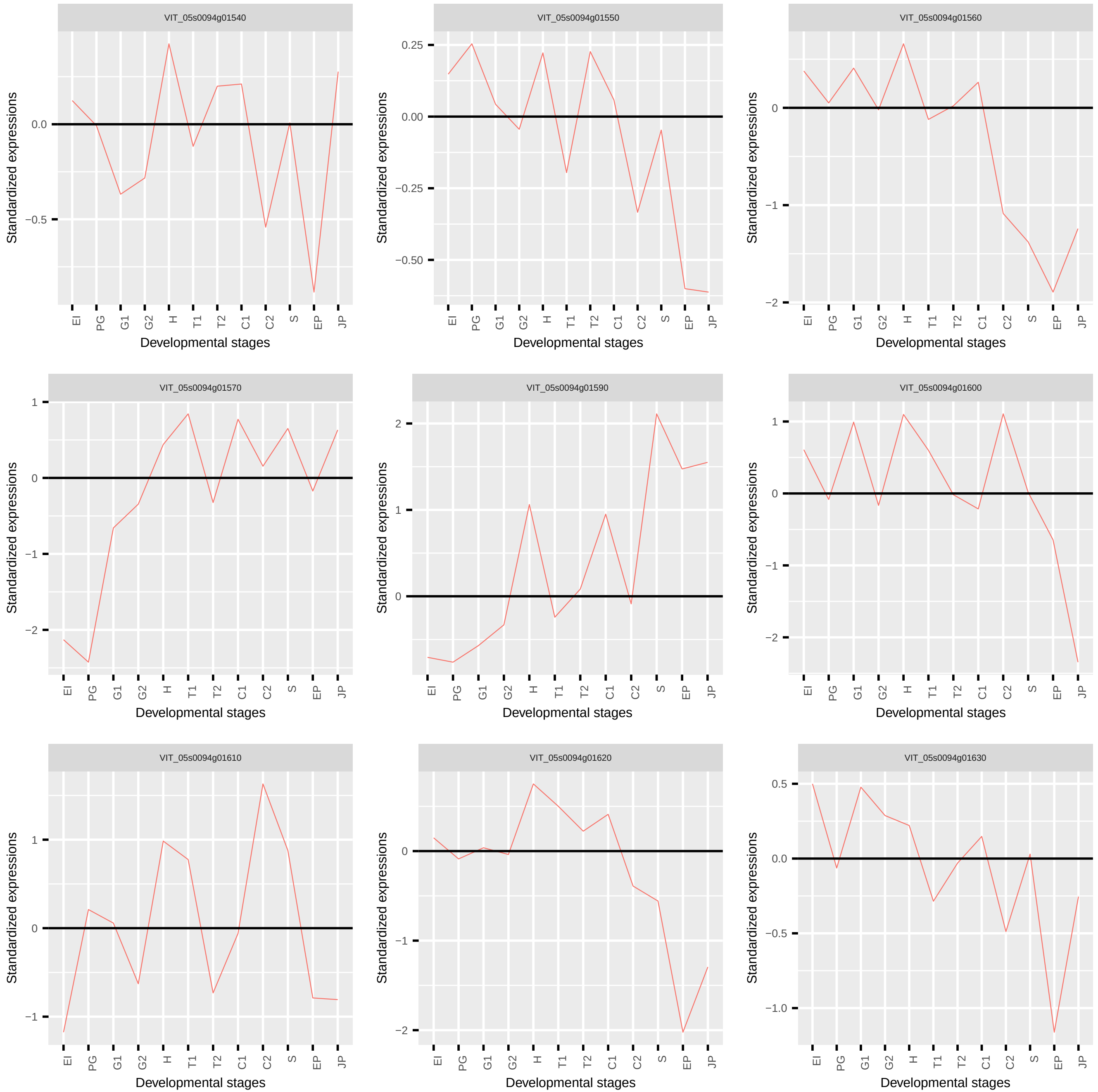
VIT_05s0094g01540
0.0
−0.5
Standardized expressions
EI PG G1 G2 H T1 T2 C1 C2 S EP JP
Developmental stages
VIT_05s0094g01550
0.25
0.00
−0.25
−0.50
Standardized expressions
EI PG G1 G2 H T1 T2 C1 C2 S EP JP
Developmental stages
VIT_05s0094g01560
0
−1
−2
Standardized expressions
EI PG G1 G2 H T1 T2 C1 C2 S EP JP
Developmental stages
VIT_05s0094g01570
1
0
−1
−2
Standardized expressions
EI PG G1 G2 H T1 T2 C1 C2 S EP JP
Developmental stages
VIT_05s0094g01590
2
1
0
Standardized expressions
EI PG G1 G2 H T1 T2 C1 C2 S EP JP
Developmental stages
VIT_05s0094g01600
1
0
−1
−2
Standardized expressions
EI PG G1 G2 H T1 T2 C1 C2 S EP JP
Developmental stages
VIT_05s0094g01610
1
0
−1
Standardized expressions
EI PG G1 G2 H T1 T2 C1 C2 S EP JP
Developmental stages
VIT_05s0094g01620
0
−1
−2
Standardized expressions
EI PG G1 G2 H T1 T2 C1 C2 S EP JP
Developmental stages
VIT_05s0094g01630
0.5
0.0
−0.5
−1.0
Standardized expressions
EI PG G1 G2 H T1 T2 C1 C2 S EP JP
Developmental stages

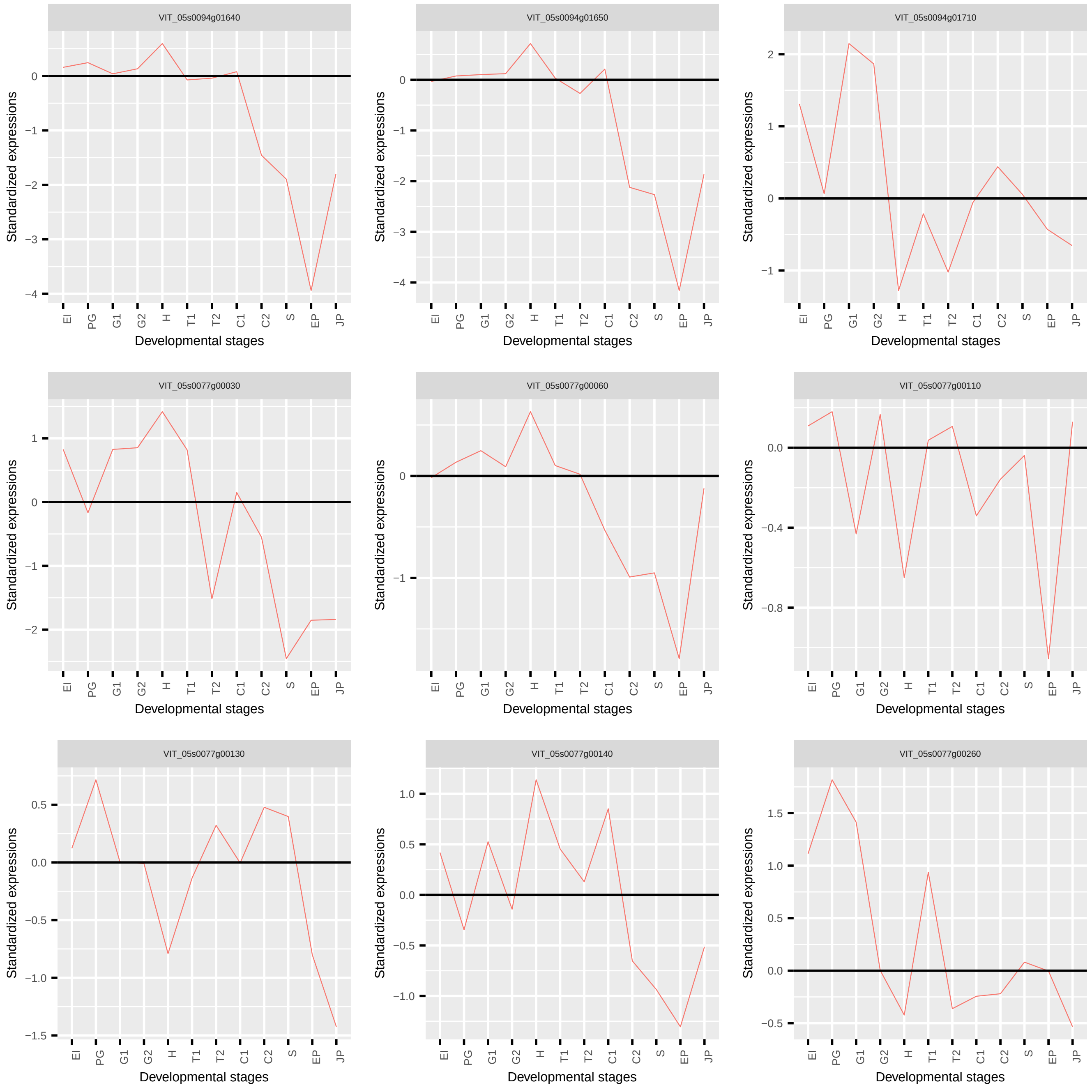
VIT_05s0094g01640
VIT_05s0094g01650
VIT_05s0094g01710
VIT_05s0077g00030
VIT_05s0077g00060
VIT_05s0077g00110
VIT_05s0077g00130
VIT_05s0077g00140
VIT_05s0077g00260
Standardized expressions
EI PG G1 G2 H T1 T2 C1 C2 S EP JP
Developmental stages

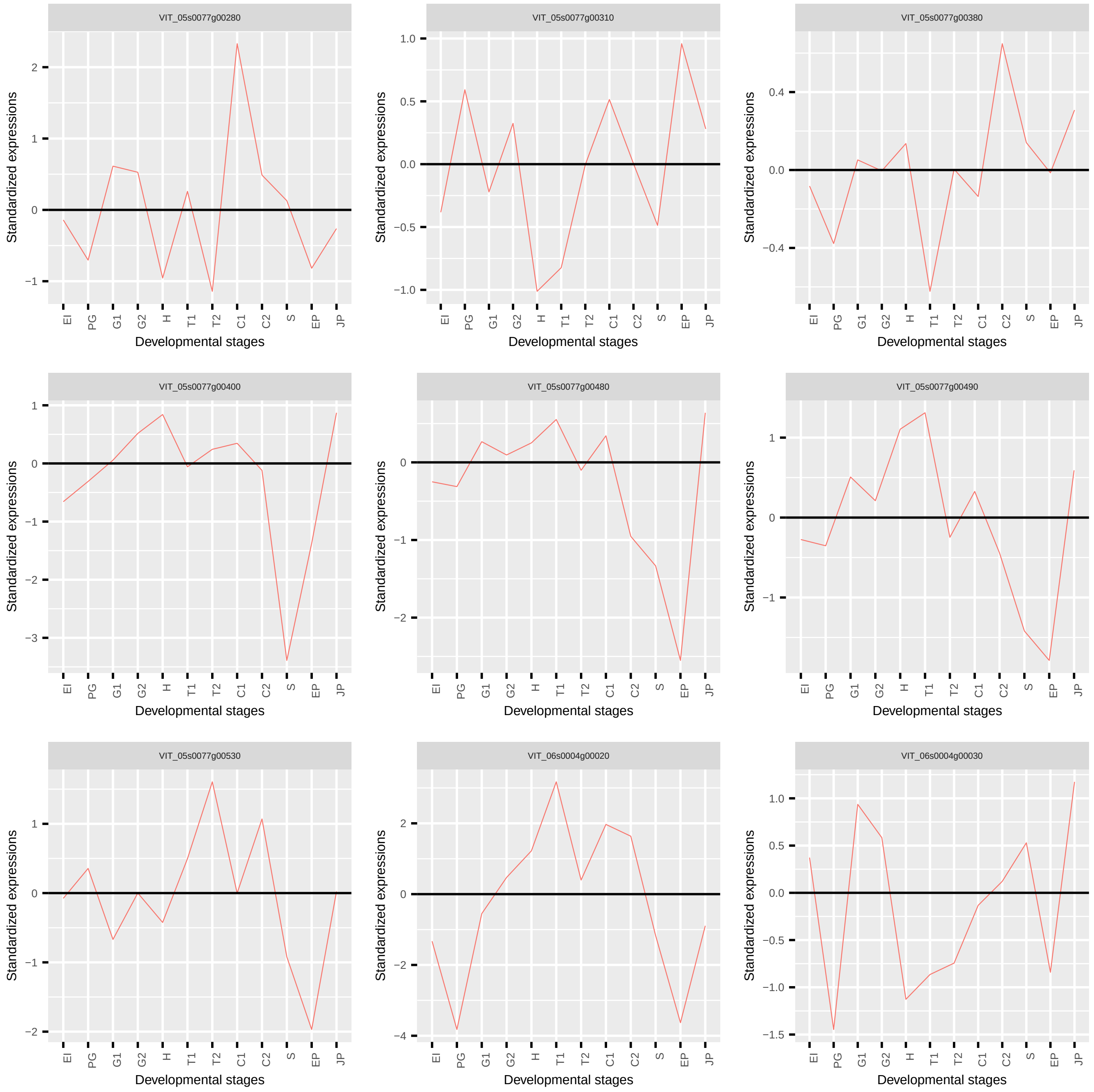
VIT_05s0077g00280
VIT_05s0077g00310
VIT_05s0077g00380
VIT_05s0077g00400
VIT_05s0077g00480
VIT_05s0077g00490
VIT_05s0077g00530
VIT_06s0004g00020
VIT_06s0004g00030
Standardized expressions
Developmental stages
EI PG G1 G2 H T1 T2 C1 C2 S EP JP

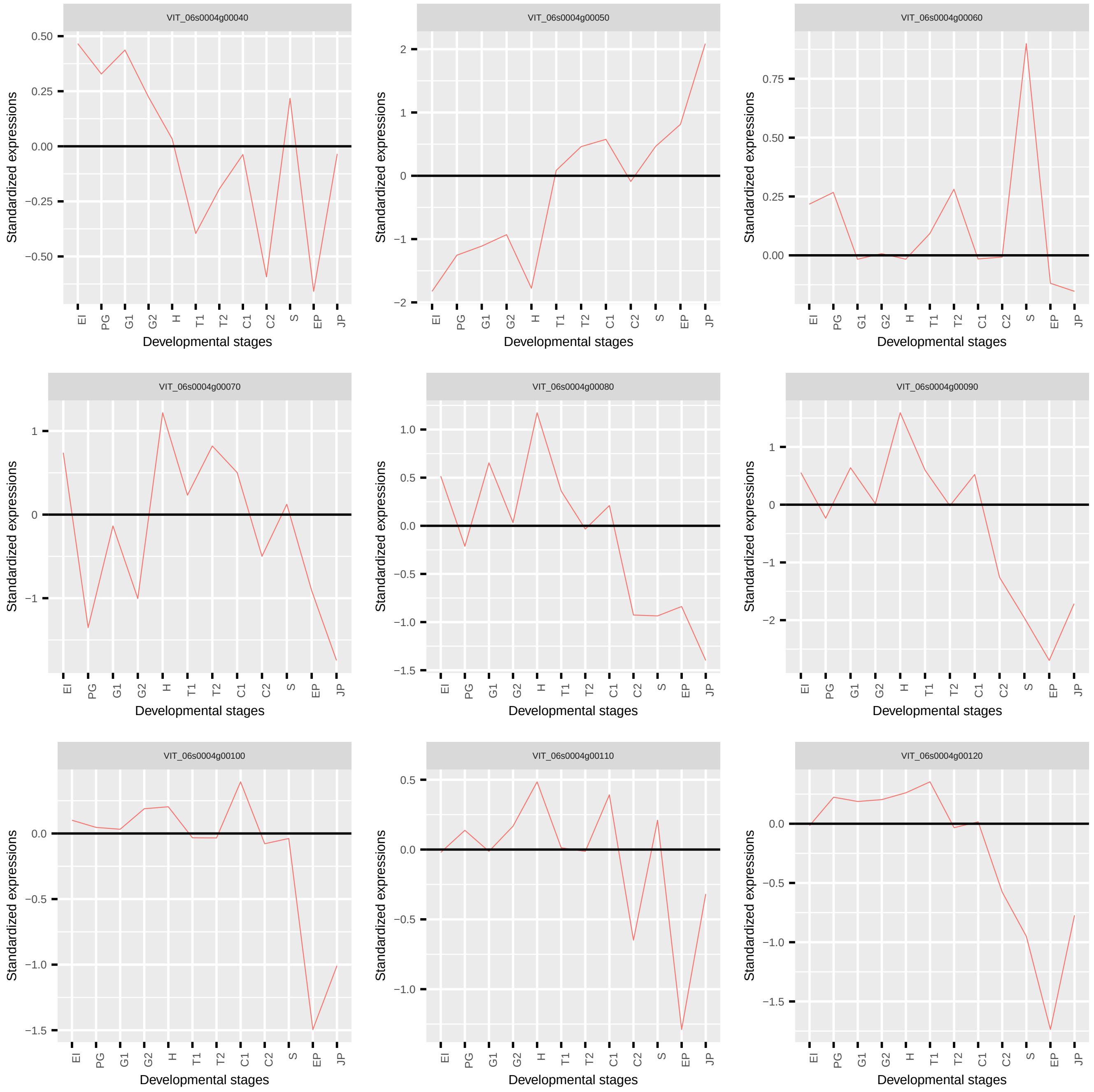
VIT_06s0004g00040
VIT_06s0004g00050
VIT_06s0004g00060
VIT_06s0004g00070
VIT_06s0004g00080
VIT_06s0004g00090
VIT_06s0004g00100
VIT_06s0004g00110
VIT_06s0004g00120
Standardized expressions
Developmental stages
EI PG G1 G2 H T1 T2 C1 C2 S EP JP

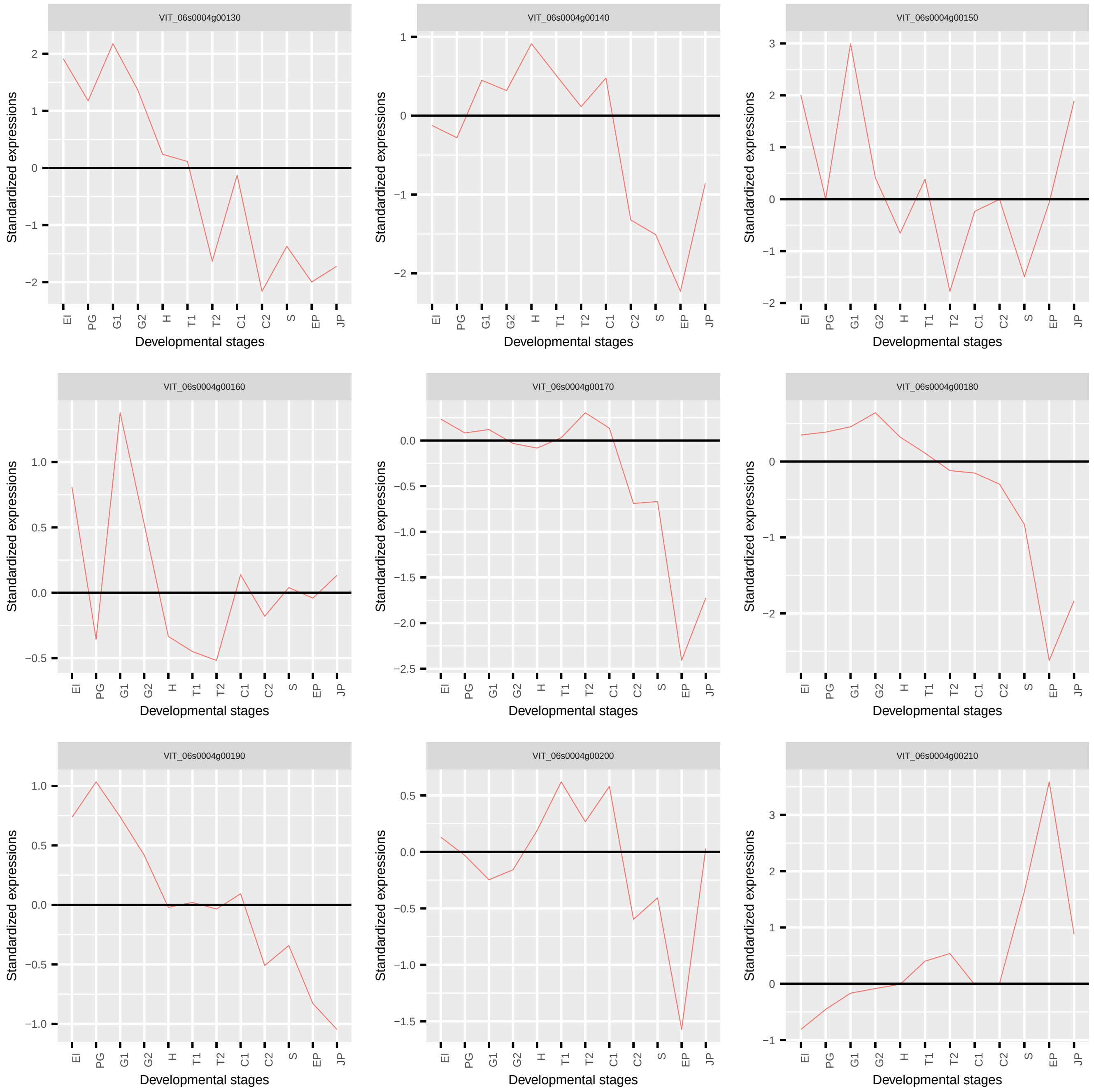
VIT_06s0004g00130
VIT_06s0004g00140
VIT_06s0004g00150
VIT_06s0004g00160
VIT_06s0004g00170
VIT_06s0004g00180
VIT_06s0004g00190
VIT_06s0004g00200
VIT_06s0004g00210
Standardized expressions
Developmental stages
EI PG G1 G2 H T1 T2 C1 C2 S EP JP

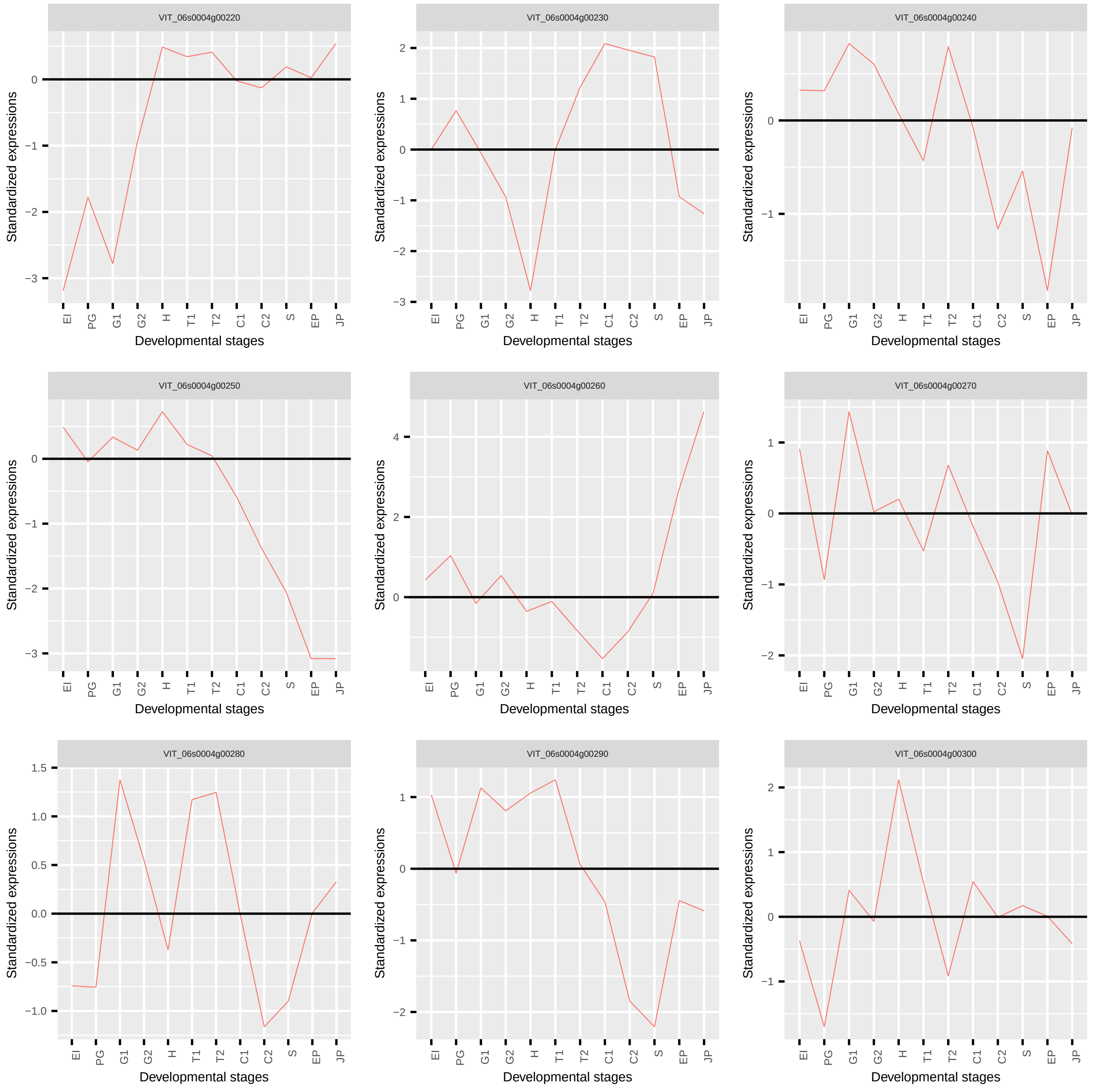
VIT_06s0004g00220
VIT_06s0004g00230
VIT_06s0004g00240
VIT_06s0004g00250
VIT_06s0004g00260
VIT_06s0004g00270
VIT_06s0004g00280
VIT_06s0004g00290
VIT_06s0004g00300
Standardized expressions
Developmental stages
EI
PG
G1
G2
H
T1
T2
C1
C2
S
EP
JP

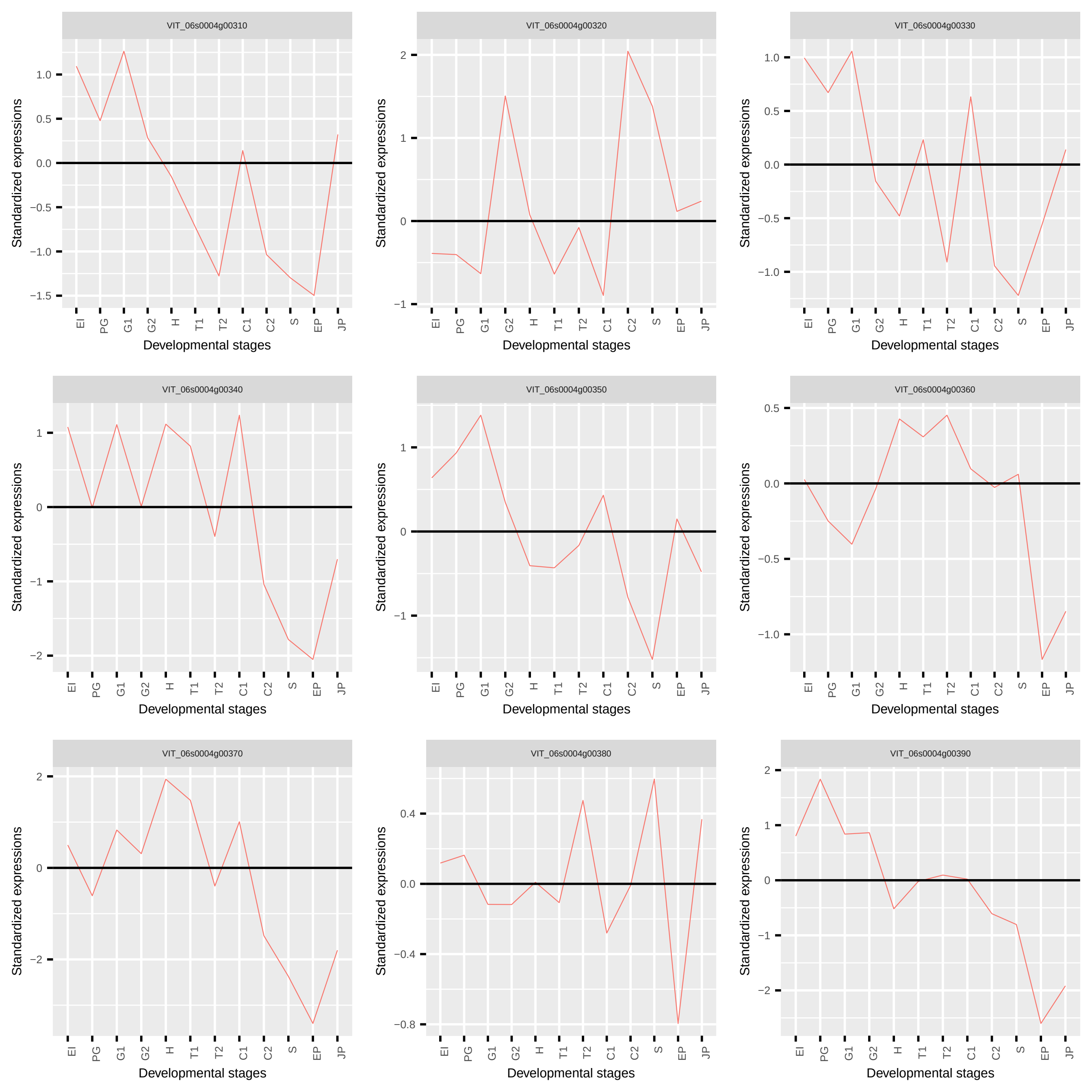
VIT_06s0004g00310
Standardized expressions
1.0
0.5
0.0
−0.5
−1.0
−1.5
EI PG G1 G2 H T1 T2 C1 C2 S EP JP
Developmental stages
VIT_06s0004g00320
Standardized expressions
2
1
0
−1
EI PG G1 G2 H T1 T2 C1 C2 S EP JP
Developmental stages
VIT_06s0004g00330
Standardized expressions
1.0
0.5
0.0
−0.5
−1.0
EI PG G1 G2 H T1 T2 C1 C2 S EP JP
Developmental stages
VIT_06s0004g00340
Standardized expressions
1
0
−1
−2
EI PG G1 G2 H T1 T2 C1 C2 S EP JP
Developmental stages
VIT_06s0004g00350
Standardized expressions
1
0
−1
EI PG G1 G2 H T1 T2 C1 C2 S EP JP
Developmental stages
VIT_06s0004g00360
Standardized expressions
0.5
0.0
−0.5
−1.0
EI PG G1 G2 H T1 T2 C1 C2 S EP JP
Developmental stages
VIT_06s0004g00370
Standardized expressions
2
0
−2
EI PG G1 G2 H T1 T2 C1 C2 S EP JP
Developmental stages
VIT_06s0004g00380
Standardized expressions
0.4
0.0
−0.4
−0.8
EI PG G1 G2 H T1 T2 C1 C2 S EP JP
Developmental stages
VIT_06s0004g00390
Standardized expressions
2
1
0
−1
−2
EI PG G1 G2 H T1 T2 C1 C2 S EP JP
Developmental stages

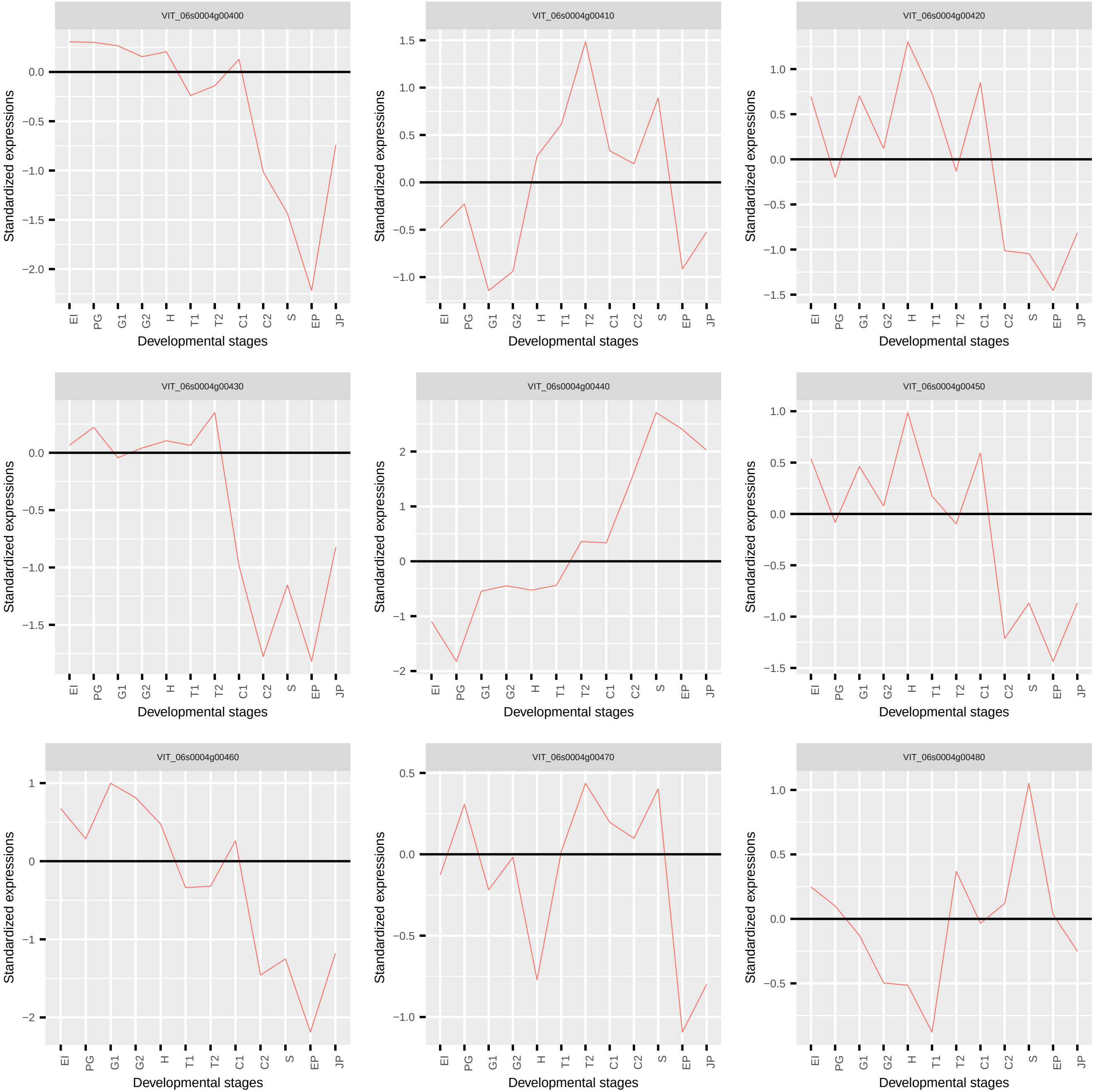
VIT_06s0004g00400
VIT_06s0004g00410
VIT_06s0004g00420
VIT_06s0004g00430
VIT_06s0004g00440
VIT_06s0004g00450
VIT_06s0004g00460
VIT_06s0004g00470
VIT_06s0004g00480
Standardized expressions
Developmental stages
EI PG G1 G2 H T1 T2 C1 C2 S EP JP

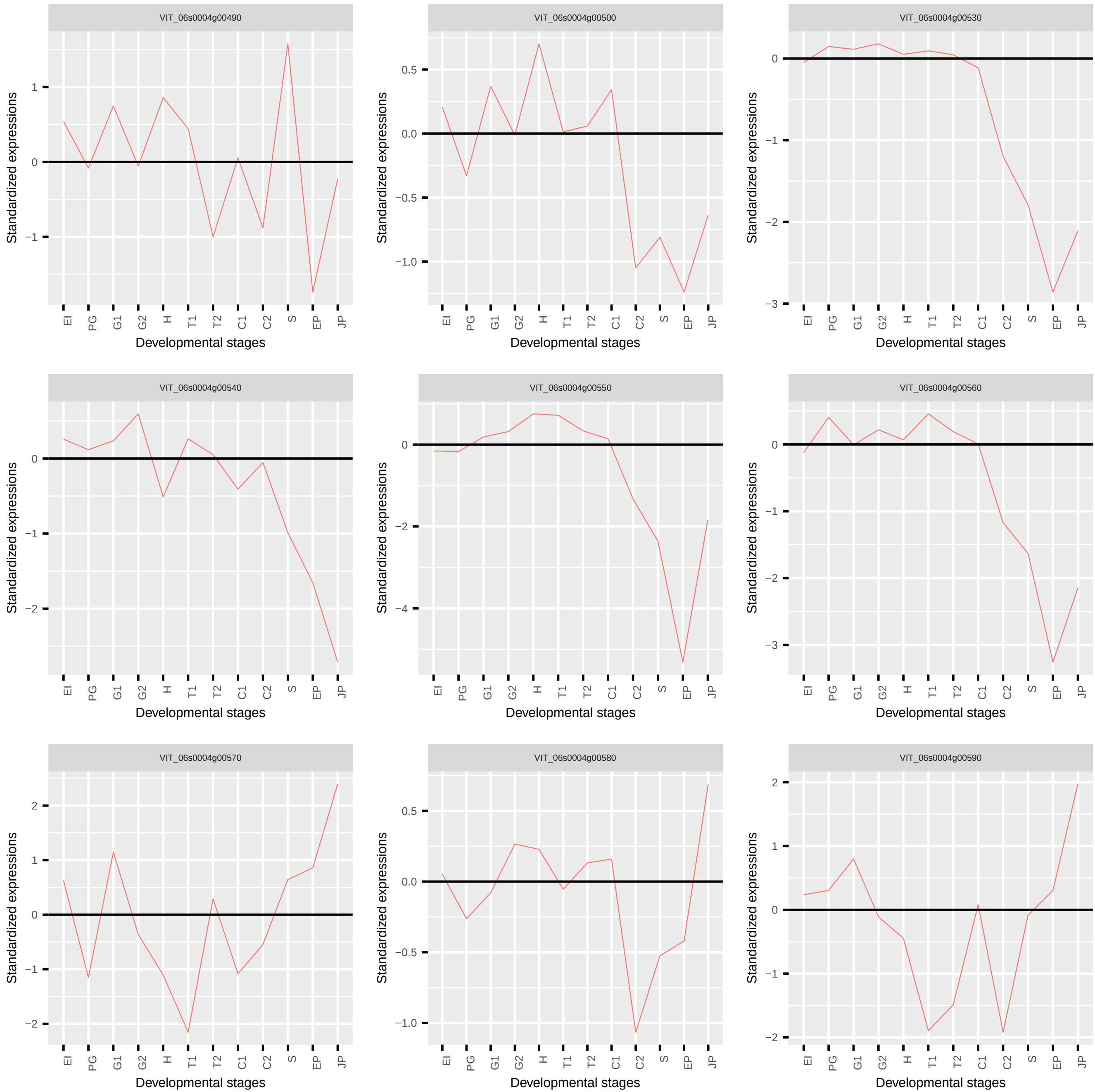
VIT_06s0004g00490
VIT_06s0004g00500
VIT_06s0004g00530
VIT_06s0004g00540
VIT_06s0004g00550
VIT_06s0004g00560
VIT_06s0004g00570
VIT_06s0004g00580
VIT_06s0004g00590
Standardized expressions
Developmental stages
EI PG G1 G2 H T1 T2 C1 C2 S EP JP

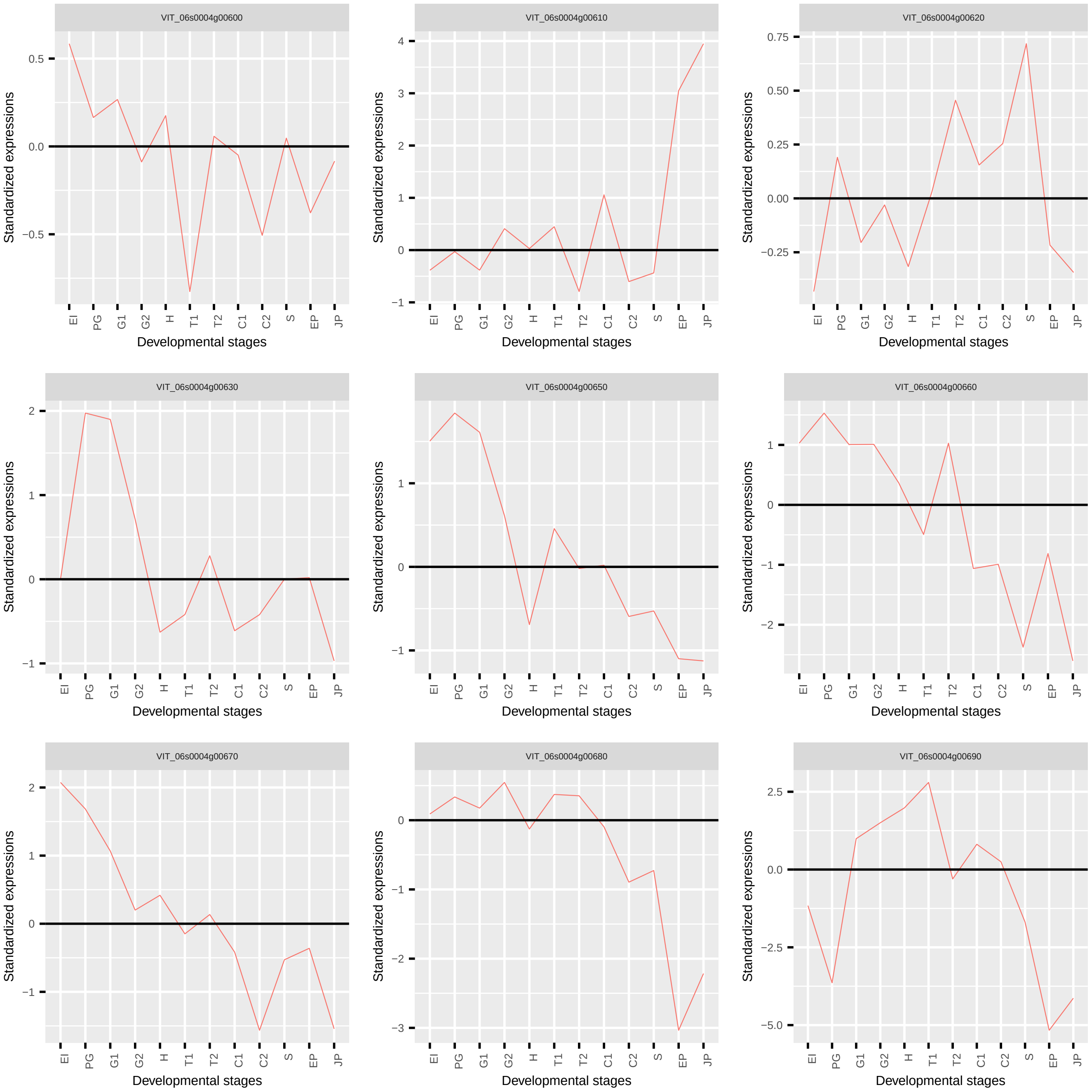
VIT_06s0004g00600
VIT_06s0004g00610
VIT_06s0004g00620
VIT_06s0004g00630
VIT_06s0004g00650
VIT_06s0004g00660
VIT_06s0004g00670
VIT_06s0004g00680
VIT_06s0004g00690
Standardized expressions
Developmental stages
EI
PG
G1
G2
H
T1
T2
C1
C2
S
EP
JP

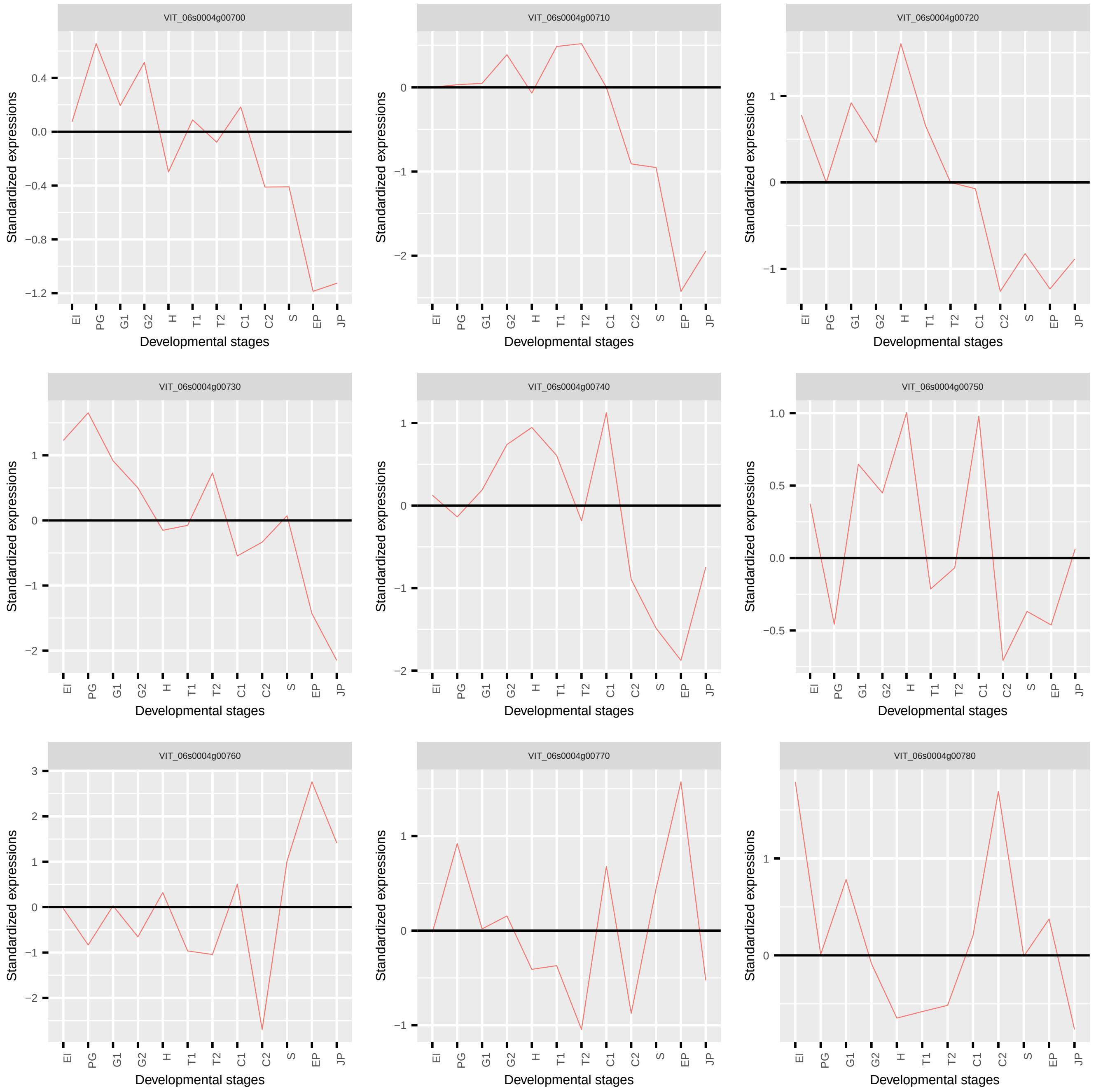
VIT_06s0004g00700
VIT_06s0004g00710
VIT_06s0004g00720
VIT_06s0004g00730
VIT_06s0004g00740
VIT_06s0004g00750
VIT_06s0004g00760
VIT_06s0004g00770
VIT_06s0004g00780
Standardized expressions
Developmental stages
EI PG G1 G2 H T1 T2 C1 C2 S EP JP

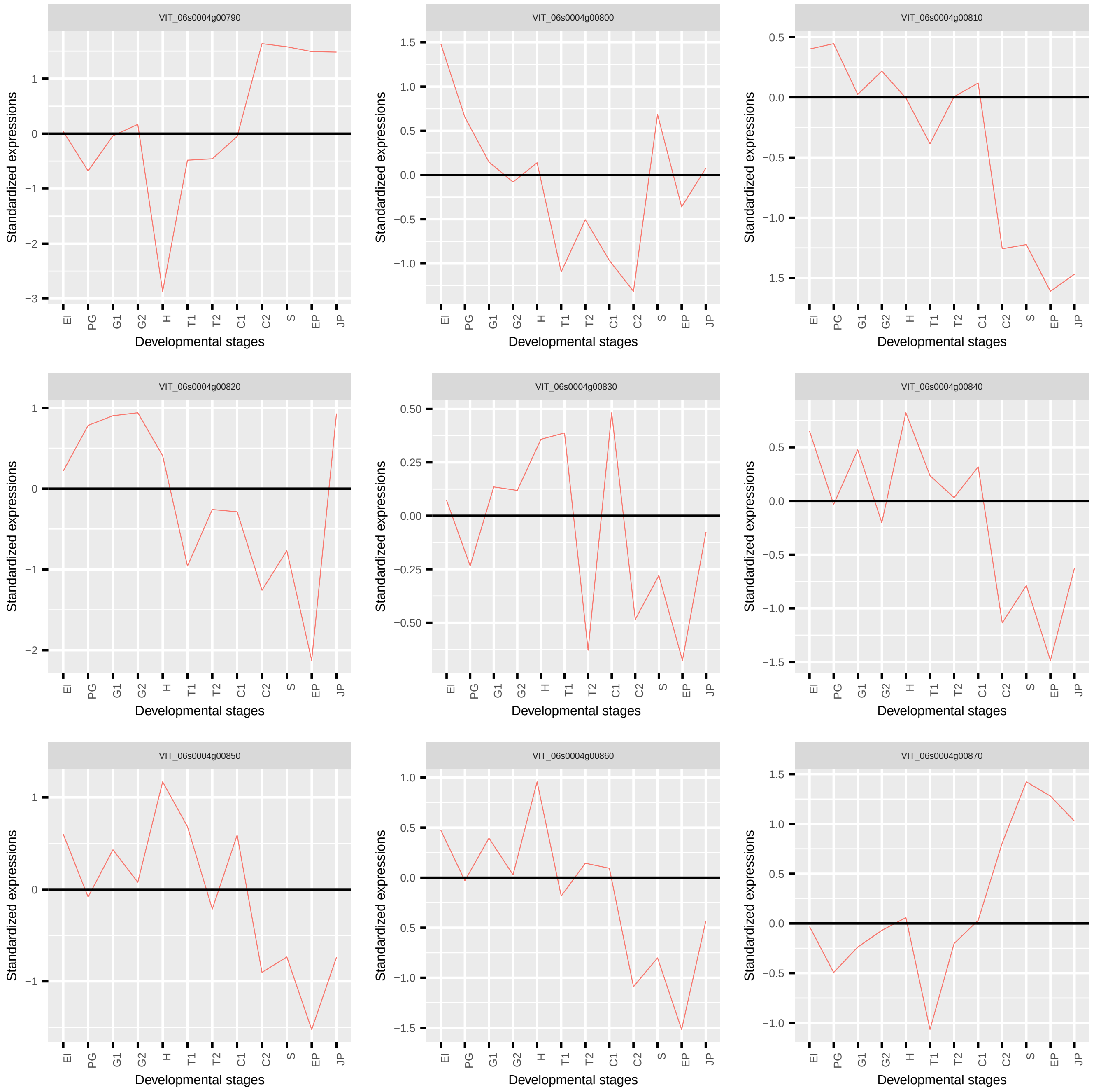

VIT_06s0004g00790
VIT_06s0004g00800
VIT_06s0004g00810
VIT_06s0004g00820
VIT_06s0004g00830
VIT_06s0004g00840
VIT_06s0004g00850
VIT_06s0004g00860
VIT_06s0004g00870
Standardized expressions
Developmental stages
EI PG G1 G2 H T1 T2 C1 C2 S EP JP

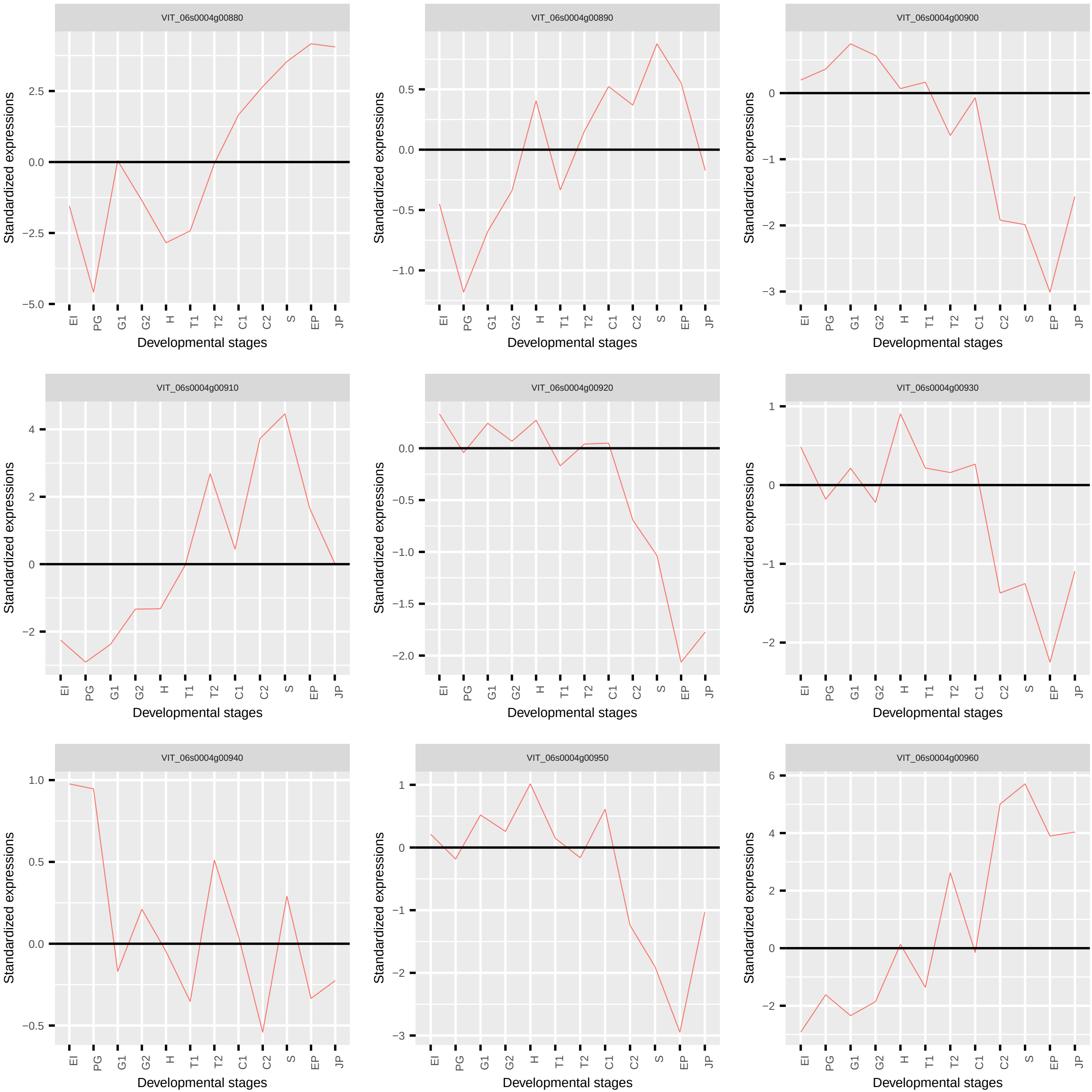
VIT_06s0004g00880
VIT_06s0004g00890
VIT_06s0004g00900
VIT_06s0004g00910
VIT_06s0004g00920
VIT_06s0004g00930
VIT_06s0004g00940
VIT_06s0004g00950
VIT_06s0004g00960
Standardized expressions
Developmental stages
EI
PG
G1
G2
H
T1
T2
C1
C2
S
EP
JP

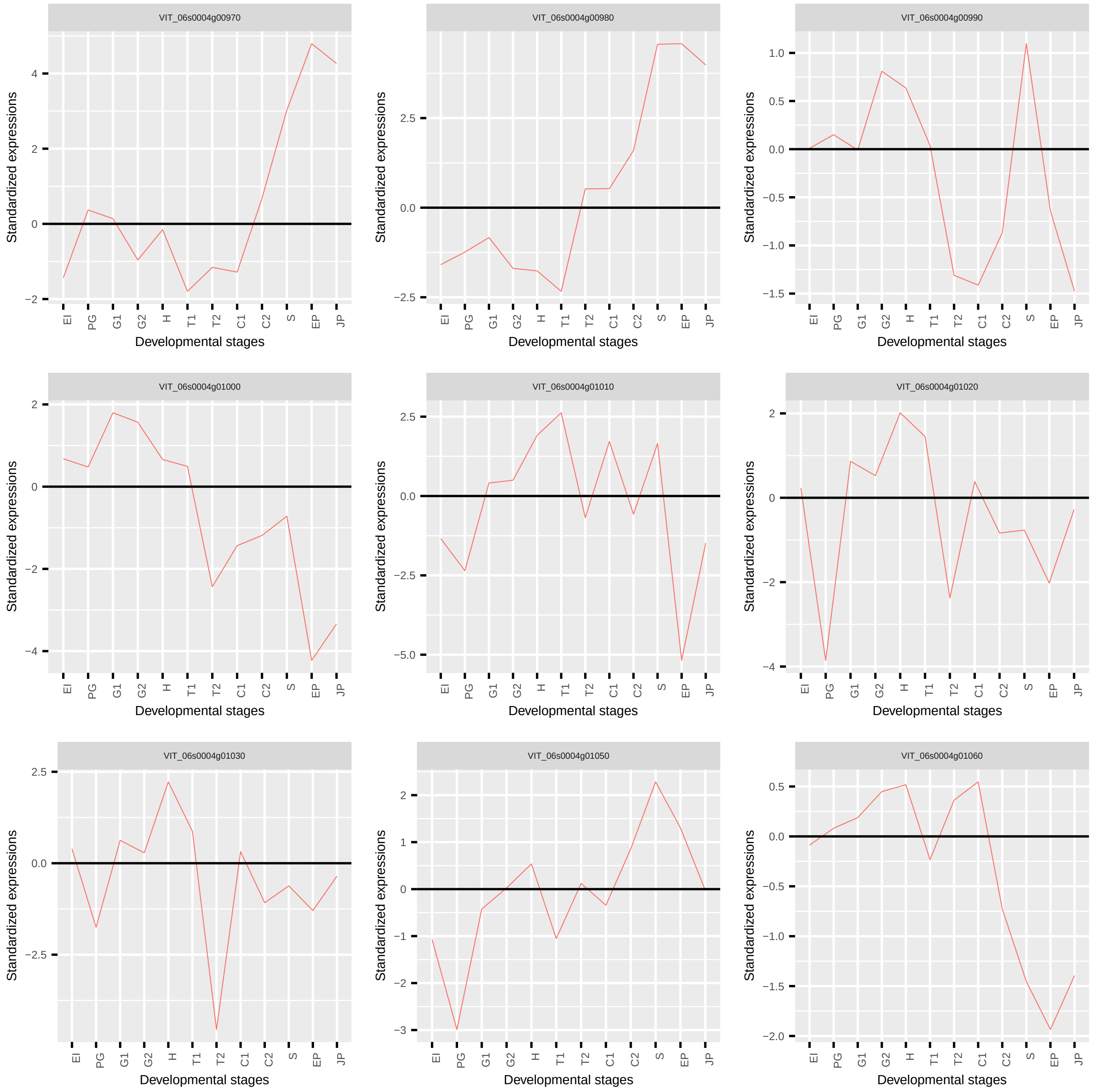
VIT_06s0004g00970
VIT_06s0004g00980
VIT_06s0004g00990
VIT_06s0004g01000
VIT_06s0004g01010
VIT_06s0004g01020
VIT_06s0004g01030
VIT_06s0004g01050
VIT_06s0004g01060
Standardized expressions
Developmental stages
EI PG G1 G2 H T1 T2 C1 C2 S EP JP

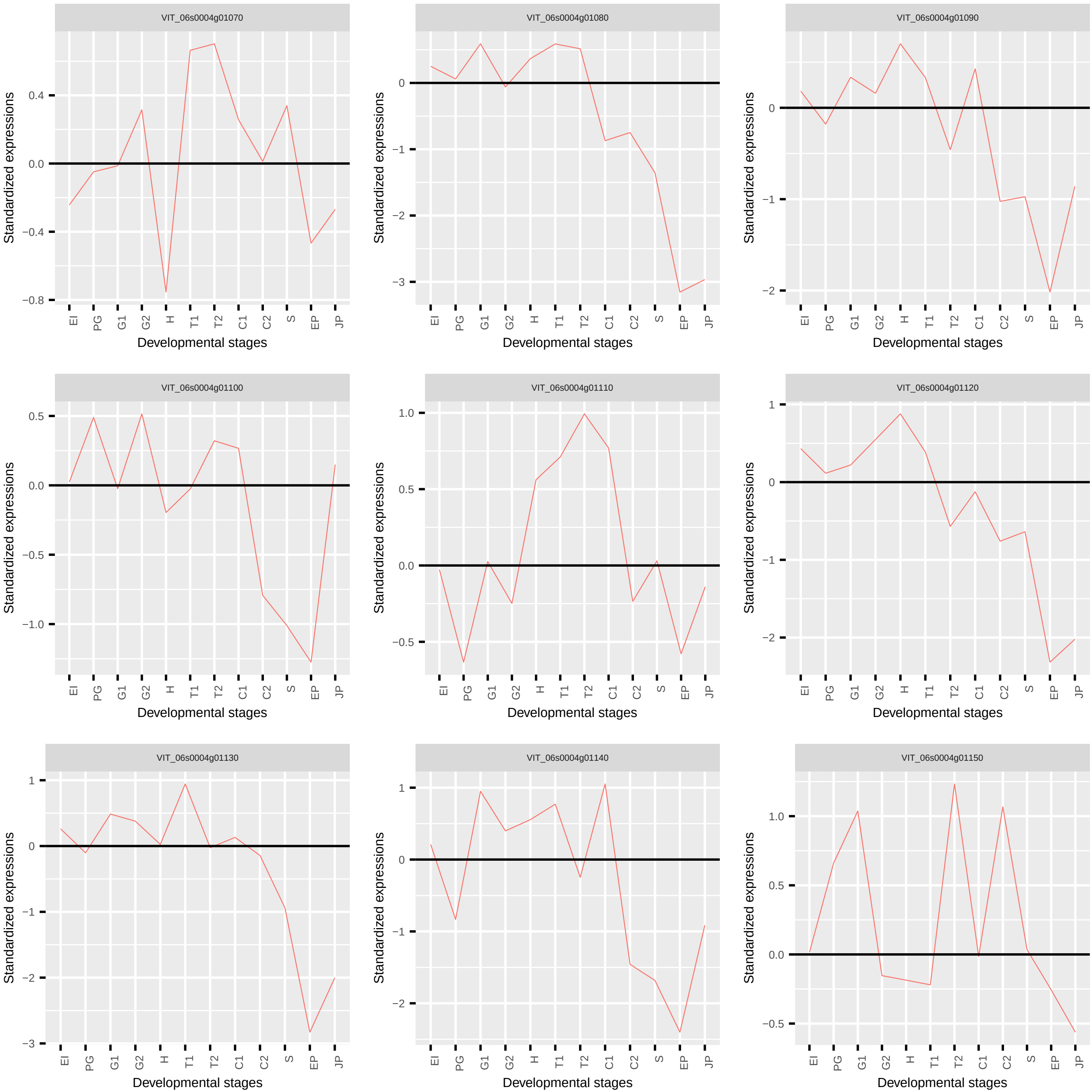

VIT_06s0004g01070
VIT_06s0004g01080
VIT_06s0004g01090
VIT_06s0004g01100
VIT_06s0004g01110
VIT_06s0004g01120
VIT_06s0004g01130
VIT_06s0004g01140
VIT_06s0004g01150
Standardized expressions
Developmental stages
EI PG G1 G2 H T1 T2 C1 C2 S EP JP

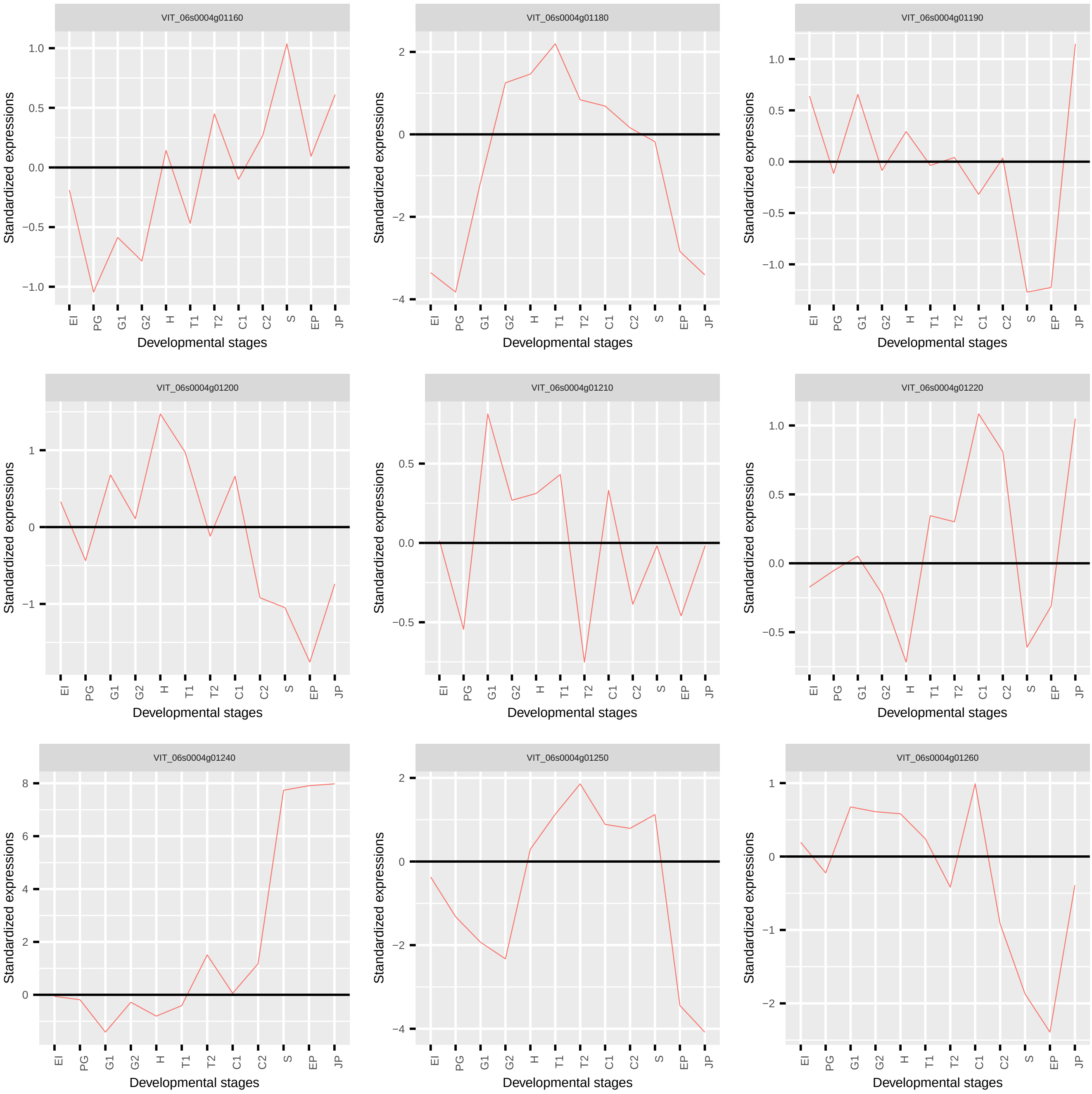
VIT_06s0004g01160
1.0
0.5
0.0
−0.5
−1.0
VIT_06s0004g01180
2
0
−2
−4
VIT_06s0004g01190
1.0
0.5
0.0
−0.5
−1.0
VIT_06s0004g01200
1
0
−1
VIT_06s0004g01210
0.5
0.0
−0.5
VIT_06s0004g01220
1.0
0.5
0.0
−0.5
VIT_06s0004g01240
8
6
4
2
0
VIT_06s0004g01250
2
0
−2
−4
VIT_06s0004g01260
1
0
−1
−2
Standardized expressions
EI
PG
G1
G2
H
T1
T2
C1
C2
S
EP
JP
Developmental stages

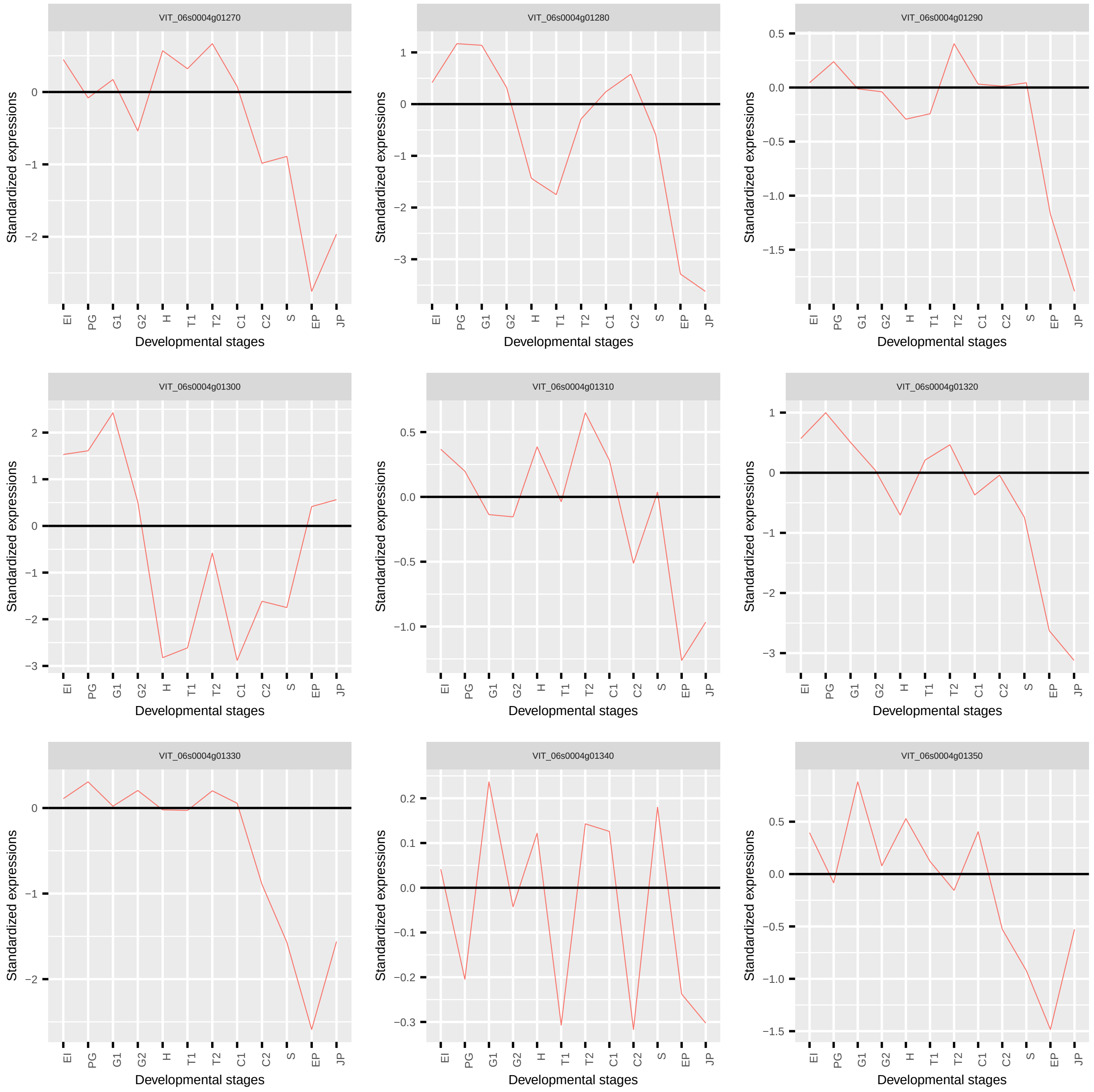
VIT_06s0004g01270
VIT_06s0004g01280
VIT_06s0004g01290
VIT_06s0004g01300
VIT_06s0004g01310
VIT_06s0004g01320
VIT_06s0004g01330
VIT_06s0004g01340
VIT_06s0004g01350
Standardized expressions
Developmental stages
EI PG G1 G2 H T1 T2 C1 C2 S EP JP

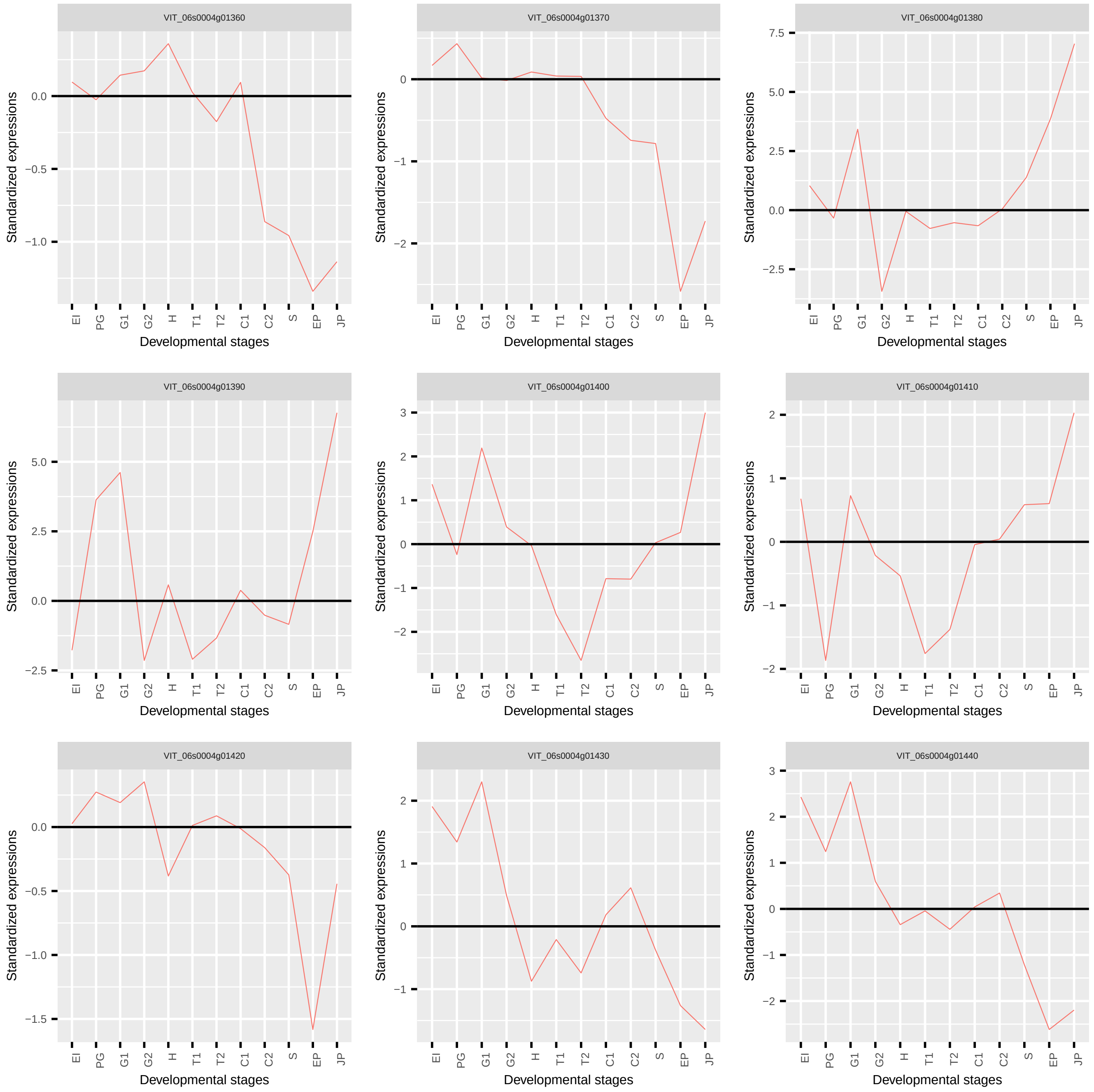
VIT_06s0004g01360
0.0
−0.5
−1.0
Standardized expressions
EI PG G1 G2 H T1 T2 C1 C2 S EP JP
Developmental stages
VIT_06s0004g01370
0
−1
−2
Standardized expressions
EI PG G1 G2 H T1 T2 C1 C2 S EP JP
Developmental stages
VIT_06s0004g01380
7.5
5.0
2.5
0.0
−2.5
Standardized expressions
EI PG G1 G2 H T1 T2 C1 C2 S EP JP
Developmental stages
VIT_06s0004g01390
5.0
2.5
0.0
−2.5
Standardized expressions
EI PG G1 G2 H T1 T2 C1 C2 S EP JP
Developmental stages
VIT_06s0004g01400
3
2
1
0
−1
−2
Standardized expressions
EI PG G1 G2 H T1 T2 C1 C2 S EP JP
Developmental stages
VIT_06s0004g01410
2
1
0
−1
−2
Standardized expressions
EI PG G1 G2 H T1 T2 C1 C2 S EP JP
Developmental stages
VIT_06s0004g01420
0.0
−0.5
−1.0
−1.5
Standardized expressions
EI PG G1 G2 H T1 T2 C1 C2 S EP JP
Developmental stages
VIT_06s0004g01430
2
1
0
−1
Standardized expressions
EI PG G1 G2 H T1 T2 C1 C2 S EP JP
Developmental stages
VIT_06s0004g01440
3
2
1
0
−1
−2
Standardized expressions
EI PG G1 G2 H T1 T2 C1 C2 S EP JP
Developmental stages

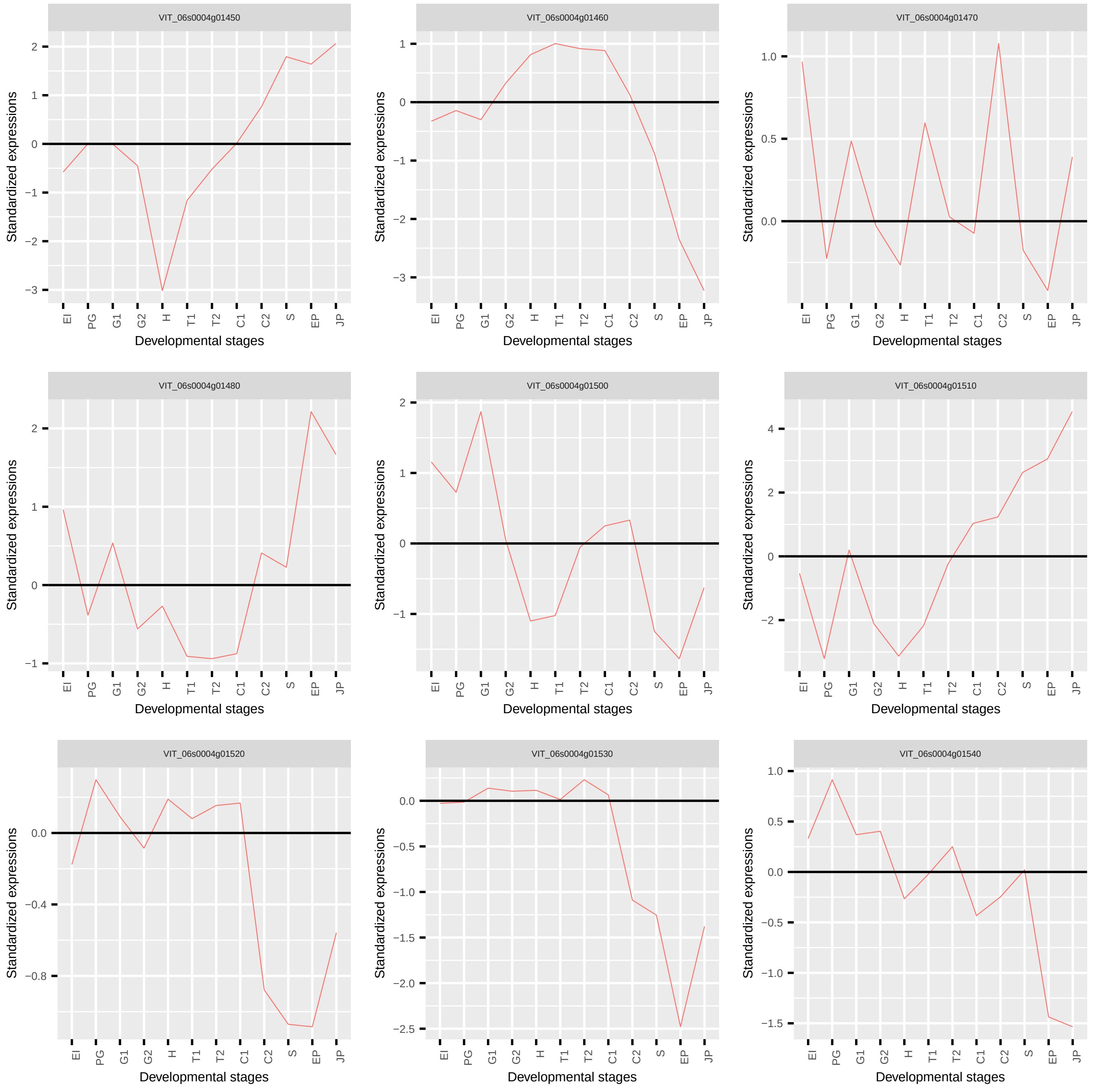
VIT_06s0004g01450
VIT_06s0004g01460
VIT_06s0004g01470
VIT_06s0004g01480
VIT_06s0004g01500
VIT_06s0004g01510
VIT_06s0004g01520
VIT_06s0004g01530
VIT_06s0004g01540
Standardized expressions
Developmental stages
EI PG G1 G2 H T1 T2 C1 C2 S EP JP

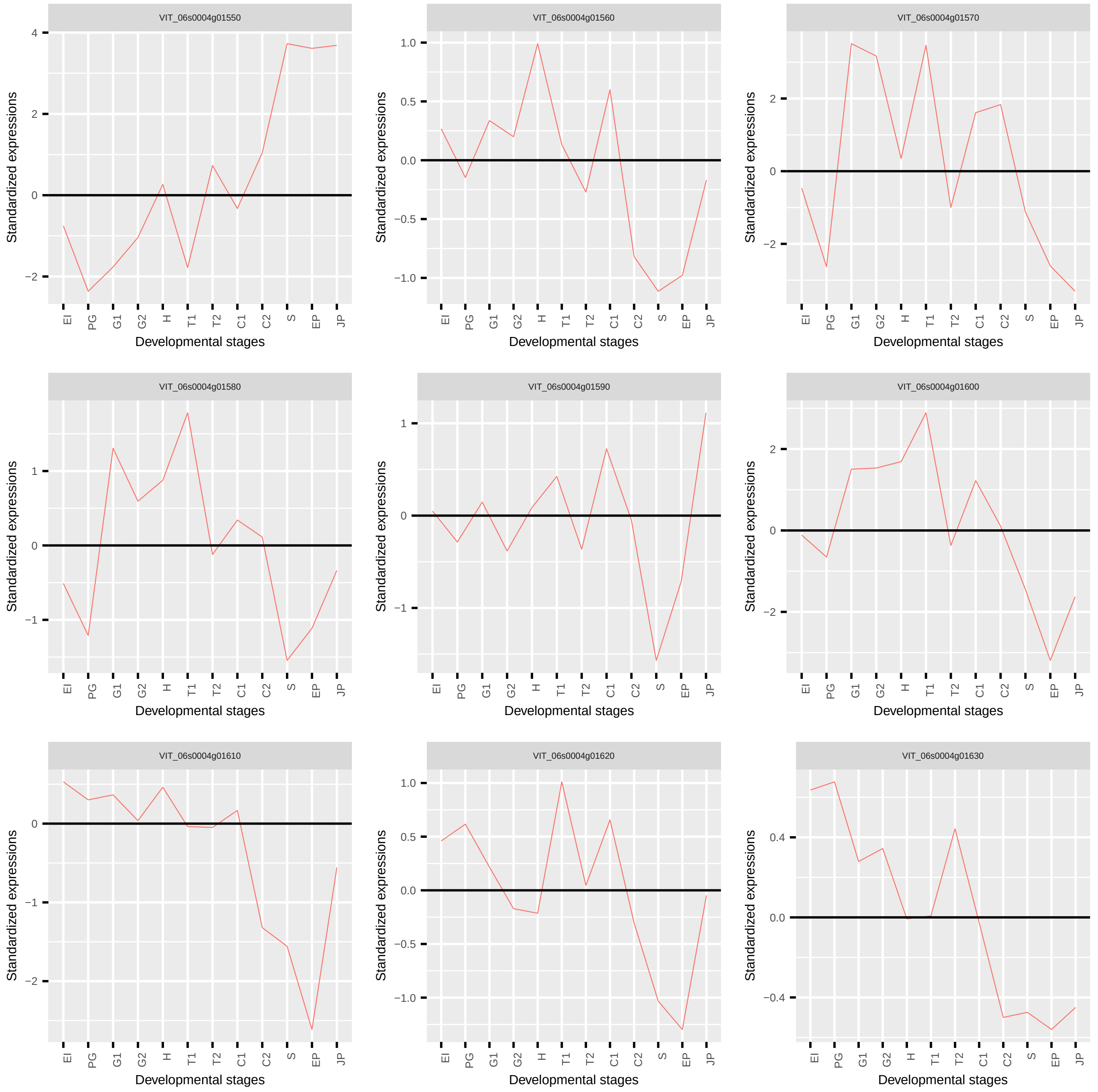
VIT_06s0004g01550
VIT_06s0004g01560
VIT_06s0004g01570
VIT_06s0004g01580
VIT_06s0004g01590
VIT_06s0004g01600
VIT_06s0004g01610
VIT_06s0004g01620
VIT_06s0004g01630
Standardized expressions
Developmental stages
EI
PG
G1
G2
H
T1
T2
C1
C2
S
EP
JP

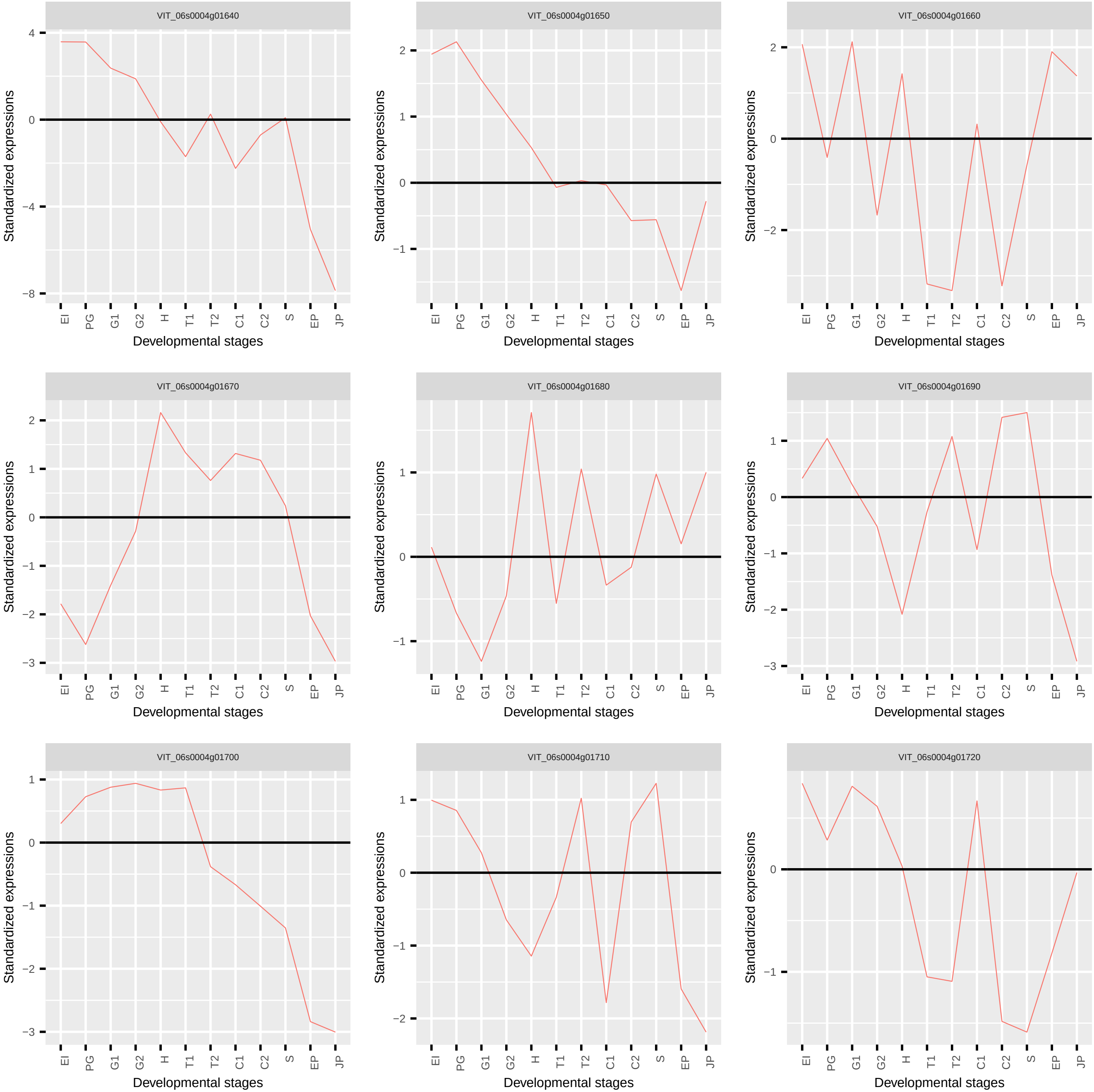
VIT_06s0004g01640
VIT_06s0004g01650
VIT_06s0004g01660
VIT_06s0004g01670
VIT_06s0004g01680
VIT_06s0004g01690
VIT_06s0004g01700
VIT_06s0004g01710
VIT_06s0004g01720
Standardized expressions
Developmental stages
EI
PG
G1
G2
H
T1
T2
C1
C2
S
EP
JP

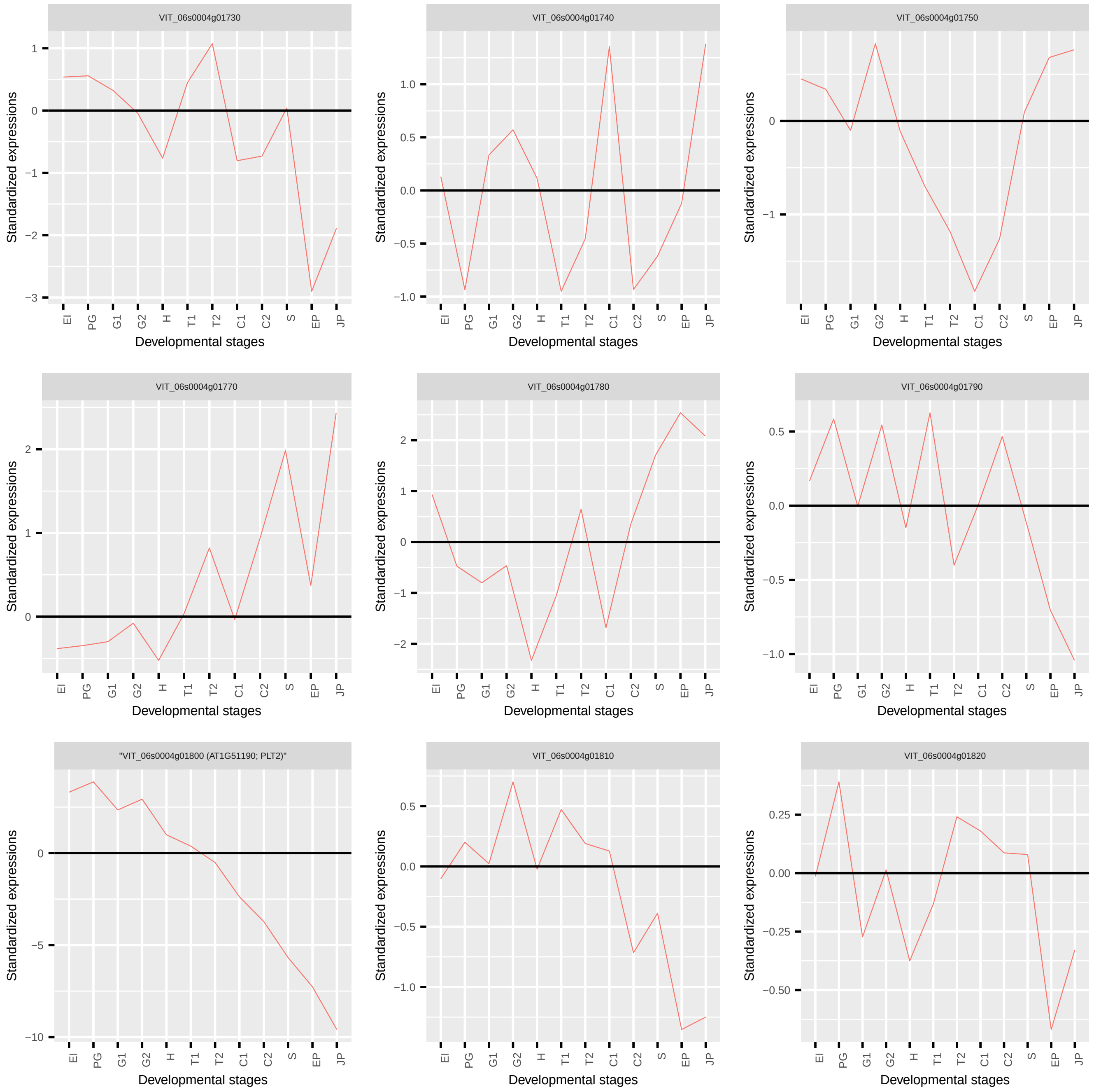
VIT_06s0004g01730
1
0
−1
−2
−3
Standardized expressions
EI PG G1 G2 H T1 T2 C1 C2 S EP JP
Developmental stages
VIT_06s0004g01740
1.0
0.5
0.0
−0.5
−1.0
Standardized expressions
EI PG G1 G2 H T1 T2 C1 C2 S EP JP
Developmental stages
VIT_06s0004g01750
0
−1
Standardized expressions
EI PG G1 G2 H T1 T2 C1 C2 S EP JP
Developmental stages
VIT_06s0004g01770
2
1
0
Standardized expressions
EI PG G1 G2 H T1 T2 C1 C2 S EP JP
Developmental stages
VIT_06s0004g01780
2
1
0
−1
−2
Standardized expressions
EI PG G1 G2 H T1 T2 C1 C2 S EP JP
Developmental stages
VIT_06s0004g01790
0.5
0.0
−0.5
−1.0
Standardized expressions
EI PG G1 G2 H T1 T2 C1 C2 S EP JP
Developmental stages
"VIT_06s0004g01800 (AT1G51190; PLT2)"
0
−5
−10
Standardized expressions
EI PG G1 G2 H T1 T2 C1 C2 S EP JP
Developmental stages
VIT_06s0004g01810
0.5
0.0
−0.5
−1.0
Standardized expressions
EI PG G1 G2 H T1 T2 C1 C2 S EP JP
Developmental stages
VIT_06s0004g01820
0.25
0.00
−0.25
−0.50
Standardized expressions
EI PG G1 G2 H T1 T2 C1 C2 S EP JP
Developmental stages

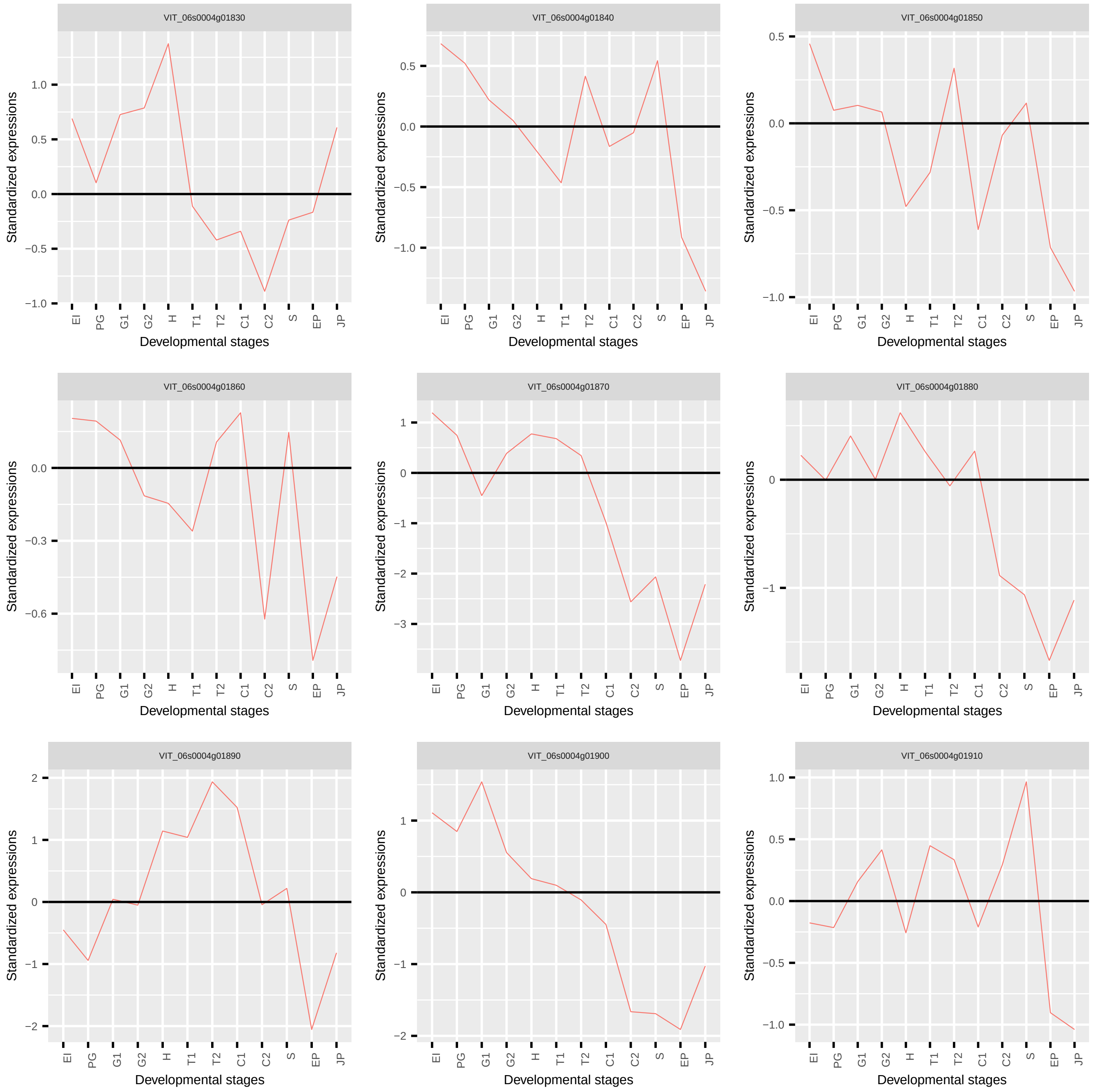
VIT_06s0004g01830
Standardized expressions
−1.0 −0.5 0.0 0.5 1.0
EI PG G1 G2 H T1 T2 C1 C2 S EP JP
Developmental stages
VIT_06s0004g01840
Standardized expressions
−1.0 −0.5 0.0 0.5
EI PG G1 G2 H T1 T2 C1 C2 S EP JP
Developmental stages
VIT_06s0004g01850
Standardized expressions
−1.0 −0.5 0.0 0.5
EI PG G1 G2 H T1 T2 C1 C2 S EP JP
Developmental stages
VIT_06s0004g01860
Standardized expressions
−0.6 −0.3 0.0
EI PG G1 G2 H T1 T2 C1 C2 S EP JP
Developmental stages
VIT_06s0004g01870
Standardized expressions
−3 −2 −1 0 1
EI PG G1 G2 H T1 T2 C1 C2 S EP JP
Developmental stages
VIT_06s0004g01880
Standardized expressions
−1 0
EI PG G1 G2 H T1 T2 C1 C2 S EP JP
Developmental stages
VIT_06s0004g01890
Standardized expressions
−2 −1 0 1 2
EI PG G1 G2 H T1 T2 C1 C2 S EP JP
Developmental stages
VIT_06s0004g01900
Standardized expressions
−2 −1 0 1
EI PG G1 G2 H T1 T2 C1 C2 S EP JP
Developmental stages
VIT_06s0004g01910
Standardized expressions
−1.0 −0.5 0.0 0.5 1.0
EI PG G1 G2 H T1 T2 C1 C2 S EP JP
Developmental stages

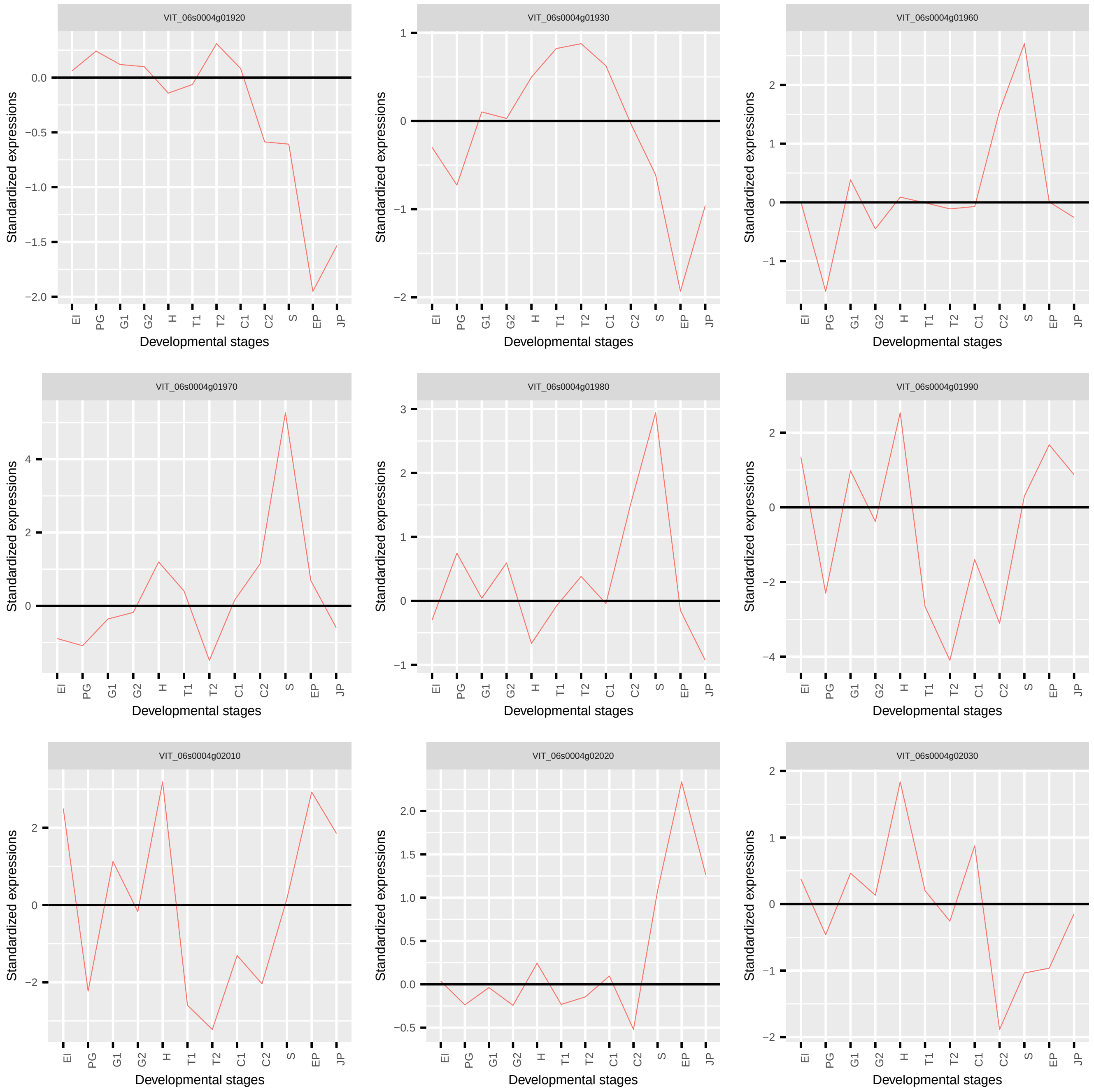

VIT_06s0004g01920
VIT_06s0004g01930
VIT_06s0004g01960
VIT_06s0004g01970
VIT_06s0004g01980
VIT_06s0004g01990
VIT_06s0004g02010
VIT_06s0004g02020
VIT_06s0004g02030
Standardized expressions
Developmental stages
EI PG G1 G2 H T1 T2 C1 C2 S EP JP

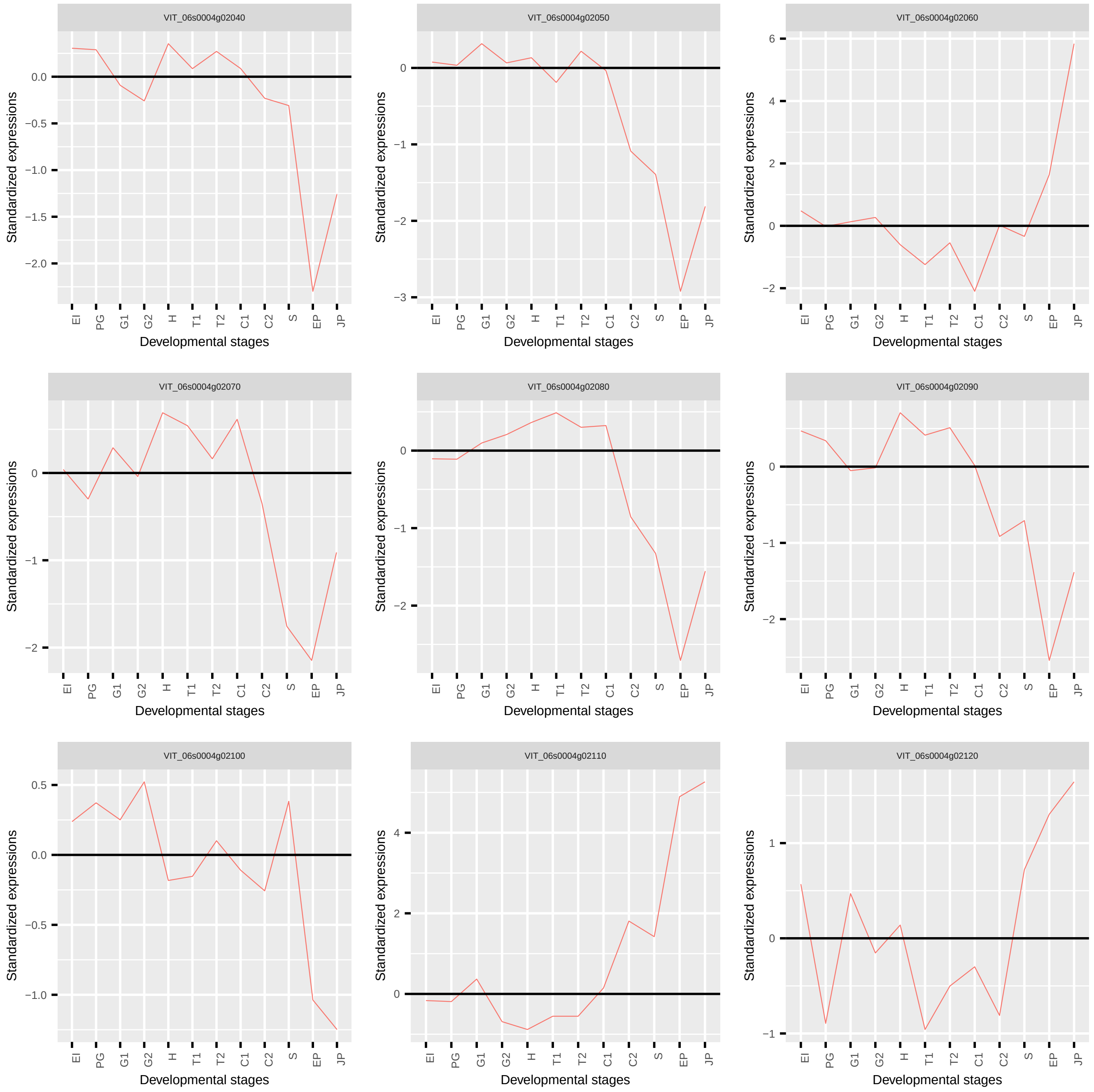
VIT_06s0004g02040
VIT_06s0004g02050
VIT_06s0004g02060
VIT_06s0004g02070
VIT_06s0004g02080
VIT_06s0004g02090
VIT_06s0004g02100
VIT_06s0004g02110
VIT_06s0004g02120
Standardized expressions
Developmental stages
EI PG G1 G2 H T1 T2 C1 C2 S EP JP

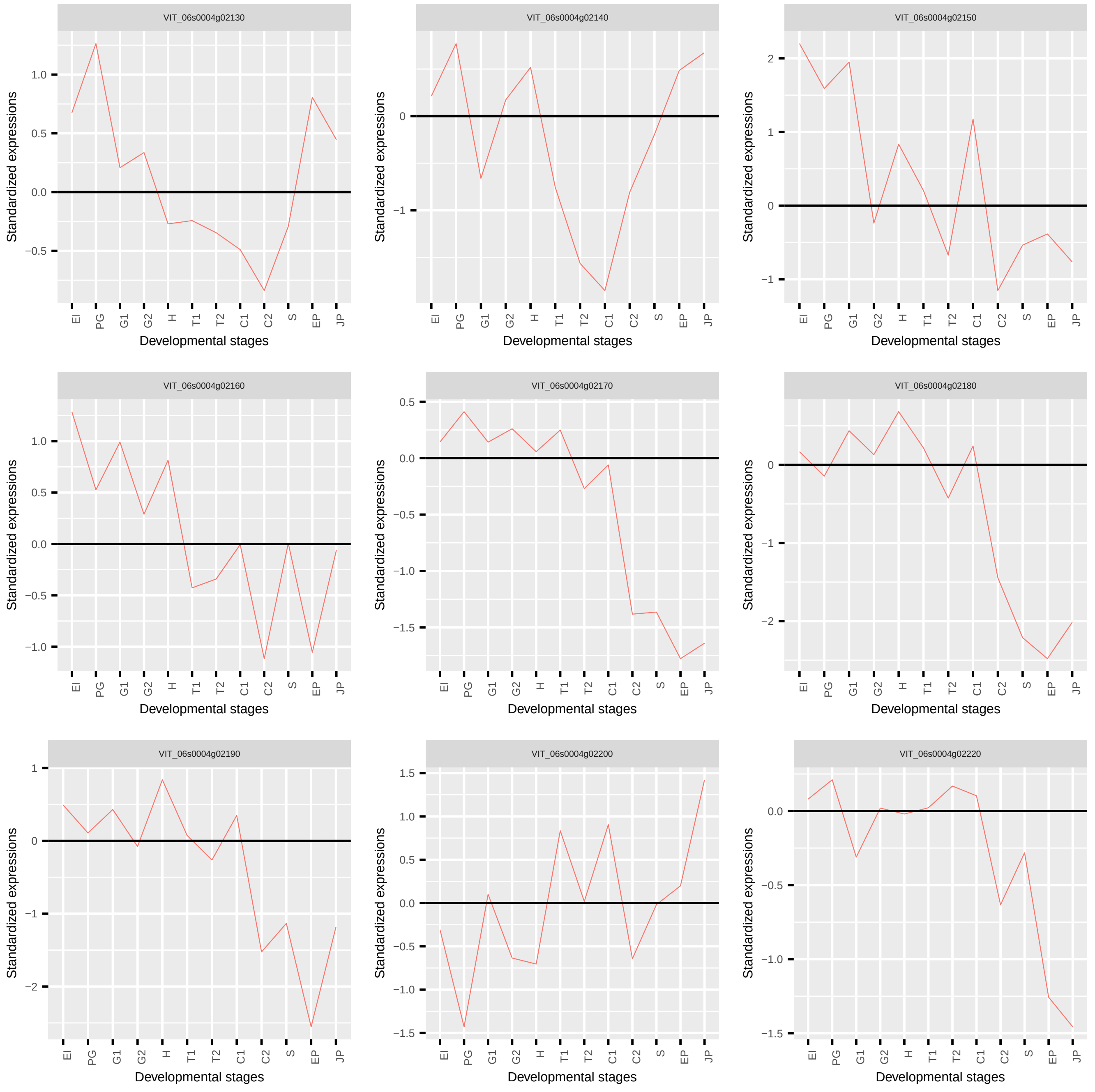
VIT_06s0004g02130
VIT_06s0004g02140
VIT_06s0004g02150
VIT_06s0004g02160
VIT_06s0004g02170
VIT_06s0004g02180
VIT_06s0004g02190
VIT_06s0004g02200
VIT_06s0004g02220
Standardized expressions
Developmental stages
EI PG G1 G2 H T1 T2 C1 C2 S EP JP

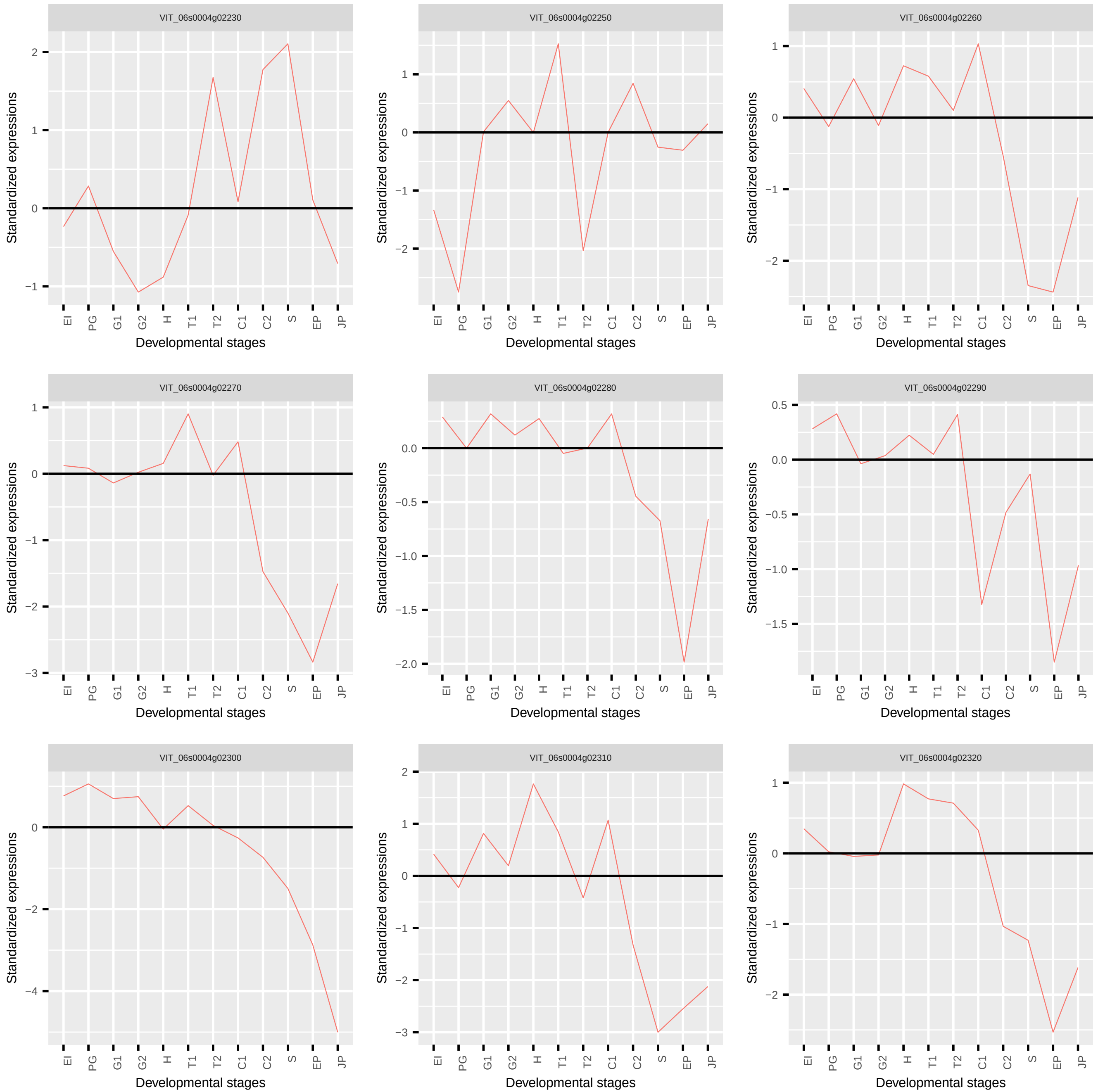
VIT_06s0004g02230
VIT_06s0004g02250
VIT_06s0004g02260
VIT_06s0004g02270
VIT_06s0004g02280
VIT_06s0004g02290
VIT_06s0004g02300
VIT_06s0004g02310
VIT_06s0004g02320
Standardized expressions
Developmental stages
EI PG G1 G2 H T1 T2 C1 C2 S EP JP

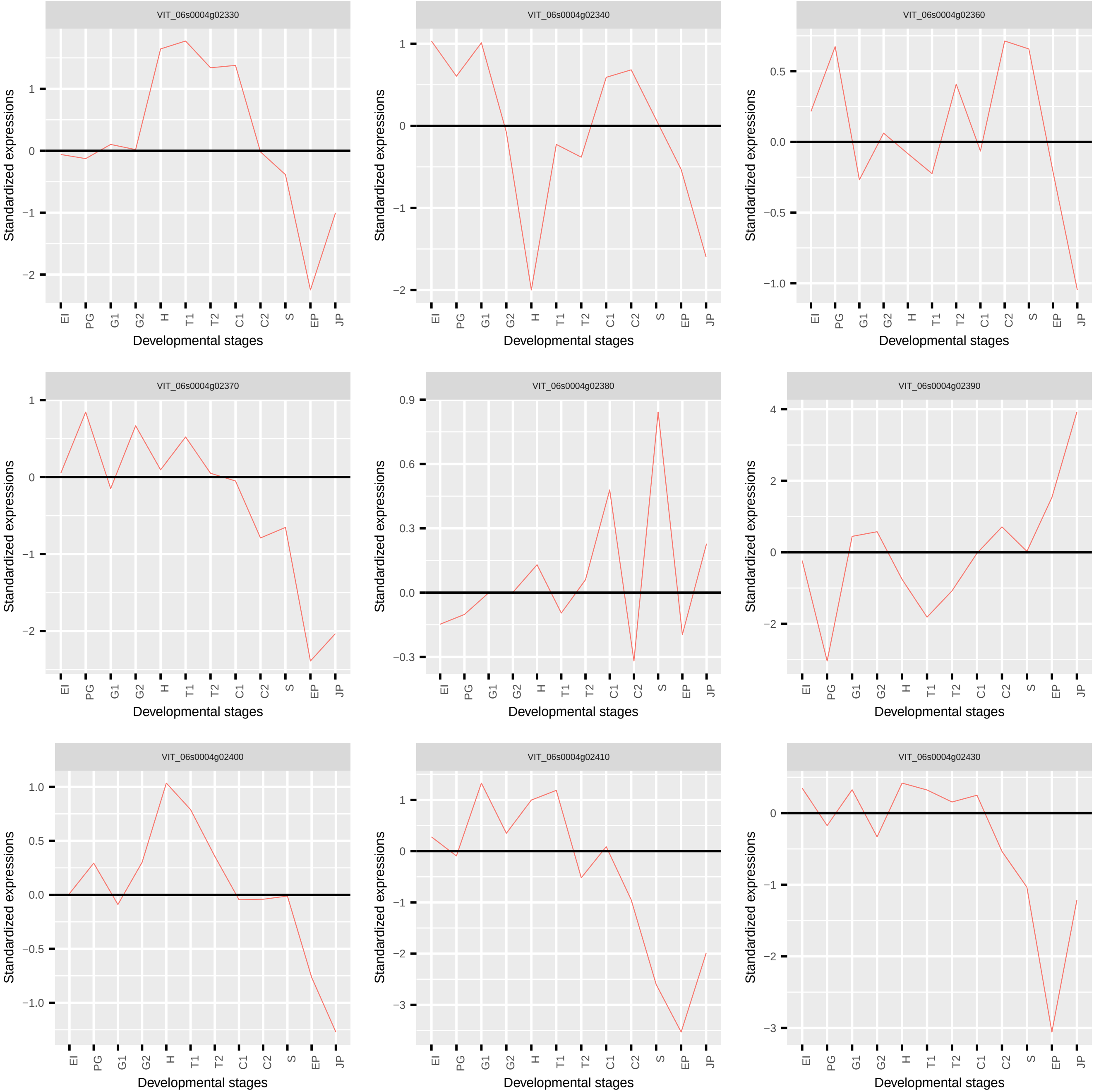
VIT_06s0004g02330
Standardized expressions
Developmental stages
EI PG G1 G2 H T1 T2 C1 C2 S EP JP
VIT_06s0004g02340
Standardized expressions
Developmental stages
EI PG G1 G2 H T1 T2 C1 C2 S EP JP
VIT_06s0004g02360
Standardized expressions
Developmental stages
EI PG G1 G2 H T1 T2 C1 C2 S EP JP
VIT_06s0004g02370
Standardized expressions
Developmental stages
EI PG G1 G2 H T1 T2 C1 C2 S EP JP
VIT_06s0004g02380
Standardized expressions
Developmental stages
EI PG G1 G2 H T1 T2 C1 C2 S EP JP
VIT_06s0004g02390
Standardized expressions
Developmental stages
EI PG G1 G2 H T1 T2 C1 C2 S EP JP
VIT_06s0004g02400
Standardized expressions
Developmental stages
EI PG G1 G2 H T1 T2 C1 C2 S EP JP
VIT_06s0004g02410
Standardized expressions
Developmental stages
EI PG G1 G2 H T1 T2 C1 C2 S EP JP
VIT_06s0004g02430
Standardized expressions
Developmental stages
EI PG G1 G2 H T1 T2 C1 C2 S EP JP

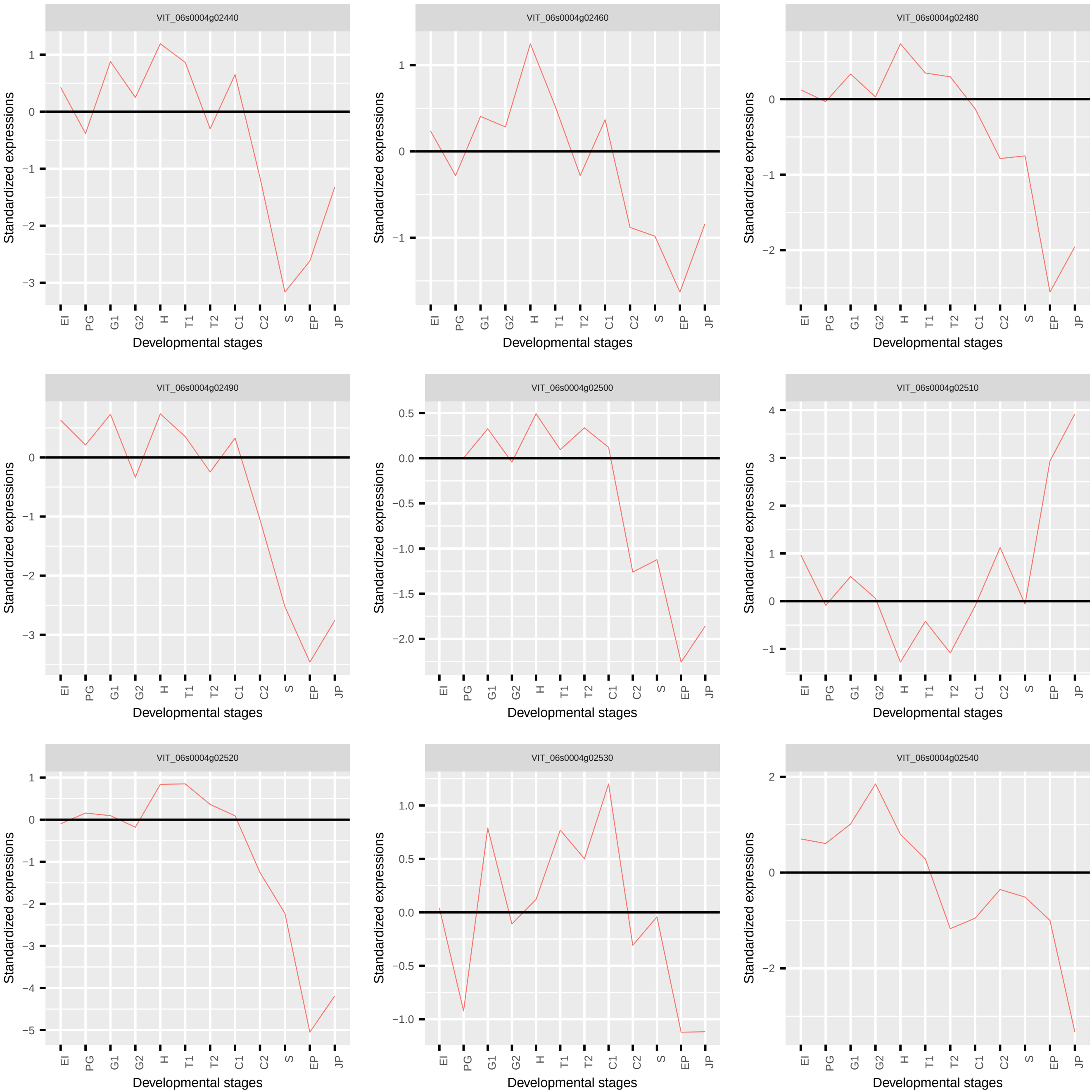
VIT_06s0004g02440
VIT_06s0004g02460
VIT_06s0004g02480
VIT_06s0004g02490
VIT_06s0004g02500
VIT_06s0004g02510
VIT_06s0004g02520
VIT_06s0004g02530
VIT_06s0004g02540
Standardized expressions
Developmental stages
EI PG G1 G2 H T1 T2 C1 C2 S EP JP

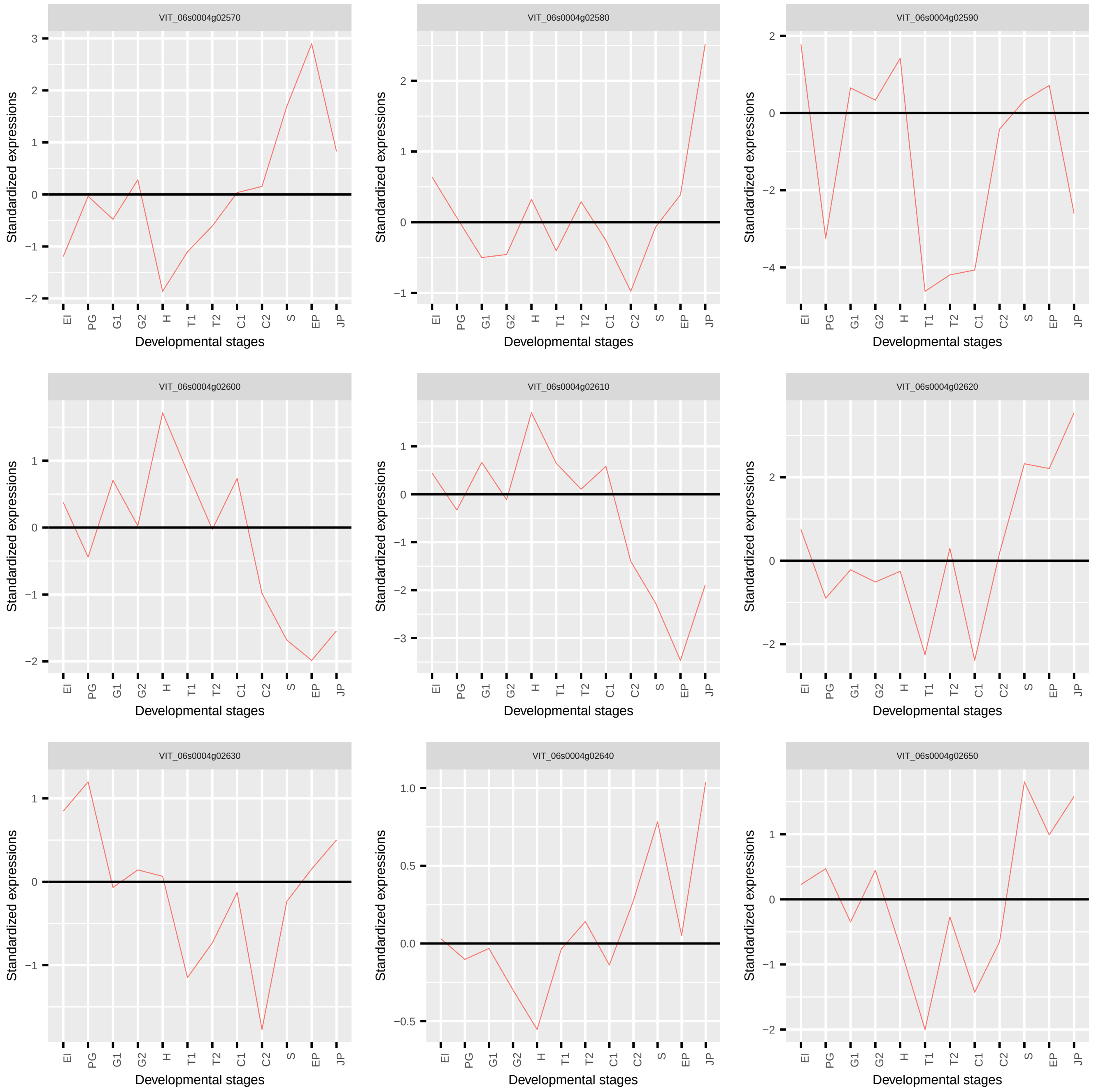

VIT_06s0004g02570
VIT_06s0004g02580
VIT_06s0004g02590
VIT_06s0004g02600
VIT_06s0004g02610
VIT_06s0004g02620
VIT_06s0004g02630
VIT_06s0004g02640
VIT_06s0004g02650
Standardized expressions
Developmental stages
EI PG G1 G2 H T1 T2 C1 C2 S EP JP

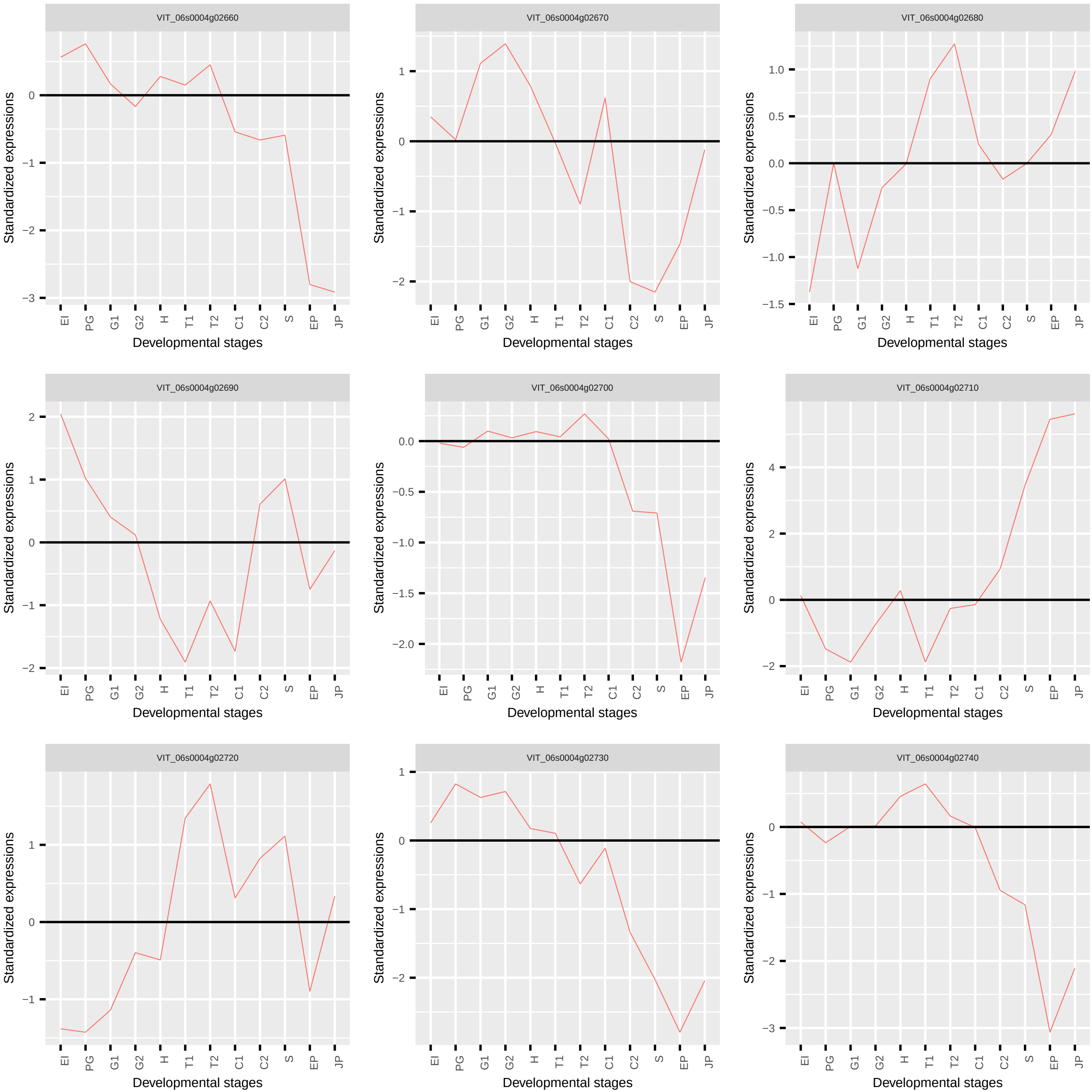
VIT_06s0004g02660
VIT_06s0004g02670
VIT_06s0004g02680
VIT_06s0004g02690
VIT_06s0004g02700
VIT_06s0004g02710
VIT_06s0004g02720
VIT_06s0004g02730
VIT_06s0004g02740
Standardized expressions
Developmental stages
EI PG G1 G2 H T1 T2 C1 C2 S EP JP

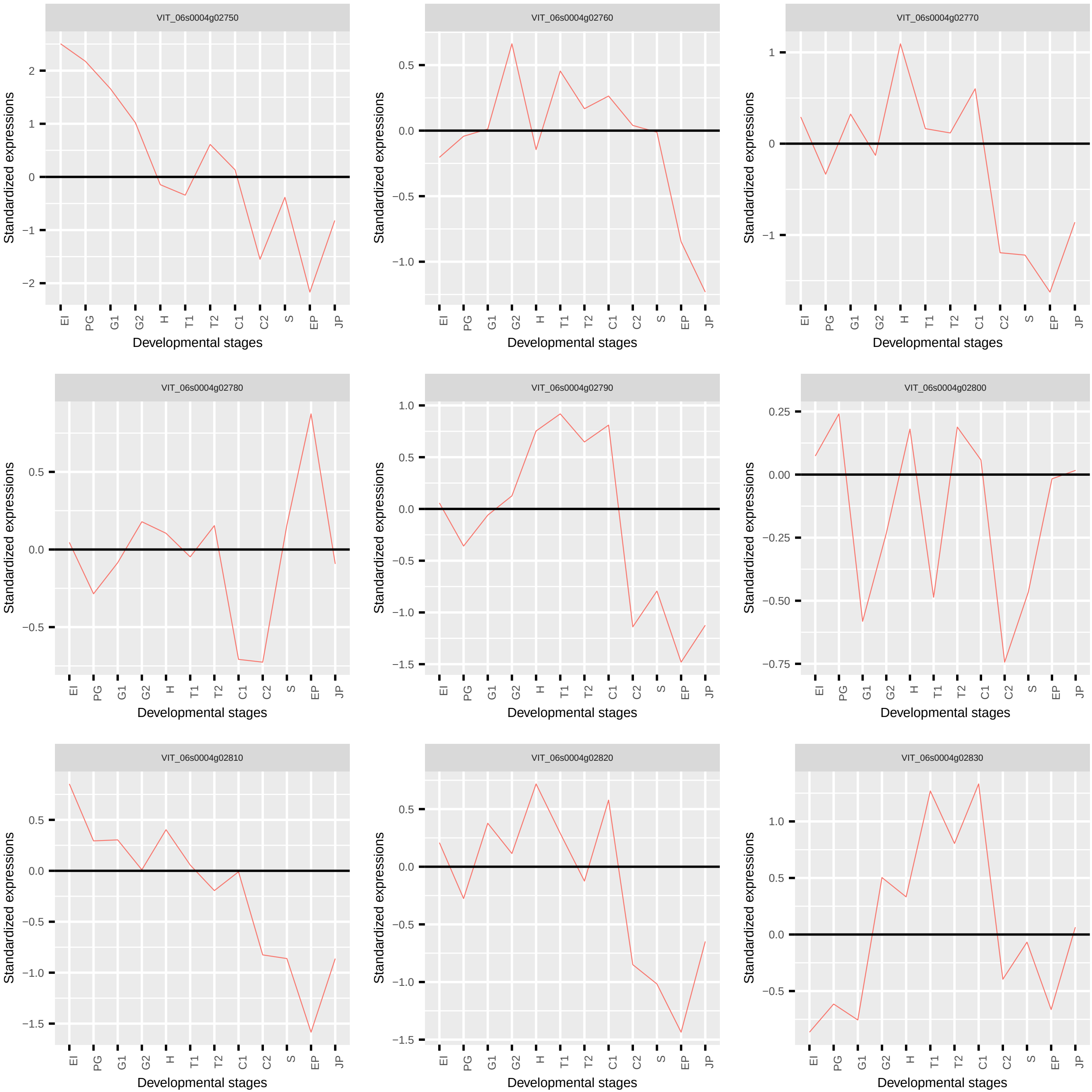
VIT_06s0004g02750
VIT_06s0004g02760
VIT_06s0004g02770
VIT_06s0004g02780
VIT_06s0004g02790
VIT_06s0004g02800
VIT_06s0004g02810
VIT_06s0004g02820
VIT_06s0004g02830
Standardized expressions
Developmental stages
EI PG G1 G2 H T1 T2 C1 C2 S EP JP

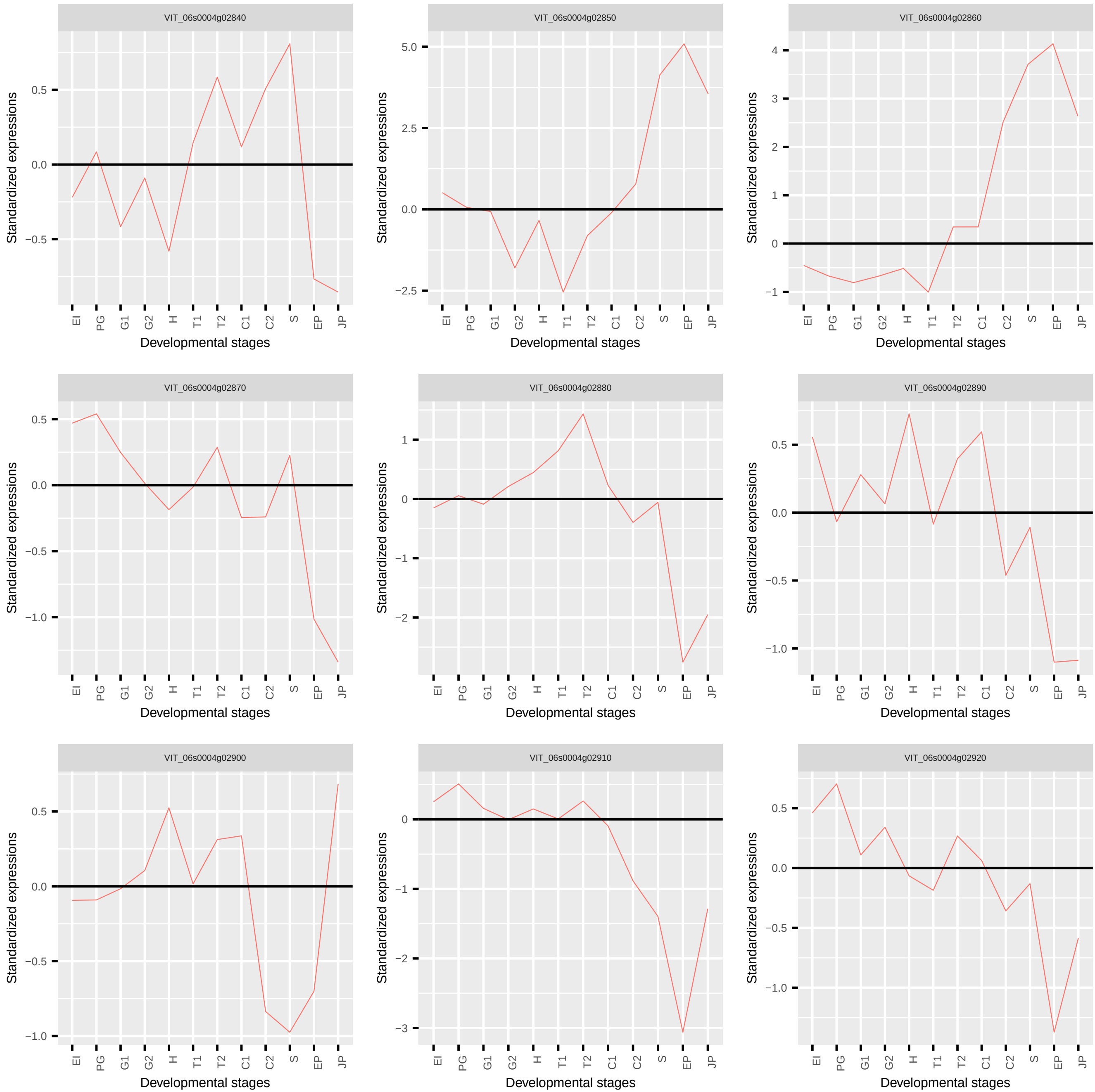
VIT_06s0004g02840
VIT_06s0004g02850
VIT_06s0004g02860
VIT_06s0004g02870
VIT_06s0004g02880
VIT_06s0004g02890
VIT_06s0004g02900
VIT_06s0004g02910
VIT_06s0004g02920
Standardized expressions
Developmental stages
EI
PG
G1
G2
H
T1
T2
C1
C2
S
EP
JP

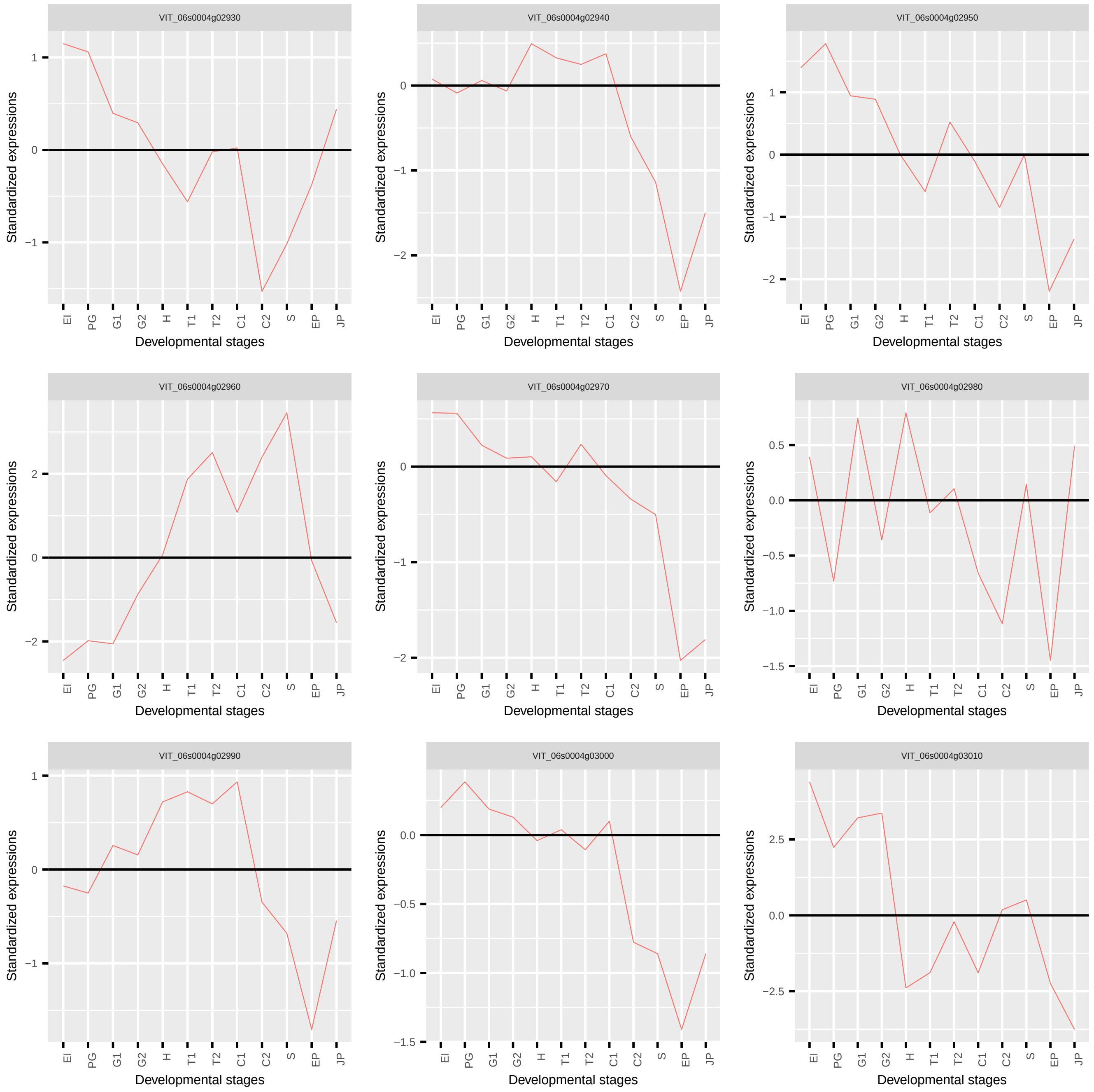
VIT_06s0004g02930
VIT_06s0004g02940
VIT_06s0004g02950
VIT_06s0004g02960
VIT_06s0004g02970
VIT_06s0004g02980
VIT_06s0004g02990
VIT_06s0004g03000
VIT_06s0004g03010
Standardized expressions
Developmental stages
EI PG G1 G2 H T1 T2 C1 C2 S EP JP

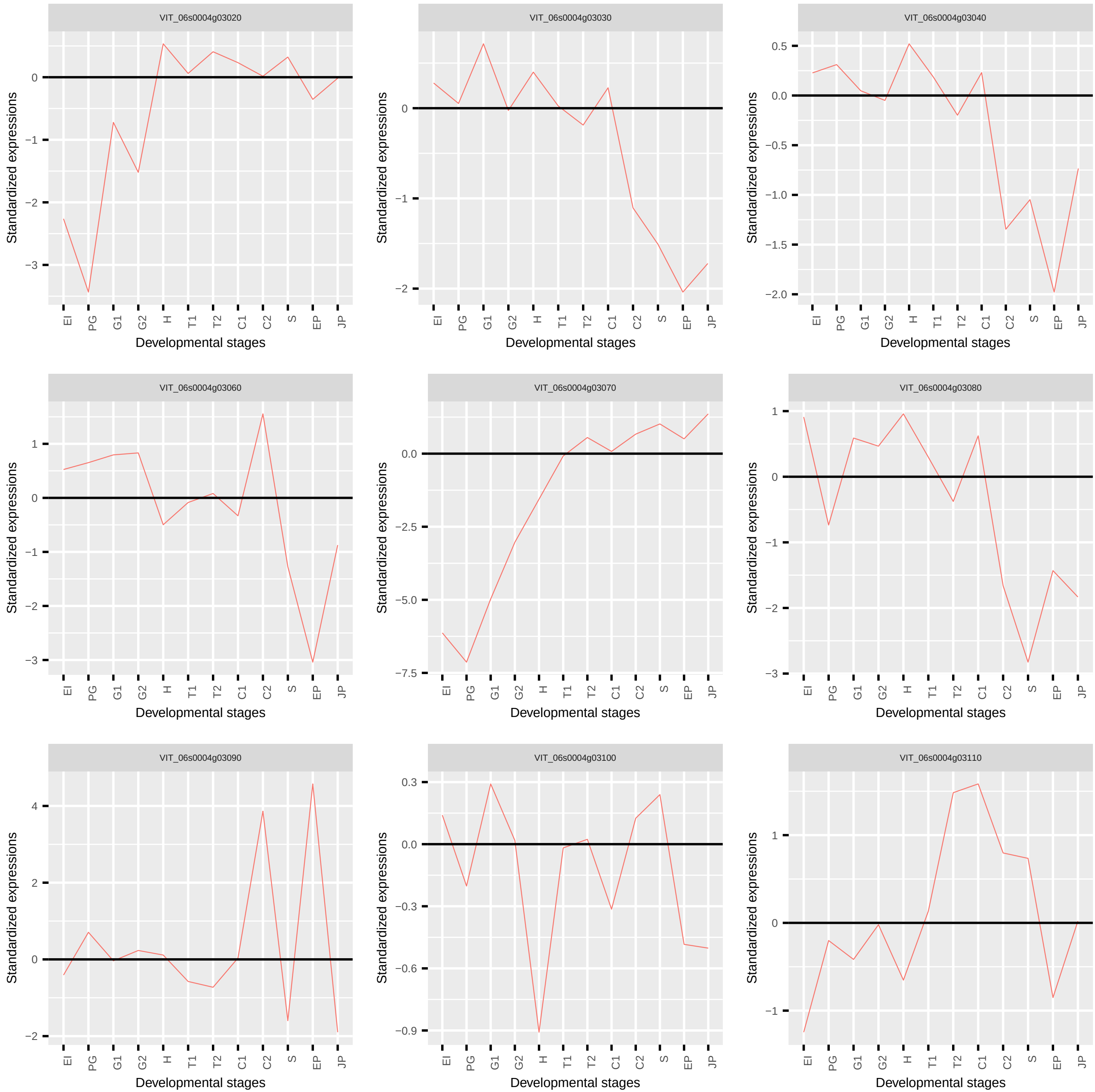
VIT_06s0004g03020
VIT_06s0004g03030
VIT_06s0004g03040
VIT_06s0004g03060
VIT_06s0004g03070
VIT_06s0004g03080
VIT_06s0004g03090
VIT_06s0004g03100
VIT_06s0004g03110
Standardized expressions
Developmental stages
EI PG G1 G2 H T1 T2 C1 C2 S EP JP

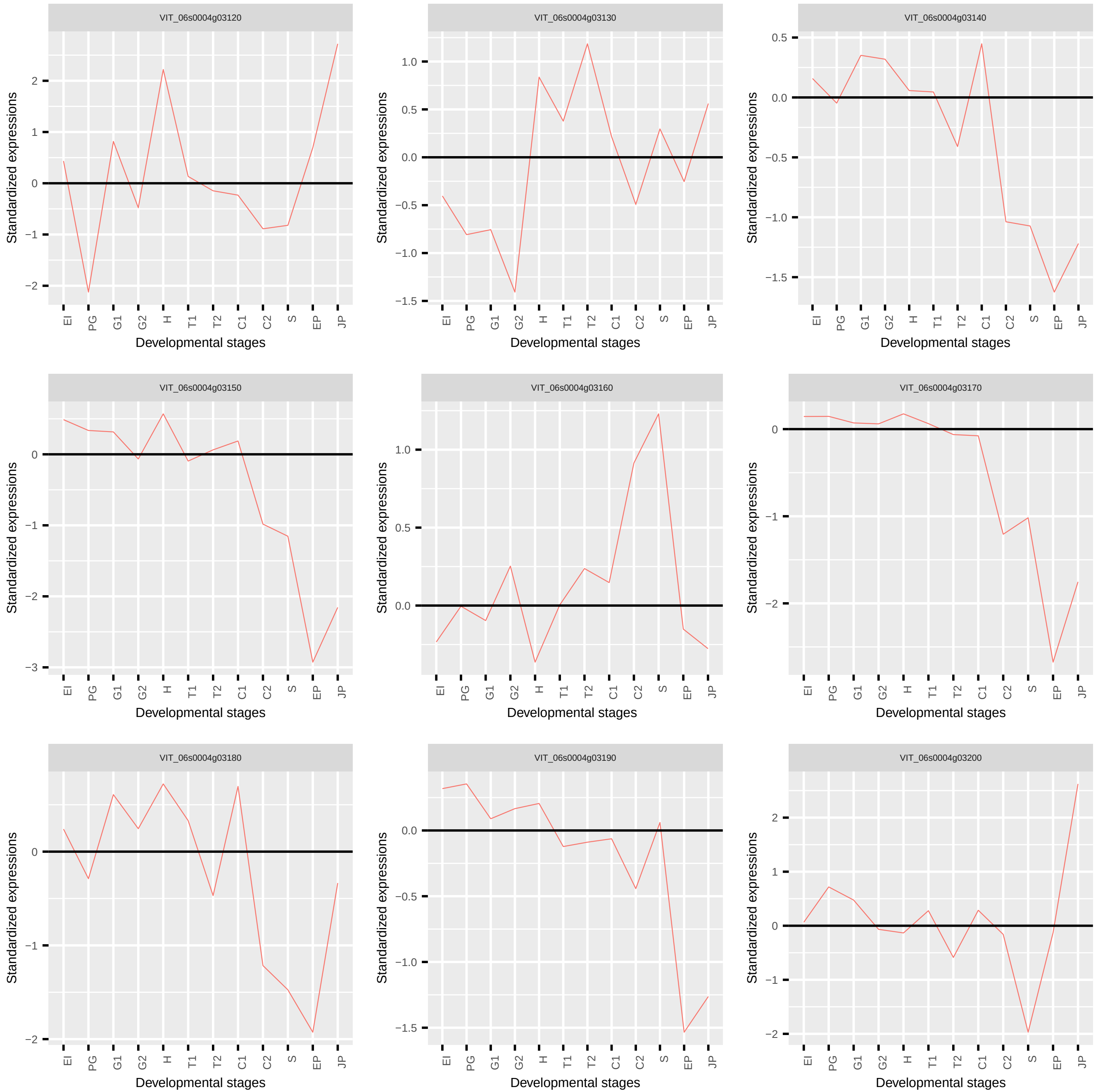
VIT_06s0004g03120
VIT_06s0004g03130
VIT_06s0004g03140
VIT_06s0004g03150
VIT_06s0004g03160
VIT_06s0004g03170
VIT_06s0004g03180
VIT_06s0004g03190
VIT_06s0004g03200
Standardized expressions
Developmental stages
EI PG G1 G2 H T1 T2 C1 C2 S EP JP

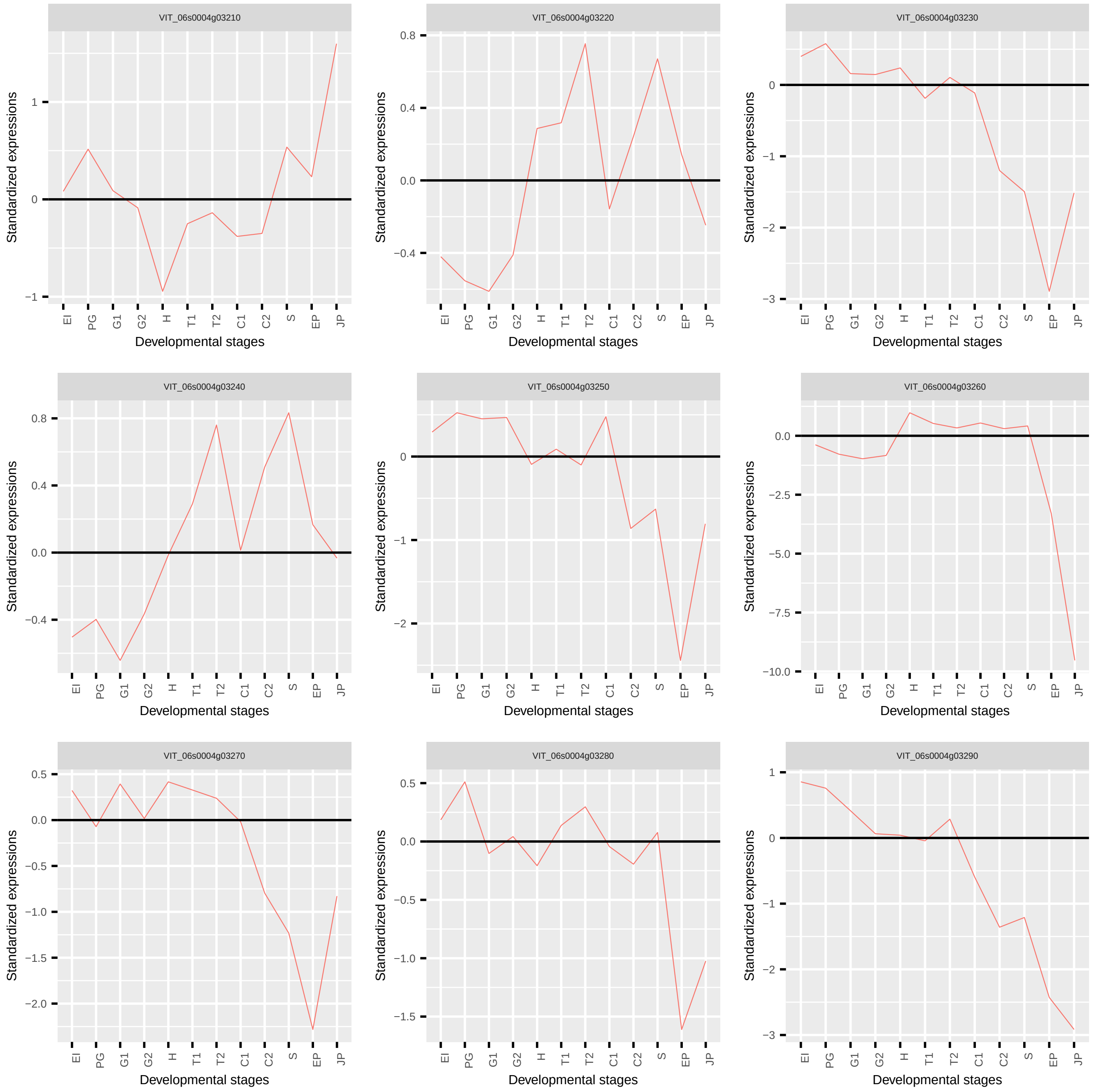

VIT_06s0004g03210
VIT_06s0004g03220
VIT_06s0004g03230
VIT_06s0004g03240
VIT_06s0004g03250
VIT_06s0004g03260
VIT_06s0004g03270
VIT_06s0004g03280
VIT_06s0004g03290
Standardized expressions
Developmental stages
EI PG G1 G2 H T1 T2 C1 C2 S EP JP

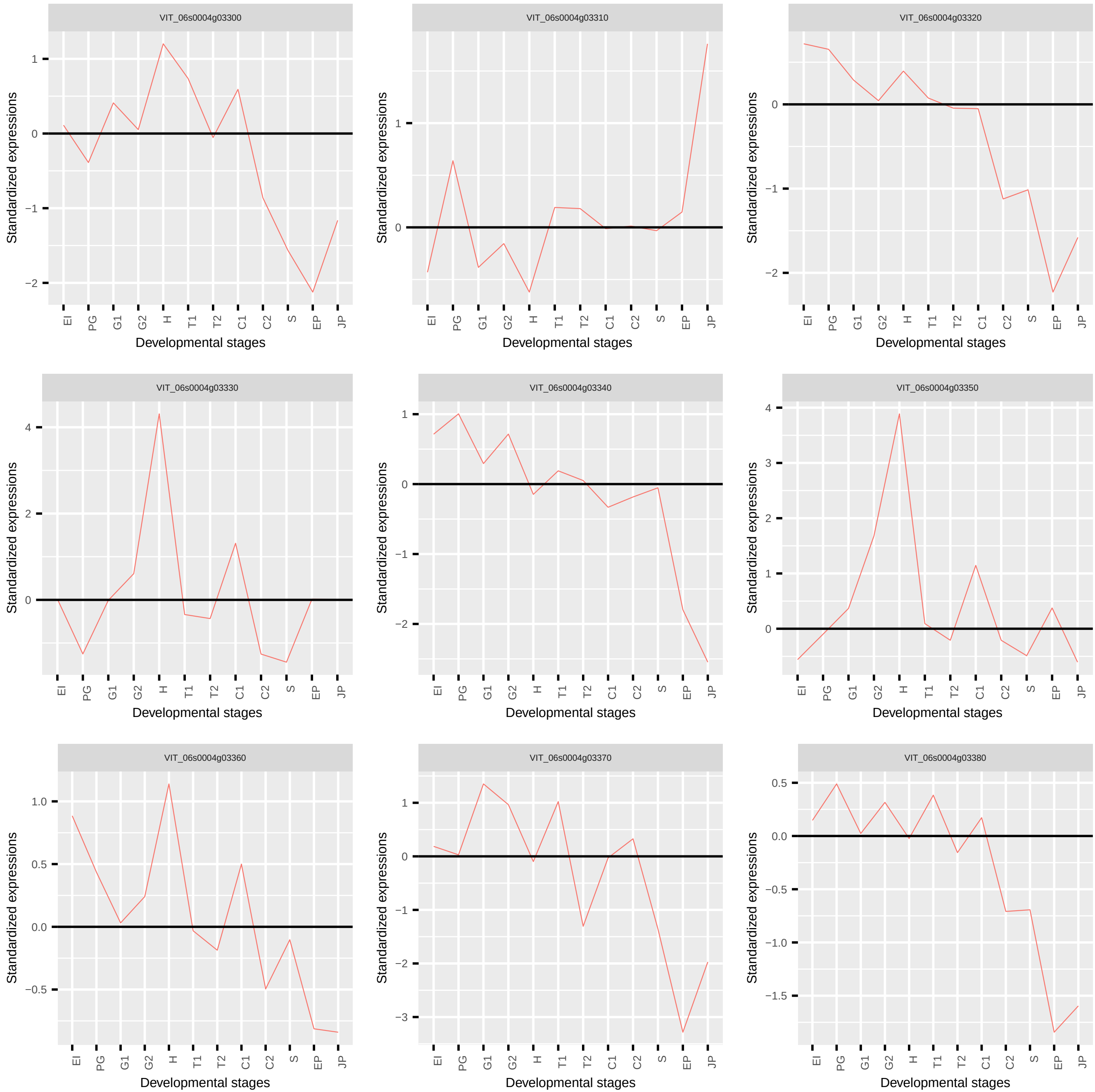
VIT_06s0004g03300
VIT_06s0004g03310
VIT_06s0004g03320
VIT_06s0004g03330
VIT_06s0004g03340
VIT_06s0004g03350
VIT_06s0004g03360
VIT_06s0004g03370
VIT_06s0004g03380
Standardized expressions
Developmental stages
EI PG G1 G2 H T1 T2 C1 C2 S EP JP

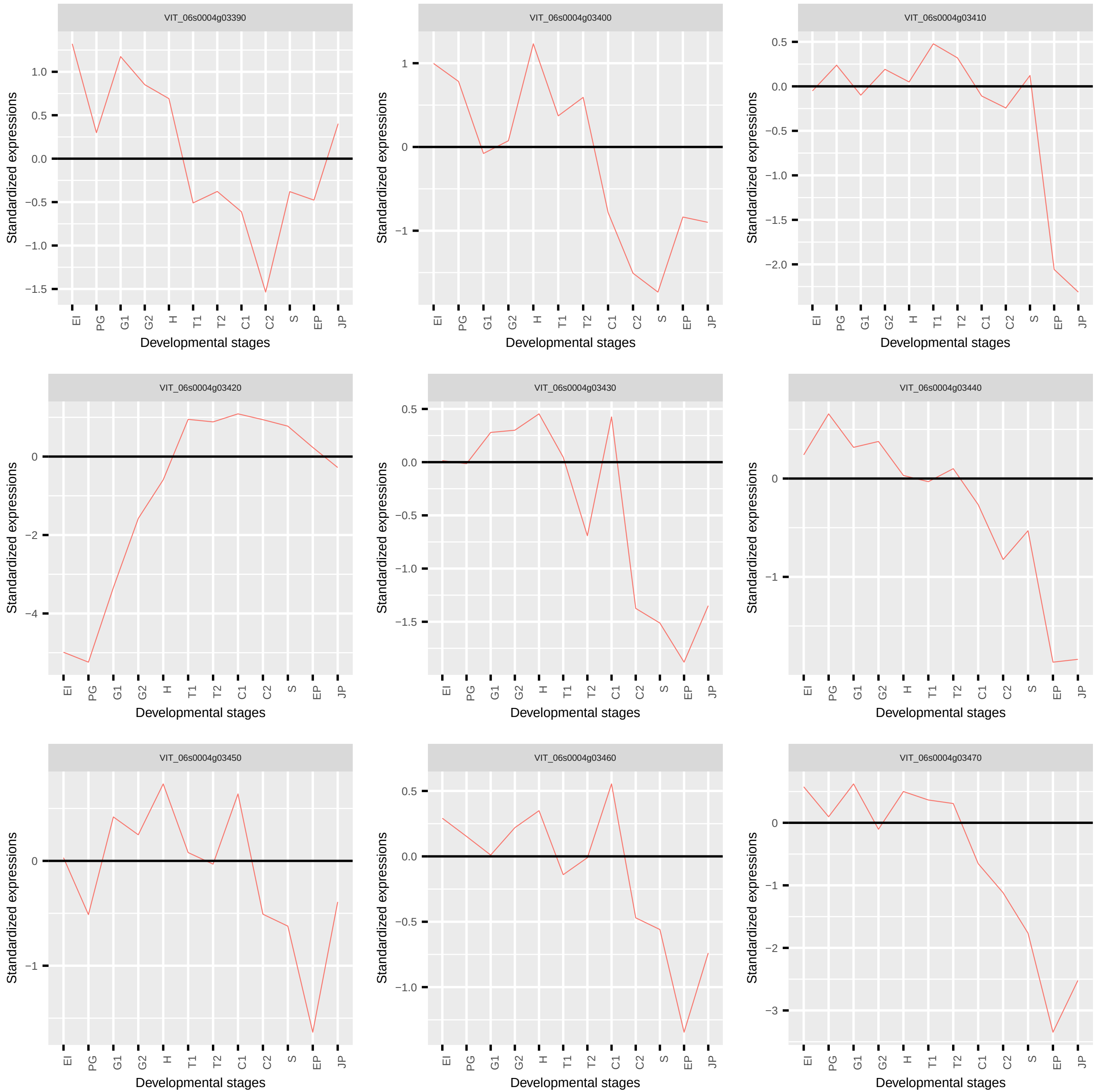
VIT_06s0004g03390
VIT_06s0004g03400
VIT_06s0004g03410
VIT_06s0004g03420
VIT_06s0004g03430
VIT_06s0004g03440
VIT_06s0004g03450
VIT_06s0004g03460
VIT_06s0004g03470
Standardized expressions
Developmental stages
EI
PG
G1
G2
H
T1
T2
C1
C2
S
EP
JP

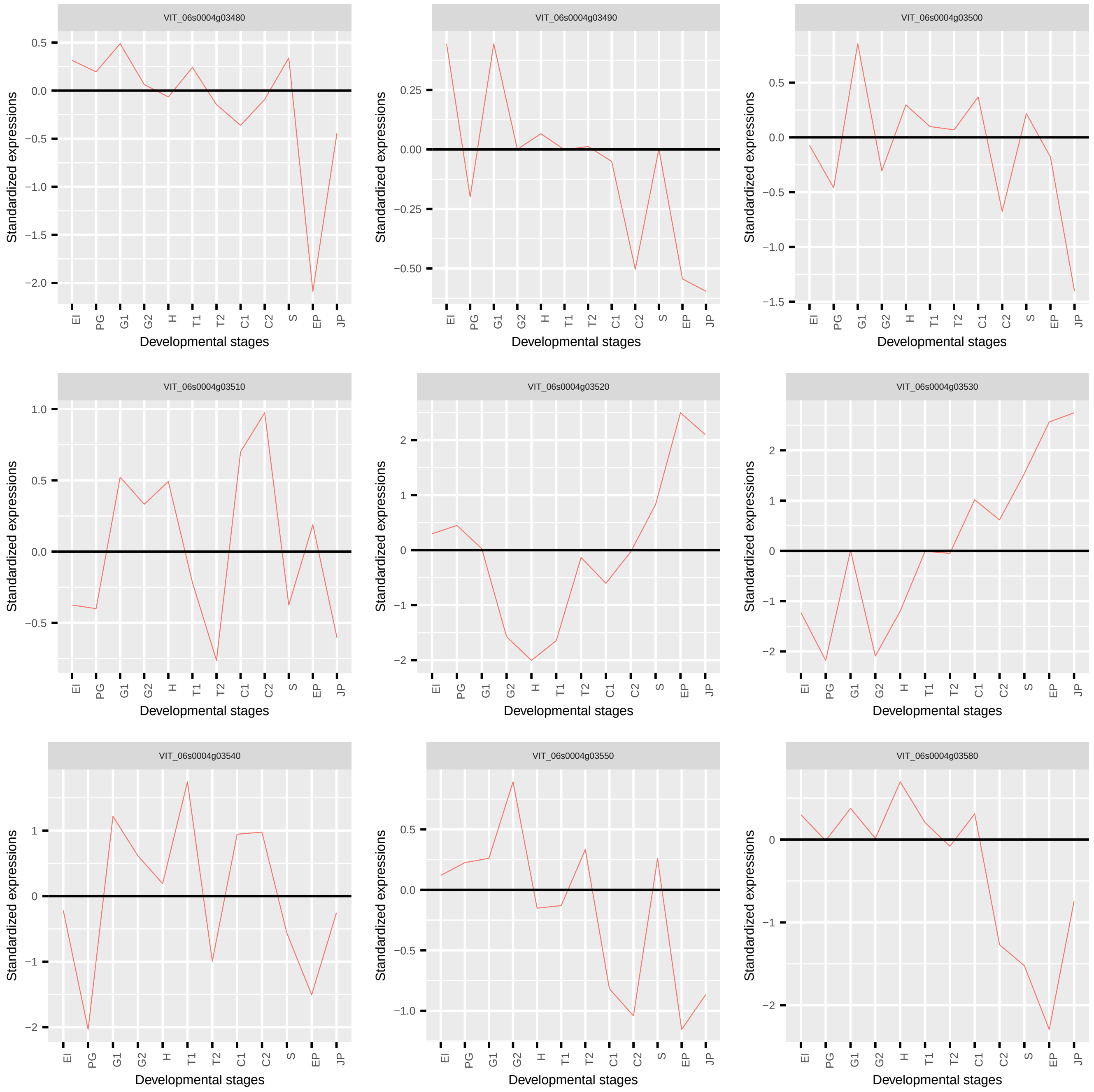
VIT_06s0004g03480
VIT_06s0004g03490
VIT_06s0004g03500
VIT_06s0004g03510
VIT_06s0004g03520
VIT_06s0004g03530
VIT_06s0004g03540
VIT_06s0004g03550
VIT_06s0004g03580
Standardized expressions
Developmental stages
EI PG G1 G2 H T1 T2 C1 C2 S EP JP

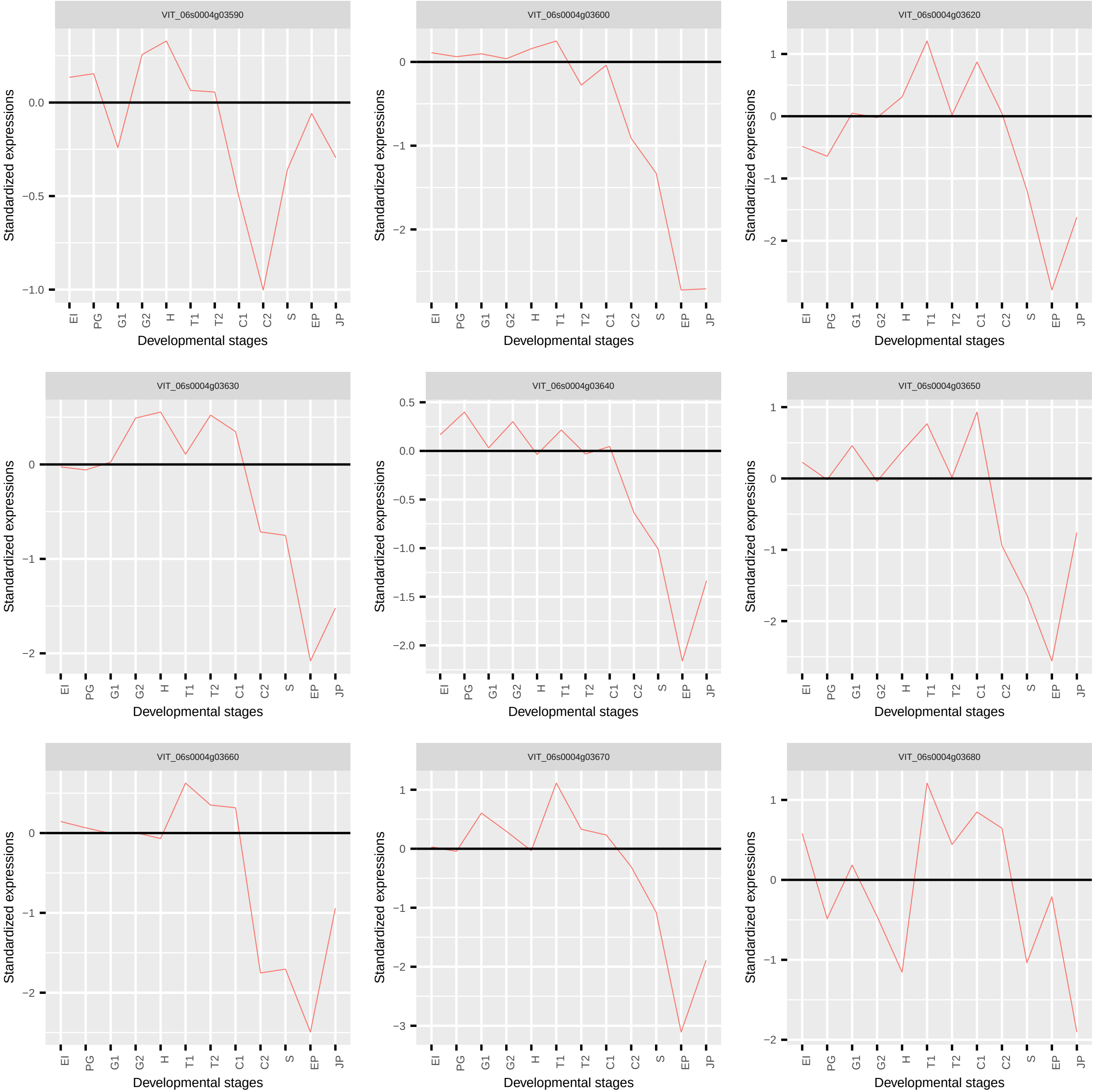

VIT_06s0004g03590
VIT_06s0004g03600
VIT_06s0004g03620
VIT_06s0004g03630
VIT_06s0004g03640
VIT_06s0004g03650
VIT_06s0004g03660
VIT_06s0004g03670
VIT_06s0004g03680
Standardized expressions
Developmental stages
EI PG G1 G2 H T1 T2 C1 C2 S EP JP

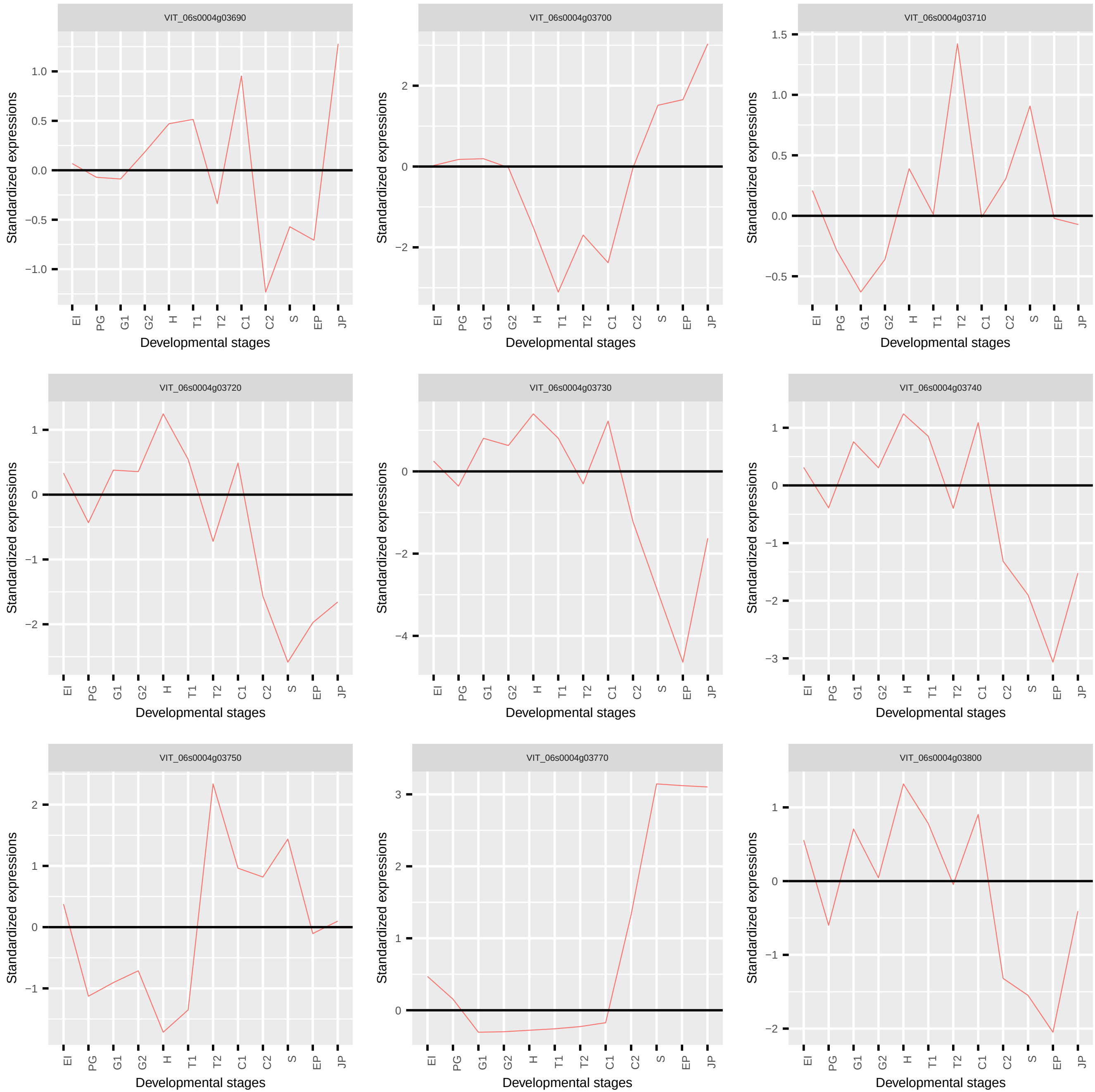
VIT_06s0004g03690
VIT_06s0004g03700
VIT_06s0004g03710
VIT_06s0004g03720
VIT_06s0004g03730
VIT_06s0004g03740
VIT_06s0004g03750
VIT_06s0004g03770
VIT_06s0004g03800
Standardized expressions
Developmental stages
EI PG G1 G2 H T1 T2 C1 C2 S EP JP

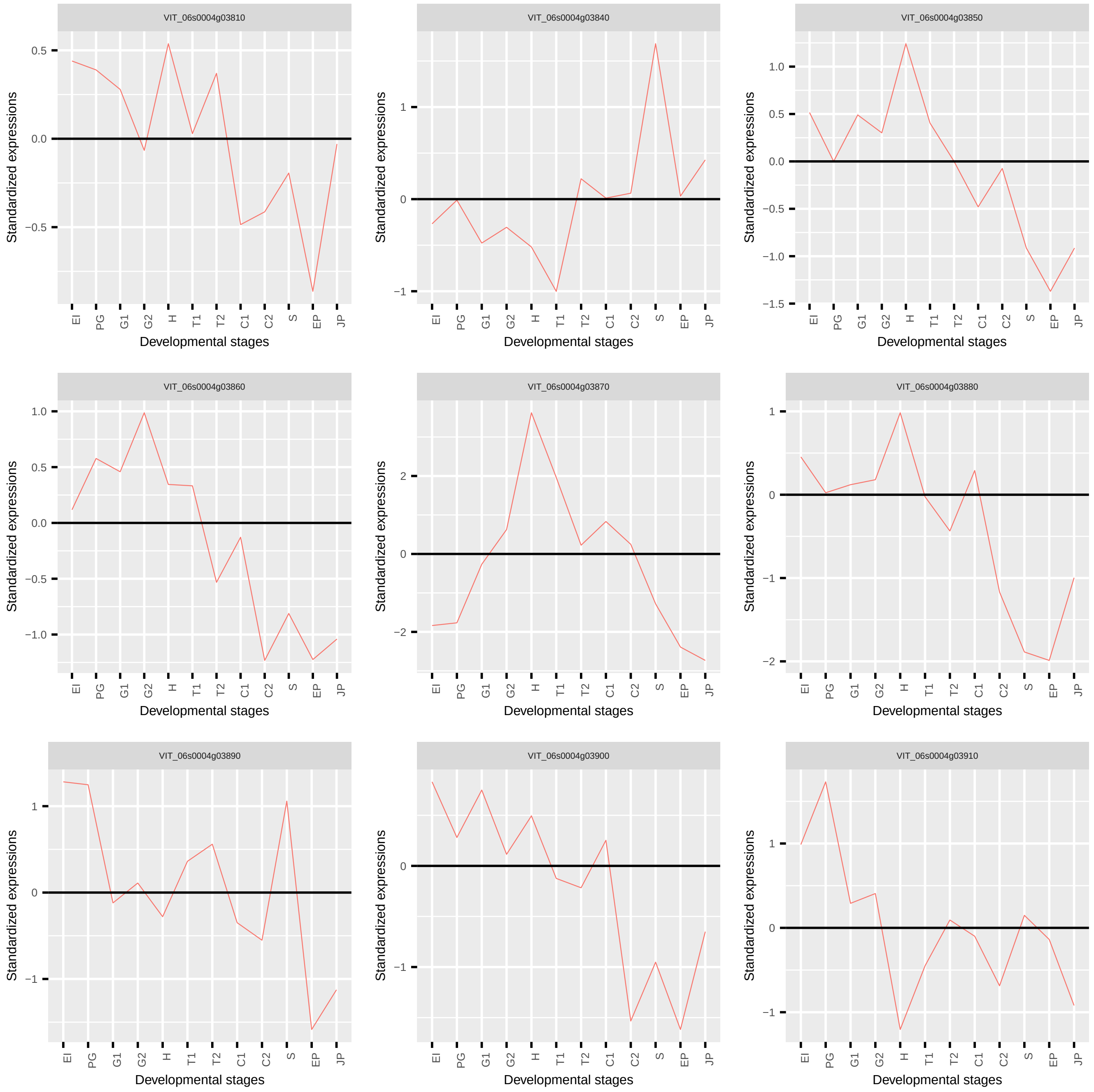
VIT_06s0004g03810
VIT_06s0004g03840
VIT_06s0004g03850
VIT_06s0004g03860
VIT_06s0004g03870
VIT_06s0004g03880
VIT_06s0004g03890
VIT_06s0004g03900
VIT_06s0004g03910
Standardized expressions
Developmental stages
EI PG G1 G2 H T1 T2 C1 C2 S EP JP

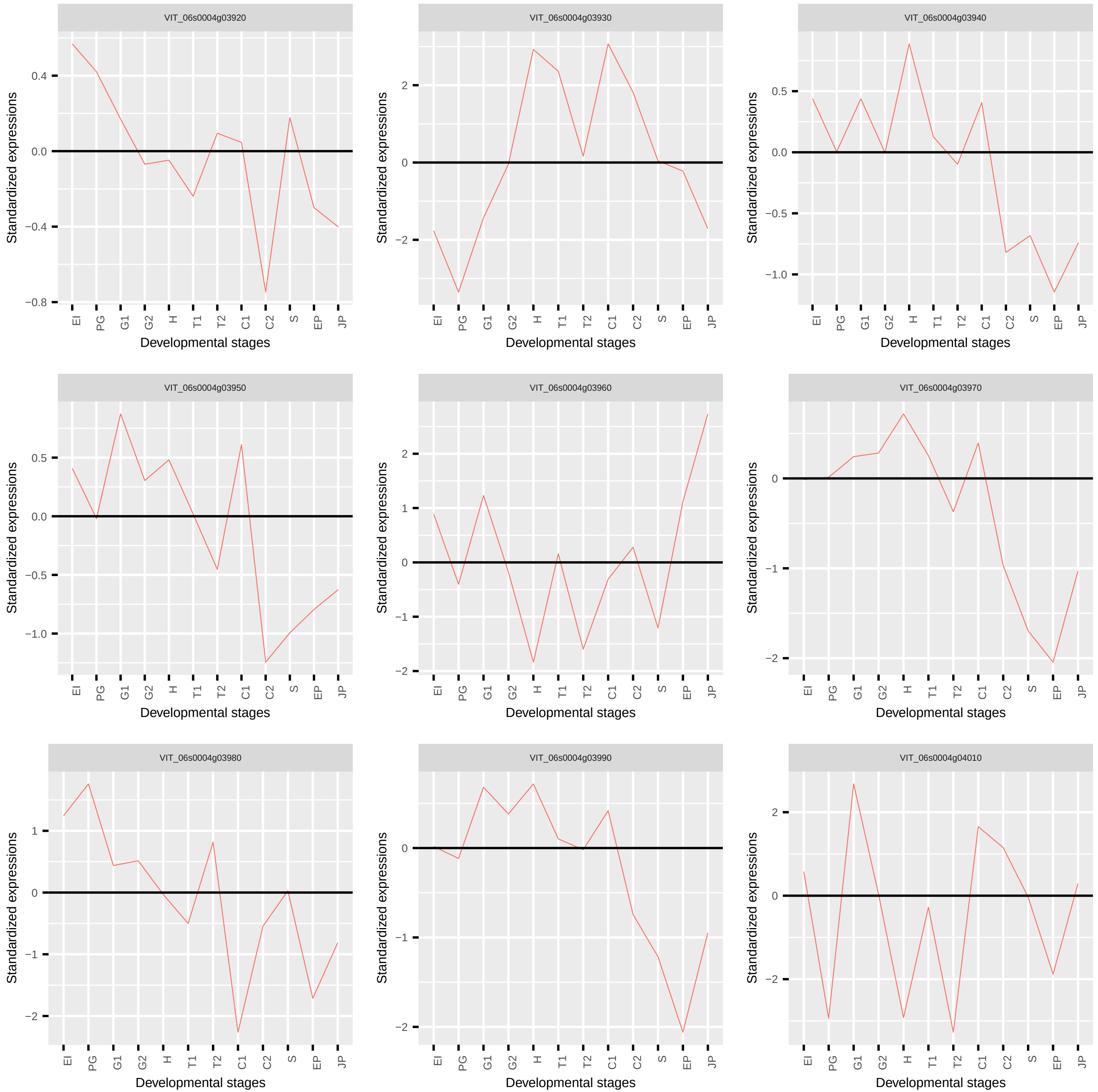
VIT_06s0004g03920
VIT_06s0004g03930
VIT_06s0004g03940
VIT_06s0004g03950
VIT_06s0004g03960
VIT_06s0004g03970
VIT_06s0004g03980
VIT_06s0004g03990
VIT_06s0004g04010
Standardized expressions
Developmental stages
EI PG G1 G2 H T1 T2 C1 C2 S EP JP

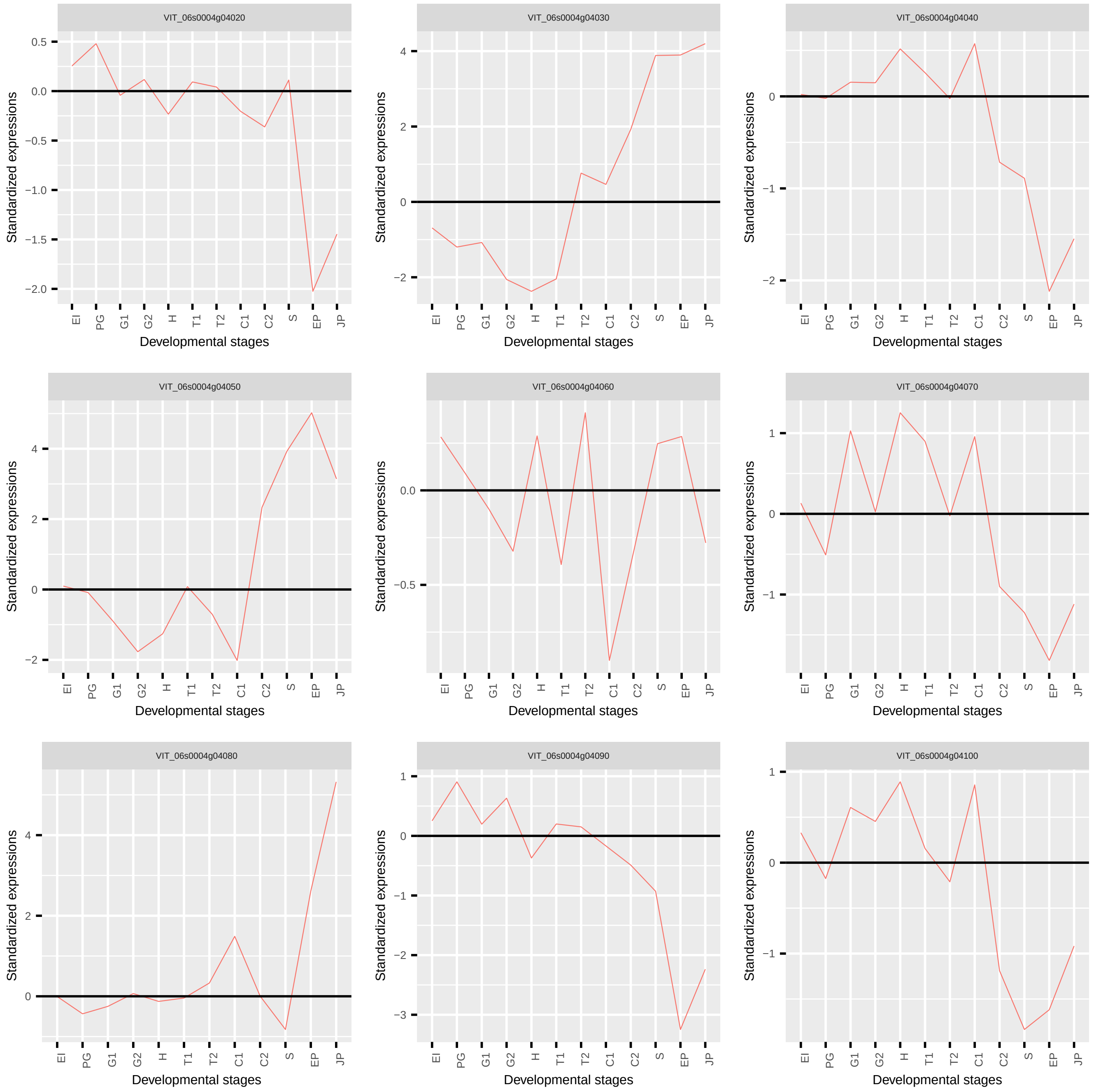

VIT_06s0004g04020
VIT_06s0004g04030
VIT_06s0004g04040
VIT_06s0004g04050
VIT_06s0004g04060
VIT_06s0004g04070
VIT_06s0004g04080
VIT_06s0004g04090
VIT_06s0004g04100
Standardized expressions
Developmental stages
EI PG G1 G2 H T1 T2 C1 C2 S EP JP

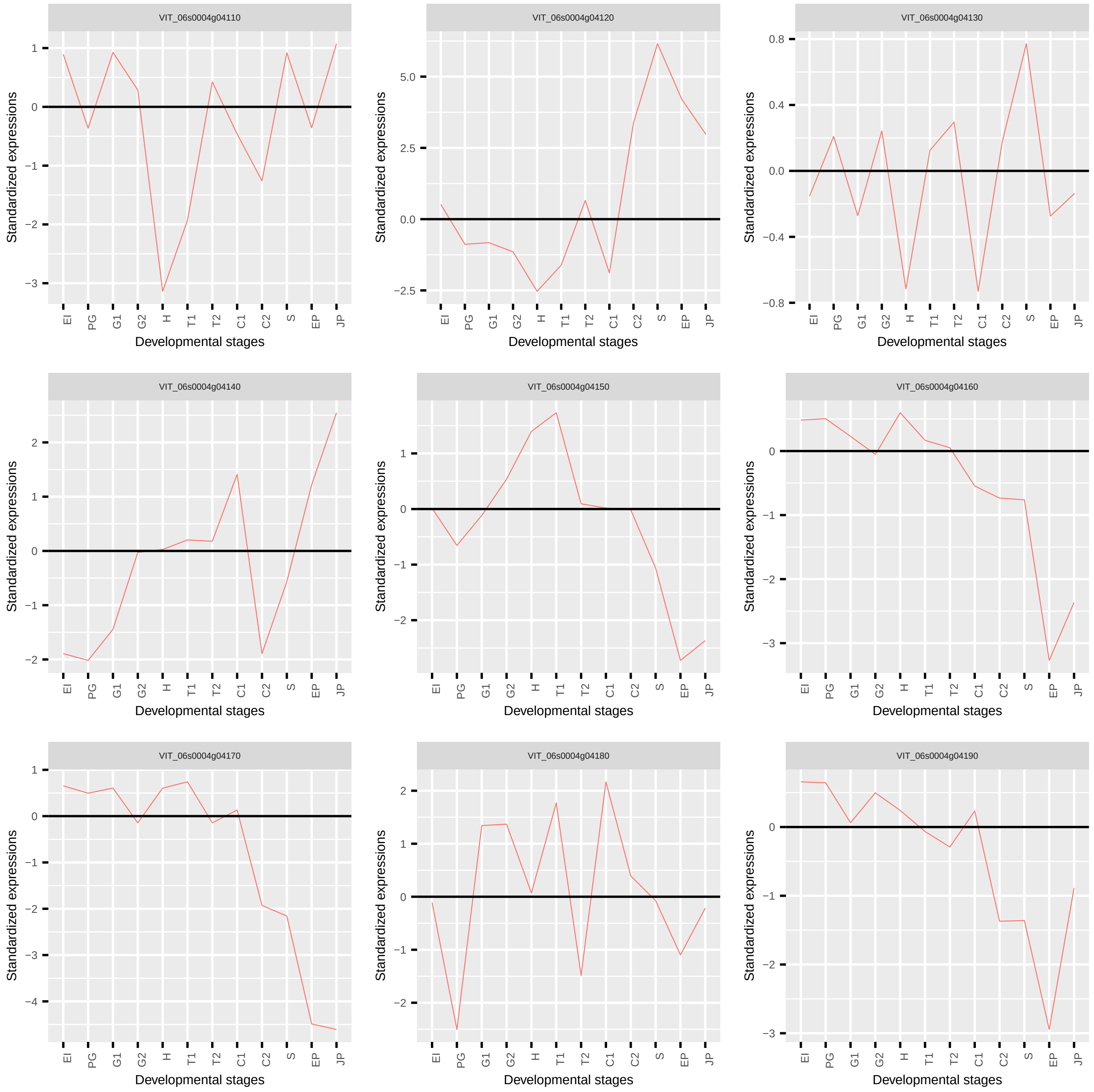
VIT_06s0004g04110
VIT_06s0004g04120
VIT_06s0004g04130
VIT_06s0004g04140
VIT_06s0004g04150
VIT_06s0004g04160
VIT_06s0004g04170
VIT_06s0004g04180
VIT_06s0004g04190
Standardized expressions
Developmental stages
EI
PG
G1
G2
H
T1
T2
C1
C2
S
EP
JP

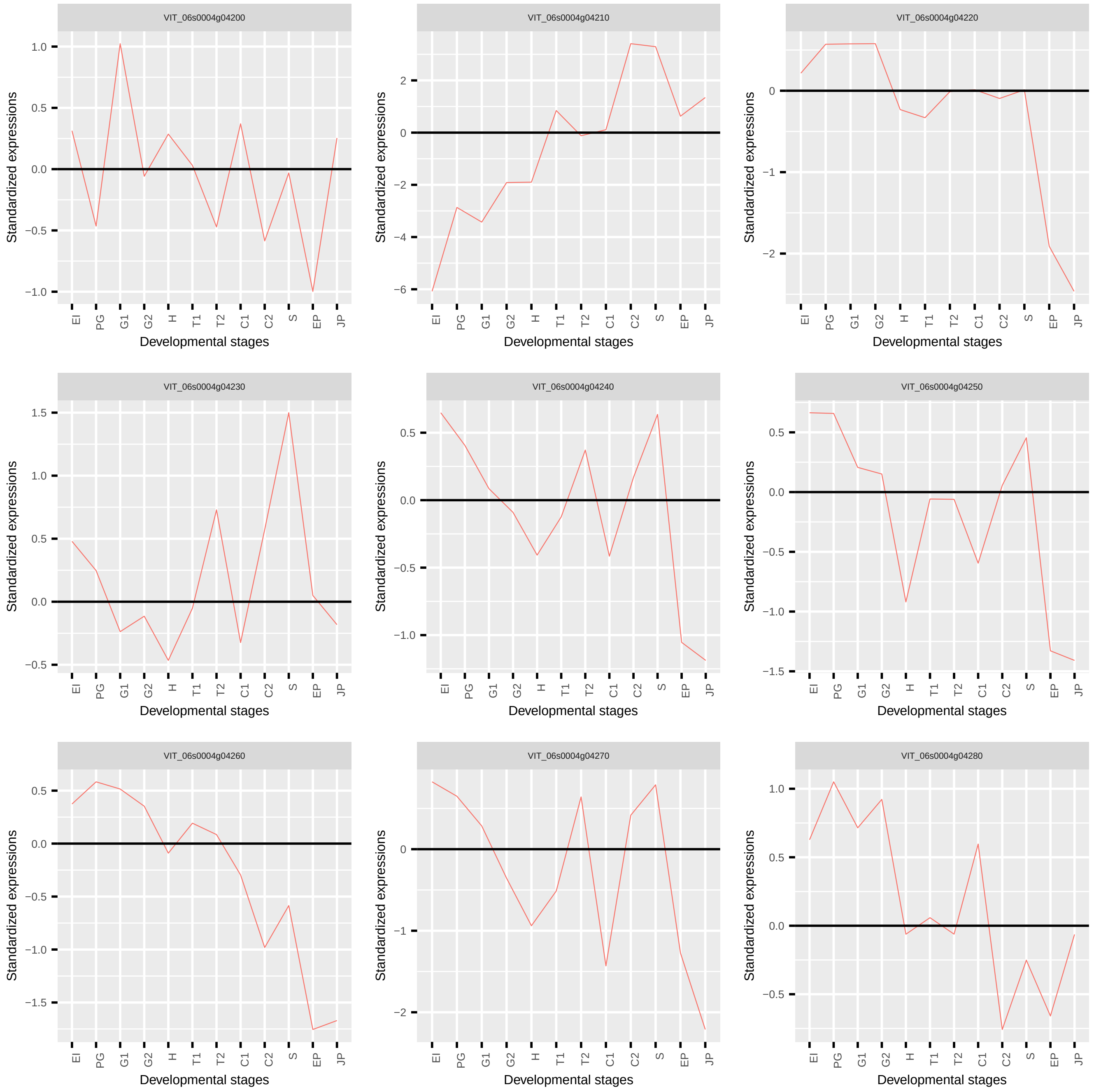

VIT_06s0004g04200
VIT_06s0004g04210
VIT_06s0004g04220
VIT_06s0004g04230
VIT_06s0004g04240
VIT_06s0004g04250
VIT_06s0004g04260
VIT_06s0004g04270
VIT_06s0004g04280
Standardized expressions
Developmental stages
EI PG G1 G2 H T1 T2 C1 C2 S EP JP

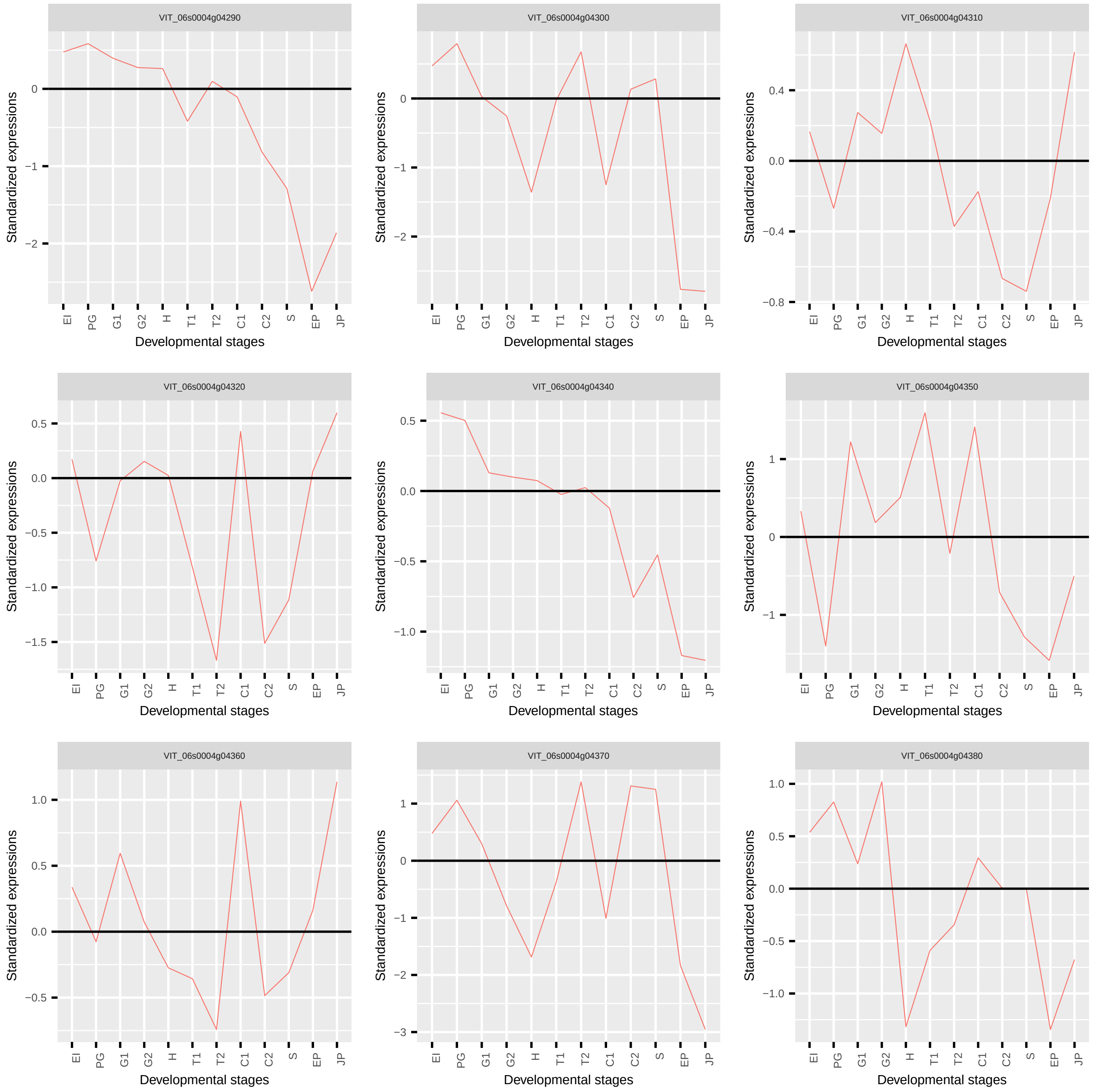
VIT_06s0004g04290
VIT_06s0004g04300
VIT_06s0004g04310
VIT_06s0004g04320
VIT_06s0004g04340
VIT_06s0004g04350
VIT_06s0004g04360
VIT_06s0004g04370
VIT_06s0004g04380
Standardized expressions
Developmental stages
EI PG G1 G2 H T1 T2 C1 C2 S EP JP

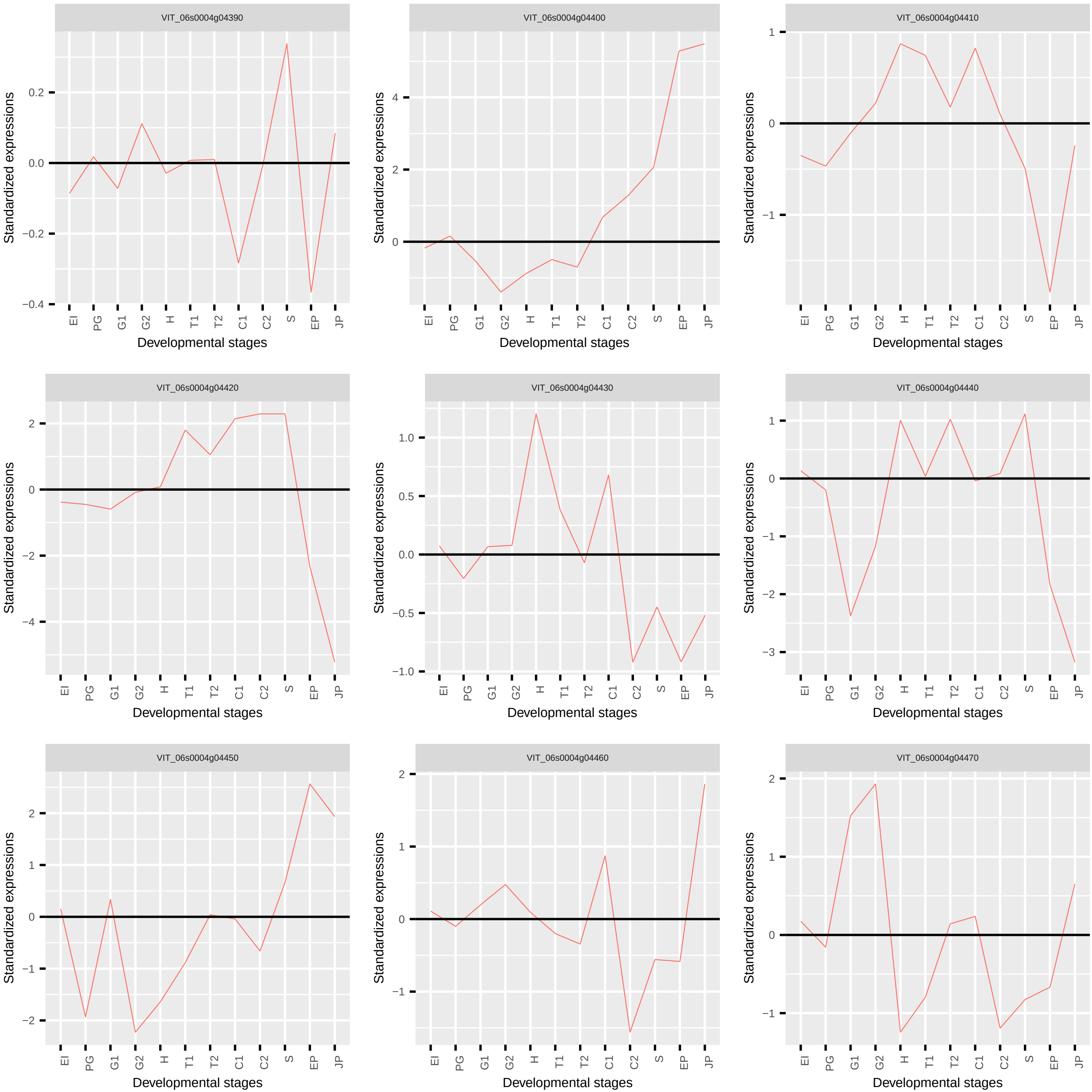
VIT_06s0004g04390
VIT_06s0004g04400
VIT_06s0004g04410
VIT_06s0004g04420
VIT_06s0004g04430
VIT_06s0004g04440
VIT_06s0004g04450
VIT_06s0004g04460
VIT_06s0004g04470
Standardized expressions
Developmental stages
EI PG G1 G2 H T1 T2 C1 C2 S EP JP

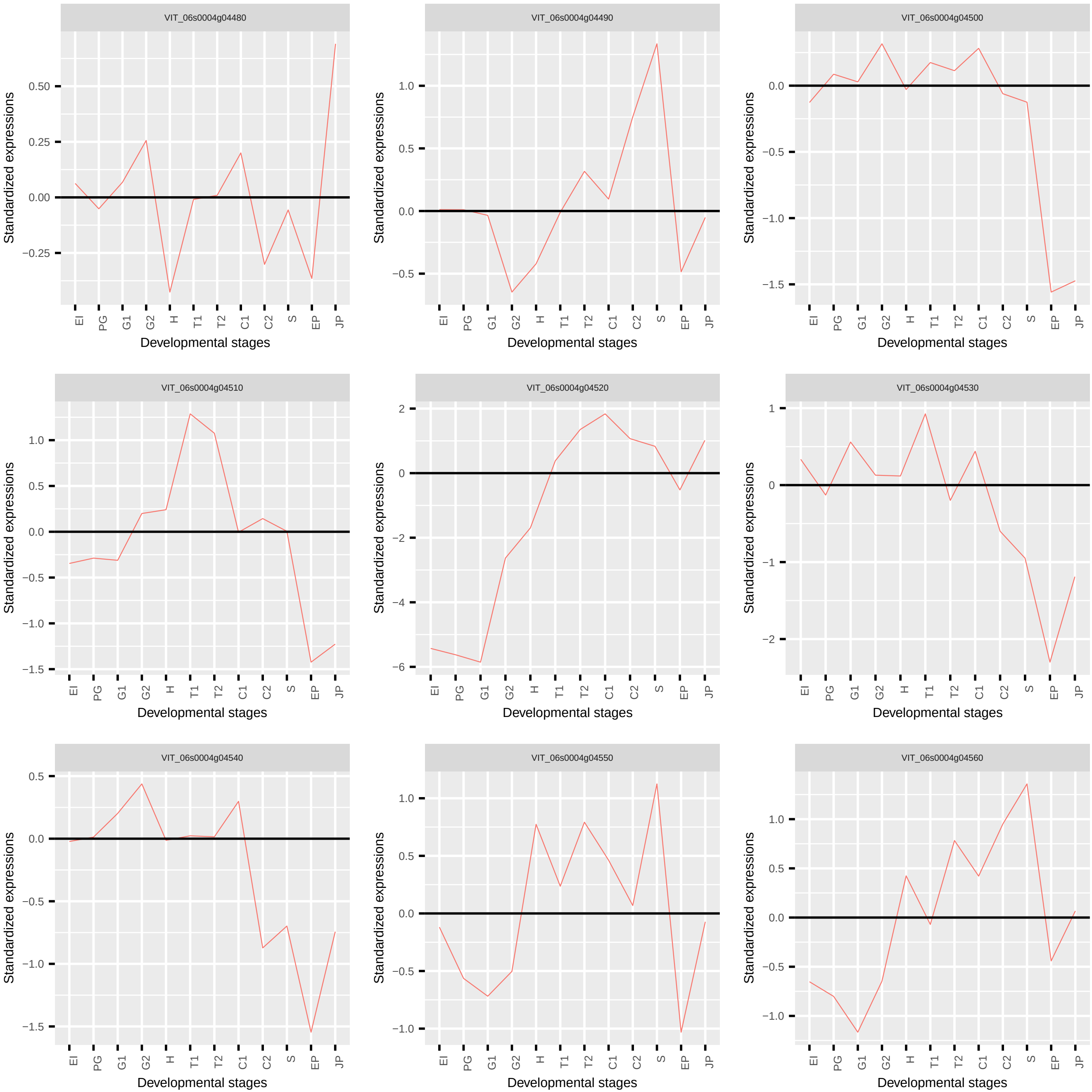
VIT_06s0004g04480
0.50
0.25
0.00
−0.25
Standardized expressions
EI PG G1 G2 H T1 T2 C1 C2 S EP JP
Developmental stages
VIT_06s0004g04490
1.0
0.5
0.0
−0.5
Standardized expressions
EI PG G1 G2 H T1 T2 C1 C2 S EP JP
Developmental stages
VIT_06s0004g04500
0.0
−0.5
−1.0
−1.5
Standardized expressions
EI PG G1 G2 H T1 T2 C1 C2 S EP JP
Developmental stages
VIT_06s0004g04510
1.0
0.5
0.0
−0.5
−1.0
−1.5
Standardized expressions
EI PG G1 G2 H T1 T2 C1 C2 S EP JP
Developmental stages
VIT_06s0004g04520
2
0
−2
−4
−6
Standardized expressions
EI PG G1 G2 H T1 T2 C1 C2 S EP JP
Developmental stages
VIT_06s0004g04530
1
0
−1
−2
Standardized expressions
EI PG G1 G2 H T1 T2 C1 C2 S EP JP
Developmental stages
VIT_06s0004g04540
0.5
0.0
−0.5
−1.0
−1.5
Standardized expressions
EI PG G1 G2 H T1 T2 C1 C2 S EP JP
Developmental stages
VIT_06s0004g04550
1.0
0.5
0.0
−0.5
−1.0
Standardized expressions
EI PG G1 G2 H T1 T2 C1 C2 S EP JP
Developmental stages
VIT_06s0004g04560
1.0
0.5
0.0
−0.5
−1.0
Standardized expressions
EI PG G1 G2 H T1 T2 C1 C2 S EP JP
Developmental stages

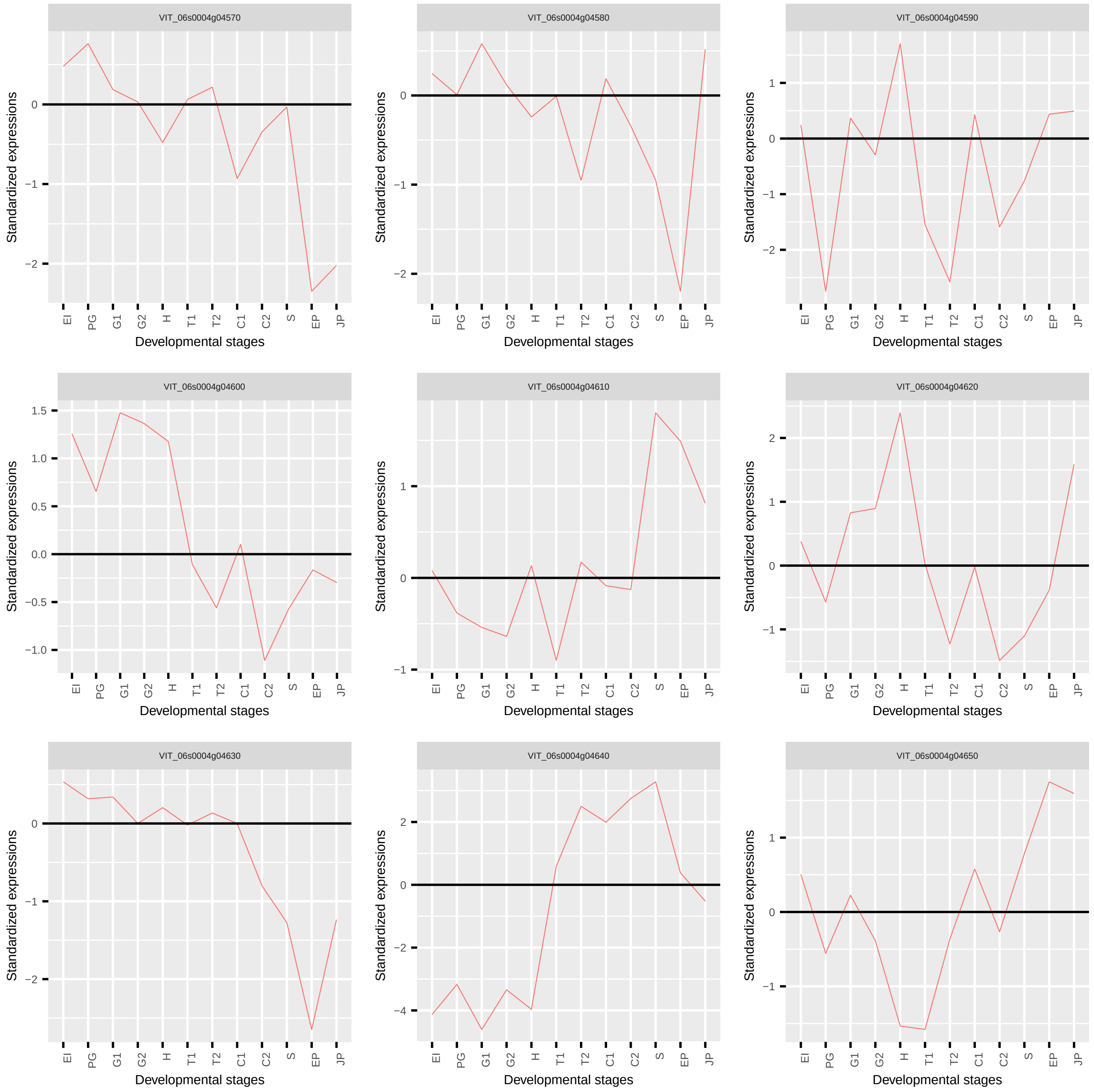
VIT_06s0004g04570
VIT_06s0004g04580
VIT_06s0004g04590
VIT_06s0004g04600
VIT_06s0004g04610
VIT_06s0004g04620
VIT_06s0004g04630
VIT_06s0004g04640
VIT_06s0004g04650
Standardized expressions
Developmental stages
EI PG G1 G2 H T1 T2 C1 C2 S EP JP

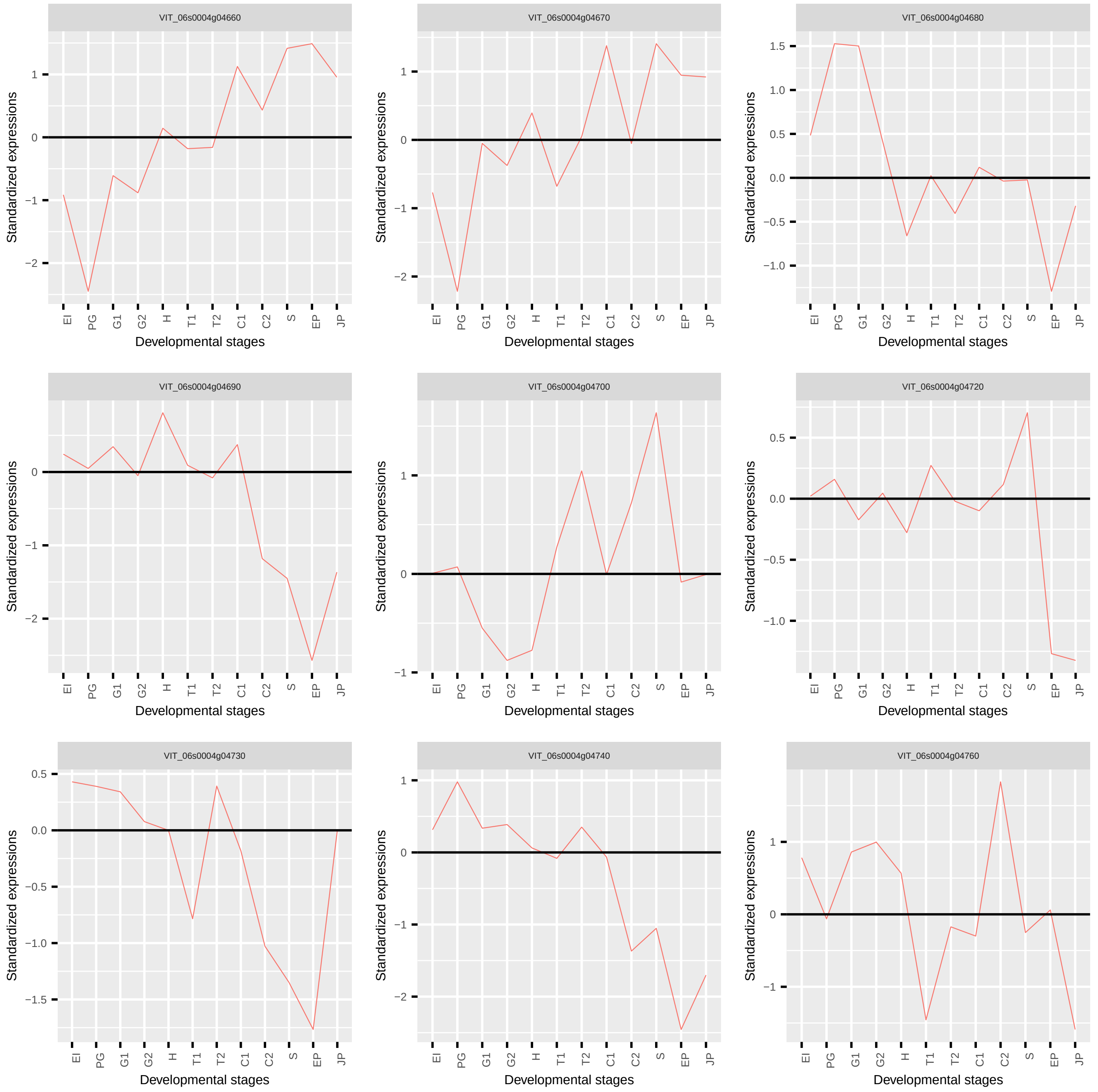

VIT_06s0004g04660
VIT_06s0004g04670
VIT_06s0004g04680
VIT_06s0004g04690
VIT_06s0004g04700
VIT_06s0004g04720
VIT_06s0004g04730
VIT_06s0004g04740
VIT_06s0004g04760
Standardized expressions
Developmental stages
EI PG G1 G2 H T1 T2 C1 C2 S EP JP

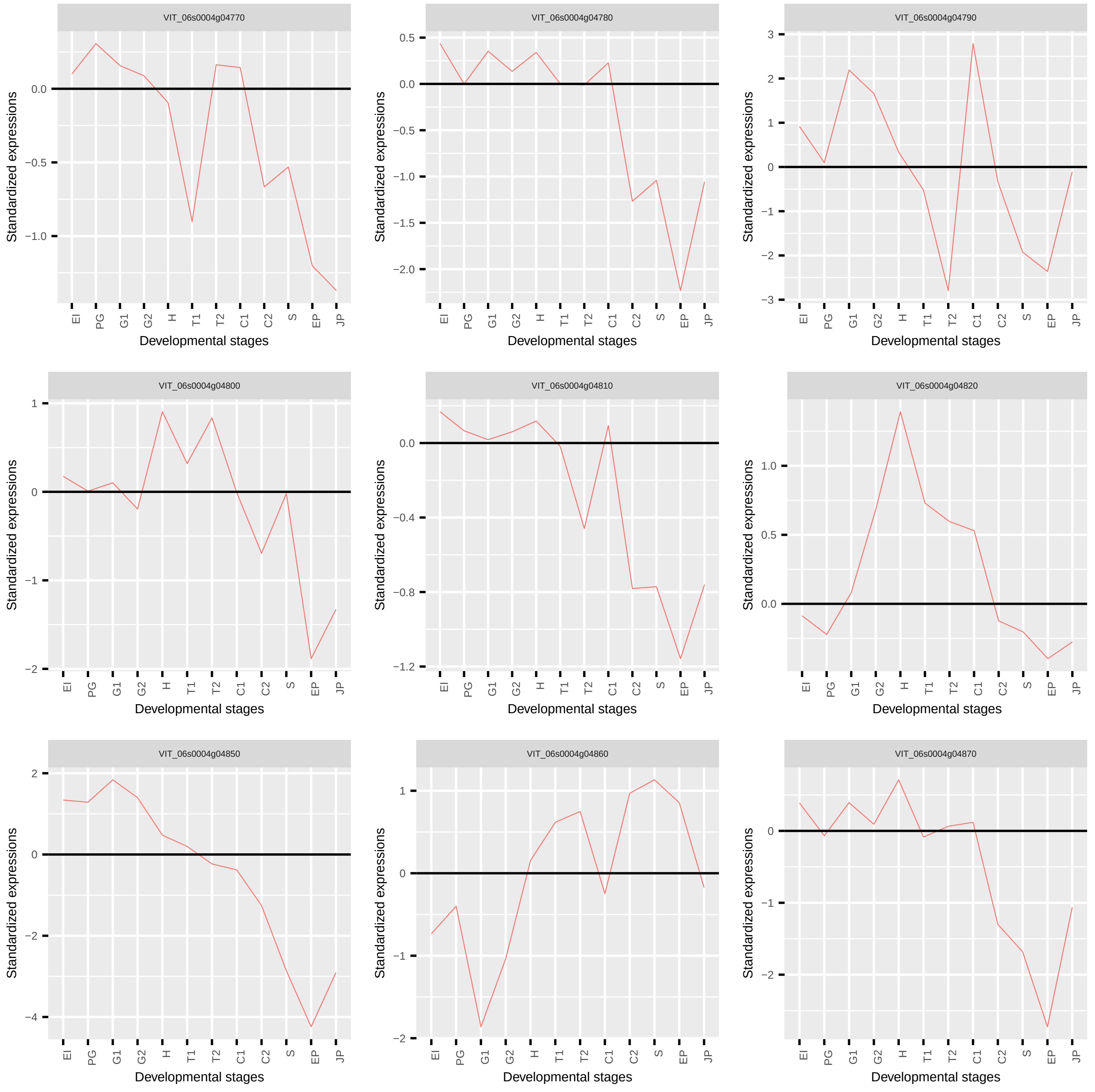
VIT_06s0004g04770
VIT_06s0004g04780
VIT_06s0004g04790
VIT_06s0004g04800
VIT_06s0004g04810
VIT_06s0004g04820
VIT_06s0004g04850
VIT_06s0004g04860
VIT_06s0004g04870
Standardized expressions
Developmental stages
EI PG G1 G2 H T1 T2 C1 C2 S EP JP

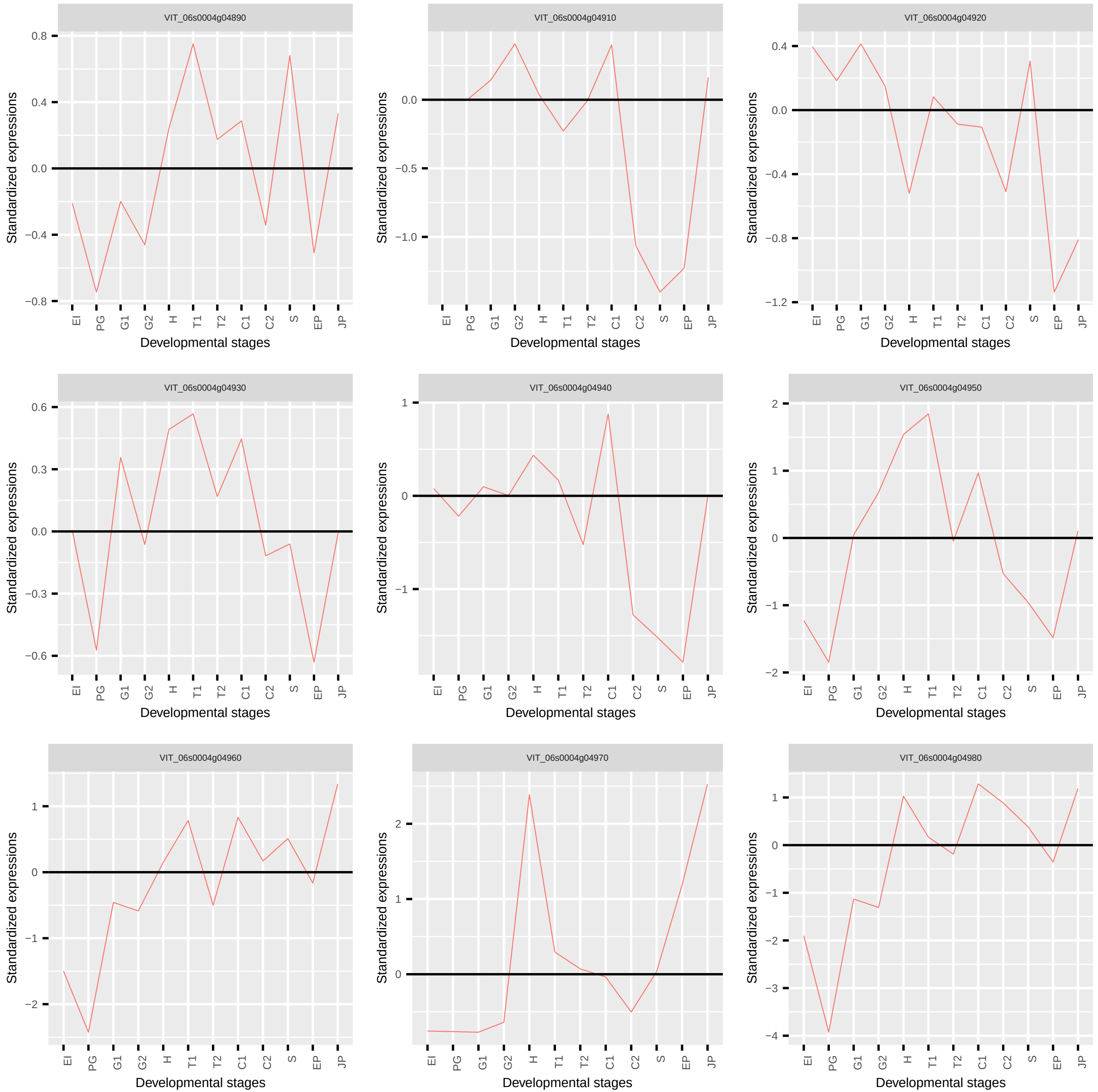
VIT_06s0004g04890
VIT_06s0004g04910
VIT_06s0004g04920
VIT_06s0004g04930
VIT_06s0004g04940
VIT_06s0004g04950
VIT_06s0004g04960
VIT_06s0004g04970
VIT_06s0004g04980
Standardized expressions
Developmental stages
EI PG G1 G2 H T1 T2 C1 C2 S EP JP

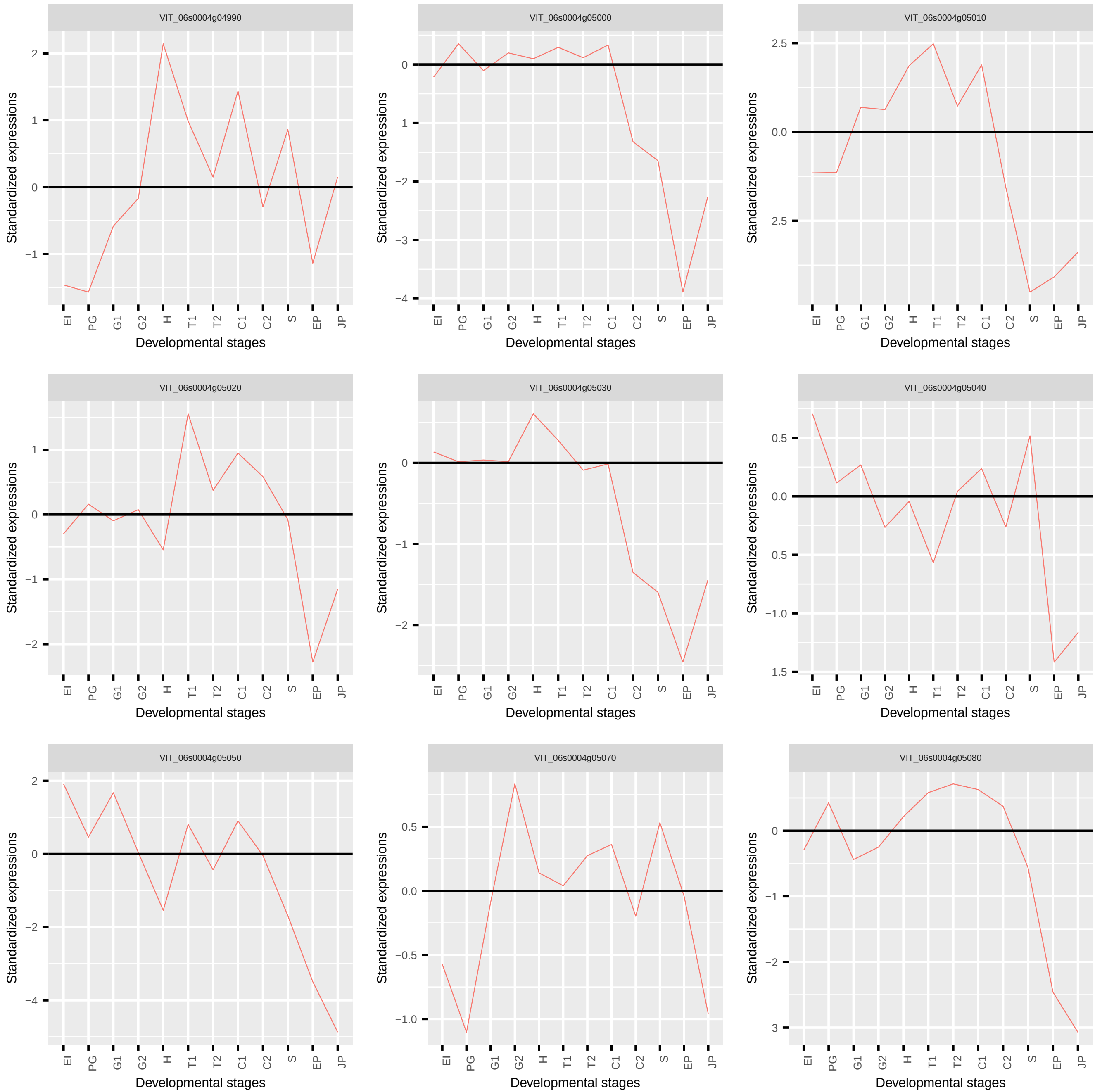
VIT_06s0004g04990
VIT_06s0004g05000
VIT_06s0004g05010
VIT_06s0004g05020
VIT_06s0004g05030
VIT_06s0004g05040
VIT_06s0004g05050
VIT_06s0004g05070
VIT_06s0004g05080
Standardized expressions
Developmental stages
EI PG G1 G2 H T1 T2 C1 C2 S EP JP

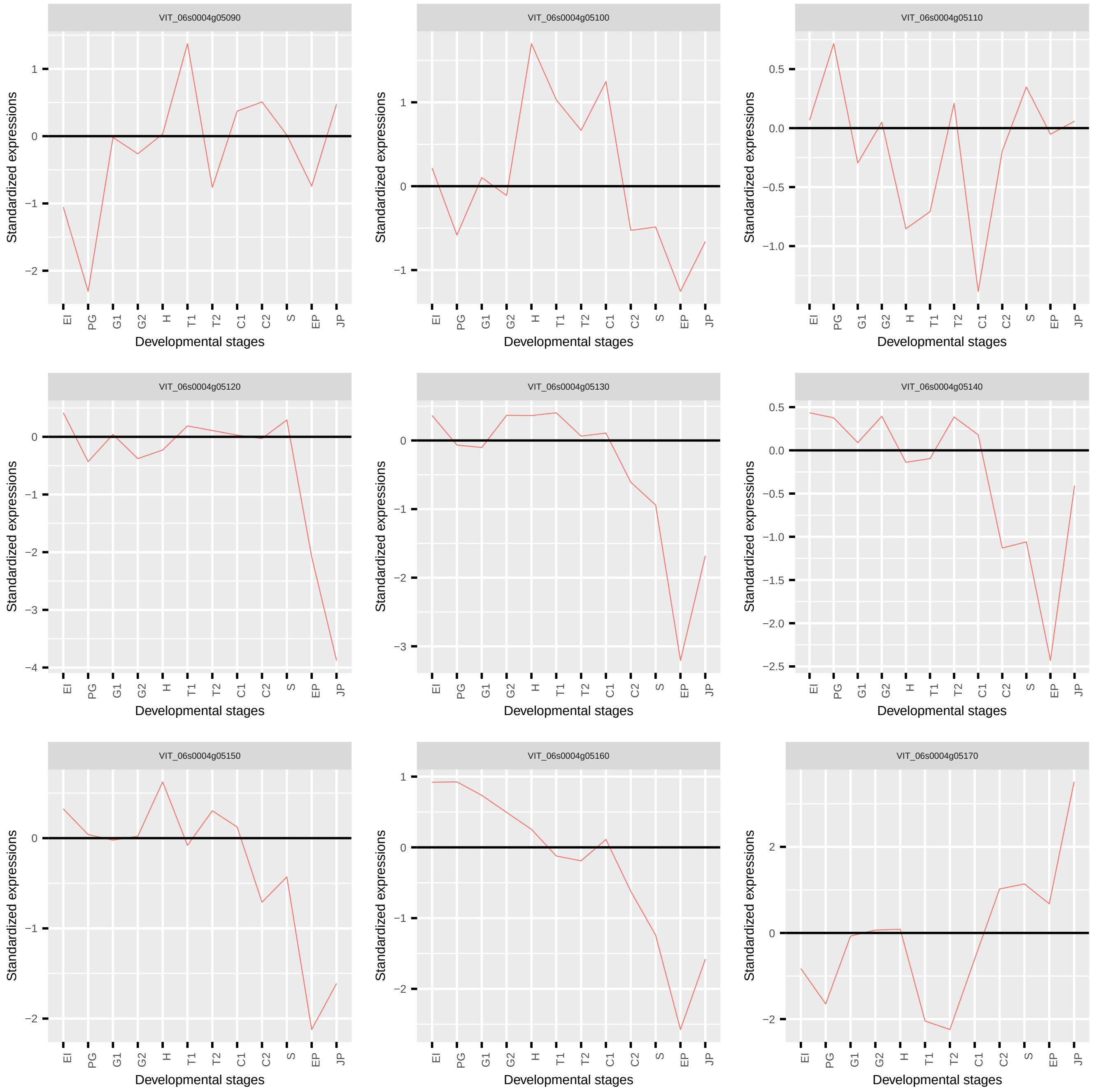
VIT_06s0004g05090
VIT_06s0004g05100
VIT_06s0004g05110
VIT_06s0004g05120
VIT_06s0004g05130
VIT_06s0004g05140
VIT_06s0004g05150
VIT_06s0004g05160
VIT_06s0004g05170
Standardized expressions
Developmental stages
EI PG G1 G2 H T1 T2 C1 C2 S EP JP

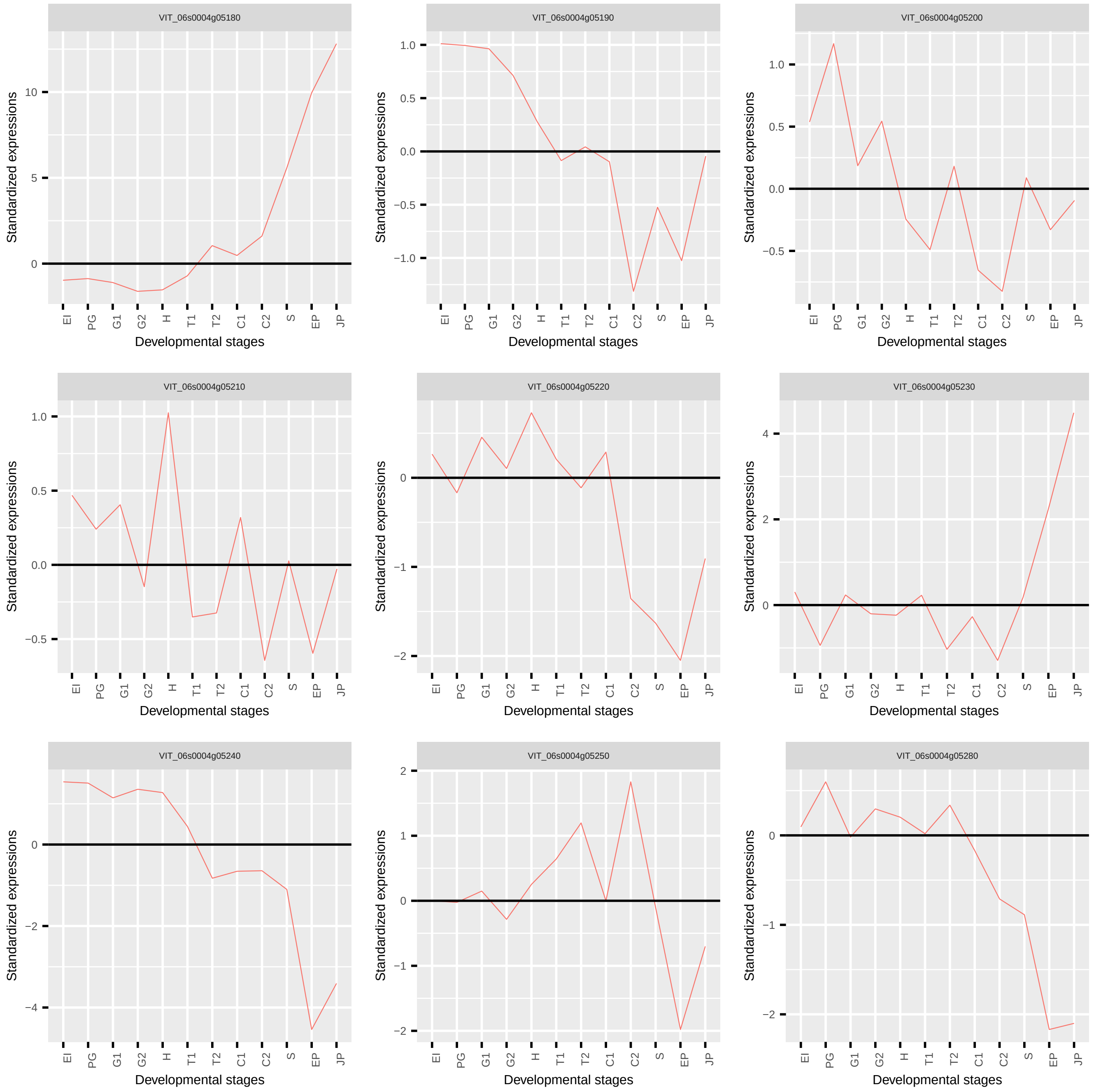
VIT_06s0004g05180
VIT_06s0004g05190
VIT_06s0004g05200
VIT_06s0004g05210
VIT_06s0004g05220
VIT_06s0004g05230
VIT_06s0004g05240
VIT_06s0004g05250
VIT_06s0004g05280
Standardized expressions
Developmental stages
EI PG G1 G2 H T1 T2 C1 C2 S EP JP

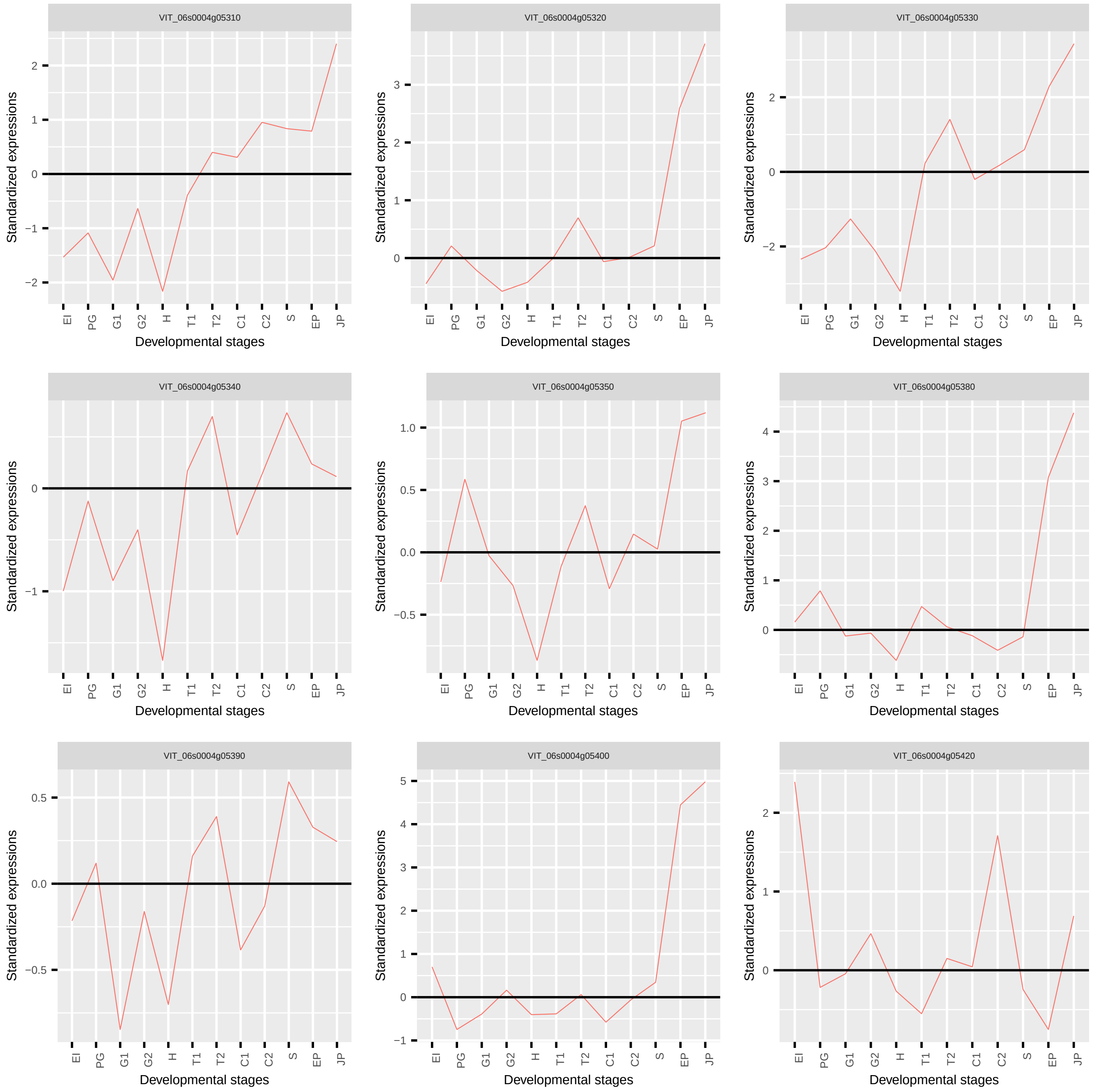
VIT_06s0004g05310
VIT_06s0004g05320
VIT_06s0004g05330
VIT_06s0004g05340
VIT_06s0004g05350
VIT_06s0004g05380
VIT_06s0004g05390
VIT_06s0004g05400
VIT_06s0004g05420
Standardized expressions
Developmental stages
EI PG G1 G2 H T1 T2 C1 C2 S EP JP

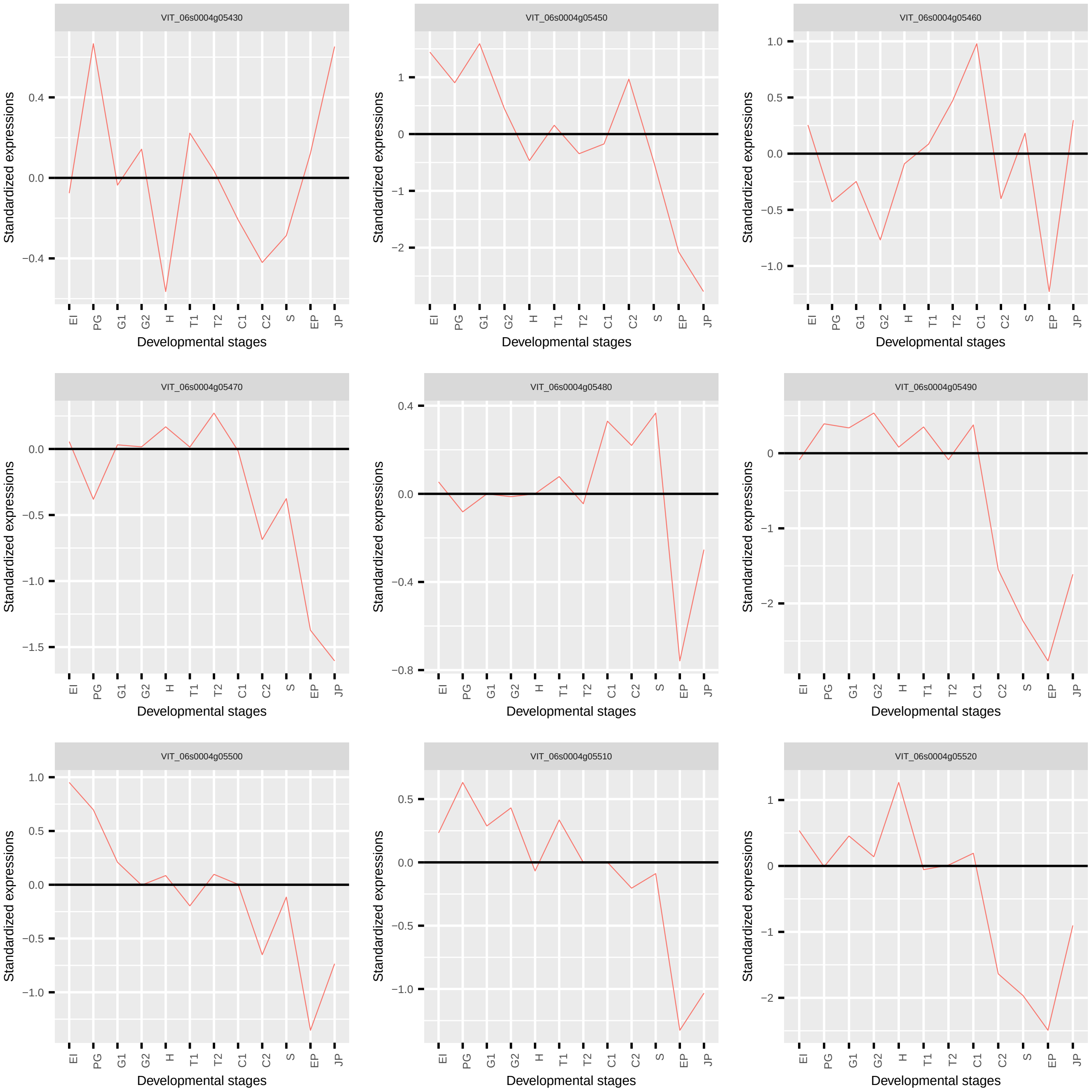
VIT_06s0004g05430
VIT_06s0004g05450
VIT_06s0004g05460
VIT_06s0004g05470
VIT_06s0004g05480
VIT_06s0004g05490
VIT_06s0004g05500
VIT_06s0004g05510
VIT_06s0004g05520
Standardized expressions
Developmental stages
EI PG G1 G2 H T1 T2 C1 C2 S EP JP
0.4
0.0
−0.4
1
0
−1
−2
1.0
0.5
0.0
−0.5
−1.0
−1.5
0.4
0.0
−0.4
−0.8
0
−1
−2

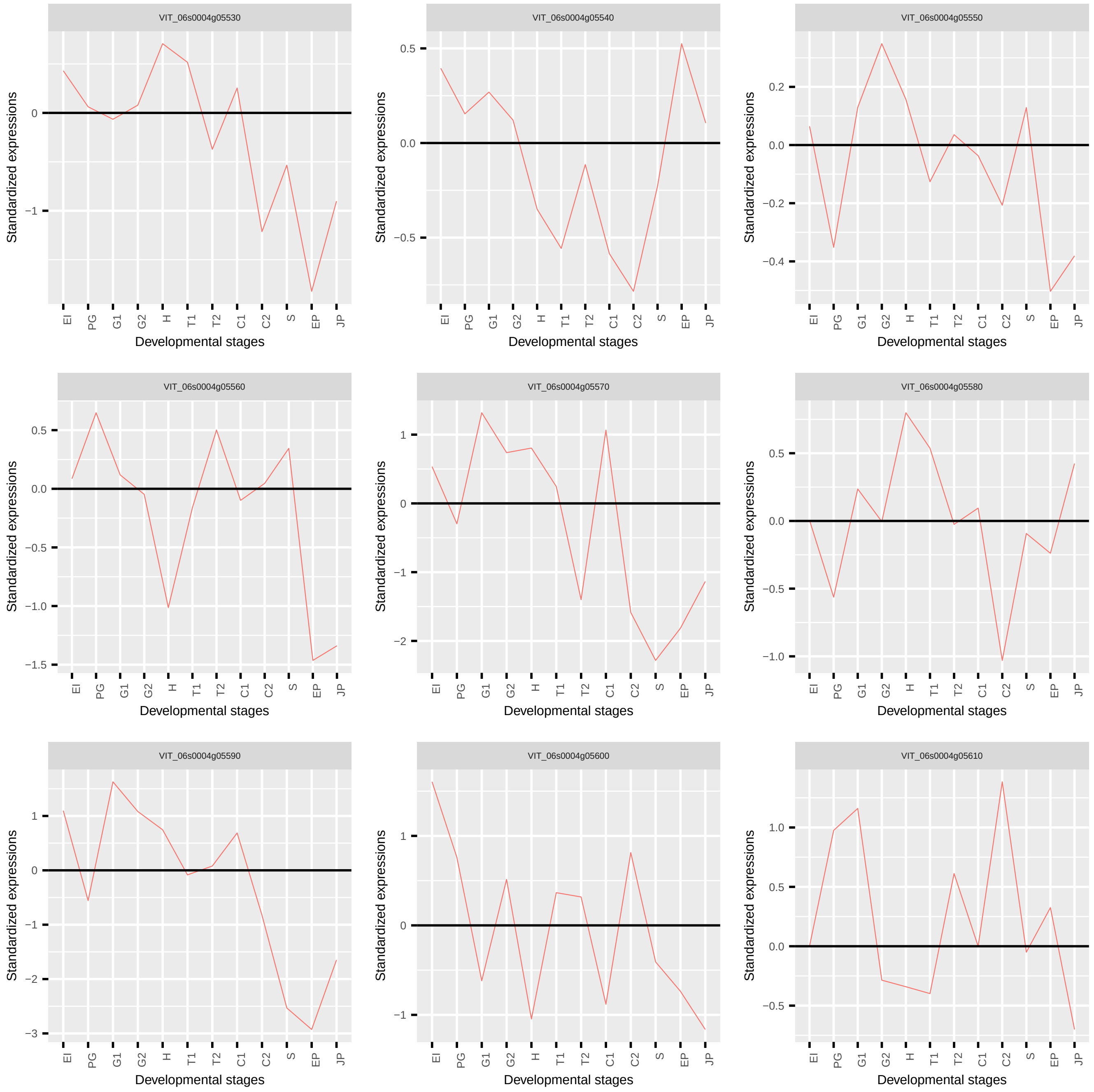
VIT_06s0004g05530
Standardized expressions
0
−1
EI PG G1 G2 H T1 T2 C1 C2 S EP JP
Developmental stages
VIT_06s0004g05540
Standardized expressions
0.5
0.0
−0.5
EI PG G1 G2 H T1 T2 C1 C2 S EP JP
Developmental stages
VIT_06s0004g05550
Standardized expressions
0.2
0.0
−0.2
−0.4
EI PG G1 G2 H T1 T2 C1 C2 S EP JP
Developmental stages
VIT_06s0004g05560
Standardized expressions
0.5
0.0
−0.5
−1.0
−1.5
EI PG G1 G2 H T1 T2 C1 C2 S EP JP
Developmental stages
VIT_06s0004g05570
Standardized expressions
1
0
−1
−2
EI PG G1 G2 H T1 T2 C1 C2 S EP JP
Developmental stages
VIT_06s0004g05580
Standardized expressions
0.5
0.0
−0.5
−1.0
EI PG G1 G2 H T1 T2 C1 C2 S EP JP
Developmental stages
VIT_06s0004g05590
Standardized expressions
1
0
−1
−2
−3
EI PG G1 G2 H T1 T2 C1 C2 S EP JP
Developmental stages
VIT_06s0004g05600
Standardized expressions
1
0
−1
EI PG G1 G2 H T1 T2 C1 C2 S EP JP
Developmental stages
VIT_06s0004g05610
Standardized expressions
1.0
0.5
0.0
−0.5
EI PG G1 G2 H T1 T2 C1 C2 S EP JP
Developmental stages

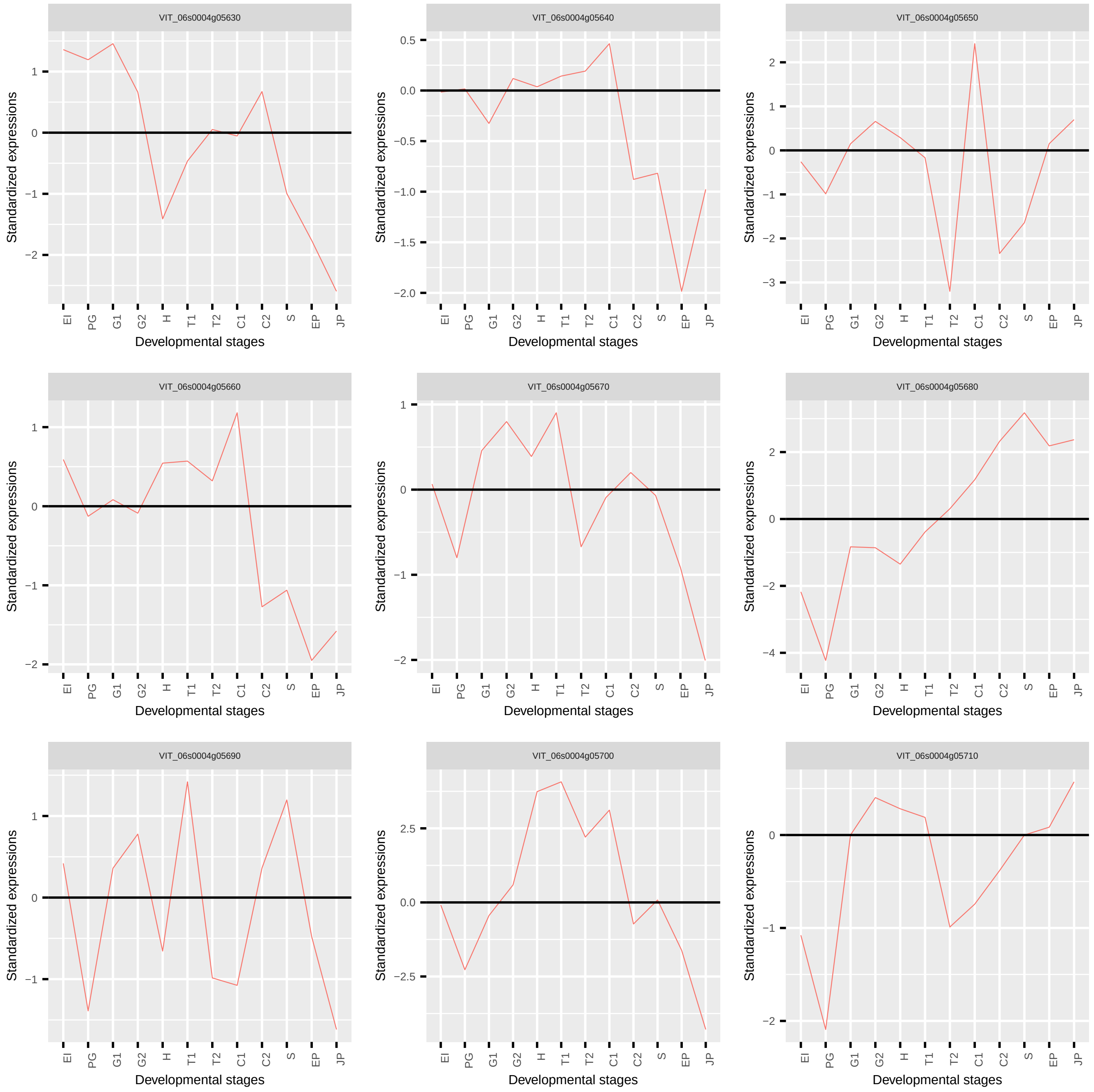
VIT_06s0004g05630
Standardized expressions
1
0
−1
−2
EI PG G1 G2 H T1 T2 C1 C2 S EP JP
Developmental stages
VIT_06s0004g05640
Standardized expressions
0.5
0.0
−0.5
−1.0
−1.5
−2.0
EI PG G1 G2 H T1 T2 C1 C2 S EP JP
Developmental stages
VIT_06s0004g05650
Standardized expressions
2
1
0
−1
−2
−3
EI PG G1 G2 H T1 T2 C1 C2 S EP JP
Developmental stages
VIT_06s0004g05660
Standardized expressions
1
0
−1
−2
EI PG G1 G2 H T1 T2 C1 C2 S EP JP
Developmental stages
VIT_06s0004g05670
Standardized expressions
1
0
−1
−2
EI PG G1 G2 H T1 T2 C1 C2 S EP JP
Developmental stages
VIT_06s0004g05680
Standardized expressions
2
0
−2
−4
EI PG G1 G2 H T1 T2 C1 C2 S EP JP
Developmental stages
VIT_06s0004g05690
Standardized expressions
1
0
−1
EI PG G1 G2 H T1 T2 C1 C2 S EP JP
Developmental stages
VIT_06s0004g05700
Standardized expressions
2.5
0.0
−2.5
EI PG G1 G2 H T1 T2 C1 C2 S EP JP
Developmental stages
VIT_06s0004g05710
Standardized expressions
0
−1
−2
EI PG G1 G2 H T1 T2 C1 C2 S EP JP
Developmental stages

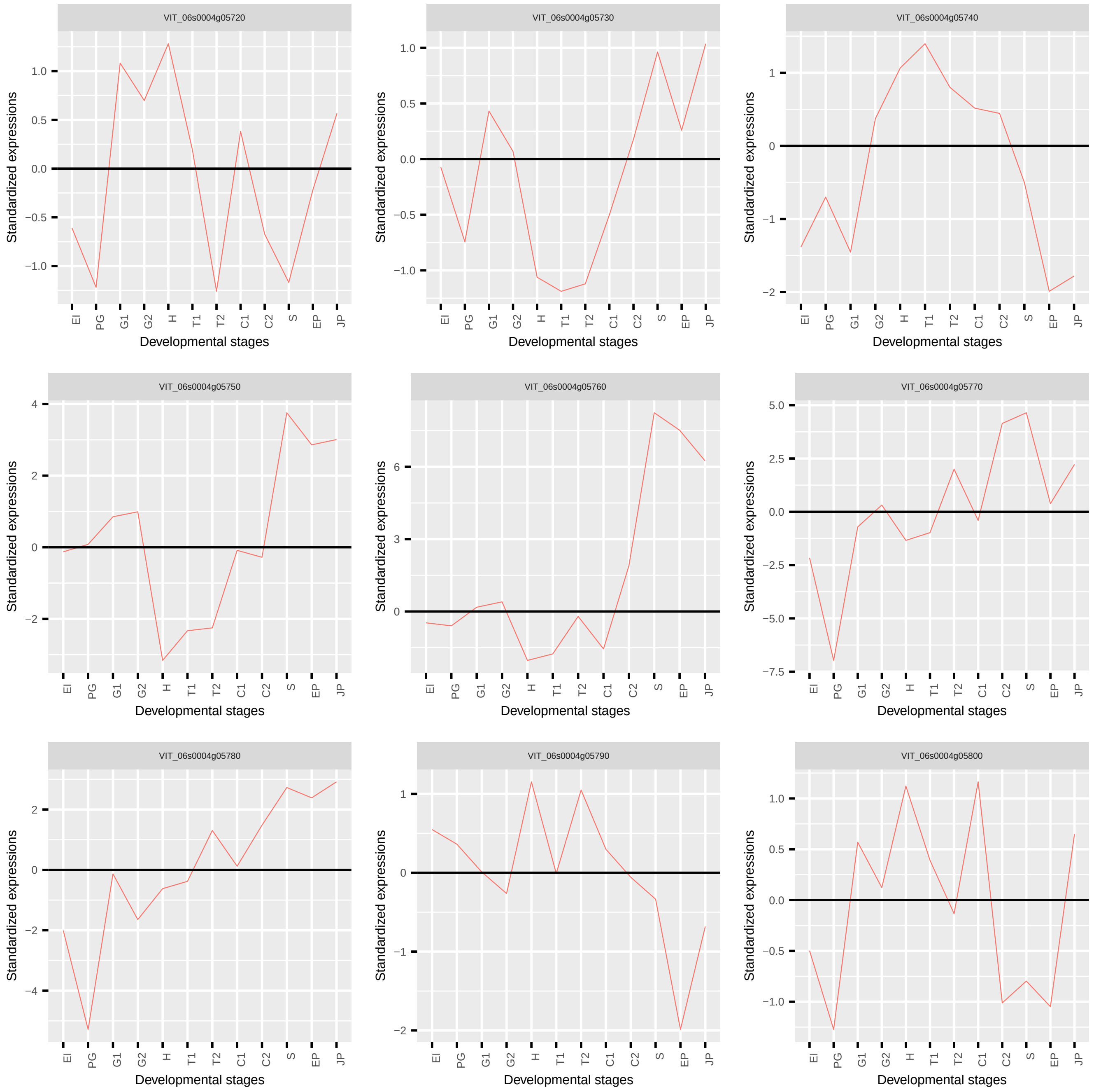

VIT_06s0004g05720
VIT_06s0004g05730
VIT_06s0004g05740
VIT_06s0004g05750
VIT_06s0004g05760
VIT_06s0004g05770
VIT_06s0004g05780
VIT_06s0004g05790
VIT_06s0004g05800
Standardized expressions
Developmental stages
EI PG G1 G2 H T1 T2 C1 C2 S EP JP

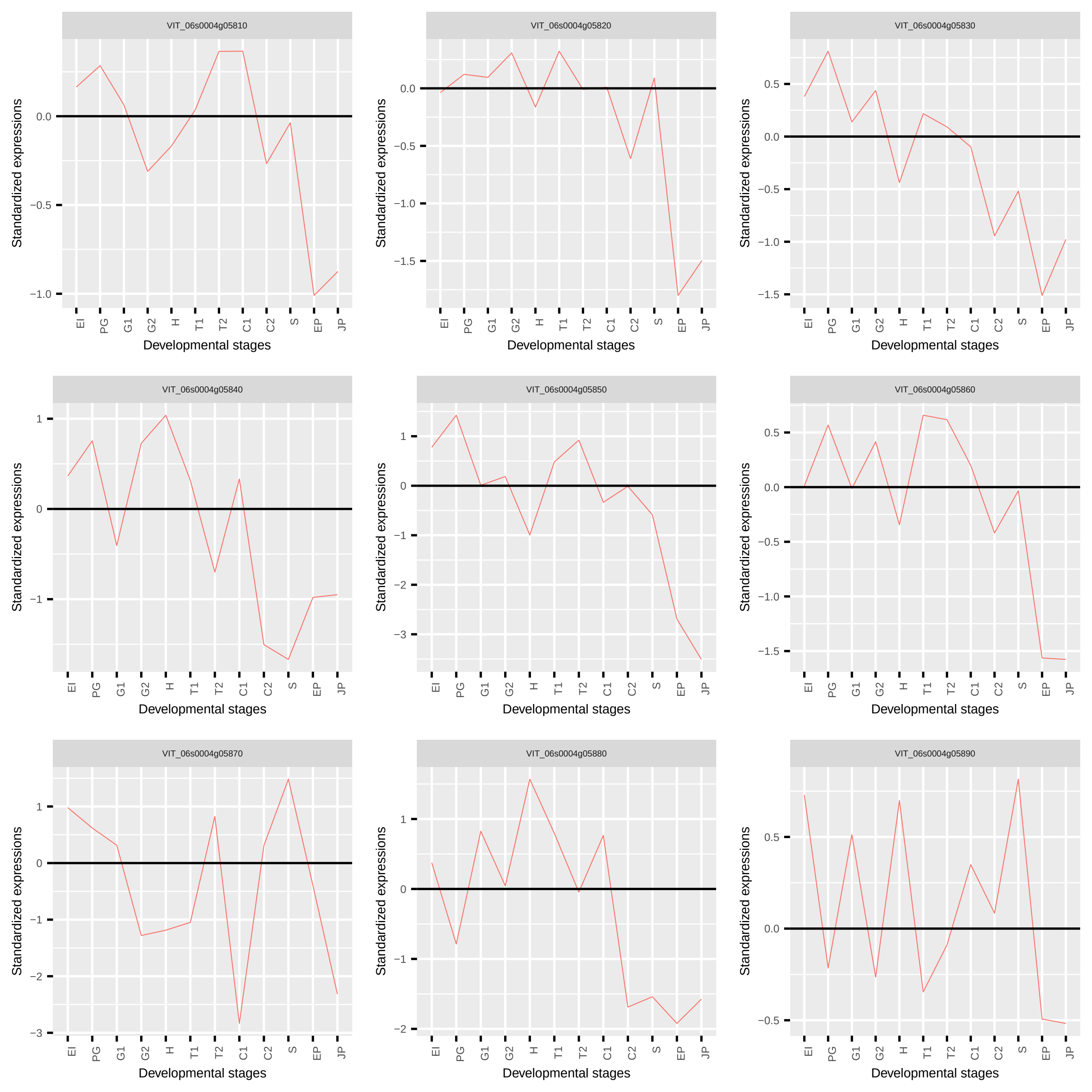

VIT_06s0004g05810
VIT_06s0004g05820
VIT_06s0004g05830
VIT_06s0004g05840
VIT_06s0004g05850
VIT_06s0004g05860
VIT_06s0004g05870
VIT_06s0004g05880
VIT_06s0004g05890
Standardized expressions
Developmental stages
EI PG G1 G2 H T1 T2 C1 C2 S EP JP

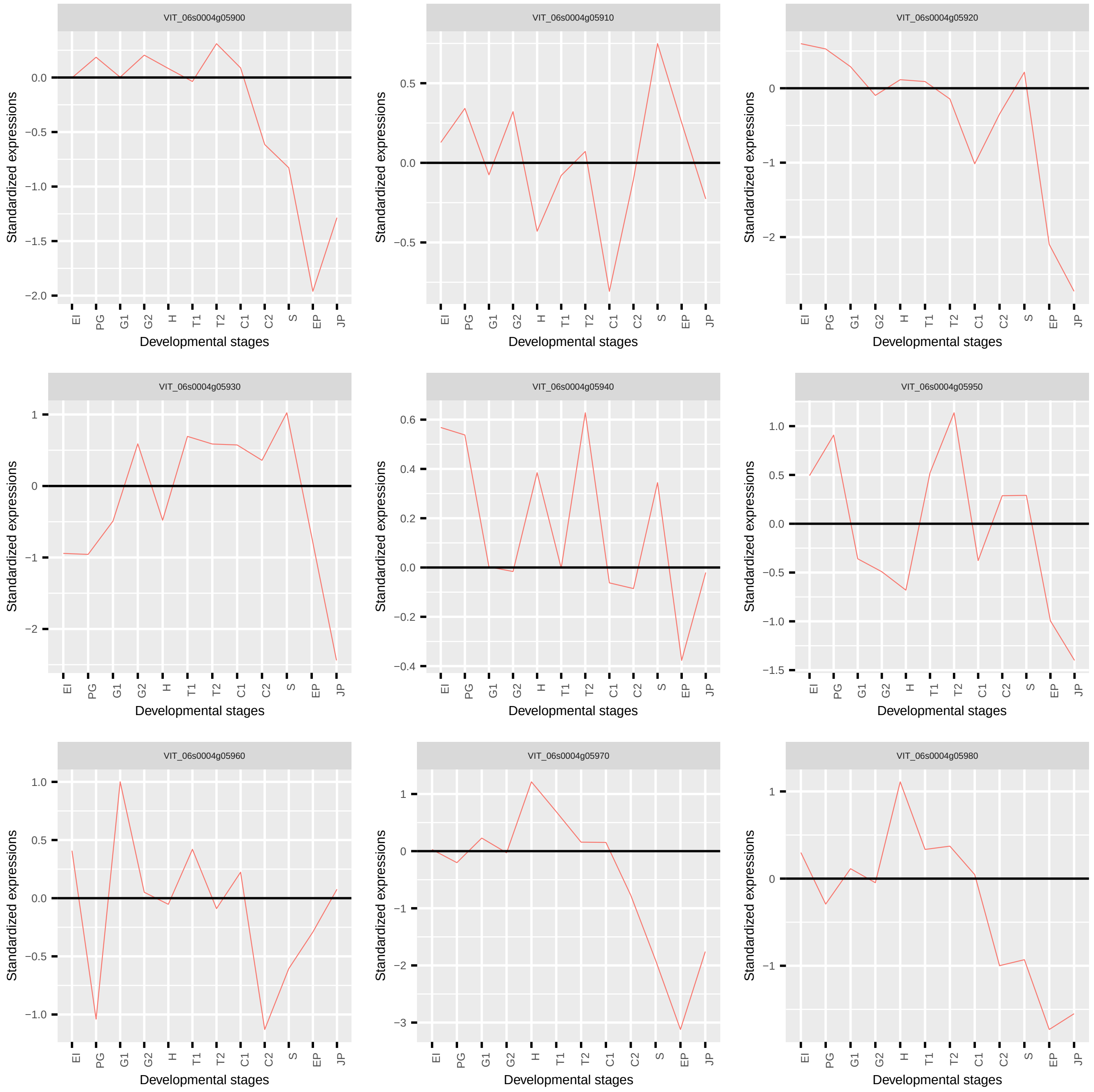
VIT_06s0004g05900
VIT_06s0004g05910
VIT_06s0004g05920
VIT_06s0004g05930
VIT_06s0004g05940
VIT_06s0004g05950
VIT_06s0004g05960
VIT_06s0004g05970
VIT_06s0004g05980
Standardized expressions
Developmental stages
EI PG G1 G2 H T1 T2 C1 C2 S EP JP

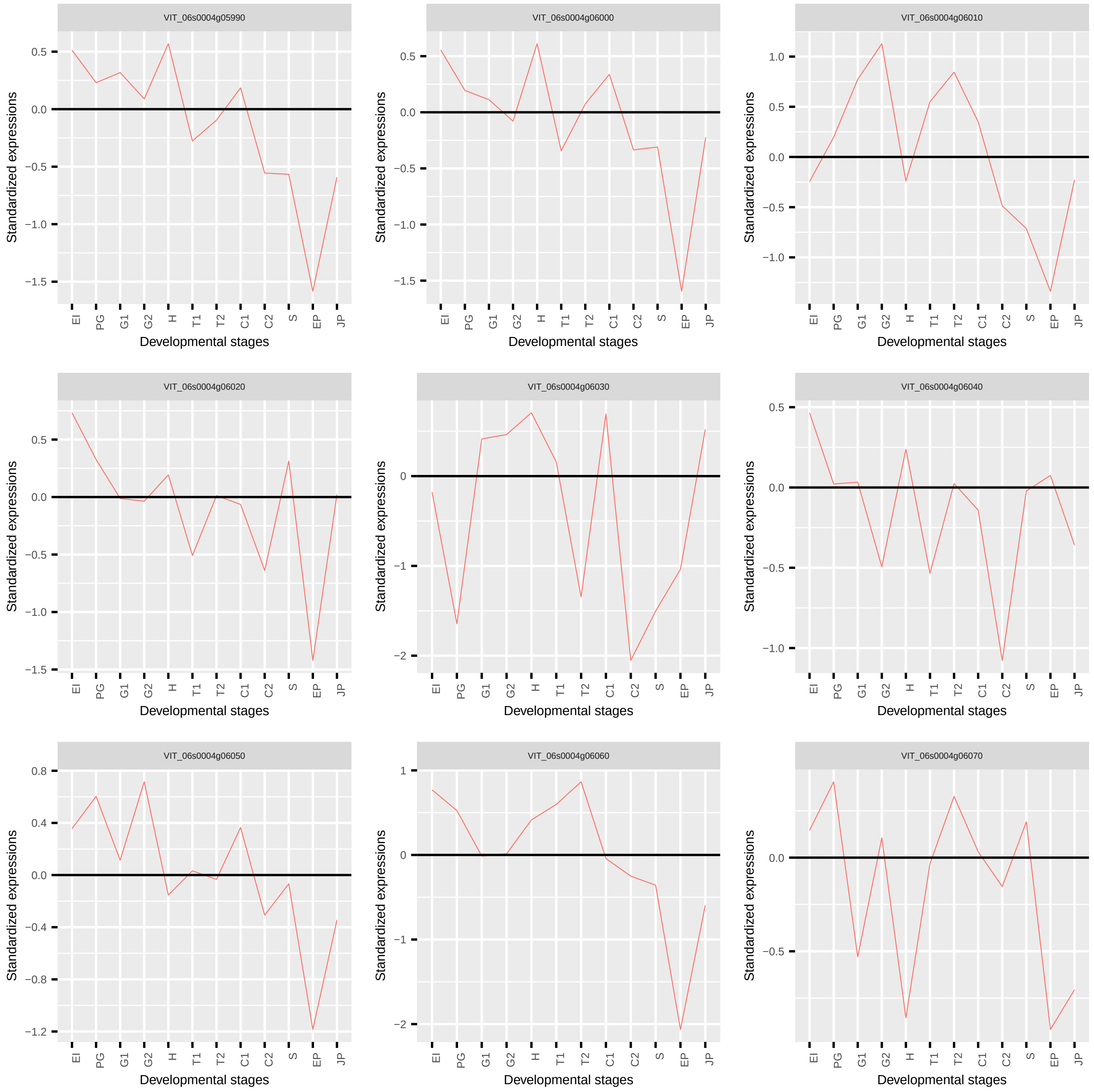
VIT_06s0004g05990
0.5
0.0
−0.5
−1.0
−1.5
Standardized expressions
EI PG G1 G2 H T1 T2 C1 C2 S EP JP
Developmental stages
VIT_06s0004g06000
0.5
0.0
−0.5
−1.0
−1.5
Standardized expressions
EI PG G1 G2 H T1 T2 C1 C2 S EP JP
Developmental stages
VIT_06s0004g06010
1.0
0.5
0.0
−0.5
−1.0
Standardized expressions
EI PG G1 G2 H T1 T2 C1 C2 S EP JP
Developmental stages
VIT_06s0004g06020
0.5
0.0
−0.5
−1.0
−1.5
Standardized expressions
EI PG G1 G2 H T1 T2 C1 C2 S EP JP
Developmental stages
VIT_06s0004g06030
0
−1
−2
Standardized expressions
EI PG G1 G2 H T1 T2 C1 C2 S EP JP
Developmental stages
VIT_06s0004g06040
0.5
0.0
−0.5
−1.0
Standardized expressions
EI PG G1 G2 H T1 T2 C1 C2 S EP JP
Developmental stages
VIT_06s0004g06050
0.8
0.4
0.0
−0.4
−0.8
−1.2
Standardized expressions
EI PG G1 G2 H T1 T2 C1 C2 S EP JP
Developmental stages
VIT_06s0004g06060
1
0
−1
−2
Standardized expressions
EI PG G1 G2 H T1 T2 C1 C2 S EP JP
Developmental stages
VIT_06s0004g06070
0.0
−0.5
Standardized expressions
EI PG G1 G2 H T1 T2 C1 C2 S EP JP
Developmental stages

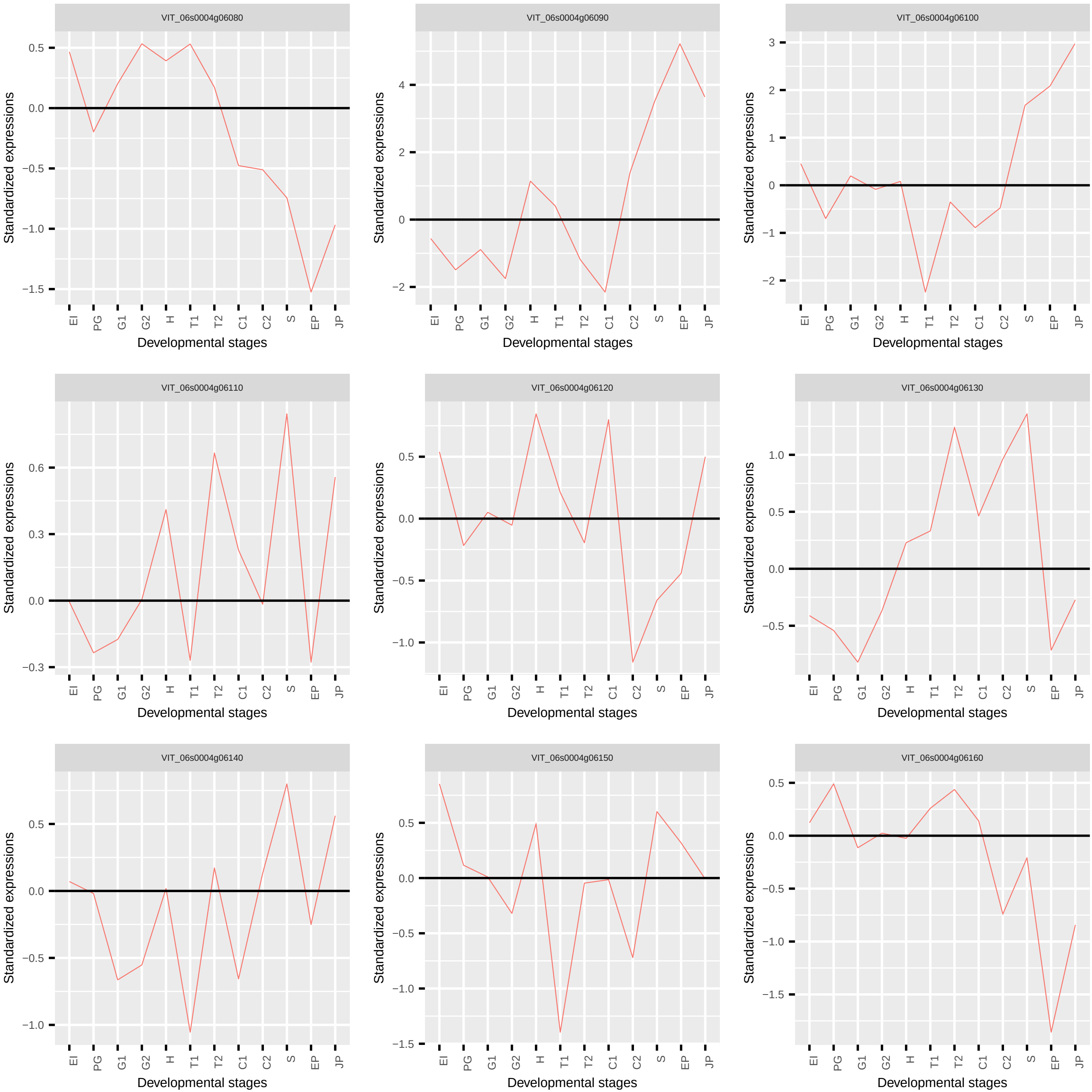
VIT_06s0004g06080
VIT_06s0004g06090
VIT_06s0004g06100
VIT_06s0004g06110
VIT_06s0004g06120
VIT_06s0004g06130
VIT_06s0004g06140
VIT_06s0004g06150
VIT_06s0004g06160
Standardized expressions
Developmental stages
EI
PG
G1
G2
H
T1
T2
C1
C2
S
EP
JP

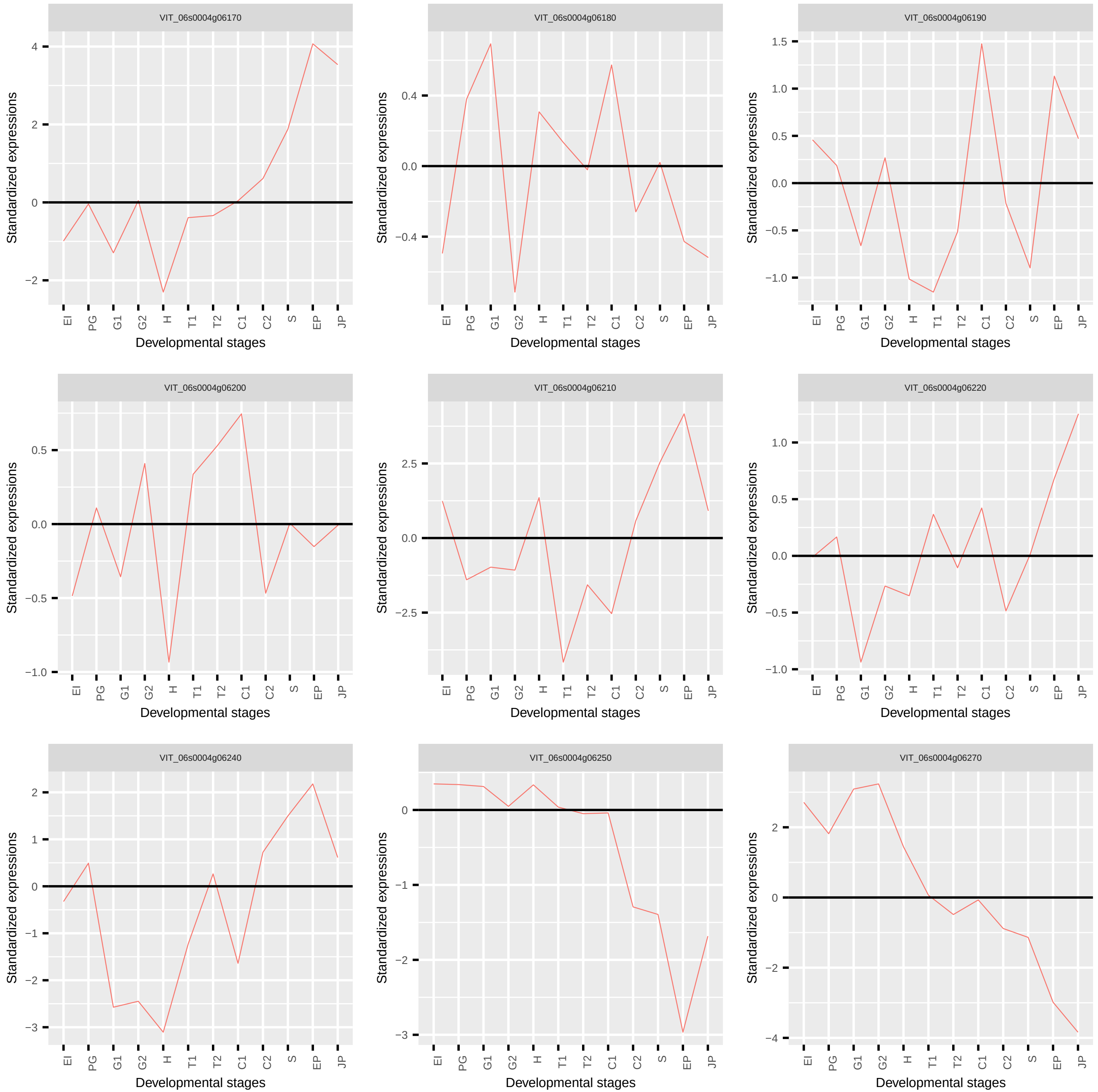
VIT_06s0004g06170
VIT_06s0004g06180
VIT_06s0004g06190
VIT_06s0004g06200
VIT_06s0004g06210
VIT_06s0004g06220
VIT_06s0004g06240
VIT_06s0004g06250
VIT_06s0004g06270
Standardized expressions
Developmental stages
EI
PG
G1
G2
H
T1
T2
C1
C2
S
EP
JP

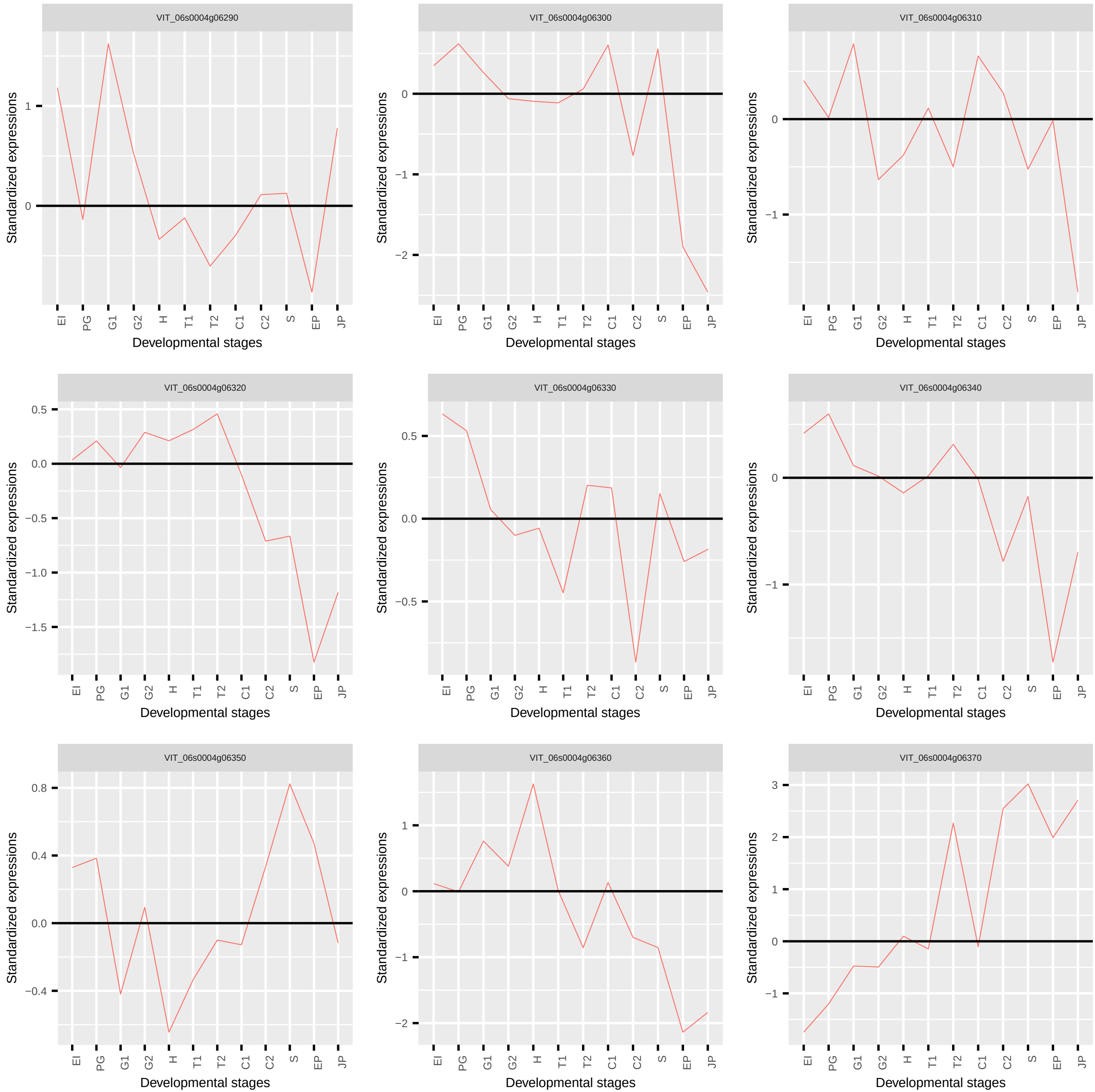

VIT_06s0004g06290
VIT_06s0004g06300
VIT_06s0004g06310
VIT_06s0004g06320
VIT_06s0004g06330
VIT_06s0004g06340
VIT_06s0004g06350
VIT_06s0004g06360
VIT_06s0004g06370
Standardized expressions
Developmental stages
EI PG G1 G2 H T1 T2 C1 C2 S EP JP

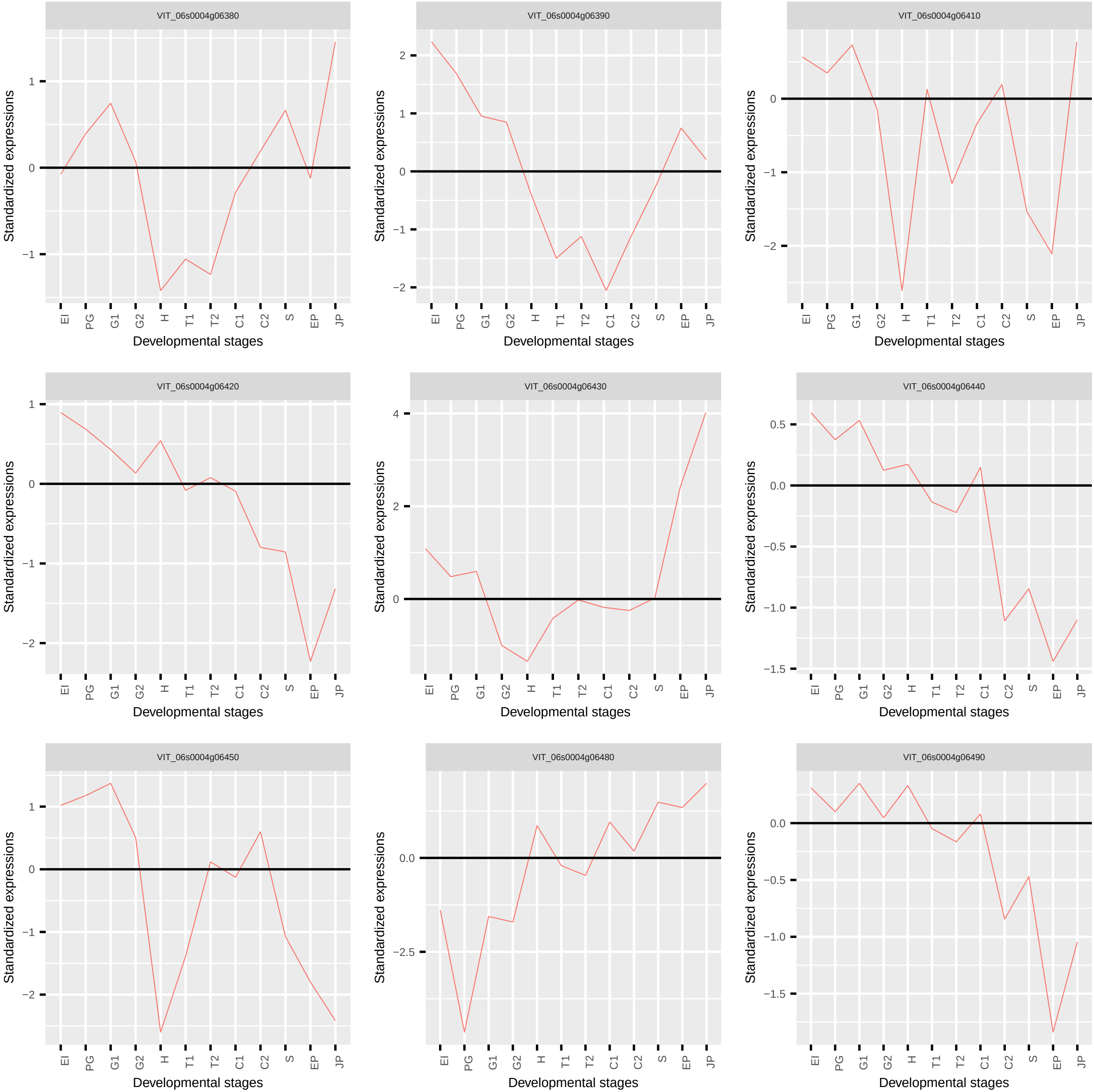

VIT_06s0004g06380
Standardized expressions
1
0
−1
EI PG G1 G2 H T1 T2 C1 C2 S EP JP
Developmental stages
VIT_06s0004g06390
Standardized expressions
2
1
0
−1
−2
EI PG G1 G2 H T1 T2 C1 C2 S EP JP
Developmental stages
VIT_06s0004g06410
Standardized expressions
0
−1
−2
EI PG G1 G2 H T1 T2 C1 C2 S EP JP
Developmental stages
VIT_06s0004g06420
Standardized expressions
1
0
−1
−2
EI PG G1 G2 H T1 T2 C1 C2 S EP JP
Developmental stages
VIT_06s0004g06430
Standardized expressions
4
2
0
EI PG G1 G2 H T1 T2 C1 C2 S EP JP
Developmental stages
VIT_06s0004g06440
Standardized expressions
0.5
0.0
−0.5
−1.0
−1.5
EI PG G1 G2 H T1 T2 C1 C2 S EP JP
Developmental stages
VIT_06s0004g06450
Standardized expressions
1
0
−1
−2
EI PG G1 G2 H T1 T2 C1 C2 S EP JP
Developmental stages
VIT_06s0004g06480
Standardized expressions
0.0
−2.5
EI PG G1 G2 H T1 T2 C1 C2 S EP JP
Developmental stages
VIT_06s0004g06490
Standardized expressions
0.0
−0.5
−1.0
−1.5
EI PG G1 G2 H T1 T2 C1 C2 S EP JP
Developmental stages

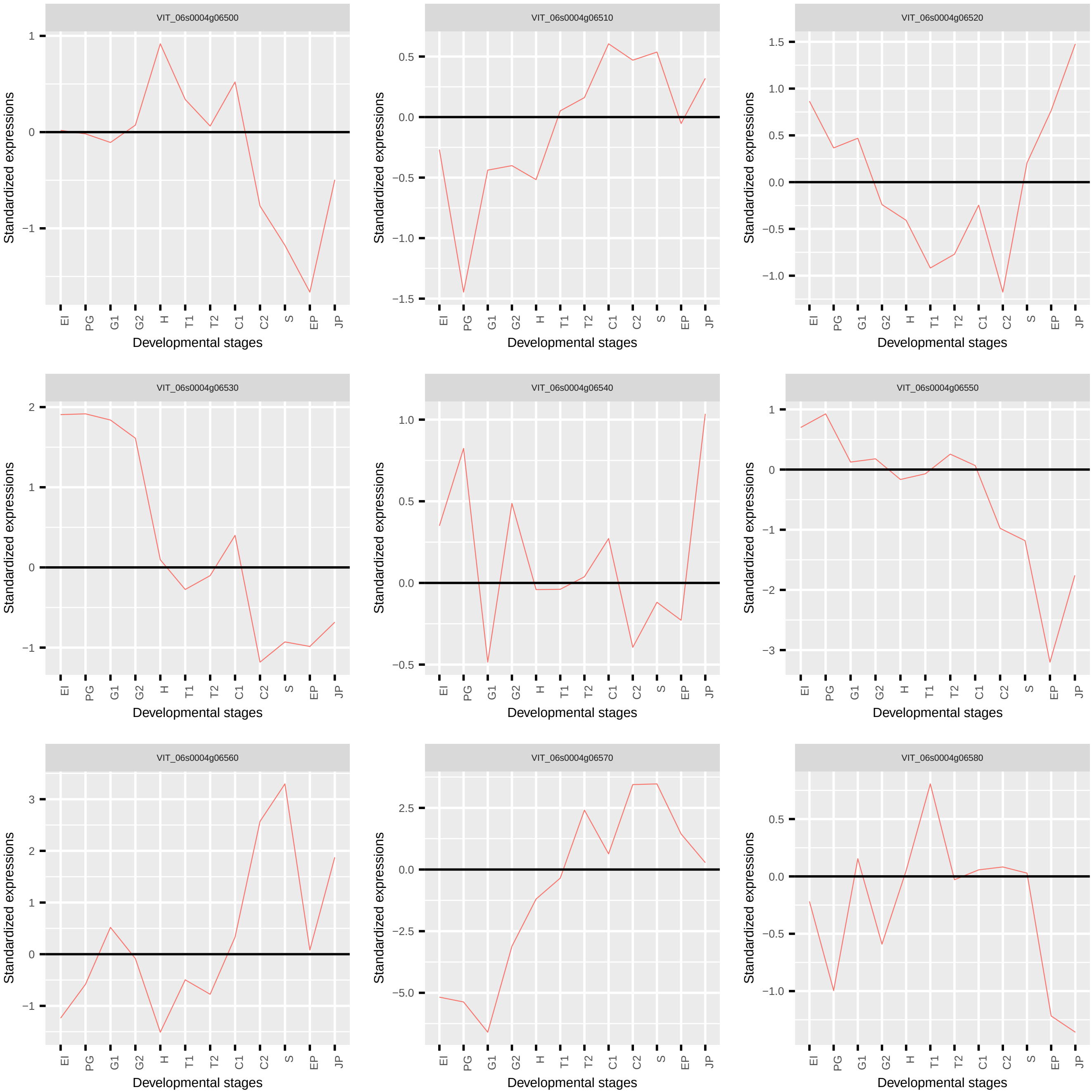
VIT_06s0004g06500
VIT_06s0004g06510
VIT_06s0004g06520
VIT_06s0004g06530
VIT_06s0004g06540
VIT_06s0004g06550
VIT_06s0004g06560
VIT_06s0004g06570
VIT_06s0004g06580
Standardized expressions
Developmental stages
EI PG G1 G2 H T1 T2 C1 C2 S EP JP

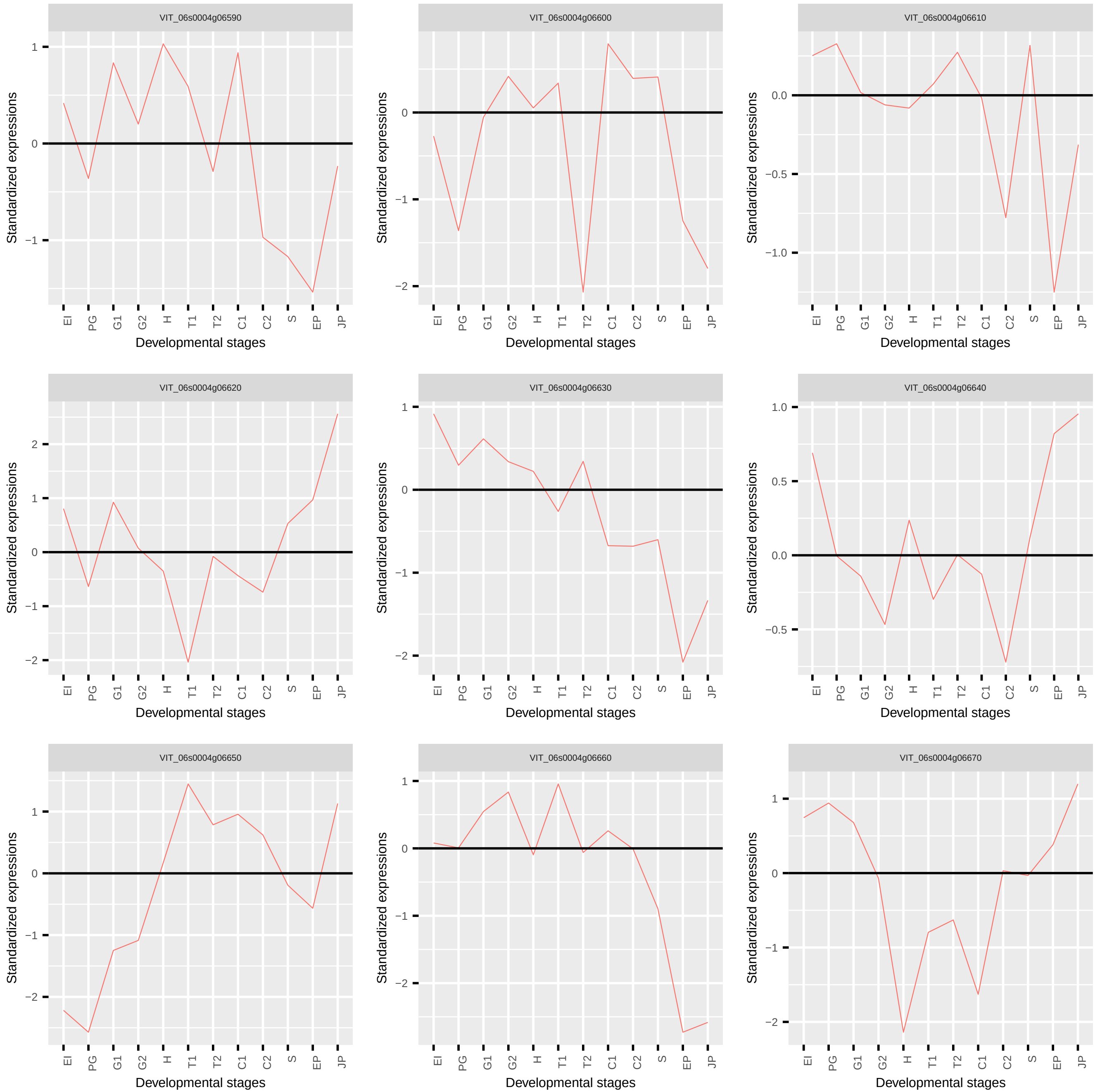
VIT_06s0004g06590
VIT_06s0004g06600
VIT_06s0004g06610
VIT_06s0004g06620
VIT_06s0004g06630
VIT_06s0004g06640
VIT_06s0004g06650
VIT_06s0004g06660
VIT_06s0004g06670
Standardized expressions
Developmental stages
EI PG G1 G2 H T1 T2 C1 C2 S EP JP

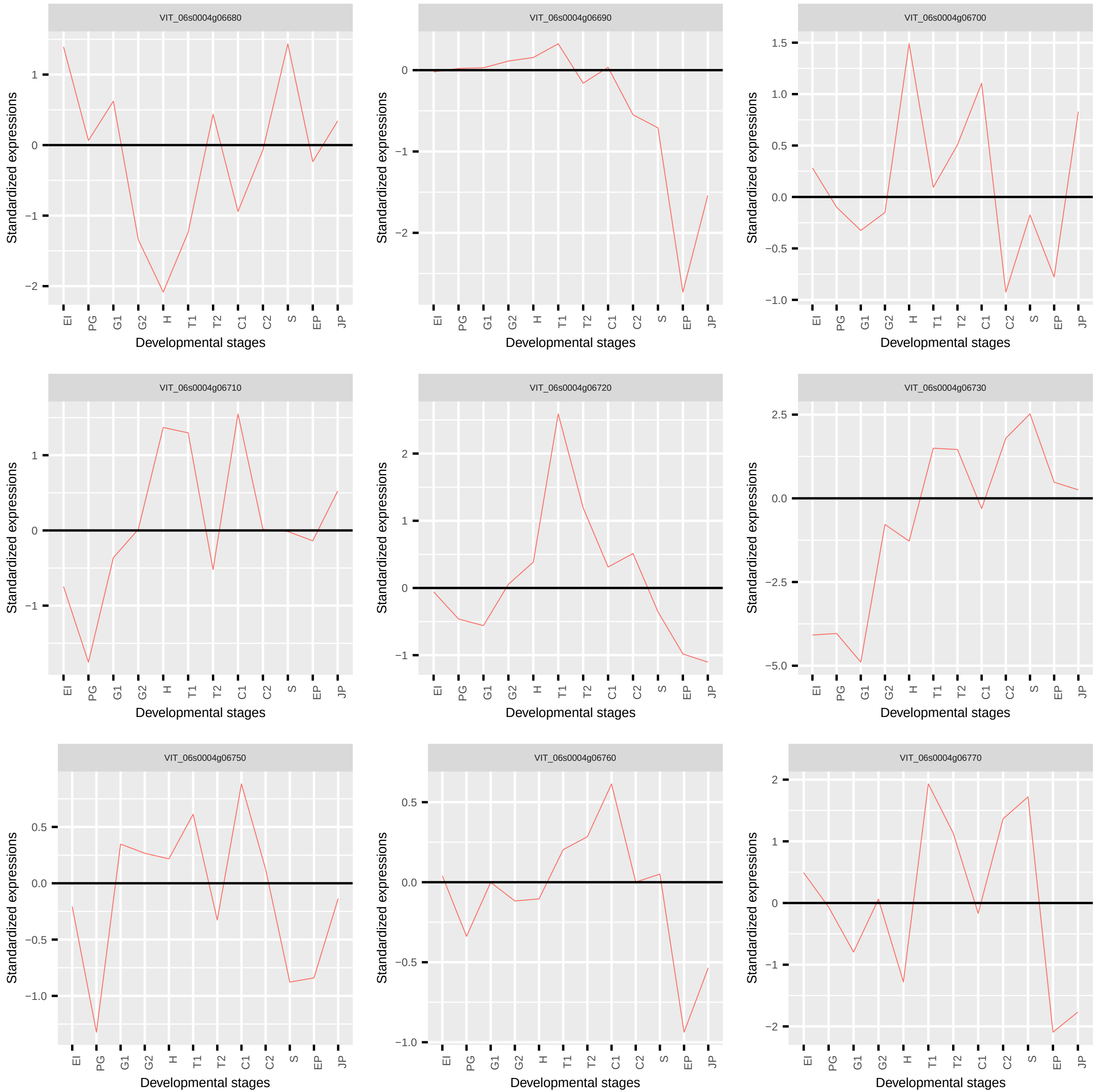

VIT_06s0004g06680
VIT_06s0004g06690
VIT_06s0004g06700
VIT_06s0004g06710
VIT_06s0004g06720
VIT_06s0004g06730
VIT_06s0004g06750
VIT_06s0004g06760
VIT_06s0004g06770
Standardized expressions
Developmental stages
EI PG G1 G2 H T1 T2 C1 C2 S EP JP

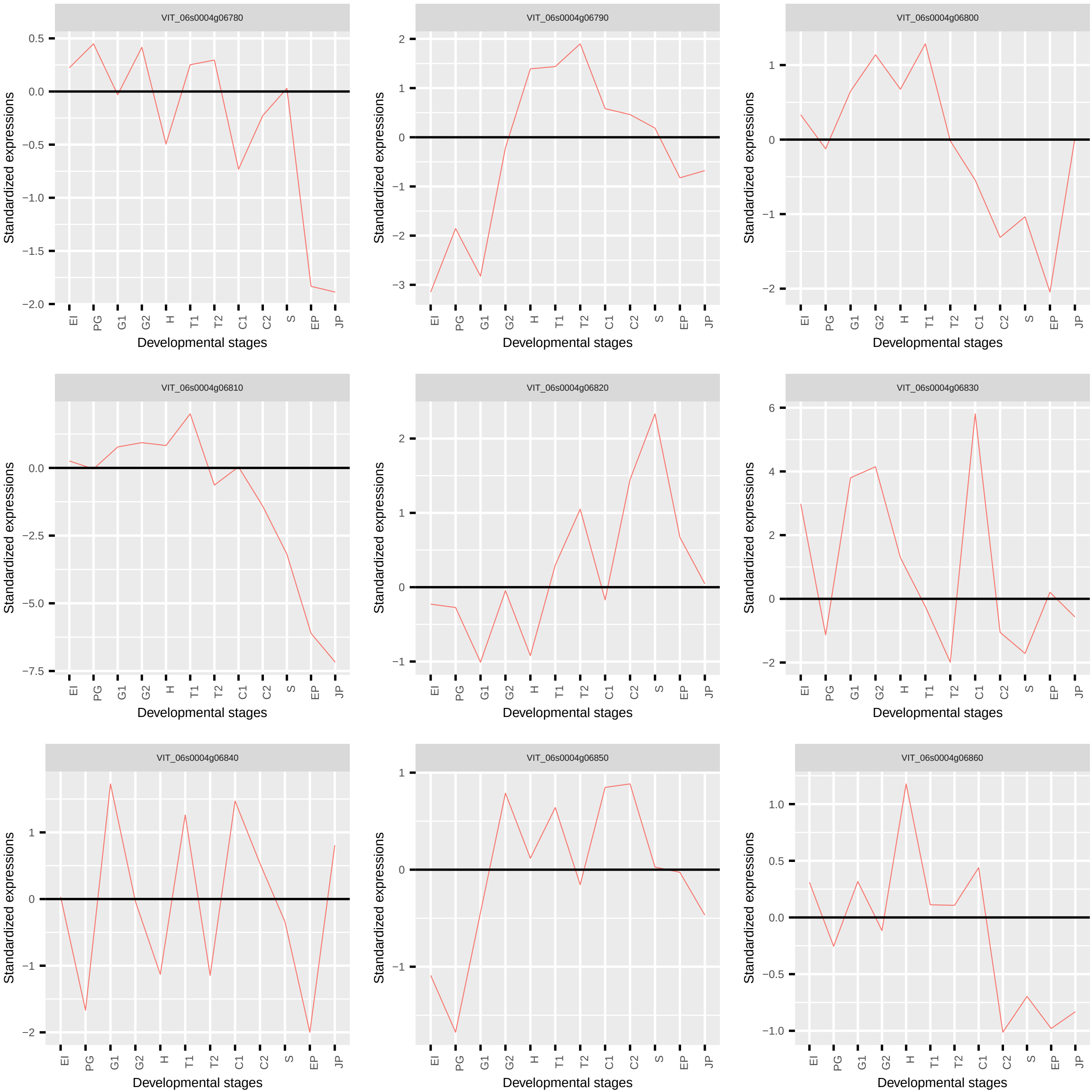
VIT_06s0004g06780
VIT_06s0004g06790
VIT_06s0004g06800
VIT_06s0004g06810
VIT_06s0004g06820
VIT_06s0004g06830
VIT_06s0004g06840
VIT_06s0004g06850
VIT_06s0004g06860
Standardized expressions
Developmental stages
EI PG G1 G2 H T1 T2 C1 C2 S EP JP

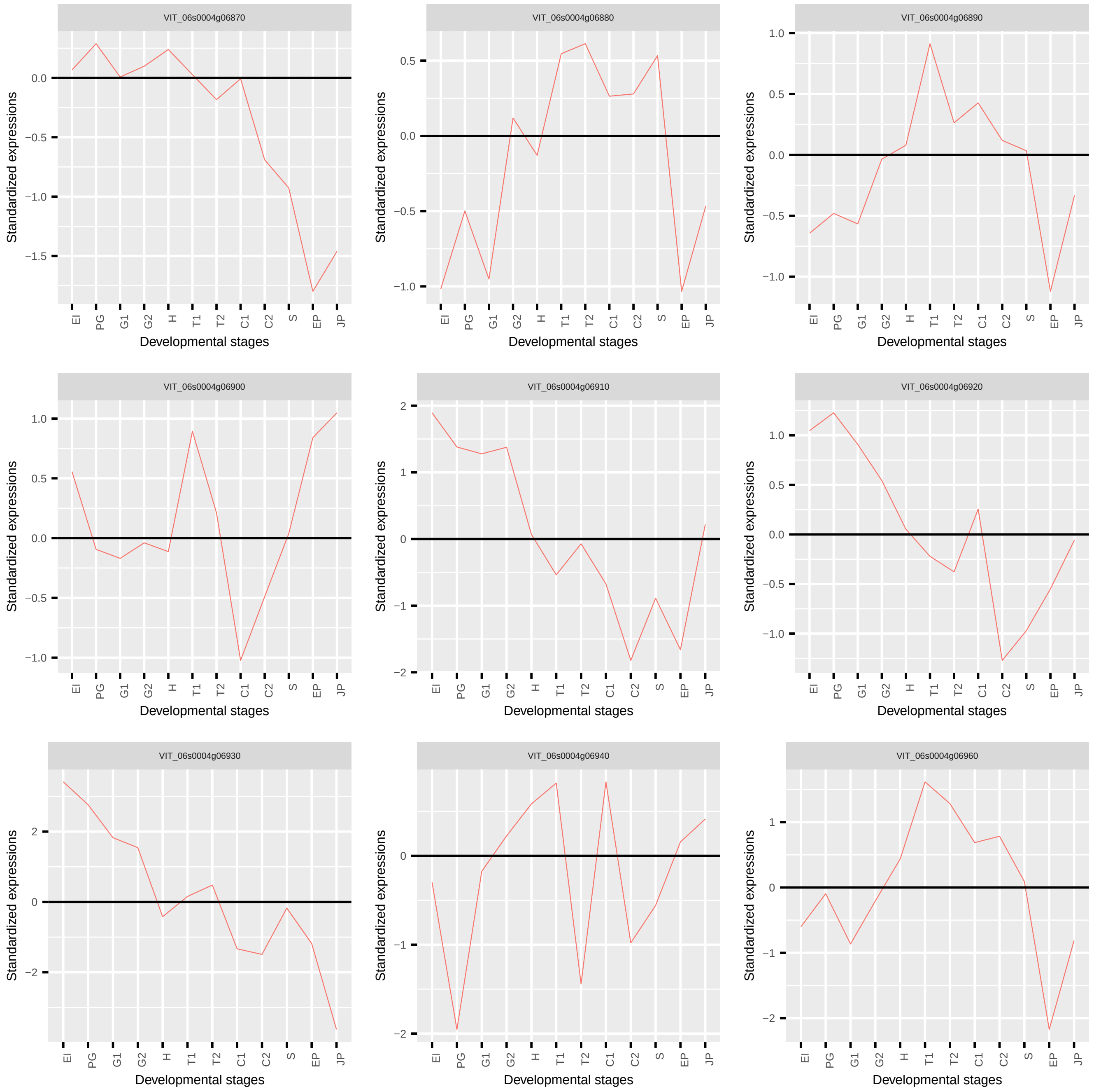
VIT_06s0004g06870
VIT_06s0004g06880
VIT_06s0004g06890
VIT_06s0004g06900
VIT_06s0004g06910
VIT_06s0004g06920
VIT_06s0004g06930
VIT_06s0004g06940
VIT_06s0004g06960
Standardized expressions
Developmental stages
EI
PG
G1
G2
H
T1
T2
C1
C2
S
EP
JP

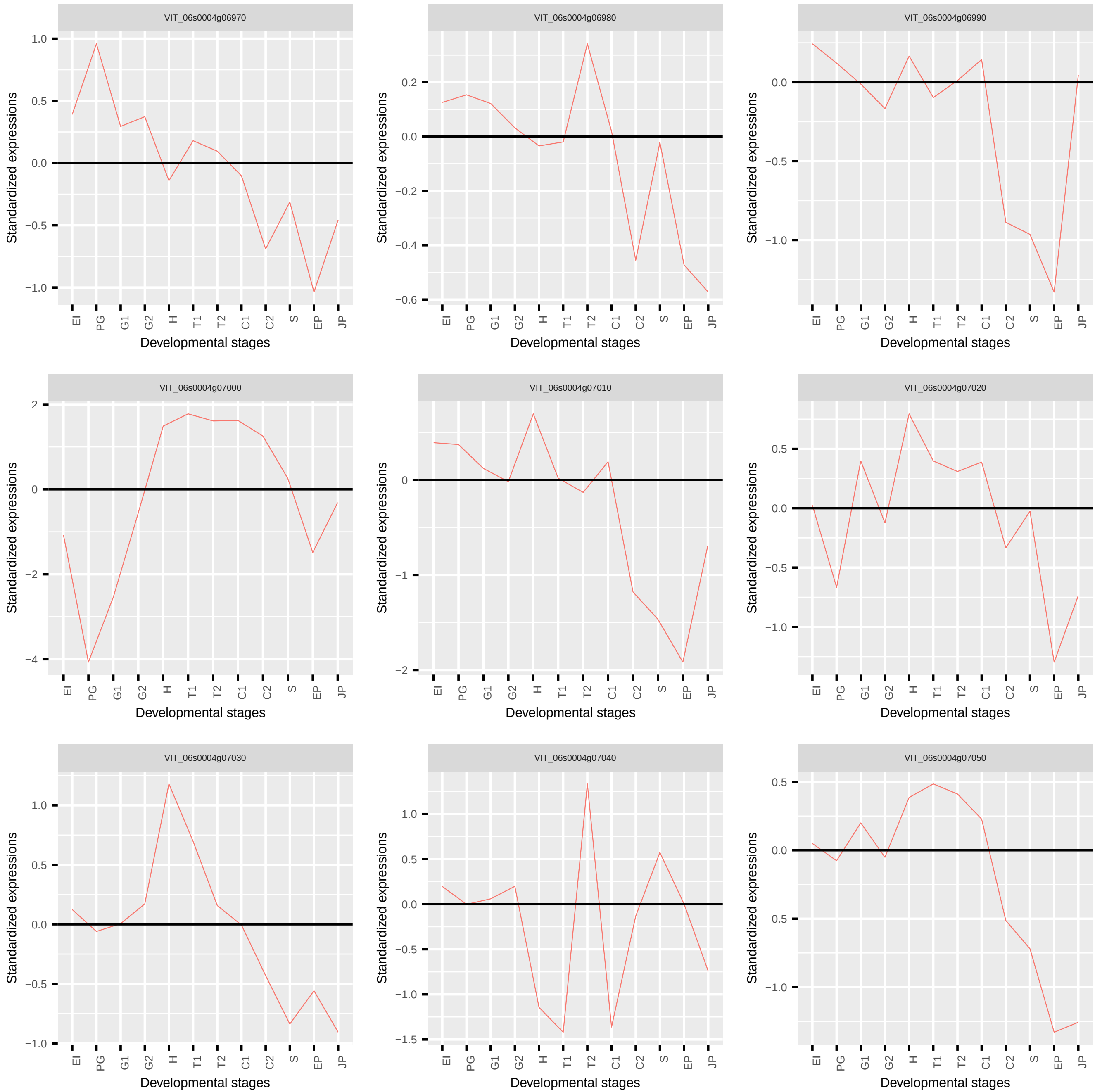
VIT_06s0004g06970
1.0
0.5
0.0
−0.5
−1.0
Standardized expressions
EI PG G1 G2 H T1 T2 C1 C2 S EP JP
Developmental stages
VIT_06s0004g06980
0.2
0.0
−0.2
−0.4
−0.6
Standardized expressions
EI PG G1 G2 H T1 T2 C1 C2 S EP JP
Developmental stages
VIT_06s0004g06990
0.0
−0.5
−1.0
Standardized expressions
EI PG G1 G2 H T1 T2 C1 C2 S EP JP
Developmental stages
VIT_06s0004g07000
2
0
−2
−4
Standardized expressions
EI PG G1 G2 H T1 T2 C1 C2 S EP JP
Developmental stages
VIT_06s0004g07010
0
−1
−2
Standardized expressions
EI PG G1 G2 H T1 T2 C1 C2 S EP JP
Developmental stages
VIT_06s0004g07020
0.5
0.0
−0.5
−1.0
Standardized expressions
EI PG G1 G2 H T1 T2 C1 C2 S EP JP
Developmental stages
VIT_06s0004g07030
1.0
0.5
0.0
−0.5
−1.0
Standardized expressions
EI PG G1 G2 H T1 T2 C1 C2 S EP JP
Developmental stages
VIT_06s0004g07040
1.0
0.5
0.0
−0.5
−1.0
−1.5
Standardized expressions
EI PG G1 G2 H T1 T2 C1 C2 S EP JP
Developmental stages
VIT_06s0004g07050
0.5
0.0
−0.5
−1.0
Standardized expressions
EI PG G1 G2 H T1 T2 C1 C2 S EP JP
Developmental stages

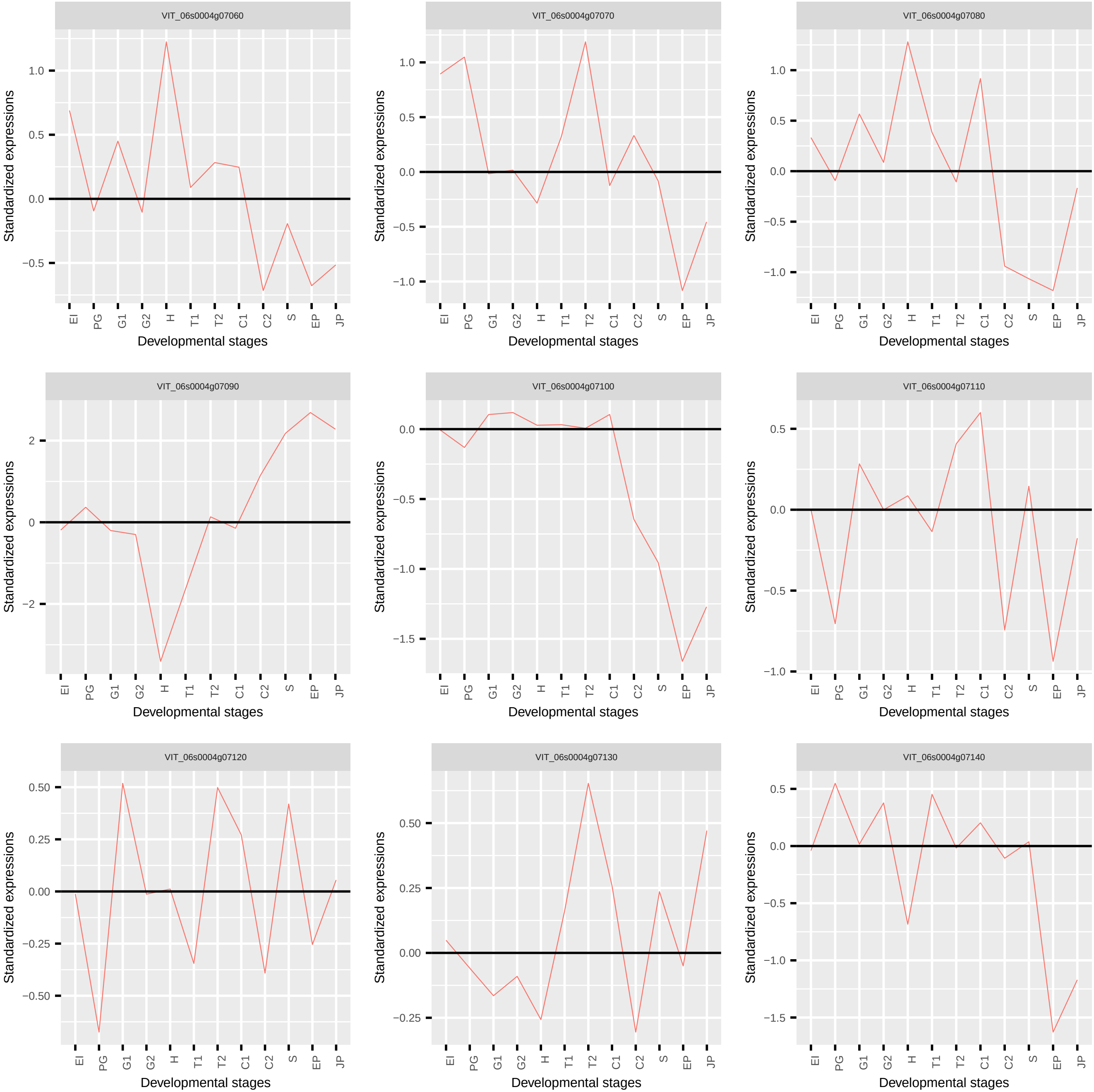
VIT_06s0004g07060
VIT_06s0004g07070
VIT_06s0004g07080
VIT_06s0004g07090
VIT_06s0004g07100
VIT_06s0004g07110
VIT_06s0004g07120
VIT_06s0004g07130
VIT_06s0004g07140
Standardized expressions
Developmental stages
EI PG G1 G2 H T1 T2 C1 C2 S EP JP
1.0 0.5 0.0 −0.5 −1.0 −1.5
2 0 −2
0.50 0.25 0.00 −0.25 −0.50

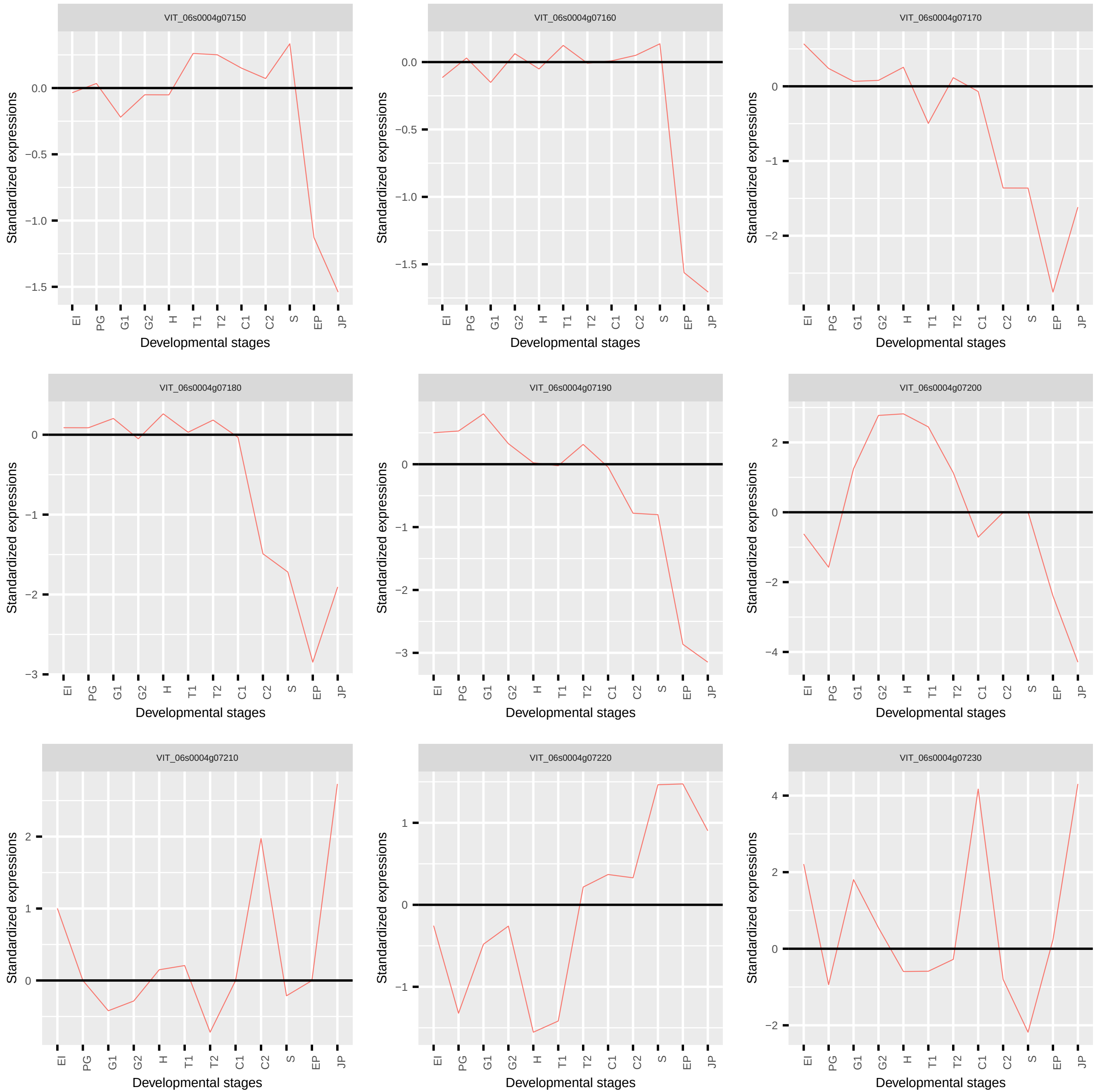
VIT_06s0004g07150
VIT_06s0004g07160
VIT_06s0004g07170
VIT_06s0004g07180
VIT_06s0004g07190
VIT_06s0004g07200
VIT_06s0004g07210
VIT_06s0004g07220
VIT_06s0004g07230
Standardized expressions
Developmental stages
EI PG G1 G2 H T1 T2 C1 C2 S EP JP

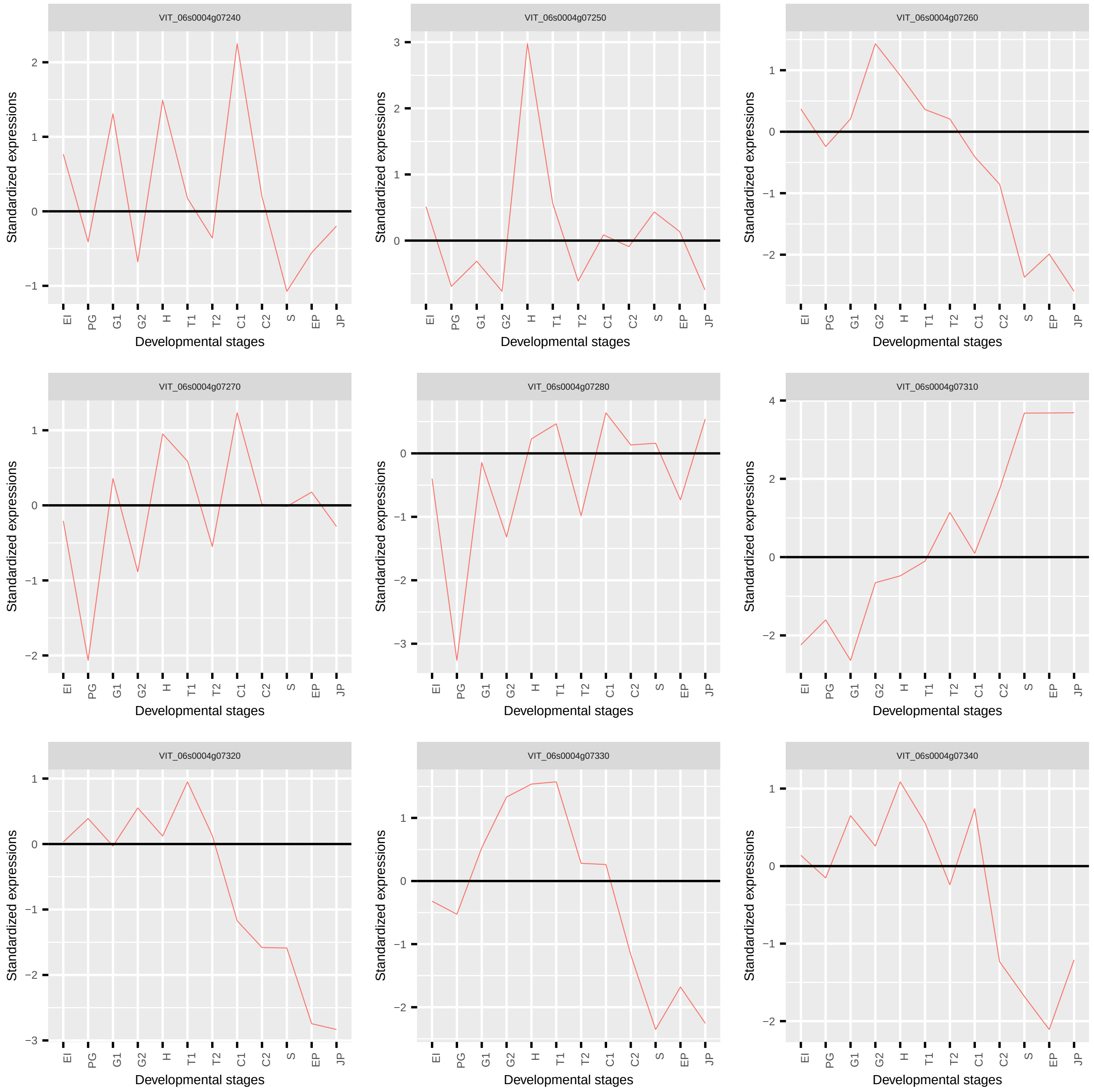

VIT_06s0004g07240
VIT_06s0004g07250
VIT_06s0004g07260
VIT_06s0004g07270
VIT_06s0004g07280
VIT_06s0004g07310
VIT_06s0004g07320
VIT_06s0004g07330
VIT_06s0004g07340
Standardized expressions
Developmental stages
EI PG G1 G2 H T1 T2 C1 C2 S EP JP

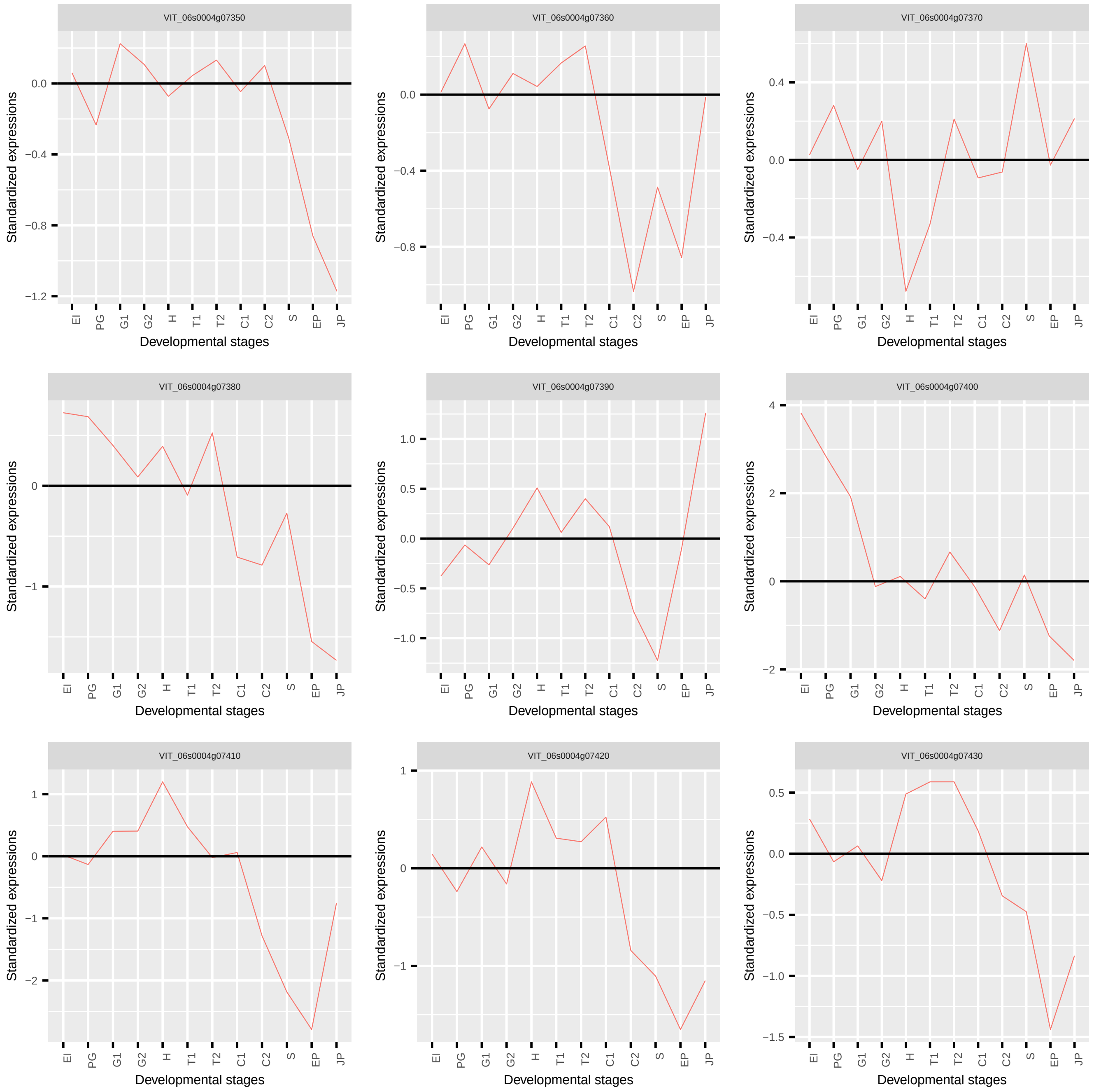
VIT_06s0004g07350
VIT_06s0004g07360
VIT_06s0004g07370
VIT_06s0004g07380
VIT_06s0004g07390
VIT_06s0004g07400
VIT_06s0004g07410
VIT_06s0004g07420
VIT_06s0004g07430
Standardized expressions
Developmental stages
EI PG G1 G2 H T1 T2 C1 C2 S EP JP

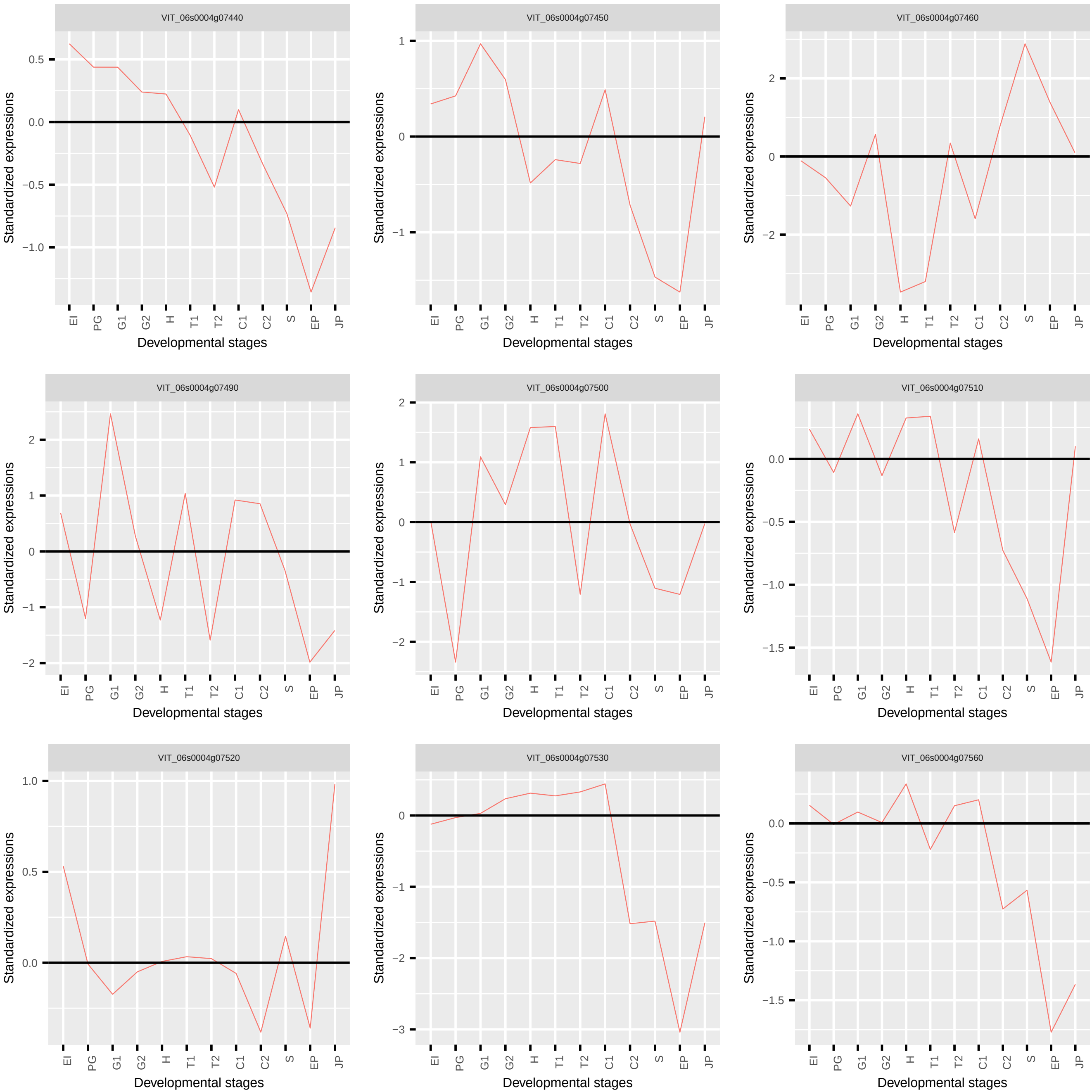
VIT_06s0004g07440
VIT_06s0004g07450
VIT_06s0004g07460
VIT_06s0004g07490
VIT_06s0004g07500
VIT_06s0004g07510
VIT_06s0004g07520
VIT_06s0004g07530
VIT_06s0004g07560
Standardized expressions
Developmental stages
EI PG G1 G2 H T1 T2 C1 C2 S EP JP

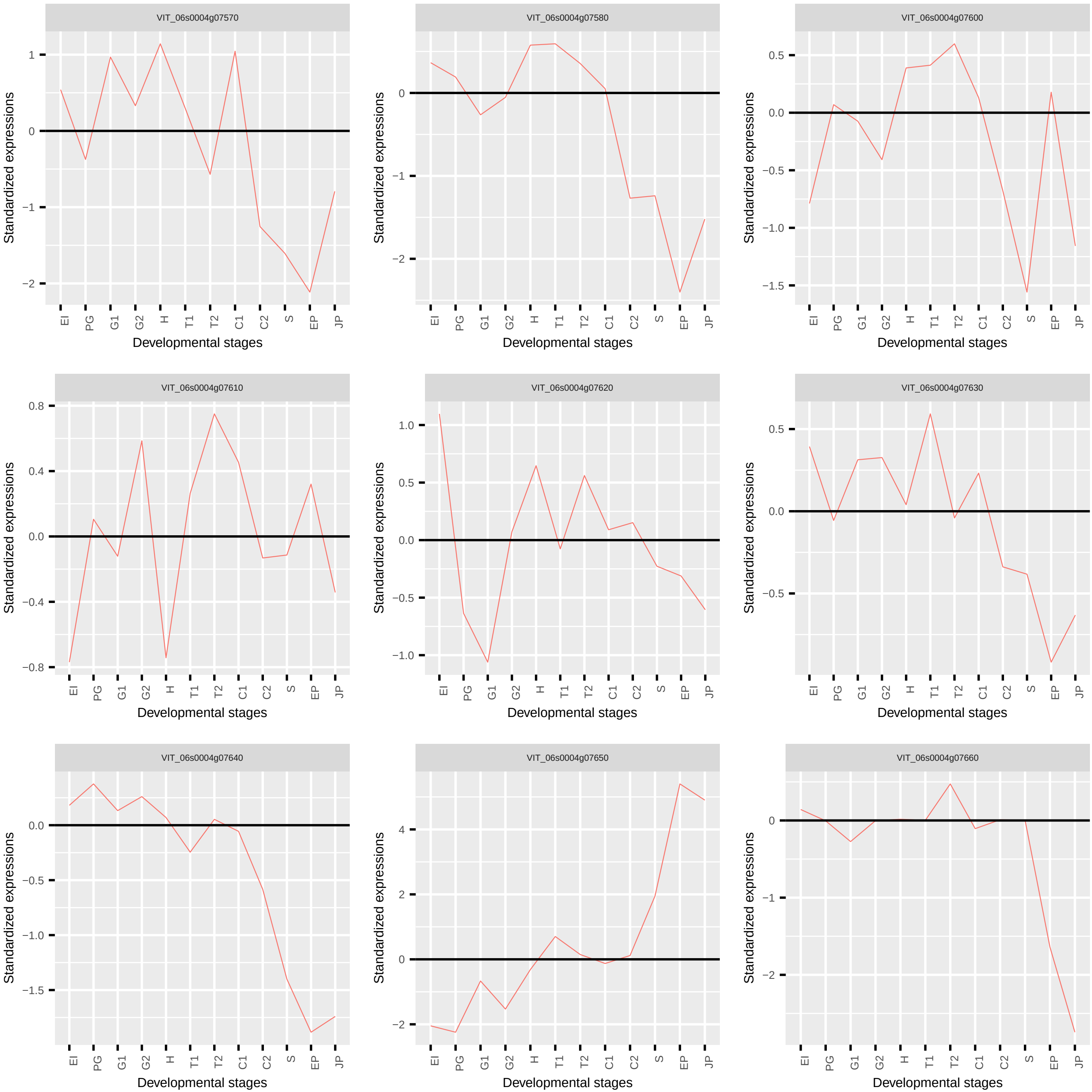
VIT_06s0004g07570
VIT_06s0004g07580
VIT_06s0004g07600
VIT_06s0004g07610
VIT_06s0004g07620
VIT_06s0004g07630
VIT_06s0004g07640
VIT_06s0004g07650
VIT_06s0004g07660
Standardized expressions
Developmental stages
EI
PG
G1
G2
H
T1
T2
C1
C2
S
EP
JP

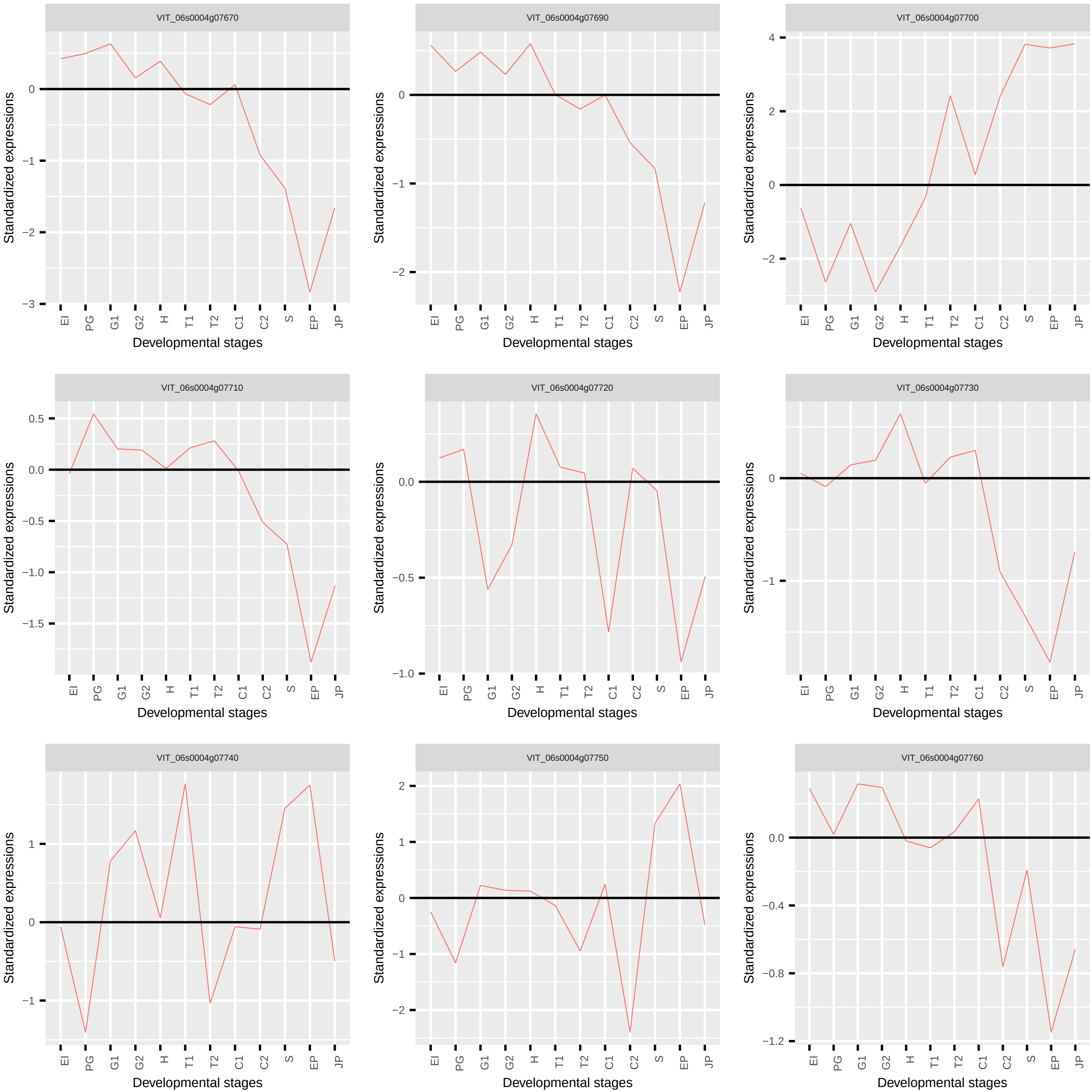
VIT_06s0004g07670
VIT_06s0004g07690
VIT_06s0004g07700
VIT_06s0004g07710
VIT_06s0004g07720
VIT_06s0004g07730
VIT_06s0004g07740
VIT_06s0004g07750
VIT_06s0004g07760
Standardized expressions
Developmental stages
EI PG G1 G2 H T1 T2 C1 C2 S EP JP

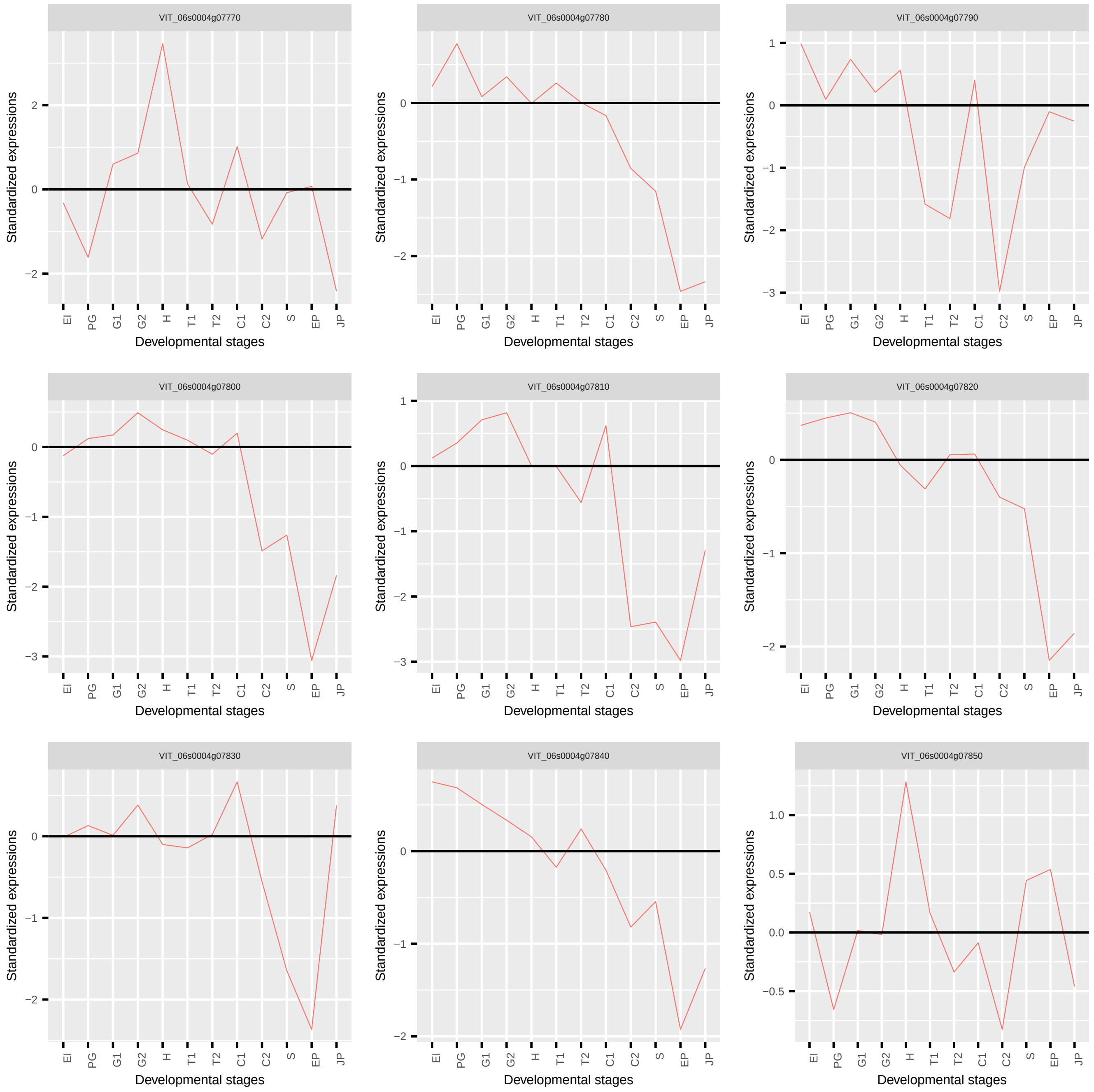

VIT_06s0004g07770
VIT_06s0004g07780
VIT_06s0004g07790
VIT_06s0004g07800
VIT_06s0004g07810
VIT_06s0004g07820
VIT_06s0004g07830
VIT_06s0004g07840
VIT_06s0004g07850
Standardized expressions
Developmental stages
EI PG G1 G2 H T1 T2 C1 C2 S EP JP

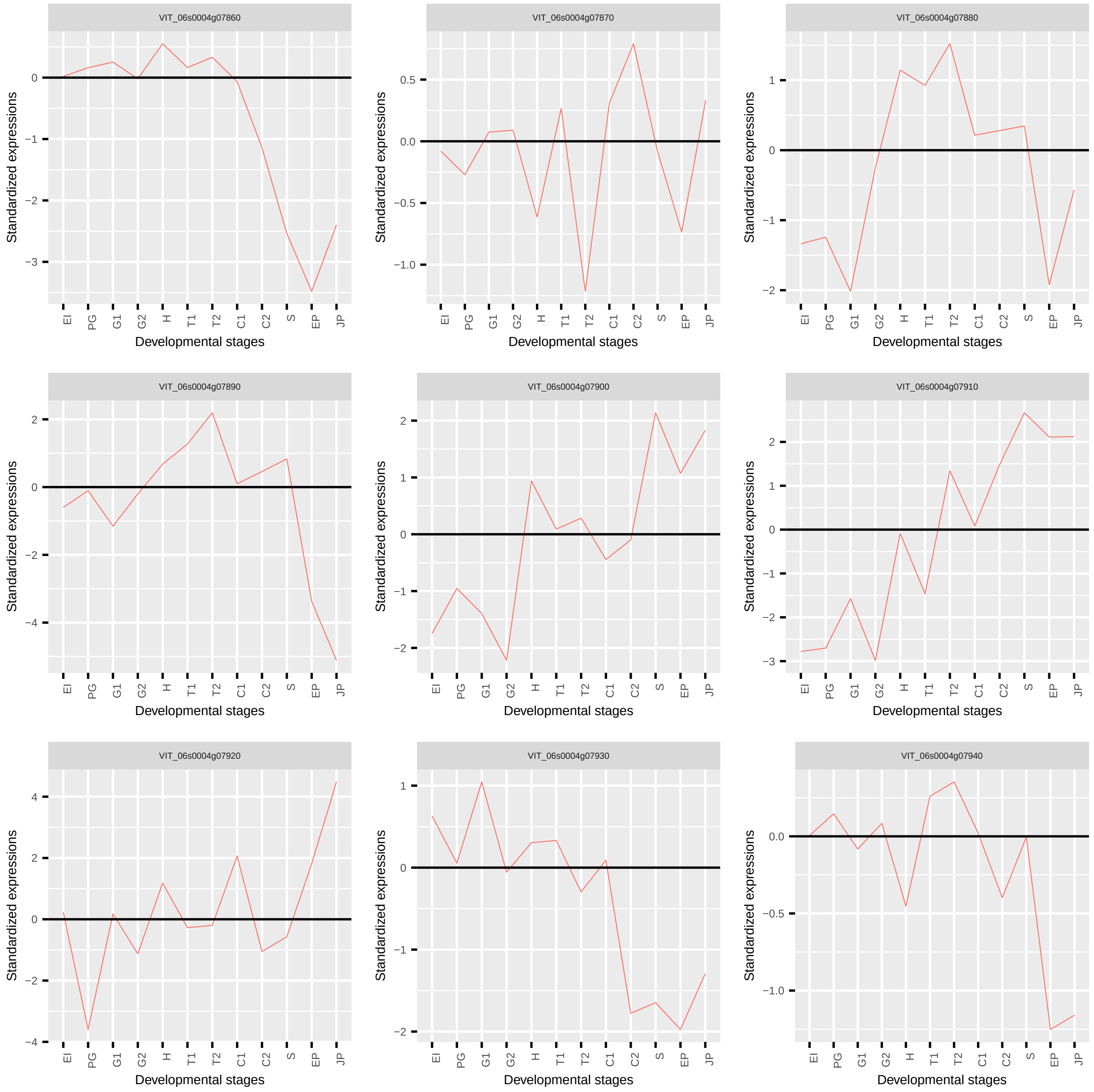
VIT_06s0004g07860
VIT_06s0004g07870
VIT_06s0004g07880
VIT_06s0004g07890
VIT_06s0004g07900
VIT_06s0004g07910
VIT_06s0004g07920
VIT_06s0004g07930
VIT_06s0004g07940
Standardized expressions
Developmental stages
EI PG G1 G2 H T1 T2 C1 C2 S EP JP
0 −1 −2 −3
0.5 0.0 −0.5 −1.0
1 0 −1 −2
2 0 −2 −4
2 1 0 −1 −2
2 1 0 −1 −2 −3
4 2 0 −2 −4
1 0 −1 −2
0.0 −0.5 −1.0

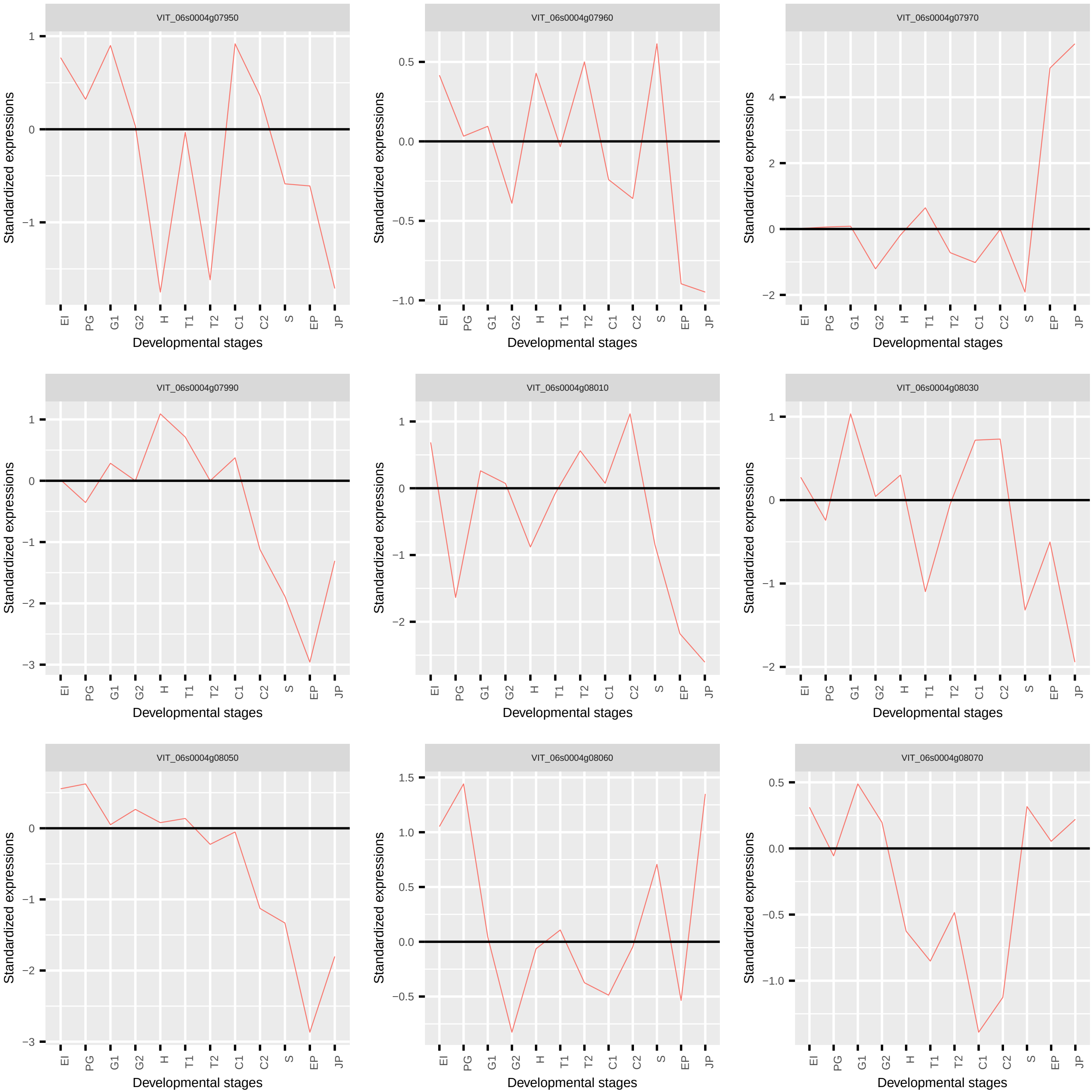
VIT_06s0004g07950
VIT_06s0004g07960
VIT_06s0004g07970
VIT_06s0004g07990
VIT_06s0004g08010
VIT_06s0004g08030
VIT_06s0004g08050
VIT_06s0004g08060
VIT_06s0004g08070
Standardized expressions
Developmental stages
EI PG G1 G2 H T1 T2 C1 C2 S EP JP

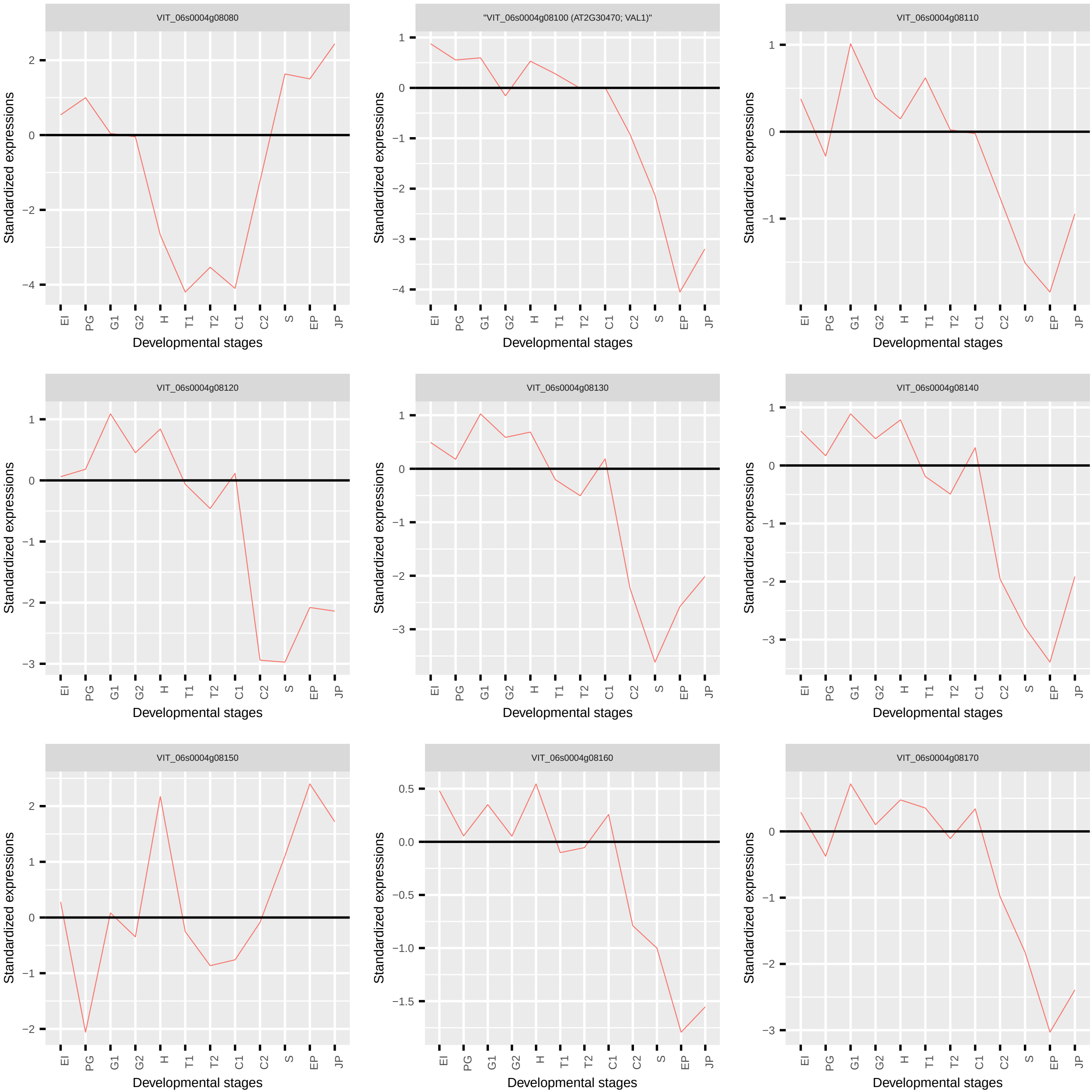
VIT_06s0004g08080
"VIT_06s0004g08100 (AT2G30470; VAL1)"
VIT_06s0004g08110
VIT_06s0004g08120
VIT_06s0004g08130
VIT_06s0004g08140
VIT_06s0004g08150
VIT_06s0004g08160
VIT_06s0004g08170
Standardized expressions
Developmental stages
EI PG G1 G2 H T1 T2 C1 C2 S EP JP

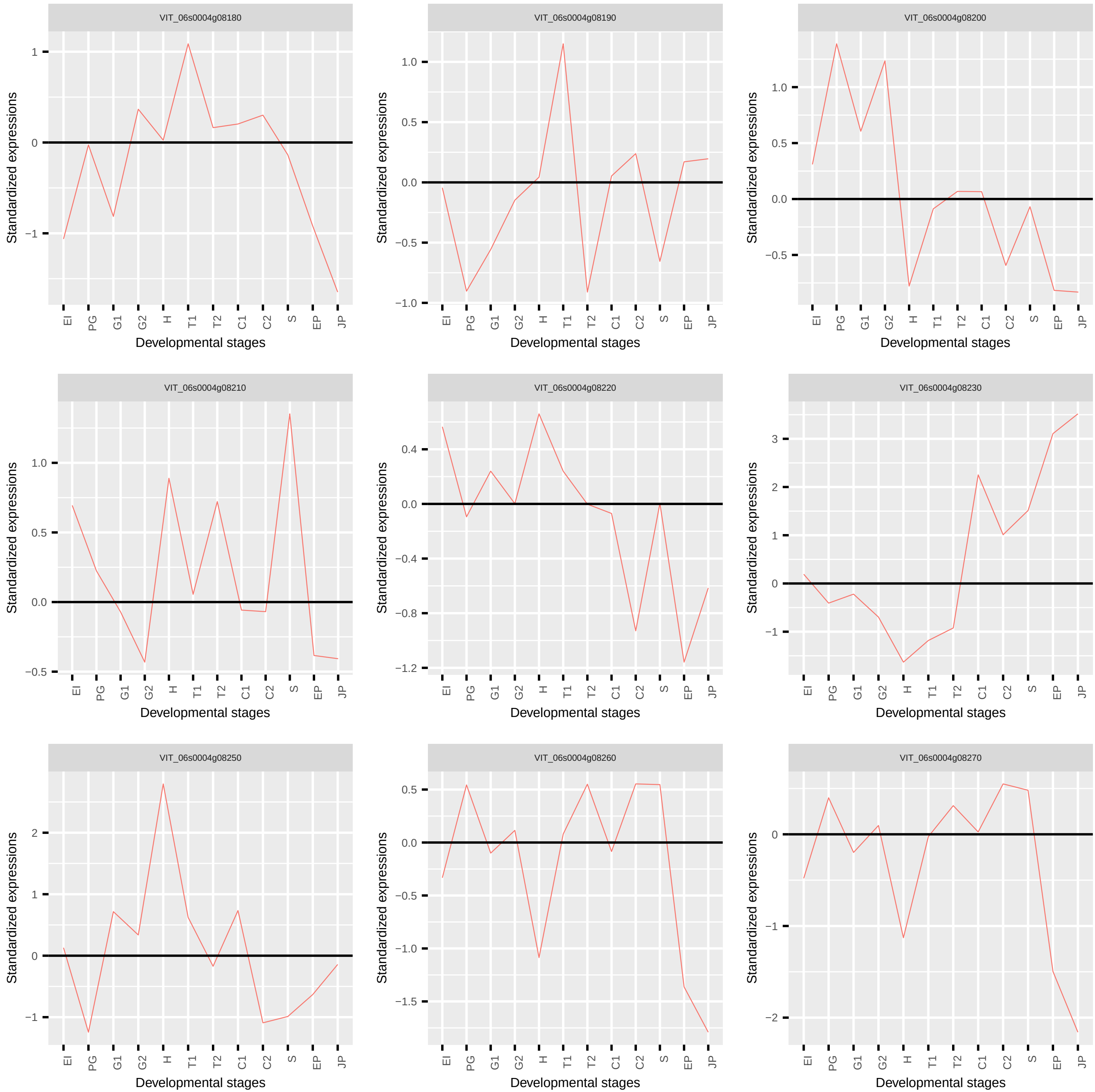
VIT_06s0004g08180
VIT_06s0004g08190
VIT_06s0004g08200
VIT_06s0004g08210
VIT_06s0004g08220
VIT_06s0004g08230
VIT_06s0004g08250
VIT_06s0004g08260
VIT_06s0004g08270
Standardized expressions
Developmental stages
EI PG G1 G2 H T1 T2 C1 C2 S EP JP

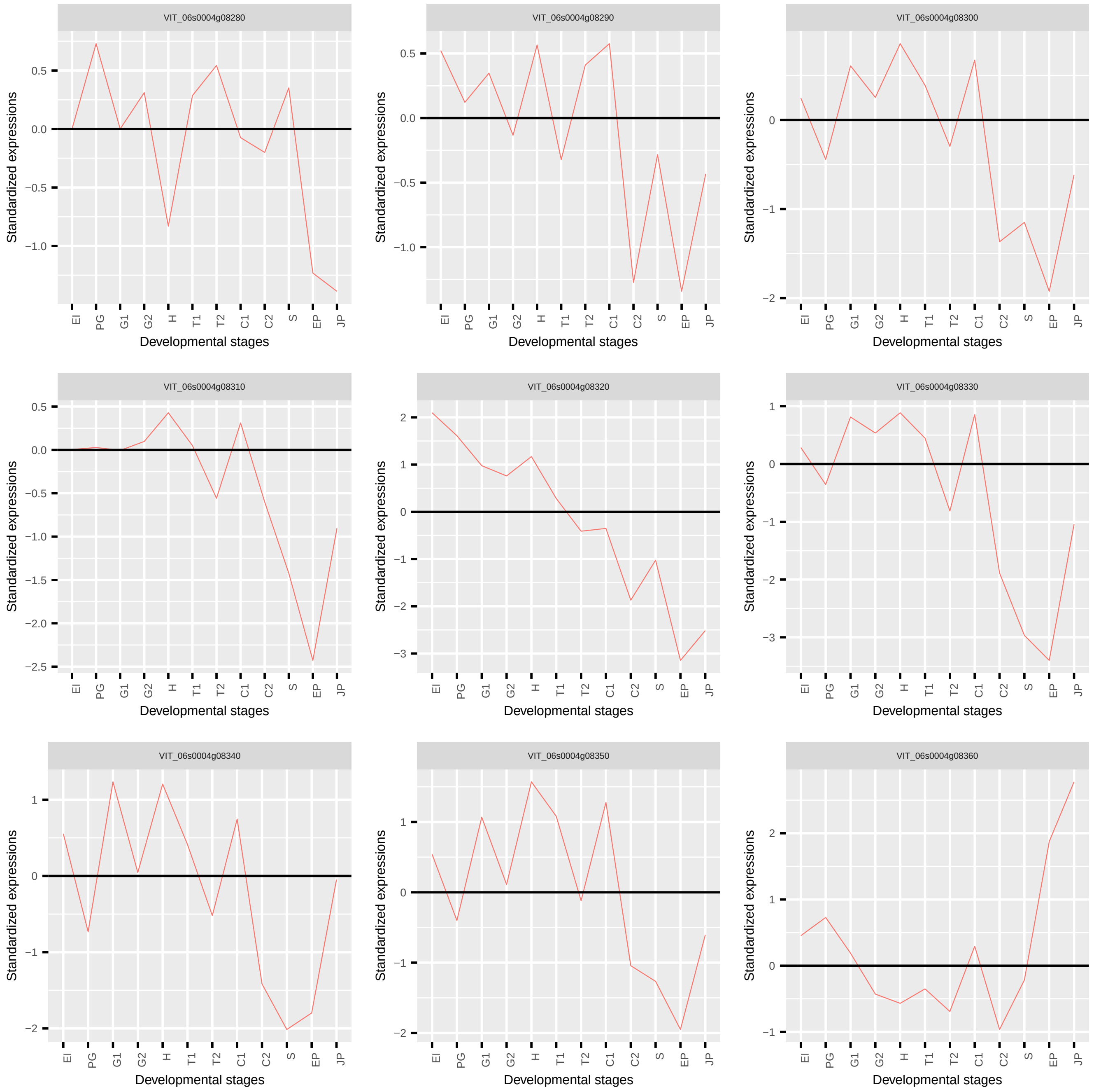
VIT_06s0004g08280
0.5
0.0
−0.5
−1.0
VIT_06s0004g08290
0.5
0.0
−0.5
−1.0
VIT_06s0004g08300
0
−1
−2
VIT_06s0004g08310
0.5
0.0
−0.5
−1.0
−1.5
−2.0
−2.5
VIT_06s0004g08320
2
1
0
−1
−2
−3
VIT_06s0004g08330
1
0
−1
−2
−3
VIT_06s0004g08340
1
0
−1
−2
VIT_06s0004g08350
1
0
−1
−2
VIT_06s0004g08360
2
1
0
−1
Standardized expressions
EI PG G1 G2 H T1 T2 C1 C2 S EP JP
Developmental stages

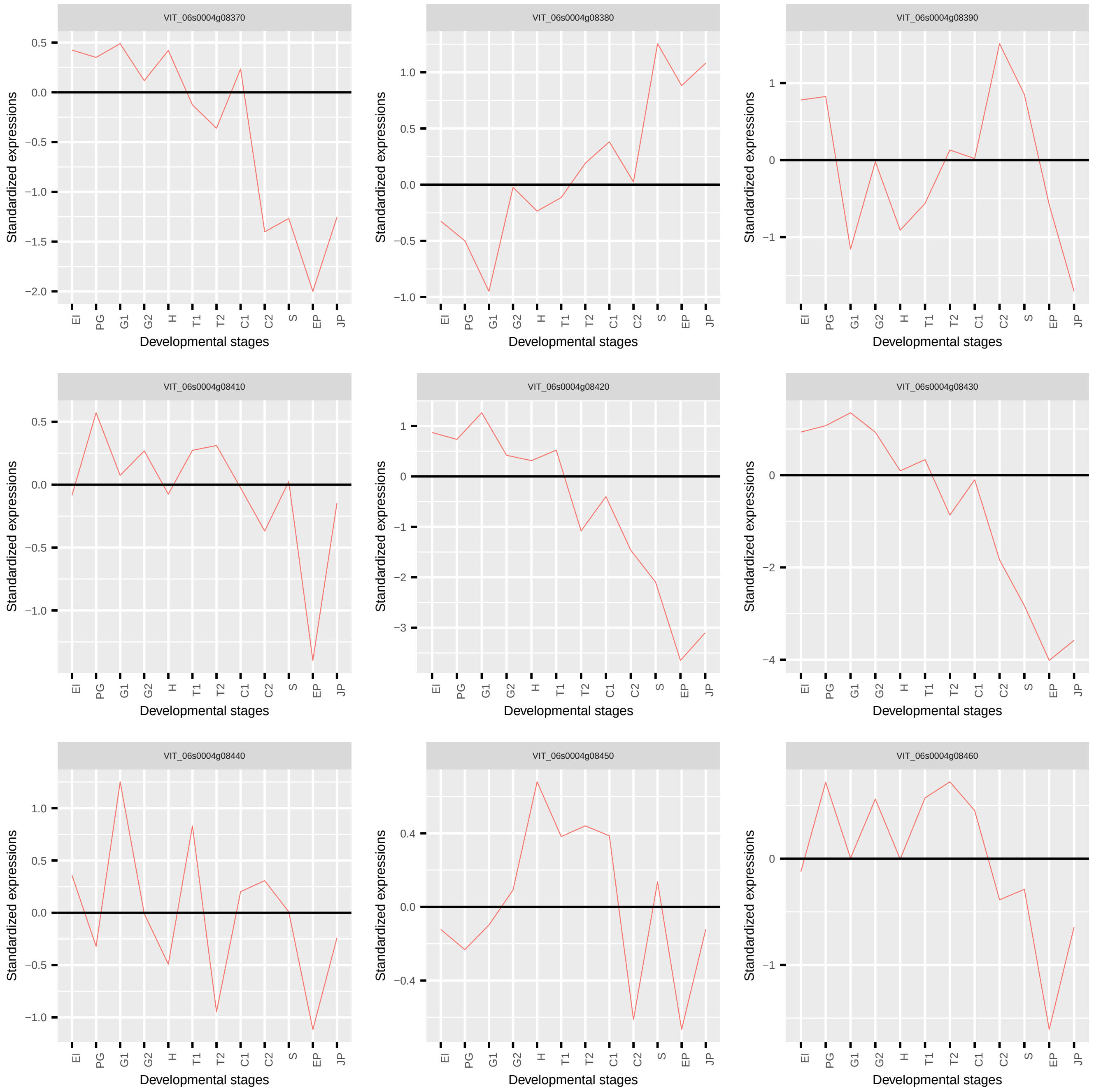
VIT_06s0004g08370
VIT_06s0004g08380
VIT_06s0004g08390
VIT_06s0004g08410
VIT_06s0004g08420
VIT_06s0004g08430
VIT_06s0004g08440
VIT_06s0004g08450
VIT_06s0004g08460
Standardized expressions
Developmental stages
EI PG G1 G2 H T1 T2 C1 C2 S EP JP

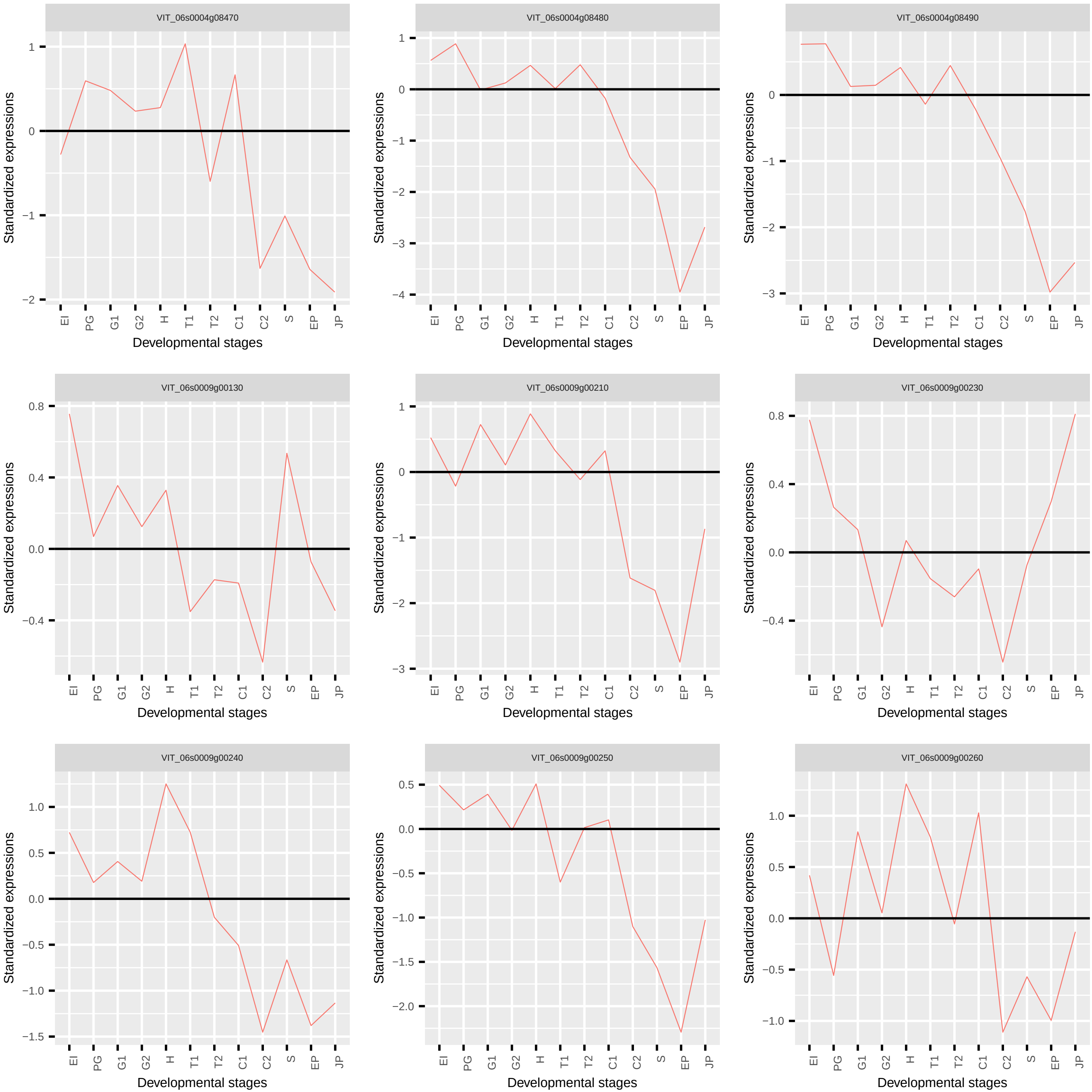
VIT_06s0004g08470
VIT_06s0004g08480
VIT_06s0004g08490
VIT_06s0009g00130
VIT_06s0009g00210
VIT_06s0009g00230
VIT_06s0009g00240
VIT_06s0009g00250
VIT_06s0009g00260
Standardized expressions
Developmental stages
EI PG G1 G2 H T1 T2 C1 C2 S EP JP

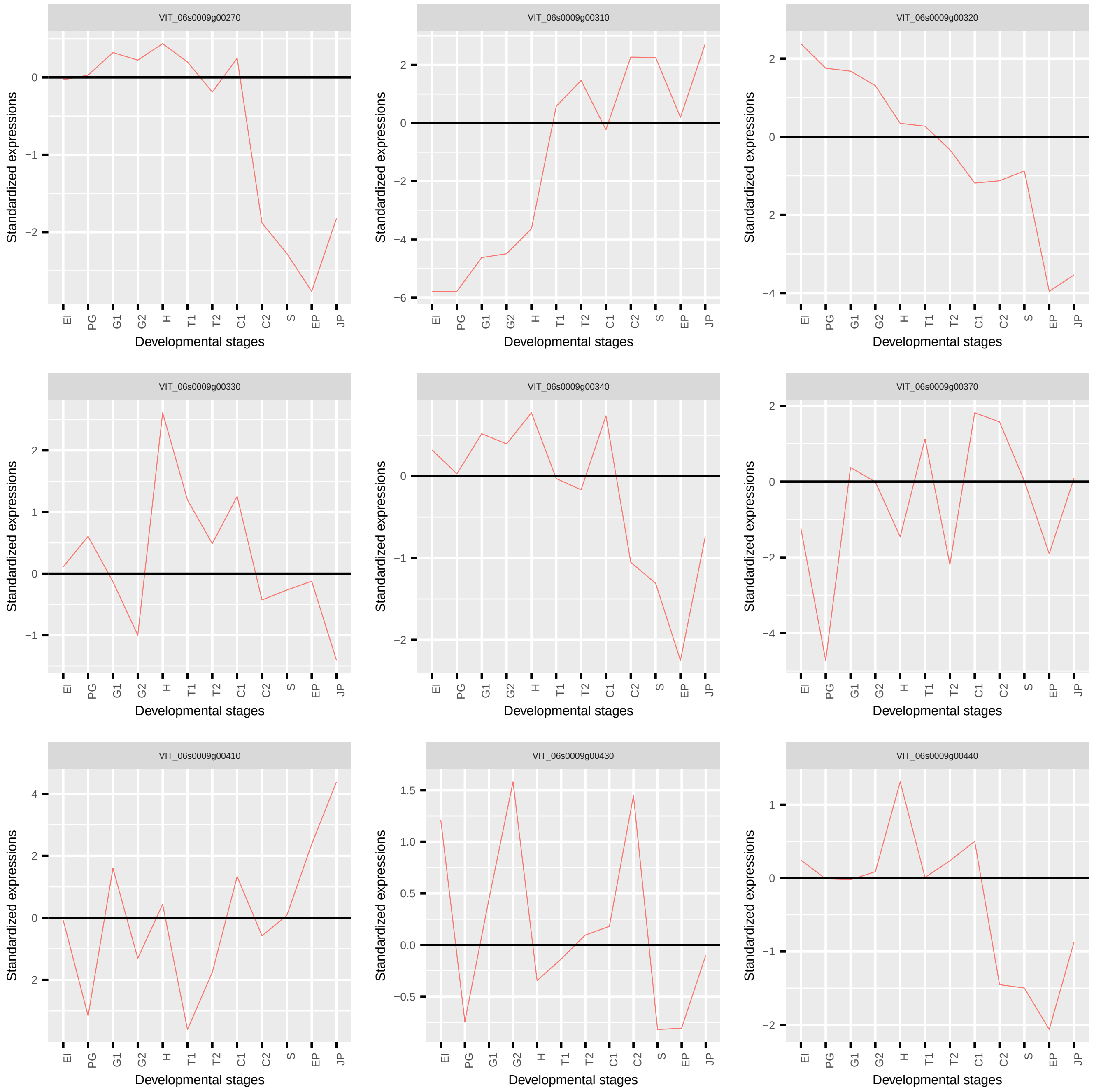

VIT_06s0009g00270
VIT_06s0009g00310
VIT_06s0009g00320
VIT_06s0009g00330
VIT_06s0009g00340
VIT_06s0009g00370
VIT_06s0009g00410
VIT_06s0009g00430
VIT_06s0009g00440
Standardized expressions
Developmental stages
EI PG G1 G2 H T1 T2 C1 C2 S EP JP

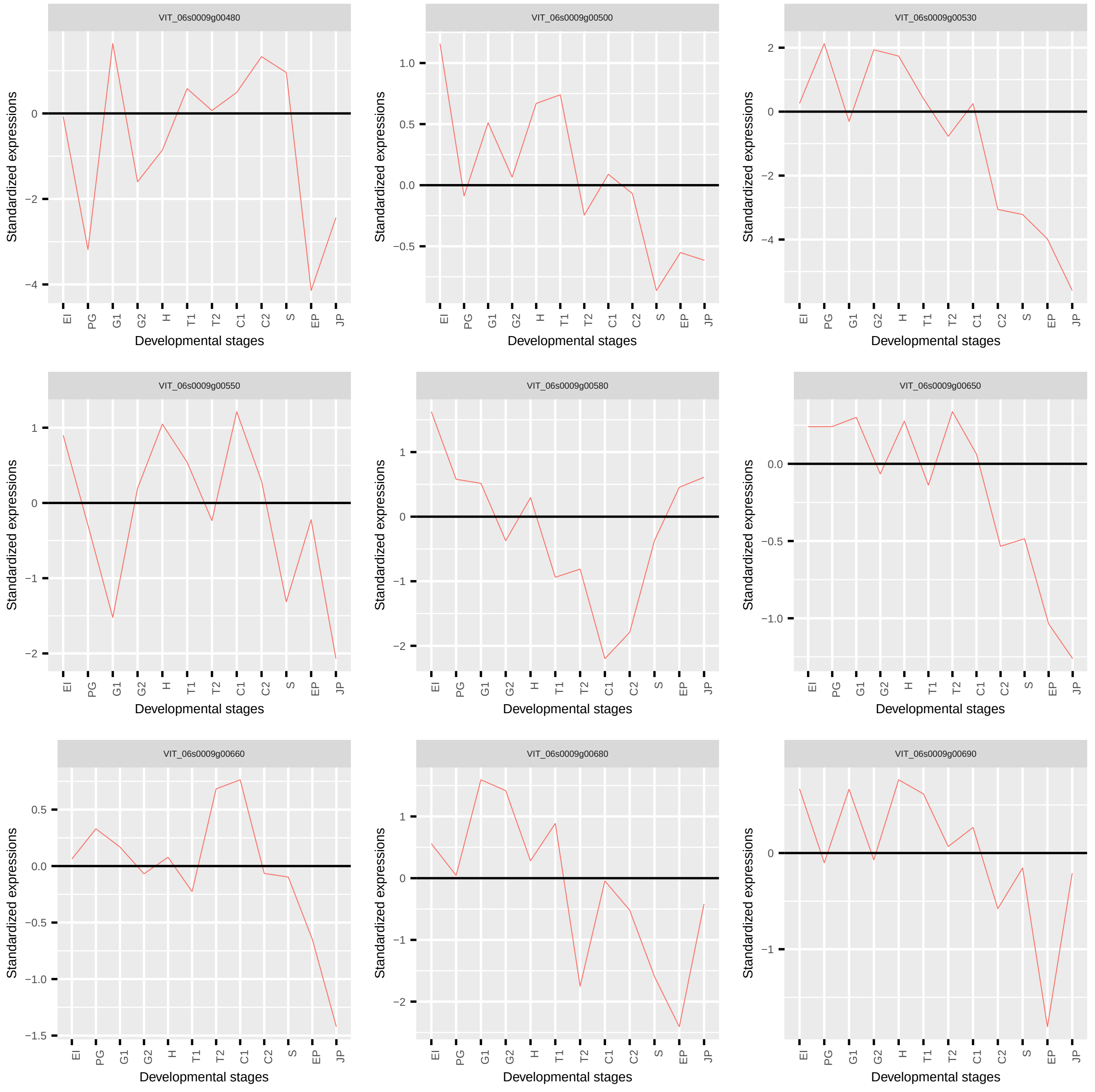

VIT_06s0009g00480
VIT_06s0009g00500
VIT_06s0009g00530
VIT_06s0009g00550
VIT_06s0009g00580
VIT_06s0009g00650
VIT_06s0009g00660
VIT_06s0009g00680
VIT_06s0009g00690
Standardized expressions
Developmental stages
EI PG G1 G2 H T1 T2 C1 C2 S EP JP

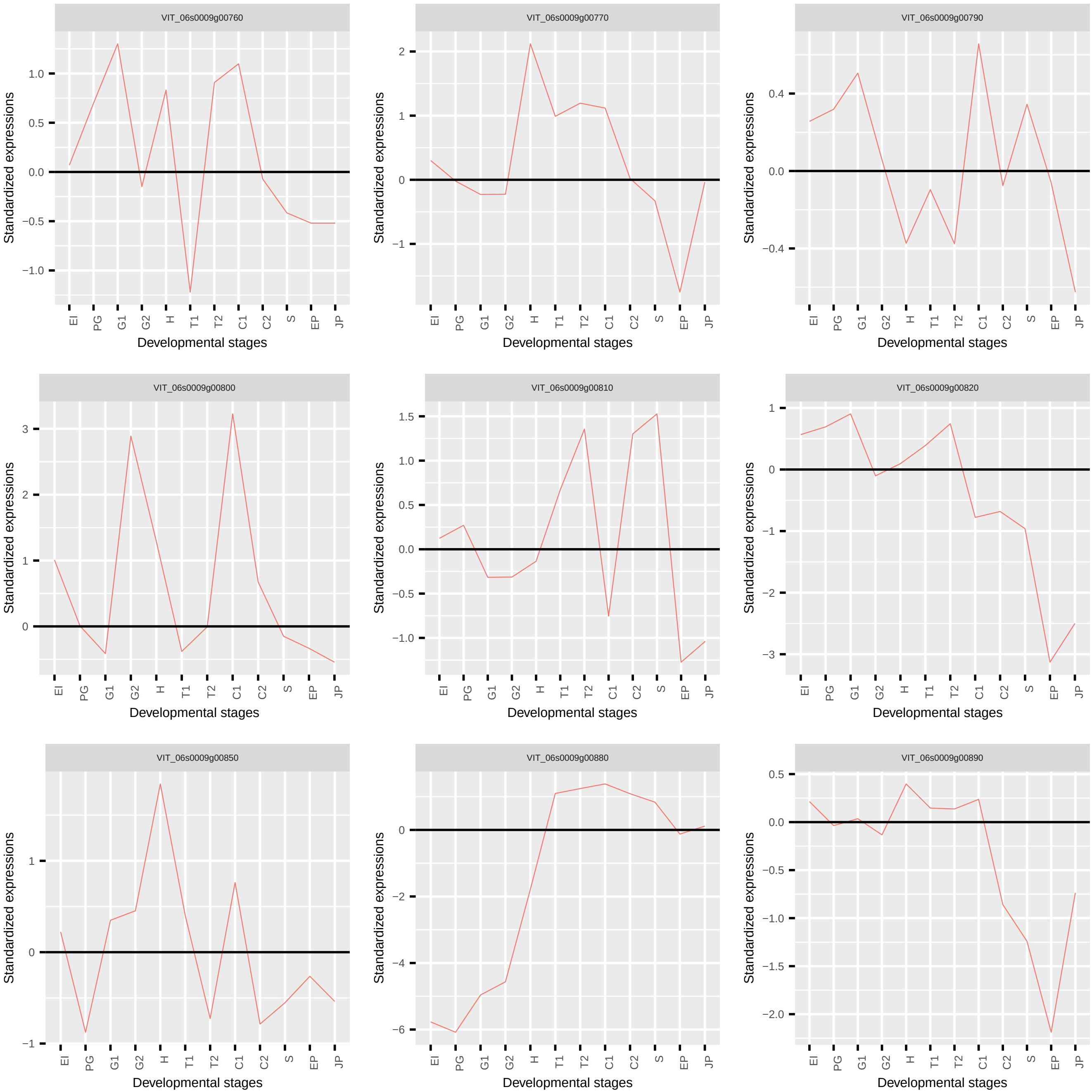

VIT_06s0009g00760
VIT_06s0009g00770
VIT_06s0009g00790
VIT_06s0009g00800
VIT_06s0009g00810
VIT_06s0009g00820
VIT_06s0009g00850
VIT_06s0009g00880
VIT_06s0009g00890
Standardized expressions
Developmental stages
EI
PG
G1
G2
H
T1
T2
C1
C2
S
EP
JP

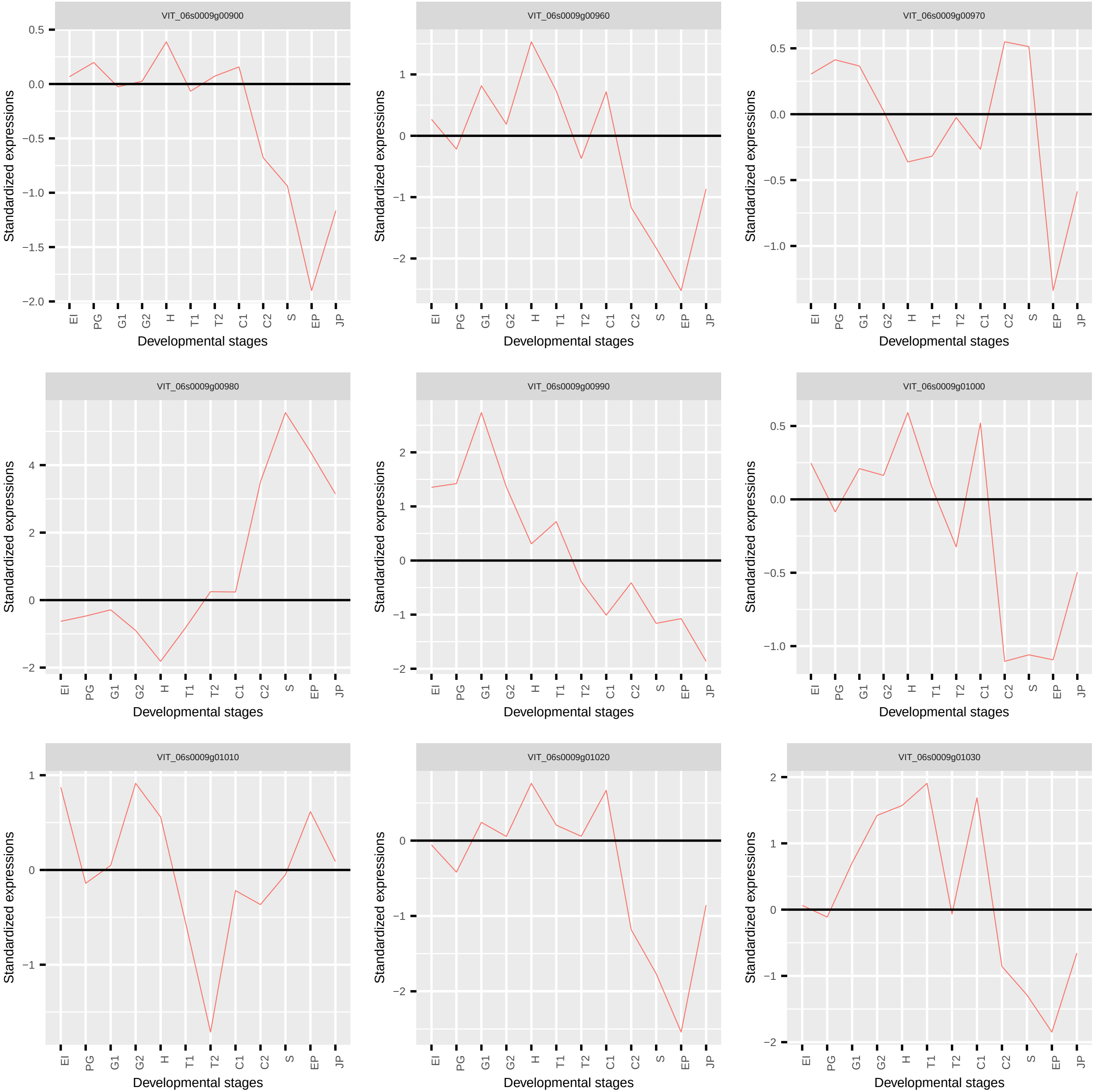
VIT_06s0009g00900
Standardized expressions
Developmental stages
EI PG G1 G2 H T1 T2 C1 C2 S EP JP
VIT_06s0009g00960
Standardized expressions
Developmental stages
EI PG G1 G2 H T1 T2 C1 C2 S EP JP
VIT_06s0009g00970
Standardized expressions
Developmental stages
EI PG G1 G2 H T1 T2 C1 C2 S EP JP
VIT_06s0009g00980
Standardized expressions
Developmental stages
EI PG G1 G2 H T1 T2 C1 C2 S EP JP
VIT_06s0009g00990
Standardized expressions
Developmental stages
EI PG G1 G2 H T1 T2 C1 C2 S EP JP
VIT_06s0009g01000
Standardized expressions
Developmental stages
EI PG G1 G2 H T1 T2 C1 C2 S EP JP
VIT_06s0009g01010
Standardized expressions
Developmental stages
EI PG G1 G2 H T1 T2 C1 C2 S EP JP
VIT_06s0009g01020
Standardized expressions
Developmental stages
EI PG G1 G2 H T1 T2 C1 C2 S EP JP
VIT_06s0009g01030
Standardized expressions
Developmental stages
EI PG G1 G2 H T1 T2 C1 C2 S EP JP

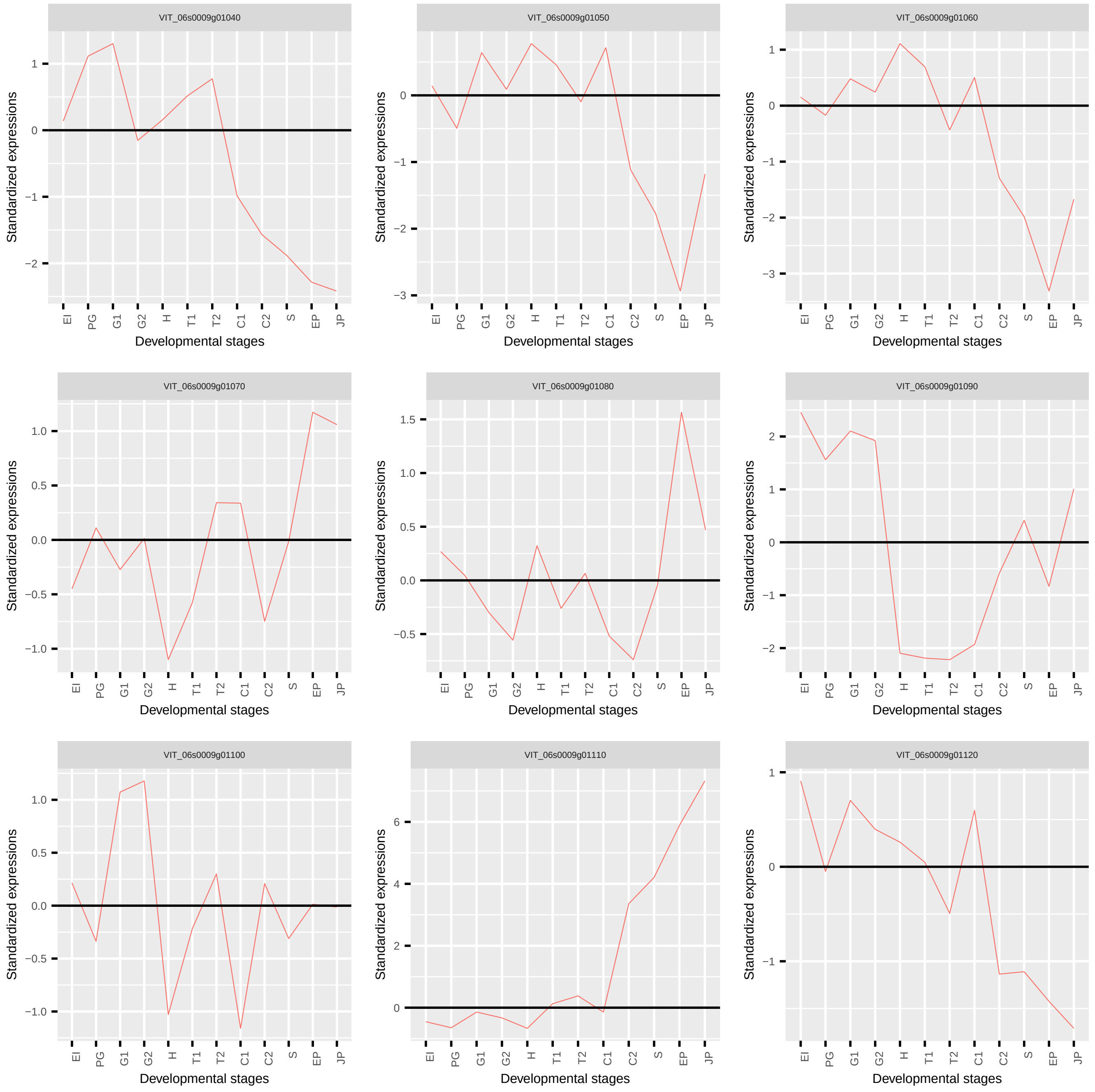
VIT_06s0009g01040
VIT_06s0009g01050
VIT_06s0009g01060
VIT_06s0009g01070
VIT_06s0009g01080
VIT_06s0009g01090
VIT_06s0009g01100
VIT_06s0009g01110
VIT_06s0009g01120
Standardized expressions
Developmental stages
EI
PG
G1
G2
H
T1
T2
C1
C2
S
EP
JP

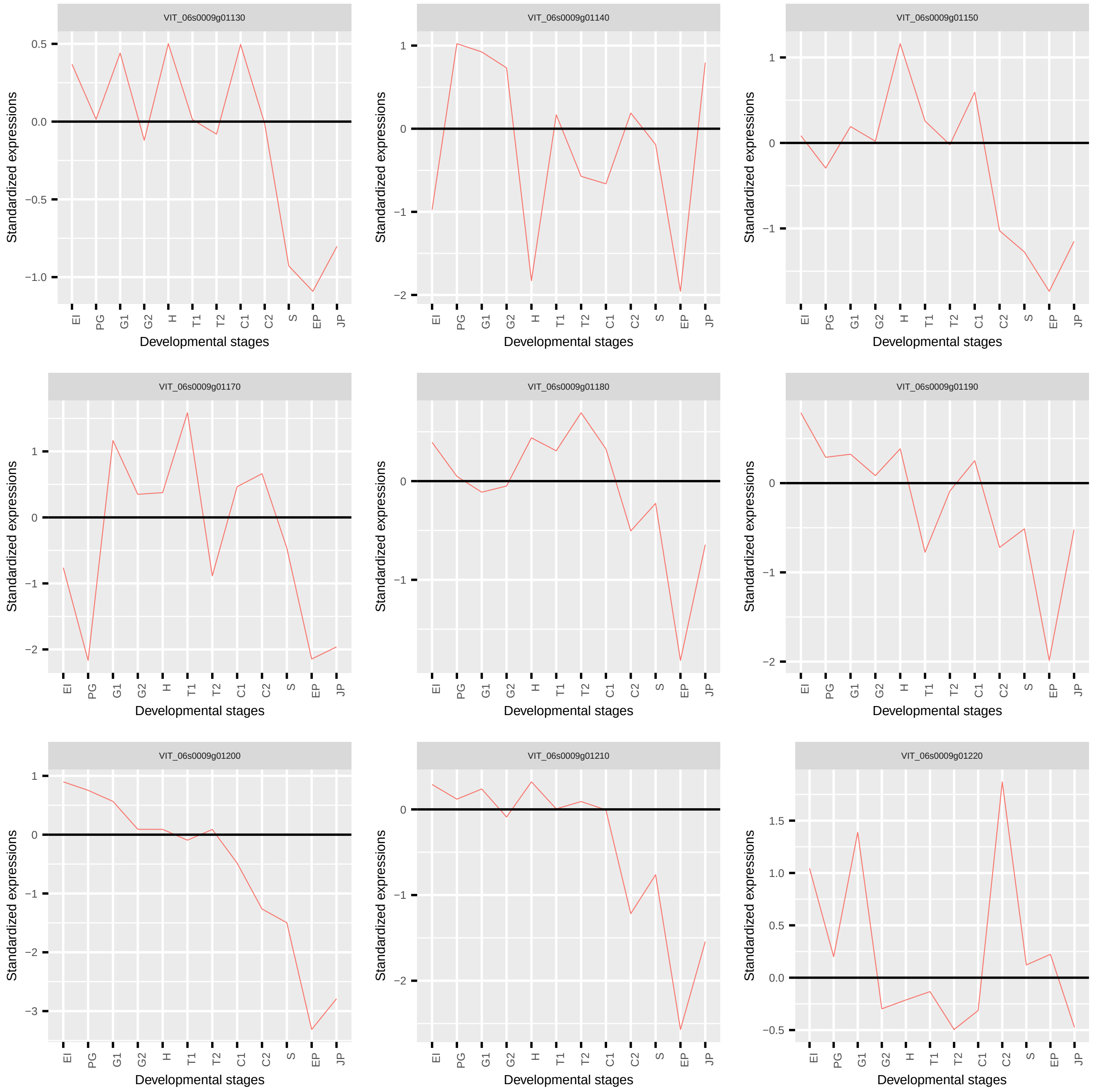
VIT_06s0009g01130
VIT_06s0009g01140
VIT_06s0009g01150
VIT_06s0009g01170
VIT_06s0009g01180
VIT_06s0009g01190
VIT_06s0009g01200
VIT_06s0009g01210
VIT_06s0009g01220
Standardized expressions
Developmental stages
EI PG G1 G2 H T1 T2 C1 C2 S EP JP

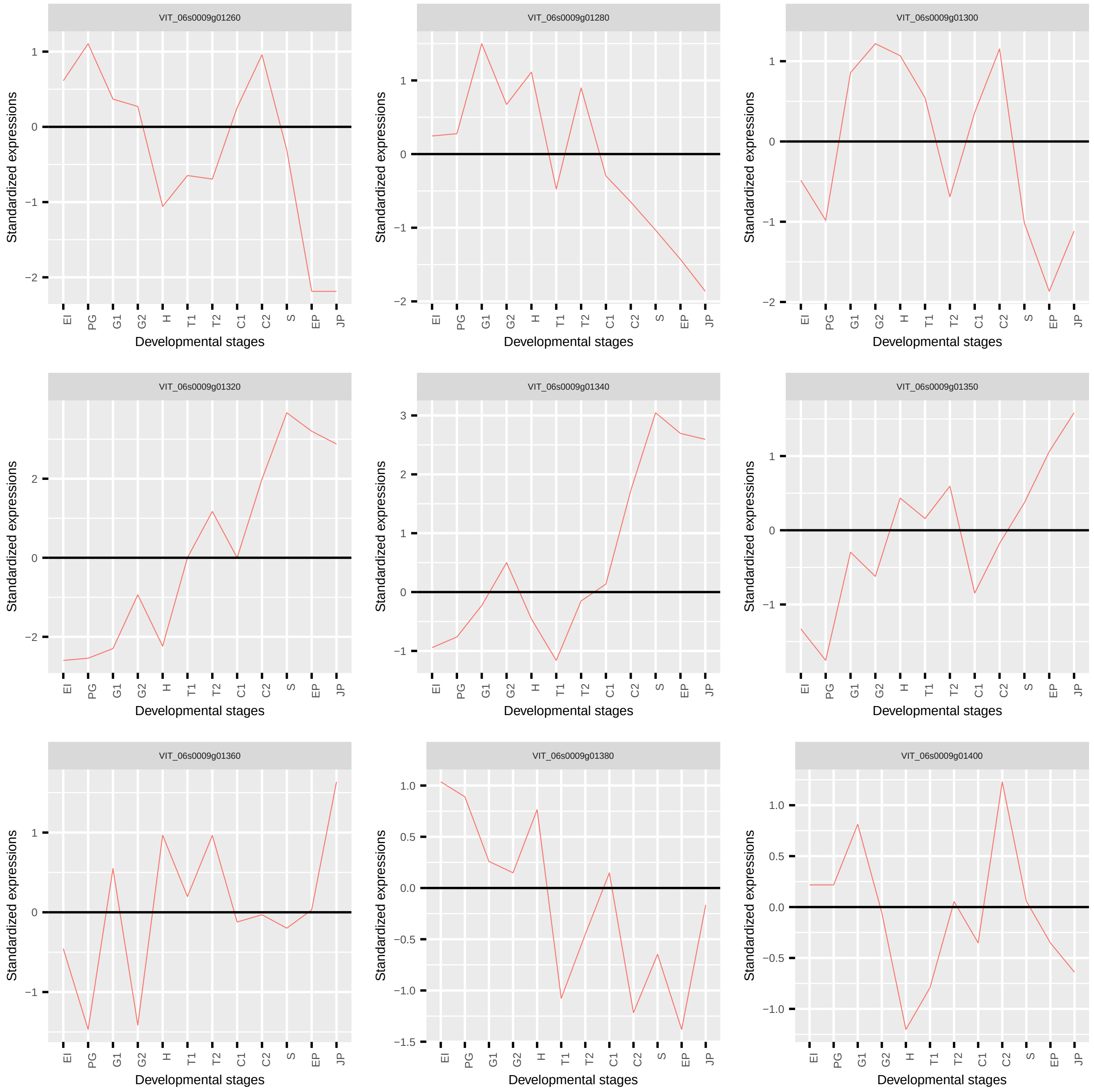
VIT_06s0009g01260
VIT_06s0009g01280
VIT_06s0009g01300
VIT_06s0009g01320
VIT_06s0009g01340
VIT_06s0009g01350
VIT_06s0009g01360
VIT_06s0009g01380
VIT_06s0009g01400
Standardized expressions
Developmental stages
EI PG G1 G2 H T1 T2 C1 C2 S EP JP

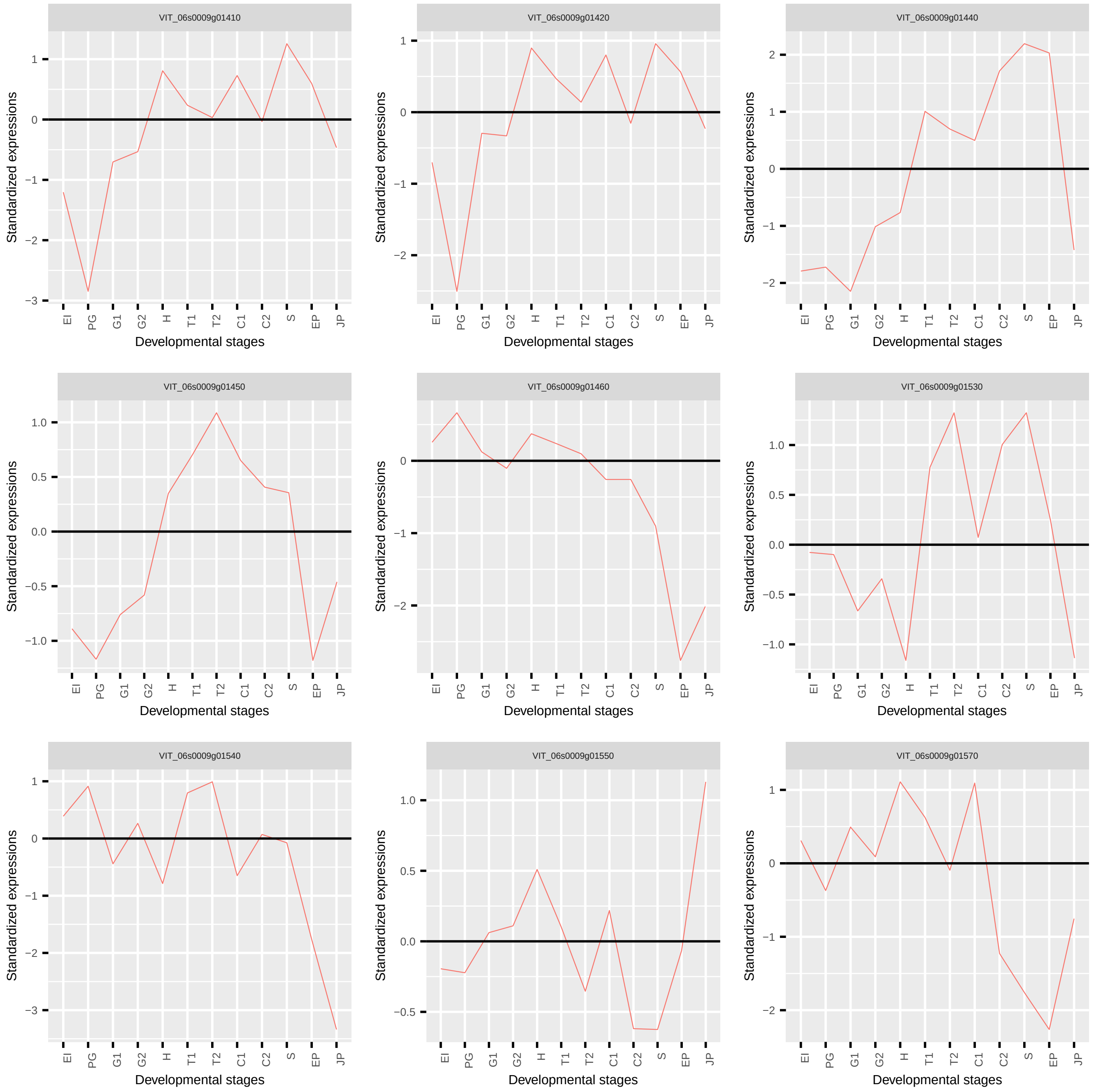
VIT_06s0009g01410
VIT_06s0009g01420
VIT_06s0009g01440
VIT_06s0009g01450
VIT_06s0009g01460
VIT_06s0009g01530
VIT_06s0009g01540
VIT_06s0009g01550
VIT_06s0009g01570
Standardized expressions
Developmental stages
EI PG G1 G2 H T1 T2 C1 C2 S EP JP

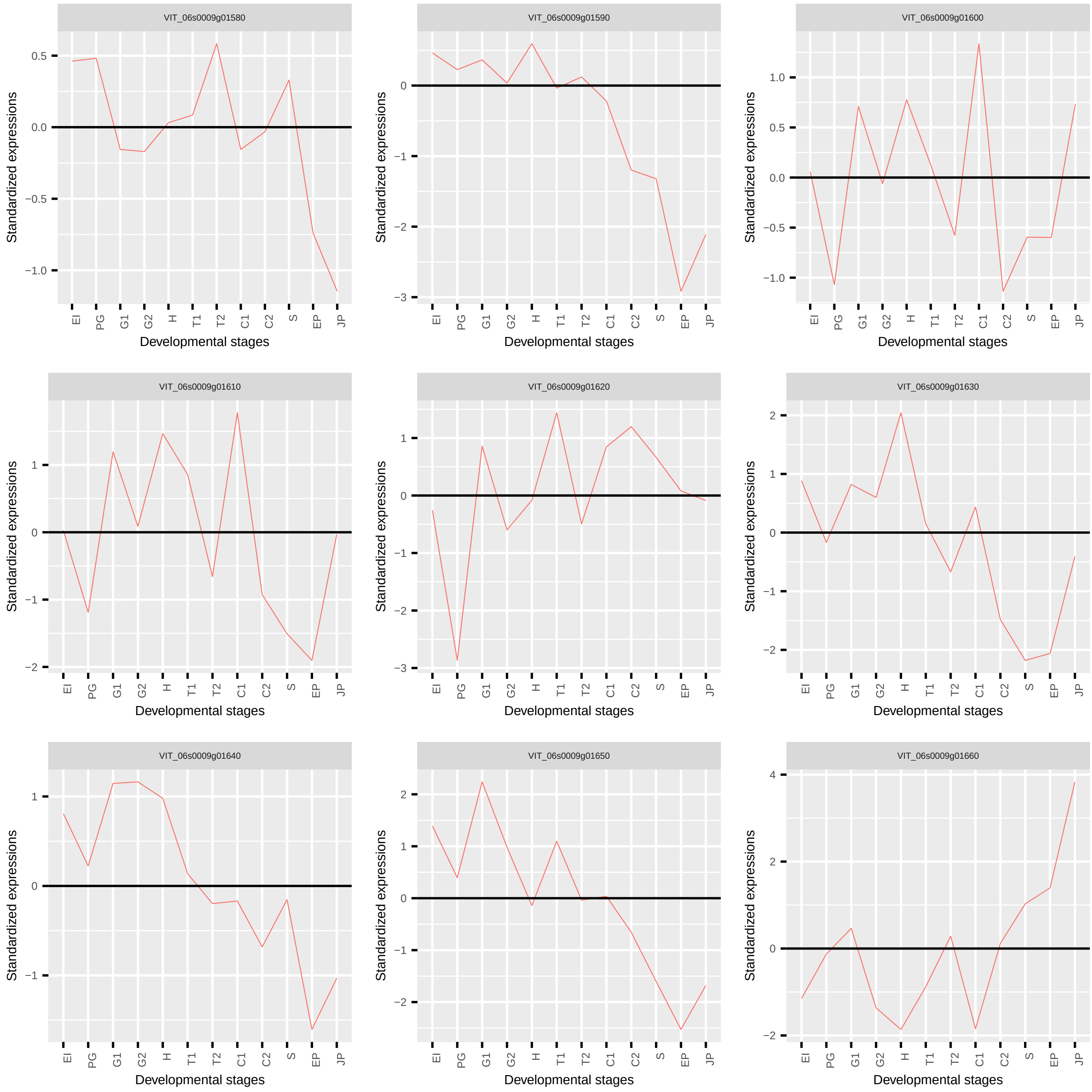

VIT_06s0009g01580
0.5
0.0
−0.5
−1.0
Standardized expressions
EI PG G1 G2 H T1 T2 C1 C2 S EP JP
Developmental stages
VIT_06s0009g01590
0
−1
−2
−3
Standardized expressions
EI PG G1 G2 H T1 T2 C1 C2 S EP JP
Developmental stages
VIT_06s0009g01600
1.0
0.5
0.0
−0.5
−1.0
Standardized expressions
EI PG G1 G2 H T1 T2 C1 C2 S EP JP
Developmental stages
VIT_06s0009g01610
1
0
−1
−2
Standardized expressions
EI PG G1 G2 H T1 T2 C1 C2 S EP JP
Developmental stages
VIT_06s0009g01620
1
0
−1
−2
−3
Standardized expressions
EI PG G1 G2 H T1 T2 C1 C2 S EP JP
Developmental stages
VIT_06s0009g01630
2
1
0
−1
−2
Standardized expressions
EI PG G1 G2 H T1 T2 C1 C2 S EP JP
Developmental stages
VIT_06s0009g01640
1
0
−1
Standardized expressions
EI PG G1 G2 H T1 T2 C1 C2 S EP JP
Developmental stages
VIT_06s0009g01650
2
1
0
−1
−2
Standardized expressions
EI PG G1 G2 H T1 T2 C1 C2 S EP JP
Developmental stages
VIT_06s0009g01660
4
2
0
−2
Standardized expressions
EI PG G1 G2 H T1 T2 C1 C2 S EP JP
Developmental stages

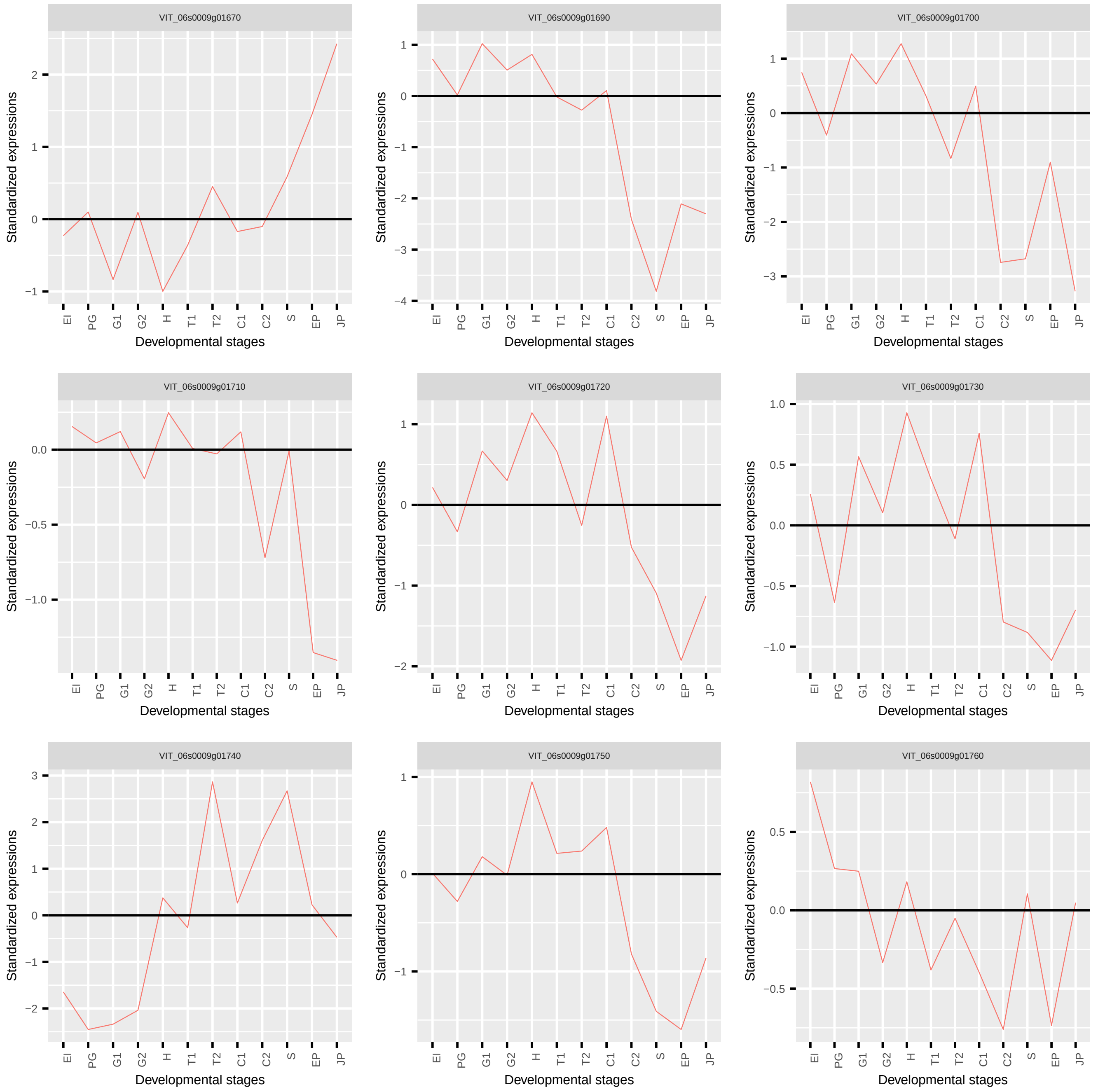
VIT_06s0009g01670
VIT_06s0009g01690
VIT_06s0009g01700
VIT_06s0009g01710
VIT_06s0009g01720
VIT_06s0009g01730
VIT_06s0009g01740
VIT_06s0009g01750
VIT_06s0009g01760
Standardized expressions
Developmental stages
EI
PG
G1
G2
H
T1
T2
C1
C2
S
EP
JP

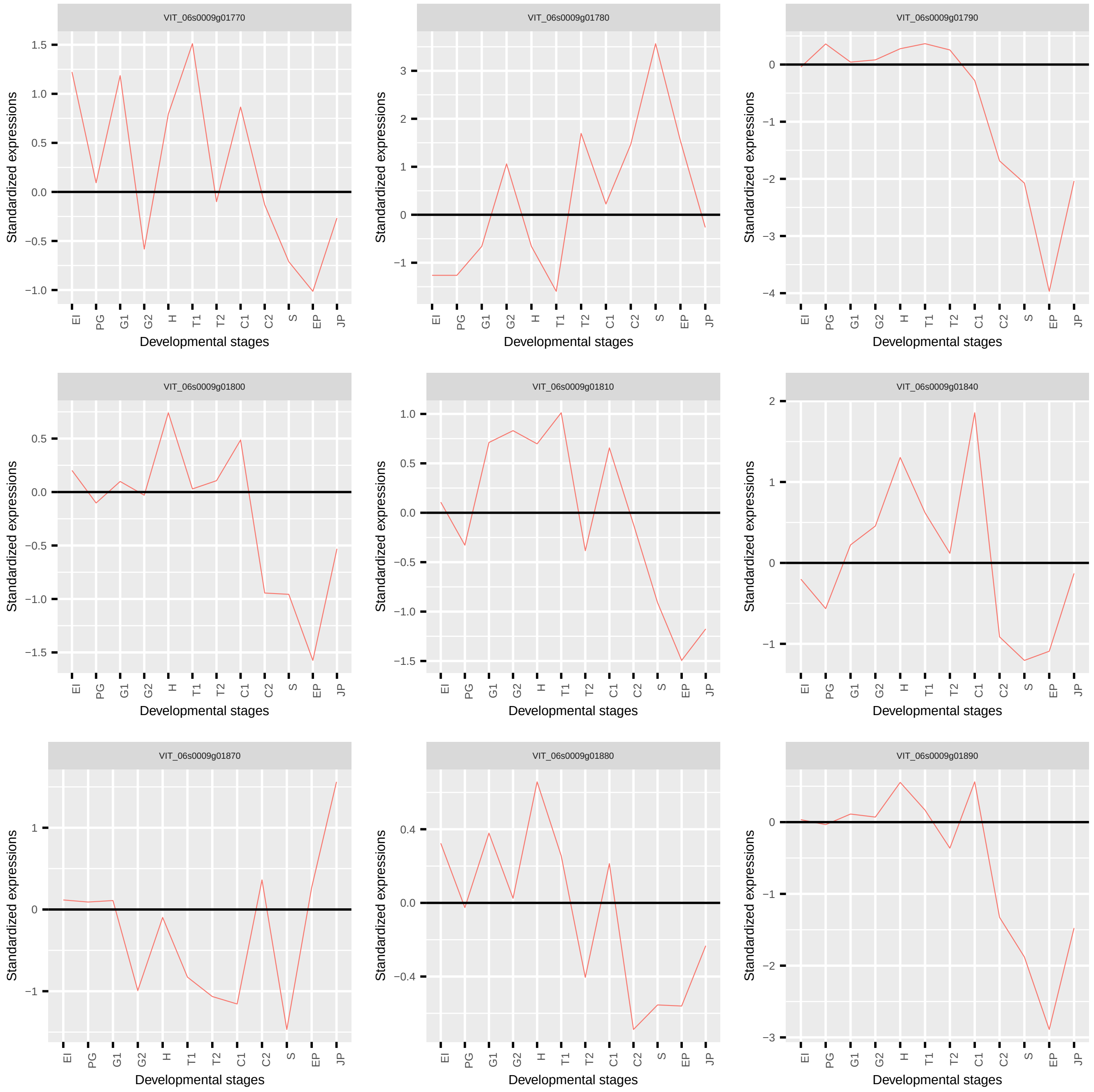
VIT_06s0009g01770
VIT_06s0009g01780
VIT_06s0009g01790
VIT_06s0009g01800
VIT_06s0009g01810
VIT_06s0009g01840
VIT_06s0009g01870
VIT_06s0009g01880
VIT_06s0009g01890
Standardized expressions
Developmental stages
EI PG G1 G2 H T1 T2 C1 C2 S EP JP

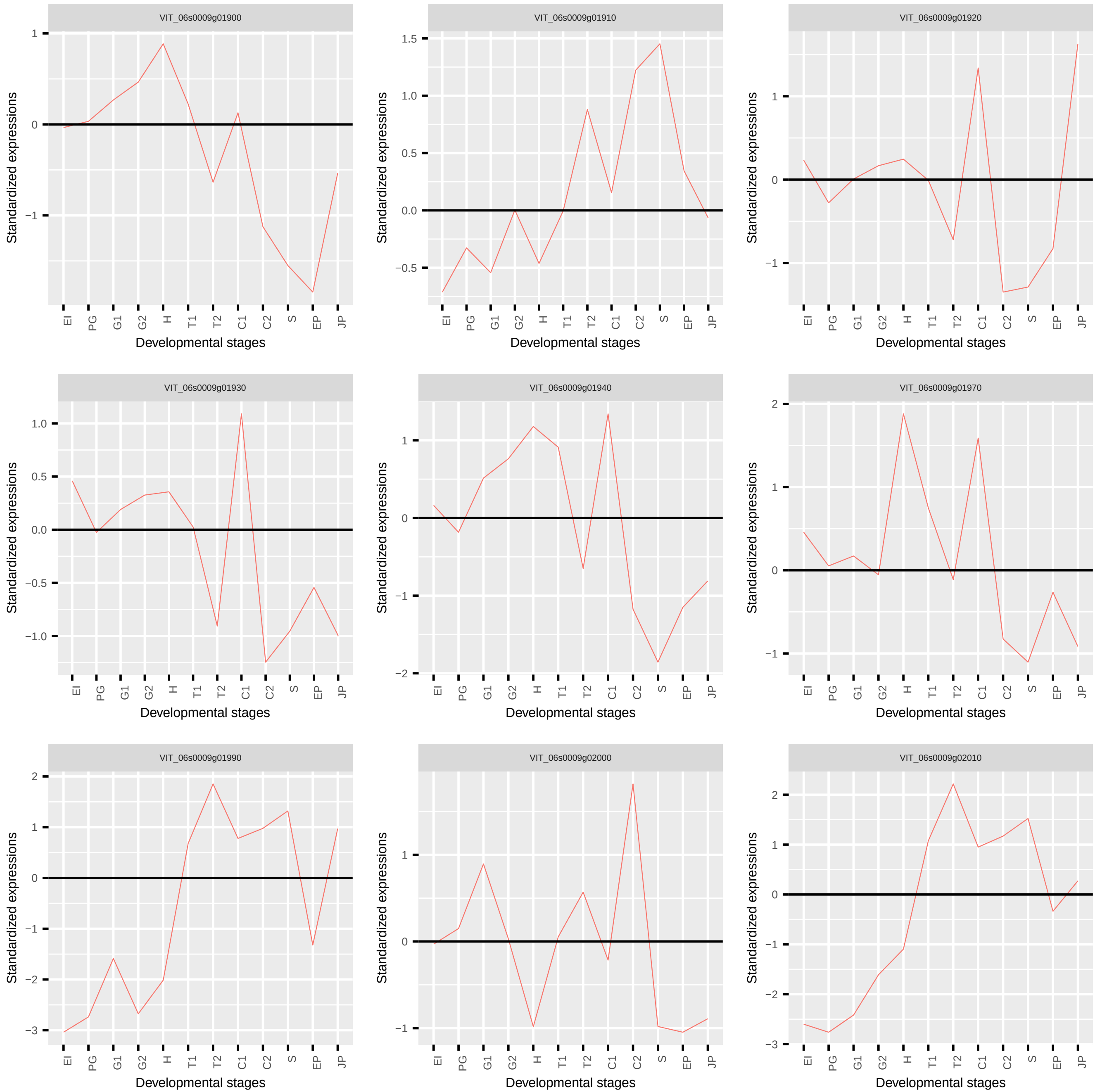
VIT_06s0009g01900
VIT_06s0009g01910
VIT_06s0009g01920
VIT_06s0009g01930
VIT_06s0009g01940
VIT_06s0009g01970
VIT_06s0009g01990
VIT_06s0009g02000
VIT_06s0009g02010
Standardized expressions
Developmental stages
EI PG G1 G2 H T1 T2 C1 C2 S EP JP

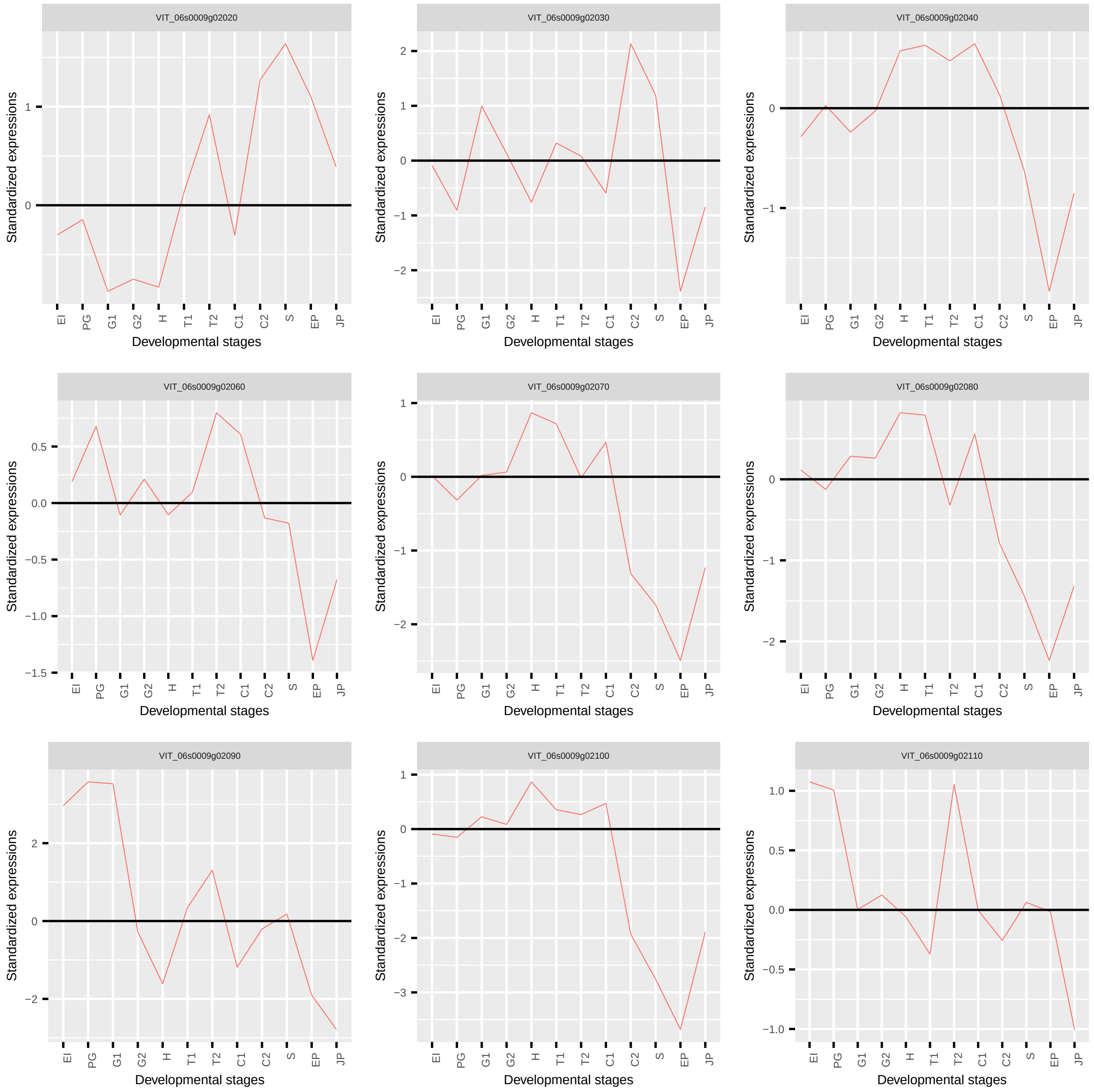
VIT_06s0009g02020
VIT_06s0009g02030
VIT_06s0009g02040
VIT_06s0009g02060
VIT_06s0009g02070
VIT_06s0009g02080
VIT_06s0009g02090
VIT_06s0009g02100
VIT_06s0009g02110
Standardized expressions
Developmental stages
EI PG G1 G2 H T1 T2 C1 C2 S EP JP

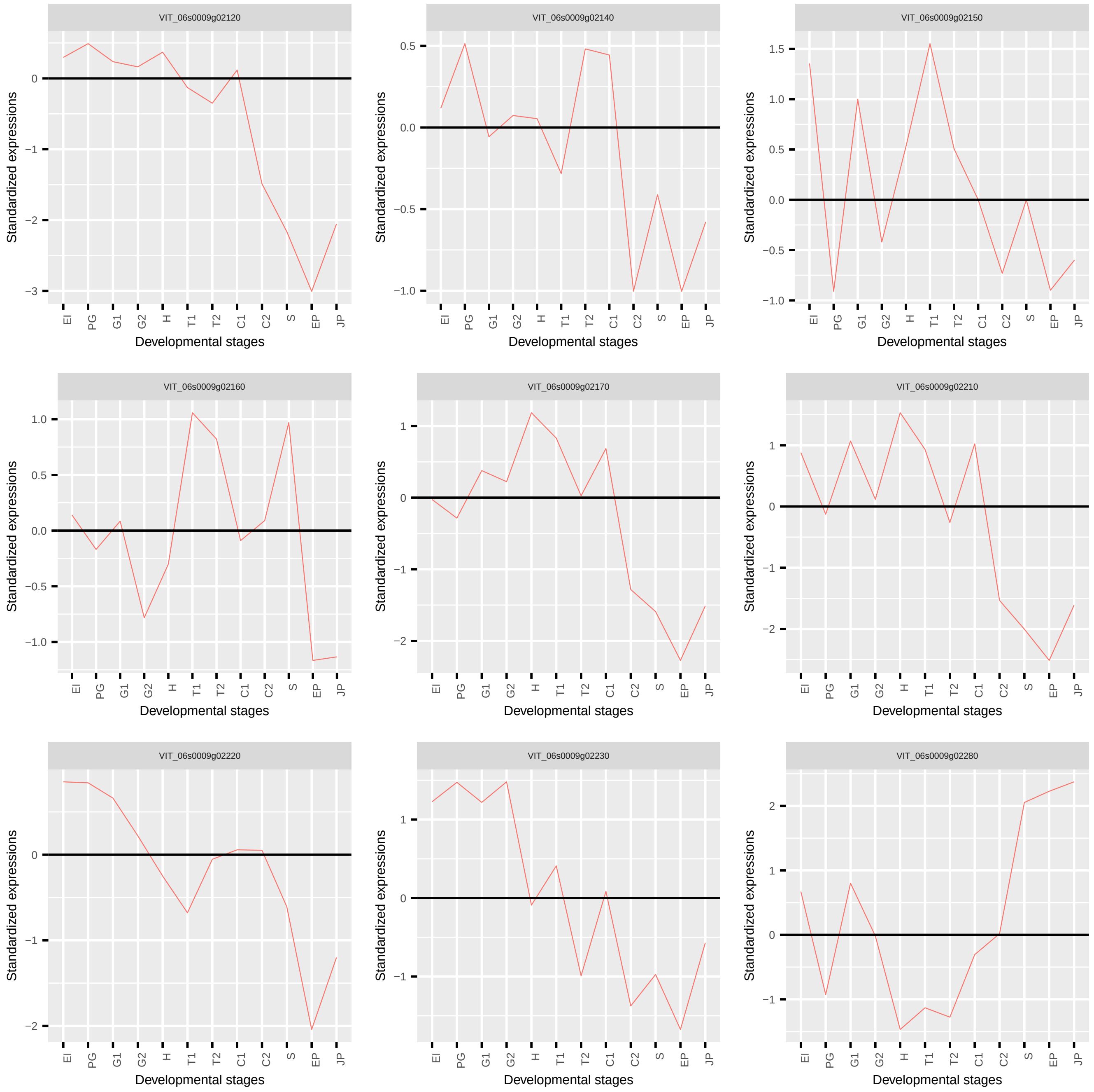
VIT_06s0009g02120
VIT_06s0009g02140
VIT_06s0009g02150
VIT_06s0009g02160
VIT_06s0009g02170
VIT_06s0009g02210
VIT_06s0009g02220
VIT_06s0009g02230
VIT_06s0009g02280
Standardized expressions
Developmental stages
EI PG G1 G2 H T1 T2 C1 C2 S EP JP

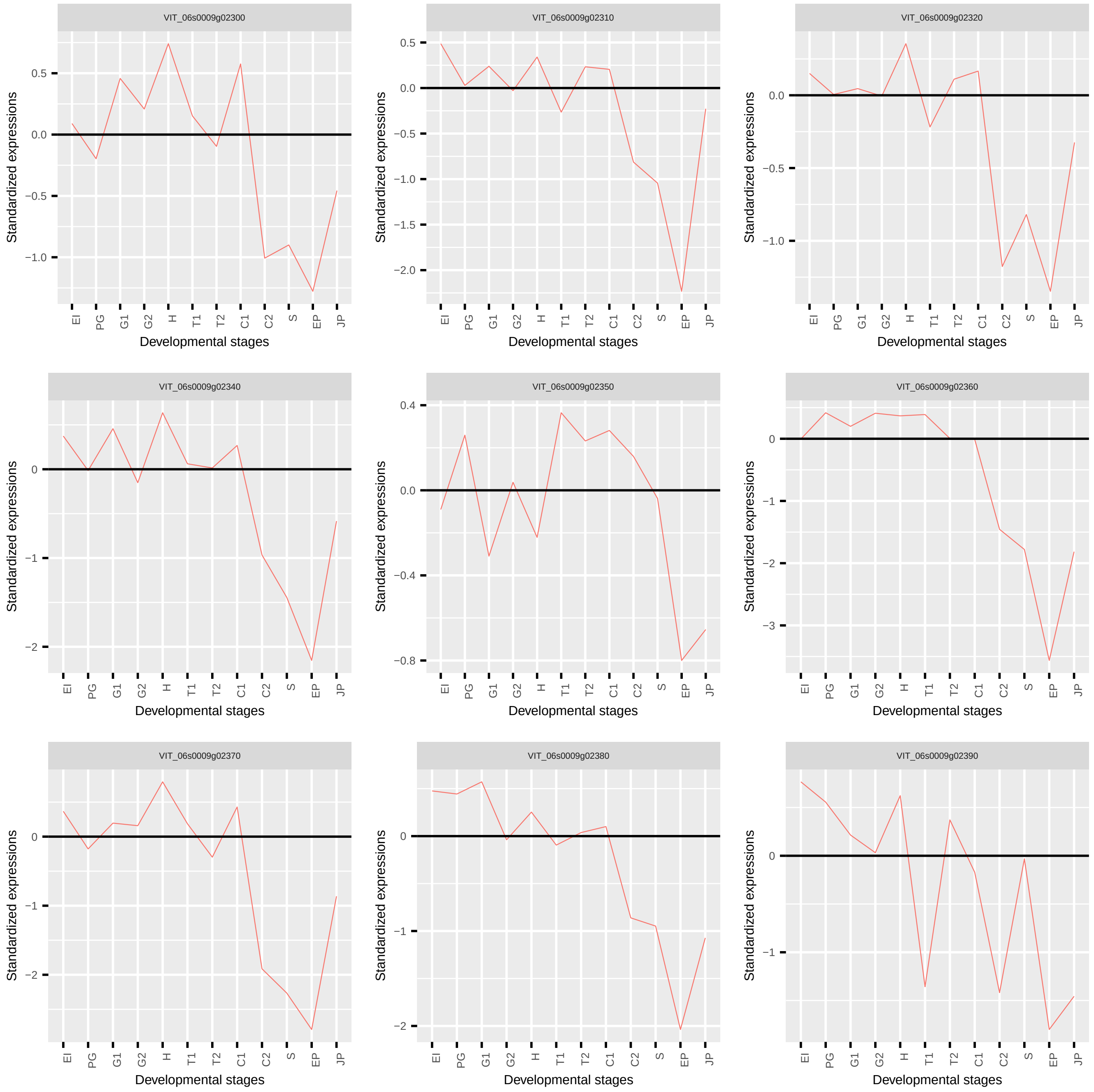
VIT_06s0009g02300
VIT_06s0009g02310
VIT_06s0009g02320
VIT_06s0009g02340
VIT_06s0009g02350
VIT_06s0009g02360
VIT_06s0009g02370
VIT_06s0009g02380
VIT_06s0009g02390
Standardized expressions
Developmental stages
EI PG G1 G2 H T1 T2 C1 C2 S EP JP
0.5
0.0
−0.5
−1.0
−1.5
−2.0
0.4
−0.4
−0.8
0
−1
−2
−3

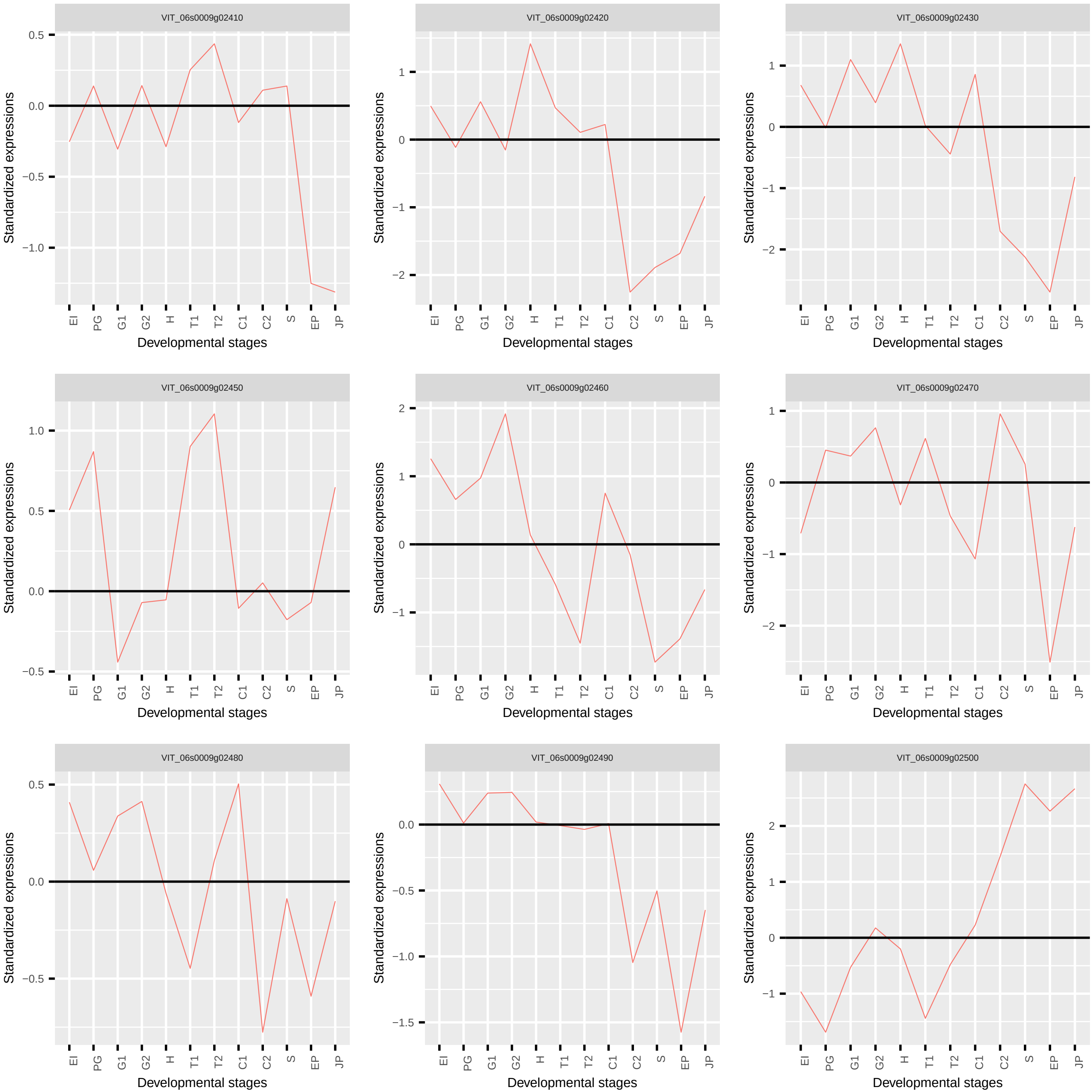
VIT_06s0009g02410
VIT_06s0009g02420
VIT_06s0009g02430
VIT_06s0009g02450
VIT_06s0009g02460
VIT_06s0009g02470
VIT_06s0009g02480
VIT_06s0009g02490
VIT_06s0009g02500
Standardized expressions
Developmental stages
EI PG G1 G2 H T1 T2 C1 C2 S EP JP

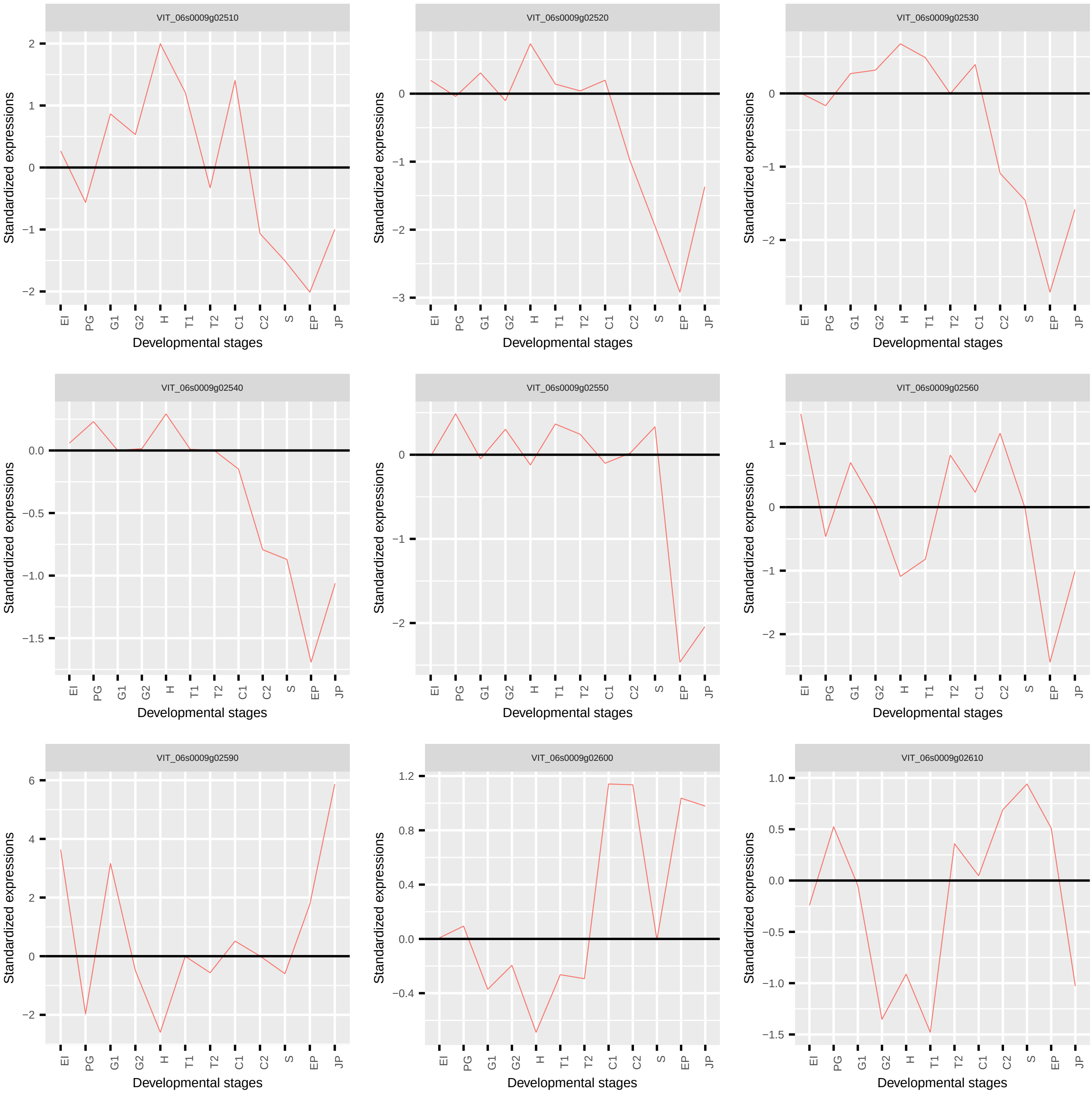
VIT_06s0009g02510
VIT_06s0009g02520
VIT_06s0009g02530
VIT_06s0009g02540
VIT_06s0009g02550
VIT_06s0009g02560
VIT_06s0009g02590
VIT_06s0009g02600
VIT_06s0009g02610
Standardized expressions
Developmental stages
EI PG G1 G2 H T1 T2 C1 C2 S EP JP

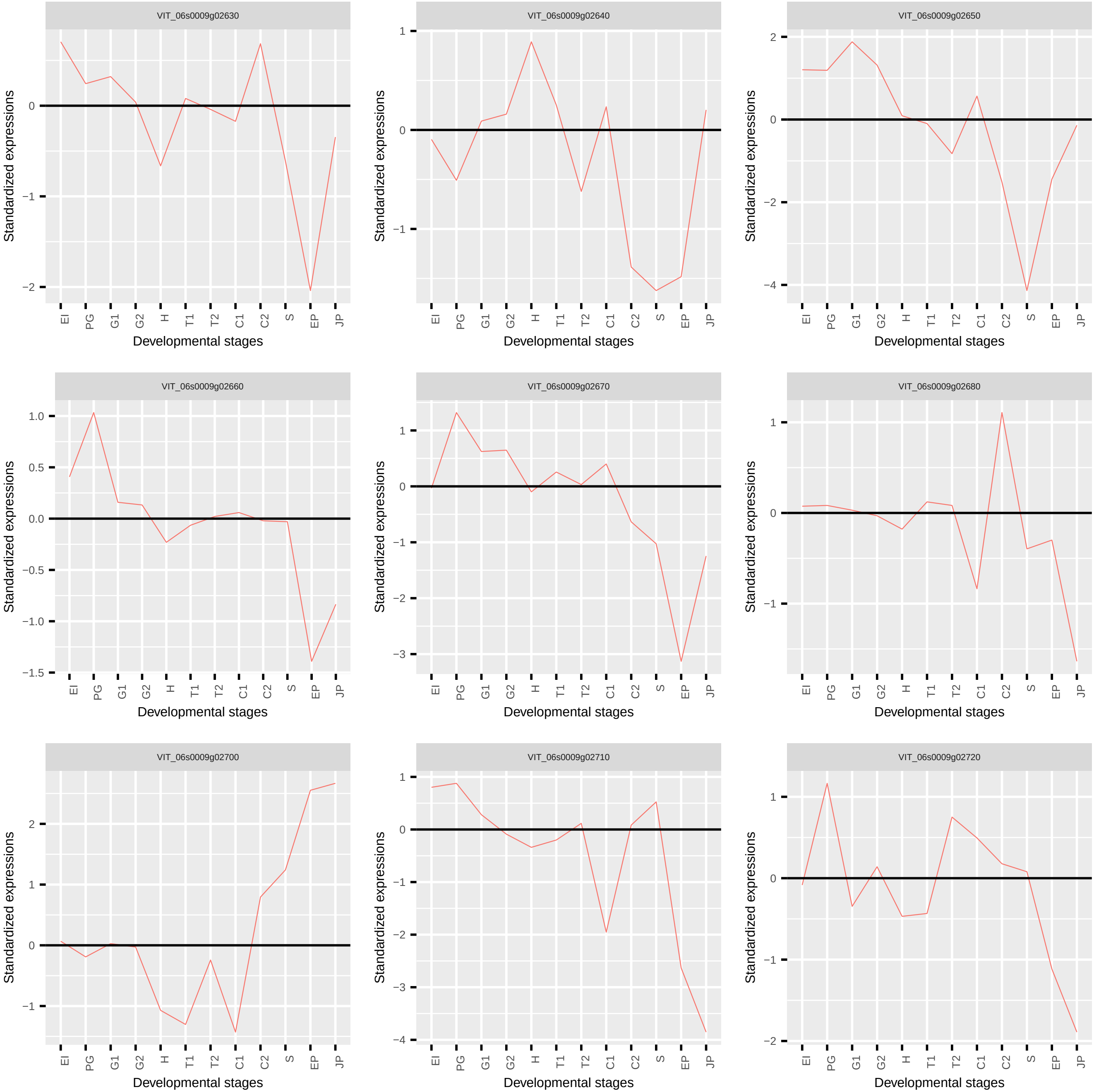
VIT_06s0009g02630
0
−1
−2
VIT_06s0009g02640
1
0
−1
VIT_06s0009g02650
2
0
−2
−4
VIT_06s0009g02660
1.0
0.5
0.0
−0.5
−1.0
−1.5
VIT_06s0009g02670
1
0
−1
−2
−3
VIT_06s0009g02680
1
0
−1
VIT_06s0009g02700
2
1
0
−1
VIT_06s0009g02710
1
0
−1
−2
−3
−4
VIT_06s0009g02720
1
0
−1
−2
Standardized expressions
EI
PG
G1
G2
H
T1
T2
C1
C2
S
EP
JP
Developmental stages

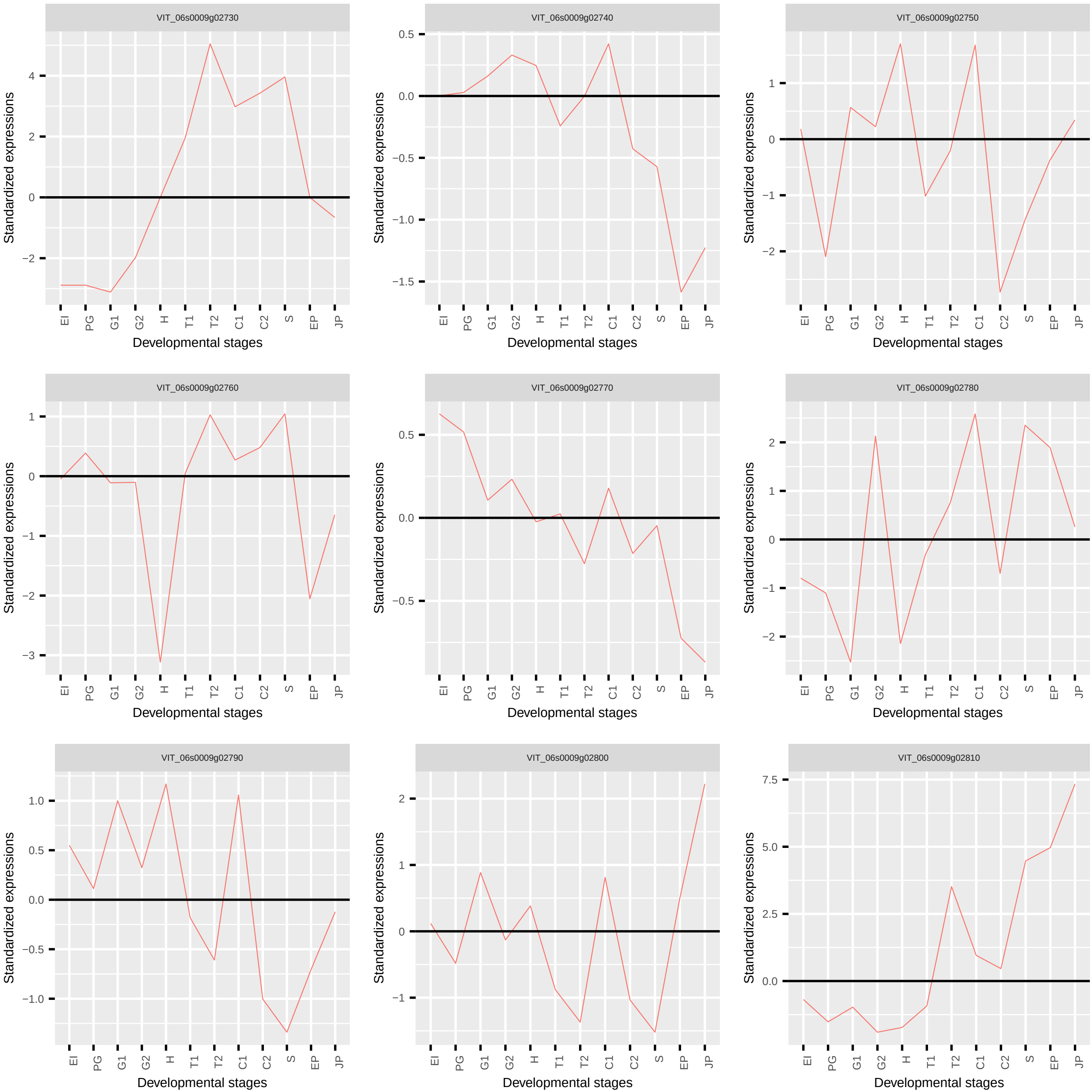

VIT_06s0009g02730
Standardized expressions
4
2
0
−2
EI PG G1 G2 H T1 T2 C1 C2 S EP JP
Developmental stages
VIT_06s0009g02740
Standardized expressions
0.5
0.0
−0.5
−1.0
−1.5
EI PG G1 G2 H T1 T2 C1 C2 S EP JP
Developmental stages
VIT_06s0009g02750
Standardized expressions
1
0
−1
−2
EI PG G1 G2 H T1 T2 C1 C2 S EP JP
Developmental stages
VIT_06s0009g02760
Standardized expressions
1
0
−1
−2
−3
EI PG G1 G2 H T1 T2 C1 C2 S EP JP
Developmental stages
VIT_06s0009g02770
Standardized expressions
0.5
0.0
−0.5
EI PG G1 G2 H T1 T2 C1 C2 S EP JP
Developmental stages
VIT_06s0009g02780
Standardized expressions
2
1
0
−1
−2
EI PG G1 G2 H T1 T2 C1 C2 S EP JP
Developmental stages
VIT_06s0009g02790
Standardized expressions
1.0
0.5
0.0
−0.5
−1.0
EI PG G1 G2 H T1 T2 C1 C2 S EP JP
Developmental stages
VIT_06s0009g02800
Standardized expressions
2
1
0
−1
EI PG G1 G2 H T1 T2 C1 C2 S EP JP
Developmental stages
VIT_06s0009g02810
Standardized expressions
7.5
5.0
2.5
0.0
EI PG G1 G2 H T1 T2 C1 C2 S EP JP
Developmental stages

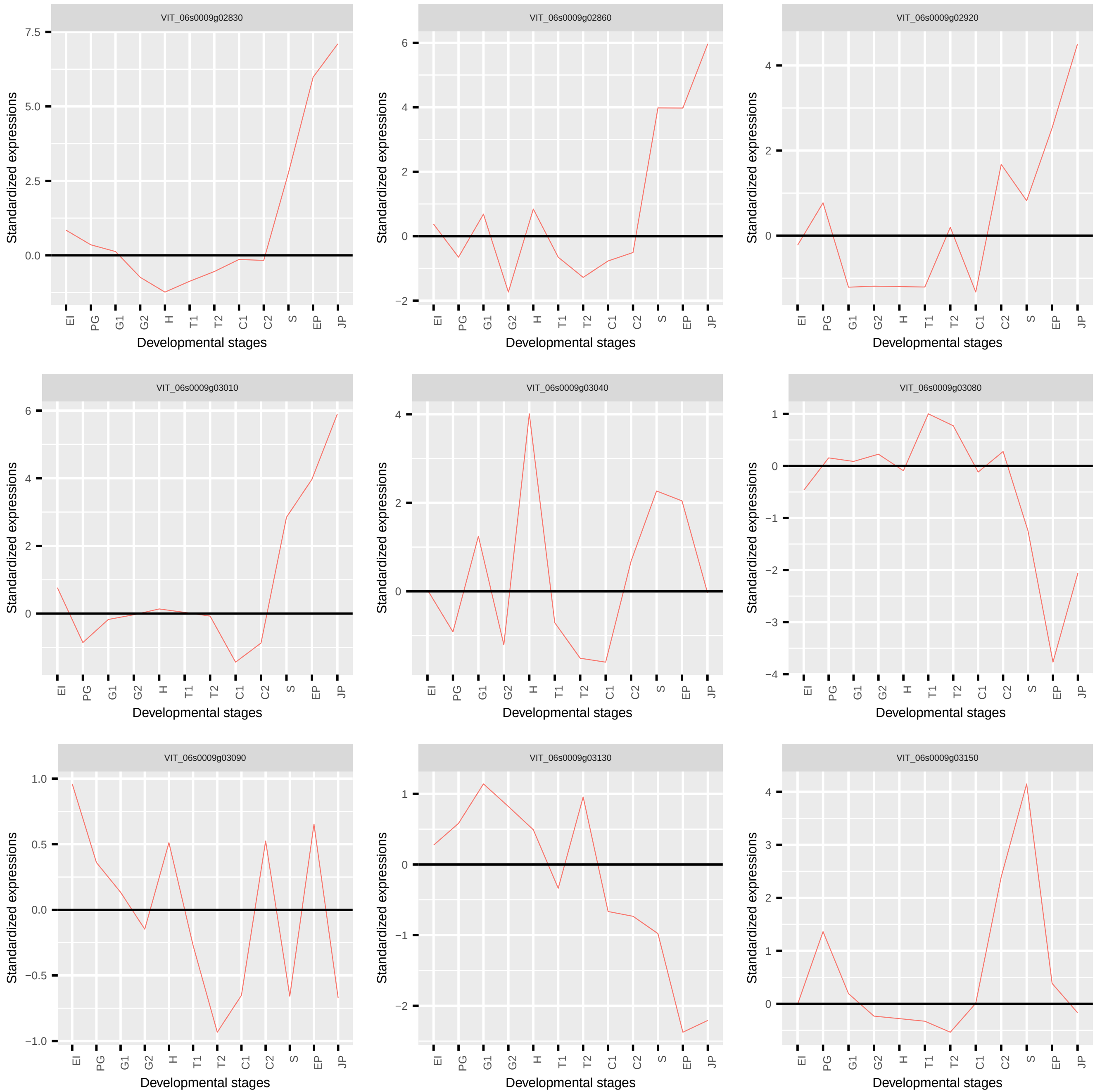
VIT_06s0009g02830
VIT_06s0009g02860
VIT_06s0009g02920
VIT_06s0009g03010
VIT_06s0009g03040
VIT_06s0009g03080
VIT_06s0009g03090
VIT_06s0009g03130
VIT_06s0009g03150
Standardized expressions
Developmental stages
EI PG G1 G2 H T1 T2 C1 C2 S EP JP

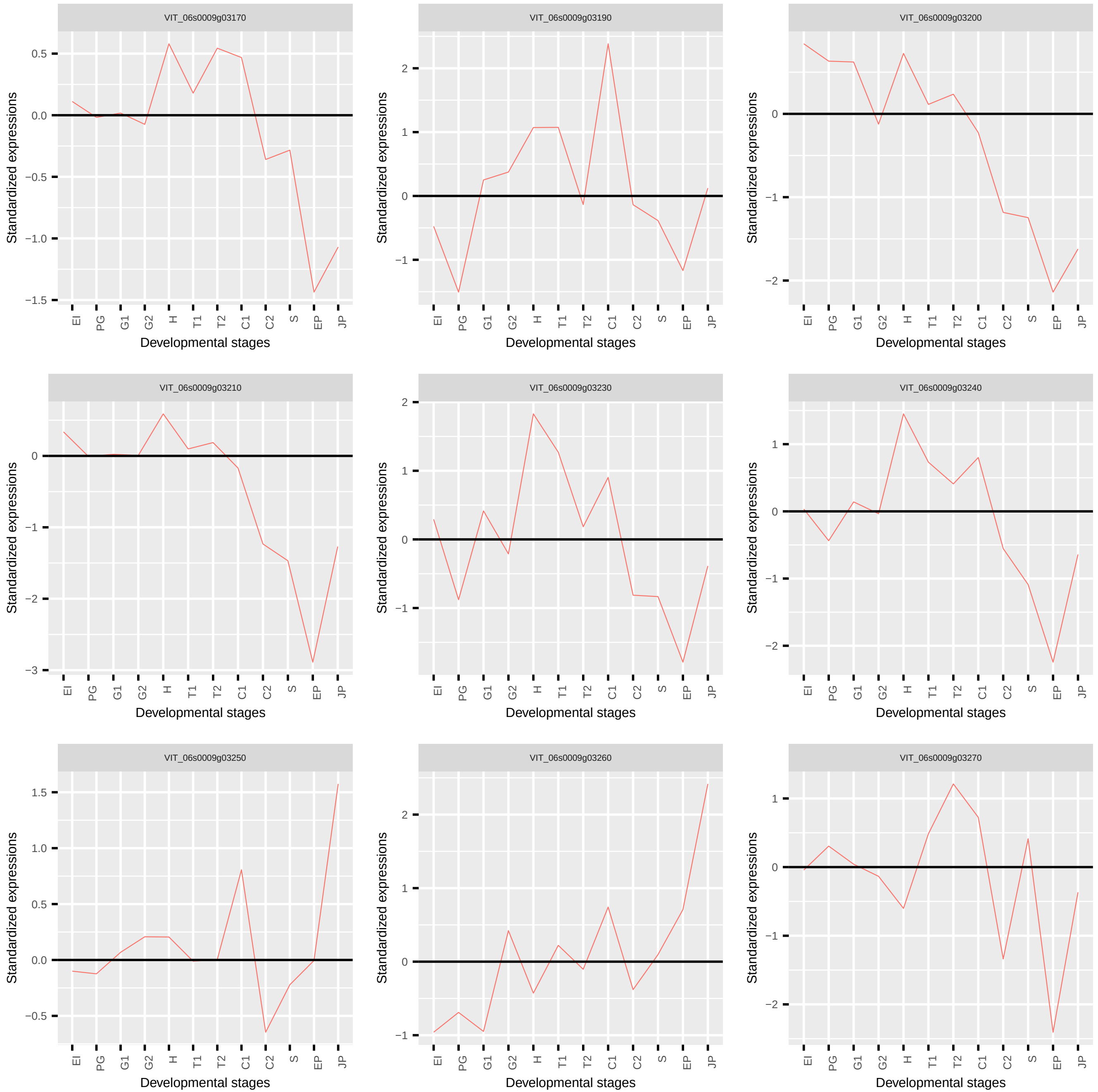
VIT_06s0009g03170
VIT_06s0009g03190
VIT_06s0009g03200
VIT_06s0009g03210
VIT_06s0009g03230
VIT_06s0009g03240
VIT_06s0009g03250
VIT_06s0009g03260
VIT_06s0009g03270
Standardized expressions
Developmental stages
EI PG G1 G2 H T1 T2 C1 C2 S EP JP

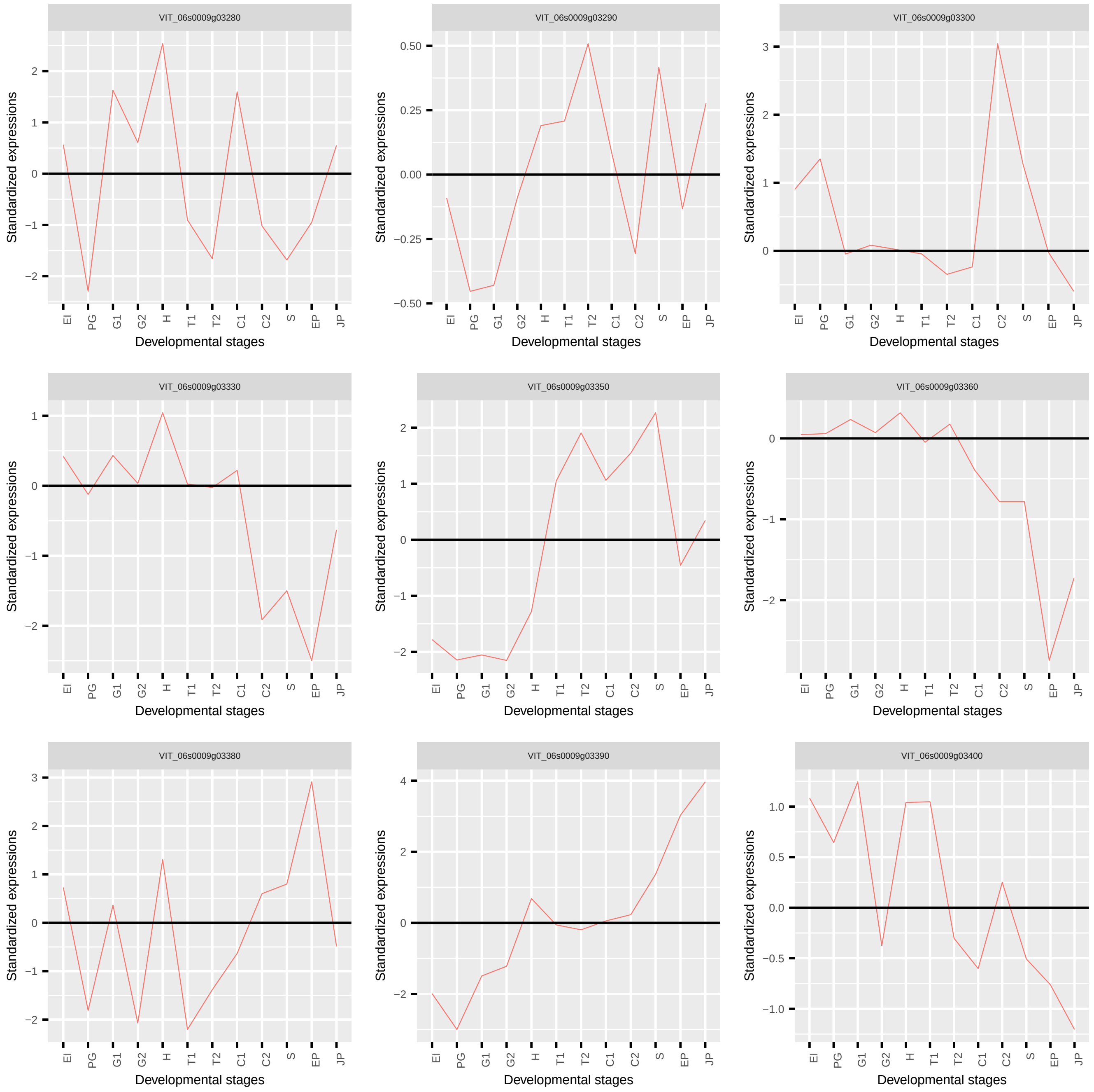
VIT_06s0009g03280
VIT_06s0009g03290
VIT_06s0009g03300
VIT_06s0009g03330
VIT_06s0009g03350
VIT_06s0009g03360
VIT_06s0009g03380
VIT_06s0009g03390
VIT_06s0009g03400
Standardized expressions
Developmental stages
EI PG G1 G2 H T1 T2 C1 C2 S EP JP

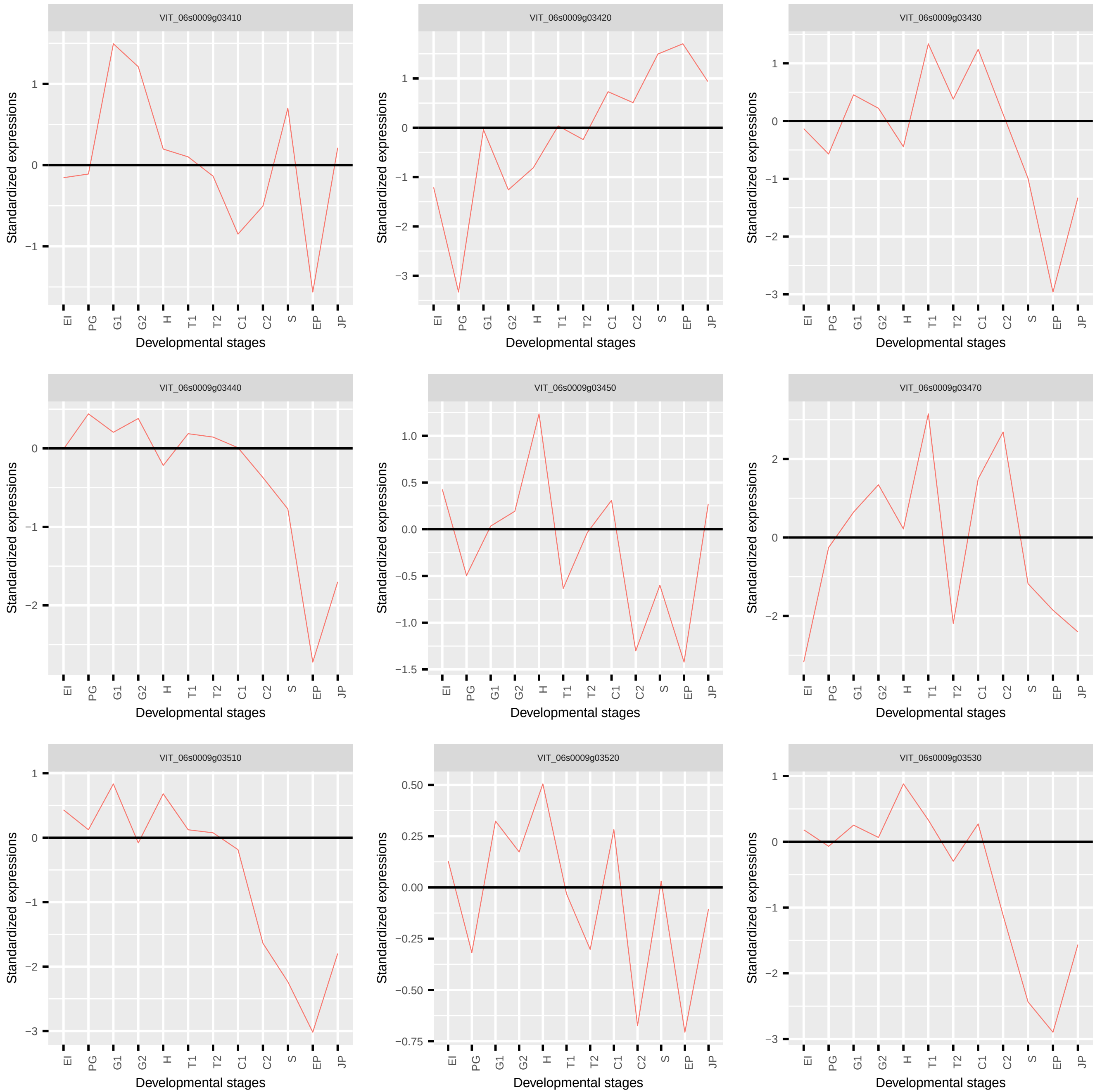

VIT_06s0009g03410
VIT_06s0009g03420
VIT_06s0009g03430
VIT_06s0009g03440
VIT_06s0009g03450
VIT_06s0009g03470
VIT_06s0009g03510
VIT_06s0009g03520
VIT_06s0009g03530
Standardized expressions
Developmental stages
EI PG G1 G2 H T1 T2 C1 C2 S EP JP

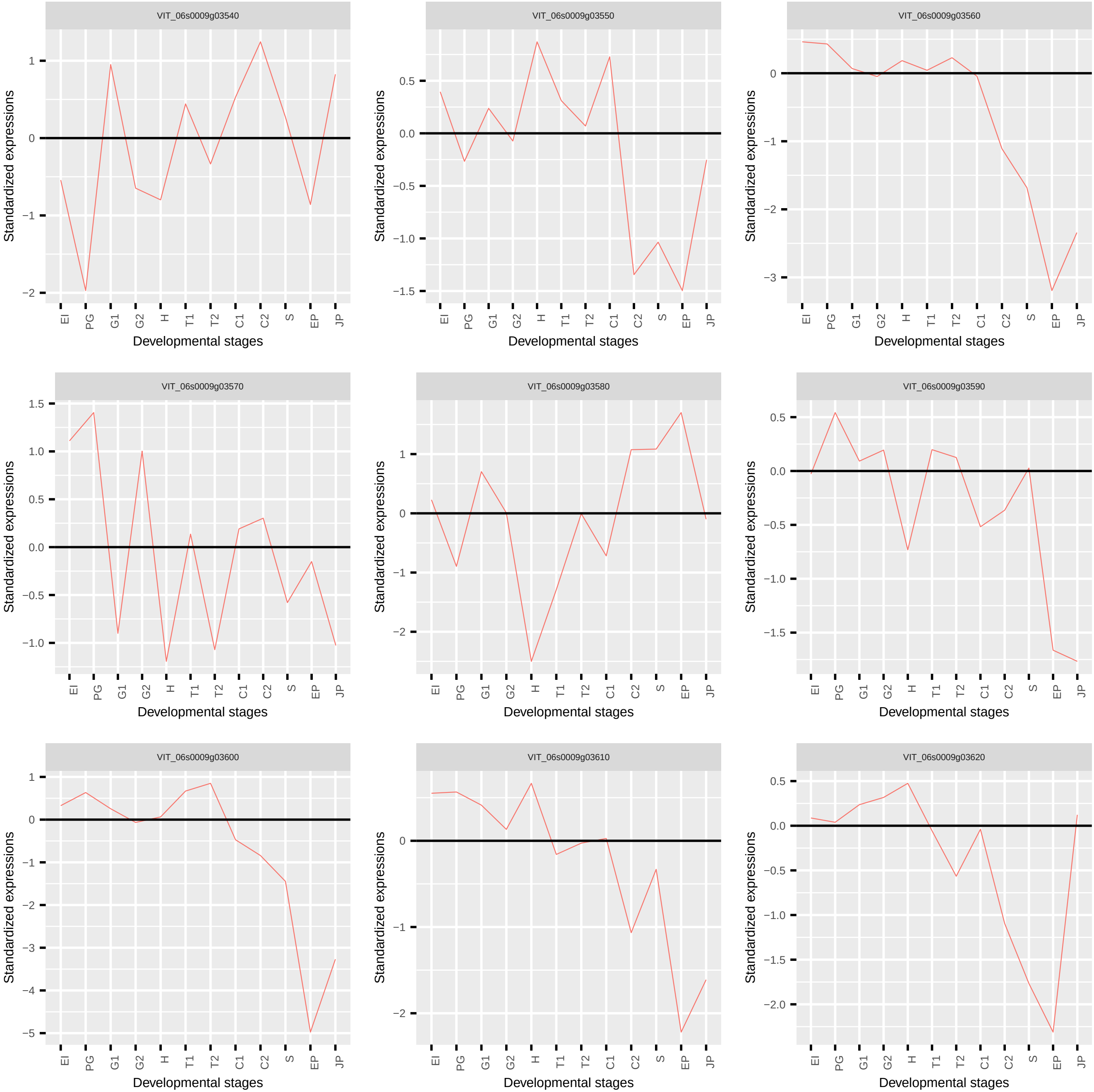

VIT_06s0009g03540
VIT_06s0009g03550
VIT_06s0009g03560
VIT_06s0009g03570
VIT_06s0009g03580
VIT_06s0009g03590
VIT_06s0009g03600
VIT_06s0009g03610
VIT_06s0009g03620
Standardized expressions
Developmental stages
EI PG G1 G2 H T1 T2 C1 C2 S EP JP

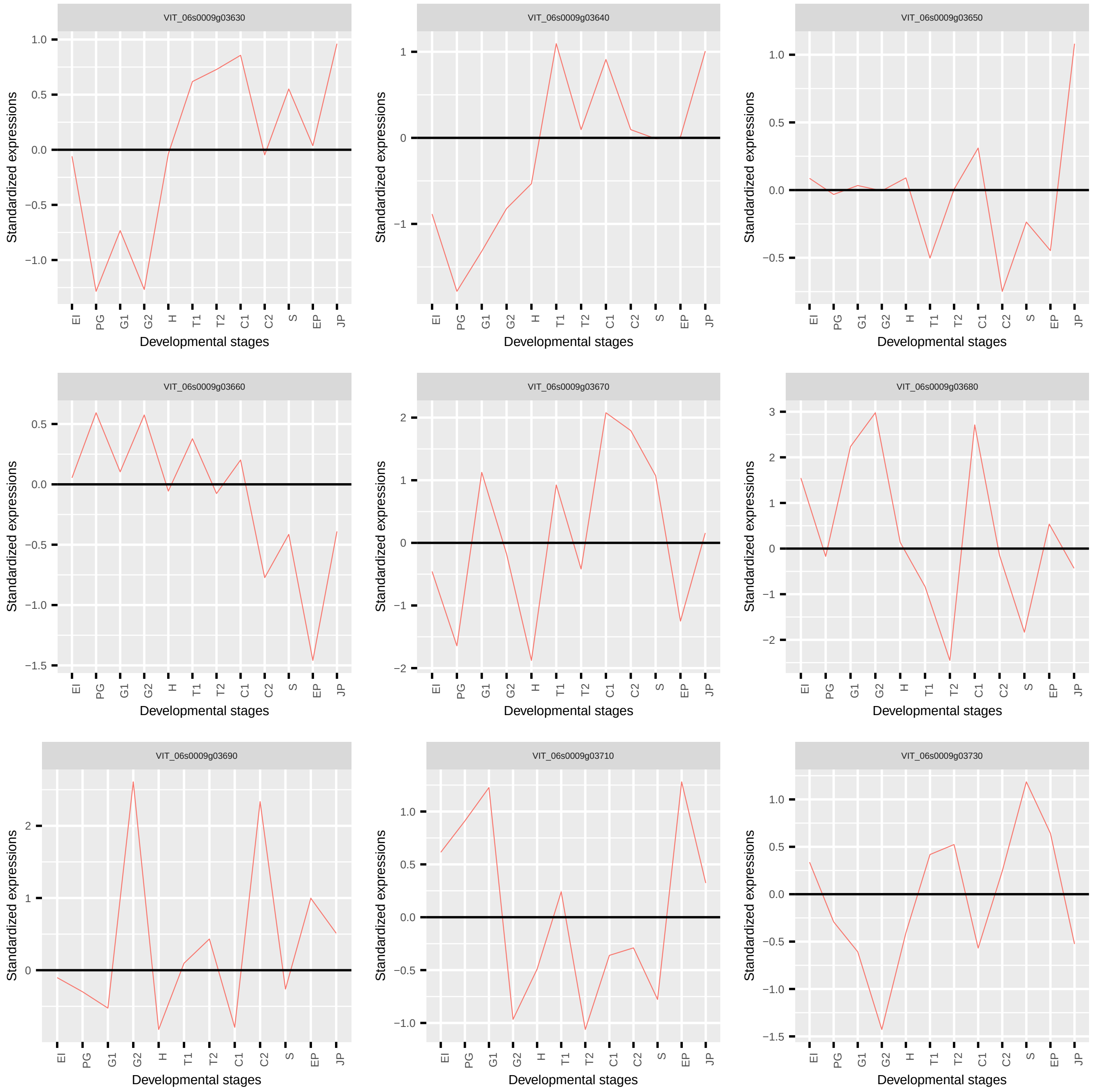
VIT_06s0009g03630
VIT_06s0009g03640
VIT_06s0009g03650
VIT_06s0009g03660
VIT_06s0009g03670
VIT_06s0009g03680
VIT_06s0009g03690
VIT_06s0009g03710
VIT_06s0009g03730
Standardized expressions
Developmental stages
EI PG G1 G2 H T1 T2 C1 C2 S EP JP

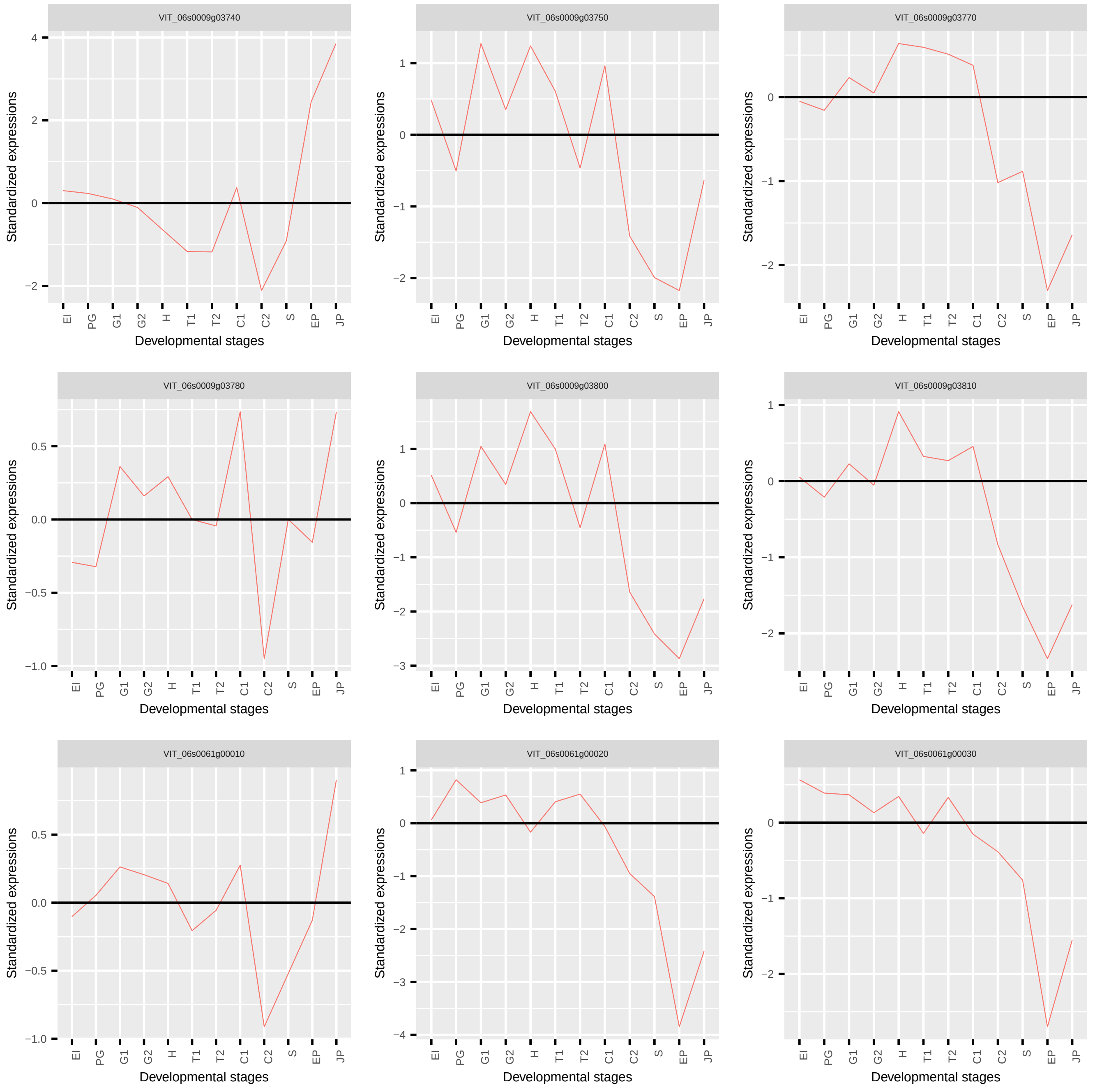
VIT_06s0009g03740
VIT_06s0009g03750
VIT_06s0009g03770
VIT_06s0009g03780
VIT_06s0009g03800
VIT_06s0009g03810
VIT_06s0061g00010
VIT_06s0061g00020
VIT_06s0061g00030
Standardized expressions
Developmental stages
EI PG G1 G2 H T1 T2 C1 C2 S EP JP

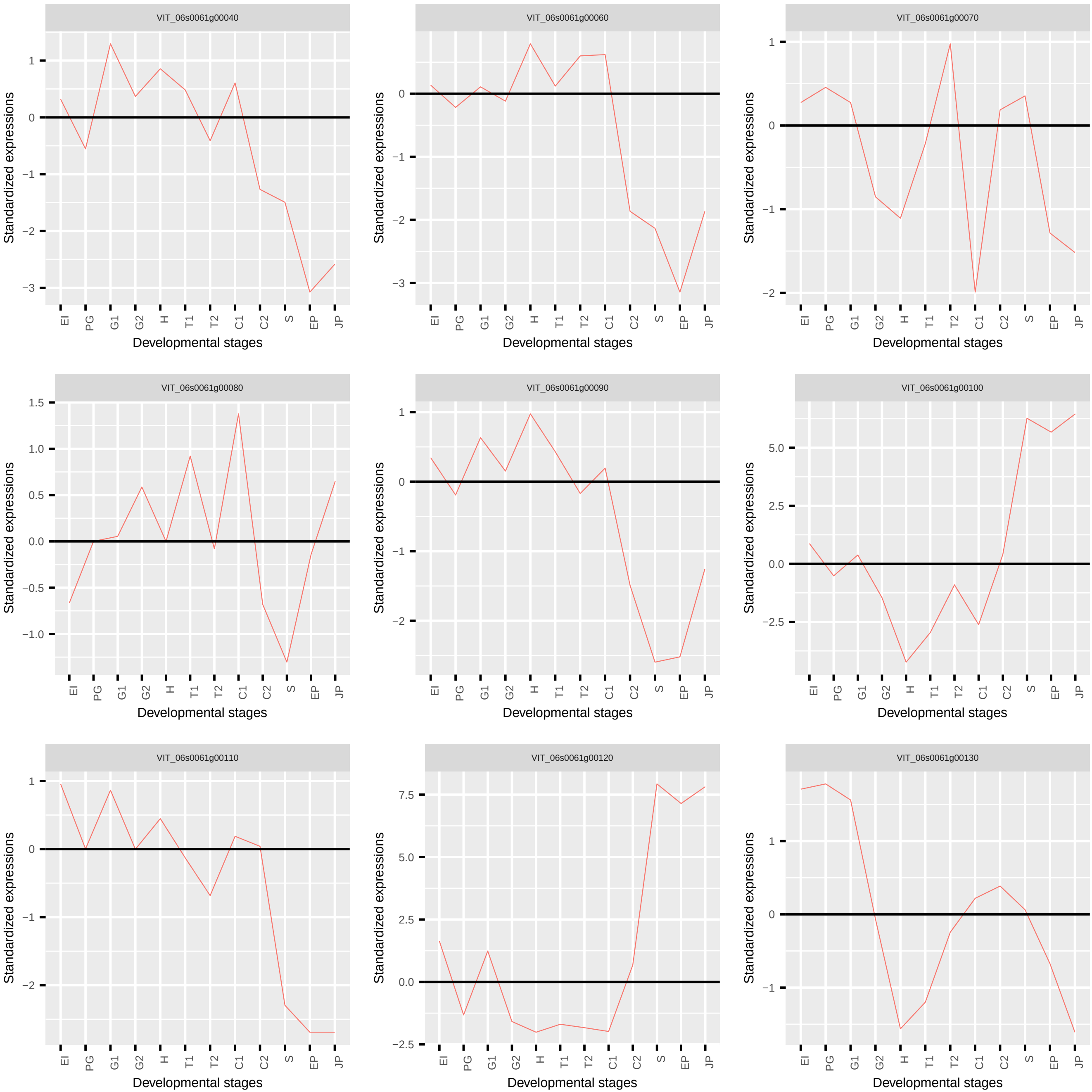
VIT_06s0061g00040
VIT_06s0061g00060
VIT_06s0061g00070
VIT_06s0061g00080
VIT_06s0061g00090
VIT_06s0061g00100
VIT_06s0061g00110
VIT_06s0061g00120
VIT_06s0061g00130
Standardized expressions
Developmental stages
EI PG G1 G2 H T1 T2 C1 C2 S EP JP

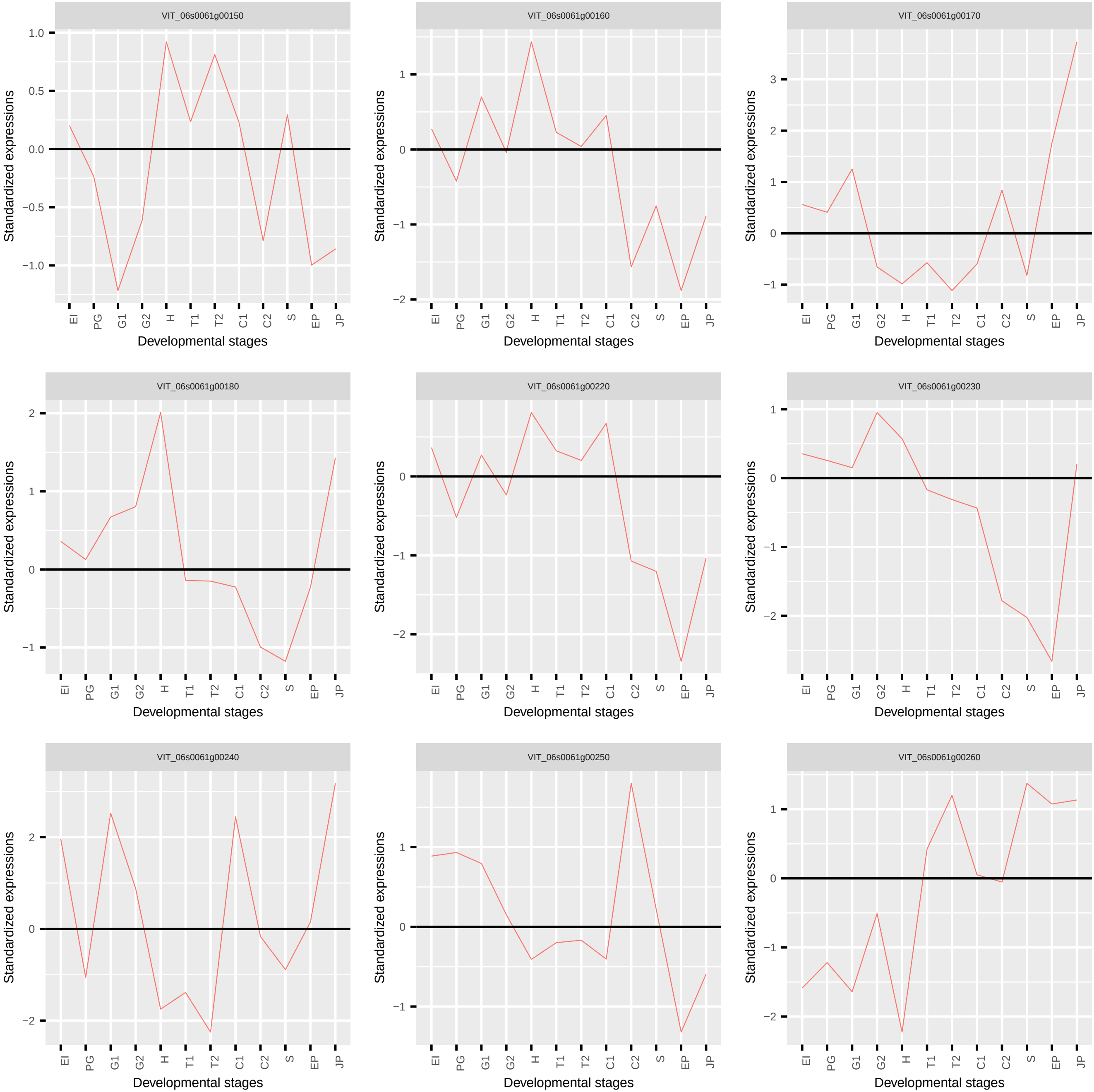
VIT_06s0061g00150
VIT_06s0061g00160
VIT_06s0061g00170
VIT_06s0061g00180
VIT_06s0061g00220
VIT_06s0061g00230
VIT_06s0061g00240
VIT_06s0061g00250
VIT_06s0061g00260
Standardized expressions
Developmental stages
EI PG G1 G2 H T1 T2 C1 C2 S EP JP

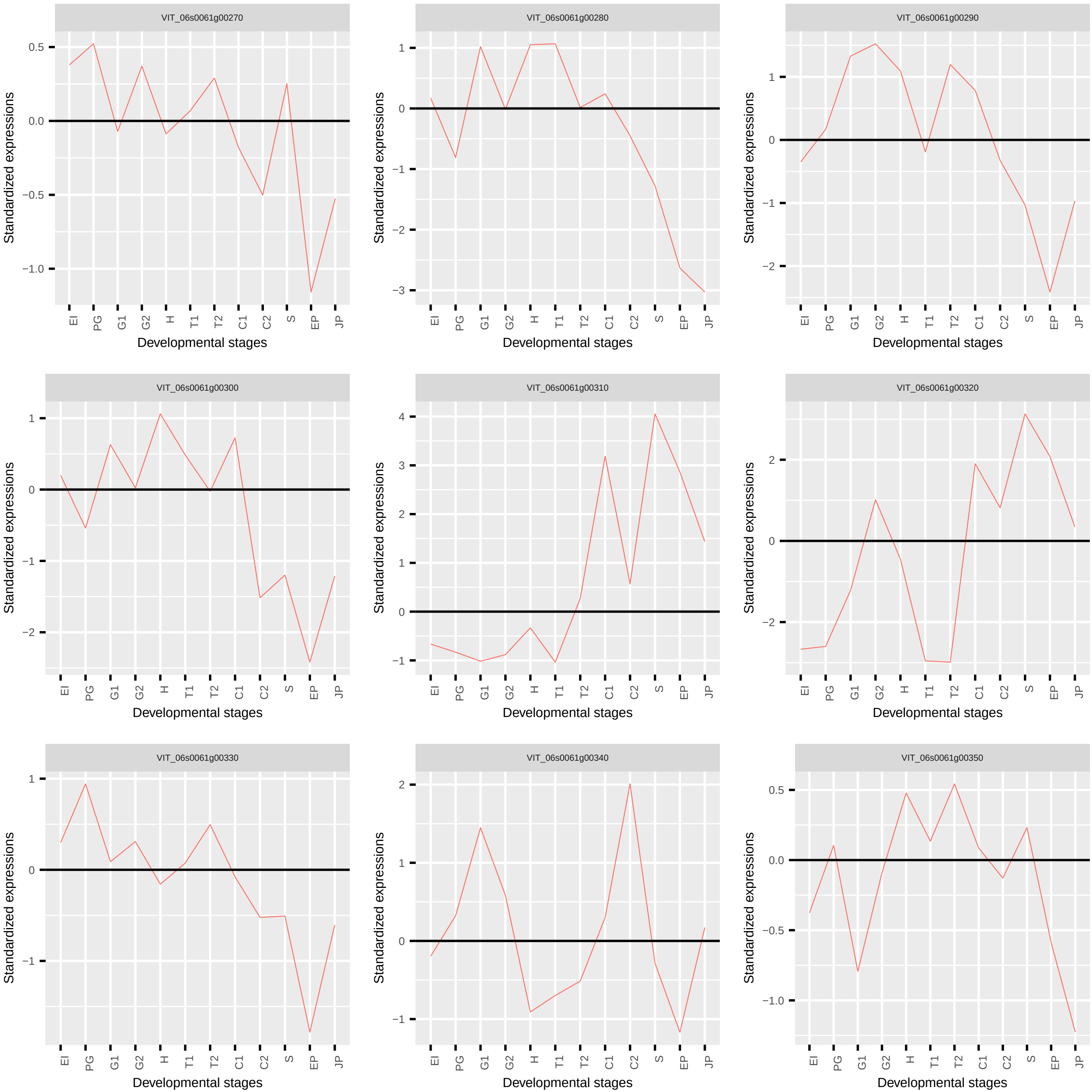

VIT_06s0061g00270
VIT_06s0061g00280
VIT_06s0061g00290
VIT_06s0061g00300
VIT_06s0061g00310
VIT_06s0061g00320
VIT_06s0061g00330
VIT_06s0061g00340
VIT_06s0061g00350
Standardized expressions
Developmental stages
EI PG G1 G2 H T1 T2 C1 C2 S EP JP

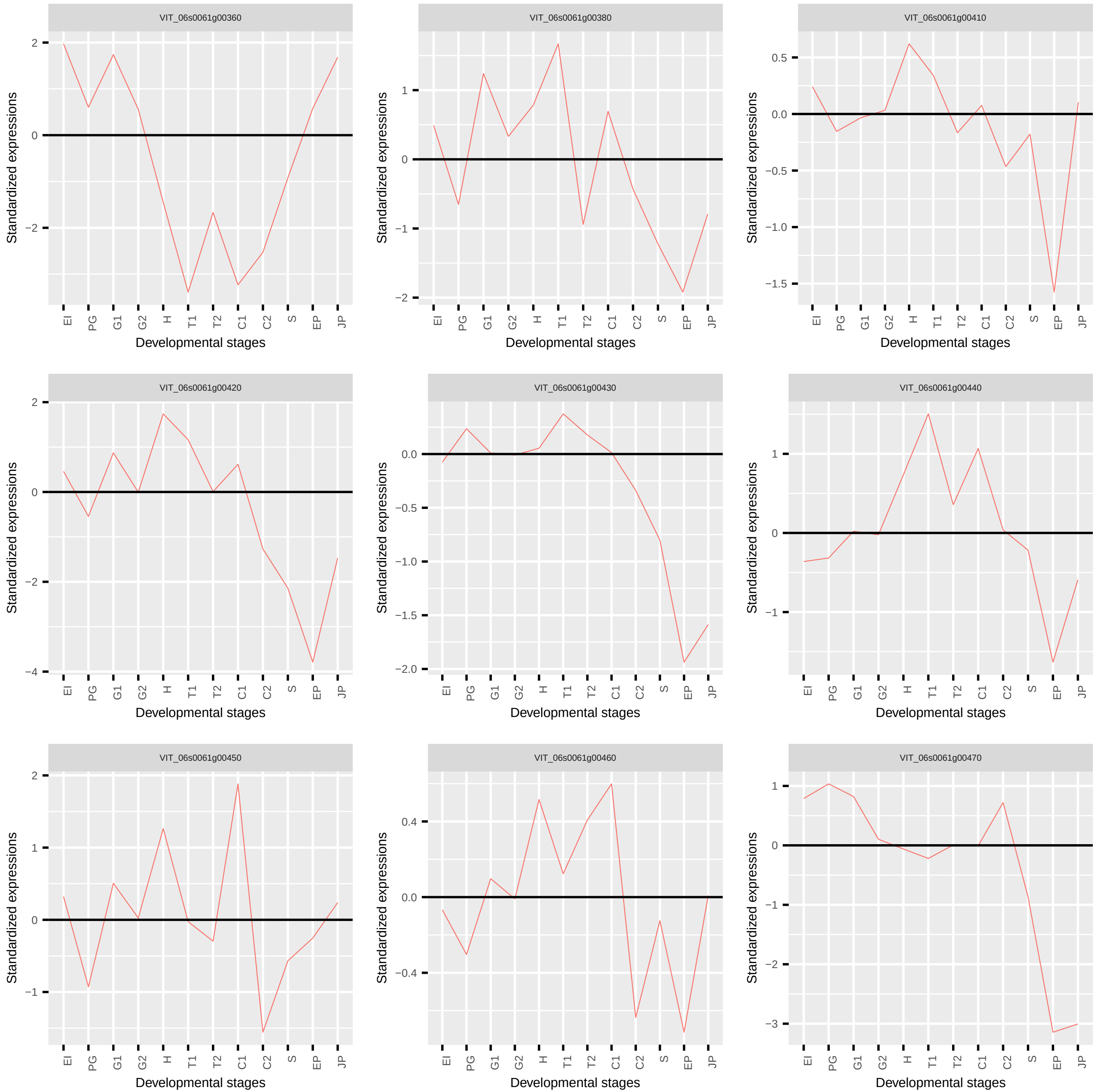
VIT_06s0061g00360
VIT_06s0061g00380
VIT_06s0061g00410
VIT_06s0061g00420
VIT_06s0061g00430
VIT_06s0061g00440
VIT_06s0061g00450
VIT_06s0061g00460
VIT_06s0061g00470
Standardized expressions
Developmental stages
EI PG G1 G2 H T1 T2 C1 C2 S EP JP
2
0
−2
1
−1
0.5
0.0
−0.5
−1.0
−1.5
−4
−2.0
0.4
−0.4
−3

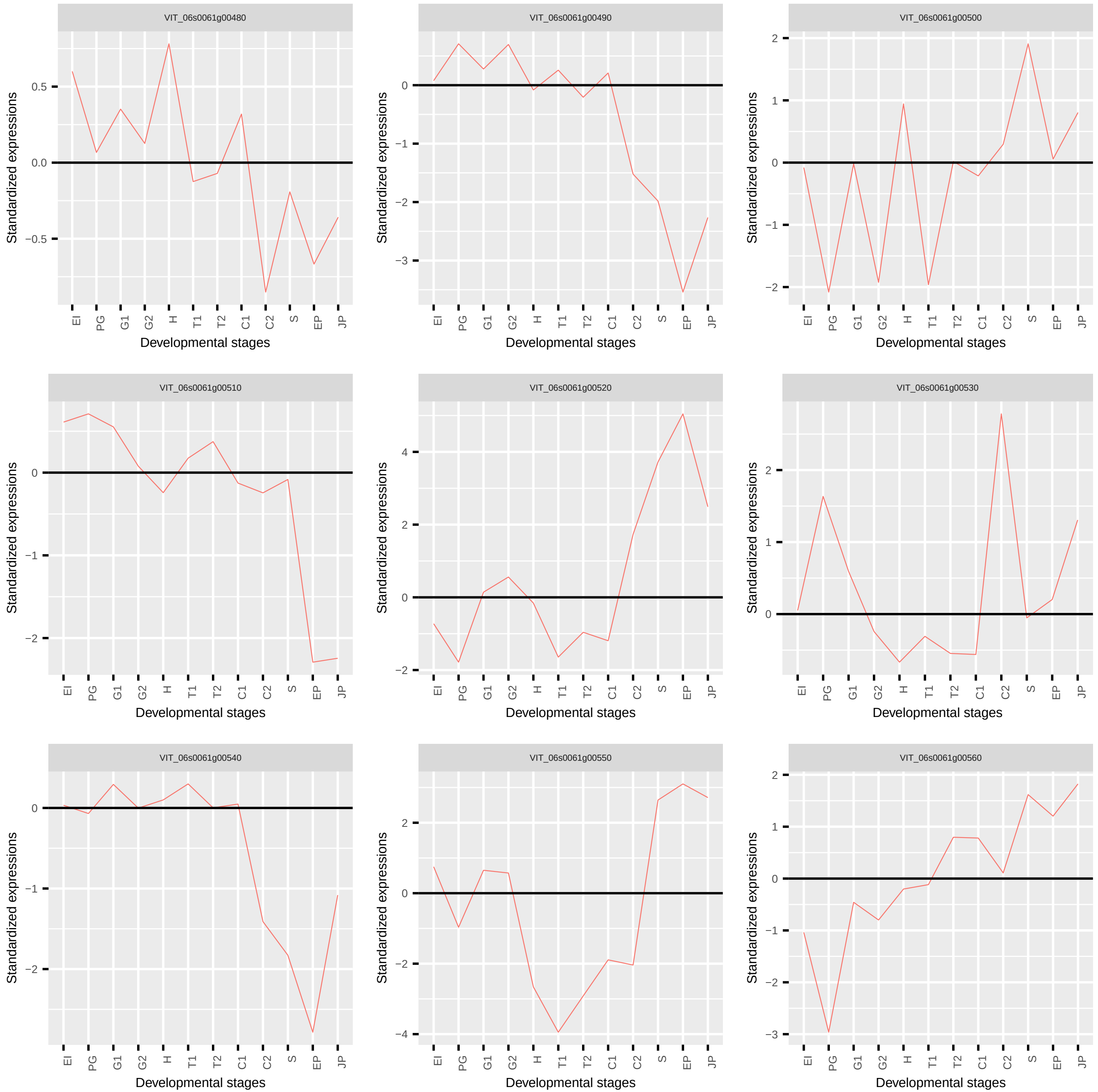
VIT_06s0061g00480
VIT_06s0061g00490
VIT_06s0061g00500
VIT_06s0061g00510
VIT_06s0061g00520
VIT_06s0061g00530
VIT_06s0061g00540
VIT_06s0061g00550
VIT_06s0061g00560
Standardized expressions
Developmental stages
EI PG G1 G2 H T1 T2 C1 C2 S EP JP

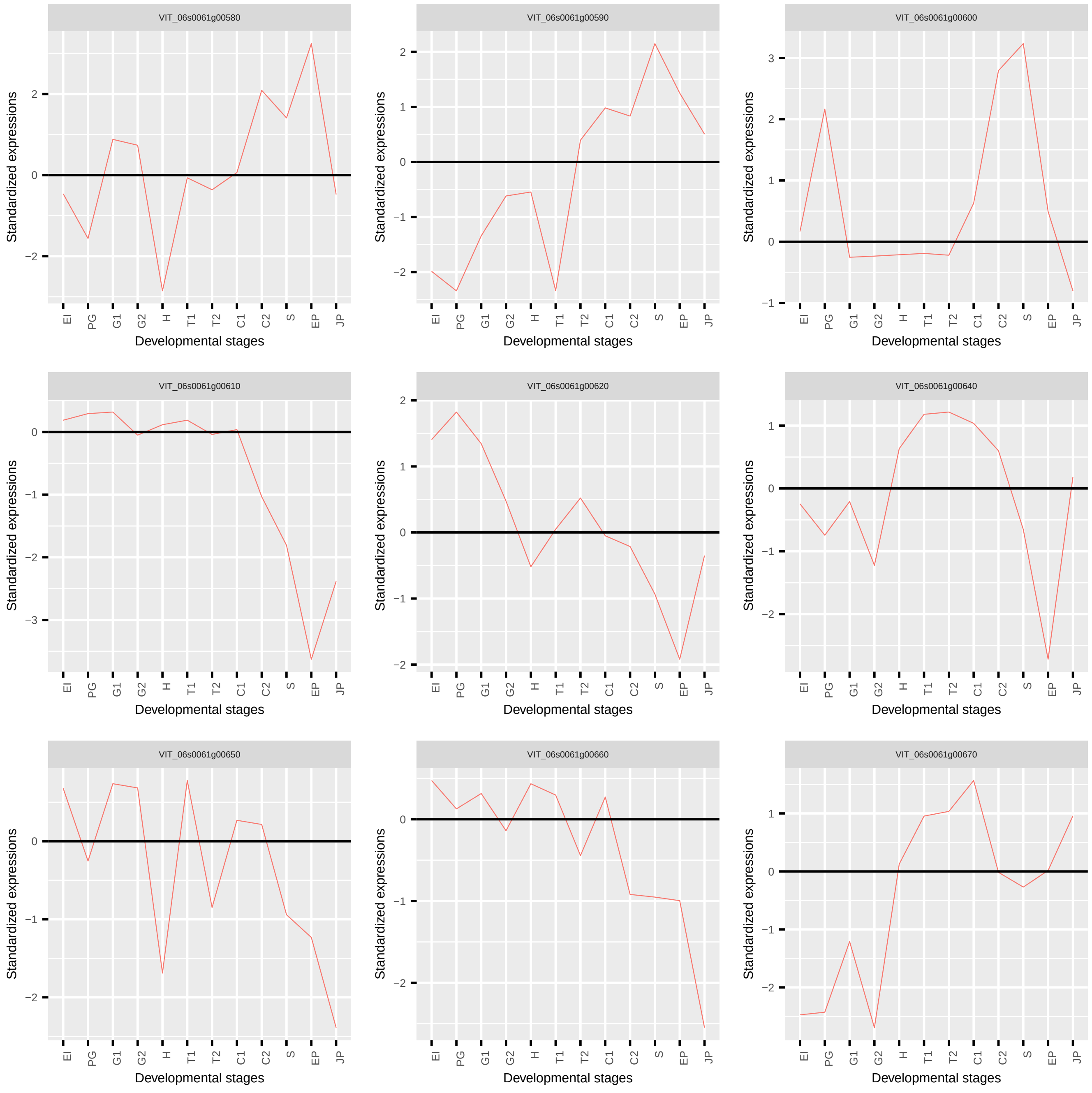
VIT_06s0061g00580
VIT_06s0061g00590
VIT_06s0061g00600
VIT_06s0061g00610
VIT_06s0061g00620
VIT_06s0061g00640
VIT_06s0061g00650
VIT_06s0061g00660
VIT_06s0061g00670
Standardized expressions
Developmental stages
EI PG G1 G2 H T1 T2 C1 C2 S EP JP

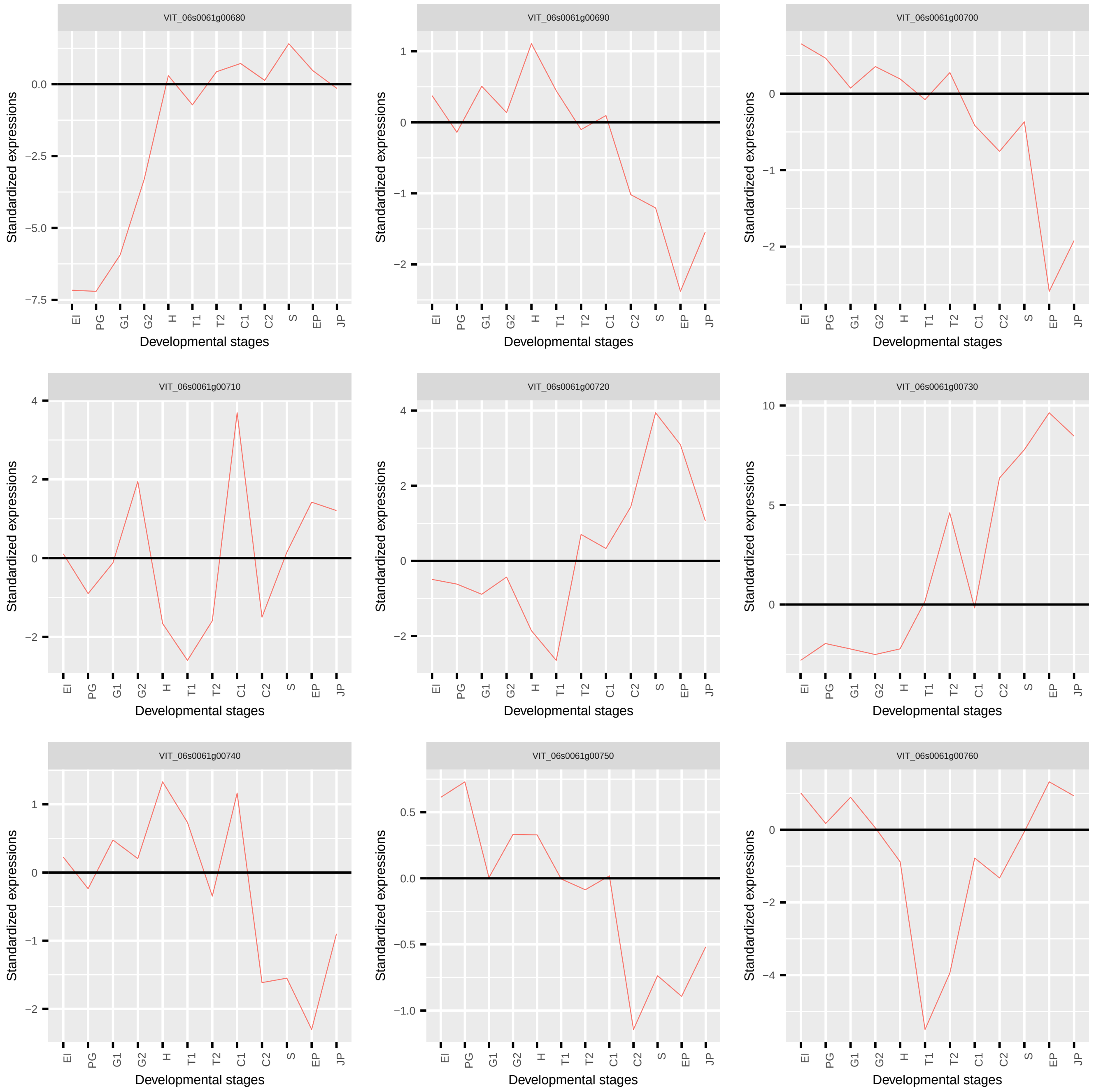
VIT_06s0061g00680
VIT_06s0061g00690
VIT_06s0061g00700
VIT_06s0061g00710
VIT_06s0061g00720
VIT_06s0061g00730
VIT_06s0061g00740
VIT_06s0061g00750
VIT_06s0061g00760
Standardized expressions
Developmental stages
EI PG G1 G2 H T1 T2 C1 C2 S EP JP

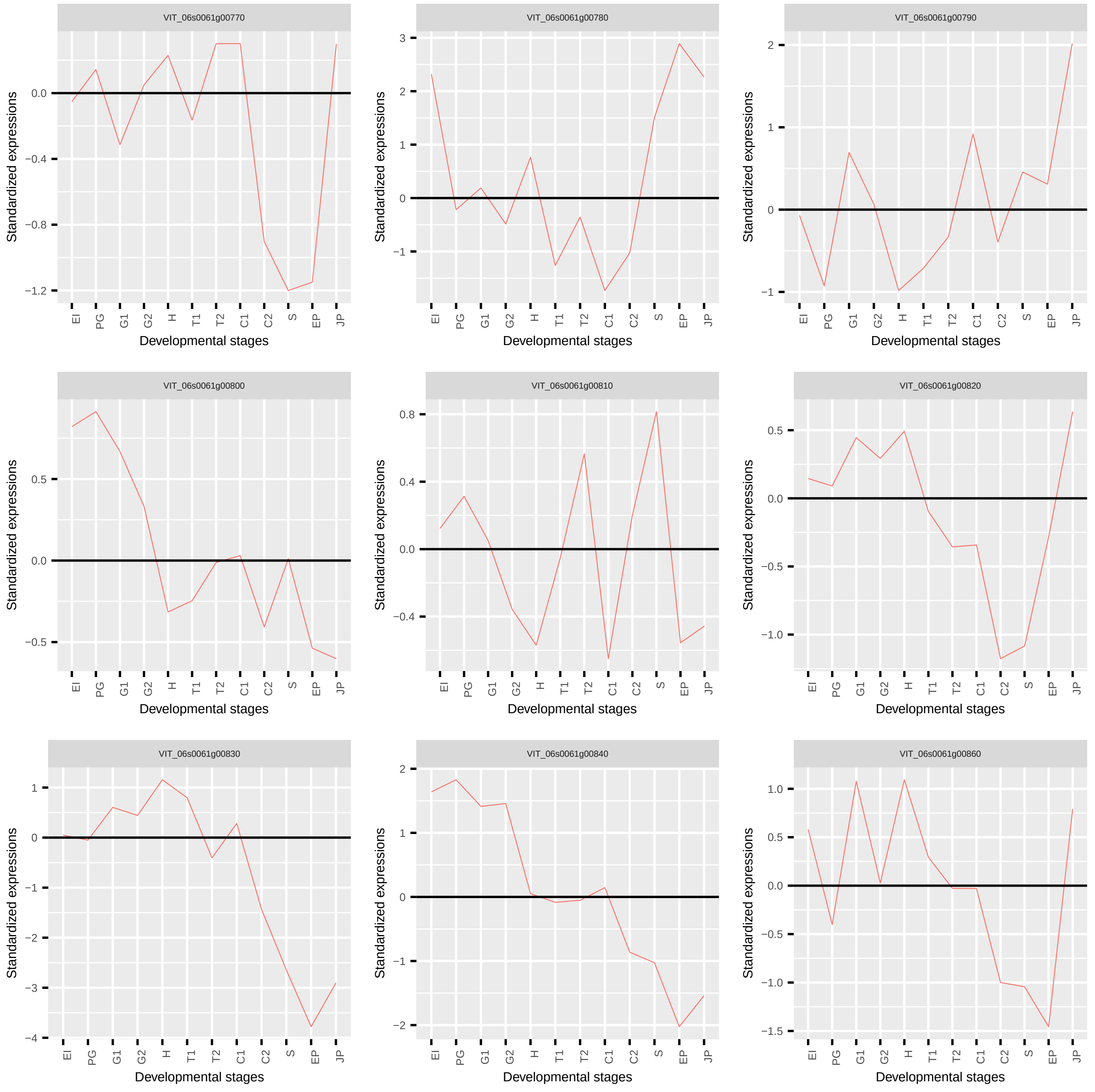
VIT_06s0061g00770
VIT_06s0061g00780
VIT_06s0061g00790
VIT_06s0061g00800
VIT_06s0061g00810
VIT_06s0061g00820
VIT_06s0061g00830
VIT_06s0061g00840
VIT_06s0061g00860
Standardized expressions
Developmental stages
EI PG G1 G2 H T1 T2 C1 C2 S EP JP

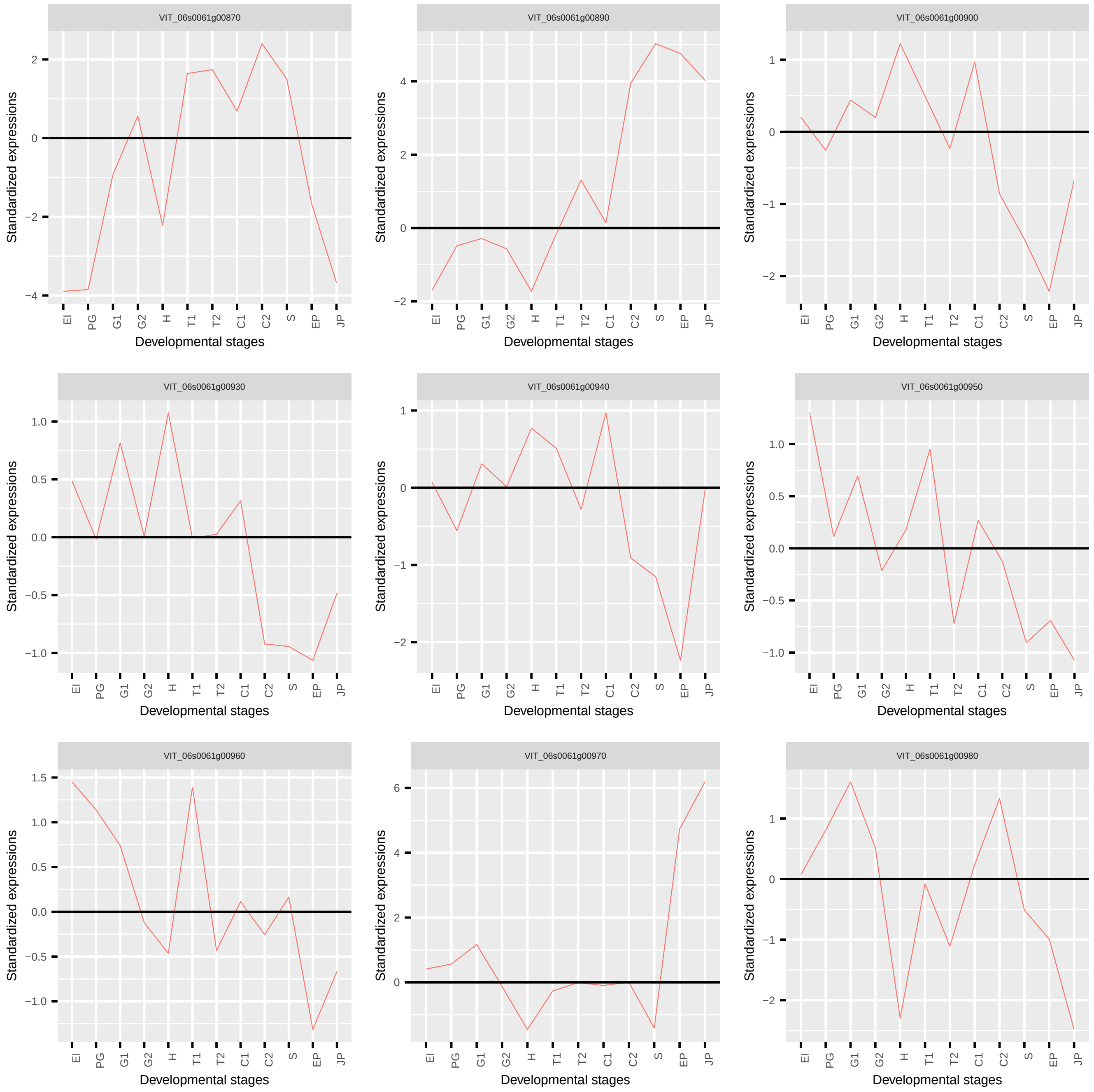
VIT_06s0061g00870
VIT_06s0061g00890
VIT_06s0061g00900
VIT_06s0061g00930
VIT_06s0061g00940
VIT_06s0061g00950
VIT_06s0061g00960
VIT_06s0061g00970
VIT_06s0061g00980
Standardized expressions
Developmental stages
EI PG G1 G2 H T1 T2 C1 C2 S EP JP

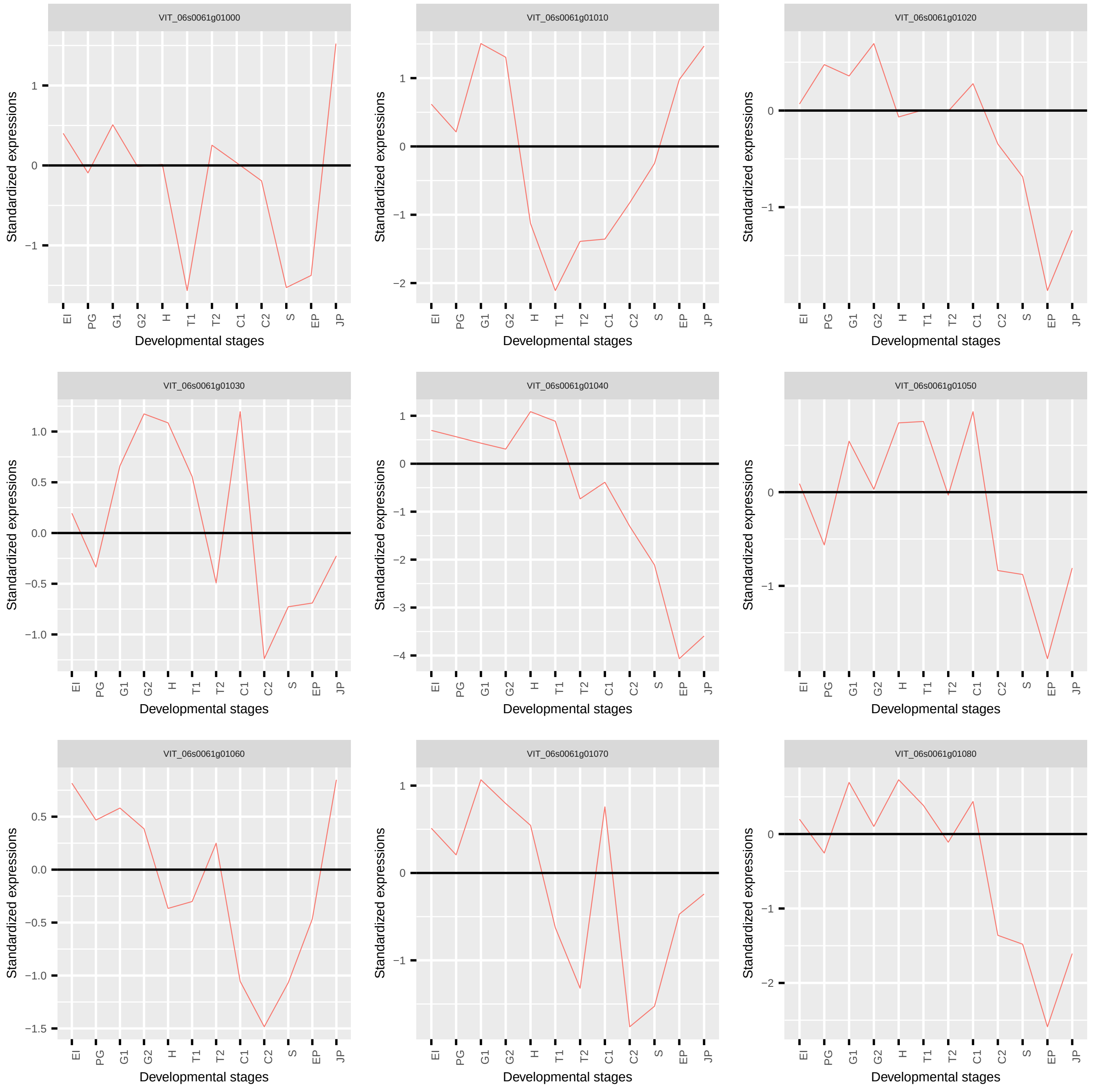

VIT_06s0061g01000
VIT_06s0061g01010
VIT_06s0061g01020
VIT_06s0061g01030
VIT_06s0061g01040
VIT_06s0061g01050
VIT_06s0061g01060
VIT_06s0061g01070
VIT_06s0061g01080
Standardized expressions
Developmental stages
EI PG G1 G2 H T1 T2 C1 C2 S EP JP

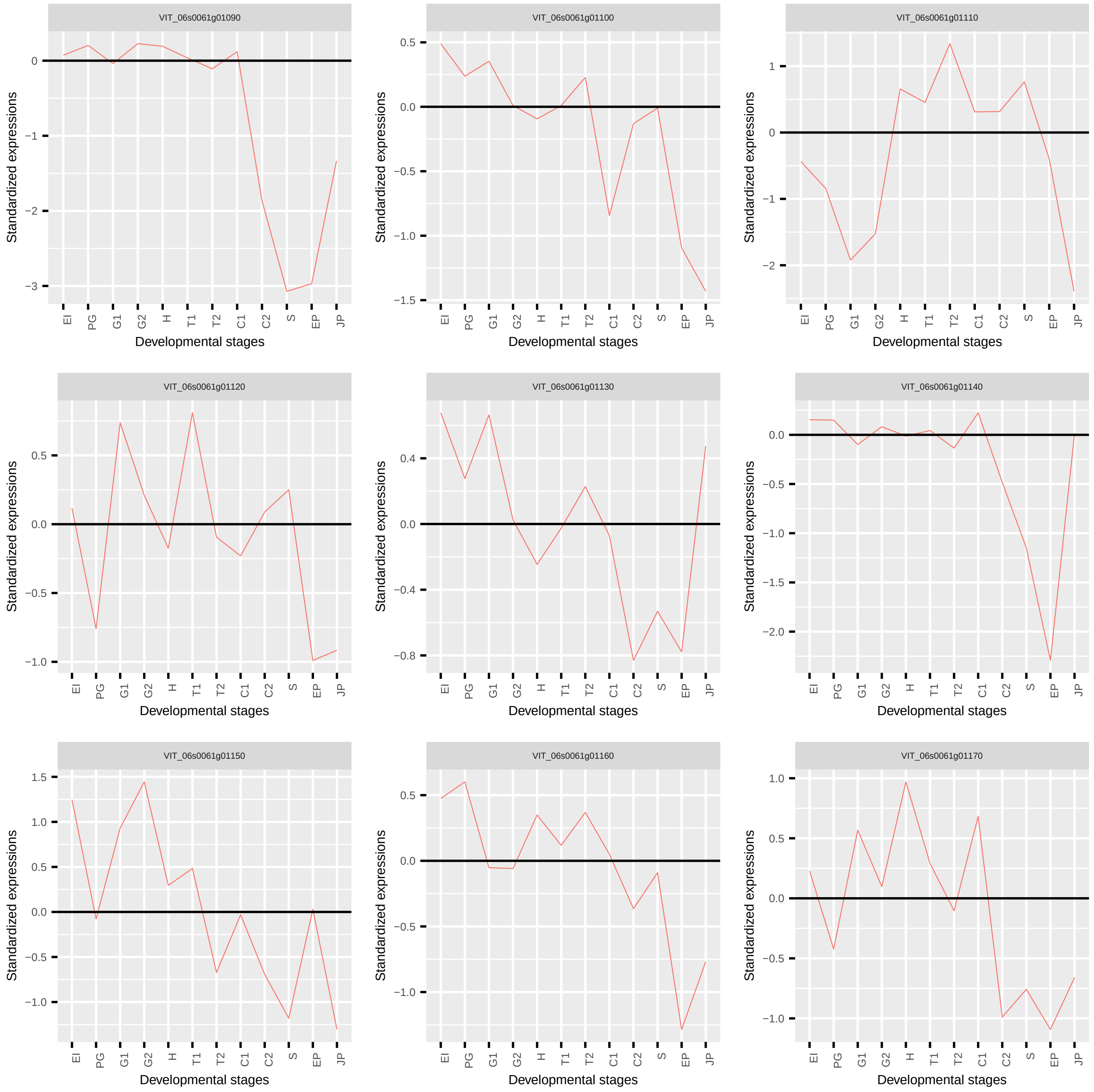
VIT_06s0061g01090
VIT_06s0061g01100
VIT_06s0061g01110
VIT_06s0061g01120
VIT_06s0061g01130
VIT_06s0061g01140
VIT_06s0061g01150
VIT_06s0061g01160
VIT_06s0061g01170
Standardized expressions
Developmental stages
EI PG G1 G2 H T1 T2 C1 C2 S EP JP

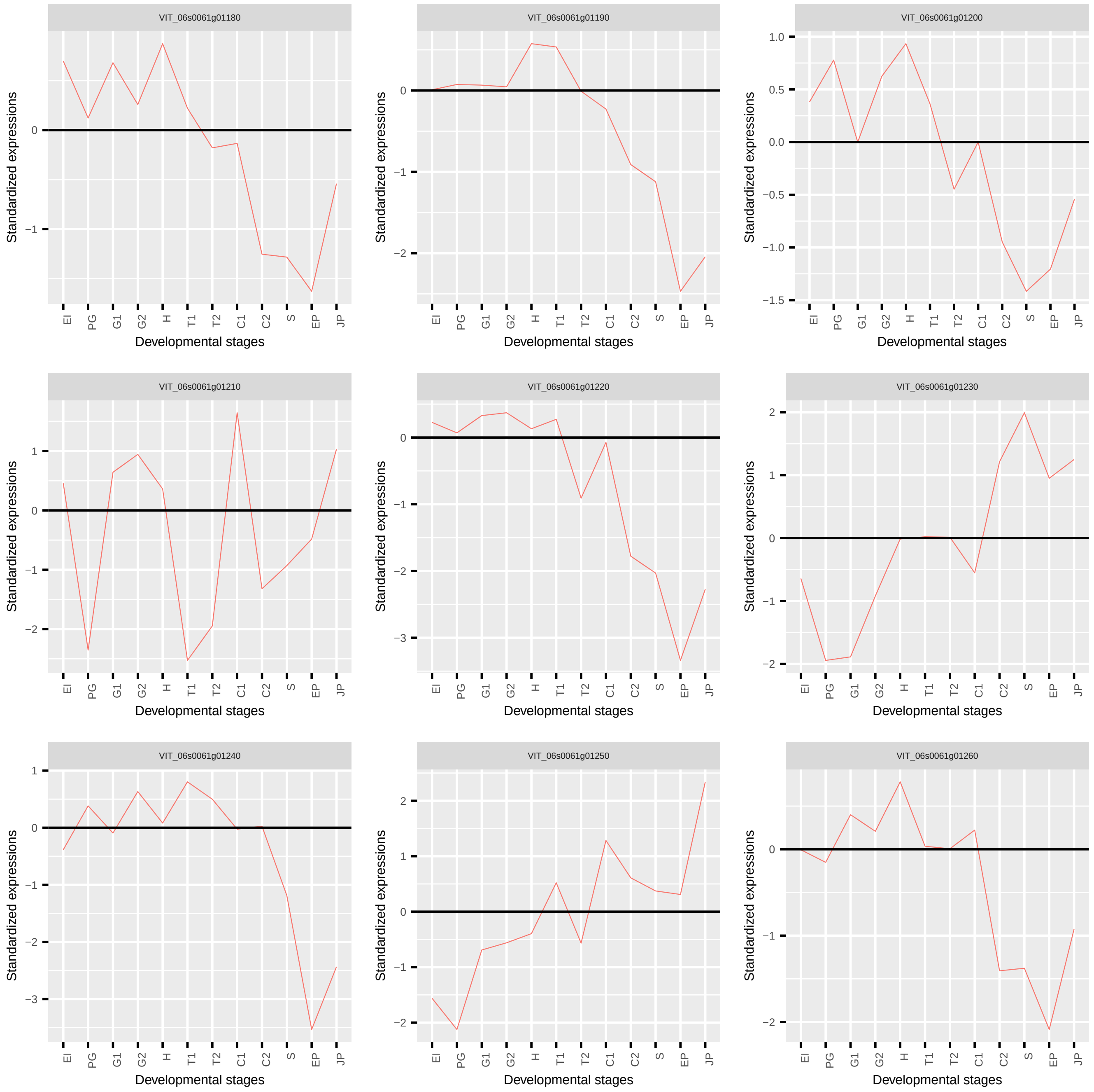
VIT_06s0061g01180
VIT_06s0061g01190
VIT_06s0061g01200
VIT_06s0061g01210
VIT_06s0061g01220
VIT_06s0061g01230
VIT_06s0061g01240
VIT_06s0061g01250
VIT_06s0061g01260
Standardized expressions
Developmental stages
EI PG G1 G2 H T1 T2 C1 C2 S EP JP

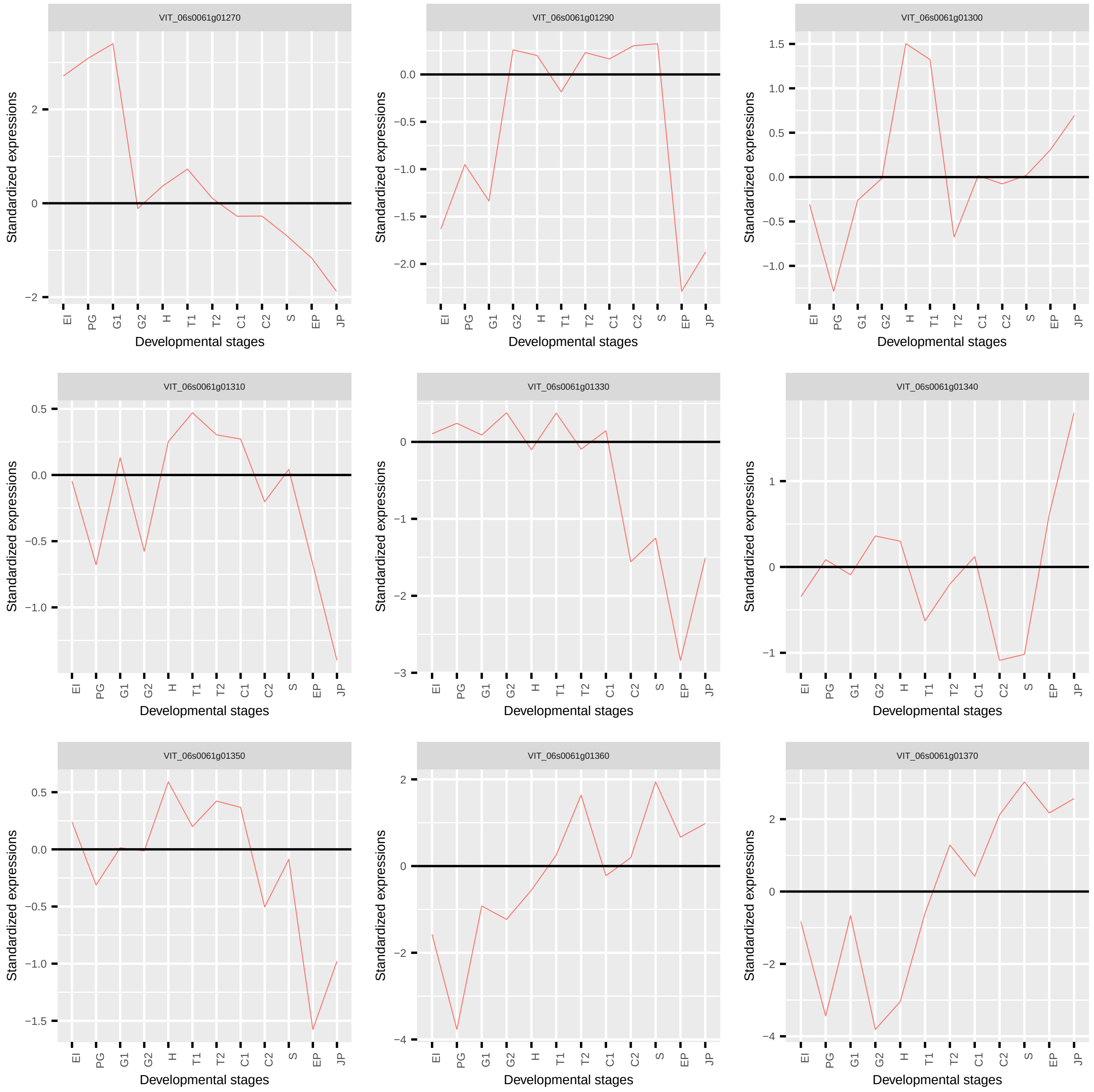
VIT_06s0061g01270
VIT_06s0061g01290
VIT_06s0061g01300
VIT_06s0061g01310
VIT_06s0061g01330
VIT_06s0061g01340
VIT_06s0061g01350
VIT_06s0061g01360
VIT_06s0061g01370
Standardized expressions
Developmental stages
EI PG G1 G2 H T1 T2 C1 C2 S EP JP
2 0 −2
0.0 −0.5 −1.0 −1.5 −2.0
1.5 1.0 0.5 0.0 −0.5 −1.0
0.5 0.0 −0.5 −1.0
0 −1 −2 −3
1 0 −1
0.5 0.0 −0.5 −1.0 −1.5
2 0 −2 −4
2 0 −2 −4

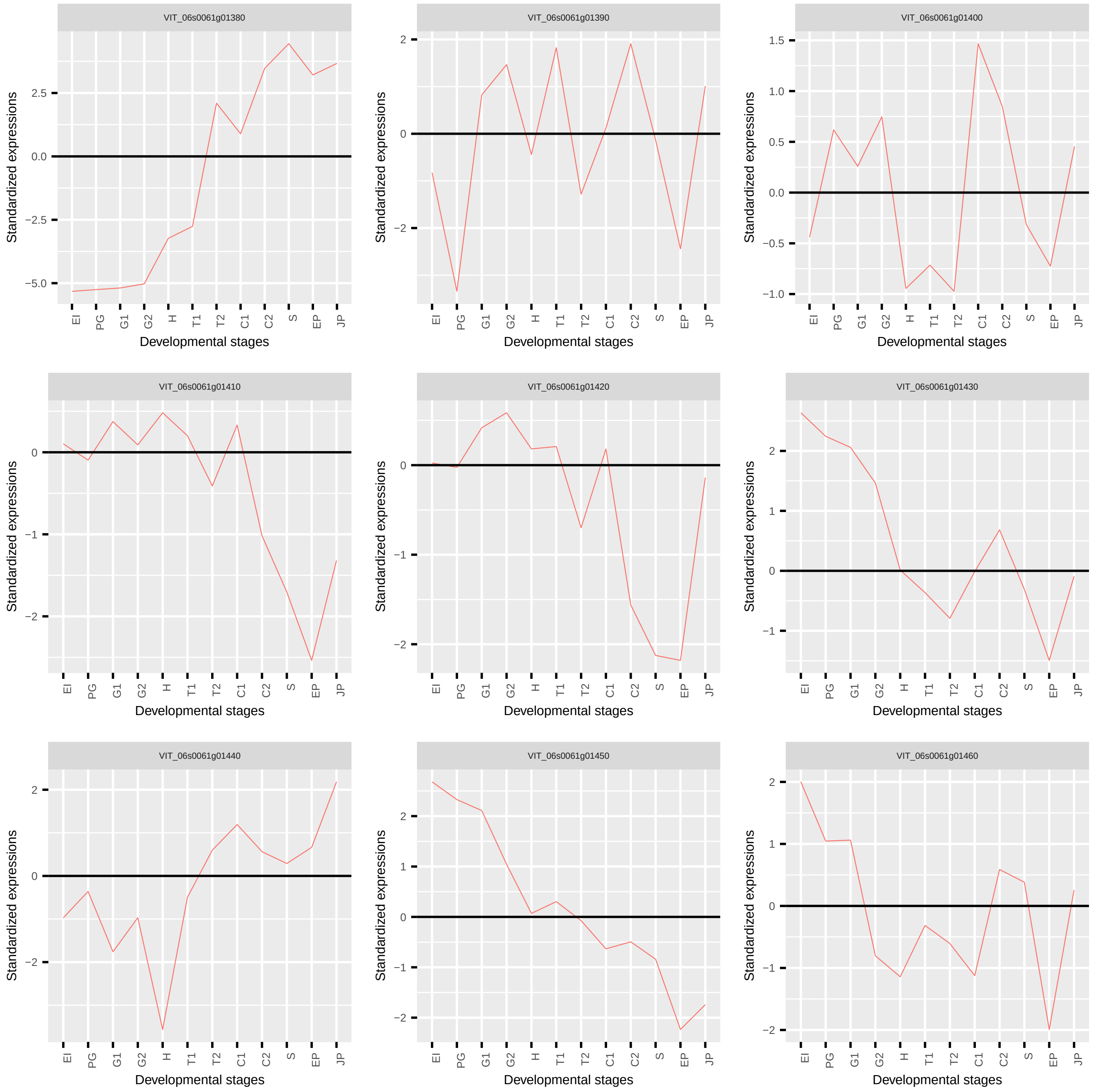
VIT_06s0061g01380
VIT_06s0061g01390
VIT_06s0061g01400
VIT_06s0061g01410
VIT_06s0061g01420
VIT_06s0061g01430
VIT_06s0061g01440
VIT_06s0061g01450
VIT_06s0061g01460
Standardized expressions
Developmental stages
EI PG G1 G2 H T1 T2 C1 C2 S EP JP

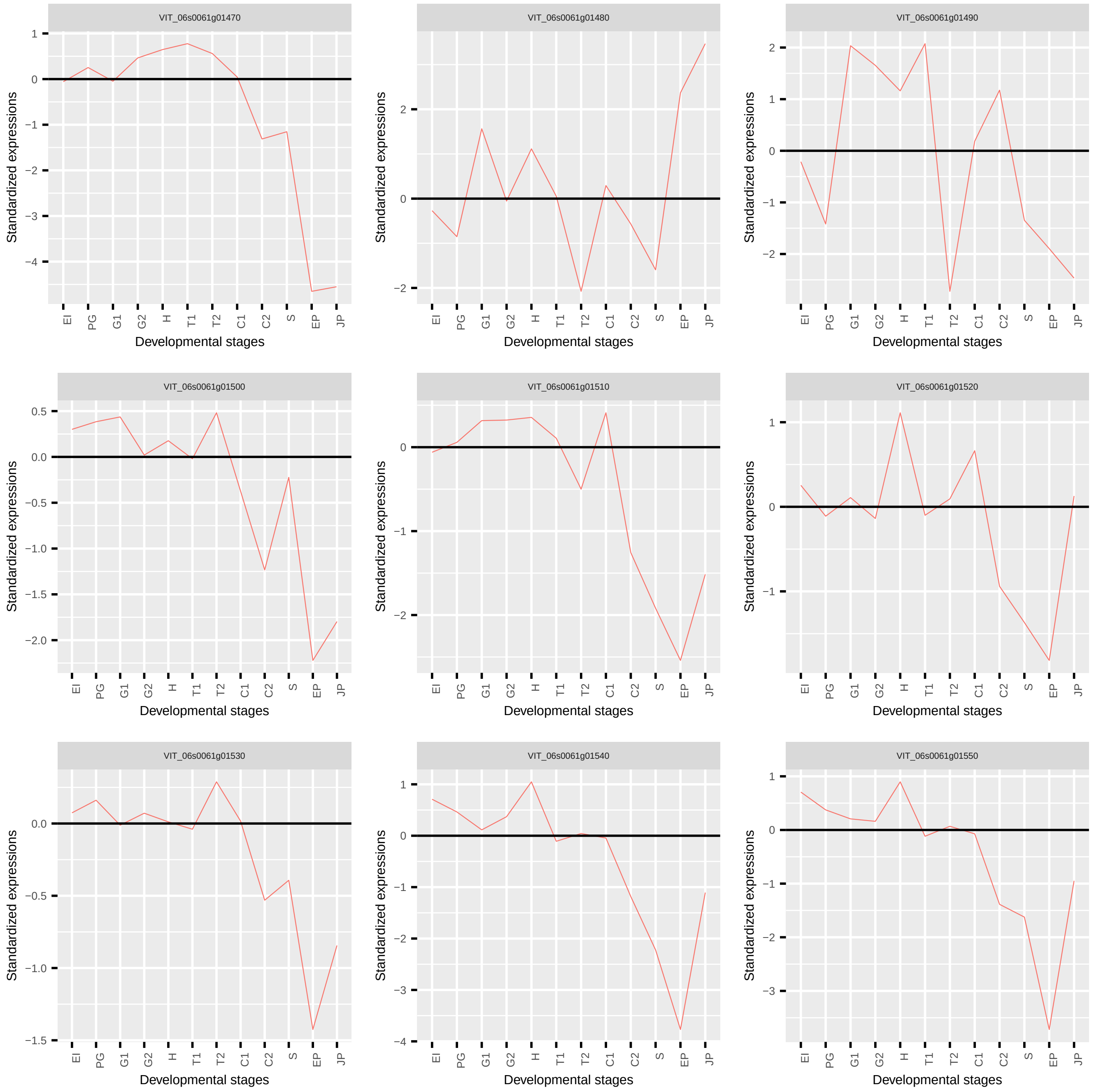
VIT_06s0061g01470
VIT_06s0061g01480
VIT_06s0061g01490
VIT_06s0061g01500
VIT_06s0061g01510
VIT_06s0061g01520
VIT_06s0061g01530
VIT_06s0061g01540
VIT_06s0061g01550
Standardized expressions
Developmental stages
EI PG G1 G2 H T1 T2 C1 C2 S EP JP

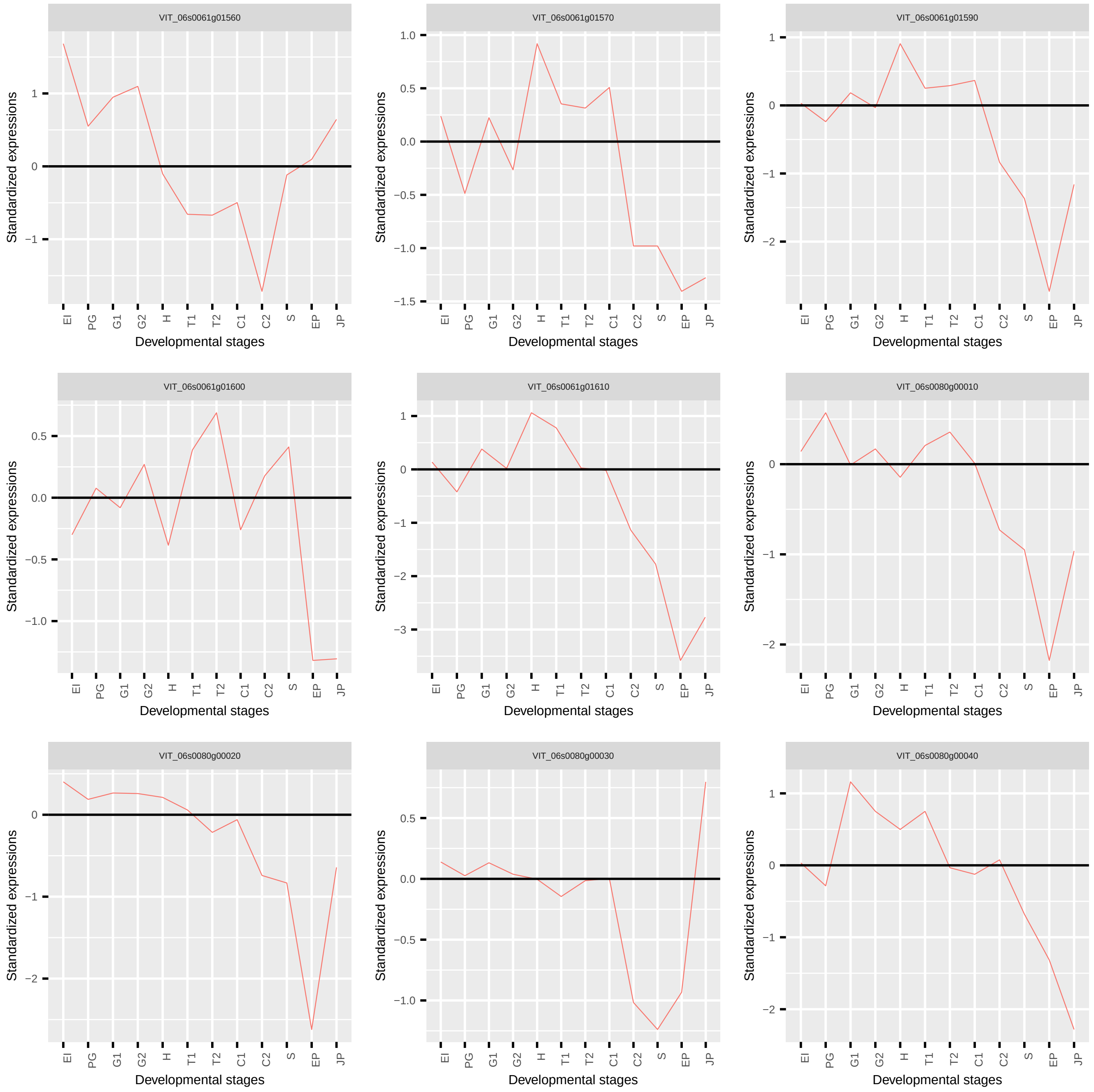
VIT_06s0061g01560
VIT_06s0061g01570
VIT_06s0061g01590
VIT_06s0061g01600
VIT_06s0061g01610
VIT_06s0080g00010
VIT_06s0080g00020
VIT_06s0080g00030
VIT_06s0080g00040
Standardized expressions
Developmental stages
EI
PG
G1
G2
H
T1
T2
C1
C2
S
EP
JP

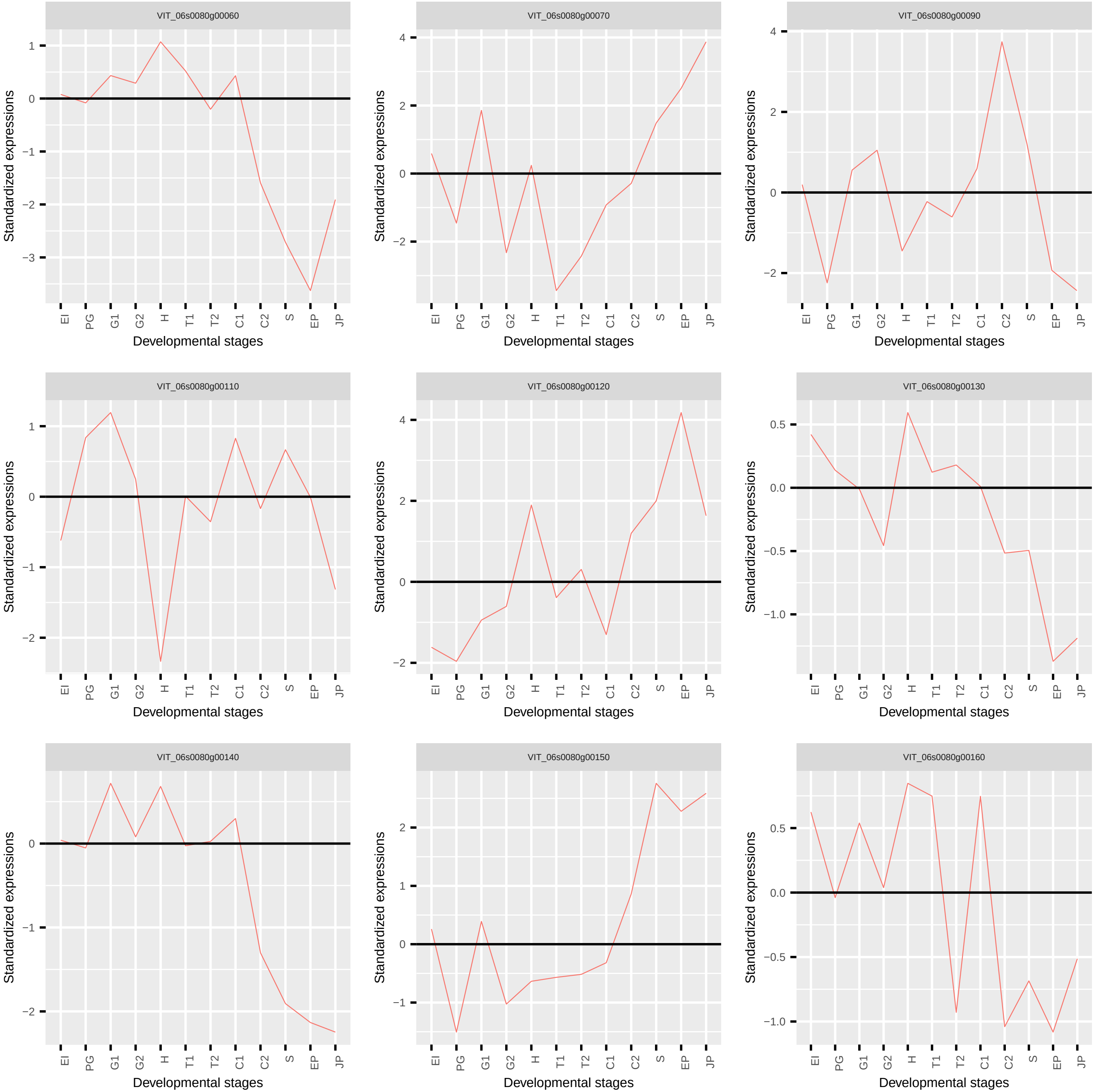
VIT_06s0080g00060
VIT_06s0080g00070
VIT_06s0080g00090
VIT_06s0080g00110
VIT_06s0080g00120
VIT_06s0080g00130
VIT_06s0080g00140
VIT_06s0080g00150
VIT_06s0080g00160
Standardized expressions
Developmental stages
EI PG G1 G2 H T1 T2 C1 C2 S EP JP

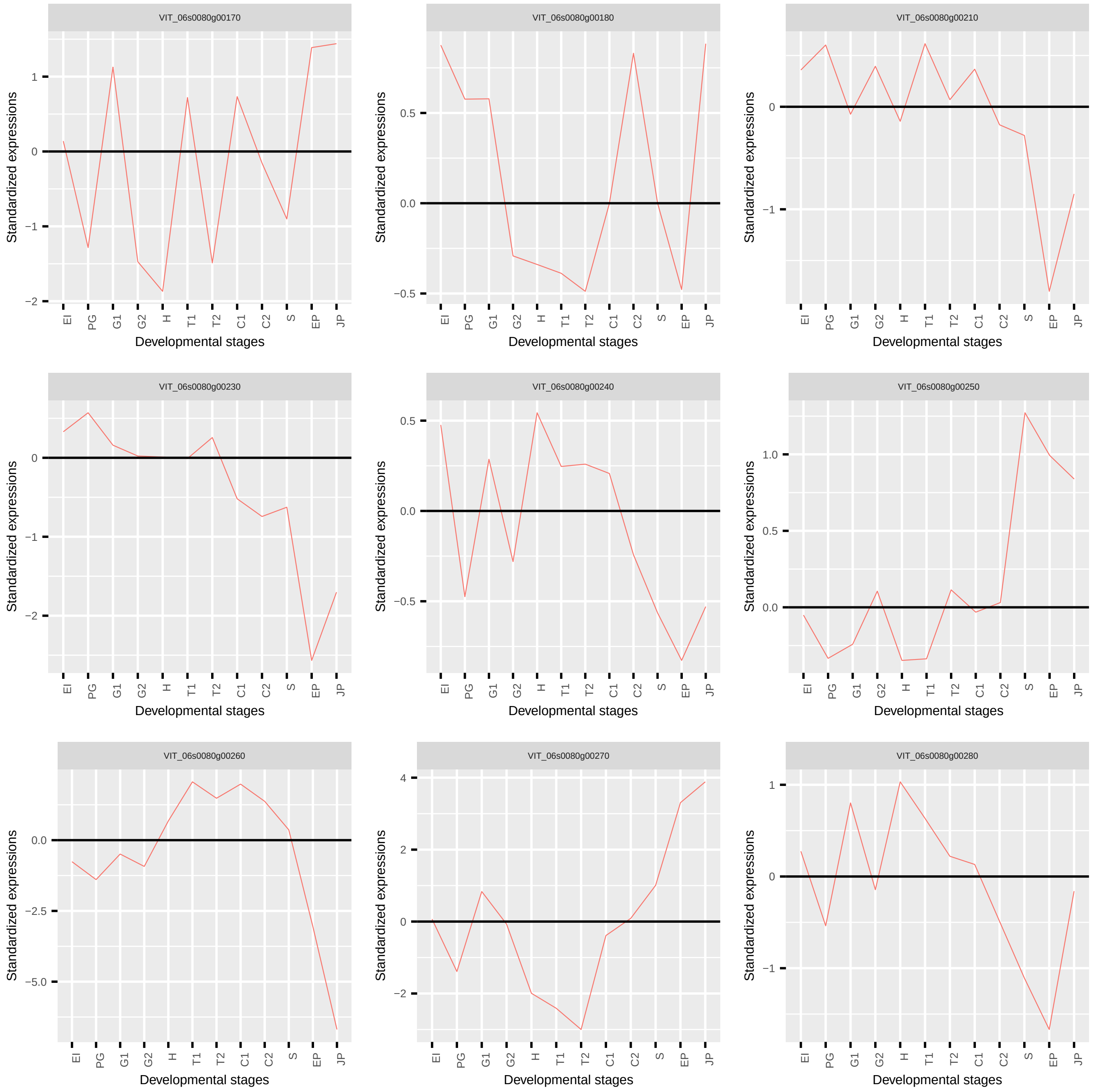
VIT_06s0080g00170
VIT_06s0080g00180
VIT_06s0080g00210
VIT_06s0080g00230
VIT_06s0080g00240
VIT_06s0080g00250
VIT_06s0080g00260
VIT_06s0080g00270
VIT_06s0080g00280
Standardized expressions
Developmental stages
EI
PG
G1
G2
H
T1
T2
C1
C2
S
EP
JP
1
0
−1
−2
0.5
0.0
−0.5
1.0
−2.5
−5.0
4
2

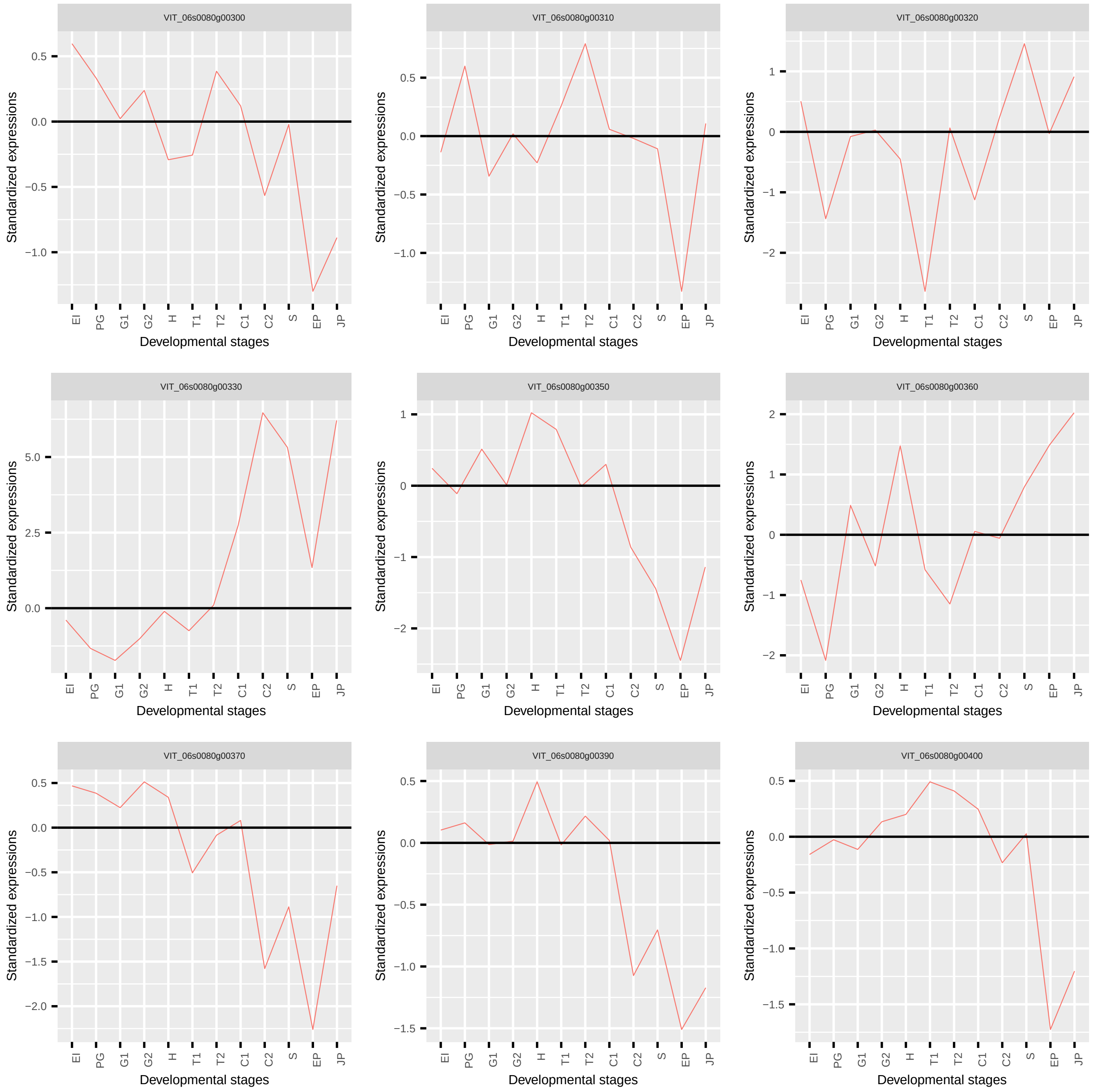

VIT_06s0080g00300
VIT_06s0080g00310
VIT_06s0080g00320
VIT_06s0080g00330
VIT_06s0080g00350
VIT_06s0080g00360
VIT_06s0080g00370
VIT_06s0080g00390
VIT_06s0080g00400
Standardized expressions
Developmental stages
EI
PG
G1
G2
H
T1
T2
C1
C2
S
EP
JP

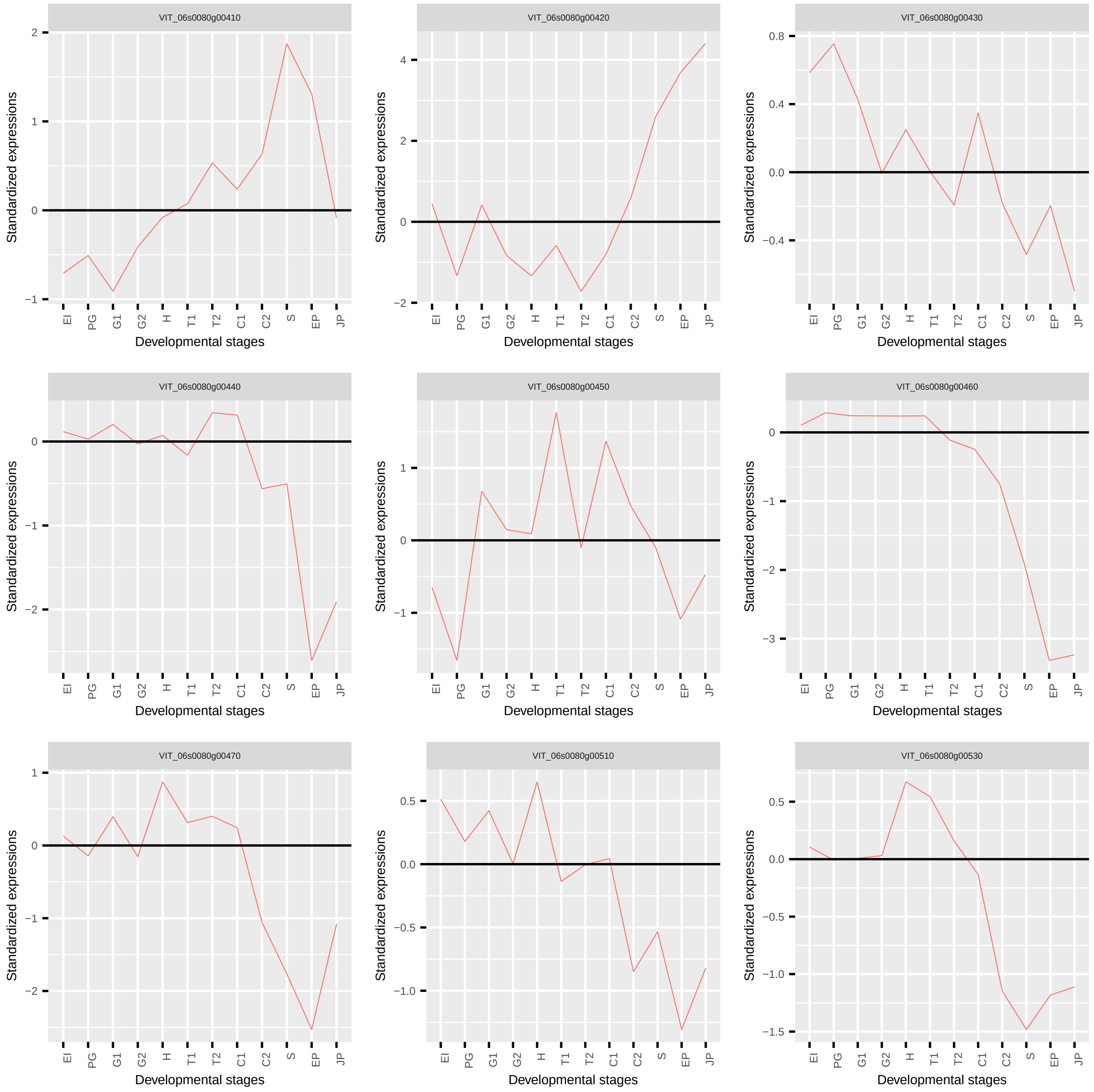
VIT_06s0080g00410
VIT_06s0080g00420
VIT_06s0080g00430
VIT_06s0080g00440
VIT_06s0080g00450
VIT_06s0080g00460
VIT_06s0080g00470
VIT_06s0080g00510
VIT_06s0080g00530
Standardized expressions
Developmental stages
EI PG G1 G2 H T1 T2 C1 C2 S EP JP

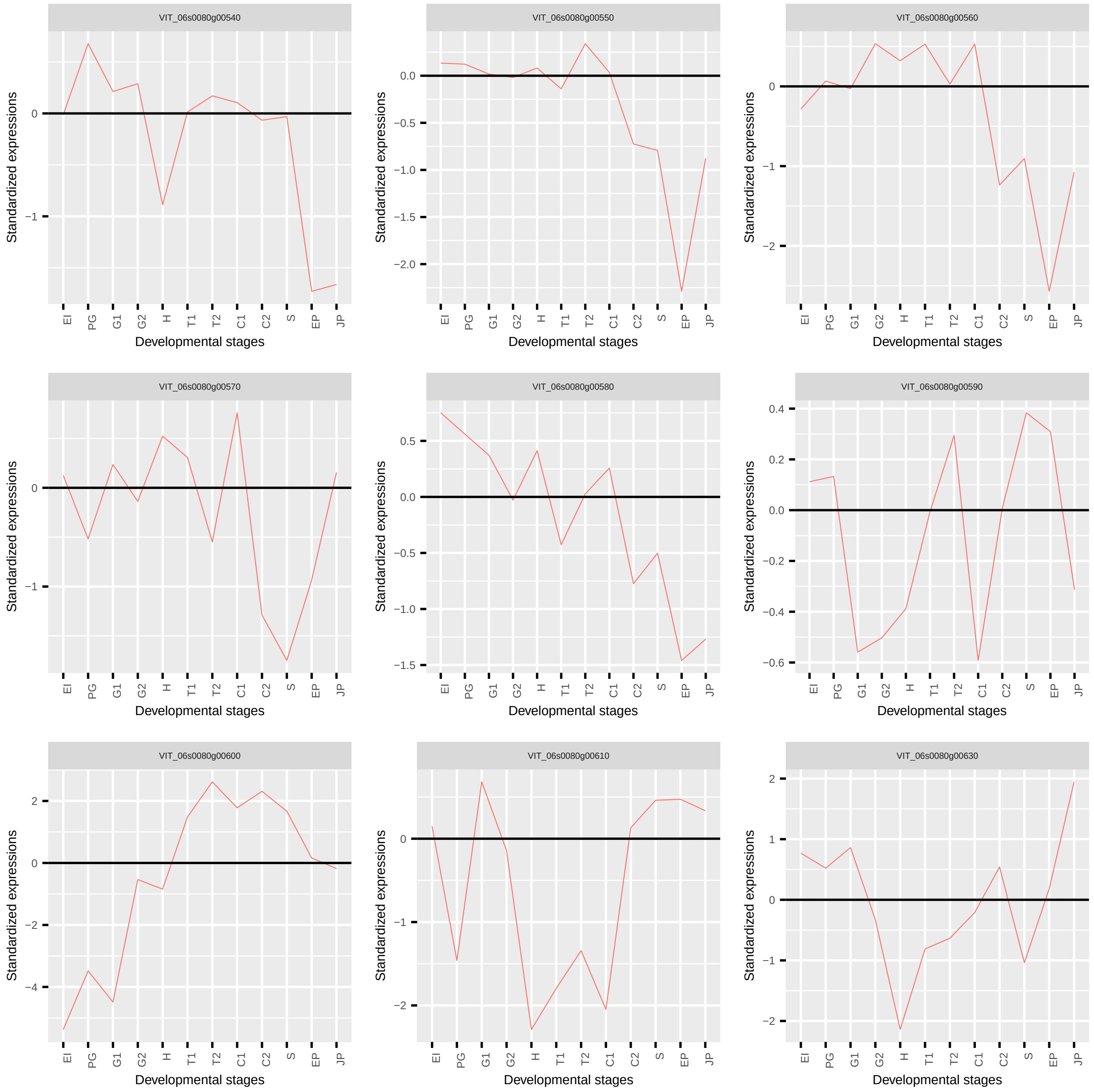
VIT_06s0080g00540
Standardized expressions
0
−1
EI PG G1 G2 H T1 T2 C1 C2 S EP JP
Developmental stages
VIT_06s0080g00550
Standardized expressions
0.0
−0.5
−1.0
−1.5
−2.0
EI PG G1 G2 H T1 T2 C1 C2 S EP JP
Developmental stages
VIT_06s0080g00560
Standardized expressions
0
−1
−2
EI PG G1 G2 H T1 T2 C1 C2 S EP JP
Developmental stages
VIT_06s0080g00570
Standardized expressions
0
−1
EI PG G1 G2 H T1 T2 C1 C2 S EP JP
Developmental stages
VIT_06s0080g00580
Standardized expressions
0.5
0.0
−0.5
−1.0
−1.5
EI PG G1 G2 H T1 T2 C1 C2 S EP JP
Developmental stages
VIT_06s0080g00590
Standardized expressions
0.4
0.2
0.0
−0.2
−0.4
−0.6
EI PG G1 G2 H T1 T2 C1 C2 S EP JP
Developmental stages
VIT_06s0080g00600
Standardized expressions
2
0
−2
−4
EI PG G1 G2 H T1 T2 C1 C2 S EP JP
Developmental stages
VIT_06s0080g00610
Standardized expressions
0
−1
−2
EI PG G1 G2 H T1 T2 C1 C2 S EP JP
Developmental stages
VIT_06s0080g00630
Standardized expressions
2
1
0
−1
−2
EI PG G1 G2 H T1 T2 C1 C2 S EP JP
Developmental stages

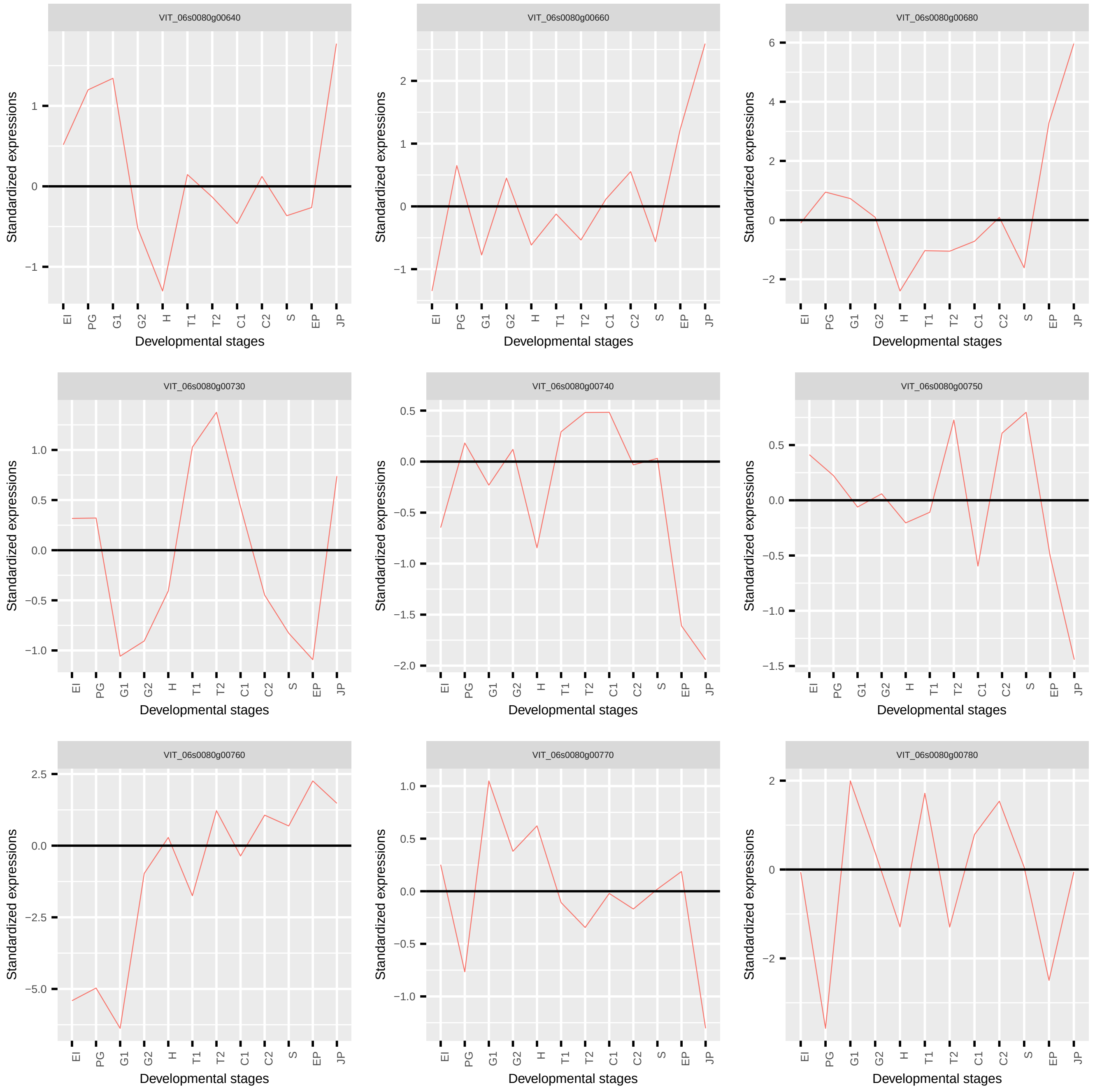
VIT_06s0080g00640
VIT_06s0080g00660
VIT_06s0080g00680
VIT_06s0080g00730
VIT_06s0080g00740
VIT_06s0080g00750
VIT_06s0080g00760
VIT_06s0080g00770
VIT_06s0080g00780
Standardized expressions
Developmental stages
EI PG G1 G2 H T1 T2 C1 C2 S EP JP

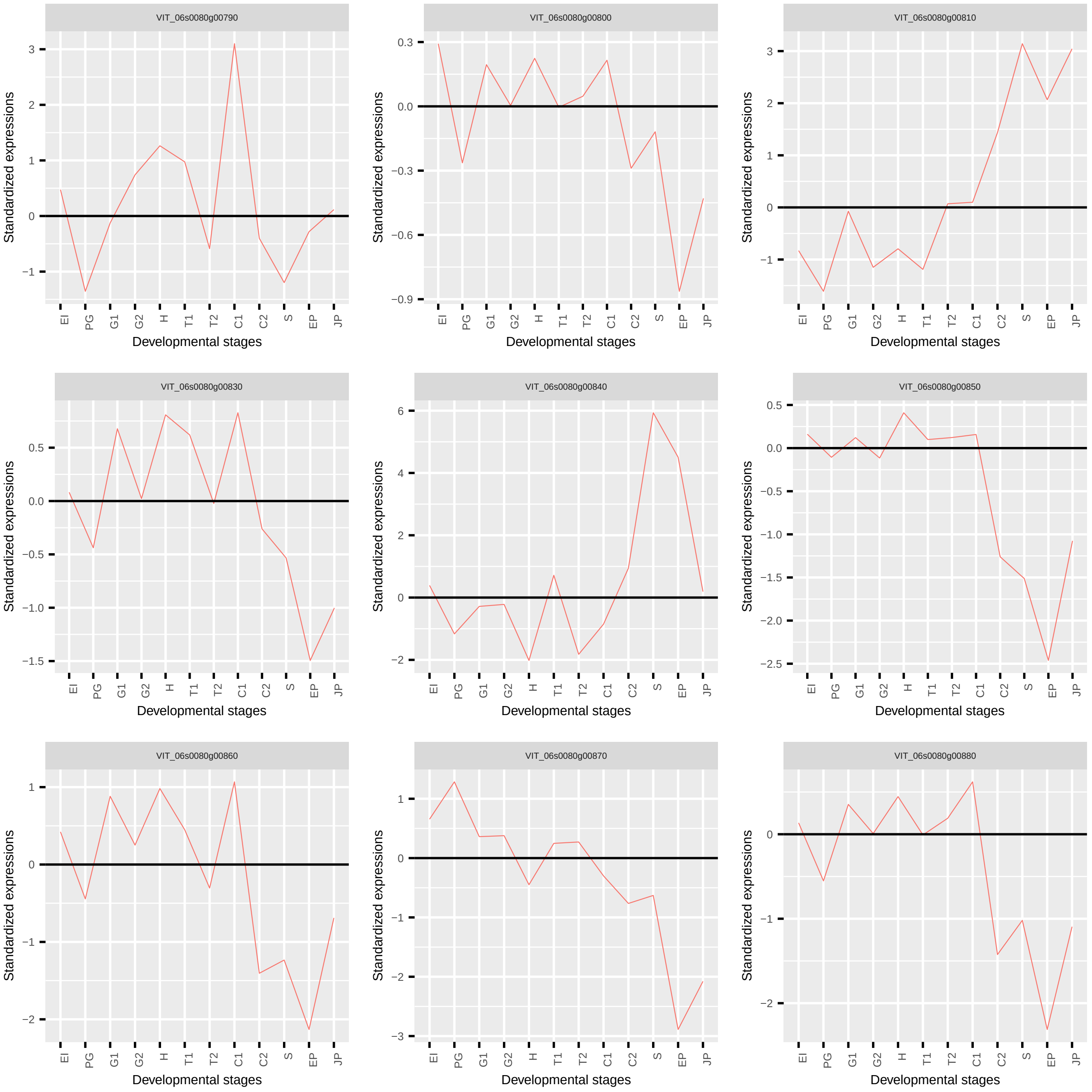
VIT_06s0080g00790
VIT_06s0080g00800
VIT_06s0080g00810
VIT_06s0080g00830
VIT_06s0080g00840
VIT_06s0080g00850
VIT_06s0080g00860
VIT_06s0080g00870
VIT_06s0080g00880
Standardized expressions
Developmental stages
EI PG G1 G2 H T1 T2 C1 C2 S EP JP

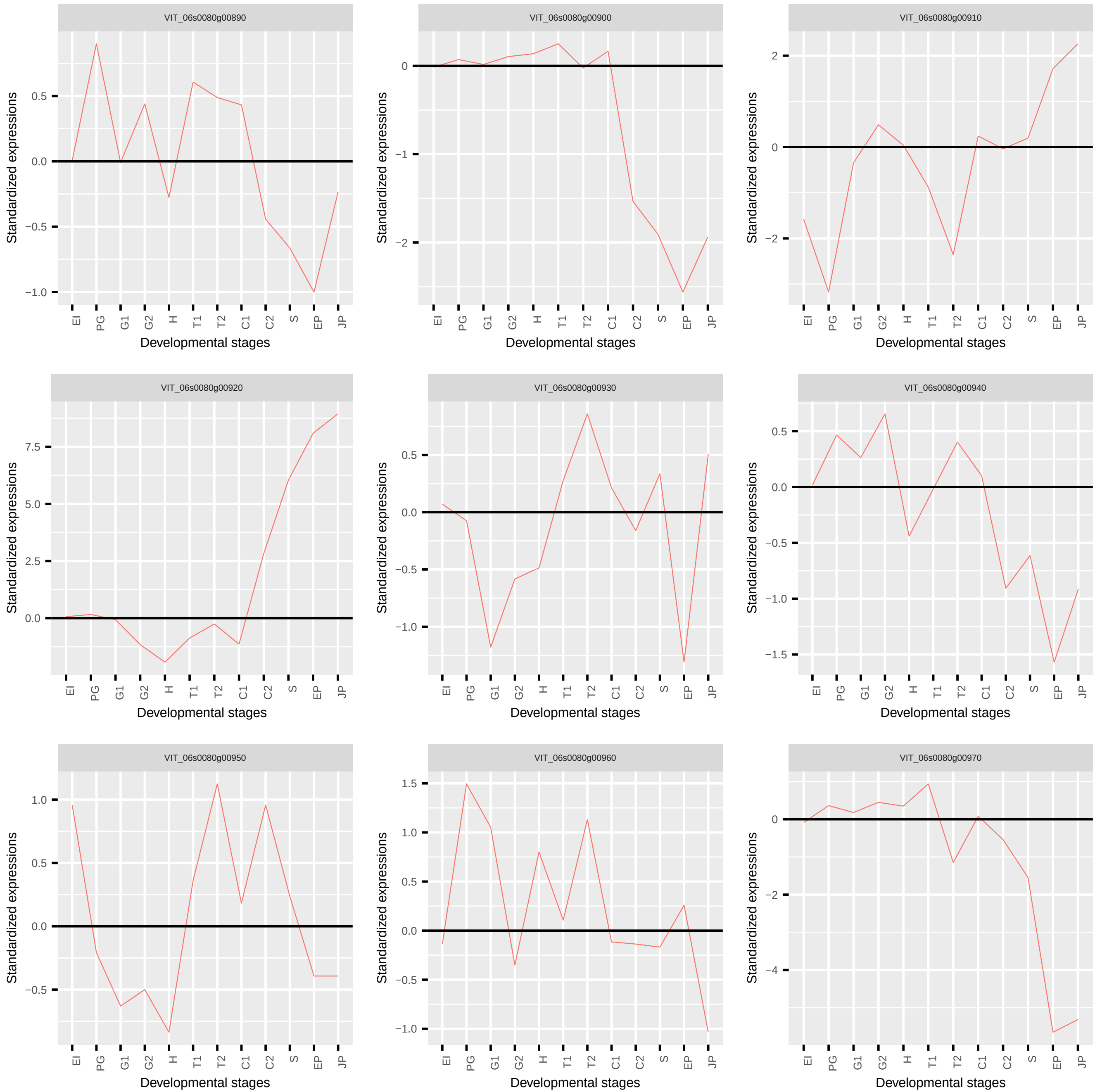
VIT_06s0080g00890
VIT_06s0080g00900
VIT_06s0080g00910
VIT_06s0080g00920
VIT_06s0080g00930
VIT_06s0080g00940
VIT_06s0080g00950
VIT_06s0080g00960
VIT_06s0080g00970
Standardized expressions
Developmental stages
EI PG G1 G2 H T1 T2 C1 C2 S EP JP

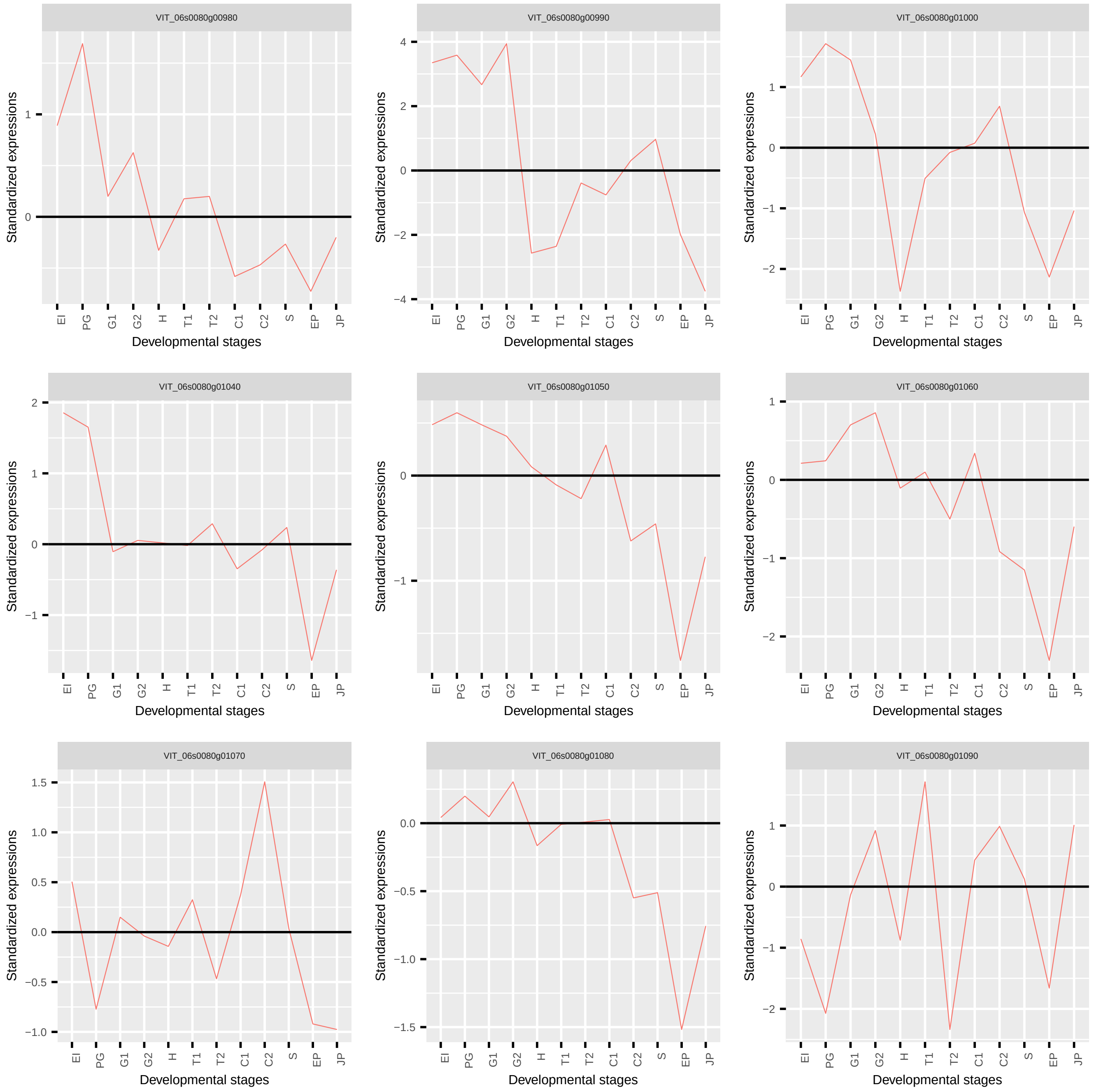
VIT_06s0080g00980
VIT_06s0080g00990
VIT_06s0080g01000
VIT_06s0080g01040
VIT_06s0080g01050
VIT_06s0080g01060
VIT_06s0080g01070
VIT_06s0080g01080
VIT_06s0080g01090
Standardized expressions
Developmental stages
EI PG G1 G2 H T1 T2 C1 C2 S EP JP

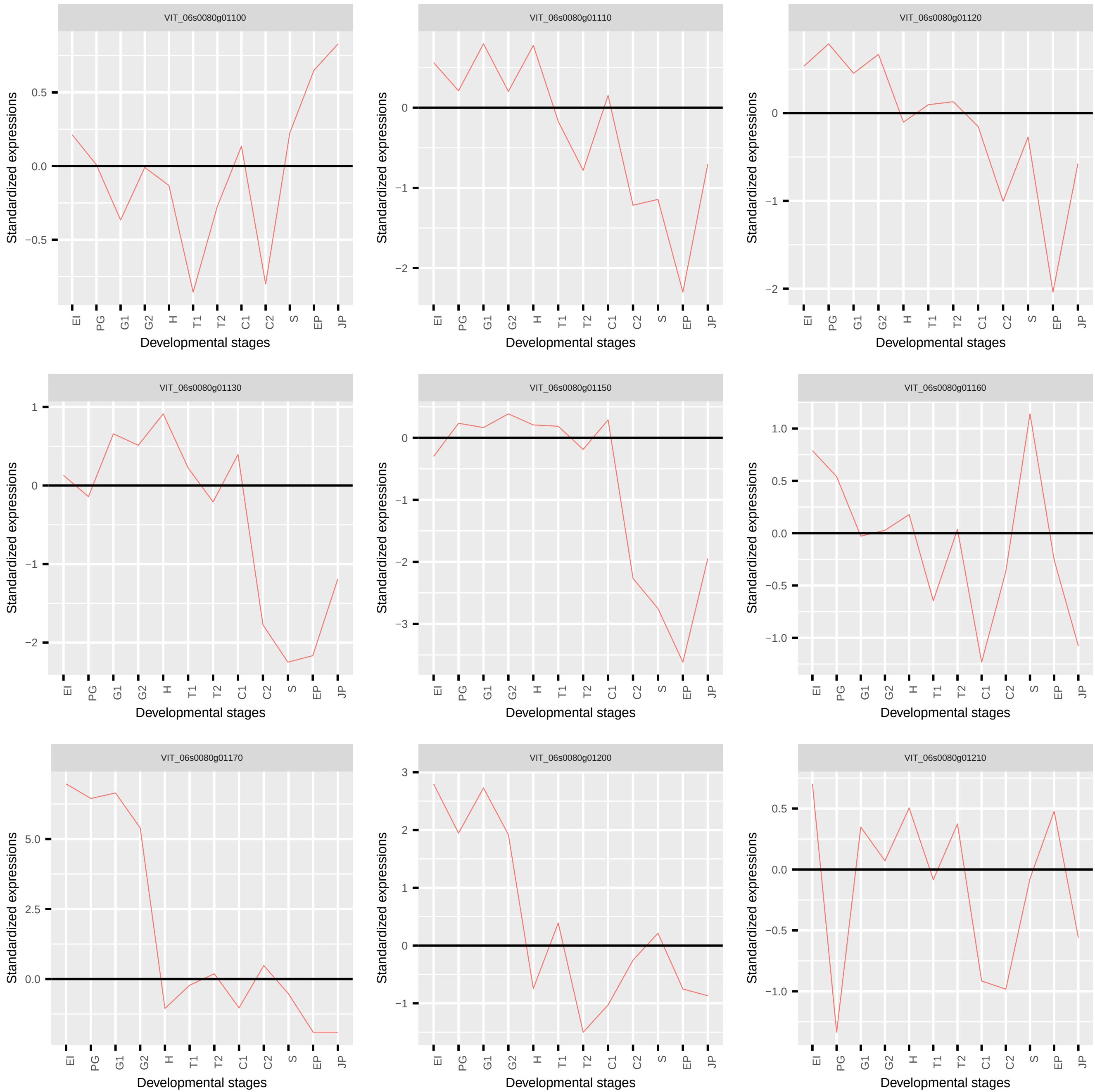

VIT_06s0080g01100
VIT_06s0080g01110
VIT_06s0080g01120
VIT_06s0080g01130
VIT_06s0080g01150
VIT_06s0080g01160
VIT_06s0080g01170
VIT_06s0080g01200
VIT_06s0080g01210
Standardized expressions
Developmental stages
EI
PG
G1
G2
H
T1
T2
C1
C2
S
EP
JP

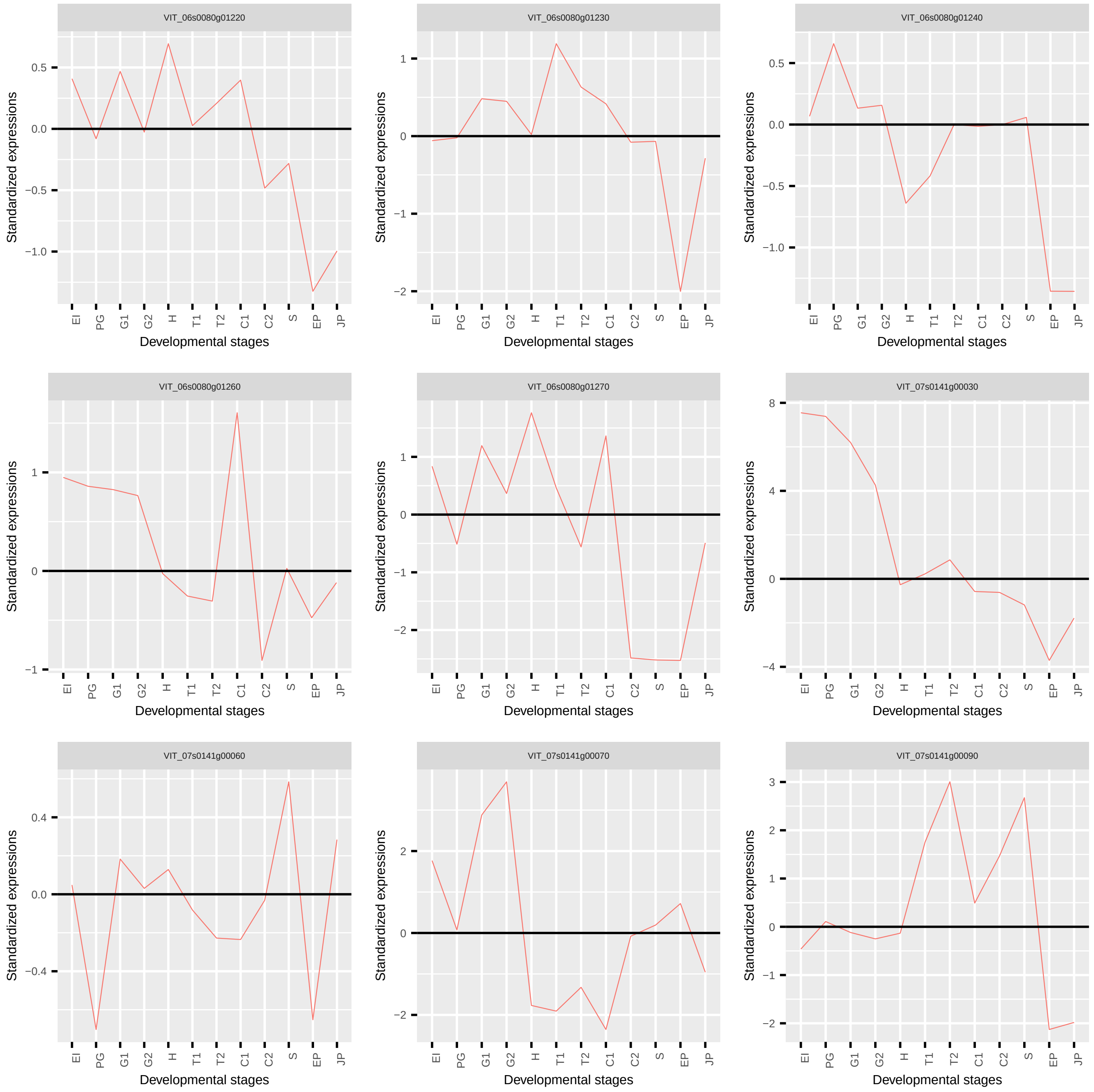
VIT_06s0080g01220
VIT_06s0080g01230
VIT_06s0080g01240
VIT_06s0080g01260
VIT_06s0080g01270
VIT_07s0141g00030
VIT_07s0141g00060
VIT_07s0141g00070
VIT_07s0141g00090
Standardized expressions
Developmental stages
EI PG G1 G2 H T1 T2 C1 C2 S EP JP

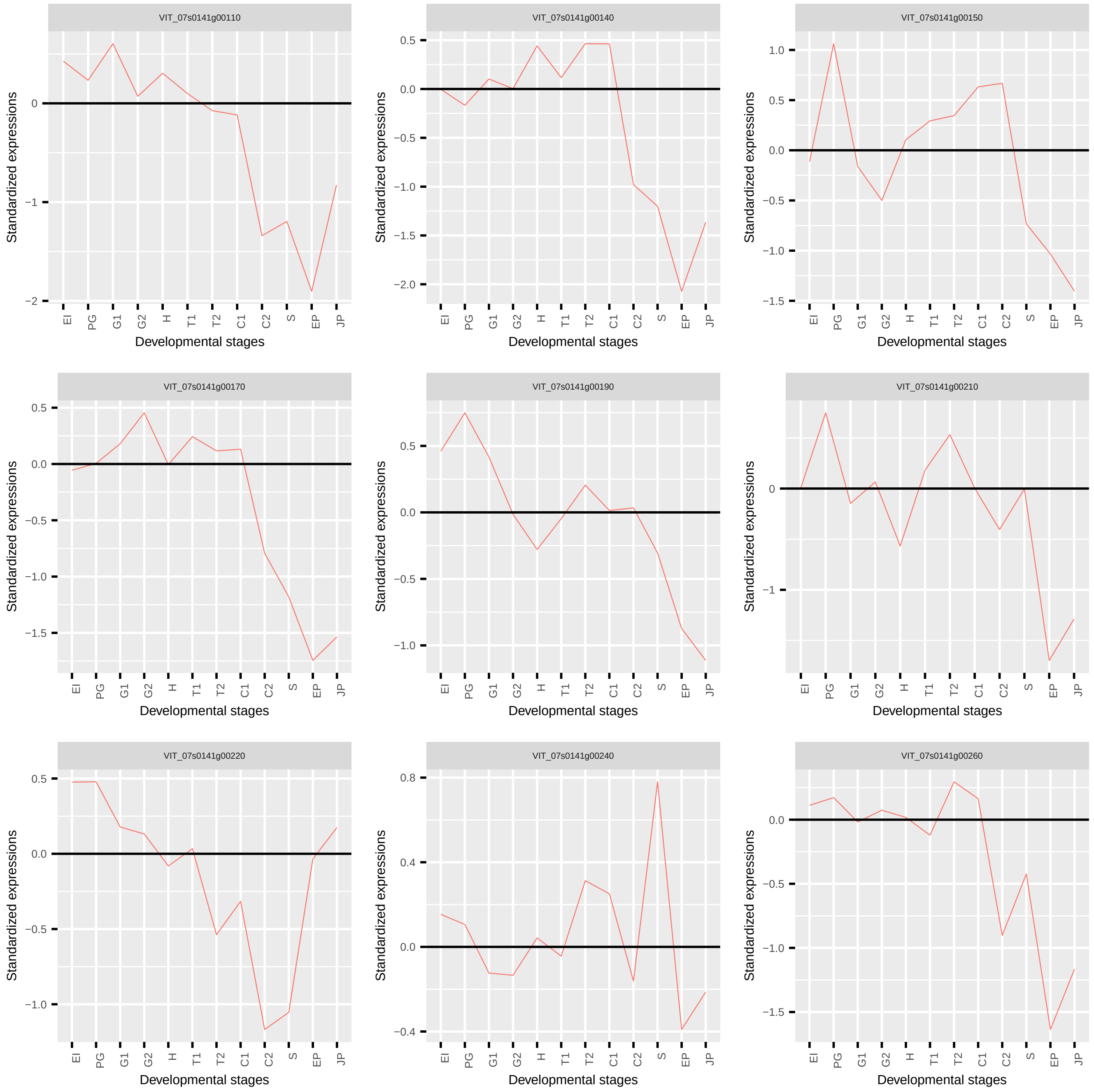
VIT_07s0141g00110
VIT_07s0141g00140
VIT_07s0141g00150
VIT_07s0141g00170
VIT_07s0141g00190
VIT_07s0141g00210
VIT_07s0141g00220
VIT_07s0141g00240
VIT_07s0141g00260
Standardized expressions
Developmental stages
EI PG G1 G2 H T1 T2 C1 C2 S EP JP

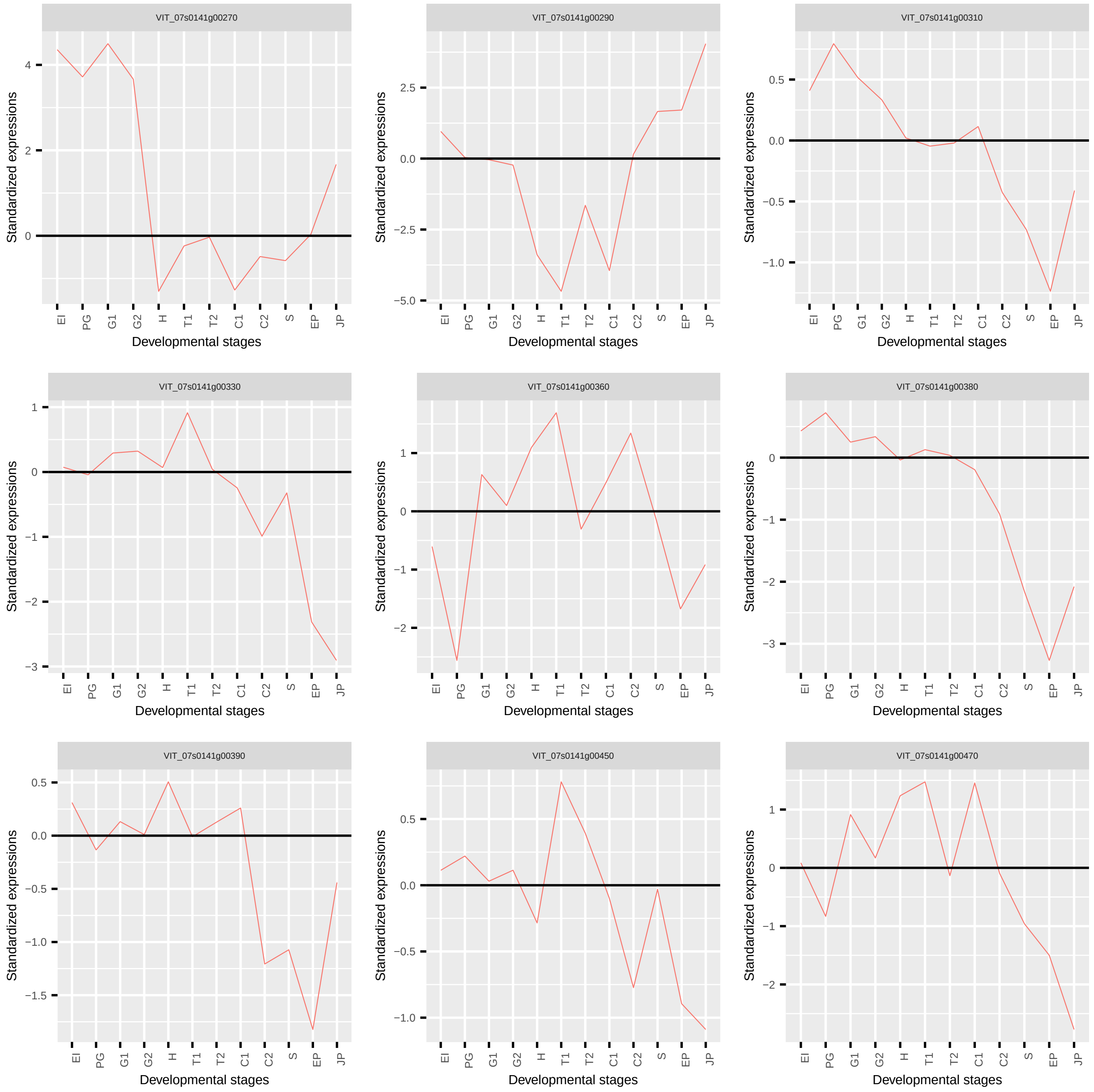
VIT_07s0141g00270
Standardized expressions
4
2
0
EI PG G1 G2 H T1 T2 C1 C2 S EP JP
Developmental stages
VIT_07s0141g00290
Standardized expressions
2.5
0.0
−2.5
−5.0
EI PG G1 G2 H T1 T2 C1 C2 S EP JP
Developmental stages
VIT_07s0141g00310
Standardized expressions
0.5
0.0
−0.5
−1.0
EI PG G1 G2 H T1 T2 C1 C2 S EP JP
Developmental stages
VIT_07s0141g00330
Standardized expressions
1
0
−1
−2
−3
EI PG G1 G2 H T1 T2 C1 C2 S EP JP
Developmental stages
VIT_07s0141g00360
Standardized expressions
1
0
−1
−2
EI PG G1 G2 H T1 T2 C1 C2 S EP JP
Developmental stages
VIT_07s0141g00380
Standardized expressions
0
−1
−2
−3
EI PG G1 G2 H T1 T2 C1 C2 S EP JP
Developmental stages
VIT_07s0141g00390
Standardized expressions
0.5
0.0
−0.5
−1.0
−1.5
EI PG G1 G2 H T1 T2 C1 C2 S EP JP
Developmental stages
VIT_07s0141g00450
Standardized expressions
0.5
0.0
−0.5
−1.0
EI PG G1 G2 H T1 T2 C1 C2 S EP JP
Developmental stages
VIT_07s0141g00470
Standardized expressions
1
0
−1
−2
EI PG G1 G2 H T1 T2 C1 C2 S EP JP
Developmental stages

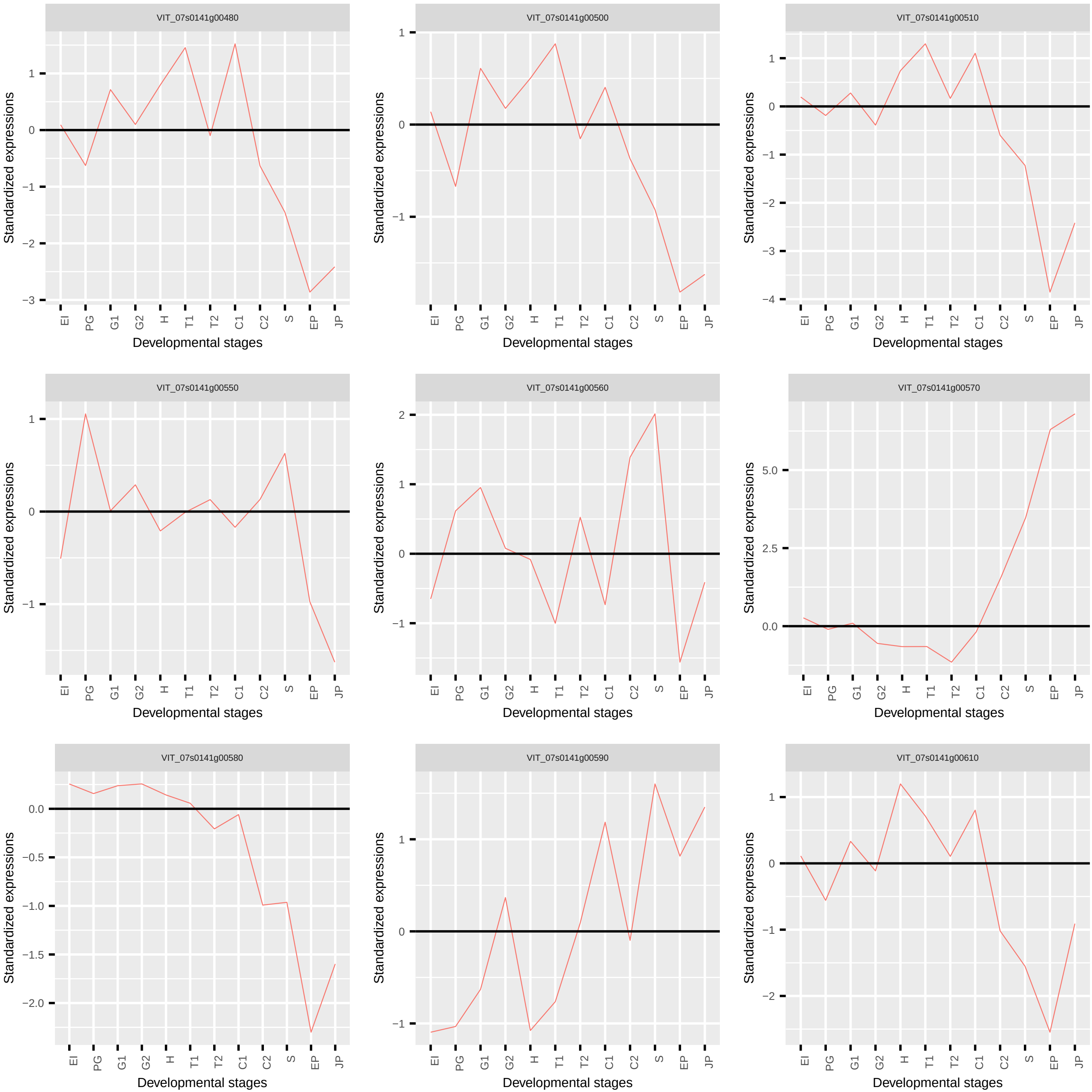
VIT_07s0141g00480
VIT_07s0141g00500
VIT_07s0141g00510
VIT_07s0141g00550
VIT_07s0141g00560
VIT_07s0141g00570
VIT_07s0141g00580
VIT_07s0141g00590
VIT_07s0141g00610
Standardized expressions
Developmental stages
EI PG G1 G2 H T1 T2 C1 C2 S EP JP

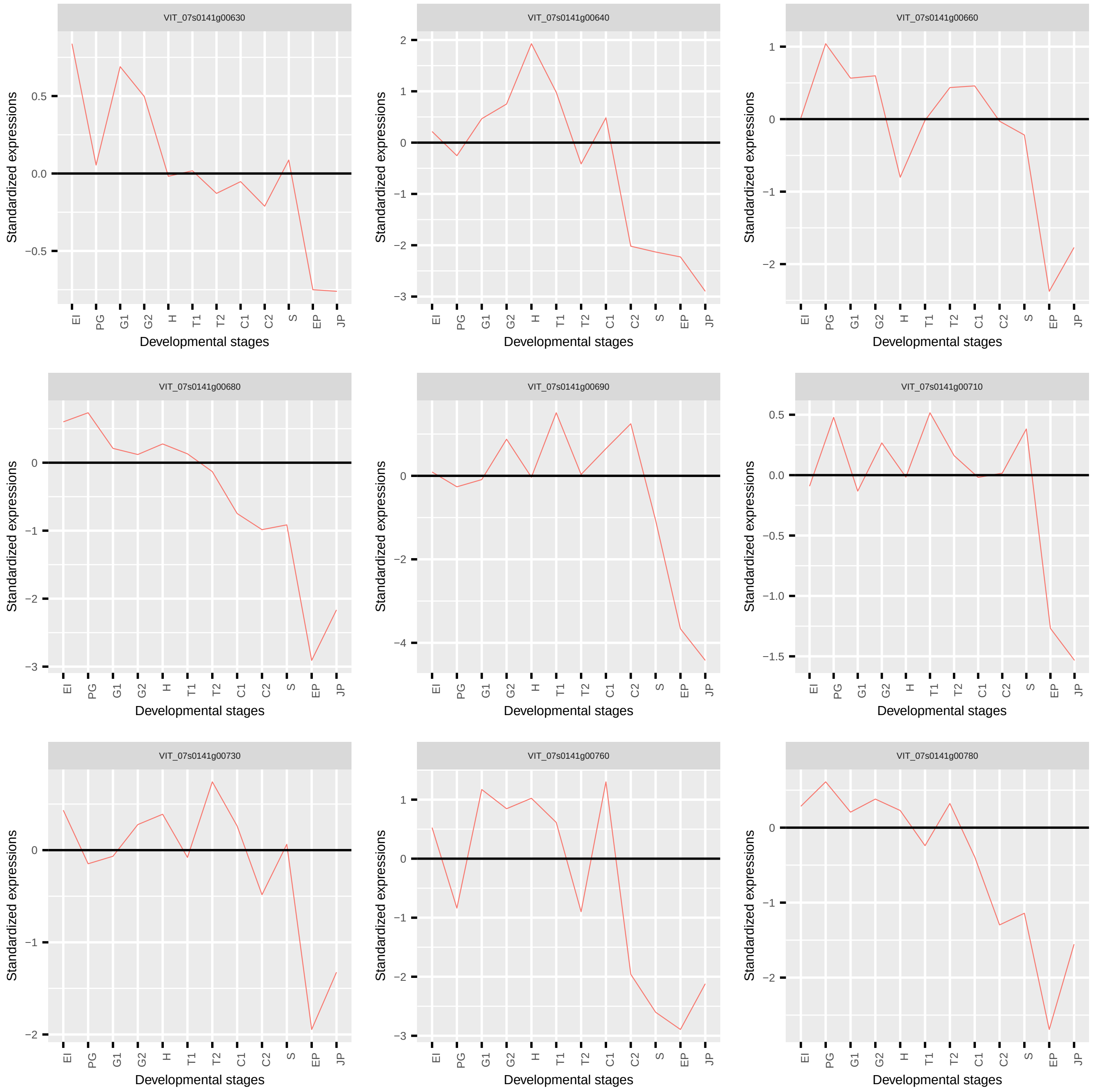
VIT_07s0141g00630
VIT_07s0141g00640
VIT_07s0141g00660
VIT_07s0141g00680
VIT_07s0141g00690
VIT_07s0141g00710
VIT_07s0141g00730
VIT_07s0141g00760
VIT_07s0141g00780
Standardized expressions
Developmental stages
EI PG G1 G2 H T1 T2 C1 C2 S EP JP

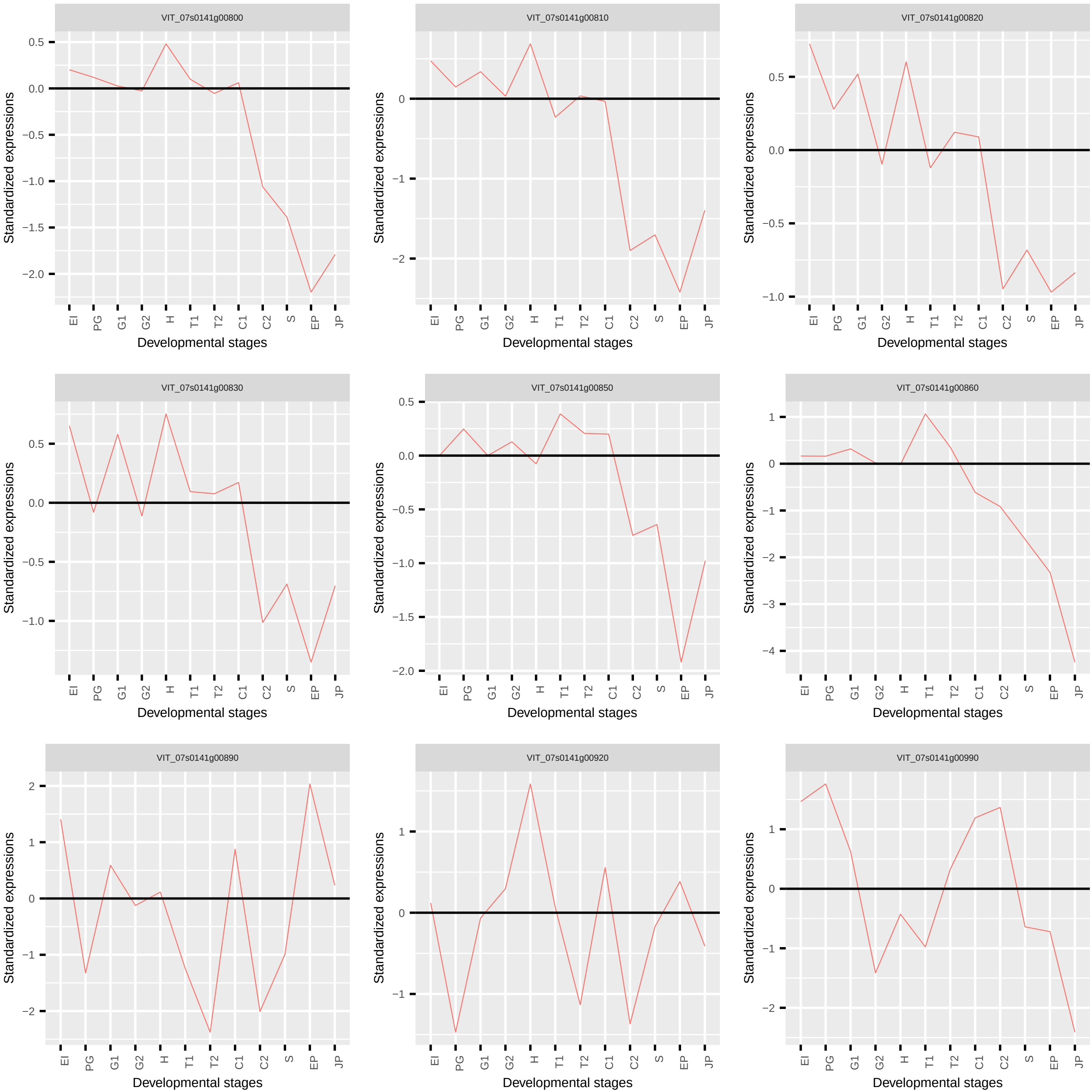

VIT_07s0141g00800
VIT_07s0141g00810
VIT_07s0141g00820
VIT_07s0141g00830
VIT_07s0141g00850
VIT_07s0141g00860
VIT_07s0141g00890
VIT_07s0141g00920
VIT_07s0141g00990
Standardized expressions
Developmental stages
EI PG G1 G2 H T1 T2 C1 C2 S EP JP

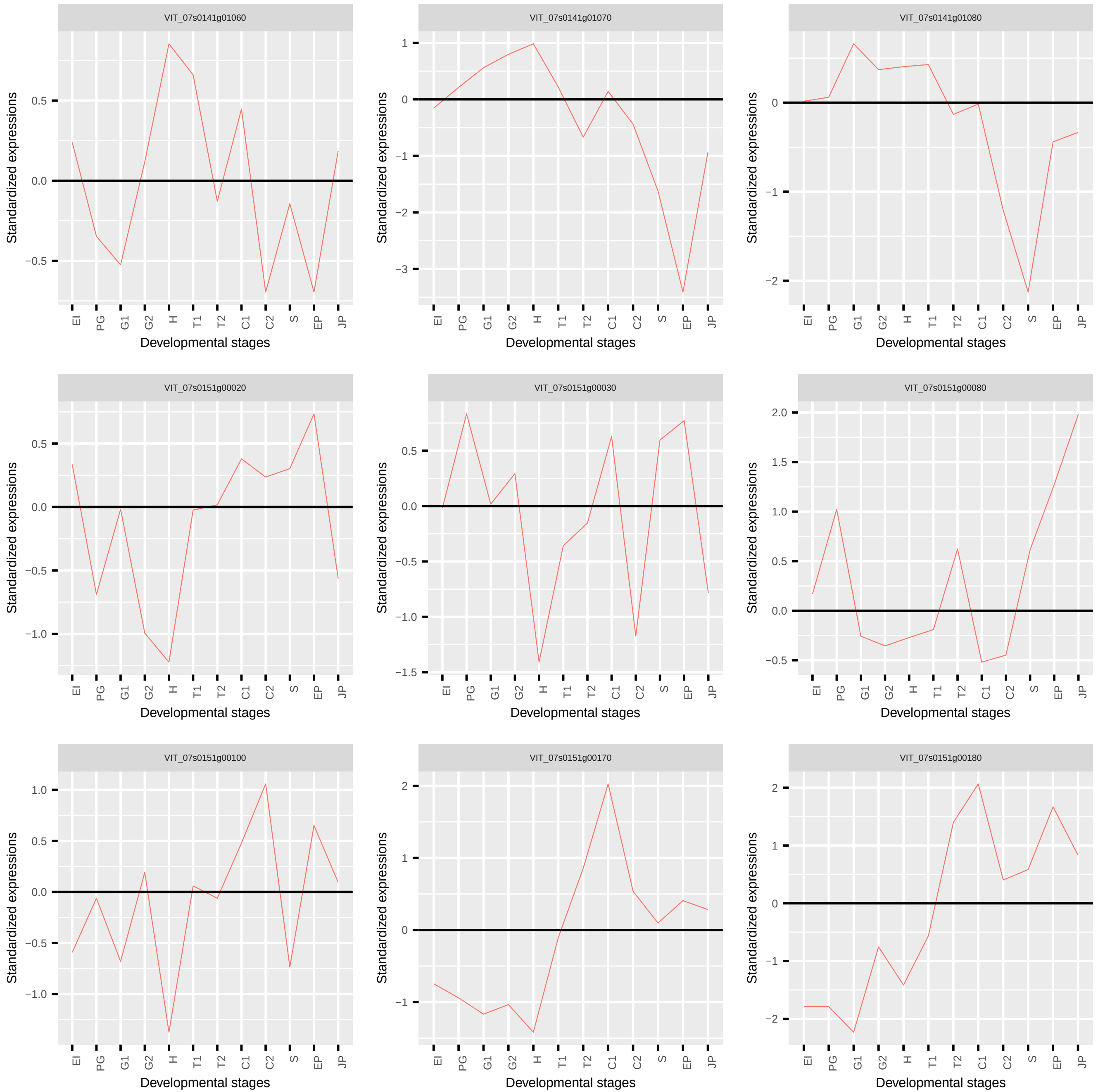
VIT_07s0141g01060
VIT_07s0141g01070
VIT_07s0141g01080
VIT_07s0151g00020
VIT_07s0151g00030
VIT_07s0151g00080
VIT_07s0151g00100
VIT_07s0151g00170
VIT_07s0151g00180
Standardized expressions
Developmental stages
EI PG G1 G2 H T1 T2 C1 C2 S EP JP

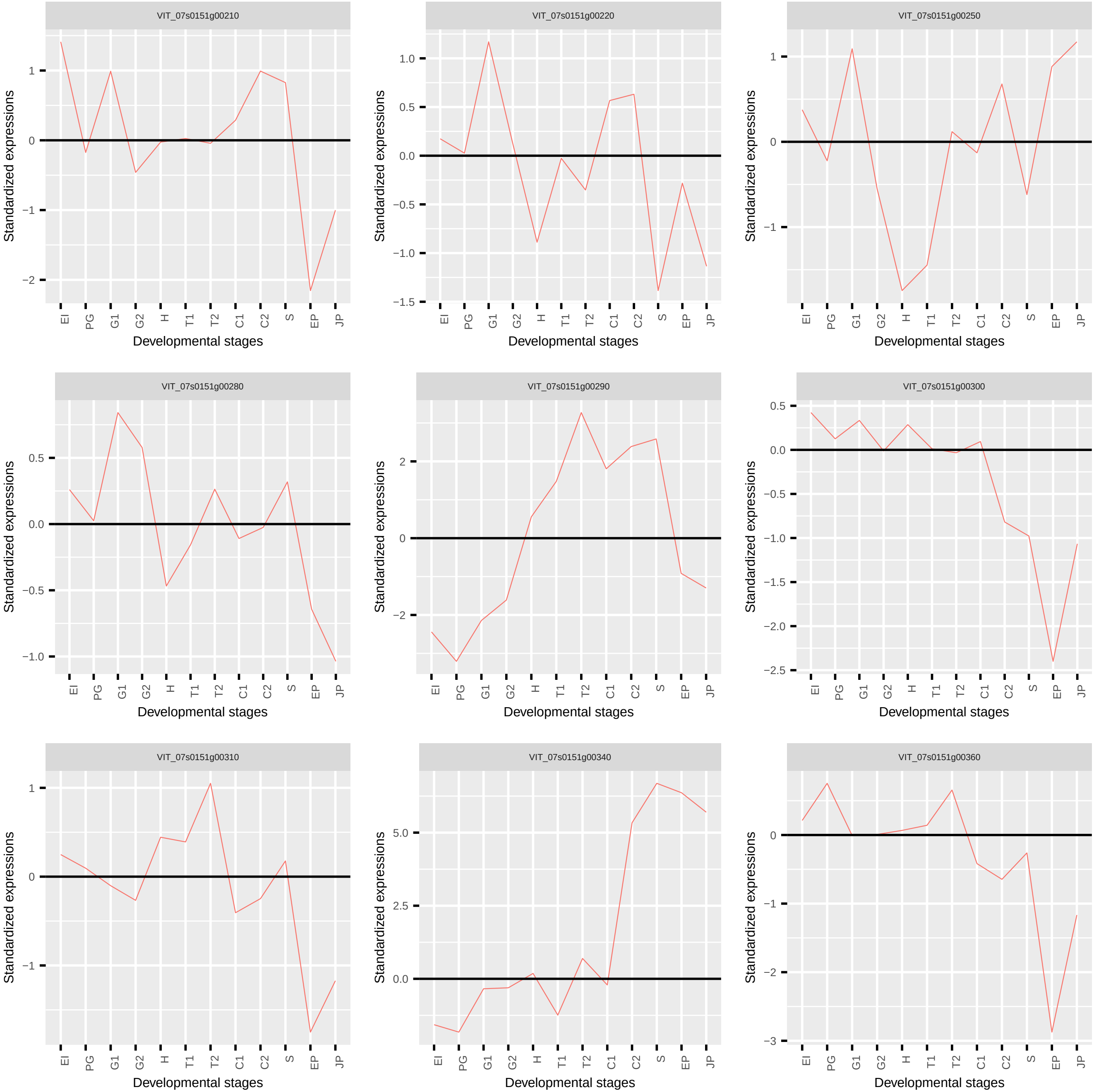
VIT_07s0151g00210
VIT_07s0151g00220
VIT_07s0151g00250
VIT_07s0151g00280
VIT_07s0151g00290
VIT_07s0151g00300
VIT_07s0151g00310
VIT_07s0151g00340
VIT_07s0151g00360
Standardized expressions
Developmental stages
EI PG G1 G2 H T1 T2 C1 C2 S EP JP

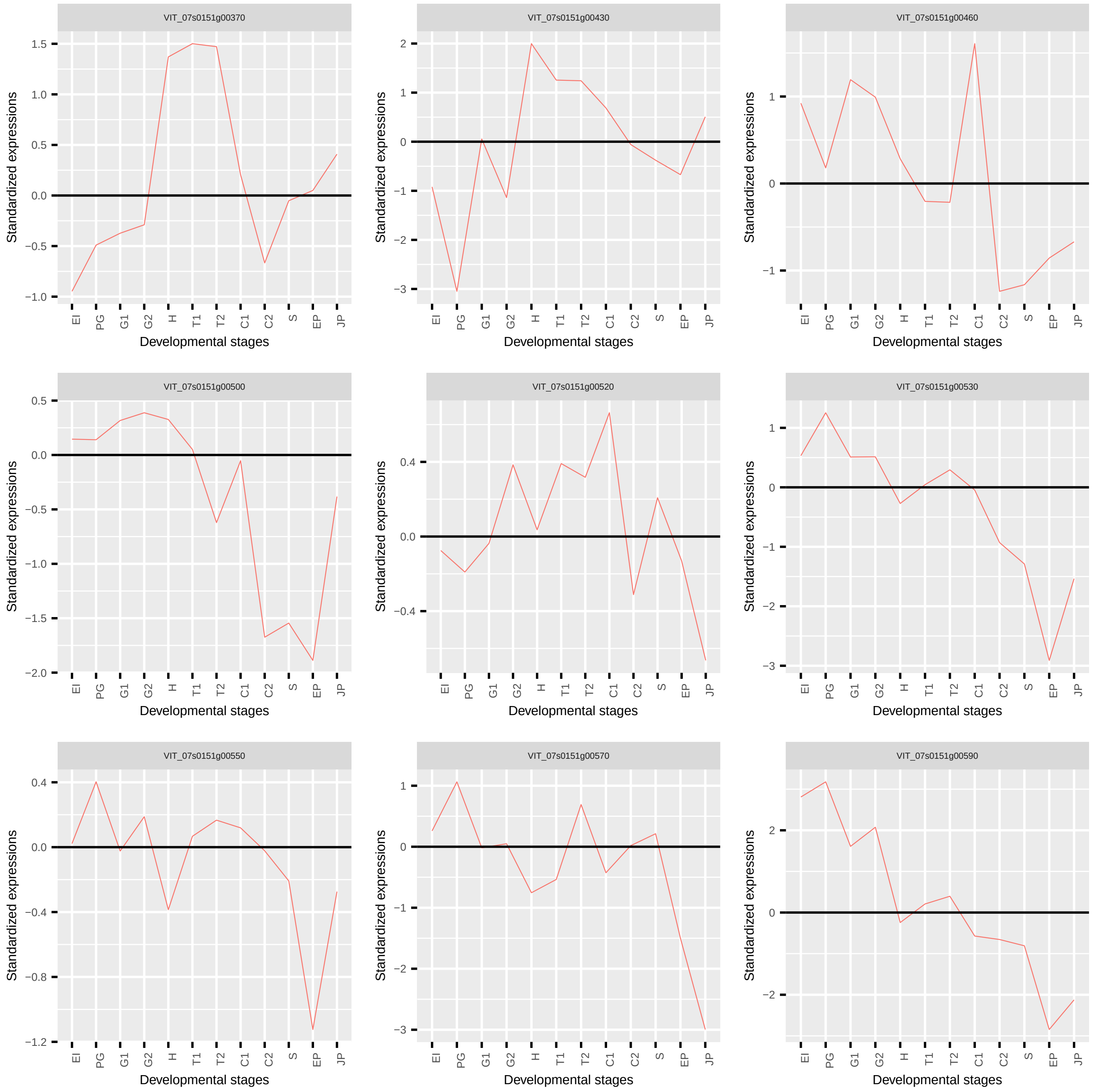

VIT_07s0151g00370
VIT_07s0151g00430
VIT_07s0151g00460
VIT_07s0151g00500
VIT_07s0151g00520
VIT_07s0151g00530
VIT_07s0151g00550
VIT_07s0151g00570
VIT_07s0151g00590
Standardized expressions
Developmental stages
EI PG G1 G2 H T1 T2 C1 C2 S EP JP

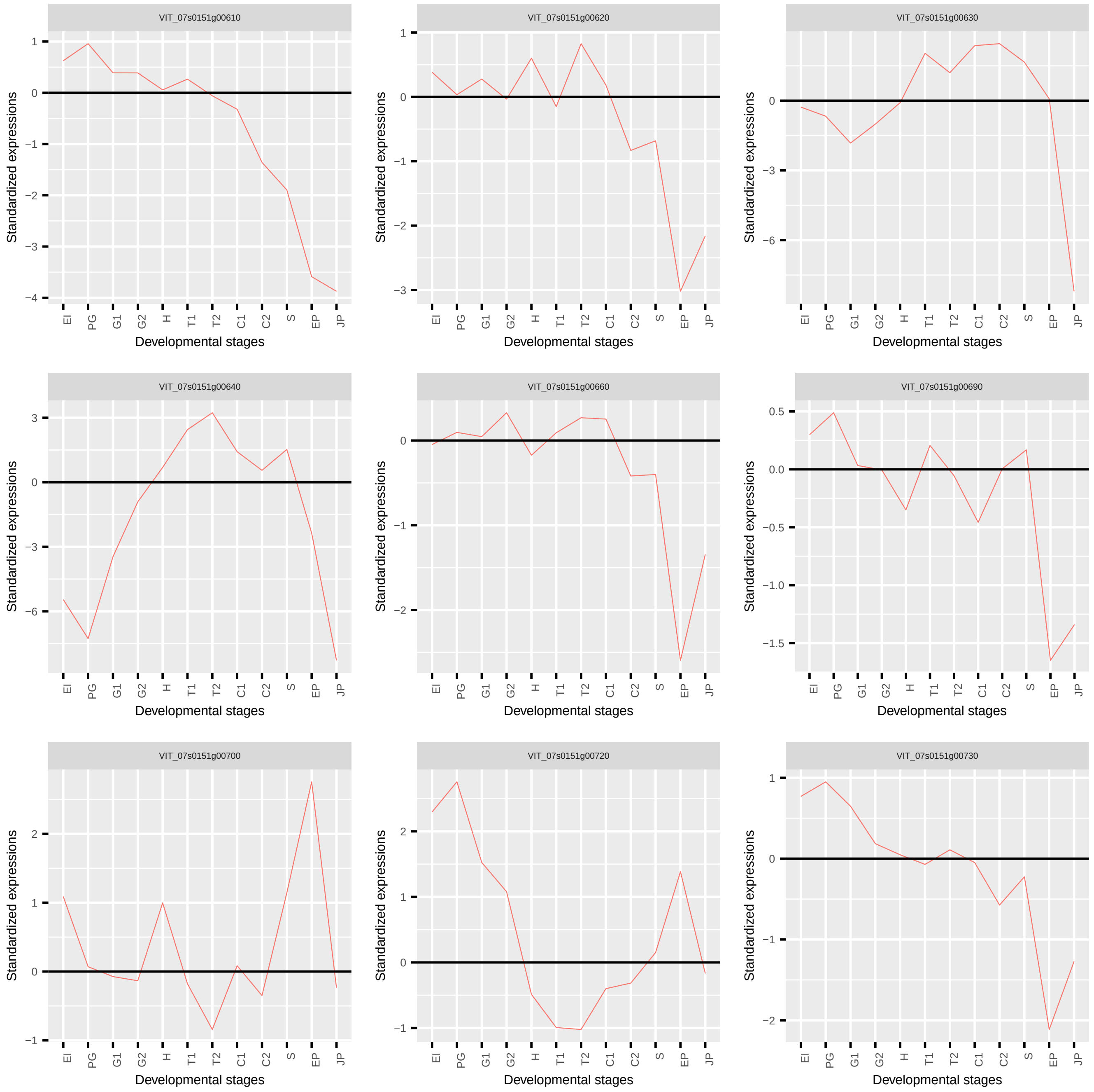
VIT_07s0151g00610
VIT_07s0151g00620
VIT_07s0151g00630
VIT_07s0151g00640
VIT_07s0151g00660
VIT_07s0151g00690
VIT_07s0151g00700
VIT_07s0151g00720
VIT_07s0151g00730
Standardized expressions
Developmental stages
EI
PG
G1
G2
H
T1
T2
C1
C2
S
EP
JP

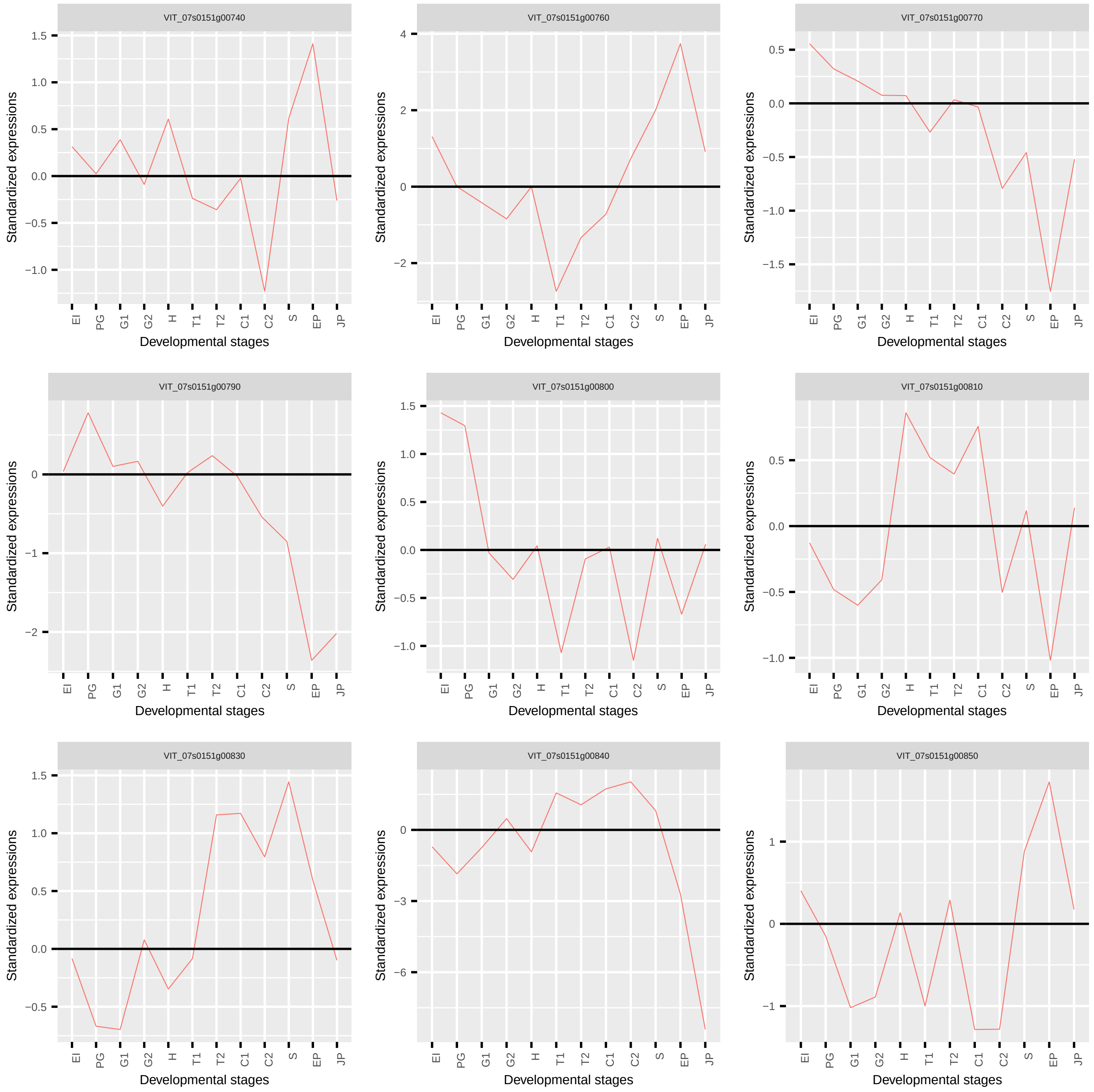
VIT_07s0151g00740
Standardized expressions
1.5
1.0
0.5
0.0
−0.5
−1.0
EI PG G1 G2 H T1 T2 C1 C2 S EP JP
Developmental stages
VIT_07s0151g00760
Standardized expressions
4
2
0
−2
EI PG G1 G2 H T1 T2 C1 C2 S EP JP
Developmental stages
VIT_07s0151g00770
Standardized expressions
0.5
0.0
−0.5
−1.0
−1.5
EI PG G1 G2 H T1 T2 C1 C2 S EP JP
Developmental stages
VIT_07s0151g00790
Standardized expressions
0
−1
−2
EI PG G1 G2 H T1 T2 C1 C2 S EP JP
Developmental stages
VIT_07s0151g00800
Standardized expressions
1.5
1.0
0.5
0.0
−0.5
−1.0
EI PG G1 G2 H T1 T2 C1 C2 S EP JP
Developmental stages
VIT_07s0151g00810
Standardized expressions
0.5
0.0
−0.5
−1.0
EI PG G1 G2 H T1 T2 C1 C2 S EP JP
Developmental stages
VIT_07s0151g00830
Standardized expressions
1.5
1.0
0.5
0.0
−0.5
EI PG G1 G2 H T1 T2 C1 C2 S EP JP
Developmental stages
VIT_07s0151g00840
Standardized expressions
0
−3
−6
EI PG G1 G2 H T1 T2 C1 C2 S EP JP
Developmental stages
VIT_07s0151g00850
Standardized expressions
1
0
−1
EI PG G1 G2 H T1 T2 C1 C2 S EP JP
Developmental stages

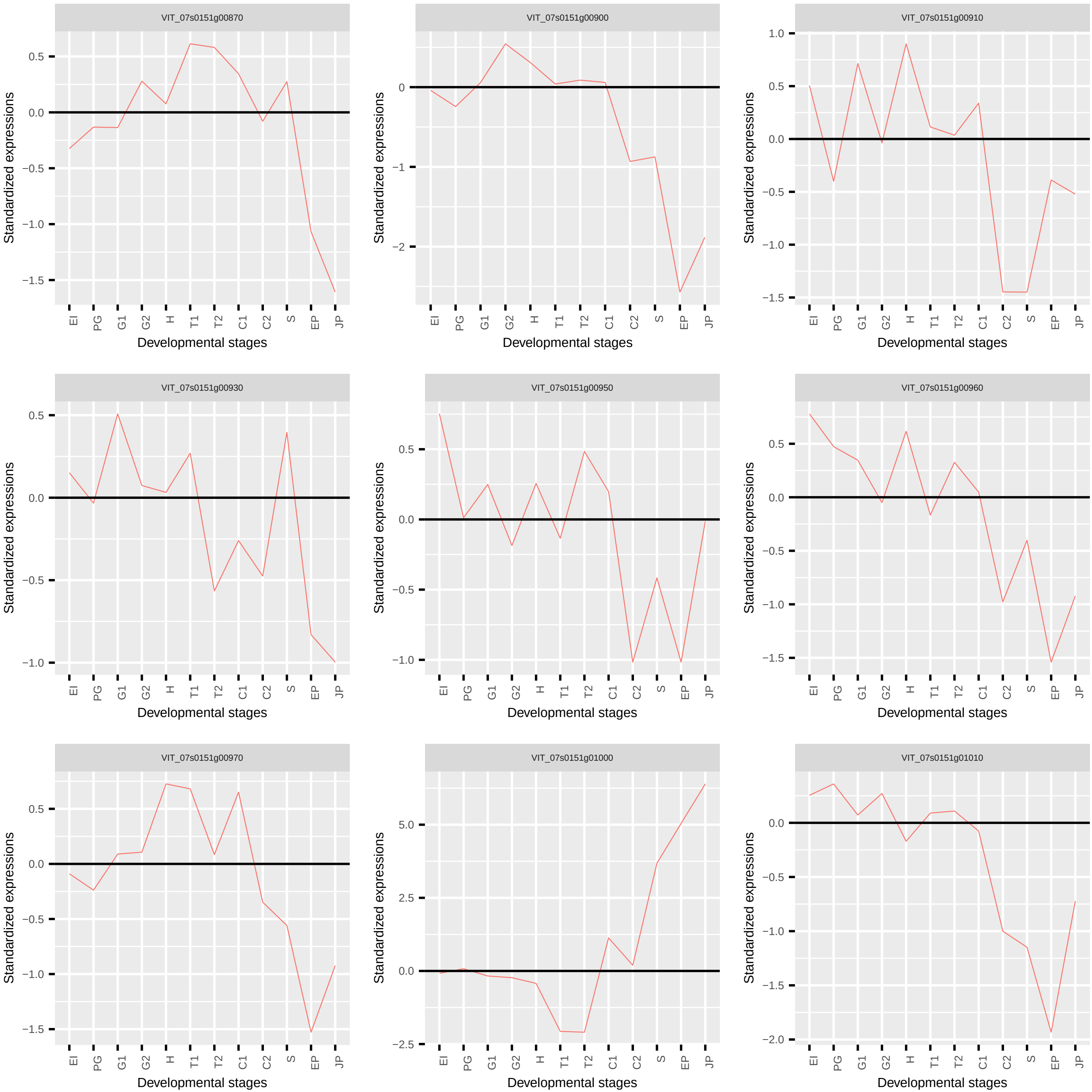
VIT_07s0151g00870
0.5
0.0
−0.5
−1.0
−1.5
Standardized expressions
EI PG G1 G2 H T1 T2 C1 C2 S EP JP
Developmental stages
VIT_07s0151g00900
0
−1
−2
Standardized expressions
EI PG G1 G2 H T1 T2 C1 C2 S EP JP
Developmental stages
VIT_07s0151g00910
1.0
0.5
0.0
−0.5
−1.0
−1.5
Standardized expressions
EI PG G1 G2 H T1 T2 C1 C2 S EP JP
Developmental stages
VIT_07s0151g00930
0.5
0.0
−0.5
−1.0
Standardized expressions
EI PG G1 G2 H T1 T2 C1 C2 S EP JP
Developmental stages
VIT_07s0151g00950
0.5
0.0
−0.5
−1.0
Standardized expressions
EI PG G1 G2 H T1 T2 C1 C2 S EP JP
Developmental stages
VIT_07s0151g00960
0.5
0.0
−0.5
−1.0
−1.5
Standardized expressions
EI PG G1 G2 H T1 T2 C1 C2 S EP JP
Developmental stages
VIT_07s0151g00970
0.5
0.0
−0.5
−1.0
−1.5
Standardized expressions
EI PG G1 G2 H T1 T2 C1 C2 S EP JP
Developmental stages
VIT_07s0151g01000
5.0
2.5
0.0
−2.5
Standardized expressions
EI PG G1 G2 H T1 T2 C1 C2 S EP JP
Developmental stages
VIT_07s0151g01010
0.0
−0.5
−1.0
−1.5
−2.0
Standardized expressions
EI PG G1 G2 H T1 T2 C1 C2 S EP JP
Developmental stages

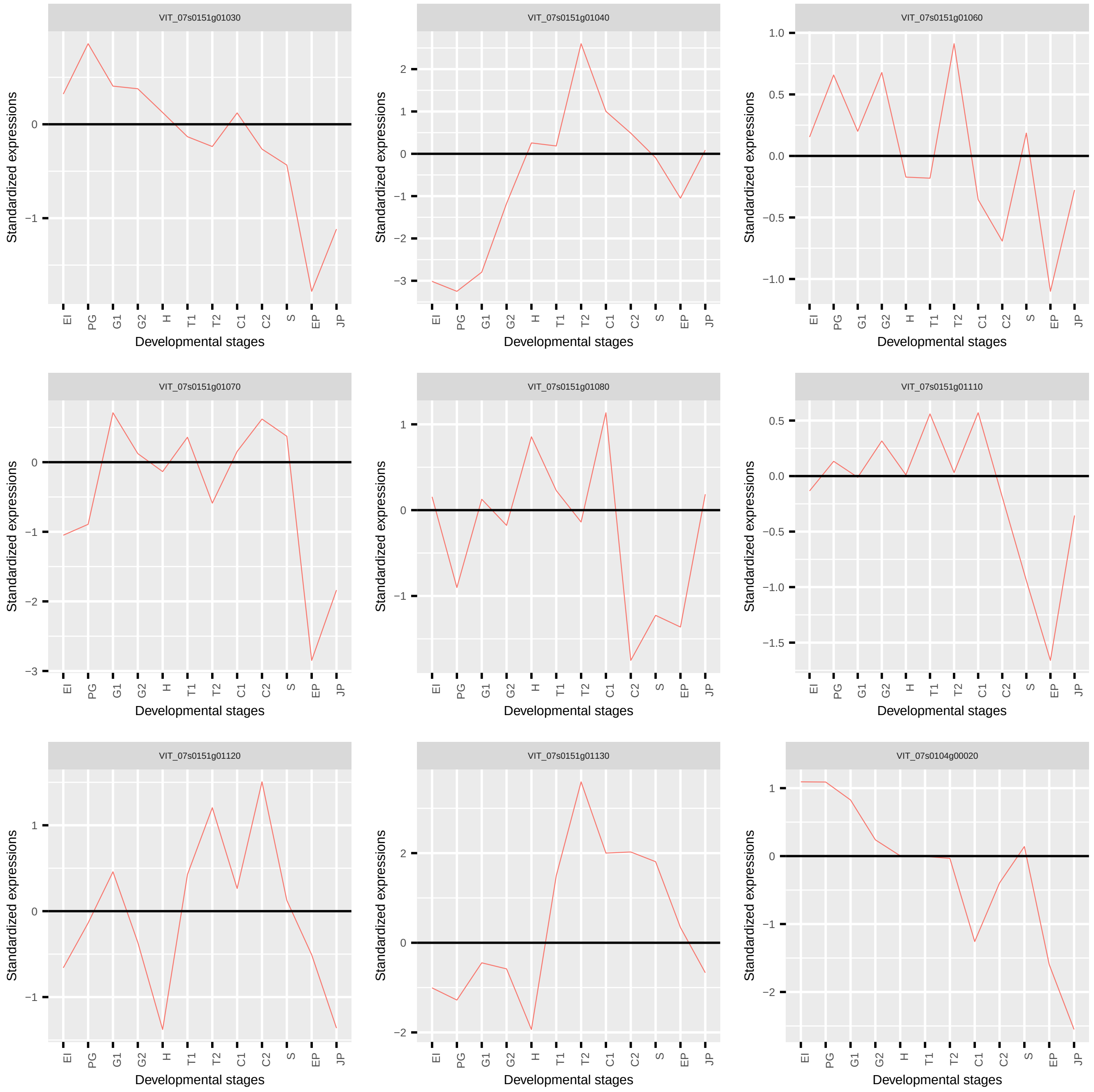
VIT_07s0151g01030
VIT_07s0151g01040
VIT_07s0151g01060
VIT_07s0151g01070
VIT_07s0151g01080
VIT_07s0151g01110
VIT_07s0151g01120
VIT_07s0151g01130
VIT_07s0104g00020
Standardized expressions
Developmental stages
EI
PG
G1
G2
H
T1
T2
C1
C2
S
EP
JP

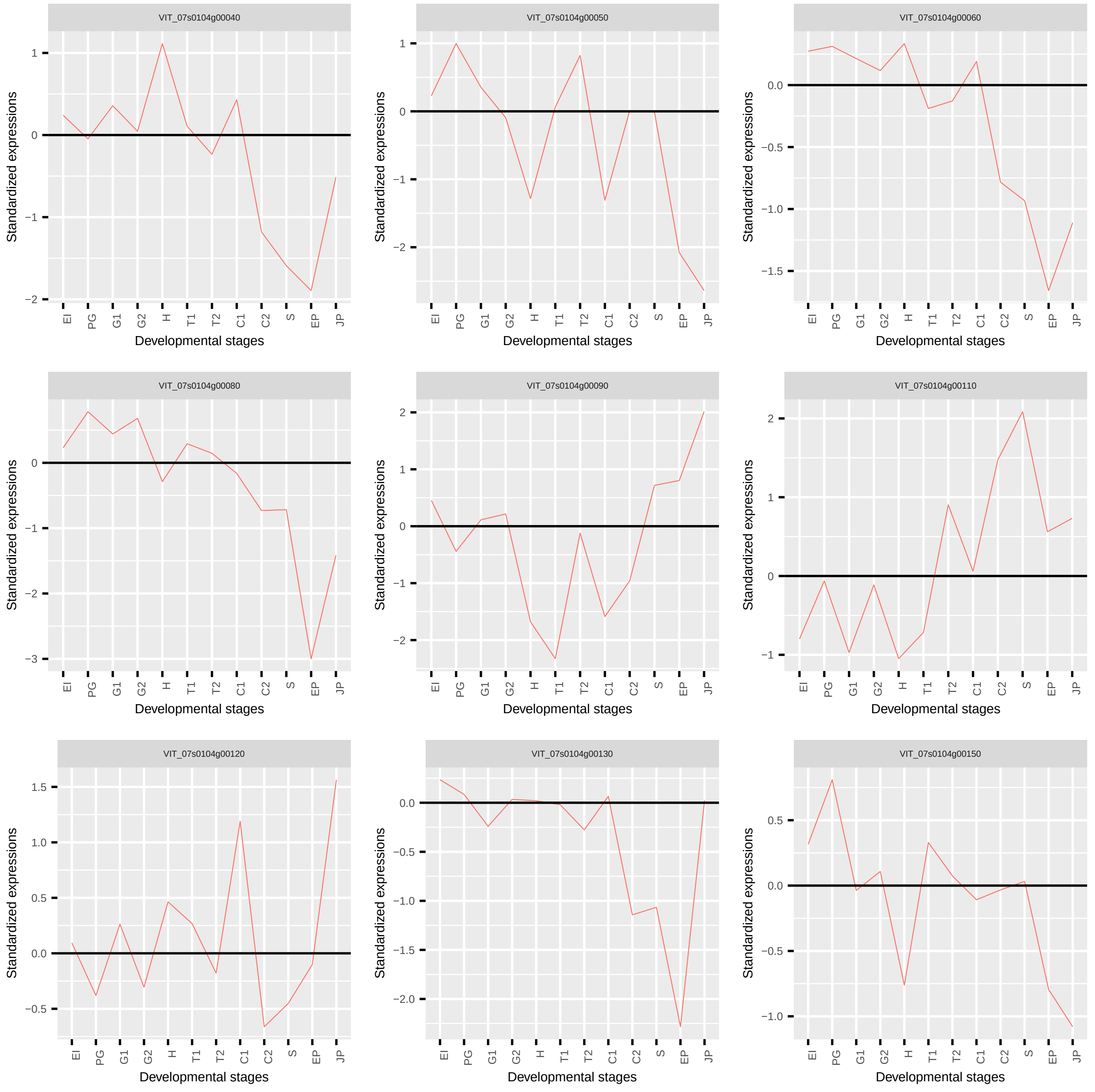
VIT_07s0104g00040
VIT_07s0104g00050
VIT_07s0104g00060
VIT_07s0104g00080
VIT_07s0104g00090
VIT_07s0104g00110
VIT_07s0104g00120
VIT_07s0104g00130
VIT_07s0104g00150
Standardized expressions
Developmental stages
EI PG G1 G2 H T1 T2 C1 C2 S EP JP

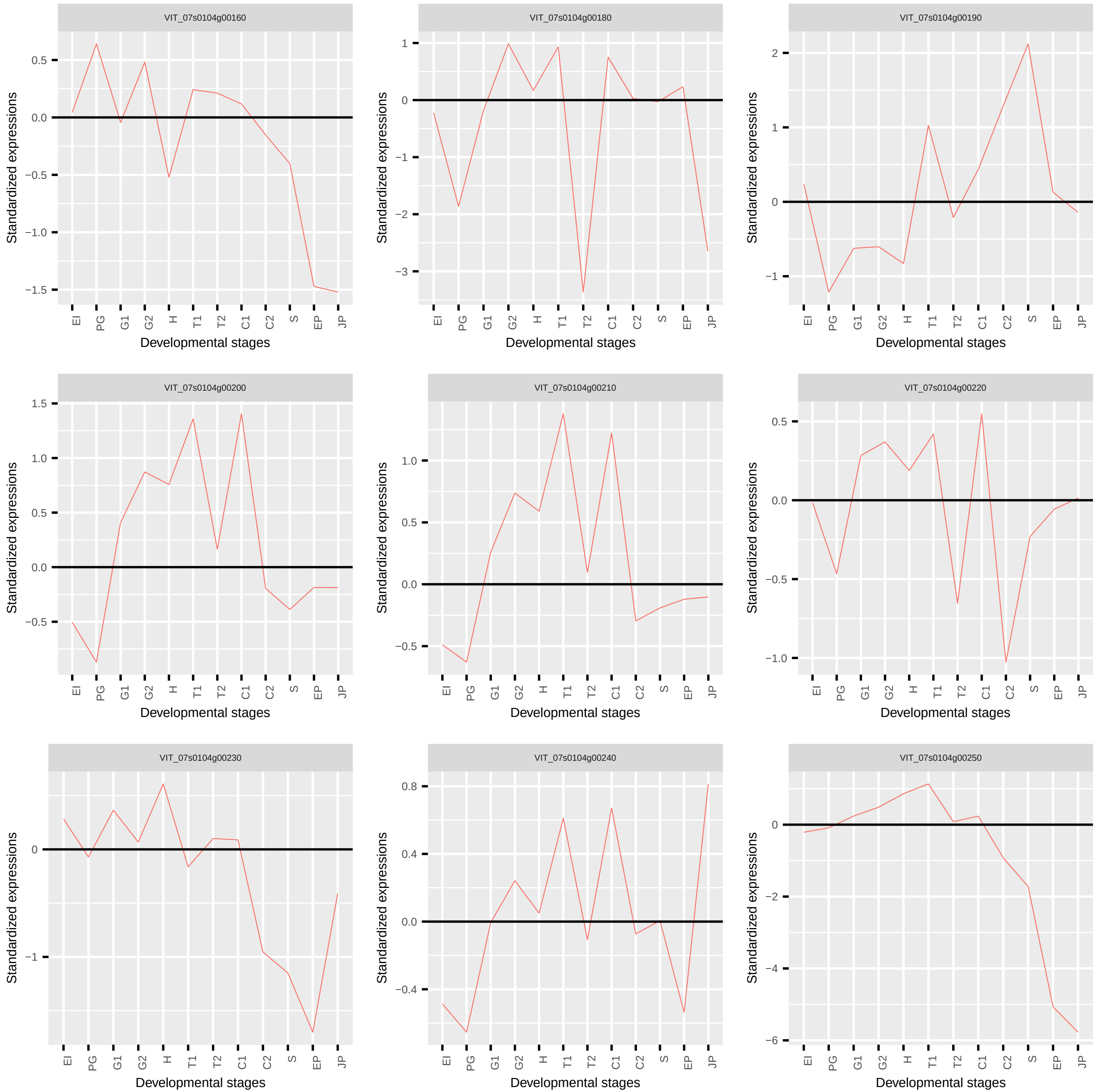

VIT_07s0104g00160
VIT_07s0104g00180
VIT_07s0104g00190
VIT_07s0104g00200
VIT_07s0104g00210
VIT_07s0104g00220
VIT_07s0104g00230
VIT_07s0104g00240
VIT_07s0104g00250
Standardized expressions
Developmental stages
EI PG G1 G2 H T1 T2 C1 C2 S EP JP

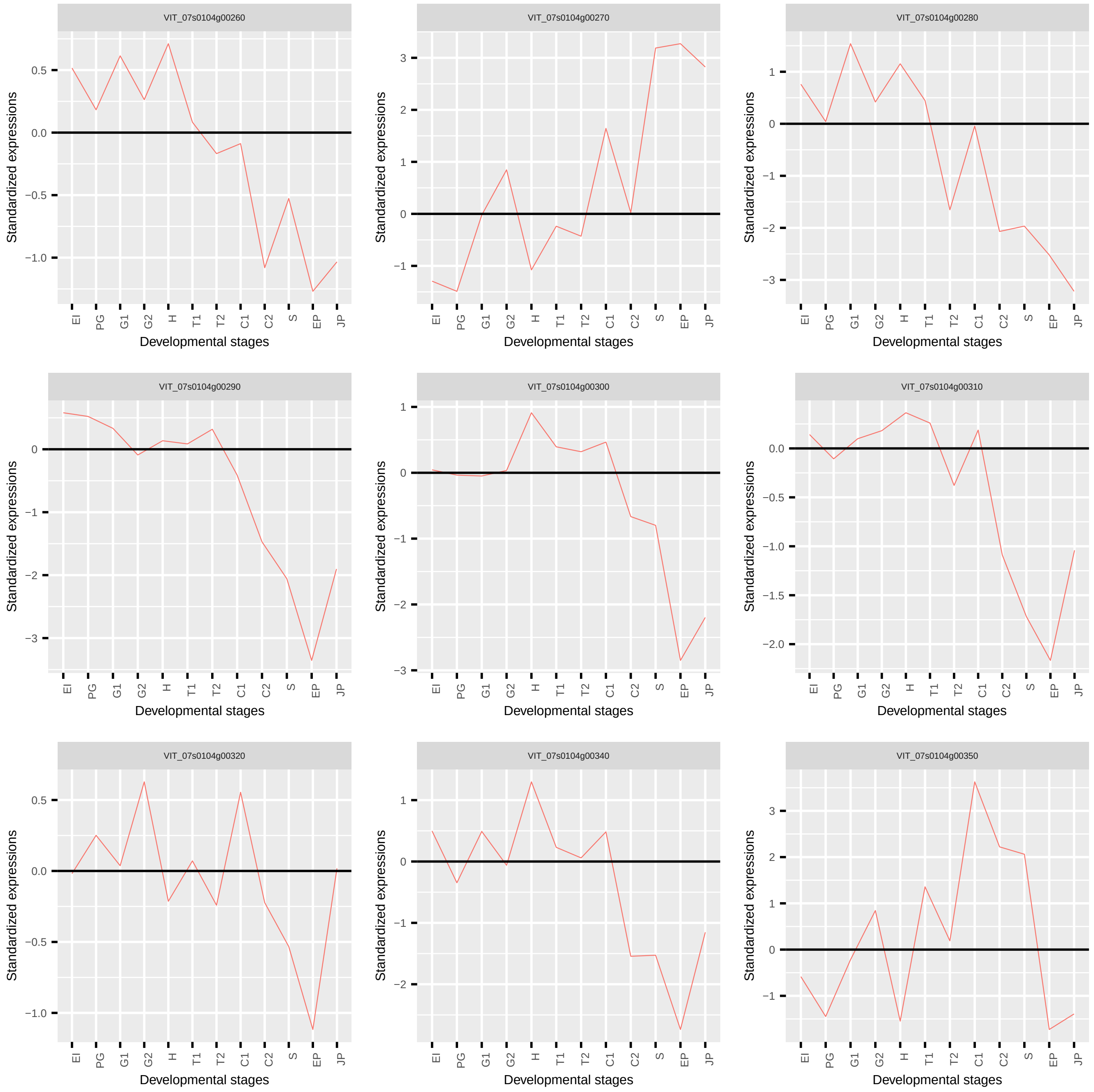

VIT_07s0104g00260
VIT_07s0104g00270
VIT_07s0104g00280
VIT_07s0104g00290
VIT_07s0104g00300
VIT_07s0104g00310
VIT_07s0104g00320
VIT_07s0104g00340
VIT_07s0104g00350
Standardized expressions
Developmental stages
EI
PG
G1
G2
H
T1
T2
C1
C2
S
EP
JP

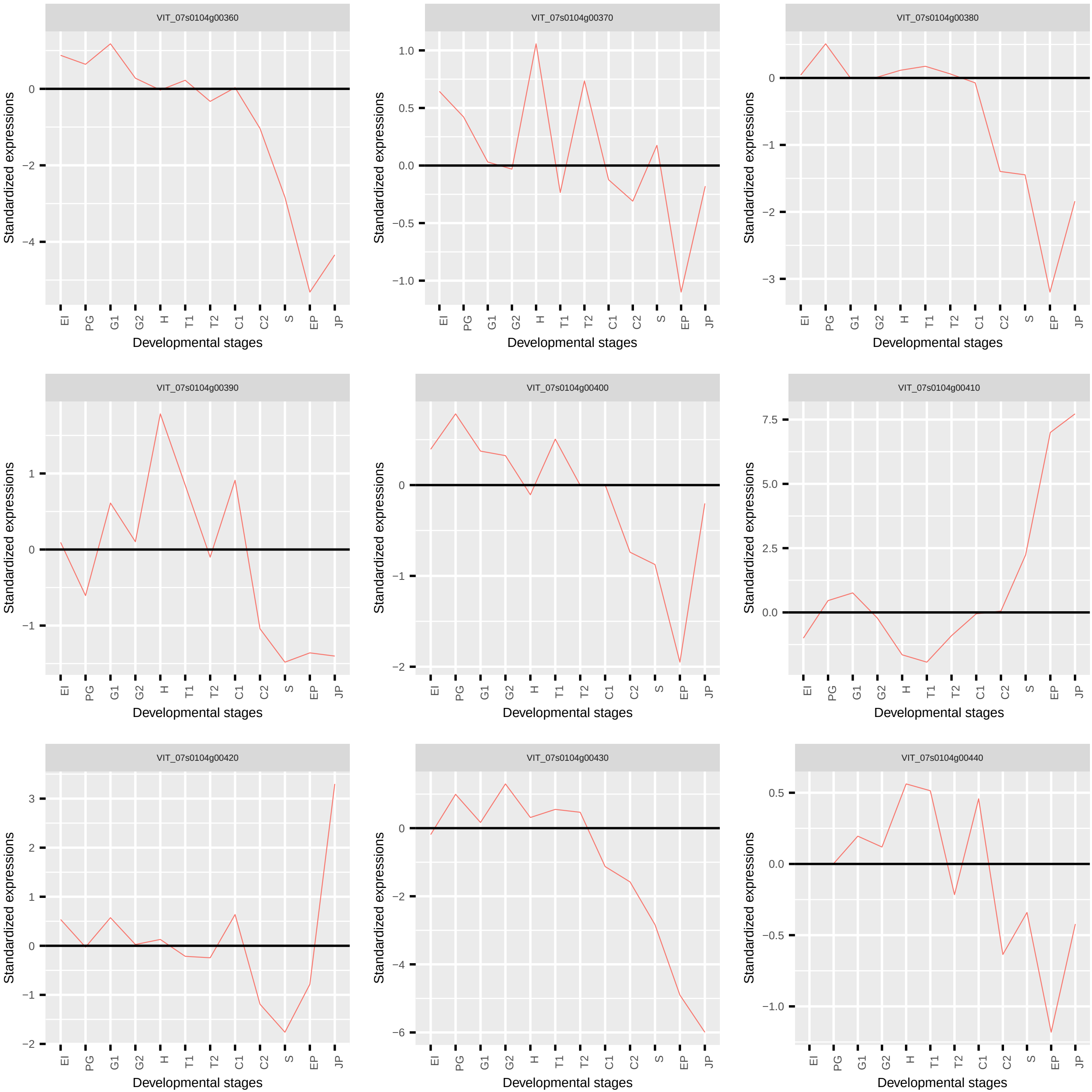
VIT_07s0104g00360
Standardized expressions
0
−2
−4
EI PG G1 G2 H T1 T2 C1 C2 S EP JP
Developmental stages
VIT_07s0104g00370
Standardized expressions
1.0
0.5
0.0
−0.5
−1.0
EI PG G1 G2 H T1 T2 C1 C2 S EP JP
Developmental stages
VIT_07s0104g00380
Standardized expressions
0
−1
−2
−3
EI PG G1 G2 H T1 T2 C1 C2 S EP JP
Developmental stages
VIT_07s0104g00390
Standardized expressions
1
0
−1
EI PG G1 G2 H T1 T2 C1 C2 S EP JP
Developmental stages
VIT_07s0104g00400
Standardized expressions
0
−1
−2
EI PG G1 G2 H T1 T2 C1 C2 S EP JP
Developmental stages
VIT_07s0104g00410
Standardized expressions
7.5
5.0
2.5
0.0
EI PG G1 G2 H T1 T2 C1 C2 S EP JP
Developmental stages
VIT_07s0104g00420
Standardized expressions
3
2
1
0
−1
−2
EI PG G1 G2 H T1 T2 C1 C2 S EP JP
Developmental stages
VIT_07s0104g00430
Standardized expressions
0
−2
−4
−6
EI PG G1 G2 H T1 T2 C1 C2 S EP JP
Developmental stages
VIT_07s0104g00440
Standardized expressions
0.5
0.0
−0.5
−1.0
EI PG G1 G2 H T1 T2 C1 C2 S EP JP
Developmental stages

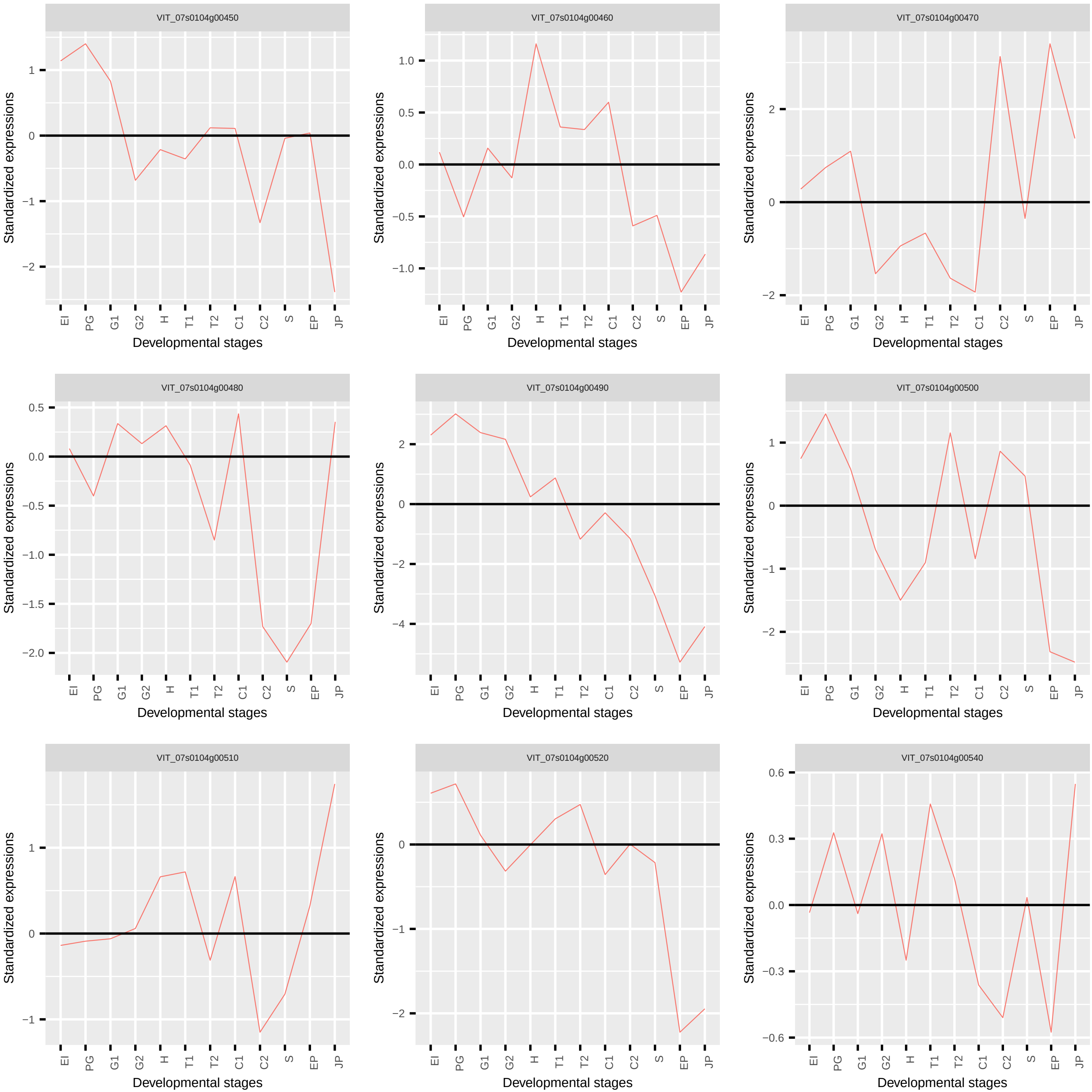
VIT_07s0104g00450
VIT_07s0104g00460
VIT_07s0104g00470
VIT_07s0104g00480
VIT_07s0104g00490
VIT_07s0104g00500
VIT_07s0104g00510
VIT_07s0104g00520
VIT_07s0104g00540
Standardized expressions
Developmental stages
EI PG G1 G2 H T1 T2 C1 C2 S EP JP

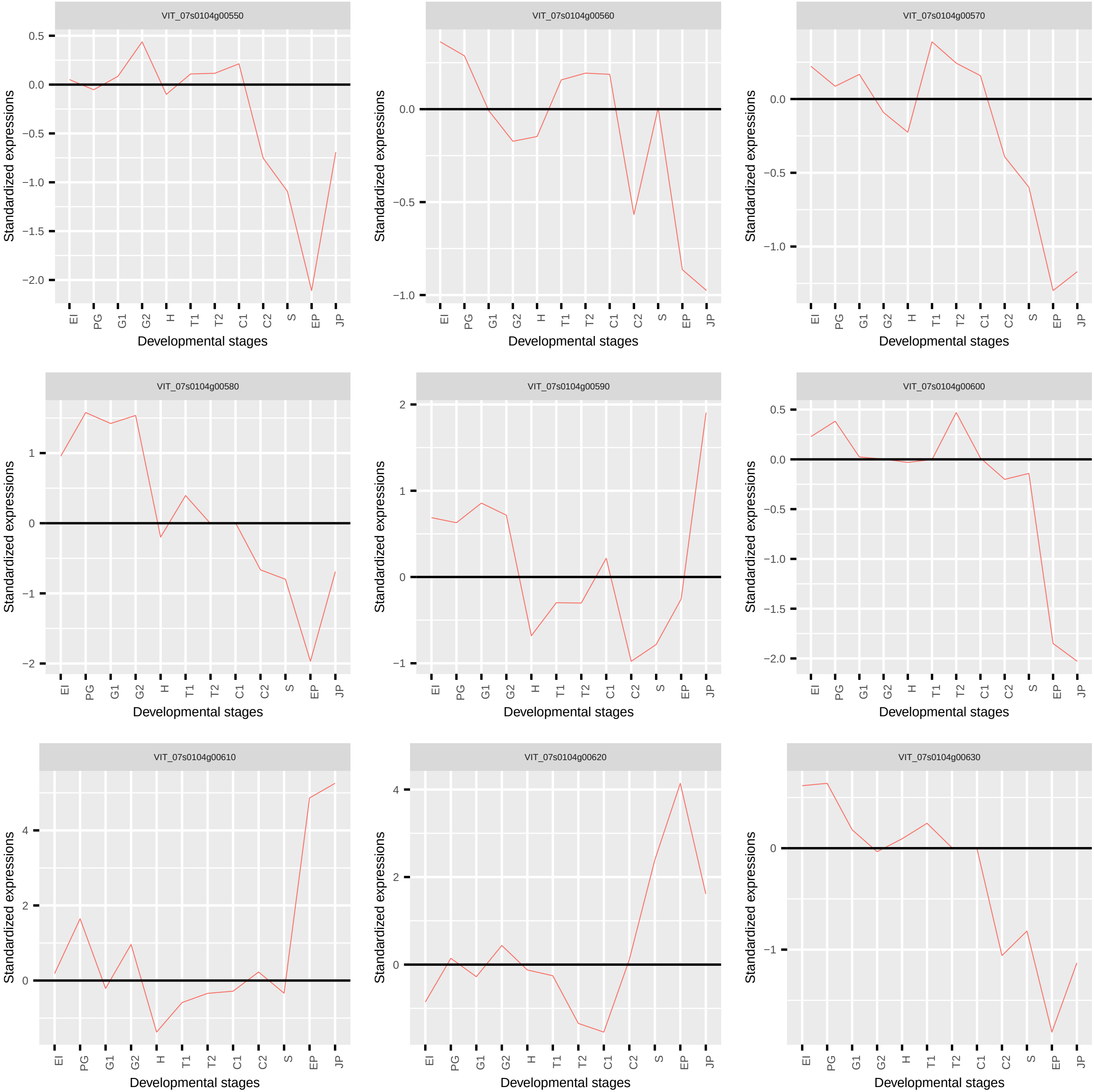
VIT_07s0104g00550
VIT_07s0104g00560
VIT_07s0104g00570
VIT_07s0104g00580
VIT_07s0104g00590
VIT_07s0104g00600
VIT_07s0104g00610
VIT_07s0104g00620
VIT_07s0104g00630
Standardized expressions
Developmental stages
EI PG G1 G2 H T1 T2 C1 C2 S EP JP

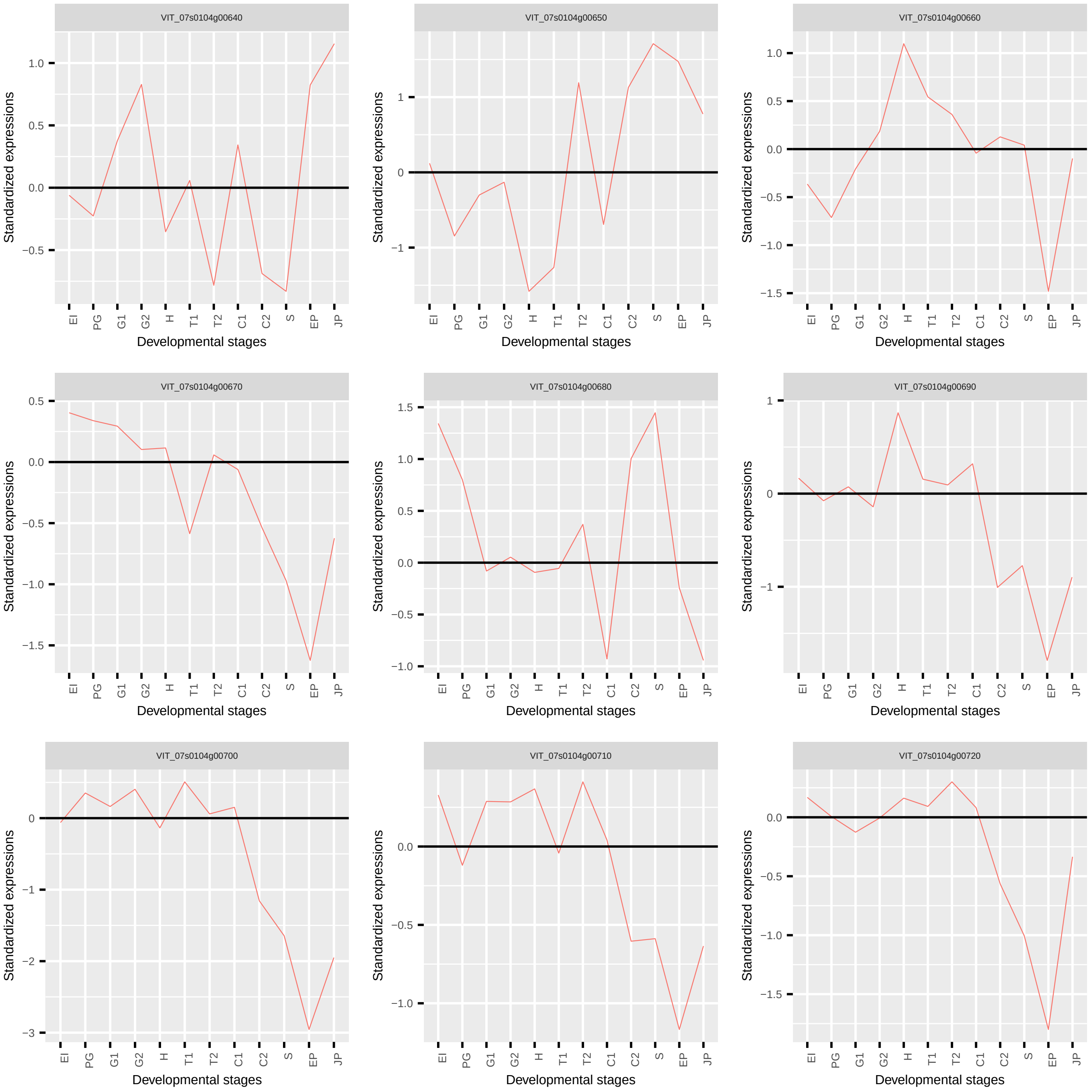

VIT_07s0104g00640
VIT_07s0104g00650
VIT_07s0104g00660
VIT_07s0104g00670
VIT_07s0104g00680
VIT_07s0104g00690
VIT_07s0104g00700
VIT_07s0104g00710
VIT_07s0104g00720
Standardized expressions
Developmental stages
EI PG G1 G2 H T1 T2 C1 C2 S EP JP

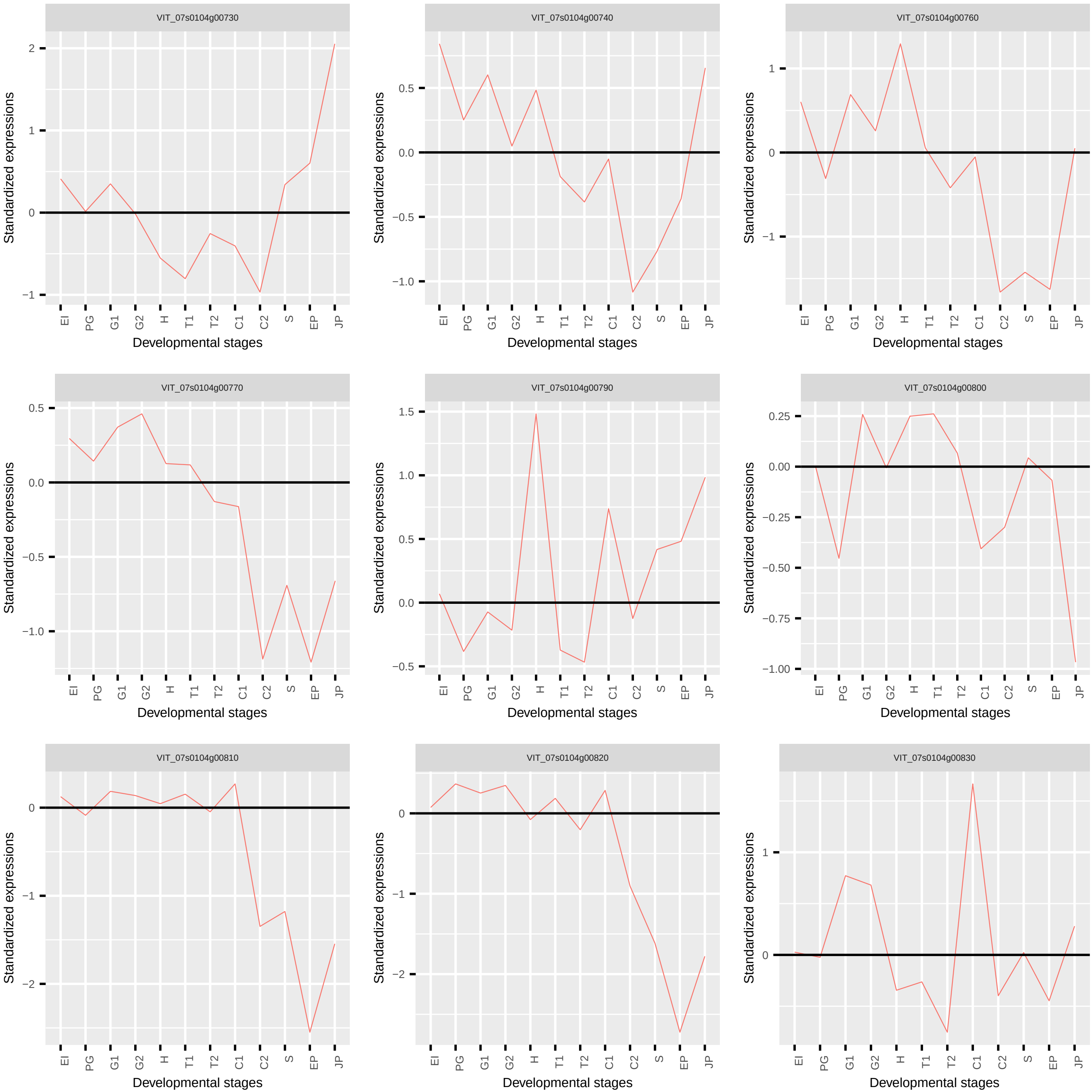

VIT_07s0104g00730
VIT_07s0104g00740
VIT_07s0104g00760
VIT_07s0104g00770
VIT_07s0104g00790
VIT_07s0104g00800
VIT_07s0104g00810
VIT_07s0104g00820
VIT_07s0104g00830
Standardized expressions
Developmental stages
EI
PG
G1
G2
H
T1
T2
C1
C2
S
EP
JP

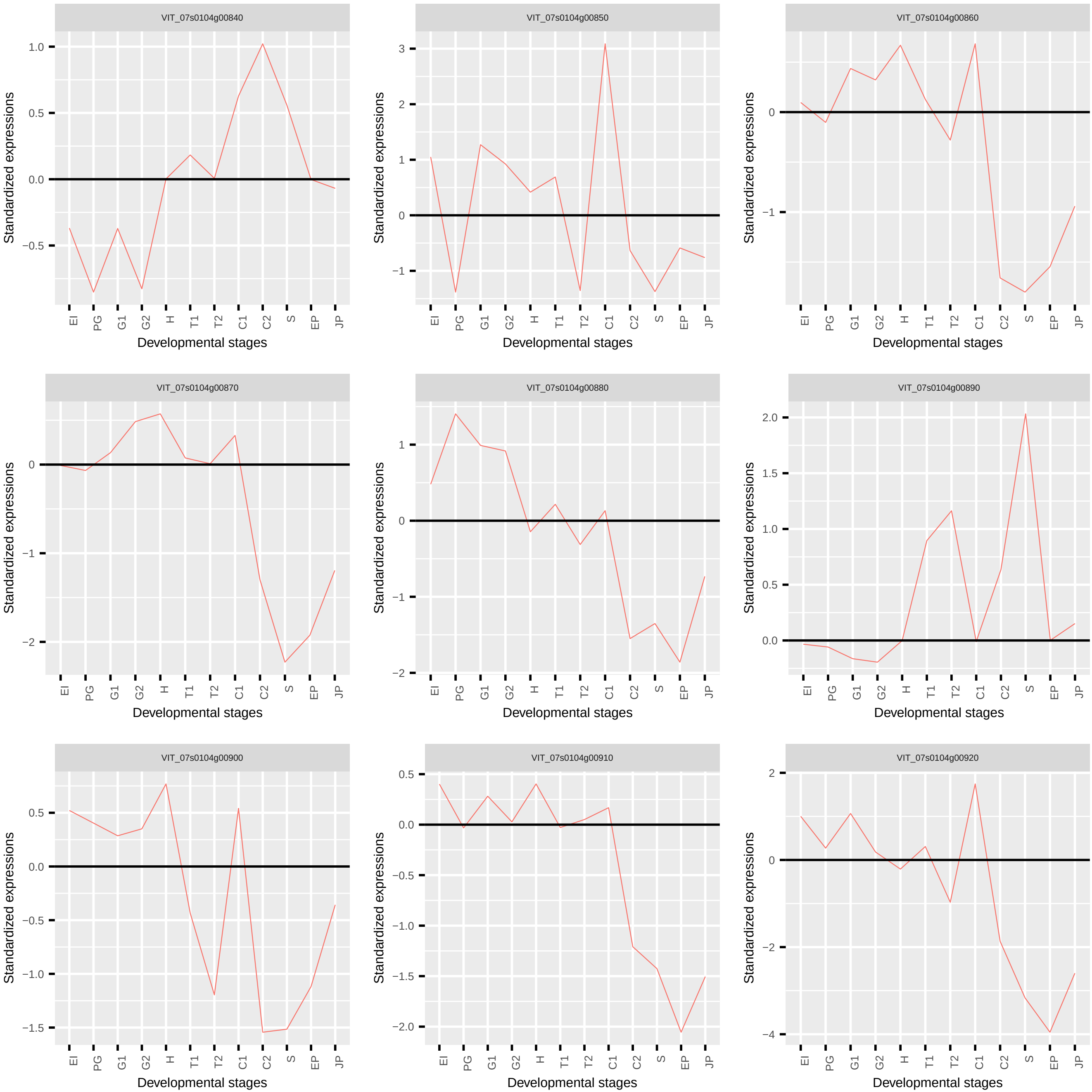
VIT_07s0104g00840
Standardized expressions
1.0
0.5
0.0
−0.5
EI
PG
G1
G2
H
T1
T2
C1
C2
S
EP
JP
Developmental stages
VIT_07s0104g00850
Standardized expressions
3
2
1
0
−1
EI
PG
G1
G2
H
T1
T2
C1
C2
S
EP
JP
Developmental stages
VIT_07s0104g00860
Standardized expressions
0
−1
EI
PG
G1
G2
H
T1
T2
C1
C2
S
EP
JP
Developmental stages
VIT_07s0104g00870
Standardized expressions
0
−1
−2
EI
PG
G1
G2
H
T1
T2
C1
C2
S
EP
JP
Developmental stages
VIT_07s0104g00880
Standardized expressions
1
0
−1
−2
EI
PG
G1
G2
H
T1
T2
C1
C2
S
EP
JP
Developmental stages
VIT_07s0104g00890
Standardized expressions
2.0
1.5
1.0
0.5
0.0
EI
PG
G1
G2
H
T1
T2
C1
C2
S
EP
JP
Developmental stages
VIT_07s0104g00900
Standardized expressions
0.5
0.0
−0.5
−1.0
−1.5
EI
PG
G1
G2
H
T1
T2
C1
C2
S
EP
JP
Developmental stages
VIT_07s0104g00910
Standardized expressions
0.5
0.0
−0.5
−1.0
−1.5
−2.0
EI
PG
G1
G2
H
T1
T2
C1
C2
S
EP
JP
Developmental stages
VIT_07s0104g00920
Standardized expressions
2
0
−2
−4
EI
PG
G1
G2
H
T1
T2
C1
C2
S
EP
JP
Developmental stages

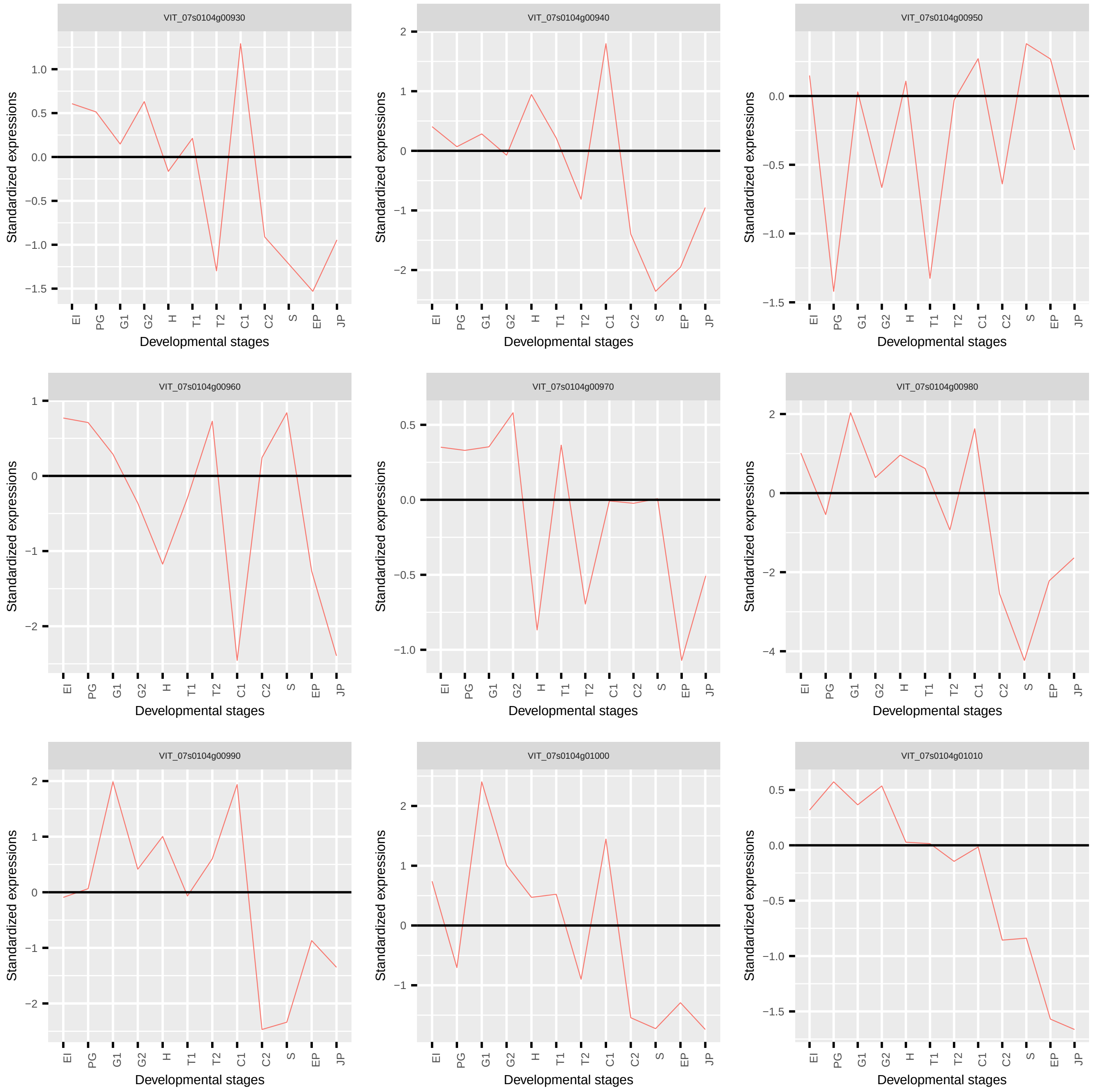
VIT_07s0104g00930
VIT_07s0104g00940
VIT_07s0104g00950
VIT_07s0104g00960
VIT_07s0104g00970
VIT_07s0104g00980
VIT_07s0104g00990
VIT_07s0104g01000
VIT_07s0104g01010
Standardized expressions
Developmental stages
EI PG G1 G2 H T1 T2 C1 C2 S EP JP

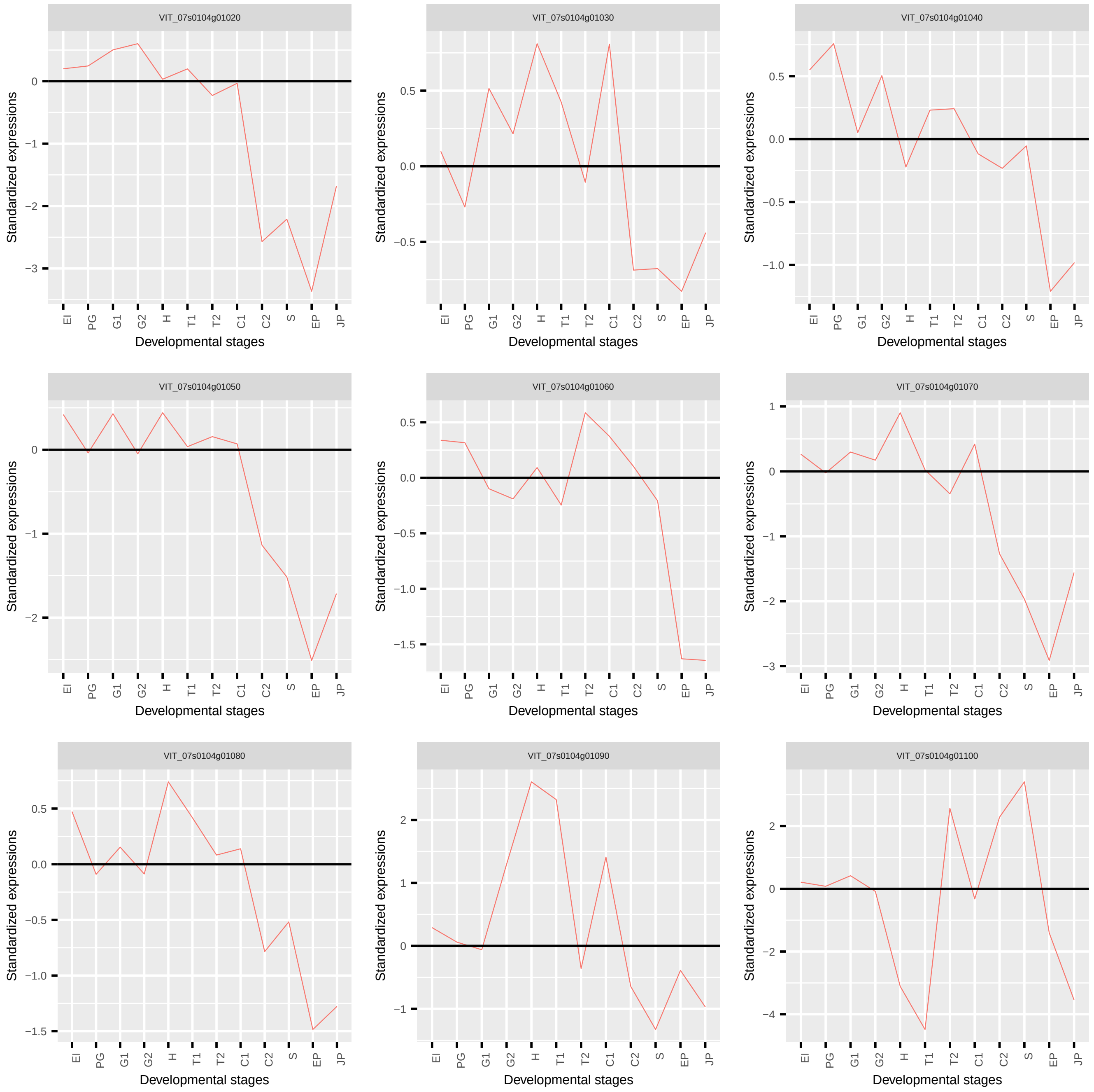
VIT_07s0104g01020
0
−1
−2
−3
Standardized expressions
EI PG G1 G2 H T1 T2 C1 C2 S EP JP
Developmental stages
VIT_07s0104g01030
0.5
0.0
−0.5
Standardized expressions
EI PG G1 G2 H T1 T2 C1 C2 S EP JP
Developmental stages
VIT_07s0104g01040
0.5
0.0
−0.5
−1.0
Standardized expressions
EI PG G1 G2 H T1 T2 C1 C2 S EP JP
Developmental stages
VIT_07s0104g01050
0
−1
−2
Standardized expressions
EI PG G1 G2 H T1 T2 C1 C2 S EP JP
Developmental stages
VIT_07s0104g01060
0.5
0.0
−0.5
−1.0
−1.5
Standardized expressions
EI PG G1 G2 H T1 T2 C1 C2 S EP JP
Developmental stages
VIT_07s0104g01070
1
0
−1
−2
−3
Standardized expressions
EI PG G1 G2 H T1 T2 C1 C2 S EP JP
Developmental stages
VIT_07s0104g01080
0.5
0.0
−0.5
−1.0
−1.5
Standardized expressions
EI PG G1 G2 H T1 T2 C1 C2 S EP JP
Developmental stages
VIT_07s0104g01090
2
1
0
−1
Standardized expressions
EI PG G1 G2 H T1 T2 C1 C2 S EP JP
Developmental stages
VIT_07s0104g01100
2
0
−2
−4
Standardized expressions
EI PG G1 G2 H T1 T2 C1 C2 S EP JP
Developmental stages

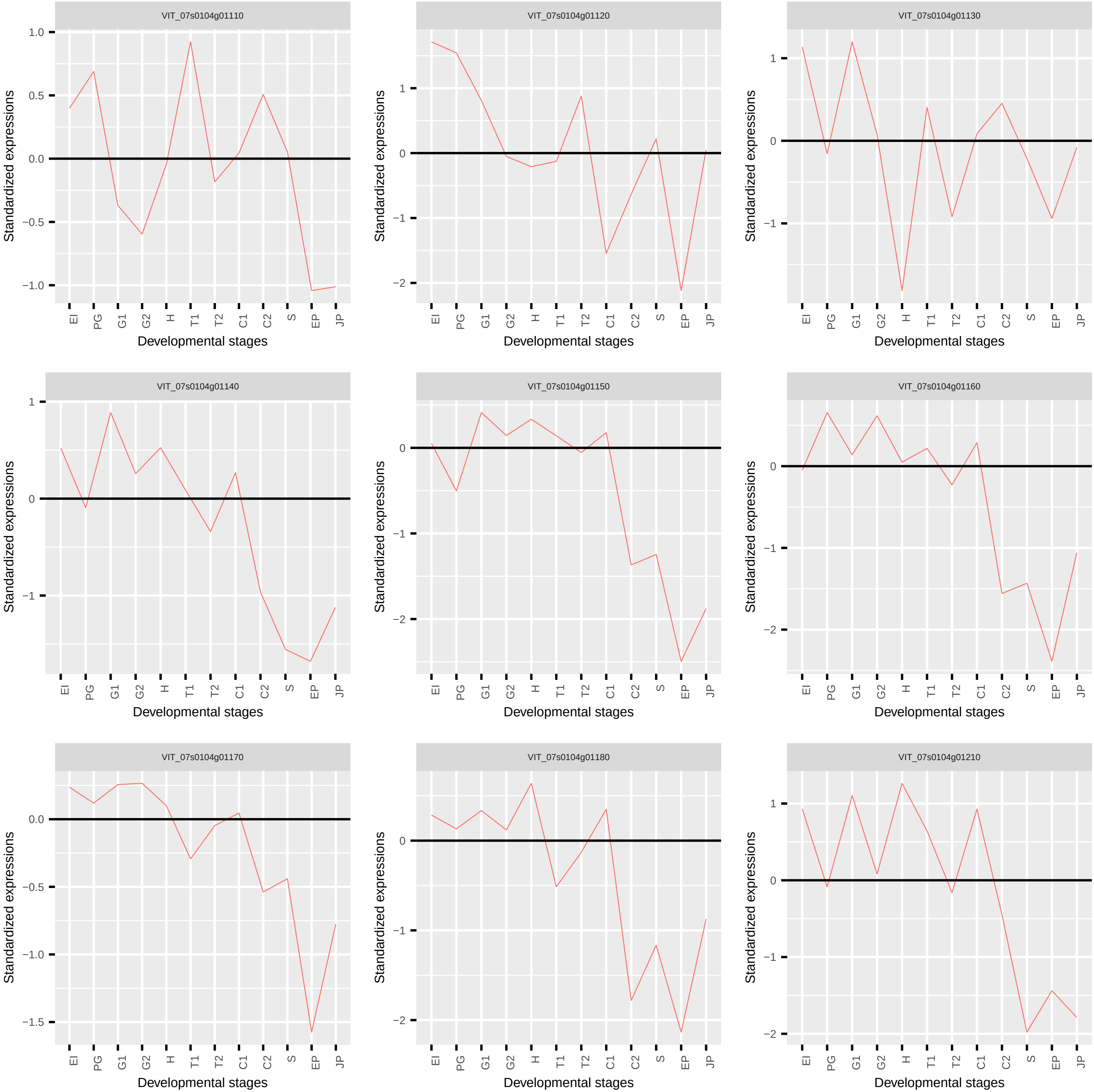
VIT_07s0104g01110
VIT_07s0104g01120
VIT_07s0104g01130
VIT_07s0104g01140
VIT_07s0104g01150
VIT_07s0104g01160
VIT_07s0104g01170
VIT_07s0104g01180
VIT_07s0104g01210
Standardized expressions
Developmental stages
EI PG G1 G2 H T1 T2 C1 C2 S EP JP

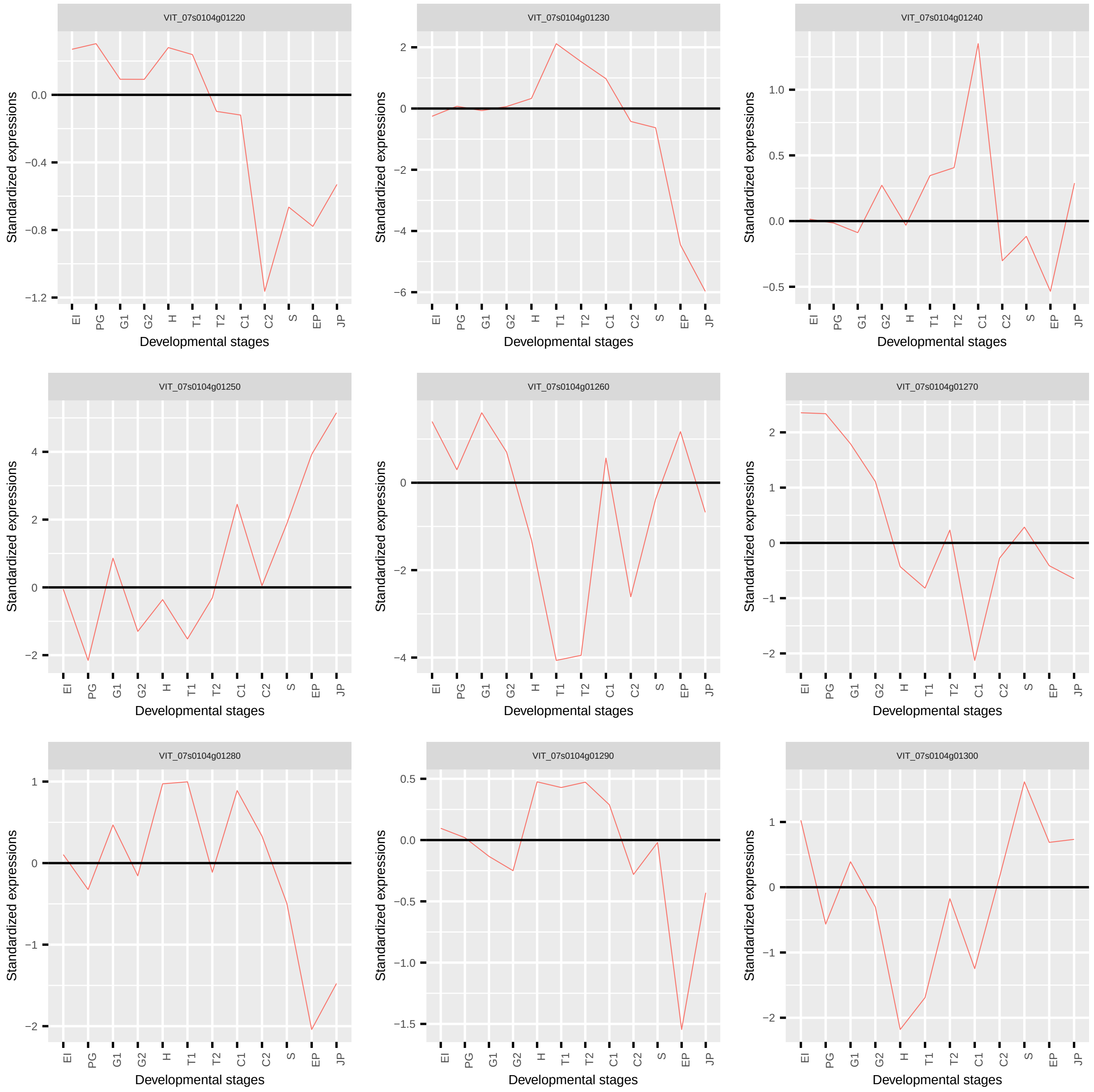
VIT_07s0104g01220
VIT_07s0104g01230
VIT_07s0104g01240
VIT_07s0104g01250
VIT_07s0104g01260
VIT_07s0104g01270
VIT_07s0104g01280
VIT_07s0104g01290
VIT_07s0104g01300
Standardized expressions
Developmental stages
EI PG G1 G2 H T1 T2 C1 C2 S EP JP

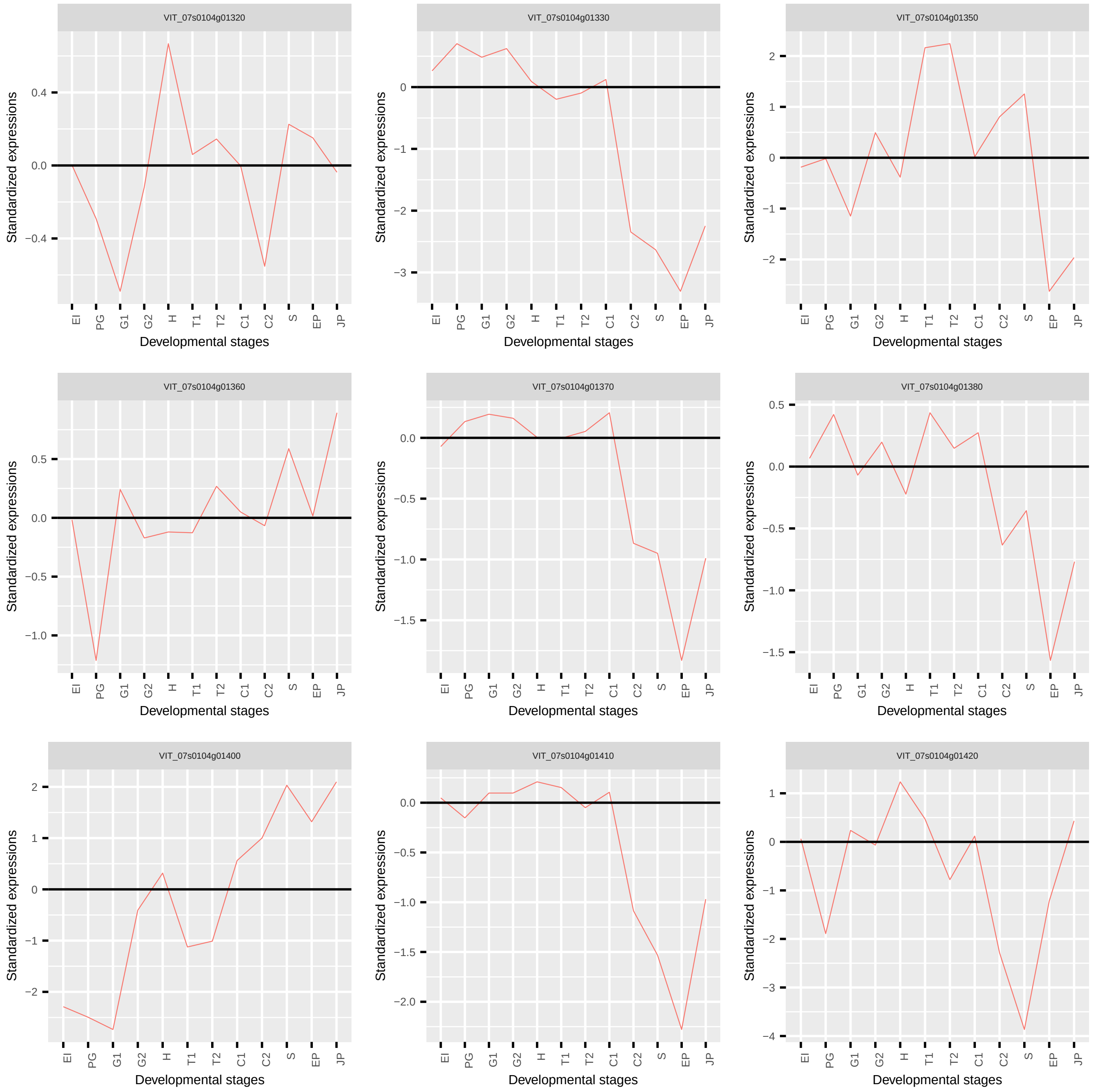
VIT_07s0104g01320
Standardized expressions
0.4
0.0
−0.4
EI PG G1 G2 H T1 T2 C1 C2 S EP JP
Developmental stages
VIT_07s0104g01330
Standardized expressions
0
−1
−2
−3
EI PG G1 G2 H T1 T2 C1 C2 S EP JP
Developmental stages
VIT_07s0104g01350
Standardized expressions
2
1
0
−1
−2
EI PG G1 G2 H T1 T2 C1 C2 S EP JP
Developmental stages
VIT_07s0104g01360
Standardized expressions
0.5
0.0
−0.5
−1.0
EI PG G1 G2 H T1 T2 C1 C2 S EP JP
Developmental stages
VIT_07s0104g01370
Standardized expressions
0.0
−0.5
−1.0
−1.5
EI PG G1 G2 H T1 T2 C1 C2 S EP JP
Developmental stages
VIT_07s0104g01380
Standardized expressions
0.5
0.0
−0.5
−1.0
−1.5
EI PG G1 G2 H T1 T2 C1 C2 S EP JP
Developmental stages
VIT_07s0104g01400
Standardized expressions
2
1
0
−1
−2
EI PG G1 G2 H T1 T2 C1 C2 S EP JP
Developmental stages
VIT_07s0104g01410
Standardized expressions
0.0
−0.5
−1.0
−1.5
−2.0
EI PG G1 G2 H T1 T2 C1 C2 S EP JP
Developmental stages
VIT_07s0104g01420
Standardized expressions
1
0
−1
−2
−3
−4
EI PG G1 G2 H T1 T2 C1 C2 S EP JP
Developmental stages

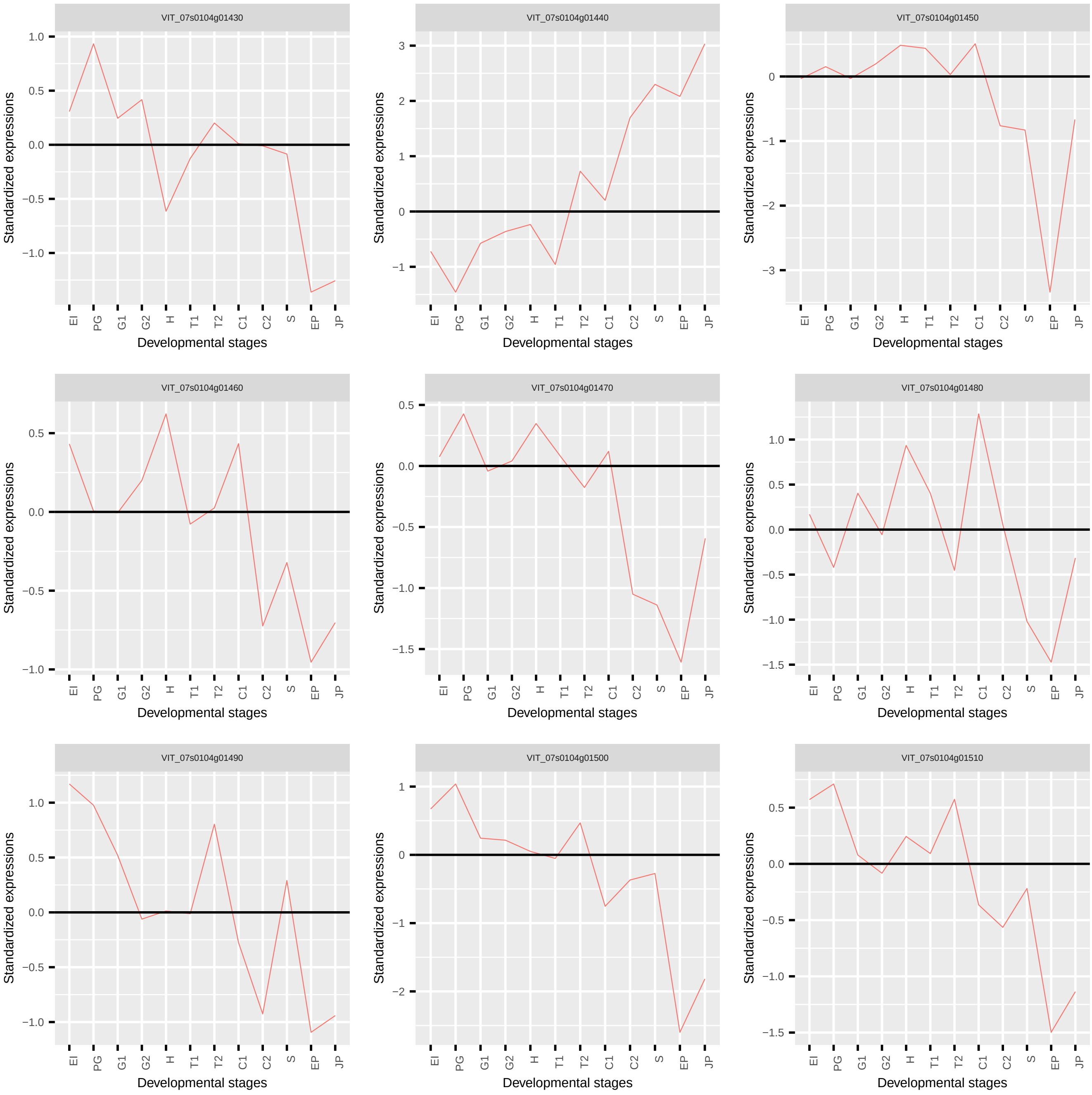

VIT_07s0104g01430
VIT_07s0104g01440
VIT_07s0104g01450
VIT_07s0104g01460
VIT_07s0104g01470
VIT_07s0104g01480
VIT_07s0104g01490
VIT_07s0104g01500
VIT_07s0104g01510
Standardized expressions
Developmental stages
EI PG G1 G2 H T1 T2 C1 C2 S EP JP
1.0 0.5 0.0 −0.5 −1.0
3 2 1 0 −1
0 −1 −2 −3
0.5 0.0 −0.5 −1.0
0.5 0.0 −0.5 −1.0 −1.5
1.0 0.5 0.0 −0.5 −1.0 −1.5
1.0 0.5 0.0 −0.5 −1.0
1 0 −1 −2
0.5 0.0 −0.5 −1.0 −1.5

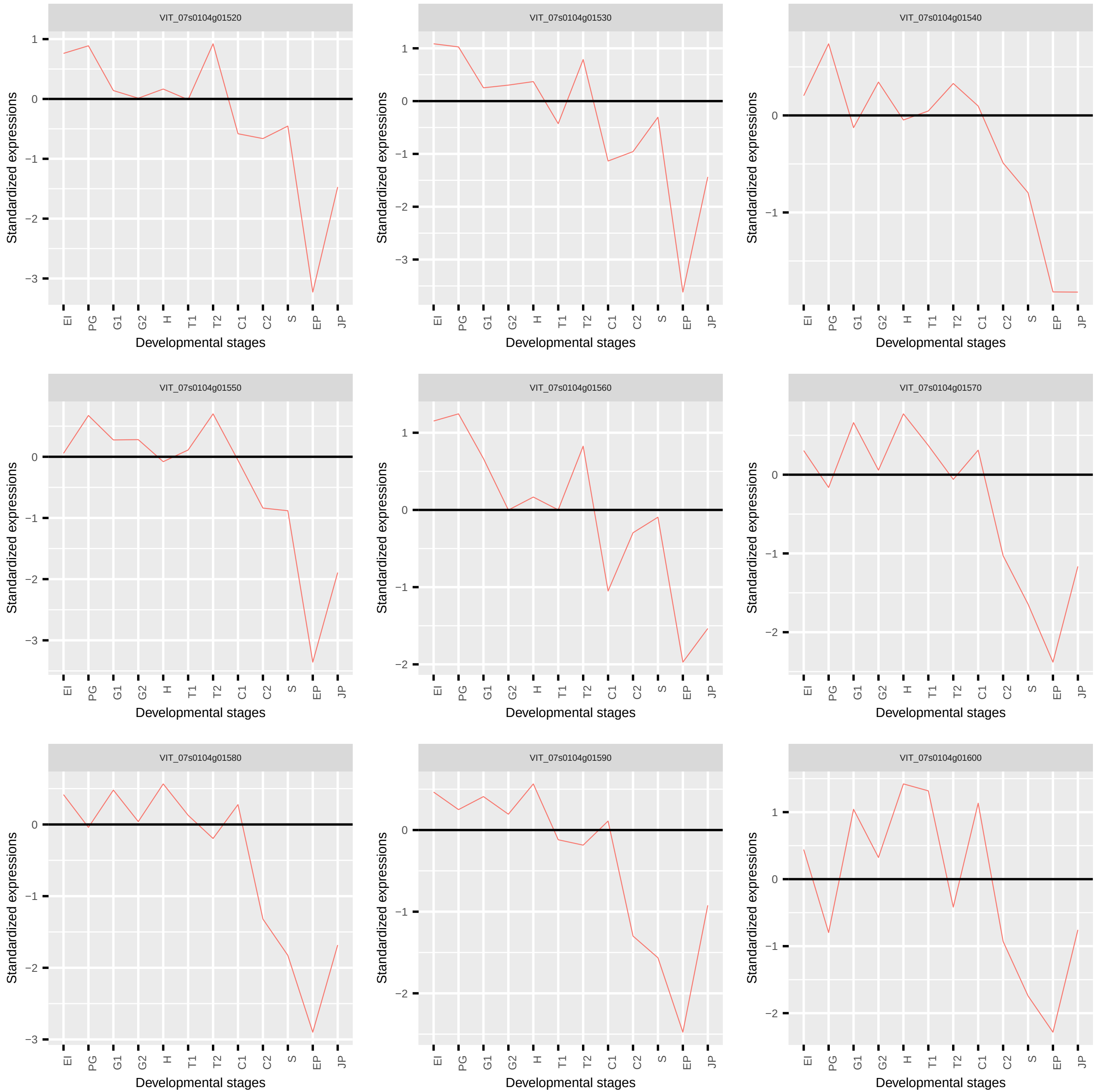
VIT_07s0104g01520
VIT_07s0104g01530
VIT_07s0104g01540
VIT_07s0104g01550
VIT_07s0104g01560
VIT_07s0104g01570
VIT_07s0104g01580
VIT_07s0104g01590
VIT_07s0104g01600
Standardized expressions
Developmental stages
EI PG G1 G2 H T1 T2 C1 C2 S EP JP

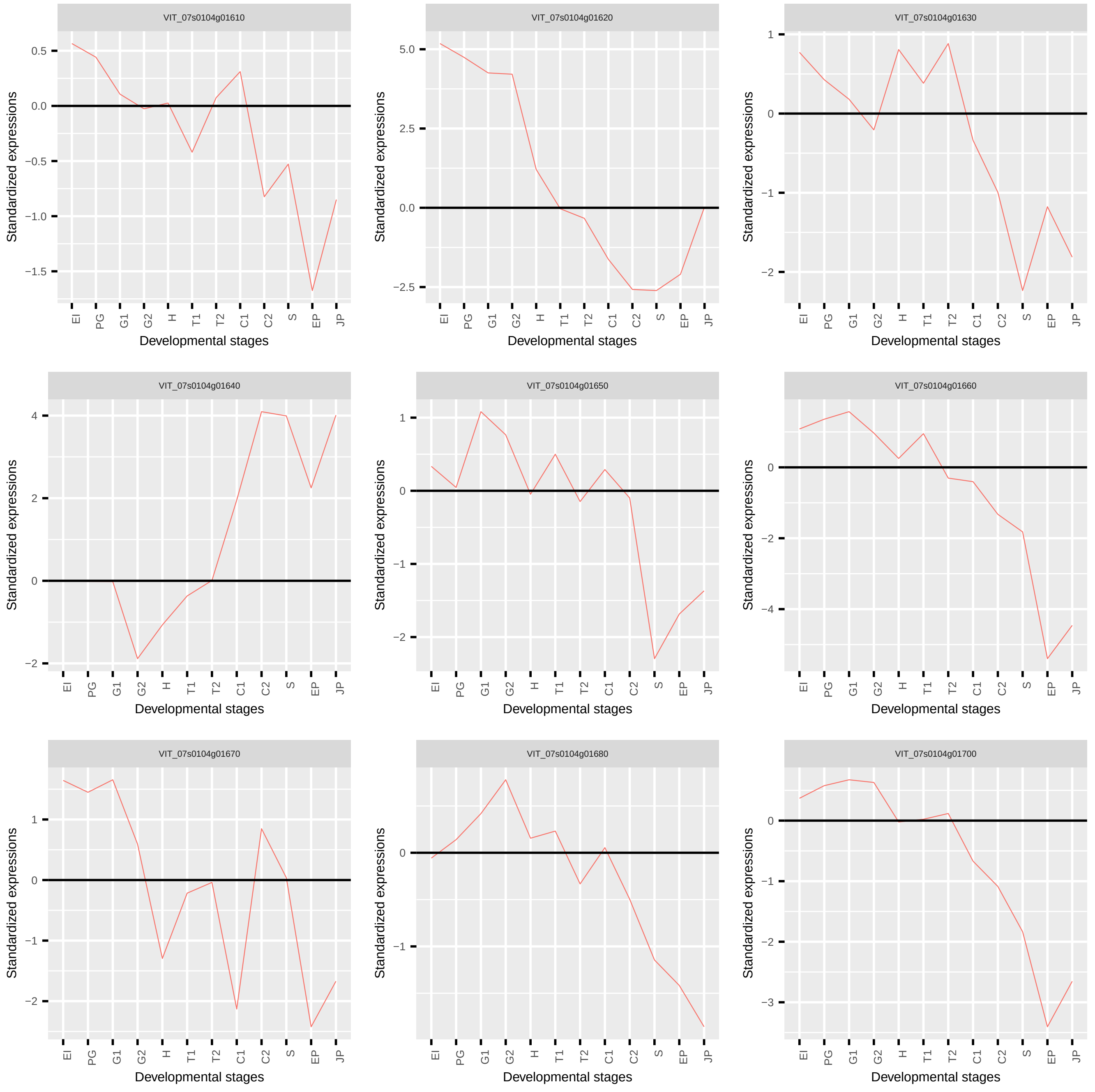
VIT_07s0104g01610
0.5
0.0
−0.5
−1.0
−1.5
VIT_07s0104g01620
5.0
2.5
0.0
−2.5
VIT_07s0104g01630
1
0
−1
−2
VIT_07s0104g01640
4
2
0
−2
VIT_07s0104g01650
1
0
−1
−2
VIT_07s0104g01660
0
−2
−4
VIT_07s0104g01670
1
0
−1
−2
VIT_07s0104g01680
0
−1
VIT_07s0104g01700
0
−1
−2
−3
Standardized expressions
EI
PG
G1
G2
H
T1
T2
C1
C2
S
EP
JP
Developmental stages

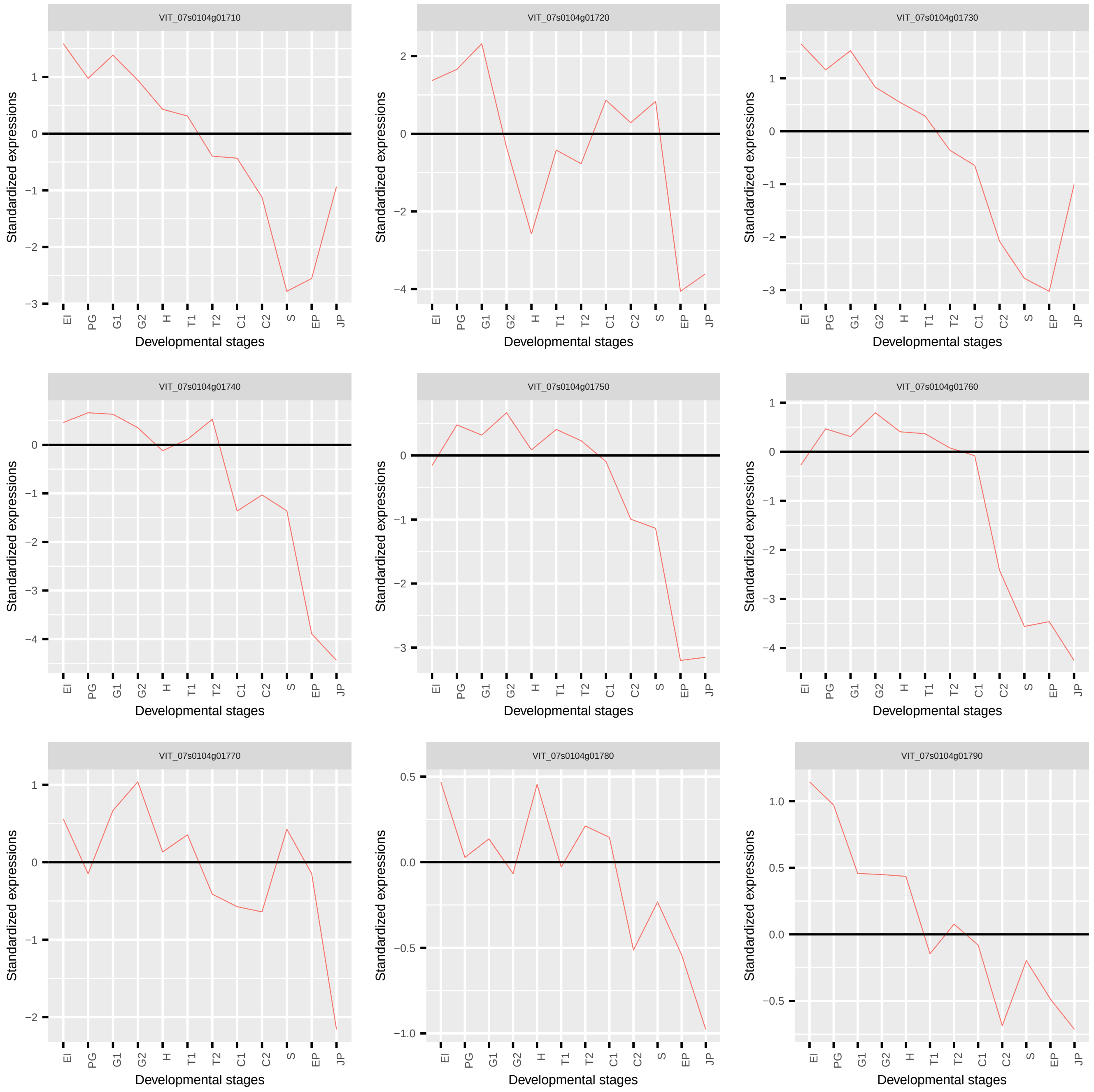
VIT_07s0104g01710
VIT_07s0104g01720
VIT_07s0104g01730
VIT_07s0104g01740
VIT_07s0104g01750
VIT_07s0104g01760
VIT_07s0104g01770
VIT_07s0104g01780
VIT_07s0104g01790
Standardized expressions
Developmental stages
EI PG G1 G2 H T1 T2 C1 C2 S EP JP

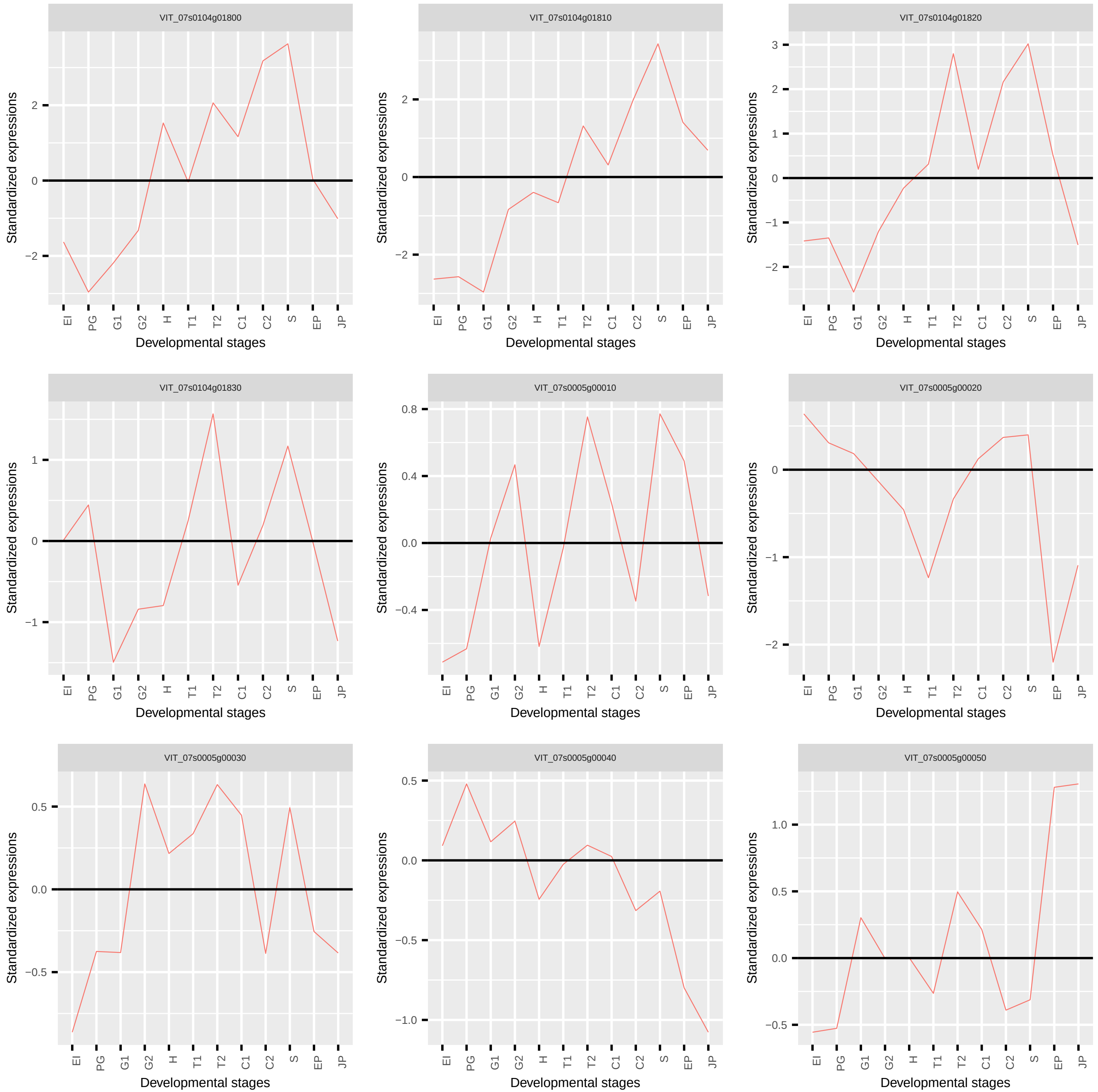
VIT_07s0104g01800
VIT_07s0104g01810
VIT_07s0104g01820
VIT_07s0104g01830
VIT_07s0005g00010
VIT_07s0005g00020
VIT_07s0005g00030
VIT_07s0005g00040
VIT_07s0005g00050
Standardized expressions
Developmental stages
EI PG G1 G2 H T1 T2 C1 C2 S EP JP

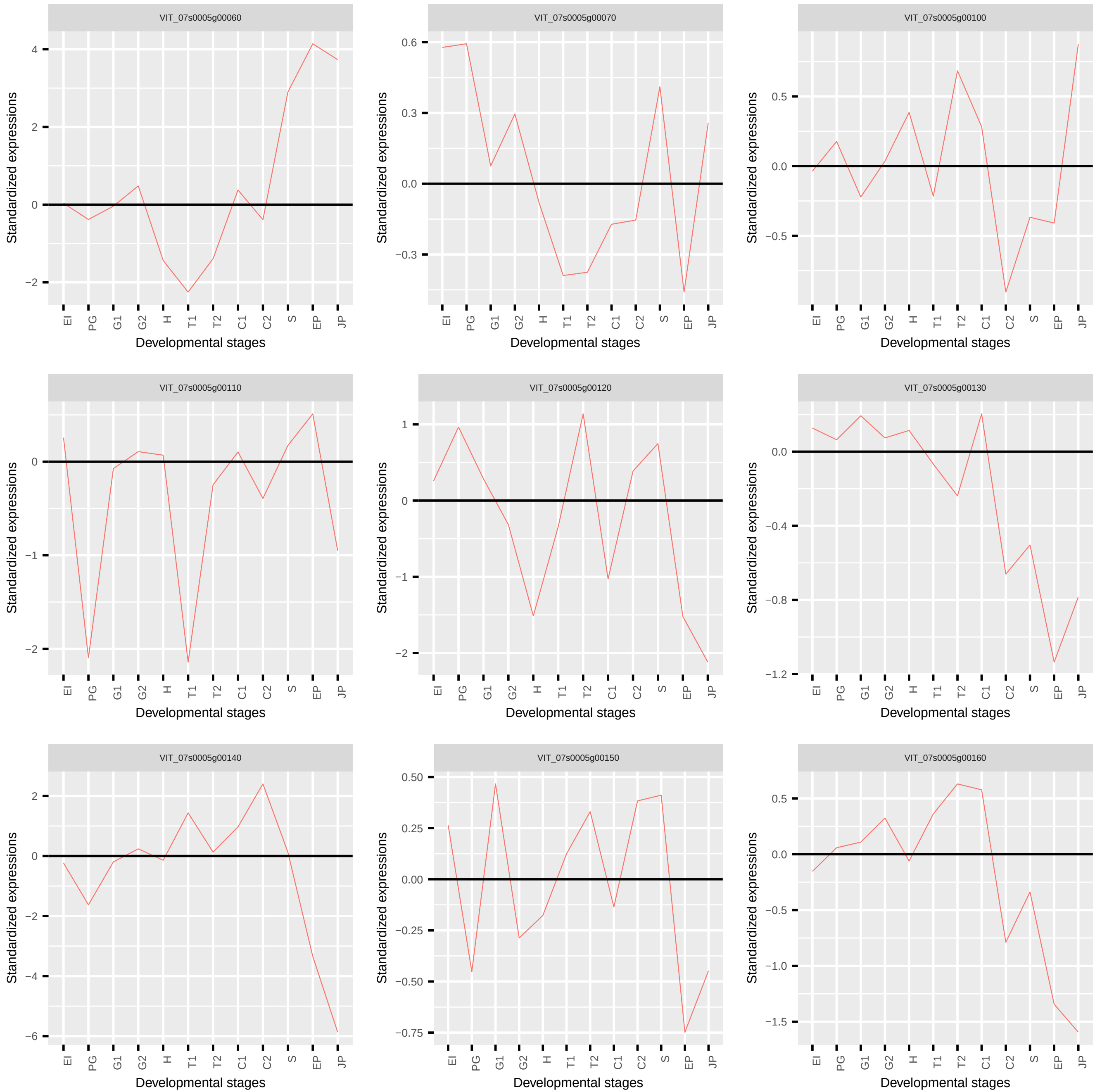
VIT_07s0005g00060
VIT_07s0005g00070
VIT_07s0005g00100
VIT_07s0005g00110
VIT_07s0005g00120
VIT_07s0005g00130
VIT_07s0005g00140
VIT_07s0005g00150
VIT_07s0005g00160
Standardized expressions
Developmental stages
EI PG G1 G2 H T1 T2 C1 C2 S EP JP

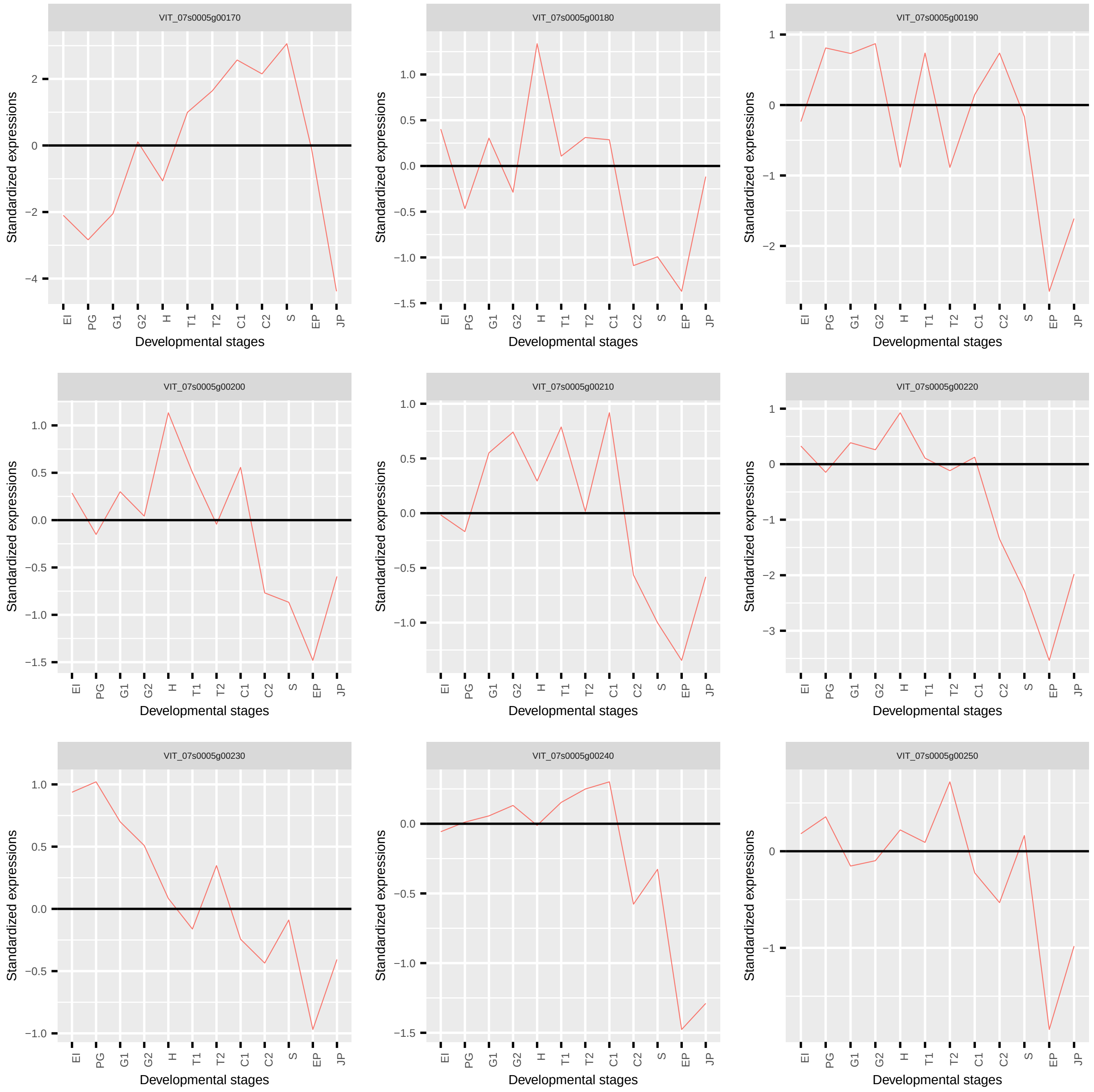

VIT_07s0005g00170
Standardized expressions
2
0
−2
−4
VIT_07s0005g00180
1.0
0.5
0.0
−0.5
−1.0
−1.5
VIT_07s0005g00190
1
0
−1
−2
VIT_07s0005g00200
1.0
0.5
0.0
−0.5
−1.0
−1.5
VIT_07s0005g00210
1.0
0.5
0.0
−0.5
−1.0
VIT_07s0005g00220
1
0
−1
−2
−3
VIT_07s0005g00230
1.0
0.5
0.0
−0.5
−1.0
VIT_07s0005g00240
0.0
−0.5
−1.0
−1.5
VIT_07s0005g00250
0
−1
EI
PG
G1
G2
H
T1
T2
C1
C2
S
EP
JP
Developmental stages

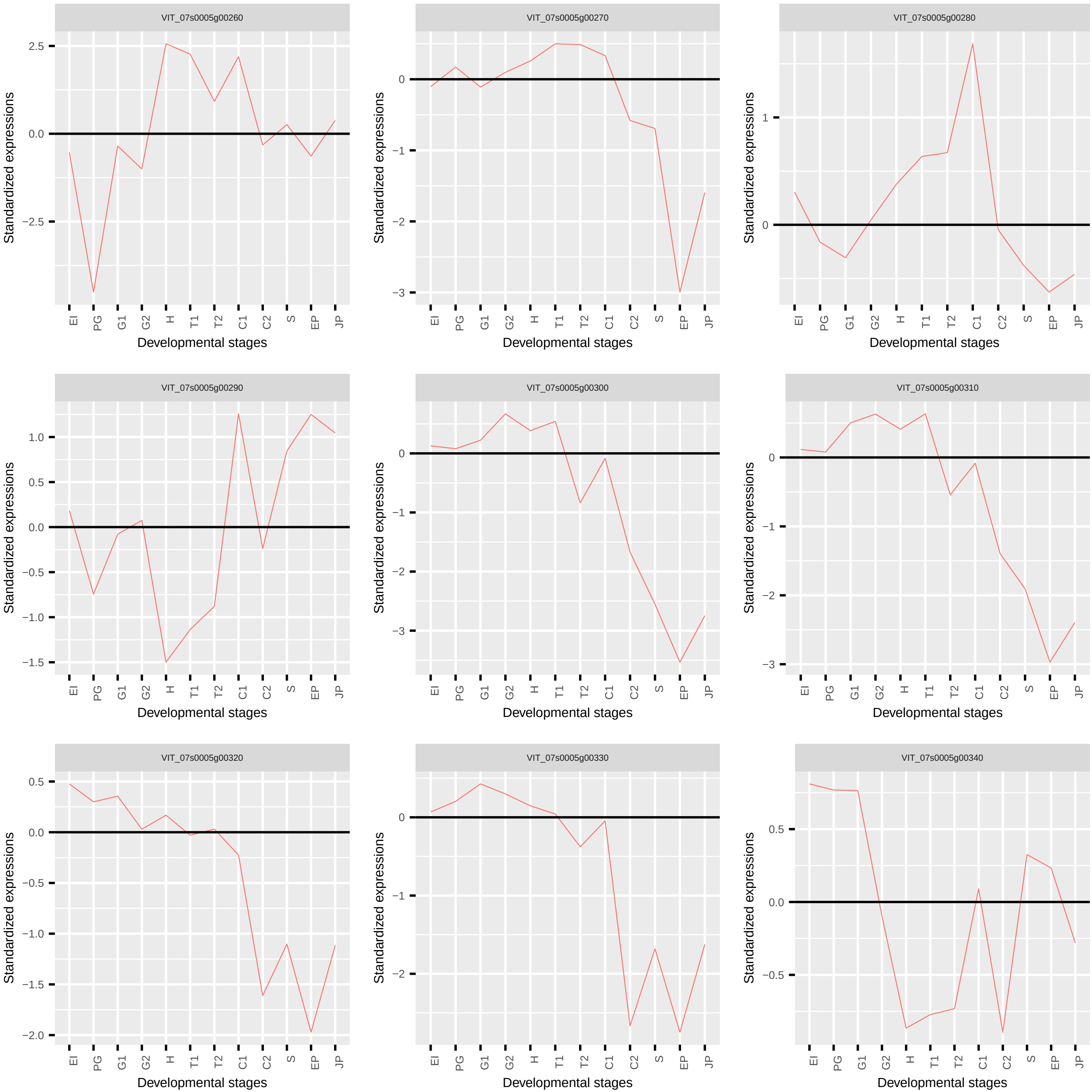
VIT_07s0005g00260
2.5
0.0
−2.5
Standardized expressions
EI PG G1 G2 H T1 T2 C1 C2 S EP JP
Developmental stages
VIT_07s0005g00270
0
−1
−2
−3
Standardized expressions
EI PG G1 G2 H T1 T2 C1 C2 S EP JP
Developmental stages
VIT_07s0005g00280
1
0
Standardized expressions
EI PG G1 G2 H T1 T2 C1 C2 S EP JP
Developmental stages
VIT_07s0005g00290
1.0
0.5
0.0
−0.5
−1.0
−1.5
Standardized expressions
EI PG G1 G2 H T1 T2 C1 C2 S EP JP
Developmental stages
VIT_07s0005g00300
0
−1
−2
−3
Standardized expressions
EI PG G1 G2 H T1 T2 C1 C2 S EP JP
Developmental stages
VIT_07s0005g00310
0
−1
−2
−3
Standardized expressions
EI PG G1 G2 H T1 T2 C1 C2 S EP JP
Developmental stages
VIT_07s0005g00320
0.5
0.0
−0.5
−1.0
−1.5
−2.0
Standardized expressions
EI PG G1 G2 H T1 T2 C1 C2 S EP JP
Developmental stages
VIT_07s0005g00330
0
−1
−2
Standardized expressions
EI PG G1 G2 H T1 T2 C1 C2 S EP JP
Developmental stages
VIT_07s0005g00340
0.5
0.0
−0.5
Standardized expressions
EI PG G1 G2 H T1 T2 C1 C2 S EP JP
Developmental stages

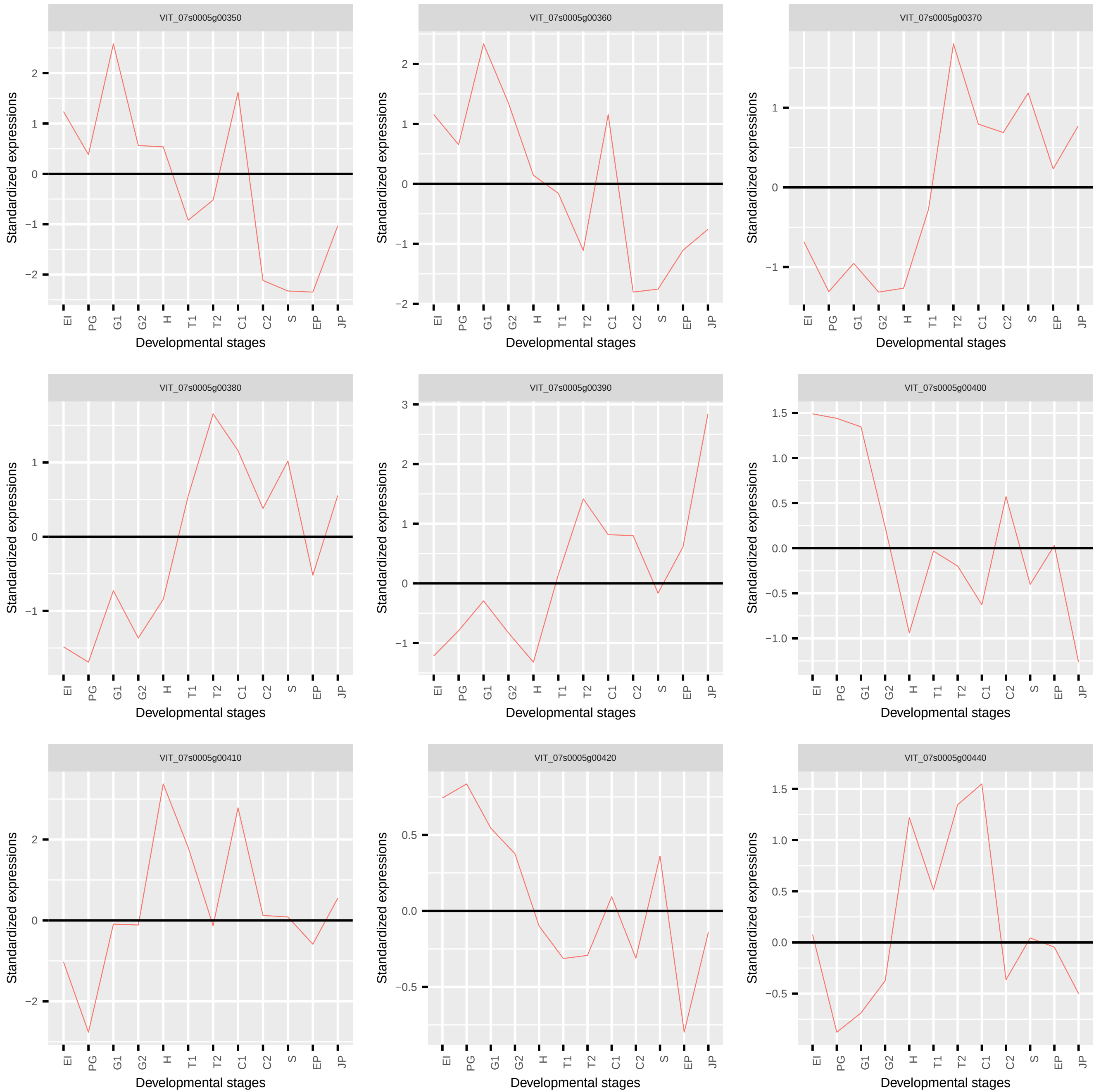
VIT_07s0005g00350
VIT_07s0005g00360
VIT_07s0005g00370
VIT_07s0005g00380
VIT_07s0005g00390
VIT_07s0005g00400
VIT_07s0005g00410
VIT_07s0005g00420
VIT_07s0005g00440
Standardized expressions
Developmental stages
EI PG G1 G2 H T1 T2 C1 C2 S EP JP

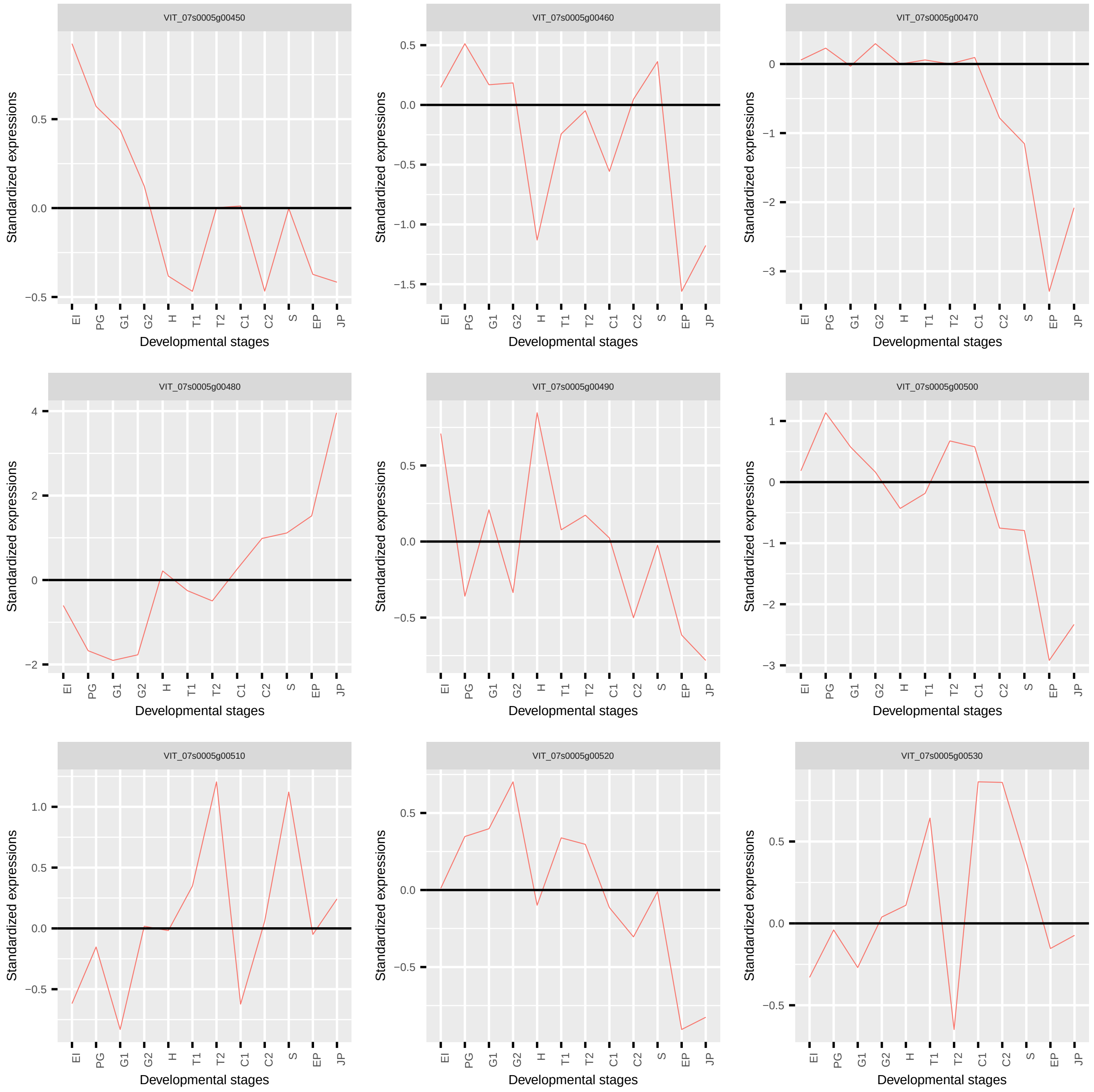
VIT_07s0005g00450
VIT_07s0005g00460
VIT_07s0005g00470
VIT_07s0005g00480
VIT_07s0005g00490
VIT_07s0005g00500
VIT_07s0005g00510
VIT_07s0005g00520
VIT_07s0005g00530
Standardized expressions
Developmental stages
EI
PG
G1
G2
H
T1
T2
C1
C2
S
EP
JP

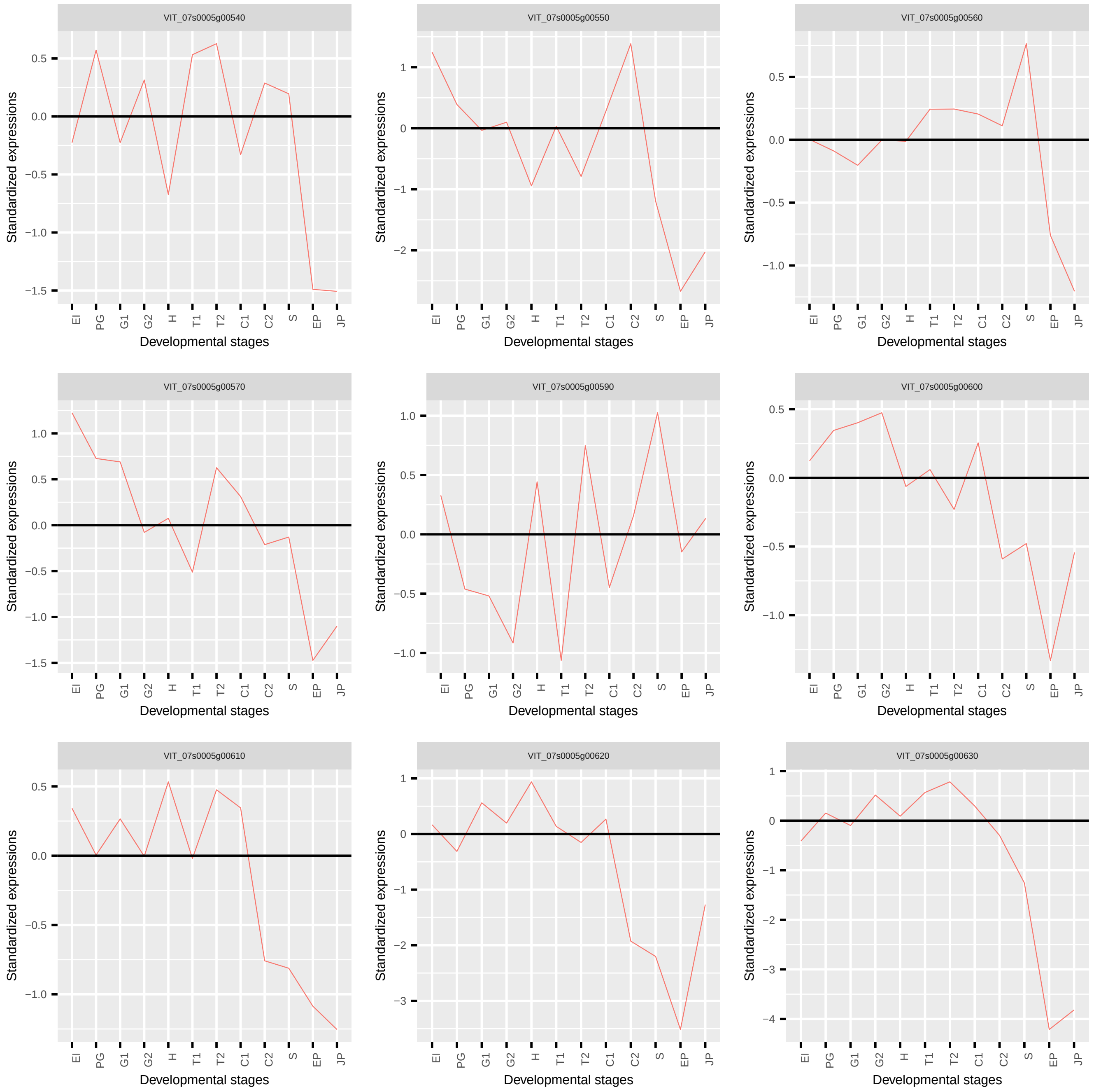
VIT_07s0005g00540
VIT_07s0005g00550
VIT_07s0005g00560
VIT_07s0005g00570
VIT_07s0005g00590
VIT_07s0005g00600
VIT_07s0005g00610
VIT_07s0005g00620
VIT_07s0005g00630
Standardized expressions
Developmental stages
EI PG G1 G2 H T1 T2 C1 C2 S EP JP

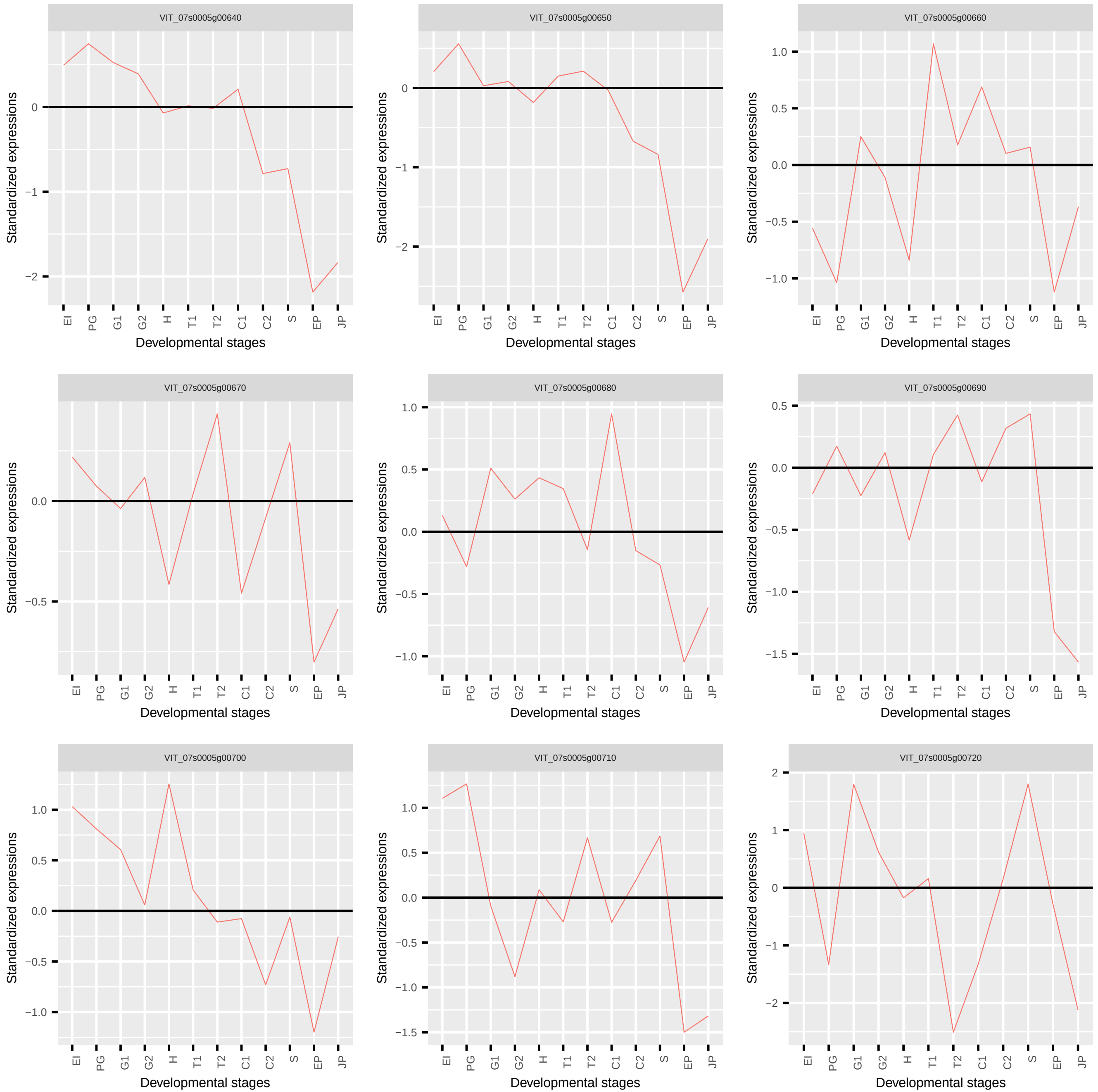
VIT_07s0005g00640
VIT_07s0005g00650
VIT_07s0005g00660
VIT_07s0005g00670
VIT_07s0005g00680
VIT_07s0005g00690
VIT_07s0005g00700
VIT_07s0005g00710
VIT_07s0005g00720
Standardized expressions
Developmental stages
EI PG G1 G2 H T1 T2 C1 C2 S EP JP

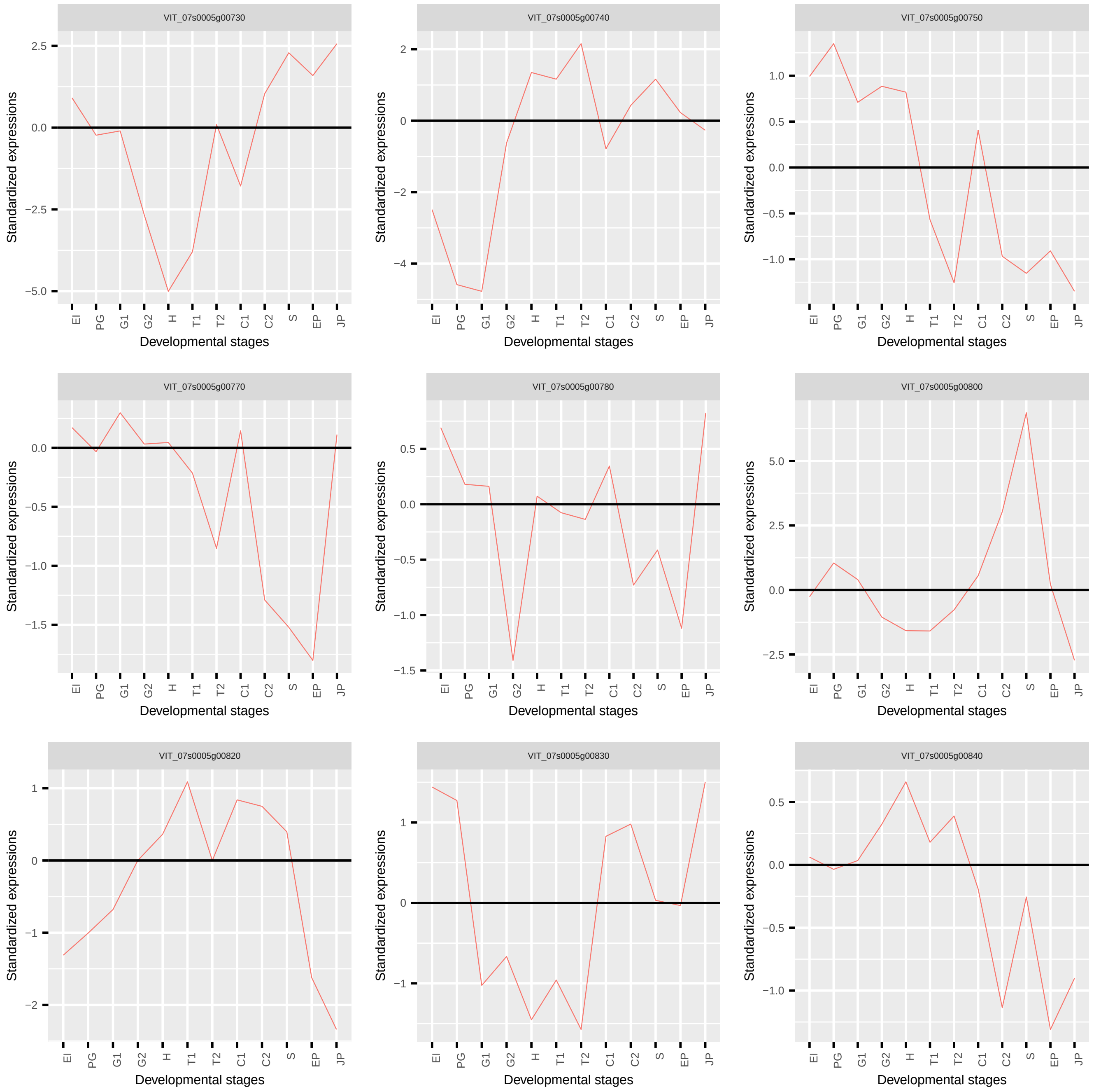
VIT_07s0005g00730
VIT_07s0005g00740
VIT_07s0005g00750
VIT_07s0005g00770
VIT_07s0005g00780
VIT_07s0005g00800
VIT_07s0005g00820
VIT_07s0005g00830
VIT_07s0005g00840
Standardized expressions
Developmental stages
EI PG G1 G2 H T1 T2 C1 C2 S EP JP

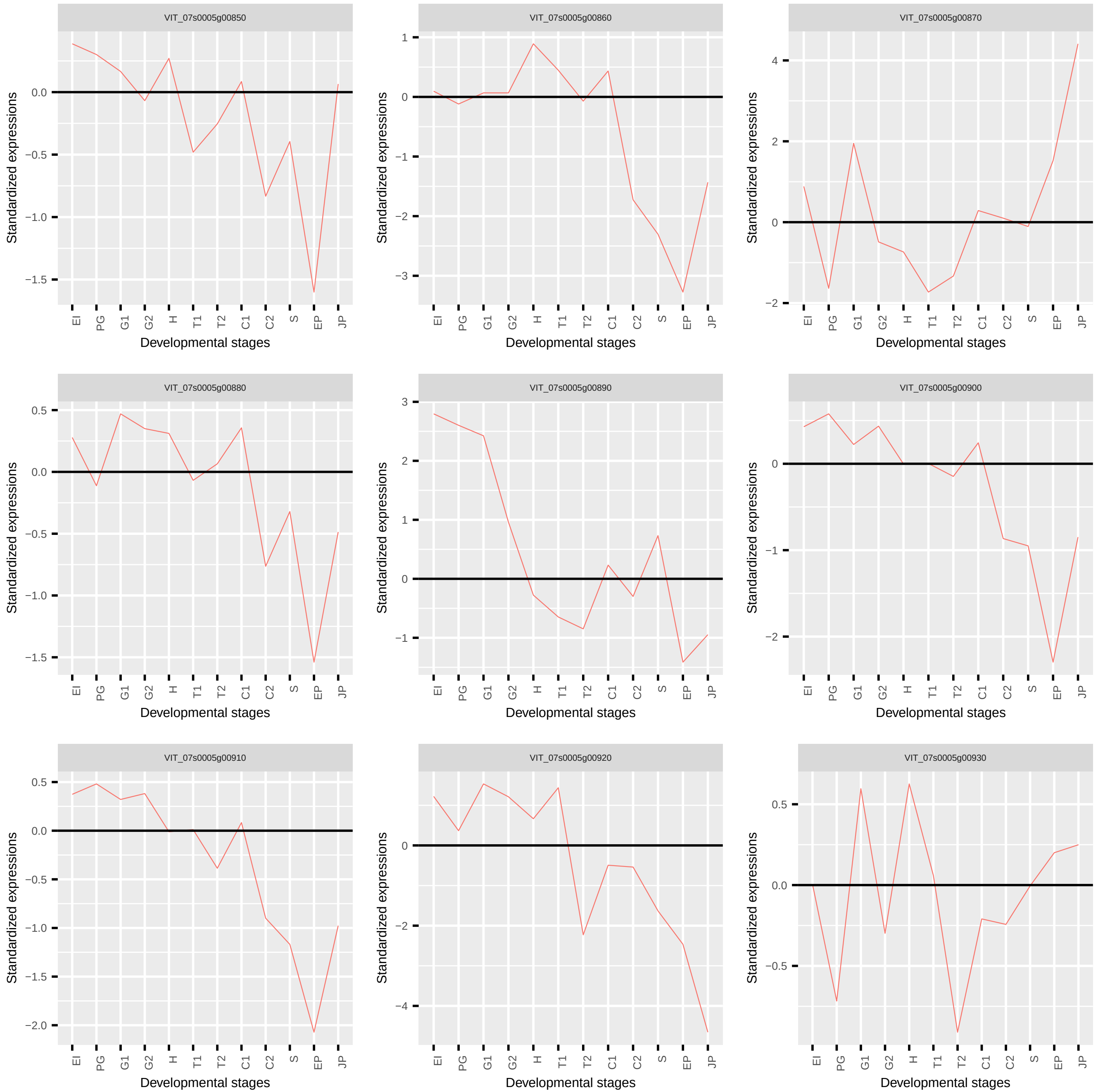
VIT_07s0005g00850
VIT_07s0005g00860
VIT_07s0005g00870
VIT_07s0005g00880
VIT_07s0005g00890
VIT_07s0005g00900
VIT_07s0005g00910
VIT_07s0005g00920
VIT_07s0005g00930
Standardized expressions
Developmental stages
EI PG G1 G2 H T1 T2 C1 C2 S EP JP

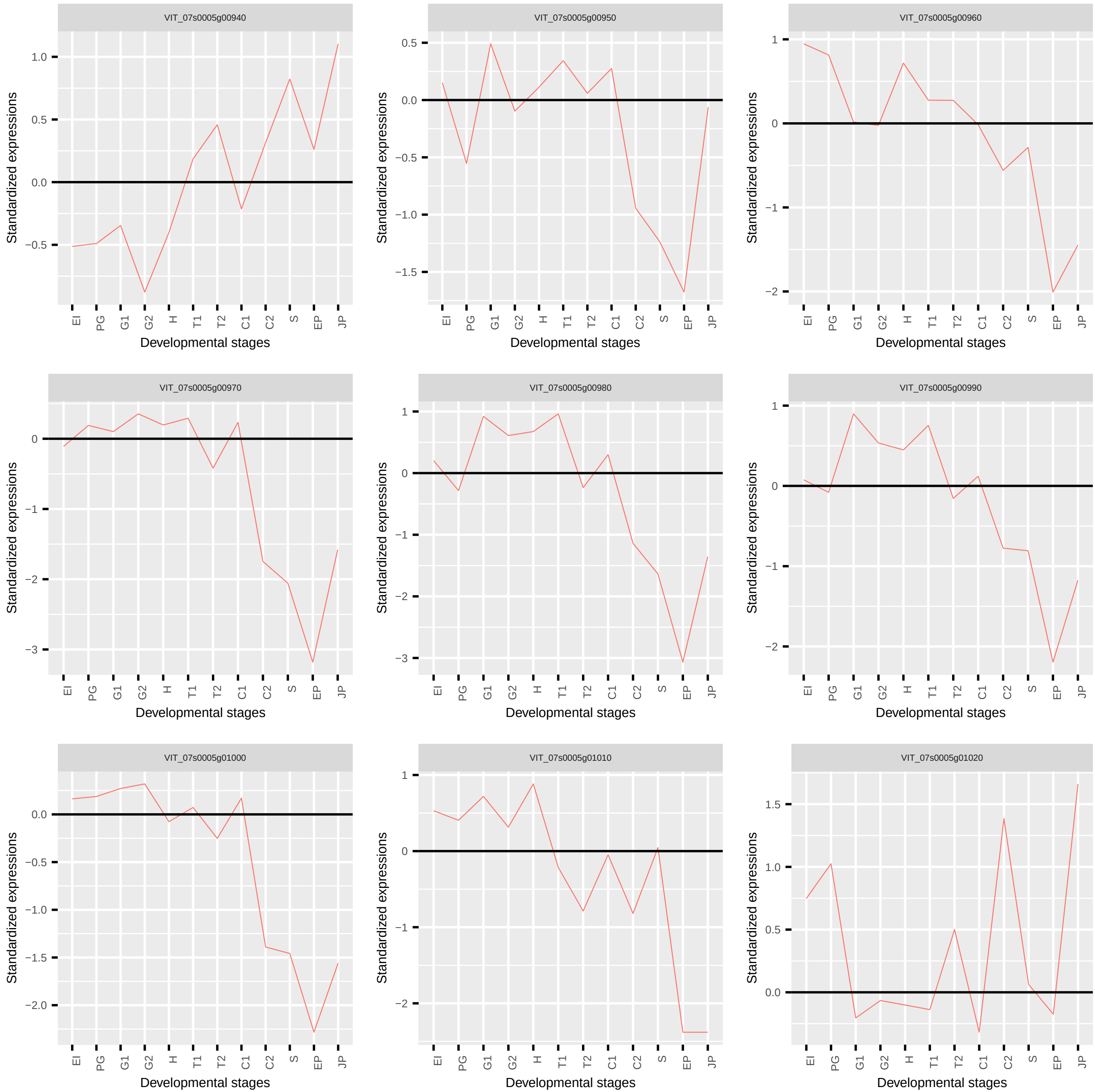

VIT_07s0005g00940
VIT_07s0005g00950
VIT_07s0005g00960
VIT_07s0005g00970
VIT_07s0005g00980
VIT_07s0005g00990
VIT_07s0005g01000
VIT_07s0005g01010
VIT_07s0005g01020
Standardized expressions
Developmental stages
EI
PG
G1
G2
H
T1
T2
C1
C2
S
EP
JP

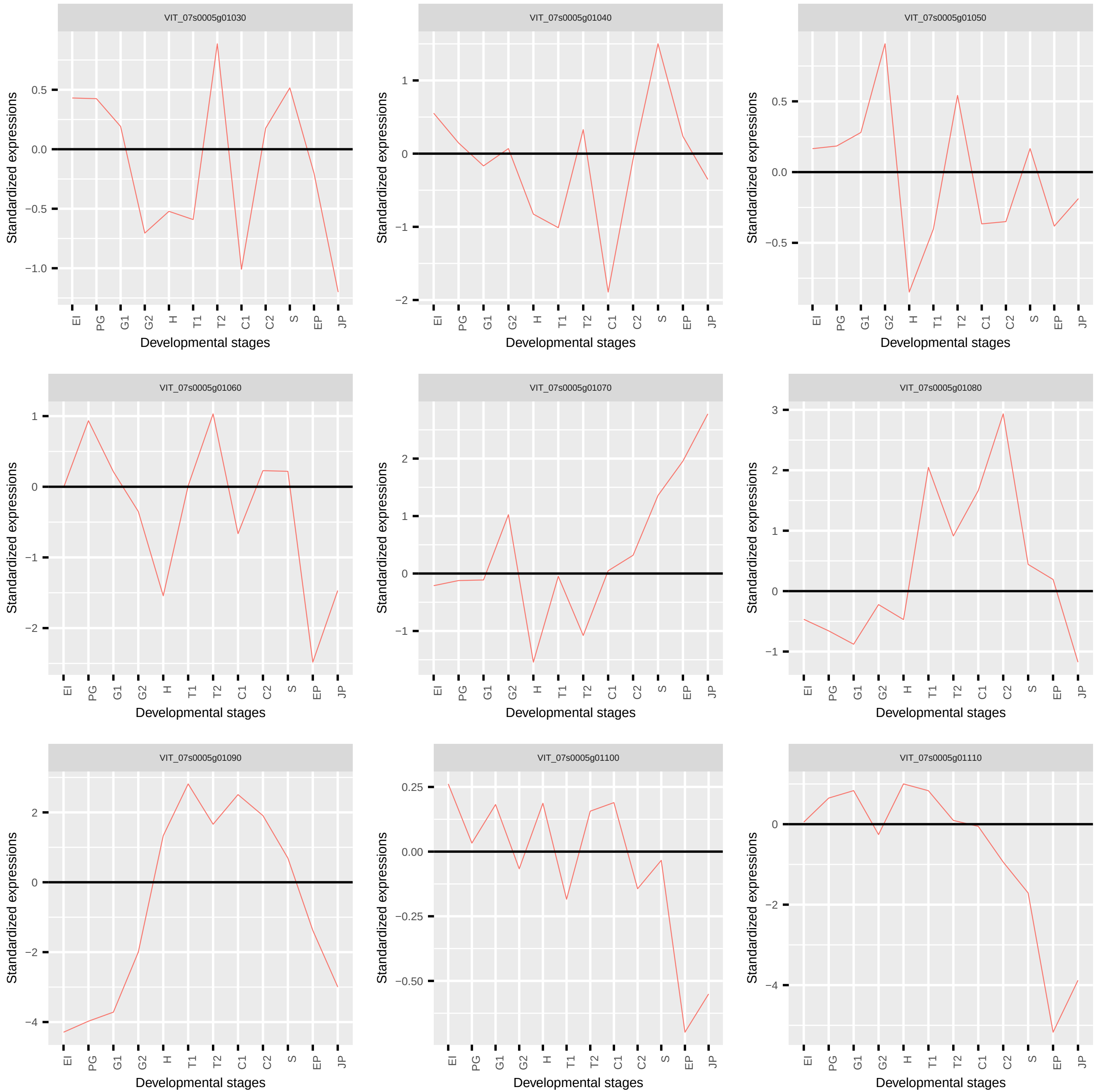
VIT_07s0005g01030
VIT_07s0005g01040
VIT_07s0005g01050
VIT_07s0005g01060
VIT_07s0005g01070
VIT_07s0005g01080
VIT_07s0005g01090
VIT_07s0005g01100
VIT_07s0005g01110
Standardized expressions
Developmental stages
EI PG G1 G2 H T1 T2 C1 C2 S EP JP

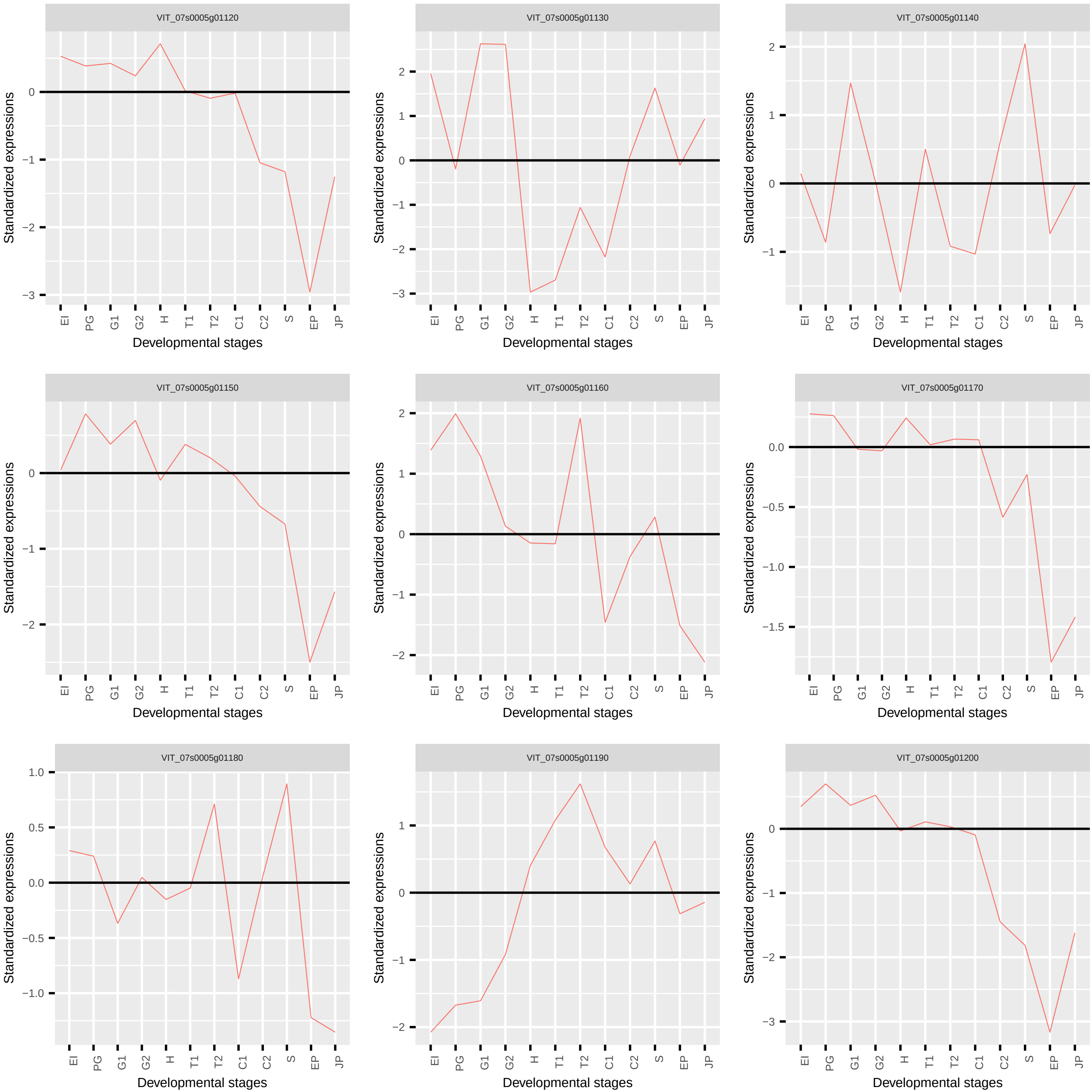
VIT_07s0005g01120
VIT_07s0005g01130
VIT_07s0005g01140
VIT_07s0005g01150
VIT_07s0005g01160
VIT_07s0005g01170
VIT_07s0005g01180
VIT_07s0005g01190
VIT_07s0005g01200
Standardized expressions
Developmental stages
EI PG G1 G2 H T1 T2 C1 C2 S EP JP

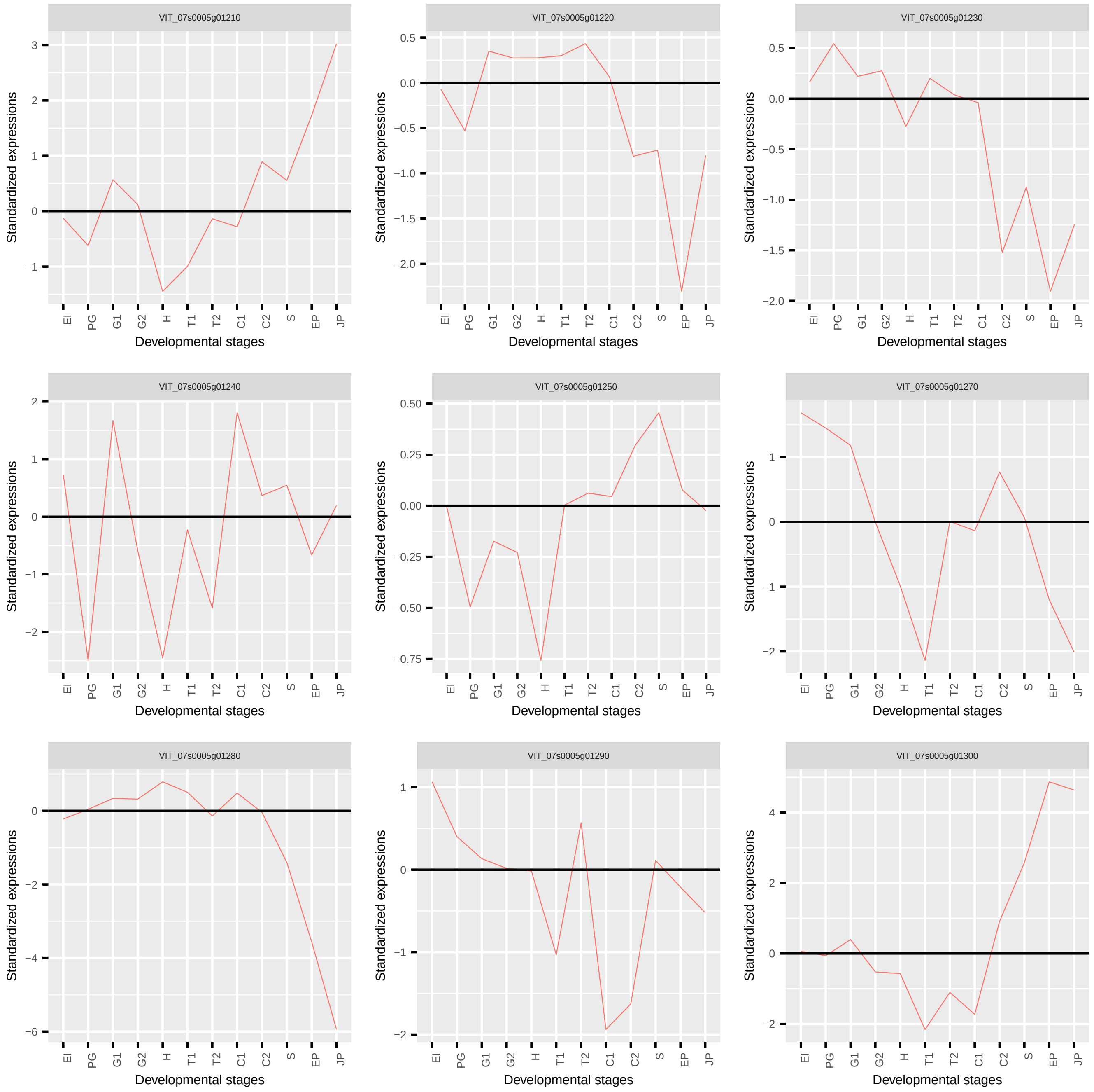

VIT_07s0005g01210
VIT_07s0005g01220
VIT_07s0005g01230
VIT_07s0005g01240
VIT_07s0005g01250
VIT_07s0005g01270
VIT_07s0005g01280
VIT_07s0005g01290
VIT_07s0005g01300
Standardized expressions
Developmental stages
EI PG G1 G2 H T1 T2 C1 C2 S EP JP

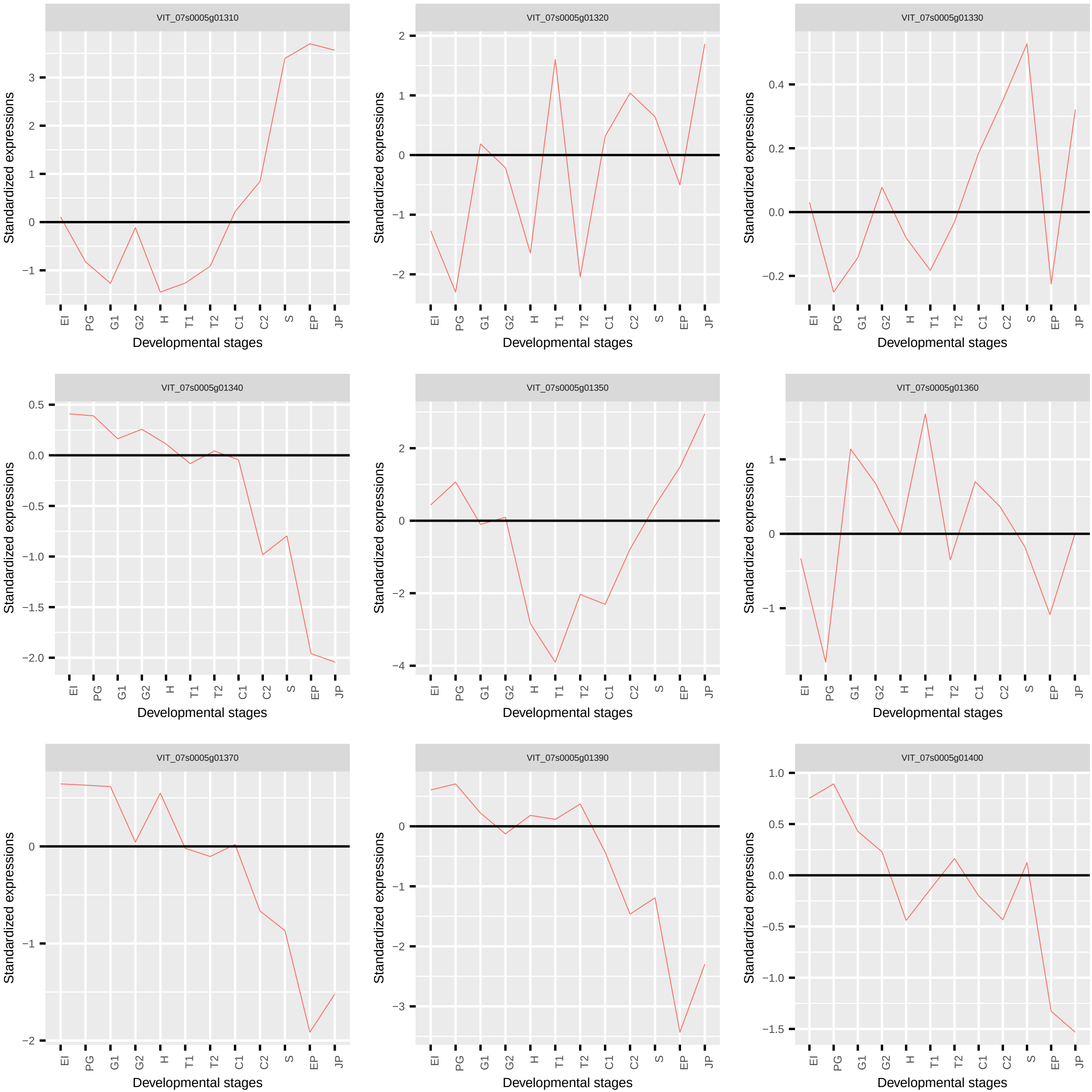
VIT_07s0005g01310
VIT_07s0005g01320
VIT_07s0005g01330
VIT_07s0005g01340
VIT_07s0005g01350
VIT_07s0005g01360
VIT_07s0005g01370
VIT_07s0005g01390
VIT_07s0005g01400
Standardized expressions
Developmental stages
EI PG G1 G2 H T1 T2 C1 C2 S EP JP

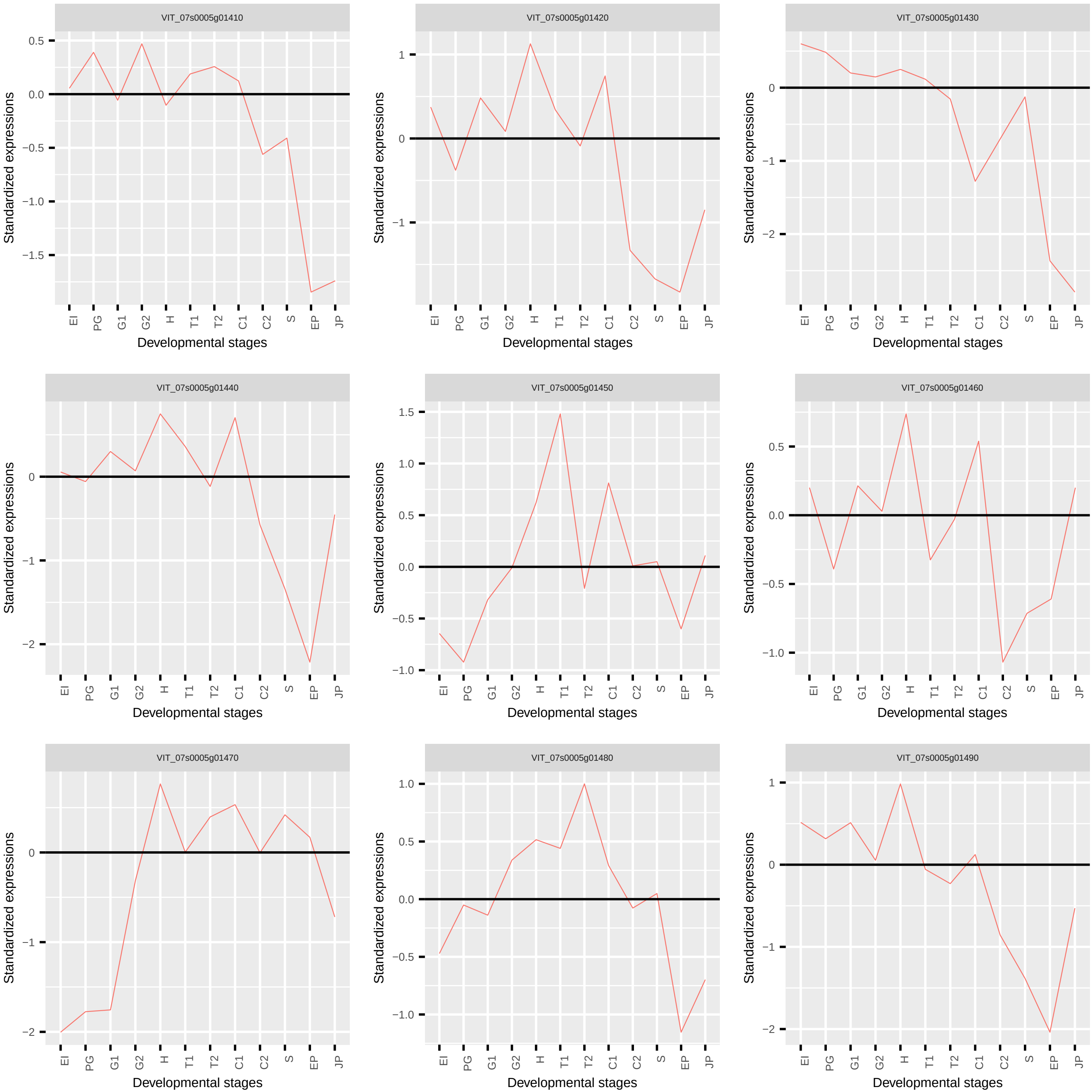
VIT_07s0005g01410
VIT_07s0005g01420
VIT_07s0005g01430
VIT_07s0005g01440
VIT_07s0005g01450
VIT_07s0005g01460
VIT_07s0005g01470
VIT_07s0005g01480
VIT_07s0005g01490
Standardized expressions
Developmental stages
EI PG G1 G2 H T1 T2 C1 C2 S EP JP
0.5
0.0
−0.5
−1.0
−1.5
1
0
−1
−2
1.5

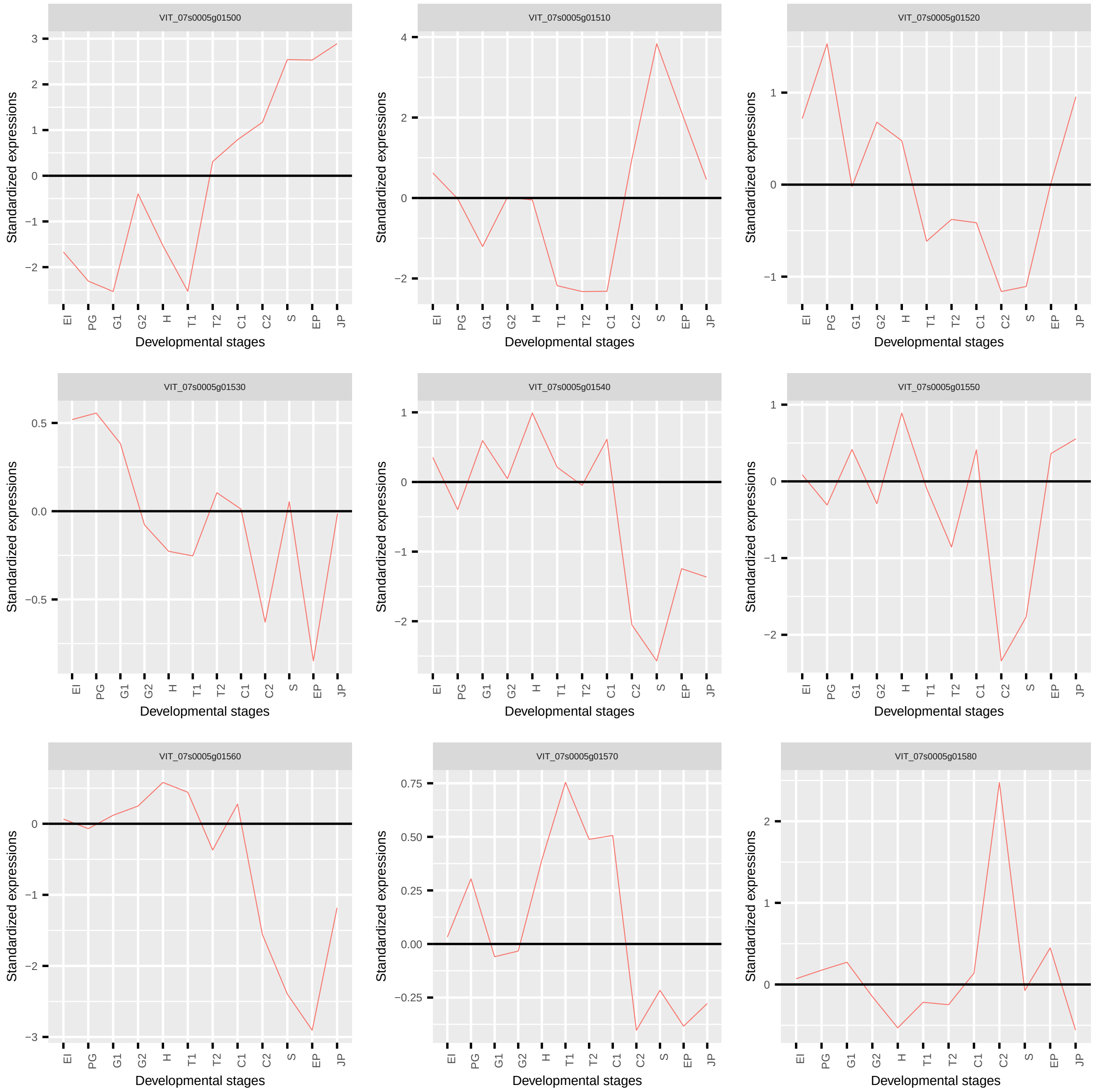
VIT_07s0005g01500
VIT_07s0005g01510
VIT_07s0005g01520
VIT_07s0005g01530
VIT_07s0005g01540
VIT_07s0005g01550
VIT_07s0005g01560
VIT_07s0005g01570
VIT_07s0005g01580
Standardized expressions
Developmental stages
EI PG G1 G2 H T1 T2 C1 C2 S EP JP

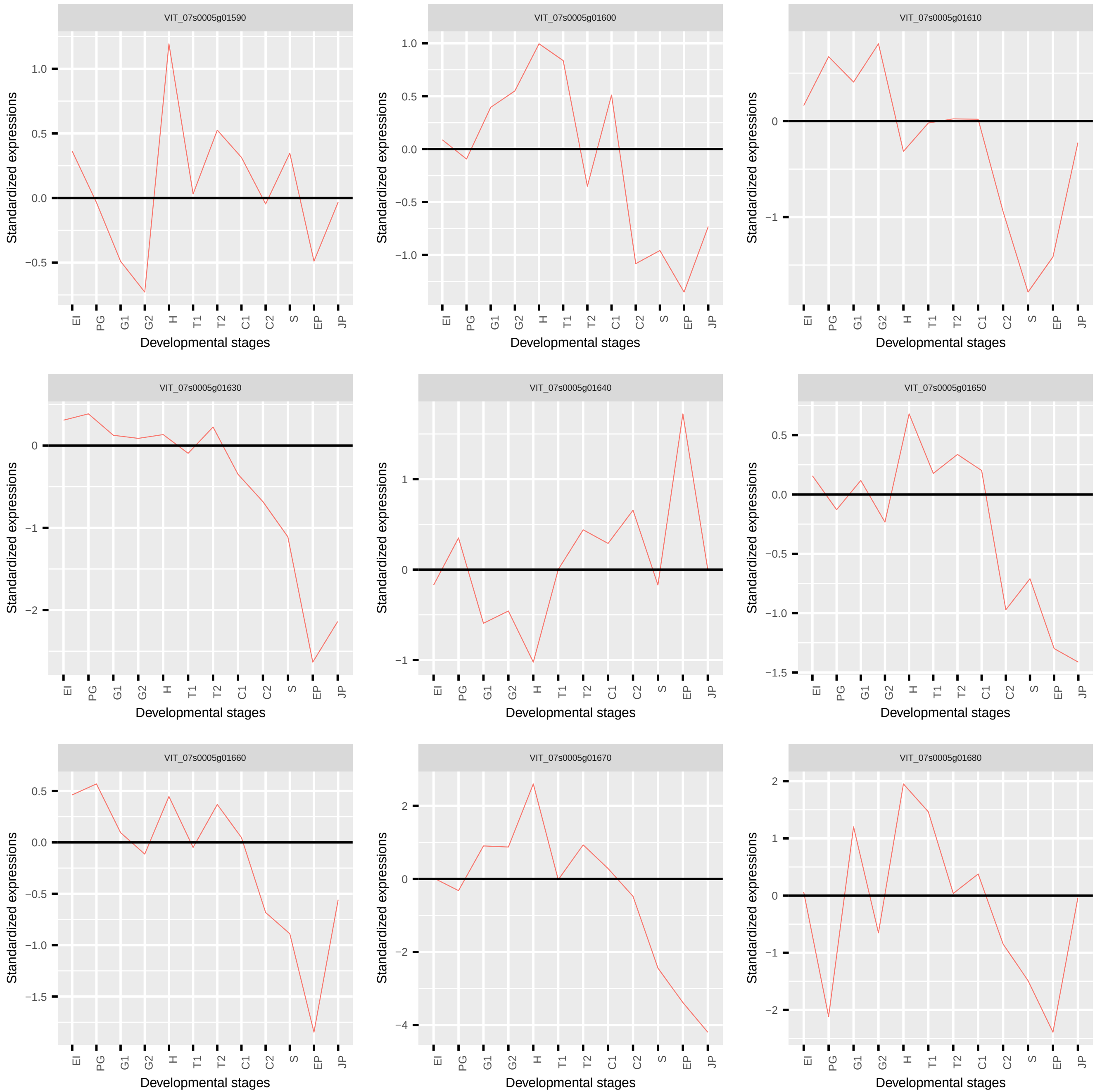
VIT_07s0005g01590
VIT_07s0005g01600
VIT_07s0005g01610
VIT_07s0005g01630
VIT_07s0005g01640
VIT_07s0005g01650
VIT_07s0005g01660
VIT_07s0005g01670
VIT_07s0005g01680
Standardized expressions
Developmental stages
EI PG G1 G2 H T1 T2 C1 C2 S EP JP

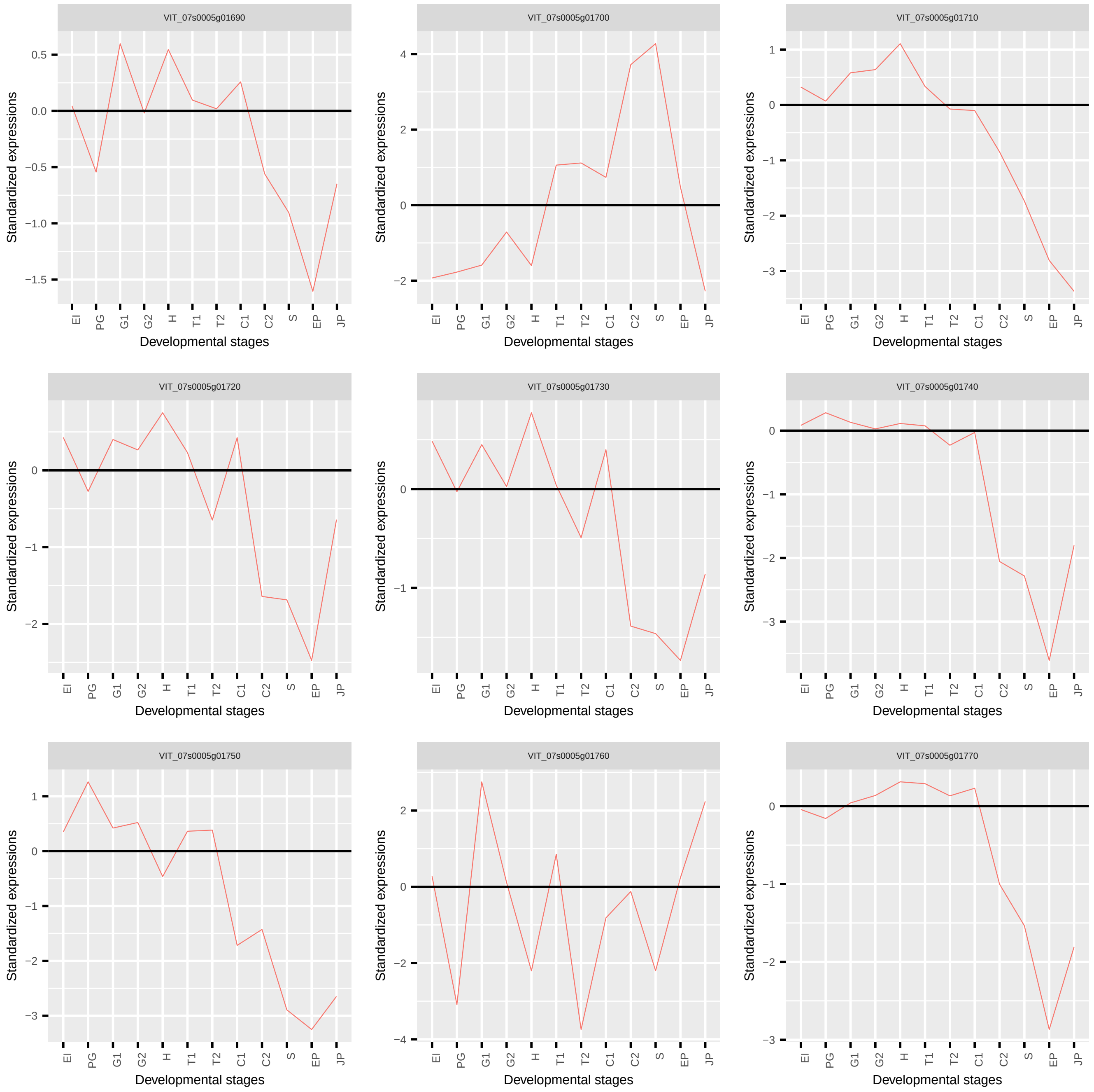

VIT_07s0005g01690
VIT_07s0005g01700
VIT_07s0005g01710
VIT_07s0005g01720
VIT_07s0005g01730
VIT_07s0005g01740
VIT_07s0005g01750
VIT_07s0005g01760
VIT_07s0005g01770
Standardized expressions
Developmental stages
EI PG G1 G2 H T1 T2 C1 C2 S EP JP
0.5 0.0 −0.5 −1.0 −1.5
4 2 0 −2
1 0 −1 −2 −3
0 −1 −2
0 −1
0 −1 −2 −3
1 0 −1 −2 −3
2 0 −2 −4
0 −1 −2 −3

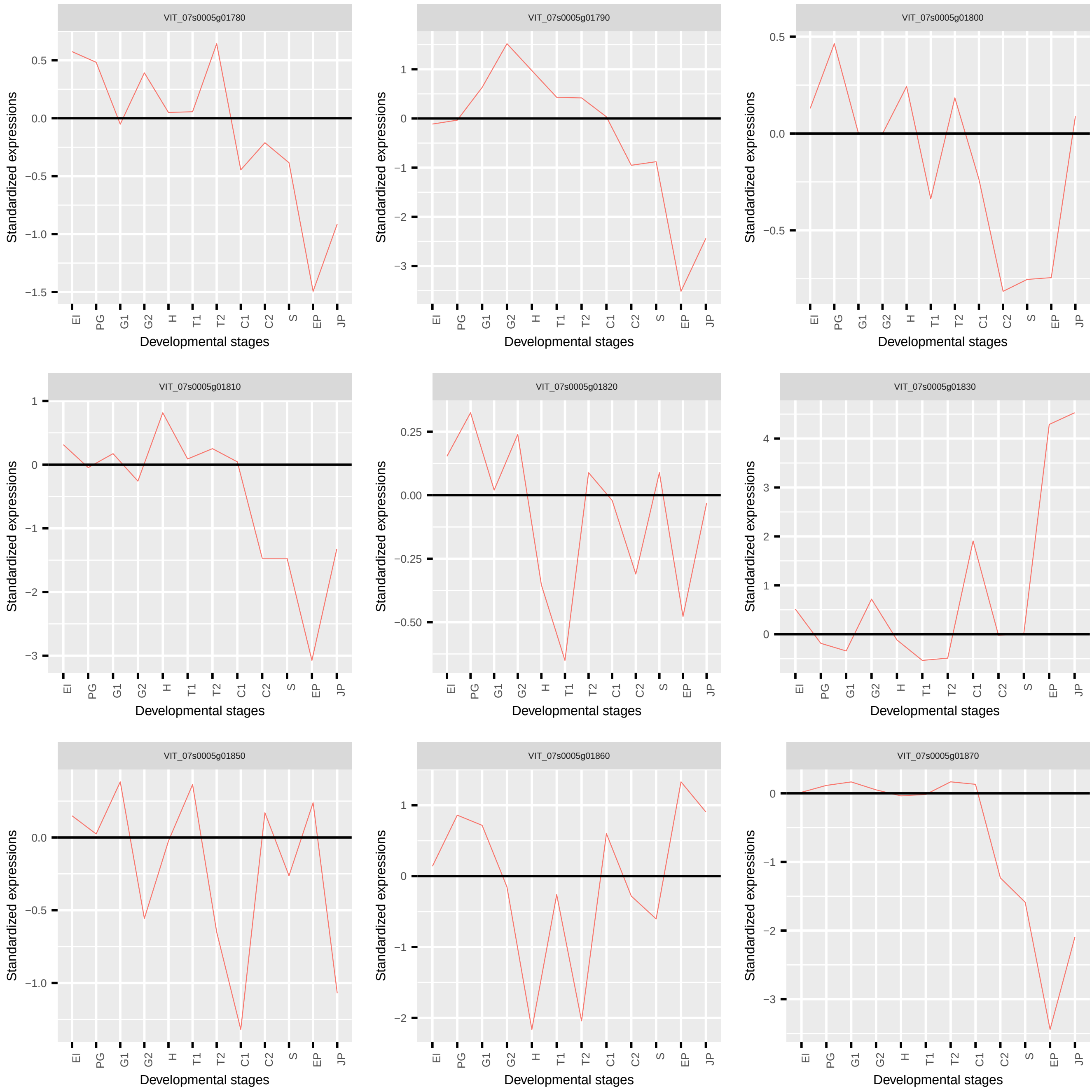

VIT_07s0005g01780
VIT_07s0005g01790
VIT_07s0005g01800
VIT_07s0005g01810
VIT_07s0005g01820
VIT_07s0005g01830
VIT_07s0005g01850
VIT_07s0005g01860
VIT_07s0005g01870
Standardized expressions
Developmental stages
EI
PG
G1
G2
H
T1
T2
C1
C2
S
EP
JP

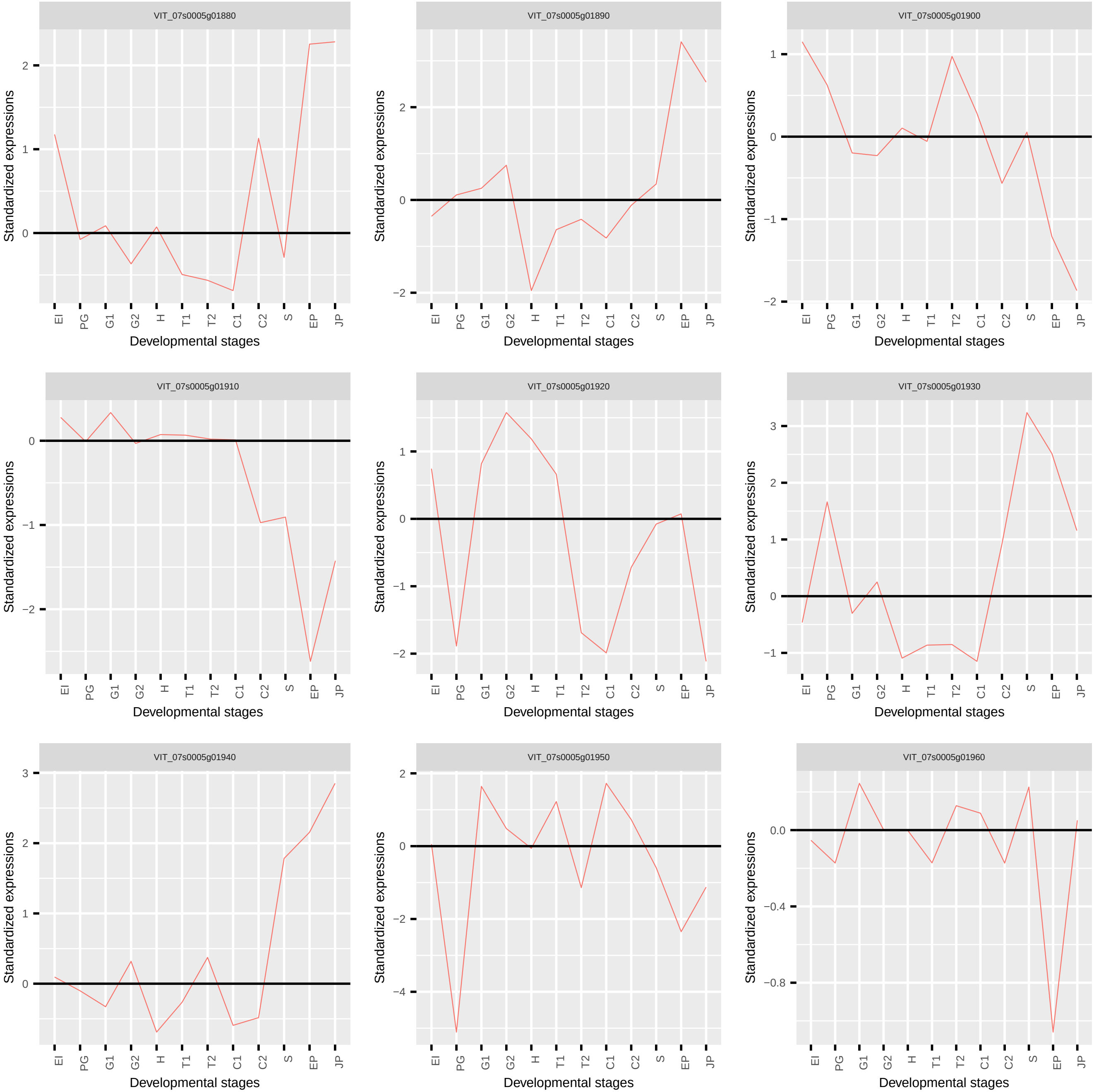
VIT_07s0005g01880
VIT_07s0005g01890
VIT_07s0005g01900
VIT_07s0005g01910
VIT_07s0005g01920
VIT_07s0005g01930
VIT_07s0005g01940
VIT_07s0005g01950
VIT_07s0005g01960
Standardized expressions
Developmental stages
EI PG G1 G2 H T1 T2 C1 C2 S EP JP

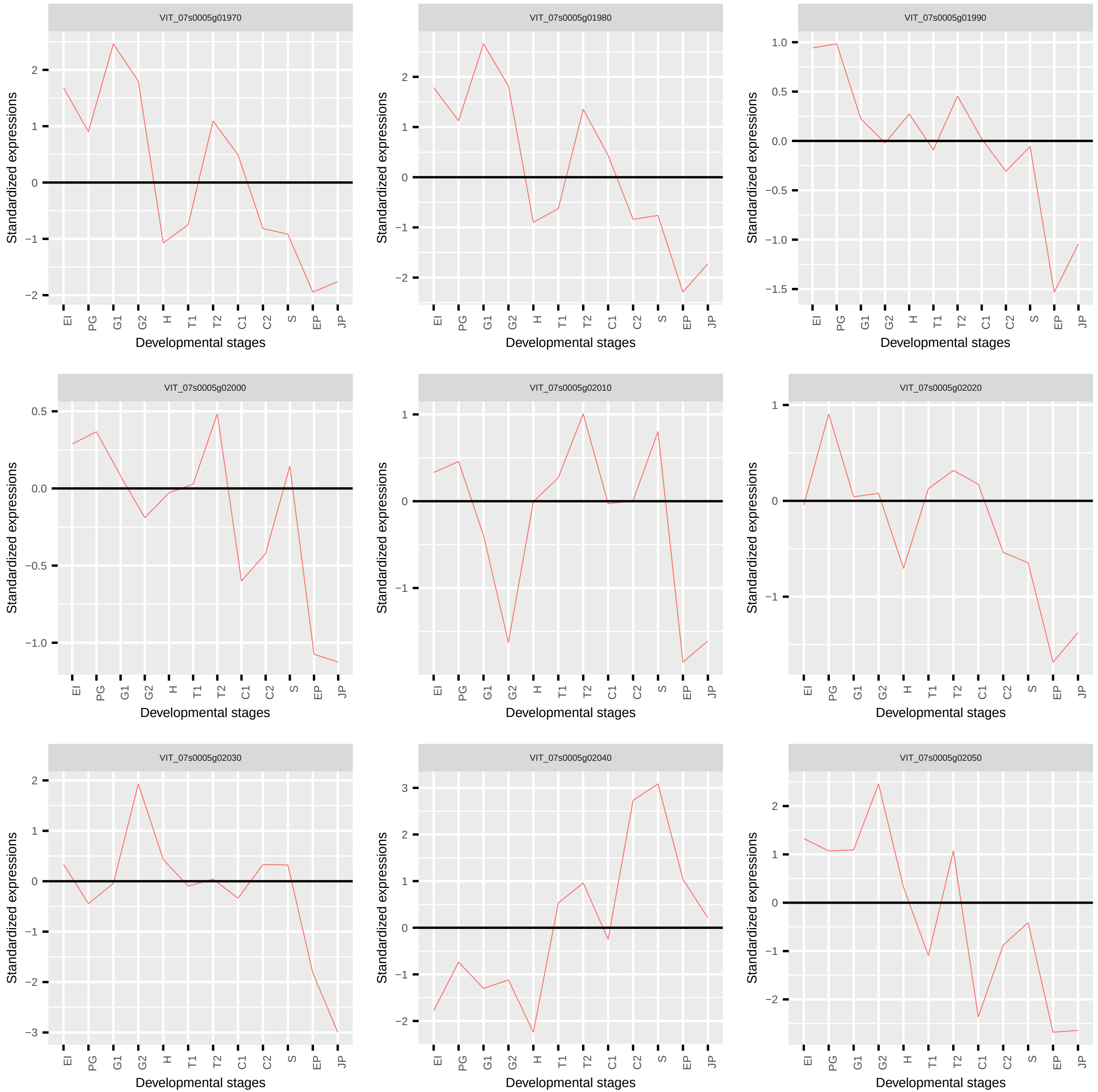
VIT_07s0005g01970
Standardized expressions
2
1
0
−1
−2
EI PG G1 G2 H T1 T2 C1 C2 S EP JP
Developmental stages
VIT_07s0005g01980
Standardized expressions
2
1
0
−1
−2
EI PG G1 G2 H T1 T2 C1 C2 S EP JP
Developmental stages
VIT_07s0005g01990
Standardized expressions
1.0
0.5
0.0
−0.5
−1.0
−1.5
EI PG G1 G2 H T1 T2 C1 C2 S EP JP
Developmental stages
VIT_07s0005g02000
Standardized expressions
0.5
0.0
−0.5
−1.0
EI PG G1 G2 H T1 T2 C1 C2 S EP JP
Developmental stages
VIT_07s0005g02010
Standardized expressions
1
0
−1
EI PG G1 G2 H T1 T2 C1 C2 S EP JP
Developmental stages
VIT_07s0005g02020
Standardized expressions
1
0
−1
EI PG G1 G2 H T1 T2 C1 C2 S EP JP
Developmental stages
VIT_07s0005g02030
Standardized expressions
2
1
0
−1
−2
−3
EI PG G1 G2 H T1 T2 C1 C2 S EP JP
Developmental stages
VIT_07s0005g02040
Standardized expressions
3
2
1
0
−1
−2
EI PG G1 G2 H T1 T2 C1 C2 S EP JP
Developmental stages
VIT_07s0005g02050
Standardized expressions
2
1
0
−1
−2
EI PG G1 G2 H T1 T2 C1 C2 S EP JP
Developmental stages

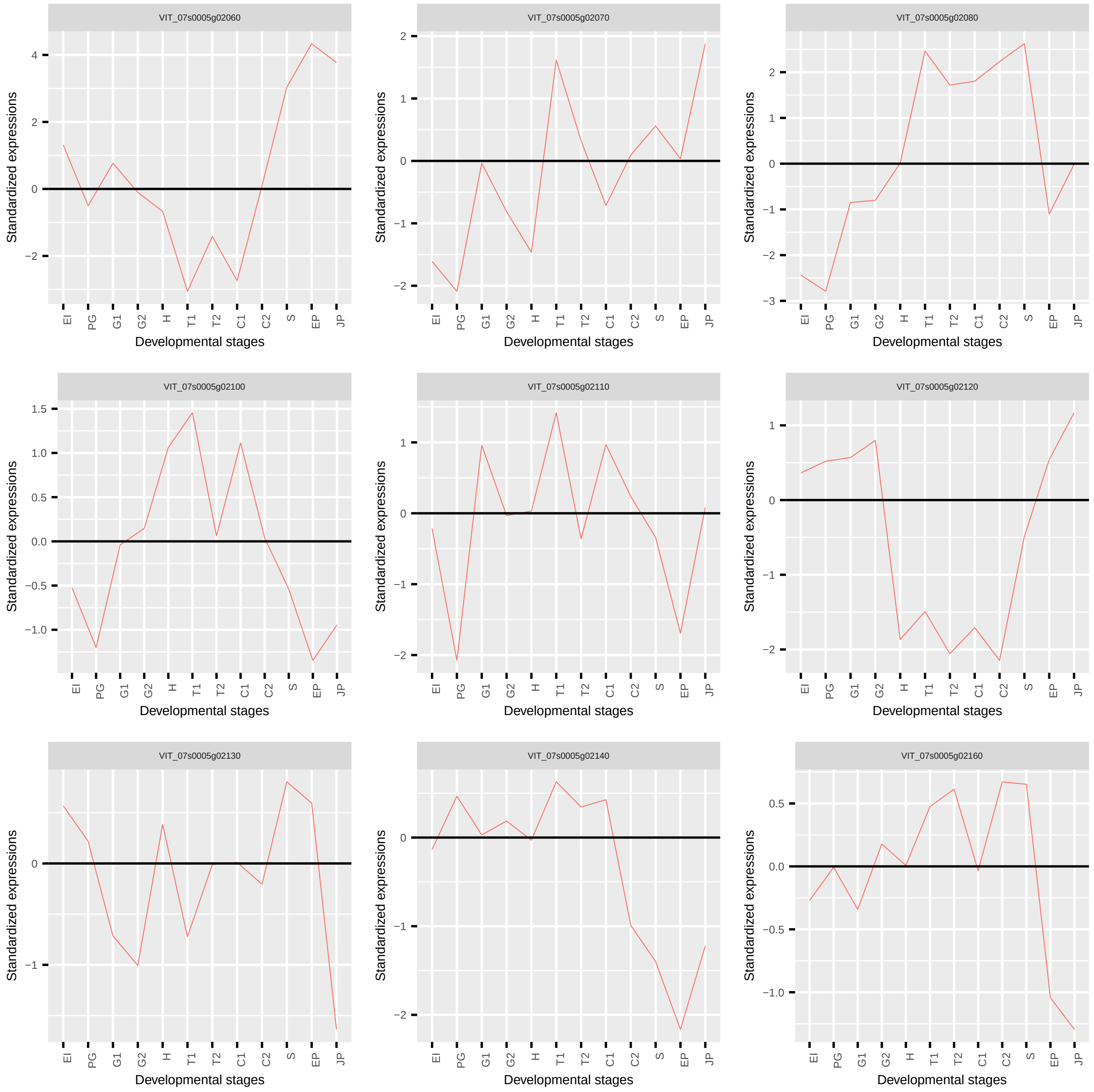

VIT_07s0005g02060
VIT_07s0005g02070
VIT_07s0005g02080
VIT_07s0005g02100
VIT_07s0005g02110
VIT_07s0005g02120
VIT_07s0005g02130
VIT_07s0005g02140
VIT_07s0005g02160
Standardized expressions
Developmental stages
EI PG G1 G2 H T1 T2 C1 C2 S EP JP

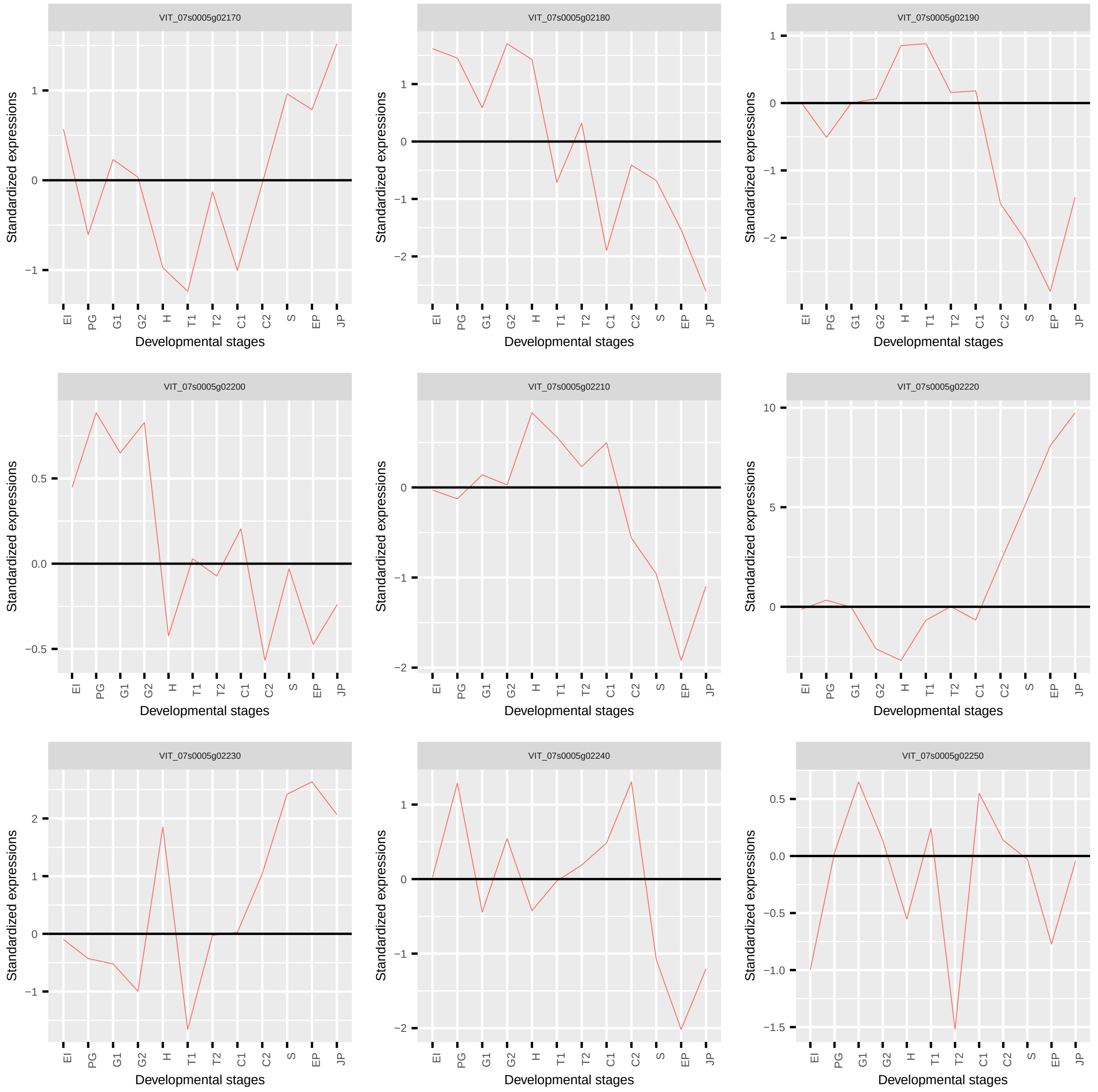
VIT_07s0005g02170
VIT_07s0005g02180
VIT_07s0005g02190
VIT_07s0005g02200
VIT_07s0005g02210
VIT_07s0005g02220
VIT_07s0005g02230
VIT_07s0005g02240
VIT_07s0005g02250
Standardized expressions
Developmental stages
EI PG G1 G2 H T1 T2 C1 C2 S EP JP

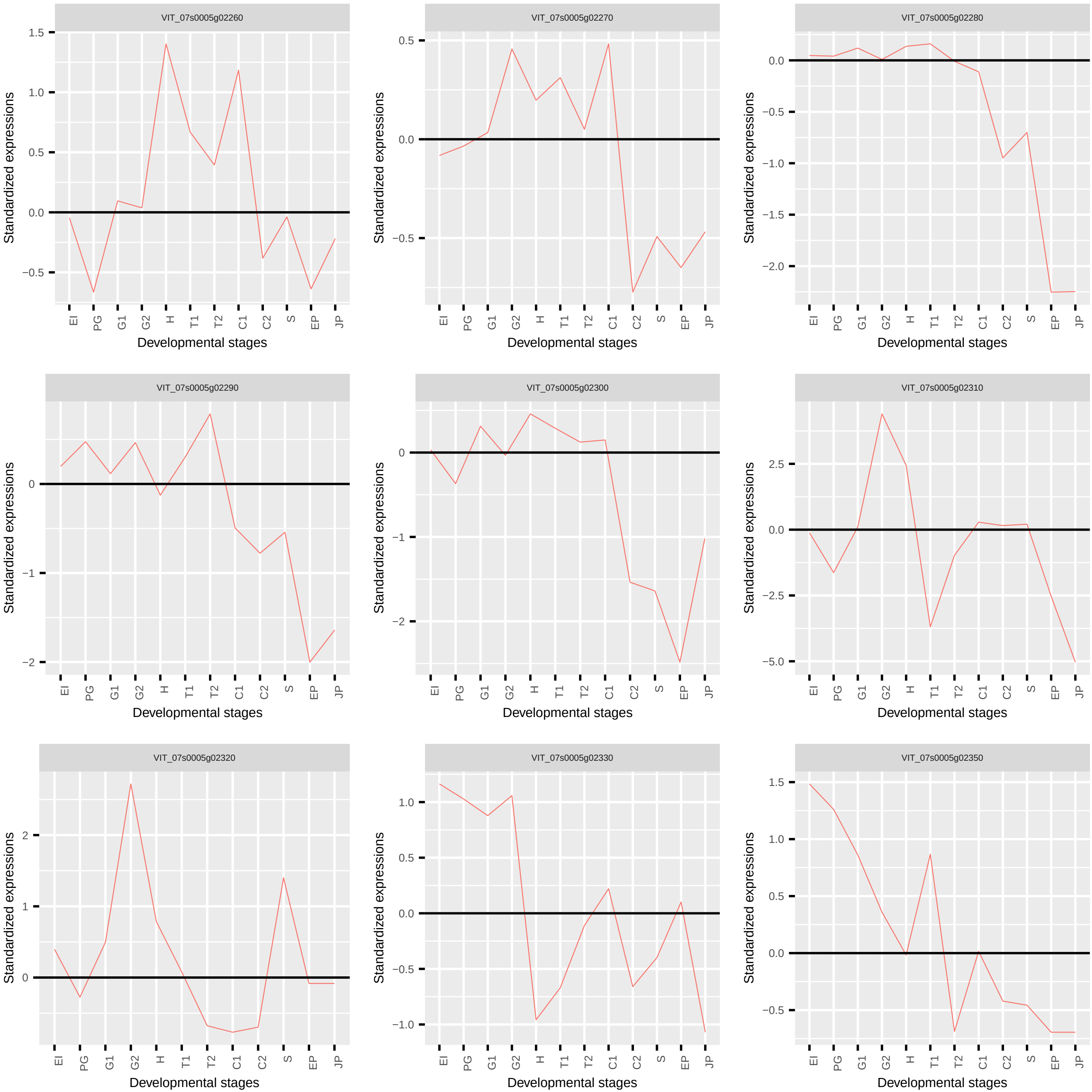
VIT_07s0005g02260
1.5
1.0
0.5
0.0
−0.5
VIT_07s0005g02270
0.5
0.0
−0.5
VIT_07s0005g02280
0.0
−0.5
−1.0
−1.5
−2.0
VIT_07s0005g02290
0
−1
−2
VIT_07s0005g02300
0
−1
−2
VIT_07s0005g02310
2.5
0.0
−2.5
−5.0
VIT_07s0005g02320
2
1
0
VIT_07s0005g02330
1.0
0.5
0.0
−0.5
−1.0
VIT_07s0005g02350
1.5
1.0
0.5
0.0
−0.5
Standardized expressions
EI PG G1 G2 H T1 T2 C1 C2 S EP JP
Developmental stages

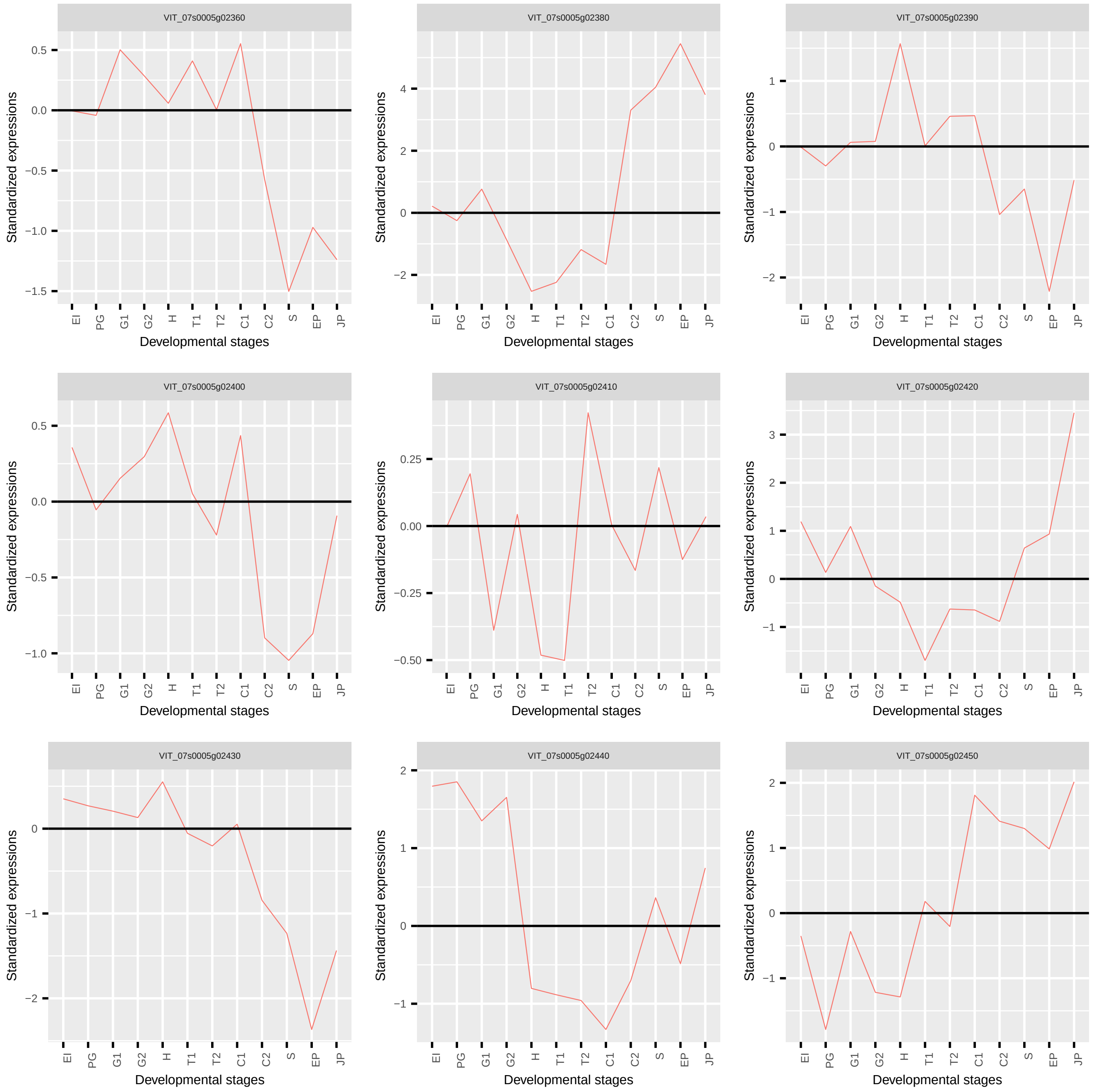
VIT_07s0005g02360
VIT_07s0005g02380
VIT_07s0005g02390
VIT_07s0005g02400
VIT_07s0005g02410
VIT_07s0005g02420
VIT_07s0005g02430
VIT_07s0005g02440
VIT_07s0005g02450
Standardized expressions
Developmental stages
EI PG G1 G2 H T1 T2 C1 C2 S EP JP

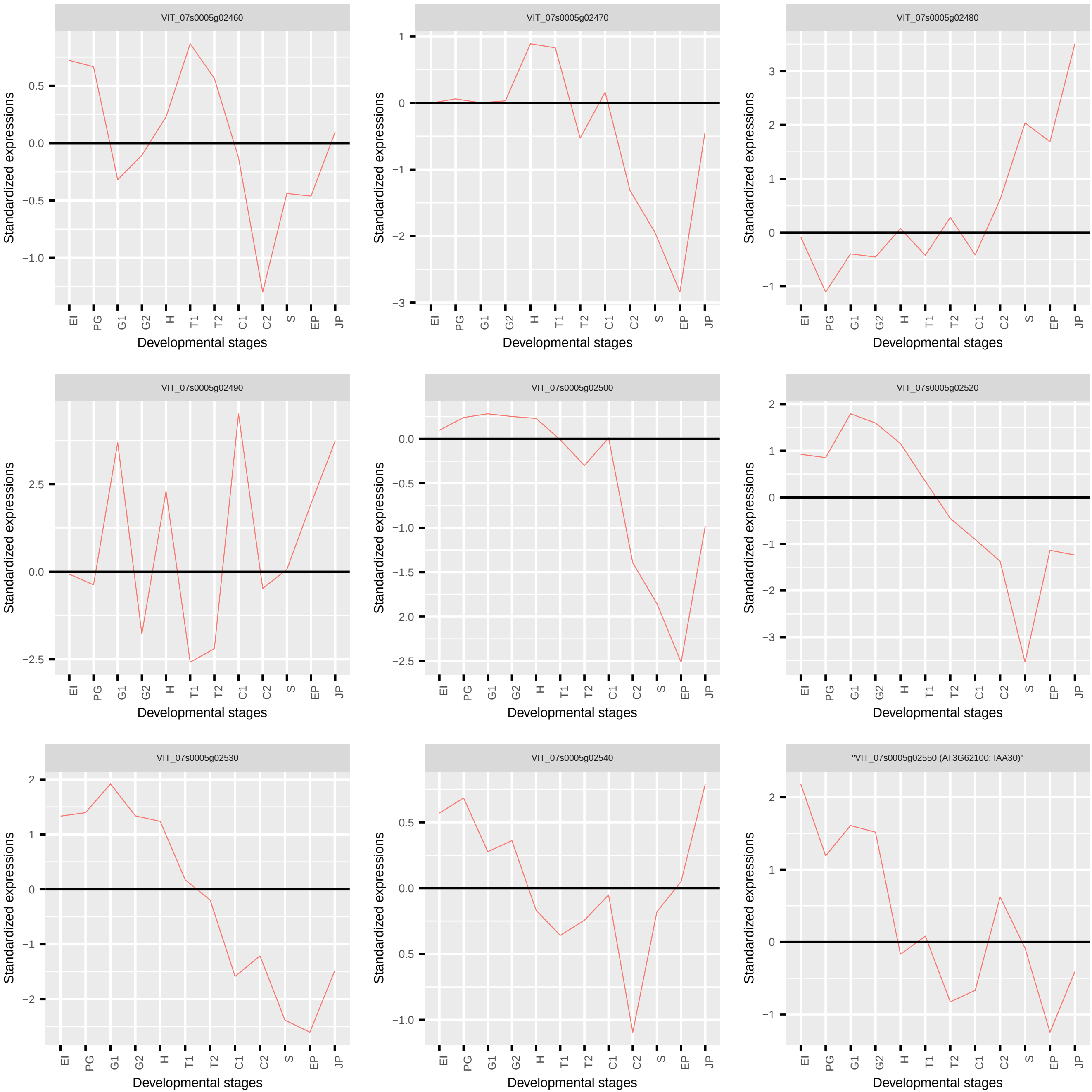
VIT_07s0005g02460
VIT_07s0005g02470
VIT_07s0005g02480
VIT_07s0005g02490
VIT_07s0005g02500
VIT_07s0005g02520
VIT_07s0005g02530
VIT_07s0005g02540
"VIT_07s0005g02550 (AT3G62100; IAA30)"
Standardized expressions
Developmental stages
EI
PG
G1
G2
H
T1
T2
C1
C2
S
EP
JP

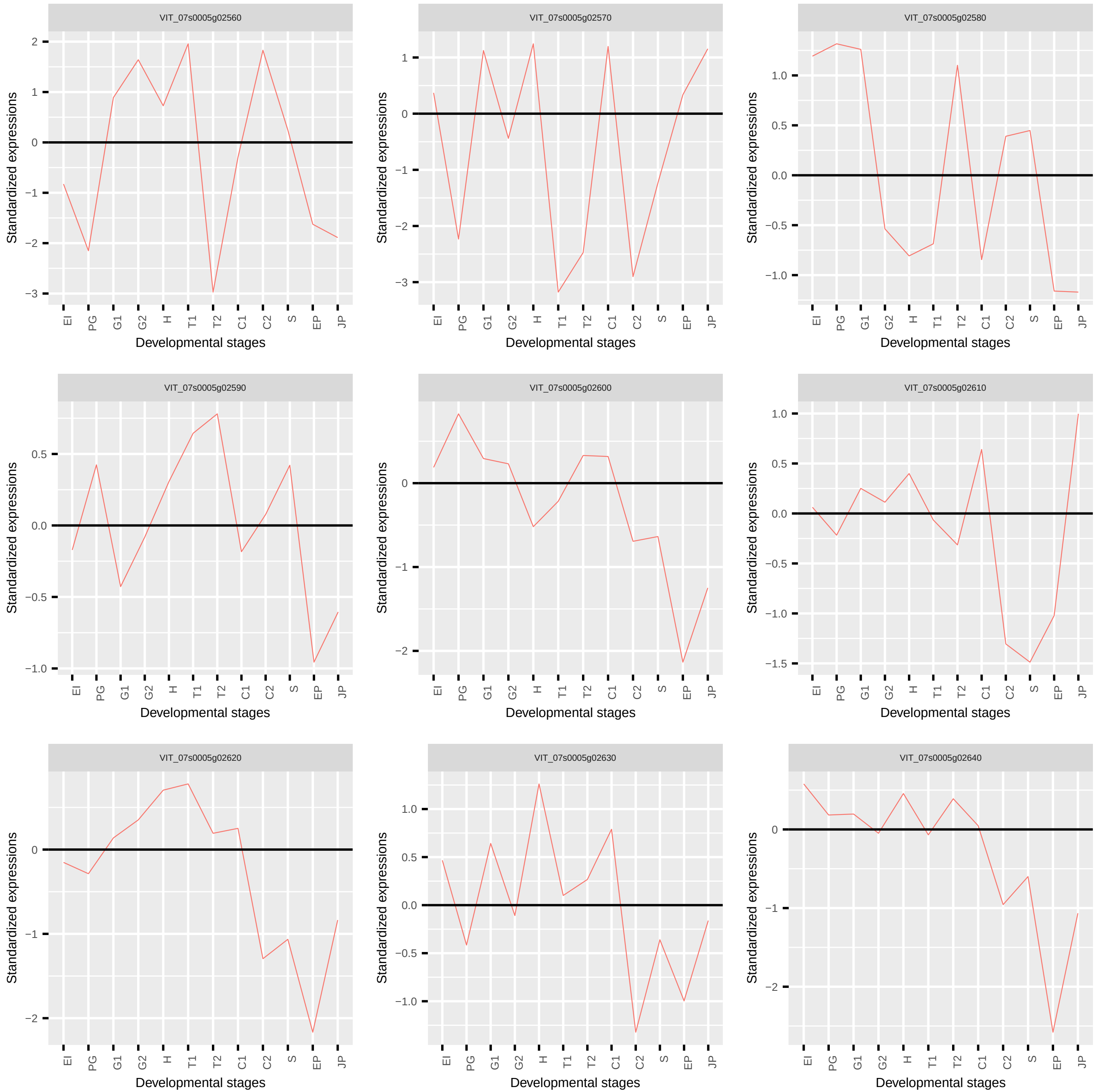
VIT_07s0005g02560
VIT_07s0005g02570
VIT_07s0005g02580
VIT_07s0005g02590
VIT_07s0005g02600
VIT_07s0005g02610
VIT_07s0005g02620
VIT_07s0005g02630
VIT_07s0005g02640
Standardized expressions
Developmental stages
EI PG G1 G2 H T1 T2 C1 C2 S EP JP

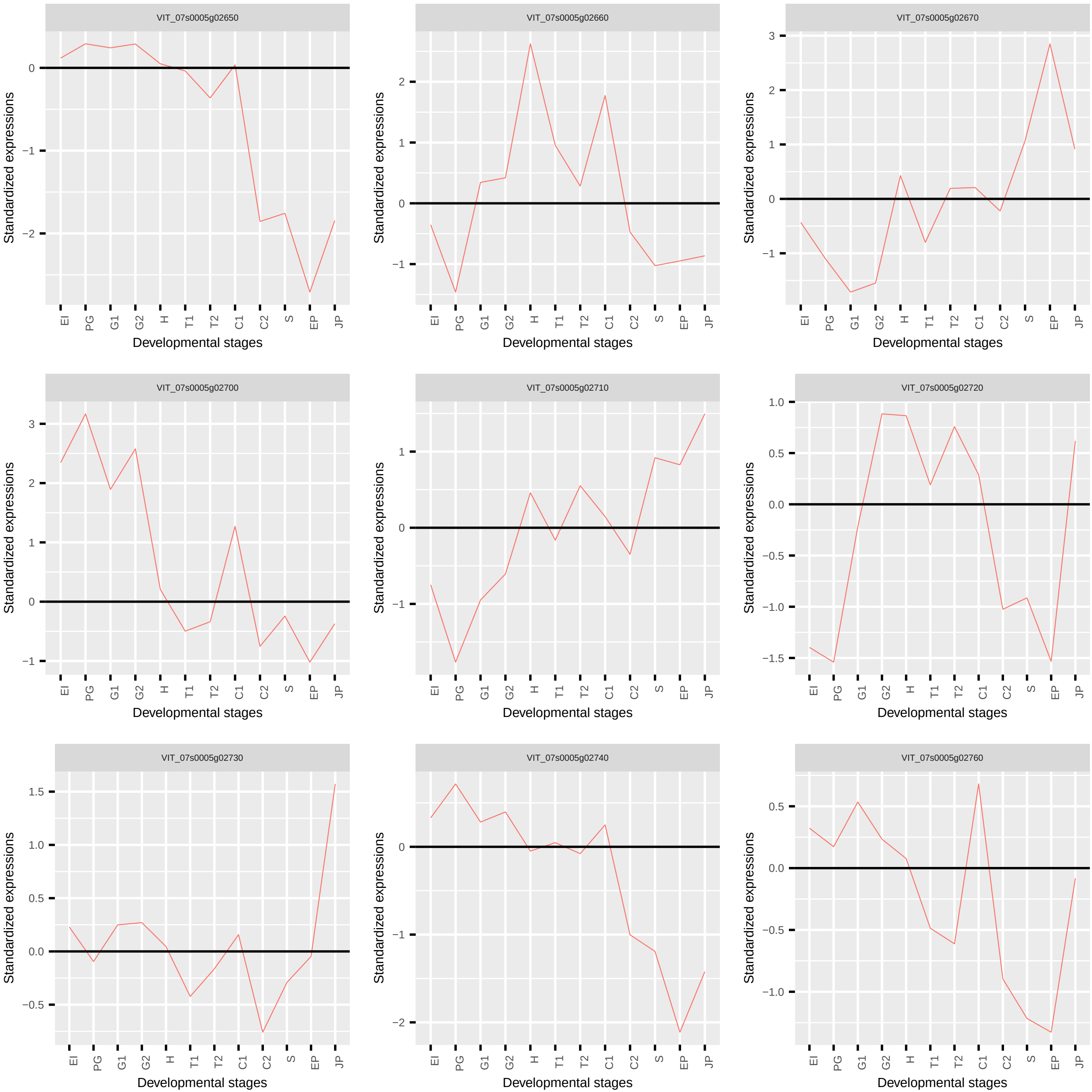
VIT_07s0005g02650
VIT_07s0005g02660
VIT_07s0005g02670
VIT_07s0005g02700
VIT_07s0005g02710
VIT_07s0005g02720
VIT_07s0005g02730
VIT_07s0005g02740
VIT_07s0005g02760
Standardized expressions
EI PG G1 G2 H T1 T2 C1 C2 S EP JP
Developmental stages

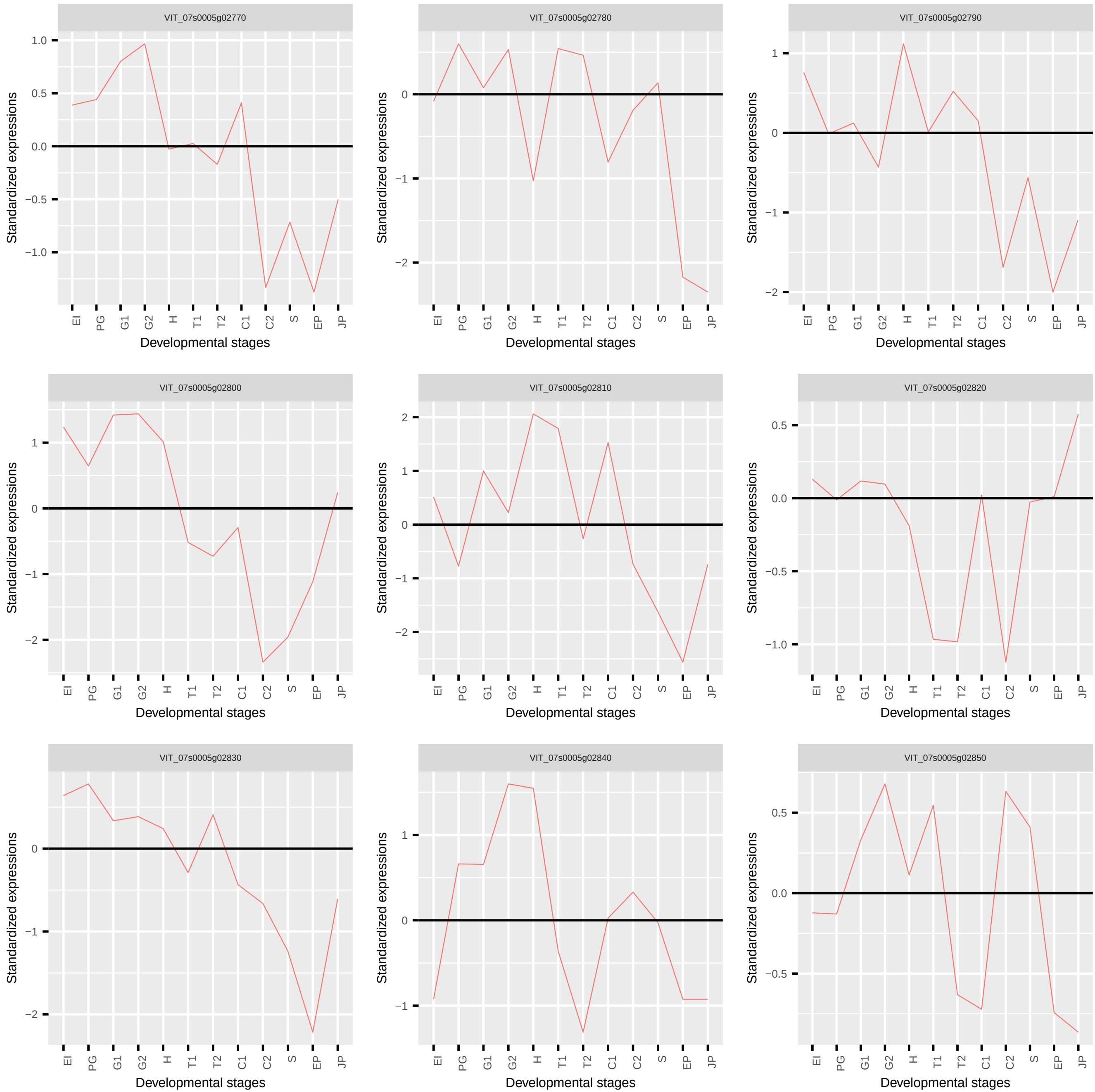
VIT_07s0005g02770
VIT_07s0005g02780
VIT_07s0005g02790
VIT_07s0005g02800
VIT_07s0005g02810
VIT_07s0005g02820
VIT_07s0005g02830
VIT_07s0005g02840
VIT_07s0005g02850
Standardized expressions
Developmental stages
EI PG G1 G2 H T1 T2 C1 C2 S EP JP

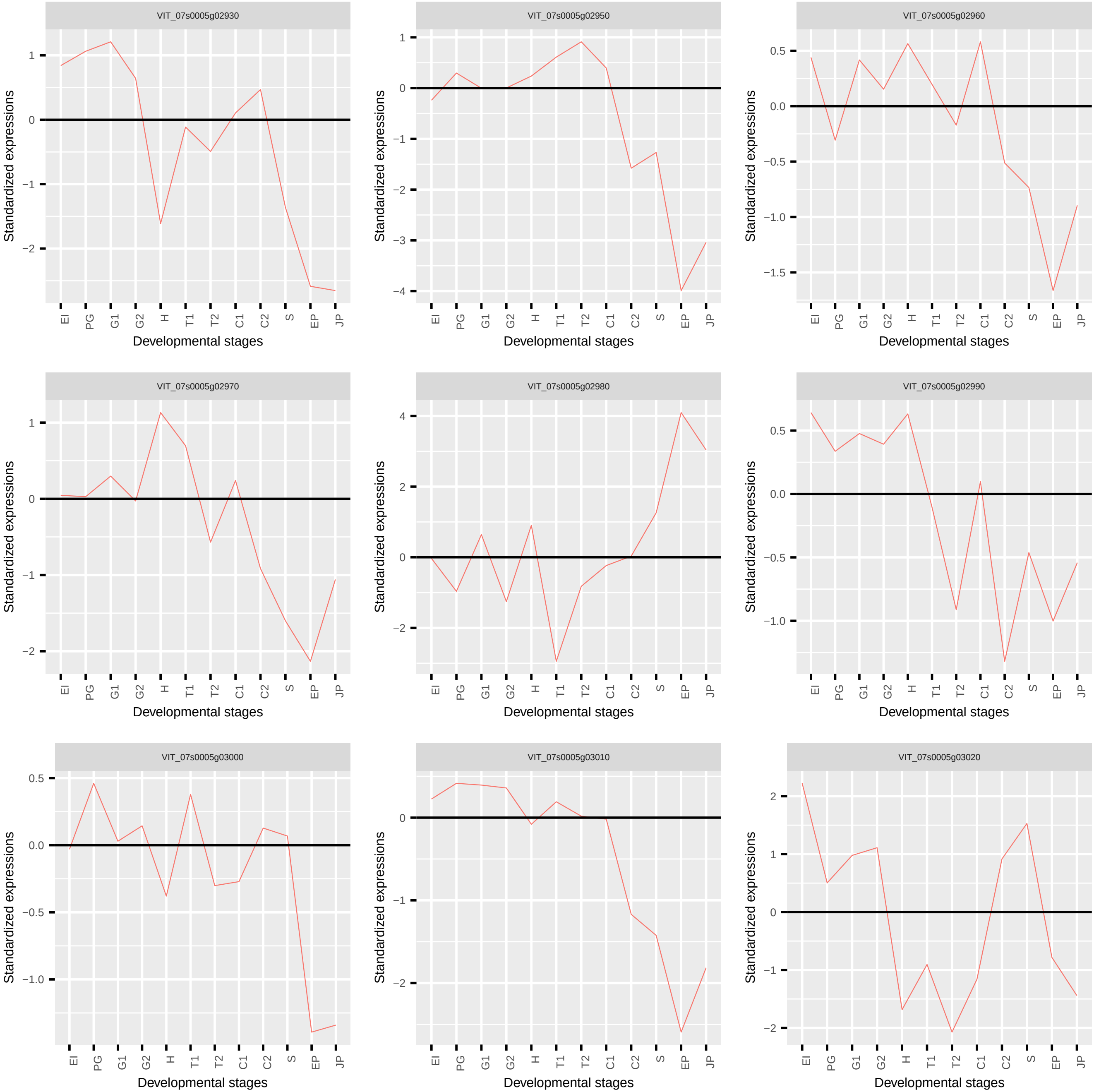
VIT_07s0005g02930
VIT_07s0005g02950
VIT_07s0005g02960
VIT_07s0005g02970
VIT_07s0005g02980
VIT_07s0005g02990
VIT_07s0005g03000
VIT_07s0005g03010
VIT_07s0005g03020
Standardized expressions
Developmental stages
EI PG G1 G2 H T1 T2 C1 C2 S EP JP

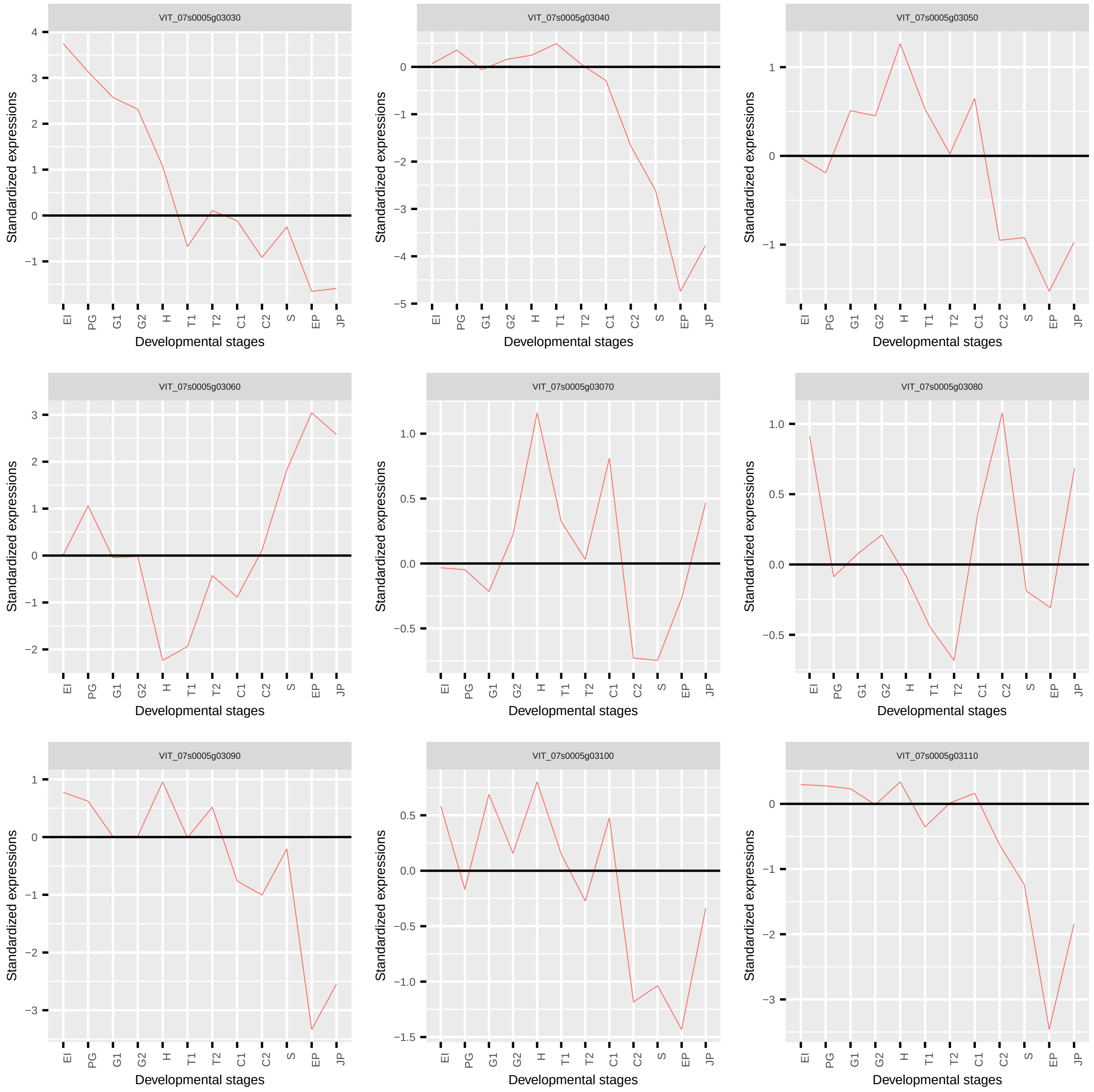
VIT_07s0005g03030
VIT_07s0005g03040
VIT_07s0005g03050
VIT_07s0005g03060
VIT_07s0005g03070
VIT_07s0005g03080
VIT_07s0005g03090
VIT_07s0005g03100
VIT_07s0005g03110
Standardized expressions
Developmental stages
EI
PG
G1
G2
H
T1
T2
C1
C2
S
EP
JP

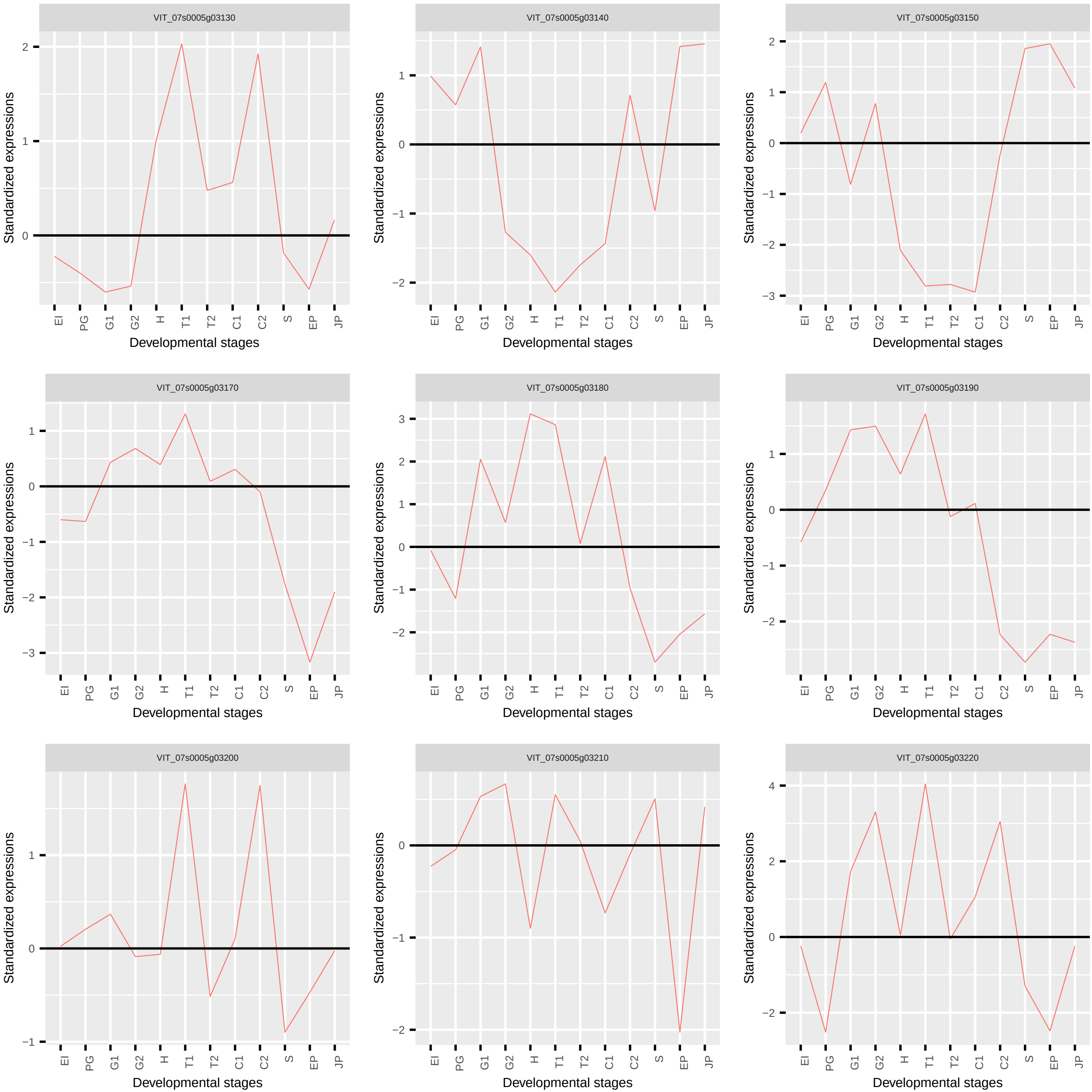
VIT_07s0005g03130
VIT_07s0005g03140
VIT_07s0005g03150
VIT_07s0005g03170
VIT_07s0005g03180
VIT_07s0005g03190
VIT_07s0005g03200
VIT_07s0005g03210
VIT_07s0005g03220
Standardized expressions
Developmental stages
EI
PG
G1
G2
H
T1
T2
C1
C2
S
EP
JP

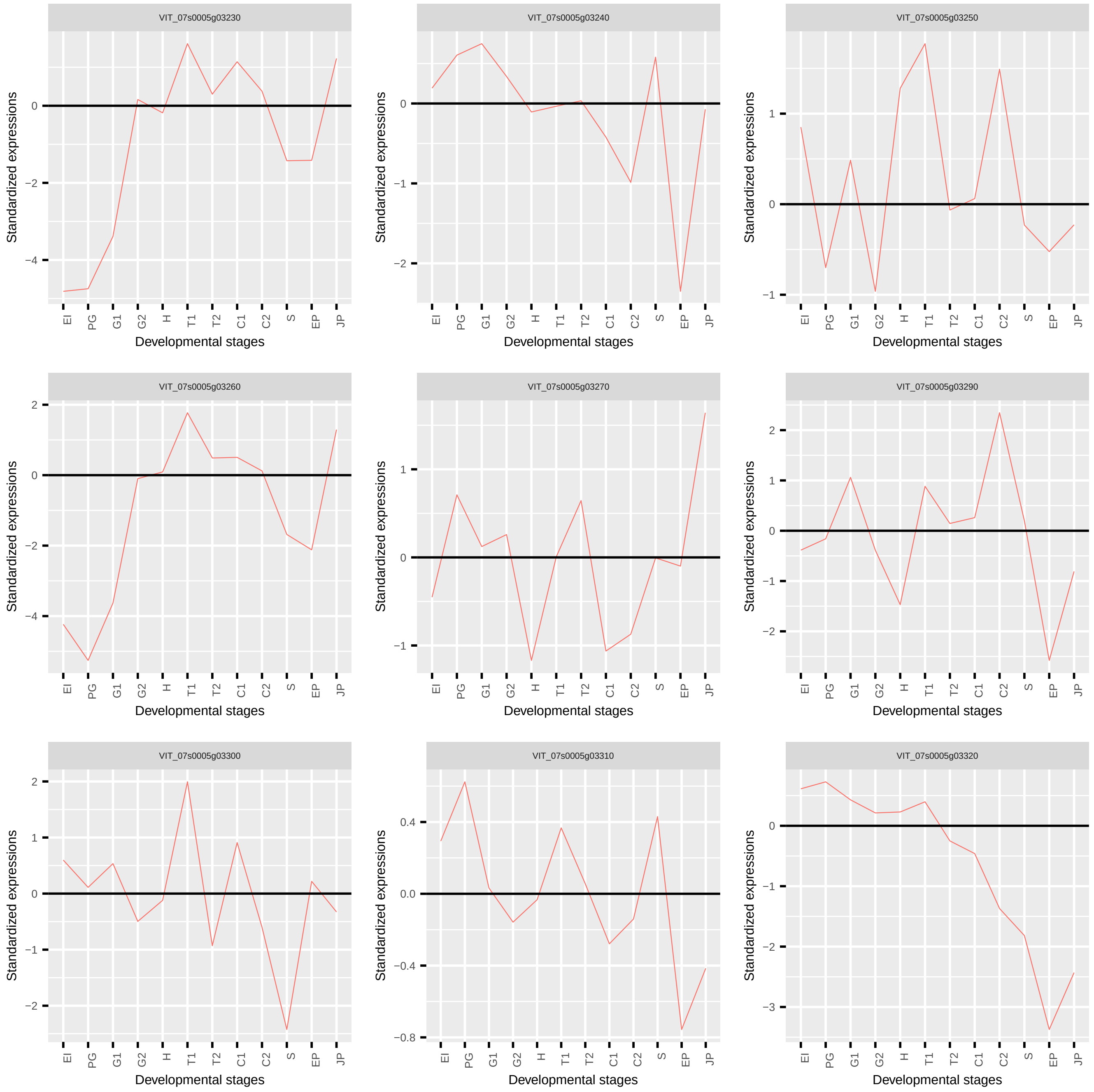
VIT_07s0005g03230
VIT_07s0005g03240
VIT_07s0005g03250
VIT_07s0005g03260
VIT_07s0005g03270
VIT_07s0005g03290
VIT_07s0005g03300
VIT_07s0005g03310
VIT_07s0005g03320
Standardized expressions
Developmental stages
EI PG G1 G2 H T1 T2 C1 C2 S EP JP

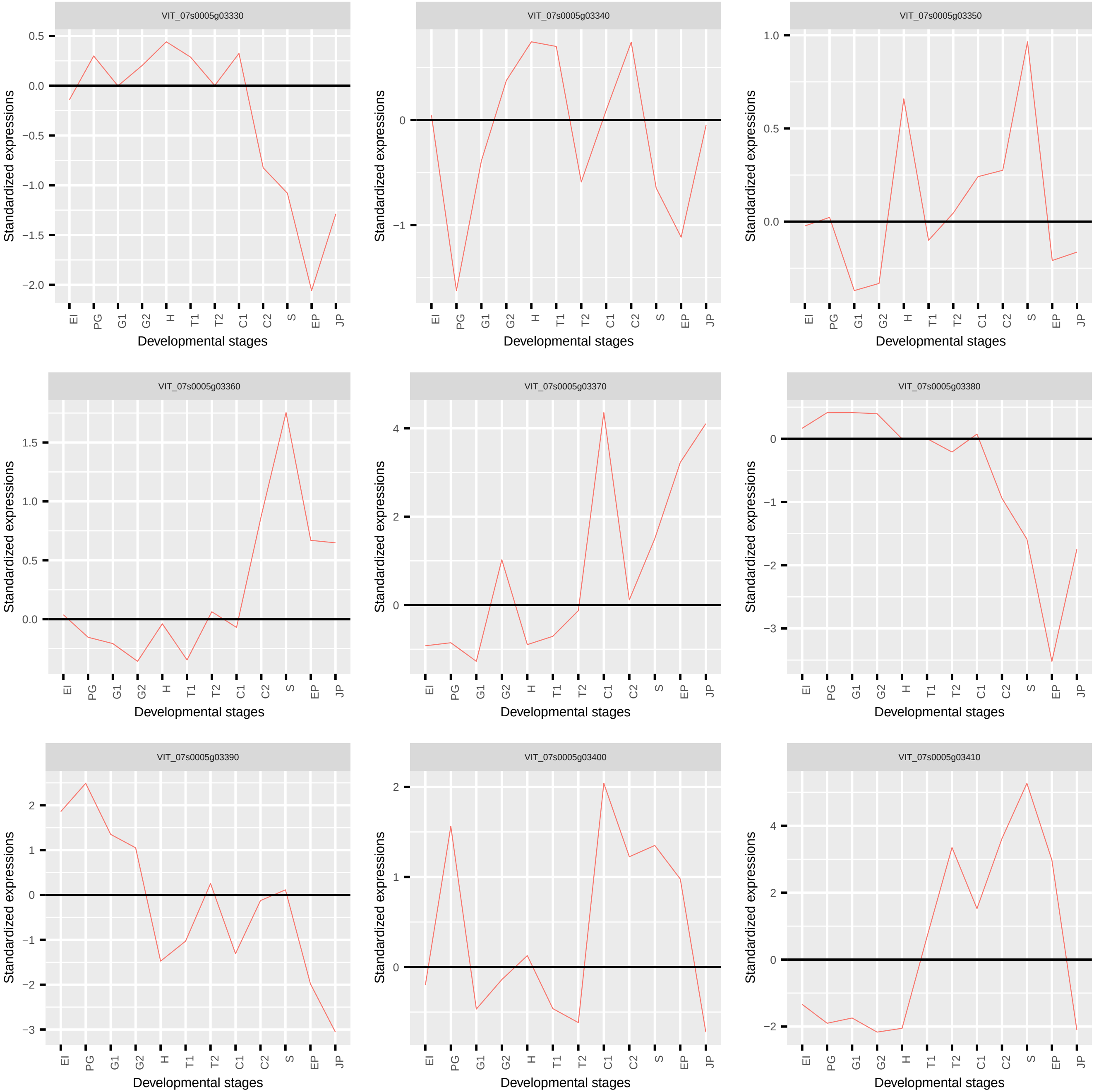
VIT_07s0005g03330
VIT_07s0005g03340
VIT_07s0005g03350
VIT_07s0005g03360
VIT_07s0005g03370
VIT_07s0005g03380
VIT_07s0005g03390
VIT_07s0005g03400
VIT_07s0005g03410
Standardized expressions
Developmental stages
EI
PG
G1
G2
H
T1
T2
C1
C2
S
EP
JP

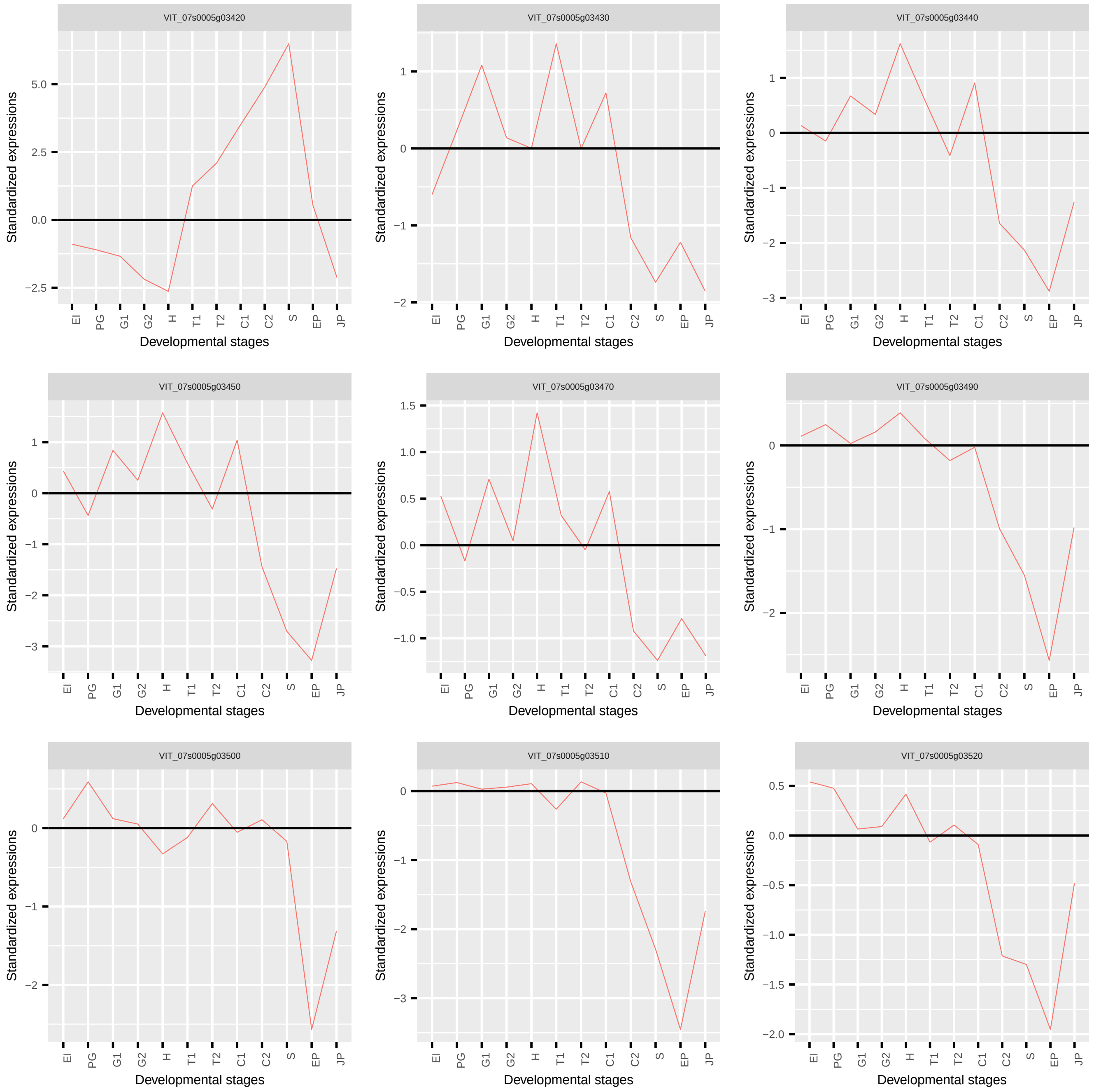
VIT_07s0005g03420
VIT_07s0005g03430
VIT_07s0005g03440
VIT_07s0005g03450
VIT_07s0005g03470
VIT_07s0005g03490
VIT_07s0005g03500
VIT_07s0005g03510
VIT_07s0005g03520
Standardized expressions
Developmental stages
EI PG G1 G2 H T1 T2 C1 C2 S EP JP

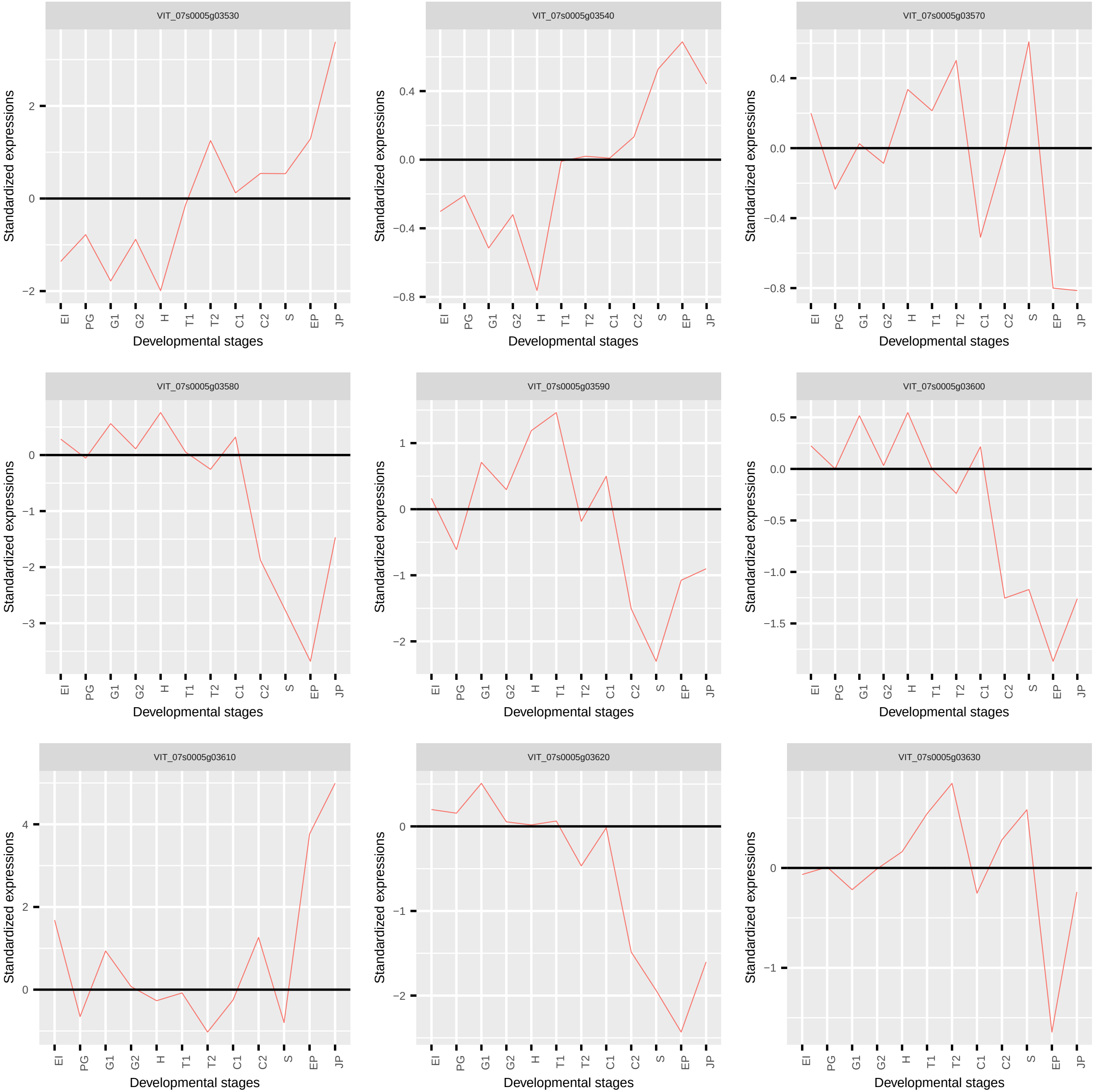

VIT_07s0005g03530
VIT_07s0005g03540
VIT_07s0005g03570
VIT_07s0005g03580
VIT_07s0005g03590
VIT_07s0005g03600
VIT_07s0005g03610
VIT_07s0005g03620
VIT_07s0005g03630
Standardized expressions
Developmental stages
EI PG G1 G2 H T1 T2 C1 C2 S EP JP

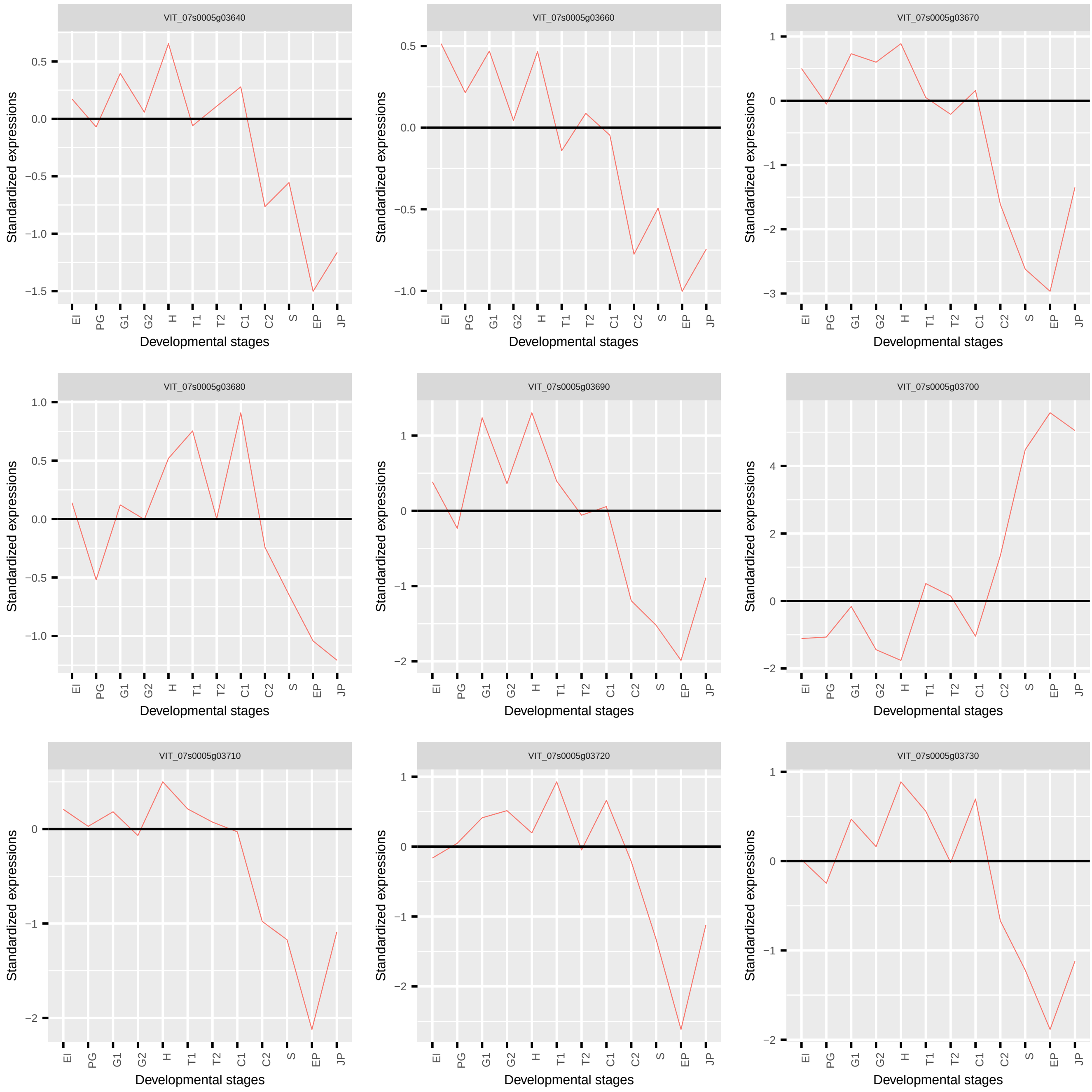
VIT_07s0005g03640
VIT_07s0005g03660
VIT_07s0005g03670
VIT_07s0005g03680
VIT_07s0005g03690
VIT_07s0005g03700
VIT_07s0005g03710
VIT_07s0005g03720
VIT_07s0005g03730
Standardized expressions
Developmental stages
EI
PG
G1
G2
H
T1
T2
C1
C2
S
EP
JP

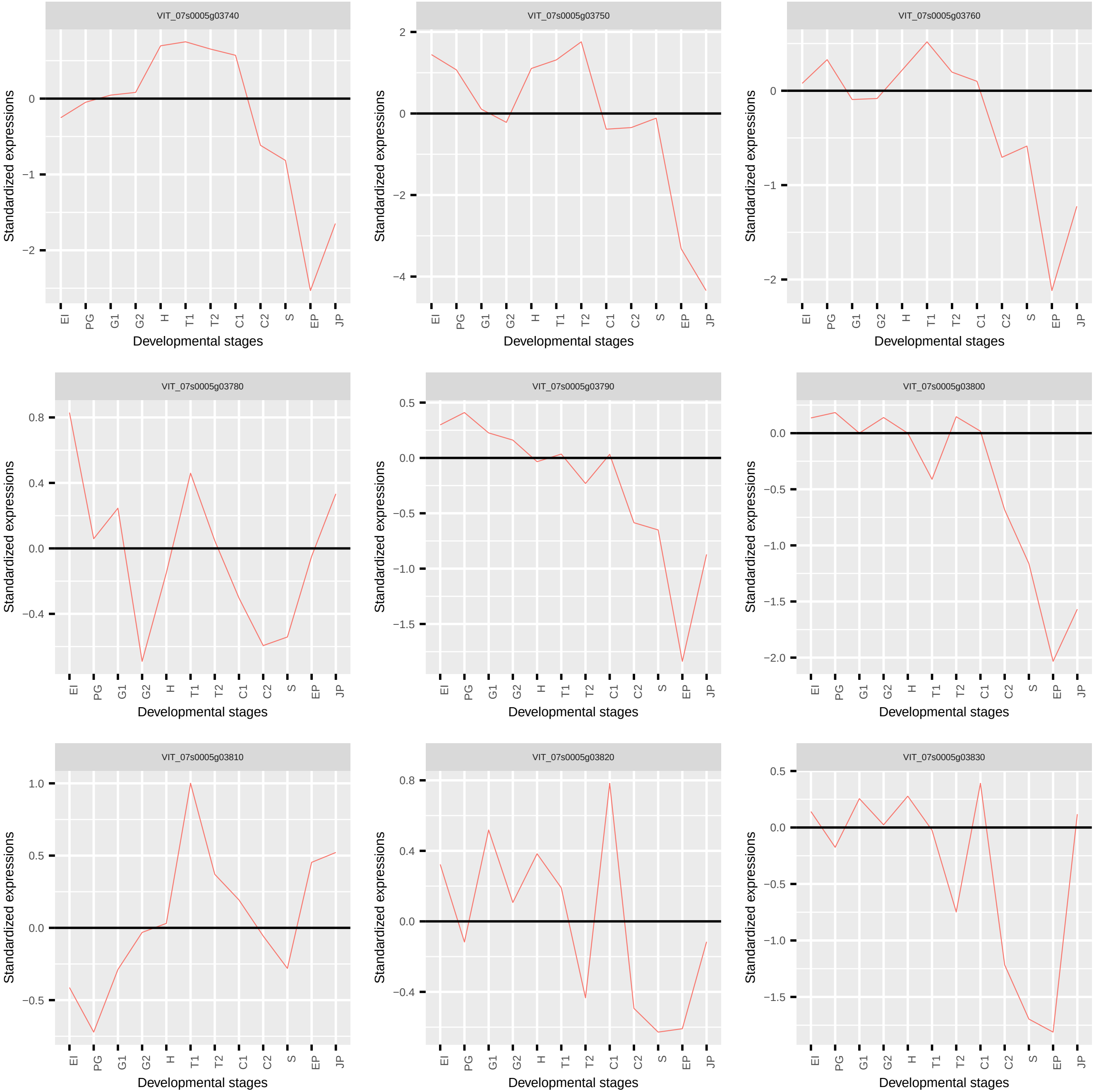
VIT_07s0005g03740
VIT_07s0005g03750
VIT_07s0005g03760
VIT_07s0005g03780
VIT_07s0005g03790
VIT_07s0005g03800
VIT_07s0005g03810
VIT_07s0005g03820
VIT_07s0005g03830
Standardized expressions
Developmental stages
EI PG G1 G2 H T1 T2 C1 C2 S EP JP

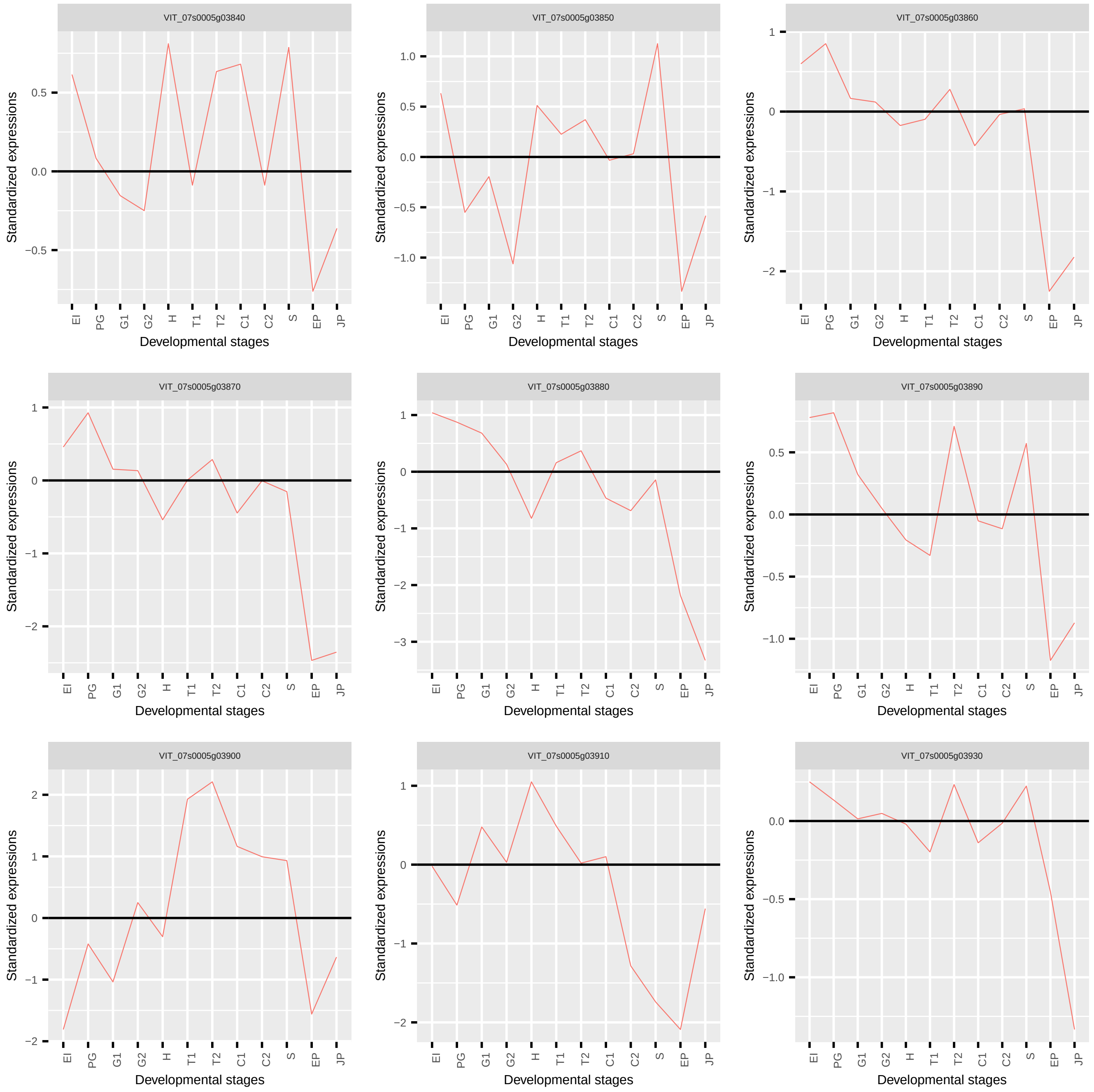
VIT_07s0005g03840
VIT_07s0005g03850
VIT_07s0005g03860
VIT_07s0005g03870
VIT_07s0005g03880
VIT_07s0005g03890
VIT_07s0005g03900
VIT_07s0005g03910
VIT_07s0005g03930
Standardized expressions
Developmental stages
EI PG G1 G2 H T1 T2 C1 C2 S EP JP

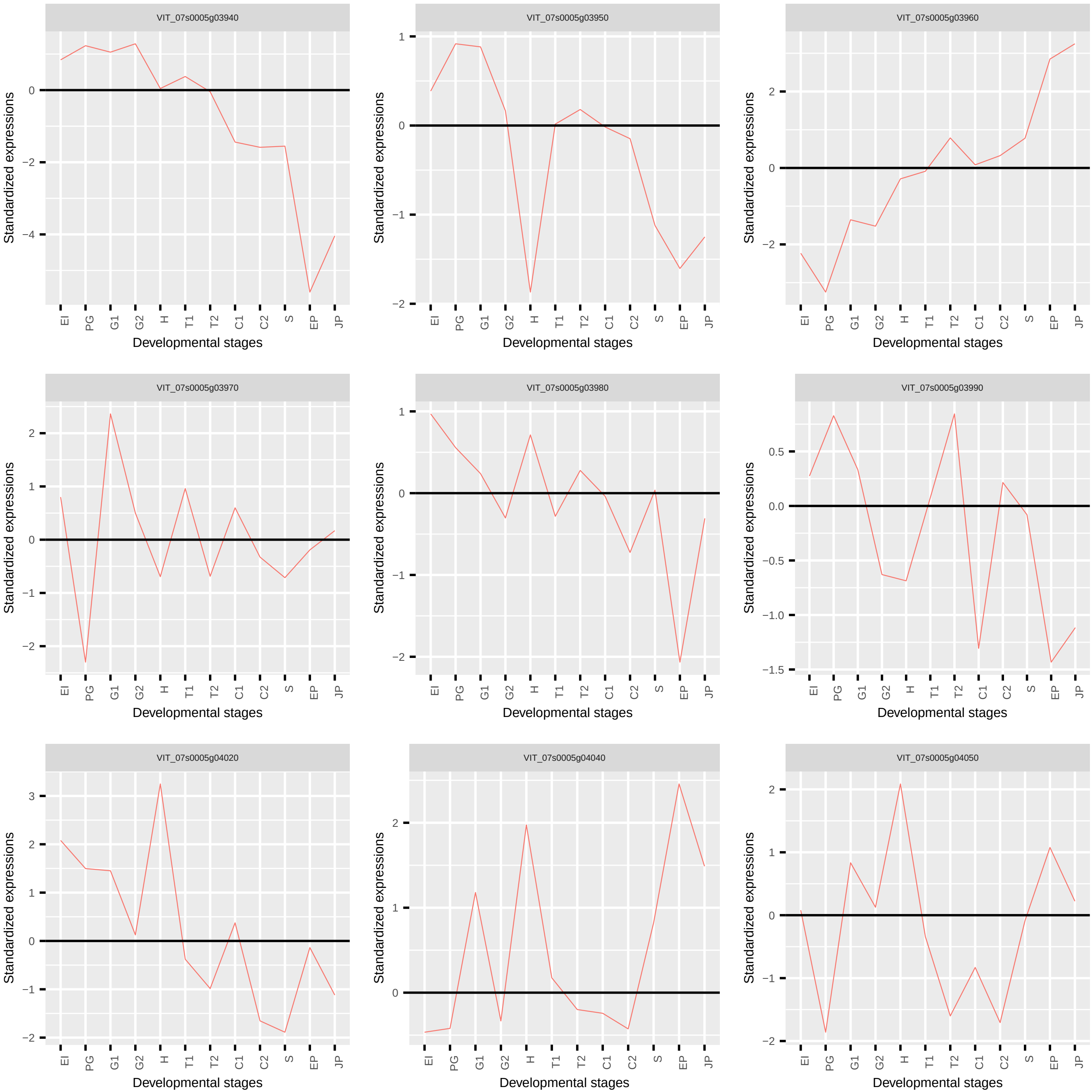
VIT_07s0005g03940
VIT_07s0005g03950
VIT_07s0005g03960
VIT_07s0005g03970
VIT_07s0005g03980
VIT_07s0005g03990
VIT_07s0005g04020
VIT_07s0005g04040
VIT_07s0005g04050
Standardized expressions
EI PG G1 G2 H T1 T2 C1 C2 S EP JP
Developmental stages

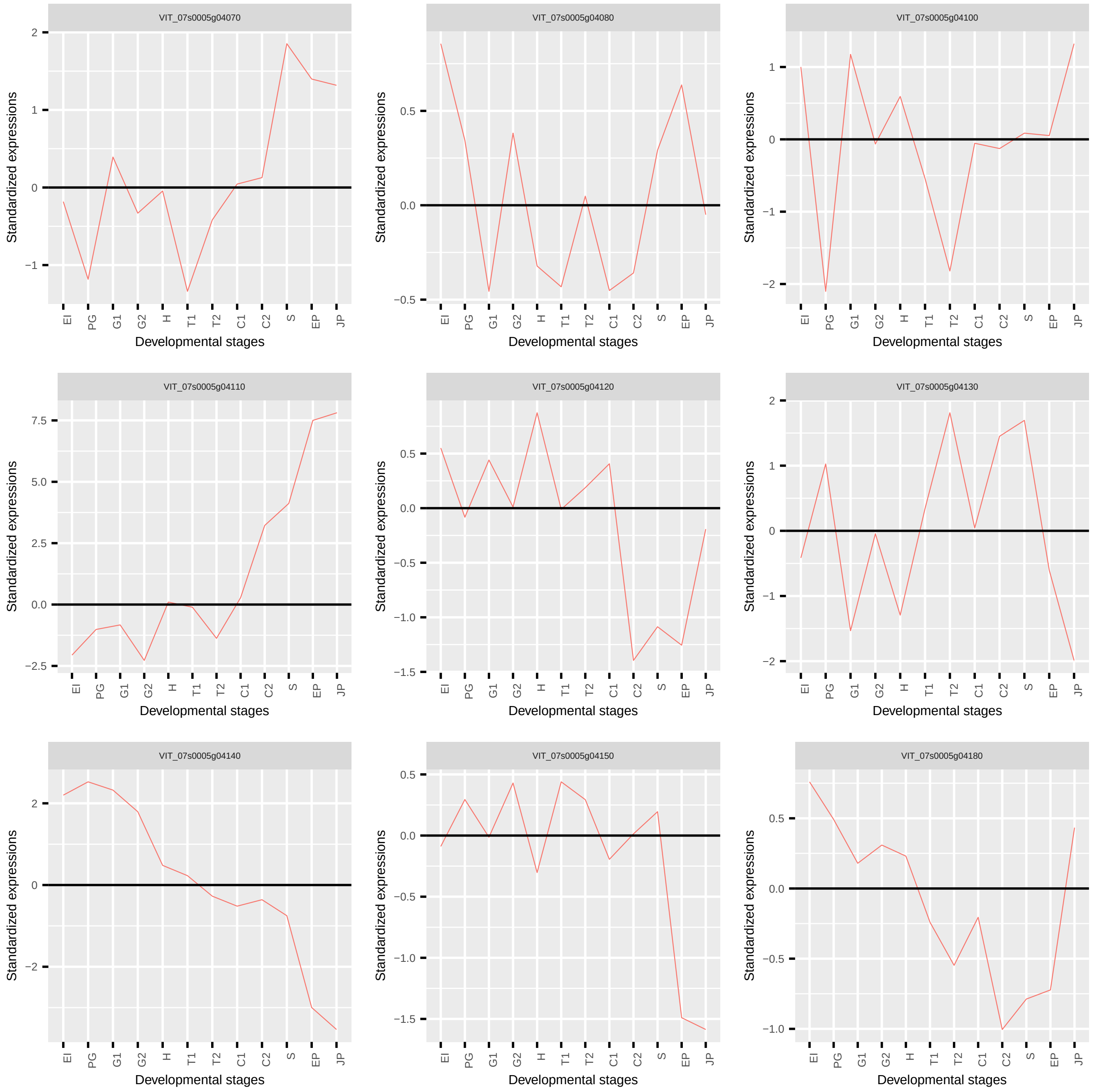
VIT_07s0005g04070
VIT_07s0005g04080
VIT_07s0005g04100
VIT_07s0005g04110
VIT_07s0005g04120
VIT_07s0005g04130
VIT_07s0005g04140
VIT_07s0005g04150
VIT_07s0005g04180
Standardized expressions
Developmental stages
EI PG G1 G2 H T1 T2 C1 C2 S EP JP

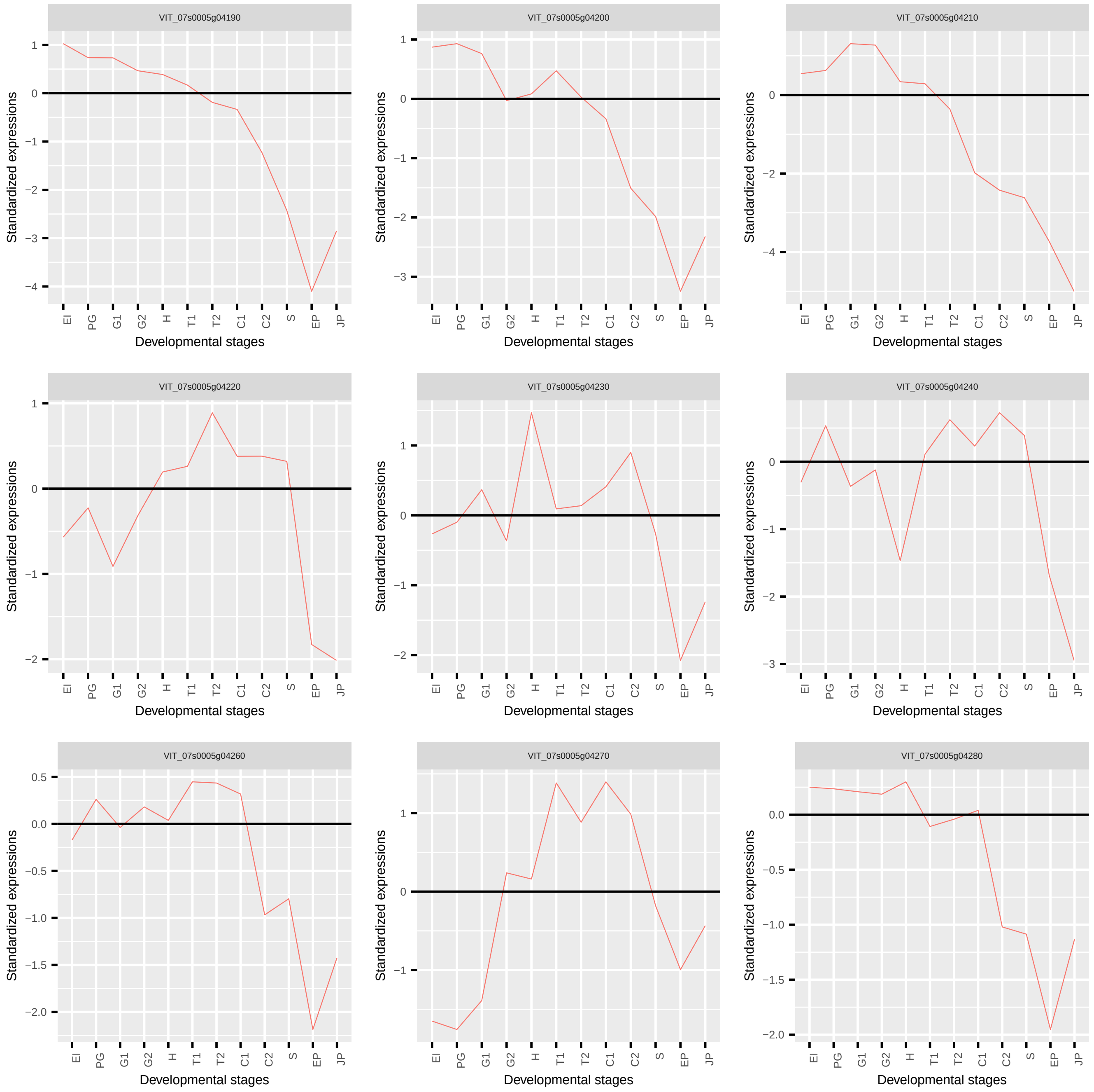

VIT_07s0005g04190
Standardized expressions
1
0
−1
−2
−3
−4
EI PG G1 G2 H T1 T2 C1 C2 S EP JP
Developmental stages
VIT_07s0005g04200
Standardized expressions
1
0
−1
−2
−3
EI PG G1 G2 H T1 T2 C1 C2 S EP JP
Developmental stages
VIT_07s0005g04210
Standardized expressions
0
−2
−4
EI PG G1 G2 H T1 T2 C1 C2 S EP JP
Developmental stages
VIT_07s0005g04220
Standardized expressions
1
0
−1
−2
EI PG G1 G2 H T1 T2 C1 C2 S EP JP
Developmental stages
VIT_07s0005g04230
Standardized expressions
1
0
−1
−2
EI PG G1 G2 H T1 T2 C1 C2 S EP JP
Developmental stages
VIT_07s0005g04240
Standardized expressions
0
−1
−2
−3
EI PG G1 G2 H T1 T2 C1 C2 S EP JP
Developmental stages
VIT_07s0005g04260
Standardized expressions
0.5
0.0
−0.5
−1.0
−1.5
−2.0
EI PG G1 G2 H T1 T2 C1 C2 S EP JP
Developmental stages
VIT_07s0005g04270
Standardized expressions
1
0
−1
EI PG G1 G2 H T1 T2 C1 C2 S EP JP
Developmental stages
VIT_07s0005g04280
Standardized expressions
0.0
−0.5
−1.0
−1.5
−2.0
EI PG G1 G2 H T1 T2 C1 C2 S EP JP
Developmental stages

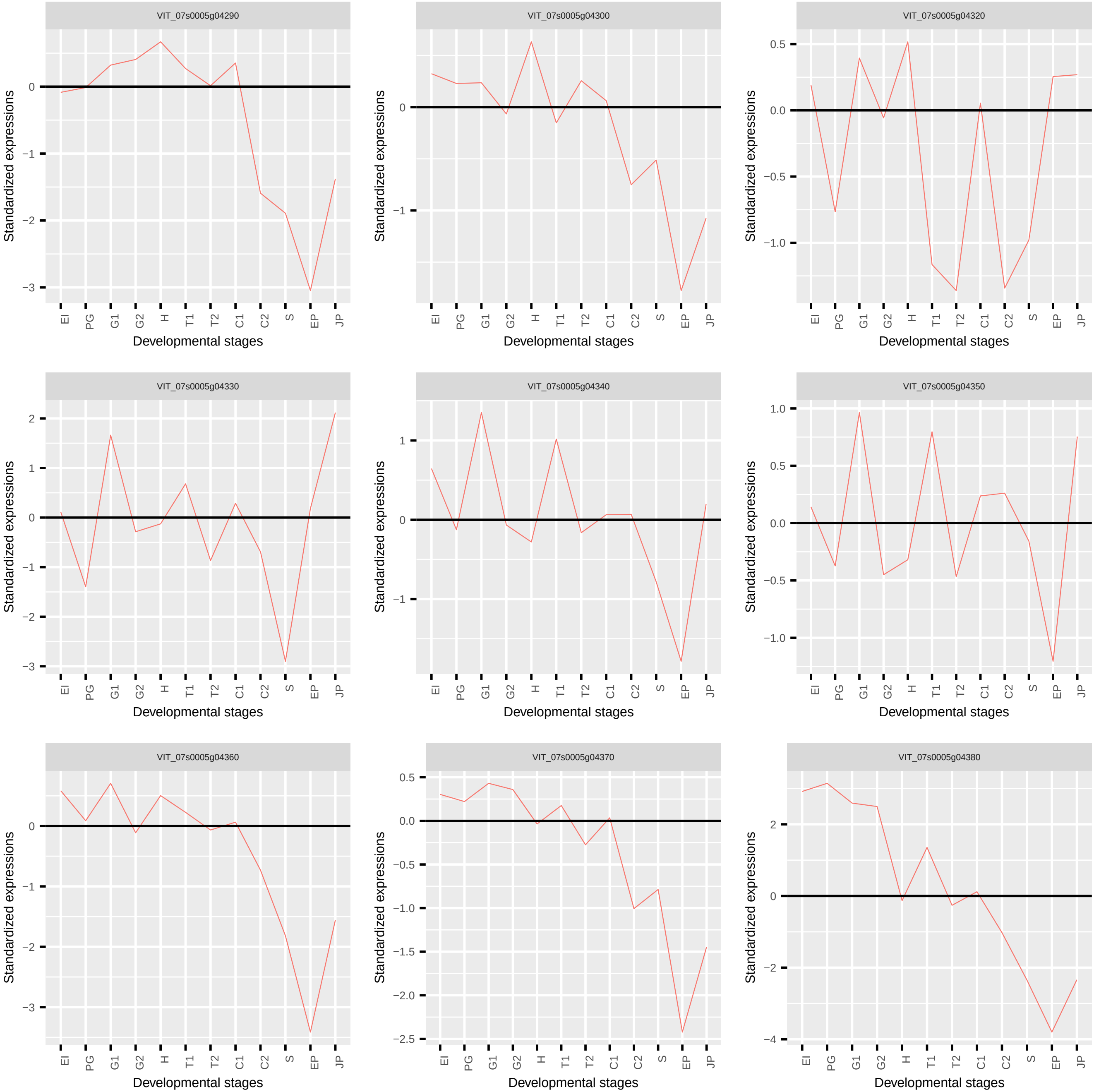
VIT_07s0005g04290
VIT_07s0005g04300
VIT_07s0005g04320
VIT_07s0005g04330
VIT_07s0005g04340
VIT_07s0005g04350
VIT_07s0005g04360
VIT_07s0005g04370
VIT_07s0005g04380
Standardized expressions
Developmental stages
EI PG G1 G2 H T1 T2 C1 C2 S EP JP

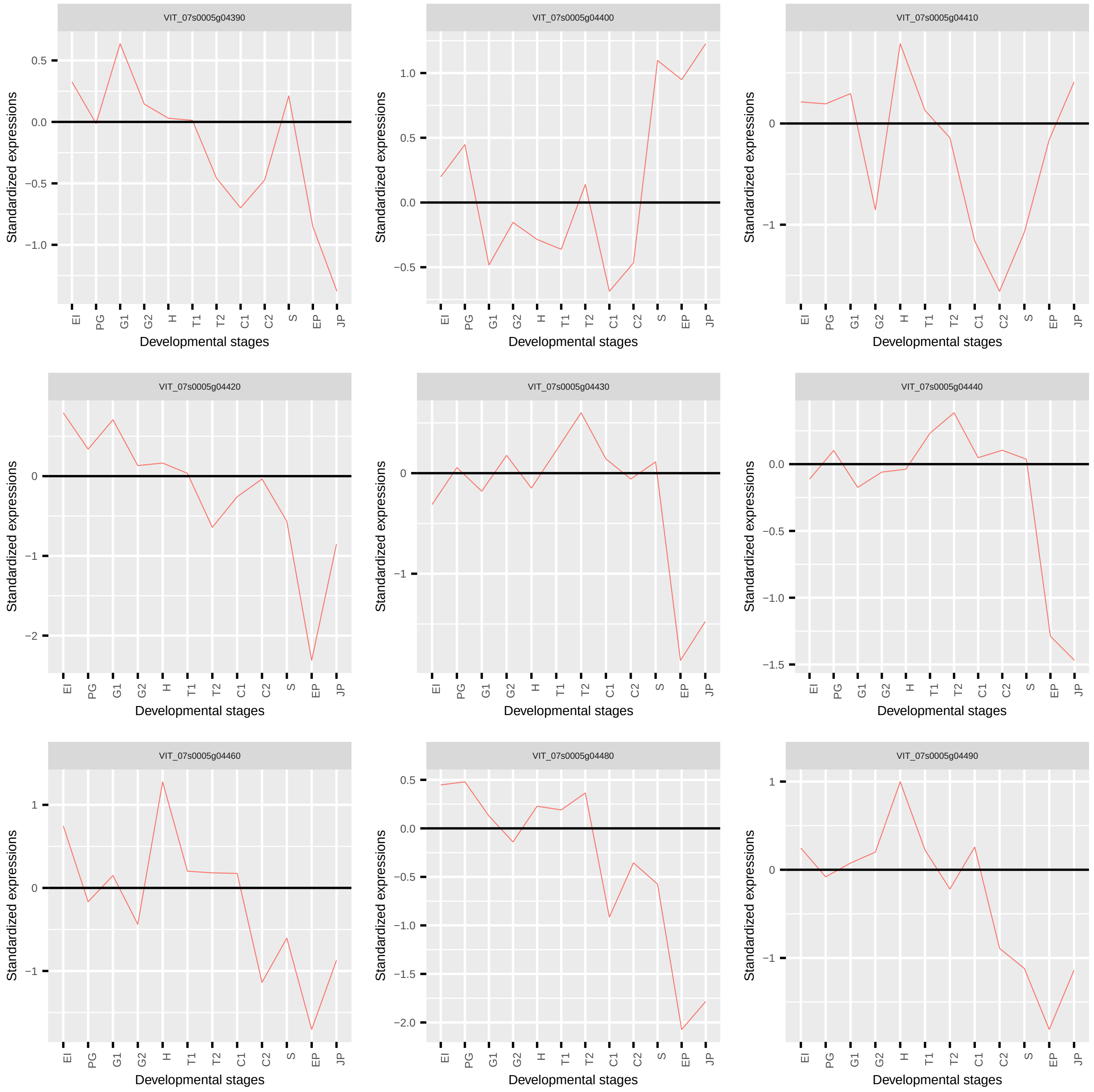
VIT_07s0005g04390
VIT_07s0005g04400
VIT_07s0005g04410
VIT_07s0005g04420
VIT_07s0005g04430
VIT_07s0005g04440
VIT_07s0005g04460
VIT_07s0005g04480
VIT_07s0005g04490
Standardized expressions
Developmental stages
EI PG G1 G2 H T1 T2 C1 C2 S EP JP

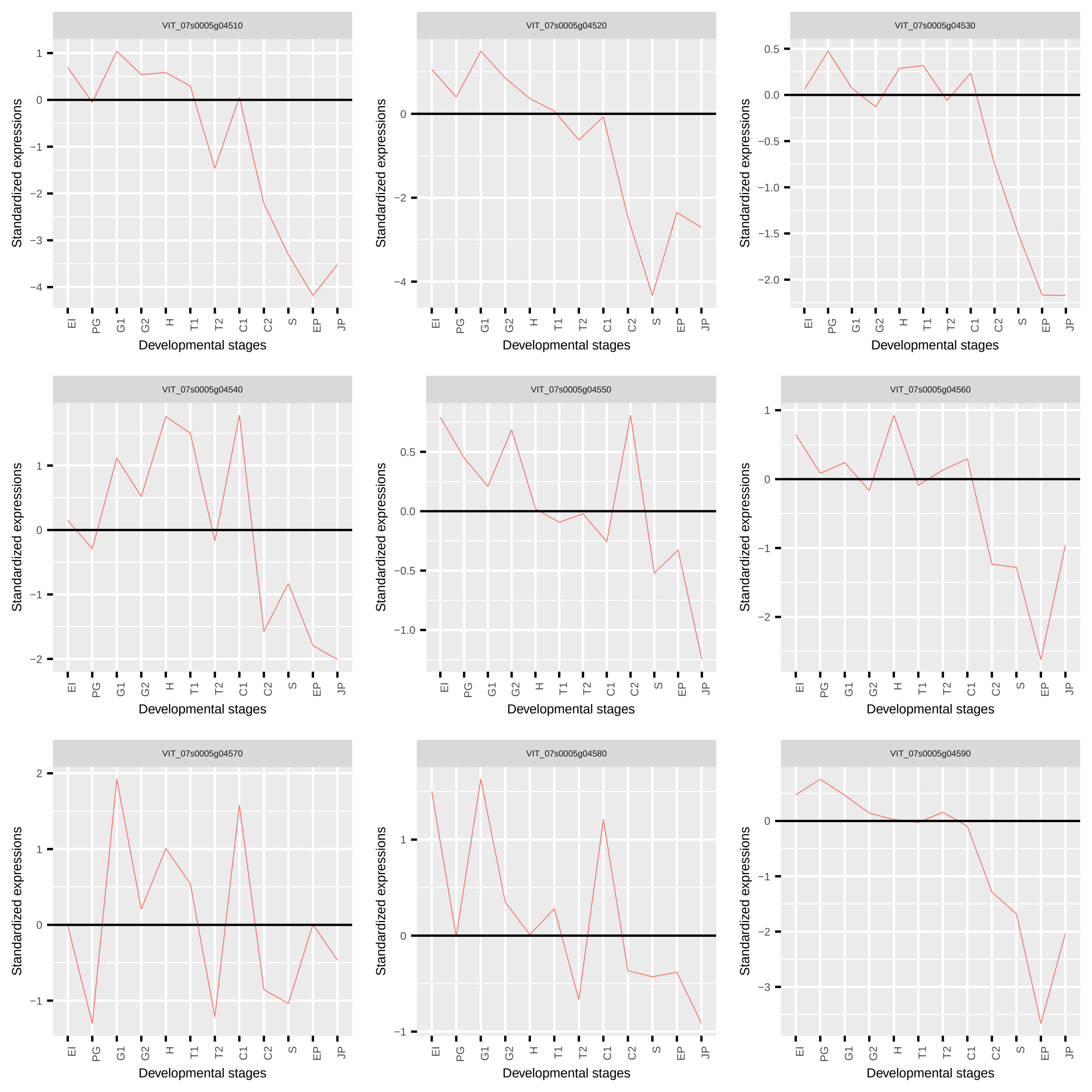

VIT_07s0005g04510
VIT_07s0005g04520
VIT_07s0005g04530
VIT_07s0005g04540
VIT_07s0005g04550
VIT_07s0005g04560
VIT_07s0005g04570
VIT_07s0005g04580
VIT_07s0005g04590
Standardized expressions
Developmental stages
EI PG G1 G2 H T1 T2 C1 C2 S EP JP

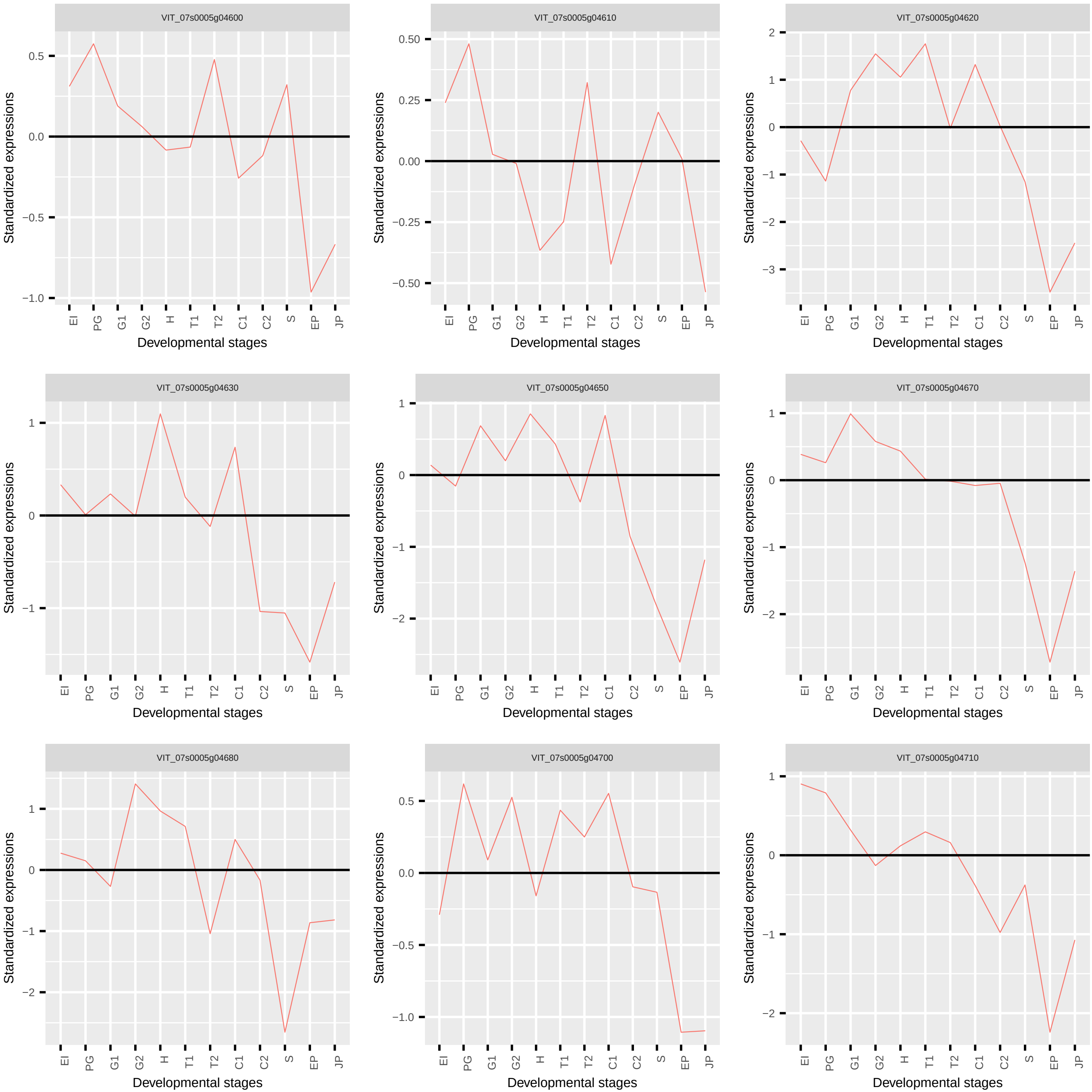
VIT_07s0005g04600
VIT_07s0005g04610
VIT_07s0005g04620
VIT_07s0005g04630
VIT_07s0005g04650
VIT_07s0005g04670
VIT_07s0005g04680
VIT_07s0005g04700
VIT_07s0005g04710
Standardized expressions
Developmental stages
EI PG G1 G2 H T1 T2 C1 C2 S EP JP

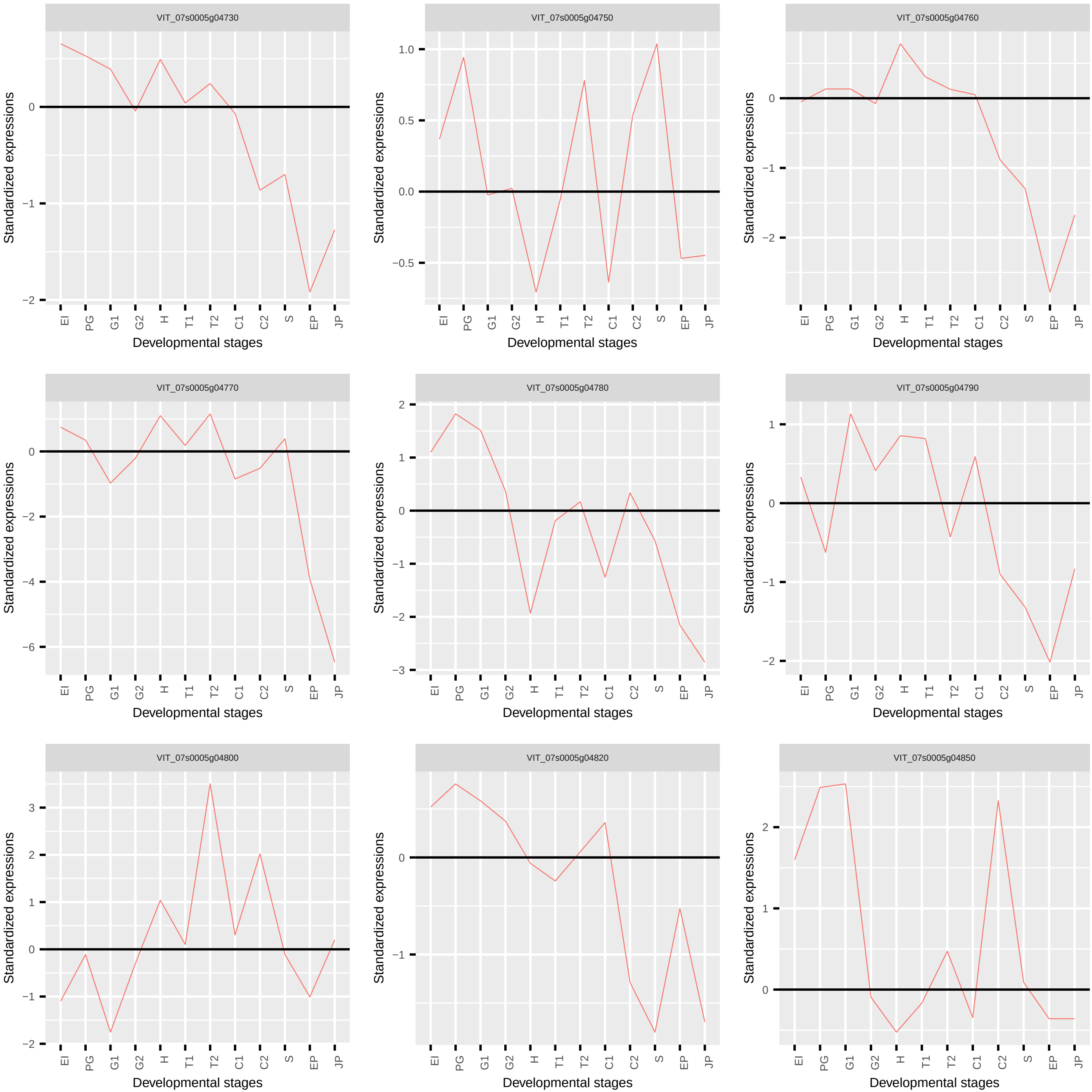
VIT_07s0005g04730
VIT_07s0005g04750
VIT_07s0005g04760
VIT_07s0005g04770
VIT_07s0005g04780
VIT_07s0005g04790
VIT_07s0005g04800
VIT_07s0005g04820
VIT_07s0005g04850
Standardized expressions
Developmental stages
EI
PG
G1
G2
H
T1
T2
C1
C2
S
EP
JP

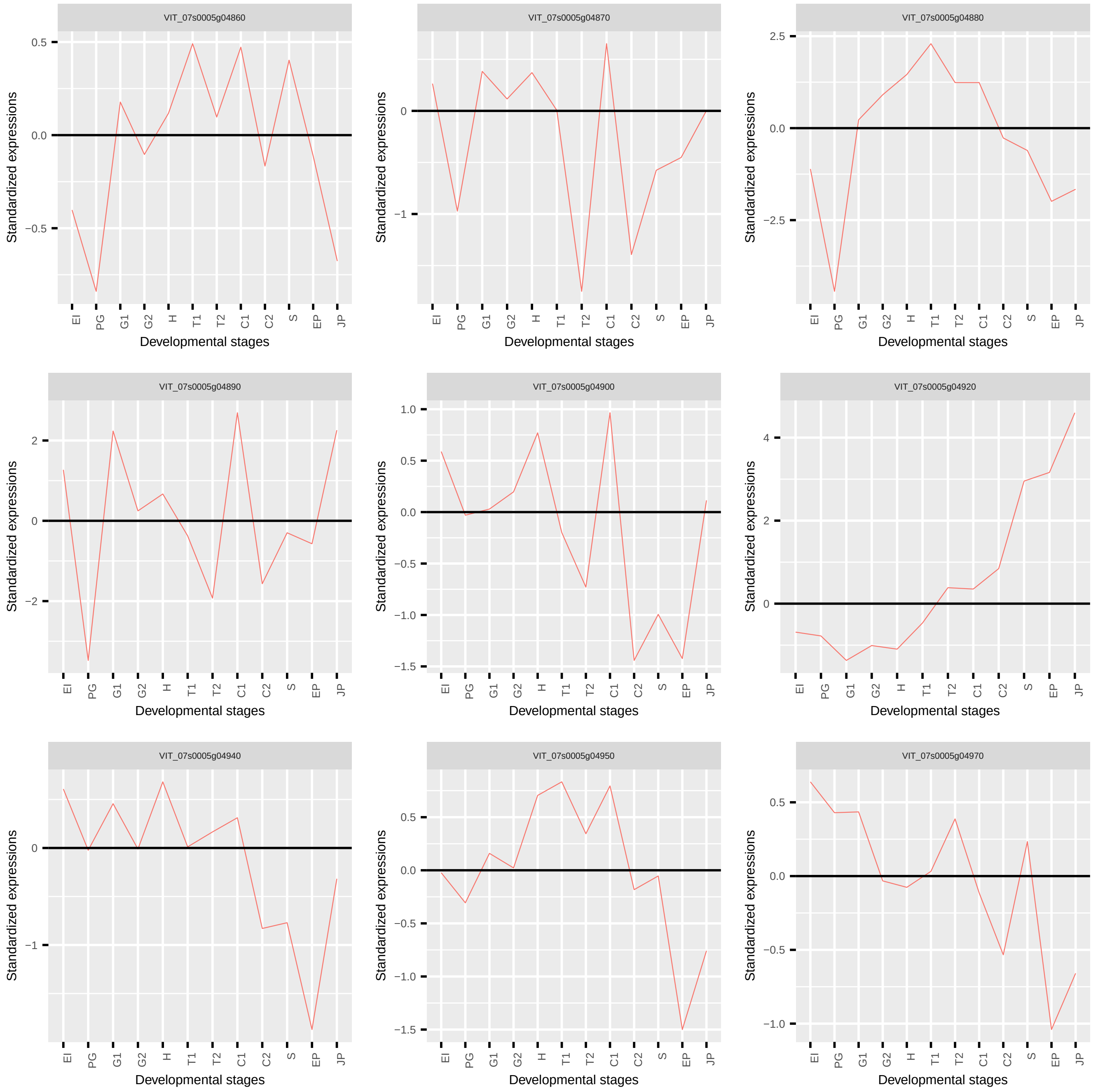

VIT_07s0005g04860
VIT_07s0005g04870
VIT_07s0005g04880
VIT_07s0005g04890
VIT_07s0005g04900
VIT_07s0005g04920
VIT_07s0005g04940
VIT_07s0005g04950
VIT_07s0005g04970
Standardized expressions
Developmental stages
EI
PG
G1
G2
H
T1
T2
C1
C2
S
EP
JP

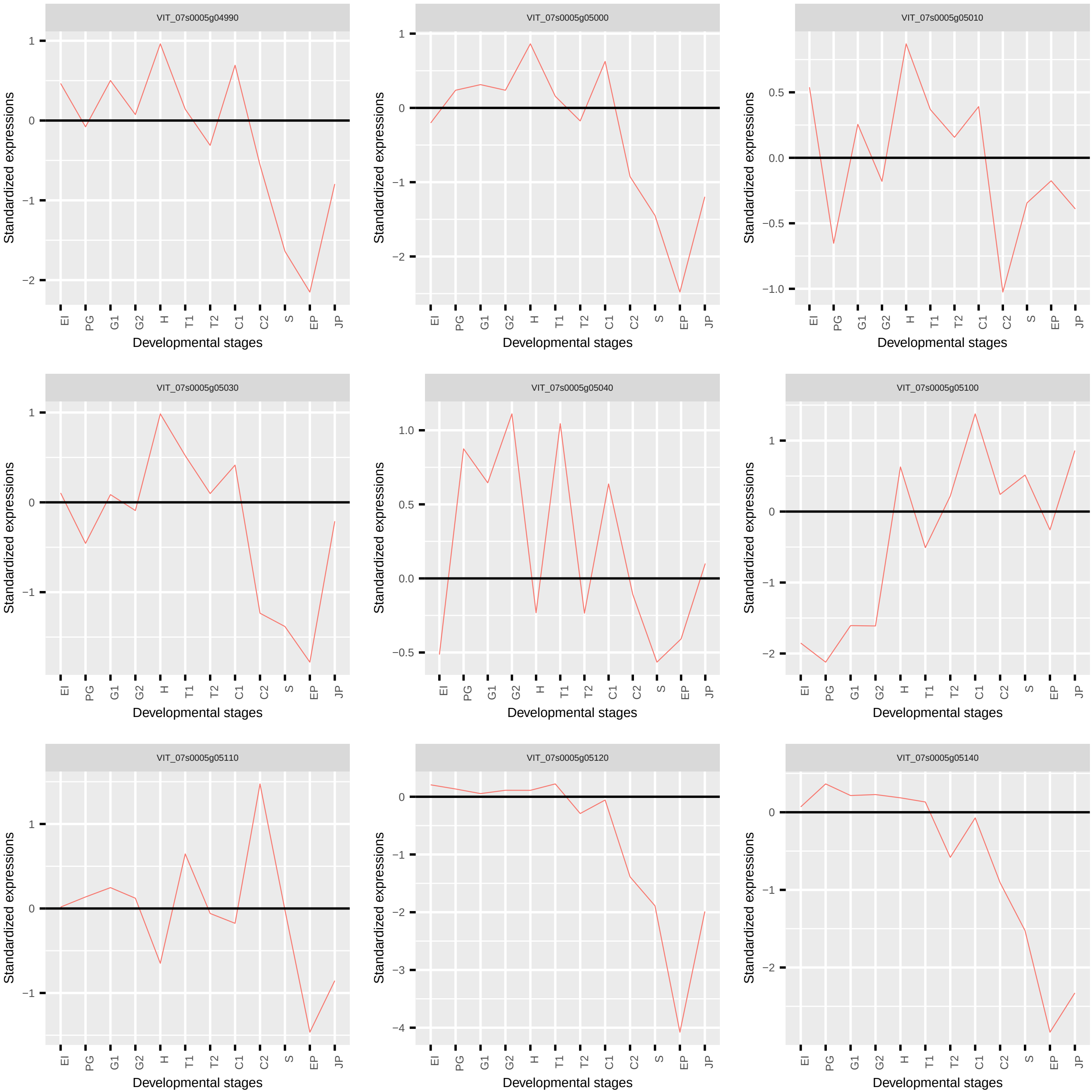

VIT_07s0005g04990
VIT_07s0005g05000
VIT_07s0005g05010
VIT_07s0005g05030
VIT_07s0005g05040
VIT_07s0005g05100
VIT_07s0005g05110
VIT_07s0005g05120
VIT_07s0005g05140
Standardized expressions
Developmental stages
EI
PG
G1
G2
H
T1
T2
C1
C2
S
EP
JP

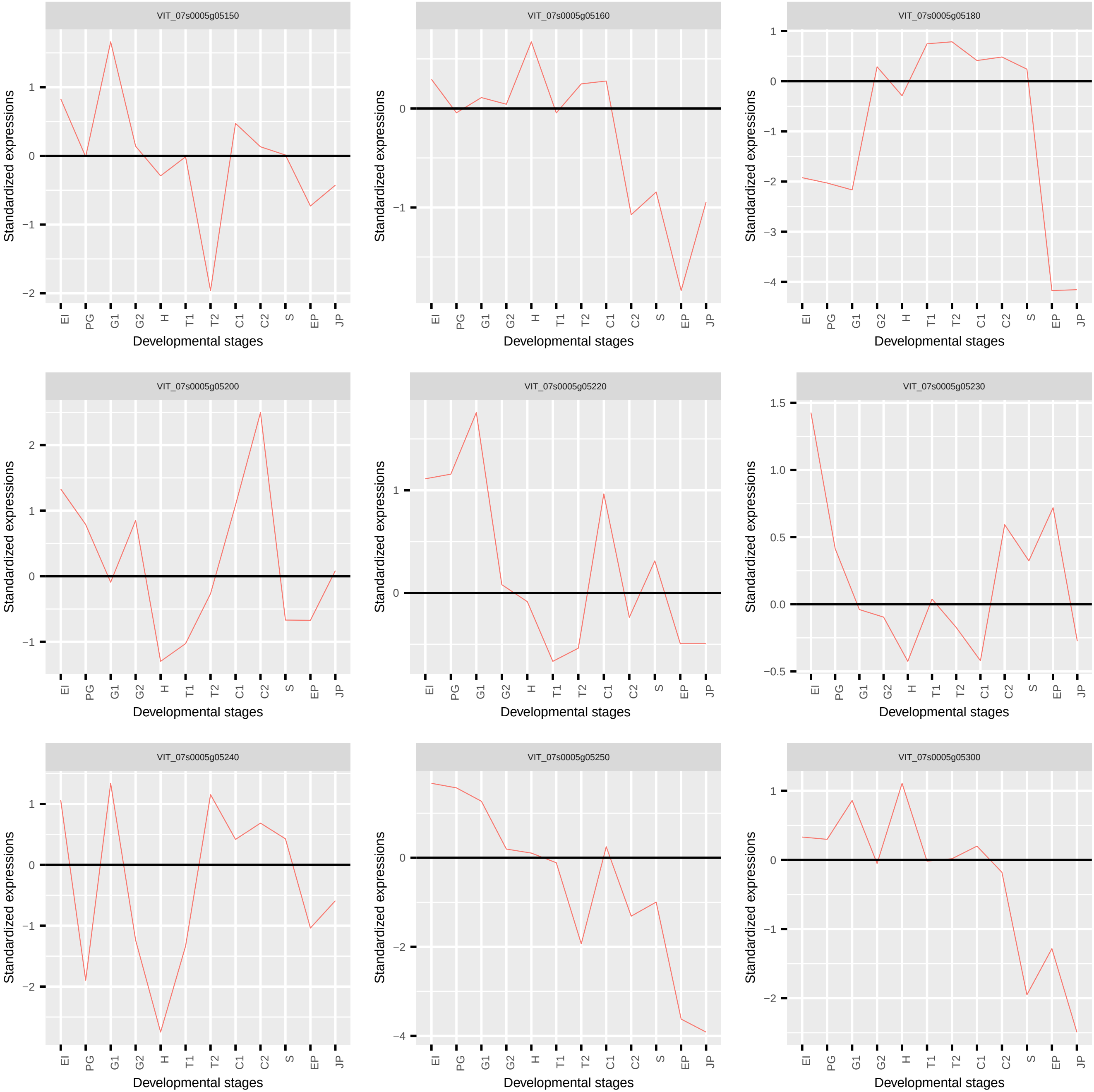
VIT_07s0005g05150
VIT_07s0005g05160
VIT_07s0005g05180
VIT_07s0005g05200
VIT_07s0005g05220
VIT_07s0005g05230
VIT_07s0005g05240
VIT_07s0005g05250
VIT_07s0005g05300
Standardized expressions
Developmental stages
EI PG G1 G2 H T1 T2 C1 C2 S EP JP

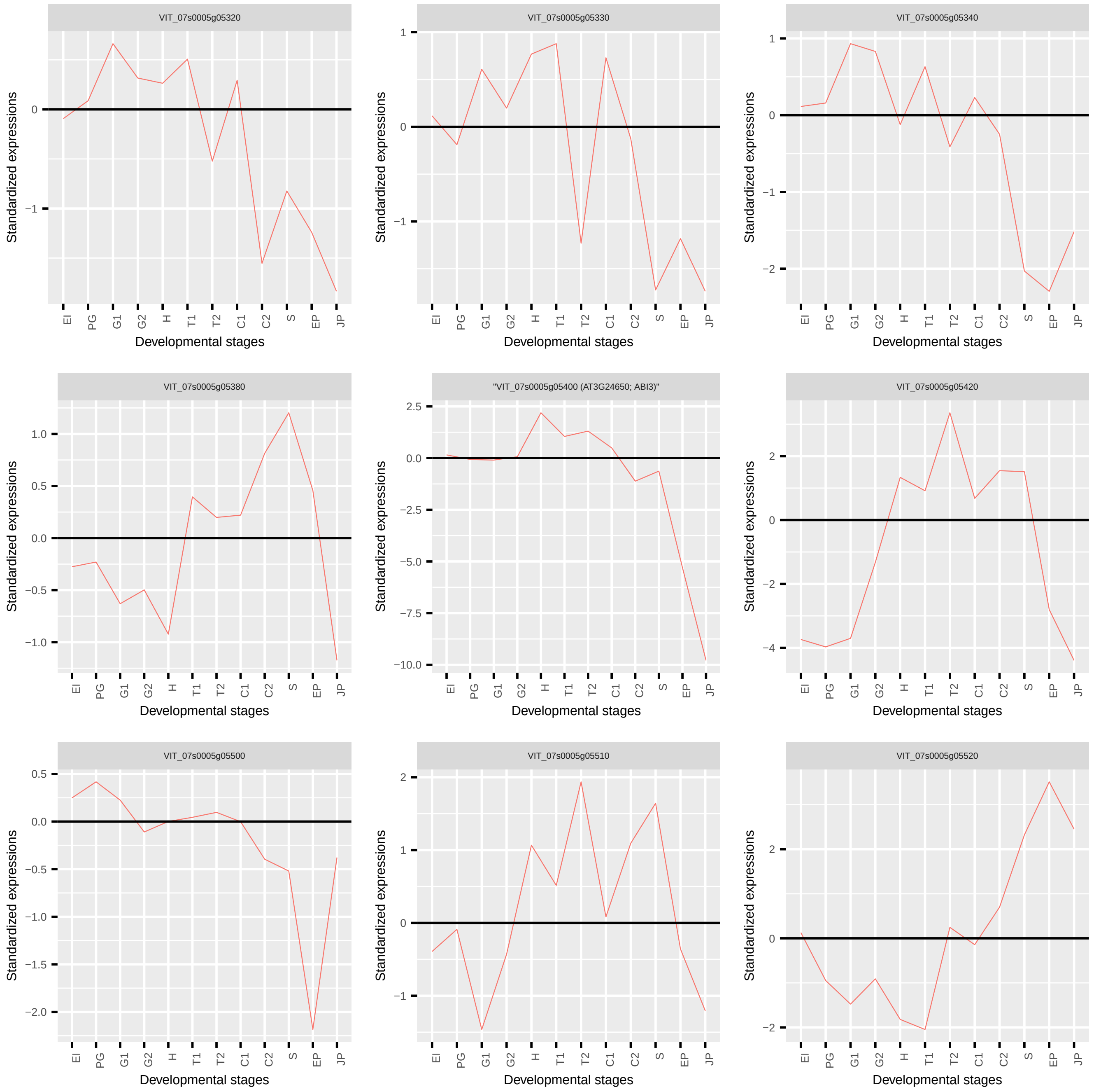
VIT_07s0005g05320
VIT_07s0005g05330
VIT_07s0005g05340
VIT_07s0005g05380
"VIT_07s0005g05400 (AT3G24650; ABI3)"
VIT_07s0005g05420
VIT_07s0005g05500
VIT_07s0005g05510
VIT_07s0005g05520
Standardized expressions
Developmental stages
EI PG G1 G2 H T1 T2 C1 C2 S EP JP
0
−1
1
−2
1.0
0.5
0.0
−0.5
−1.0
2.5
−2.5
−5.0
−7.5
−10.0
2
−4
−1.5
−2.0

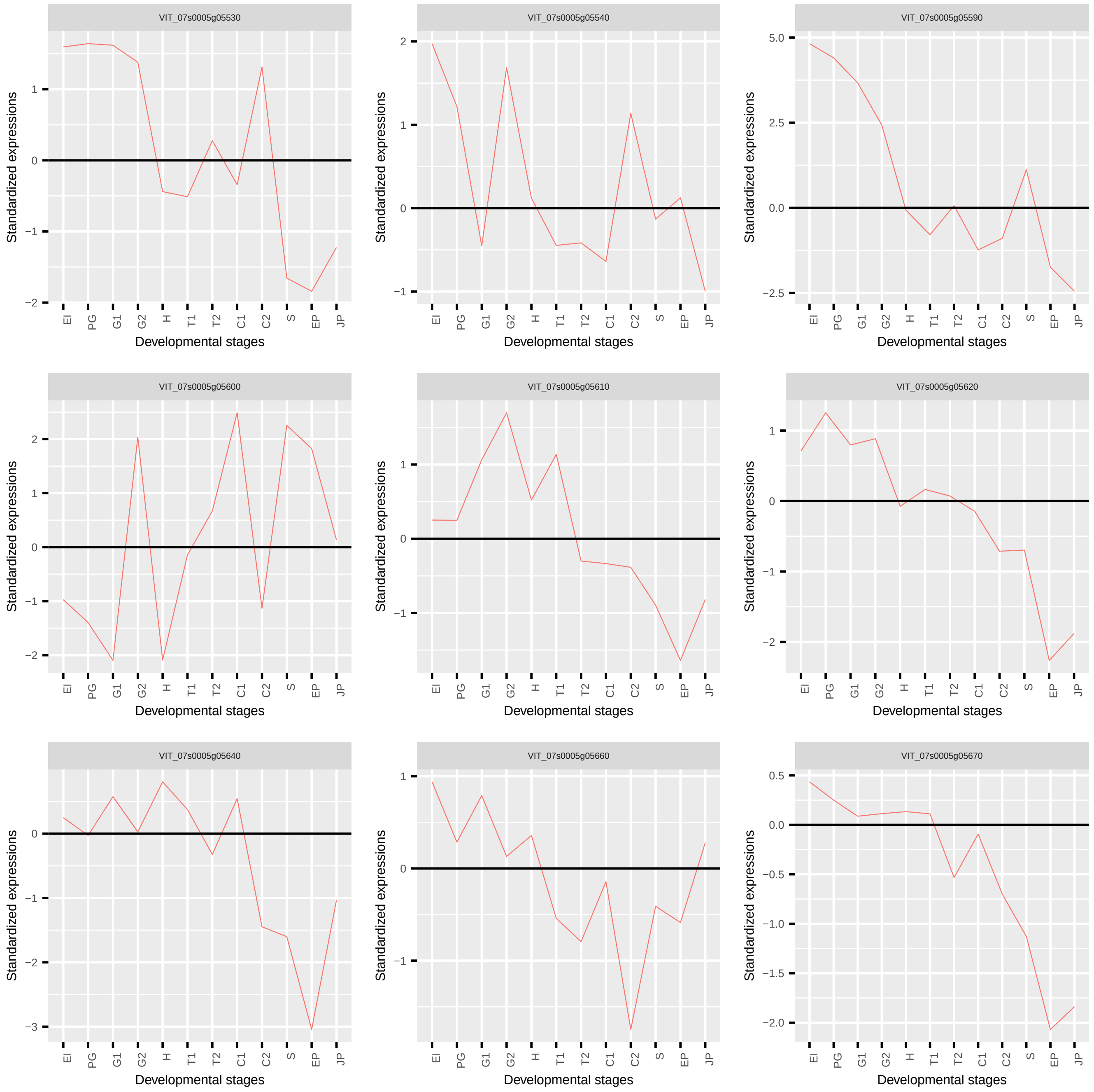
VIT_07s0005g05530
VIT_07s0005g05540
VIT_07s0005g05590
VIT_07s0005g05600
VIT_07s0005g05610
VIT_07s0005g05620
VIT_07s0005g05640
VIT_07s0005g05660
VIT_07s0005g05670
Standardized expressions
Developmental stages
EI PG G1 G2 H T1 T2 C1 C2 S EP JP

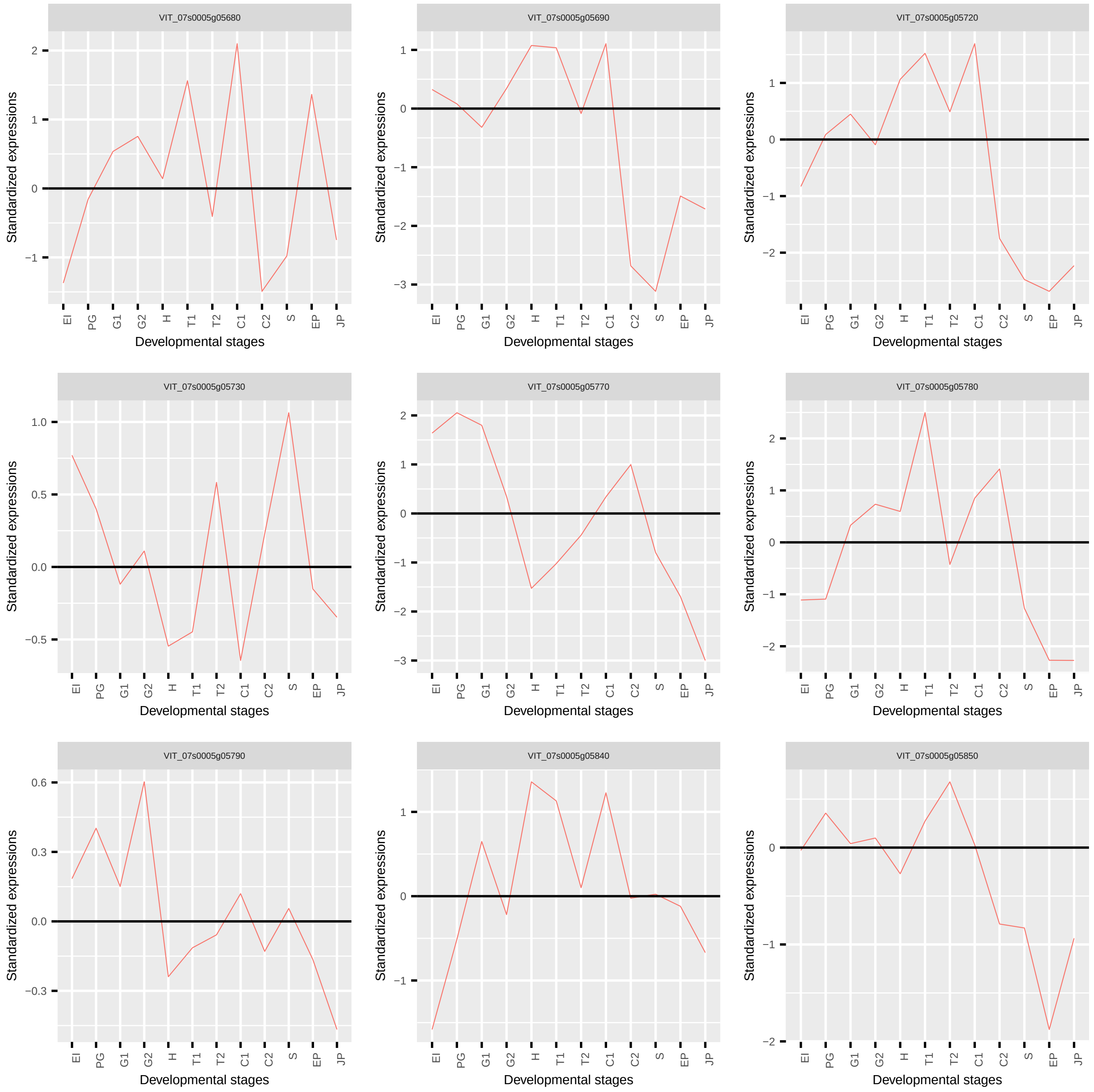
VIT_07s0005g05680
VIT_07s0005g05690
VIT_07s0005g05720
VIT_07s0005g05730
VIT_07s0005g05770
VIT_07s0005g05780
VIT_07s0005g05790
VIT_07s0005g05840
VIT_07s0005g05850
Standardized expressions
Developmental stages
EI
PG
G1
G2
H
T1
T2
C1
C2
S
EP
JP

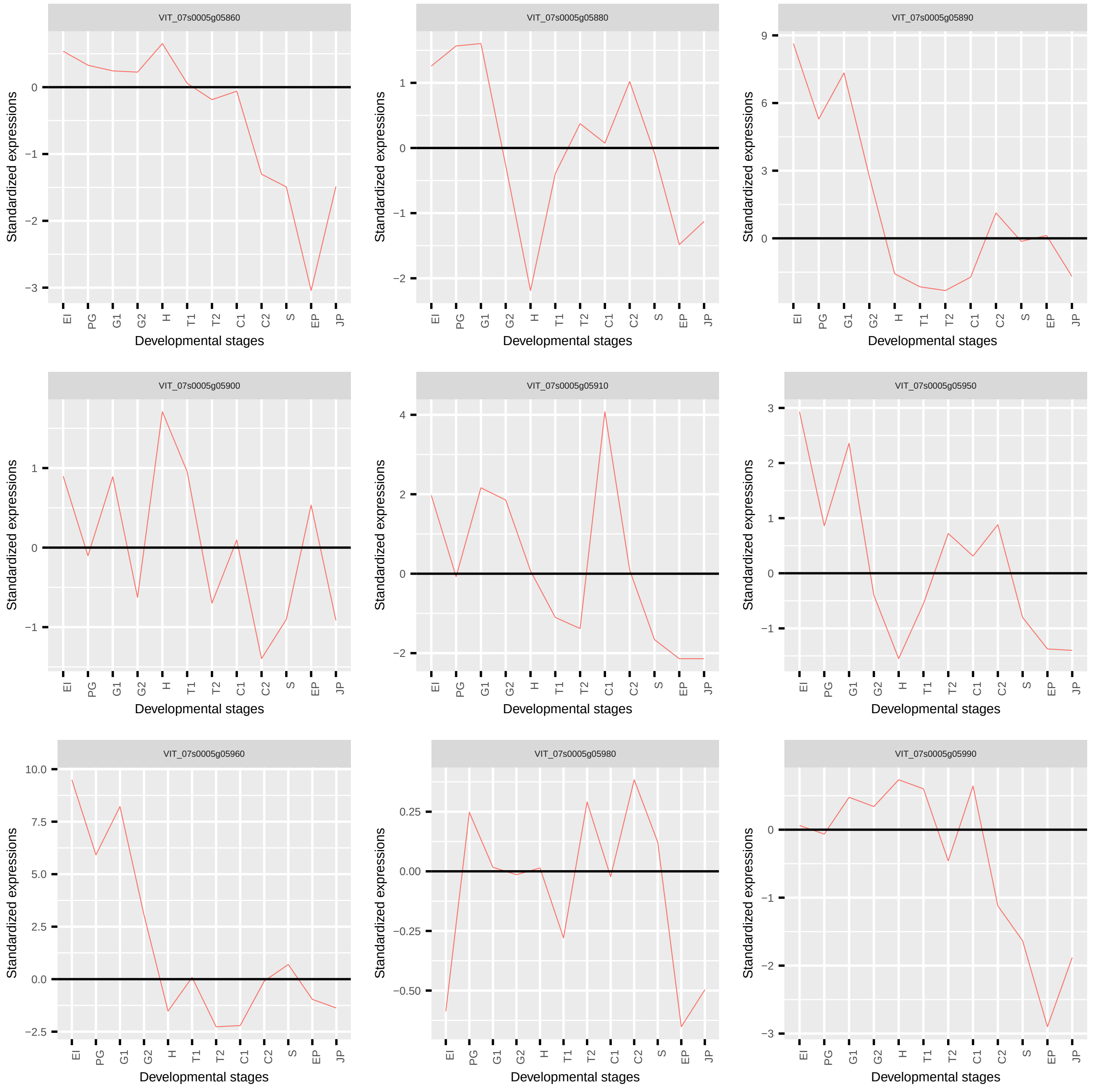
VIT_07s0005g05860
VIT_07s0005g05880
VIT_07s0005g05890
VIT_07s0005g05900
VIT_07s0005g05910
VIT_07s0005g05950
VIT_07s0005g05960
VIT_07s0005g05980
VIT_07s0005g05990
Standardized expressions
Developmental stages
EI PG G1 G2 H T1 T2 C1 C2 S EP JP

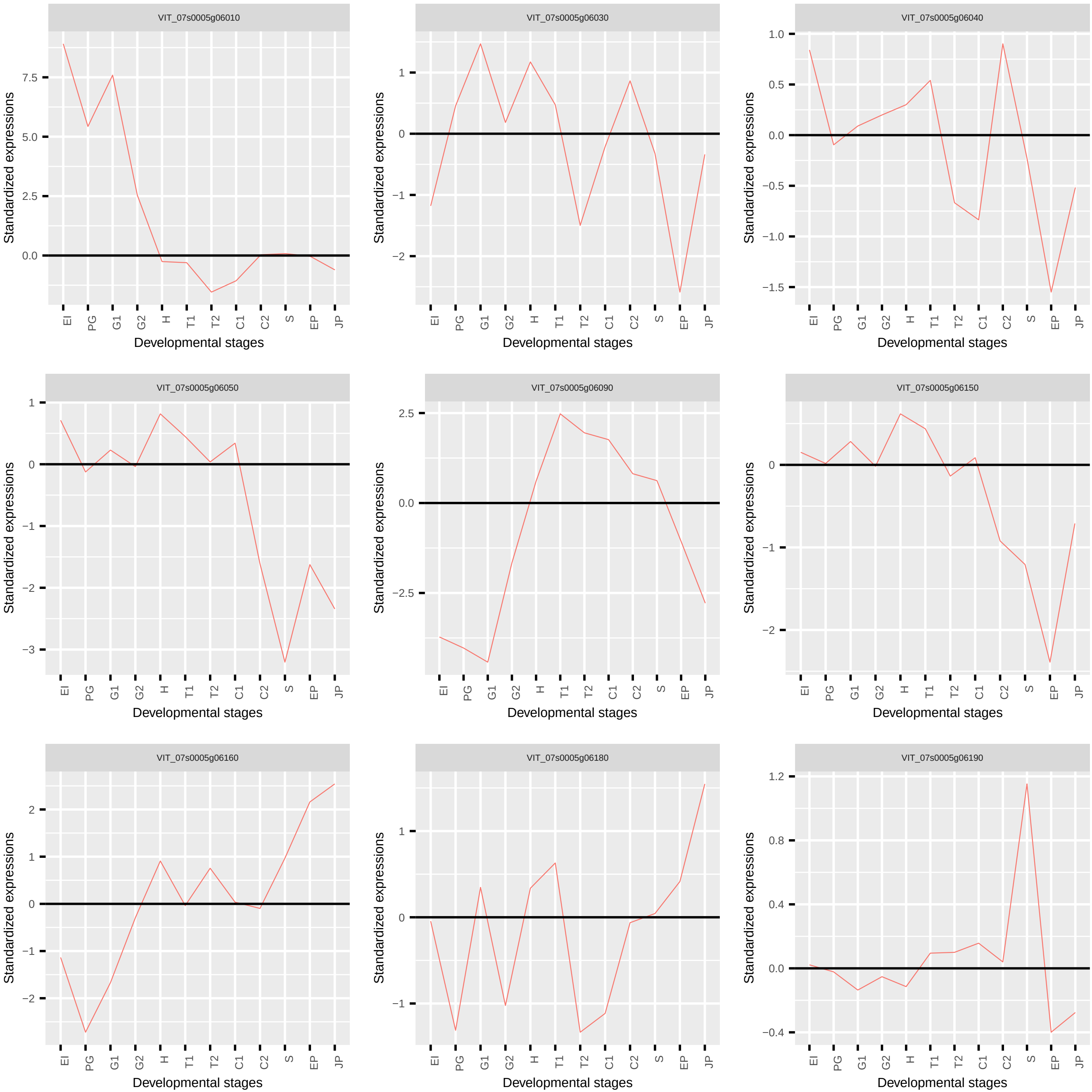
VIT_07s0005g06010
VIT_07s0005g06030
VIT_07s0005g06040
VIT_07s0005g06050
VIT_07s0005g06090
VIT_07s0005g06150
VIT_07s0005g06160
VIT_07s0005g06180
VIT_07s0005g06190
Standardized expressions
Developmental stages
EI PG G1 G2 H T1 T2 C1 C2 S EP JP
0.0 2.5 5.0 7.5
−2 −1 0 1
−1.5 −1.0 −0.5 0.0 0.5 1.0
−3 −2 −1 0 1
−2.5 0.0 2.5
−2 −1 0
−2 −1 0 1 2
−1 0 1
−0.4 0.0 0.4 0.8 1.2

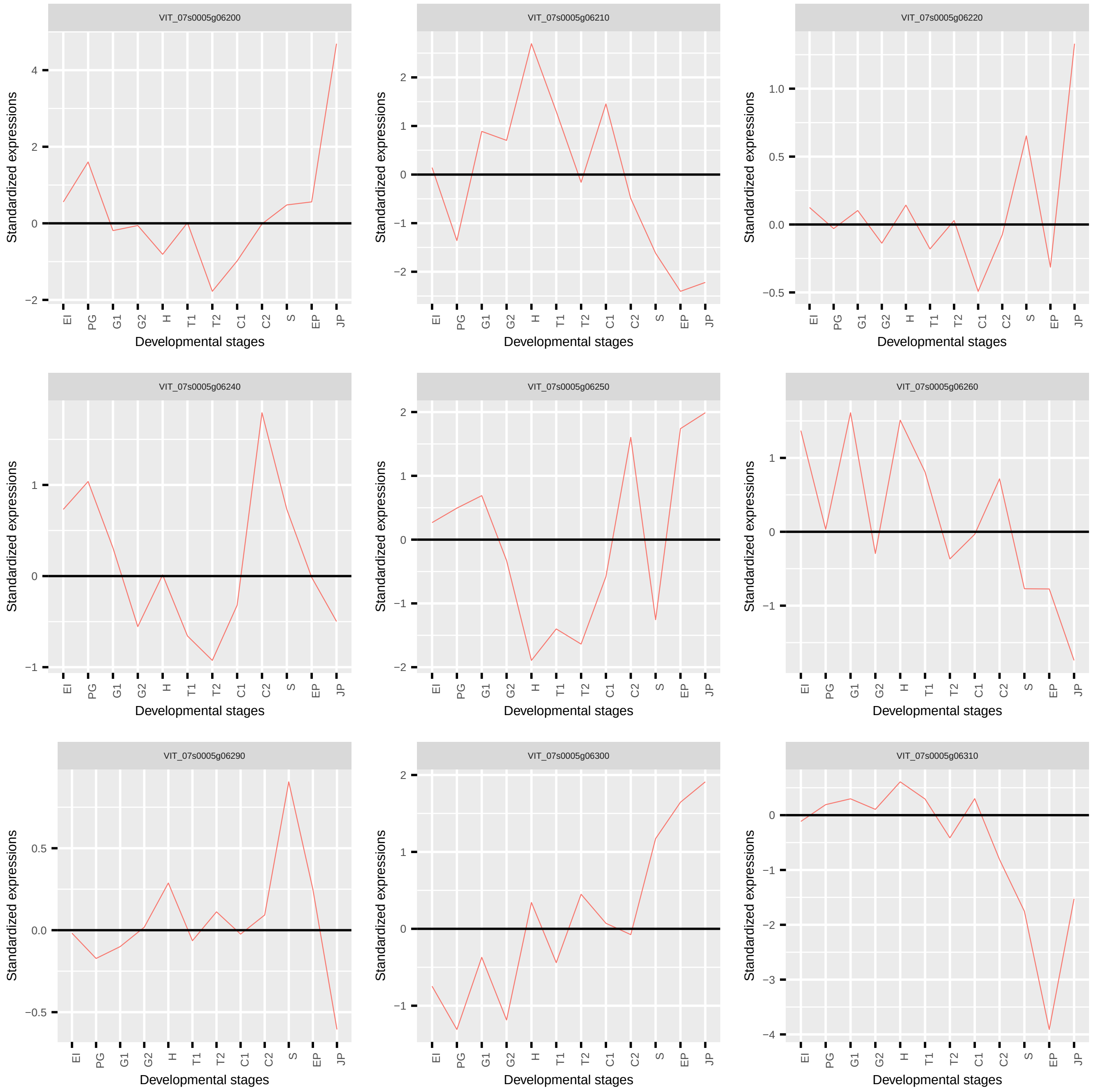
VIT_07s0005g06200
VIT_07s0005g06210
VIT_07s0005g06220
VIT_07s0005g06240
VIT_07s0005g06250
VIT_07s0005g06260
VIT_07s0005g06290
VIT_07s0005g06300
VIT_07s0005g06310
Standardized expressions
Developmental stages
EI PG G1 G2 H T1 T2 C1 C2 S EP JP

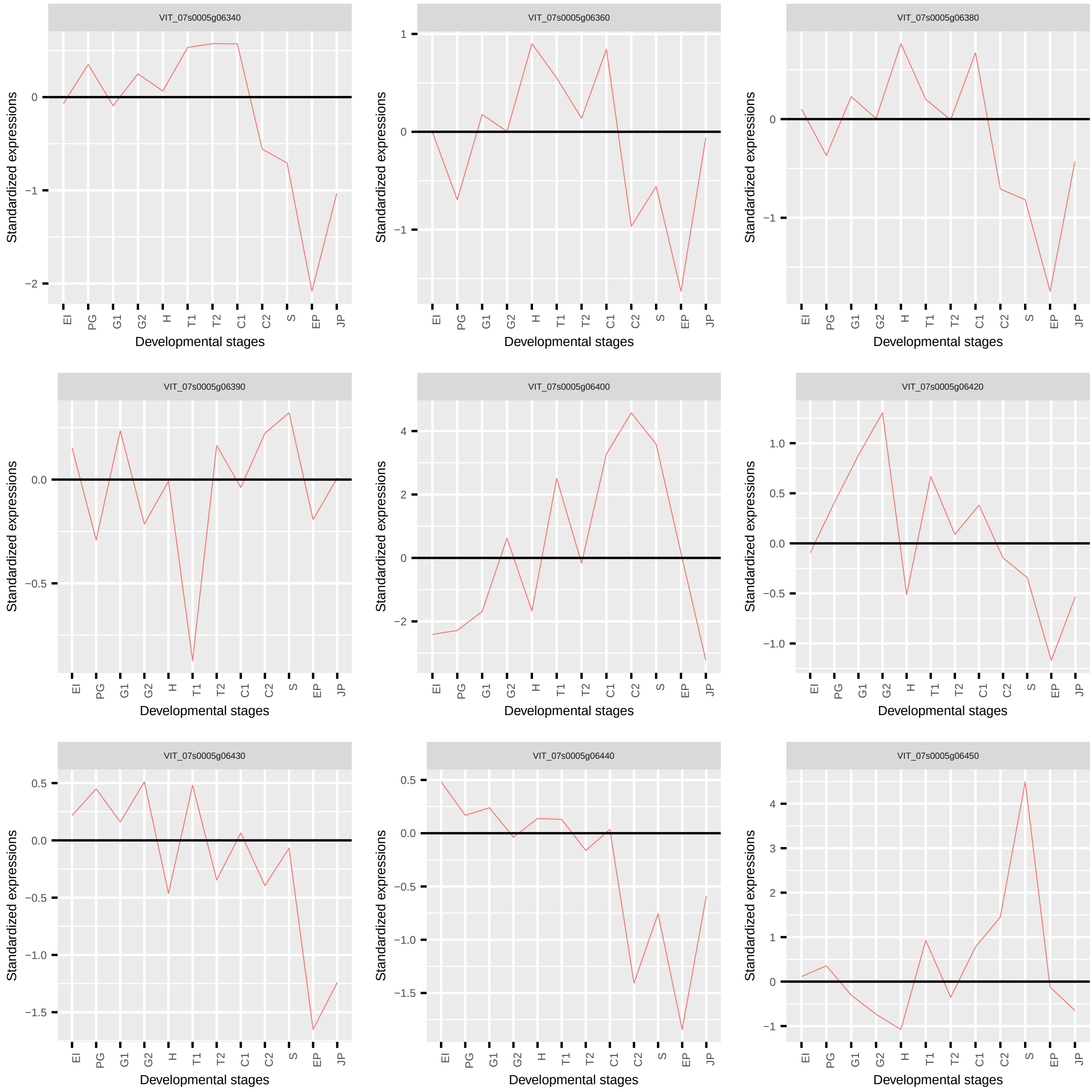
VIT_07s0005g06340
VIT_07s0005g06360
VIT_07s0005g06380
VIT_07s0005g06390
VIT_07s0005g06400
VIT_07s0005g06420
VIT_07s0005g06430
VIT_07s0005g06440
VIT_07s0005g06450
Standardized expressions
Developmental stages
EI
PG
G1
G2
H
T1
T2
C1
C2
S
EP
JP

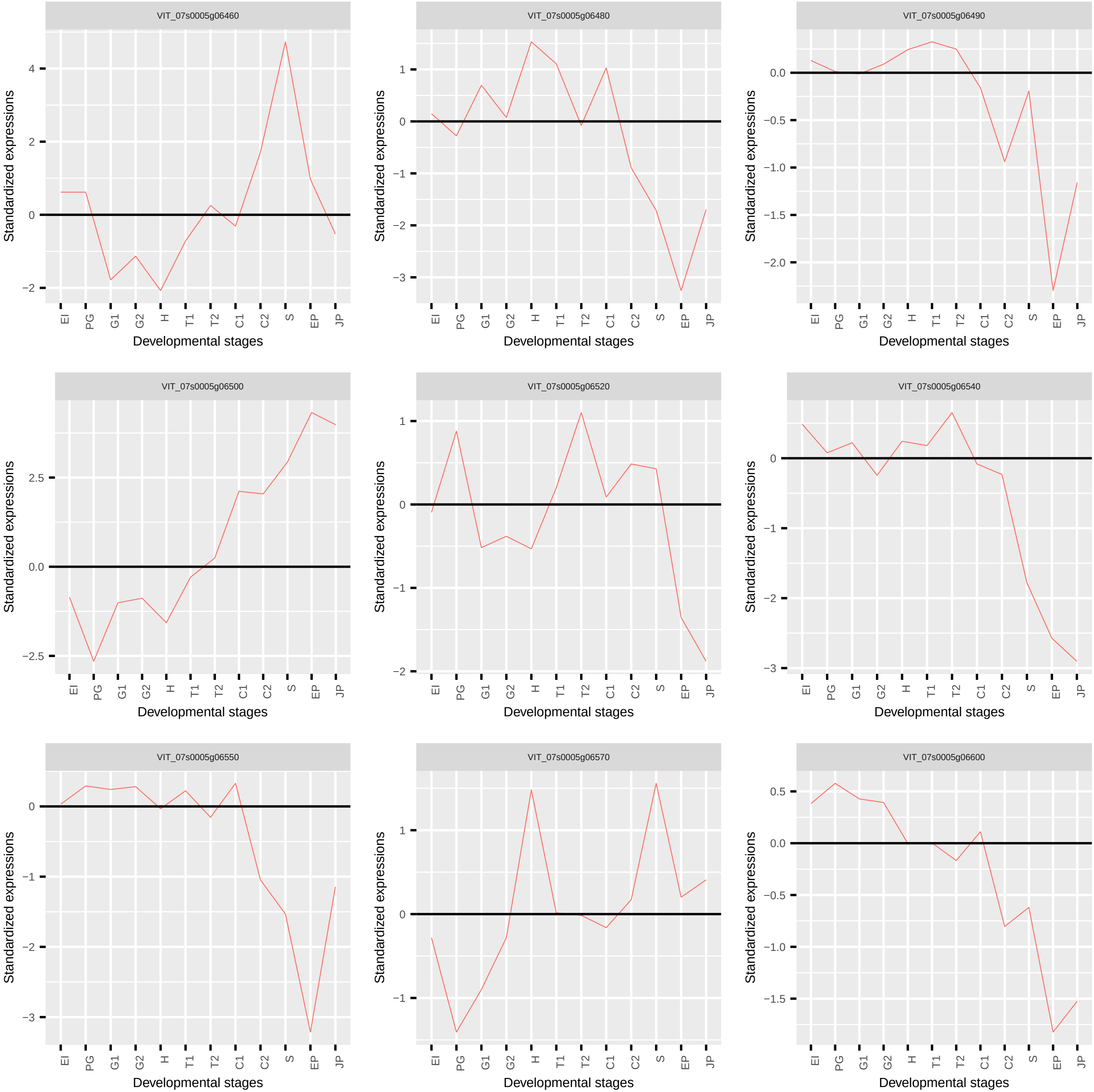
VIT_07s0005g06460
VIT_07s0005g06480
VIT_07s0005g06490
VIT_07s0005g06500
VIT_07s0005g06520
VIT_07s0005g06540
VIT_07s0005g06550
VIT_07s0005g06570
VIT_07s0005g06600
Standardized expressions
Developmental stages
EI PG G1 G2 H T1 T2 C1 C2 S EP JP
4 2 0 −2
1 0 −1 −2 −3
0.0 −0.5 −1.0 −1.5 −2.0
2.5 0.0 −2.5
1 0 −1 −2
0 −1 −2 −3
0 −1 −2 −3
1 0 −1
0.5 0.0 −0.5 −1.0 −1.5

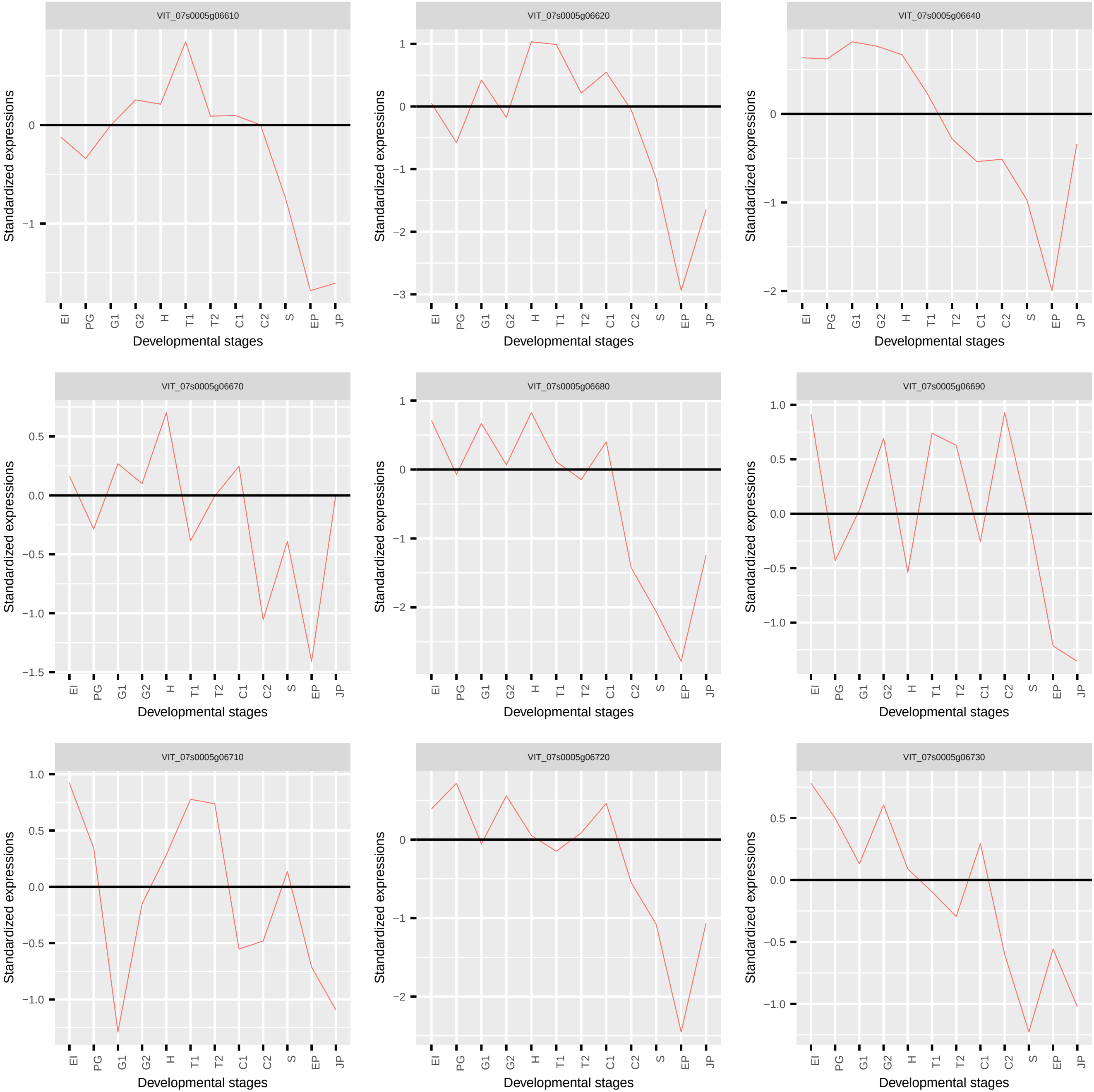

VIT_07s0005g06610
VIT_07s0005g06620
VIT_07s0005g06640
VIT_07s0005g06670
VIT_07s0005g06680
VIT_07s0005g06690
VIT_07s0005g06710
VIT_07s0005g06720
VIT_07s0005g06730
Standardized expressions
Developmental stages
EI
PG
G1
G2
H
T1
T2
C1
C2
S
EP
JP
1
0
−1
−2
−3
1.0
0.5
0.0
−0.5
−1.0
−1.5

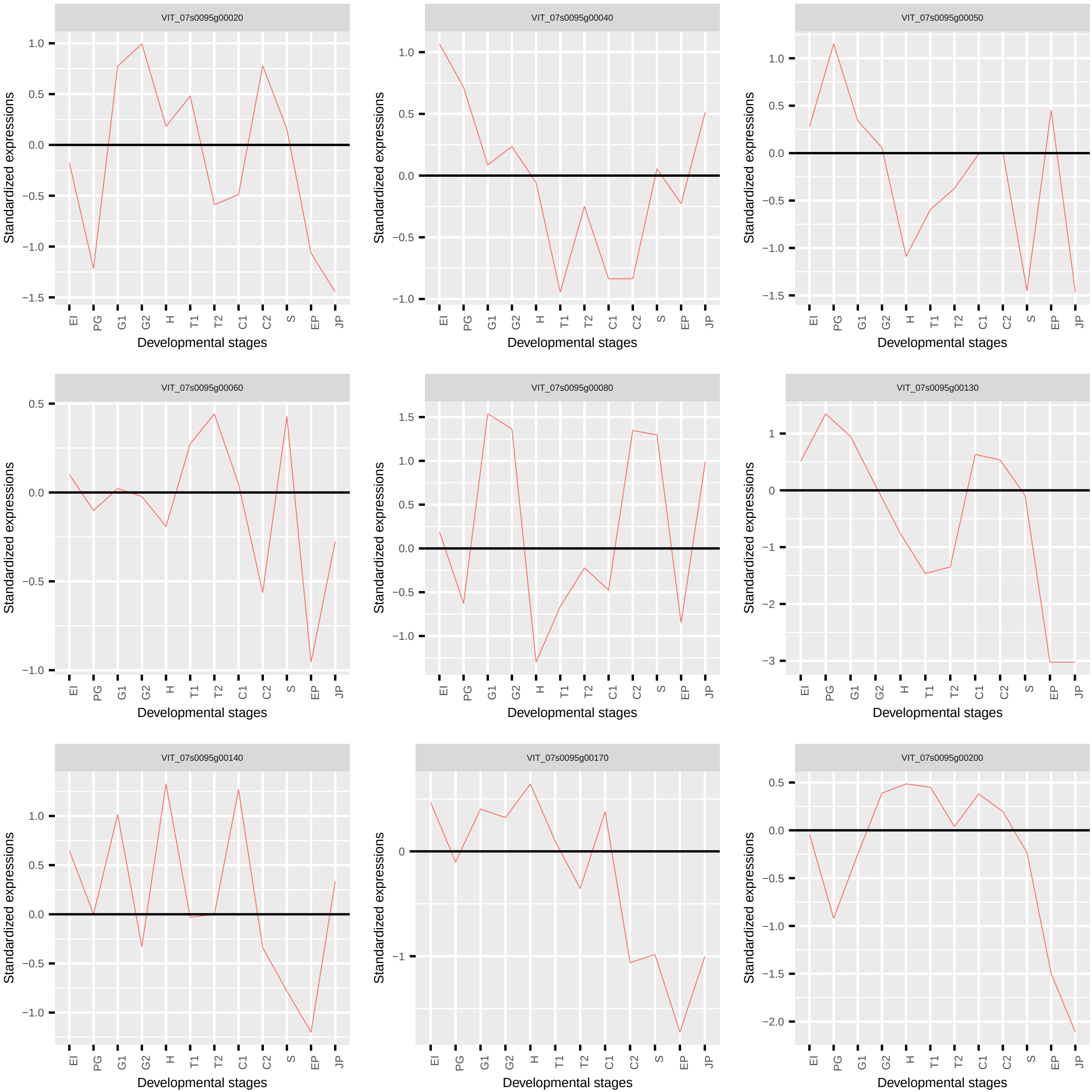
VIT_07s0095g00020
VIT_07s0095g00040
VIT_07s0095g00050
VIT_07s0095g00060
VIT_07s0095g00080
VIT_07s0095g00130
VIT_07s0095g00140
VIT_07s0095g00170
VIT_07s0095g00200
Standardized expressions
Developmental stages
EI PG G1 G2 H T1 T2 C1 C2 S EP JP

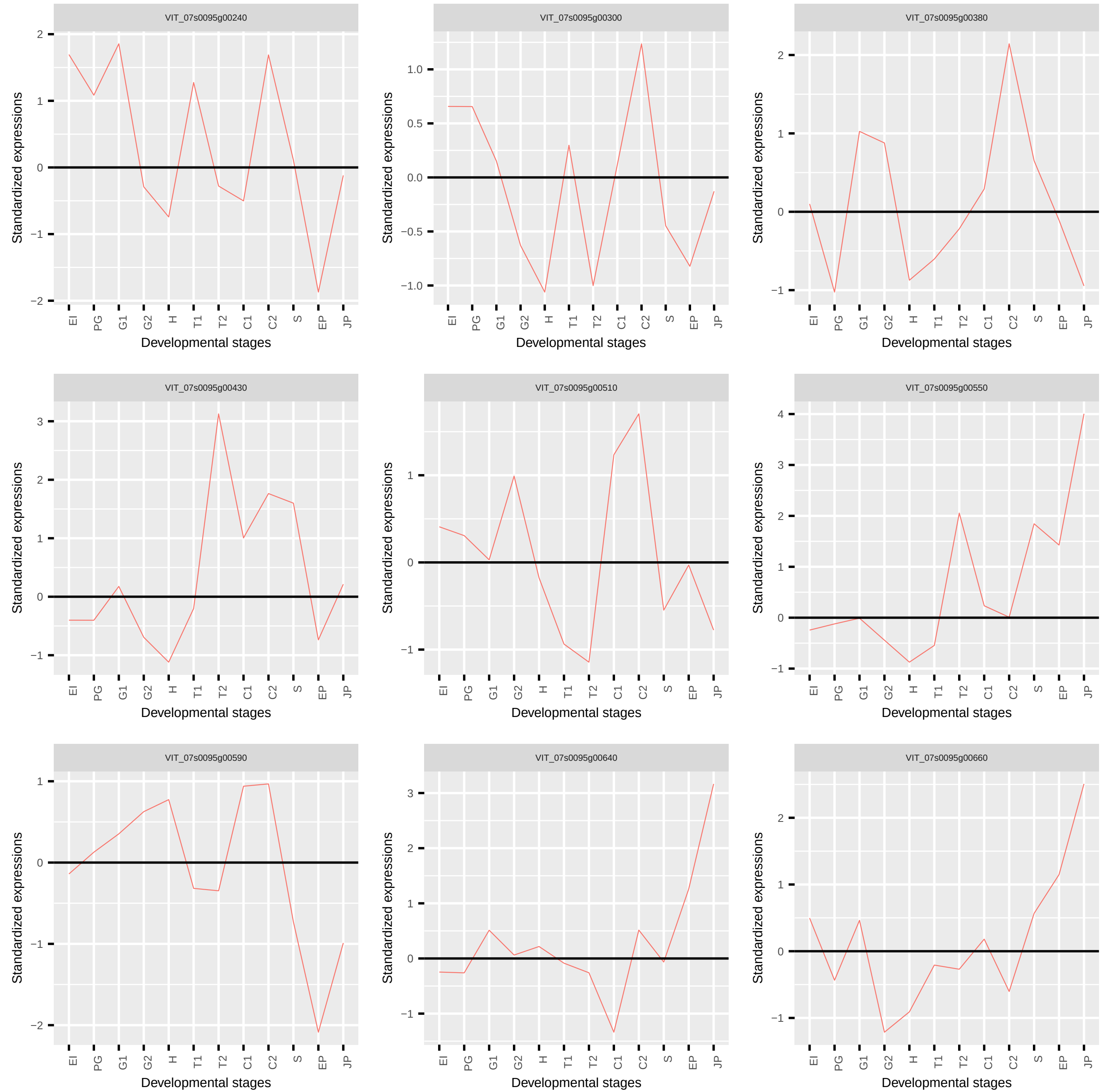
VIT_07s0095g00240
Standardized expressions
EI PG G1 G2 H T1 T2 C1 C2 S EP JP
Developmental stages
VIT_07s0095g00300
Standardized expressions
EI PG G1 G2 H T1 T2 C1 C2 S EP JP
Developmental stages
VIT_07s0095g00380
Standardized expressions
EI PG G1 G2 H T1 T2 C1 C2 S EP JP
Developmental stages
VIT_07s0095g00430
Standardized expressions
EI PG G1 G2 H T1 T2 C1 C2 S EP JP
Developmental stages
VIT_07s0095g00510
Standardized expressions
EI PG G1 G2 H T1 T2 C1 C2 S EP JP
Developmental stages
VIT_07s0095g00550
Standardized expressions
EI PG G1 G2 H T1 T2 C1 C2 S EP JP
Developmental stages
VIT_07s0095g00590
Standardized expressions
EI PG G1 G2 H T1 T2 C1 C2 S EP JP
Developmental stages
VIT_07s0095g00640
Standardized expressions
EI PG G1 G2 H T1 T2 C1 C2 S EP JP
Developmental stages
VIT_07s0095g00660
Standardized expressions
EI PG G1 G2 H T1 T2 C1 C2 S EP JP
Developmental stages

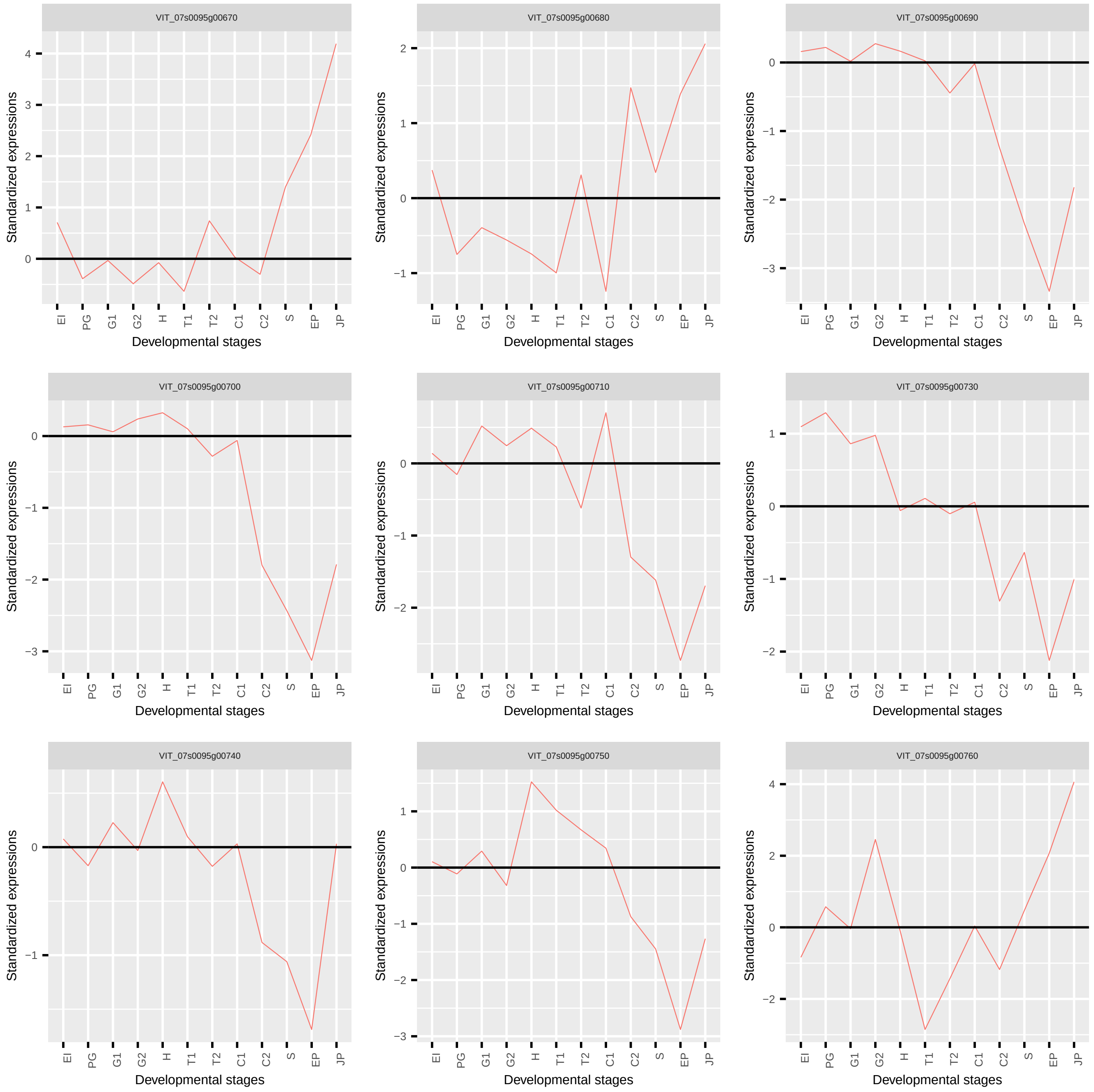
VIT_07s0095g00670
VIT_07s0095g00680
VIT_07s0095g00690
VIT_07s0095g00700
VIT_07s0095g00710
VIT_07s0095g00730
VIT_07s0095g00740
VIT_07s0095g00750
VIT_07s0095g00760
Standardized expressions
Developmental stages
EI PG G1 G2 H T1 T2 C1 C2 S EP JP

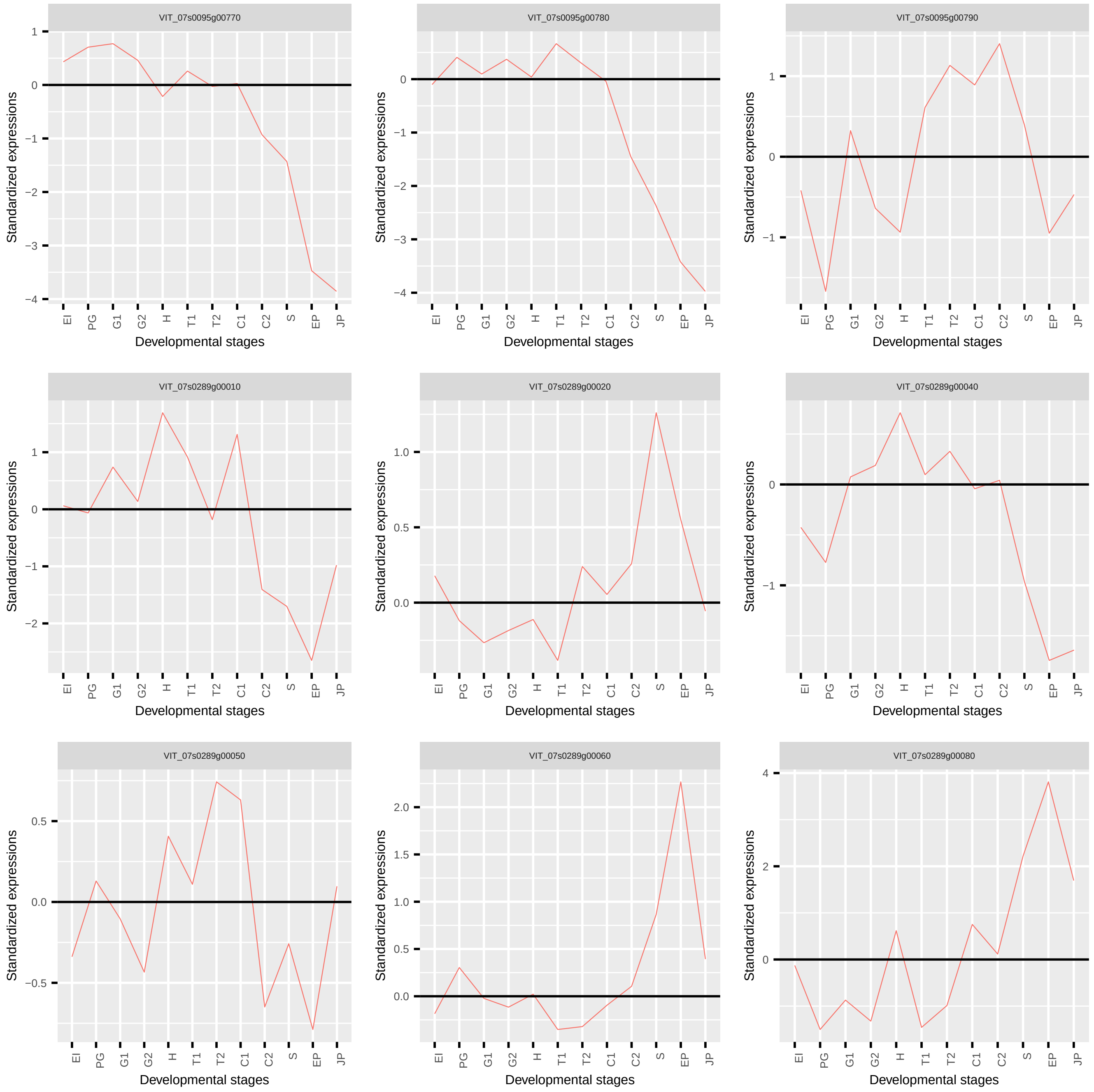

VIT_07s0095g00770
VIT_07s0095g00780
VIT_07s0095g00790
VIT_07s0289g00010
VIT_07s0289g00020
VIT_07s0289g00040
VIT_07s0289g00050
VIT_07s0289g00060
VIT_07s0289g00080
Standardized expressions
Developmental stages
EI PG G1 G2 H T1 T2 C1 C2 S EP JP

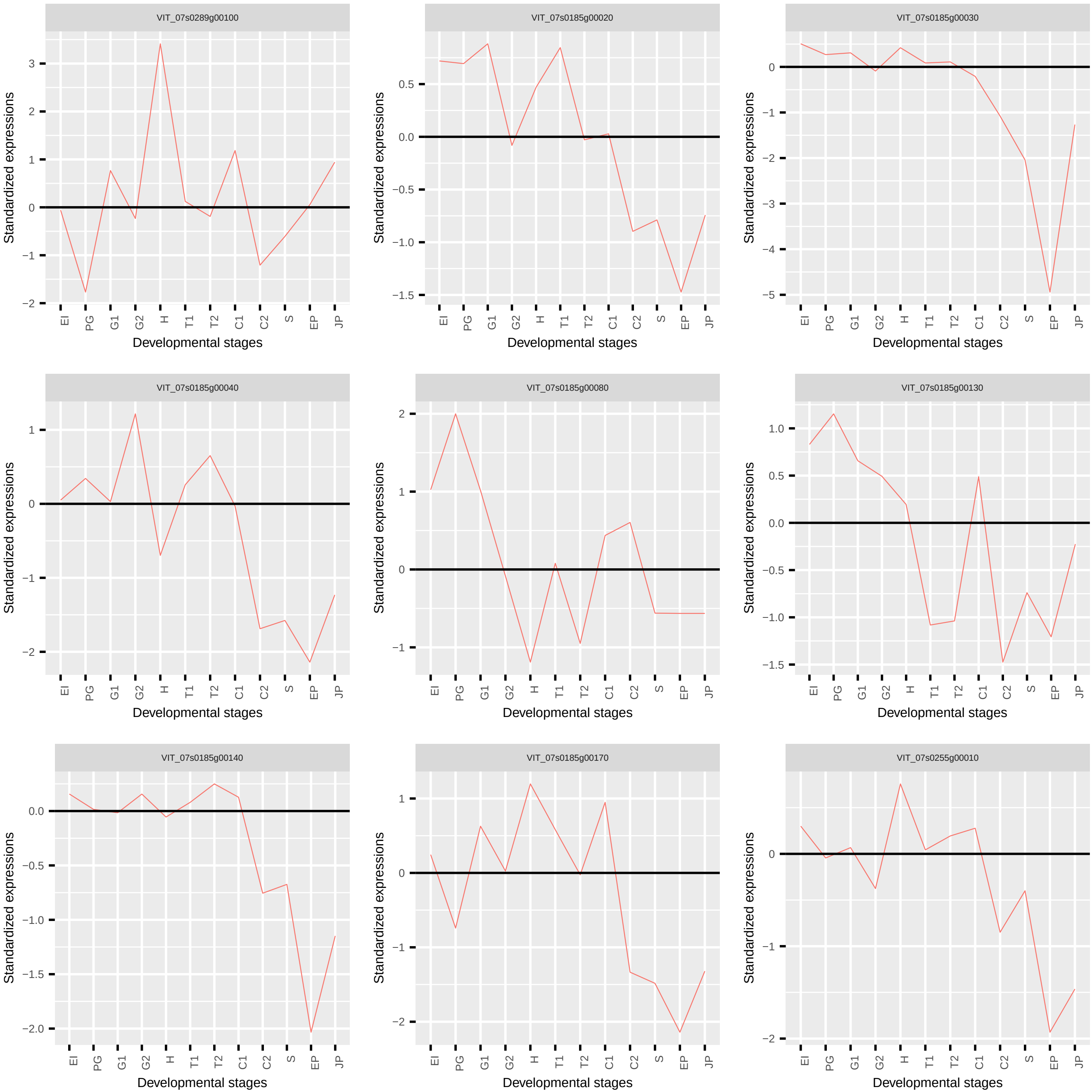
VIT_07s0289g00100
VIT_07s0185g00020
VIT_07s0185g00030
VIT_07s0185g00040
VIT_07s0185g00080
VIT_07s0185g00130
VIT_07s0185g00140
VIT_07s0185g00170
VIT_07s0255g00010
Standardized expressions
Developmental stages
EI PG G1 G2 H T1 T2 C1 C2 S EP JP

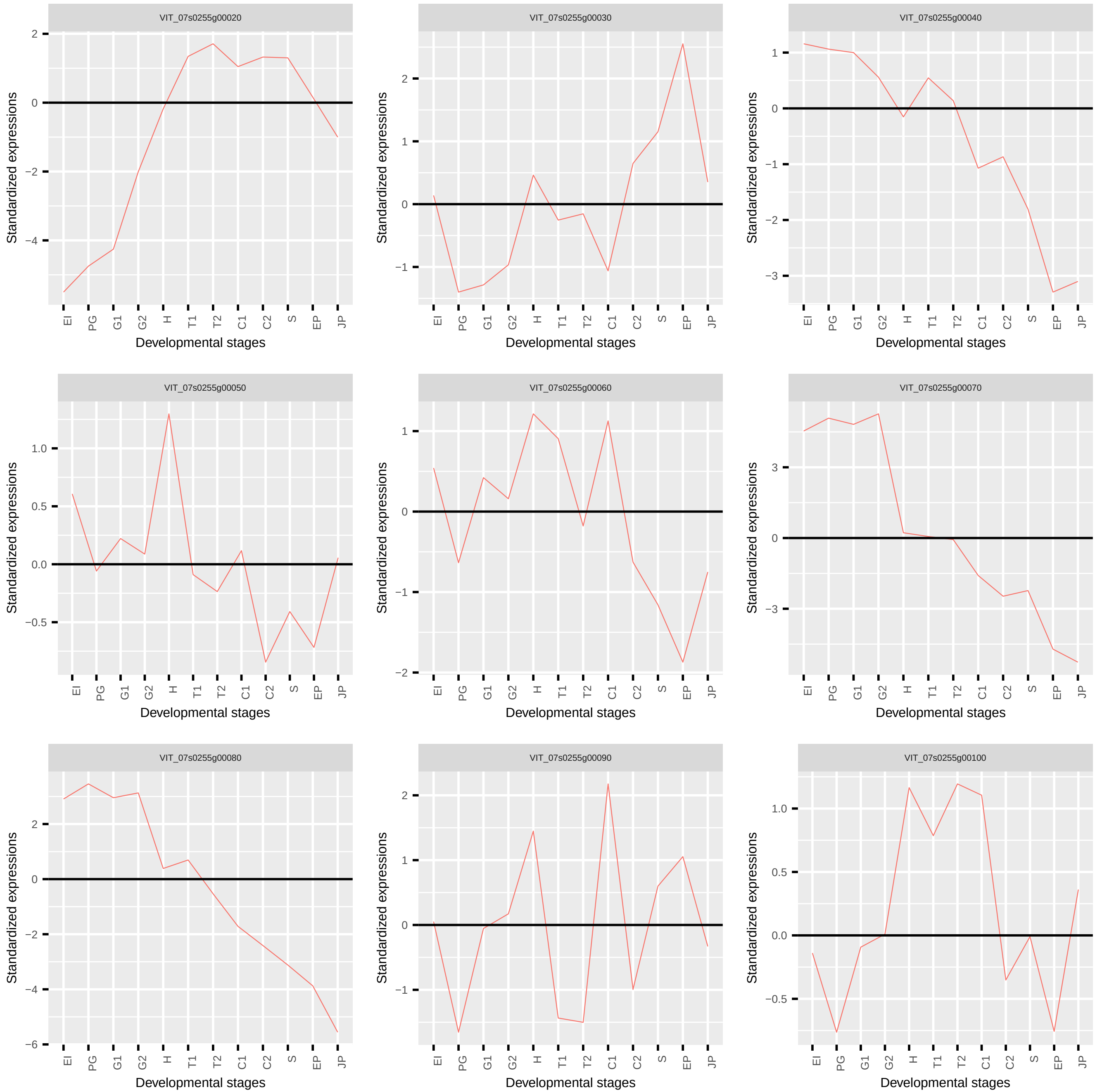
VIT_07s0255g00020
VIT_07s0255g00030
VIT_07s0255g00040
VIT_07s0255g00050
VIT_07s0255g00060
VIT_07s0255g00070
VIT_07s0255g00080
VIT_07s0255g00090
VIT_07s0255g00100
Standardized expressions
Developmental stages
EI PG G1 G2 H T1 T2 C1 C2 S EP JP

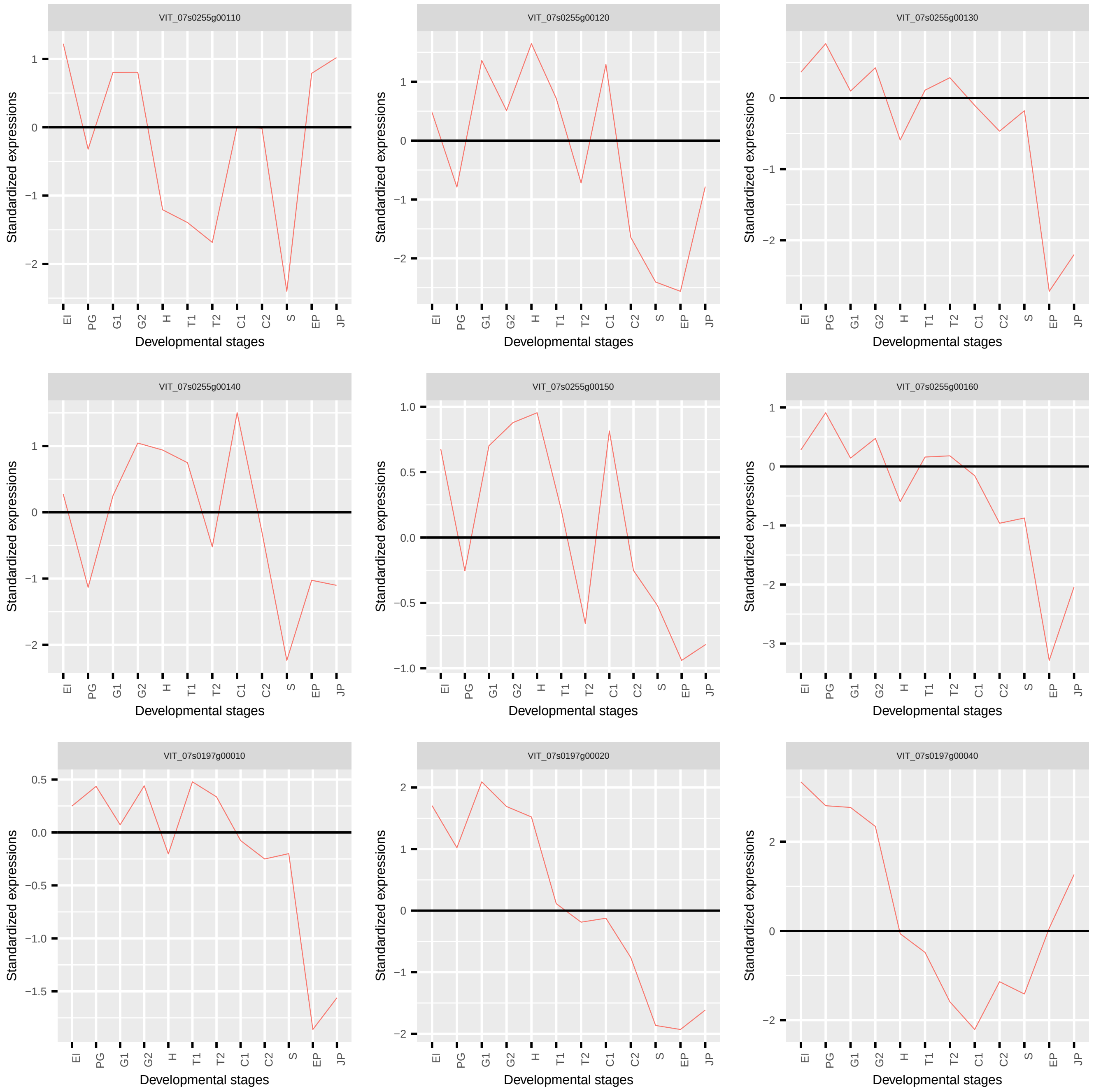
VIT_07s0255g00110
VIT_07s0255g00120
VIT_07s0255g00130
VIT_07s0255g00140
VIT_07s0255g00150
VIT_07s0255g00160
VIT_07s0197g00010
VIT_07s0197g00020
VIT_07s0197g00040
Standardized expressions
Developmental stages
EI PG G1 G2 H T1 T2 C1 C2 S EP JP

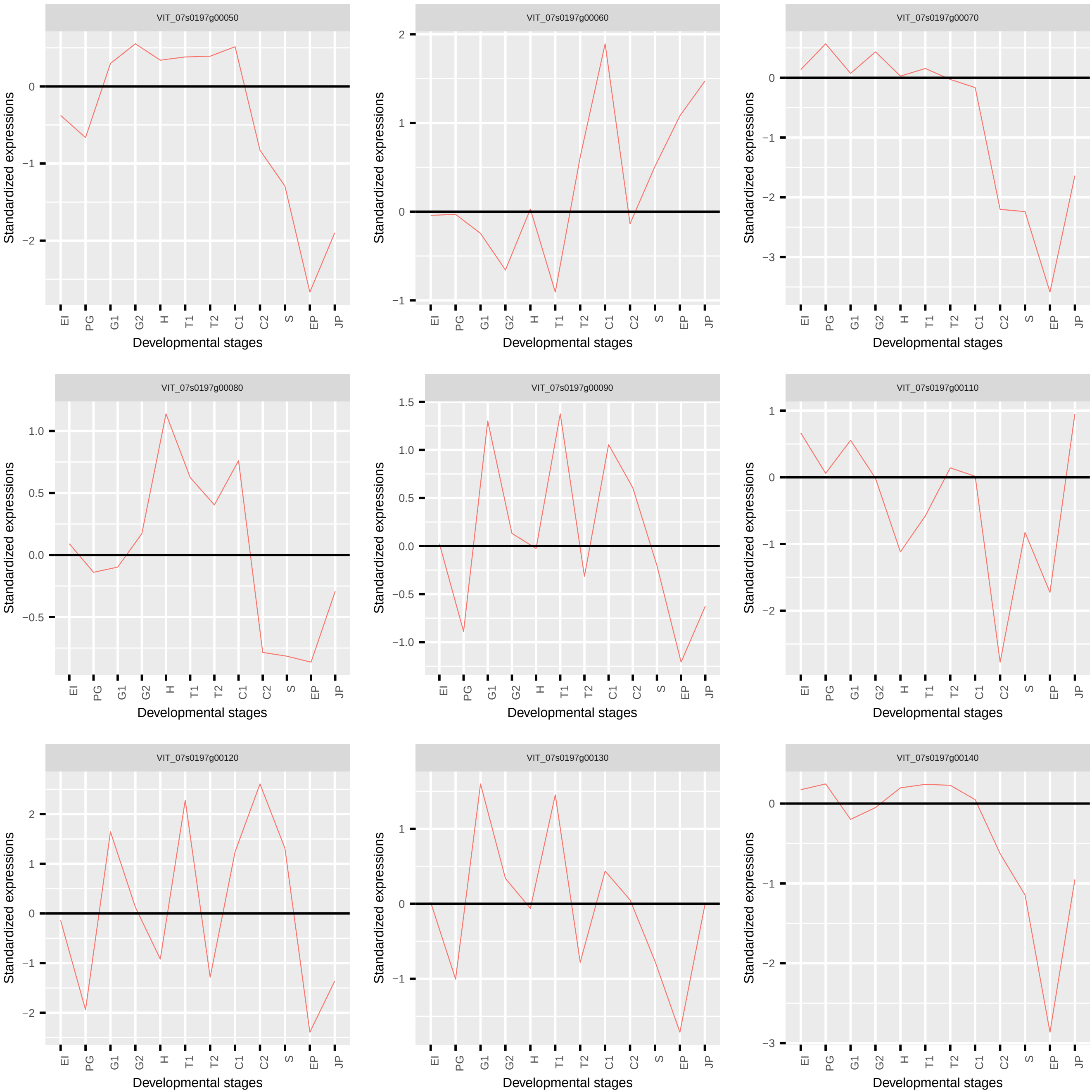
VIT_07s0197g00050
VIT_07s0197g00060
VIT_07s0197g00070
VIT_07s0197g00080
VIT_07s0197g00090
VIT_07s0197g00110
VIT_07s0197g00120
VIT_07s0197g00130
VIT_07s0197g00140
Standardized expressions
Developmental stages
EI PG G1 G2 H T1 T2 C1 C2 S EP JP

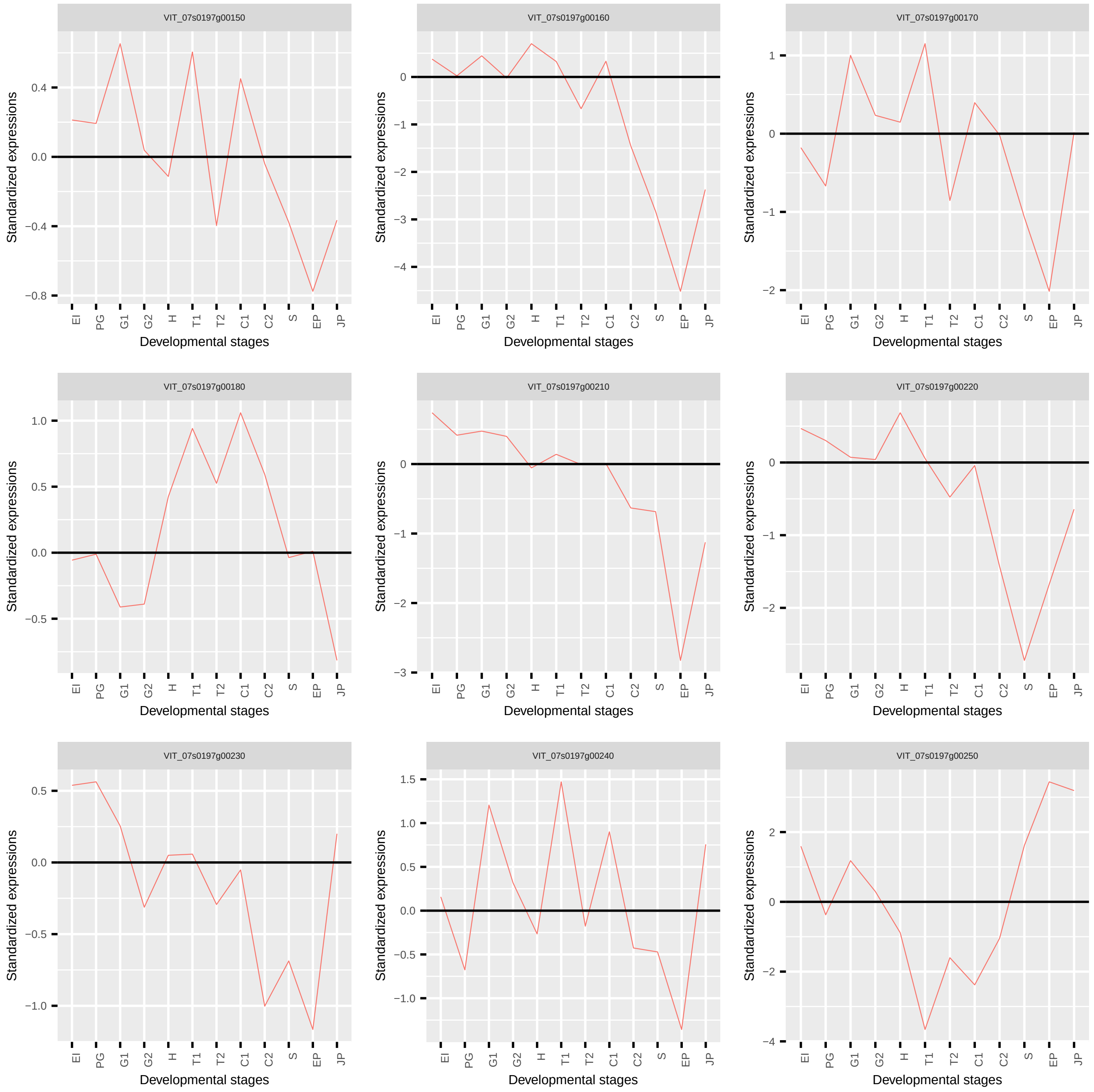

VIT_07s0197g00150
VIT_07s0197g00160
VIT_07s0197g00170
VIT_07s0197g00180
VIT_07s0197g00210
VIT_07s0197g00220
VIT_07s0197g00230
VIT_07s0197g00240
VIT_07s0197g00250
Standardized expressions
Developmental stages
EI PG G1 G2 H T1 T2 C1 C2 S EP JP

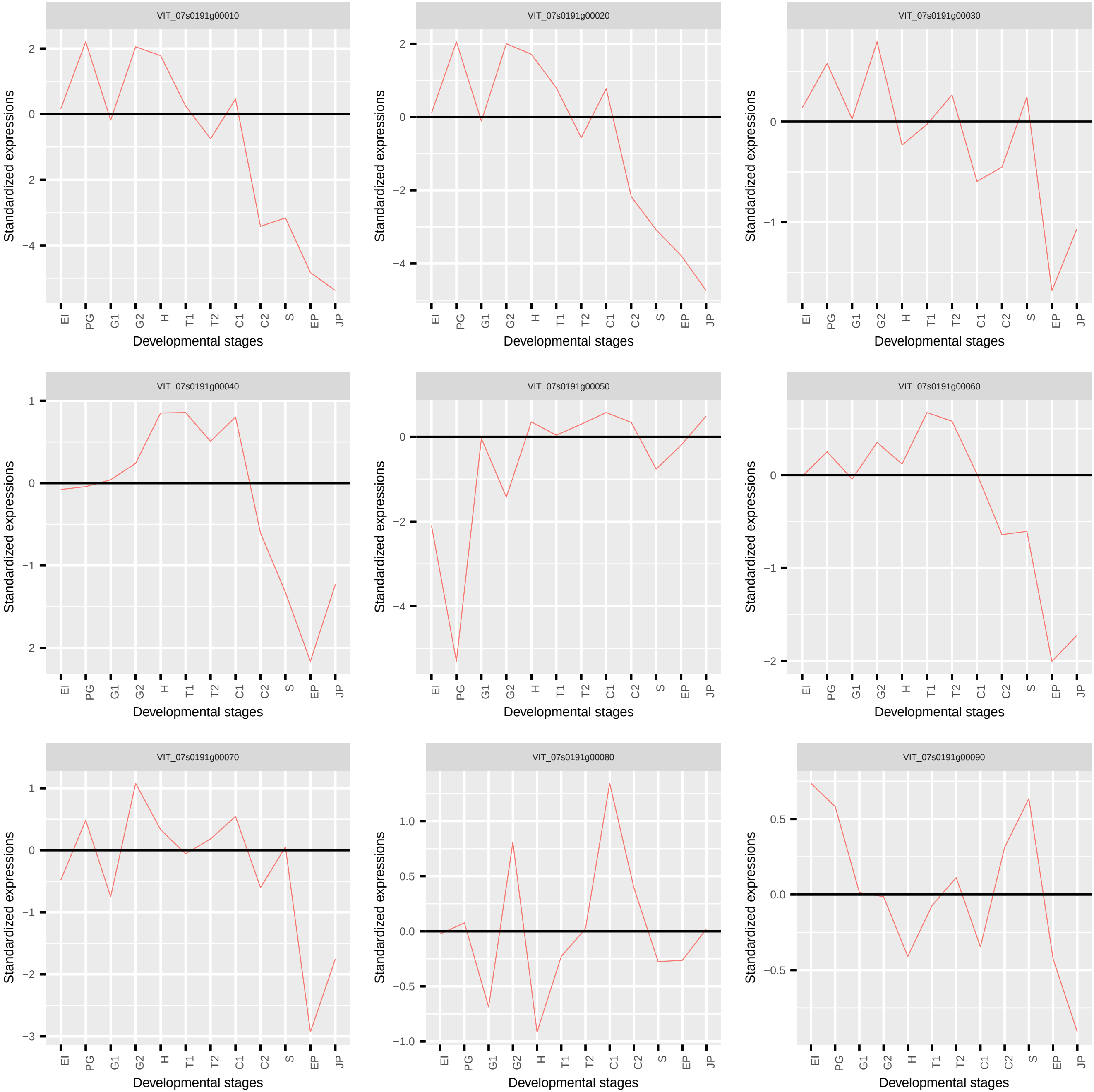
VIT_07s0191g00010
VIT_07s0191g00020
VIT_07s0191g00030
VIT_07s0191g00040
VIT_07s0191g00050
VIT_07s0191g00060
VIT_07s0191g00070
VIT_07s0191g00080
VIT_07s0191g00090
Standardized expressions
Developmental stages
EI PG G1 G2 H T1 T2 C1 C2 S EP JP
2 0 −2 −4
−1 1
1.0 0.5 0.0 −0.5 −1.0
−3

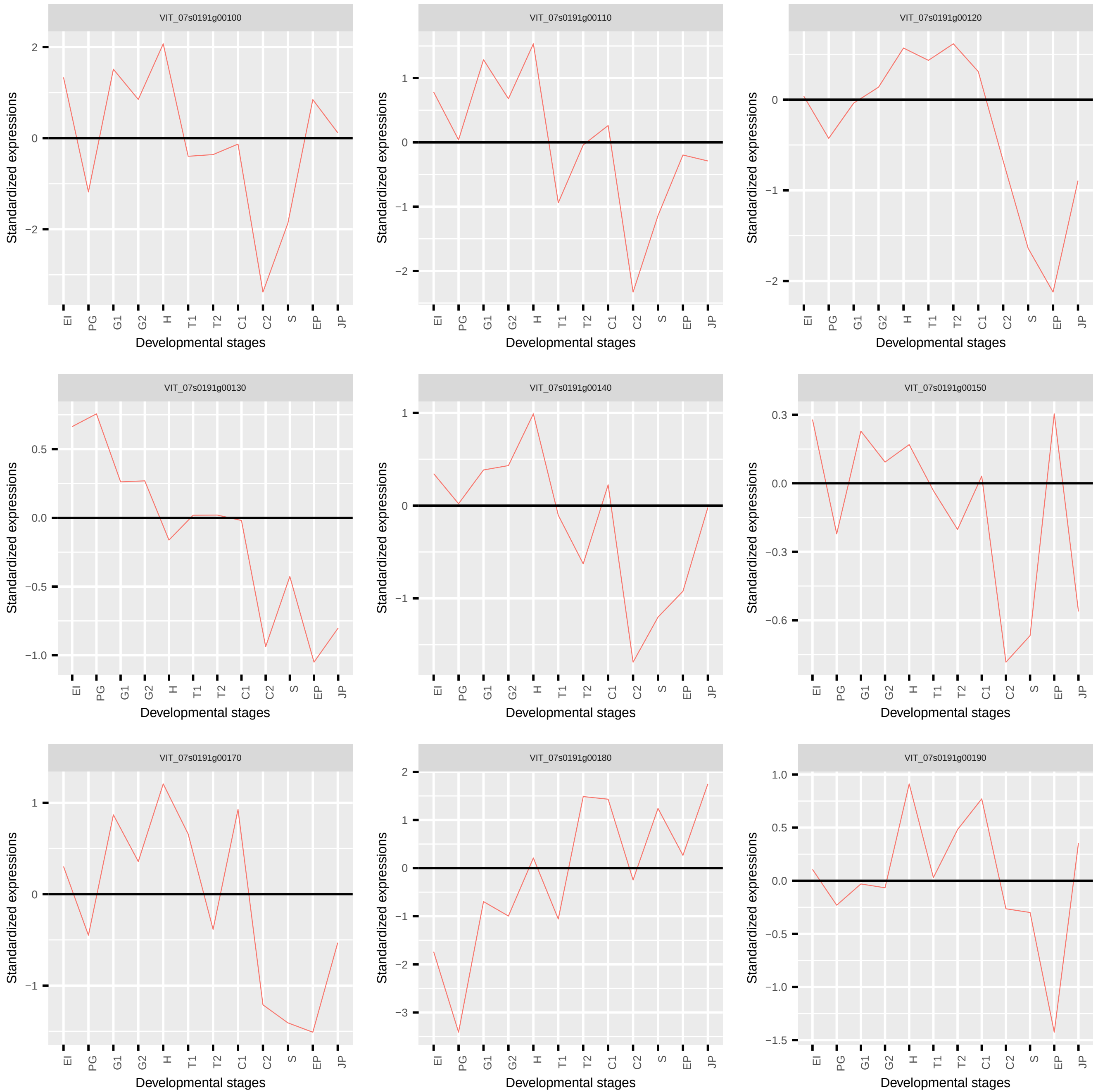
VIT_07s0191g00100
VIT_07s0191g00110
VIT_07s0191g00120
VIT_07s0191g00130
VIT_07s0191g00140
VIT_07s0191g00150
VIT_07s0191g00170
VIT_07s0191g00180
VIT_07s0191g00190
Standardized expressions
Developmental stages
EI PG G1 G2 H T1 T2 C1 C2 S EP JP

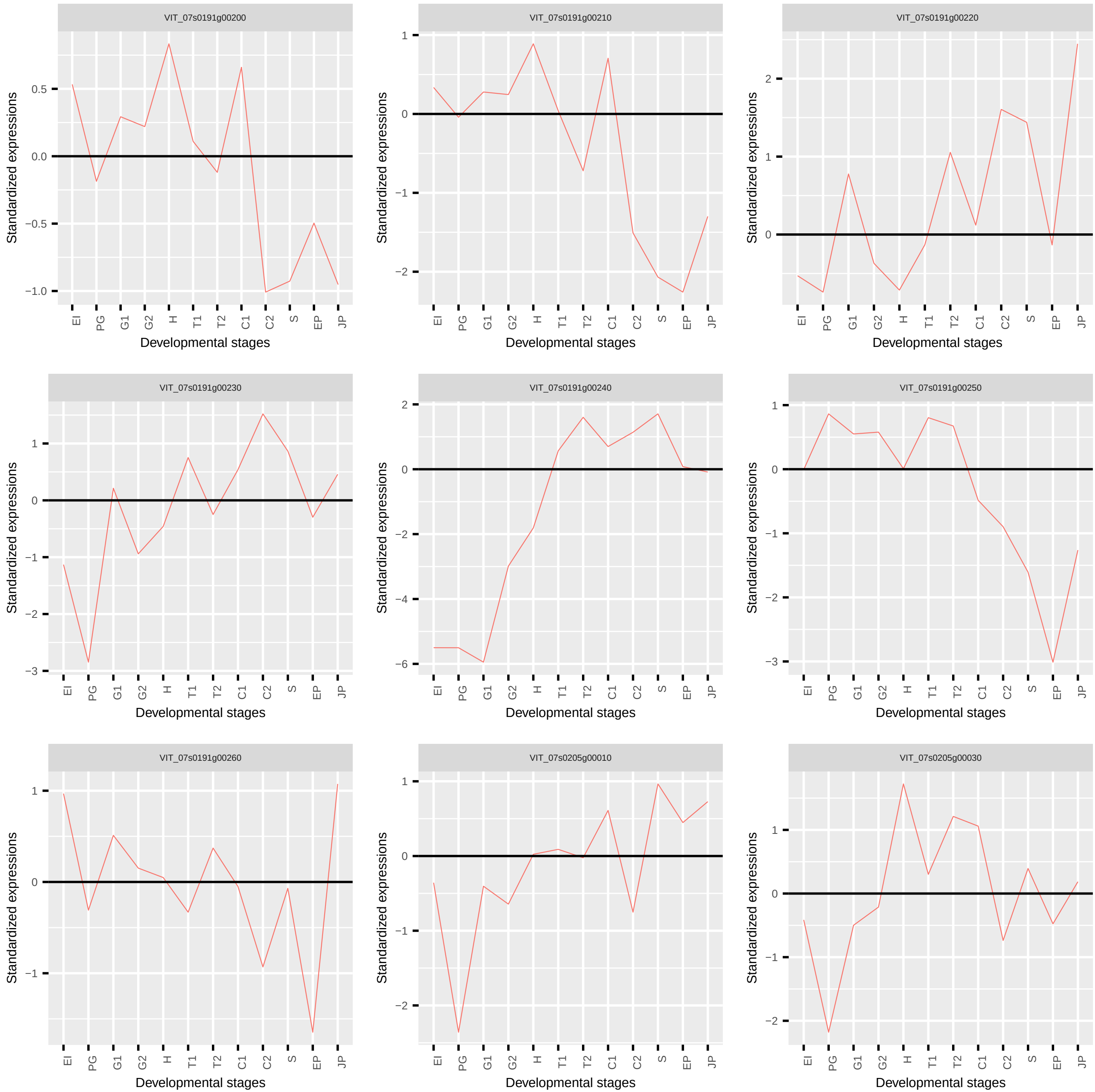
VIT_07s0191g00200
VIT_07s0191g00210
VIT_07s0191g00220
VIT_07s0191g00230
VIT_07s0191g00240
VIT_07s0191g00250
VIT_07s0191g00260
VIT_07s0205g00010
VIT_07s0205g00030
Standardized expressions
Developmental stages
EI PG G1 G2 H T1 T2 C1 C2 S EP JP

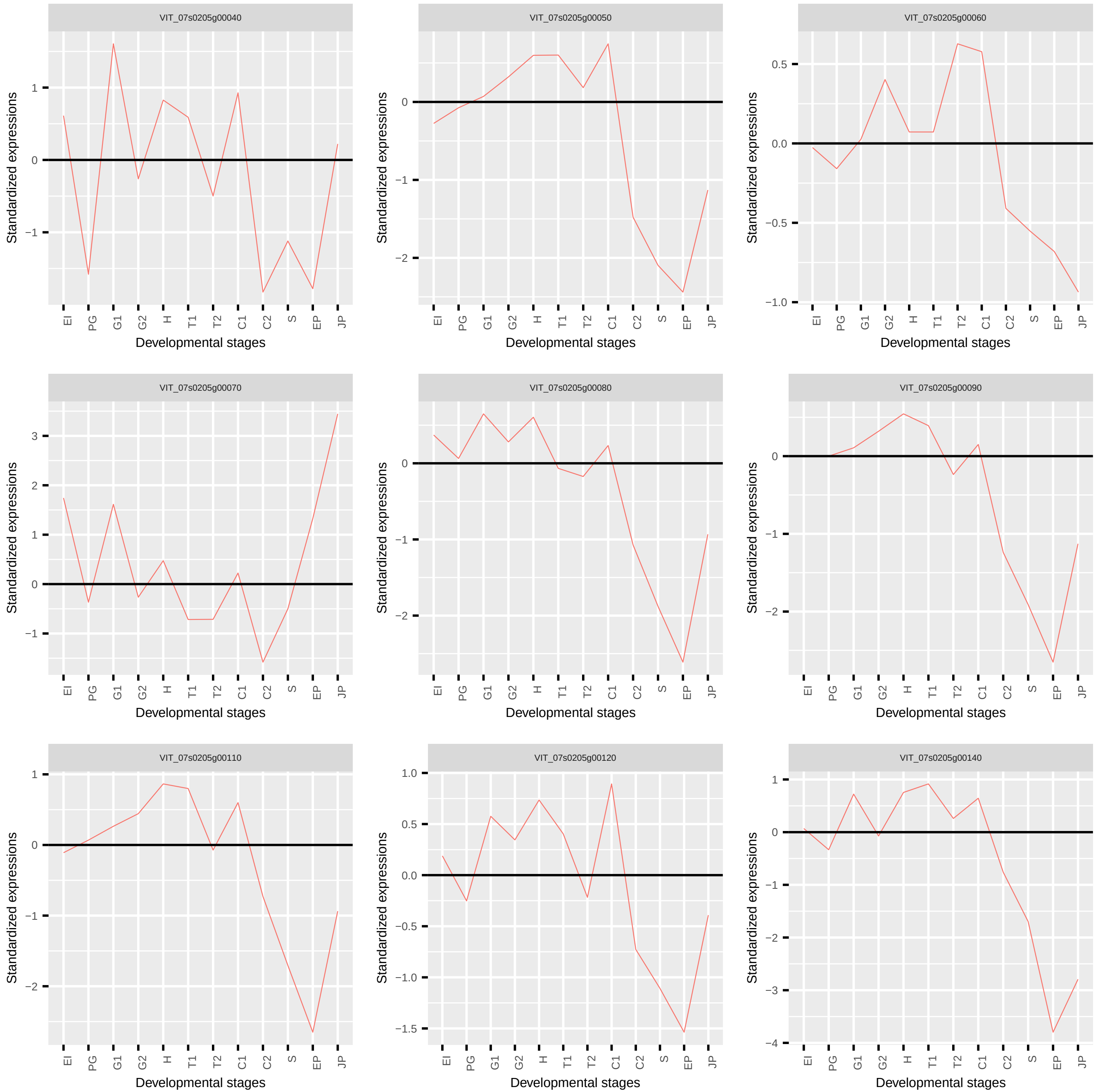
VIT_07s0205g00040
VIT_07s0205g00050
VIT_07s0205g00060
VIT_07s0205g00070
VIT_07s0205g00080
VIT_07s0205g00090
VIT_07s0205g00110
VIT_07s0205g00120
VIT_07s0205g00140
Standardized expressions
Developmental stages
EI
PG
G1
G2
H
T1
T2
C1
C2
S
EP
JP

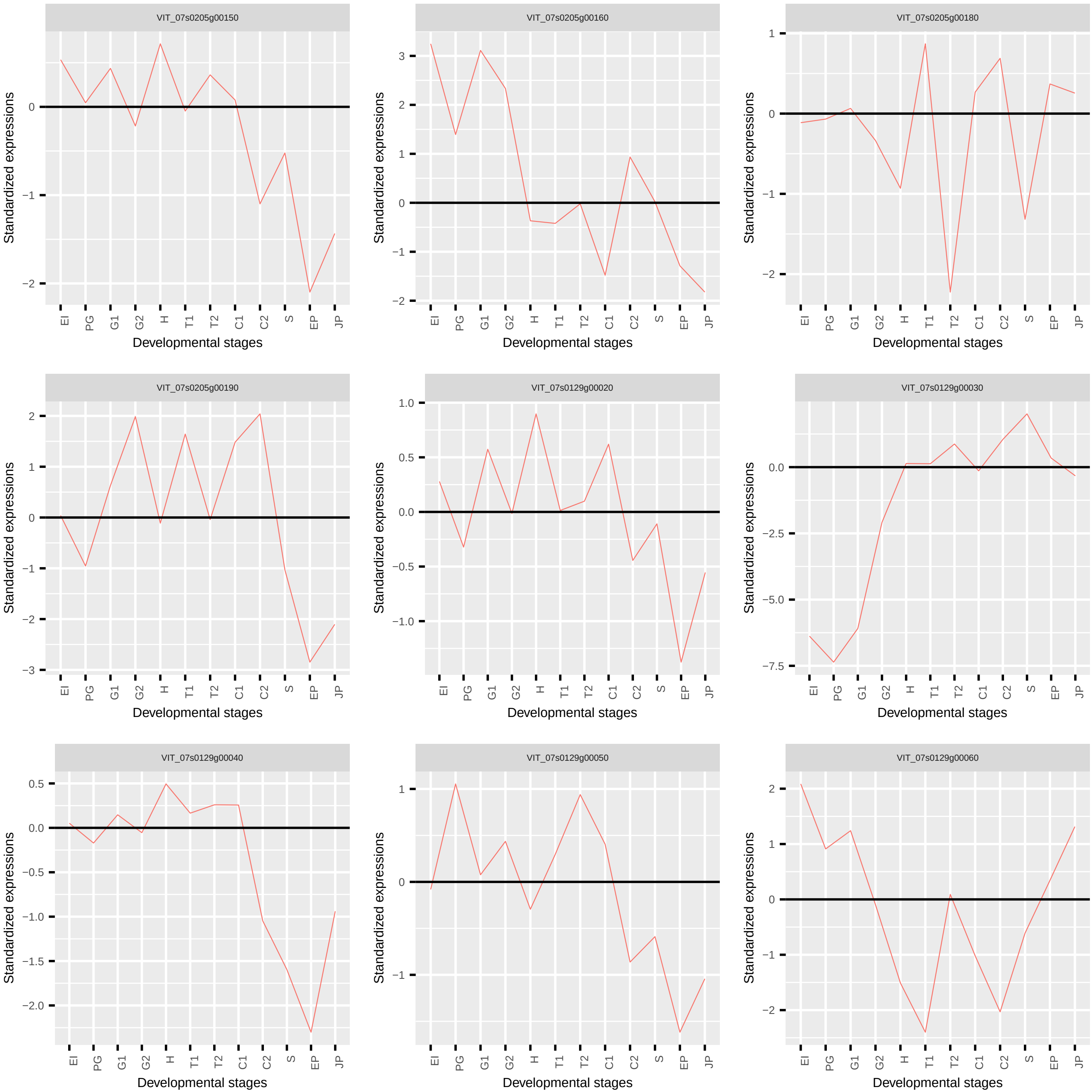

VIT_07s0205g00150
VIT_07s0205g00160
VIT_07s0205g00180
VIT_07s0205g00190
VIT_07s0129g00020
VIT_07s0129g00030
VIT_07s0129g00040
VIT_07s0129g00050
VIT_07s0129g00060
Standardized expressions
Developmental stages
EI PG G1 G2 H T1 T2 C1 C2 S EP JP
3 2 1 0 −1 −2 −3
1.0 0.5 0.0 −0.5 −1.0
0.0 −2.5 −5.0 −7.5
0.5 0.0 −0.5 −1.0 −1.5 −2.0

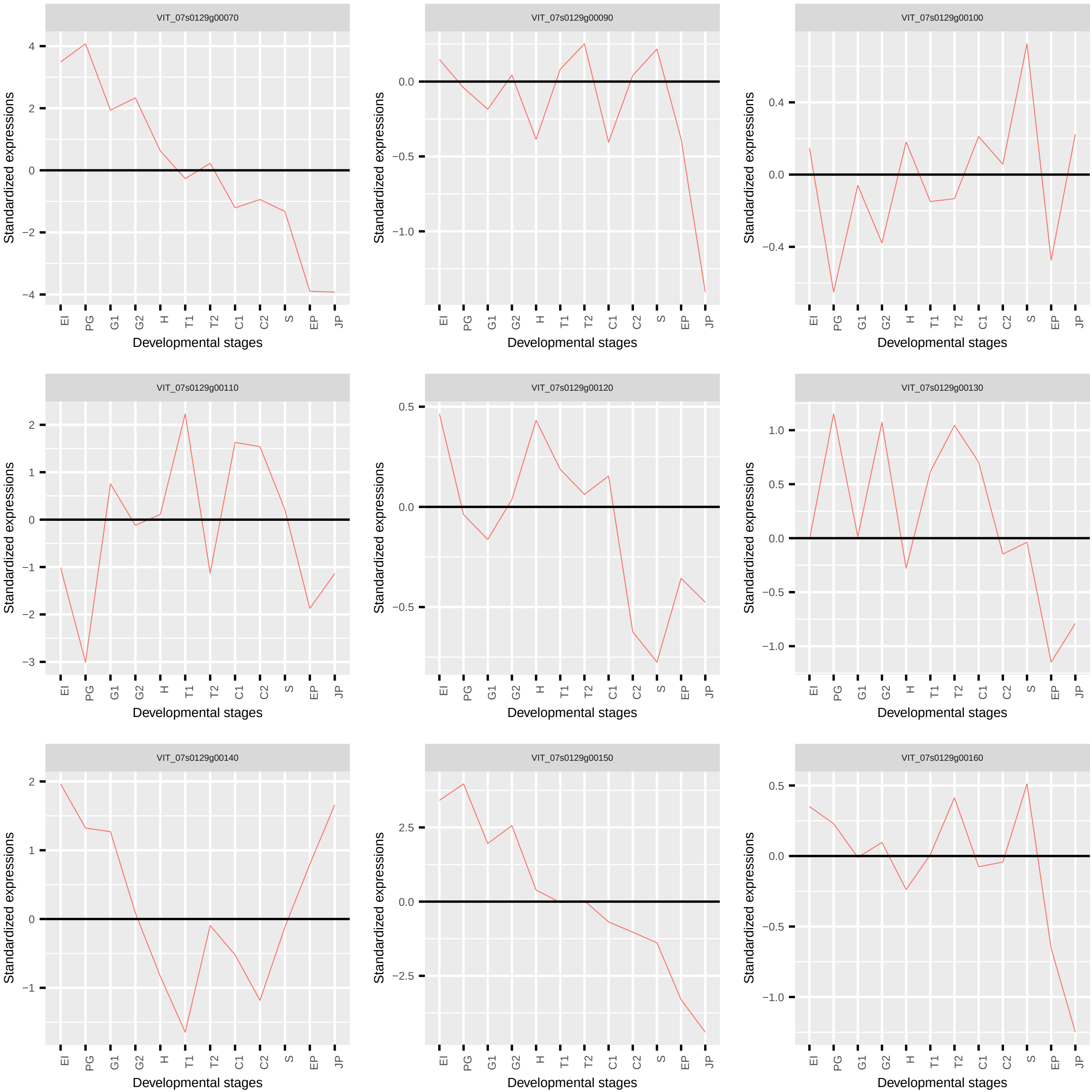
VIT_07s0129g00070
VIT_07s0129g00090
VIT_07s0129g00100
VIT_07s0129g00110
VIT_07s0129g00120
VIT_07s0129g00130
VIT_07s0129g00140
VIT_07s0129g00150
VIT_07s0129g00160
Standardized expressions
Developmental stages
EI PG G1 G2 H T1 T2 C1 C2 S EP JP
4 2 0 −2 −4
0.0 −0.5 −1.0
0.4 0.0 −0.4
2 1 0 −1 −2 −3
0.5 0.0 −0.5
1.0 0.5 0.0 −0.5 −1.0
2 1 0 −1
2.5 0.0 −2.5
0.5 0.0 −0.5 −1.0

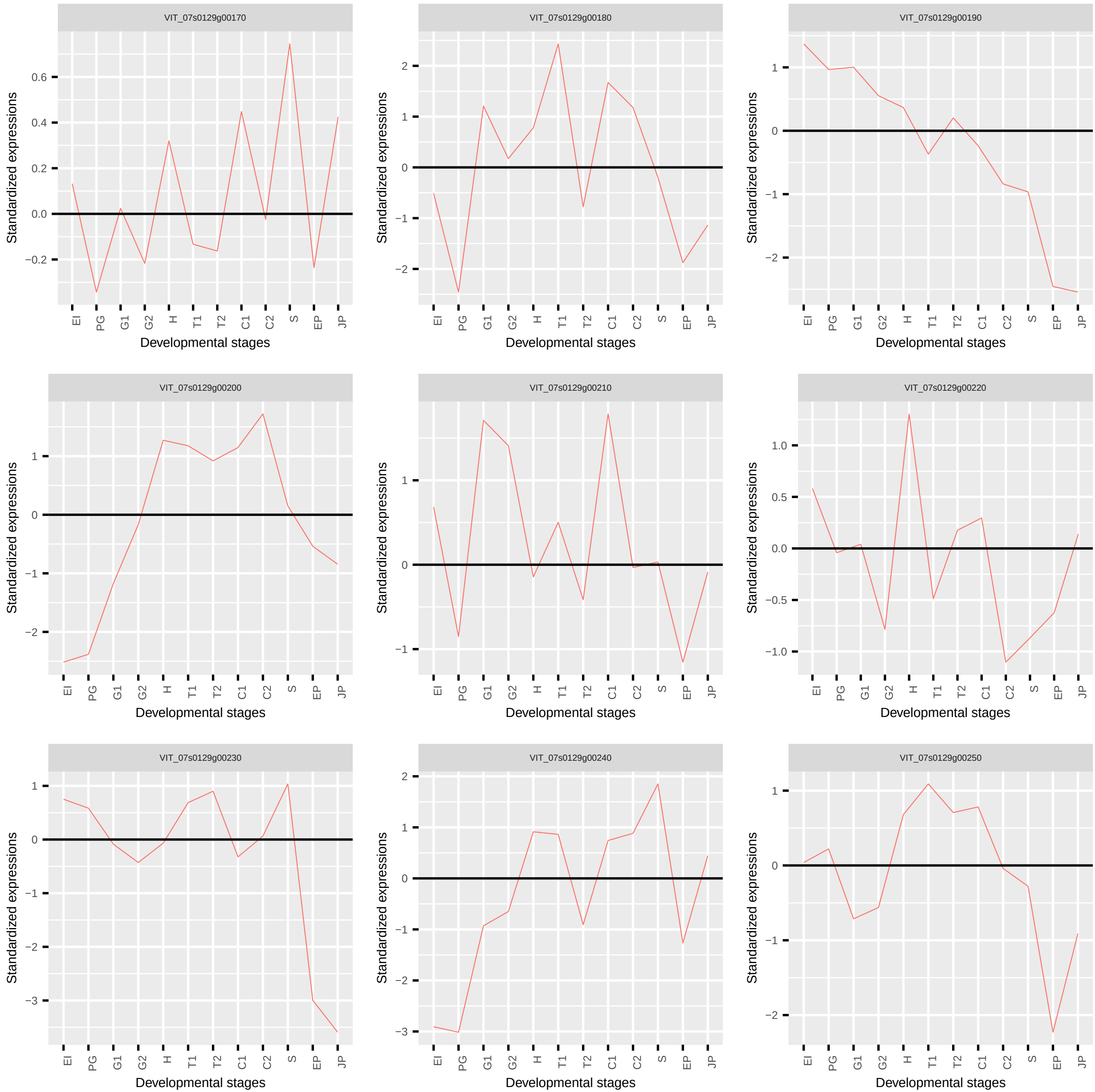
VIT_07s0129g00170
VIT_07s0129g00180
VIT_07s0129g00190
VIT_07s0129g00200
VIT_07s0129g00210
VIT_07s0129g00220
VIT_07s0129g00230
VIT_07s0129g00240
VIT_07s0129g00250
Standardized expressions
Developmental stages
EI
PG
G1
G2
H
T1
T2
C1
C2
S
EP
JP

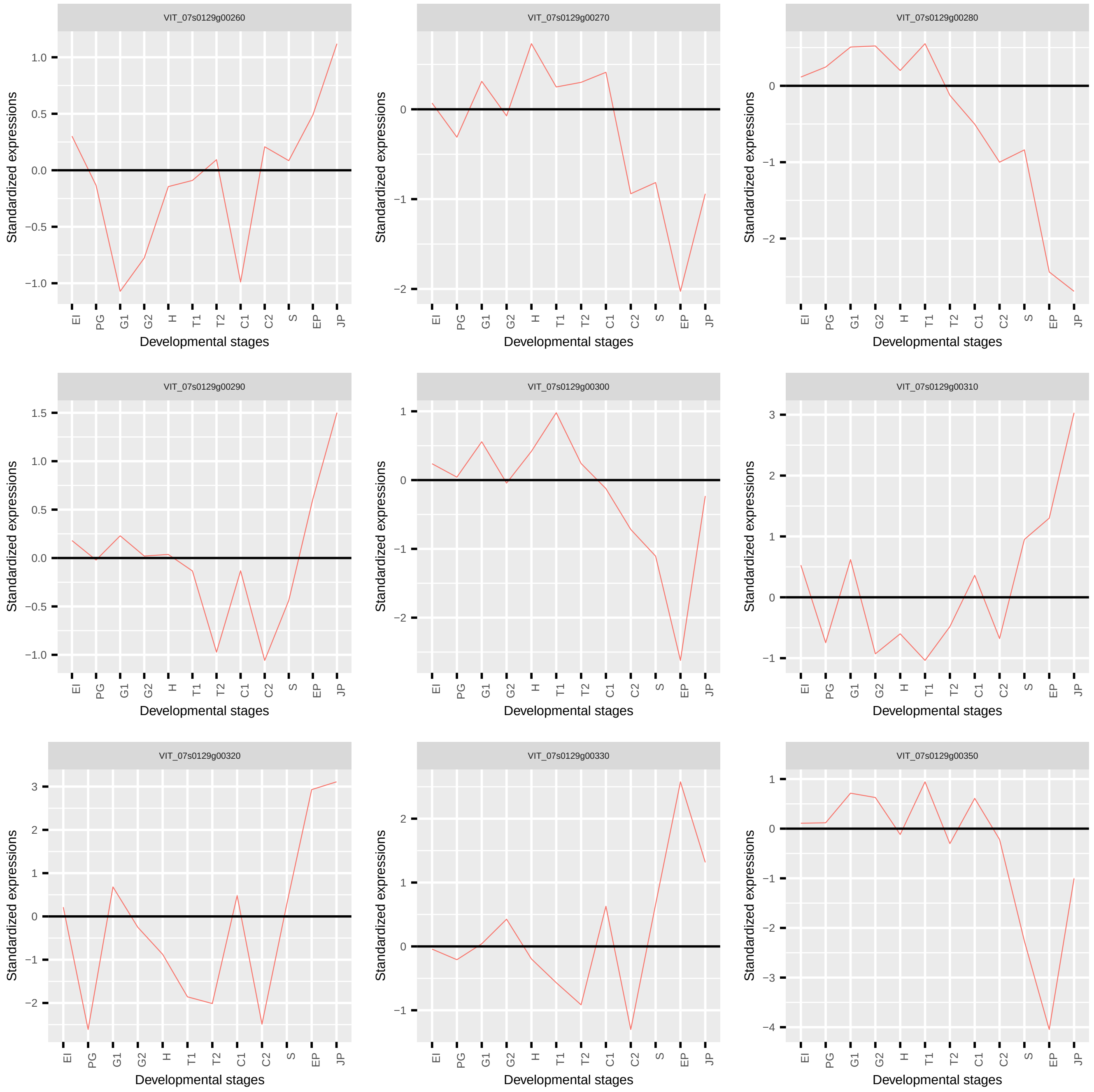
VIT_07s0129g00260
VIT_07s0129g00270
VIT_07s0129g00280
VIT_07s0129g00290
VIT_07s0129g00300
VIT_07s0129g00310
VIT_07s0129g00320
VIT_07s0129g00330
VIT_07s0129g00350
Standardized expressions
Developmental stages
EI PG G1 G2 H T1 T2 C1 C2 S EP JP

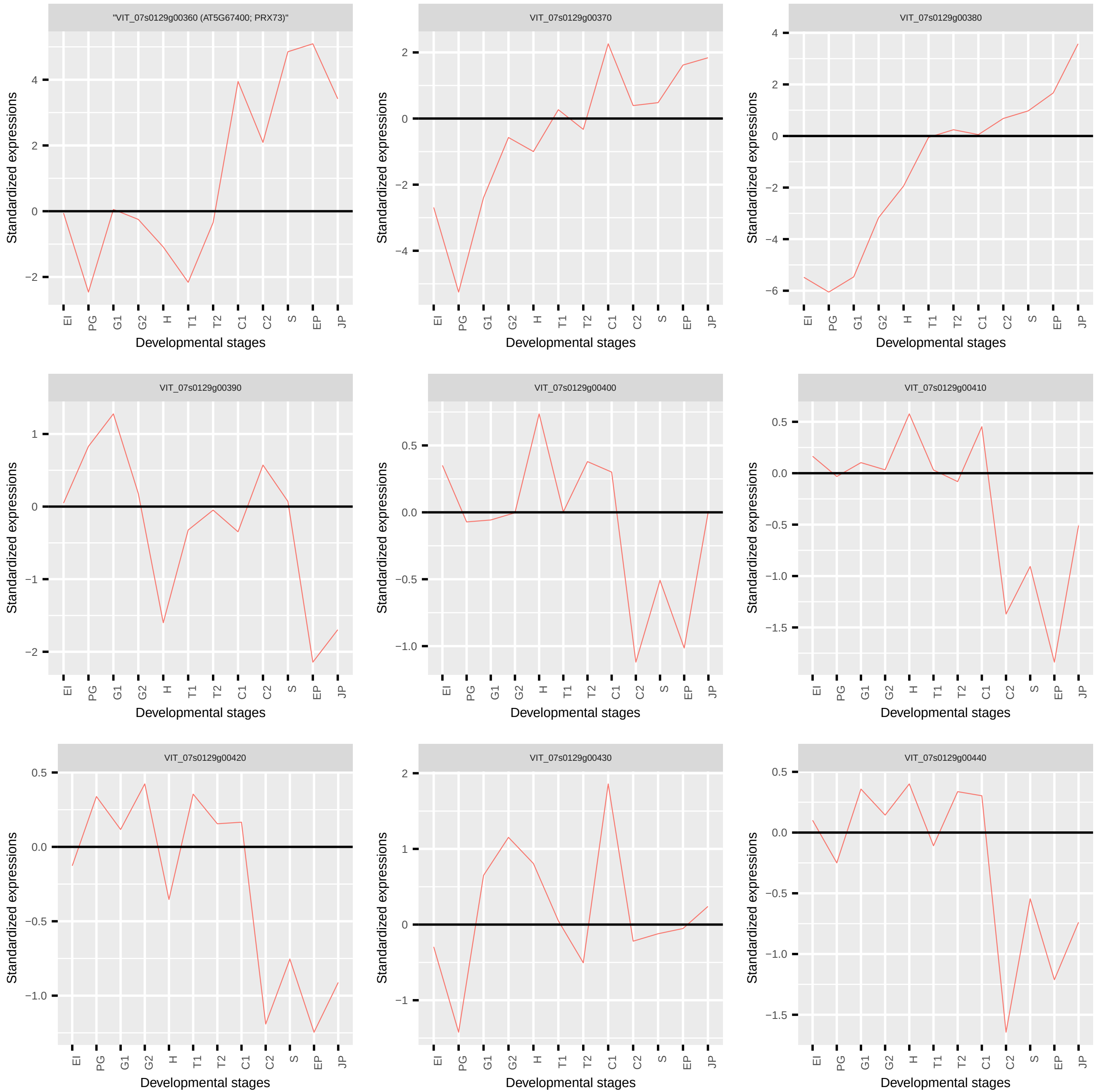
"VIT_07s0129g00360 (AT5G67400; PRX73)"
VIT_07s0129g00370
VIT_07s0129g00380
VIT_07s0129g00390
VIT_07s0129g00400
VIT_07s0129g00410
VIT_07s0129g00420
VIT_07s0129g00430
VIT_07s0129g00440
Standardized expressions
Developmental stages
EI PG G1 G2 H T1 T2 C1 C2 S EP JP

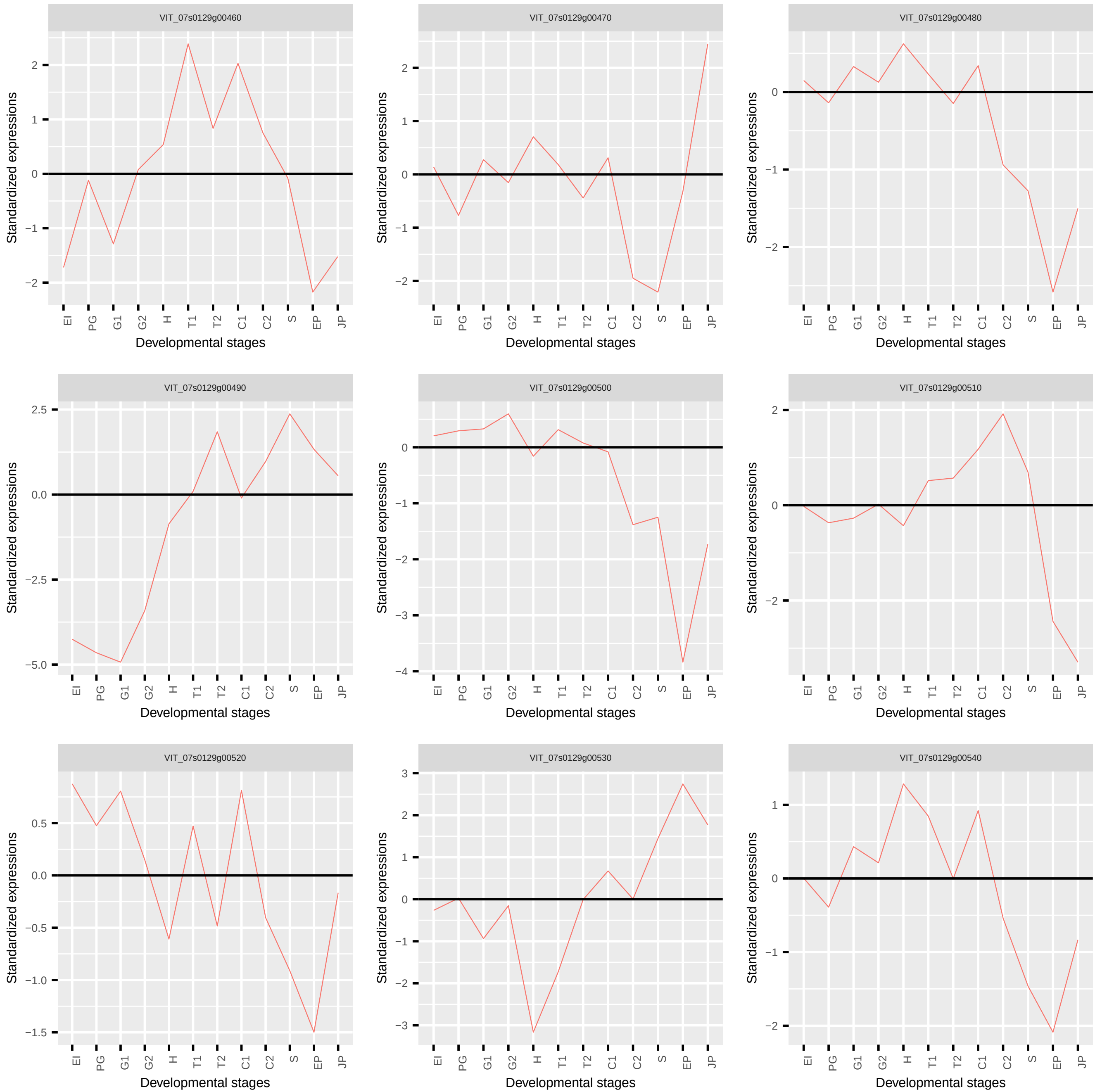
VIT_07s0129g00460
VIT_07s0129g00470
VIT_07s0129g00480
VIT_07s0129g00490
VIT_07s0129g00500
VIT_07s0129g00510
VIT_07s0129g00520
VIT_07s0129g00530
VIT_07s0129g00540
Standardized expressions
Developmental stages
EI PG G1 G2 H T1 T2 C1 C2 S EP JP

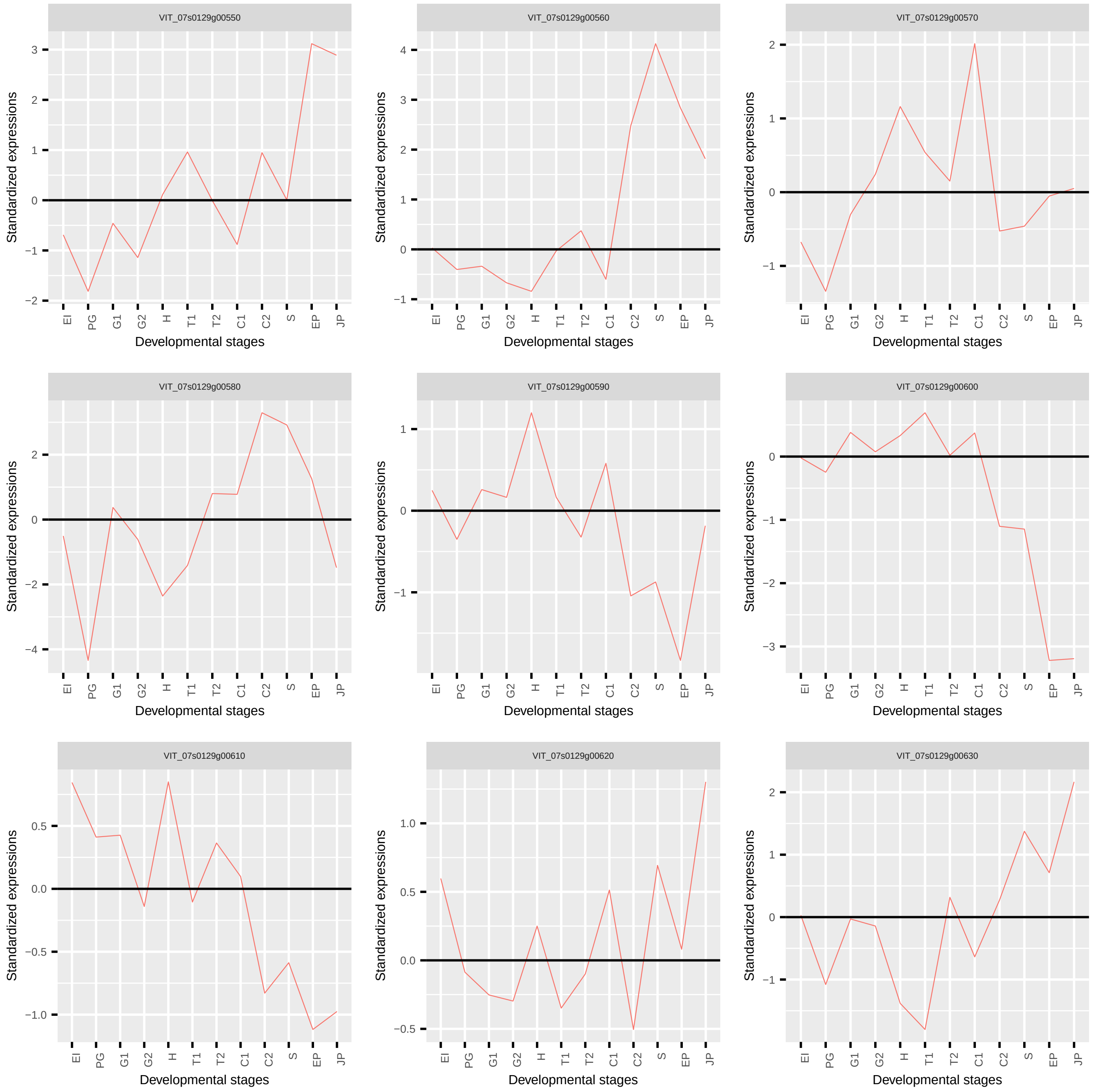
VIT_07s0129g00550
VIT_07s0129g00560
VIT_07s0129g00570
VIT_07s0129g00580
VIT_07s0129g00590
VIT_07s0129g00600
VIT_07s0129g00610
VIT_07s0129g00620
VIT_07s0129g00630
Standardized expressions
Developmental stages
EI
PG
G1
G2
H
T1
T2
C1
C2
S
EP
JP

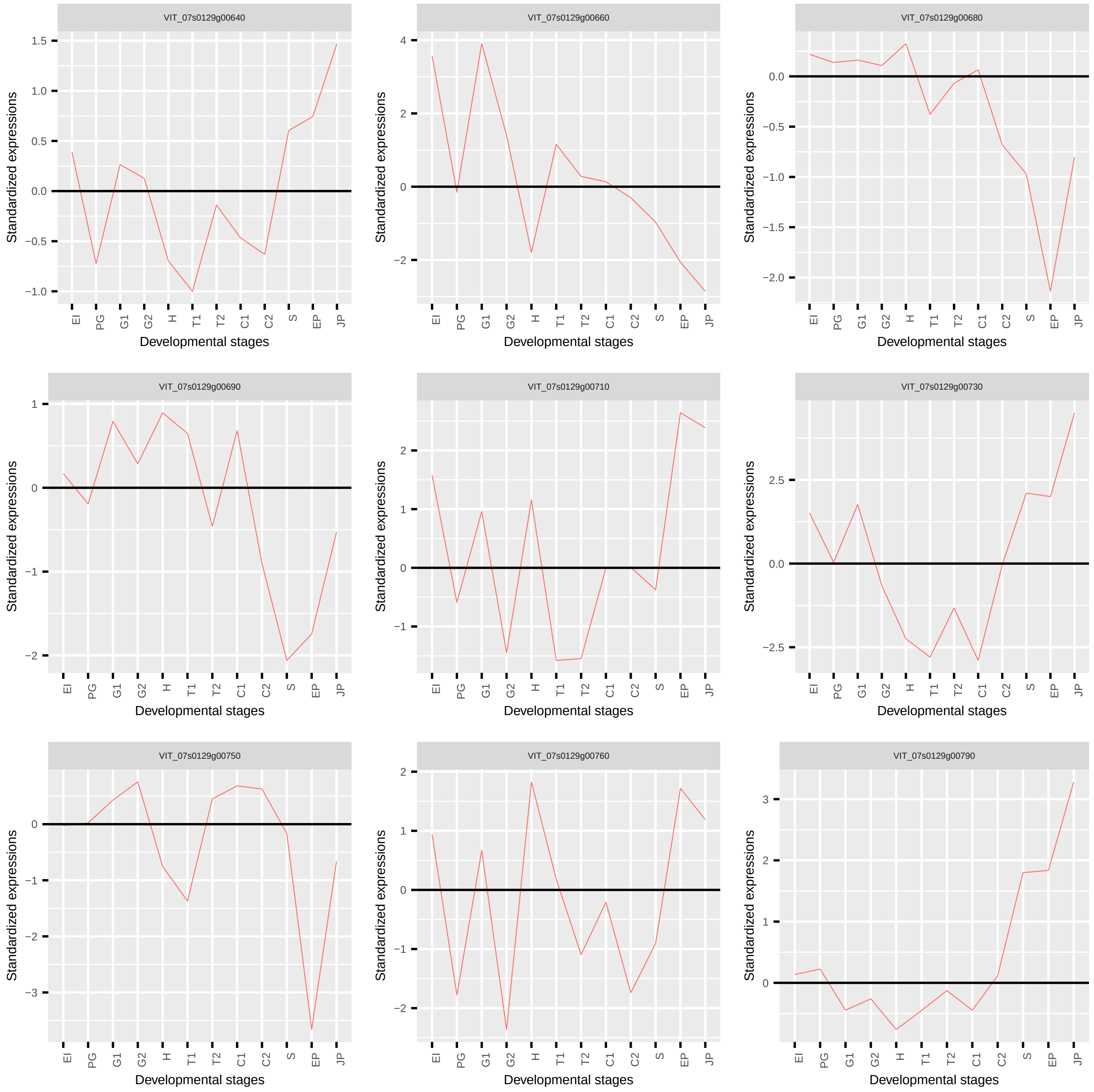
VIT_07s0129g00640
VIT_07s0129g00660
VIT_07s0129g00680
VIT_07s0129g00690
VIT_07s0129g00710
VIT_07s0129g00730
VIT_07s0129g00750
VIT_07s0129g00760
VIT_07s0129g00790
Standardized expressions
Developmental stages
EI PG G1 G2 H T1 T2 C1 C2 S EP JP
1.5 1.0 0.5 0.0 −0.5 −1.0
4 2 0 −2
0.0 −0.5 −1.0 −1.5 −2.0
1 0 −1 −2
2 1 0 −1
2.5 0.0 −2.5
0 −1 −2 −3
2 1 0 −1 −2
3 2 1 0

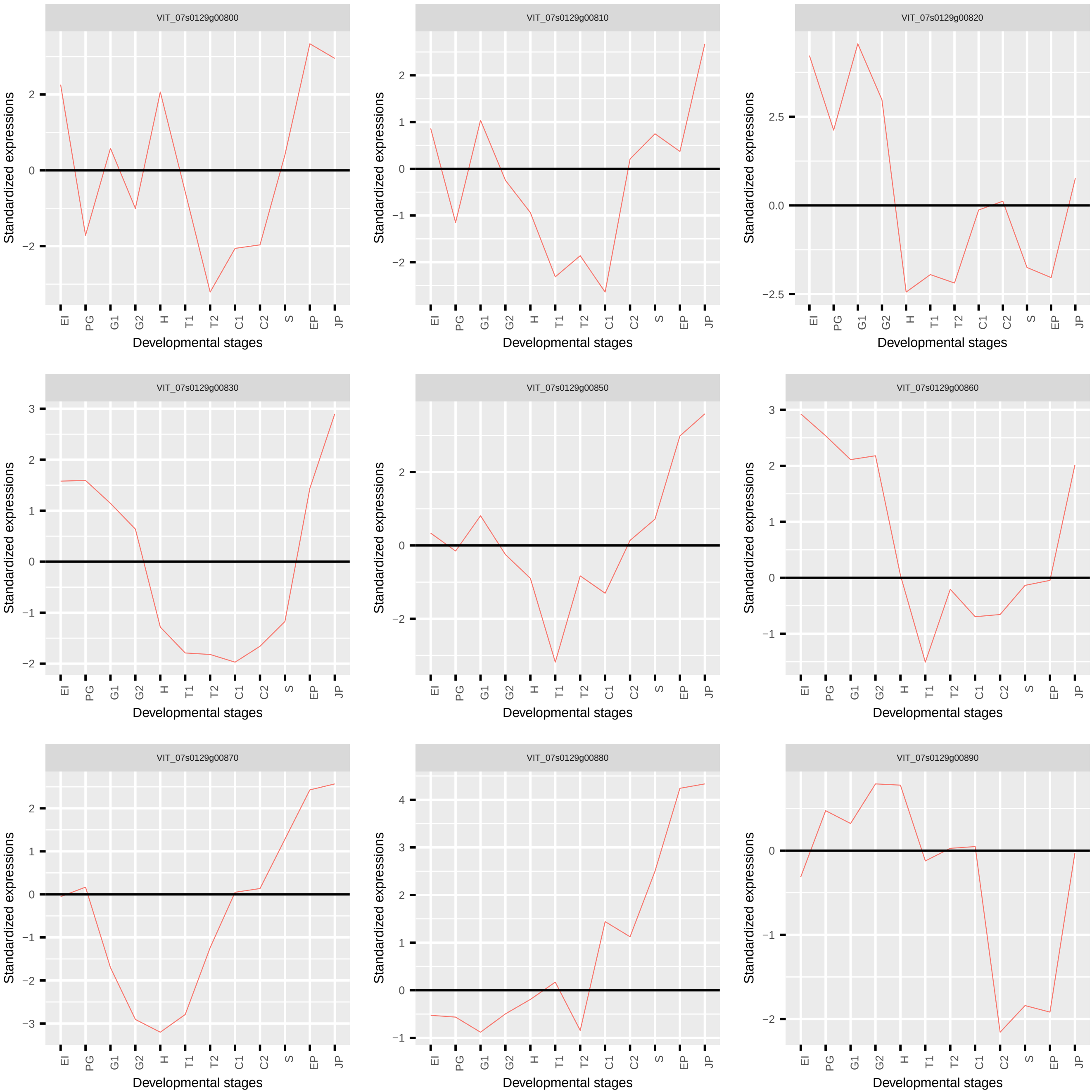
VIT_07s0129g00800
VIT_07s0129g00810
VIT_07s0129g00820
VIT_07s0129g00830
VIT_07s0129g00850
VIT_07s0129g00860
VIT_07s0129g00870
VIT_07s0129g00880
VIT_07s0129g00890
Standardized expressions
Developmental stages
EI
PG
G1
G2
H
T1
T2
C1
C2
S
EP
JP

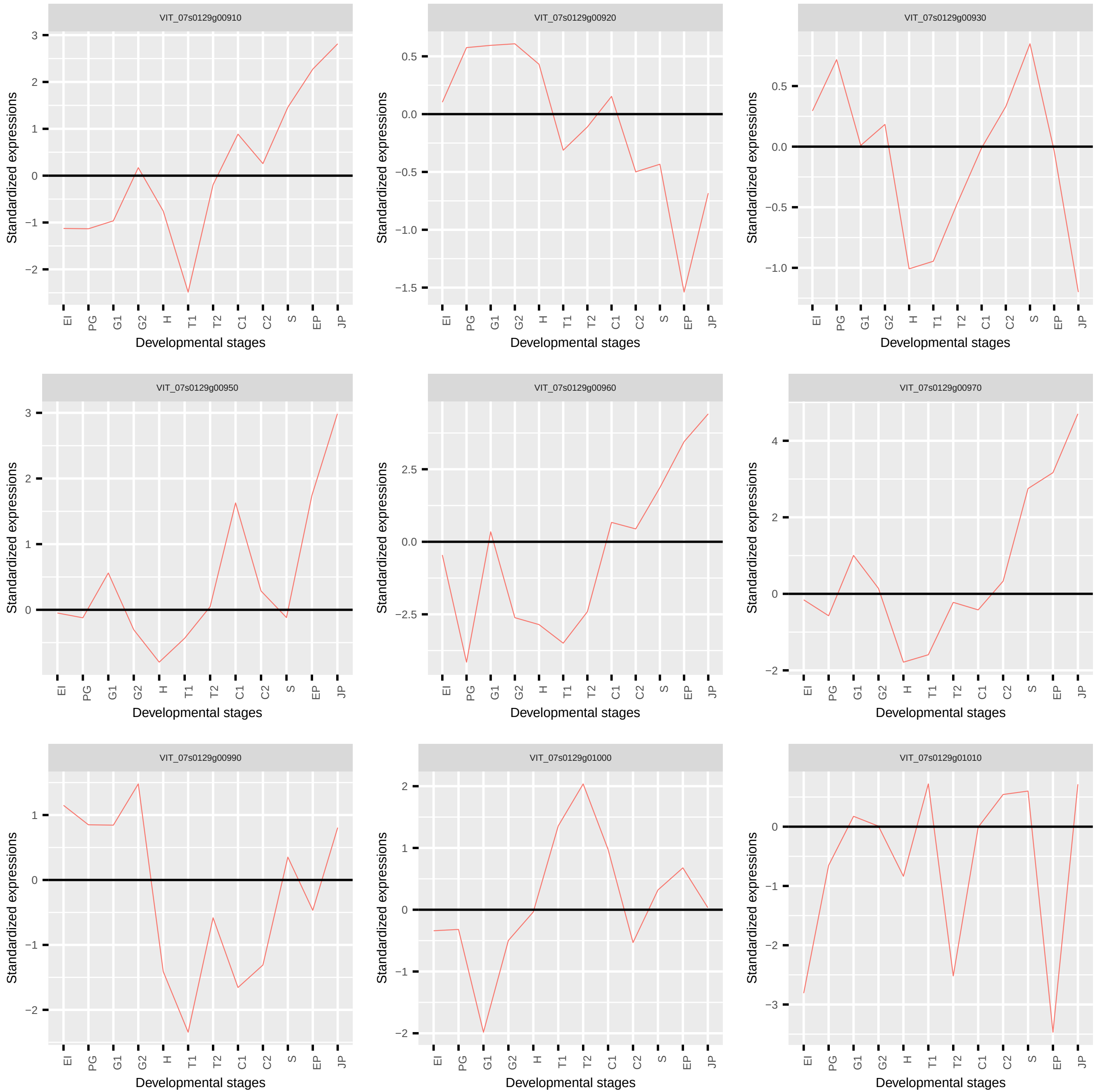
VIT_07s0129g00910
Standardized expressions
3
2
1
0
−1
−2
EI PG G1 G2 H T1 T2 C1 C2 S EP JP
Developmental stages
VIT_07s0129g00920
Standardized expressions
0.5
0.0
−0.5
−1.0
−1.5
EI PG G1 G2 H T1 T2 C1 C2 S EP JP
Developmental stages
VIT_07s0129g00930
Standardized expressions
0.5
0.0
−0.5
−1.0
EI PG G1 G2 H T1 T2 C1 C2 S EP JP
Developmental stages
VIT_07s0129g00950
Standardized expressions
3
2
1
0
EI PG G1 G2 H T1 T2 C1 C2 S EP JP
Developmental stages
VIT_07s0129g00960
Standardized expressions
2.5
0.0
−2.5
EI PG G1 G2 H T1 T2 C1 C2 S EP JP
Developmental stages
VIT_07s0129g00970
Standardized expressions
4
2
0
−2
EI PG G1 G2 H T1 T2 C1 C2 S EP JP
Developmental stages
VIT_07s0129g00990
Standardized expressions
1
0
−1
−2
EI PG G1 G2 H T1 T2 C1 C2 S EP JP
Developmental stages
VIT_07s0129g01000
Standardized expressions
2
1
0
−1
−2
EI PG G1 G2 H T1 T2 C1 C2 S EP JP
Developmental stages
VIT_07s0129g01010
Standardized expressions
0
−1
−2
−3
EI PG G1 G2 H T1 T2 C1 C2 S EP JP
Developmental stages

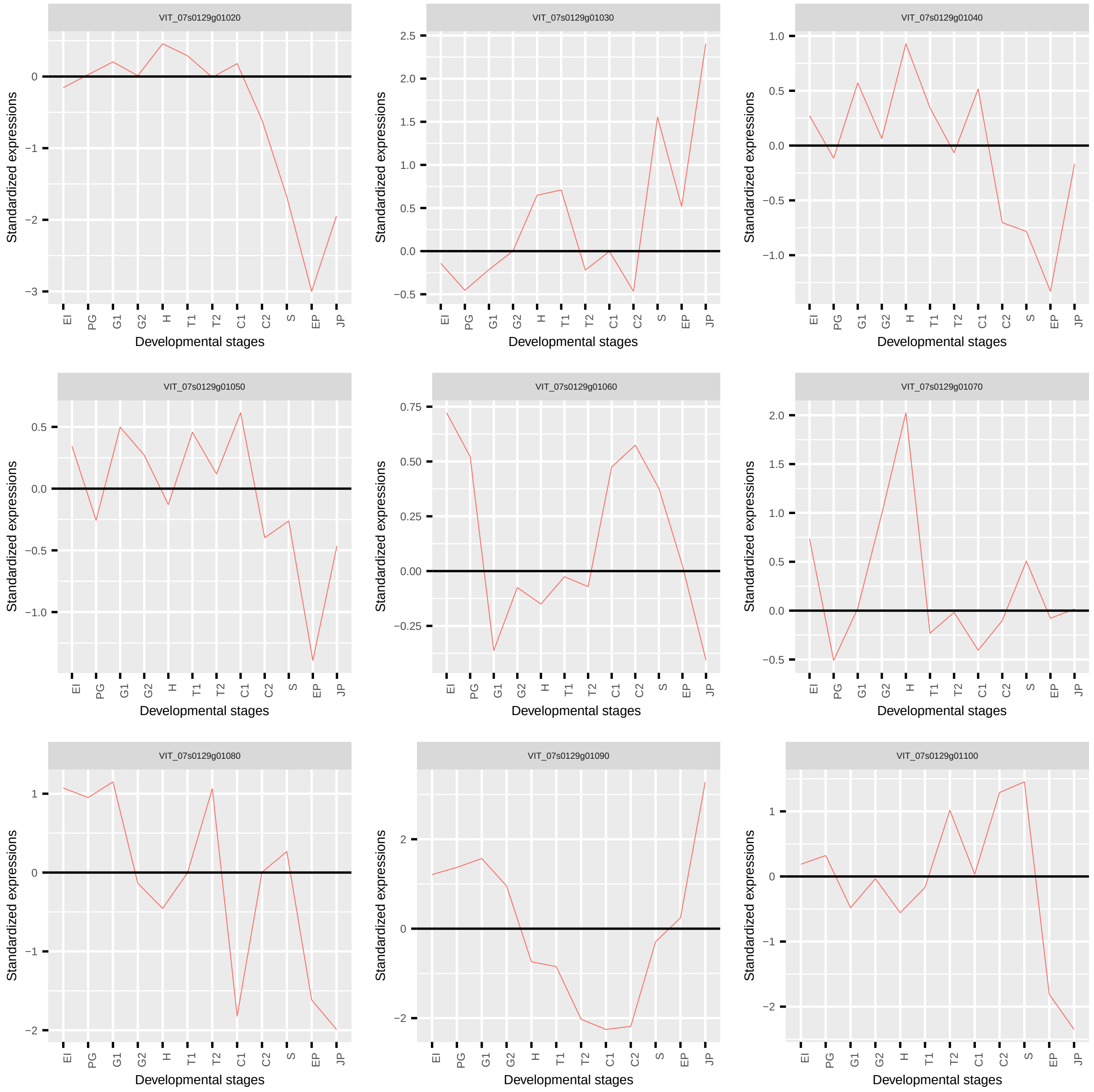

VIT_07s0129g01020
VIT_07s0129g01030
VIT_07s0129g01040
VIT_07s0129g01050
VIT_07s0129g01060
VIT_07s0129g01070
VIT_07s0129g01080
VIT_07s0129g01090
VIT_07s0129g01100
Standardized expressions
Developmental stages
EI PG G1 G2 H T1 T2 C1 C2 S EP JP

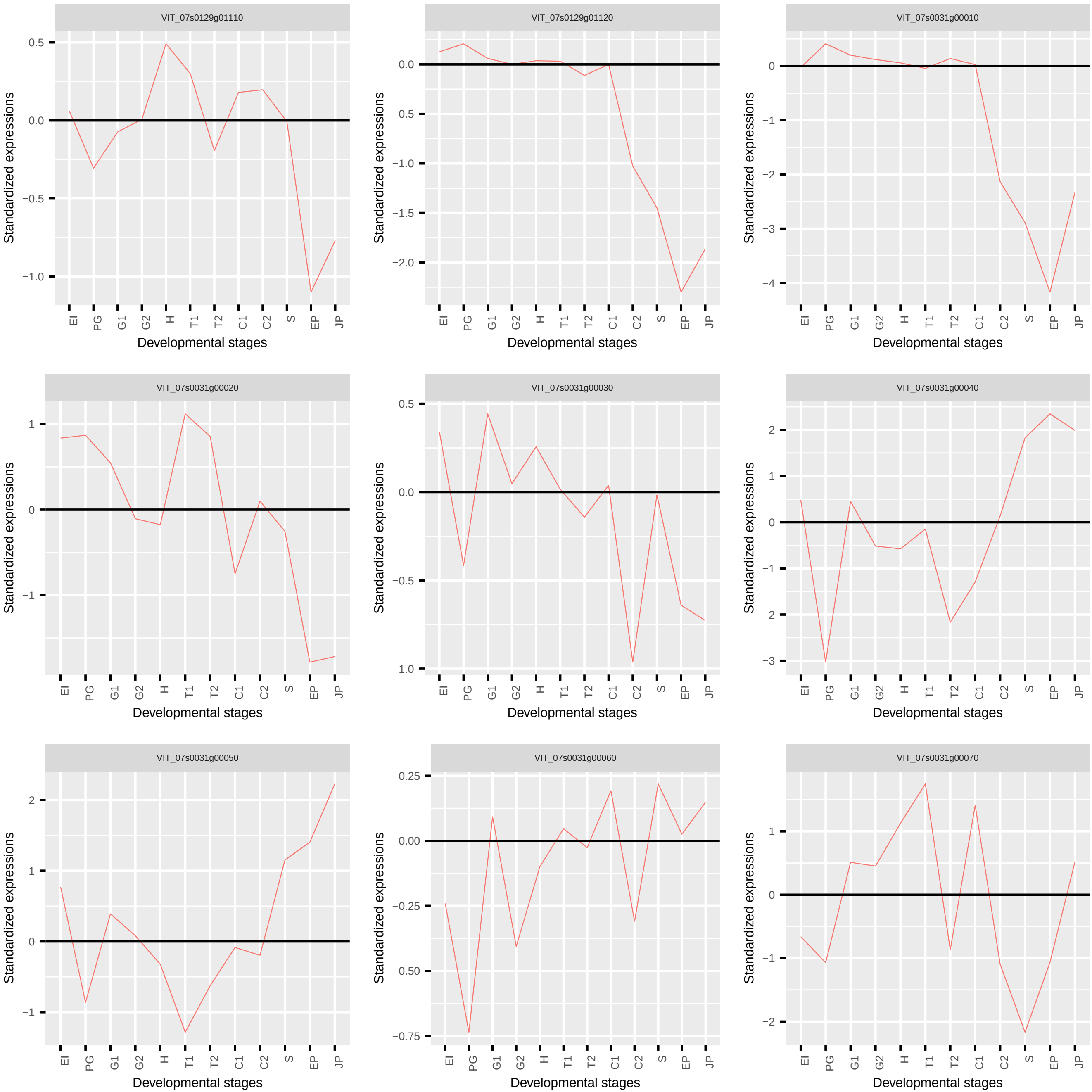
VIT_07s0129g01110
VIT_07s0129g01120
VIT_07s0031g00010
VIT_07s0031g00020
VIT_07s0031g00030
VIT_07s0031g00040
VIT_07s0031g00050
VIT_07s0031g00060
VIT_07s0031g00070
Standardized expressions
Developmental stages
EI
PG
G1
G2
H
T1
T2
C1
C2
S
EP
JP

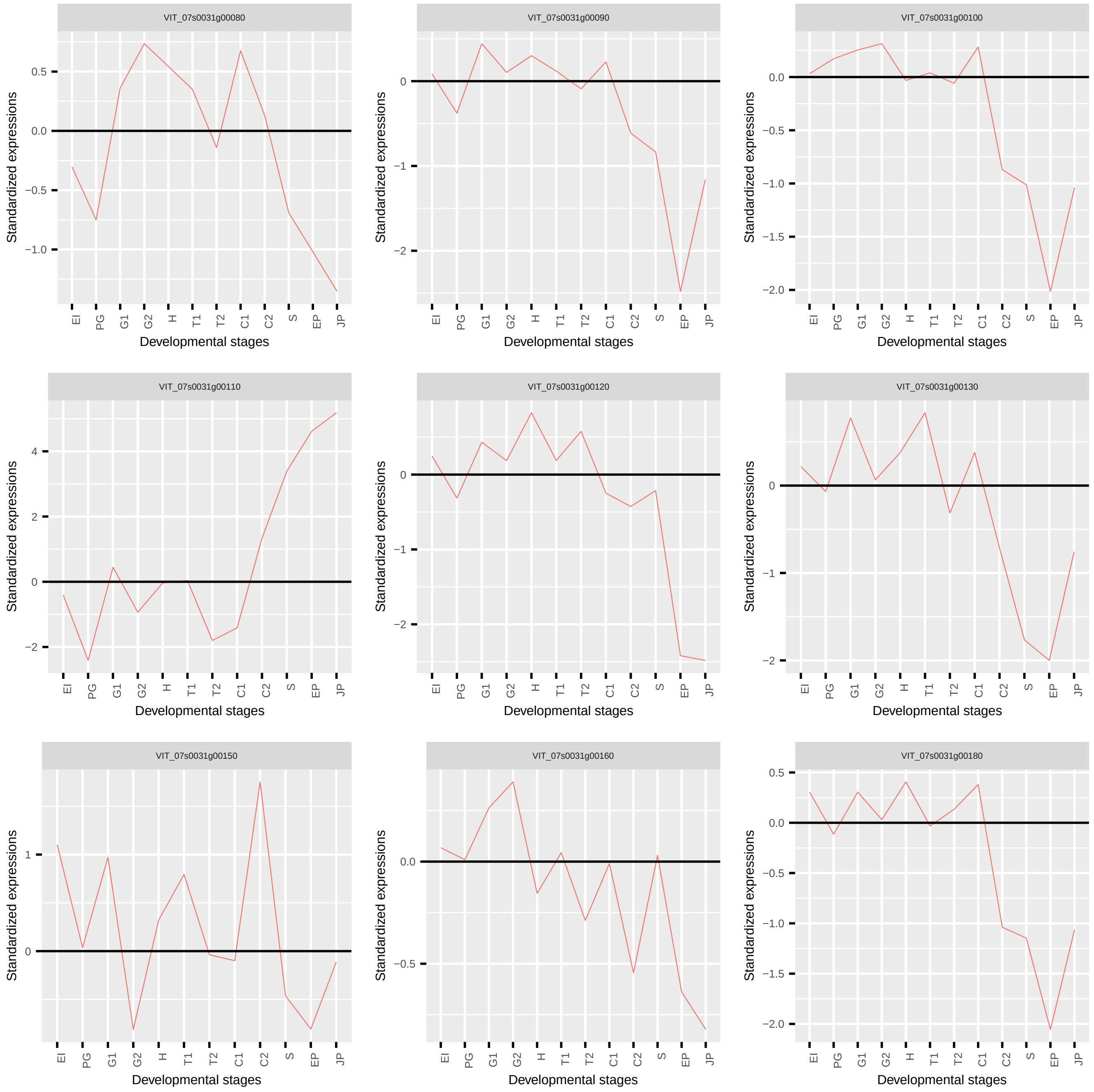
VIT_07s0031g00080
VIT_07s0031g00090
VIT_07s0031g00100
VIT_07s0031g00110
VIT_07s0031g00120
VIT_07s0031g00130
VIT_07s0031g00150
VIT_07s0031g00160
VIT_07s0031g00180
Standardized expressions
Developmental stages
EI
PG
G1
G2
H
T1
T2
C1
C2
S
EP
JP

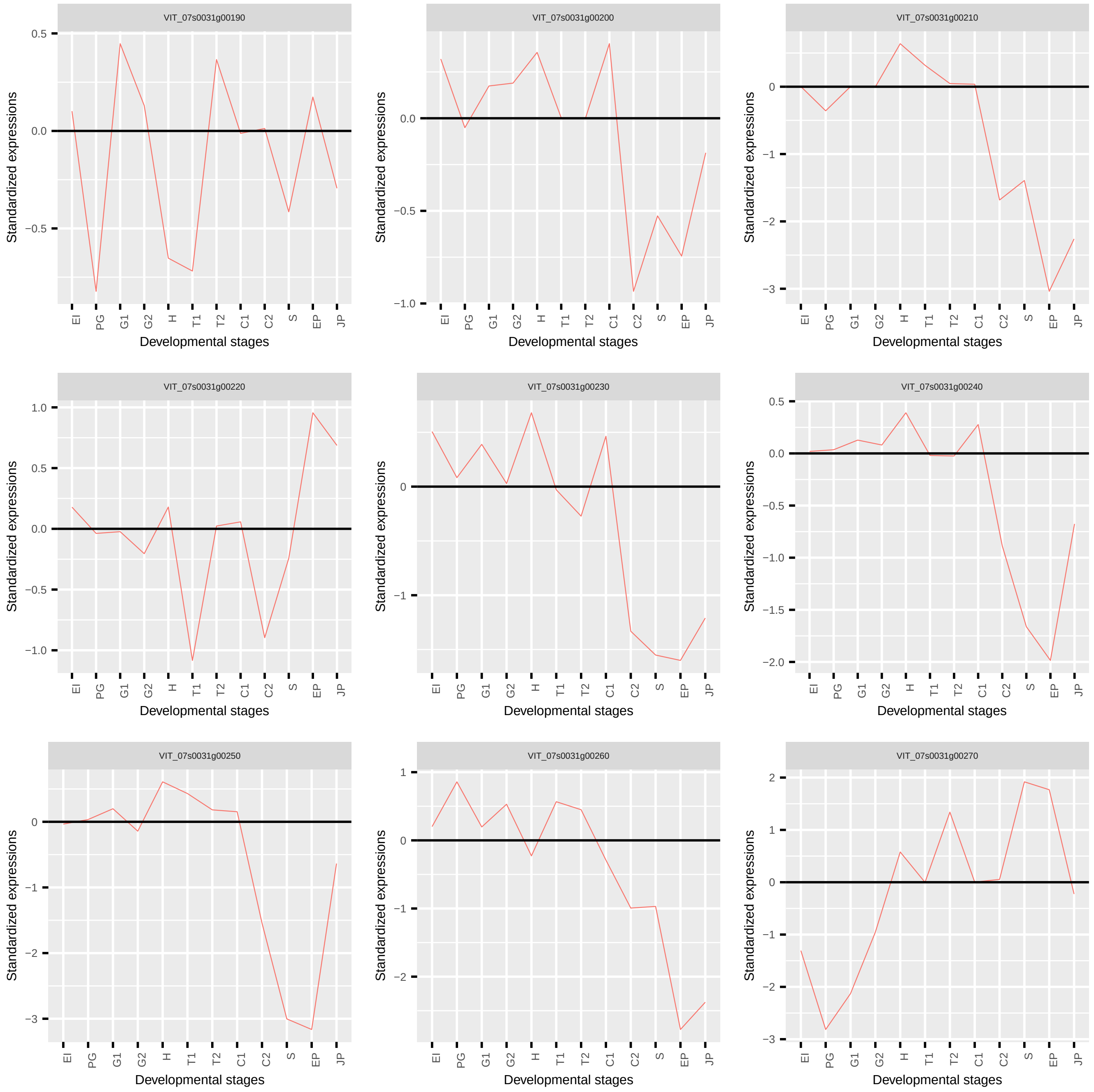
VIT_07s0031g00190
VIT_07s0031g00200
VIT_07s0031g00210
VIT_07s0031g00220
VIT_07s0031g00230
VIT_07s0031g00240
VIT_07s0031g00250
VIT_07s0031g00260
VIT_07s0031g00270
Standardized expressions
Developmental stages
EI PG G1 G2 H T1 T2 C1 C2 S EP JP

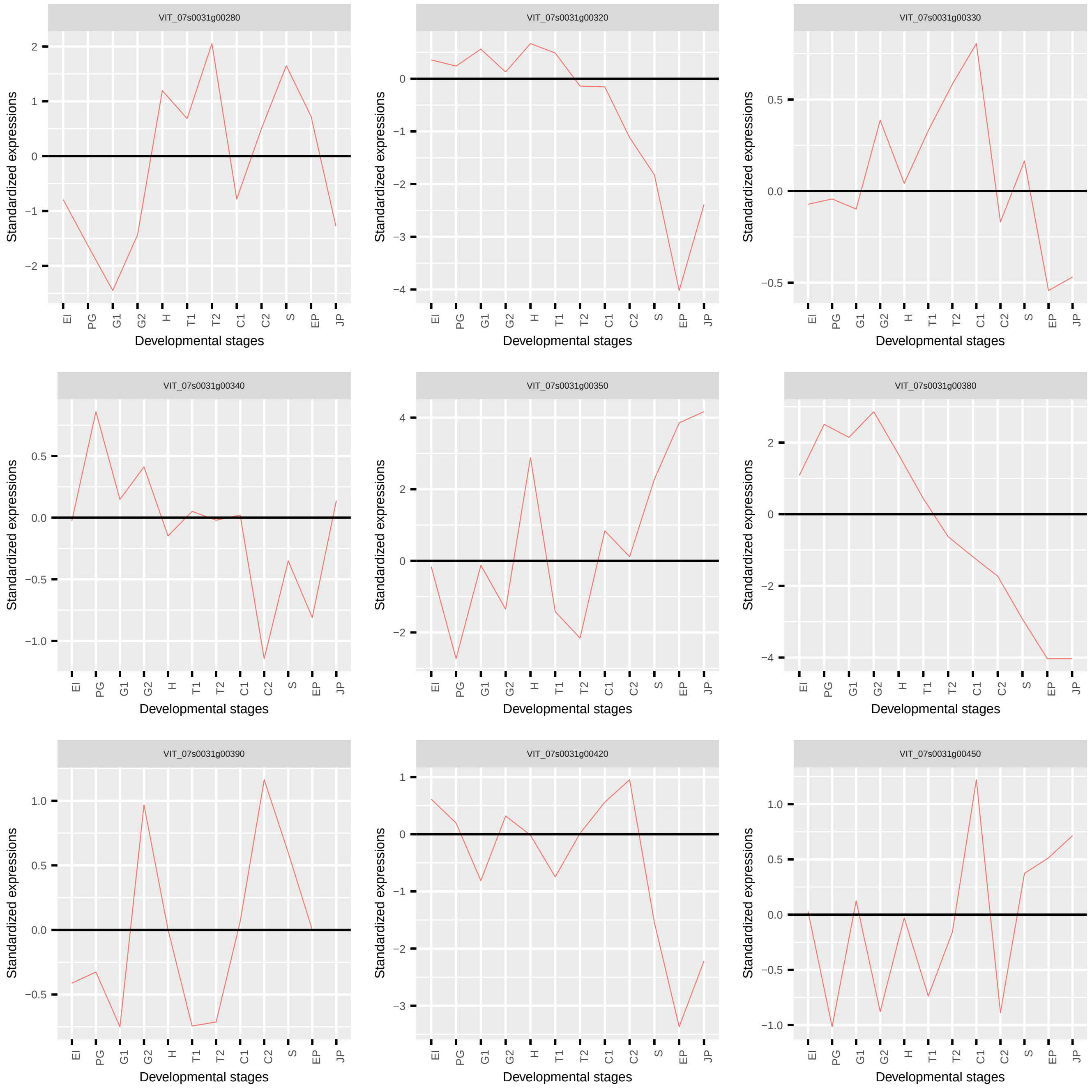
VIT_07s0031g00280
VIT_07s0031g00320
VIT_07s0031g00330
VIT_07s0031g00340
VIT_07s0031g00350
VIT_07s0031g00380
VIT_07s0031g00390
VIT_07s0031g00420
VIT_07s0031g00450
Standardized expressions
Developmental stages
EI
PG
G1
G2
H
T1
T2
C1
C2
S
EP
JP

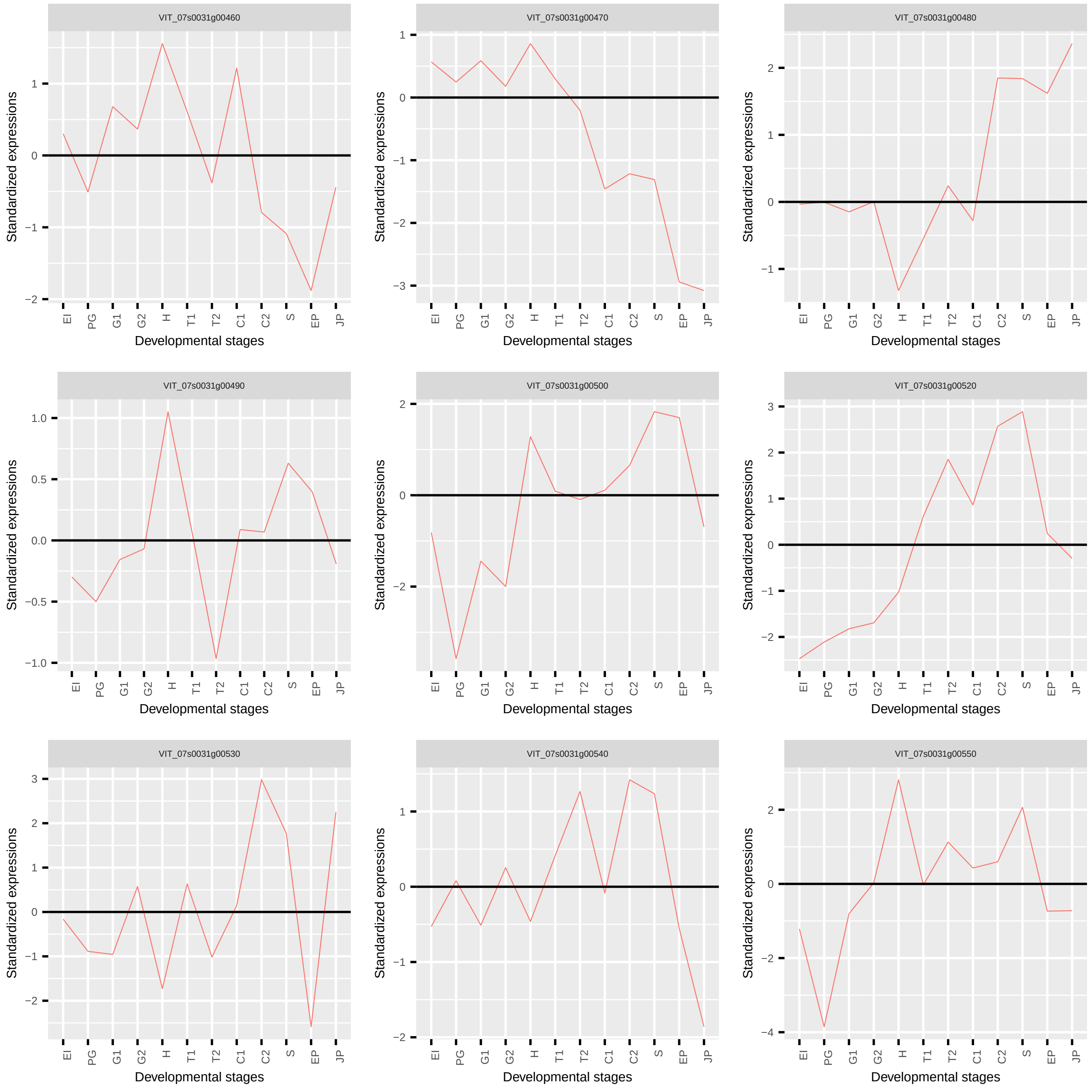
VIT_07s0031g00460
VIT_07s0031g00470
VIT_07s0031g00480
VIT_07s0031g00490
VIT_07s0031g00500
VIT_07s0031g00520
VIT_07s0031g00530
VIT_07s0031g00540
VIT_07s0031g00550
Standardized expressions
Developmental stages
EI PG G1 G2 H T1 T2 C1 C2 S EP JP

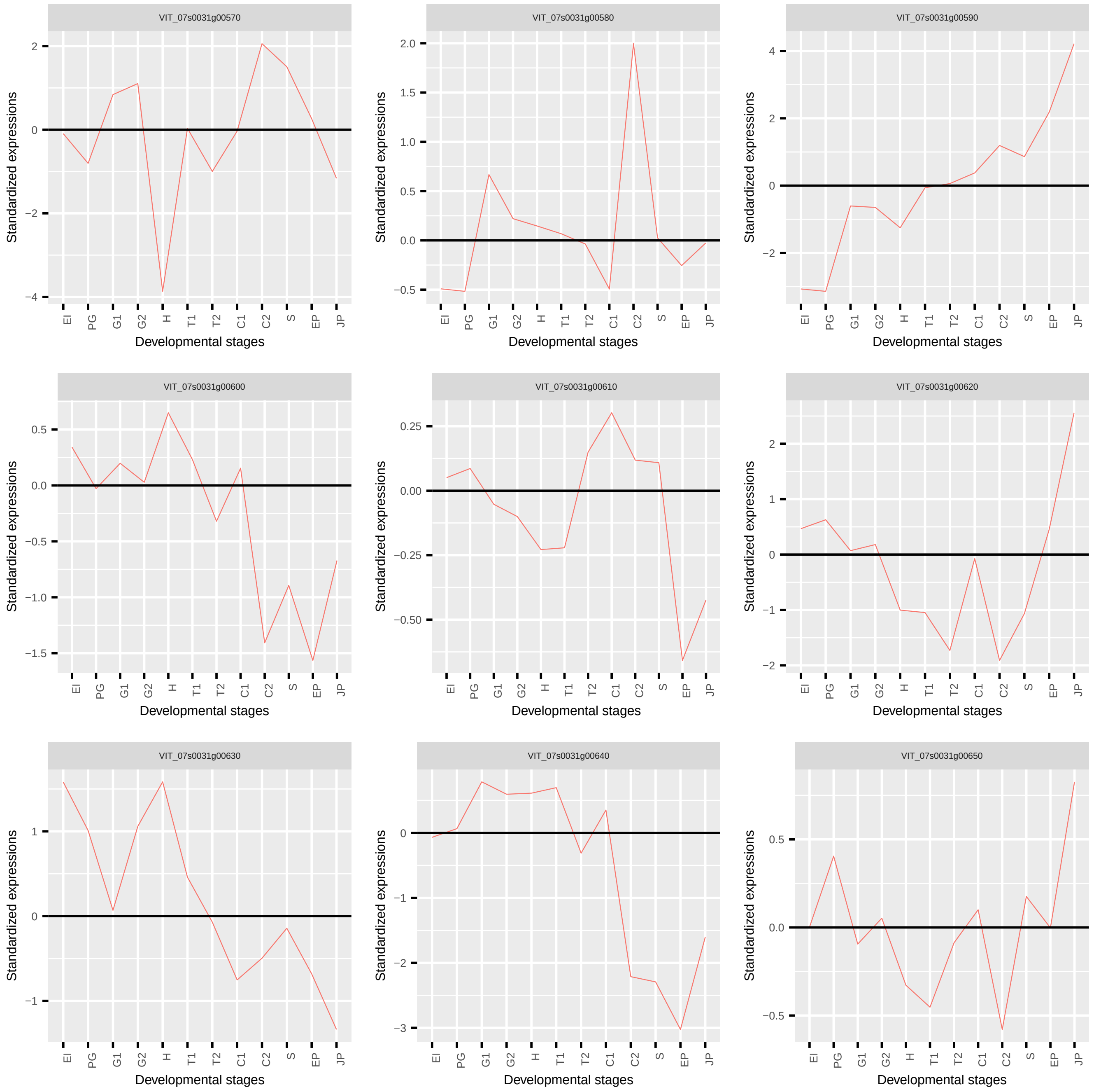
VIT_07s0031g00570
VIT_07s0031g00580
VIT_07s0031g00590
VIT_07s0031g00600
VIT_07s0031g00610
VIT_07s0031g00620
VIT_07s0031g00630
VIT_07s0031g00640
VIT_07s0031g00650
Standardized expressions
Developmental stages
EI PG G1 G2 H T1 T2 C1 C2 S EP JP

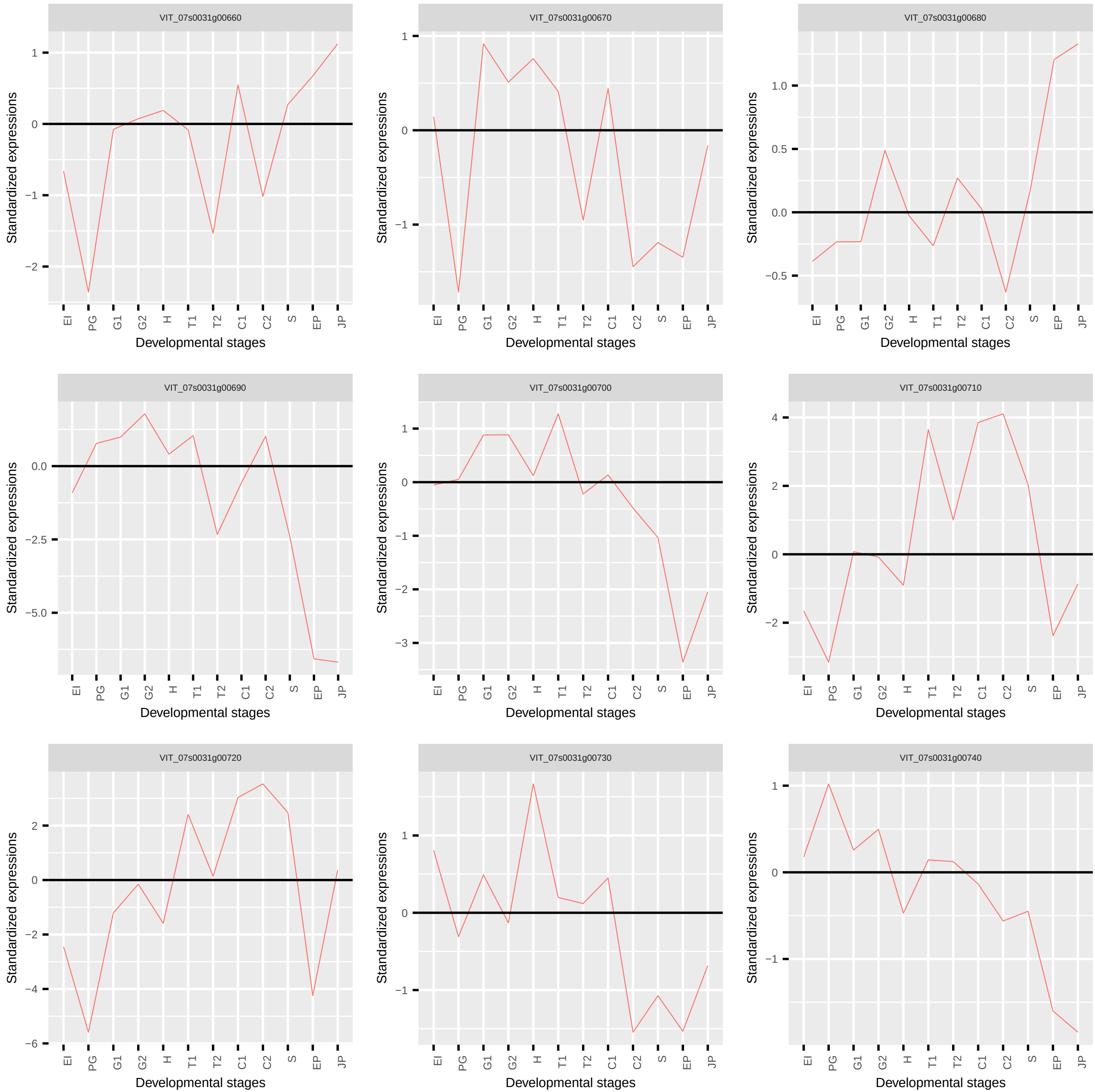
VIT_07s0031g00660
VIT_07s0031g00670
VIT_07s0031g00680
VIT_07s0031g00690
VIT_07s0031g00700
VIT_07s0031g00710
VIT_07s0031g00720
VIT_07s0031g00730
VIT_07s0031g00740
Standardized expressions
Developmental stages
EI PG G1 G2 H T1 T2 C1 C2 S EP JP

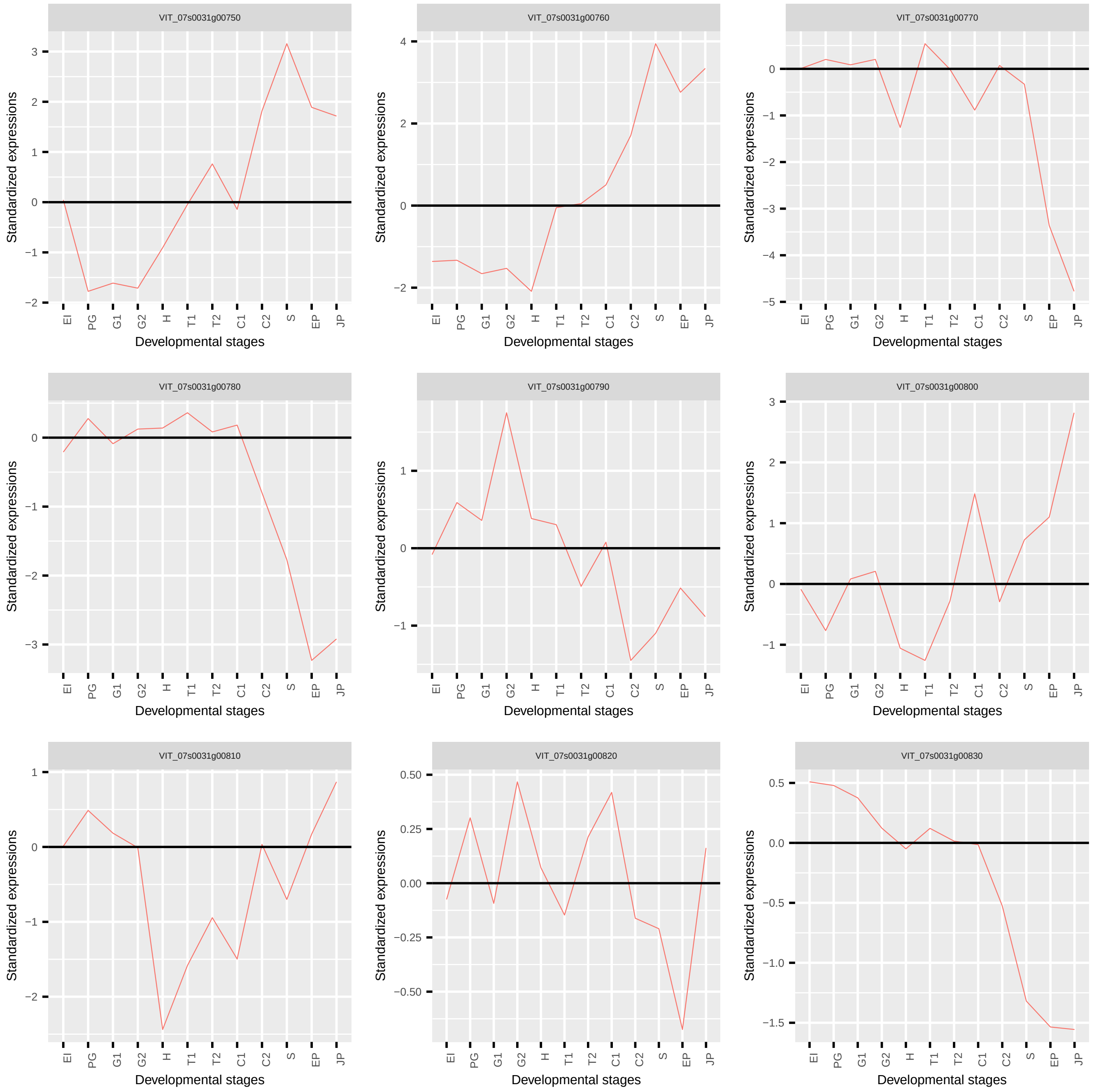
VIT_07s0031g00750
VIT_07s0031g00760
VIT_07s0031g00770
VIT_07s0031g00780
VIT_07s0031g00790
VIT_07s0031g00800
VIT_07s0031g00810
VIT_07s0031g00820
VIT_07s0031g00830
Standardized expressions
Developmental stages
EI PG G1 G2 H T1 T2 C1 C2 S EP JP

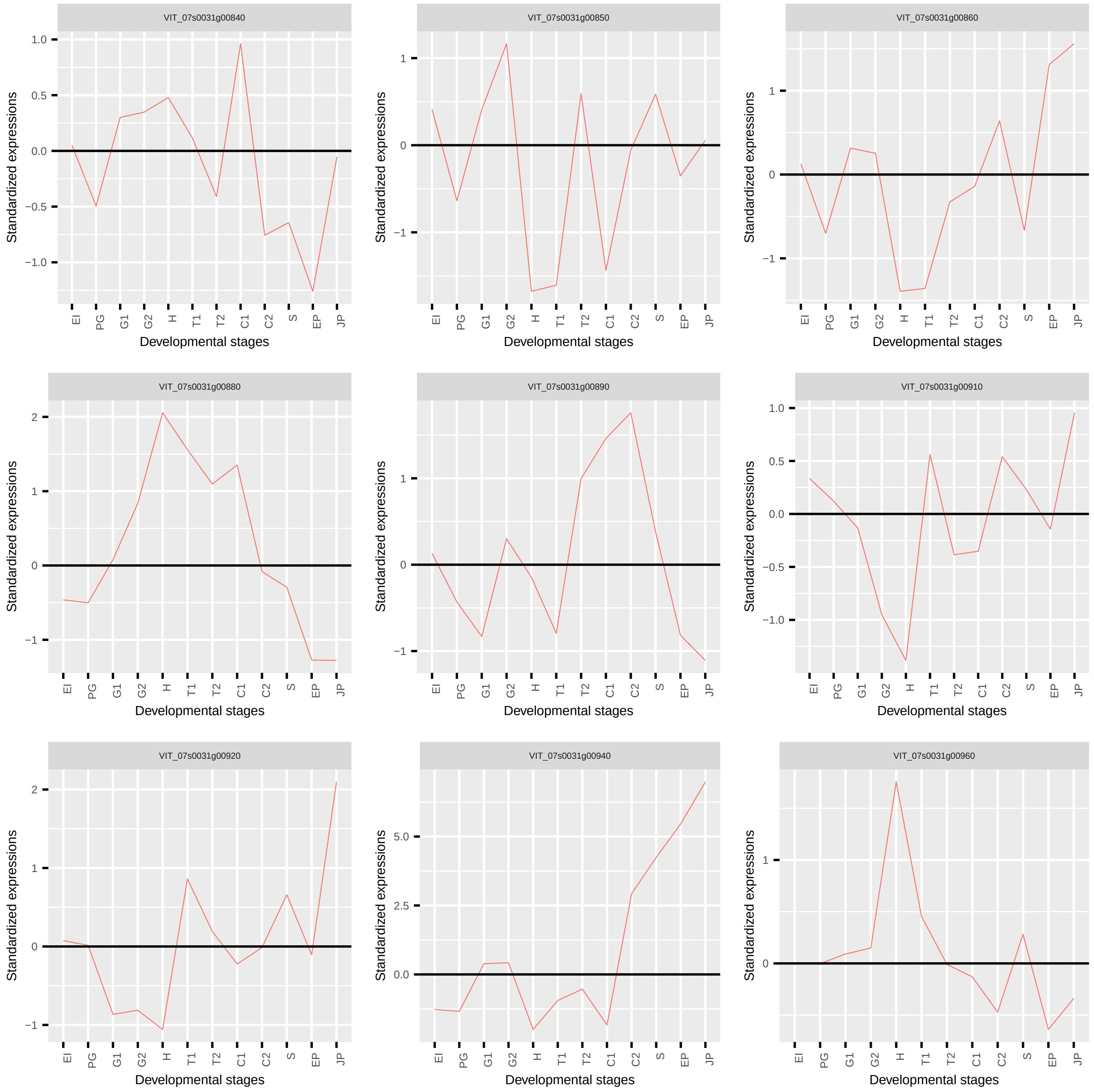

VIT_07s0031g00840
VIT_07s0031g00850
VIT_07s0031g00860
VIT_07s0031g00880
VIT_07s0031g00890
VIT_07s0031g00910
VIT_07s0031g00920
VIT_07s0031g00940
VIT_07s0031g00960
Standardized expressions
Developmental stages
EI
PG
G1
G2
H
T1
T2
C1
C2
S
EP
JP

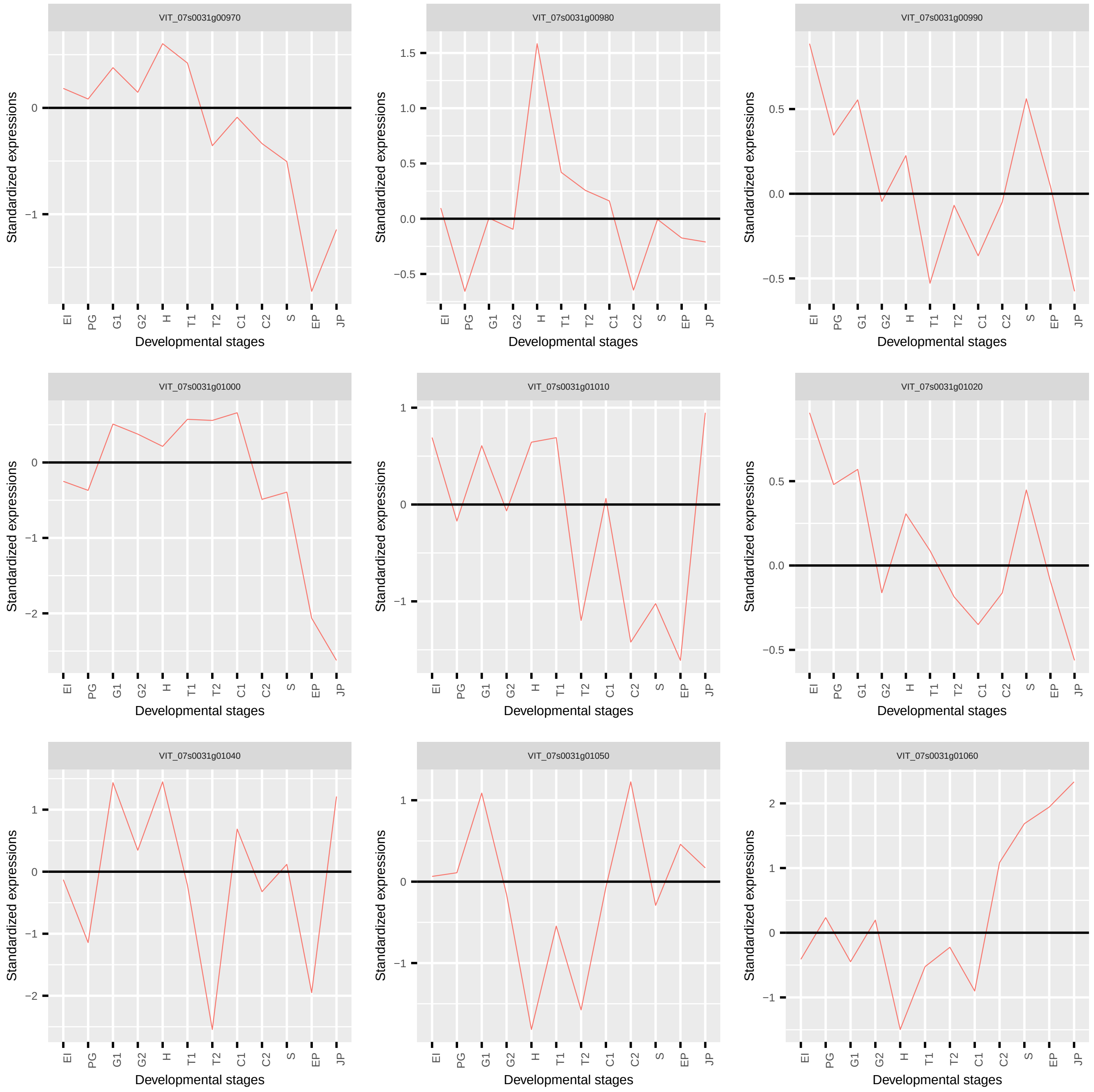

VIT_07s0031g00970
VIT_07s0031g00980
VIT_07s0031g00990
VIT_07s0031g01000
VIT_07s0031g01010
VIT_07s0031g01020
VIT_07s0031g01040
VIT_07s0031g01050
VIT_07s0031g01060
Standardized expressions
Developmental stages
EI
PG
G1
G2
H
T1
T2
C1
C2
S
EP
JP

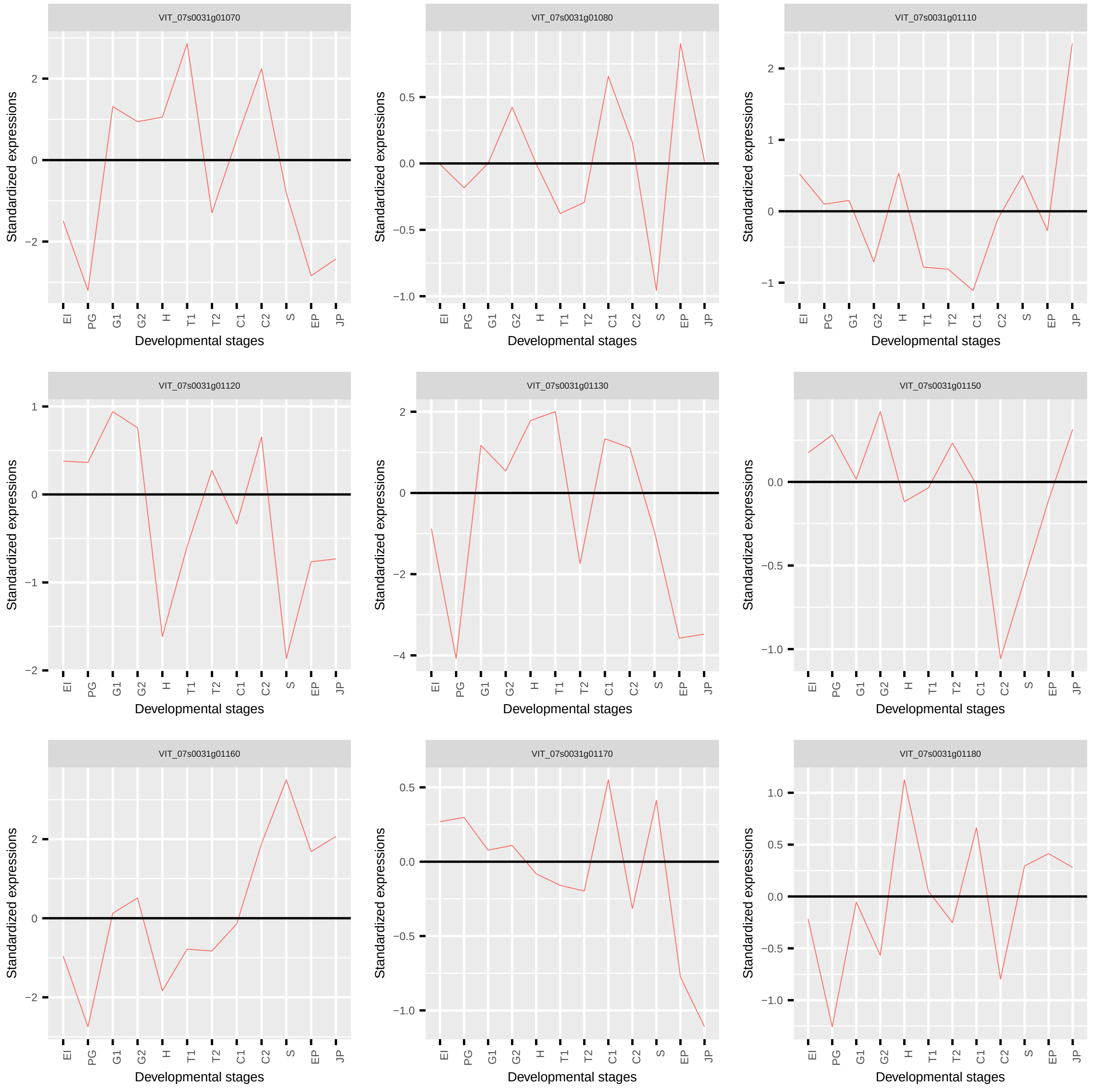
VIT_07s0031g01070
VIT_07s0031g01080
VIT_07s0031g01110
VIT_07s0031g01120
VIT_07s0031g01130
VIT_07s0031g01150
VIT_07s0031g01160
VIT_07s0031g01170
VIT_07s0031g01180
Standardized expressions
Developmental stages
EI PG G1 G2 H T1 T2 C1 C2 S EP JP

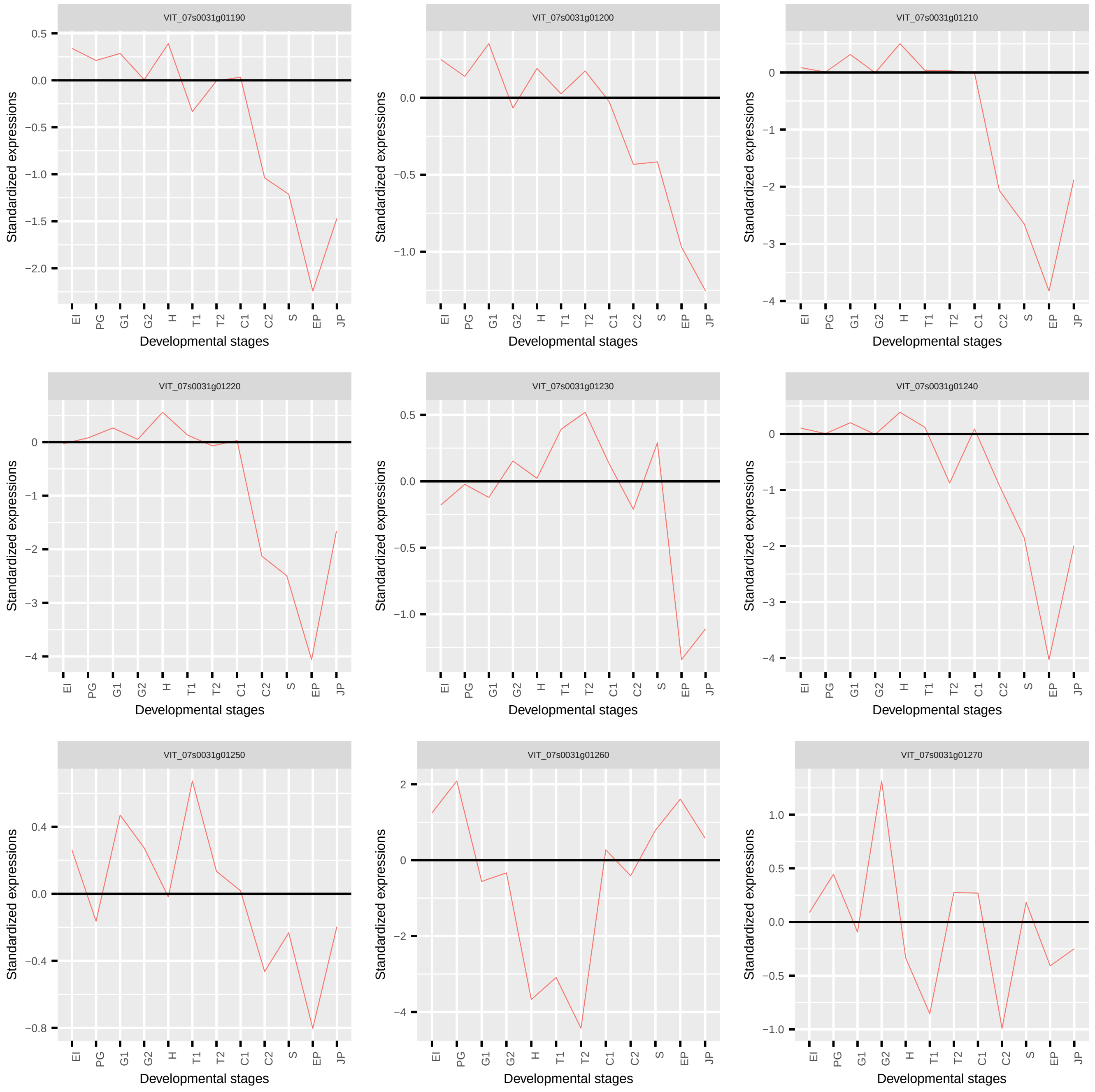
VIT_07s0031g01190
VIT_07s0031g01200
VIT_07s0031g01210
VIT_07s0031g01220
VIT_07s0031g01230
VIT_07s0031g01240
VIT_07s0031g01250
VIT_07s0031g01260
VIT_07s0031g01270
Standardized expressions
Developmental stages
EI PG G1 G2 H T1 T2 C1 C2 S EP JP

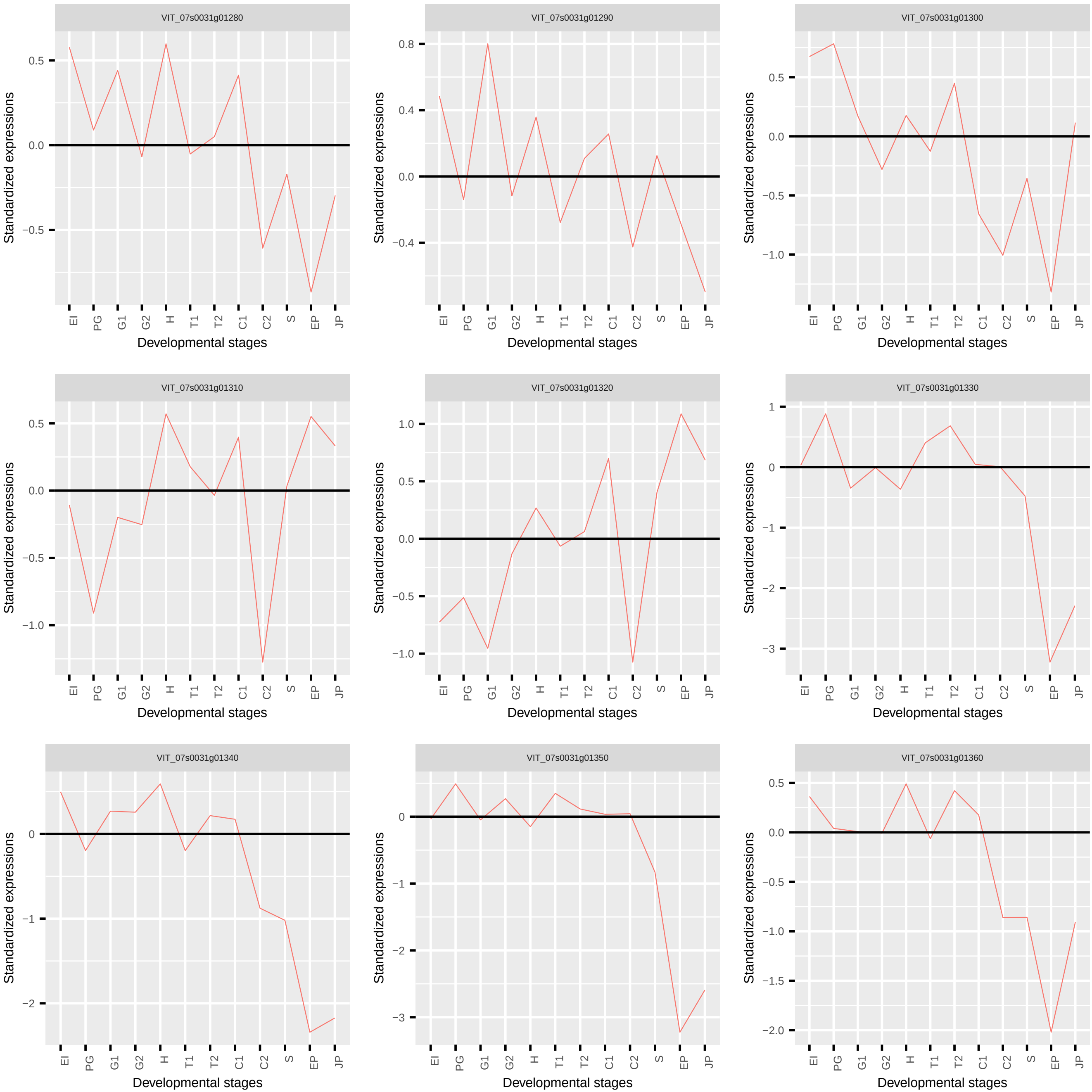
VIT_07s0031g01280
VIT_07s0031g01290
VIT_07s0031g01300
VIT_07s0031g01310
VIT_07s0031g01320
VIT_07s0031g01330
VIT_07s0031g01340
VIT_07s0031g01350
VIT_07s0031g01360
Standardized expressions
Developmental stages
EI
PG
G1
G2
H
T1
T2
C1
C2
S
EP
JP

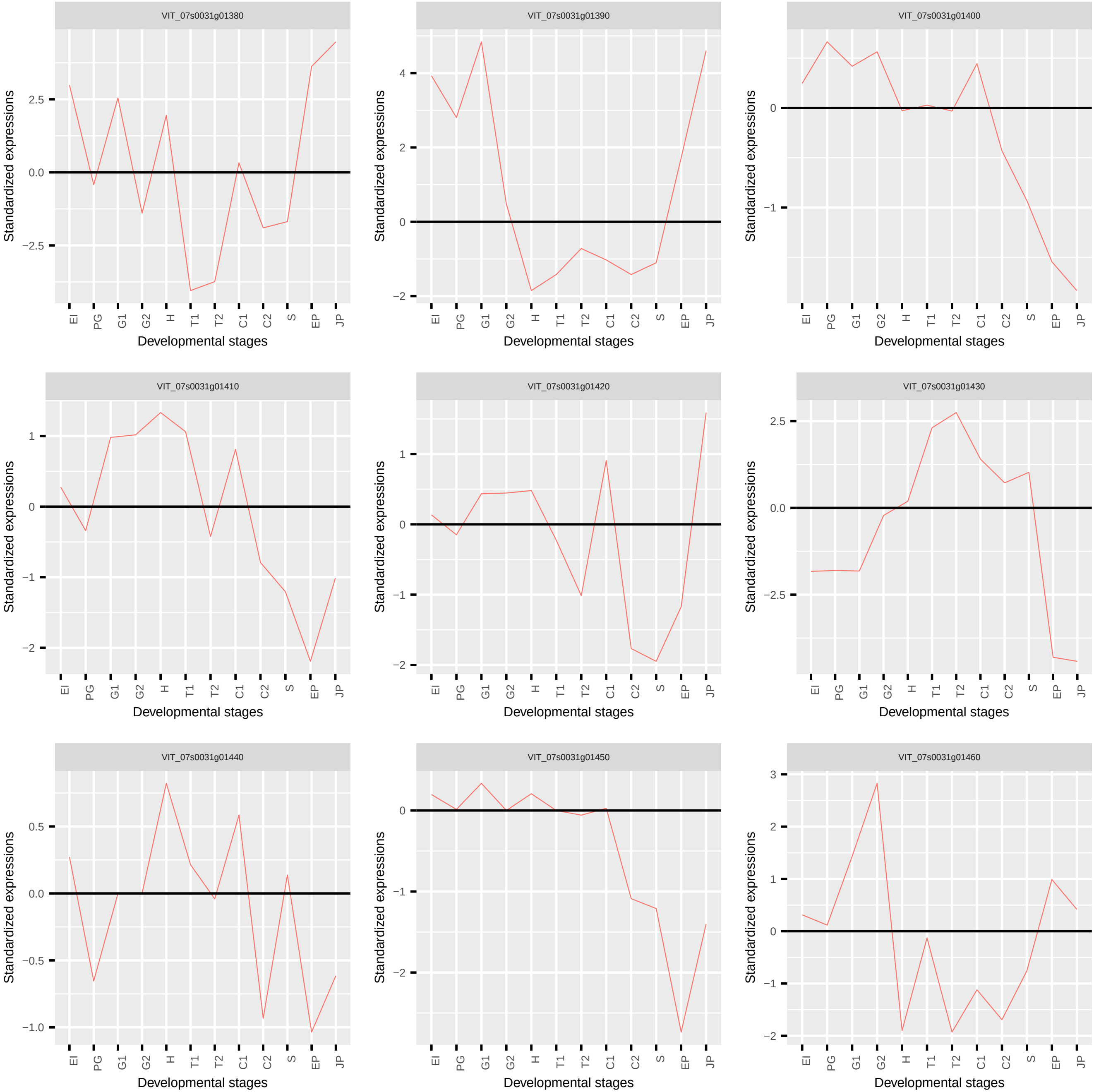
VIT_07s0031g01380
VIT_07s0031g01390
VIT_07s0031g01400
VIT_07s0031g01410
VIT_07s0031g01420
VIT_07s0031g01430
VIT_07s0031g01440
VIT_07s0031g01450
VIT_07s0031g01460
Standardized expressions
Developmental stages
EI PG G1 G2 H T1 T2 C1 C2 S EP JP

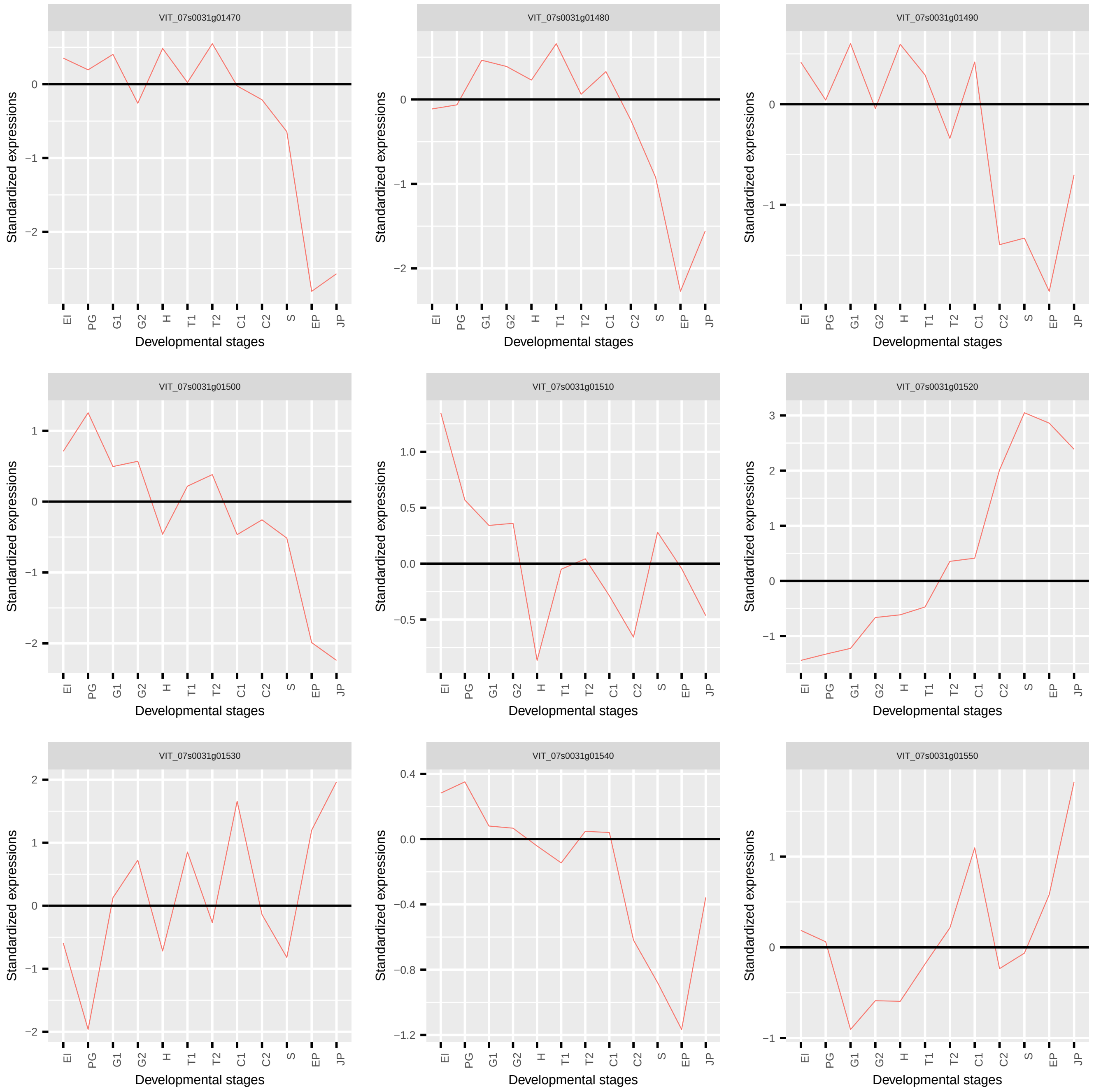
VIT_07s0031g01470
VIT_07s0031g01480
VIT_07s0031g01490
VIT_07s0031g01500
VIT_07s0031g01510
VIT_07s0031g01520
VIT_07s0031g01530
VIT_07s0031g01540
VIT_07s0031g01550
Standardized expressions
Developmental stages
EI PG G1 G2 H T1 T2 C1 C2 S EP JP

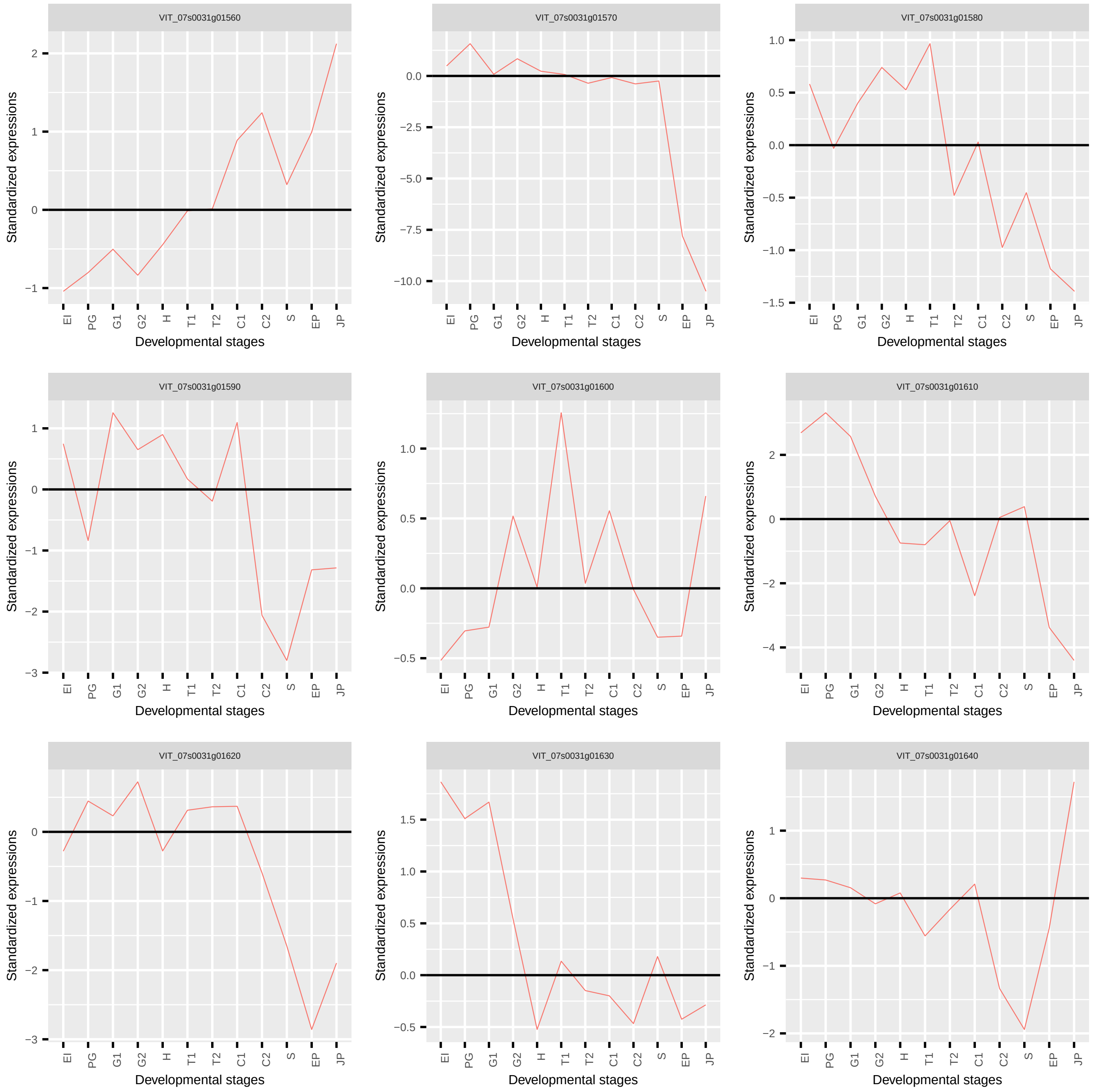
VIT_07s0031g01560
VIT_07s0031g01570
VIT_07s0031g01580
VIT_07s0031g01590
VIT_07s0031g01600
VIT_07s0031g01610
VIT_07s0031g01620
VIT_07s0031g01630
VIT_07s0031g01640
Standardized expressions
Developmental stages
EI
PG
G1
G2
H
T1
T2
C1
C2
S
EP
JP

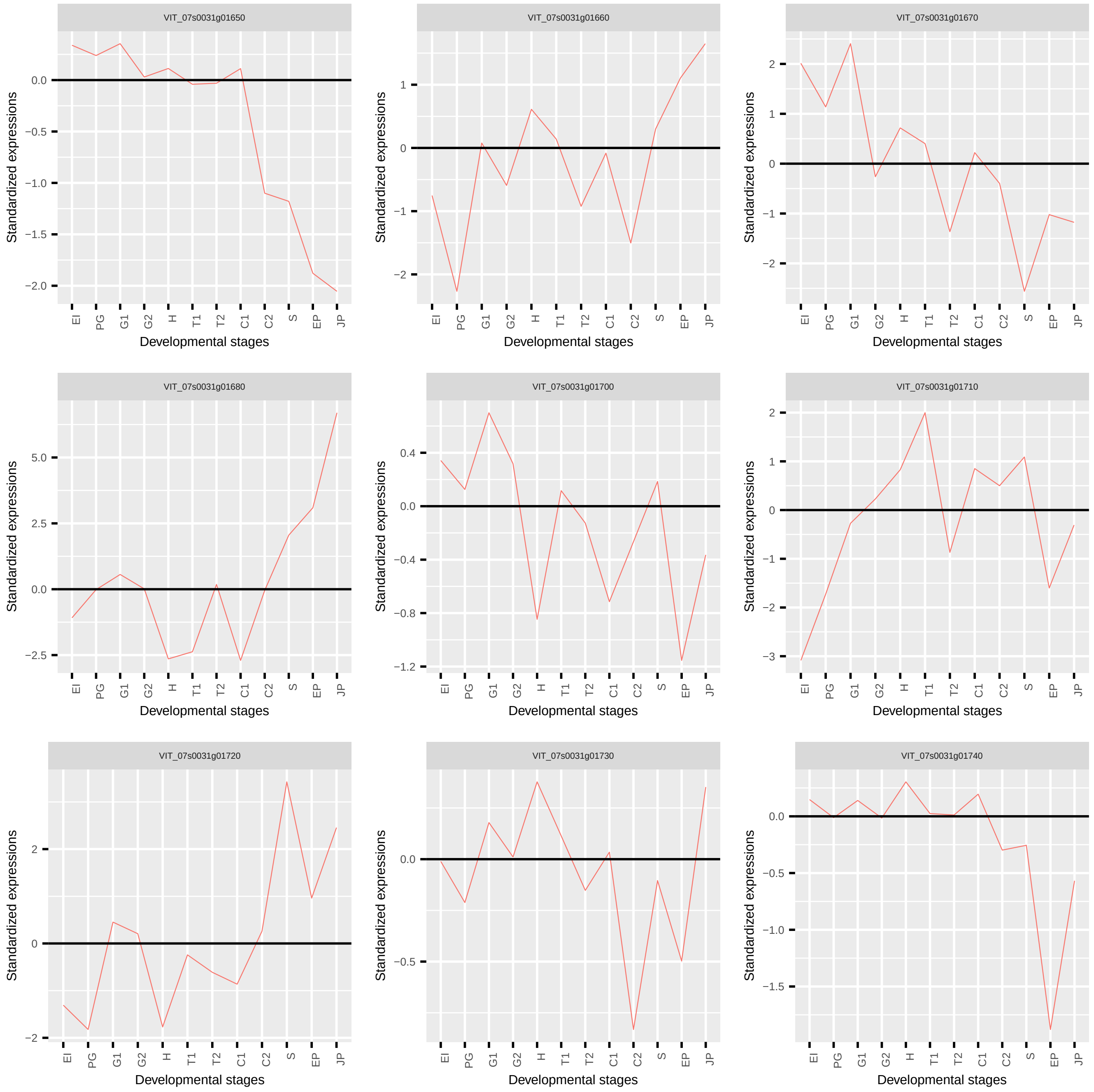
VIT_07s0031g01650
0.0
−0.5
−1.0
−1.5
−2.0
Standardized expressions
EI PG G1 G2 H T1 T2 C1 C2 S EP JP
Developmental stages
VIT_07s0031g01660
1
0
−1
−2
Standardized expressions
EI PG G1 G2 H T1 T2 C1 C2 S EP JP
Developmental stages
VIT_07s0031g01670
2
1
0
−1
−2
Standardized expressions
EI PG G1 G2 H T1 T2 C1 C2 S EP JP
Developmental stages
VIT_07s0031g01680
5.0
2.5
0.0
−2.5
Standardized expressions
EI PG G1 G2 H T1 T2 C1 C2 S EP JP
Developmental stages
VIT_07s0031g01700
0.4
0.0
−0.4
−0.8
−1.2
Standardized expressions
EI PG G1 G2 H T1 T2 C1 C2 S EP JP
Developmental stages
VIT_07s0031g01710
2
1
0
−1
−2
−3
Standardized expressions
EI PG G1 G2 H T1 T2 C1 C2 S EP JP
Developmental stages
VIT_07s0031g01720
2
0
−2
Standardized expressions
EI PG G1 G2 H T1 T2 C1 C2 S EP JP
Developmental stages
VIT_07s0031g01730
0.0
−0.5
Standardized expressions
EI PG G1 G2 H T1 T2 C1 C2 S EP JP
Developmental stages
VIT_07s0031g01740
0.0
−0.5
−1.0
−1.5
Standardized expressions
EI PG G1 G2 H T1 T2 C1 C2 S EP JP
Developmental stages

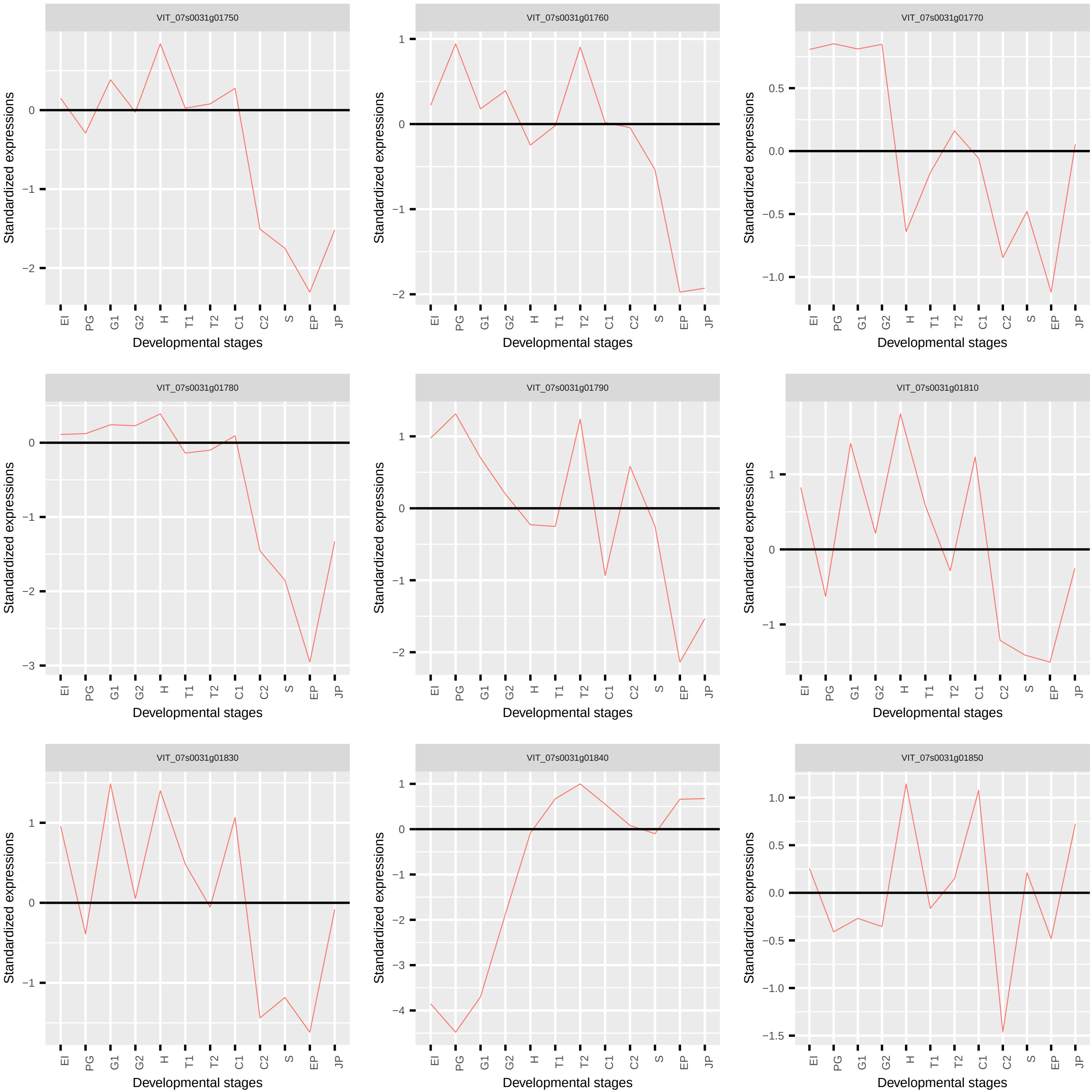
VIT_07s0031g01750
VIT_07s0031g01760
VIT_07s0031g01770
VIT_07s0031g01780
VIT_07s0031g01790
VIT_07s0031g01810
VIT_07s0031g01830
VIT_07s0031g01840
VIT_07s0031g01850
Standardized expressions
EI PG G1 G2 H T1 T2 C1 C2 S EP JP
Developmental stages

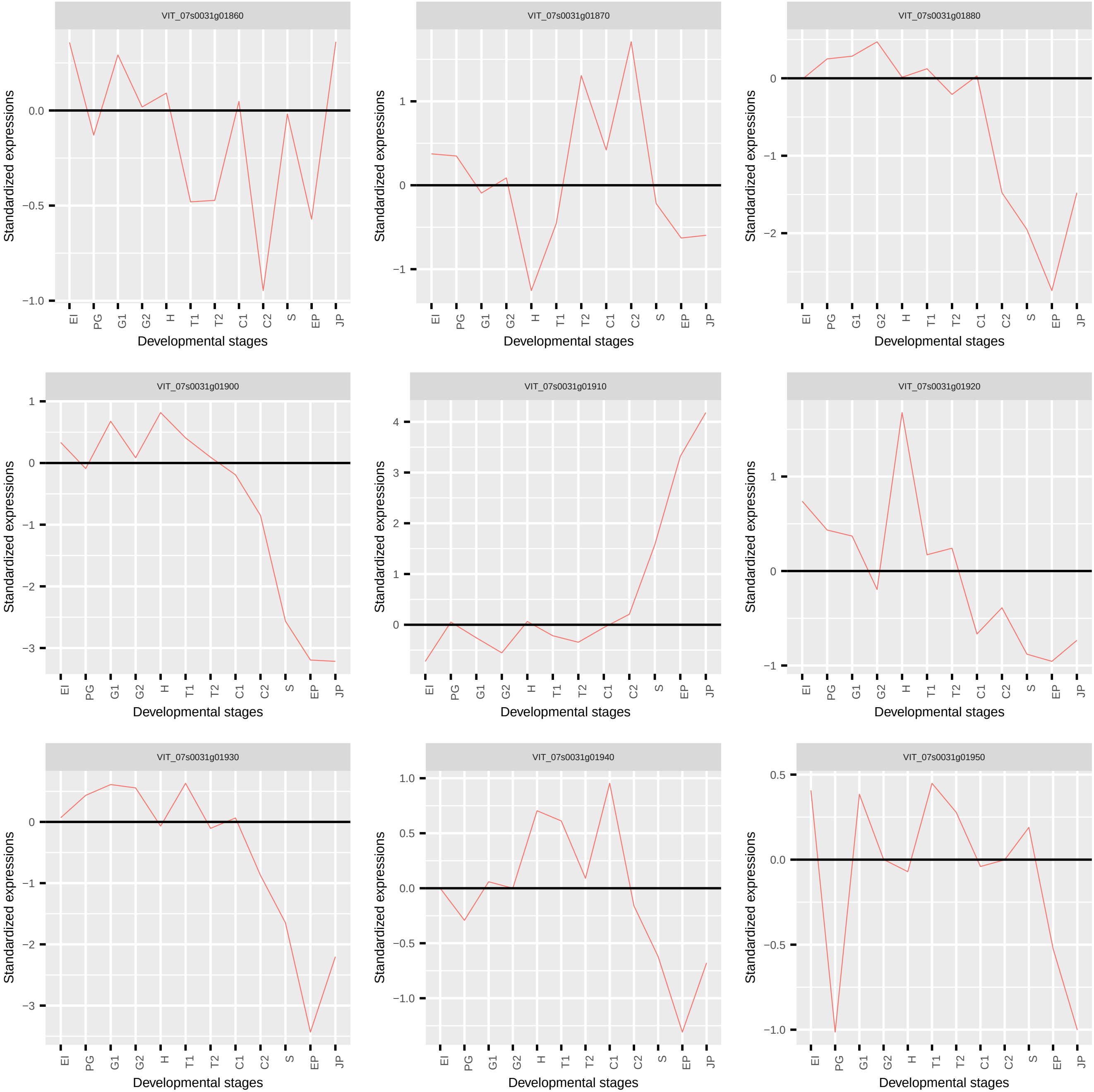
VIT_07s0031g01860
VIT_07s0031g01870
VIT_07s0031g01880
VIT_07s0031g01900
VIT_07s0031g01910
VIT_07s0031g01920
VIT_07s0031g01930
VIT_07s0031g01940
VIT_07s0031g01950
Standardized expressions
Developmental stages
EI PG G1 G2 H T1 T2 C1 C2 S EP JP

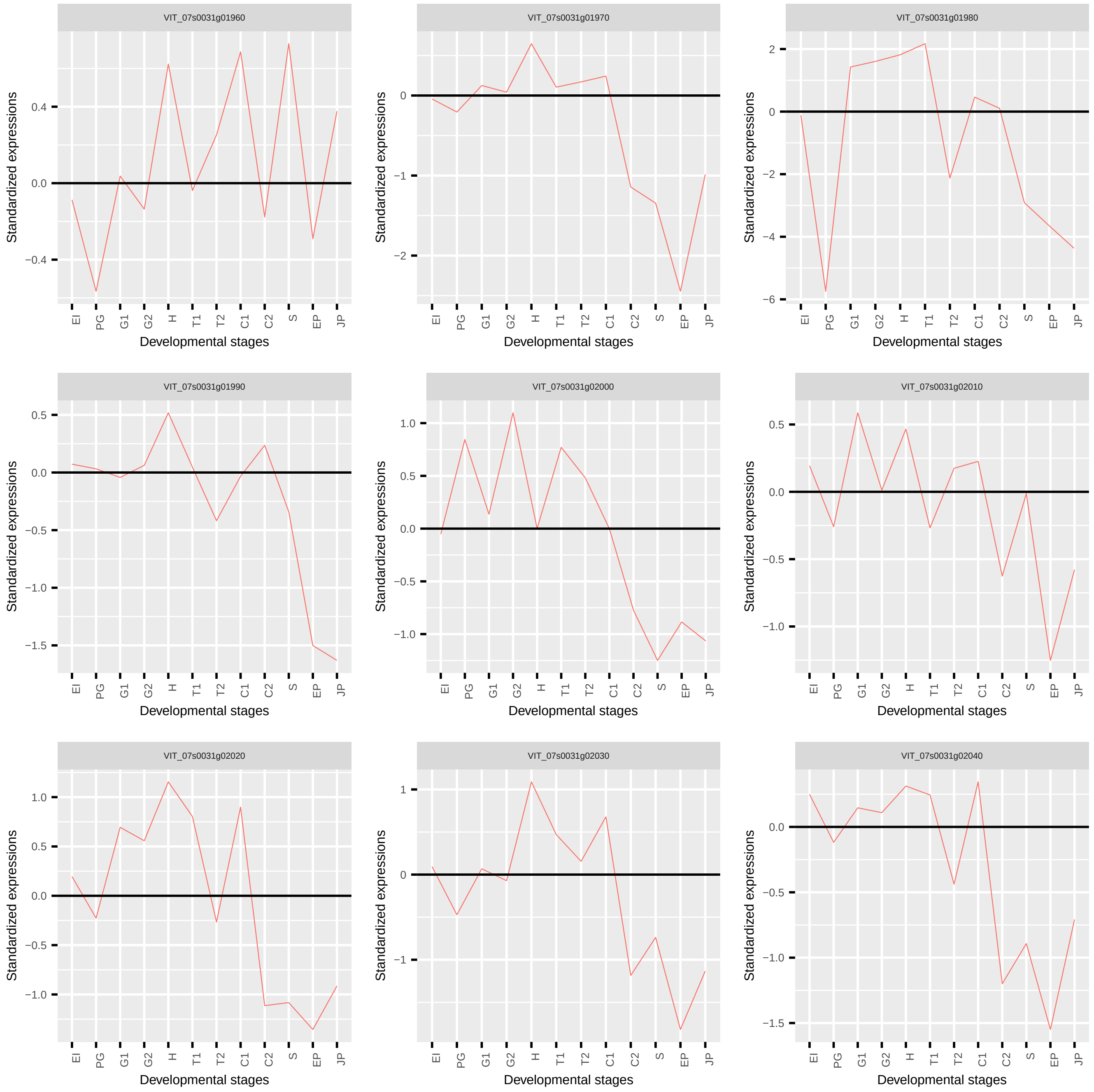
VIT_07s0031g01960
0.4
0.0
−0.4
Standardized expressions
EI PG G1 G2 H T1 T2 C1 C2 S EP JP
Developmental stages
VIT_07s0031g01970
0
−1
−2
Standardized expressions
EI PG G1 G2 H T1 T2 C1 C2 S EP JP
Developmental stages
VIT_07s0031g01980
2
0
−2
−4
−6
Standardized expressions
EI PG G1 G2 H T1 T2 C1 C2 S EP JP
Developmental stages
VIT_07s0031g01990
0.5
0.0
−0.5
−1.0
−1.5
Standardized expressions
EI PG G1 G2 H T1 T2 C1 C2 S EP JP
Developmental stages
VIT_07s0031g02000
1.0
0.5
0.0
−0.5
−1.0
Standardized expressions
EI PG G1 G2 H T1 T2 C1 C2 S EP JP
Developmental stages
VIT_07s0031g02010
0.5
0.0
−0.5
−1.0
Standardized expressions
EI PG G1 G2 H T1 T2 C1 C2 S EP JP
Developmental stages
VIT_07s0031g02020
1.0
0.5
0.0
−0.5
−1.0
Standardized expressions
EI PG G1 G2 H T1 T2 C1 C2 S EP JP
Developmental stages
VIT_07s0031g02030
1
0
−1
Standardized expressions
EI PG G1 G2 H T1 T2 C1 C2 S EP JP
Developmental stages
VIT_07s0031g02040
0.0
−0.5
−1.0
−1.5
Standardized expressions
EI PG G1 G2 H T1 T2 C1 C2 S EP JP
Developmental stages

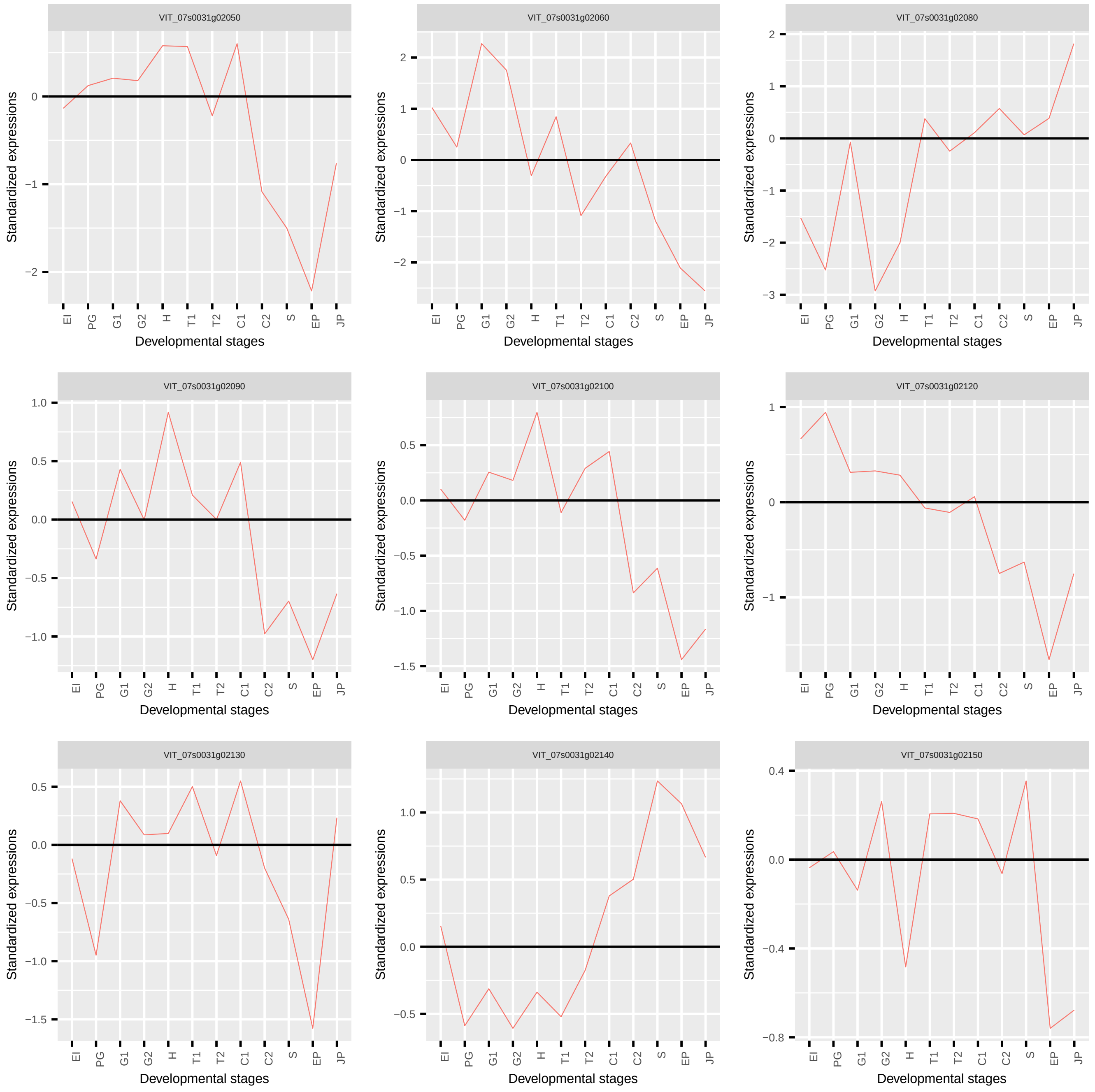

VIT_07s0031g02050
VIT_07s0031g02060
VIT_07s0031g02080
VIT_07s0031g02090
VIT_07s0031g02100
VIT_07s0031g02120
VIT_07s0031g02130
VIT_07s0031g02140
VIT_07s0031g02150
Standardized expressions
Developmental stages
EI PG G1 G2 H T1 T2 C1 C2 S EP JP

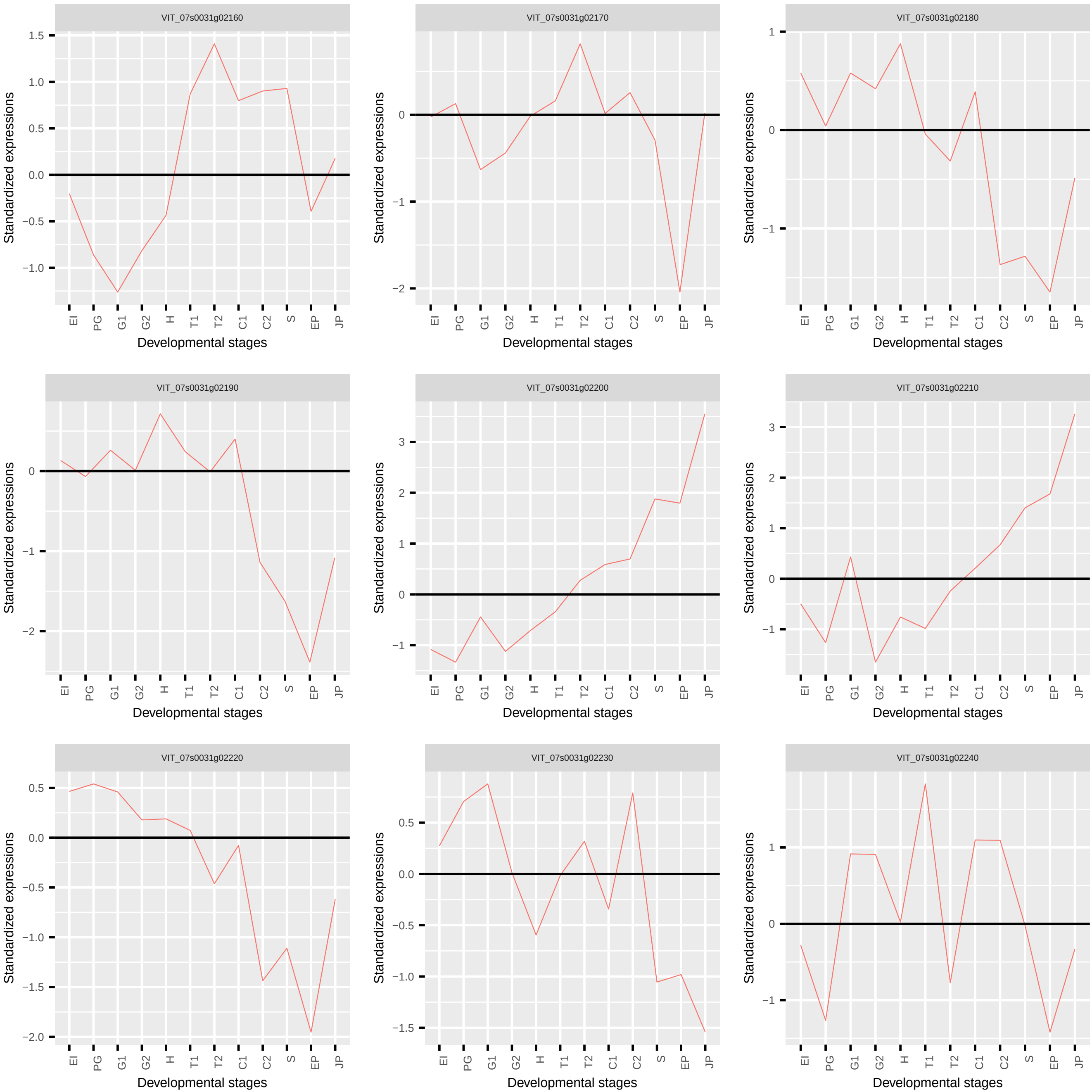

VIT_07s0031g02160
VIT_07s0031g02170
VIT_07s0031g02180
VIT_07s0031g02190
VIT_07s0031g02200
VIT_07s0031g02210
VIT_07s0031g02220
VIT_07s0031g02230
VIT_07s0031g02240
Standardized expressions
Developmental stages
EI PG G1 G2 H T1 T2 C1 C2 S EP JP

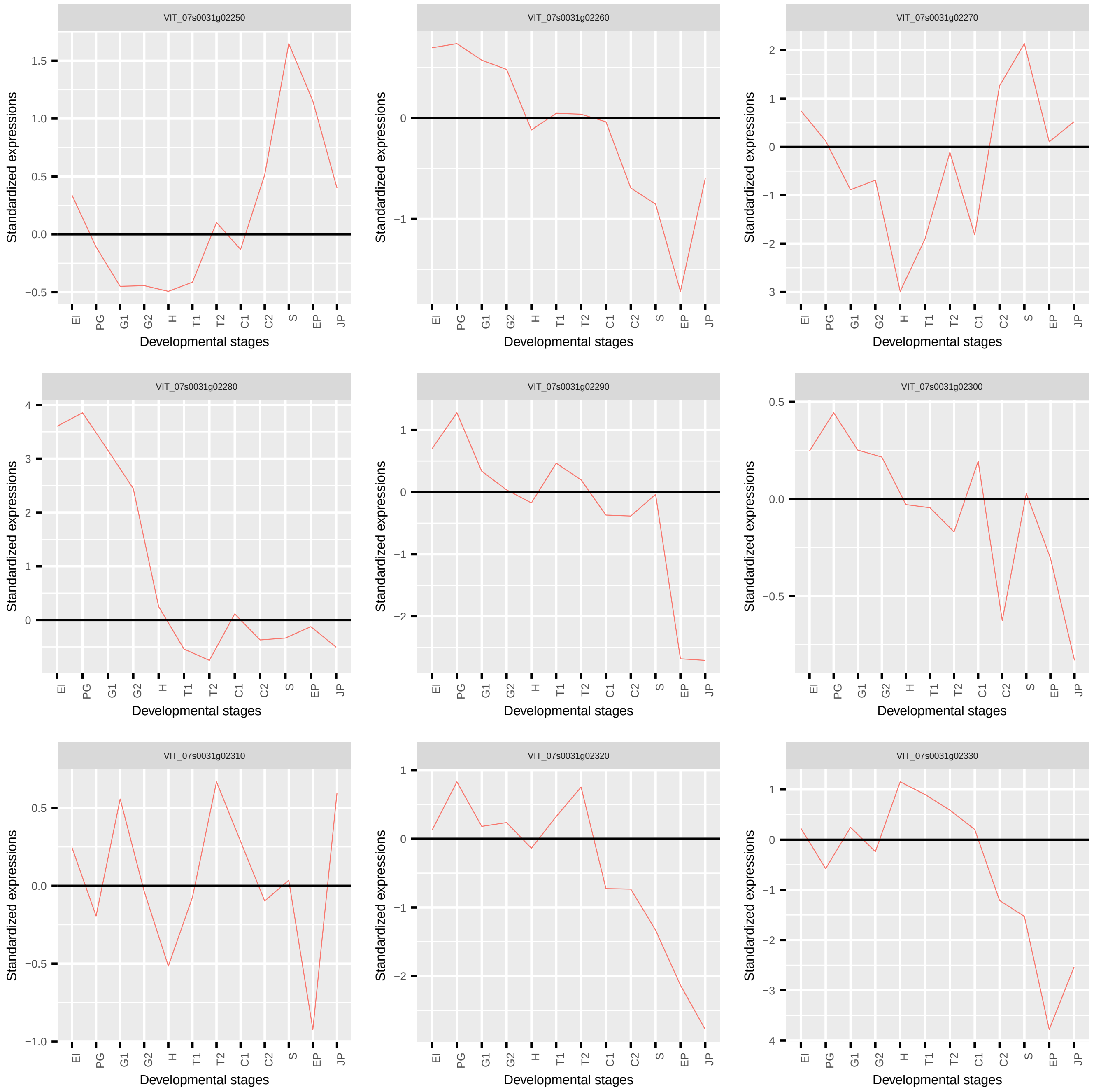
VIT_07s0031g02250
VIT_07s0031g02260
VIT_07s0031g02270
VIT_07s0031g02280
VIT_07s0031g02290
VIT_07s0031g02300
VIT_07s0031g02310
VIT_07s0031g02320
VIT_07s0031g02330
Standardized expressions
Developmental stages
EI
PG
G1
G2
H
T1
T2
C1
C2
S
EP
JP

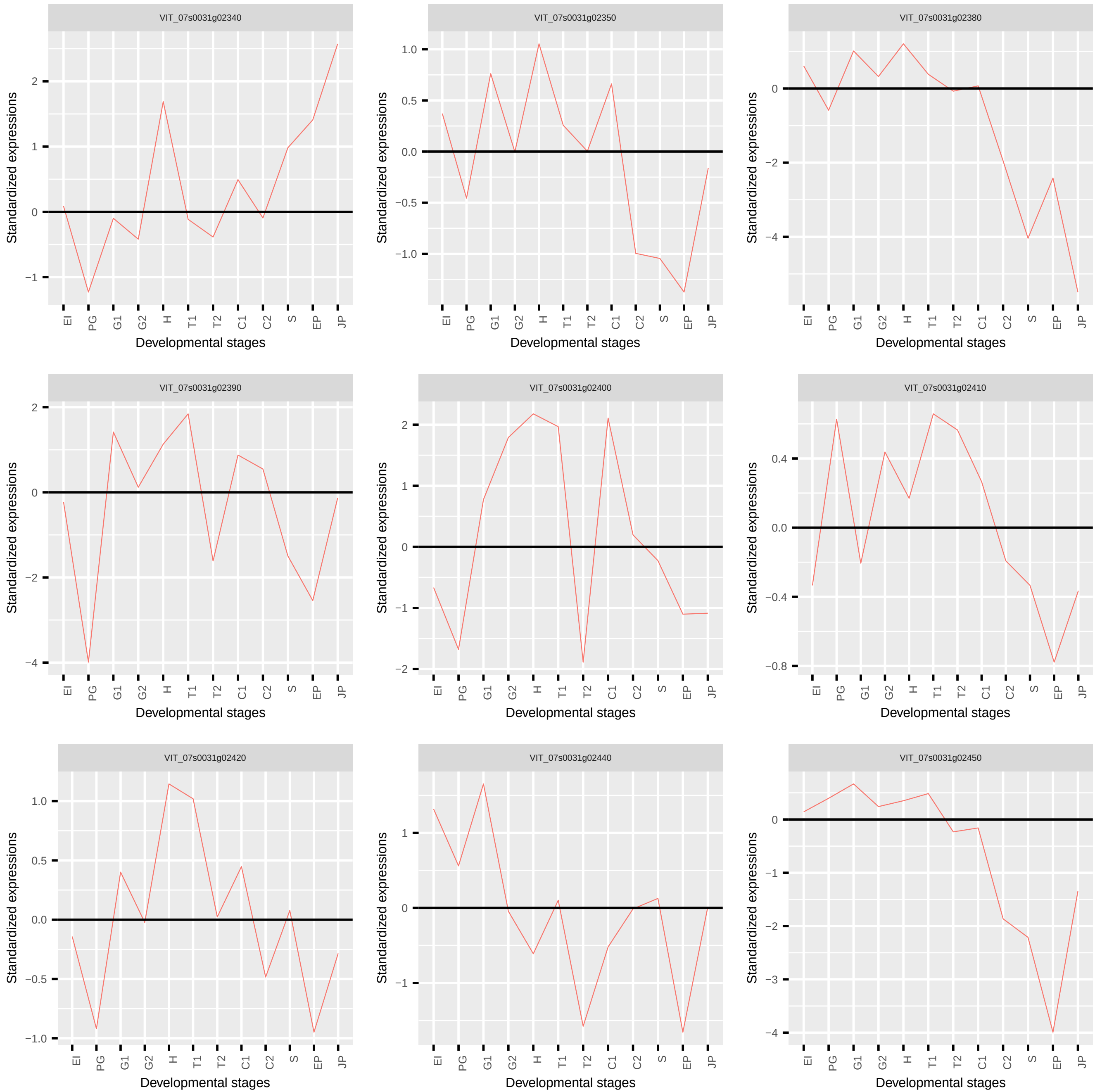
VIT_07s0031g02340
VIT_07s0031g02350
VIT_07s0031g02380
VIT_07s0031g02390
VIT_07s0031g02400
VIT_07s0031g02410
VIT_07s0031g02420
VIT_07s0031g02440
VIT_07s0031g02450
Standardized expressions
Developmental stages
EI PG G1 G2 H T1 T2 C1 C2 S EP JP

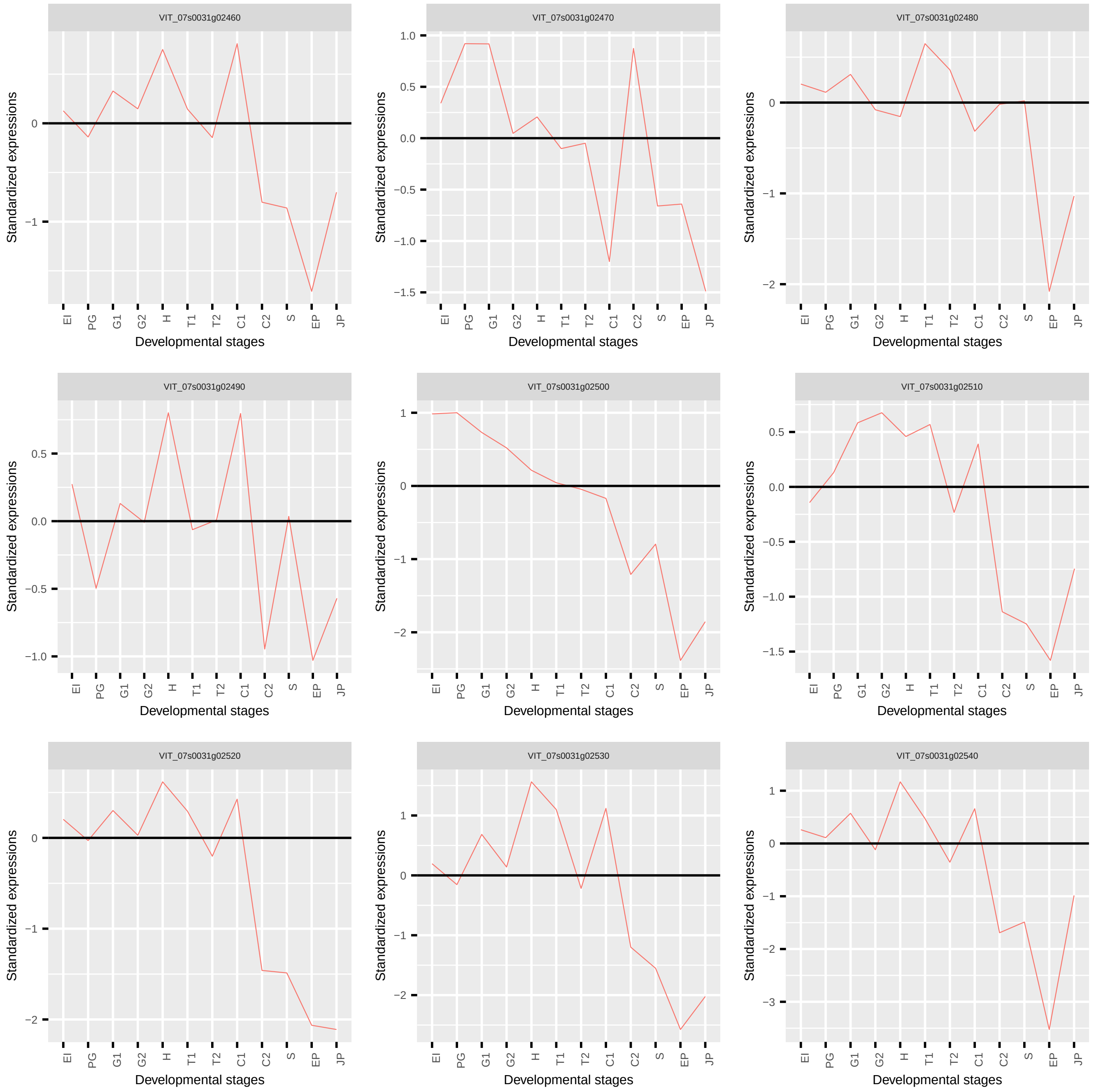

VIT_07s0031g02460
VIT_07s0031g02470
VIT_07s0031g02480
VIT_07s0031g02490
VIT_07s0031g02500
VIT_07s0031g02510
VIT_07s0031g02520
VIT_07s0031g02530
VIT_07s0031g02540
Standardized expressions
Developmental stages
EI PG G1 G2 H T1 T2 C1 C2 S EP JP

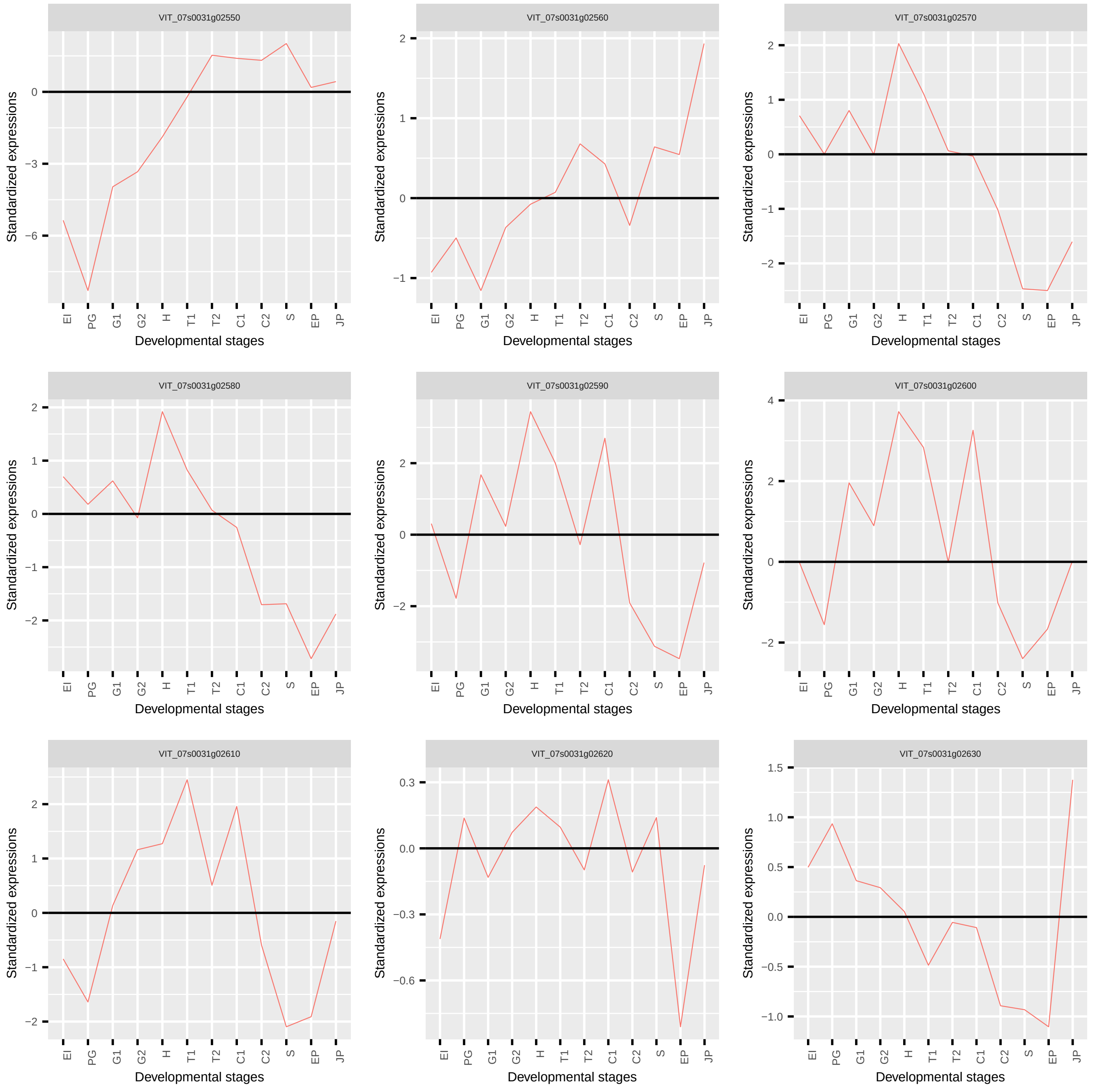
VIT_07s0031g02550
VIT_07s0031g02560
VIT_07s0031g02570
VIT_07s0031g02580
VIT_07s0031g02590
VIT_07s0031g02600
VIT_07s0031g02610
VIT_07s0031g02620
VIT_07s0031g02630
Standardized expressions
Developmental stages
EI PG G1 G2 H T1 T2 C1 C2 S EP JP

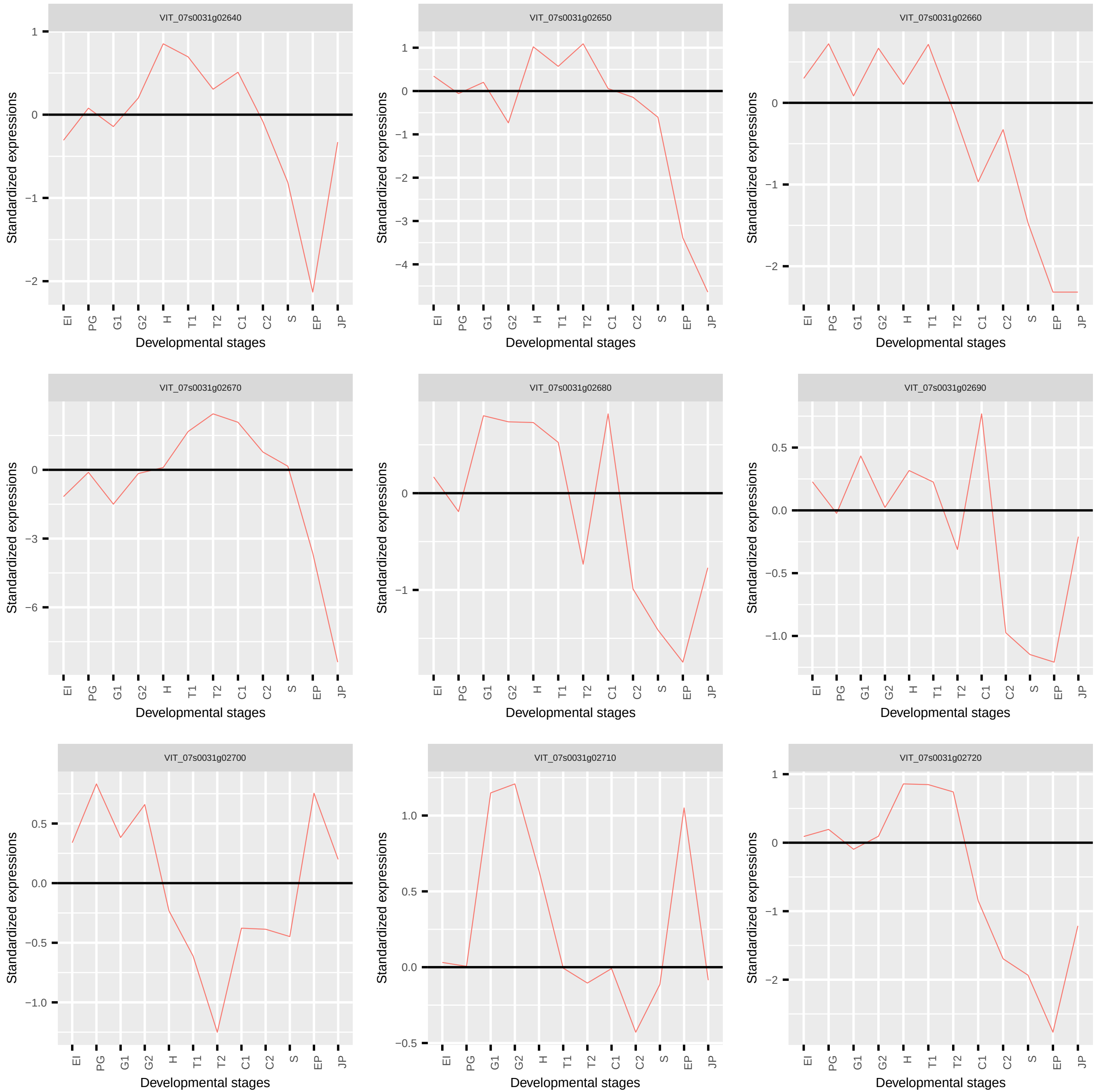

VIT_07s0031g02640
VIT_07s0031g02650
VIT_07s0031g02660
VIT_07s0031g02670
VIT_07s0031g02680
VIT_07s0031g02690
VIT_07s0031g02700
VIT_07s0031g02710
VIT_07s0031g02720
Standardized expressions
Developmental stages
EI
PG
G1
G2
H
T1
T2
C1
C2
S
EP
JP

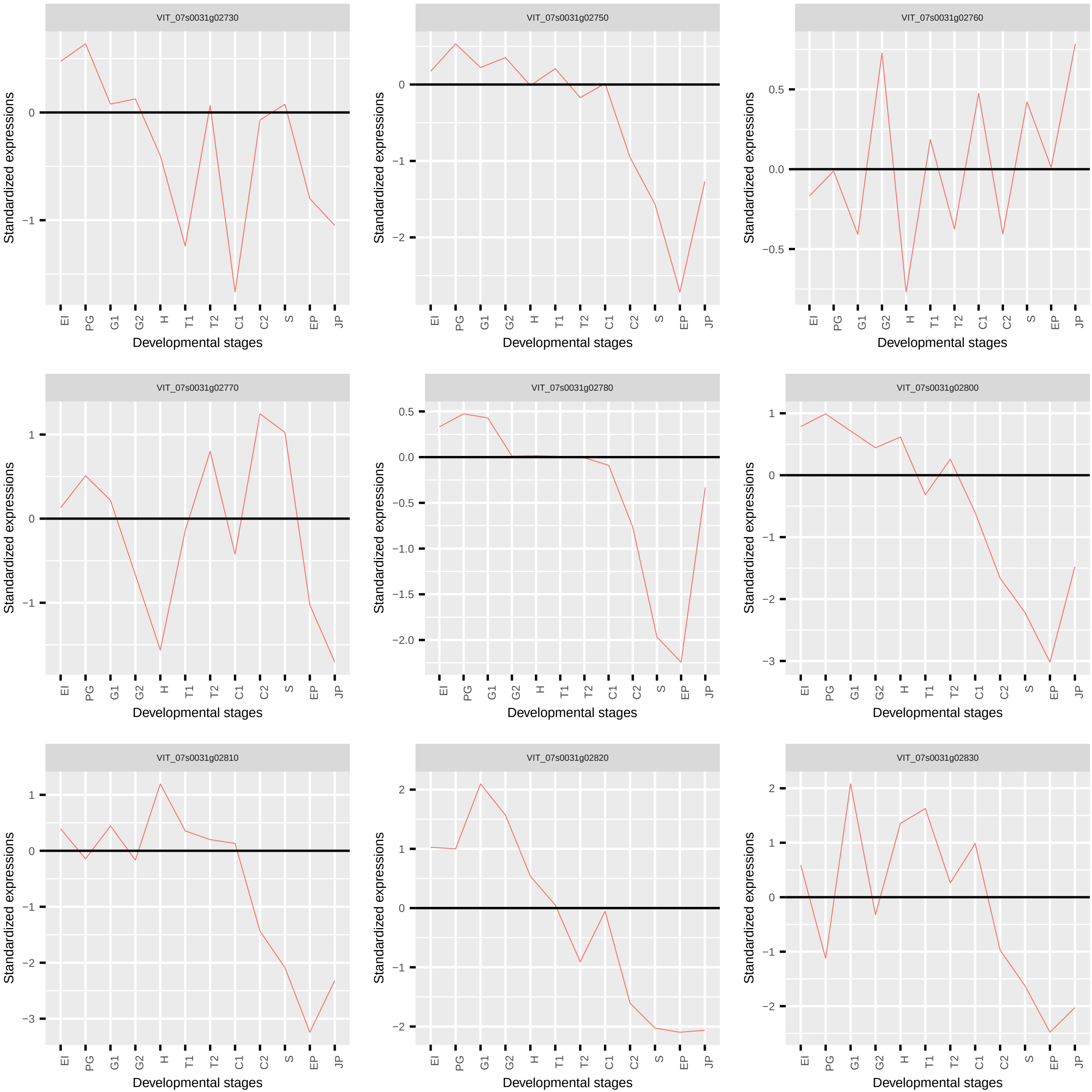
VIT_07s0031g02730
VIT_07s0031g02750
VIT_07s0031g02760
VIT_07s0031g02770
VIT_07s0031g02780
VIT_07s0031g02800
VIT_07s0031g02810
VIT_07s0031g02820
VIT_07s0031g02830
Standardized expressions
Developmental stages
EI
PG
G1
G2
H
T1
T2
C1
C2
S
EP
JP

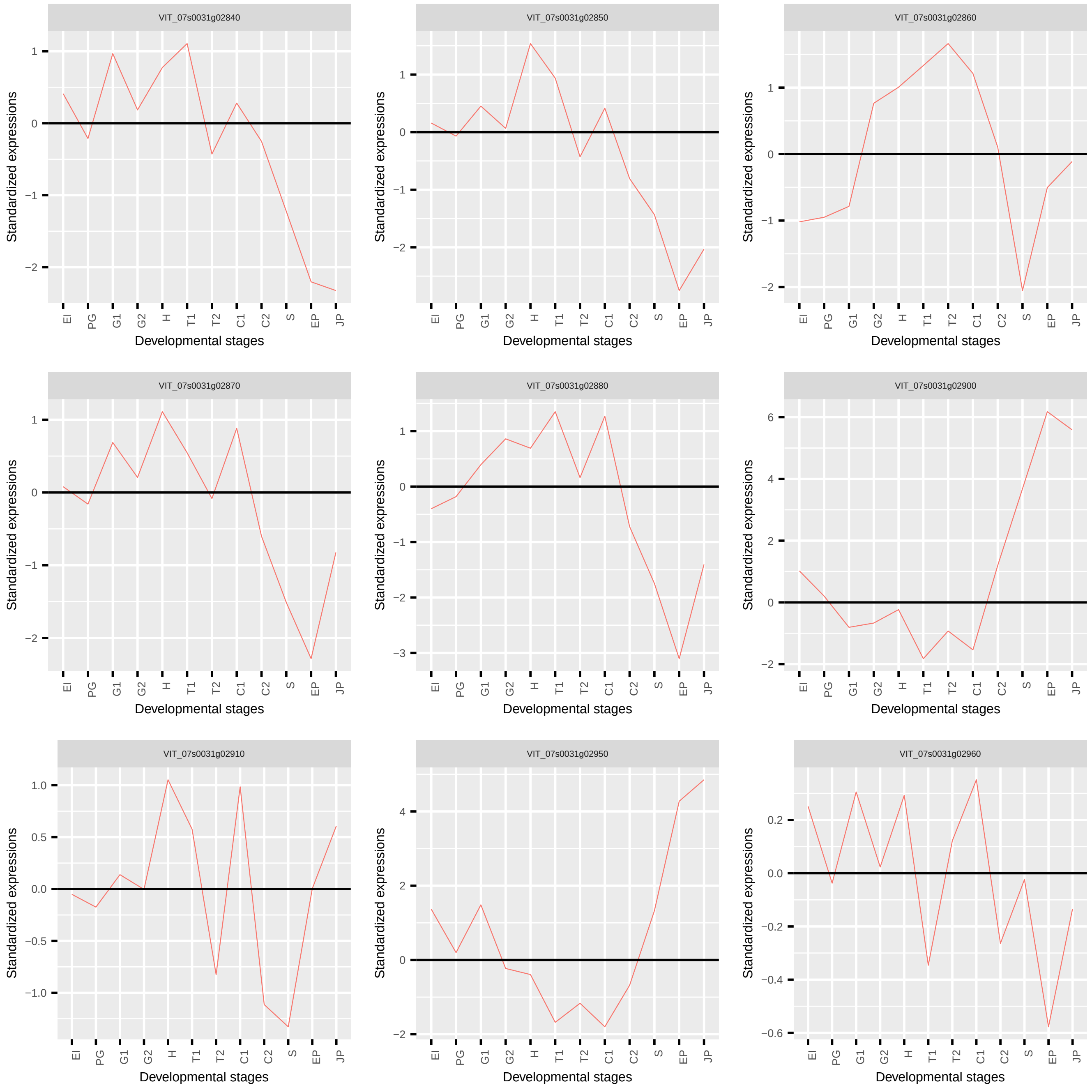
VIT_07s0031g02840
VIT_07s0031g02850
VIT_07s0031g02860
VIT_07s0031g02870
VIT_07s0031g02880
VIT_07s0031g02900
VIT_07s0031g02910
VIT_07s0031g02950
VIT_07s0031g02960
Standardized expressions
Developmental stages
EI PG G1 G2 H T1 T2 C1 C2 S EP JP

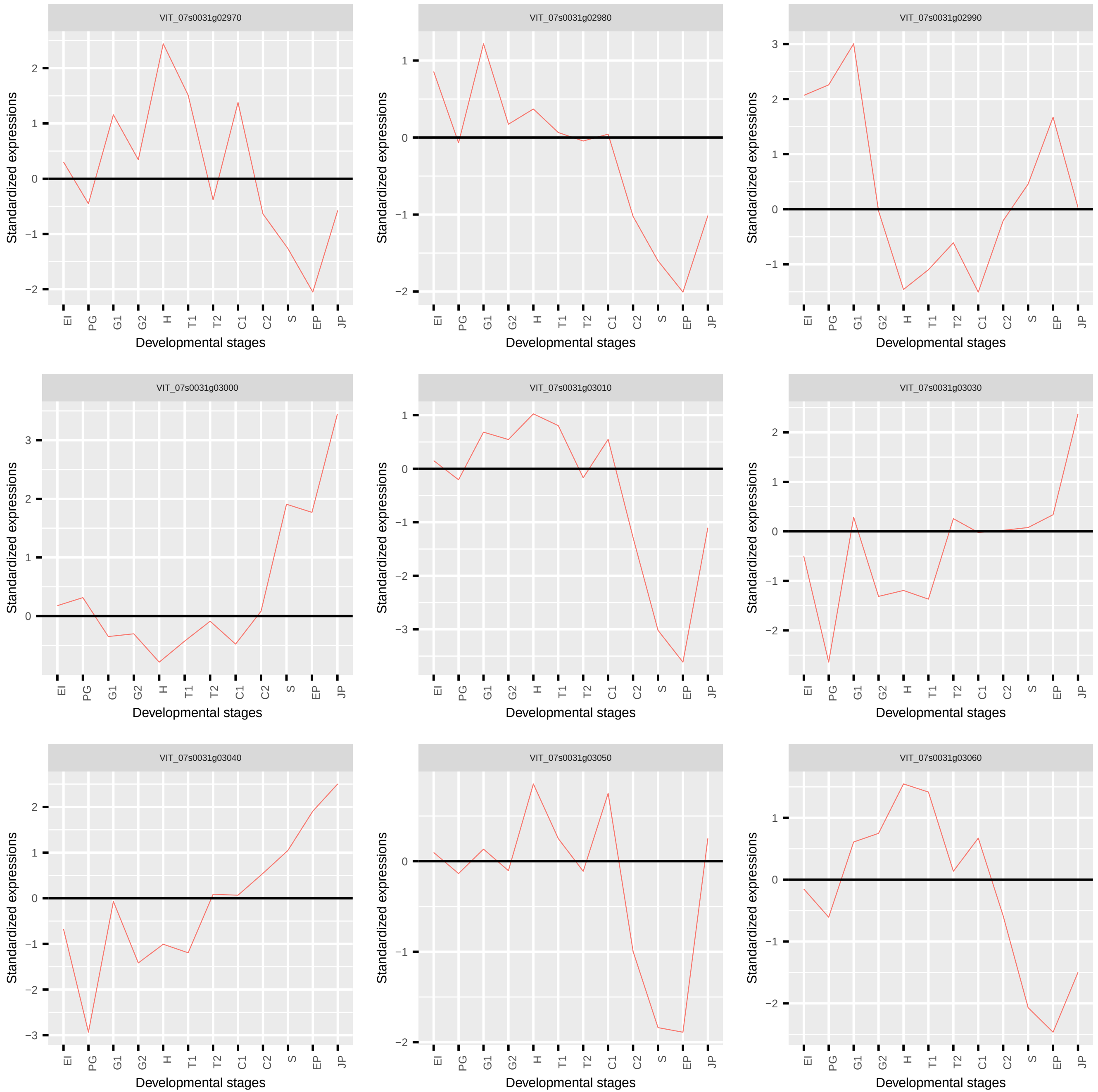
VIT_07s0031g02970
VIT_07s0031g02980
VIT_07s0031g02990
VIT_07s0031g03000
VIT_07s0031g03010
VIT_07s0031g03030
VIT_07s0031g03040
VIT_07s0031g03050
VIT_07s0031g03060
Standardized expressions
Developmental stages
EI
PG
G1
G2
H
T1
T2
C1
C2
S
EP
JP

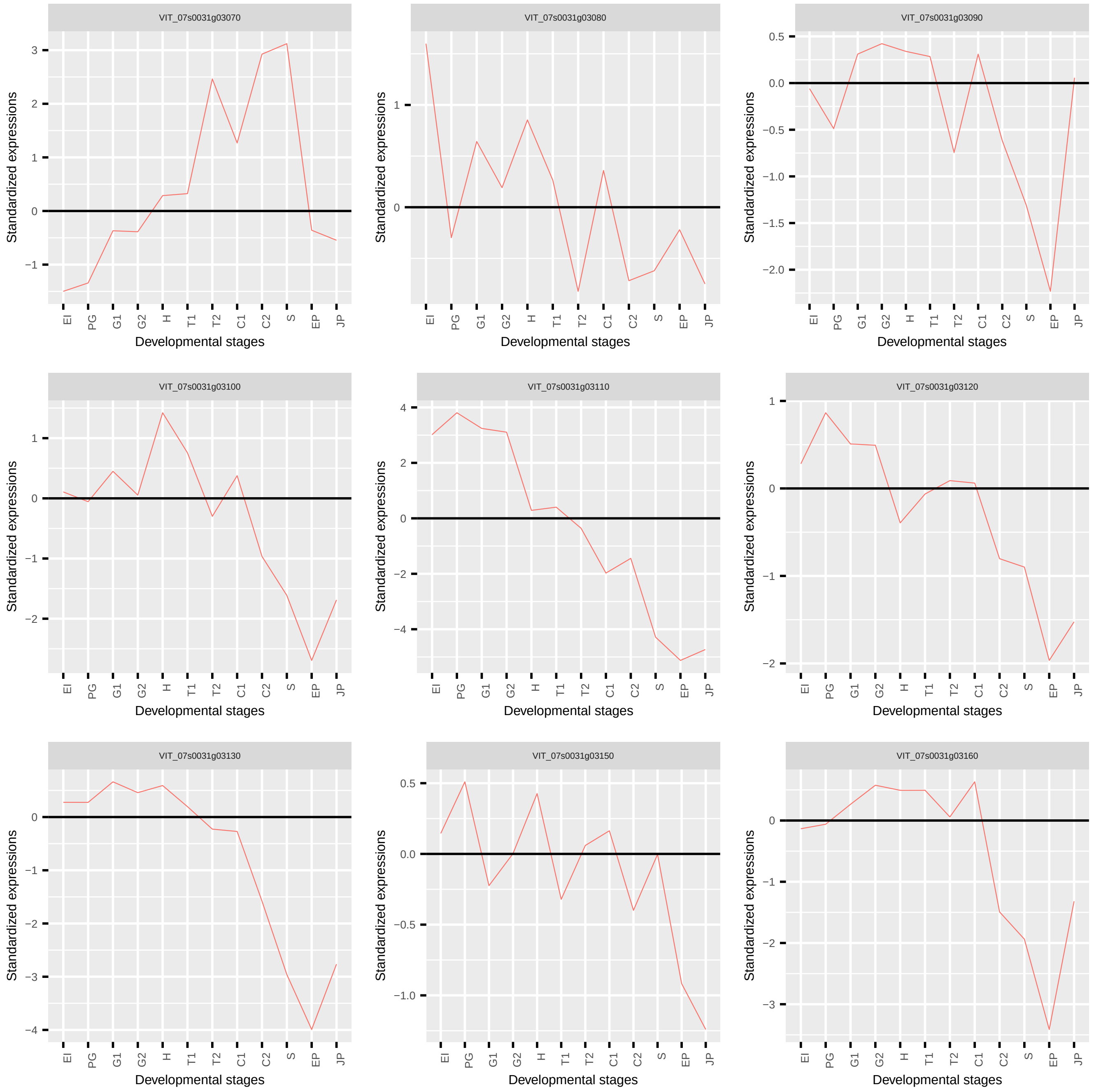
VIT_07s0031g03070
VIT_07s0031g03080
VIT_07s0031g03090
VIT_07s0031g03100
VIT_07s0031g03110
VIT_07s0031g03120
VIT_07s0031g03130
VIT_07s0031g03150
VIT_07s0031g03160
Standardized expressions
Developmental stages
EI PG G1 G2 H T1 T2 C1 C2 S EP JP

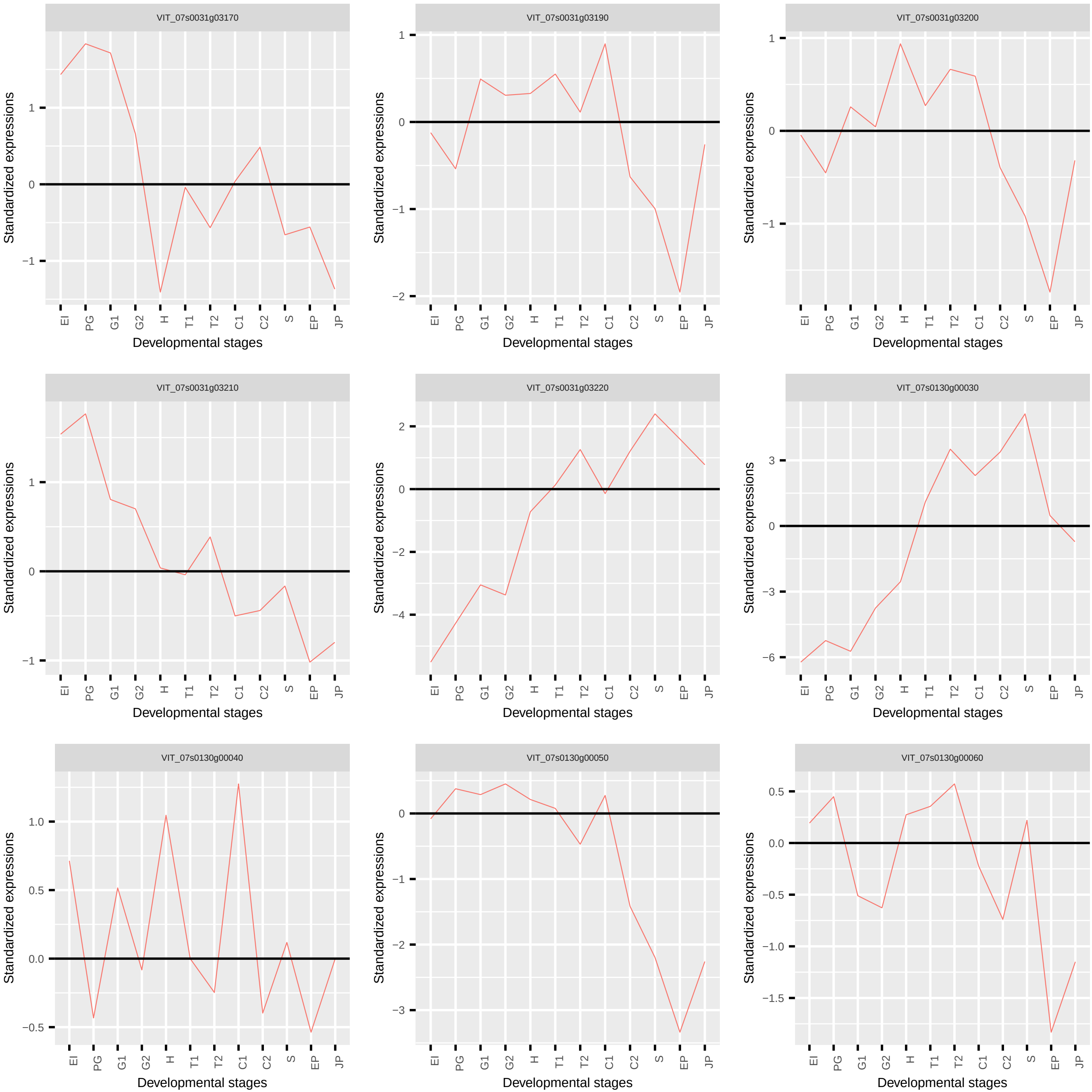

VIT_07s0031g03170
VIT_07s0031g03190
VIT_07s0031g03200
VIT_07s0031g03210
VIT_07s0031g03220
VIT_07s0130g00030
VIT_07s0130g00040
VIT_07s0130g00050
VIT_07s0130g00060
Standardized expressions
Developmental stages
EI PG G1 G2 H T1 T2 C1 C2 S EP JP

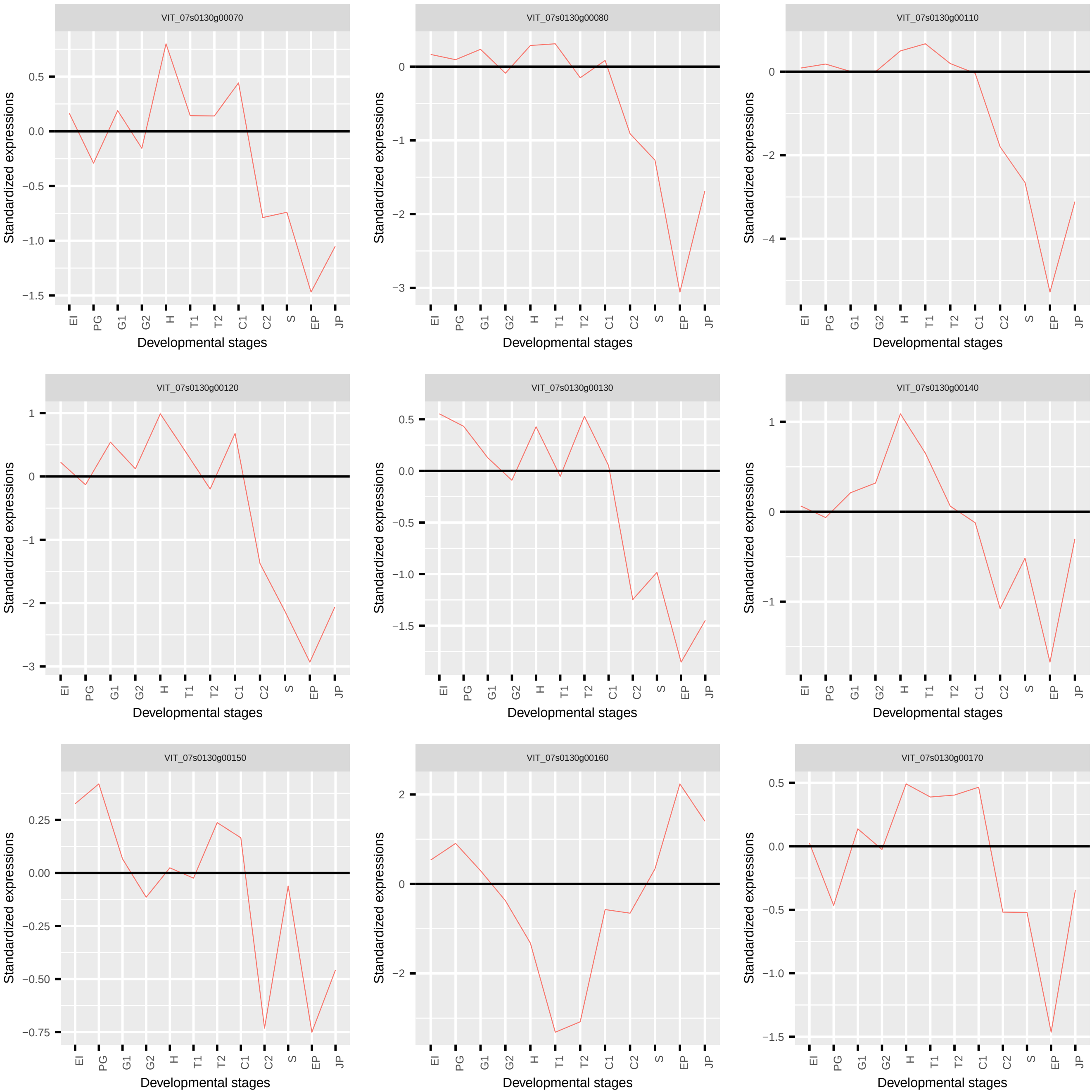
VIT_07s0130g00070
0.5
0.0
−0.5
−1.0
−1.5
Standardized expressions
EI PG G1 G2 H T1 T2 C1 C2 S EP JP
Developmental stages
VIT_07s0130g00080
0
−1
−2
−3
Standardized expressions
EI PG G1 G2 H T1 T2 C1 C2 S EP JP
Developmental stages
VIT_07s0130g00110
0
−2
−4
Standardized expressions
EI PG G1 G2 H T1 T2 C1 C2 S EP JP
Developmental stages
VIT_07s0130g00120
1
0
−1
−2
−3
Standardized expressions
EI PG G1 G2 H T1 T2 C1 C2 S EP JP
Developmental stages
VIT_07s0130g00130
0.5
0.0
−0.5
−1.0
−1.5
Standardized expressions
EI PG G1 G2 H T1 T2 C1 C2 S EP JP
Developmental stages
VIT_07s0130g00140
1
0
−1
Standardized expressions
EI PG G1 G2 H T1 T2 C1 C2 S EP JP
Developmental stages
VIT_07s0130g00150
0.25
0.00
−0.25
−0.50
−0.75
Standardized expressions
EI PG G1 G2 H T1 T2 C1 C2 S EP JP
Developmental stages
VIT_07s0130g00160
2
0
−2
Standardized expressions
EI PG G1 G2 H T1 T2 C1 C2 S EP JP
Developmental stages
VIT_07s0130g00170
0.5
0.0
−0.5
−1.0
−1.5
Standardized expressions
EI PG G1 G2 H T1 T2 C1 C2 S EP JP
Developmental stages

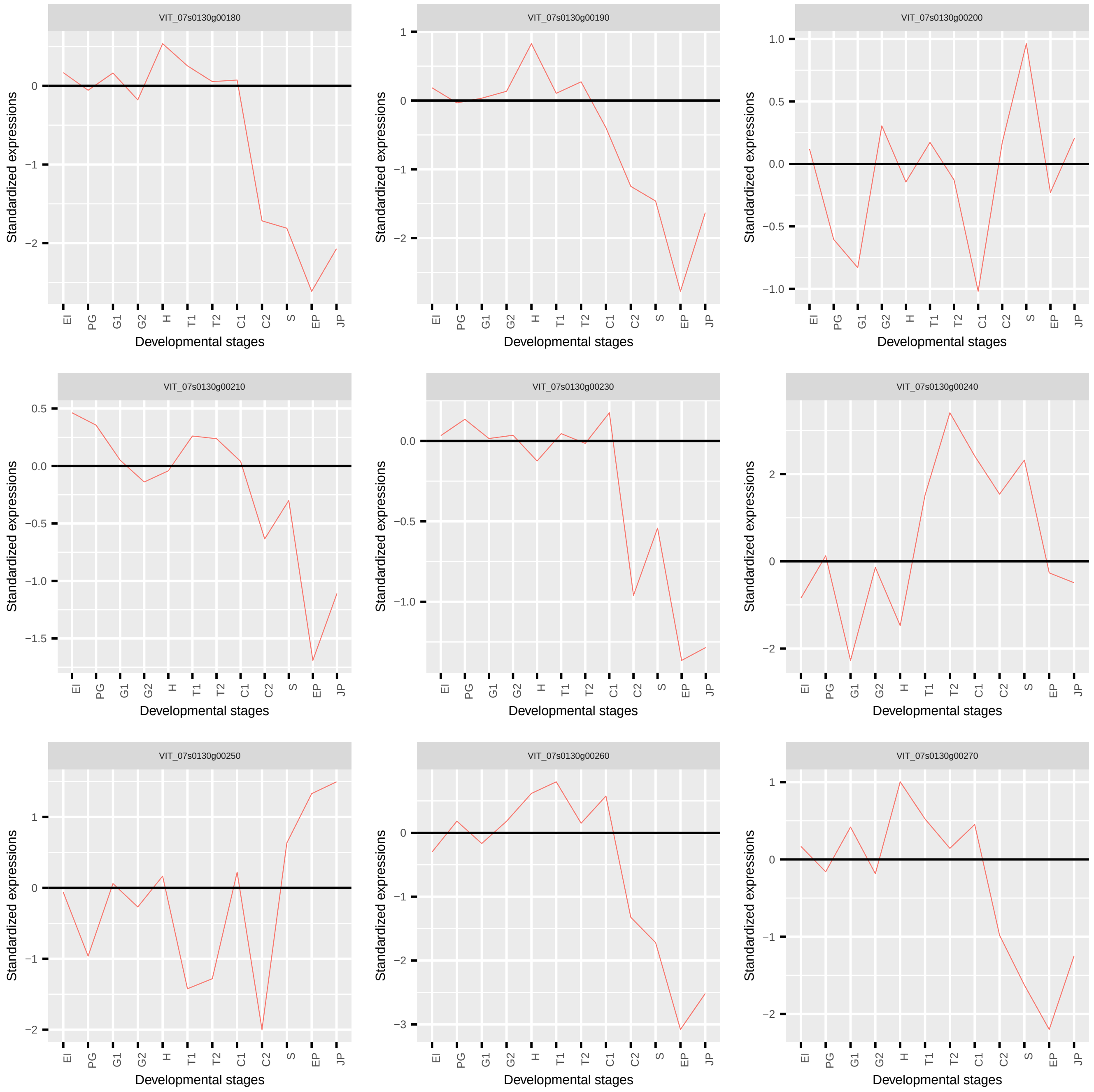
VIT_07s0130g00180
VIT_07s0130g00190
VIT_07s0130g00200
VIT_07s0130g00210
VIT_07s0130g00230
VIT_07s0130g00240
VIT_07s0130g00250
VIT_07s0130g00260
VIT_07s0130g00270
Standardized expressions
Developmental stages
EI PG G1 G2 H T1 T2 C1 C2 S EP JP

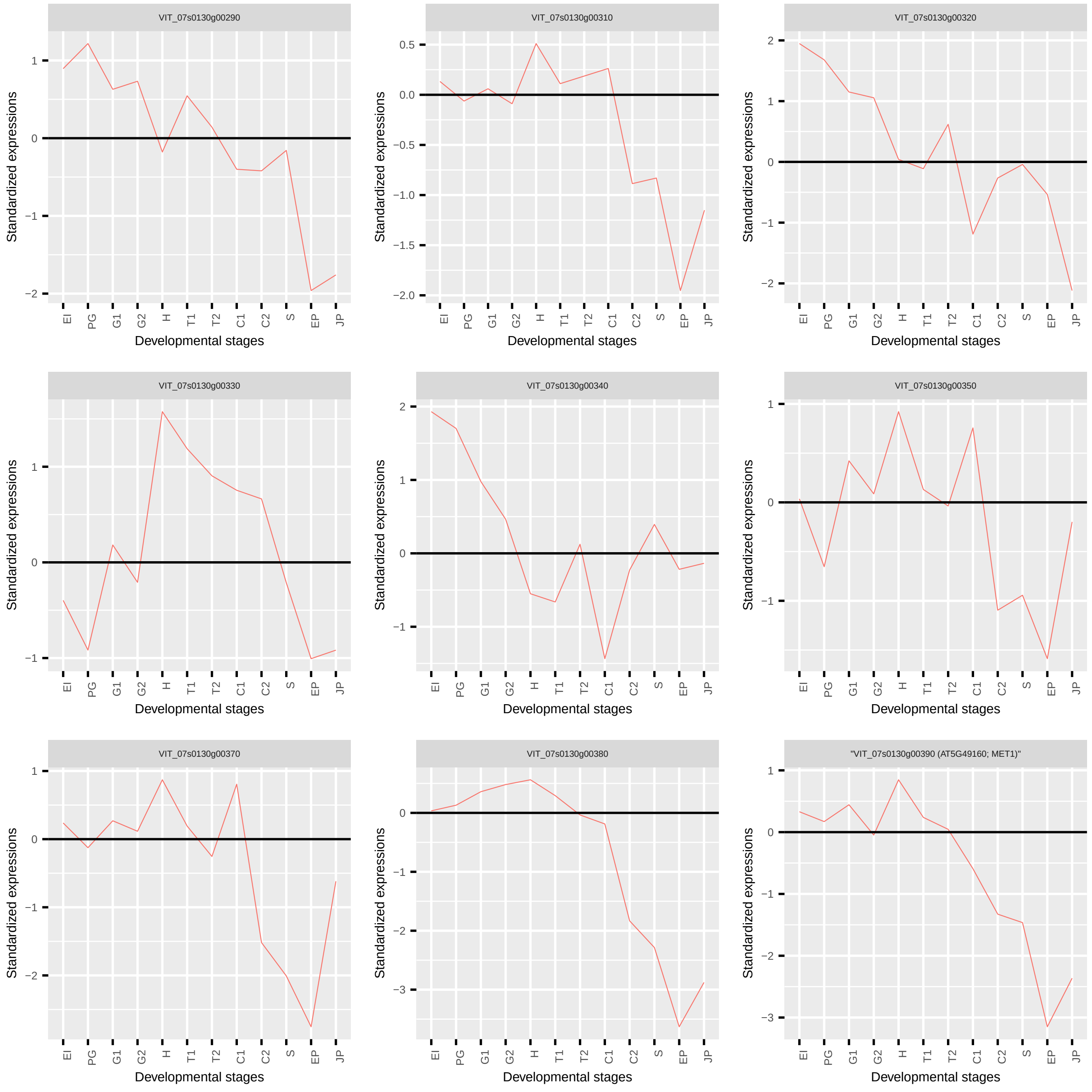

VIT_07s0130g00290
VIT_07s0130g00310
VIT_07s0130g00320
VIT_07s0130g00330
VIT_07s0130g00340
VIT_07s0130g00350
VIT_07s0130g00370
VIT_07s0130g00380
"VIT_07s0130g00390 (AT5G49160; MET1)"
Standardized expressions
Developmental stages
EI PG G1 G2 H T1 T2 C1 C2 S EP JP

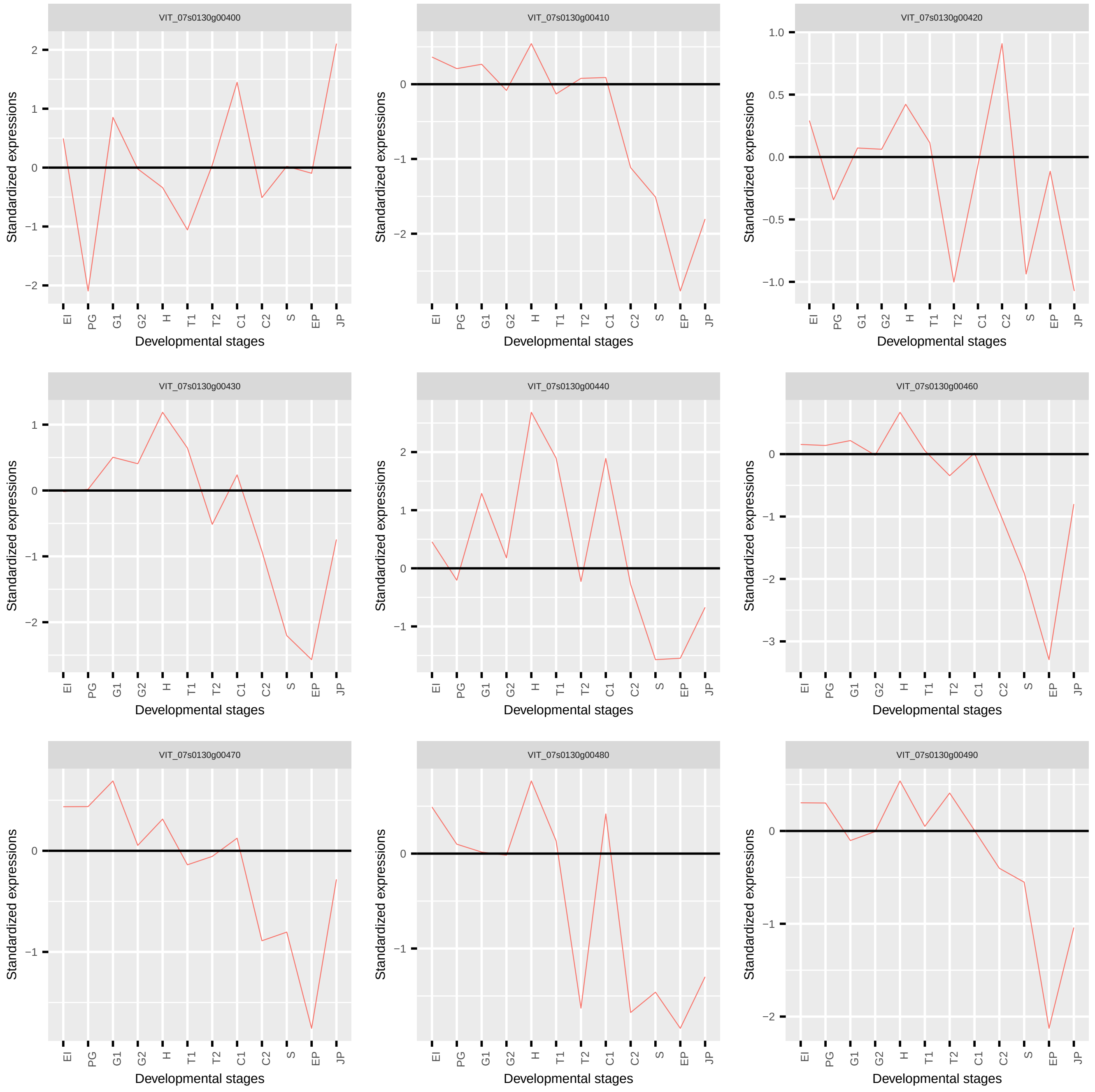
VIT_07s0130g00400
VIT_07s0130g00410
VIT_07s0130g00420
VIT_07s0130g00430
VIT_07s0130g00440
VIT_07s0130g00460
VIT_07s0130g00470
VIT_07s0130g00480
VIT_07s0130g00490
Standardized expressions
Developmental stages
EI PG G1 G2 H T1 T2 C1 C2 S EP JP

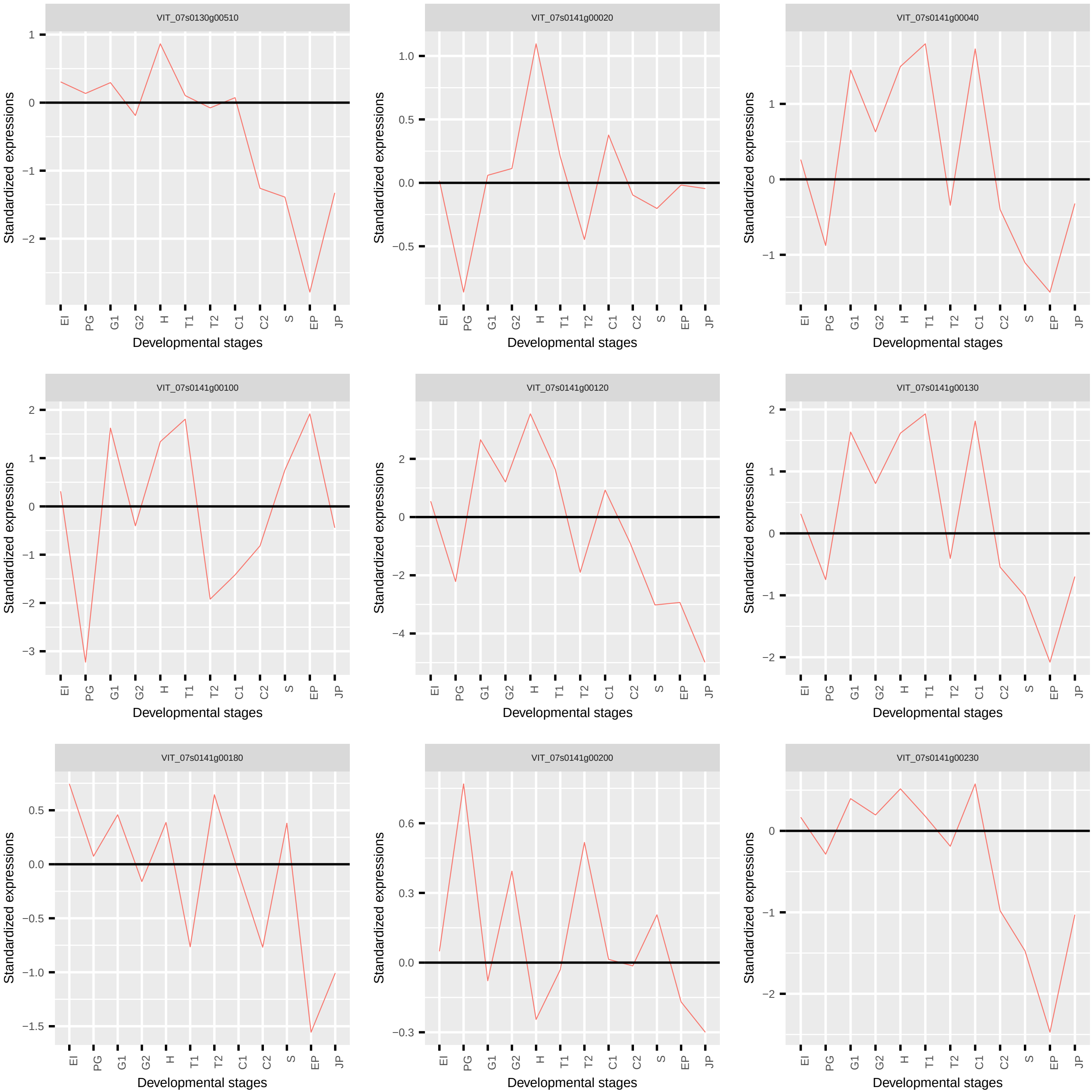
VIT_07s0130g00510
VIT_07s0141g00020
VIT_07s0141g00040
VIT_07s0141g00100
VIT_07s0141g00120
VIT_07s0141g00130
VIT_07s0141g00180
VIT_07s0141g00200
VIT_07s0141g00230
Standardized expressions
Developmental stages
EI
PG
G1
G2
H
T1
T2
C1
C2
S
EP
JP

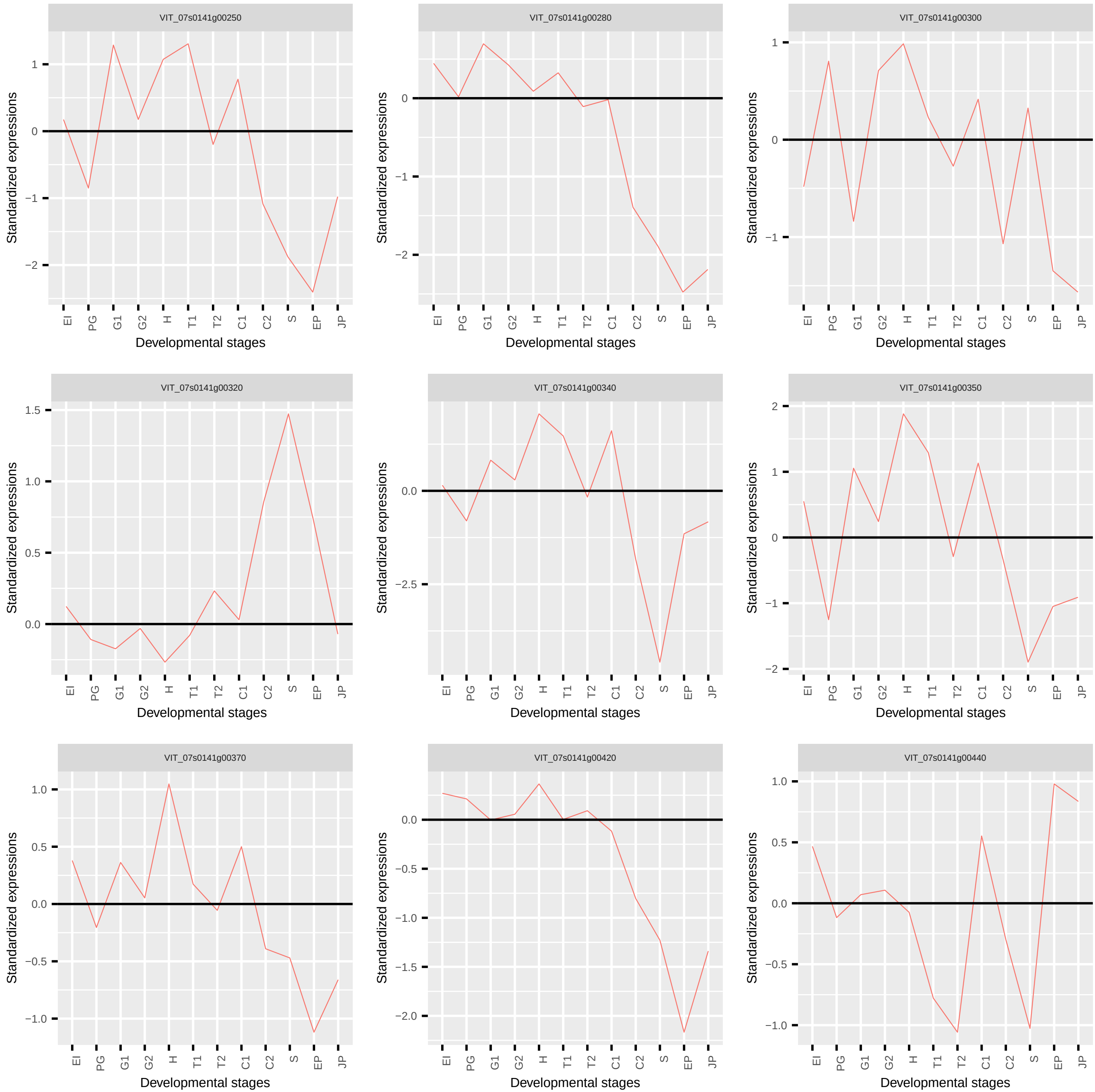
VIT_07s0141g00250
Standardized expressions
1
0
−1
−2
EI PG G1 G2 H T1 T2 C1 C2 S EP JP
Developmental stages
VIT_07s0141g00280
Standardized expressions
0
−1
−2
EI PG G1 G2 H T1 T2 C1 C2 S EP JP
Developmental stages
VIT_07s0141g00300
Standardized expressions
1
0
−1
EI PG G1 G2 H T1 T2 C1 C2 S EP JP
Developmental stages
VIT_07s0141g00320
Standardized expressions
1.5
1.0
0.5
0.0
EI PG G1 G2 H T1 T2 C1 C2 S EP JP
Developmental stages
VIT_07s0141g00340
Standardized expressions
0.0
−2.5
EI PG G1 G2 H T1 T2 C1 C2 S EP JP
Developmental stages
VIT_07s0141g00350
Standardized expressions
2
1
0
−1
−2
EI PG G1 G2 H T1 T2 C1 C2 S EP JP
Developmental stages
VIT_07s0141g00370
Standardized expressions
1.0
0.5
0.0
−0.5
−1.0
EI PG G1 G2 H T1 T2 C1 C2 S EP JP
Developmental stages
VIT_07s0141g00420
Standardized expressions
0.0
−0.5
−1.0
−1.5
−2.0
EI PG G1 G2 H T1 T2 C1 C2 S EP JP
Developmental stages
VIT_07s0141g00440
Standardized expressions
1.0
0.5
0.0
−0.5
−1.0
EI PG G1 G2 H T1 T2 C1 C2 S EP JP
Developmental stages

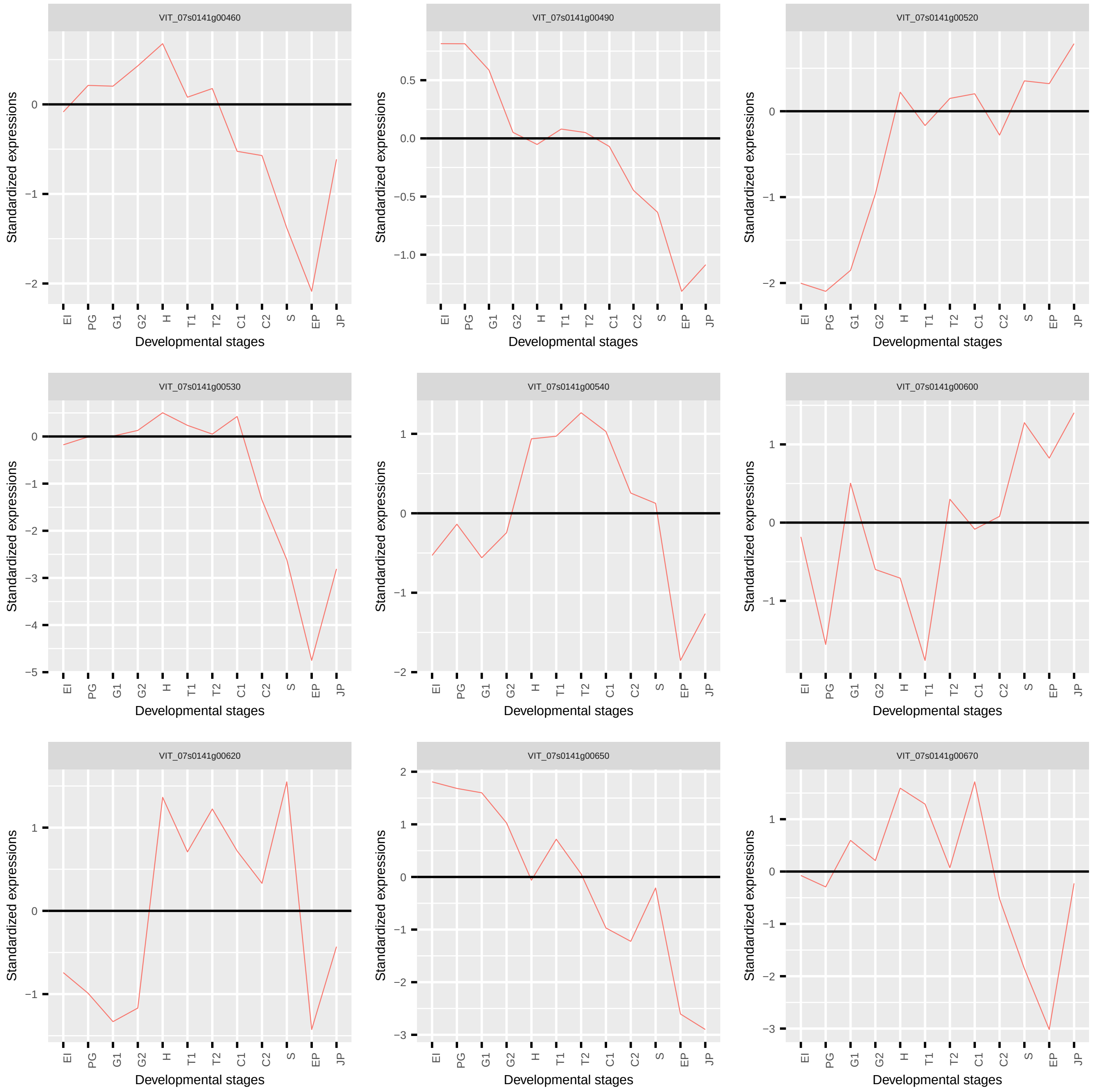
VIT_07s0141g00460
VIT_07s0141g00490
VIT_07s0141g00520
VIT_07s0141g00530
VIT_07s0141g00540
VIT_07s0141g00600
VIT_07s0141g00620
VIT_07s0141g00650
VIT_07s0141g00670
Standardized expressions
Developmental stages
EI PG G1 G2 H T1 T2 C1 C2 S EP JP

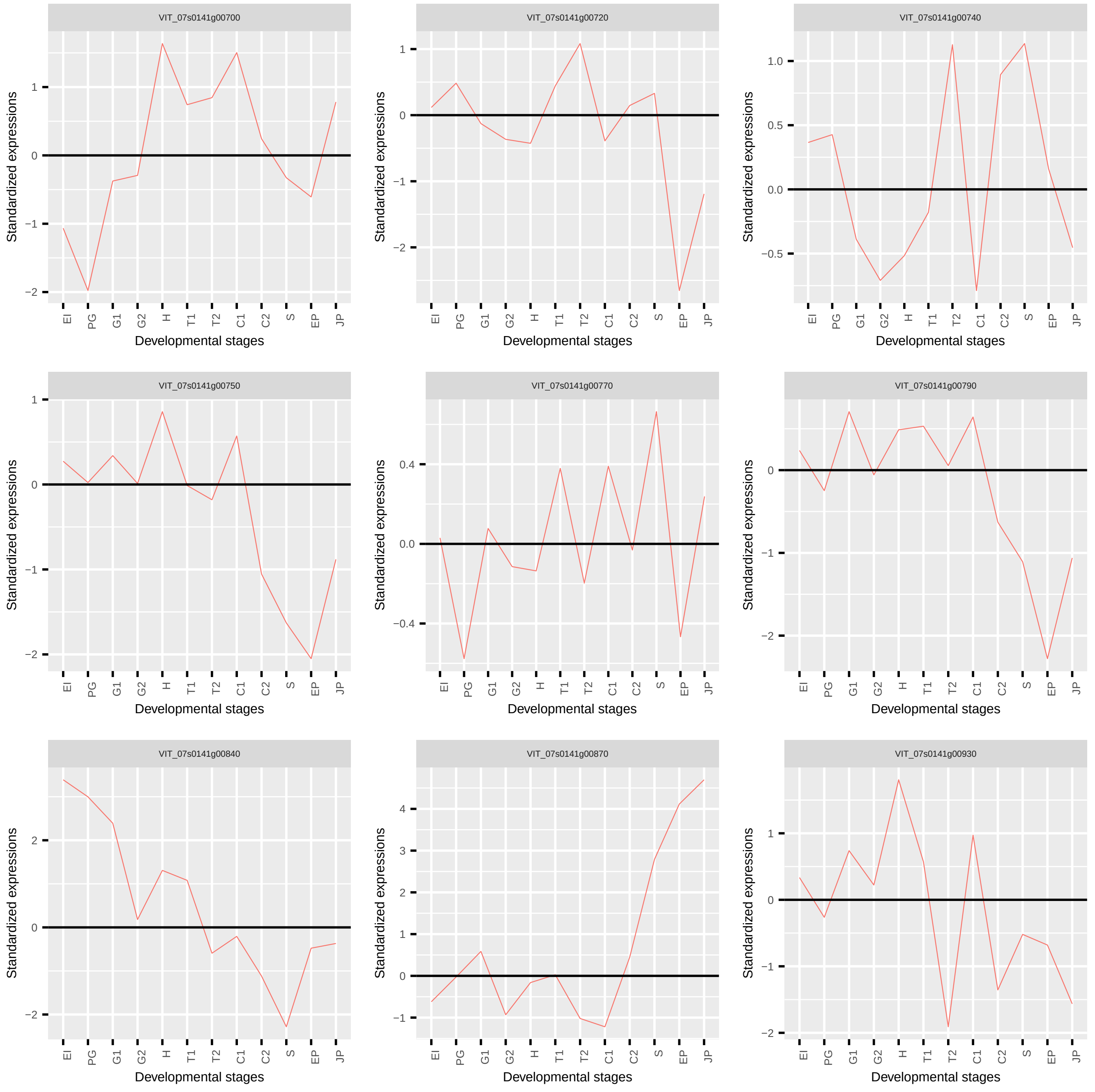

VIT_07s0141g00700
VIT_07s0141g00720
VIT_07s0141g00740
VIT_07s0141g00750
VIT_07s0141g00770
VIT_07s0141g00790
VIT_07s0141g00840
VIT_07s0141g00870
VIT_07s0141g00930
Standardized expressions
Developmental stages
EI PG G1 G2 H T1 T2 C1 C2 S EP JP

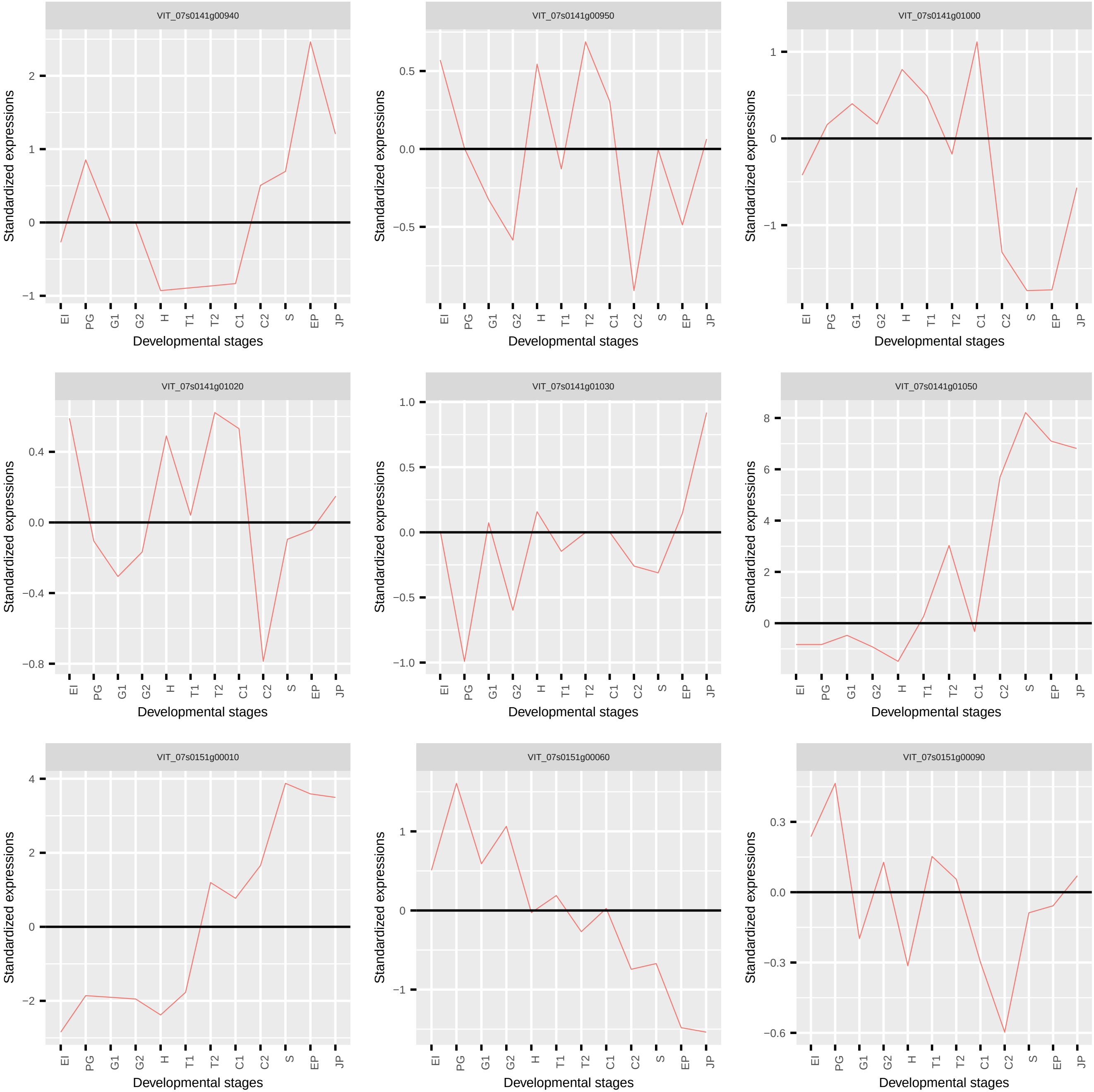
VIT_07s0141g00940
VIT_07s0141g00950
VIT_07s0141g01000
VIT_07s0141g01020
VIT_07s0141g01030
VIT_07s0141g01050
VIT_07s0151g00010
VIT_07s0151g00060
VIT_07s0151g00090
Standardized expressions
Developmental stages
EI PG G1 G2 H T1 T2 C1 C2 S EP JP

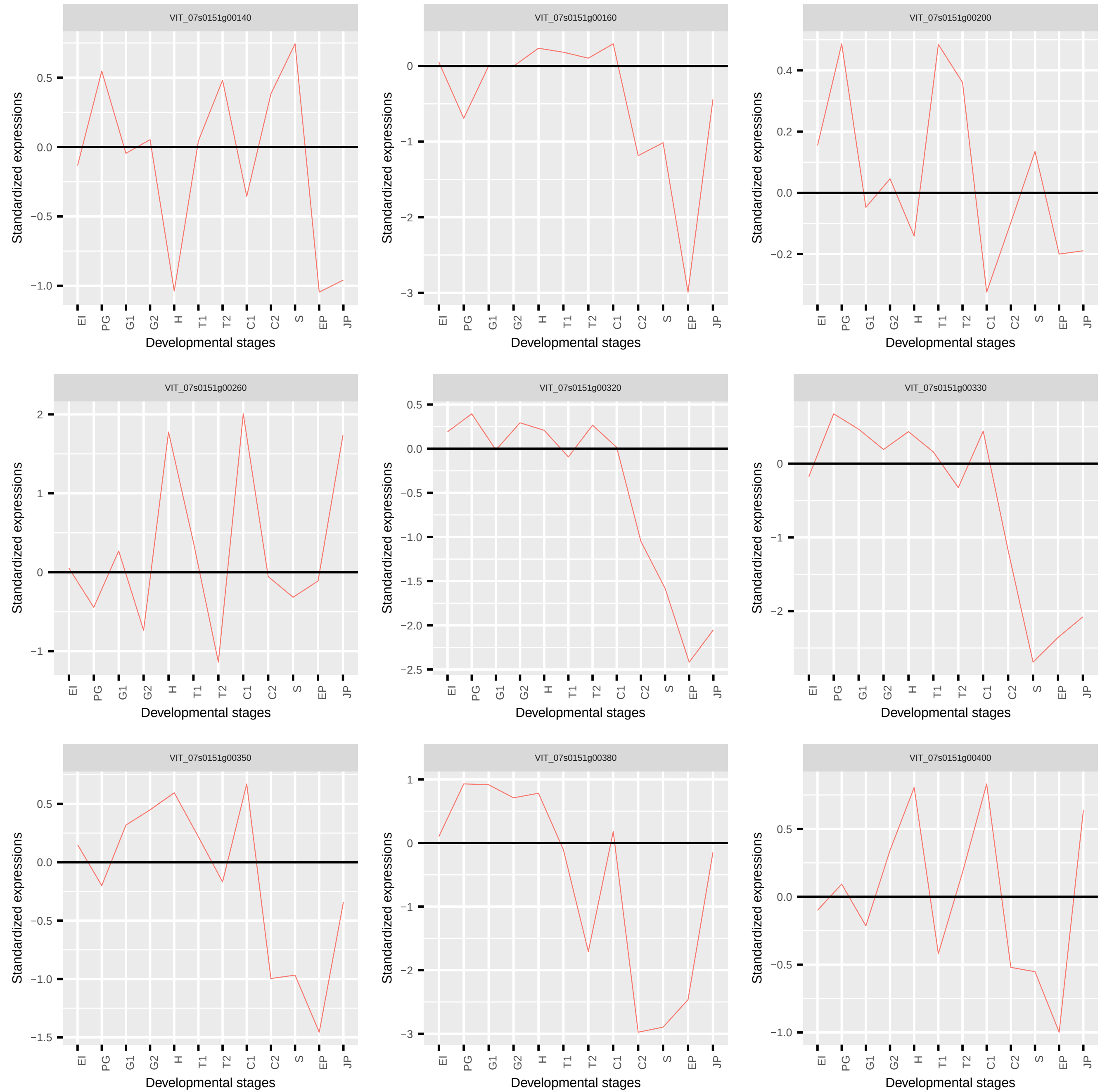
VIT_07s0151g00140
VIT_07s0151g00160
VIT_07s0151g00200
VIT_07s0151g00260
VIT_07s0151g00320
VIT_07s0151g00330
VIT_07s0151g00350
VIT_07s0151g00380
VIT_07s0151g00400
Standardized expressions
Developmental stages
EI PG G1 G2 H T1 T2 C1 C2 S EP JP

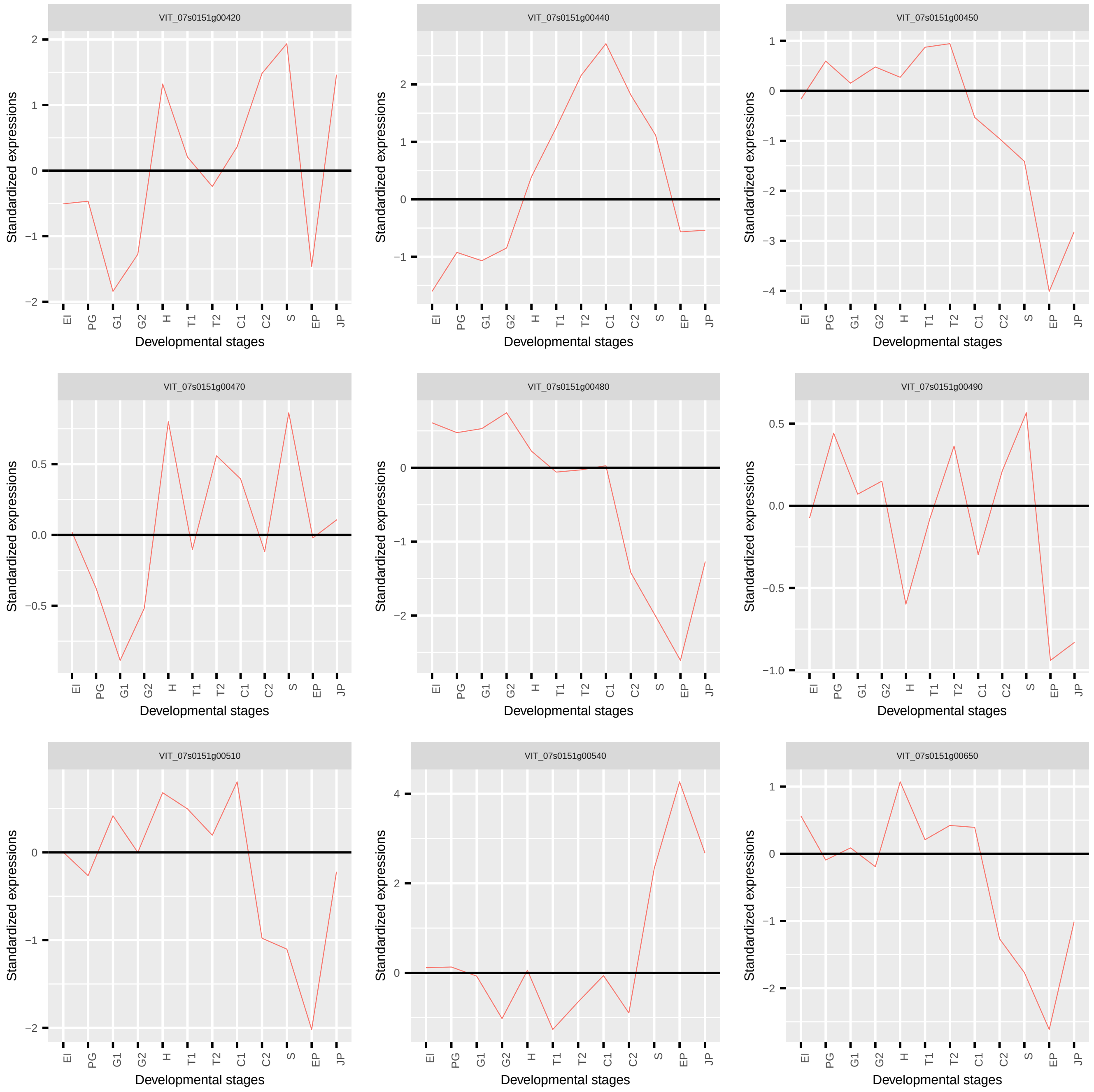
VIT_07s0151g00420
VIT_07s0151g00440
VIT_07s0151g00450
VIT_07s0151g00470
VIT_07s0151g00480
VIT_07s0151g00490
VIT_07s0151g00510
VIT_07s0151g00540
VIT_07s0151g00650
Standardized expressions
Developmental stages
EI PG G1 G2 H T1 T2 C1 C2 S EP JP

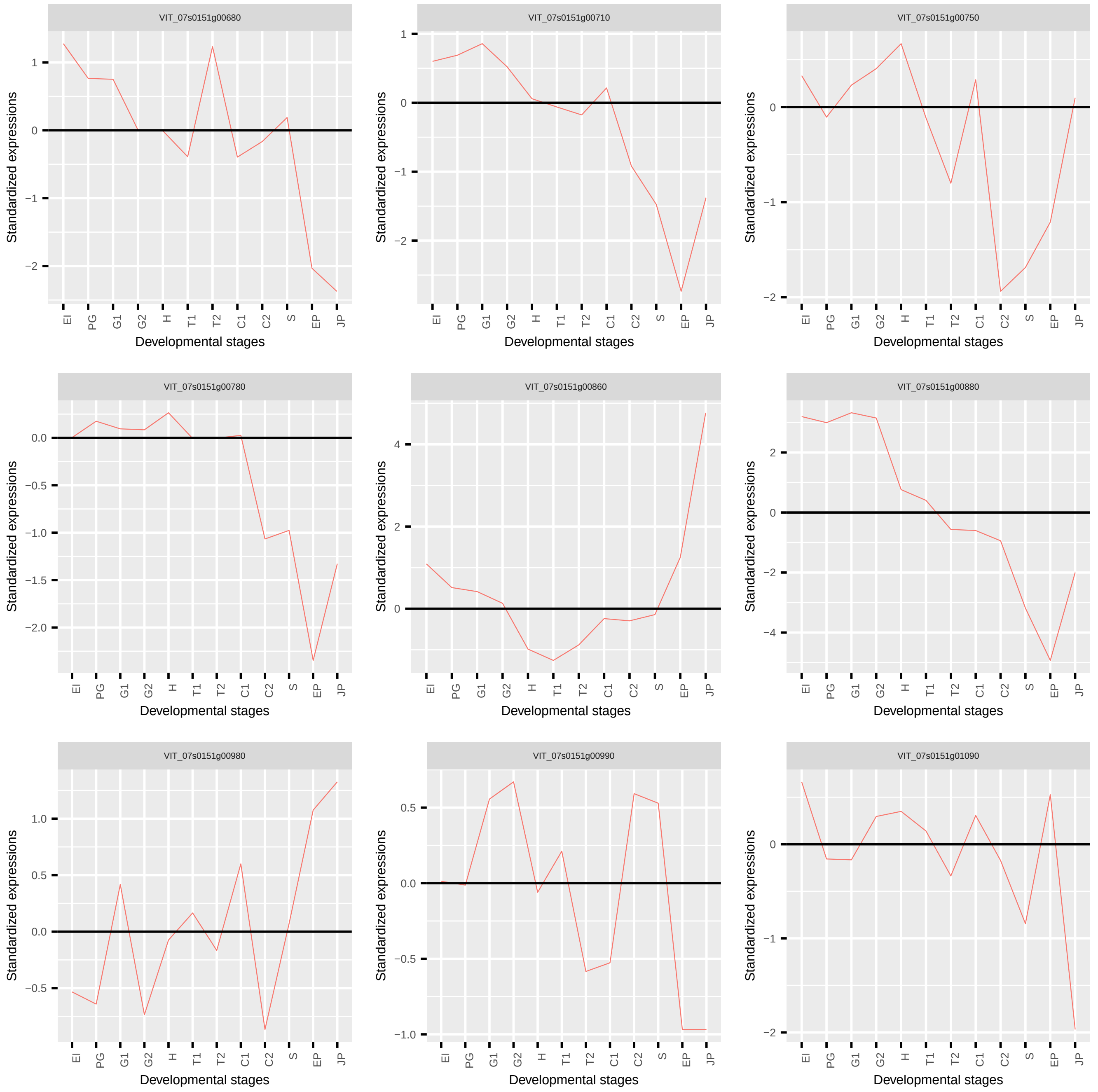
VIT_07s0151g00680
VIT_07s0151g00710
VIT_07s0151g00750
VIT_07s0151g00780
VIT_07s0151g00860
VIT_07s0151g00880
VIT_07s0151g00980
VIT_07s0151g00990
VIT_07s0151g01090
Standardized expressions
Developmental stages
EI
PG
G1
G2
H
T1
T2
C1
C2
S
EP
JP

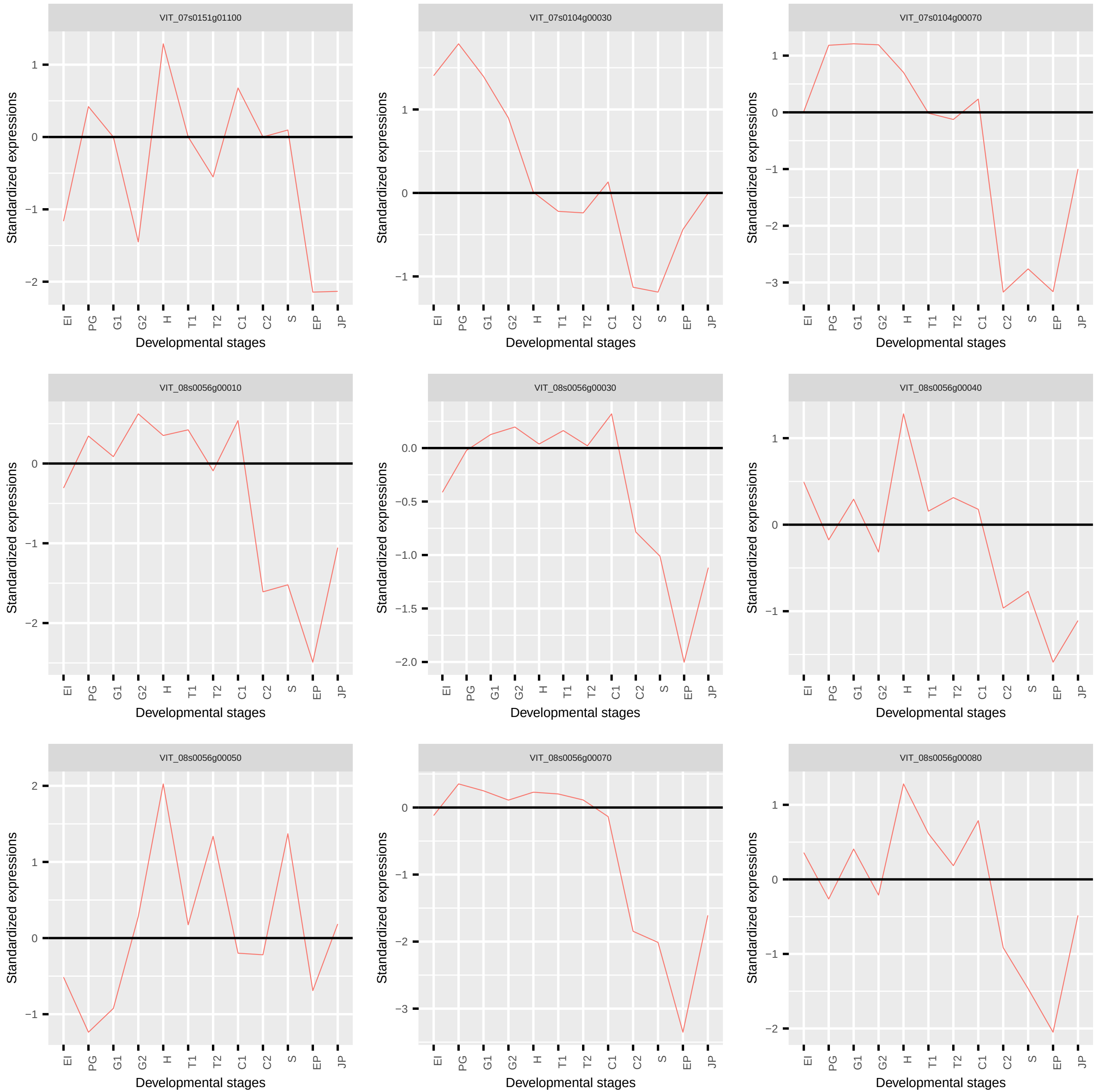
VIT_07s0151g01100
VIT_07s0104g00030
VIT_07s0104g00070
VIT_08s0056g00010
VIT_08s0056g00030
VIT_08s0056g00040
VIT_08s0056g00050
VIT_08s0056g00070
VIT_08s0056g00080
Standardized expressions
Developmental stages
EI PG G1 G2 H T1 T2 C1 C2 S EP JP
1 0 −1 −2
0.0 −0.5 −1.0 −1.5 −2.0
2 1 0 −1
0 −1 −2 −3

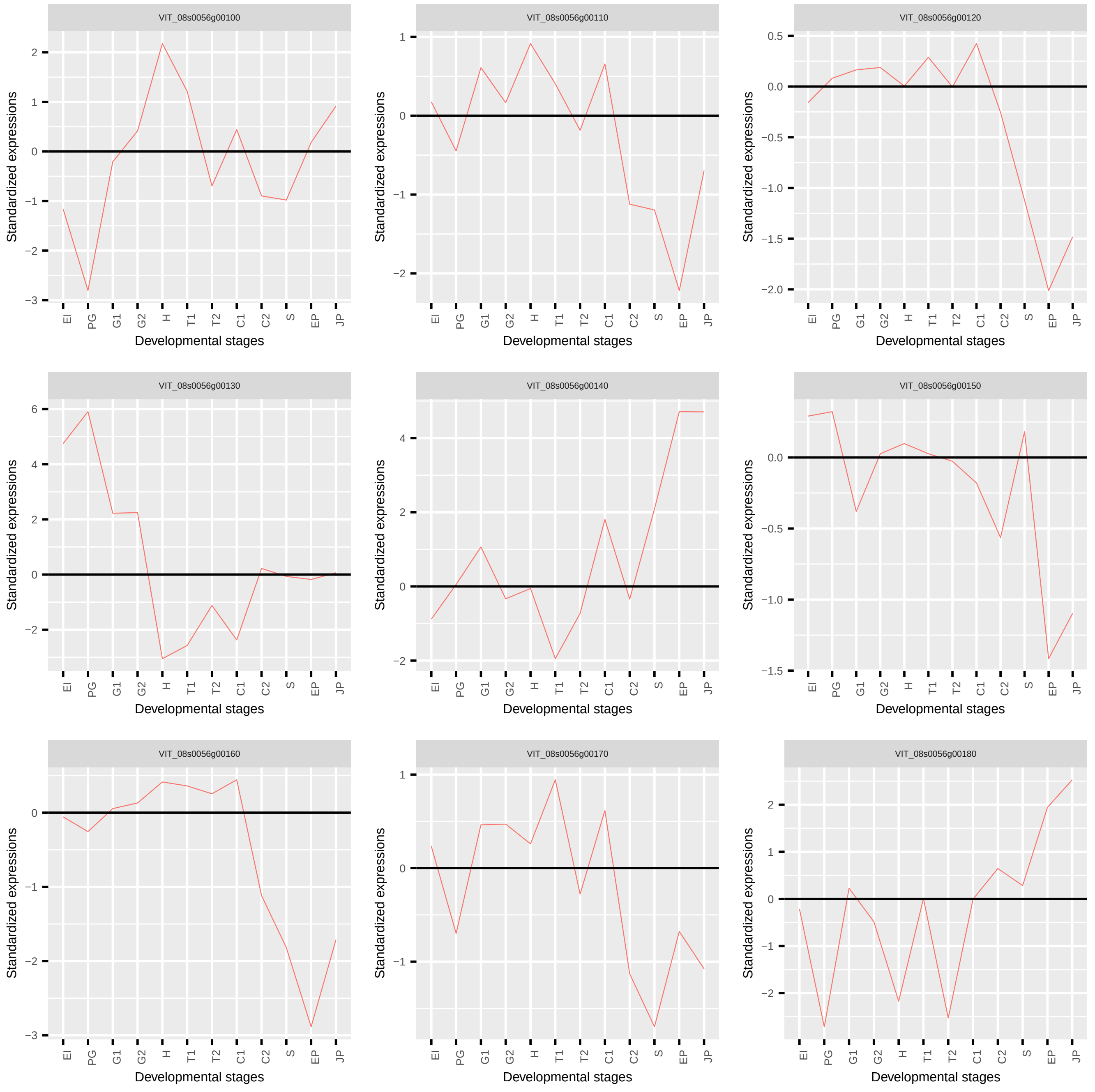
VIT_08s0056g00100
VIT_08s0056g00110
VIT_08s0056g00120
VIT_08s0056g00130
VIT_08s0056g00140
VIT_08s0056g00150
VIT_08s0056g00160
VIT_08s0056g00170
VIT_08s0056g00180
Standardized expressions
Developmental stages
EI
PG
G1
G2
H
T1
T2
C1
C2
S
EP
JP

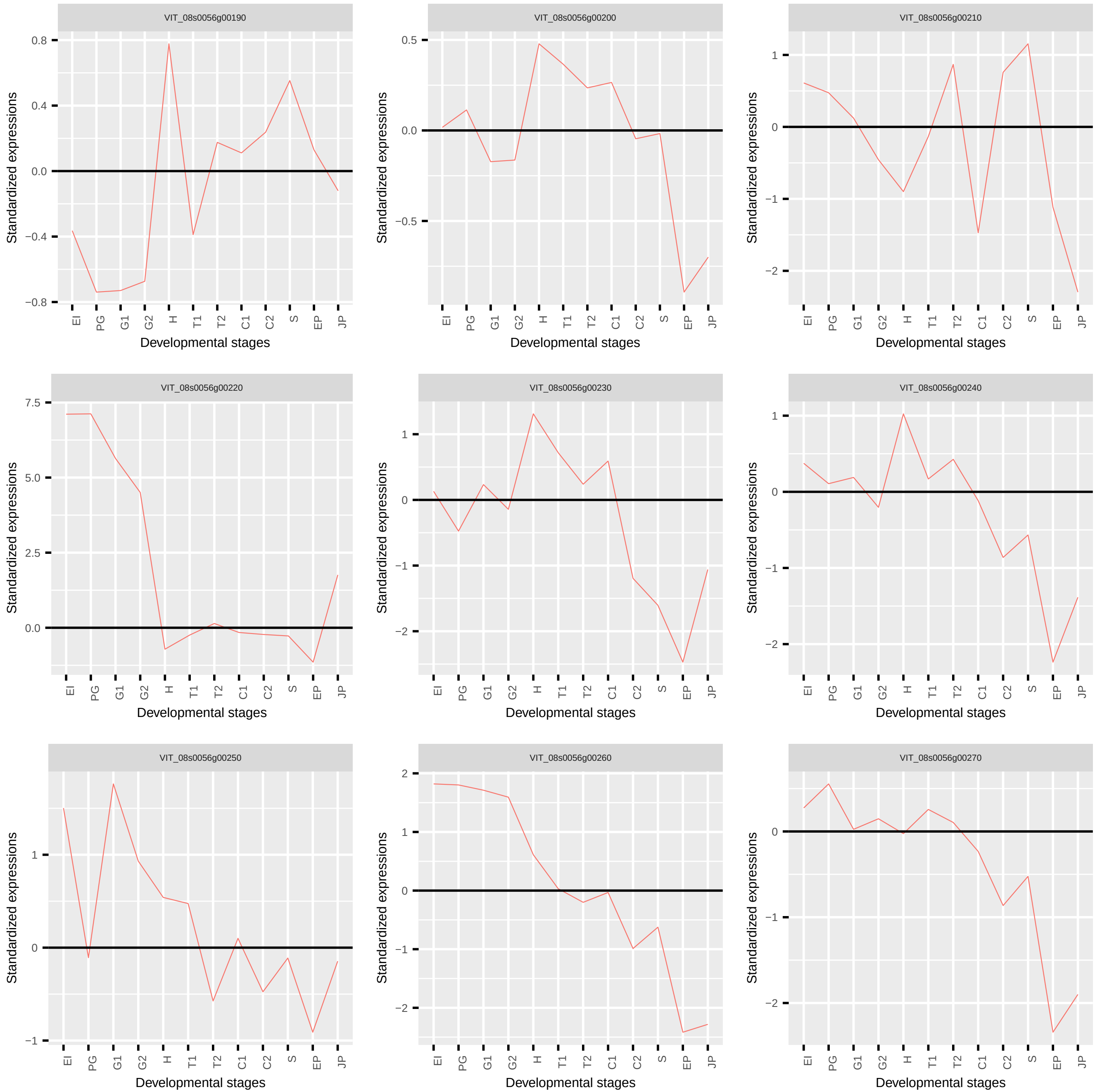
VIT_08s0056g00190
VIT_08s0056g00200
VIT_08s0056g00210
VIT_08s0056g00220
VIT_08s0056g00230
VIT_08s0056g00240
VIT_08s0056g00250
VIT_08s0056g00260
VIT_08s0056g00270
Standardized expressions
Developmental stages
EI PG G1 G2 H T1 T2 C1 C2 S EP JP
0.8 0.4 0.0 −0.4 −0.8
0.5 0.0 −0.5
1 0 −1 −2
7.5 5.0 2.5 0.0
2 1 0 −1 −2

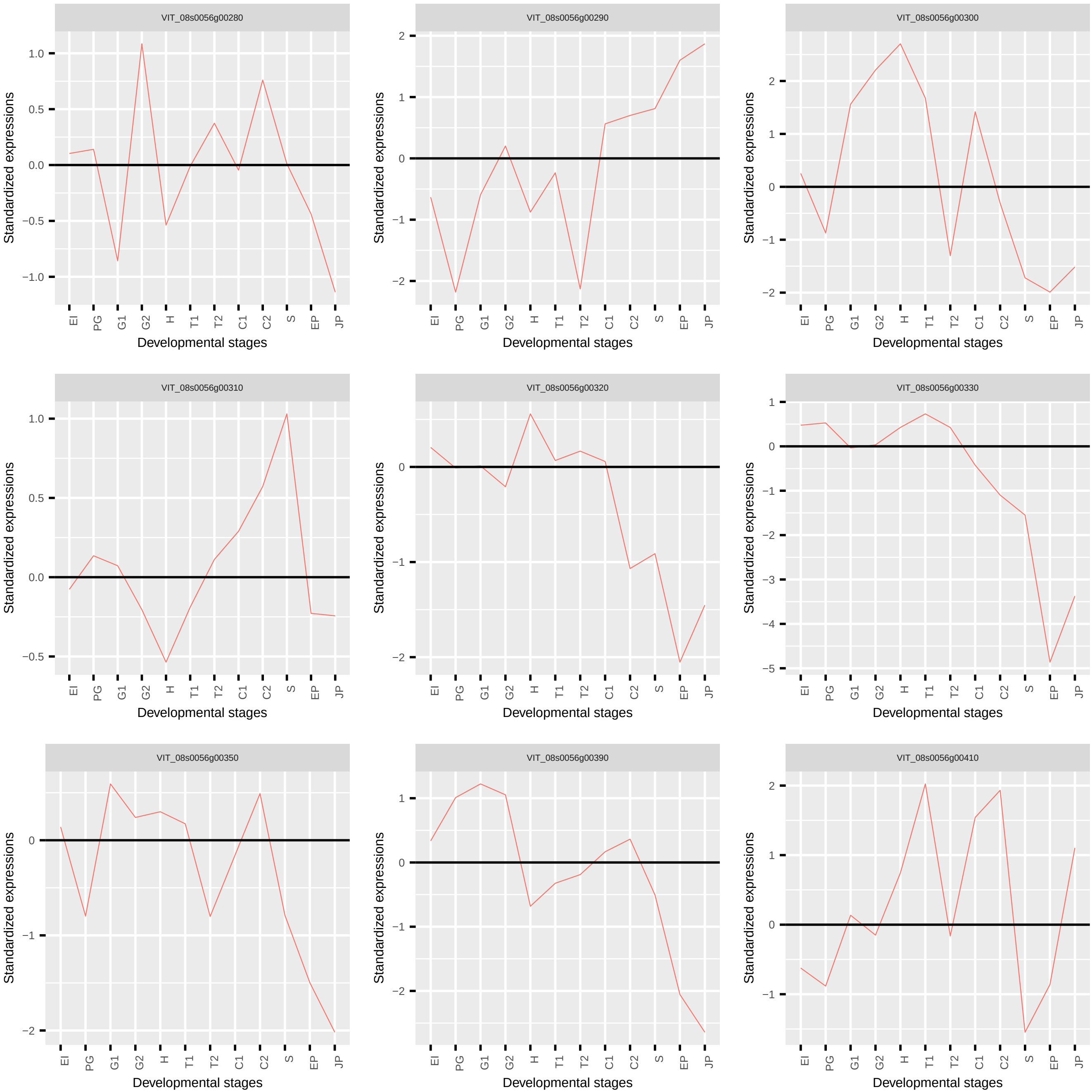
VIT_08s0056g00280
VIT_08s0056g00290
VIT_08s0056g00300
VIT_08s0056g00310
VIT_08s0056g00320
VIT_08s0056g00330
VIT_08s0056g00350
VIT_08s0056g00390
VIT_08s0056g00410
Standardized expressions
Developmental stages
EI PG G1 G2 H T1 T2 C1 C2 S EP JP

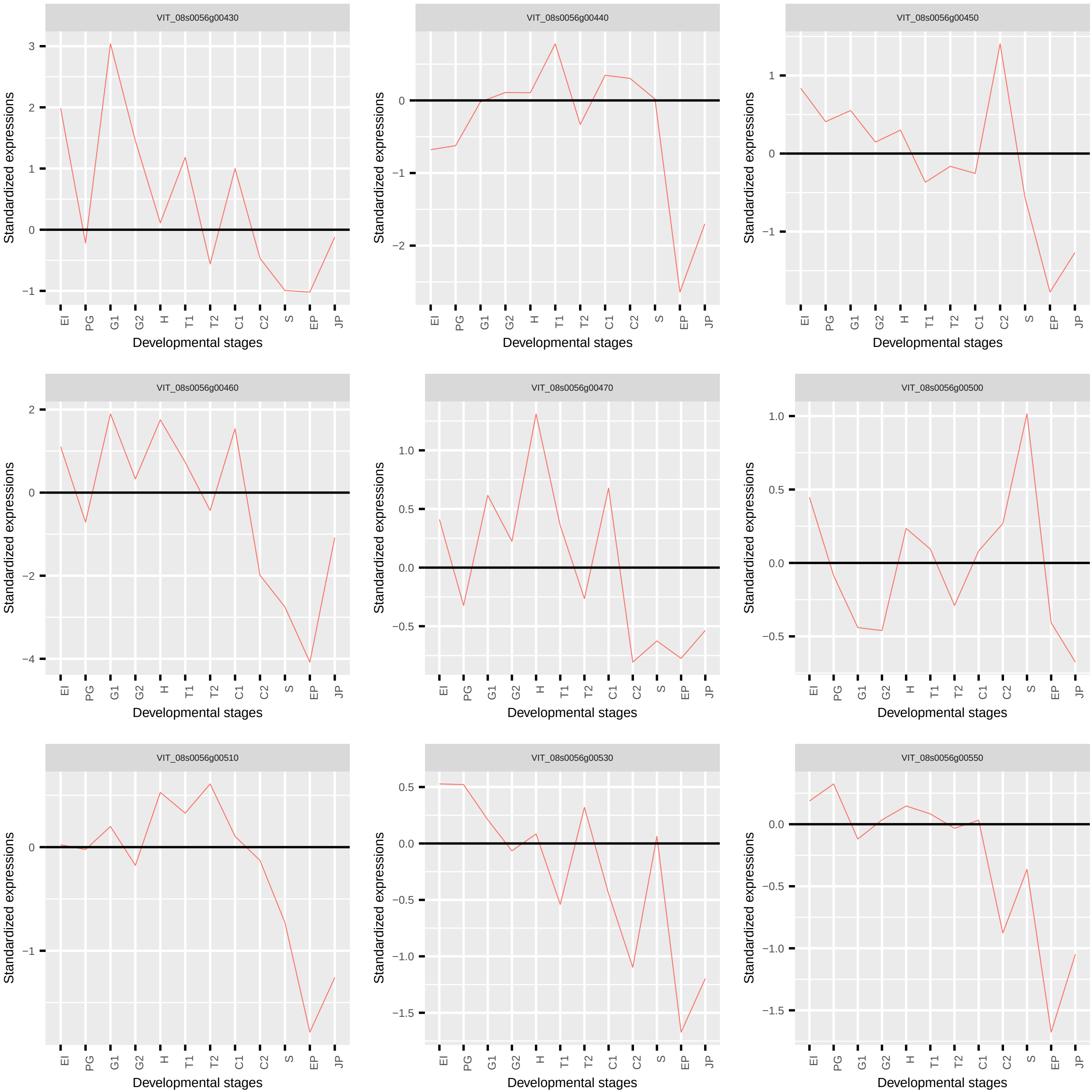
VIT_08s0056g00430
VIT_08s0056g00440
VIT_08s0056g00450
VIT_08s0056g00460
VIT_08s0056g00470
VIT_08s0056g00500
VIT_08s0056g00510
VIT_08s0056g00530
VIT_08s0056g00550
Standardized expressions
Developmental stages
EI
PG
G1
G2
H
T1
T2
C1
C2
S
EP
JP

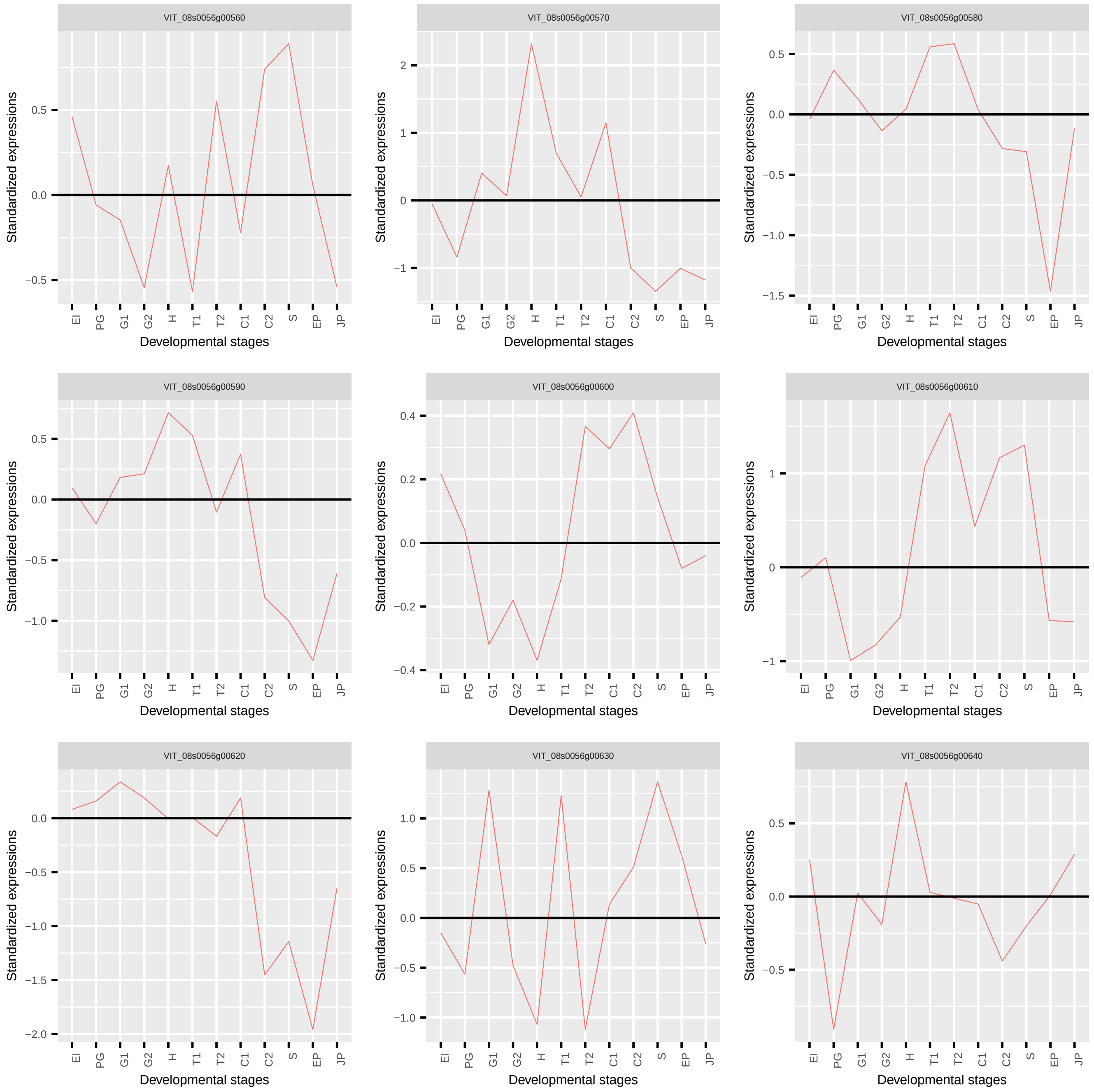
VIT_08s0056g00560
VIT_08s0056g00570
VIT_08s0056g00580
VIT_08s0056g00590
VIT_08s0056g00600
VIT_08s0056g00610
VIT_08s0056g00620
VIT_08s0056g00630
VIT_08s0056g00640
Standardized expressions
Developmental stages
EI PG G1 G2 H T1 T2 C1 C2 S EP JP
0.5
0.0
−0.5
−1.0
−1.5
−2.0
2
1
0
−1
0.4
0.2
0.0
−0.2
−0.4
1.0

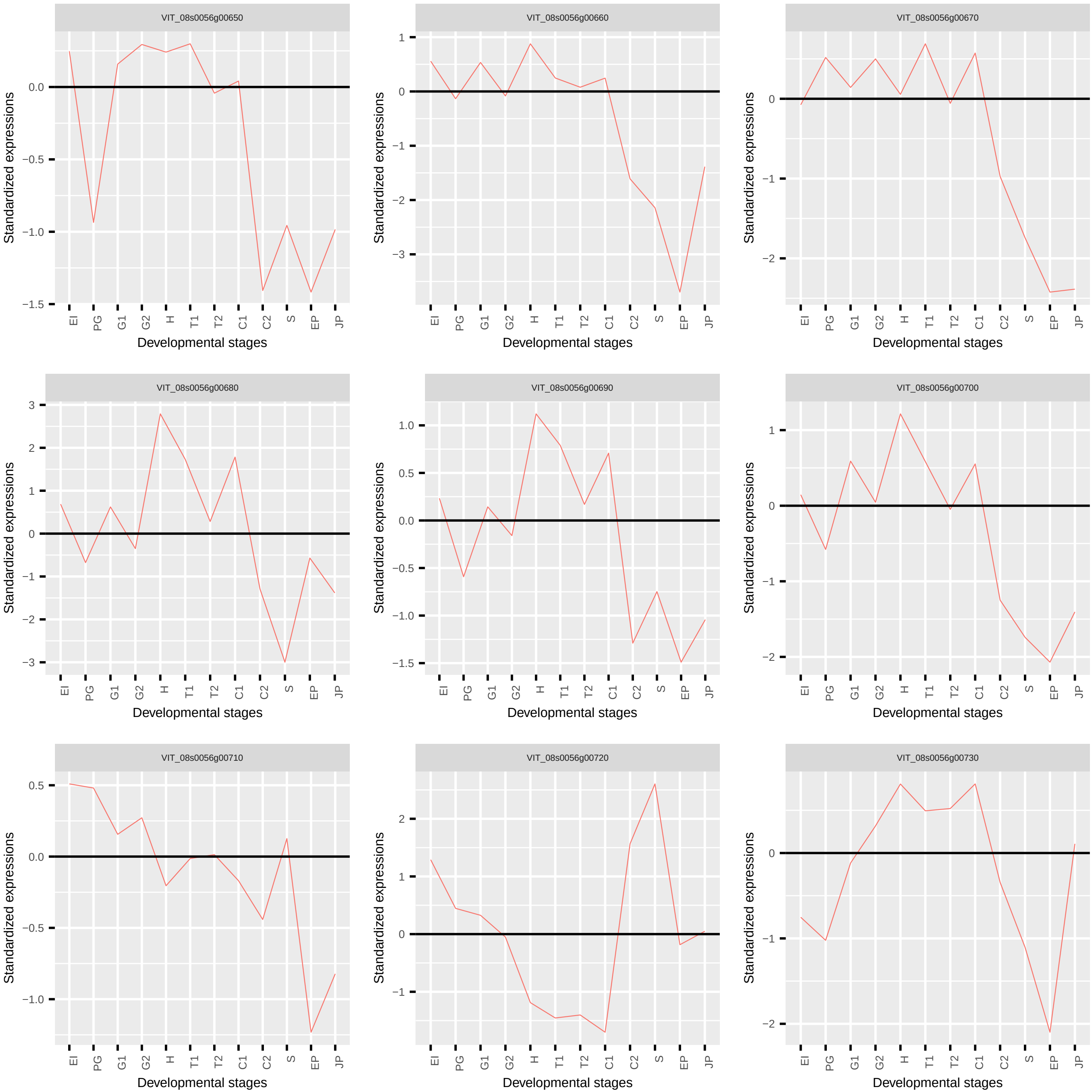
VIT_08s0056g00650
VIT_08s0056g00660
VIT_08s0056g00670
VIT_08s0056g00680
VIT_08s0056g00690
VIT_08s0056g00700
VIT_08s0056g00710
VIT_08s0056g00720
VIT_08s0056g00730
Standardized expressions
Developmental stages
EI PG G1 G2 H T1 T2 C1 C2 S EP JP

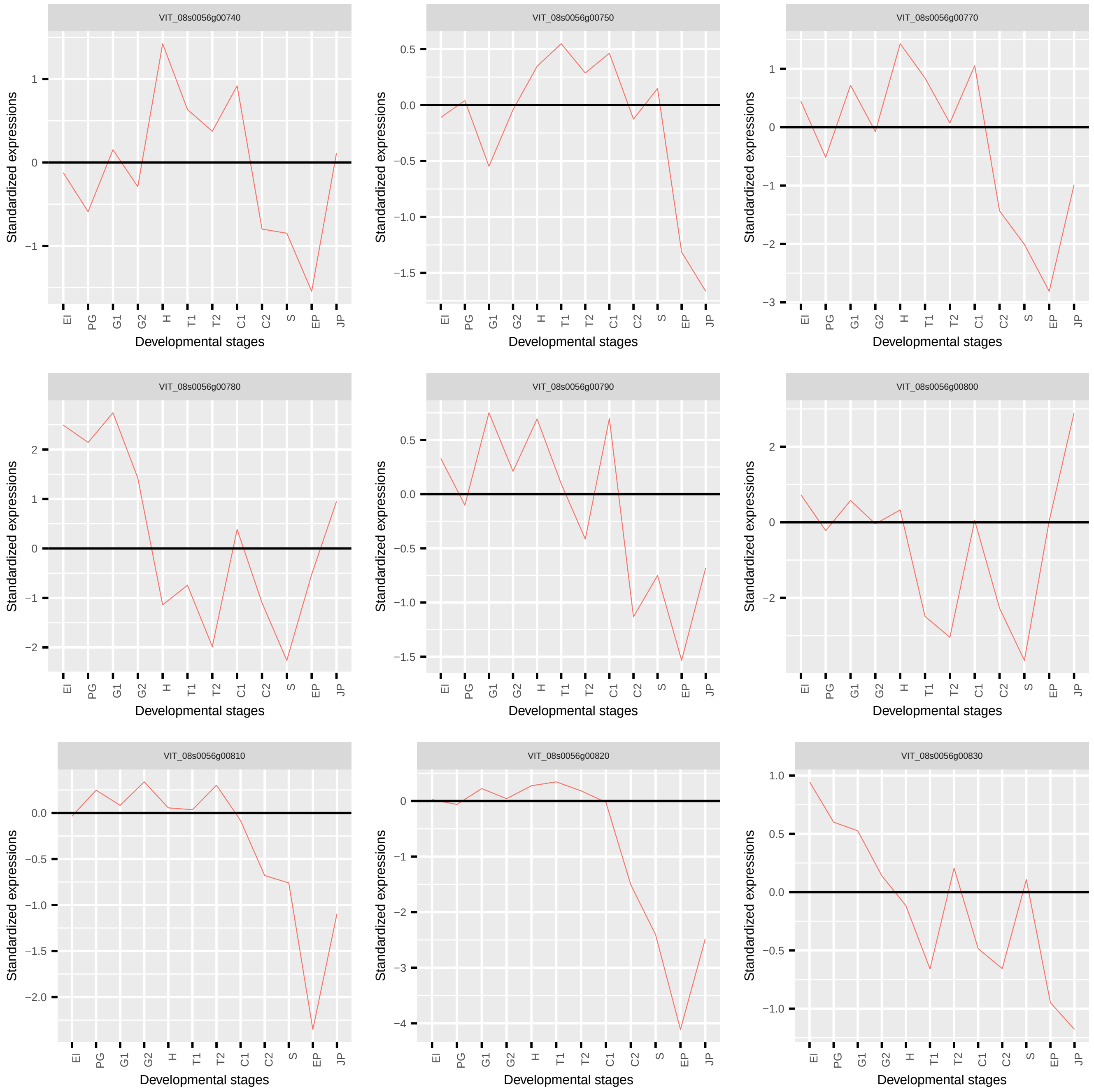

VIT_08s0056g00740
VIT_08s0056g00750
VIT_08s0056g00770
VIT_08s0056g00780
VIT_08s0056g00790
VIT_08s0056g00800
VIT_08s0056g00810
VIT_08s0056g00820
VIT_08s0056g00830
Standardized expressions
Developmental stages
EI PG G1 G2 H T1 T2 C1 C2 S EP JP

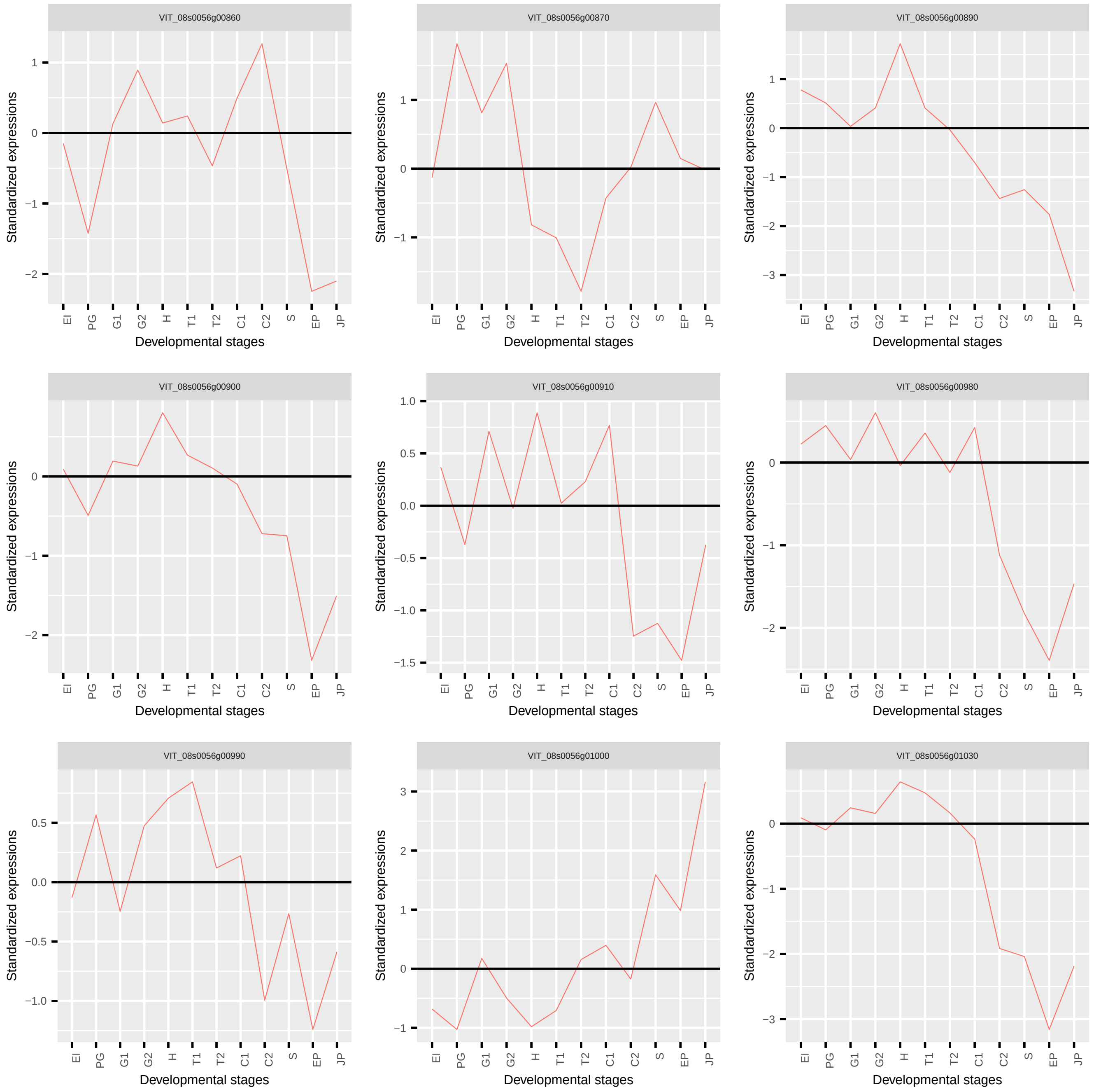
VIT_08s0056g00860
VIT_08s0056g00870
VIT_08s0056g00890
VIT_08s0056g00900
VIT_08s0056g00910
VIT_08s0056g00980
VIT_08s0056g00990
VIT_08s0056g01000
VIT_08s0056g01030
Standardized expressions
Developmental stages
EI
PG
G1
G2
H
T1
T2
C1
C2
S
EP
JP

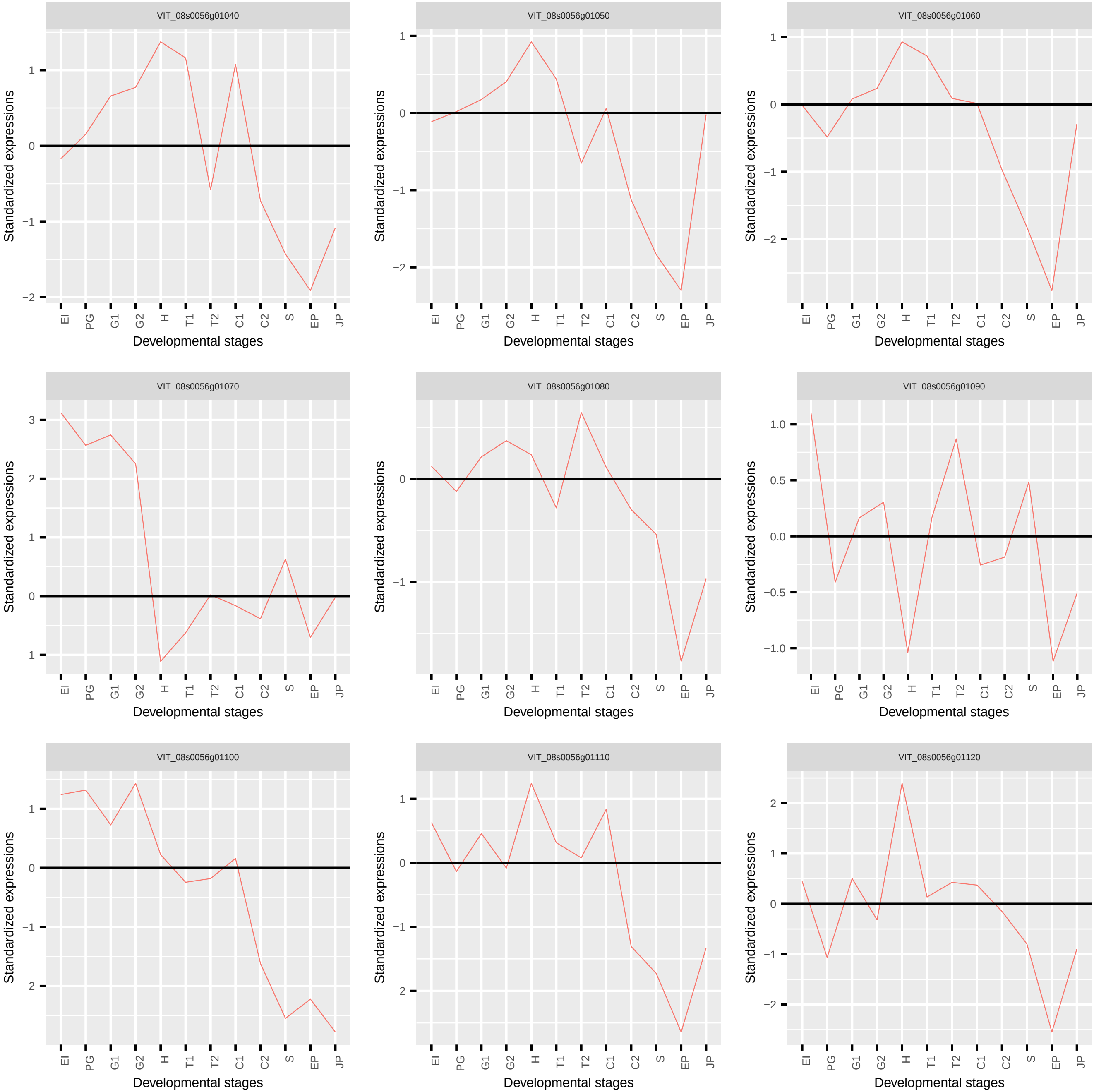
VIT_08s0056g01040
VIT_08s0056g01050
VIT_08s0056g01060
VIT_08s0056g01070
VIT_08s0056g01080
VIT_08s0056g01090
VIT_08s0056g01100
VIT_08s0056g01110
VIT_08s0056g01120
Standardized expressions
Developmental stages
EI PG G1 G2 H T1 T2 C1 C2 S EP JP

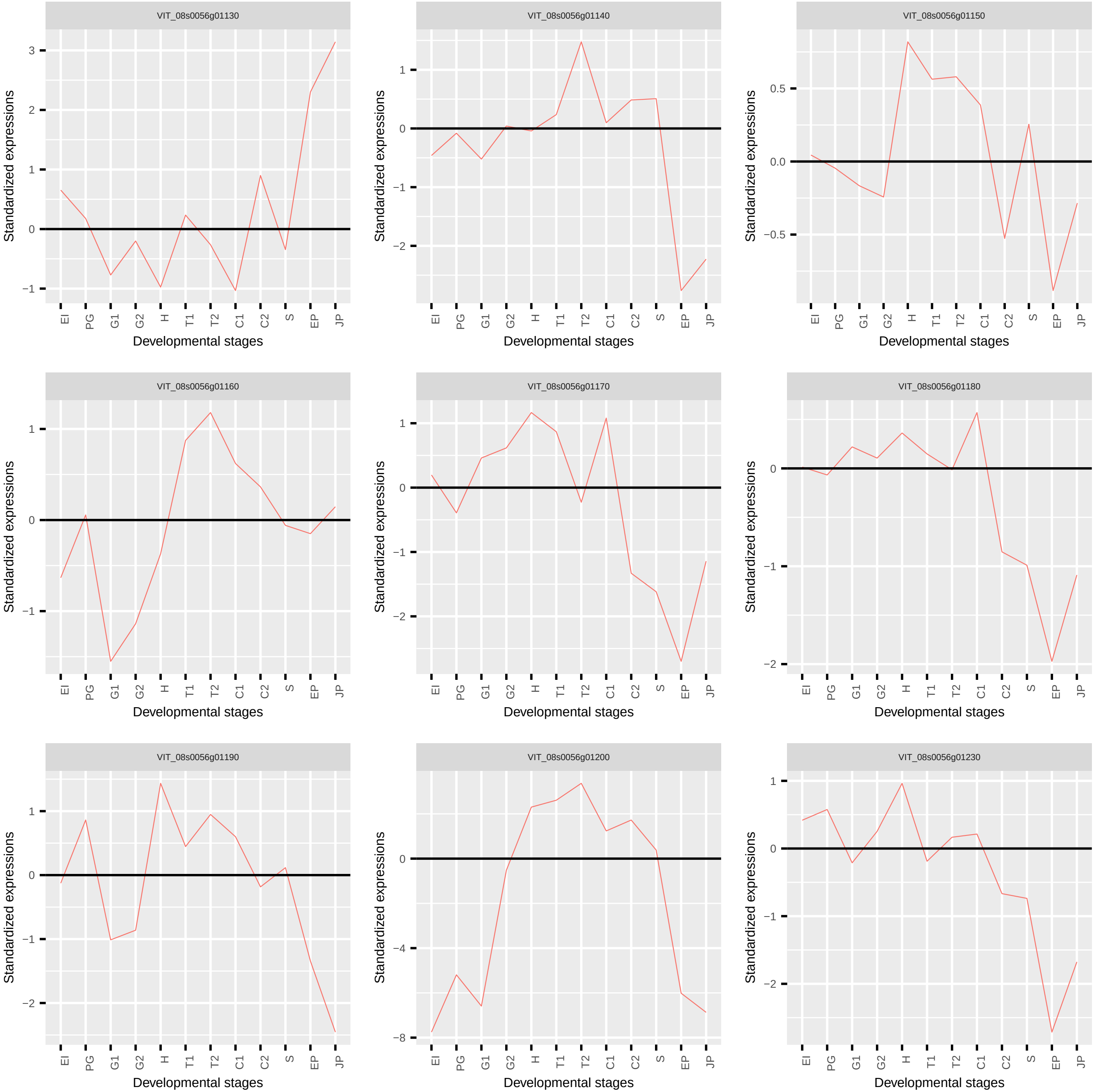
VIT_08s0056g01130
VIT_08s0056g01140
VIT_08s0056g01150
VIT_08s0056g01160
VIT_08s0056g01170
VIT_08s0056g01180
VIT_08s0056g01190
VIT_08s0056g01200
VIT_08s0056g01230
Standardized expressions
Developmental stages
EI
PG
G1
G2
H
T1
T2
C1
C2
S
EP
JP

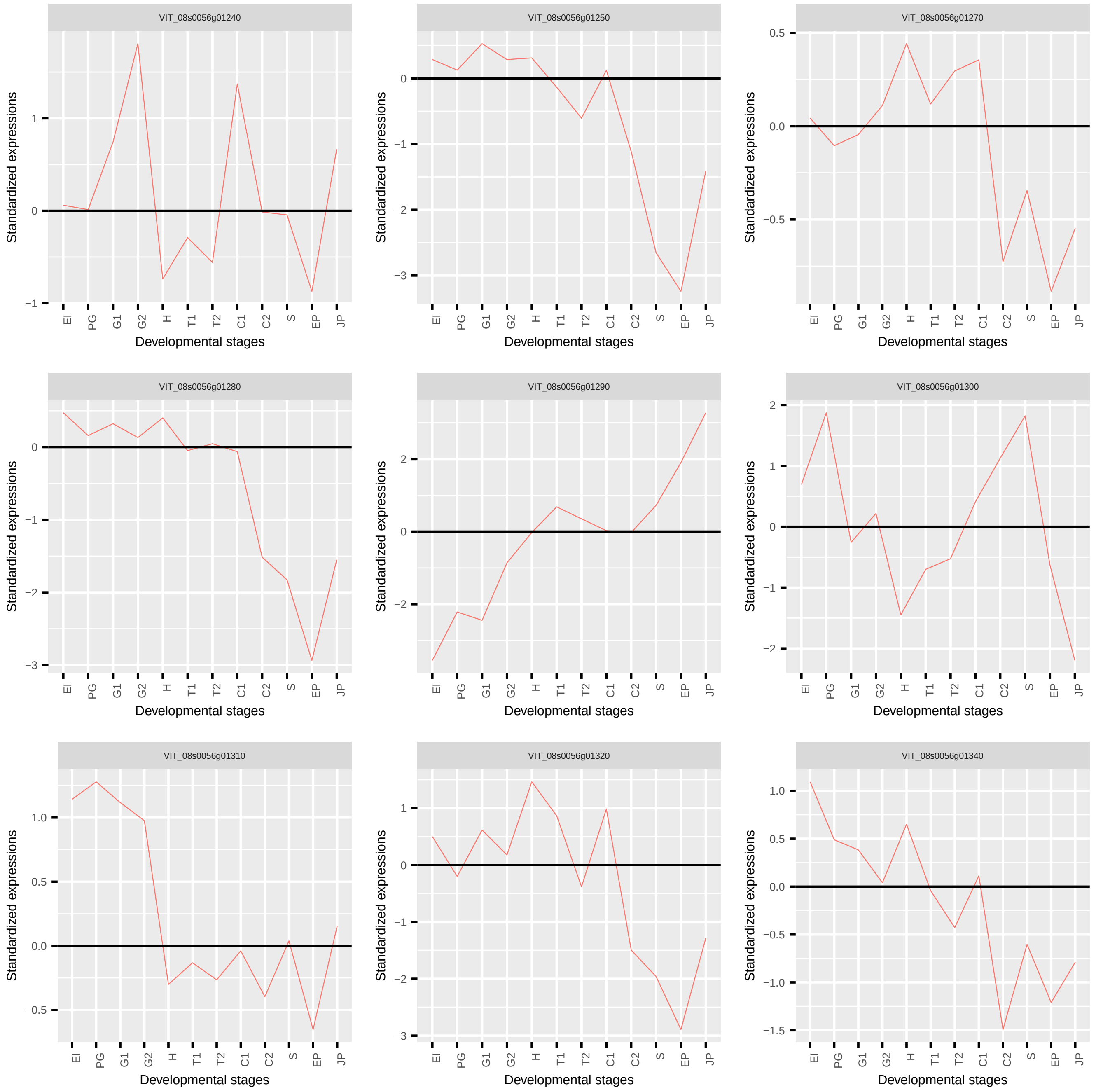
VIT_08s0056g01240
VIT_08s0056g01250
VIT_08s0056g01270
VIT_08s0056g01280
VIT_08s0056g01290
VIT_08s0056g01300
VIT_08s0056g01310
VIT_08s0056g01320
VIT_08s0056g01340
Standardized expressions
Developmental stages
EI PG G1 G2 H T1 T2 C1 C2 S EP JP

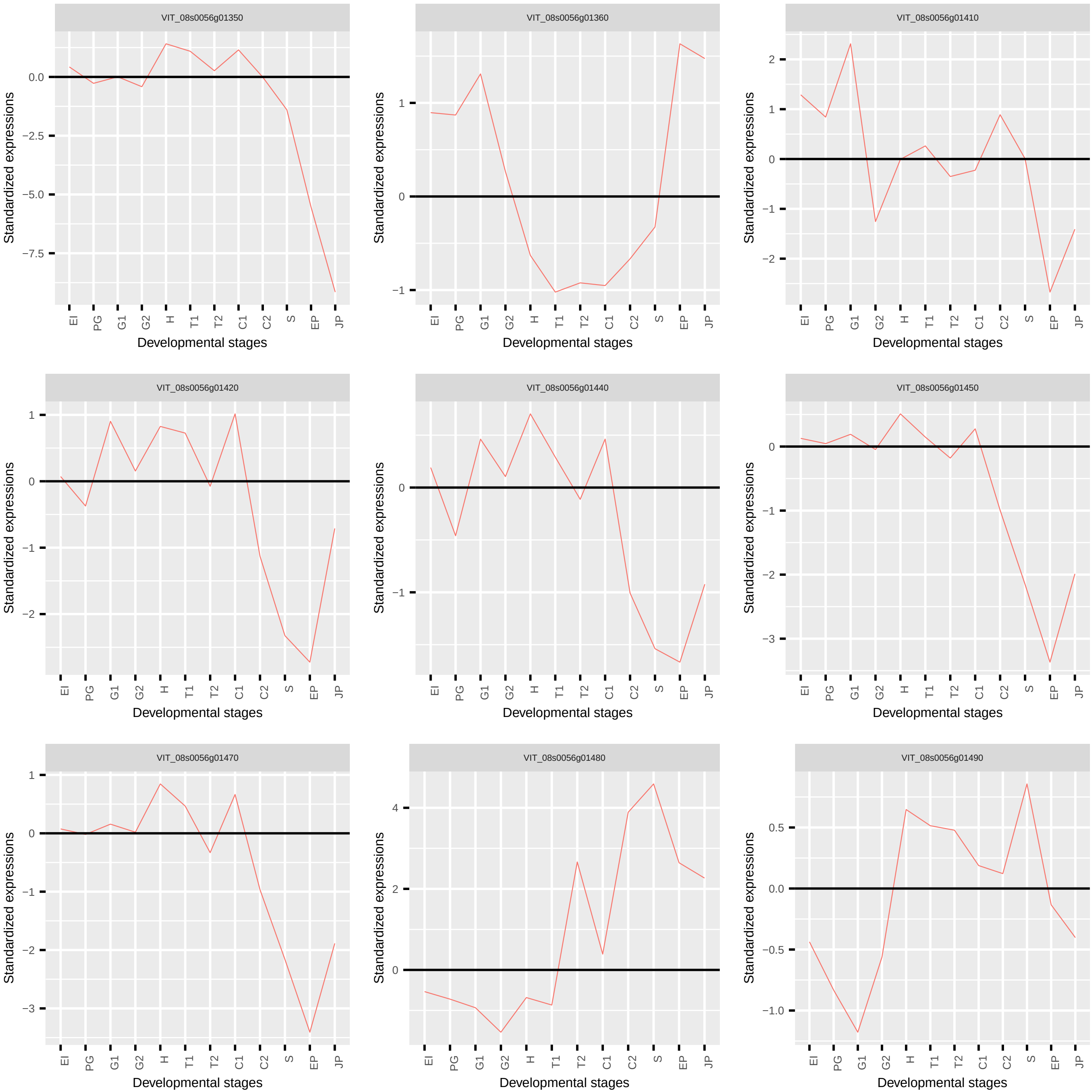
VIT_08s0056g01350
VIT_08s0056g01360
VIT_08s0056g01410
VIT_08s0056g01420
VIT_08s0056g01440
VIT_08s0056g01450
VIT_08s0056g01470
VIT_08s0056g01480
VIT_08s0056g01490
Standardized expressions
Developmental stages
EI PG G1 G2 H T1 T2 C1 C2 S EP JP
0.0 −2.5 −5.0 −7.5
1 0 −1
2 1 0 −1 −2
1 0 −1 −2
0 −1
0 −1 −2 −3
1 0 −1 −2 −3
4 2 0
0.5 0.0 −0.5 −1.0

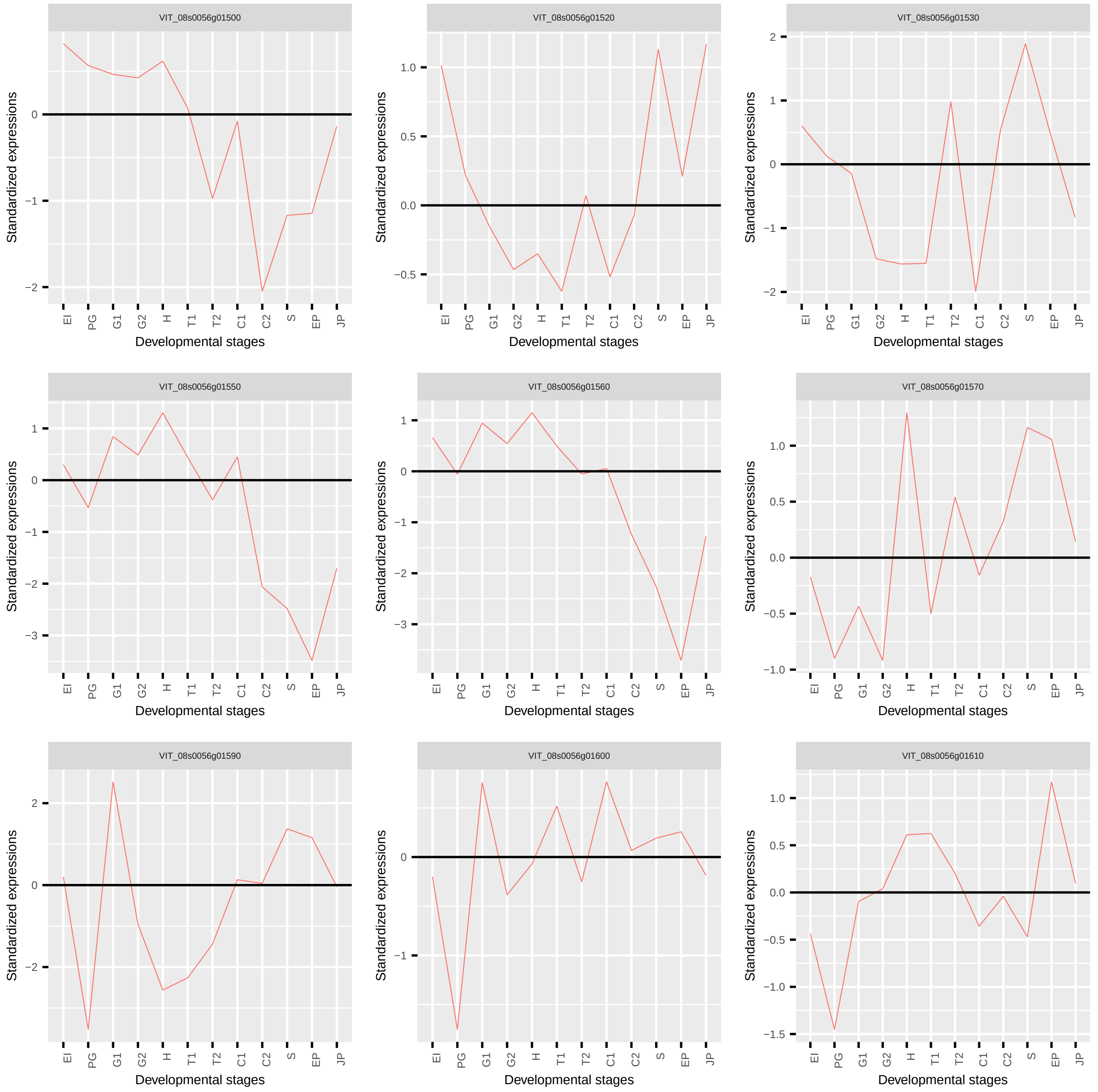
VIT_08s0056g01500
VIT_08s0056g01520
VIT_08s0056g01530
VIT_08s0056g01550
VIT_08s0056g01560
VIT_08s0056g01570
VIT_08s0056g01590
VIT_08s0056g01600
VIT_08s0056g01610
Standardized expressions
Developmental stages
EI
PG
G1
G2
H
T1
T2
C1
C2
S
EP
JP

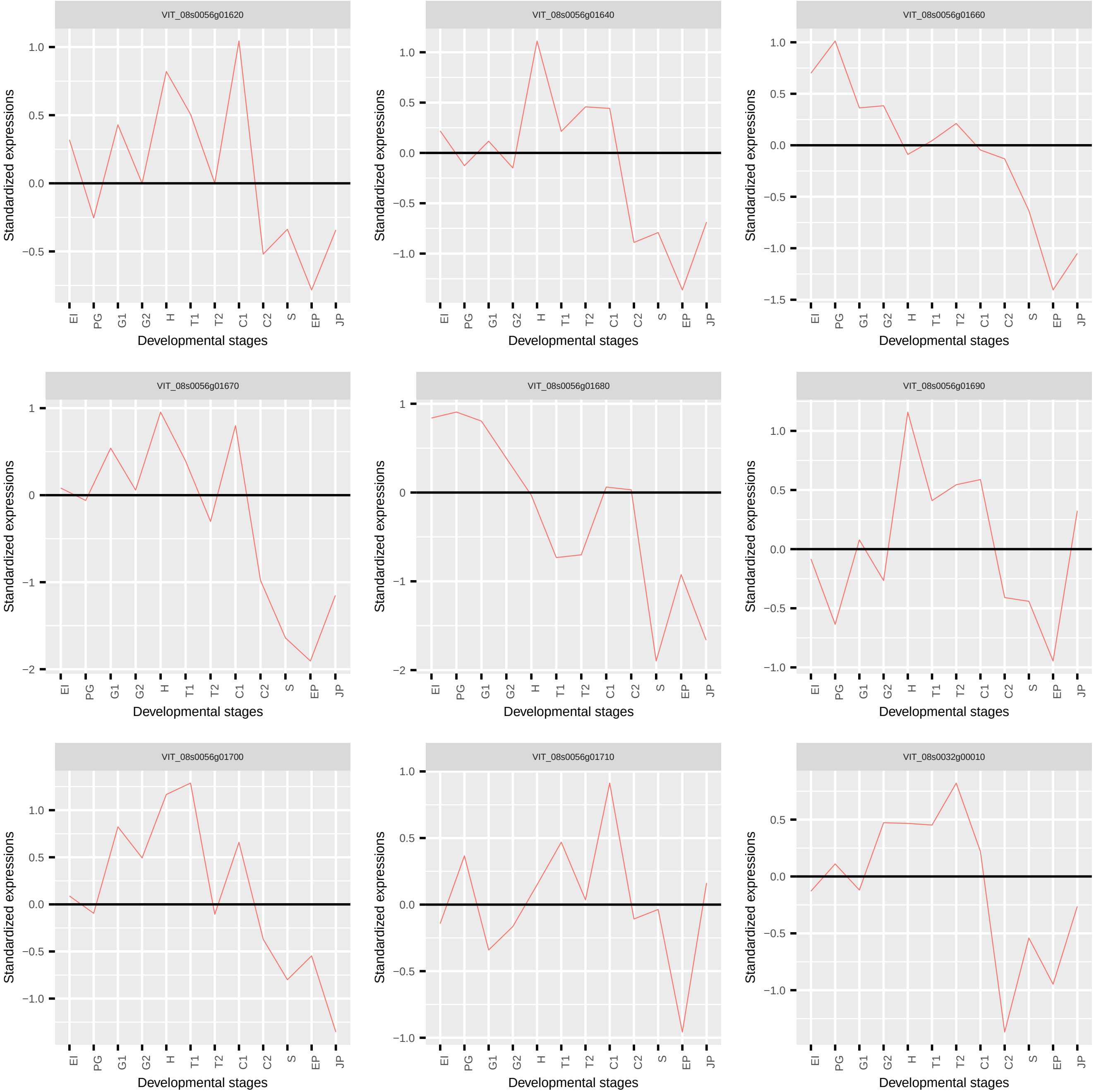

VIT_08s0056g01620
VIT_08s0056g01640
VIT_08s0056g01660
VIT_08s0056g01670
VIT_08s0056g01680
VIT_08s0056g01690
VIT_08s0056g01700
VIT_08s0056g01710
VIT_08s0032g00010
Standardized expressions
Developmental stages
EI PG G1 G2 H T1 T2 C1 C2 S EP JP
1.0
0.5
0.0
−0.5
−1.0
−1.5
1
0
−1
−2

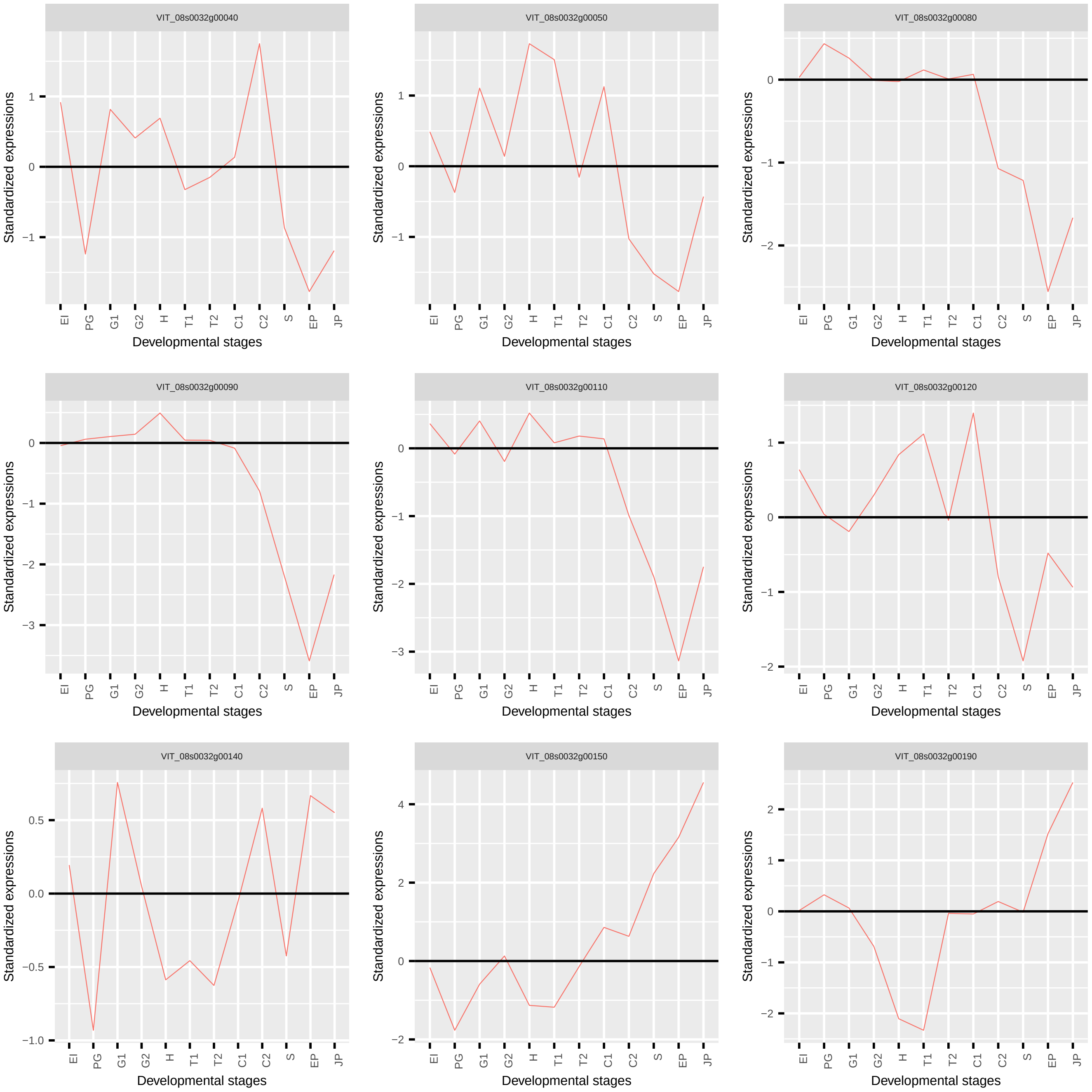
VIT_08s0032g00040
VIT_08s0032g00050
VIT_08s0032g00080
VIT_08s0032g00090
VIT_08s0032g00110
VIT_08s0032g00120
VIT_08s0032g00140
VIT_08s0032g00150
VIT_08s0032g00190
Standardized expressions
Developmental stages
EI PG G1 G2 H T1 T2 C1 C2 S EP JP

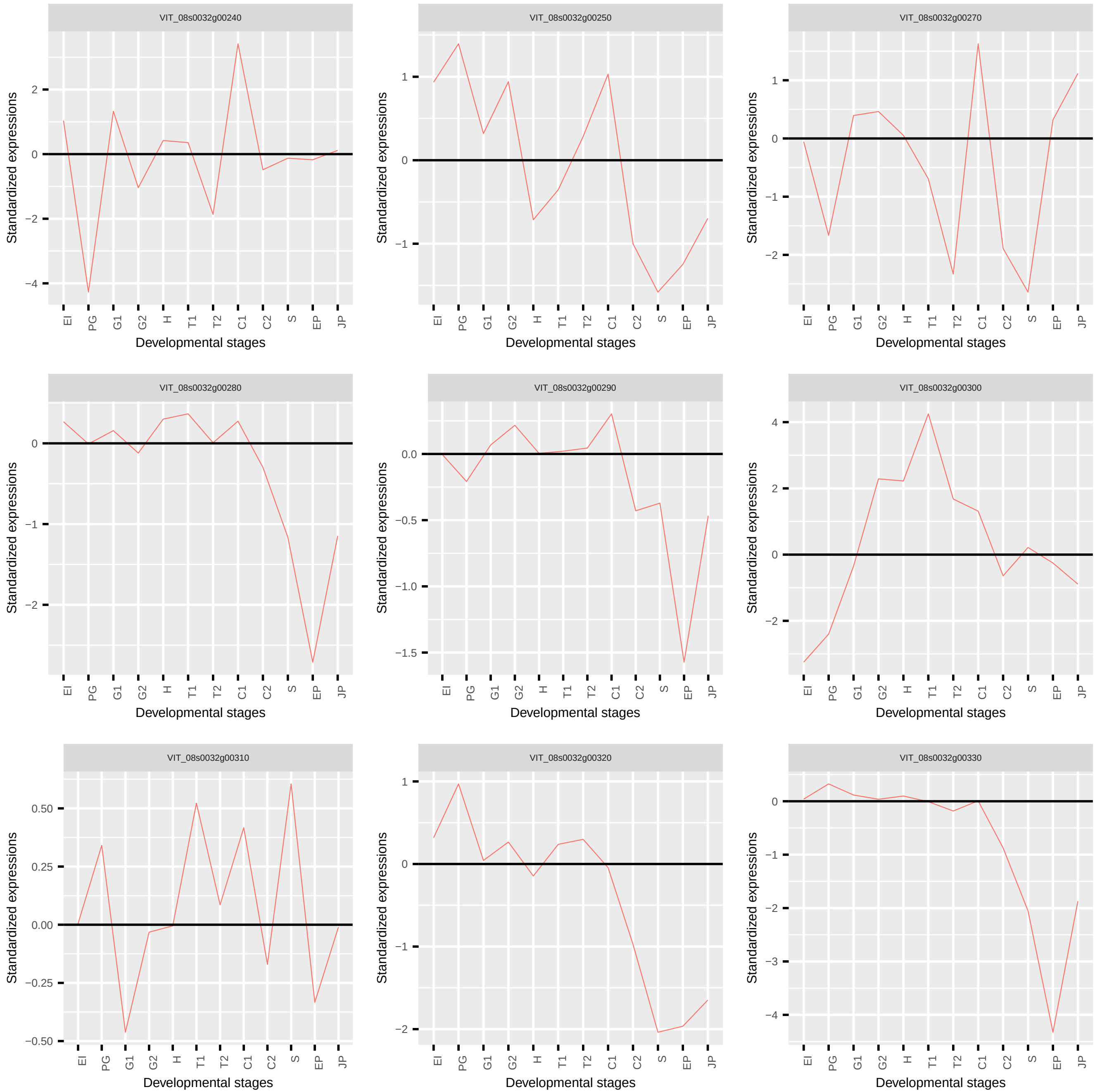
VIT_08s0032g00240
VIT_08s0032g00250
VIT_08s0032g00270
VIT_08s0032g00280
VIT_08s0032g00290
VIT_08s0032g00300
VIT_08s0032g00310
VIT_08s0032g00320
VIT_08s0032g00330
Standardized expressions
Developmental stages
EI PG G1 G2 H T1 T2 C1 C2 S EP JP

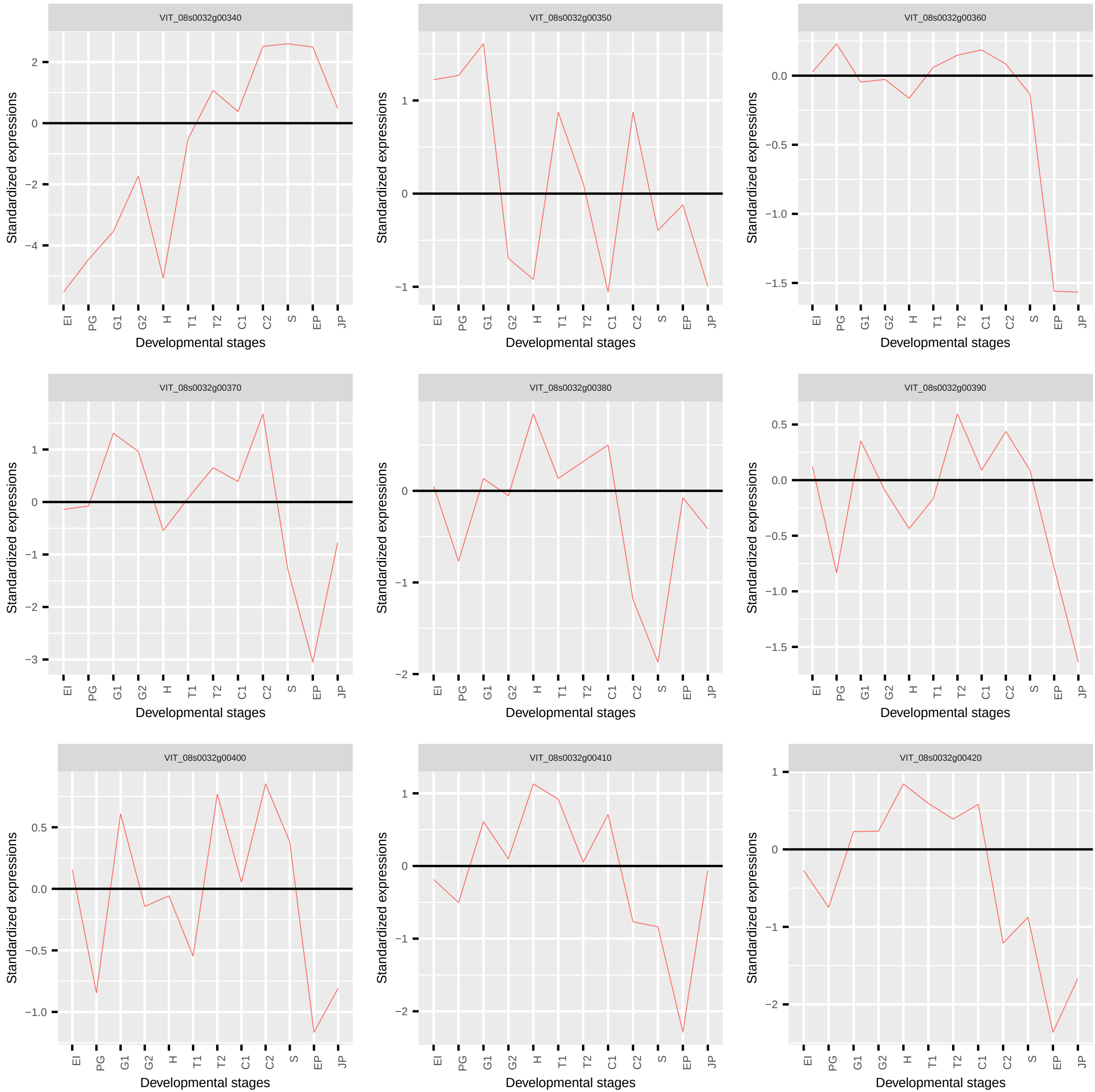
VIT_08s0032g00340
VIT_08s0032g00350
VIT_08s0032g00360
VIT_08s0032g00370
VIT_08s0032g00380
VIT_08s0032g00390
VIT_08s0032g00400
VIT_08s0032g00410
VIT_08s0032g00420
Standardized expressions
Developmental stages
EI PG G1 G2 H T1 T2 C1 C2 S EP JP
2 0 −2 −4
1 0 −1
0.0 −0.5 −1.0 −1.5
1 0 −1 −2 −3
0 −1 −2
0.5 0.0 −0.5 −1.0 −1.5
0.5 0.0 −0.5 −1.0
1 0 −1 −2

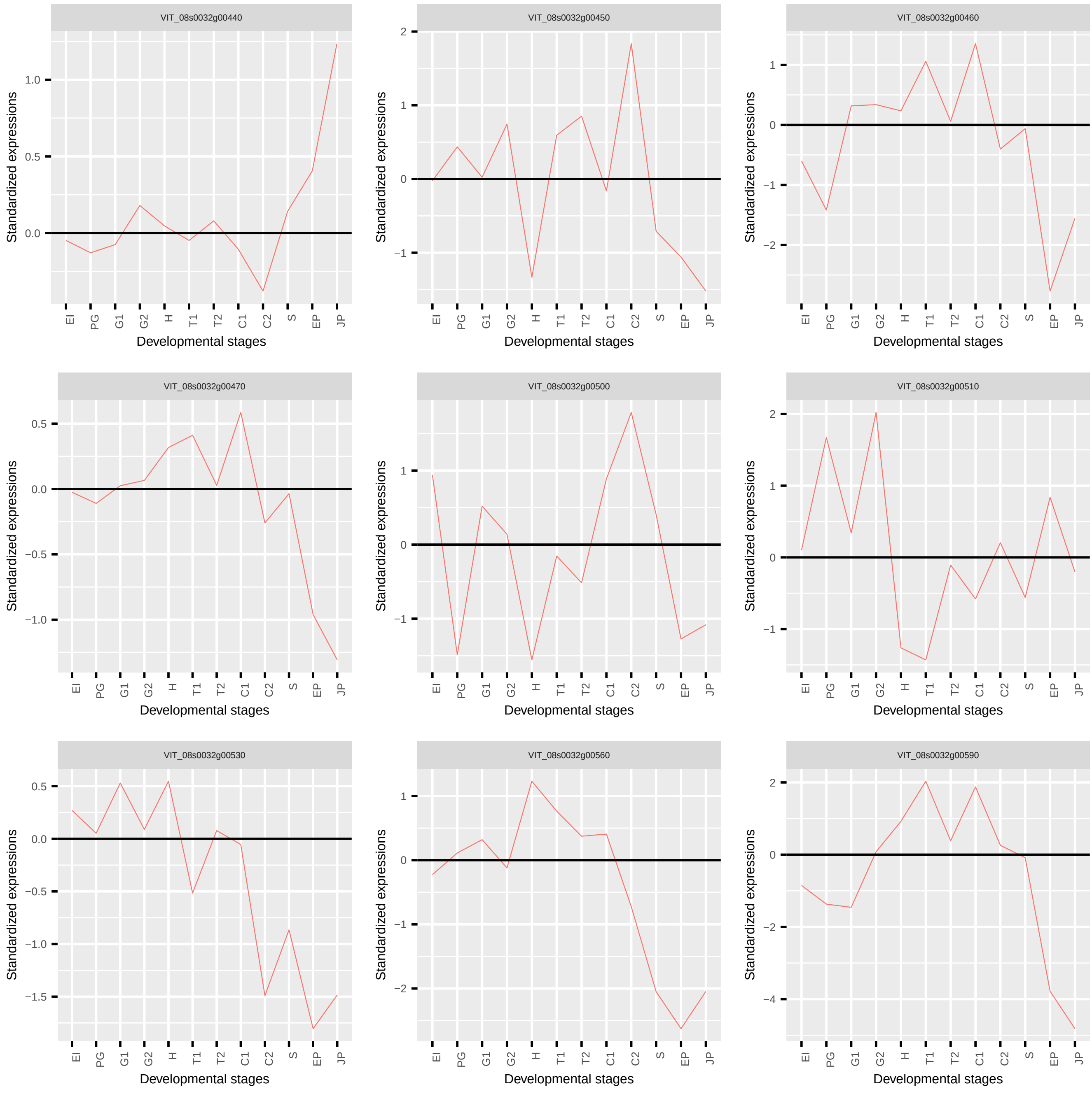
VIT_08s0032g00440
VIT_08s0032g00450
VIT_08s0032g00460
VIT_08s0032g00470
VIT_08s0032g00500
VIT_08s0032g00510
VIT_08s0032g00530
VIT_08s0032g00560
VIT_08s0032g00590
Standardized expressions
EI PG G1 G2 H T1 T2 C1 C2 S EP JP
Developmental stages

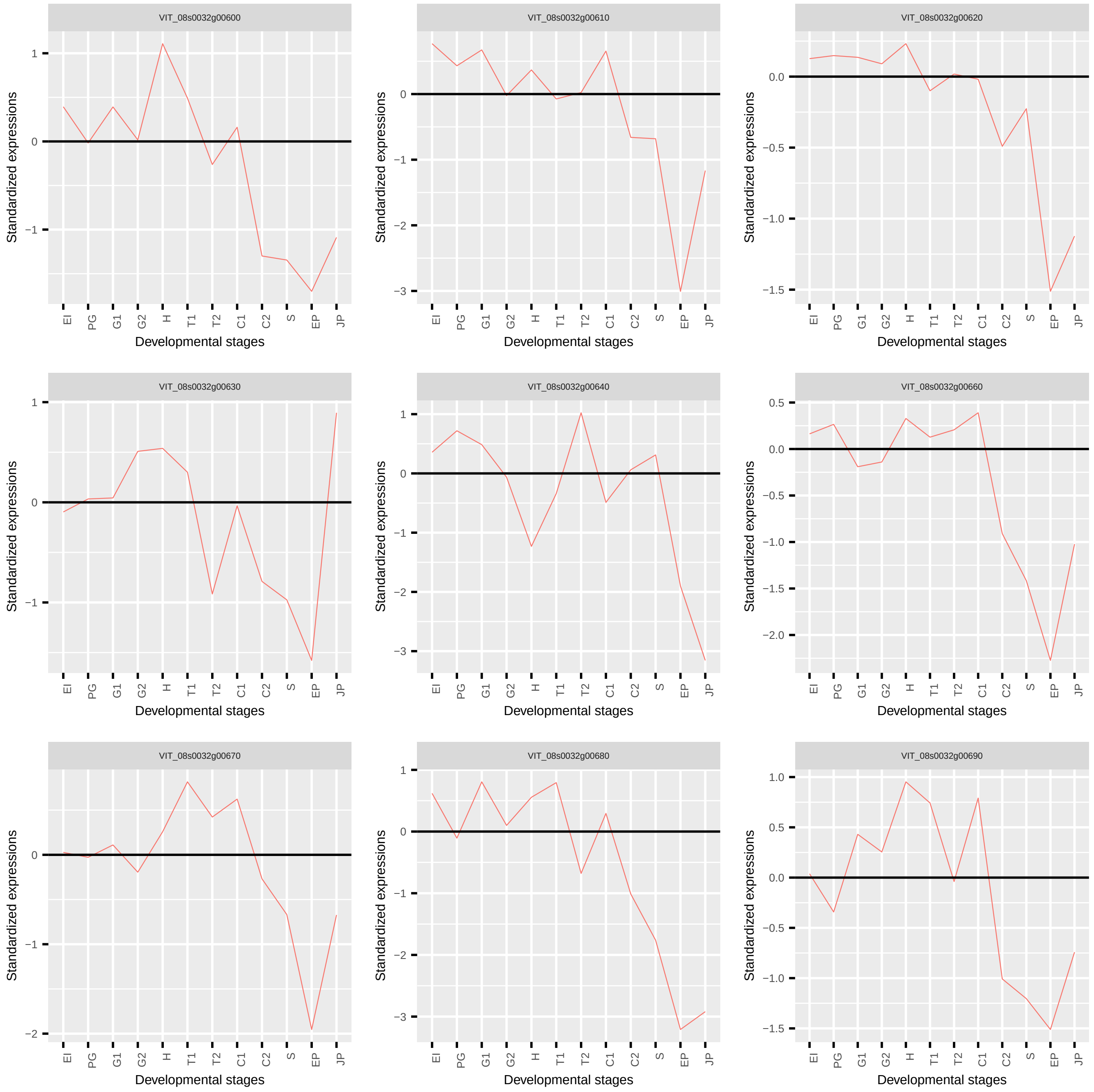
VIT_08s0032g00600
VIT_08s0032g00610
VIT_08s0032g00620
VIT_08s0032g00630
VIT_08s0032g00640
VIT_08s0032g00660
VIT_08s0032g00670
VIT_08s0032g00680
VIT_08s0032g00690
Standardized expressions
Developmental stages
EI
PG
G1
G2
H
T1
T2
C1
C2
S
EP
JP

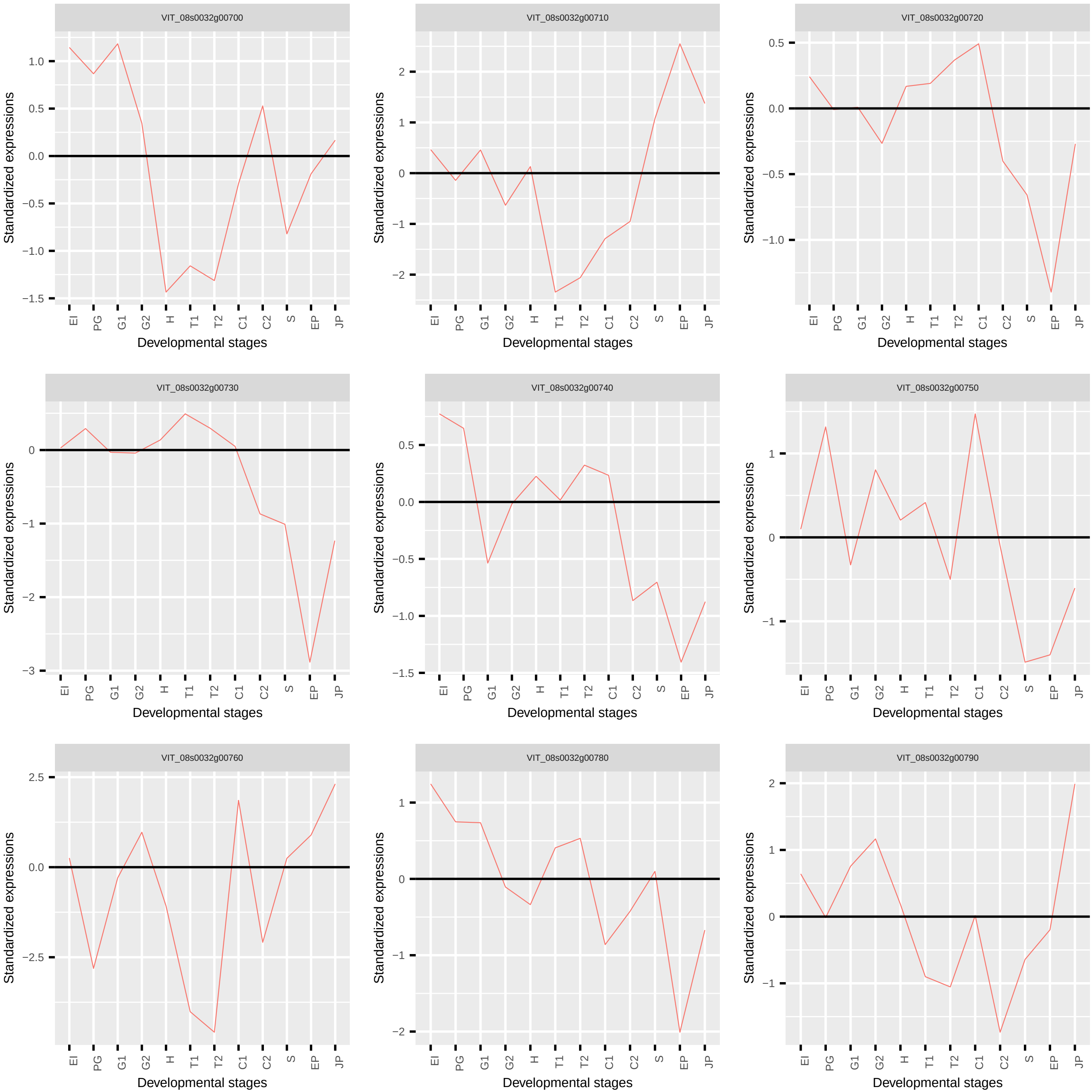

VIT_08s0032g00700
VIT_08s0032g00710
VIT_08s0032g00720
VIT_08s0032g00730
VIT_08s0032g00740
VIT_08s0032g00750
VIT_08s0032g00760
VIT_08s0032g00780
VIT_08s0032g00790
Standardized expressions
Developmental stages
EI
PG
G1
G2
H
T1
T2
C1
C2
S
EP
JP

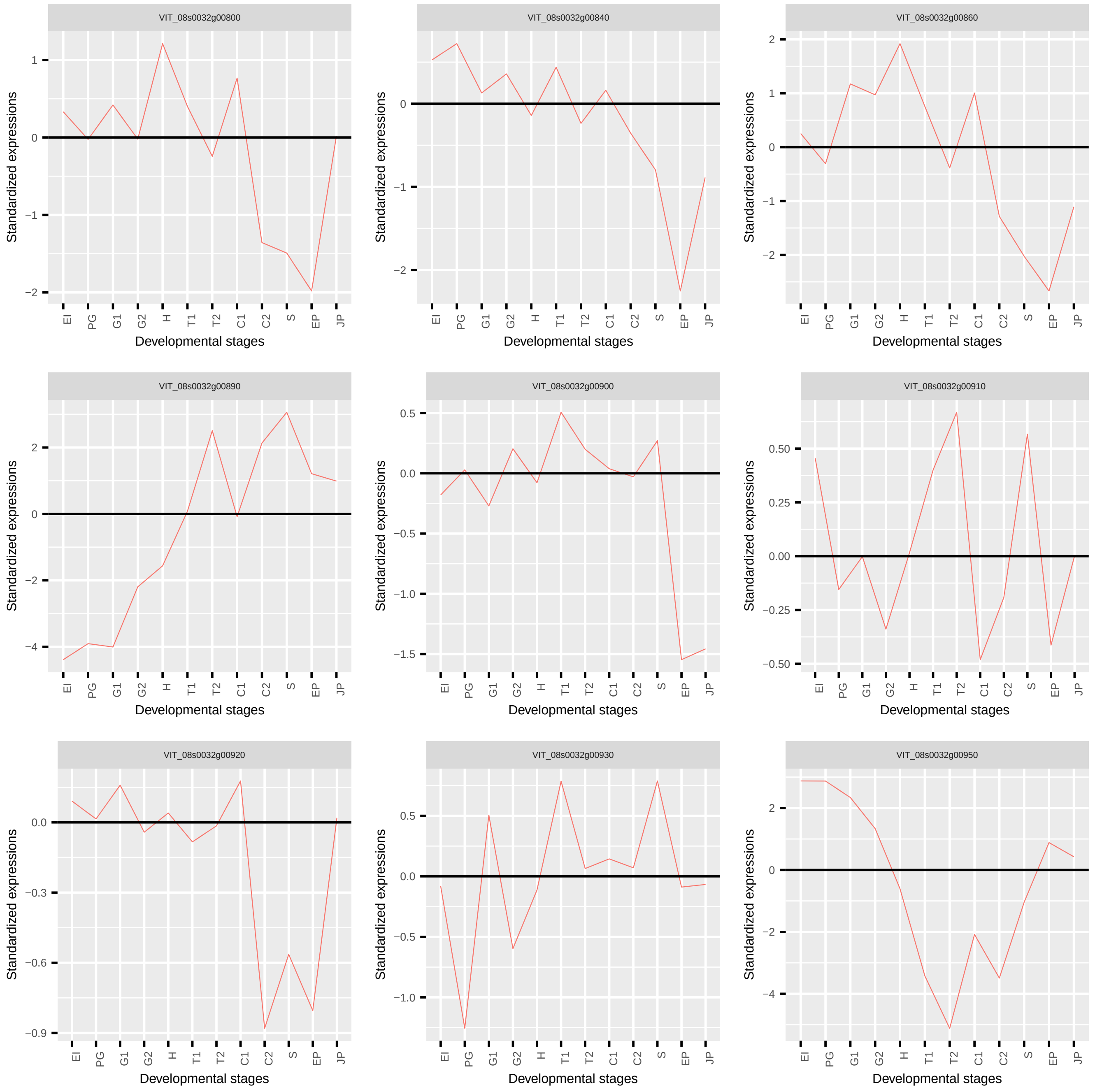
VIT_08s0032g00800
VIT_08s0032g00840
VIT_08s0032g00860
VIT_08s0032g00890
VIT_08s0032g00900
VIT_08s0032g00910
VIT_08s0032g00920
VIT_08s0032g00930
VIT_08s0032g00950
Standardized expressions
Developmental stages
EI PG G1 G2 H T1 T2 C1 C2 S EP JP

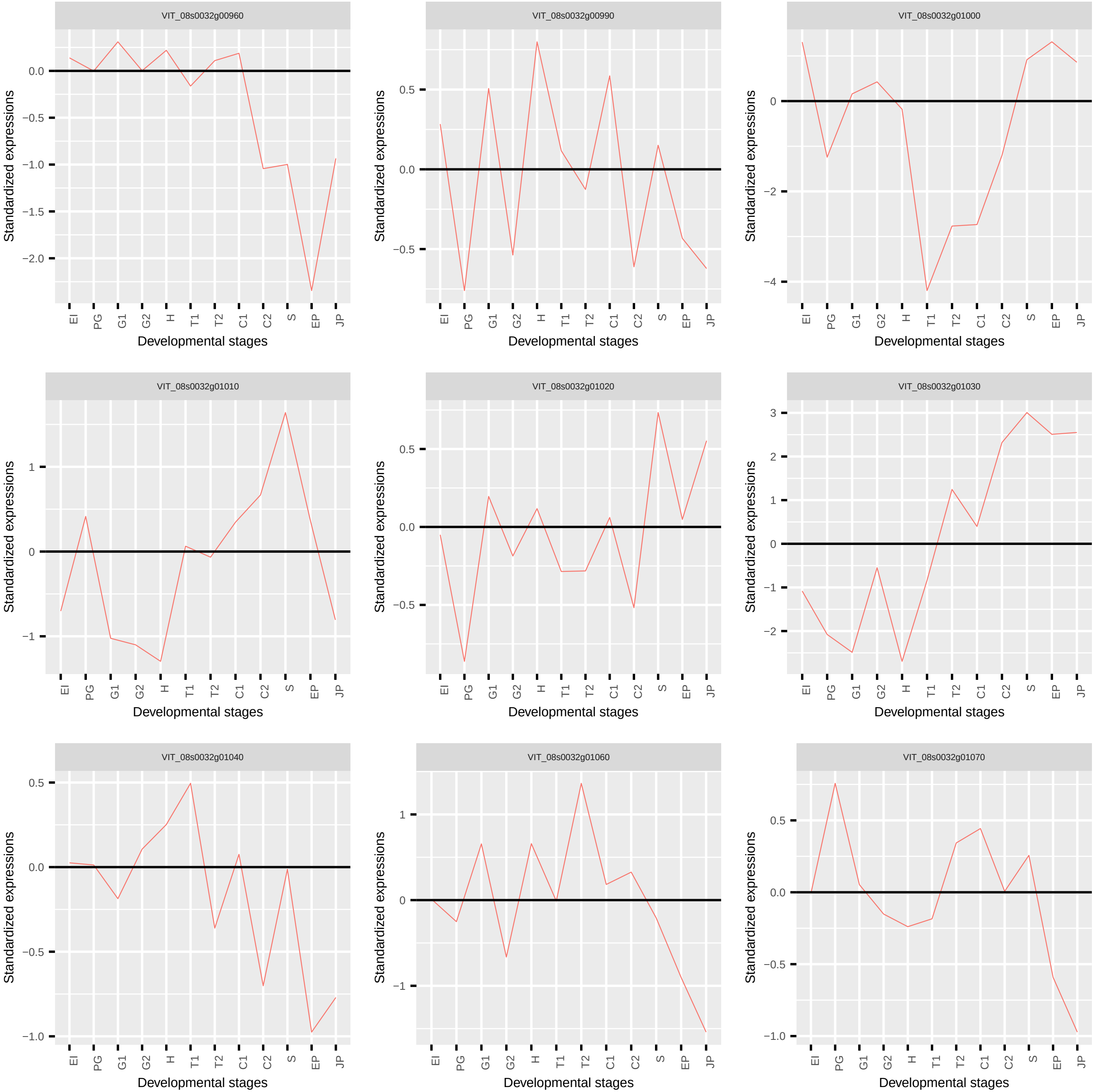
VIT_08s0032g00960
Standardized expressions
0.0
−0.5
−1.0
−1.5
−2.0
EI PG G1 G2 H T1 T2 C1 C2 S EP JP
Developmental stages
VIT_08s0032g00990
Standardized expressions
0.5
0.0
−0.5
EI PG G1 G2 H T1 T2 C1 C2 S EP JP
Developmental stages
VIT_08s0032g01000
Standardized expressions
0
−2
−4
EI PG G1 G2 H T1 T2 C1 C2 S EP JP
Developmental stages
VIT_08s0032g01010
Standardized expressions
1
0
−1
EI PG G1 G2 H T1 T2 C1 C2 S EP JP
Developmental stages
VIT_08s0032g01020
Standardized expressions
0.5
0.0
−0.5
EI PG G1 G2 H T1 T2 C1 C2 S EP JP
Developmental stages
VIT_08s0032g01030
Standardized expressions
3
2
1
0
−1
−2
EI PG G1 G2 H T1 T2 C1 C2 S EP JP
Developmental stages
VIT_08s0032g01040
Standardized expressions
0.5
0.0
−0.5
−1.0
EI PG G1 G2 H T1 T2 C1 C2 S EP JP
Developmental stages
VIT_08s0032g01060
Standardized expressions
1
0
−1
EI PG G1 G2 H T1 T2 C1 C2 S EP JP
Developmental stages
VIT_08s0032g01070
Standardized expressions
0.5
0.0
−0.5
−1.0
EI PG G1 G2 H T1 T2 C1 C2 S EP JP
Developmental stages

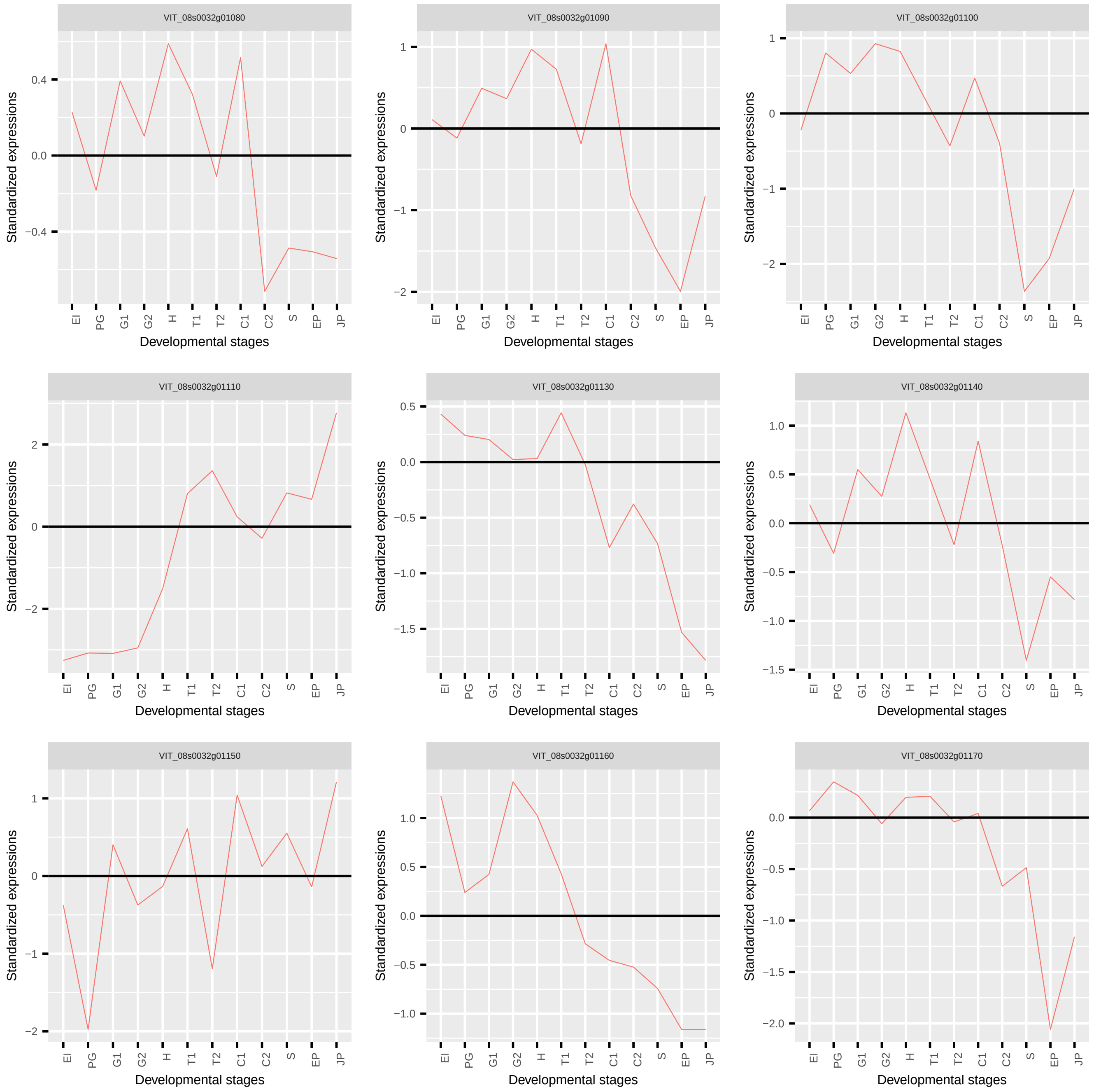

VIT_08s0032g01080
VIT_08s0032g01090
VIT_08s0032g01100
VIT_08s0032g01110
VIT_08s0032g01130
VIT_08s0032g01140
VIT_08s0032g01150
VIT_08s0032g01160
VIT_08s0032g01170
Standardized expressions
Developmental stages
EI PG G1 G2 H T1 T2 C1 C2 S EP JP

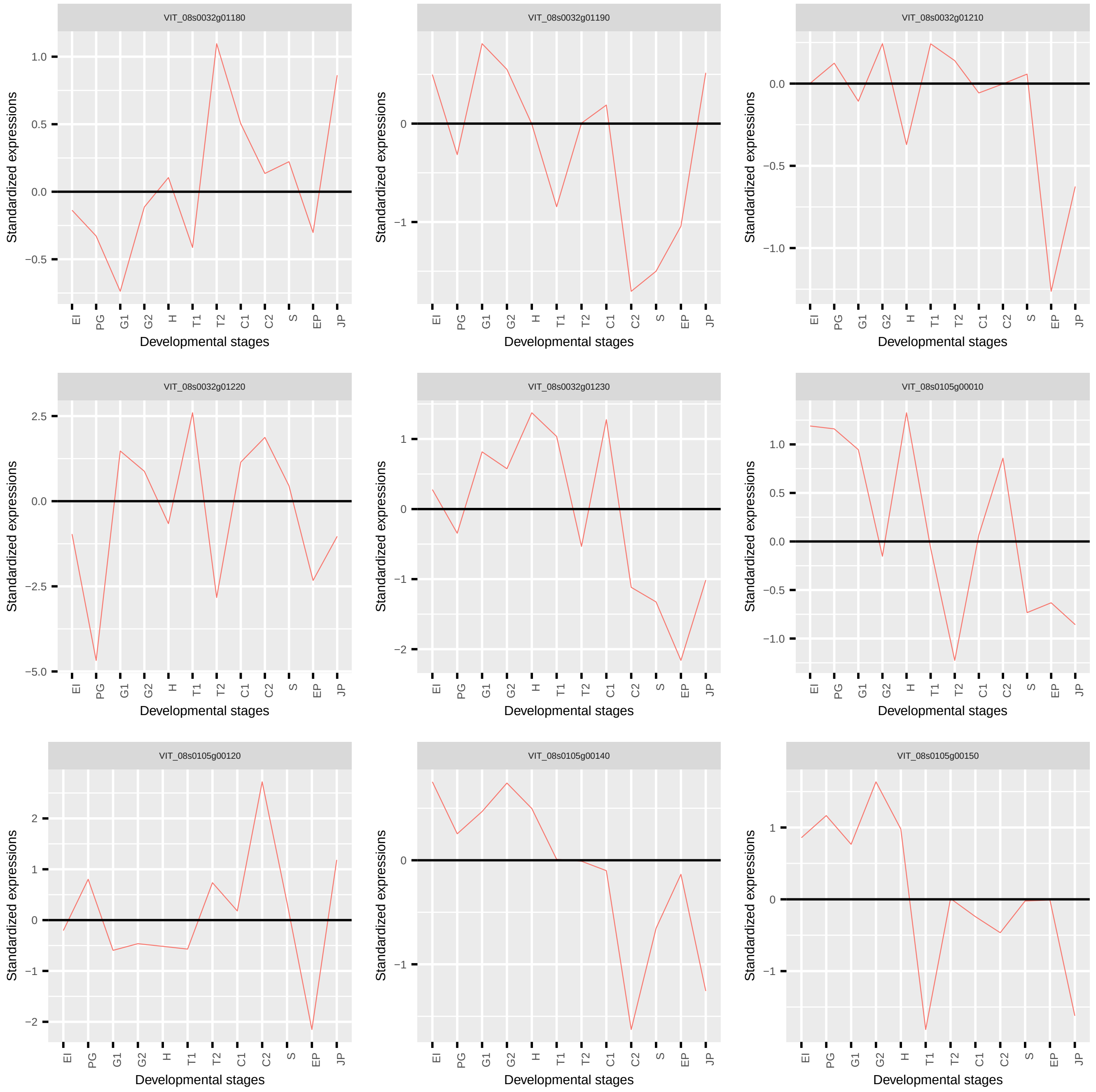

VIT_08s0032g01180
VIT_08s0032g01190
VIT_08s0032g01210
VIT_08s0032g01220
VIT_08s0032g01230
VIT_08s0105g00010
VIT_08s0105g00120
VIT_08s0105g00140
VIT_08s0105g00150
Standardized expressions
Developmental stages
EI PG G1 G2 H T1 T2 C1 C2 S EP JP

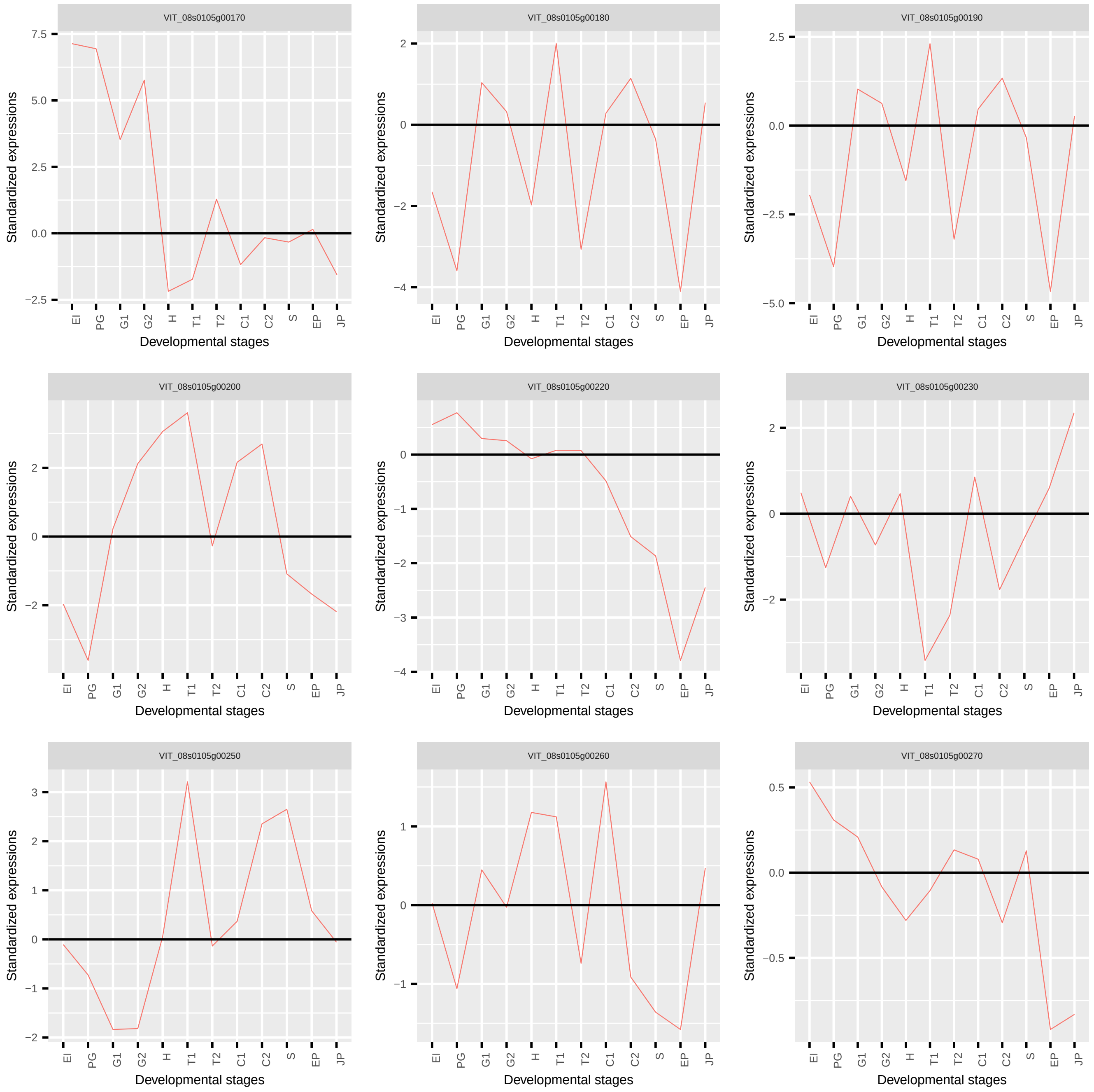
VIT_08s0105g00170
7.5
5.0
2.5
0.0
−2.5
Standardized expressions
EI PG G1 G2 H T1 T2 C1 C2 S EP JP
Developmental stages
VIT_08s0105g00180
2
0
−2
−4
Standardized expressions
EI PG G1 G2 H T1 T2 C1 C2 S EP JP
Developmental stages
VIT_08s0105g00190
2.5
0.0
−2.5
−5.0
Standardized expressions
EI PG G1 G2 H T1 T2 C1 C2 S EP JP
Developmental stages
VIT_08s0105g00200
2
0
−2
Standardized expressions
EI PG G1 G2 H T1 T2 C1 C2 S EP JP
Developmental stages
VIT_08s0105g00220
0
−1
−2
−3
−4
Standardized expressions
EI PG G1 G2 H T1 T2 C1 C2 S EP JP
Developmental stages
VIT_08s0105g00230
2
0
−2
Standardized expressions
EI PG G1 G2 H T1 T2 C1 C2 S EP JP
Developmental stages
VIT_08s0105g00250
3
2
1
0
−1
−2
Standardized expressions
EI PG G1 G2 H T1 T2 C1 C2 S EP JP
Developmental stages
VIT_08s0105g00260
1
0
−1
Standardized expressions
EI PG G1 G2 H T1 T2 C1 C2 S EP JP
Developmental stages
VIT_08s0105g00270
0.5
0.0
−0.5
Standardized expressions
EI PG G1 G2 H T1 T2 C1 C2 S EP JP
Developmental stages

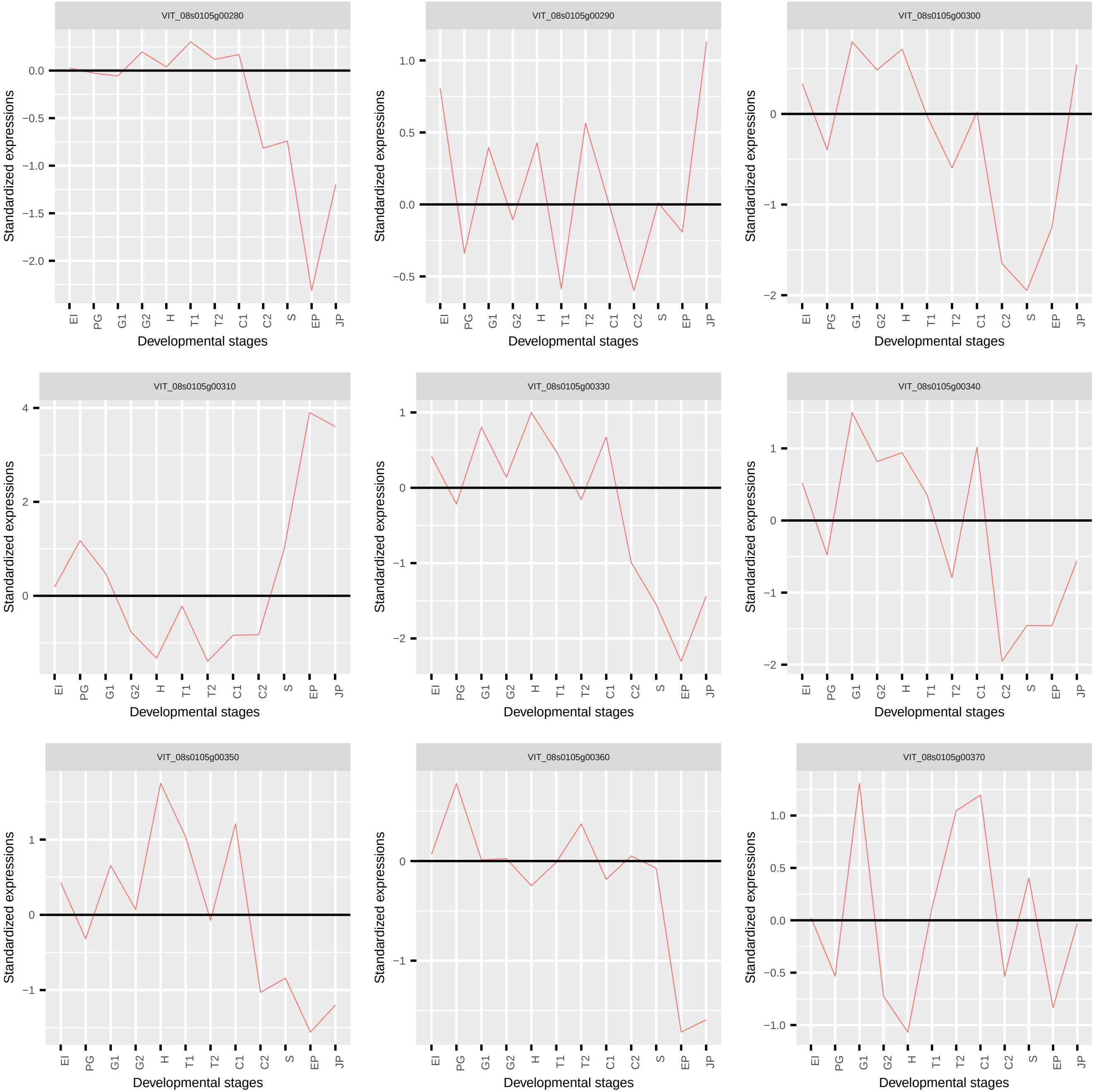

VIT_08s0105g00280
0.0
−0.5
−1.0
−1.5
−2.0
Standardized expressions
EI PG G1 G2 H T1 T2 C1 C2 S EP JP
Developmental stages
VIT_08s0105g00290
1.0
0.5
0.0
−0.5
Standardized expressions
EI PG G1 G2 H T1 T2 C1 C2 S EP JP
Developmental stages
VIT_08s0105g00300
0
−1
−2
Standardized expressions
EI PG G1 G2 H T1 T2 C1 C2 S EP JP
Developmental stages
VIT_08s0105g00310
4
2
0
Standardized expressions
EI PG G1 G2 H T1 T2 C1 C2 S EP JP
Developmental stages
VIT_08s0105g00330
1
0
−1
−2
Standardized expressions
EI PG G1 G2 H T1 T2 C1 C2 S EP JP
Developmental stages
VIT_08s0105g00340
1
0
−1
−2
Standardized expressions
EI PG G1 G2 H T1 T2 C1 C2 S EP JP
Developmental stages
VIT_08s0105g00350
1
0
−1
Standardized expressions
EI PG G1 G2 H T1 T2 C1 C2 S EP JP
Developmental stages
VIT_08s0105g00360
0
−1
Standardized expressions
EI PG G1 G2 H T1 T2 C1 C2 S EP JP
Developmental stages
VIT_08s0105g00370
1.0
0.5
0.0
−0.5
−1.0
Standardized expressions
EI PG G1 G2 H T1 T2 C1 C2 S EP JP
Developmental stages

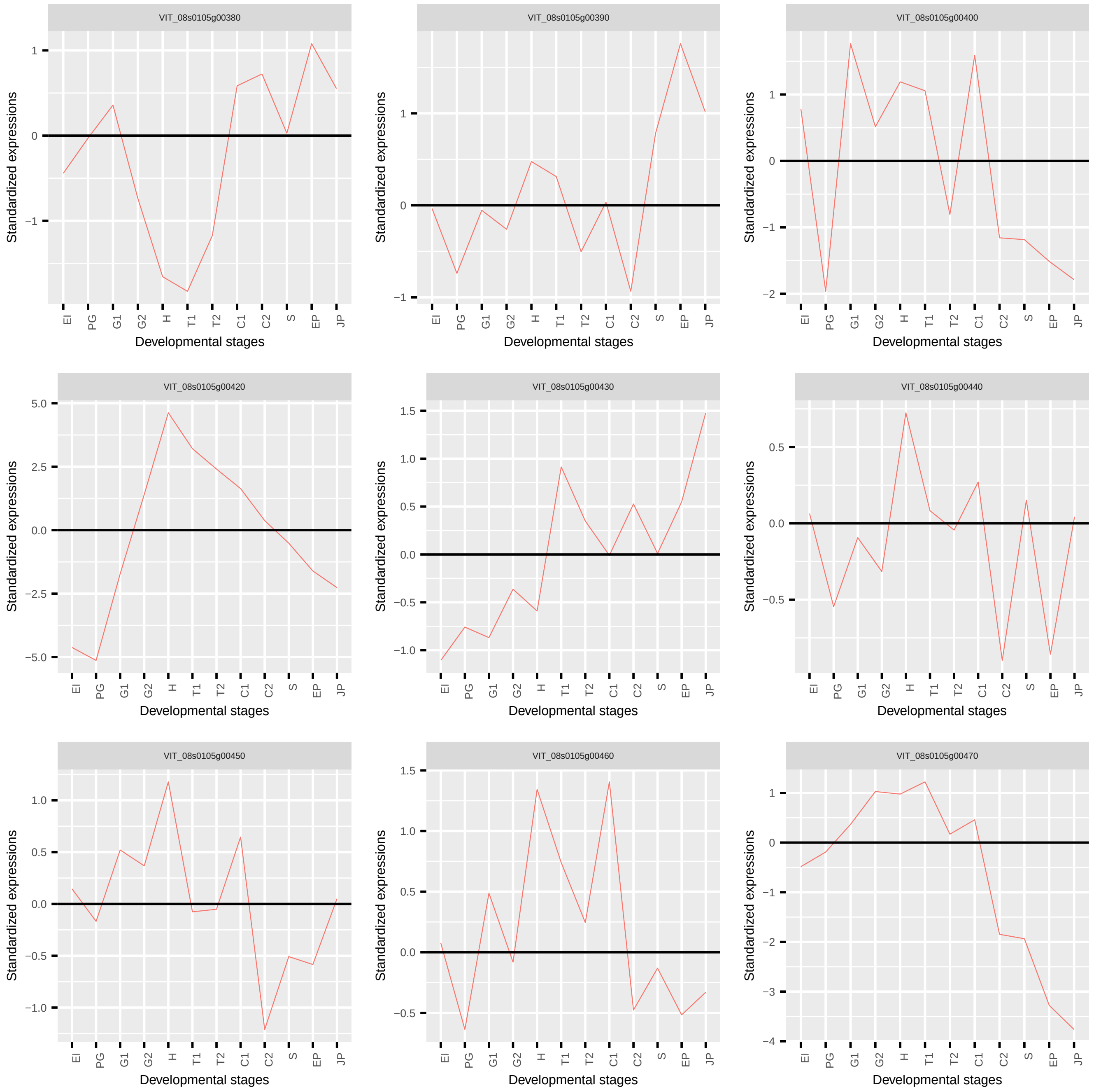
VIT_08s0105g00380
VIT_08s0105g00390
VIT_08s0105g00400
VIT_08s0105g00420
VIT_08s0105g00430
VIT_08s0105g00440
VIT_08s0105g00450
VIT_08s0105g00460
VIT_08s0105g00470
Standardized expressions
Developmental stages
EI PG G1 G2 H T1 T2 C1 C2 S EP JP

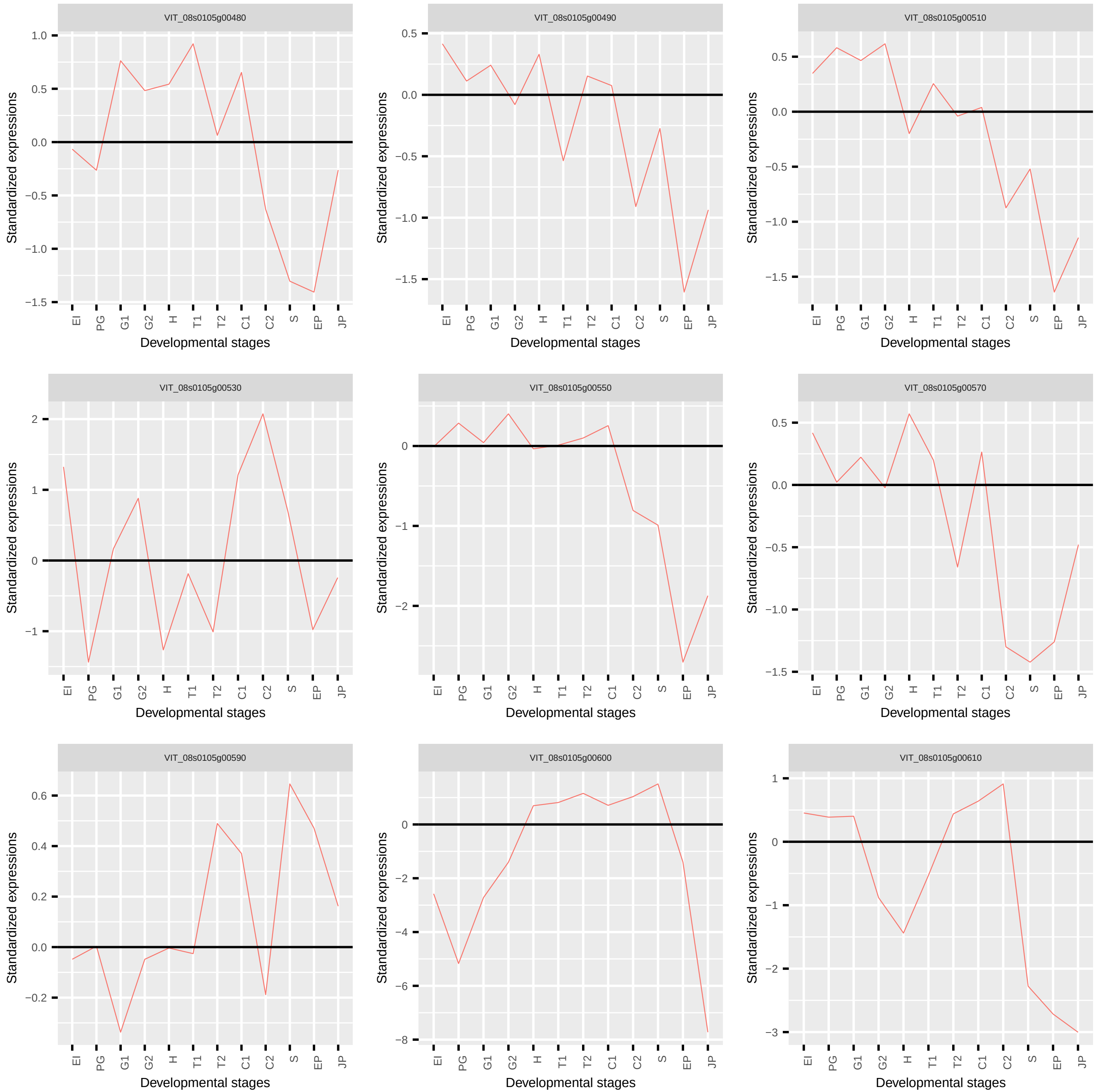
VIT_08s0105g00480
VIT_08s0105g00490
VIT_08s0105g00510
VIT_08s0105g00530
VIT_08s0105g00550
VIT_08s0105g00570
VIT_08s0105g00590
VIT_08s0105g00600
VIT_08s0105g00610
Standardized expressions
Developmental stages
EI PG G1 G2 H T1 T2 C1 C2 S EP JP

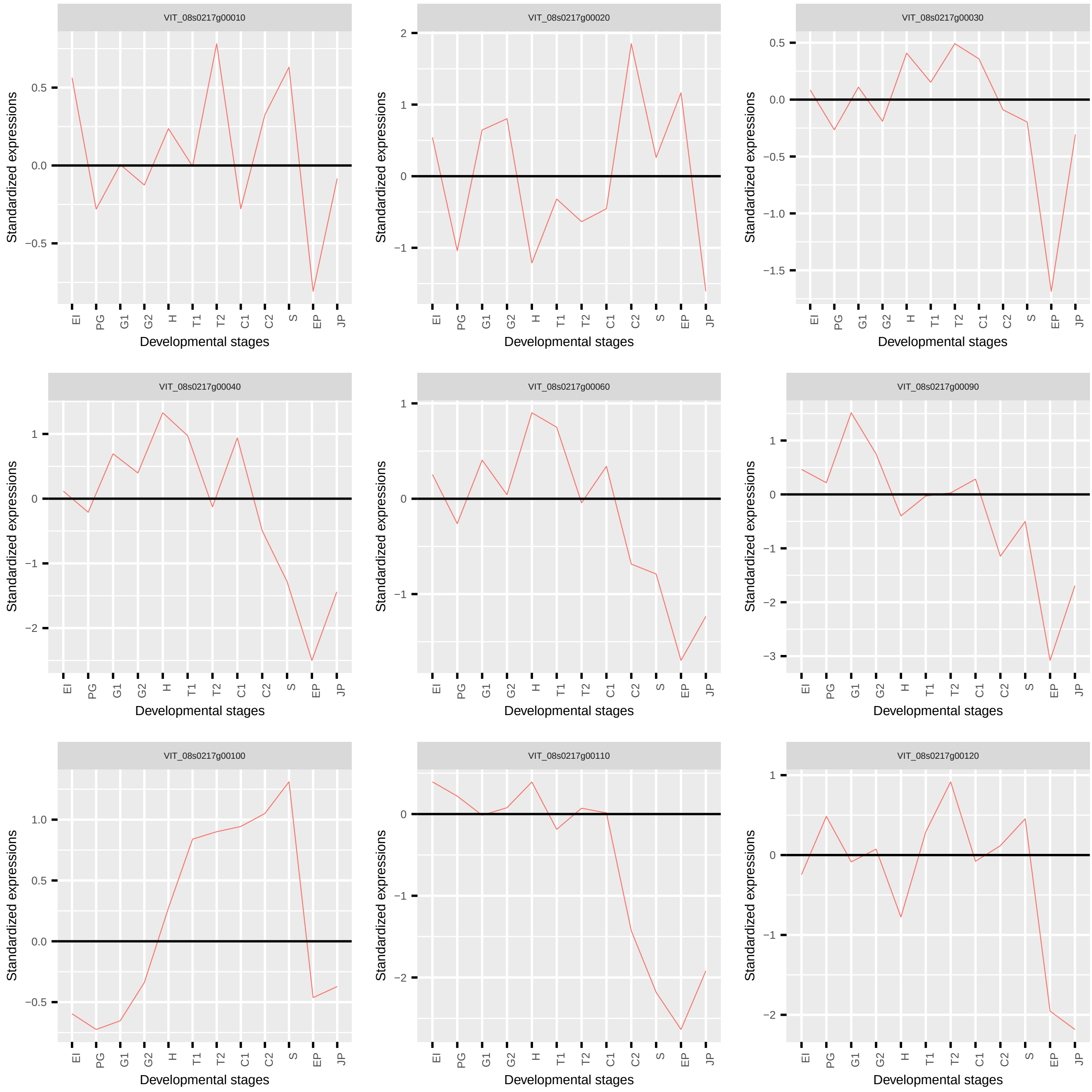
VIT_08s0217g00010
VIT_08s0217g00020
VIT_08s0217g00030
VIT_08s0217g00040
VIT_08s0217g00060
VIT_08s0217g00090
VIT_08s0217g00100
VIT_08s0217g00110
VIT_08s0217g00120
Standardized expressions
Developmental stages
EI PG G1 G2 H T1 T2 C1 C2 S EP JP

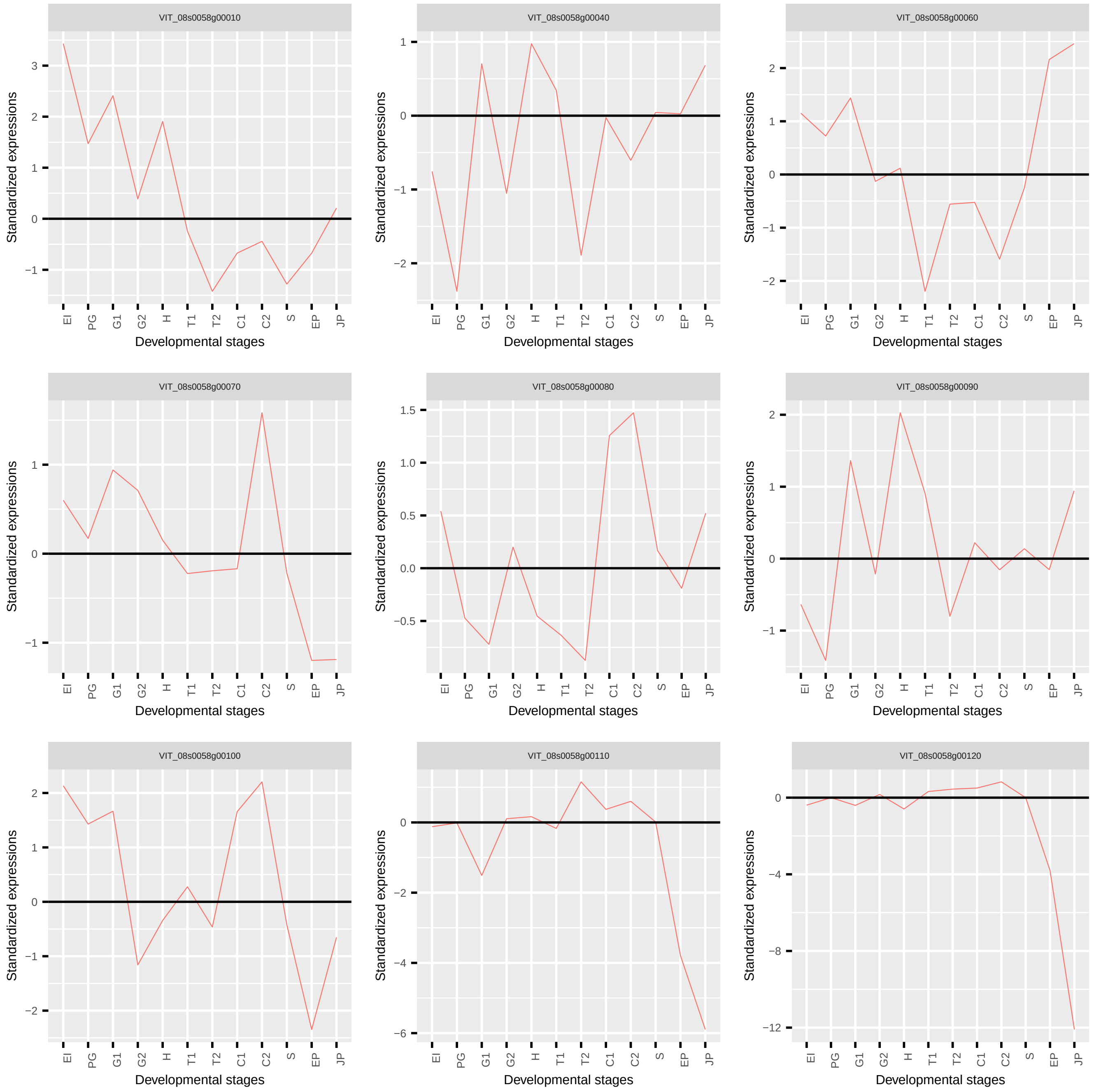
VIT_08s0058g00010
Standardized expressions
3
2
1
0
−1
EI PG G1 G2 H T1 T2 C1 C2 S EP JP
Developmental stages
VIT_08s0058g00040
Standardized expressions
1
0
−1
−2
EI PG G1 G2 H T1 T2 C1 C2 S EP JP
Developmental stages
VIT_08s0058g00060
Standardized expressions
2
1
0
−1
−2
EI PG G1 G2 H T1 T2 C1 C2 S EP JP
Developmental stages
VIT_08s0058g00070
Standardized expressions
1
0
−1
EI PG G1 G2 H T1 T2 C1 C2 S EP JP
Developmental stages
VIT_08s0058g00080
Standardized expressions
1.5
1.0
0.5
0.0
−0.5
EI PG G1 G2 H T1 T2 C1 C2 S EP JP
Developmental stages
VIT_08s0058g00090
Standardized expressions
2
1
0
−1
EI PG G1 G2 H T1 T2 C1 C2 S EP JP
Developmental stages
VIT_08s0058g00100
Standardized expressions
2
1
0
−1
−2
EI PG G1 G2 H T1 T2 C1 C2 S EP JP
Developmental stages
VIT_08s0058g00110
Standardized expressions
0
−2
−4
−6
EI PG G1 G2 H T1 T2 C1 C2 S EP JP
Developmental stages
VIT_08s0058g00120
Standardized expressions
0
−4
−8
−12
EI PG G1 G2 H T1 T2 C1 C2 S EP JP
Developmental stages

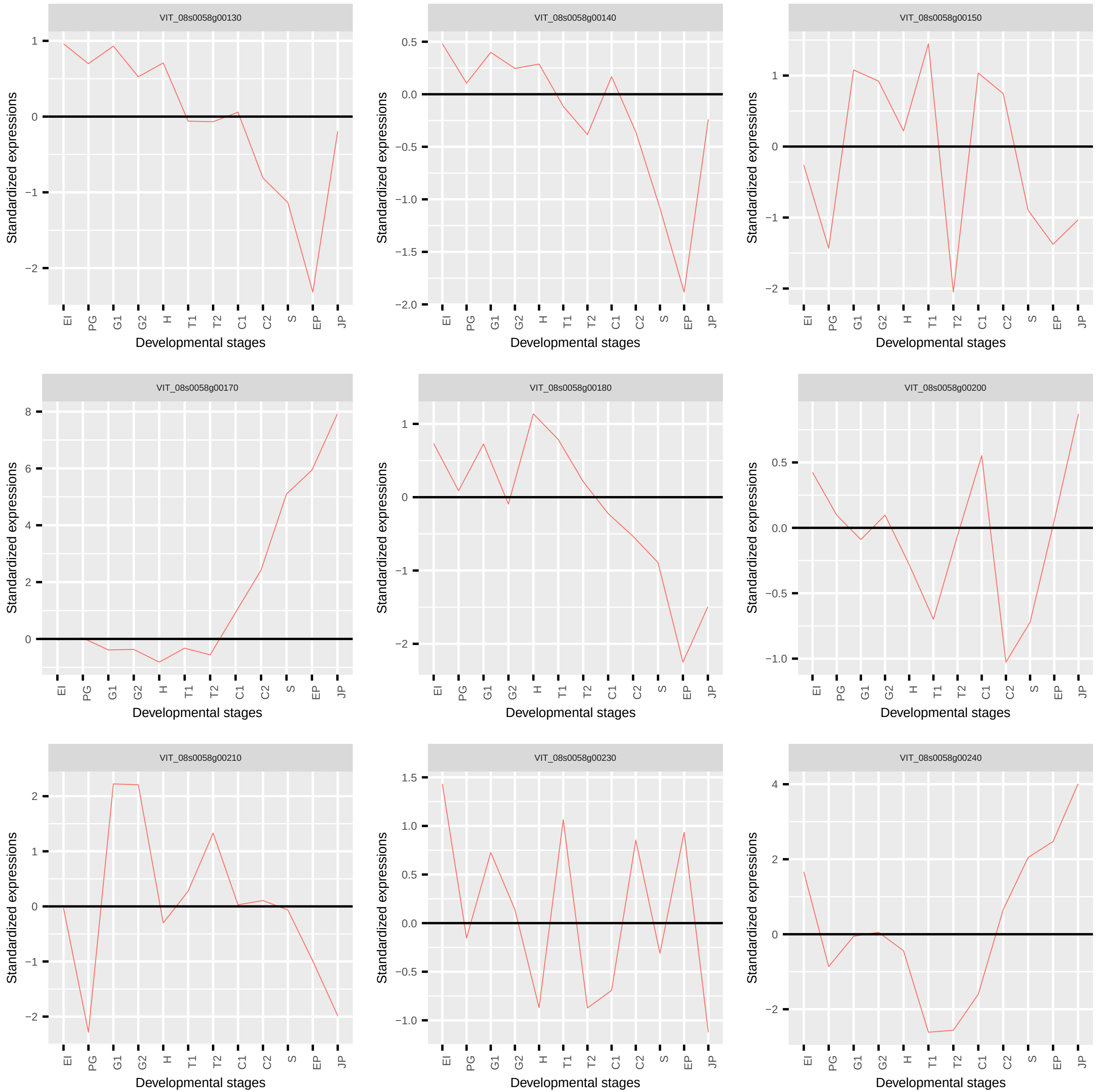
VIT_08s0058g00130
VIT_08s0058g00140
VIT_08s0058g00150
VIT_08s0058g00170
VIT_08s0058g00180
VIT_08s0058g00200
VIT_08s0058g00210
VIT_08s0058g00230
VIT_08s0058g00240
Standardized expressions
Developmental stages
EI
PG
G1
G2
H
T1
T2
C1
C2
S
EP
JP

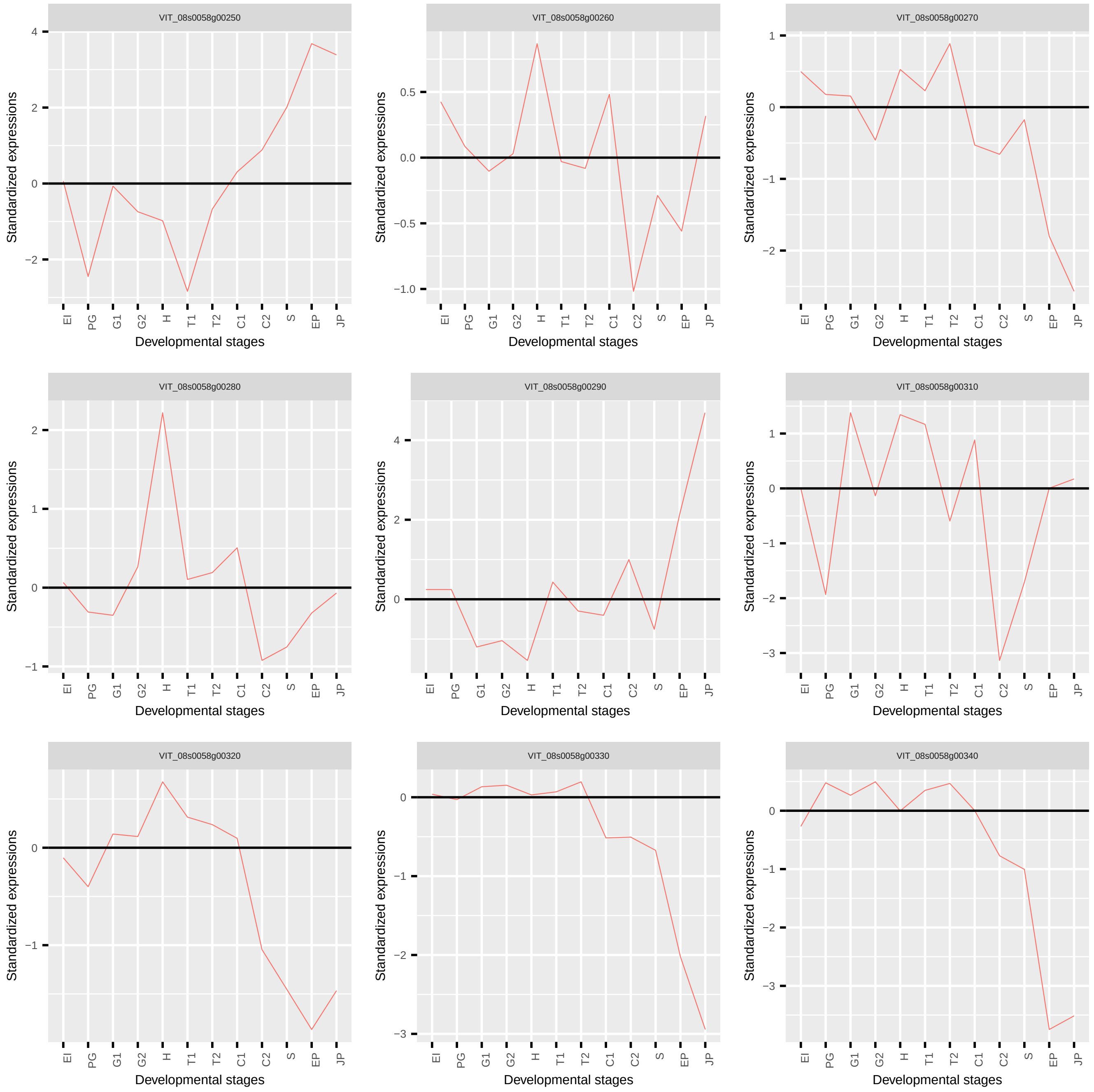
VIT_08s0058g00250
VIT_08s0058g00260
VIT_08s0058g00270
VIT_08s0058g00280
VIT_08s0058g00290
VIT_08s0058g00310
VIT_08s0058g00320
VIT_08s0058g00330
VIT_08s0058g00340
Standardized expressions
Developmental stages
EI PG G1 G2 H T1 T2 C1 C2 S EP JP

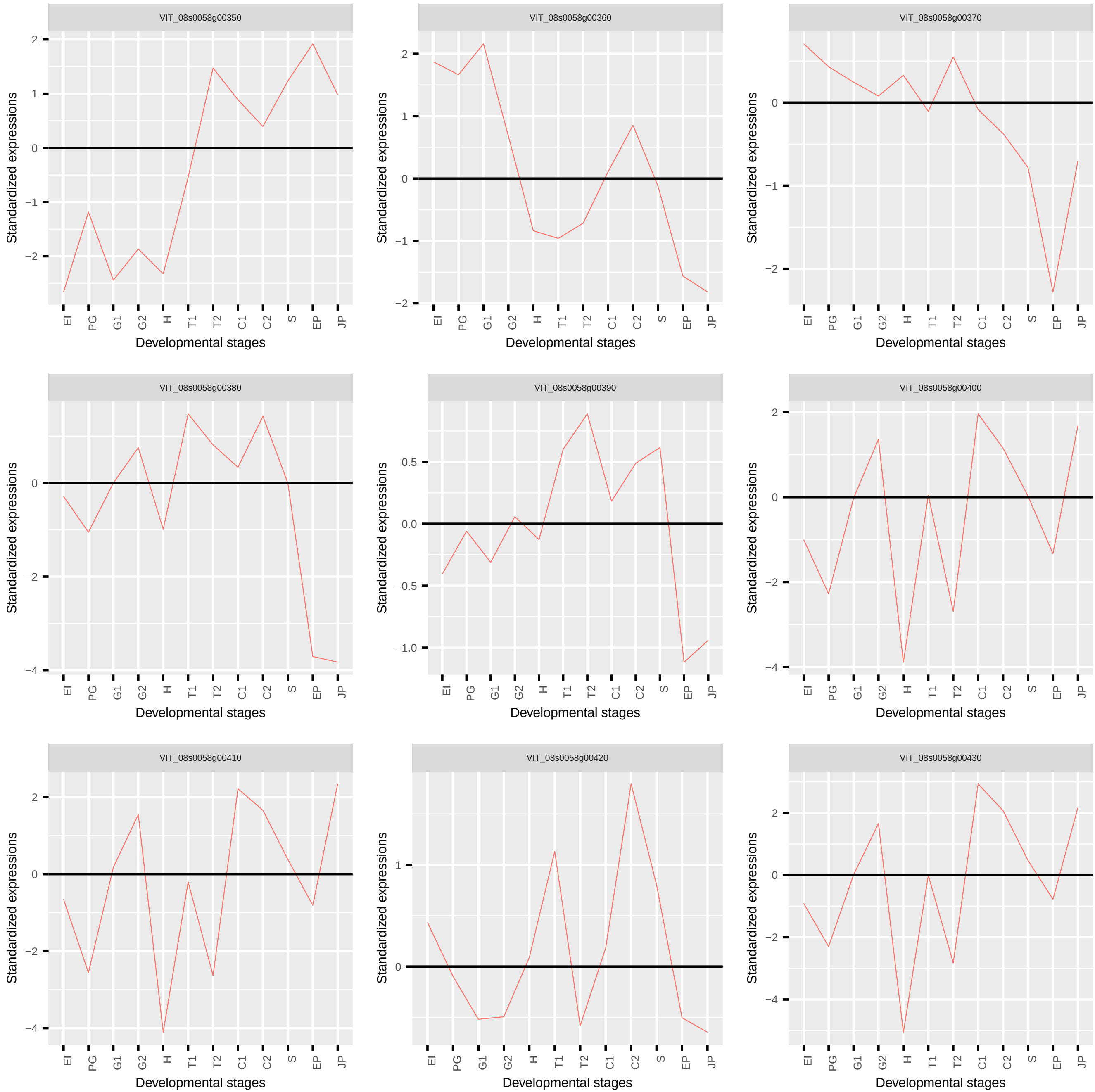
VIT_08s0058g00350
VIT_08s0058g00360
VIT_08s0058g00370
VIT_08s0058g00380
VIT_08s0058g00390
VIT_08s0058g00400
VIT_08s0058g00410
VIT_08s0058g00420
VIT_08s0058g00430
Standardized expressions
Developmental stages
EI PG G1 G2 H T1 T2 C1 C2 S EP JP

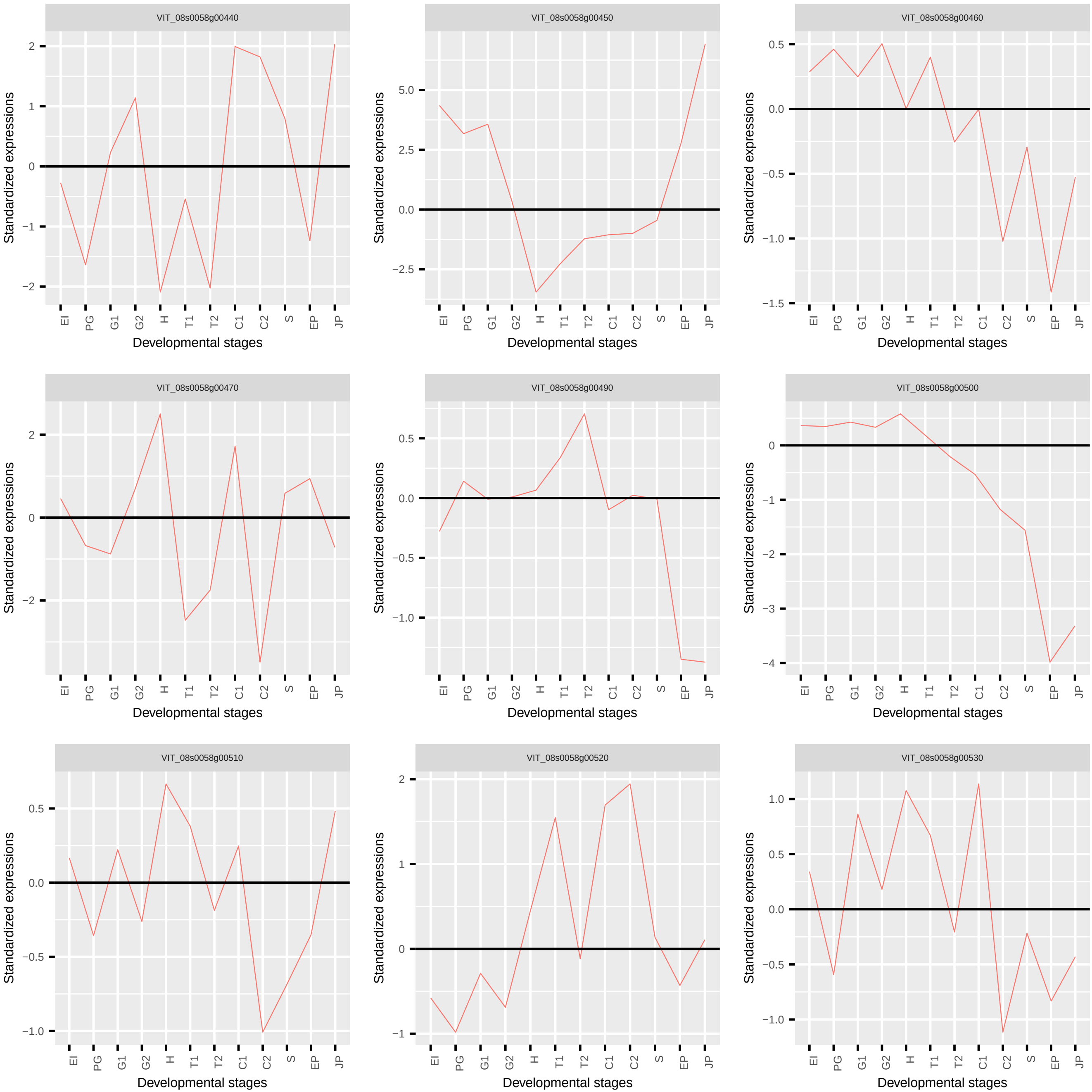
VIT_08s0058g00440
VIT_08s0058g00450
VIT_08s0058g00460
VIT_08s0058g00470
VIT_08s0058g00490
VIT_08s0058g00500
VIT_08s0058g00510
VIT_08s0058g00520
VIT_08s0058g00530
Standardized expressions
Developmental stages
EI PG G1 G2 H T1 T2 C1 C2 S EP JP

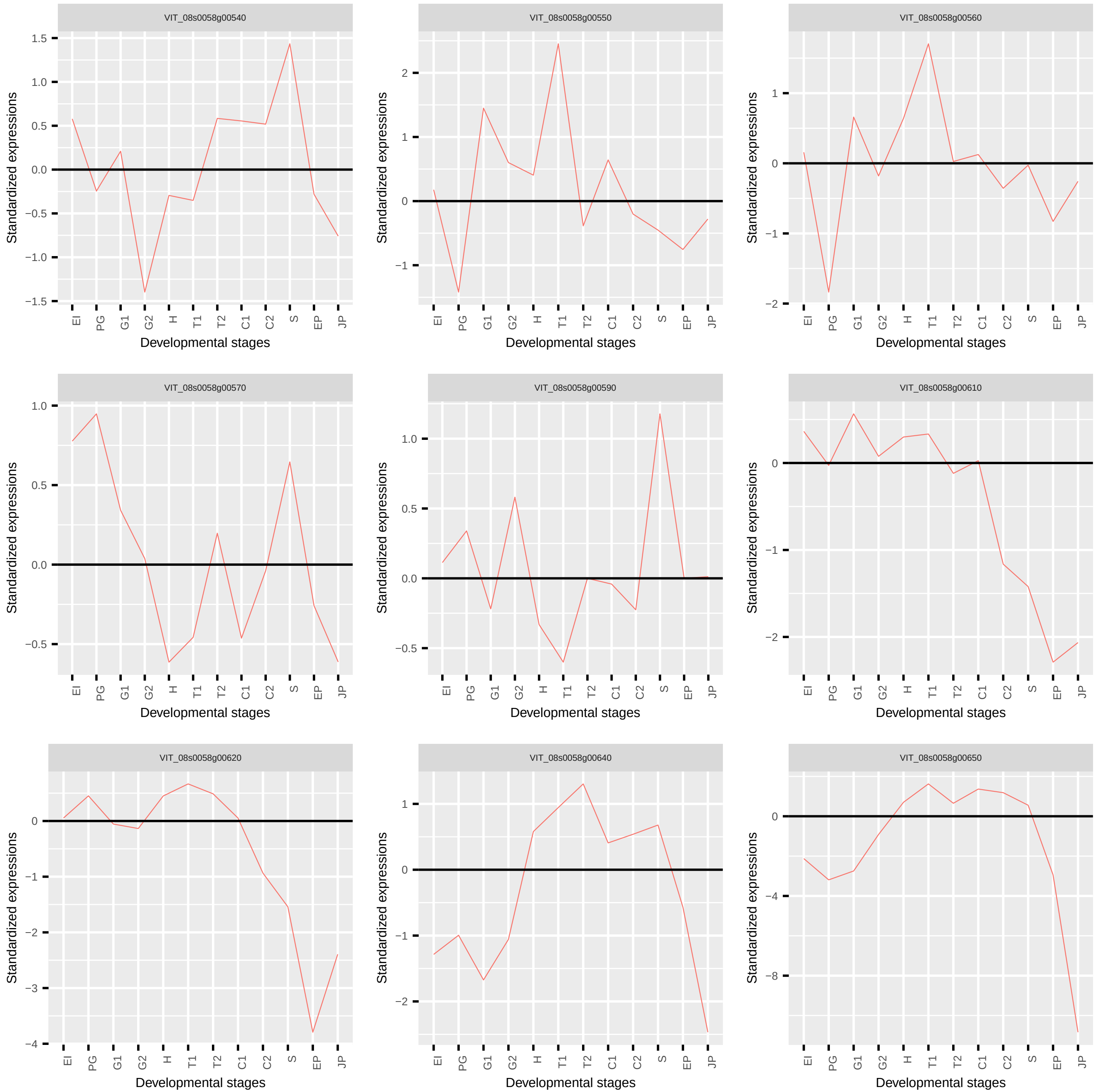
VIT_08s0058g00540
VIT_08s0058g00550
VIT_08s0058g00560
VIT_08s0058g00570
VIT_08s0058g00590
VIT_08s0058g00610
VIT_08s0058g00620
VIT_08s0058g00640
VIT_08s0058g00650
Standardized expressions
Developmental stages
EI PG G1 G2 H T1 T2 C1 C2 S EP JP

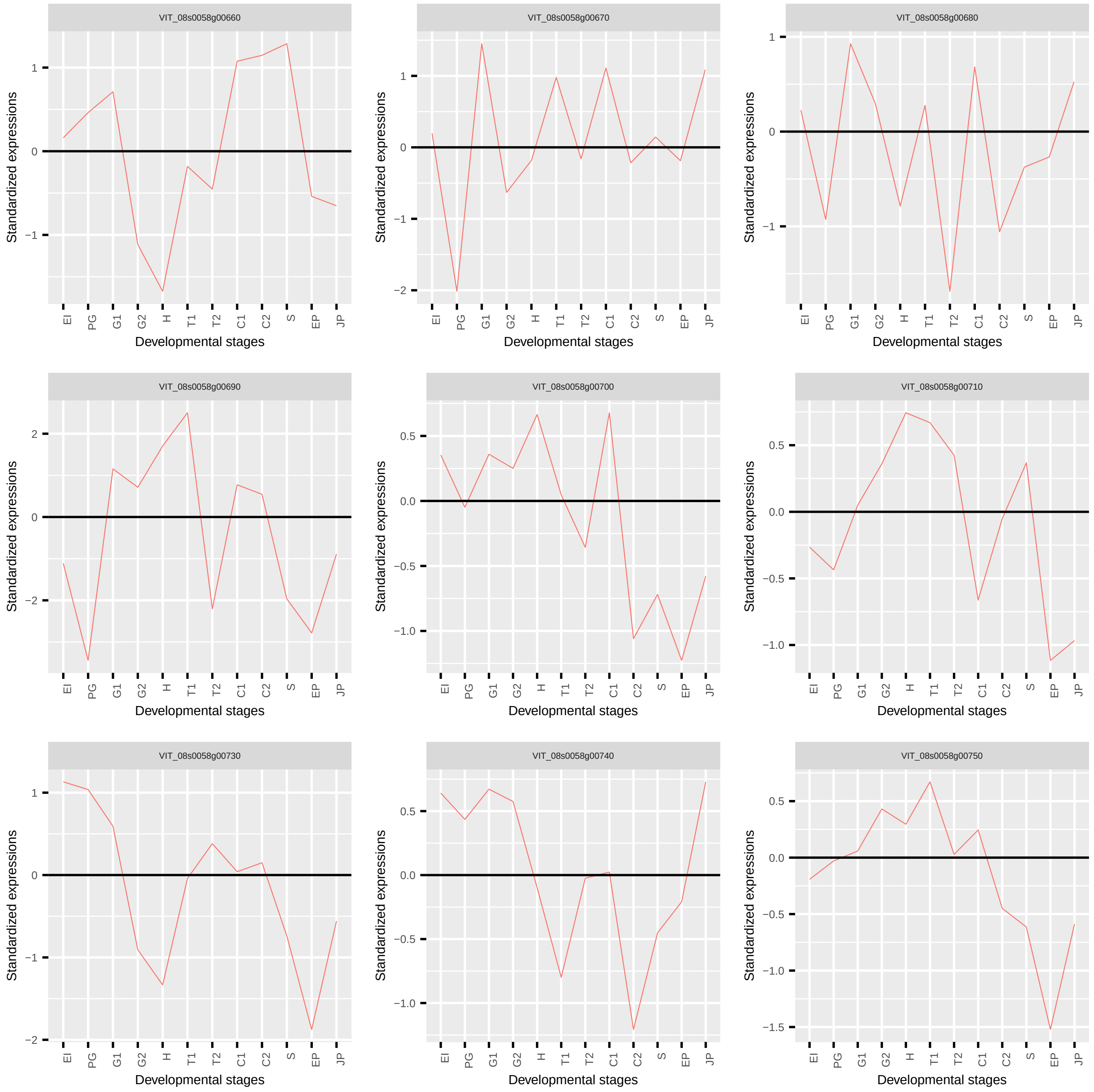
VIT_08s0058g00660
VIT_08s0058g00670
VIT_08s0058g00680
VIT_08s0058g00690
VIT_08s0058g00700
VIT_08s0058g00710
VIT_08s0058g00730
VIT_08s0058g00740
VIT_08s0058g00750
Standardized expressions
Developmental stages
EI
PG
G1
G2
H
T1
T2
C1
C2
S
EP
JP

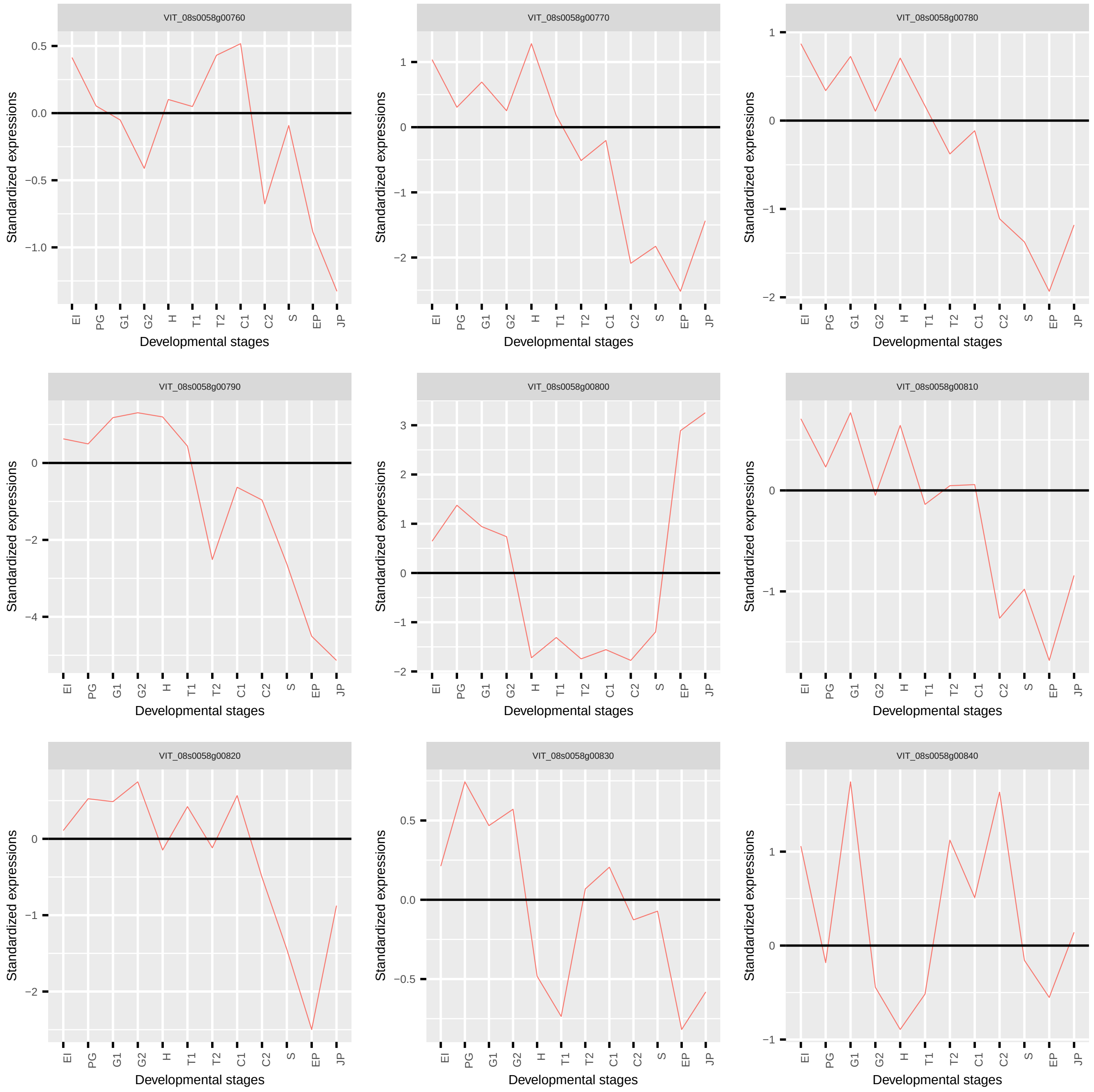
VIT_08s0058g00760
VIT_08s0058g00770
VIT_08s0058g00780
VIT_08s0058g00790
VIT_08s0058g00800
VIT_08s0058g00810
VIT_08s0058g00820
VIT_08s0058g00830
VIT_08s0058g00840
Standardized expressions
Developmental stages
EI PG G1 G2 H T1 T2 C1 C2 S EP JP

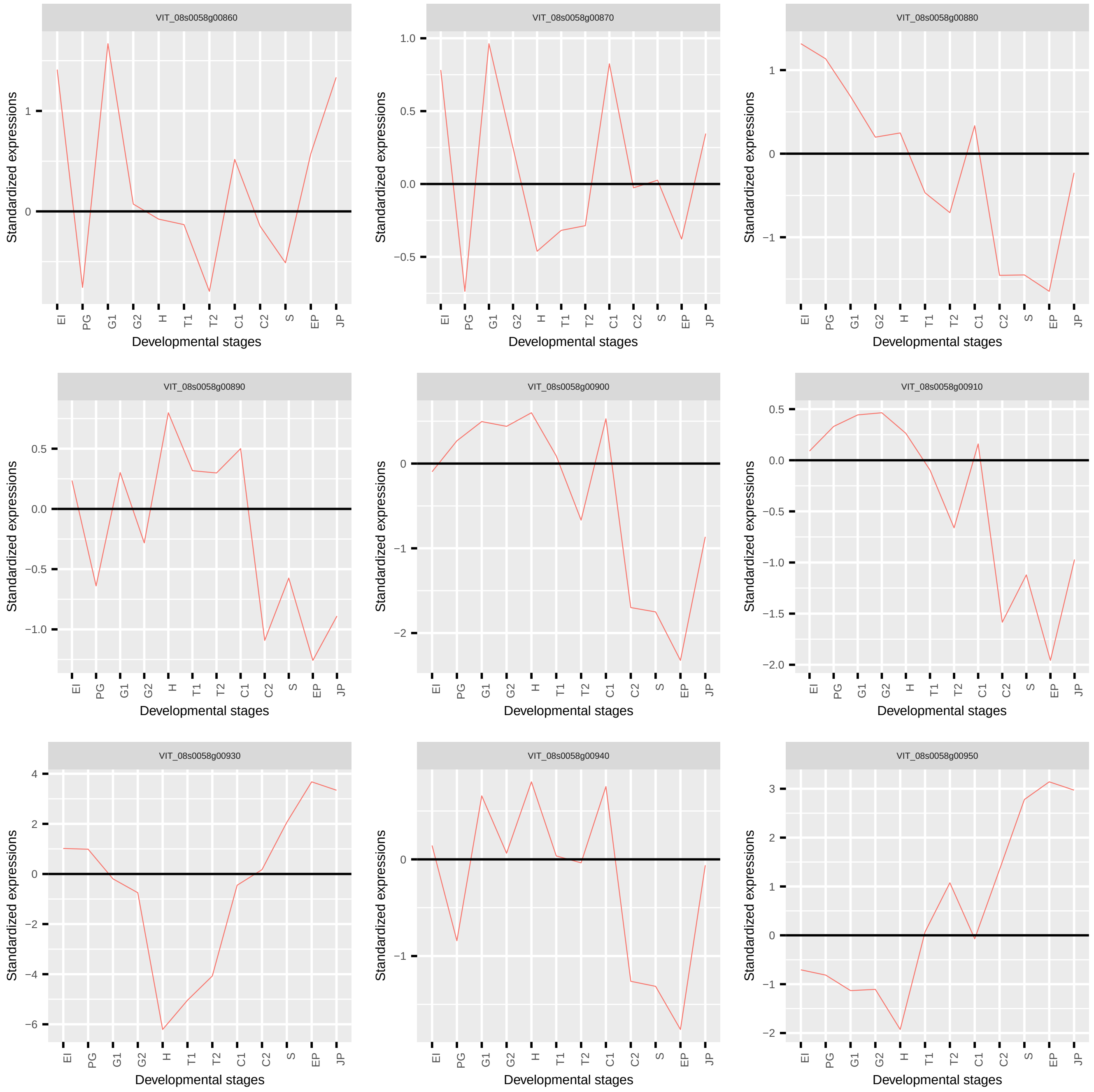
VIT_08s0058g00860
VIT_08s0058g00870
VIT_08s0058g00880
VIT_08s0058g00890
VIT_08s0058g00900
VIT_08s0058g00910
VIT_08s0058g00930
VIT_08s0058g00940
VIT_08s0058g00950
Standardized expressions
Developmental stages
EI PG G1 G2 H T1 T2 C1 C2 S EP JP

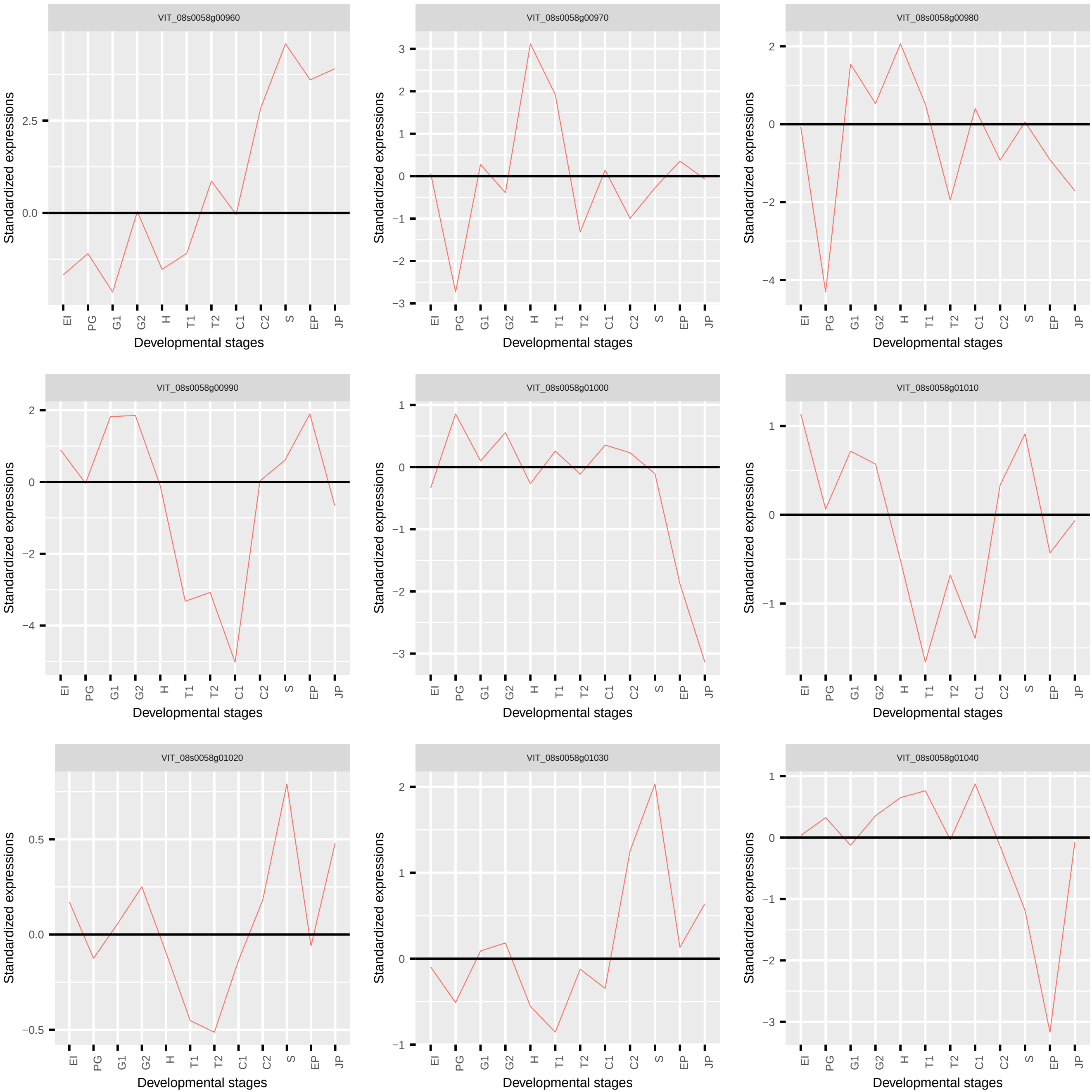
VIT_08s0058g00960
VIT_08s0058g00970
VIT_08s0058g00980
VIT_08s0058g00990
VIT_08s0058g01000
VIT_08s0058g01010
VIT_08s0058g01020
VIT_08s0058g01030
VIT_08s0058g01040
Standardized expressions
Developmental stages
EI PG G1 G2 H T1 T2 C1 C2 S EP JP

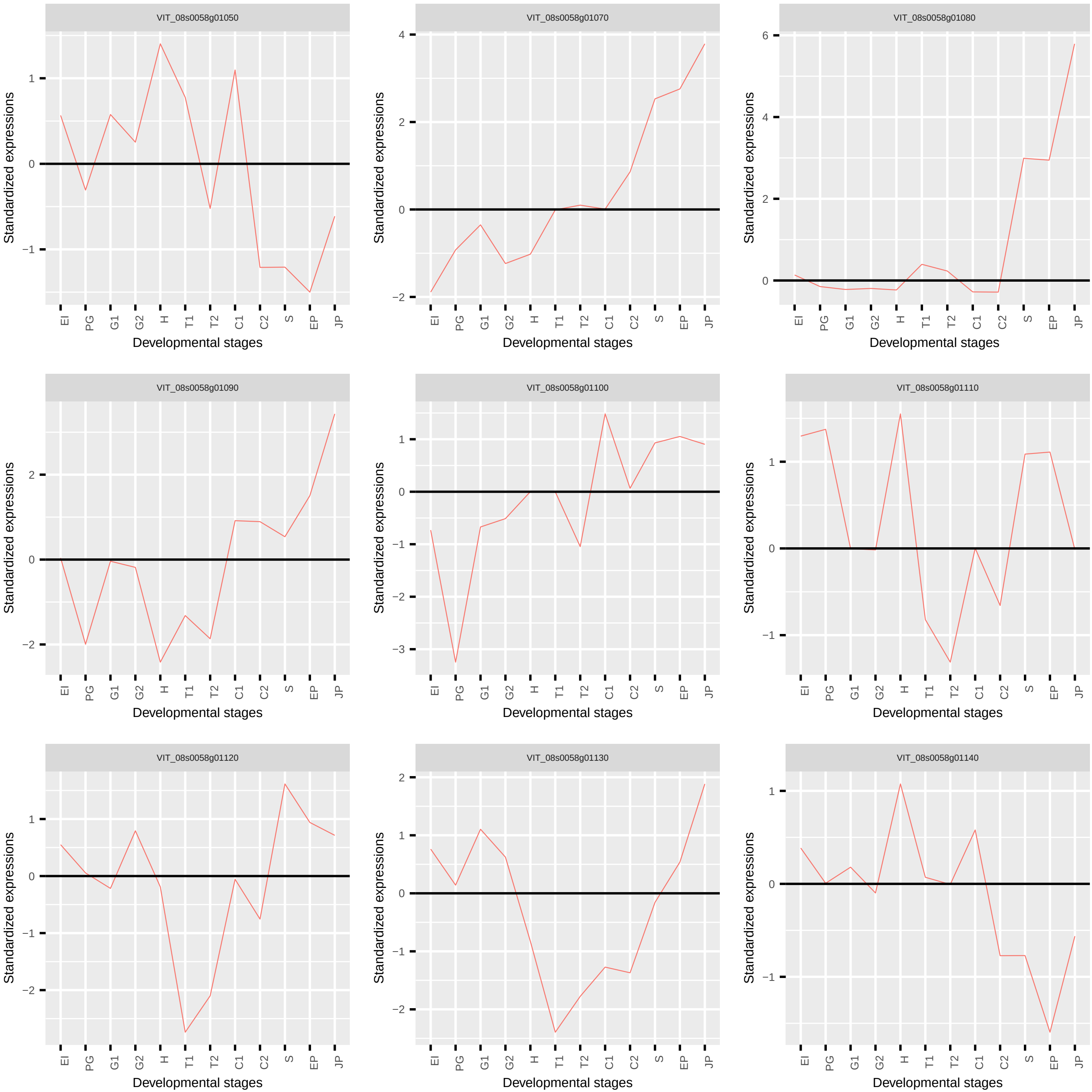
VIT_08s0058g01050
VIT_08s0058g01070
VIT_08s0058g01080
VIT_08s0058g01090
VIT_08s0058g01100
VIT_08s0058g01110
VIT_08s0058g01120
VIT_08s0058g01130
VIT_08s0058g01140
Standardized expressions
Developmental stages
EI PG G1 G2 H T1 T2 C1 C2 S EP JP

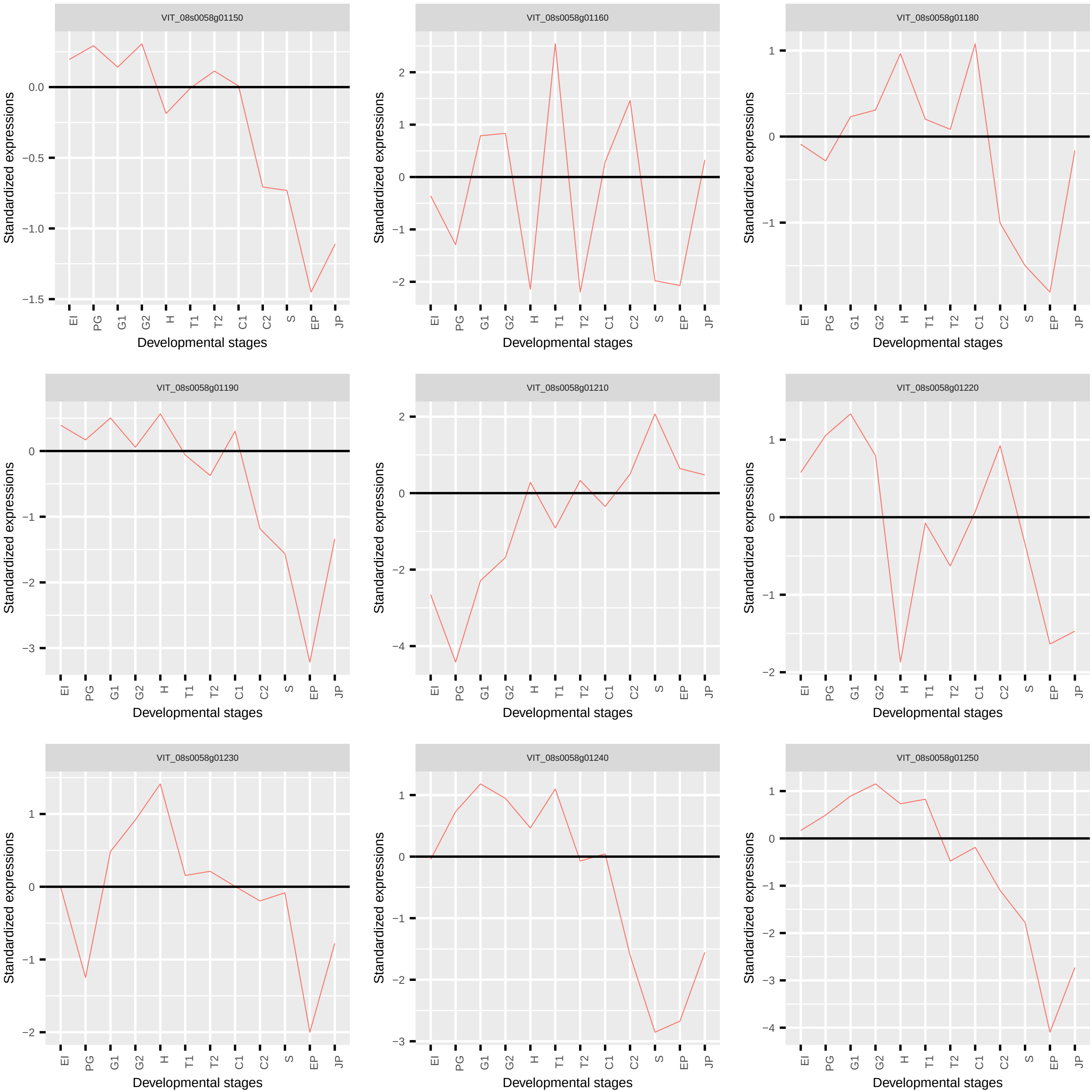
VIT_08s0058g01150
VIT_08s0058g01160
VIT_08s0058g01180
VIT_08s0058g01190
VIT_08s0058g01210
VIT_08s0058g01220
VIT_08s0058g01230
VIT_08s0058g01240
VIT_08s0058g01250
Standardized expressions
Developmental stages
EI PG G1 G2 H T1 T2 C1 C2 S EP JP

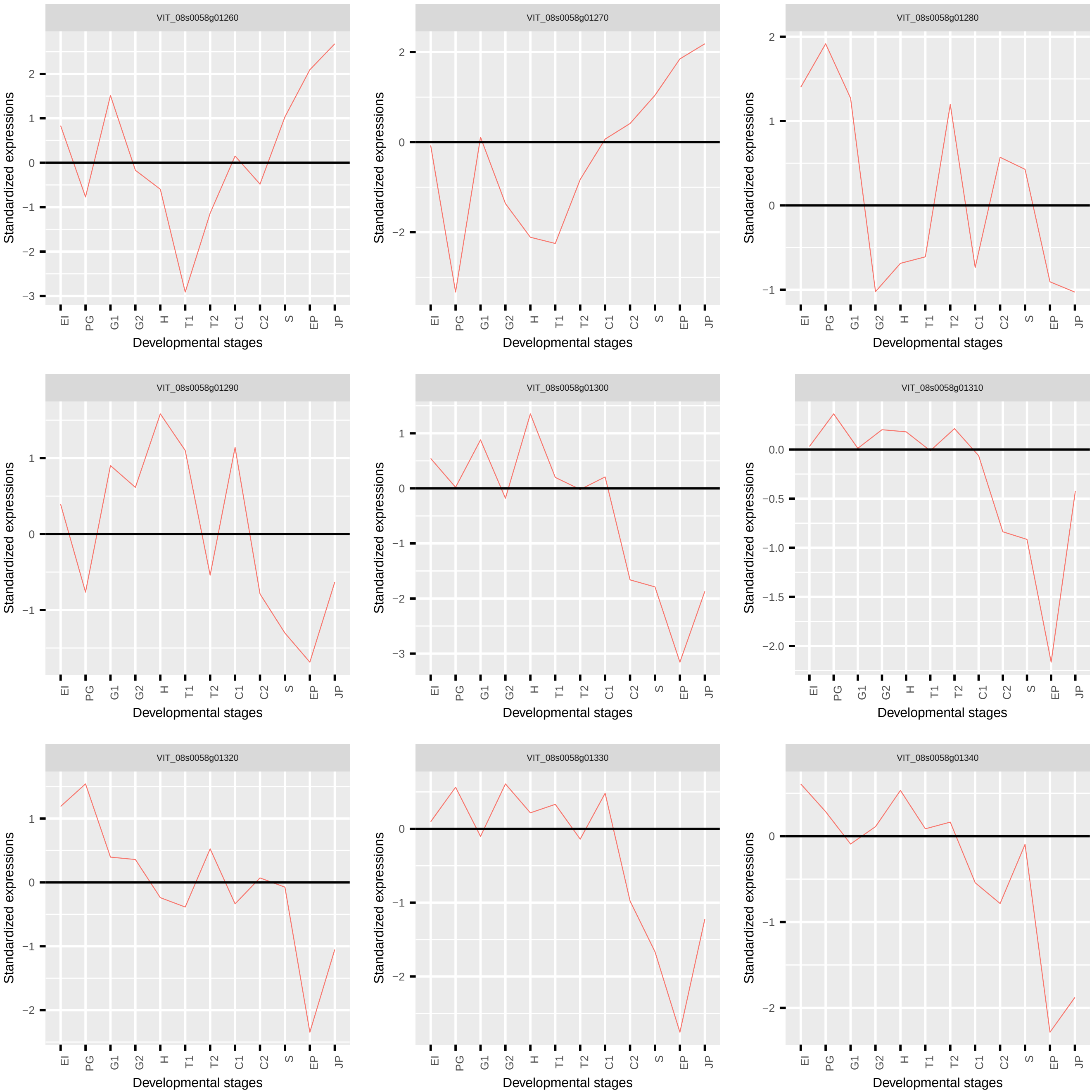
VIT_08s0058g01260
VIT_08s0058g01270
VIT_08s0058g01280
VIT_08s0058g01290
VIT_08s0058g01300
VIT_08s0058g01310
VIT_08s0058g01320
VIT_08s0058g01330
VIT_08s0058g01340
Standardized expressions
Developmental stages
EI
PG
G1
G2
H
T1
T2
C1
C2
S
EP
JP

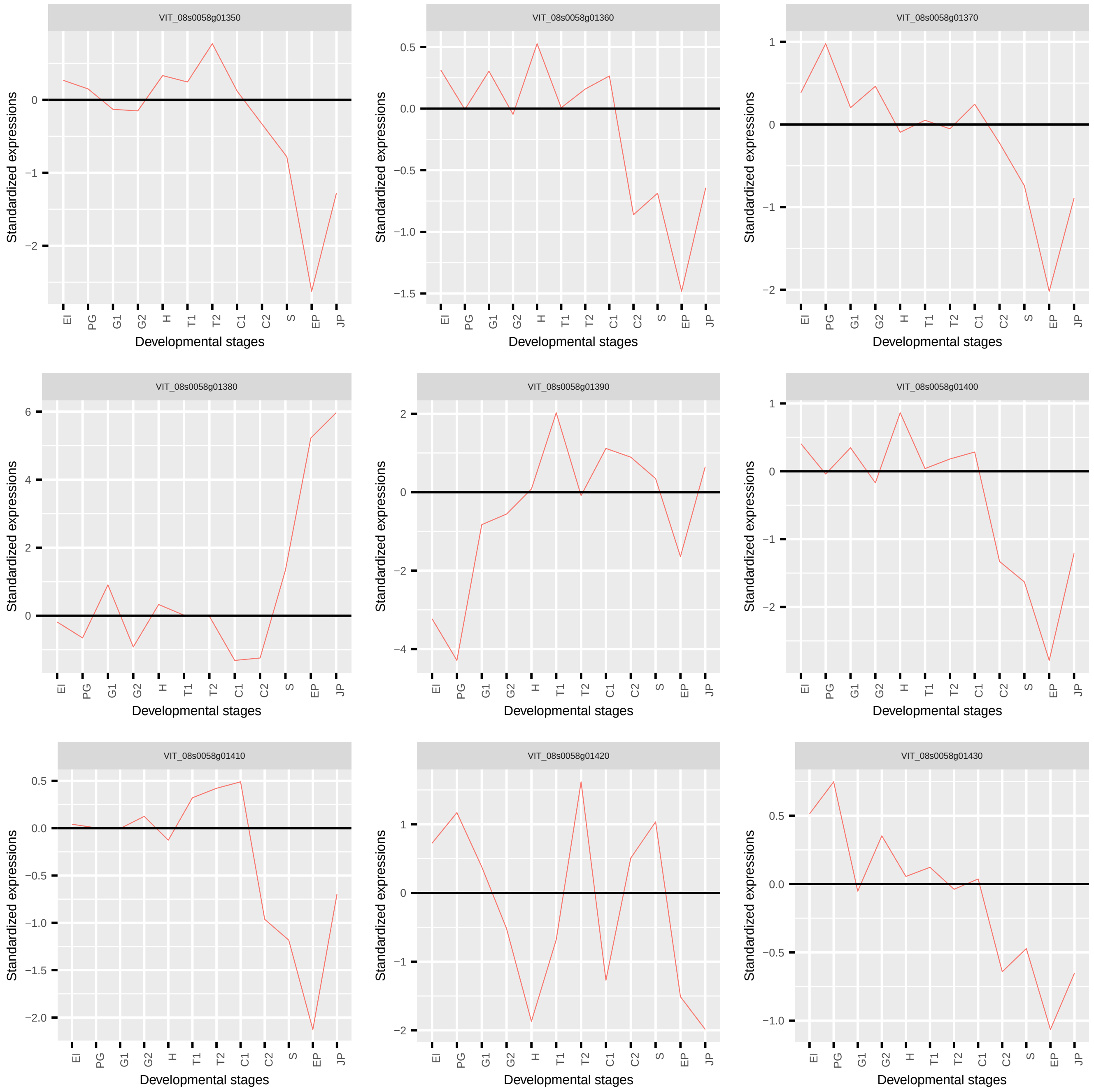
VIT_08s0058g01350
VIT_08s0058g01360
VIT_08s0058g01370
VIT_08s0058g01380
VIT_08s0058g01390
VIT_08s0058g01400
VIT_08s0058g01410
VIT_08s0058g01420
VIT_08s0058g01430
Standardized expressions
Developmental stages
EI PG G1 G2 H T1 T2 C1 C2 S EP JP

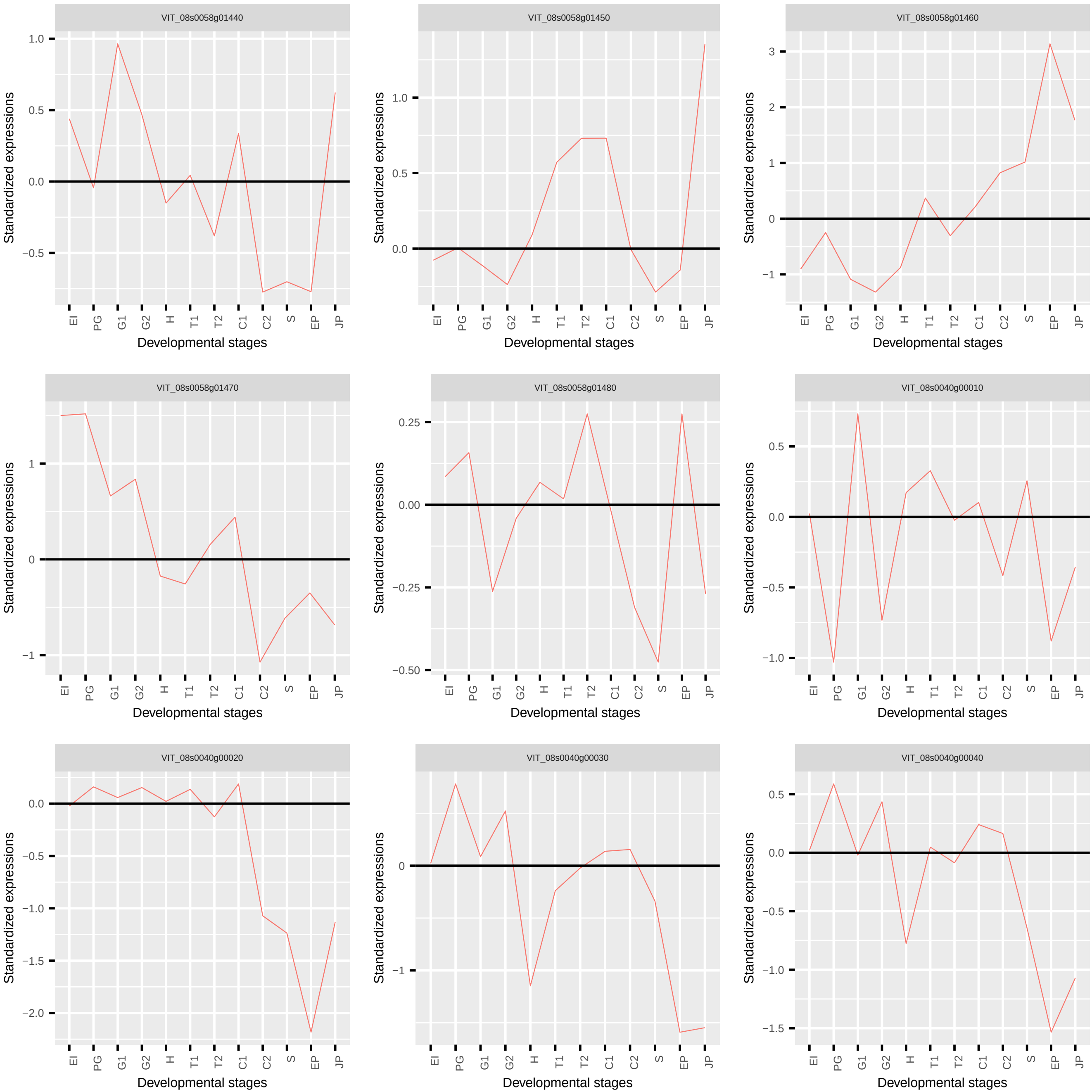
VIT_08s0058g01440
VIT_08s0058g01450
VIT_08s0058g01460
VIT_08s0058g01470
VIT_08s0058g01480
VIT_08s0040g00010
VIT_08s0040g00020
VIT_08s0040g00030
VIT_08s0040g00040
Standardized expressions
Developmental stages
EI PG G1 G2 H T1 T2 C1 C2 S EP JP

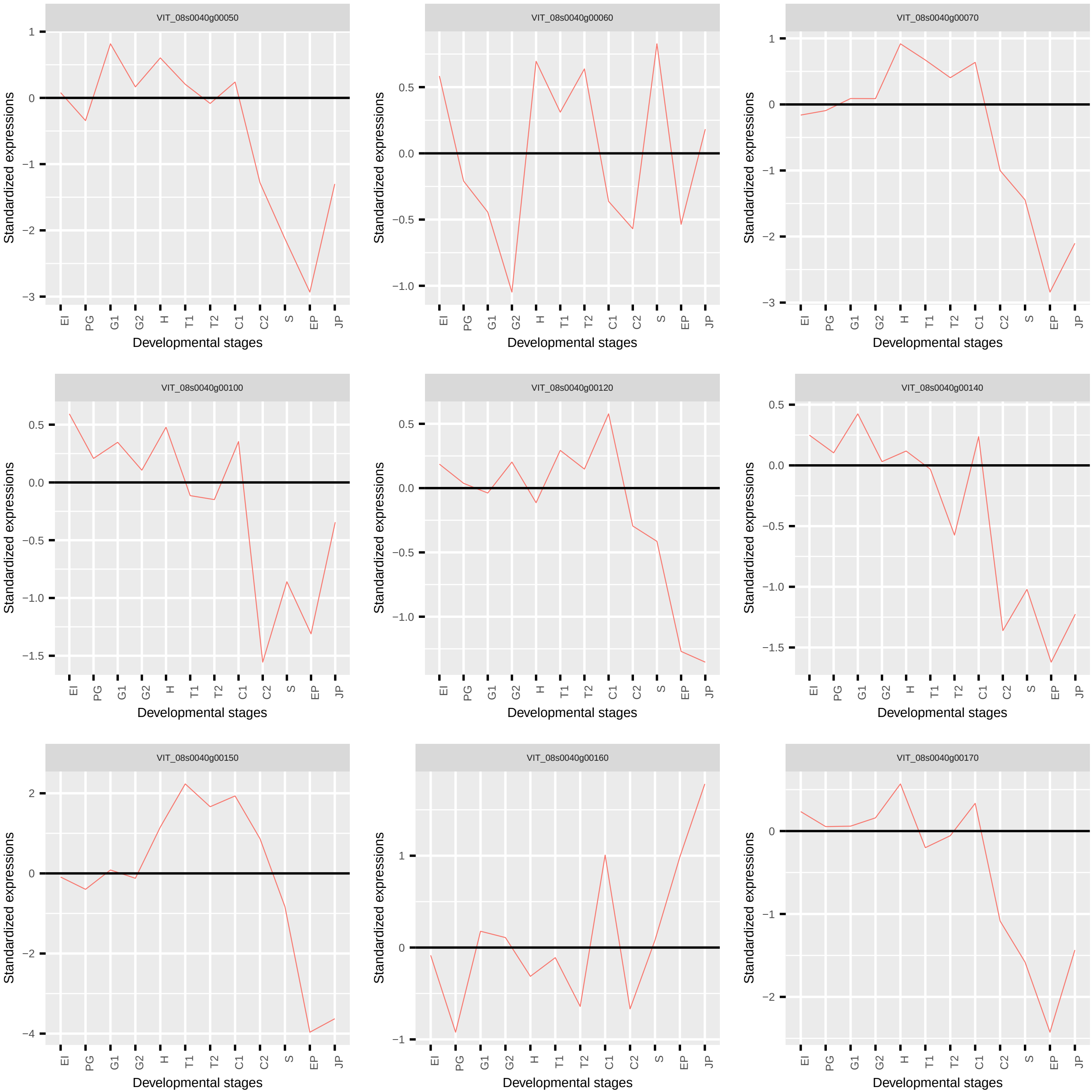

VIT_08s0040g00050
1 0 −1 −2 −3
Standardized expressions
EI PG G1 G2 H T1 T2 C1 C2 S EP JP
Developmental stages
VIT_08s0040g00060
0.5 0.0 −0.5 −1.0
Standardized expressions
EI PG G1 G2 H T1 T2 C1 C2 S EP JP
Developmental stages
VIT_08s0040g00070
1 0 −1 −2 −3
Standardized expressions
EI PG G1 G2 H T1 T2 C1 C2 S EP JP
Developmental stages
VIT_08s0040g00100
0.5 0.0 −0.5 −1.0 −1.5
Standardized expressions
EI PG G1 G2 H T1 T2 C1 C2 S EP JP
Developmental stages
VIT_08s0040g00120
0.5 0.0 −0.5 −1.0
Standardized expressions
EI PG G1 G2 H T1 T2 C1 C2 S EP JP
Developmental stages
VIT_08s0040g00140
0.5 0.0 −0.5 −1.0 −1.5
Standardized expressions
EI PG G1 G2 H T1 T2 C1 C2 S EP JP
Developmental stages
VIT_08s0040g00150
2 0 −2 −4
Standardized expressions
EI PG G1 G2 H T1 T2 C1 C2 S EP JP
Developmental stages
VIT_08s0040g00160
1 0 −1
Standardized expressions
EI PG G1 G2 H T1 T2 C1 C2 S EP JP
Developmental stages
VIT_08s0040g00170
0 −1 −2
Standardized expressions
EI PG G1 G2 H T1 T2 C1 C2 S EP JP
Developmental stages

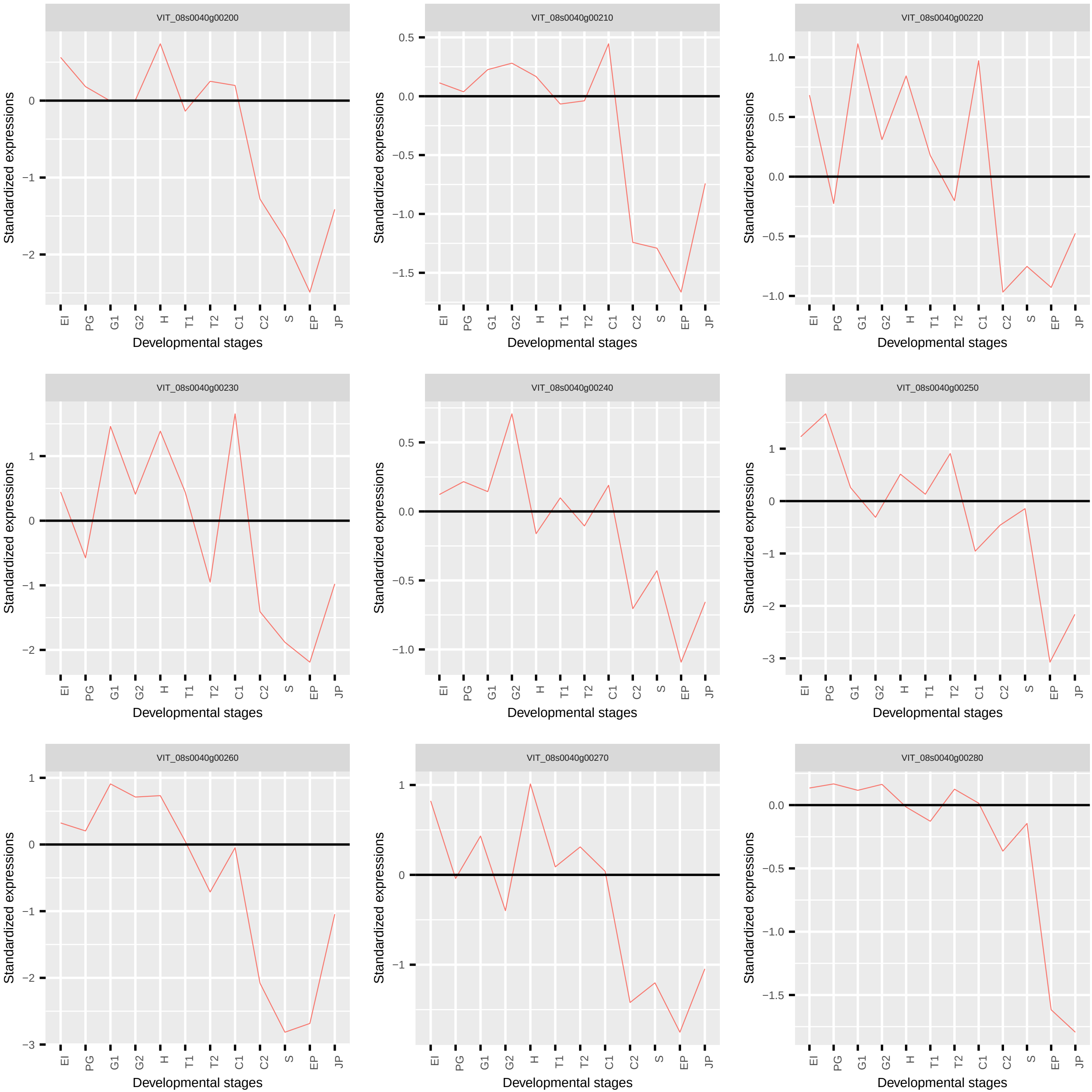
VIT_08s0040g00200
VIT_08s0040g00210
VIT_08s0040g00220
VIT_08s0040g00230
VIT_08s0040g00240
VIT_08s0040g00250
VIT_08s0040g00260
VIT_08s0040g00270
VIT_08s0040g00280
Standardized expressions
Developmental stages
EI PG G1 G2 H T1 T2 C1 C2 S EP JP
0 −1 −2
0.5 0.0 −0.5 −1.0 −1.5
1.0 0.5 0.0 −0.5 −1.0
1 0 −1 −2
0.5 0.0 −0.5 −1.0
1 0 −1 −2 −3
1 0 −1 −2 −3
1 0 −1
0.0 −0.5 −1.0 −1.5

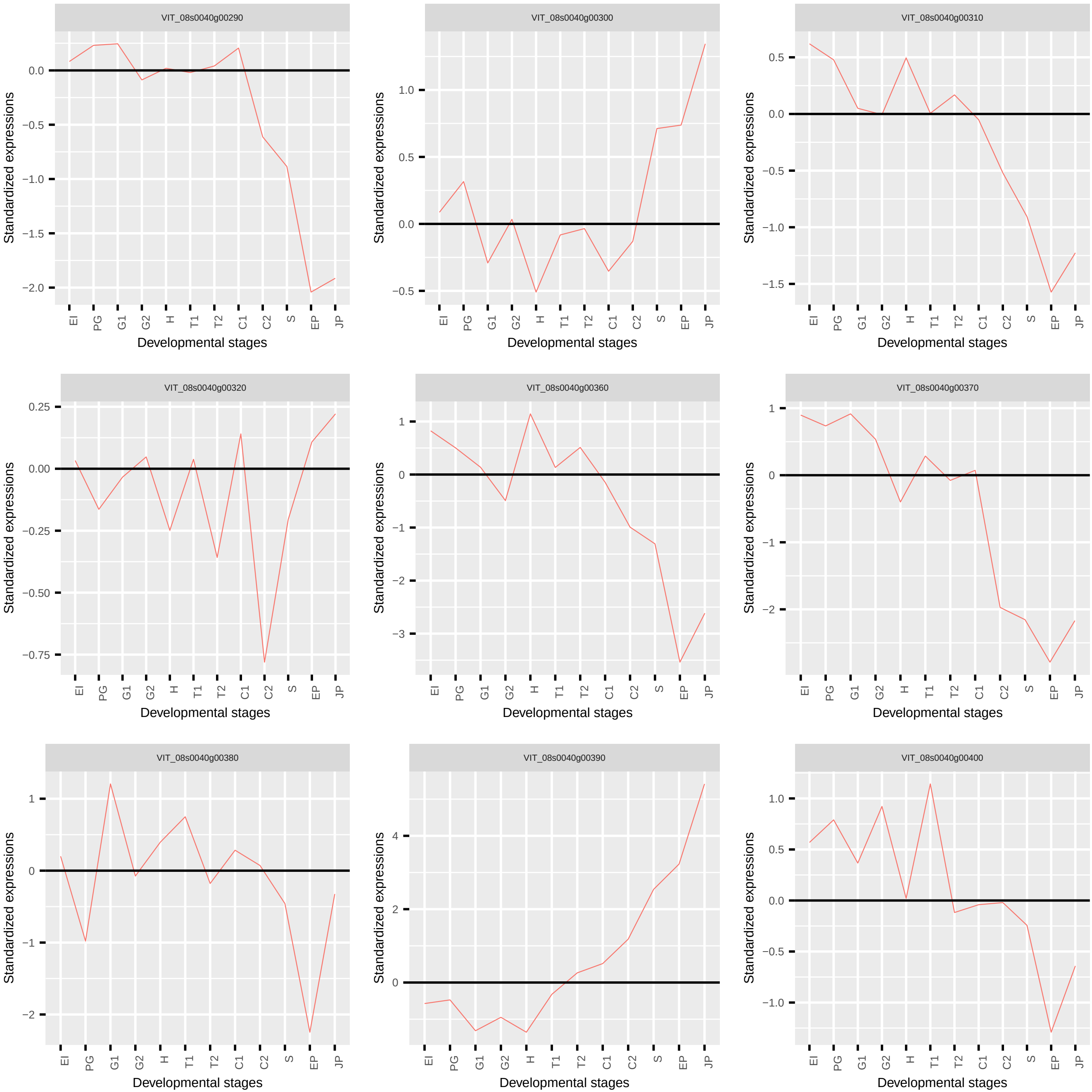
VIT_08s0040g00290
VIT_08s0040g00300
VIT_08s0040g00310
VIT_08s0040g00320
VIT_08s0040g00360
VIT_08s0040g00370
VIT_08s0040g00380
VIT_08s0040g00390
VIT_08s0040g00400
Standardized expressions
Developmental stages
EI PG G1 G2 H T1 T2 C1 C2 S EP JP

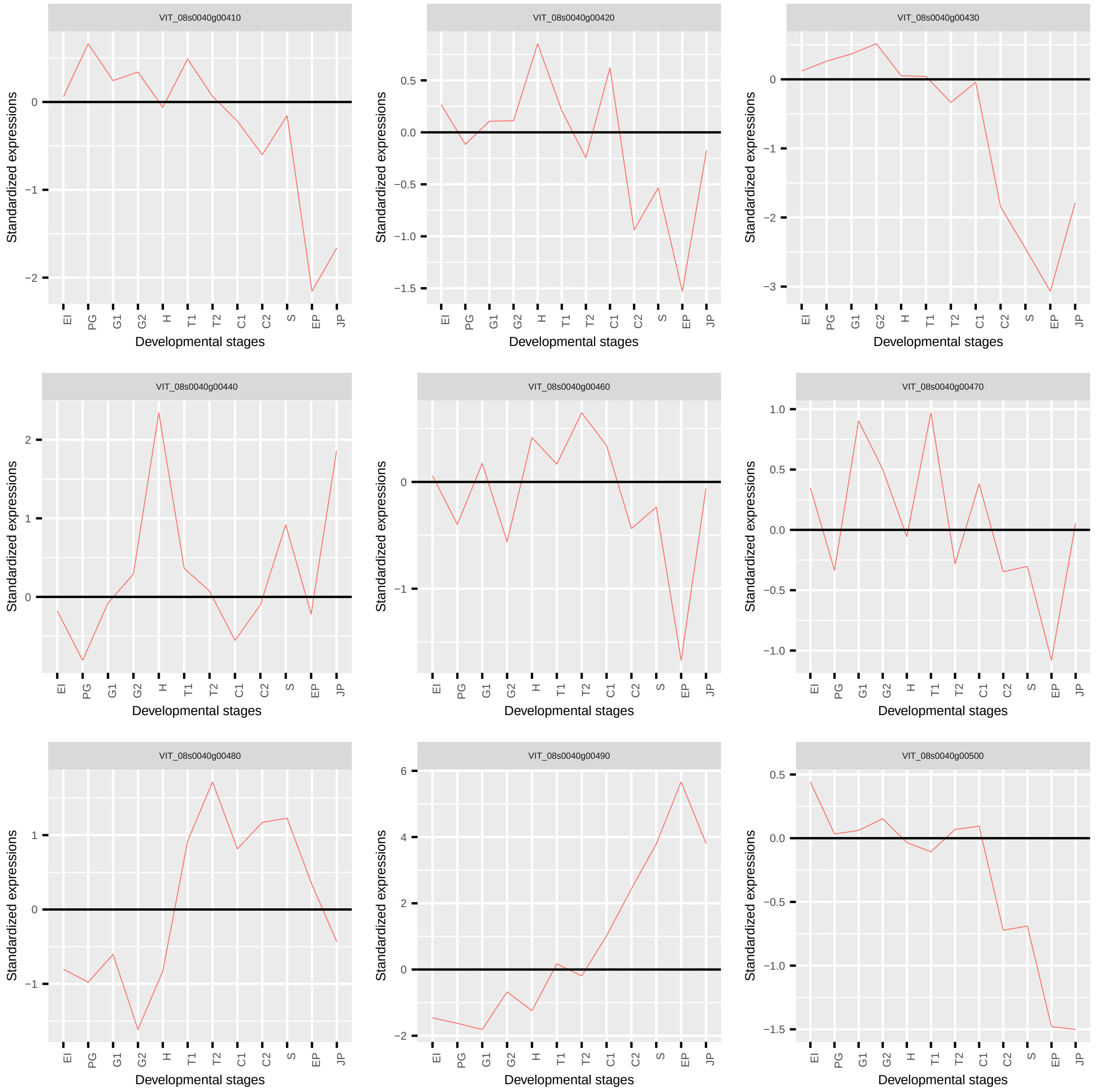

VIT_08s0040g00410
VIT_08s0040g00420
VIT_08s0040g00430
VIT_08s0040g00440
VIT_08s0040g00460
VIT_08s0040g00470
VIT_08s0040g00480
VIT_08s0040g00490
VIT_08s0040g00500
Standardized expressions
Developmental stages
EI
PG
G1
G2
H
T1
T2
C1
C2
S
EP
JP

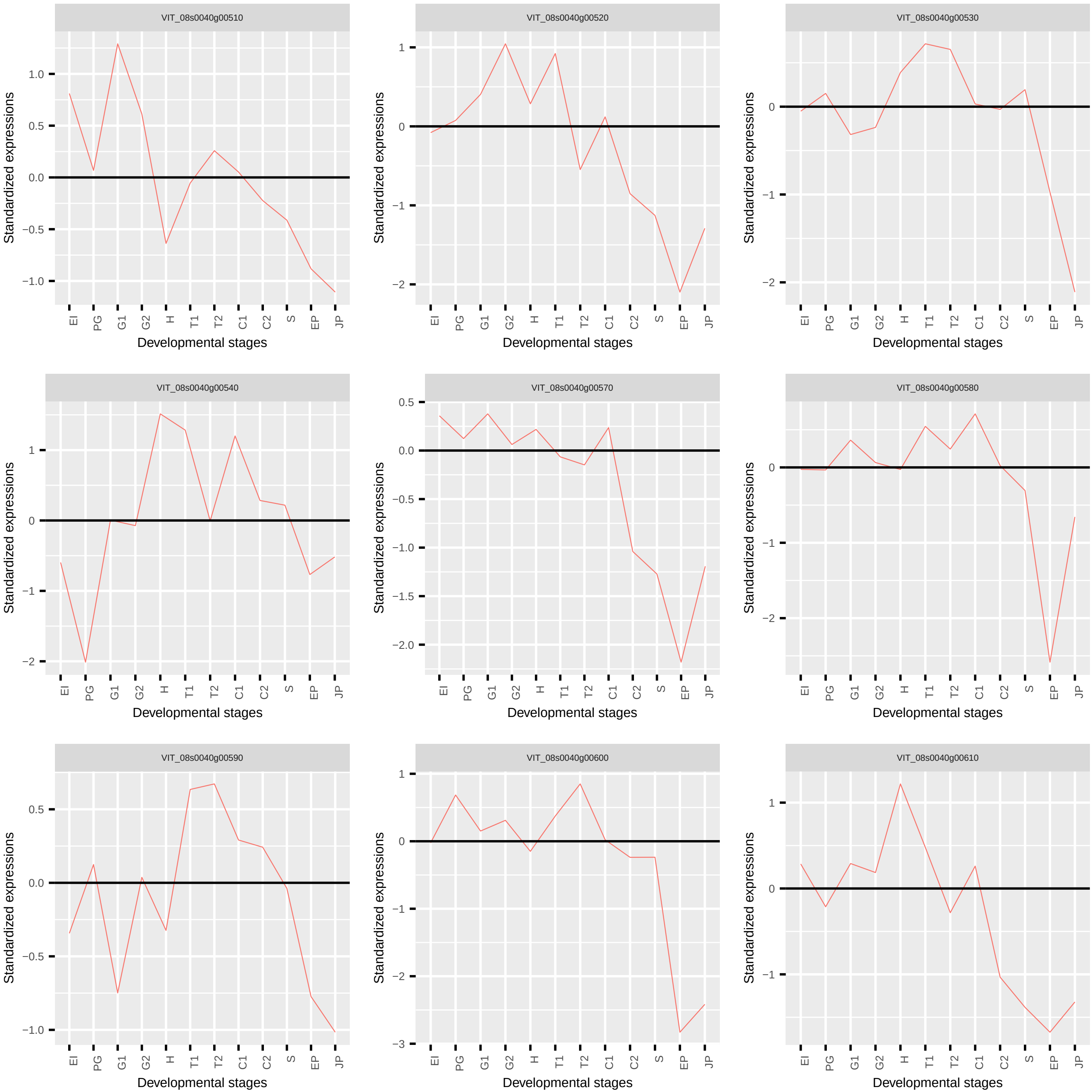

VIT_08s0040g00510
VIT_08s0040g00520
VIT_08s0040g00530
VIT_08s0040g00540
VIT_08s0040g00570
VIT_08s0040g00580
VIT_08s0040g00590
VIT_08s0040g00600
VIT_08s0040g00610
Standardized expressions
Developmental stages
EI PG G1 G2 H T1 T2 C1 C2 S EP JP

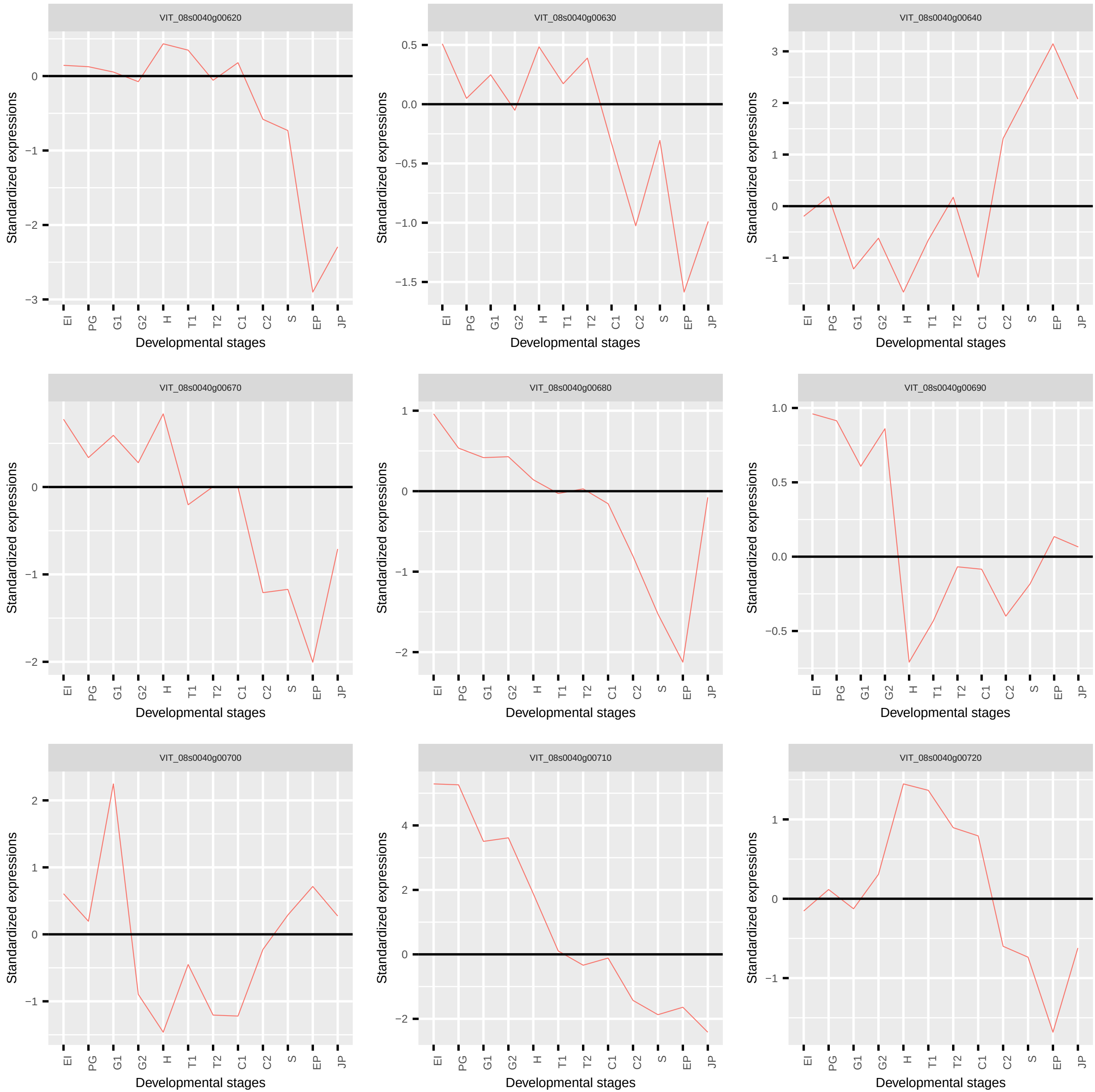
VIT_08s0040g00620
VIT_08s0040g00630
VIT_08s0040g00640
VIT_08s0040g00670
VIT_08s0040g00680
VIT_08s0040g00690
VIT_08s0040g00700
VIT_08s0040g00710
VIT_08s0040g00720
Standardized expressions
Developmental stages
EI PG G1 G2 H T1 T2 C1 C2 S EP JP

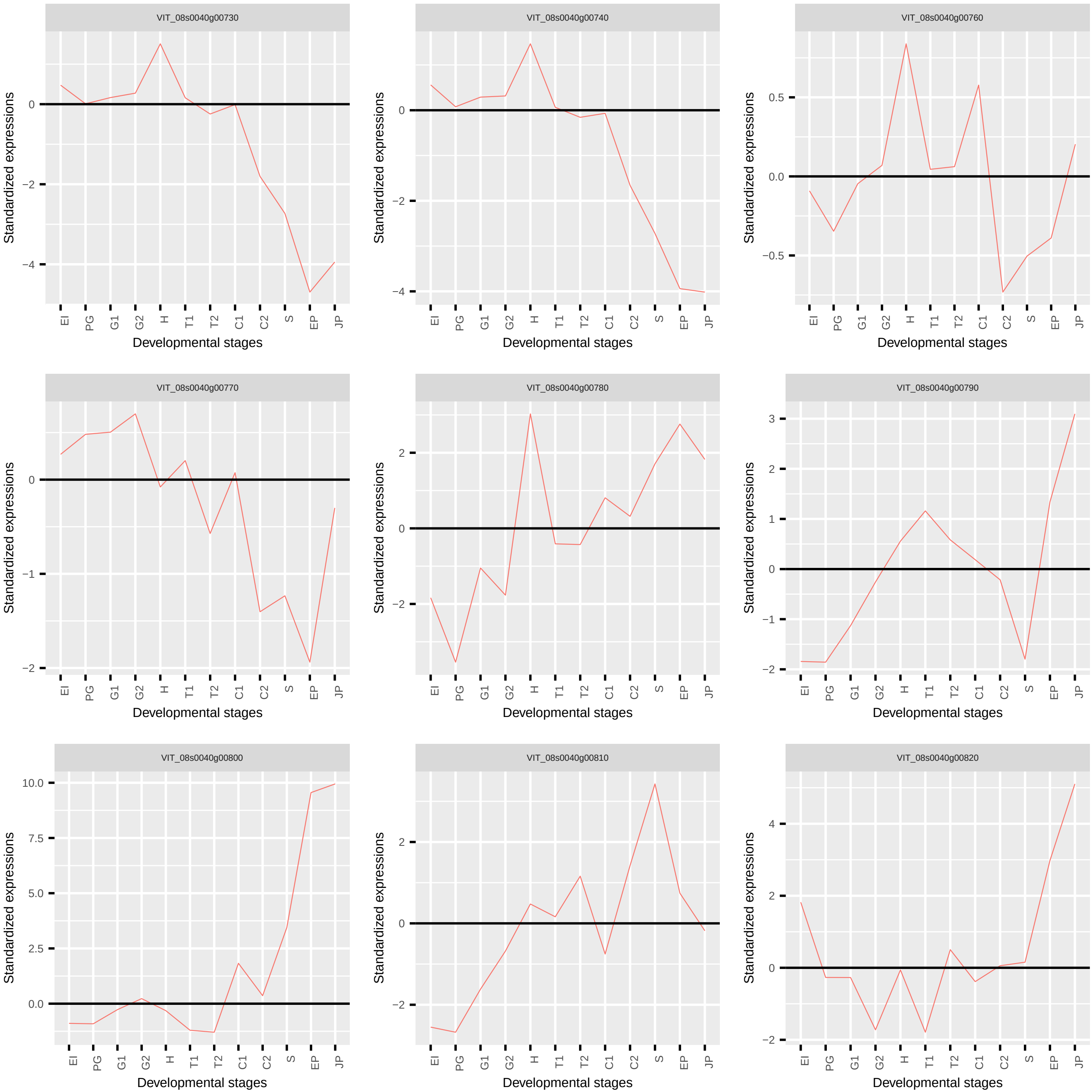
VIT_08s0040g00730
VIT_08s0040g00740
VIT_08s0040g00760
VIT_08s0040g00770
VIT_08s0040g00780
VIT_08s0040g00790
VIT_08s0040g00800
VIT_08s0040g00810
VIT_08s0040g00820
Standardized expressions
Developmental stages
EI PG G1 G2 H T1 T2 C1 C2 S EP JP
0
−2
−4
0.5
0.0
−0.5
−1
2
3
1
10.0
7.5
5.0
2.5
4

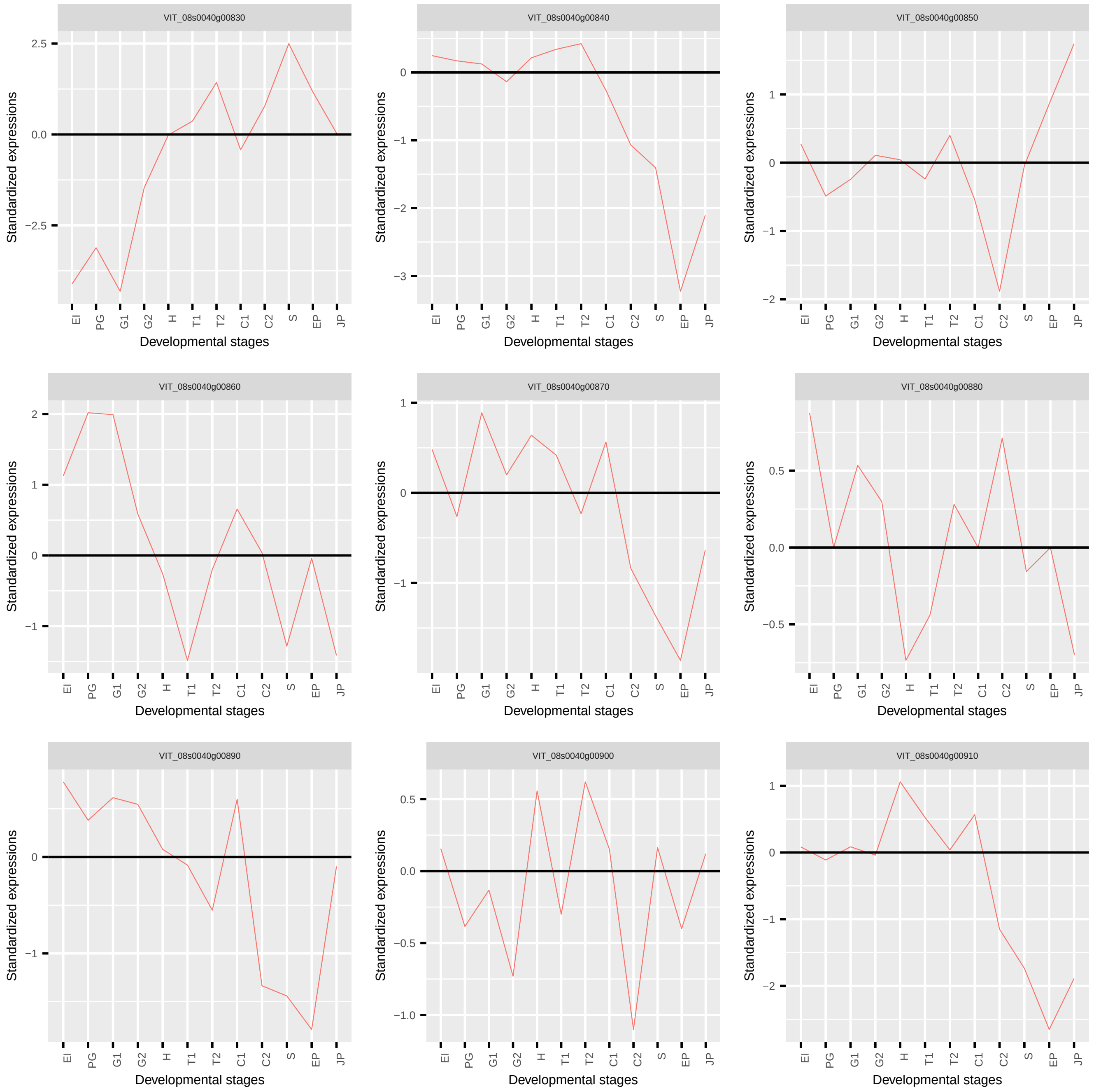
VIT_08s0040g00830
2.5
0.0
−2.5
Standardized expressions
EI PG G1 G2 H T1 T2 C1 C2 S EP JP
Developmental stages
VIT_08s0040g00840
0
−1
−2
−3
Standardized expressions
EI PG G1 G2 H T1 T2 C1 C2 S EP JP
Developmental stages
VIT_08s0040g00850
1
0
−1
−2
Standardized expressions
EI PG G1 G2 H T1 T2 C1 C2 S EP JP
Developmental stages
VIT_08s0040g00860
2
1
0
−1
Standardized expressions
EI PG G1 G2 H T1 T2 C1 C2 S EP JP
Developmental stages
VIT_08s0040g00870
1
0
−1
Standardized expressions
EI PG G1 G2 H T1 T2 C1 C2 S EP JP
Developmental stages
VIT_08s0040g00880
0.5
0.0
−0.5
Standardized expressions
EI PG G1 G2 H T1 T2 C1 C2 S EP JP
Developmental stages
VIT_08s0040g00890
0
−1
Standardized expressions
EI PG G1 G2 H T1 T2 C1 C2 S EP JP
Developmental stages
VIT_08s0040g00900
0.5
0.0
−0.5
−1.0
Standardized expressions
EI PG G1 G2 H T1 T2 C1 C2 S EP JP
Developmental stages
VIT_08s0040g00910
1
0
−1
−2
Standardized expressions
EI PG G1 G2 H T1 T2 C1 C2 S EP JP
Developmental stages

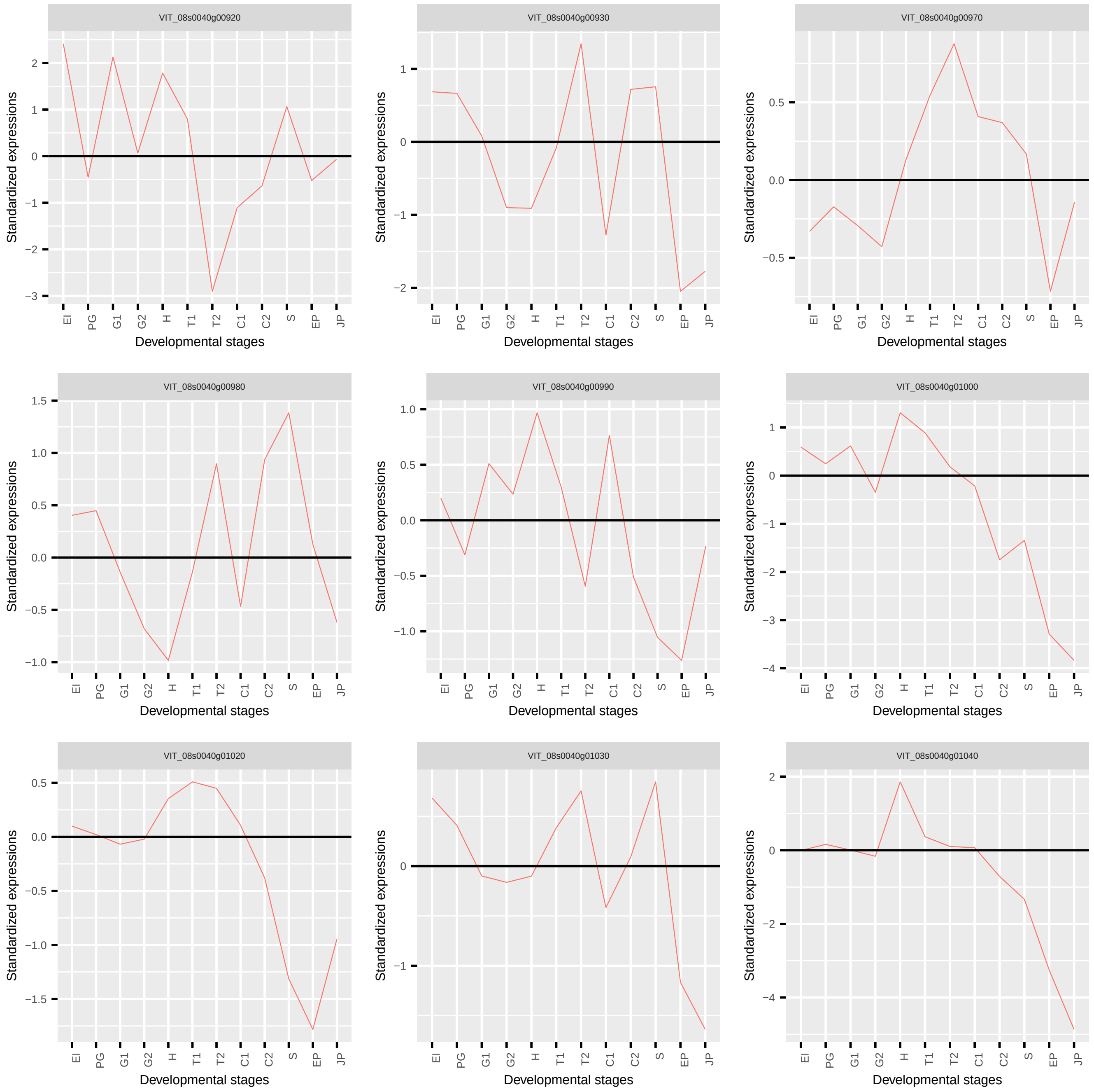

VIT_08s0040g00920
Standardized expressions
2
1
0
−1
−2
−3
EI PG G1 G2 H T1 T2 C1 C2 S EP JP
Developmental stages
VIT_08s0040g00930
Standardized expressions
1
0
−1
−2
EI PG G1 G2 H T1 T2 C1 C2 S EP JP
Developmental stages
VIT_08s0040g00970
Standardized expressions
0.5
0.0
−0.5
EI PG G1 G2 H T1 T2 C1 C2 S EP JP
Developmental stages
VIT_08s0040g00980
Standardized expressions
1.5
1.0
0.5
0.0
−0.5
−1.0
EI PG G1 G2 H T1 T2 C1 C2 S EP JP
Developmental stages
VIT_08s0040g00990
Standardized expressions
1.0
0.5
0.0
−0.5
−1.0
EI PG G1 G2 H T1 T2 C1 C2 S EP JP
Developmental stages
VIT_08s0040g01000
Standardized expressions
1
0
−1
−2
−3
−4
EI PG G1 G2 H T1 T2 C1 C2 S EP JP
Developmental stages
VIT_08s0040g01020
Standardized expressions
0.5
0.0
−0.5
−1.0
−1.5
EI PG G1 G2 H T1 T2 C1 C2 S EP JP
Developmental stages
VIT_08s0040g01030
Standardized expressions
0
−1
EI PG G1 G2 H T1 T2 C1 C2 S EP JP
Developmental stages
VIT_08s0040g01040
Standardized expressions
2
0
−2
−4
EI PG G1 G2 H T1 T2 C1 C2 S EP JP
Developmental stages

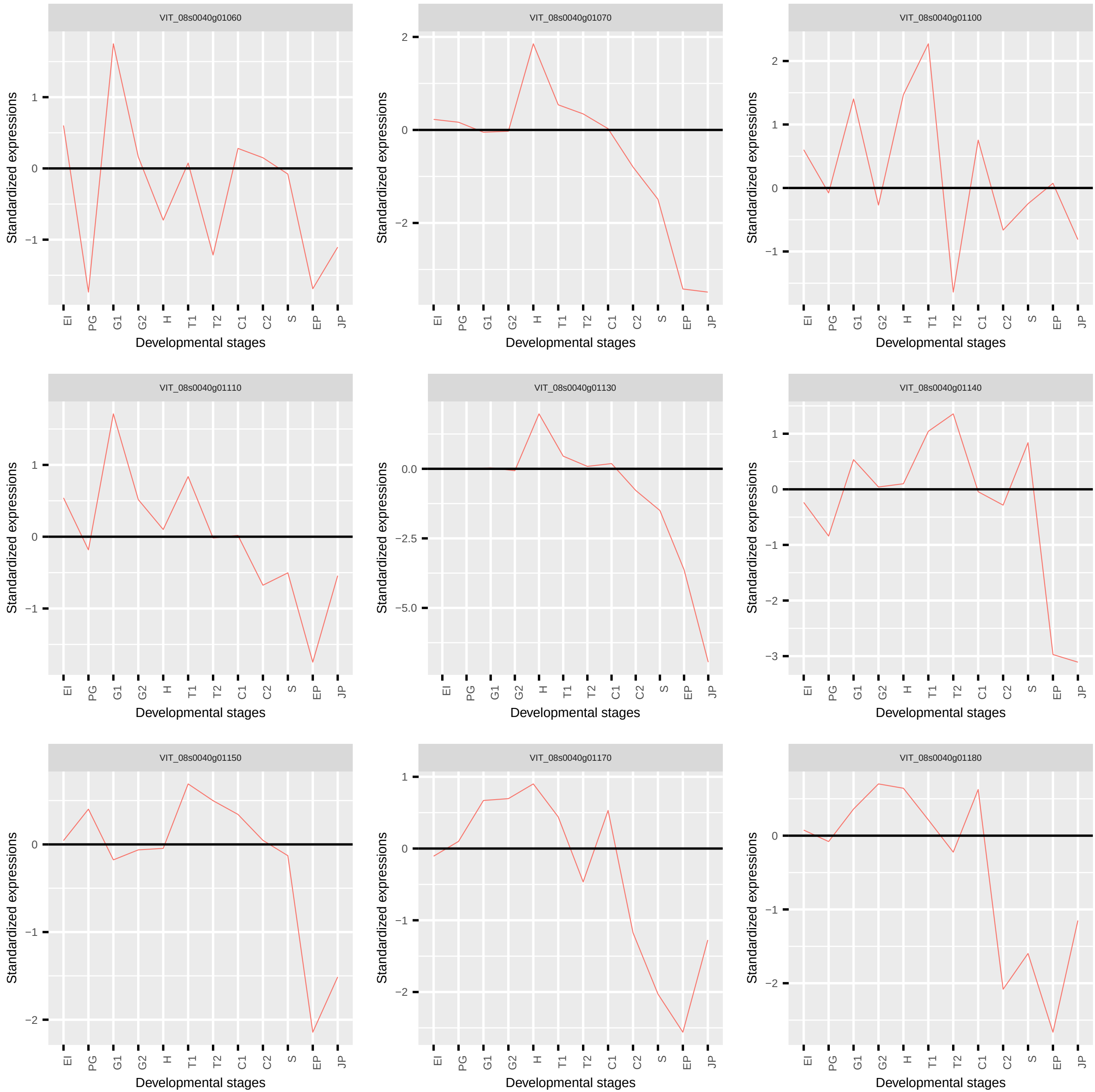
VIT_08s0040g01060
VIT_08s0040g01070
VIT_08s0040g01100
VIT_08s0040g01110
VIT_08s0040g01130
VIT_08s0040g01140
VIT_08s0040g01150
VIT_08s0040g01170
VIT_08s0040g01180
Standardized expressions
Developmental stages
EI PG G1 G2 H T1 T2 C1 C2 S EP JP

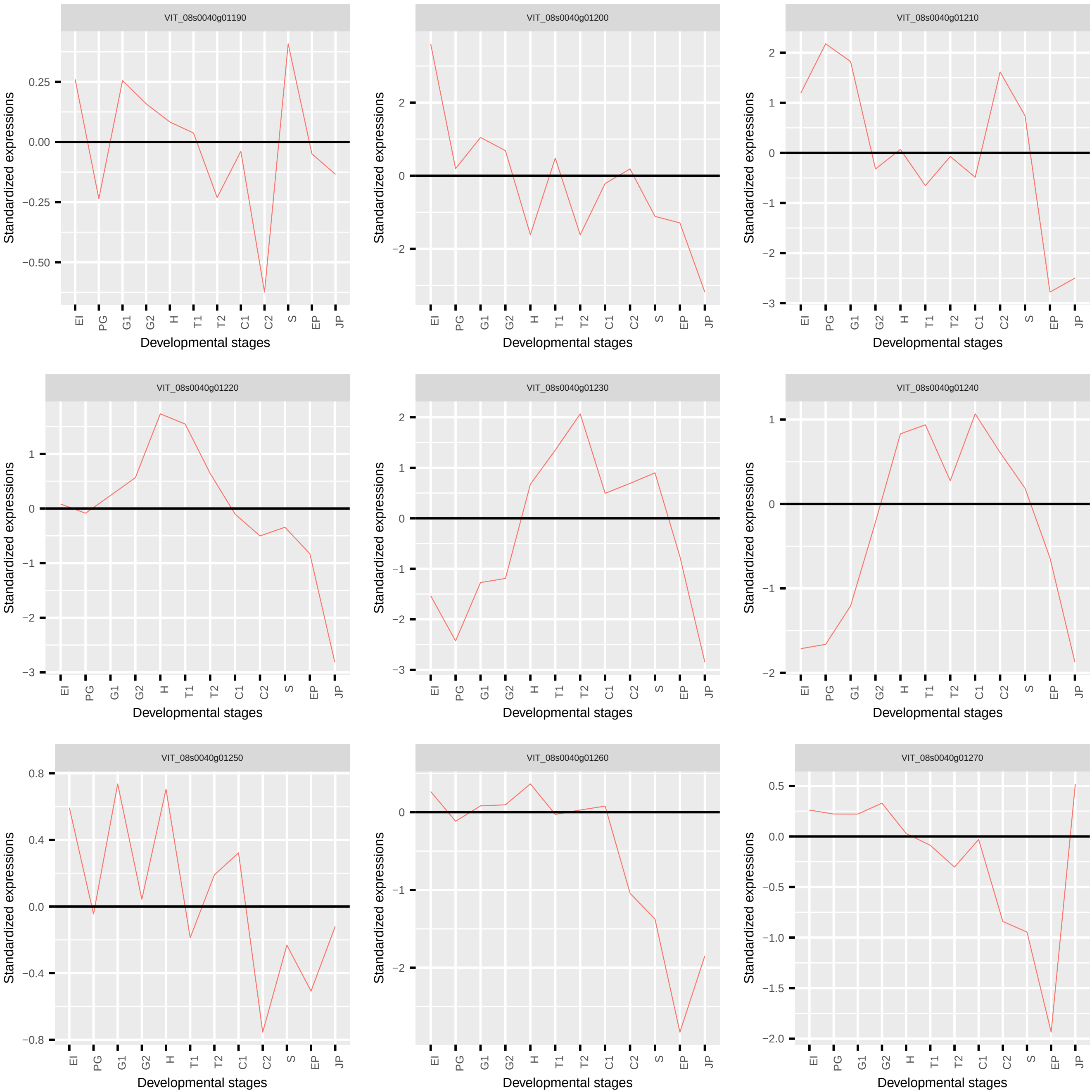
VIT_08s0040g01190
0.25
0.00
−0.25
−0.50
Standardized expressions
EI PG G1 G2 H T1 T2 C1 C2 S EP JP
Developmental stages
VIT_08s0040g01200
2
0
−2
Standardized expressions
EI PG G1 G2 H T1 T2 C1 C2 S EP JP
Developmental stages
VIT_08s0040g01210
2
1
0
−1
−2
−3
Standardized expressions
EI PG G1 G2 H T1 T2 C1 C2 S EP JP
Developmental stages
VIT_08s0040g01220
1
0
−1
−2
−3
Standardized expressions
EI PG G1 G2 H T1 T2 C1 C2 S EP JP
Developmental stages
VIT_08s0040g01230
2
1
0
−1
−2
−3
Standardized expressions
EI PG G1 G2 H T1 T2 C1 C2 S EP JP
Developmental stages
VIT_08s0040g01240
1
0
−1
−2
Standardized expressions
EI PG G1 G2 H T1 T2 C1 C2 S EP JP
Developmental stages
VIT_08s0040g01250
0.8
0.4
0.0
−0.4
−0.8
Standardized expressions
EI PG G1 G2 H T1 T2 C1 C2 S EP JP
Developmental stages
VIT_08s0040g01260
0
−1
−2
Standardized expressions
EI PG G1 G2 H T1 T2 C1 C2 S EP JP
Developmental stages
VIT_08s0040g01270
0.5
0.0
−0.5
−1.0
−1.5
−2.0
Standardized expressions
EI PG G1 G2 H T1 T2 C1 C2 S EP JP
Developmental stages

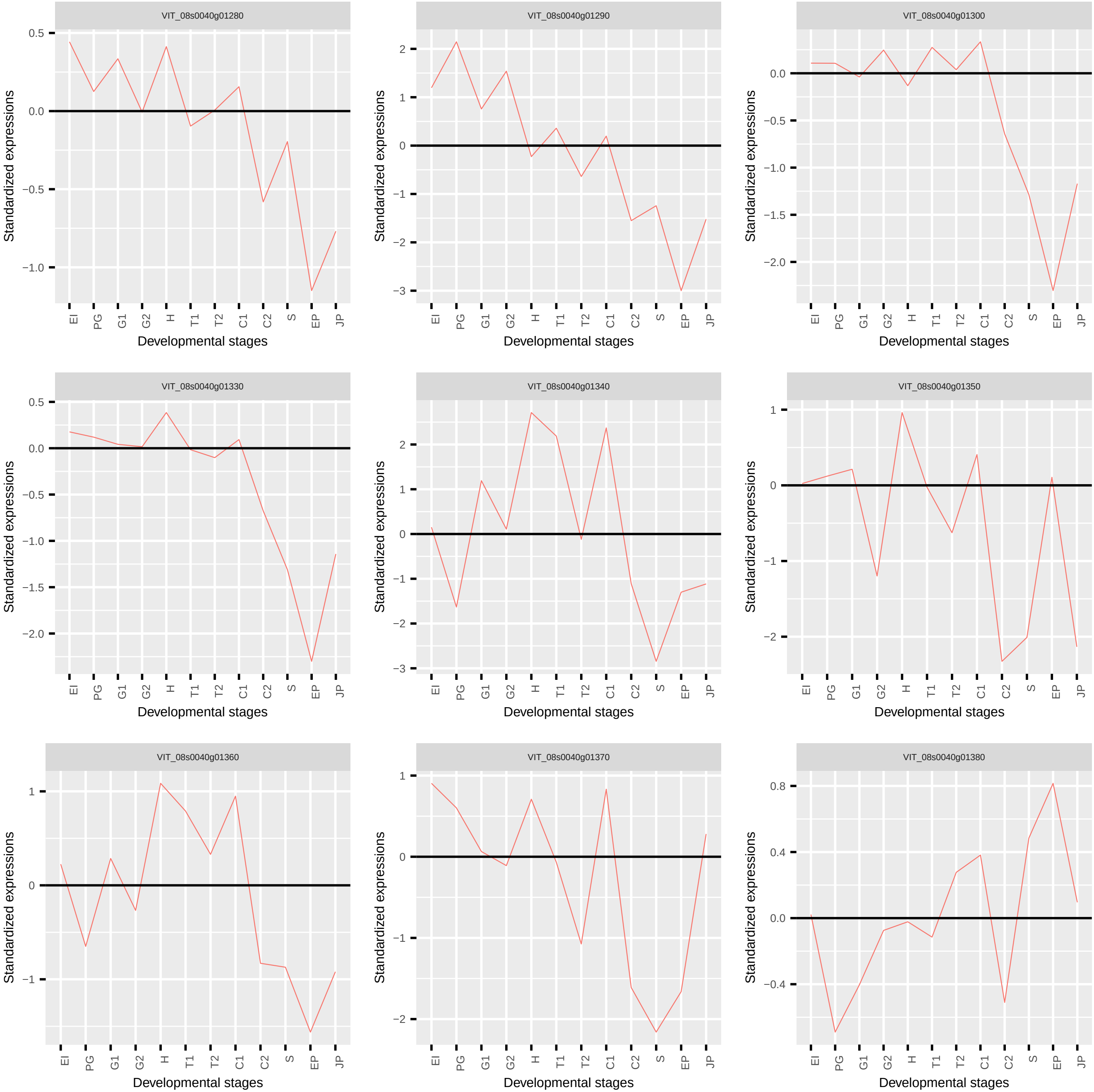
VIT_08s0040g01280
VIT_08s0040g01290
VIT_08s0040g01300
VIT_08s0040g01330
VIT_08s0040g01340
VIT_08s0040g01350
VIT_08s0040g01360
VIT_08s0040g01370
VIT_08s0040g01380
Standardized expressions
Developmental stages
EI PG G1 G2 H T1 T2 C1 C2 S EP JP

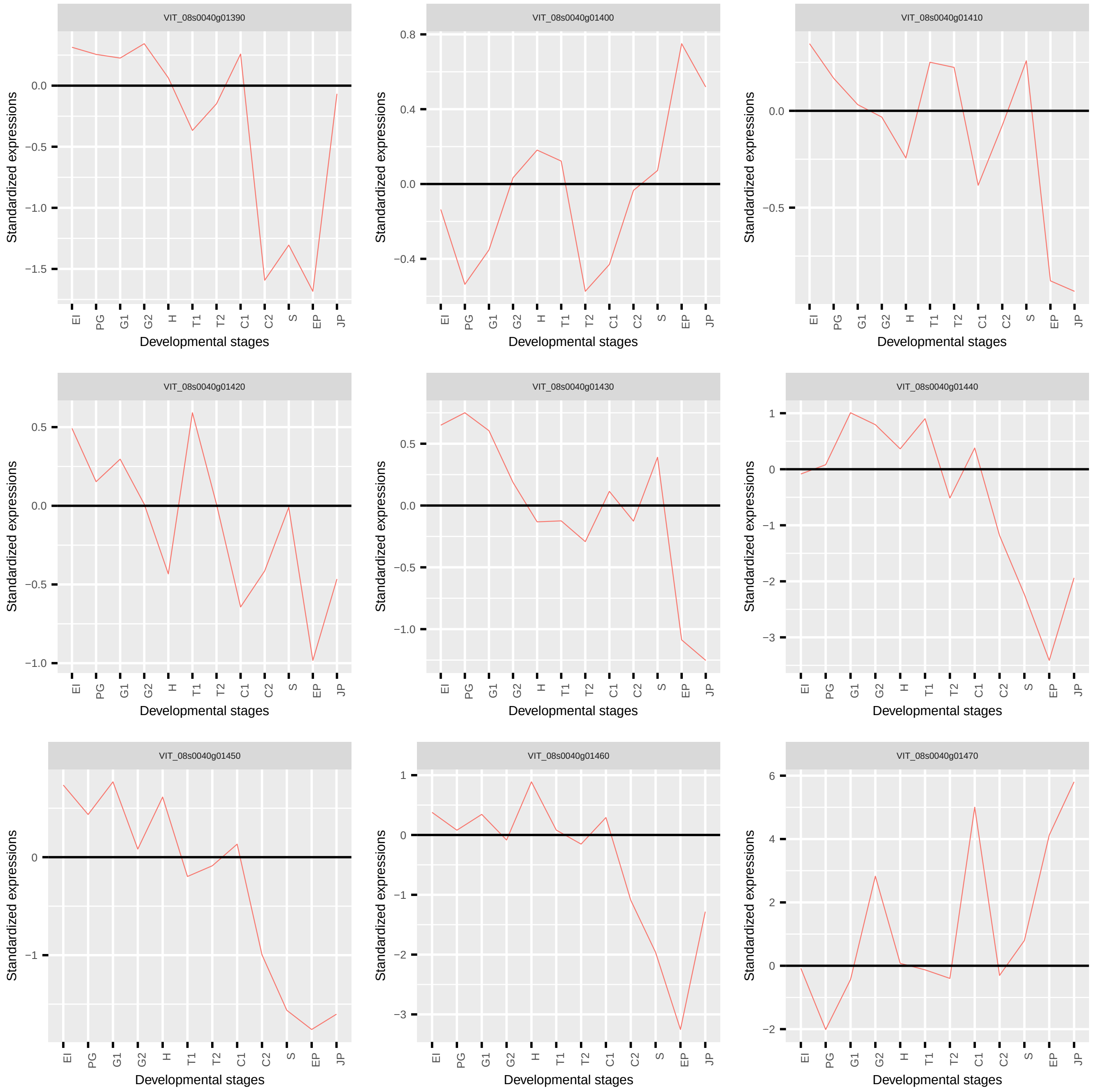
VIT_08s0040g01390
VIT_08s0040g01400
VIT_08s0040g01410
VIT_08s0040g01420
VIT_08s0040g01430
VIT_08s0040g01440
VIT_08s0040g01450
VIT_08s0040g01460
VIT_08s0040g01470
Standardized expressions
Developmental stages
EI PG G1 G2 H T1 T2 C1 C2 S EP JP

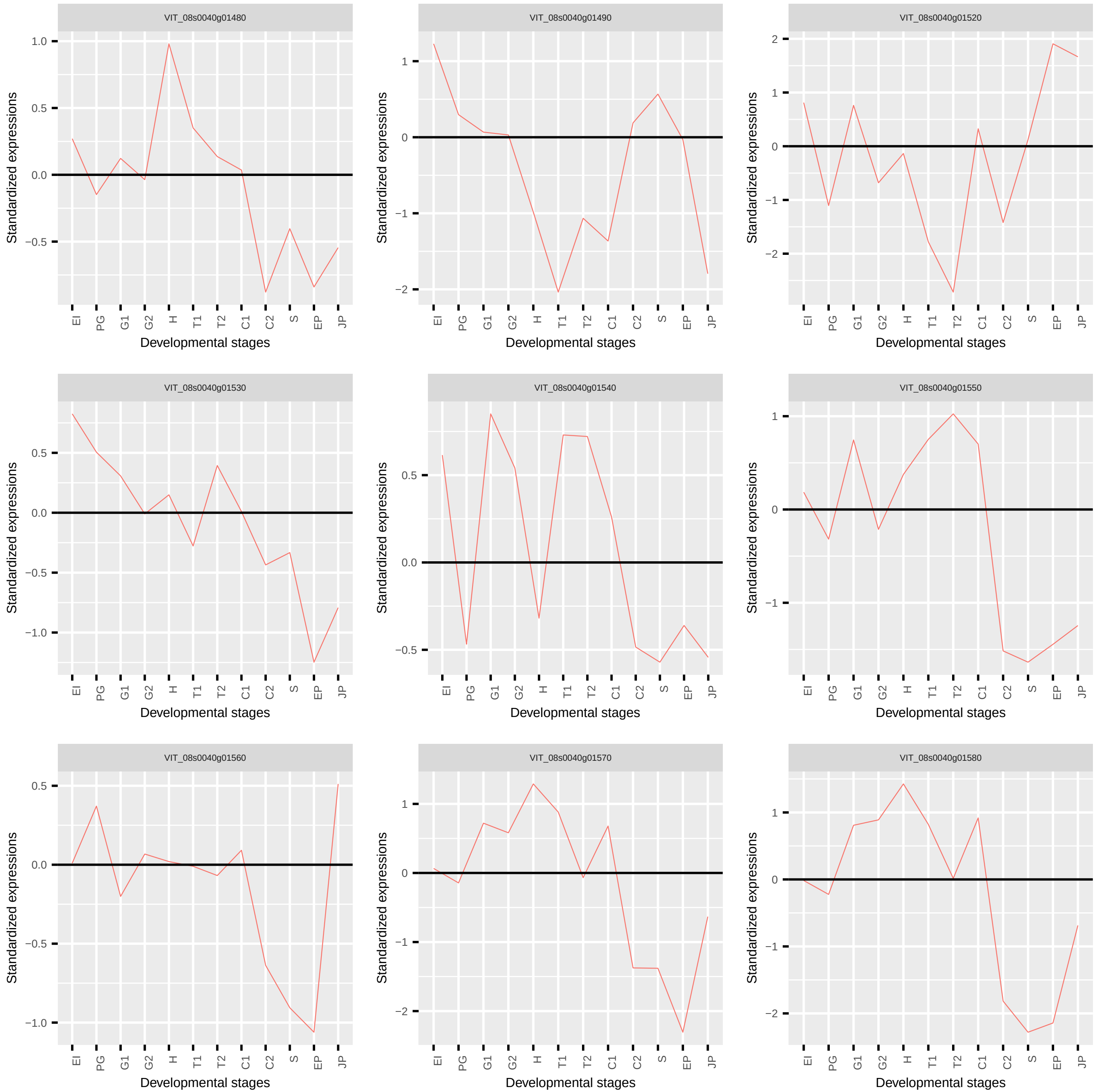
VIT_08s0040g01480
VIT_08s0040g01490
VIT_08s0040g01520
VIT_08s0040g01530
VIT_08s0040g01540
VIT_08s0040g01550
VIT_08s0040g01560
VIT_08s0040g01570
VIT_08s0040g01580
Standardized expressions
Developmental stages
EI PG G1 G2 H T1 T2 C1 C2 S EP JP

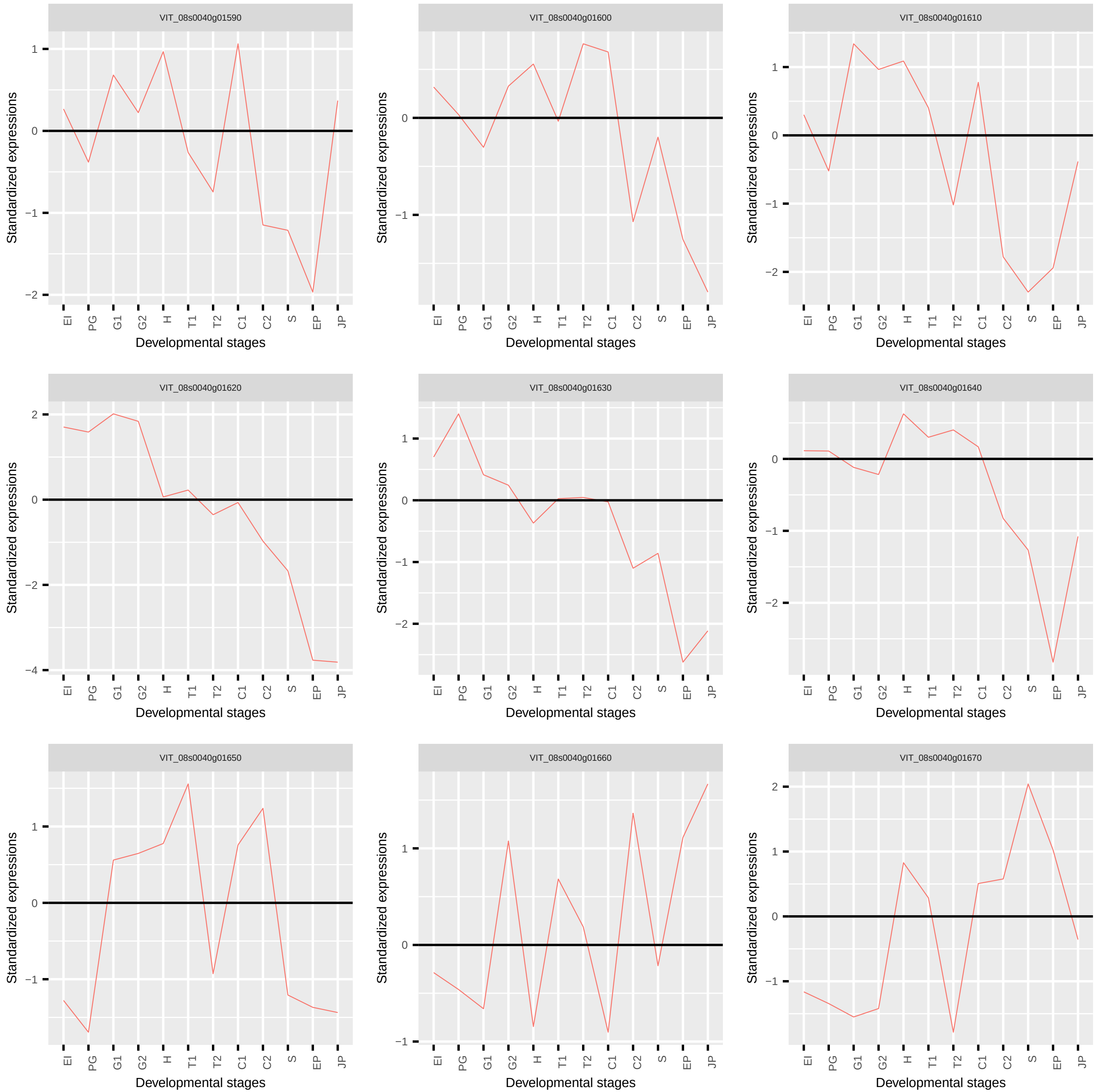
VIT_08s0040g01590
VIT_08s0040g01600
VIT_08s0040g01610
VIT_08s0040g01620
VIT_08s0040g01630
VIT_08s0040g01640
VIT_08s0040g01650
VIT_08s0040g01660
VIT_08s0040g01670
Standardized expressions
Developmental stages
EI PG G1 G2 H T1 T2 C1 C2 S EP JP

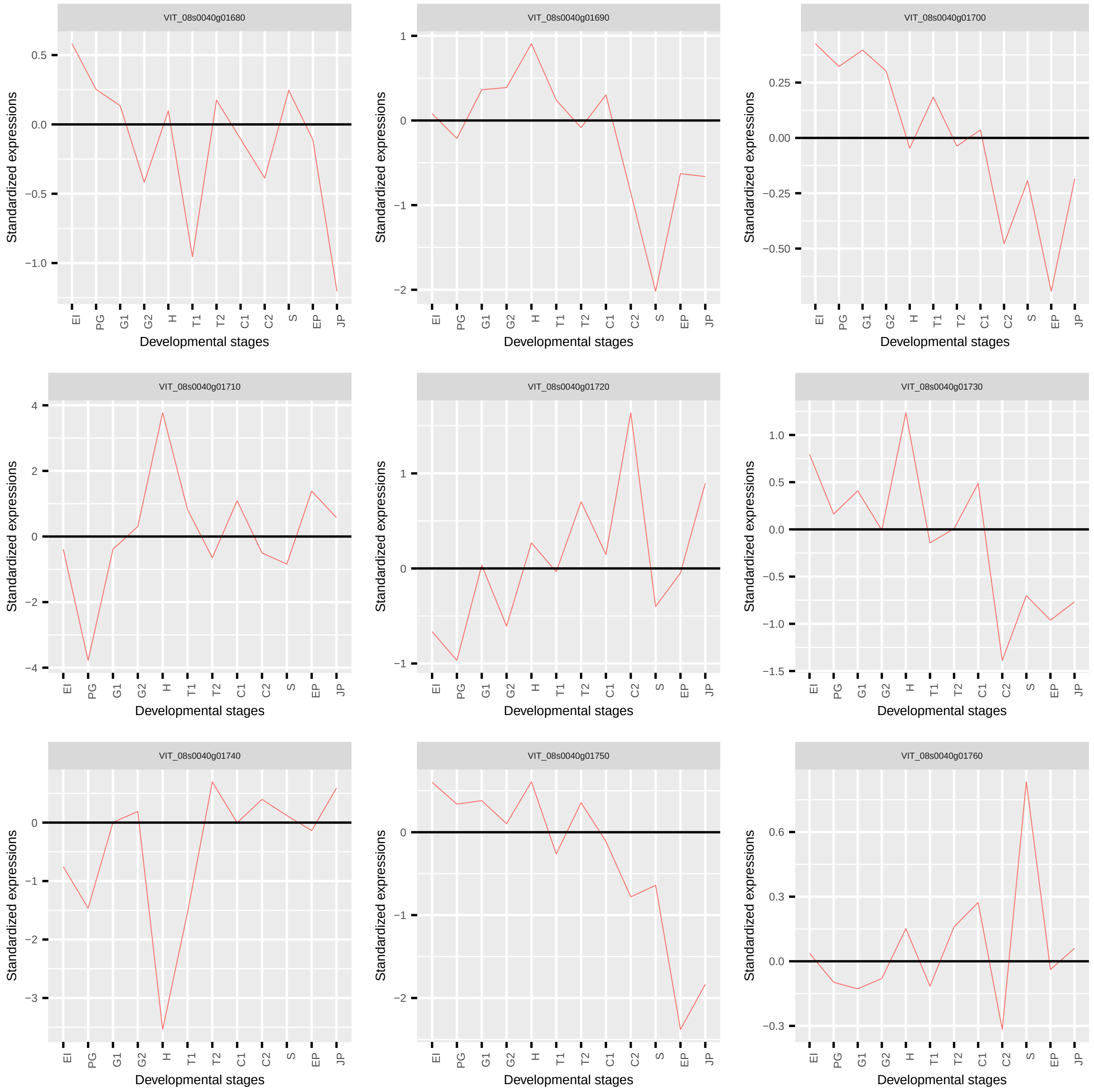
VIT_08s0040g01680
0.5
0.0
−0.5
−1.0
Standardized expressions
EI PG G1 G2 H T1 T2 C1 C2 S EP JP
Developmental stages
VIT_08s0040g01690
1
0
−1
−2
Standardized expressions
EI PG G1 G2 H T1 T2 C1 C2 S EP JP
Developmental stages
VIT_08s0040g01700
0.25
0.00
−0.25
−0.50
Standardized expressions
EI PG G1 G2 H T1 T2 C1 C2 S EP JP
Developmental stages
VIT_08s0040g01710
4
2
0
−2
−4
Standardized expressions
EI PG G1 G2 H T1 T2 C1 C2 S EP JP
Developmental stages
VIT_08s0040g01720
1
0
−1
Standardized expressions
EI PG G1 G2 H T1 T2 C1 C2 S EP JP
Developmental stages
VIT_08s0040g01730
1.0
0.5
0.0
−0.5
−1.0
−1.5
Standardized expressions
EI PG G1 G2 H T1 T2 C1 C2 S EP JP
Developmental stages
VIT_08s0040g01740
0
−1
−2
−3
Standardized expressions
EI PG G1 G2 H T1 T2 C1 C2 S EP JP
Developmental stages
VIT_08s0040g01750
0
−1
−2
Standardized expressions
EI PG G1 G2 H T1 T2 C1 C2 S EP JP
Developmental stages
VIT_08s0040g01760
0.6
0.3
0.0
−0.3
Standardized expressions
EI PG G1 G2 H T1 T2 C1 C2 S EP JP
Developmental stages

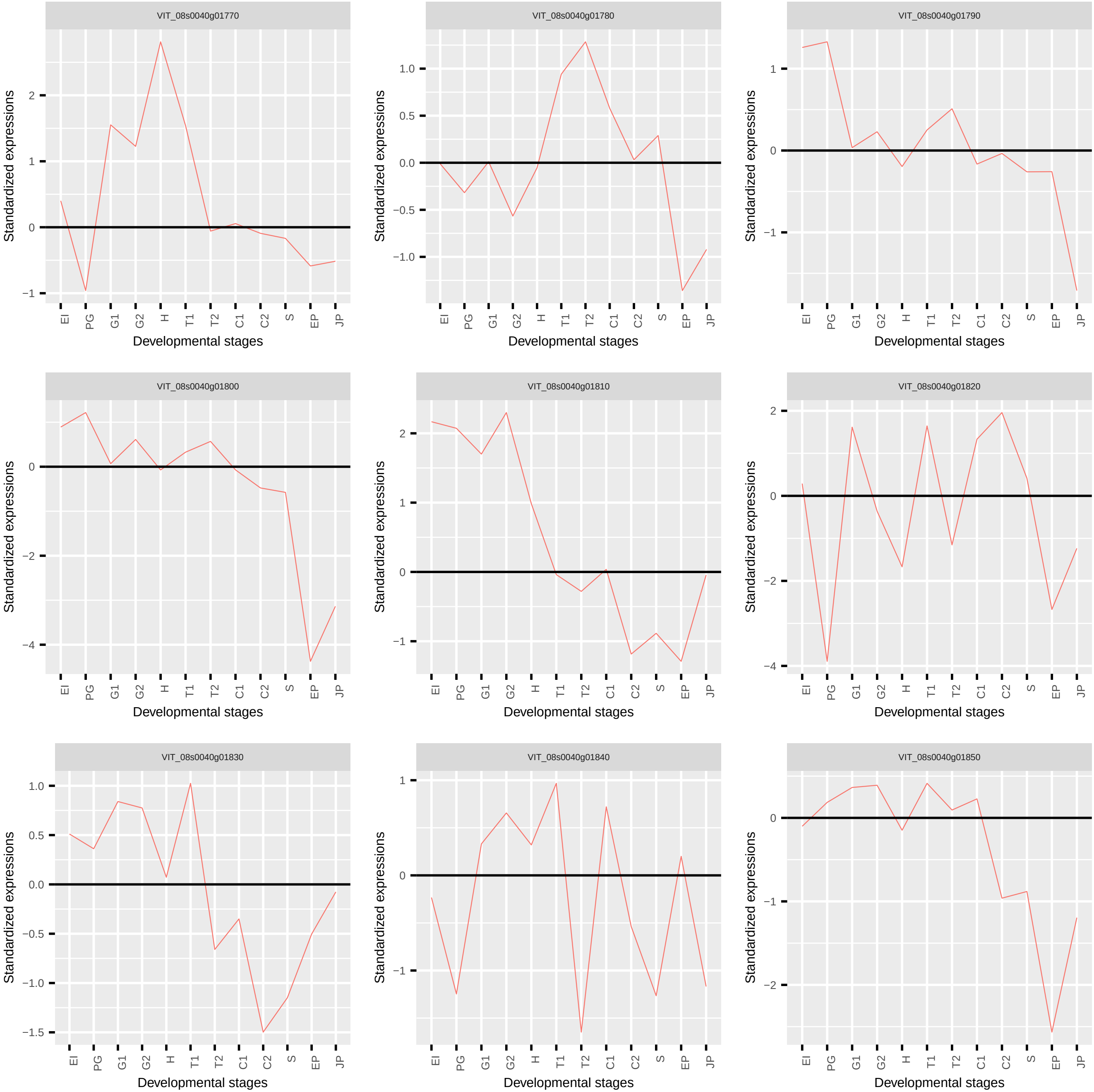
VIT_08s0040g01770
VIT_08s0040g01780
VIT_08s0040g01790
VIT_08s0040g01800
VIT_08s0040g01810
VIT_08s0040g01820
VIT_08s0040g01830
VIT_08s0040g01840
VIT_08s0040g01850
Standardized expressions
Developmental stages
EI PG G1 G2 H T1 T2 C1 C2 S EP JP

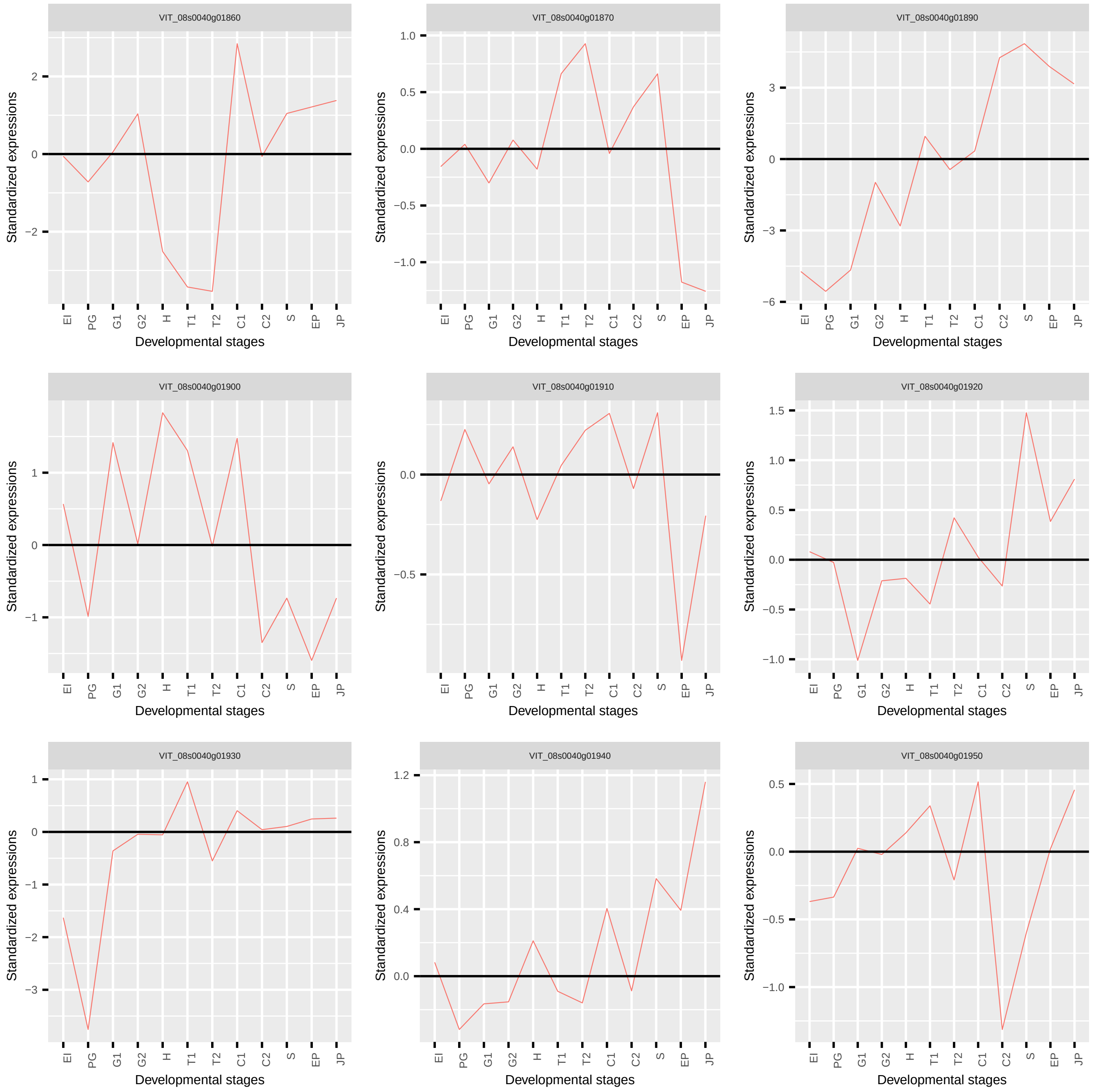

VIT_08s0040g01860
VIT_08s0040g01870
VIT_08s0040g01890
VIT_08s0040g01900
VIT_08s0040g01910
VIT_08s0040g01920
VIT_08s0040g01930
VIT_08s0040g01940
VIT_08s0040g01950
Standardized expressions
Developmental stages
EI PG G1 G2 H T1 T2 C1 C2 S EP JP

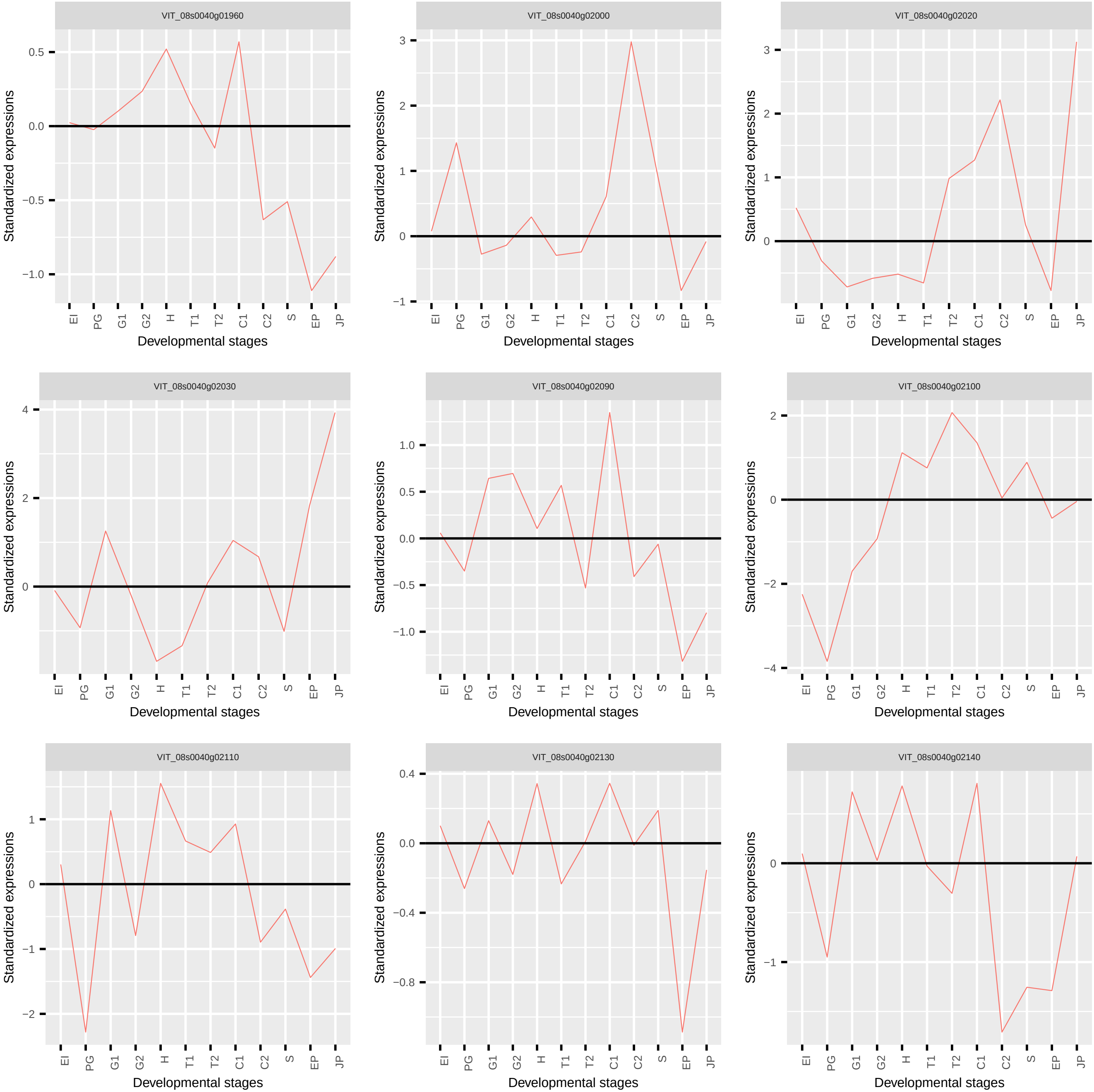
VIT_08s0040g01960
VIT_08s0040g02000
VIT_08s0040g02020
VIT_08s0040g02030
VIT_08s0040g02090
VIT_08s0040g02100
VIT_08s0040g02110
VIT_08s0040g02130
VIT_08s0040g02140
Standardized expressions
Developmental stages
EI PG G1 G2 H T1 T2 C1 C2 S EP JP

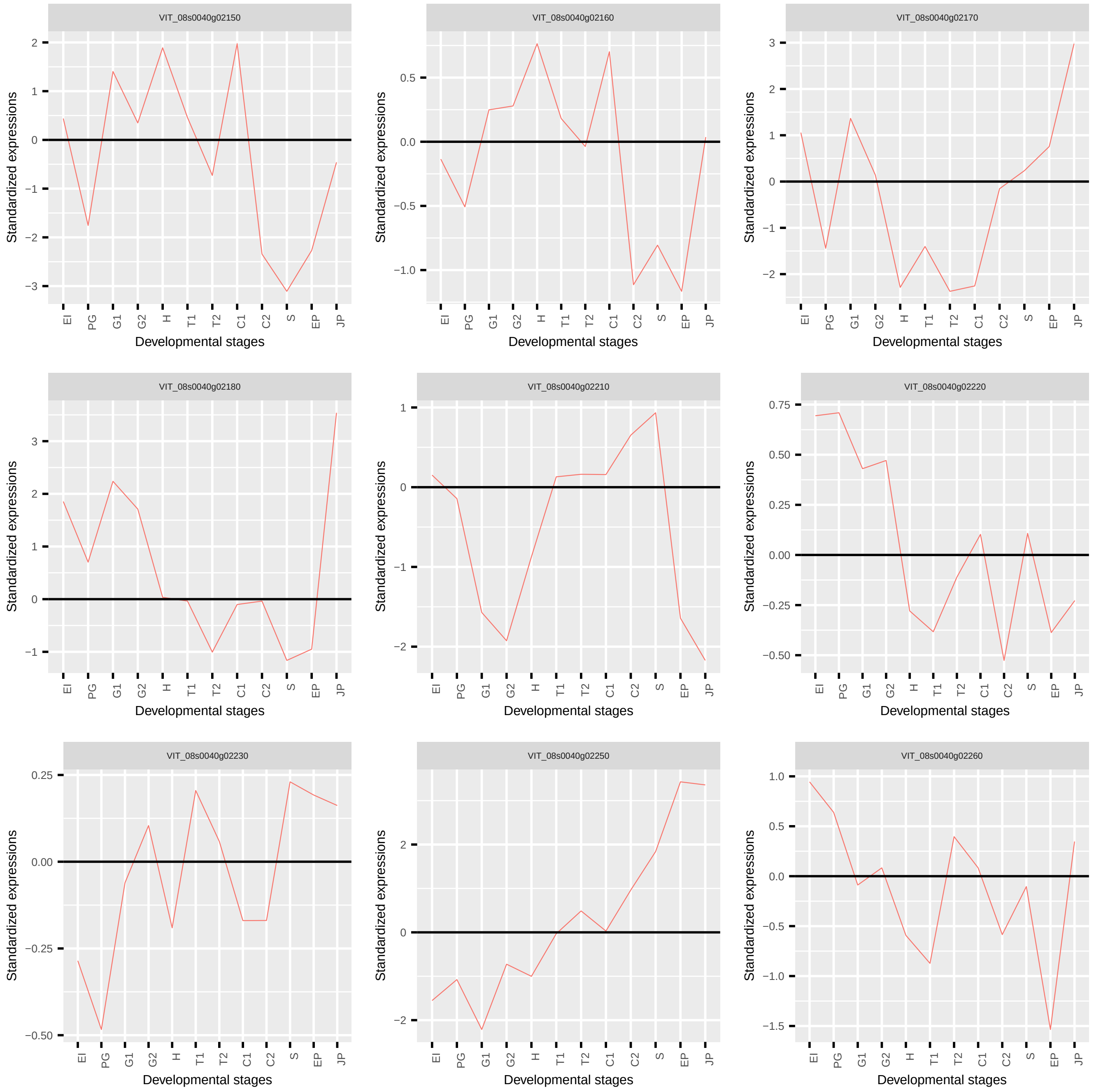
VIT_08s0040g02150
VIT_08s0040g02160
VIT_08s0040g02170
VIT_08s0040g02180
VIT_08s0040g02210
VIT_08s0040g02220
VIT_08s0040g02230
VIT_08s0040g02250
VIT_08s0040g02260
Standardized expressions
Developmental stages
EI PG G1 G2 H T1 T2 C1 C2 S EP JP

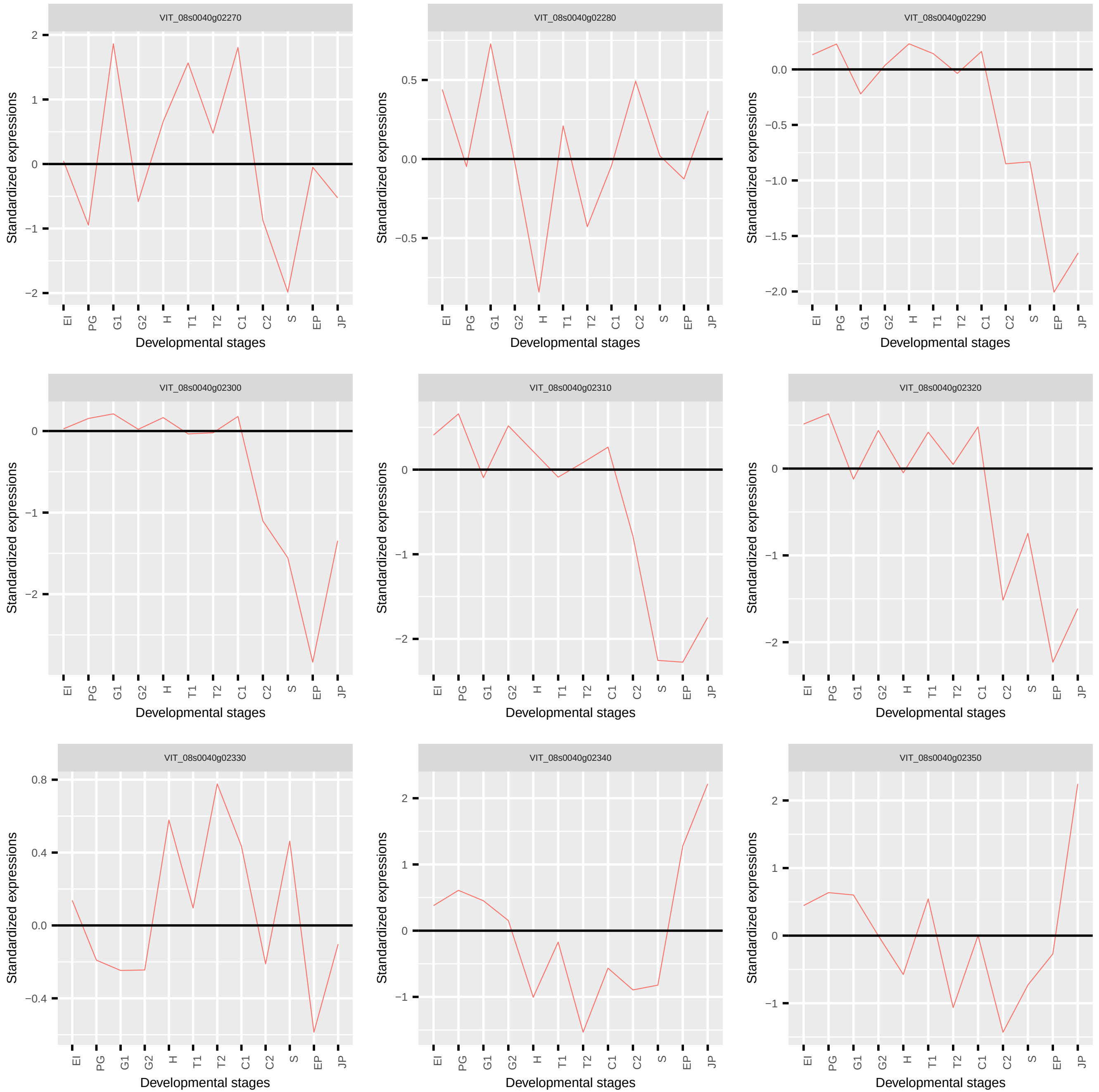
VIT_08s0040g02270
VIT_08s0040g02280
VIT_08s0040g02290
VIT_08s0040g02300
VIT_08s0040g02310
VIT_08s0040g02320
VIT_08s0040g02330
VIT_08s0040g02340
VIT_08s0040g02350
Standardized expressions
Developmental stages
EI PG G1 G2 H T1 T2 C1 C2 S EP JP

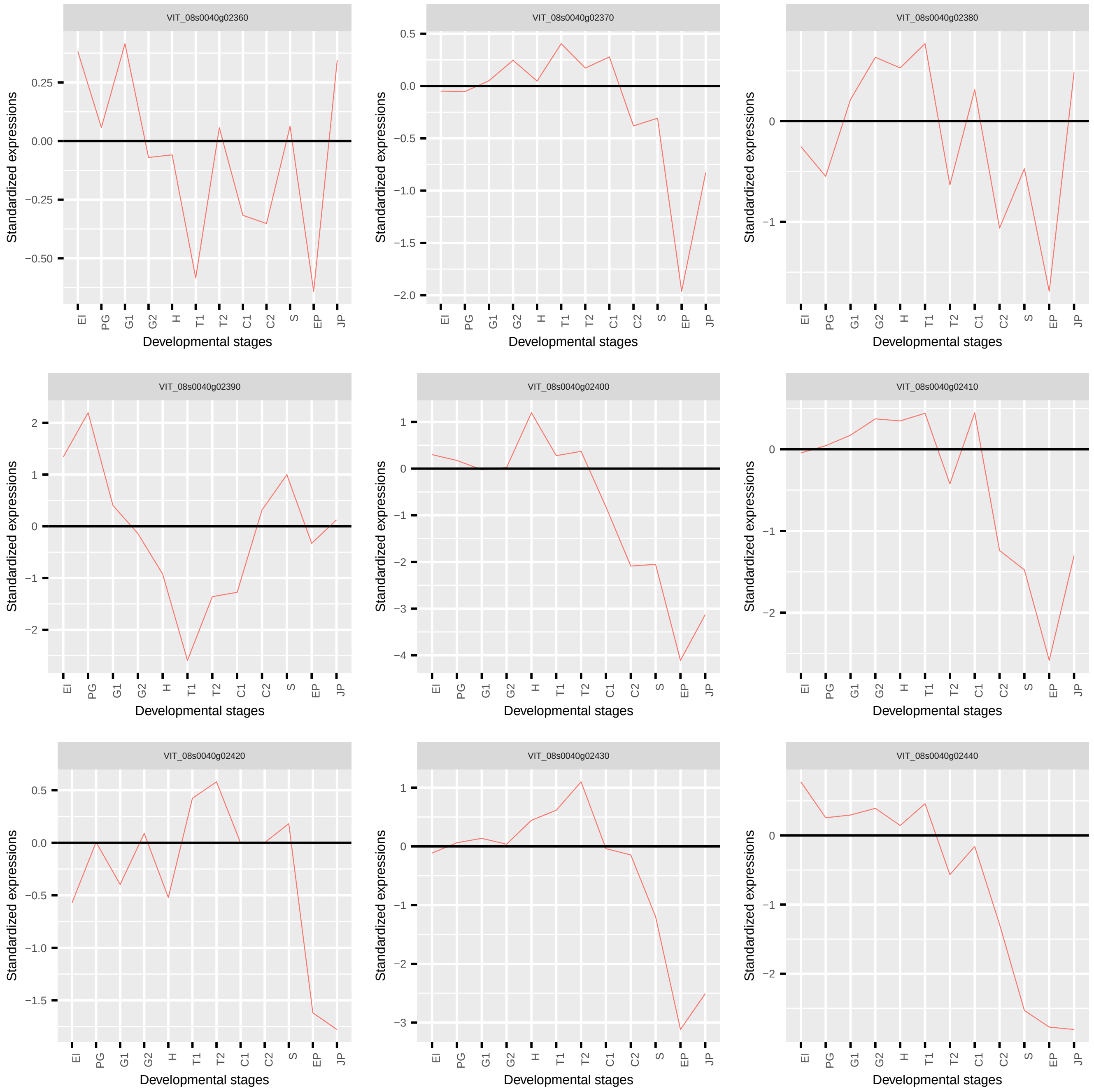
VIT_08s0040g02360
VIT_08s0040g02370
VIT_08s0040g02380
VIT_08s0040g02390
VIT_08s0040g02400
VIT_08s0040g02410
VIT_08s0040g02420
VIT_08s0040g02430
VIT_08s0040g02440
Standardized expressions
Developmental stages
EI PG G1 G2 H T1 T2 C1 C2 S EP JP
0.25 0.00 −0.25 −0.50
0.5 0.0 −0.5 −1.0 −1.5 −2.0
0 −1
2 1 0 −1 −2
1 0 −1 −2 −3 −4
0 −1 −2
0.5 0.0 −0.5 −1.0 −1.5
1 0 −1 −2 −3
0 −1 −2

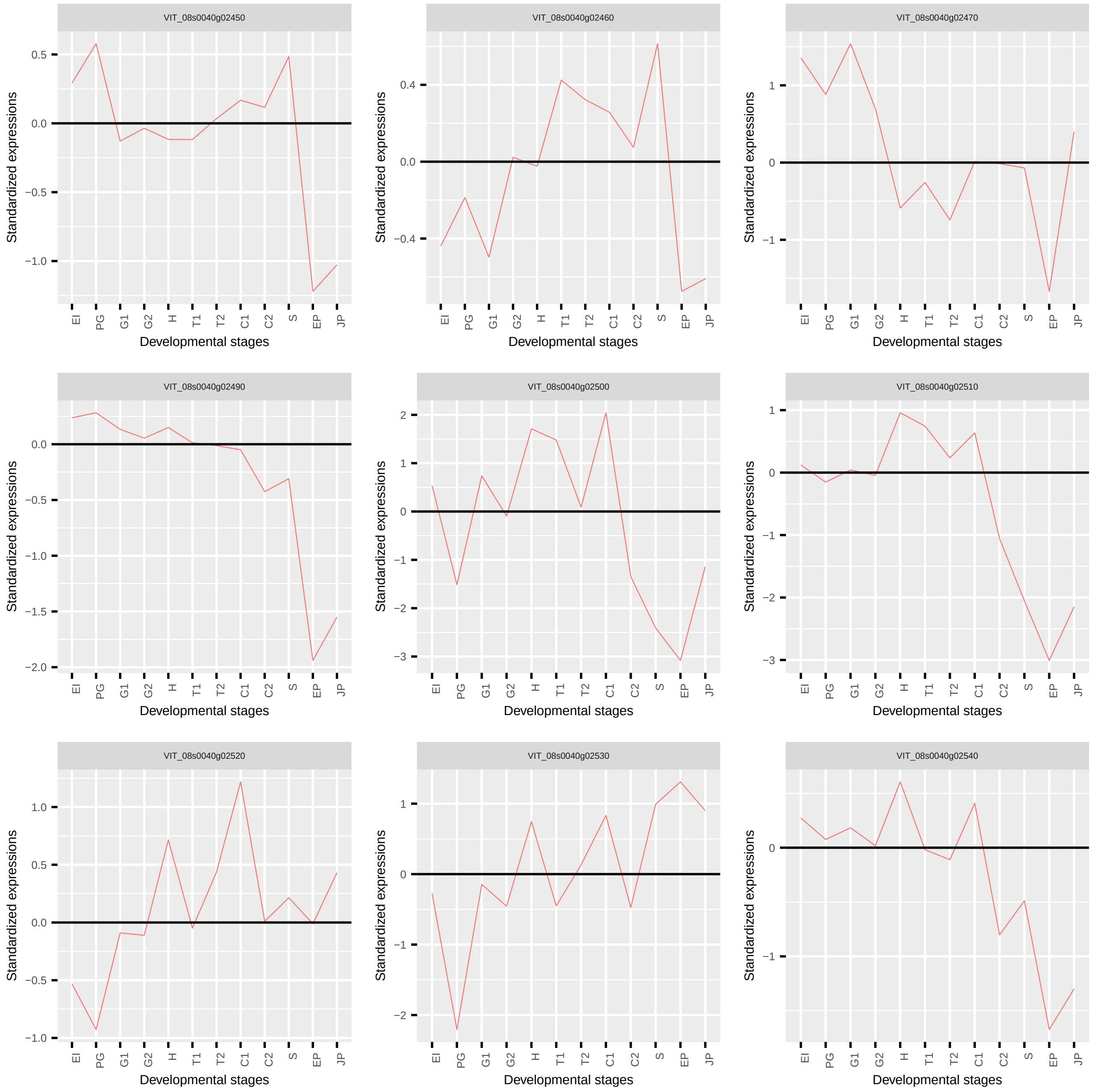
VIT_08s0040g02450
0.5
0.0
−0.5
−1.0
Standardized expressions
EI PG G1 G2 H T1 T2 C1 C2 S EP JP
Developmental stages
VIT_08s0040g02460
0.4
0.0
−0.4
Standardized expressions
EI PG G1 G2 H T1 T2 C1 C2 S EP JP
Developmental stages
VIT_08s0040g02470
1
0
−1
Standardized expressions
EI PG G1 G2 H T1 T2 C1 C2 S EP JP
Developmental stages
VIT_08s0040g02490
0.0
−0.5
−1.0
−1.5
−2.0
Standardized expressions
EI PG G1 G2 H T1 T2 C1 C2 S EP JP
Developmental stages
VIT_08s0040g02500
2
1
0
−1
−2
−3
Standardized expressions
EI PG G1 G2 H T1 T2 C1 C2 S EP JP
Developmental stages
VIT_08s0040g02510
1
0
−1
−2
−3
Standardized expressions
EI PG G1 G2 H T1 T2 C1 C2 S EP JP
Developmental stages
VIT_08s0040g02520
1.0
0.5
0.0
−0.5
−1.0
Standardized expressions
EI PG G1 G2 H T1 T2 C1 C2 S EP JP
Developmental stages
VIT_08s0040g02530
1
0
−1
−2
Standardized expressions
EI PG G1 G2 H T1 T2 C1 C2 S EP JP
Developmental stages
VIT_08s0040g02540
0
−1
Standardized expressions
EI PG G1 G2 H T1 T2 C1 C2 S EP JP
Developmental stages

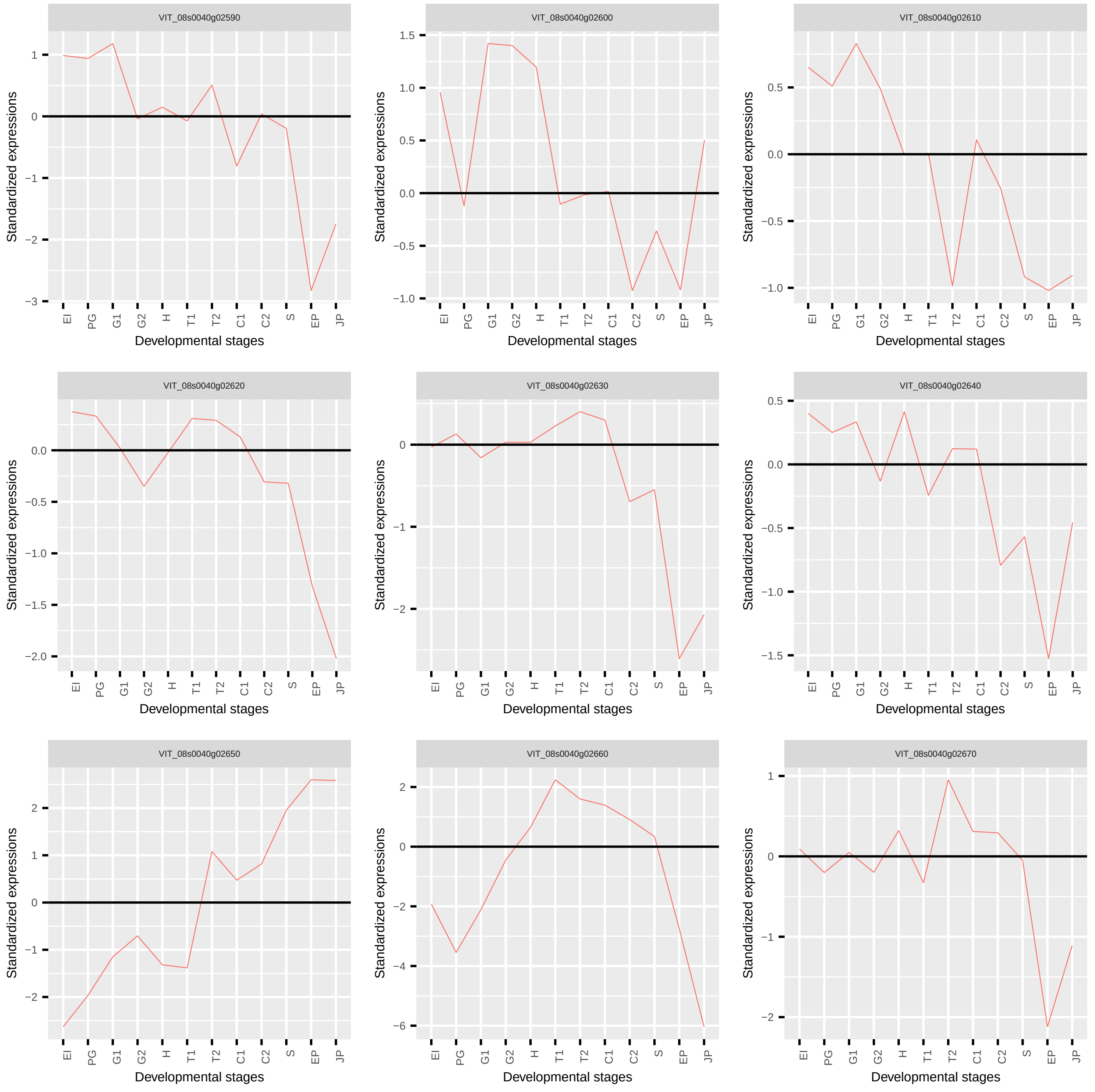

VIT_08s0040g02590
Standardized expressions
1
0
−1
−2
−3
EI PG G1 G2 H T1 T2 C1 C2 S EP JP
Developmental stages
VIT_08s0040g02600
Standardized expressions
1.5
1.0
0.5
0.0
−0.5
−1.0
EI PG G1 G2 H T1 T2 C1 C2 S EP JP
Developmental stages
VIT_08s0040g02610
Standardized expressions
0.5
0.0
−0.5
−1.0
EI PG G1 G2 H T1 T2 C1 C2 S EP JP
Developmental stages
VIT_08s0040g02620
Standardized expressions
0.0
−0.5
−1.0
−1.5
−2.0
EI PG G1 G2 H T1 T2 C1 C2 S EP JP
Developmental stages
VIT_08s0040g02630
Standardized expressions
0
−1
−2
EI PG G1 G2 H T1 T2 C1 C2 S EP JP
Developmental stages
VIT_08s0040g02640
Standardized expressions
0.5
0.0
−0.5
−1.0
−1.5
EI PG G1 G2 H T1 T2 C1 C2 S EP JP
Developmental stages
VIT_08s0040g02650
Standardized expressions
2
1
0
−1
−2
EI PG G1 G2 H T1 T2 C1 C2 S EP JP
Developmental stages
VIT_08s0040g02660
Standardized expressions
2
0
−2
−4
−6
EI PG G1 G2 H T1 T2 C1 C2 S EP JP
Developmental stages
VIT_08s0040g02670
Standardized expressions
1
0
−1
−2
EI PG G1 G2 H T1 T2 C1 C2 S EP JP
Developmental stages

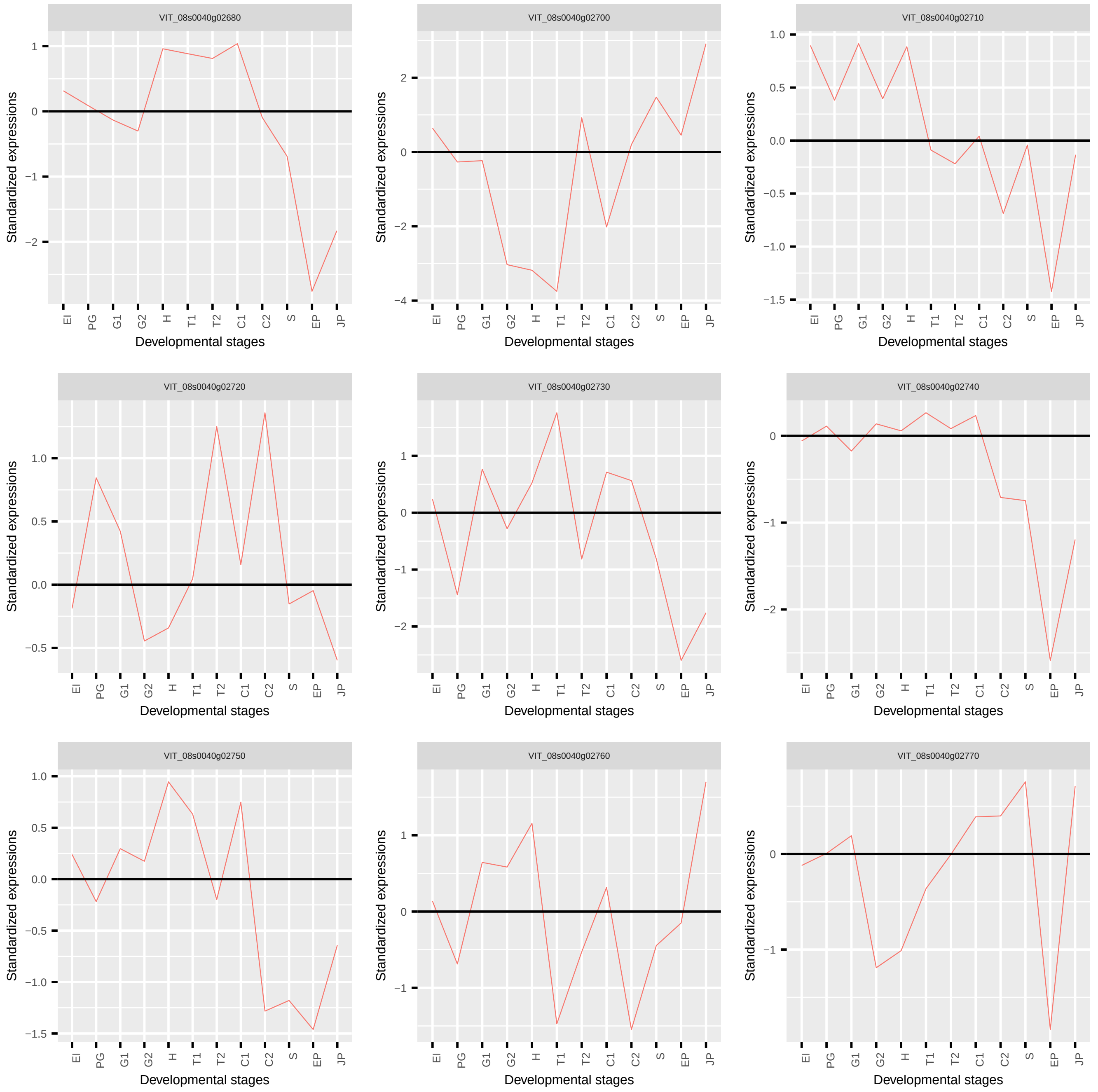
VIT_08s0040g02680
VIT_08s0040g02700
VIT_08s0040g02710
VIT_08s0040g02720
VIT_08s0040g02730
VIT_08s0040g02740
VIT_08s0040g02750
VIT_08s0040g02760
VIT_08s0040g02770
Standardized expressions
Developmental stages
EI PG G1 G2 H T1 T2 C1 C2 S EP JP

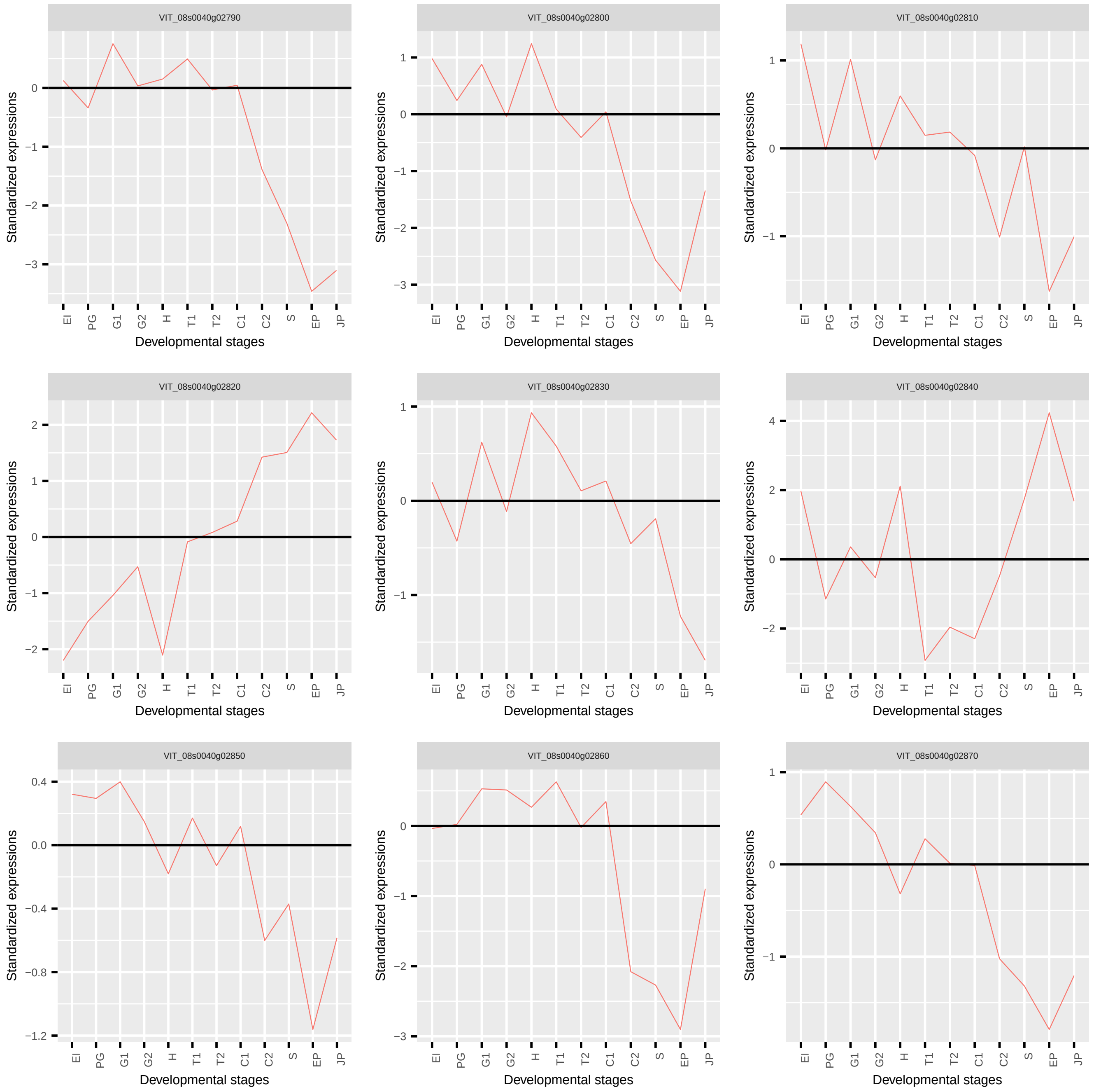
VIT_08s0040g02790
VIT_08s0040g02800
VIT_08s0040g02810
VIT_08s0040g02820
VIT_08s0040g02830
VIT_08s0040g02840
VIT_08s0040g02850
VIT_08s0040g02860
VIT_08s0040g02870
Standardized expressions
Developmental stages
EI PG G1 G2 H T1 T2 C1 C2 S EP JP

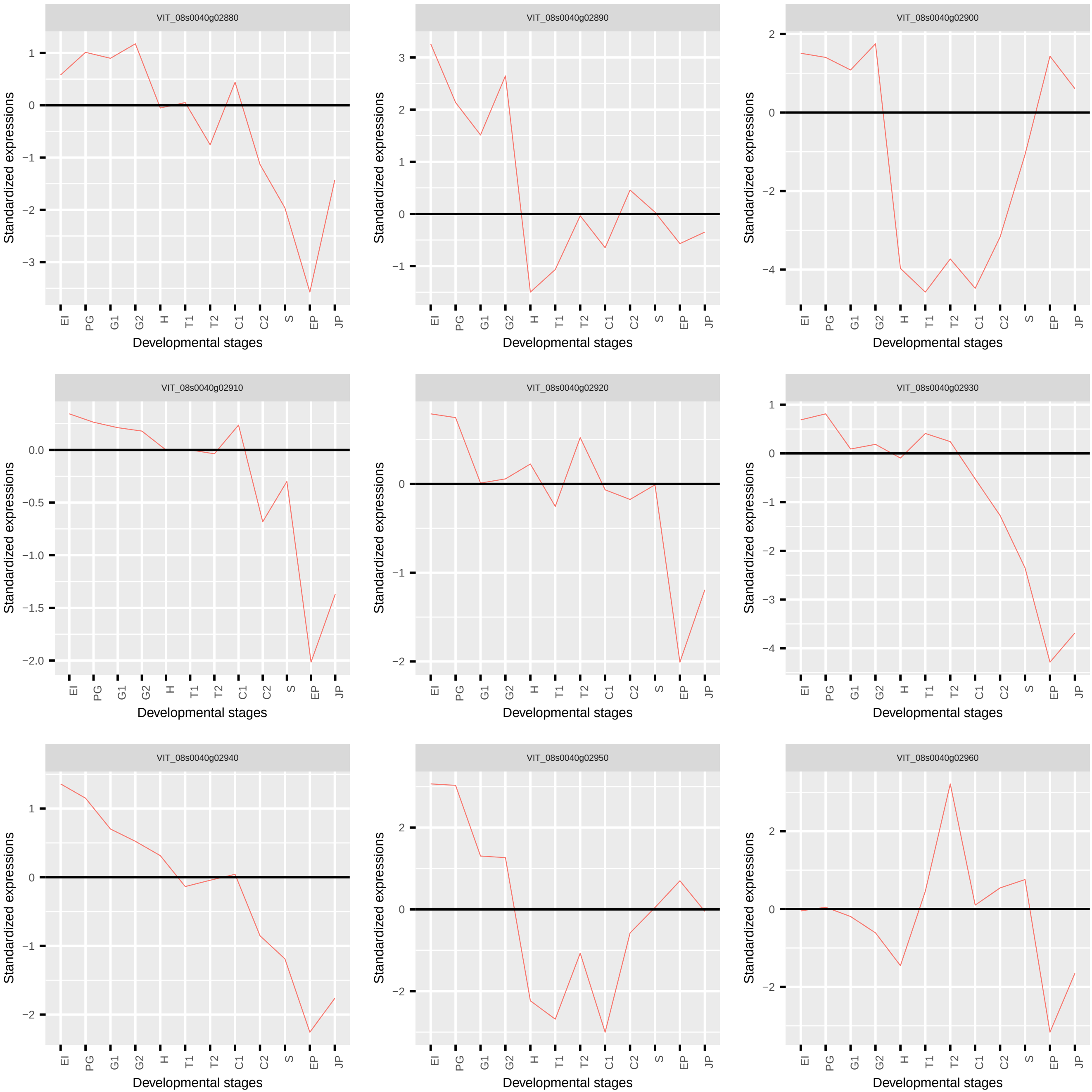

VIT_08s0040g02880
VIT_08s0040g02890
VIT_08s0040g02900
VIT_08s0040g02910
VIT_08s0040g02920
VIT_08s0040g02930
VIT_08s0040g02940
VIT_08s0040g02950
VIT_08s0040g02960
Standardized expressions
Developmental stages
EI PG G1 G2 H T1 T2 C1 C2 S EP JP

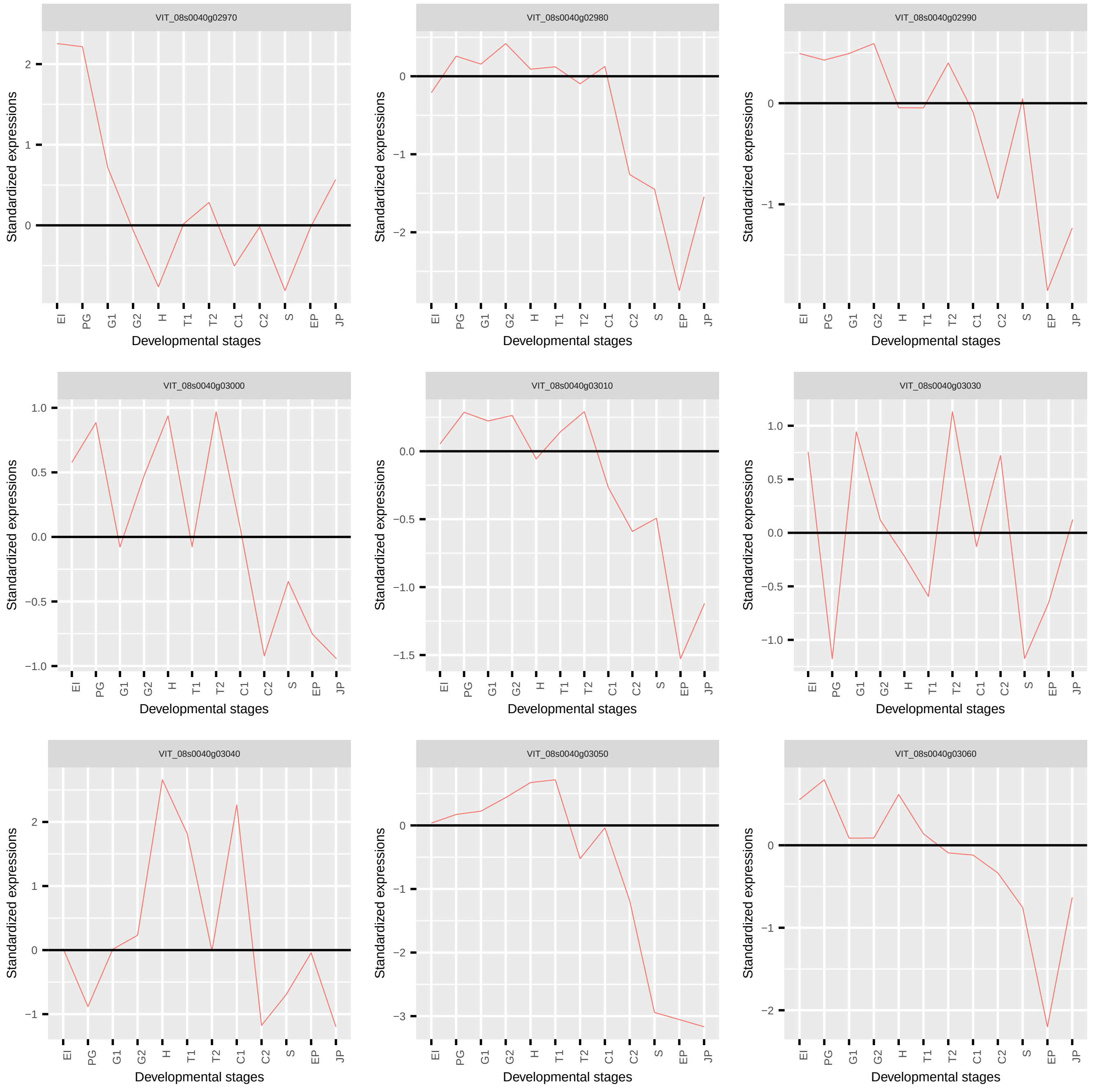
VIT_08s0040g02970
VIT_08s0040g02980
VIT_08s0040g02990
VIT_08s0040g03000
VIT_08s0040g03010
VIT_08s0040g03030
VIT_08s0040g03040
VIT_08s0040g03050
VIT_08s0040g03060
Standardized expressions
Developmental stages
EI PG G1 G2 H T1 T2 C1 C2 S EP JP

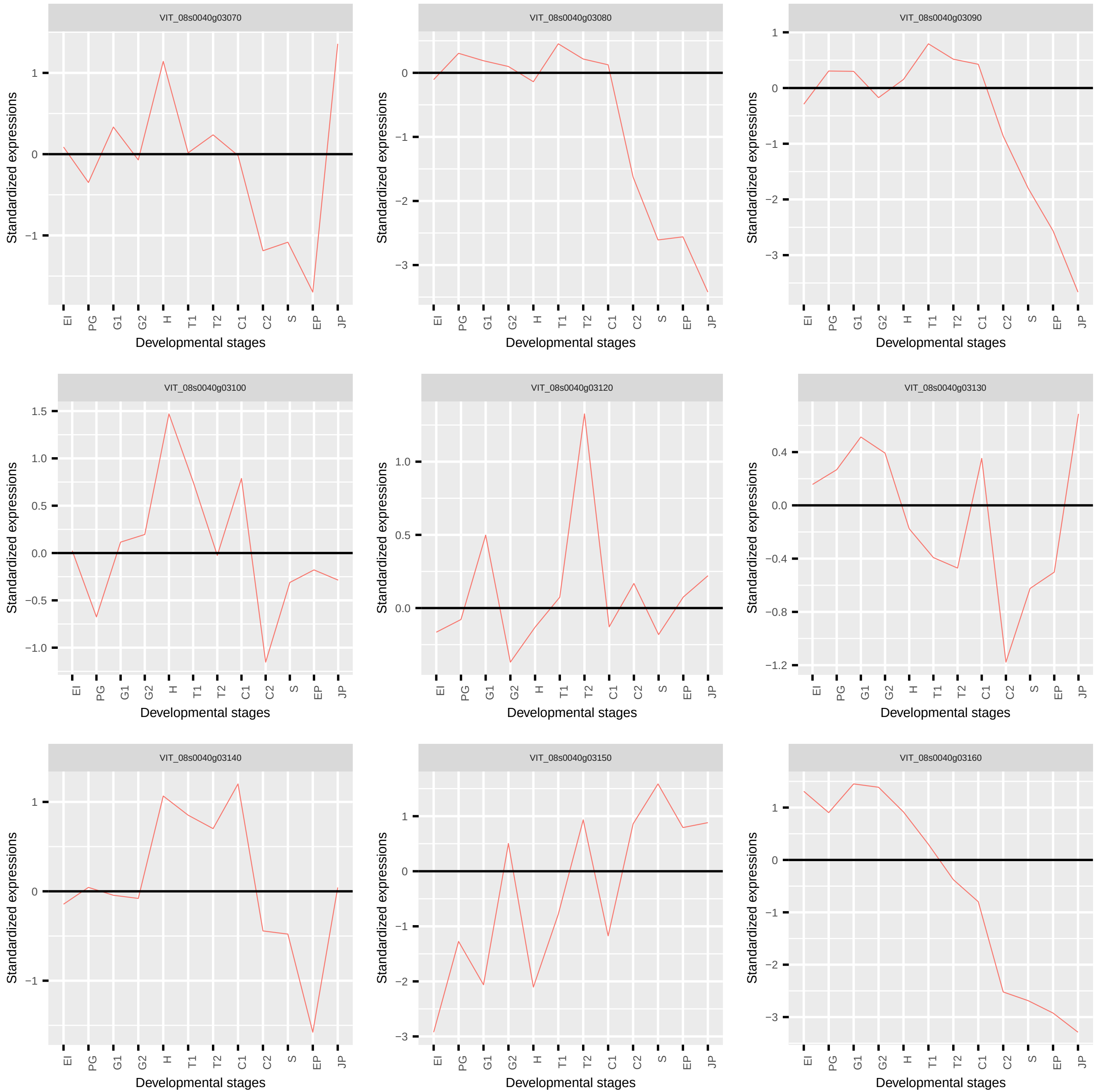
VIT_08s0040g03070
VIT_08s0040g03080
VIT_08s0040g03090
VIT_08s0040g03100
VIT_08s0040g03120
VIT_08s0040g03130
VIT_08s0040g03140
VIT_08s0040g03150
VIT_08s0040g03160
Standardized expressions
Developmental stages
EI
PG
G1
G2
H
T1
T2
C1
C2
S
EP
JP

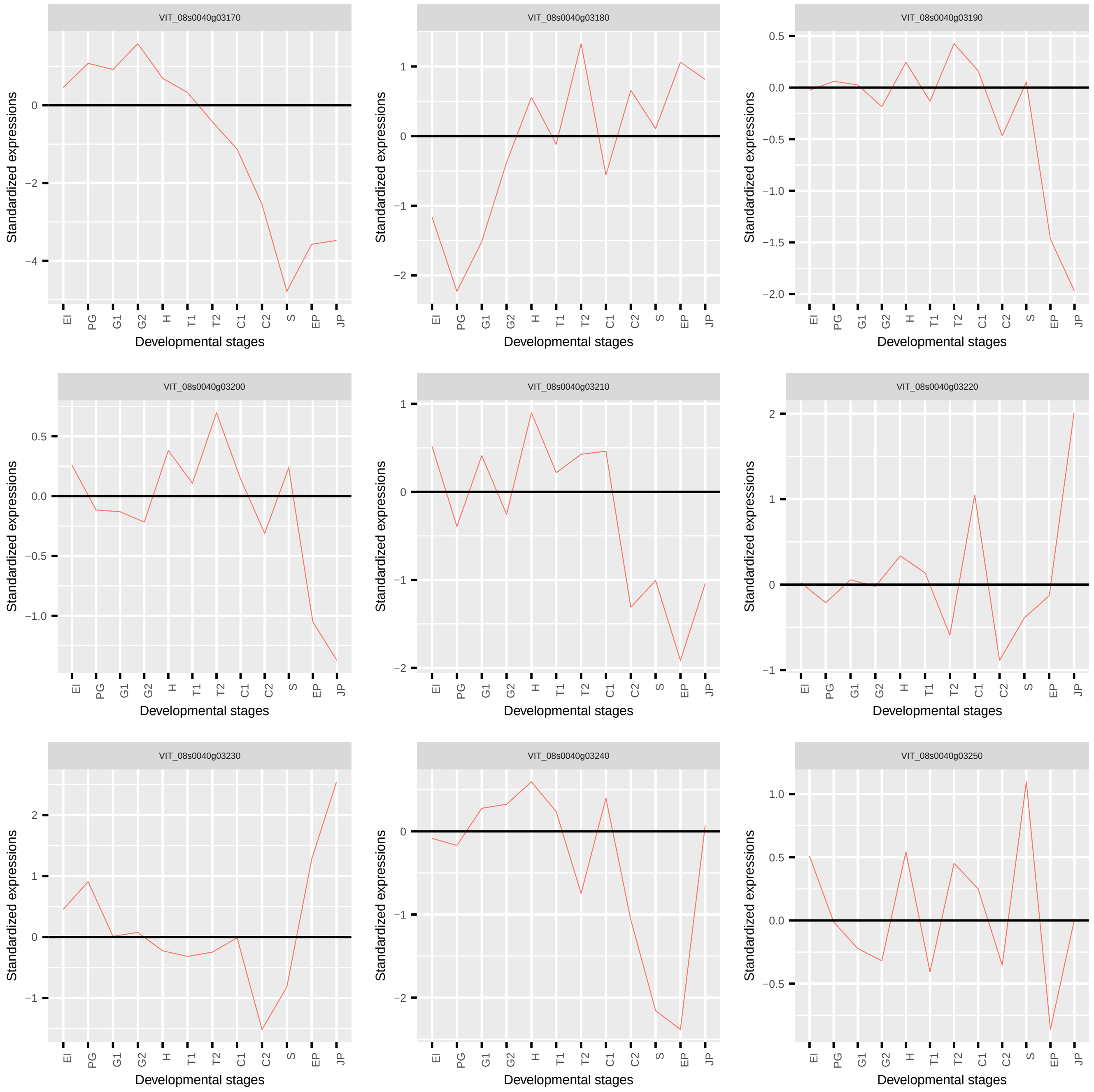
VIT_08s0040g03170
VIT_08s0040g03180
VIT_08s0040g03190
VIT_08s0040g03200
VIT_08s0040g03210
VIT_08s0040g03220
VIT_08s0040g03230
VIT_08s0040g03240
VIT_08s0040g03250
Standardized expressions
Developmental stages
EI PG G1 G2 H T1 T2 C1 C2 S EP JP

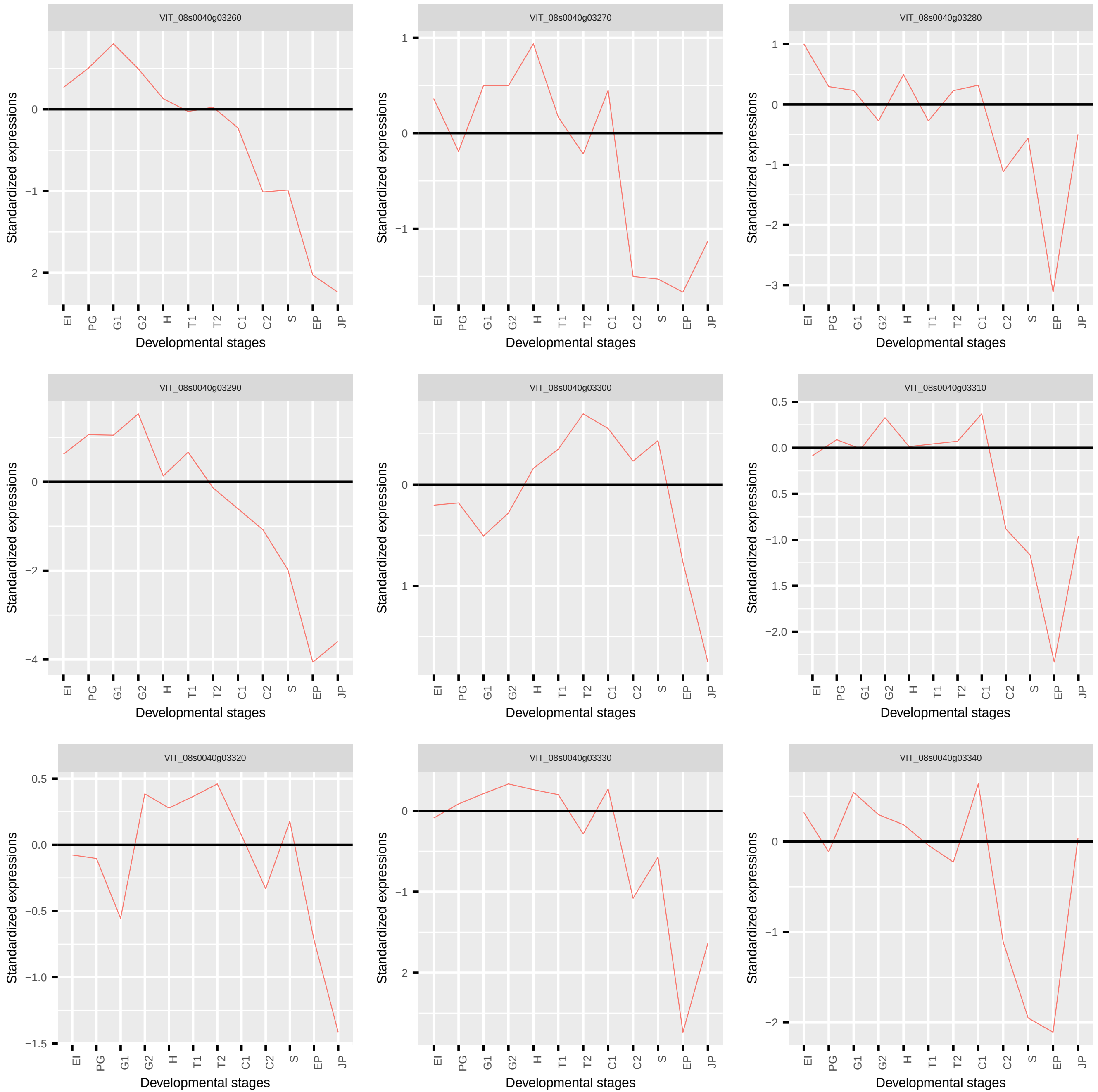
VIT_08s0040g03260
VIT_08s0040g03270
VIT_08s0040g03280
VIT_08s0040g03290
VIT_08s0040g03300
VIT_08s0040g03310
VIT_08s0040g03320
VIT_08s0040g03330
VIT_08s0040g03340
Standardized expressions
Developmental stages
EI PG G1 G2 H T1 T2 C1 C2 S EP JP

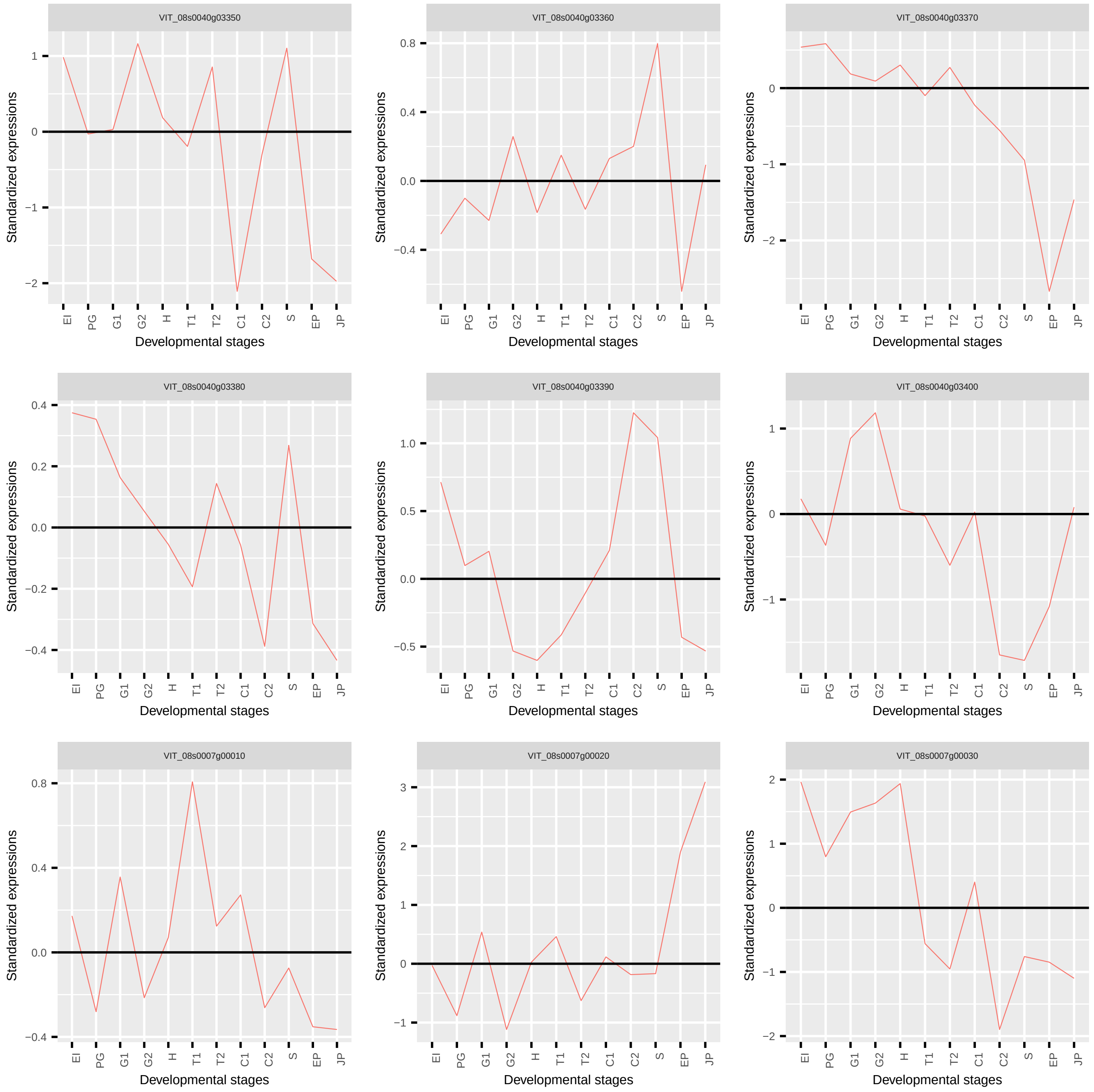
VIT_08s0040g03350
VIT_08s0040g03360
VIT_08s0040g03370
VIT_08s0040g03380
VIT_08s0040g03390
VIT_08s0040g03400
VIT_08s0007g00010
VIT_08s0007g00020
VIT_08s0007g00030
Standardized expressions
Developmental stages
EI PG G1 G2 H T1 T2 C1 C2 S EP JP
1 0 −1 −2
0.8 0.4 0.0 −0.4
0 −1 −2
0.4 0.2 0.0 −0.2 −0.4
1.0 0.5 0.0 −0.5
1 0 −1
0.8 0.4 0.0 −0.4
3 2 1 0 −1
2 1 0 −1 −2

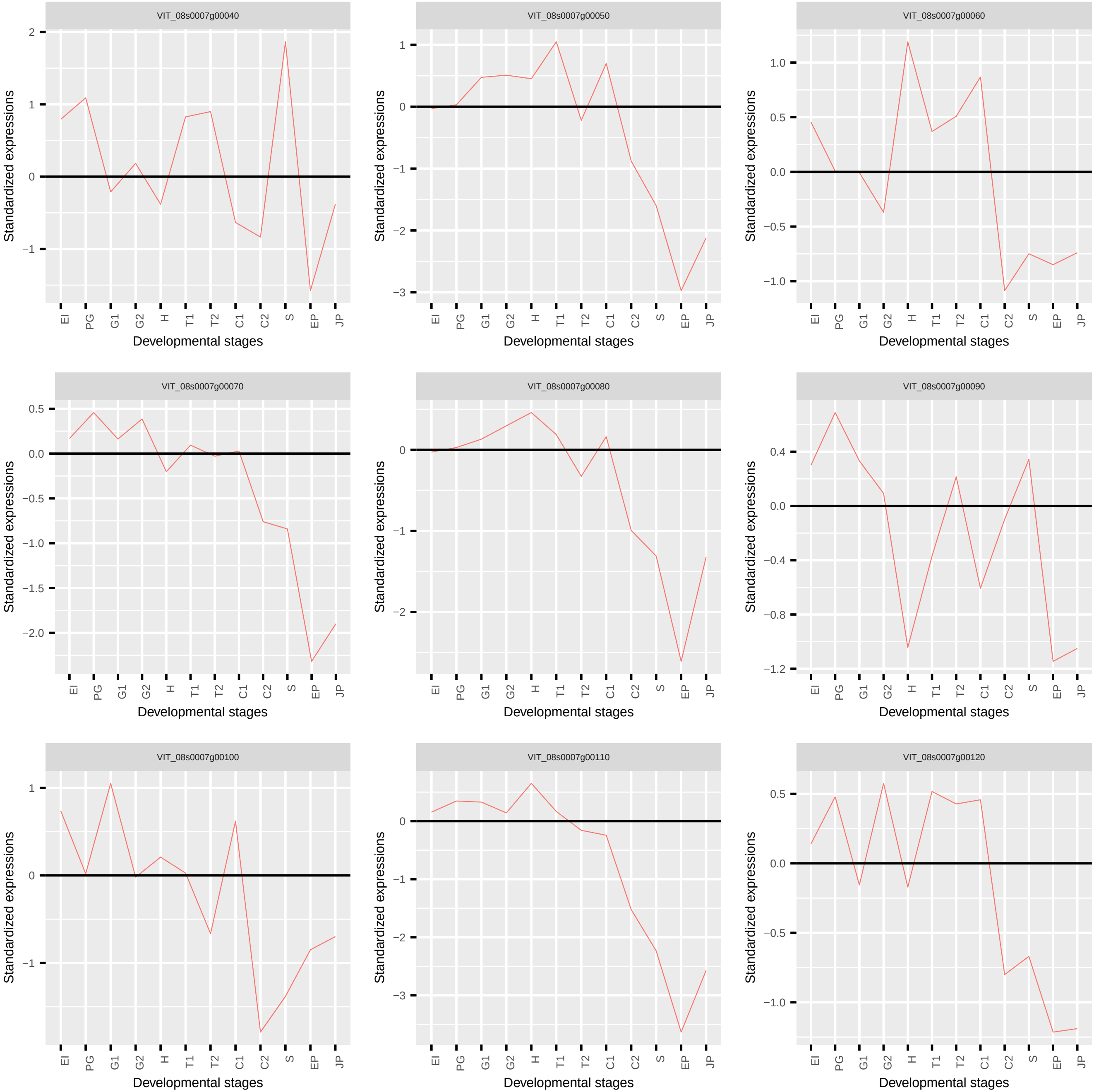
VIT_08s0007g00040
VIT_08s0007g00050
VIT_08s0007g00060
VIT_08s0007g00070
VIT_08s0007g00080
VIT_08s0007g00090
VIT_08s0007g00100
VIT_08s0007g00110
VIT_08s0007g00120
Standardized expressions
Developmental stages
EI PG G1 G2 H T1 T2 C1 C2 S EP JP

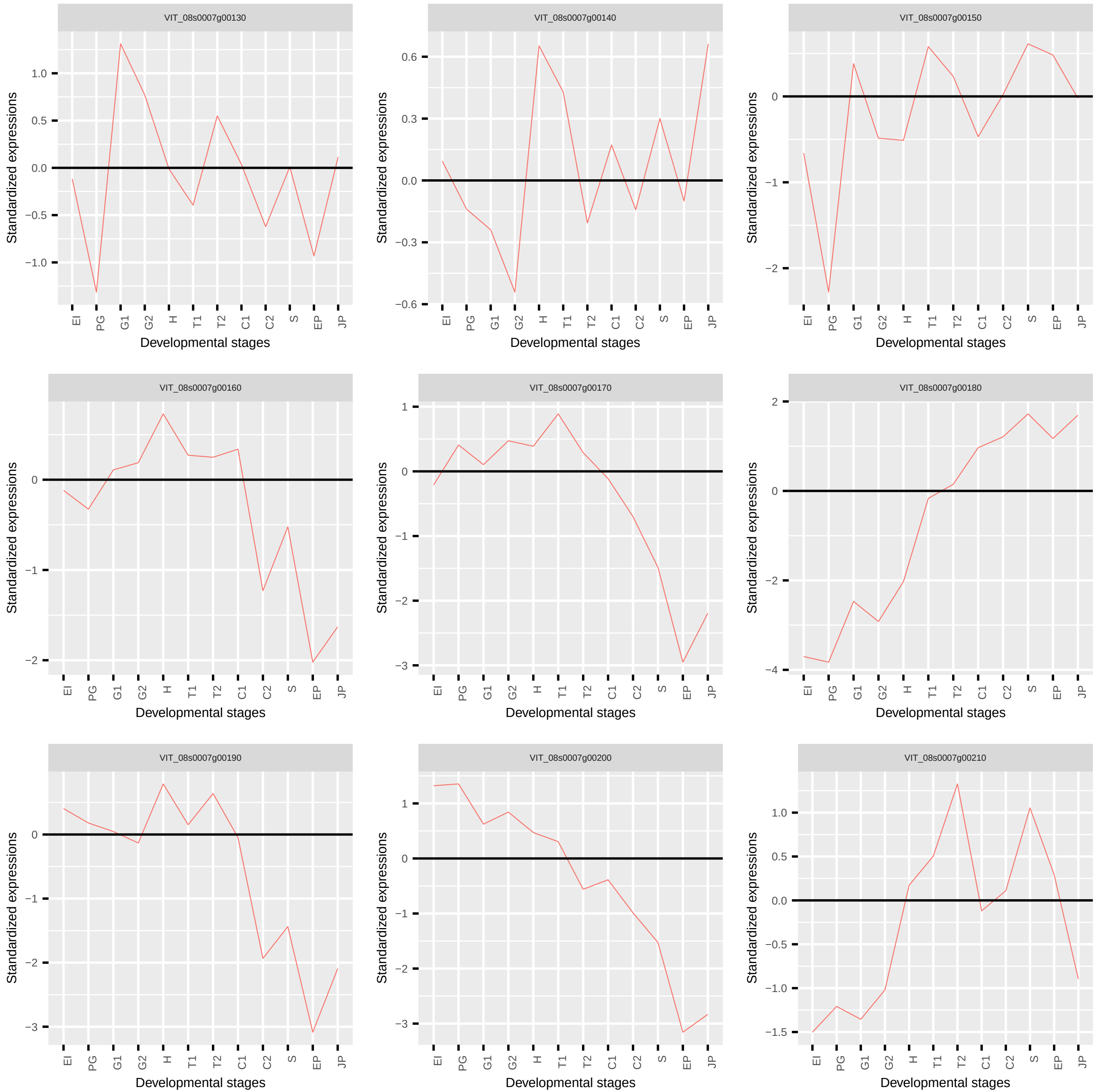
VIT_08s0007g00130
Standardized expressions
1.0
0.5
0.0
−0.5
−1.0
EI PG G1 G2 H T1 T2 C1 C2 S EP JP
Developmental stages
VIT_08s0007g00140
Standardized expressions
0.6
0.3
0.0
−0.3
−0.6
EI PG G1 G2 H T1 T2 C1 C2 S EP JP
Developmental stages
VIT_08s0007g00150
Standardized expressions
0
−1
−2
EI PG G1 G2 H T1 T2 C1 C2 S EP JP
Developmental stages
VIT_08s0007g00160
Standardized expressions
0
−1
−2
EI PG G1 G2 H T1 T2 C1 C2 S EP JP
Developmental stages
VIT_08s0007g00170
Standardized expressions
1
0
−1
−2
−3
EI PG G1 G2 H T1 T2 C1 C2 S EP JP
Developmental stages
VIT_08s0007g00180
Standardized expressions
2
0
−2
−4
EI PG G1 G2 H T1 T2 C1 C2 S EP JP
Developmental stages
VIT_08s0007g00190
Standardized expressions
0
−1
−2
−3
EI PG G1 G2 H T1 T2 C1 C2 S EP JP
Developmental stages
VIT_08s0007g00200
Standardized expressions
1
0
−1
−2
−3
EI PG G1 G2 H T1 T2 C1 C2 S EP JP
Developmental stages
VIT_08s0007g00210
Standardized expressions
1.0
0.5
0.0
−0.5
−1.0
−1.5
EI PG G1 G2 H T1 T2 C1 C2 S EP JP
Developmental stages

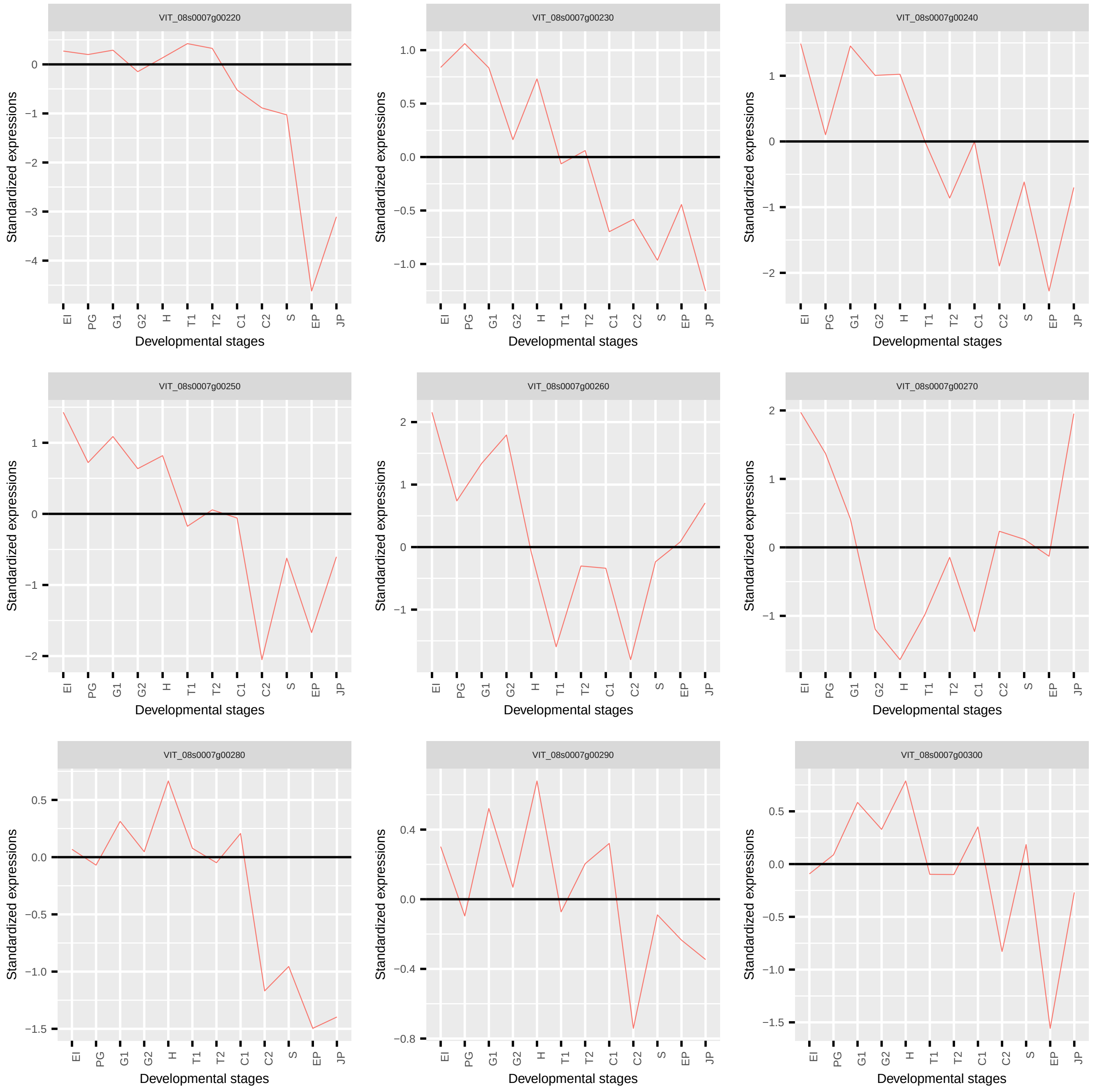
VIT_08s0007g00220
VIT_08s0007g00230
VIT_08s0007g00240
VIT_08s0007g00250
VIT_08s0007g00260
VIT_08s0007g00270
VIT_08s0007g00280
VIT_08s0007g00290
VIT_08s0007g00300
Standardized expressions
Developmental stages
EI PG G1 G2 H T1 T2 C1 C2 S EP JP

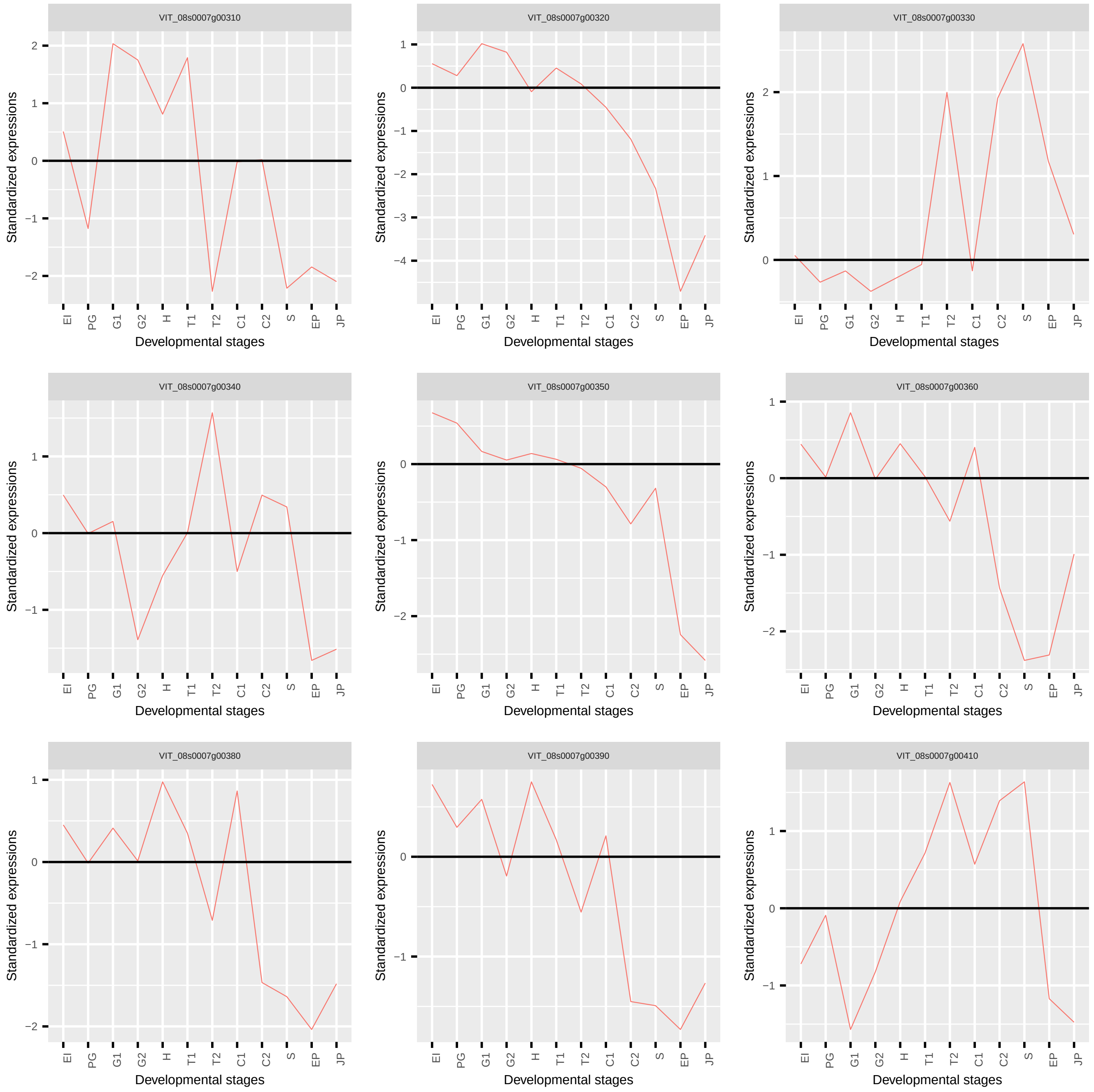

VIT_08s0007g00310
VIT_08s0007g00320
VIT_08s0007g00330
VIT_08s0007g00340
VIT_08s0007g00350
VIT_08s0007g00360
VIT_08s0007g00380
VIT_08s0007g00390
VIT_08s0007g00410
Standardized expressions
Developmental stages
EI PG G1 G2 H T1 T2 C1 C2 S EP JP

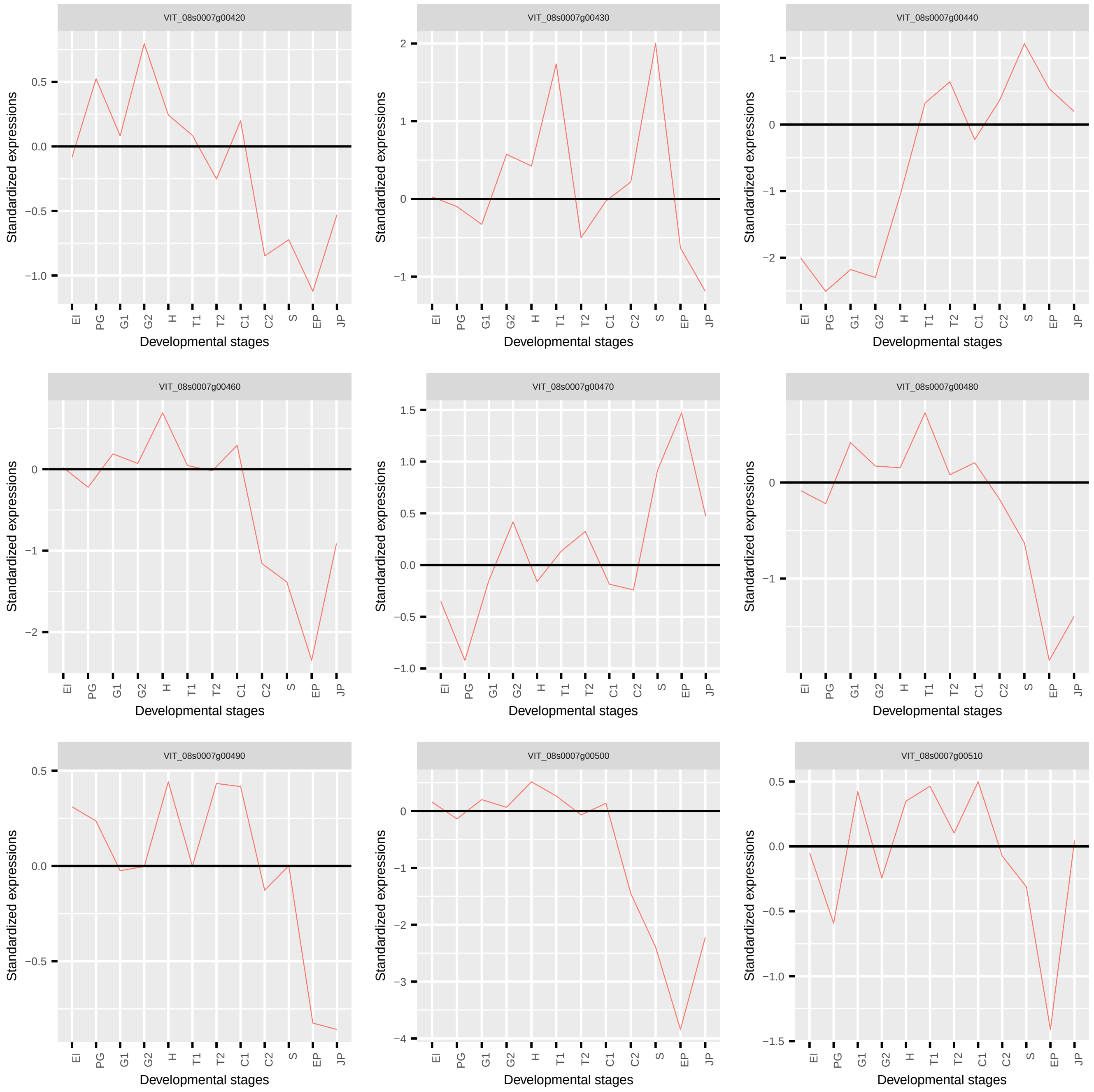

VIT_08s0007g00420
VIT_08s0007g00430
VIT_08s0007g00440
VIT_08s0007g00460
VIT_08s0007g00470
VIT_08s0007g00480
VIT_08s0007g00490
VIT_08s0007g00500
VIT_08s0007g00510
Standardized expressions
Developmental stages
EI PG G1 G2 H T1 T2 C1 C2 S EP JP

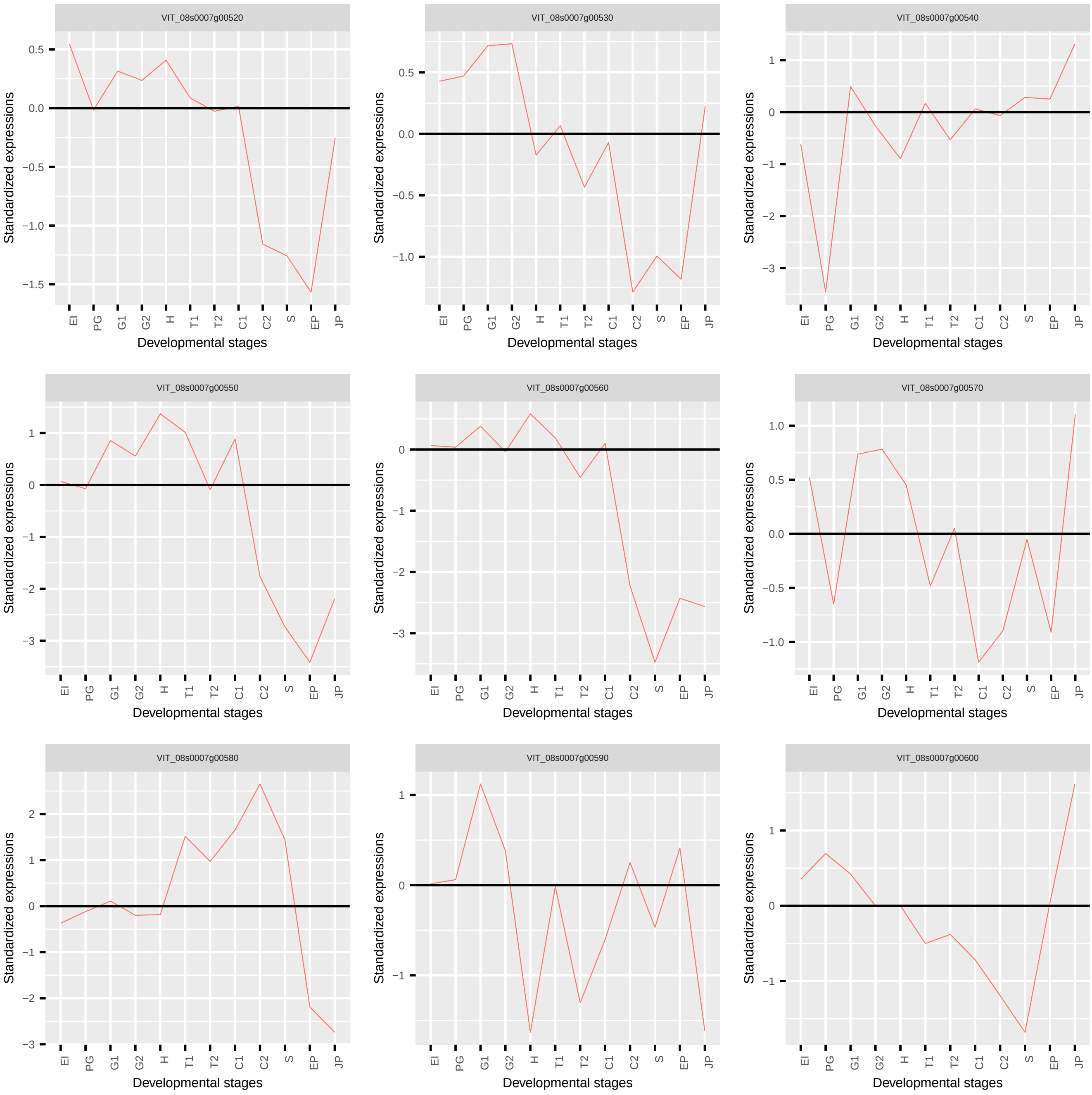
VIT_08s0007g00520
VIT_08s0007g00530
VIT_08s0007g00540
VIT_08s0007g00550
VIT_08s0007g00560
VIT_08s0007g00570
VIT_08s0007g00580
VIT_08s0007g00590
VIT_08s0007g00600
Standardized expressions
Developmental stages
EI PG G1 G2 H T1 T2 C1 C2 S EP JP

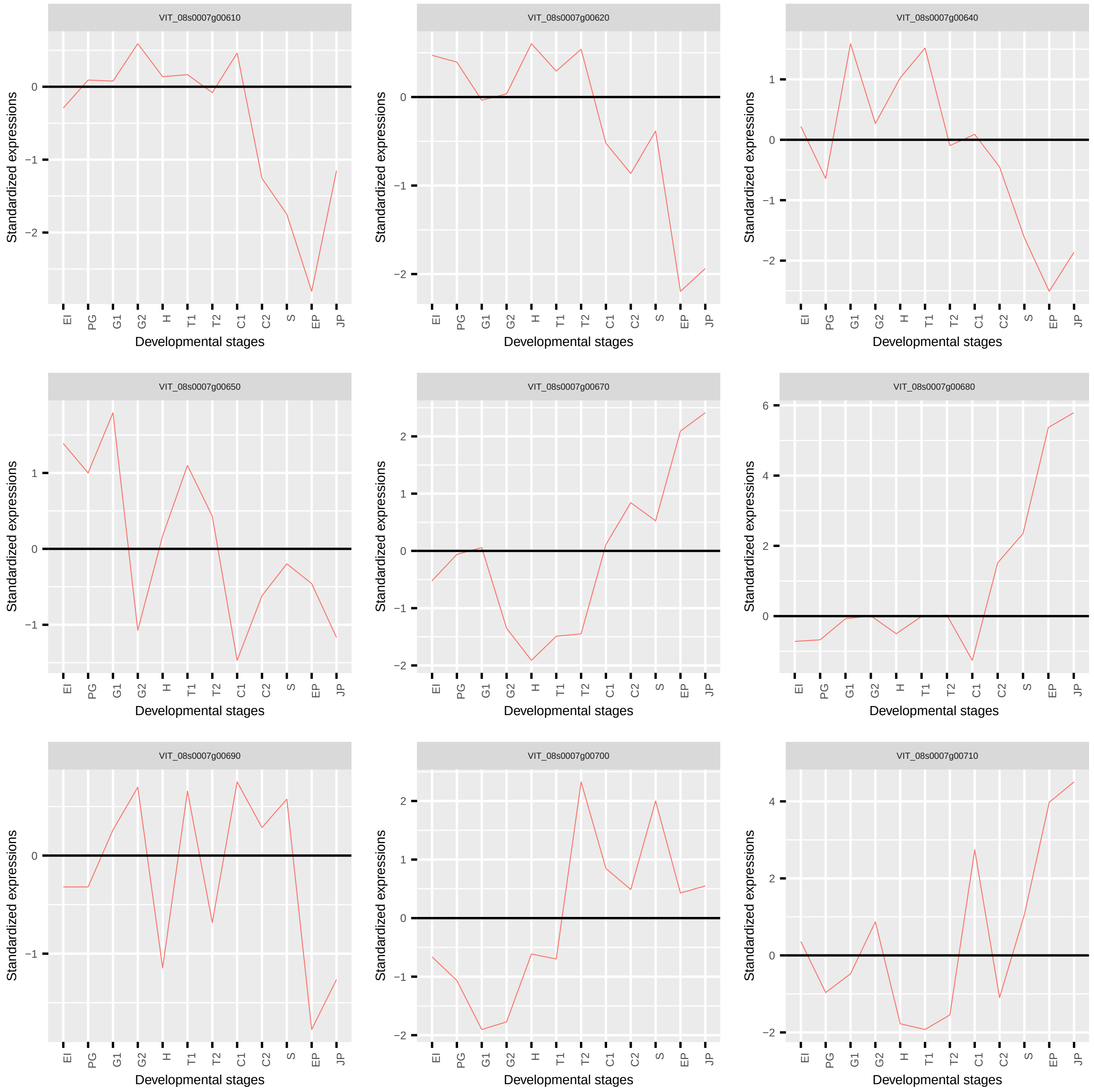
VIT_08s0007g00610
VIT_08s0007g00620
VIT_08s0007g00640
VIT_08s0007g00650
VIT_08s0007g00670
VIT_08s0007g00680
VIT_08s0007g00690
VIT_08s0007g00700
VIT_08s0007g00710
Standardized expressions
Developmental stages
EI PG G1 G2 H T1 T2 C1 C2 S EP JP

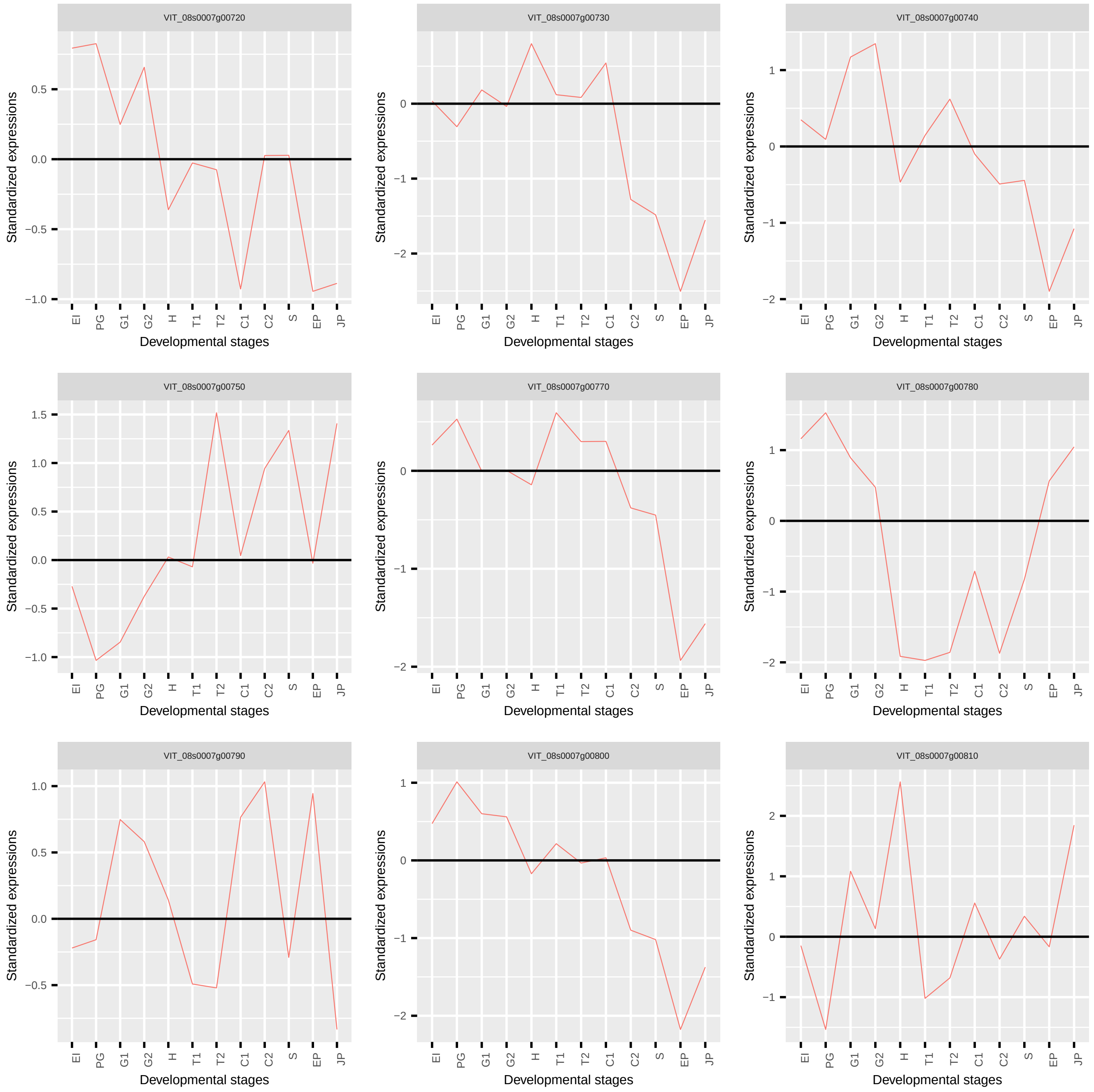
VIT_08s0007g00720
VIT_08s0007g00730
VIT_08s0007g00740
VIT_08s0007g00750
VIT_08s0007g00770
VIT_08s0007g00780
VIT_08s0007g00790
VIT_08s0007g00800
VIT_08s0007g00810
Standardized expressions
Developmental stages
EI PG G1 G2 H T1 T2 C1 C2 S EP JP

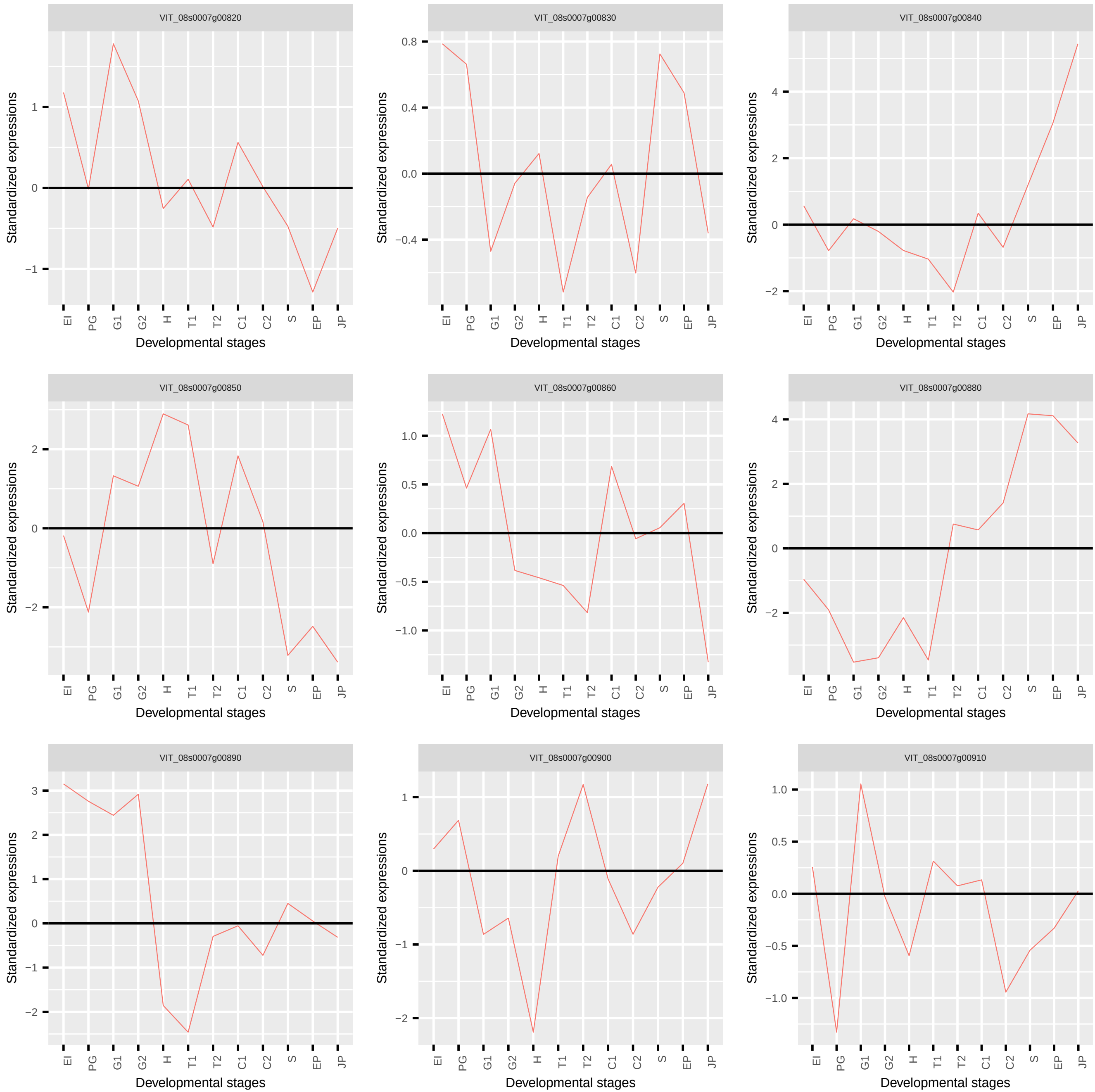
VIT_08s0007g00820
VIT_08s0007g00830
VIT_08s0007g00840
VIT_08s0007g00850
VIT_08s0007g00860
VIT_08s0007g00880
VIT_08s0007g00890
VIT_08s0007g00900
VIT_08s0007g00910
Standardized expressions
Developmental stages
EI PG G1 G2 H T1 T2 C1 C2 S EP JP

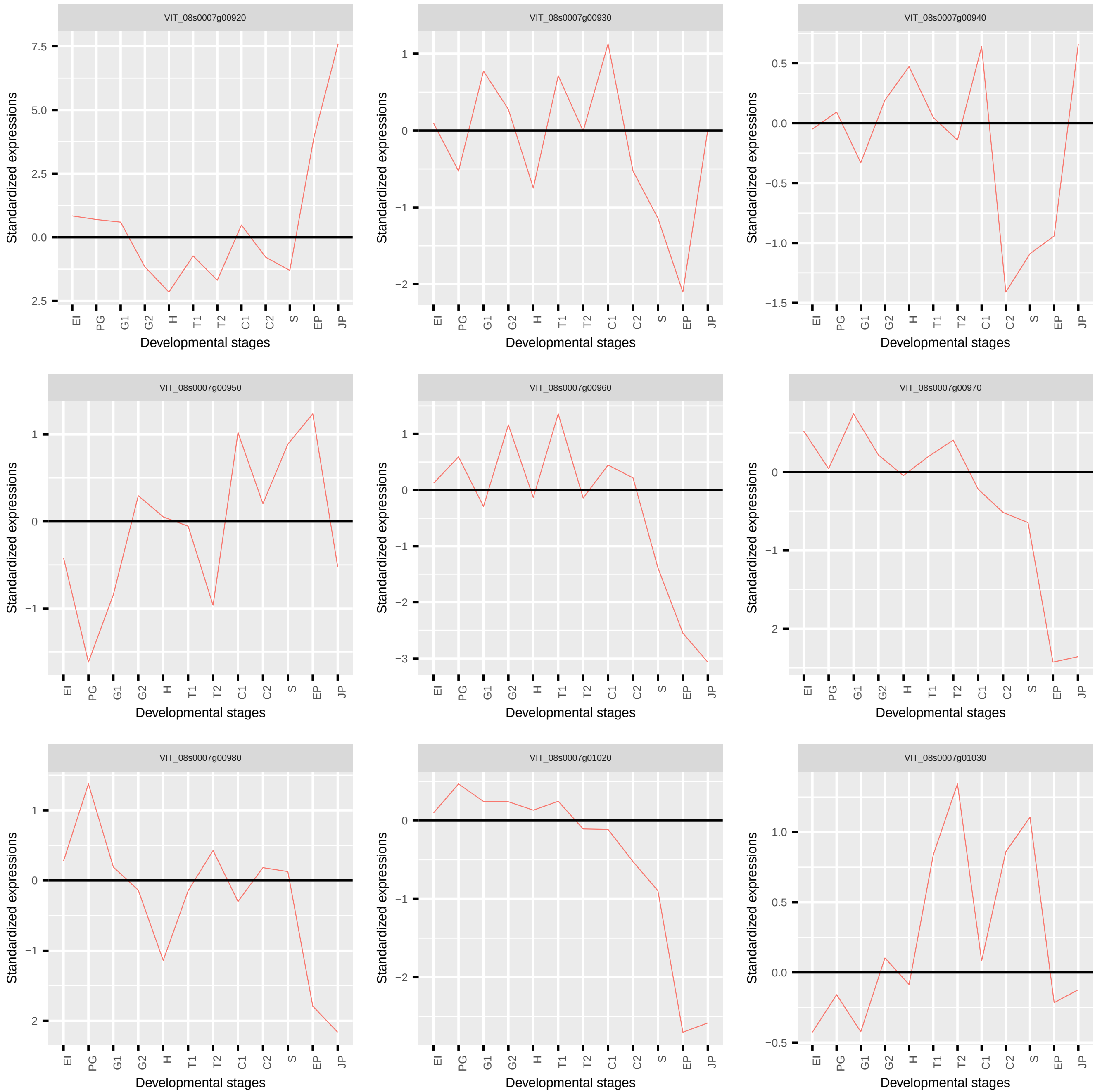
VIT_08s0007g00920
VIT_08s0007g00930
VIT_08s0007g00940
VIT_08s0007g00950
VIT_08s0007g00960
VIT_08s0007g00970
VIT_08s0007g00980
VIT_08s0007g01020
VIT_08s0007g01030
Standardized expressions
Developmental stages
EI PG G1 G2 H T1 T2 C1 C2 S EP JP

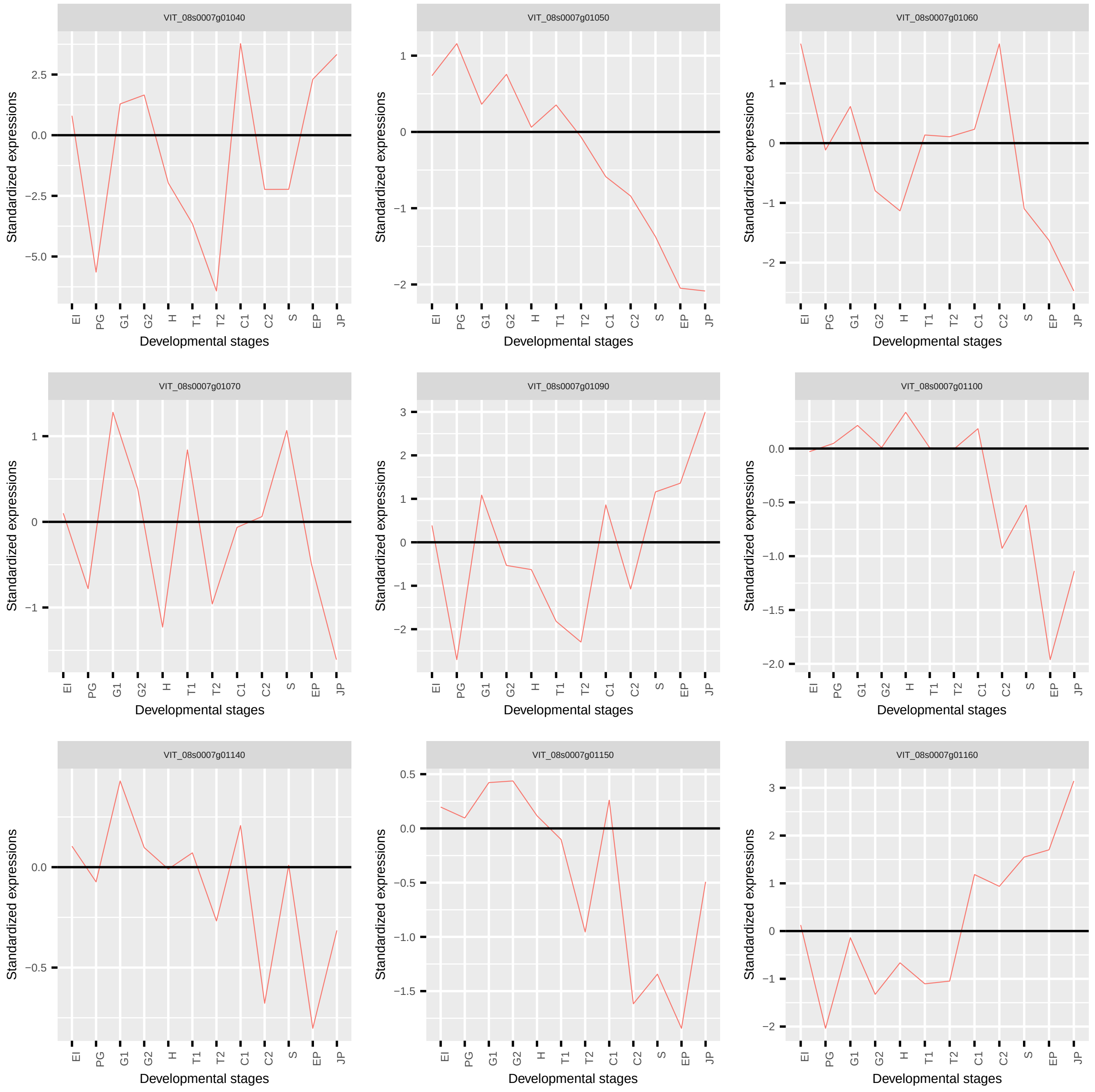
VIT_08s0007g01040
VIT_08s0007g01050
VIT_08s0007g01060
VIT_08s0007g01070
VIT_08s0007g01090
VIT_08s0007g01100
VIT_08s0007g01140
VIT_08s0007g01150
VIT_08s0007g01160
Standardized expressions
Developmental stages
EI PG G1 G2 H T1 T2 C1 C2 S EP JP

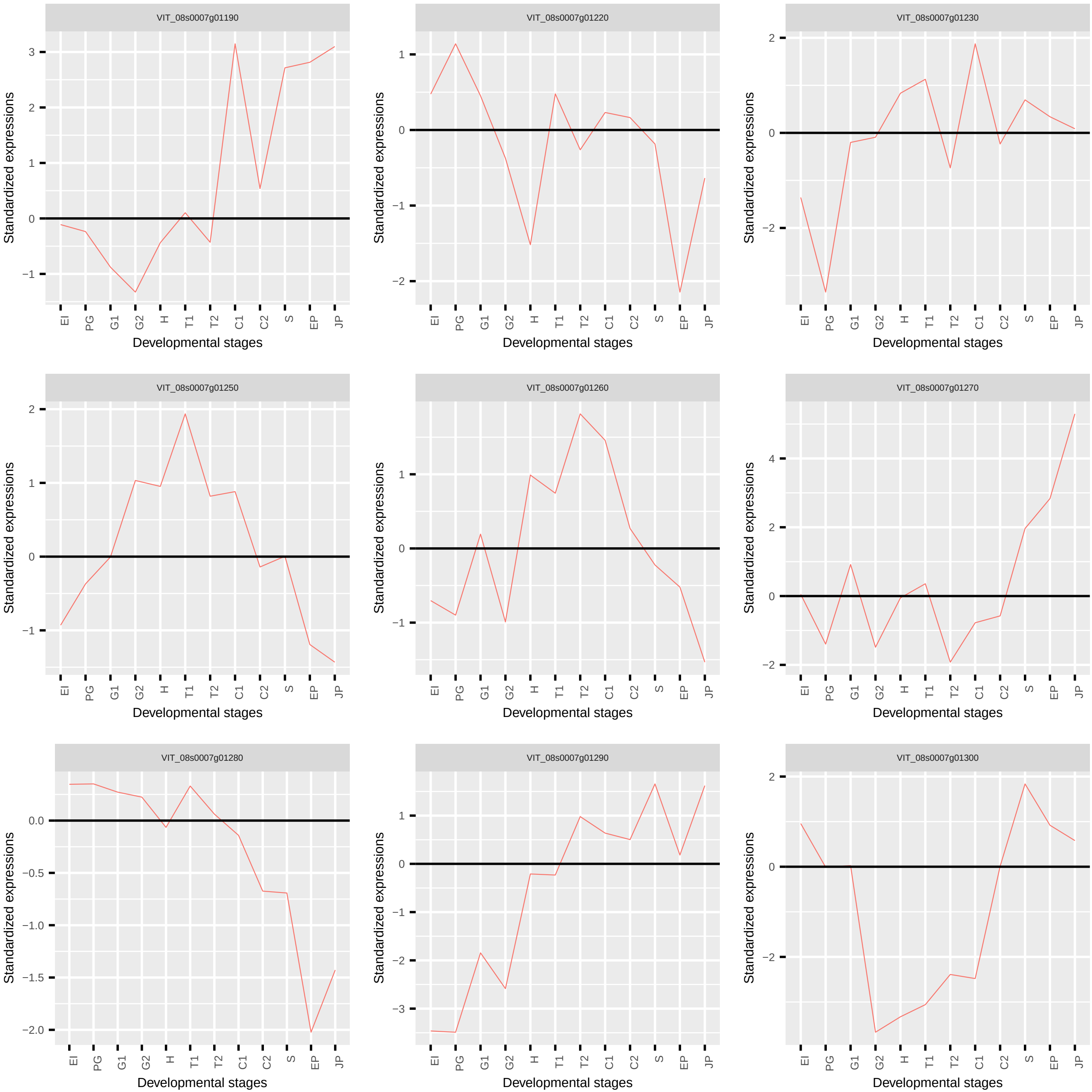

VIT_08s0007g01190
VIT_08s0007g01220
VIT_08s0007g01230
VIT_08s0007g01250
VIT_08s0007g01260
VIT_08s0007g01270
VIT_08s0007g01280
VIT_08s0007g01290
VIT_08s0007g01300
Standardized expressions
Developmental stages
EI PG G1 G2 H T1 T2 C1 C2 S EP JP

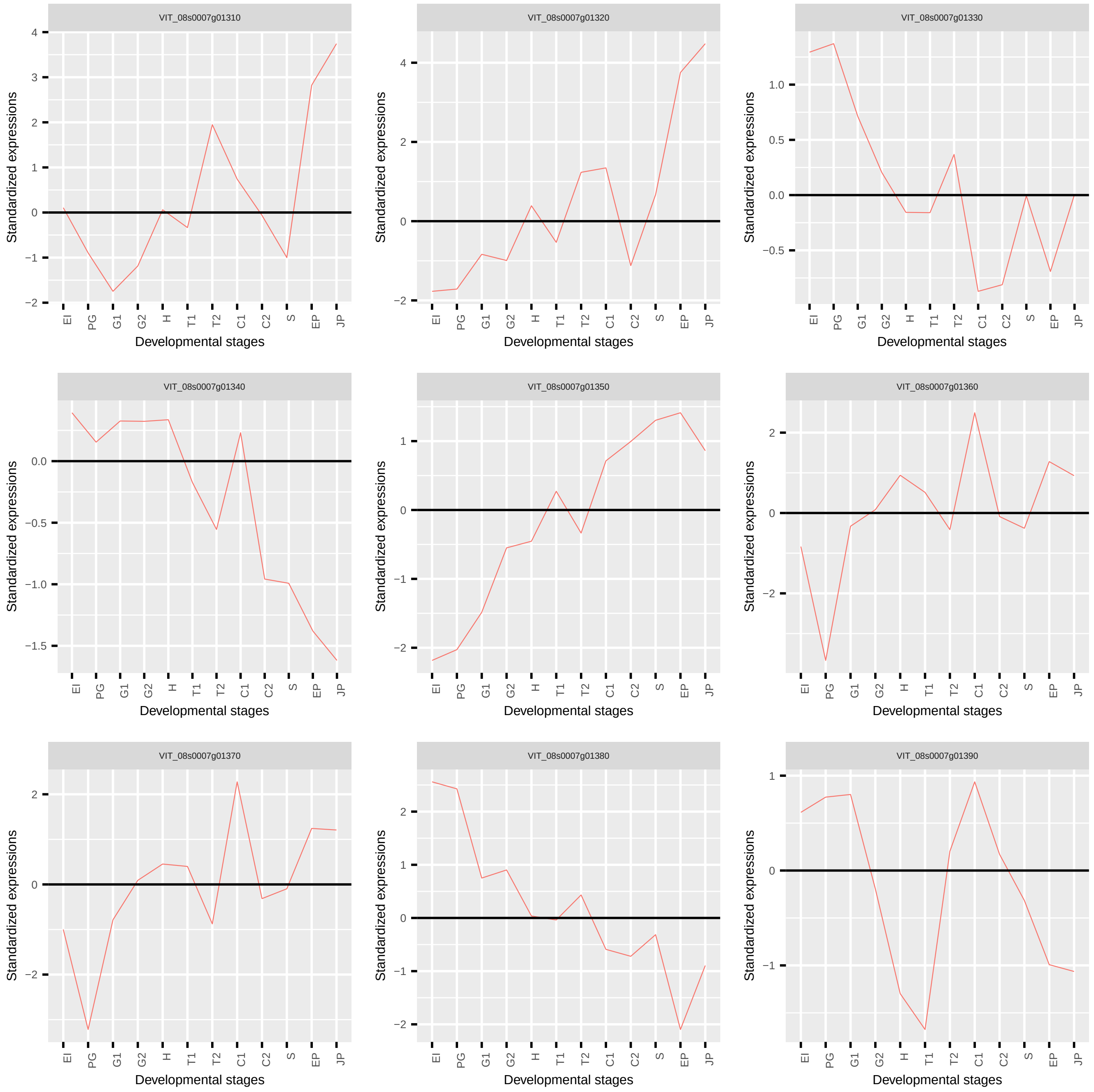
VIT_08s0007g01310
VIT_08s0007g01320
VIT_08s0007g01330
VIT_08s0007g01340
VIT_08s0007g01350
VIT_08s0007g01360
VIT_08s0007g01370
VIT_08s0007g01380
VIT_08s0007g01390
Standardized expressions
Developmental stages
EI
PG
G1
G2
H
T1
T2
C1
C2
S
EP
JP

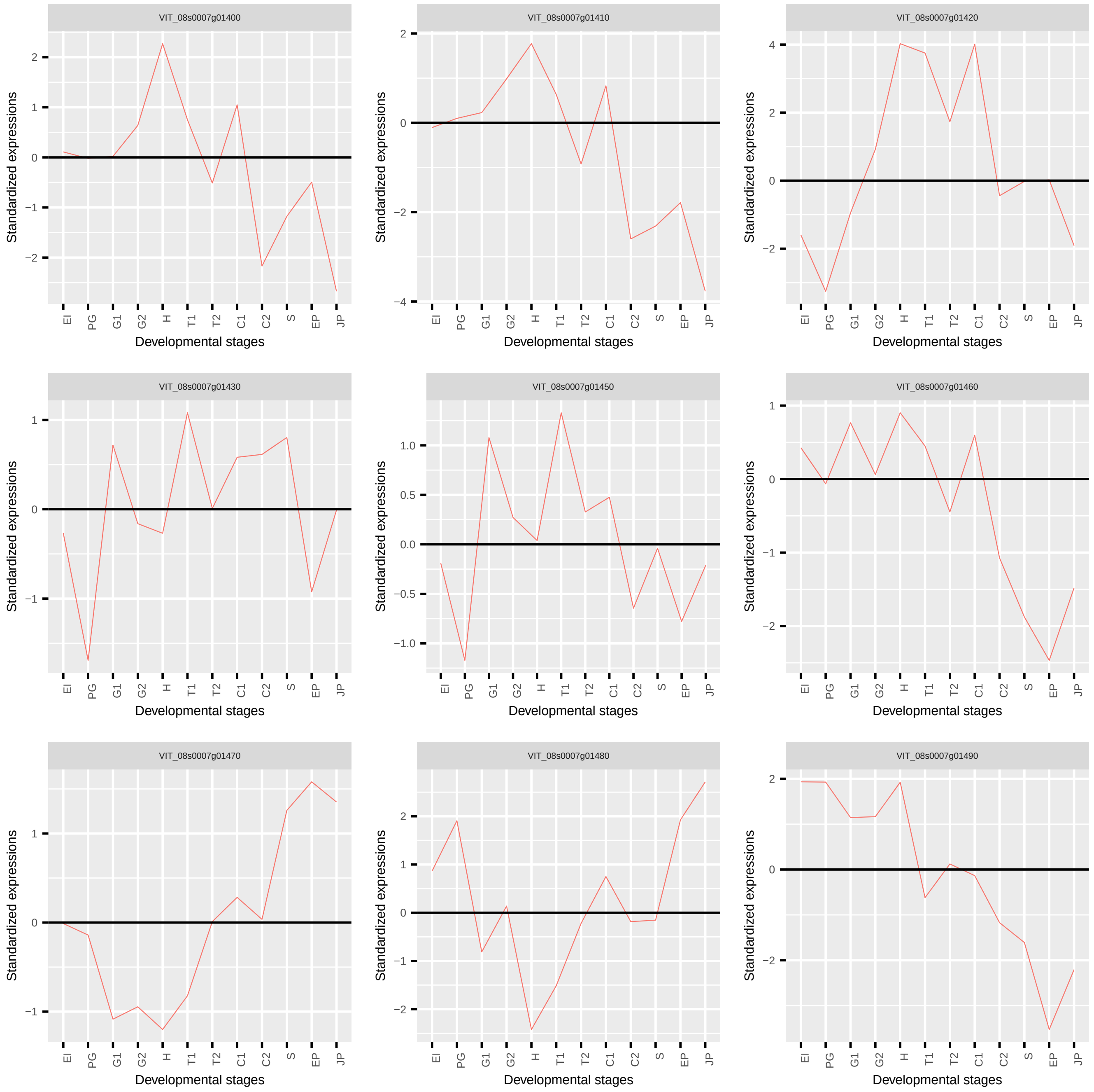
VIT_08s0007g01400
VIT_08s0007g01410
VIT_08s0007g01420
VIT_08s0007g01430
VIT_08s0007g01450
VIT_08s0007g01460
VIT_08s0007g01470
VIT_08s0007g01480
VIT_08s0007g01490
Standardized expressions
Developmental stages
EI PG G1 G2 H T1 T2 C1 C2 S EP JP

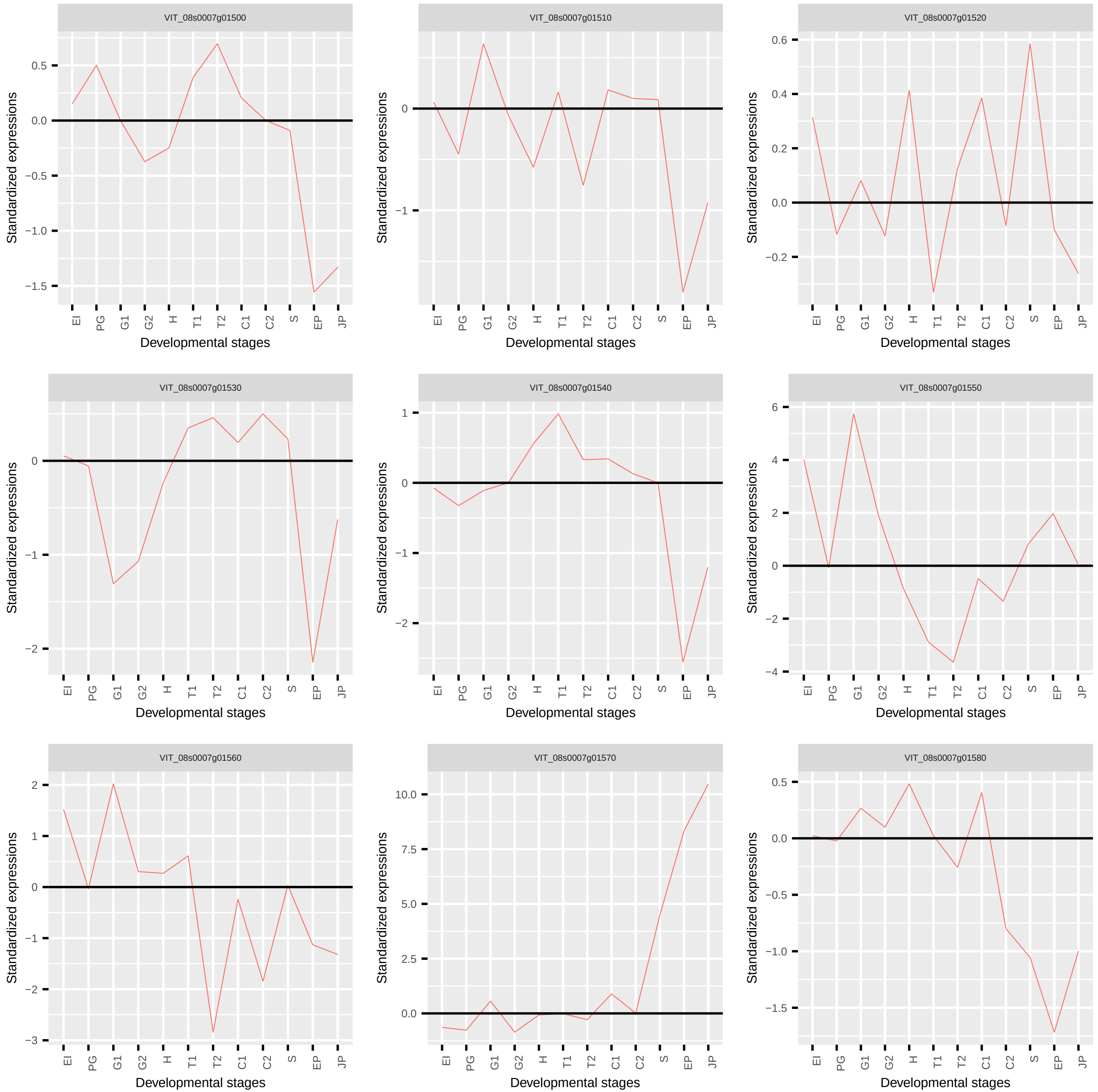
VIT_08s0007g01500
VIT_08s0007g01510
VIT_08s0007g01520
VIT_08s0007g01530
VIT_08s0007g01540
VIT_08s0007g01550
VIT_08s0007g01560
VIT_08s0007g01570
VIT_08s0007g01580
Standardized expressions
Developmental stages
EI PG G1 G2 H T1 T2 C1 C2 S EP JP

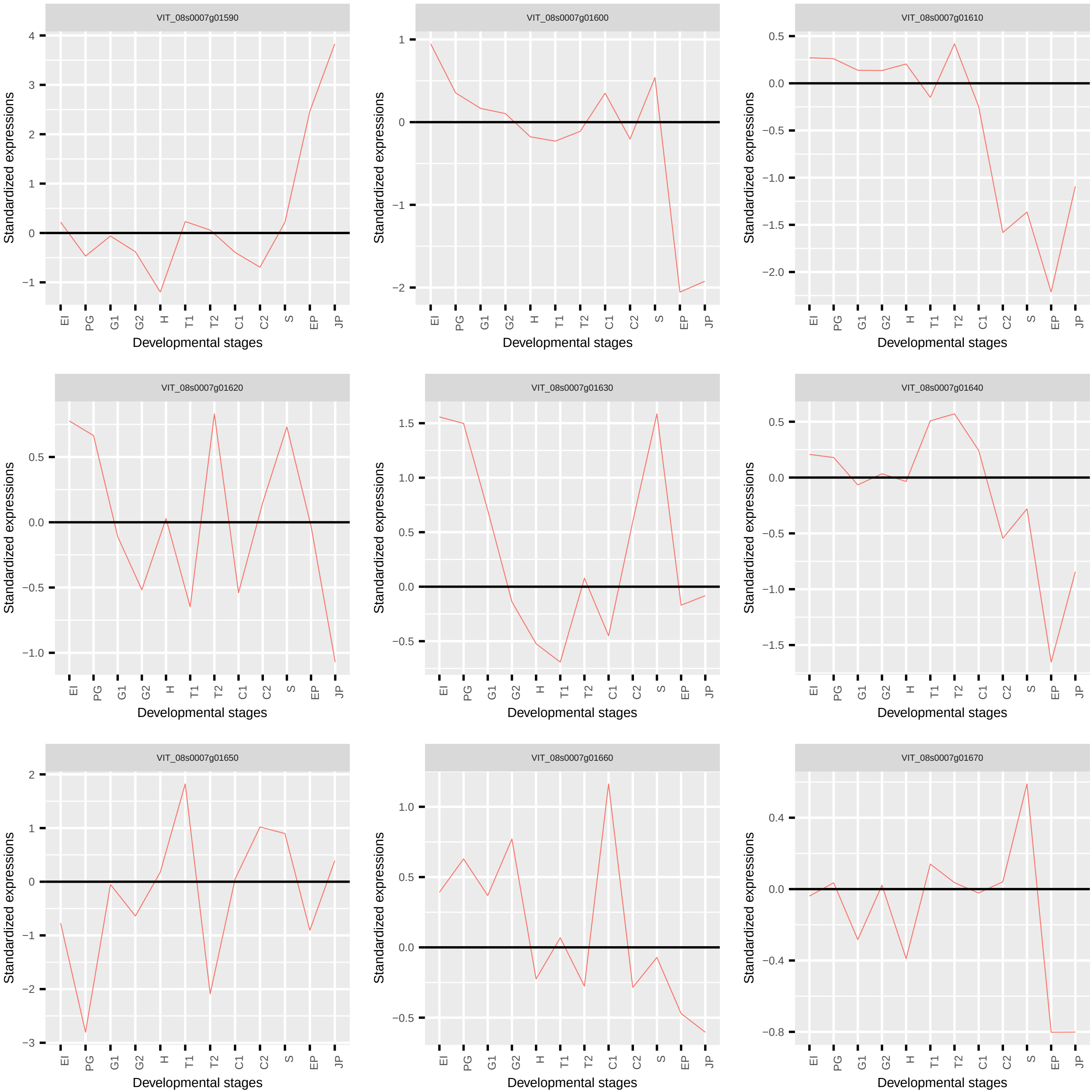
VIT_08s0007g01590
VIT_08s0007g01600
VIT_08s0007g01610
VIT_08s0007g01620
VIT_08s0007g01630
VIT_08s0007g01640
VIT_08s0007g01650
VIT_08s0007g01660
VIT_08s0007g01670
Standardized expressions
Developmental stages
EI PG G1 G2 H T1 T2 C1 C2 S EP JP

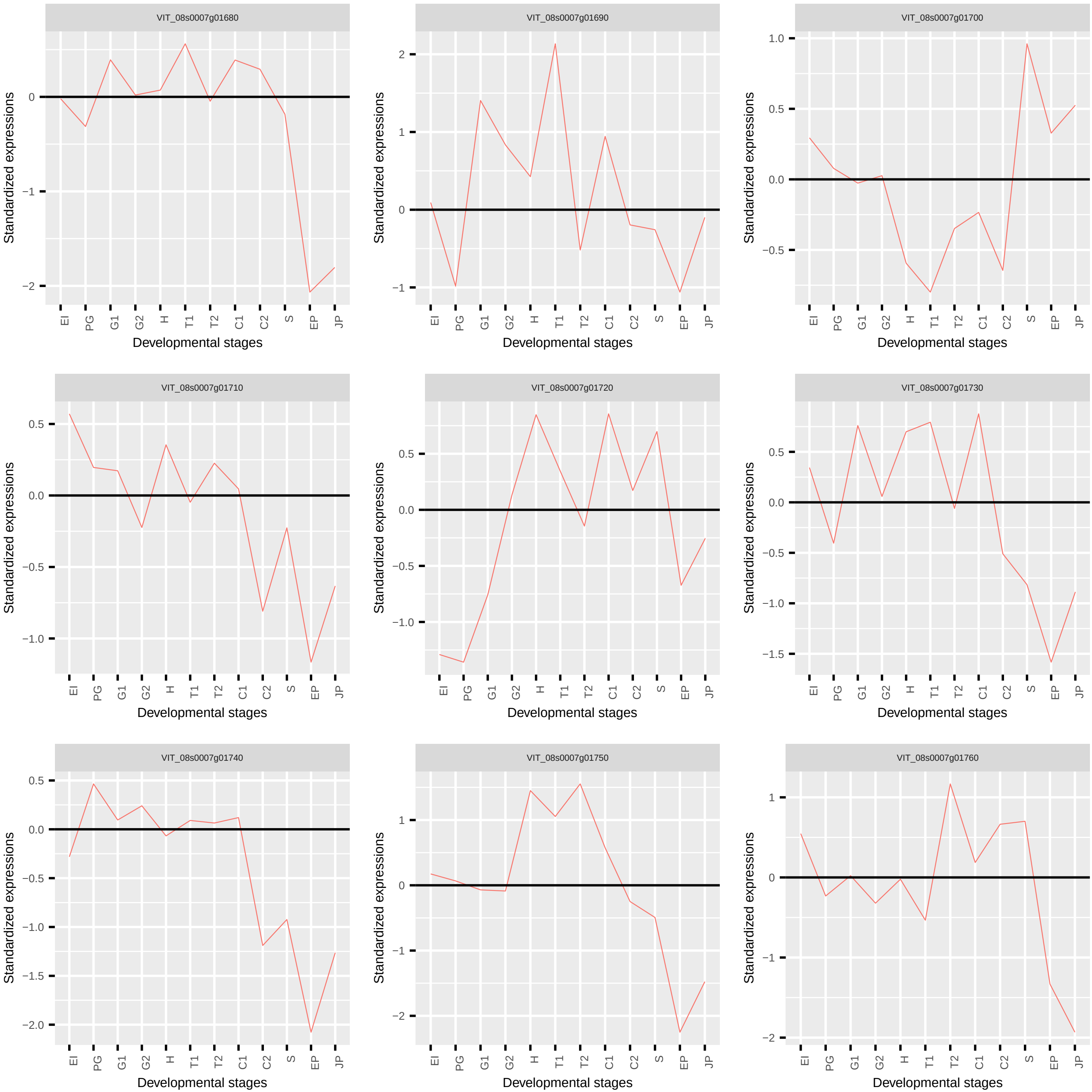

VIT_08s0007g01680
VIT_08s0007g01690
VIT_08s0007g01700
VIT_08s0007g01710
VIT_08s0007g01720
VIT_08s0007g01730
VIT_08s0007g01740
VIT_08s0007g01750
VIT_08s0007g01760
Standardized expressions
Developmental stages
EI
PG
G1
G2
H
T1
T2
C1
C2
S
EP
JP

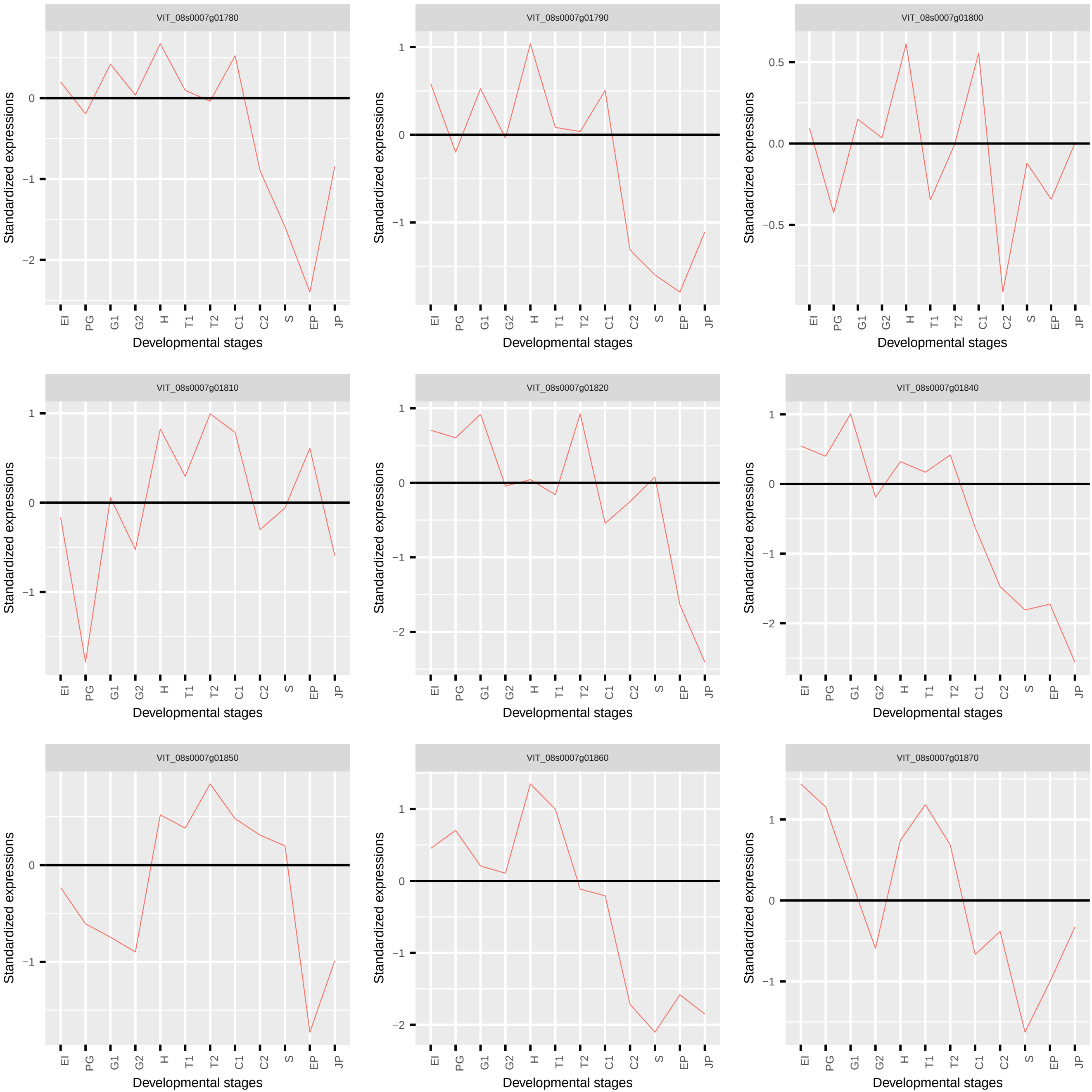
VIT_08s0007g01780
VIT_08s0007g01790
VIT_08s0007g01800
VIT_08s0007g01810
VIT_08s0007g01820
VIT_08s0007g01840
VIT_08s0007g01850
VIT_08s0007g01860
VIT_08s0007g01870
Standardized expressions
Developmental stages
EI
PG
G1
G2
H
T1
T2
C1
C2
S
EP
JP

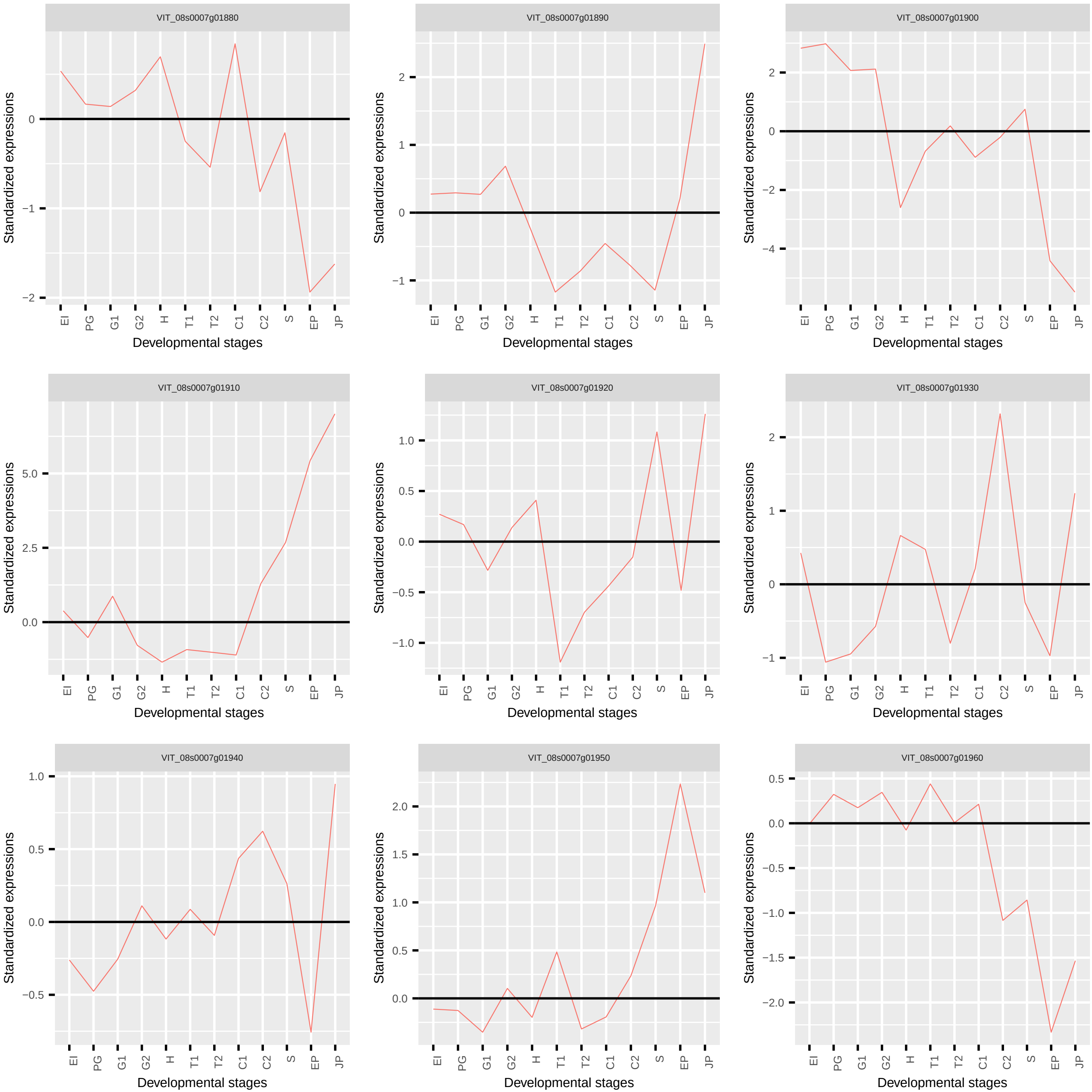
VIT_08s0007g01880
VIT_08s0007g01890
VIT_08s0007g01900
VIT_08s0007g01910
VIT_08s0007g01920
VIT_08s0007g01930
VIT_08s0007g01940
VIT_08s0007g01950
VIT_08s0007g01960
Standardized expressions
Developmental stages
EI PG G1 G2 H T1 T2 C1 C2 S EP JP

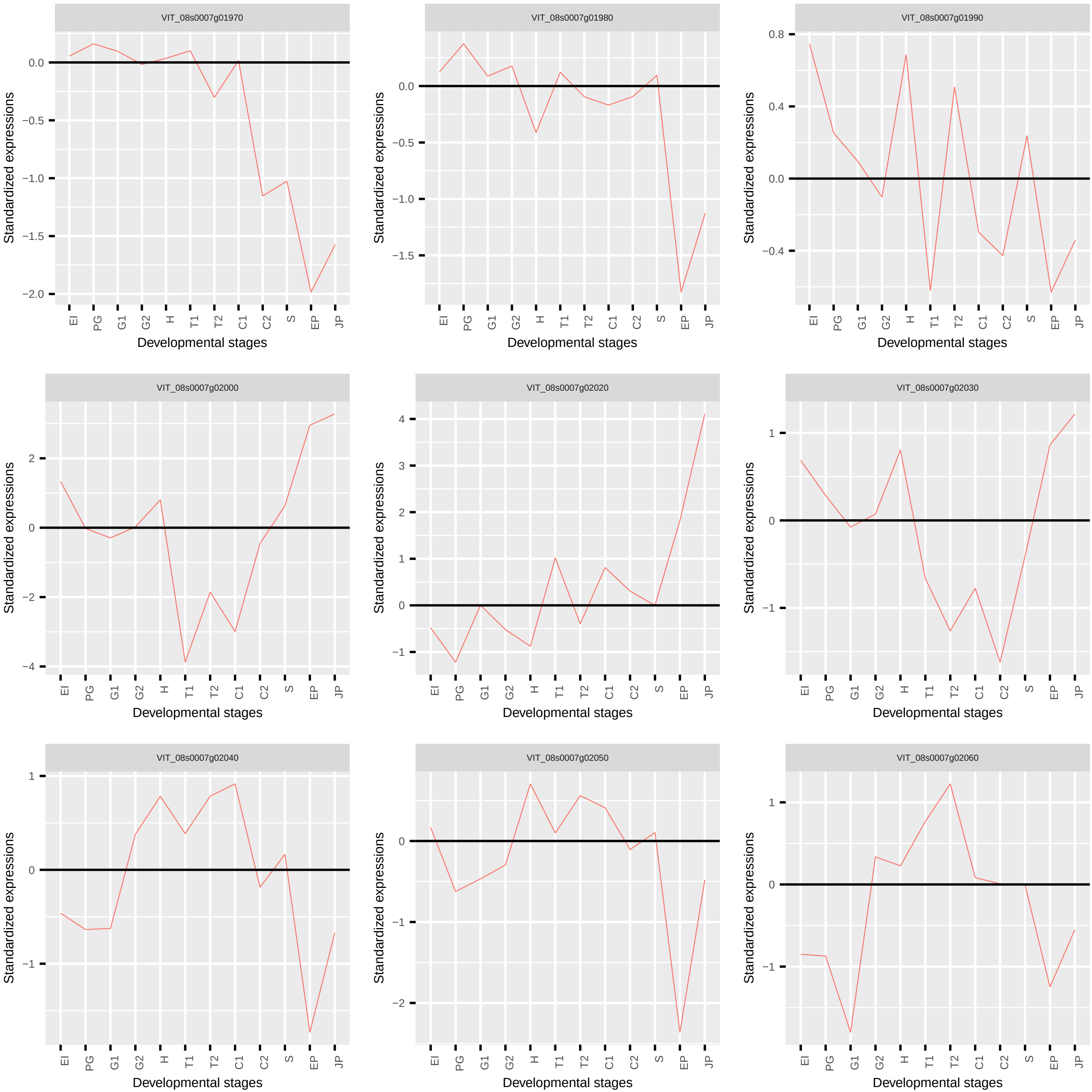
VIT_08s0007g01970
VIT_08s0007g01980
VIT_08s0007g01990
VIT_08s0007g02000
VIT_08s0007g02020
VIT_08s0007g02030
VIT_08s0007g02040
VIT_08s0007g02050
VIT_08s0007g02060
Standardized expressions
Developmental stages
EI PG G1 G2 H T1 T2 C1 C2 S EP JP

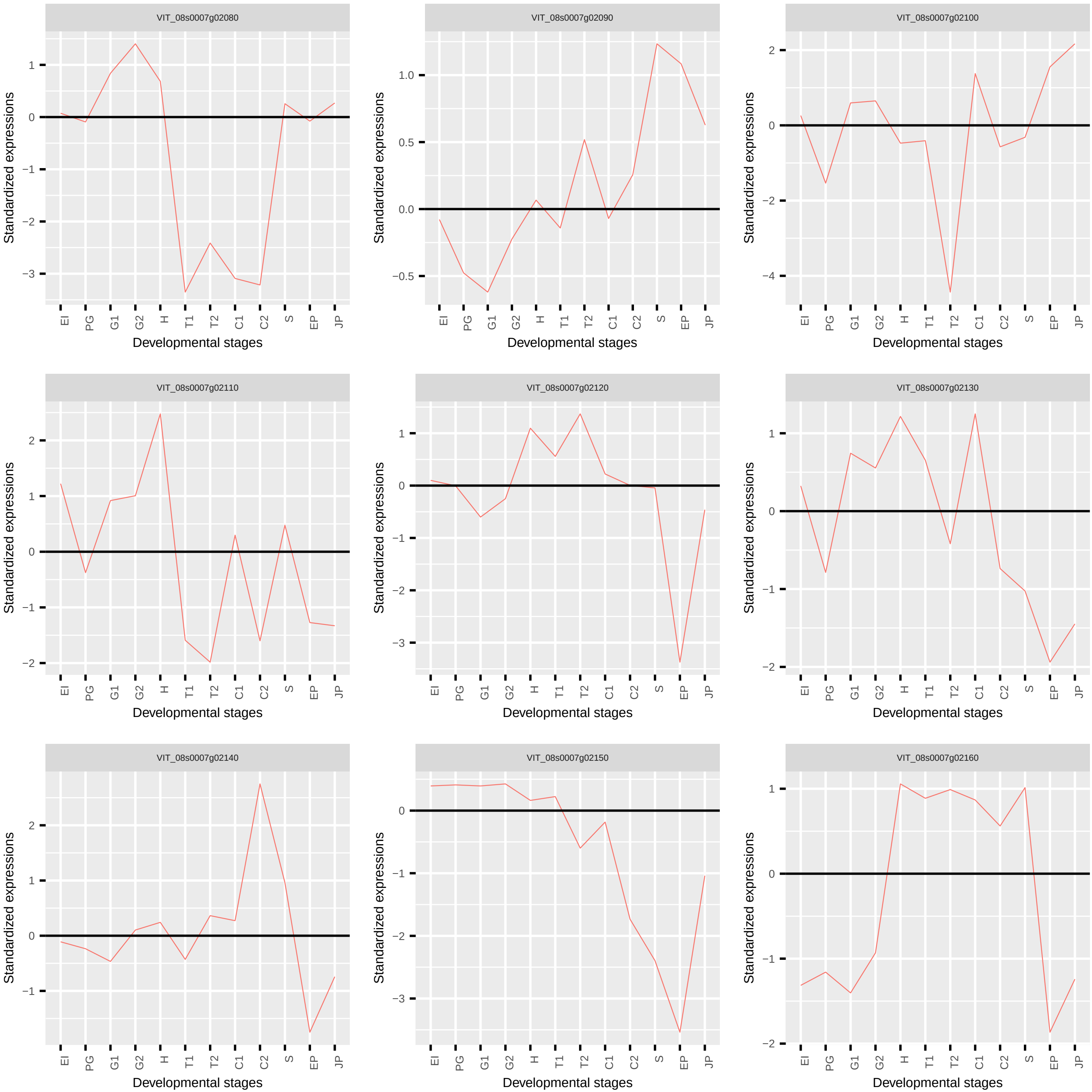
VIT_08s0007g02080
VIT_08s0007g02090
VIT_08s0007g02100
VIT_08s0007g02110
VIT_08s0007g02120
VIT_08s0007g02130
VIT_08s0007g02140
VIT_08s0007g02150
VIT_08s0007g02160
Standardized expressions
Developmental stages
EI
PG
G1
G2
H
T1
T2
C1
C2
S
EP
JP

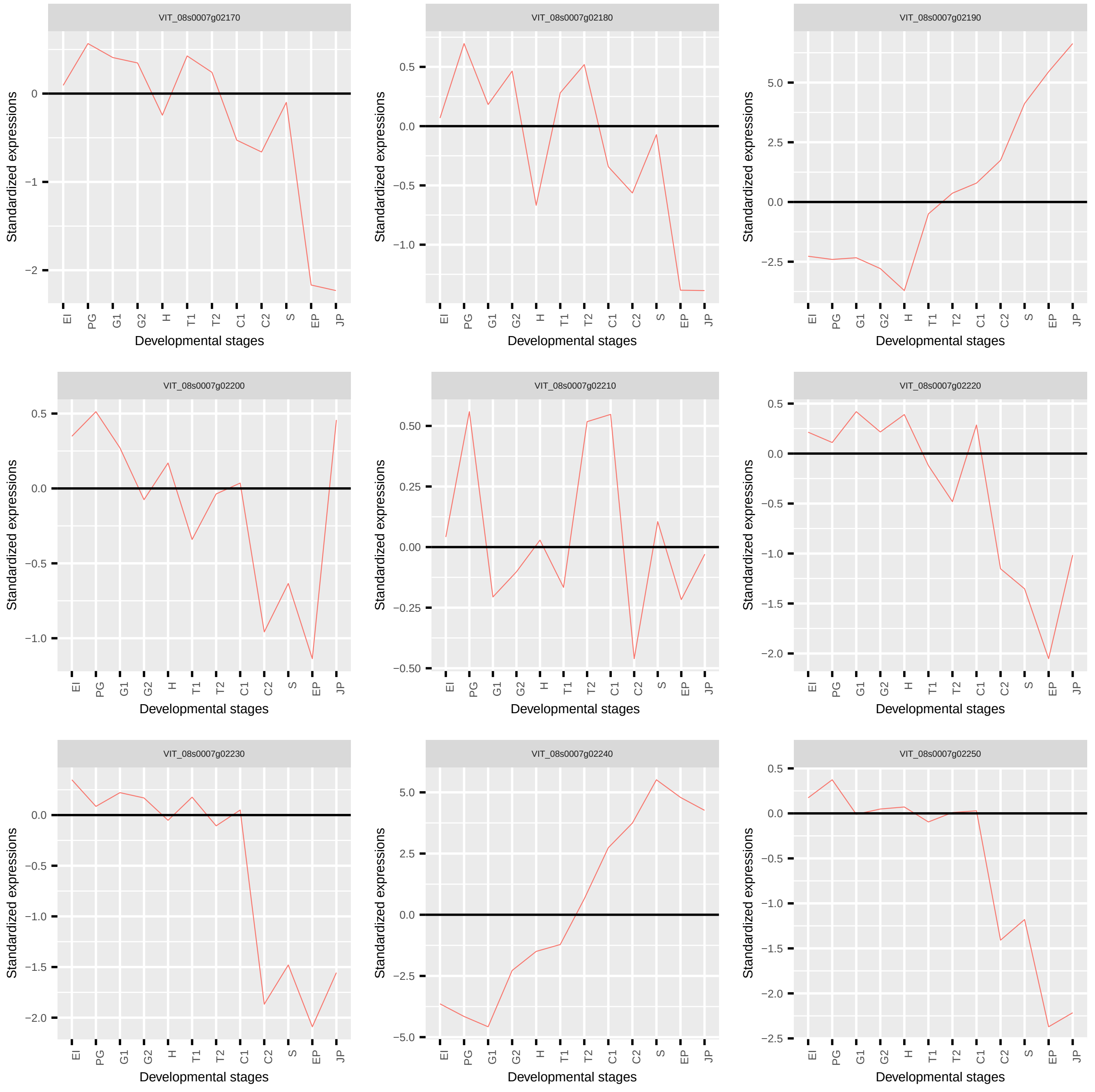

VIT_08s0007g02170
VIT_08s0007g02180
VIT_08s0007g02190
VIT_08s0007g02200
VIT_08s0007g02210
VIT_08s0007g02220
VIT_08s0007g02230
VIT_08s0007g02240
VIT_08s0007g02250
Standardized expressions
Developmental stages
EI PG G1 G2 H T1 T2 C1 C2 S EP JP

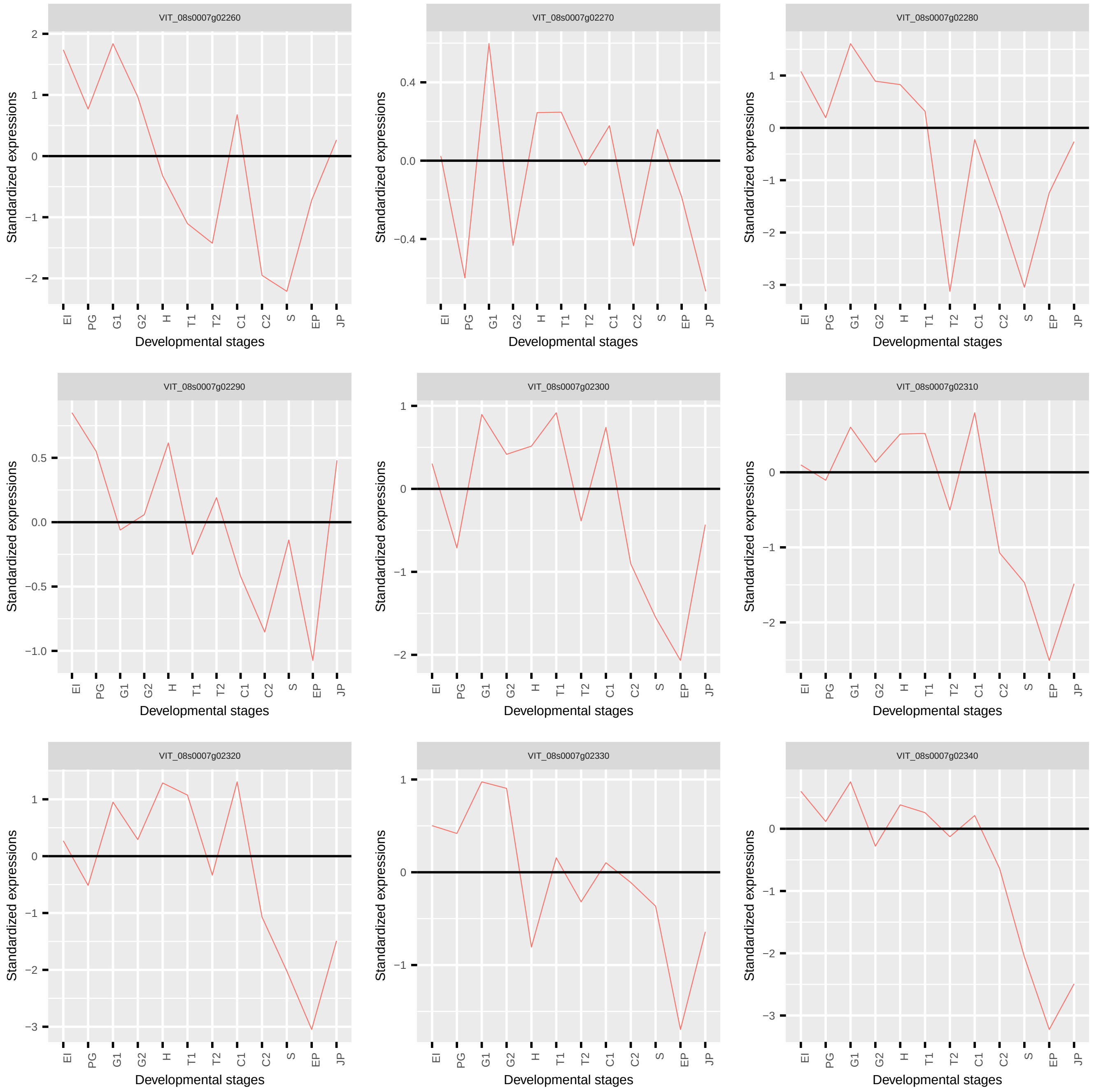
VIT_08s0007g02260
VIT_08s0007g02270
VIT_08s0007g02280
VIT_08s0007g02290
VIT_08s0007g02300
VIT_08s0007g02310
VIT_08s0007g02320
VIT_08s0007g02330
VIT_08s0007g02340
Standardized expressions
Developmental stages
EI PG G1 G2 H T1 T2 C1 C2 S EP JP

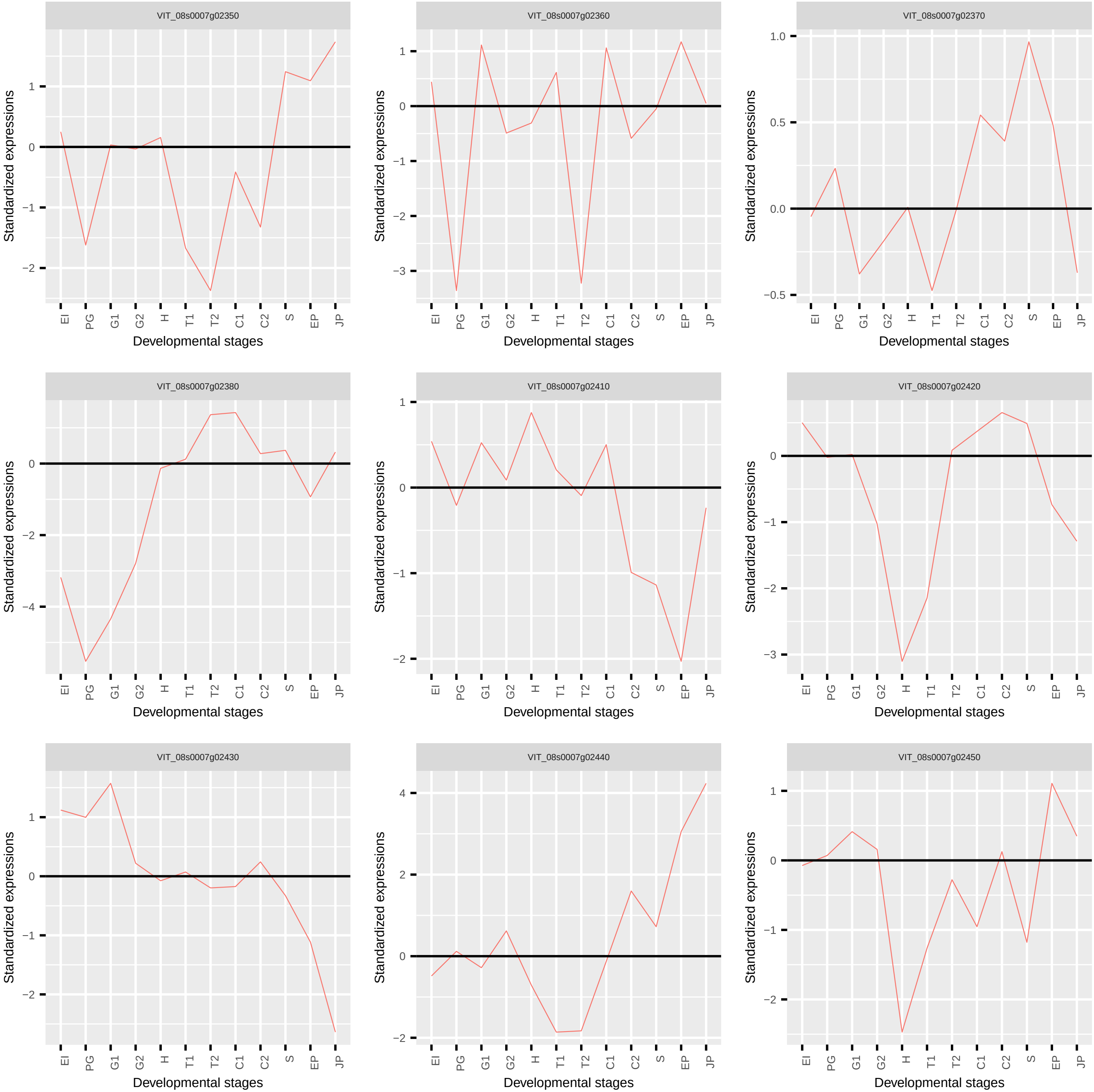
VIT_08s0007g02350
VIT_08s0007g02360
VIT_08s0007g02370
VIT_08s0007g02380
VIT_08s0007g02410
VIT_08s0007g02420
VIT_08s0007g02430
VIT_08s0007g02440
VIT_08s0007g02450
Standardized expressions
Developmental stages
EI PG G1 G2 H T1 T2 C1 C2 S EP JP

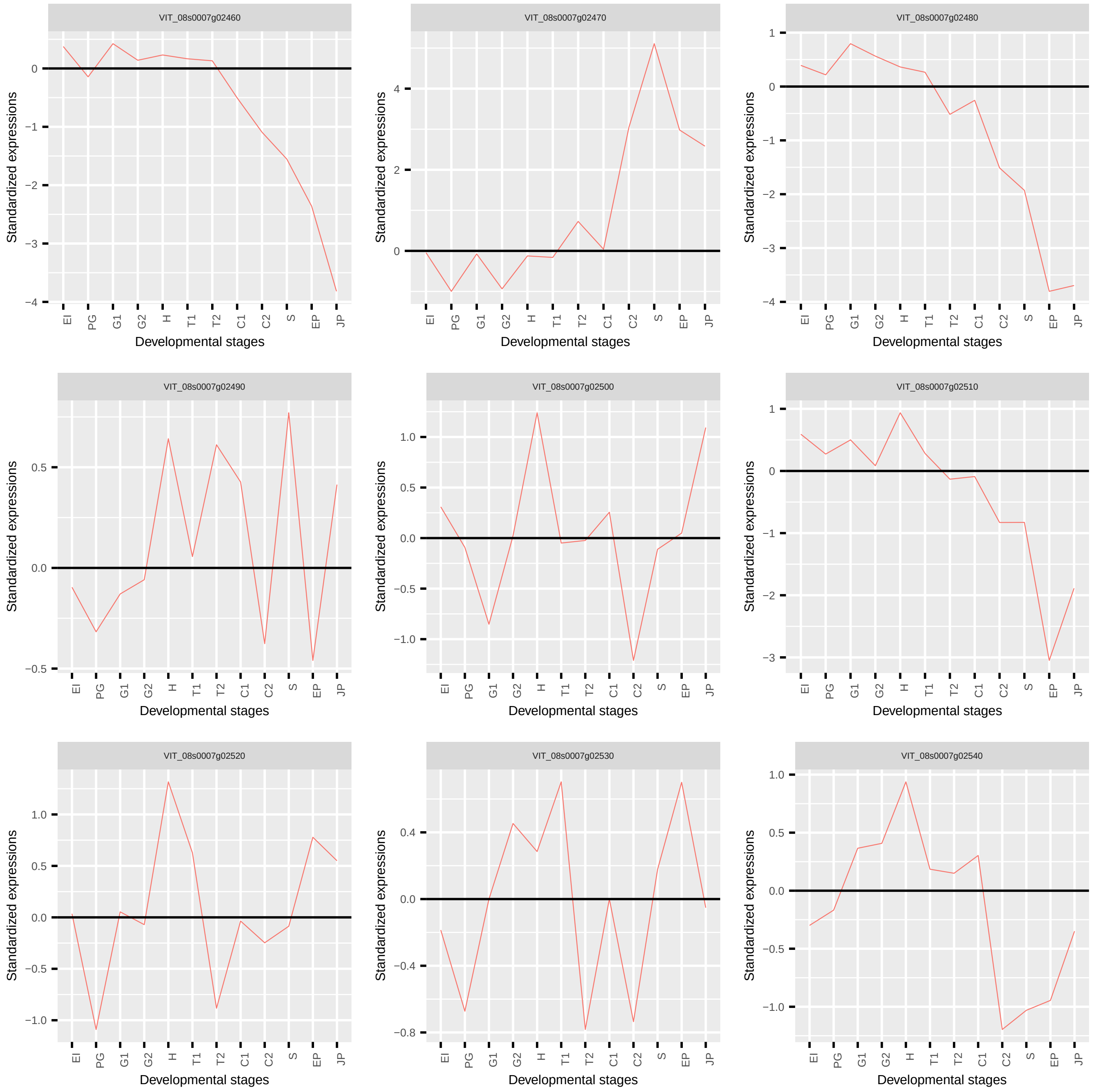
VIT_08s0007g02460
0
−1
−2
−3
−4
Standardized expressions
EI PG G1 G2 H T1 T2 C1 C2 S EP JP
Developmental stages
VIT_08s0007g02470
4
2
0
Standardized expressions
EI PG G1 G2 H T1 T2 C1 C2 S EP JP
Developmental stages
VIT_08s0007g02480
1
0
−1
−2
−3
−4
Standardized expressions
EI PG G1 G2 H T1 T2 C1 C2 S EP JP
Developmental stages
VIT_08s0007g02490
0.5
0.0
−0.5
Standardized expressions
EI PG G1 G2 H T1 T2 C1 C2 S EP JP
Developmental stages
VIT_08s0007g02500
1.0
0.5
0.0
−0.5
−1.0
Standardized expressions
EI PG G1 G2 H T1 T2 C1 C2 S EP JP
Developmental stages
VIT_08s0007g02510
1
0
−1
−2
−3
Standardized expressions
EI PG G1 G2 H T1 T2 C1 C2 S EP JP
Developmental stages
VIT_08s0007g02520
1.0
0.5
0.0
−0.5
−1.0
Standardized expressions
EI PG G1 G2 H T1 T2 C1 C2 S EP JP
Developmental stages
VIT_08s0007g02530
0.4
0.0
−0.4
−0.8
Standardized expressions
EI PG G1 G2 H T1 T2 C1 C2 S EP JP
Developmental stages
VIT_08s0007g02540
1.0
0.5
0.0
−0.5
−1.0
Standardized expressions
EI PG G1 G2 H T1 T2 C1 C2 S EP JP
Developmental stages

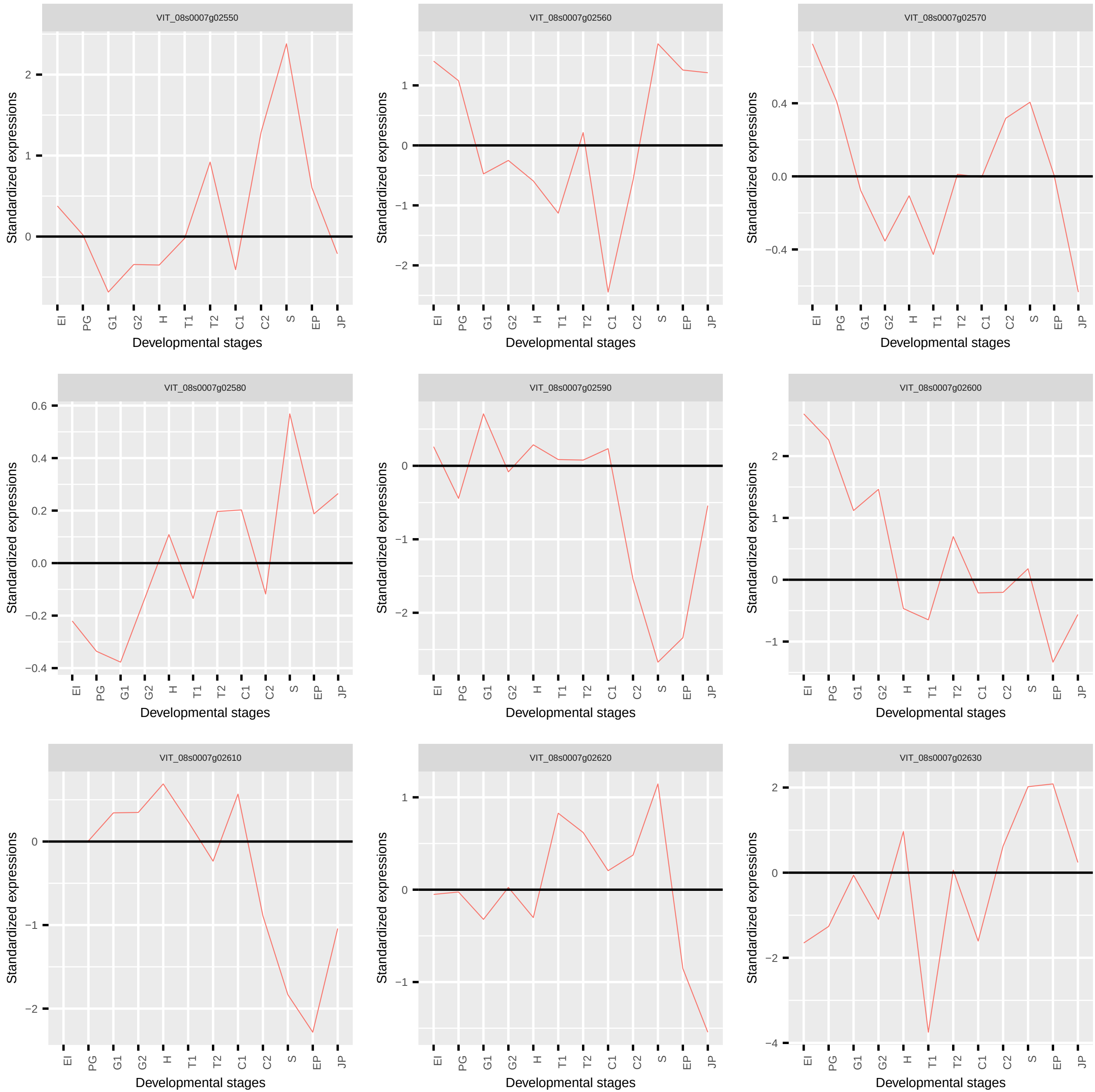
VIT_08s0007g02550
VIT_08s0007g02560
VIT_08s0007g02570
VIT_08s0007g02580
VIT_08s0007g02590
VIT_08s0007g02600
VIT_08s0007g02610
VIT_08s0007g02620
VIT_08s0007g02630
Standardized expressions
Developmental stages
EI
PG
G1
G2
H
T1
T2
C1
C2
S
EP
JP

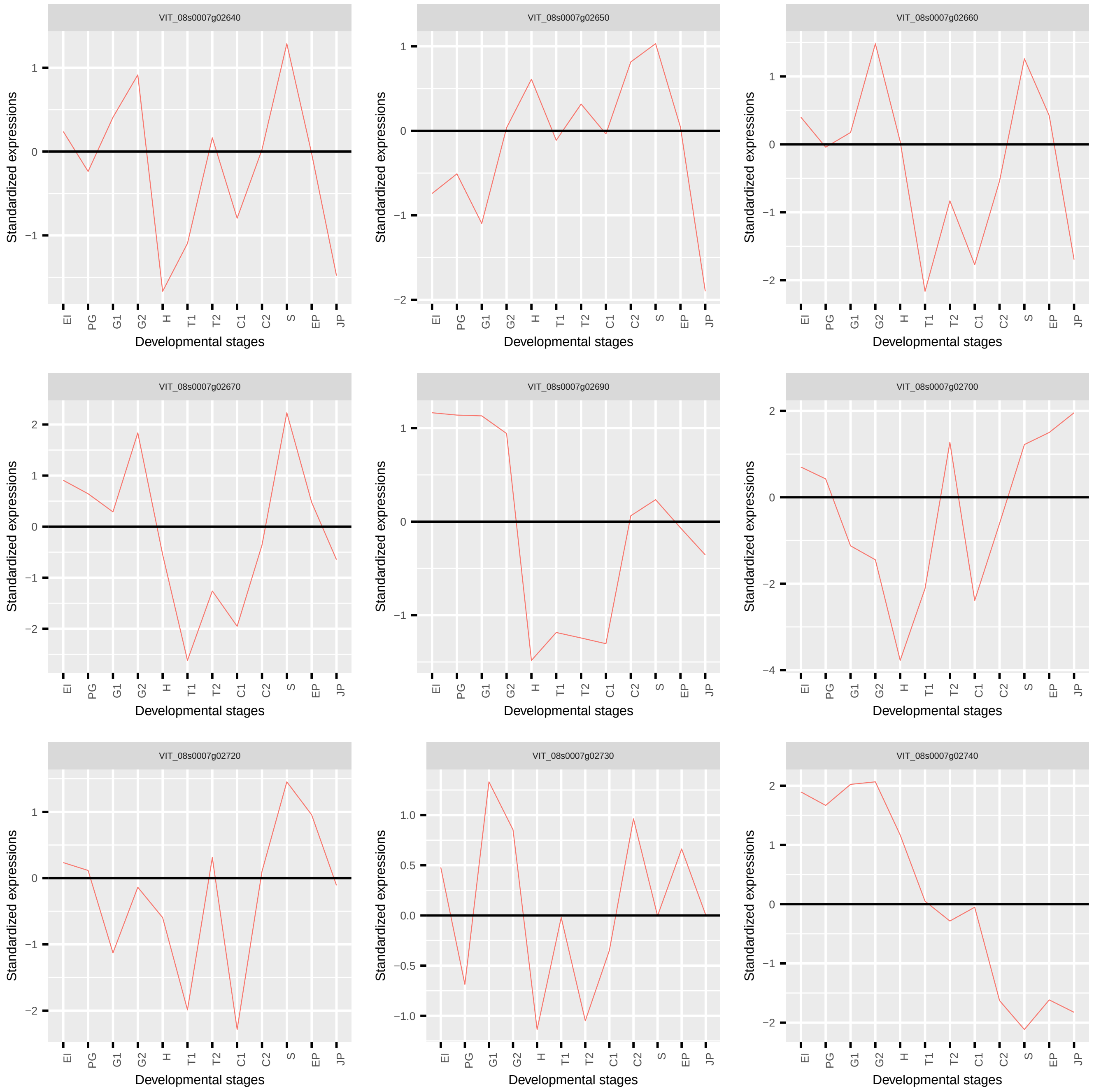
VIT_08s0007g02640
VIT_08s0007g02650
VIT_08s0007g02660
VIT_08s0007g02670
VIT_08s0007g02690
VIT_08s0007g02700
VIT_08s0007g02720
VIT_08s0007g02730
VIT_08s0007g02740
Standardized expressions
Developmental stages
EI
PG
G1
G2
H
T1
T2
C1
C2
S
EP
JP

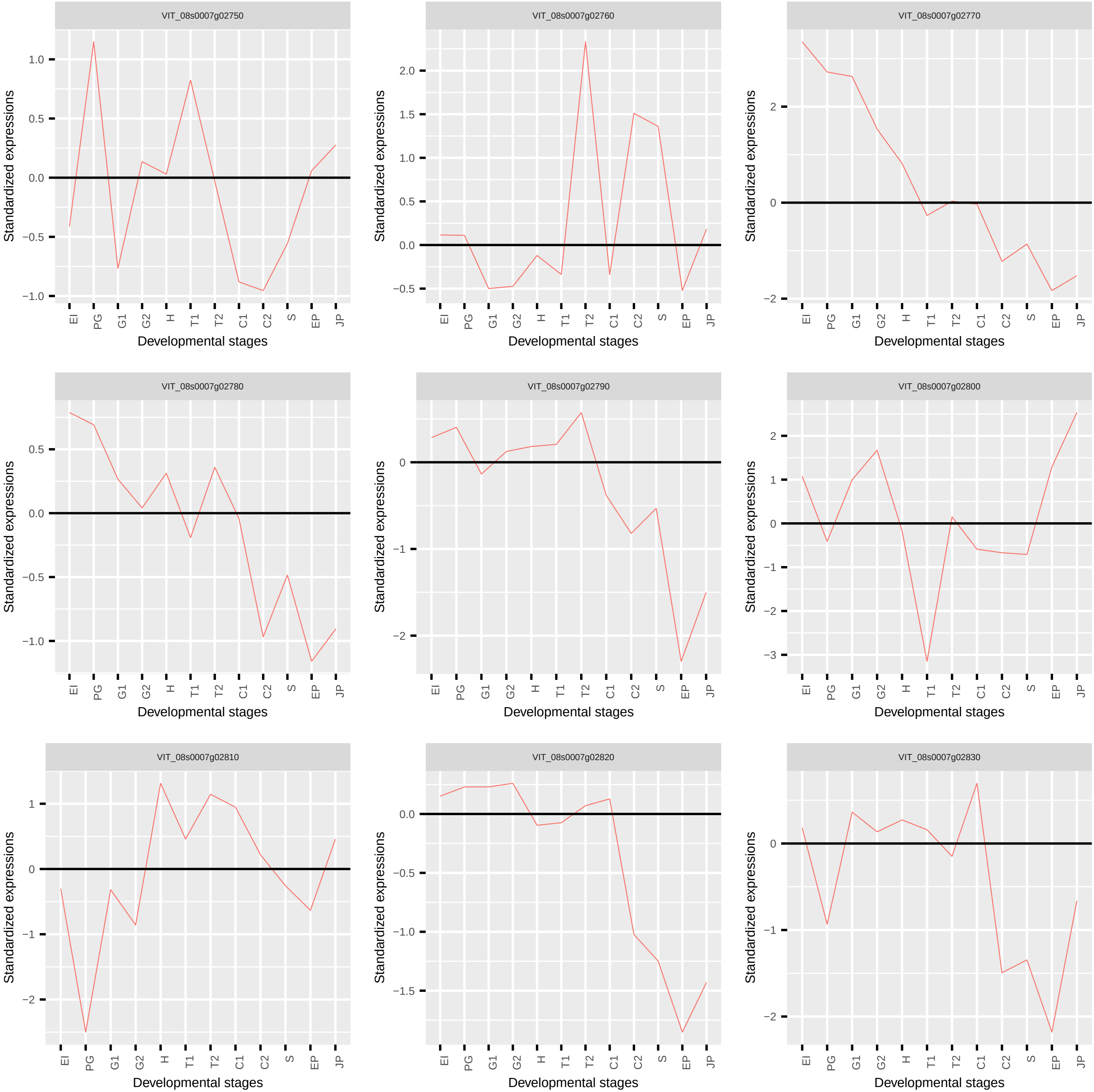
VIT_08s0007g02750
VIT_08s0007g02760
VIT_08s0007g02770
VIT_08s0007g02780
VIT_08s0007g02790
VIT_08s0007g02800
VIT_08s0007g02810
VIT_08s0007g02820
VIT_08s0007g02830
Standardized expressions
Developmental stages
EI PG G1 G2 H T1 T2 C1 C2 S EP JP

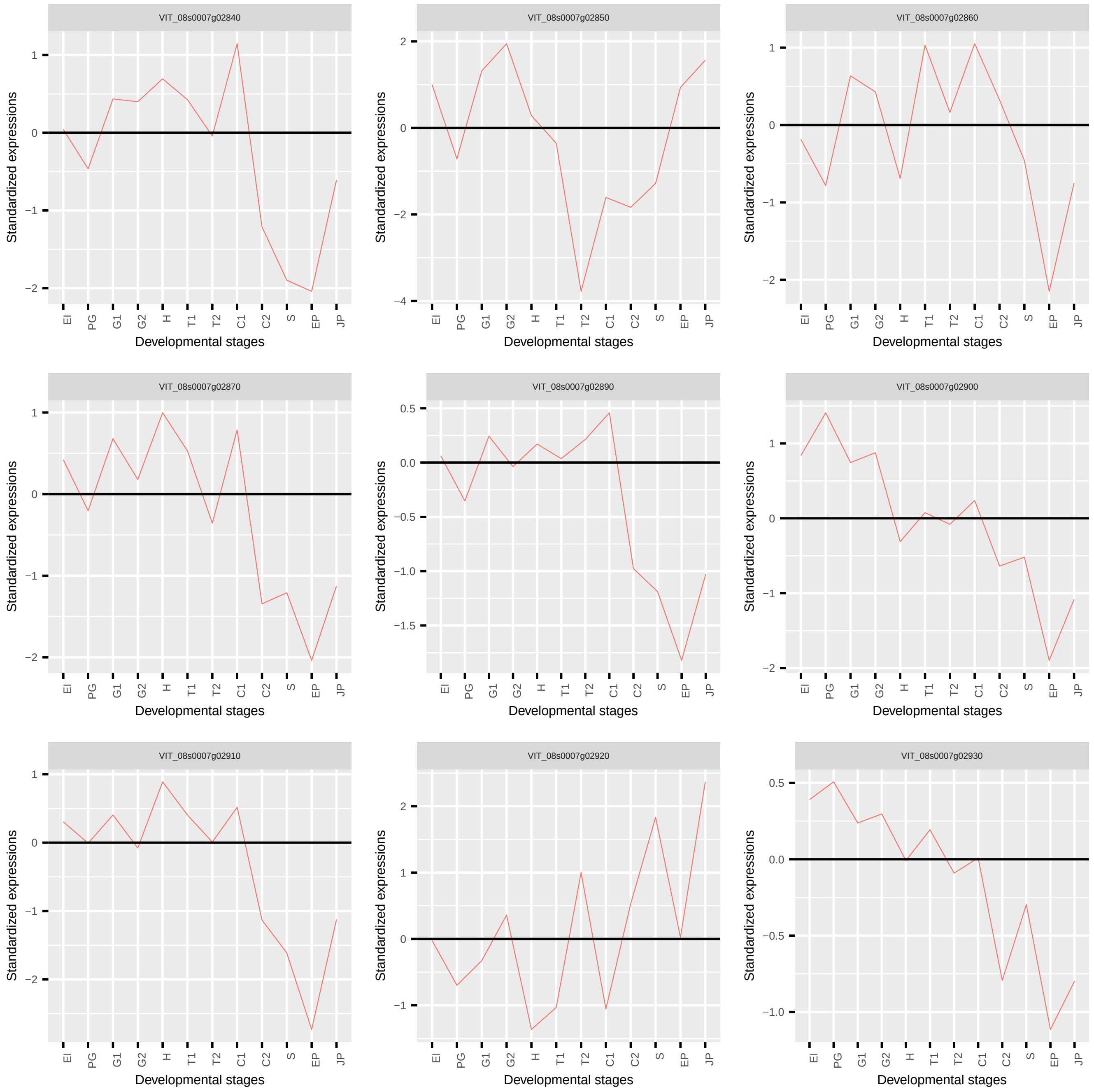

VIT_08s0007g02840
VIT_08s0007g02850
VIT_08s0007g02860
VIT_08s0007g02870
VIT_08s0007g02890
VIT_08s0007g02900
VIT_08s0007g02910
VIT_08s0007g02920
VIT_08s0007g02930
Standardized expressions
Developmental stages
EI PG G1 G2 H T1 T2 C1 C2 S EP JP

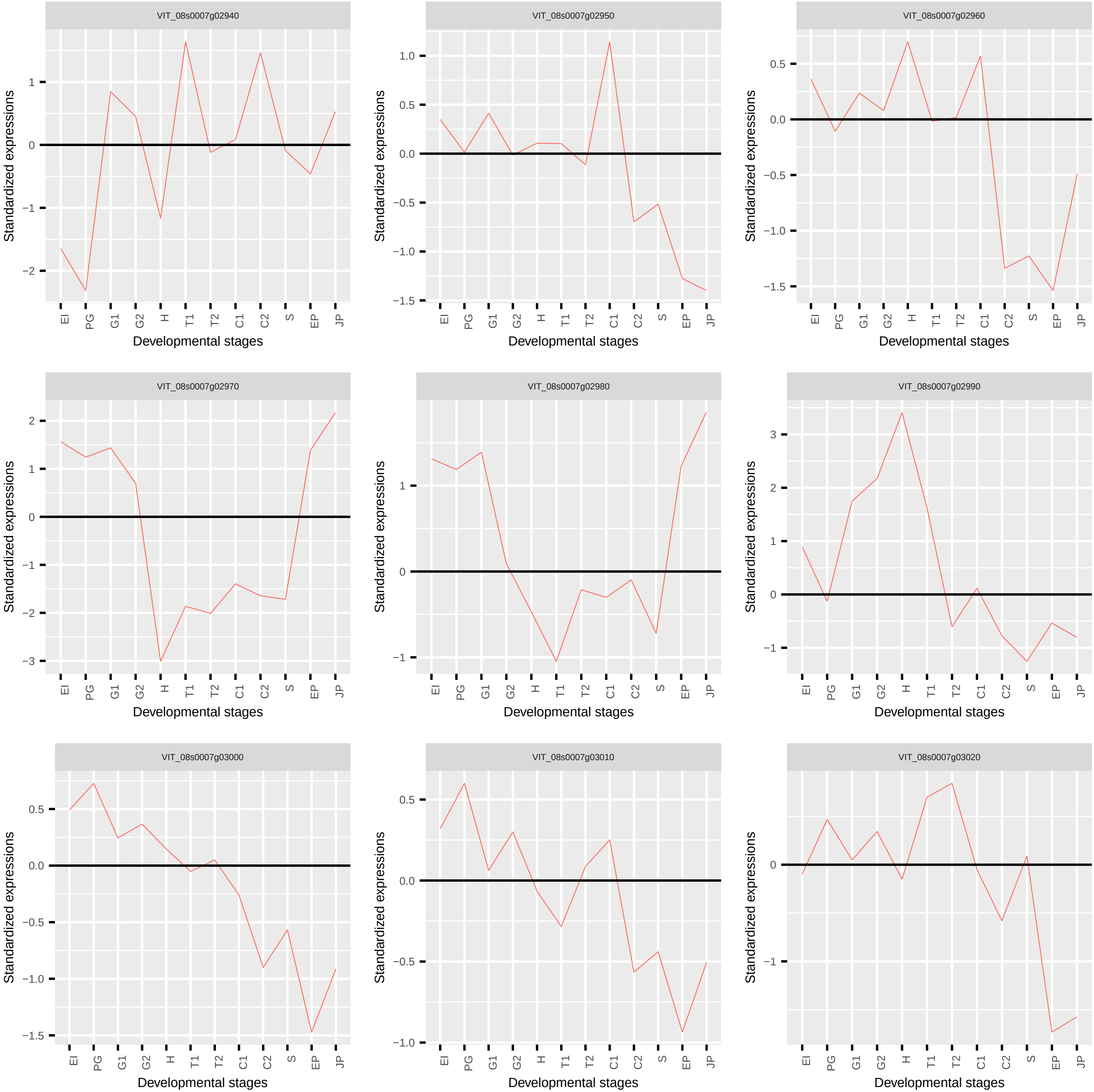
VIT_08s0007g02940
Standardized expressions
EI PG G1 G2 H T1 T2 C1 C2 S EP JP
Developmental stages
VIT_08s0007g02950
Standardized expressions
EI PG G1 G2 H T1 T2 C1 C2 S EP JP
Developmental stages
VIT_08s0007g02960
Standardized expressions
EI PG G1 G2 H T1 T2 C1 C2 S EP JP
Developmental stages
VIT_08s0007g02970
Standardized expressions
EI PG G1 G2 H T1 T2 C1 C2 S EP JP
Developmental stages
VIT_08s0007g02980
Standardized expressions
EI PG G1 G2 H T1 T2 C1 C2 S EP JP
Developmental stages
VIT_08s0007g02990
Standardized expressions
EI PG G1 G2 H T1 T2 C1 C2 S EP JP
Developmental stages
VIT_08s0007g03000
Standardized expressions
EI PG G1 G2 H T1 T2 C1 C2 S EP JP
Developmental stages
VIT_08s0007g03010
Standardized expressions
EI PG G1 G2 H T1 T2 C1 C2 S EP JP
Developmental stages
VIT_08s0007g03020
Standardized expressions
EI PG G1 G2 H T1 T2 C1 C2 S EP JP
Developmental stages

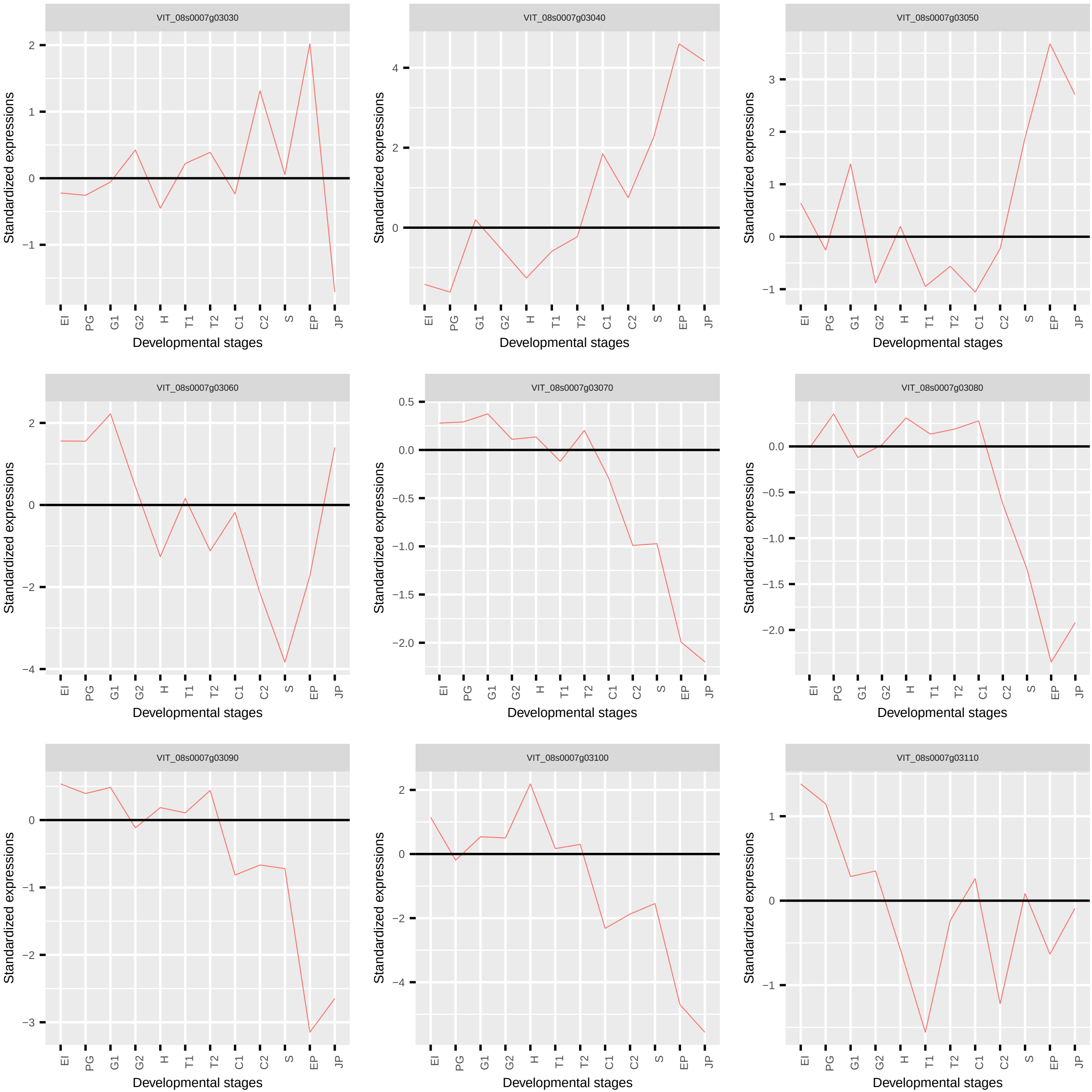
VIT_08s0007g03030
Standardized expressions
2
1
0
−1
EI PG G1 G2 H T1 T2 C1 C2 S EP JP
Developmental stages
VIT_08s0007g03040
Standardized expressions
4
2
0
EI PG G1 G2 H T1 T2 C1 C2 S EP JP
Developmental stages
VIT_08s0007g03050
Standardized expressions
3
2
1
0
−1
EI PG G1 G2 H T1 T2 C1 C2 S EP JP
Developmental stages
VIT_08s0007g03060
Standardized expressions
2
0
−2
−4
EI PG G1 G2 H T1 T2 C1 C2 S EP JP
Developmental stages
VIT_08s0007g03070
Standardized expressions
0.5
0.0
−0.5
−1.0
−1.5
−2.0
EI PG G1 G2 H T1 T2 C1 C2 S EP JP
Developmental stages
VIT_08s0007g03080
Standardized expressions
0.0
−0.5
−1.0
−1.5
−2.0
EI PG G1 G2 H T1 T2 C1 C2 S EP JP
Developmental stages
VIT_08s0007g03090
Standardized expressions
0
−1
−2
−3
EI PG G1 G2 H T1 T2 C1 C2 S EP JP
Developmental stages
VIT_08s0007g03100
Standardized expressions
2
0
−2
−4
EI PG G1 G2 H T1 T2 C1 C2 S EP JP
Developmental stages
VIT_08s0007g03110
Standardized expressions
1
0
−1
EI PG G1 G2 H T1 T2 C1 C2 S EP JP
Developmental stages

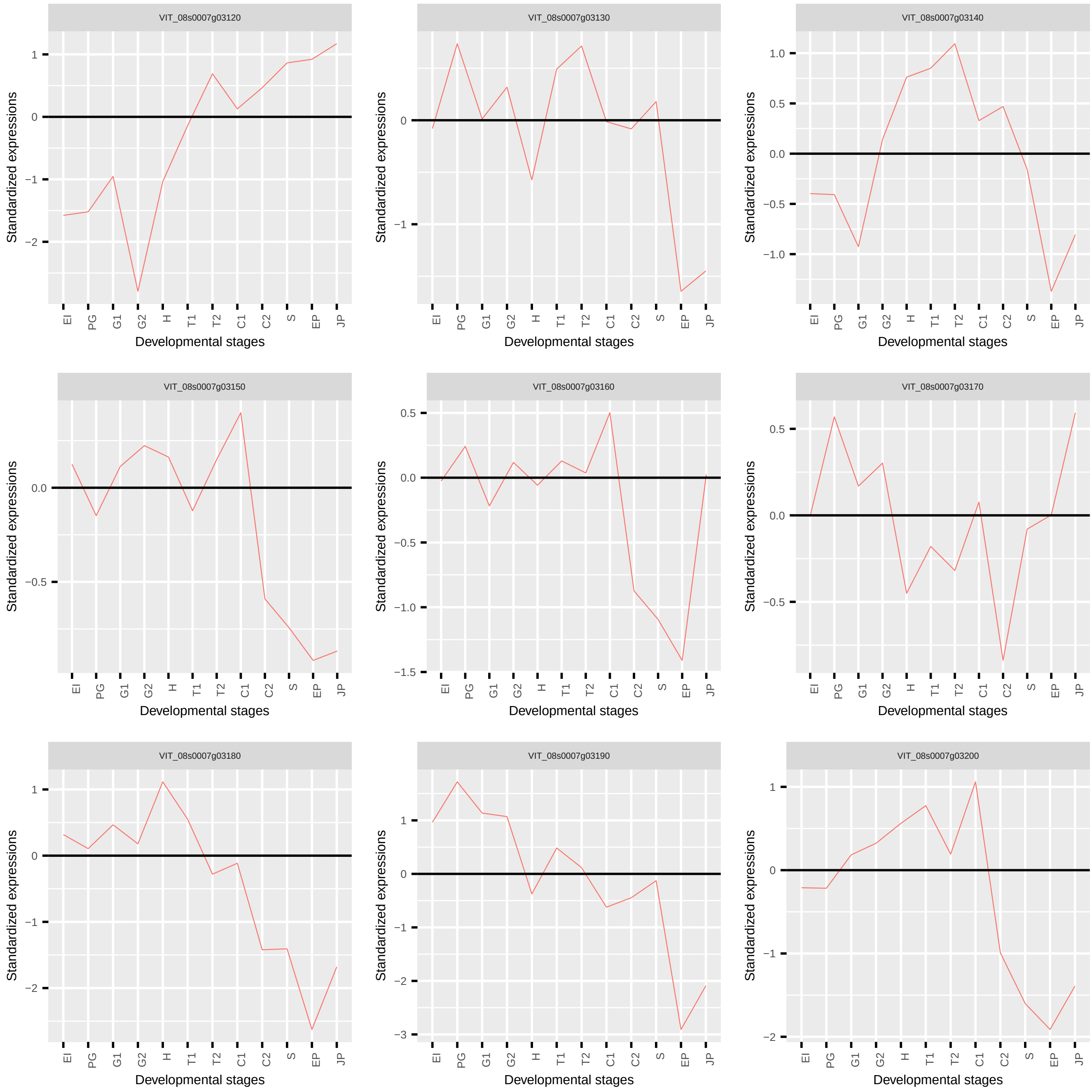
VIT_08s0007g03120
VIT_08s0007g03130
VIT_08s0007g03140
VIT_08s0007g03150
VIT_08s0007g03160
VIT_08s0007g03170
VIT_08s0007g03180
VIT_08s0007g03190
VIT_08s0007g03200
Standardized expressions
Developmental stages
EI PG G1 G2 H T1 T2 C1 C2 S EP JP

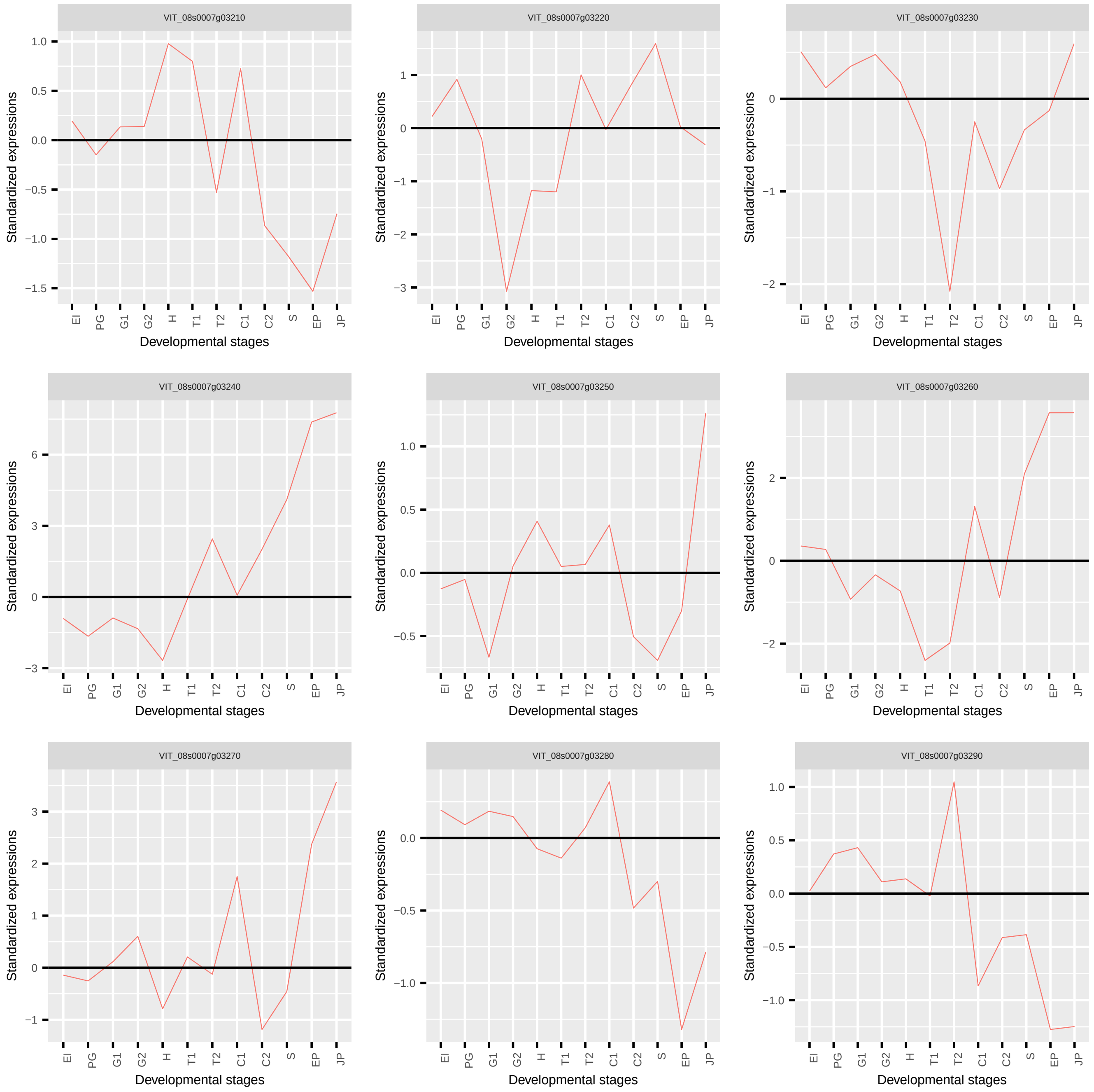
VIT_08s0007g03210
VIT_08s0007g03220
VIT_08s0007g03230
VIT_08s0007g03240
VIT_08s0007g03250
VIT_08s0007g03260
VIT_08s0007g03270
VIT_08s0007g03280
VIT_08s0007g03290
Standardized expressions
Developmental stages
EI PG G1 G2 H T1 T2 C1 C2 S EP JP

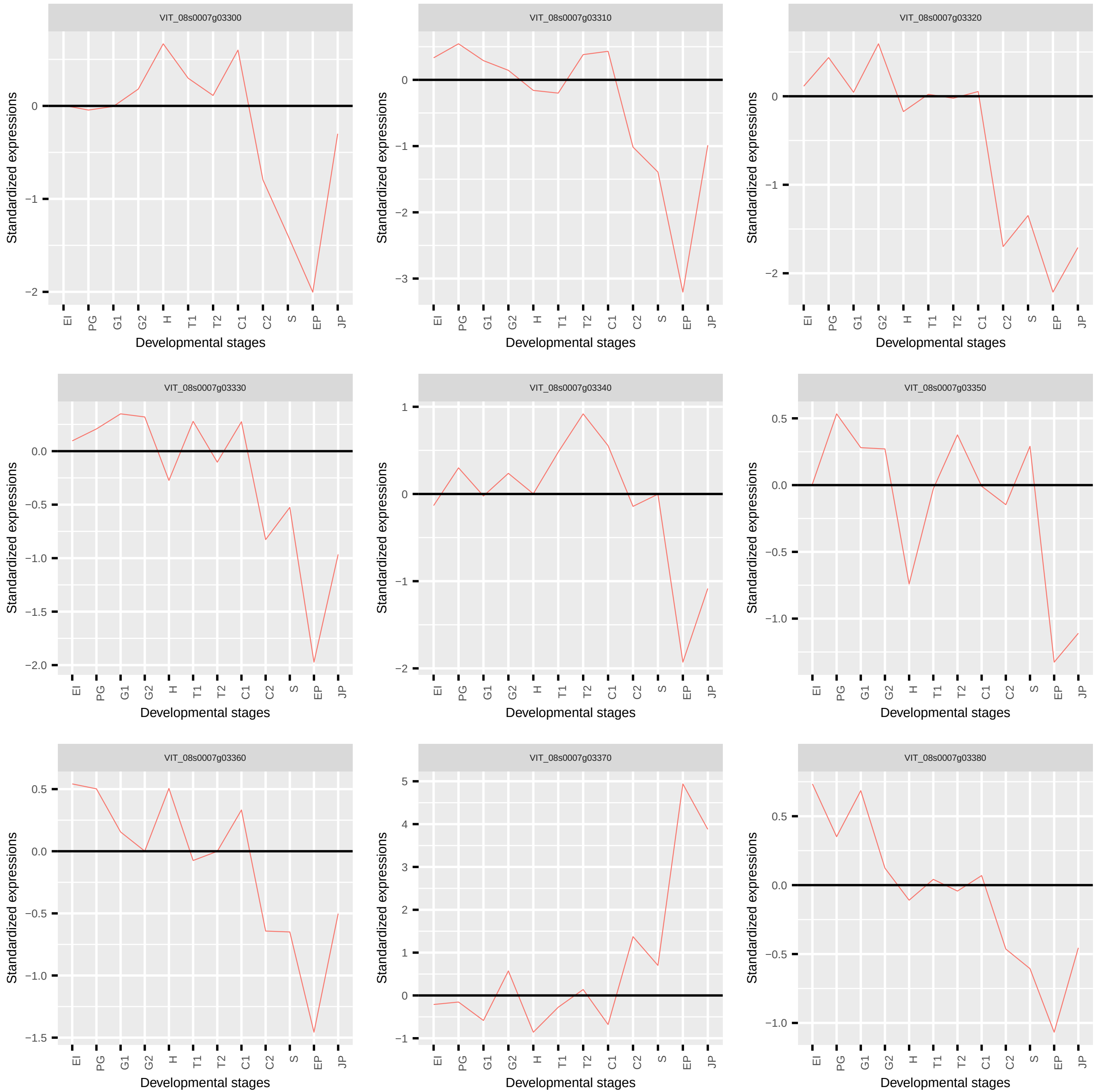
VIT_08s0007g03300
VIT_08s0007g03310
VIT_08s0007g03320
VIT_08s0007g03330
VIT_08s0007g03340
VIT_08s0007g03350
VIT_08s0007g03360
VIT_08s0007g03370
VIT_08s0007g03380
Standardized expressions
Developmental stages
EI PG G1 G2 H T1 T2 C1 C2 S EP JP

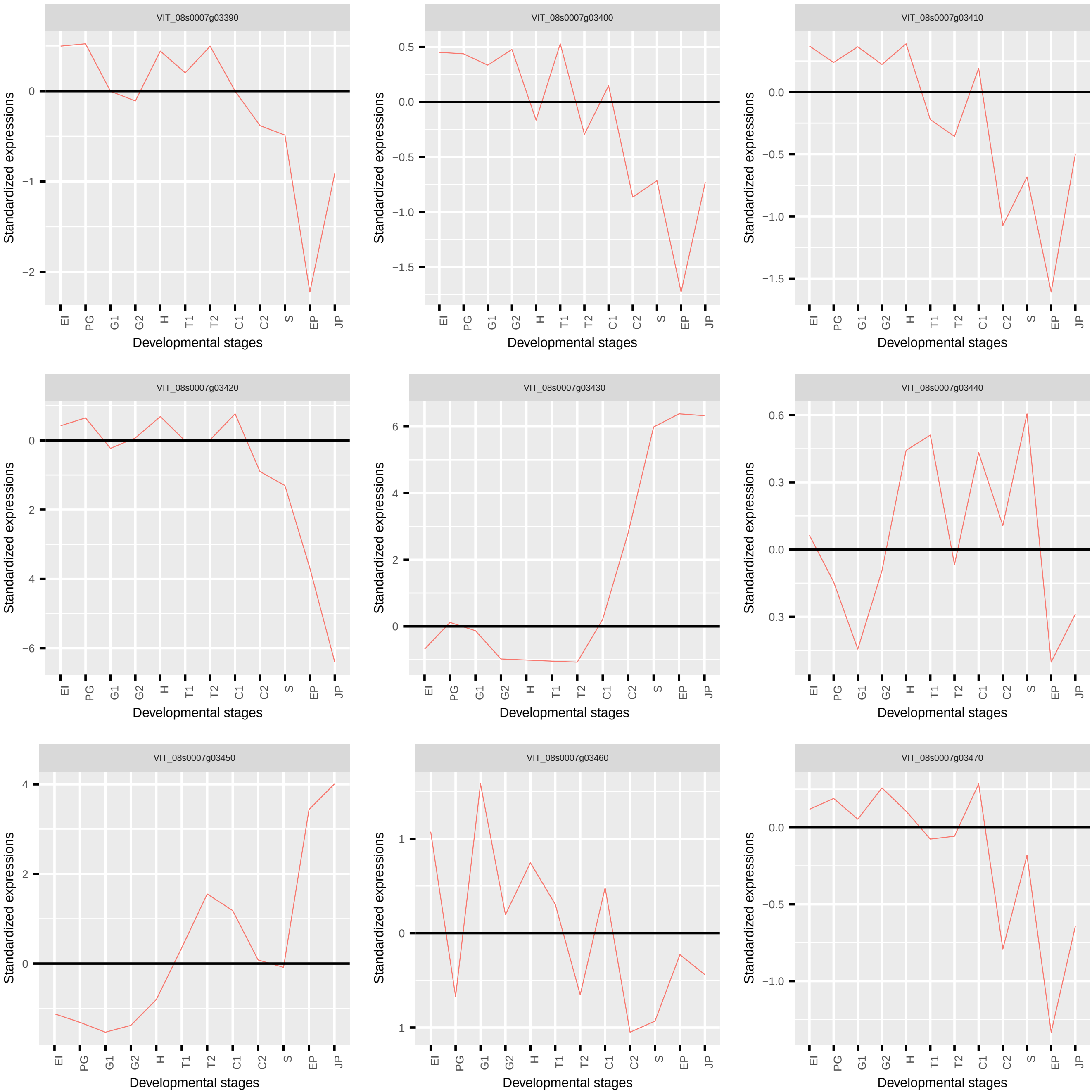

VIT_08s0007g03390
VIT_08s0007g03400
VIT_08s0007g03410
VIT_08s0007g03420
VIT_08s0007g03430
VIT_08s0007g03440
VIT_08s0007g03450
VIT_08s0007g03460
VIT_08s0007g03470
Standardized expressions
Developmental stages
EI PG G1 G2 H T1 T2 C1 C2 S EP JP
0 −1 −2
0.5 0.0 −0.5 −1.0 −1.5
0.0 −0.5 −1.0 −1.5
0 −2 −4 −6
6 4 2 0
0.6 0.3 0.0 −0.3
4 2 0
1 0 −1
0.0 −0.5 −1.0

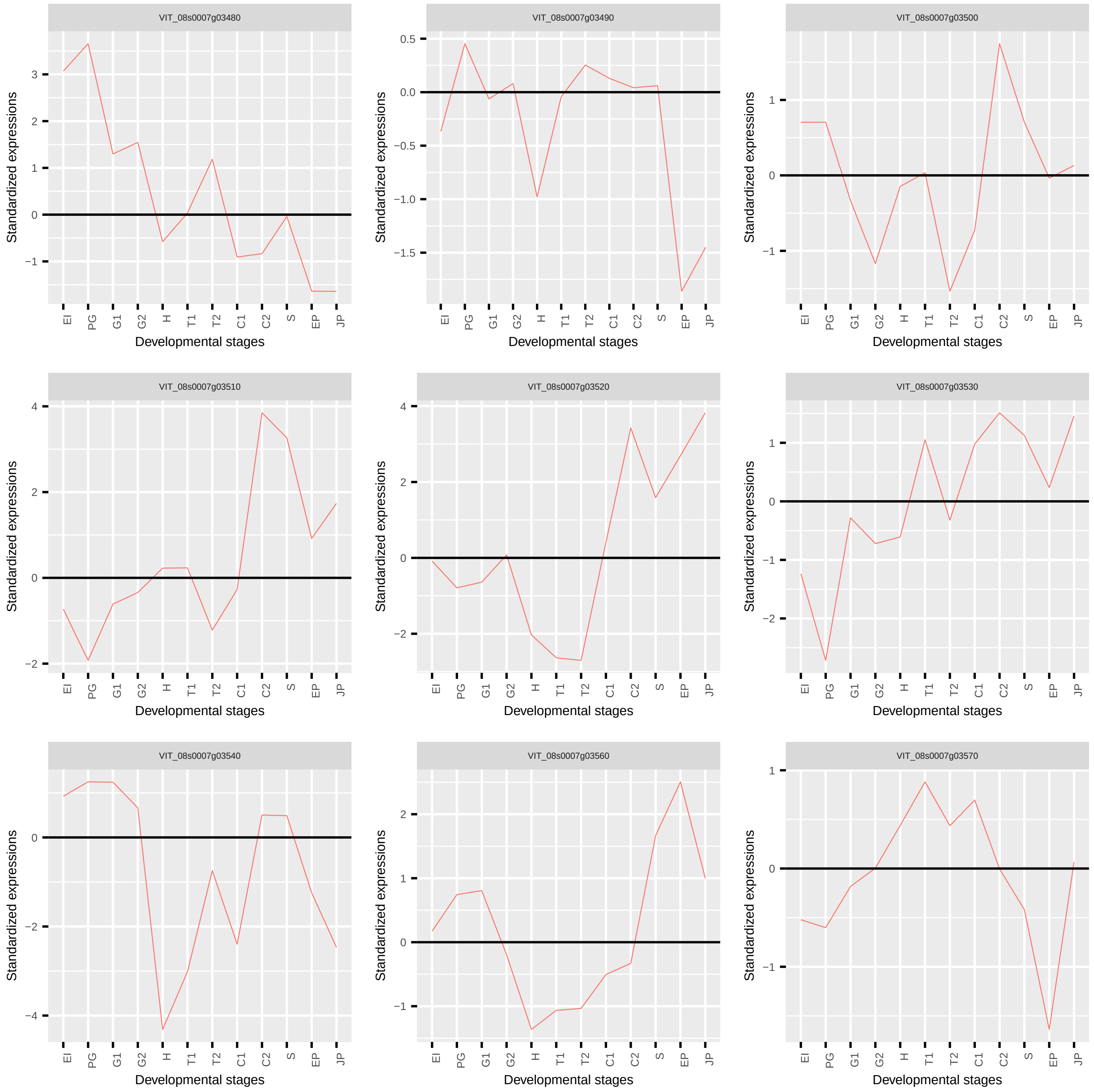

VIT_08s0007g03480
VIT_08s0007g03490
VIT_08s0007g03500
VIT_08s0007g03510
VIT_08s0007g03520
VIT_08s0007g03530
VIT_08s0007g03540
VIT_08s0007g03560
VIT_08s0007g03570
Standardized expressions
Developmental stages
EI PG G1 G2 H T1 T2 C1 C2 S EP JP

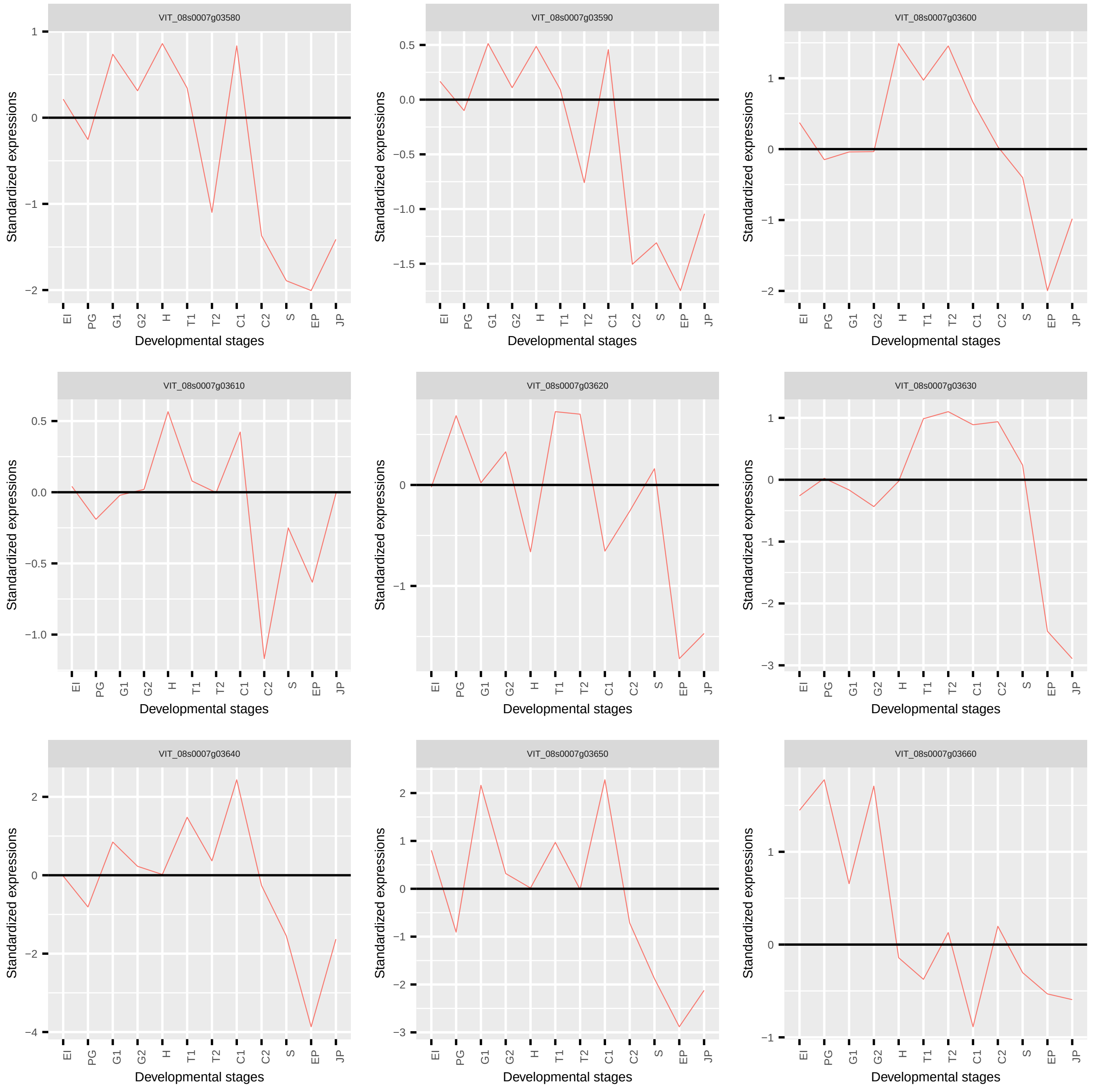

VIT_08s0007g03580
VIT_08s0007g03590
VIT_08s0007g03600
VIT_08s0007g03610
VIT_08s0007g03620
VIT_08s0007g03630
VIT_08s0007g03640
VIT_08s0007g03650
VIT_08s0007g03660
Standardized expressions
Developmental stages
EI PG G1 G2 H T1 T2 C1 C2 S EP JP

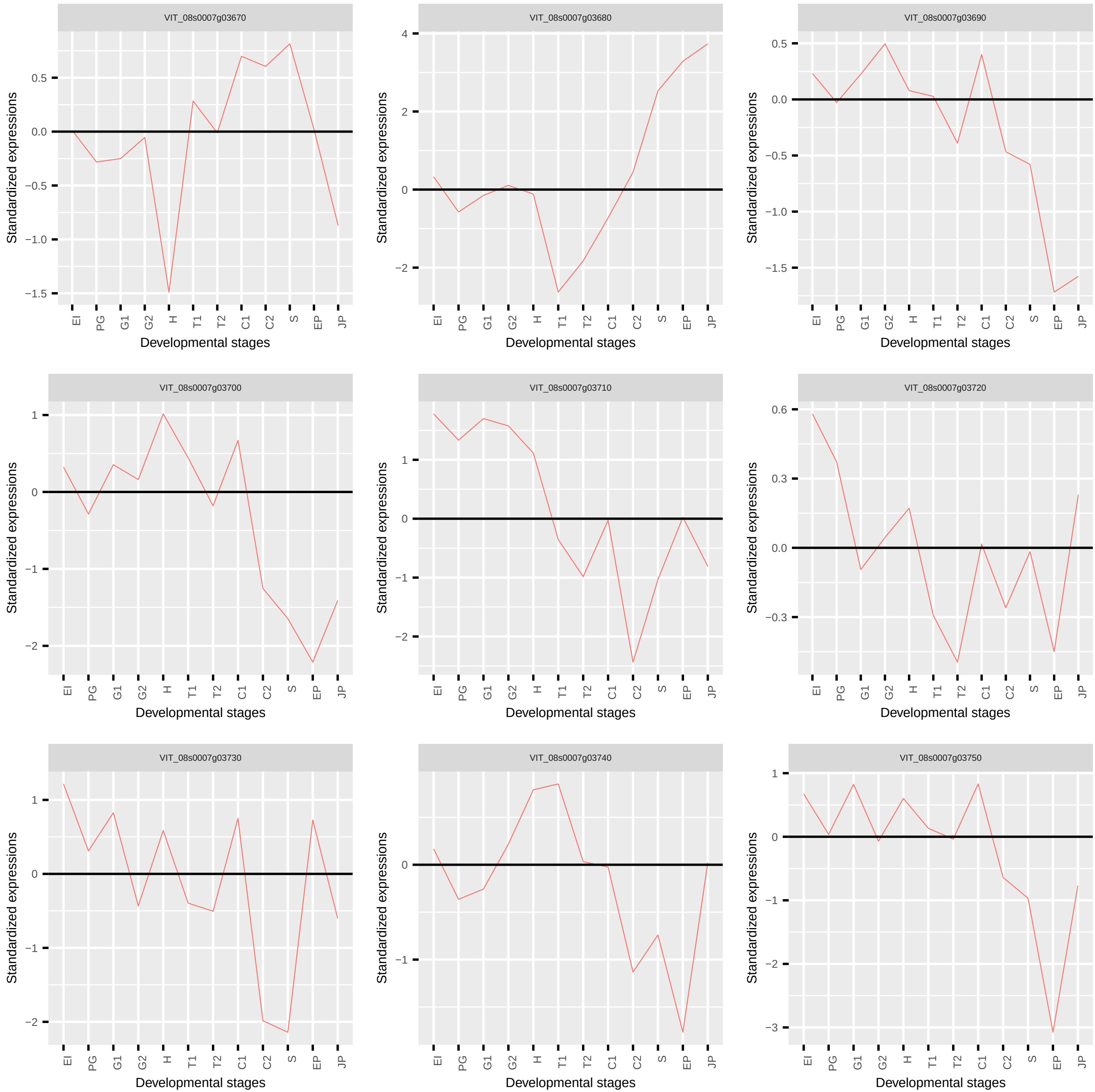
VIT_08s0007g03670
VIT_08s0007g03680
VIT_08s0007g03690
VIT_08s0007g03700
VIT_08s0007g03710
VIT_08s0007g03720
VIT_08s0007g03730
VIT_08s0007g03740
VIT_08s0007g03750
Standardized expressions
Developmental stages
EI PG G1 G2 H T1 T2 C1 C2 S EP JP
0.5 0.0 −0.5 −1.0 −1.5
4 2 0 −2
0.5 0.0 −0.5 −1.0 −1.5
1 0 −1 −2
1 0 −1 −2
0.6 0.3 0.0 −0.3
1 0 −1 −2
0 −1
1 0 −1 −2 −3

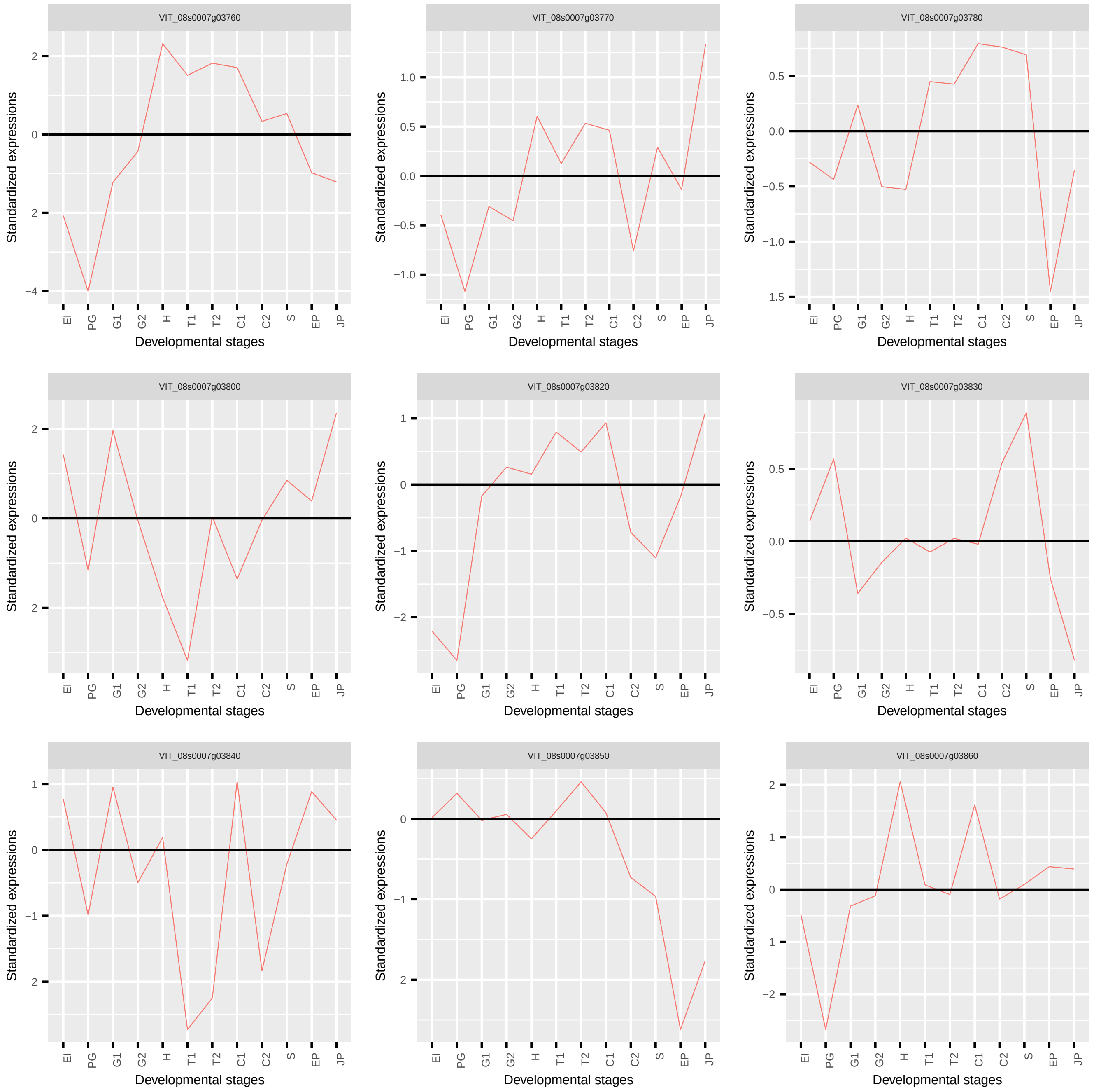
VIT_08s0007g03760
VIT_08s0007g03770
VIT_08s0007g03780
VIT_08s0007g03800
VIT_08s0007g03820
VIT_08s0007g03830
VIT_08s0007g03840
VIT_08s0007g03850
VIT_08s0007g03860
Standardized expressions
Developmental stages
EI PG G1 G2 H T1 T2 C1 C2 S EP JP

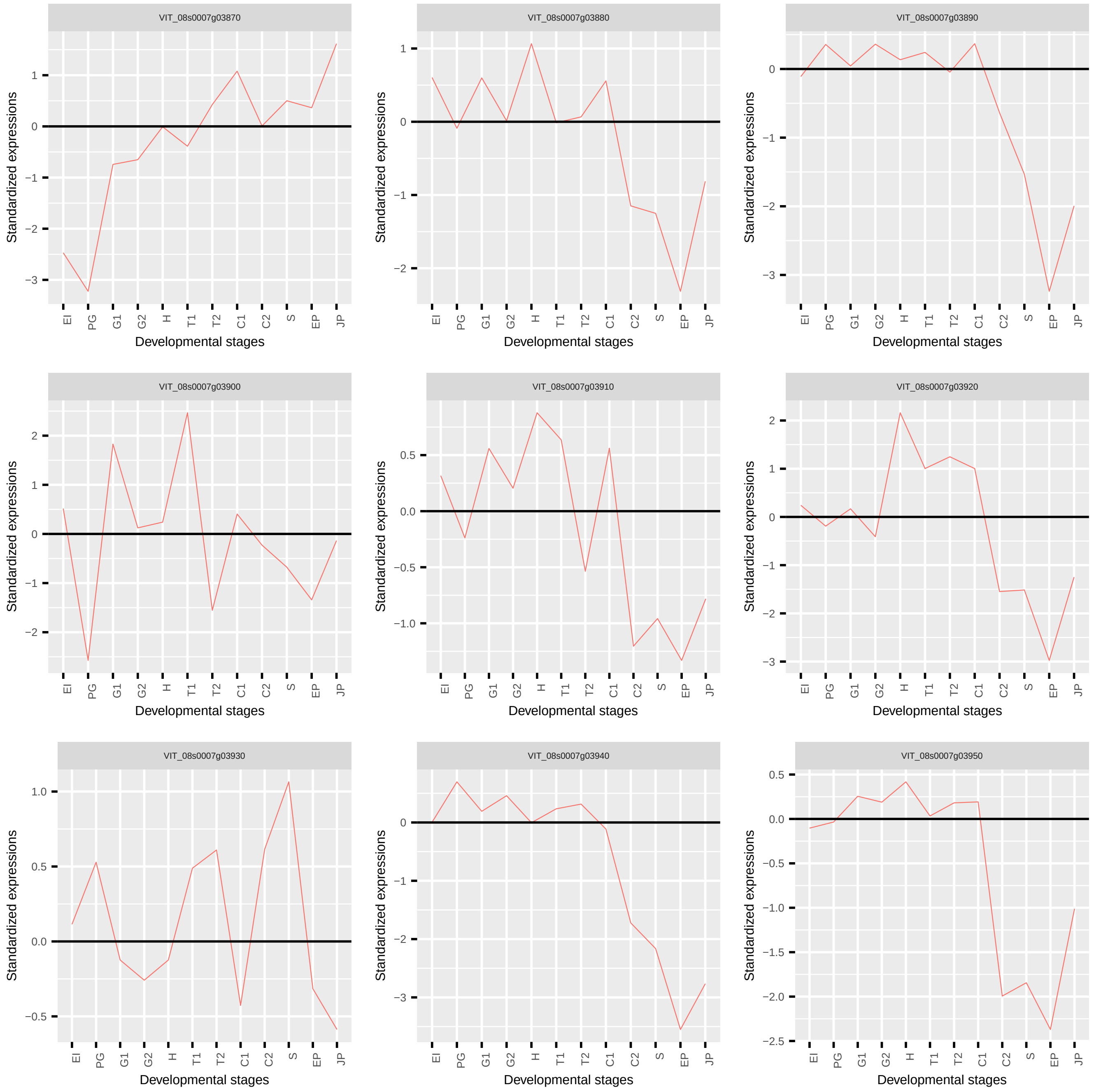
VIT_08s0007g03870
VIT_08s0007g03880
VIT_08s0007g03890
VIT_08s0007g03900
VIT_08s0007g03910
VIT_08s0007g03920
VIT_08s0007g03930
VIT_08s0007g03940
VIT_08s0007g03950
Standardized expressions
Developmental stages
EI PG G1 G2 H T1 T2 C1 C2 S EP JP

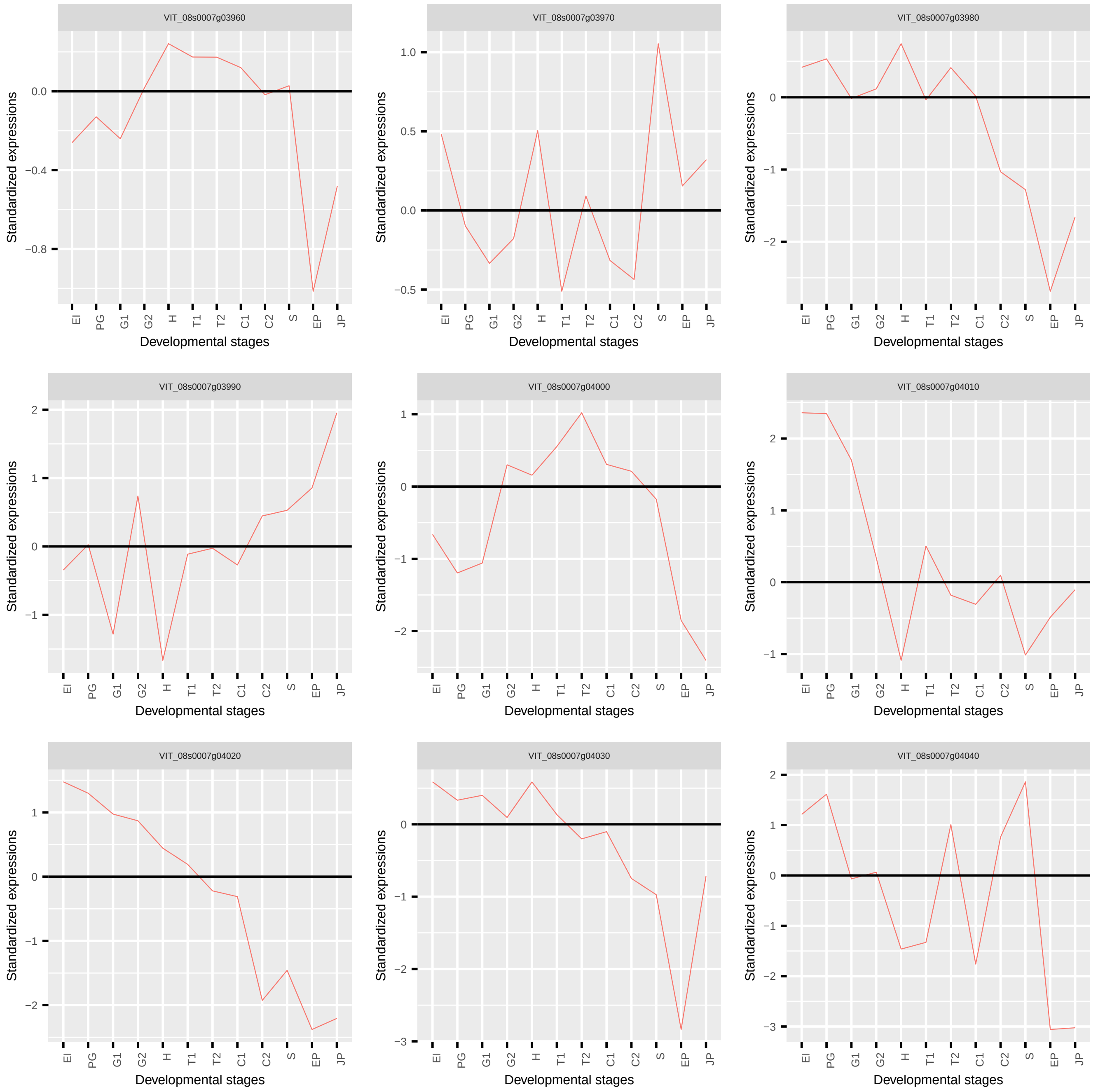

VIT_08s0007g03960
VIT_08s0007g03970
VIT_08s0007g03980
VIT_08s0007g03990
VIT_08s0007g04000
VIT_08s0007g04010
VIT_08s0007g04020
VIT_08s0007g04030
VIT_08s0007g04040
Standardized expressions
Developmental stages
EI
PG
G1
G2
H
T1
T2
C1
C2
S
EP
JP

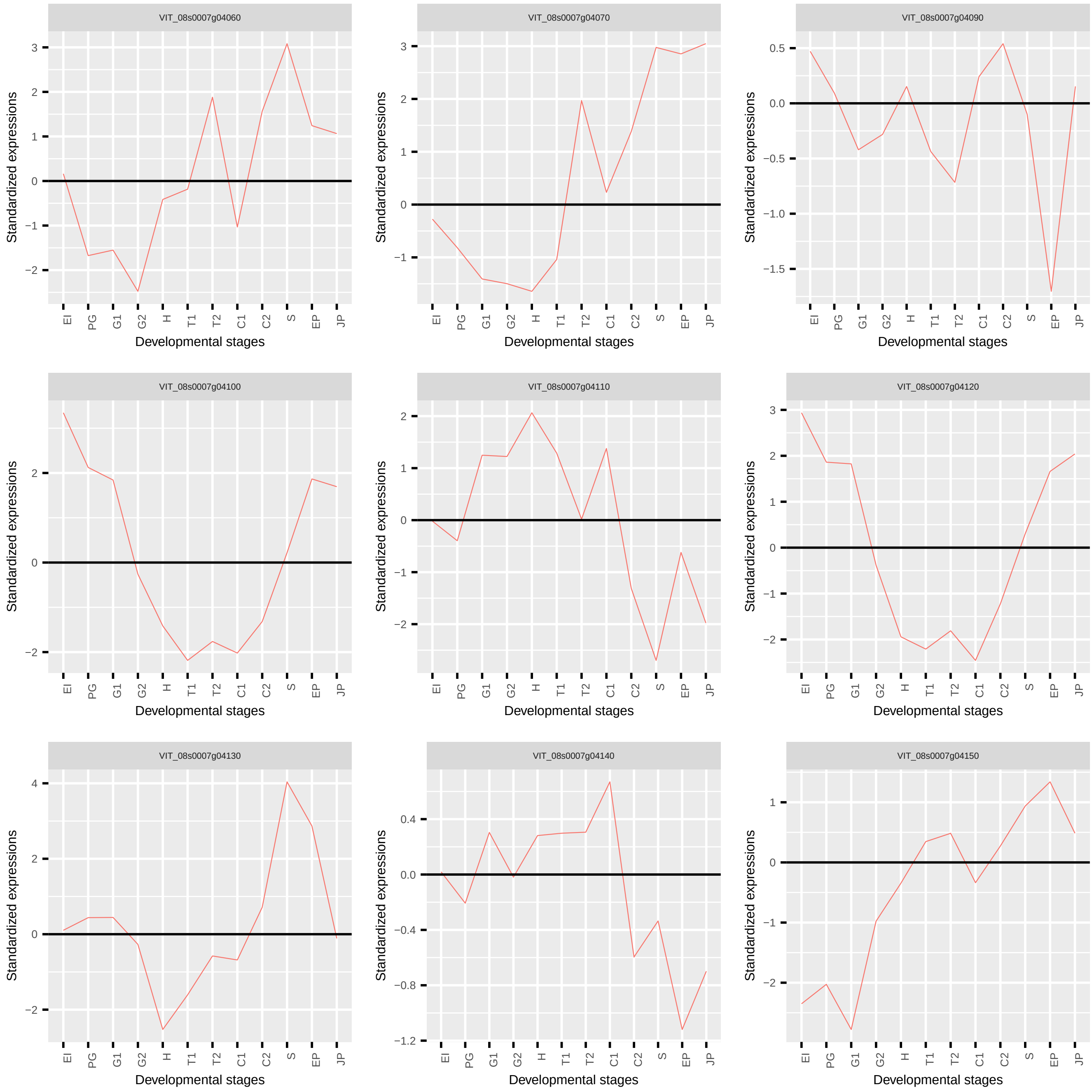

VIT_08s0007g04060
VIT_08s0007g04070
VIT_08s0007g04090
VIT_08s0007g04100
VIT_08s0007g04110
VIT_08s0007g04120
VIT_08s0007g04130
VIT_08s0007g04140
VIT_08s0007g04150
Standardized expressions
Developmental stages
EI PG G1 G2 H T1 T2 C1 C2 S EP JP

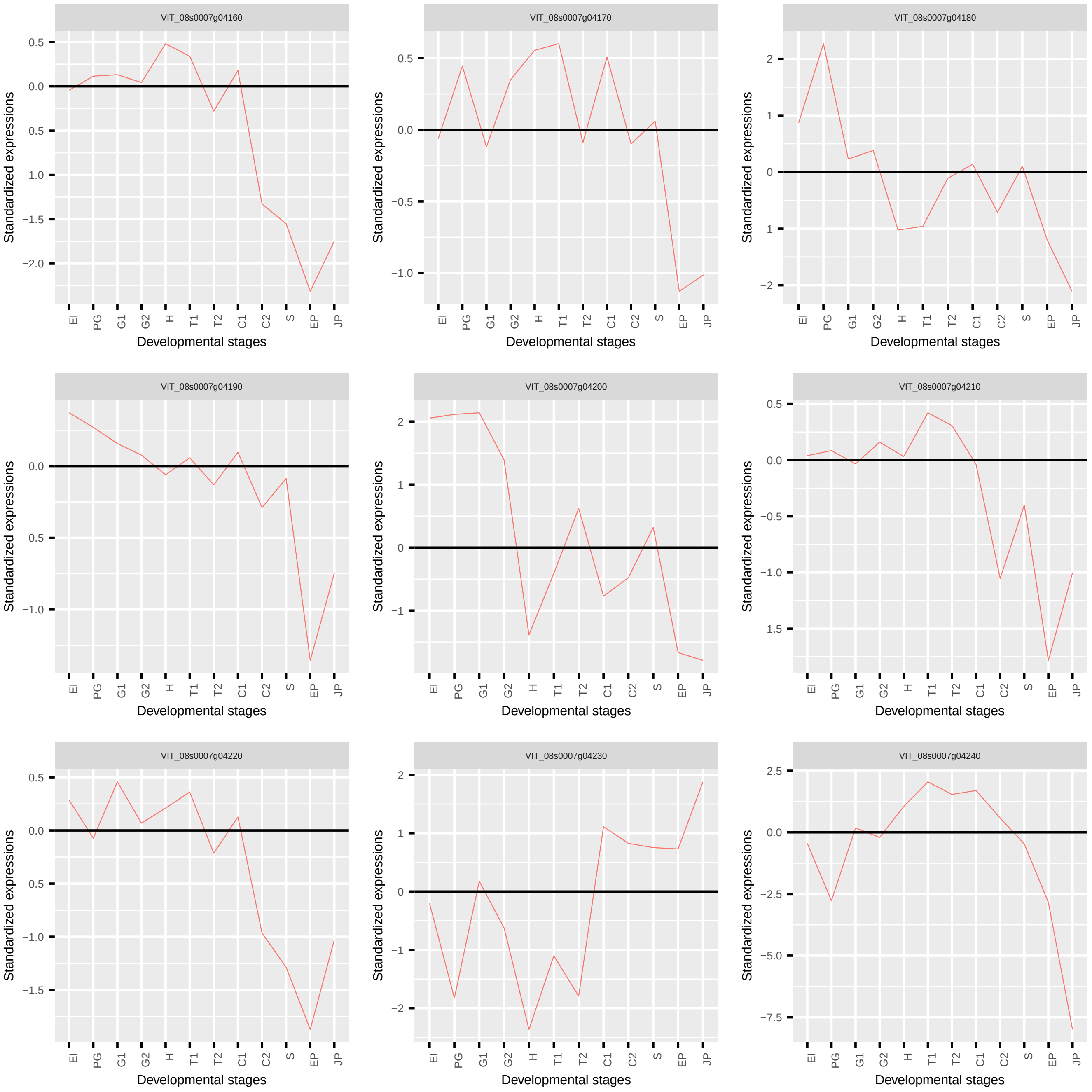

VIT_08s0007g04160
VIT_08s0007g04170
VIT_08s0007g04180
VIT_08s0007g04190
VIT_08s0007g04200
VIT_08s0007g04210
VIT_08s0007g04220
VIT_08s0007g04230
VIT_08s0007g04240
Standardized expressions
Developmental stages
EI PG G1 G2 H T1 T2 C1 C2 S EP JP

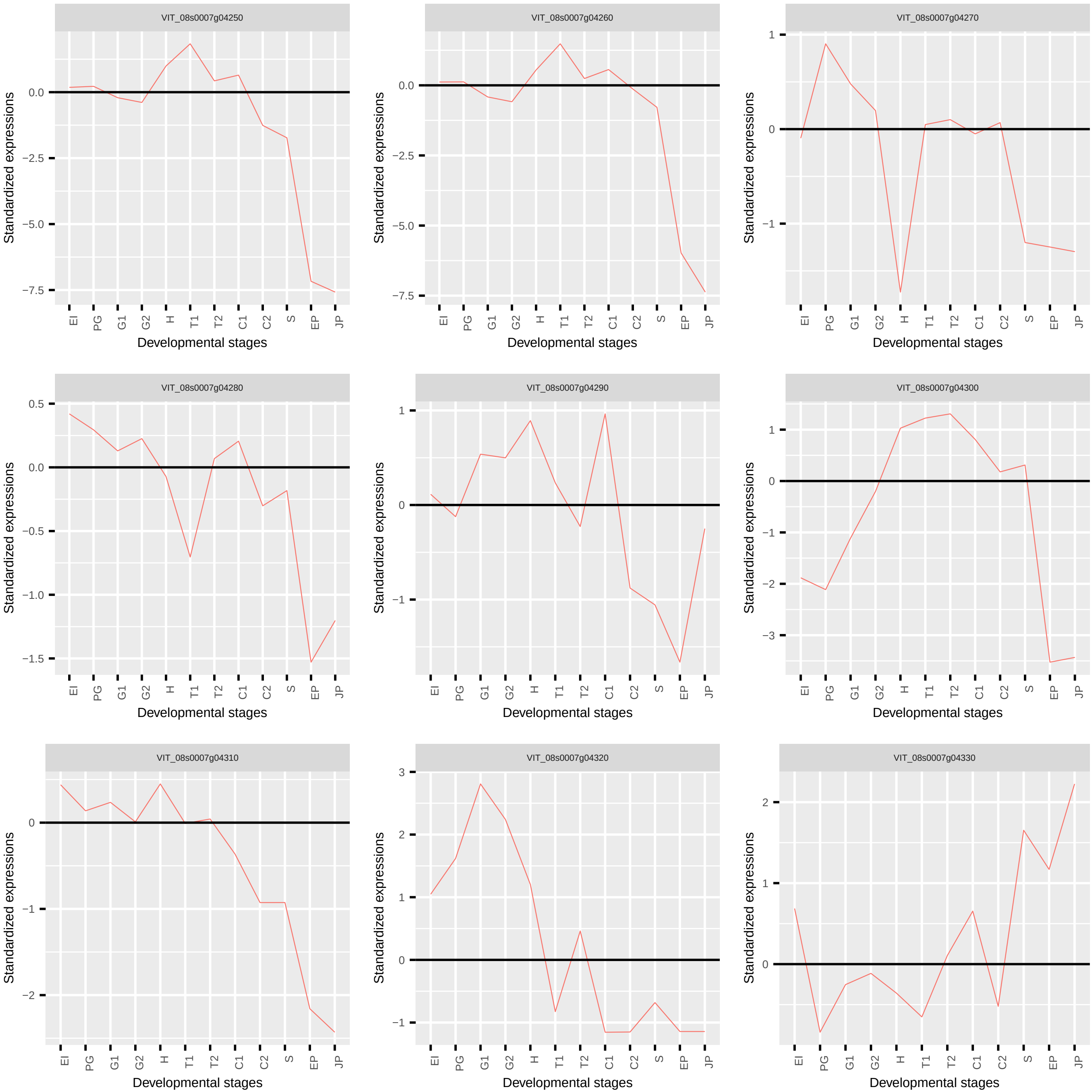

VIT_08s0007g04250
VIT_08s0007g04260
VIT_08s0007g04270
VIT_08s0007g04280
VIT_08s0007g04290
VIT_08s0007g04300
VIT_08s0007g04310
VIT_08s0007g04320
VIT_08s0007g04330
Standardized expressions
Developmental stages
EI
PG
G1
G2
H
T1
T2
C1
C2
S
EP
JP

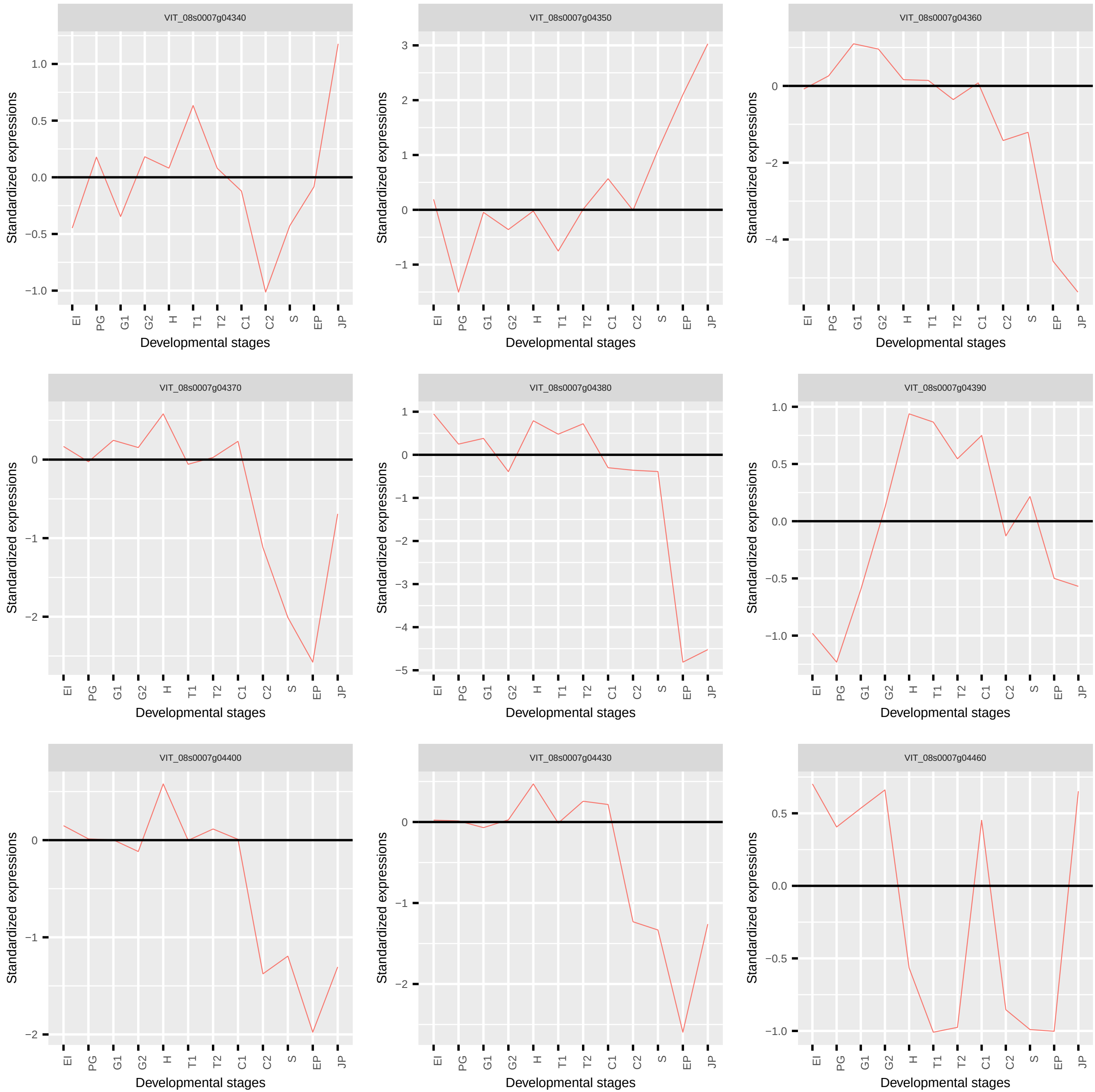
VIT_08s0007g04340
VIT_08s0007g04350
VIT_08s0007g04360
VIT_08s0007g04370
VIT_08s0007g04380
VIT_08s0007g04390
VIT_08s0007g04400
VIT_08s0007g04430
VIT_08s0007g04460
Standardized expressions
Developmental stages
EI
PG
G1
G2
H
T1
T2
C1
C2
S
EP
JP

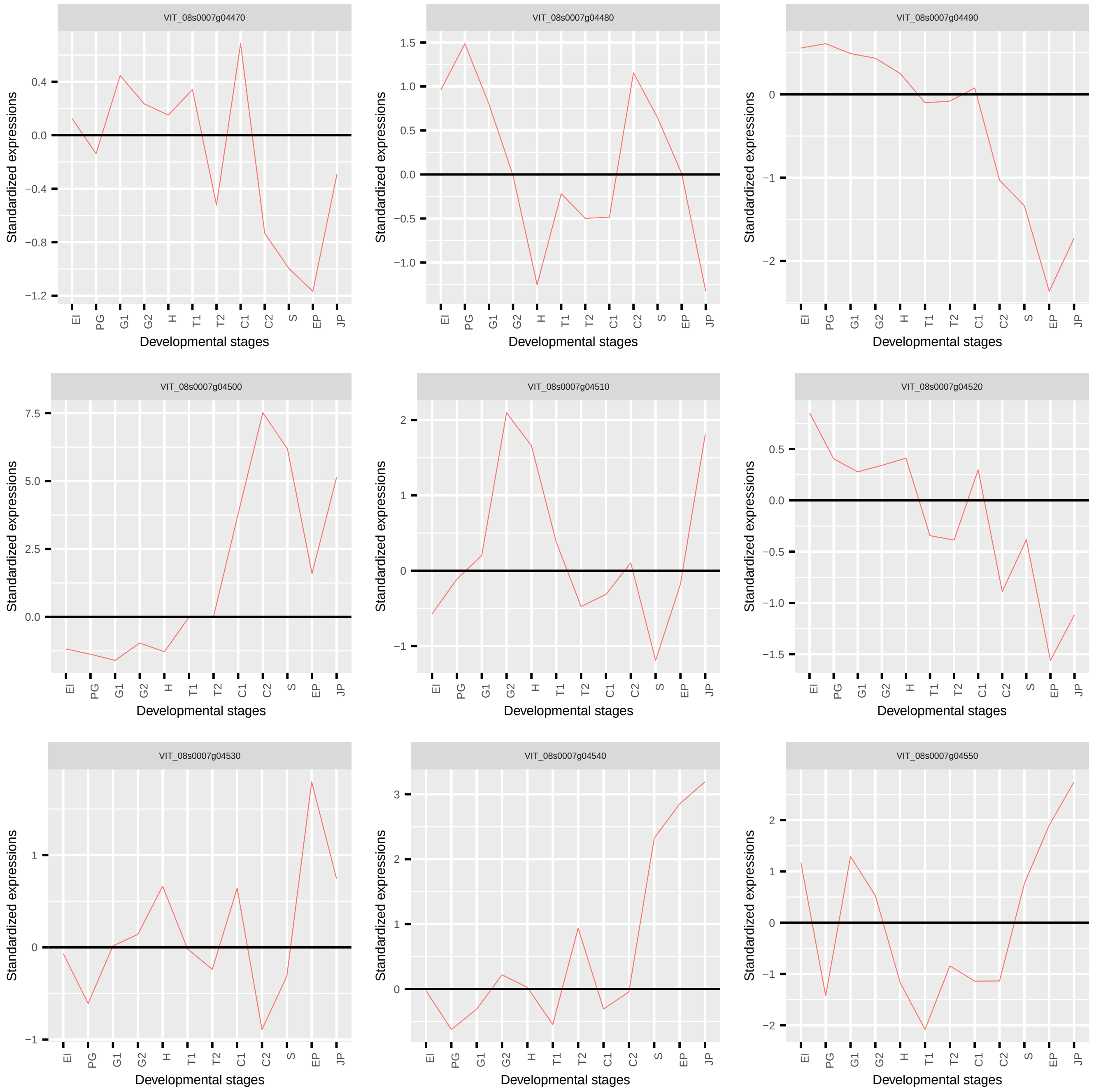

VIT_08s0007g04470
VIT_08s0007g04480
VIT_08s0007g04490
VIT_08s0007g04500
VIT_08s0007g04510
VIT_08s0007g04520
VIT_08s0007g04530
VIT_08s0007g04540
VIT_08s0007g04550
Standardized expressions
Developmental stages
EI PG G1 G2 H T1 T2 C1 C2 S EP JP
0.4 0.0 −0.4 −0.8 −1.2
1.5 1.0 0.5 0.0 −0.5 −1.0
0 −1 −2
7.5 5.0 2.5 0.0
2 1 0 −1
0.5 0.0 −0.5 −1.0 −1.5
1 0 −1
3 2 1 0
2 1 0 −1 −2

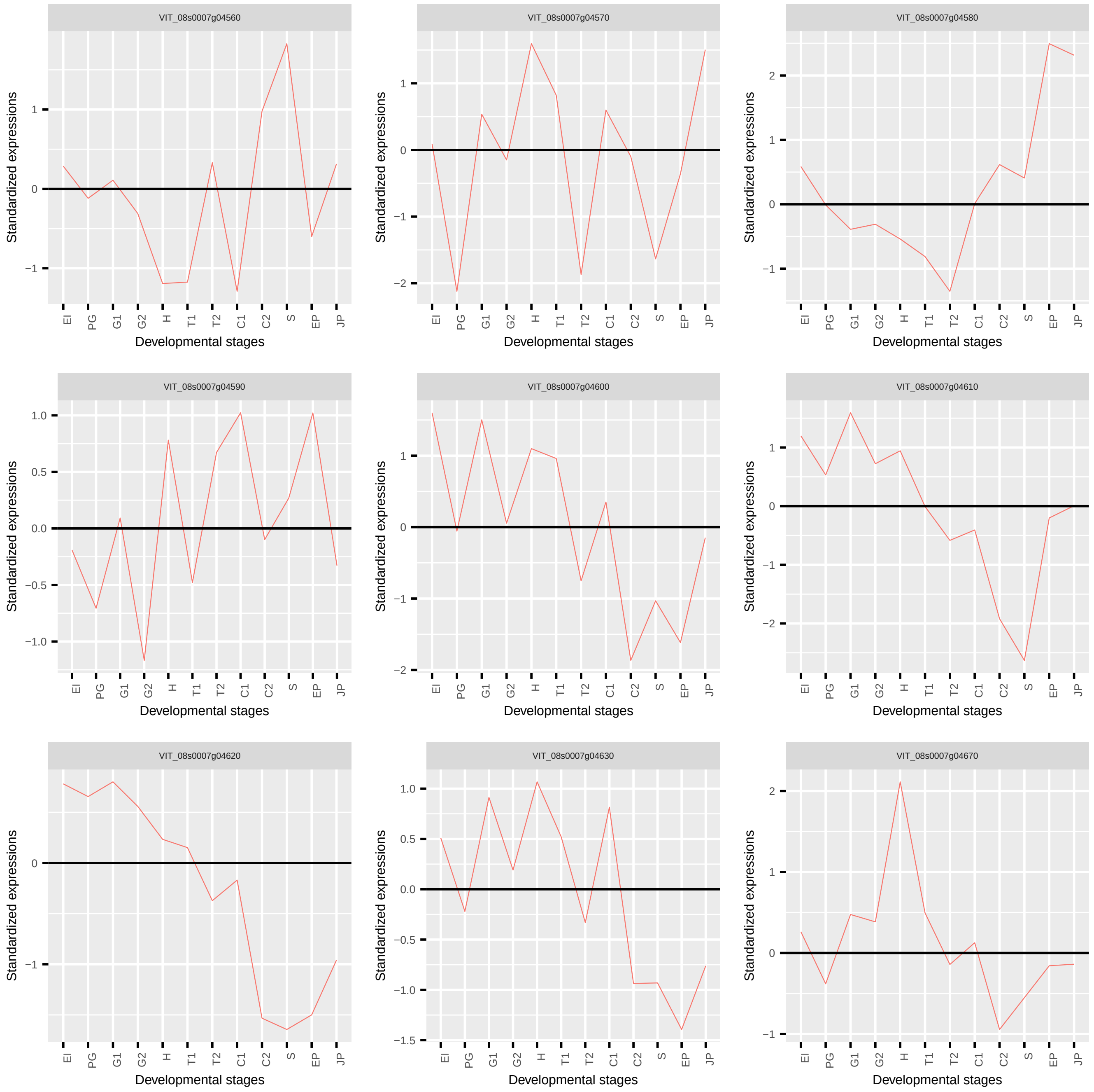
VIT_08s0007g04560
VIT_08s0007g04570
VIT_08s0007g04580
VIT_08s0007g04590
VIT_08s0007g04600
VIT_08s0007g04610
VIT_08s0007g04620
VIT_08s0007g04630
VIT_08s0007g04670
Standardized expressions
Developmental stages
EI PG G1 G2 H T1 T2 C1 C2 S EP JP

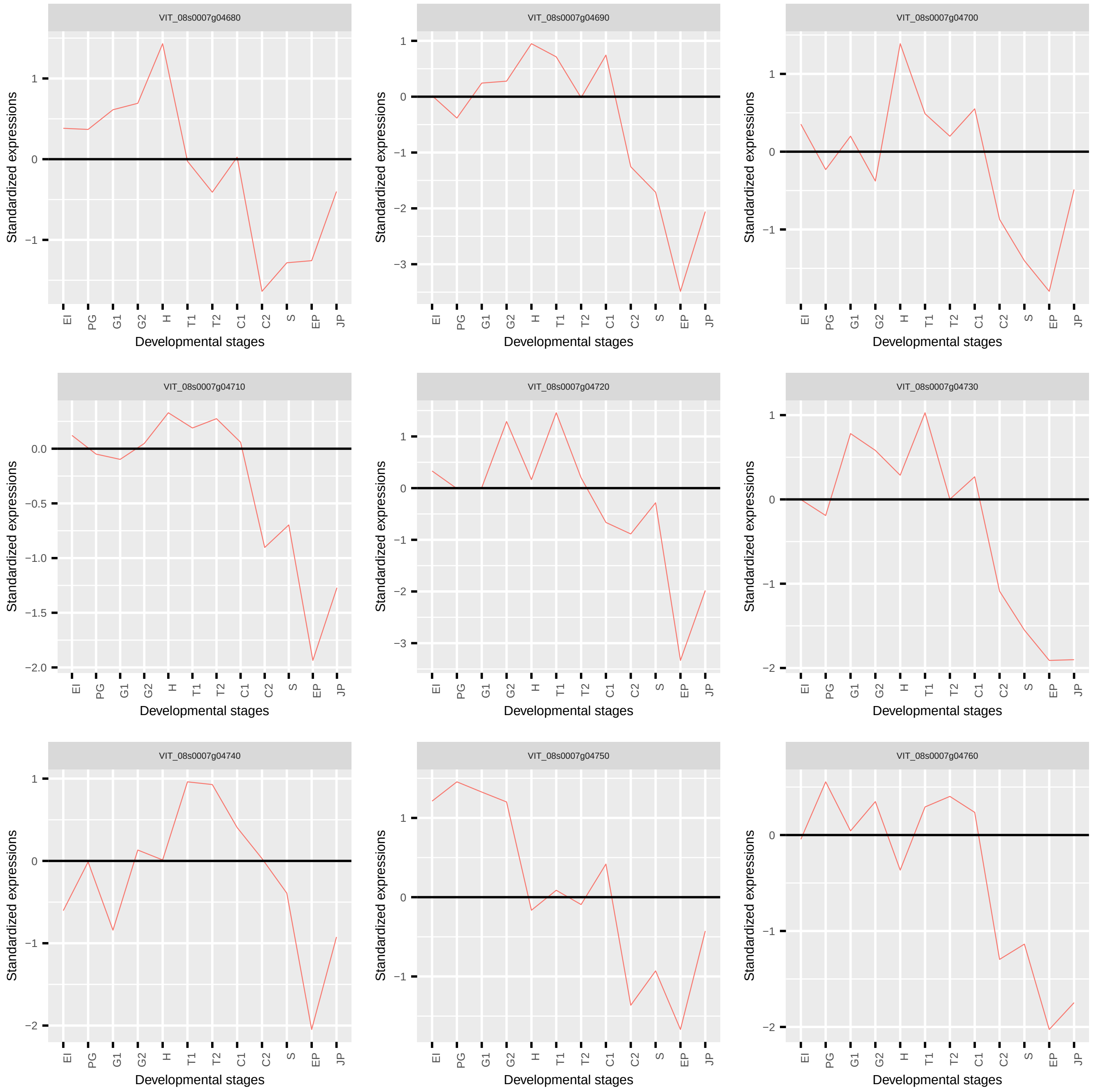
VIT_08s0007g04680
VIT_08s0007g04690
VIT_08s0007g04700
VIT_08s0007g04710
VIT_08s0007g04720
VIT_08s0007g04730
VIT_08s0007g04740
VIT_08s0007g04750
VIT_08s0007g04760
Standardized expressions
Developmental stages
EI
PG
G1
G2
H
T1
T2
C1
C2
S
EP
JP

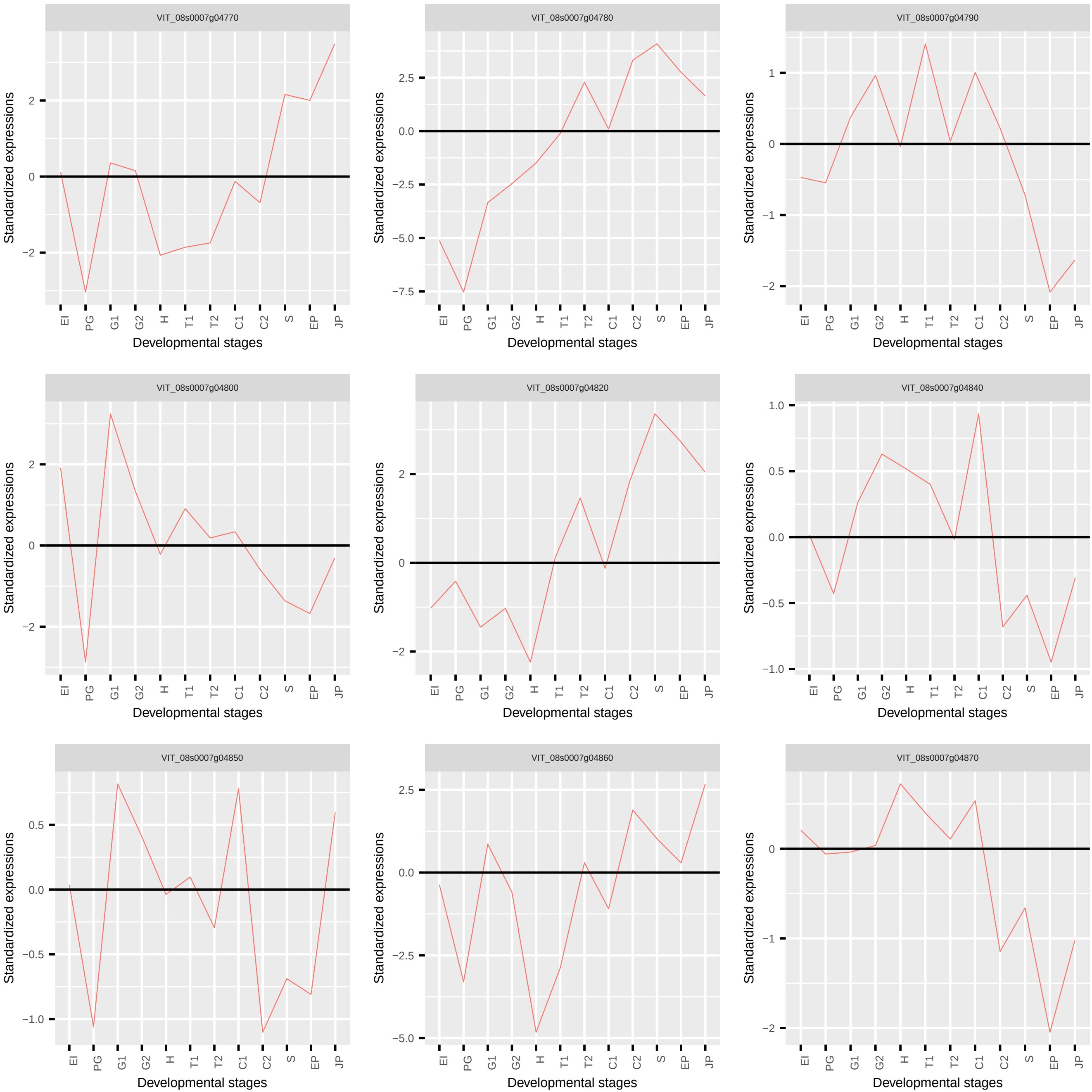
VIT_08s0007g04770
VIT_08s0007g04780
VIT_08s0007g04790
VIT_08s0007g04800
VIT_08s0007g04820
VIT_08s0007g04840
VIT_08s0007g04850
VIT_08s0007g04860
VIT_08s0007g04870
Standardized expressions
Developmental stages
EI PG G1 G2 H T1 T2 C1 C2 S EP JP

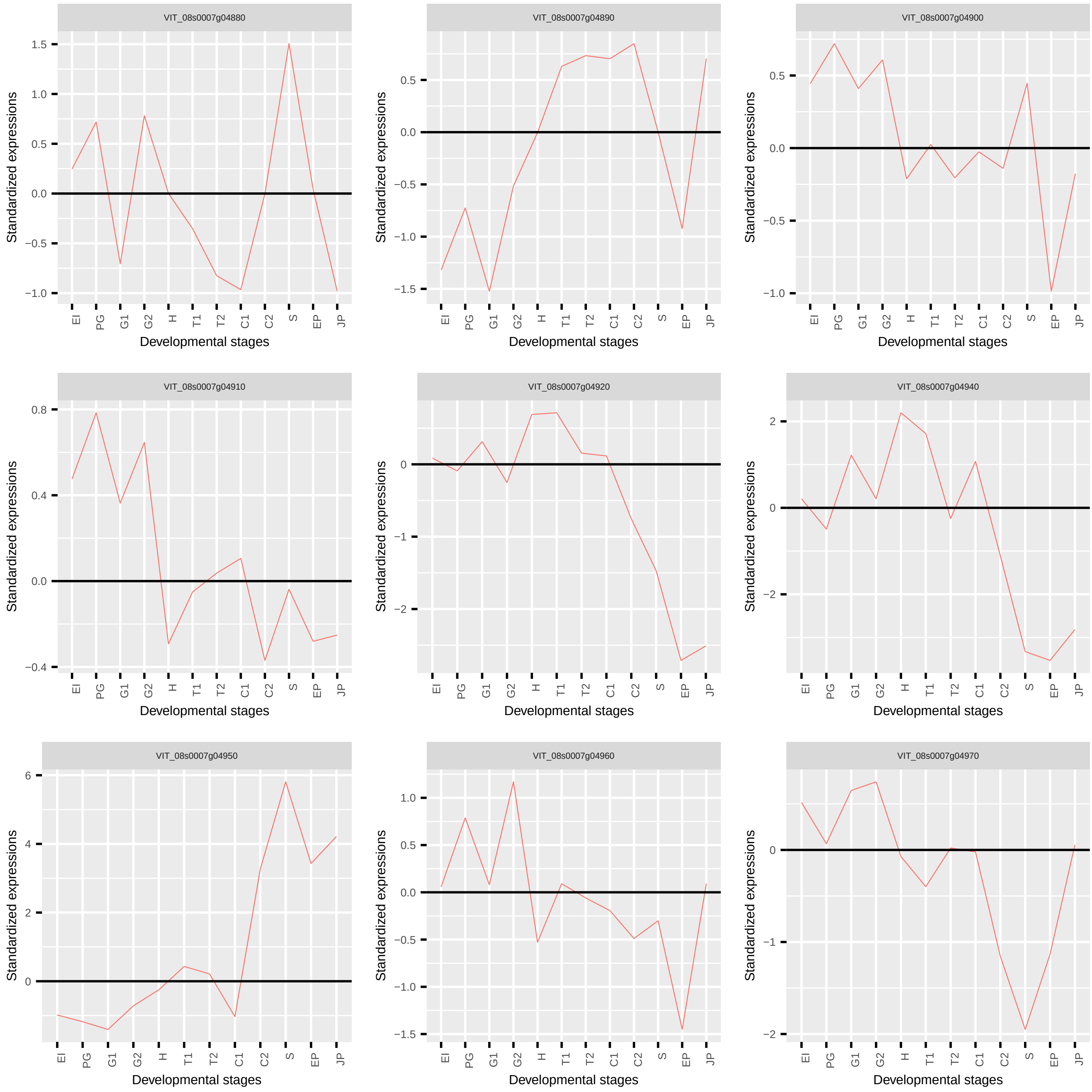
VIT_08s0007g04880
VIT_08s0007g04890
VIT_08s0007g04900
VIT_08s0007g04910
VIT_08s0007g04920
VIT_08s0007g04940
VIT_08s0007g04950
VIT_08s0007g04960
VIT_08s0007g04970
Standardized expressions
Developmental stages
EI PG G1 G2 H T1 T2 C1 C2 S EP JP

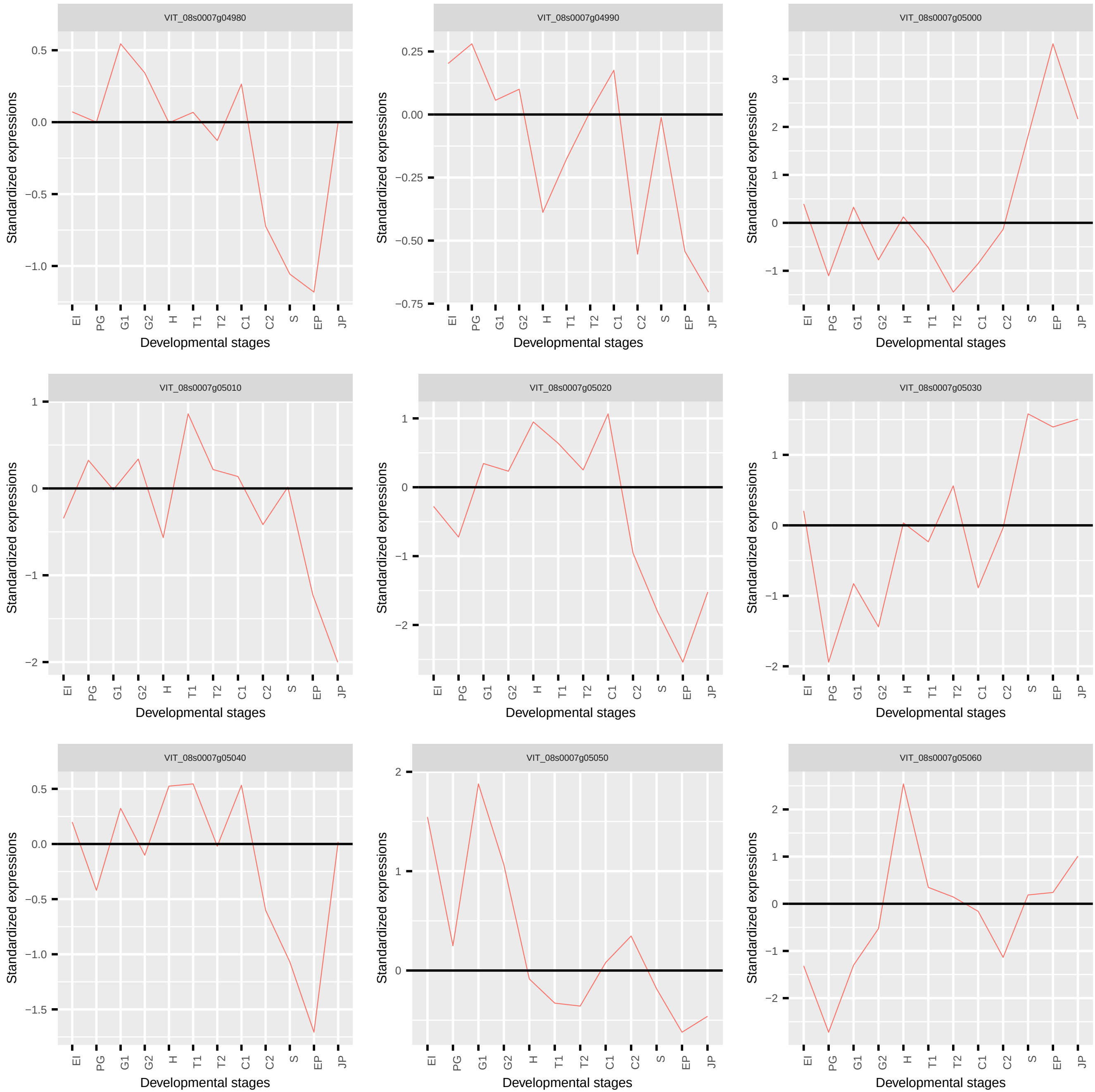

VIT_08s0007g04980
VIT_08s0007g04990
VIT_08s0007g05000
VIT_08s0007g05010
VIT_08s0007g05020
VIT_08s0007g05030
VIT_08s0007g05040
VIT_08s0007g05050
VIT_08s0007g05060
Standardized expressions
Developmental stages
EI
PG
G1
G2
H
T1
T2
C1
C2
S
EP
JP

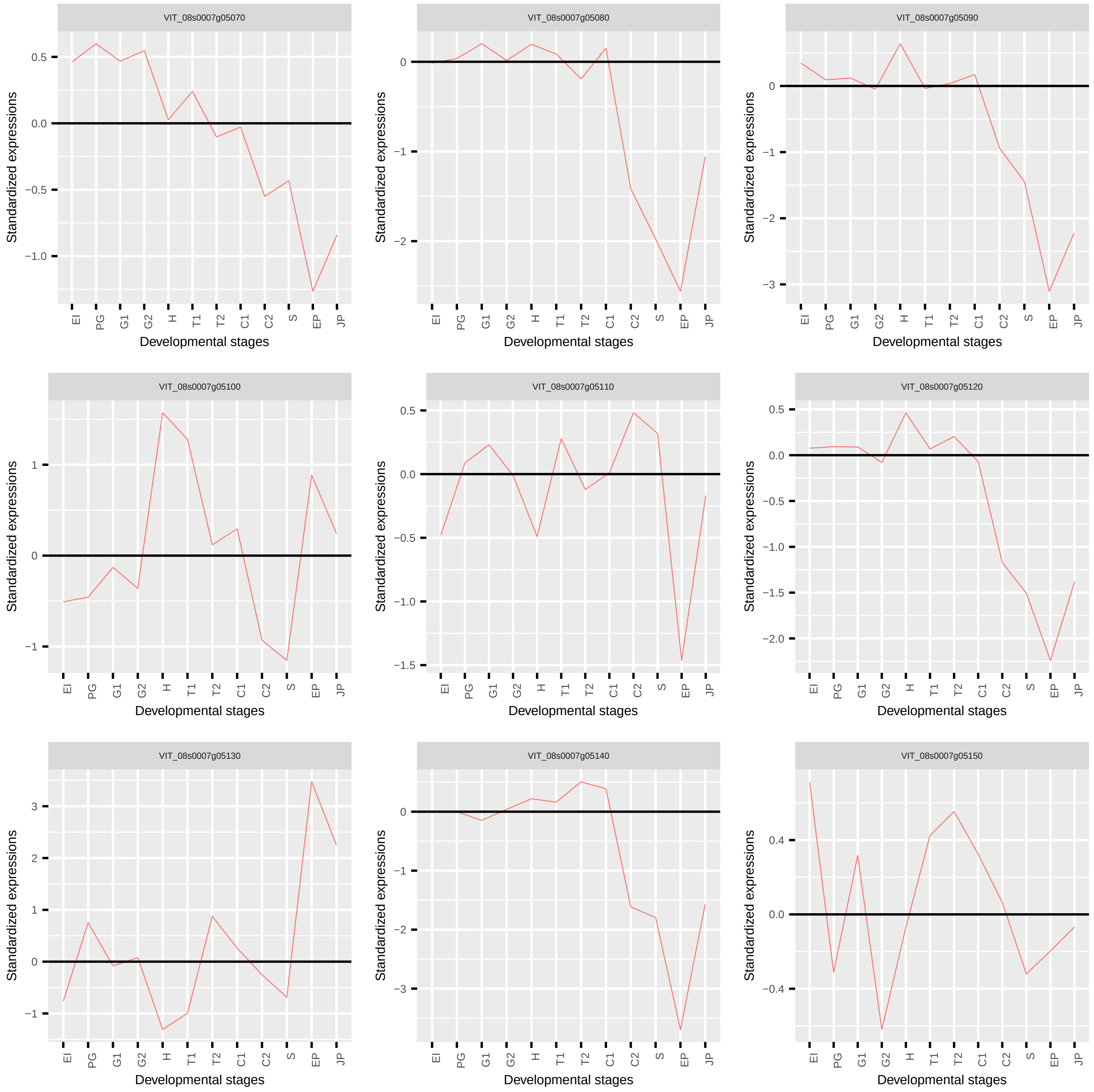

VIT_08s0007g05070
VIT_08s0007g05080
VIT_08s0007g05090
VIT_08s0007g05100
VIT_08s0007g05110
VIT_08s0007g05120
VIT_08s0007g05130
VIT_08s0007g05140
VIT_08s0007g05150
Standardized expressions
Developmental stages
EI
PG
G1
G2
H
T1
T2
C1
C2
S
EP
JP

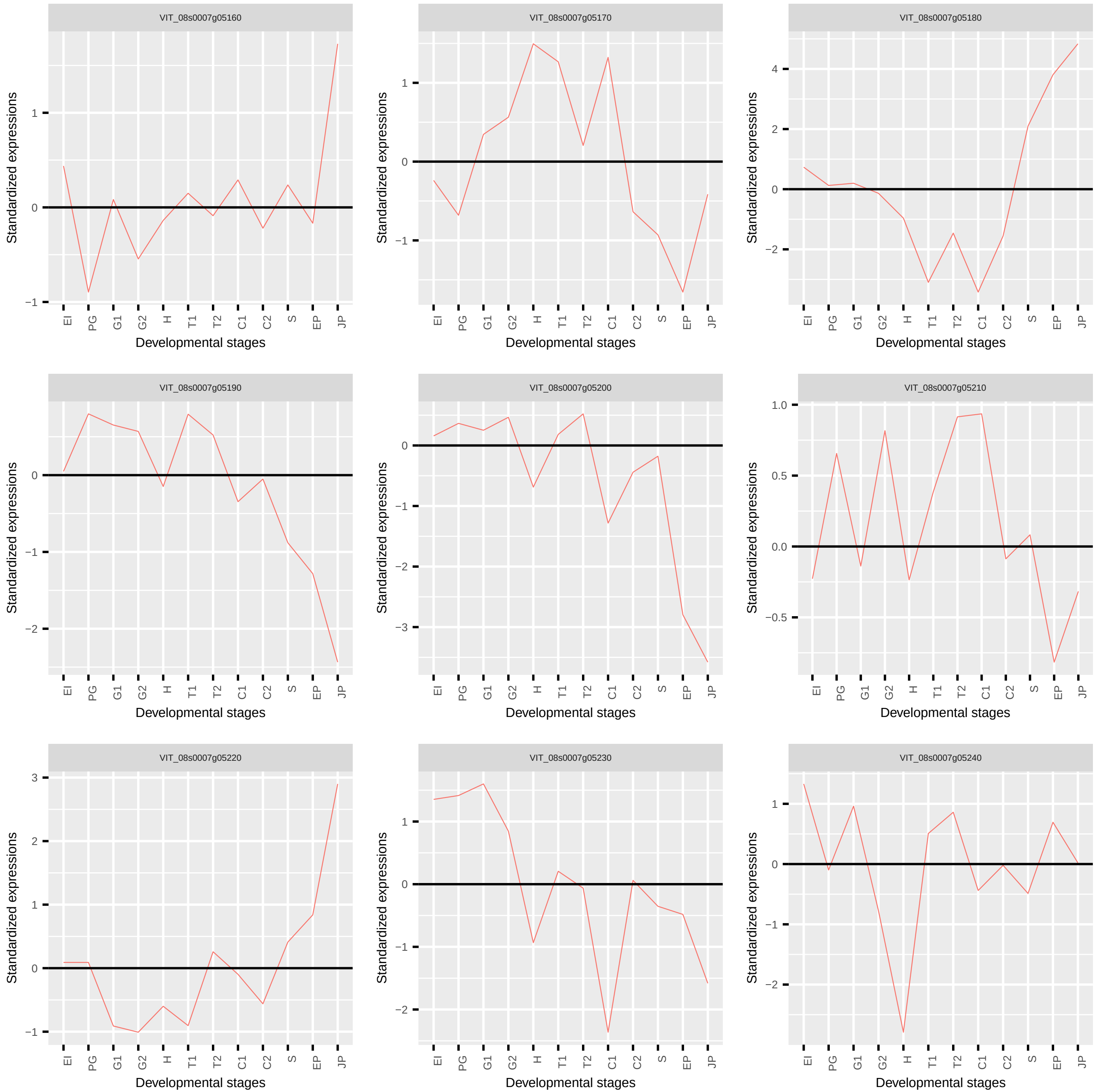

VIT_08s0007g05160
VIT_08s0007g05170
VIT_08s0007g05180
VIT_08s0007g05190
VIT_08s0007g05200
VIT_08s0007g05210
VIT_08s0007g05220
VIT_08s0007g05230
VIT_08s0007g05240
Standardized expressions
Developmental stages
EI
PG
G1
G2
H
T1
T2
C1
C2
S
EP
JP

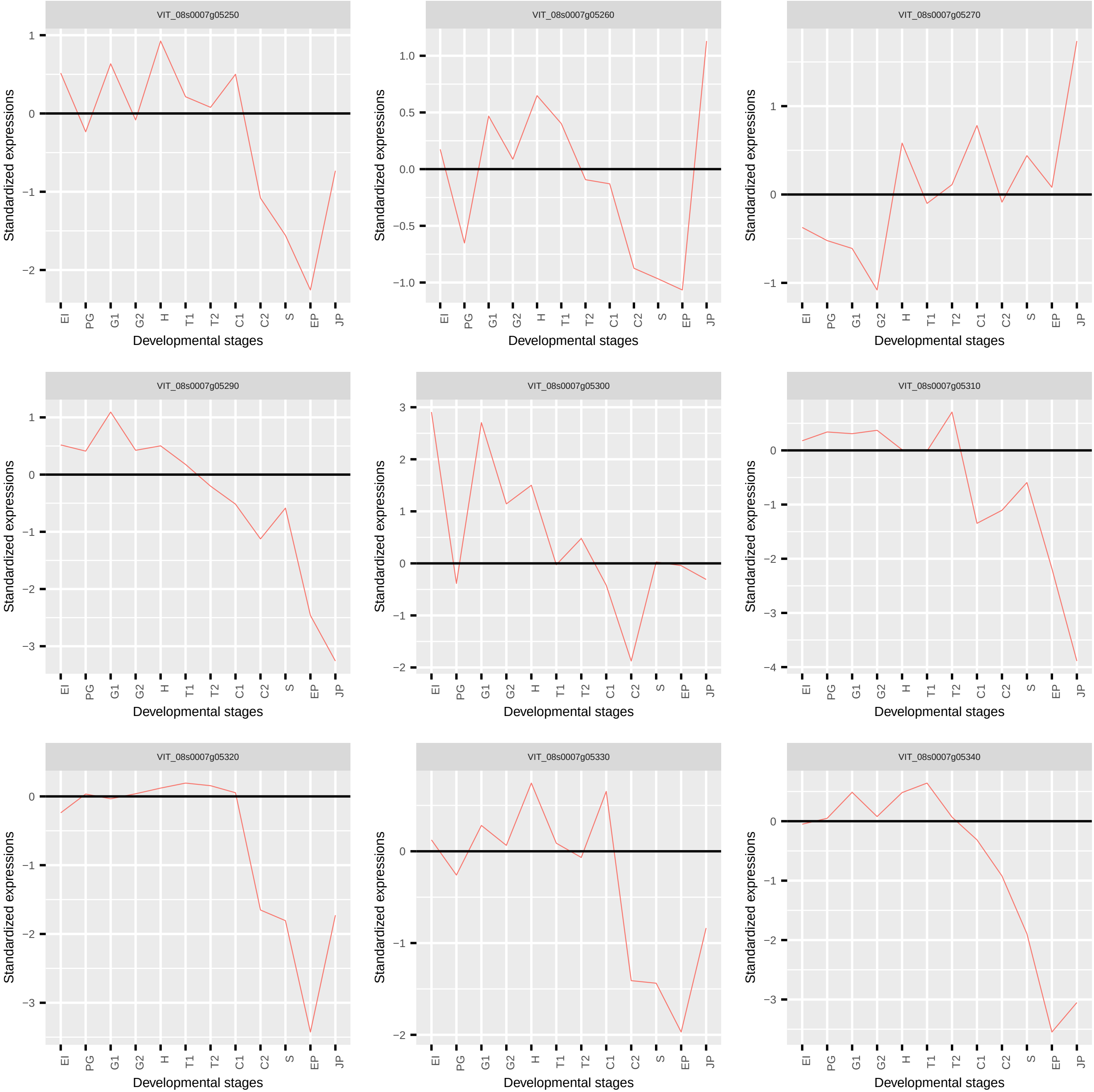
VIT_08s0007g05250
VIT_08s0007g05260
VIT_08s0007g05270
VIT_08s0007g05290
VIT_08s0007g05300
VIT_08s0007g05310
VIT_08s0007g05320
VIT_08s0007g05330
VIT_08s0007g05340
Standardized expressions
Developmental stages
EI PG G1 G2 H T1 T2 C1 C2 S EP JP

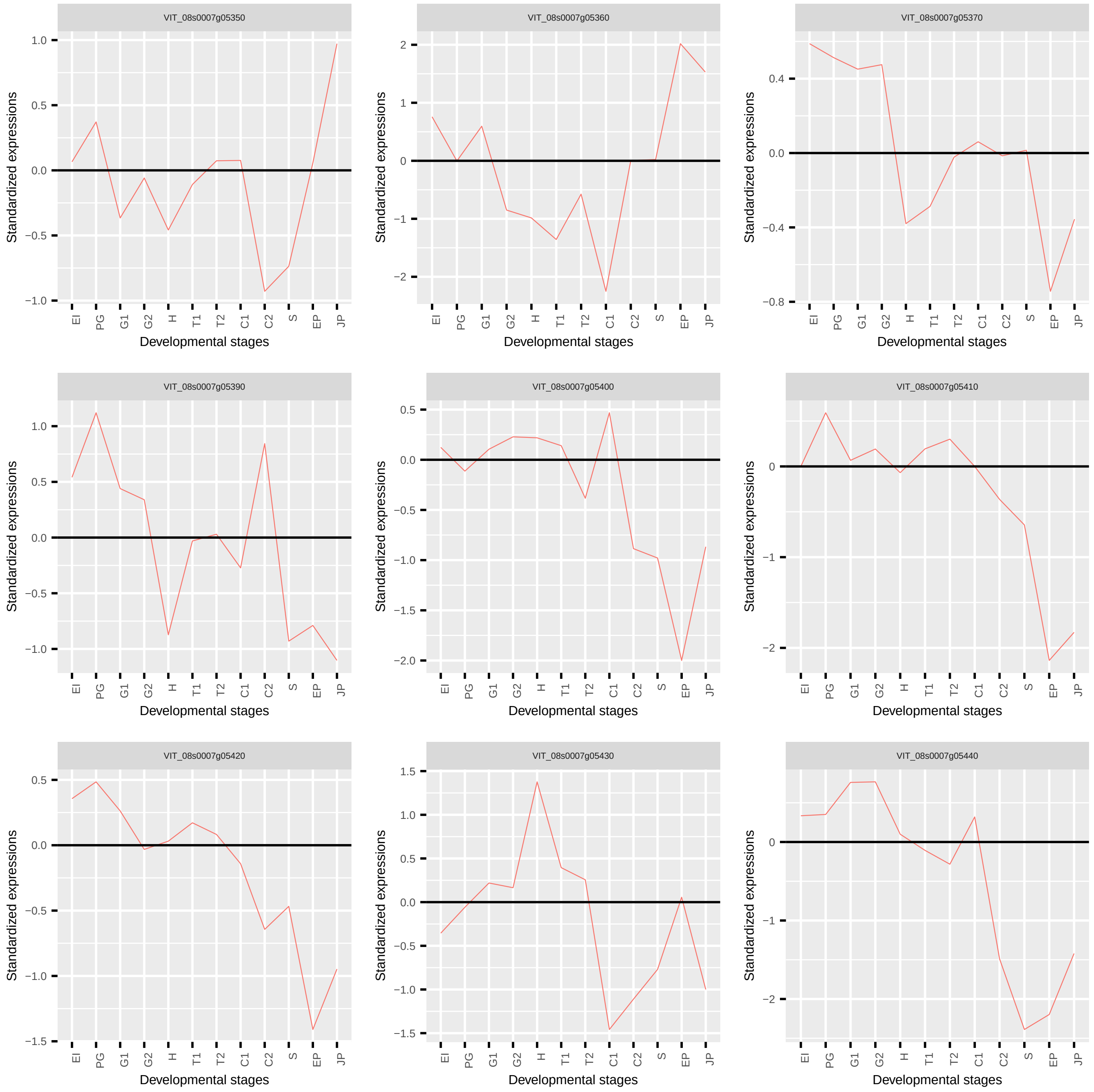
VIT_08s0007g05350
VIT_08s0007g05360
VIT_08s0007g05370
VIT_08s0007g05390
VIT_08s0007g05400
VIT_08s0007g05410
VIT_08s0007g05420
VIT_08s0007g05430
VIT_08s0007g05440
Standardized expressions
Developmental stages
EI PG G1 G2 H T1 T2 C1 C2 S EP JP
1.0 0.5 0.0 −0.5 −1.0
2 1 0 −1 −2
0.4 0.0 −0.4 −0.8
1.0 0.5 0.0 −0.5 −1.0
0.5 0.0 −0.5 −1.0 −1.5 −2.0
0 −1 −2
0.5 0.0 −0.5 −1.0 −1.5
1.5 1.0 0.5 0.0 −0.5 −1.0 −1.5
0 −1 −2

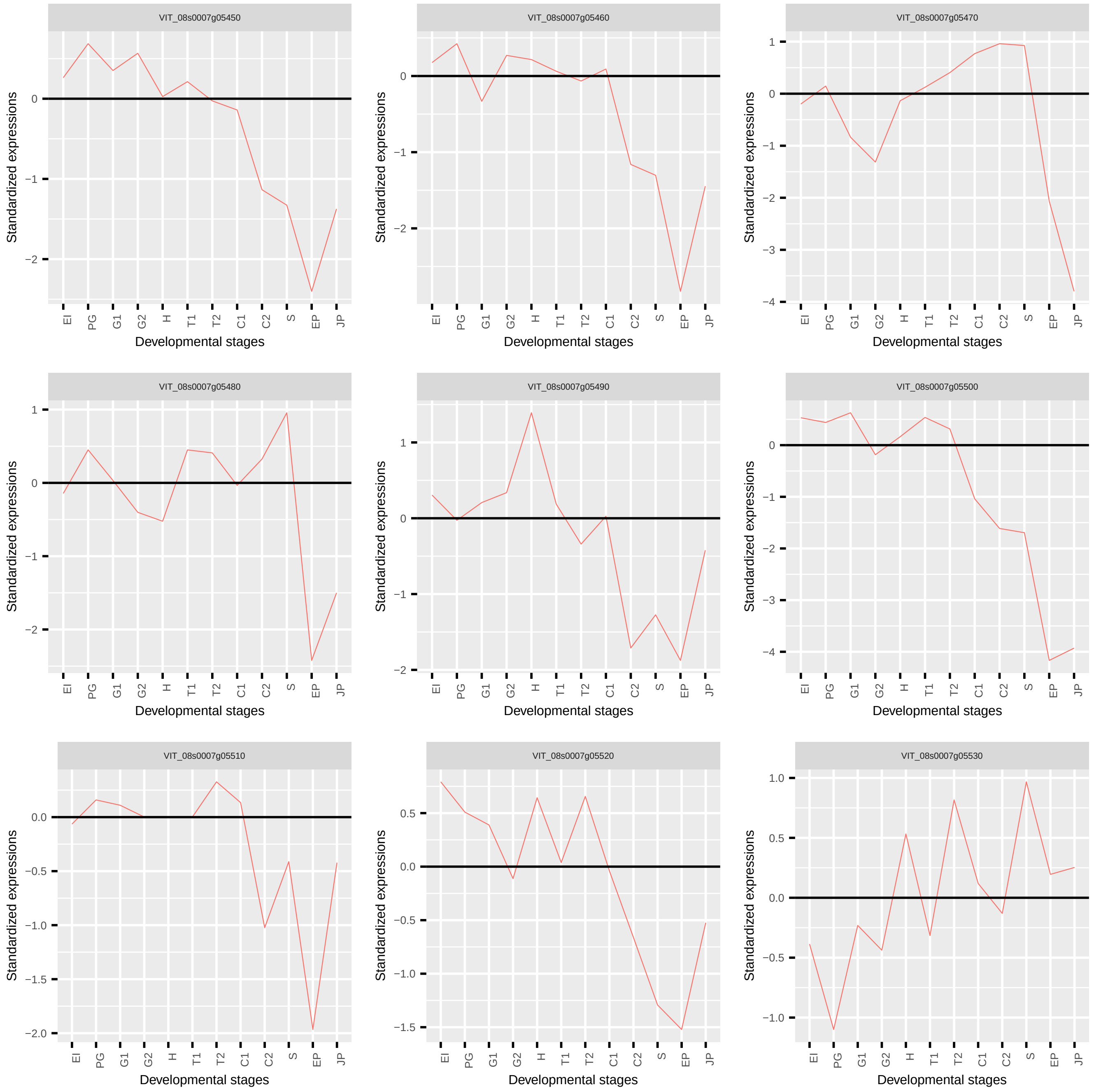

VIT_08s0007g05450
VIT_08s0007g05460
VIT_08s0007g05470
VIT_08s0007g05480
VIT_08s0007g05490
VIT_08s0007g05500
VIT_08s0007g05510
VIT_08s0007g05520
VIT_08s0007g05530
Standardized expressions
Developmental stages
EI PG G1 G2 H T1 T2 C1 C2 S EP JP

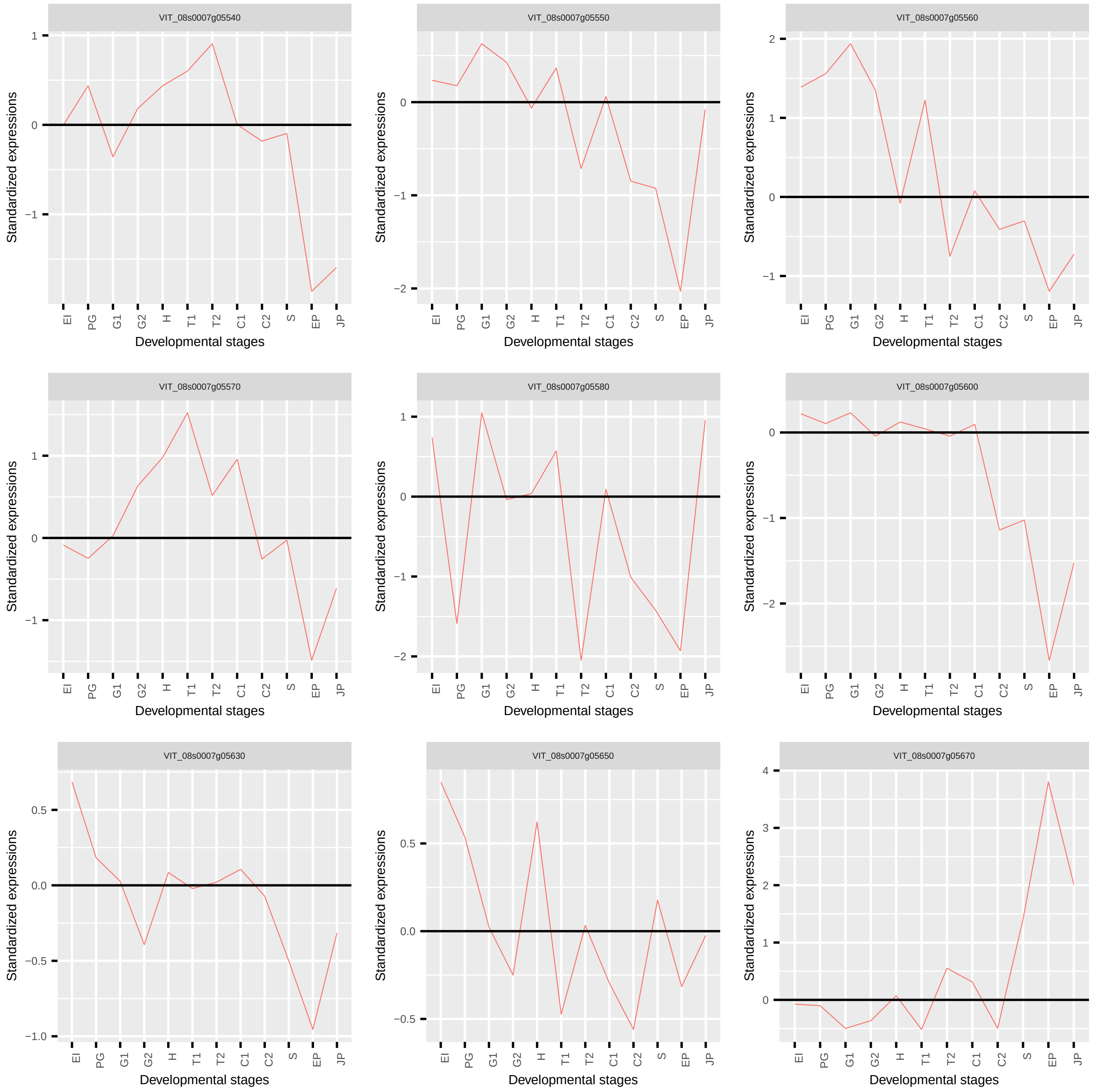
VIT_08s0007g05540
VIT_08s0007g05550
VIT_08s0007g05560
VIT_08s0007g05570
VIT_08s0007g05580
VIT_08s0007g05600
VIT_08s0007g05630
VIT_08s0007g05650
VIT_08s0007g05670
Standardized expressions
Developmental stages
EI
PG
G1
G2
H
T1
T2
C1
C2
S
EP
JP

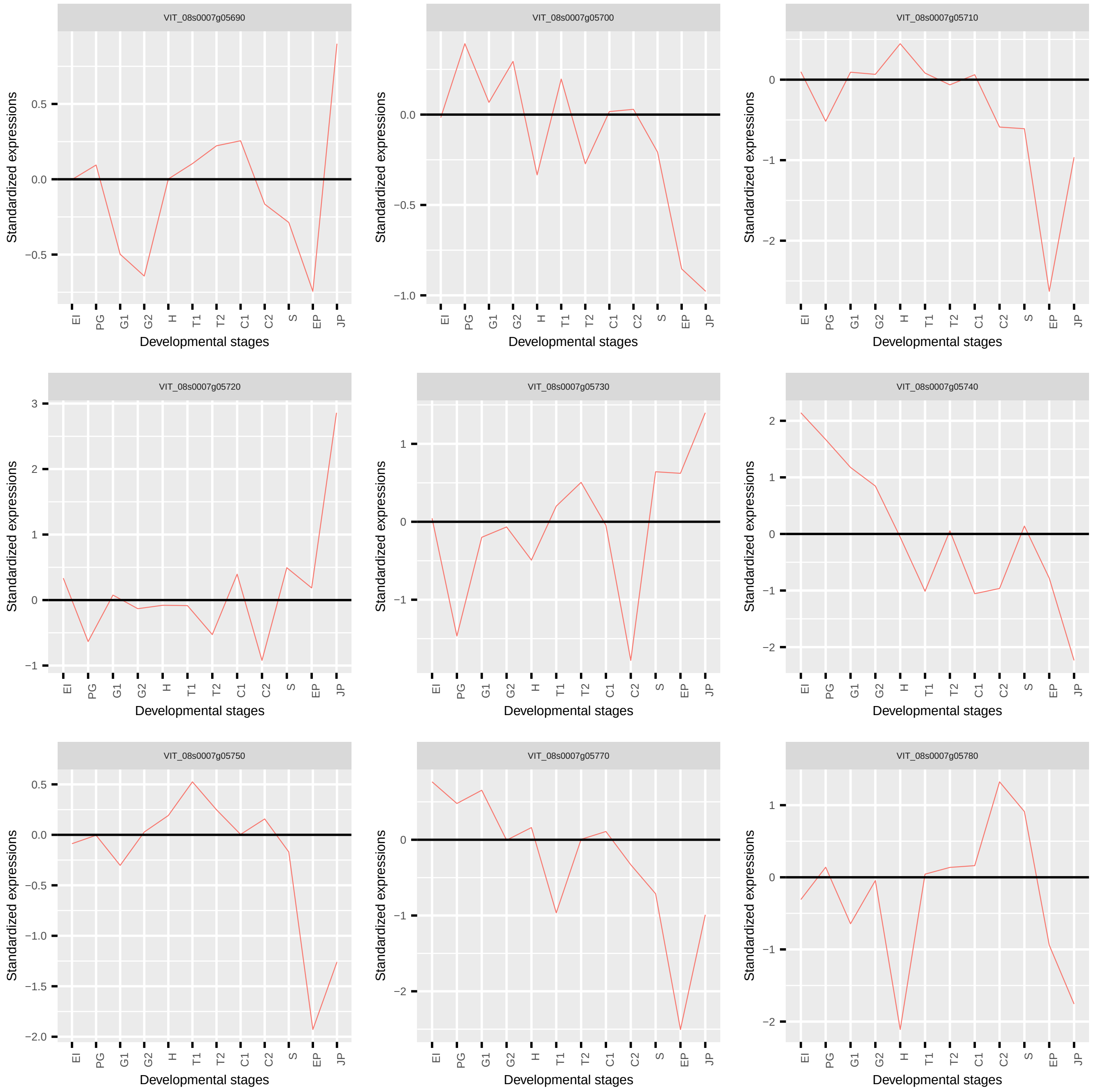
VIT_08s0007g05690
VIT_08s0007g05700
VIT_08s0007g05710
VIT_08s0007g05720
VIT_08s0007g05730
VIT_08s0007g05740
VIT_08s0007g05750
VIT_08s0007g05770
VIT_08s0007g05780
Standardized expressions
Developmental stages
EI PG G1 G2 H T1 T2 C1 C2 S EP JP

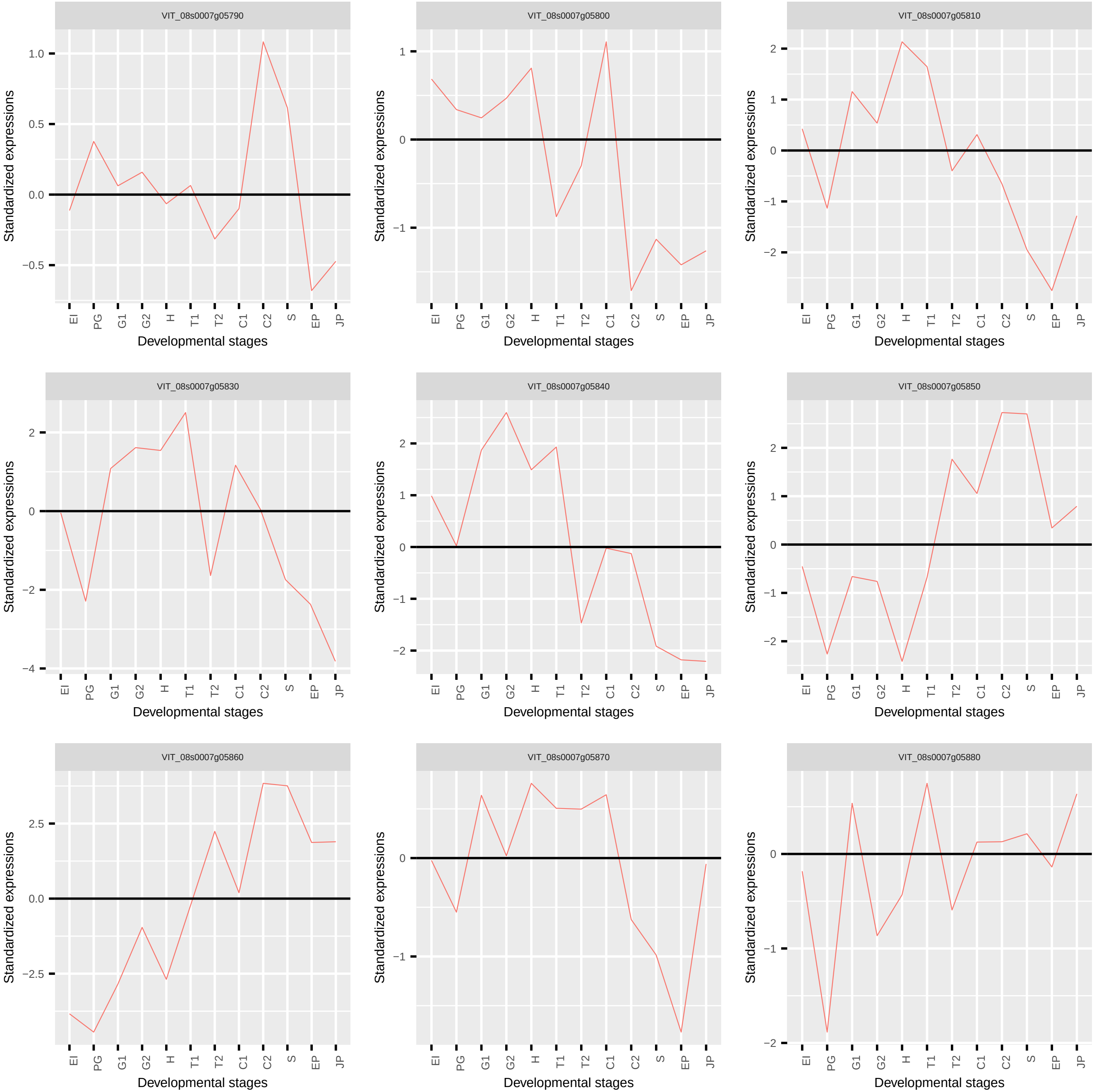

VIT_08s0007g05790
VIT_08s0007g05800
VIT_08s0007g05810
VIT_08s0007g05830
VIT_08s0007g05840
VIT_08s0007g05850
VIT_08s0007g05860
VIT_08s0007g05870
VIT_08s0007g05880
Standardized expressions
Developmental stages
EI
PG
G1
G2
H
T1
T2
C1
C2
S
EP
JP

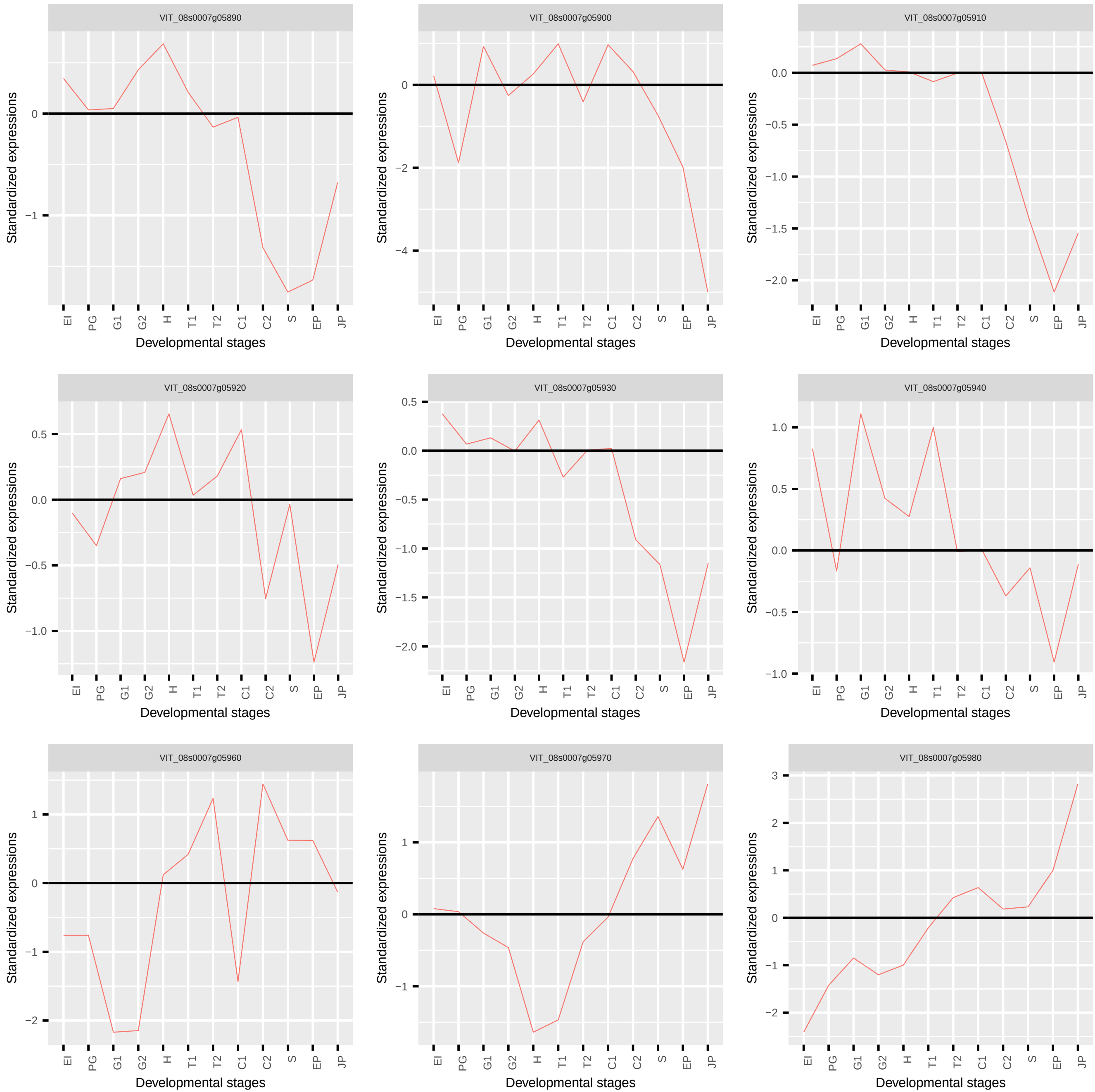

VIT_08s0007g05890
VIT_08s0007g05900
VIT_08s0007g05910
VIT_08s0007g05920
VIT_08s0007g05930
VIT_08s0007g05940
VIT_08s0007g05960
VIT_08s0007g05970
VIT_08s0007g05980
Standardized expressions
Developmental stages
EI PG G1 G2 H T1 T2 C1 C2 S EP JP

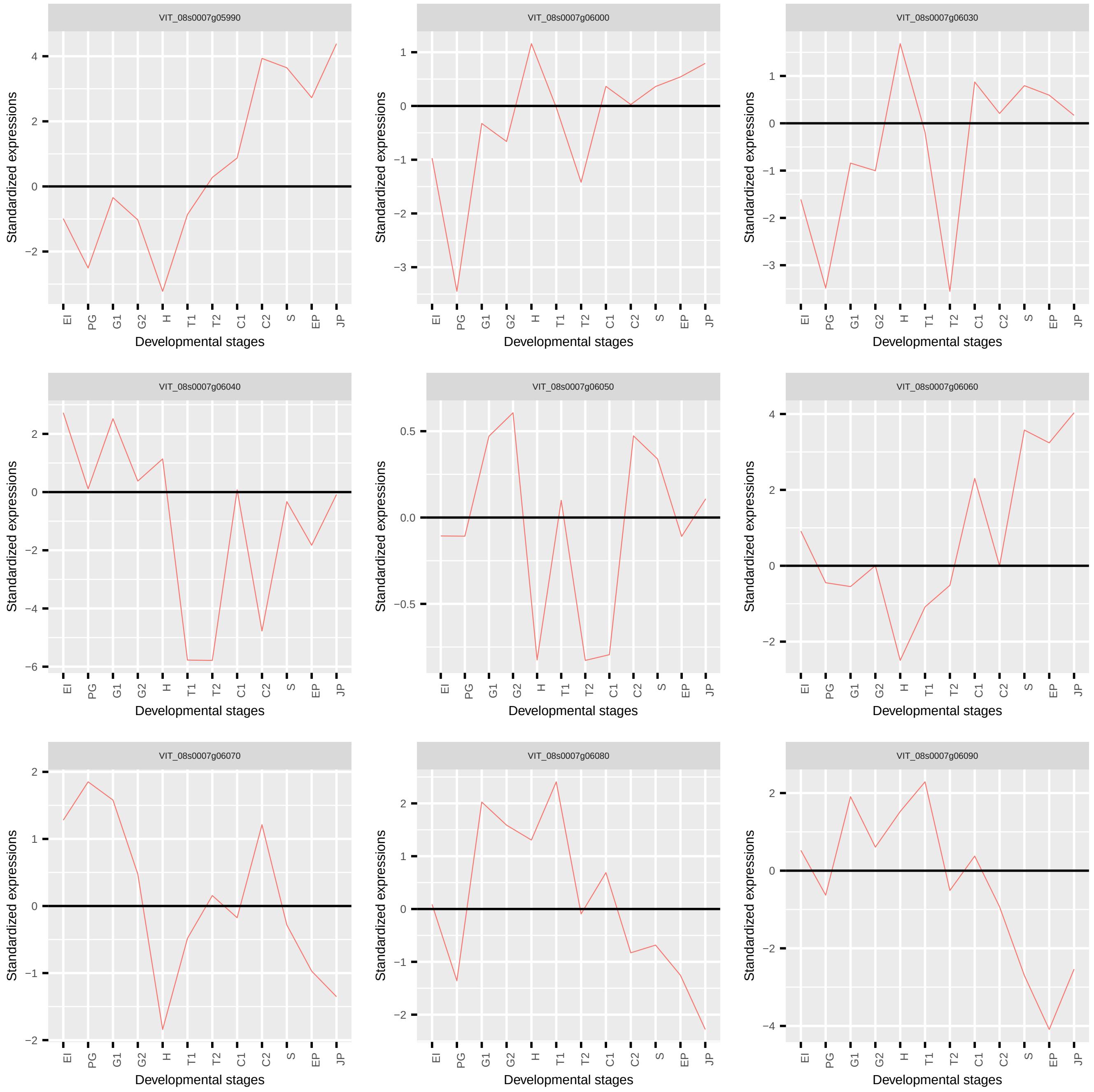

VIT_08s0007g05990
VIT_08s0007g06000
VIT_08s0007g06030
VIT_08s0007g06040
VIT_08s0007g06050
VIT_08s0007g06060
VIT_08s0007g06070
VIT_08s0007g06080
VIT_08s0007g06090
Standardized expressions
Developmental stages
EI PG G1 G2 H T1 T2 C1 C2 S EP JP

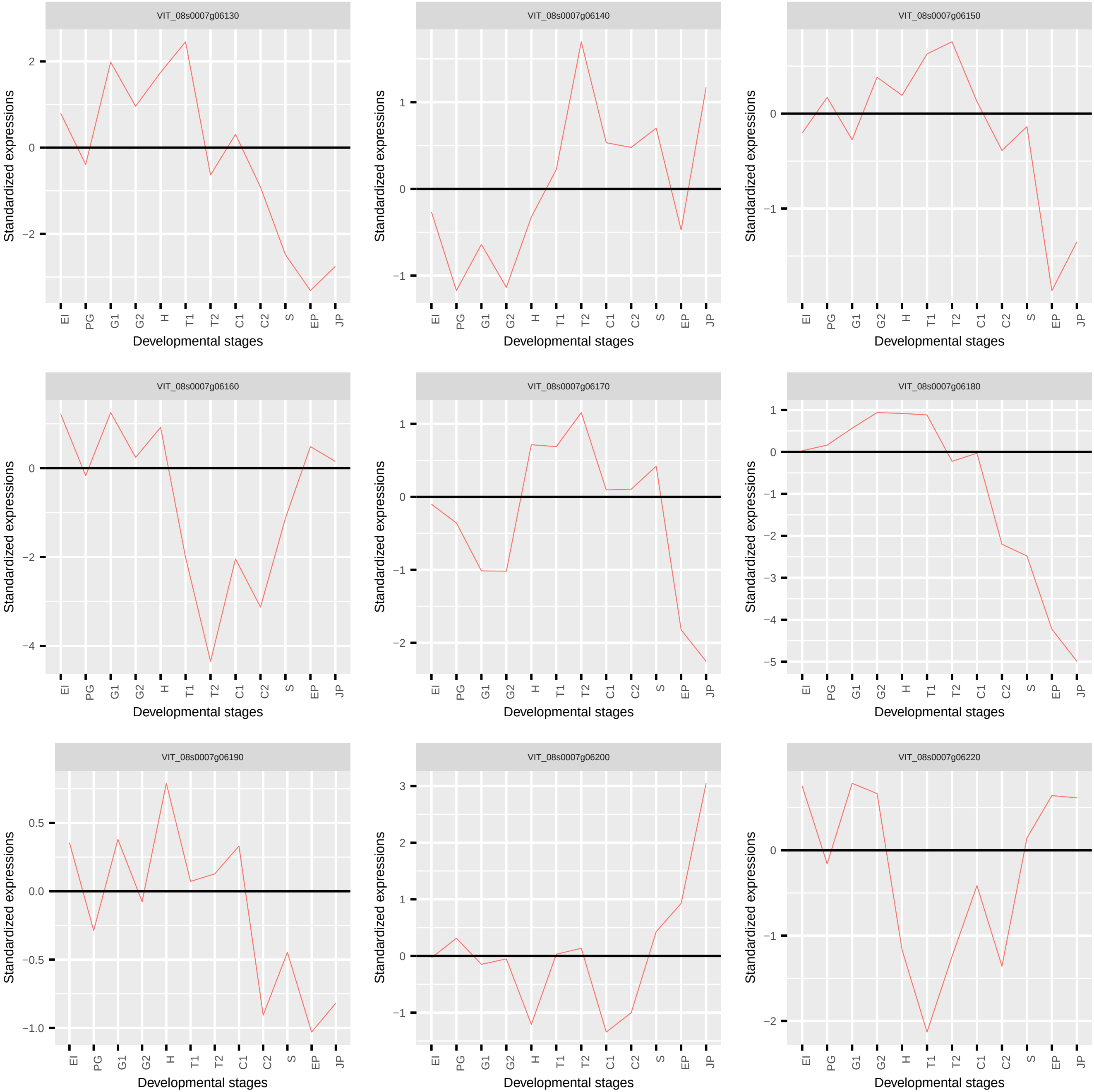
VIT_08s0007g06130
VIT_08s0007g06140
VIT_08s0007g06150
VIT_08s0007g06160
VIT_08s0007g06170
VIT_08s0007g06180
VIT_08s0007g06190
VIT_08s0007g06200
VIT_08s0007g06220
Standardized expressions
Developmental stages
EI PG G1 G2 H T1 T2 C1 C2 S EP JP

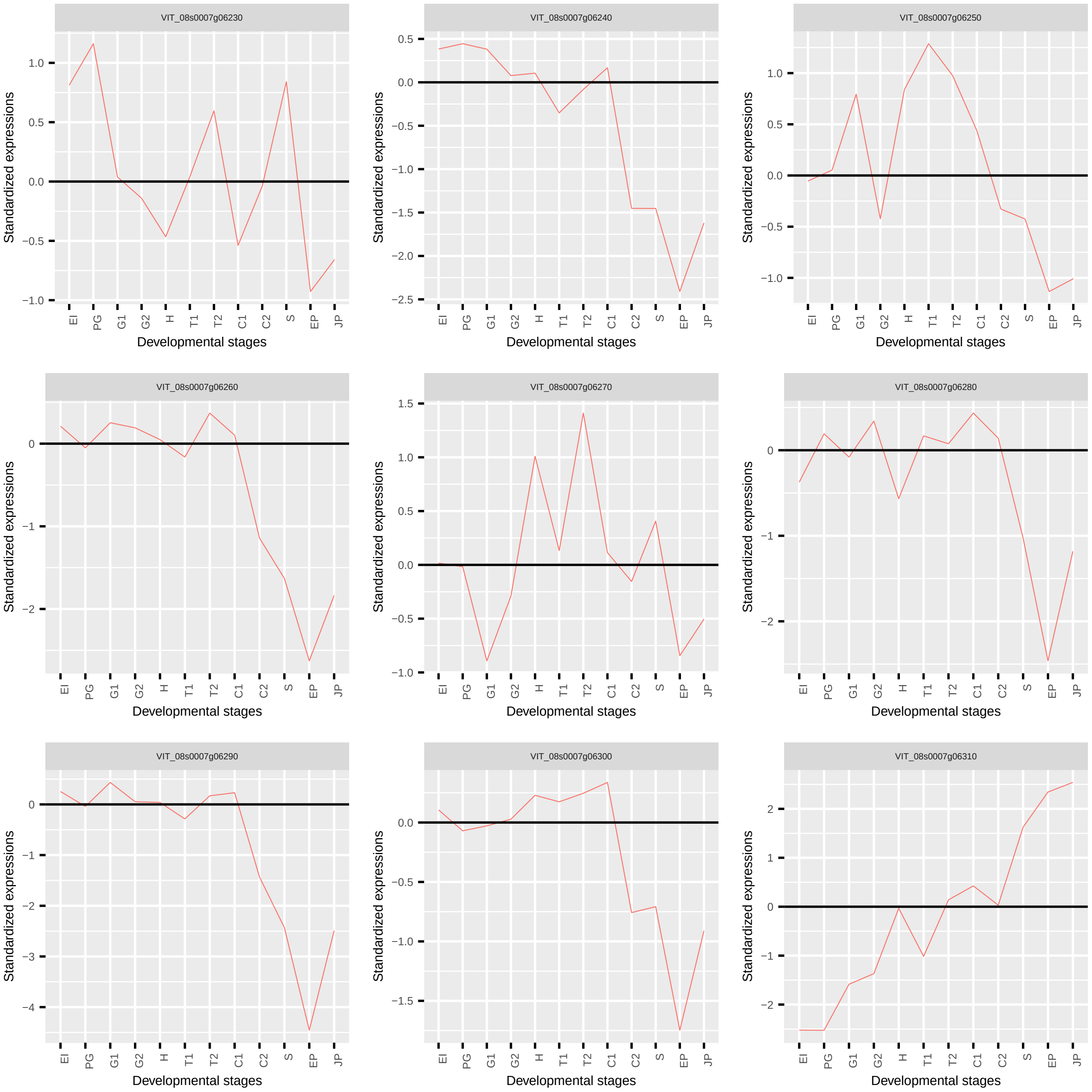
VIT_08s0007g06230
VIT_08s0007g06240
VIT_08s0007g06250
VIT_08s0007g06260
VIT_08s0007g06270
VIT_08s0007g06280
VIT_08s0007g06290
VIT_08s0007g06300
VIT_08s0007g06310
Standardized expressions
Developmental stages
EI PG G1 G2 H T1 T2 C1 C2 S EP JP

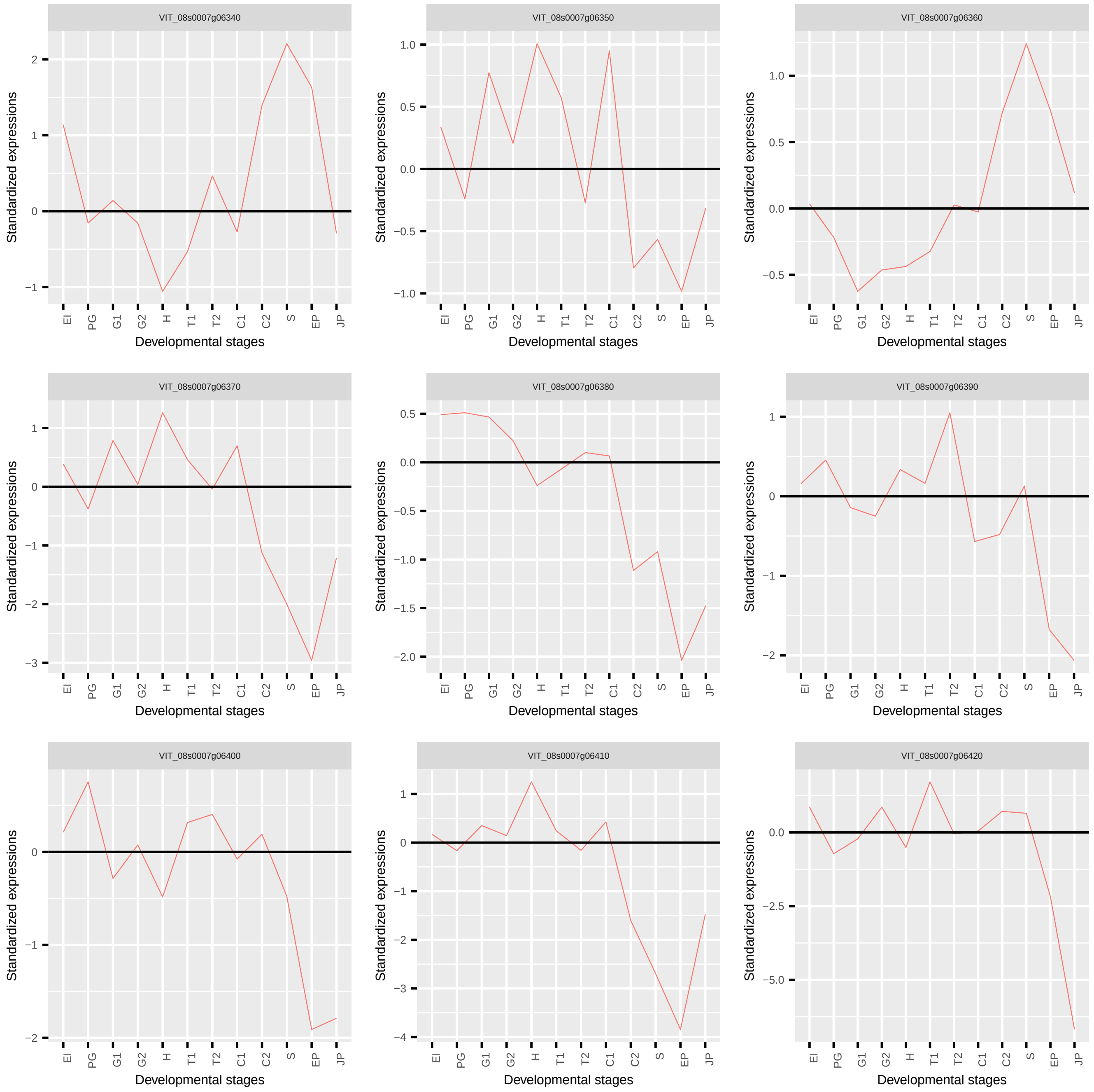
VIT_08s0007g06340
VIT_08s0007g06350
VIT_08s0007g06360
VIT_08s0007g06370
VIT_08s0007g06380
VIT_08s0007g06390
VIT_08s0007g06400
VIT_08s0007g06410
VIT_08s0007g06420
Standardized expressions
Developmental stages
EI PG G1 G2 H T1 T2 C1 C2 S EP JP

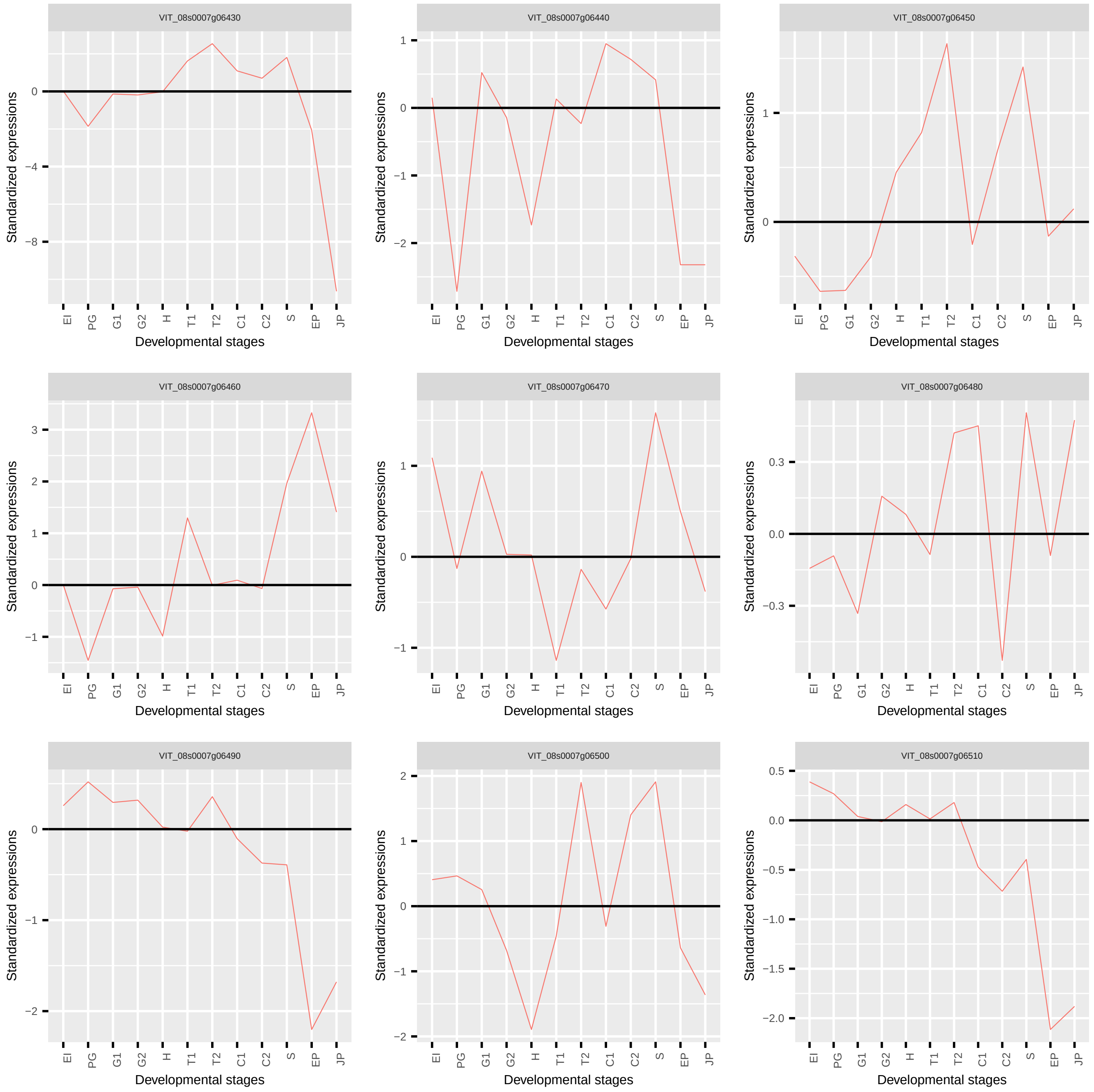
VIT_08s0007g06430
VIT_08s0007g06440
VIT_08s0007g06450
VIT_08s0007g06460
VIT_08s0007g06470
VIT_08s0007g06480
VIT_08s0007g06490
VIT_08s0007g06500
VIT_08s0007g06510
Standardized expressions
Developmental stages
EI PG G1 G2 H T1 T2 C1 C2 S EP JP

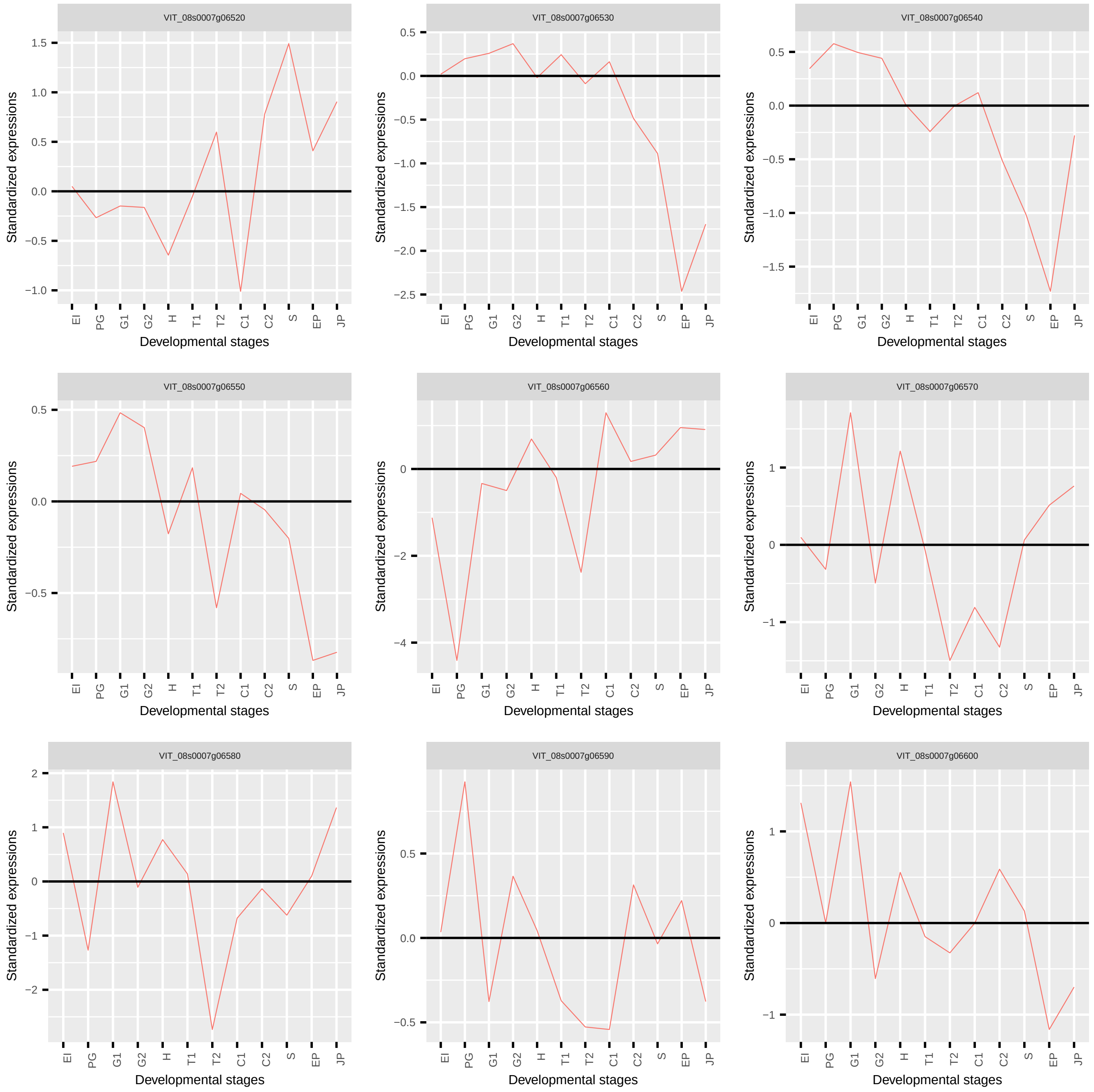
VIT_08s0007g06520
1.5
1.0
0.5
0.0
−0.5
−1.0
Standardized expressions
EI PG G1 G2 H T1 T2 C1 C2 S EP JP
Developmental stages
VIT_08s0007g06530
0.5
0.0
−0.5
−1.0
−1.5
−2.0
−2.5
Standardized expressions
EI PG G1 G2 H T1 T2 C1 C2 S EP JP
Developmental stages
VIT_08s0007g06540
0.5
0.0
−0.5
−1.0
−1.5
Standardized expressions
EI PG G1 G2 H T1 T2 C1 C2 S EP JP
Developmental stages
VIT_08s0007g06550
0.5
0.0
−0.5
Standardized expressions
EI PG G1 G2 H T1 T2 C1 C2 S EP JP
Developmental stages
VIT_08s0007g06560
0
−2
−4
Standardized expressions
EI PG G1 G2 H T1 T2 C1 C2 S EP JP
Developmental stages
VIT_08s0007g06570
1
0
−1
Standardized expressions
EI PG G1 G2 H T1 T2 C1 C2 S EP JP
Developmental stages
VIT_08s0007g06580
2
1
0
−1
−2
Standardized expressions
EI PG G1 G2 H T1 T2 C1 C2 S EP JP
Developmental stages
VIT_08s0007g06590
0.5
0.0
−0.5
Standardized expressions
EI PG G1 G2 H T1 T2 C1 C2 S EP JP
Developmental stages
VIT_08s0007g06600
1
0
−1
Standardized expressions
EI PG G1 G2 H T1 T2 C1 C2 S EP JP
Developmental stages

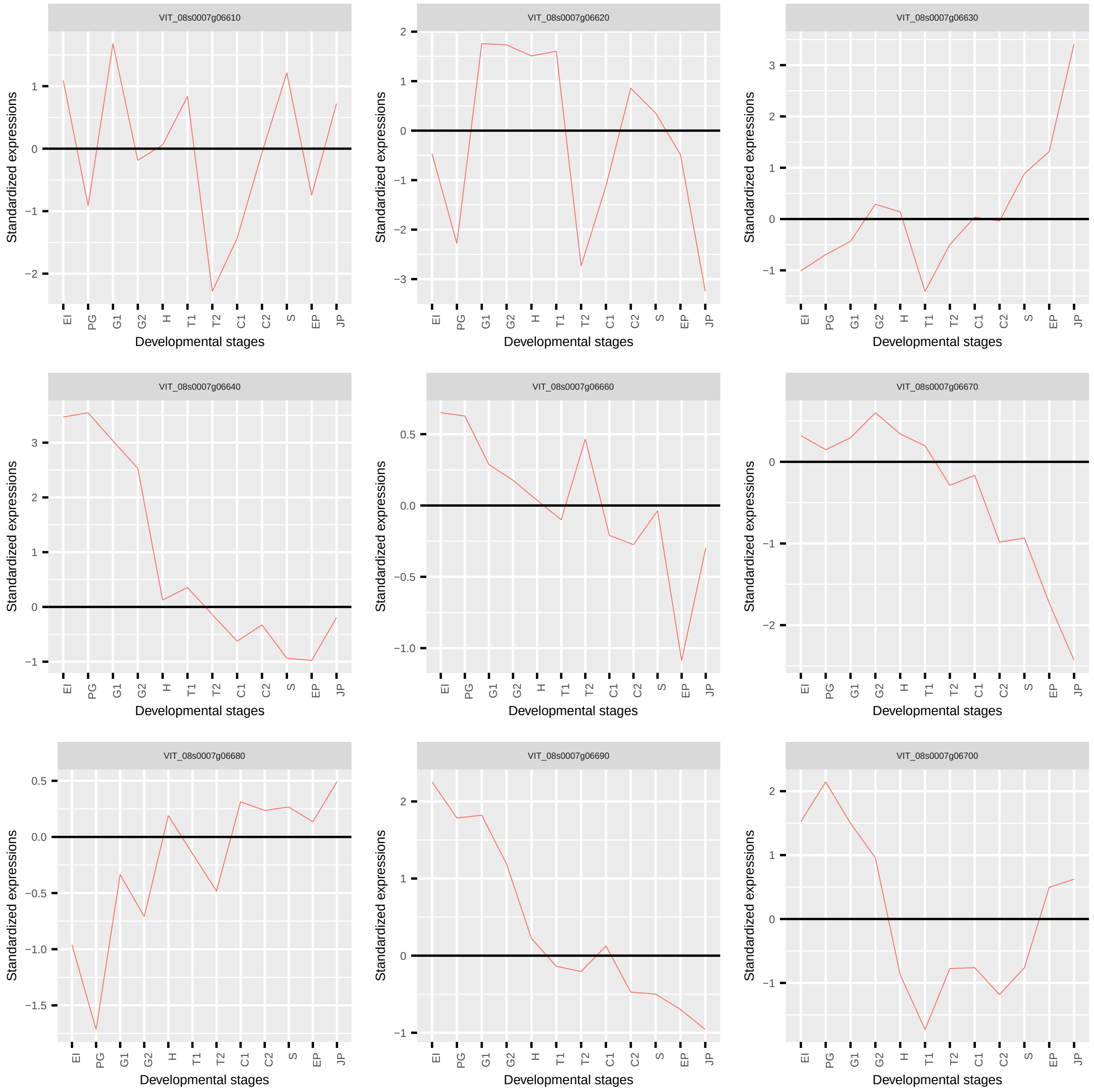
VIT_08s0007g06610
VIT_08s0007g06620
VIT_08s0007g06630
VIT_08s0007g06640
VIT_08s0007g06660
VIT_08s0007g06670
VIT_08s0007g06680
VIT_08s0007g06690
VIT_08s0007g06700
Standardized expressions
Developmental stages
EI PG G1 G2 H T1 T2 C1 C2 S EP JP

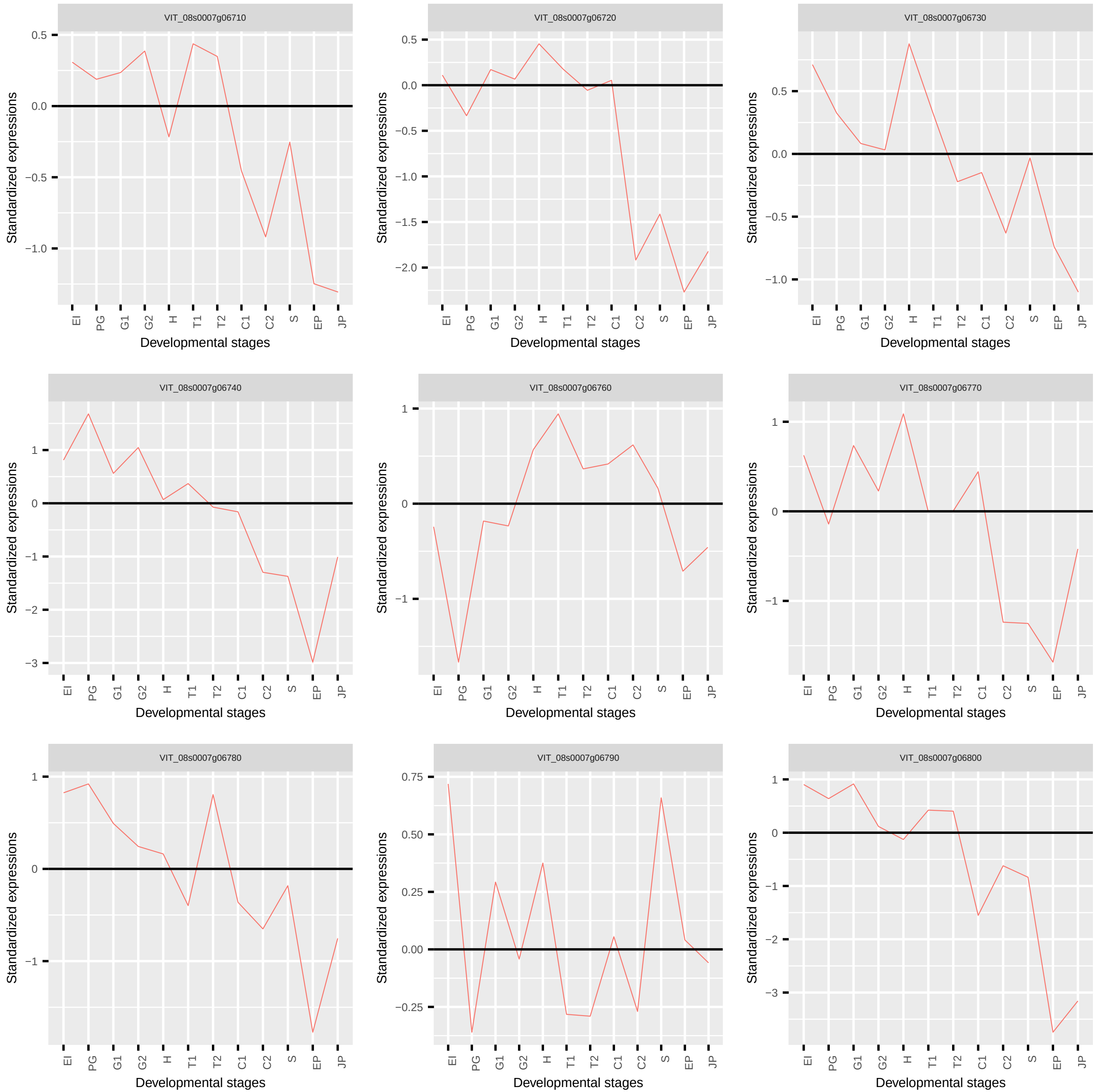

VIT_08s0007g06710
VIT_08s0007g06720
VIT_08s0007g06730
VIT_08s0007g06740
VIT_08s0007g06760
VIT_08s0007g06770
VIT_08s0007g06780
VIT_08s0007g06790
VIT_08s0007g06800
Standardized expressions
Developmental stages
EI
PG
G1
G2
H
T1
T2
C1
C2
S
EP
JP

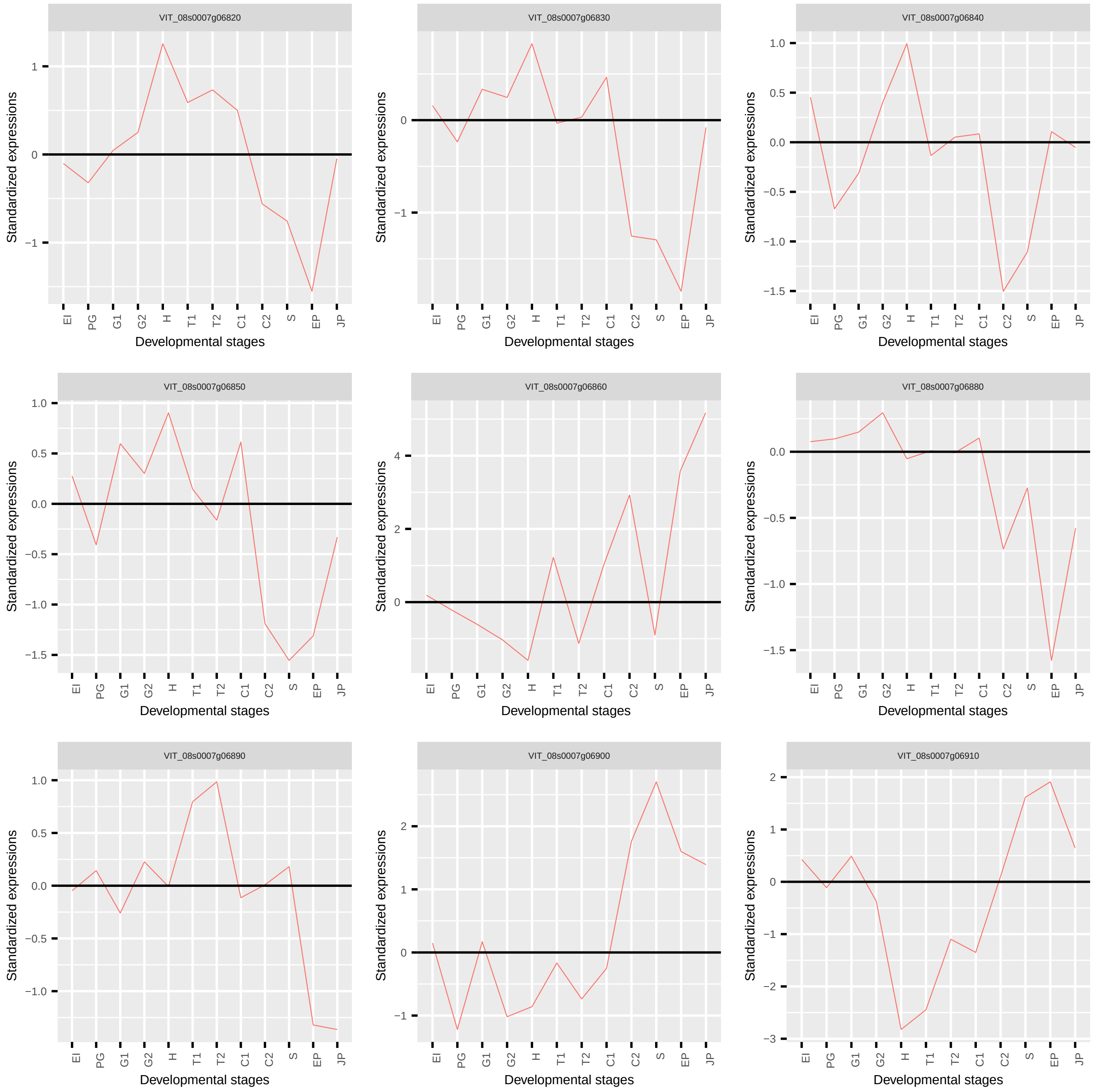
VIT_08s0007g06820
VIT_08s0007g06830
VIT_08s0007g06840
VIT_08s0007g06850
VIT_08s0007g06860
VIT_08s0007g06880
VIT_08s0007g06890
VIT_08s0007g06900
VIT_08s0007g06910
Standardized expressions
Developmental stages
EI PG G1 G2 H T1 T2 C1 C2 S EP JP

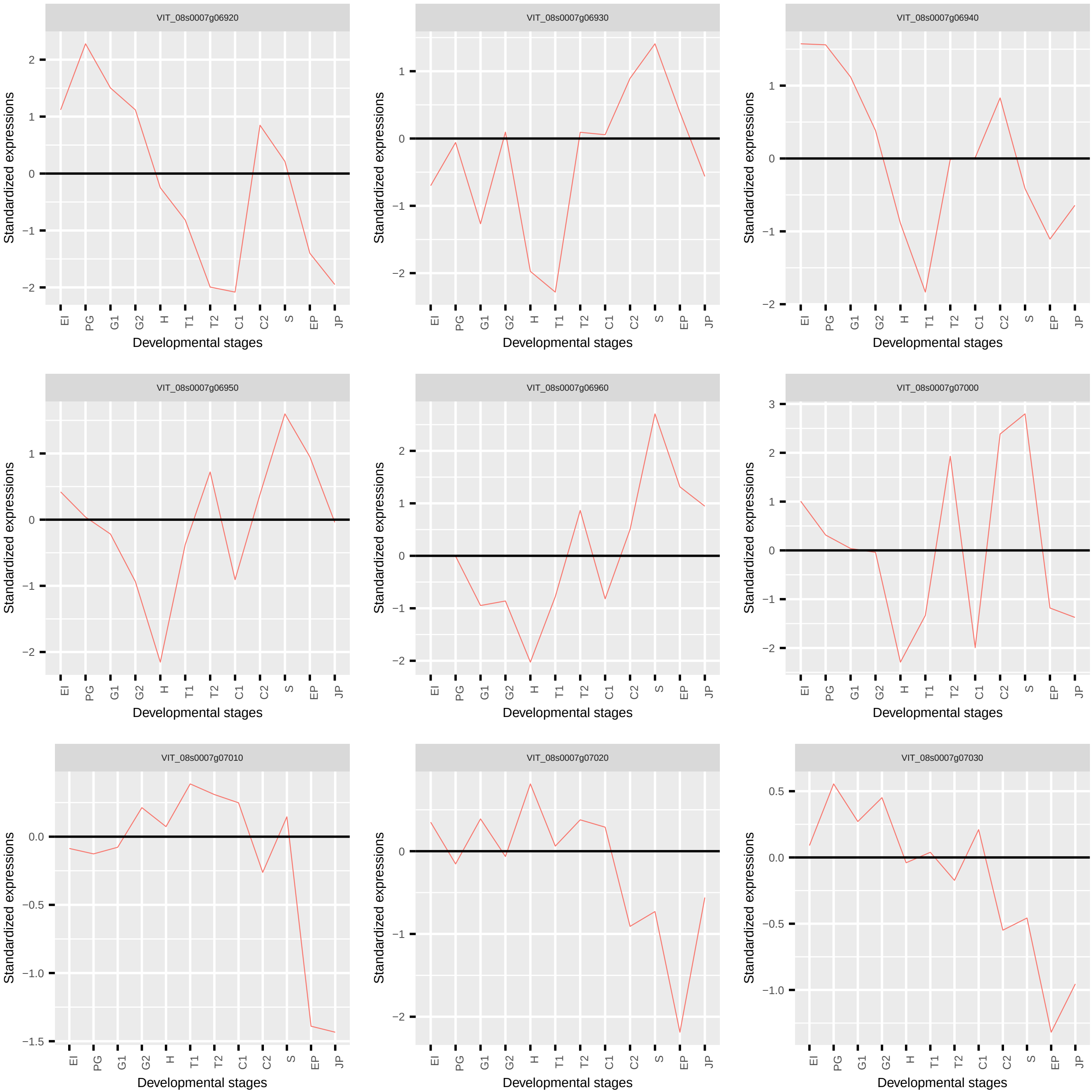
VIT_08s0007g06920
VIT_08s0007g06930
VIT_08s0007g06940
VIT_08s0007g06950
VIT_08s0007g06960
VIT_08s0007g07000
VIT_08s0007g07010
VIT_08s0007g07020
VIT_08s0007g07030
Standardized expressions
Developmental stages
EI PG G1 G2 H T1 T2 C1 C2 S EP JP

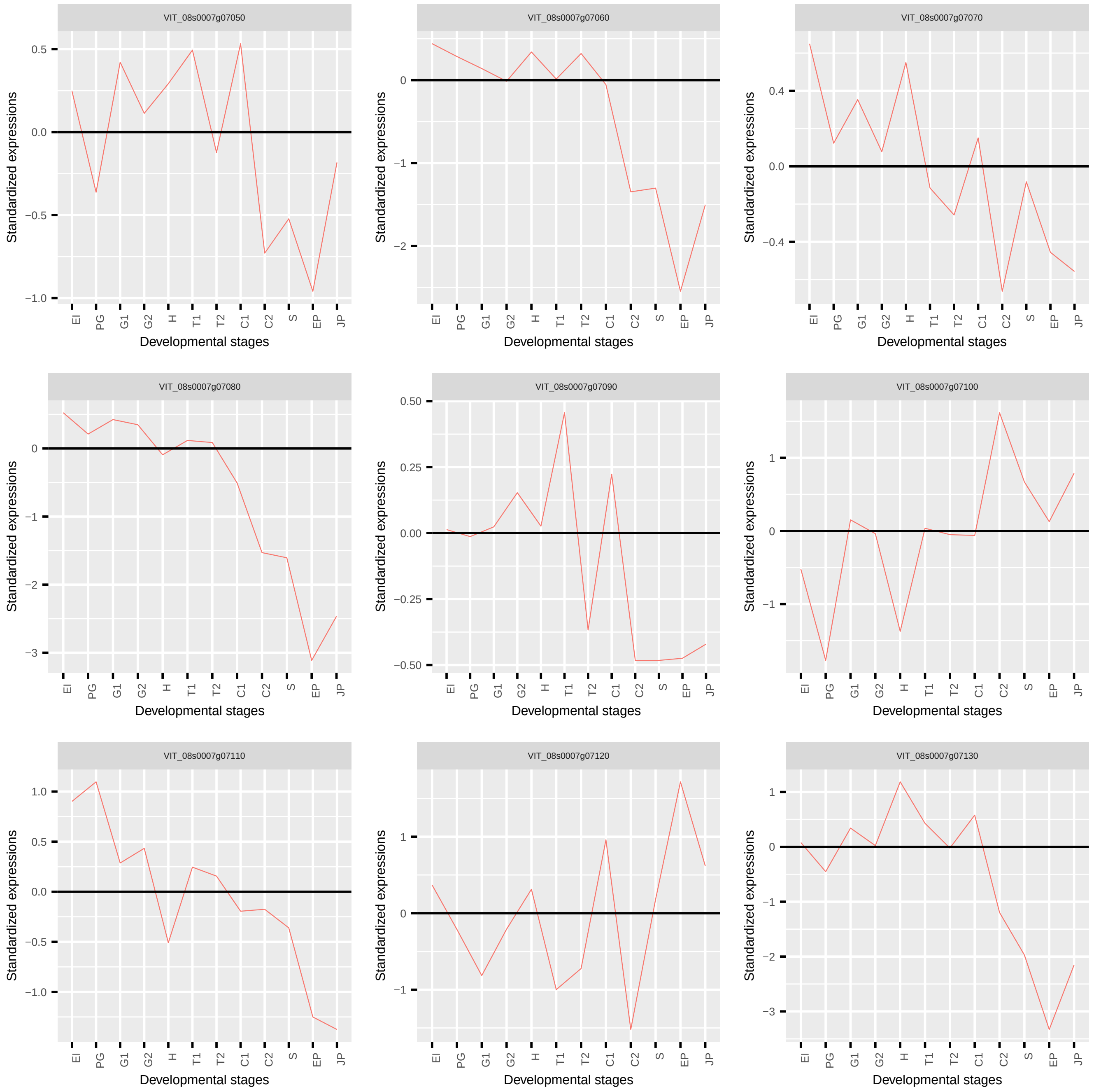

VIT_08s0007g07050
Standardized expressions
0.5
0.0
−0.5
−1.0
EI PG G1 G2 H T1 T2 C1 C2 S EP JP
Developmental stages
VIT_08s0007g07060
Standardized expressions
0
−1
−2
EI PG G1 G2 H T1 T2 C1 C2 S EP JP
Developmental stages
VIT_08s0007g07070
Standardized expressions
0.4
0.0
−0.4
EI PG G1 G2 H T1 T2 C1 C2 S EP JP
Developmental stages
VIT_08s0007g07080
Standardized expressions
0
−1
−2
−3
EI PG G1 G2 H T1 T2 C1 C2 S EP JP
Developmental stages
VIT_08s0007g07090
Standardized expressions
0.50
0.25
0.00
−0.25
−0.50
EI PG G1 G2 H T1 T2 C1 C2 S EP JP
Developmental stages
VIT_08s0007g07100
Standardized expressions
1
0
−1
EI PG G1 G2 H T1 T2 C1 C2 S EP JP
Developmental stages
VIT_08s0007g07110
Standardized expressions
1.0
0.5
0.0
−0.5
−1.0
EI PG G1 G2 H T1 T2 C1 C2 S EP JP
Developmental stages
VIT_08s0007g07120
Standardized expressions
1
0
−1
EI PG G1 G2 H T1 T2 C1 C2 S EP JP
Developmental stages
VIT_08s0007g07130
Standardized expressions
1
0
−1
−2
−3
EI PG G1 G2 H T1 T2 C1 C2 S EP JP
Developmental stages

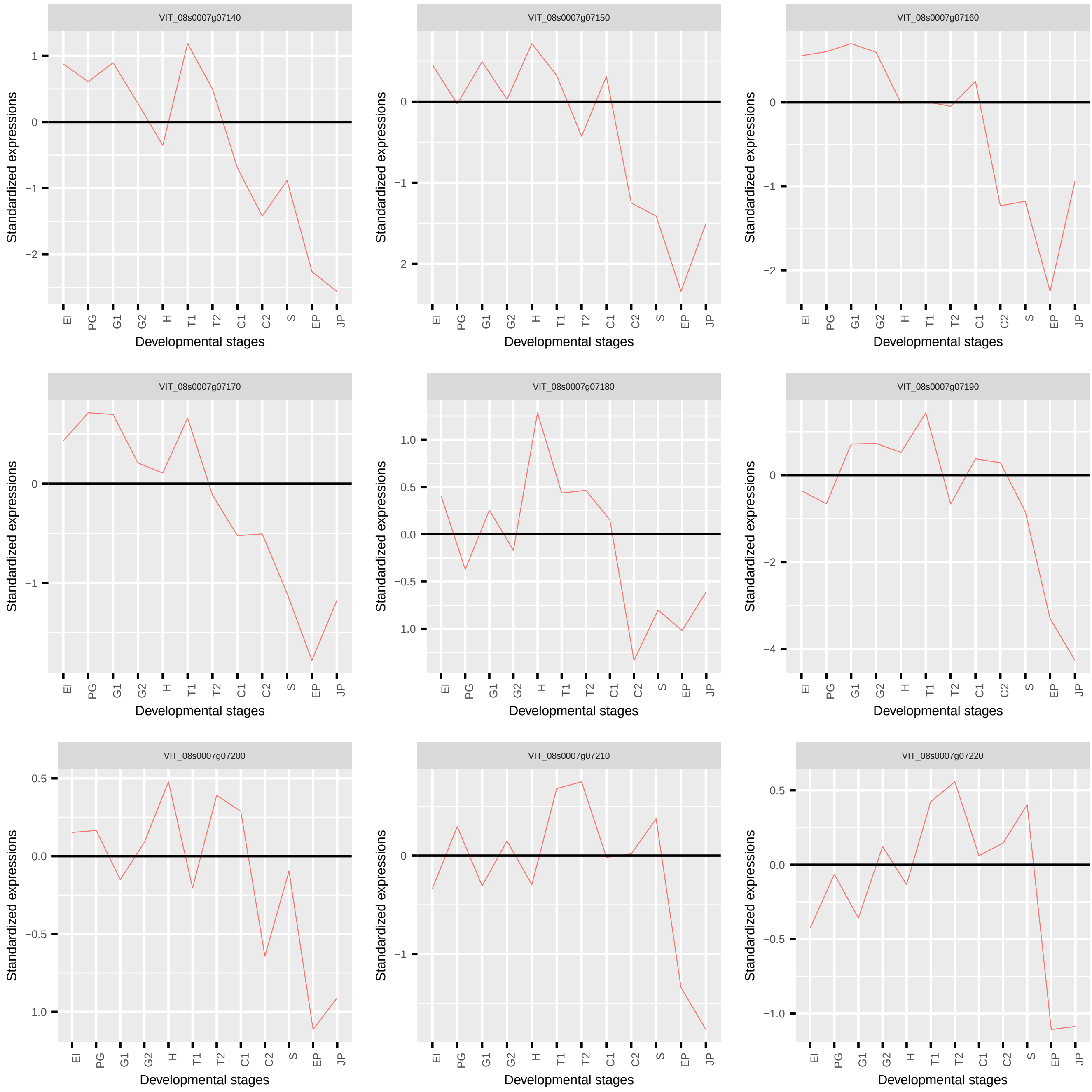
VIT_08s0007g07140
1
0
−1
−2
VIT_08s0007g07150
0
−1
−2
VIT_08s0007g07160
0
−1
−2
VIT_08s0007g07170
0
−1
VIT_08s0007g07180
1.0
0.5
0.0
−0.5
−1.0
VIT_08s0007g07190
0
−2
−4
VIT_08s0007g07200
0.5
0.0
−0.5
−1.0
VIT_08s0007g07210
0
−1
VIT_08s0007g07220
0.5
0.0
−0.5
−1.0
Standardized expressions
EI
PG
G1
G2
H
T1
T2
C1
C2
S
EP
JP
Developmental stages

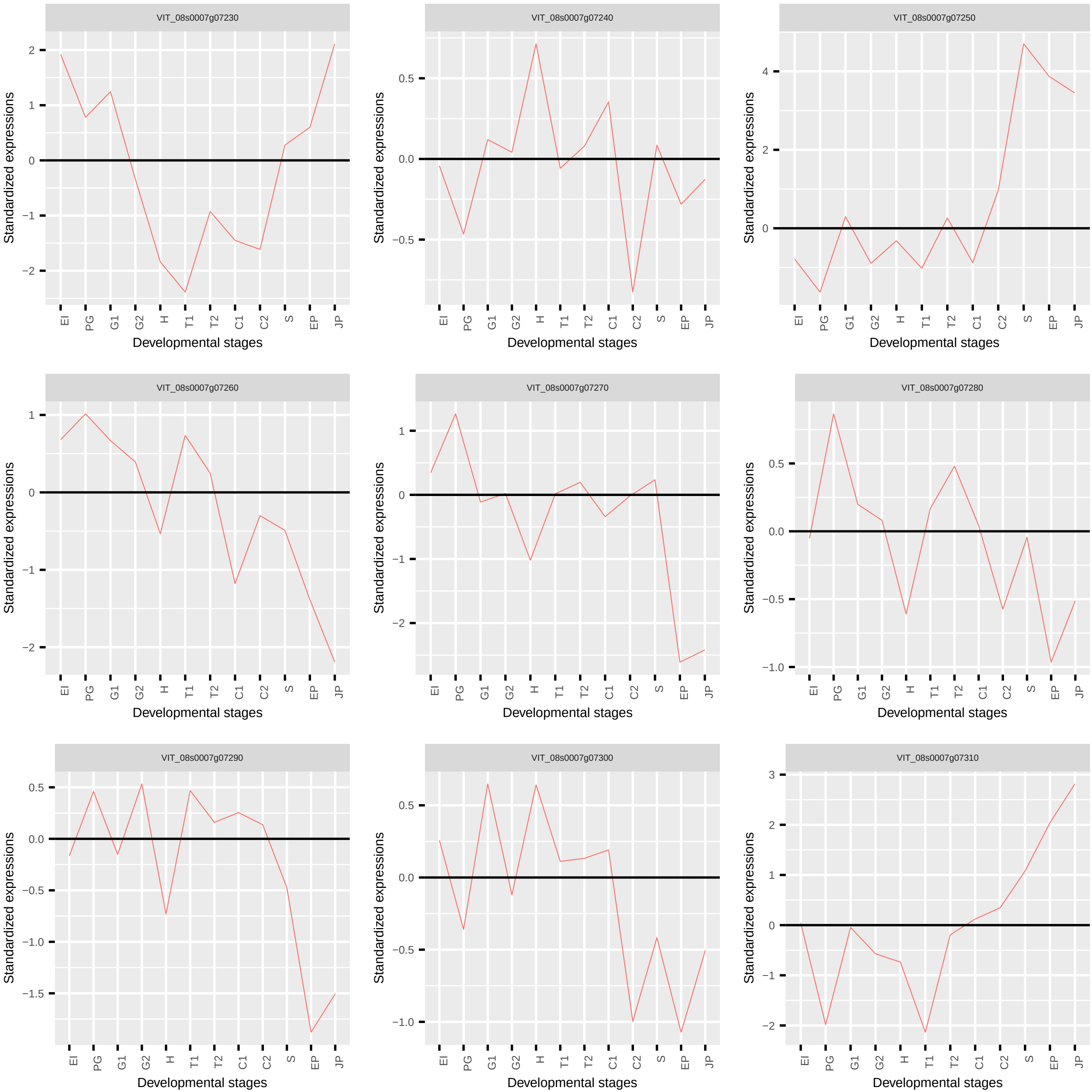
VIT_08s0007g07230
VIT_08s0007g07240
VIT_08s0007g07250
VIT_08s0007g07260
VIT_08s0007g07270
VIT_08s0007g07280
VIT_08s0007g07290
VIT_08s0007g07300
VIT_08s0007g07310
Standardized expressions
Developmental stages
EI PG G1 G2 H T1 T2 C1 C2 S EP JP

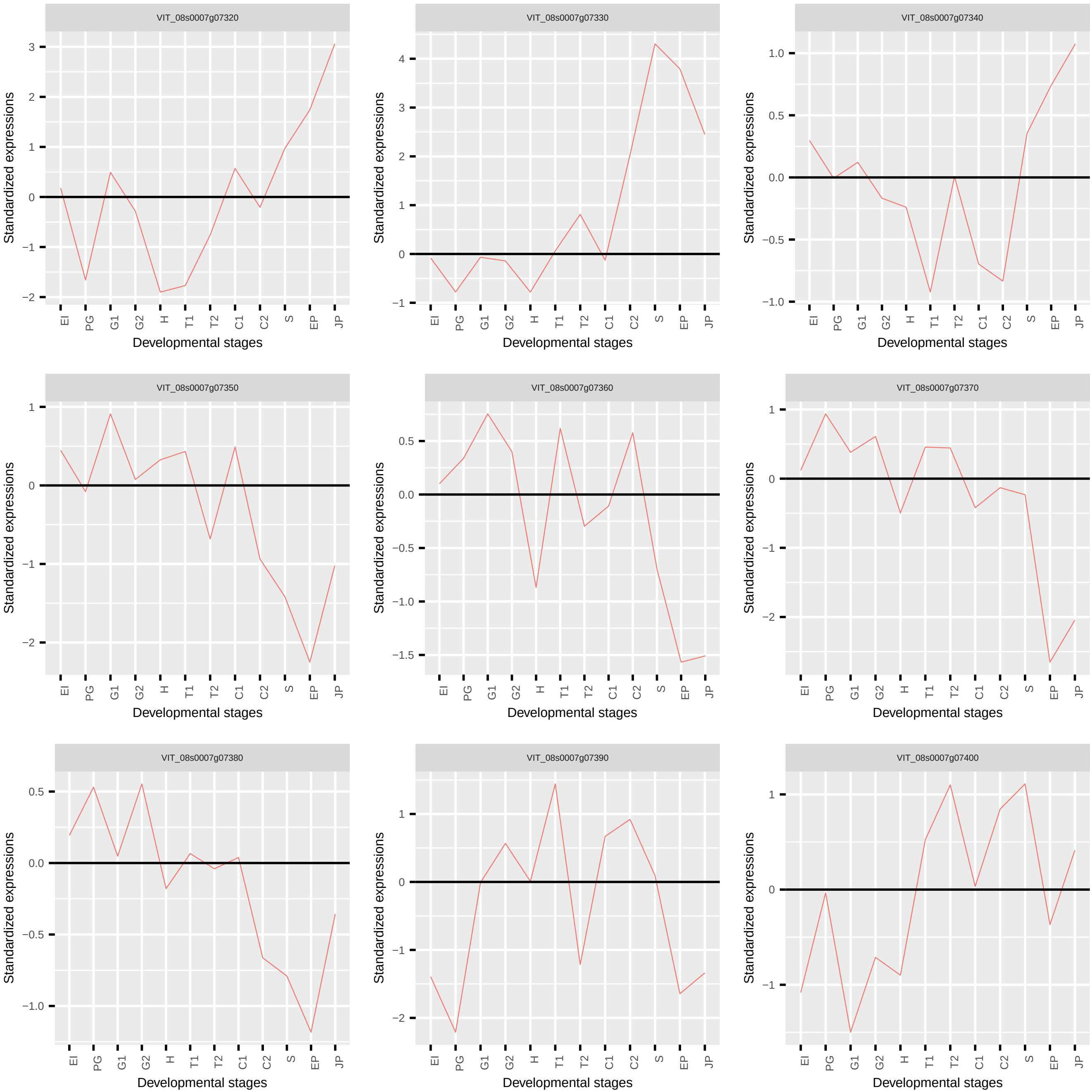
VIT_08s0007g07320
VIT_08s0007g07330
VIT_08s0007g07340
VIT_08s0007g07350
VIT_08s0007g07360
VIT_08s0007g07370
VIT_08s0007g07380
VIT_08s0007g07390
VIT_08s0007g07400
Standardized expressions
Developmental stages
EI PG G1 G2 H T1 T2 C1 C2 S EP JP

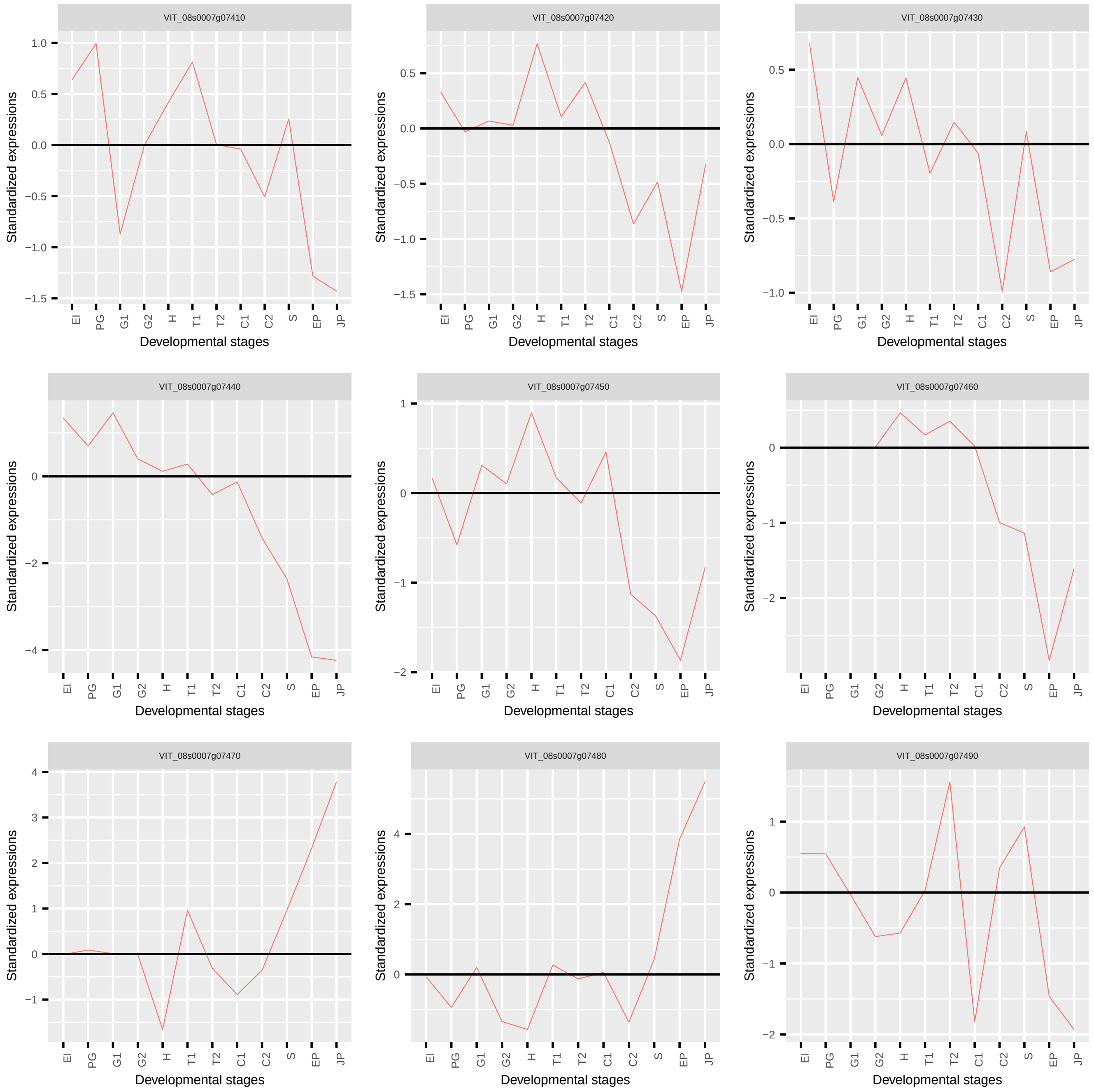

VIT_08s0007g07410
VIT_08s0007g07420
VIT_08s0007g07430
VIT_08s0007g07440
VIT_08s0007g07450
VIT_08s0007g07460
VIT_08s0007g07470
VIT_08s0007g07480
VIT_08s0007g07490
Standardized expressions
Developmental stages
EI PG G1 G2 H T1 T2 C1 C2 S EP JP

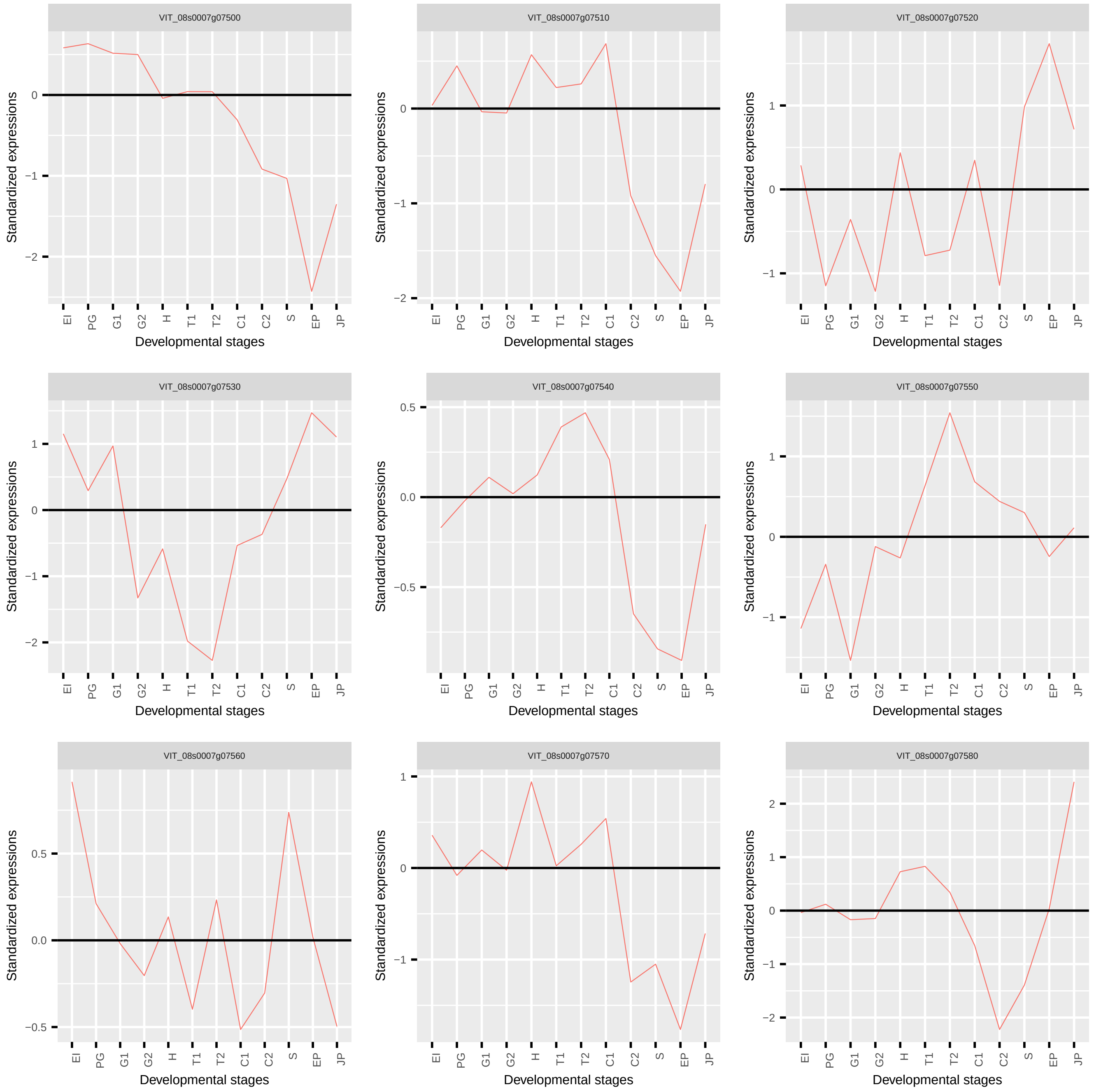

VIT_08s0007g07500
VIT_08s0007g07510
VIT_08s0007g07520
VIT_08s0007g07530
VIT_08s0007g07540
VIT_08s0007g07550
VIT_08s0007g07560
VIT_08s0007g07570
VIT_08s0007g07580
Standardized expressions
Developmental stages
EI
PG
G1
G2
H
T1
T2
C1
C2
S
EP
JP

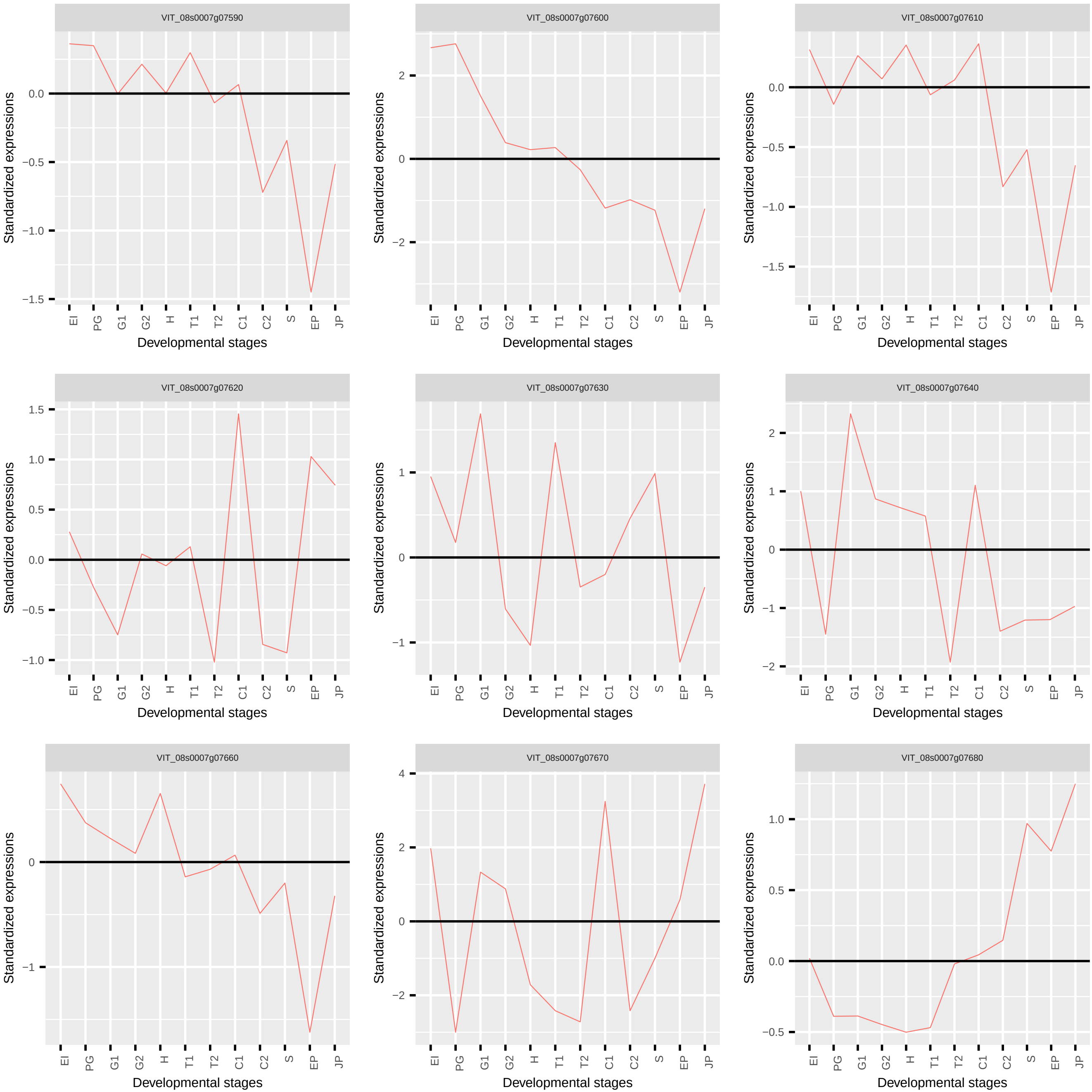
VIT_08s0007g07590
VIT_08s0007g07600
VIT_08s0007g07610
VIT_08s0007g07620
VIT_08s0007g07630
VIT_08s0007g07640
VIT_08s0007g07660
VIT_08s0007g07670
VIT_08s0007g07680
Standardized expressions
Developmental stages
EI
PG
G1
G2
H
T1
T2
C1
C2
S
EP
JP

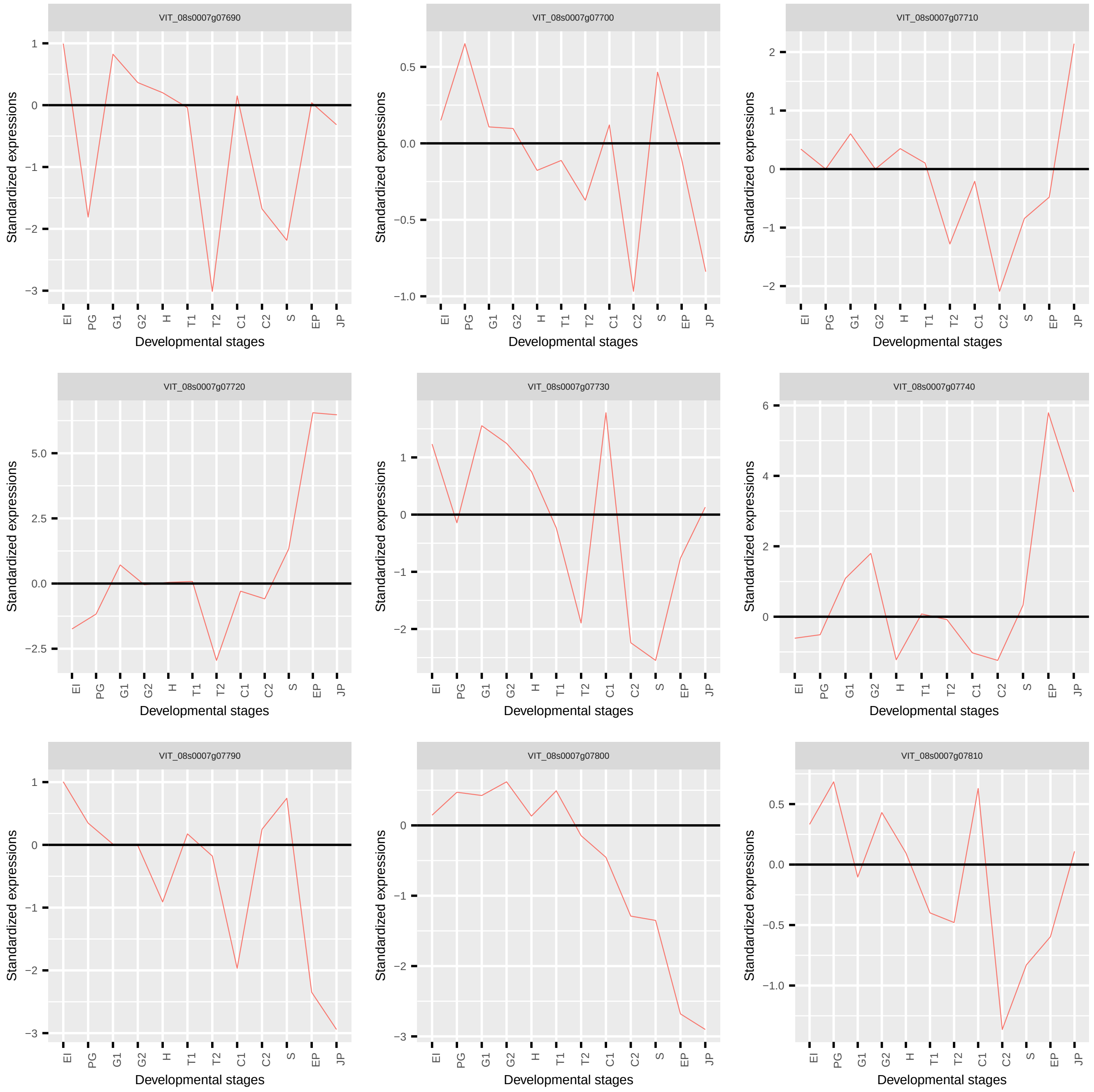
VIT_08s0007g07690
VIT_08s0007g07700
VIT_08s0007g07710
VIT_08s0007g07720
VIT_08s0007g07730
VIT_08s0007g07740
VIT_08s0007g07790
VIT_08s0007g07800
VIT_08s0007g07810
Standardized expressions
Developmental stages
EI PG G1 G2 H T1 T2 C1 C2 S EP JP

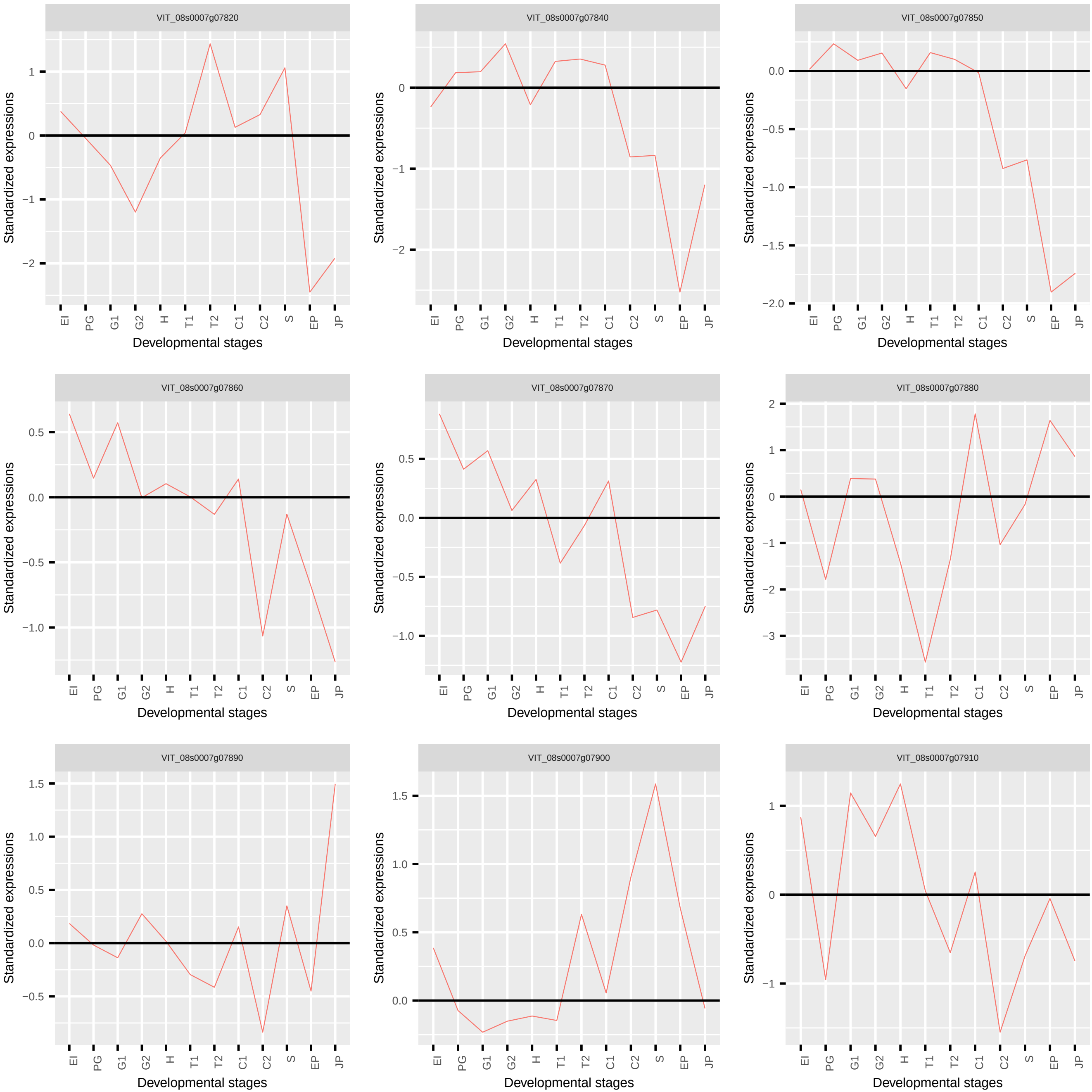
VIT_08s0007g07820
1
0
−1
−2
Standardized expressions
EI PG G1 G2 H T1 T2 C1 C2 S EP JP
Developmental stages
VIT_08s0007g07840
0
−1
−2
Standardized expressions
EI PG G1 G2 H T1 T2 C1 C2 S EP JP
Developmental stages
VIT_08s0007g07850
0.0
−0.5
−1.0
−1.5
−2.0
Standardized expressions
EI PG G1 G2 H T1 T2 C1 C2 S EP JP
Developmental stages
VIT_08s0007g07860
0.5
0.0
−0.5
−1.0
Standardized expressions
EI PG G1 G2 H T1 T2 C1 C2 S EP JP
Developmental stages
VIT_08s0007g07870
0.5
0.0
−0.5
−1.0
Standardized expressions
EI PG G1 G2 H T1 T2 C1 C2 S EP JP
Developmental stages
VIT_08s0007g07880
2
1
0
−1
−2
−3
Standardized expressions
EI PG G1 G2 H T1 T2 C1 C2 S EP JP
Developmental stages
VIT_08s0007g07890
1.5
1.0
0.5
0.0
−0.5
Standardized expressions
EI PG G1 G2 H T1 T2 C1 C2 S EP JP
Developmental stages
VIT_08s0007g07900
1.5
1.0
0.5
0.0
Standardized expressions
EI PG G1 G2 H T1 T2 C1 C2 S EP JP
Developmental stages
VIT_08s0007g07910
1
0
−1
Standardized expressions
EI PG G1 G2 H T1 T2 C1 C2 S EP JP
Developmental stages

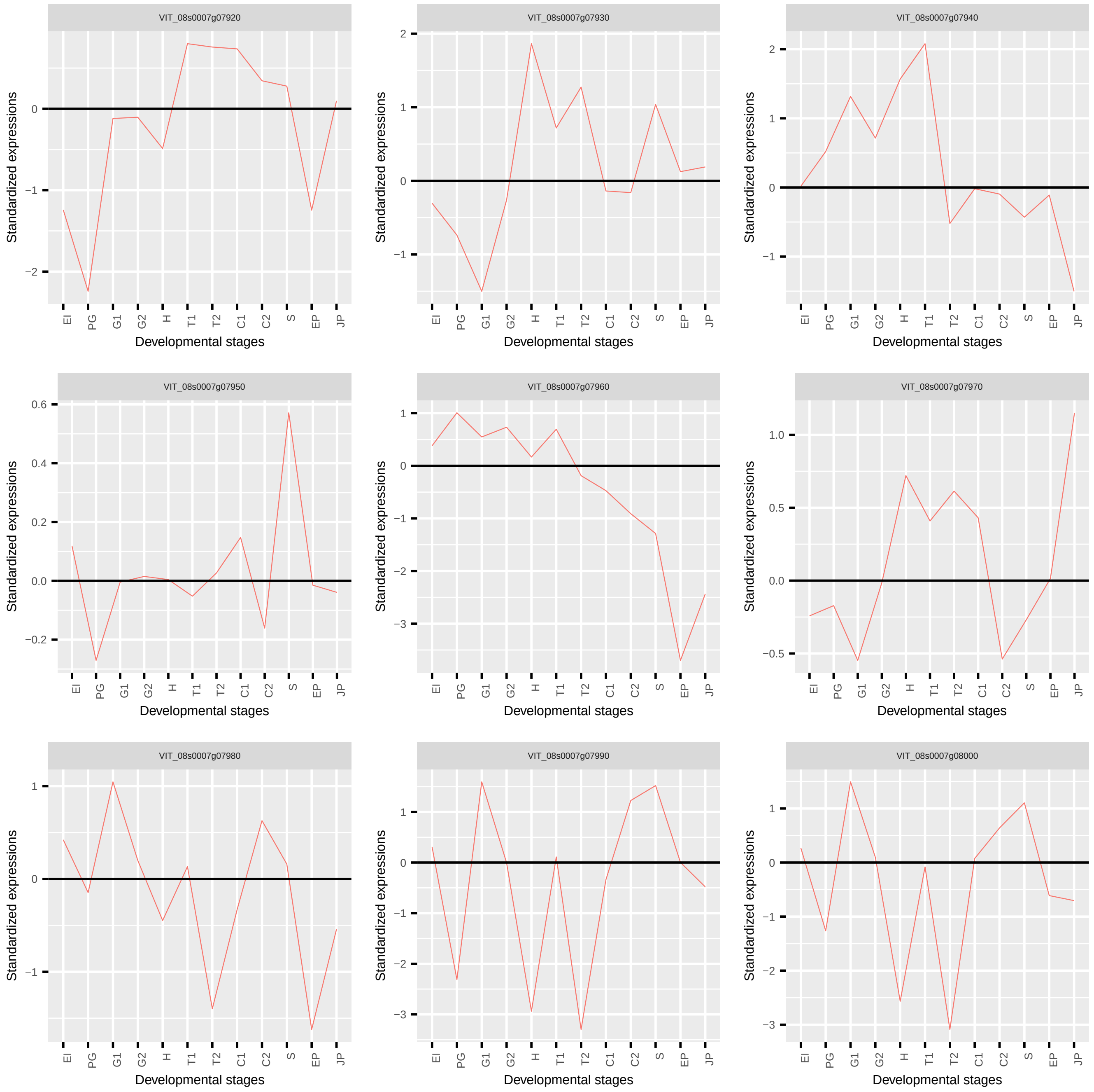

VIT_08s0007g07920
0
−1
−2
Standardized expressions
EI PG G1 G2 H T1 T2 C1 C2 S EP JP
Developmental stages
VIT_08s0007g07930
2
1
0
−1
Standardized expressions
EI PG G1 G2 H T1 T2 C1 C2 S EP JP
Developmental stages
VIT_08s0007g07940
2
1
0
−1
Standardized expressions
EI PG G1 G2 H T1 T2 C1 C2 S EP JP
Developmental stages
VIT_08s0007g07950
0.6
0.4
0.2
0.0
−0.2
Standardized expressions
EI PG G1 G2 H T1 T2 C1 C2 S EP JP
Developmental stages
VIT_08s0007g07960
1
0
−1
−2
−3
Standardized expressions
EI PG G1 G2 H T1 T2 C1 C2 S EP JP
Developmental stages
VIT_08s0007g07970
1.0
0.5
0.0
−0.5
Standardized expressions
EI PG G1 G2 H T1 T2 C1 C2 S EP JP
Developmental stages
VIT_08s0007g07980
1
0
−1
Standardized expressions
EI PG G1 G2 H T1 T2 C1 C2 S EP JP
Developmental stages
VIT_08s0007g07990
1
0
−1
−2
−3
Standardized expressions
EI PG G1 G2 H T1 T2 C1 C2 S EP JP
Developmental stages
VIT_08s0007g08000
1
0
−1
−2
−3
Standardized expressions
EI PG G1 G2 H T1 T2 C1 C2 S EP JP
Developmental stages

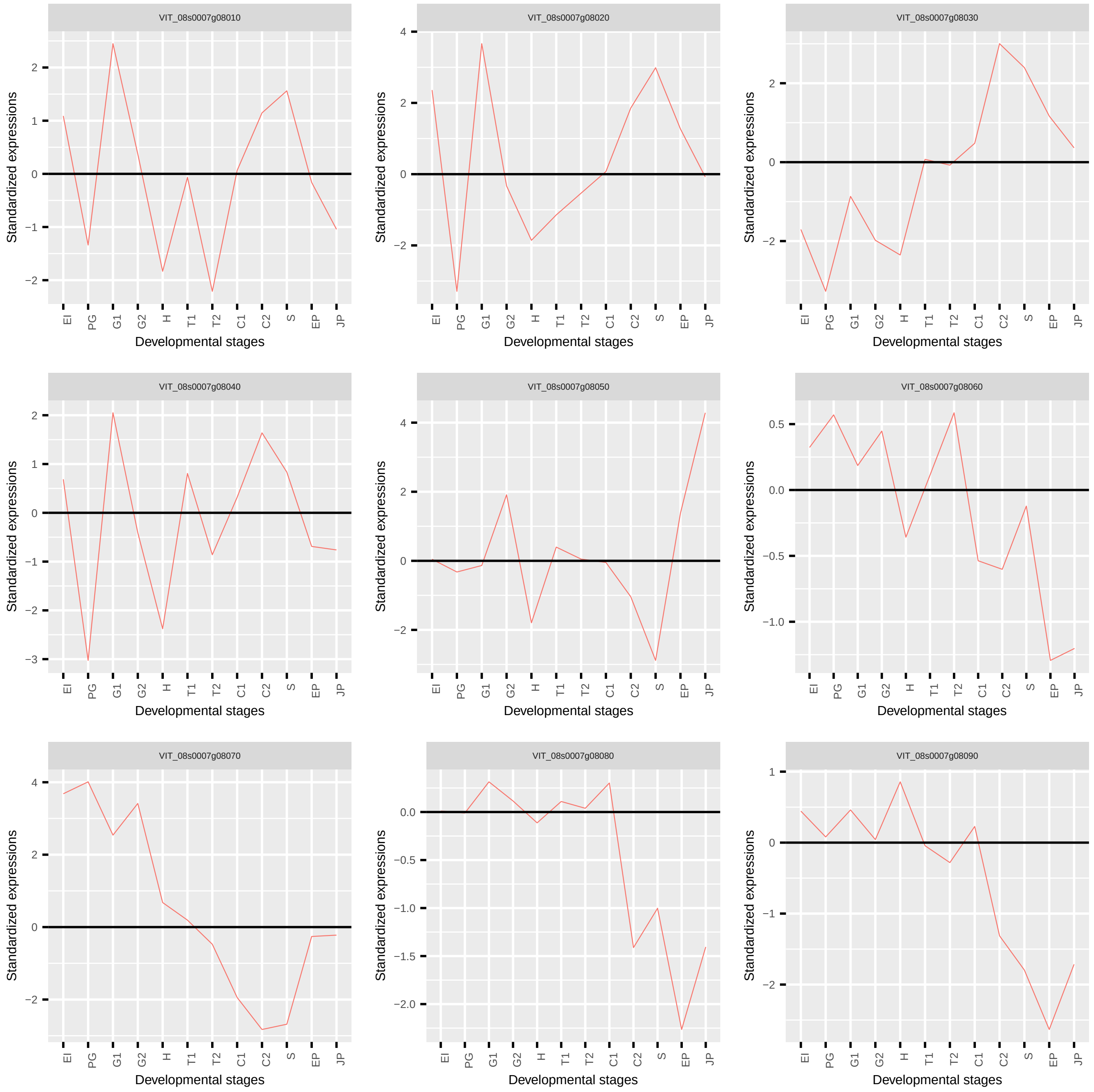

VIT_08s0007g08010
VIT_08s0007g08020
VIT_08s0007g08030
VIT_08s0007g08040
VIT_08s0007g08050
VIT_08s0007g08060
VIT_08s0007g08070
VIT_08s0007g08080
VIT_08s0007g08090
Standardized expressions
Developmental stages
EI
PG
G1
G2
H
T1
T2
C1
C2
S
EP
JP

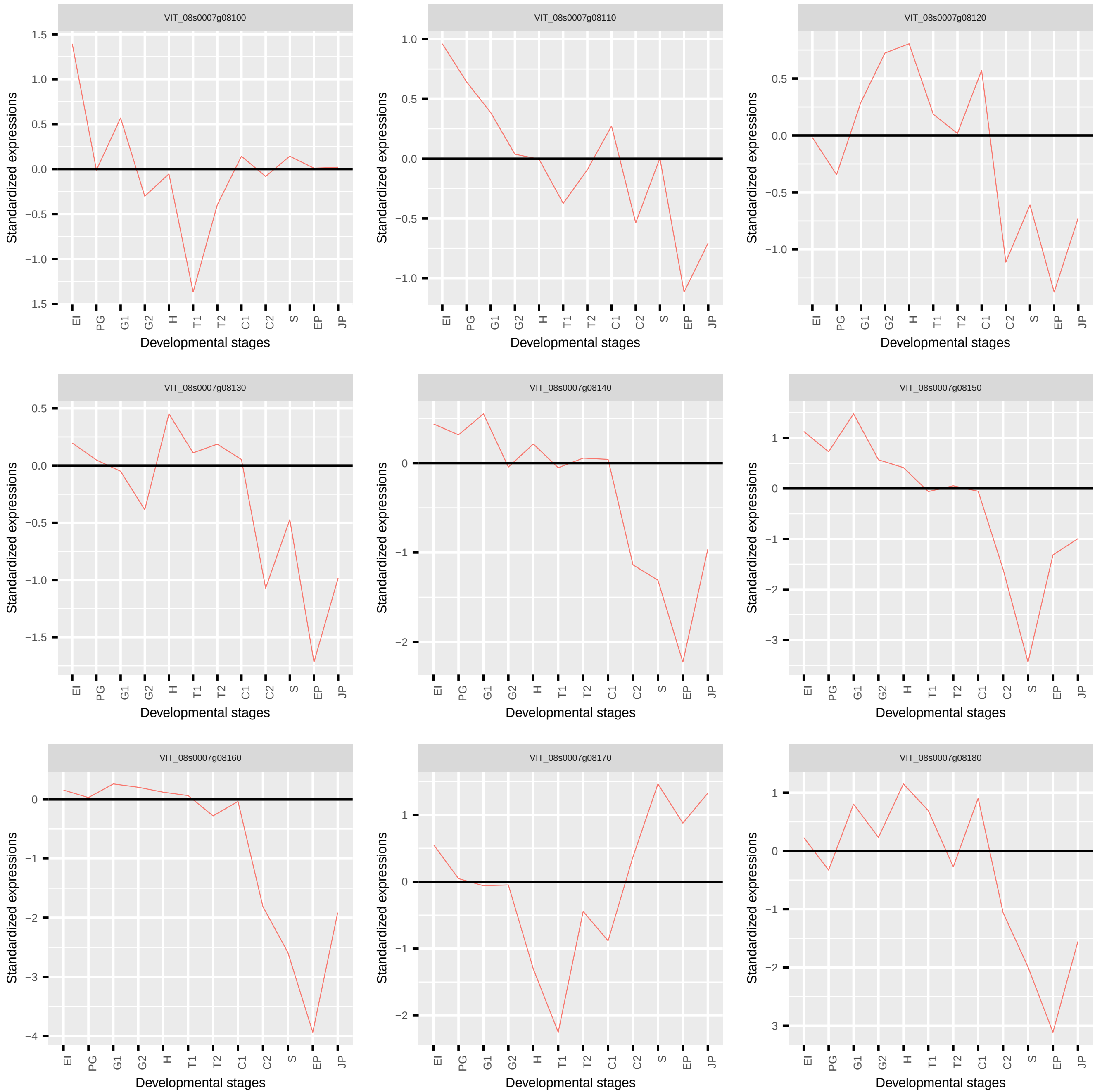
VIT_08s0007g08100
VIT_08s0007g08110
VIT_08s0007g08120
VIT_08s0007g08130
VIT_08s0007g08140
VIT_08s0007g08150
VIT_08s0007g08160
VIT_08s0007g08170
VIT_08s0007g08180
Standardized expressions
Developmental stages
EI
PG
G1
G2
H
T1
T2
C1
C2
S
EP
JP
1.5
1.0
0.5
0.0
−0.5
−1.0
−1.5
1
0
−1
−2
−3
−4

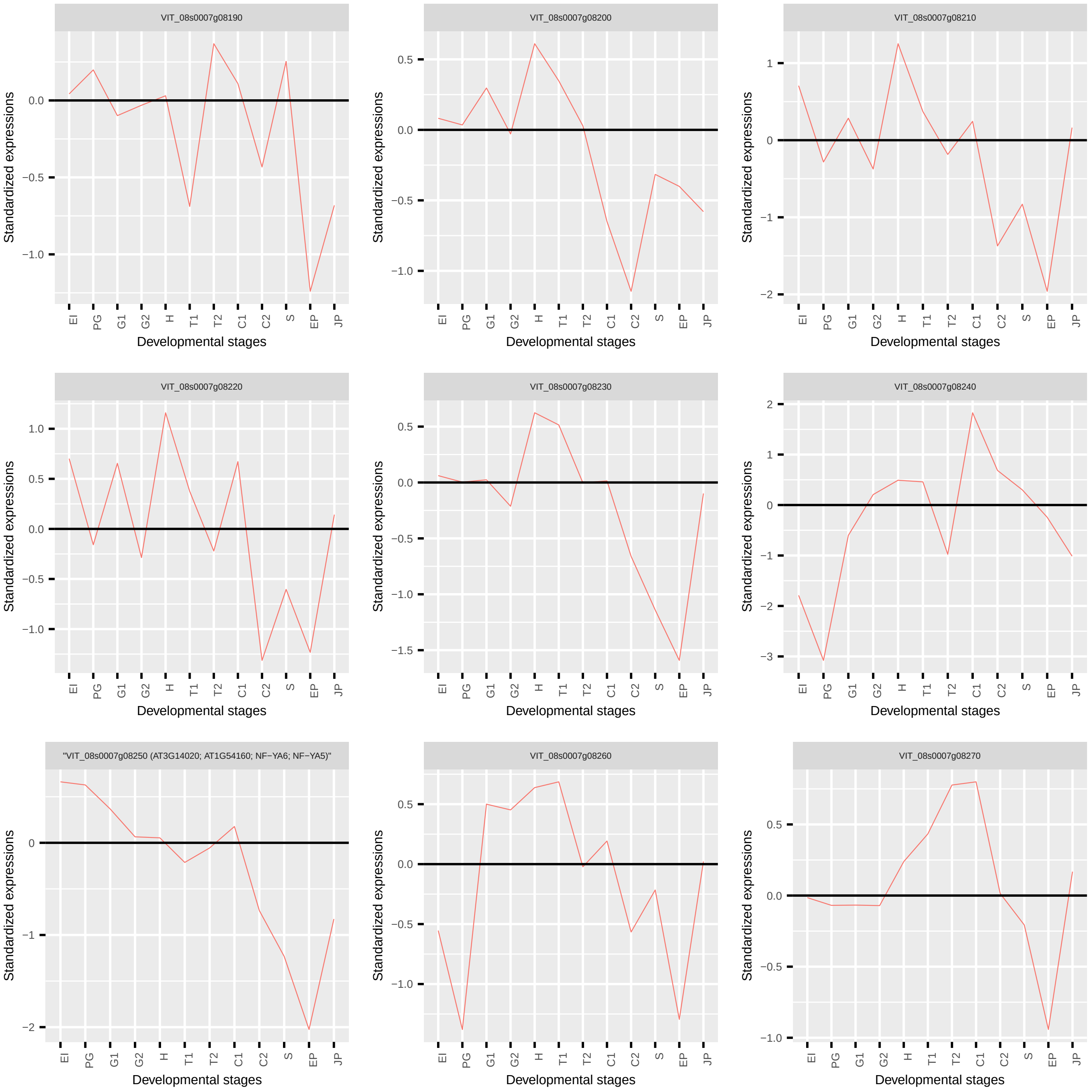
VIT_08s0007g08190
VIT_08s0007g08200
VIT_08s0007g08210
VIT_08s0007g08220
VIT_08s0007g08230
VIT_08s0007g08240
"VIT_08s0007g08250 (AT3G14020; AT1G54160; NF−YA6; NF−YA5)"
VIT_08s0007g08260
VIT_08s0007g08270
Standardized expressions
Developmental stages
EI PG G1 G2 H T1 T2 C1 C2 S EP JP

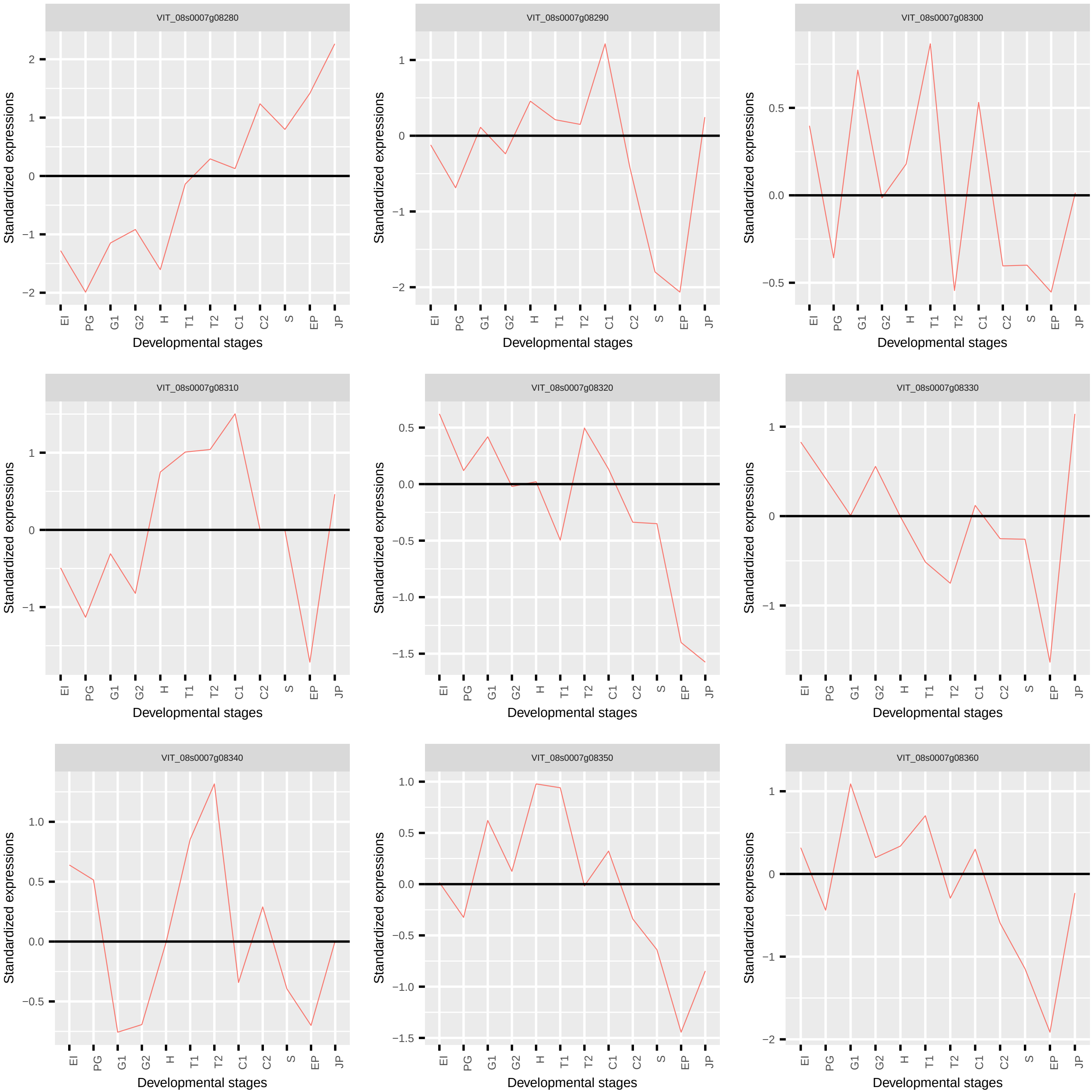
VIT_08s0007g08280
VIT_08s0007g08290
VIT_08s0007g08300
VIT_08s0007g08310
VIT_08s0007g08320
VIT_08s0007g08330
VIT_08s0007g08340
VIT_08s0007g08350
VIT_08s0007g08360
Standardized expressions
Developmental stages
EI PG G1 G2 H T1 T2 C1 C2 S EP JP

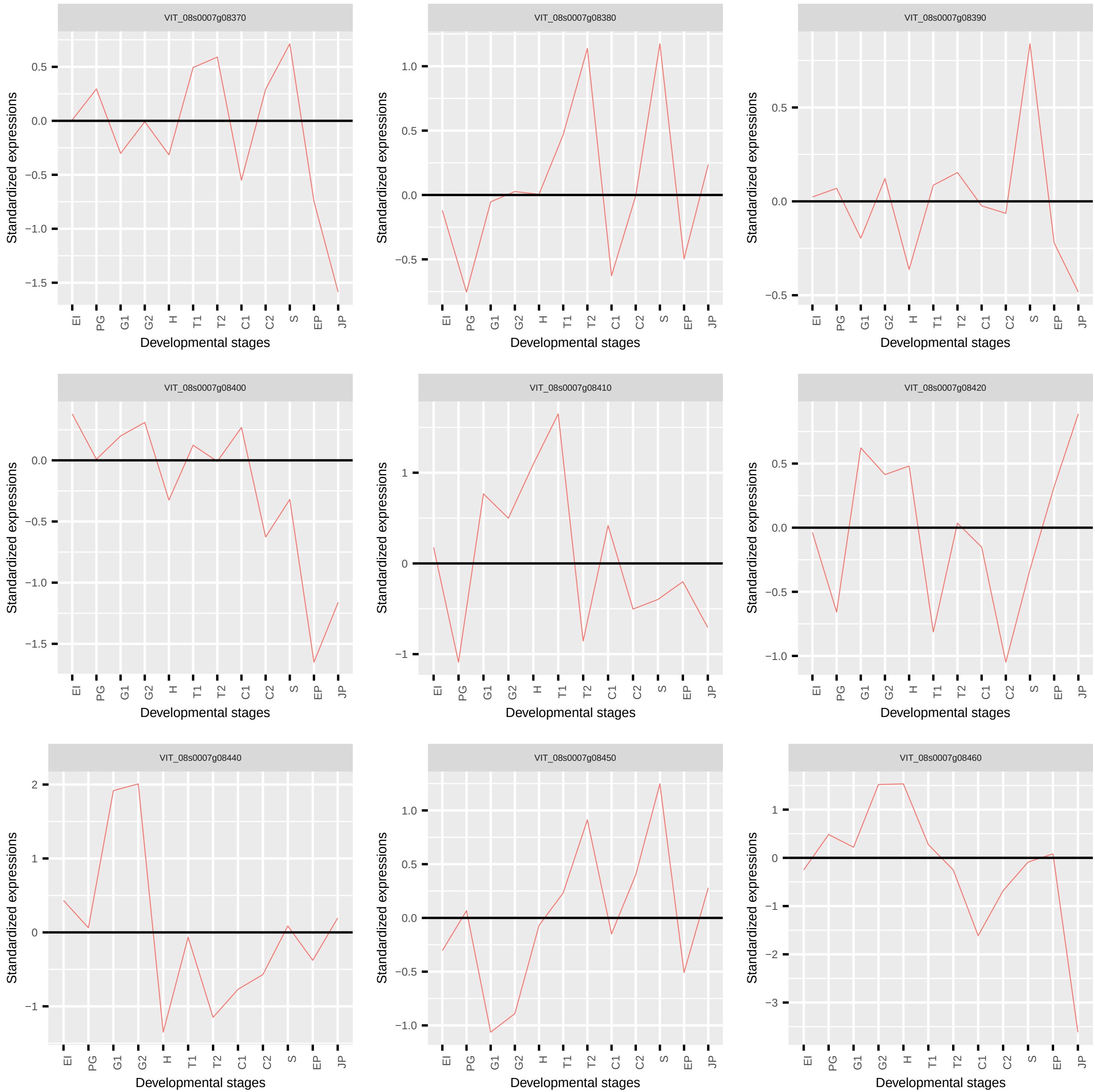
VIT_08s0007g08370
VIT_08s0007g08380
VIT_08s0007g08390
VIT_08s0007g08400
VIT_08s0007g08410
VIT_08s0007g08420
VIT_08s0007g08440
VIT_08s0007g08450
VIT_08s0007g08460
Standardized expressions
Developmental stages
EI
PG
G1
G2
H
T1
T2
C1
C2
S
EP
JP

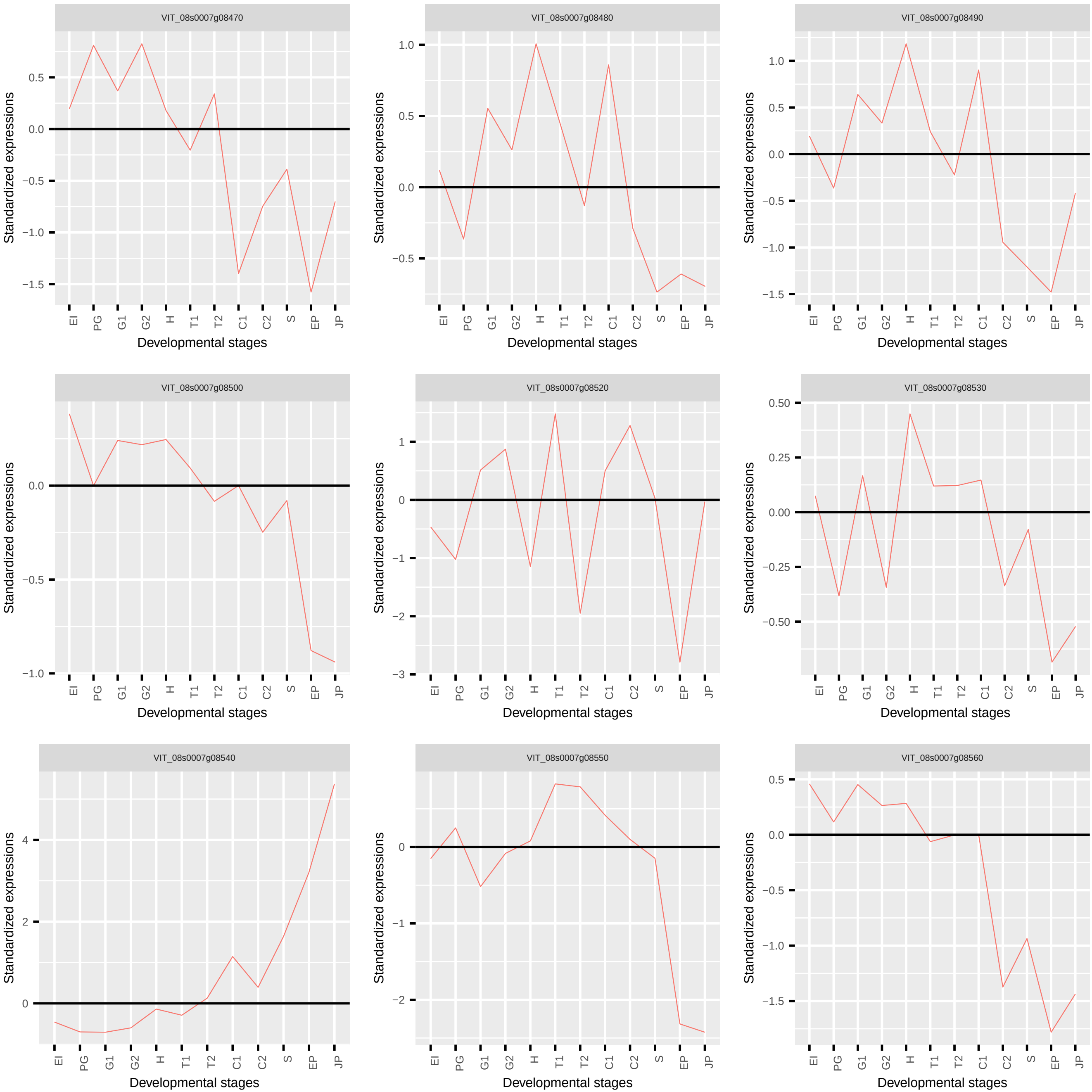

VIT_08s0007g08470
0.5
0.0
−0.5
−1.0
−1.5
Standardized expressions
EI PG G1 G2 H T1 T2 C1 C2 S EP JP
Developmental stages
VIT_08s0007g08480
1.0
0.5
0.0
−0.5
Standardized expressions
EI PG G1 G2 H T1 T2 C1 C2 S EP JP
Developmental stages
VIT_08s0007g08490
1.0
0.5
0.0
−0.5
−1.0
−1.5
Standardized expressions
EI PG G1 G2 H T1 T2 C1 C2 S EP JP
Developmental stages
VIT_08s0007g08500
0.0
−0.5
−1.0
Standardized expressions
EI PG G1 G2 H T1 T2 C1 C2 S EP JP
Developmental stages
VIT_08s0007g08520
1
0
−1
−2
−3
Standardized expressions
EI PG G1 G2 H T1 T2 C1 C2 S EP JP
Developmental stages
VIT_08s0007g08530
0.50
0.25
0.00
−0.25
−0.50
Standardized expressions
EI PG G1 G2 H T1 T2 C1 C2 S EP JP
Developmental stages
VIT_08s0007g08540
4
2
0
Standardized expressions
EI PG G1 G2 H T1 T2 C1 C2 S EP JP
Developmental stages
VIT_08s0007g08550
0
−1
−2
Standardized expressions
EI PG G1 G2 H T1 T2 C1 C2 S EP JP
Developmental stages
VIT_08s0007g08560
0.5
0.0
−0.5
−1.0
−1.5
Standardized expressions
EI PG G1 G2 H T1 T2 C1 C2 S EP JP
Developmental stages

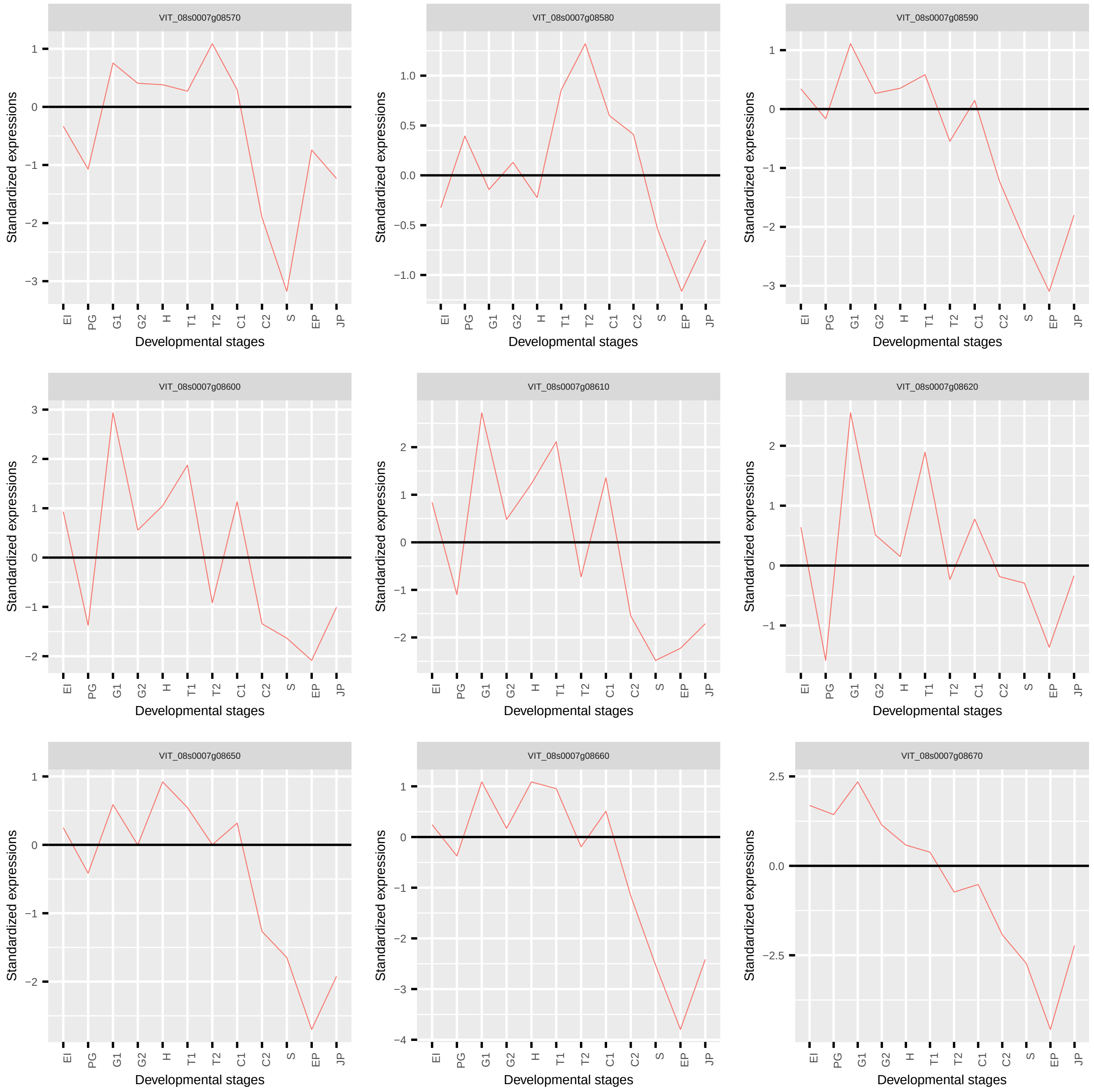
VIT_08s0007g08570
VIT_08s0007g08580
VIT_08s0007g08590
VIT_08s0007g08600
VIT_08s0007g08610
VIT_08s0007g08620
VIT_08s0007g08650
VIT_08s0007g08660
VIT_08s0007g08670
Standardized expressions
Developmental stages
EI PG G1 G2 H T1 T2 C1 C2 S EP JP

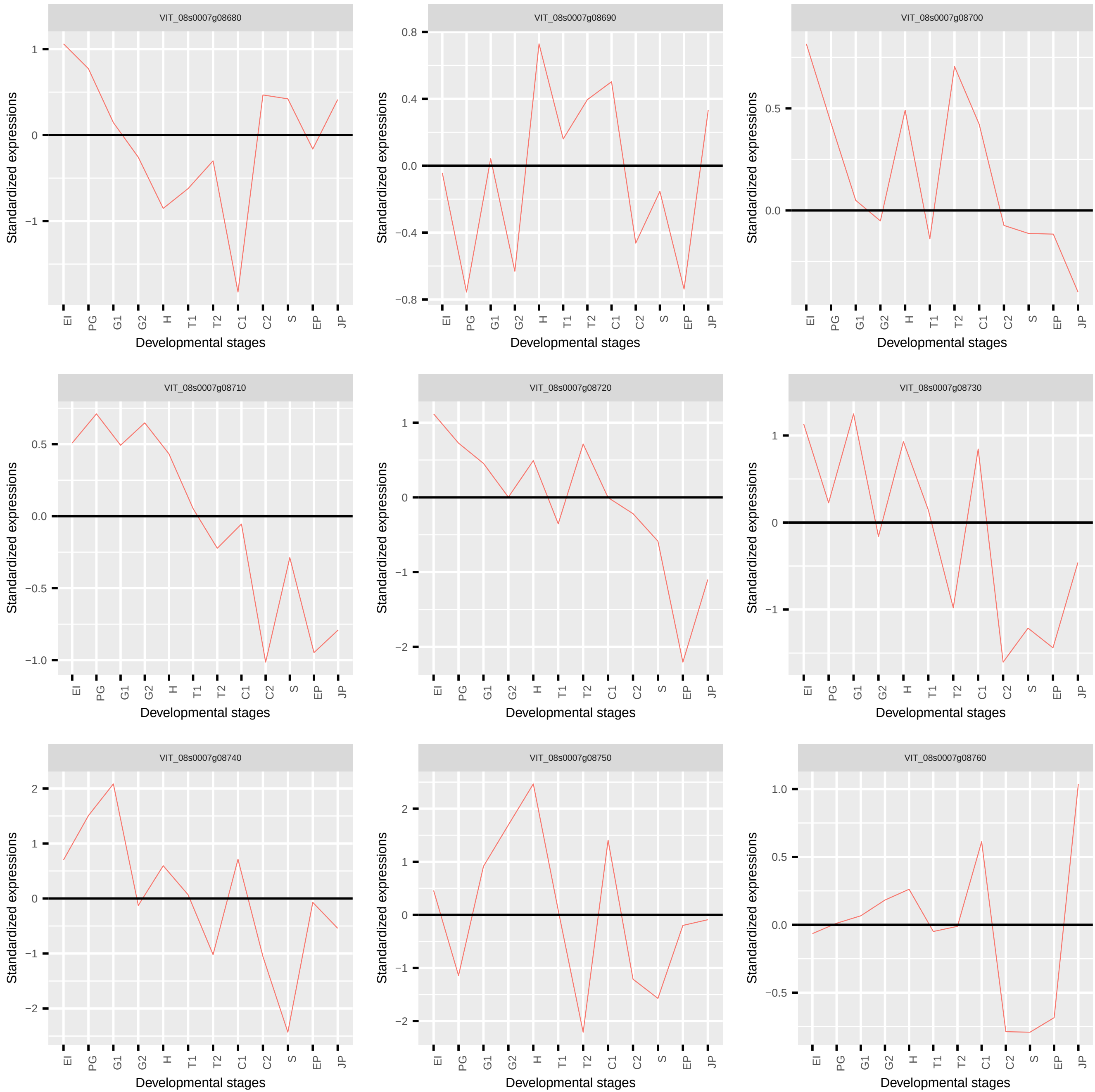

VIT_08s0007g08680
1
0
−1
Standardized expressions
EI PG G1 G2 H T1 T2 C1 C2 S EP JP
Developmental stages
VIT_08s0007g08690
0.8
0.4
0.0
−0.4
−0.8
Standardized expressions
EI PG G1 G2 H T1 T2 C1 C2 S EP JP
Developmental stages
VIT_08s0007g08700
0.5
0.0
Standardized expressions
EI PG G1 G2 H T1 T2 C1 C2 S EP JP
Developmental stages
VIT_08s0007g08710
0.5
0.0
−0.5
−1.0
Standardized expressions
EI PG G1 G2 H T1 T2 C1 C2 S EP JP
Developmental stages
VIT_08s0007g08720
1
0
−1
−2
Standardized expressions
EI PG G1 G2 H T1 T2 C1 C2 S EP JP
Developmental stages
VIT_08s0007g08730
1
0
−1
Standardized expressions
EI PG G1 G2 H T1 T2 C1 C2 S EP JP
Developmental stages
VIT_08s0007g08740
2
1
0
−1
−2
Standardized expressions
EI PG G1 G2 H T1 T2 C1 C2 S EP JP
Developmental stages
VIT_08s0007g08750
2
1
0
−1
−2
Standardized expressions
EI PG G1 G2 H T1 T2 C1 C2 S EP JP
Developmental stages
VIT_08s0007g08760
1.0
0.5
0.0
−0.5
Standardized expressions
EI PG G1 G2 H T1 T2 C1 C2 S EP JP
Developmental stages

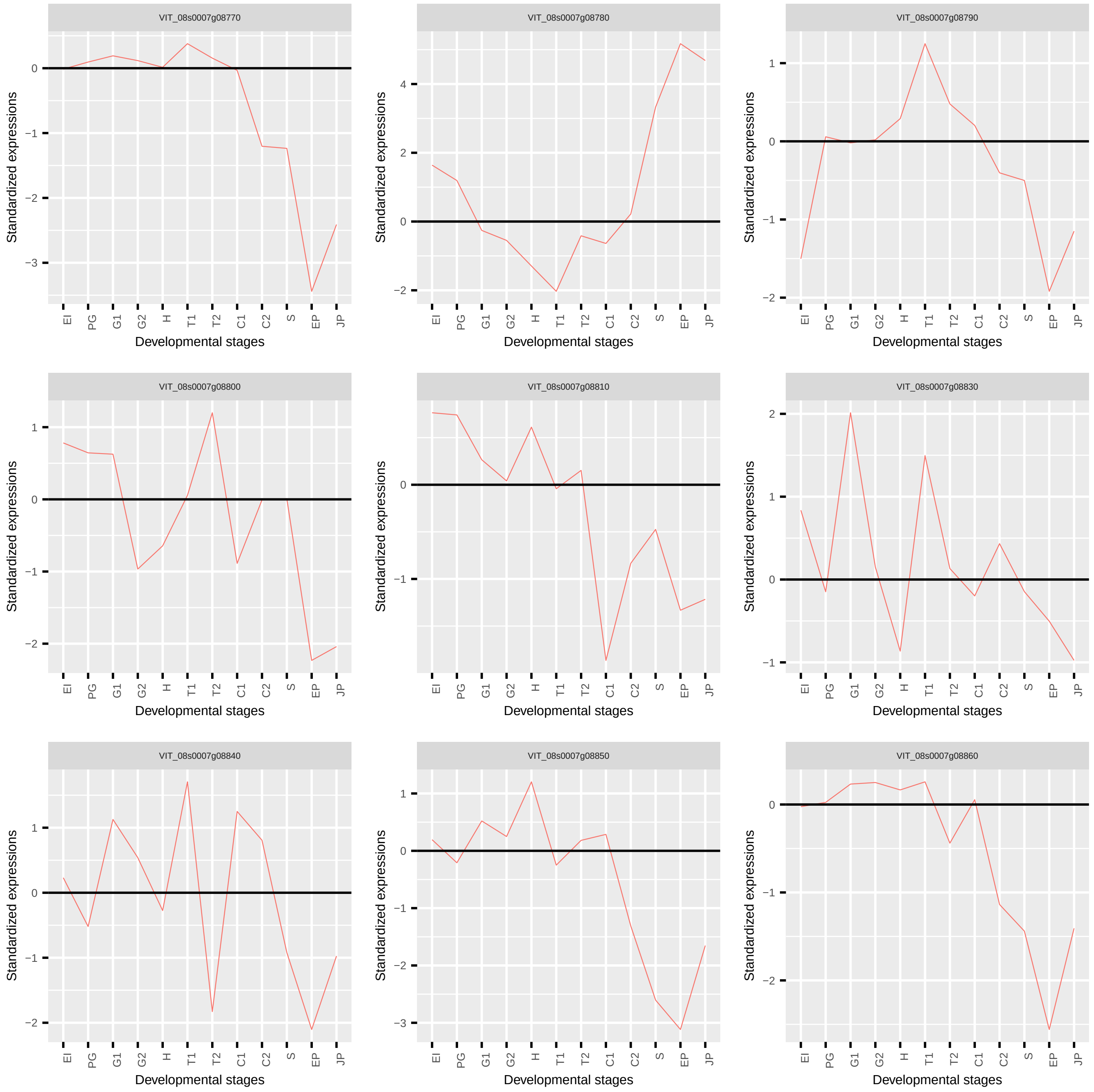
VIT_08s0007g08770
VIT_08s0007g08780
VIT_08s0007g08790
VIT_08s0007g08800
VIT_08s0007g08810
VIT_08s0007g08830
VIT_08s0007g08840
VIT_08s0007g08850
VIT_08s0007g08860
Standardized expressions
Developmental stages
EI PG G1 G2 H T1 T2 C1 C2 S EP JP

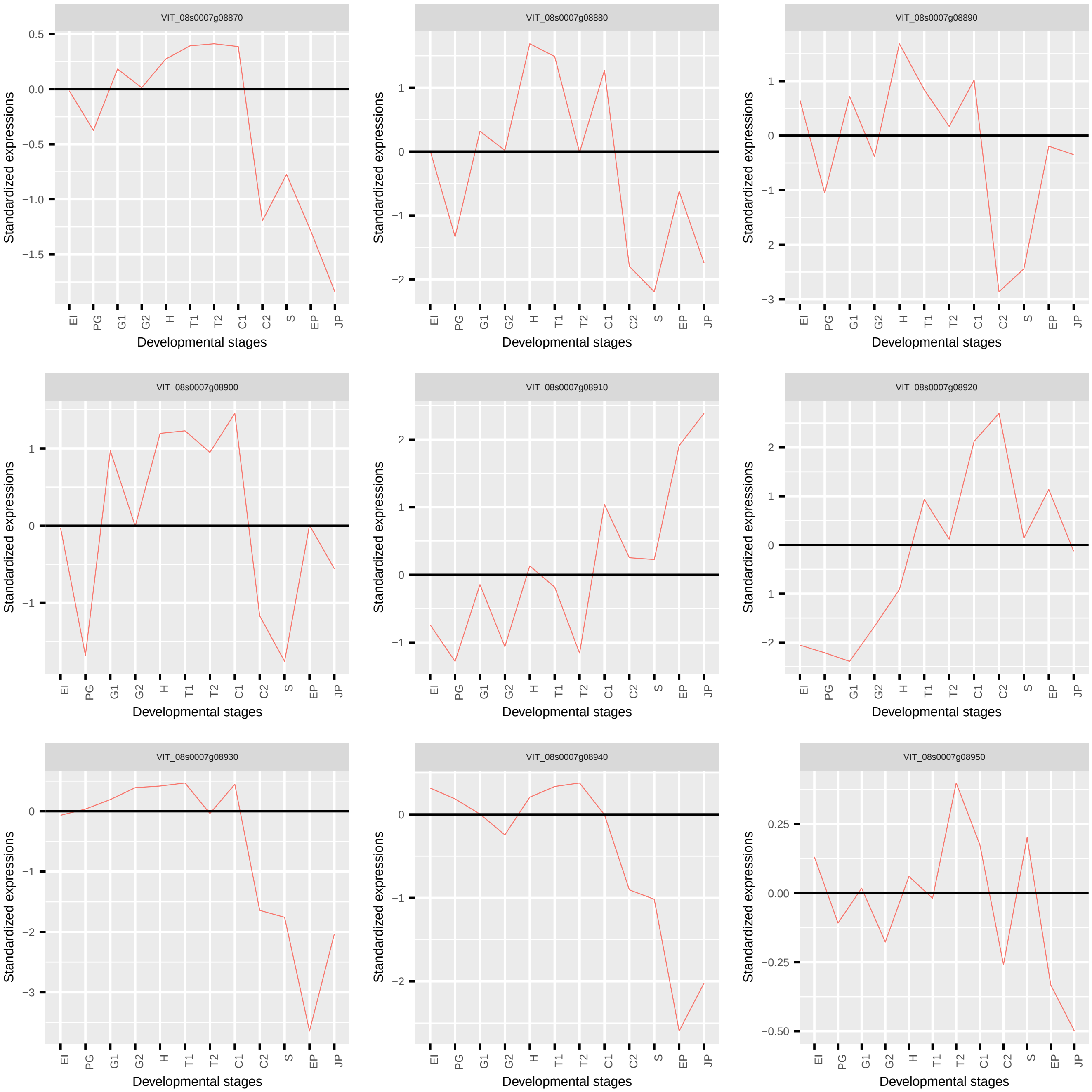
VIT_08s0007g08870
VIT_08s0007g08880
VIT_08s0007g08890
VIT_08s0007g08900
VIT_08s0007g08910
VIT_08s0007g08920
VIT_08s0007g08930
VIT_08s0007g08940
VIT_08s0007g08950
Standardized expressions
Developmental stages
EI
PG
G1
G2
H
T1
T2
C1
C2
S
EP
JP

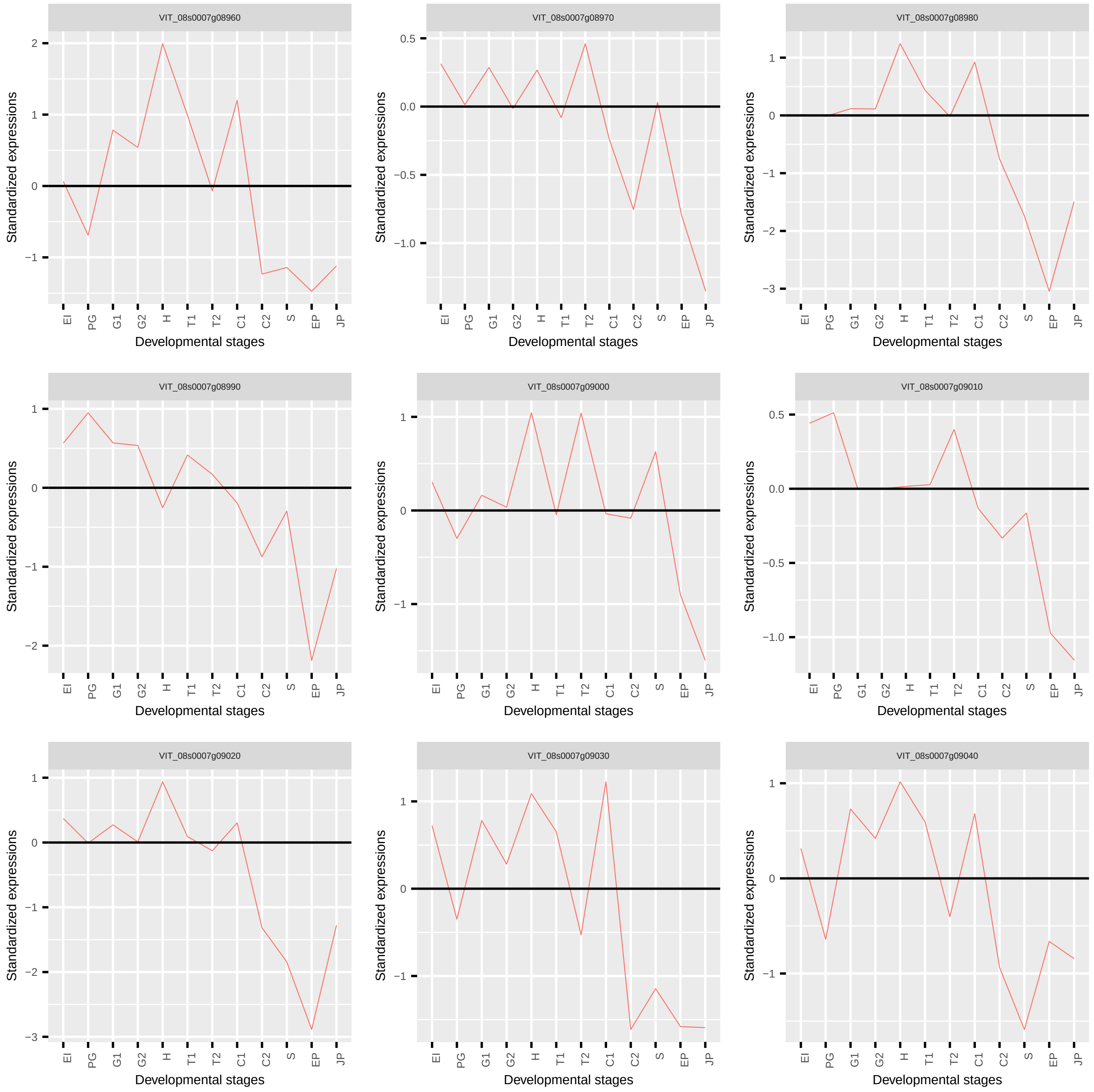

VIT_08s0007g08960
VIT_08s0007g08970
VIT_08s0007g08980
VIT_08s0007g08990
VIT_08s0007g09000
VIT_08s0007g09010
VIT_08s0007g09020
VIT_08s0007g09030
VIT_08s0007g09040
Standardized expressions
Developmental stages
EI
PG
G1
G2
H
T1
T2
C1
C2
S
EP
JP

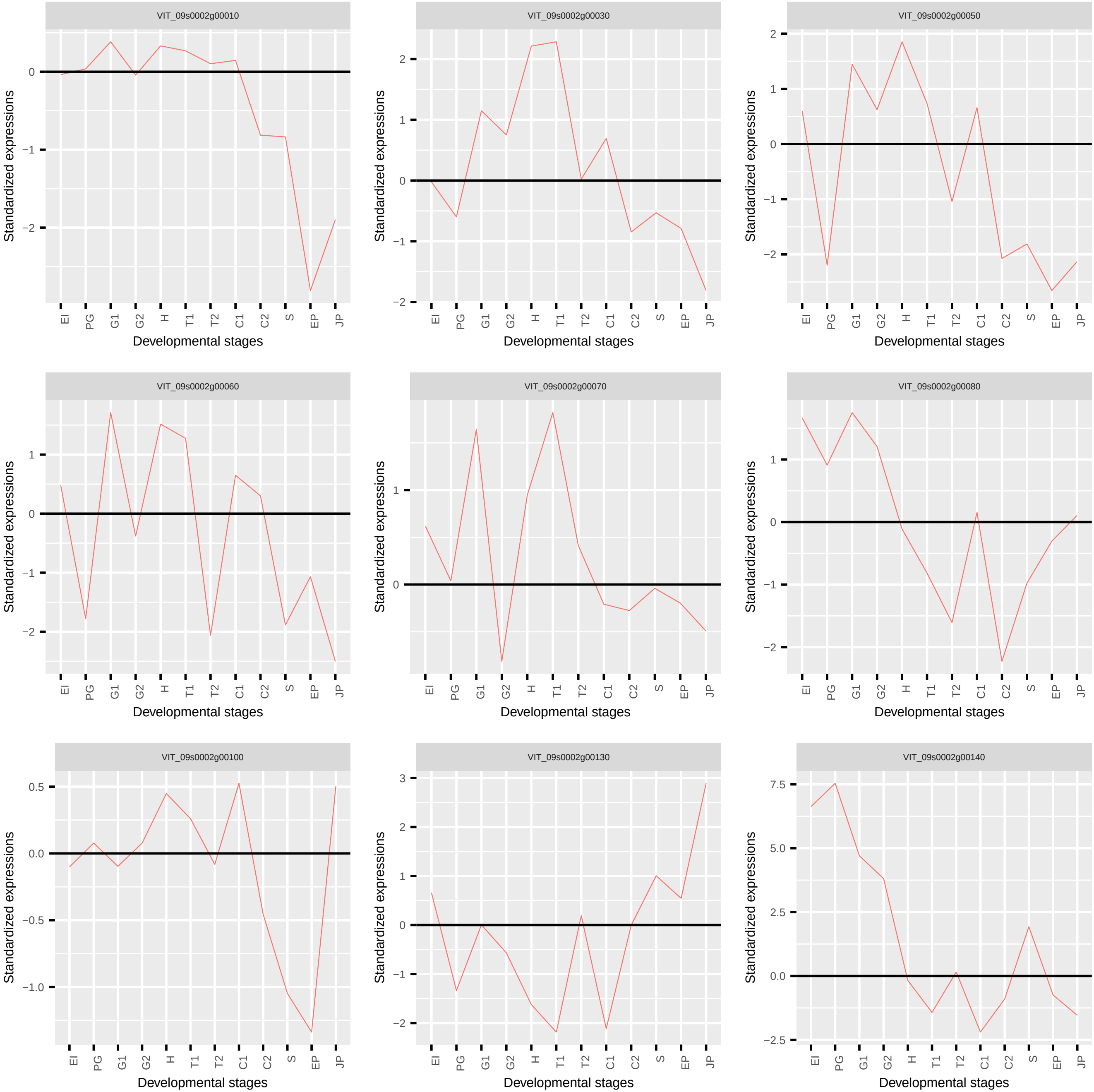
VIT_09s0002g00010
VIT_09s0002g00030
VIT_09s0002g00050
VIT_09s0002g00060
VIT_09s0002g00070
VIT_09s0002g00080
VIT_09s0002g00100
VIT_09s0002g00130
VIT_09s0002g00140
Standardized expressions
Developmental stages
EI PG G1 G2 H T1 T2 C1 C2 S EP JP

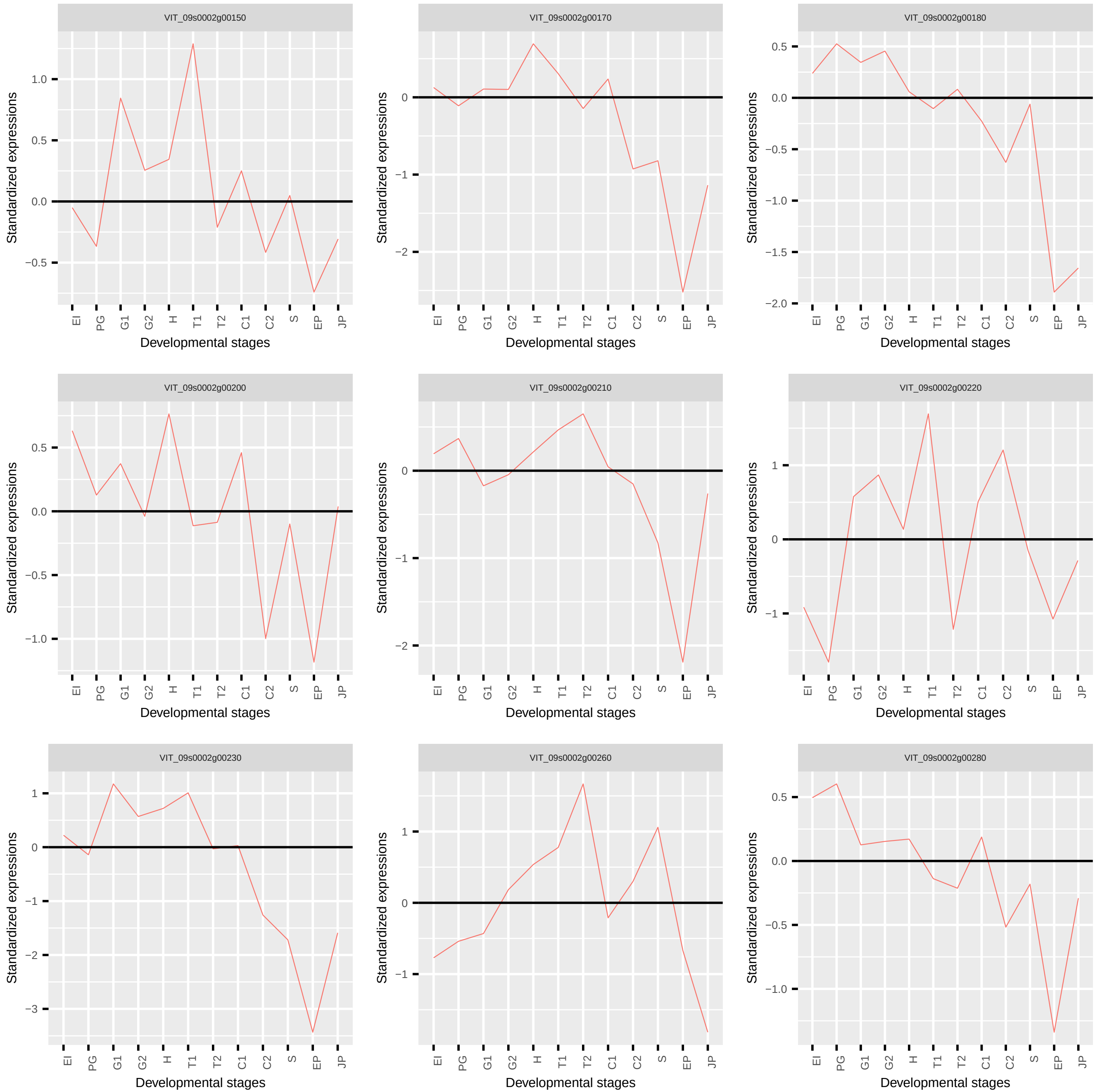
VIT_09s0002g00150
VIT_09s0002g00170
VIT_09s0002g00180
VIT_09s0002g00200
VIT_09s0002g00210
VIT_09s0002g00220
VIT_09s0002g00230
VIT_09s0002g00260
VIT_09s0002g00280
Standardized expressions
Developmental stages
EI PG G1 G2 H T1 T2 C1 C2 S EP JP

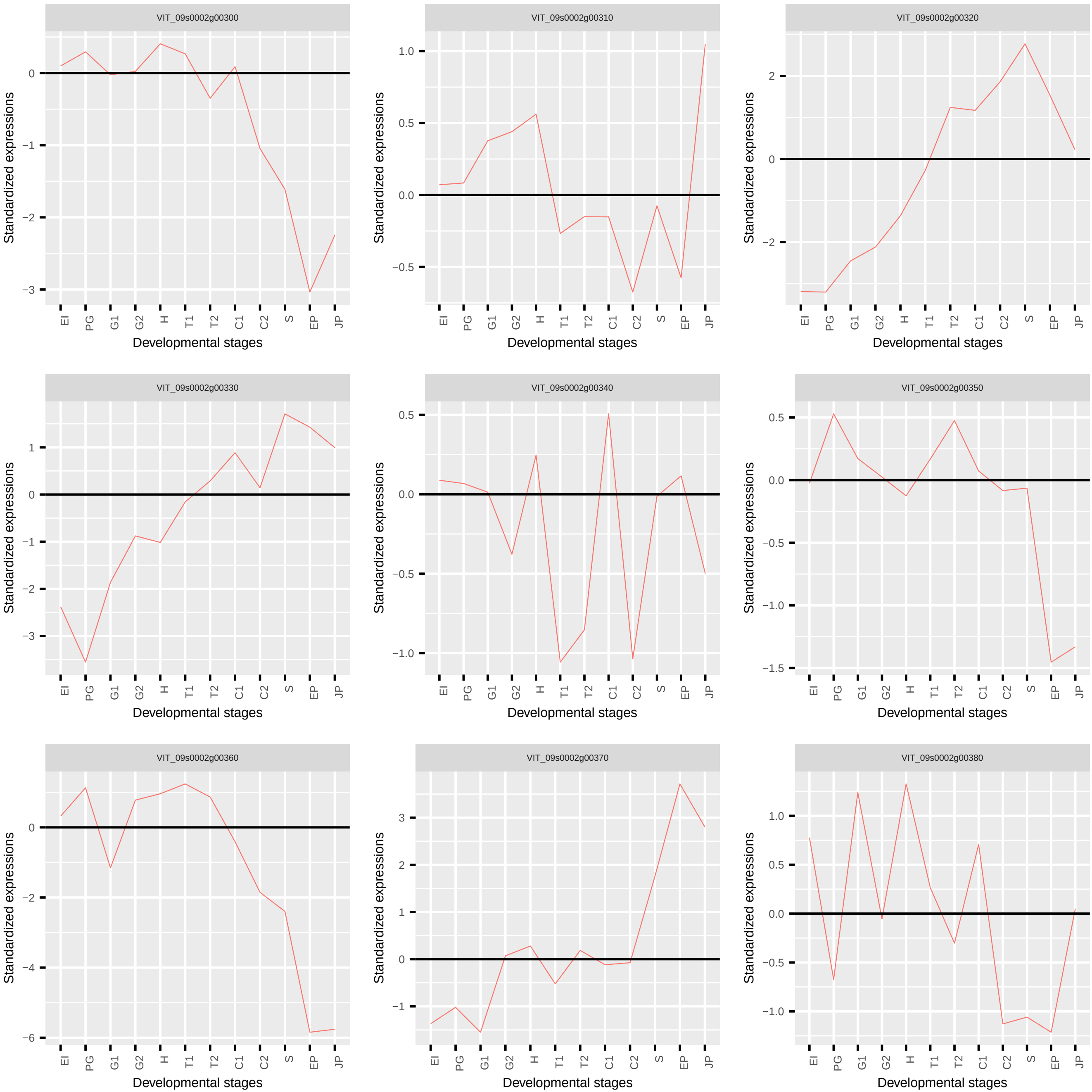

VIT_09s0002g00300
VIT_09s0002g00310
VIT_09s0002g00320
VIT_09s0002g00330
VIT_09s0002g00340
VIT_09s0002g00350
VIT_09s0002g00360
VIT_09s0002g00370
VIT_09s0002g00380
Standardized expressions
Developmental stages
EI PG G1 G2 H T1 T2 C1 C2 S EP JP
0 −1 −2 −3
1.0 0.5 0.0 −0.5
2 0 −2
1 0 −1 −2 −3
0.5 0.0 −0.5 −1.0
0.5 0.0 −0.5 −1.0 −1.5
0 −2 −4 −6
3 2 1 0 −1
1.0 0.5 0.0 −0.5 −1.0

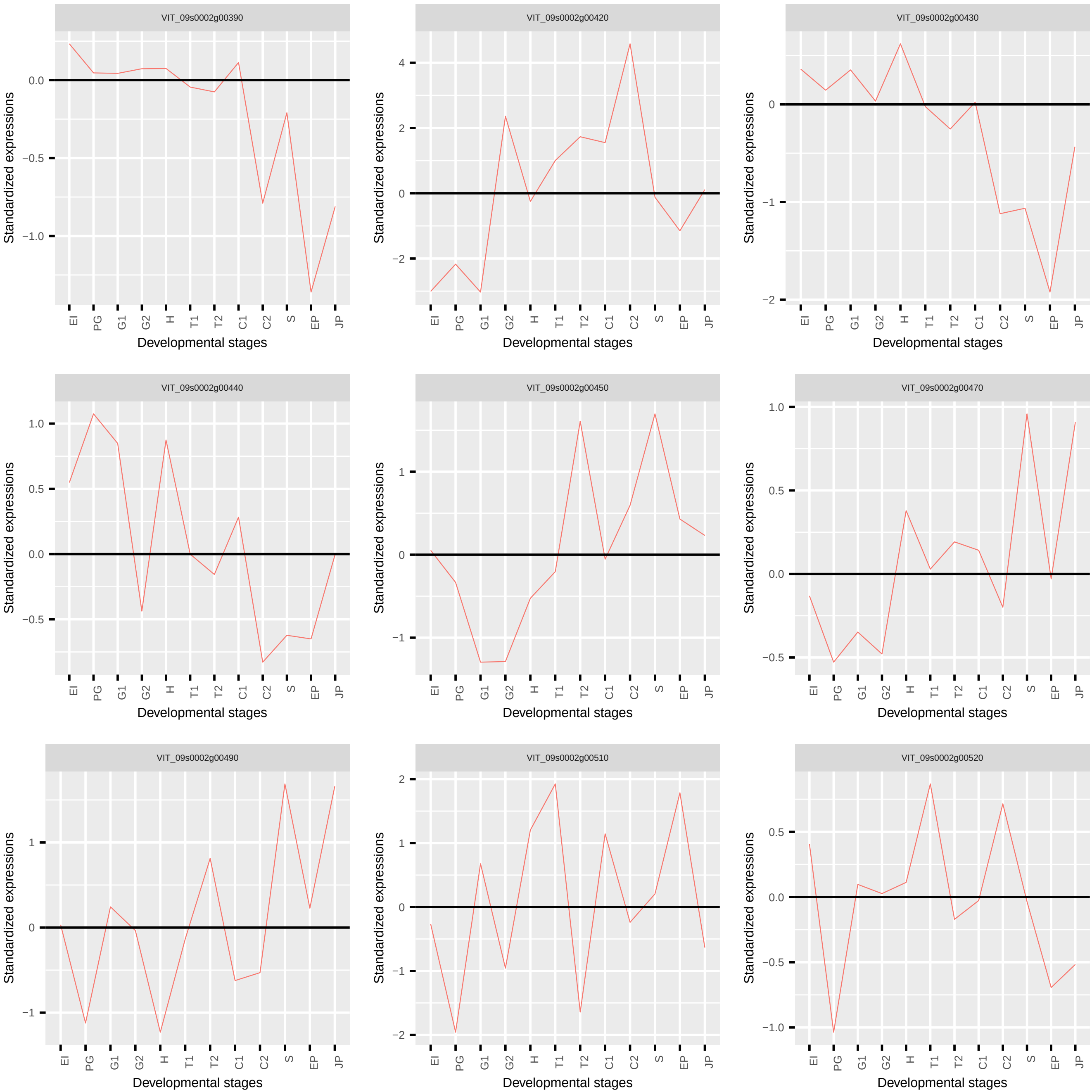

VIT_09s0002g00390
VIT_09s0002g00420
VIT_09s0002g00430
VIT_09s0002g00440
VIT_09s0002g00450
VIT_09s0002g00470
VIT_09s0002g00490
VIT_09s0002g00510
VIT_09s0002g00520
Standardized expressions
Developmental stages
EI
PG
G1
G2
H
T1
T2
C1
C2
S
EP
JP

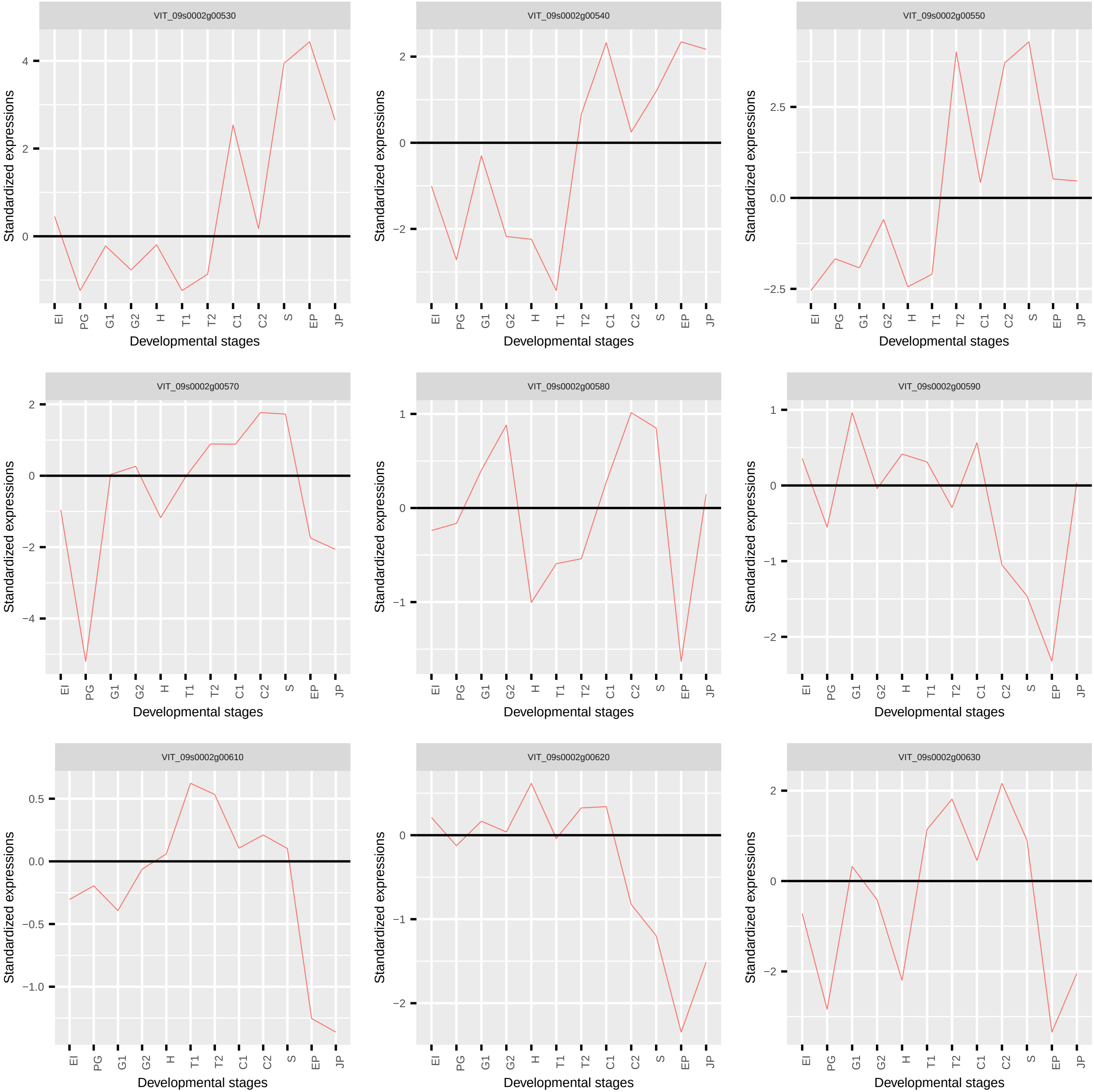
VIT_09s0002g00530
VIT_09s0002g00540
VIT_09s0002g00550
VIT_09s0002g00570
VIT_09s0002g00580
VIT_09s0002g00590
VIT_09s0002g00610
VIT_09s0002g00620
VIT_09s0002g00630
Standardized expressions
Developmental stages
EI
PG
G1
G2
H
T1
T2
C1
C2
S
EP
JP

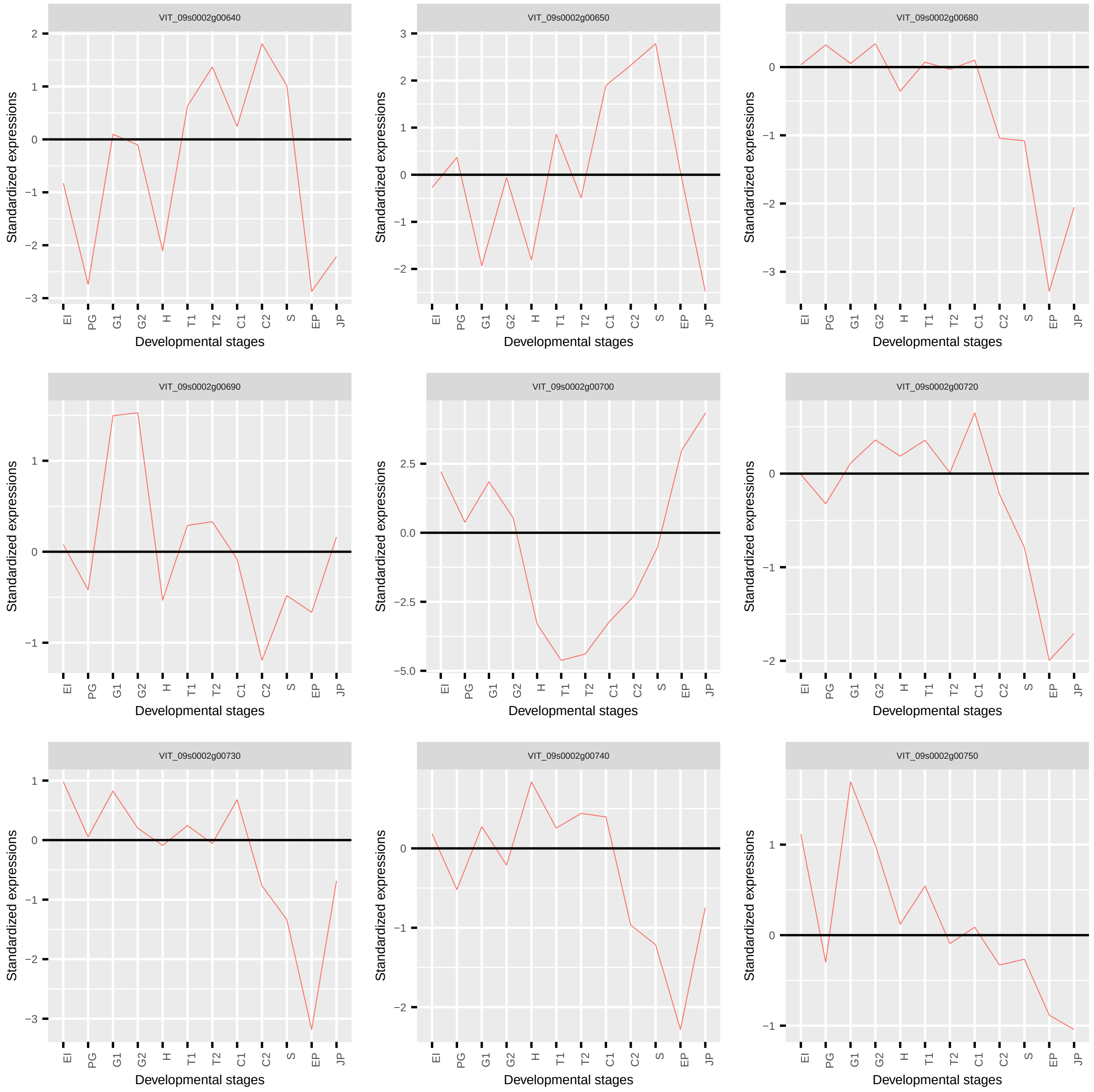
VIT_09s0002g00640
VIT_09s0002g00650
VIT_09s0002g00680
VIT_09s0002g00690
VIT_09s0002g00700
VIT_09s0002g00720
VIT_09s0002g00730
VIT_09s0002g00740
VIT_09s0002g00750
Standardized expressions
Developmental stages
EI
PG
G1
G2
H
T1
T2
C1
C2
S
EP
JP

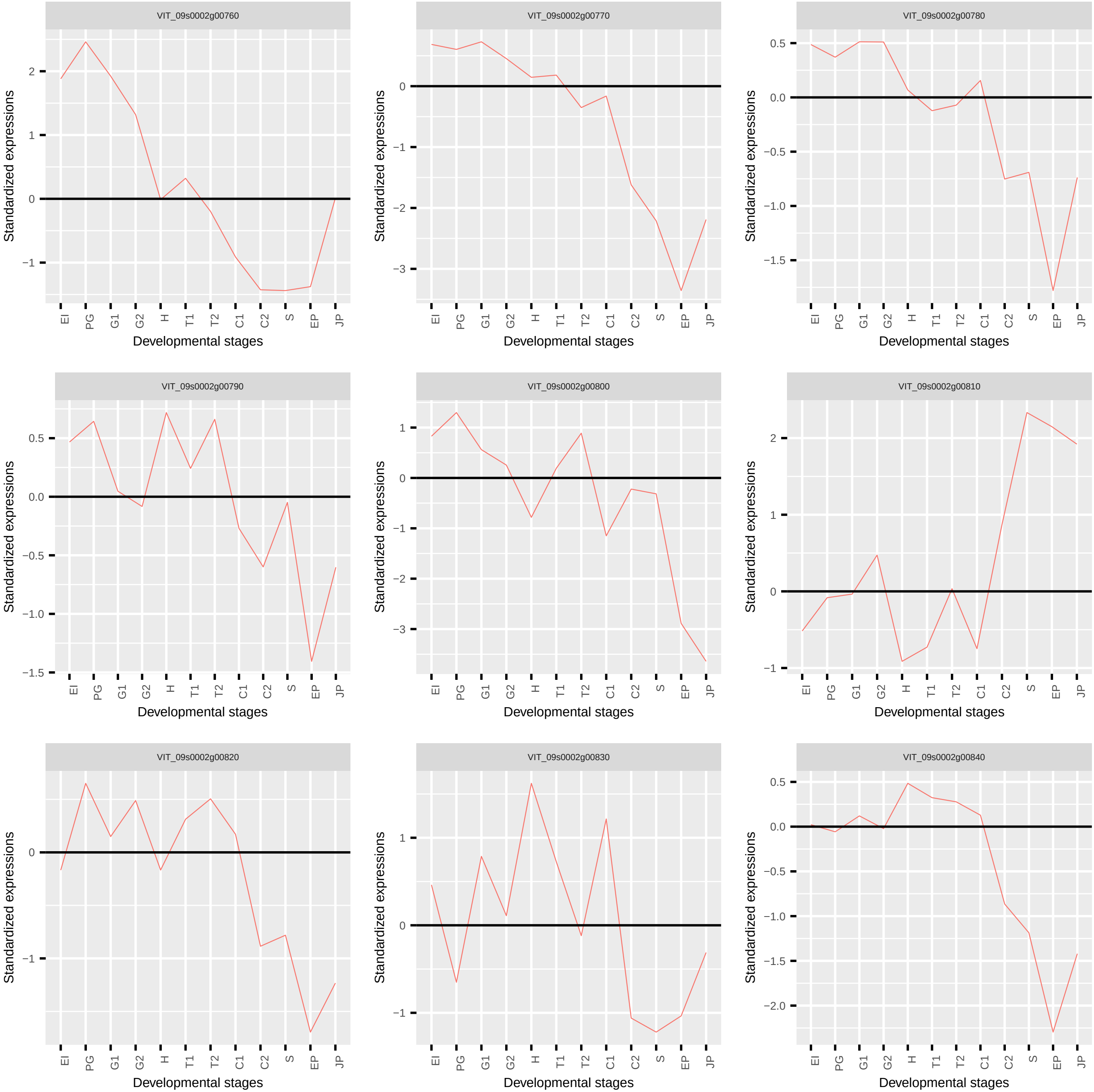
VIT_09s0002g00760
2
1
0
−1
Standardized expressions
EI PG G1 G2 H T1 T2 C1 C2 S EP JP
Developmental stages
VIT_09s0002g00770
0
−1
−2
−3
Standardized expressions
EI PG G1 G2 H T1 T2 C1 C2 S EP JP
Developmental stages
VIT_09s0002g00780
0.5
0.0
−0.5
−1.0
−1.5
Standardized expressions
EI PG G1 G2 H T1 T2 C1 C2 S EP JP
Developmental stages
VIT_09s0002g00790
0.5
0.0
−0.5
−1.0
−1.5
Standardized expressions
EI PG G1 G2 H T1 T2 C1 C2 S EP JP
Developmental stages
VIT_09s0002g00800
1
0
−1
−2
−3
Standardized expressions
EI PG G1 G2 H T1 T2 C1 C2 S EP JP
Developmental stages
VIT_09s0002g00810
2
1
0
−1
Standardized expressions
EI PG G1 G2 H T1 T2 C1 C2 S EP JP
Developmental stages
VIT_09s0002g00820
0
−1
Standardized expressions
EI PG G1 G2 H T1 T2 C1 C2 S EP JP
Developmental stages
VIT_09s0002g00830
1
0
−1
Standardized expressions
EI PG G1 G2 H T1 T2 C1 C2 S EP JP
Developmental stages
VIT_09s0002g00840
0.5
0.0
−0.5
−1.0
−1.5
−2.0
Standardized expressions
EI PG G1 G2 H T1 T2 C1 C2 S EP JP
Developmental stages

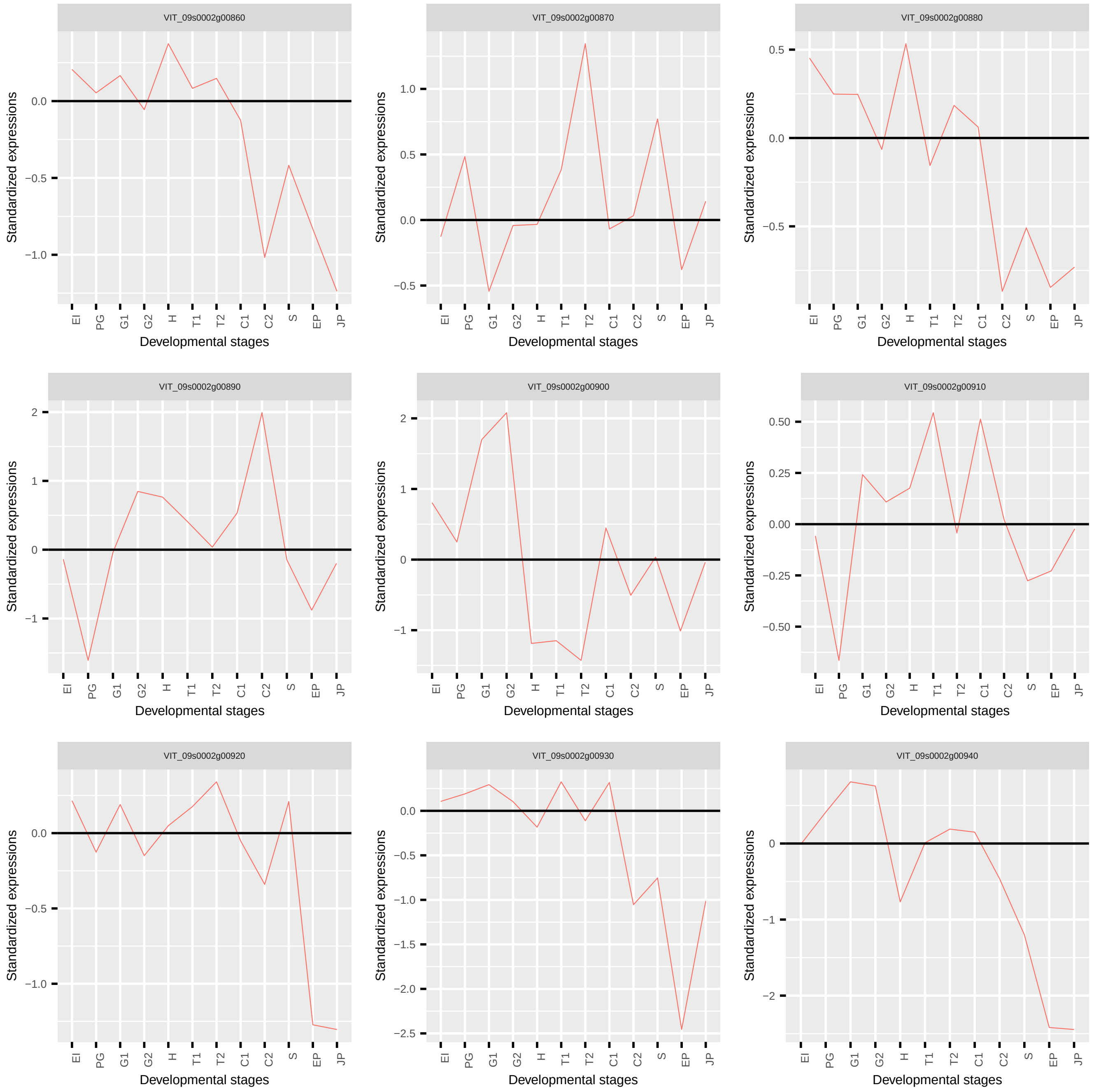
VIT_09s0002g00860
Standardized expressions
0.0
−0.5
−1.0
EI
PG
G1
G2
H
T1
T2
C1
C2
S
EP
JP
Developmental stages
VIT_09s0002g00870
Standardized expressions
1.0
0.5
0.0
−0.5
EI
PG
G1
G2
H
T1
T2
C1
C2
S
EP
JP
Developmental stages
VIT_09s0002g00880
Standardized expressions
0.5
0.0
−0.5
EI
PG
G1
G2
H
T1
T2
C1
C2
S
EP
JP
Developmental stages
VIT_09s0002g00890
Standardized expressions
2
1
0
−1
EI
PG
G1
G2
H
T1
T2
C1
C2
S
EP
JP
Developmental stages
VIT_09s0002g00900
Standardized expressions
2
1
0
−1
EI
PG
G1
G2
H
T1
T2
C1
C2
S
EP
JP
Developmental stages
VIT_09s0002g00910
Standardized expressions
0.50
0.25
0.00
−0.25
−0.50
EI
PG
G1
G2
H
T1
T2
C1
C2
S
EP
JP
Developmental stages
VIT_09s0002g00920
Standardized expressions
0.0
−0.5
−1.0
EI
PG
G1
G2
H
T1
T2
C1
C2
S
EP
JP
Developmental stages
VIT_09s0002g00930
Standardized expressions
0.0
−0.5
−1.0
−1.5
−2.0
−2.5
EI
PG
G1
G2
H
T1
T2
C1
C2
S
EP
JP
Developmental stages
VIT_09s0002g00940
Standardized expressions
0
−1
−2
EI
PG
G1
G2
H
T1
T2
C1
C2
S
EP
JP
Developmental stages

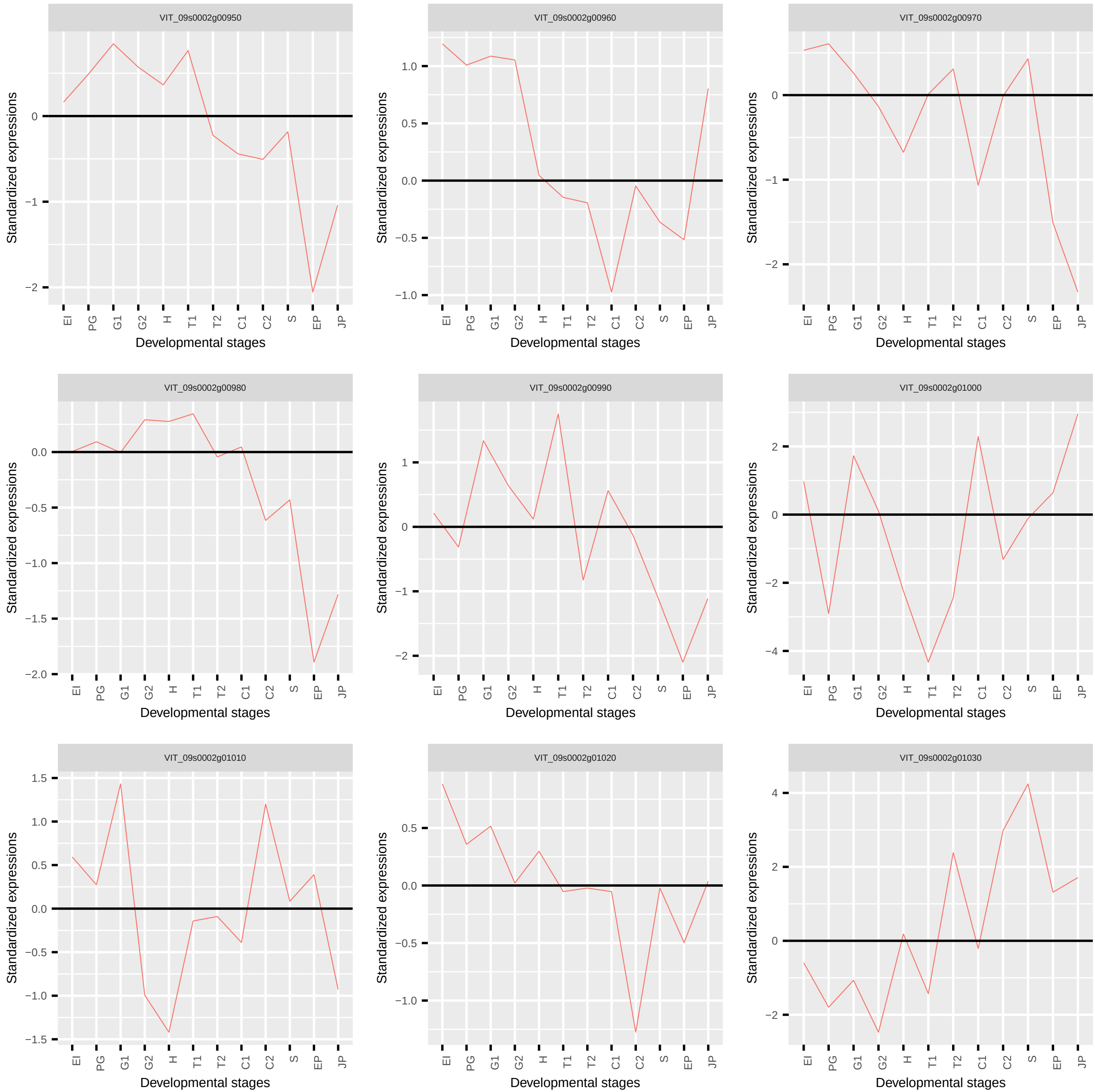
VIT_09s0002g00950
VIT_09s0002g00960
VIT_09s0002g00970
VIT_09s0002g00980
VIT_09s0002g00990
VIT_09s0002g01000
VIT_09s0002g01010
VIT_09s0002g01020
VIT_09s0002g01030
Standardized expressions
Developmental stages
EI
PG
G1
G2
H
T1
T2
C1
C2
S
EP
JP

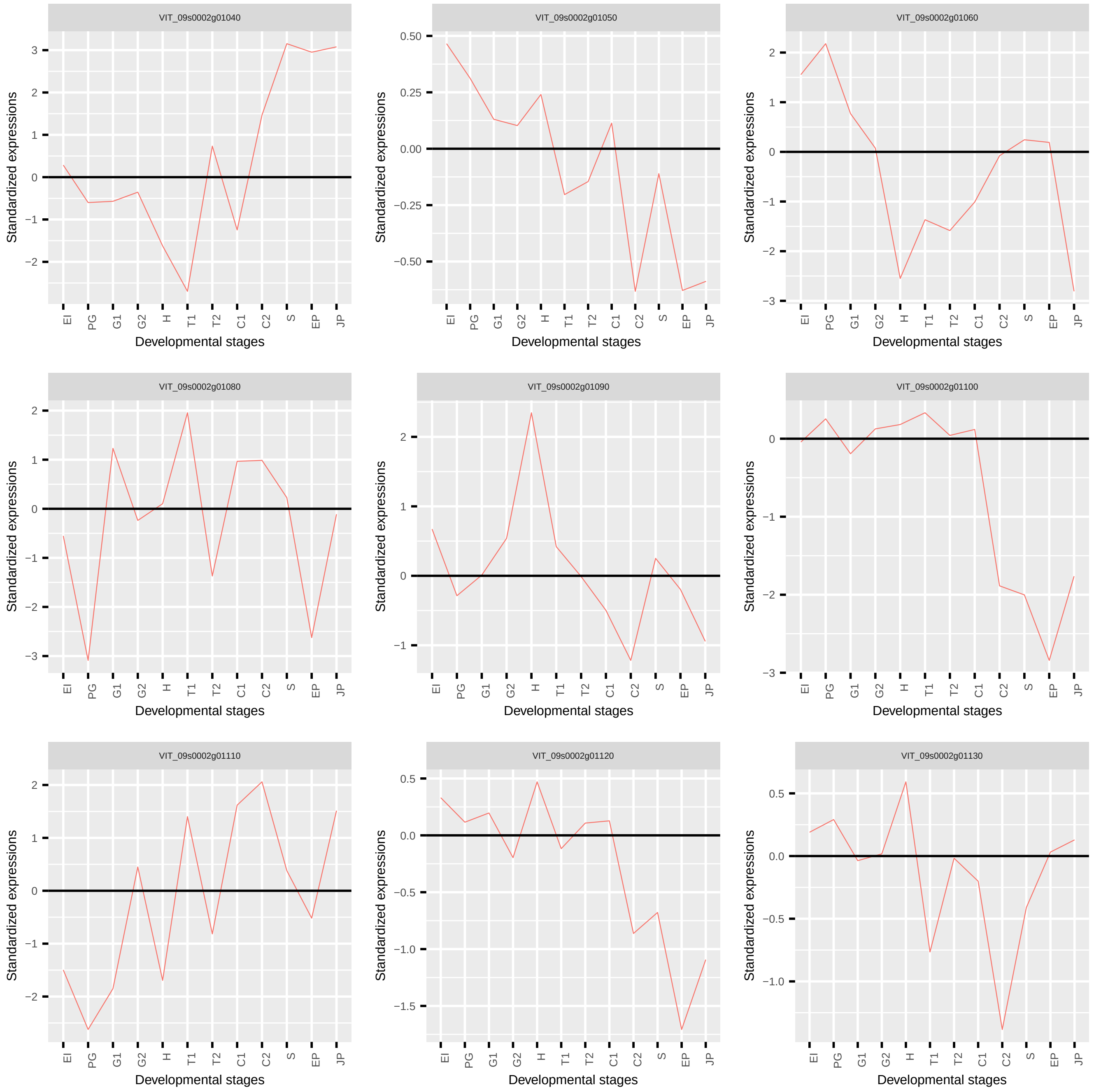
VIT_09s0002g01040
VIT_09s0002g01050
VIT_09s0002g01060
VIT_09s0002g01080
VIT_09s0002g01090
VIT_09s0002g01100
VIT_09s0002g01110
VIT_09s0002g01120
VIT_09s0002g01130
Standardized expressions
Developmental stages
EI PG G1 G2 H T1 T2 C1 C2 S EP JP

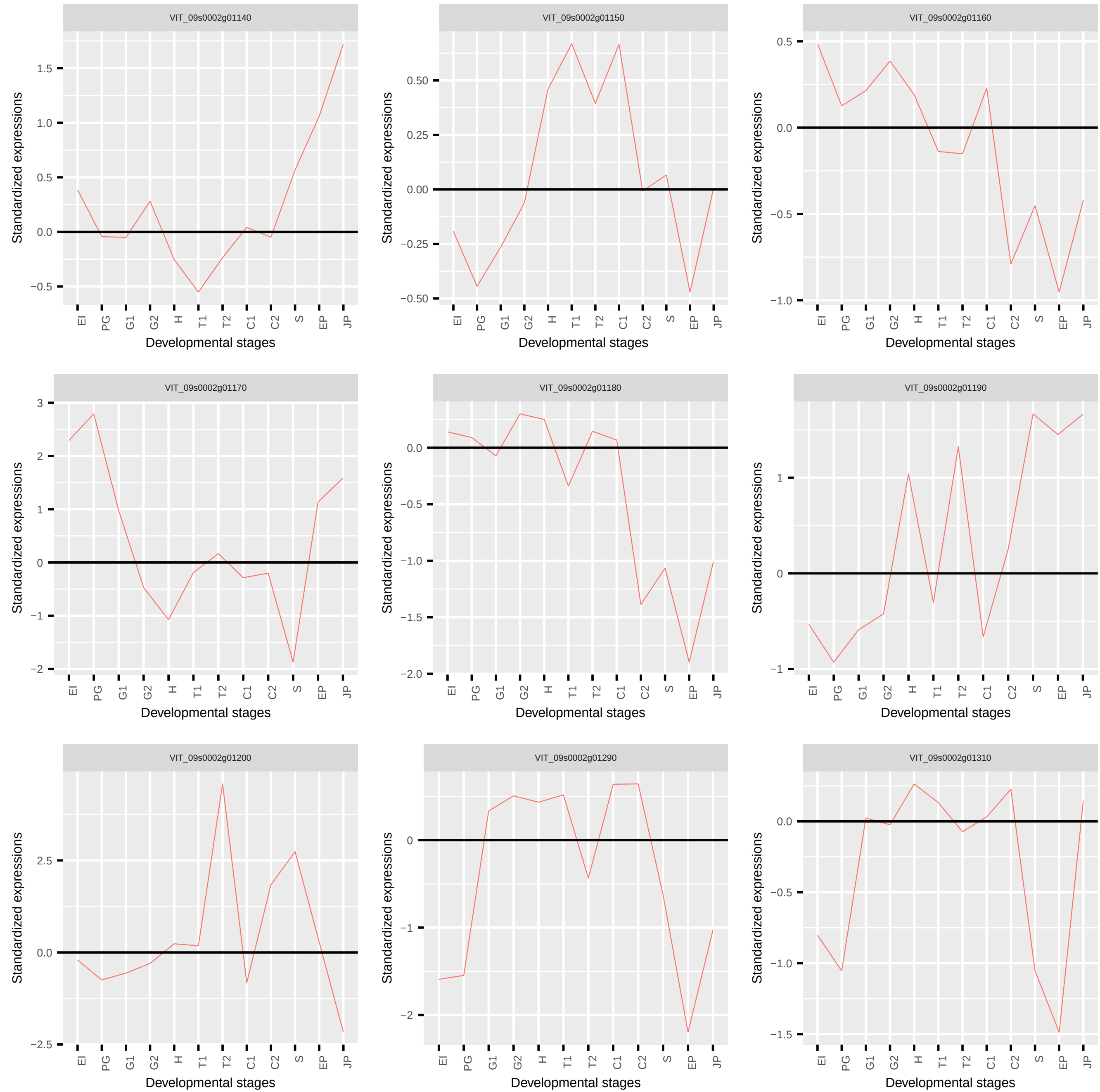
VIT_09s0002g01140
VIT_09s0002g01150
VIT_09s0002g01160
VIT_09s0002g01170
VIT_09s0002g01180
VIT_09s0002g01190
VIT_09s0002g01200
VIT_09s0002g01290
VIT_09s0002g01310
Standardized expressions
Developmental stages
EI PG G1 G2 H T1 T2 C1 C2 S EP JP

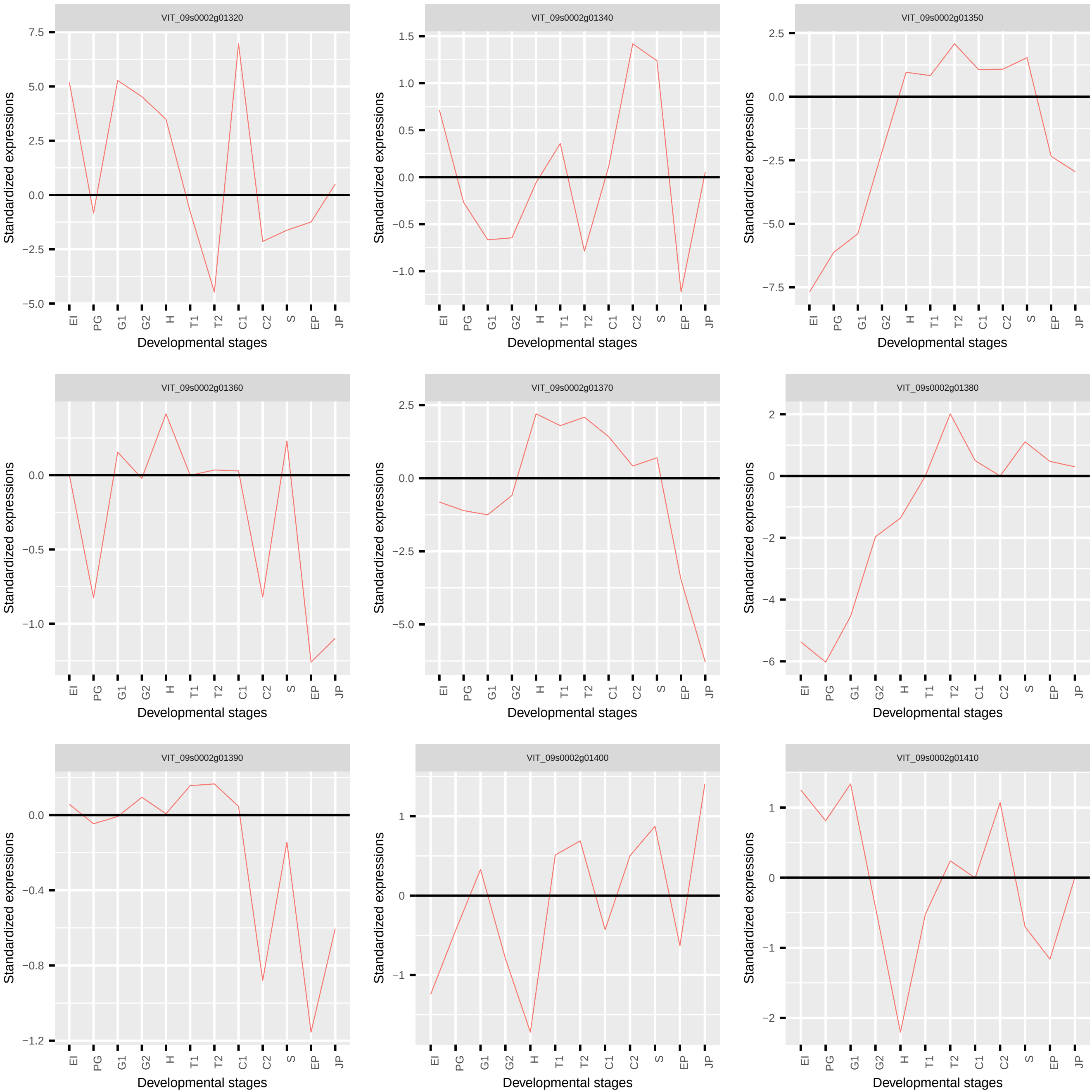
VIT_09s0002g01320
VIT_09s0002g01340
VIT_09s0002g01350
VIT_09s0002g01360
VIT_09s0002g01370
VIT_09s0002g01380
VIT_09s0002g01390
VIT_09s0002g01400
VIT_09s0002g01410
Standardized expressions
Developmental stages
EI PG G1 G2 H T1 T2 C1 C2 S EP JP

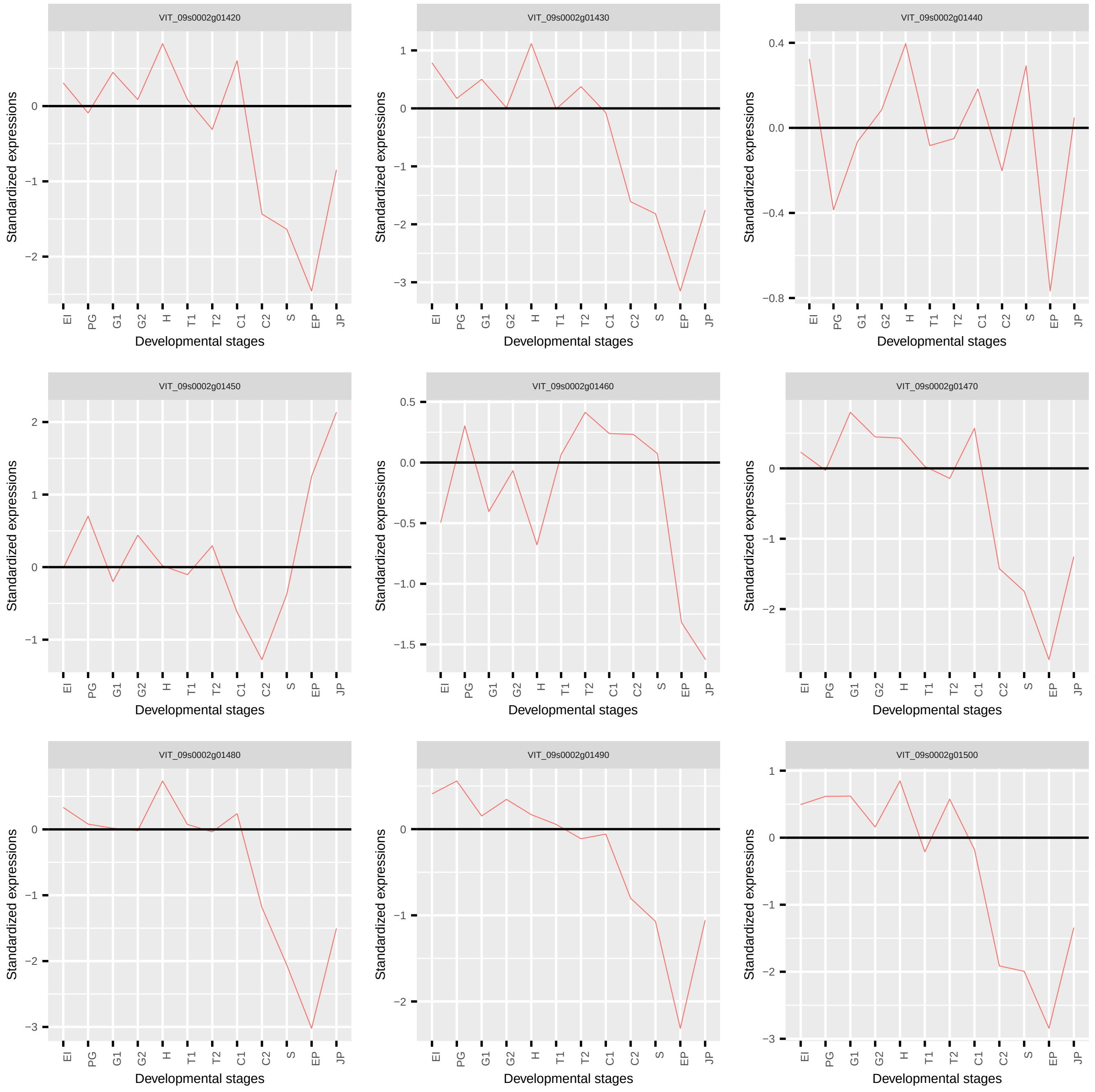
VIT_09s0002g01420
VIT_09s0002g01430
VIT_09s0002g01440
VIT_09s0002g01450
VIT_09s0002g01460
VIT_09s0002g01470
VIT_09s0002g01480
VIT_09s0002g01490
VIT_09s0002g01500
Standardized expressions
Developmental stages
EI PG G1 G2 H T1 T2 C1 C2 S EP JP

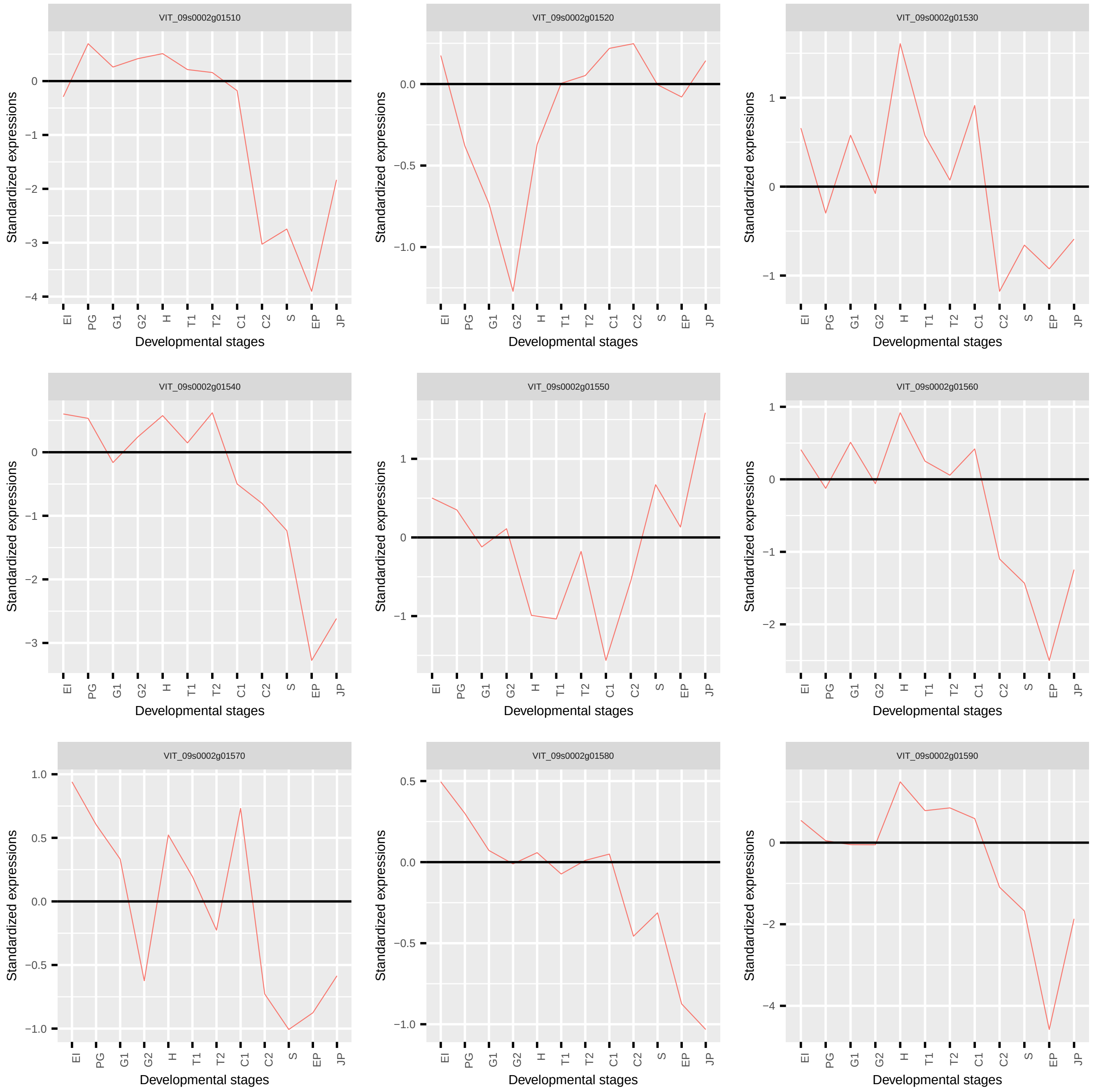
VIT_09s0002g01510
VIT_09s0002g01520
VIT_09s0002g01530
VIT_09s0002g01540
VIT_09s0002g01550
VIT_09s0002g01560
VIT_09s0002g01570
VIT_09s0002g01580
VIT_09s0002g01590
Standardized expressions
Developmental stages
EI PG G1 G2 H T1 T2 C1 C2 S EP JP

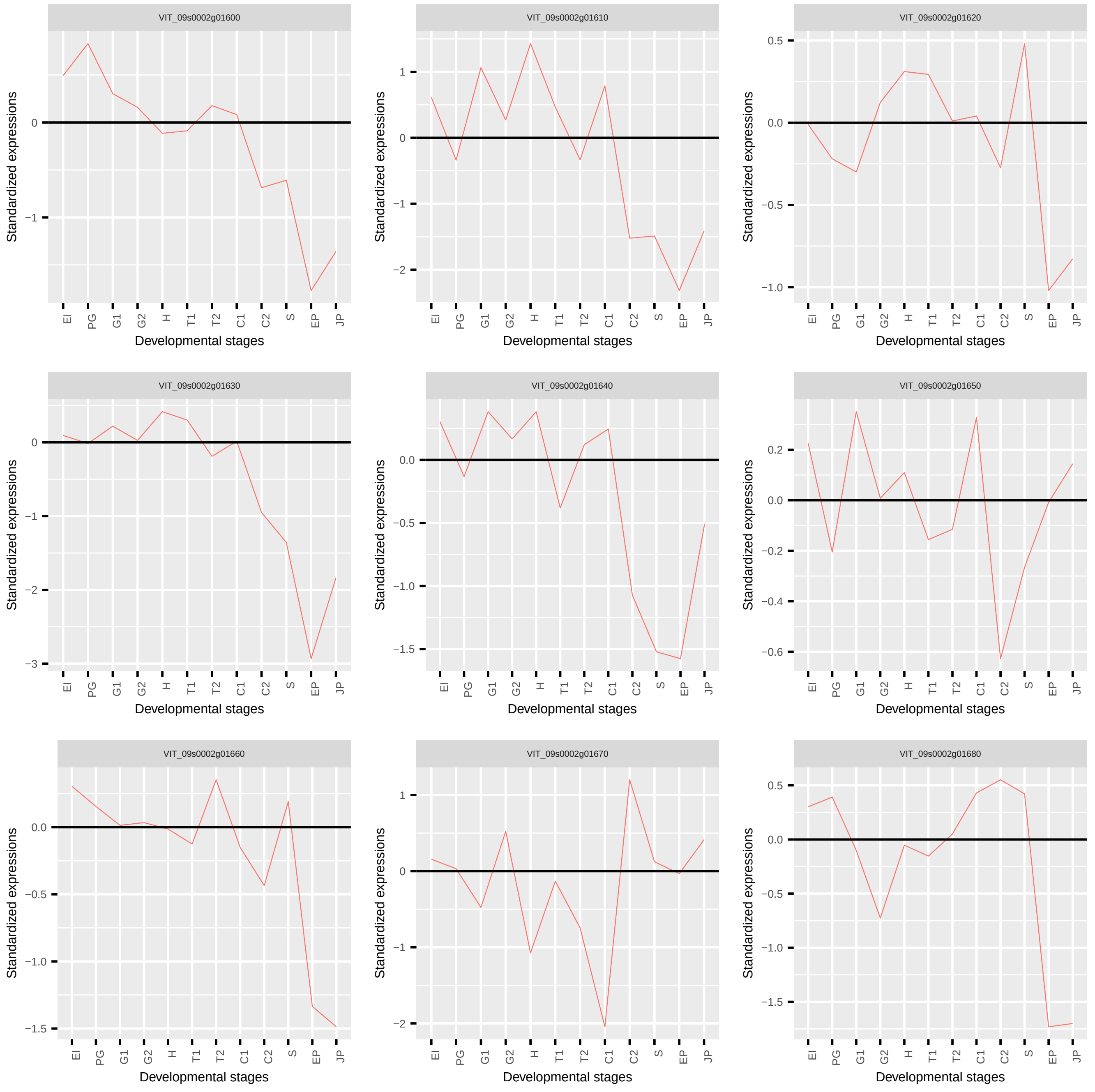

VIT_09s0002g01600
Standardized expressions
0
−1
EI PG G1 G2 H T1 T2 C1 C2 S EP JP
Developmental stages
VIT_09s0002g01610
Standardized expressions
1
0
−1
−2
EI PG G1 G2 H T1 T2 C1 C2 S EP JP
Developmental stages
VIT_09s0002g01620
Standardized expressions
0.5
0.0
−0.5
−1.0
EI PG G1 G2 H T1 T2 C1 C2 S EP JP
Developmental stages
VIT_09s0002g01630
Standardized expressions
0
−1
−2
−3
EI PG G1 G2 H T1 T2 C1 C2 S EP JP
Developmental stages
VIT_09s0002g01640
Standardized expressions
0.0
−0.5
−1.0
−1.5
EI PG G1 G2 H T1 T2 C1 C2 S EP JP
Developmental stages
VIT_09s0002g01650
Standardized expressions
0.2
0.0
−0.2
−0.4
−0.6
EI PG G1 G2 H T1 T2 C1 C2 S EP JP
Developmental stages
VIT_09s0002g01660
Standardized expressions
0.0
−0.5
−1.0
−1.5
EI PG G1 G2 H T1 T2 C1 C2 S EP JP
Developmental stages
VIT_09s0002g01670
Standardized expressions
1
0
−1
−2
EI PG G1 G2 H T1 T2 C1 C2 S EP JP
Developmental stages
VIT_09s0002g01680
Standardized expressions
0.5
0.0
−0.5
−1.0
−1.5
EI PG G1 G2 H T1 T2 C1 C2 S EP JP
Developmental stages

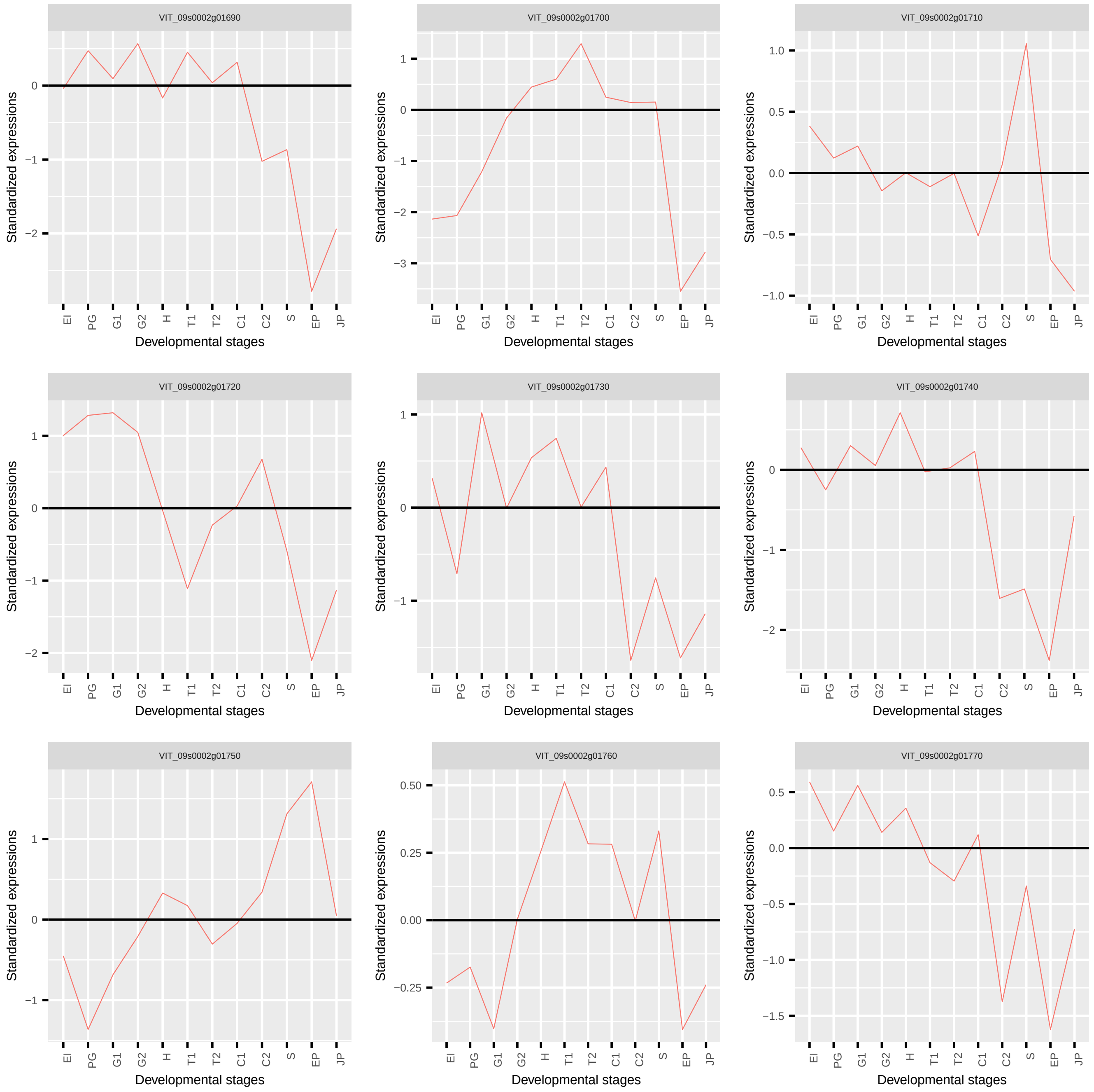
VIT_09s0002g01690
VIT_09s0002g01700
VIT_09s0002g01710
VIT_09s0002g01720
VIT_09s0002g01730
VIT_09s0002g01740
VIT_09s0002g01750
VIT_09s0002g01760
VIT_09s0002g01770
Standardized expressions
Developmental stages
EI PG G1 G2 H T1 T2 C1 C2 S EP JP

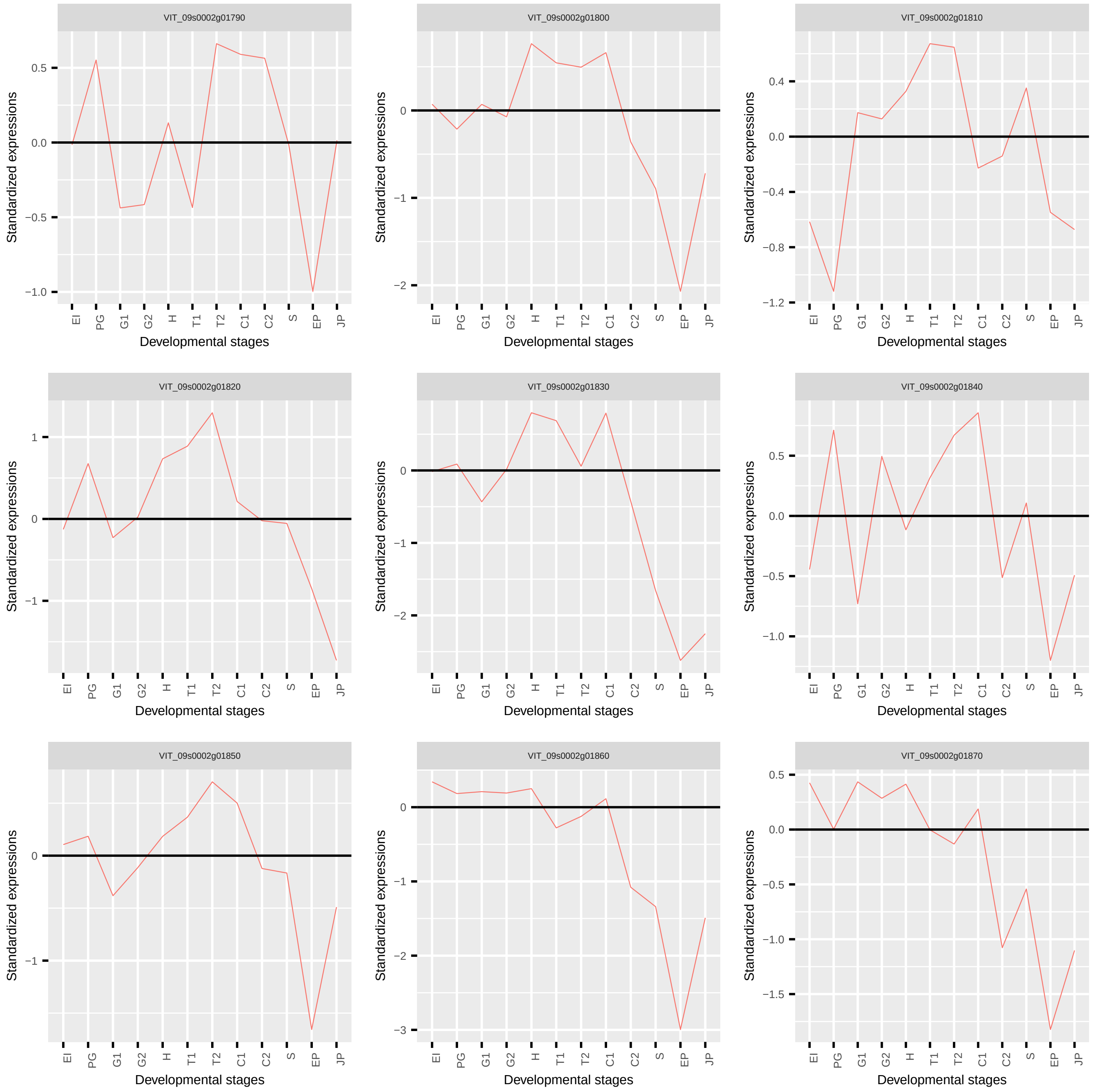
VIT_09s0002g01790
VIT_09s0002g01800
VIT_09s0002g01810
VIT_09s0002g01820
VIT_09s0002g01830
VIT_09s0002g01840
VIT_09s0002g01850
VIT_09s0002g01860
VIT_09s0002g01870
Standardized expressions
Developmental stages
EI
PG
G1
G2
H
T1
T2
C1
C2
S
EP
JP

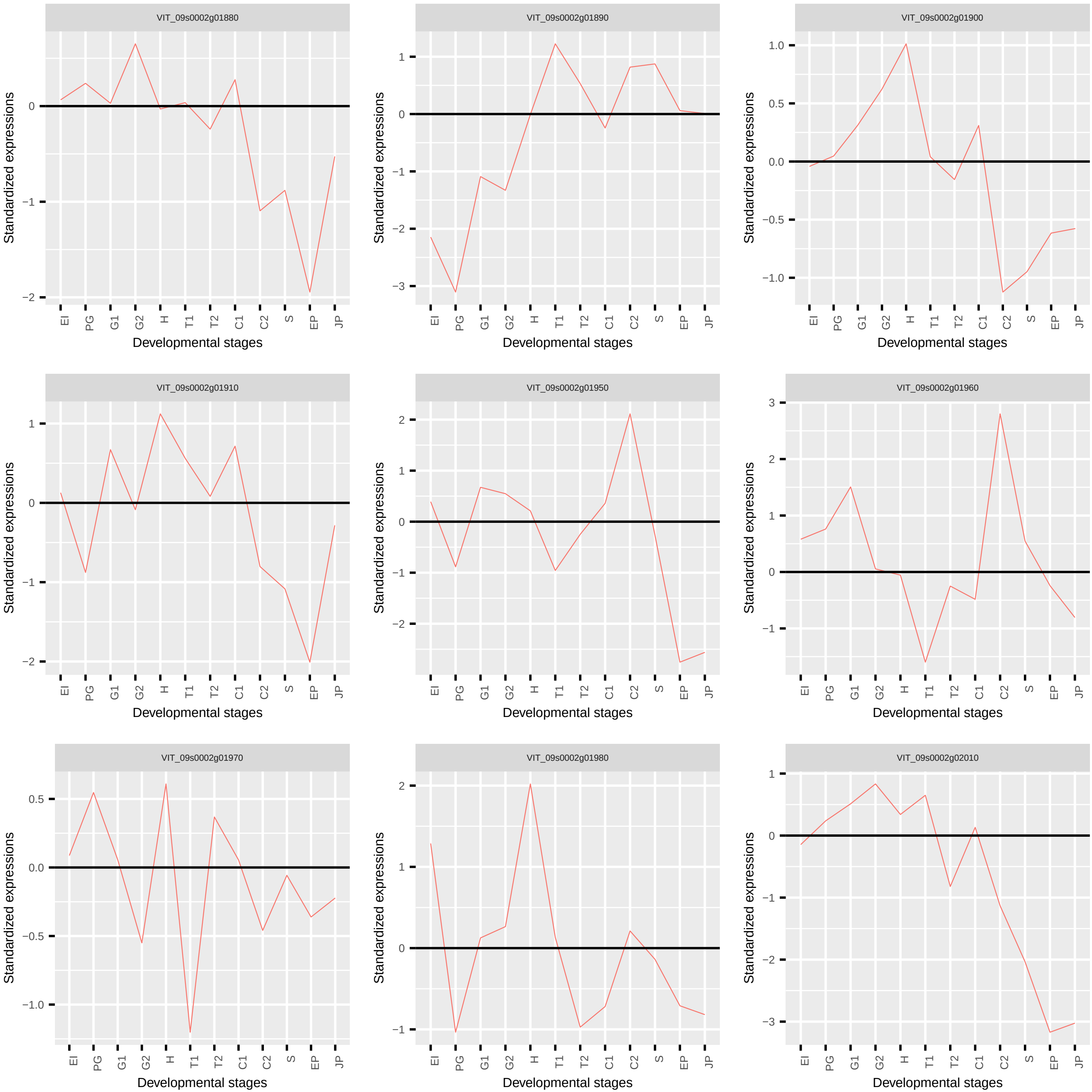

VIT_09s0002g01880
VIT_09s0002g01890
VIT_09s0002g01900
VIT_09s0002g01910
VIT_09s0002g01950
VIT_09s0002g01960
VIT_09s0002g01970
VIT_09s0002g01980
VIT_09s0002g02010
Standardized expressions
Developmental stages
EI PG G1 G2 H T1 T2 C1 C2 S EP JP

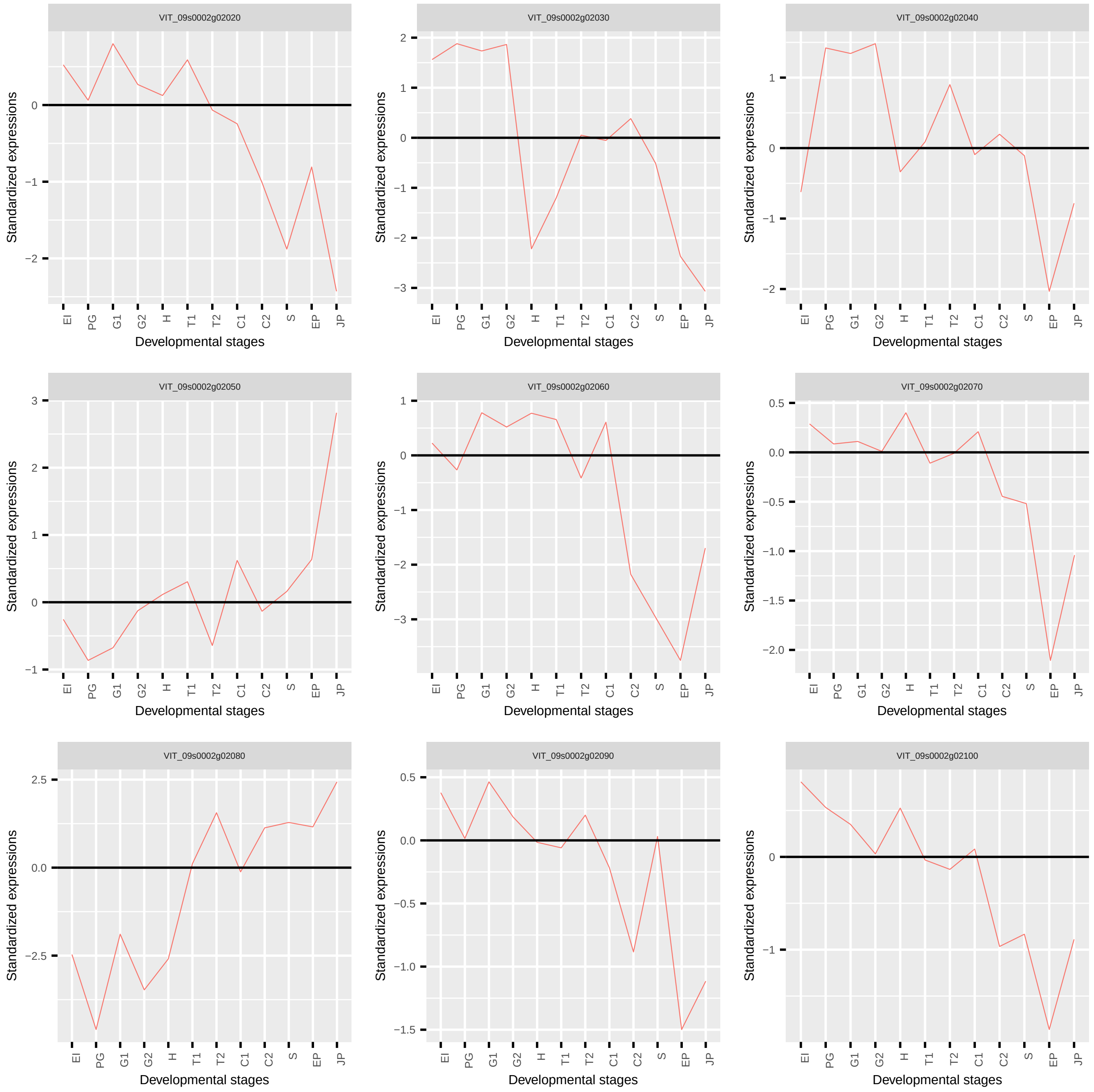
VIT_09s0002g02020
VIT_09s0002g02030
VIT_09s0002g02040
VIT_09s0002g02050
VIT_09s0002g02060
VIT_09s0002g02070
VIT_09s0002g02080
VIT_09s0002g02090
VIT_09s0002g02100
Standardized expressions
Developmental stages
EI
PG
G1
G2
H
T1
T2
C1
C2
S
EP
JP

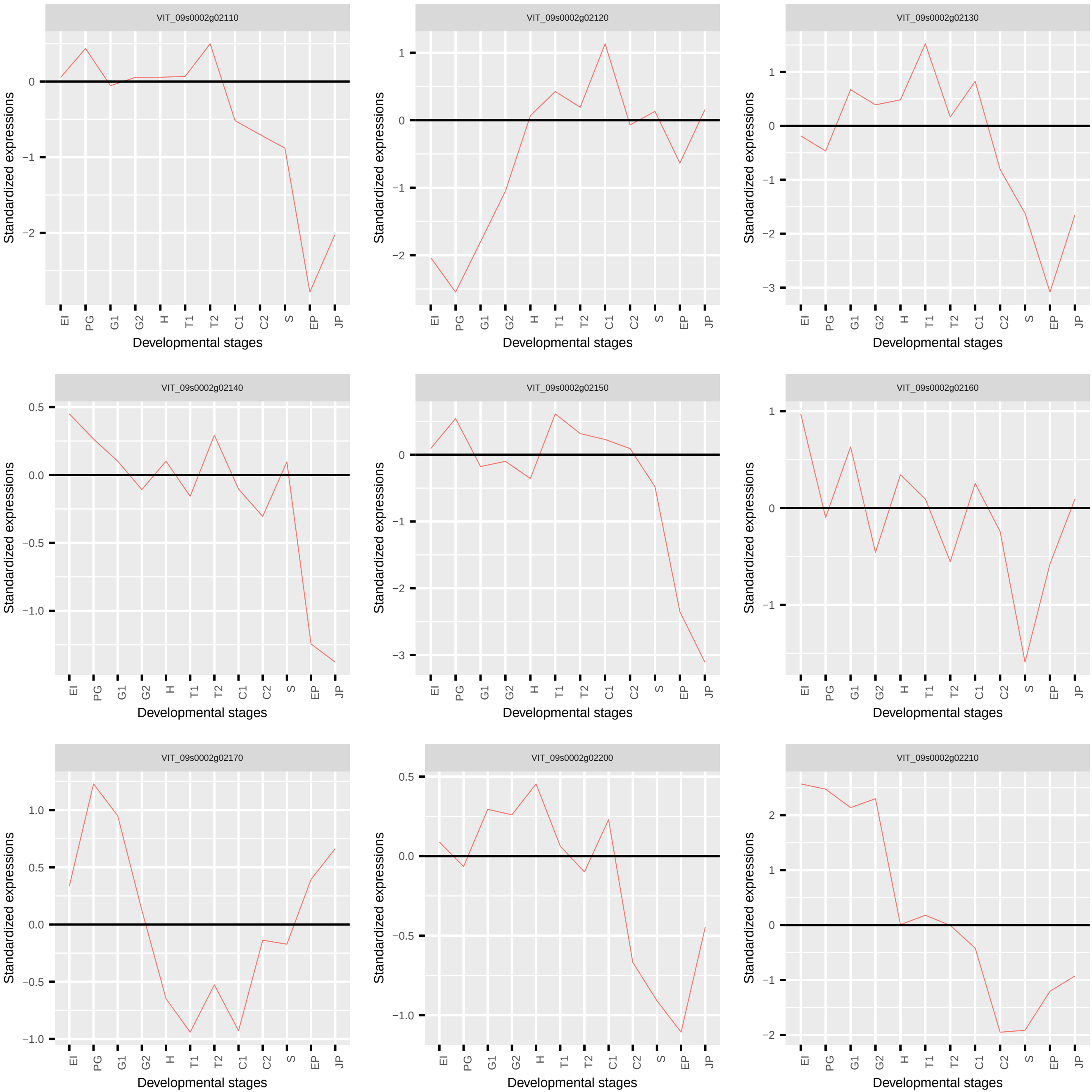

VIT_09s0002g02110
VIT_09s0002g02120
VIT_09s0002g02130
VIT_09s0002g02140
VIT_09s0002g02150
VIT_09s0002g02160
VIT_09s0002g02170
VIT_09s0002g02200
VIT_09s0002g02210
Standardized expressions
Developmental stages
EI PG G1 G2 H T1 T2 C1 C2 S EP JP

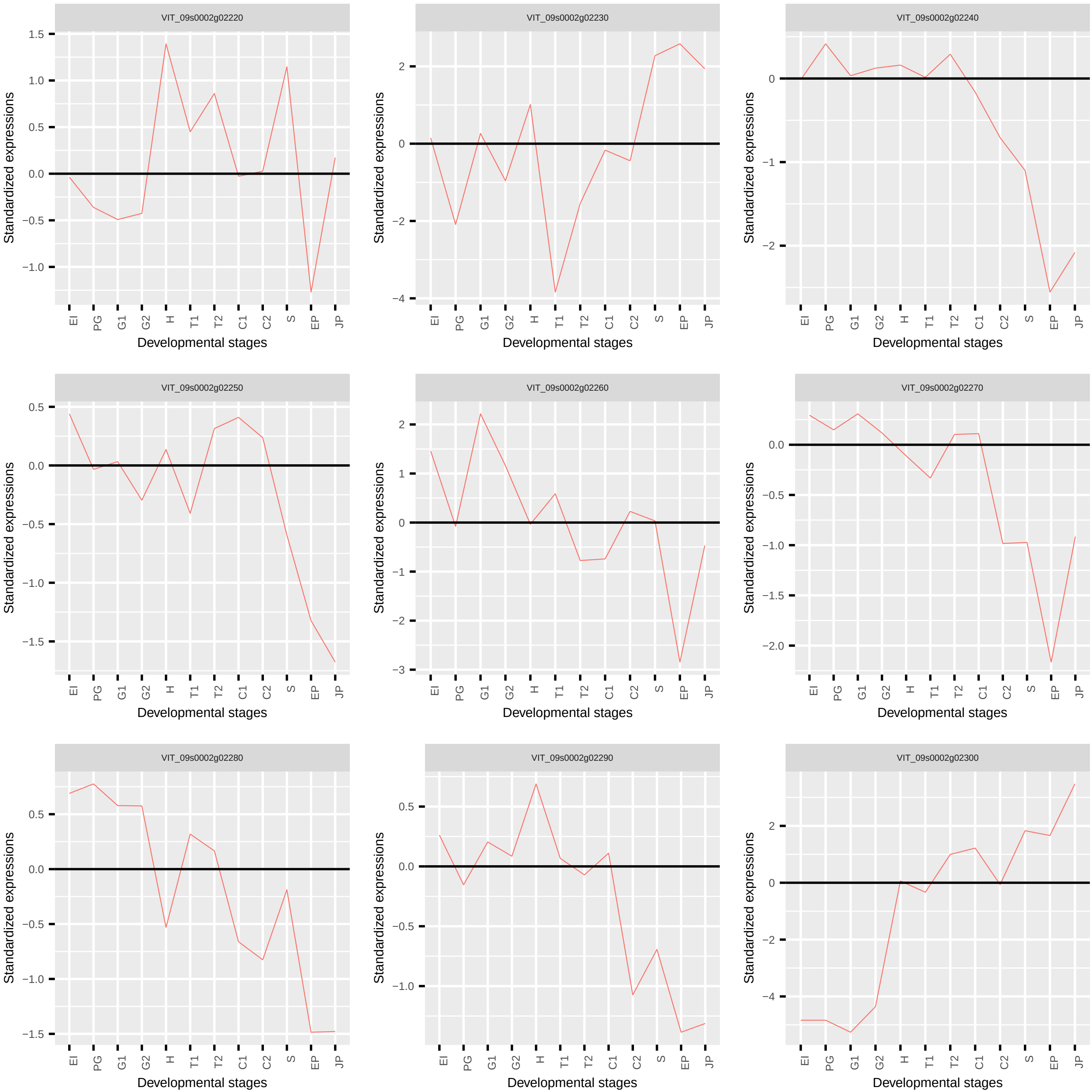
VIT_09s0002g02220
VIT_09s0002g02230
VIT_09s0002g02240
VIT_09s0002g02250
VIT_09s0002g02260
VIT_09s0002g02270
VIT_09s0002g02280
VIT_09s0002g02290
VIT_09s0002g02300
Standardized expressions
Developmental stages
EI
PG
G1
G2
H
T1
T2
C1
C2
S
EP
JP

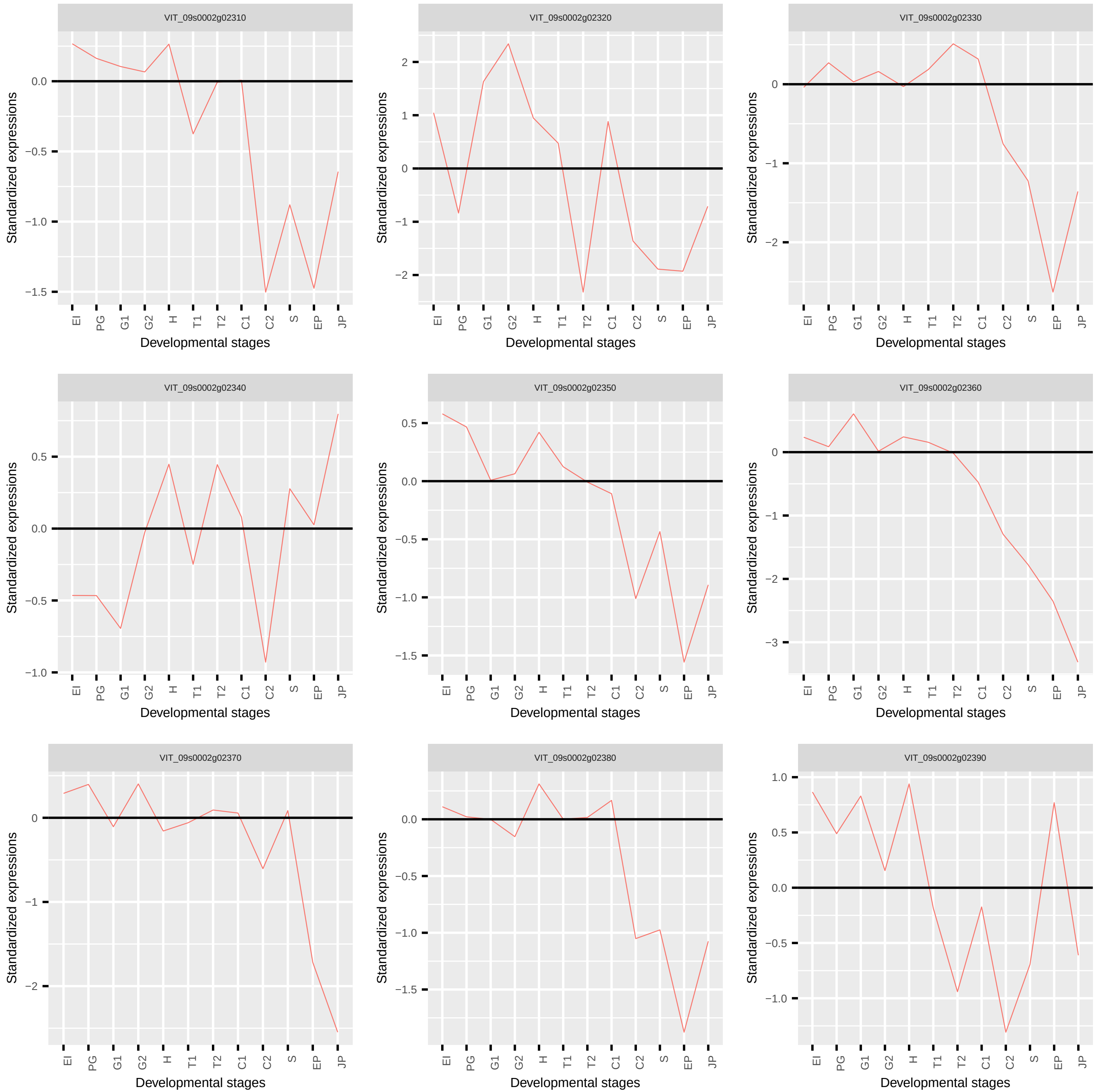
VIT_09s0002g02310
VIT_09s0002g02320
VIT_09s0002g02330
VIT_09s0002g02340
VIT_09s0002g02350
VIT_09s0002g02360
VIT_09s0002g02370
VIT_09s0002g02380
VIT_09s0002g02390
Standardized expressions
Developmental stages
EI PG G1 G2 H T1 T2 C1 C2 S EP JP

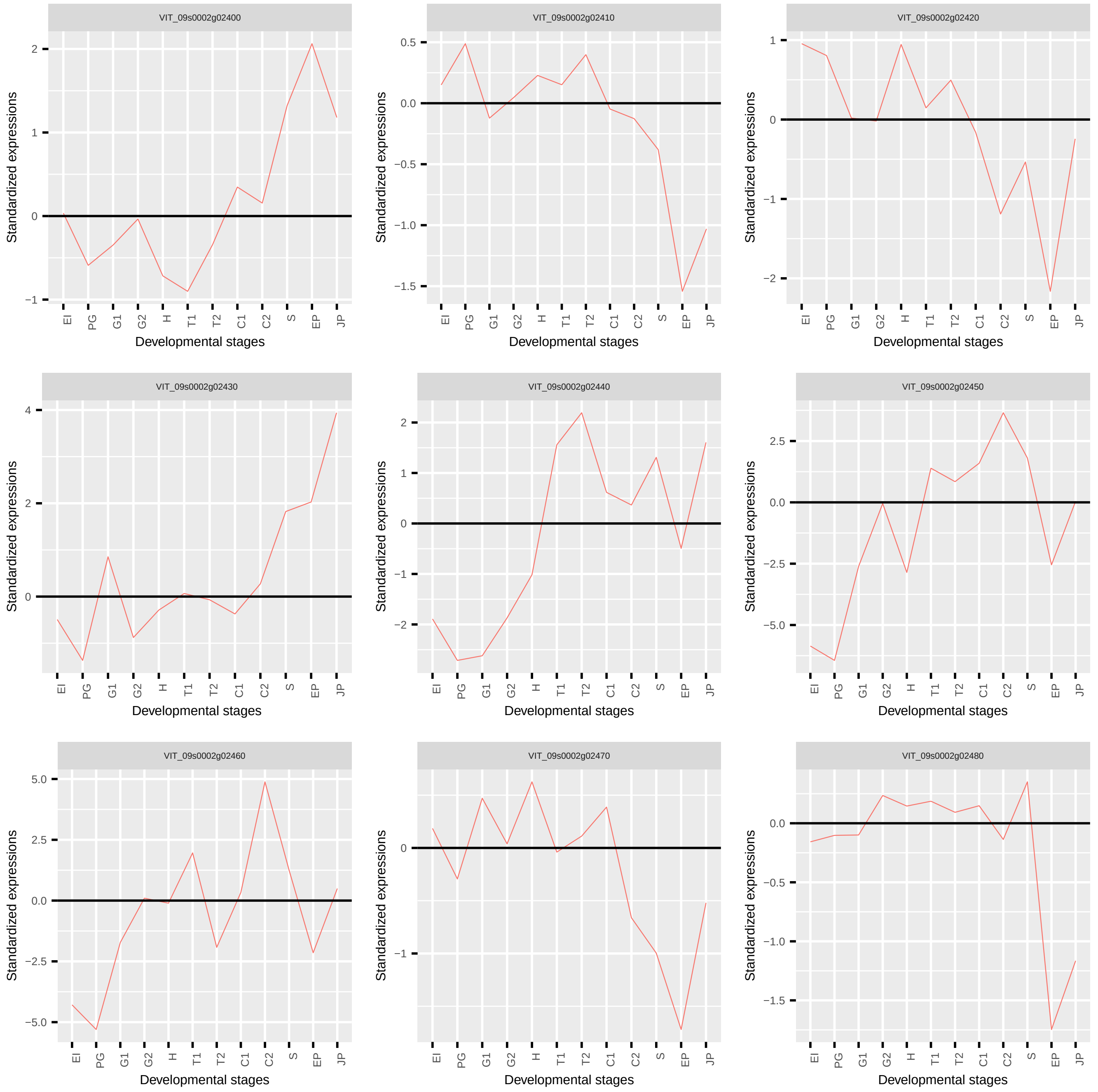
VIT_09s0002g02400
VIT_09s0002g02410
VIT_09s0002g02420
VIT_09s0002g02430
VIT_09s0002g02440
VIT_09s0002g02450
VIT_09s0002g02460
VIT_09s0002g02470
VIT_09s0002g02480
Standardized expressions
Developmental stages
EI PG G1 G2 H T1 T2 C1 C2 S EP JP

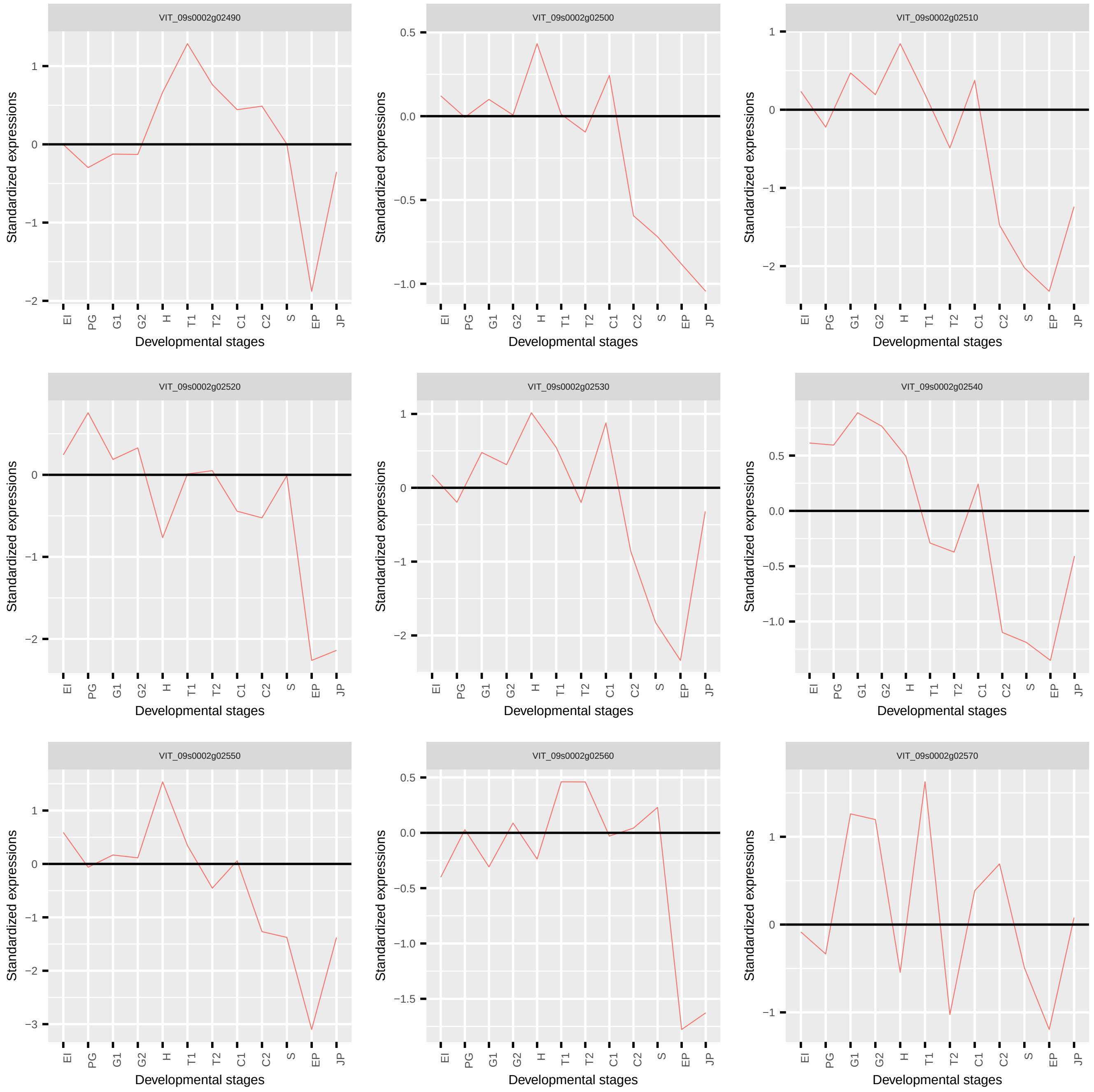
VIT_09s0002g02490
VIT_09s0002g02500
VIT_09s0002g02510
VIT_09s0002g02520
VIT_09s0002g02530
VIT_09s0002g02540
VIT_09s0002g02550
VIT_09s0002g02560
VIT_09s0002g02570
Standardized expressions
Developmental stages
EI PG G1 G2 H T1 T2 C1 C2 S EP JP

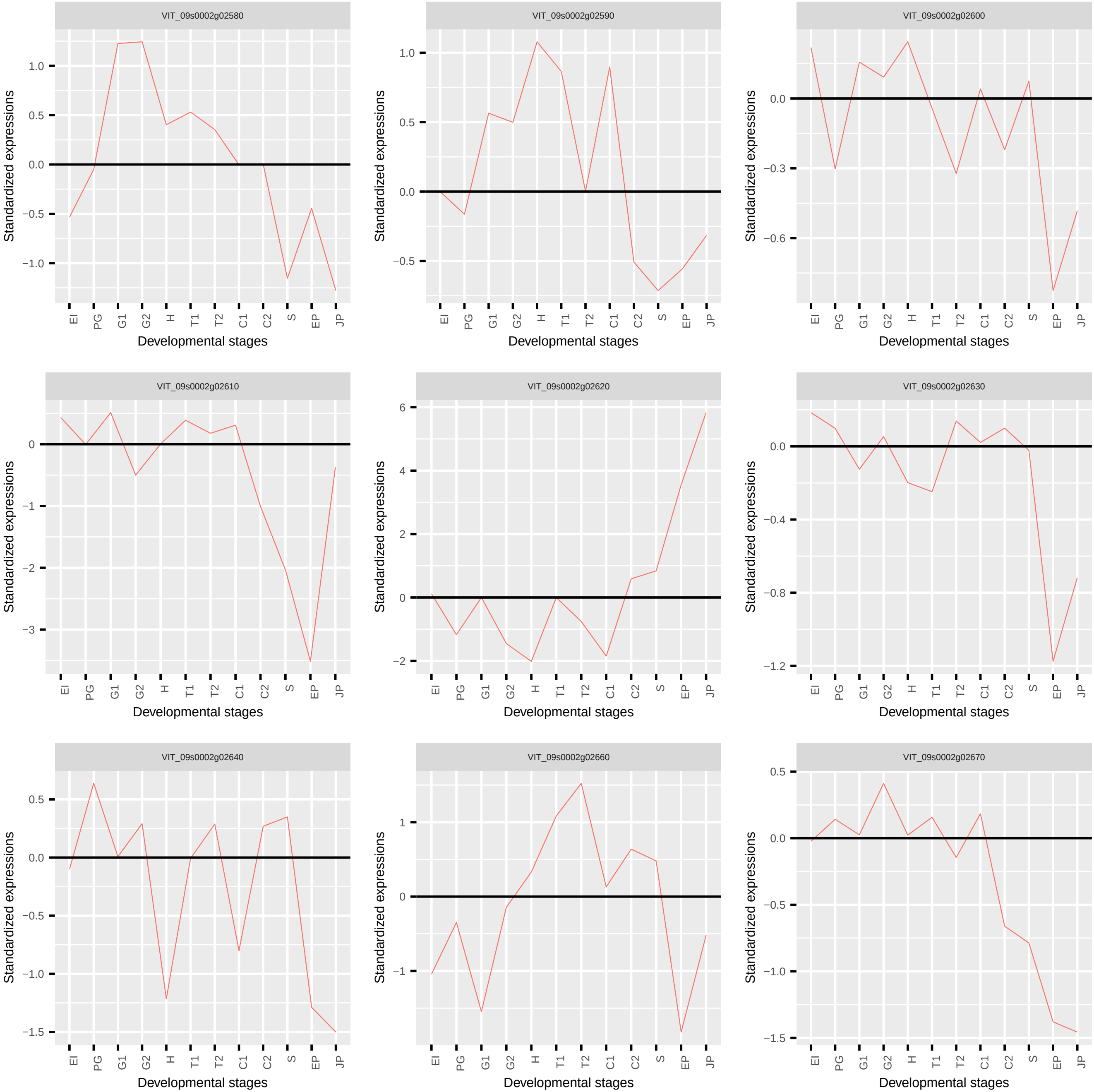

VIT_09s0002g02580
VIT_09s0002g02590
VIT_09s0002g02600
VIT_09s0002g02610
VIT_09s0002g02620
VIT_09s0002g02630
VIT_09s0002g02640
VIT_09s0002g02660
VIT_09s0002g02670
Standardized expressions
Developmental stages
EI PG G1 G2 H T1 T2 C1 C2 S EP JP

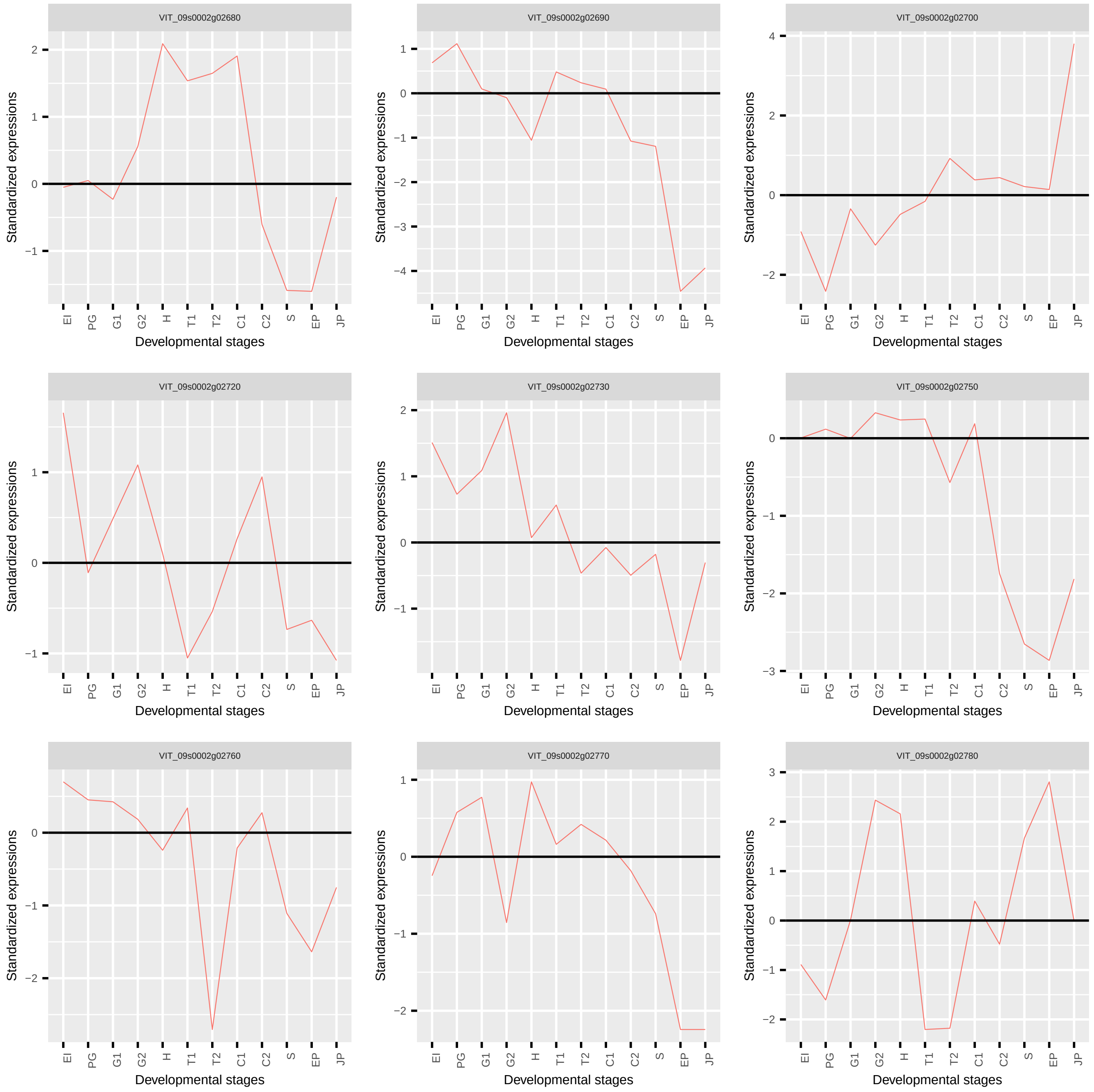
VIT_09s0002g02680
VIT_09s0002g02690
VIT_09s0002g02700
VIT_09s0002g02720
VIT_09s0002g02730
VIT_09s0002g02750
VIT_09s0002g02760
VIT_09s0002g02770
VIT_09s0002g02780
Standardized expressions
Developmental stages
EI PG G1 G2 H T1 T2 C1 C2 S EP JP

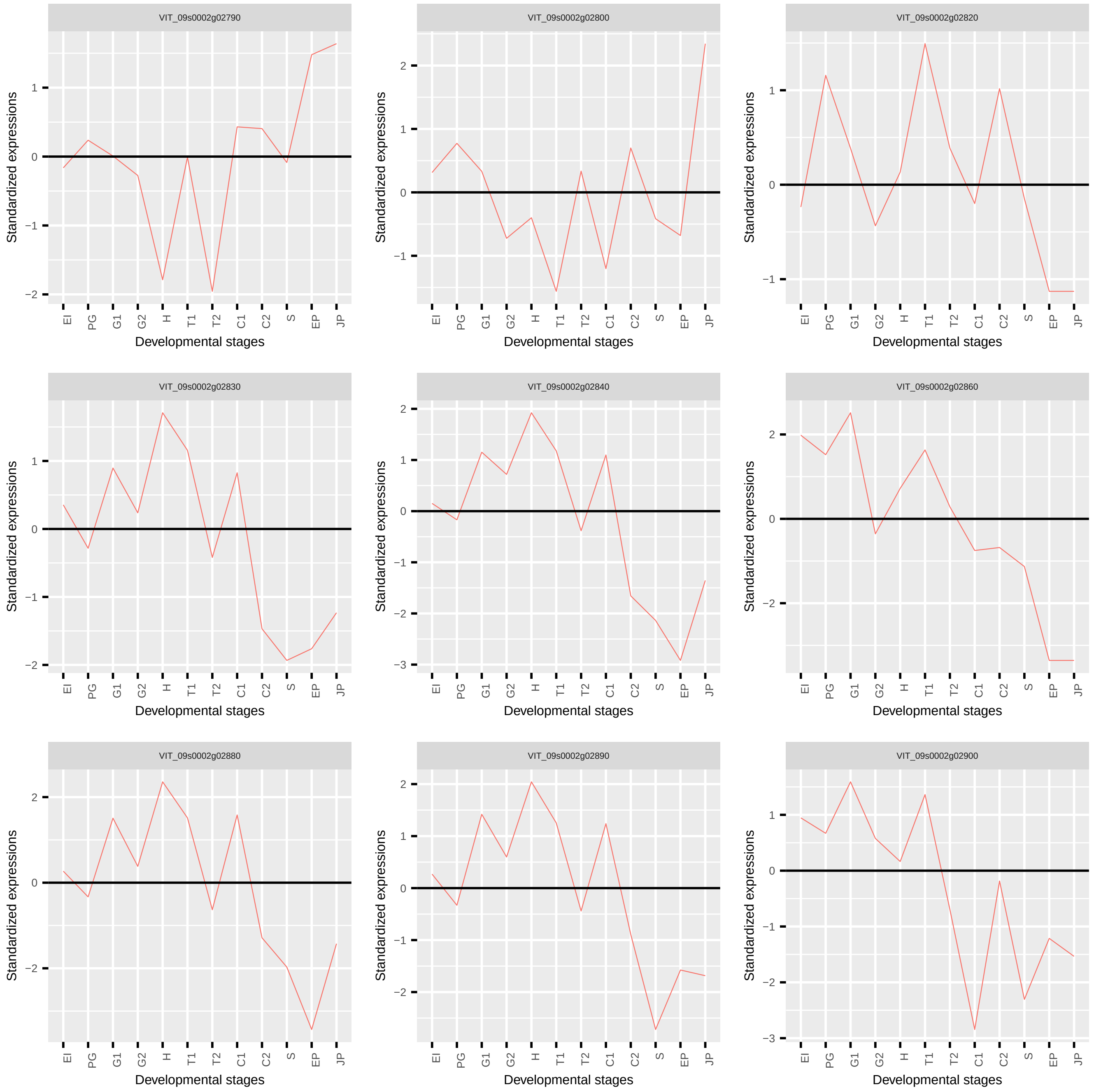
VIT_09s0002g02790
VIT_09s0002g02800
VIT_09s0002g02820
VIT_09s0002g02830
VIT_09s0002g02840
VIT_09s0002g02860
VIT_09s0002g02880
VIT_09s0002g02890
VIT_09s0002g02900
Standardized expressions
Developmental stages
EI PG G1 G2 H T1 T2 C1 C2 S EP JP

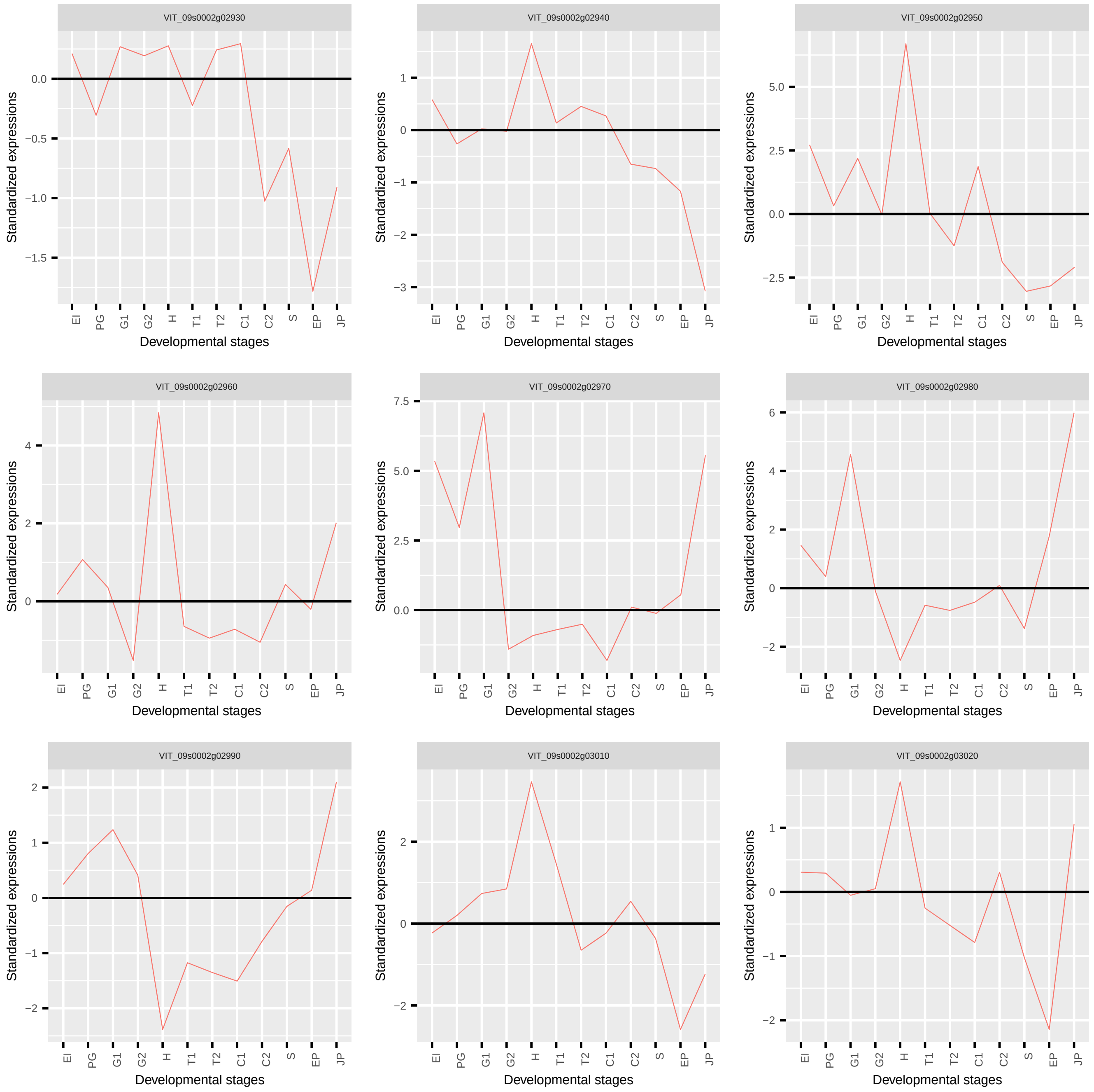
VIT_09s0002g02930
0.0
−0.5
−1.0
−1.5
Standardized expressions
EI PG G1 G2 H T1 T2 C1 C2 S EP JP
Developmental stages
VIT_09s0002g02940
1
0
−1
−2
−3
Standardized expressions
EI PG G1 G2 H T1 T2 C1 C2 S EP JP
Developmental stages
VIT_09s0002g02950
5.0
2.5
0.0
−2.5
Standardized expressions
EI PG G1 G2 H T1 T2 C1 C2 S EP JP
Developmental stages
VIT_09s0002g02960
4
2
0
Standardized expressions
EI PG G1 G2 H T1 T2 C1 C2 S EP JP
Developmental stages
VIT_09s0002g02970
7.5
5.0
2.5
0.0
Standardized expressions
EI PG G1 G2 H T1 T2 C1 C2 S EP JP
Developmental stages
VIT_09s0002g02980
6
4
2
0
−2
Standardized expressions
EI PG G1 G2 H T1 T2 C1 C2 S EP JP
Developmental stages
VIT_09s0002g02990
2
1
0
−1
−2
Standardized expressions
EI PG G1 G2 H T1 T2 C1 C2 S EP JP
Developmental stages
VIT_09s0002g03010
2
0
−2
Standardized expressions
EI PG G1 G2 H T1 T2 C1 C2 S EP JP
Developmental stages
VIT_09s0002g03020
1
0
−1
−2
Standardized expressions
EI PG G1 G2 H T1 T2 C1 C2 S EP JP
Developmental stages

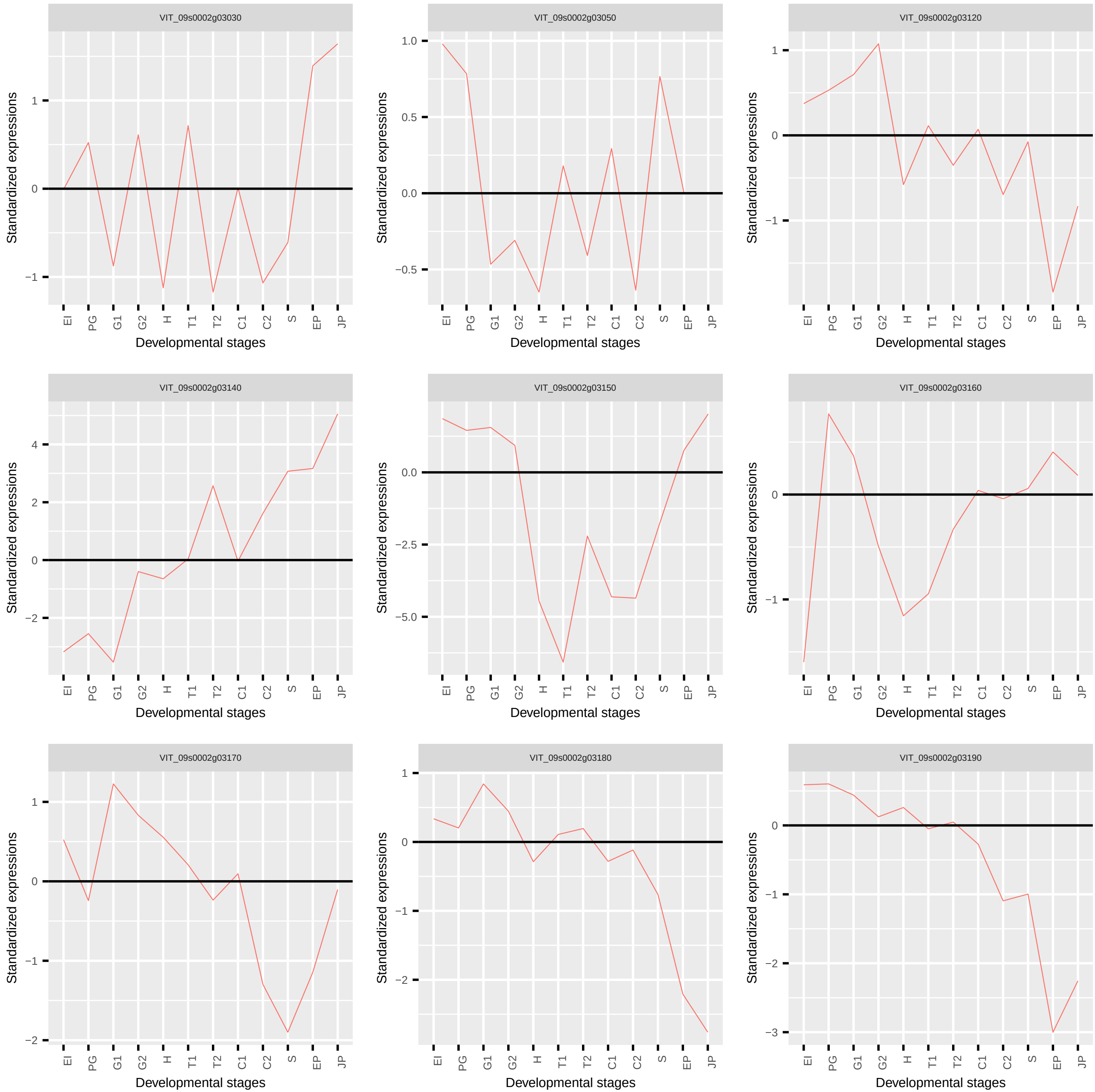
VIT_09s0002g03030
VIT_09s0002g03050
VIT_09s0002g03120
VIT_09s0002g03140
VIT_09s0002g03150
VIT_09s0002g03160
VIT_09s0002g03170
VIT_09s0002g03180
VIT_09s0002g03190
Standardized expressions
Developmental stages
EI
PG
G1
G2
H
T1
T2
C1
C2
S
EP
JP
1
0
−1
1.0
0.5
0.0
−0.5
4
2
−2
−2.5
−5.0
−3

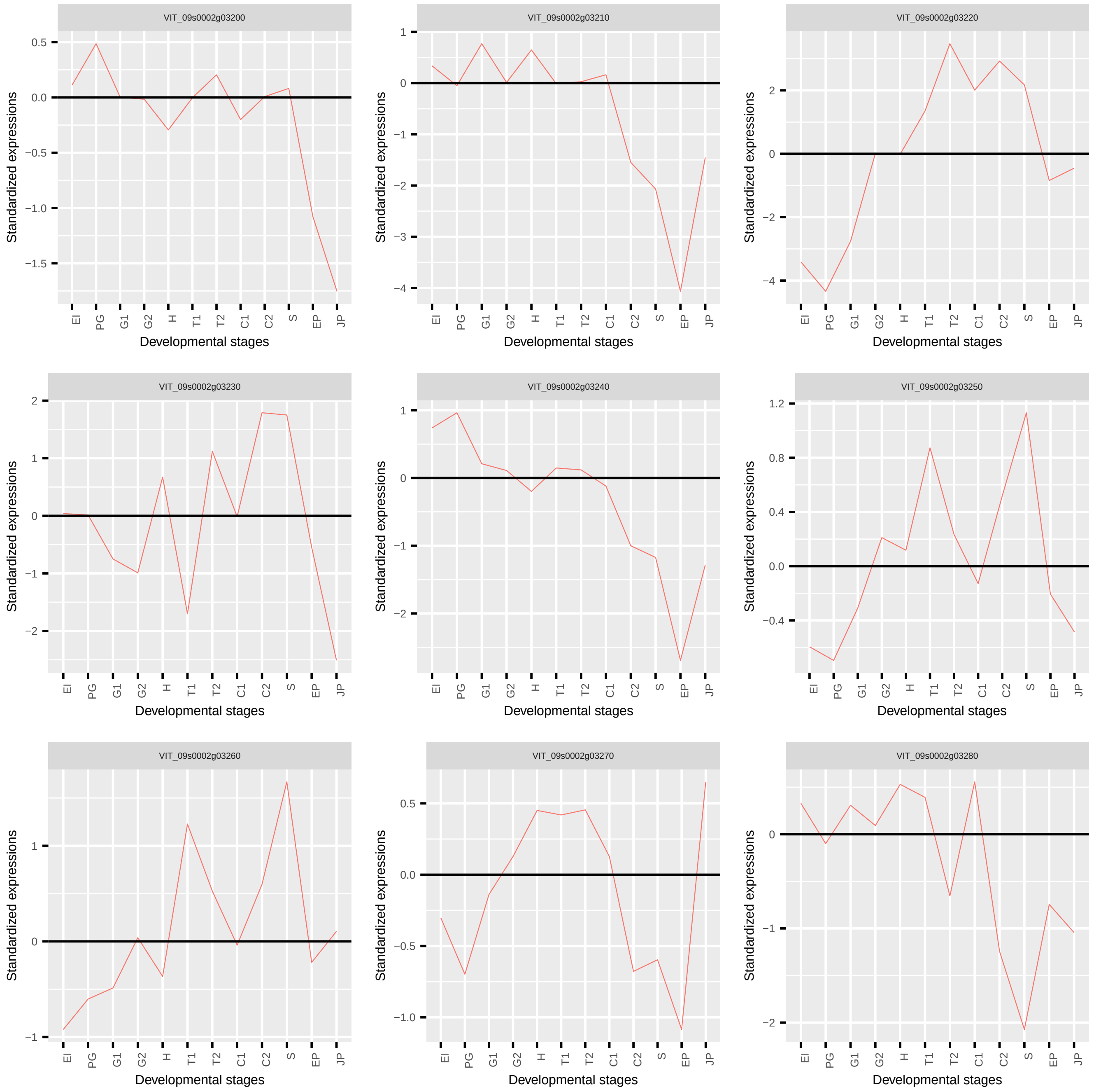
VIT_09s0002g03200
VIT_09s0002g03210
VIT_09s0002g03220
VIT_09s0002g03230
VIT_09s0002g03240
VIT_09s0002g03250
VIT_09s0002g03260
VIT_09s0002g03270
VIT_09s0002g03280
Standardized expressions
Developmental stages
EI PG G1 G2 H T1 T2 C1 C2 S EP JP

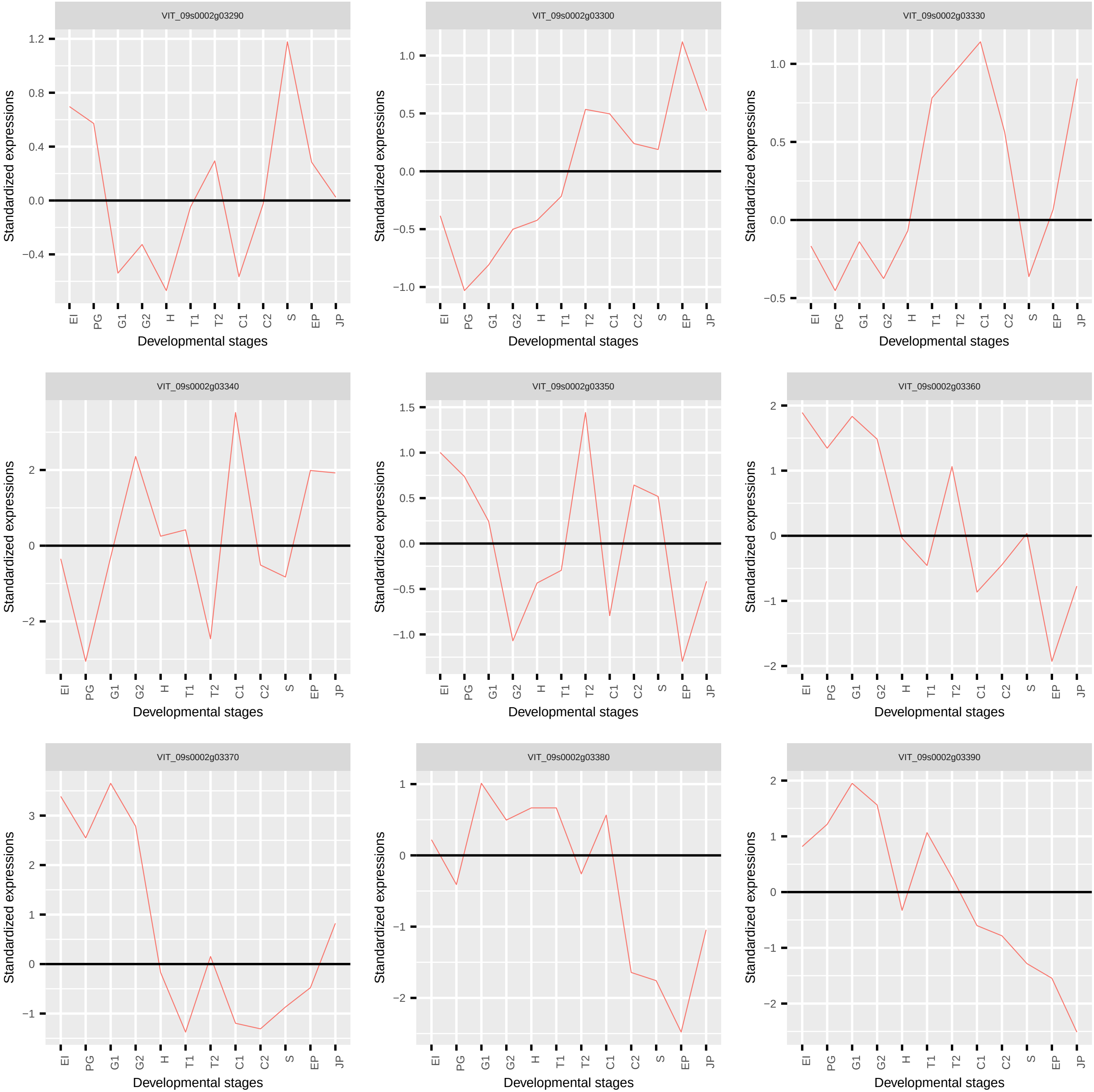
VIT_09s0002g03290
VIT_09s0002g03300
VIT_09s0002g03330
VIT_09s0002g03340
VIT_09s0002g03350
VIT_09s0002g03360
VIT_09s0002g03370
VIT_09s0002g03380
VIT_09s0002g03390
Standardized expressions
Developmental stages
EI
PG
G1
G2
H
T1
T2
C1
C2
S
EP
JP
1.2
0.8
0.4
0.0
-0.4
1.0
0.5
0.0
−0.5
−1.0
1.5
2
1
0
−1
−2
3

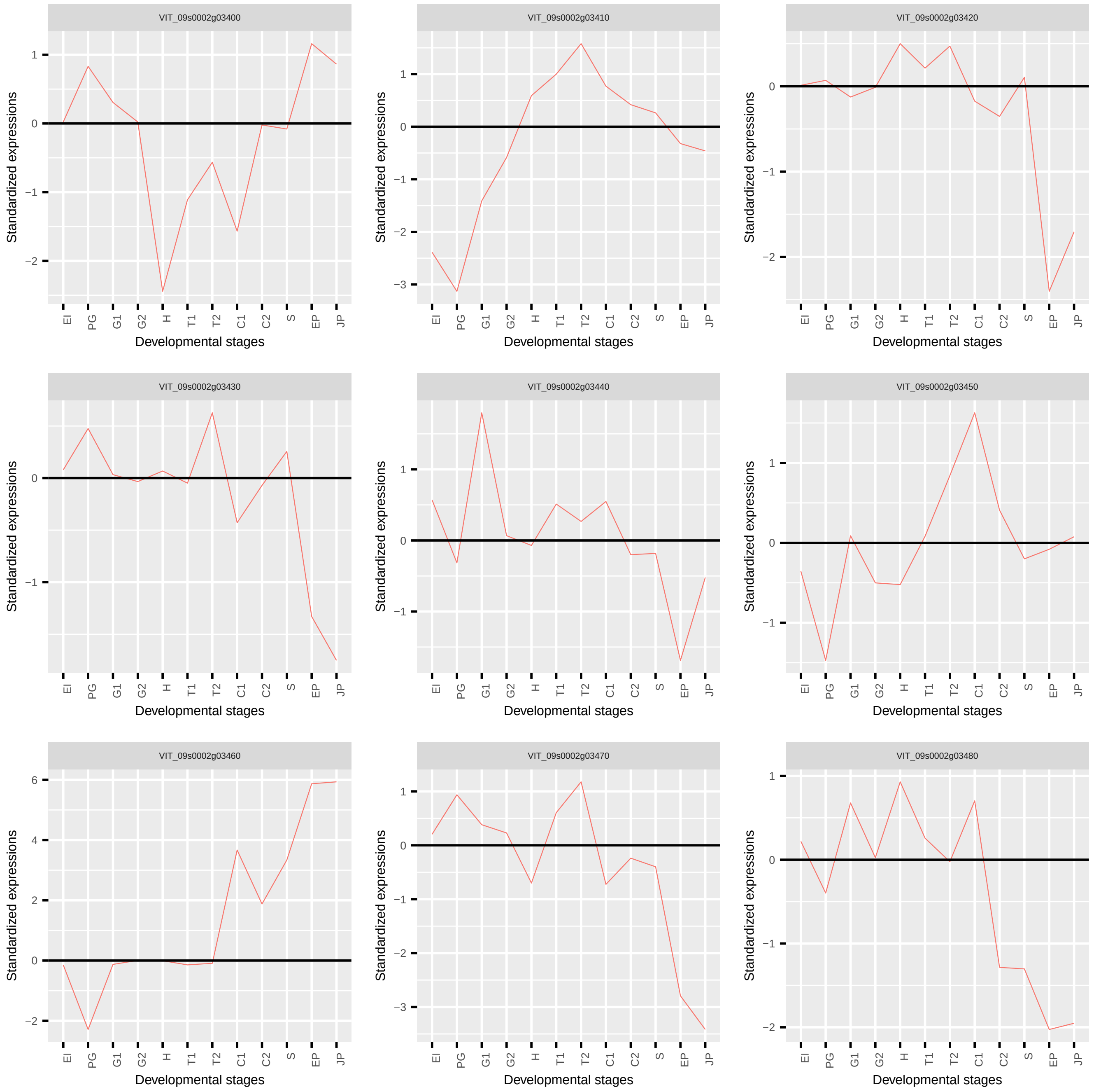
VIT_09s0002g03400
VIT_09s0002g03410
VIT_09s0002g03420
VIT_09s0002g03430
VIT_09s0002g03440
VIT_09s0002g03450
VIT_09s0002g03460
VIT_09s0002g03470
VIT_09s0002g03480
Standardized expressions
Developmental stages
EI PG G1 G2 H T1 T2 C1 C2 S EP JP

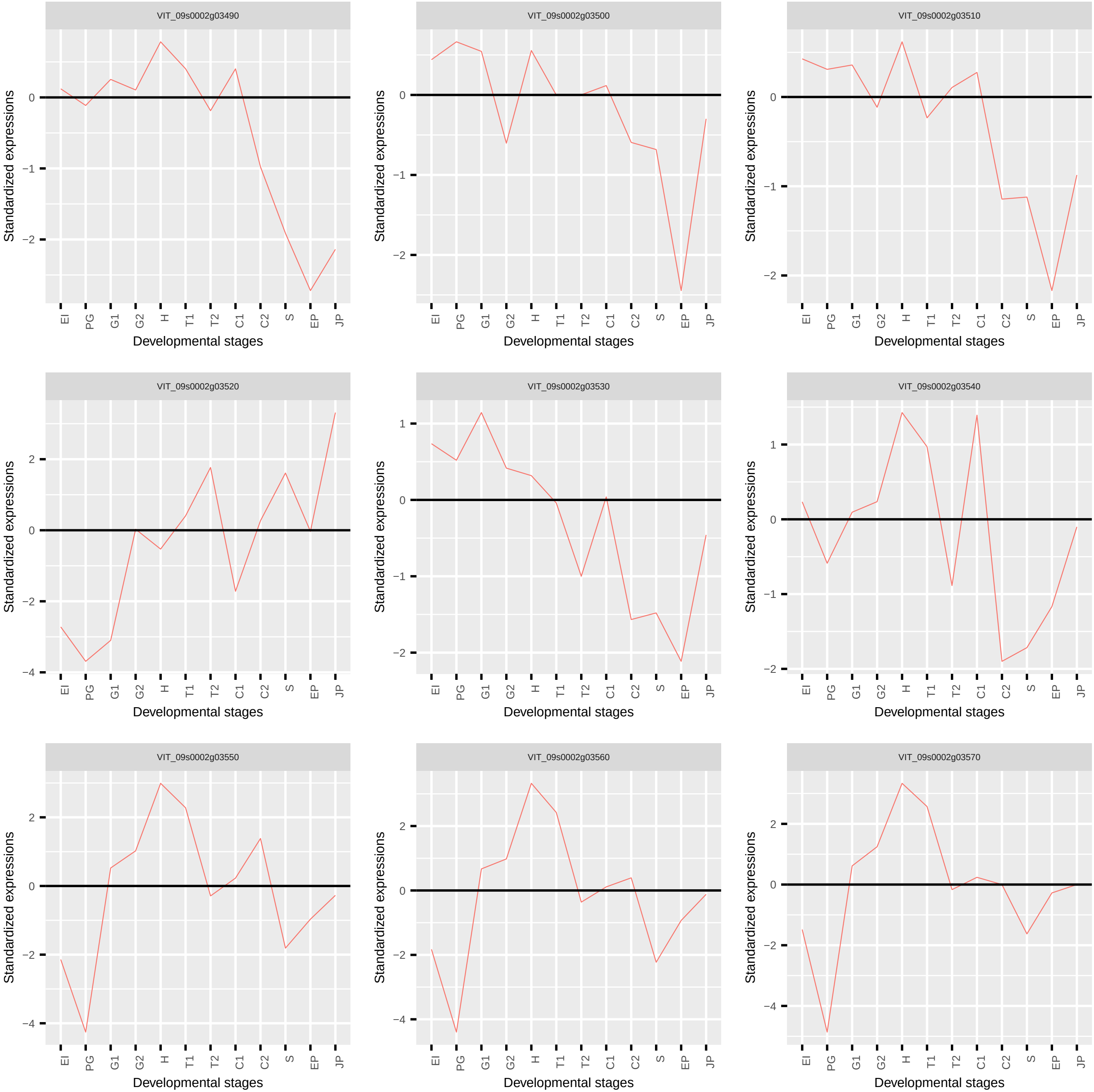

VIT_09s0002g03490
VIT_09s0002g03500
VIT_09s0002g03510
VIT_09s0002g03520
VIT_09s0002g03530
VIT_09s0002g03540
VIT_09s0002g03550
VIT_09s0002g03560
VIT_09s0002g03570
Standardized expressions
Developmental stages
EI PG G1 G2 H T1 T2 C1 C2 S EP JP

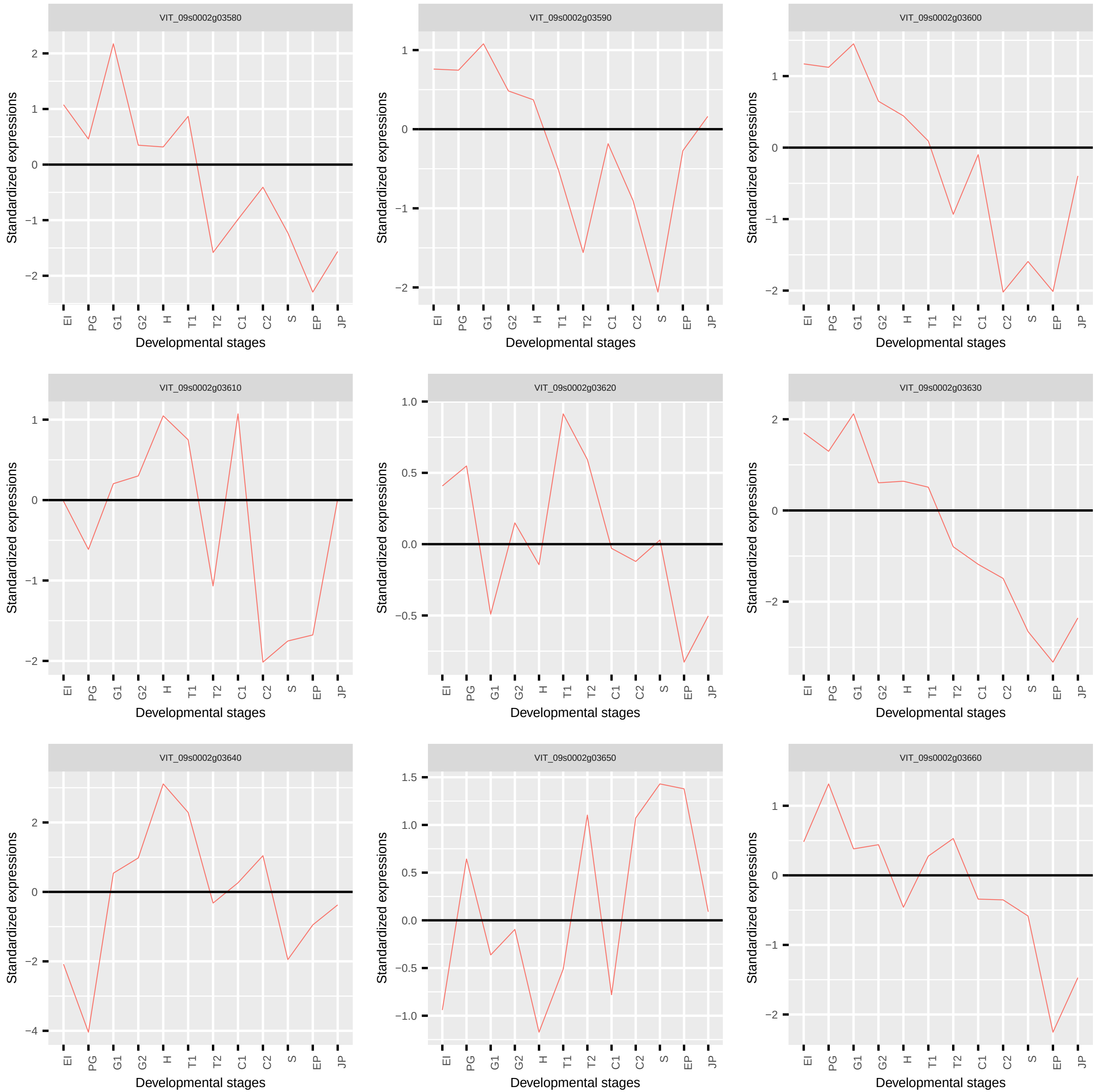

VIT_09s0002g03580
VIT_09s0002g03590
VIT_09s0002g03600
VIT_09s0002g03610
VIT_09s0002g03620
VIT_09s0002g03630
VIT_09s0002g03640
VIT_09s0002g03650
VIT_09s0002g03660
Standardized expressions
Developmental stages
EI
PG
G1
G2
H
T1
T2
C1
C2
S
EP
JP

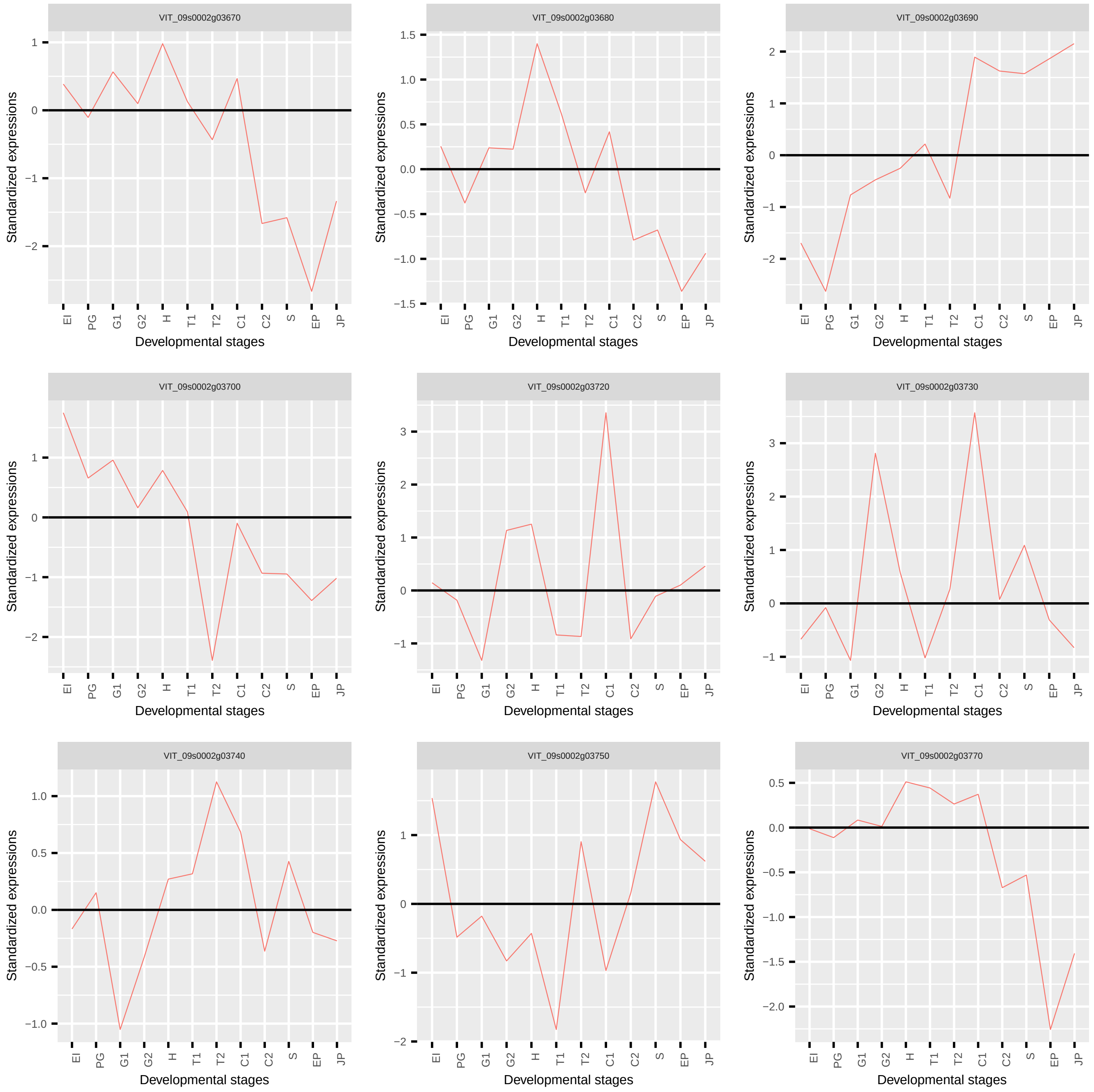
VIT_09s0002g03670
VIT_09s0002g03680
VIT_09s0002g03690
VIT_09s0002g03700
VIT_09s0002g03720
VIT_09s0002g03730
VIT_09s0002g03740
VIT_09s0002g03750
VIT_09s0002g03770
Standardized expressions
Developmental stages
EI PG G1 G2 H T1 T2 C1 C2 S EP JP

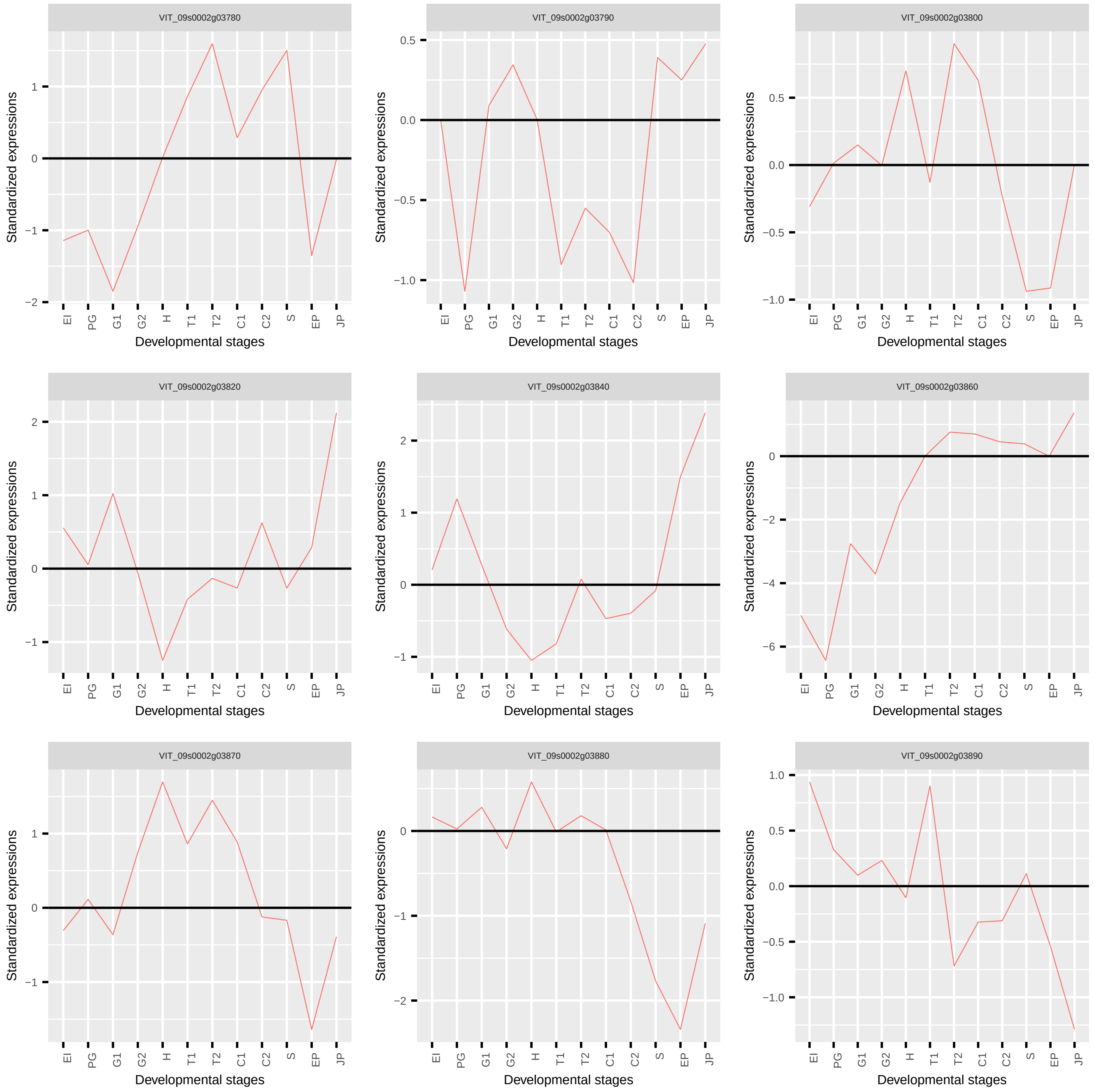
VIT_09s0002g03780
VIT_09s0002g03790
VIT_09s0002g03800
VIT_09s0002g03820
VIT_09s0002g03840
VIT_09s0002g03860
VIT_09s0002g03870
VIT_09s0002g03880
VIT_09s0002g03890
Standardized expressions
Developmental stages
EI PG G1 G2 H T1 T2 C1 C2 S EP JP

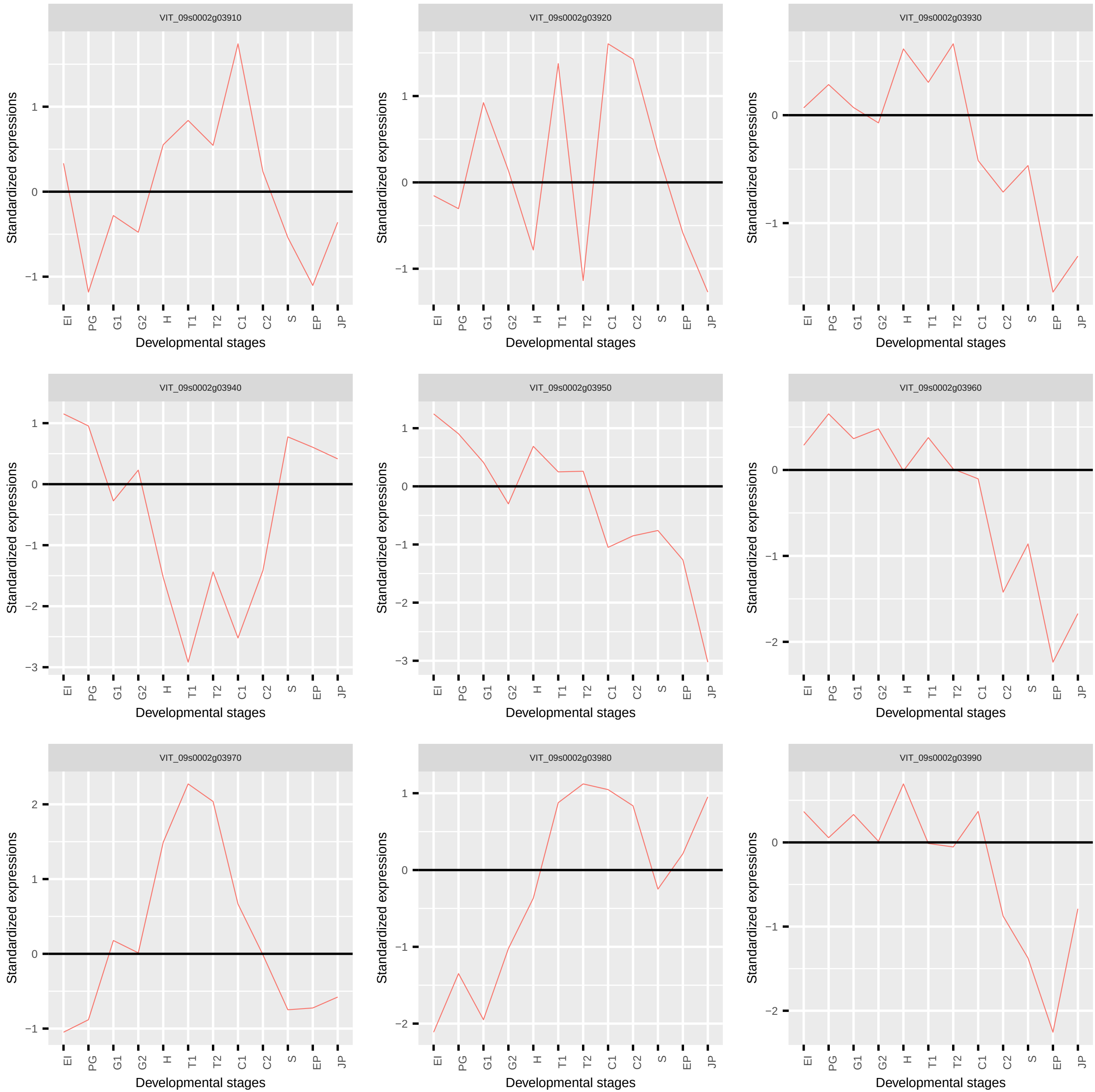
VIT_09s0002g03910
VIT_09s0002g03920
VIT_09s0002g03930
VIT_09s0002g03940
VIT_09s0002g03950
VIT_09s0002g03960
VIT_09s0002g03970
VIT_09s0002g03980
VIT_09s0002g03990
Standardized expressions
EI PG G1 G2 H T1 T2 C1 C2 S EP JP
Developmental stages

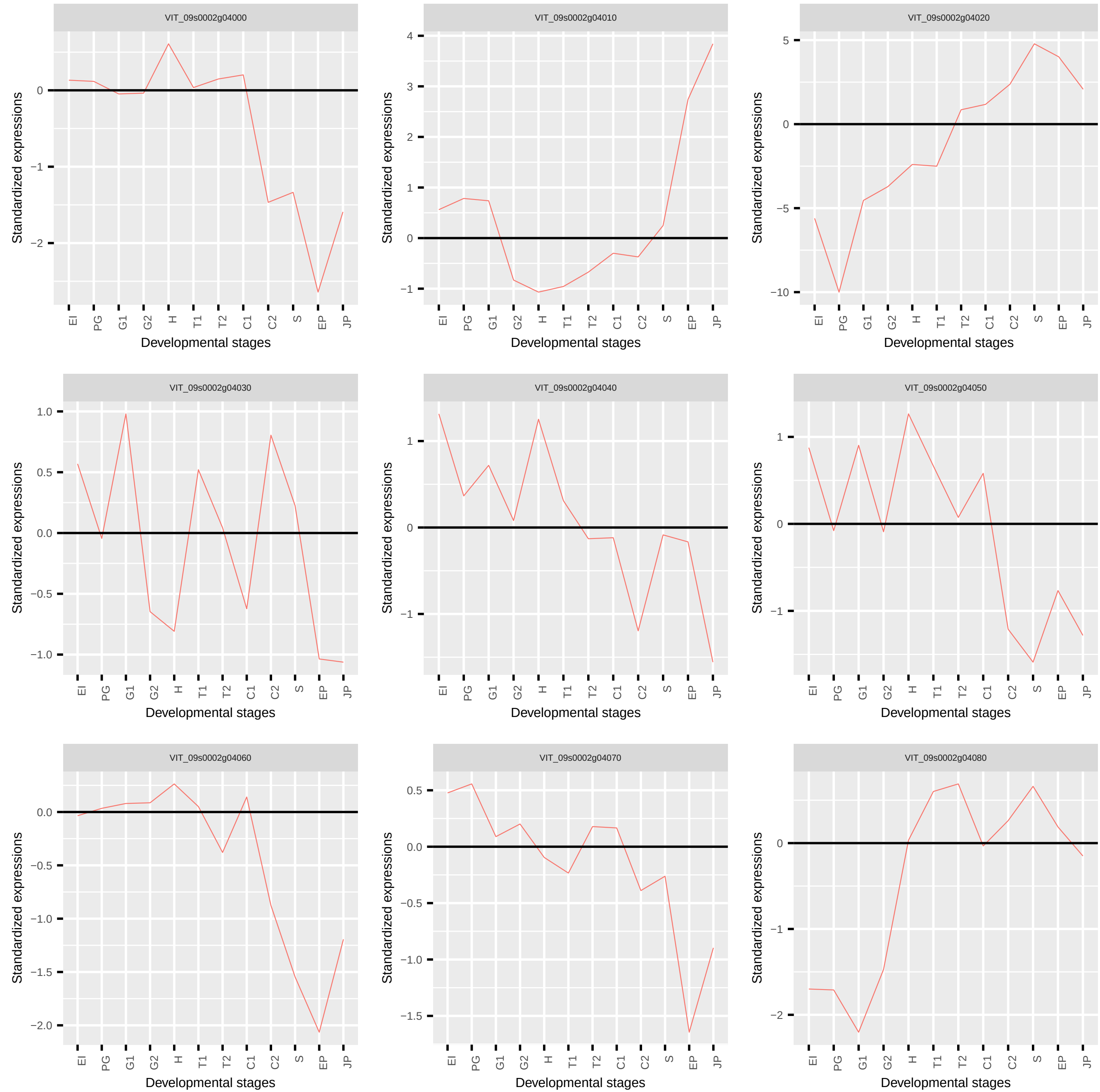
VIT_09s0002g04000
0
−1
−2
VIT_09s0002g04010
4
3
2
1
0
−1
VIT_09s0002g04020
5
0
−5
−10
VIT_09s0002g04030
1.0
0.5
0.0
−0.5
−1.0
VIT_09s0002g04040
1
0
−1
VIT_09s0002g04050
1
0
−1
VIT_09s0002g04060
0.0
−0.5
−1.0
−1.5
−2.0
VIT_09s0002g04070
0.5
0.0
−0.5
−1.0
−1.5
VIT_09s0002g04080
0
−1
−2
Standardized expressions
EI
PG
G1
G2
H
T1
T2
C1
C2
S
EP
JP
Developmental stages

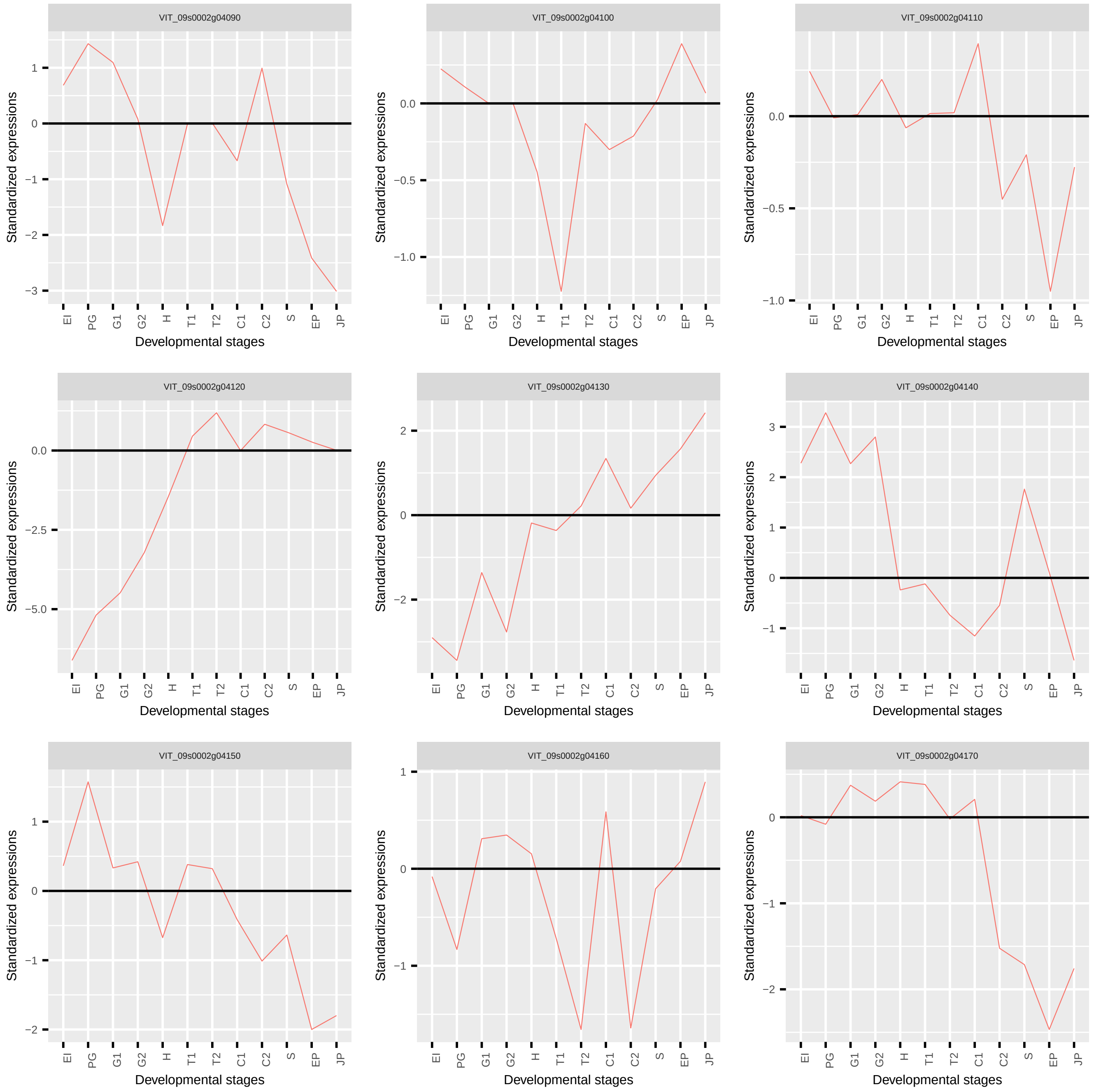
VIT_09s0002g04090
VIT_09s0002g04100
VIT_09s0002g04110
VIT_09s0002g04120
VIT_09s0002g04130
VIT_09s0002g04140
VIT_09s0002g04150
VIT_09s0002g04160
VIT_09s0002g04170
Standardized expressions
Developmental stages
EI PG G1 G2 H T1 T2 C1 C2 S EP JP

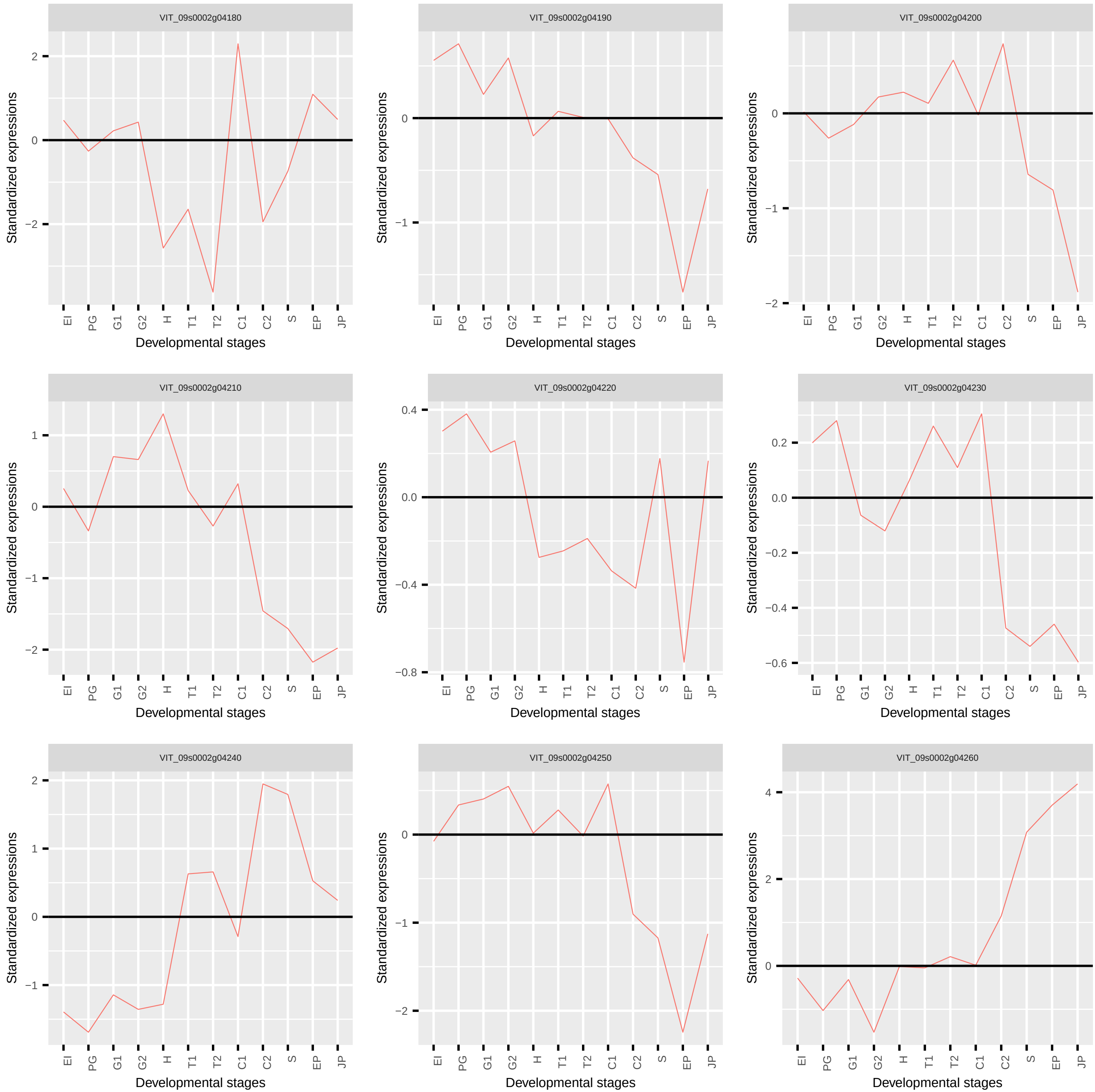
VIT_09s0002g04180
VIT_09s0002g04190
VIT_09s0002g04200
VIT_09s0002g04210
VIT_09s0002g04220
VIT_09s0002g04230
VIT_09s0002g04240
VIT_09s0002g04250
VIT_09s0002g04260
Standardized expressions
Developmental stages
EI PG G1 G2 H T1 T2 C1 C2 S EP JP

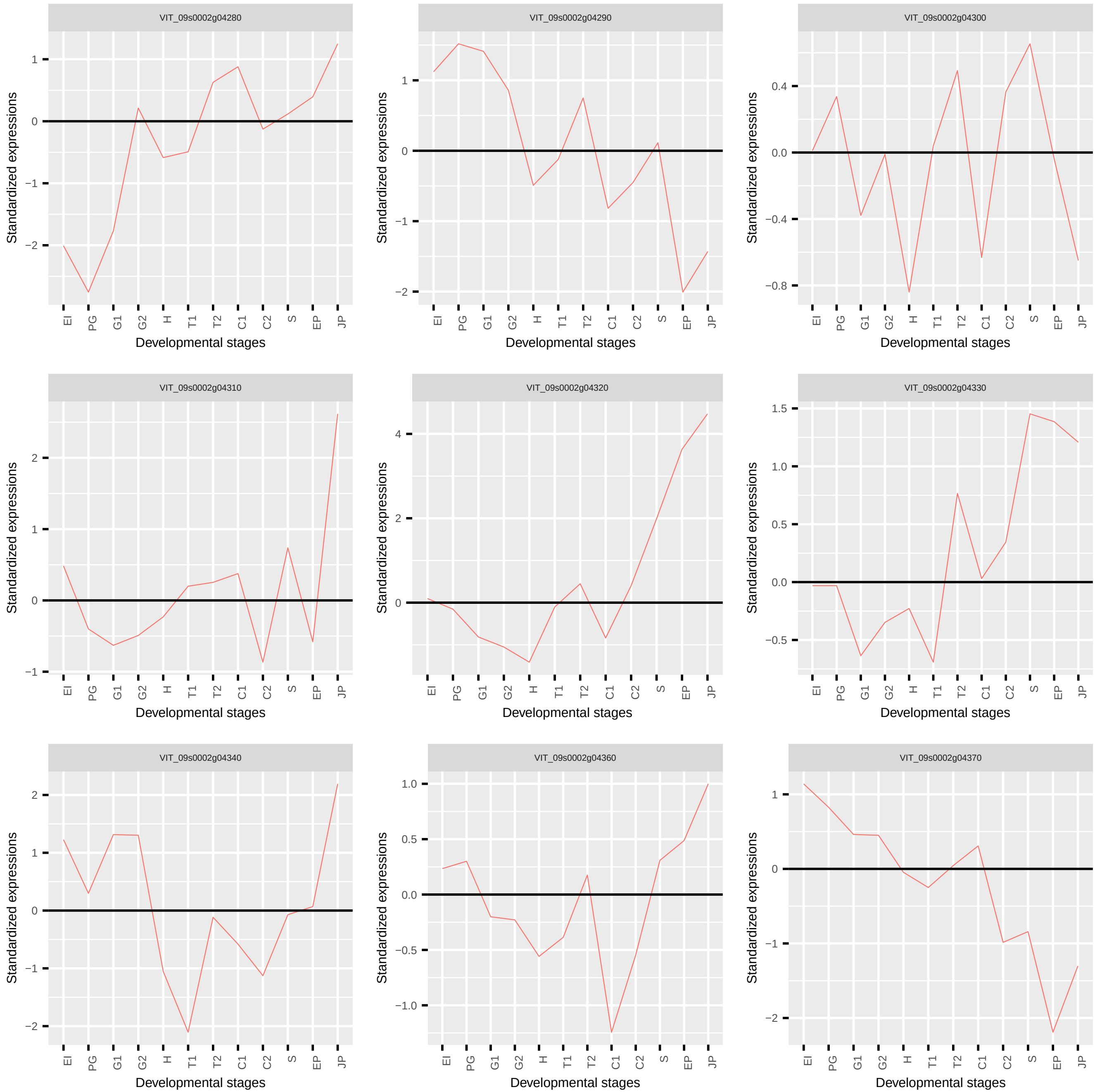
VIT_09s0002g04280
1
0
−1
−2
Standardized expressions
EI PG G1 G2 H T1 T2 C1 C2 S EP JP
Developmental stages
VIT_09s0002g04290
1
0
−1
−2
Standardized expressions
EI PG G1 G2 H T1 T2 C1 C2 S EP JP
Developmental stages
VIT_09s0002g04300
0.4
0.0
−0.4
−0.8
Standardized expressions
EI PG G1 G2 H T1 T2 C1 C2 S EP JP
Developmental stages
VIT_09s0002g04310
2
1
0
−1
Standardized expressions
EI PG G1 G2 H T1 T2 C1 C2 S EP JP
Developmental stages
VIT_09s0002g04320
4
2
0
Standardized expressions
EI PG G1 G2 H T1 T2 C1 C2 S EP JP
Developmental stages
VIT_09s0002g04330
1.5
1.0
0.5
0.0
−0.5
Standardized expressions
EI PG G1 G2 H T1 T2 C1 C2 S EP JP
Developmental stages
VIT_09s0002g04340
2
1
0
−1
−2
Standardized expressions
EI PG G1 G2 H T1 T2 C1 C2 S EP JP
Developmental stages
VIT_09s0002g04360
1.0
0.5
0.0
−0.5
−1.0
Standardized expressions
EI PG G1 G2 H T1 T2 C1 C2 S EP JP
Developmental stages
VIT_09s0002g04370
1
0
−1
−2
Standardized expressions
EI PG G1 G2 H T1 T2 C1 C2 S EP JP
Developmental stages

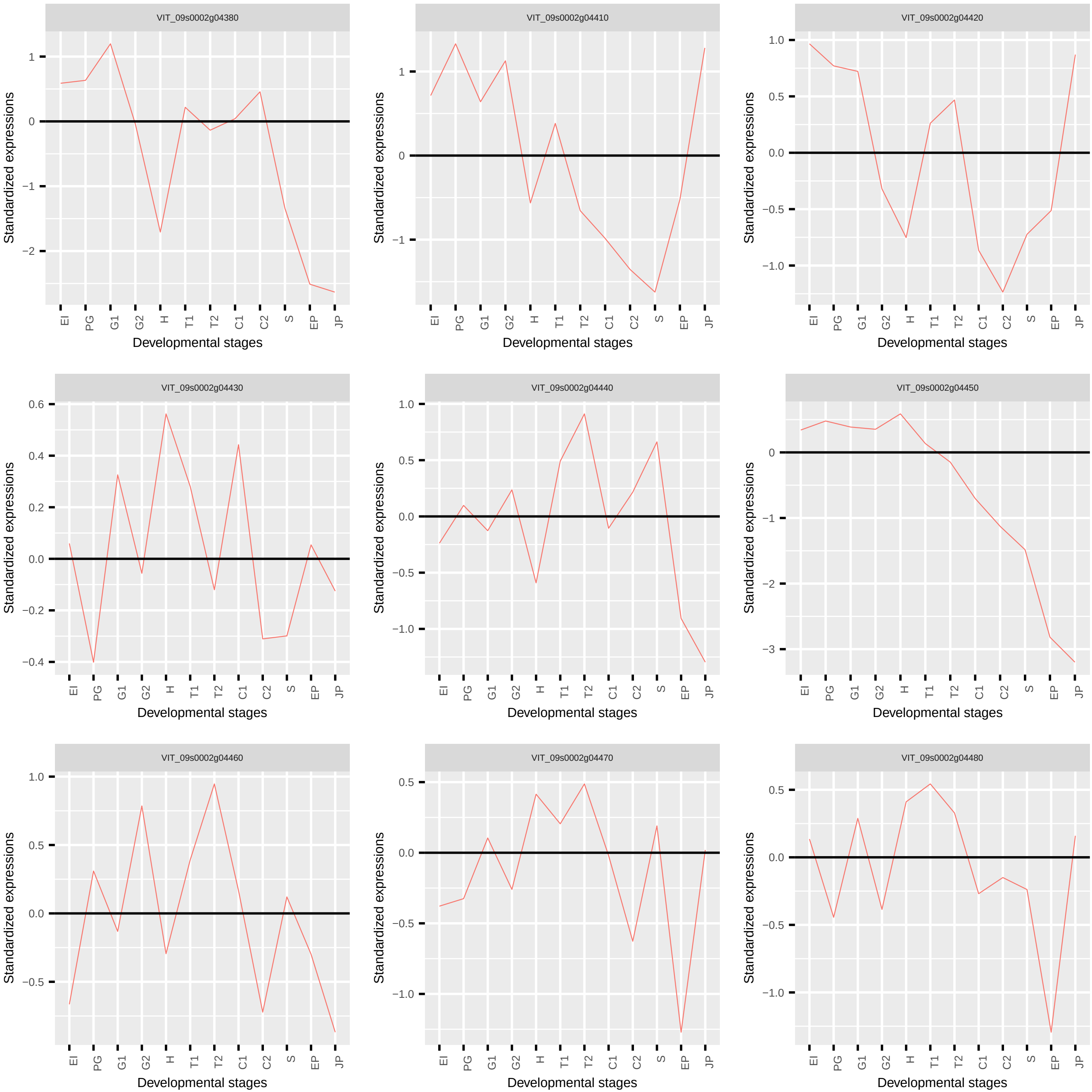
VIT_09s0002g04380
VIT_09s0002g04410
VIT_09s0002g04420
VIT_09s0002g04430
VIT_09s0002g04440
VIT_09s0002g04450
VIT_09s0002g04460
VIT_09s0002g04470
VIT_09s0002g04480
Standardized expressions
Developmental stages
EI PG G1 G2 H T1 T2 C1 C2 S EP JP

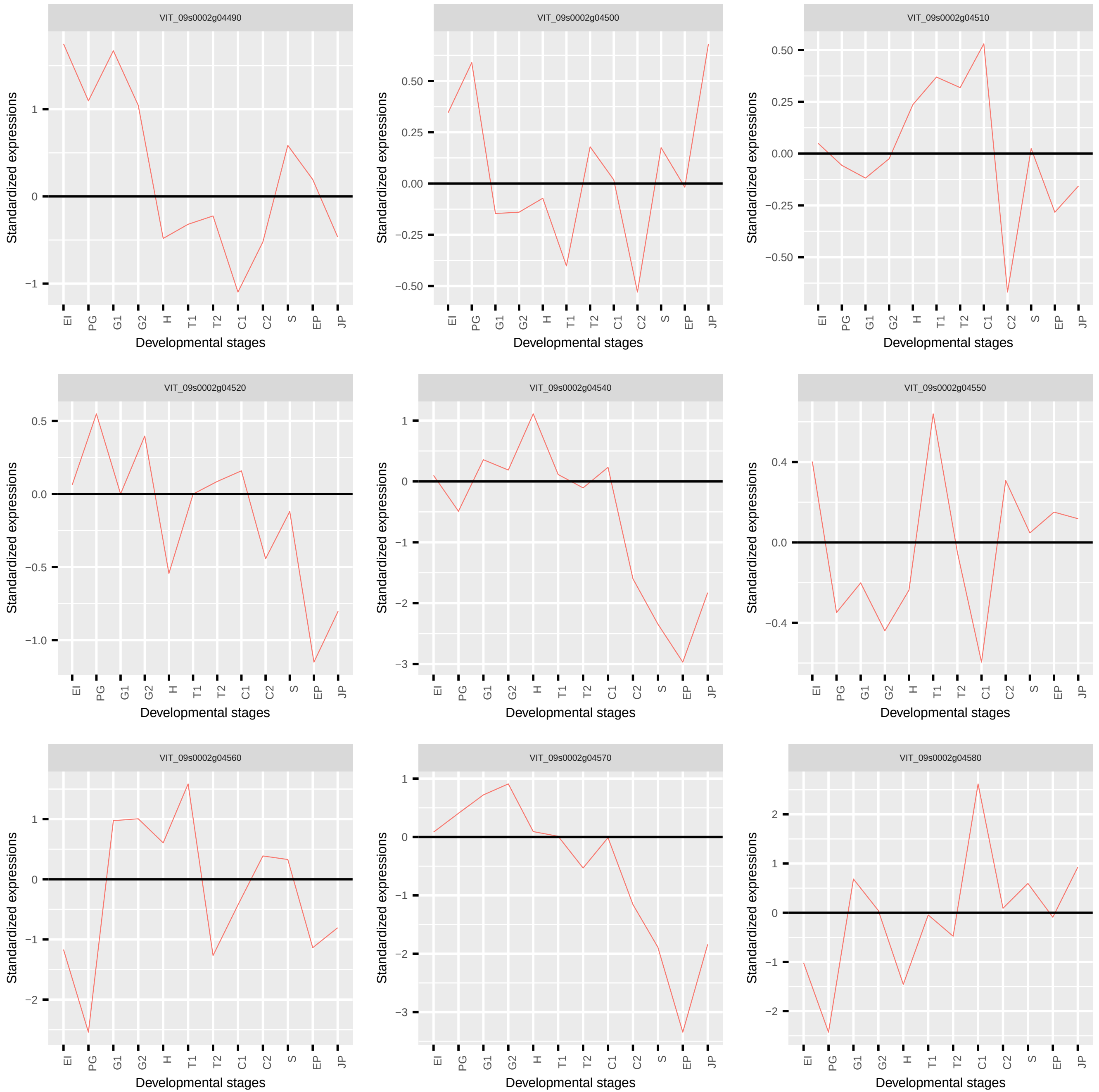
VIT_09s0002g04490
VIT_09s0002g04500
VIT_09s0002g04510
VIT_09s0002g04520
VIT_09s0002g04540
VIT_09s0002g04550
VIT_09s0002g04560
VIT_09s0002g04570
VIT_09s0002g04580
Standardized expressions
Developmental stages
EI
PG
G1
G2
H
T1
T2
C1
C2
S
EP
JP

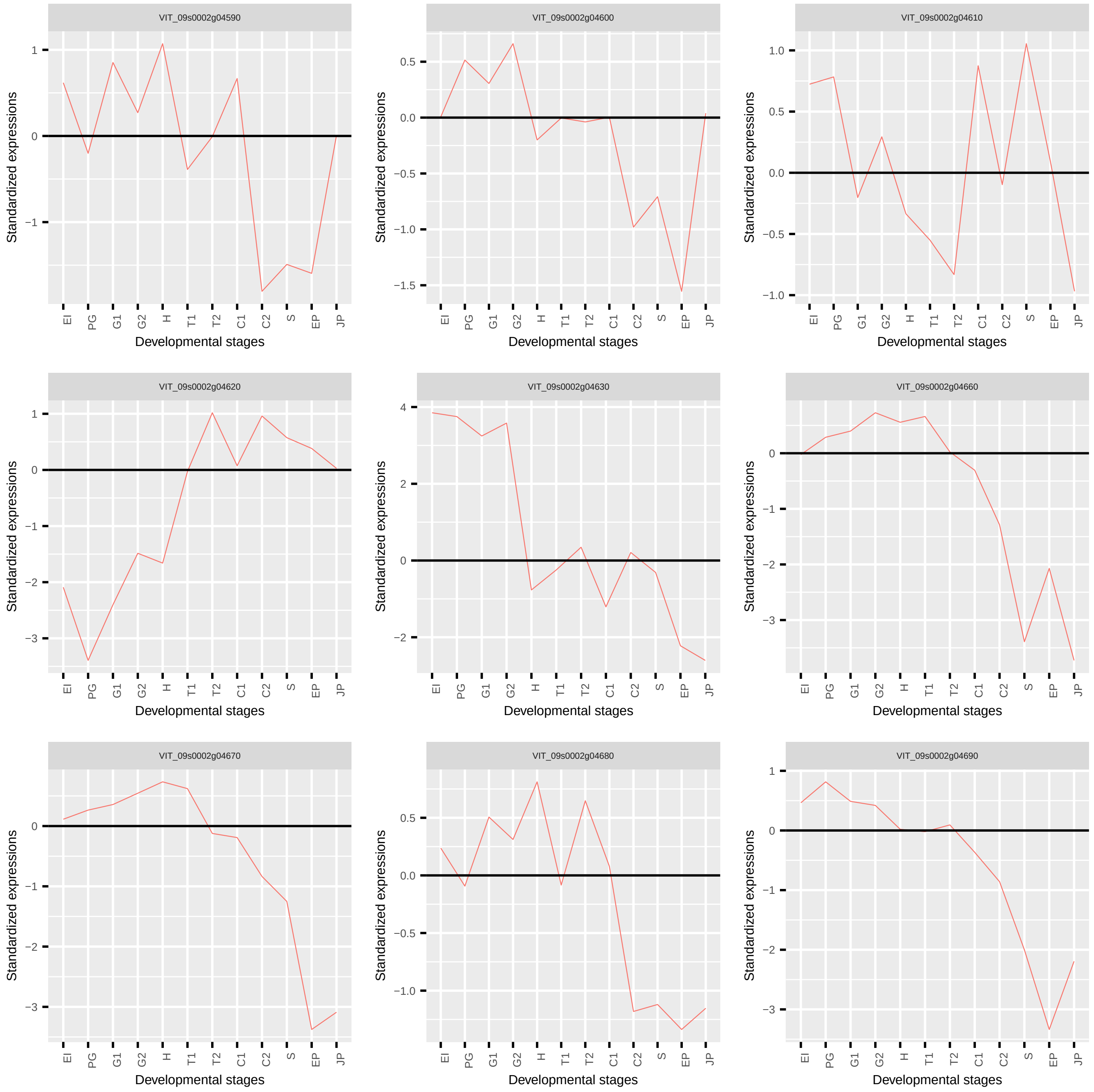
VIT_09s0002g04590
VIT_09s0002g04600
VIT_09s0002g04610
VIT_09s0002g04620
VIT_09s0002g04630
VIT_09s0002g04660
VIT_09s0002g04670
VIT_09s0002g04680
VIT_09s0002g04690
Standardized expressions
Developmental stages
EI PG G1 G2 H T1 T2 C1 C2 S EP JP

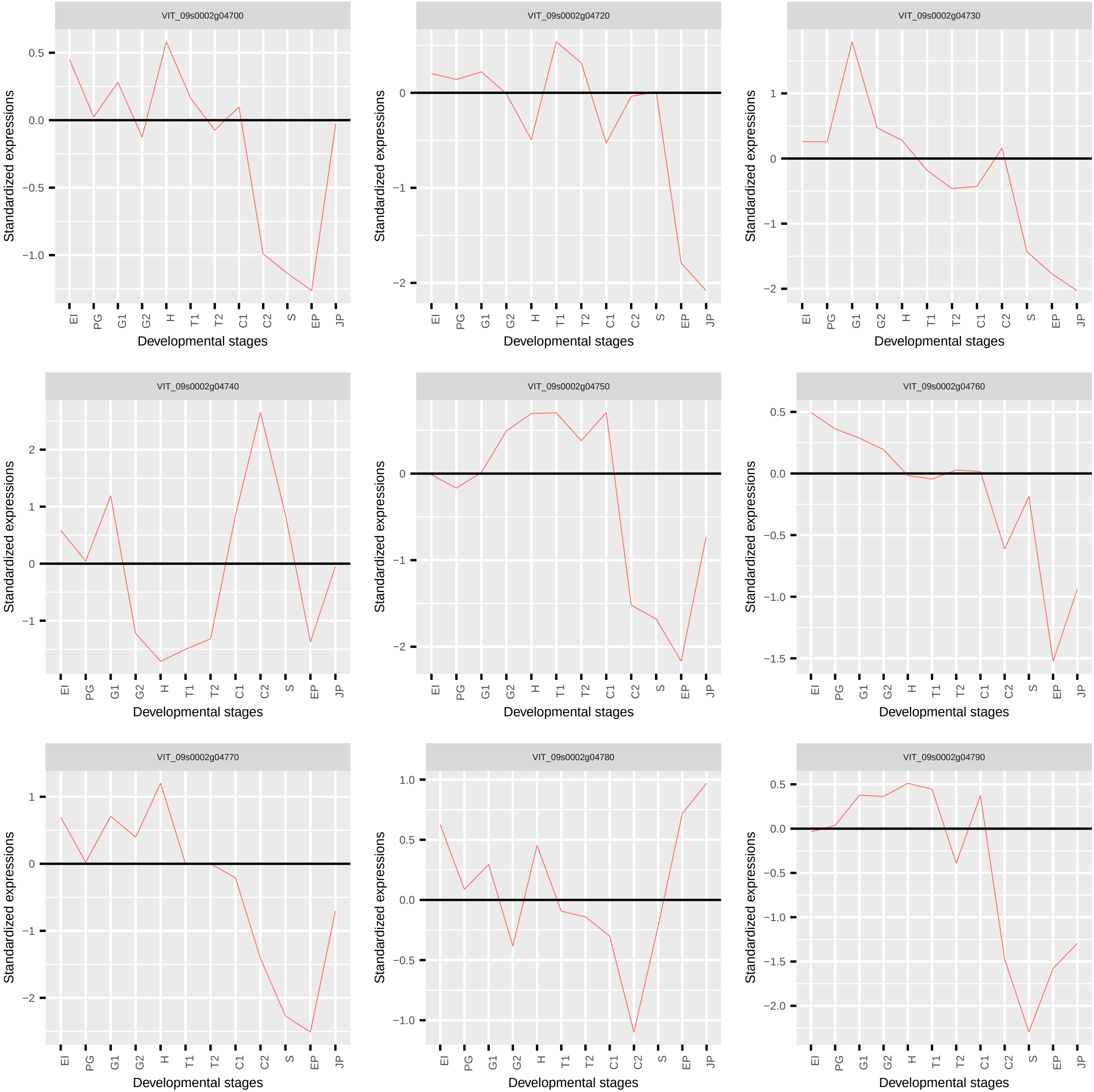
VIT_09s0002g04700
0.5
0.0
−0.5
−1.0
VIT_09s0002g04720
0
−1
−2
VIT_09s0002g04730
1
0
−1
−2
VIT_09s0002g04740
2
1
0
−1
VIT_09s0002g04750
0
−1
−2
VIT_09s0002g04760
0.5
0.0
−0.5
−1.0
−1.5
VIT_09s0002g04770
1
0
−1
−2
VIT_09s0002g04780
1.0
0.5
0.0
−0.5
−1.0
VIT_09s0002g04790
0.5
0.0
−0.5
−1.0
−1.5
−2.0
Standardized expressions
EI PG G1 G2 H T1 T2 C1 C2 S EP JP
Developmental stages

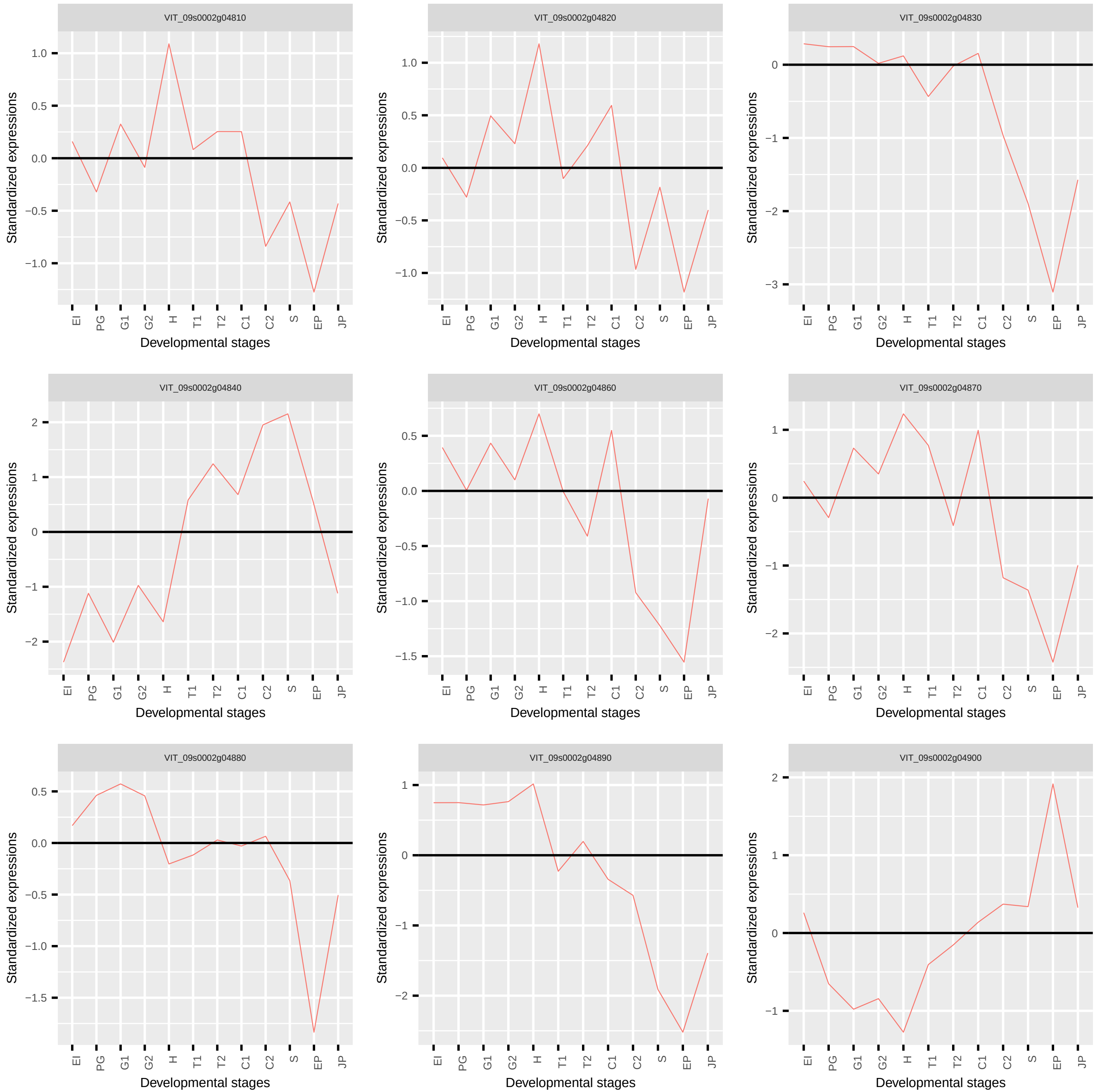

VIT_09s0002g04810
VIT_09s0002g04820
VIT_09s0002g04830
VIT_09s0002g04840
VIT_09s0002g04860
VIT_09s0002g04870
VIT_09s0002g04880
VIT_09s0002g04890
VIT_09s0002g04900
Standardized expressions
Developmental stages
EI PG G1 G2 H T1 T2 C1 C2 S EP JP

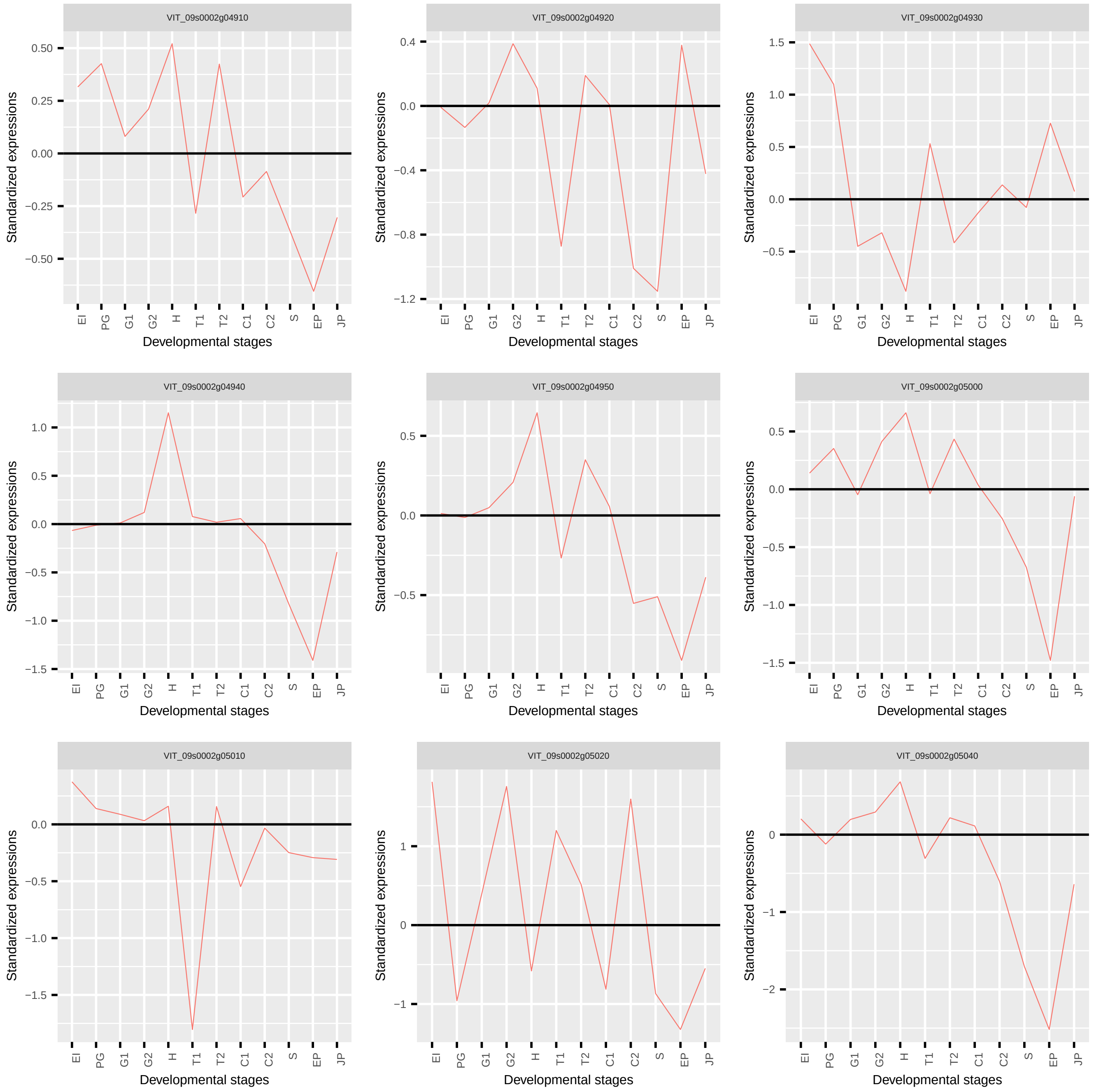
VIT_09s0002g04910
0.50
0.25
0.00
−0.25
−0.50
Standardized expressions
EI PG G1 G2 H T1 T2 C1 C2 S EP JP
Developmental stages
VIT_09s0002g04920
0.4
0.0
−0.4
−0.8
−1.2
Standardized expressions
EI PG G1 G2 H T1 T2 C1 C2 S EP JP
Developmental stages
VIT_09s0002g04930
1.5
1.0
0.5
0.0
−0.5
Standardized expressions
EI PG G1 G2 H T1 T2 C1 C2 S EP JP
Developmental stages
VIT_09s0002g04940
1.0
0.5
0.0
−0.5
−1.0
−1.5
Standardized expressions
EI PG G1 G2 H T1 T2 C1 C2 S EP JP
Developmental stages
VIT_09s0002g04950
0.5
0.0
−0.5
Standardized expressions
EI PG G1 G2 H T1 T2 C1 C2 S EP JP
Developmental stages
VIT_09s0002g05000
0.5
0.0
−0.5
−1.0
−1.5
Standardized expressions
EI PG G1 G2 H T1 T2 C1 C2 S EP JP
Developmental stages
VIT_09s0002g05010
0.0
−0.5
−1.0
−1.5
Standardized expressions
EI PG G1 G2 H T1 T2 C1 C2 S EP JP
Developmental stages
VIT_09s0002g05020
1
0
−1
Standardized expressions
EI PG G1 G2 H T1 T2 C1 C2 S EP JP
Developmental stages
VIT_09s0002g05040
0
−1
−2
Standardized expressions
EI PG G1 G2 H T1 T2 C1 C2 S EP JP
Developmental stages

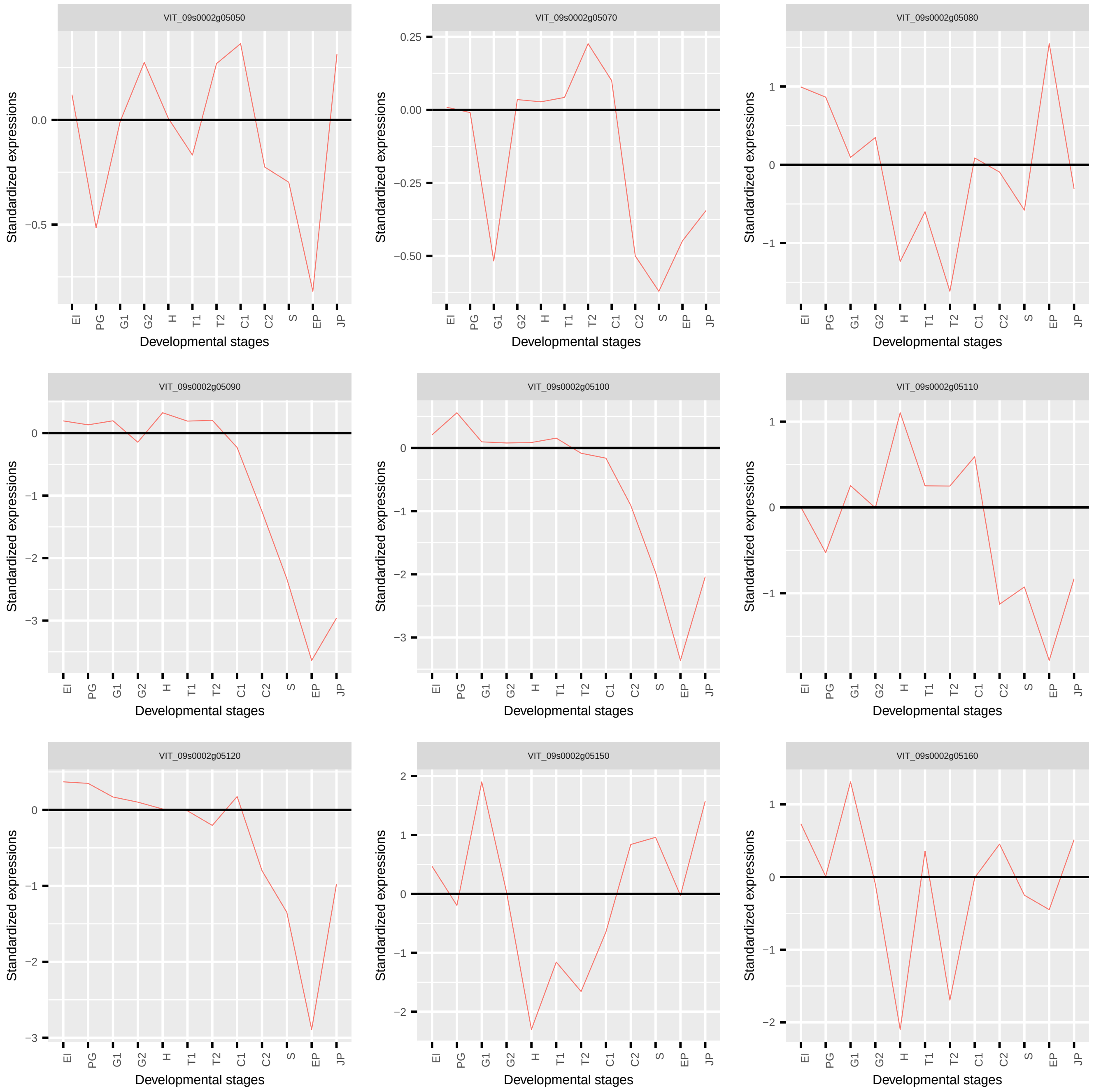
VIT_09s0002g05050
VIT_09s0002g05070
VIT_09s0002g05080
VIT_09s0002g05090
VIT_09s0002g05100
VIT_09s0002g05110
VIT_09s0002g05120
VIT_09s0002g05150
VIT_09s0002g05160
Standardized expressions
Developmental stages
EI PG G1 G2 H T1 T2 C1 C2 S EP JP

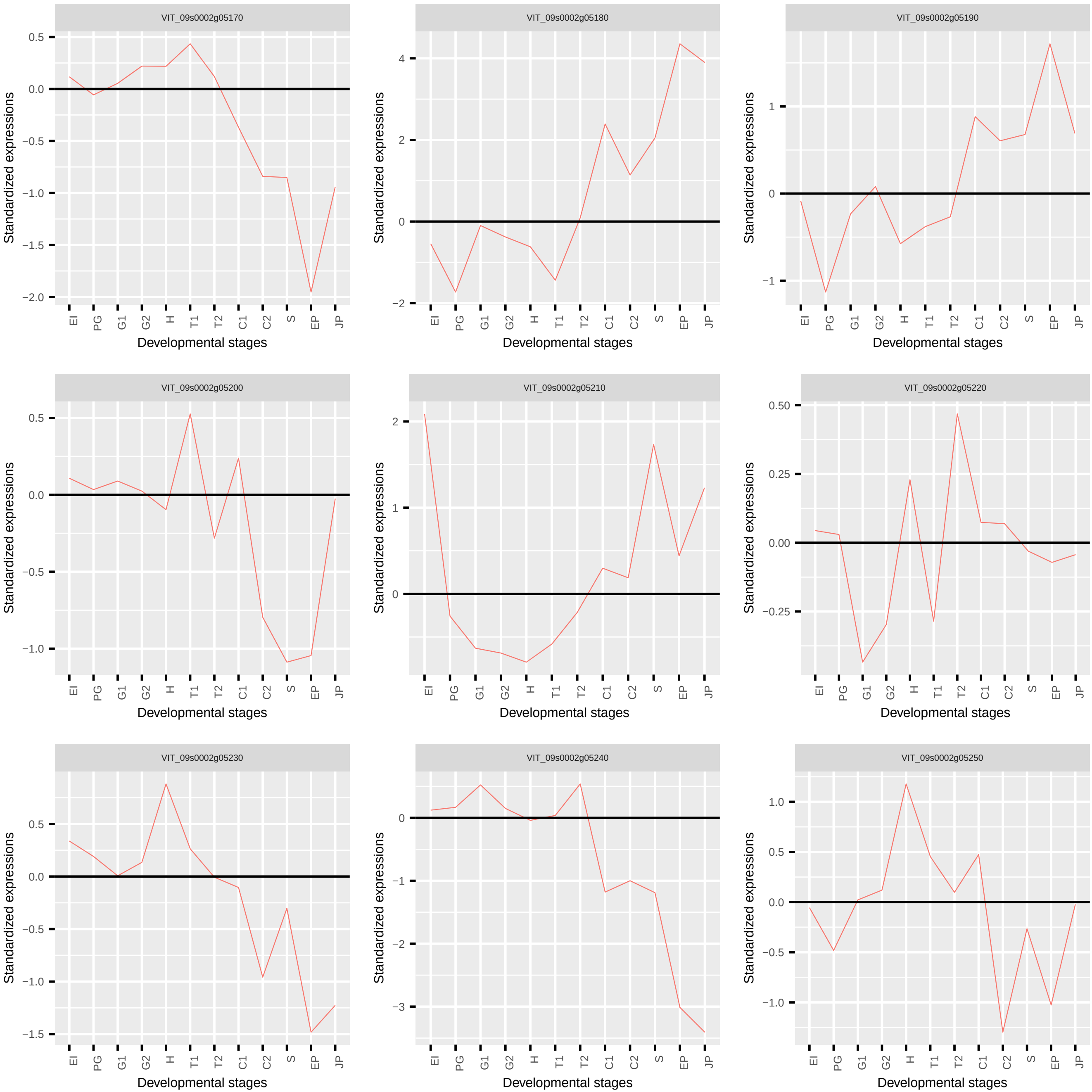
VIT_09s0002g05170
VIT_09s0002g05180
VIT_09s0002g05190
VIT_09s0002g05200
VIT_09s0002g05210
VIT_09s0002g05220
VIT_09s0002g05230
VIT_09s0002g05240
VIT_09s0002g05250
Standardized expressions
Developmental stages
EI PG G1 G2 H T1 T2 C1 C2 S EP JP
0.5 0.0 −0.5 −1.0 −1.5 −2.0
4 2 0 −2
1 0 −1
0.5 0.0 −0.5 −1.0
2 1 0
0.50 0.25 0.00 −0.25
0.5 0.0 −0.5 −1.0 −1.5
0 −1 −2 −3
1.0 0.5 0.0 −0.5 −1.0

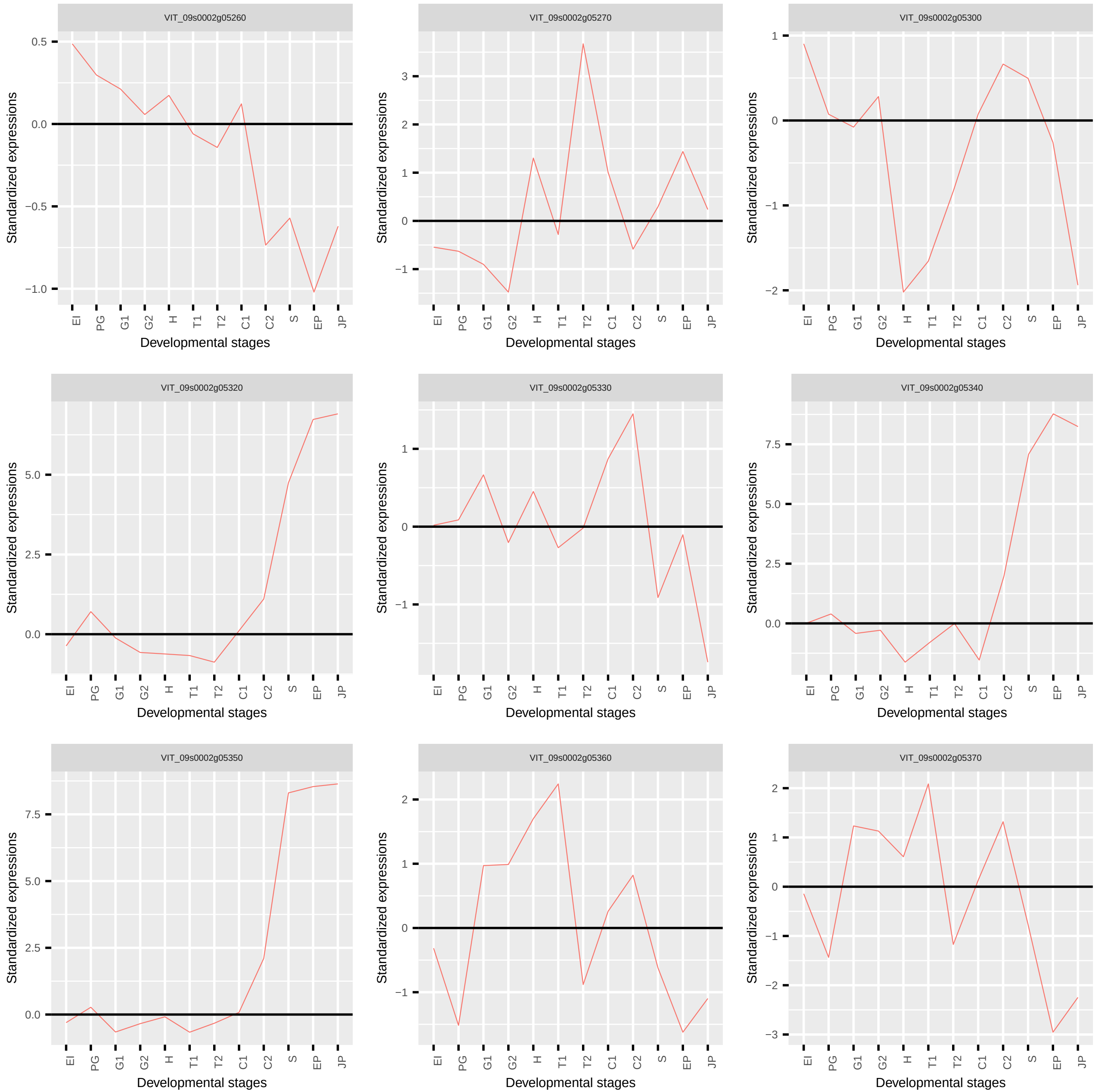
VIT_09s0002g05260
VIT_09s0002g05270
VIT_09s0002g05300
VIT_09s0002g05320
VIT_09s0002g05330
VIT_09s0002g05340
VIT_09s0002g05350
VIT_09s0002g05360
VIT_09s0002g05370
Standardized expressions
Developmental stages
EI PG G1 G2 H T1 T2 C1 C2 S EP JP

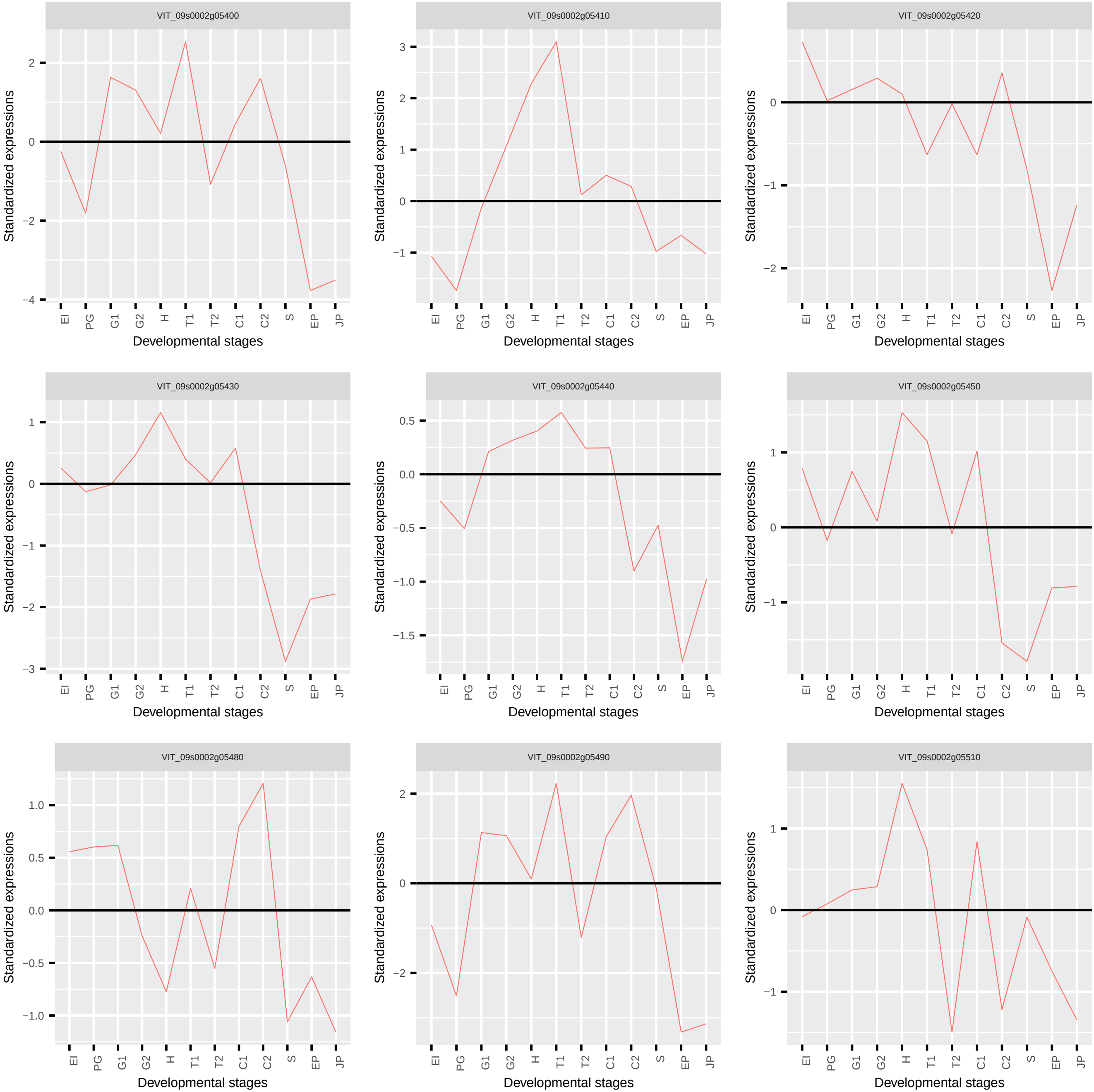

VIT_09s0002g05400
VIT_09s0002g05410
VIT_09s0002g05420
VIT_09s0002g05430
VIT_09s0002g05440
VIT_09s0002g05450
VIT_09s0002g05480
VIT_09s0002g05490
VIT_09s0002g05510
Standardized expressions
Developmental stages
EI
PG
G1
G2
H
T1
T2
C1
C2
S
EP
JP

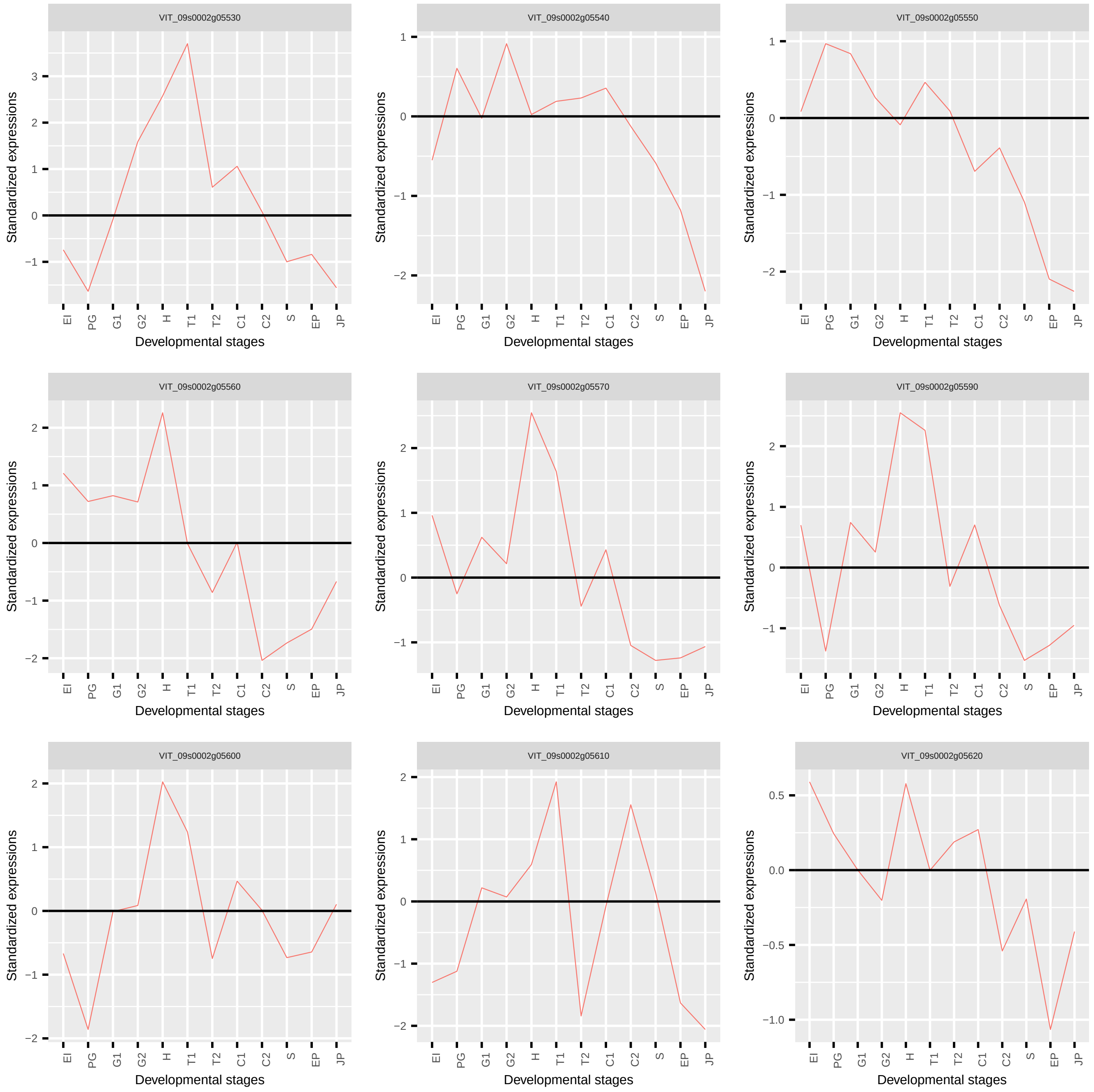
VIT_09s0002g05530
VIT_09s0002g05540
VIT_09s0002g05550
VIT_09s0002g05560
VIT_09s0002g05570
VIT_09s0002g05590
VIT_09s0002g05600
VIT_09s0002g05610
VIT_09s0002g05620
Standardized expressions
Developmental stages
EI PG G1 G2 H T1 T2 C1 C2 S EP JP

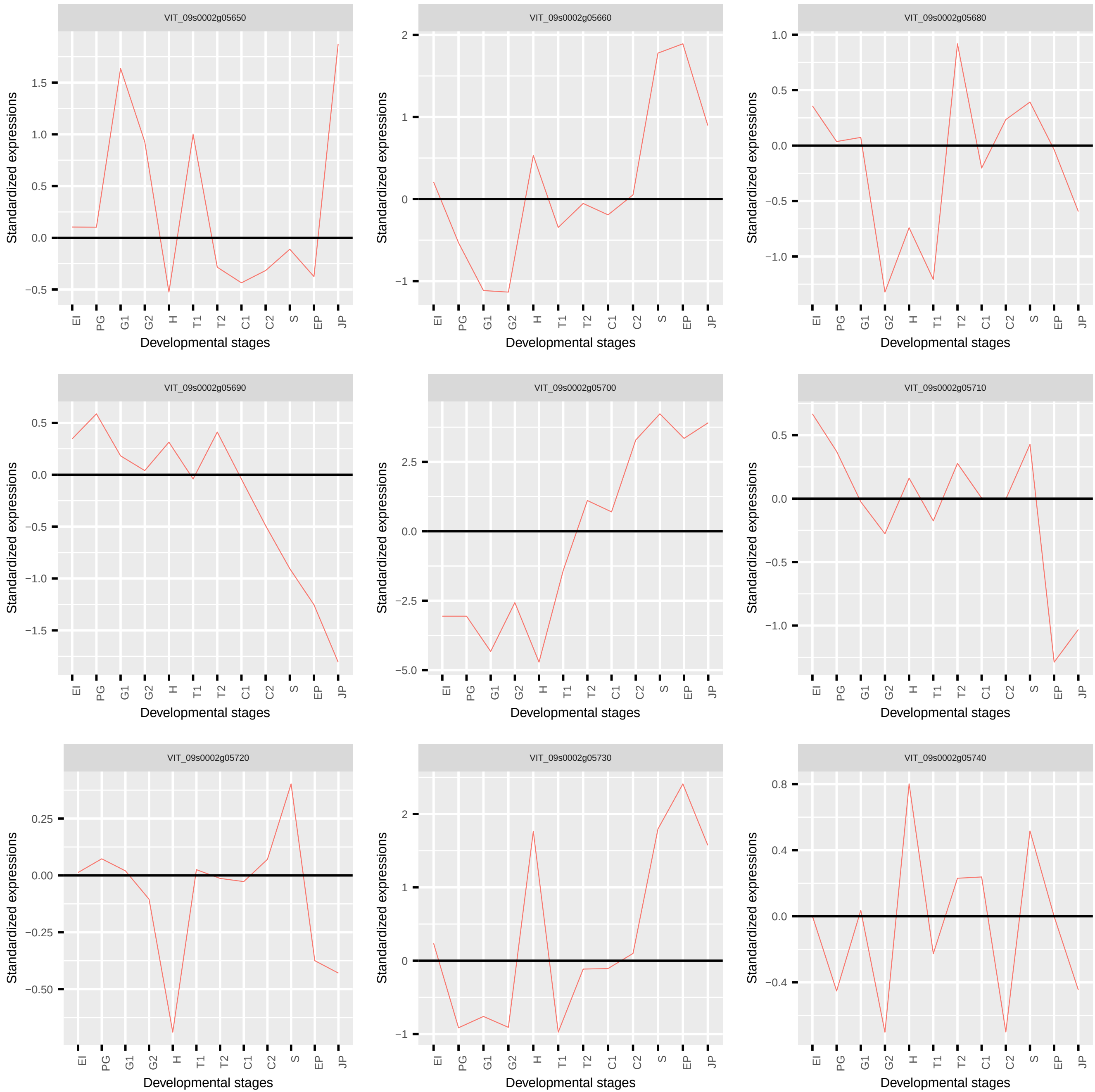
VIT_09s0002g05650
VIT_09s0002g05660
VIT_09s0002g05680
VIT_09s0002g05690
VIT_09s0002g05700
VIT_09s0002g05710
VIT_09s0002g05720
VIT_09s0002g05730
VIT_09s0002g05740
Standardized expressions
Developmental stages
EI
PG
G1
G2
H
T1
T2
C1
C2
S
EP
JP

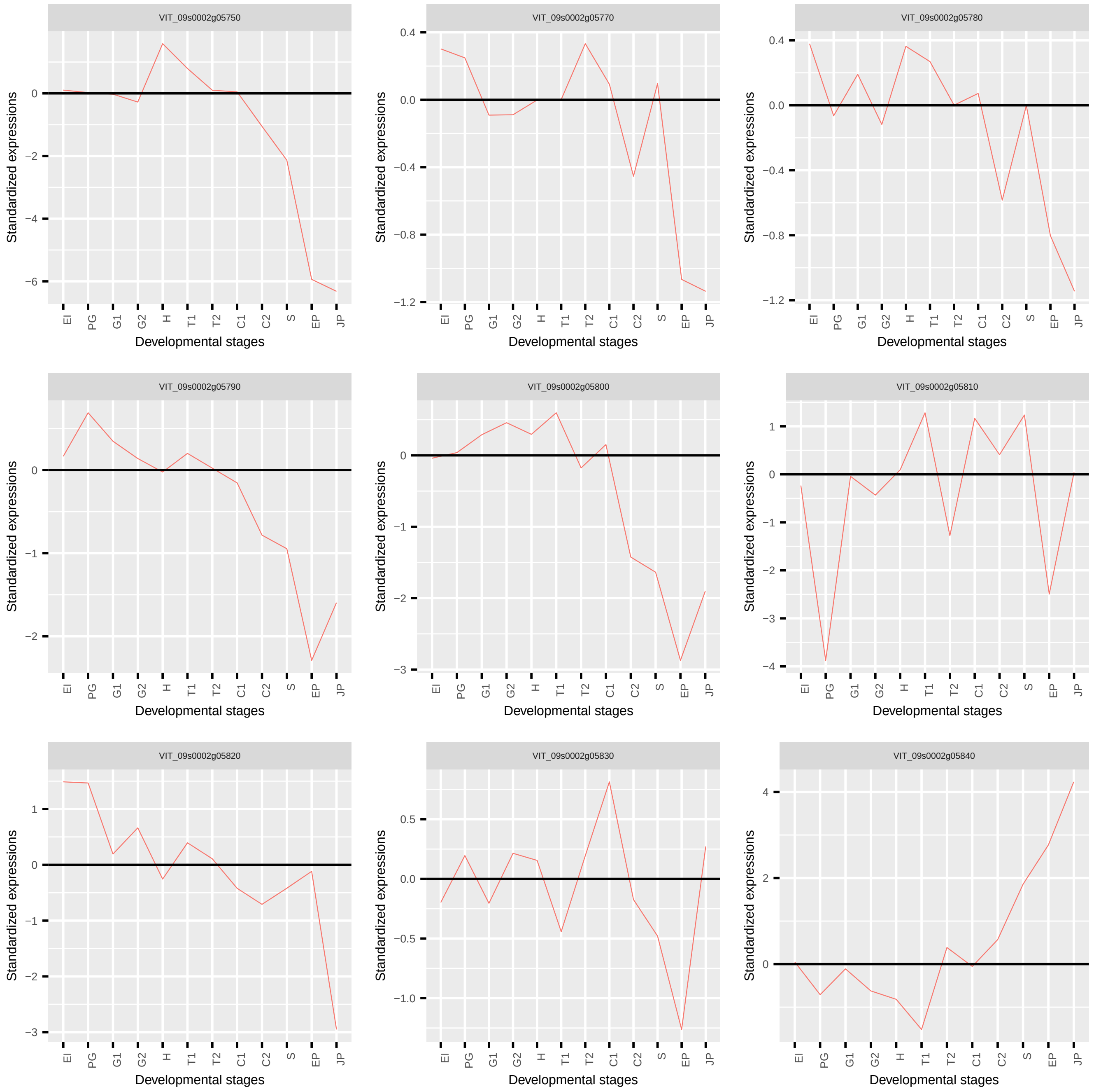
VIT_09s0002g05750
VIT_09s0002g05770
VIT_09s0002g05780
VIT_09s0002g05790
VIT_09s0002g05800
VIT_09s0002g05810
VIT_09s0002g05820
VIT_09s0002g05830
VIT_09s0002g05840
Standardized expressions
Developmental stages
EI
PG
G1
G2
H
T1
T2
C1
C2
S
EP
JP

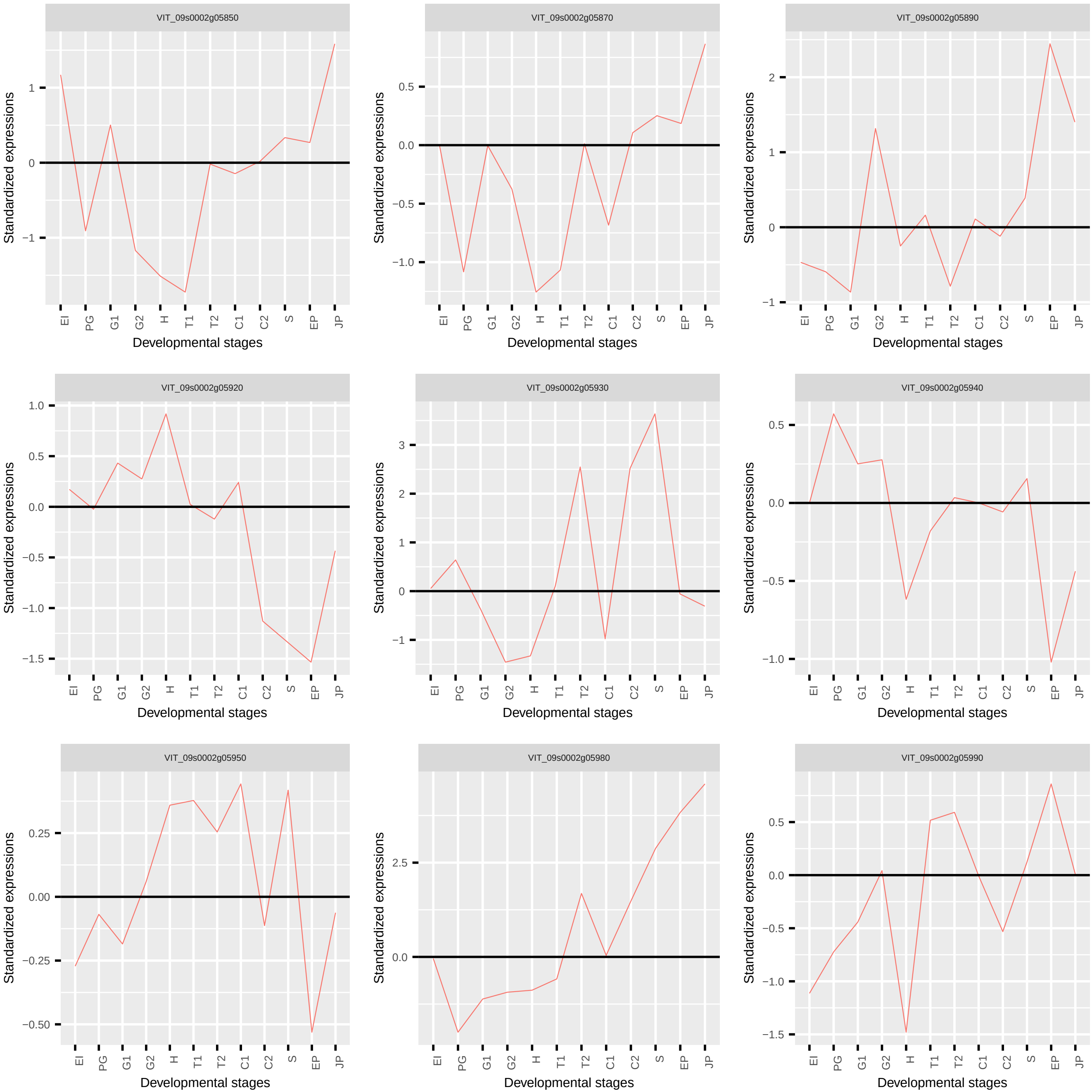
VIT_09s0002g05850
VIT_09s0002g05870
VIT_09s0002g05890
VIT_09s0002g05920
VIT_09s0002g05930
VIT_09s0002g05940
VIT_09s0002g05950
VIT_09s0002g05980
VIT_09s0002g05990
Standardized expressions
Developmental stages
EI PG G1 G2 H T1 T2 C1 C2 S EP JP

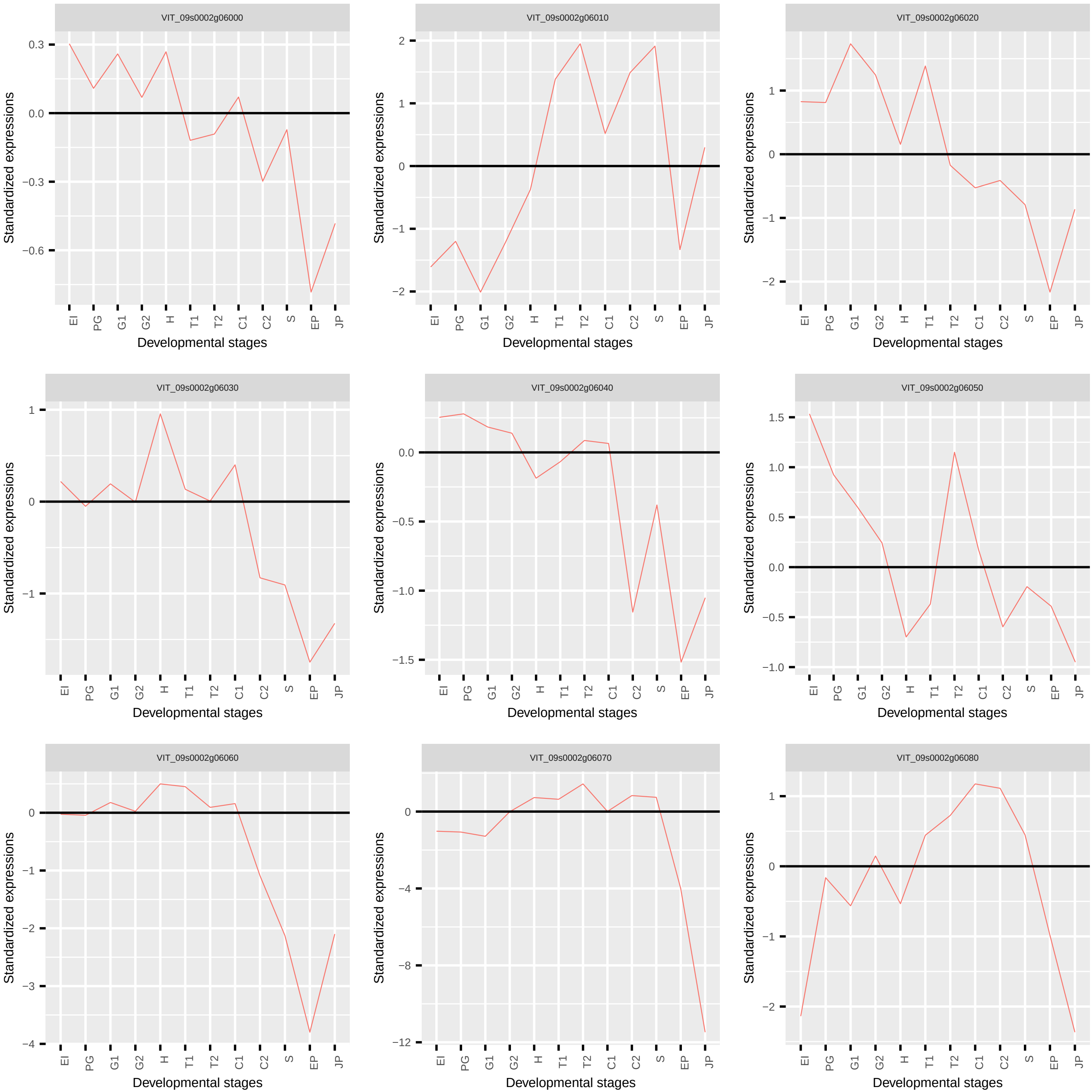
VIT_09s0002g06000
VIT_09s0002g06010
VIT_09s0002g06020
VIT_09s0002g06030
VIT_09s0002g06040
VIT_09s0002g06050
VIT_09s0002g06060
VIT_09s0002g06070
VIT_09s0002g06080
Standardized expressions
Developmental stages
EI PG G1 G2 H T1 T2 C1 C2 S EP JP

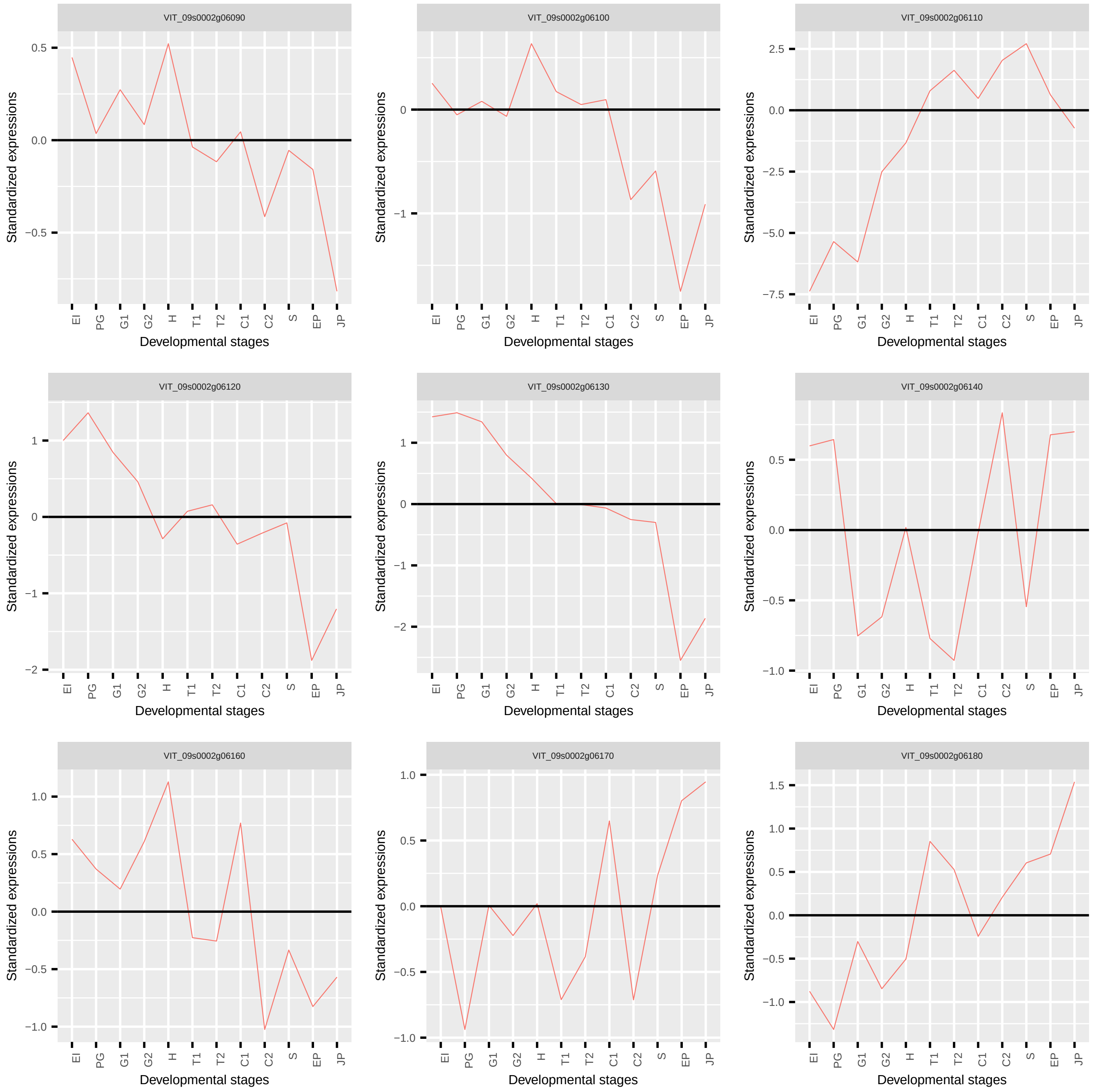
VIT_09s0002g06090
VIT_09s0002g06100
VIT_09s0002g06110
VIT_09s0002g06120
VIT_09s0002g06130
VIT_09s0002g06140
VIT_09s0002g06160
VIT_09s0002g06170
VIT_09s0002g06180
Standardized expressions
Developmental stages
EI PG G1 G2 H T1 T2 C1 C2 S EP JP

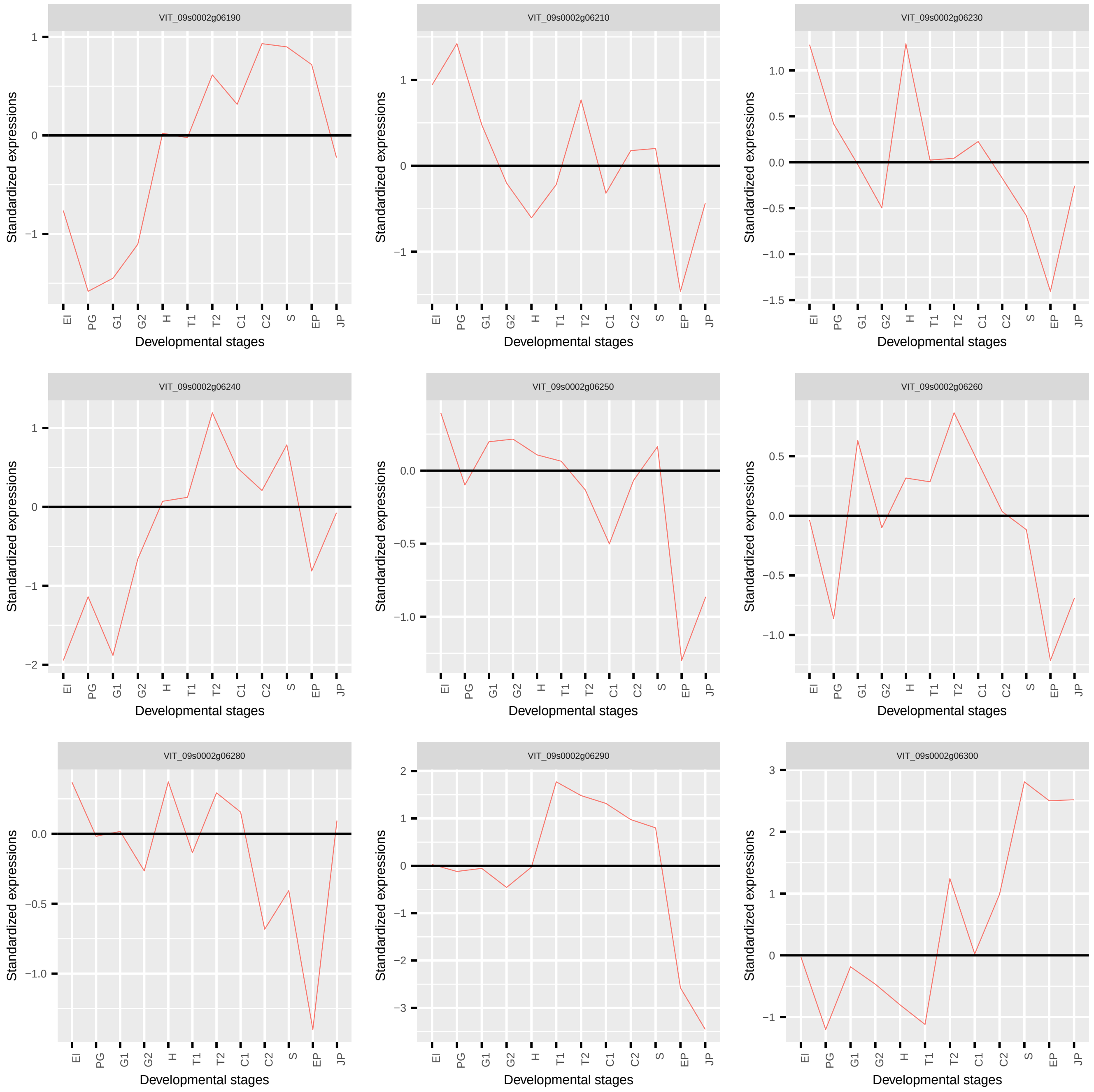

VIT_09s0002g06190
VIT_09s0002g06210
VIT_09s0002g06230
VIT_09s0002g06240
VIT_09s0002g06250
VIT_09s0002g06260
VIT_09s0002g06280
VIT_09s0002g06290
VIT_09s0002g06300
Standardized expressions
Developmental stages
EI PG G1 G2 H T1 T2 C1 C2 S EP JP

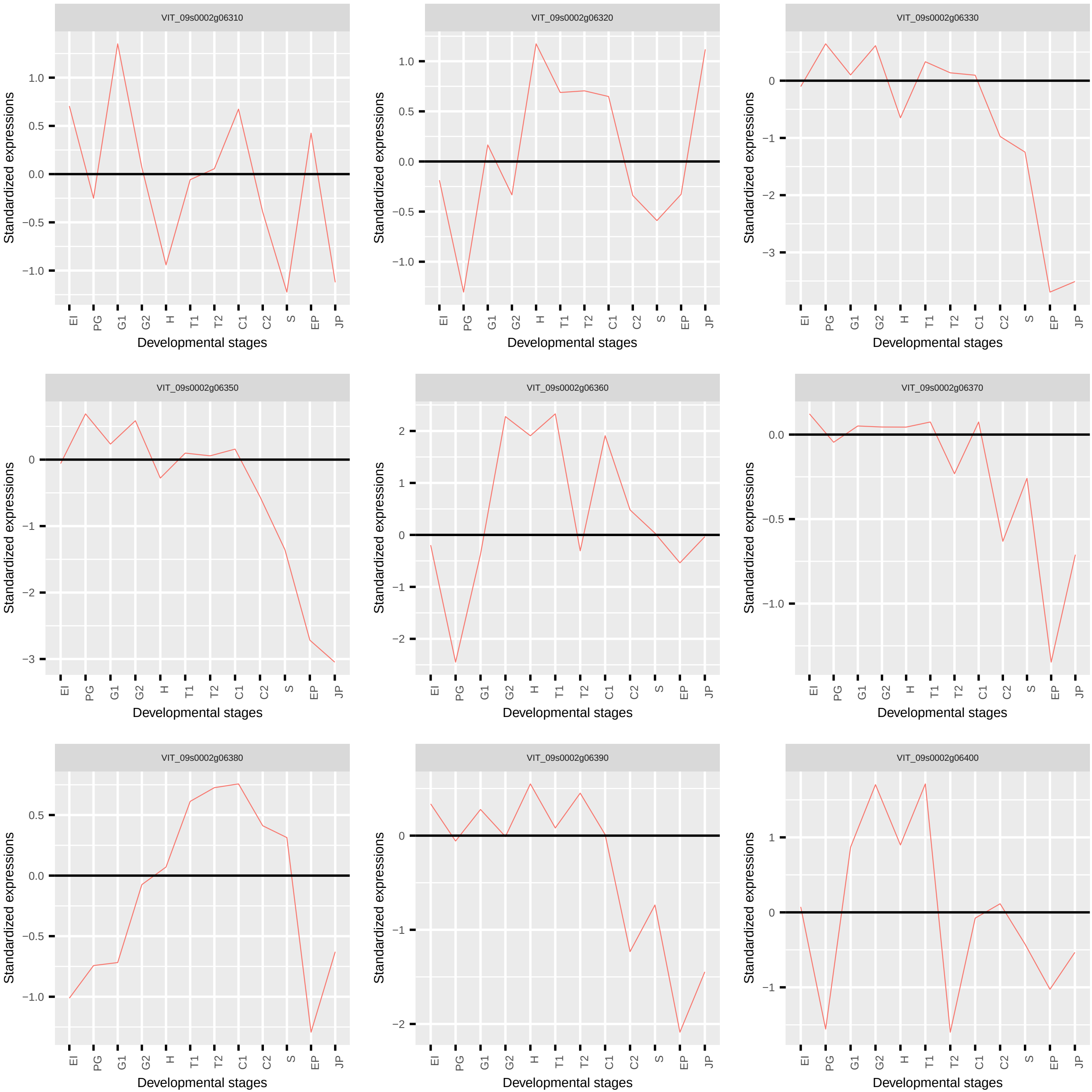
VIT_09s0002g06310
VIT_09s0002g06320
VIT_09s0002g06330
VIT_09s0002g06350
VIT_09s0002g06360
VIT_09s0002g06370
VIT_09s0002g06380
VIT_09s0002g06390
VIT_09s0002g06400
Standardized expressions
Developmental stages
EI PG G1 G2 H T1 T2 C1 C2 S EP JP

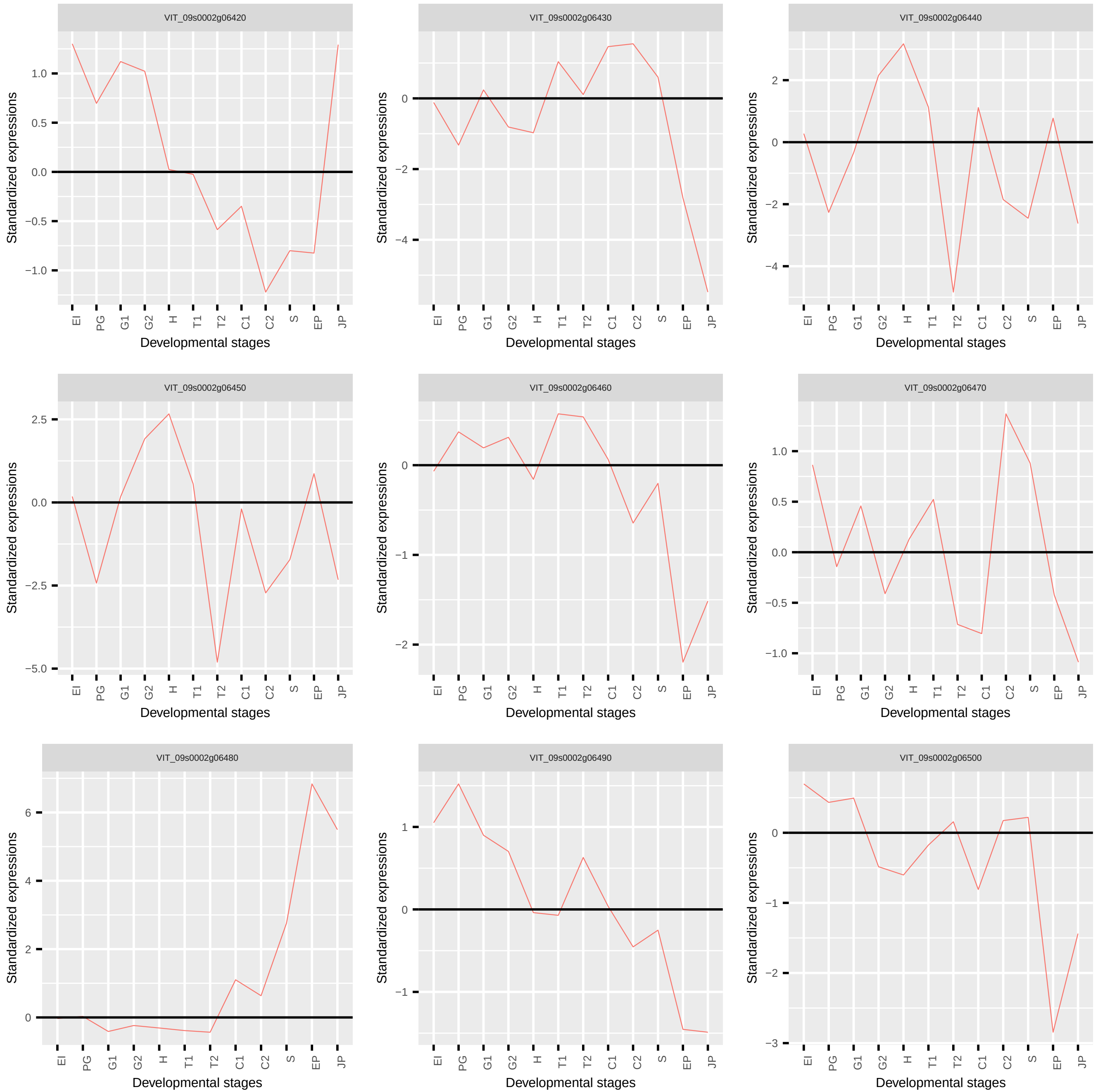
VIT_09s0002g06420
VIT_09s0002g06430
VIT_09s0002g06440
VIT_09s0002g06450
VIT_09s0002g06460
VIT_09s0002g06470
VIT_09s0002g06480
VIT_09s0002g06490
VIT_09s0002g06500
Standardized expressions
Developmental stages
EI PG G1 G2 H T1 T2 C1 C2 S EP JP

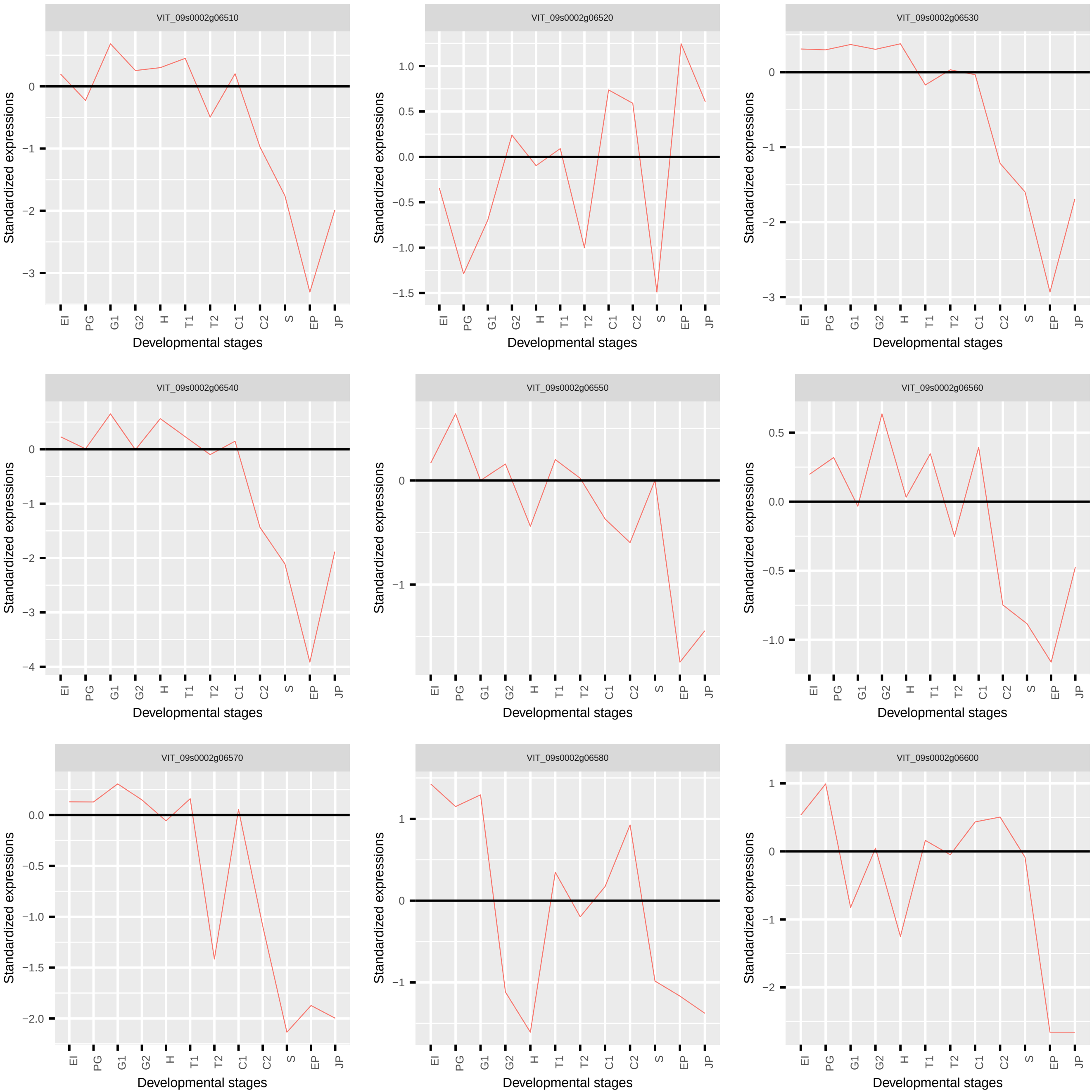
VIT_09s0002g06510
VIT_09s0002g06520
VIT_09s0002g06530
VIT_09s0002g06540
VIT_09s0002g06550
VIT_09s0002g06560
VIT_09s0002g06570
VIT_09s0002g06580
VIT_09s0002g06600
Standardized expressions
Developmental stages
EI PG G1 G2 H T1 T2 C1 C2 S EP JP

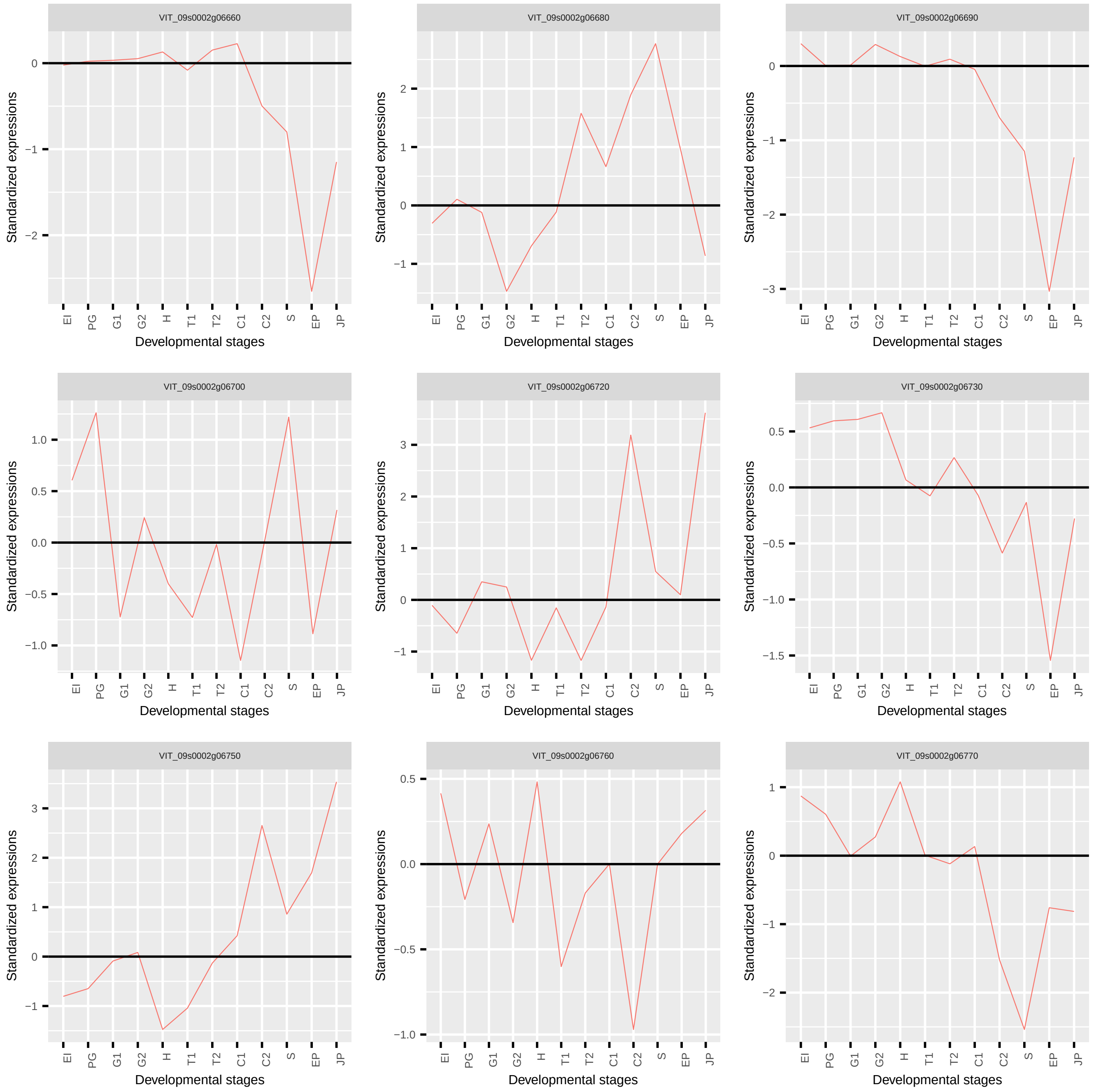
VIT_09s0002g06660
VIT_09s0002g06680
VIT_09s0002g06690
VIT_09s0002g06700
VIT_09s0002g06720
VIT_09s0002g06730
VIT_09s0002g06750
VIT_09s0002g06760
VIT_09s0002g06770
Standardized expressions
Developmental stages
EI PG G1 G2 H T1 T2 C1 C2 S EP JP

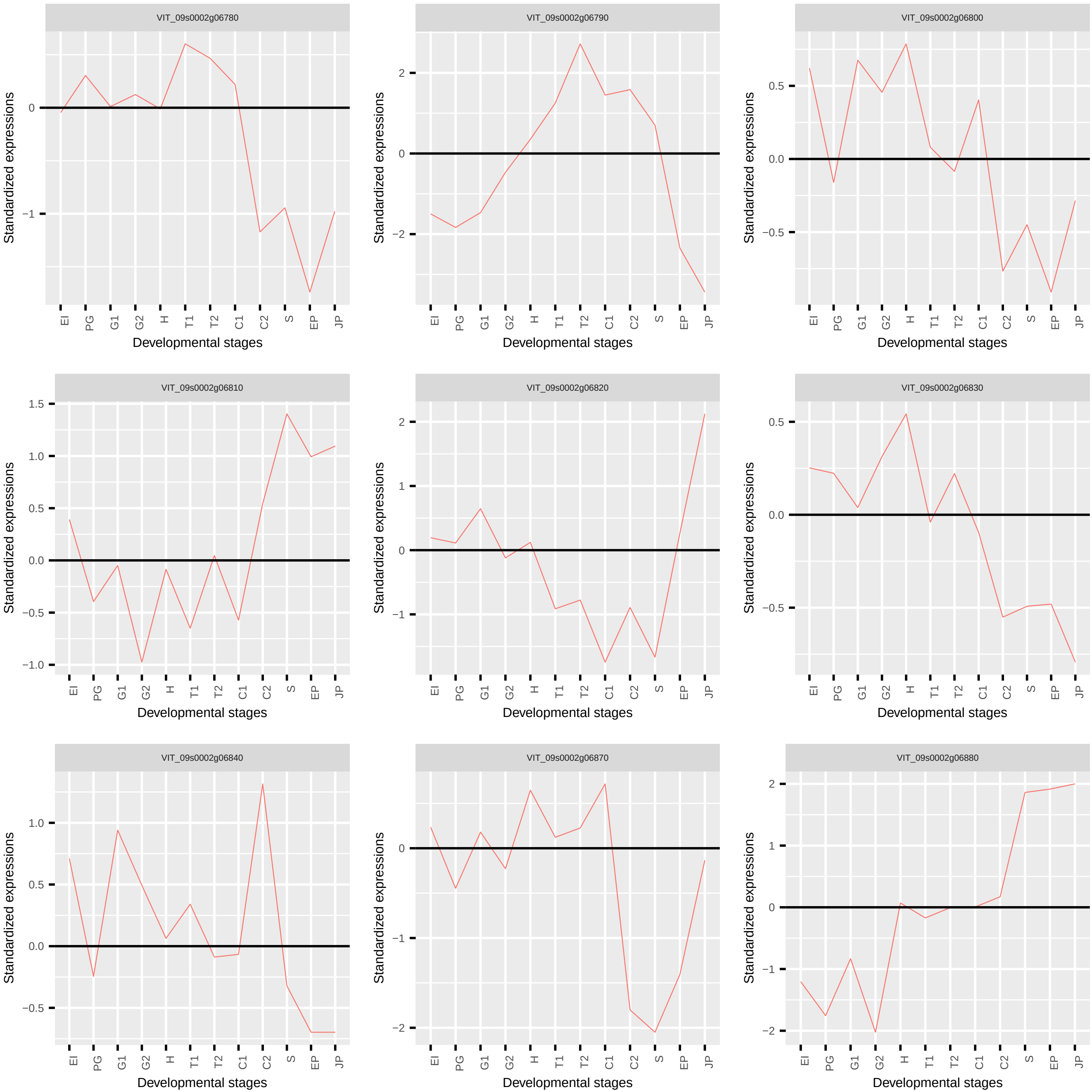
VIT_09s0002g06780
VIT_09s0002g06790
VIT_09s0002g06800
VIT_09s0002g06810
VIT_09s0002g06820
VIT_09s0002g06830
VIT_09s0002g06840
VIT_09s0002g06870
VIT_09s0002g06880
Standardized expressions
Developmental stages
EI
PG
G1
G2
H
T1
T2
C1
C2
S
EP
JP

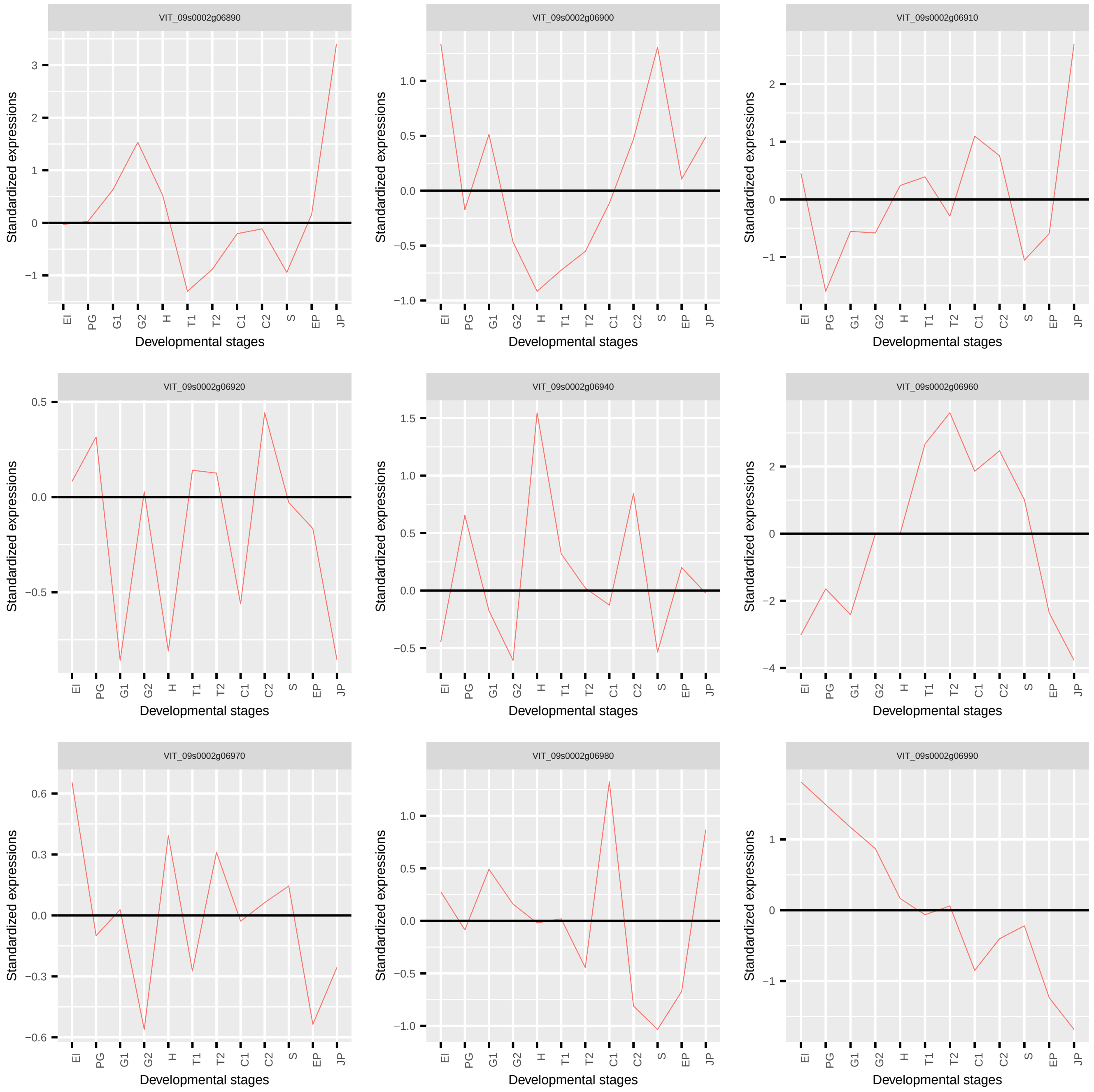
VIT_09s0002g06890
VIT_09s0002g06900
VIT_09s0002g06910
VIT_09s0002g06920
VIT_09s0002g06940
VIT_09s0002g06960
VIT_09s0002g06970
VIT_09s0002g06980
VIT_09s0002g06990
Standardized expressions
Developmental stages
EI
PG
G1
G2
H
T1
T2
C1
C2
S
EP
JP

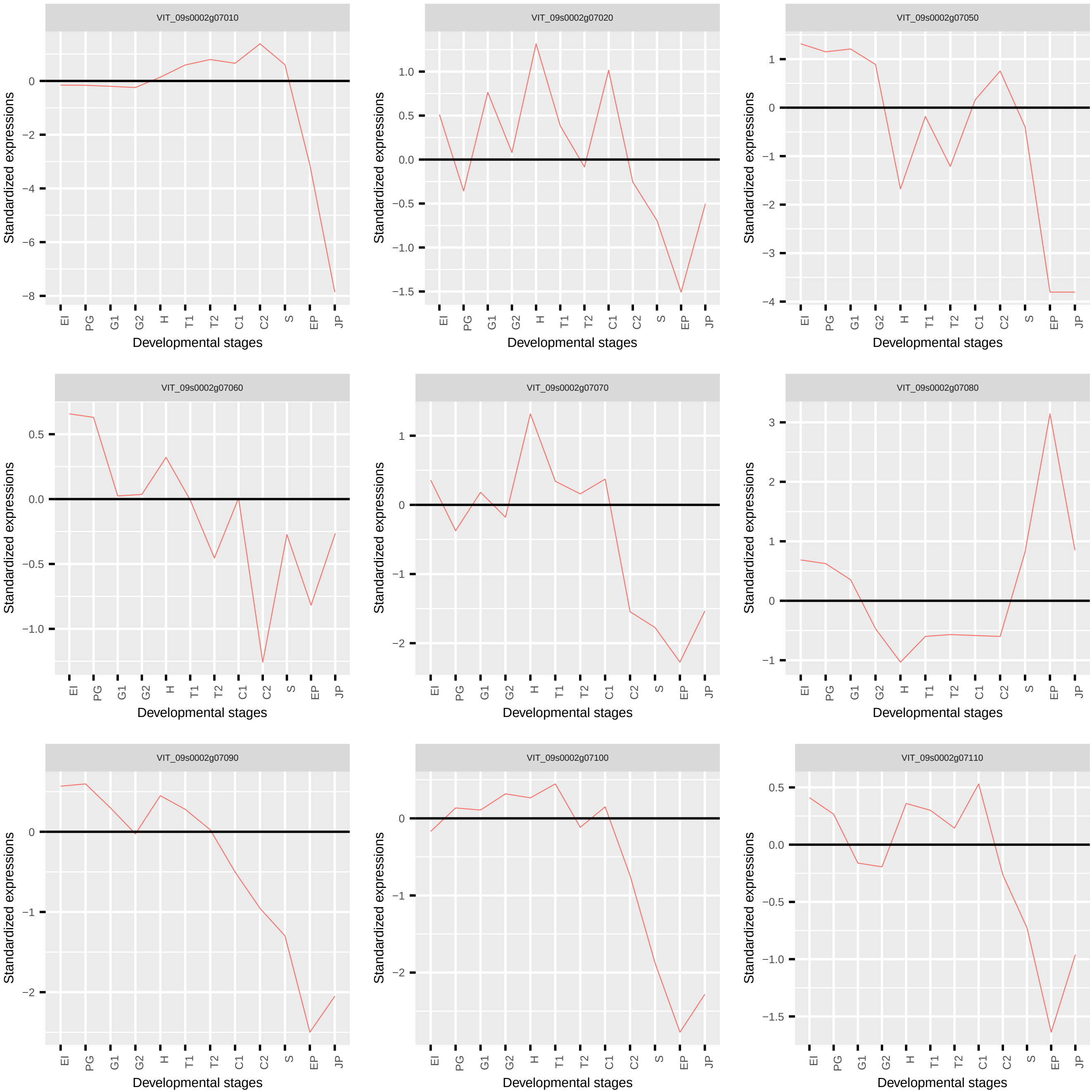
VIT_09s0002g07010
VIT_09s0002g07020
VIT_09s0002g07050
VIT_09s0002g07060
VIT_09s0002g07070
VIT_09s0002g07080
VIT_09s0002g07090
VIT_09s0002g07100
VIT_09s0002g07110
Standardized expressions
Developmental stages
EI PG G1 G2 H T1 T2 C1 C2 S EP JP

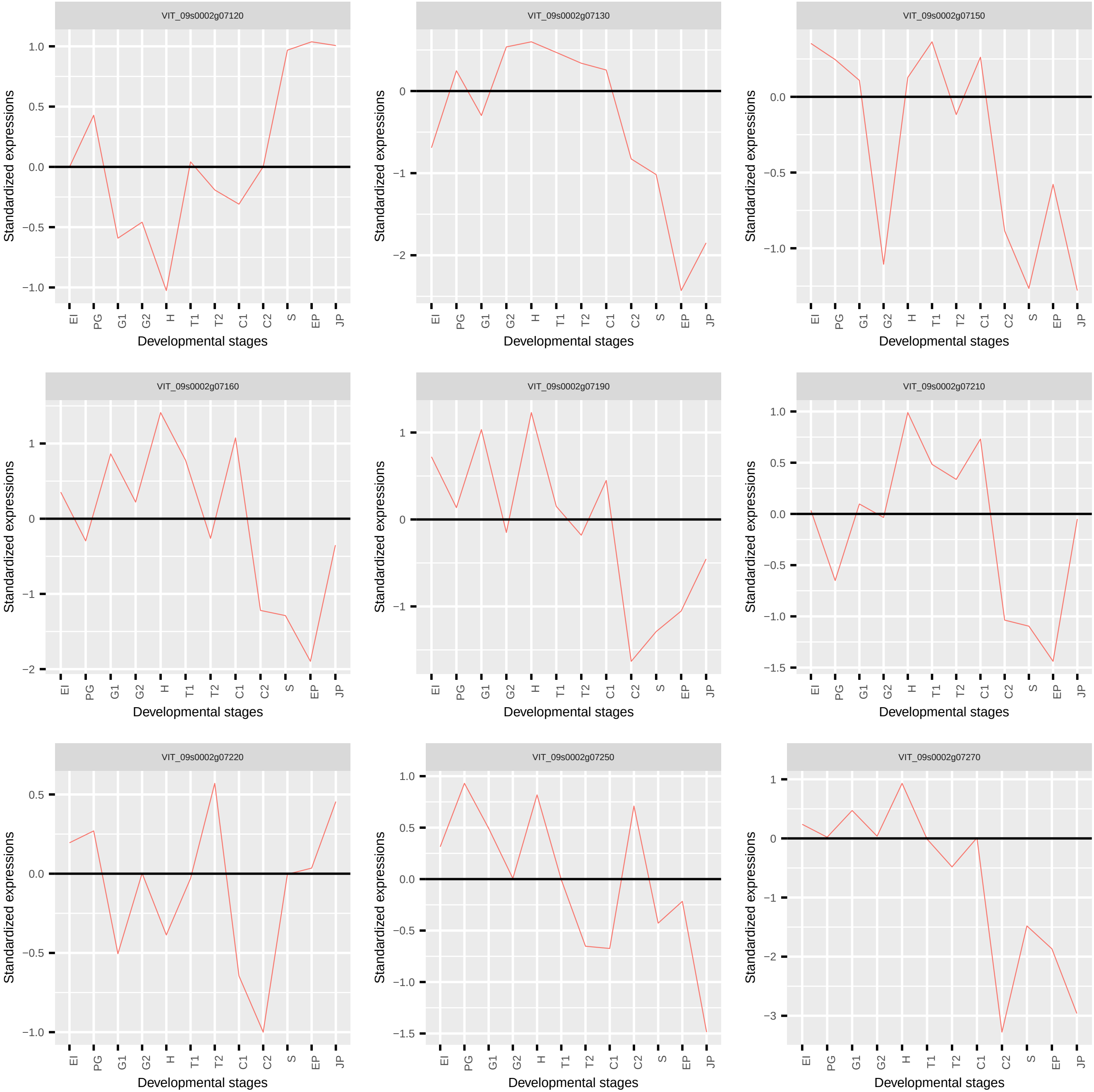

VIT_09s0002g07120
Standardized expressions
1.0
0.5
0.0
−0.5
−1.0
EI
PG
G1
G2
H
T1
T2
C1
C2
S
EP
JP
Developmental stages
VIT_09s0002g07130
Standardized expressions
0
−1
−2
EI
PG
G1
G2
H
T1
T2
C1
C2
S
EP
JP
Developmental stages
VIT_09s0002g07150
Standardized expressions
0.0
−0.5
−1.0
EI
PG
G1
G2
H
T1
T2
C1
C2
S
EP
JP
Developmental stages
VIT_09s0002g07160
Standardized expressions
1
0
−1
−2
EI
PG
G1
G2
H
T1
T2
C1
C2
S
EP
JP
Developmental stages
VIT_09s0002g07190
Standardized expressions
1
0
−1
EI
PG
G1
G2
H
T1
T2
C1
C2
S
EP
JP
Developmental stages
VIT_09s0002g07210
Standardized expressions
1.0
0.5
0.0
−0.5
−1.0
−1.5
EI
PG
G1
G2
H
T1
T2
C1
C2
S
EP
JP
Developmental stages
VIT_09s0002g07220
Standardized expressions
0.5
0.0
−0.5
−1.0
EI
PG
G1
G2
H
T1
T2
C1
C2
S
EP
JP
Developmental stages
VIT_09s0002g07250
Standardized expressions
1.0
0.5
0.0
−0.5
−1.0
−1.5
EI
PG
G1
G2
H
T1
T2
C1
C2
S
EP
JP
Developmental stages
VIT_09s0002g07270
Standardized expressions
1
0
−1
−2
−3
EI
PG
G1
G2
H
T1
T2
C1
C2
S
EP
JP
Developmental stages

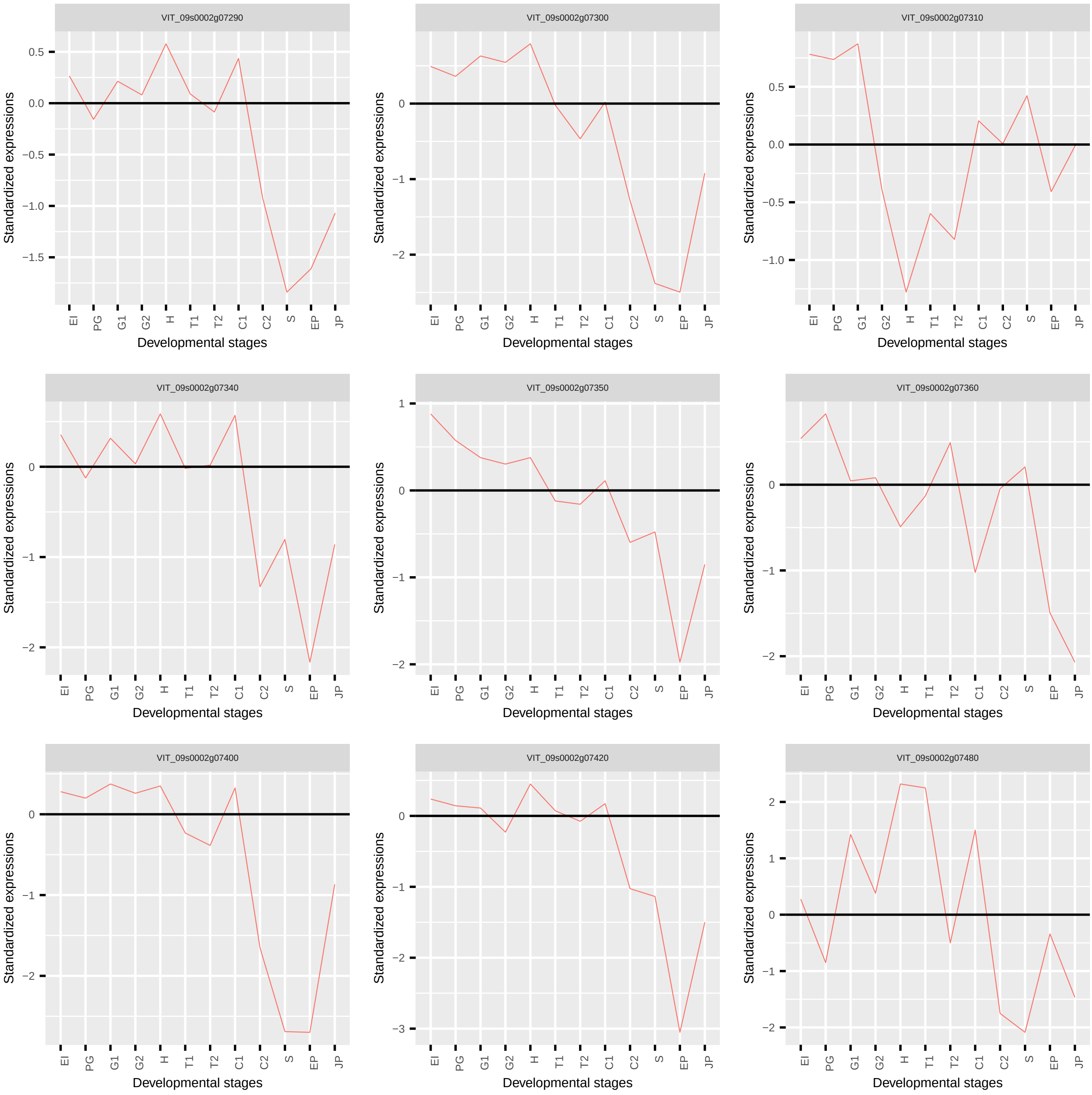
VIT_09s0002g07290
VIT_09s0002g07300
VIT_09s0002g07310
VIT_09s0002g07340
VIT_09s0002g07350
VIT_09s0002g07360
VIT_09s0002g07400
VIT_09s0002g07420
VIT_09s0002g07480
Standardized expressions
Developmental stages
EI
PG
G1
G2
H
T1
T2
C1
C2
S
EP
JP

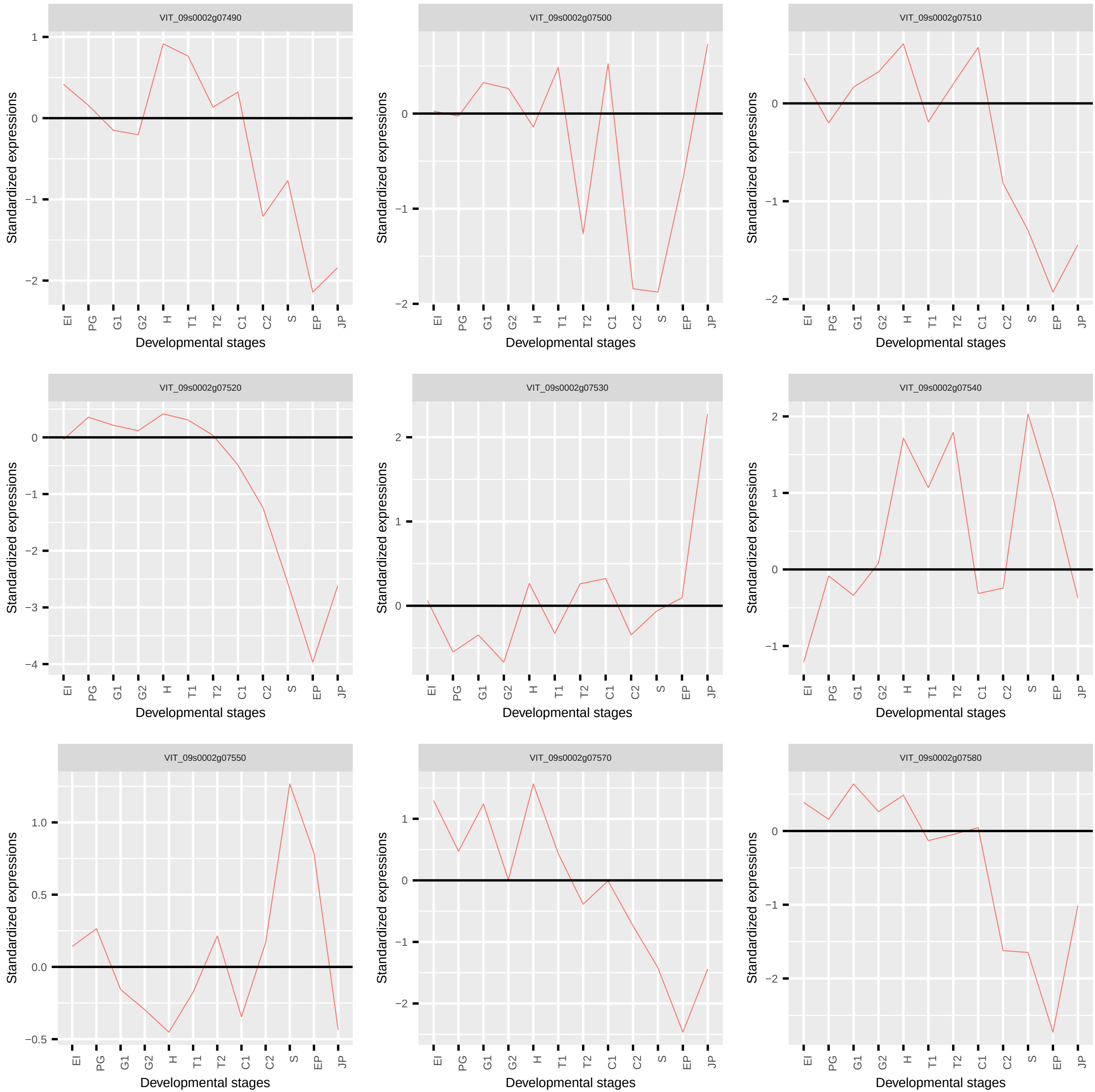
VIT_09s0002g07490
VIT_09s0002g07500
VIT_09s0002g07510
VIT_09s0002g07520
VIT_09s0002g07530
VIT_09s0002g07540
VIT_09s0002g07550
VIT_09s0002g07570
VIT_09s0002g07580
Standardized expressions
Developmental stages
EI PG G1 G2 H T1 T2 C1 C2 S EP JP

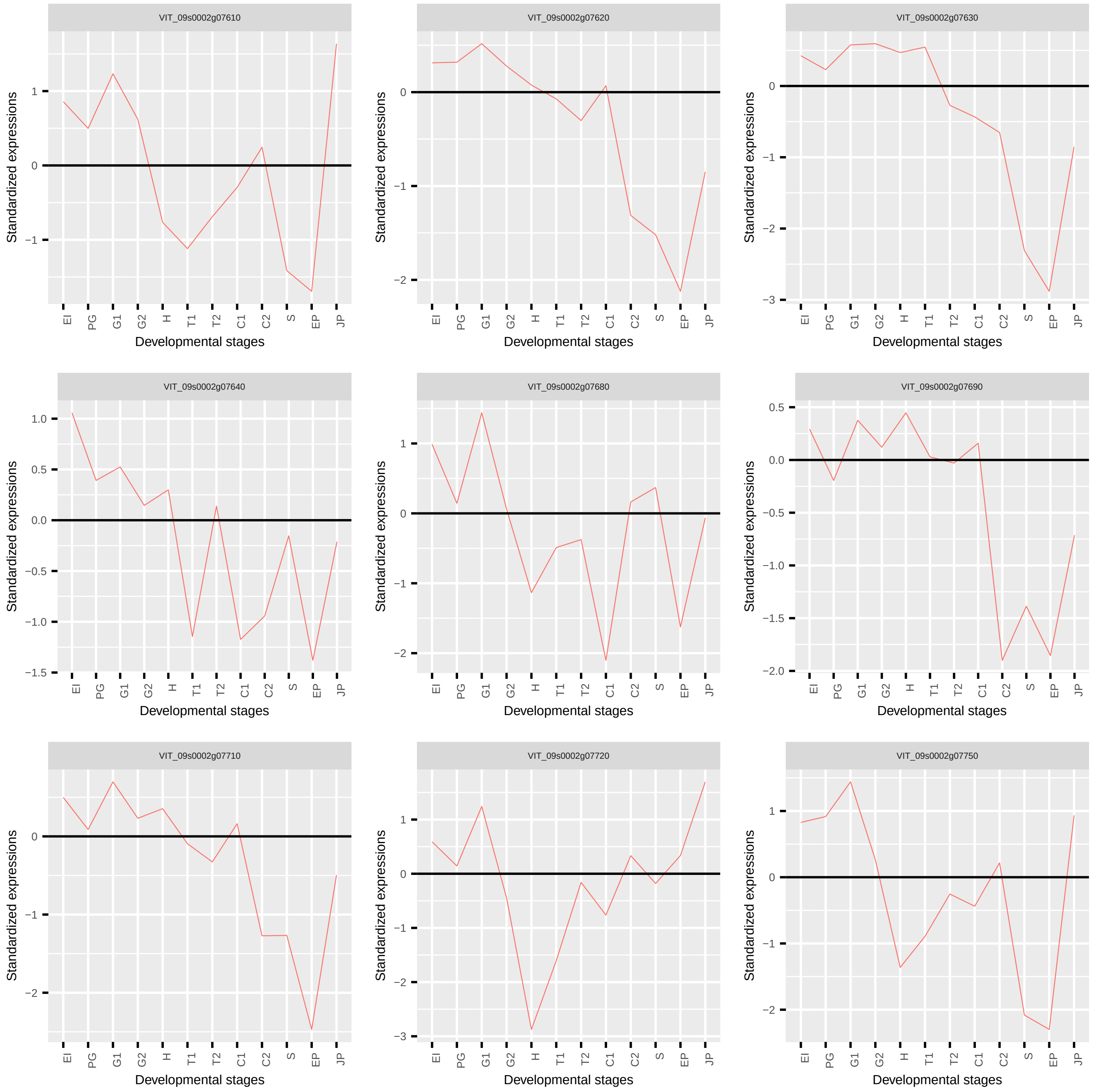
VIT_09s0002g07610
VIT_09s0002g07620
VIT_09s0002g07630
VIT_09s0002g07640
VIT_09s0002g07680
VIT_09s0002g07690
VIT_09s0002g07710
VIT_09s0002g07720
VIT_09s0002g07750
Standardized expressions
Developmental stages
EI PG G1 G2 H T1 T2 C1 C2 S EP JP

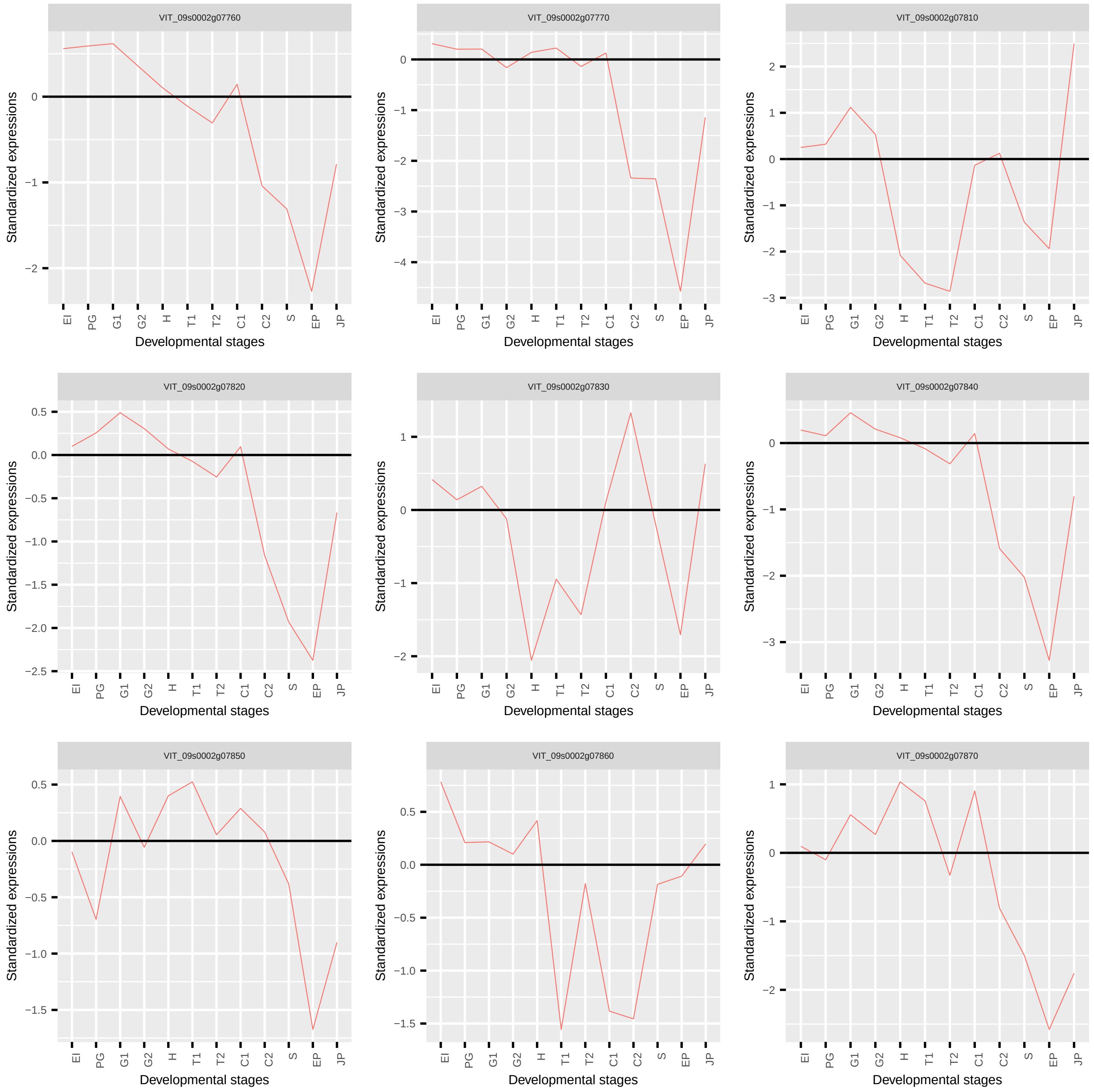
VIT_09s0002g07760
VIT_09s0002g07770
VIT_09s0002g07810
VIT_09s0002g07820
VIT_09s0002g07830
VIT_09s0002g07840
VIT_09s0002g07850
VIT_09s0002g07860
VIT_09s0002g07870
Standardized expressions
Developmental stages
EI PG G1 G2 H T1 T2 C1 C2 S EP JP

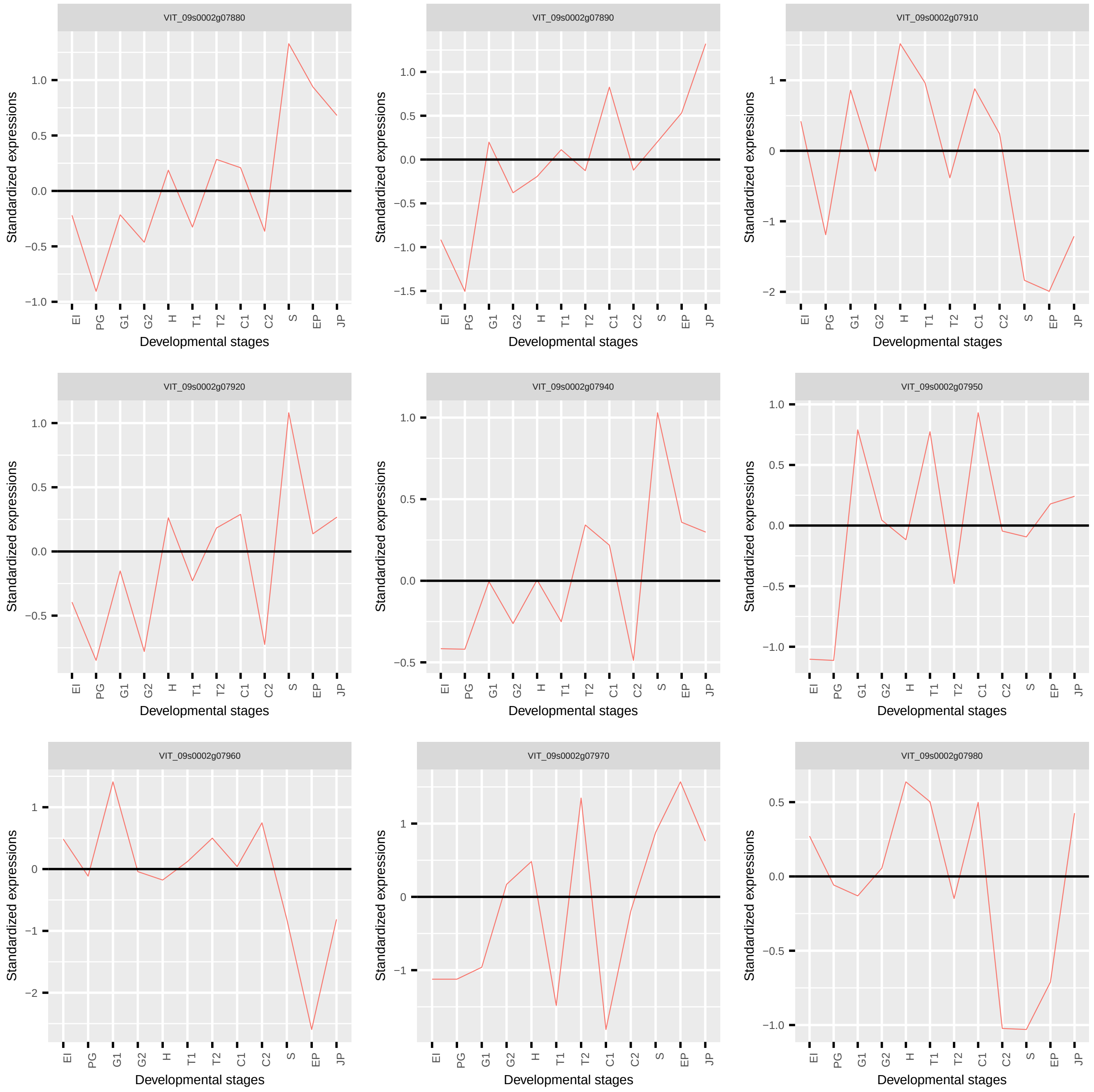
VIT_09s0002g07880
VIT_09s0002g07890
VIT_09s0002g07910
VIT_09s0002g07920
VIT_09s0002g07940
VIT_09s0002g07950
VIT_09s0002g07960
VIT_09s0002g07970
VIT_09s0002g07980
Standardized expressions
Developmental stages
EI PG G1 G2 H T1 T2 C1 C2 S EP JP

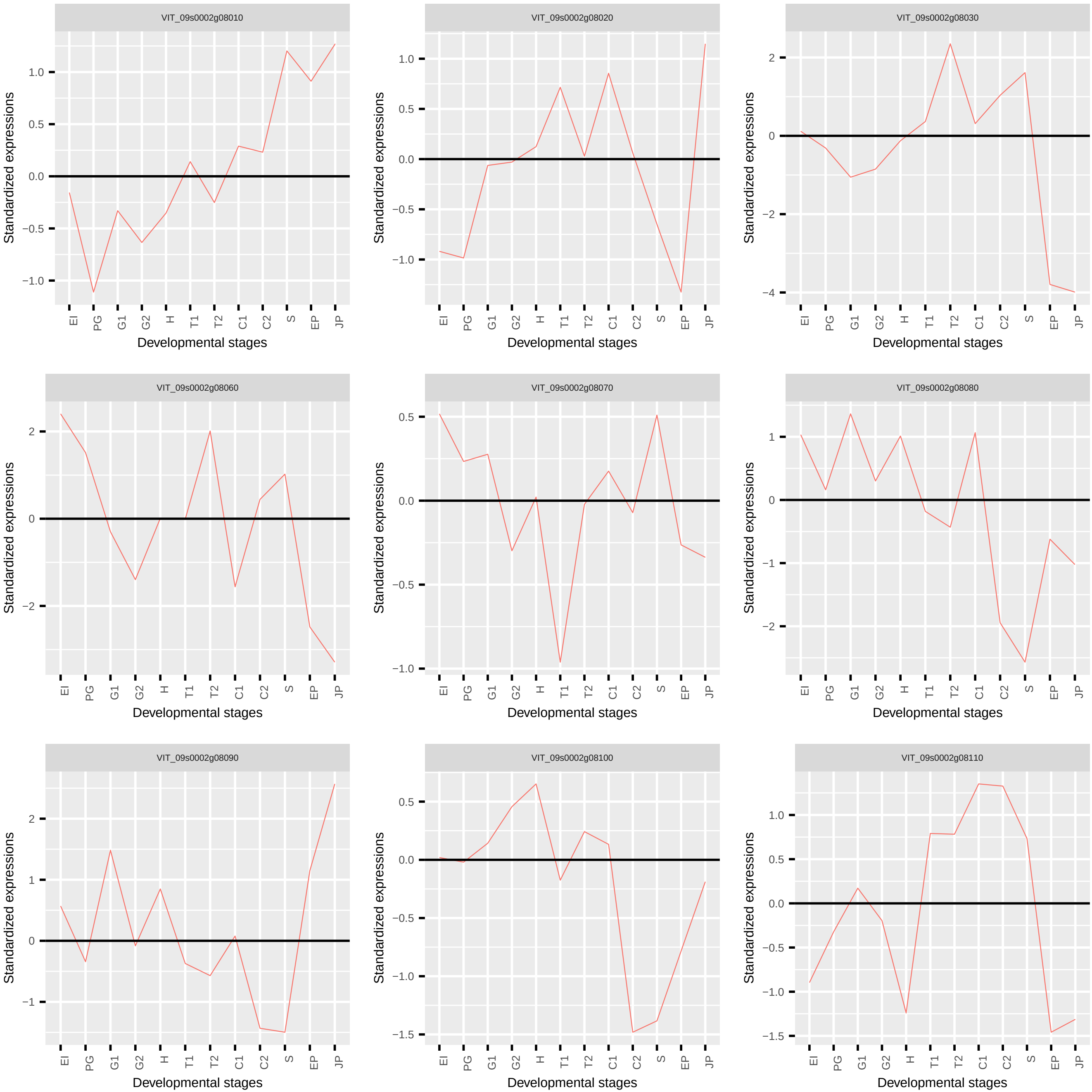
VIT_09s0002g08010
VIT_09s0002g08020
VIT_09s0002g08030
VIT_09s0002g08060
VIT_09s0002g08070
VIT_09s0002g08080
VIT_09s0002g08090
VIT_09s0002g08100
VIT_09s0002g08110
Standardized expressions
Developmental stages
EI PG G1 G2 H T1 T2 C1 C2 S EP JP

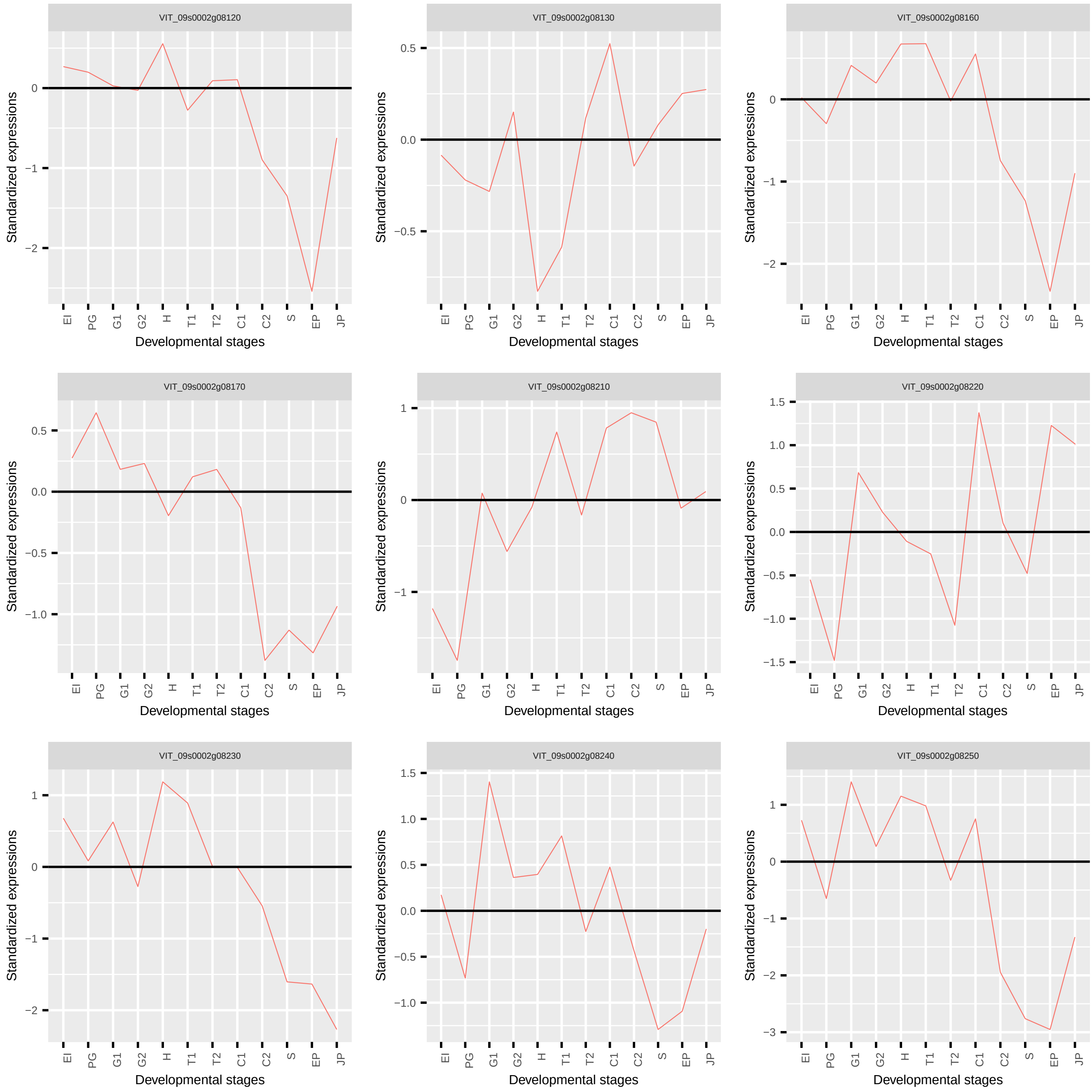
VIT_09s0002g08120
Standardized expressions
0
−1
−2
EI PG G1 G2 H T1 T2 C1 C2 S EP JP
Developmental stages
VIT_09s0002g08130
Standardized expressions
0.5
0.0
−0.5
EI PG G1 G2 H T1 T2 C1 C2 S EP JP
Developmental stages
VIT_09s0002g08160
Standardized expressions
0
−1
−2
EI PG G1 G2 H T1 T2 C1 C2 S EP JP
Developmental stages
VIT_09s0002g08170
Standardized expressions
0.5
0.0
−0.5
−1.0
EI PG G1 G2 H T1 T2 C1 C2 S EP JP
Developmental stages
VIT_09s0002g08210
Standardized expressions
1
0
−1
EI PG G1 G2 H T1 T2 C1 C2 S EP JP
Developmental stages
VIT_09s0002g08220
Standardized expressions
1.5
1.0
0.5
0.0
−0.5
−1.0
−1.5
EI PG G1 G2 H T1 T2 C1 C2 S EP JP
Developmental stages
VIT_09s0002g08230
Standardized expressions
1
0
−1
−2
EI PG G1 G2 H T1 T2 C1 C2 S EP JP
Developmental stages
VIT_09s0002g08240
Standardized expressions
1.5
1.0
0.5
0.0
−0.5
−1.0
EI PG G1 G2 H T1 T2 C1 C2 S EP JP
Developmental stages
VIT_09s0002g08250
Standardized expressions
1
0
−1
−2
−3
EI PG G1 G2 H T1 T2 C1 C2 S EP JP
Developmental stages

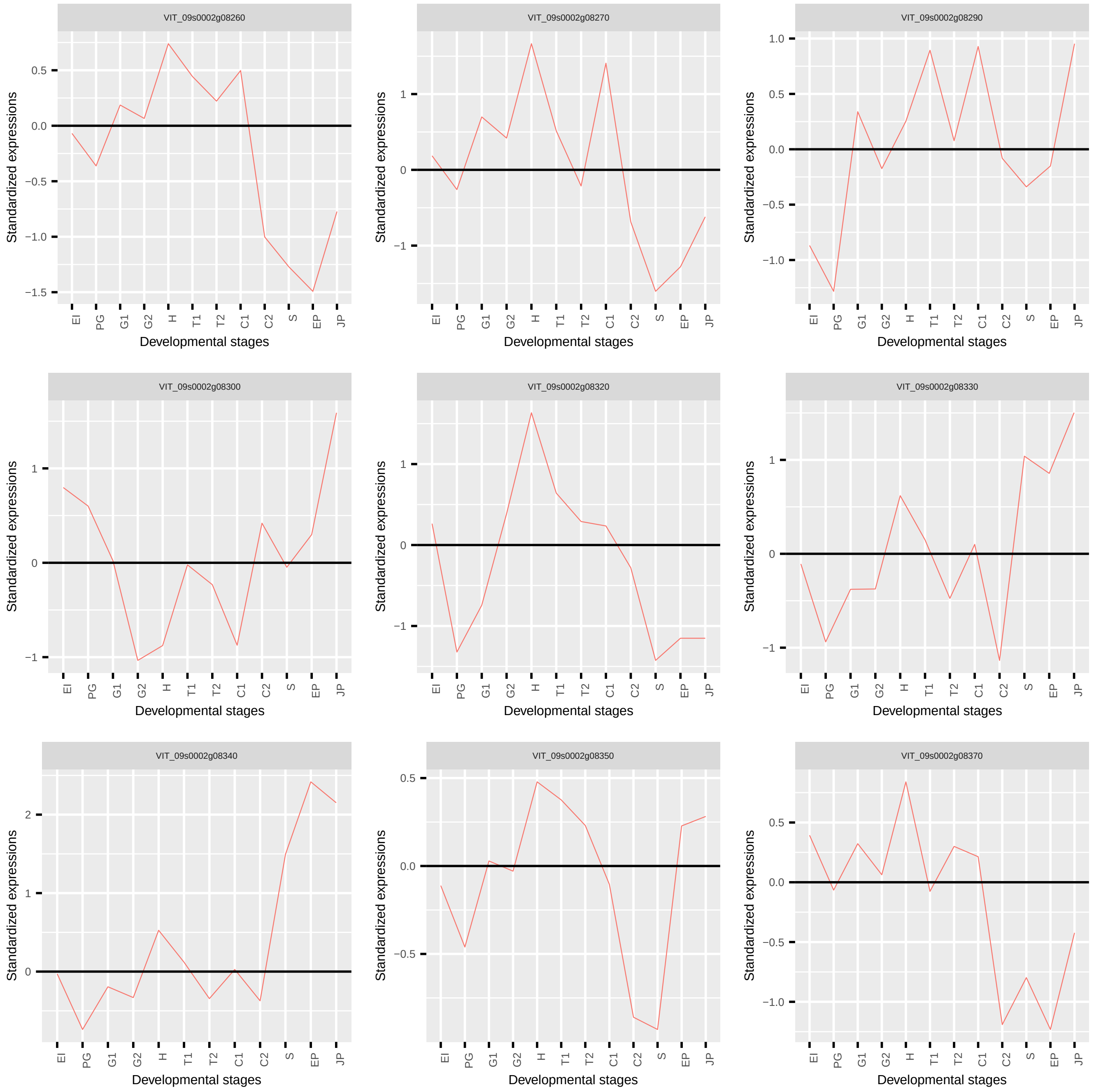
VIT_09s0002g08260
VIT_09s0002g08270
VIT_09s0002g08290
VIT_09s0002g08300
VIT_09s0002g08320
VIT_09s0002g08330
VIT_09s0002g08340
VIT_09s0002g08350
VIT_09s0002g08370
Standardized expressions
Developmental stages
EI PG G1 G2 H T1 T2 C1 C2 S EP JP

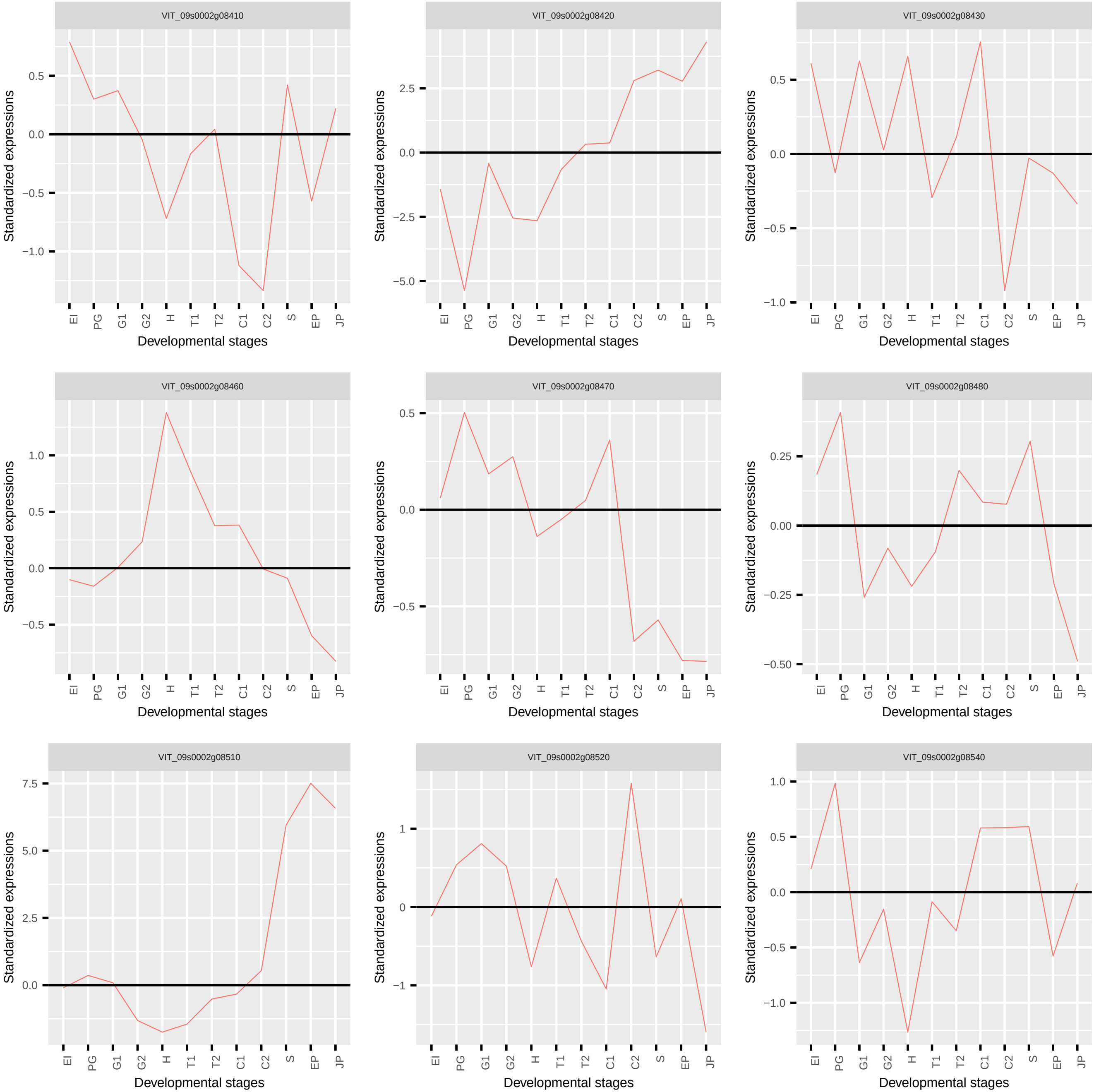
VIT_09s0002g08410
0.5
0.0
−0.5
−1.0
Standardized expressions
EI PG G1 G2 H T1 T2 C1 C2 S EP JP
Developmental stages
VIT_09s0002g08420
2.5
0.0
−2.5
−5.0
Standardized expressions
EI PG G1 G2 H T1 T2 C1 C2 S EP JP
Developmental stages
VIT_09s0002g08430
0.5
0.0
−0.5
−1.0
Standardized expressions
EI PG G1 G2 H T1 T2 C1 C2 S EP JP
Developmental stages
VIT_09s0002g08460
1.0
0.5
0.0
−0.5
Standardized expressions
EI PG G1 G2 H T1 T2 C1 C2 S EP JP
Developmental stages
VIT_09s0002g08470
0.5
0.0
−0.5
Standardized expressions
EI PG G1 G2 H T1 T2 C1 C2 S EP JP
Developmental stages
VIT_09s0002g08480
0.25
0.00
−0.25
−0.50
Standardized expressions
EI PG G1 G2 H T1 T2 C1 C2 S EP JP
Developmental stages
VIT_09s0002g08510
7.5
5.0
2.5
0.0
Standardized expressions
EI PG G1 G2 H T1 T2 C1 C2 S EP JP
Developmental stages
VIT_09s0002g08520
1
0
−1
Standardized expressions
EI PG G1 G2 H T1 T2 C1 C2 S EP JP
Developmental stages
VIT_09s0002g08540
1.0
0.5
0.0
−0.5
−1.0
Standardized expressions
EI PG G1 G2 H T1 T2 C1 C2 S EP JP
Developmental stages

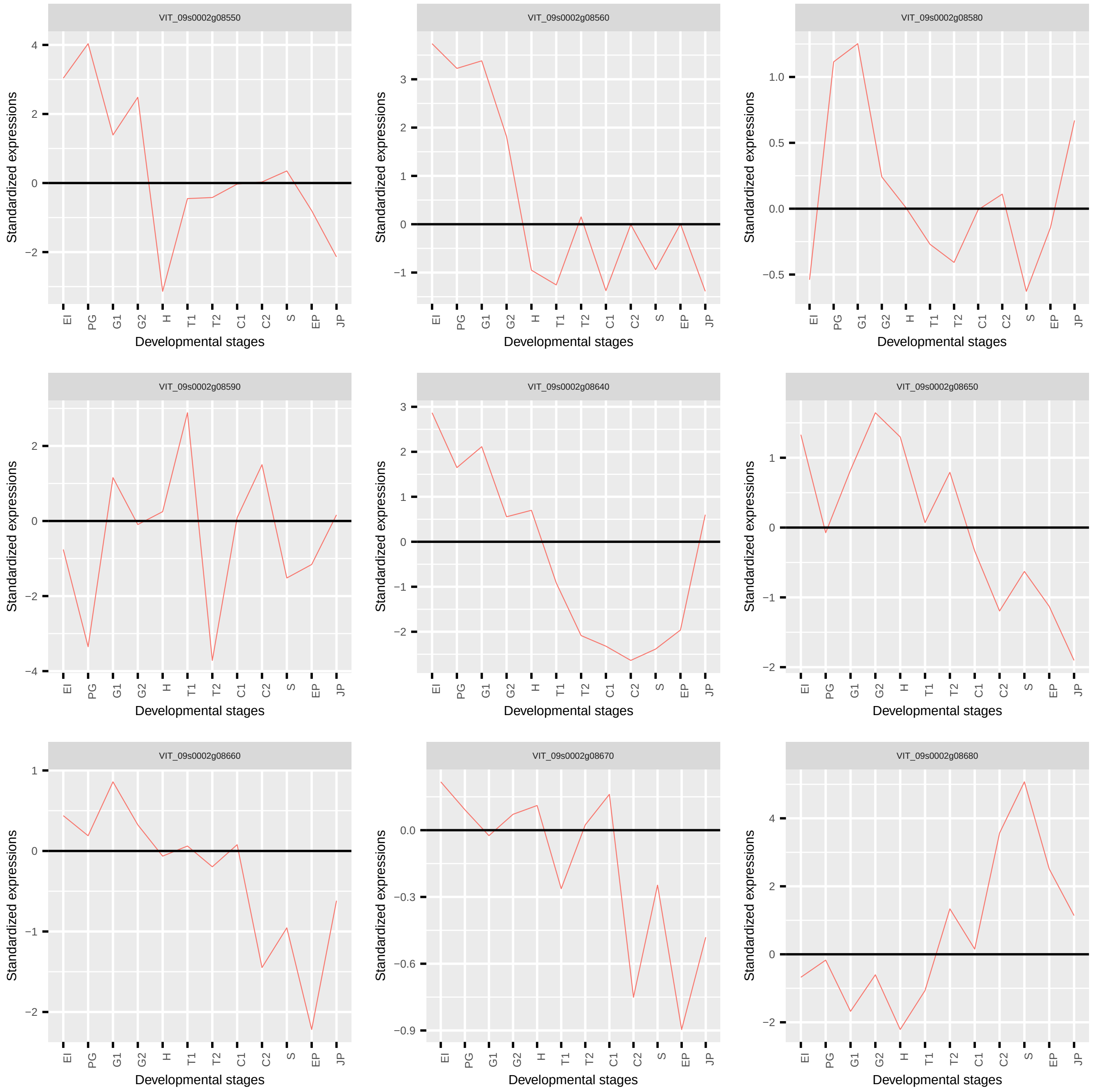
VIT_09s0002g08550
4
2
0
−2
Standardized expressions
EI PG G1 G2 H T1 T2 C1 C2 S EP JP
Developmental stages
VIT_09s0002g08560
3
2
1
0
−1
Standardized expressions
EI PG G1 G2 H T1 T2 C1 C2 S EP JP
Developmental stages
VIT_09s0002g08580
1.0
0.5
0.0
−0.5
Standardized expressions
EI PG G1 G2 H T1 T2 C1 C2 S EP JP
Developmental stages
VIT_09s0002g08590
2
0
−2
−4
Standardized expressions
EI PG G1 G2 H T1 T2 C1 C2 S EP JP
Developmental stages
VIT_09s0002g08640
3
2
1
0
−1
−2
Standardized expressions
EI PG G1 G2 H T1 T2 C1 C2 S EP JP
Developmental stages
VIT_09s0002g08650
1
0
−1
−2
Standardized expressions
EI PG G1 G2 H T1 T2 C1 C2 S EP JP
Developmental stages
VIT_09s0002g08660
1
0
−1
−2
Standardized expressions
EI PG G1 G2 H T1 T2 C1 C2 S EP JP
Developmental stages
VIT_09s0002g08670
0.0
−0.3
−0.6
−0.9
Standardized expressions
EI PG G1 G2 H T1 T2 C1 C2 S EP JP
Developmental stages
VIT_09s0002g08680
4
2
0
−2
Standardized expressions
EI PG G1 G2 H T1 T2 C1 C2 S EP JP
Developmental stages

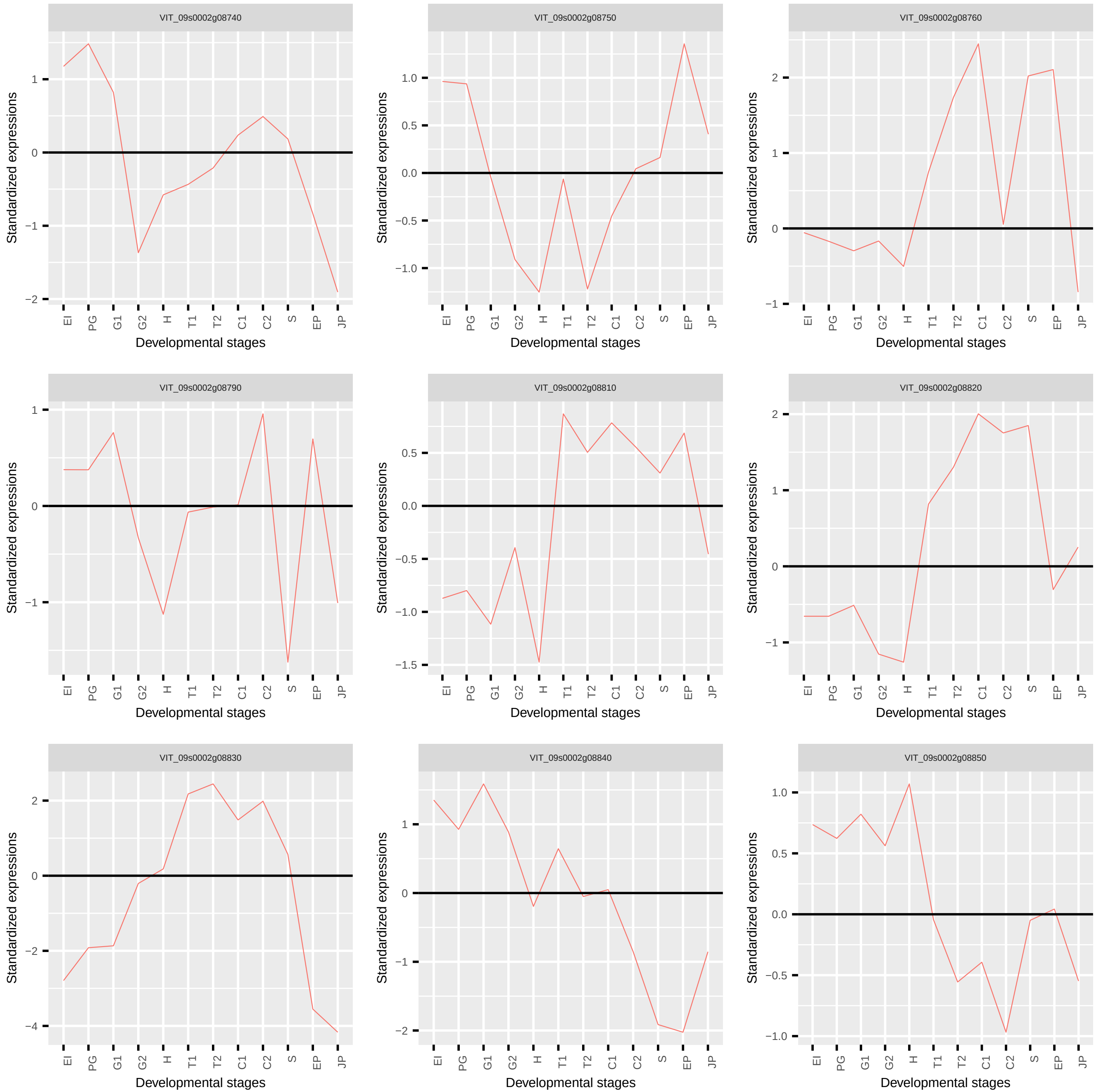
VIT_09s0002g08740
VIT_09s0002g08750
VIT_09s0002g08760
VIT_09s0002g08790
VIT_09s0002g08810
VIT_09s0002g08820
VIT_09s0002g08830
VIT_09s0002g08840
VIT_09s0002g08850
Standardized expressions
Developmental stages
EI PG G1 G2 H T1 T2 C1 C2 S EP JP

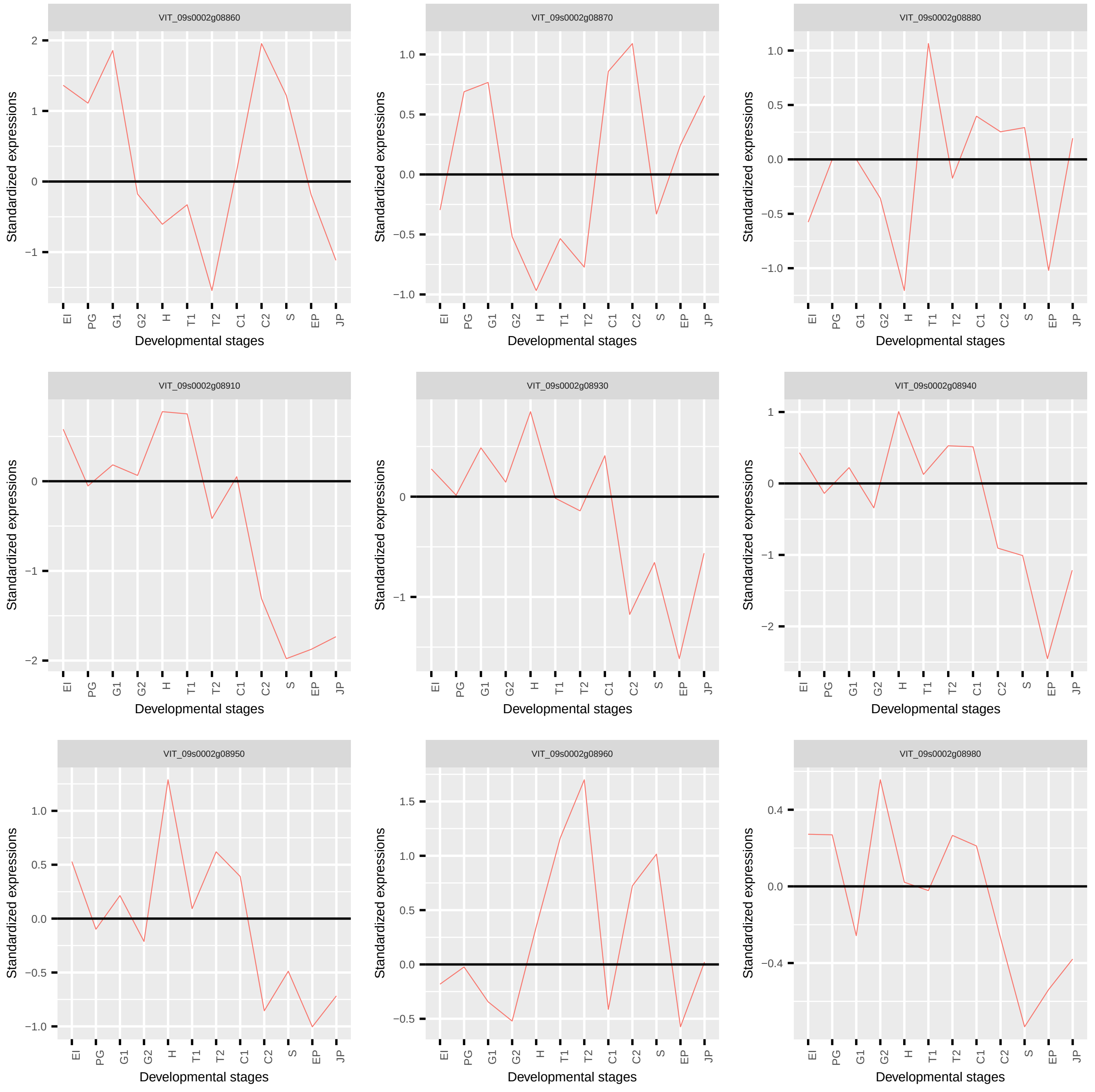
VIT_09s0002g08860
VIT_09s0002g08870
VIT_09s0002g08880
VIT_09s0002g08910
VIT_09s0002g08930
VIT_09s0002g08940
VIT_09s0002g08950
VIT_09s0002g08960
VIT_09s0002g08980
Standardized expressions
Developmental stages
EI
PG
G1
G2
H
T1
T2
C1
C2
S
EP
JP

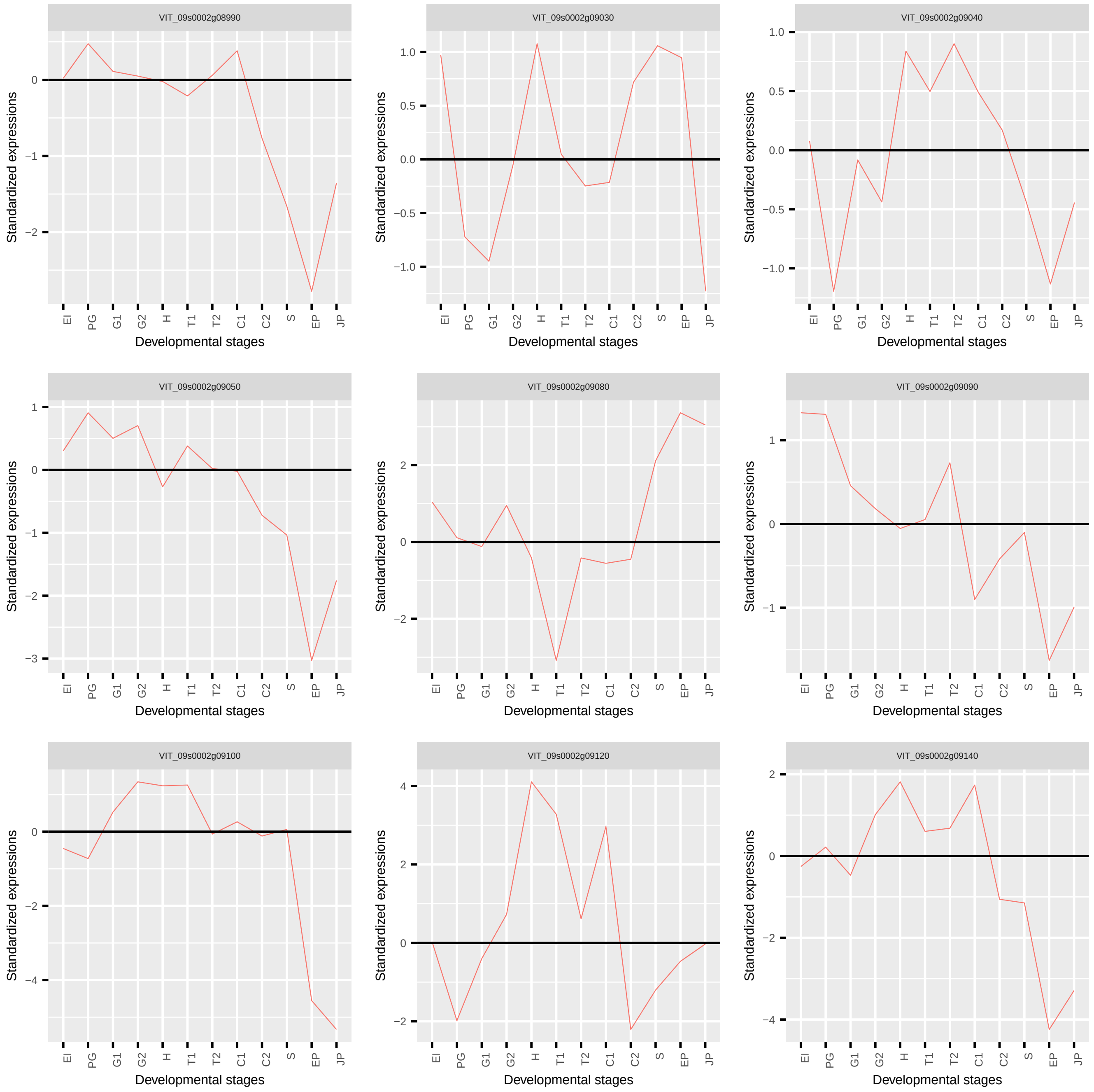
VIT_09s0002g08990
VIT_09s0002g09030
VIT_09s0002g09040
VIT_09s0002g09050
VIT_09s0002g09080
VIT_09s0002g09090
VIT_09s0002g09100
VIT_09s0002g09120
VIT_09s0002g09140
Standardized expressions
Developmental stages
EI PG G1 G2 H T1 T2 C1 C2 S EP JP

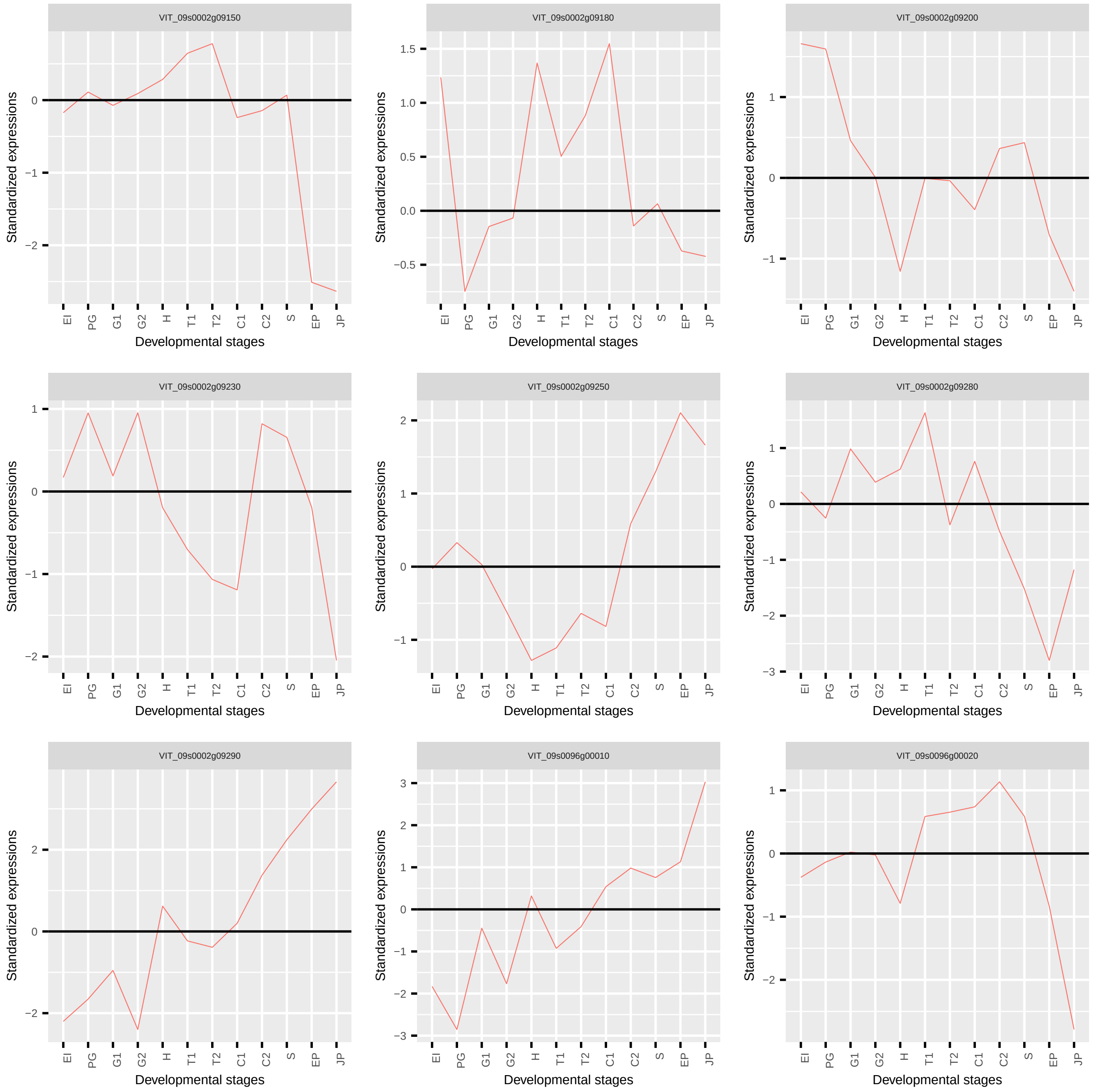
VIT_09s0002g09150
VIT_09s0002g09180
VIT_09s0002g09200
VIT_09s0002g09230
VIT_09s0002g09250
VIT_09s0002g09280
VIT_09s0002g09290
VIT_09s0096g00010
VIT_09s0096g00020
Standardized expressions
Developmental stages
EI PG G1 G2 H T1 T2 C1 C2 S EP JP

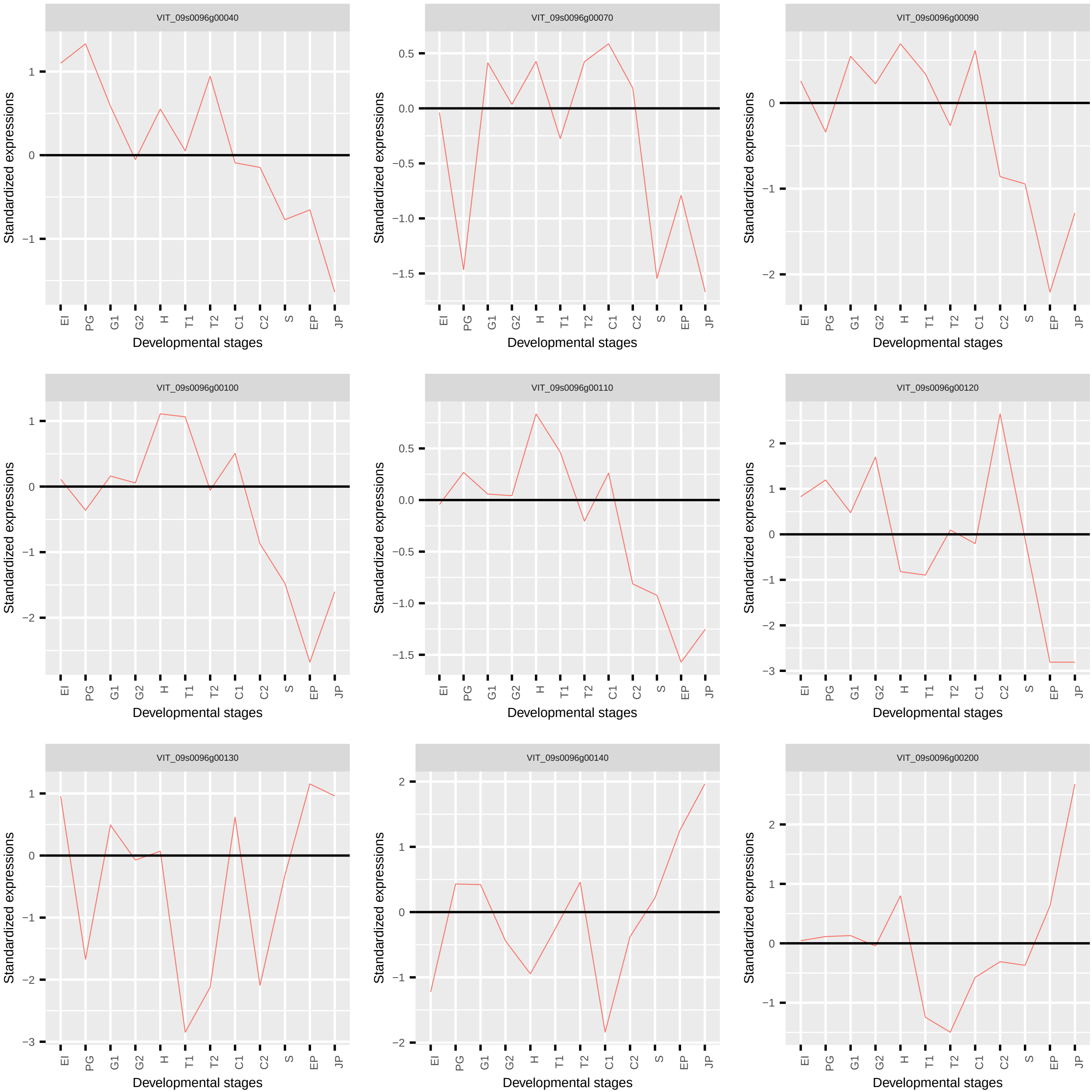
VIT_09s0096g00040
VIT_09s0096g00070
VIT_09s0096g00090
VIT_09s0096g00100
VIT_09s0096g00110
VIT_09s0096g00120
VIT_09s0096g00130
VIT_09s0096g00140
VIT_09s0096g00200
Standardized expressions
Developmental stages
EI PG G1 G2 H T1 T2 C1 C2 S EP JP

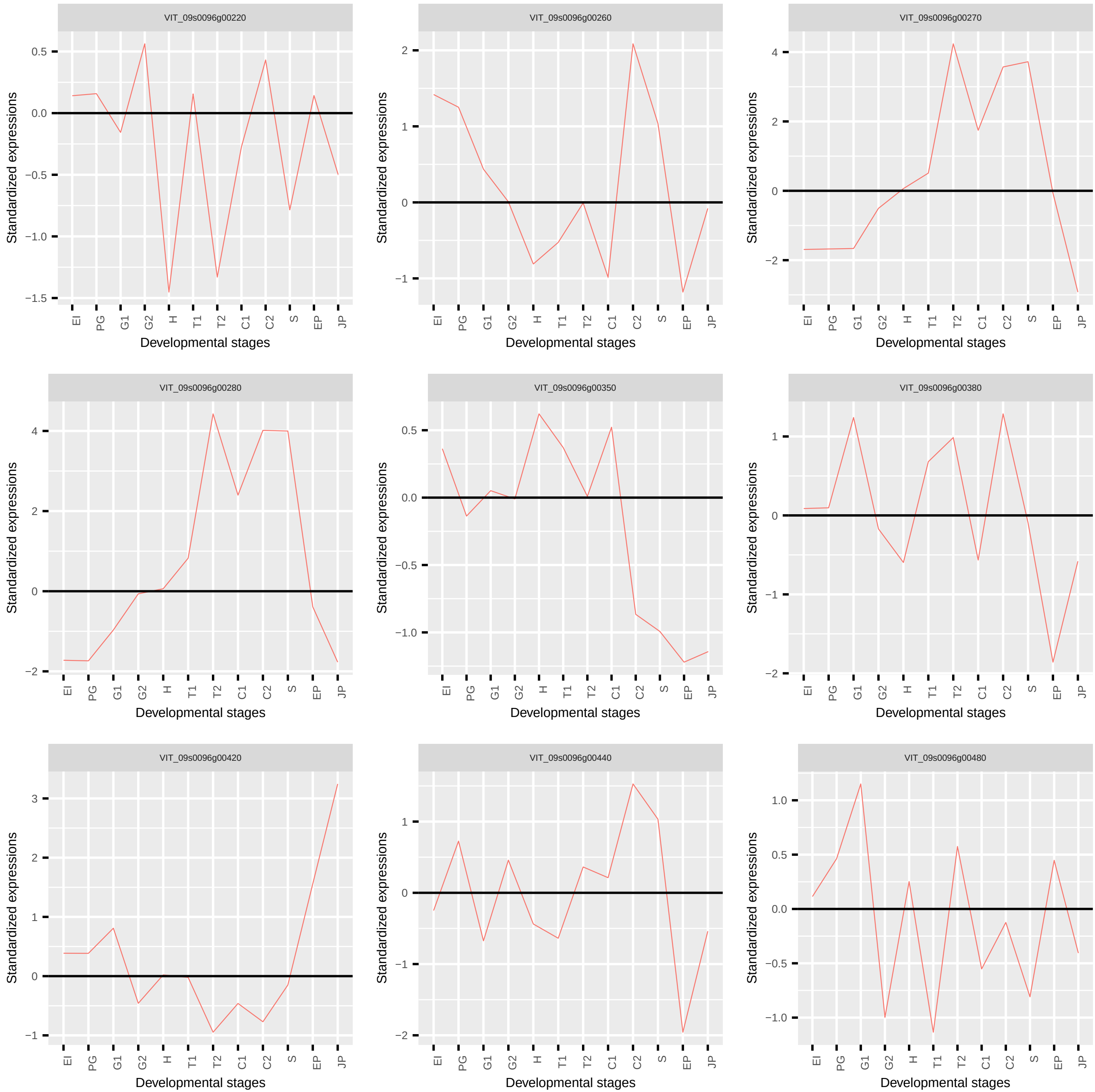
VIT_09s0096g00220
VIT_09s0096g00260
VIT_09s0096g00270
VIT_09s0096g00280
VIT_09s0096g00350
VIT_09s0096g00380
VIT_09s0096g00420
VIT_09s0096g00440
VIT_09s0096g00480
Standardized expressions
Developmental stages
EI
PG
G1
G2
H
T1
T2
C1
C2
S
EP
JP
0.5
0.0
−0.5
−1.0
−1.5
2
1
0
−1
4
2
0
−2
1.0
−2

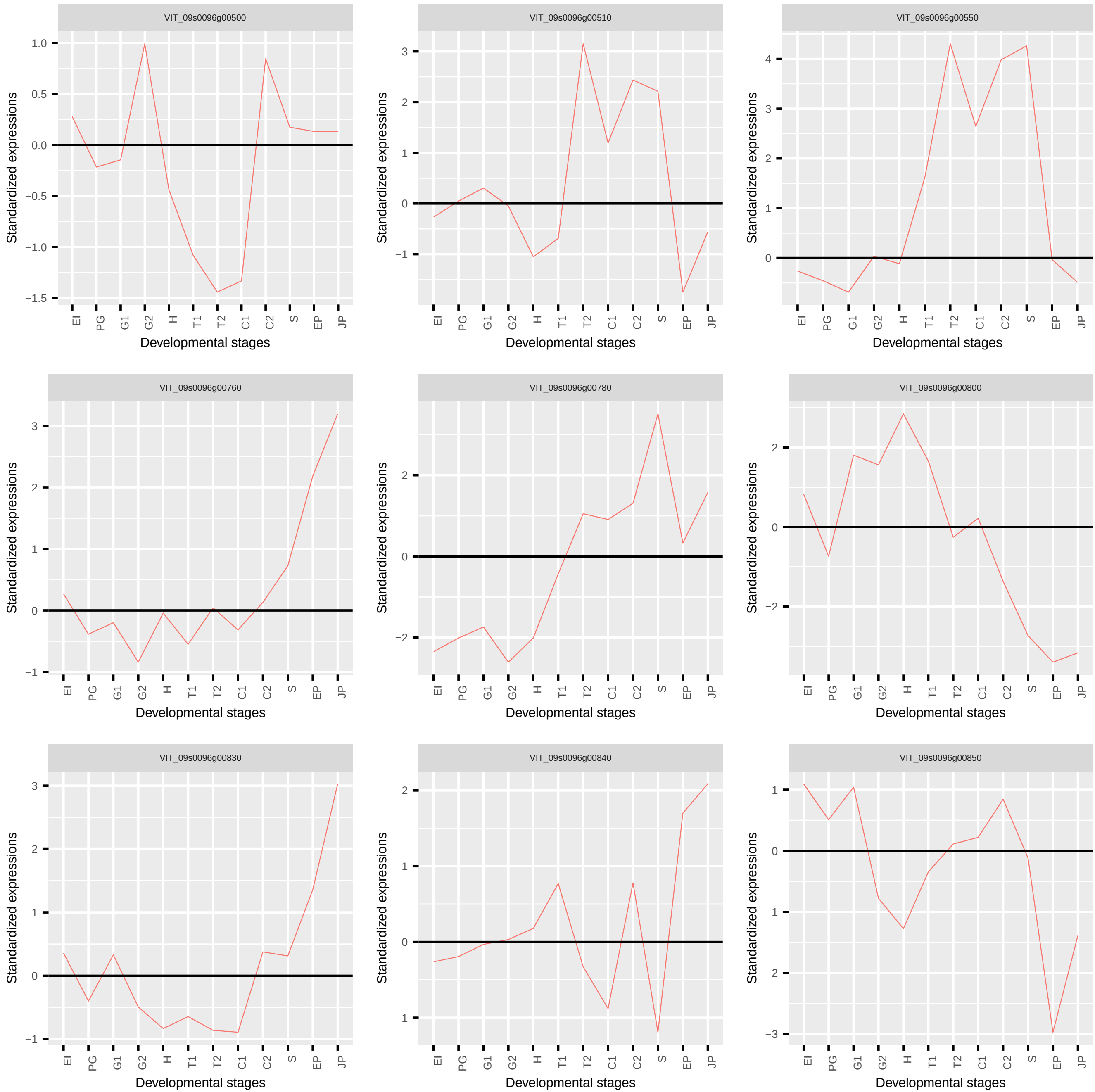
VIT_09s0096g00500
VIT_09s0096g00510
VIT_09s0096g00550
VIT_09s0096g00760
VIT_09s0096g00780
VIT_09s0096g00800
VIT_09s0096g00830
VIT_09s0096g00840
VIT_09s0096g00850
Standardized expressions
Developmental stages
EI PG G1 G2 H T1 T2 C1 C2 S EP JP

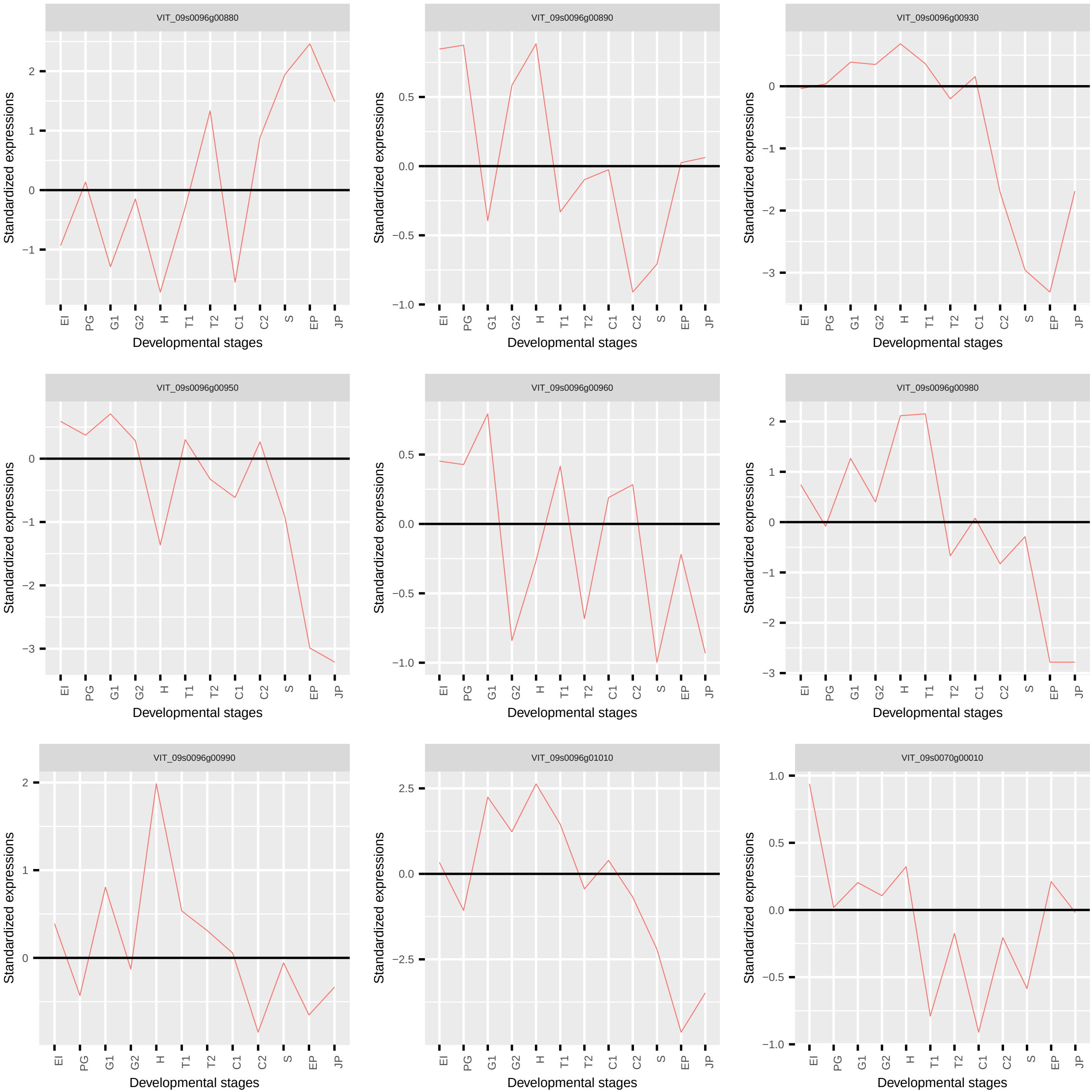
VIT_09s0096g00880
VIT_09s0096g00890
VIT_09s0096g00930
VIT_09s0096g00950
VIT_09s0096g00960
VIT_09s0096g00980
VIT_09s0096g00990
VIT_09s0096g01010
VIT_09s0070g00010
Standardized expressions
Developmental stages
EI PG G1 G2 H T1 T2 C1 C2 S EP JP

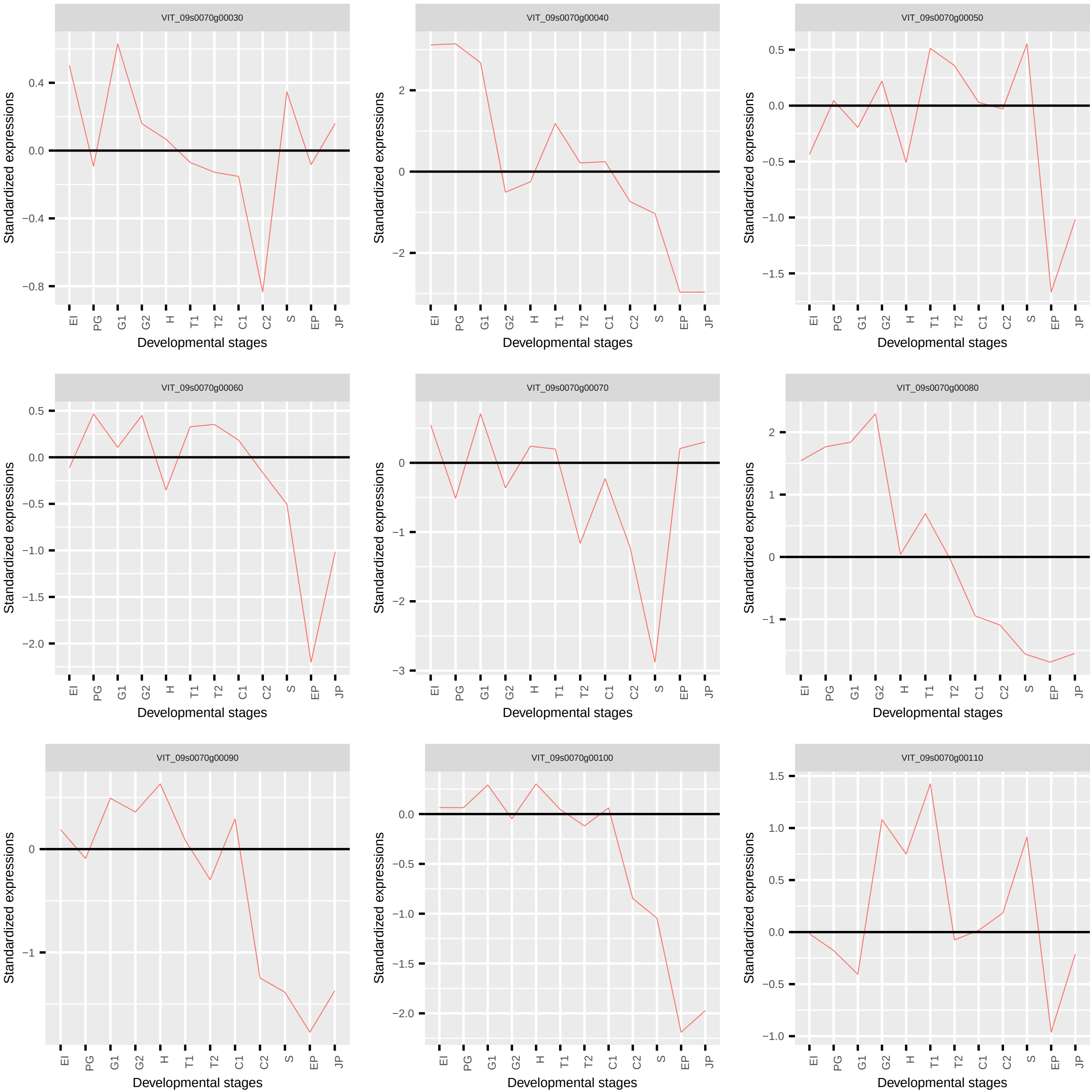
VIT_09s0070g00030
VIT_09s0070g00040
VIT_09s0070g00050
VIT_09s0070g00060
VIT_09s0070g00070
VIT_09s0070g00080
VIT_09s0070g00090
VIT_09s0070g00100
VIT_09s0070g00110
Standardized expressions
Developmental stages
EI PG G1 G2 H T1 T2 C1 C2 S EP JP

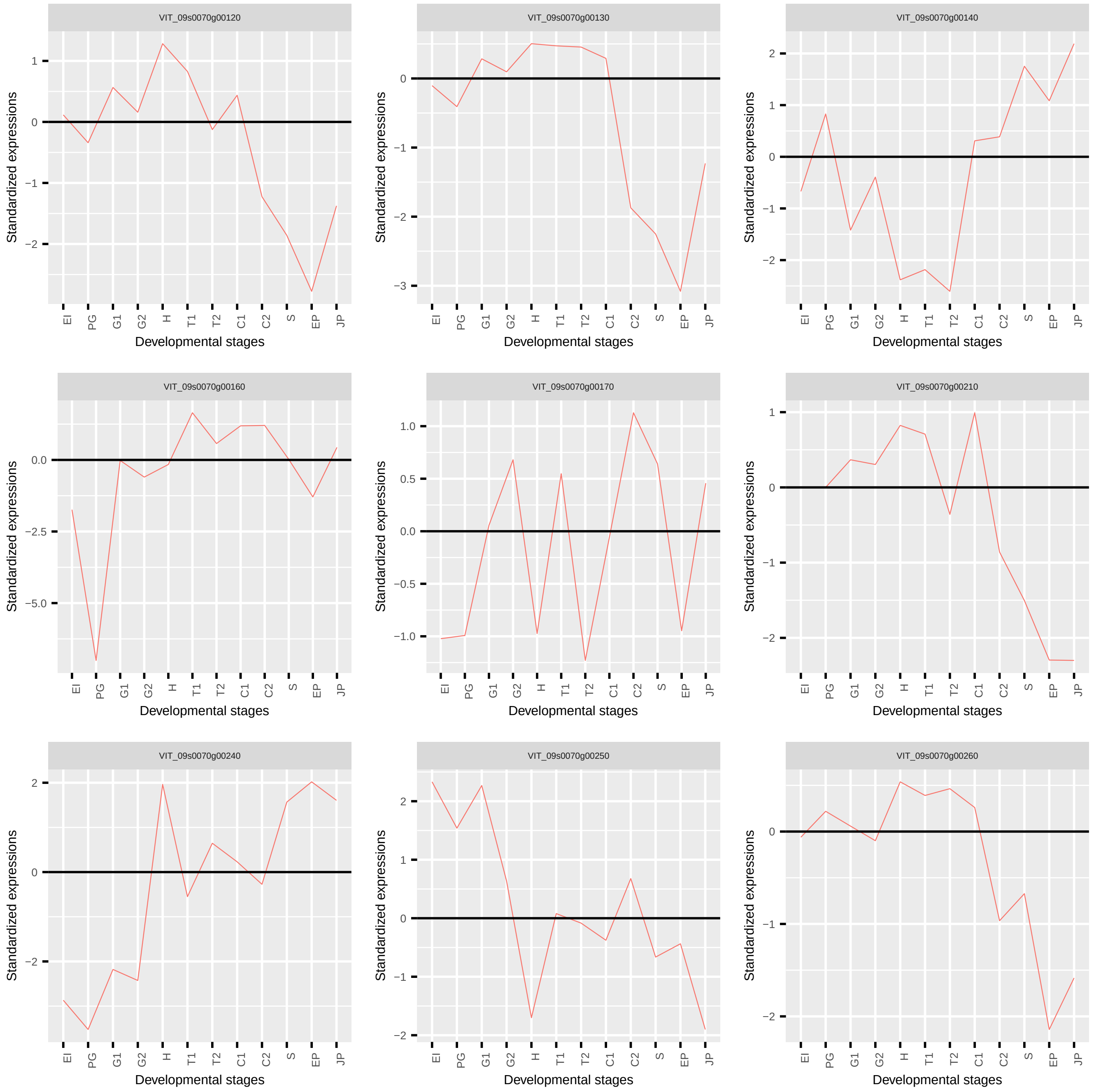
VIT_09s0070g00120
VIT_09s0070g00130
VIT_09s0070g00140
VIT_09s0070g00160
VIT_09s0070g00170
VIT_09s0070g00210
VIT_09s0070g00240
VIT_09s0070g00250
VIT_09s0070g00260
Standardized expressions
Developmental stages
EI
PG
G1
G2
H
T1
T2
C1
C2
S
EP
JP

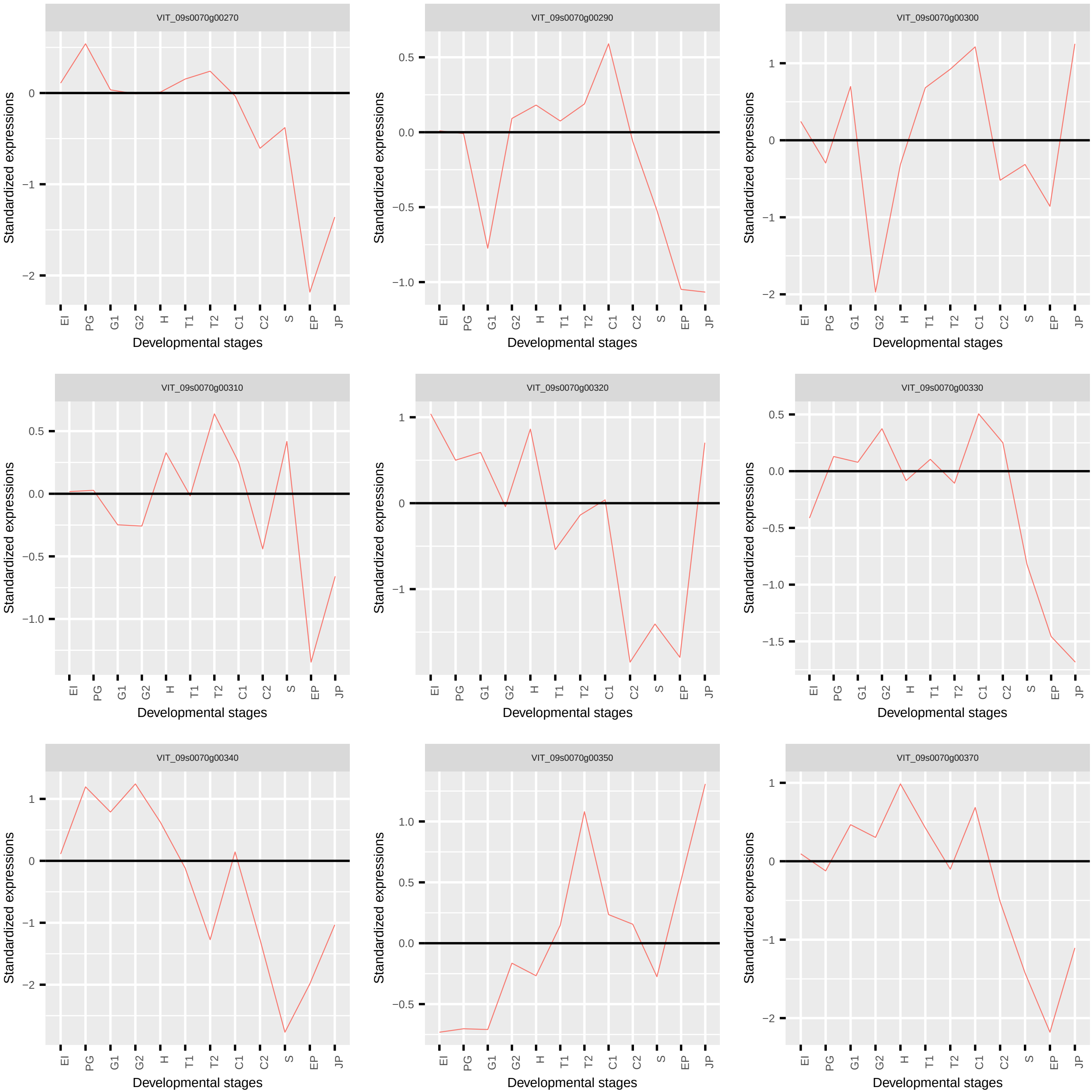
VIT_09s0070g00270
VIT_09s0070g00290
VIT_09s0070g00300
VIT_09s0070g00310
VIT_09s0070g00320
VIT_09s0070g00330
VIT_09s0070g00340
VIT_09s0070g00350
VIT_09s0070g00370
Standardized expressions
Developmental stages
EI
PG
G1
G2
H
T1
T2
C1
C2
S
EP
JP

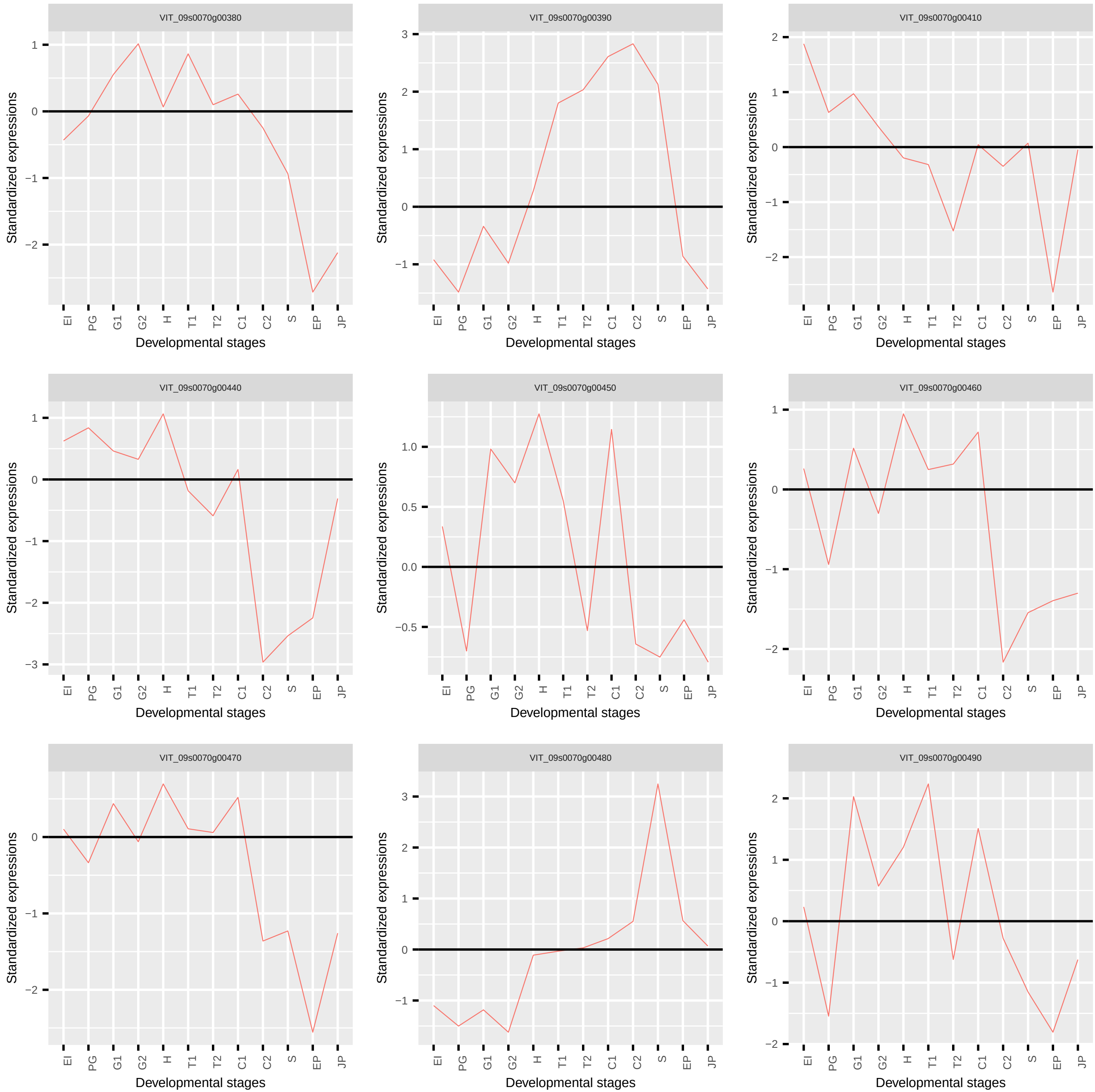
VIT_09s0070g00380
VIT_09s0070g00390
VIT_09s0070g00410
VIT_09s0070g00440
VIT_09s0070g00450
VIT_09s0070g00460
VIT_09s0070g00470
VIT_09s0070g00480
VIT_09s0070g00490
Standardized expressions
Developmental stages
EI PG G1 G2 H T1 T2 C1 C2 S EP JP

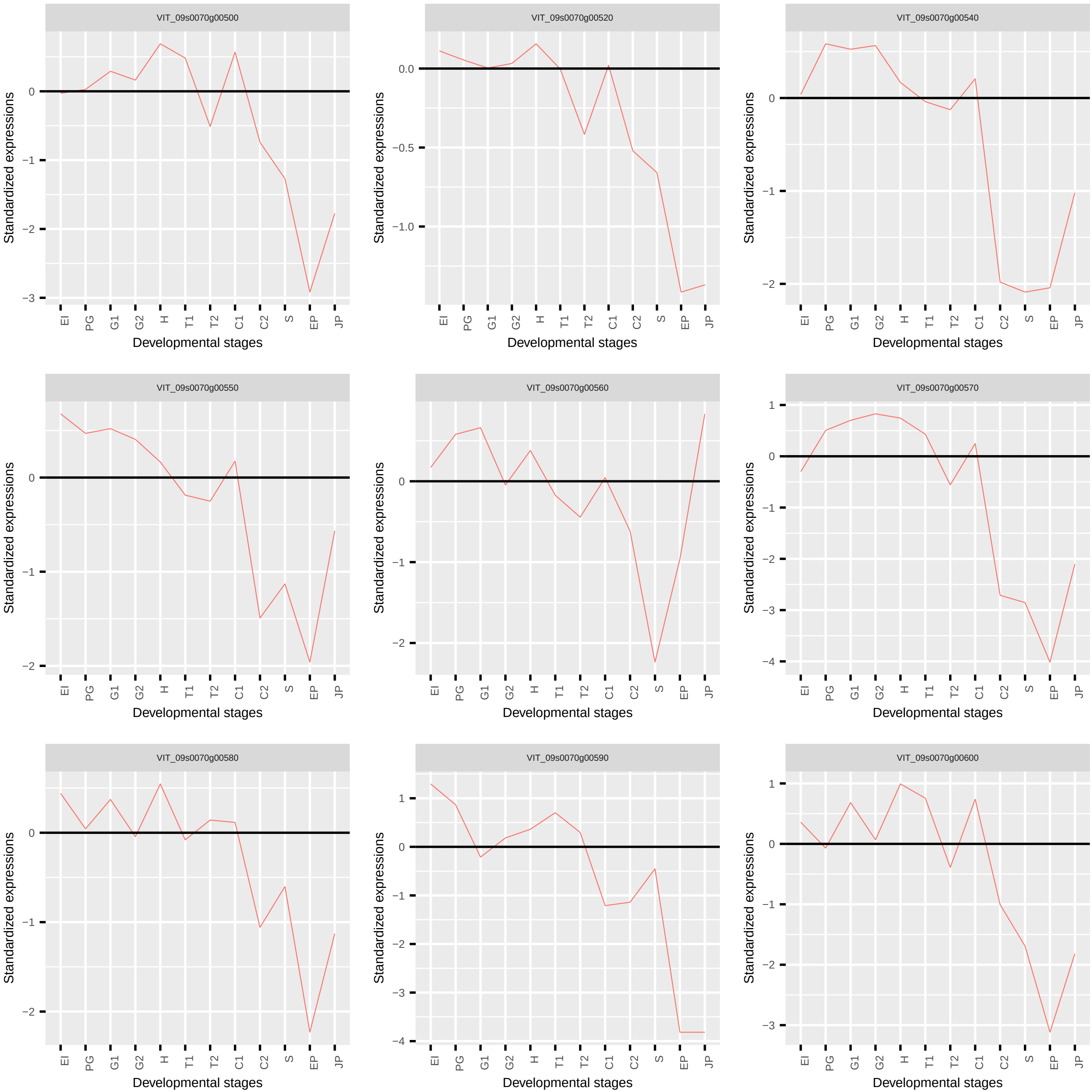

VIT_09s0070g00500
Standardized expressions
0
−1
−2
−3
EI PG G1 G2 H T1 T2 C1 C2 S EP JP
Developmental stages
VIT_09s0070g00520
Standardized expressions
0.0
−0.5
−1.0
EI PG G1 G2 H T1 T2 C1 C2 S EP JP
Developmental stages
VIT_09s0070g00540
Standardized expressions
0
−1
−2
EI PG G1 G2 H T1 T2 C1 C2 S EP JP
Developmental stages
VIT_09s0070g00550
Standardized expressions
0
−1
−2
EI PG G1 G2 H T1 T2 C1 C2 S EP JP
Developmental stages
VIT_09s0070g00560
Standardized expressions
0
−1
−2
EI PG G1 G2 H T1 T2 C1 C2 S EP JP
Developmental stages
VIT_09s0070g00570
Standardized expressions
1
0
−1
−2
−3
−4
EI PG G1 G2 H T1 T2 C1 C2 S EP JP
Developmental stages
VIT_09s0070g00580
Standardized expressions
0
−1
−2
EI PG G1 G2 H T1 T2 C1 C2 S EP JP
Developmental stages
VIT_09s0070g00590
Standardized expressions
1
0
−1
−2
−3
−4
EI PG G1 G2 H T1 T2 C1 C2 S EP JP
Developmental stages
VIT_09s0070g00600
Standardized expressions
1
0
−1
−2
−3
EI PG G1 G2 H T1 T2 C1 C2 S EP JP
Developmental stages

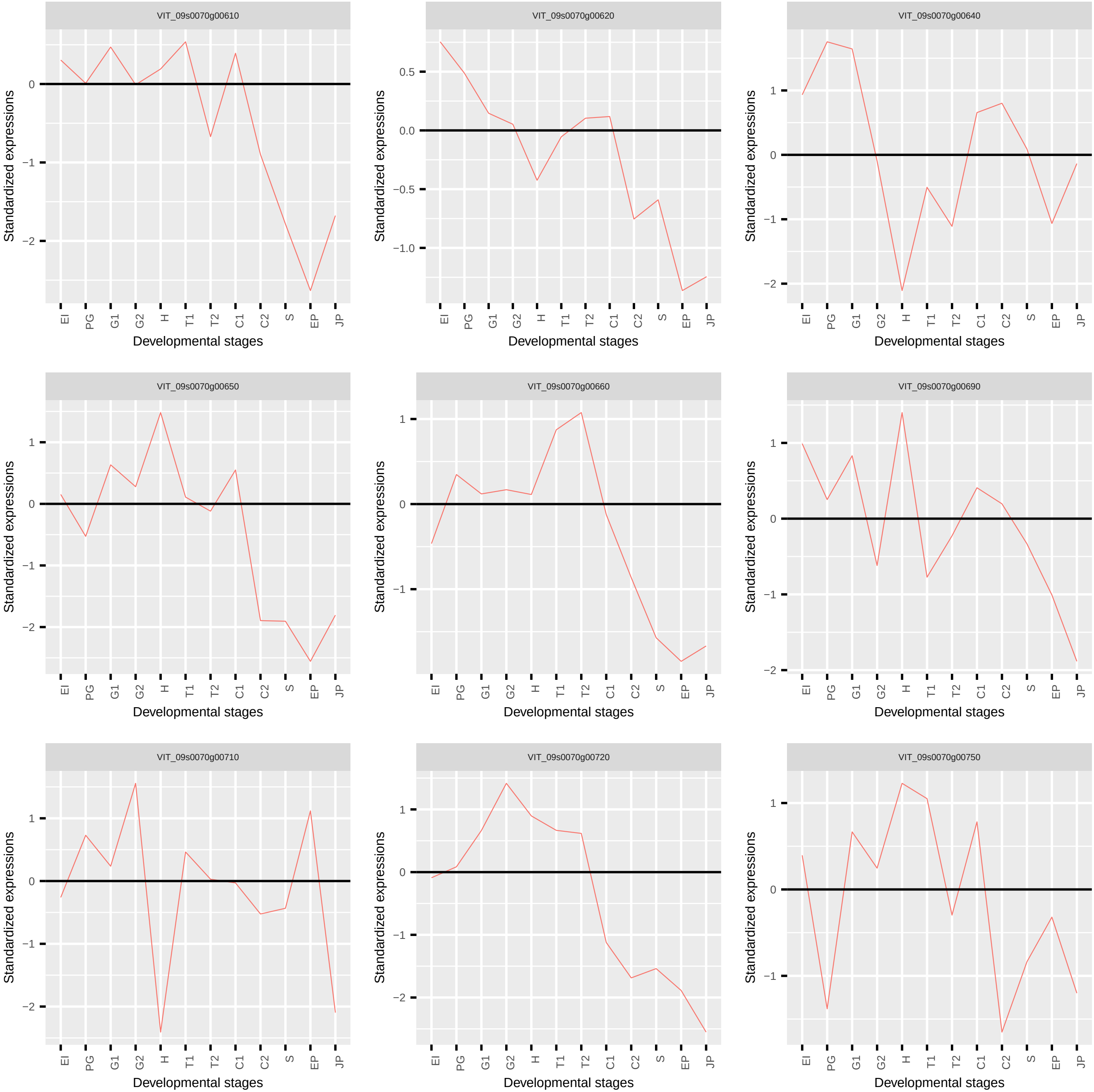

VIT_09s0070g00610
0
−1
−2
VIT_09s0070g00620
0.5
0.0
−0.5
−1.0
VIT_09s0070g00640
1
0
−1
−2
VIT_09s0070g00650
1
0
−1
−2
VIT_09s0070g00660
1
0
−1
VIT_09s0070g00690
1
0
−1
−2
VIT_09s0070g00710
1
0
−1
−2
VIT_09s0070g00720
1
0
−1
−2
VIT_09s0070g00750
1
0
−1
Standardized expressions
EI
PG
G1
G2
H
T1
T2
C1
C2
S
EP
JP
Developmental stages

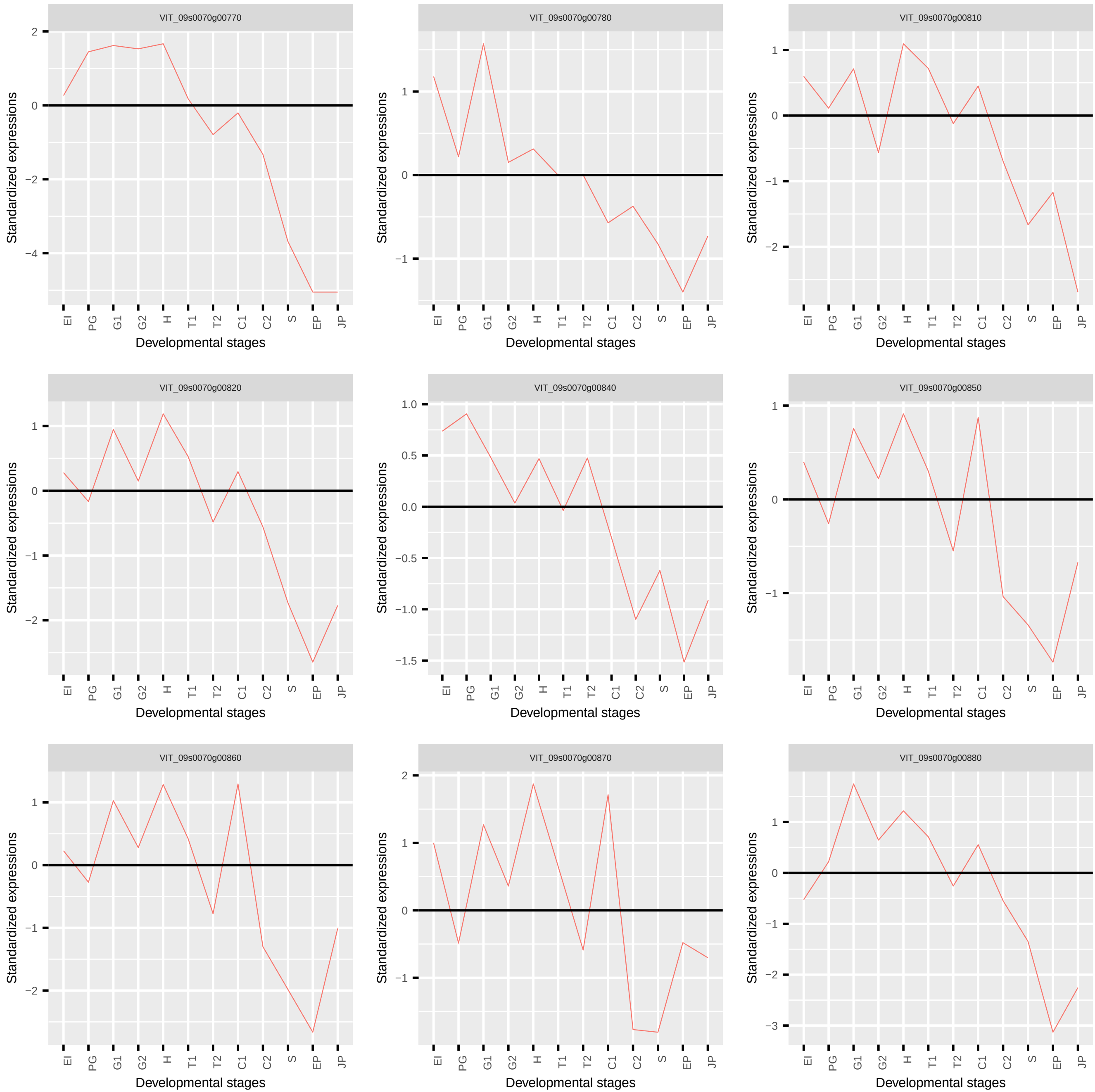
VIT_09s0070g00770
VIT_09s0070g00780
VIT_09s0070g00810
VIT_09s0070g00820
VIT_09s0070g00840
VIT_09s0070g00850
VIT_09s0070g00860
VIT_09s0070g00870
VIT_09s0070g00880
Standardized expressions
Developmental stages
EI PG G1 G2 H T1 T2 C1 C2 S EP JP

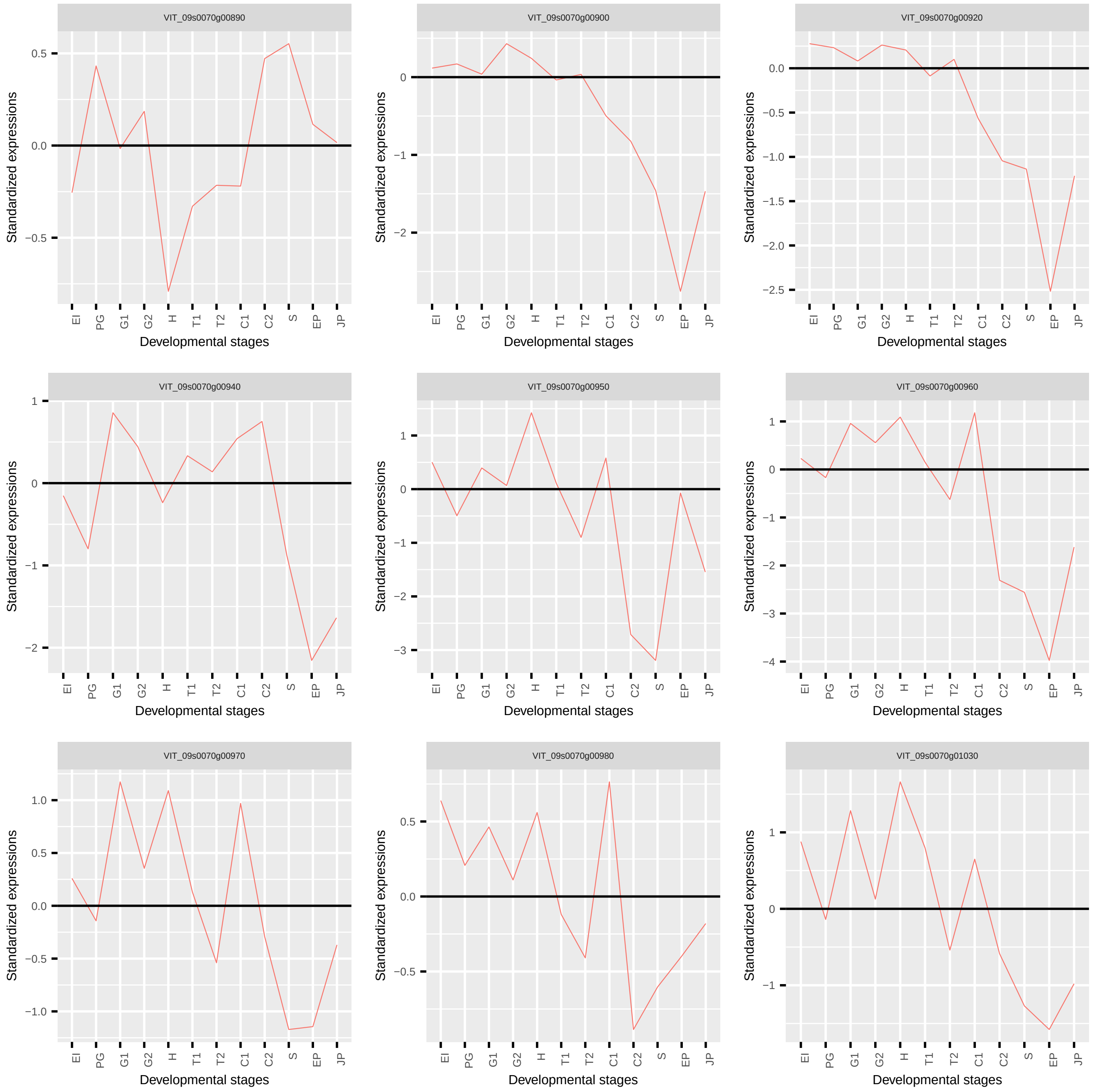
VIT_09s0070g00890
VIT_09s0070g00900
VIT_09s0070g00920
VIT_09s0070g00940
VIT_09s0070g00950
VIT_09s0070g00960
VIT_09s0070g00970
VIT_09s0070g00980
VIT_09s0070g01030
Standardized expressions
Developmental stages
EI PG G1 G2 H T1 T2 C1 C2 S EP JP

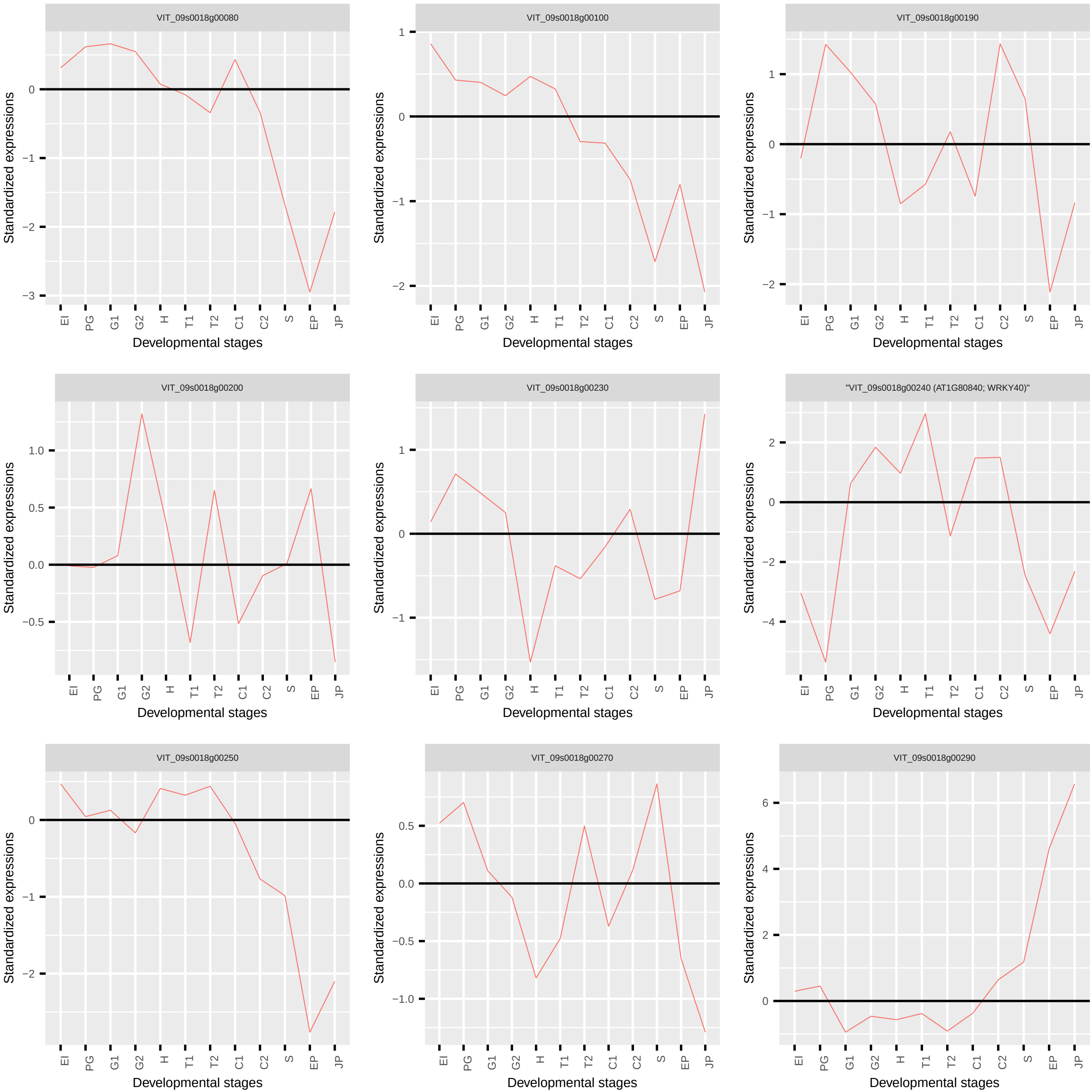

VIT_09s0018g00080
VIT_09s0018g00100
VIT_09s0018g00190
VIT_09s0018g00200
VIT_09s0018g00230
"VIT_09s0018g00240 (AT1G80840; WRKY40)"
VIT_09s0018g00250
VIT_09s0018g00270
VIT_09s0018g00290
Standardized expressions
Developmental stages
EI
PG
G1
G2
H
T1
T2
C1
C2
S
EP
JP

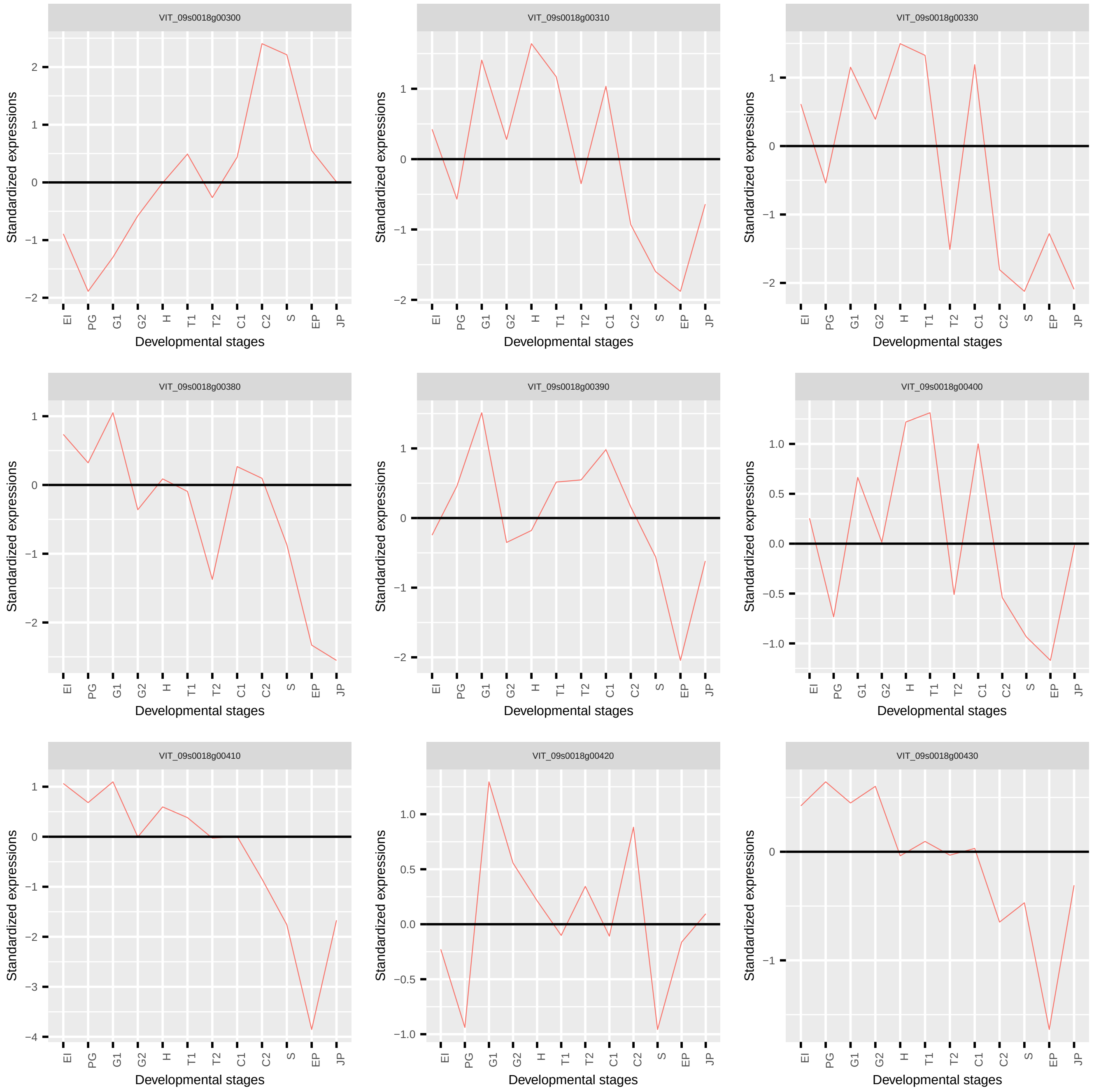

VIT_09s0018g00300
VIT_09s0018g00310
VIT_09s0018g00330
VIT_09s0018g00380
VIT_09s0018g00390
VIT_09s0018g00400
VIT_09s0018g00410
VIT_09s0018g00420
VIT_09s0018g00430
Standardized expressions
Developmental stages
EI PG G1 G2 H T1 T2 C1 C2 S EP JP

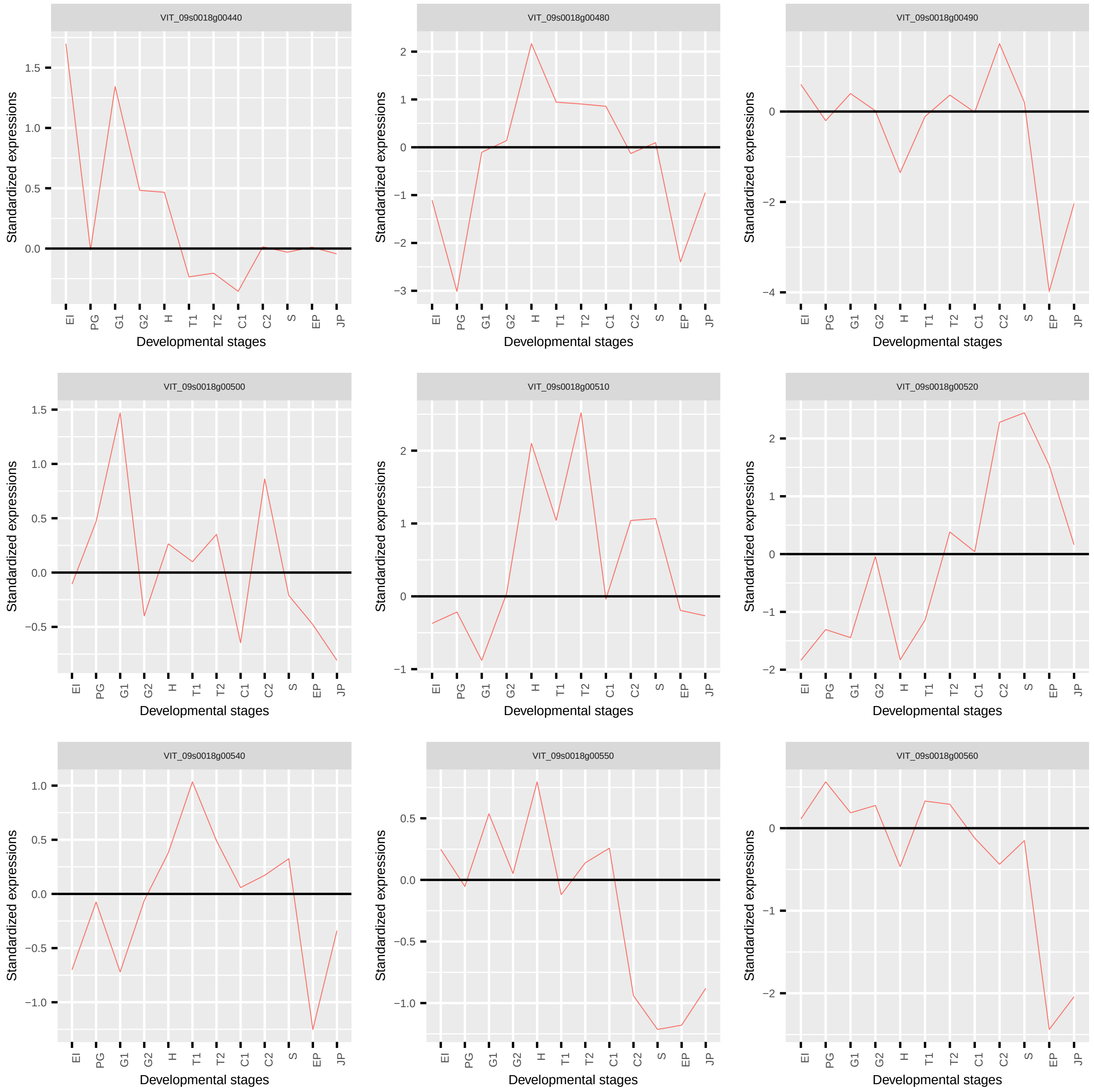
VIT_09s0018g00440
VIT_09s0018g00480
VIT_09s0018g00490
VIT_09s0018g00500
VIT_09s0018g00510
VIT_09s0018g00520
VIT_09s0018g00540
VIT_09s0018g00550
VIT_09s0018g00560
Standardized expressions
Developmental stages
EI
PG
G1
G2
H
T1
T2
C1
C2
S
EP
JP

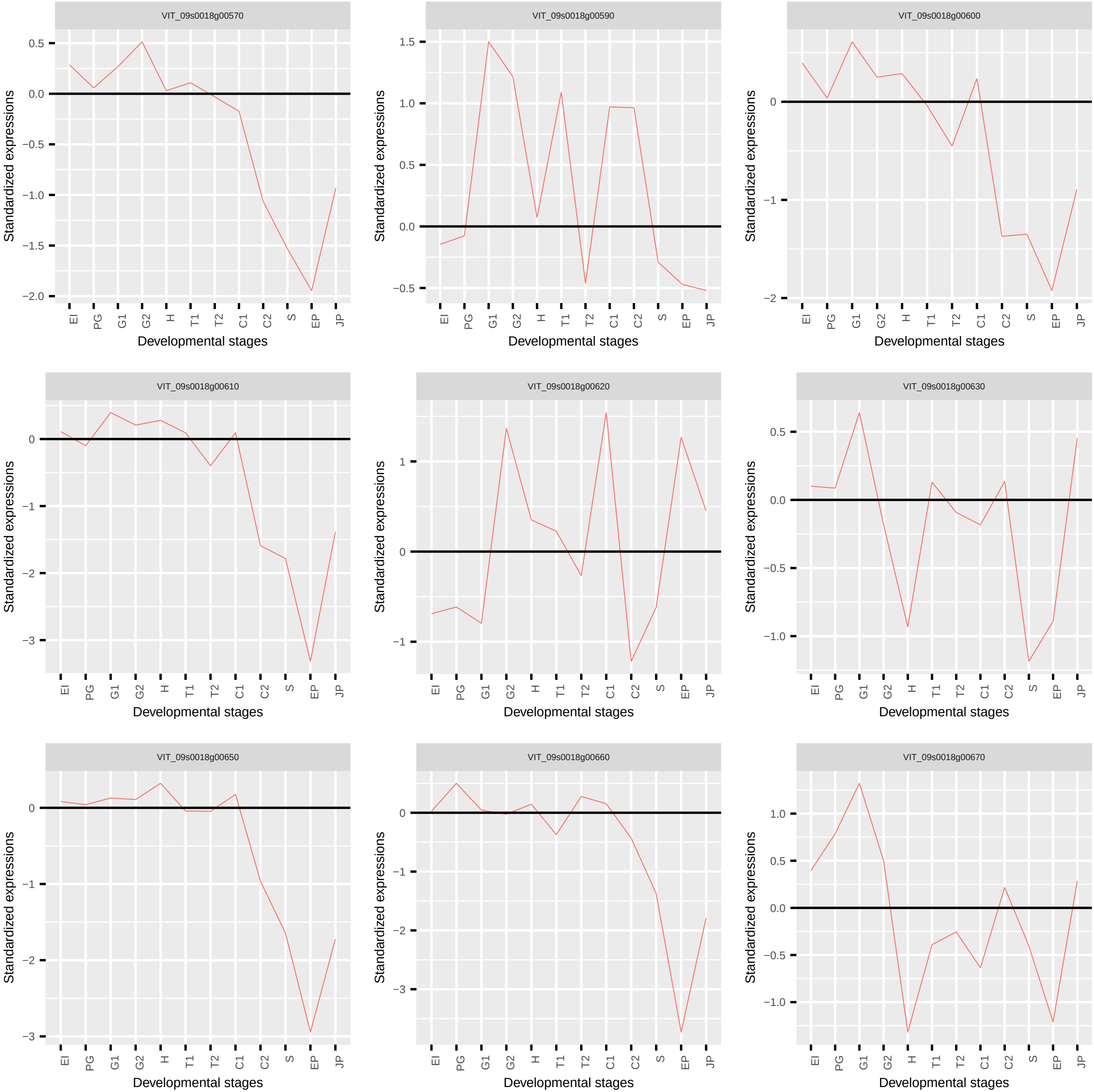
VIT_09s0018g00570
VIT_09s0018g00590
VIT_09s0018g00600
VIT_09s0018g00610
VIT_09s0018g00620
VIT_09s0018g00630
VIT_09s0018g00650
VIT_09s0018g00660
VIT_09s0018g00670
Standardized expressions
Developmental stages
EI PG G1 G2 H T1 T2 C1 C2 S EP JP

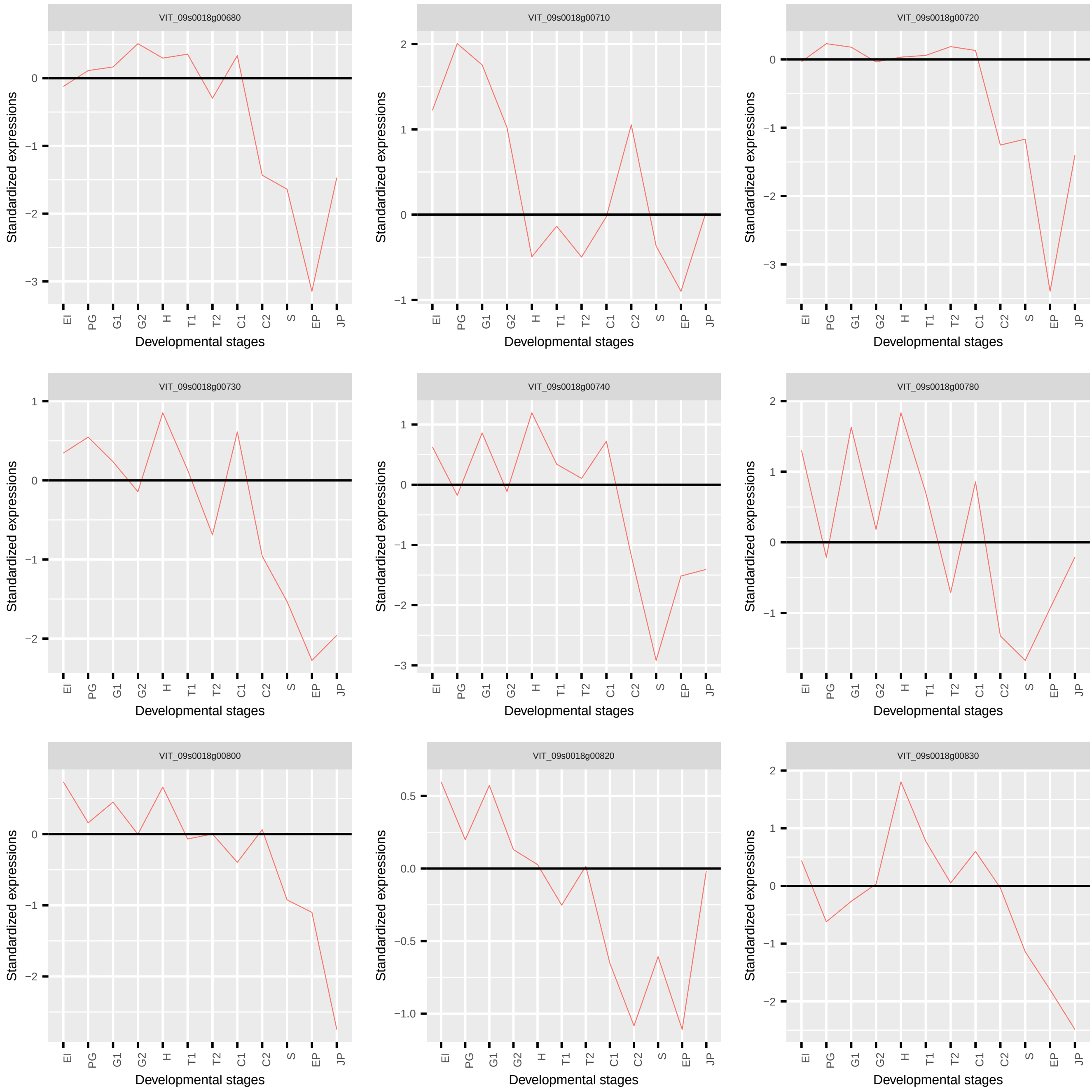
VIT_09s0018g00680
VIT_09s0018g00710
VIT_09s0018g00720
VIT_09s0018g00730
VIT_09s0018g00740
VIT_09s0018g00780
VIT_09s0018g00800
VIT_09s0018g00820
VIT_09s0018g00830
Standardized expressions
Developmental stages
EI PG G1 G2 H T1 T2 C1 C2 S EP JP

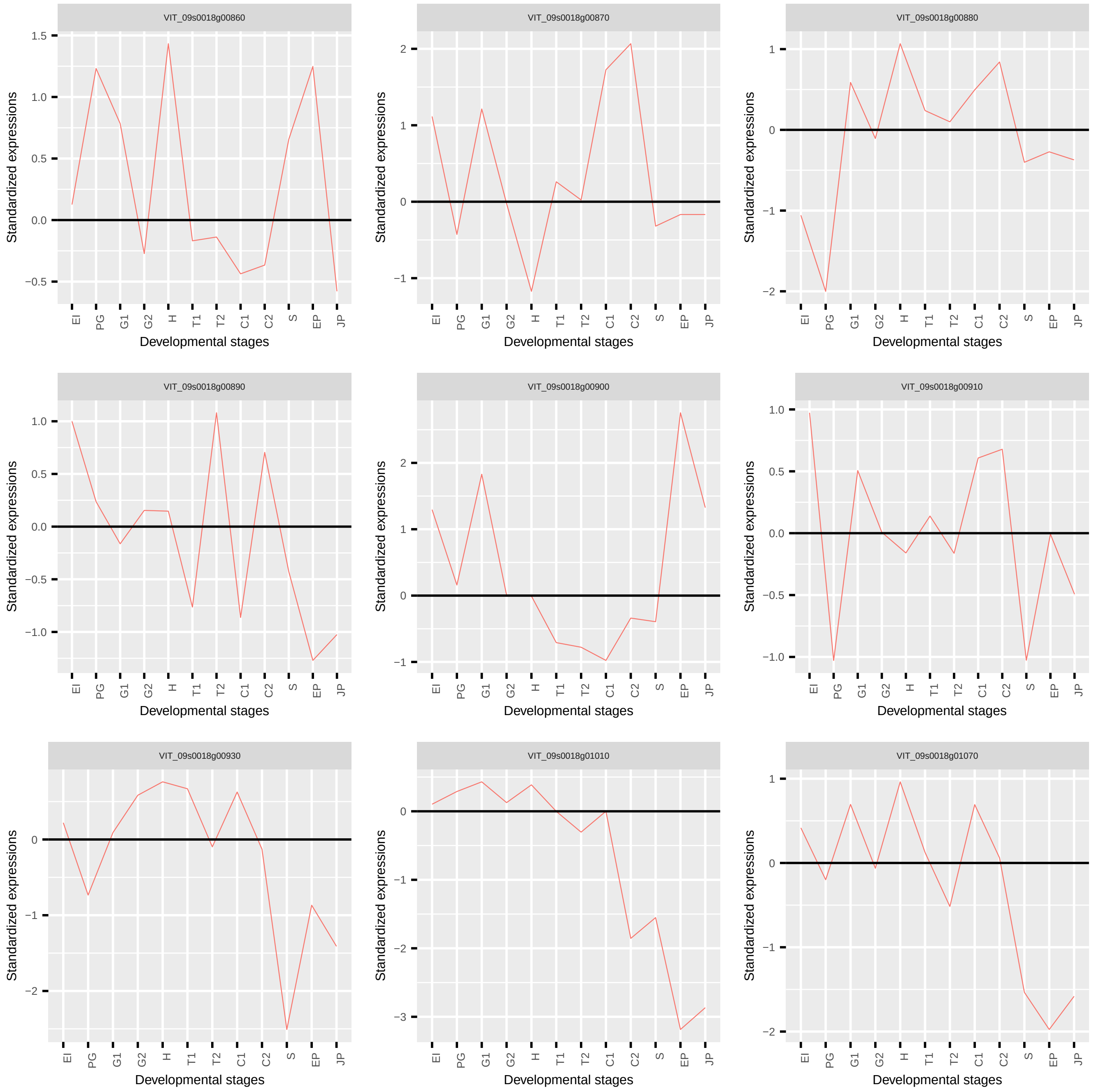
VIT_09s0018g00860
Standardized expressions
Developmental stages
EI PG G1 G2 H T1 T2 C1 C2 S EP JP
VIT_09s0018g00870
Standardized expressions
Developmental stages
EI PG G1 G2 H T1 T2 C1 C2 S EP JP
VIT_09s0018g00880
Standardized expressions
Developmental stages
EI PG G1 G2 H T1 T2 C1 C2 S EP JP
VIT_09s0018g00890
Standardized expressions
Developmental stages
EI PG G1 G2 H T1 T2 C1 C2 S EP JP
VIT_09s0018g00900
Standardized expressions
Developmental stages
EI PG G1 G2 H T1 T2 C1 C2 S EP JP
VIT_09s0018g00910
Standardized expressions
Developmental stages
EI PG G1 G2 H T1 T2 C1 C2 S EP JP
VIT_09s0018g00930
Standardized expressions
Developmental stages
EI PG G1 G2 H T1 T2 C1 C2 S EP JP
VIT_09s0018g01010
Standardized expressions
Developmental stages
EI PG G1 G2 H T1 T2 C1 C2 S EP JP
VIT_09s0018g01070
Standardized expressions
Developmental stages
EI PG G1 G2 H T1 T2 C1 C2 S EP JP

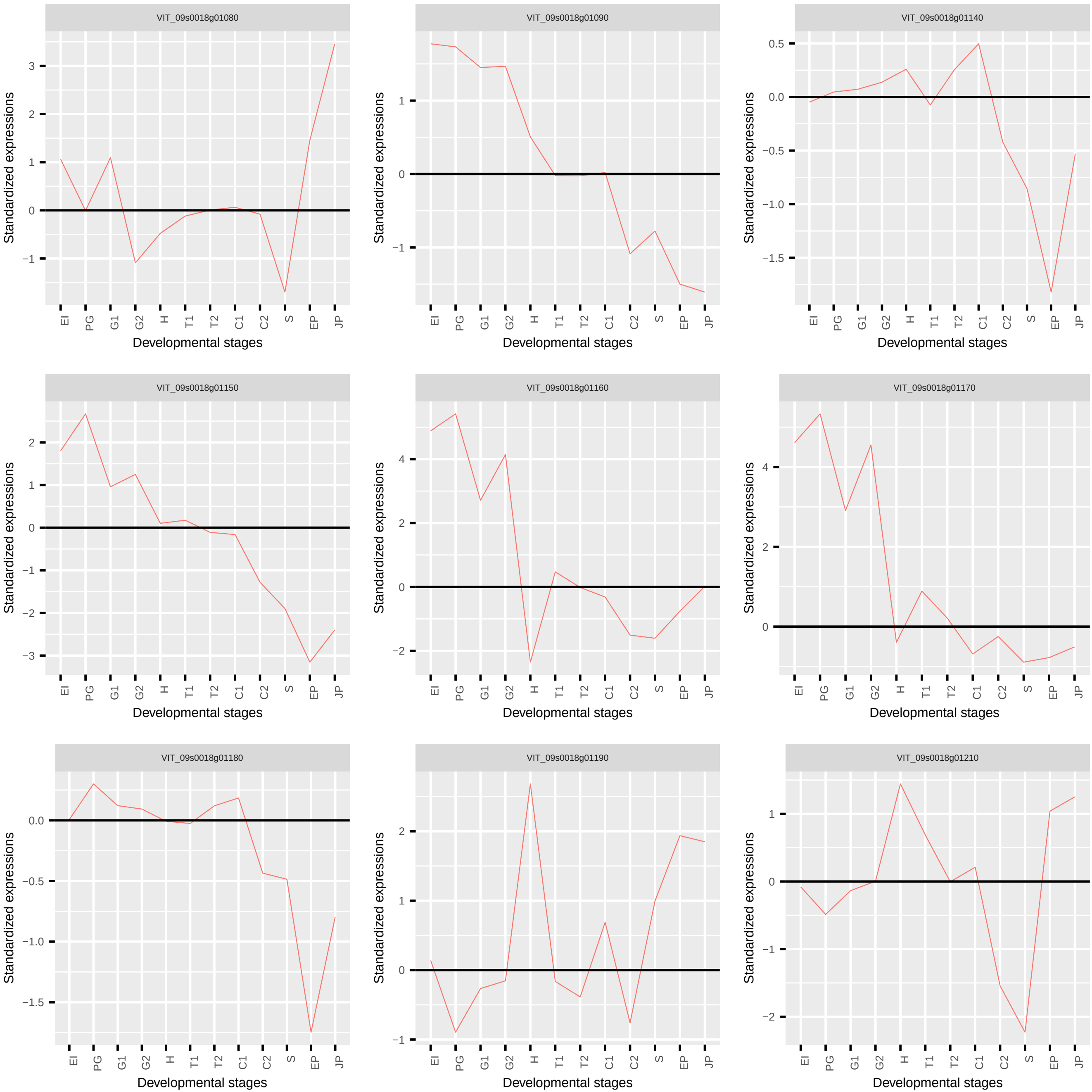
VIT_09s0018g01080
VIT_09s0018g01090
VIT_09s0018g01140
VIT_09s0018g01150
VIT_09s0018g01160
VIT_09s0018g01170
VIT_09s0018g01180
VIT_09s0018g01190
VIT_09s0018g01210
Standardized expressions
Developmental stages
EI PG G1 G2 H T1 T2 C1 C2 S EP JP

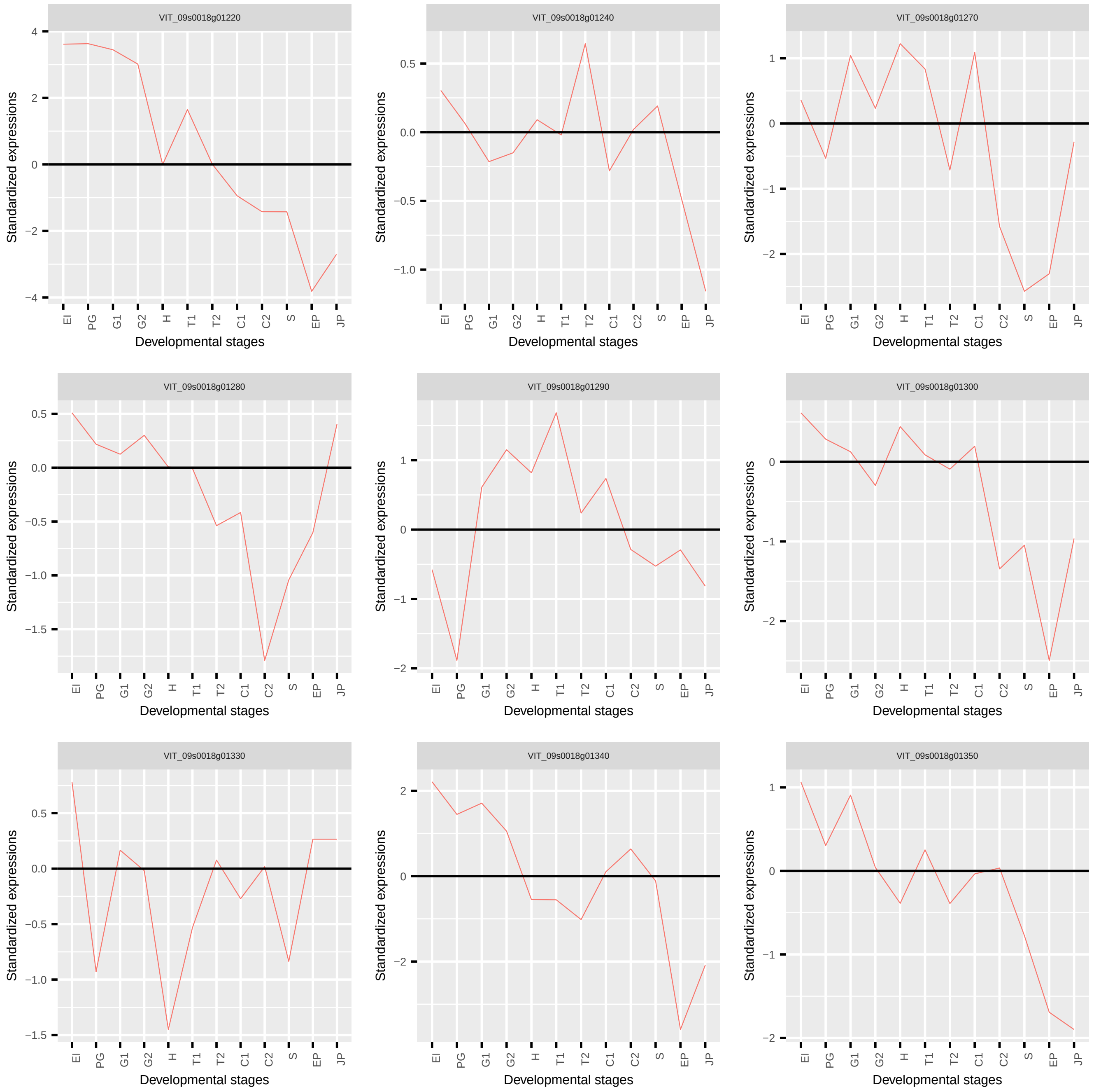
VIT_09s0018g01220
VIT_09s0018g01240
VIT_09s0018g01270
VIT_09s0018g01280
VIT_09s0018g01290
VIT_09s0018g01300
VIT_09s0018g01330
VIT_09s0018g01340
VIT_09s0018g01350
Standardized expressions
Developmental stages
EI PG G1 G2 H T1 T2 C1 C2 S EP JP

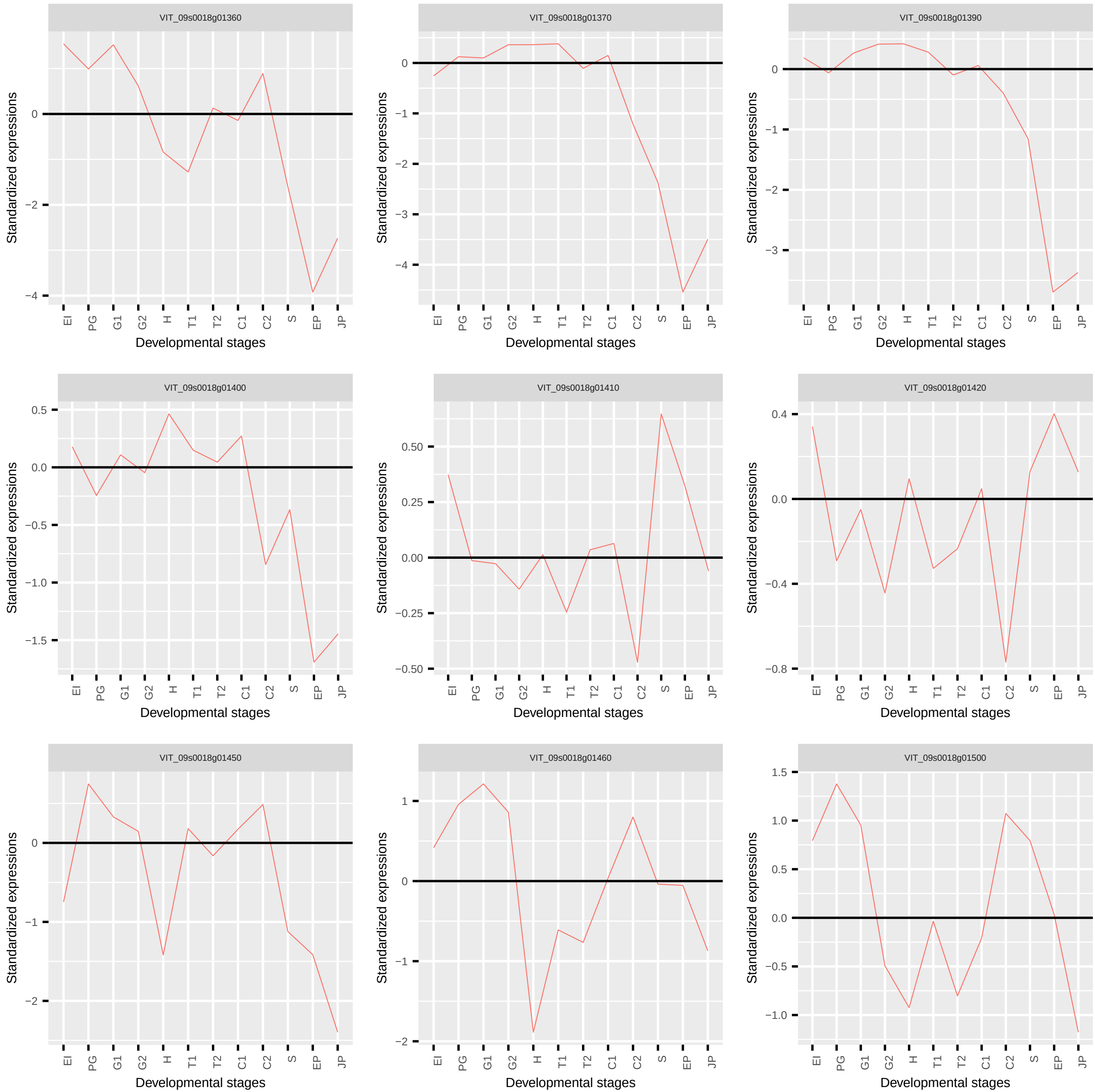

VIT_09s0018g01360
VIT_09s0018g01370
VIT_09s0018g01390
VIT_09s0018g01400
VIT_09s0018g01410
VIT_09s0018g01420
VIT_09s0018g01450
VIT_09s0018g01460
VIT_09s0018g01500
Standardized expressions
Developmental stages
EI PG G1 G2 H T1 T2 C1 C2 S EP JP
0
−2
−4
−1
−3
0.5
0.0
−0.5
−1.0
−1.5
0.50
0.25
0.00
−0.25
−0.50
0.4
−0.4
−0.8
1
1.5

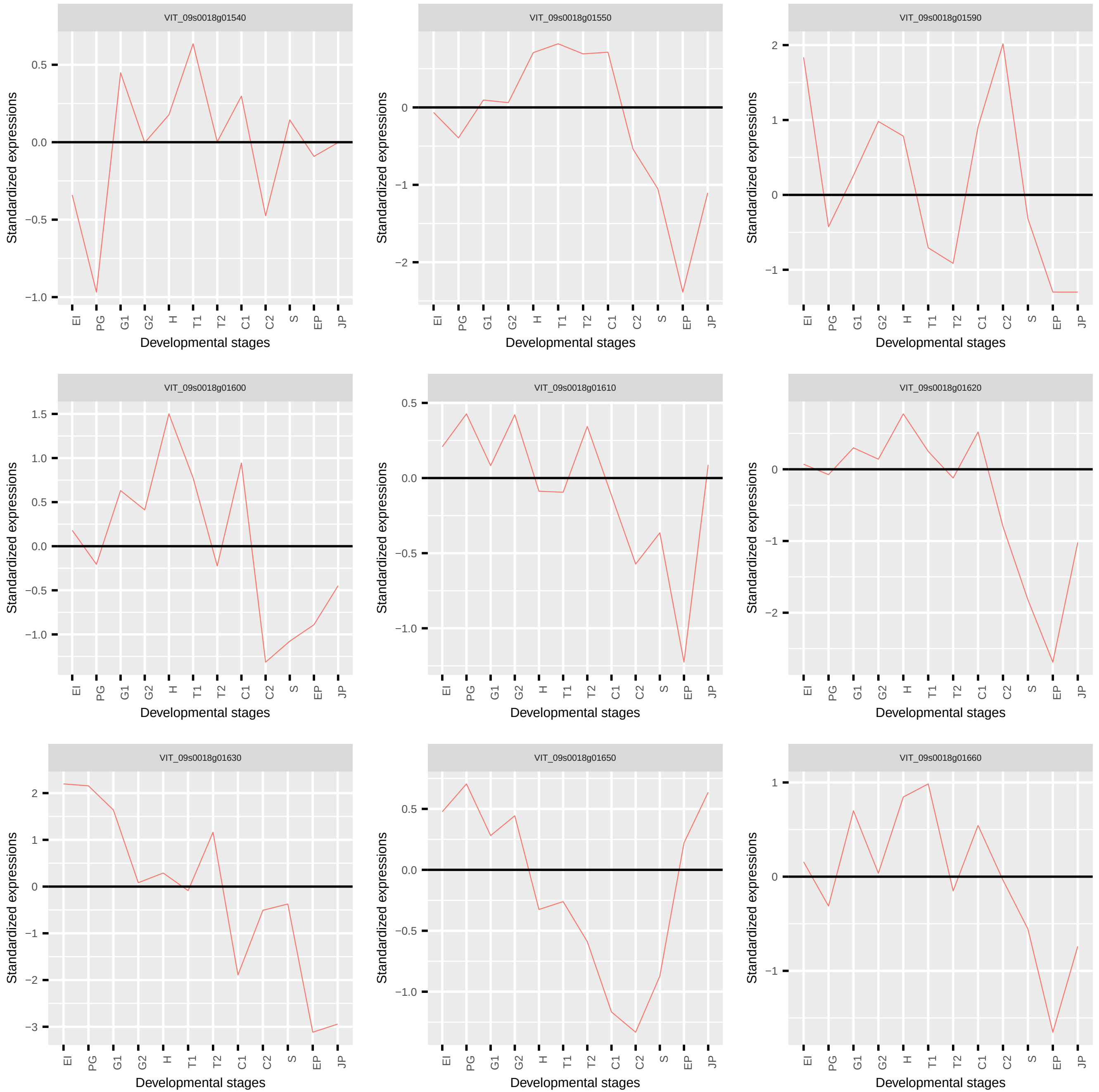
VIT_09s0018g01540
VIT_09s0018g01550
VIT_09s0018g01590
VIT_09s0018g01600
VIT_09s0018g01610
VIT_09s0018g01620
VIT_09s0018g01630
VIT_09s0018g01650
VIT_09s0018g01660
Standardized expressions
Developmental stages
EI PG G1 G2 H T1 T2 C1 C2 S EP JP

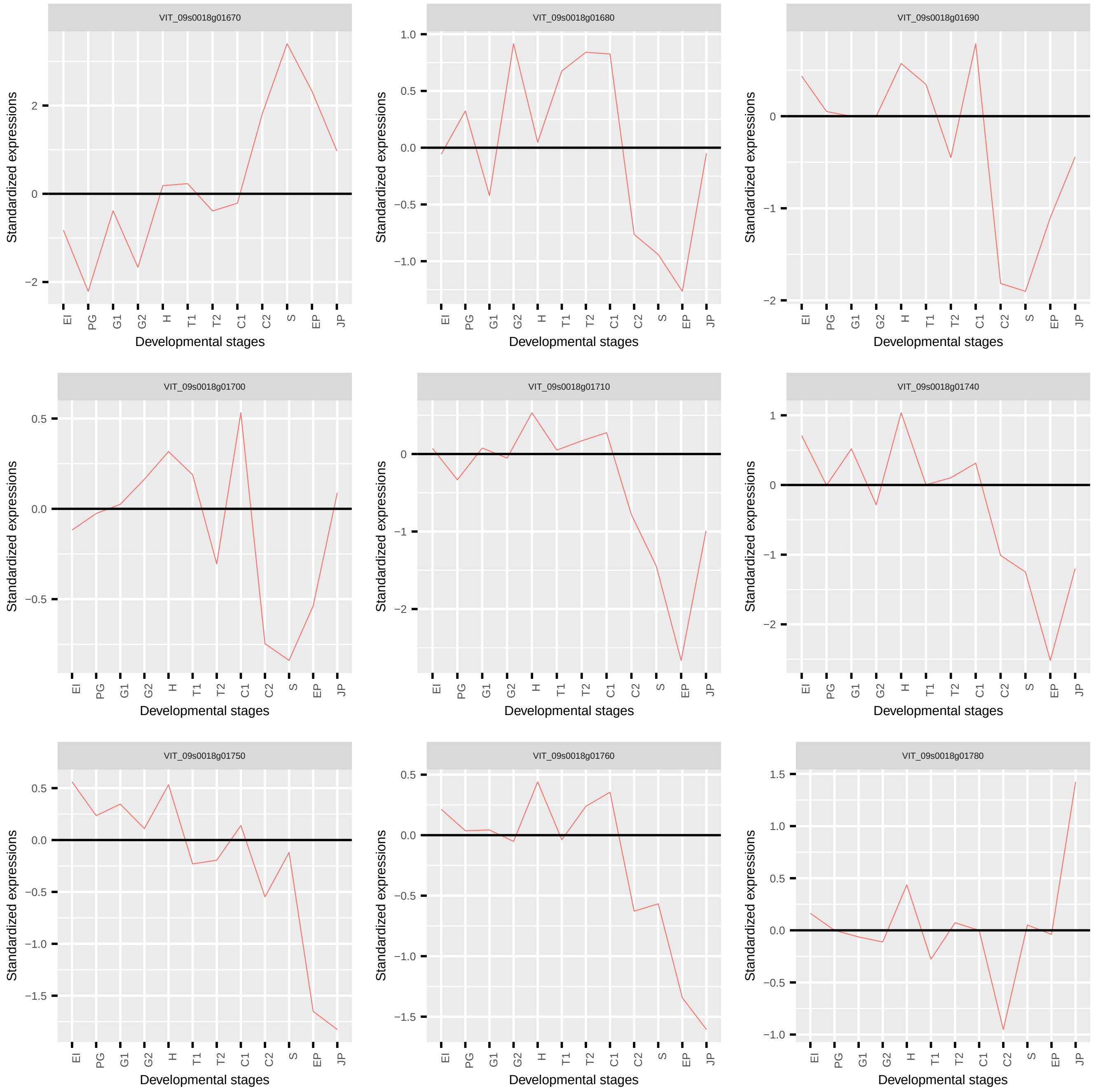
VIT_09s0018g01670
VIT_09s0018g01680
VIT_09s0018g01690
VIT_09s0018g01700
VIT_09s0018g01710
VIT_09s0018g01740
VIT_09s0018g01750
VIT_09s0018g01760
VIT_09s0018g01780
Standardized expressions
Developmental stages
EI PG G1 G2 H T1 T2 C1 C2 S EP JP

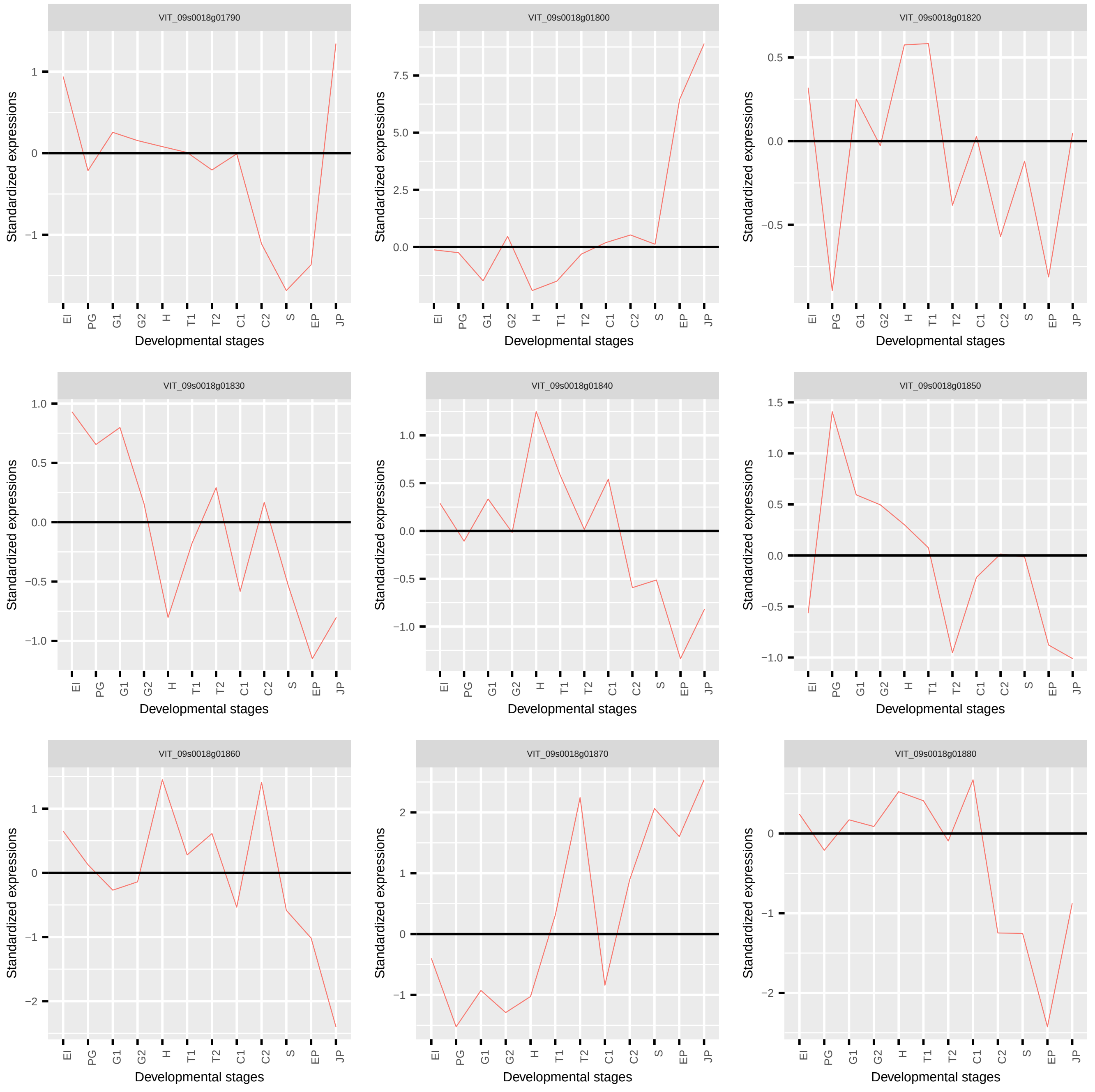
VIT_09s0018g01790
VIT_09s0018g01800
VIT_09s0018g01820
VIT_09s0018g01830
VIT_09s0018g01840
VIT_09s0018g01850
VIT_09s0018g01860
VIT_09s0018g01870
VIT_09s0018g01880
Standardized expressions
Developmental stages
EI PG G1 G2 H T1 T2 C1 C2 S EP JP

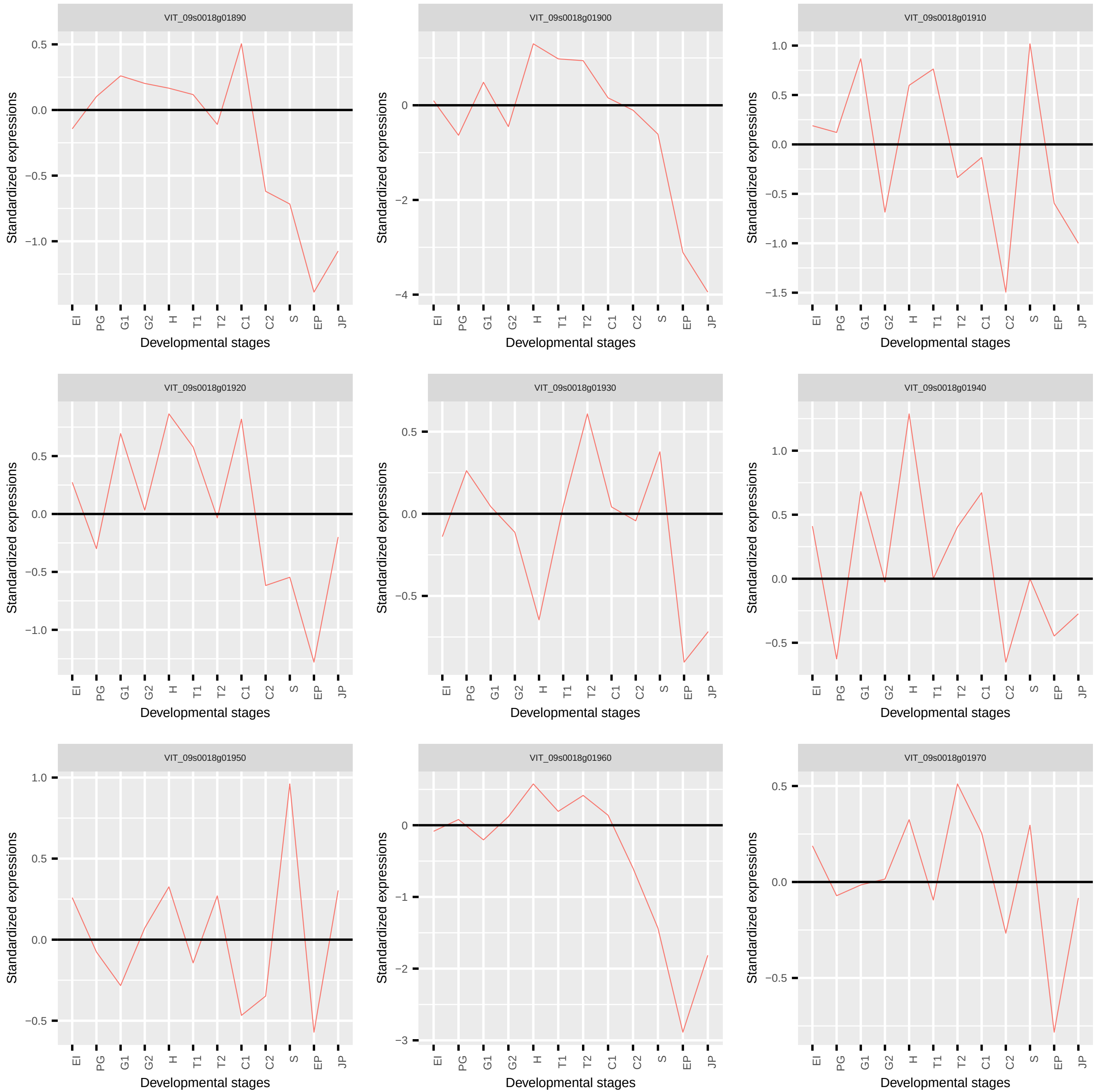

VIT_09s0018g01890
VIT_09s0018g01900
VIT_09s0018g01910
VIT_09s0018g01920
VIT_09s0018g01930
VIT_09s0018g01940
VIT_09s0018g01950
VIT_09s0018g01960
VIT_09s0018g01970
Standardized expressions
Developmental stages
EI PG G1 G2 H T1 T2 C1 C2 S EP JP

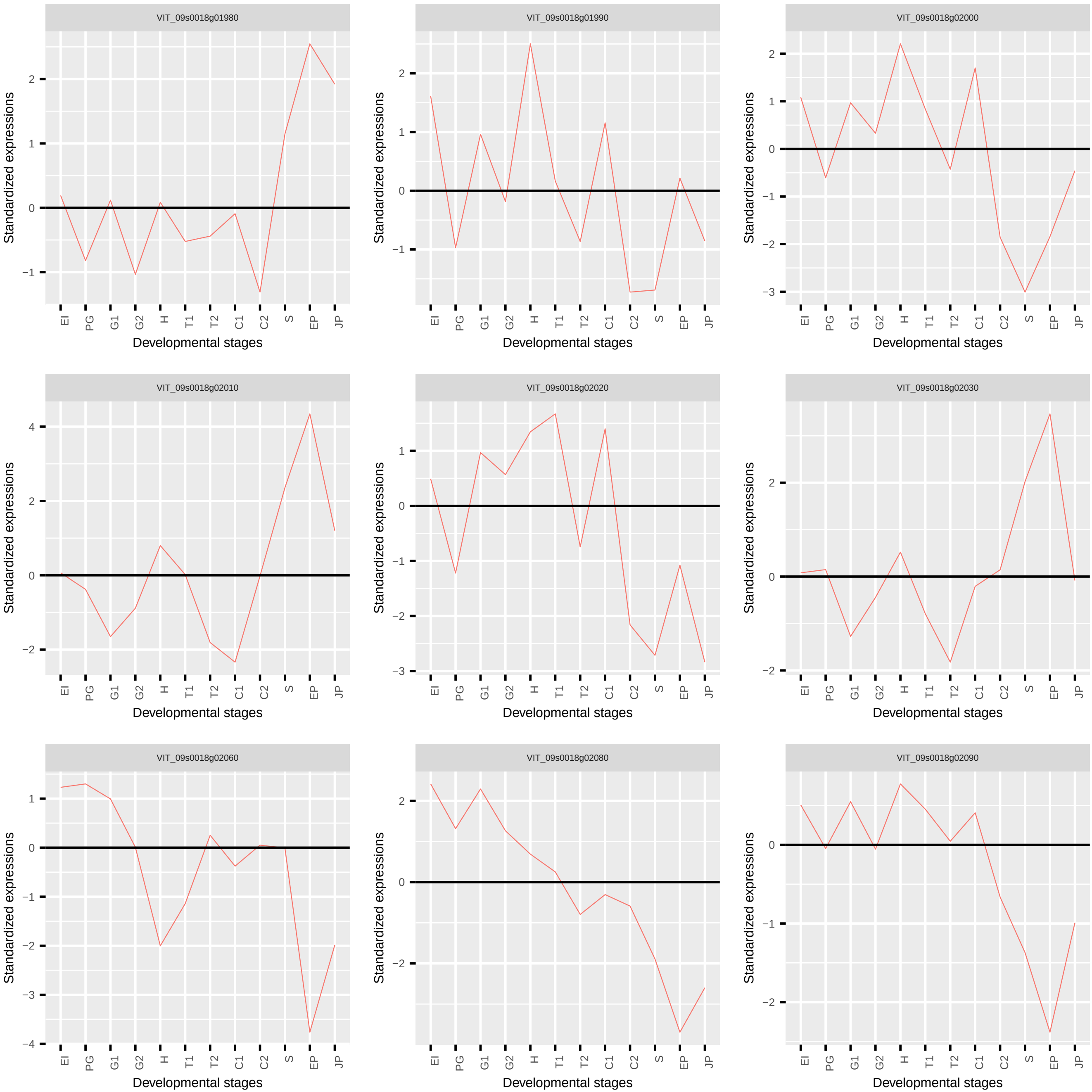
VIT_09s0018g01980
Standardized expressions
2
1
0
−1
EI
PG
G1
G2
H
T1
T2
C1
C2
S
EP
JP
Developmental stages
VIT_09s0018g01990
Standardized expressions
2
1
0
−1
EI
PG
G1
G2
H
T1
T2
C1
C2
S
EP
JP
Developmental stages
VIT_09s0018g02000
Standardized expressions
2
1
0
−1
−2
−3
EI
PG
G1
G2
H
T1
T2
C1
C2
S
EP
JP
Developmental stages
VIT_09s0018g02010
Standardized expressions
4
2
0
−2
EI
PG
G1
G2
H
T1
T2
C1
C2
S
EP
JP
Developmental stages
VIT_09s0018g02020
Standardized expressions
1
0
−1
−2
−3
EI
PG
G1
G2
H
T1
T2
C1
C2
S
EP
JP
Developmental stages
VIT_09s0018g02030
Standardized expressions
2
0
−2
EI
PG
G1
G2
H
T1
T2
C1
C2
S
EP
JP
Developmental stages
VIT_09s0018g02060
Standardized expressions
1
0
−1
−2
−3
−4
EI
PG
G1
G2
H
T1
T2
C1
C2
S
EP
JP
Developmental stages
VIT_09s0018g02080
Standardized expressions
2
0
−2
EI
PG
G1
G2
H
T1
T2
C1
C2
S
EP
JP
Developmental stages
VIT_09s0018g02090
Standardized expressions
0
−1
−2
EI
PG
G1
G2
H
T1
T2
C1
C2
S
EP
JP
Developmental stages

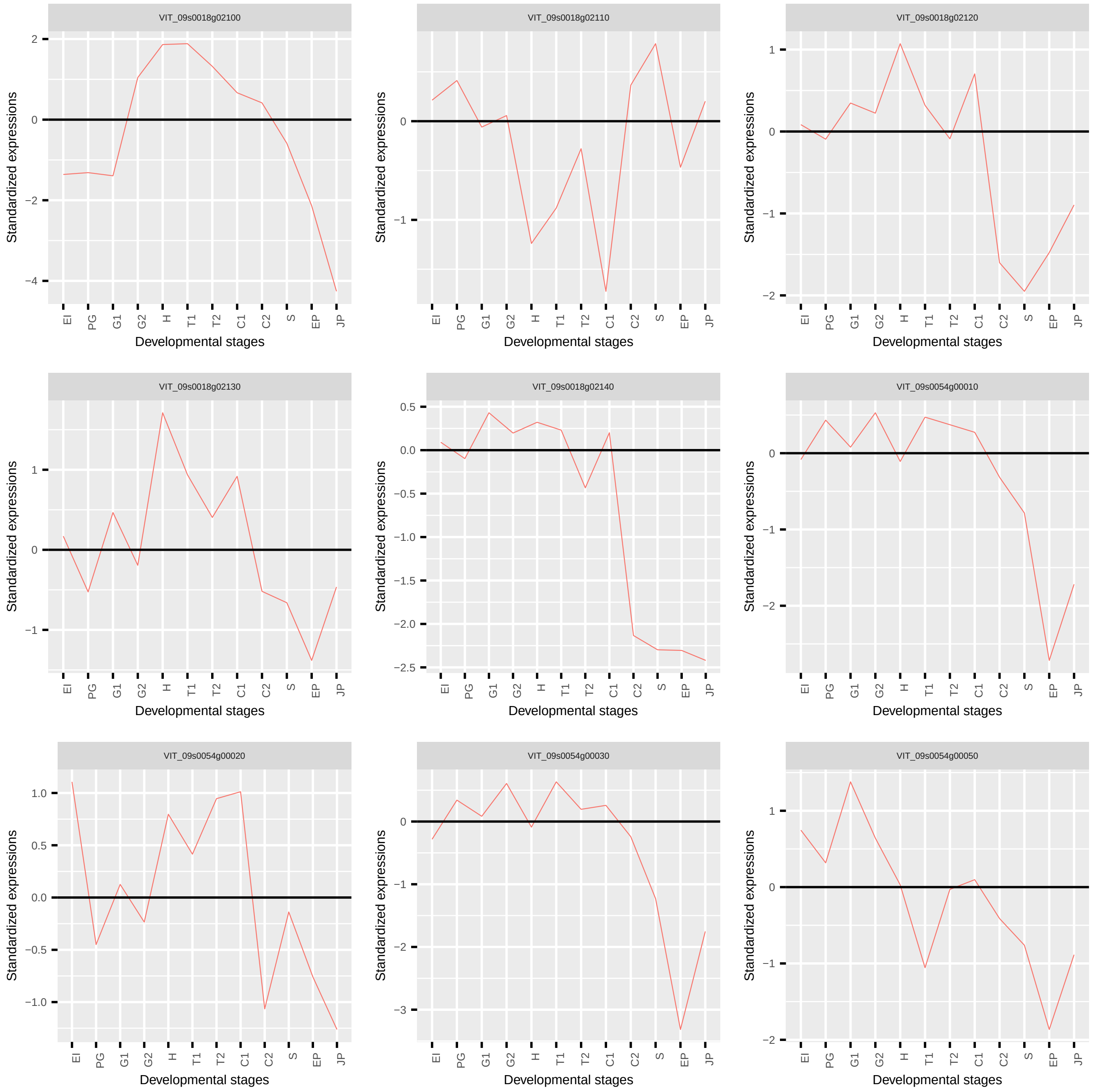

VIT_09s0018g02100
VIT_09s0018g02110
VIT_09s0018g02120
VIT_09s0018g02130
VIT_09s0018g02140
VIT_09s0054g00010
VIT_09s0054g00020
VIT_09s0054g00030
VIT_09s0054g00050
Standardized expressions
Developmental stages
EI PG G1 G2 H T1 T2 C1 C2 S EP JP

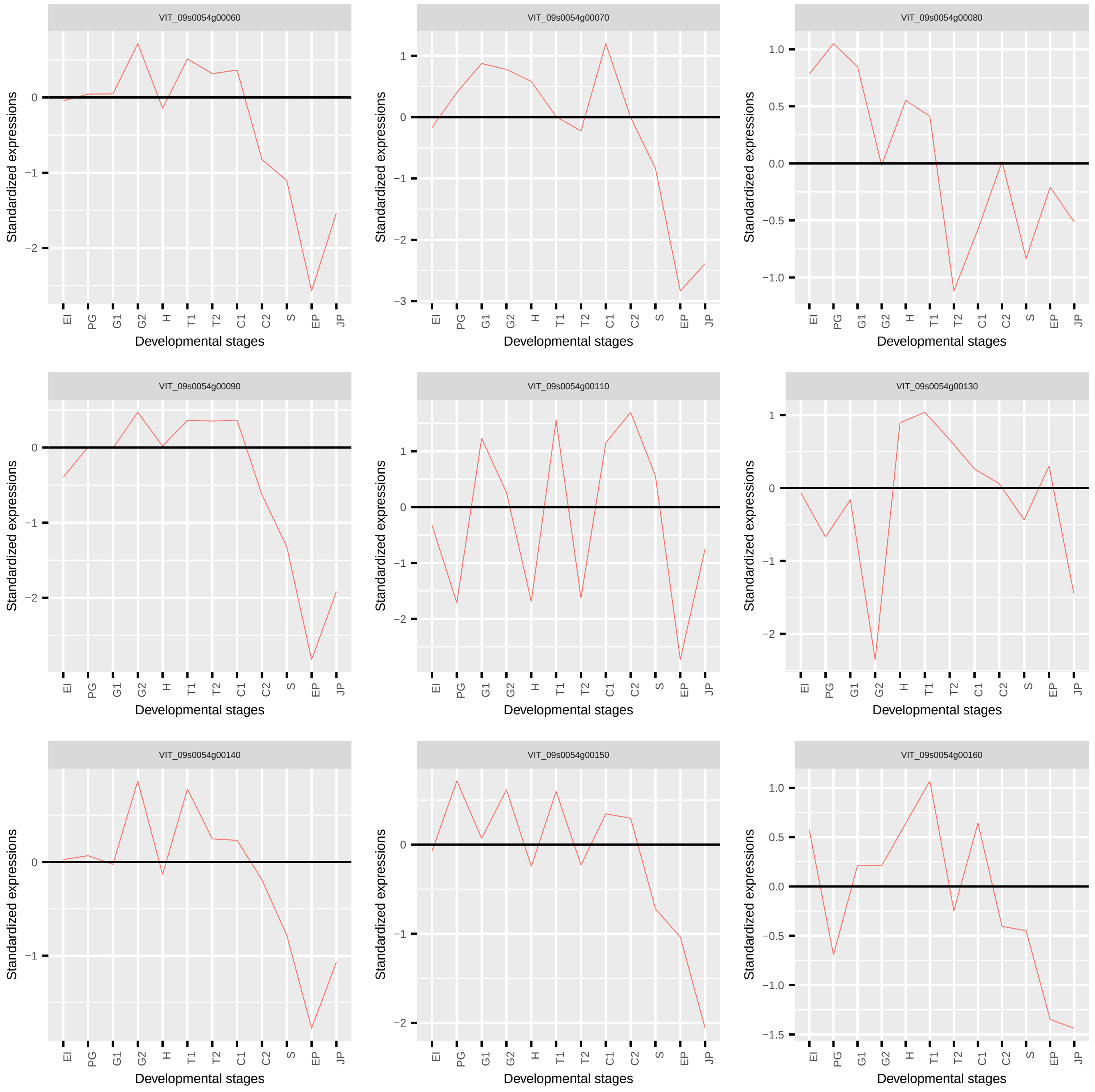
VIT_09s0054g00060
VIT_09s0054g00070
VIT_09s0054g00080
VIT_09s0054g00090
VIT_09s0054g00110
VIT_09s0054g00130
VIT_09s0054g00140
VIT_09s0054g00150
VIT_09s0054g00160
Standardized expressions
Developmental stages
EI PG G1 G2 H T1 T2 C1 C2 S EP JP

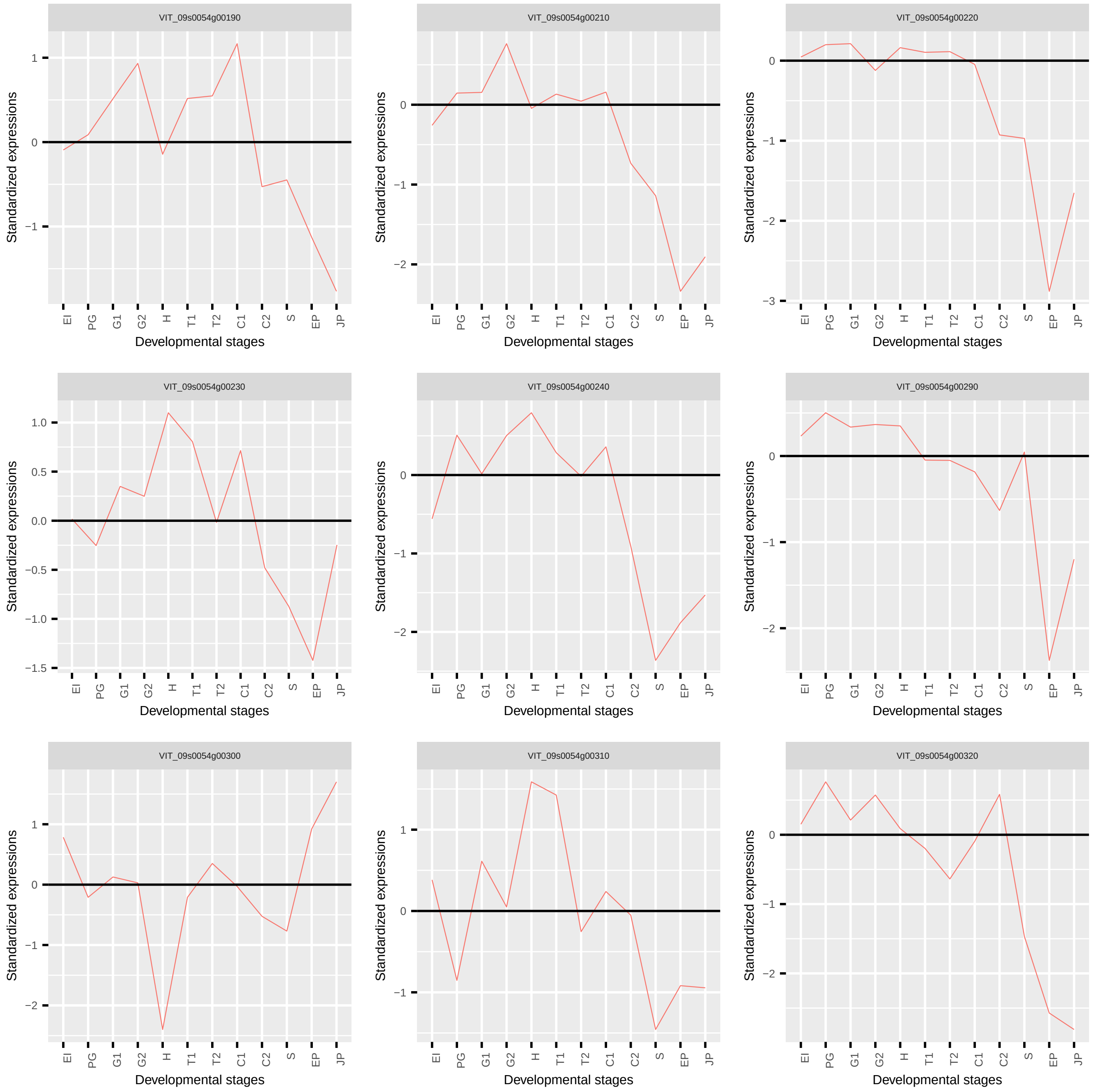

VIT_09s0054g00190
VIT_09s0054g00210
VIT_09s0054g00220
VIT_09s0054g00230
VIT_09s0054g00240
VIT_09s0054g00290
VIT_09s0054g00300
VIT_09s0054g00310
VIT_09s0054g00320
Standardized expressions
Developmental stages
EI PG G1 G2 H T1 T2 C1 C2 S EP JP

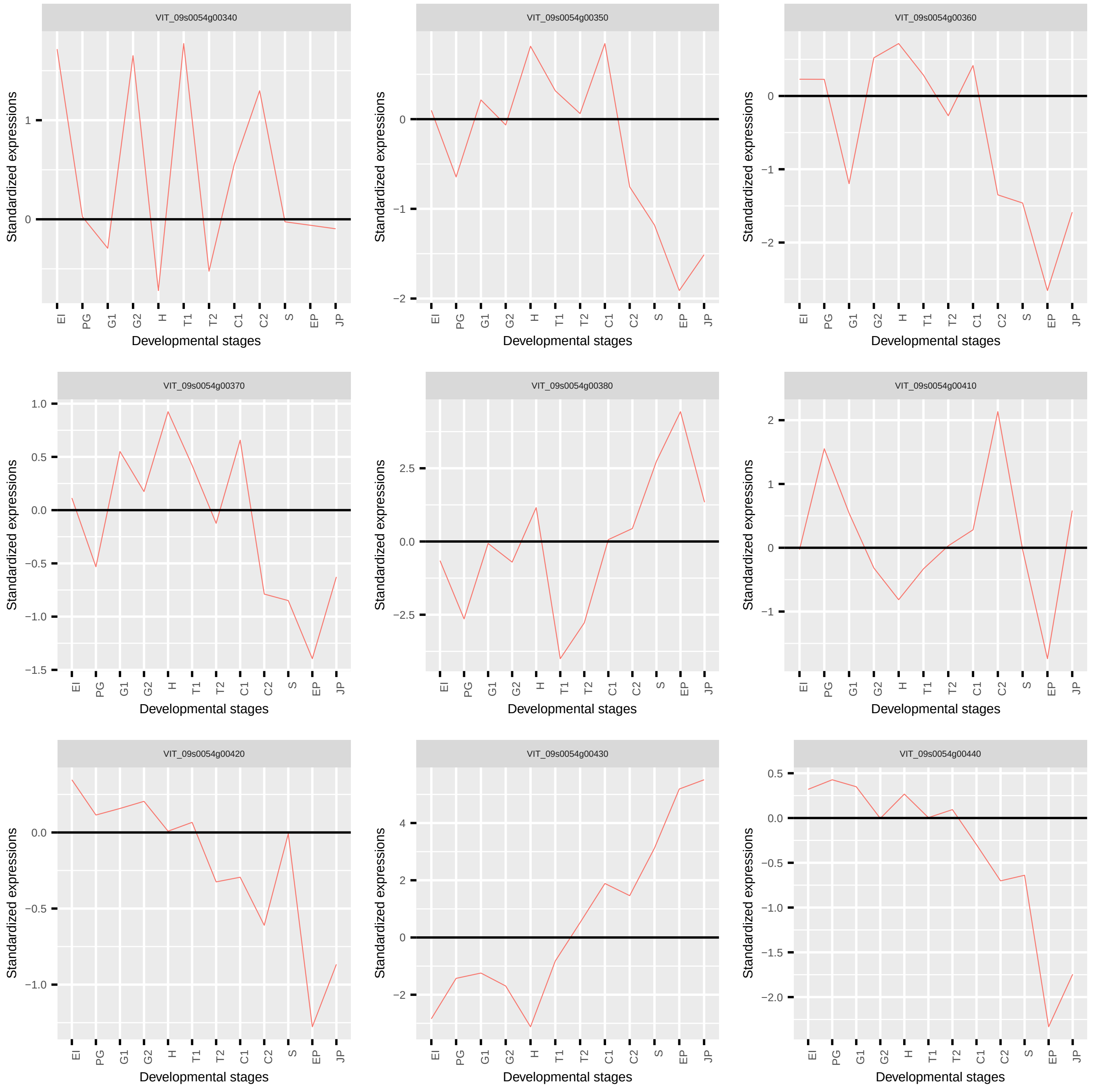
VIT_09s0054g00340
VIT_09s0054g00350
VIT_09s0054g00360
VIT_09s0054g00370
VIT_09s0054g00380
VIT_09s0054g00410
VIT_09s0054g00420
VIT_09s0054g00430
VIT_09s0054g00440
Standardized expressions
Developmental stages
EI PG G1 G2 H T1 T2 C1 C2 S EP JP

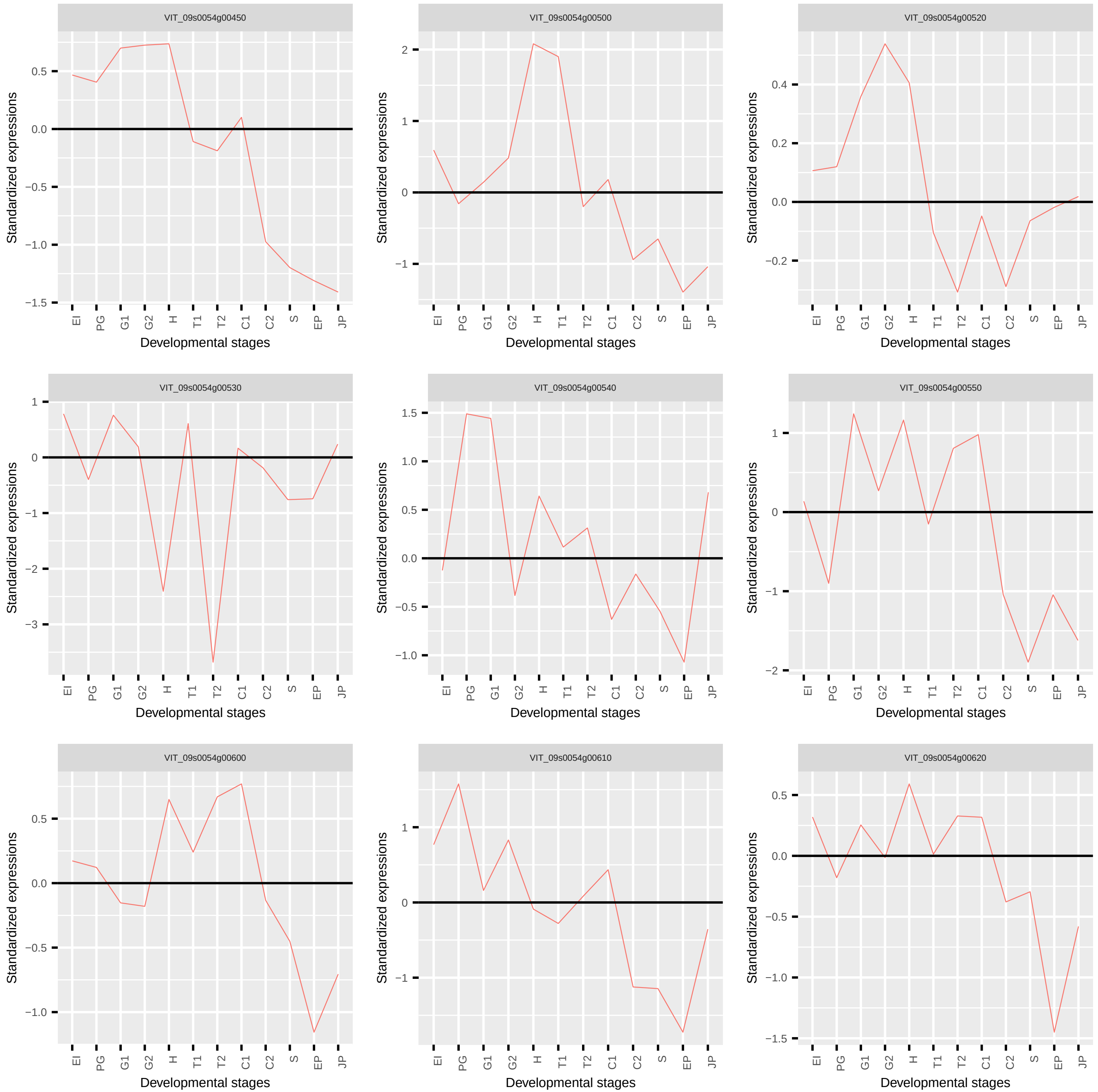
VIT_09s0054g00450
VIT_09s0054g00500
VIT_09s0054g00520
VIT_09s0054g00530
VIT_09s0054g00540
VIT_09s0054g00550
VIT_09s0054g00600
VIT_09s0054g00610
VIT_09s0054g00620
Standardized expressions
Developmental stages
EI PG G1 G2 H T1 T2 C1 C2 S EP JP

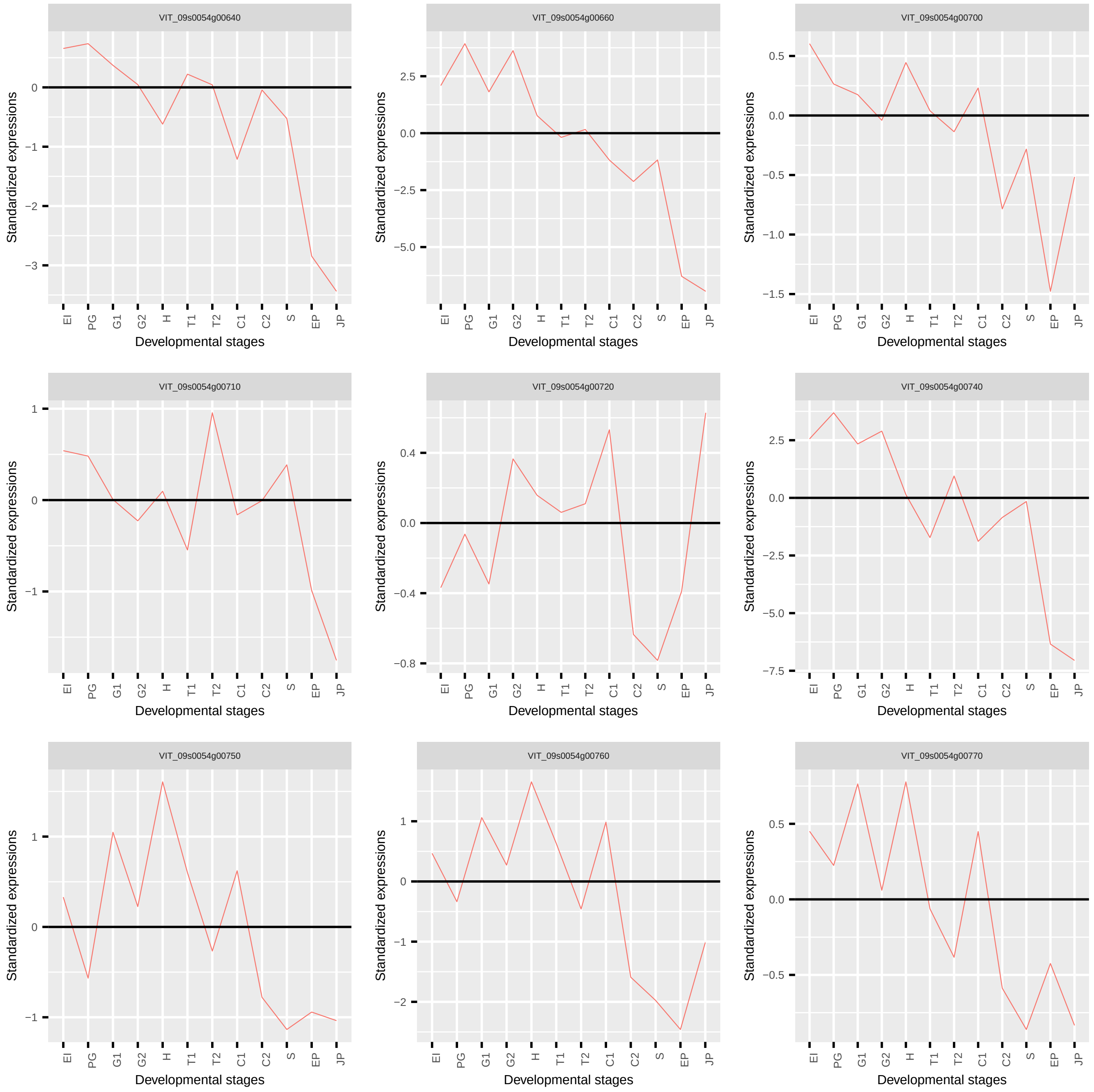
VIT_09s0054g00640
VIT_09s0054g00660
VIT_09s0054g00700
VIT_09s0054g00710
VIT_09s0054g00720
VIT_09s0054g00740
VIT_09s0054g00750
VIT_09s0054g00760
VIT_09s0054g00770
Standardized expressions
Developmental stages
EI PG G1 G2 H T1 T2 C1 C2 S EP JP

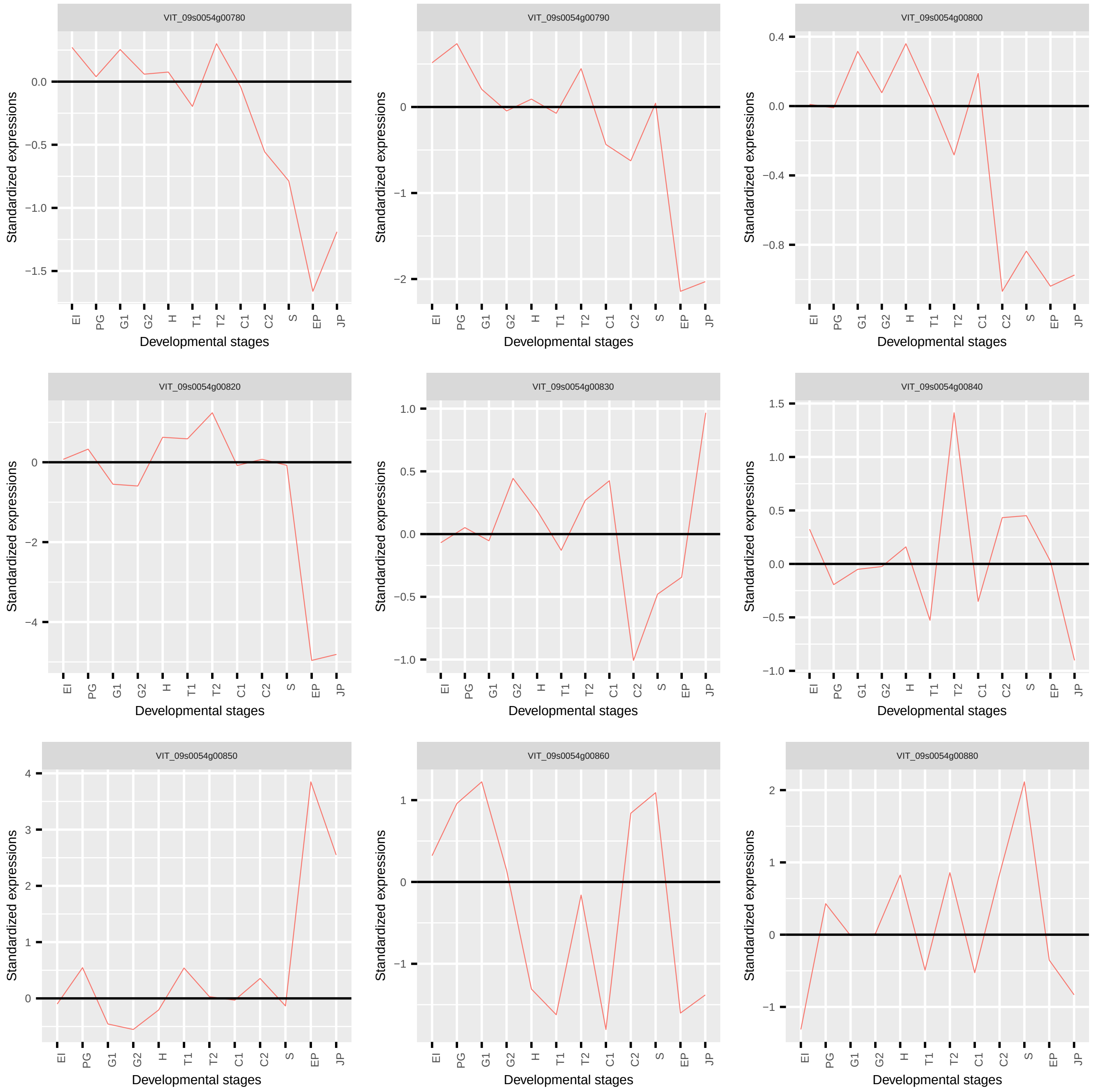
VIT_09s0054g00780
VIT_09s0054g00790
VIT_09s0054g00800
VIT_09s0054g00820
VIT_09s0054g00830
VIT_09s0054g00840
VIT_09s0054g00850
VIT_09s0054g00860
VIT_09s0054g00880
Standardized expressions
Developmental stages
EI PG G1 G2 H T1 T2 C1 C2 S EP JP

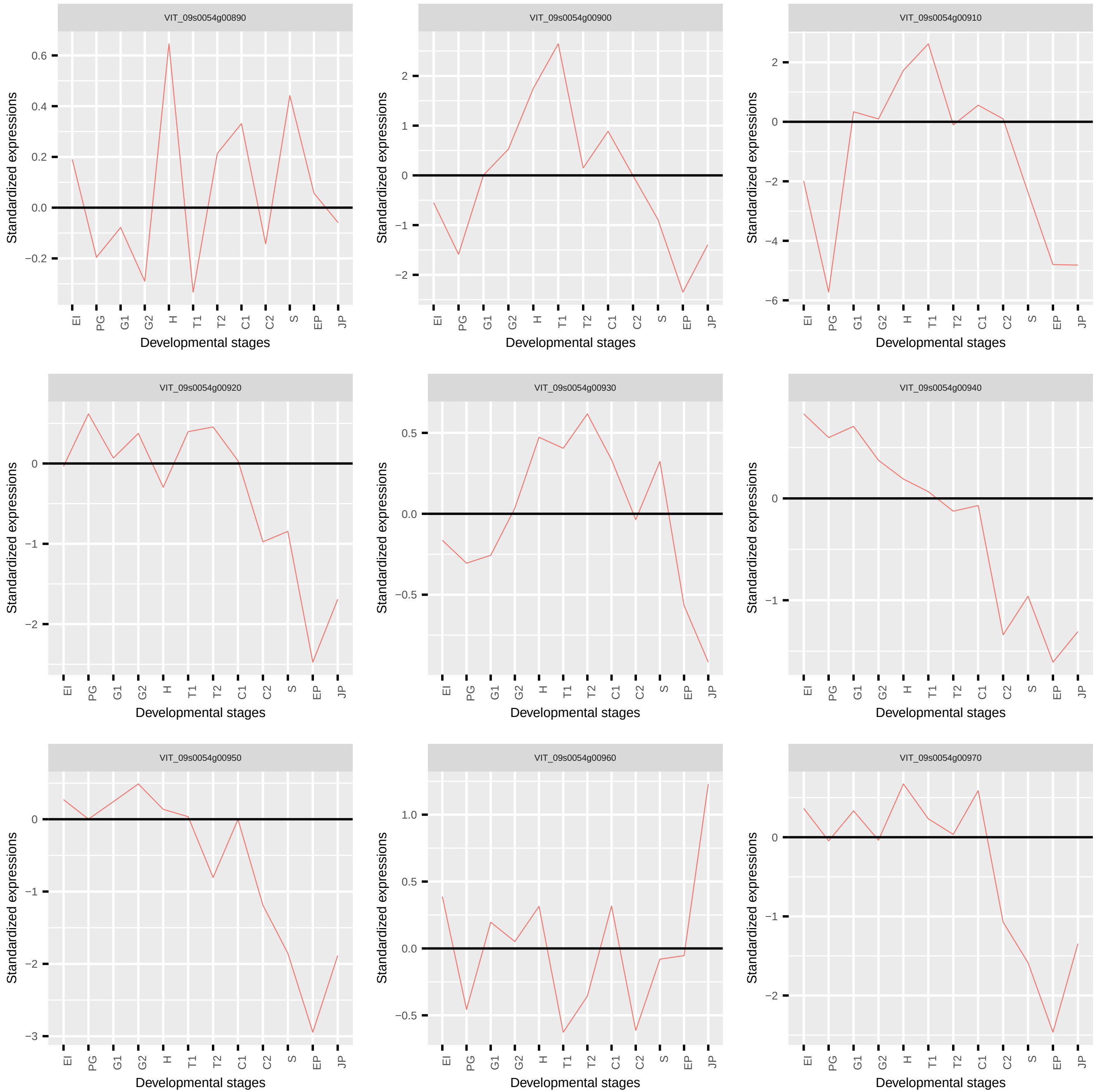
VIT_09s0054g00890
VIT_09s0054g00900
VIT_09s0054g00910
VIT_09s0054g00920
VIT_09s0054g00930
VIT_09s0054g00940
VIT_09s0054g00950
VIT_09s0054g00960
VIT_09s0054g00970
Standardized expressions
Developmental stages
EI PG G1 G2 H T1 T2 C1 C2 S EP JP

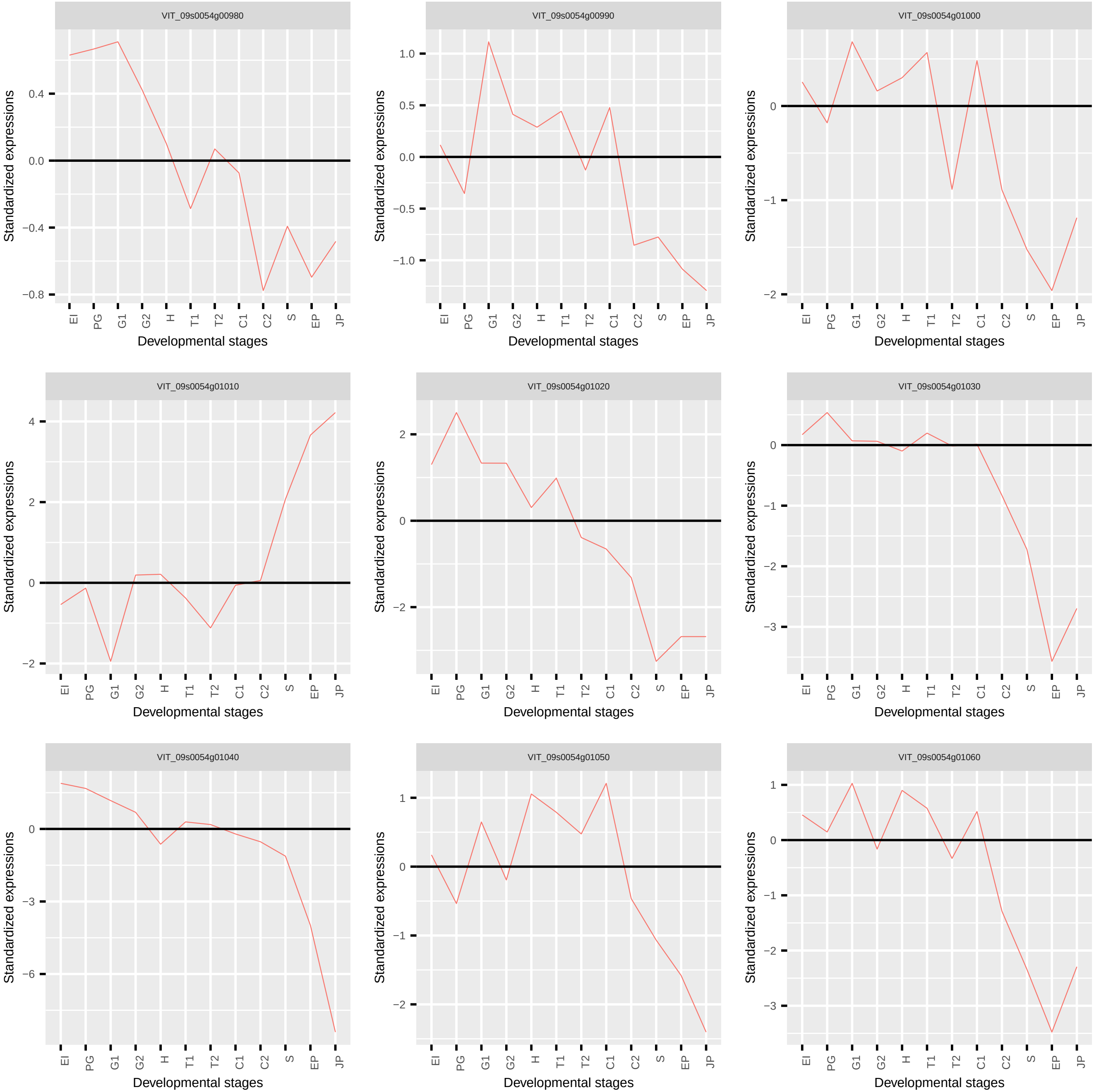
VIT_09s0054g00980
VIT_09s0054g00990
VIT_09s0054g01000
VIT_09s0054g01010
VIT_09s0054g01020
VIT_09s0054g01030
VIT_09s0054g01040
VIT_09s0054g01050
VIT_09s0054g01060
Standardized expressions
Developmental stages
EI PG G1 G2 H T1 T2 C1 C2 S EP JP

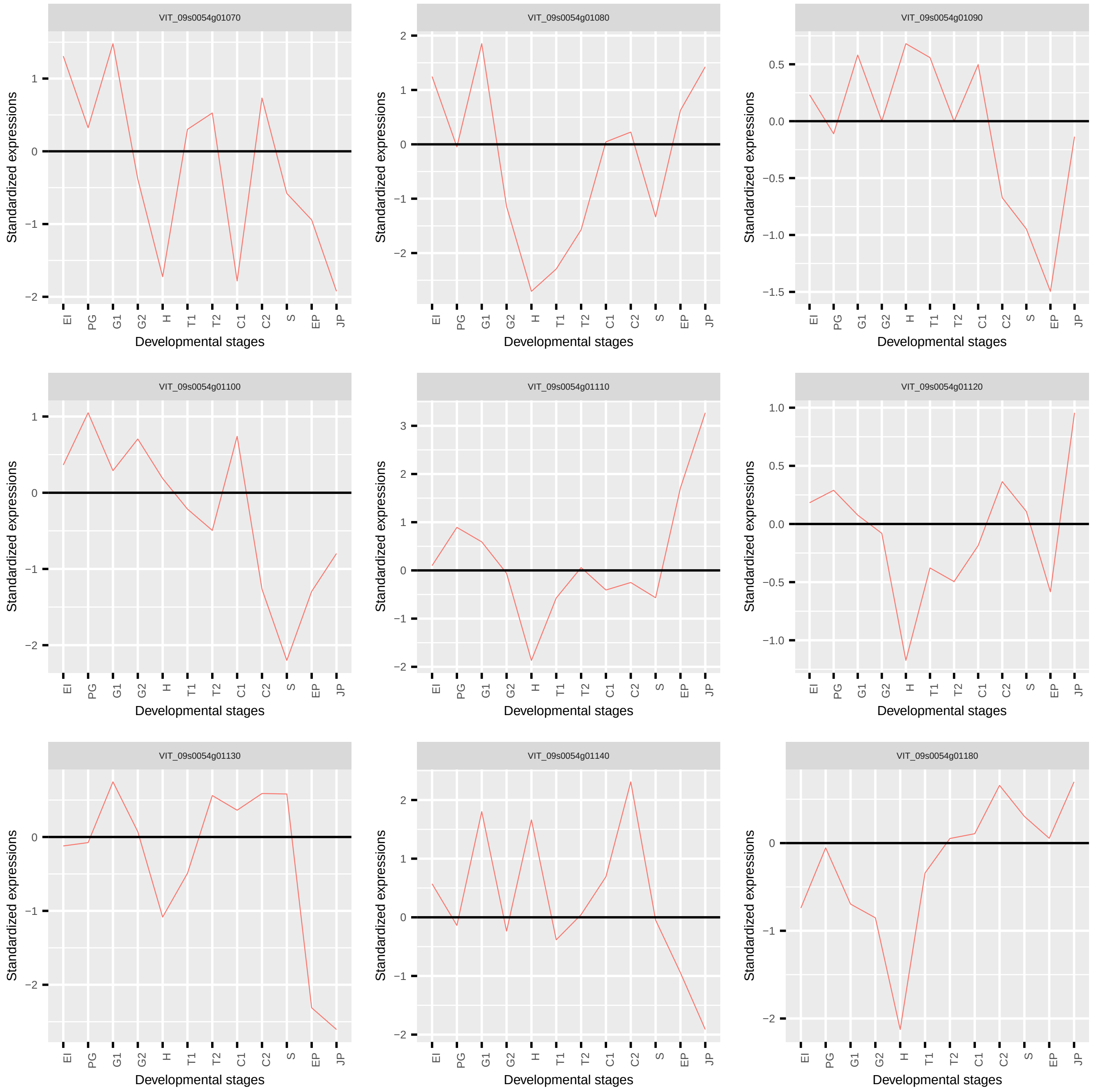
VIT_09s0054g01070
VIT_09s0054g01080
VIT_09s0054g01090
VIT_09s0054g01100
VIT_09s0054g01110
VIT_09s0054g01120
VIT_09s0054g01130
VIT_09s0054g01140
VIT_09s0054g01180
Standardized expressions
Developmental stages
EI PG G1 G2 H T1 T2 C1 C2 S EP JP

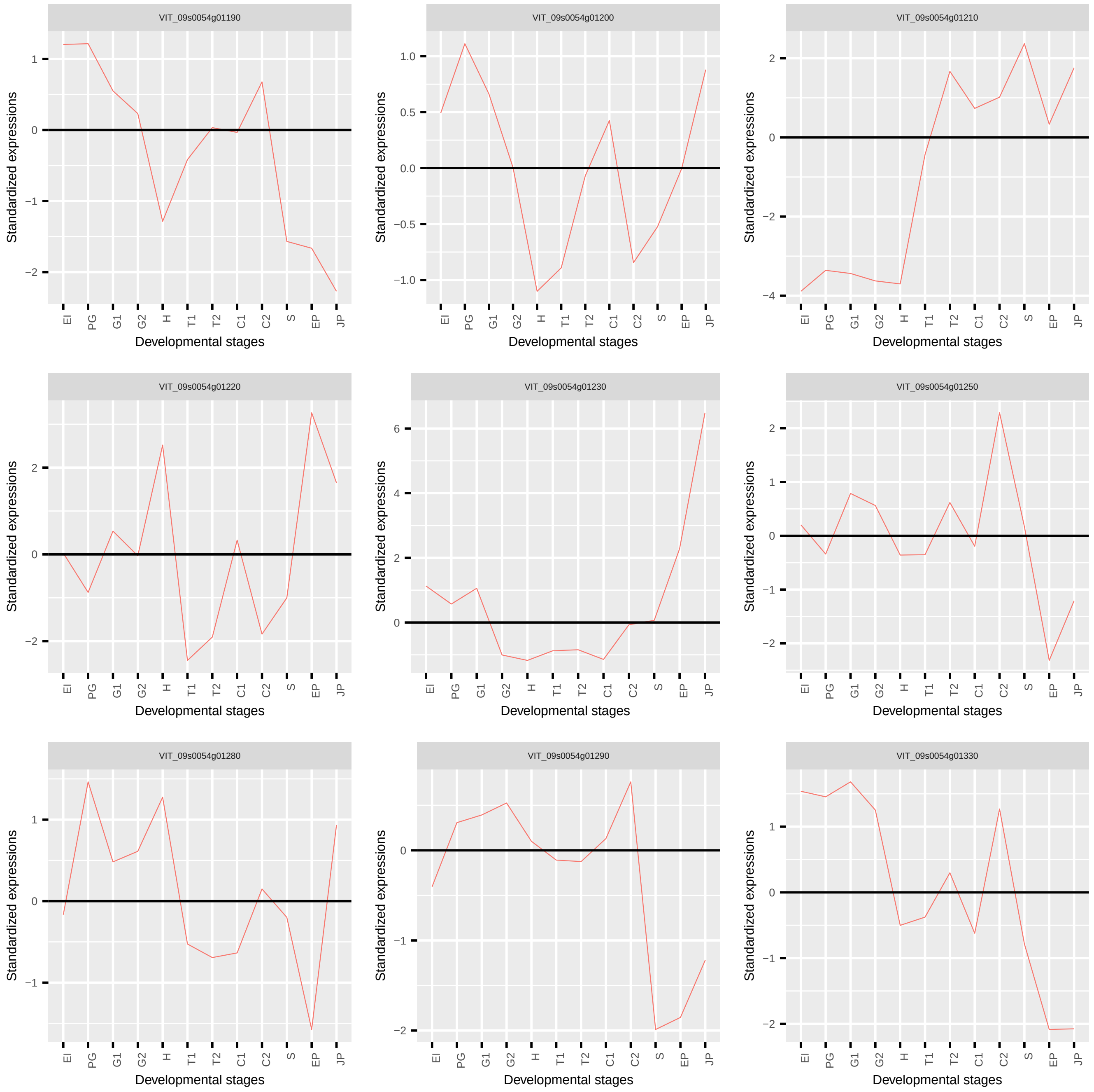

VIT_09s0054g01190
VIT_09s0054g01200
VIT_09s0054g01210
VIT_09s0054g01220
VIT_09s0054g01230
VIT_09s0054g01250
VIT_09s0054g01280
VIT_09s0054g01290
VIT_09s0054g01330
Standardized expressions
Developmental stages
EI PG G1 G2 H T1 T2 C1 C2 S EP JP

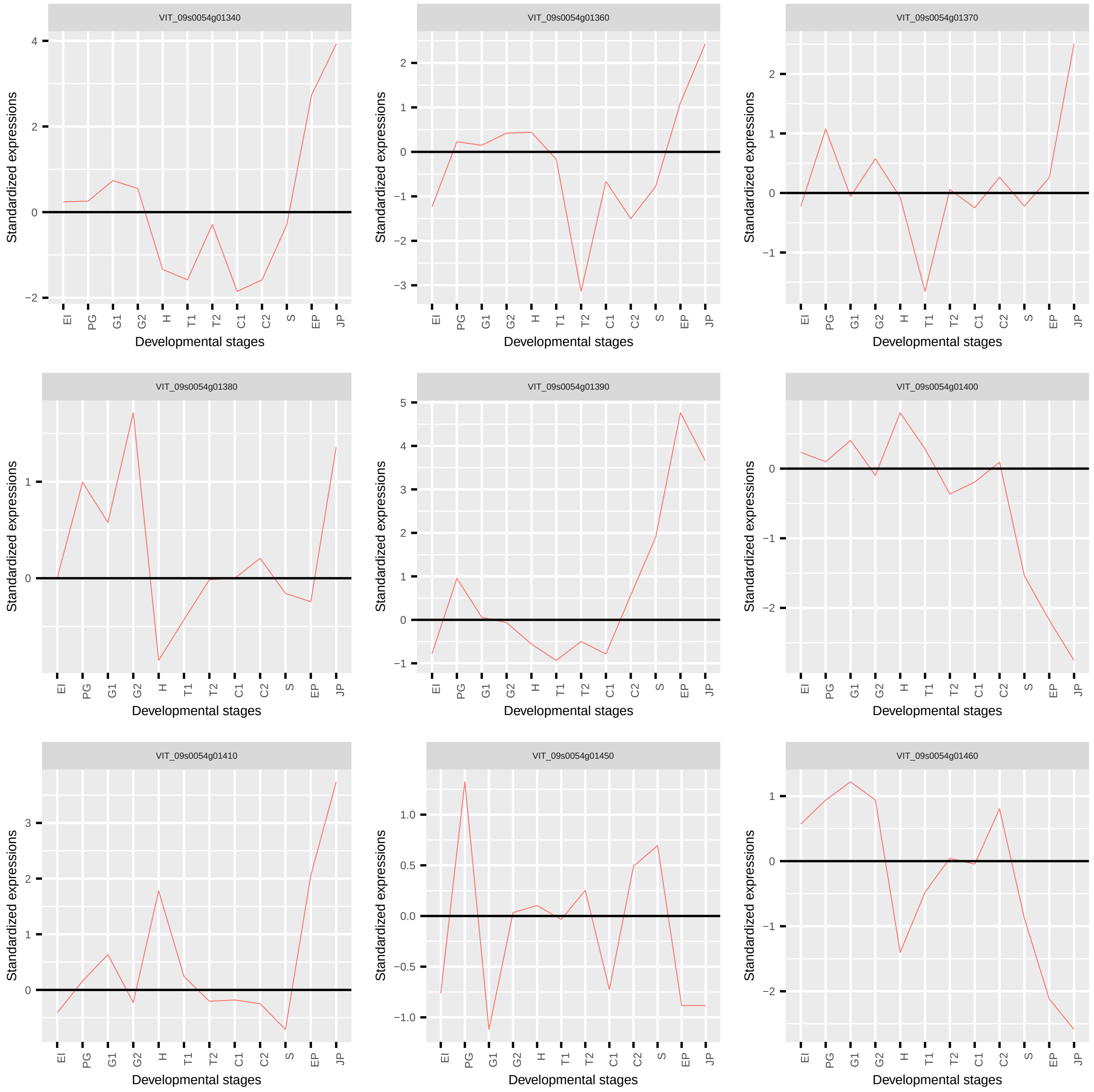

VIT_09s0054g01340
VIT_09s0054g01360
VIT_09s0054g01370
VIT_09s0054g01380
VIT_09s0054g01390
VIT_09s0054g01400
VIT_09s0054g01410
VIT_09s0054g01450
VIT_09s0054g01460
Standardized expressions
Developmental stages
EI PG G1 G2 H T1 T2 C1 C2 S EP JP

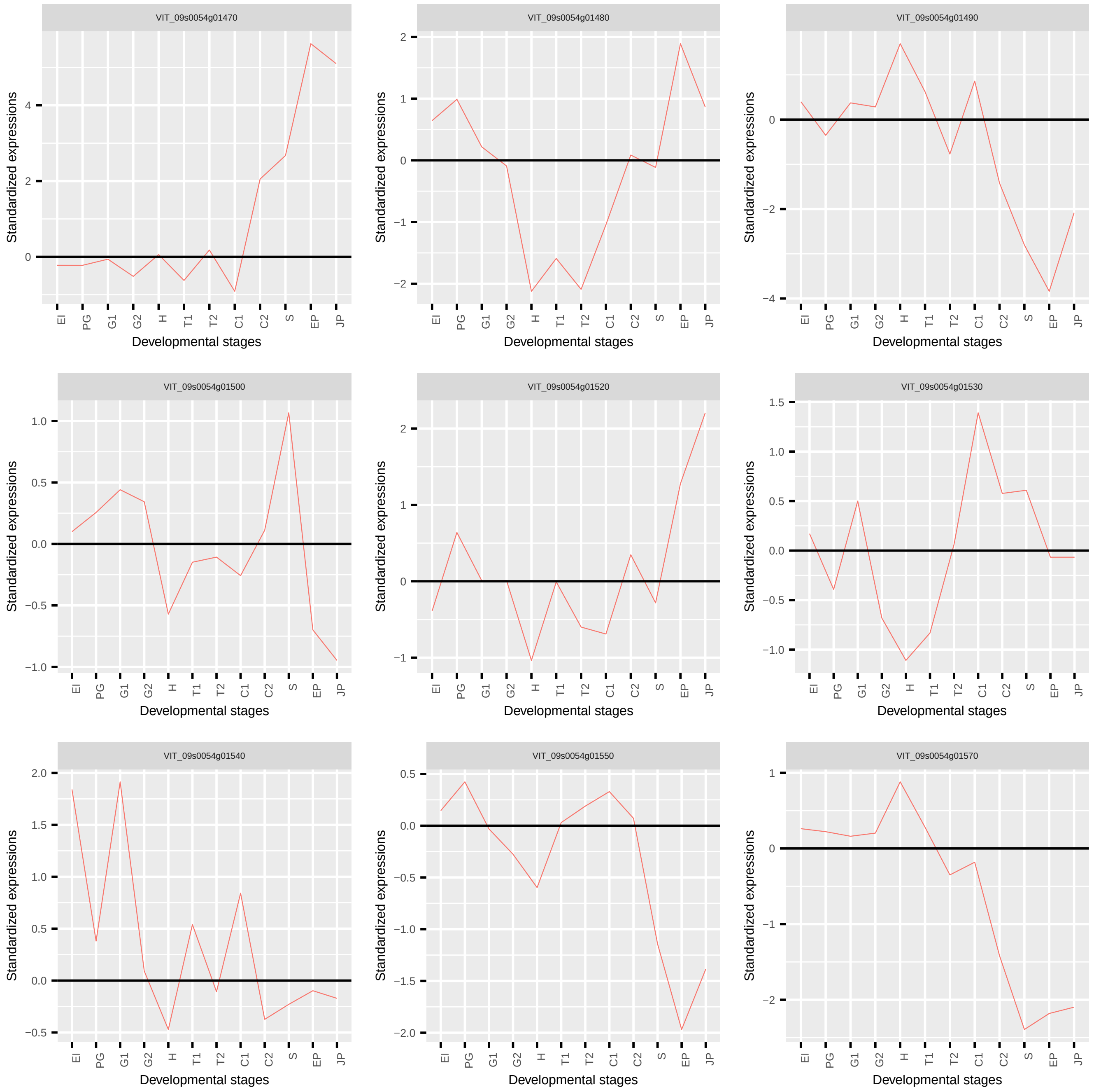
VIT_09s0054g01470
VIT_09s0054g01480
VIT_09s0054g01490
VIT_09s0054g01500
VIT_09s0054g01520
VIT_09s0054g01530
VIT_09s0054g01540
VIT_09s0054g01550
VIT_09s0054g01570
Standardized expressions
Developmental stages
EI PG G1 G2 H T1 T2 C1 C2 S EP JP

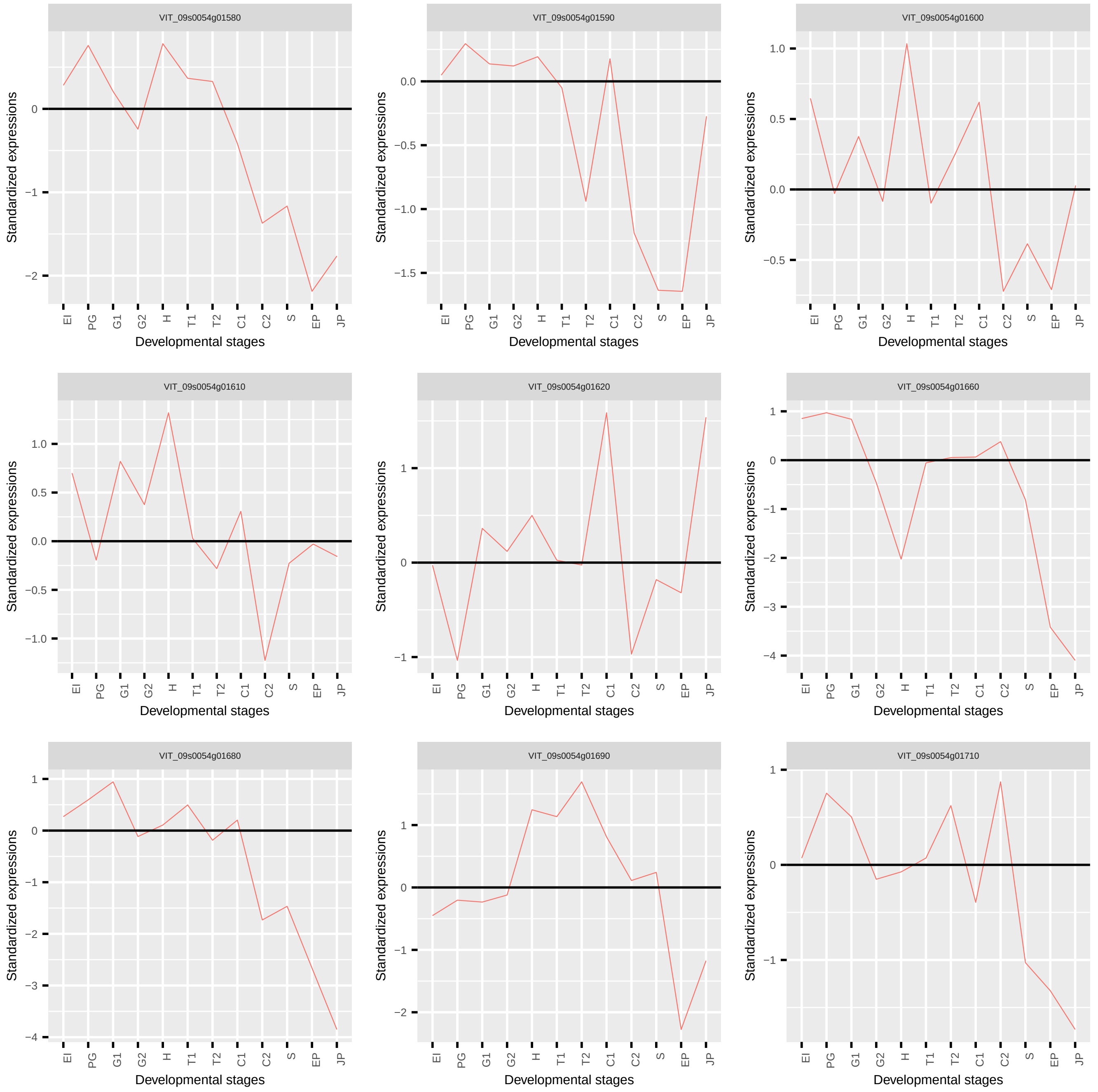
VIT_09s0054g01580
VIT_09s0054g01590
VIT_09s0054g01600
VIT_09s0054g01610
VIT_09s0054g01620
VIT_09s0054g01660
VIT_09s0054g01680
VIT_09s0054g01690
VIT_09s0054g01710
Standardized expressions
Developmental stages
EI PG G1 G2 H T1 T2 C1 C2 S EP JP

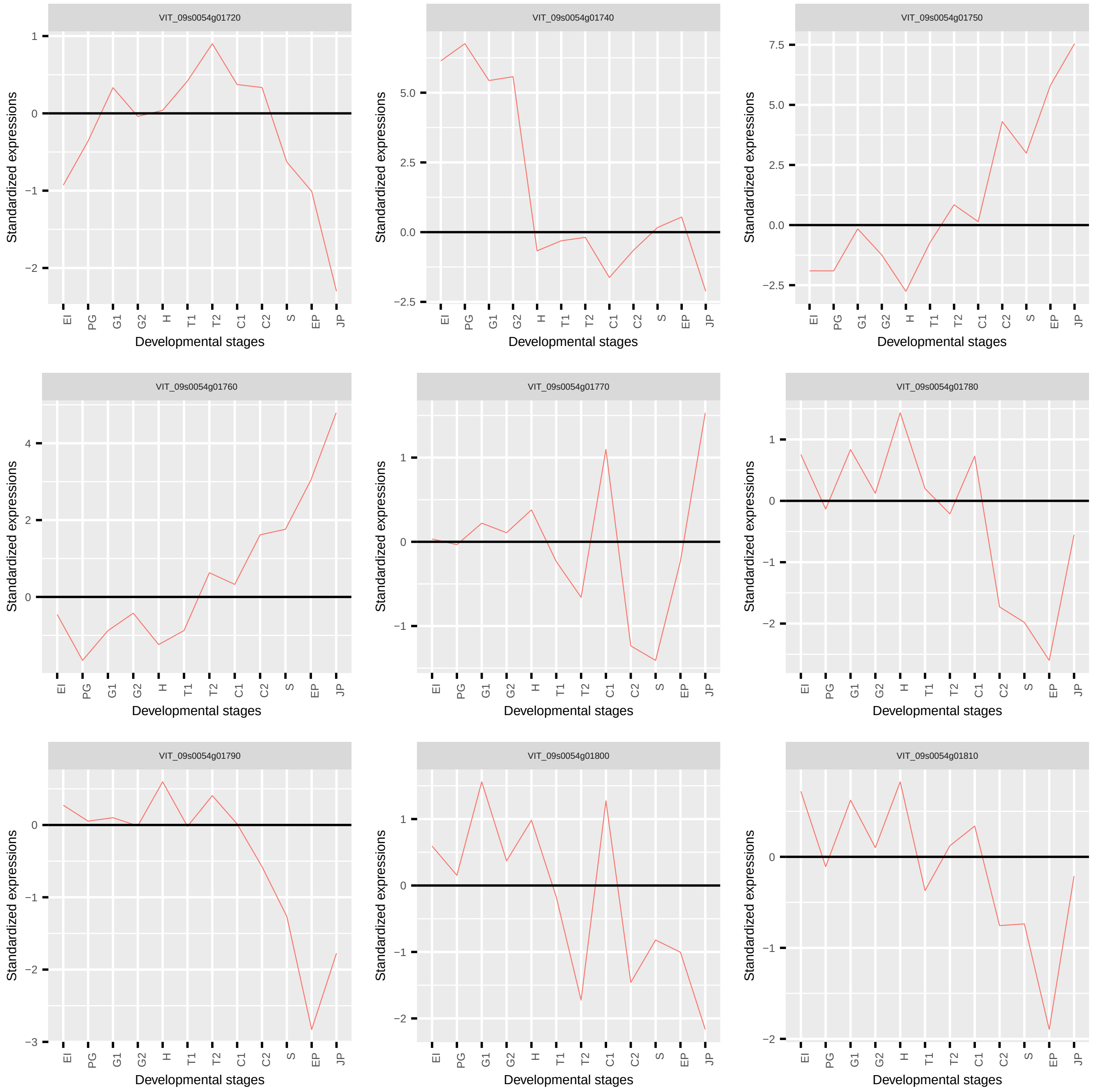
VIT_09s0054g01720
Standardized expressions
1
0
−1
−2
EI PG G1 G2 H T1 T2 C1 C2 S EP JP
Developmental stages
VIT_09s0054g01740
Standardized expressions
5.0
2.5
0.0
−2.5
EI PG G1 G2 H T1 T2 C1 C2 S EP JP
Developmental stages
VIT_09s0054g01750
Standardized expressions
7.5
5.0
2.5
0.0
−2.5
EI PG G1 G2 H T1 T2 C1 C2 S EP JP
Developmental stages
VIT_09s0054g01760
Standardized expressions
4
2
0
EI PG G1 G2 H T1 T2 C1 C2 S EP JP
Developmental stages
VIT_09s0054g01770
Standardized expressions
1
0
−1
EI PG G1 G2 H T1 T2 C1 C2 S EP JP
Developmental stages
VIT_09s0054g01780
Standardized expressions
1
0
−1
−2
EI PG G1 G2 H T1 T2 C1 C2 S EP JP
Developmental stages
VIT_09s0054g01790
Standardized expressions
0
−1
−2
−3
EI PG G1 G2 H T1 T2 C1 C2 S EP JP
Developmental stages
VIT_09s0054g01800
Standardized expressions
1
0
−1
−2
EI PG G1 G2 H T1 T2 C1 C2 S EP JP
Developmental stages
VIT_09s0054g01810
Standardized expressions
0
−1
−2
EI PG G1 G2 H T1 T2 C1 C2 S EP JP
Developmental stages

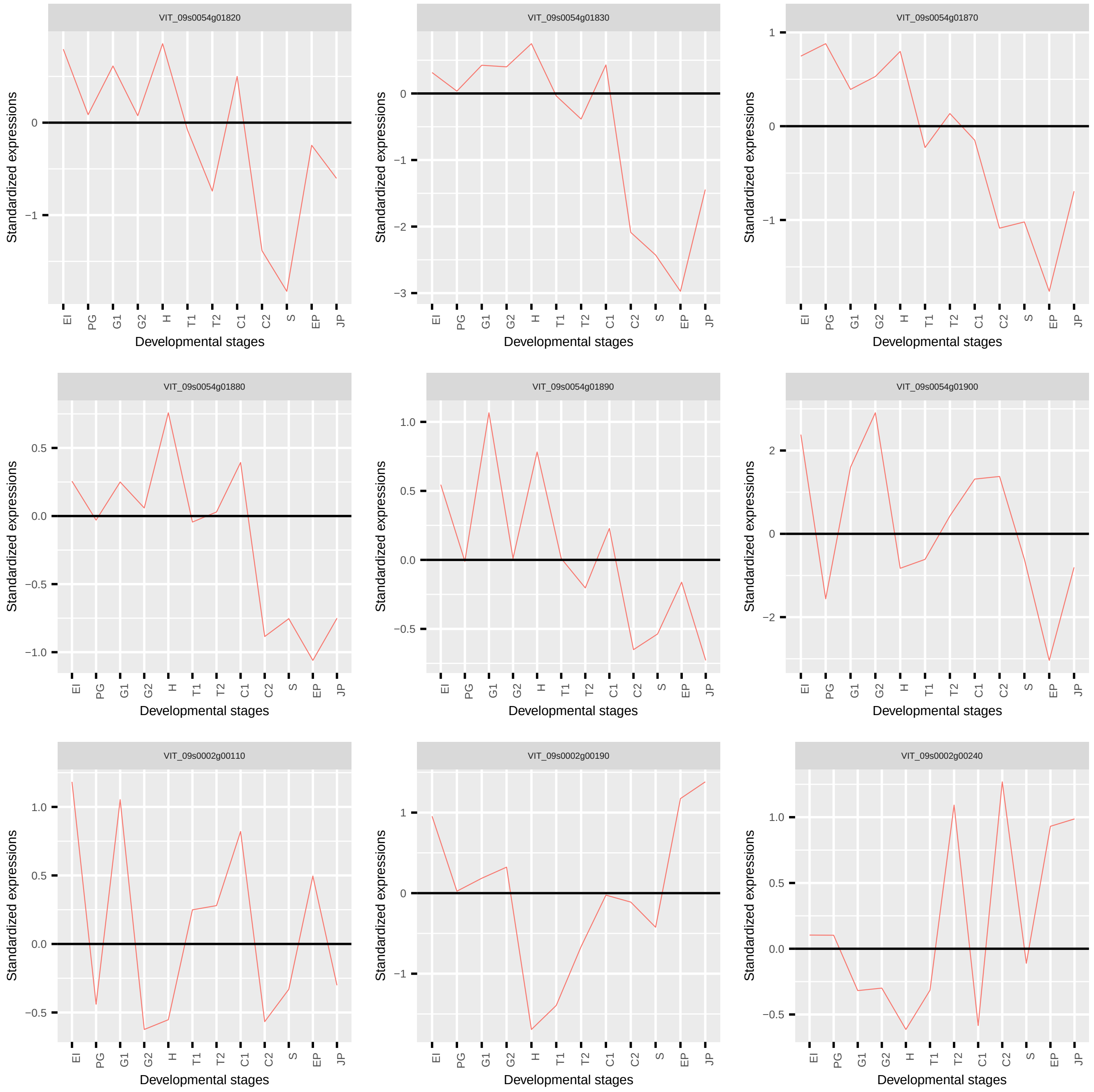
VIT_09s0054g01820
VIT_09s0054g01830
VIT_09s0054g01870
VIT_09s0054g01880
VIT_09s0054g01890
VIT_09s0054g01900
VIT_09s0002g00110
VIT_09s0002g00190
VIT_09s0002g00240
Standardized expressions
Developmental stages
EI PG G1 G2 H T1 T2 C1 C2 S EP JP

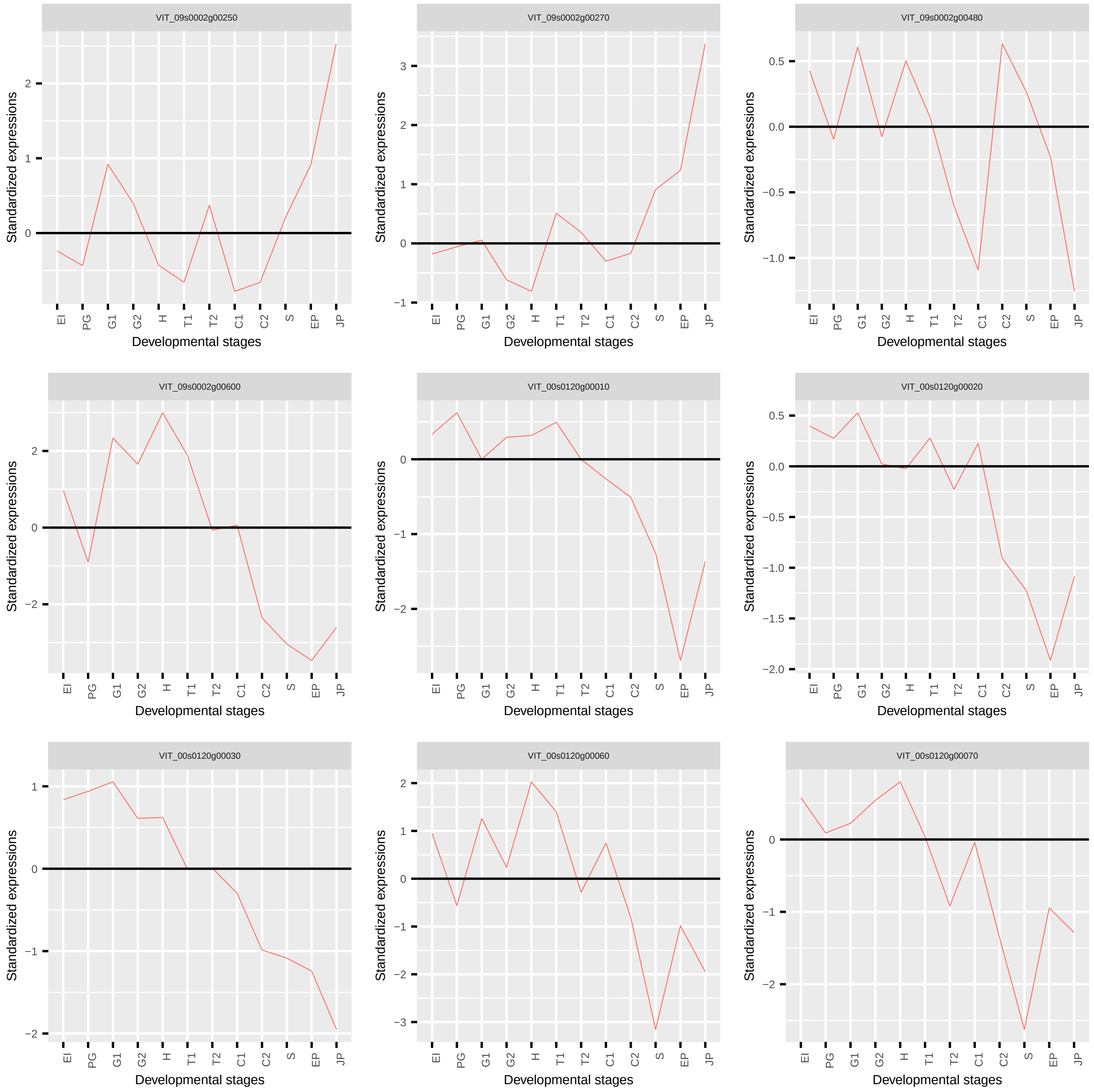
VIT_09s0002g00250
VIT_09s0002g00270
VIT_09s0002g00480
VIT_09s0002g00600
VIT_00s0120g00010
VIT_00s0120g00020
VIT_00s0120g00030
VIT_00s0120g00060
VIT_00s0120g00070
Standardized expressions
Developmental stages
EI PG G1 G2 H T1 T2 C1 C2 S EP JP

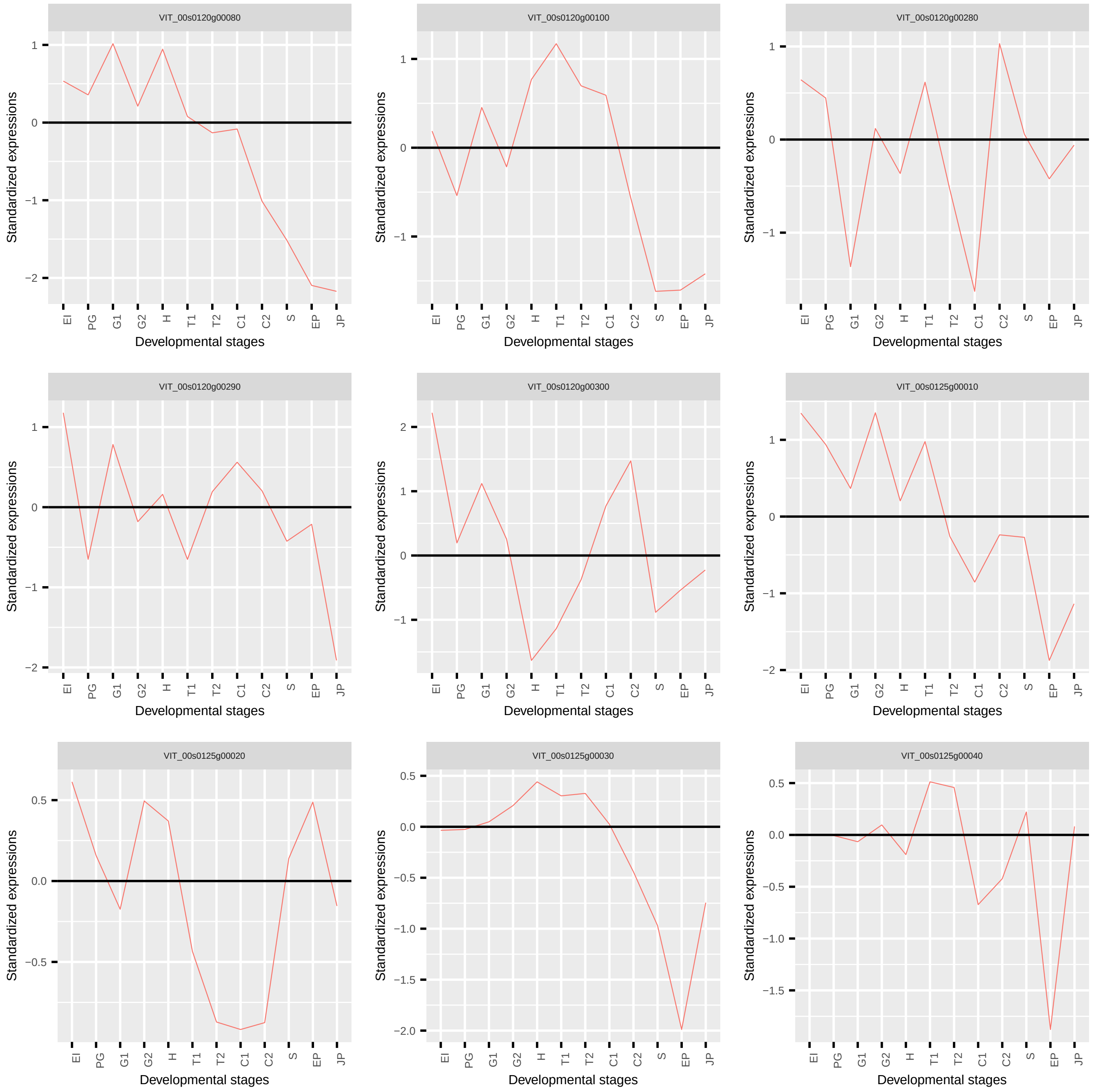
VIT_00s0120g00080
VIT_00s0120g00100
VIT_00s0120g00280
VIT_00s0120g00290
VIT_00s0120g00300
VIT_00s0125g00010
VIT_00s0125g00020
VIT_00s0125g00030
VIT_00s0125g00040
Standardized expressions
Developmental stages
EI
PG
G1
G2
H
T1
T2
C1
C2
S
EP
JP

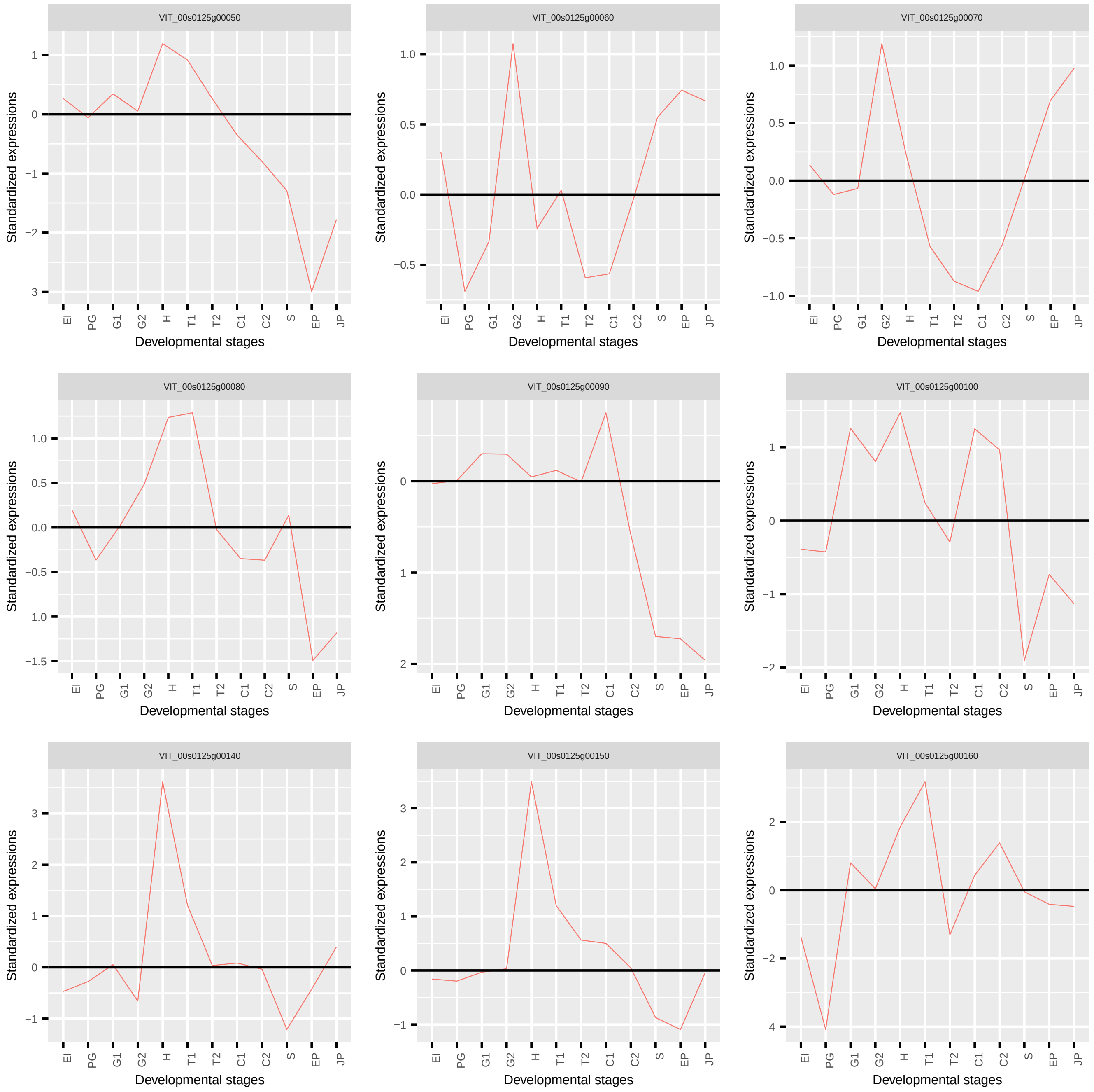
VIT_00s0125g00050
VIT_00s0125g00060
VIT_00s0125g00070
VIT_00s0125g00080
VIT_00s0125g00090
VIT_00s0125g00100
VIT_00s0125g00140
VIT_00s0125g00150
VIT_00s0125g00160
Standardized expressions
Developmental stages
EI PG G1 G2 H T1 T2 C1 C2 S EP JP

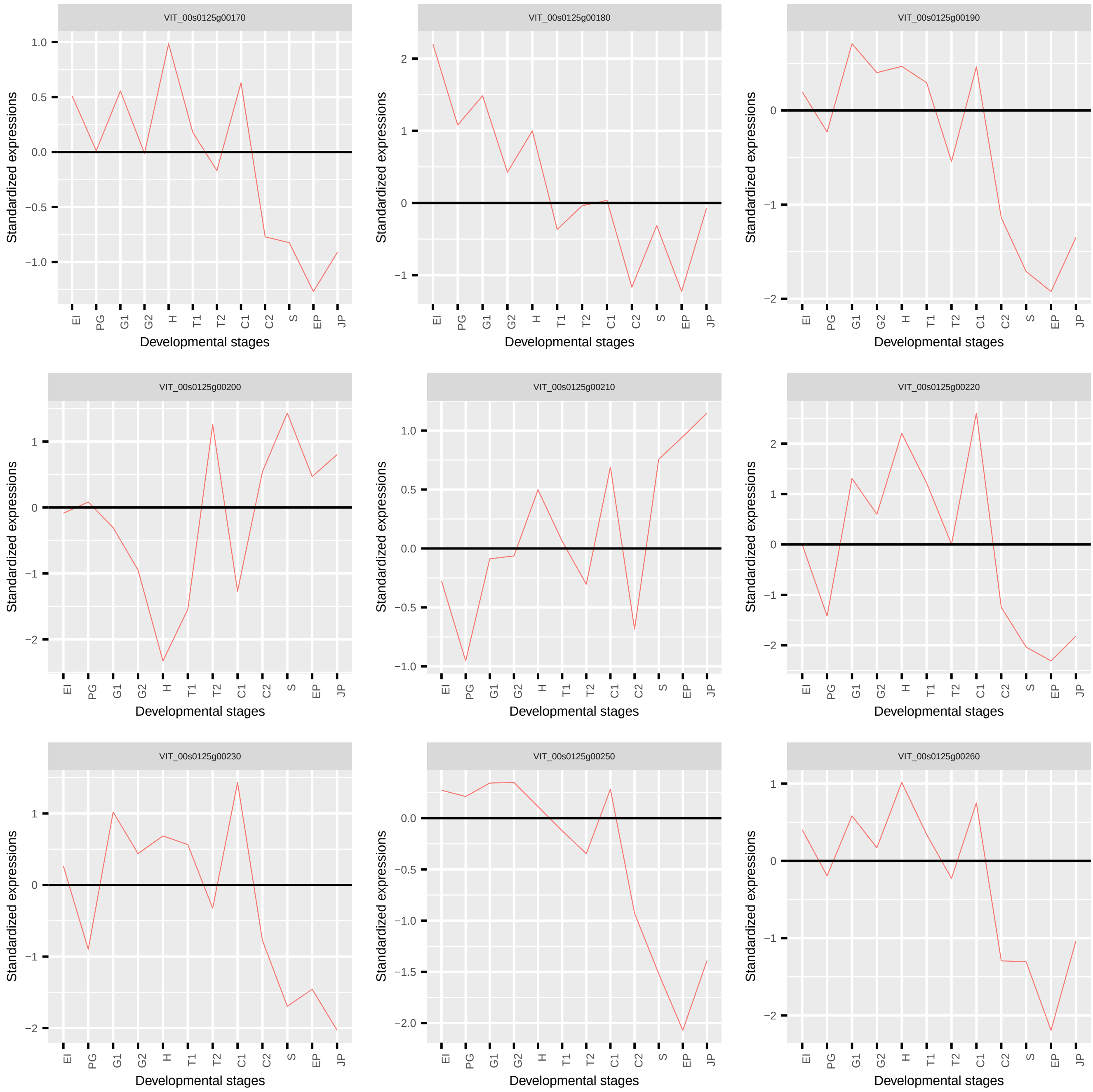

VIT_00s0125g00170
VIT_00s0125g00180
VIT_00s0125g00190
VIT_00s0125g00200
VIT_00s0125g00210
VIT_00s0125g00220
VIT_00s0125g00230
VIT_00s0125g00250
VIT_00s0125g00260
Standardized expressions
Developmental stages
EI
PG
G1
G2
H
T1
T2
C1
C2
S
EP
JP
1.0
0.5
0.0
−0.5
−1.0
−1.5
−2.0
2
1
0
−1
−2

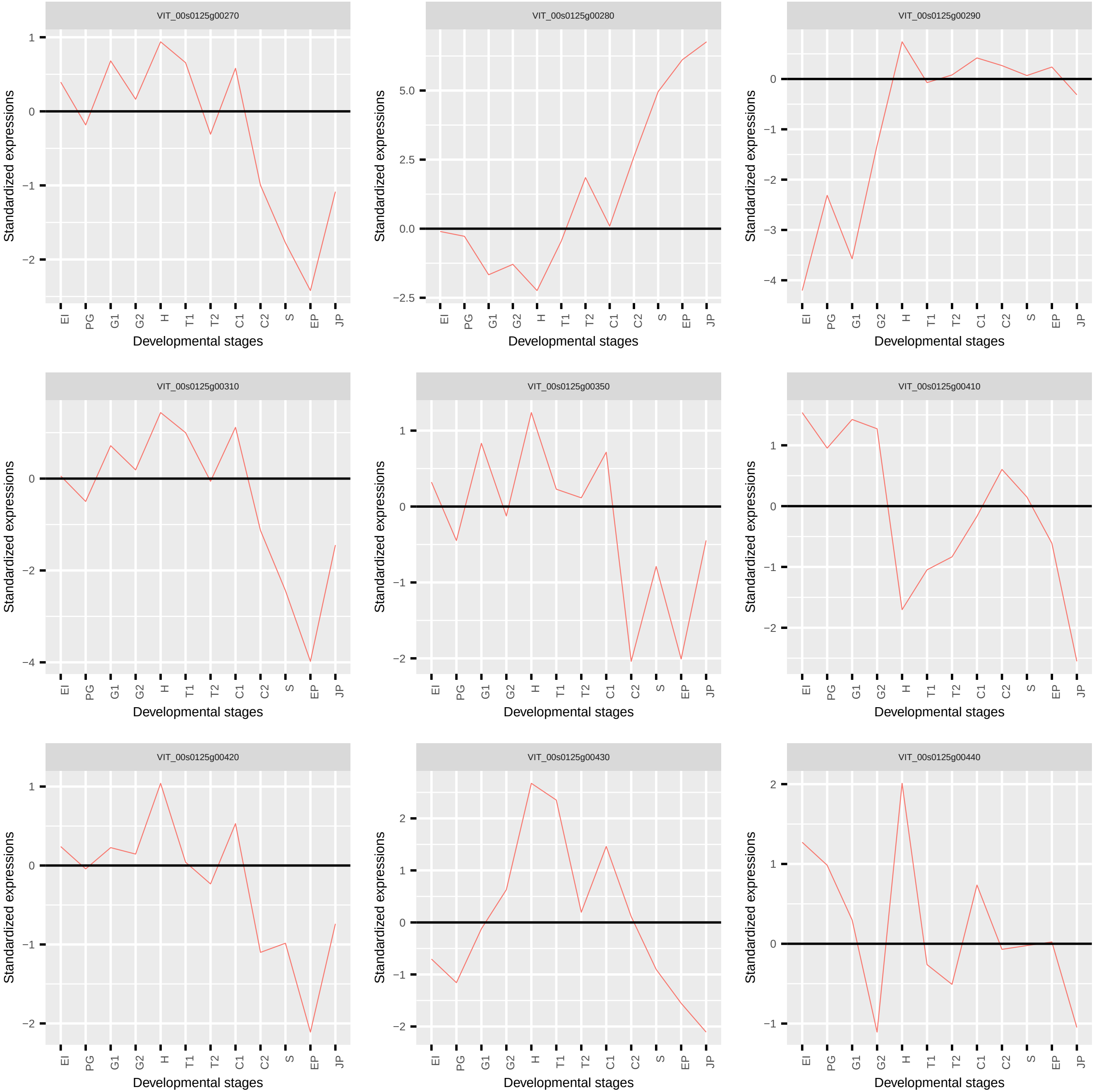

VIT_00s0125g00270
1
0
−1
−2
Standardized expressions
EI
PG
G1
G2
H
T1
T2
C1
C2
S
EP
JP
Developmental stages
VIT_00s0125g00280
5.0
2.5
0.0
−2.5
Standardized expressions
EI
PG
G1
G2
H
T1
T2
C1
C2
S
EP
JP
Developmental stages
VIT_00s0125g00290
0
−1
−2
−3
−4
Standardized expressions
EI
PG
G1
G2
H
T1
T2
C1
C2
S
EP
JP
Developmental stages
VIT_00s0125g00310
0
−2
−4
Standardized expressions
EI
PG
G1
G2
H
T1
T2
C1
C2
S
EP
JP
Developmental stages
VIT_00s0125g00350
1
0
−1
−2
Standardized expressions
EI
PG
G1
G2
H
T1
T2
C1
C2
S
EP
JP
Developmental stages
VIT_00s0125g00410
1
0
−1
−2
Standardized expressions
EI
PG
G1
G2
H
T1
T2
C1
C2
S
EP
JP
Developmental stages
VIT_00s0125g00420
1
0
−1
−2
Standardized expressions
EI
PG
G1
G2
H
T1
T2
C1
C2
S
EP
JP
Developmental stages
VIT_00s0125g00430
2
1
0
−1
−2
Standardized expressions
EI
PG
G1
G2
H
T1
T2
C1
C2
S
EP
JP
Developmental stages
VIT_00s0125g00440
2
1
0
−1
Standardized expressions
EI
PG
G1
G2
H
T1
T2
C1
C2
S
EP
JP
Developmental stages

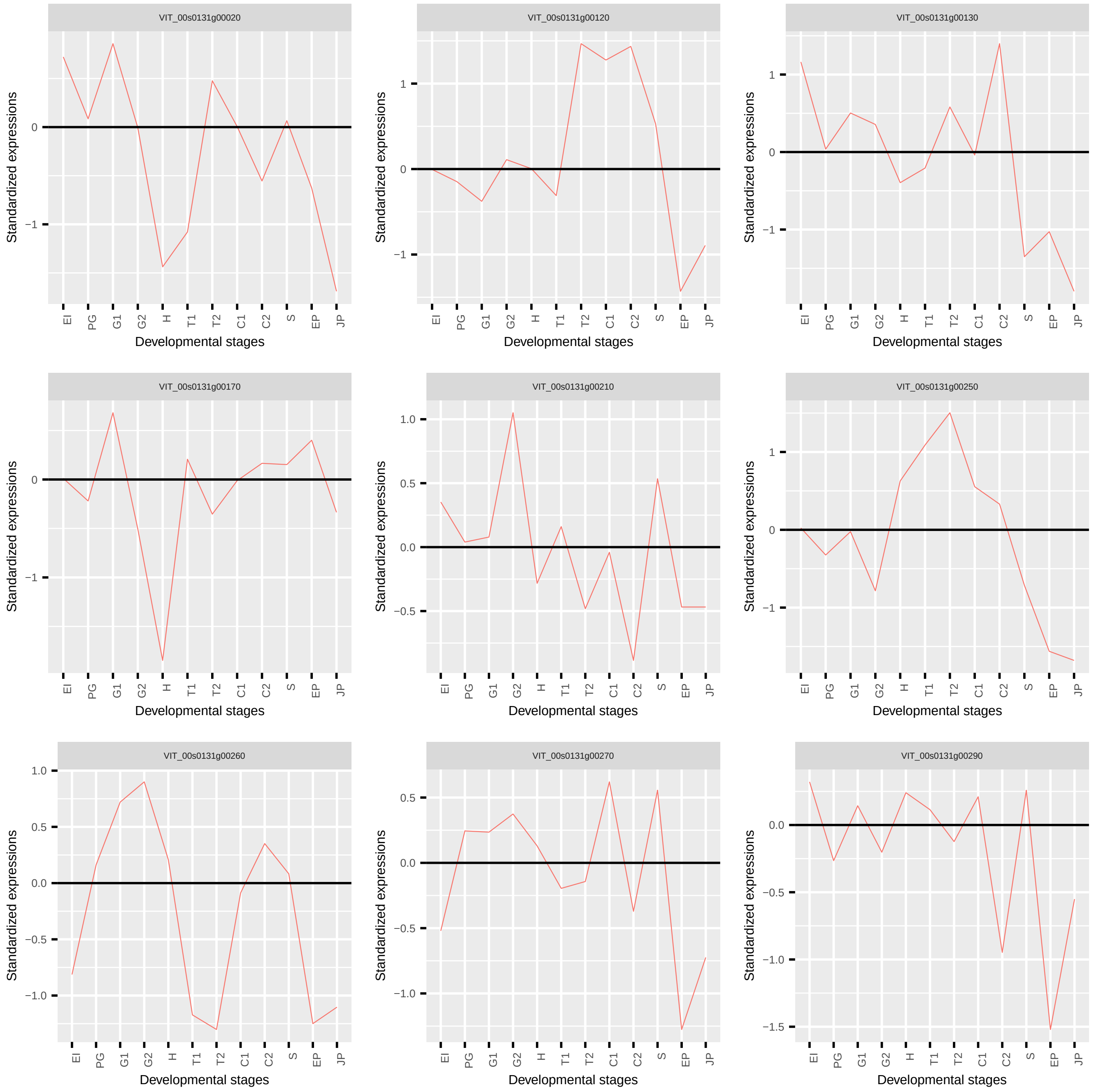
VIT_00s0131g00020
VIT_00s0131g00120
VIT_00s0131g00130
VIT_00s0131g00170
VIT_00s0131g00210
VIT_00s0131g00250
VIT_00s0131g00260
VIT_00s0131g00270
VIT_00s0131g00290
Standardized expressions
Developmental stages
EI
PG
G1
G2
H
T1
T2
C1
C2
S
EP
JP
1
0
−1
1.0
0.5
0.0
−0.5
−1.0
−1.5

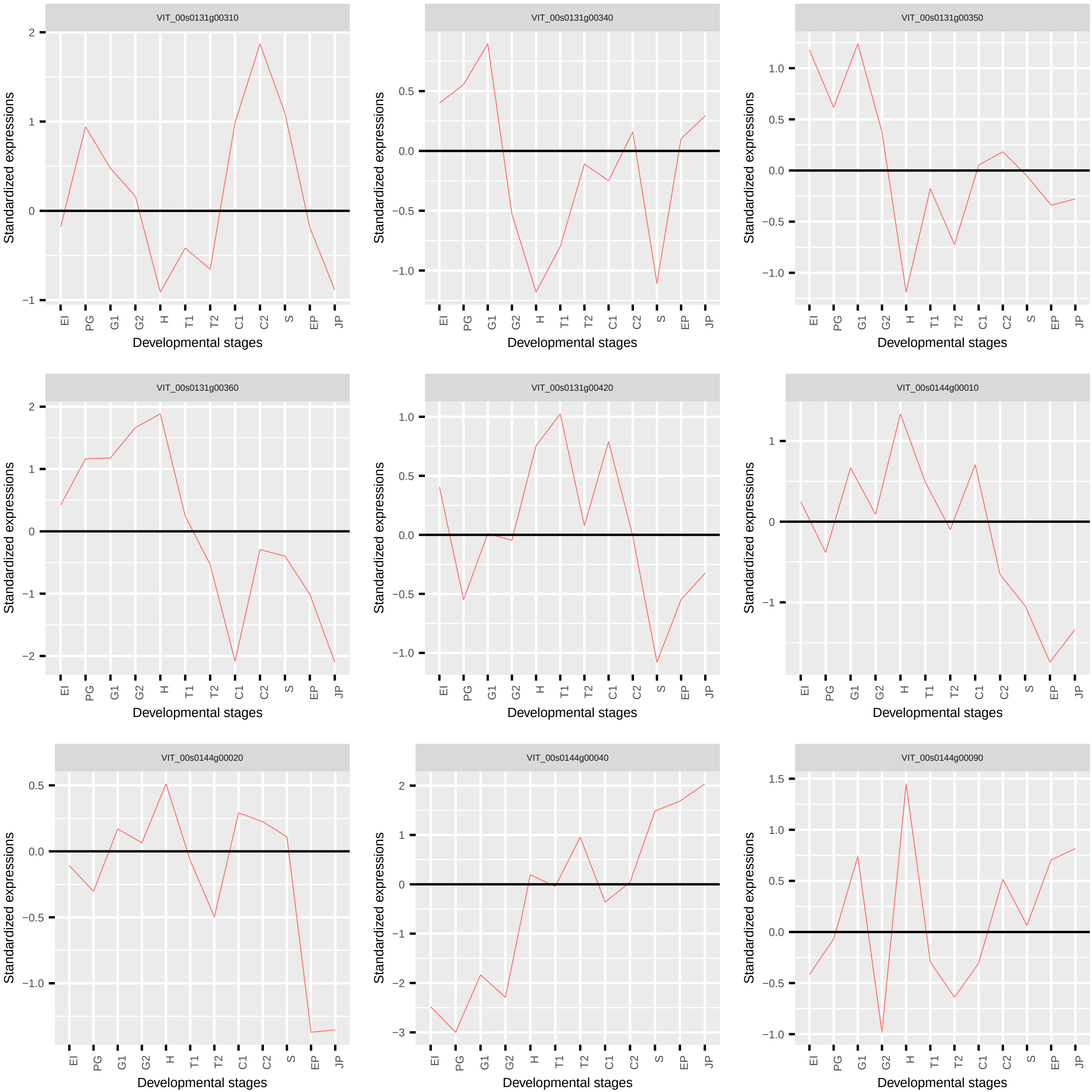
VIT_00s0131g00310
VIT_00s0131g00340
VIT_00s0131g00350
VIT_00s0131g00360
VIT_00s0131g00420
VIT_00s0144g00010
VIT_00s0144g00020
VIT_00s0144g00040
VIT_00s0144g00090
Standardized expressions
Developmental stages
EI PG G1 G2 H T1 T2 C1 C2 S EP JP

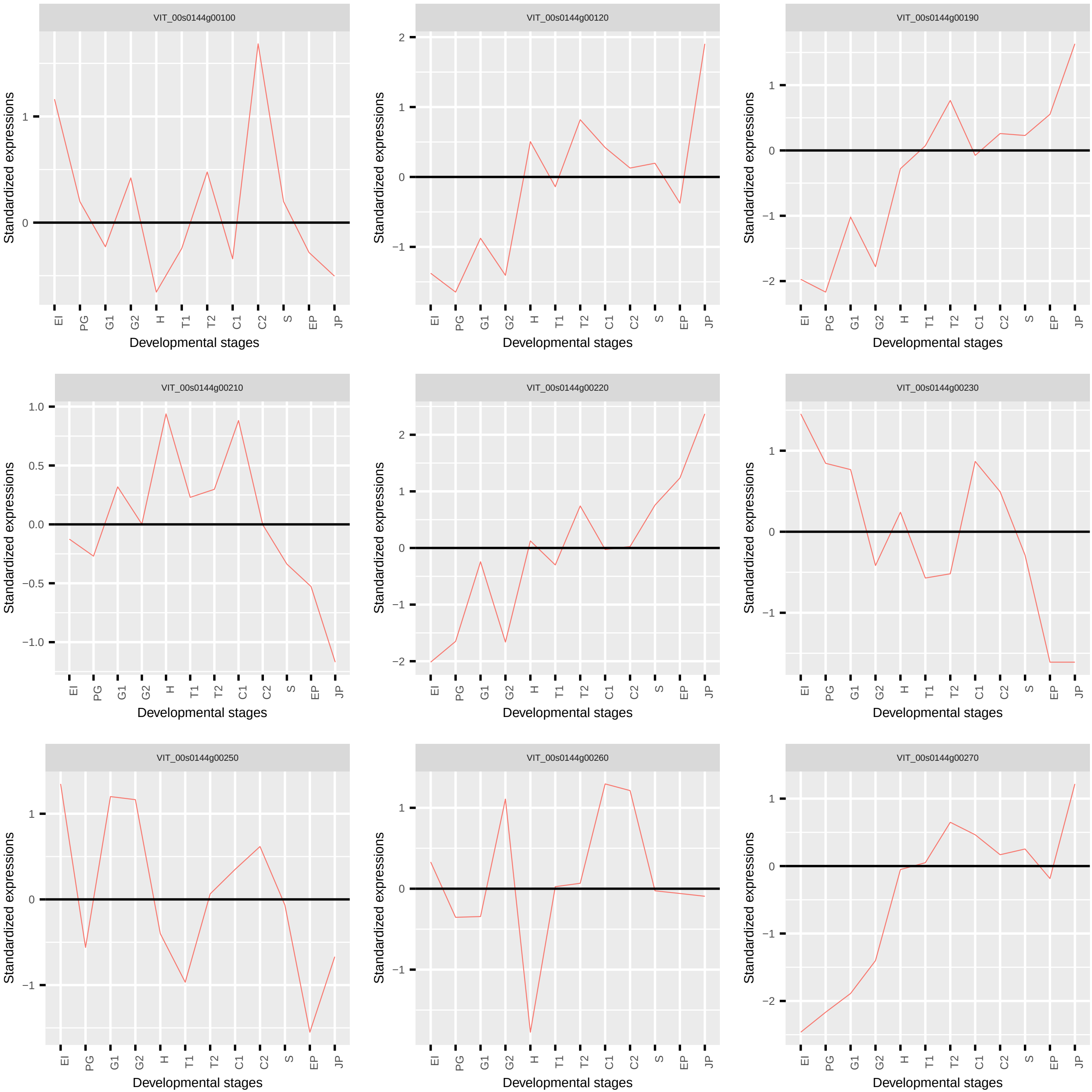
VIT_00s0144g00100
VIT_00s0144g00120
VIT_00s0144g00190
VIT_00s0144g00210
VIT_00s0144g00220
VIT_00s0144g00230
VIT_00s0144g00250
VIT_00s0144g00260
VIT_00s0144g00270
Standardized expressions
Developmental stages
EI PG G1 G2 H T1 T2 C1 C2 S EP JP

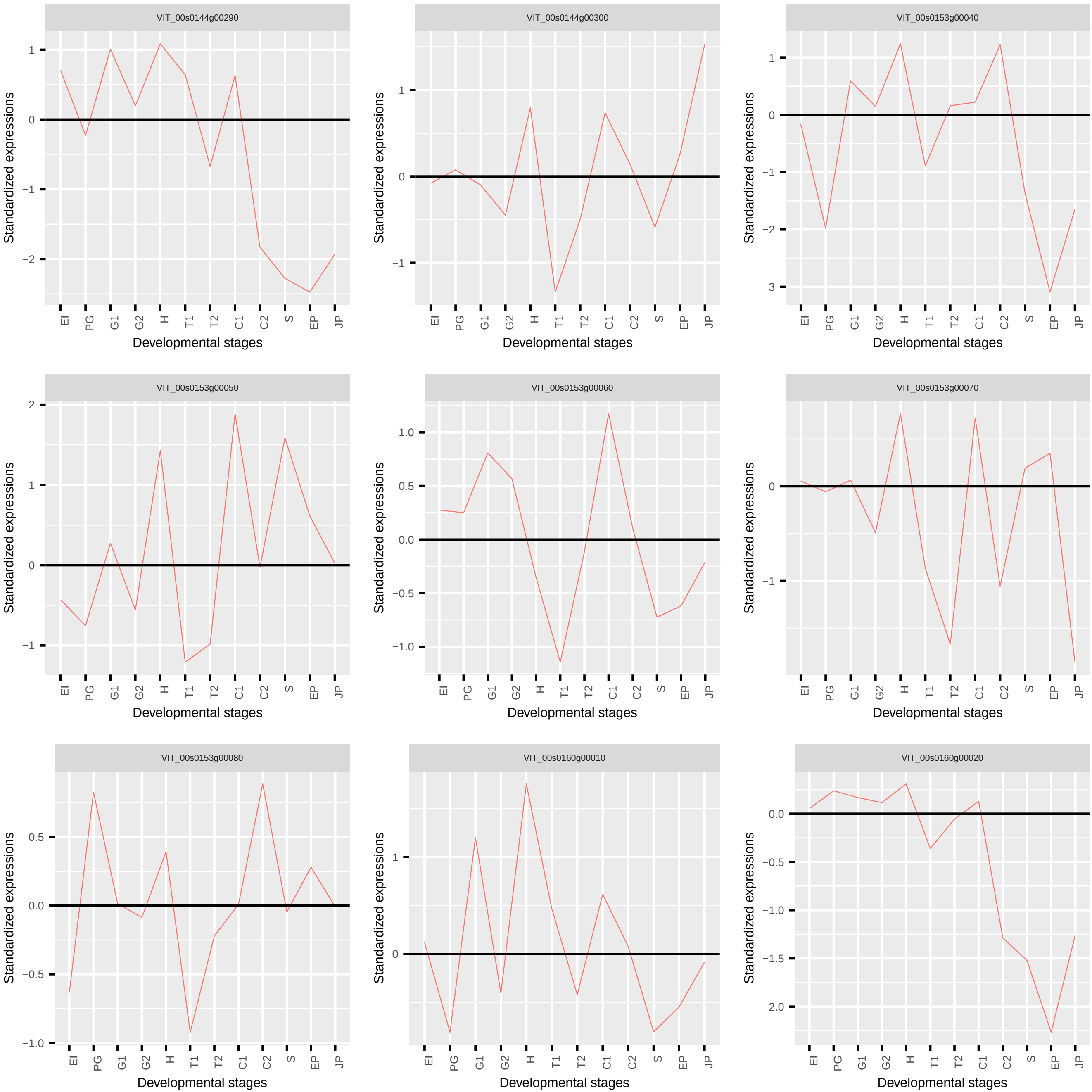
VIT_00s0144g00290
VIT_00s0144g00300
VIT_00s0153g00040
VIT_00s0153g00050
VIT_00s0153g00060
VIT_00s0153g00070
VIT_00s0153g00080
VIT_00s0160g00010
VIT_00s0160g00020
Standardized expressions
Developmental stages
EI PG G1 G2 H T1 T2 C1 C2 S EP JP

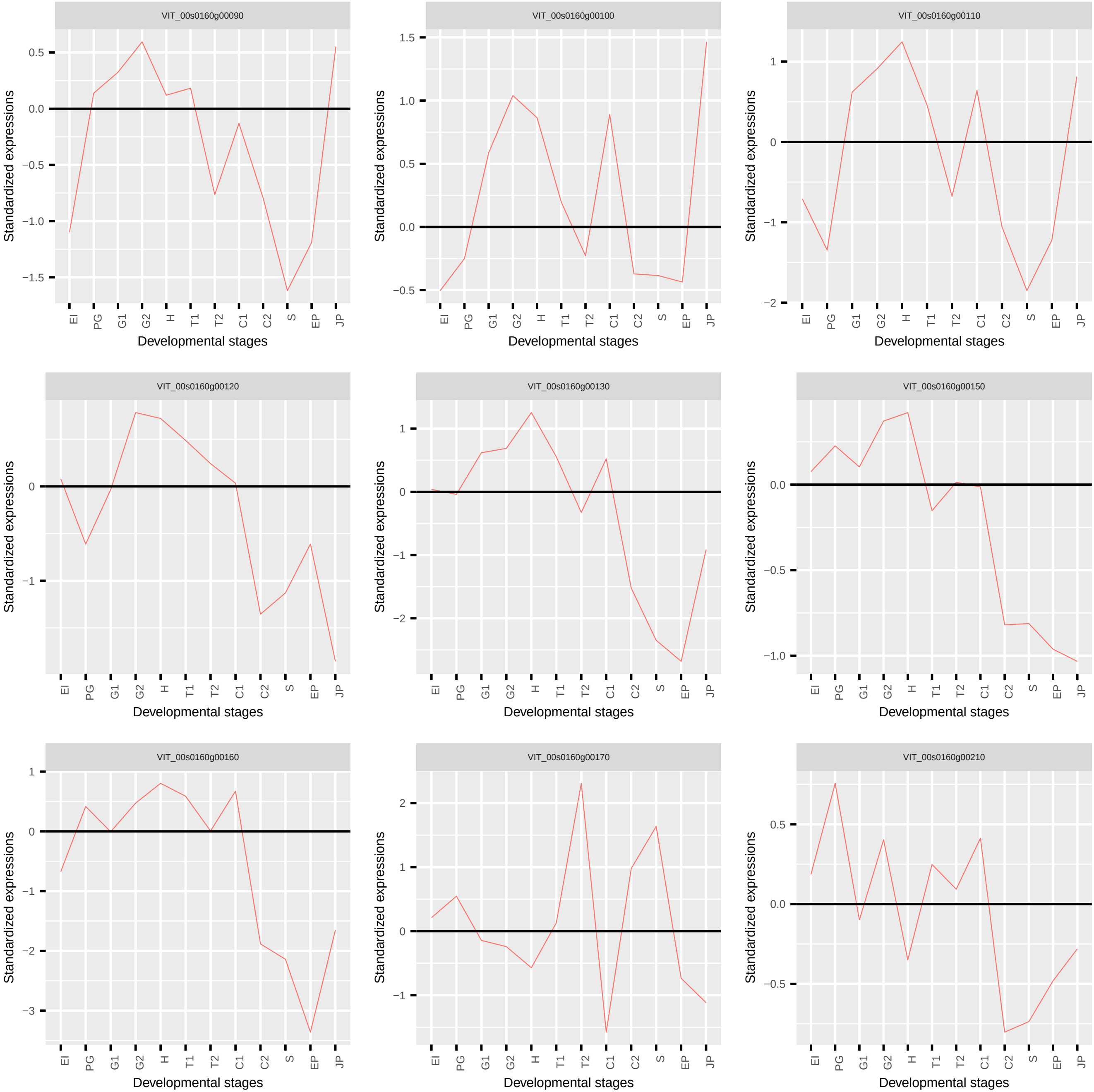
VIT_00s0160g00090
0.5
0.0
−0.5
−1.0
−1.5
Standardized expressions
EI PG G1 G2 H T1 T2 C1 C2 S EP JP
Developmental stages
VIT_00s0160g00100
1.5
1.0
0.5
0.0
−0.5
Standardized expressions
EI PG G1 G2 H T1 T2 C1 C2 S EP JP
Developmental stages
VIT_00s0160g00110
1
0
−1
−2
Standardized expressions
EI PG G1 G2 H T1 T2 C1 C2 S EP JP
Developmental stages
VIT_00s0160g00120
0
−1
Standardized expressions
EI PG G1 G2 H T1 T2 C1 C2 S EP JP
Developmental stages
VIT_00s0160g00130
1
0
−1
−2
Standardized expressions
EI PG G1 G2 H T1 T2 C1 C2 S EP JP
Developmental stages
VIT_00s0160g00150
0.0
−0.5
−1.0
Standardized expressions
EI PG G1 G2 H T1 T2 C1 C2 S EP JP
Developmental stages
VIT_00s0160g00160
1
0
−1
−2
−3
Standardized expressions
EI PG G1 G2 H T1 T2 C1 C2 S EP JP
Developmental stages
VIT_00s0160g00170
2
1
0
−1
Standardized expressions
EI PG G1 G2 H T1 T2 C1 C2 S EP JP
Developmental stages
VIT_00s0160g00210
0.5
0.0
−0.5
Standardized expressions
EI PG G1 G2 H T1 T2 C1 C2 S EP JP
Developmental stages

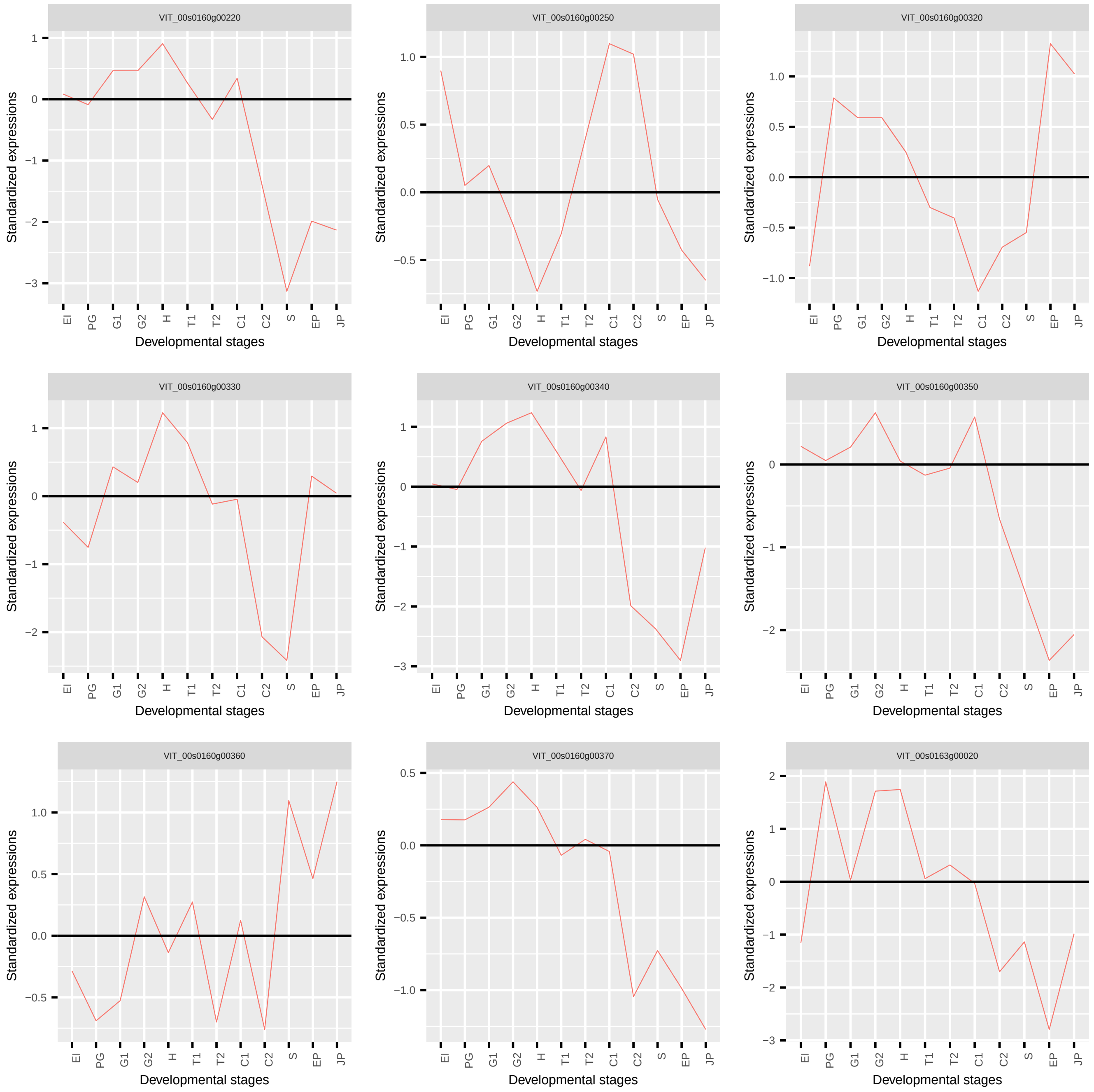
VIT_00s0160g00220
VIT_00s0160g00250
VIT_00s0160g00320
VIT_00s0160g00330
VIT_00s0160g00340
VIT_00s0160g00350
VIT_00s0160g00360
VIT_00s0160g00370
VIT_00s0163g00020
Standardized expressions
Developmental stages
EI
PG
G1
G2
H
T1
T2
C1
C2
S
EP
JP

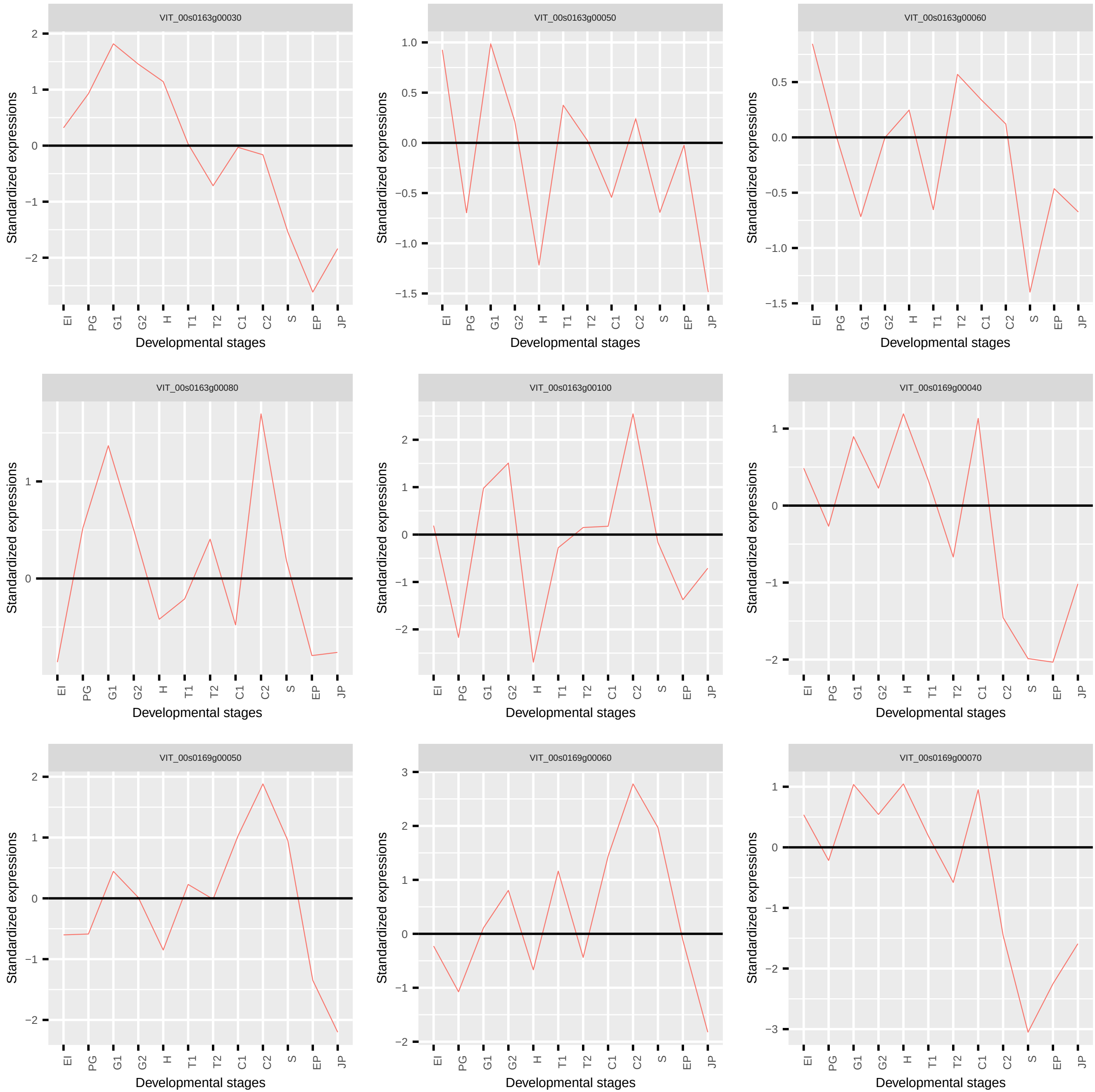
VIT_00s0163g00030
VIT_00s0163g00050
VIT_00s0163g00060
VIT_00s0163g00080
VIT_00s0163g00100
VIT_00s0169g00040
VIT_00s0169g00050
VIT_00s0169g00060
VIT_00s0169g00070
Standardized expressions
Developmental stages
EI PG G1 G2 H T1 T2 C1 C2 S EP JP

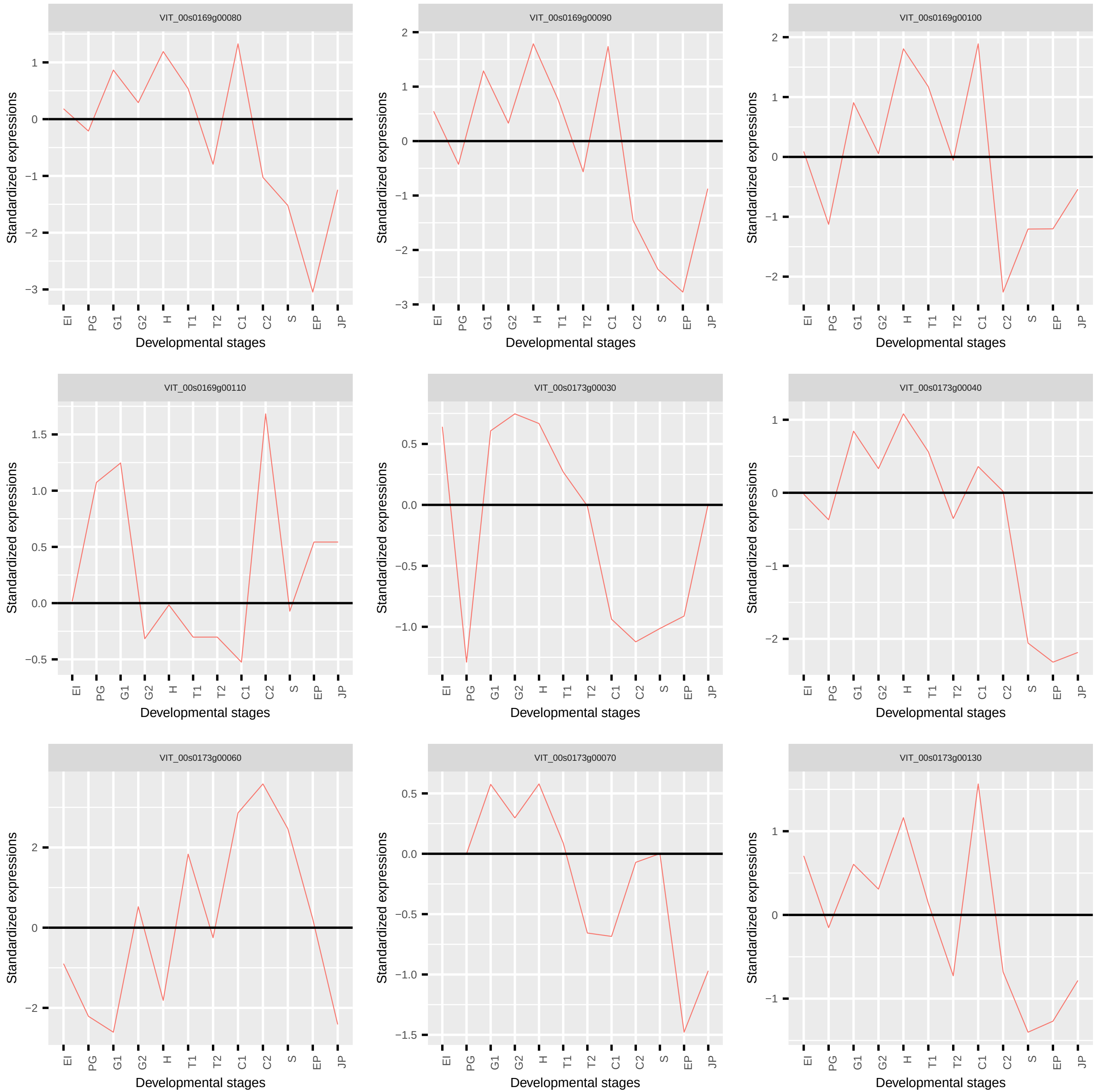
VIT_00s0169g00080
VIT_00s0169g00090
VIT_00s0169g00100
VIT_00s0169g00110
VIT_00s0173g00030
VIT_00s0173g00040
VIT_00s0173g00060
VIT_00s0173g00070
VIT_00s0173g00130
Standardized expressions
Developmental stages
EI PG G1 G2 H T1 T2 C1 C2 S EP JP

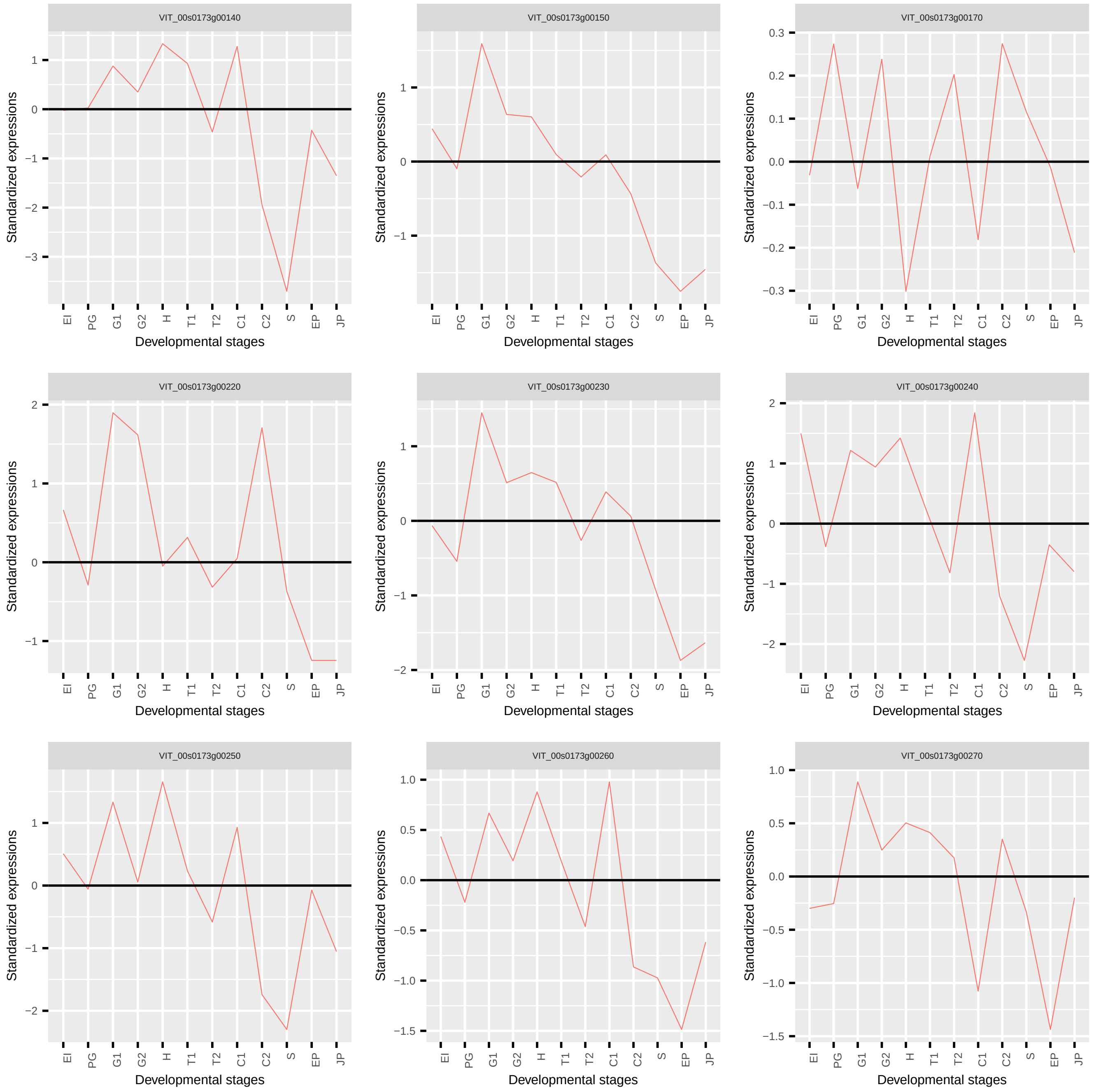
VIT_00s0173g00140
VIT_00s0173g00150
VIT_00s0173g00170
VIT_00s0173g00220
VIT_00s0173g00230
VIT_00s0173g00240
VIT_00s0173g00250
VIT_00s0173g00260
VIT_00s0173g00270
Standardized expressions
Developmental stages
EI
PG
G1
G2
H
T1
T2
C1
C2
S
EP
JP

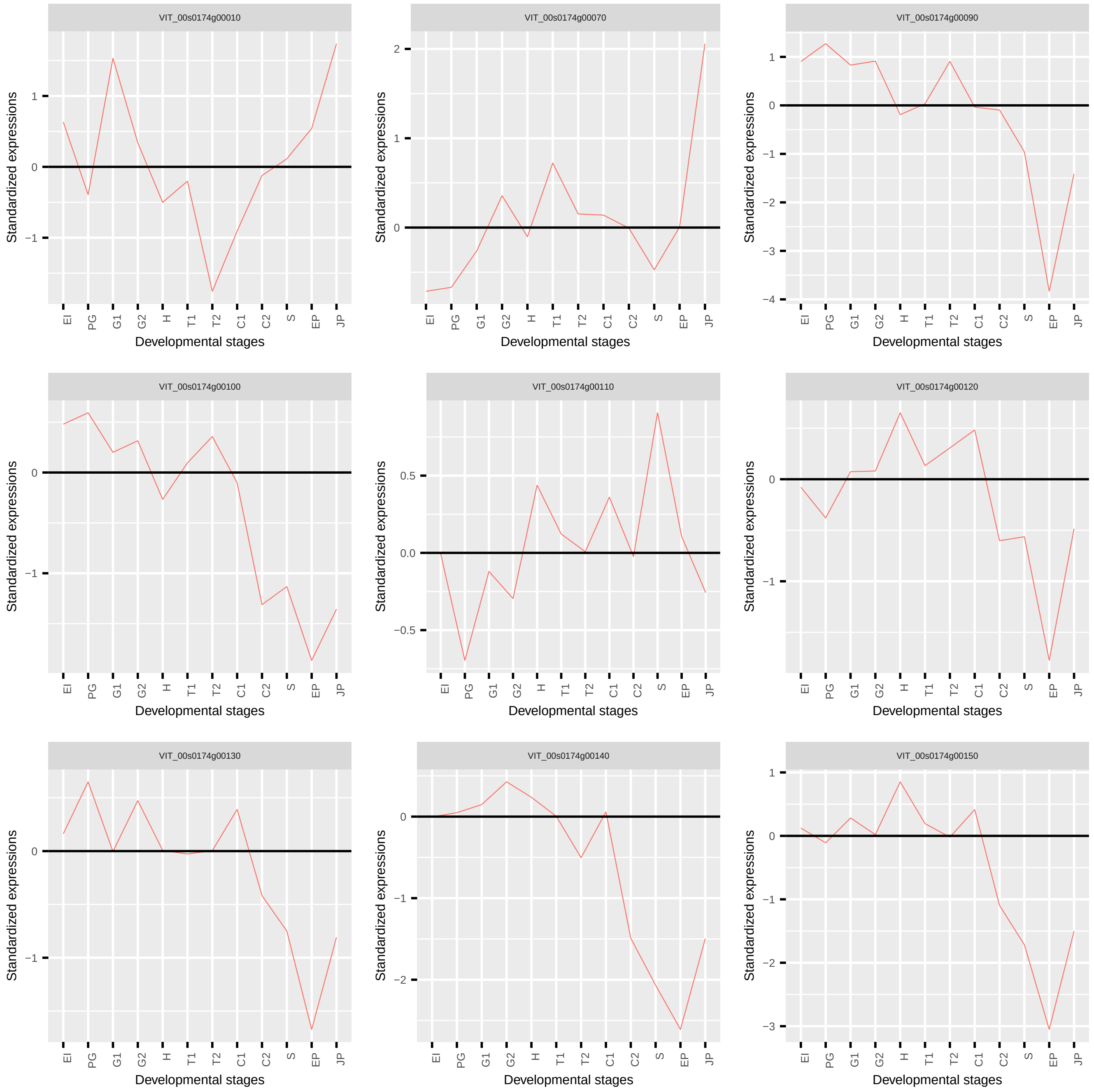
VIT_00s0174g00010
VIT_00s0174g00070
VIT_00s0174g00090
VIT_00s0174g00100
VIT_00s0174g00110
VIT_00s0174g00120
VIT_00s0174g00130
VIT_00s0174g00140
VIT_00s0174g00150
Standardized expressions
Developmental stages
EI
PG
G1
G2
H
T1
T2
C1
C2
S
EP
JP
1
0
−1
2
−2
−3
−4
0.5
0.0
−0.5

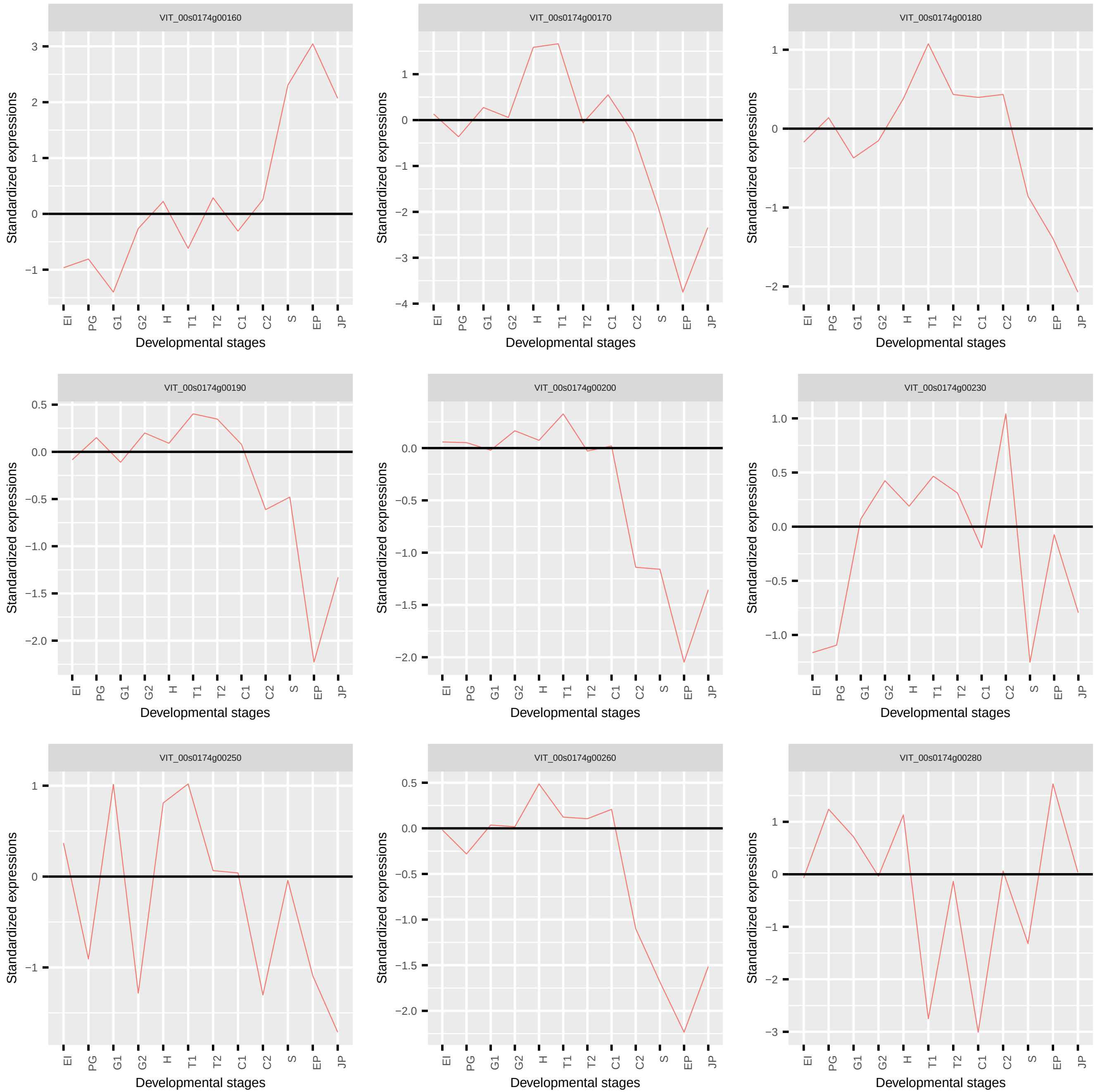
VIT_00s0174g00160
VIT_00s0174g00170
VIT_00s0174g00180
VIT_00s0174g00190
VIT_00s0174g00200
VIT_00s0174g00230
VIT_00s0174g00250
VIT_00s0174g00260
VIT_00s0174g00280
Standardized expressions
Developmental stages
EI
PG
G1
G2
H
T1
T2
C1
C2
S
EP
JP

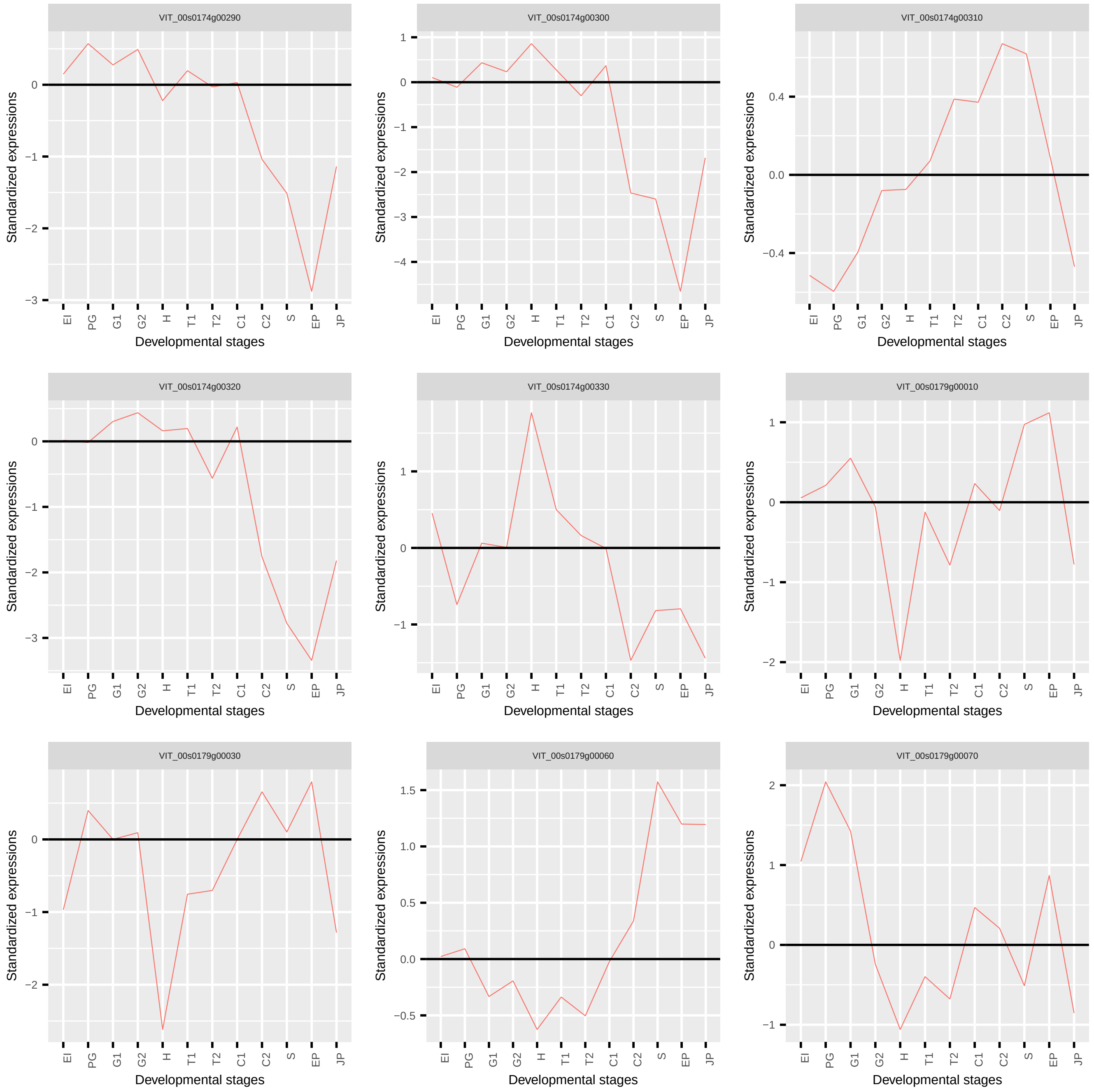
VIT_00s0174g00290
VIT_00s0174g00300
VIT_00s0174g00310
VIT_00s0174g00320
VIT_00s0174g00330
VIT_00s0179g00010
VIT_00s0179g00030
VIT_00s0179g00060
VIT_00s0179g00070
Standardized expressions
Developmental stages
EI PG G1 G2 H T1 T2 C1 C2 S EP JP

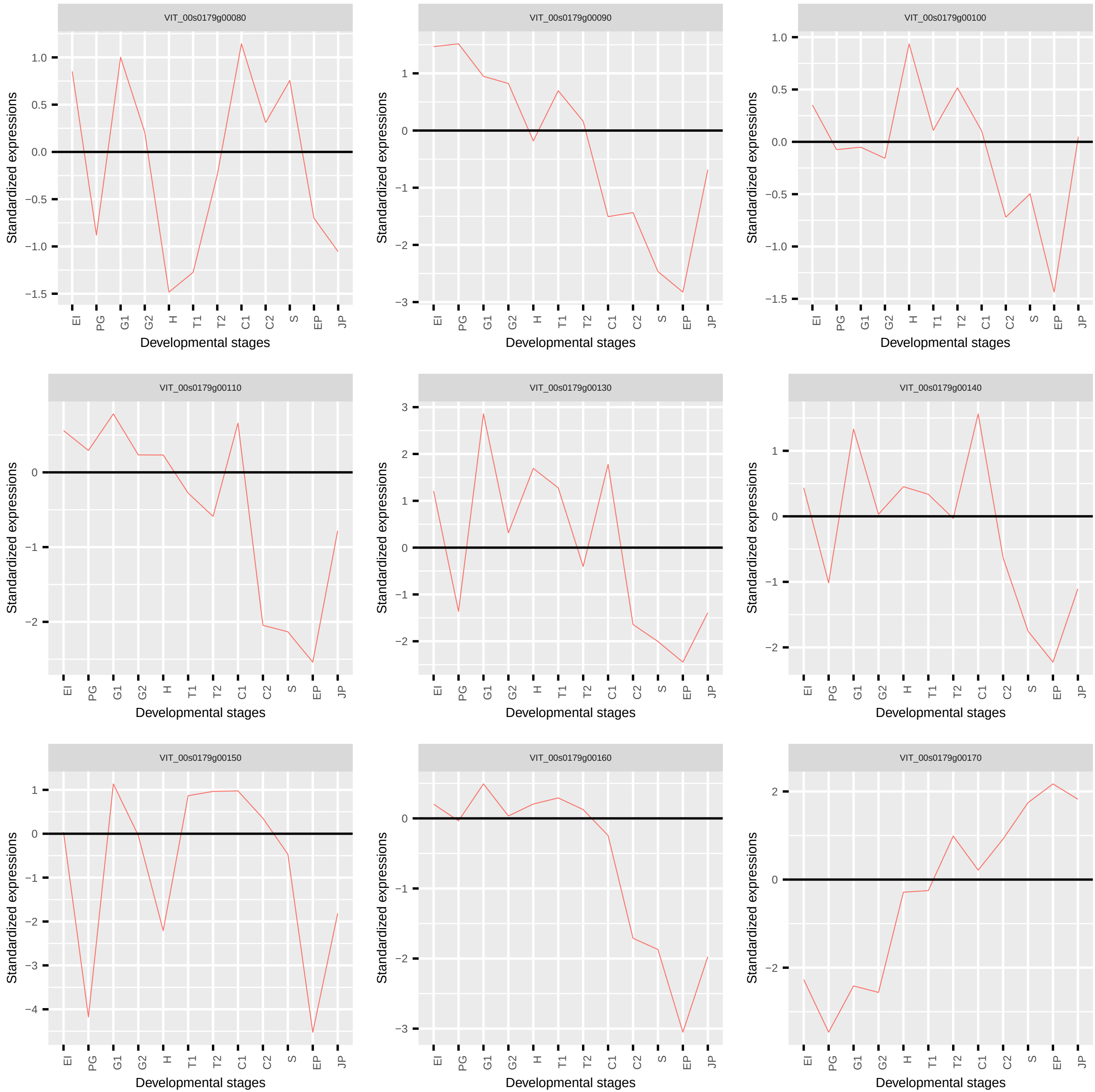
VIT_00s0179g00080
VIT_00s0179g00090
VIT_00s0179g00100
VIT_00s0179g00110
VIT_00s0179g00130
VIT_00s0179g00140
VIT_00s0179g00150
VIT_00s0179g00160
VIT_00s0179g00170
Standardized expressions
Developmental stages
EI PG G1 G2 H T1 T2 C1 C2 S EP JP

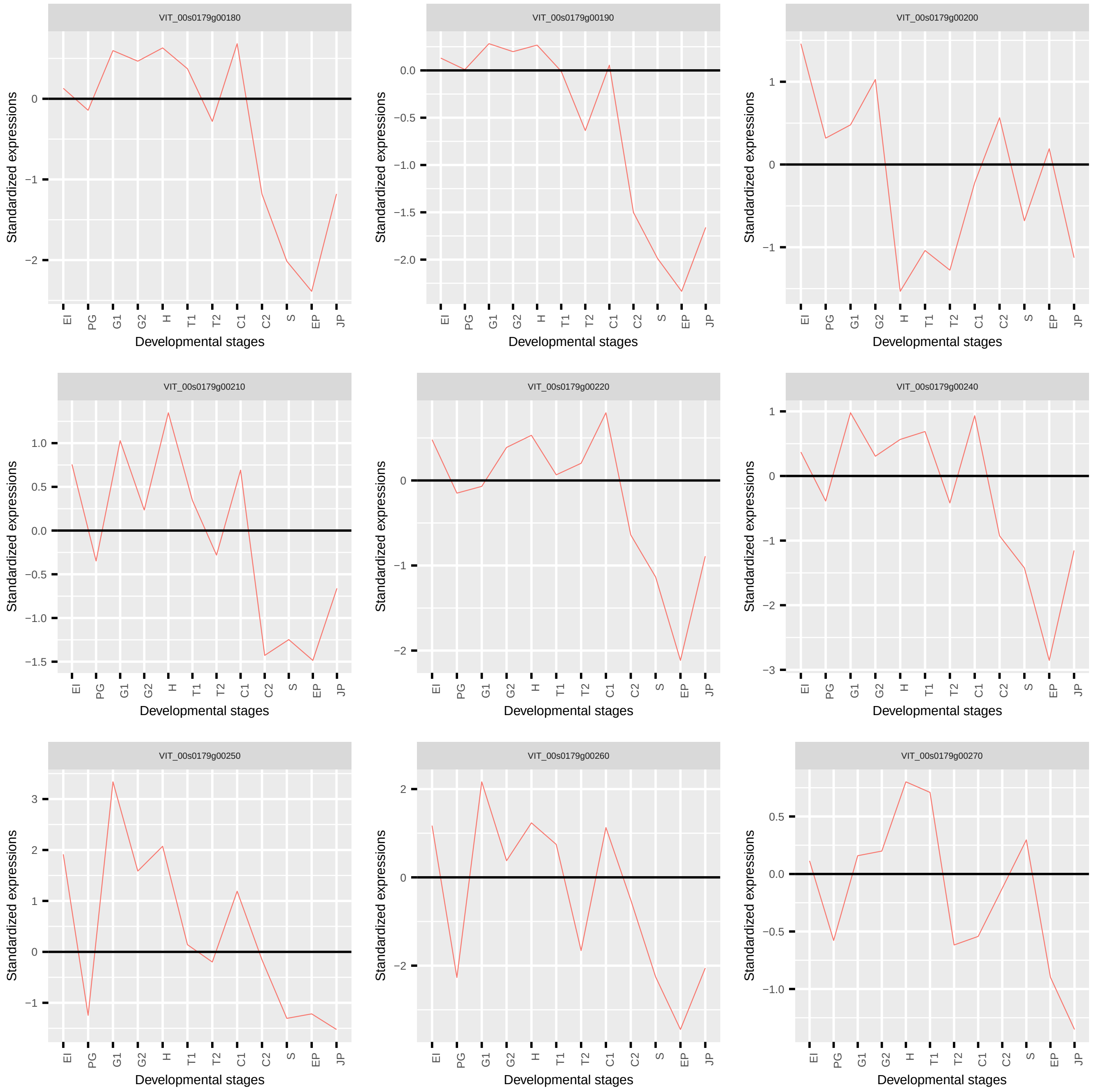
VIT_00s0179g00180
VIT_00s0179g00190
VIT_00s0179g00200
VIT_00s0179g00210
VIT_00s0179g00220
VIT_00s0179g00240
VIT_00s0179g00250
VIT_00s0179g00260
VIT_00s0179g00270
Standardized expressions
Developmental stages
EI PG G1 G2 H T1 T2 C1 C2 S EP JP

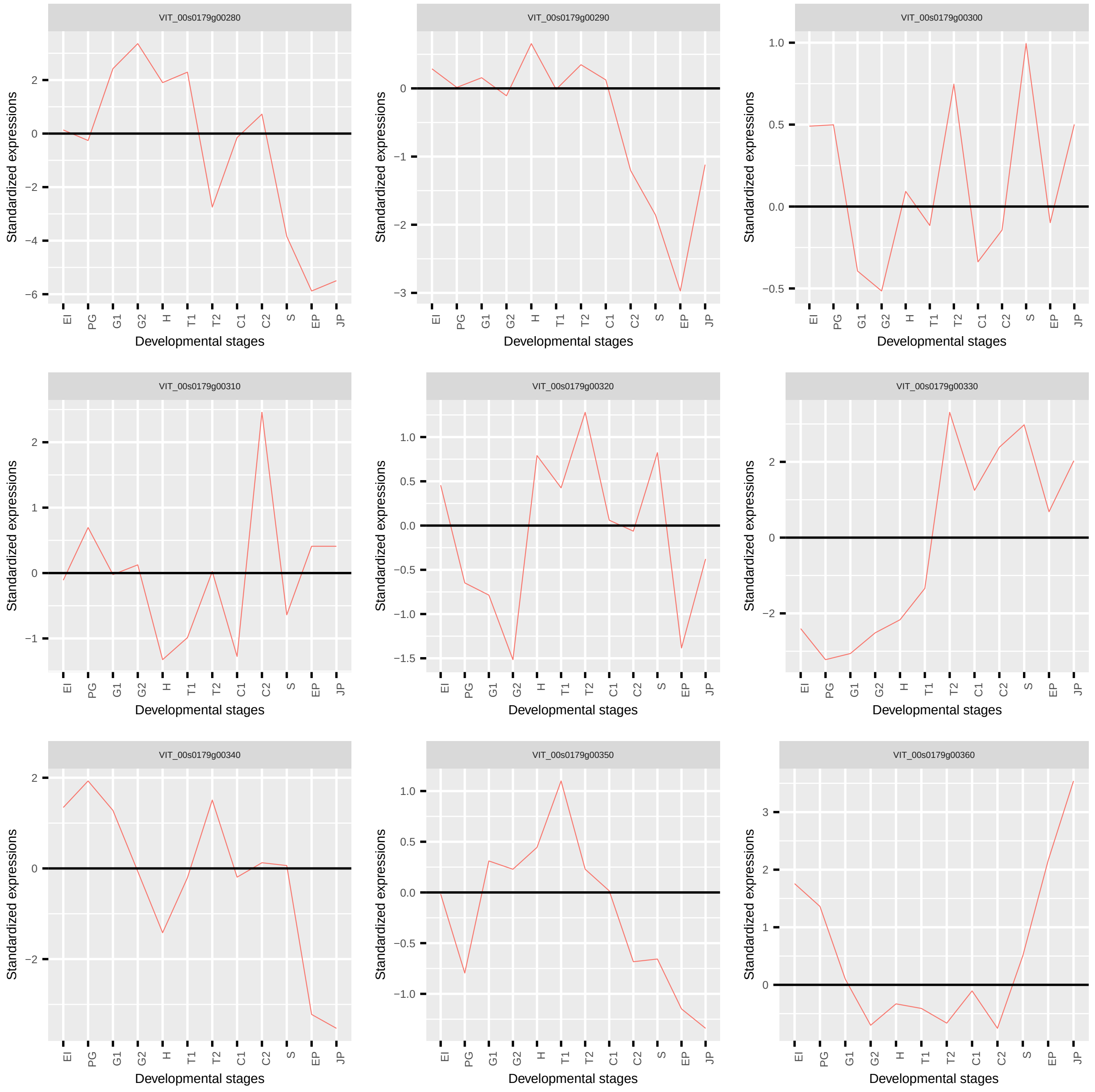

VIT_00s0179g00280
VIT_00s0179g00290
VIT_00s0179g00300
VIT_00s0179g00310
VIT_00s0179g00320
VIT_00s0179g00330
VIT_00s0179g00340
VIT_00s0179g00350
VIT_00s0179g00360
Standardized expressions
Developmental stages
EI PG G1 G2 H T1 T2 C1 C2 S EP JP

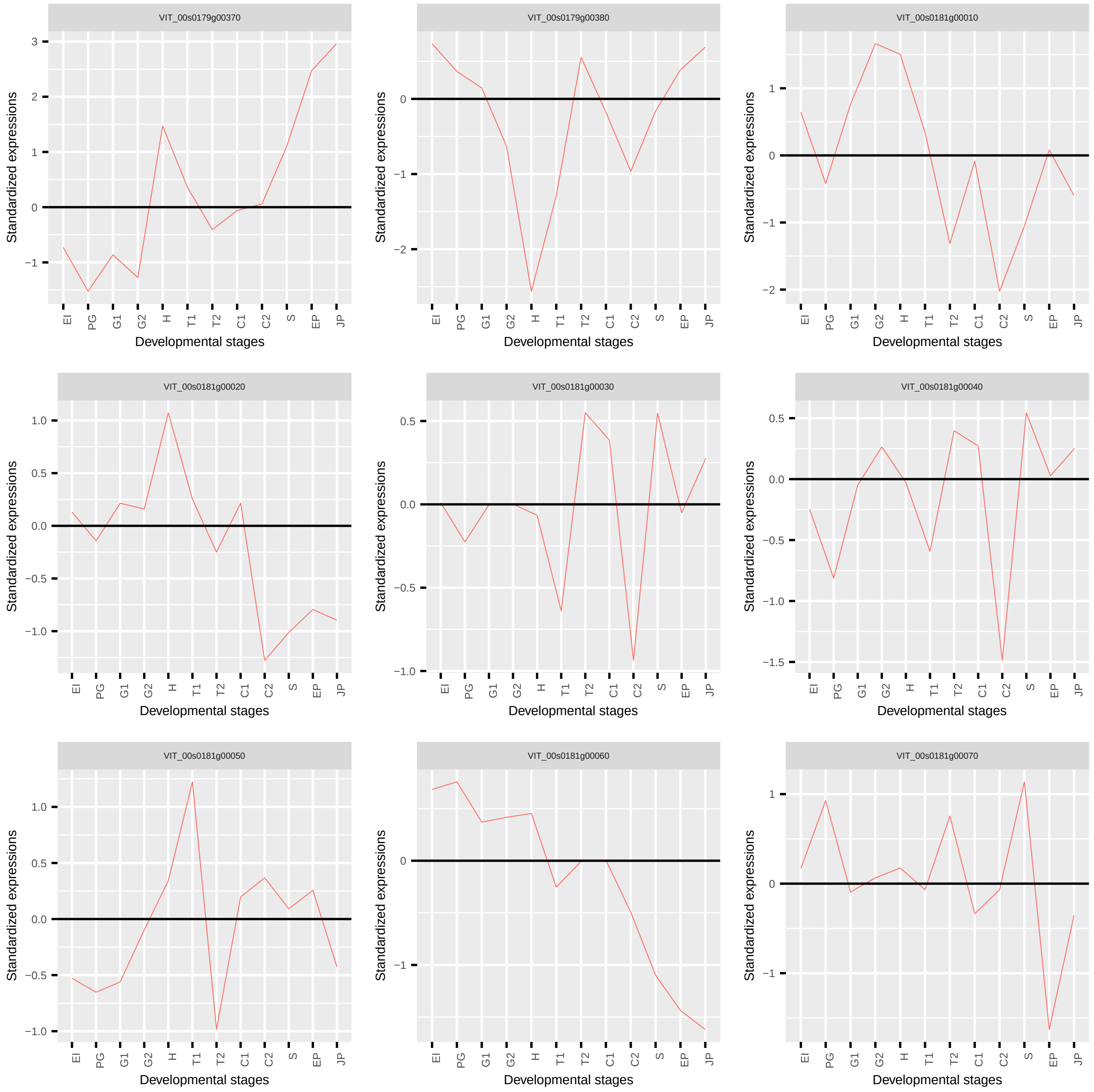
VIT_00s0179g00370
VIT_00s0179g00380
VIT_00s0181g00010
VIT_00s0181g00020
VIT_00s0181g00030
VIT_00s0181g00040
VIT_00s0181g00050
VIT_00s0181g00060
VIT_00s0181g00070
Standardized expressions
Developmental stages
EI PG G1 G2 H T1 T2 C1 C2 S EP JP

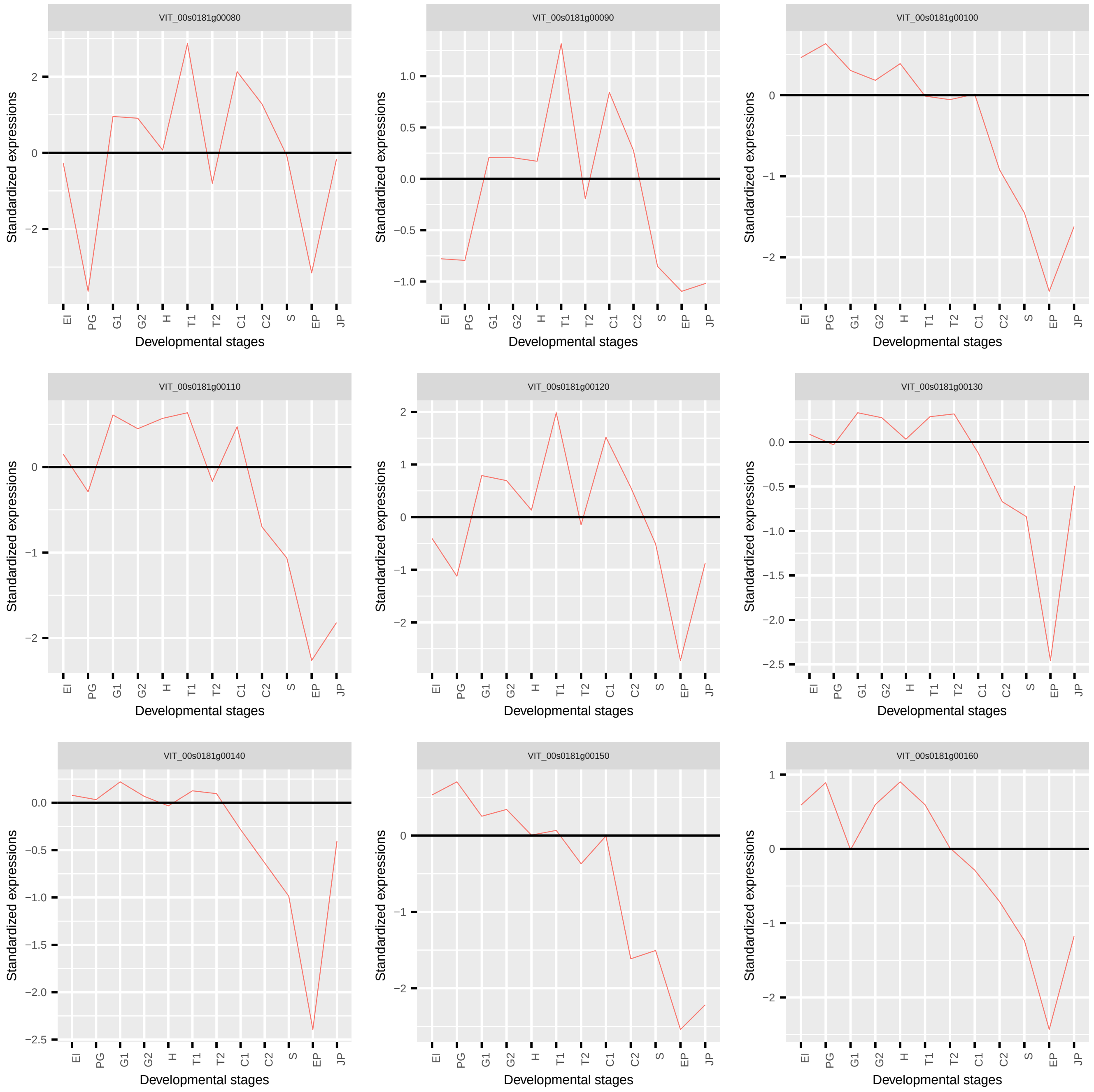
VIT_00s0181g00080
VIT_00s0181g00090
VIT_00s0181g00100
VIT_00s0181g00110
VIT_00s0181g00120
VIT_00s0181g00130
VIT_00s0181g00140
VIT_00s0181g00150
VIT_00s0181g00160
Standardized expressions
Developmental stages
EI PG G1 G2 H T1 T2 C1 C2 S EP JP

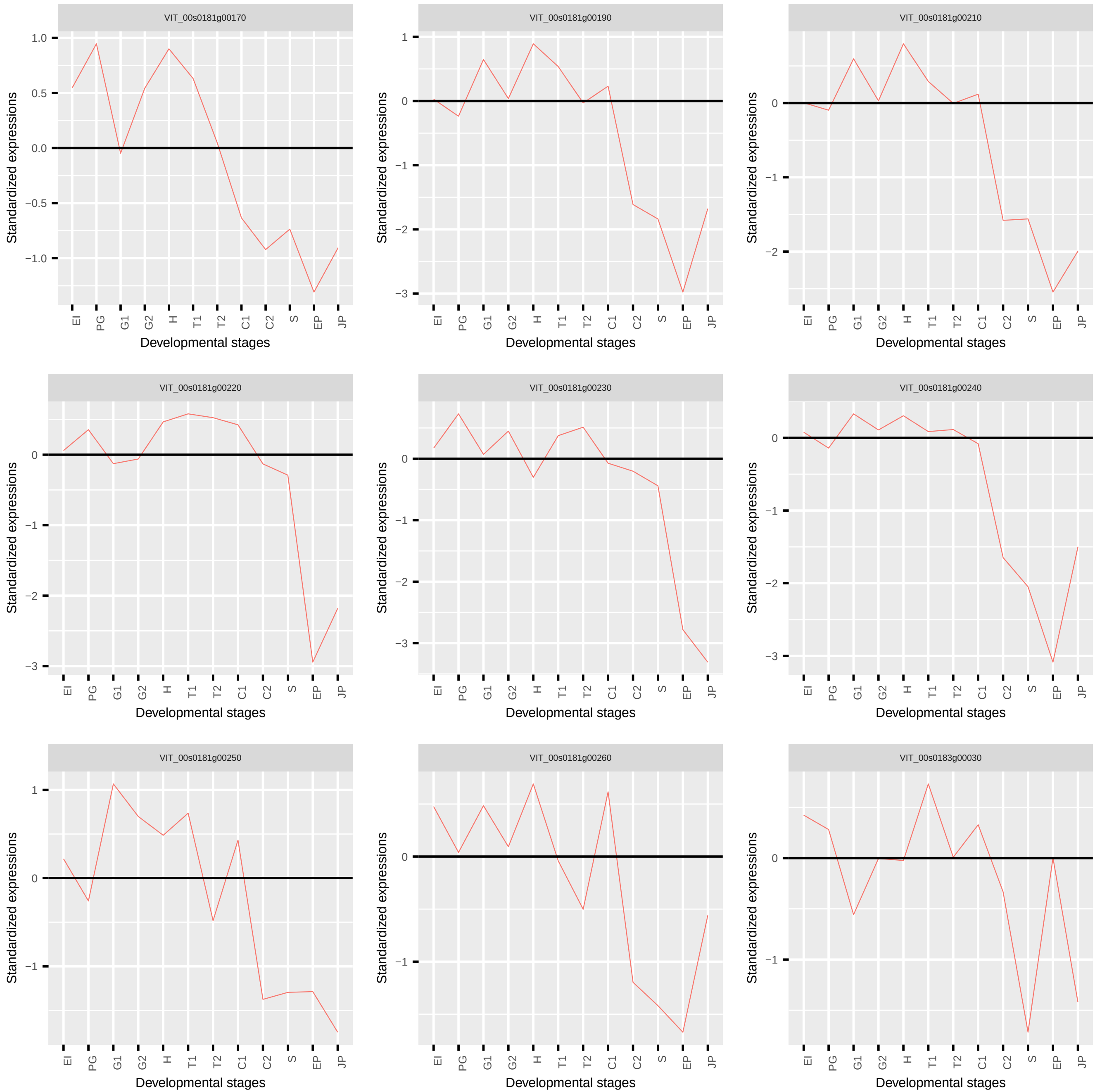
VIT_00s0181g00170
VIT_00s0181g00190
VIT_00s0181g00210
VIT_00s0181g00220
VIT_00s0181g00230
VIT_00s0181g00240
VIT_00s0181g00250
VIT_00s0181g00260
VIT_00s0183g00030
Standardized expressions
Developmental stages
EI
PG
G1
G2
H
T1
T2
C1
C2
S
EP
JP
1.0
0.5
0.0
−0.5
−1.0
1
0
−1
−2
−3

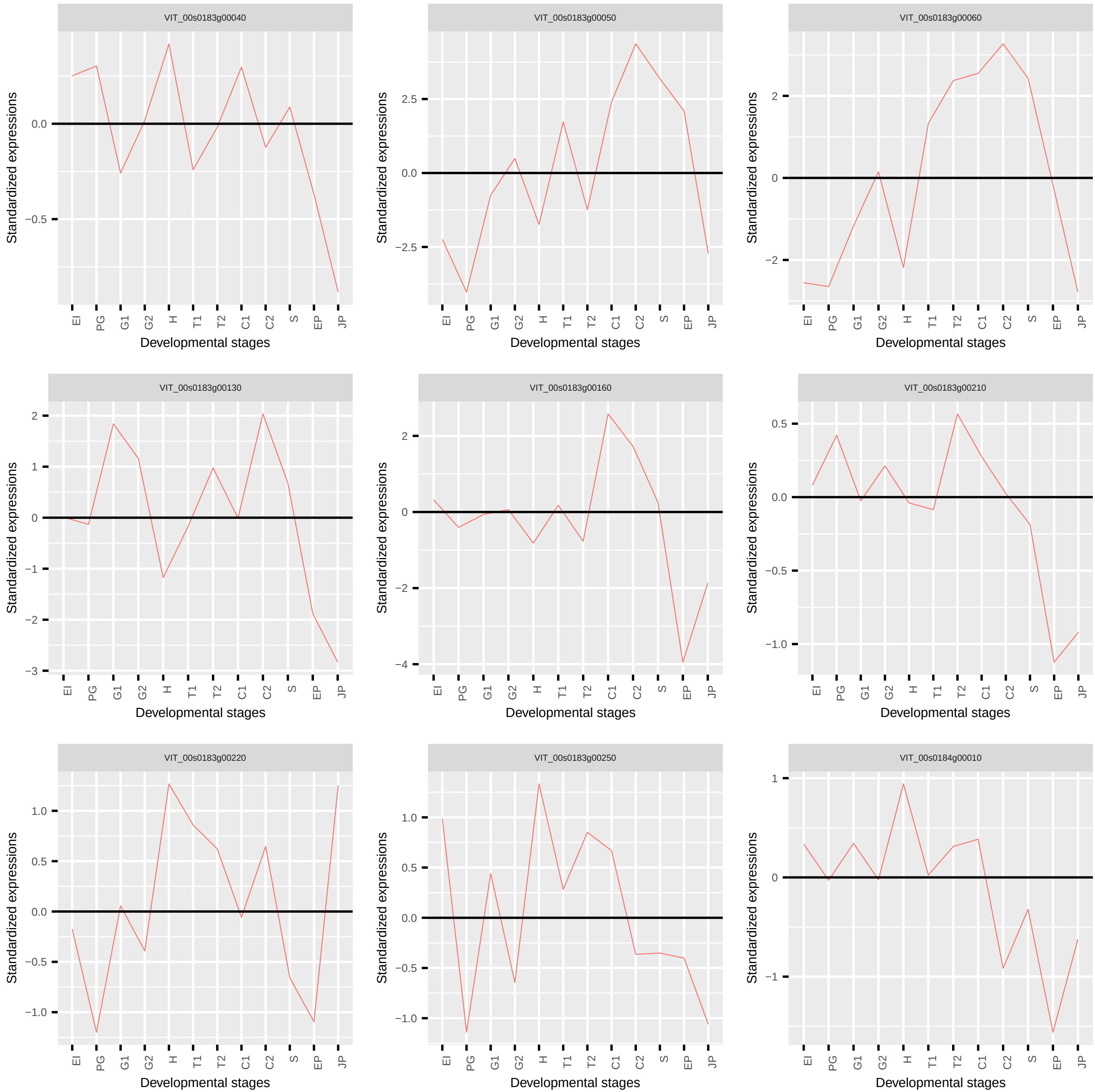
VIT_00s0183g00040
VIT_00s0183g00050
VIT_00s0183g00060
VIT_00s0183g00130
VIT_00s0183g00160
VIT_00s0183g00210
VIT_00s0183g00220
VIT_00s0183g00250
VIT_00s0184g00010
Standardized expressions
Developmental stages
EI
PG
G1
G2
H
T1
T2
C1
C2
S
EP
JP

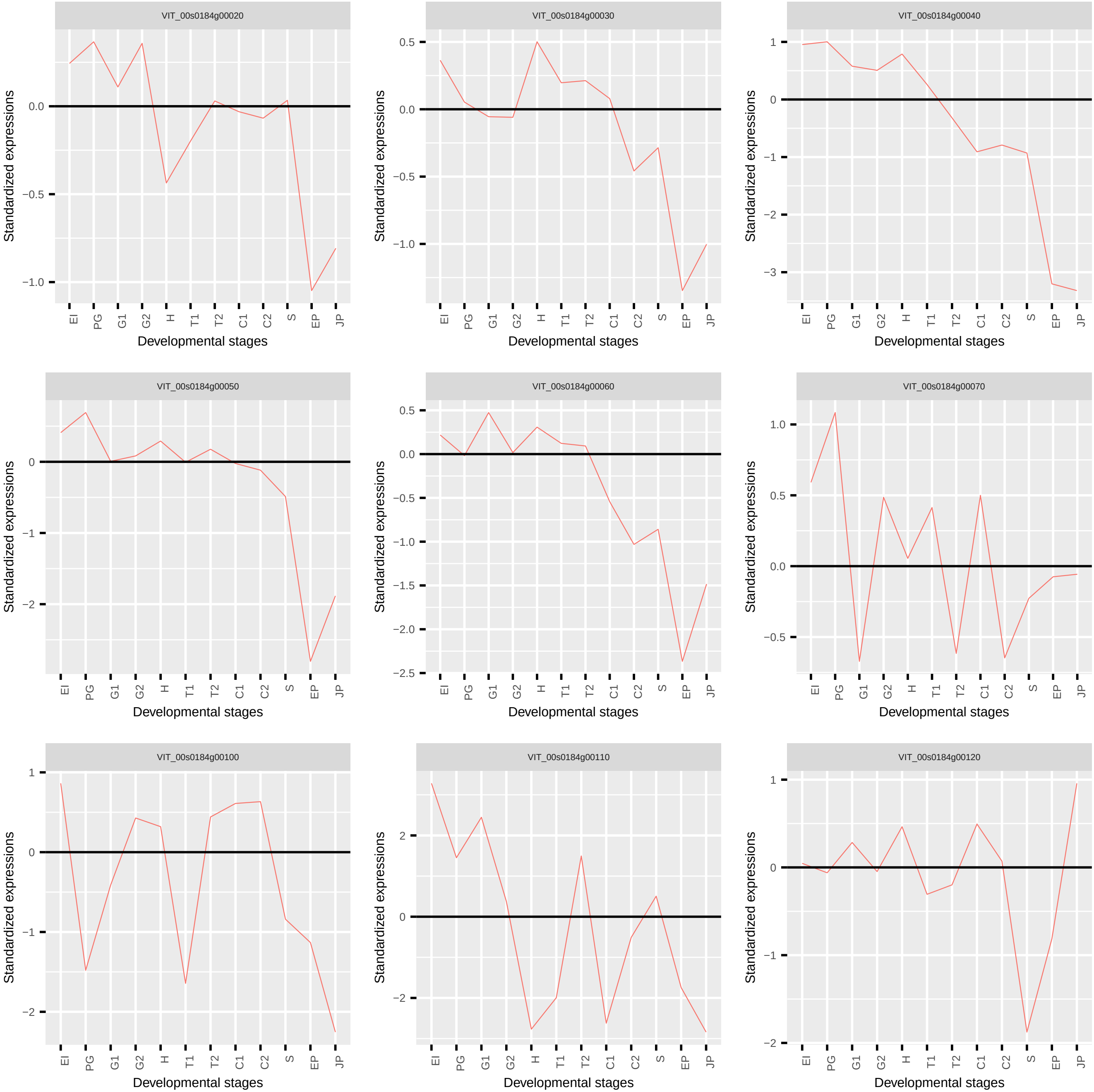
VIT_00s0184g00020
VIT_00s0184g00030
VIT_00s0184g00040
VIT_00s0184g00050
VIT_00s0184g00060
VIT_00s0184g00070
VIT_00s0184g00100
VIT_00s0184g00110
VIT_00s0184g00120
Standardized expressions
Developmental stages
EI PG G1 G2 H T1 T2 C1 C2 S EP JP

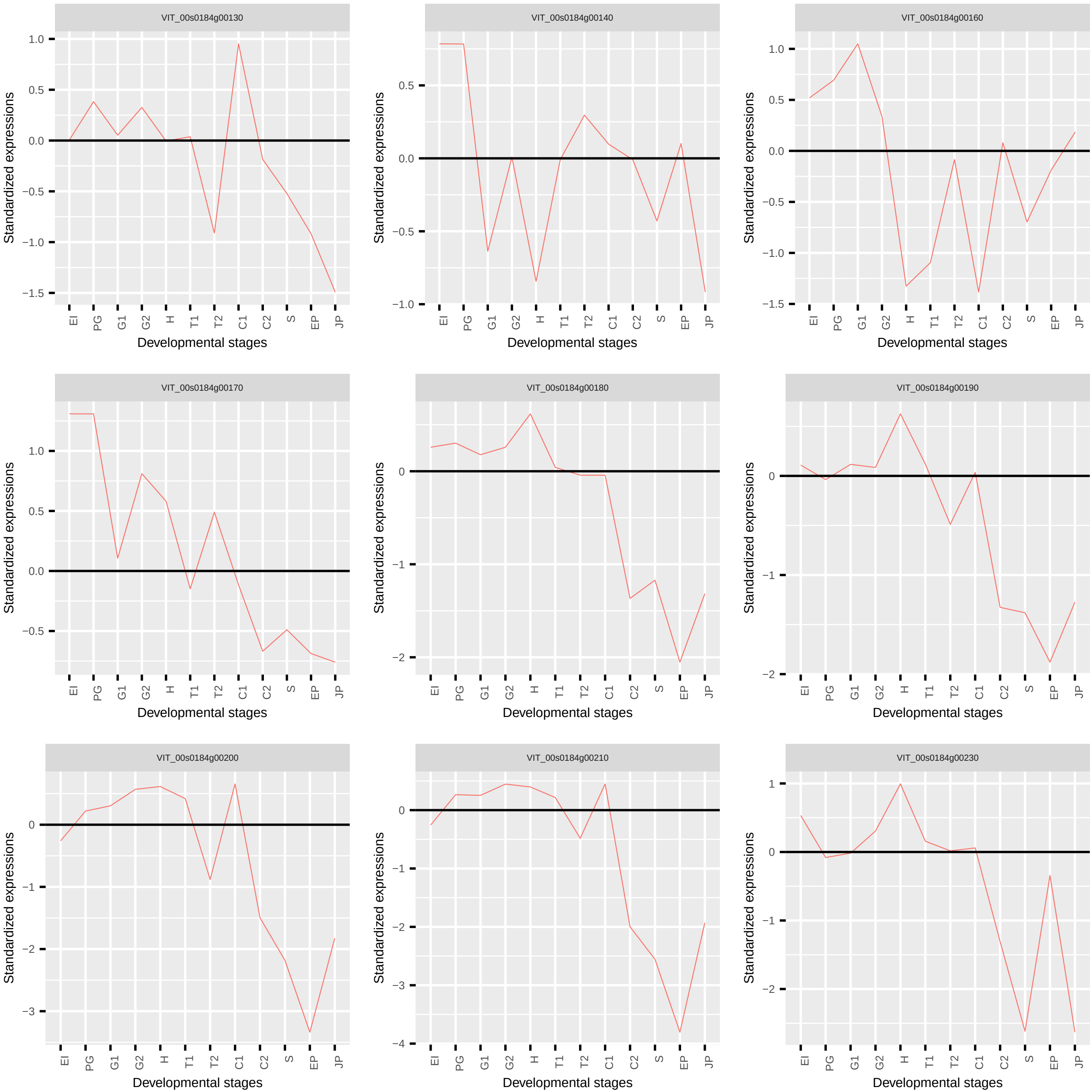

VIT_00s0184g00130
VIT_00s0184g00140
VIT_00s0184g00160
VIT_00s0184g00170
VIT_00s0184g00180
VIT_00s0184g00190
VIT_00s0184g00200
VIT_00s0184g00210
VIT_00s0184g00230
Standardized expressions
Developmental stages
EI
PG
G1
G2
H
T1
T2
C1
C2
S
EP
JP

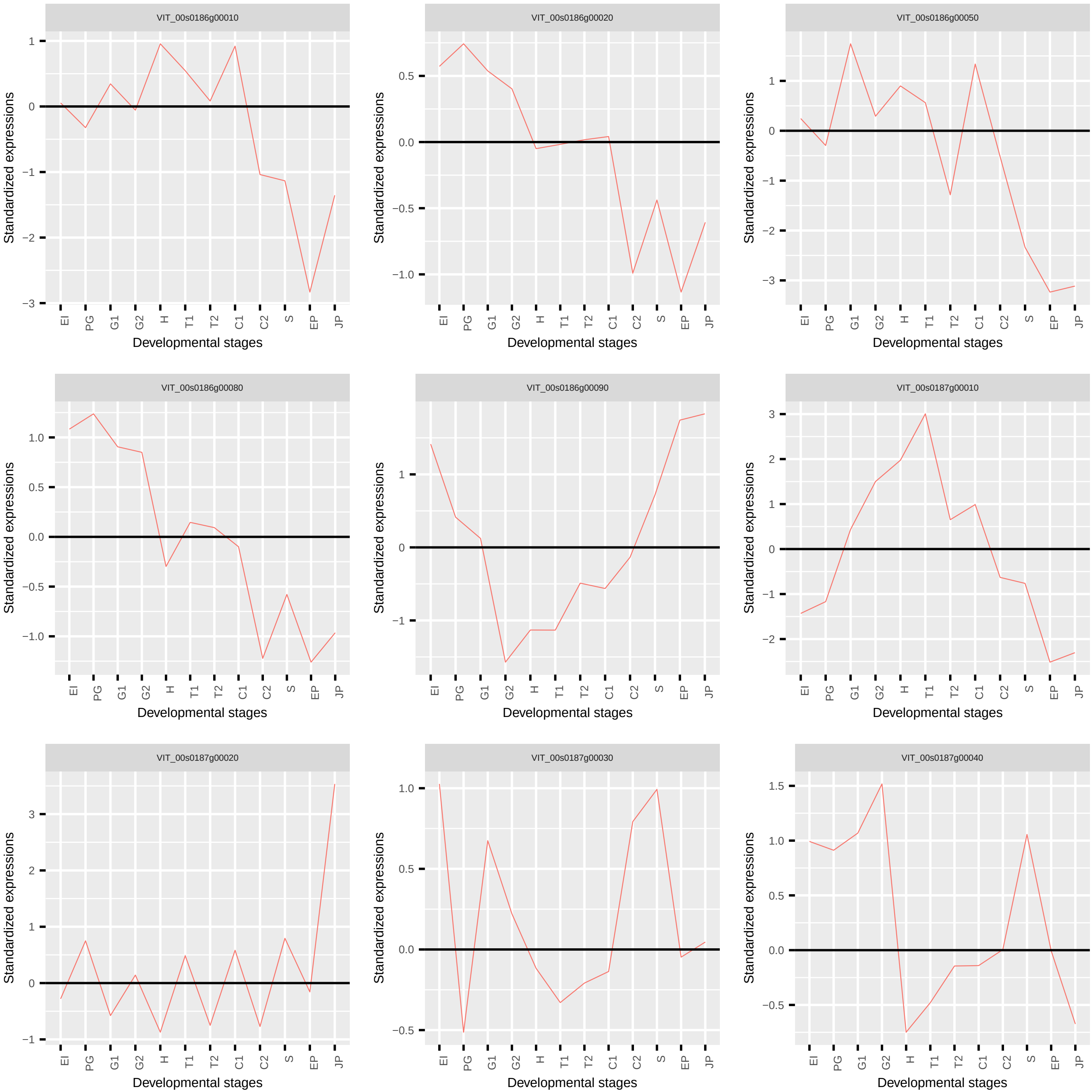
VIT_00s0186g00010
VIT_00s0186g00020
VIT_00s0186g00050
VIT_00s0186g00080
VIT_00s0186g00090
VIT_00s0187g00010
VIT_00s0187g00020
VIT_00s0187g00030
VIT_00s0187g00040
Standardized expressions
Developmental stages
EI PG G1 G2 H T1 T2 C1 C2 S EP JP

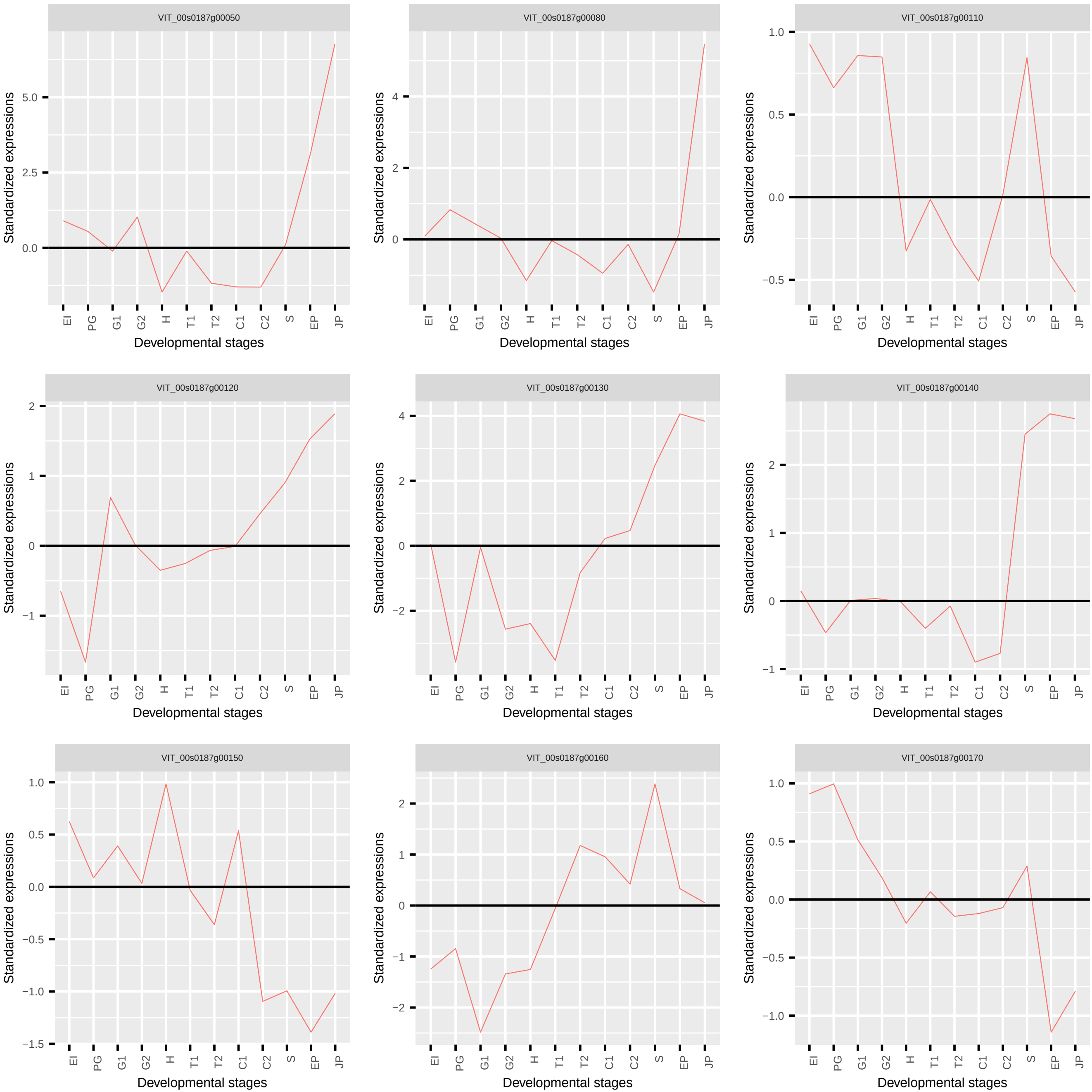
VIT_00s0187g00050
VIT_00s0187g00080
VIT_00s0187g00110
VIT_00s0187g00120
VIT_00s0187g00130
VIT_00s0187g00140
VIT_00s0187g00150
VIT_00s0187g00160
VIT_00s0187g00170
Standardized expressions
Developmental stages
EI PG G1 G2 H T1 T2 C1 C2 S EP JP

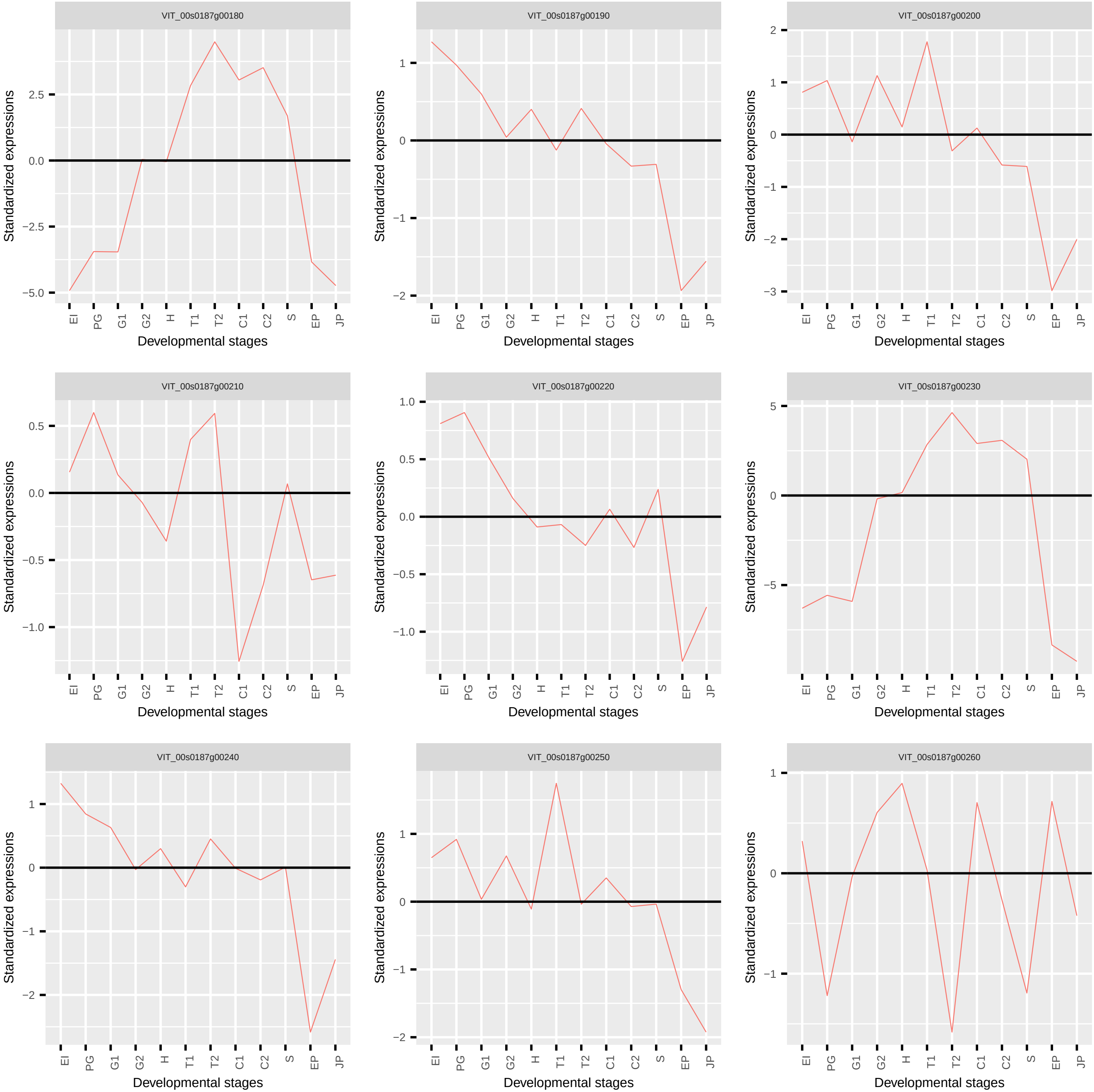
VIT_00s0187g00180
VIT_00s0187g00190
VIT_00s0187g00200
VIT_00s0187g00210
VIT_00s0187g00220
VIT_00s0187g00230
VIT_00s0187g00240
VIT_00s0187g00250
VIT_00s0187g00260
Standardized expressions
Developmental stages
EI PG G1 G2 H T1 T2 C1 C2 S EP JP

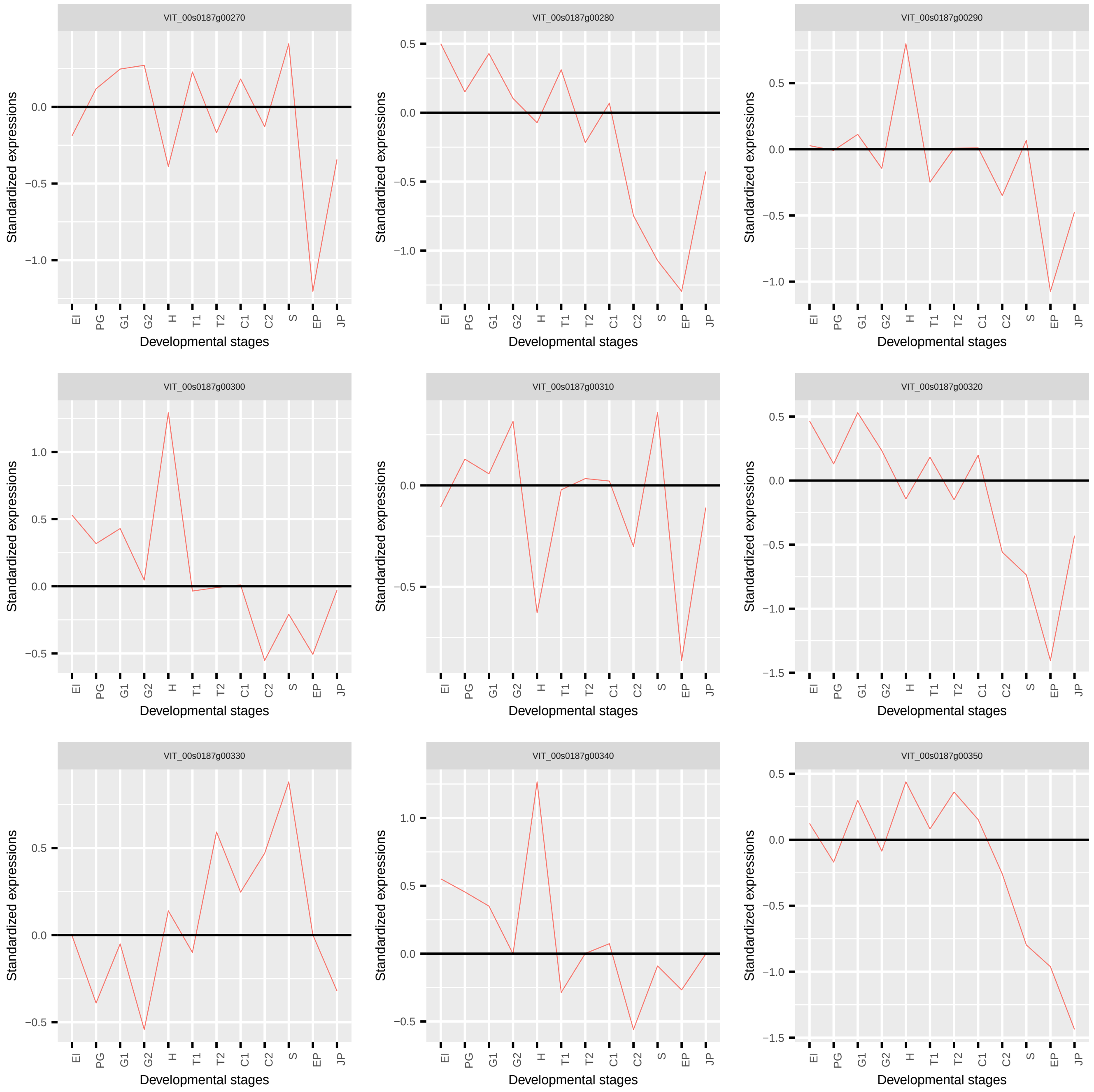
VIT_00s0187g00270
VIT_00s0187g00280
VIT_00s0187g00290
VIT_00s0187g00300
VIT_00s0187g00310
VIT_00s0187g00320
VIT_00s0187g00330
VIT_00s0187g00340
VIT_00s0187g00350
Standardized expressions
Developmental stages
EI PG G1 G2 H T1 T2 C1 C2 S EP JP

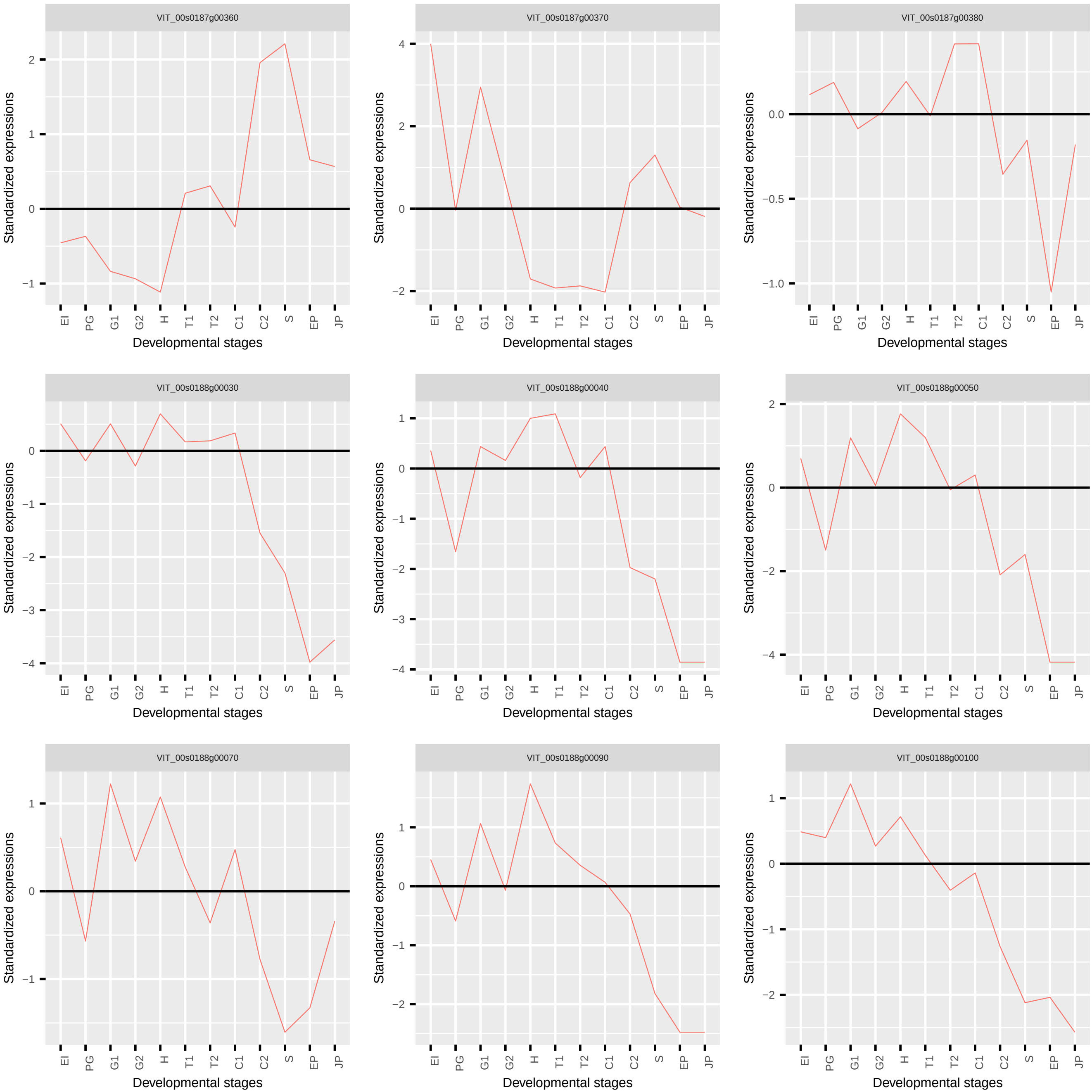
VIT_00s0187g00360
VIT_00s0187g00370
VIT_00s0187g00380
VIT_00s0188g00030
VIT_00s0188g00040
VIT_00s0188g00050
VIT_00s0188g00070
VIT_00s0188g00090
VIT_00s0188g00100
Standardized expressions
Developmental stages
EI
PG
G1
G2
H
T1
T2
C1
C2
S
EP
JP

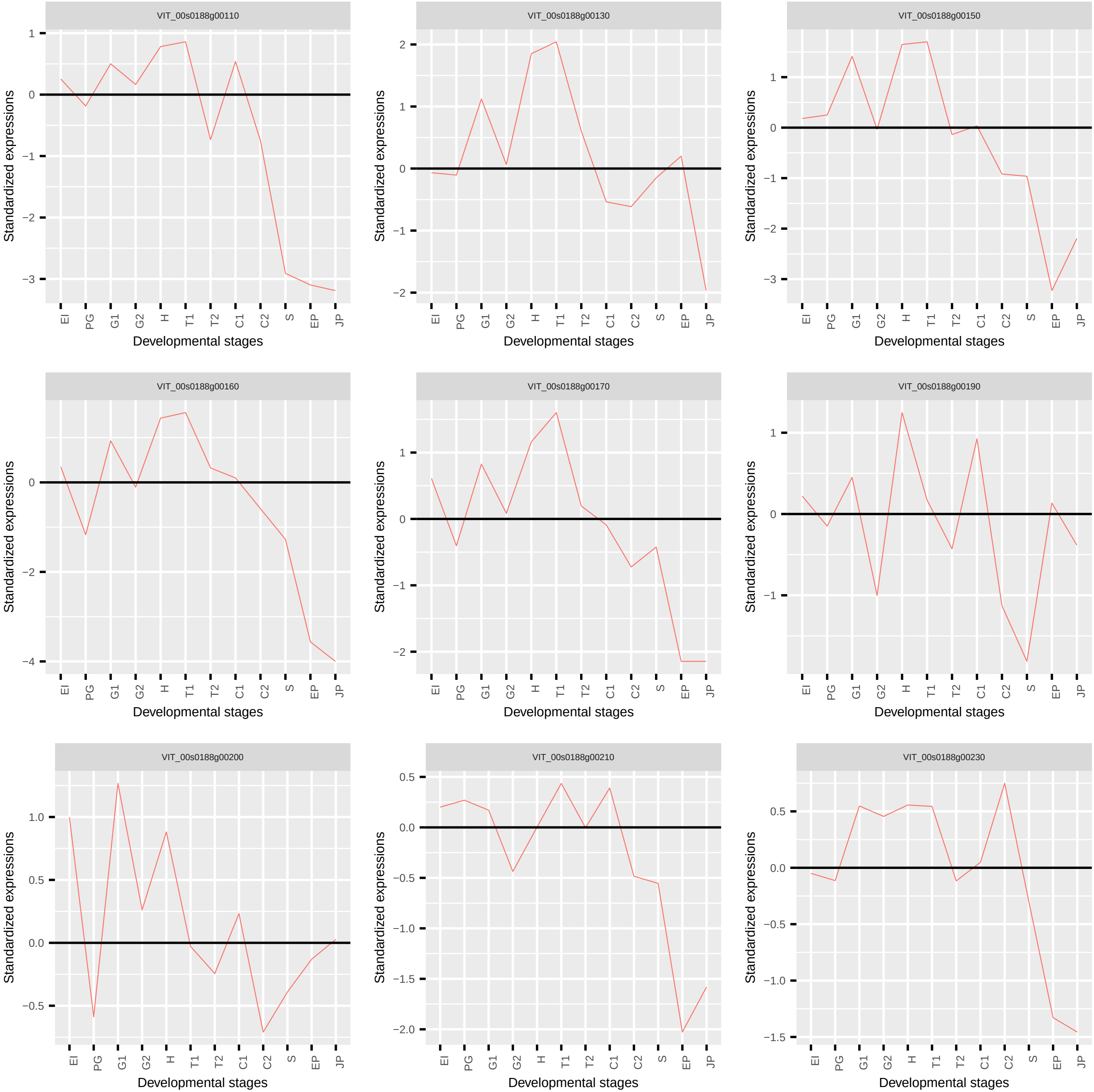
VIT_00s0188g00110
VIT_00s0188g00130
VIT_00s0188g00150
VIT_00s0188g00160
VIT_00s0188g00170
VIT_00s0188g00190
VIT_00s0188g00200
VIT_00s0188g00210
VIT_00s0188g00230
Standardized expressions
Developmental stages
EI PG G1 G2 H T1 T2 C1 C2 S EP JP
1 0 −1 −2 −3
2 1 0 −1 −2
0 −2 −4
1.0 0.5 0.0 −0.5
0.5 0.0 −0.5 −1.0 −1.5 −2.0

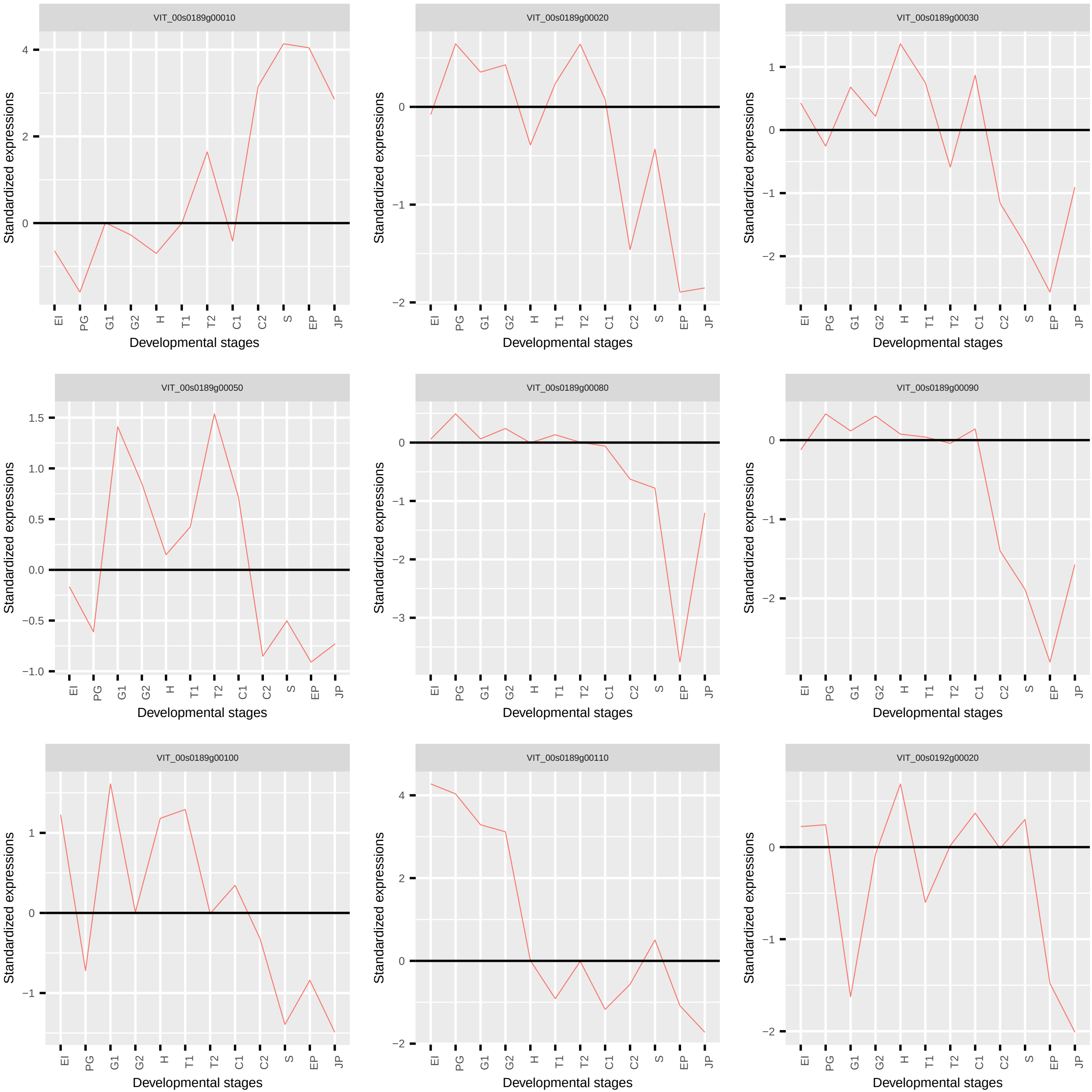
VIT_00s0189g00010
VIT_00s0189g00020
VIT_00s0189g00030
VIT_00s0189g00050
VIT_00s0189g00080
VIT_00s0189g00090
VIT_00s0189g00100
VIT_00s0189g00110
VIT_00s0192g00020
Standardized expressions
Developmental stages
EI PG G1 G2 H T1 T2 C1 C2 S EP JP

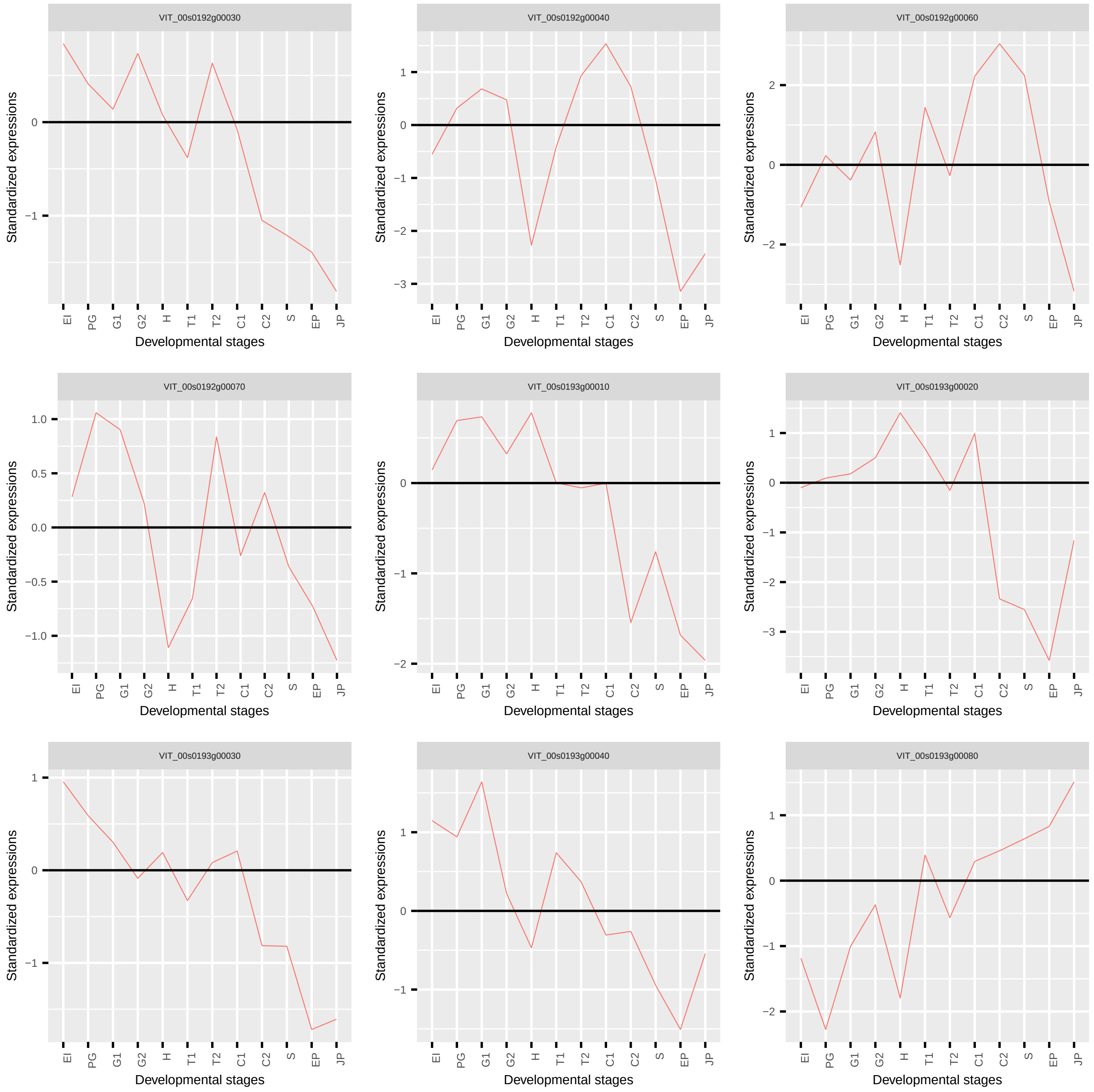

VIT_00s0192g00030
VIT_00s0192g00040
VIT_00s0192g00060
VIT_00s0192g00070
VIT_00s0193g00010
VIT_00s0193g00020
VIT_00s0193g00030
VIT_00s0193g00040
VIT_00s0193g00080
Standardized expressions
Developmental stages
EI PG G1 G2 H T1 T2 C1 C2 S EP JP

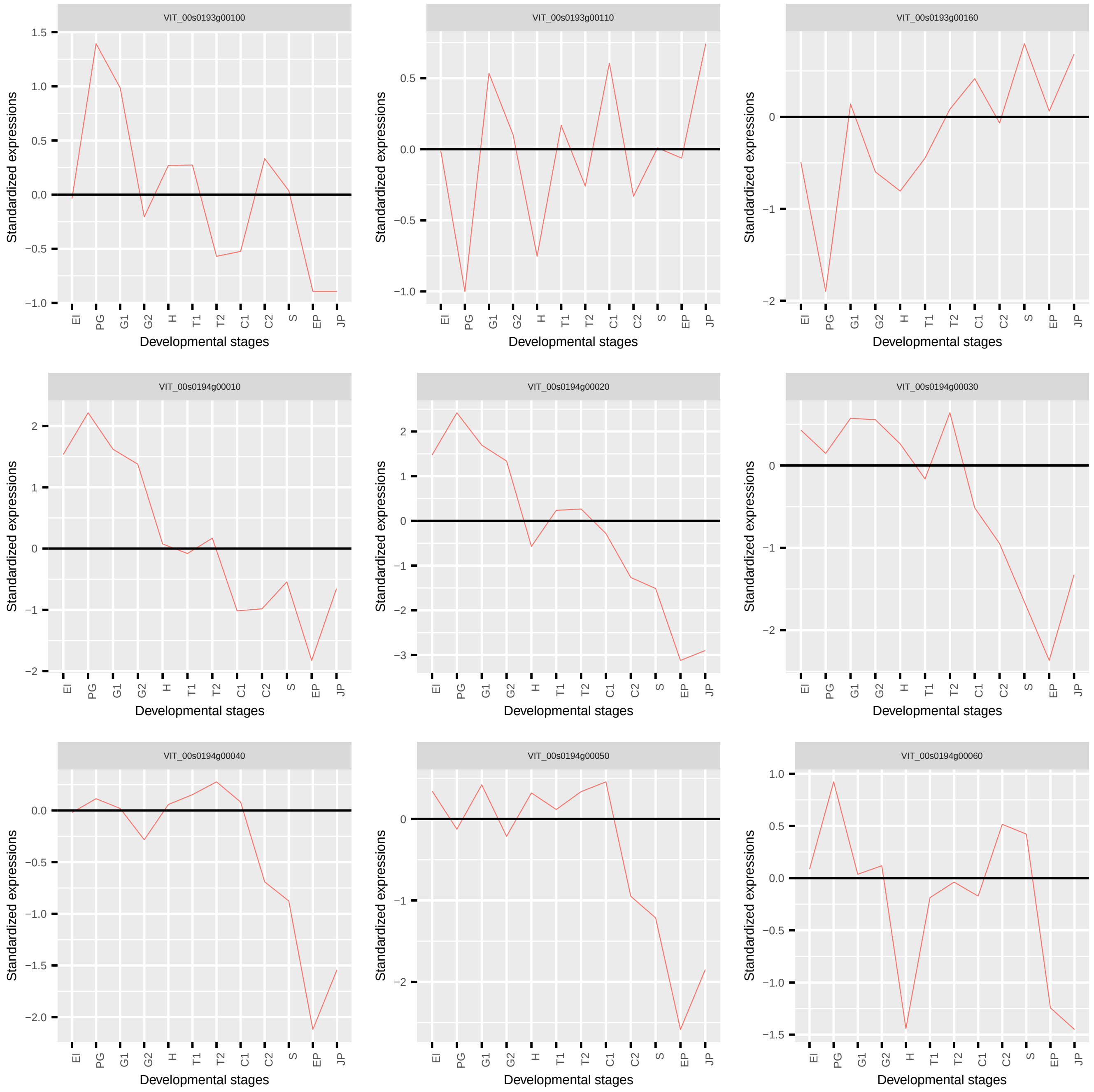
VIT_00s0193g00100
VIT_00s0193g00110
VIT_00s0193g00160
VIT_00s0194g00010
VIT_00s0194g00020
VIT_00s0194g00030
VIT_00s0194g00040
VIT_00s0194g00050
VIT_00s0194g00060
Standardized expressions
Developmental stages
EI PG G1 G2 H T1 T2 C1 C2 S EP JP

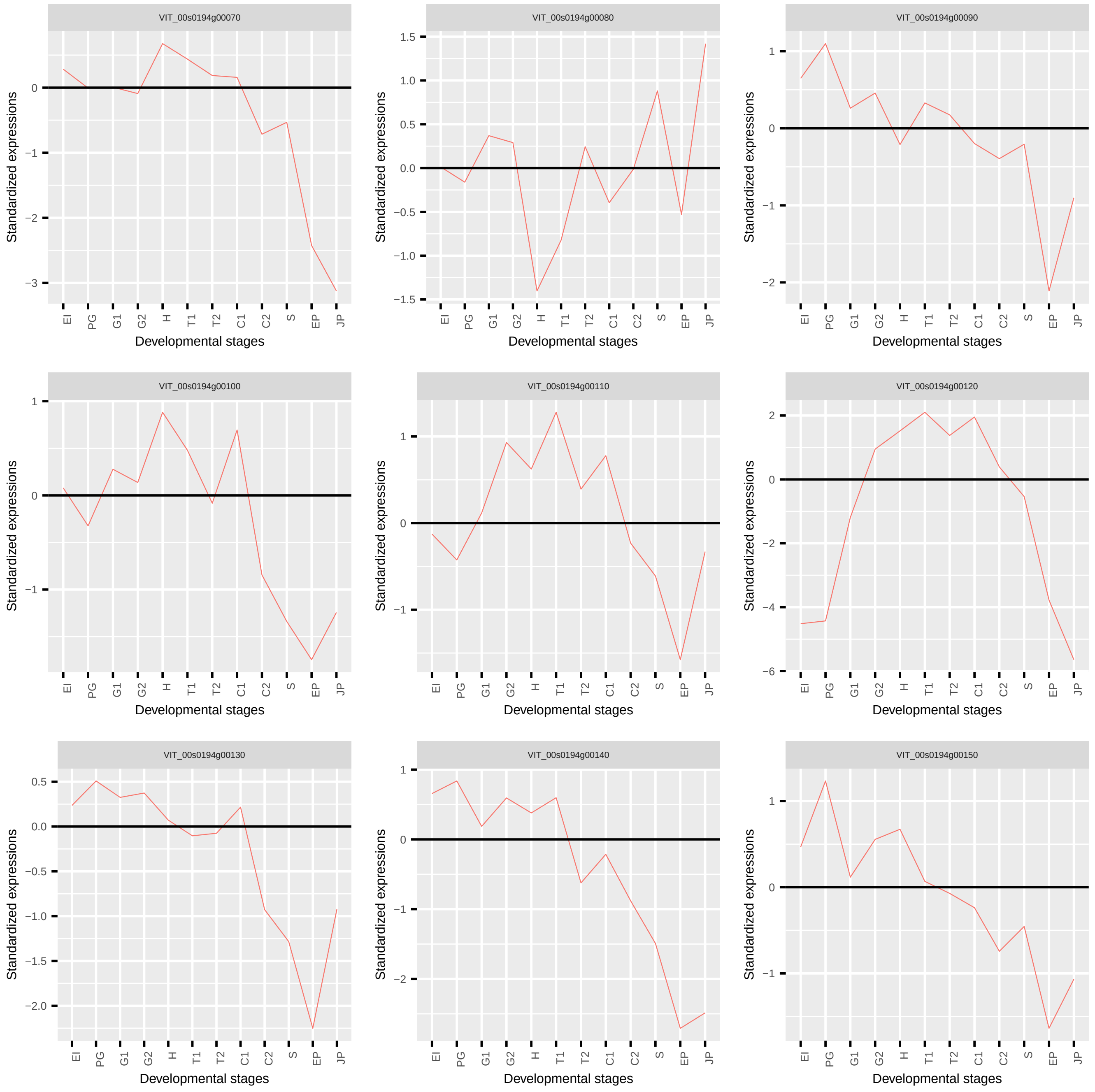
VIT_00s0194g00070
0
−1
−2
−3
Standardized expressions
EI PG G1 G2 H T1 T2 C1 C2 S EP JP
Developmental stages
VIT_00s0194g00080
1.5
1.0
0.5
0.0
−0.5
−1.0
−1.5
Standardized expressions
EI PG G1 G2 H T1 T2 C1 C2 S EP JP
Developmental stages
VIT_00s0194g00090
1
0
−1
−2
Standardized expressions
EI PG G1 G2 H T1 T2 C1 C2 S EP JP
Developmental stages
VIT_00s0194g00100
1
0
−1
Standardized expressions
EI PG G1 G2 H T1 T2 C1 C2 S EP JP
Developmental stages
VIT_00s0194g00110
1
0
−1
Standardized expressions
EI PG G1 G2 H T1 T2 C1 C2 S EP JP
Developmental stages
VIT_00s0194g00120
2
0
−2
−4
−6
Standardized expressions
EI PG G1 G2 H T1 T2 C1 C2 S EP JP
Developmental stages
VIT_00s0194g00130
0.5
0.0
−0.5
−1.0
−1.5
−2.0
Standardized expressions
EI PG G1 G2 H T1 T2 C1 C2 S EP JP
Developmental stages
VIT_00s0194g00140
1
0
−1
−2
Standardized expressions
EI PG G1 G2 H T1 T2 C1 C2 S EP JP
Developmental stages
VIT_00s0194g00150
1
0
−1
Standardized expressions
EI PG G1 G2 H T1 T2 C1 C2 S EP JP
Developmental stages

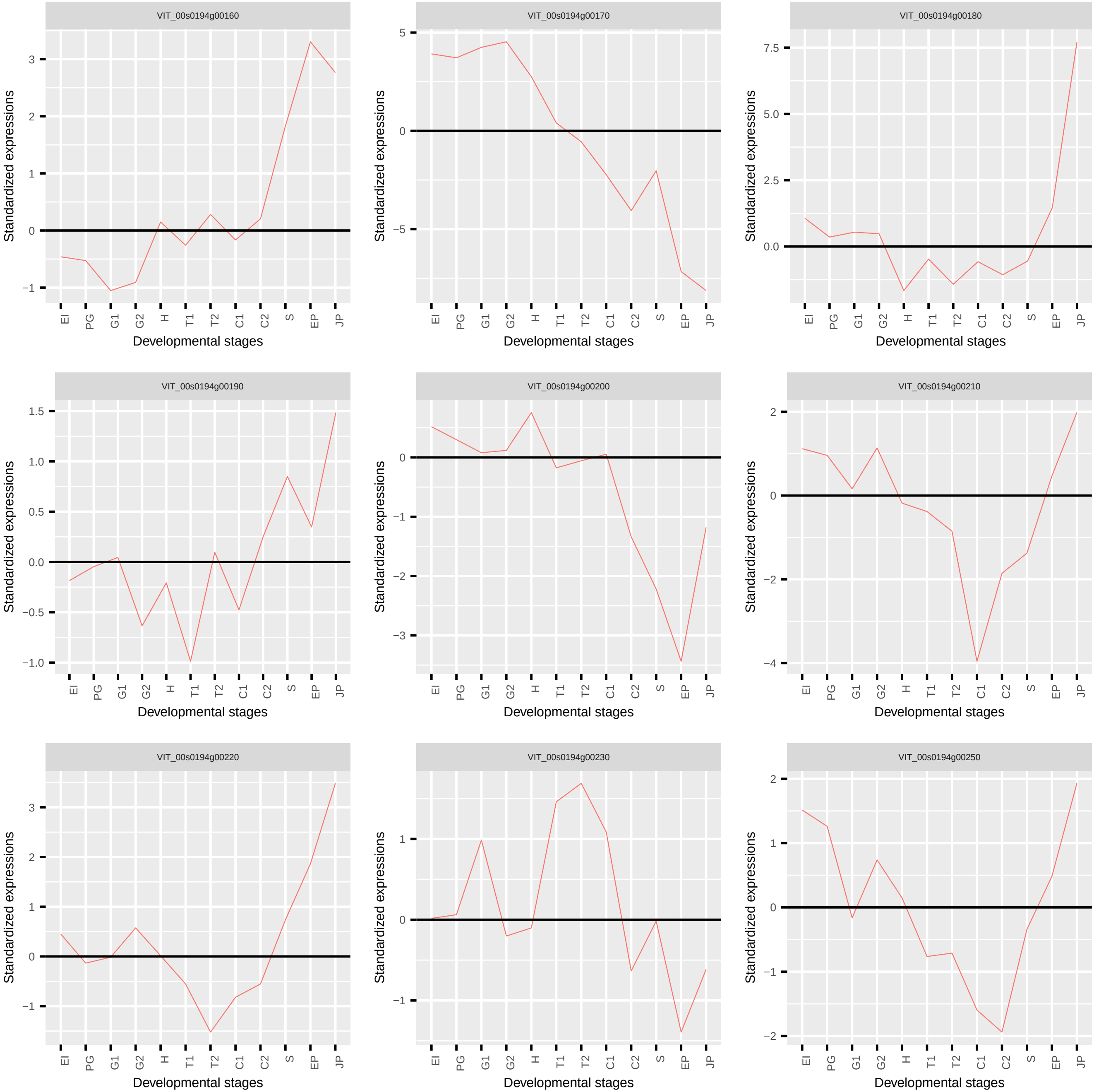
VIT_00s0194g00160
VIT_00s0194g00170
VIT_00s0194g00180
VIT_00s0194g00190
VIT_00s0194g00200
VIT_00s0194g00210
VIT_00s0194g00220
VIT_00s0194g00230
VIT_00s0194g00250
Standardized expressions
Developmental stages
EI
PG
G1
G2
H
T1
T2
C1
C2
S
EP
JP

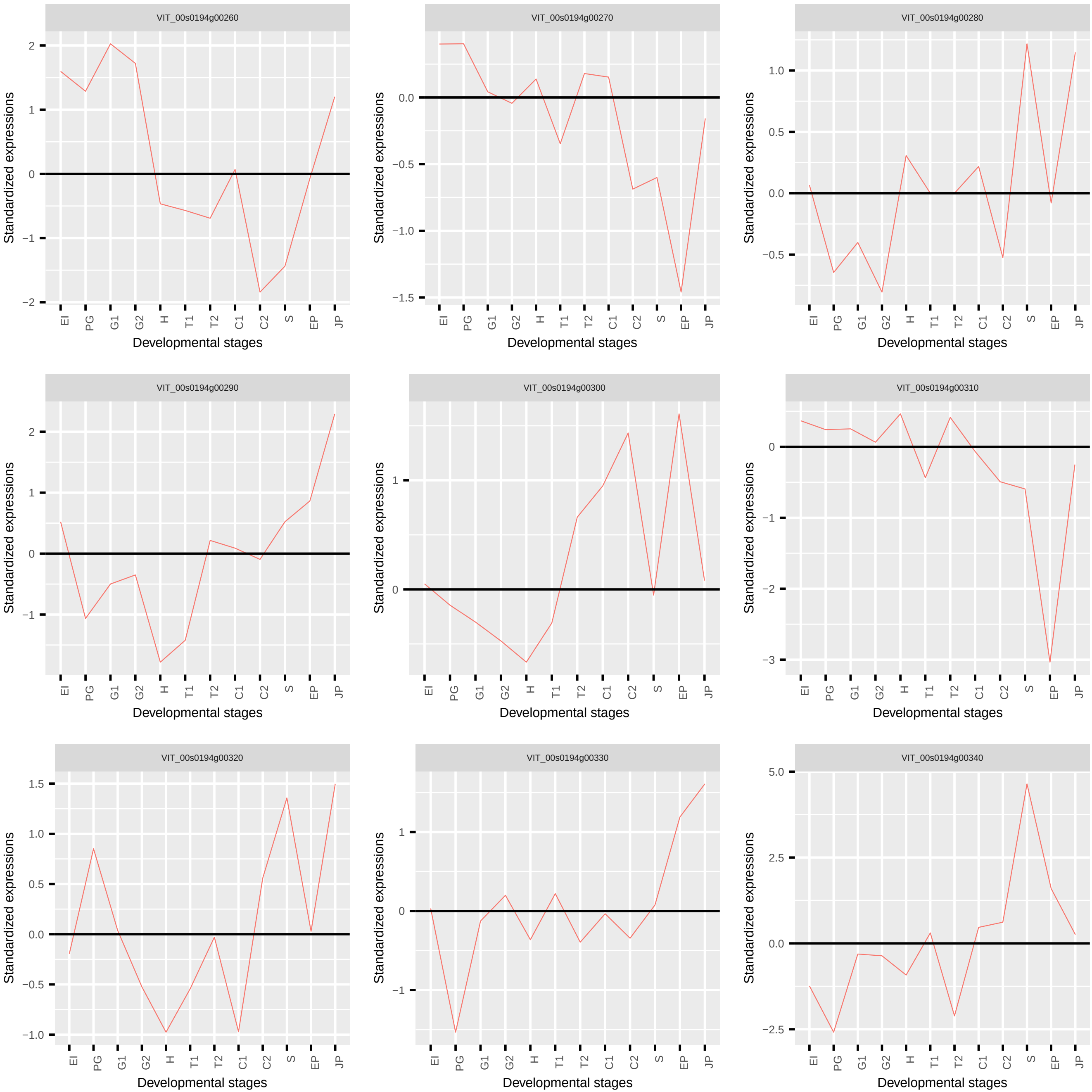

VIT_00s0194g00260
Standardized expressions
2
1
0
−1
−2
EI PG G1 G2 H T1 T2 C1 C2 S EP JP
Developmental stages
VIT_00s0194g00270
Standardized expressions
0.0
−0.5
−1.0
−1.5
EI PG G1 G2 H T1 T2 C1 C2 S EP JP
Developmental stages
VIT_00s0194g00280
Standardized expressions
1.0
0.5
0.0
−0.5
EI PG G1 G2 H T1 T2 C1 C2 S EP JP
Developmental stages
VIT_00s0194g00290
Standardized expressions
2
1
0
−1
EI PG G1 G2 H T1 T2 C1 C2 S EP JP
Developmental stages
VIT_00s0194g00300
Standardized expressions
1
0
EI PG G1 G2 H T1 T2 C1 C2 S EP JP
Developmental stages
VIT_00s0194g00310
Standardized expressions
0
−1
−2
−3
EI PG G1 G2 H T1 T2 C1 C2 S EP JP
Developmental stages
VIT_00s0194g00320
Standardized expressions
1.5
1.0
0.5
0.0
−0.5
−1.0
EI PG G1 G2 H T1 T2 C1 C2 S EP JP
Developmental stages
VIT_00s0194g00330
Standardized expressions
1
0
−1
EI PG G1 G2 H T1 T2 C1 C2 S EP JP
Developmental stages
VIT_00s0194g00340
Standardized expressions
5.0
2.5
0.0
−2.5
EI PG G1 G2 H T1 T2 C1 C2 S EP JP
Developmental stages

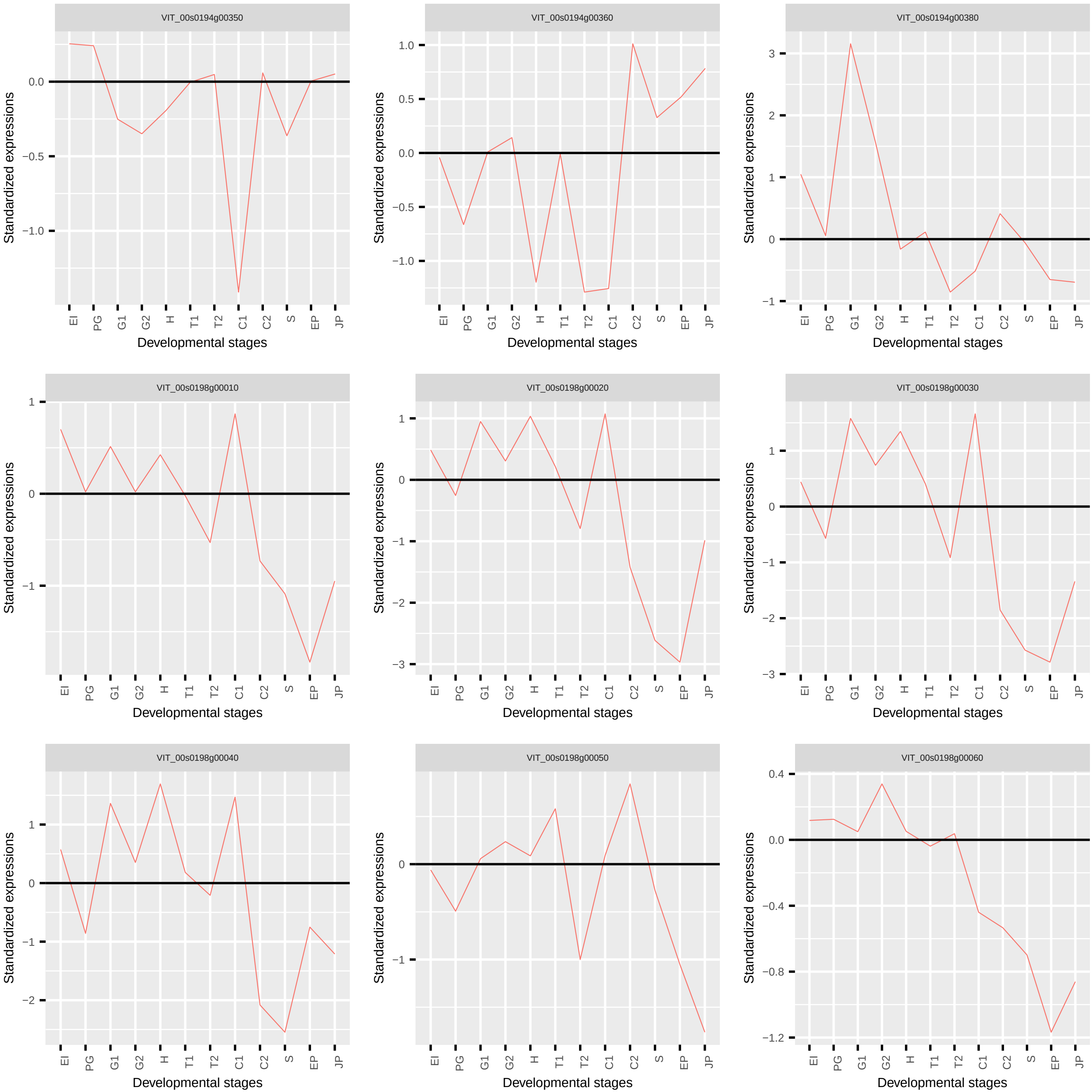
VIT_00s0194g00350
VIT_00s0194g00360
VIT_00s0194g00380
VIT_00s0198g00010
VIT_00s0198g00020
VIT_00s0198g00030
VIT_00s0198g00040
VIT_00s0198g00050
VIT_00s0198g00060
Standardized expressions
Developmental stages
EI PG G1 G2 H T1 T2 C1 C2 S EP JP

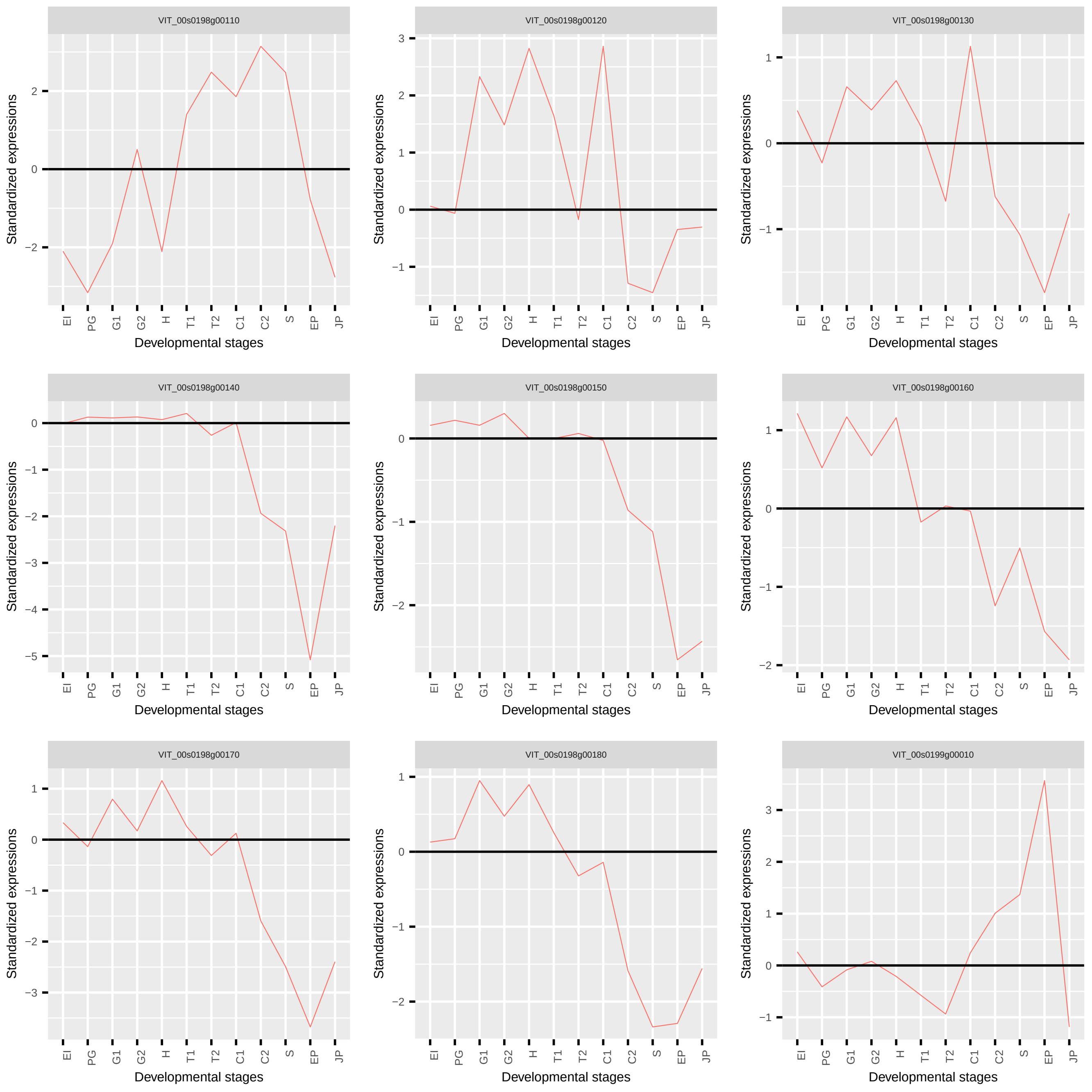
VIT_00s0198g00110
VIT_00s0198g00120
VIT_00s0198g00130
VIT_00s0198g00140
VIT_00s0198g00150
VIT_00s0198g00160
VIT_00s0198g00170
VIT_00s0198g00180
VIT_00s0199g00010
Standardized expressions
Developmental stages
EI PG G1 G2 H T1 T2 C1 C2 S EP JP

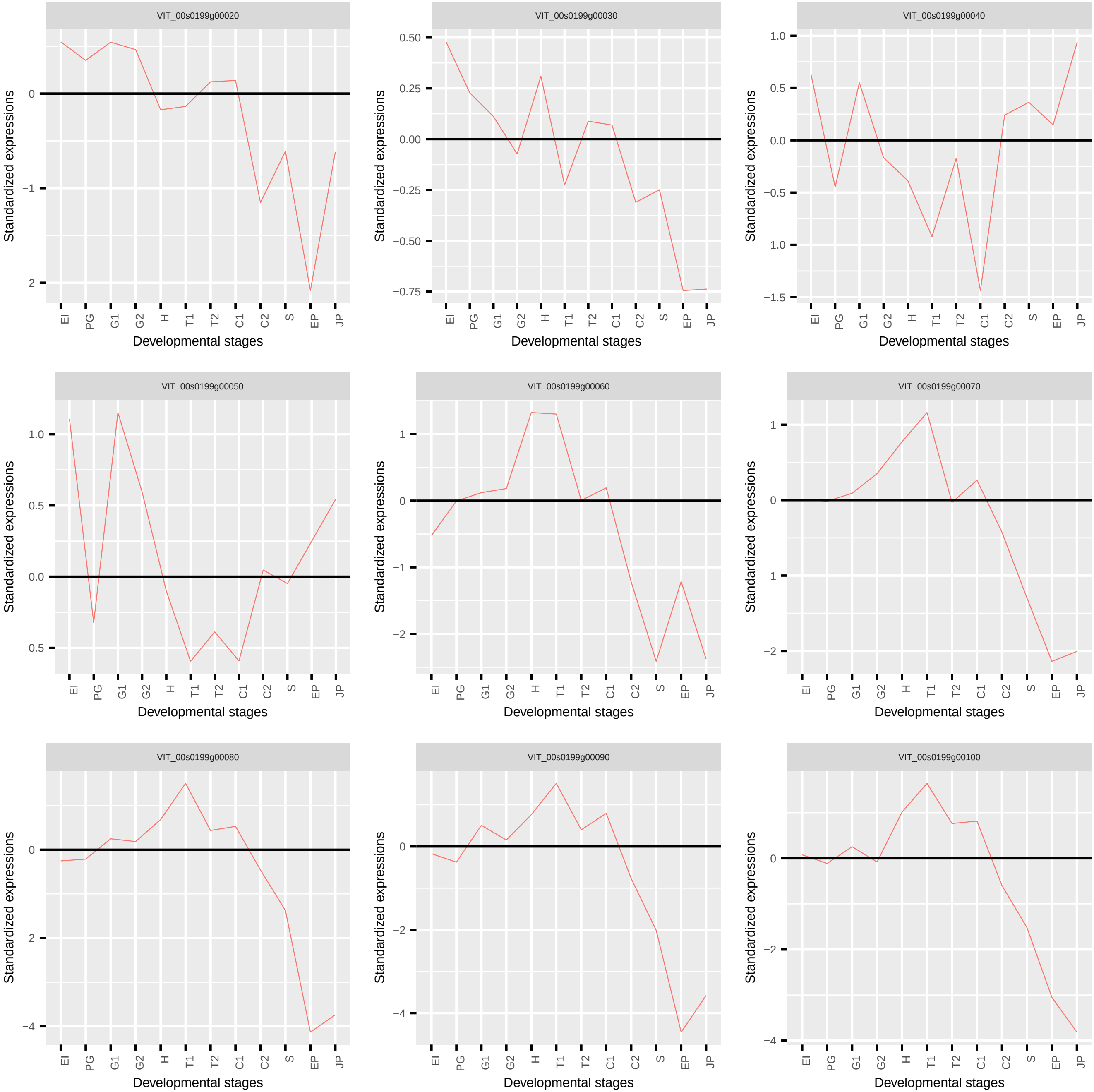

VIT_00s0199g00020
VIT_00s0199g00030
VIT_00s0199g00040
VIT_00s0199g00050
VIT_00s0199g00060
VIT_00s0199g00070
VIT_00s0199g00080
VIT_00s0199g00090
VIT_00s0199g00100
Standardized expressions
Developmental stages
EI PG G1 G2 H T1 T2 C1 C2 S EP JP
0
−1
−2
0.50
0.25
0.00
−0.25
−0.50
−0.75
1.0
0.5
0.0
−0.5
−1.0
−1.5
1
−4

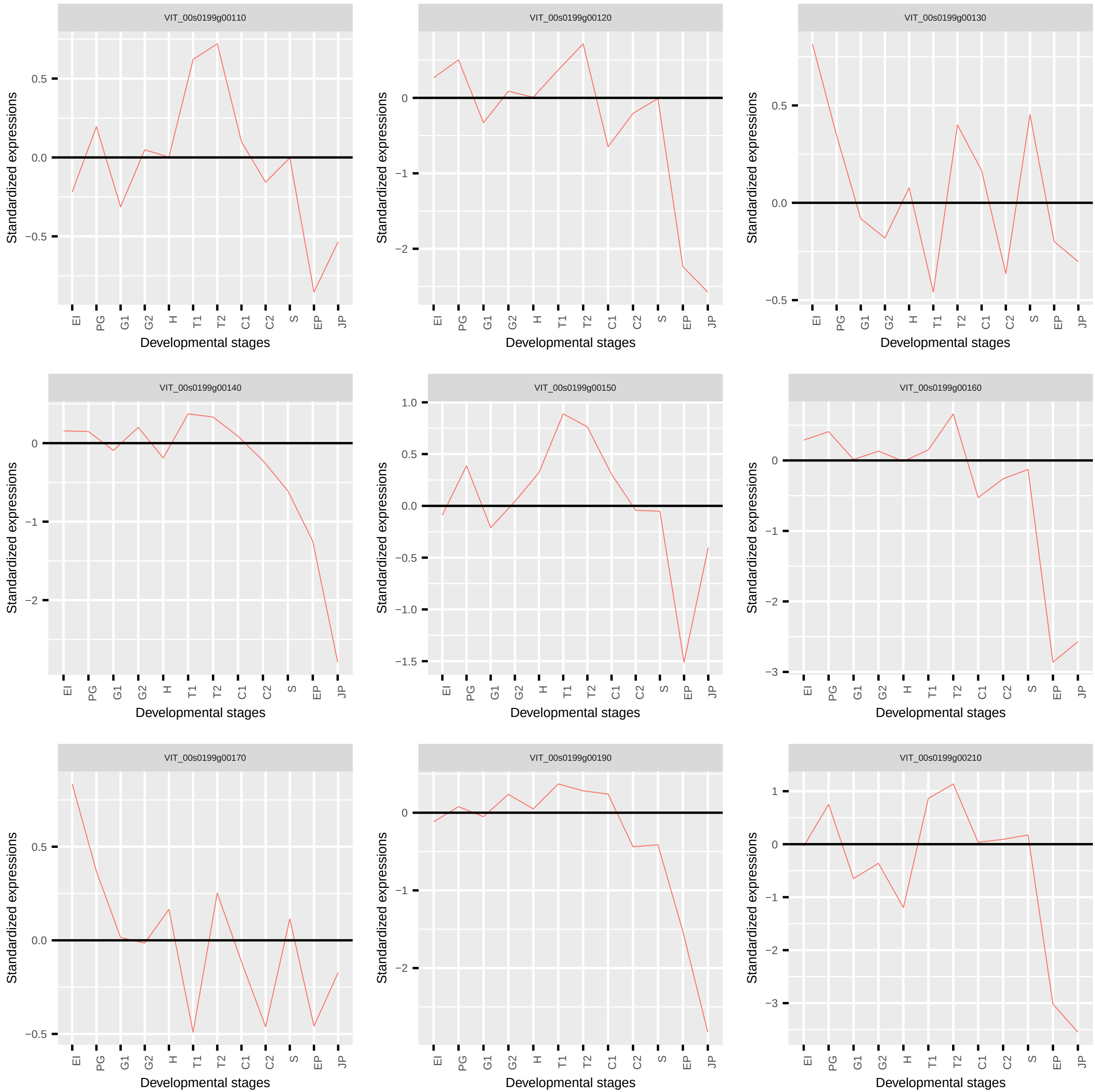
VIT_00s0199g00110
VIT_00s0199g00120
VIT_00s0199g00130
VIT_00s0199g00140
VIT_00s0199g00150
VIT_00s0199g00160
VIT_00s0199g00170
VIT_00s0199g00190
VIT_00s0199g00210
Standardized expressions
Developmental stages
EI PG G1 G2 H T1 T2 C1 C2 S EP JP

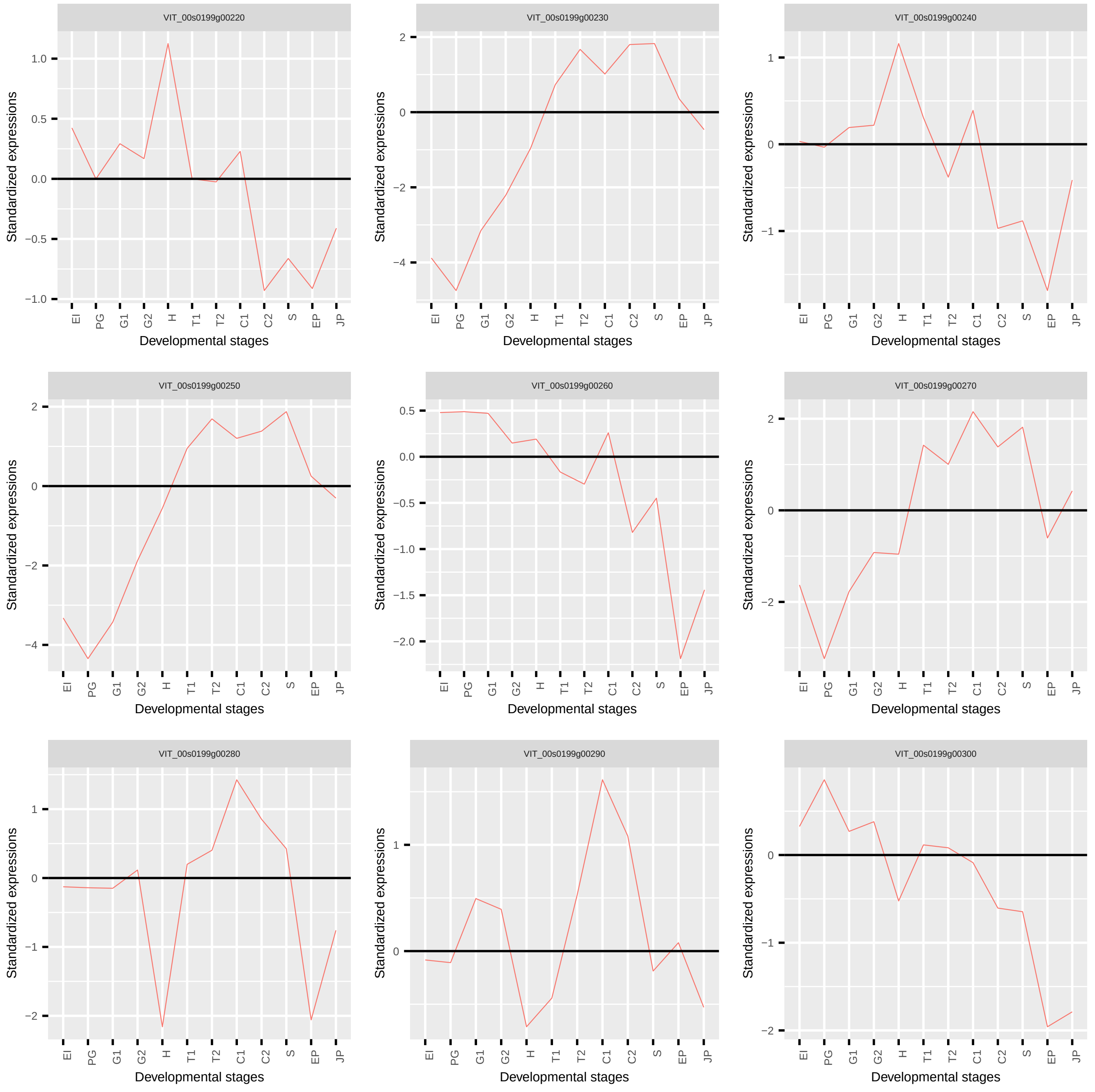
VIT_00s0199g00220
VIT_00s0199g00230
VIT_00s0199g00240
VIT_00s0199g00250
VIT_00s0199g00260
VIT_00s0199g00270
VIT_00s0199g00280
VIT_00s0199g00290
VIT_00s0199g00300
Standardized expressions
Developmental stages
EI PG G1 G2 H T1 T2 C1 C2 S EP JP

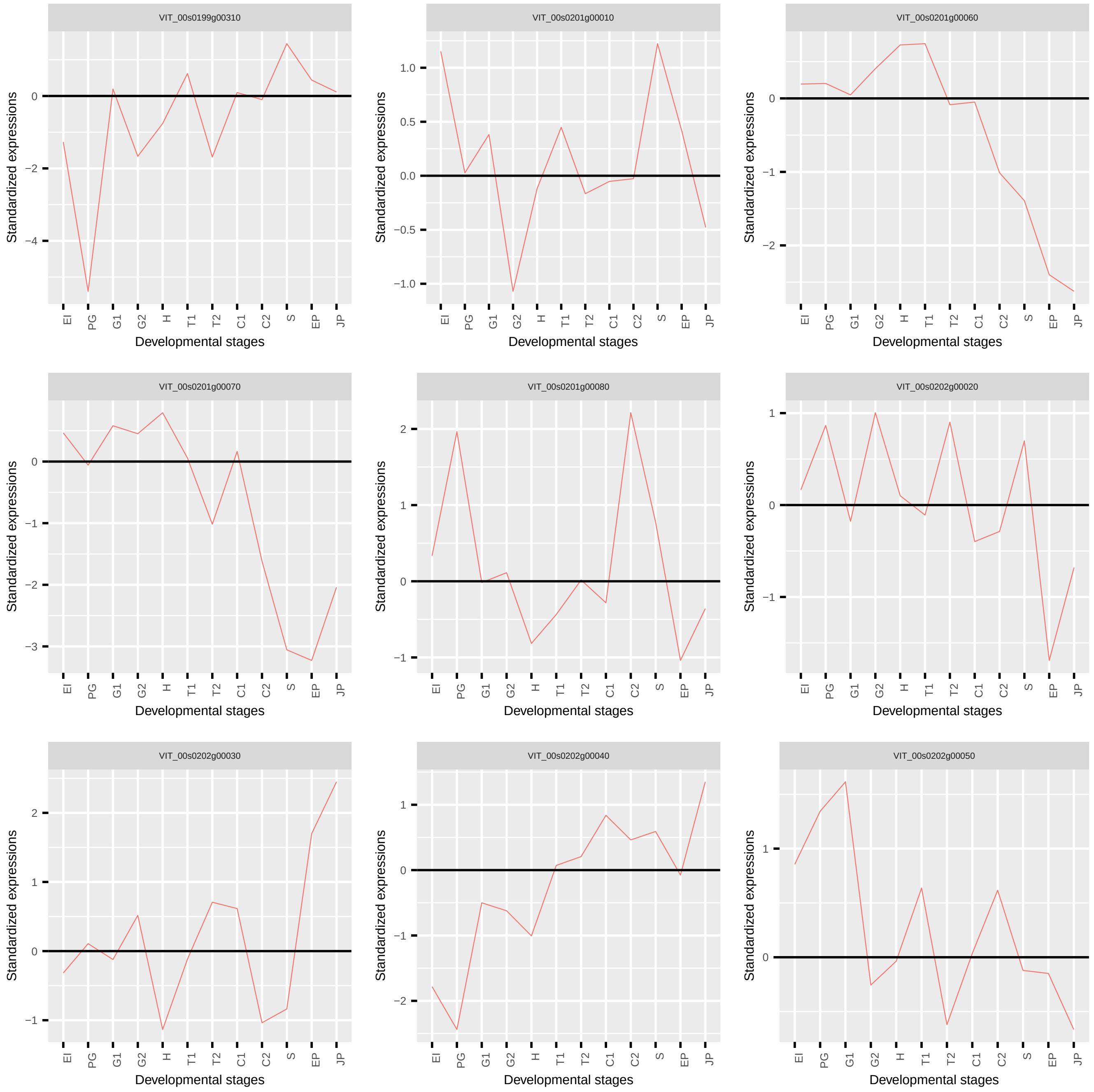
VIT_00s0199g00310
Standardized expressions
0
−2
−4
EI PG G1 G2 H T1 T2 C1 C2 S EP JP
Developmental stages
VIT_00s0201g00010
1.0
0.5
0.0
−0.5
−1.0
VIT_00s0201g00060
0
−1
−2
VIT_00s0201g00070
0
−1
−2
−3
VIT_00s0201g00080
2
1
0
−1
VIT_00s0202g00020
1
0
−1
VIT_00s0202g00030
2
1
0
−1
VIT_00s0202g00040
1
0
−1
−2
VIT_00s0202g00050
1
0

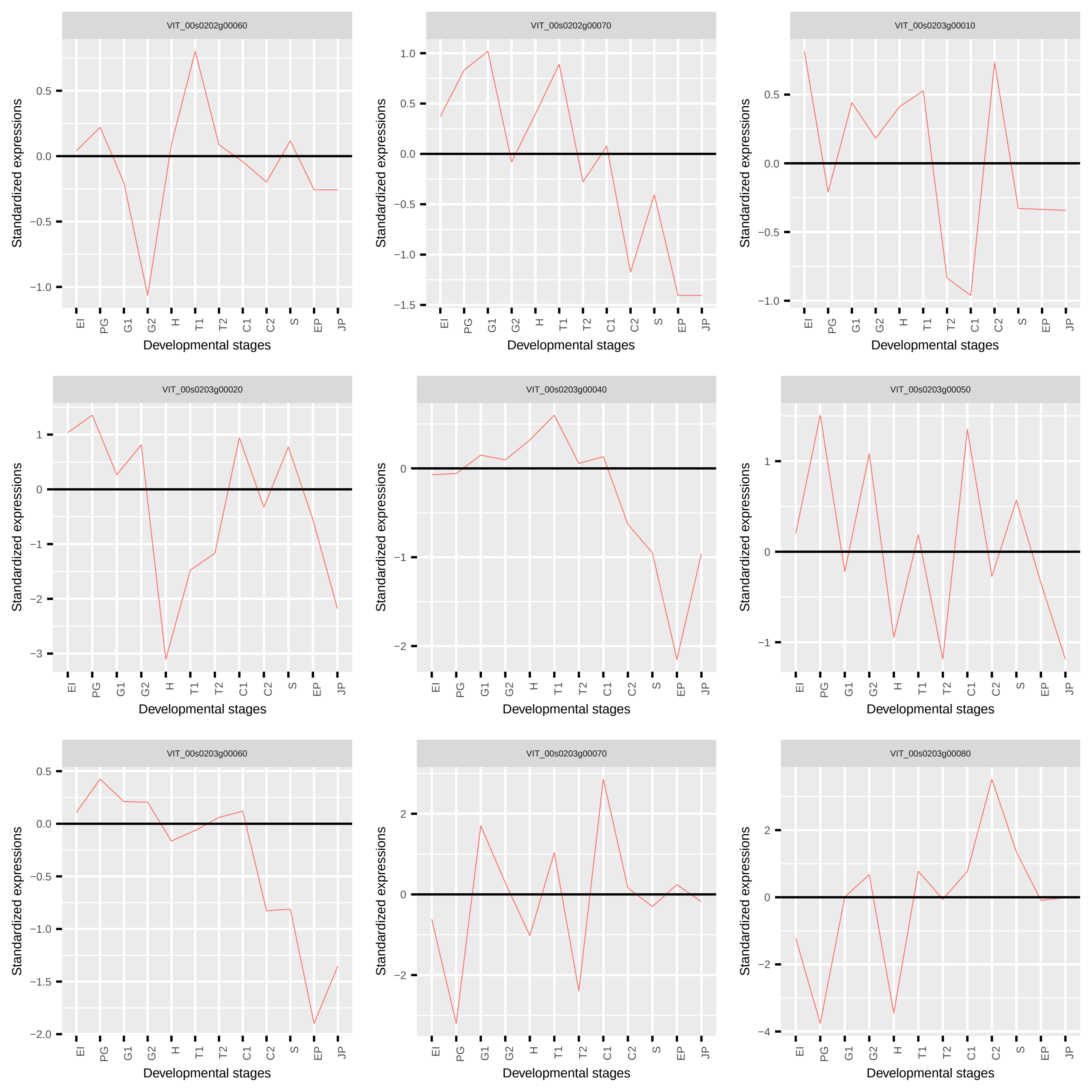

VIT_00s0202g00060
VIT_00s0202g00070
VIT_00s0203g00010
VIT_00s0203g00020
VIT_00s0203g00040
VIT_00s0203g00050
VIT_00s0203g00060
VIT_00s0203g00070
VIT_00s0203g00080
Standardized expressions
Developmental stages
EI PG G1 G2 H T1 T2 C1 C2 S EP JP

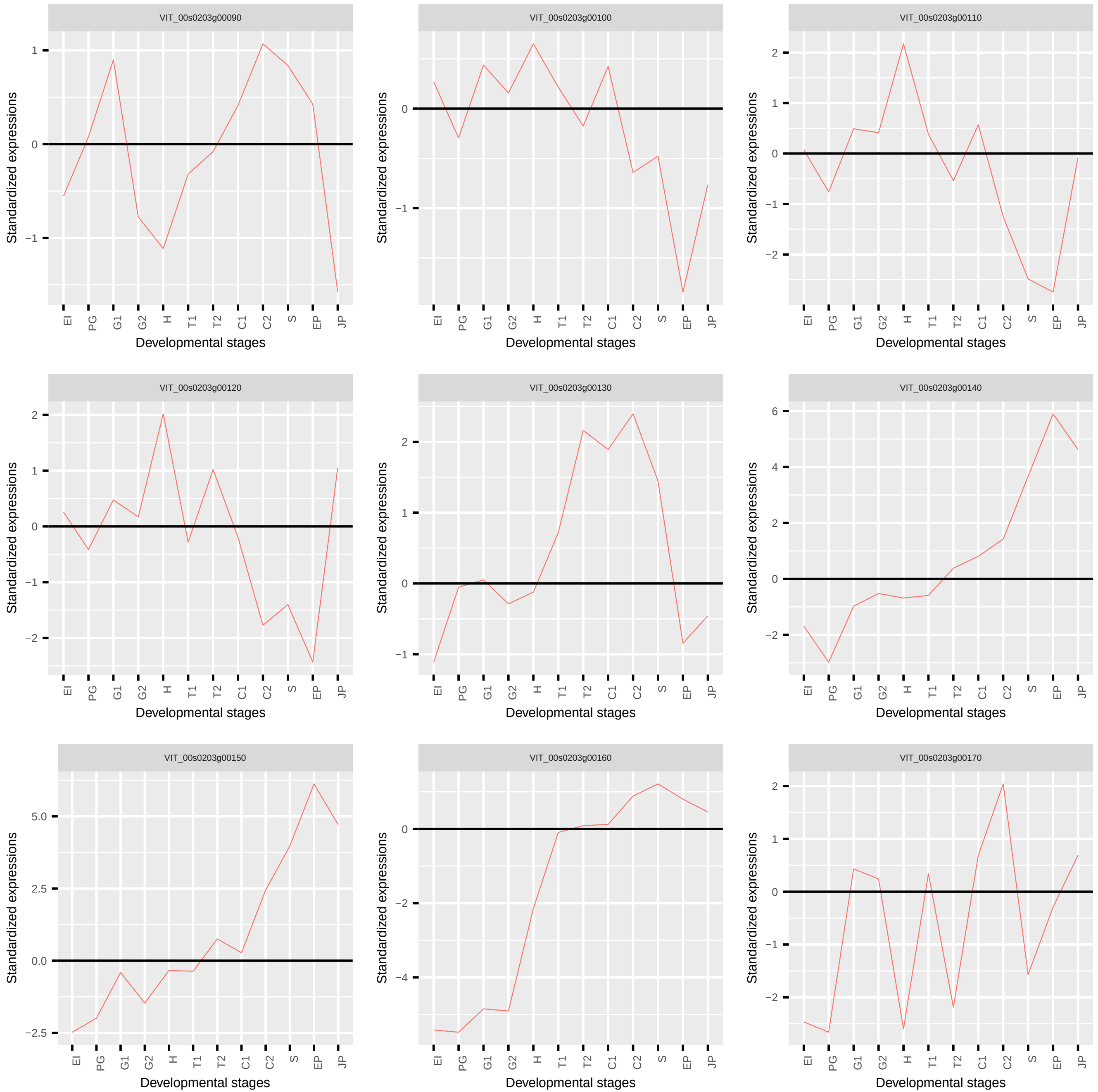
VIT_00s0203g00090
VIT_00s0203g00100
VIT_00s0203g00110
VIT_00s0203g00120
VIT_00s0203g00130
VIT_00s0203g00140
VIT_00s0203g00150
VIT_00s0203g00160
VIT_00s0203g00170
Standardized expressions
Developmental stages
EI
PG
G1
G2
H
T1
T2
C1
C2
S
EP
JP

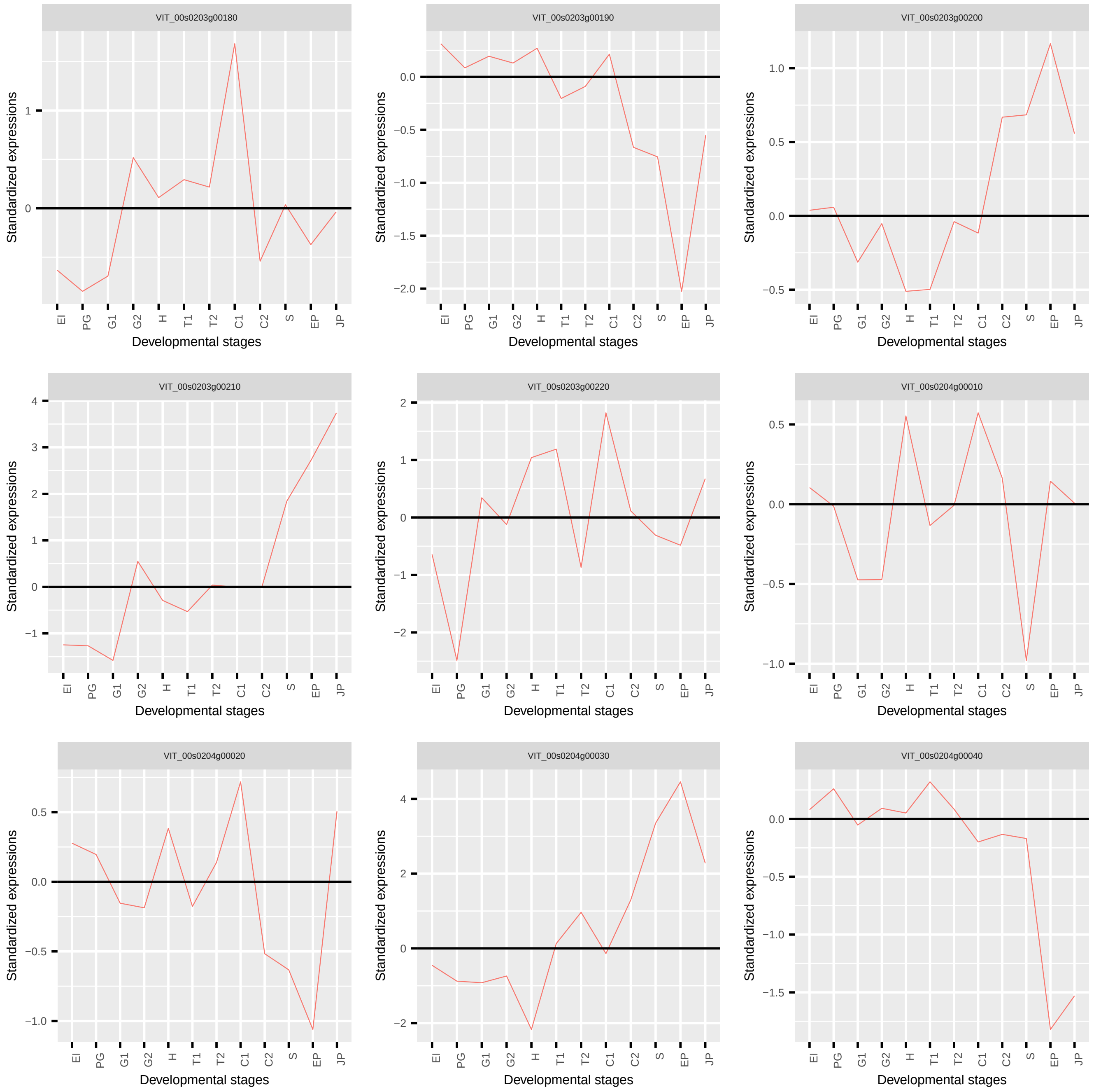
VIT_00s0203g00180
VIT_00s0203g00190
VIT_00s0203g00200
VIT_00s0203g00210
VIT_00s0203g00220
VIT_00s0204g00010
VIT_00s0204g00020
VIT_00s0204g00030
VIT_00s0204g00040
Standardized expressions
Developmental stages
EI PG G1 G2 H T1 T2 C1 C2 S EP JP

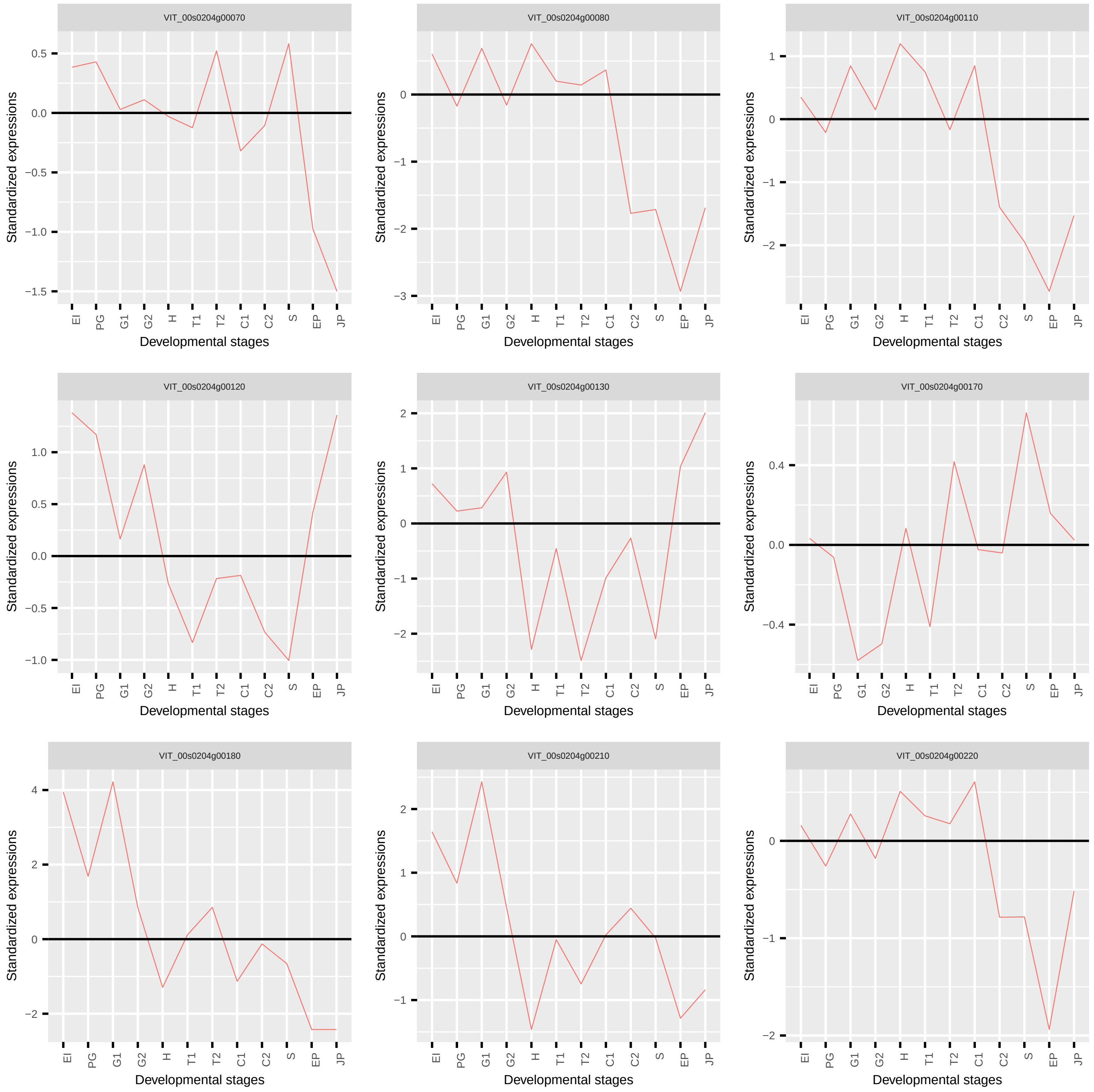
VIT_00s0204g00070
VIT_00s0204g00080
VIT_00s0204g00110
VIT_00s0204g00120
VIT_00s0204g00130
VIT_00s0204g00170
VIT_00s0204g00180
VIT_00s0204g00210
VIT_00s0204g00220
Standardized expressions
Developmental stages
EI PG G1 G2 H T1 T2 C1 C2 S EP JP

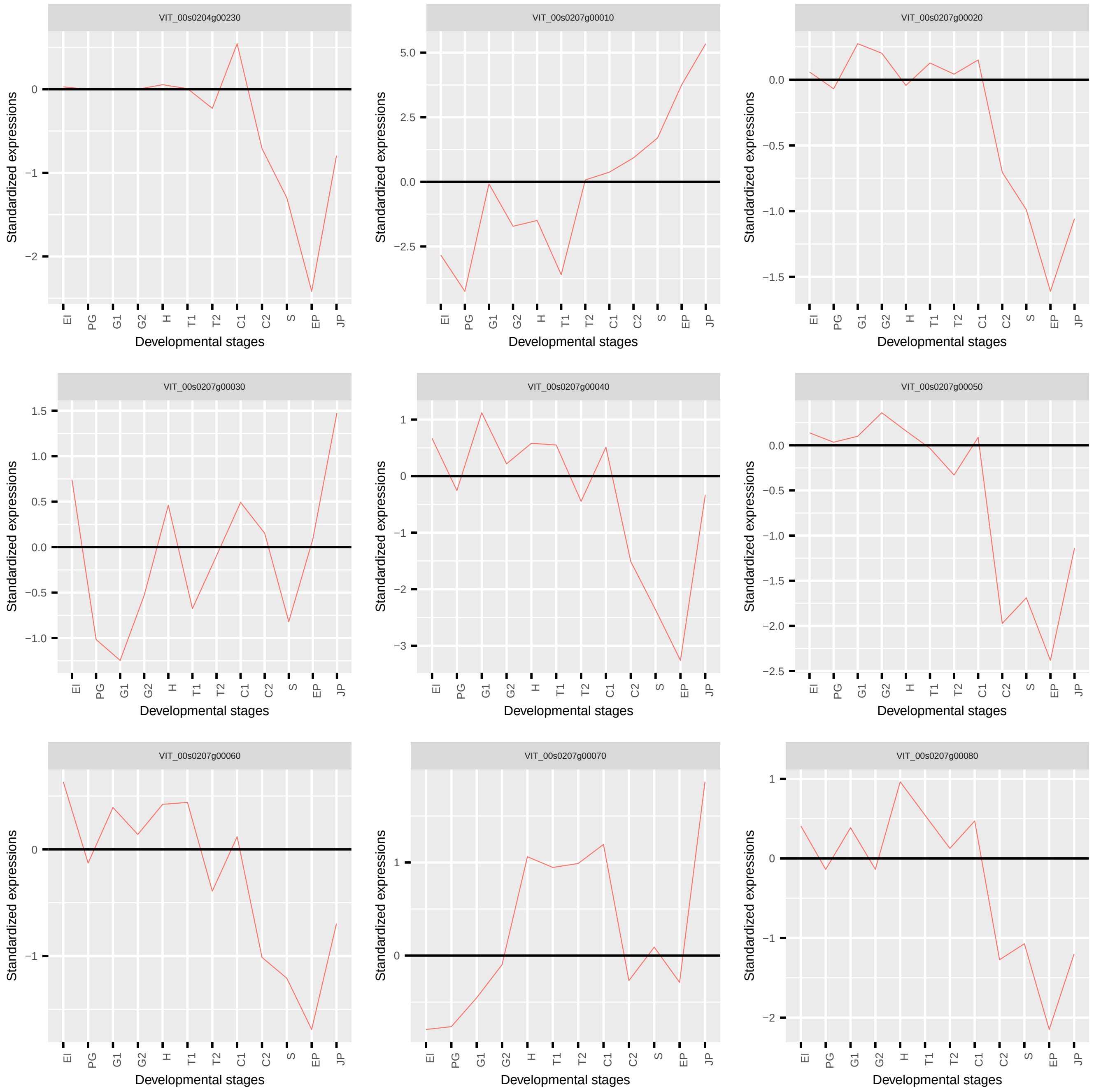

VIT_00s0204g00230
VIT_00s0207g00010
VIT_00s0207g00020
VIT_00s0207g00030
VIT_00s0207g00040
VIT_00s0207g00050
VIT_00s0207g00060
VIT_00s0207g00070
VIT_00s0207g00080
Standardized expressions
Developmental stages
EI PG G1 G2 H T1 T2 C1 C2 S EP JP

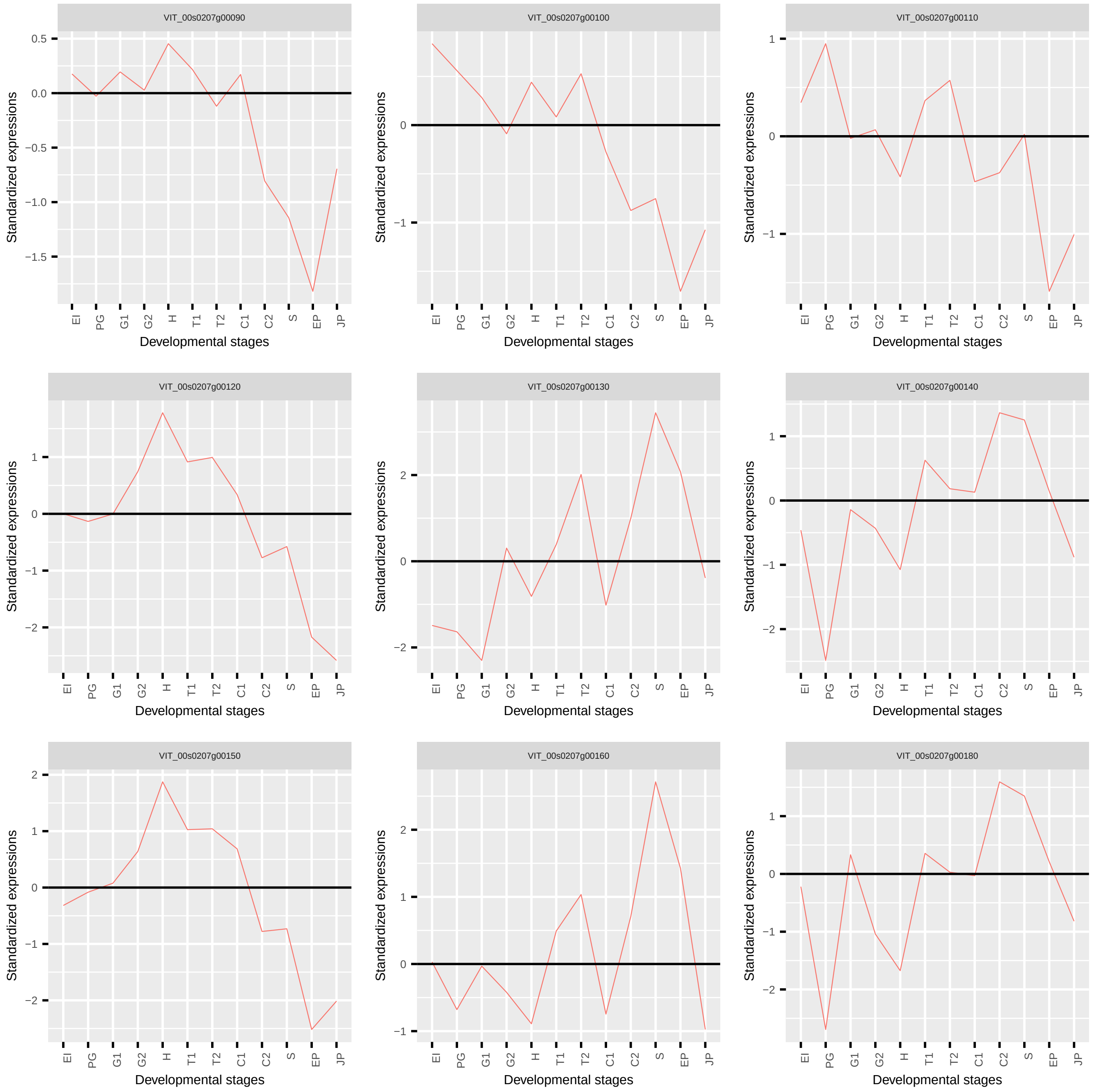
VIT_00s0207g00090
VIT_00s0207g00100
VIT_00s0207g00110
VIT_00s0207g00120
VIT_00s0207g00130
VIT_00s0207g00140
VIT_00s0207g00150
VIT_00s0207g00160
VIT_00s0207g00180
Standardized expressions
Developmental stages
EI PG G1 G2 H T1 T2 C1 C2 S EP JP

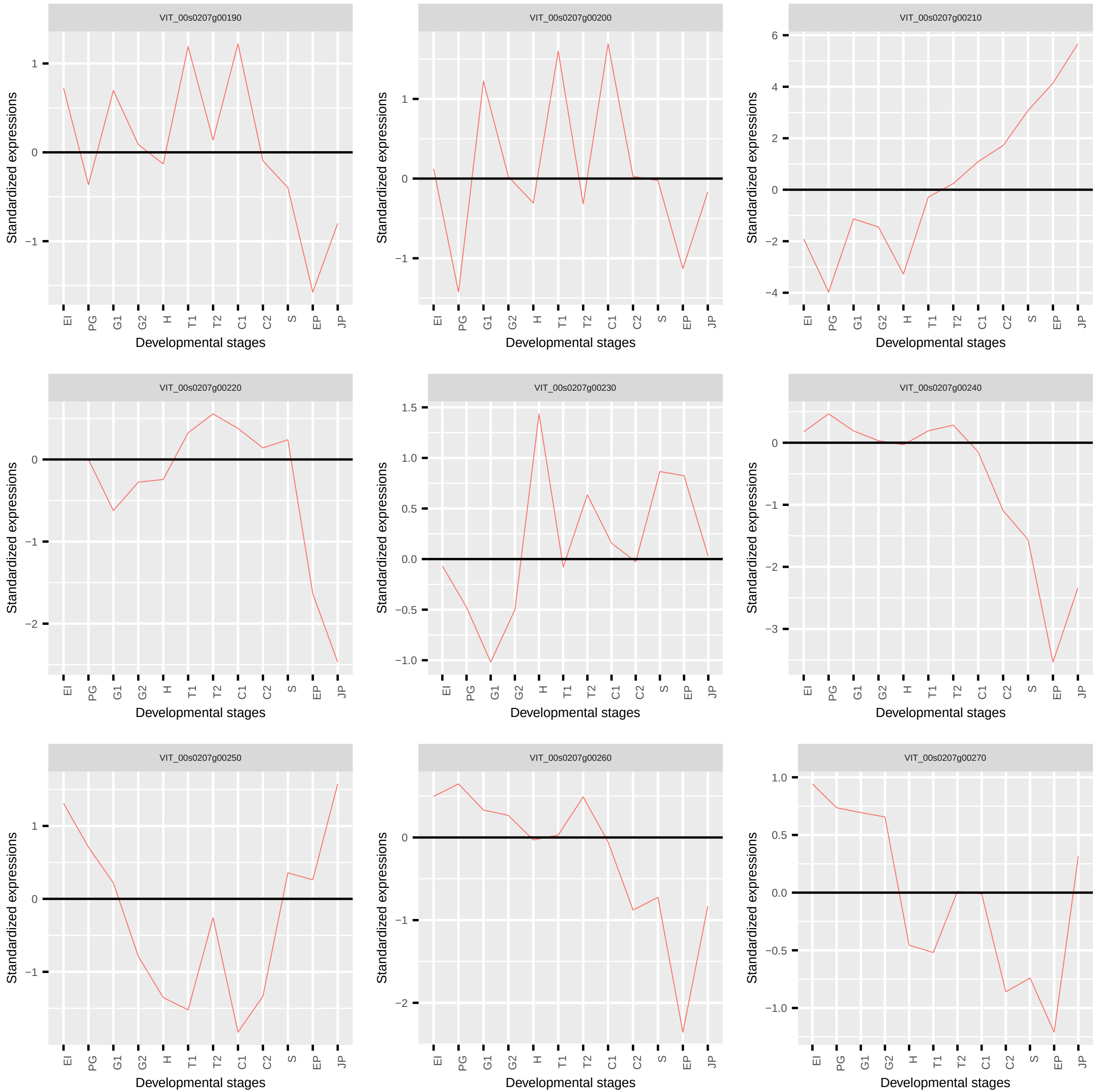
VIT_00s0207g00190
VIT_00s0207g00200
VIT_00s0207g00210
VIT_00s0207g00220
VIT_00s0207g00230
VIT_00s0207g00240
VIT_00s0207g00250
VIT_00s0207g00260
VIT_00s0207g00270
Standardized expressions
Developmental stages
EI
PG
G1
G2
H
T1
T2
C1
C2
S
EP
JP

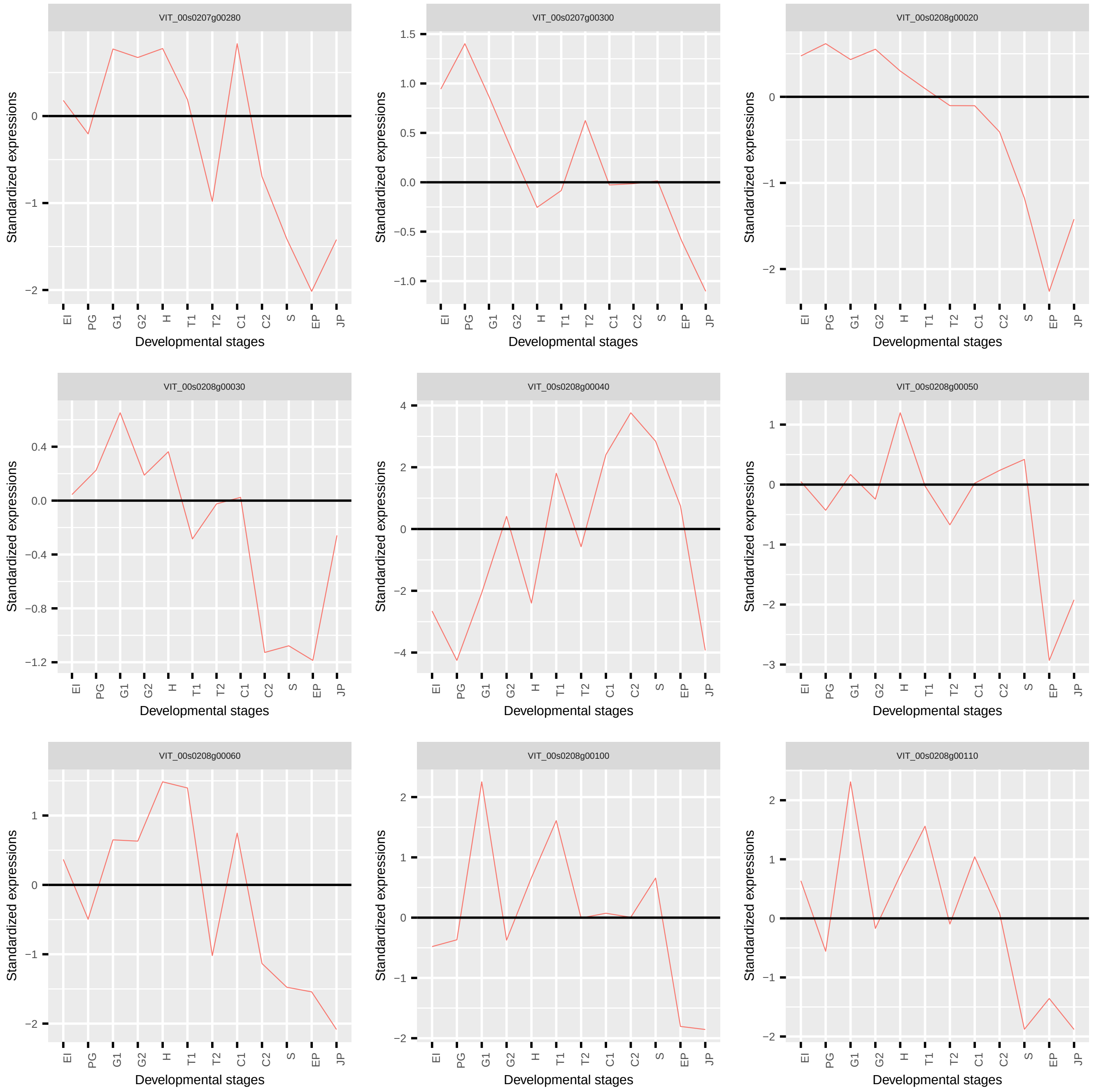
VIT_00s0207g00280
VIT_00s0207g00300
VIT_00s0208g00020
VIT_00s0208g00030
VIT_00s0208g00040
VIT_00s0208g00050
VIT_00s0208g00060
VIT_00s0208g00100
VIT_00s0208g00110
Standardized expressions
Developmental stages
EI
PG
G1
G2
H
T1
T2
C1
C2
S
EP
JP

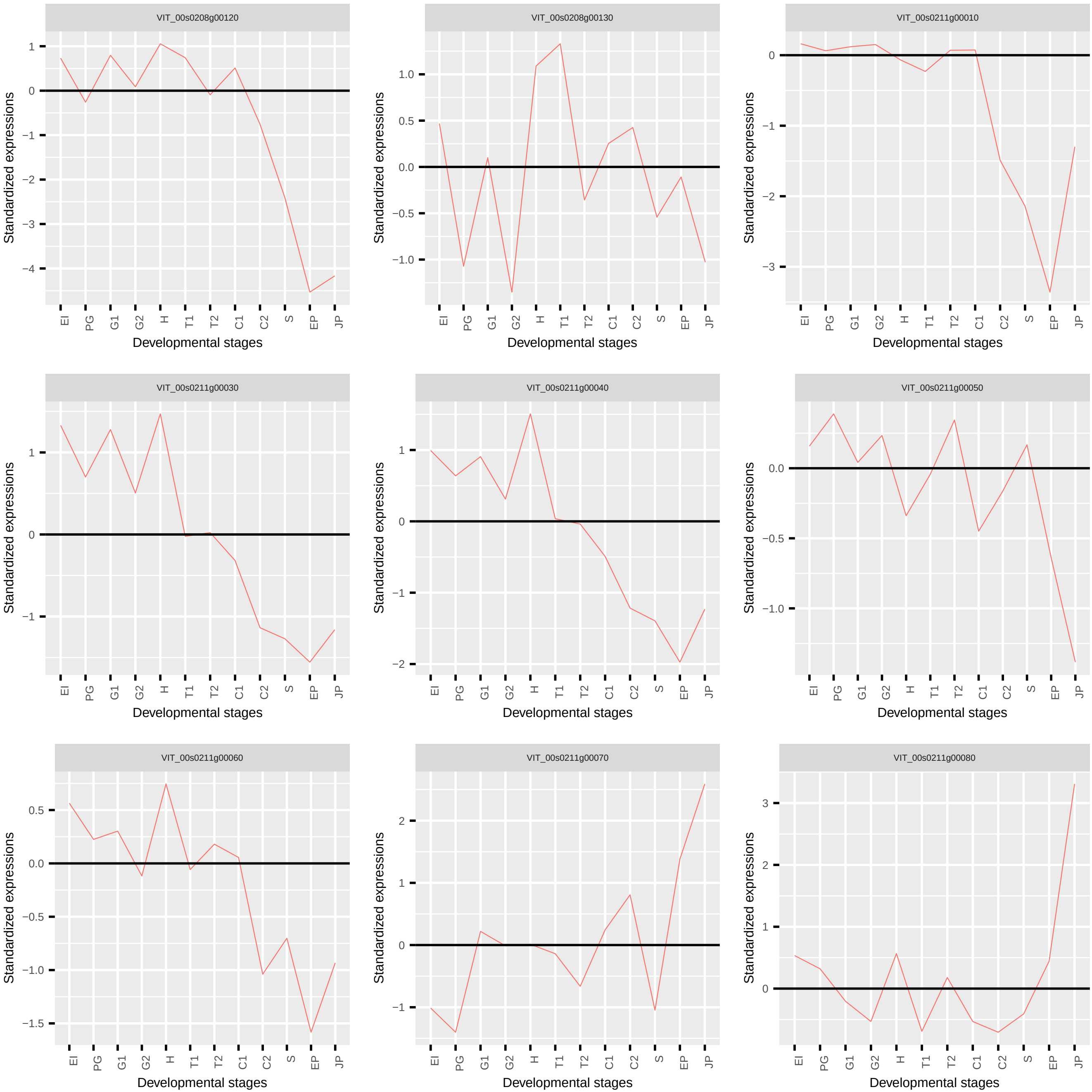
VIT_00s0208g00120
VIT_00s0208g00130
VIT_00s0211g00010
VIT_00s0211g00030
VIT_00s0211g00040
VIT_00s0211g00050
VIT_00s0211g00060
VIT_00s0211g00070
VIT_00s0211g00080
Standardized expressions
Developmental stages
EI PG G1 G2 H T1 T2 C1 C2 S EP JP

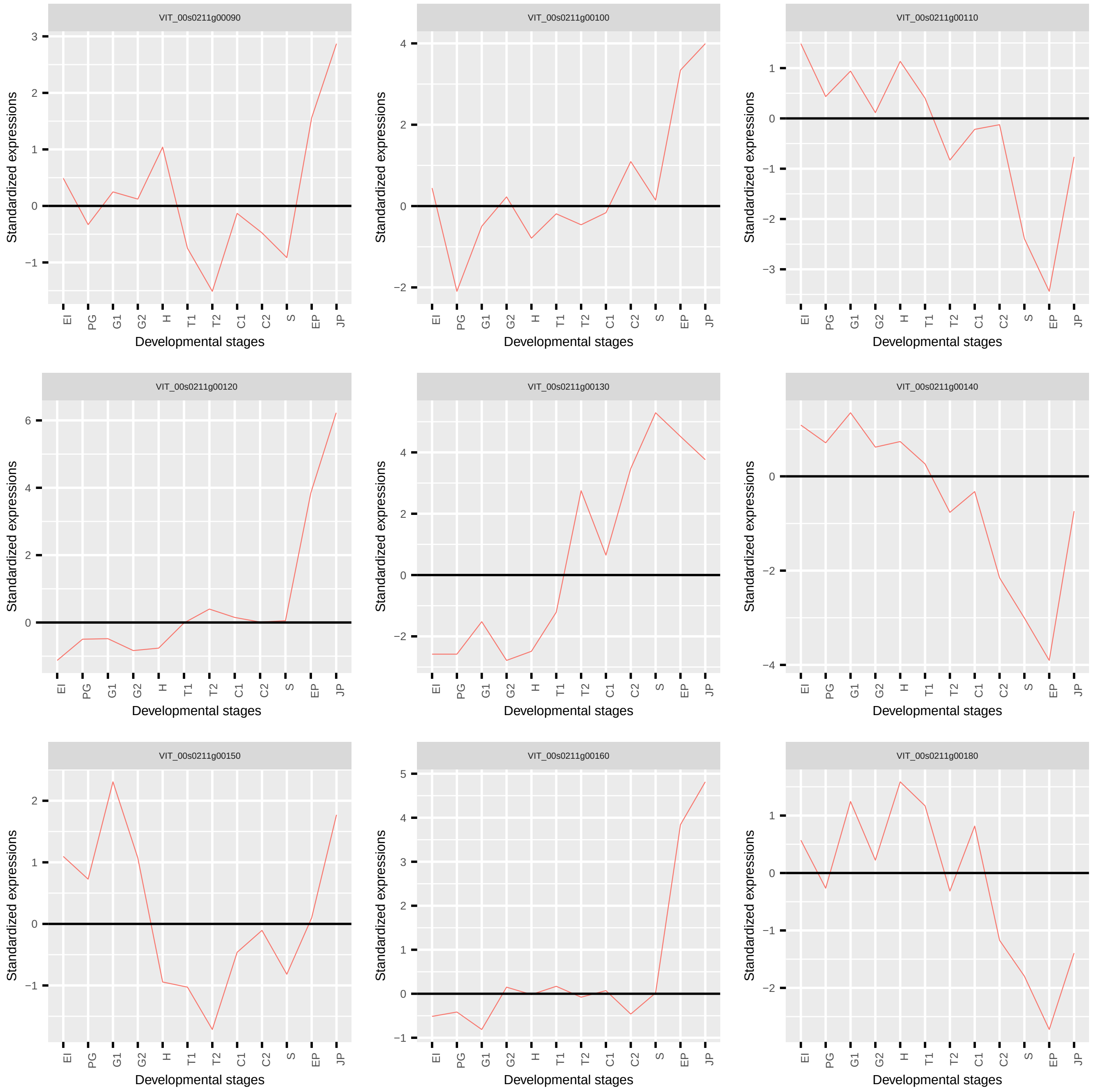

VIT_00s0211g00090
VIT_00s0211g00100
VIT_00s0211g00110
VIT_00s0211g00120
VIT_00s0211g00130
VIT_00s0211g00140
VIT_00s0211g00150
VIT_00s0211g00160
VIT_00s0211g00180
Standardized expressions
Developmental stages
EI PG G1 G2 H T1 T2 C1 C2 S EP JP

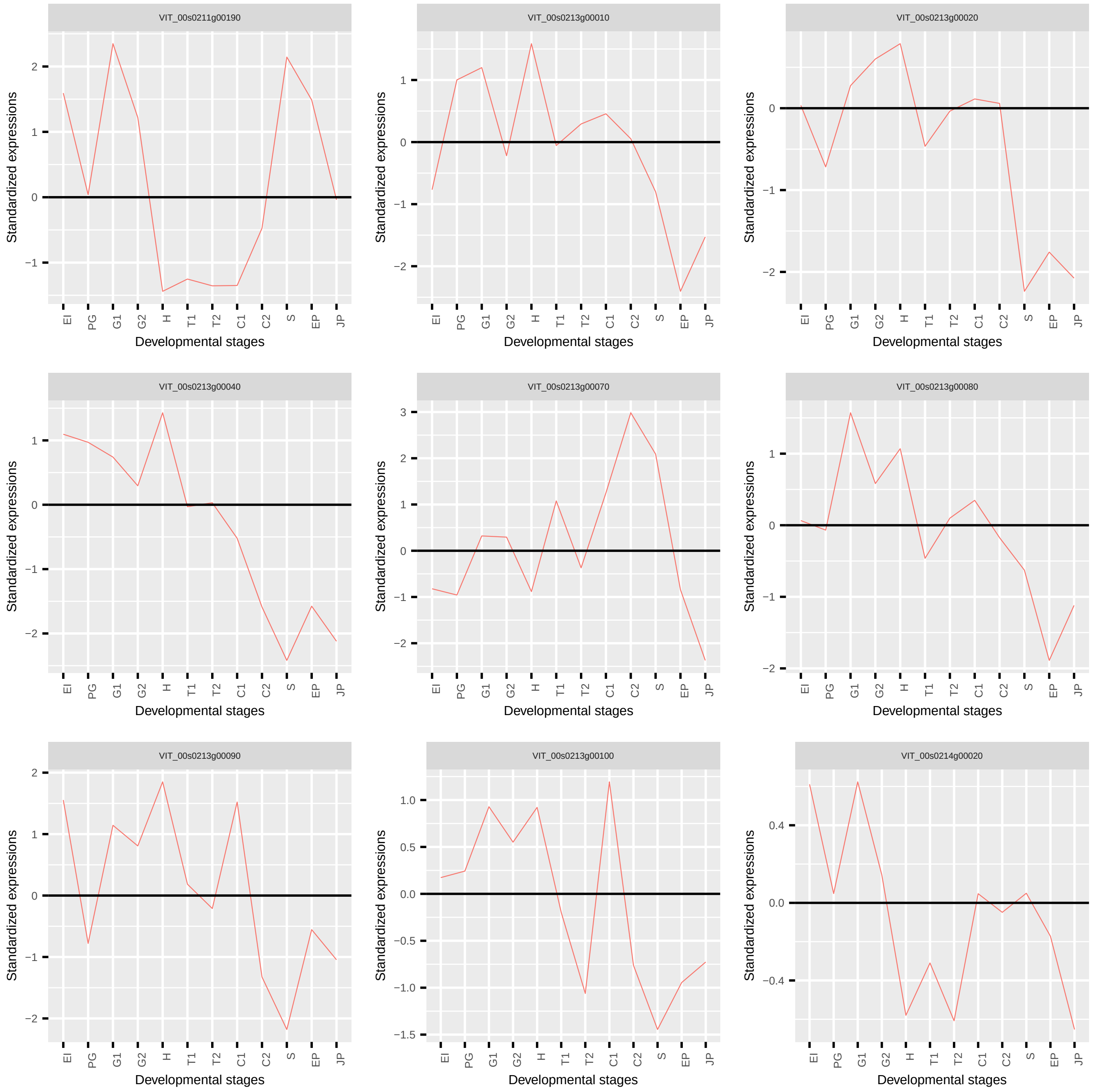
VIT_00s0211g00190
VIT_00s0213g00010
VIT_00s0213g00020
VIT_00s0213g00040
VIT_00s0213g00070
VIT_00s0213g00080
VIT_00s0213g00090
VIT_00s0213g00100
VIT_00s0214g00020
Standardized expressions
Developmental stages
EI PG G1 G2 H T1 T2 C1 C2 S EP JP

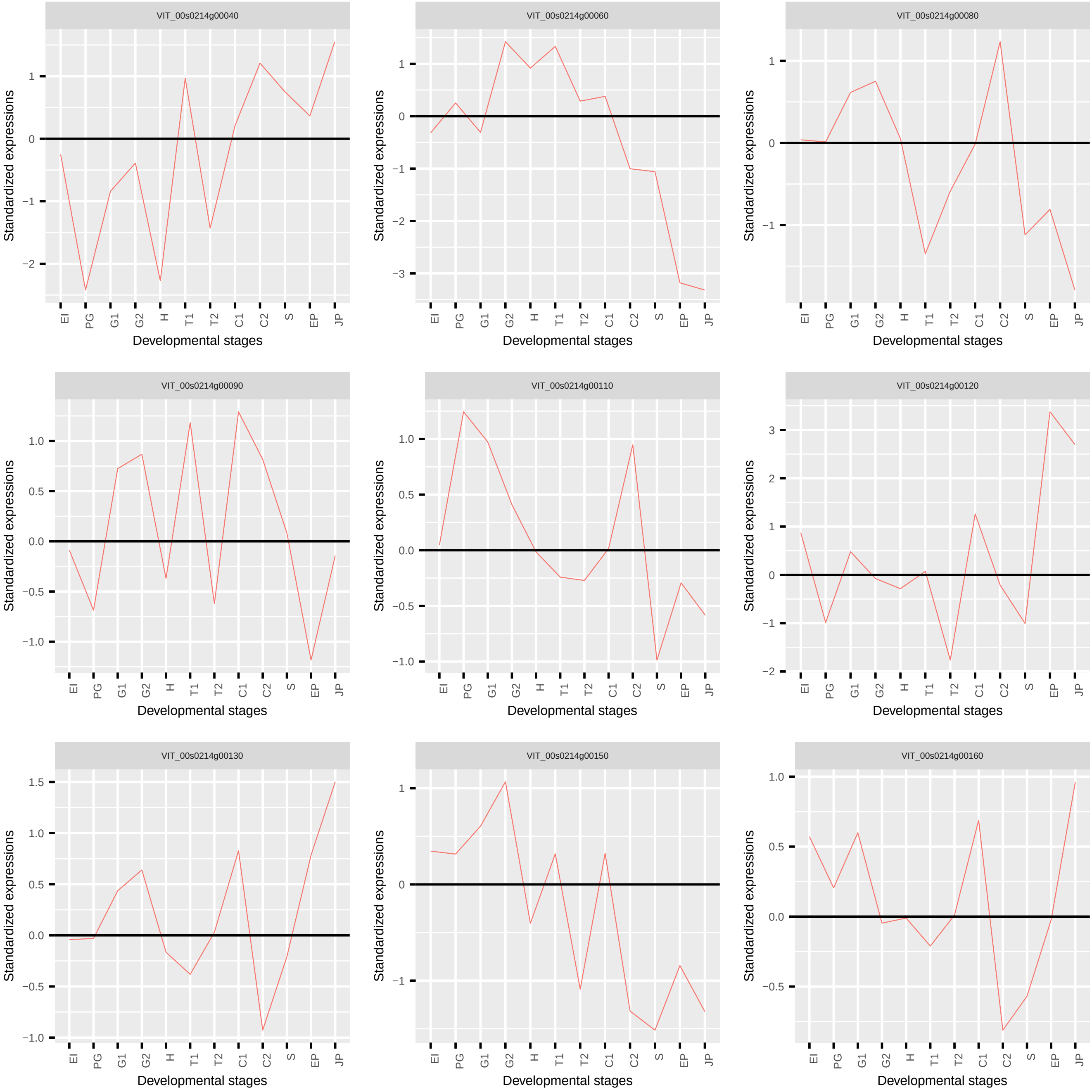
VIT_00s0214g00040
VIT_00s0214g00060
VIT_00s0214g00080
VIT_00s0214g00090
VIT_00s0214g00110
VIT_00s0214g00120
VIT_00s0214g00130
VIT_00s0214g00150
VIT_00s0214g00160
Standardized expressions
Developmental stages
EI PG G1 G2 H T1 T2 C1 C2 S EP JP

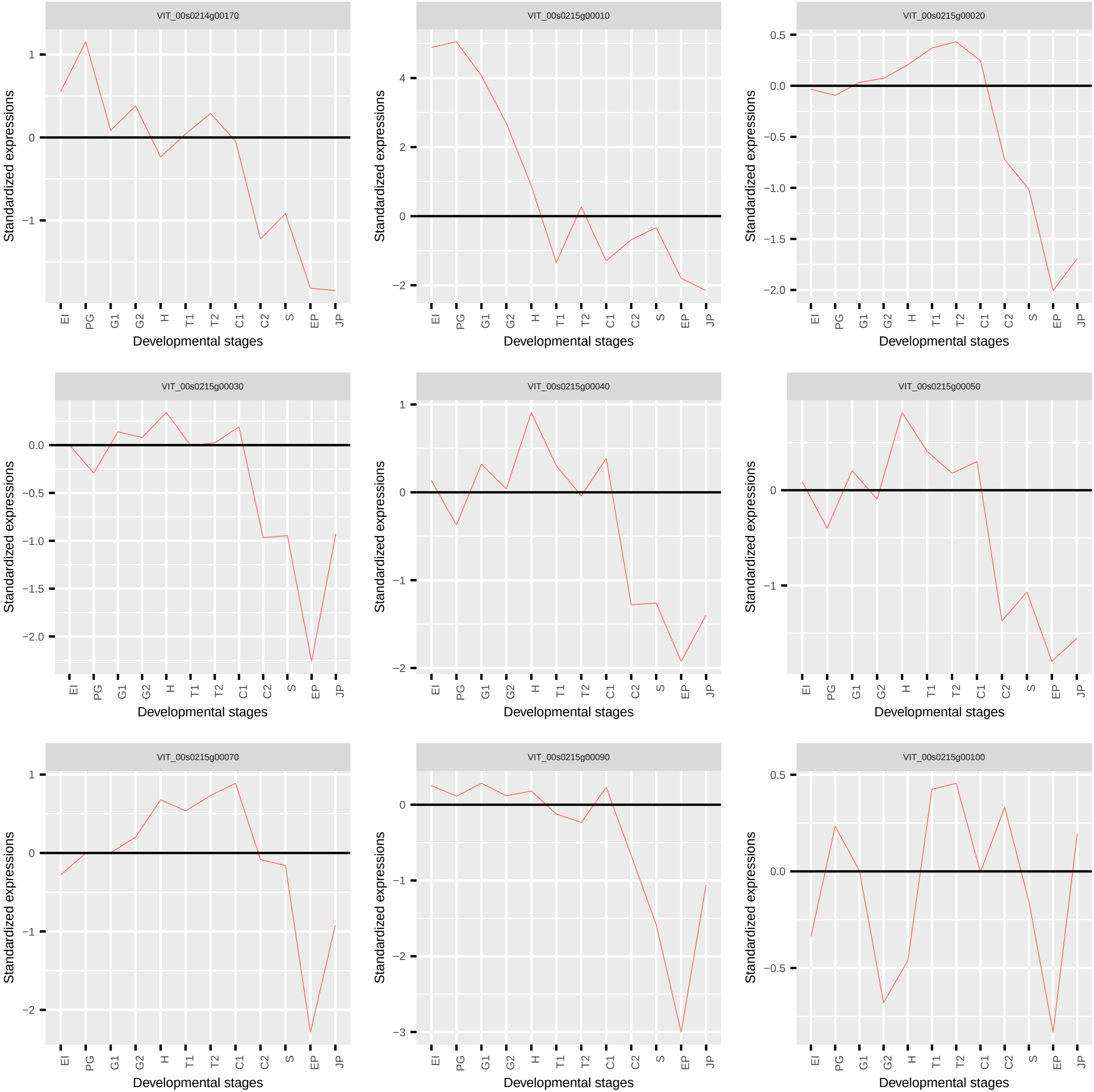
VIT_00s0214g00170
VIT_00s0215g00010
VIT_00s0215g00020
VIT_00s0215g00030
VIT_00s0215g00040
VIT_00s0215g00050
VIT_00s0215g00070
VIT_00s0215g00090
VIT_00s0215g00100
Standardized expressions
Developmental stages
EI PG G1 G2 H T1 T2 C1 C2 S EP JP

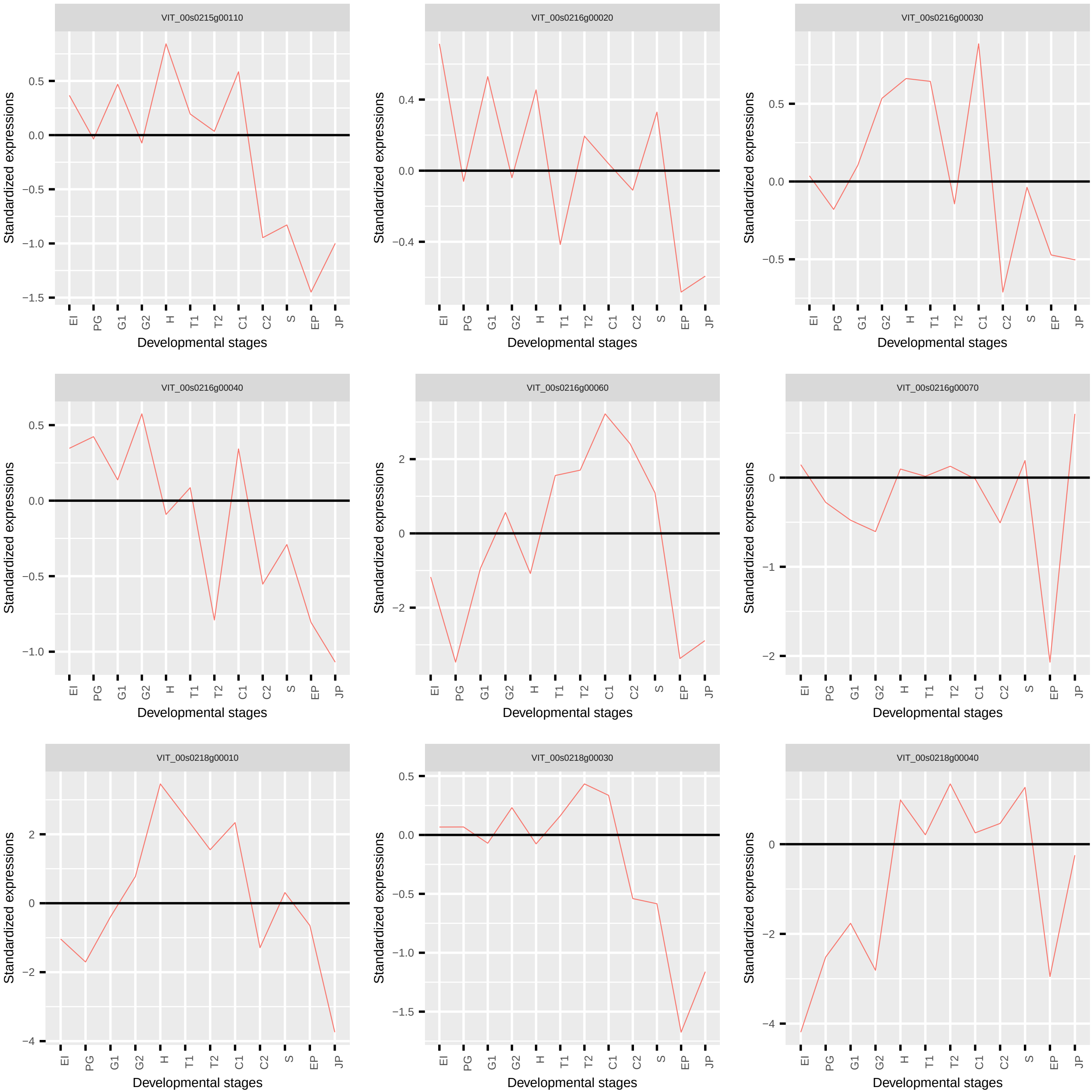
VIT_00s0215g00110
VIT_00s0216g00020
VIT_00s0216g00030
VIT_00s0216g00040
VIT_00s0216g00060
VIT_00s0216g00070
VIT_00s0218g00010
VIT_00s0218g00030
VIT_00s0218g00040
Standardized expressions
Developmental stages
EI PG G1 G2 H T1 T2 C1 C2 S EP JP

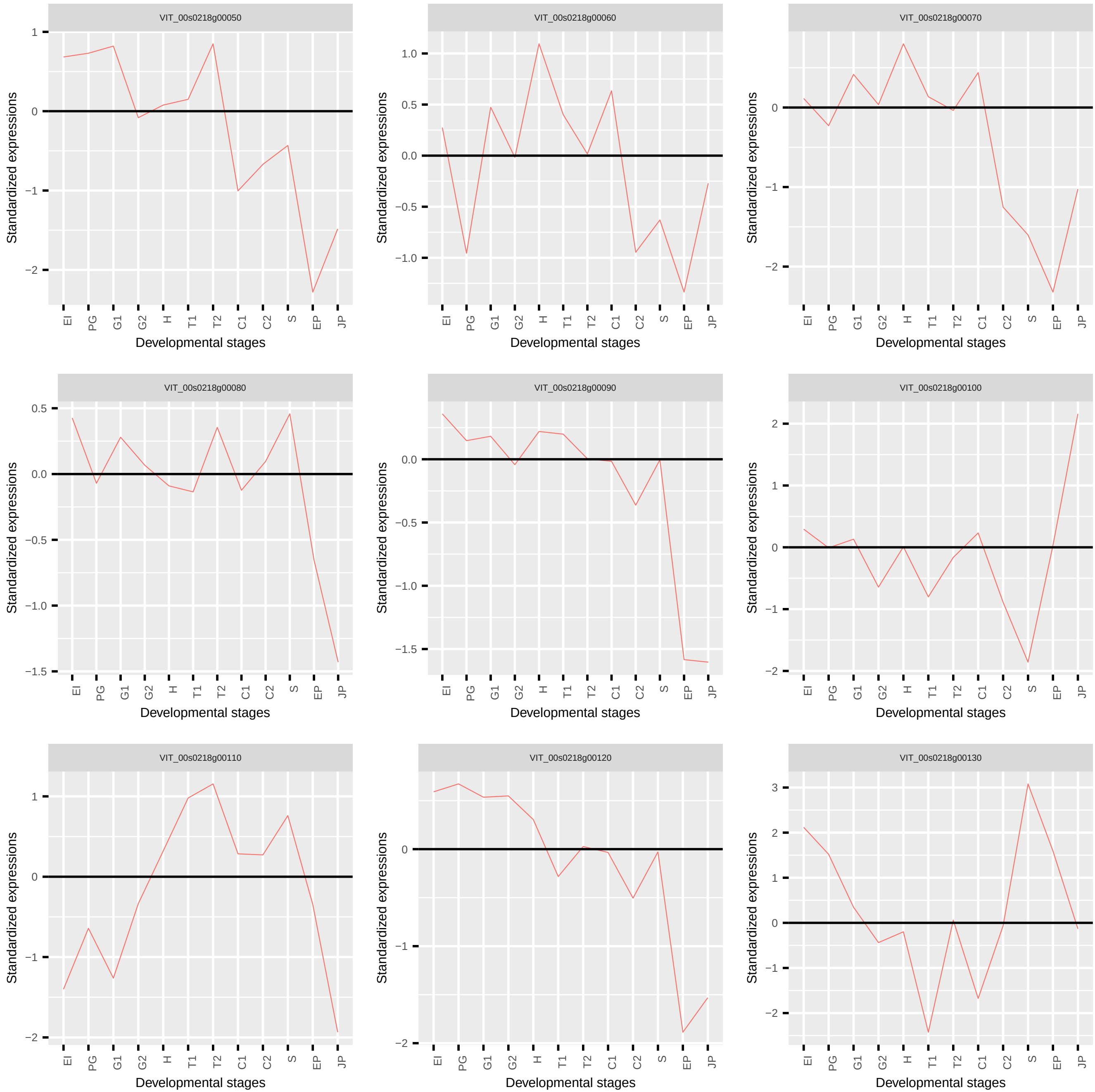

VIT_00s0218g00050
VIT_00s0218g00060
VIT_00s0218g00070
VIT_00s0218g00080
VIT_00s0218g00090
VIT_00s0218g00100
VIT_00s0218g00110
VIT_00s0218g00120
VIT_00s0218g00130
Standardized expressions
Developmental stages
EI PG G1 G2 H T1 T2 C1 C2 S EP JP

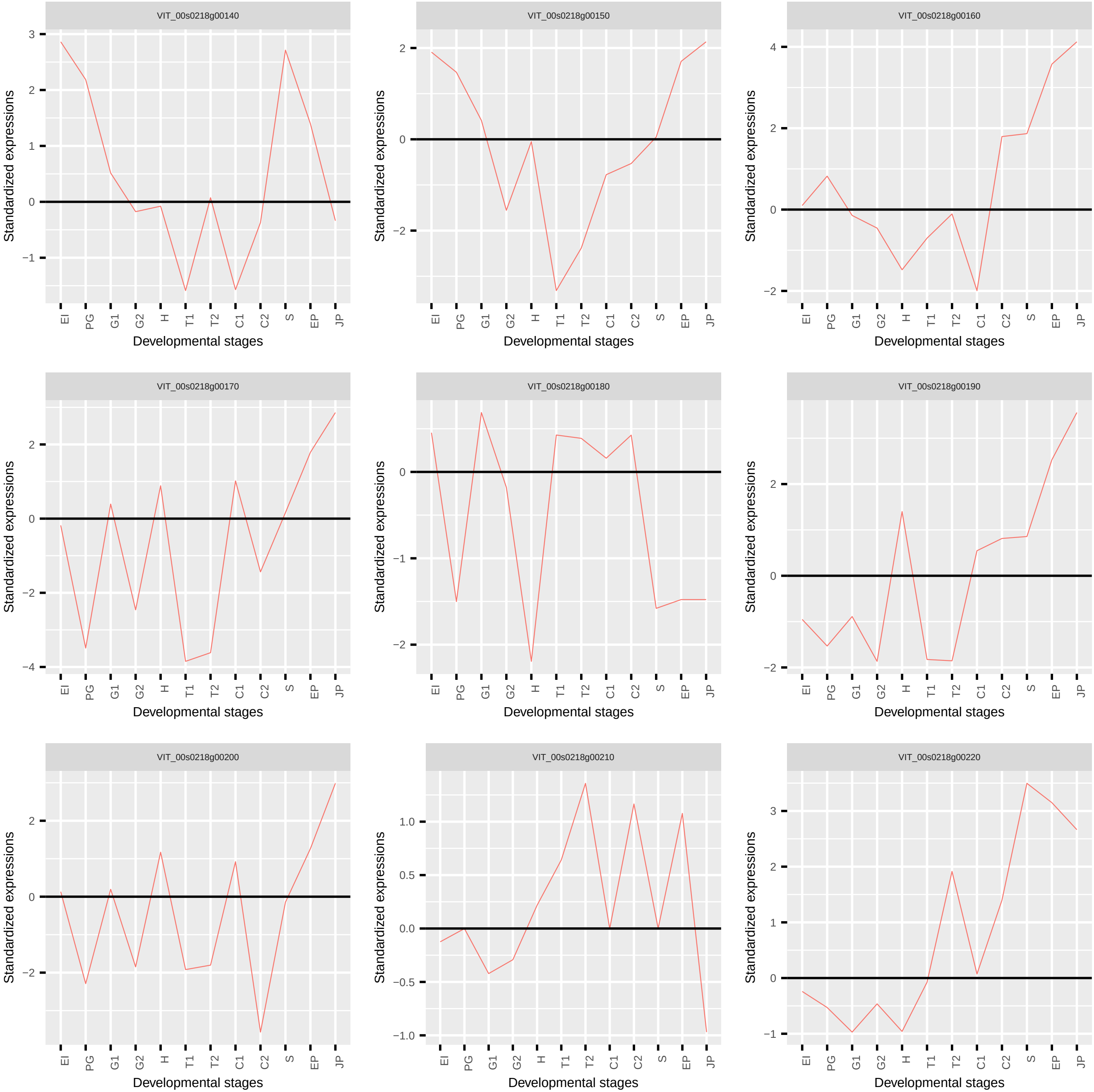
VIT_00s0218g00140
VIT_00s0218g00150
VIT_00s0218g00160
VIT_00s0218g00170
VIT_00s0218g00180
VIT_00s0218g00190
VIT_00s0218g00200
VIT_00s0218g00210
VIT_00s0218g00220
Standardized expressions
Developmental stages
EI
PG
G1
G2
H
T1
T2
C1
C2
S
EP
JP

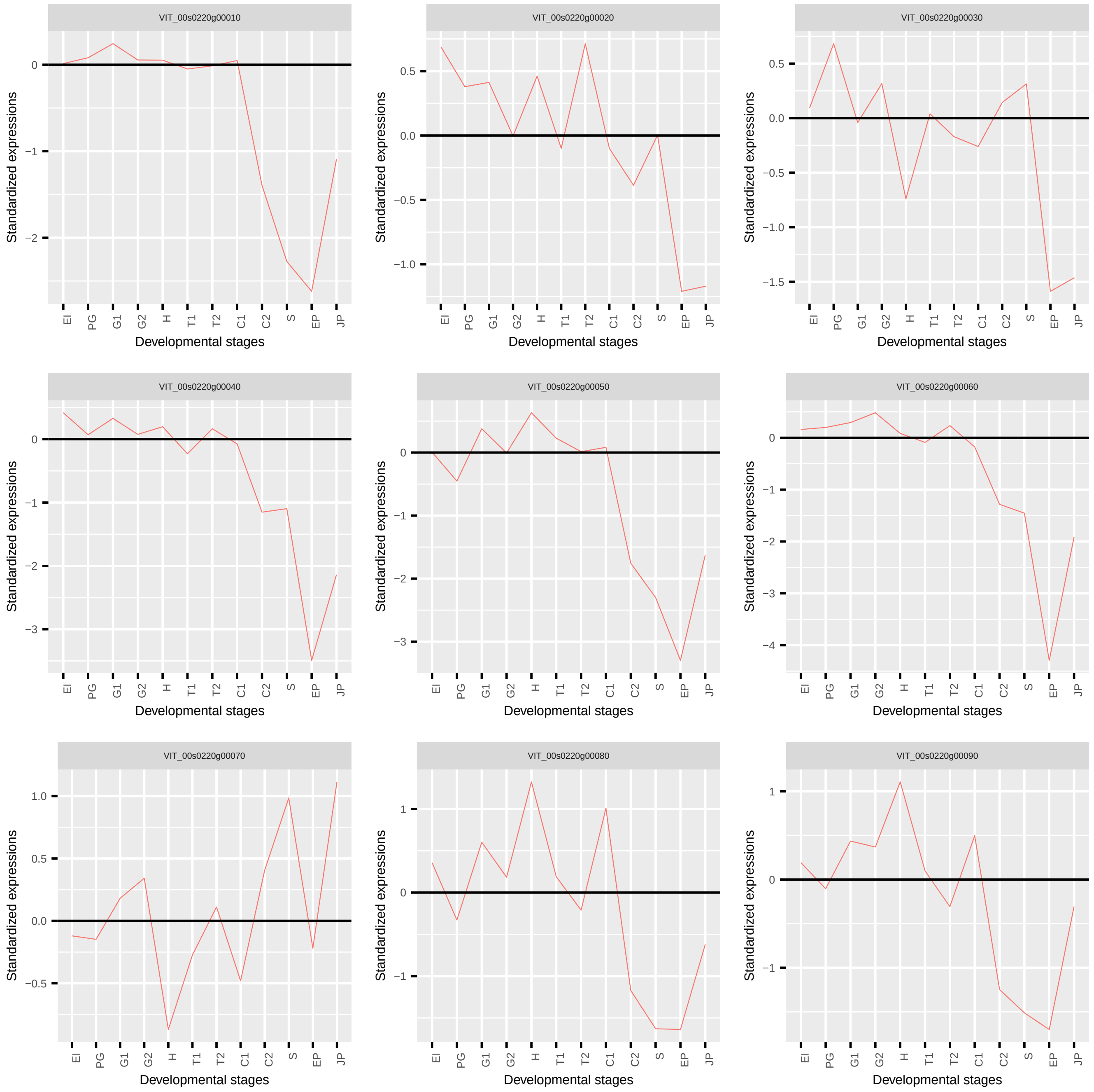

VIT_00s0220g00010
VIT_00s0220g00020
VIT_00s0220g00030
VIT_00s0220g00040
VIT_00s0220g00050
VIT_00s0220g00060
VIT_00s0220g00070
VIT_00s0220g00080
VIT_00s0220g00090
Standardized expressions
Developmental stages
EI PG G1 G2 H T1 T2 C1 C2 S EP JP

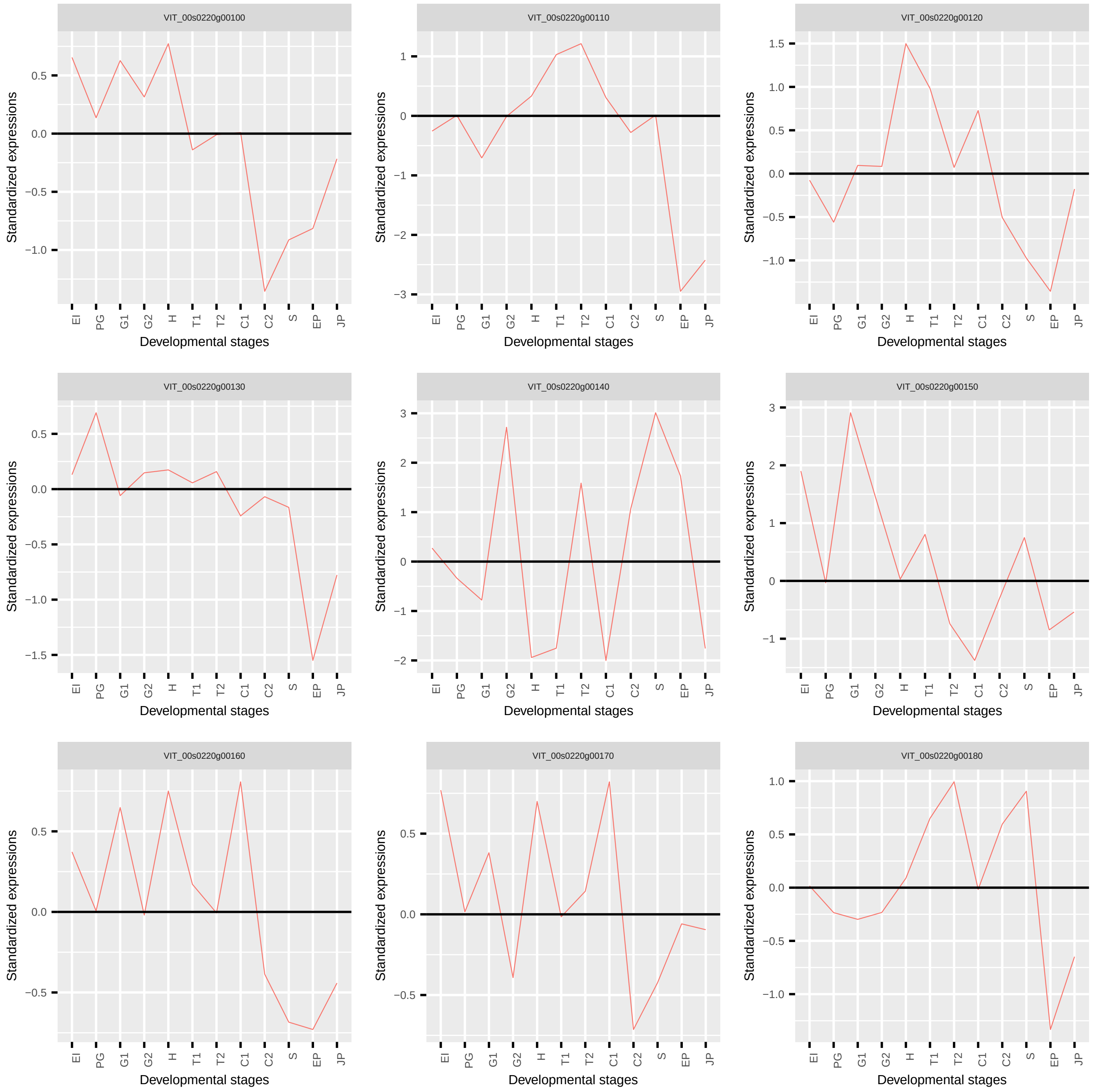
VIT_00s0220g00100
VIT_00s0220g00110
VIT_00s0220g00120
VIT_00s0220g00130
VIT_00s0220g00140
VIT_00s0220g00150
VIT_00s0220g00160
VIT_00s0220g00170
VIT_00s0220g00180
Standardized expressions
Developmental stages
EI PG G1 G2 H T1 T2 C1 C2 S EP JP

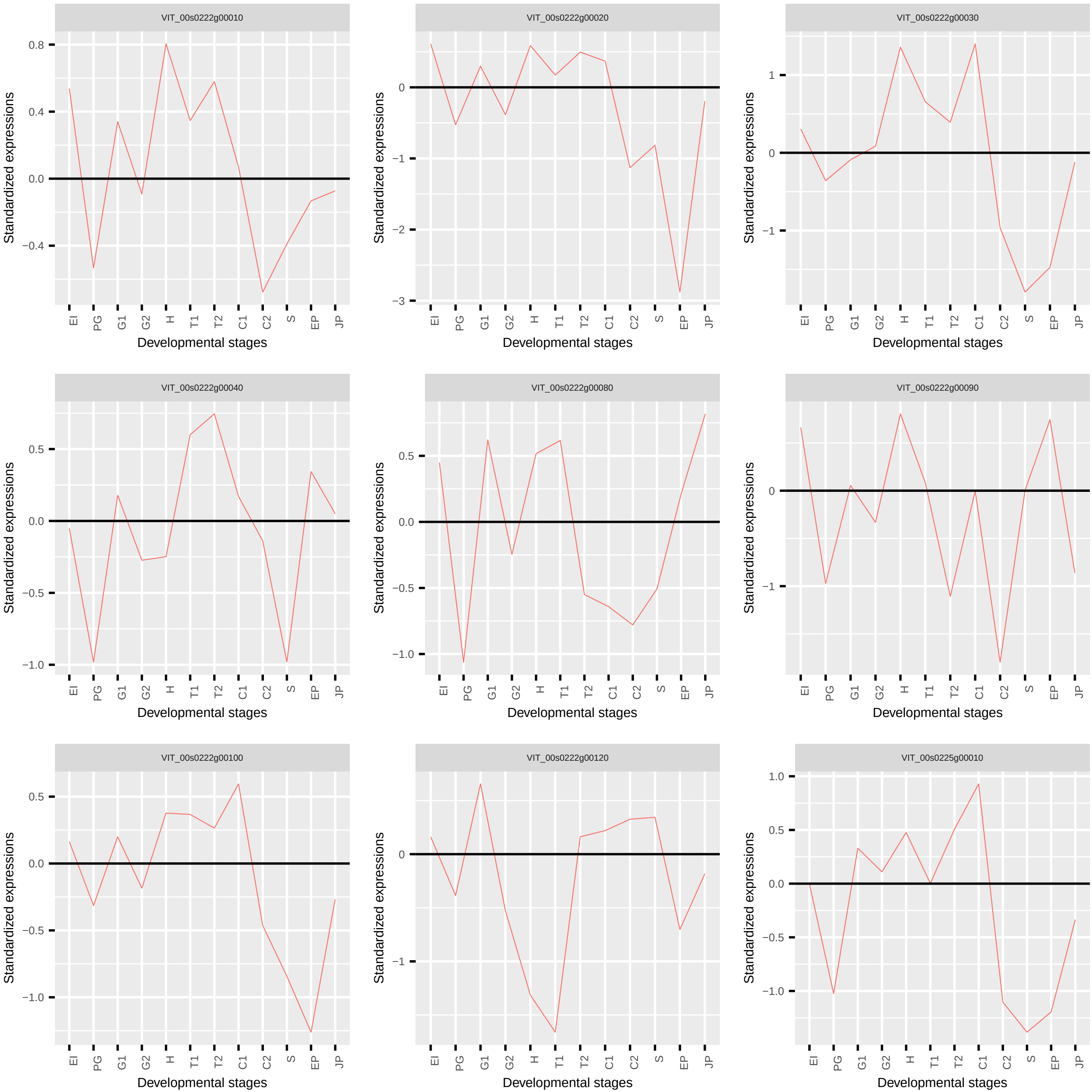
VIT_00s0222g00010
VIT_00s0222g00020
VIT_00s0222g00030
VIT_00s0222g00040
VIT_00s0222g00080
VIT_00s0222g00090
VIT_00s0222g00100
VIT_00s0222g00120
VIT_00s0225g00010
Standardized expressions
Developmental stages
EI PG G1 G2 H T1 T2 C1 C2 S EP JP

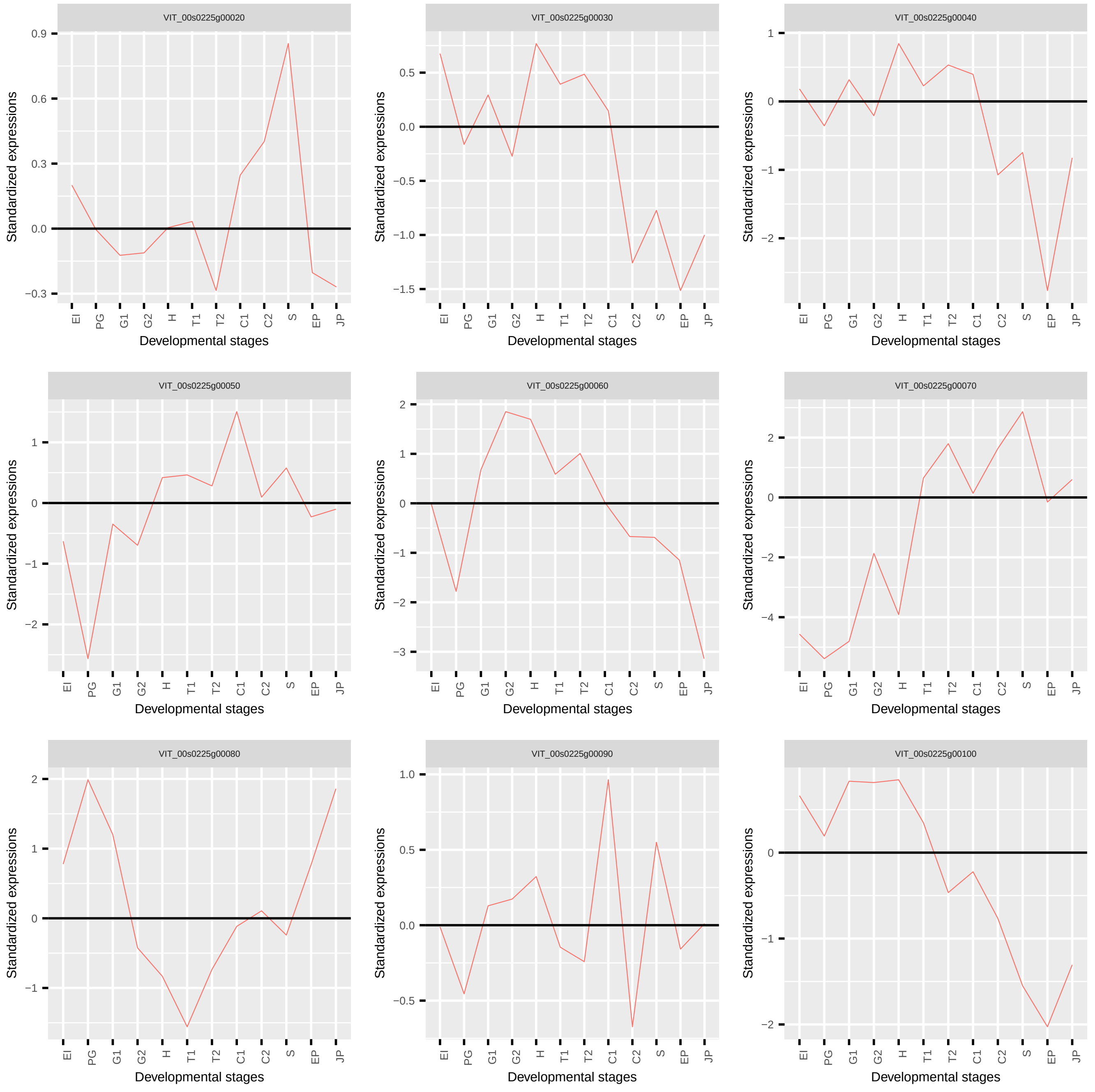
VIT_00s0225g00020
VIT_00s0225g00030
VIT_00s0225g00040
VIT_00s0225g00050
VIT_00s0225g00060
VIT_00s0225g00070
VIT_00s0225g00080
VIT_00s0225g00090
VIT_00s0225g00100
Standardized expressions
Developmental stages
EI PG G1 G2 H T1 T2 C1 C2 S EP JP
0.9 0.6 0.3 0.0 −0.3
0.5 0.0 −0.5 −1.0 −1.5
1 0 −1 −2
2 1 0 −1 −2 −3
2 0 −2 −4
1.0 0.5 0.0 −0.5

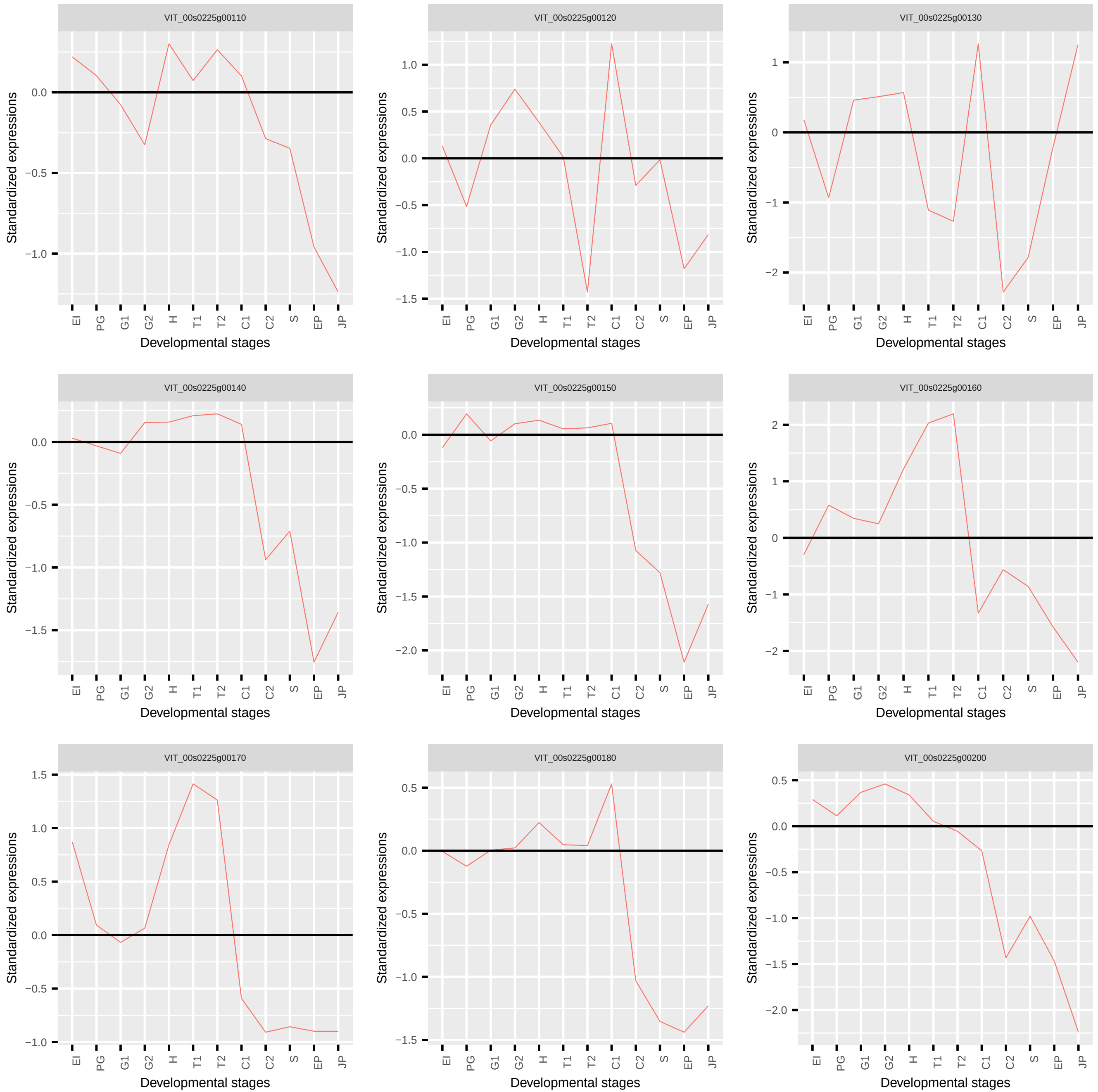
VIT_00s0225g00110
VIT_00s0225g00120
VIT_00s0225g00130
VIT_00s0225g00140
VIT_00s0225g00150
VIT_00s0225g00160
VIT_00s0225g00170
VIT_00s0225g00180
VIT_00s0225g00200
Standardized expressions
Developmental stages
EI PG G1 G2 H T1 T2 C1 C2 S EP JP

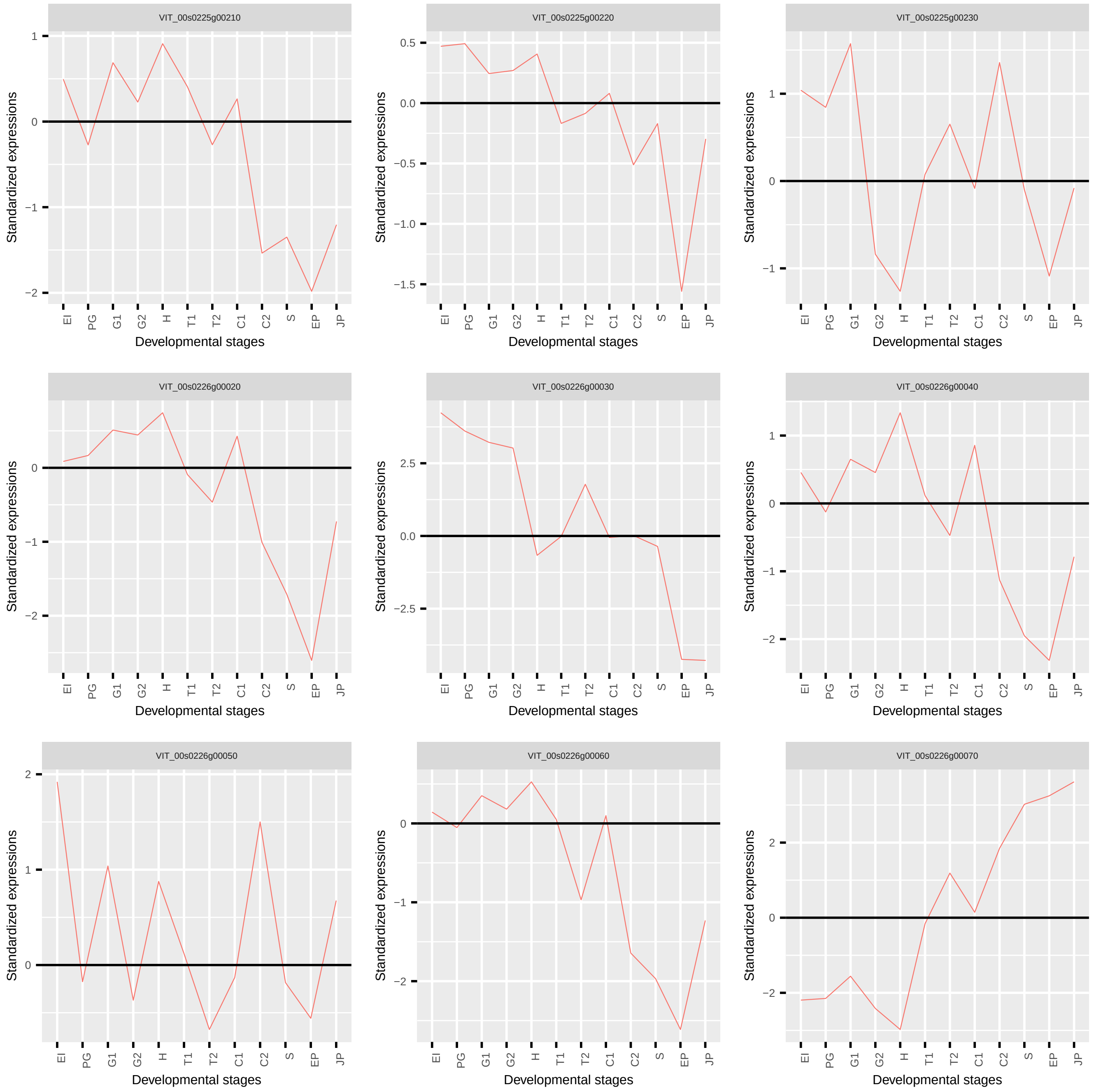
VIT_00s0225g00210
VIT_00s0225g00220
VIT_00s0225g00230
VIT_00s0226g00020
VIT_00s0226g00030
VIT_00s0226g00040
VIT_00s0226g00050
VIT_00s0226g00060
VIT_00s0226g00070
Standardized expressions
Developmental stages
EI PG G1 G2 H T1 T2 C1 C2 S EP JP
1 0 −1 −2
0.5 0.0 −0.5 −1.0 −1.5
1 0 −1
0 −1 −2
2.5 0.0 −2.5
1 0 −1 −2
2 1 0
0 −1 −2
2 0 −2

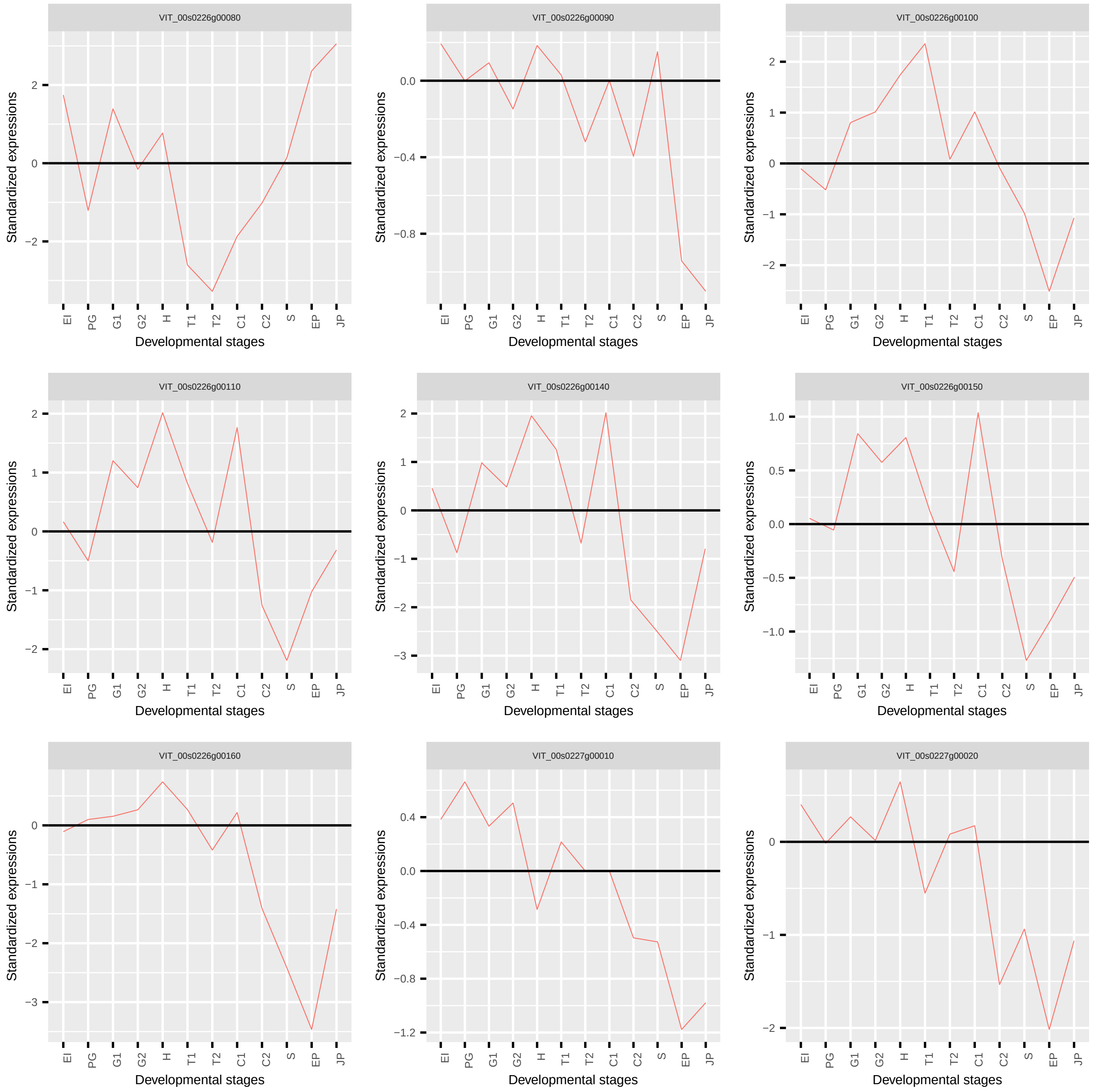
VIT_00s0226g00080
VIT_00s0226g00090
VIT_00s0226g00100
VIT_00s0226g00110
VIT_00s0226g00140
VIT_00s0226g00150
VIT_00s0226g00160
VIT_00s0227g00010
VIT_00s0227g00020
Standardized expressions
Developmental stages
EI PG G1 G2 H T1 T2 C1 C2 S EP JP

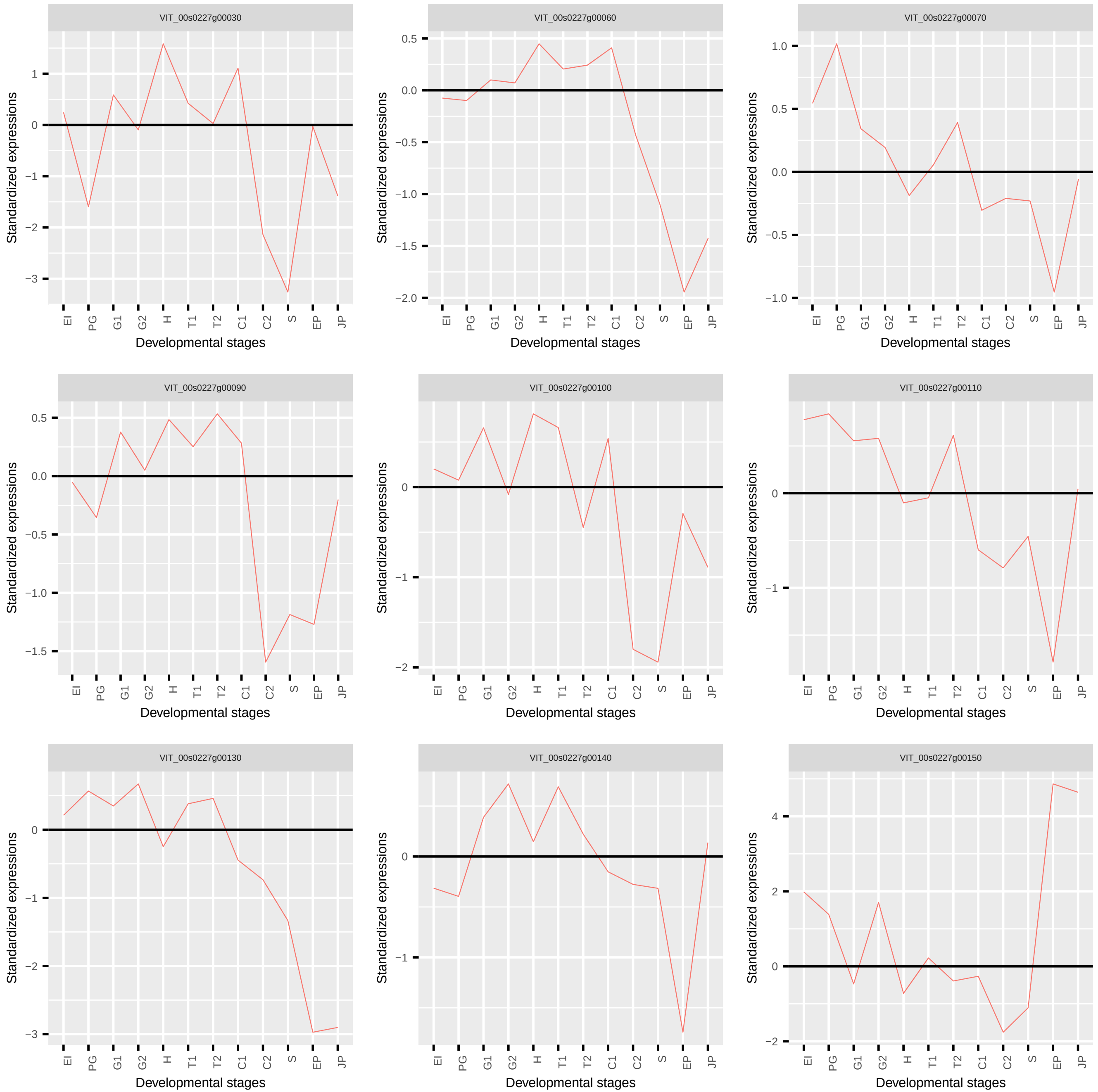
VIT_00s0227g00030
Standardized expressions
1
0
−1
−2
−3
EI PG G1 G2 H T1 T2 C1 C2 S EP JP
Developmental stages
VIT_00s0227g00060
Standardized expressions
0.5
0.0
−0.5
−1.0
−1.5
−2.0
EI PG G1 G2 H T1 T2 C1 C2 S EP JP
Developmental stages
VIT_00s0227g00070
Standardized expressions
1.0
0.5
0.0
−0.5
−1.0
EI PG G1 G2 H T1 T2 C1 C2 S EP JP
Developmental stages
VIT_00s0227g00090
Standardized expressions
0.5
0.0
−0.5
−1.0
−1.5
EI PG G1 G2 H T1 T2 C1 C2 S EP JP
Developmental stages
VIT_00s0227g00100
Standardized expressions
0
−1
−2
EI PG G1 G2 H T1 T2 C1 C2 S EP JP
Developmental stages
VIT_00s0227g00110
Standardized expressions
0
−1
EI PG G1 G2 H T1 T2 C1 C2 S EP JP
Developmental stages
VIT_00s0227g00130
Standardized expressions
0
−1
−2
−3
EI PG G1 G2 H T1 T2 C1 C2 S EP JP
Developmental stages
VIT_00s0227g00140
Standardized expressions
0
−1
EI PG G1 G2 H T1 T2 C1 C2 S EP JP
Developmental stages
VIT_00s0227g00150
Standardized expressions
4
2
0
−2
EI PG G1 G2 H T1 T2 C1 C2 S EP JP
Developmental stages

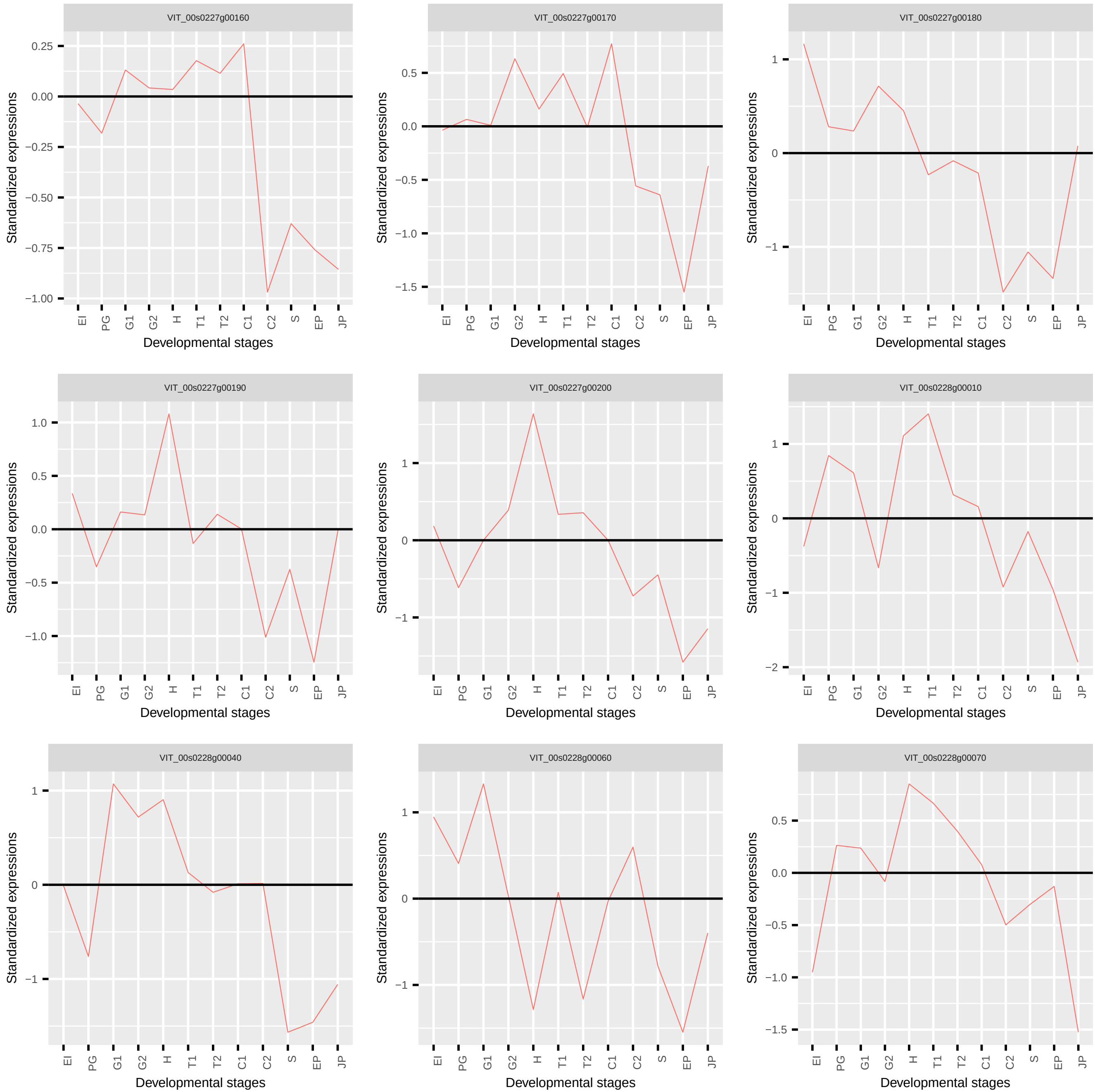

VIT_00s0227g00160
VIT_00s0227g00170
VIT_00s0227g00180
VIT_00s0227g00190
VIT_00s0227g00200
VIT_00s0228g00010
VIT_00s0228g00040
VIT_00s0228g00060
VIT_00s0228g00070
Standardized expressions
Developmental stages
EI PG G1 G2 H T1 T2 C1 C2 S EP JP
0.25 0.00 −0.25 −0.50 −0.75 −1.00
0.5 0.0 −0.5 −1.0 −1.5
1 0 −1 −2

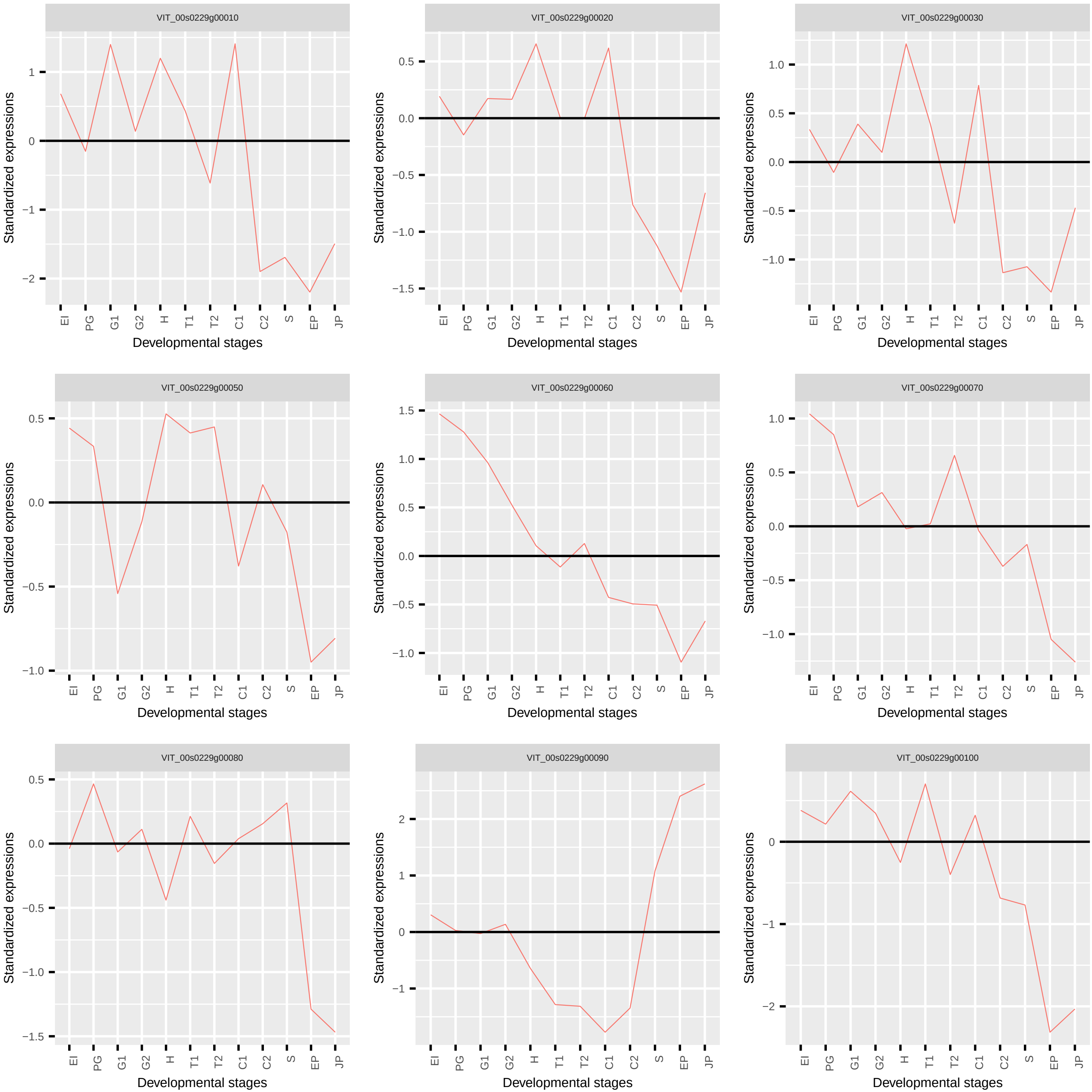

VIT_00s0229g00010
1
0
−1
−2
Standardized expressions
EI PG G1 G2 H T1 T2 C1 C2 S EP JP
Developmental stages
VIT_00s0229g00020
0.5
0.0
−0.5
−1.0
−1.5
Standardized expressions
EI PG G1 G2 H T1 T2 C1 C2 S EP JP
Developmental stages
VIT_00s0229g00030
1.0
0.5
0.0
−0.5
−1.0
Standardized expressions
EI PG G1 G2 H T1 T2 C1 C2 S EP JP
Developmental stages
VIT_00s0229g00050
0.5
0.0
−0.5
−1.0
Standardized expressions
EI PG G1 G2 H T1 T2 C1 C2 S EP JP
Developmental stages
VIT_00s0229g00060
1.5
1.0
0.5
0.0
−0.5
−1.0
Standardized expressions
EI PG G1 G2 H T1 T2 C1 C2 S EP JP
Developmental stages
VIT_00s0229g00070
1.0
0.5
0.0
−0.5
−1.0
Standardized expressions
EI PG G1 G2 H T1 T2 C1 C2 S EP JP
Developmental stages
VIT_00s0229g00080
0.5
0.0
−0.5
−1.0
−1.5
Standardized expressions
EI PG G1 G2 H T1 T2 C1 C2 S EP JP
Developmental stages
VIT_00s0229g00090
2
1
0
−1
Standardized expressions
EI PG G1 G2 H T1 T2 C1 C2 S EP JP
Developmental stages
VIT_00s0229g00100
0
−1
−2
Standardized expressions
EI PG G1 G2 H T1 T2 C1 C2 S EP JP
Developmental stages

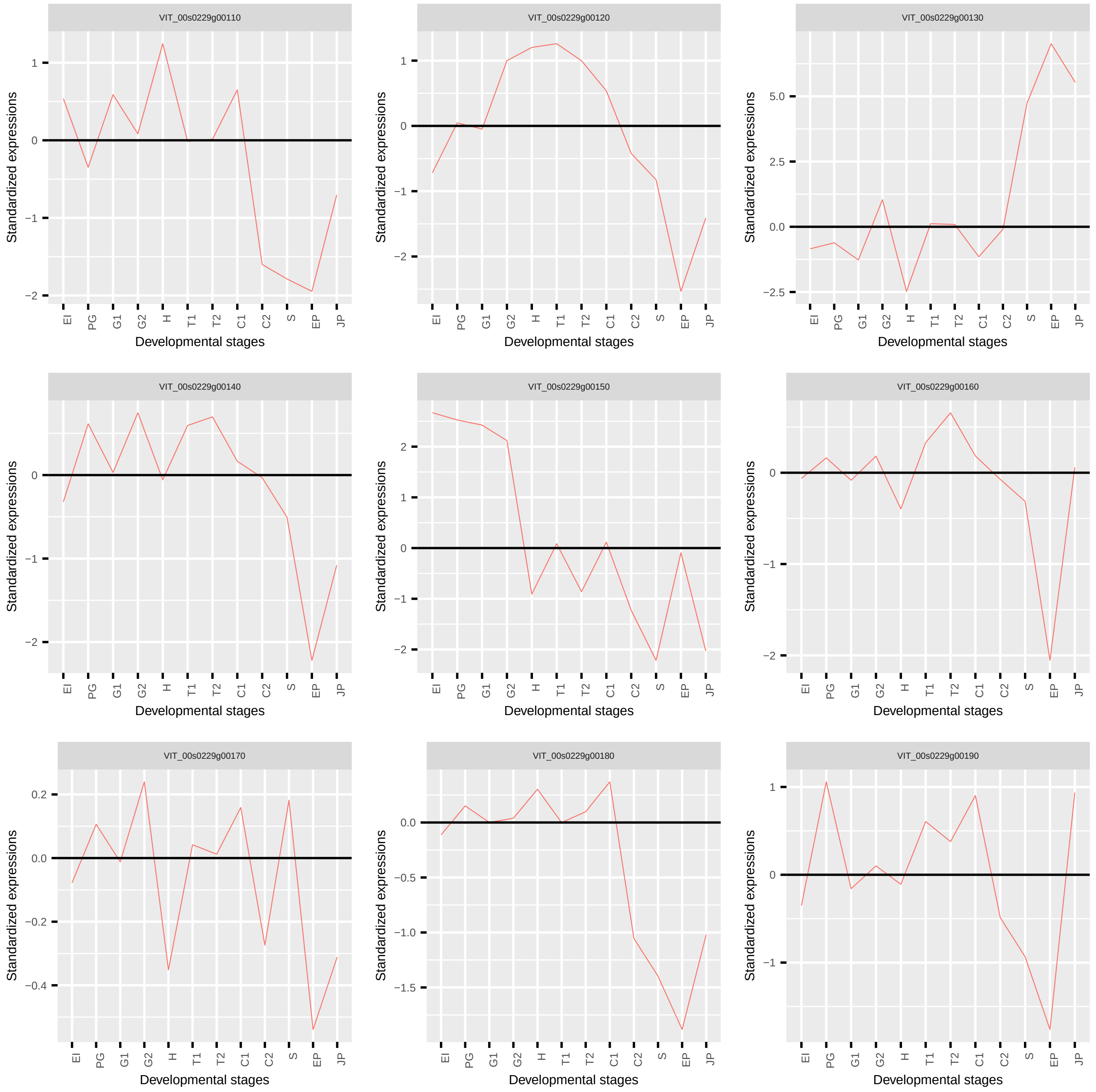
VIT_00s0229g00110
VIT_00s0229g00120
VIT_00s0229g00130
VIT_00s0229g00140
VIT_00s0229g00150
VIT_00s0229g00160
VIT_00s0229g00170
VIT_00s0229g00180
VIT_00s0229g00190
Standardized expressions
Developmental stages
EI PG G1 G2 H T1 T2 C1 C2 S EP JP

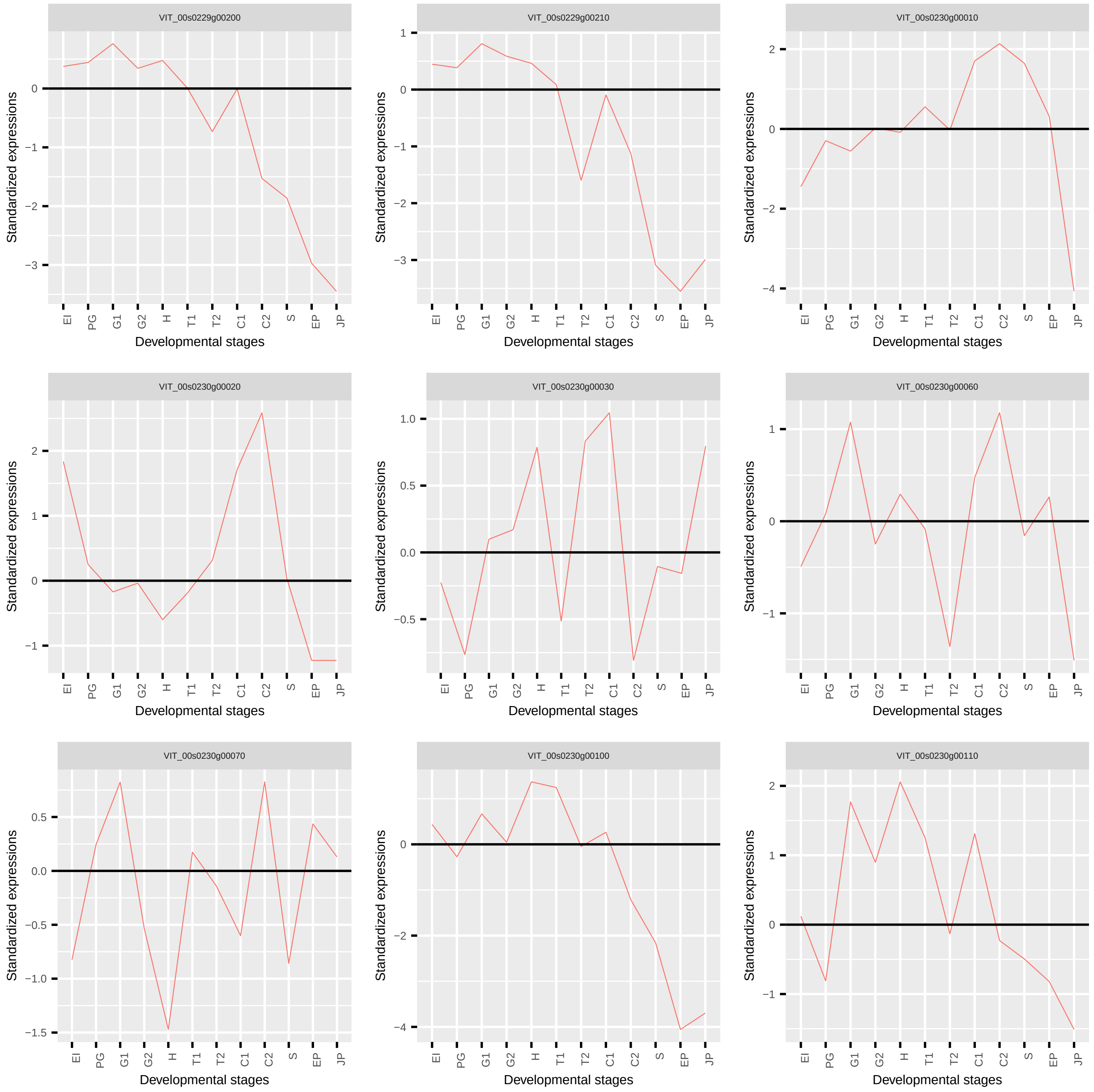

VIT_00s0229g00200
VIT_00s0229g00210
VIT_00s0230g00010
VIT_00s0230g00020
VIT_00s0230g00030
VIT_00s0230g00060
VIT_00s0230g00070
VIT_00s0230g00100
VIT_00s0230g00110
Standardized expressions
Developmental stages
EI
PG
G1
G2
H
T1
T2
C1
C2
S
EP
JP

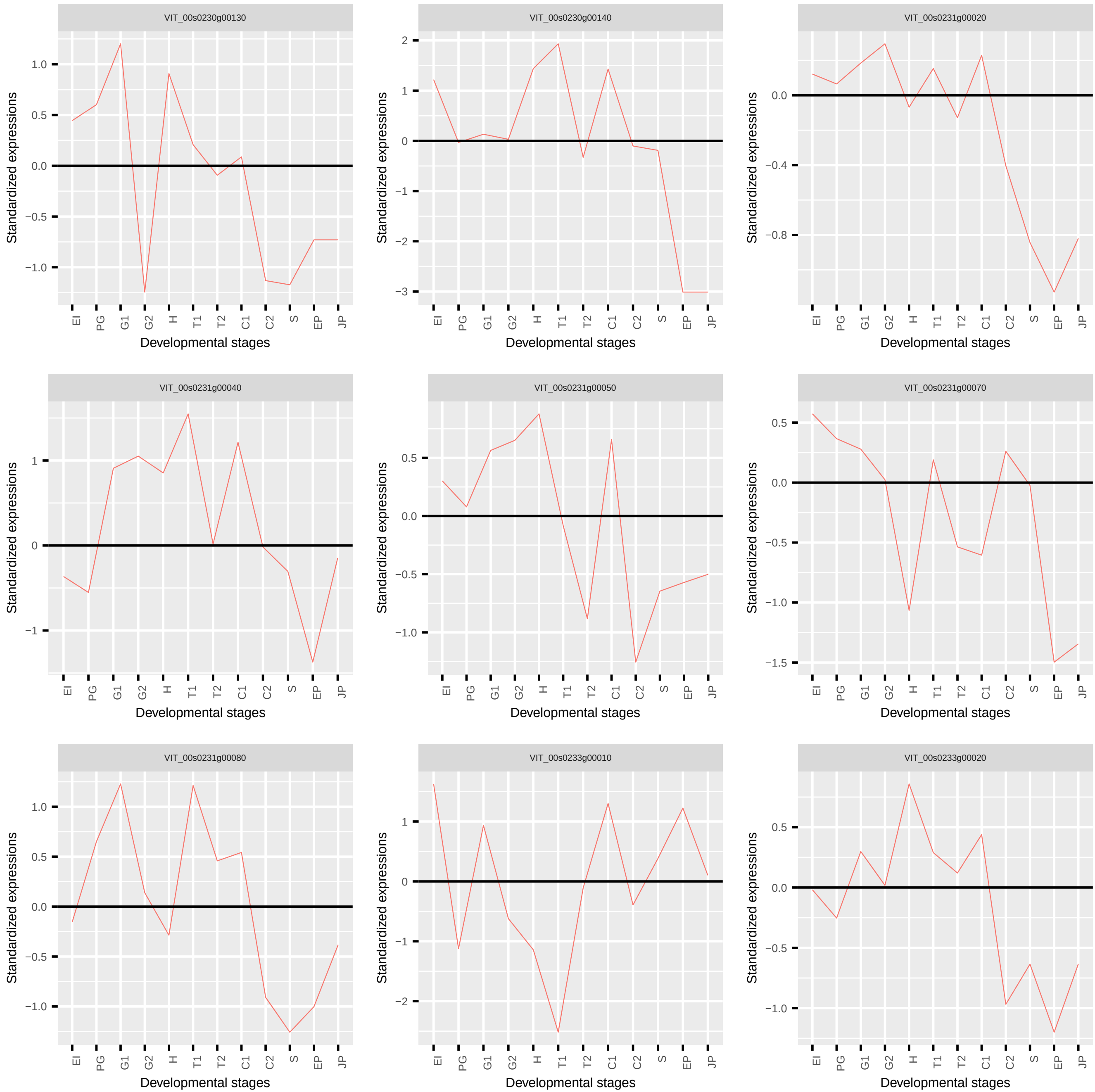
VIT_00s0230g00130
VIT_00s0230g00140
VIT_00s0231g00020
VIT_00s0231g00040
VIT_00s0231g00050
VIT_00s0231g00070
VIT_00s0231g00080
VIT_00s0233g00010
VIT_00s0233g00020
Standardized expressions
Developmental stages
EI
PG
G1
G2
H
T1
T2
C1
C2
S
EP
JP

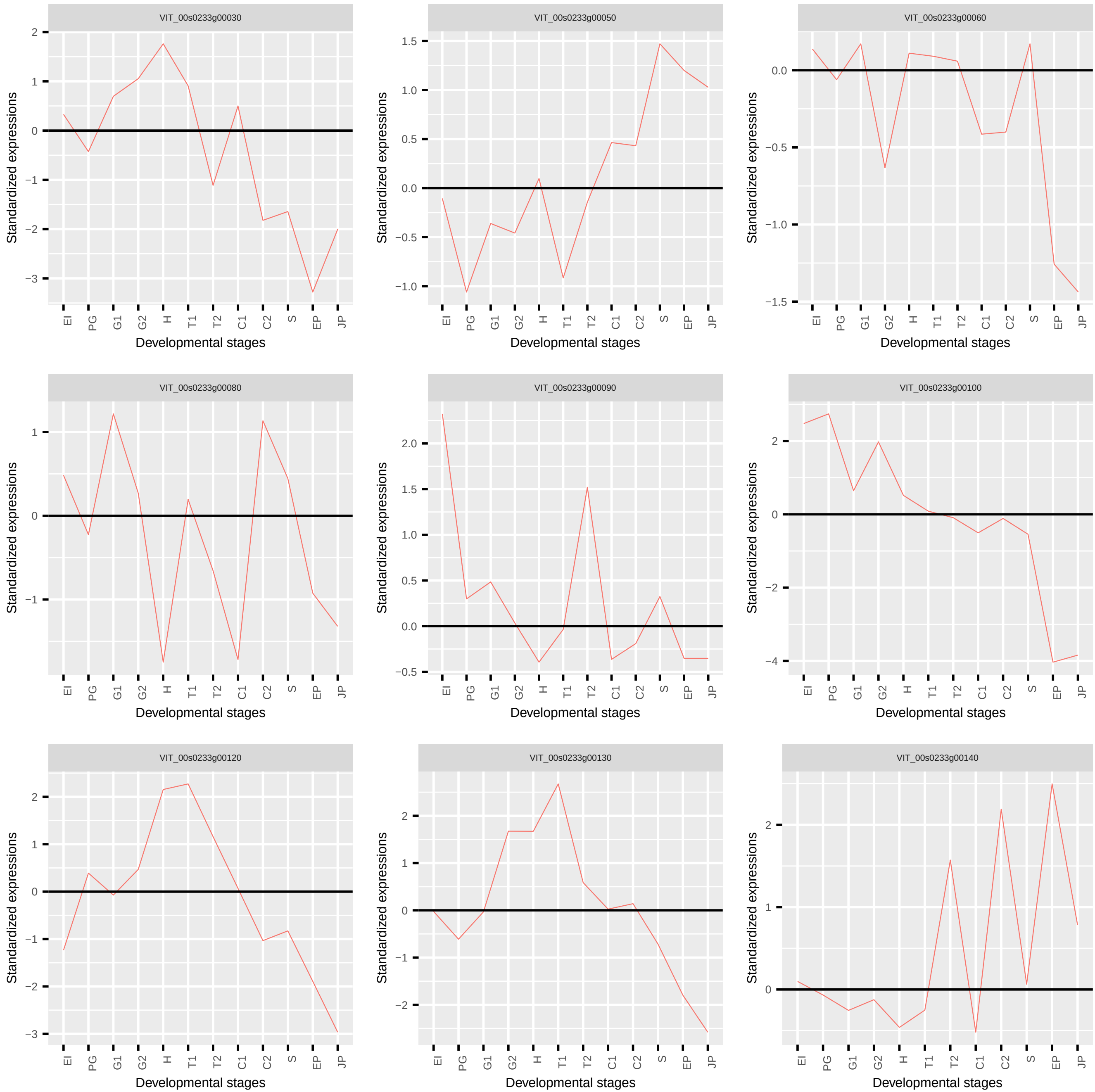
VIT_00s0233g00030
VIT_00s0233g00050
VIT_00s0233g00060
VIT_00s0233g00080
VIT_00s0233g00090
VIT_00s0233g00100
VIT_00s0233g00120
VIT_00s0233g00130
VIT_00s0233g00140
Standardized expressions
Developmental stages
EI PG G1 G2 H T1 T2 C1 C2 S EP JP

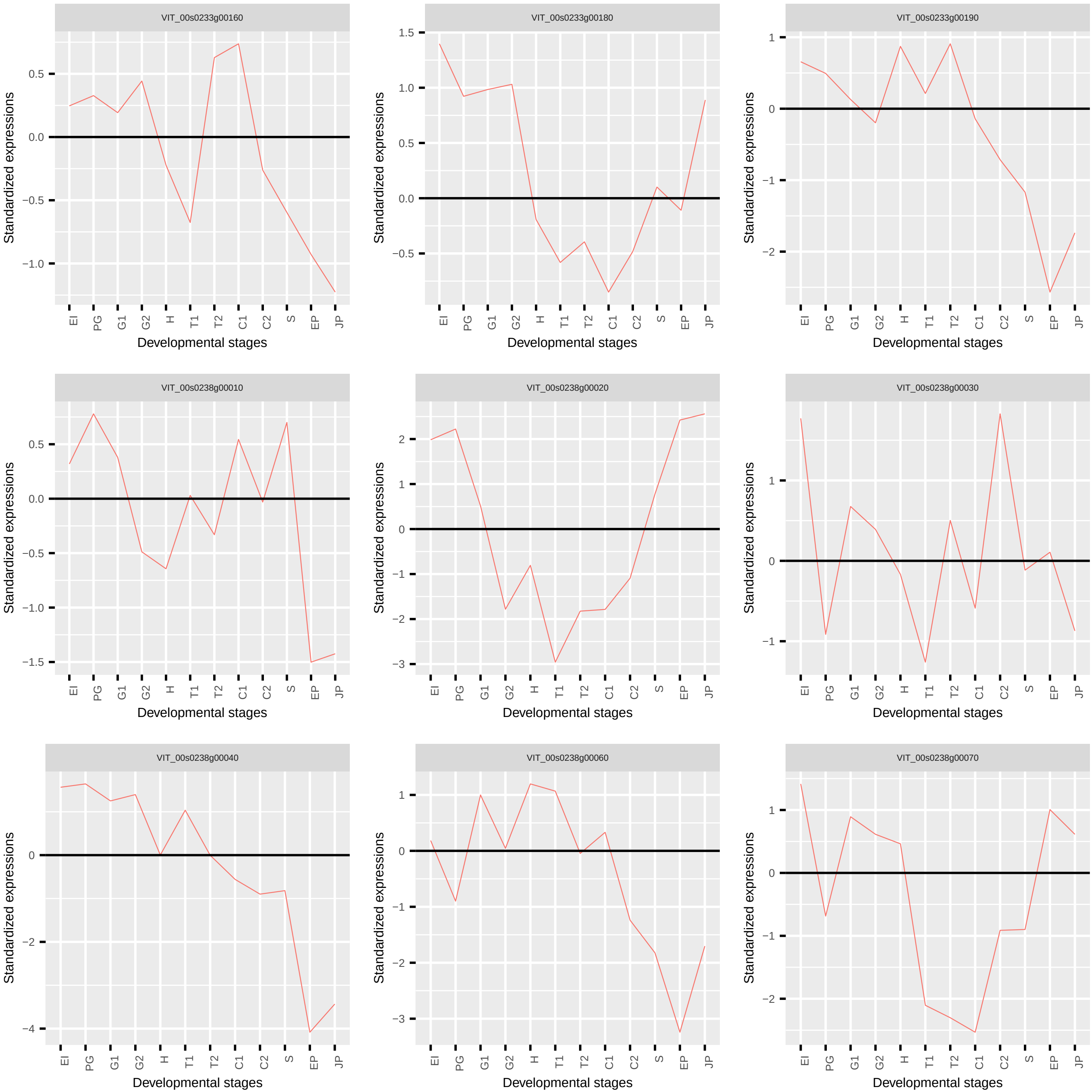

VIT_00s0233g00160
VIT_00s0233g00180
VIT_00s0233g00190
VIT_00s0238g00010
VIT_00s0238g00020
VIT_00s0238g00030
VIT_00s0238g00040
VIT_00s0238g00060
VIT_00s0238g00070
Standardized expressions
Developmental stages
EI PG G1 G2 H T1 T2 C1 C2 S EP JP

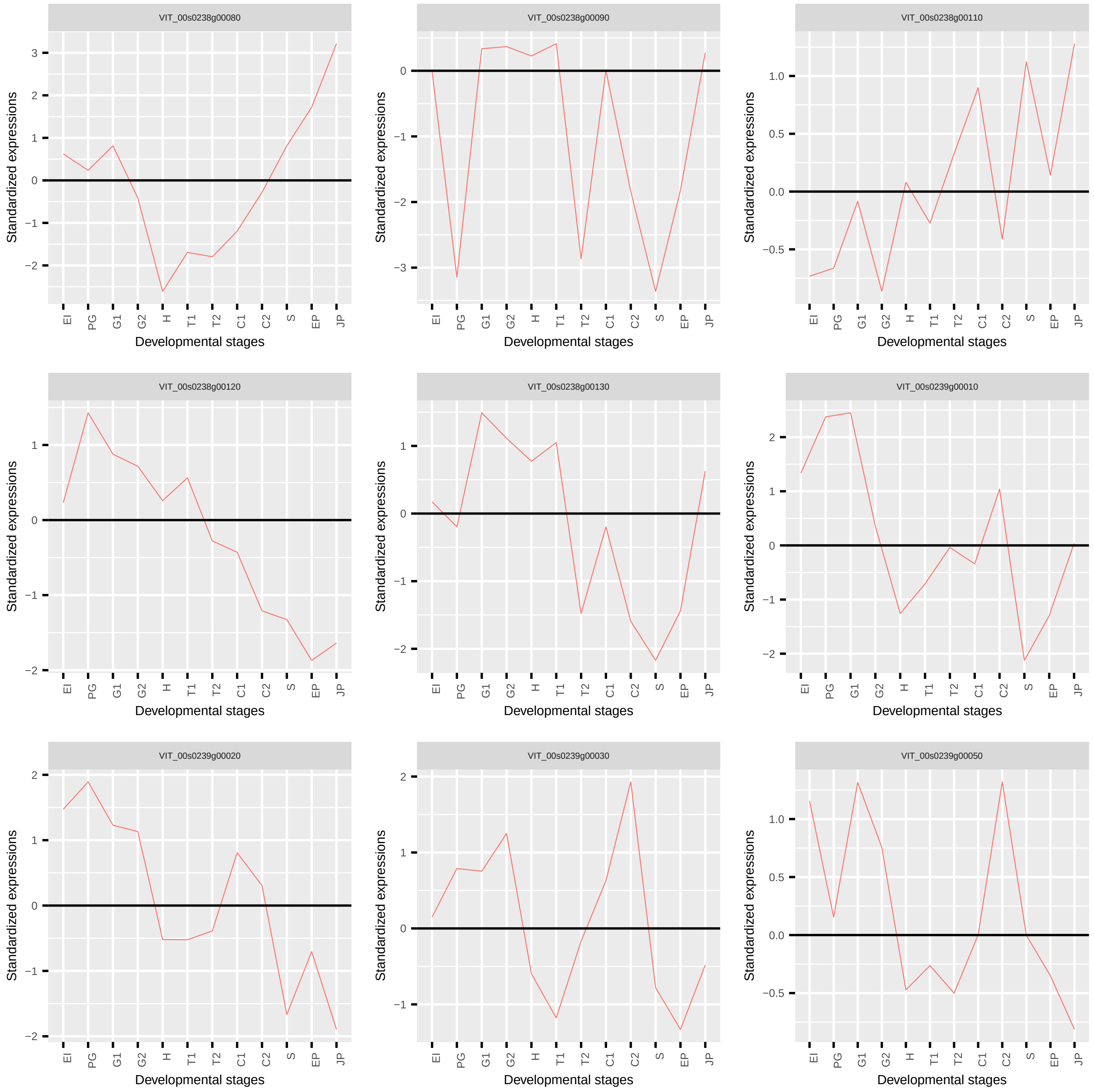
VIT_00s0238g00080
VIT_00s0238g00090
VIT_00s0238g00110
VIT_00s0238g00120
VIT_00s0238g00130
VIT_00s0239g00010
VIT_00s0239g00020
VIT_00s0239g00030
VIT_00s0239g00050
Standardized expressions
Developmental stages
EI PG G1 G2 H T1 T2 C1 C2 S EP JP

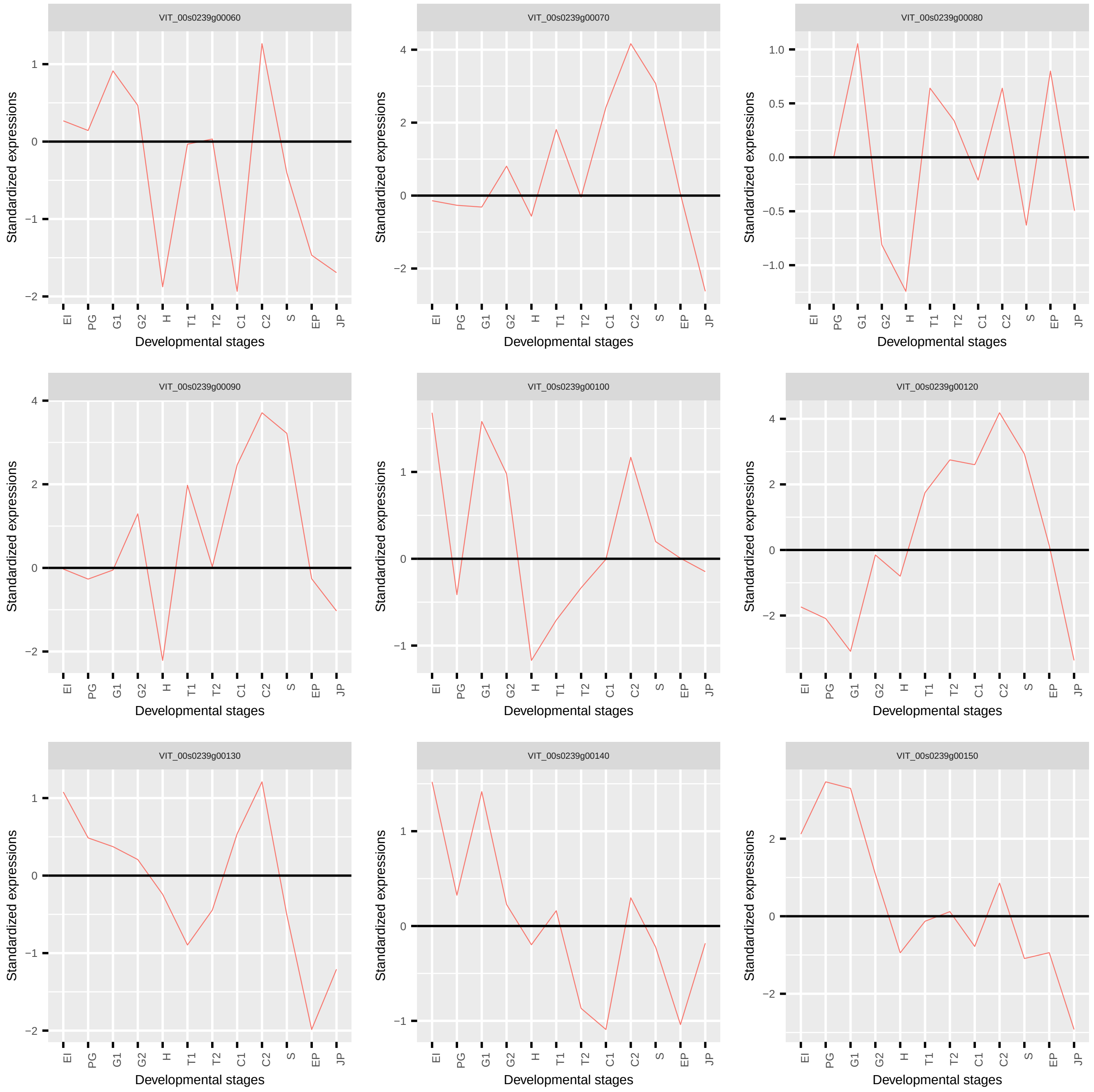
VIT_00s0239g00060
VIT_00s0239g00070
VIT_00s0239g00080
VIT_00s0239g00090
VIT_00s0239g00100
VIT_00s0239g00120
VIT_00s0239g00130
VIT_00s0239g00140
VIT_00s0239g00150
Standardized expressions
Developmental stages
EI PG G1 G2 H T1 T2 C1 C2 S EP JP

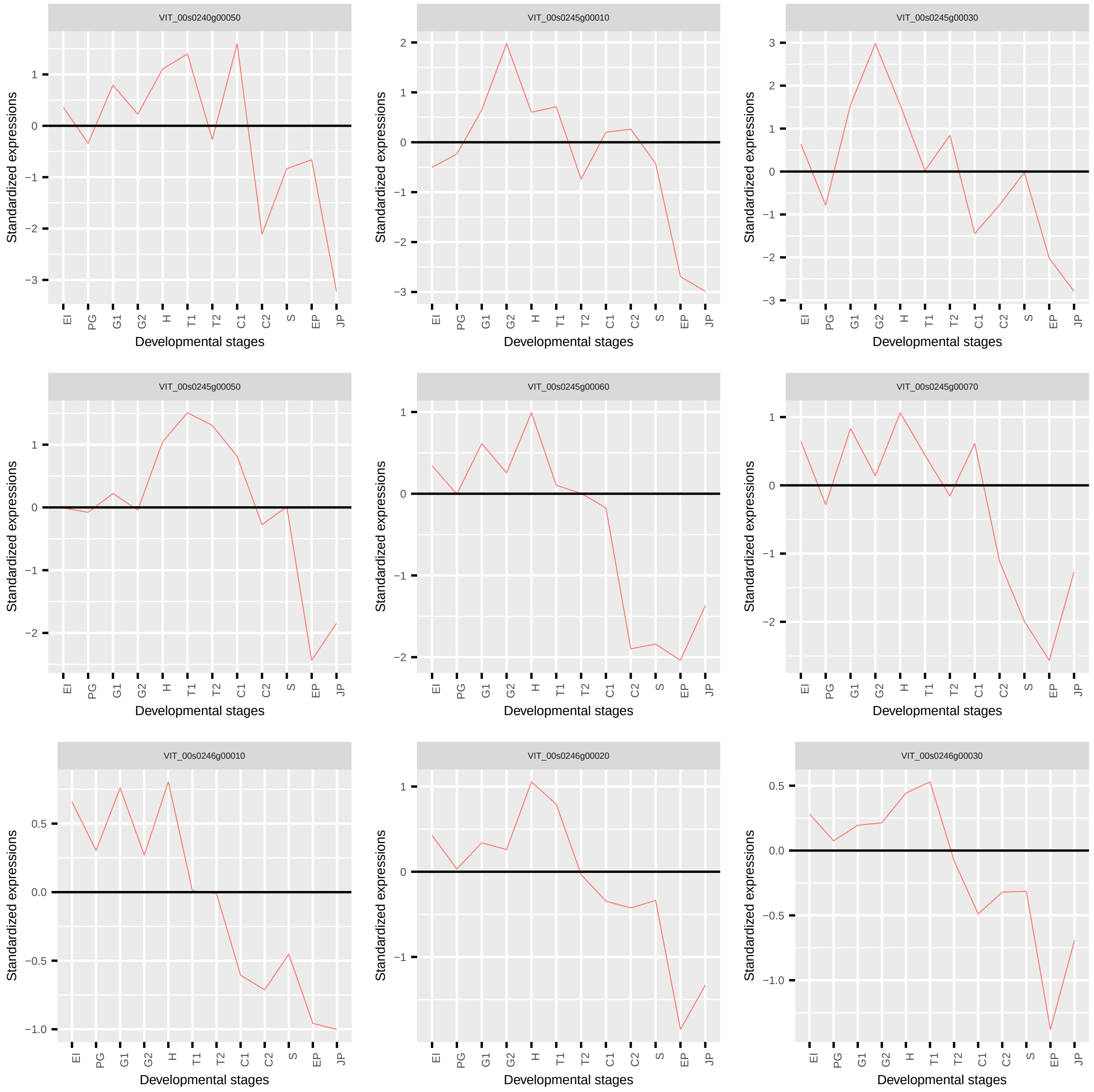
VIT_00s0240g00050
VIT_00s0245g00010
VIT_00s0245g00030
VIT_00s0245g00050
VIT_00s0245g00060
VIT_00s0245g00070
VIT_00s0246g00010
VIT_00s0246g00020
VIT_00s0246g00030
Standardized expressions
Developmental stages
EI PG G1 G2 H T1 T2 C1 C2 S EP JP

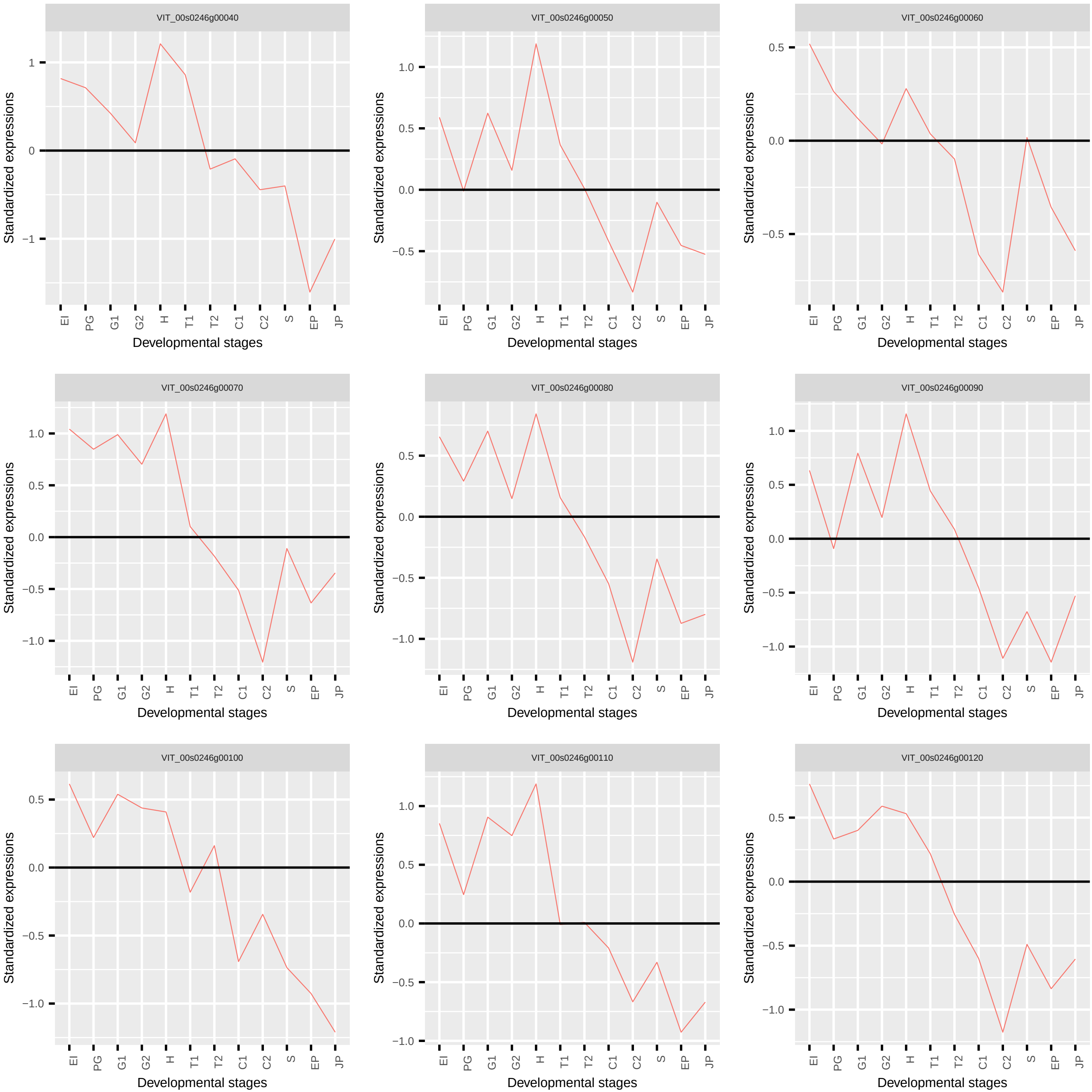

VIT_00s0246g00040
Standardized expressions
1
0
−1
EI PG G1 G2 H T1 T2 C1 C2 S EP JP
Developmental stages
VIT_00s0246g00050
Standardized expressions
1.0
0.5
0.0
−0.5
EI PG G1 G2 H T1 T2 C1 C2 S EP JP
Developmental stages
VIT_00s0246g00060
Standardized expressions
0.5
0.0
−0.5
EI PG G1 G2 H T1 T2 C1 C2 S EP JP
Developmental stages
VIT_00s0246g00070
Standardized expressions
1.0
0.5
0.0
−0.5
−1.0
EI PG G1 G2 H T1 T2 C1 C2 S EP JP
Developmental stages
VIT_00s0246g00080
Standardized expressions
0.5
0.0
−0.5
−1.0
EI PG G1 G2 H T1 T2 C1 C2 S EP JP
Developmental stages
VIT_00s0246g00090
Standardized expressions
1.0
0.5
0.0
−0.5
−1.0
EI PG G1 G2 H T1 T2 C1 C2 S EP JP
Developmental stages
VIT_00s0246g00100
Standardized expressions
0.5
0.0
−0.5
−1.0
EI PG G1 G2 H T1 T2 C1 C2 S EP JP
Developmental stages
VIT_00s0246g00110
Standardized expressions
1.0
0.5
0.0
−0.5
−1.0
EI PG G1 G2 H T1 T2 C1 C2 S EP JP
Developmental stages
VIT_00s0246g00120
Standardized expressions
0.5
0.0
−0.5
−1.0
EI PG G1 G2 H T1 T2 C1 C2 S EP JP
Developmental stages

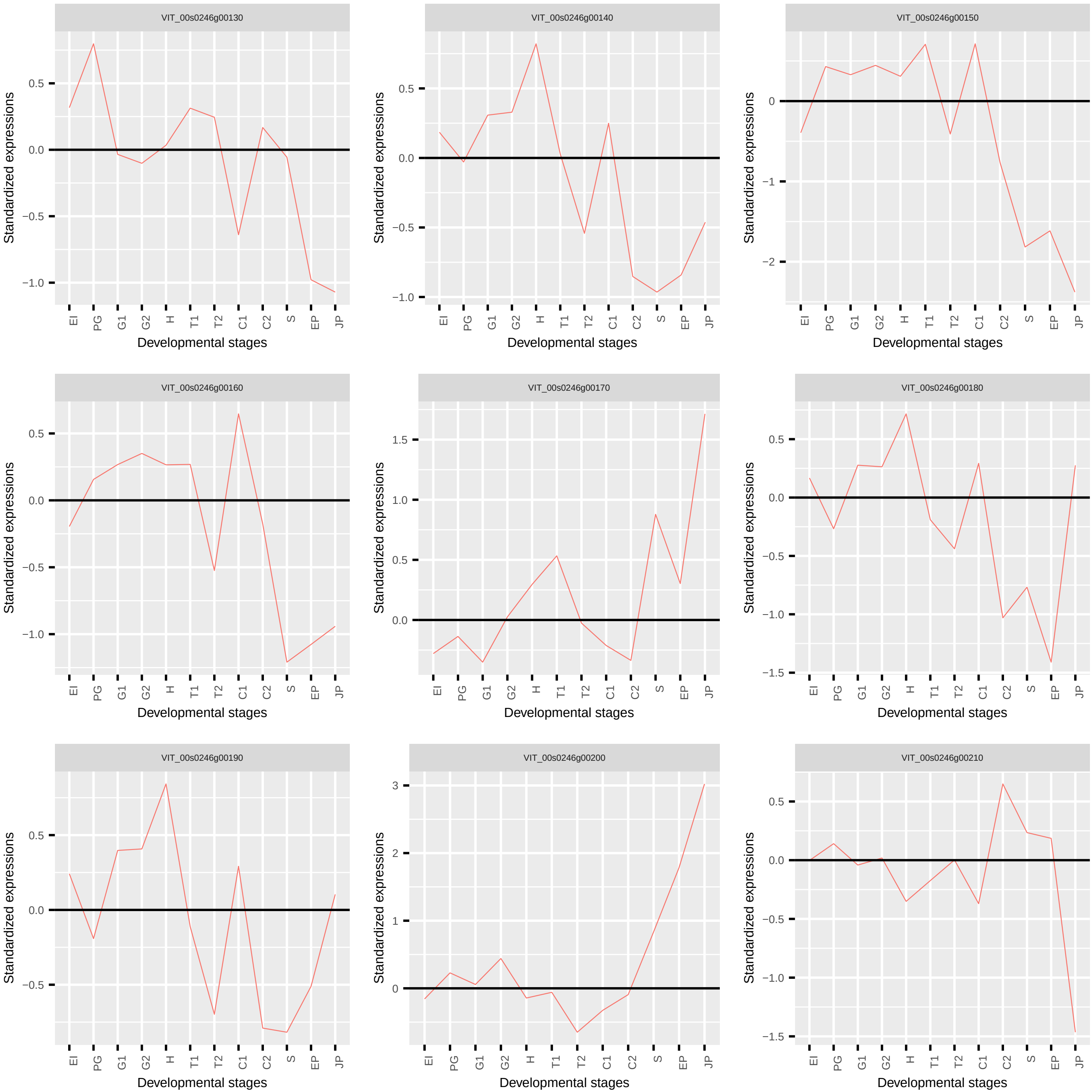
VIT_00s0246g00130
VIT_00s0246g00140
VIT_00s0246g00150
VIT_00s0246g00160
VIT_00s0246g00170
VIT_00s0246g00180
VIT_00s0246g00190
VIT_00s0246g00200
VIT_00s0246g00210
Standardized expressions
Developmental stages
EI PG G1 G2 H T1 T2 C1 C2 S EP JP

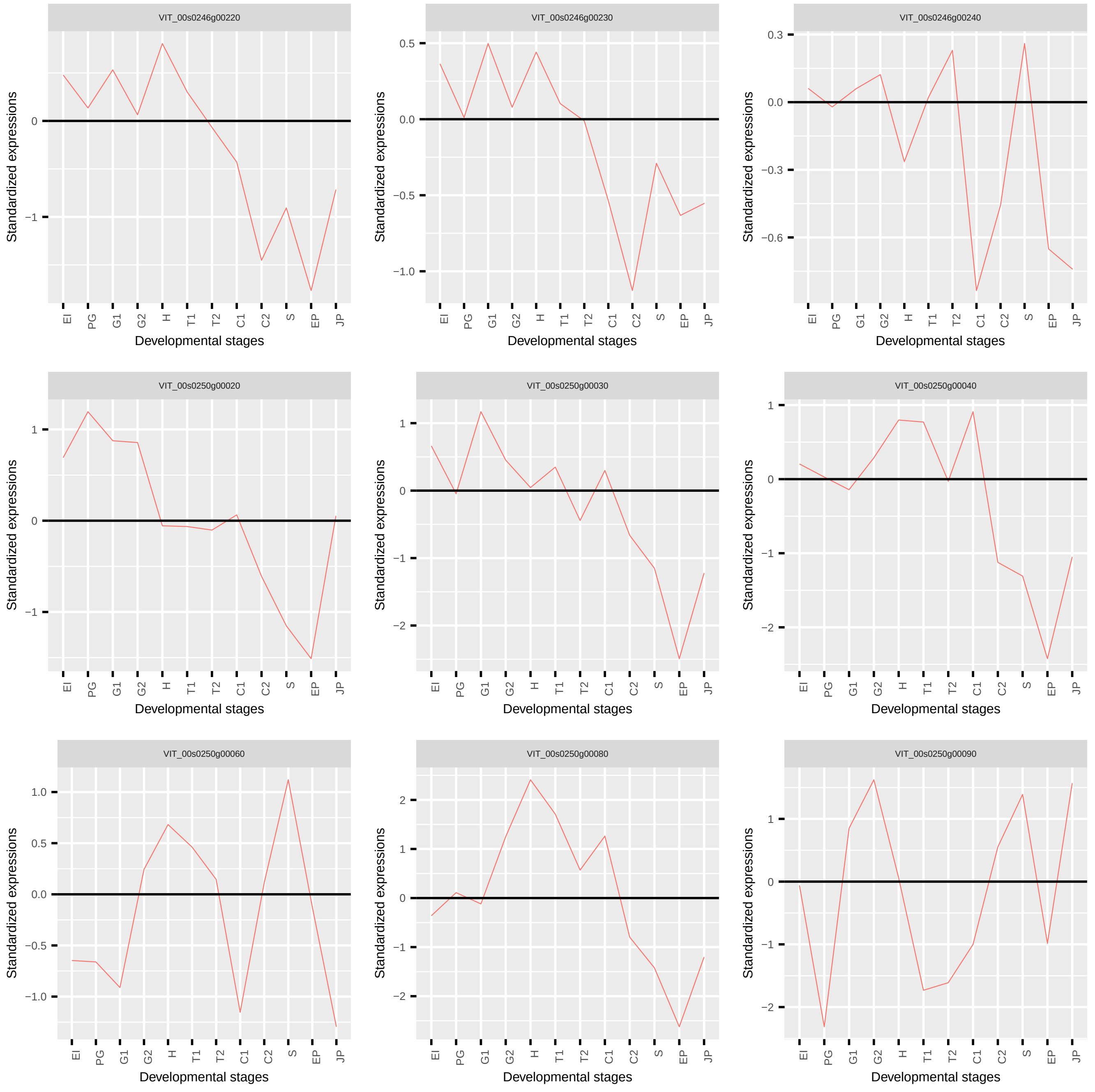
VIT_00s0246g00220
VIT_00s0246g00230
VIT_00s0246g00240
VIT_00s0250g00020
VIT_00s0250g00030
VIT_00s0250g00040
VIT_00s0250g00060
VIT_00s0250g00080
VIT_00s0250g00090
Standardized expressions
Developmental stages
EI PG G1 G2 H T1 T2 C1 C2 S EP JP

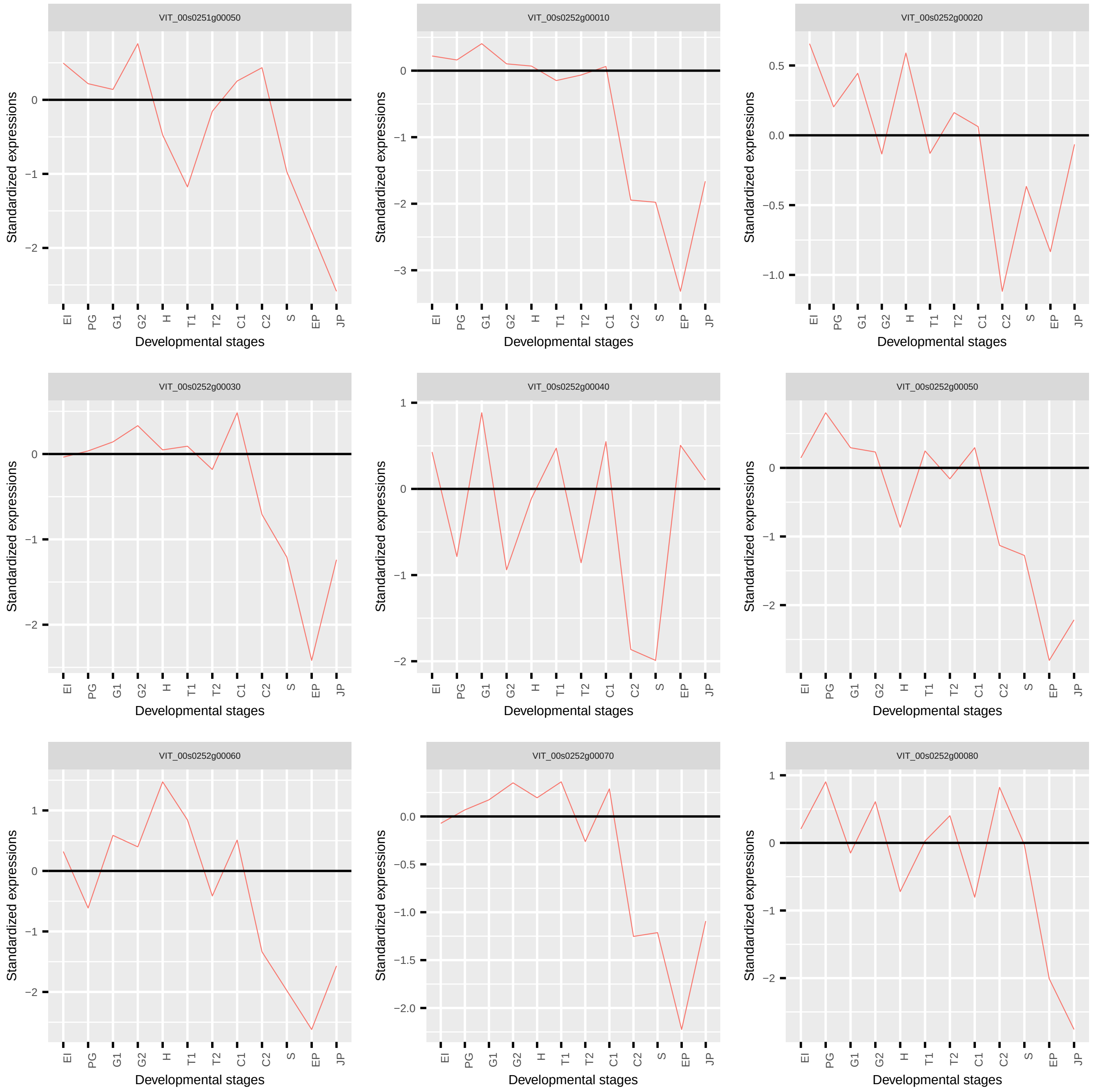
VIT_00s0251g00050
VIT_00s0252g00010
VIT_00s0252g00020
VIT_00s0252g00030
VIT_00s0252g00040
VIT_00s0252g00050
VIT_00s0252g00060
VIT_00s0252g00070
VIT_00s0252g00080
Standardized expressions
Developmental stages
EI PG G1 G2 H T1 T2 C1 C2 S EP JP

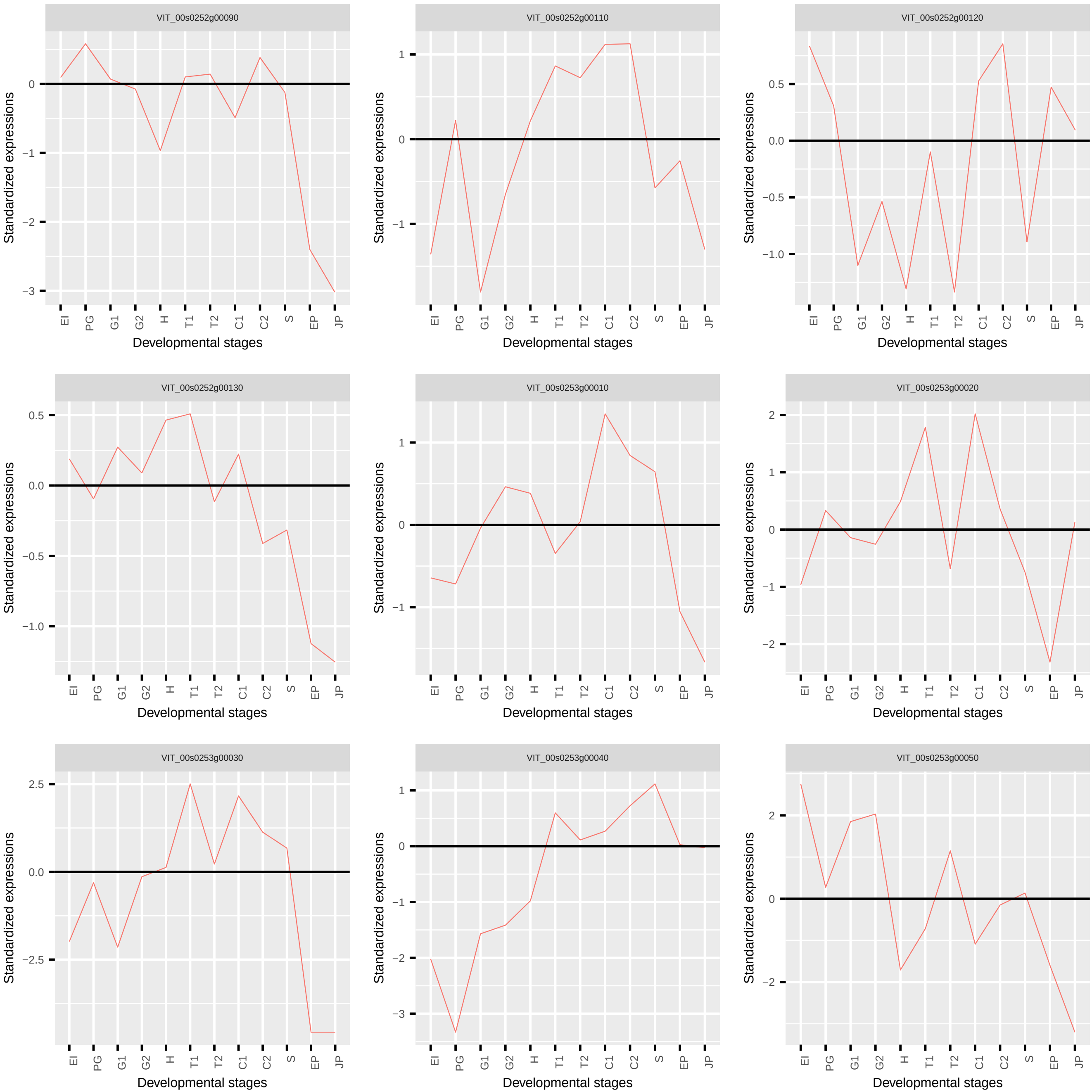
VIT_00s0252g00090
VIT_00s0252g00110
VIT_00s0252g00120
VIT_00s0252g00130
VIT_00s0253g00010
VIT_00s0253g00020
VIT_00s0253g00030
VIT_00s0253g00040
VIT_00s0253g00050
Standardized expressions
Developmental stages
EI PG G1 G2 H T1 T2 C1 C2 S EP JP

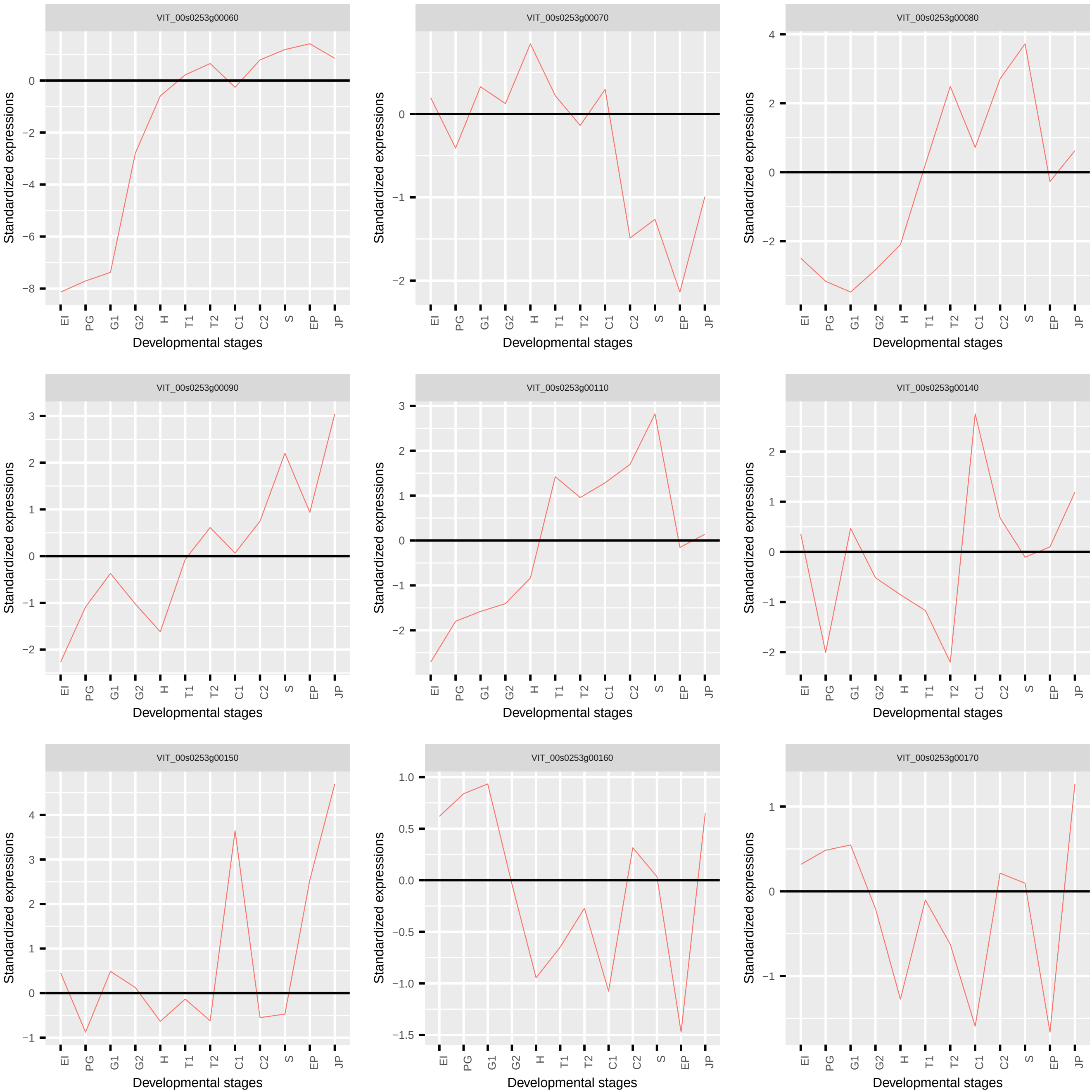
VIT_00s0253g00060
VIT_00s0253g00070
VIT_00s0253g00080
VIT_00s0253g00090
VIT_00s0253g00110
VIT_00s0253g00140
VIT_00s0253g00150
VIT_00s0253g00160
VIT_00s0253g00170
Standardized expressions
Developmental stages
EI PG G1 G2 H T1 T2 C1 C2 S EP JP

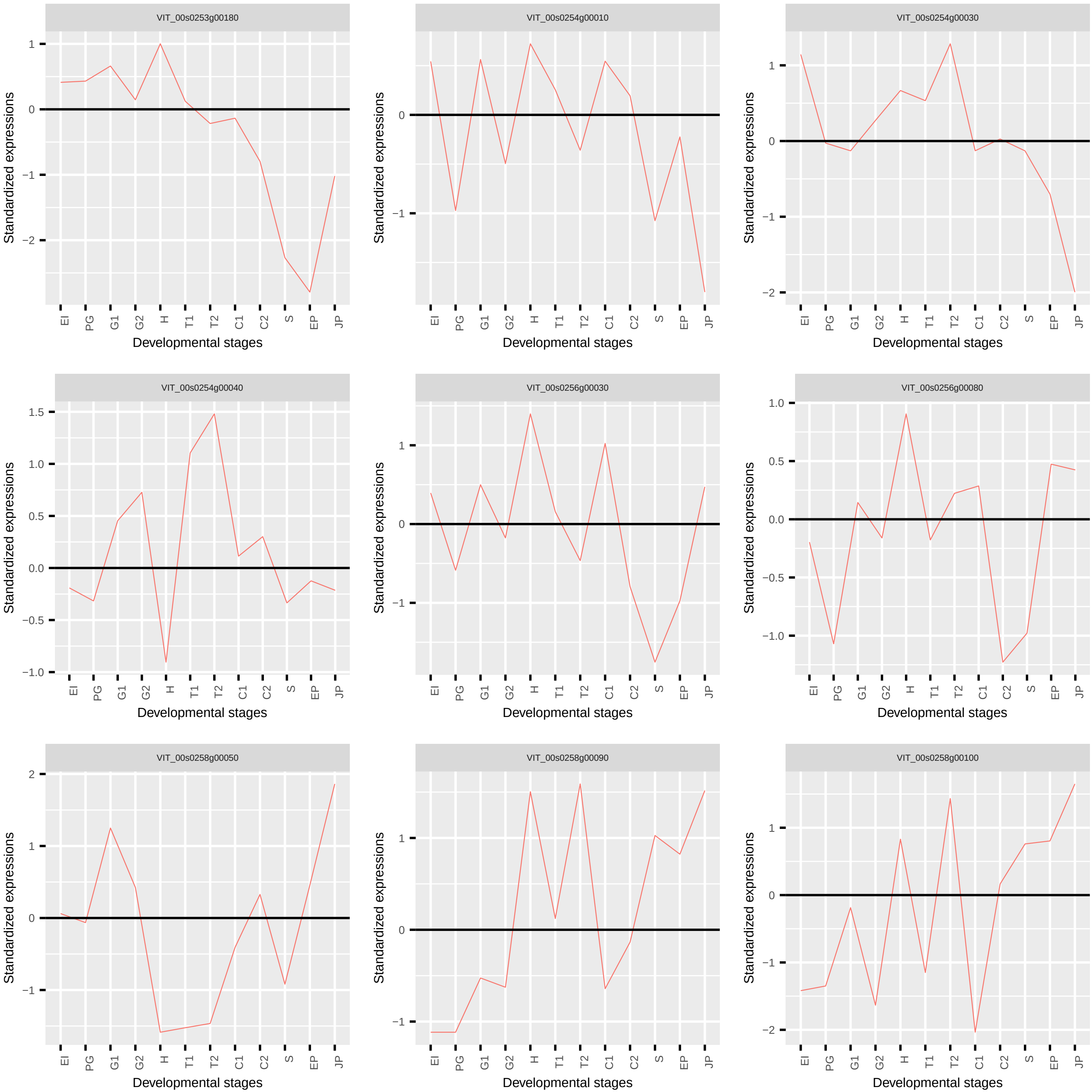
VIT_00s0253g00180
Standardized expressions
1
0
−1
−2
EI PG G1 G2 H T1 T2 C1 C2 S EP JP
Developmental stages
VIT_00s0254g00010
Standardized expressions
0
−1
EI PG G1 G2 H T1 T2 C1 C2 S EP JP
Developmental stages
VIT_00s0254g00030
Standardized expressions
1
0
−1
−2
EI PG G1 G2 H T1 T2 C1 C2 S EP JP
Developmental stages
VIT_00s0254g00040
Standardized expressions
1.5
1.0
0.5
0.0
−0.5
−1.0
EI PG G1 G2 H T1 T2 C1 C2 S EP JP
Developmental stages
VIT_00s0256g00030
Standardized expressions
1
0
−1
EI PG G1 G2 H T1 T2 C1 C2 S EP JP
Developmental stages
VIT_00s0256g00080
Standardized expressions
1.0
0.5
0.0
−0.5
−1.0
EI PG G1 G2 H T1 T2 C1 C2 S EP JP
Developmental stages
VIT_00s0258g00050
Standardized expressions
2
1
0
−1
EI PG G1 G2 H T1 T2 C1 C2 S EP JP
Developmental stages
VIT_00s0258g00090
Standardized expressions
1
0
−1
EI PG G1 G2 H T1 T2 C1 C2 S EP JP
Developmental stages
VIT_00s0258g00100
Standardized expressions
1
0
−1
−2
EI PG G1 G2 H T1 T2 C1 C2 S EP JP
Developmental stages

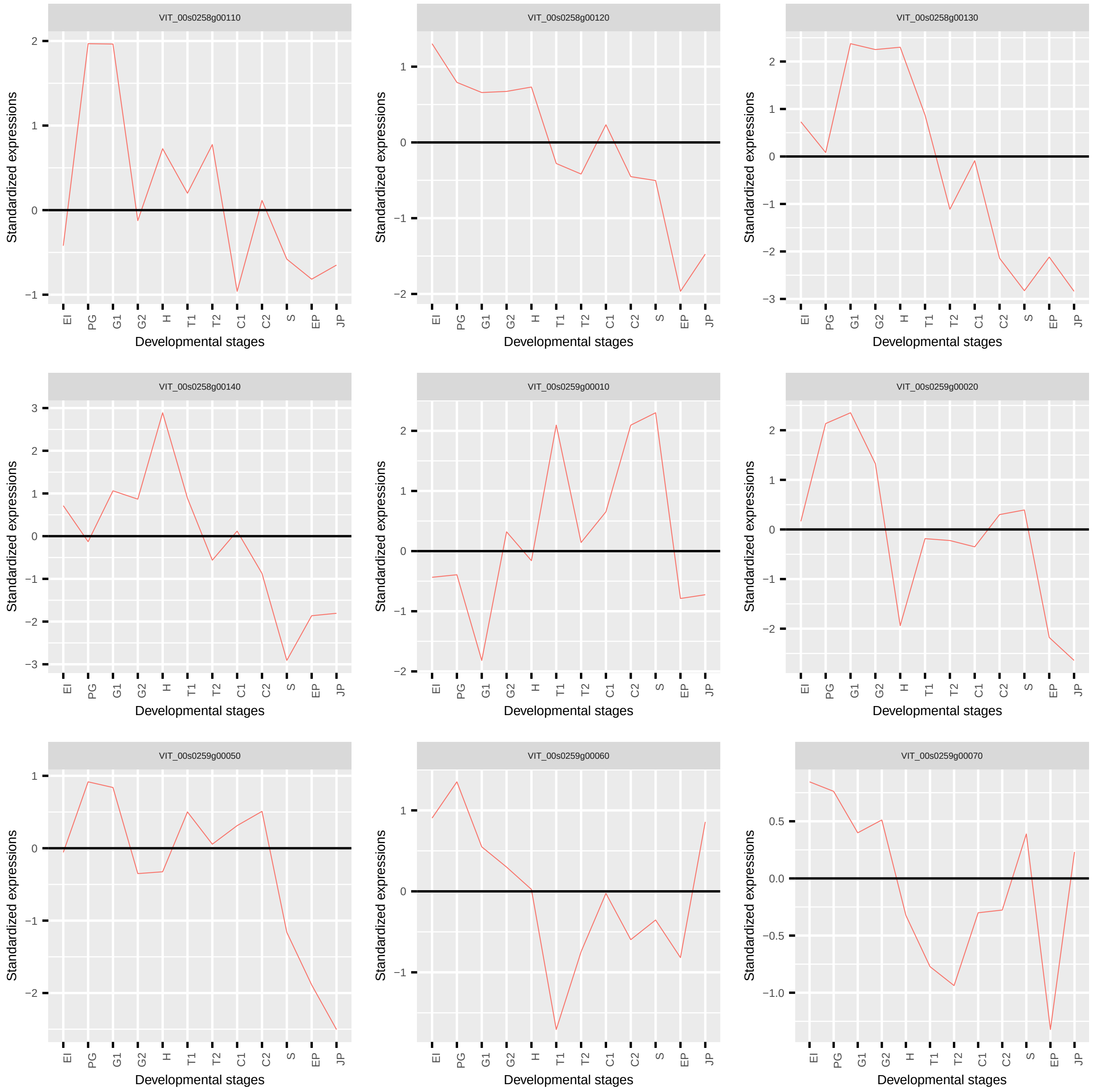

VIT_00s0258g00110
VIT_00s0258g00120
VIT_00s0258g00130
VIT_00s0258g00140
VIT_00s0259g00010
VIT_00s0259g00020
VIT_00s0259g00050
VIT_00s0259g00060
VIT_00s0259g00070
Standardized expressions
Developmental stages
EI
PG
G1
G2
H
T1
T2
C1
C2
S
EP
JP

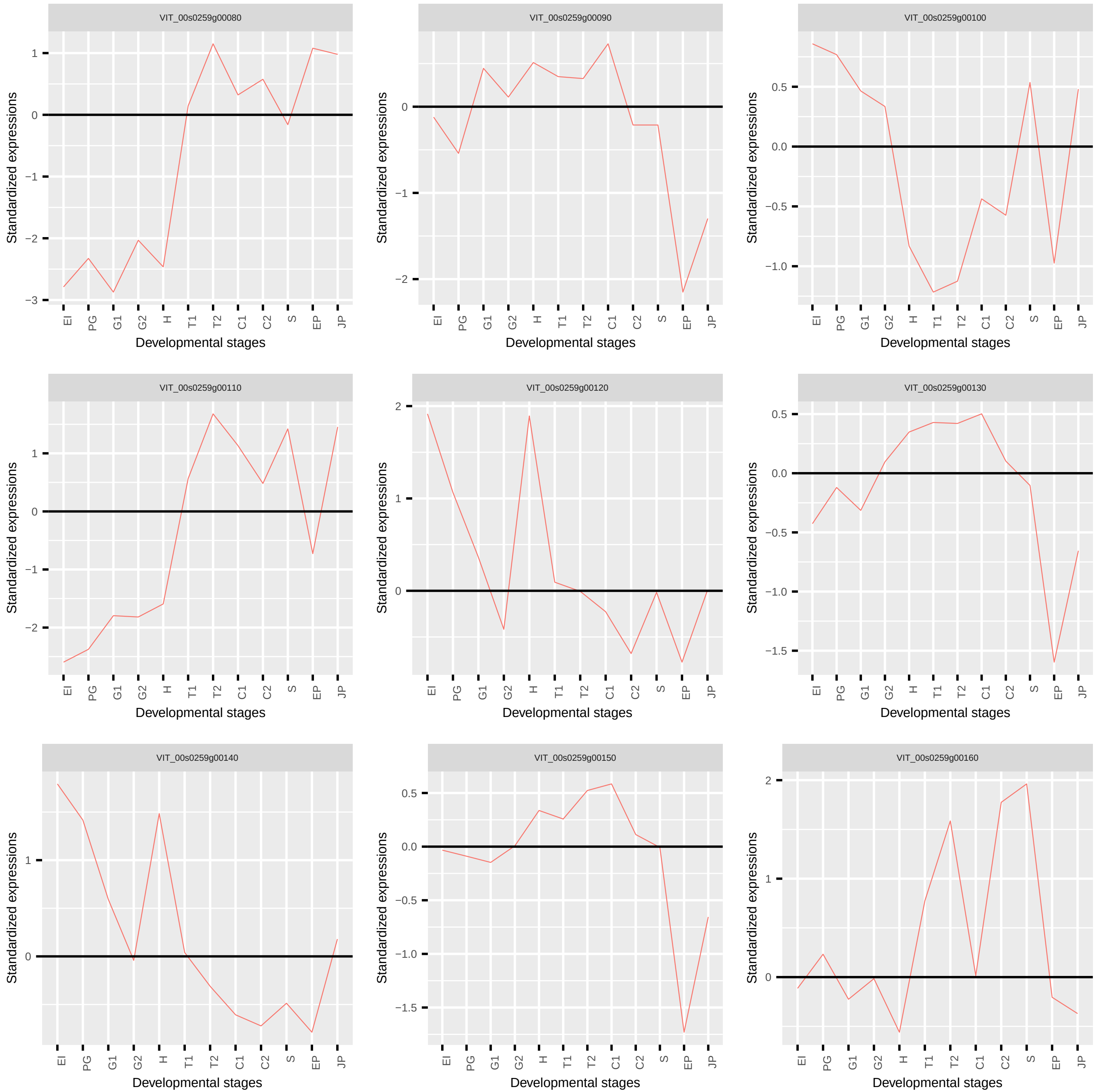
VIT_00s0259g00080
Standardized expressions
1
0
−1
−2
−3
EI PG G1 G2 H T1 T2 C1 C2 S EP JP
Developmental stages
VIT_00s0259g00090
Standardized expressions
0
−1
−2
EI PG G1 G2 H T1 T2 C1 C2 S EP JP
Developmental stages
VIT_00s0259g00100
Standardized expressions
0.5
0.0
−0.5
−1.0
EI PG G1 G2 H T1 T2 C1 C2 S EP JP
Developmental stages
VIT_00s0259g00110
Standardized expressions
1
0
−1
−2
EI PG G1 G2 H T1 T2 C1 C2 S EP JP
Developmental stages
VIT_00s0259g00120
Standardized expressions
2
1
0
EI PG G1 G2 H T1 T2 C1 C2 S EP JP
Developmental stages
VIT_00s0259g00130
Standardized expressions
0.5
0.0
−0.5
−1.0
−1.5
EI PG G1 G2 H T1 T2 C1 C2 S EP JP
Developmental stages
VIT_00s0259g00140
Standardized expressions
1
0
EI PG G1 G2 H T1 T2 C1 C2 S EP JP
Developmental stages
VIT_00s0259g00150
Standardized expressions
0.5
0.0
−0.5
−1.0
−1.5
EI PG G1 G2 H T1 T2 C1 C2 S EP JP
Developmental stages
VIT_00s0259g00160
Standardized expressions
2
1
0
EI PG G1 G2 H T1 T2 C1 C2 S EP JP
Developmental stages

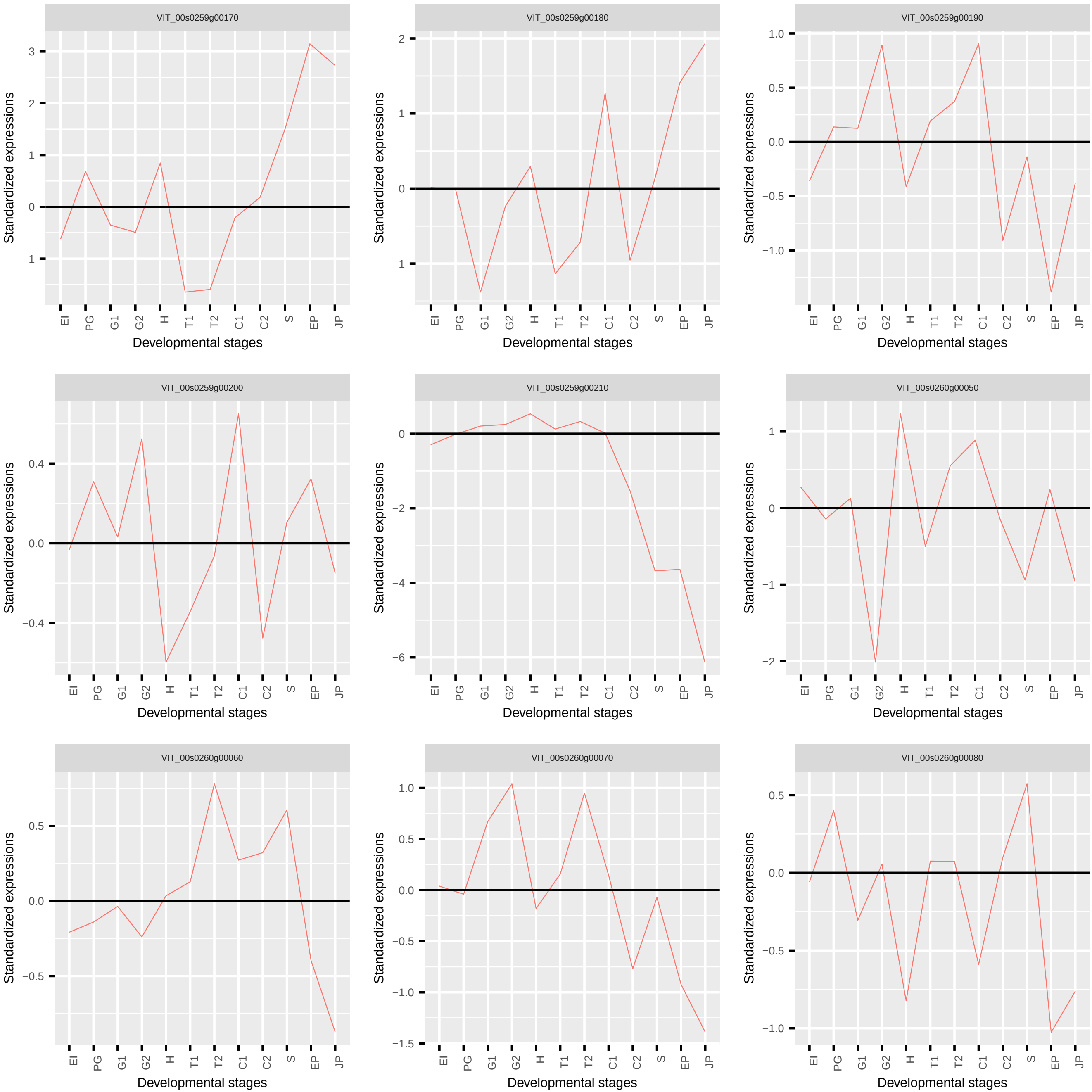
VIT_00s0259g00170
VIT_00s0259g00180
VIT_00s0259g00190
VIT_00s0259g00200
VIT_00s0259g00210
VIT_00s0260g00050
VIT_00s0260g00060
VIT_00s0260g00070
VIT_00s0260g00080
Standardized expressions
Developmental stages
EI PG G1 G2 H T1 T2 C1 C2 S EP JP

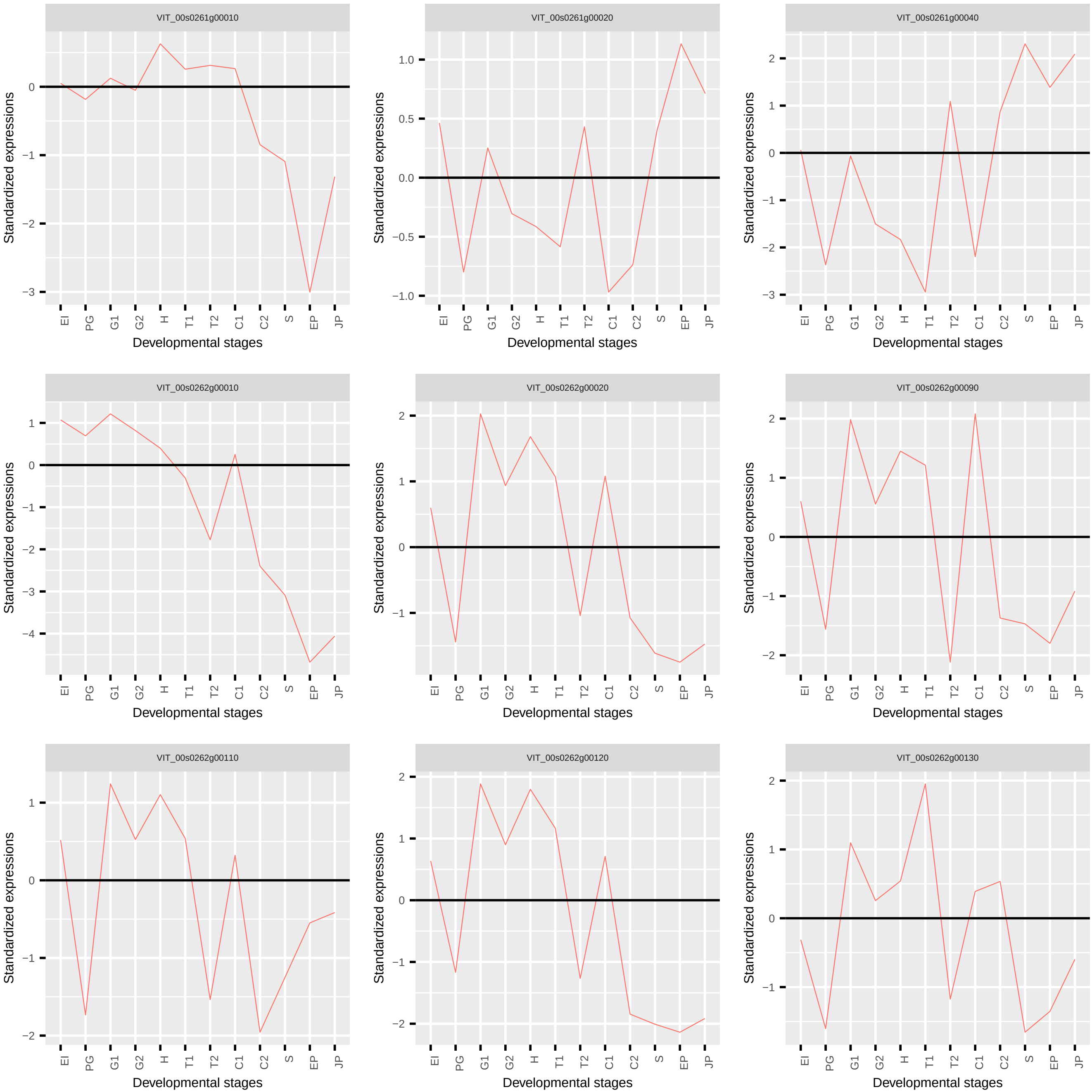
VIT_00s0261g00010
VIT_00s0261g00020
VIT_00s0261g00040
VIT_00s0262g00010
VIT_00s0262g00020
VIT_00s0262g00090
VIT_00s0262g00110
VIT_00s0262g00120
VIT_00s0262g00130
Standardized expressions
Developmental stages
EI PG G1 G2 H T1 T2 C1 C2 S EP JP

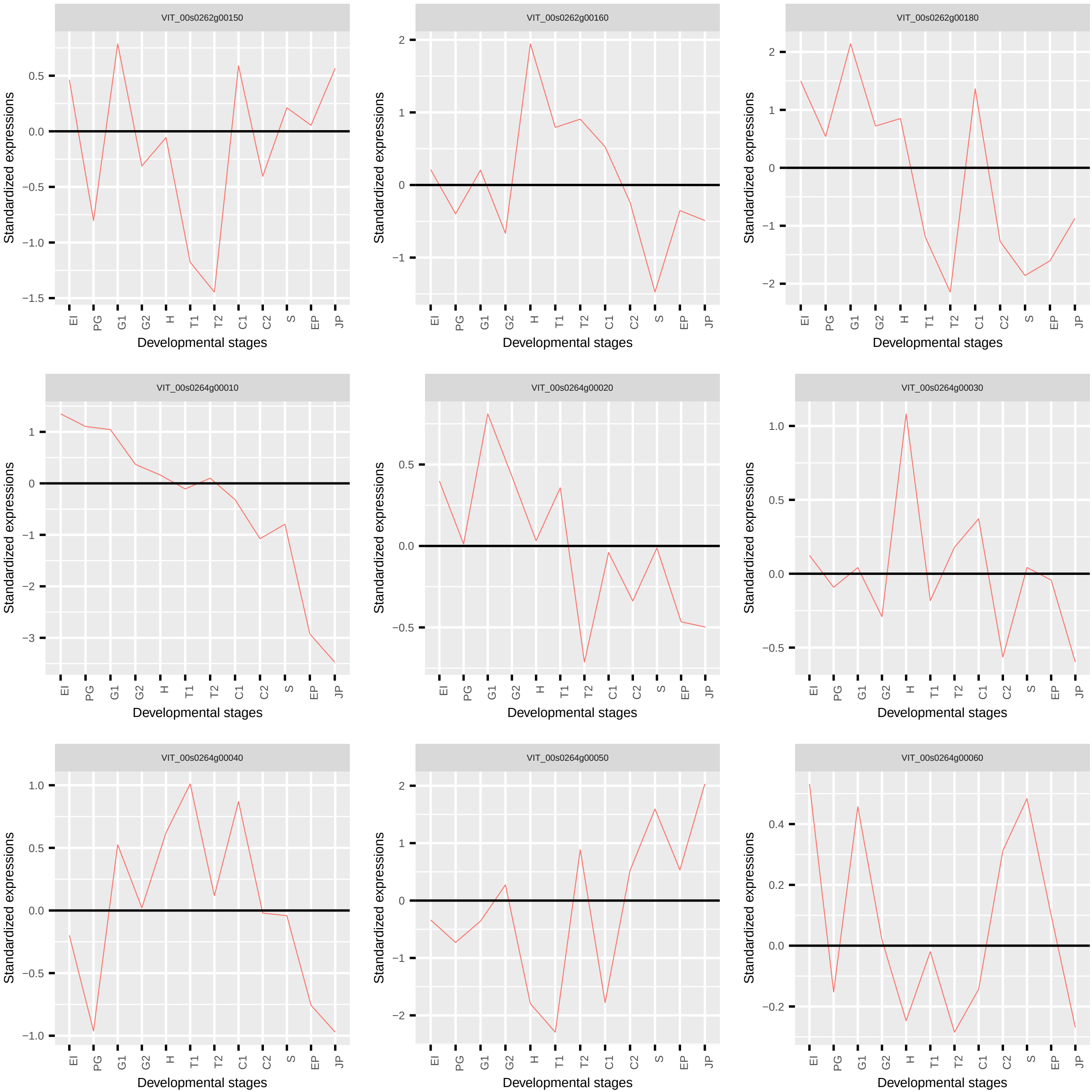
VIT_00s0262g00150
VIT_00s0262g00160
VIT_00s0262g00180
VIT_00s0264g00010
VIT_00s0264g00020
VIT_00s0264g00030
VIT_00s0264g00040
VIT_00s0264g00050
VIT_00s0264g00060
Standardized expressions
Developmental stages
EI PG G1 G2 H T1 T2 C1 C2 S EP JP

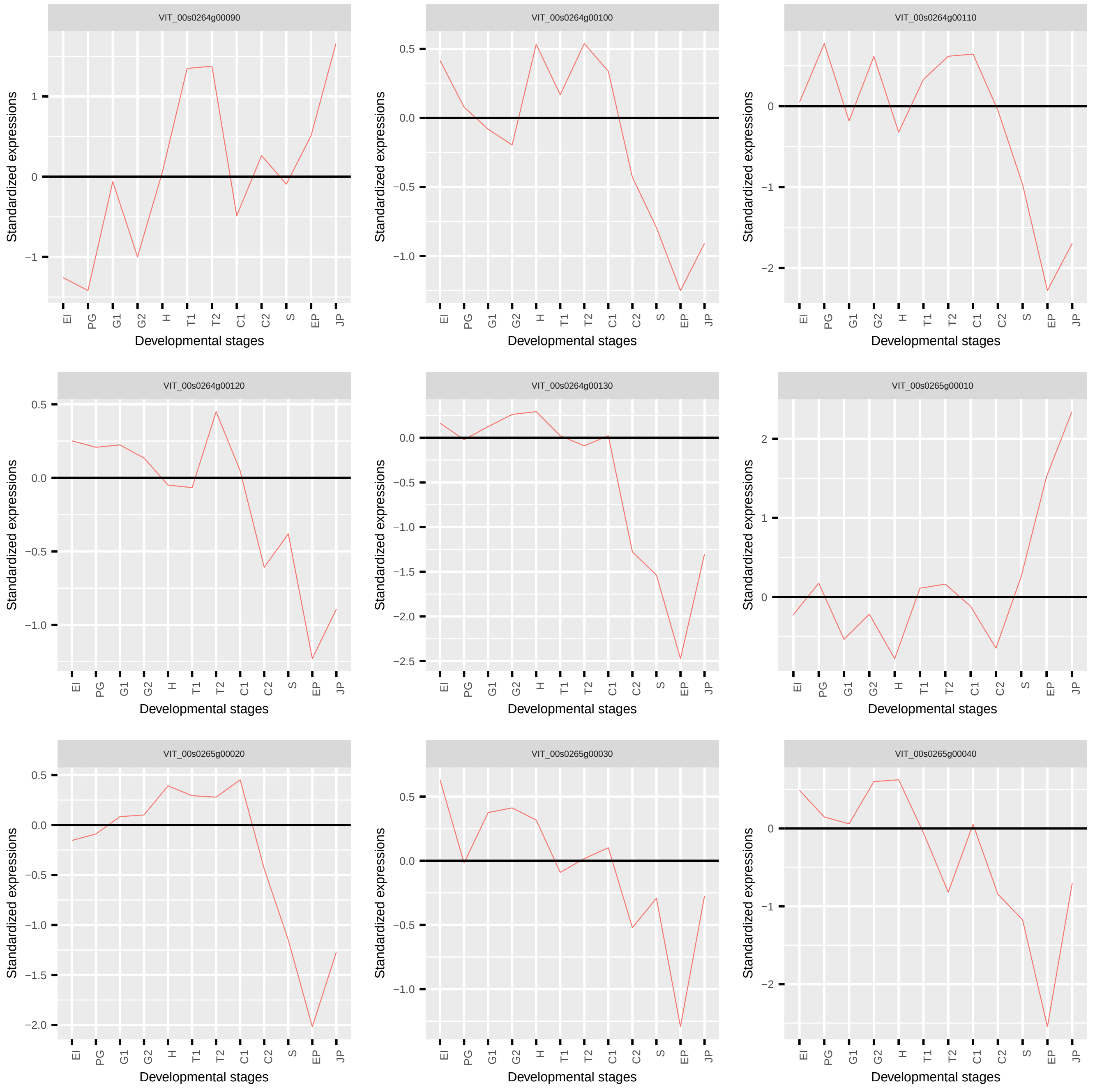
VIT_00s0264g00090
Standardized expressions
1
0
−1
EI PG G1 G2 H T1 T2 C1 C2 S EP JP
Developmental stages
VIT_00s0264g00100
Standardized expressions
0.5
0.0
−0.5
−1.0
EI PG G1 G2 H T1 T2 C1 C2 S EP JP
Developmental stages
VIT_00s0264g00110
Standardized expressions
0
−1
−2
EI PG G1 G2 H T1 T2 C1 C2 S EP JP
Developmental stages
VIT_00s0264g00120
Standardized expressions
0.5
0.0
−0.5
−1.0
EI PG G1 G2 H T1 T2 C1 C2 S EP JP
Developmental stages
VIT_00s0264g00130
Standardized expressions
0.0
−0.5
−1.0
−1.5
−2.0
−2.5
EI PG G1 G2 H T1 T2 C1 C2 S EP JP
Developmental stages
VIT_00s0265g00010
Standardized expressions
2
1
0
EI PG G1 G2 H T1 T2 C1 C2 S EP JP
Developmental stages
VIT_00s0265g00020
Standardized expressions
0.5
0.0
−0.5
−1.0
−1.5
−2.0
EI PG G1 G2 H T1 T2 C1 C2 S EP JP
Developmental stages
VIT_00s0265g00030
Standardized expressions
0.5
0.0
−0.5
−1.0
EI PG G1 G2 H T1 T2 C1 C2 S EP JP
Developmental stages
VIT_00s0265g00040
Standardized expressions
0
−1
−2
EI PG G1 G2 H T1 T2 C1 C2 S EP JP
Developmental stages

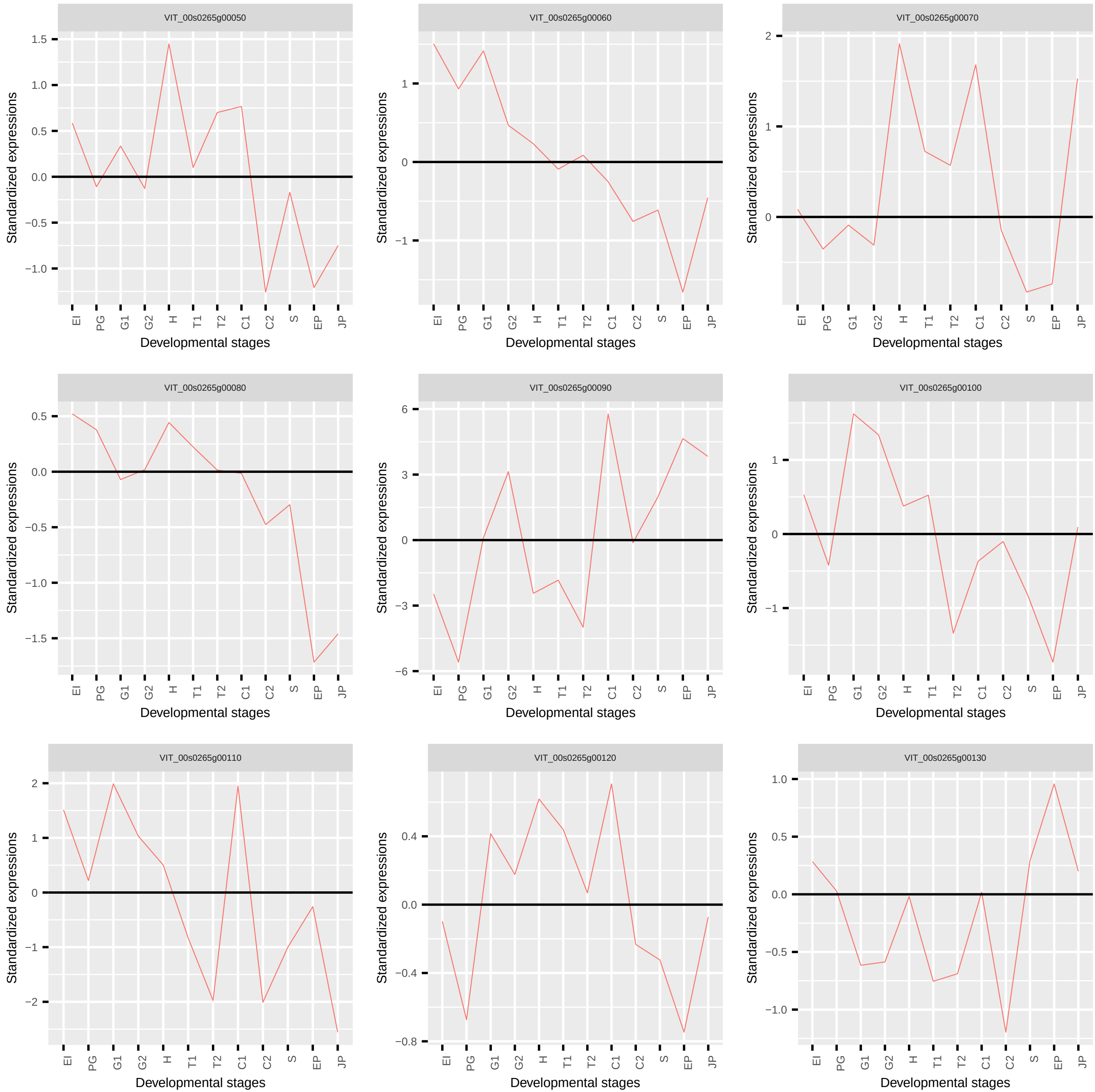
VIT_00s0265g00050
VIT_00s0265g00060
VIT_00s0265g00070
VIT_00s0265g00080
VIT_00s0265g00090
VIT_00s0265g00100
VIT_00s0265g00110
VIT_00s0265g00120
VIT_00s0265g00130
Standardized expressions
Developmental stages
EI PG G1 G2 H T1 T2 C1 C2 S EP JP
1.5 1.0 0.5 0.0 −0.5 −1.0
1 0 −1
2 1 0
0.5 0.0 −0.5 −1.0 −1.5
6 3 0 −3 −6
1 0 −1
2 1 0 −1 −2
0.4 0.0 −0.4 −0.8
1.0 0.5 0.0 −0.5 −1.0

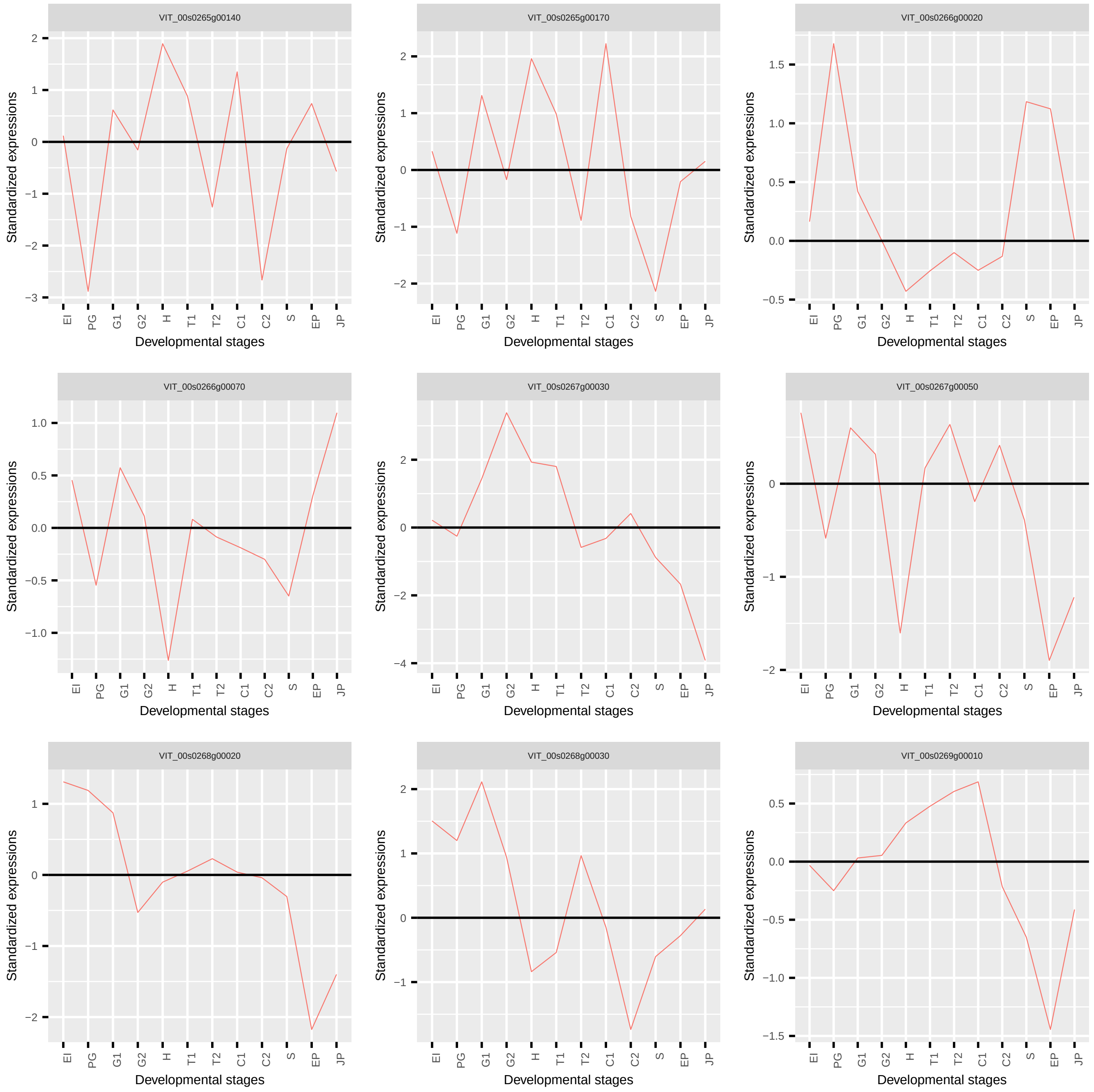
VIT_00s0265g00140
VIT_00s0265g00170
VIT_00s0266g00020
VIT_00s0266g00070
VIT_00s0267g00030
VIT_00s0267g00050
VIT_00s0268g00020
VIT_00s0268g00030
VIT_00s0269g00010
Standardized expressions
Developmental stages
EI
PG
G1
G2
H
T1
T2
C1
C2
S
EP
JP

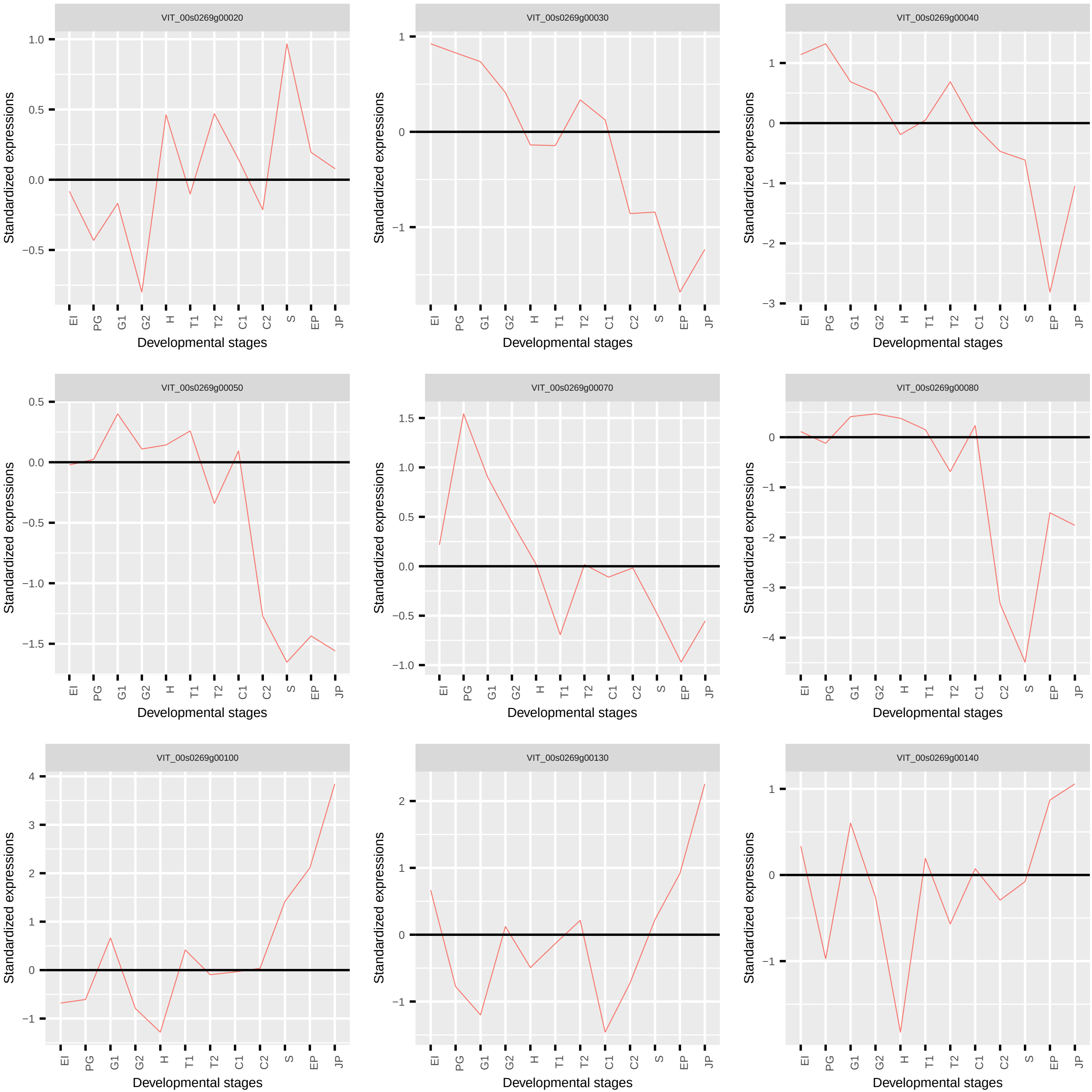

VIT_00s0269g00020
VIT_00s0269g00030
VIT_00s0269g00040
VIT_00s0269g00050
VIT_00s0269g00070
VIT_00s0269g00080
VIT_00s0269g00100
VIT_00s0269g00130
VIT_00s0269g00140
Standardized expressions
Developmental stages
EI
PG
G1
G2
H
T1
T2
C1
C2
S
EP
JP

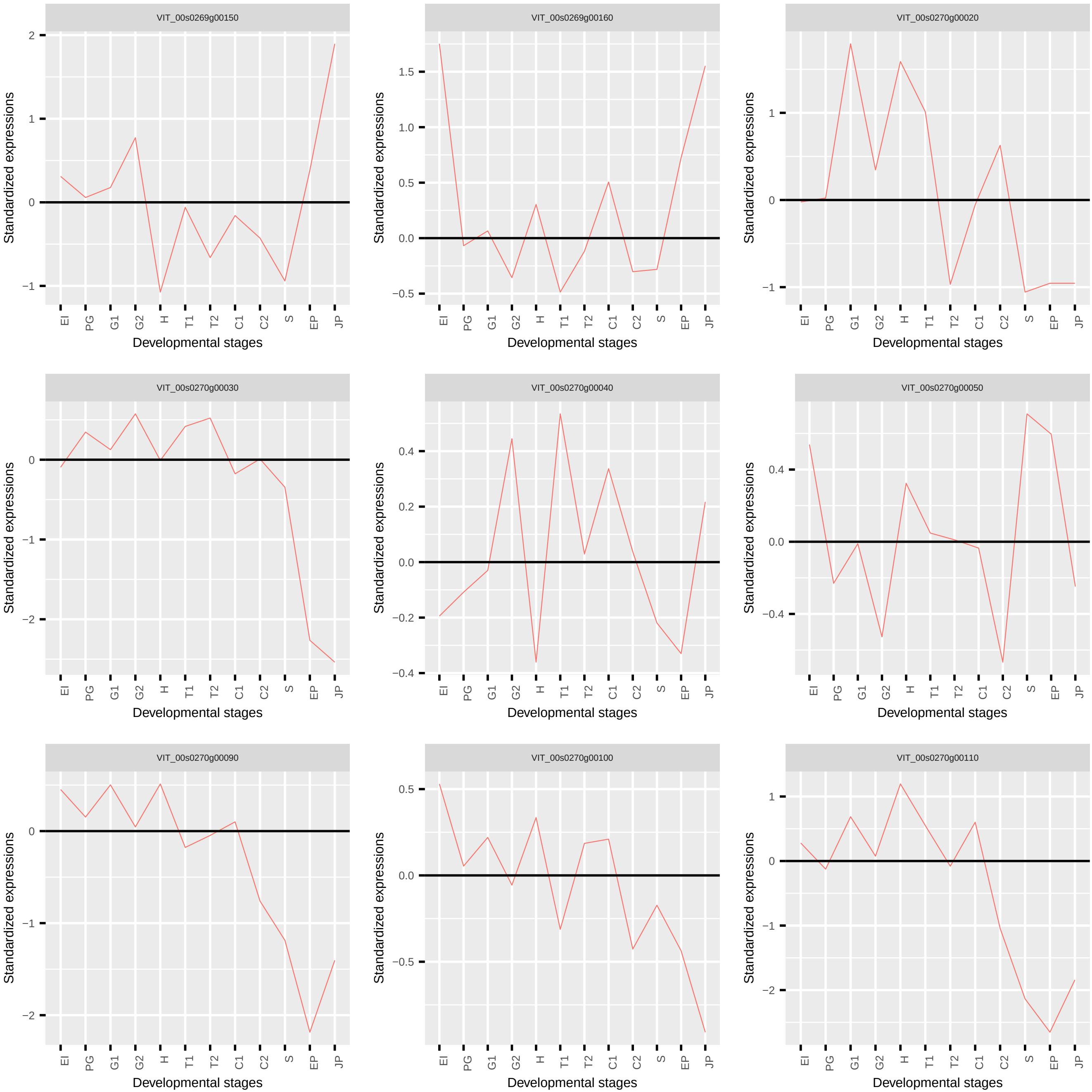
VIT_00s0269g00150
VIT_00s0269g00160
VIT_00s0270g00020
VIT_00s0270g00030
VIT_00s0270g00040
VIT_00s0270g00050
VIT_00s0270g00090
VIT_00s0270g00100
VIT_00s0270g00110
Standardized expressions
Developmental stages
EI PG G1 G2 H T1 T2 C1 C2 S EP JP

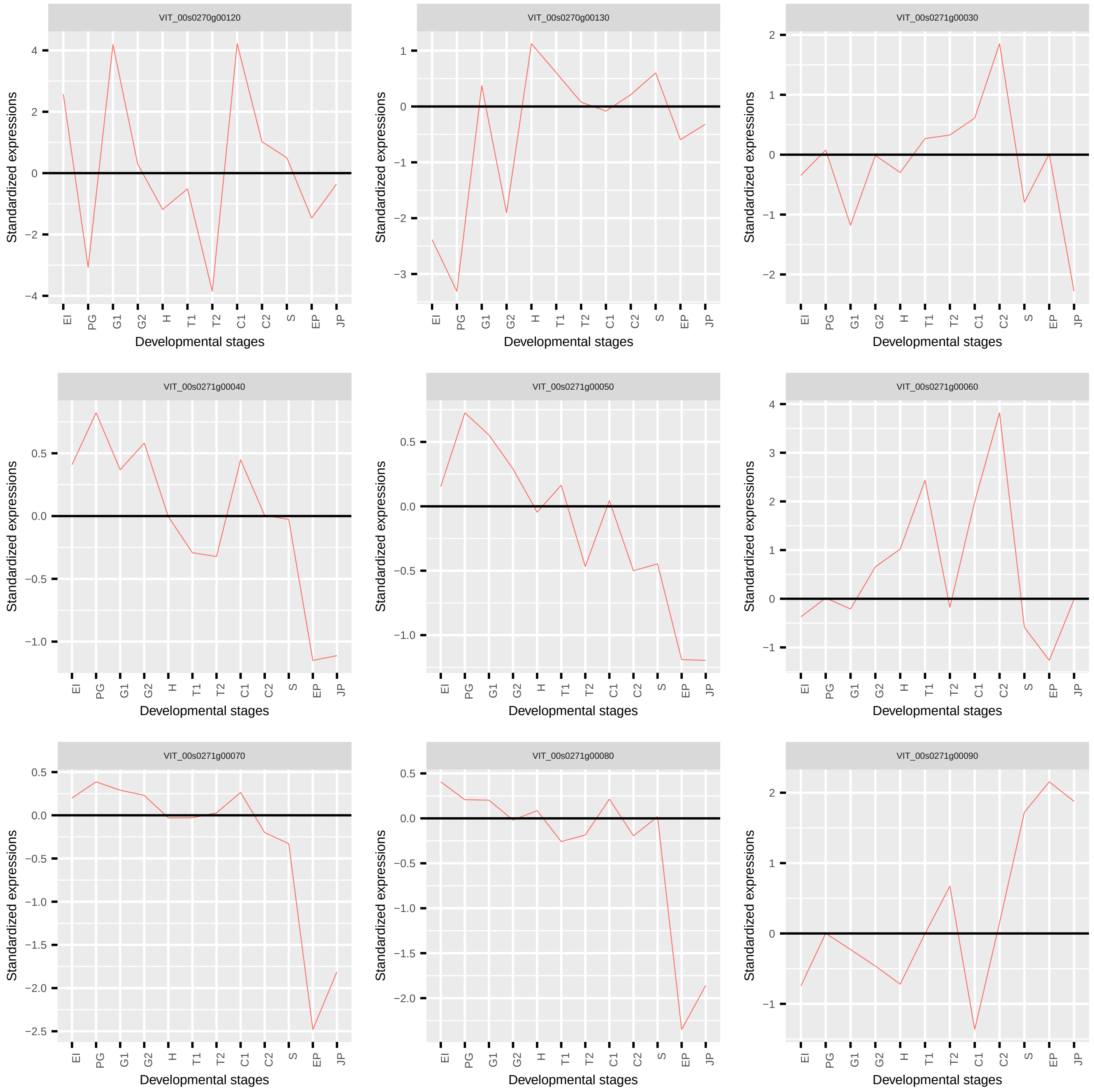
VIT_00s0270g00120
VIT_00s0270g00130
VIT_00s0271g00030
VIT_00s0271g00040
VIT_00s0271g00050
VIT_00s0271g00060
VIT_00s0271g00070
VIT_00s0271g00080
VIT_00s0271g00090
Standardized expressions
Developmental stages
EI PG G1 G2 H T1 T2 C1 C2 S EP JP

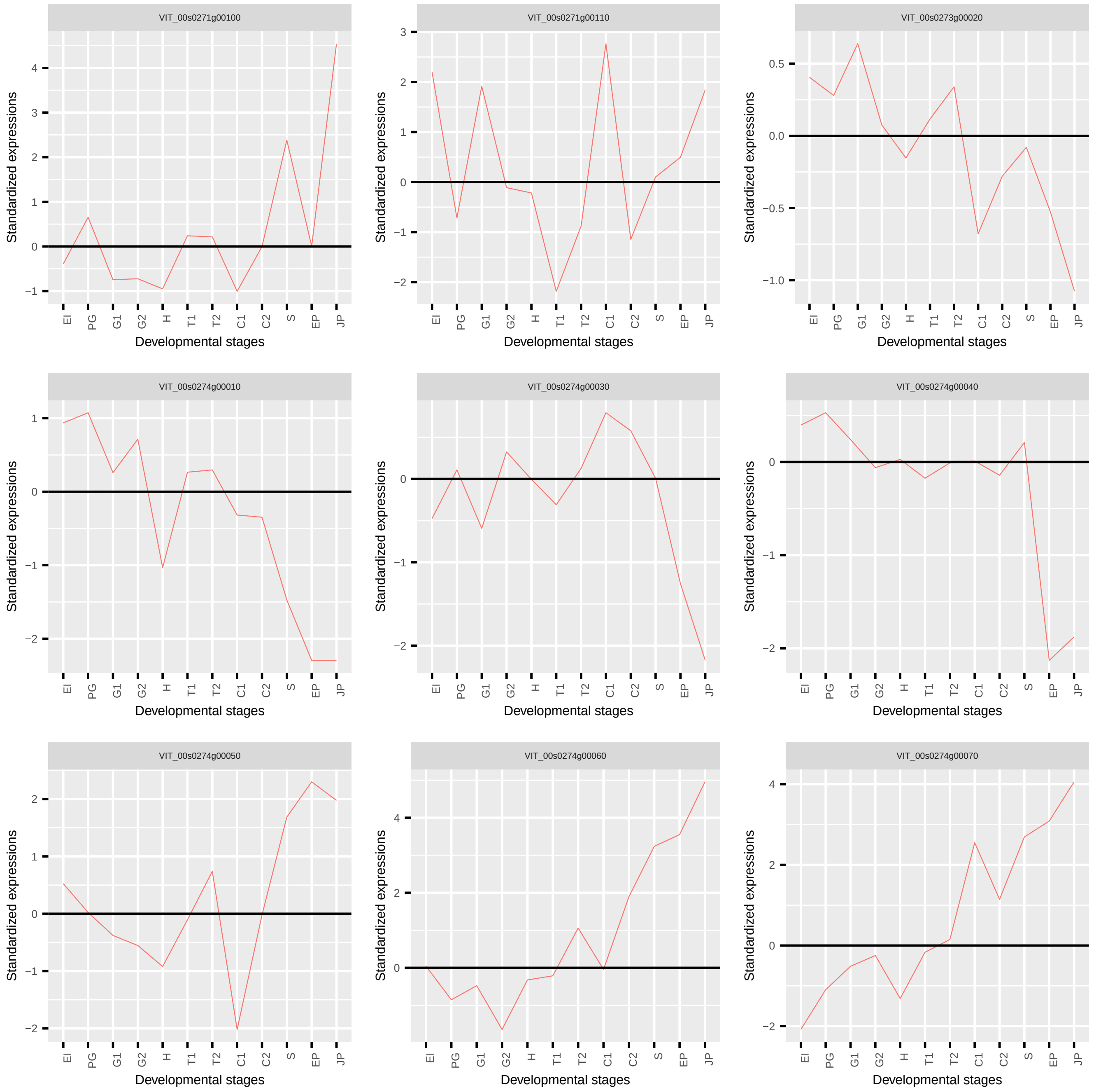
VIT_00s0271g00100
VIT_00s0271g00110
VIT_00s0273g00020
VIT_00s0274g00010
VIT_00s0274g00030
VIT_00s0274g00040
VIT_00s0274g00050
VIT_00s0274g00060
VIT_00s0274g00070
Standardized expressions
Developmental stages
EI PG G1 G2 H T1 T2 C1 C2 S EP JP

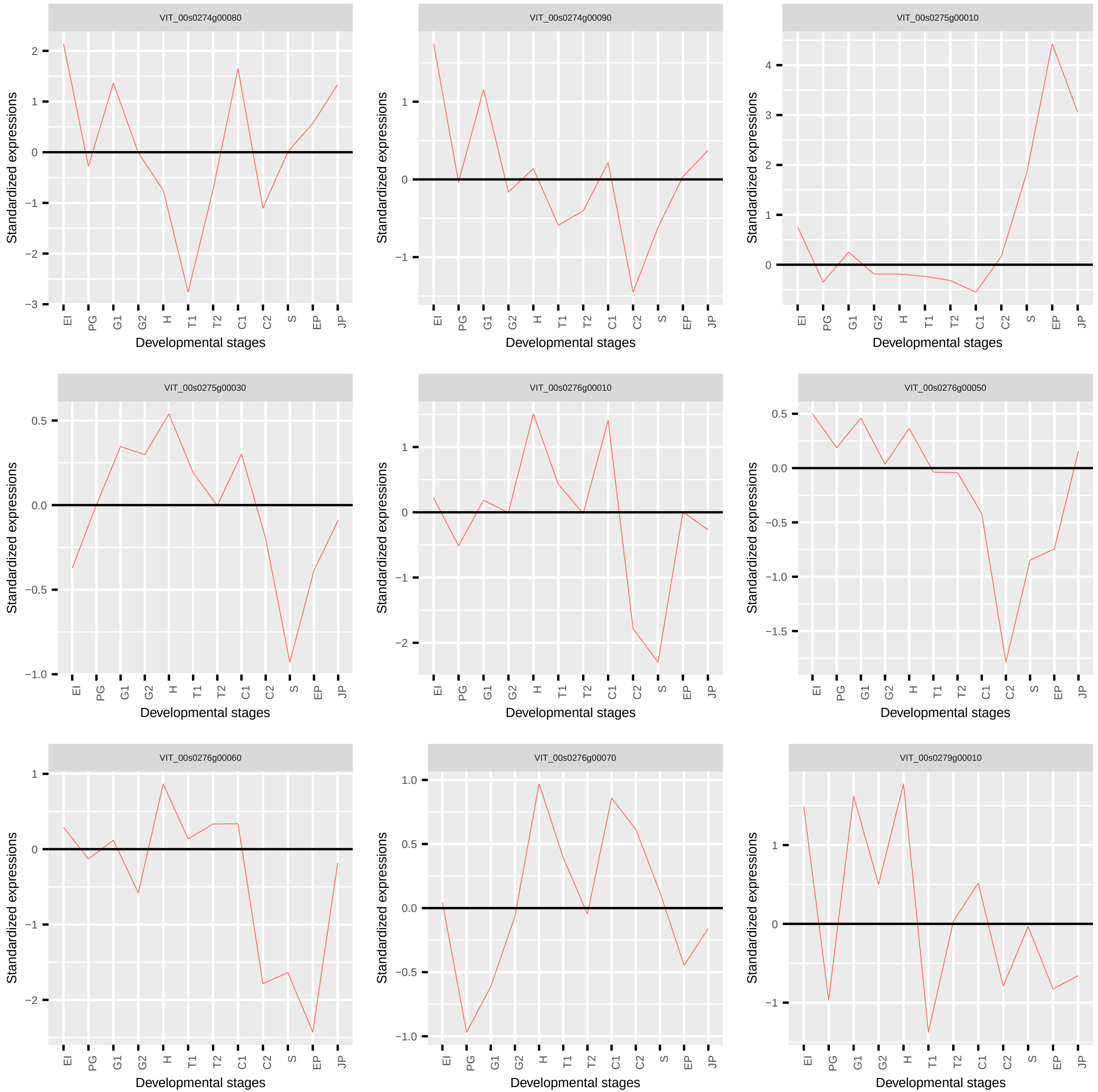

VIT_00s0274g00080
VIT_00s0274g00090
VIT_00s0275g00010
VIT_00s0275g00030
VIT_00s0276g00010
VIT_00s0276g00050
VIT_00s0276g00060
VIT_00s0276g00070
VIT_00s0279g00010
Standardized expressions
Developmental stages
EI
PG
G1
G2
H
T1
T2
C1
C2
S
EP
JP

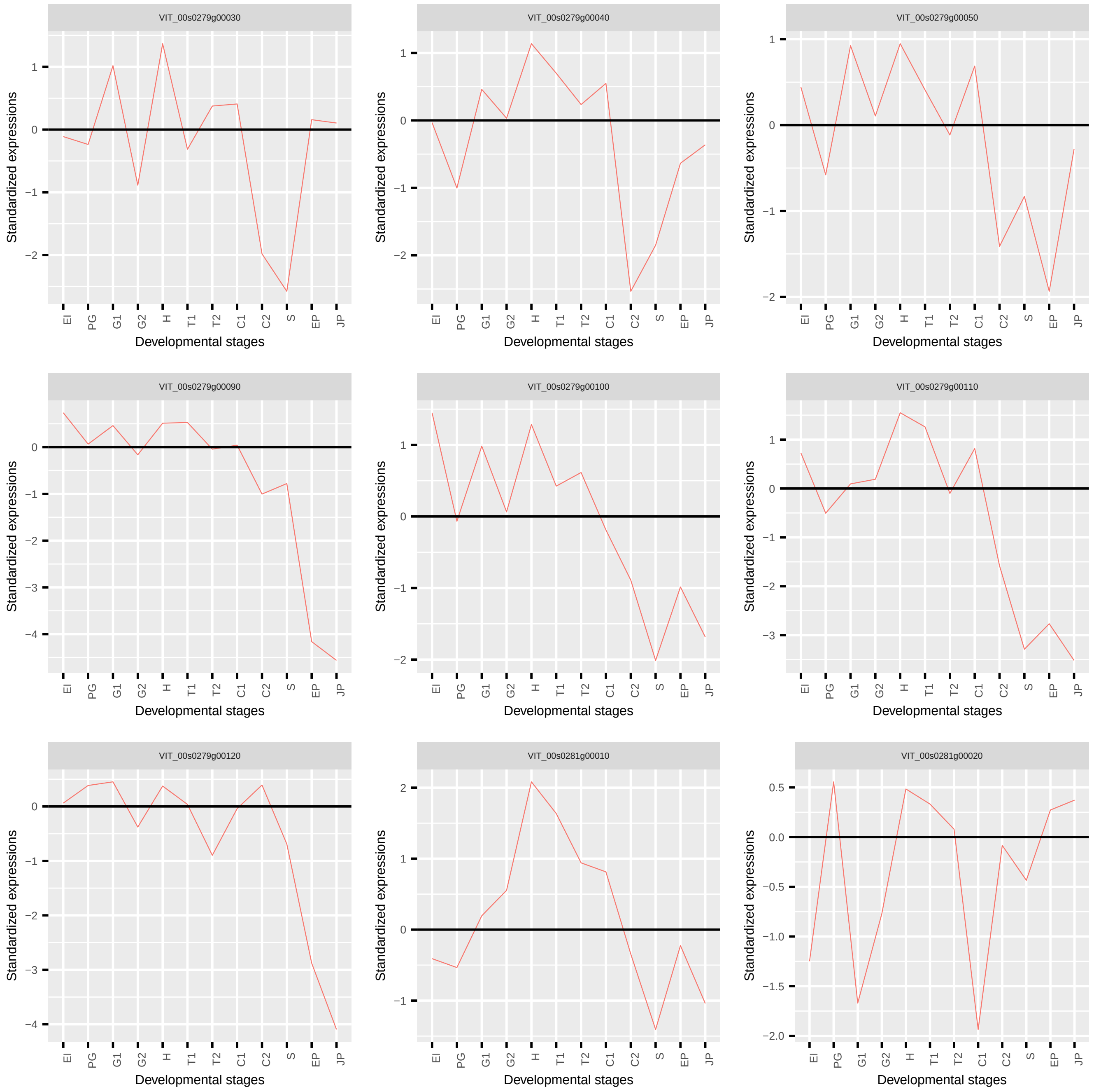
VIT_00s0279g00030
VIT_00s0279g00040
VIT_00s0279g00050
VIT_00s0279g00090
VIT_00s0279g00100
VIT_00s0279g00110
VIT_00s0279g00120
VIT_00s0281g00010
VIT_00s0281g00020
Standardized expressions
Developmental stages
EI PG G1 G2 H T1 T2 C1 C2 S EP JP

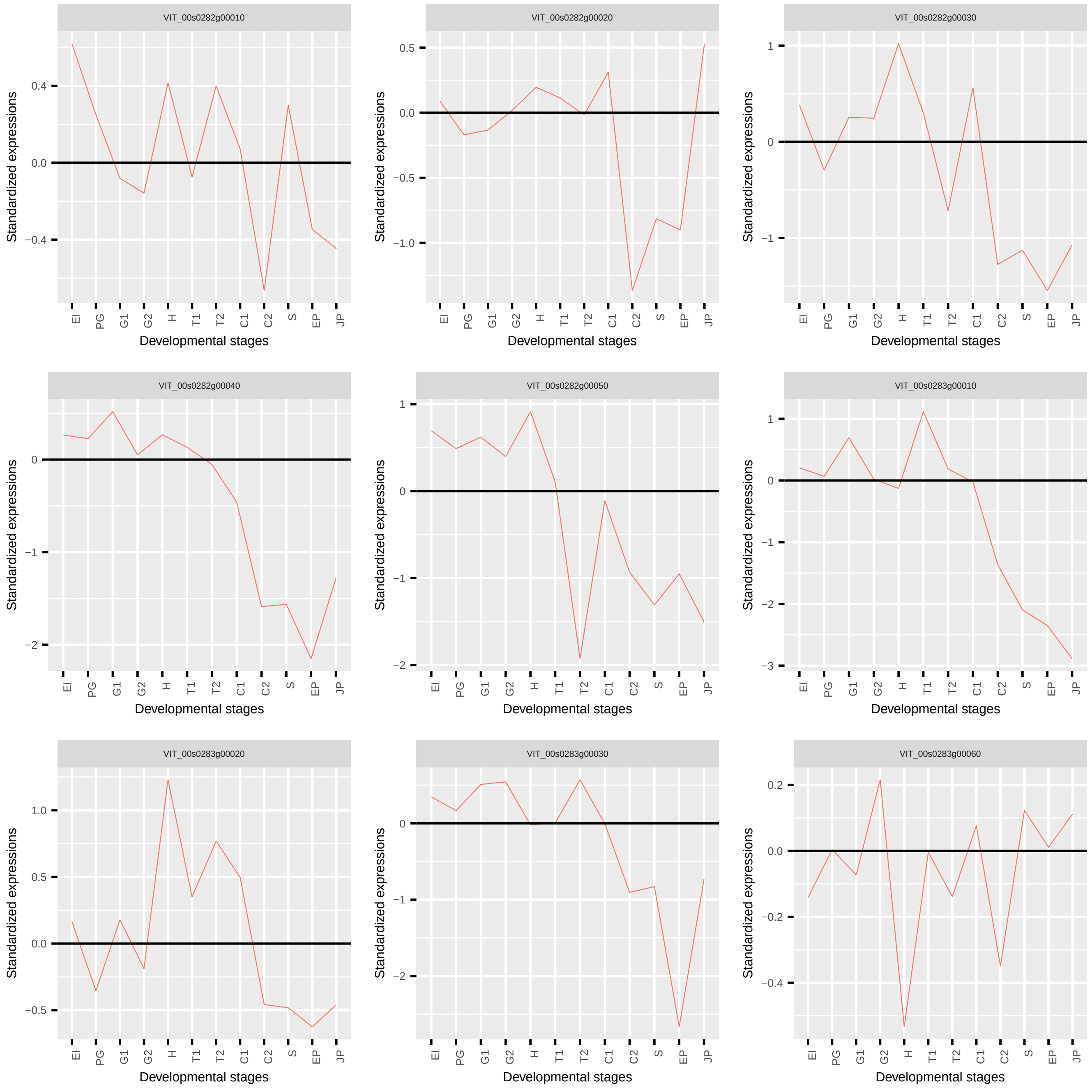
VIT_00s0282g00010
0.4
0.0
−0.4
VIT_00s0282g00020
0.5
0.0
−0.5
−1.0
VIT_00s0282g00030
1
0
−1
VIT_00s0282g00040
0
−1
−2
VIT_00s0282g00050
1
0
−1
−2
VIT_00s0283g00010
1
0
−1
−2
−3
VIT_00s0283g00020
1.0
0.5
0.0
−0.5
VIT_00s0283g00030
0
−1
−2
VIT_00s0283g00060
0.2
0.0
−0.2
−0.4
Standardized expressions
EI PG G1 G2 H T1 T2 C1 C2 S EP JP
Developmental stages

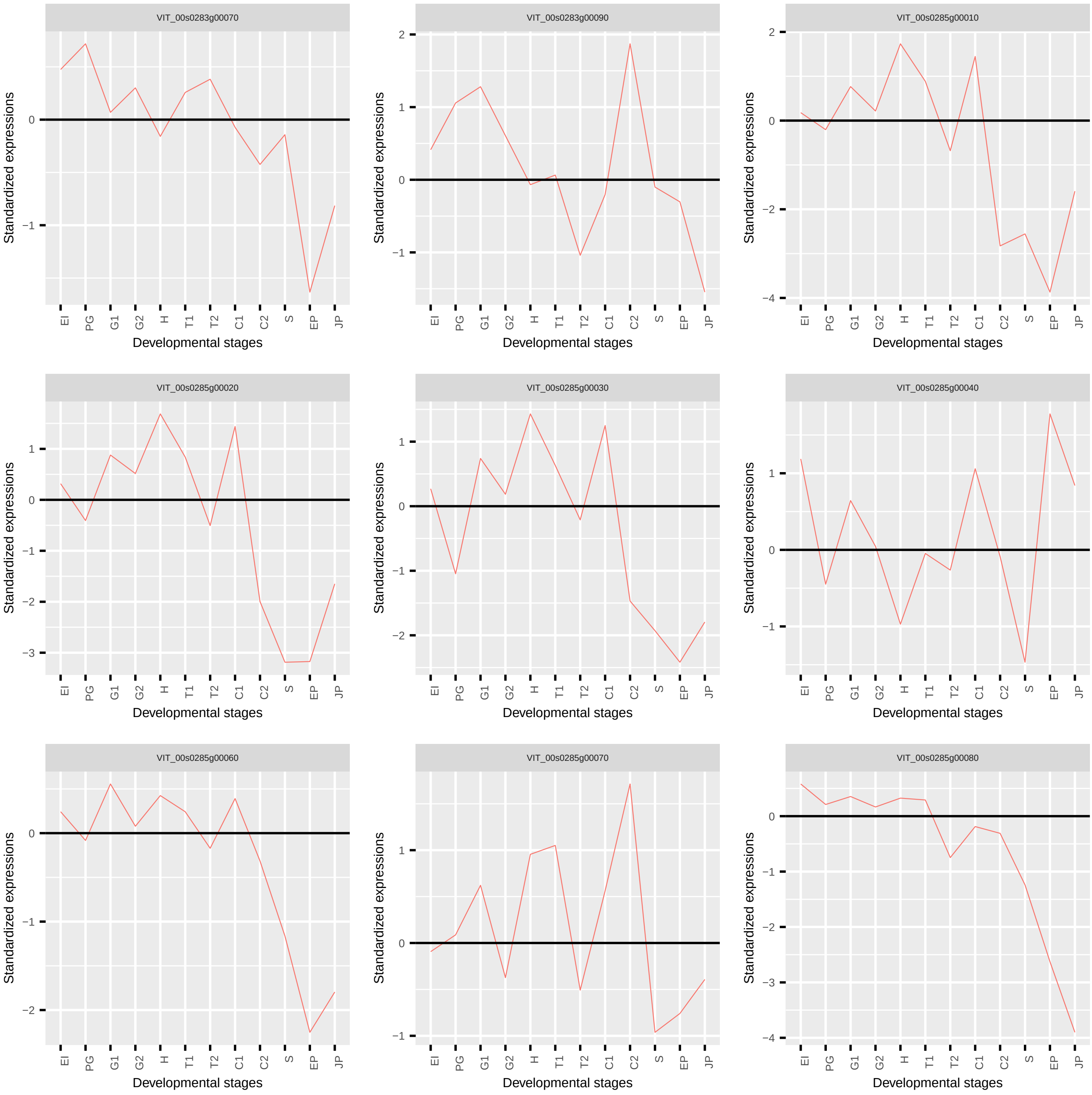
VIT_00s0283g00070
VIT_00s0283g00090
VIT_00s0285g00010
VIT_00s0285g00020
VIT_00s0285g00030
VIT_00s0285g00040
VIT_00s0285g00060
VIT_00s0285g00070
VIT_00s0285g00080
Standardized expressions
Developmental stages
EI PG G1 G2 H T1 T2 C1 C2 S EP JP

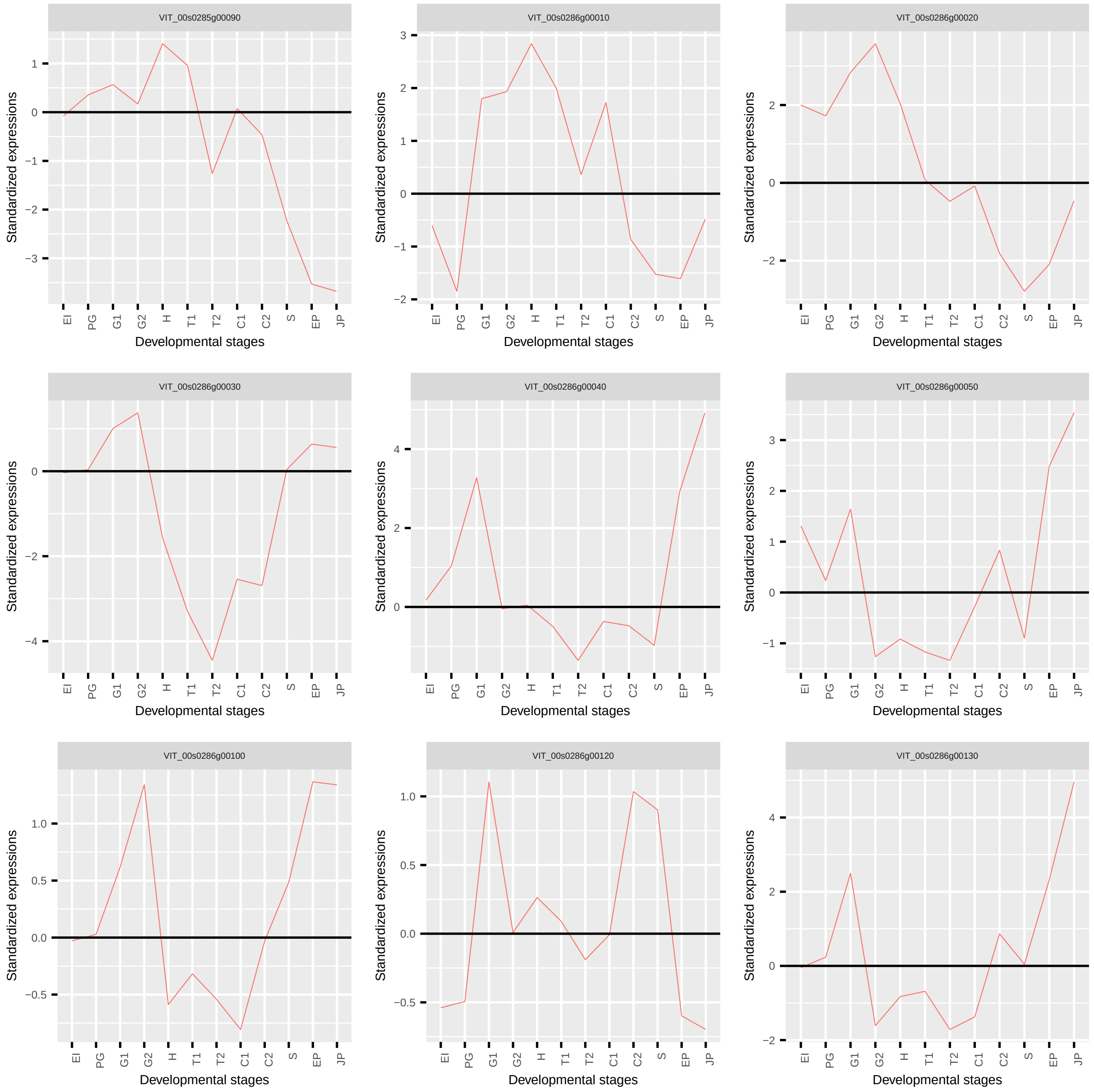
VIT_00s0285g00090
VIT_00s0286g00010
VIT_00s0286g00020
VIT_00s0286g00030
VIT_00s0286g00040
VIT_00s0286g00050
VIT_00s0286g00100
VIT_00s0286g00120
VIT_00s0286g00130
Standardized expressions
Developmental stages
EI PG G1 G2 H T1 T2 C1 C2 S EP JP

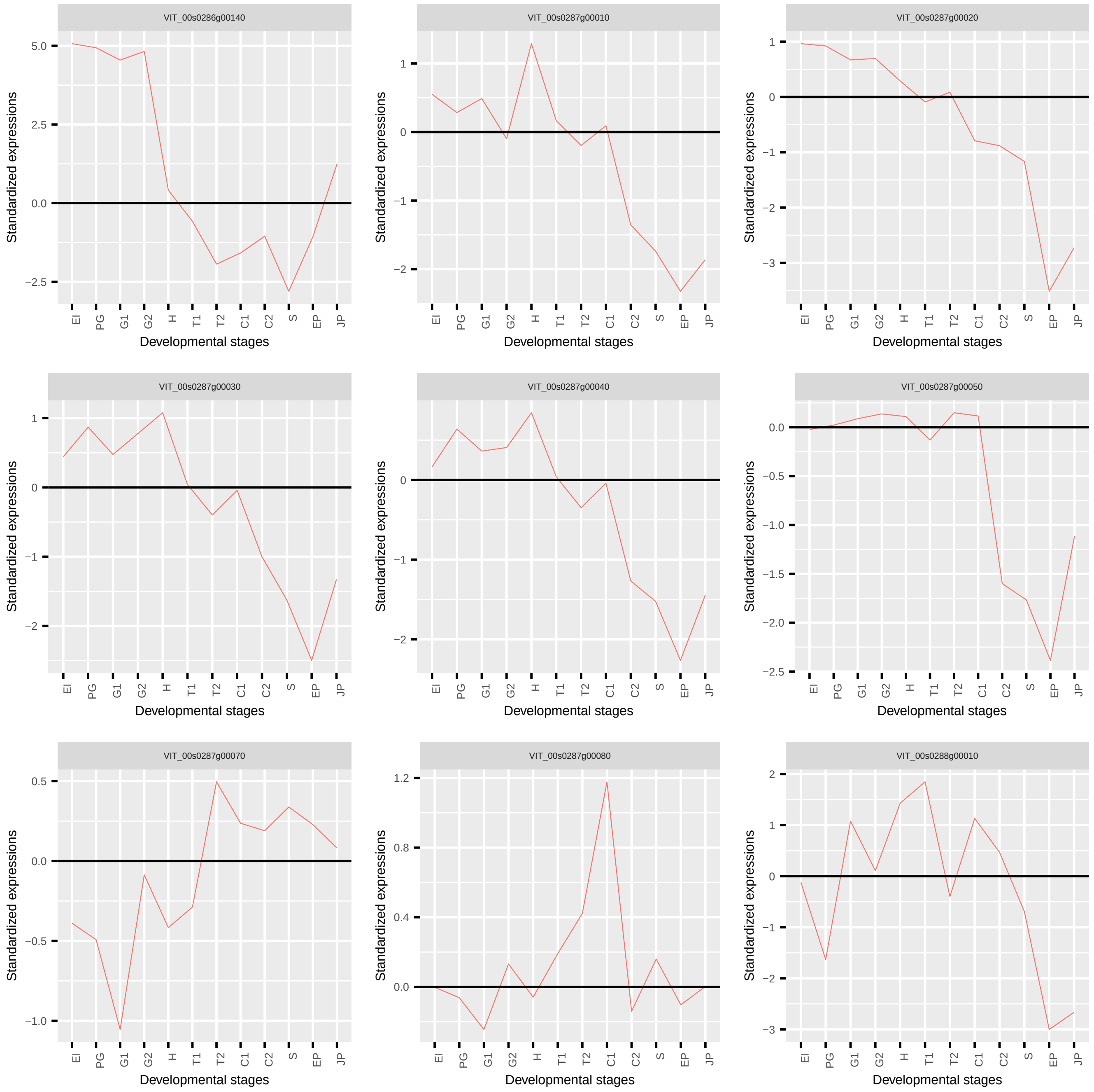
VIT_00s0286g00140
VIT_00s0287g00010
VIT_00s0287g00020
VIT_00s0287g00030
VIT_00s0287g00040
VIT_00s0287g00050
VIT_00s0287g00070
VIT_00s0287g00080
VIT_00s0288g00010
Standardized expressions
Developmental stages
EI PG G1 G2 H T1 T2 C1 C2 S EP JP

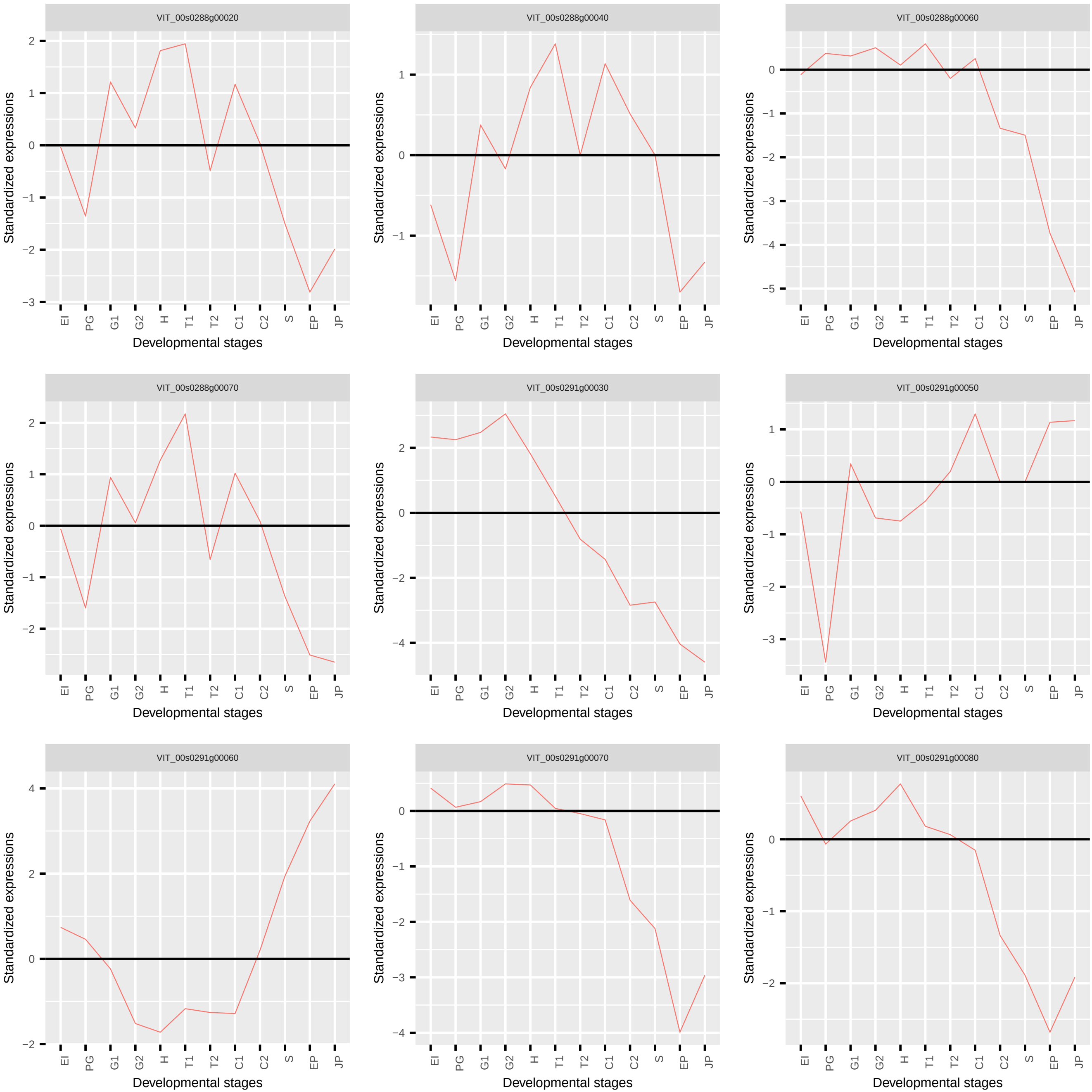
VIT_00s0288g00020
VIT_00s0288g00040
VIT_00s0288g00060
VIT_00s0288g00070
VIT_00s0291g00030
VIT_00s0291g00050
VIT_00s0291g00060
VIT_00s0291g00070
VIT_00s0291g00080
Standardized expressions
Developmental stages
EI
PG
G1
G2
H
T1
T2
C1
C2
S
EP
JP

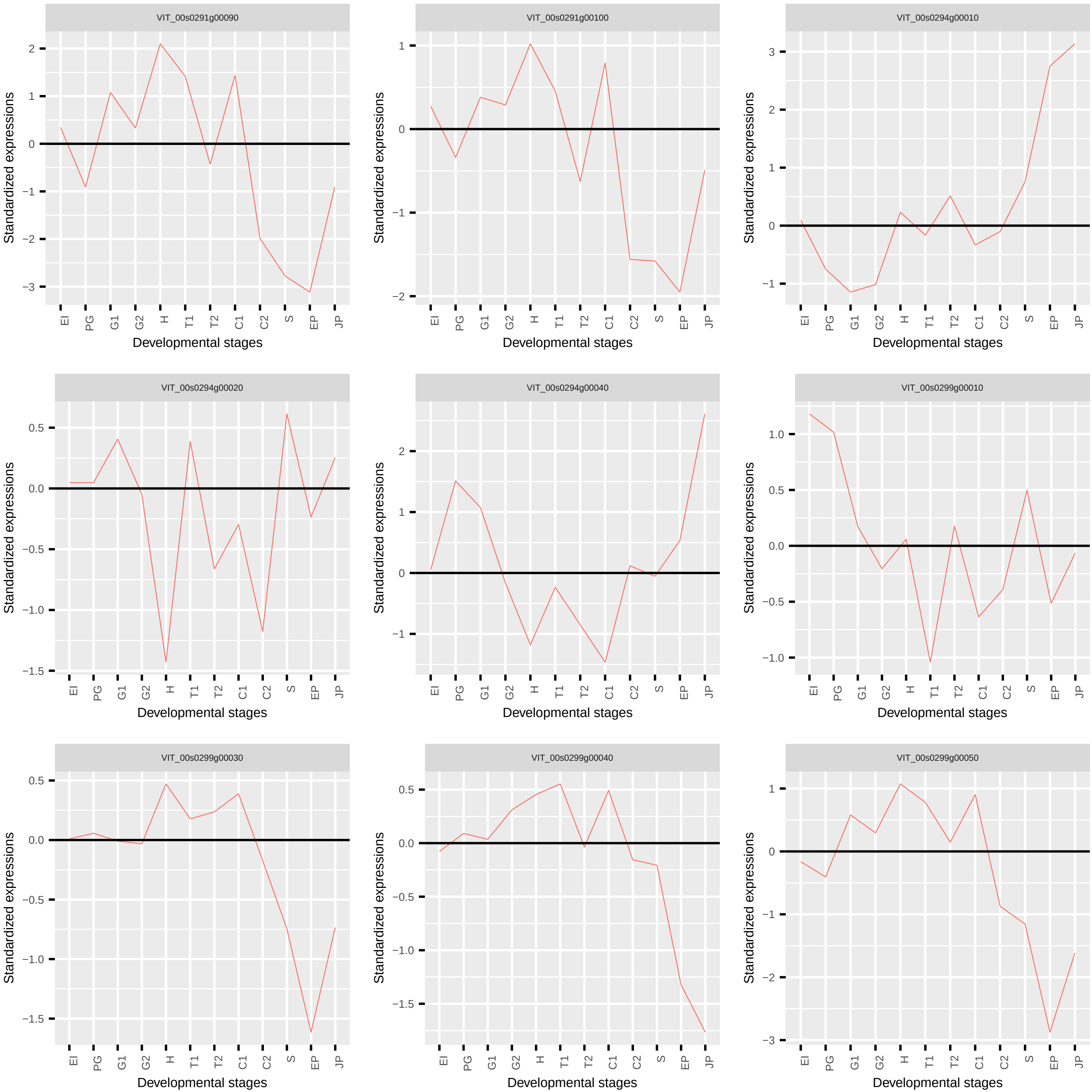
VIT_00s0291g00090
VIT_00s0291g00100
VIT_00s0294g00010
VIT_00s0294g00020
VIT_00s0294g00040
VIT_00s0299g00010
VIT_00s0299g00030
VIT_00s0299g00040
VIT_00s0299g00050
Standardized expressions
Developmental stages
EI PG G1 G2 H T1 T2 C1 C2 S EP JP

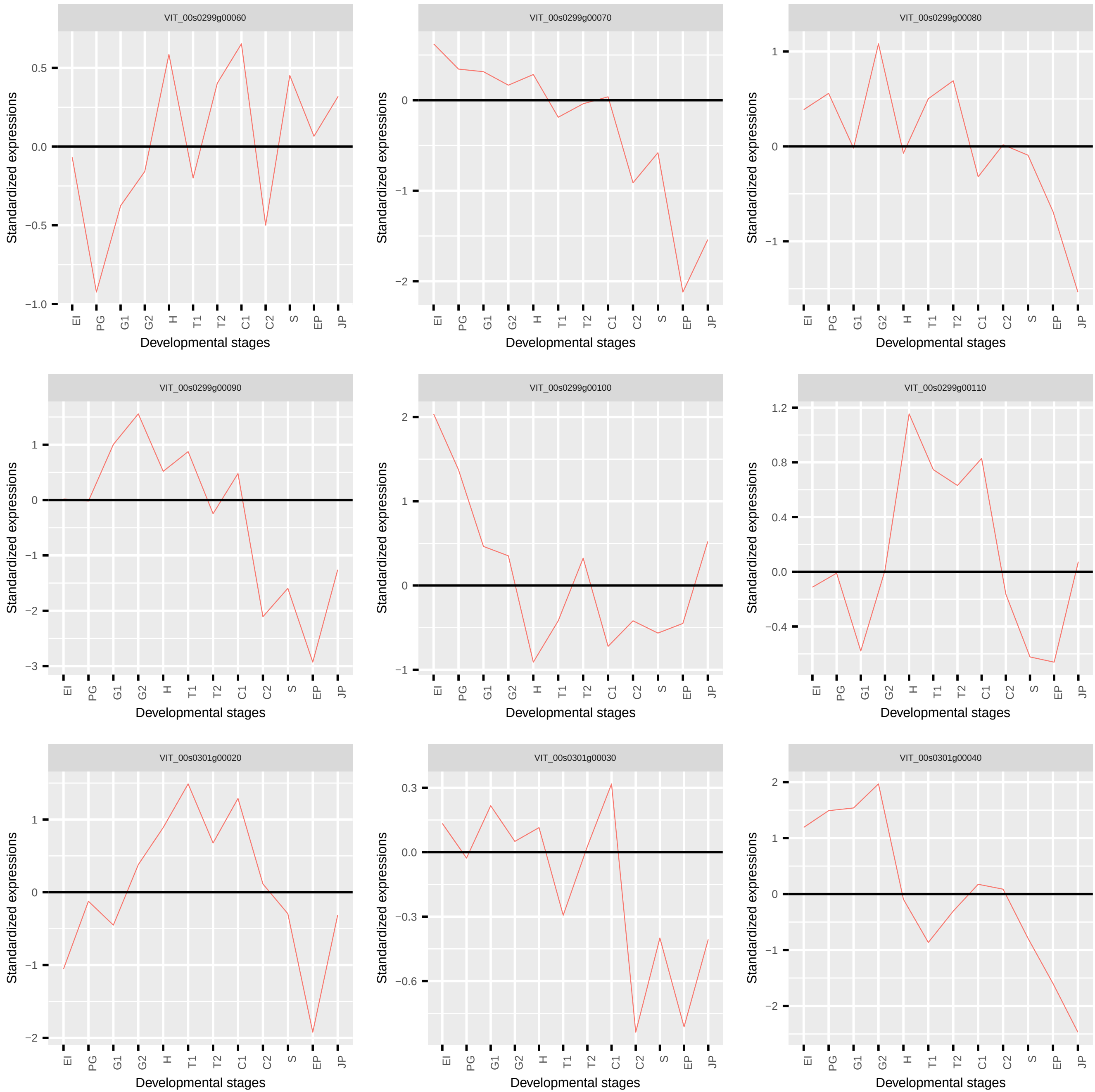
VIT_00s0299g00060
VIT_00s0299g00070
VIT_00s0299g00080
VIT_00s0299g00090
VIT_00s0299g00100
VIT_00s0299g00110
VIT_00s0301g00020
VIT_00s0301g00030
VIT_00s0301g00040
Standardized expressions
Developmental stages
EI PG G1 G2 H T1 T2 C1 C2 S EP JP
0.5
0.0
−0.5
−1.0
0
−1
−2
1
2
1.2
0.8
0.4
−0.4
−3
0.3
−0.3
−0.6

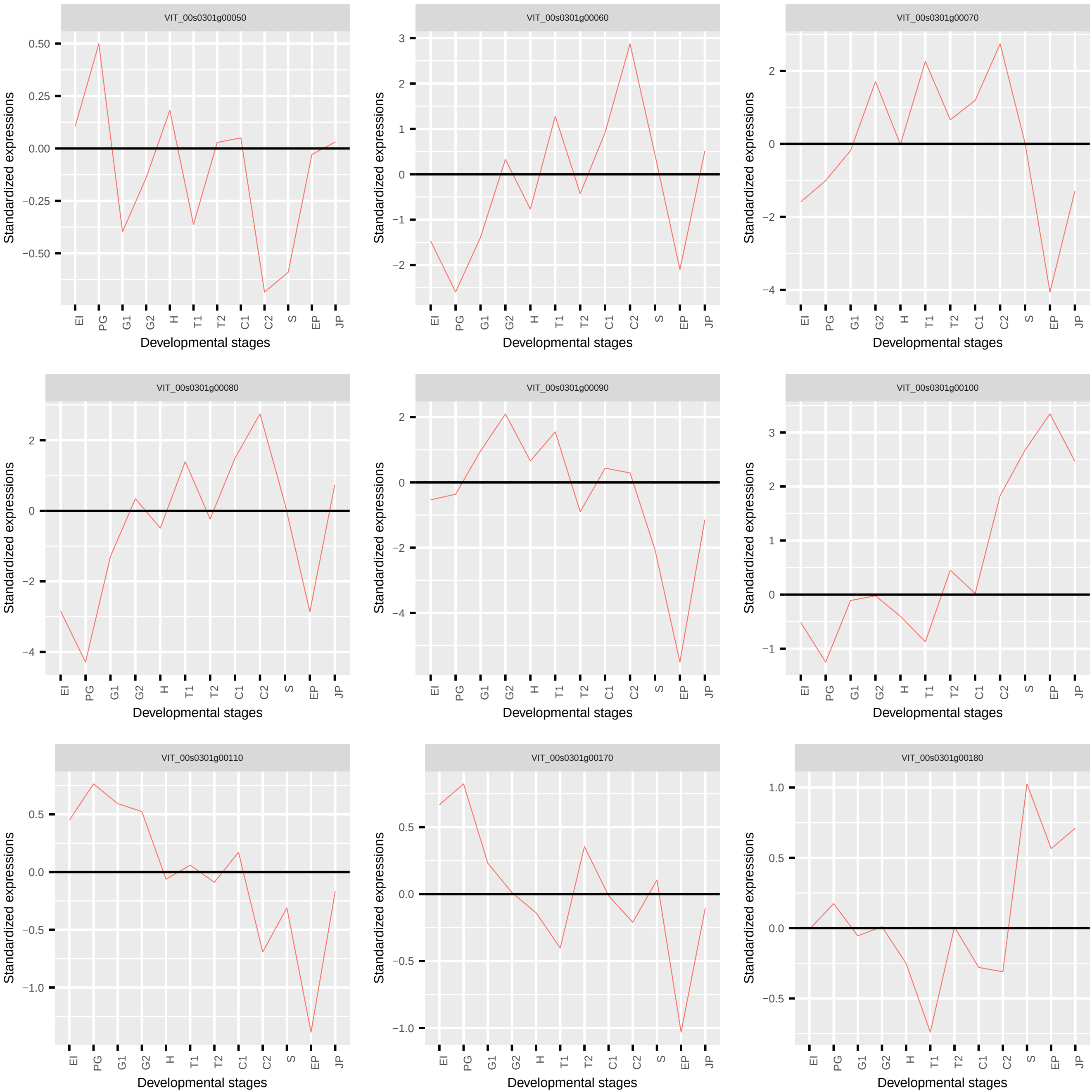
VIT_00s0301g00050
Standardized expressions
0.50
0.25
0.00
−0.25
−0.50
EI PG G1 G2 H T1 T2 C1 C2 S EP JP
Developmental stages
VIT_00s0301g00060
Standardized expressions
3
2
1
0
−1
−2
EI PG G1 G2 H T1 T2 C1 C2 S EP JP
Developmental stages
VIT_00s0301g00070
Standardized expressions
2
0
−2
−4
EI PG G1 G2 H T1 T2 C1 C2 S EP JP
Developmental stages
VIT_00s0301g00080
Standardized expressions
2
0
−2
−4
EI PG G1 G2 H T1 T2 C1 C2 S EP JP
Developmental stages
VIT_00s0301g00090
Standardized expressions
2
0
−2
−4
EI PG G1 G2 H T1 T2 C1 C2 S EP JP
Developmental stages
VIT_00s0301g00100
Standardized expressions
3
2
1
0
−1
EI PG G1 G2 H T1 T2 C1 C2 S EP JP
Developmental stages
VIT_00s0301g00110
Standardized expressions
0.5
0.0
−0.5
−1.0
EI PG G1 G2 H T1 T2 C1 C2 S EP JP
Developmental stages
VIT_00s0301g00170
Standardized expressions
0.5
0.0
−0.5
−1.0
EI PG G1 G2 H T1 T2 C1 C2 S EP JP
Developmental stages
VIT_00s0301g00180
Standardized expressions
1.0
0.5
0.0
−0.5
EI PG G1 G2 H T1 T2 C1 C2 S EP JP
Developmental stages

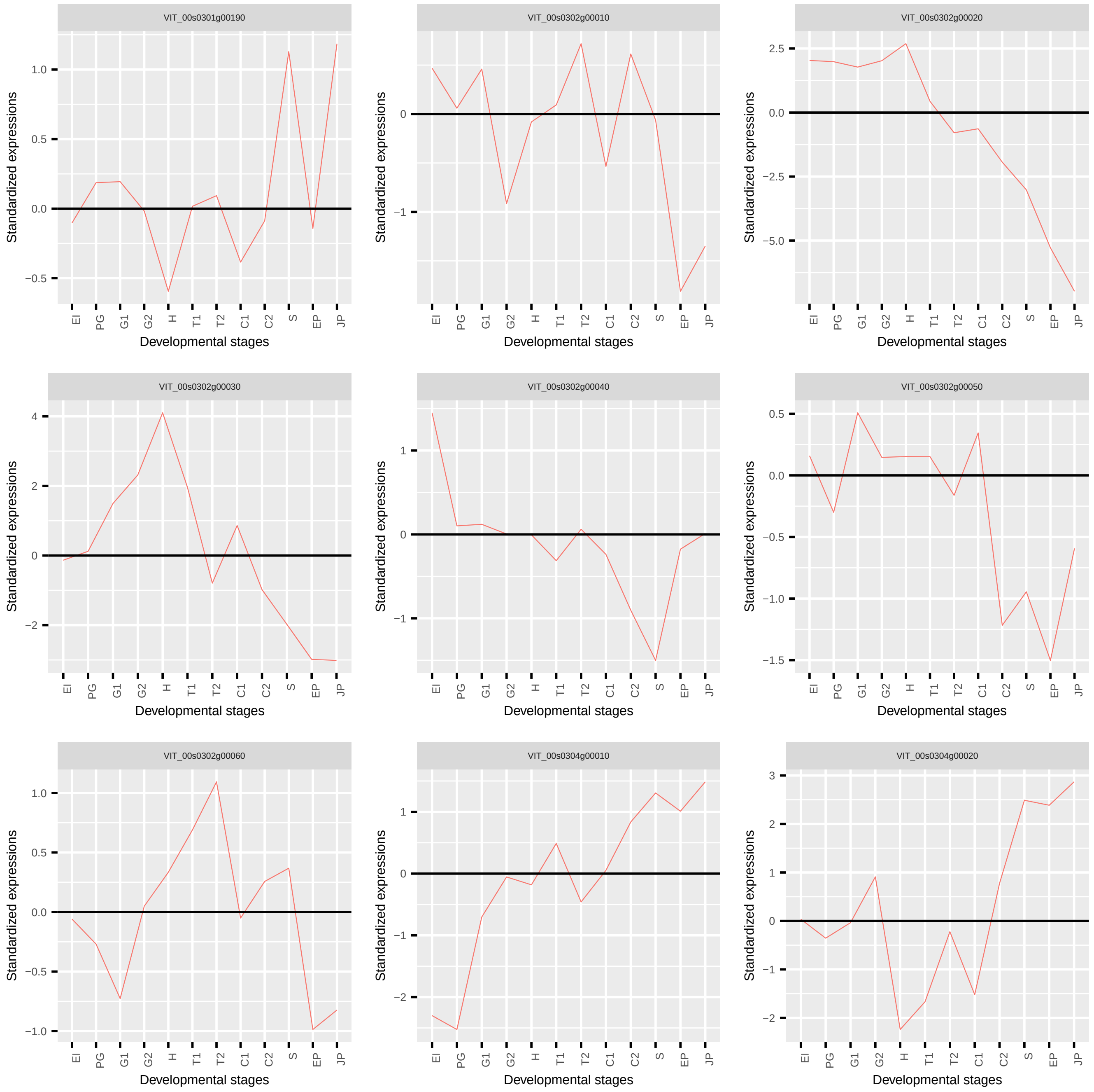
VIT_00s0301g00190
VIT_00s0302g00010
VIT_00s0302g00020
VIT_00s0302g00030
VIT_00s0302g00040
VIT_00s0302g00050
VIT_00s0302g00060
VIT_00s0304g00010
VIT_00s0304g00020
Standardized expressions
Developmental stages
EI PG G1 G2 H T1 T2 C1 C2 S EP JP
1.0 0.5 0.0 −0.5
0 −1
2.5 0.0 −2.5 −5.0
4 2 0 −2
1 0 −1
0.5 0.0 −0.5 −1.0 −1.5
1.0 0.5 0.0 −0.5 −1.0
1 0 −1 −2
3 2 1 0 −1 −2

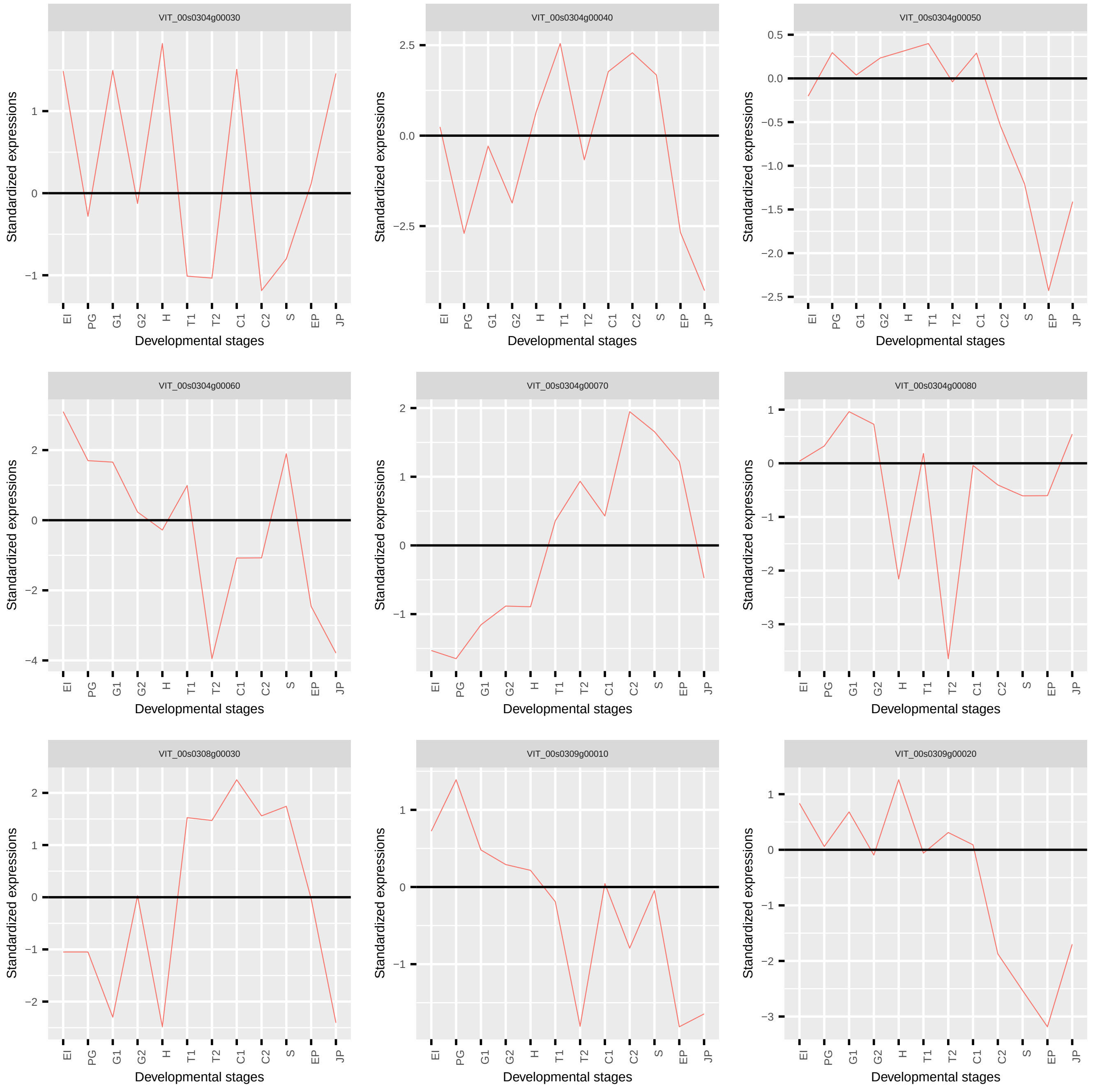
VIT_00s0304g00030
VIT_00s0304g00040
VIT_00s0304g00050
VIT_00s0304g00060
VIT_00s0304g00070
VIT_00s0304g00080
VIT_00s0308g00030
VIT_00s0309g00010
VIT_00s0309g00020
Standardized expressions
Developmental stages
EI PG G1 G2 H T1 T2 C1 C2 S EP JP

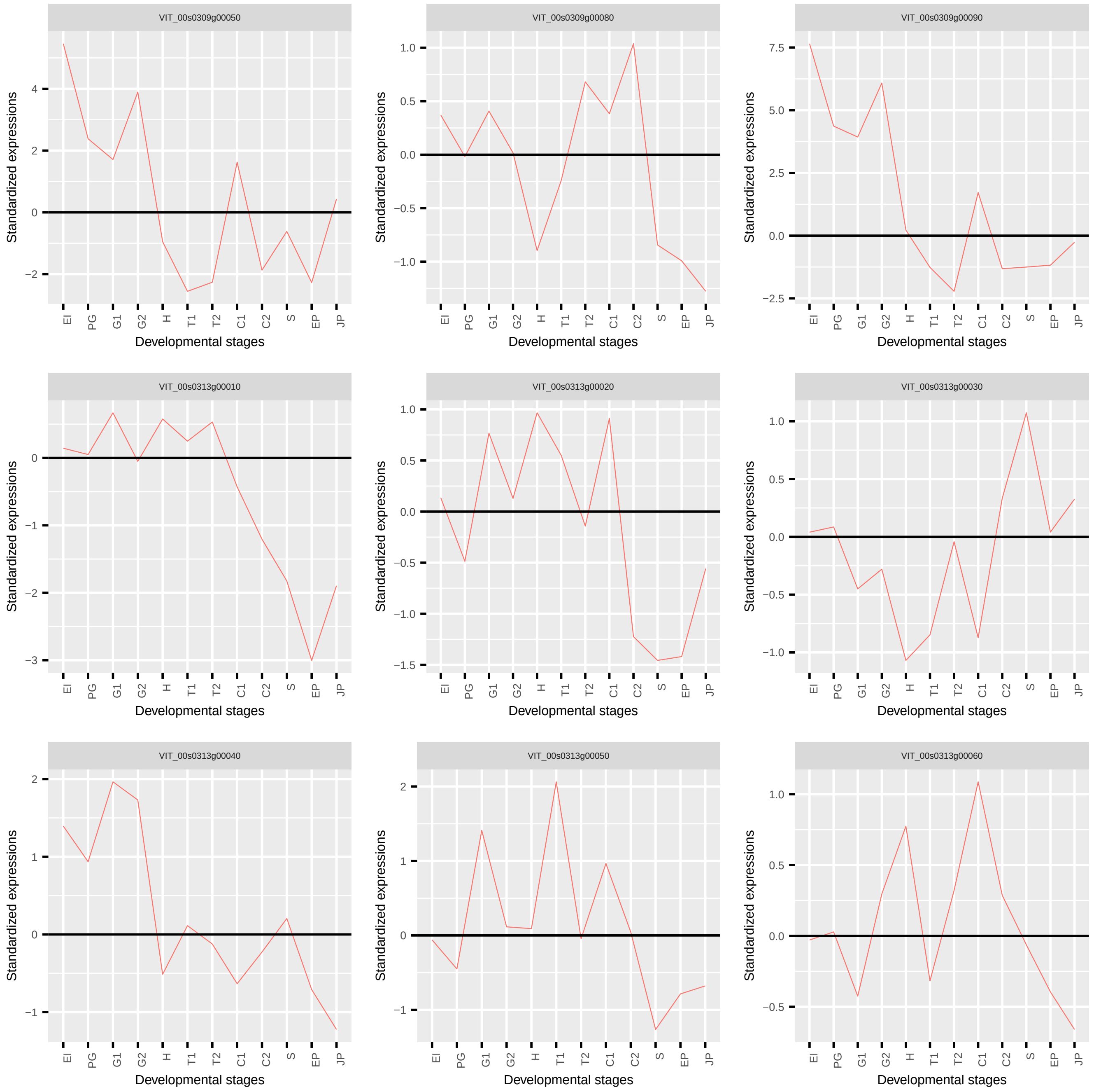

VIT_00s0309g00050
VIT_00s0309g00080
VIT_00s0309g00090
VIT_00s0313g00010
VIT_00s0313g00020
VIT_00s0313g00030
VIT_00s0313g00040
VIT_00s0313g00050
VIT_00s0313g00060
Standardized expressions
Developmental stages
EI
PG
G1
G2
H
T1
T2
C1
C2
S
EP
JP

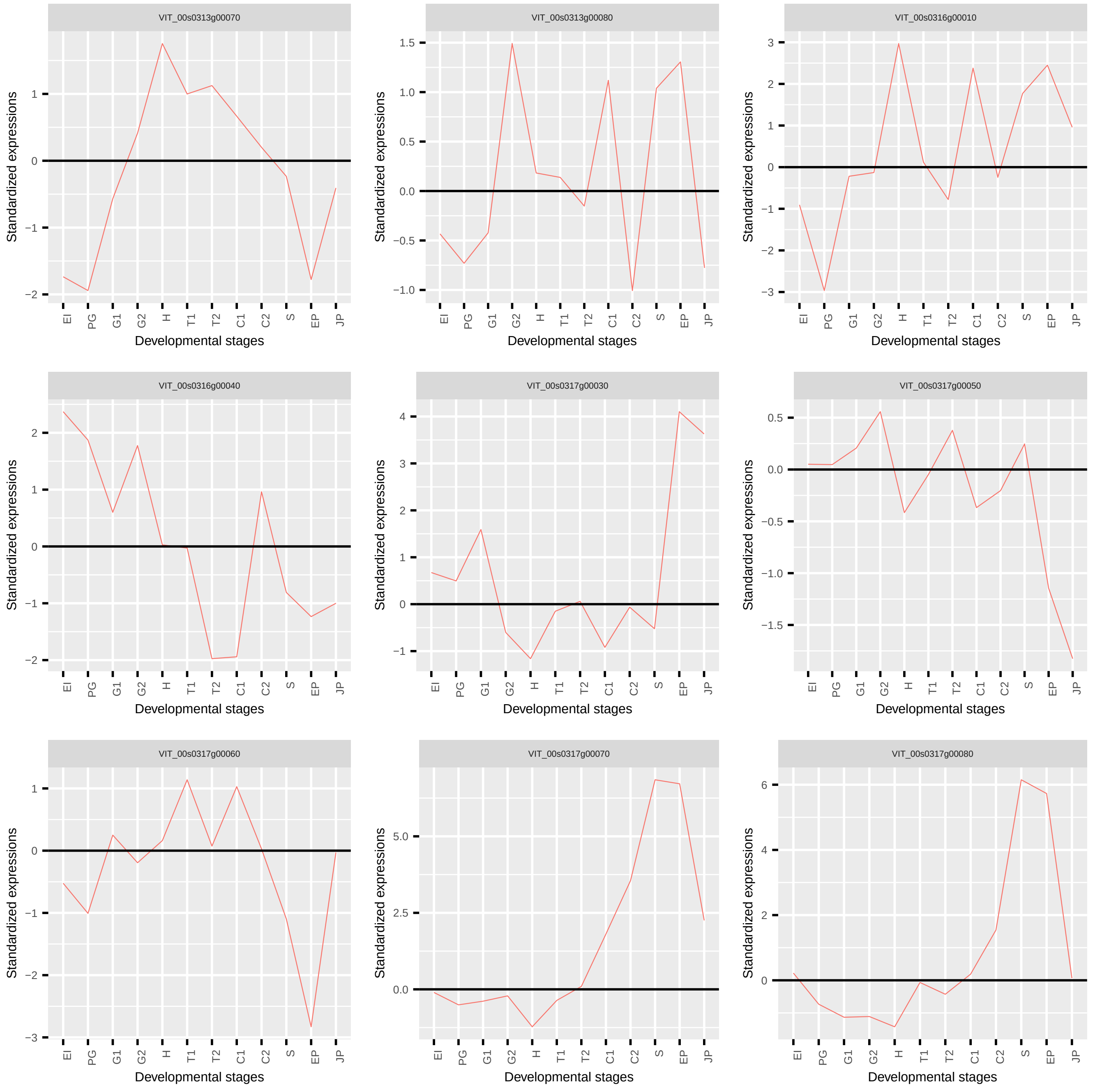
VIT_00s0313g00070
VIT_00s0313g00080
VIT_00s0316g00010
VIT_00s0316g00040
VIT_00s0317g00030
VIT_00s0317g00050
VIT_00s0317g00060
VIT_00s0317g00070
VIT_00s0317g00080
Standardized expressions
Developmental stages
EI PG G1 G2 H T1 T2 C1 C2 S EP JP

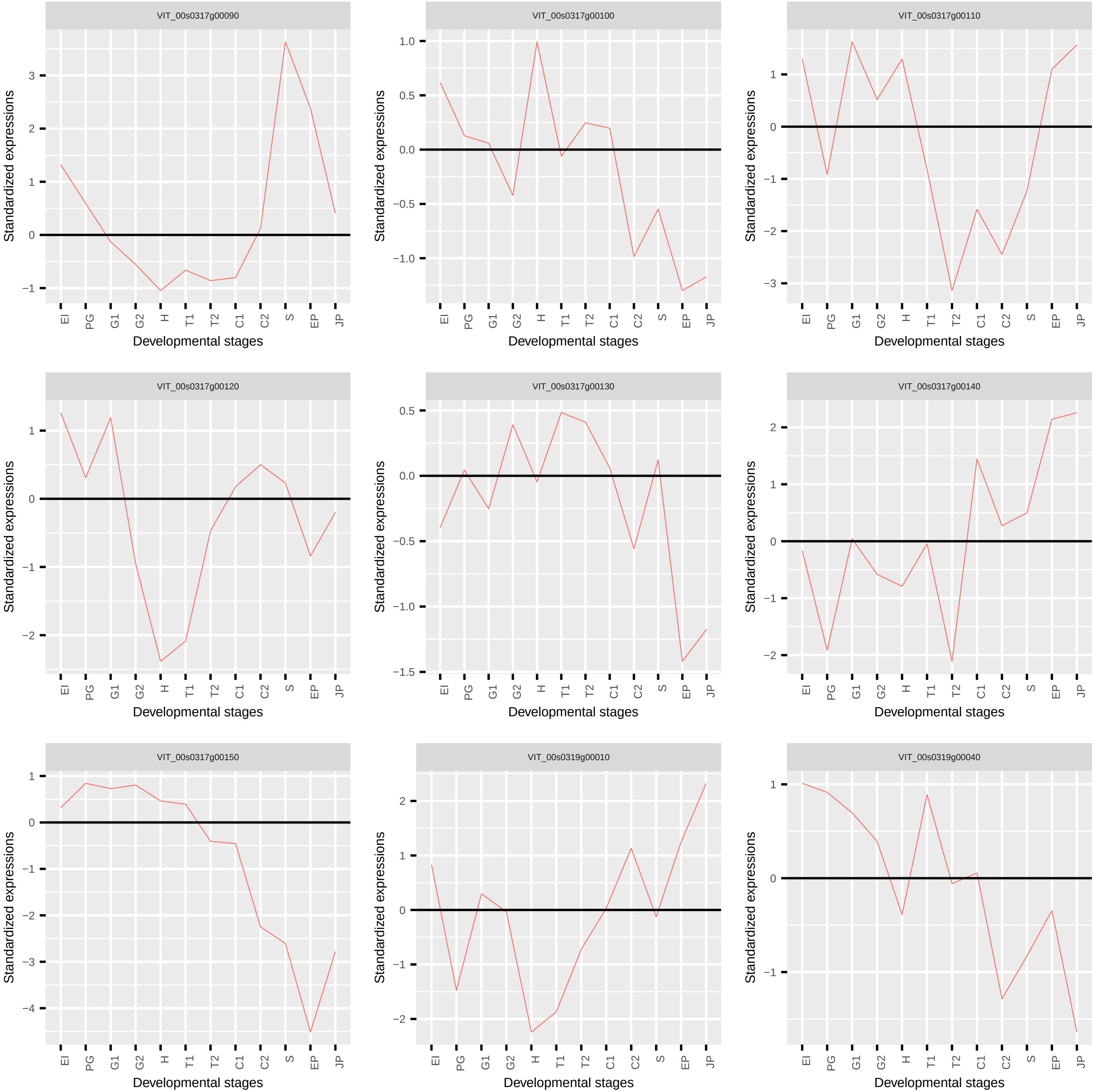
VIT_00s0317g00090
VIT_00s0317g00100
VIT_00s0317g00110
VIT_00s0317g00120
VIT_00s0317g00130
VIT_00s0317g00140
VIT_00s0317g00150
VIT_00s0319g00010
VIT_00s0319g00040
Standardized expressions
Developmental stages
EI PG G1 G2 H T1 T2 C1 C2 S EP JP

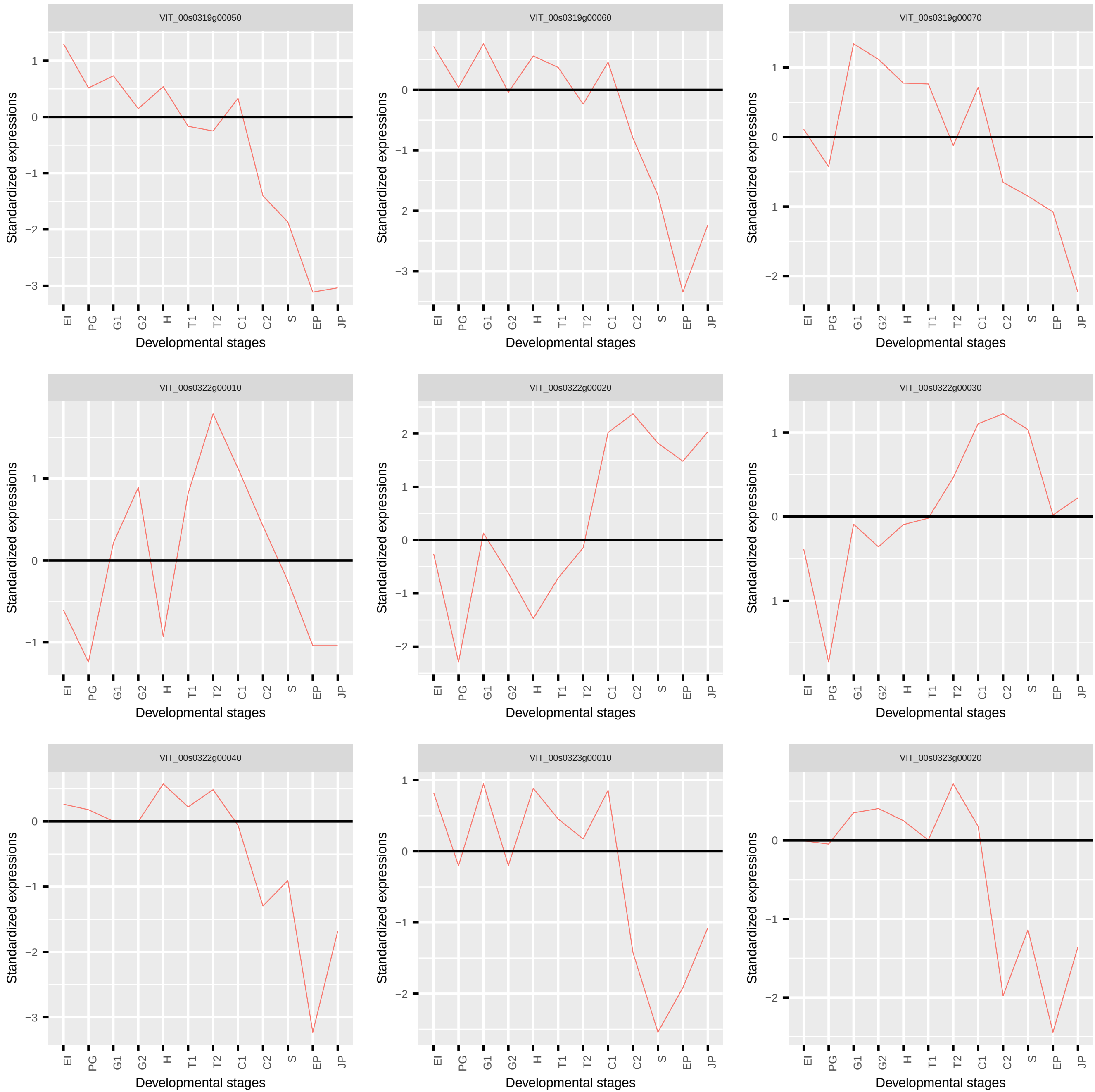
VIT_00s0319g00050
VIT_00s0319g00060
VIT_00s0319g00070
VIT_00s0322g00010
VIT_00s0322g00020
VIT_00s0322g00030
VIT_00s0322g00040
VIT_00s0323g00010
VIT_00s0323g00020
Standardized expressions
Developmental stages
EI PG G1 G2 H T1 T2 C1 C2 S EP JP

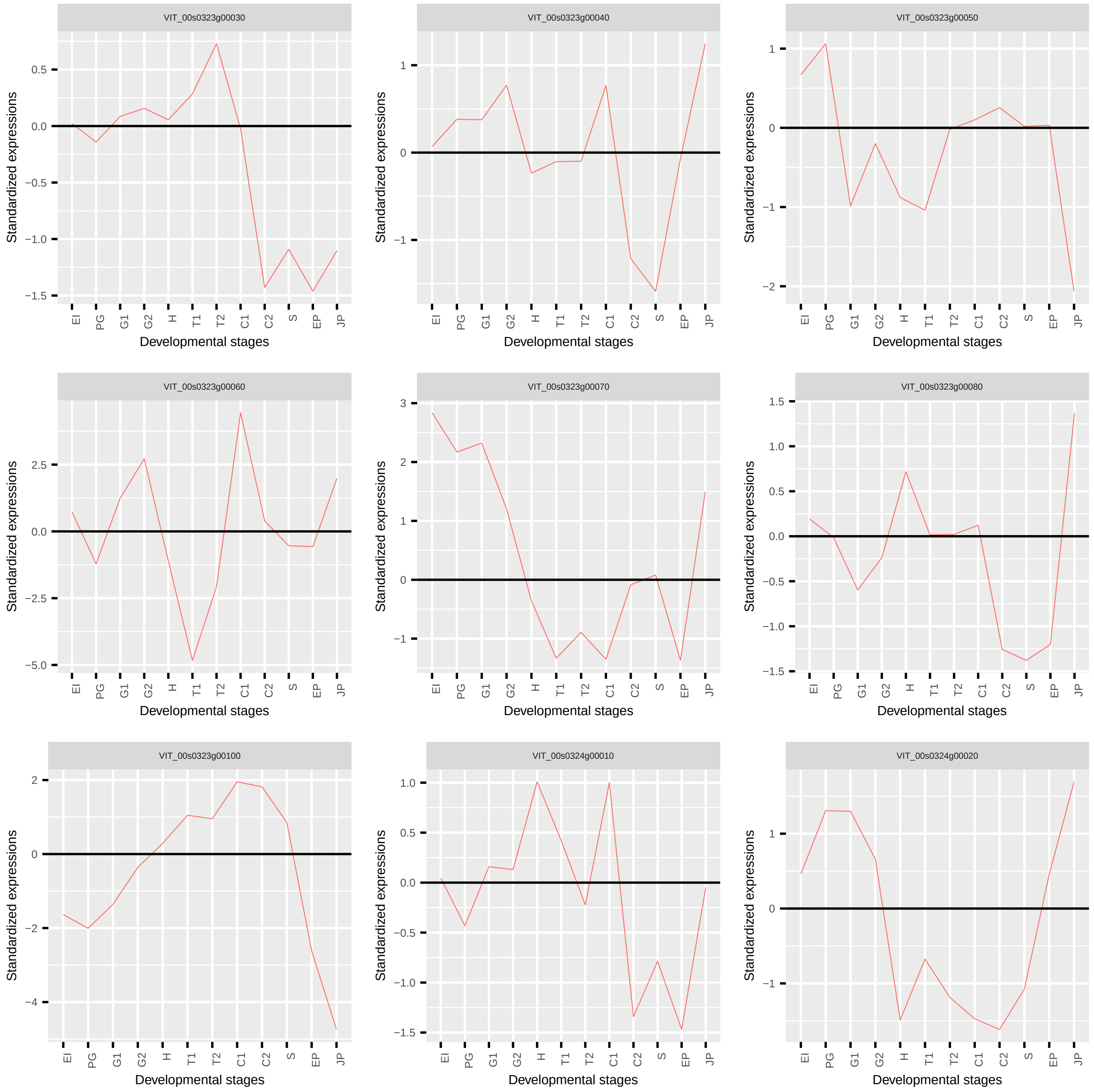

VIT_00s0323g00030
VIT_00s0323g00040
VIT_00s0323g00050
VIT_00s0323g00060
VIT_00s0323g00070
VIT_00s0323g00080
VIT_00s0323g00100
VIT_00s0324g00010
VIT_00s0324g00020
Standardized expressions
Developmental stages
EI
PG
G1
G2
H
T1
T2
C1
C2
S
EP
JP

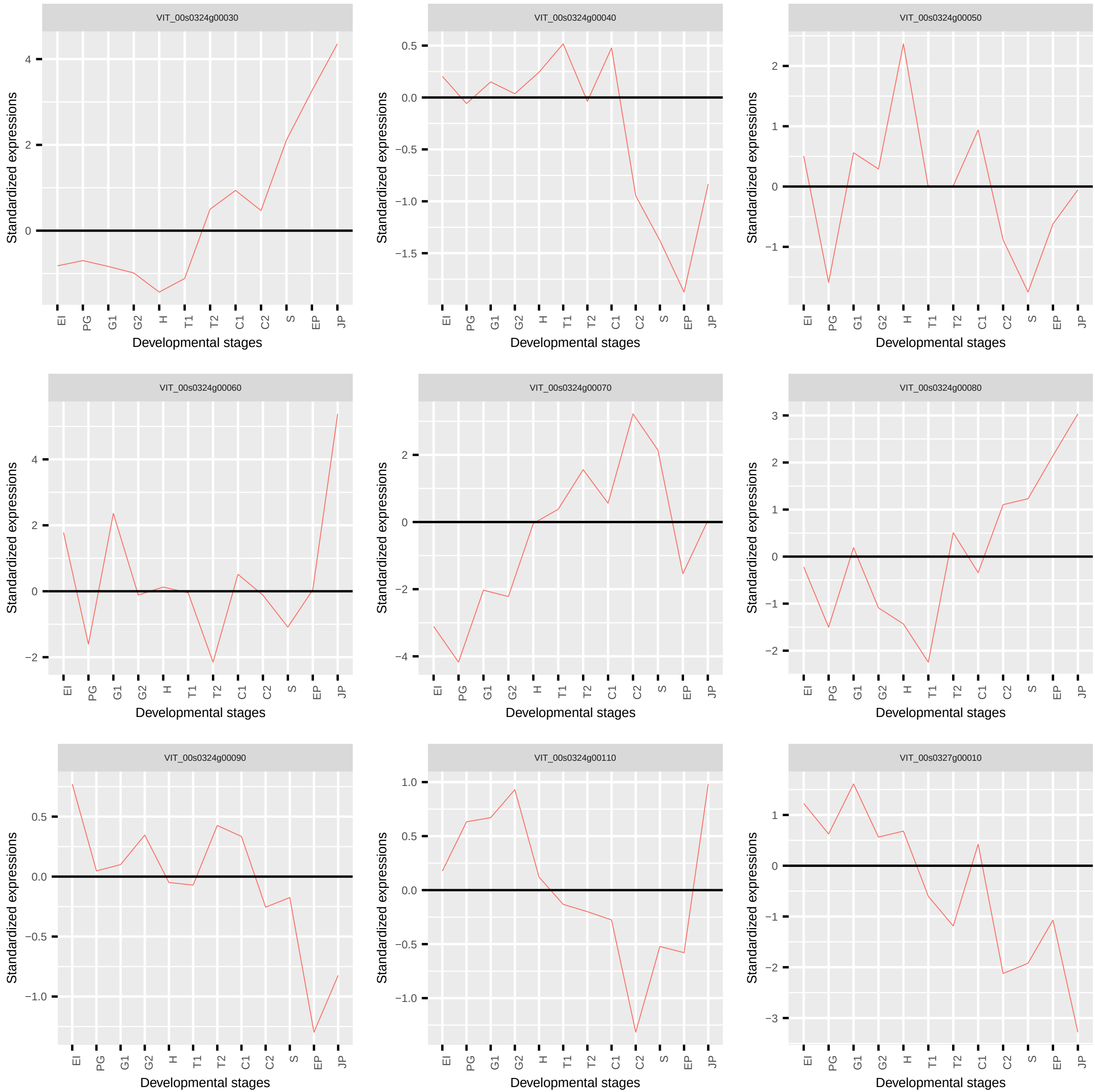
VIT_00s0324g00030
VIT_00s0324g00040
VIT_00s0324g00050
VIT_00s0324g00060
VIT_00s0324g00070
VIT_00s0324g00080
VIT_00s0324g00090
VIT_00s0324g00110
VIT_00s0327g00010
Standardized expressions
Developmental stages
EI
PG
G1
G2
H
T1
T2
C1
C2
S
EP
JP

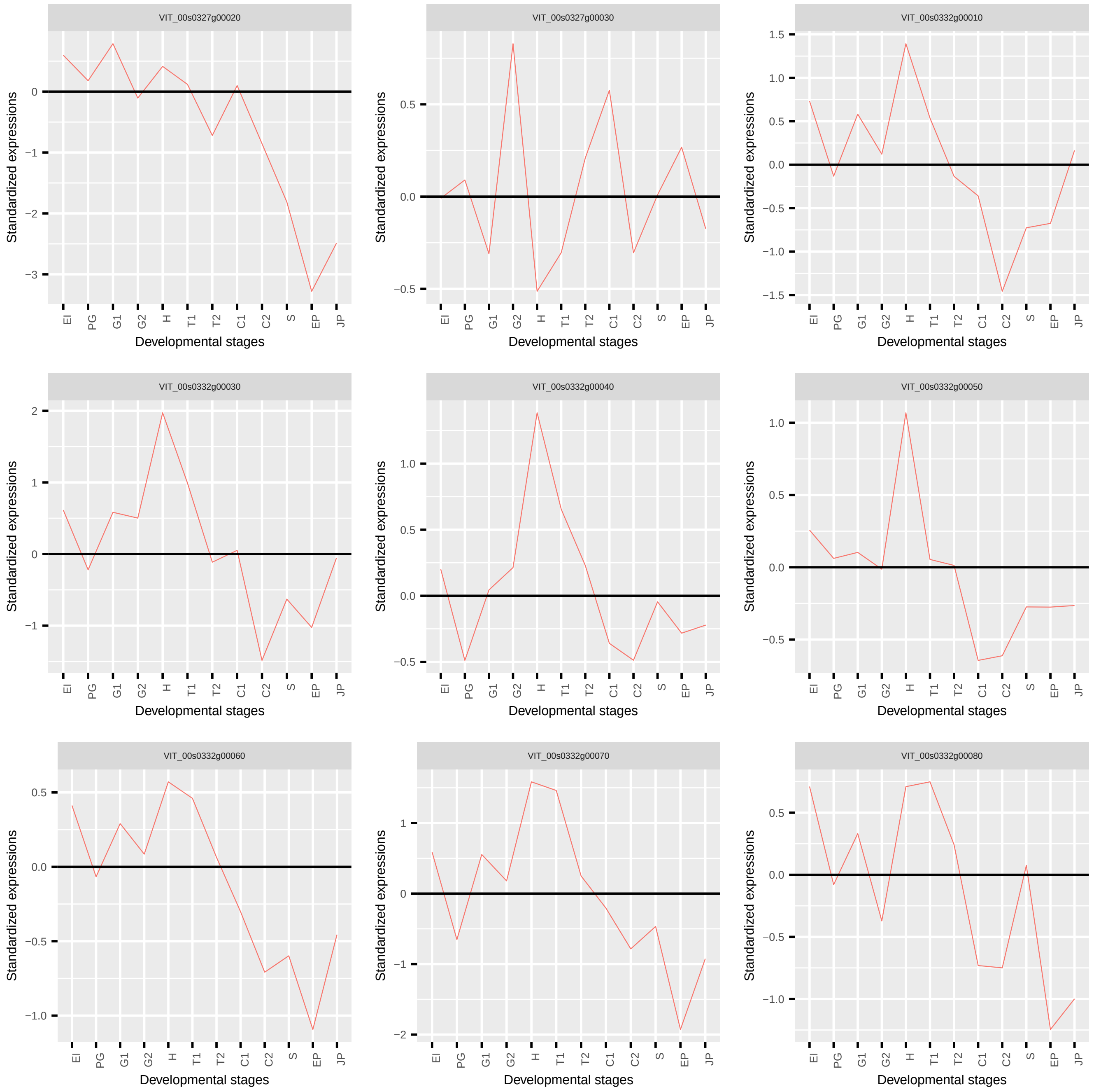
VIT_00s0327g00020
VIT_00s0327g00030
VIT_00s0332g00010
VIT_00s0332g00030
VIT_00s0332g00040
VIT_00s0332g00050
VIT_00s0332g00060
VIT_00s0332g00070
VIT_00s0332g00080
Standardized expressions
Developmental stages
EI PG G1 G2 H T1 T2 C1 C2 S EP JP

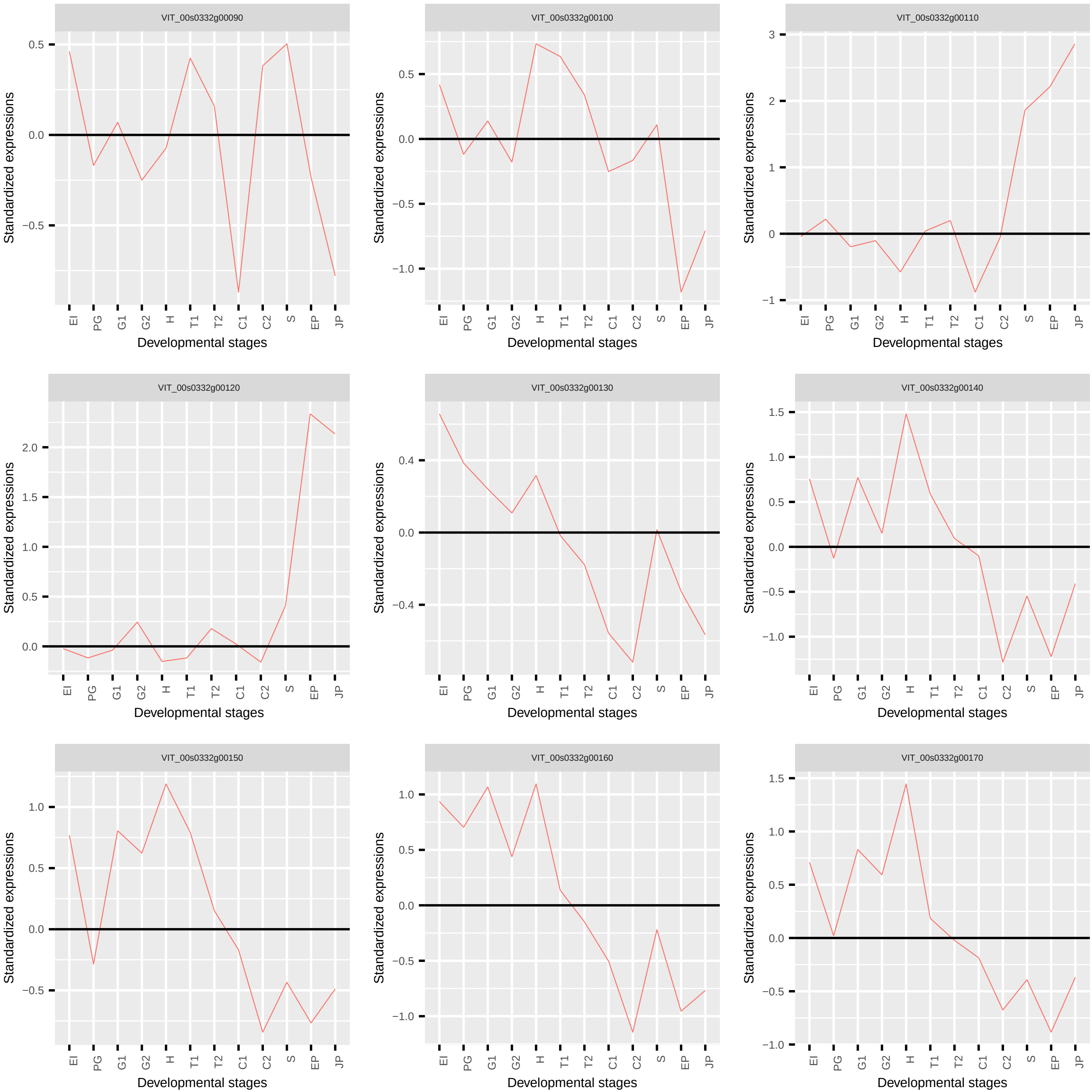
VIT_00s0332g00090
VIT_00s0332g00100
VIT_00s0332g00110
VIT_00s0332g00120
VIT_00s0332g00130
VIT_00s0332g00140
VIT_00s0332g00150
VIT_00s0332g00160
VIT_00s0332g00170
Standardized expressions
Developmental stages
EI PG G1 G2 H T1 T2 C1 C2 S EP JP

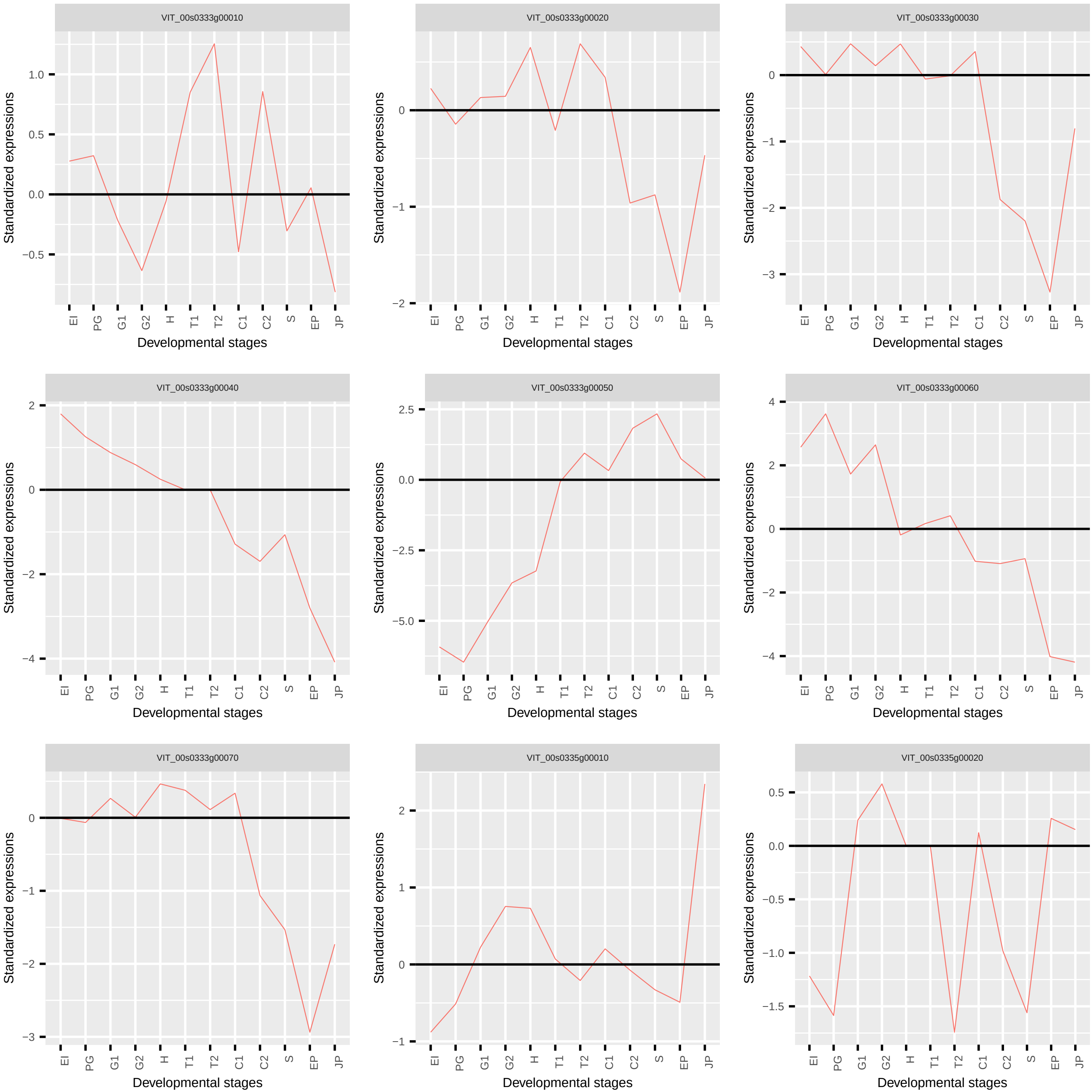

VIT_00s0333g00010
1.0
0.5
0.0
−0.5
Standardized expressions
EI PG G1 G2 H T1 T2 C1 C2 S EP JP
Developmental stages
VIT_00s0333g00020
0
−1
−2
Standardized expressions
EI PG G1 G2 H T1 T2 C1 C2 S EP JP
Developmental stages
VIT_00s0333g00030
0
−1
−2
−3
Standardized expressions
EI PG G1 G2 H T1 T2 C1 C2 S EP JP
Developmental stages
VIT_00s0333g00040
2
0
−2
−4
Standardized expressions
EI PG G1 G2 H T1 T2 C1 C2 S EP JP
Developmental stages
VIT_00s0333g00050
2.5
0.0
−2.5
−5.0
Standardized expressions
EI PG G1 G2 H T1 T2 C1 C2 S EP JP
Developmental stages
VIT_00s0333g00060
4
2
0
−2
−4
Standardized expressions
EI PG G1 G2 H T1 T2 C1 C2 S EP JP
Developmental stages
VIT_00s0333g00070
0
−1
−2
−3
Standardized expressions
EI PG G1 G2 H T1 T2 C1 C2 S EP JP
Developmental stages
VIT_00s0335g00010
2
1
0
−1
Standardized expressions
EI PG G1 G2 H T1 T2 C1 C2 S EP JP
Developmental stages
VIT_00s0335g00020
0.5
0.0
−0.5
−1.0
−1.5
Standardized expressions
EI PG G1 G2 H T1 T2 C1 C2 S EP JP
Developmental stages

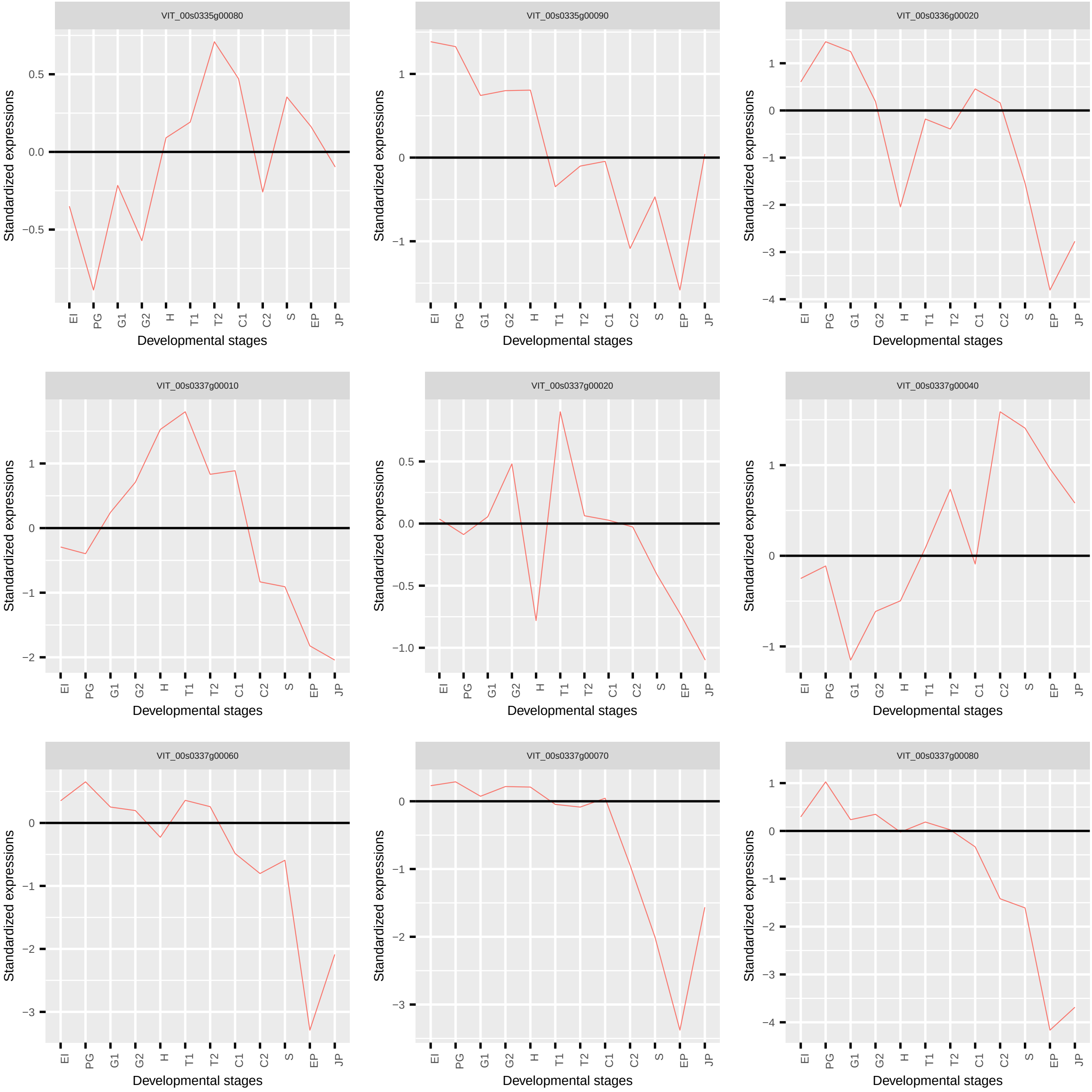
VIT_00s0335g00080
VIT_00s0335g00090
VIT_00s0336g00020
VIT_00s0337g00010
VIT_00s0337g00020
VIT_00s0337g00040
VIT_00s0337g00060
VIT_00s0337g00070
VIT_00s0337g00080
Standardized expressions
Developmental stages
EI PG G1 G2 H T1 T2 C1 C2 S EP JP

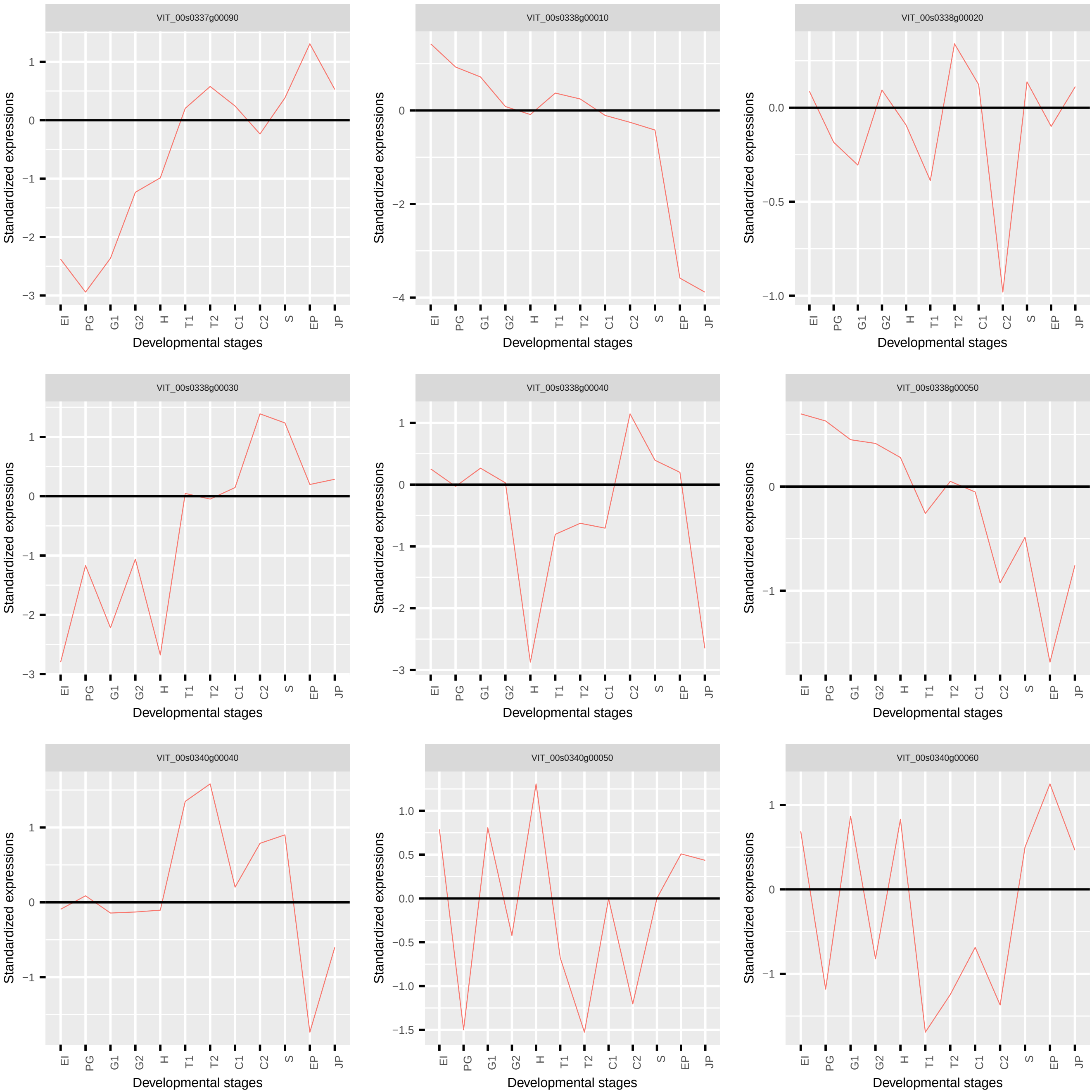
VIT_00s0337g00090
VIT_00s0338g00010
VIT_00s0338g00020
VIT_00s0338g00030
VIT_00s0338g00040
VIT_00s0338g00050
VIT_00s0340g00040
VIT_00s0340g00050
VIT_00s0340g00060
Standardized expressions
Developmental stages
EI PG G1 G2 H T1 T2 C1 C2 S EP JP

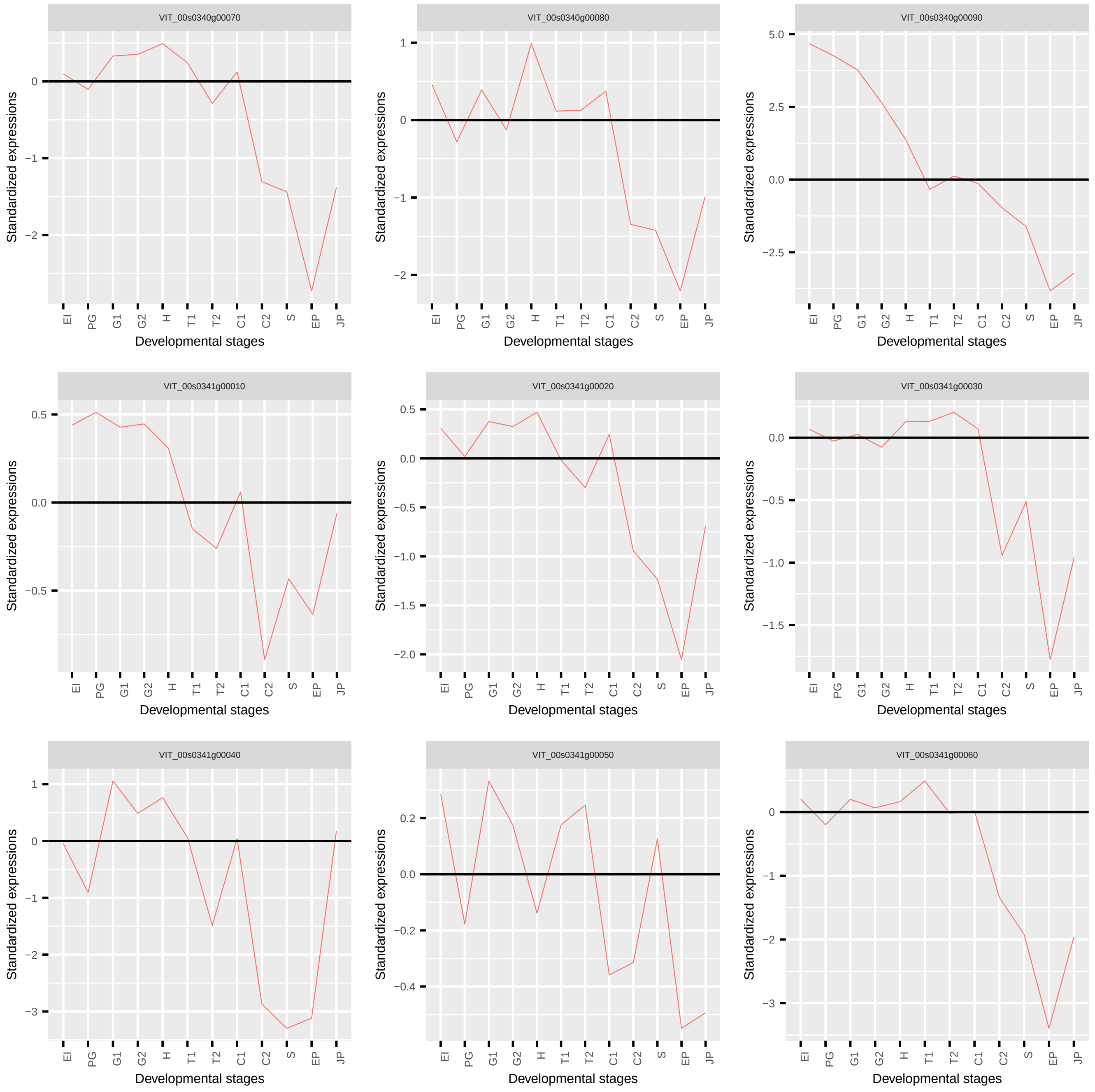
VIT_00s0340g00070
VIT_00s0340g00080
VIT_00s0340g00090
VIT_00s0341g00010
VIT_00s0341g00020
VIT_00s0341g00030
VIT_00s0341g00040
VIT_00s0341g00050
VIT_00s0341g00060
Standardized expressions
Developmental stages
EI
PG
G1
G2
H
T1
T2
C1
C2
S
EP
JP

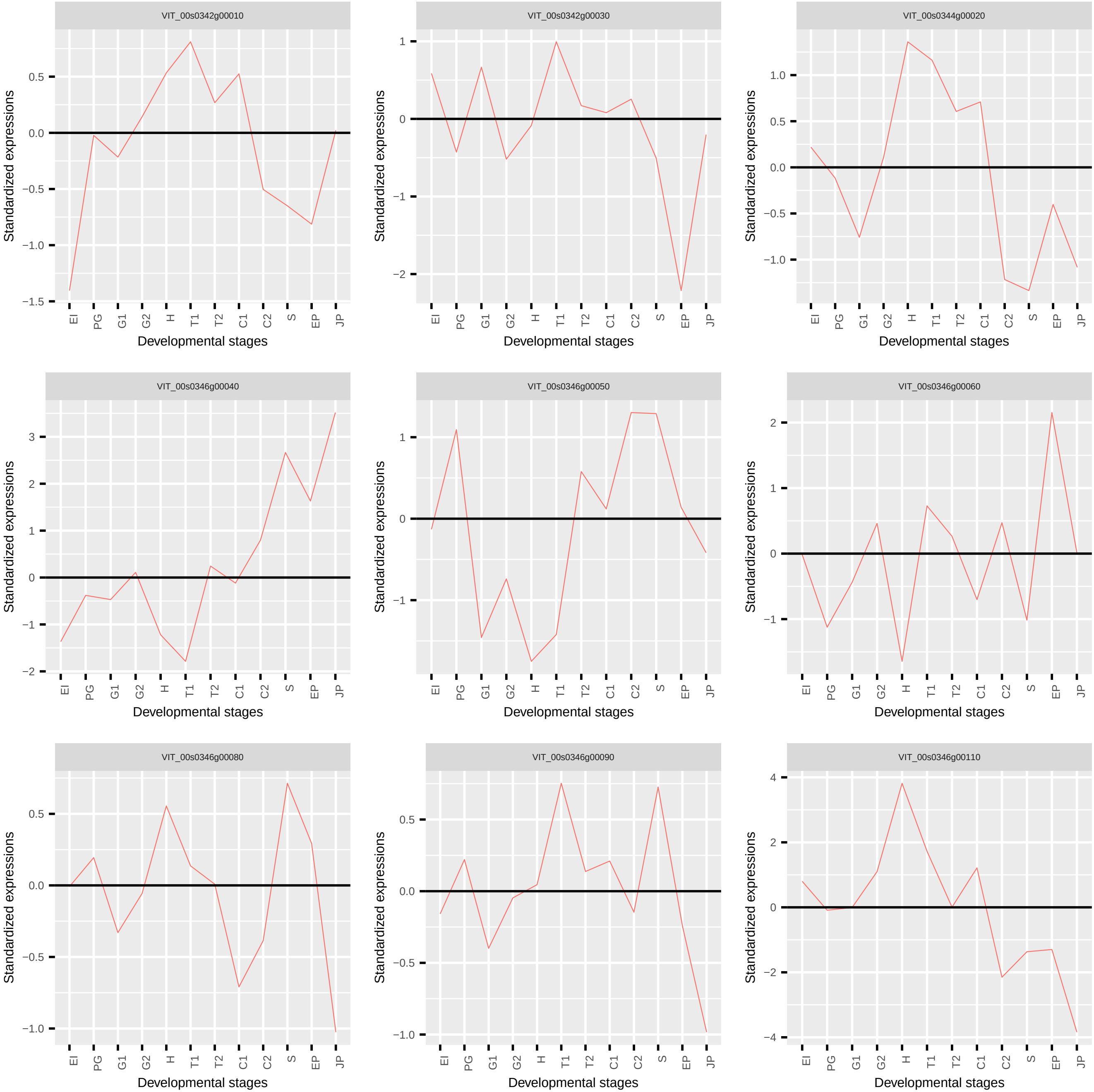
VIT_00s0342g00010
VIT_00s0342g00030
VIT_00s0344g00020
VIT_00s0346g00040
VIT_00s0346g00050
VIT_00s0346g00060
VIT_00s0346g00080
VIT_00s0346g00090
VIT_00s0346g00110
Standardized expressions
EI PG G1 G2 H T1 T2 C1 C2 S EP JP
Developmental stages

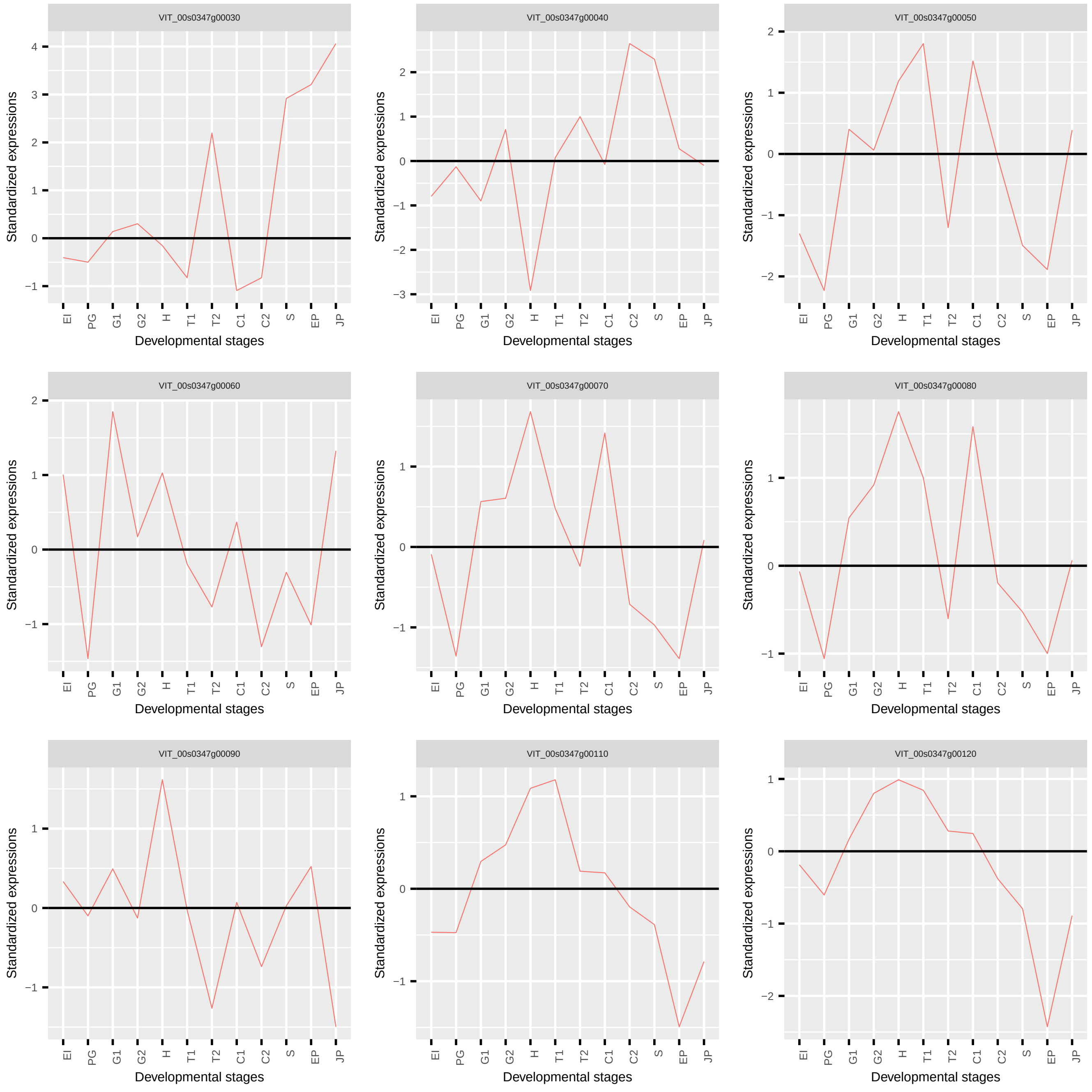

VIT_00s0347g00030
VIT_00s0347g00040
VIT_00s0347g00050
VIT_00s0347g00060
VIT_00s0347g00070
VIT_00s0347g00080
VIT_00s0347g00090
VIT_00s0347g00110
VIT_00s0347g00120
Standardized expressions
Developmental stages
EI PG G1 G2 H T1 T2 C1 C2 S EP JP

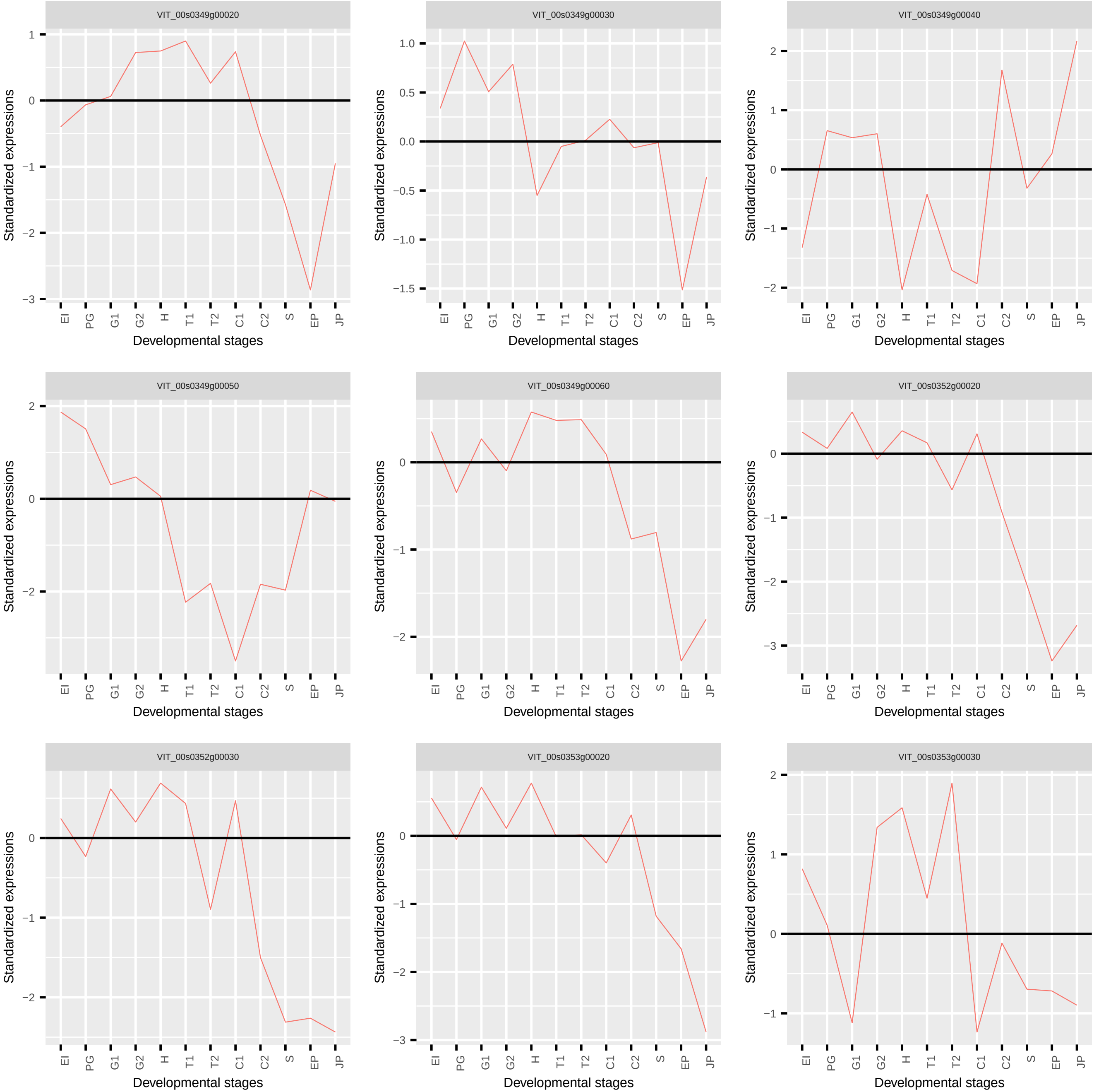

VIT_00s0349g00020
VIT_00s0349g00030
VIT_00s0349g00040
VIT_00s0349g00050
VIT_00s0349g00060
VIT_00s0352g00020
VIT_00s0352g00030
VIT_00s0353g00020
VIT_00s0353g00030
Standardized expressions
Developmental stages
EI PG G1 G2 H T1 T2 C1 C2 S EP JP

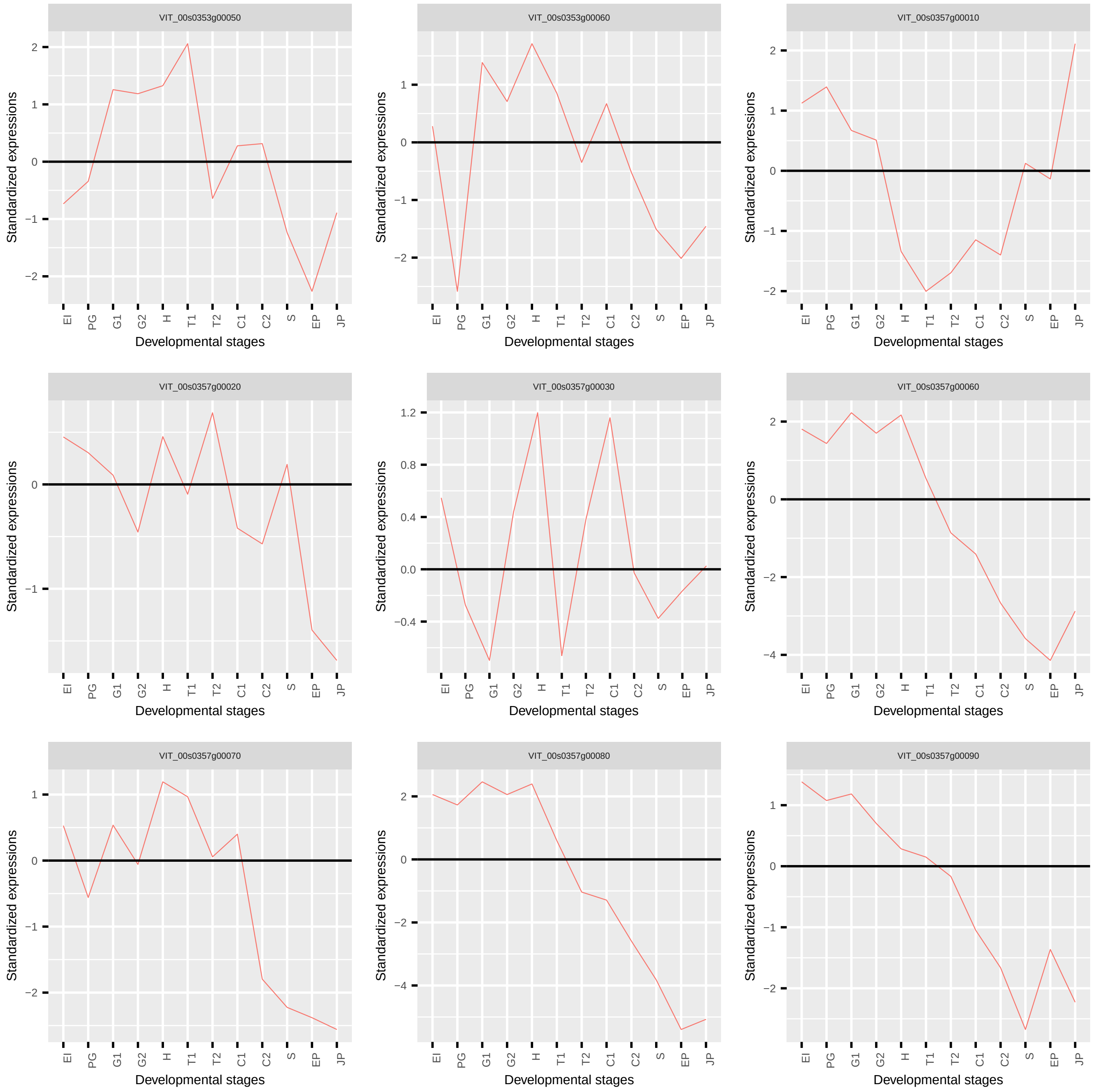

VIT_00s0353g00050
VIT_00s0353g00060
VIT_00s0357g00010
VIT_00s0357g00020
VIT_00s0357g00030
VIT_00s0357g00060
VIT_00s0357g00070
VIT_00s0357g00080
VIT_00s0357g00090
Standardized expressions
Developmental stages
EI PG G1 G2 H T1 T2 C1 C2 S EP JP

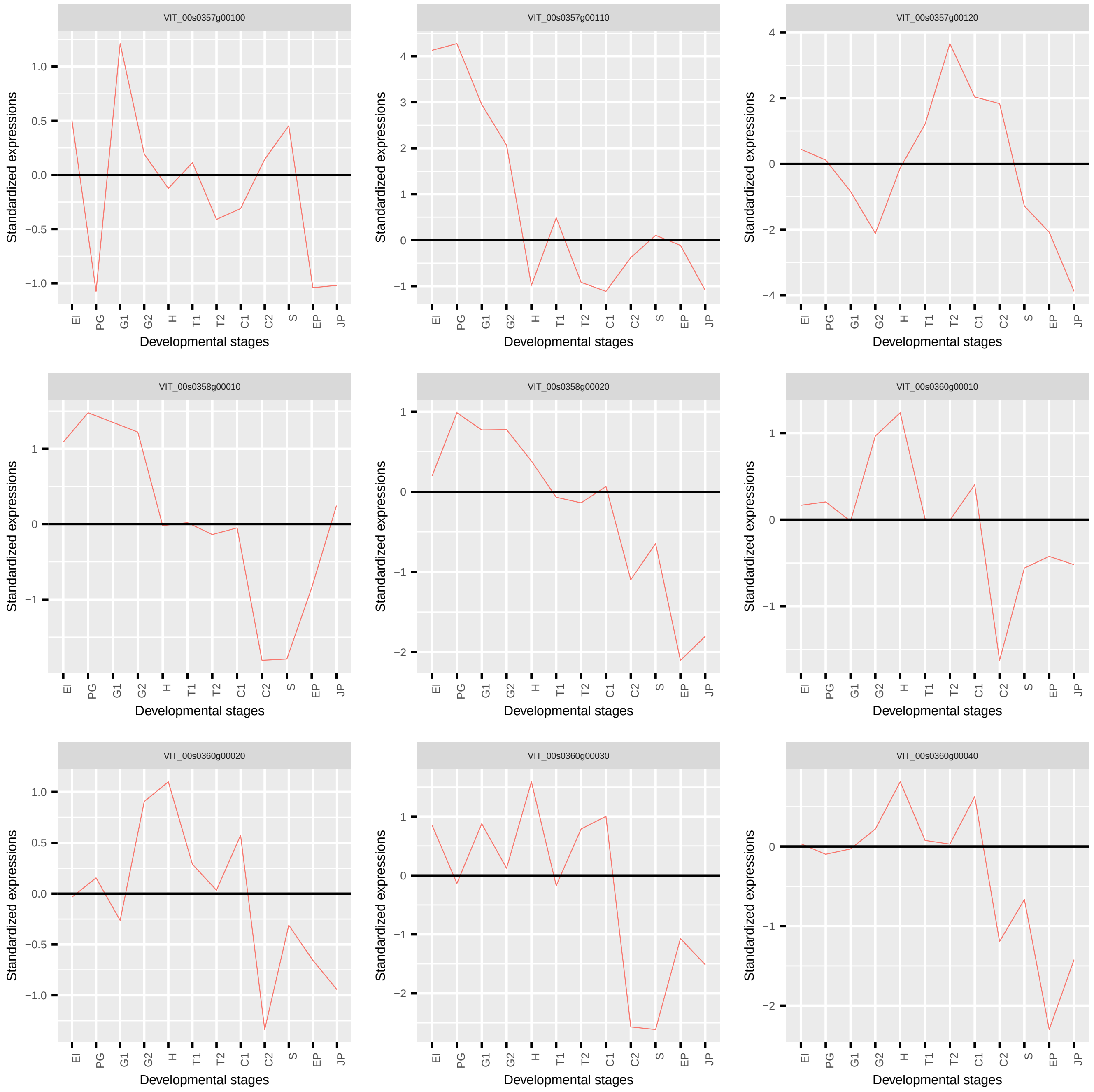
VIT_00s0357g00100
VIT_00s0357g00110
VIT_00s0357g00120
VIT_00s0358g00010
VIT_00s0358g00020
VIT_00s0360g00010
VIT_00s0360g00020
VIT_00s0360g00030
VIT_00s0360g00040
Standardized expressions
Developmental stages
EI PG G1 G2 H T1 T2 C1 C2 S EP JP

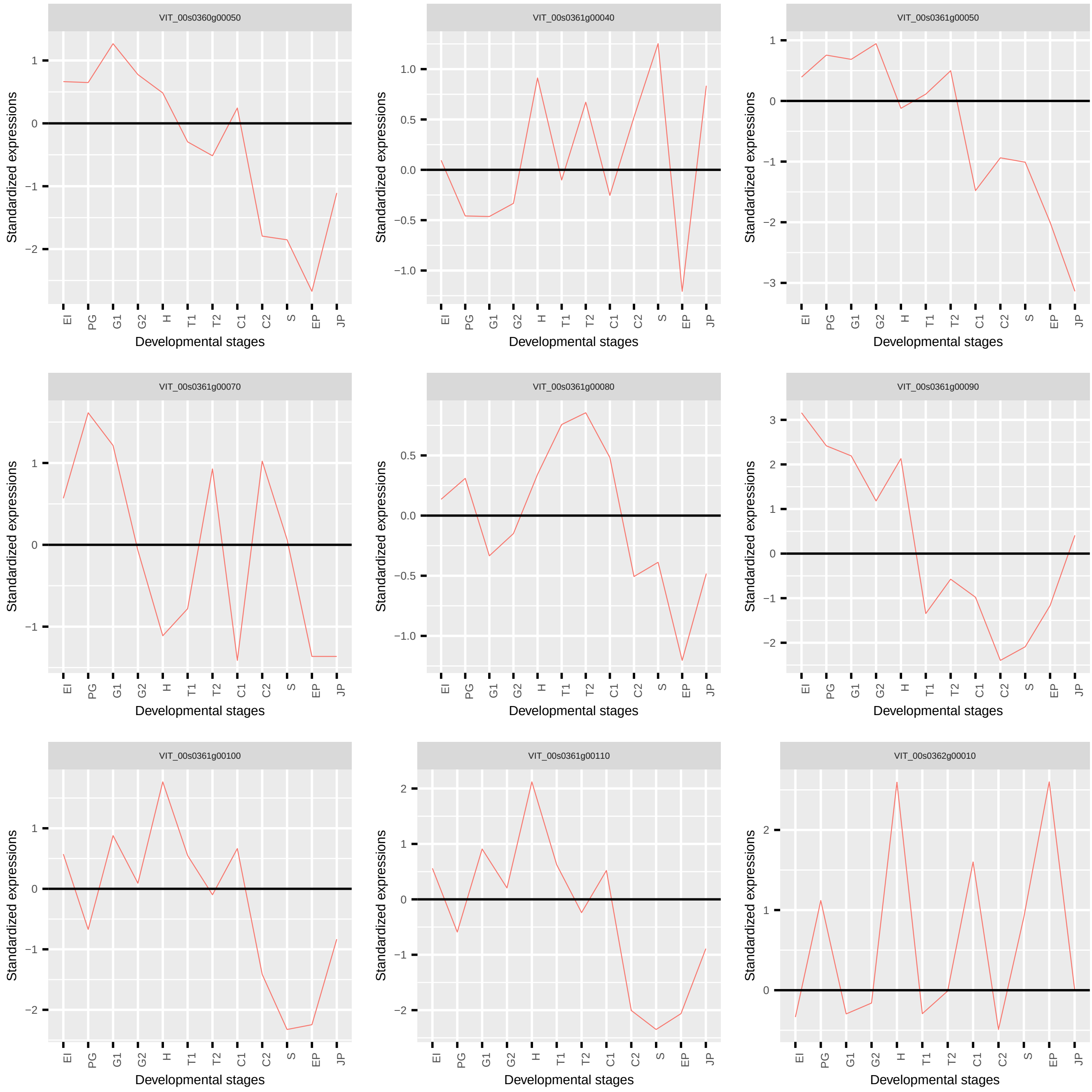
VIT_00s0360g00050
VIT_00s0361g00040
VIT_00s0361g00050
VIT_00s0361g00070
VIT_00s0361g00080
VIT_00s0361g00090
VIT_00s0361g00100
VIT_00s0361g00110
VIT_00s0362g00010
Standardized expressions
Developmental stages
EI PG G1 G2 H T1 T2 C1 C2 S EP JP

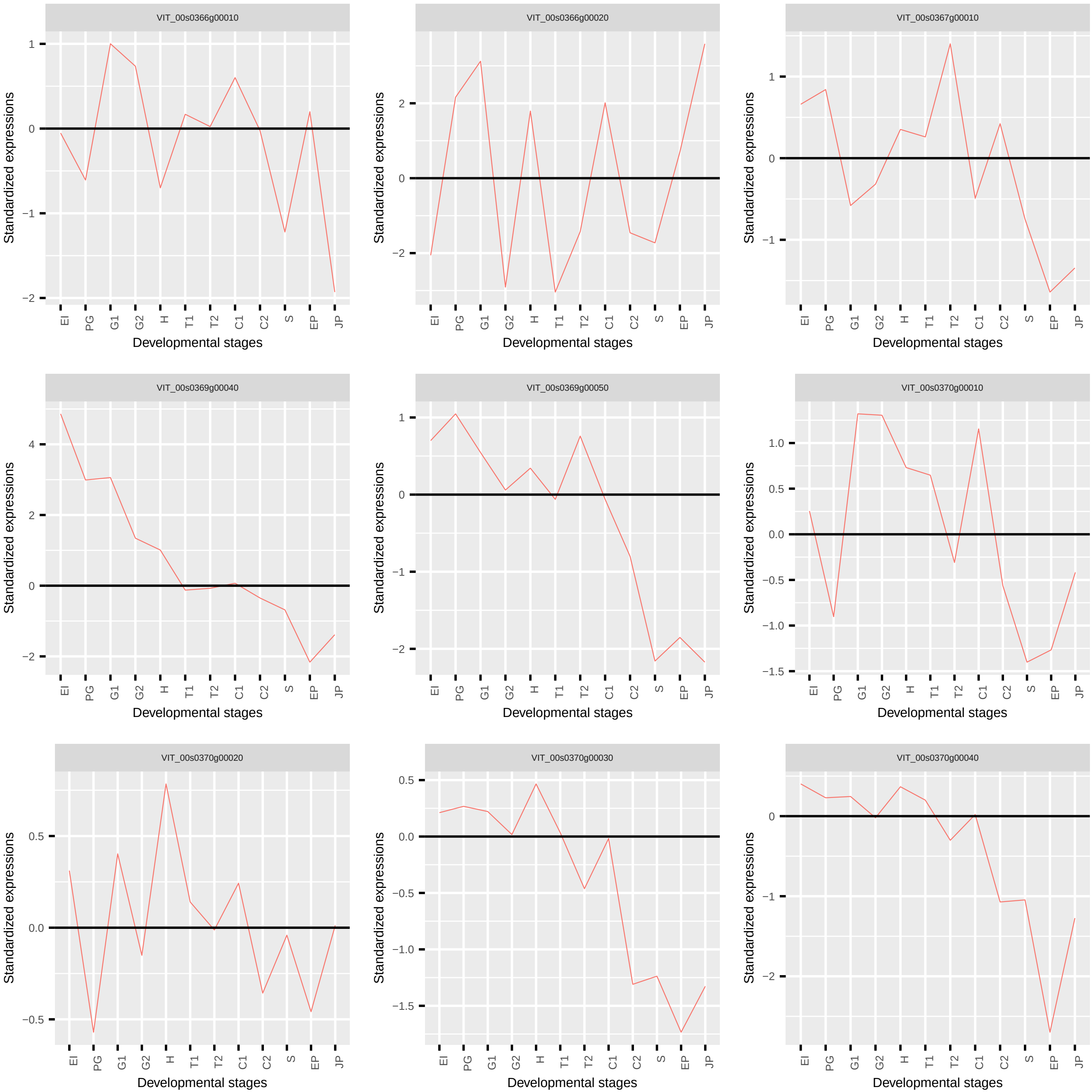
VIT_00s0366g00010
VIT_00s0366g00020
VIT_00s0367g00010
VIT_00s0369g00040
VIT_00s0369g00050
VIT_00s0370g00010
VIT_00s0370g00020
VIT_00s0370g00030
VIT_00s0370g00040
Standardized expressions
Developmental stages
EI PG G1 G2 H T1 T2 C1 C2 S EP JP

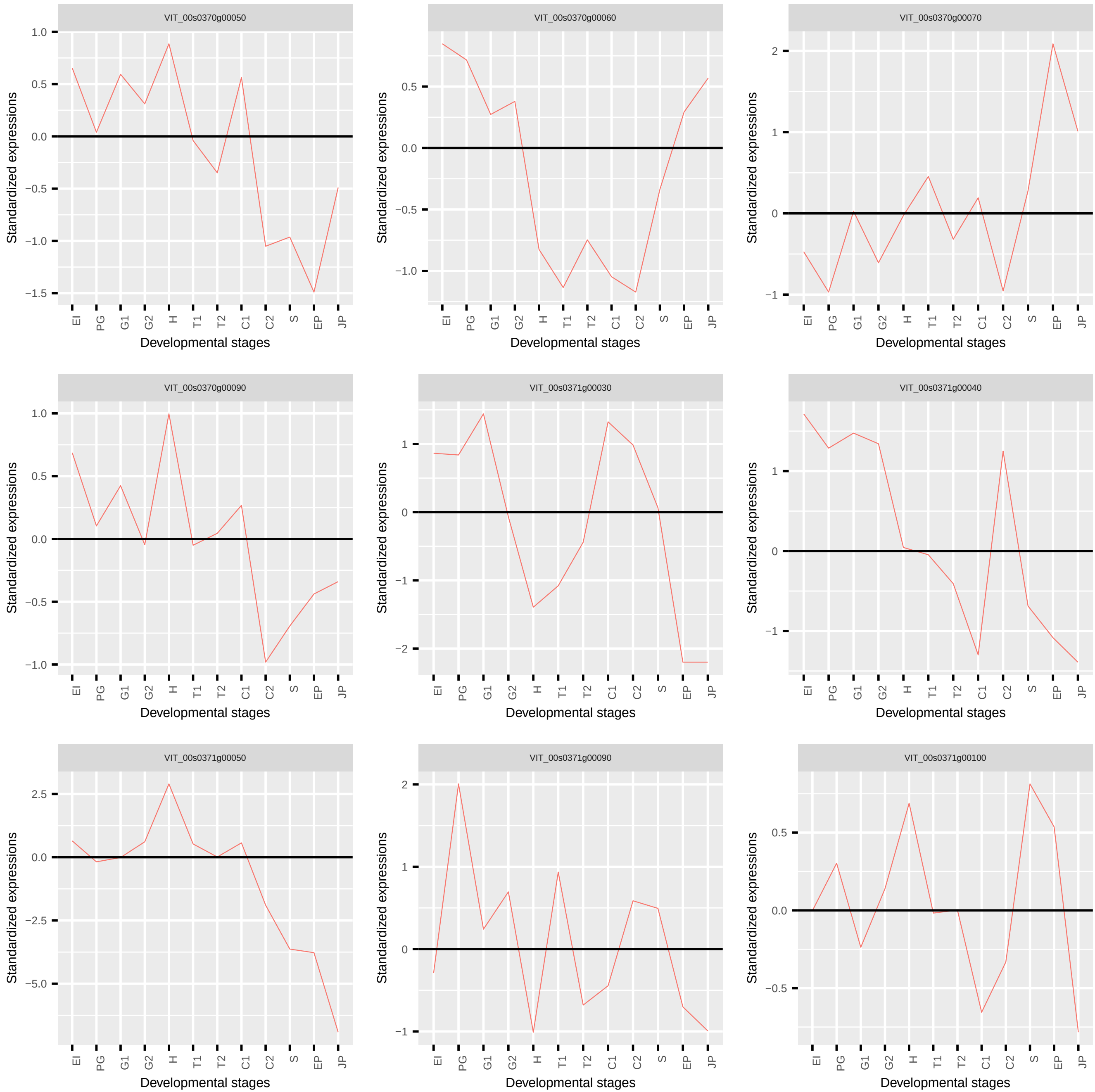
VIT_00s0370g00050
VIT_00s0370g00060
VIT_00s0370g00070
VIT_00s0370g00090
VIT_00s0371g00030
VIT_00s0371g00040
VIT_00s0371g00050
VIT_00s0371g00090
VIT_00s0371g00100
Standardized expressions
Developmental stages
EI PG G1 G2 H T1 T2 C1 C2 S EP JP

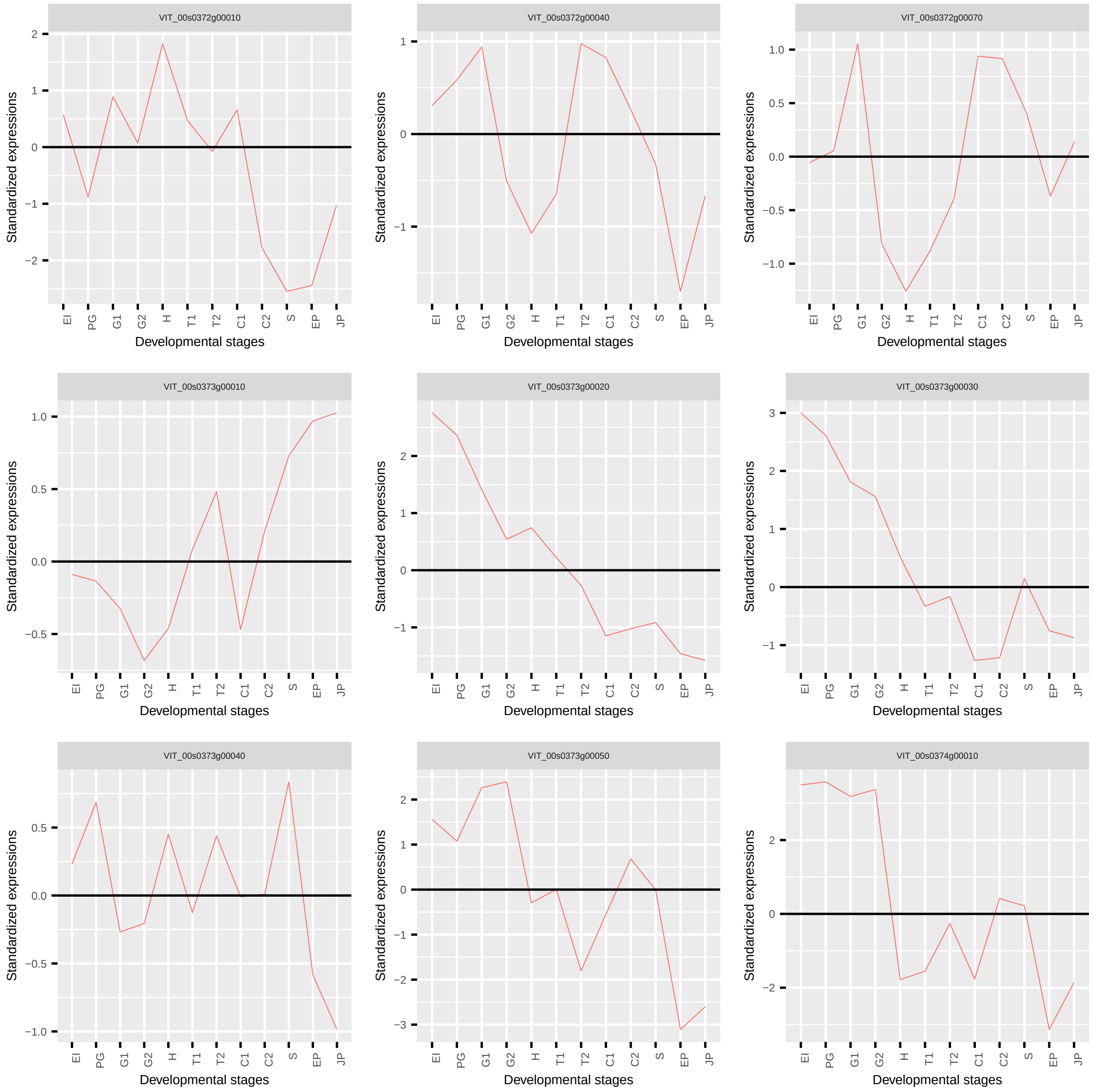
VIT_00s0372g00010
VIT_00s0372g00040
VIT_00s0372g00070
VIT_00s0373g00010
VIT_00s0373g00020
VIT_00s0373g00030
VIT_00s0373g00040
VIT_00s0373g00050
VIT_00s0374g00010
Standardized expressions
Developmental stages
EI PG G1 G2 H T1 T2 C1 C2 S EP JP

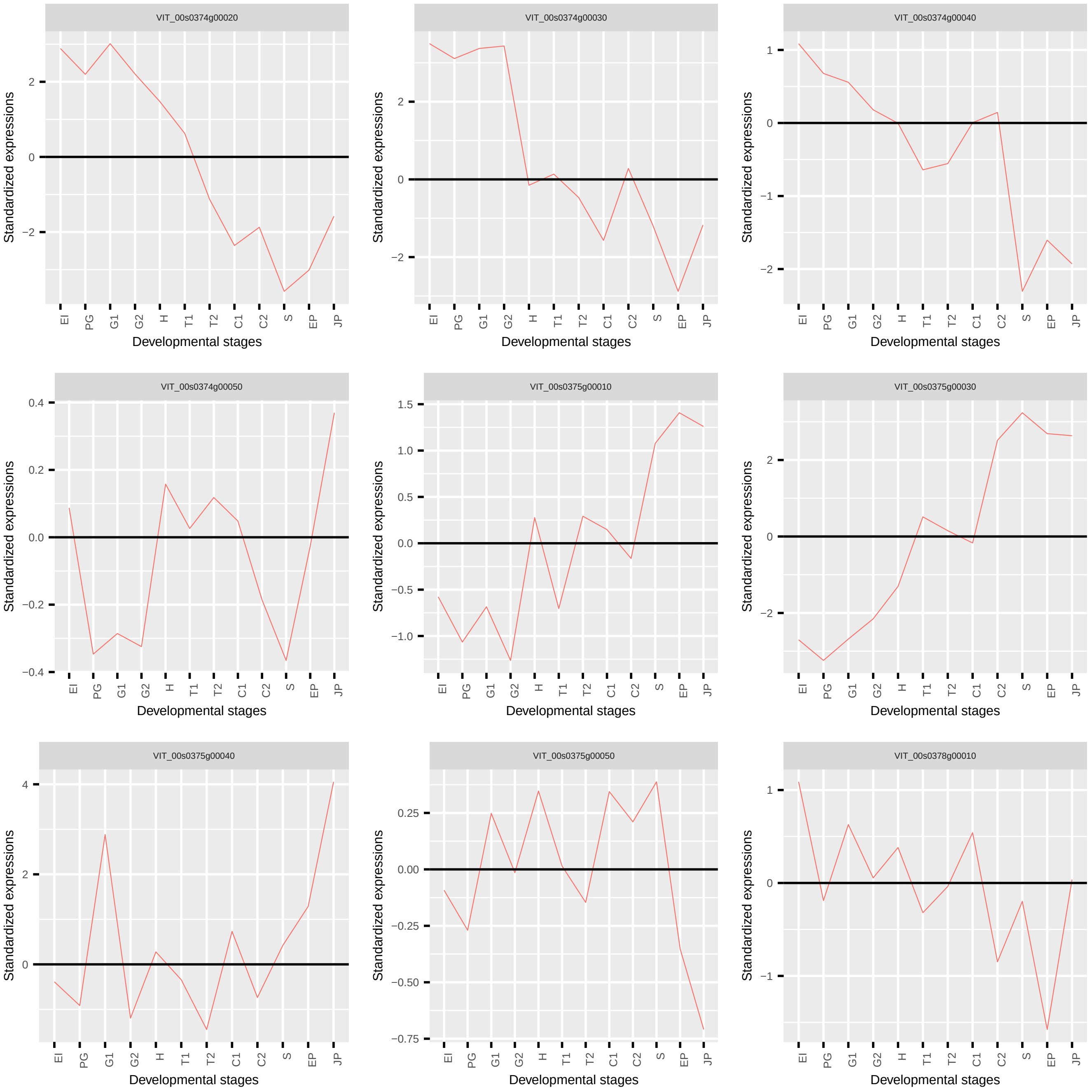

VIT_00s0374g00020
VIT_00s0374g00030
VIT_00s0374g00040
VIT_00s0374g00050
VIT_00s0375g00010
VIT_00s0375g00030
VIT_00s0375g00040
VIT_00s0375g00050
VIT_00s0378g00010
Standardized expressions
Developmental stages
EI PG G1 G2 H T1 T2 C1 C2 S EP JP
2
0
−2
−1
1
0.4
0.2
0.0
−0.2
−0.4
1.5
1.0
0.5
−0.5
−1.0
4
0.25
0.00
−0.25
−0.50
−0.75

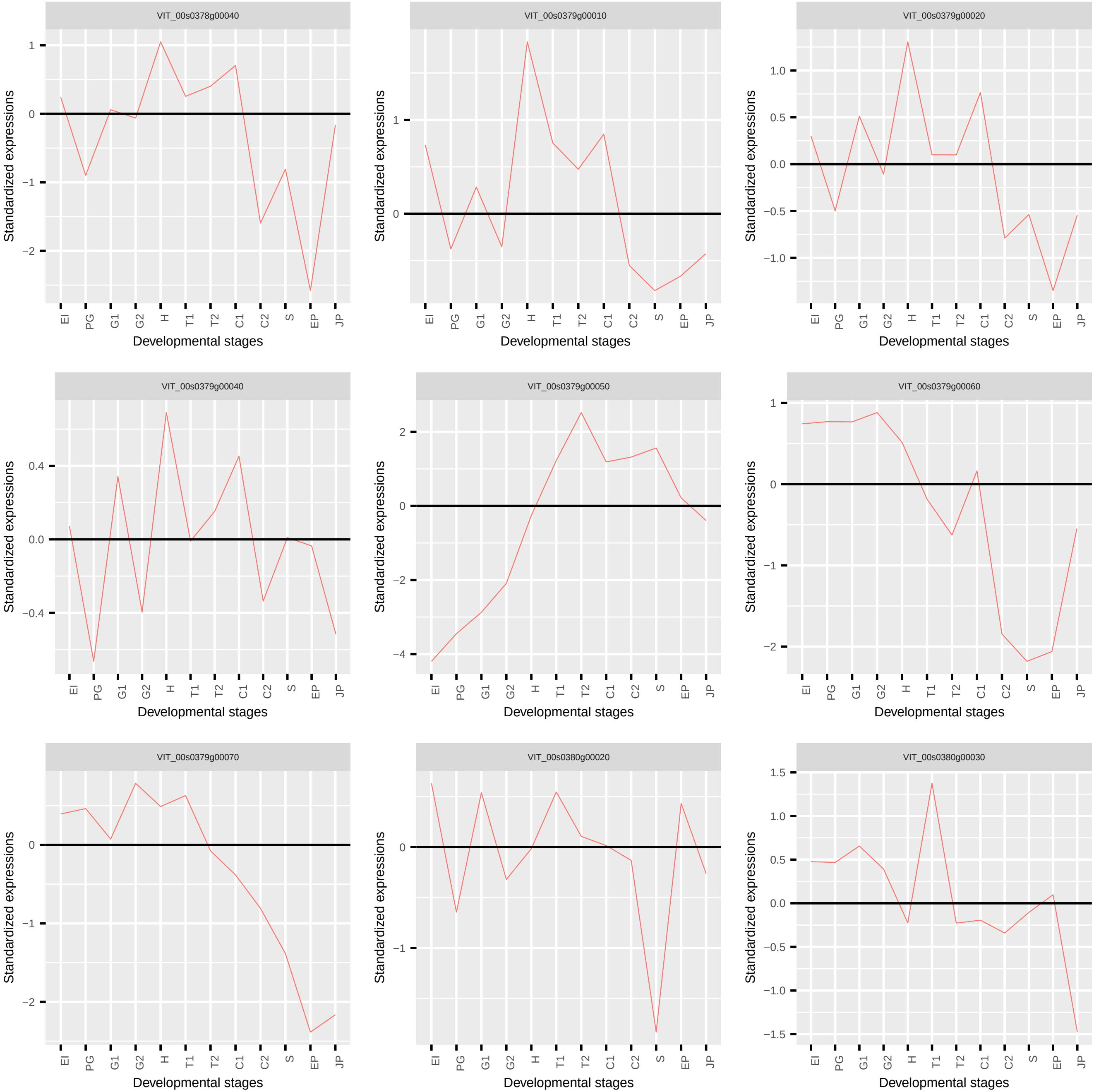
VIT_00s0378g00040
VIT_00s0379g00010
VIT_00s0379g00020
VIT_00s0379g00040
VIT_00s0379g00050
VIT_00s0379g00060
VIT_00s0379g00070
VIT_00s0380g00020
VIT_00s0380g00030
Standardized expressions
Developmental stages
EI PG G1 G2 H T1 T2 C1 C2 S EP JP

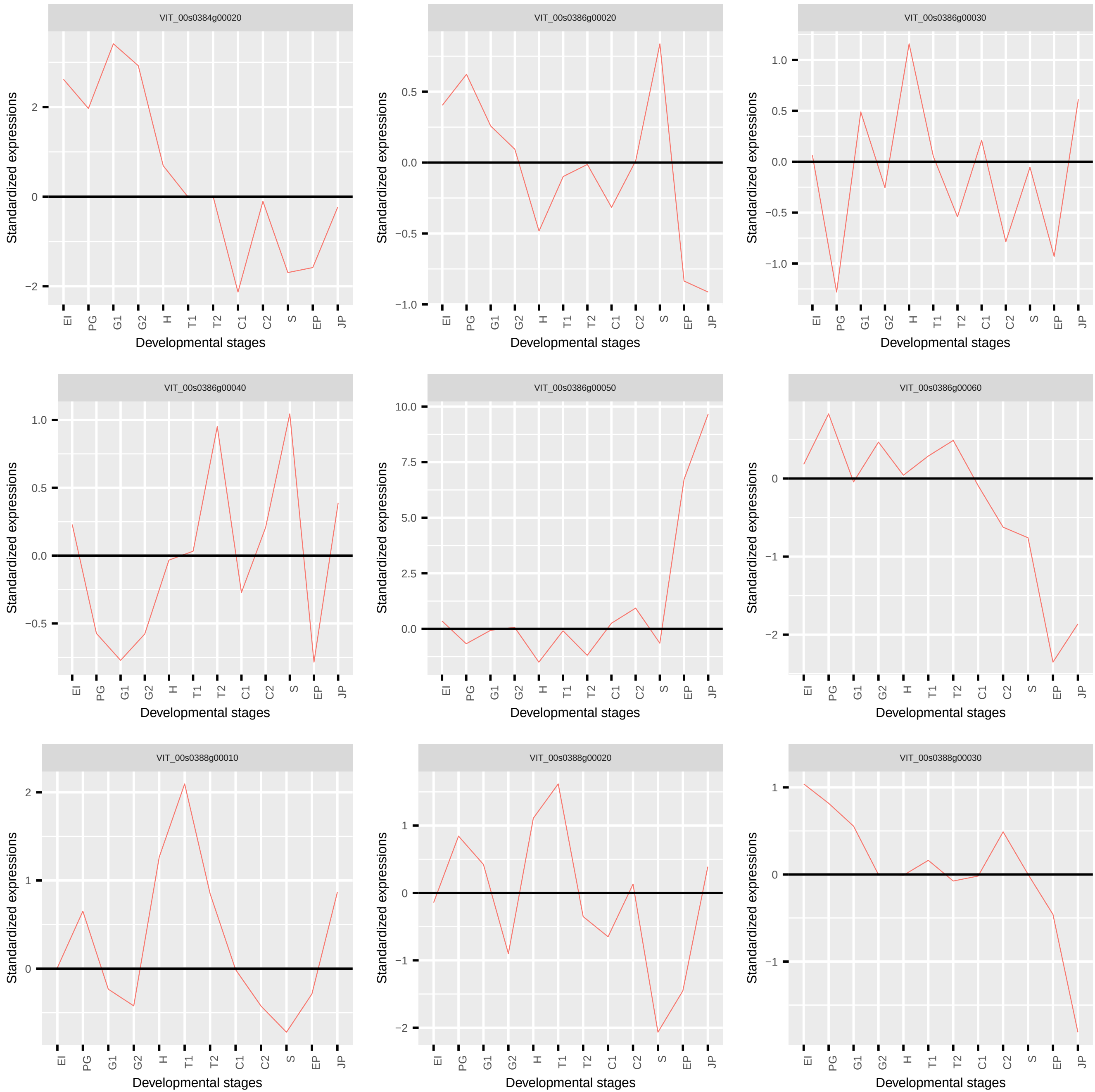

VIT_00s0384g00020
VIT_00s0386g00020
VIT_00s0386g00030
VIT_00s0386g00040
VIT_00s0386g00050
VIT_00s0386g00060
VIT_00s0388g00010
VIT_00s0388g00020
VIT_00s0388g00030
Standardized expressions
Developmental stages
EI
PG
G1
G2
H
T1
T2
C1
C2
S
EP
JP

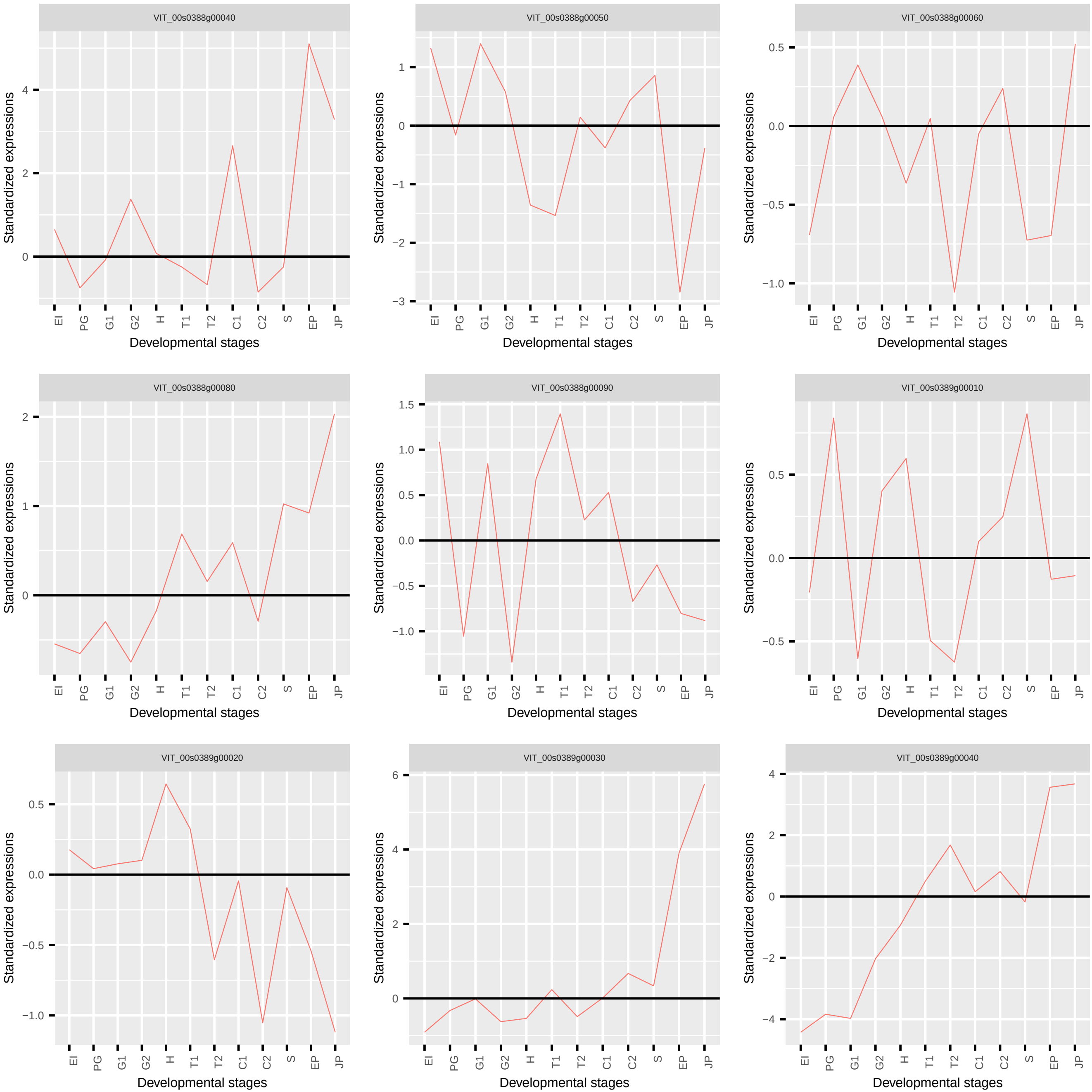
VIT_00s0388g00040
4
2
0
Standardized expressions
EI PG G1 G2 H T1 T2 C1 C2 S EP JP
Developmental stages
VIT_00s0388g00050
1
0
−1
−2
−3
Standardized expressions
EI PG G1 G2 H T1 T2 C1 C2 S EP JP
Developmental stages
VIT_00s0388g00060
0.5
0.0
−0.5
−1.0
Standardized expressions
EI PG G1 G2 H T1 T2 C1 C2 S EP JP
Developmental stages
VIT_00s0388g00080
2
1
0
Standardized expressions
EI PG G1 G2 H T1 T2 C1 C2 S EP JP
Developmental stages
VIT_00s0388g00090
1.5
1.0
0.5
0.0
−0.5
−1.0
Standardized expressions
EI PG G1 G2 H T1 T2 C1 C2 S EP JP
Developmental stages
VIT_00s0389g00010
0.5
0.0
−0.5
Standardized expressions
EI PG G1 G2 H T1 T2 C1 C2 S EP JP
Developmental stages
VIT_00s0389g00020
0.5
0.0
−0.5
−1.0
Standardized expressions
EI PG G1 G2 H T1 T2 C1 C2 S EP JP
Developmental stages
VIT_00s0389g00030
6
4
2
0
Standardized expressions
EI PG G1 G2 H T1 T2 C1 C2 S EP JP
Developmental stages
VIT_00s0389g00040
4
2
0
−2
−4
Standardized expressions
EI PG G1 G2 H T1 T2 C1 C2 S EP JP
Developmental stages

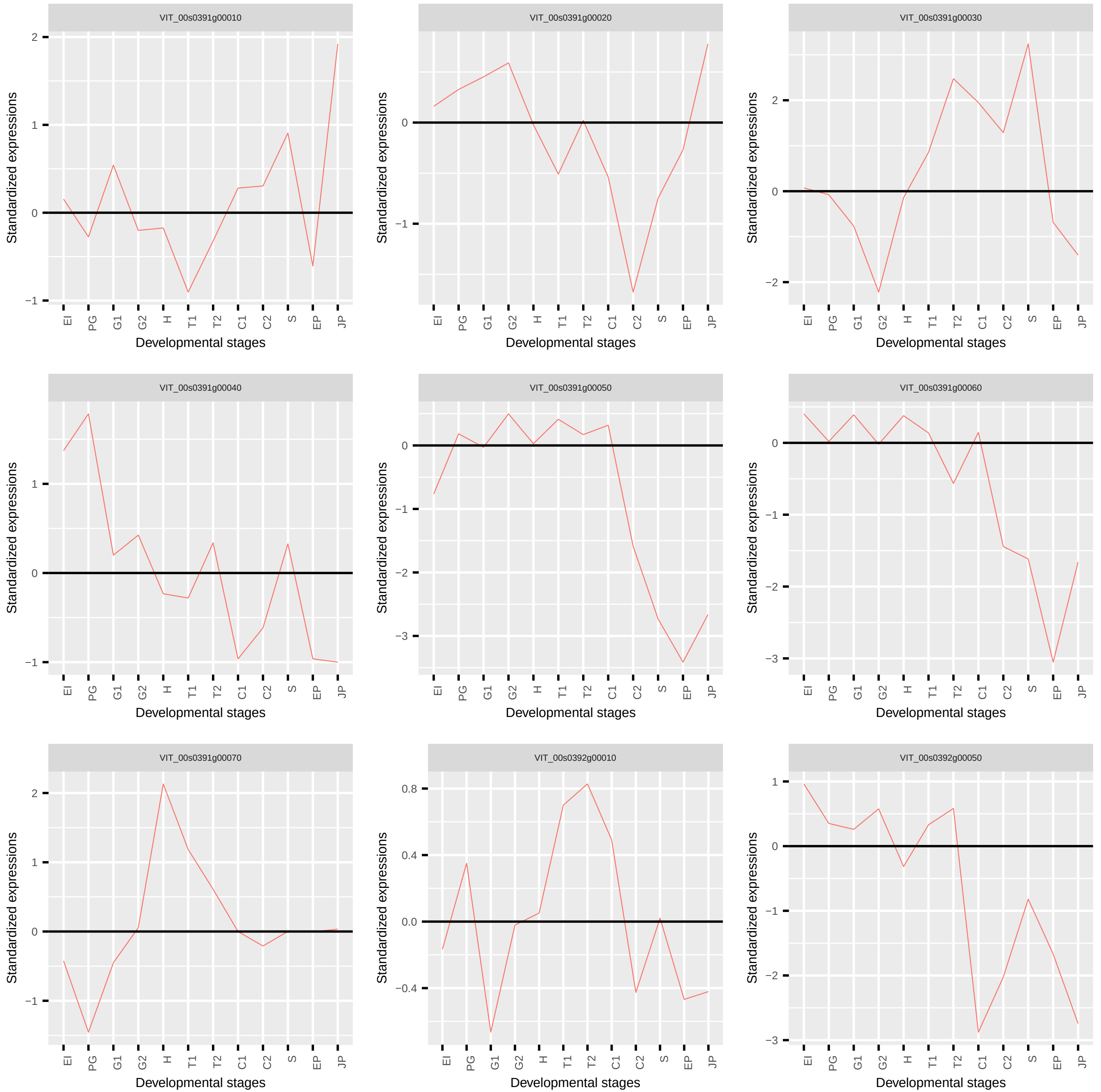

VIT_00s0391g00010
VIT_00s0391g00020
VIT_00s0391g00030
VIT_00s0391g00040
VIT_00s0391g00050
VIT_00s0391g00060
VIT_00s0391g00070
VIT_00s0392g00010
VIT_00s0392g00050
Standardized expressions
Developmental stages
EI
PG
G1
G2
H
T1
T2
C1
C2
S
EP
JP

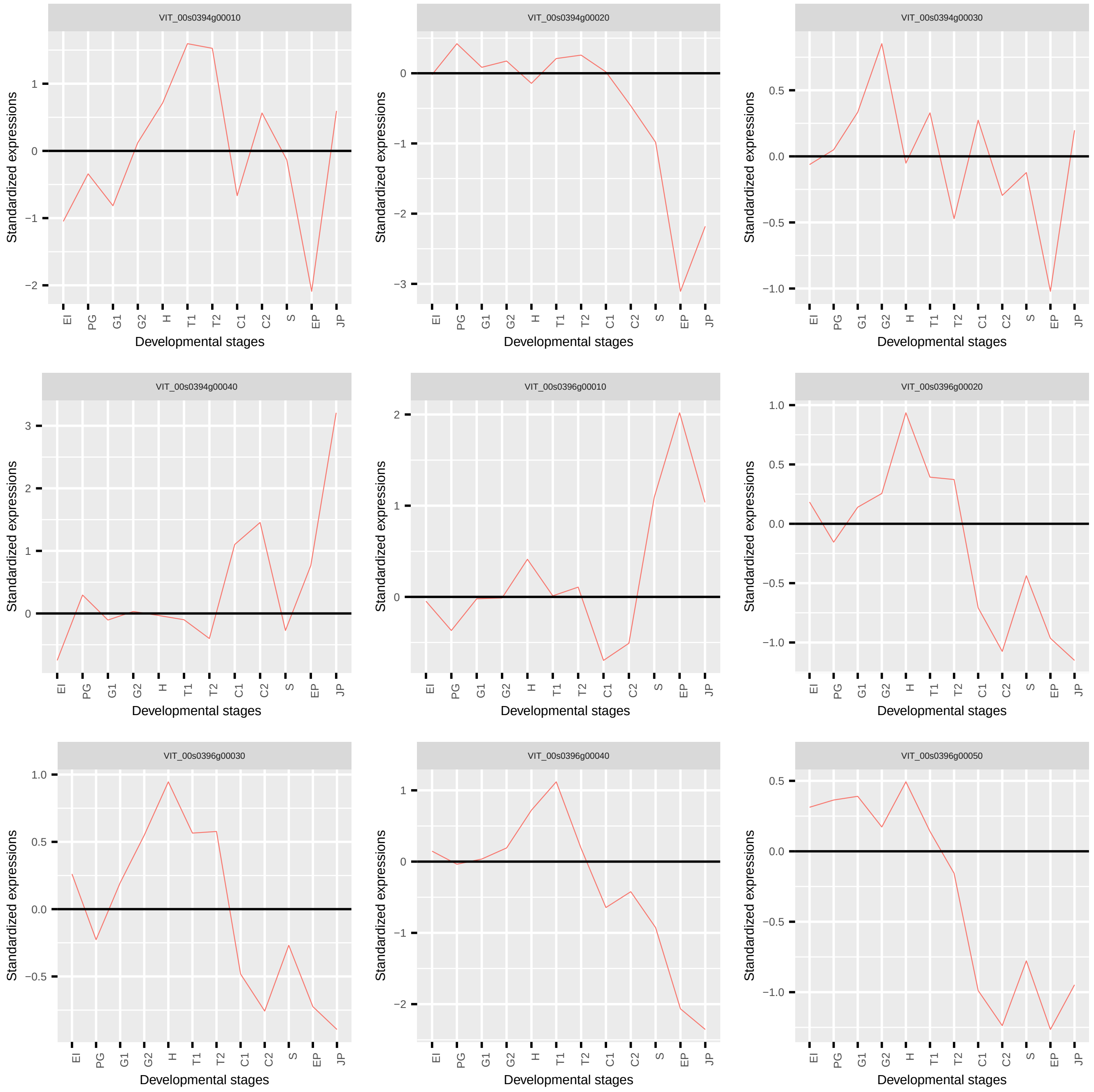
VIT_00s0394g00010
VIT_00s0394g00020
VIT_00s0394g00030
VIT_00s0394g00040
VIT_00s0396g00010
VIT_00s0396g00020
VIT_00s0396g00030
VIT_00s0396g00040
VIT_00s0396g00050
Standardized expressions
Developmental stages
EI
PG
G1
G2
H
T1
T2
C1
C2
S
EP
JP

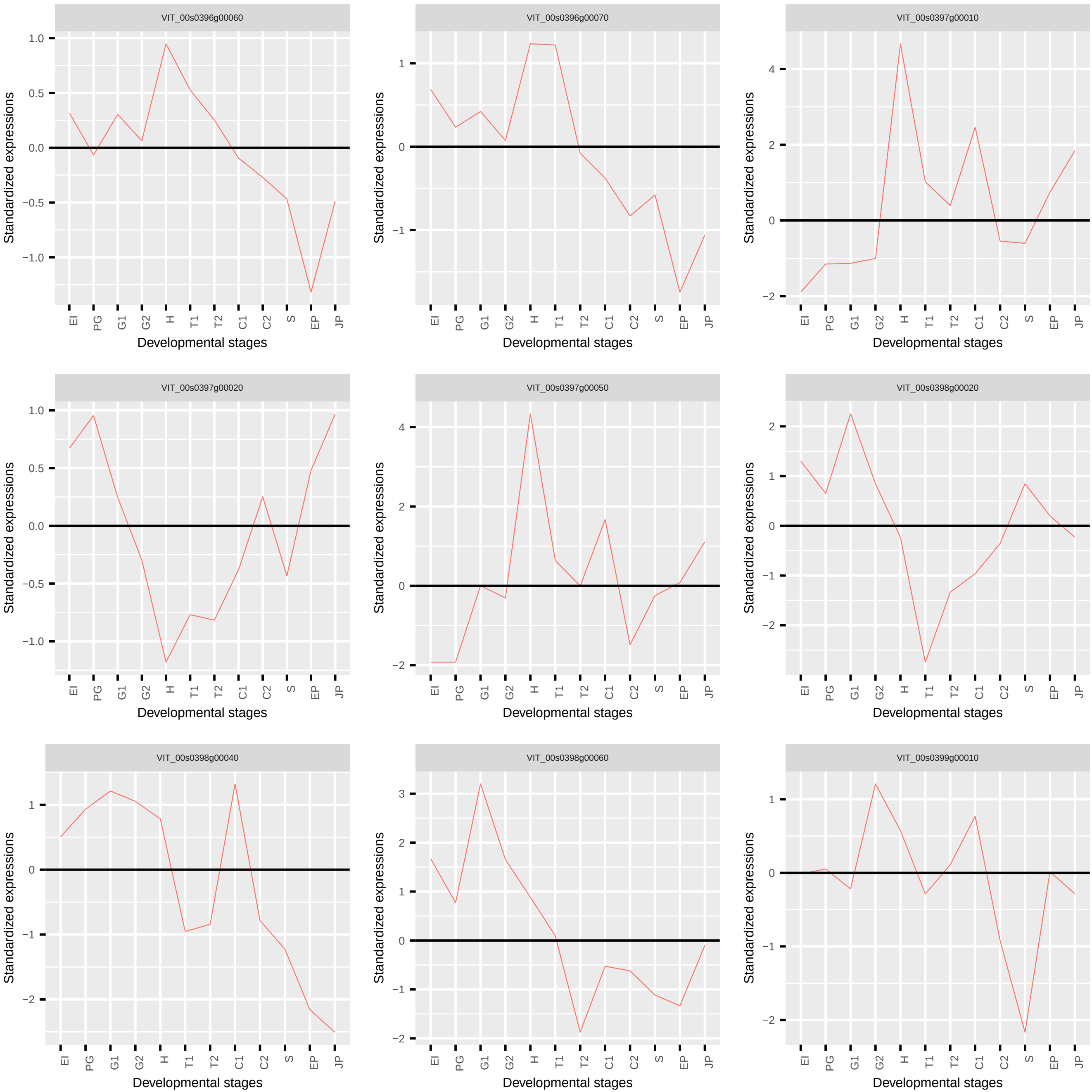
VIT_00s0396g00060
VIT_00s0396g00070
VIT_00s0397g00010
VIT_00s0397g00020
VIT_00s0397g00050
VIT_00s0398g00020
VIT_00s0398g00040
VIT_00s0398g00060
VIT_00s0399g00010
Standardized expressions
Developmental stages
EI PG G1 G2 H T1 T2 C1 C2 S EP JP

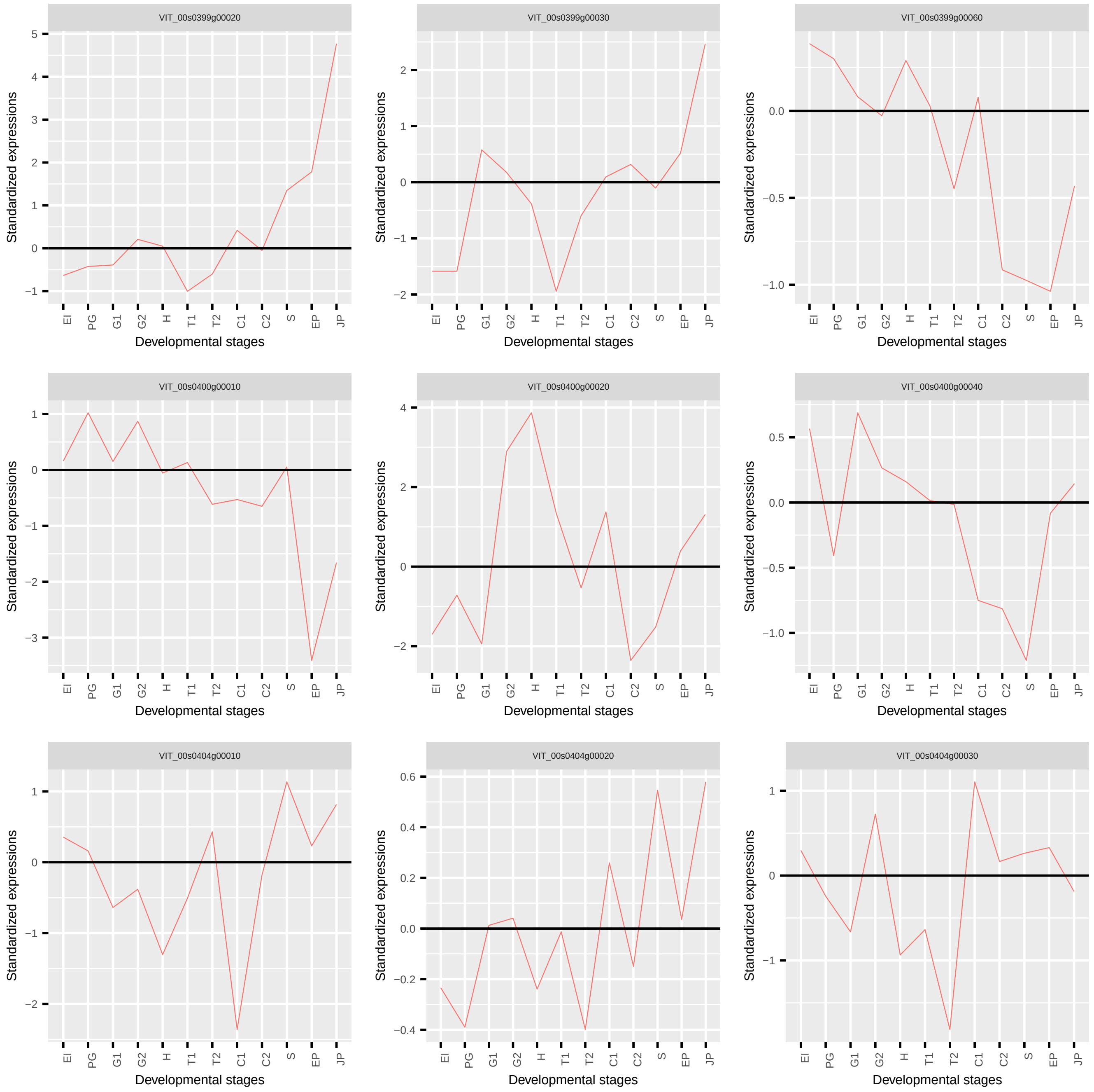
VIT_00s0399g00020
VIT_00s0399g00030
VIT_00s0399g00060
VIT_00s0400g00010
VIT_00s0400g00020
VIT_00s0400g00040
VIT_00s0404g00010
VIT_00s0404g00020
VIT_00s0404g00030
Standardized expressions
Developmental stages
EI
PG
G1
G2
H
T1
T2
C1
C2
S
EP
JP

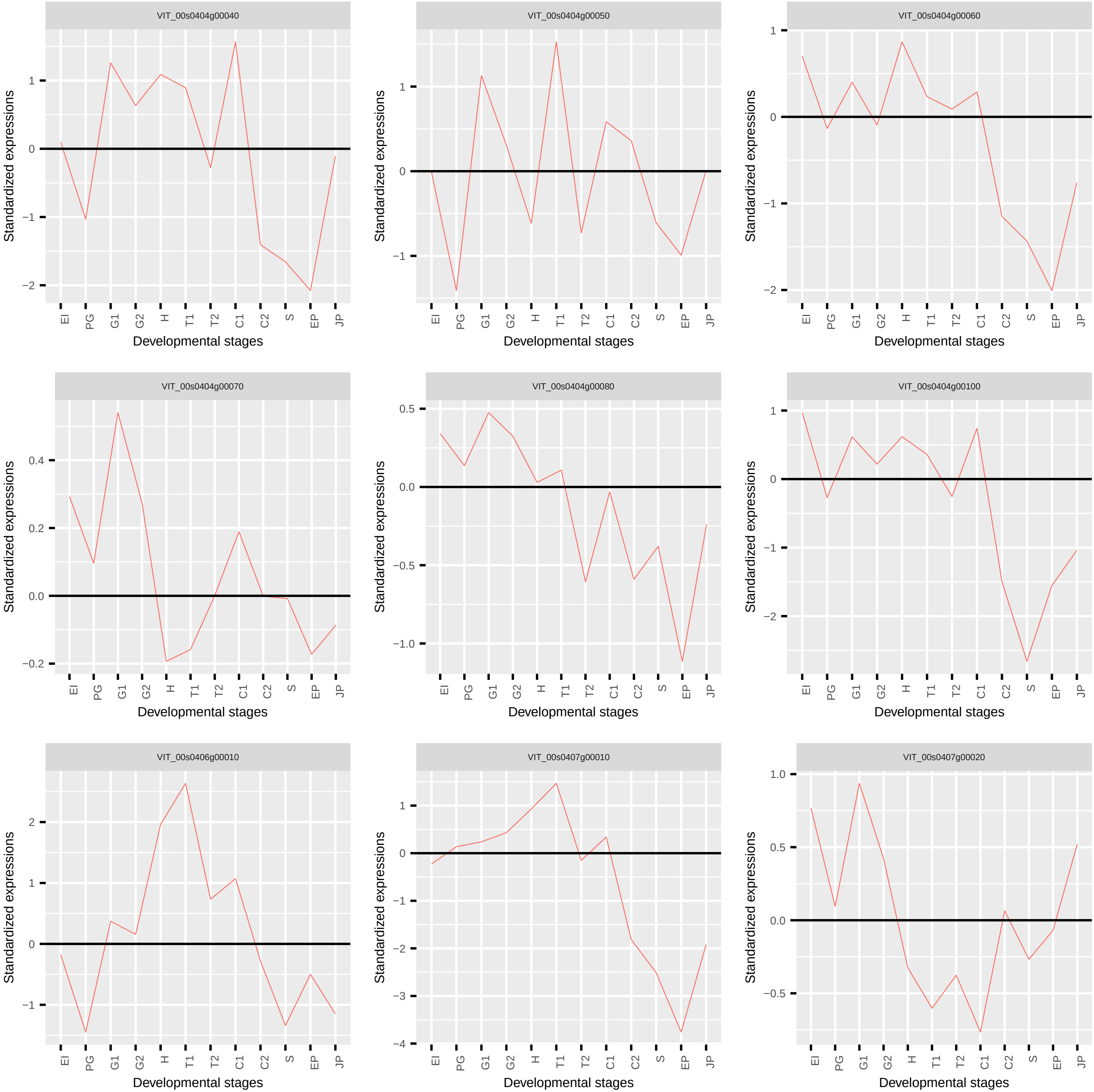
VIT_00s0404g00040
VIT_00s0404g00050
VIT_00s0404g00060
VIT_00s0404g00070
VIT_00s0404g00080
VIT_00s0404g00100
VIT_00s0406g00010
VIT_00s0407g00010
VIT_00s0407g00020
Standardized expressions
Developmental stages
EI
PG
G1
G2
H
T1
T2
C1
C2
S
EP
JP

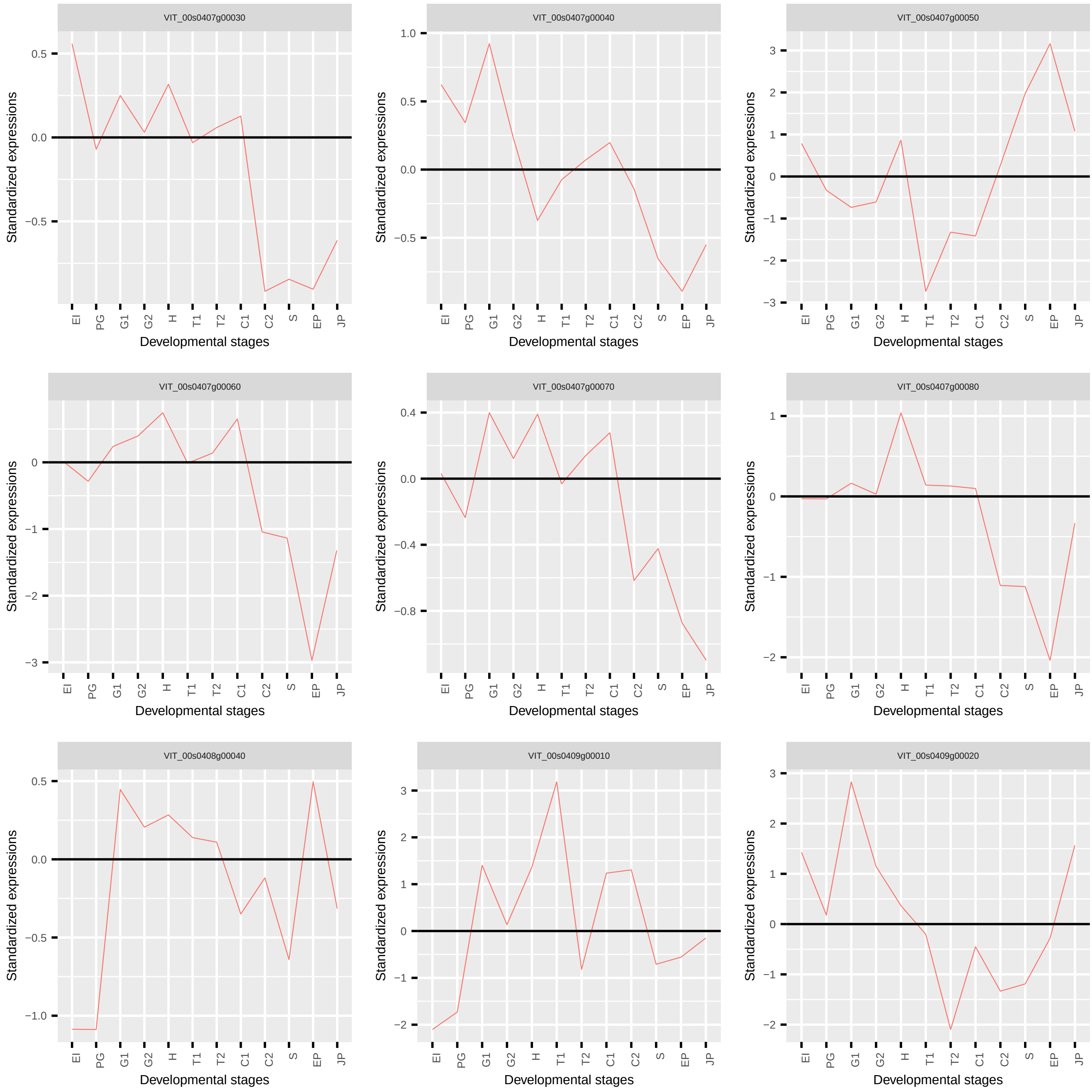
VIT_00s0407g00030
VIT_00s0407g00040
VIT_00s0407g00050
VIT_00s0407g00060
VIT_00s0407g00070
VIT_00s0407g00080
VIT_00s0408g00040
VIT_00s0409g00010
VIT_00s0409g00020
Standardized expressions
Developmental stages
EI
PG
G1
G2
H
T1
T2
C1
C2
S
EP
JP

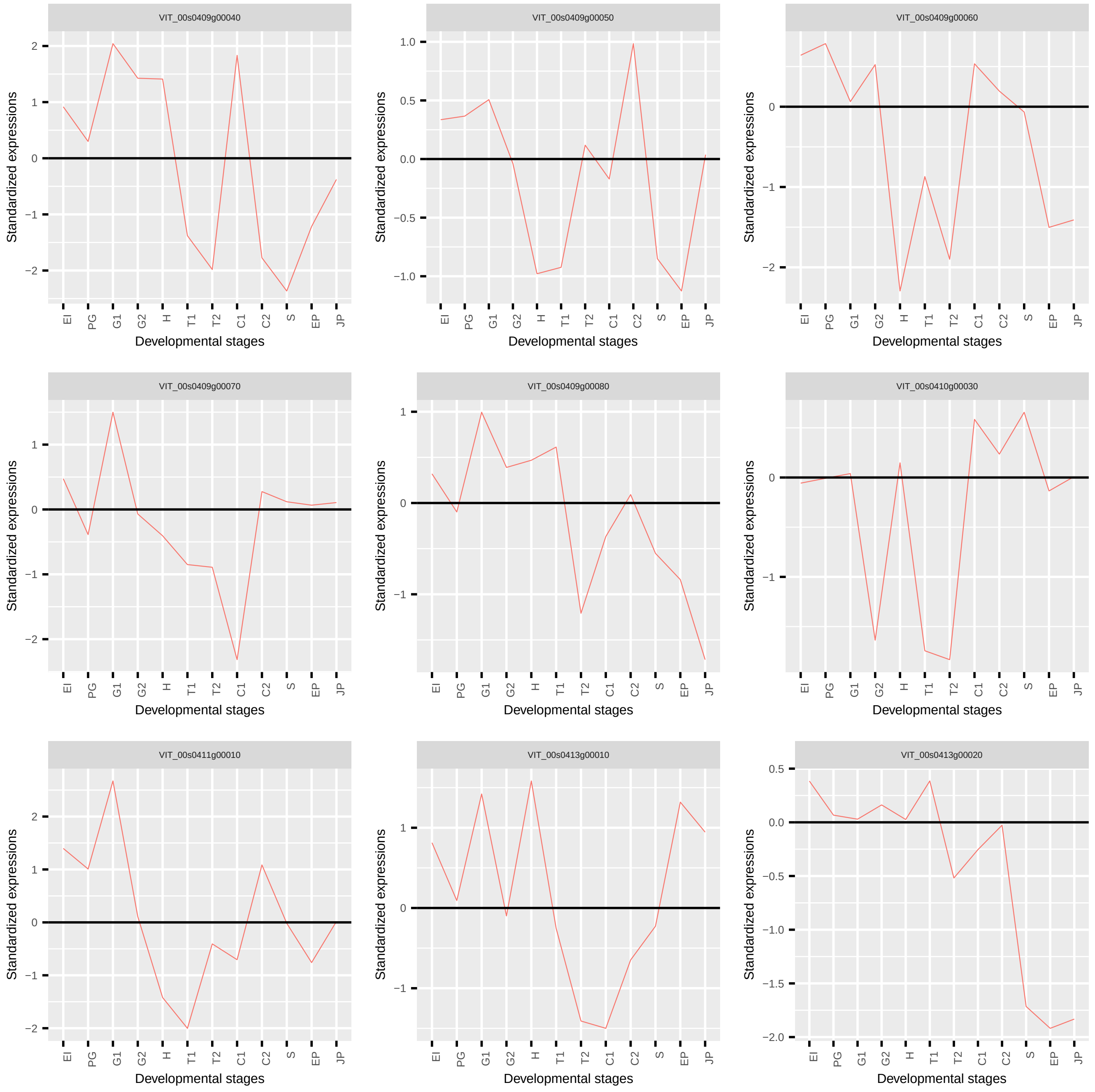

VIT_00s0409g00040
VIT_00s0409g00050
VIT_00s0409g00060
VIT_00s0409g00070
VIT_00s0409g00080
VIT_00s0410g00030
VIT_00s0411g00010
VIT_00s0413g00010
VIT_00s0413g00020
Standardized expressions
Developmental stages
EI PG G1 G2 H T1 T2 C1 C2 S EP JP

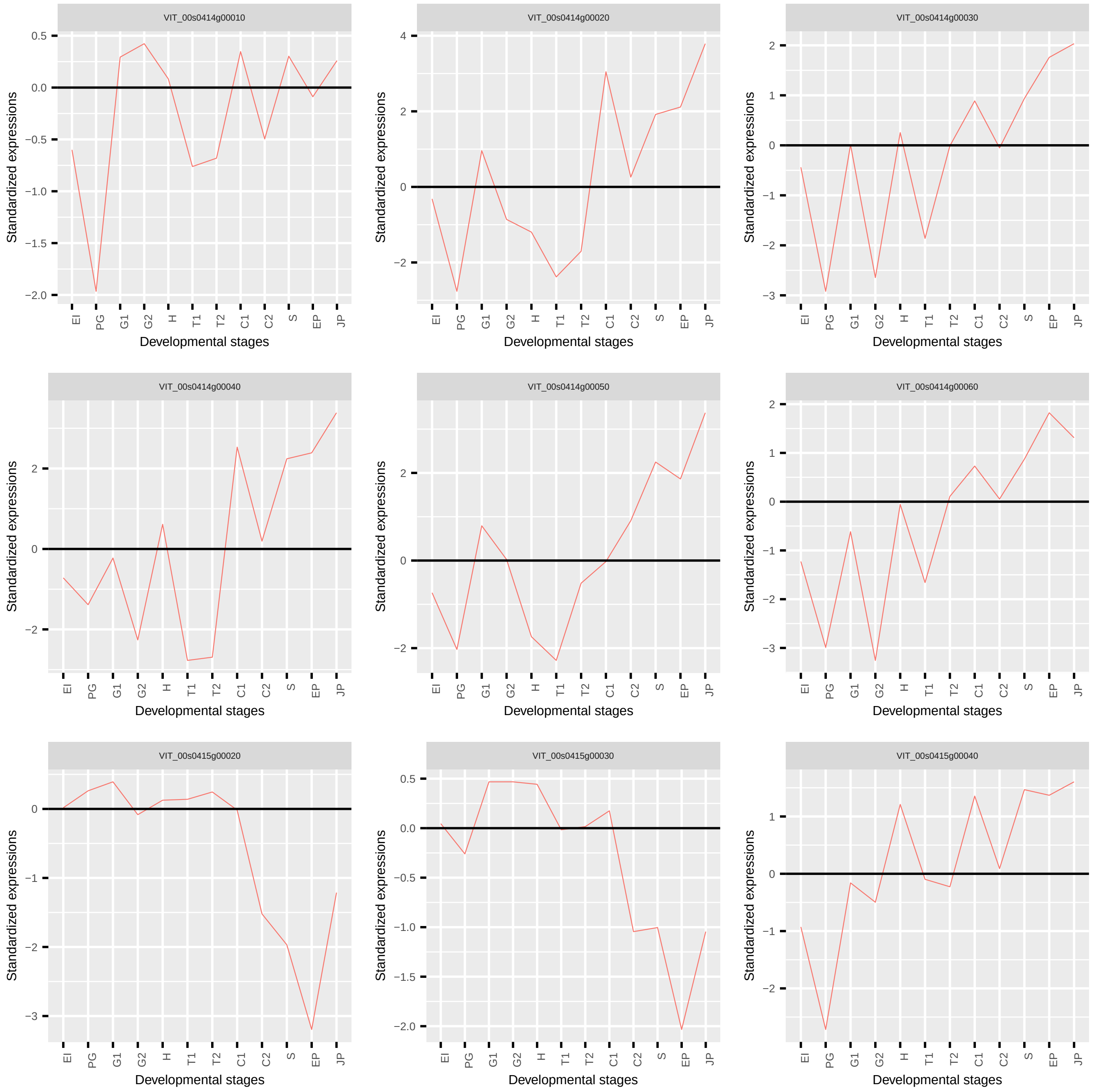
VIT_00s0414g00010
Standardized expressions
0.5
0.0
−0.5
−1.0
−1.5
−2.0
EI PG G1 G2 H T1 T2 C1 C2 S EP JP
Developmental stages
VIT_00s0414g00020
Standardized expressions
4
2
0
−2
EI PG G1 G2 H T1 T2 C1 C2 S EP JP
Developmental stages
VIT_00s0414g00030
Standardized expressions
2
1
0
−1
−2
−3
EI PG G1 G2 H T1 T2 C1 C2 S EP JP
Developmental stages
VIT_00s0414g00040
Standardized expressions
2
0
−2
EI PG G1 G2 H T1 T2 C1 C2 S EP JP
Developmental stages
VIT_00s0414g00050
Standardized expressions
2
0
−2
EI PG G1 G2 H T1 T2 C1 C2 S EP JP
Developmental stages
VIT_00s0414g00060
Standardized expressions
2
1
0
−1
−2
−3
EI PG G1 G2 H T1 T2 C1 C2 S EP JP
Developmental stages
VIT_00s0415g00020
Standardized expressions
0
−1
−2
−3
EI PG G1 G2 H T1 T2 C1 C2 S EP JP
Developmental stages
VIT_00s0415g00030
Standardized expressions
0.5
0.0
−0.5
−1.0
−1.5
−2.0
EI PG G1 G2 H T1 T2 C1 C2 S EP JP
Developmental stages
VIT_00s0415g00040
Standardized expressions
1
0
−1
−2
EI PG G1 G2 H T1 T2 C1 C2 S EP JP
Developmental stages

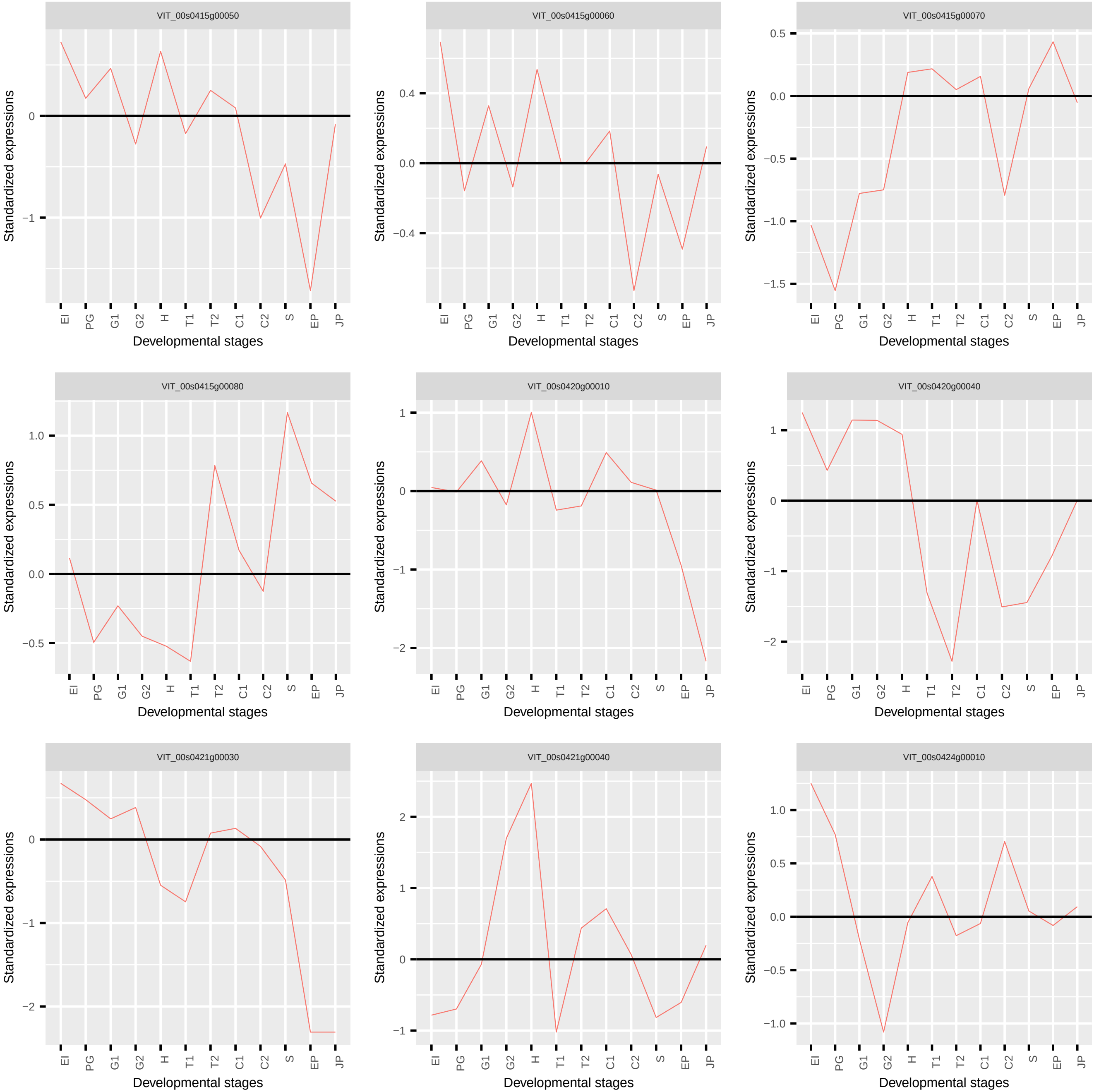
VIT_00s0415g00050
VIT_00s0415g00060
VIT_00s0415g00070
VIT_00s0415g00080
VIT_00s0420g00010
VIT_00s0420g00040
VIT_00s0421g00030
VIT_00s0421g00040
VIT_00s0424g00010
Standardized expressions
Developmental stages
EI PG G1 G2 H T1 T2 C1 C2 S EP JP

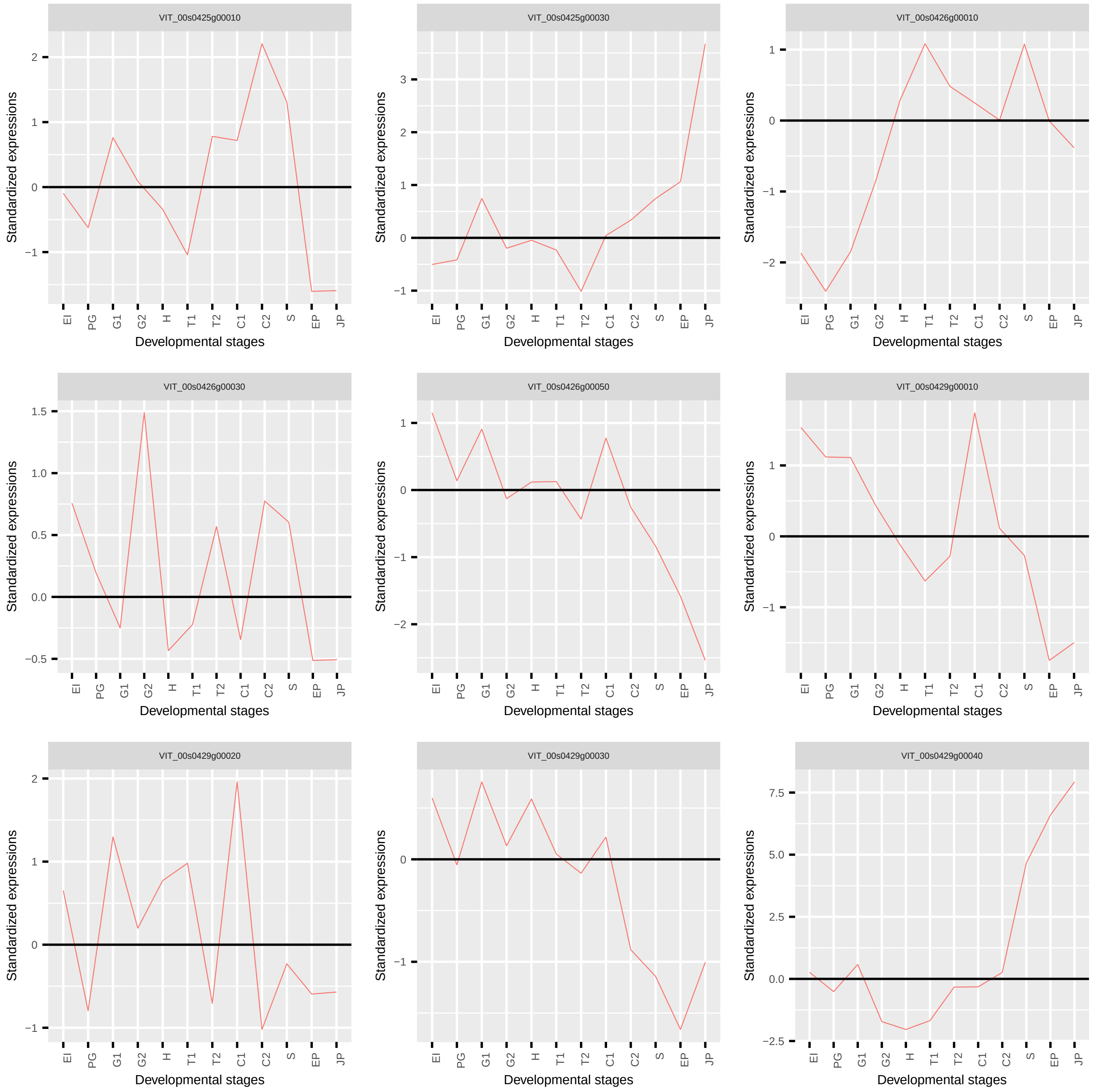
VIT_00s0425g00010
VIT_00s0425g00030
VIT_00s0426g00010
VIT_00s0426g00030
VIT_00s0426g00050
VIT_00s0429g00010
VIT_00s0429g00020
VIT_00s0429g00030
VIT_00s0429g00040
Standardized expressions
Developmental stages
EI PG G1 G2 H T1 T2 C1 C2 S EP JP

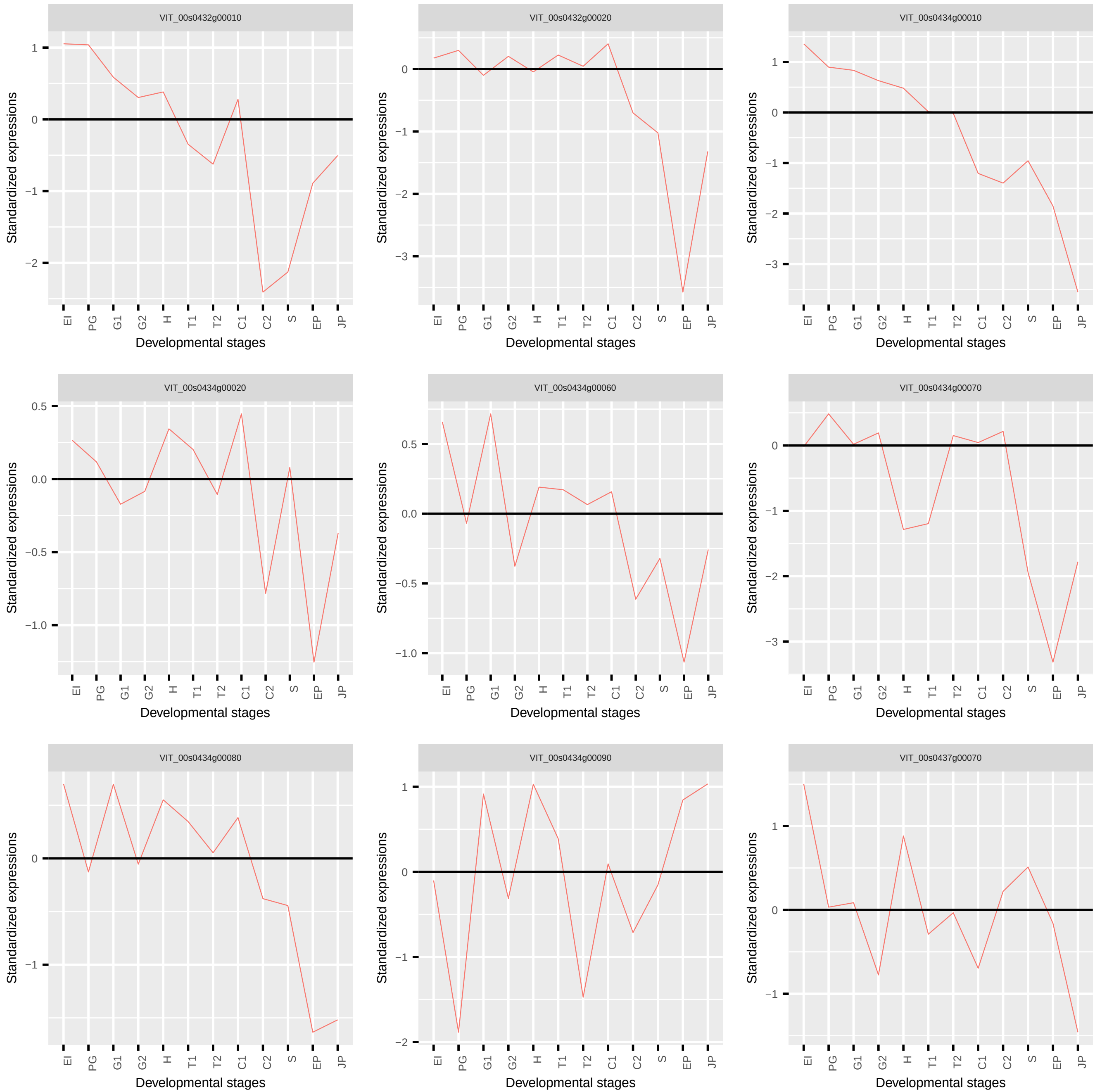
VIT_00s0432g00010
VIT_00s0432g00020
VIT_00s0434g00010
VIT_00s0434g00020
VIT_00s0434g00060
VIT_00s0434g00070
VIT_00s0434g00080
VIT_00s0434g00090
VIT_00s0437g00070
Standardized expressions
Developmental stages
EI
PG
G1
G2
H
T1
T2
C1
C2
S
EP
JP

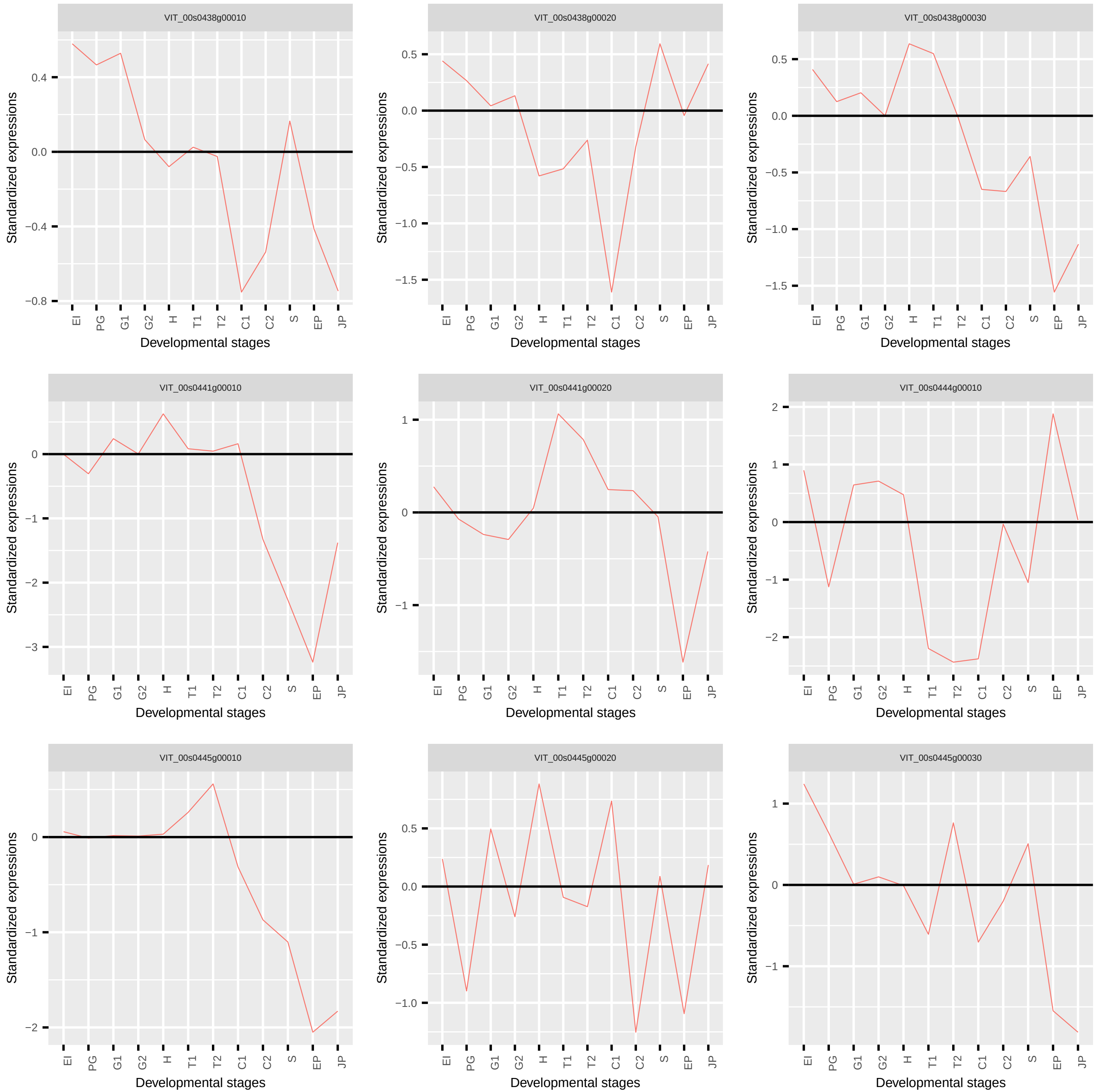

VIT_00s0438g00010
Standardized expressions
0.4
0.0
−0.4
−0.8
EI PG G1 G2 H T1 T2 C1 C2 S EP JP
Developmental stages
VIT_00s0438g00020
Standardized expressions
0.5
0.0
−0.5
−1.0
−1.5
EI PG G1 G2 H T1 T2 C1 C2 S EP JP
Developmental stages
VIT_00s0438g00030
Standardized expressions
0.5
0.0
−0.5
−1.0
−1.5
EI PG G1 G2 H T1 T2 C1 C2 S EP JP
Developmental stages
VIT_00s0441g00010
Standardized expressions
0
−1
−2
−3
EI PG G1 G2 H T1 T2 C1 C2 S EP JP
Developmental stages
VIT_00s0441g00020
Standardized expressions
1
0
−1
EI PG G1 G2 H T1 T2 C1 C2 S EP JP
Developmental stages
VIT_00s0444g00010
Standardized expressions
2
1
0
−1
−2
EI PG G1 G2 H T1 T2 C1 C2 S EP JP
Developmental stages
VIT_00s0445g00010
Standardized expressions
0
−1
−2
EI PG G1 G2 H T1 T2 C1 C2 S EP JP
Developmental stages
VIT_00s0445g00020
Standardized expressions
0.5
0.0
−0.5
−1.0
EI PG G1 G2 H T1 T2 C1 C2 S EP JP
Developmental stages
VIT_00s0445g00030
Standardized expressions
1
0
−1
EI PG G1 G2 H T1 T2 C1 C2 S EP JP
Developmental stages

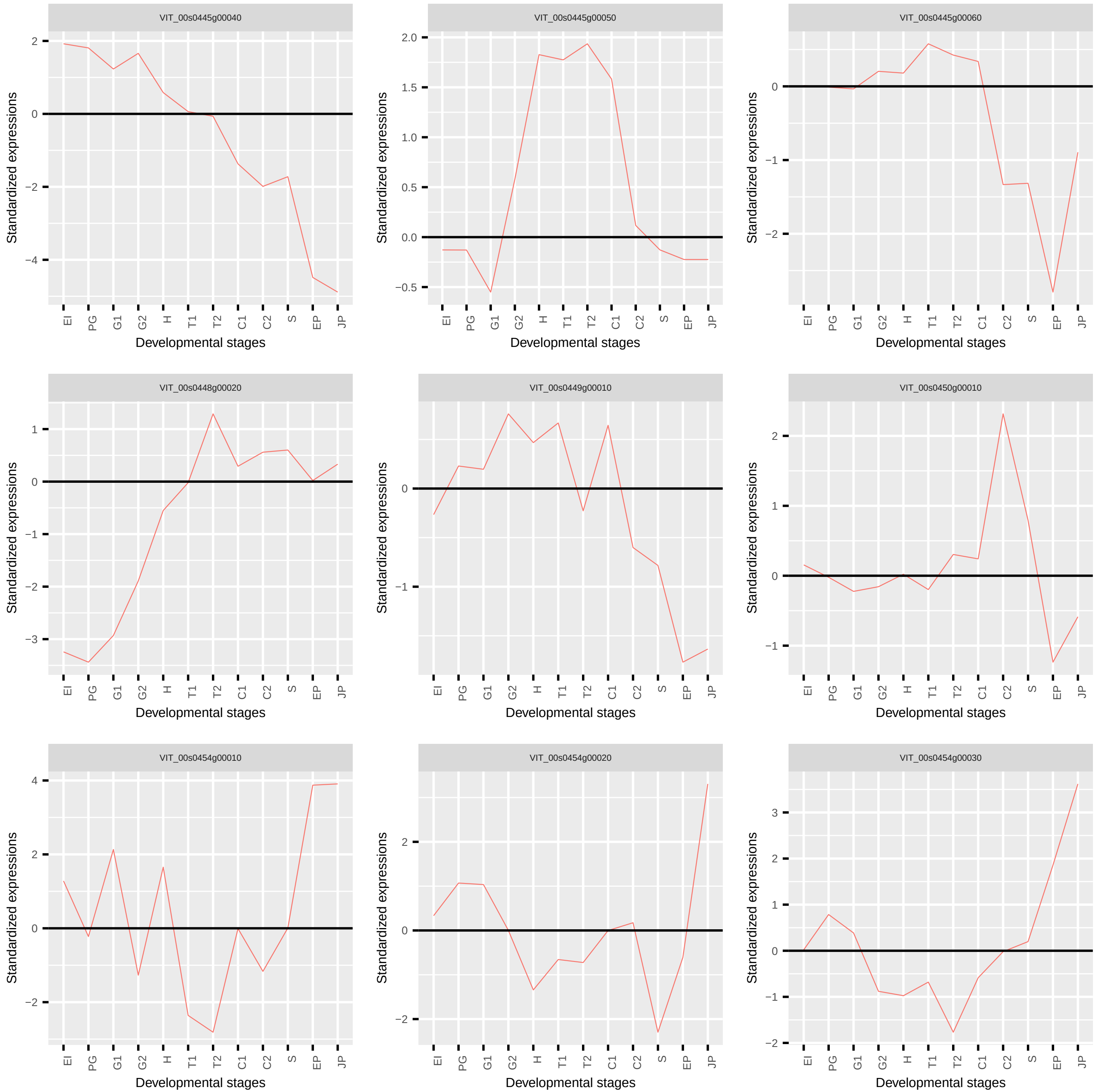
VIT_00s0445g00040
VIT_00s0445g00050
VIT_00s0445g00060
VIT_00s0448g00020
VIT_00s0449g00010
VIT_00s0450g00010
VIT_00s0454g00010
VIT_00s0454g00020
VIT_00s0454g00030
Standardized expressions
Developmental stages
EI PG G1 G2 H T1 T2 C1 C2 S EP JP

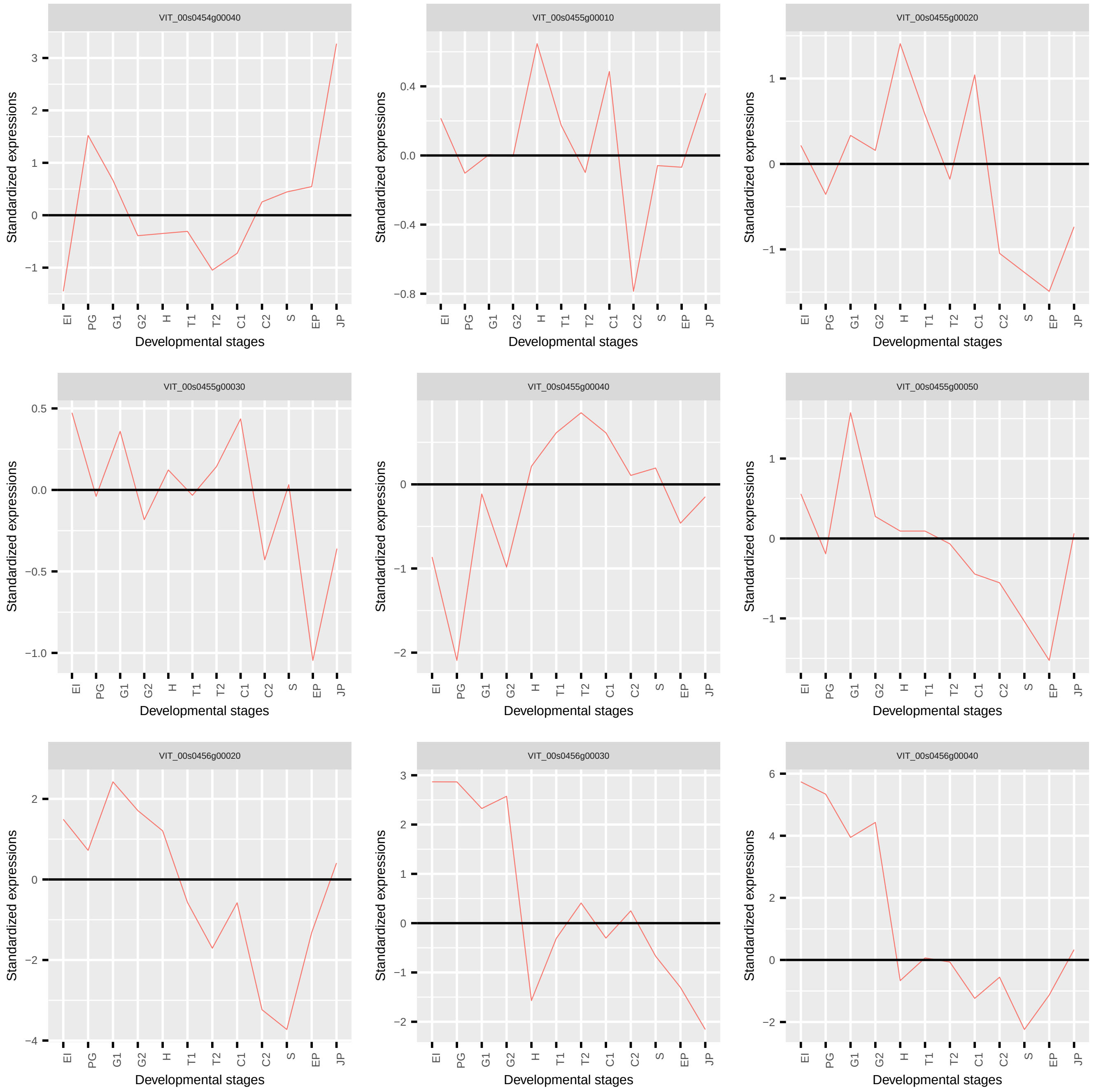
VIT_00s0454g00040
VIT_00s0455g00010
VIT_00s0455g00020
VIT_00s0455g00030
VIT_00s0455g00040
VIT_00s0455g00050
VIT_00s0456g00020
VIT_00s0456g00030
VIT_00s0456g00040
Standardized expressions
Developmental stages
EI
PG
G1
G2
H
T1
T2
C1
C2
S
EP
JP

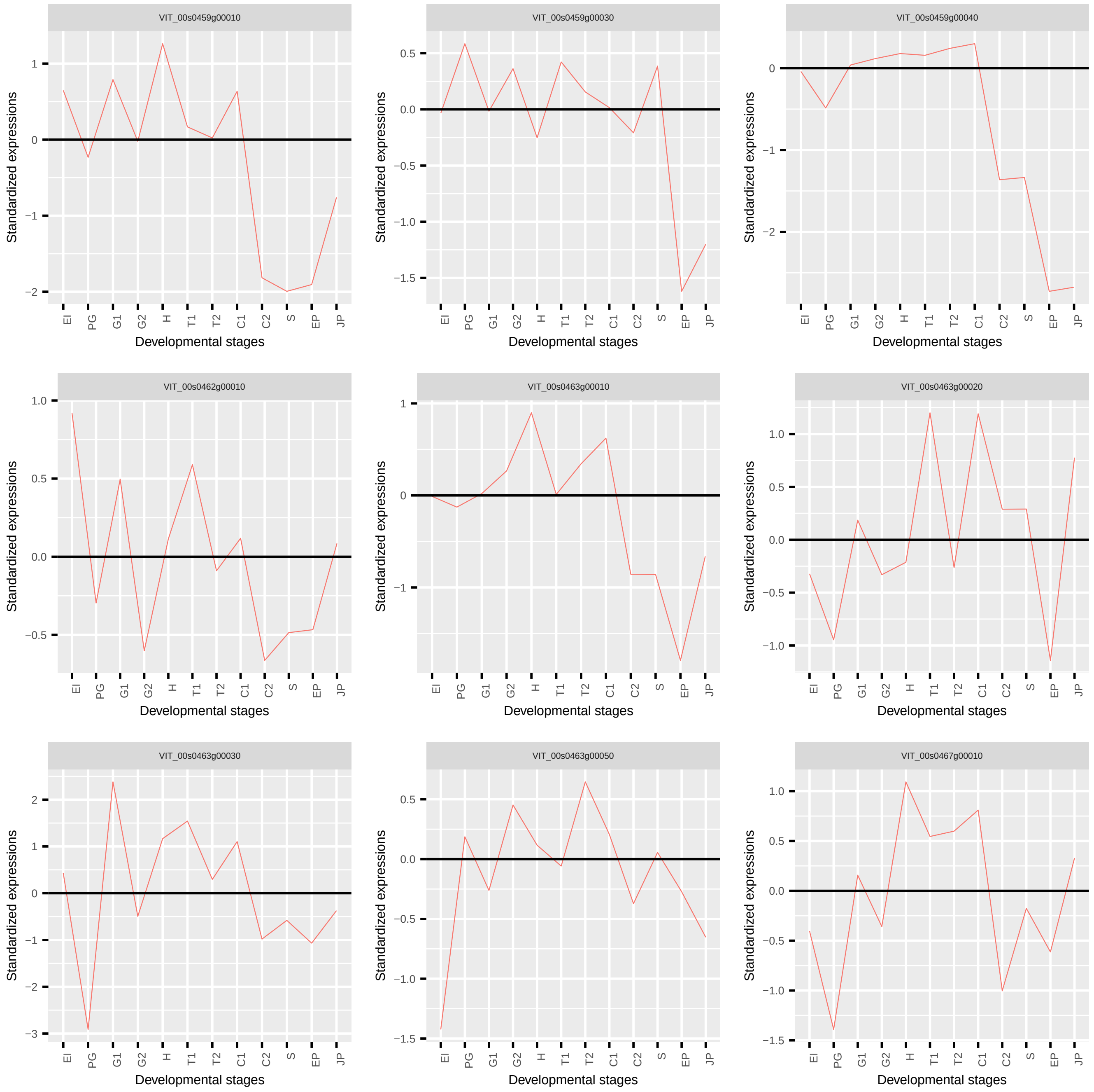
VIT_00s0459g00010
VIT_00s0459g00030
VIT_00s0459g00040
VIT_00s0462g00010
VIT_00s0463g00010
VIT_00s0463g00020
VIT_00s0463g00030
VIT_00s0463g00050
VIT_00s0467g00010
Standardized expressions
Developmental stages
EI
PG
G1
G2
H
T1
T2
C1
C2
S
EP
JP

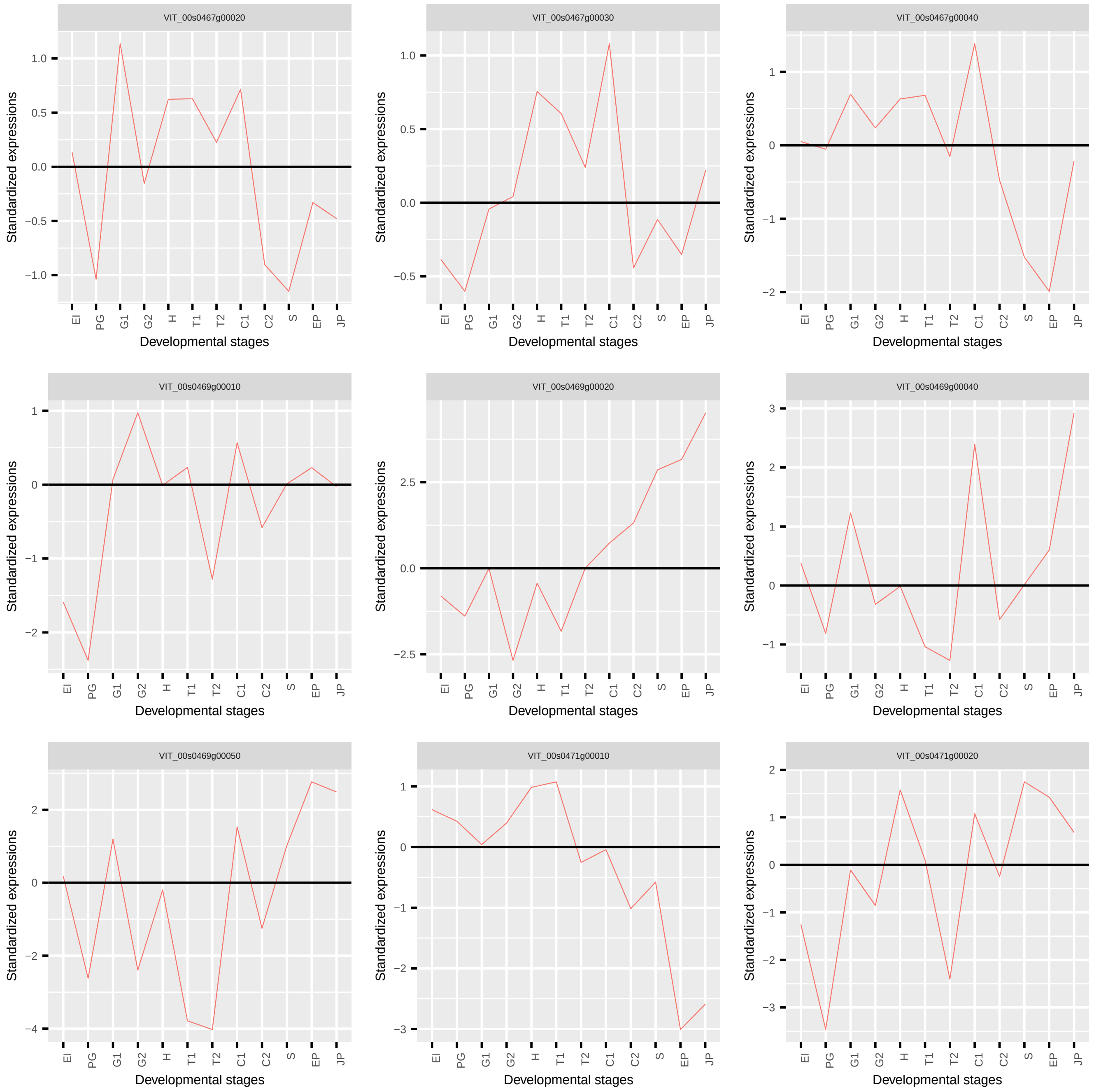
VIT_00s0467g00020
VIT_00s0467g00030
VIT_00s0467g00040
VIT_00s0469g00010
VIT_00s0469g00020
VIT_00s0469g00040
VIT_00s0469g00050
VIT_00s0471g00010
VIT_00s0471g00020
Standardized expressions
Developmental stages
EI PG G1 G2 H T1 T2 C1 C2 S EP JP

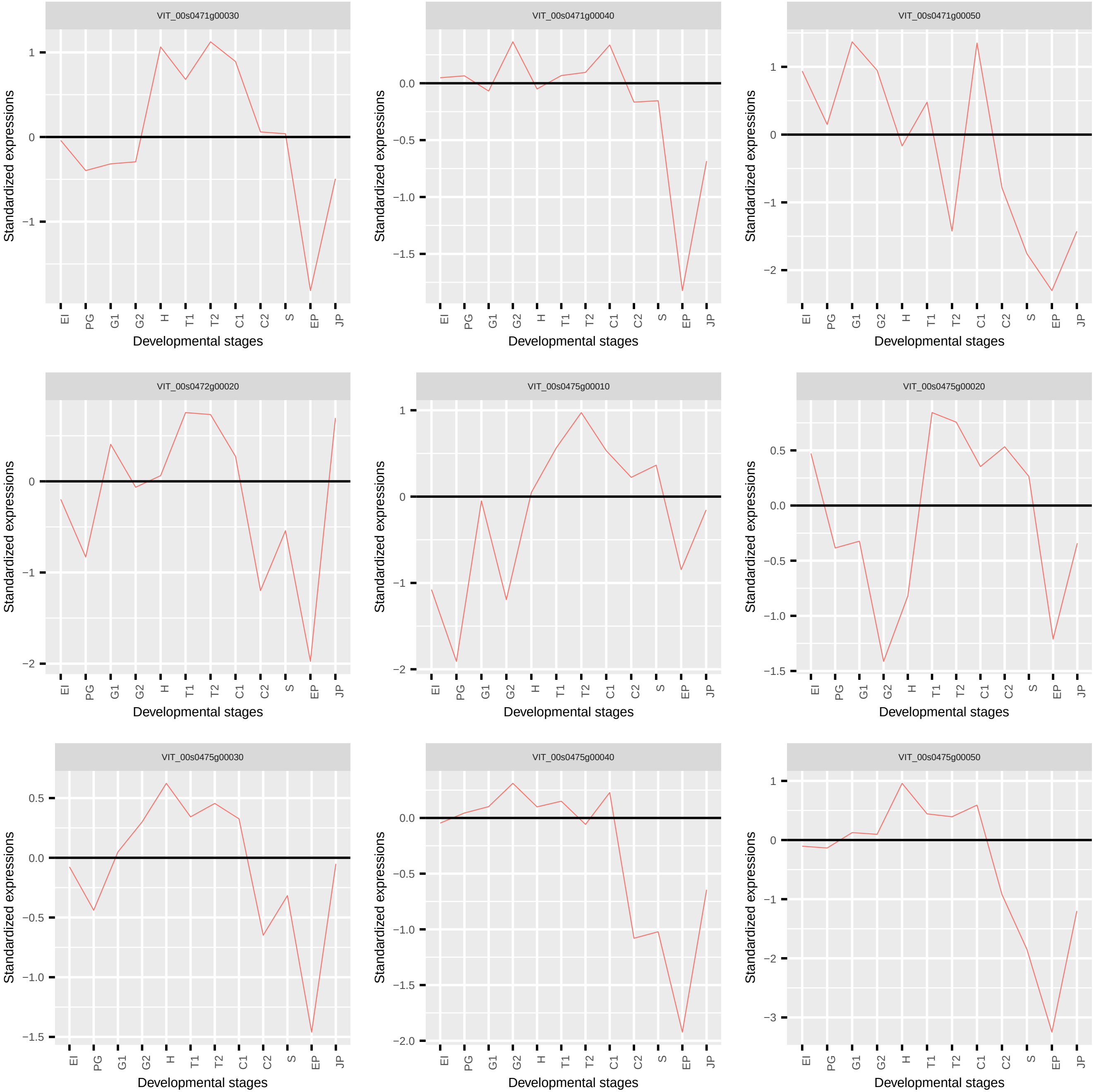
VIT_00s0471g00030
VIT_00s0471g00040
VIT_00s0471g00050
VIT_00s0472g00020
VIT_00s0475g00010
VIT_00s0475g00020
VIT_00s0475g00030
VIT_00s0475g00040
VIT_00s0475g00050
Standardized expressions
Developmental stages
EI PG G1 G2 H T1 T2 C1 C2 S EP JP

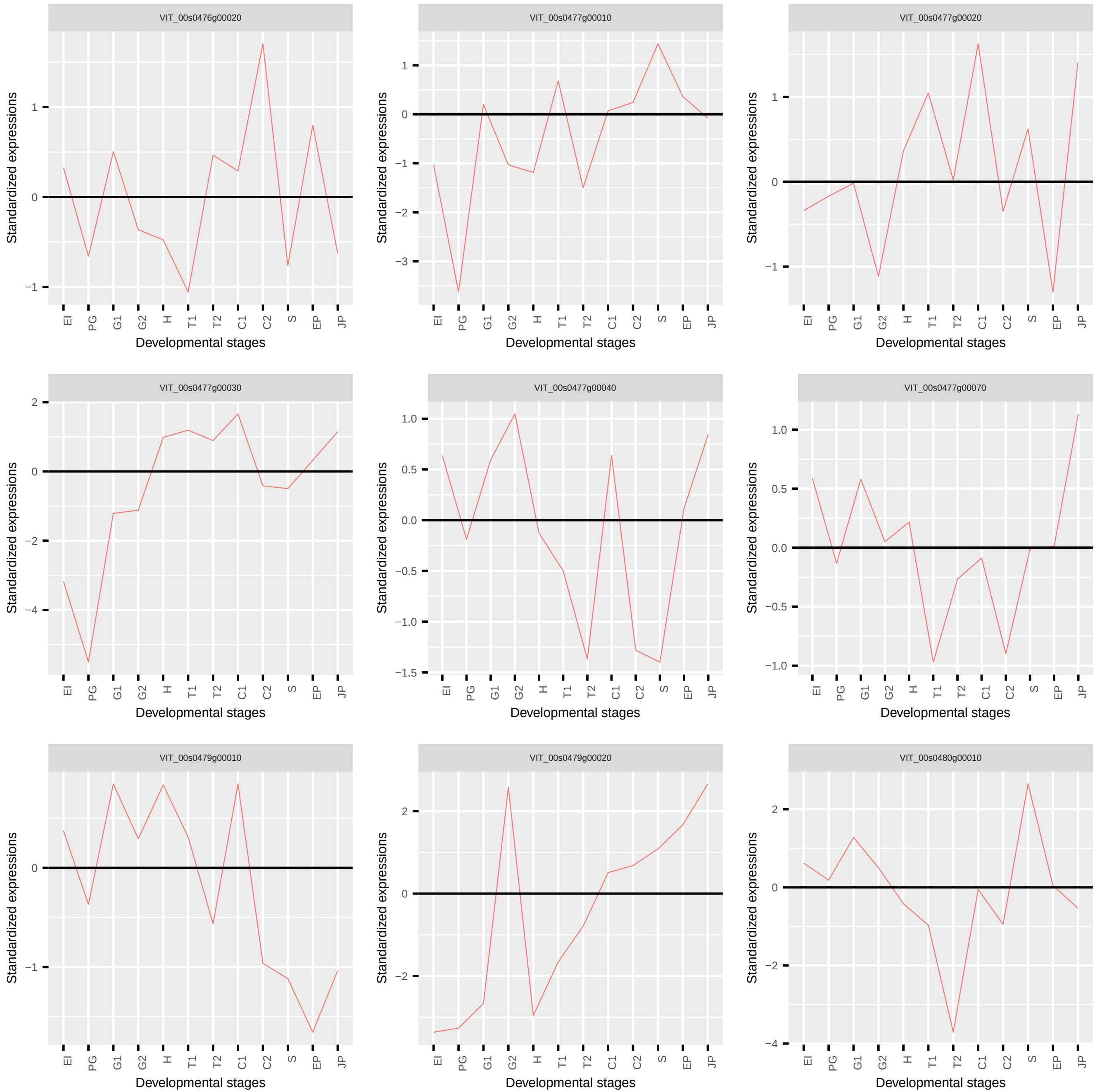
VIT_00s0476g00020
VIT_00s0477g00010
VIT_00s0477g00020
VIT_00s0477g00030
VIT_00s0477g00040
VIT_00s0477g00070
VIT_00s0479g00010
VIT_00s0479g00020
VIT_00s0480g00010
Standardized expressions
Developmental stages
EI PG G1 G2 H T1 T2 C1 C2 S EP JP

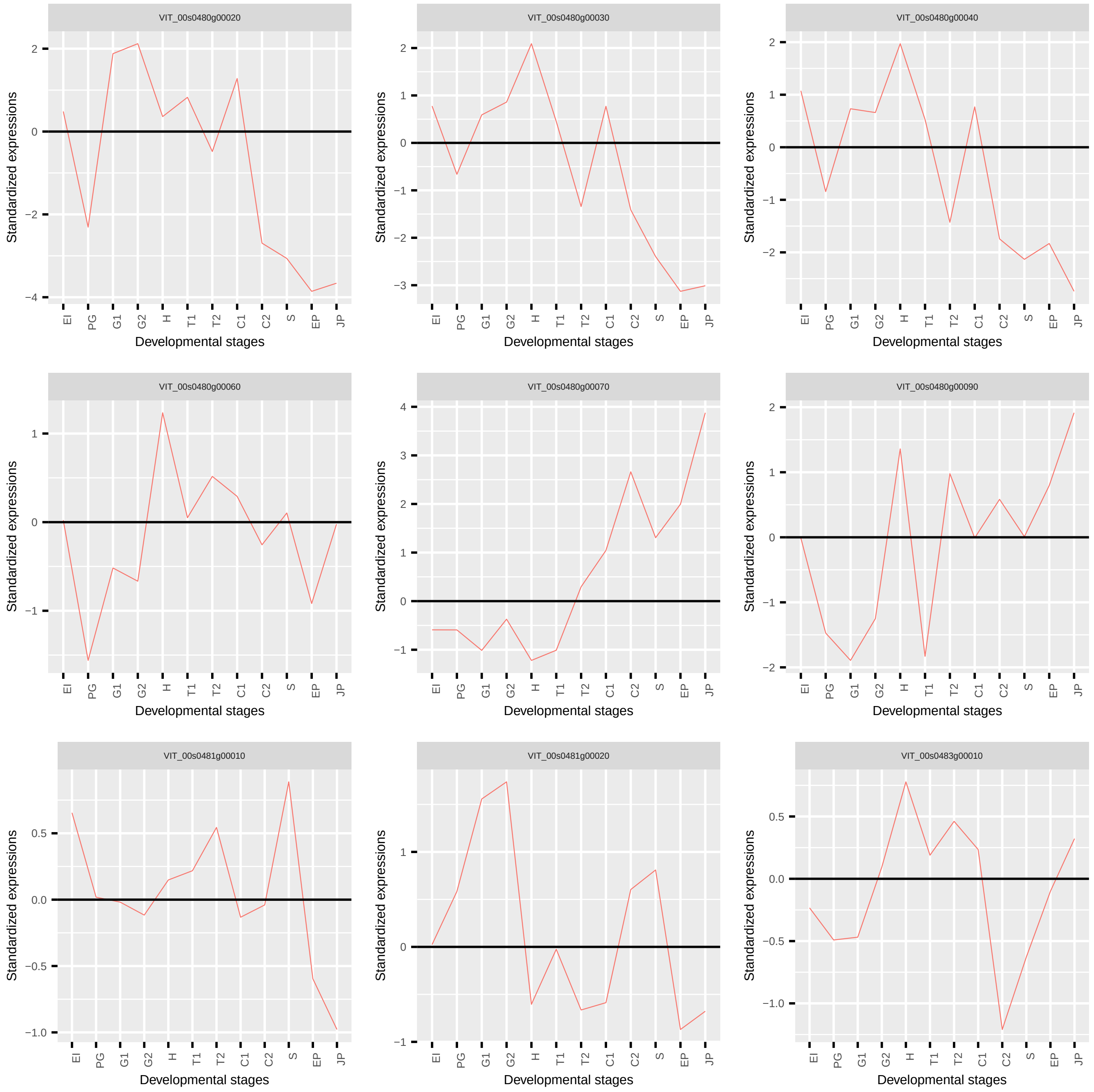

VIT_00s0480g00020
VIT_00s0480g00030
VIT_00s0480g00040
VIT_00s0480g00060
VIT_00s0480g00070
VIT_00s0480g00090
VIT_00s0481g00010
VIT_00s0481g00020
VIT_00s0483g00010
Standardized expressions
Developmental stages
EI PG G1 G2 H T1 T2 C1 C2 S EP JP

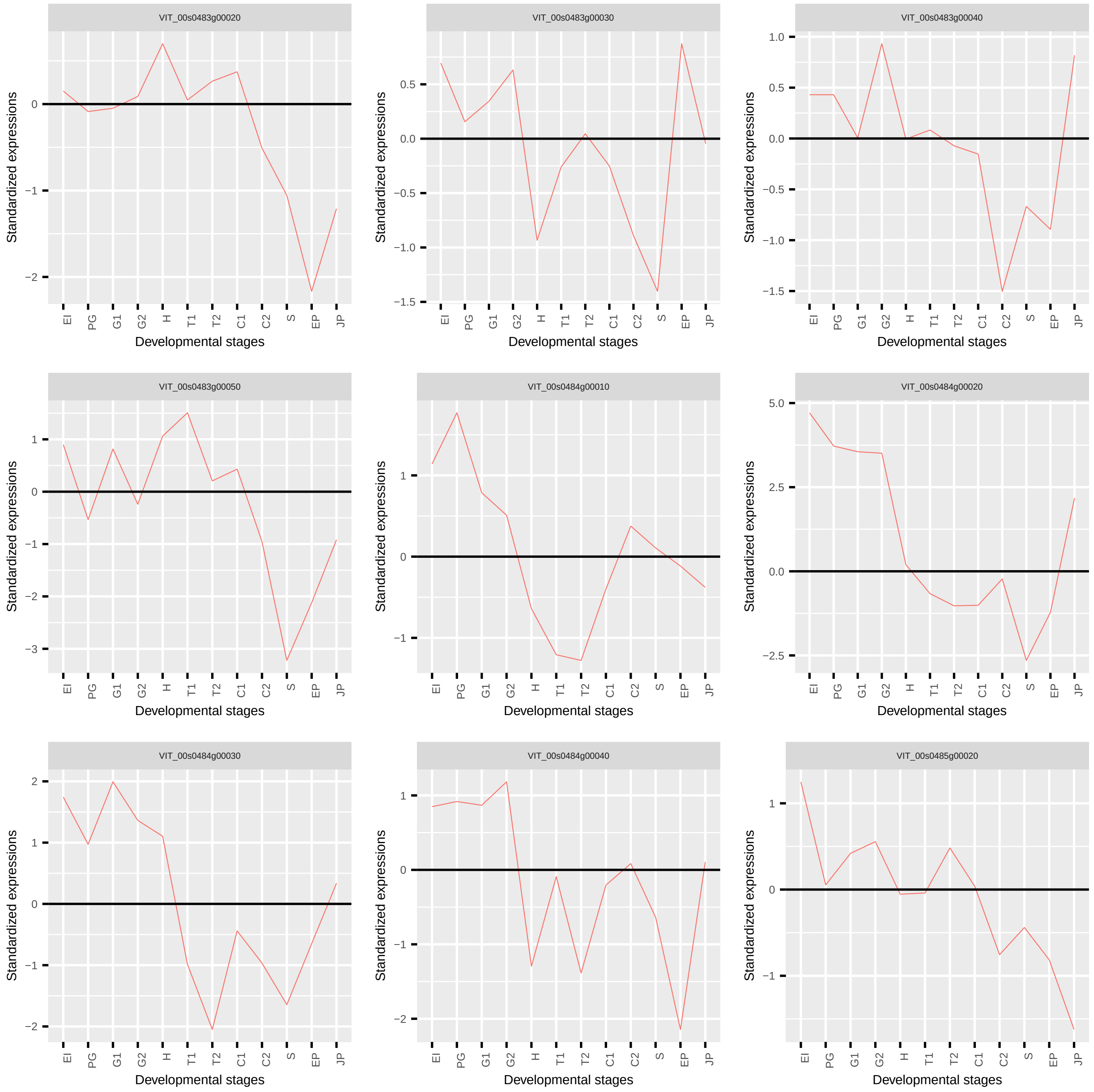
VIT_00s0483g00020
0
−1
−2
Standardized expressions
EI PG G1 G2 H T1 T2 C1 C2 S EP JP
Developmental stages
VIT_00s0483g00030
0.5
0.0
−0.5
−1.0
−1.5
Standardized expressions
EI PG G1 G2 H T1 T2 C1 C2 S EP JP
Developmental stages
VIT_00s0483g00040
1.0
0.5
0.0
−0.5
−1.0
−1.5
Standardized expressions
EI PG G1 G2 H T1 T2 C1 C2 S EP JP
Developmental stages
VIT_00s0483g00050
1
0
−1
−2
−3
Standardized expressions
EI PG G1 G2 H T1 T2 C1 C2 S EP JP
Developmental stages
VIT_00s0484g00010
1
0
−1
Standardized expressions
EI PG G1 G2 H T1 T2 C1 C2 S EP JP
Developmental stages
VIT_00s0484g00020
5.0
2.5
0.0
−2.5
Standardized expressions
EI PG G1 G2 H T1 T2 C1 C2 S EP JP
Developmental stages
VIT_00s0484g00030
2
1
0
−1
−2
Standardized expressions
EI PG G1 G2 H T1 T2 C1 C2 S EP JP
Developmental stages
VIT_00s0484g00040
1
0
−1
−2
Standardized expressions
EI PG G1 G2 H T1 T2 C1 C2 S EP JP
Developmental stages
VIT_00s0485g00020
1
0
−1
Standardized expressions
EI PG G1 G2 H T1 T2 C1 C2 S EP JP
Developmental stages

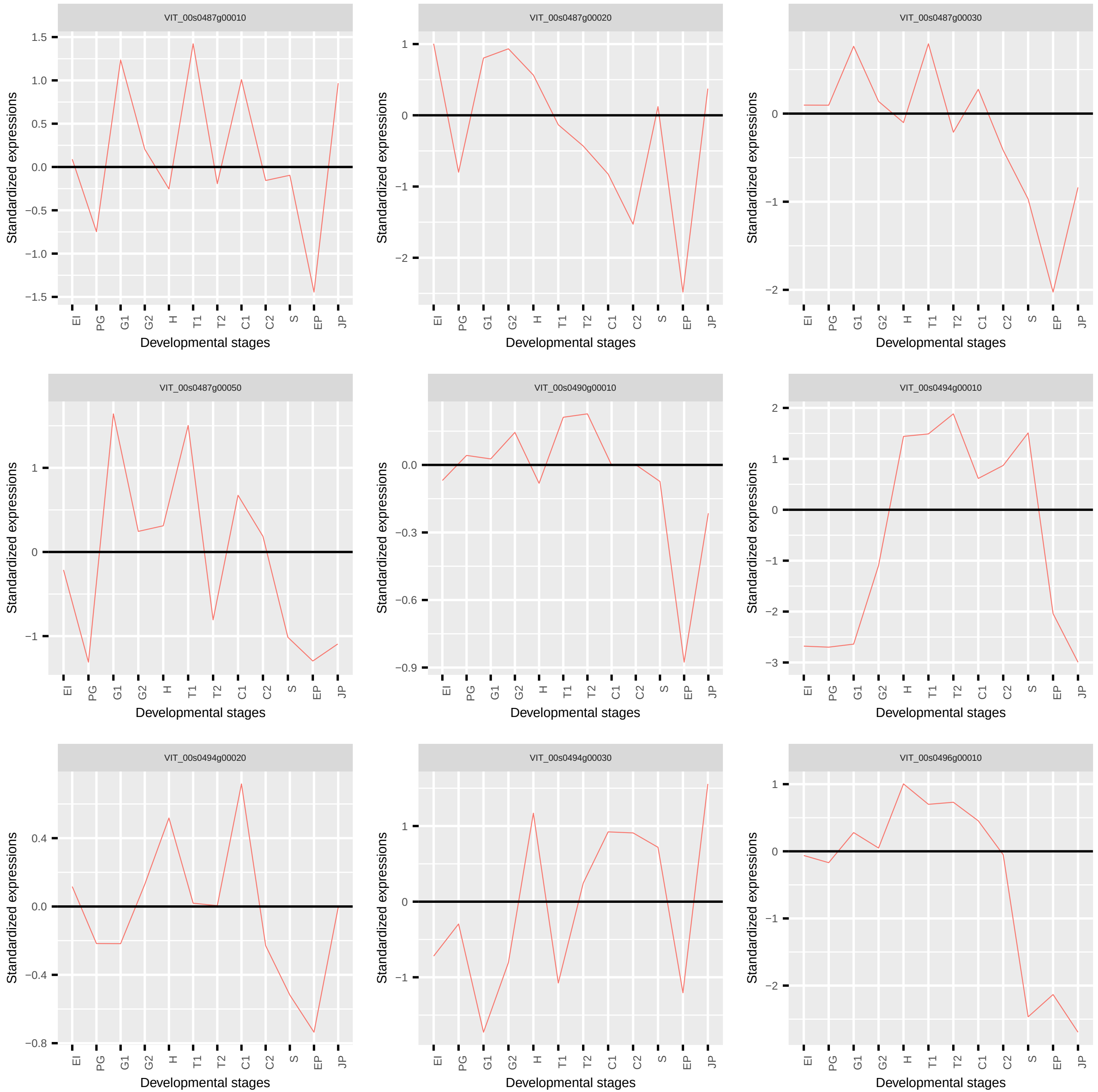
VIT_00s0487g00010
VIT_00s0487g00020
VIT_00s0487g00030
VIT_00s0487g00050
VIT_00s0490g00010
VIT_00s0494g00010
VIT_00s0494g00020
VIT_00s0494g00030
VIT_00s0496g00010
Standardized expressions
Developmental stages
EI
PG
G1
G2
H
T1
T2
C1
C2
S
EP
JP

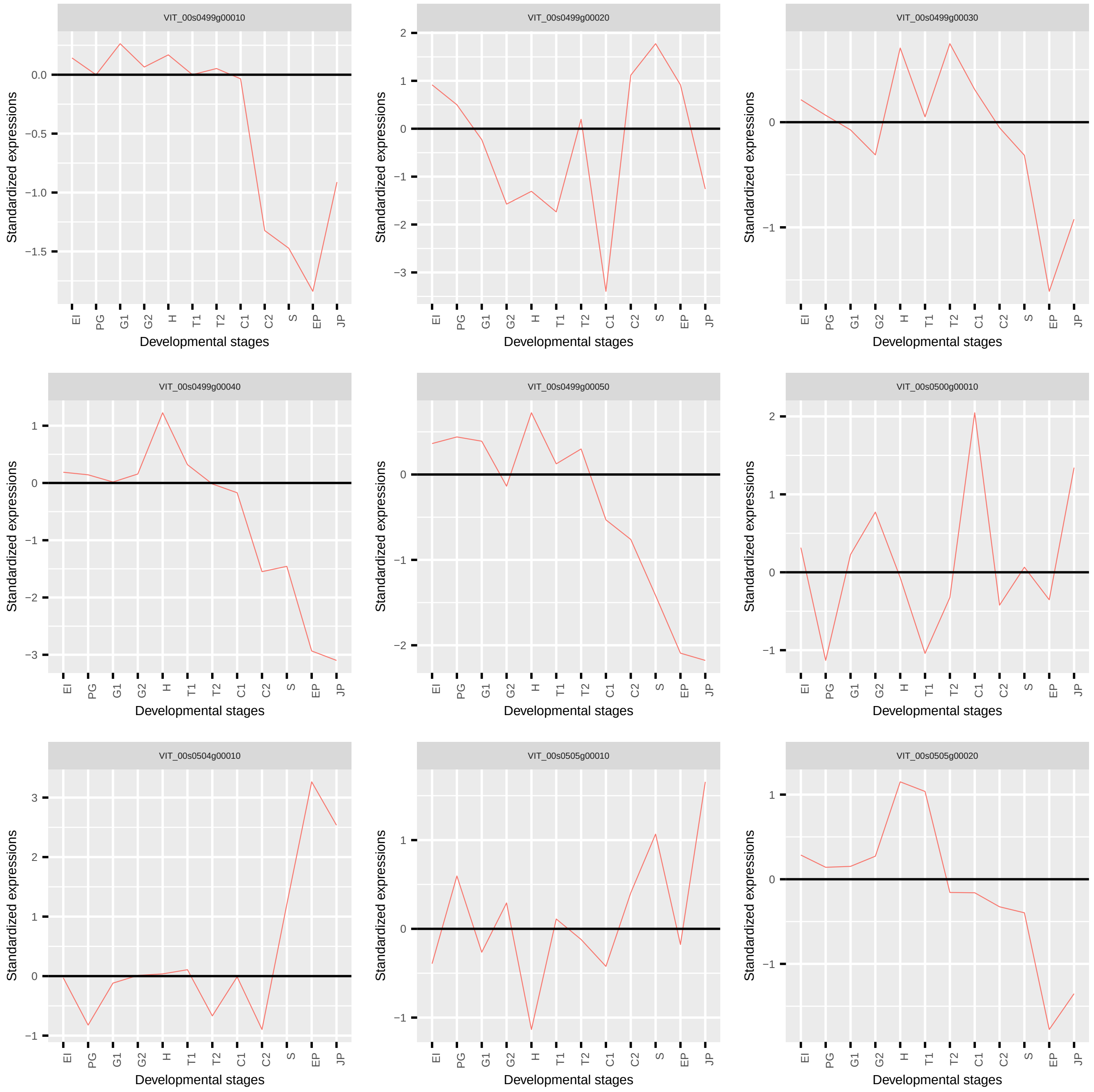

VIT_00s0499g00010
VIT_00s0499g00020
VIT_00s0499g00030
VIT_00s0499g00040
VIT_00s0499g00050
VIT_00s0500g00010
VIT_00s0504g00010
VIT_00s0505g00010
VIT_00s0505g00020
Standardized expressions
Developmental stages
EI PG G1 G2 H T1 T2 C1 C2 S EP JP

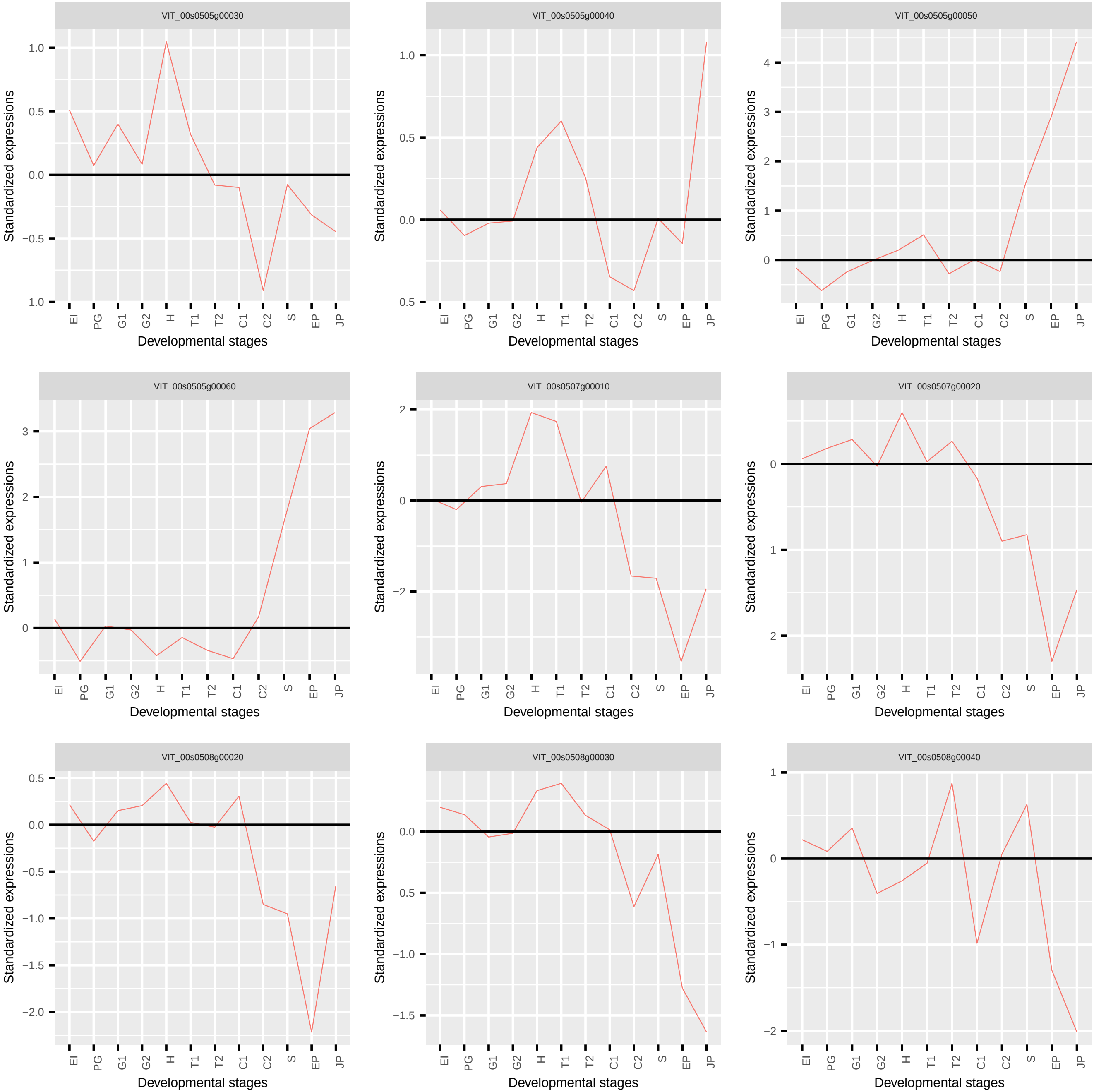

VIT_00s0505g00030
VIT_00s0505g00040
VIT_00s0505g00050
VIT_00s0505g00060
VIT_00s0507g00010
VIT_00s0507g00020
VIT_00s0508g00020
VIT_00s0508g00030
VIT_00s0508g00040
Standardized expressions
Developmental stages
EI PG G1 G2 H T1 T2 C1 C2 S EP JP

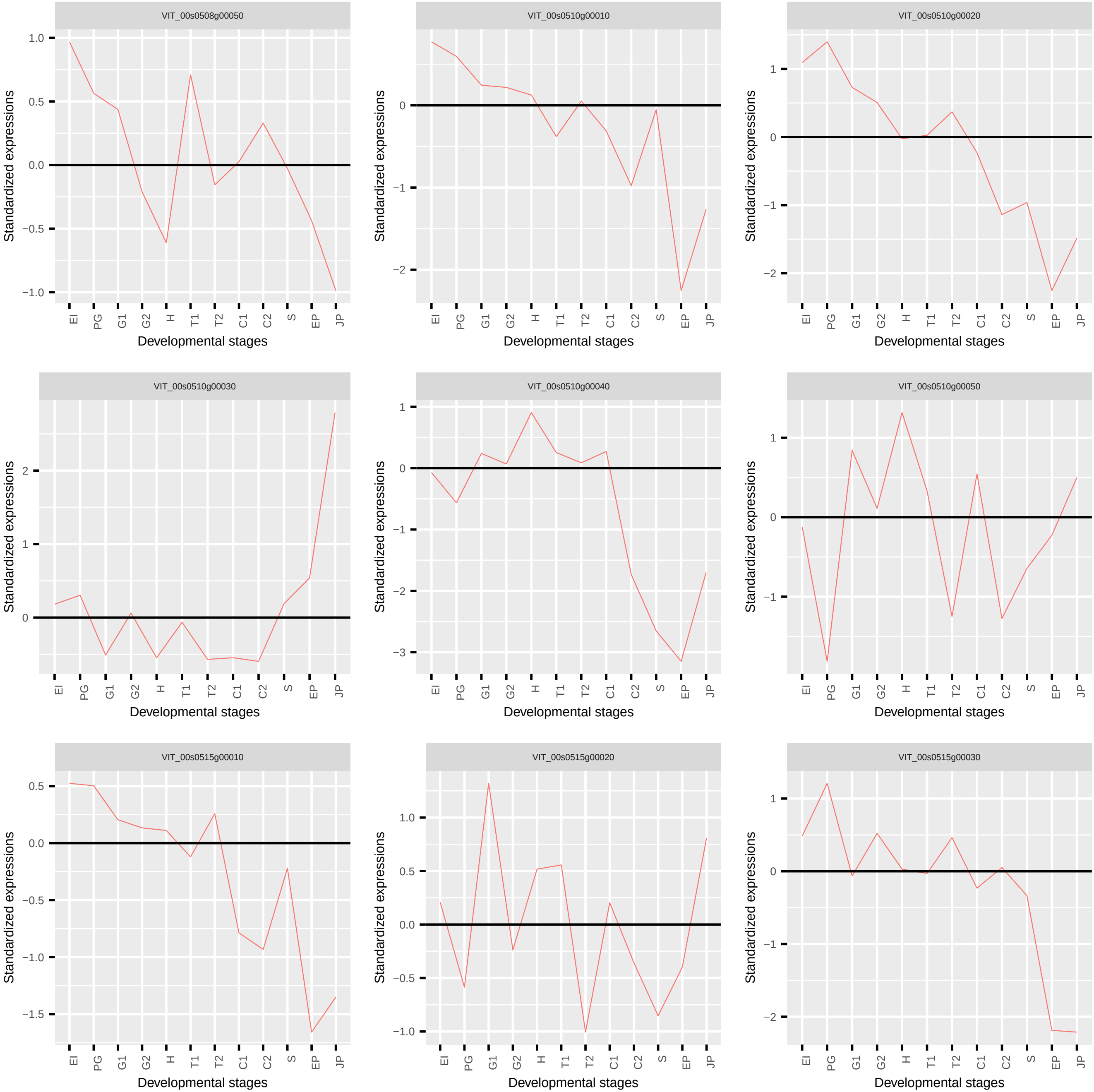
VIT_00s0508g00050
VIT_00s0510g00010
VIT_00s0510g00020
VIT_00s0510g00030
VIT_00s0510g00040
VIT_00s0510g00050
VIT_00s0515g00010
VIT_00s0515g00020
VIT_00s0515g00030
Standardized expressions
Developmental stages
EI
PG
G1
G2
H
T1
T2
C1
C2
S
EP
JP

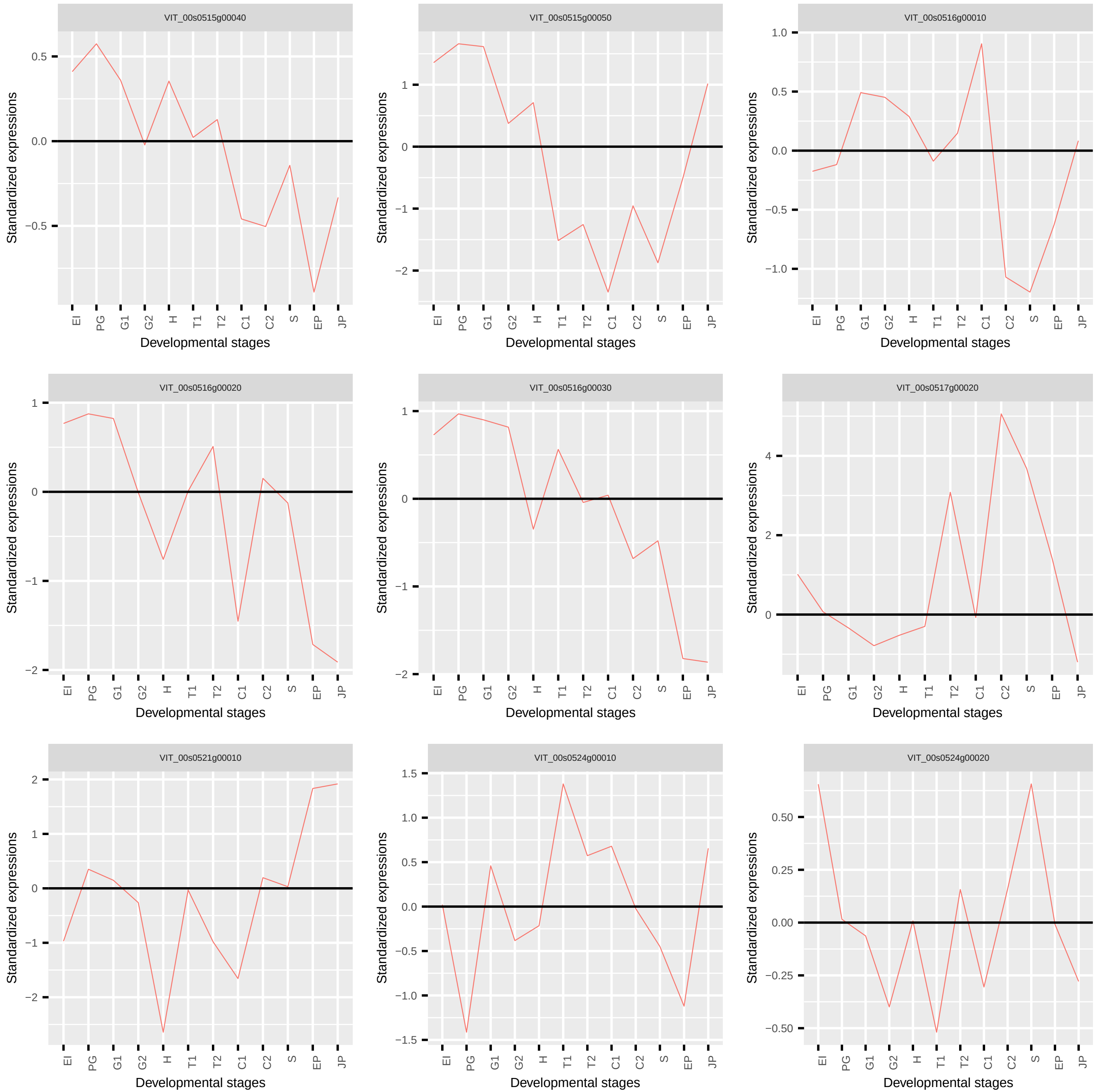
VIT_00s0515g00040
VIT_00s0515g00050
VIT_00s0516g00010
VIT_00s0516g00020
VIT_00s0516g00030
VIT_00s0517g00020
VIT_00s0521g00010
VIT_00s0524g00010
VIT_00s0524g00020
Standardized expressions
Developmental stages
EI
PG
G1
G2
H
T1
T2
C1
C2
S
EP
JP

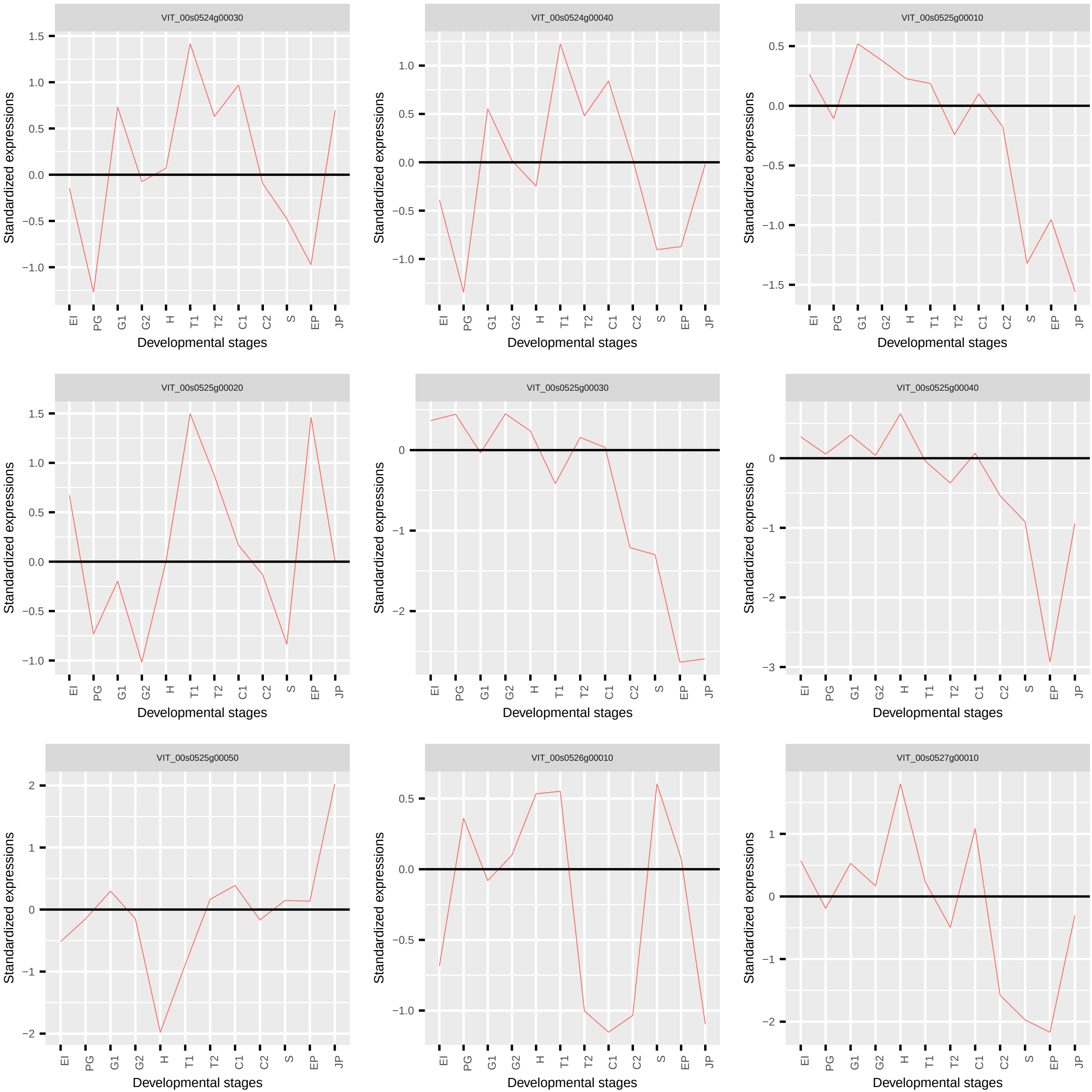
VIT_00s0524g00030
VIT_00s0524g00040
VIT_00s0525g00010
VIT_00s0525g00020
VIT_00s0525g00030
VIT_00s0525g00040
VIT_00s0525g00050
VIT_00s0526g00010
VIT_00s0527g00010
Standardized expressions
Developmental stages
EI PG G1 G2 H T1 T2 C1 C2 S EP JP

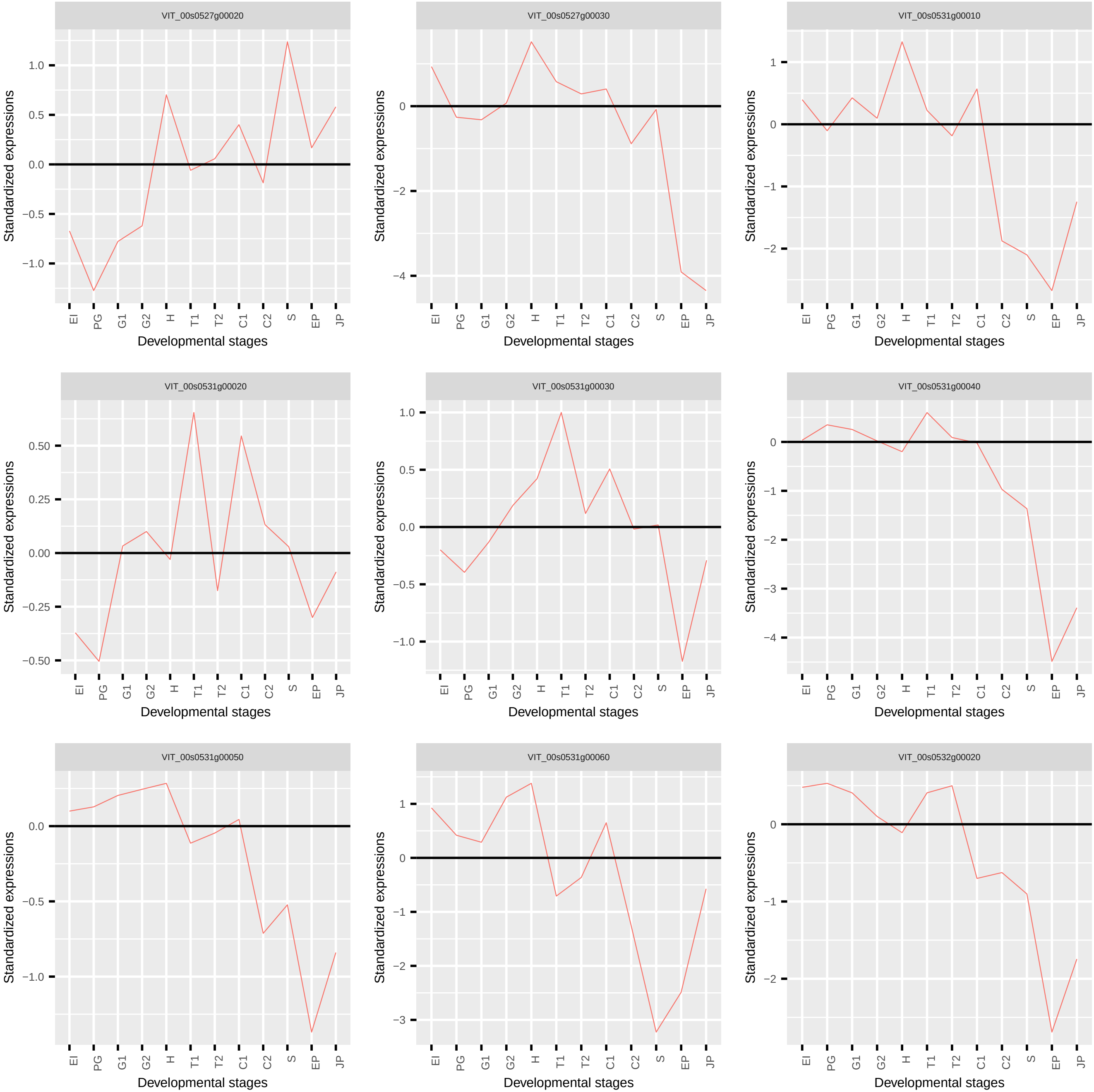

VIT_00s0527g00020
Standardized expressions
1.0
0.5
0.0
−0.5
−1.0
EI PG G1 G2 H T1 T2 C1 C2 S EP JP
Developmental stages
VIT_00s0527g00030
Standardized expressions
0
−2
−4
EI PG G1 G2 H T1 T2 C1 C2 S EP JP
Developmental stages
VIT_00s0531g00010
Standardized expressions
1
0
−1
−2
EI PG G1 G2 H T1 T2 C1 C2 S EP JP
Developmental stages
VIT_00s0531g00020
Standardized expressions
0.50
0.25
0.00
−0.25
−0.50
EI PG G1 G2 H T1 T2 C1 C2 S EP JP
Developmental stages
VIT_00s0531g00030
Standardized expressions
1.0
0.5
0.0
−0.5
−1.0
EI PG G1 G2 H T1 T2 C1 C2 S EP JP
Developmental stages
VIT_00s0531g00040
Standardized expressions
0
−1
−2
−3
−4
EI PG G1 G2 H T1 T2 C1 C2 S EP JP
Developmental stages
VIT_00s0531g00050
Standardized expressions
0.0
−0.5
−1.0
EI PG G1 G2 H T1 T2 C1 C2 S EP JP
Developmental stages
VIT_00s0531g00060
Standardized expressions
1
0
−1
−2
−3
EI PG G1 G2 H T1 T2 C1 C2 S EP JP
Developmental stages
VIT_00s0532g00020
Standardized expressions
0
−1
−2
EI PG G1 G2 H T1 T2 C1 C2 S EP JP
Developmental stages

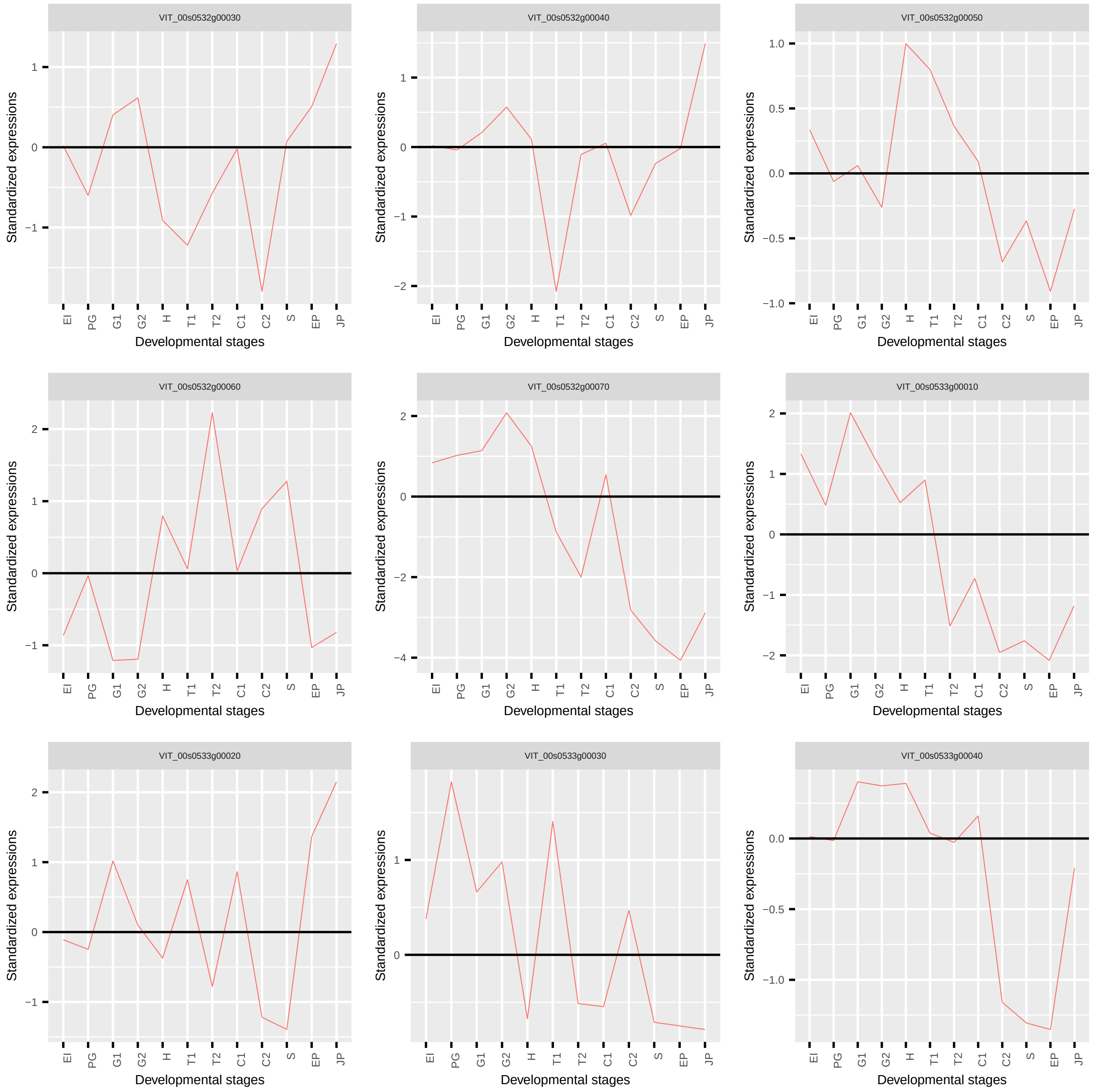
VIT_00s0532g00030
VIT_00s0532g00040
VIT_00s0532g00050
VIT_00s0532g00060
VIT_00s0532g00070
VIT_00s0533g00010
VIT_00s0533g00020
VIT_00s0533g00030
VIT_00s0533g00040
Standardized expressions
Developmental stages
EI
PG
G1
G2
H
T1
T2
C1
C2
S
EP
JP

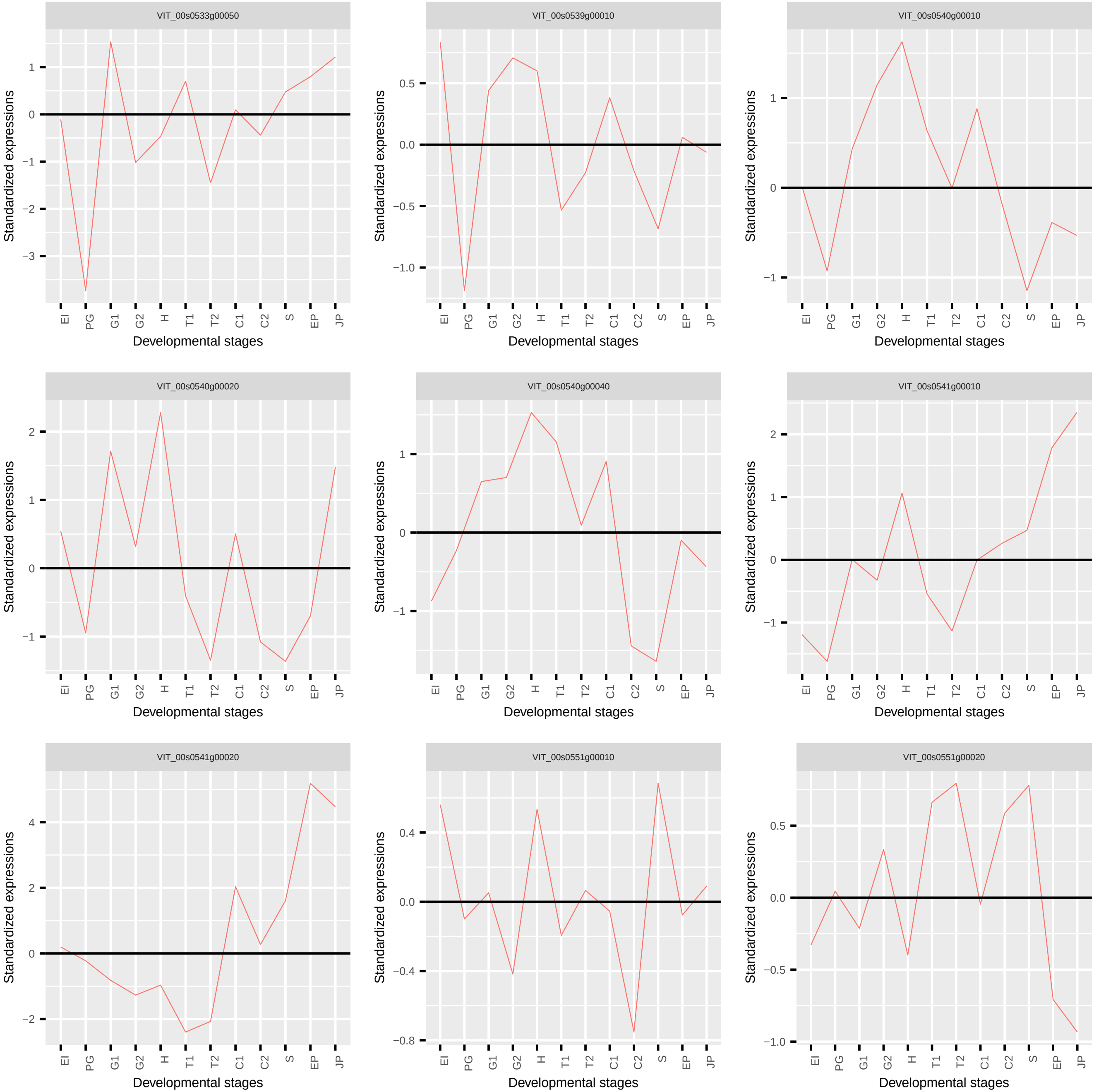
VIT_00s0533g00050
1
0
−1
−2
−3
Standardized expressions
EI PG G1 G2 H T1 T2 C1 C2 S EP JP
Developmental stages
VIT_00s0539g00010
0.5
0.0
−0.5
−1.0
Standardized expressions
EI PG G1 G2 H T1 T2 C1 C2 S EP JP
Developmental stages
VIT_00s0540g00010
1
0
−1
Standardized expressions
EI PG G1 G2 H T1 T2 C1 C2 S EP JP
Developmental stages
VIT_00s0540g00020
2
1
0
−1
Standardized expressions
EI PG G1 G2 H T1 T2 C1 C2 S EP JP
Developmental stages
VIT_00s0540g00040
1
0
−1
Standardized expressions
EI PG G1 G2 H T1 T2 C1 C2 S EP JP
Developmental stages
VIT_00s0541g00010
2
1
0
−1
Standardized expressions
EI PG G1 G2 H T1 T2 C1 C2 S EP JP
Developmental stages
VIT_00s0541g00020
4
2
0
−2
Standardized expressions
EI PG G1 G2 H T1 T2 C1 C2 S EP JP
Developmental stages
VIT_00s0551g00010
0.4
0.0
−0.4
−0.8
Standardized expressions
EI PG G1 G2 H T1 T2 C1 C2 S EP JP
Developmental stages
VIT_00s0551g00020
0.5
0.0
−0.5
−1.0
Standardized expressions
EI PG G1 G2 H T1 T2 C1 C2 S EP JP
Developmental stages

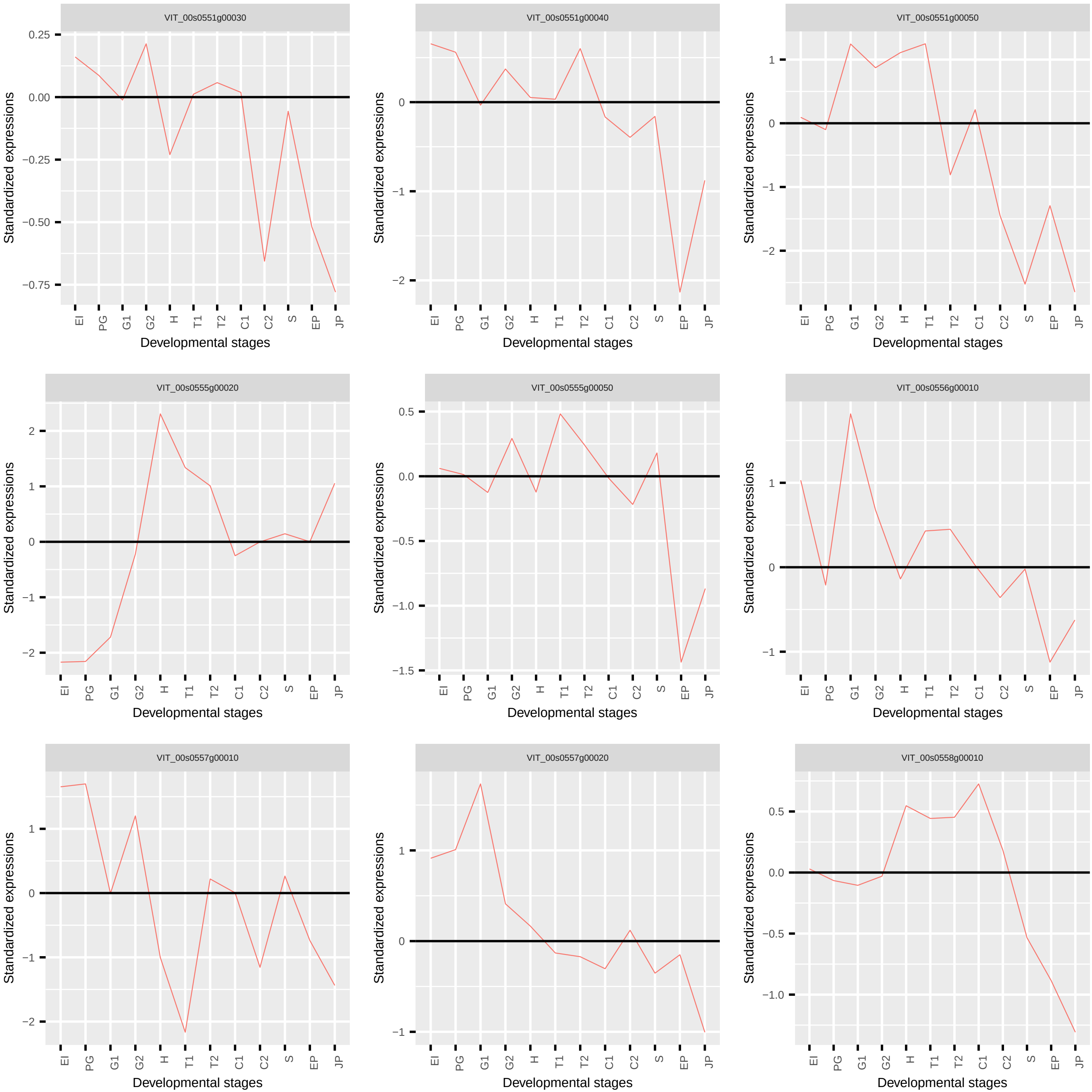

VIT_00s0551g00030
VIT_00s0551g00040
VIT_00s0551g00050
VIT_00s0555g00020
VIT_00s0555g00050
VIT_00s0556g00010
VIT_00s0557g00010
VIT_00s0557g00020
VIT_00s0558g00010
Standardized expressions
Developmental stages
EI PG G1 G2 H T1 T2 C1 C2 S EP JP

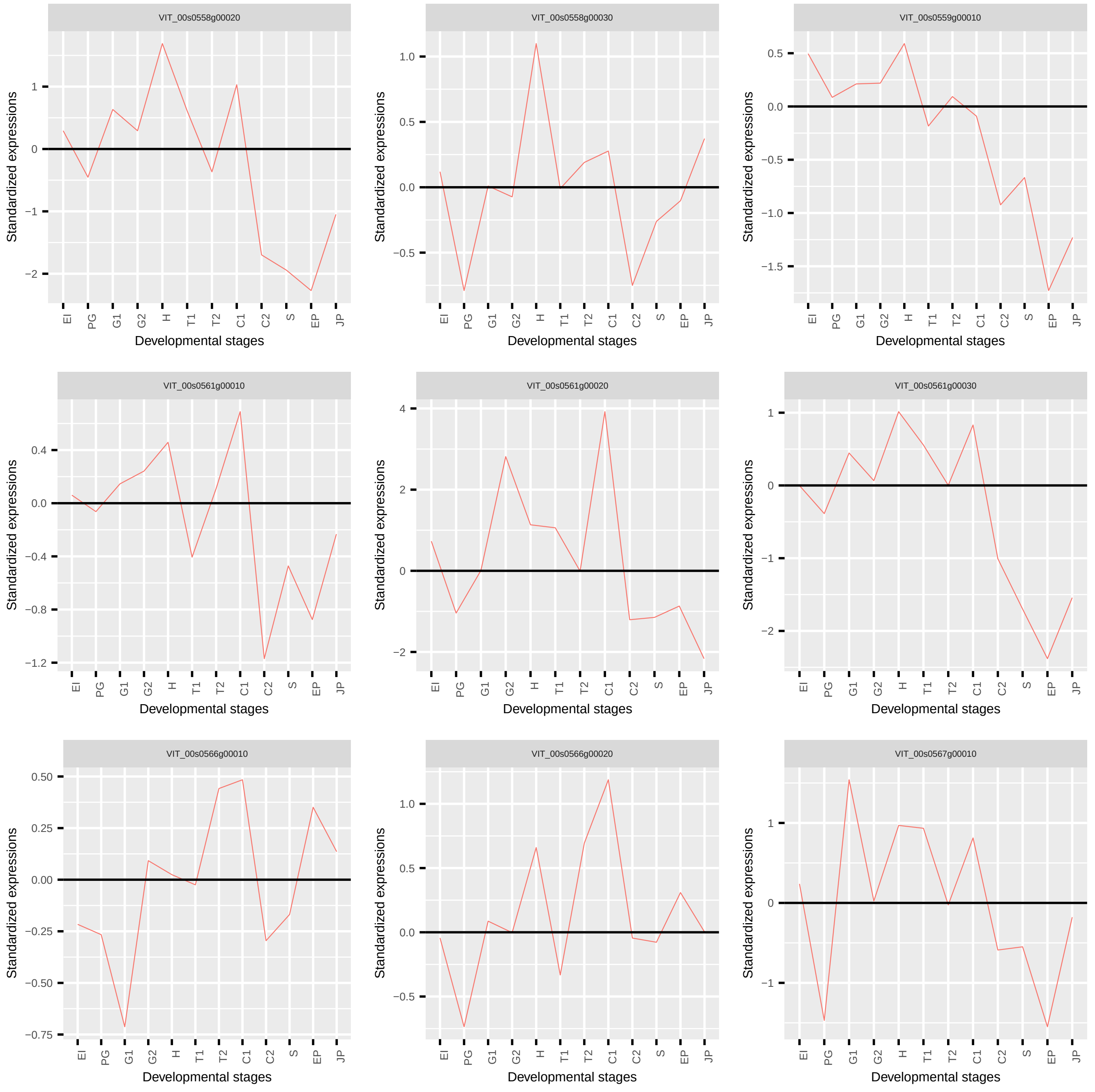
VIT_00s0558g00020
VIT_00s0558g00030
VIT_00s0559g00010
VIT_00s0561g00010
VIT_00s0561g00020
VIT_00s0561g00030
VIT_00s0566g00010
VIT_00s0566g00020
VIT_00s0567g00010
Standardized expressions
Developmental stages
EI PG G1 G2 H T1 T2 C1 C2 S EP JP

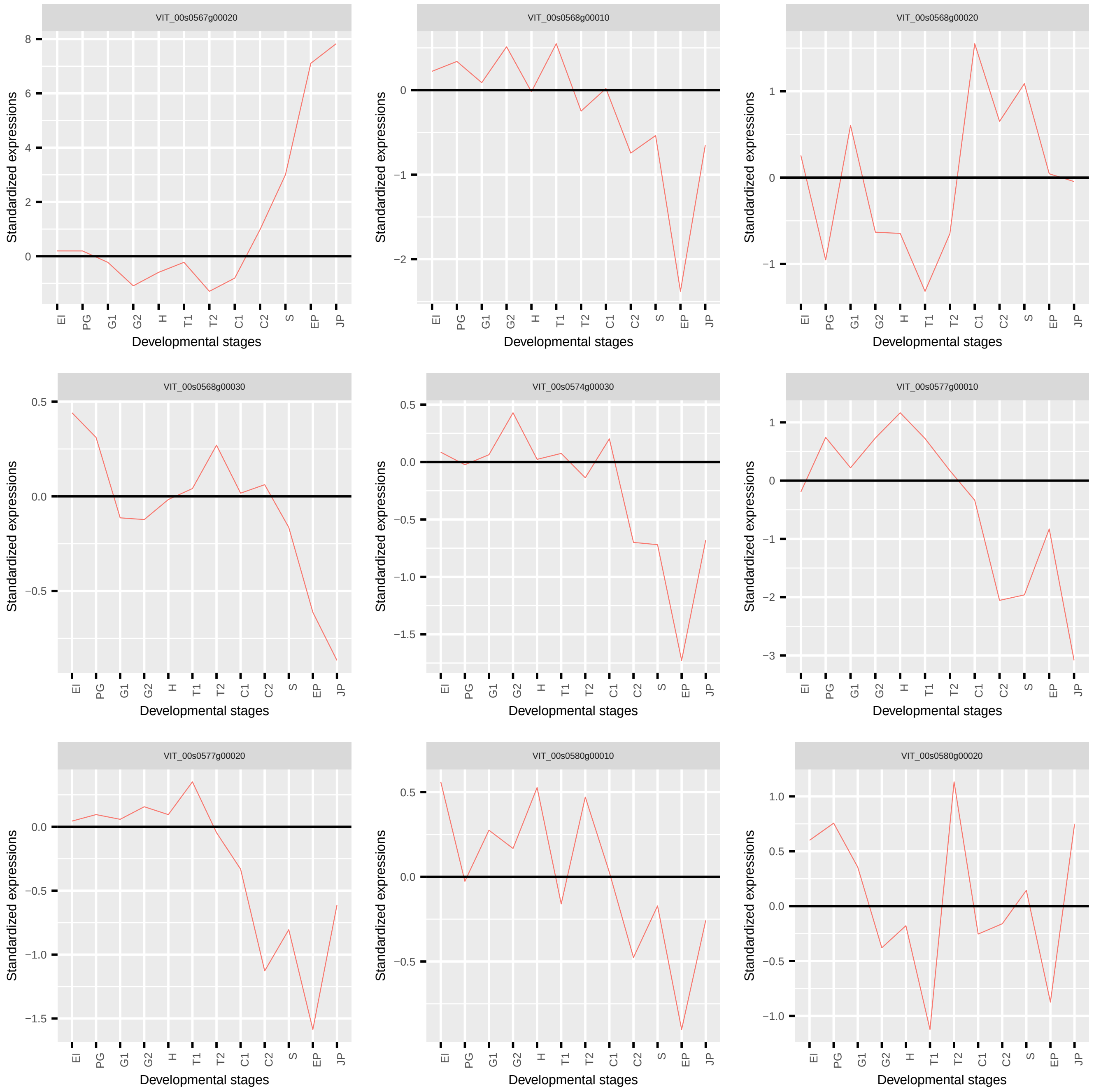
VIT_00s0567g00020
8
6
4
2
0
Standardized expressions
EI PG G1 G2 H T1 T2 C1 C2 S EP JP
Developmental stages
VIT_00s0568g00010
0
−1
−2
Standardized expressions
EI PG G1 G2 H T1 T2 C1 C2 S EP JP
Developmental stages
VIT_00s0568g00020
1
0
−1
Standardized expressions
EI PG G1 G2 H T1 T2 C1 C2 S EP JP
Developmental stages
VIT_00s0568g00030
0.5
0.0
−0.5
Standardized expressions
EI PG G1 G2 H T1 T2 C1 C2 S EP JP
Developmental stages
VIT_00s0574g00030
0.5
0.0
−0.5
−1.0
−1.5
Standardized expressions
EI PG G1 G2 H T1 T2 C1 C2 S EP JP
Developmental stages
VIT_00s0577g00010
1
0
−1
−2
−3
Standardized expressions
EI PG G1 G2 H T1 T2 C1 C2 S EP JP
Developmental stages
VIT_00s0577g00020
0.0
−0.5
−1.0
−1.5
Standardized expressions
EI PG G1 G2 H T1 T2 C1 C2 S EP JP
Developmental stages
VIT_00s0580g00010
0.5
0.0
−0.5
Standardized expressions
EI PG G1 G2 H T1 T2 C1 C2 S EP JP
Developmental stages
VIT_00s0580g00020
1.0
0.5
0.0
−0.5
−1.0
Standardized expressions
EI PG G1 G2 H T1 T2 C1 C2 S EP JP
Developmental stages

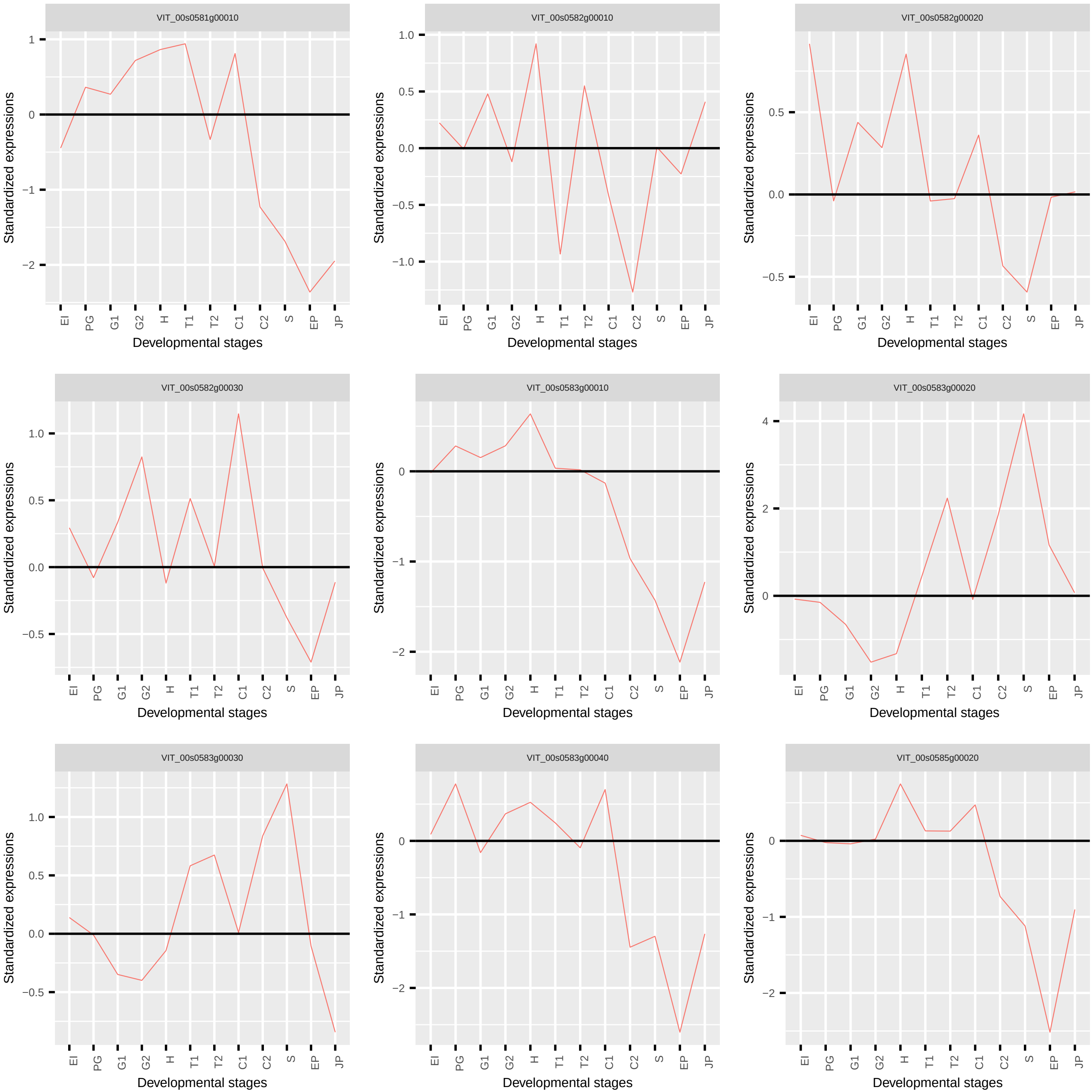
VIT_00s0581g00010
VIT_00s0582g00010
VIT_00s0582g00020
VIT_00s0582g00030
VIT_00s0583g00010
VIT_00s0583g00020
VIT_00s0583g00030
VIT_00s0583g00040
VIT_00s0585g00020
Standardized expressions
Developmental stages
EI PG G1 G2 H T1 T2 C1 C2 S EP JP

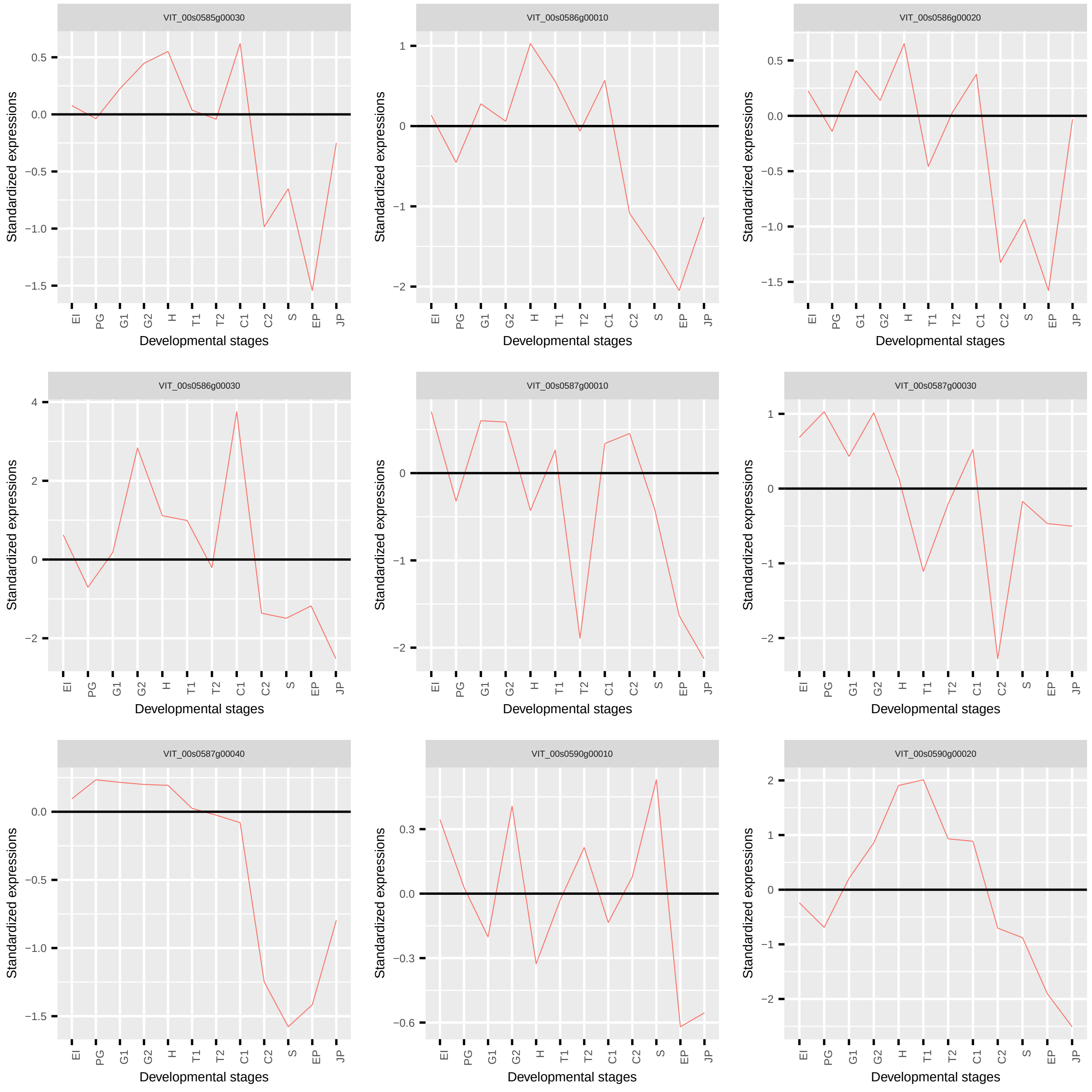

VIT_00s0585g00030
VIT_00s0586g00010
VIT_00s0586g00020
VIT_00s0586g00030
VIT_00s0587g00010
VIT_00s0587g00030
VIT_00s0587g00040
VIT_00s0590g00010
VIT_00s0590g00020
Standardized expressions
Developmental stages
EI
PG
G1
G2
H
T1
T2
C1
C2
S
EP
JP

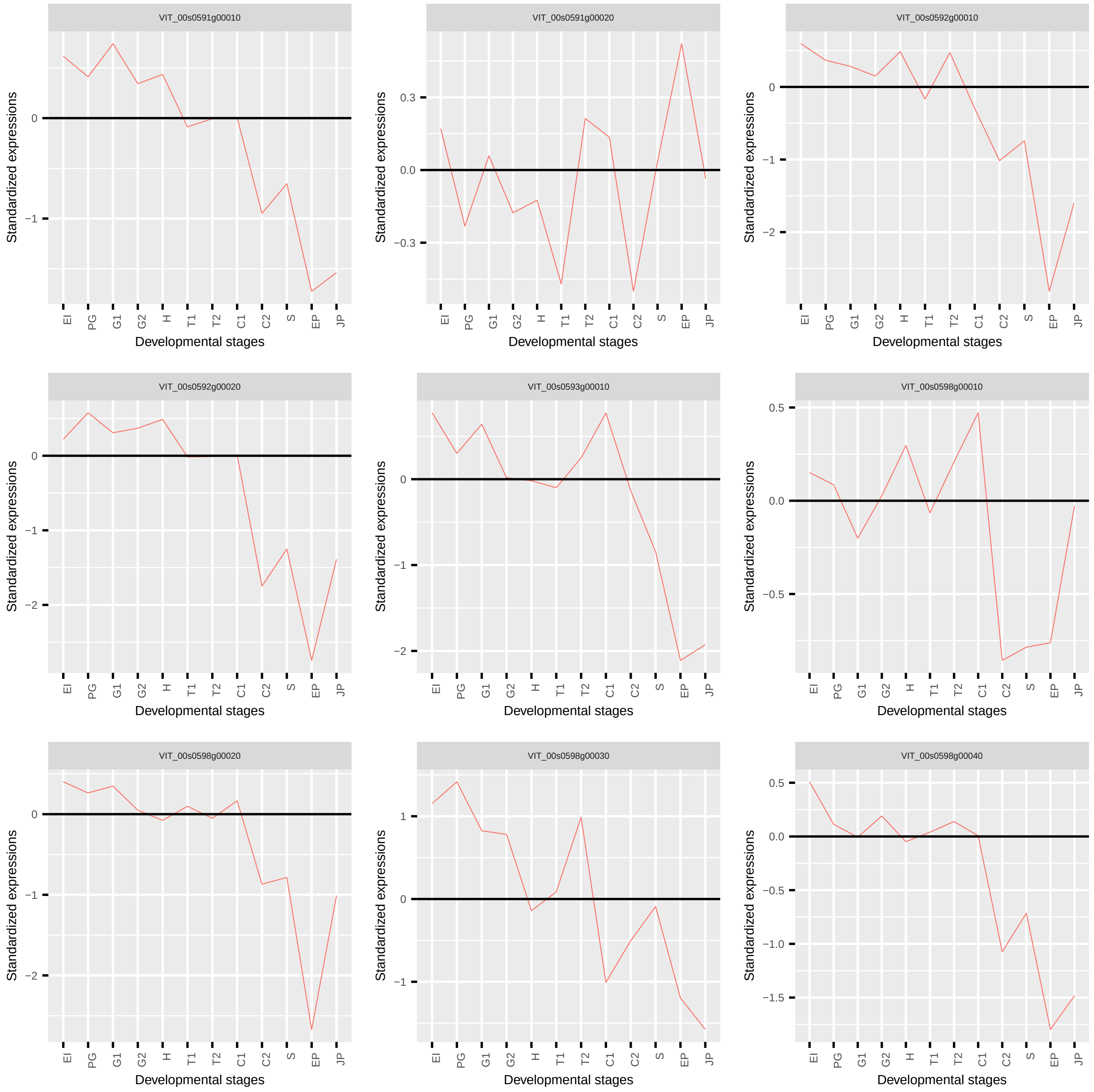
VIT_00s0591g00010
VIT_00s0591g00020
VIT_00s0592g00010
VIT_00s0592g00020
VIT_00s0593g00010
VIT_00s0598g00010
VIT_00s0598g00020
VIT_00s0598g00030
VIT_00s0598g00040
Standardized expressions
Developmental stages
EI PG G1 G2 H T1 T2 C1 C2 S EP JP

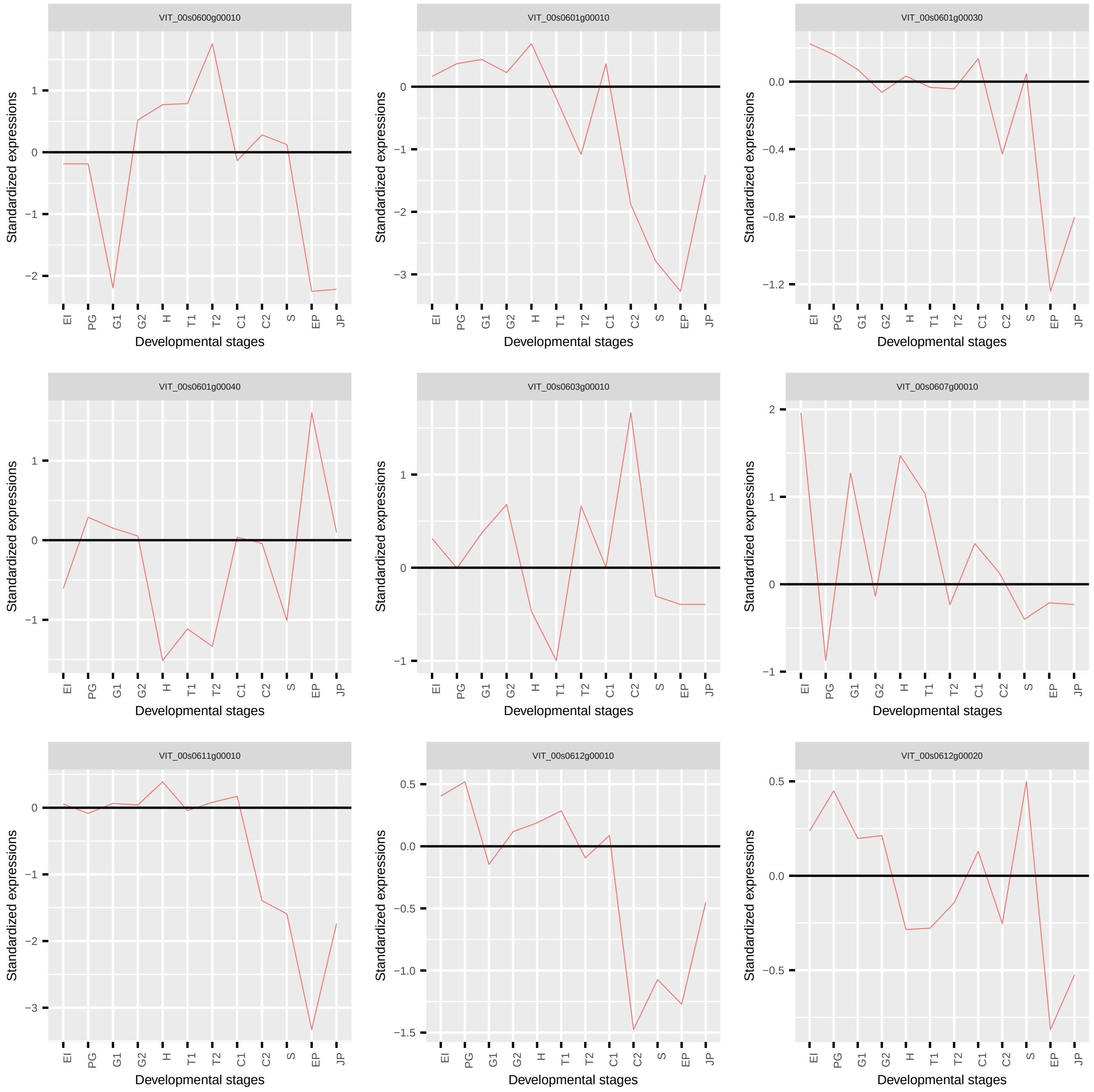
VIT_00s0600g00010
VIT_00s0601g00010
VIT_00s0601g00030
VIT_00s0601g00040
VIT_00s0603g00010
VIT_00s0607g00010
VIT_00s0611g00010
VIT_00s0612g00010
VIT_00s0612g00020
Standardized expressions
Developmental stages
EI PG G1 G2 H T1 T2 C1 C2 S EP JP

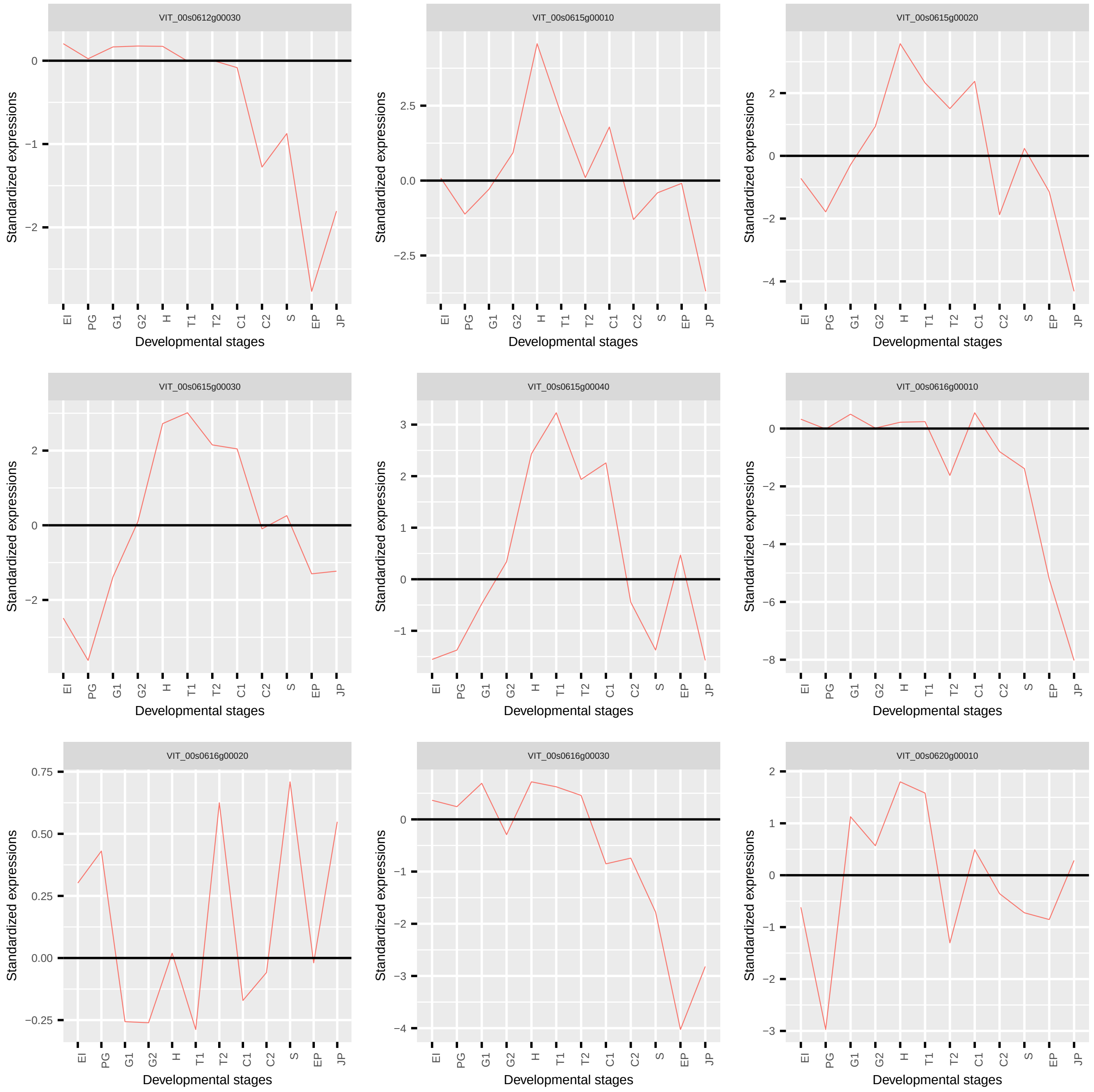
VIT_00s0612g00030
VIT_00s0615g00010
VIT_00s0615g00020
VIT_00s0615g00030
VIT_00s0615g00040
VIT_00s0616g00010
VIT_00s0616g00020
VIT_00s0616g00030
VIT_00s0620g00010
Standardized expressions
Developmental stages
EI PG G1 G2 H T1 T2 C1 C2 S EP JP

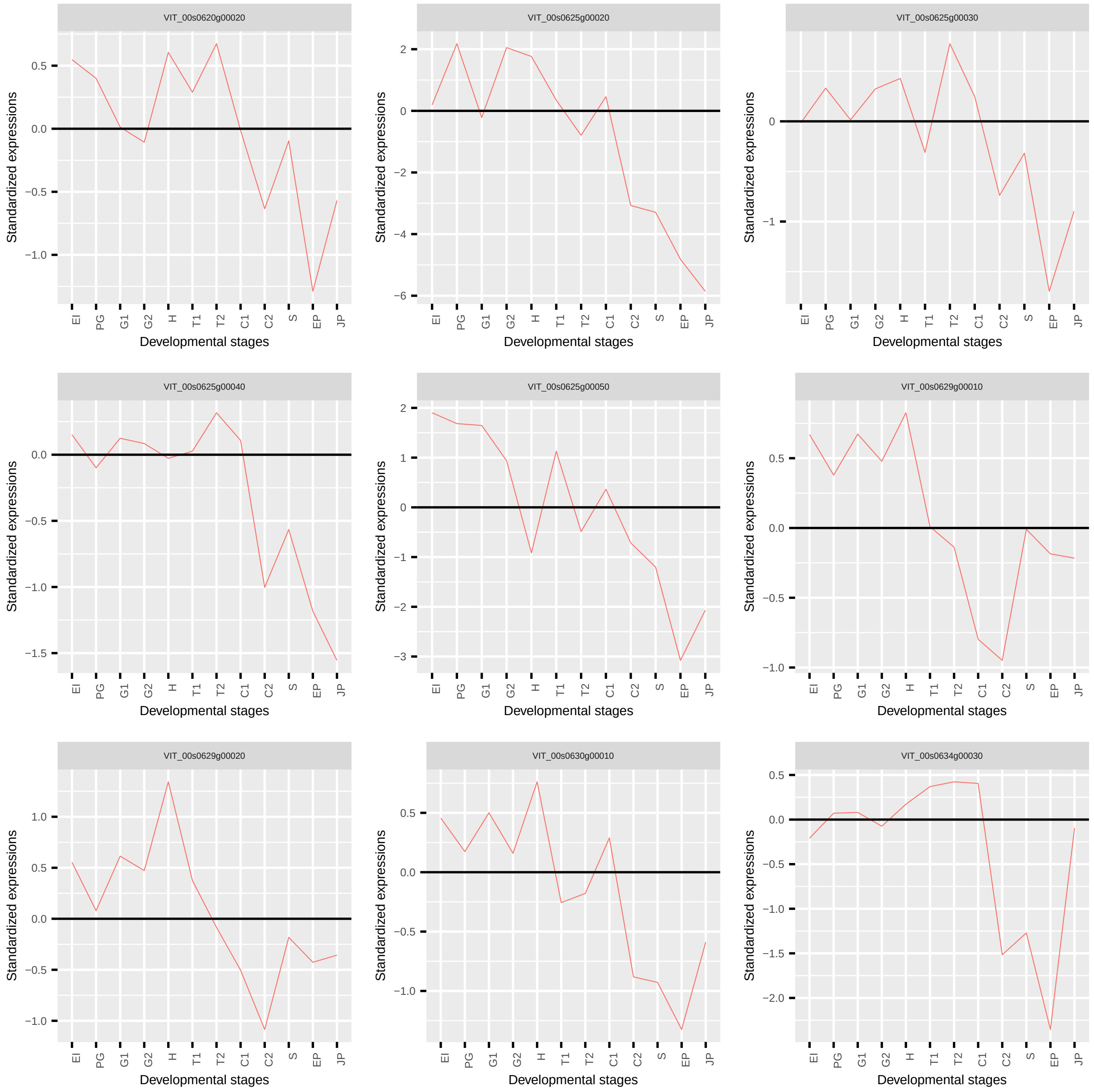
VIT_00s0620g00020
VIT_00s0625g00020
VIT_00s0625g00030
VIT_00s0625g00040
VIT_00s0625g00050
VIT_00s0629g00010
VIT_00s0629g00020
VIT_00s0630g00010
VIT_00s0634g00030
Standardized expressions
Developmental stages
EI PG G1 G2 H T1 T2 C1 C2 S EP JP
0.5 0.0 −0.5 −1.0
2 0 −2 −4 −6
0 −1
0.0 −0.5 −1.0 −1.5
2 1 0 −1 −2 −3
0.5 0.0 −0.5 −1.0
1.0 0.5 0.0 −0.5 −1.0
0.5 0.0 −0.5 −1.0
0.5 0.0 −0.5 −1.0 −1.5 −2.0

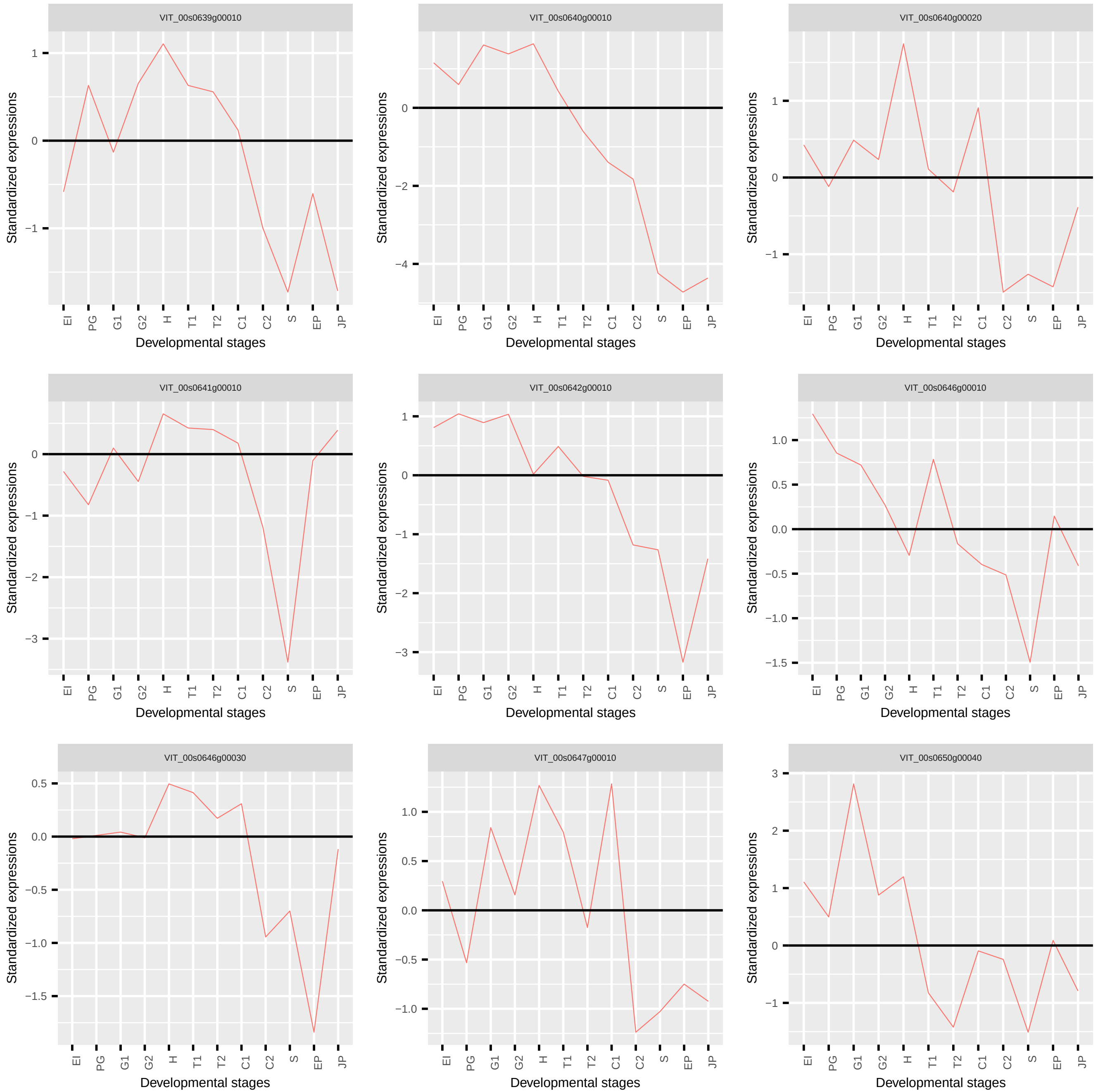

VIT_00s0639g00010
VIT_00s0640g00010
VIT_00s0640g00020
VIT_00s0641g00010
VIT_00s0642g00010
VIT_00s0646g00010
VIT_00s0646g00030
VIT_00s0647g00010
VIT_00s0650g00040
Standardized expressions
Developmental stages
EI
PG
G1
G2
H
T1
T2
C1
C2
S
EP
JP

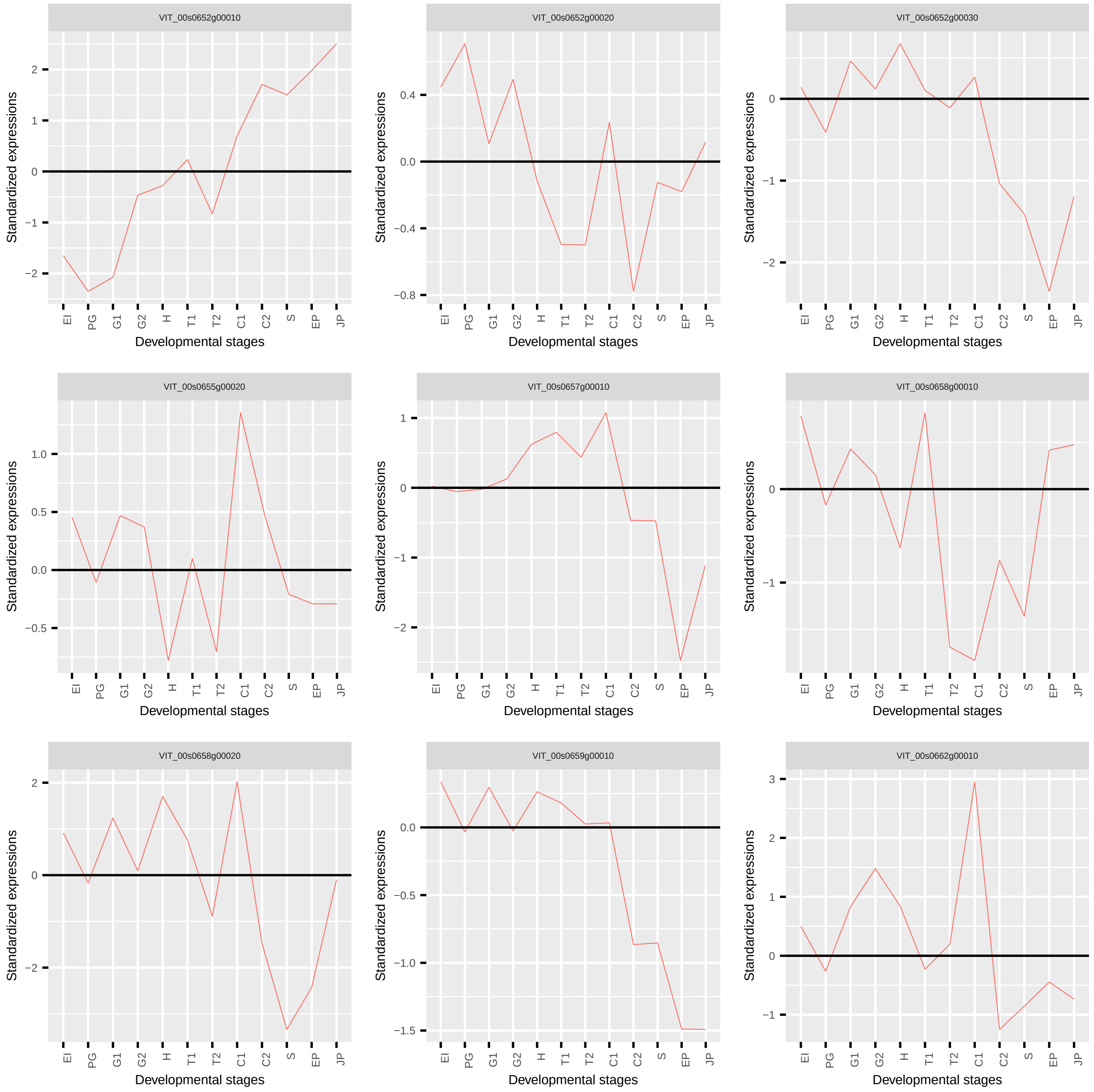
VIT_00s0652g00010
VIT_00s0652g00020
VIT_00s0652g00030
VIT_00s0655g00020
VIT_00s0657g00010
VIT_00s0658g00010
VIT_00s0658g00020
VIT_00s0659g00010
VIT_00s0662g00010
Standardized expressions
Developmental stages
EI PG G1 G2 H T1 T2 C1 C2 S EP JP

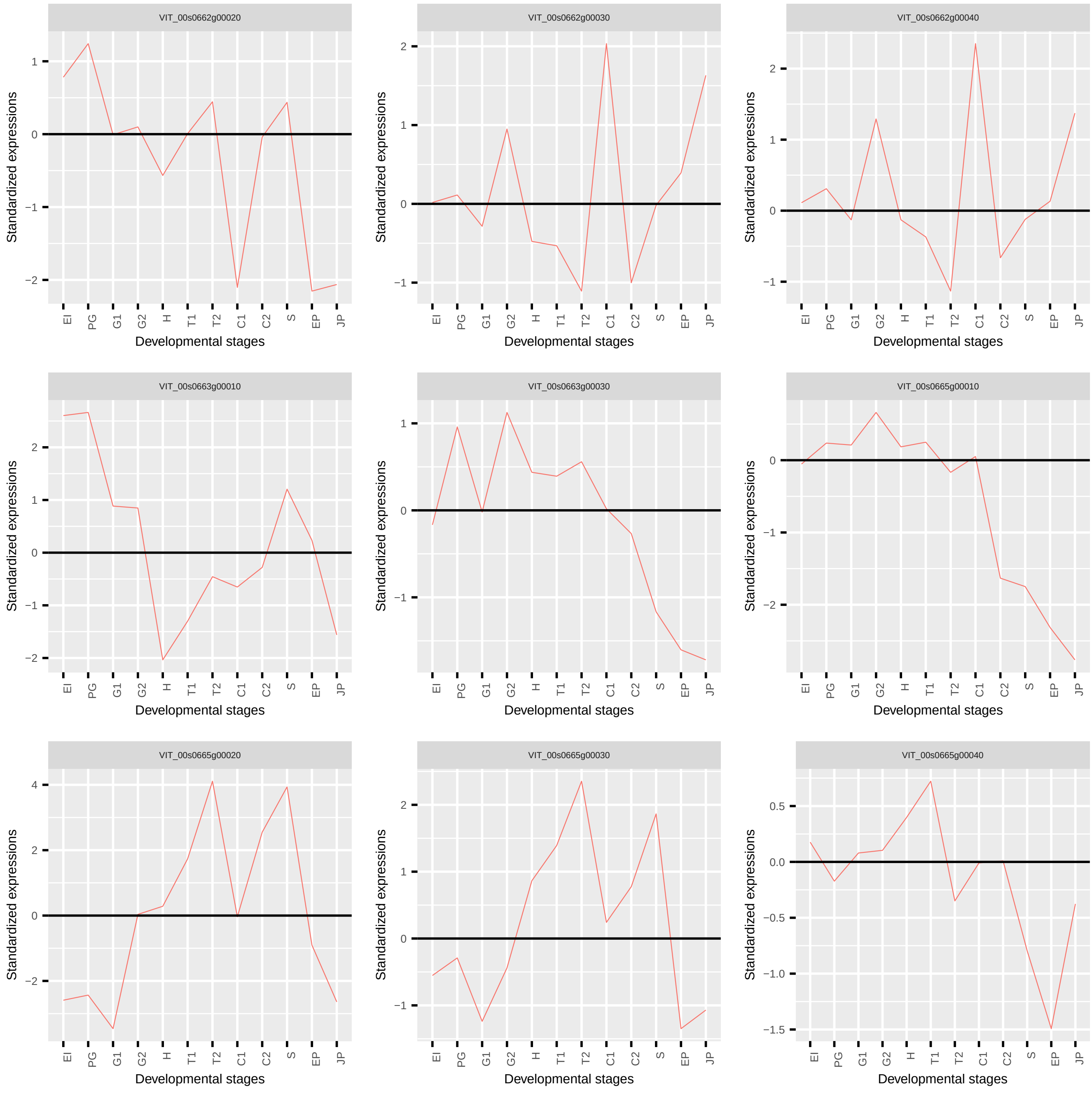
VIT_00s0662g00020
VIT_00s0662g00030
VIT_00s0662g00040
VIT_00s0663g00010
VIT_00s0663g00030
VIT_00s0665g00010
VIT_00s0665g00020
VIT_00s0665g00030
VIT_00s0665g00040
Standardized expressions
Developmental stages
EI
PG
G1
G2
H
T1
T2
C1
C2
S
EP
JP

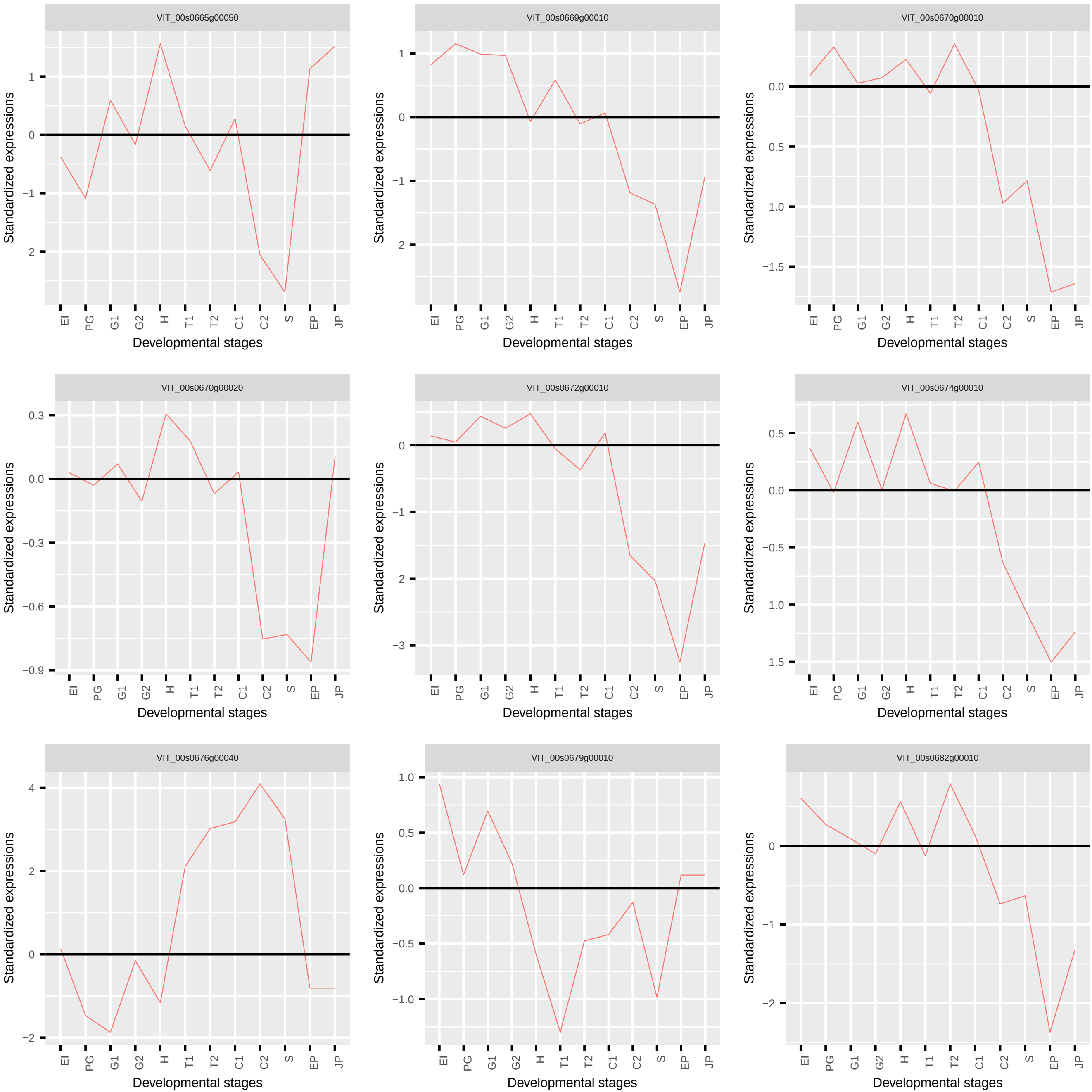
VIT_00s0665g00050
VIT_00s0669g00010
VIT_00s0670g00010
VIT_00s0670g00020
VIT_00s0672g00010
VIT_00s0674g00010
VIT_00s0676g00040
VIT_00s0679g00010
VIT_00s0682g00010
Standardized expressions
EI PG G1 G2 H T1 T2 C1 C2 S EP JP
Developmental stages

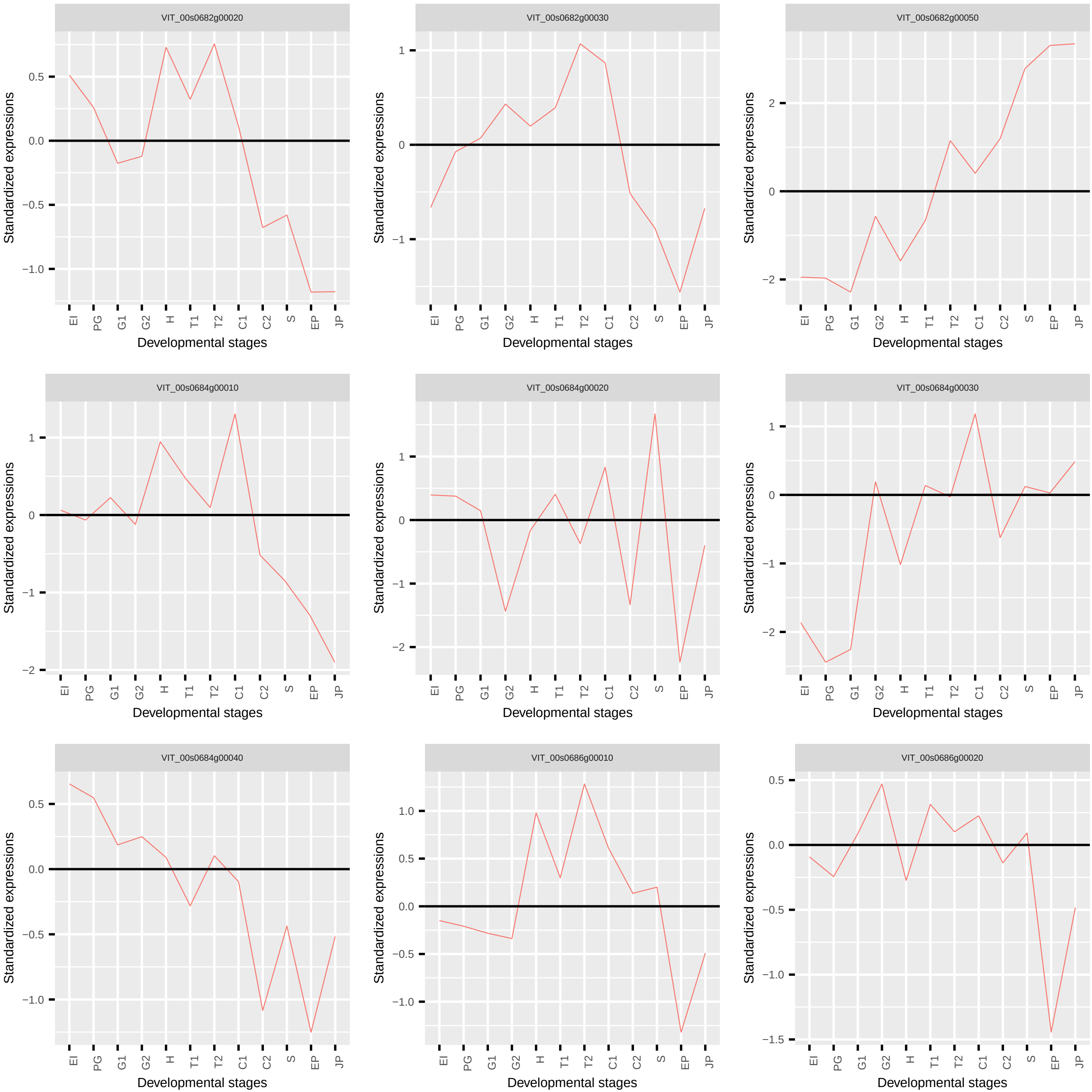
VIT_00s0682g00020
VIT_00s0682g00030
VIT_00s0682g00050
VIT_00s0684g00010
VIT_00s0684g00020
VIT_00s0684g00030
VIT_00s0684g00040
VIT_00s0686g00010
VIT_00s0686g00020
Standardized expressions
Developmental stages
EI PG G1 G2 H T1 T2 C1 C2 S EP JP

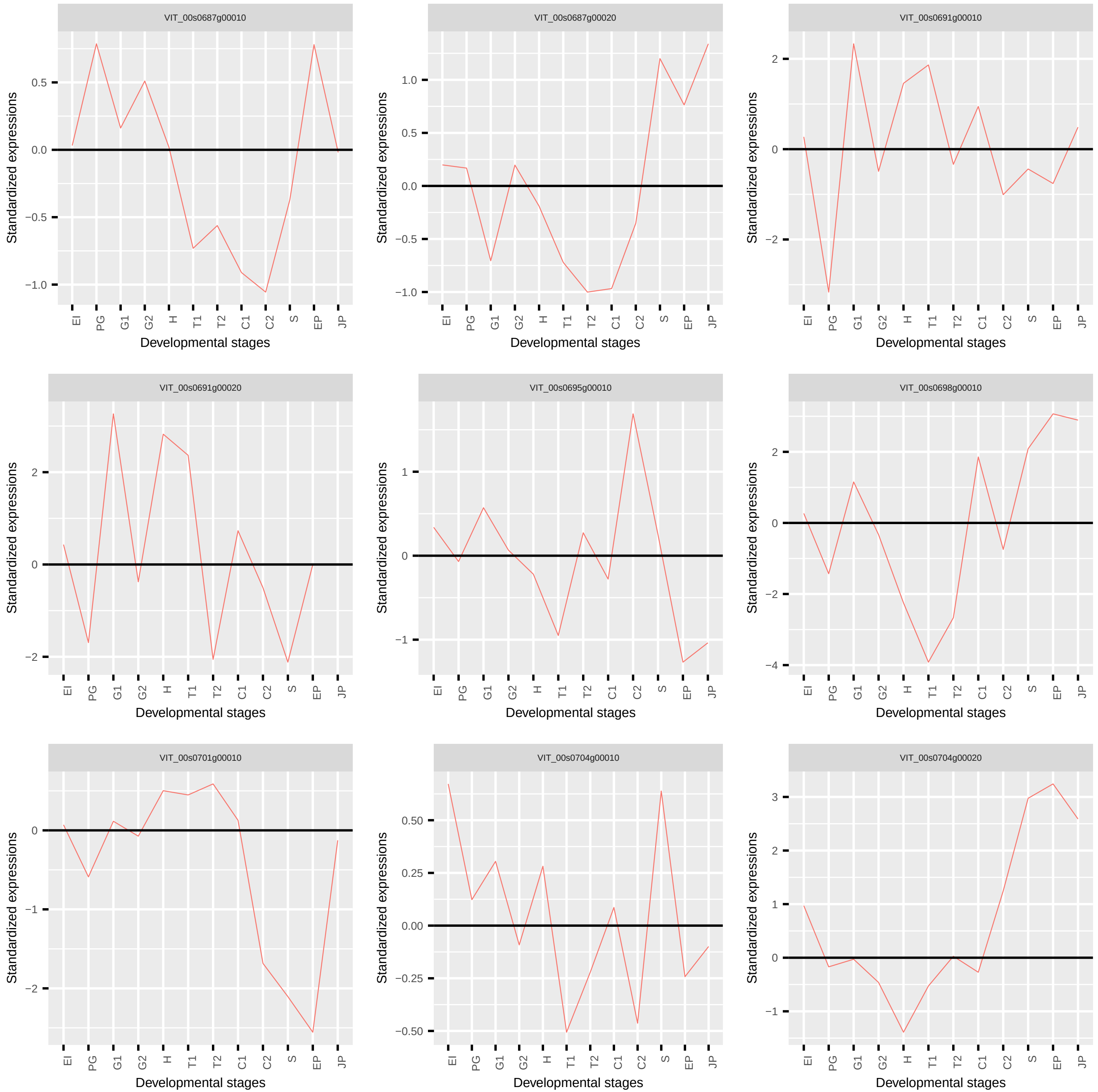
VIT_00s0687g00010
VIT_00s0687g00020
VIT_00s0691g00010
VIT_00s0691g00020
VIT_00s0695g00010
VIT_00s0698g00010
VIT_00s0701g00010
VIT_00s0704g00010
VIT_00s0704g00020
Standardized expressions
Developmental stages
EI PG G1 G2 H T1 T2 C1 C2 S EP JP

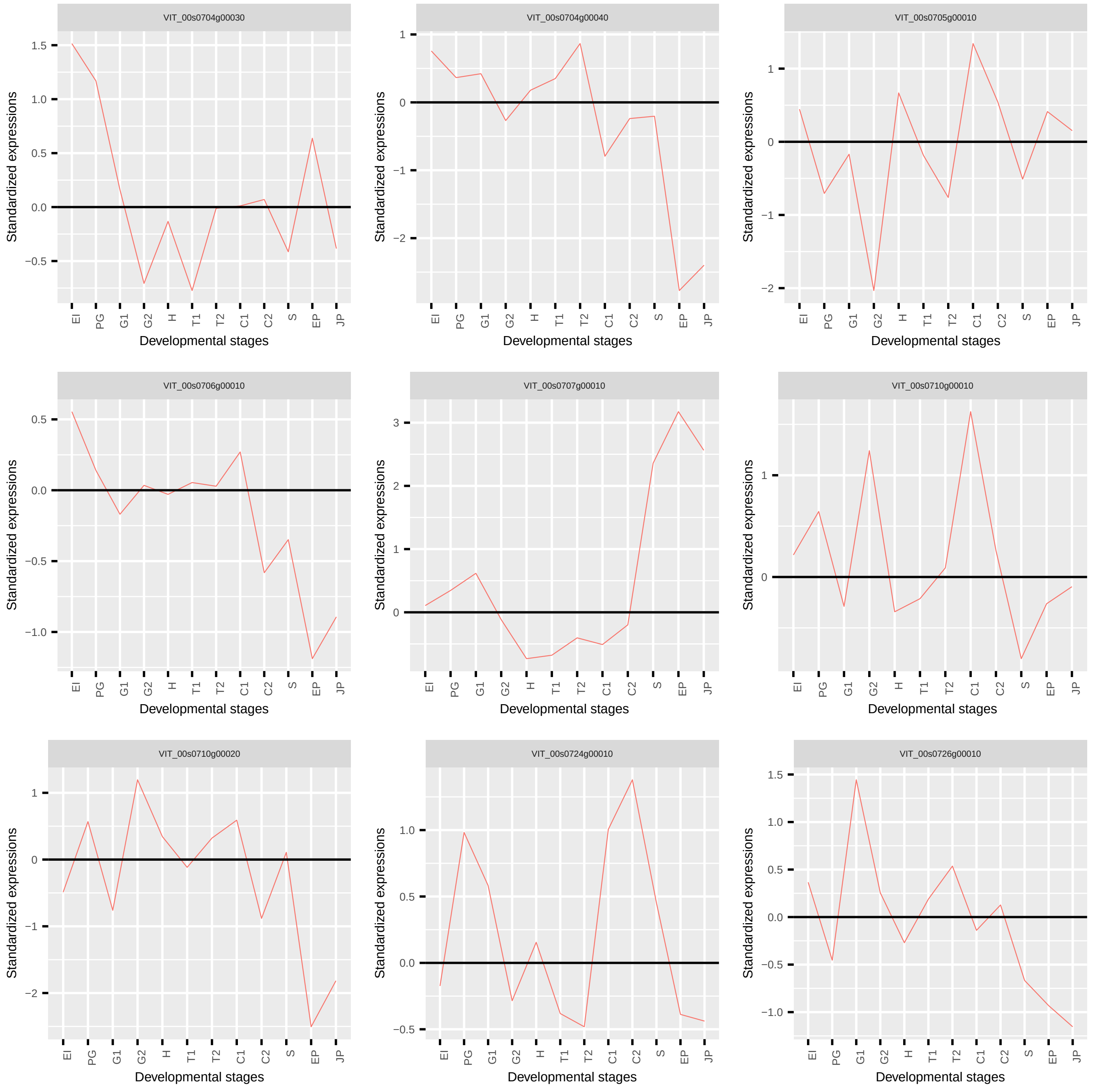
VIT_00s0704g00030
VIT_00s0704g00040
VIT_00s0705g00010
VIT_00s0706g00010
VIT_00s0707g00010
VIT_00s0710g00010
VIT_00s0710g00020
VIT_00s0724g00010
VIT_00s0726g00010
Standardized expressions
Developmental stages
EI PG G1 G2 H T1 T2 C1 C2 S EP JP

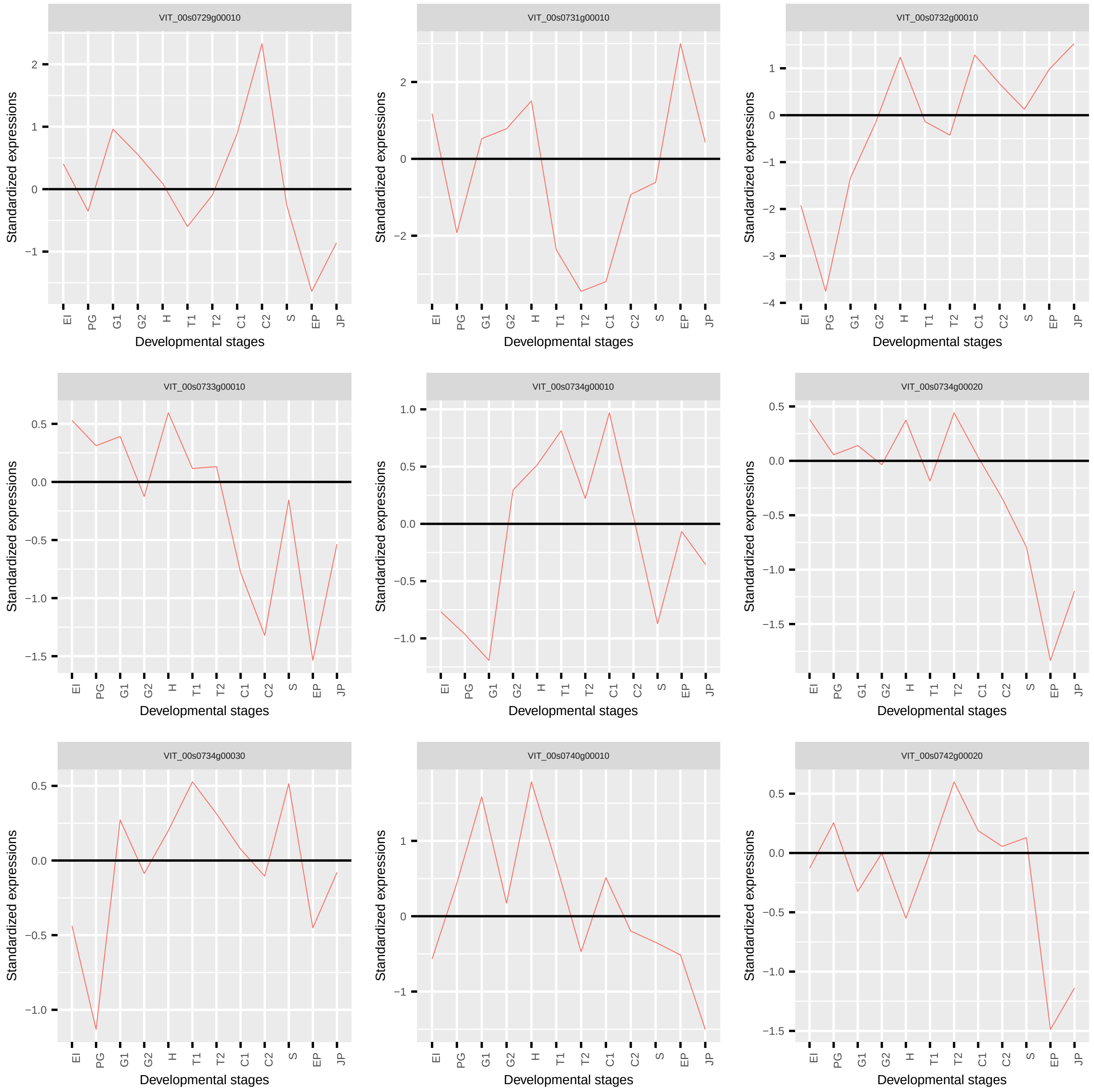
VIT_00s0729g00010
VIT_00s0731g00010
VIT_00s0732g00010
VIT_00s0733g00010
VIT_00s0734g00010
VIT_00s0734g00020
VIT_00s0734g00030
VIT_00s0740g00010
VIT_00s0742g00020
Standardized expressions
Developmental stages
EI PG G1 G2 H T1 T2 C1 C2 S EP JP

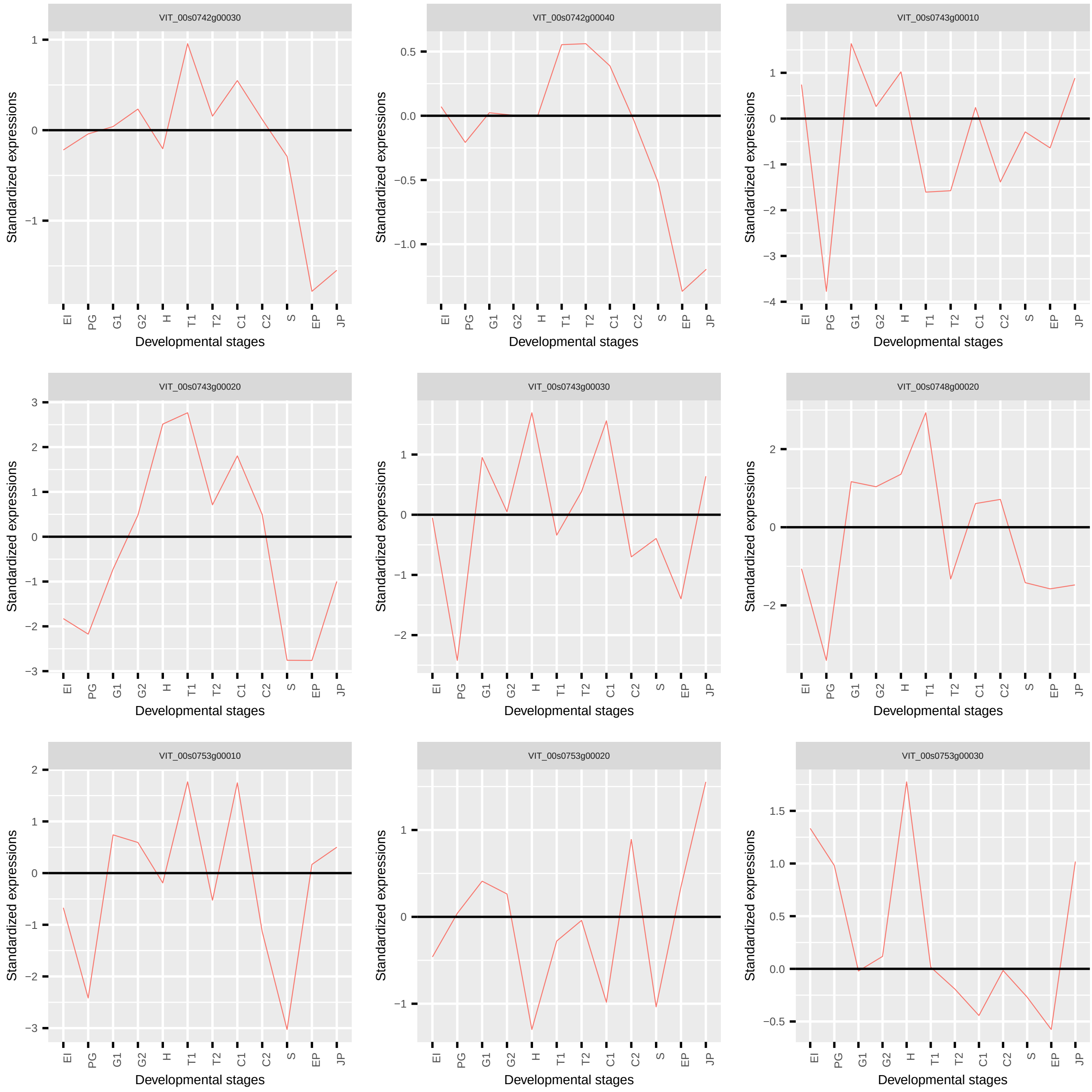
VIT_00s0742g00030
VIT_00s0742g00040
VIT_00s0743g00010
VIT_00s0743g00020
VIT_00s0743g00030
VIT_00s0748g00020
VIT_00s0753g00010
VIT_00s0753g00020
VIT_00s0753g00030
Standardized expressions
Developmental stages
EI
PG
G1
G2
H
T1
T2
C1
C2
S
EP
JP

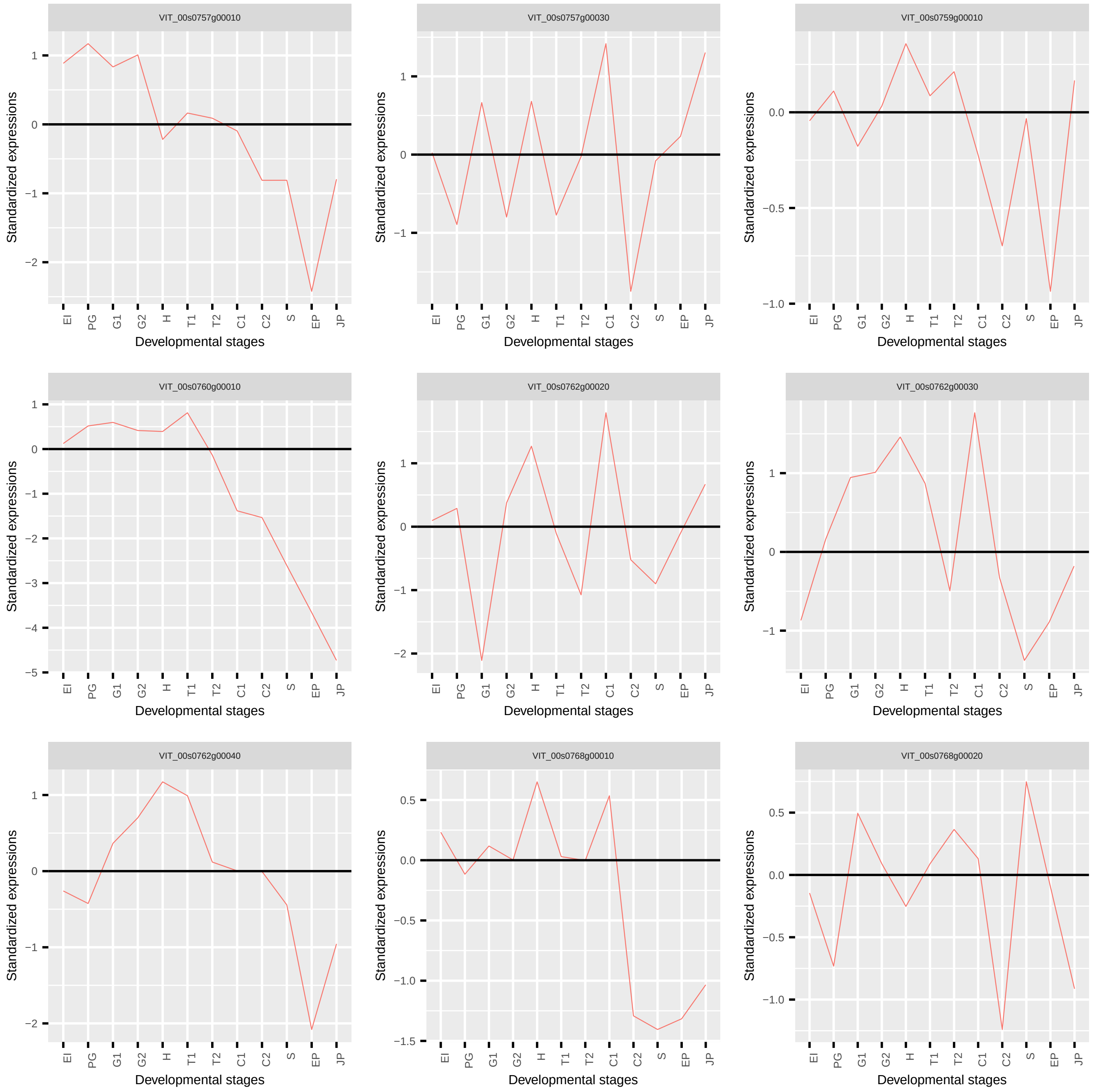

VIT_00s0757g00010
VIT_00s0757g00030
VIT_00s0759g00010
VIT_00s0760g00010
VIT_00s0762g00020
VIT_00s0762g00030
VIT_00s0762g00040
VIT_00s0768g00010
VIT_00s0768g00020
Standardized expressions
Developmental stages
EI PG G1 G2 H T1 T2 C1 C2 S EP JP

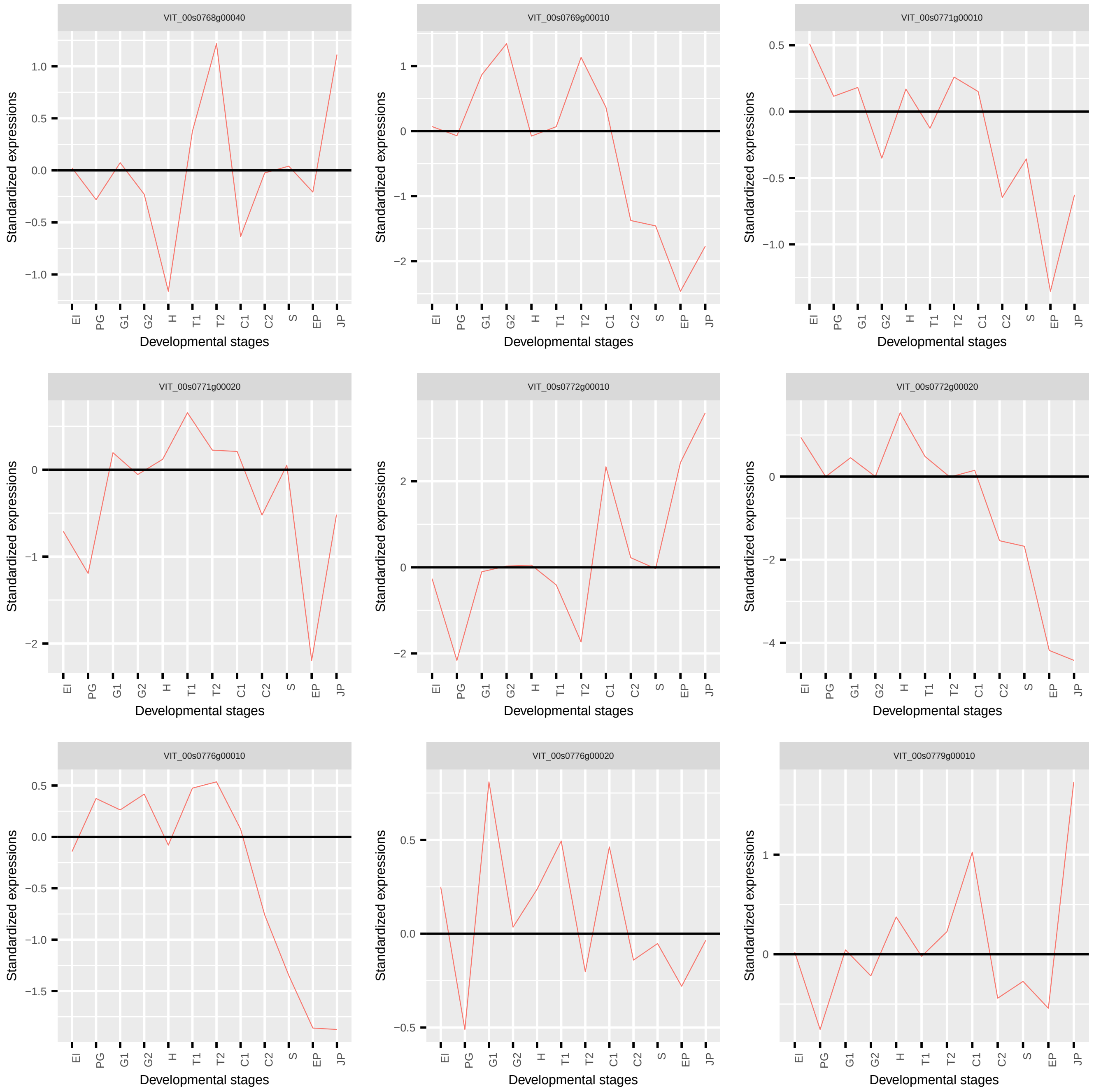
VIT_00s0768g00040
VIT_00s0769g00010
VIT_00s0771g00010
VIT_00s0771g00020
VIT_00s0772g00010
VIT_00s0772g00020
VIT_00s0776g00010
VIT_00s0776g00020
VIT_00s0779g00010
Standardized expressions
Developmental stages
EI PG G1 G2 H T1 T2 C1 C2 S EP JP

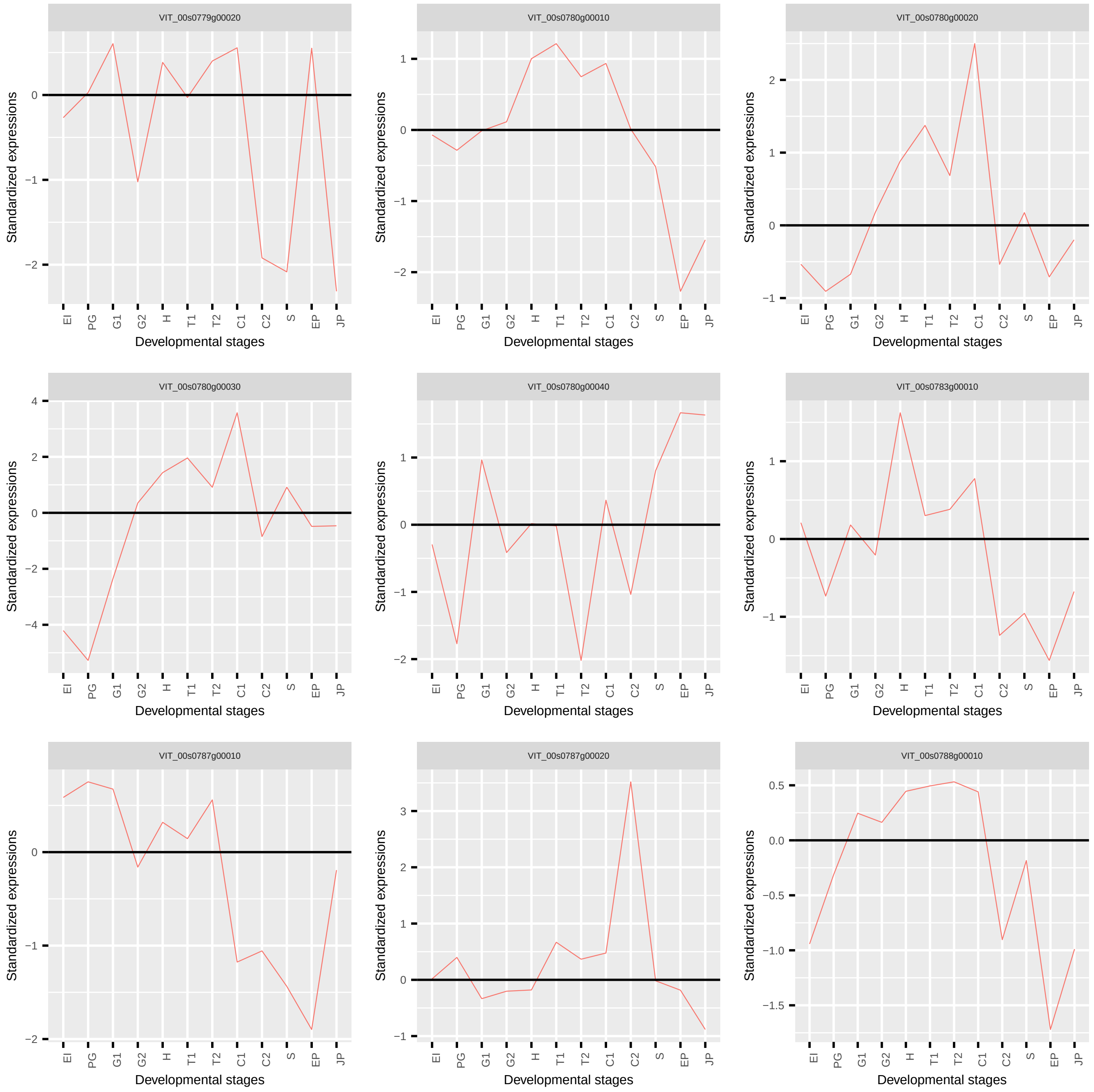
VIT_00s0779g00020
VIT_00s0780g00010
VIT_00s0780g00020
VIT_00s0780g00030
VIT_00s0780g00040
VIT_00s0783g00010
VIT_00s0787g00010
VIT_00s0787g00020
VIT_00s0788g00010
Standardized expressions
Developmental stages
EI PG G1 G2 H T1 T2 C1 C2 S EP JP

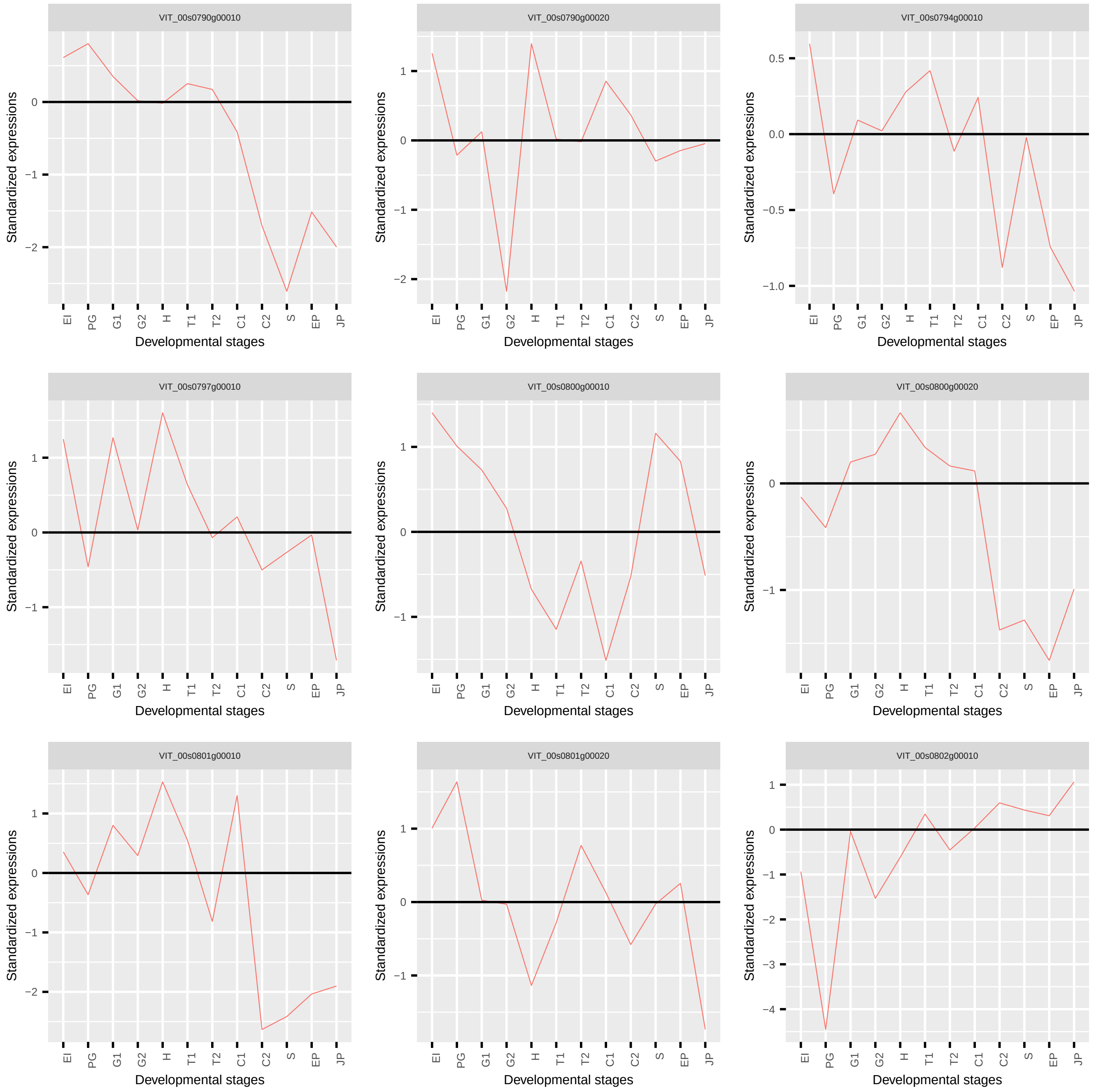

VIT_00s0790g00010
VIT_00s0790g00020
VIT_00s0794g00010
VIT_00s0797g00010
VIT_00s0800g00010
VIT_00s0800g00020
VIT_00s0801g00010
VIT_00s0801g00020
VIT_00s0802g00010
Standardized expressions
Developmental stages
EI PG G1 G2 H T1 T2 C1 C2 S EP JP

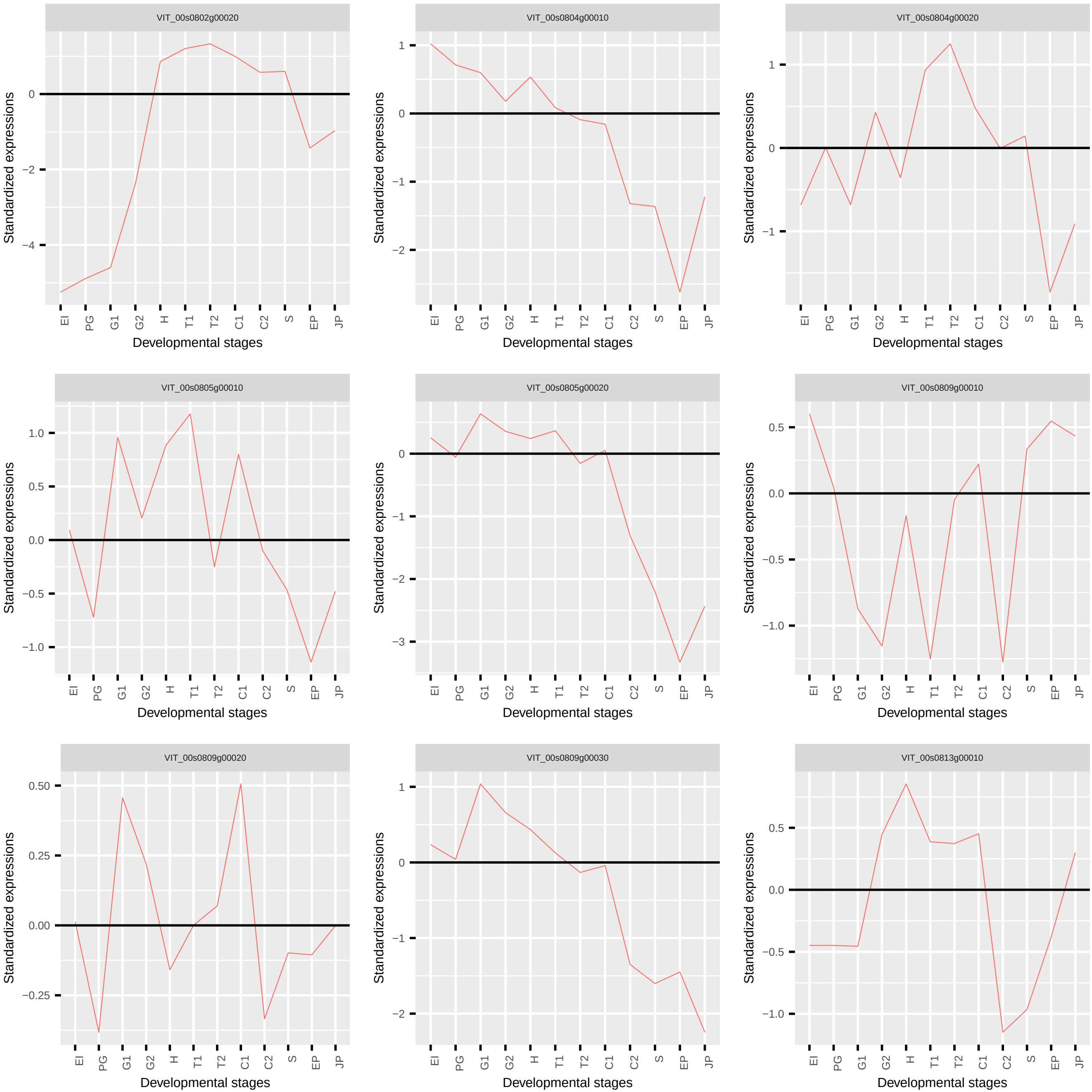

VIT_00s0802g00020
VIT_00s0804g00010
VIT_00s0804g00020
VIT_00s0805g00010
VIT_00s0805g00020
VIT_00s0809g00010
VIT_00s0809g00020
VIT_00s0809g00030
VIT_00s0813g00010
Standardized expressions
Developmental stages
EI PG G1 G2 H T1 T2 C1 C2 S EP JP

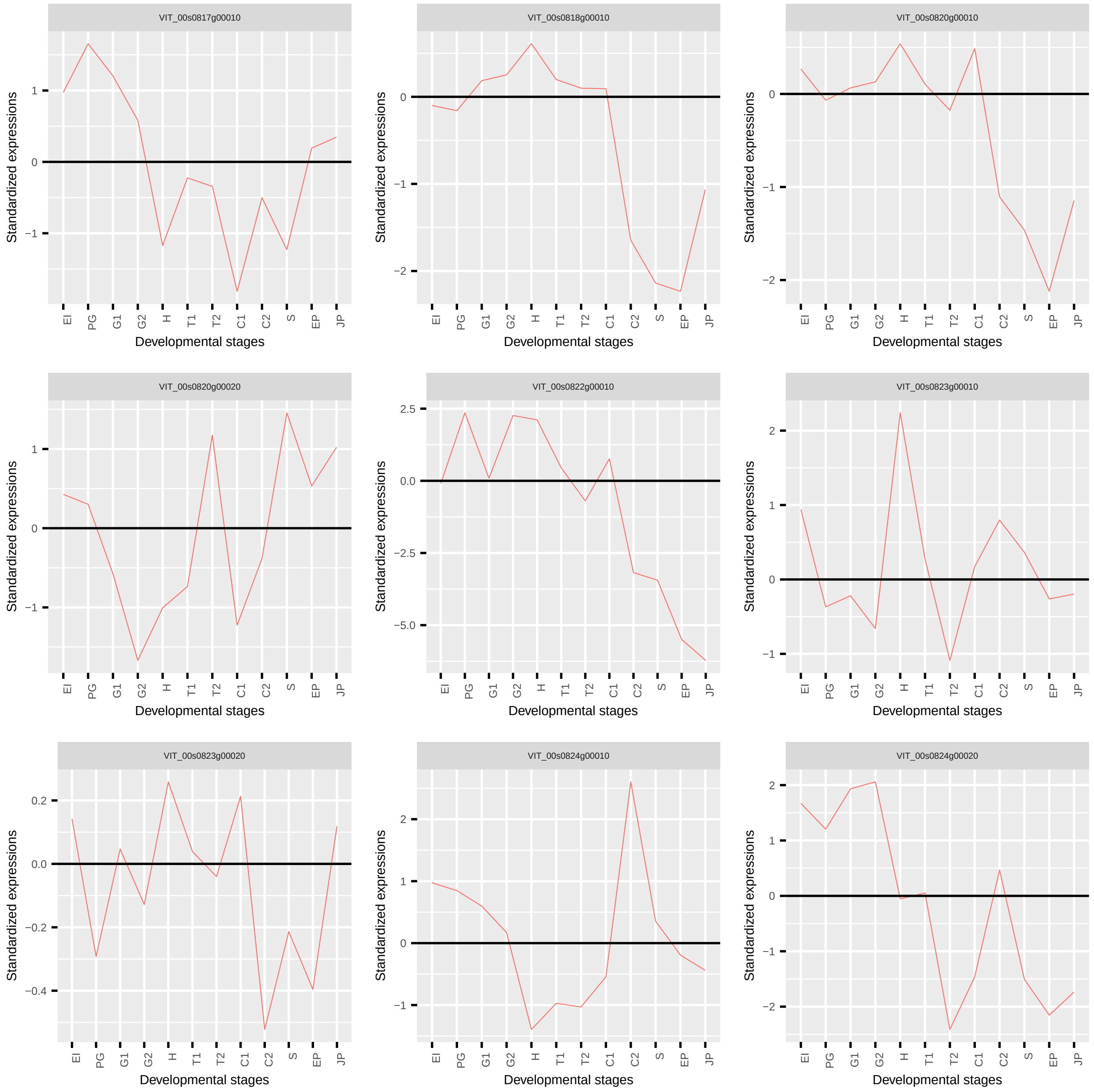
VIT_00s0817g00010
VIT_00s0818g00010
VIT_00s0820g00010
VIT_00s0820g00020
VIT_00s0822g00010
VIT_00s0823g00010
VIT_00s0823g00020
VIT_00s0824g00010
VIT_00s0824g00020
Standardized expressions
Developmental stages
EI
PG
G1
G2
H
T1
T2
C1
C2
S
EP
JP

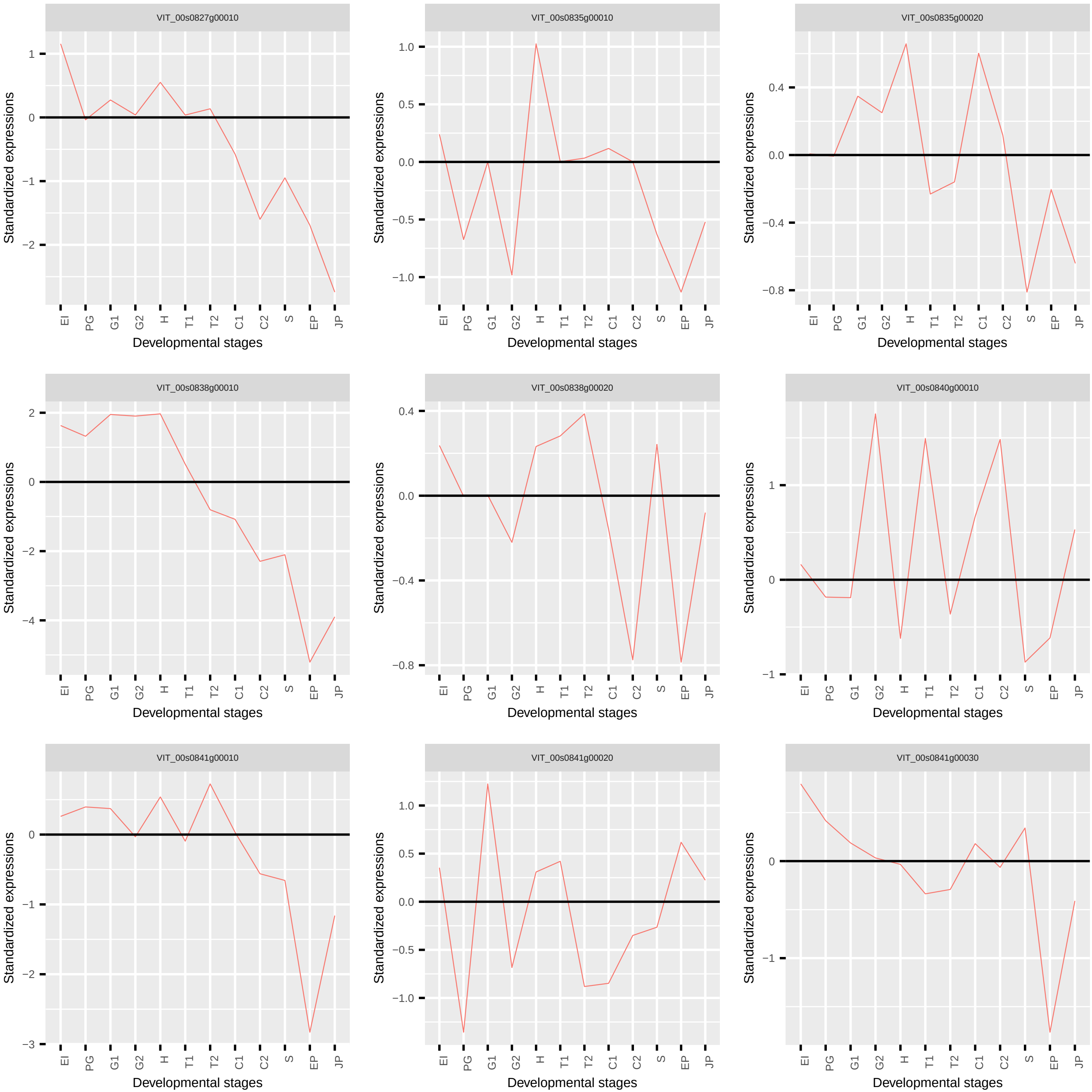
VIT_00s0827g00010
VIT_00s0835g00010
VIT_00s0835g00020
VIT_00s0838g00010
VIT_00s0838g00020
VIT_00s0840g00010
VIT_00s0841g00010
VIT_00s0841g00020
VIT_00s0841g00030
Standardized expressions
Developmental stages
EI
PG
G1
G2
H
T1
T2
C1
C2
S
EP
JP

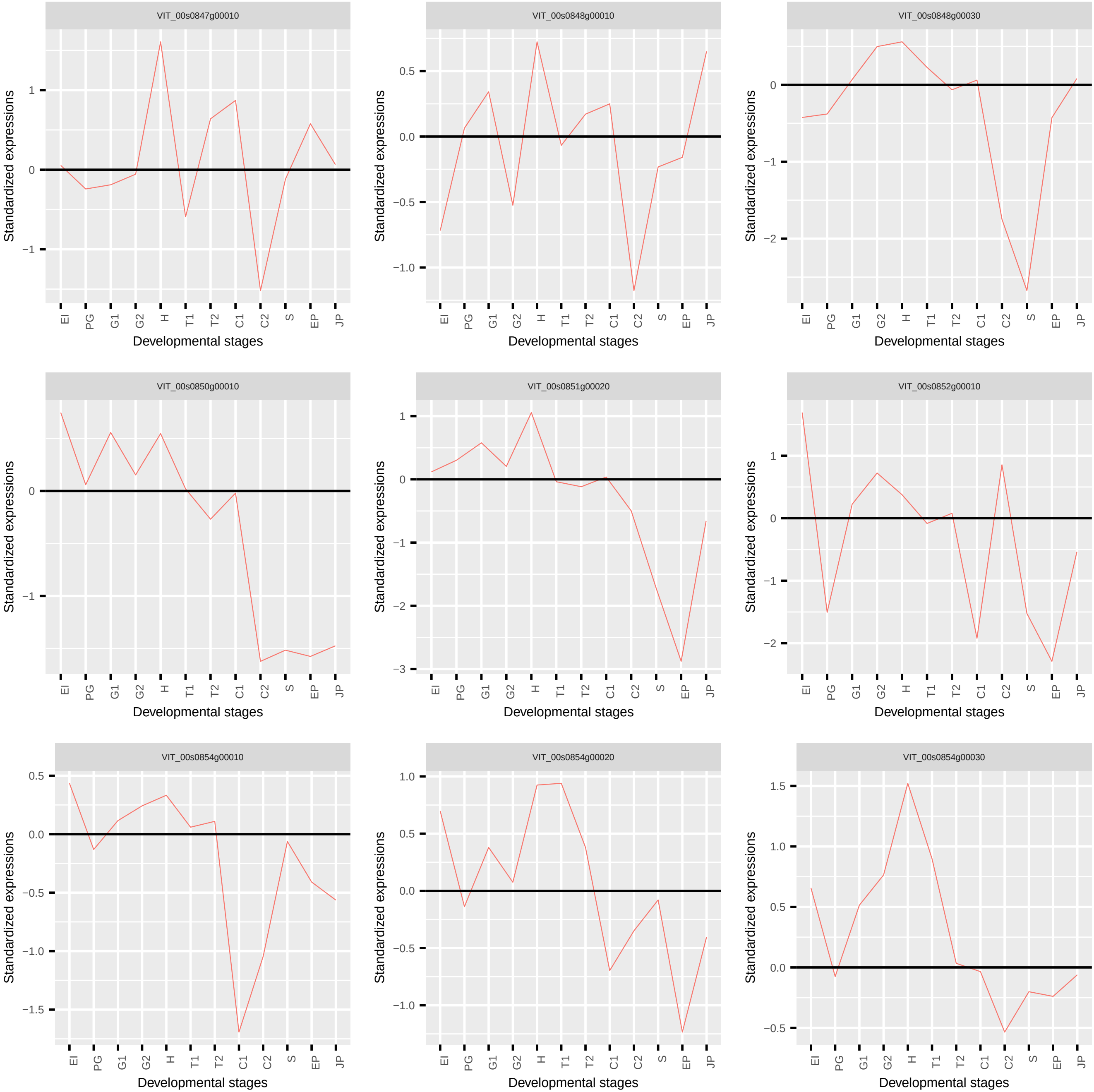

VIT_00s0847g00010
VIT_00s0848g00010
VIT_00s0848g00030
VIT_00s0850g00010
VIT_00s0851g00020
VIT_00s0852g00010
VIT_00s0854g00010
VIT_00s0854g00020
VIT_00s0854g00030
Standardized expressions
Developmental stages
EI
PG
G1
G2
H
T1
T2
C1
C2
S
EP
JP

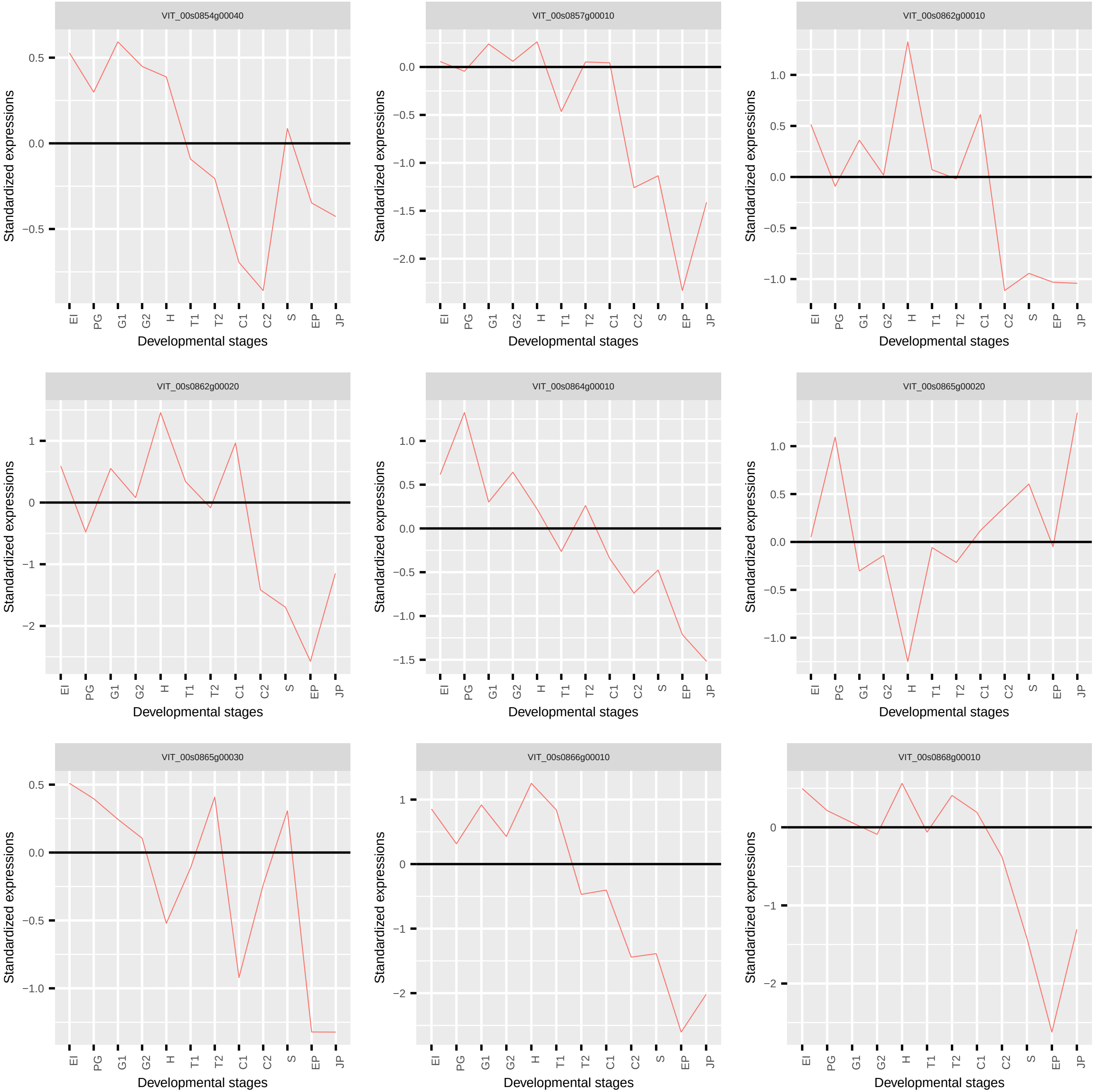
VIT_00s0854g00040
VIT_00s0857g00010
VIT_00s0862g00010
VIT_00s0862g00020
VIT_00s0864g00010
VIT_00s0865g00020
VIT_00s0865g00030
VIT_00s0866g00010
VIT_00s0868g00010
Standardized expressions
Developmental stages
EI PG G1 G2 H T1 T2 C1 C2 S EP JP

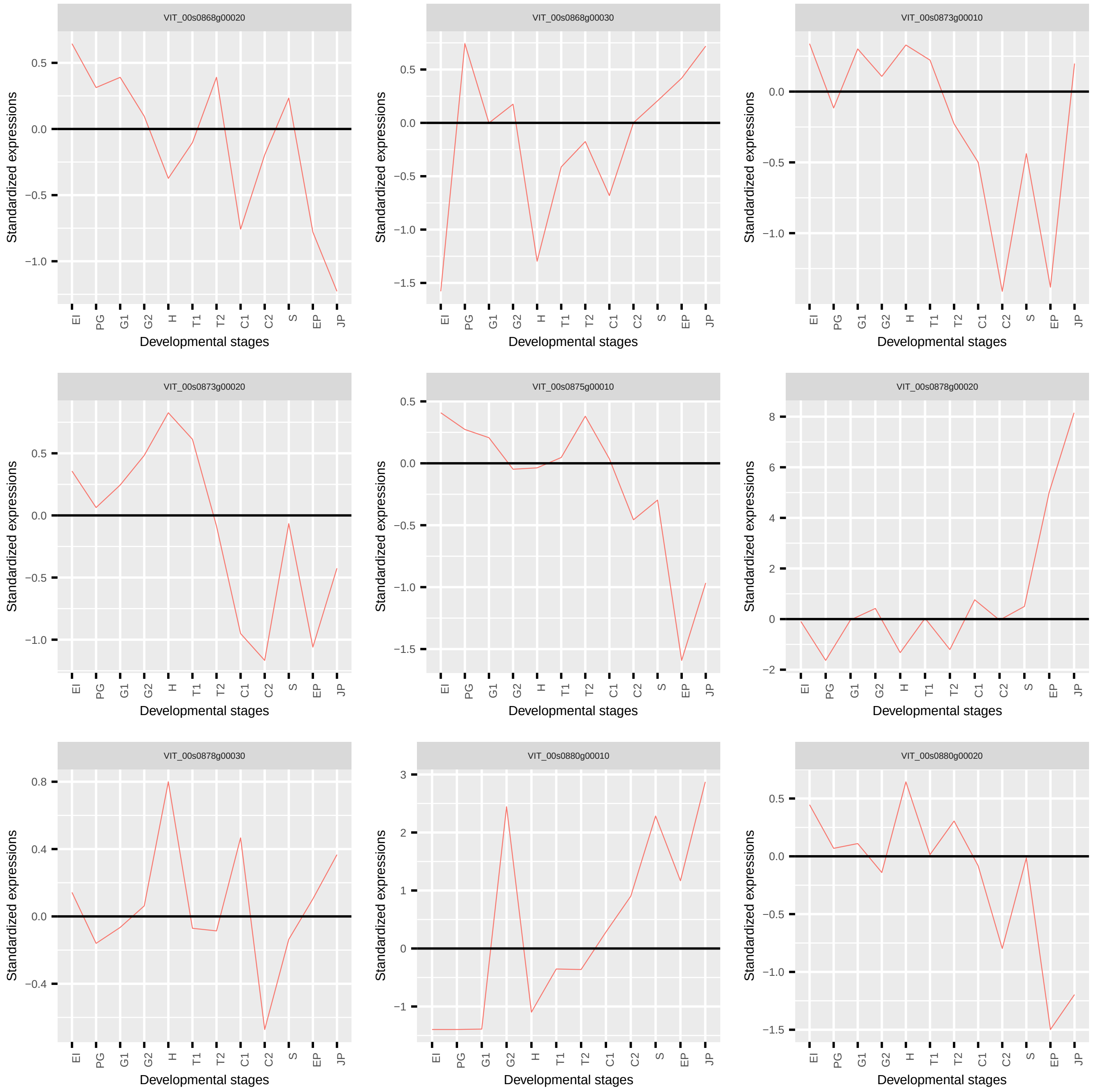
VIT_00s0868g00020
VIT_00s0868g00030
VIT_00s0873g00010
VIT_00s0873g00020
VIT_00s0875g00010
VIT_00s0878g00020
VIT_00s0878g00030
VIT_00s0880g00010
VIT_00s0880g00020
Standardized expressions
Developmental stages
EI PG G1 G2 H T1 T2 C1 C2 S EP JP

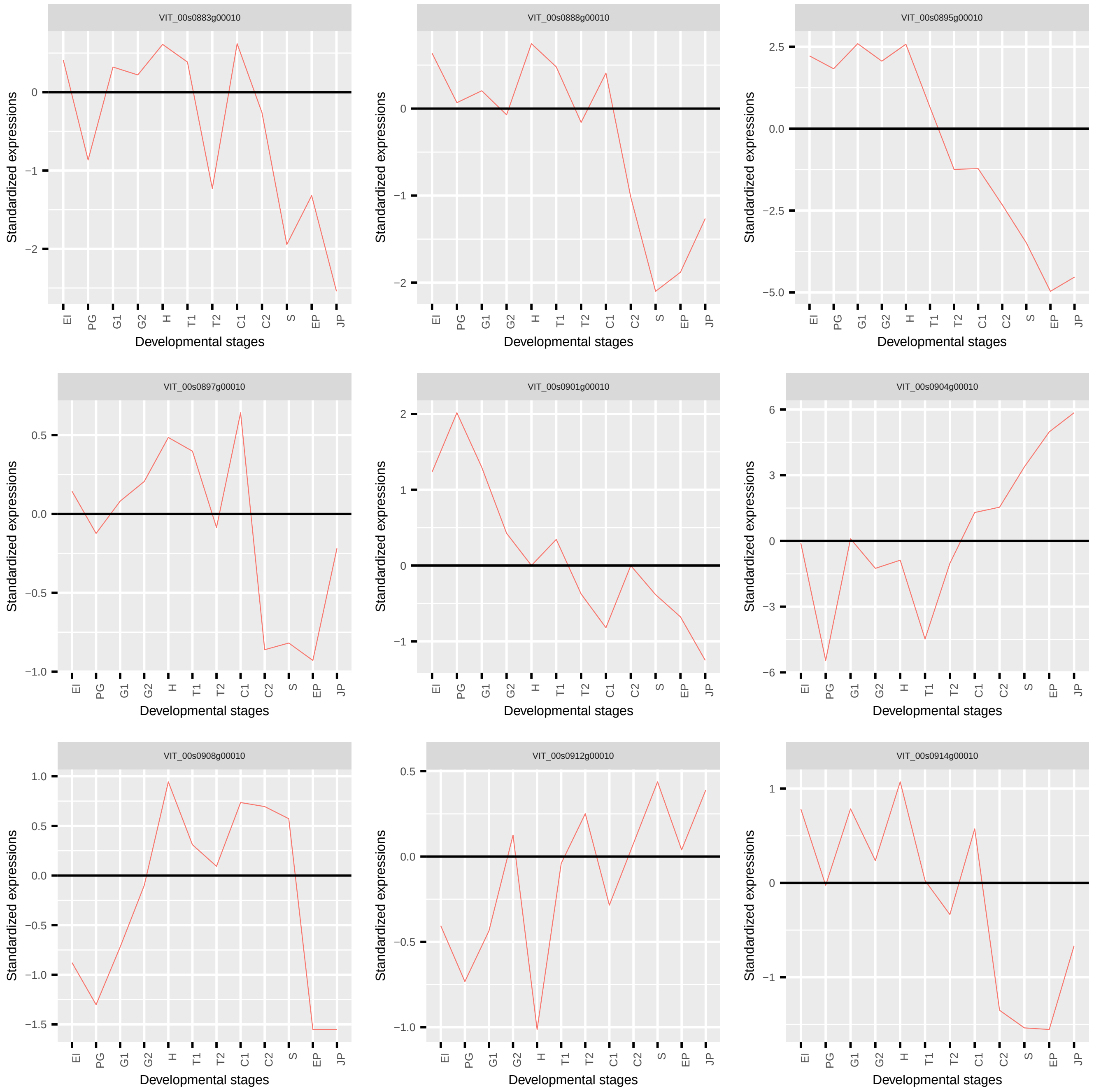
VIT_00s0883g00010
VIT_00s0888g00010
VIT_00s0895g00010
VIT_00s0897g00010
VIT_00s0901g00010
VIT_00s0904g00010
VIT_00s0908g00010
VIT_00s0912g00010
VIT_00s0914g00010
Standardized expressions
Developmental stages
EI
PG
G1
G2
H
T1
T2
C1
C2
S
EP
JP
0
−1
−2
2.5
0.0
−2.5
−5.0
0.5
−0.5
−1.0
2
1
6
3
−3
−6
1.0
−1.5

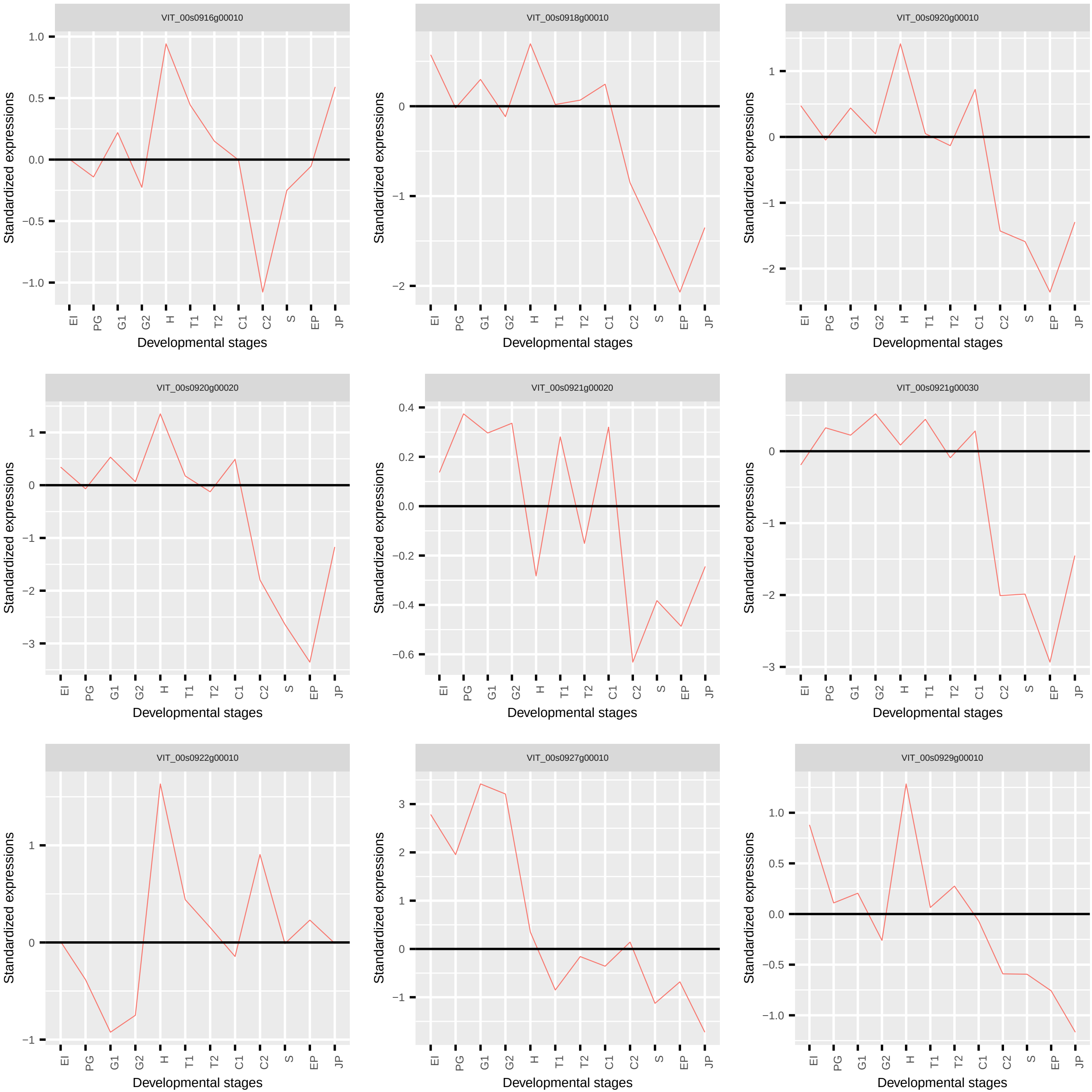
VIT_00s0916g00010
VIT_00s0918g00010
VIT_00s0920g00010
VIT_00s0920g00020
VIT_00s0921g00020
VIT_00s0921g00030
VIT_00s0922g00010
VIT_00s0927g00010
VIT_00s0929g00010
Standardized expressions
EI
PG
G1
G2
H
T1
T2
C1
C2
S
EP
JP
Developmental stages

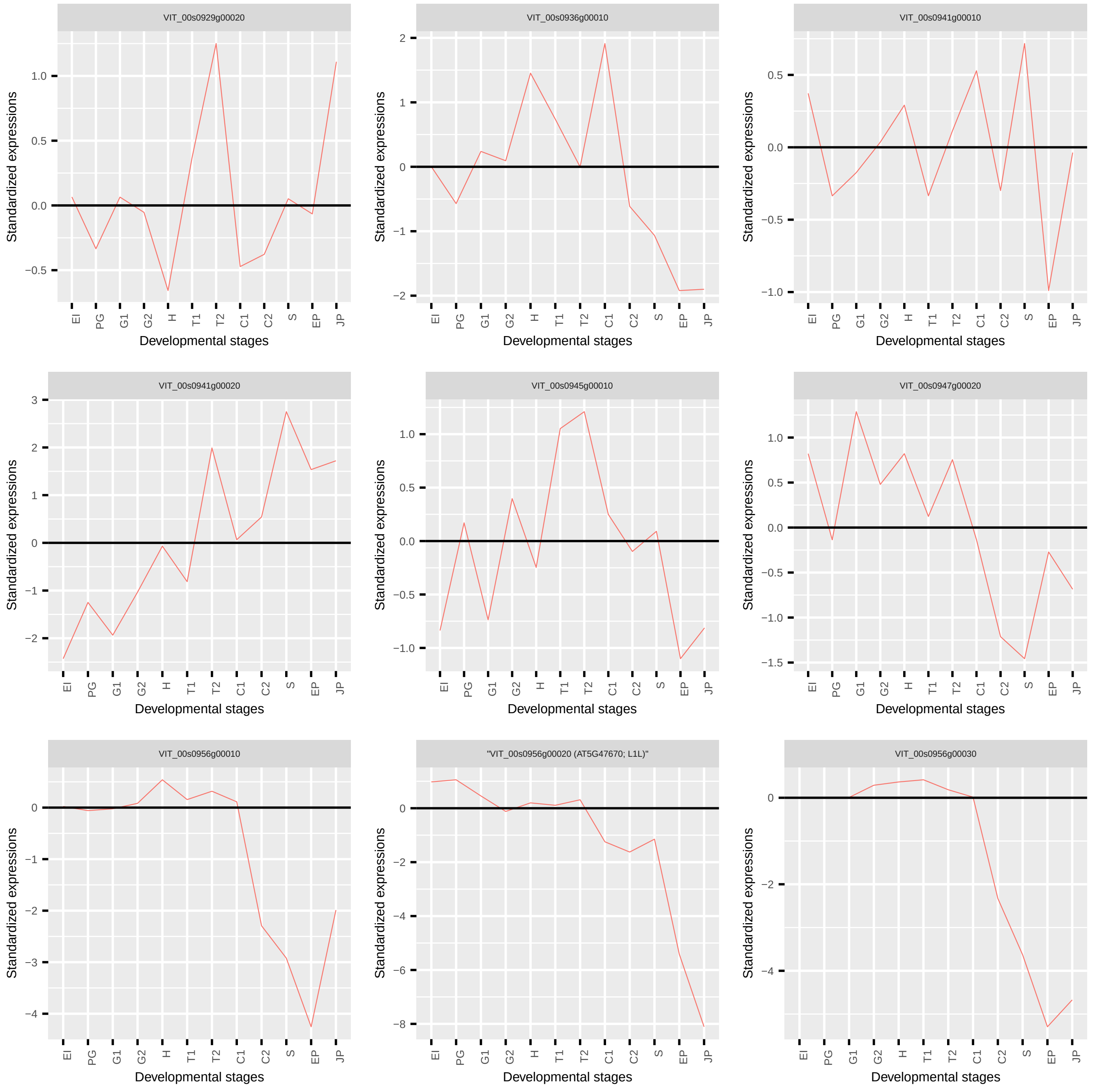
VIT_00s0929g00020
VIT_00s0936g00010
VIT_00s0941g00010
VIT_00s0941g00020
VIT_00s0945g00010
VIT_00s0947g00020
VIT_00s0956g00010
"VIT_00s0956g00020 (AT5G47670; L1L)"
VIT_00s0956g00030
Standardized expressions
Developmental stages
EI PG G1 G2 H T1 T2 C1 C2 S EP JP

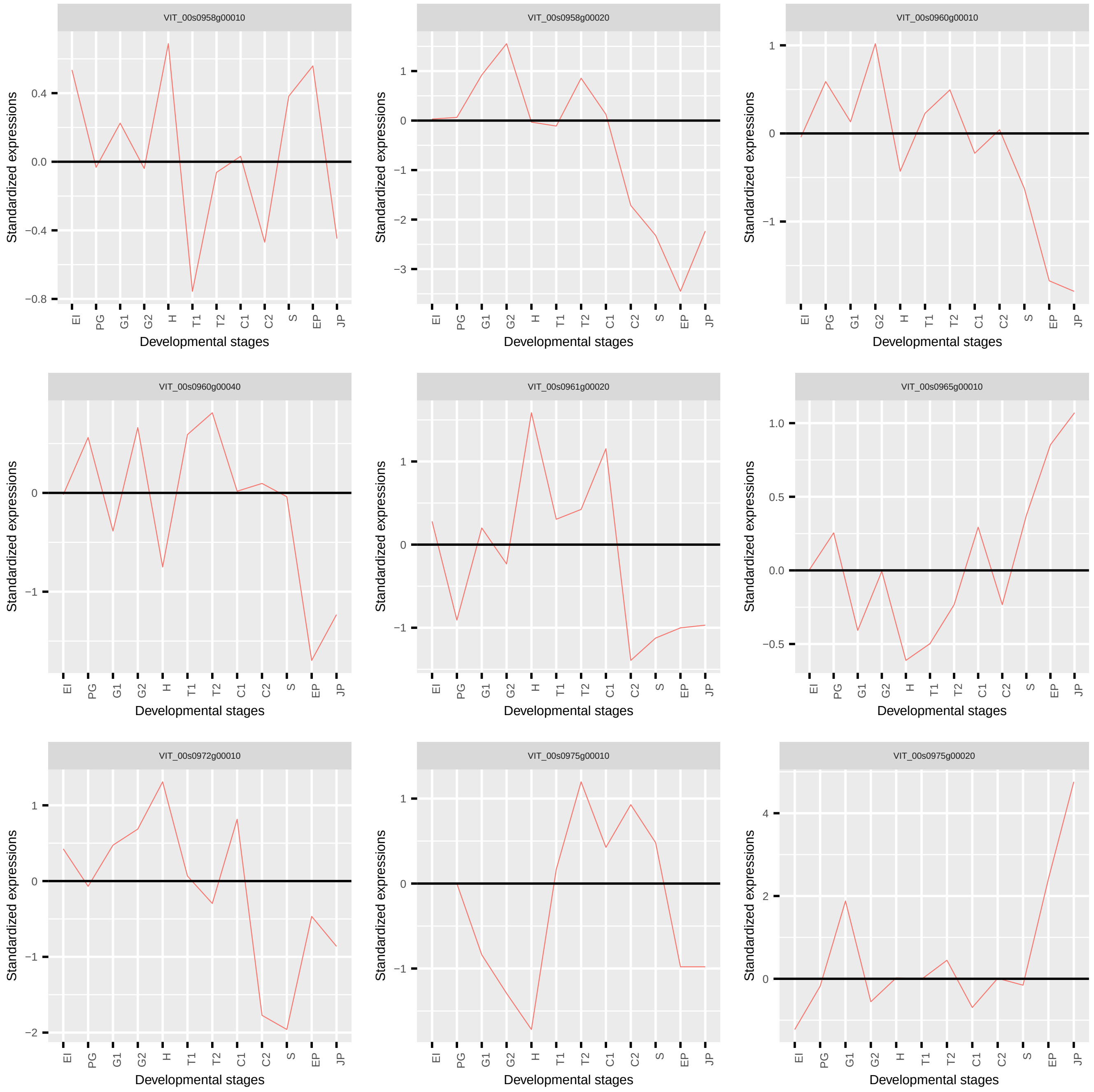
VIT_00s0958g00010
VIT_00s0958g00020
VIT_00s0960g00010
VIT_00s0960g00040
VIT_00s0961g00020
VIT_00s0965g00010
VIT_00s0972g00010
VIT_00s0975g00010
VIT_00s0975g00020
Standardized expressions
Developmental stages
EI PG G1 G2 H T1 T2 C1 C2 S EP JP

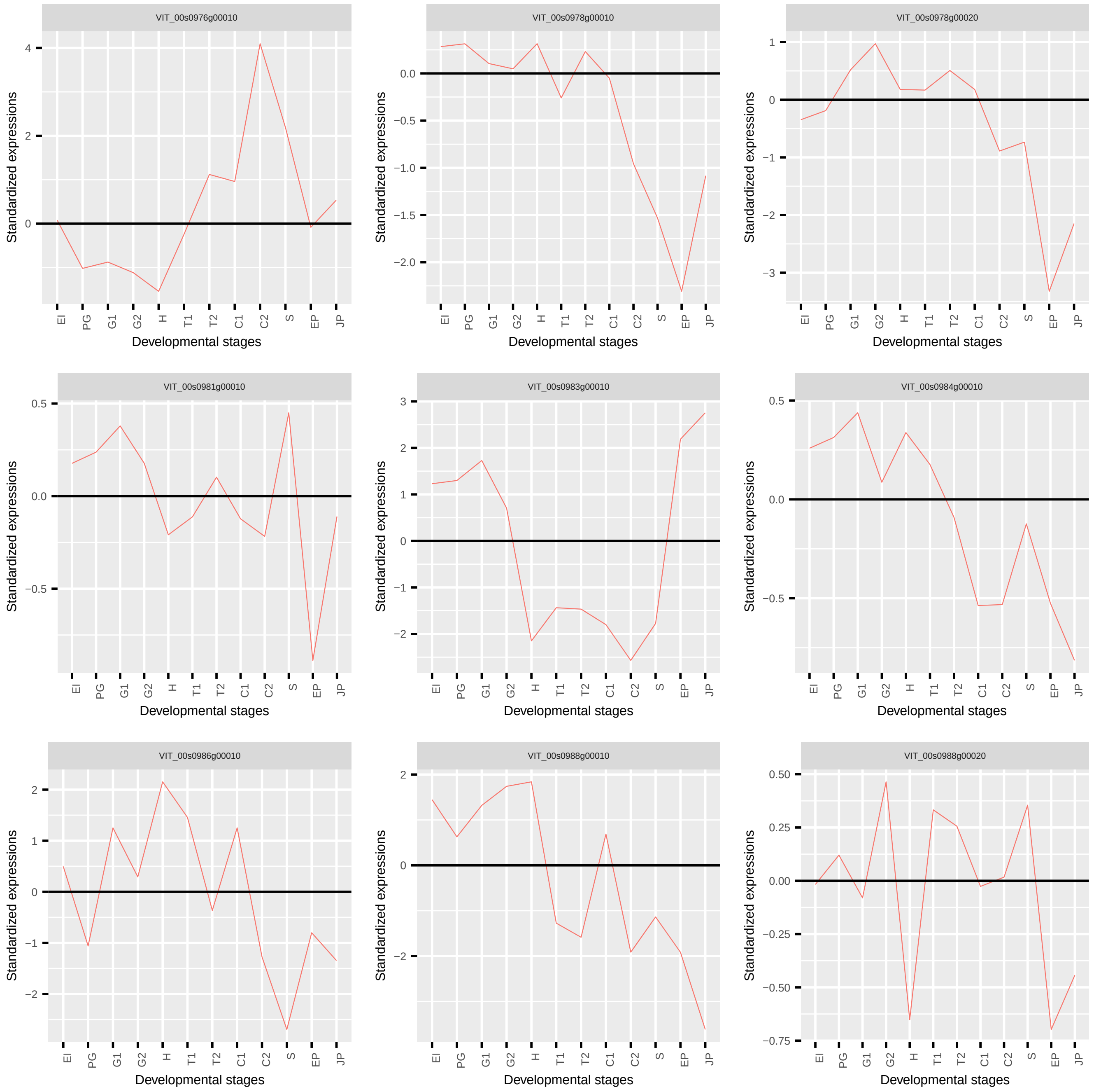
VIT_00s0976g00010
Standardized expressions
4
2
0
EI PG G1 G2 H T1 T2 C1 C2 S EP JP
Developmental stages
VIT_00s0978g00010
Standardized expressions
0.0
−0.5
−1.0
−1.5
−2.0
EI PG G1 G2 H T1 T2 C1 C2 S EP JP
Developmental stages
VIT_00s0978g00020
Standardized expressions
1
0
−1
−2
−3
EI PG G1 G2 H T1 T2 C1 C2 S EP JP
Developmental stages
VIT_00s0981g00010
Standardized expressions
0.5
0.0
−0.5
EI PG G1 G2 H T1 T2 C1 C2 S EP JP
Developmental stages
VIT_00s0983g00010
Standardized expressions
3
2
1
0
−1
−2
EI PG G1 G2 H T1 T2 C1 C2 S EP JP
Developmental stages
VIT_00s0984g00010
Standardized expressions
0.5
0.0
−0.5
EI PG G1 G2 H T1 T2 C1 C2 S EP JP
Developmental stages
VIT_00s0986g00010
Standardized expressions
2
1
0
−1
−2
EI PG G1 G2 H T1 T2 C1 C2 S EP JP
Developmental stages
VIT_00s0988g00010
Standardized expressions
2
0
−2
EI PG G1 G2 H T1 T2 C1 C2 S EP JP
Developmental stages
VIT_00s0988g00020
Standardized expressions
0.50
0.25
0.00
−0.25
−0.50
−0.75
EI PG G1 G2 H T1 T2 C1 C2 S EP JP
Developmental stages

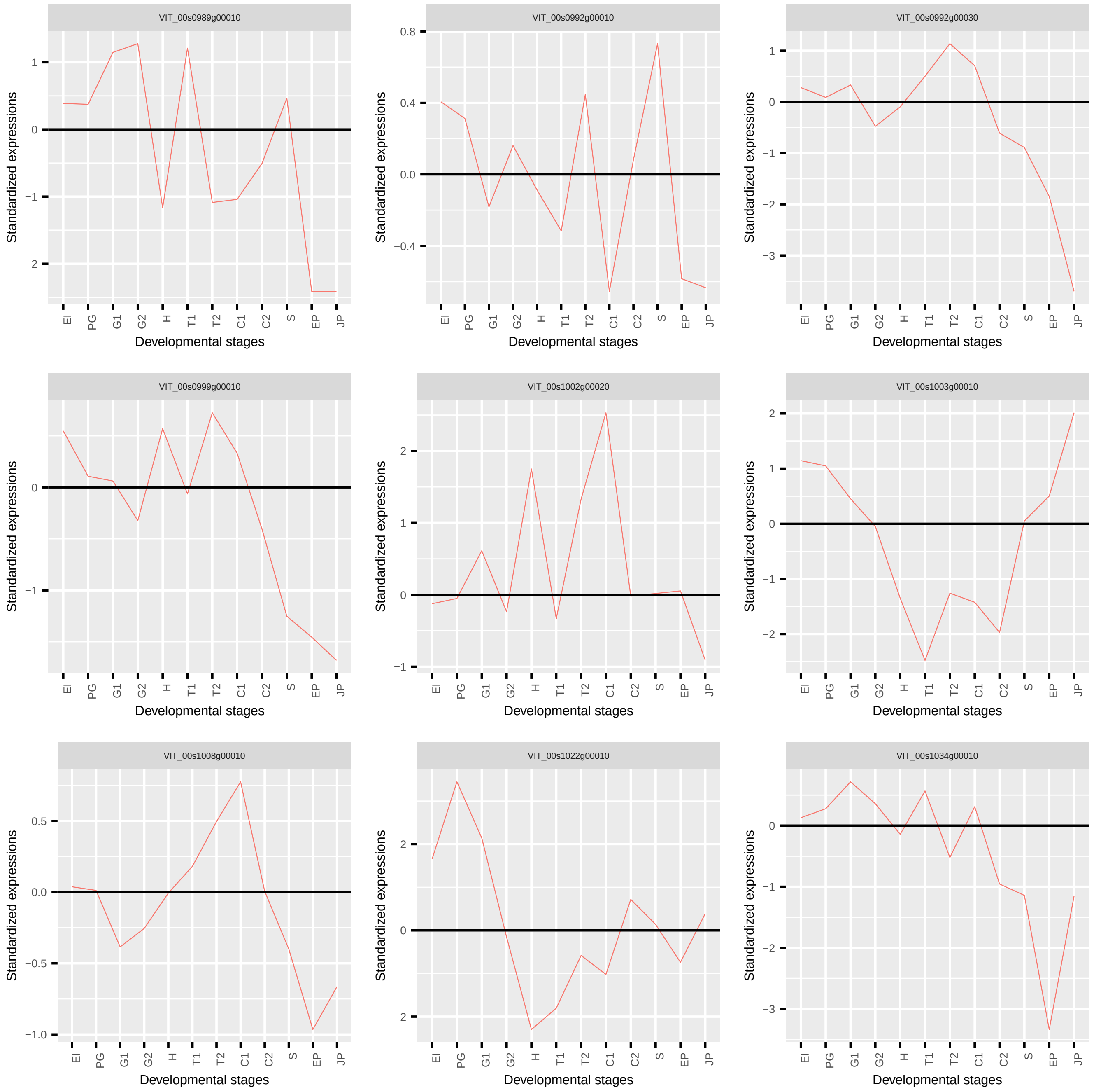
VIT_00s0989g00010
Standardized expressions
1
0
−1
−2
EI PG G1 G2 H T1 T2 C1 C2 S EP JP
Developmental stages
VIT_00s0992g00010
Standardized expressions
0.8
0.4
0.0
−0.4
EI PG G1 G2 H T1 T2 C1 C2 S EP JP
Developmental stages
VIT_00s0992g00030
Standardized expressions
1
0
−1
−2
−3
EI PG G1 G2 H T1 T2 C1 C2 S EP JP
Developmental stages
VIT_00s0999g00010
Standardized expressions
0
−1
EI PG G1 G2 H T1 T2 C1 C2 S EP JP
Developmental stages
VIT_00s1002g00020
Standardized expressions
2
1
0
−1
EI PG G1 G2 H T1 T2 C1 C2 S EP JP
Developmental stages
VIT_00s1003g00010
Standardized expressions
2
1
0
−1
−2
EI PG G1 G2 H T1 T2 C1 C2 S EP JP
Developmental stages
VIT_00s1008g00010
Standardized expressions
0.5
0.0
−0.5
−1.0
EI PG G1 G2 H T1 T2 C1 C2 S EP JP
Developmental stages
VIT_00s1022g00010
Standardized expressions
2
0
−2
EI PG G1 G2 H T1 T2 C1 C2 S EP JP
Developmental stages
VIT_00s1034g00010
Standardized expressions
0
−1
−2
−3
EI PG G1 G2 H T1 T2 C1 C2 S EP JP
Developmental stages

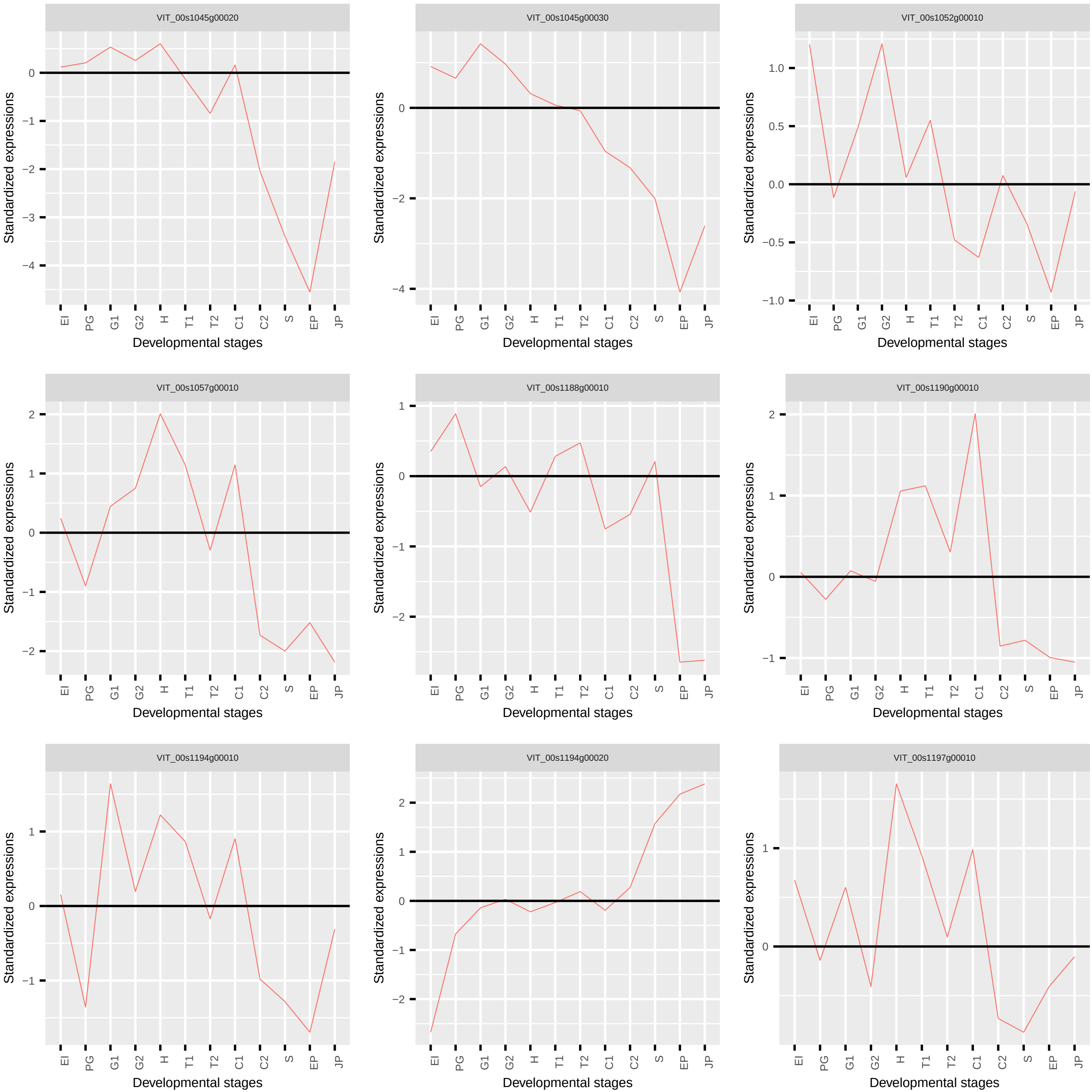
VIT_00s1045g00020
VIT_00s1045g00030
VIT_00s1052g00010
VIT_00s1057g00010
VIT_00s1188g00010
VIT_00s1190g00010
VIT_00s1194g00010
VIT_00s1194g00020
VIT_00s1197g00010
Standardized expressions
Developmental stages
EI PG G1 G2 H T1 T2 C1 C2 S EP JP
0 −1 −2 −3 −4
0 −2 −4
1.0 0.5 0.0 −0.5 −1.0
2 1 0 −1 −2
1 0 −1 −2
2 1 0 −1
1 0 −1
2 1 0 −1 −2
1 0

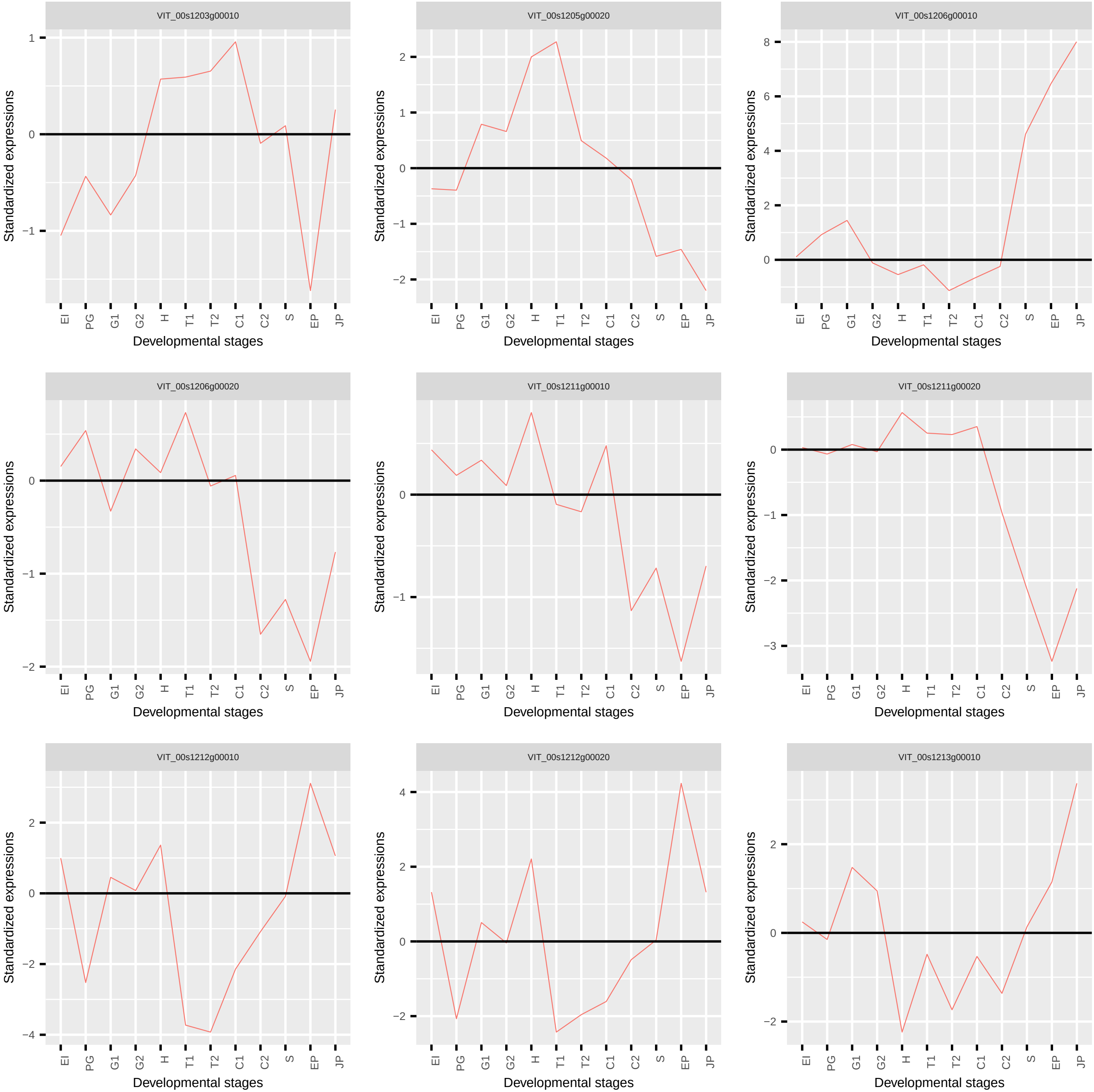

VIT_00s1203g00010
VIT_00s1205g00020
VIT_00s1206g00010
VIT_00s1206g00020
VIT_00s1211g00010
VIT_00s1211g00020
VIT_00s1212g00010
VIT_00s1212g00020
VIT_00s1213g00010
Standardized expressions
Developmental stages
EI
PG
G1
G2
H
T1
T2
C1
C2
S
EP
JP

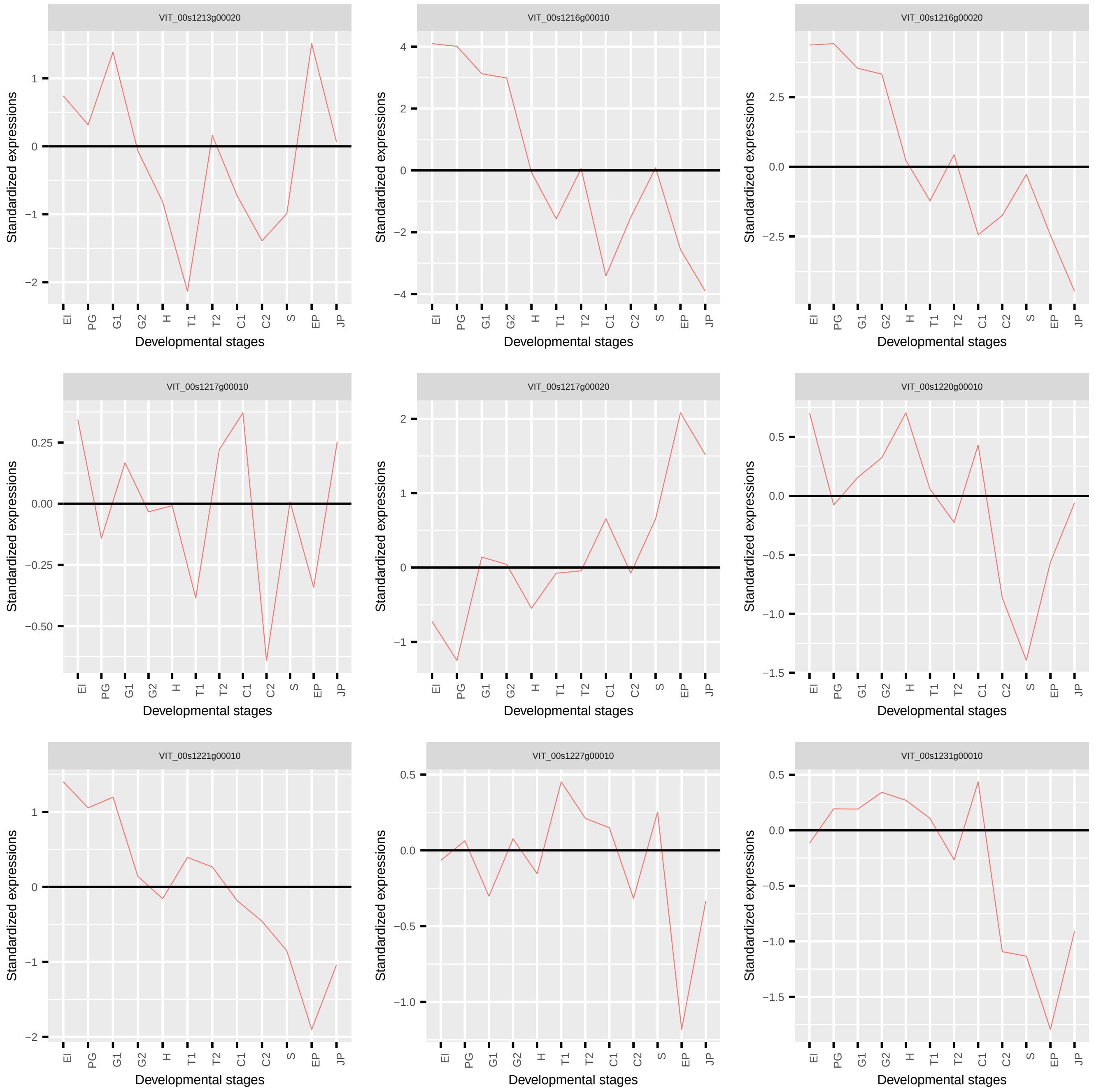
VIT_00s1213g00020
VIT_00s1216g00010
VIT_00s1216g00020
VIT_00s1217g00010
VIT_00s1217g00020
VIT_00s1220g00010
VIT_00s1221g00010
VIT_00s1227g00010
VIT_00s1231g00010
Standardized expressions
Developmental stages
EI PG G1 G2 H T1 T2 C1 C2 S EP JP

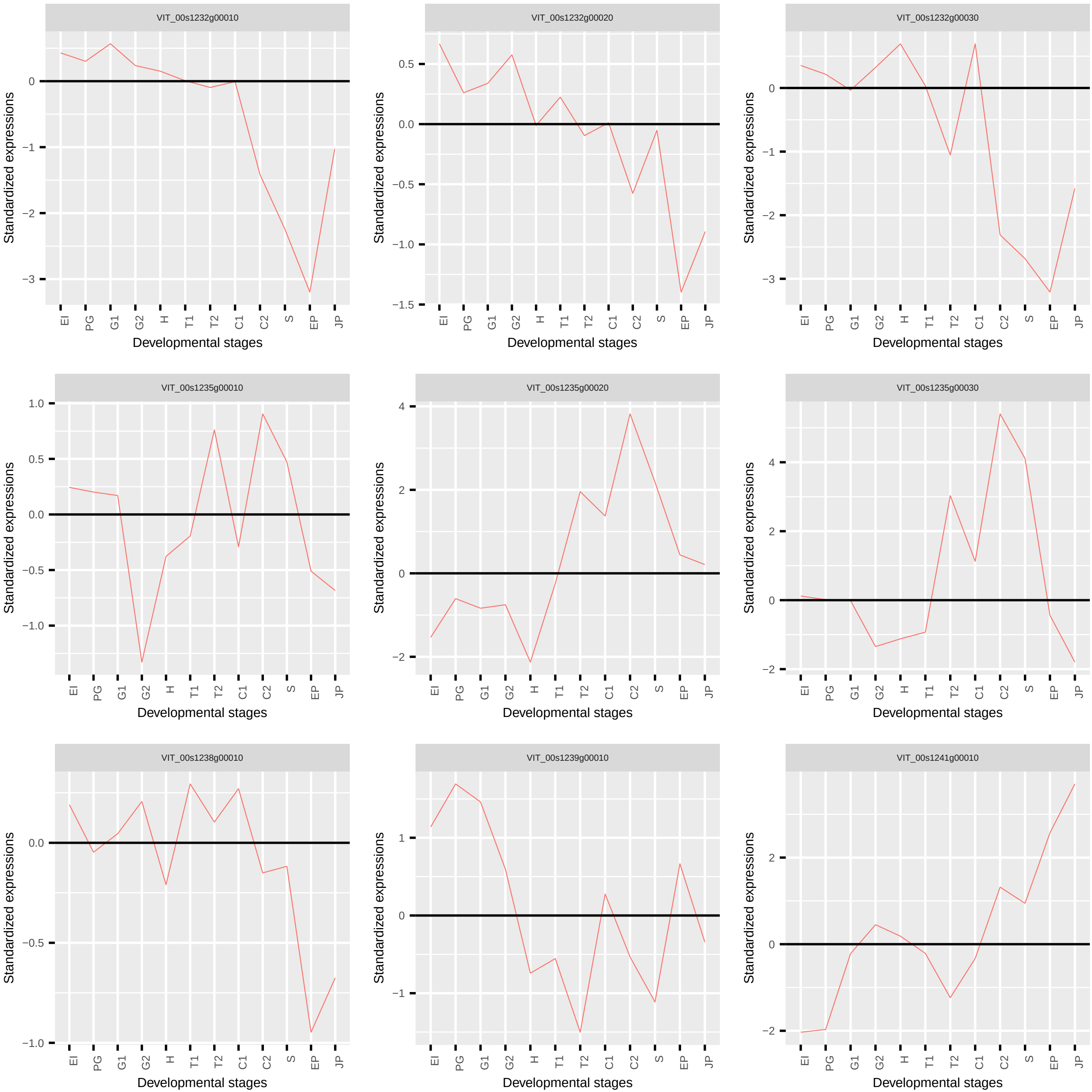
VIT_00s1232g00010
VIT_00s1232g00020
VIT_00s1232g00030
VIT_00s1235g00010
VIT_00s1235g00020
VIT_00s1235g00030
VIT_00s1238g00010
VIT_00s1239g00010
VIT_00s1241g00010
Standardized expressions
Developmental stages
EI PG G1 G2 H T1 T2 C1 C2 S EP JP
0 −1 −2 −3
0.5 0.0 −0.5 −1.0 −1.5
1.0 0.5 0.0 −0.5 −1.0
4 2 0 −2
1 0 −1

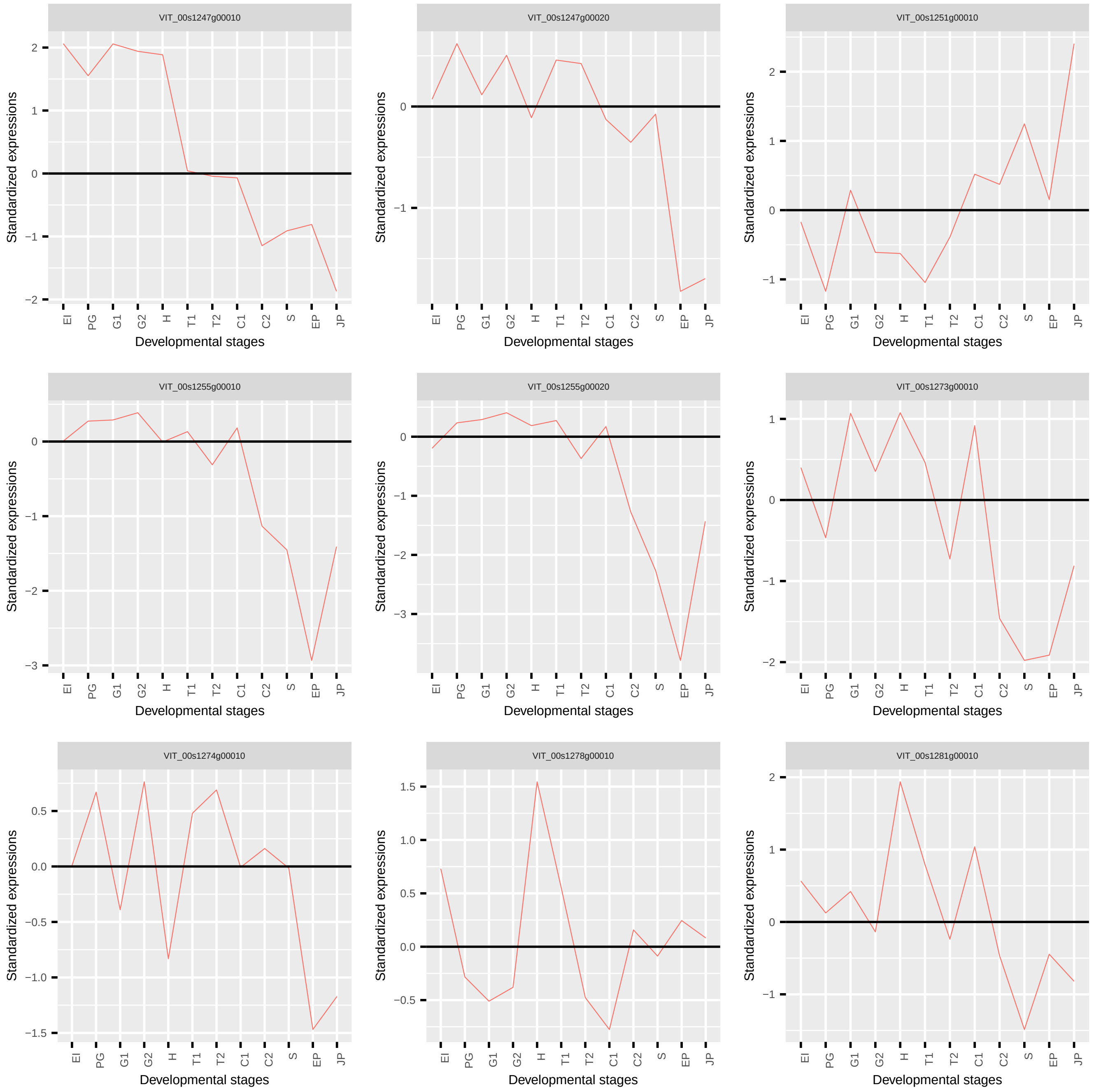

VIT_00s1247g00010
VIT_00s1247g00020
VIT_00s1251g00010
VIT_00s1255g00010
VIT_00s1255g00020
VIT_00s1273g00010
VIT_00s1274g00010
VIT_00s1278g00010
VIT_00s1281g00010
Standardized expressions
Developmental stages
EI PG G1 G2 H T1 T2 C1 C2 S EP JP
2 1 0 −1 −2
0 −1
2 1 0 −1
0 −1 −2 −3
0 −1 −2 −3
1 0 −1 −2
0.5 0.0 −0.5 −1.0 −1.5
1.5 1.0 0.5 0.0 −0.5
2 1 0 −1

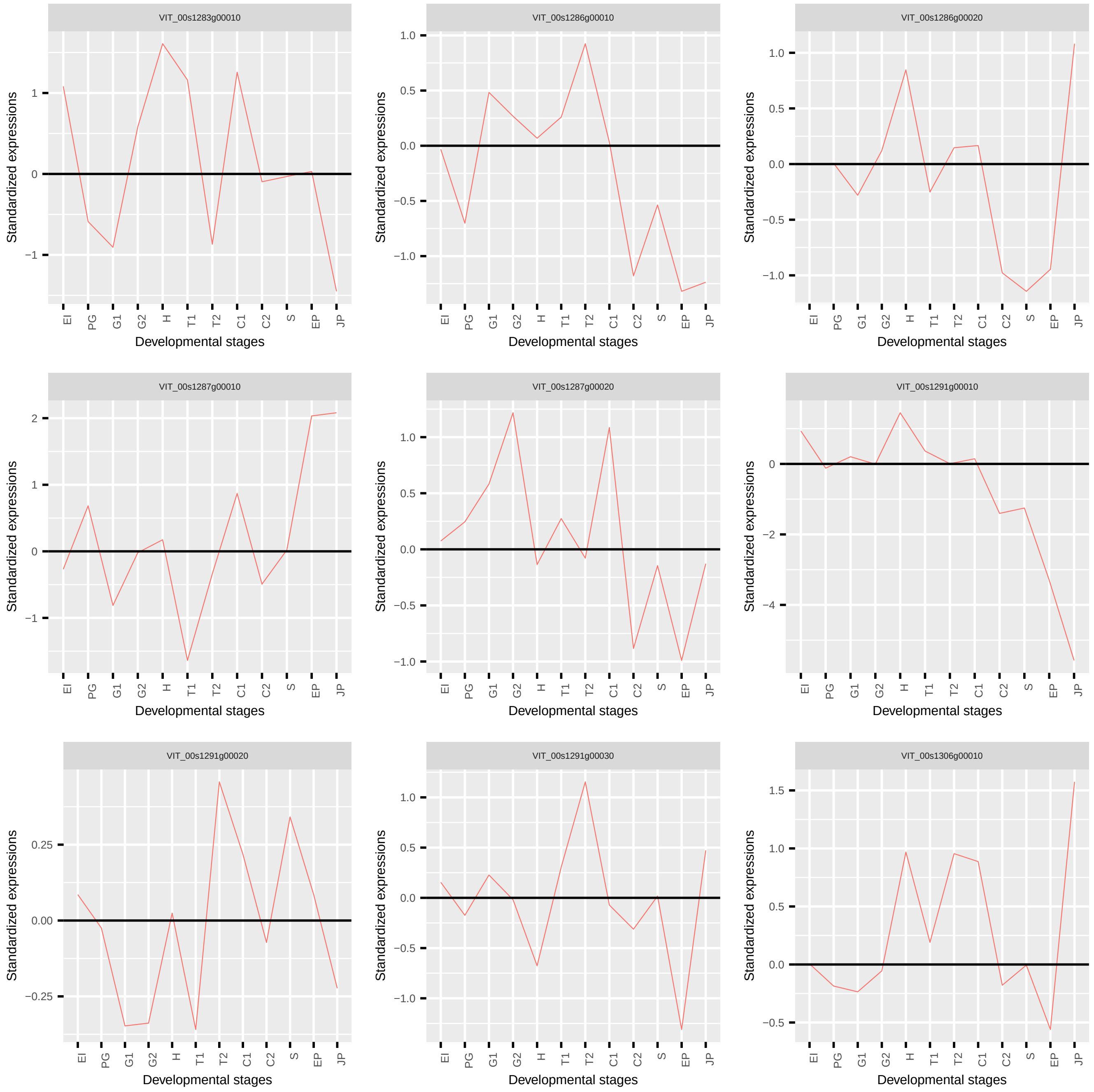
VIT_00s1283g00010
VIT_00s1286g00010
VIT_00s1286g00020
VIT_00s1287g00010
VIT_00s1287g00020
VIT_00s1291g00010
VIT_00s1291g00020
VIT_00s1291g00030
VIT_00s1306g00010
Standardized expressions
Developmental stages
EI
PG
G1
G2
H
T1
T2
C1
C2
S
EP
JP
1
0
−1
2
−2
−4
1.5
1.0
0.5
0.0
−0.5
−1.0
0.25
0.00
−0.25

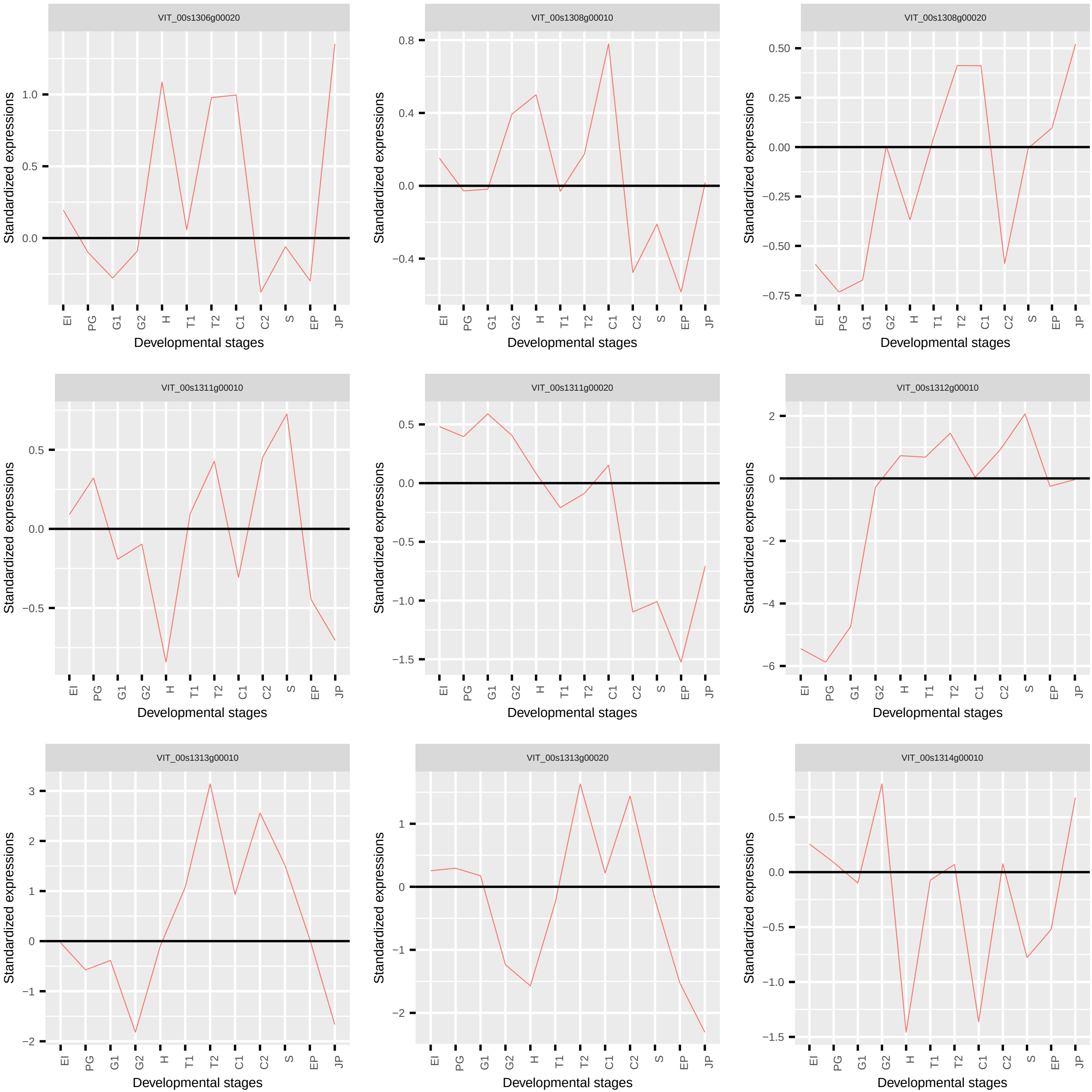
VIT_00s1306g00020
VIT_00s1308g00010
VIT_00s1308g00020
VIT_00s1311g00010
VIT_00s1311g00020
VIT_00s1312g00010
VIT_00s1313g00010
VIT_00s1313g00020
VIT_00s1314g00010
Standardized expressions
Developmental stages
EI PG G1 G2 H T1 T2 C1 C2 S EP JP

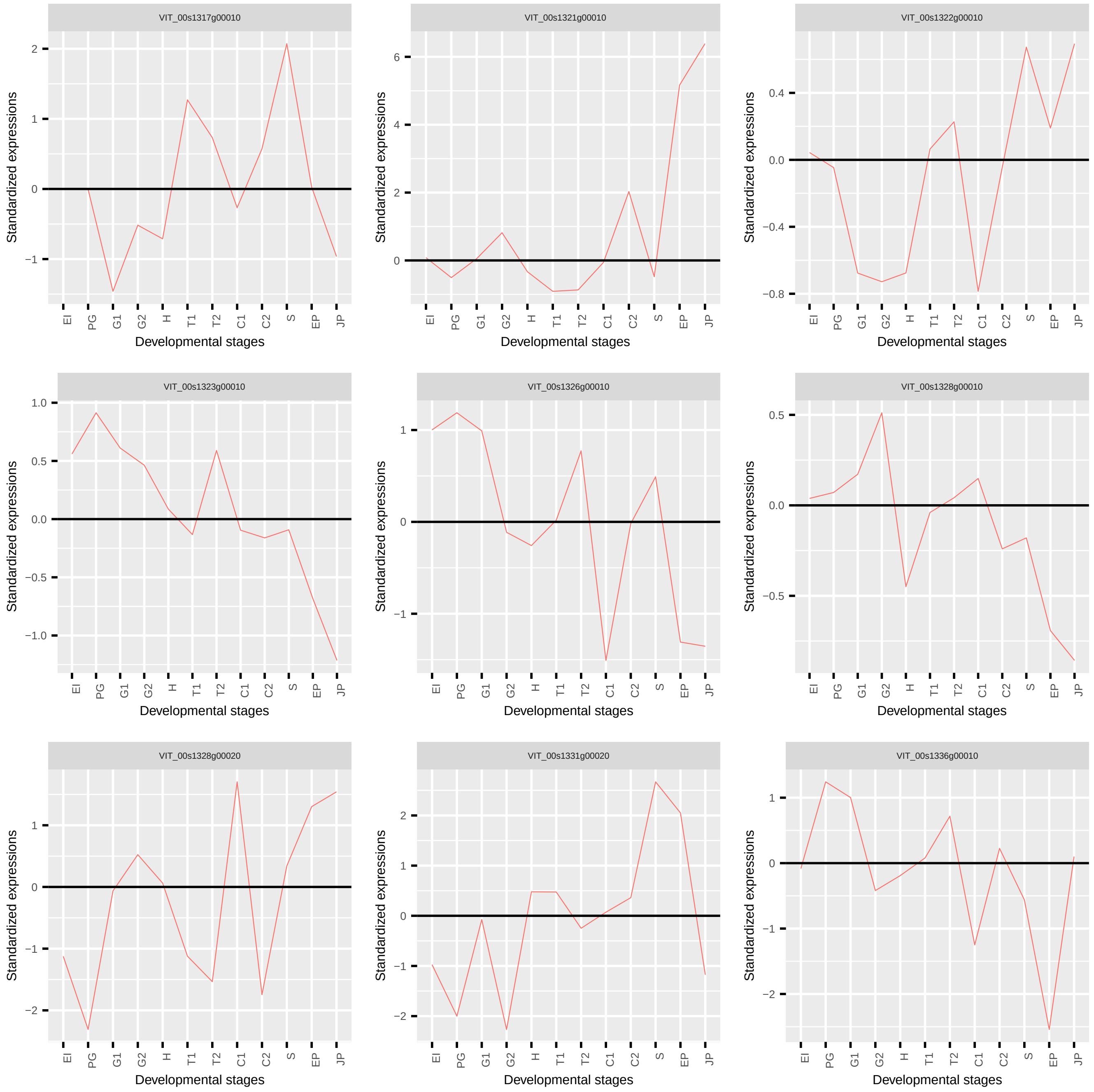
VIT_00s1317g00010
VIT_00s1321g00010
VIT_00s1322g00010
VIT_00s1323g00010
VIT_00s1326g00010
VIT_00s1328g00010
VIT_00s1328g00020
VIT_00s1331g00020
VIT_00s1336g00010
Standardized expressions
Developmental stages
EI PG G1 G2 H T1 T2 C1 C2 S EP JP

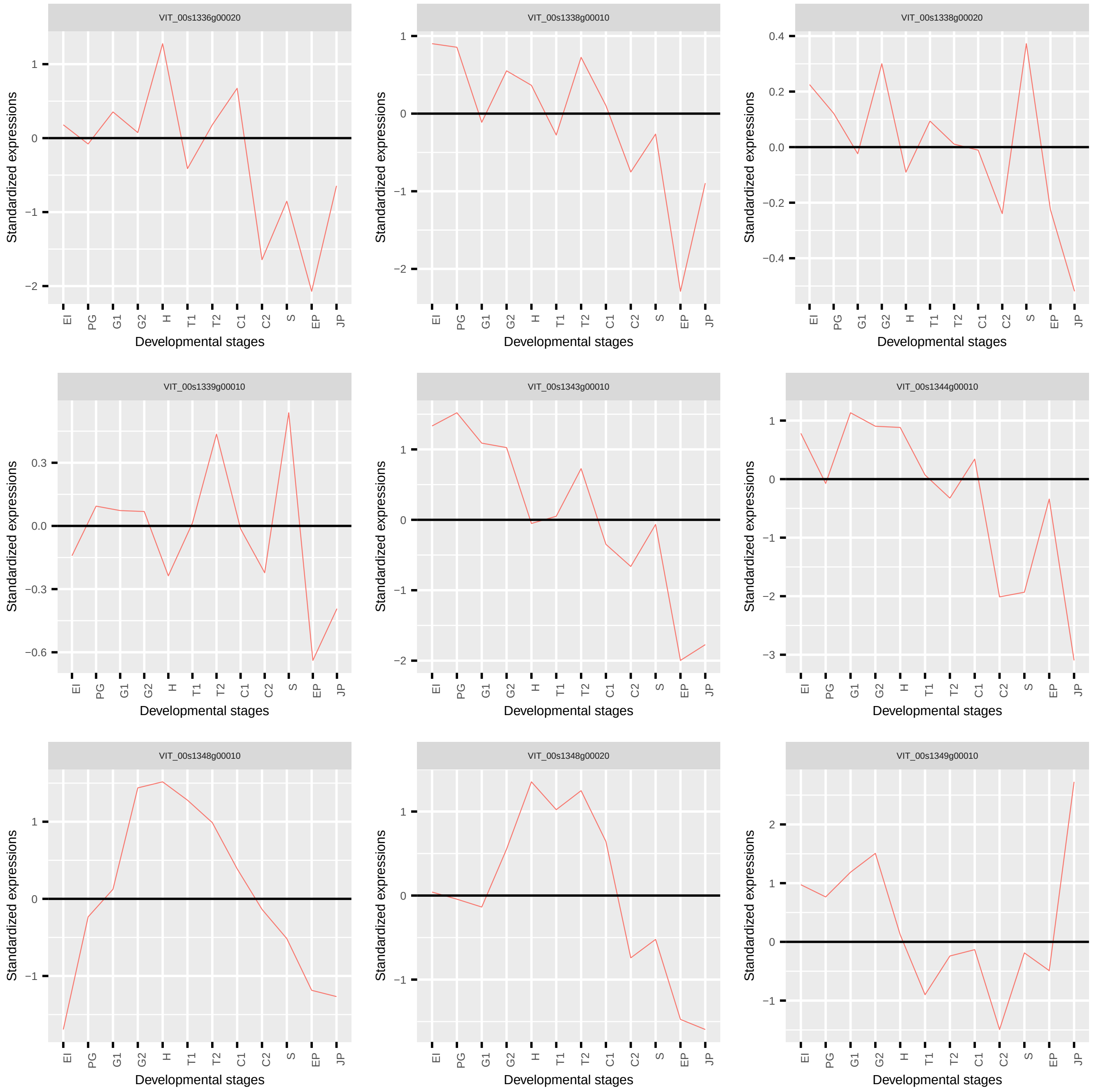

VIT_00s1336g00020
Standardized expressions
1
0
−1
−2
EI
PG
G1
G2
H
T1
T2
C1
C2
S
EP
JP
Developmental stages
VIT_00s1338g00010
Standardized expressions
1
0
−1
−2
EI
PG
G1
G2
H
T1
T2
C1
C2
S
EP
JP
Developmental stages
VIT_00s1338g00020
Standardized expressions
0.4
0.2
0.0
−0.2
−0.4
EI
PG
G1
G2
H
T1
T2
C1
C2
S
EP
JP
Developmental stages
VIT_00s1339g00010
Standardized expressions
0.3
0.0
−0.3
−0.6
EI
PG
G1
G2
H
T1
T2
C1
C2
S
EP
JP
Developmental stages
VIT_00s1343g00010
Standardized expressions
1
0
−1
−2
EI
PG
G1
G2
H
T1
T2
C1
C2
S
EP
JP
Developmental stages
VIT_00s1344g00010
Standardized expressions
1
0
−1
−2
−3
EI
PG
G1
G2
H
T1
T2
C1
C2
S
EP
JP
Developmental stages
VIT_00s1348g00010
Standardized expressions
1
0
−1
EI
PG
G1
G2
H
T1
T2
C1
C2
S
EP
JP
Developmental stages
VIT_00s1348g00020
Standardized expressions
1
0
−1
EI
PG
G1
G2
H
T1
T2
C1
C2
S
EP
JP
Developmental stages
VIT_00s1349g00010
Standardized expressions
2
1
0
−1
EI
PG
G1
G2
H
T1
T2
C1
C2
S
EP
JP
Developmental stages

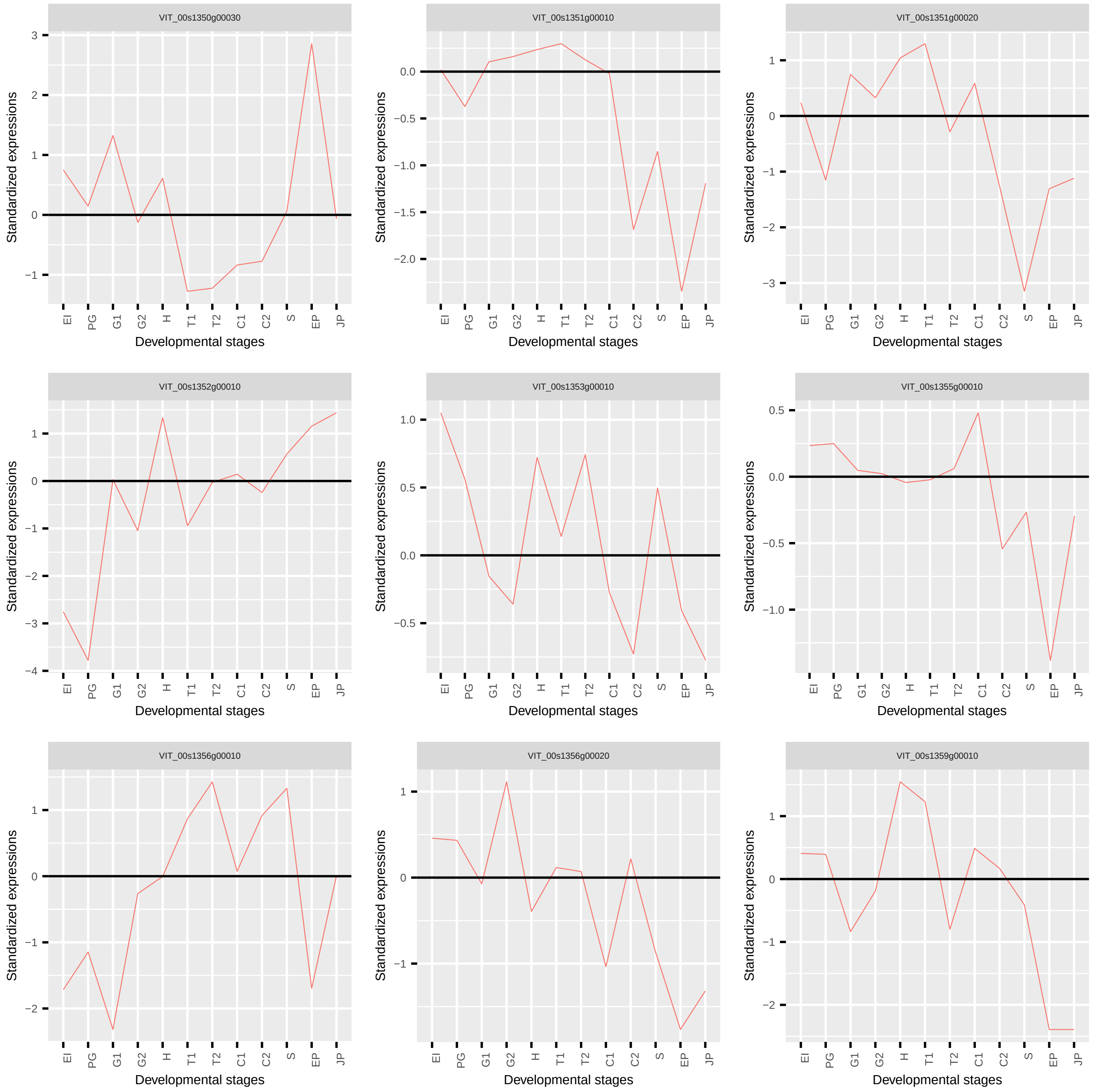
VIT_00s1350g00030
Standardized expressions
3 2 1 0 −1
EI PG G1 G2 H T1 T2 C1 C2 S EP JP
Developmental stages
VIT_00s1351g00010
Standardized expressions
0.0 −0.5 −1.0 −1.5 −2.0
EI PG G1 G2 H T1 T2 C1 C2 S EP JP
Developmental stages
VIT_00s1351g00020
Standardized expressions
1 0 −1 −2 −3
EI PG G1 G2 H T1 T2 C1 C2 S EP JP
Developmental stages
VIT_00s1352g00010
Standardized expressions
1 0 −1 −2 −3 −4
EI PG G1 G2 H T1 T2 C1 C2 S EP JP
Developmental stages
VIT_00s1353g00010
Standardized expressions
1.0 0.5 0.0 −0.5
EI PG G1 G2 H T1 T2 C1 C2 S EP JP
Developmental stages
VIT_00s1355g00010
Standardized expressions
0.5 0.0 −0.5 −1.0
EI PG G1 G2 H T1 T2 C1 C2 S EP JP
Developmental stages
VIT_00s1356g00010
Standardized expressions
1 0 −1 −2
EI PG G1 G2 H T1 T2 C1 C2 S EP JP
Developmental stages
VIT_00s1356g00020
Standardized expressions
1 0 −1
EI PG G1 G2 H T1 T2 C1 C2 S EP JP
Developmental stages
VIT_00s1359g00010
Standardized expressions
1 0 −1 −2
EI PG G1 G2 H T1 T2 C1 C2 S EP JP
Developmental stages

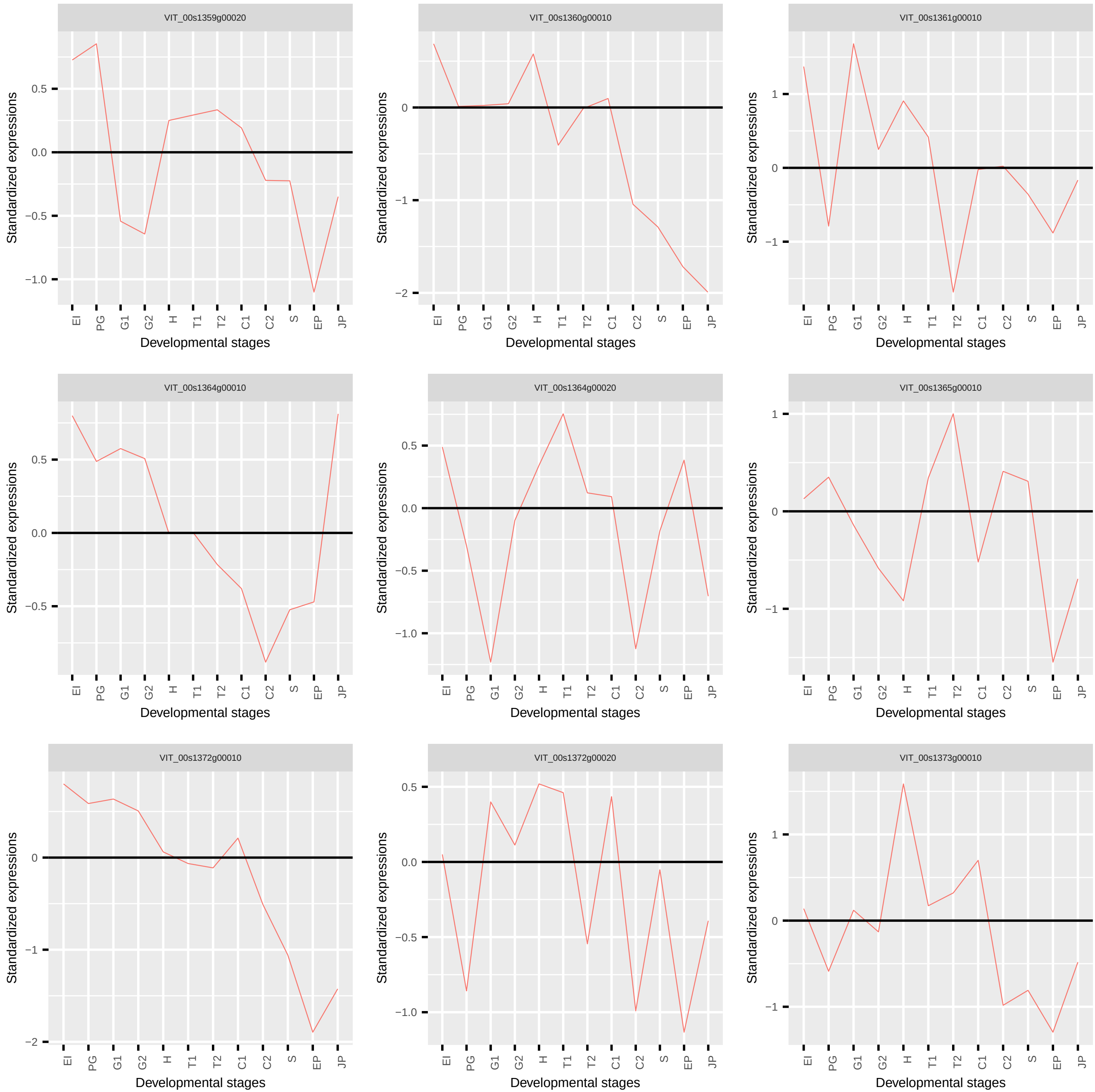
VIT_00s1359g00020
VIT_00s1360g00010
VIT_00s1361g00010
VIT_00s1364g00010
VIT_00s1364g00020
VIT_00s1365g00010
VIT_00s1372g00010
VIT_00s1372g00020
VIT_00s1373g00010
Standardized expressions
Developmental stages
EI PG G1 G2 H T1 T2 C1 C2 S EP JP

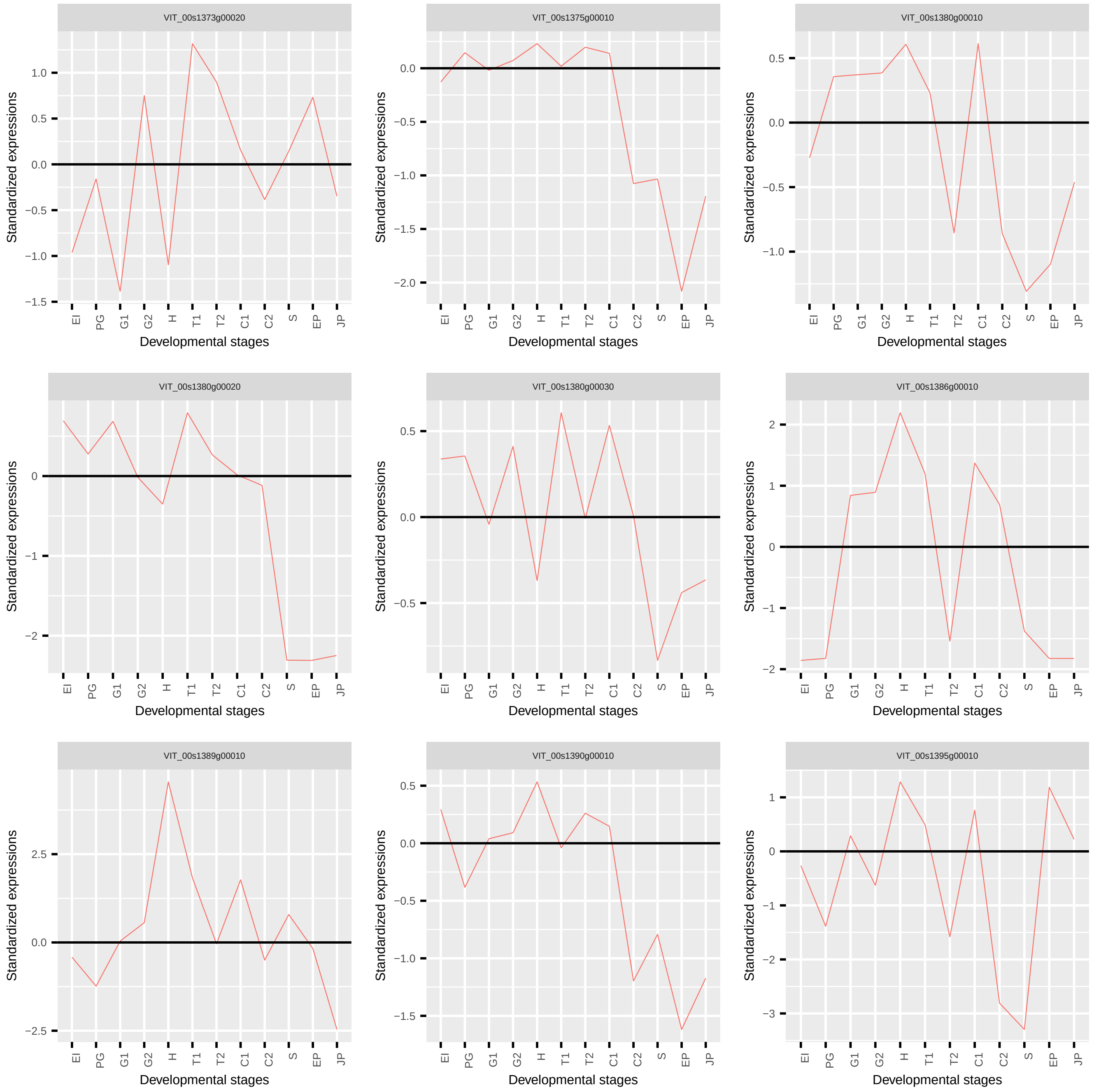
VIT_00s1373g00020
VIT_00s1375g00010
VIT_00s1380g00010
VIT_00s1380g00020
VIT_00s1380g00030
VIT_00s1386g00010
VIT_00s1389g00010
VIT_00s1390g00010
VIT_00s1395g00010
Standardized expressions
Developmental stages
EI
PG
G1
G2
H
T1
T2
C1
C2
S
EP
JP
1.0
0.5
0.0
−0.5
−1.0
−1.5
−2.0
0
−1
−2
2
1
−3
2.5
−2.5

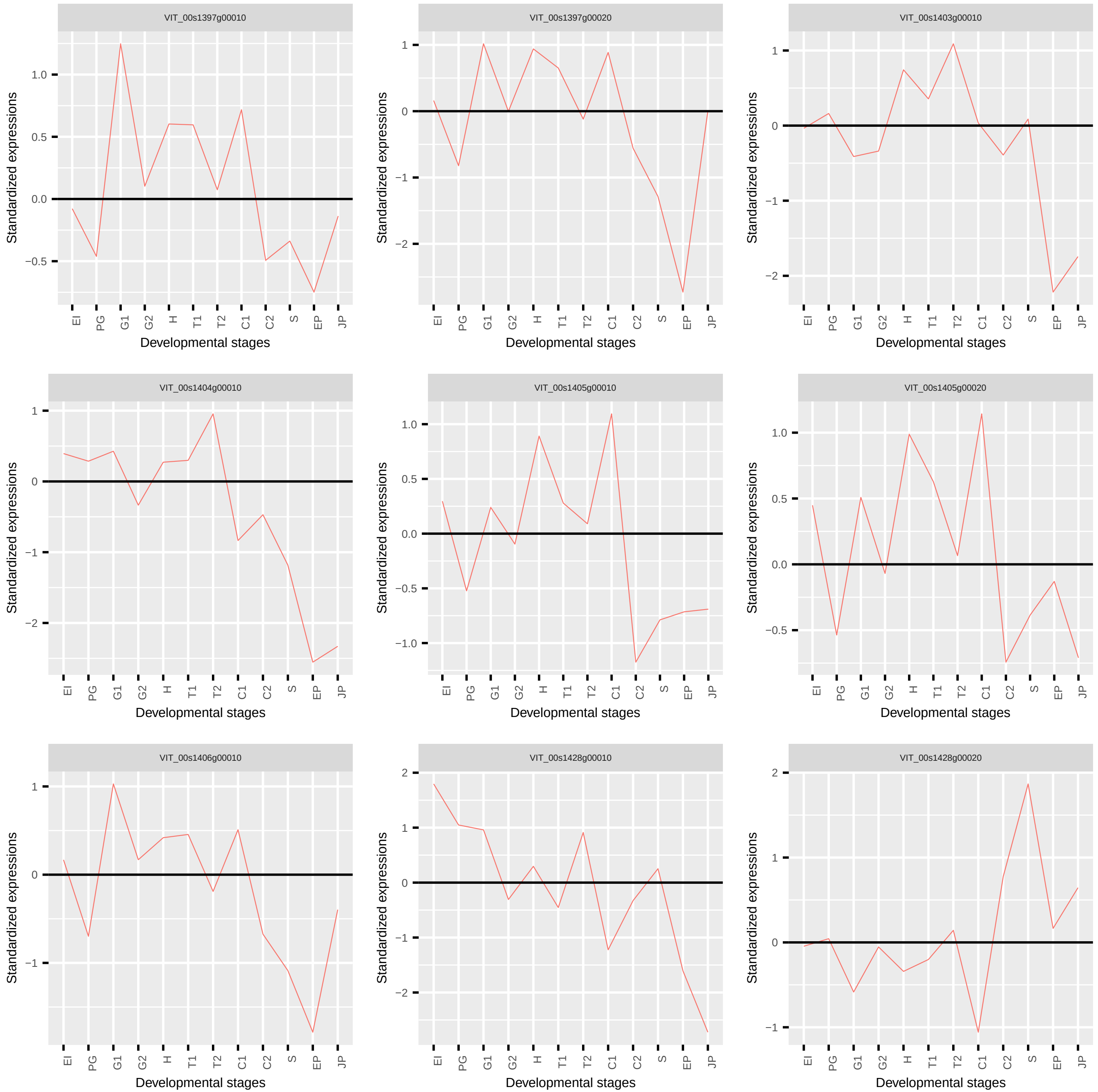
VIT_00s1397g00010
Standardized expressions
1.0
0.5
0.0
−0.5
EI PG G1 G2 H T1 T2 C1 C2 S EP JP
Developmental stages
VIT_00s1397g00020
Standardized expressions
1
0
−1
−2
EI PG G1 G2 H T1 T2 C1 C2 S EP JP
Developmental stages
VIT_00s1403g00010
Standardized expressions
1
0
−1
−2
EI PG G1 G2 H T1 T2 C1 C2 S EP JP
Developmental stages
VIT_00s1404g00010
Standardized expressions
1
0
−1
−2
EI PG G1 G2 H T1 T2 C1 C2 S EP JP
Developmental stages
VIT_00s1405g00010
Standardized expressions
1.0
0.5
0.0
−0.5
−1.0
EI PG G1 G2 H T1 T2 C1 C2 S EP JP
Developmental stages
VIT_00s1405g00020
Standardized expressions
1.0
0.5
0.0
−0.5
EI PG G1 G2 H T1 T2 C1 C2 S EP JP
Developmental stages
VIT_00s1406g00010
Standardized expressions
1
0
−1
EI PG G1 G2 H T1 T2 C1 C2 S EP JP
Developmental stages
VIT_00s1428g00010
Standardized expressions
2
1
0
−1
−2
EI PG G1 G2 H T1 T2 C1 C2 S EP JP
Developmental stages
VIT_00s1428g00020
Standardized expressions
2
1
0
−1
EI PG G1 G2 H T1 T2 C1 C2 S EP JP
Developmental stages

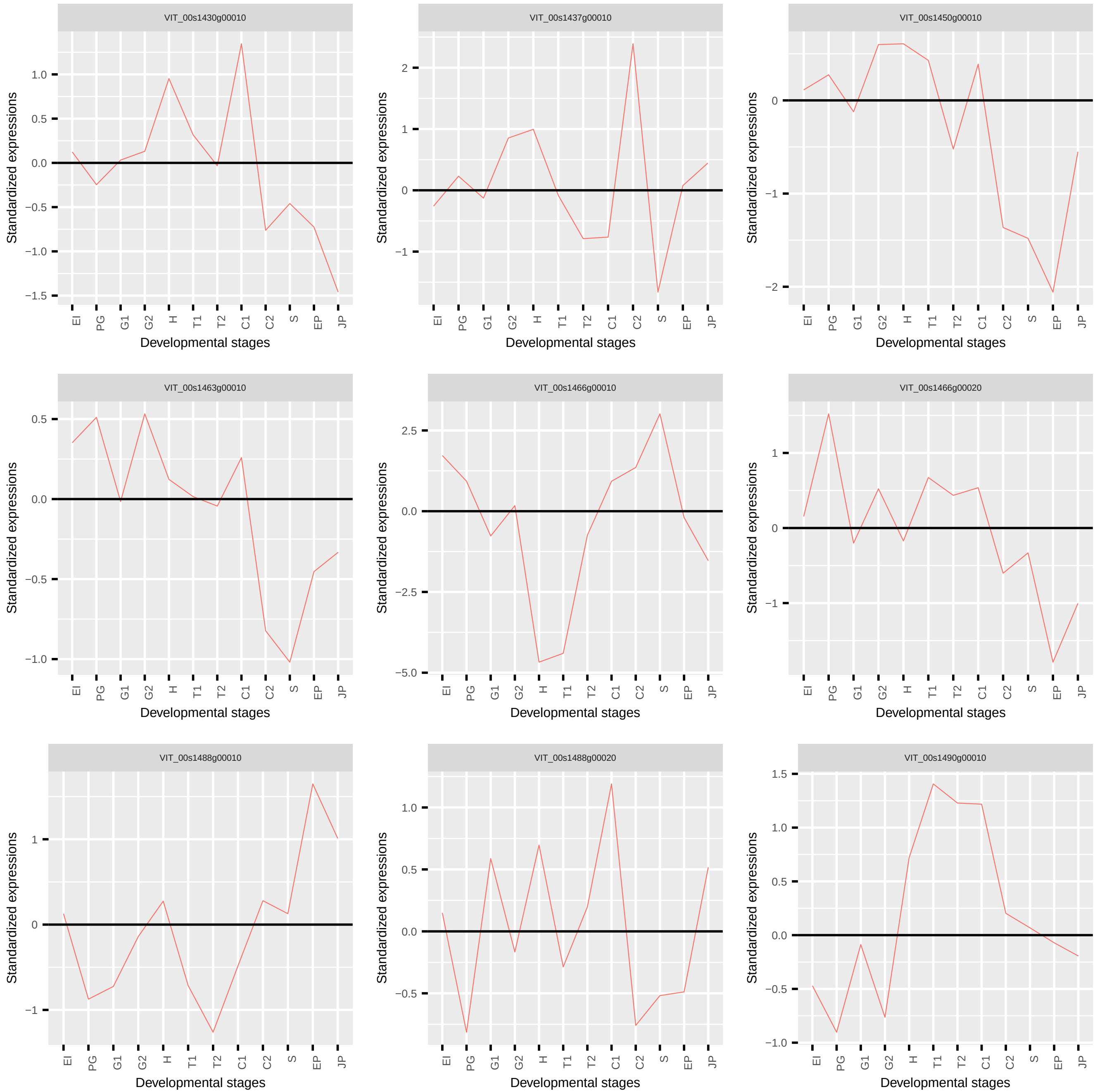

VIT_00s1430g00010
Standardized expressions
1.0
0.5
0.0
−0.5
−1.0
−1.5
EI
PG
G1
G2
H
T1
T2
C1
C2
S
EP
JP
Developmental stages
VIT_00s1437g00010
Standardized expressions
2
1
0
−1
EI
PG
G1
G2
H
T1
T2
C1
C2
S
EP
JP
Developmental stages
VIT_00s1450g00010
Standardized expressions
0
−1
−2
EI
PG
G1
G2
H
T1
T2
C1
C2
S
EP
JP
Developmental stages
VIT_00s1463g00010
Standardized expressions
0.5
0.0
−0.5
−1.0
EI
PG
G1
G2
H
T1
T2
C1
C2
S
EP
JP
Developmental stages
VIT_00s1466g00010
Standardized expressions
2.5
0.0
-2.5
-5.0
EI
PG
G1
G2
H
T1
T2
C1
C2
S
EP
JP
Developmental stages
VIT_00s1466g00020
Standardized expressions
1
0
−1
EI
PG
G1
G2
H
T1
T2
C1
C2
S
EP
JP
Developmental stages
VIT_00s1488g00010
Standardized expressions
1
0
−1
EI
PG
G1
G2
H
T1
T2
C1
C2
S
EP
JP
Developmental stages
VIT_00s1488g00020
Standardized expressions
1.0
0.5
0.0
−0.5
EI
PG
G1
G2
H
T1
T2
C1
C2
S
EP
JP
Developmental stages
VIT_00s1490g00010
Standardized expressions
1.5
1.0
0.5
0.0
−0.5
−1.0
EI
PG
G1
G2
H
T1
T2
C1
C2
S
EP
JP
Developmental stages

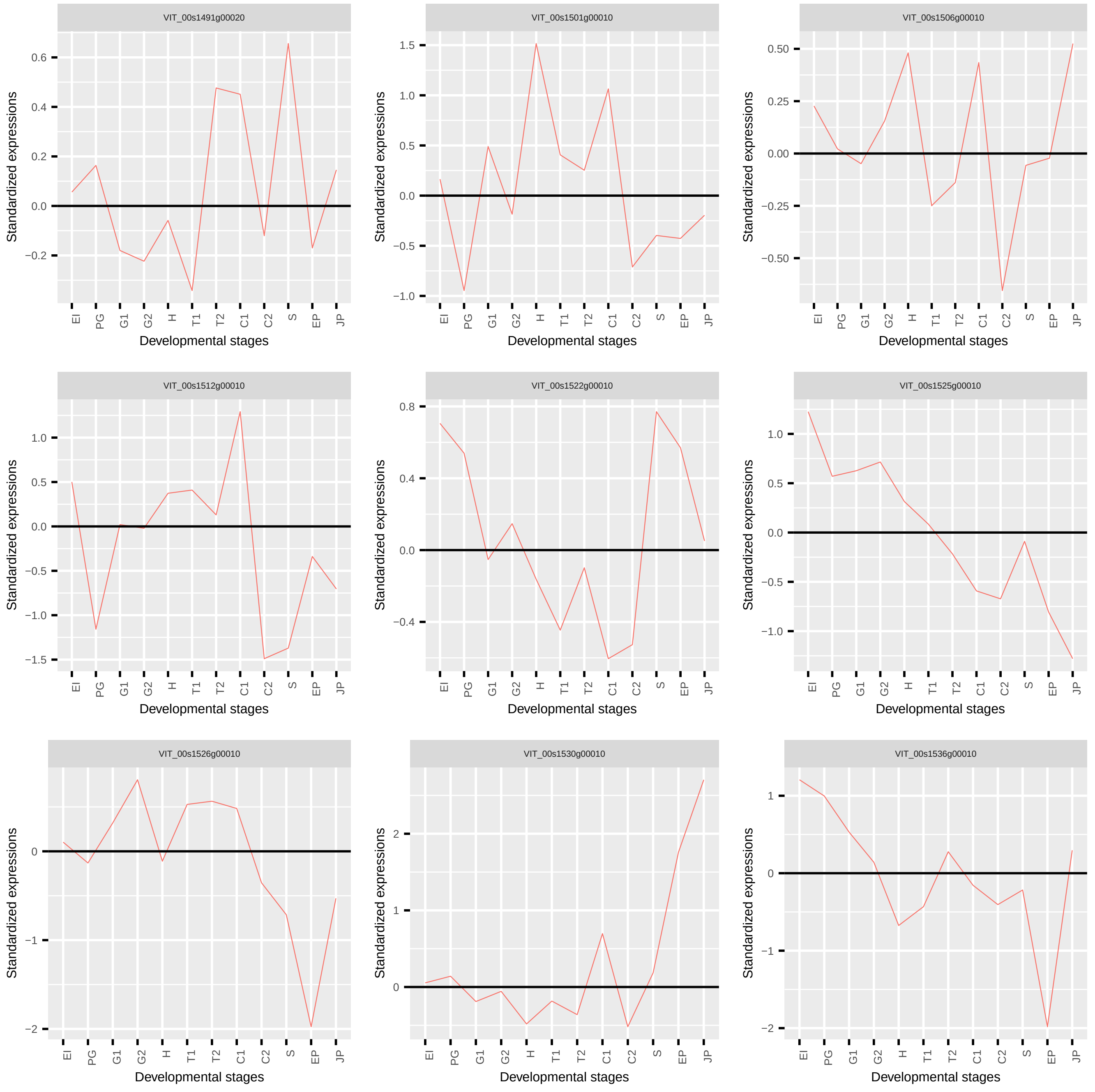
VIT_00s1491g00020
VIT_00s1501g00010
VIT_00s1506g00010
VIT_00s1512g00010
VIT_00s1522g00010
VIT_00s1525g00010
VIT_00s1526g00010
VIT_00s1530g00010
VIT_00s1536g00010
Standardized expressions
Developmental stages
EI PG G1 G2 H T1 T2 C1 C2 S EP JP

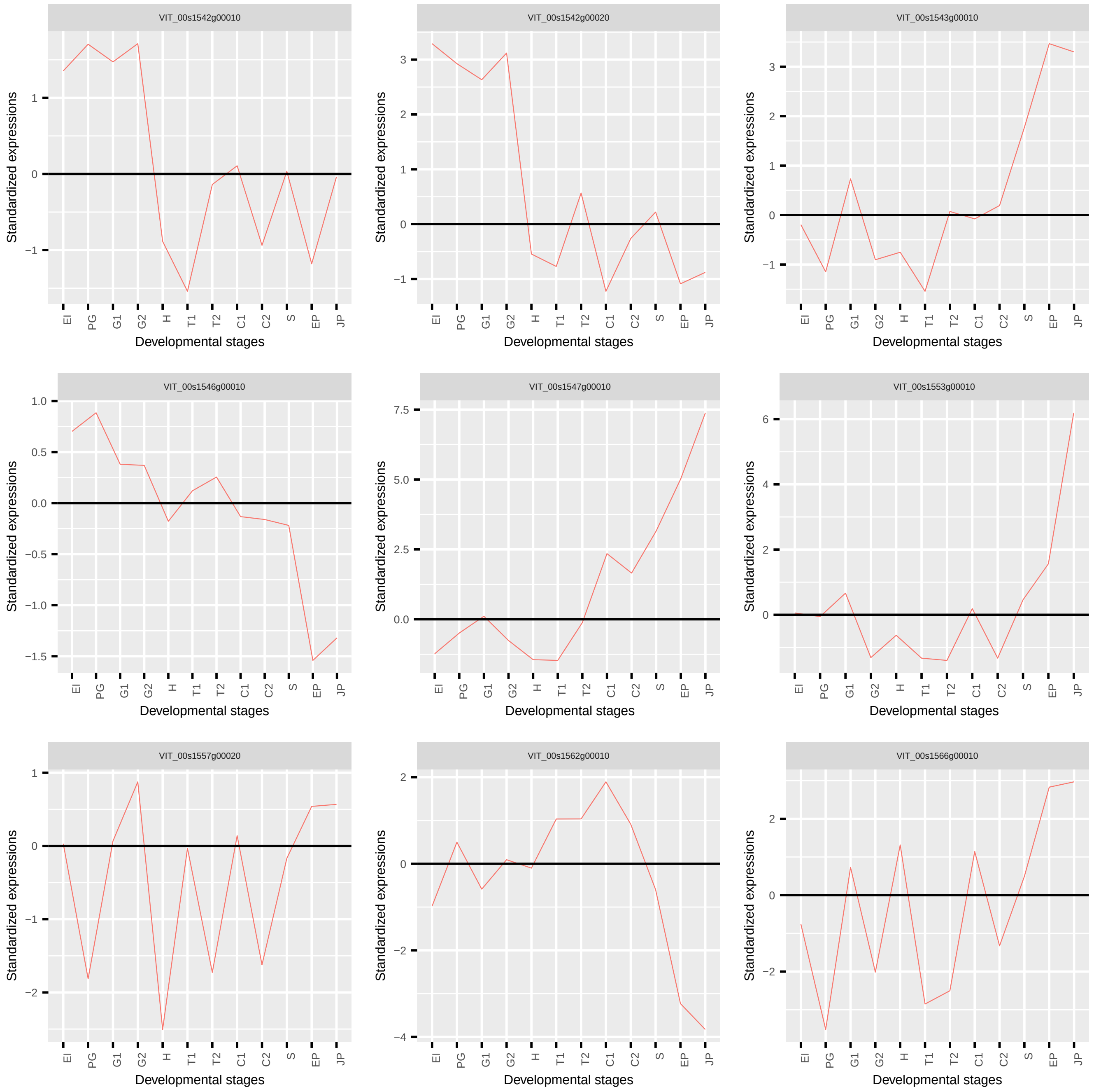
VIT_00s1542g00010
VIT_00s1542g00020
VIT_00s1543g00010
VIT_00s1546g00010
VIT_00s1547g00010
VIT_00s1553g00010
VIT_00s1557g00020
VIT_00s1562g00010
VIT_00s1566g00010
Standardized expressions
Developmental stages
EI PG G1 G2 H T1 T2 C1 C2 S EP JP

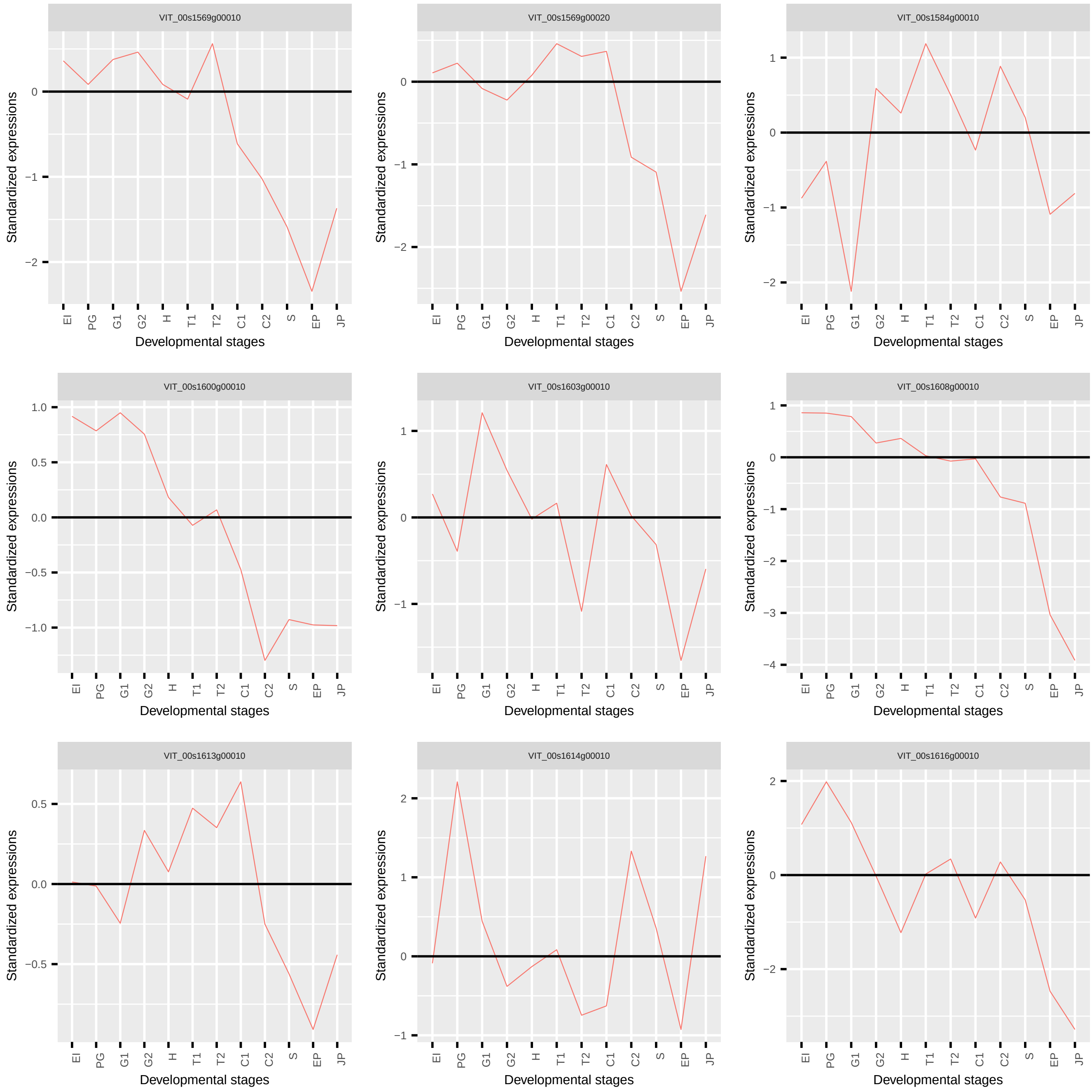
VIT_00s1569g00010
VIT_00s1569g00020
VIT_00s1584g00010
VIT_00s1600g00010
VIT_00s1603g00010
VIT_00s1608g00010
VIT_00s1613g00010
VIT_00s1614g00010
VIT_00s1616g00010
Standardized expressions
Developmental stages
EI PG G1 G2 H T1 T2 C1 C2 S EP JP

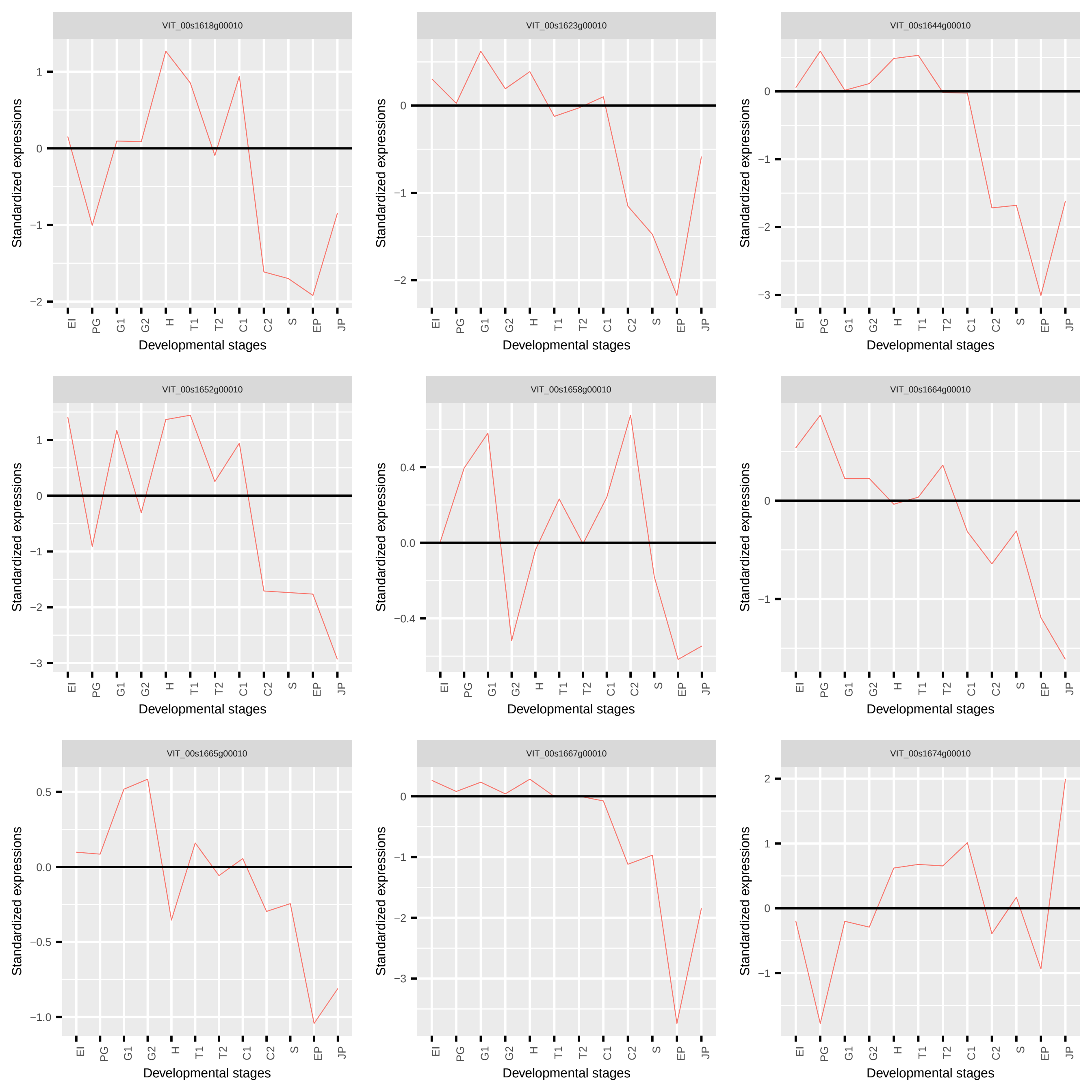
VIT_00s1618g00010
VIT_00s1623g00010
VIT_00s1644g00010
VIT_00s1652g00010
VIT_00s1658g00010
VIT_00s1664g00010
VIT_00s1665g00010
VIT_00s1667g00010
VIT_00s1674g00010
Standardized expressions
Developmental stages
EI PG G1 G2 H T1 T2 C1 C2 S EP JP

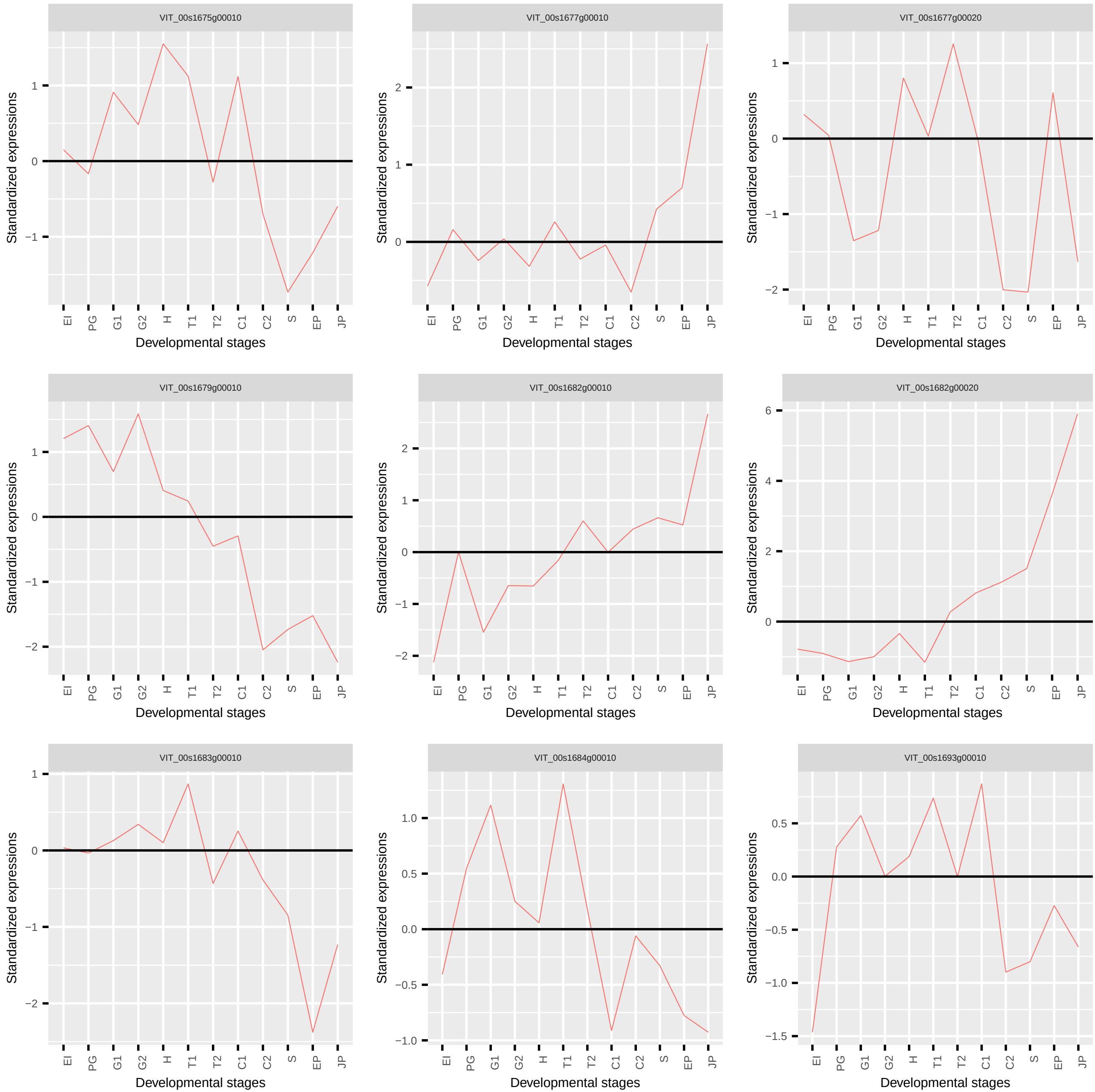

VIT_00s1675g00010
VIT_00s1677g00010
VIT_00s1677g00020
VIT_00s1679g00010
VIT_00s1682g00010
VIT_00s1682g00020
VIT_00s1683g00010
VIT_00s1684g00010
VIT_00s1693g00010
Standardized expressions
Developmental stages
EI PG G1 G2 H T1 T2 C1 C2 S EP JP

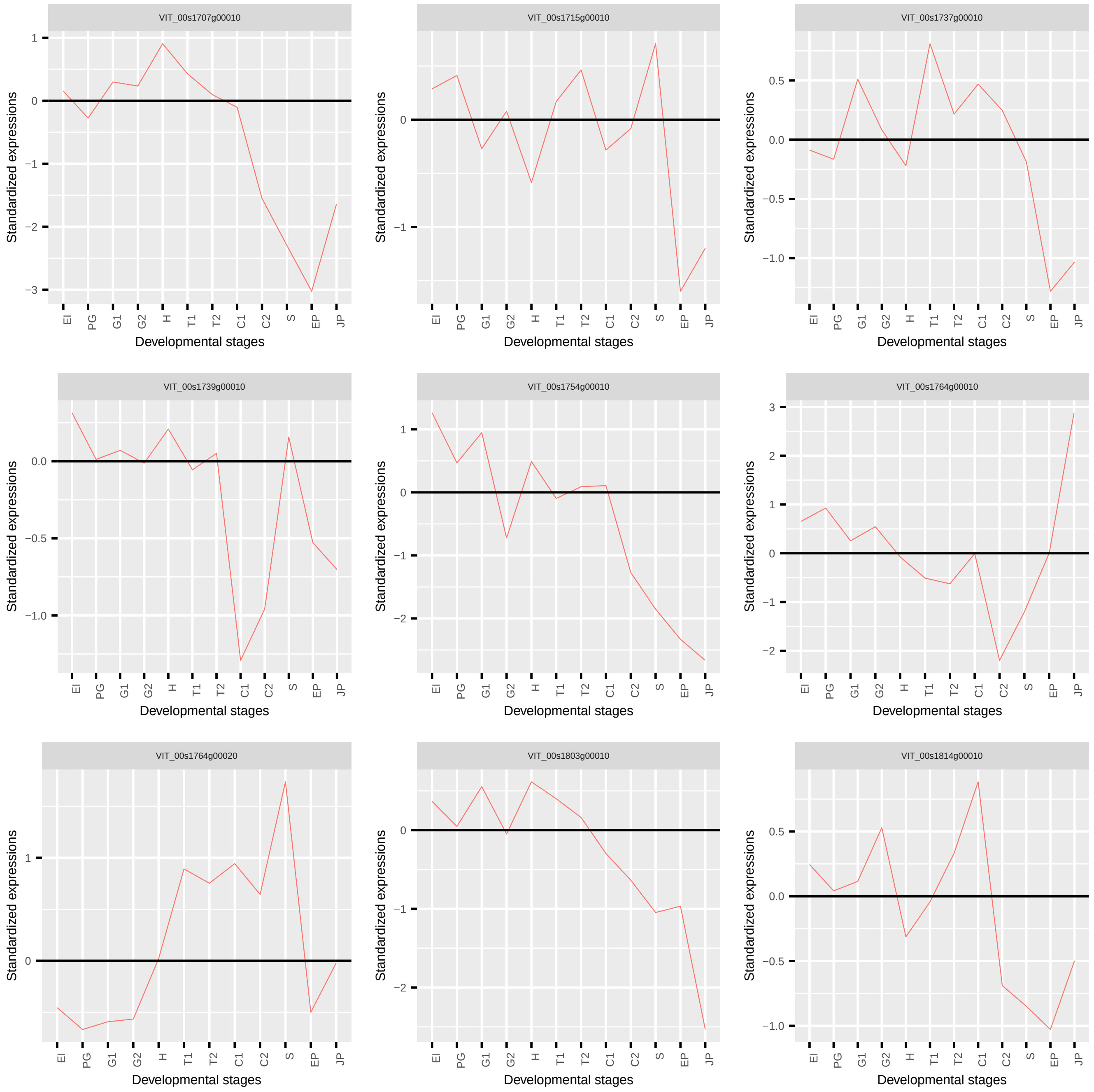
VIT_00s1707g00010
VIT_00s1715g00010
VIT_00s1737g00010
VIT_00s1739g00010
VIT_00s1754g00010
VIT_00s1764g00010
VIT_00s1764g00020
VIT_00s1803g00010
VIT_00s1814g00010
Standardized expressions
Developmental stages
EI PG G1 G2 H T1 T2 C1 C2 S EP JP

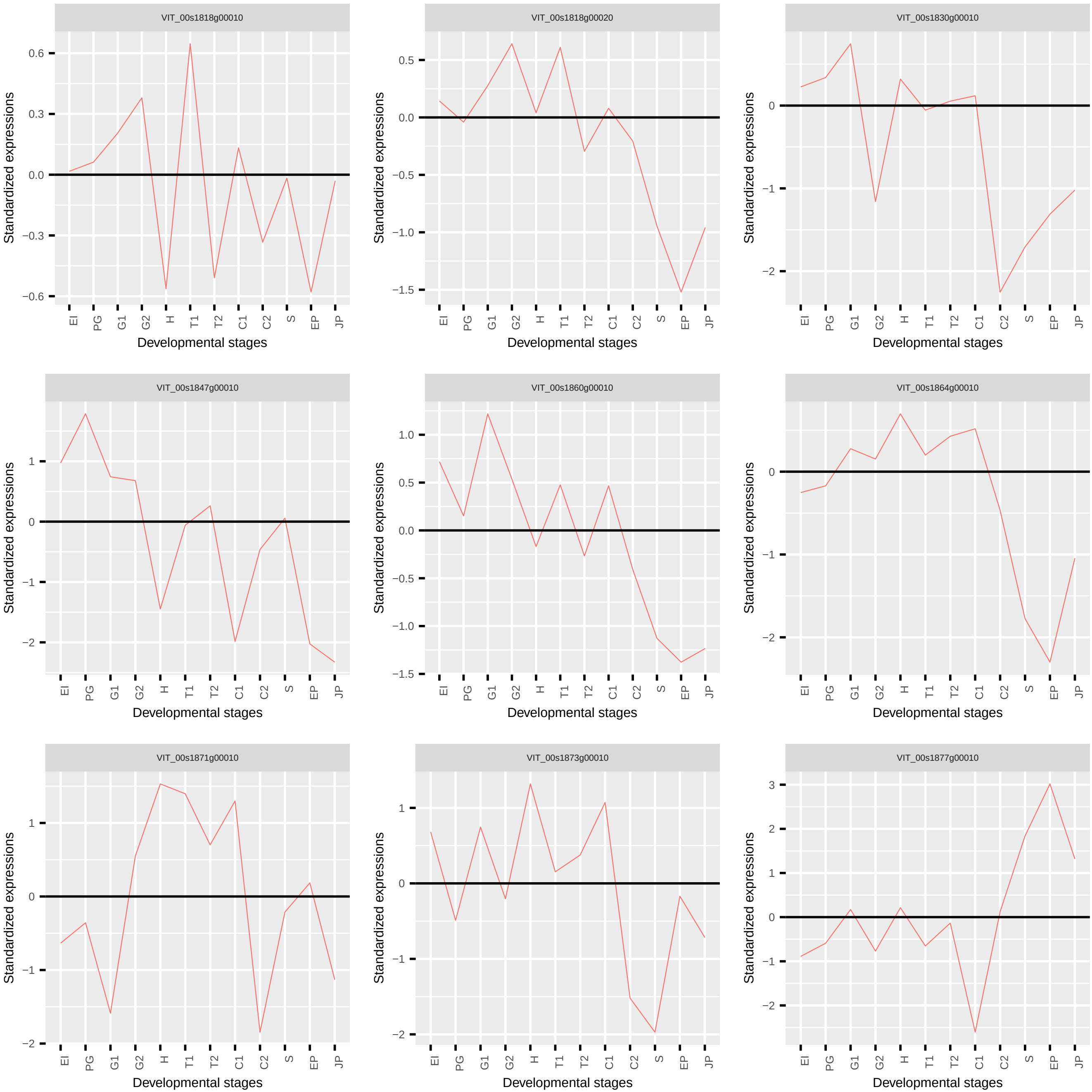
VIT_00s1818g00010
Standardized expressions
0.6
0.3
0.0
−0.3
−0.6
EI PG G1 G2 H T1 T2 C1 C2 S EP JP
Developmental stages
VIT_00s1818g00020
Standardized expressions
0.5
0.0
−0.5
−1.0
−1.5
EI PG G1 G2 H T1 T2 C1 C2 S EP JP
Developmental stages
VIT_00s1830g00010
Standardized expressions
0
−1
−2
EI PG G1 G2 H T1 T2 C1 C2 S EP JP
Developmental stages
VIT_00s1847g00010
Standardized expressions
1
0
−1
−2
EI PG G1 G2 H T1 T2 C1 C2 S EP JP
Developmental stages
VIT_00s1860g00010
Standardized expressions
1.0
0.5
0.0
−0.5
−1.0
−1.5
EI PG G1 G2 H T1 T2 C1 C2 S EP JP
Developmental stages
VIT_00s1864g00010
Standardized expressions
0
−1
−2
EI PG G1 G2 H T1 T2 C1 C2 S EP JP
Developmental stages
VIT_00s1871g00010
Standardized expressions
1
0
−1
−2
EI PG G1 G2 H T1 T2 C1 C2 S EP JP
Developmental stages
VIT_00s1873g00010
Standardized expressions
1
0
−1
−2
EI PG G1 G2 H T1 T2 C1 C2 S EP JP
Developmental stages
VIT_00s1877g00010
Standardized expressions
3
2
1
0
−1
−2
EI PG G1 G2 H T1 T2 C1 C2 S EP JP
Developmental stages

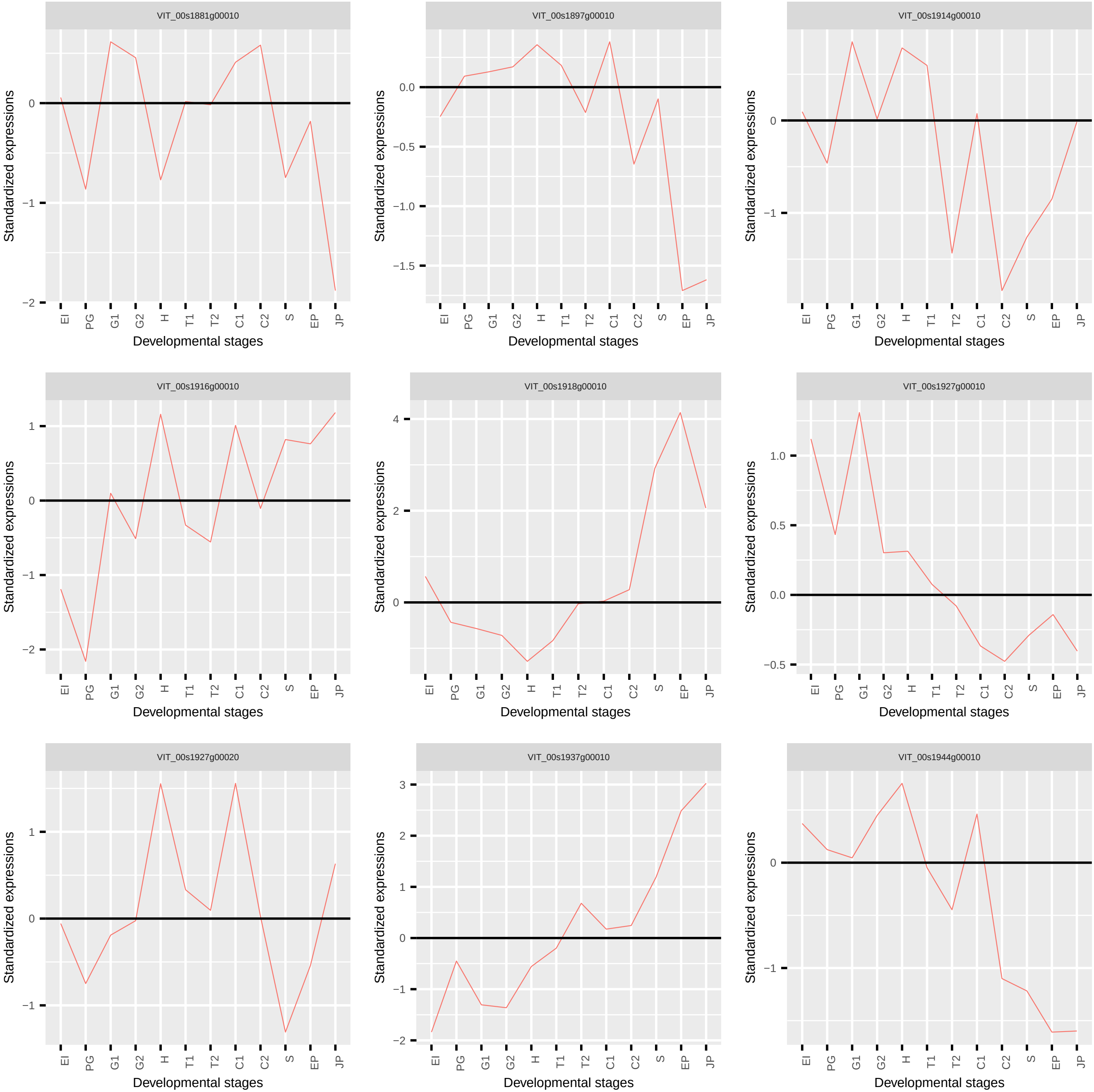
VIT_00s1881g00010
VIT_00s1897g00010
VIT_00s1914g00010
VIT_00s1916g00010
VIT_00s1918g00010
VIT_00s1927g00010
VIT_00s1927g00020
VIT_00s1937g00010
VIT_00s1944g00010
Standardized expressions
Developmental stages
EI PG G1 G2 H T1 T2 C1 C2 S EP JP

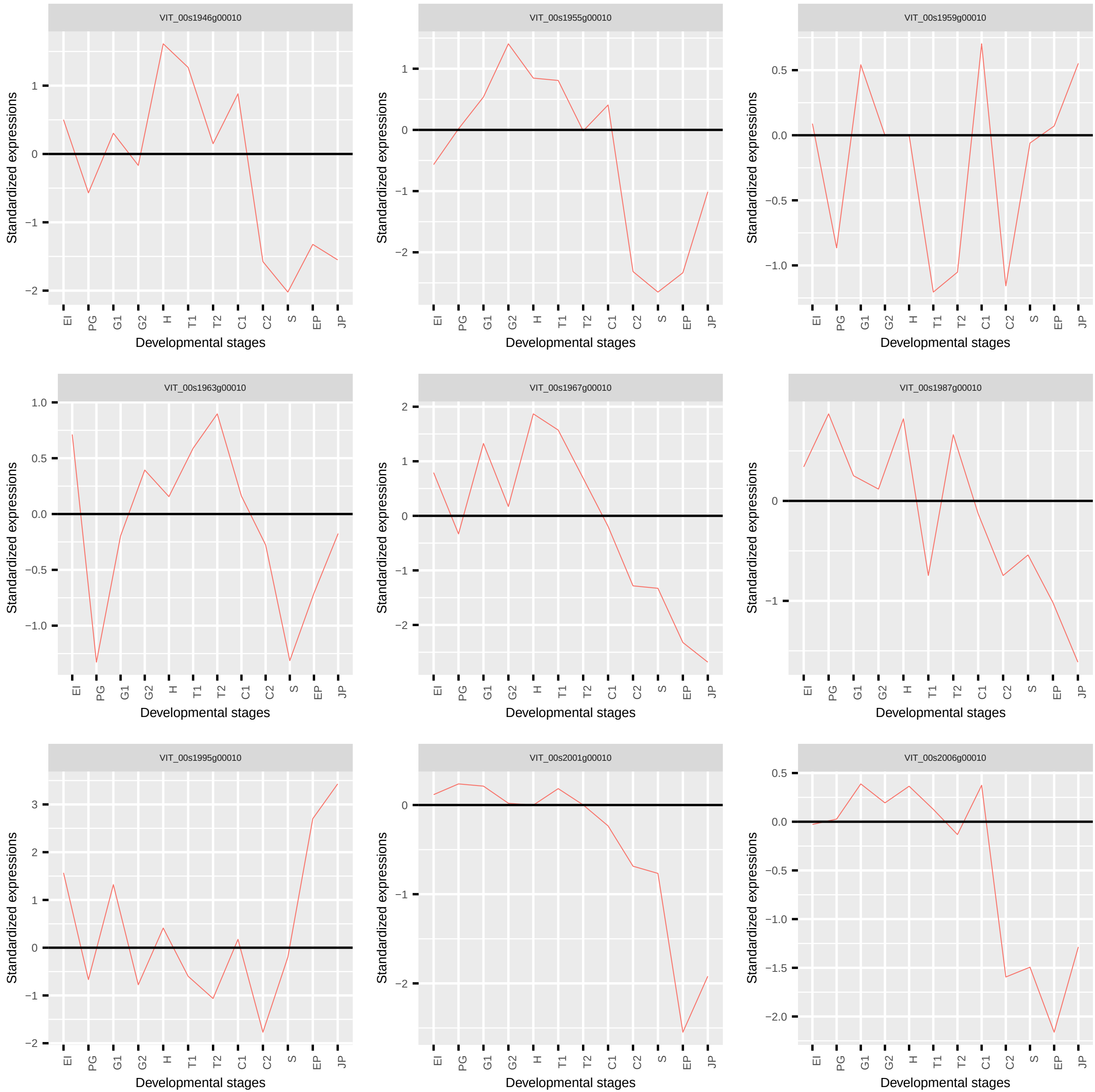
VIT_00s1946g00010
Standardized expressions
1
0
−1
−2
EI PG G1 G2 H T1 T2 C1 C2 S EP JP
Developmental stages
VIT_00s1955g00010
Standardized expressions
1
0
−1
−2
EI PG G1 G2 H T1 T2 C1 C2 S EP JP
Developmental stages
VIT_00s1959g00010
Standardized expressions
0.5
0.0
−0.5
−1.0
EI PG G1 G2 H T1 T2 C1 C2 S EP JP
Developmental stages
VIT_00s1963g00010
Standardized expressions
1.0
0.5
0.0
−0.5
−1.0
EI PG G1 G2 H T1 T2 C1 C2 S EP JP
Developmental stages
VIT_00s1967g00010
Standardized expressions
2
1
0
−1
−2
EI PG G1 G2 H T1 T2 C1 C2 S EP JP
Developmental stages
VIT_00s1987g00010
Standardized expressions
0
−1
EI PG G1 G2 H T1 T2 C1 C2 S EP JP
Developmental stages
VIT_00s1995g00010
Standardized expressions
3
2
1
0
−1
−2
EI PG G1 G2 H T1 T2 C1 C2 S EP JP
Developmental stages
VIT_00s2001g00010
Standardized expressions
0
−1
−2
EI PG G1 G2 H T1 T2 C1 C2 S EP JP
Developmental stages
VIT_00s2006g00010
Standardized expressions
0.5
0.0
−0.5
−1.0
−1.5
−2.0
EI PG G1 G2 H T1 T2 C1 C2 S EP JP
Developmental stages

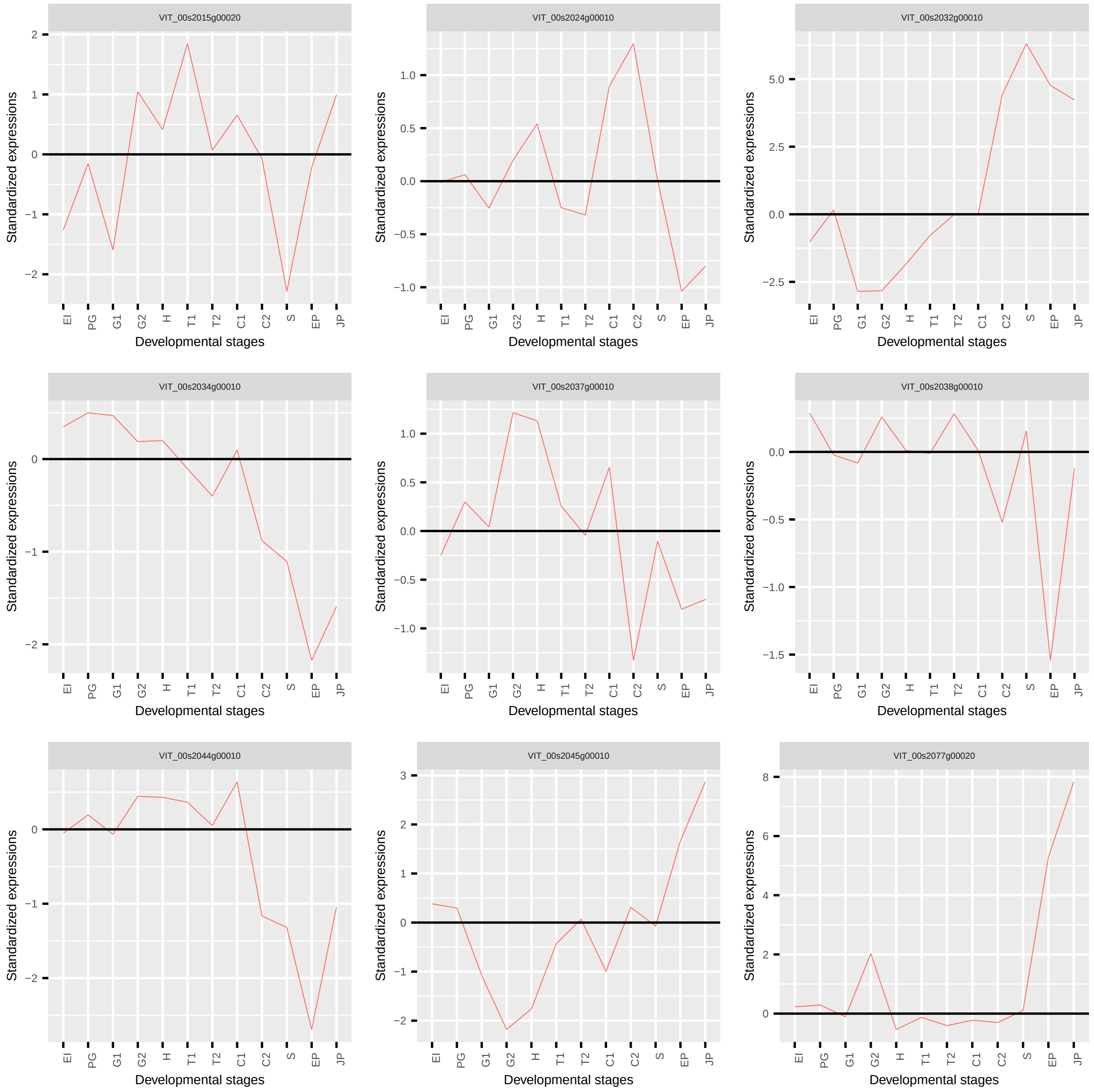

VIT_00s2015g00020
VIT_00s2024g00010
VIT_00s2032g00010
VIT_00s2034g00010
VIT_00s2037g00010
VIT_00s2038g00010
VIT_00s2044g00010
VIT_00s2045g00010
VIT_00s2077g00020
Standardized expressions
Developmental stages
EI PG G1 G2 H T1 T2 C1 C2 S EP JP
2 1 0 −1 −2
1.0 0.5 0.0 −0.5 −1.0
5.0 2.5 0.0 −2.5
0 −1 −2
1.0 0.5 0.0 −0.5 −1.0
0.0 −0.5 −1.0 −1.5
0 −1 −2
3 2 1 0 −1 −2
8 6 4 2 0

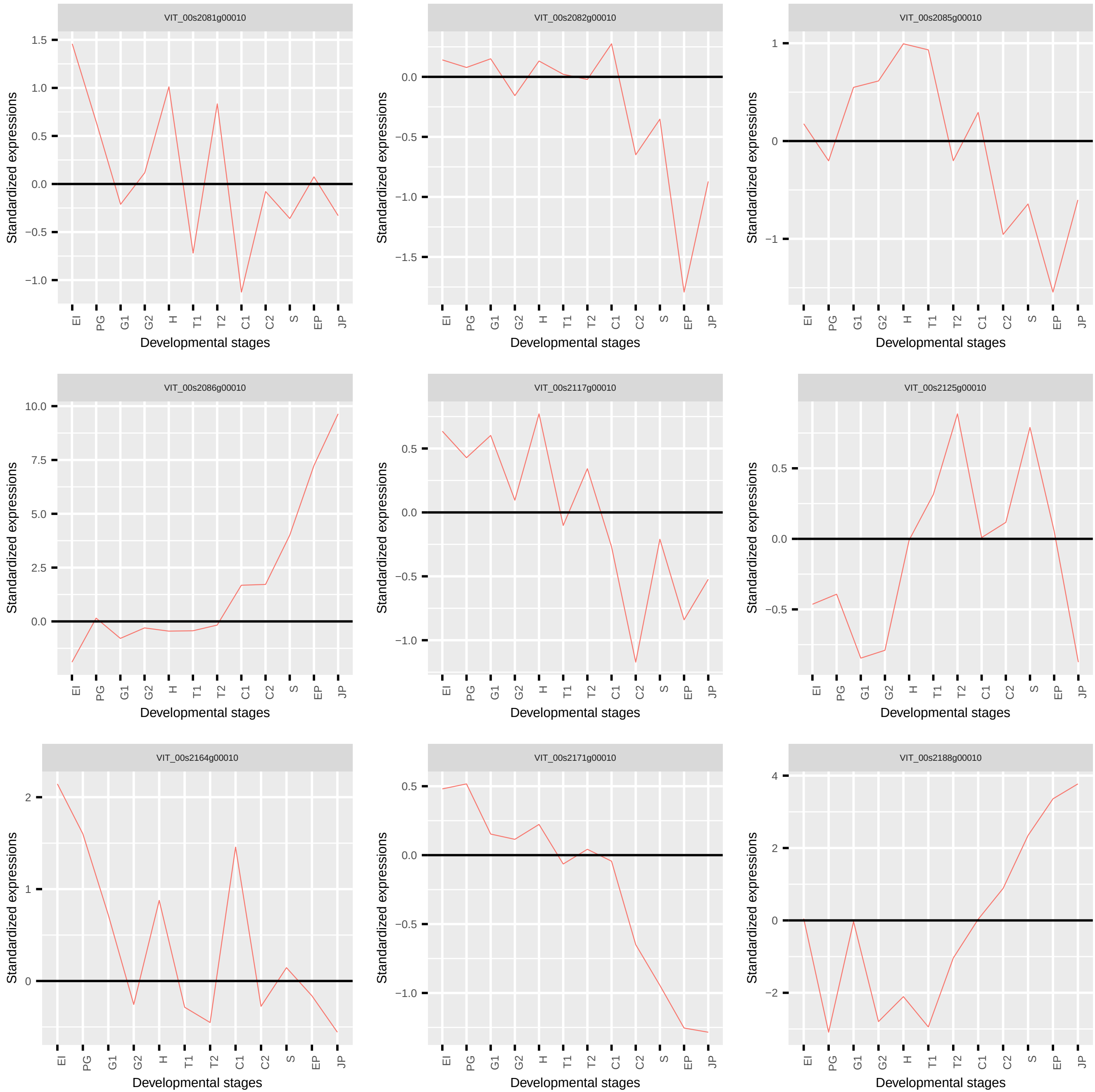
VIT_00s2081g00010
VIT_00s2082g00010
VIT_00s2085g00010
VIT_00s2086g00010
VIT_00s2117g00010
VIT_00s2125g00010
VIT_00s2164g00010
VIT_00s2171g00010
VIT_00s2188g00010
Standardized expressions
Developmental stages
EI PG G1 G2 H T1 T2 C1 C2 S EP JP

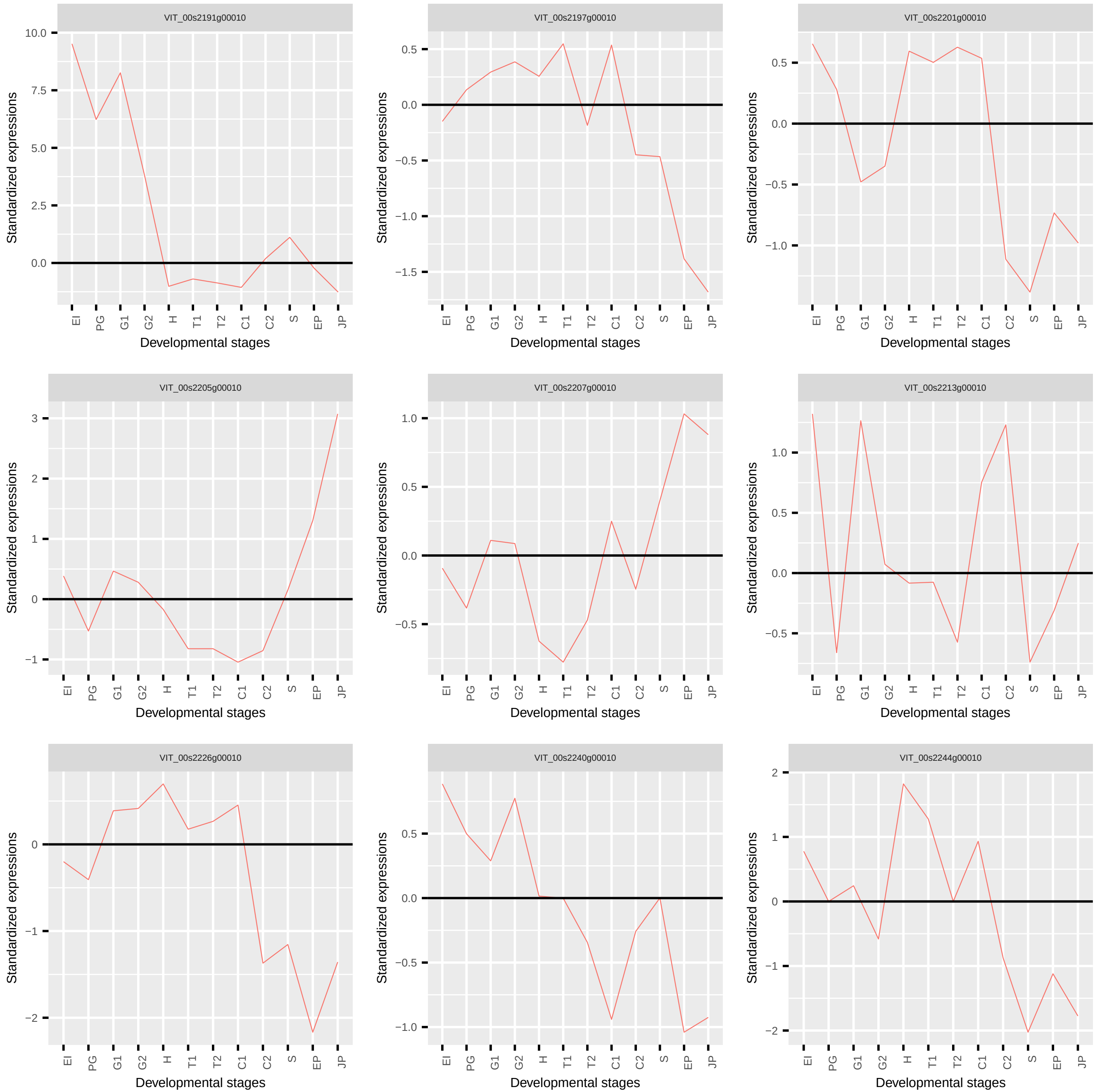
VIT_00s2191g00010
VIT_00s2197g00010
VIT_00s2201g00010
VIT_00s2205g00010
VIT_00s2207g00010
VIT_00s2213g00010
VIT_00s2226g00010
VIT_00s2240g00010
VIT_00s2244g00010
Standardized expressions
Developmental stages
EI PG G1 G2 H T1 T2 C1 C2 S EP JP

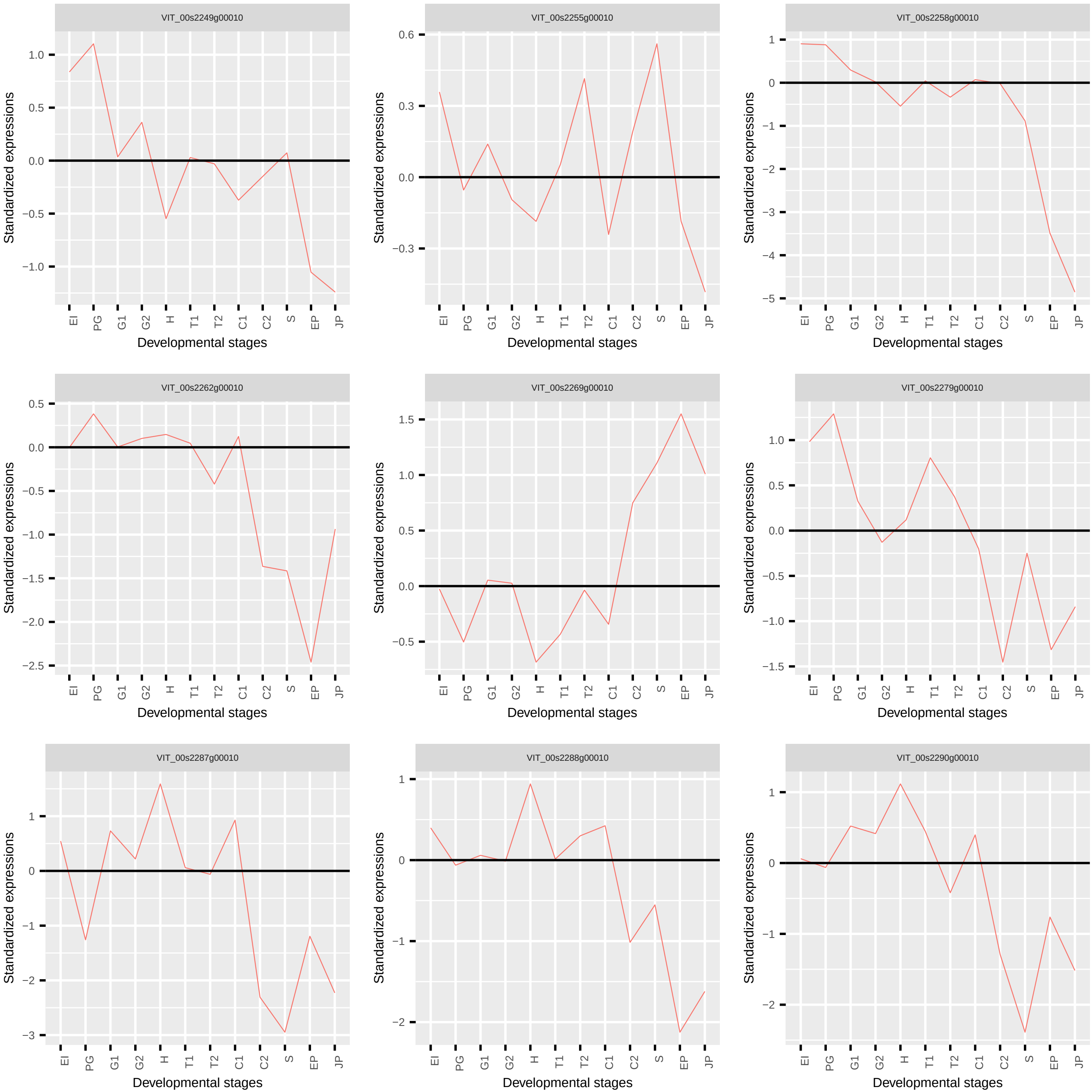
VIT_00s2249g00010
VIT_00s2255g00010
VIT_00s2258g00010
VIT_00s2262g00010
VIT_00s2269g00010
VIT_00s2279g00010
VIT_00s2287g00010
VIT_00s2288g00010
VIT_00s2290g00010
Standardized expressions
Developmental stages
EI PG G1 G2 H T1 T2 C1 C2 S EP JP

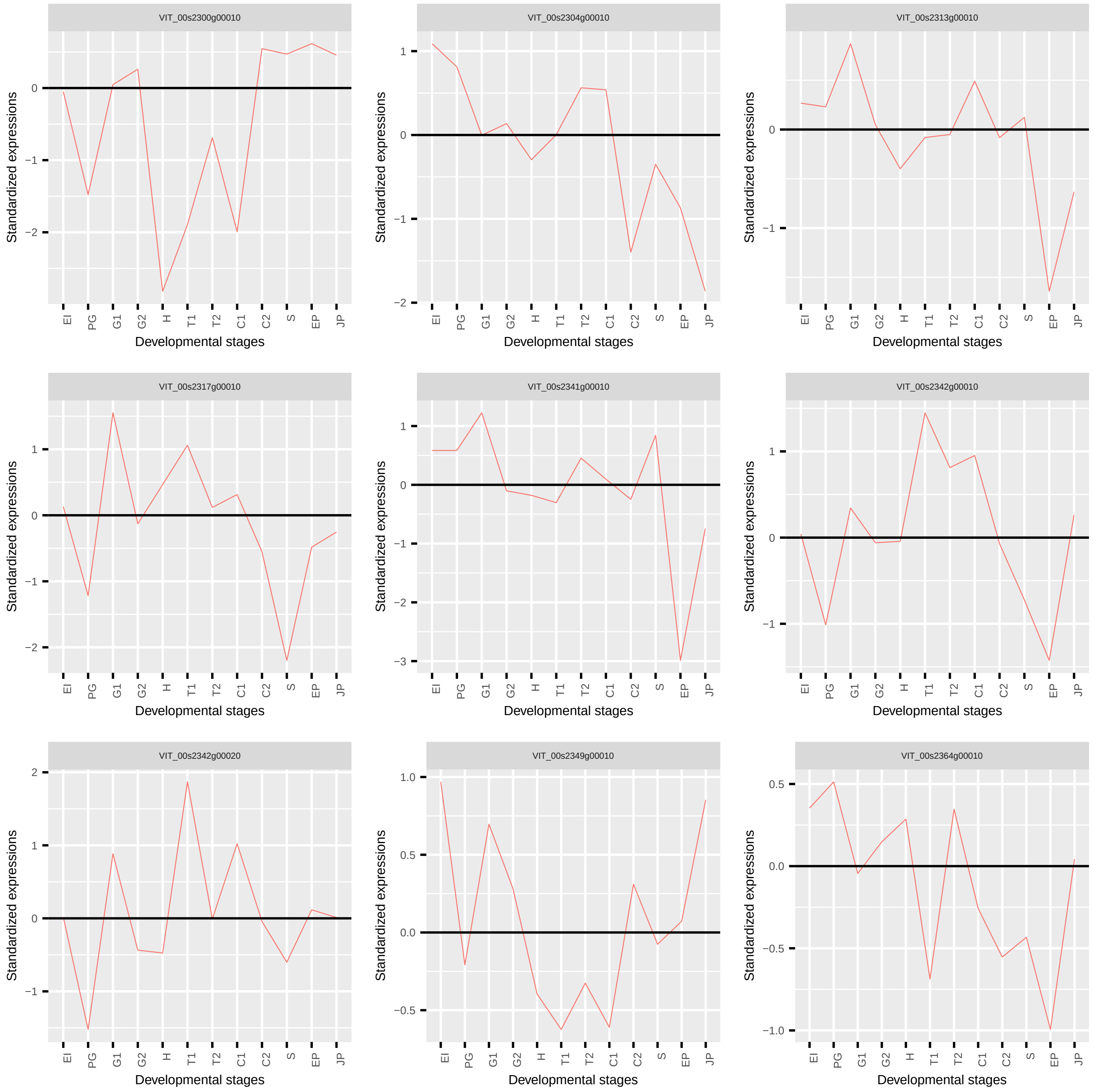
VIT_00s2300g00010
VIT_00s2304g00010
VIT_00s2313g00010
VIT_00s2317g00010
VIT_00s2341g00010
VIT_00s2342g00010
VIT_00s2342g00020
VIT_00s2349g00010
VIT_00s2364g00010
Standardized expressions
Developmental stages
EI
PG
G1
G2
H
T1
T2
C1
C2
S
EP
JP

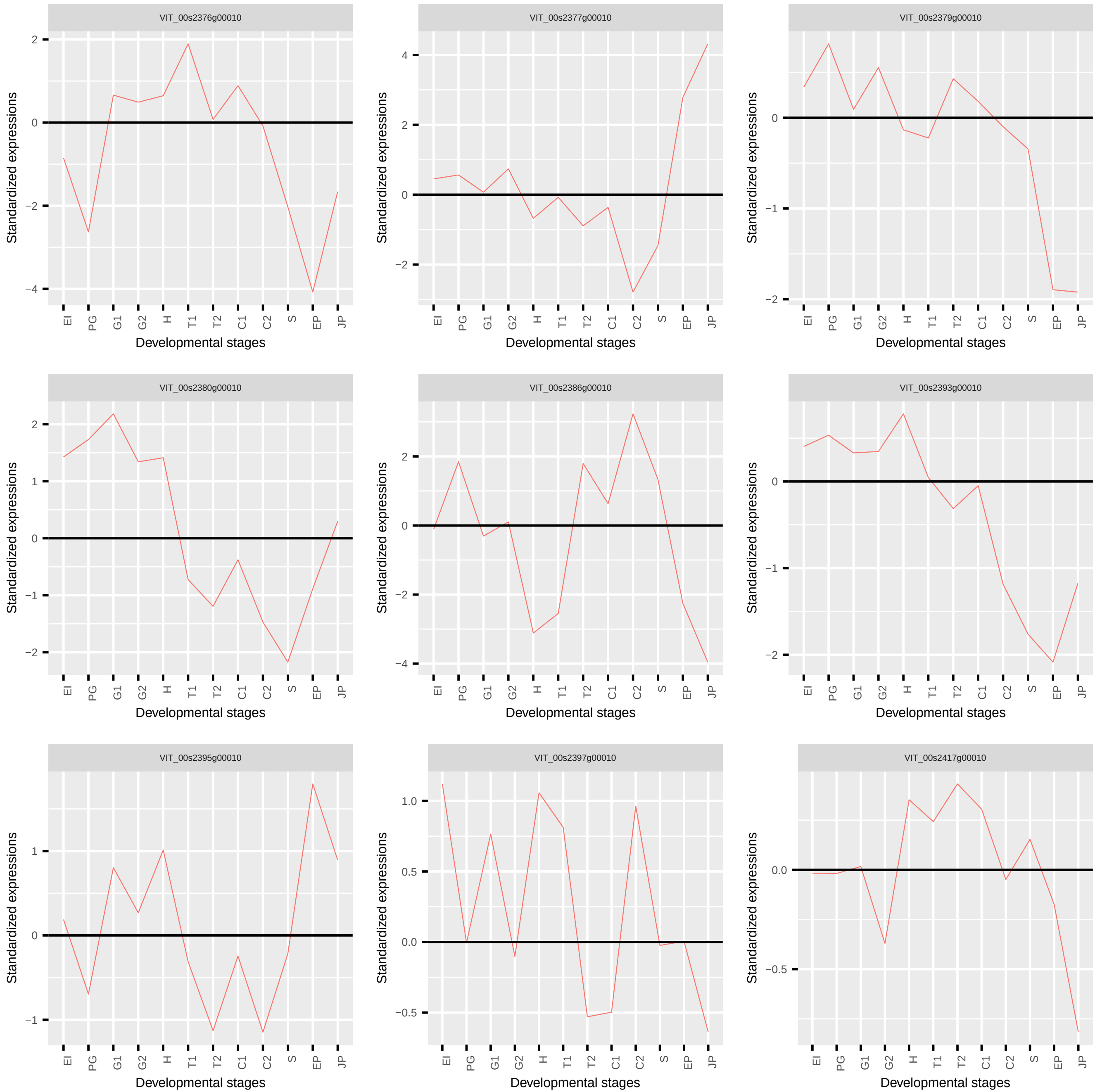

VIT_00s2376g00010
VIT_00s2377g00010
VIT_00s2379g00010
VIT_00s2380g00010
VIT_00s2386g00010
VIT_00s2393g00010
VIT_00s2395g00010
VIT_00s2397g00010
VIT_00s2417g00010
Standardized expressions
Developmental stages
EI PG G1 G2 H T1 T2 C1 C2 S EP JP

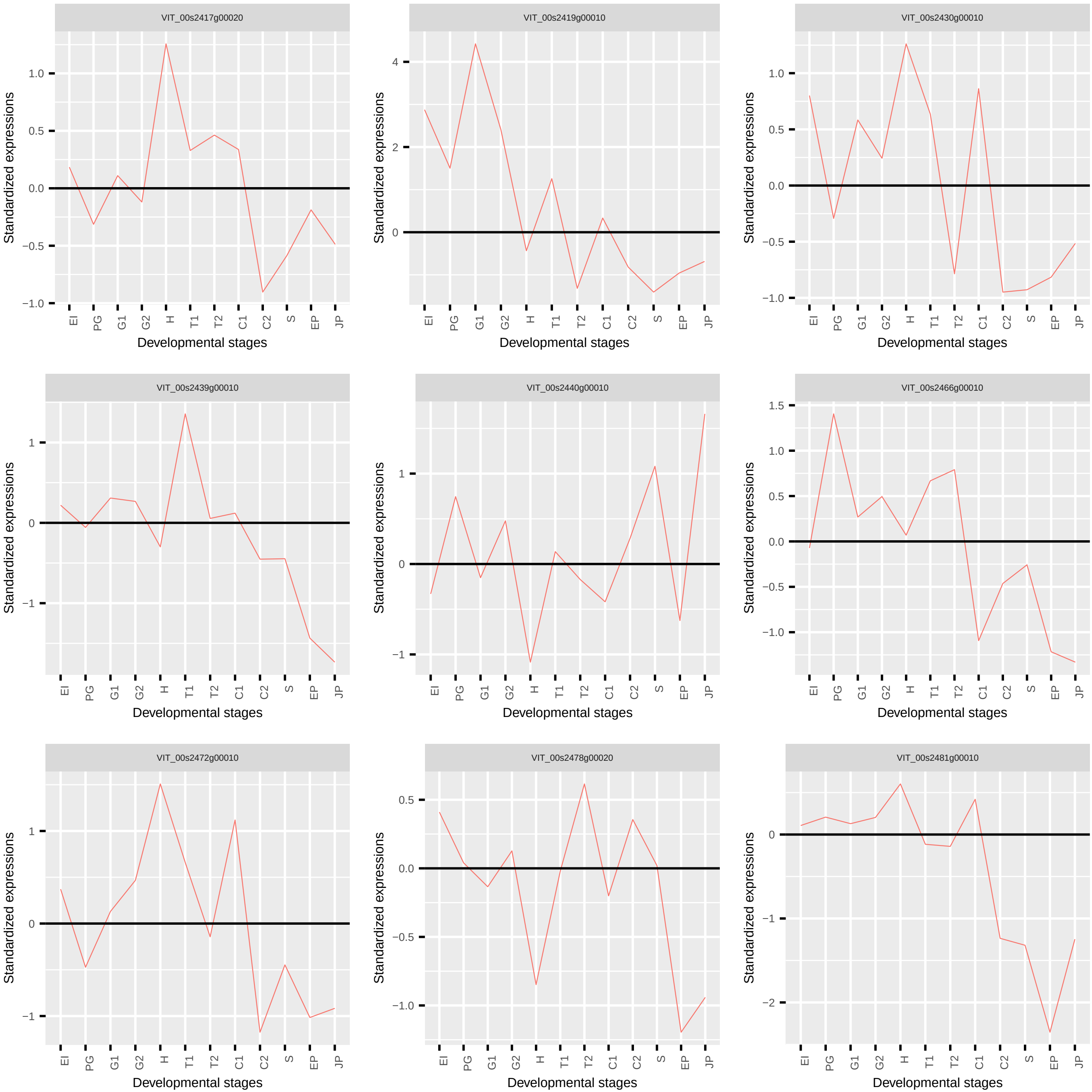

VIT_00s2417g00020
VIT_00s2419g00010
VIT_00s2430g00010
VIT_00s2439g00010
VIT_00s2440g00010
VIT_00s2466g00010
VIT_00s2472g00010
VIT_00s2478g00020
VIT_00s2481g00010
Standardized expressions
Developmental stages
EI PG G1 G2 H T1 T2 C1 C2 S EP JP

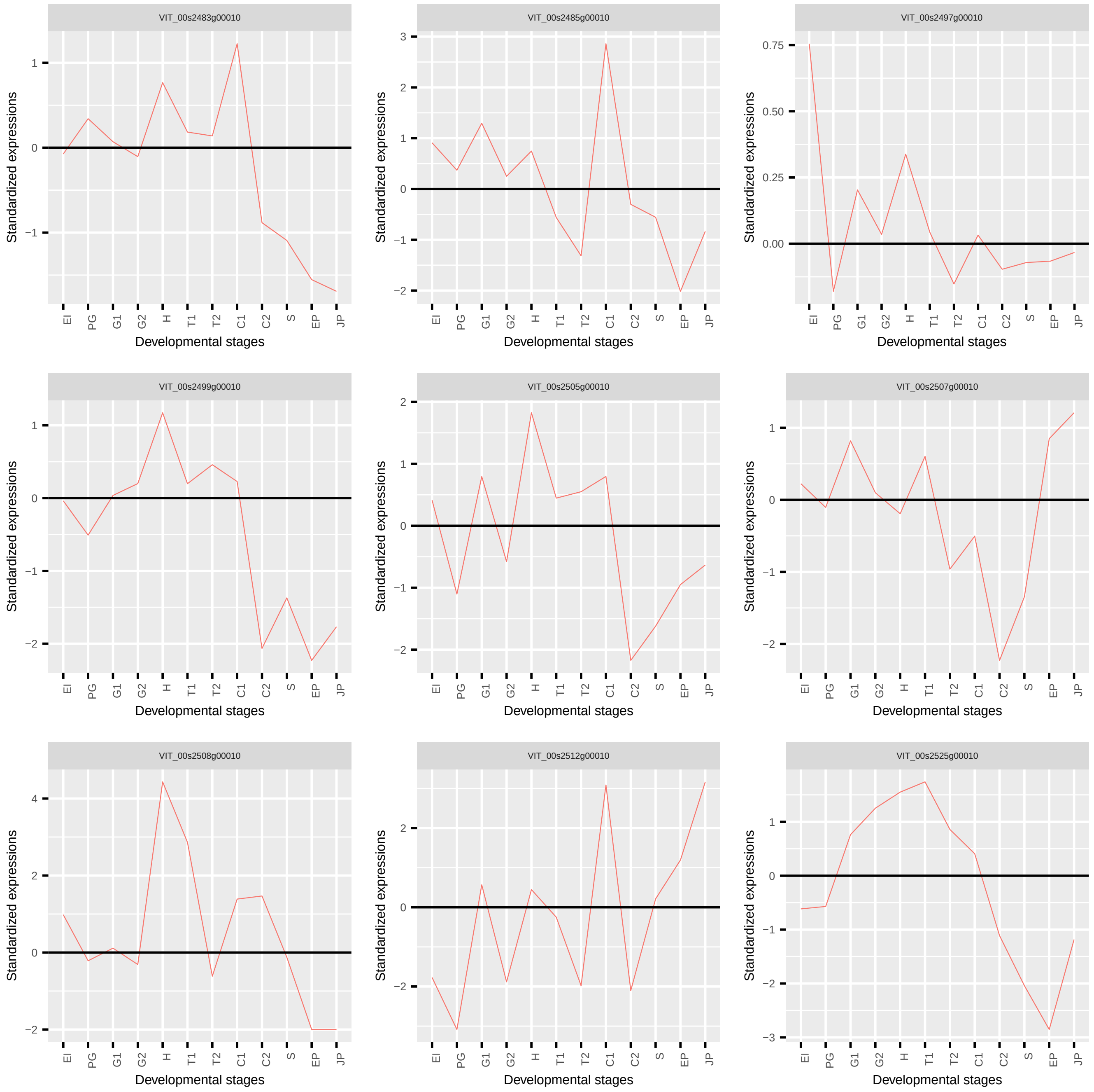
VIT_00s2483g00010
VIT_00s2485g00010
VIT_00s2497g00010
VIT_00s2499g00010
VIT_00s2505g00010
VIT_00s2507g00010
VIT_00s2508g00010
VIT_00s2512g00010
VIT_00s2525g00010
Standardized expressions
Developmental stages
EI PG G1 G2 H T1 T2 C1 C2 S EP JP

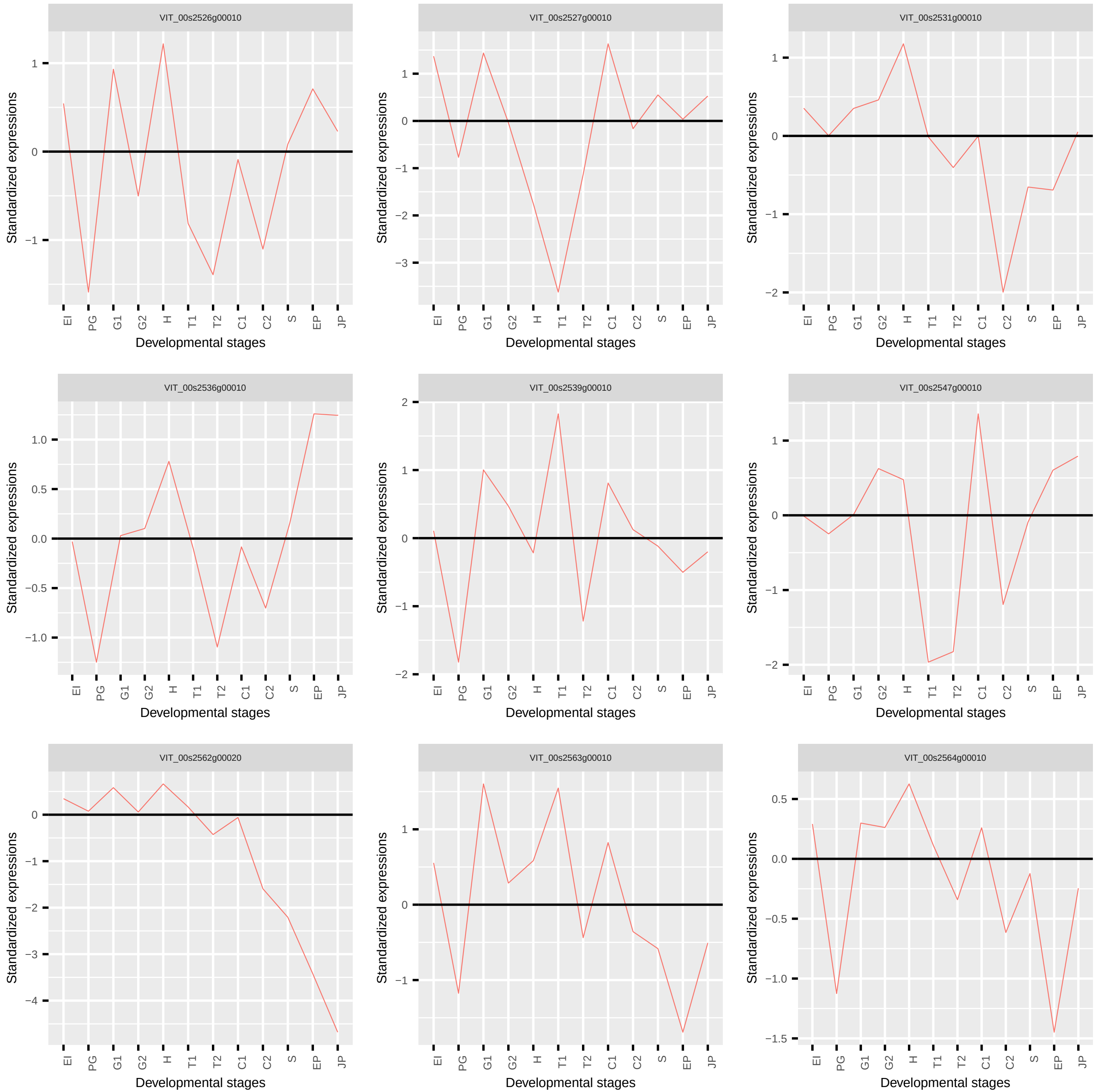
VIT_00s2526g00010
VIT_00s2527g00010
VIT_00s2531g00010
VIT_00s2536g00010
VIT_00s2539g00010
VIT_00s2547g00010
VIT_00s2562g00020
VIT_00s2563g00010
VIT_00s2564g00010
Standardized expressions
Developmental stages
EI
PG
G1
G2
H
T1
T2
C1
C2
S
EP
JP

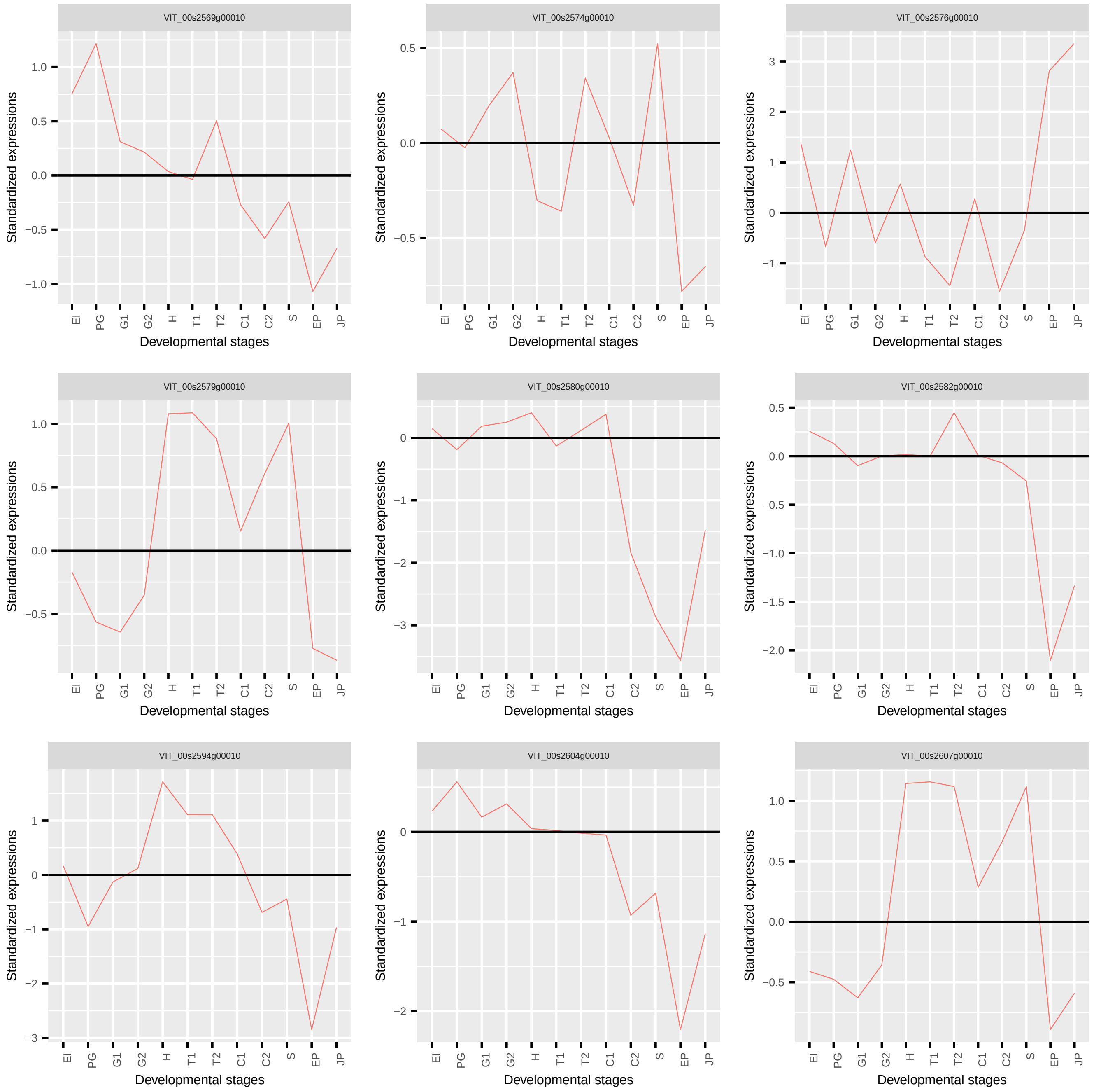
VIT_00s2569g00010
VIT_00s2574g00010
VIT_00s2576g00010
VIT_00s2579g00010
VIT_00s2580g00010
VIT_00s2582g00010
VIT_00s2594g00010
VIT_00s2604g00010
VIT_00s2607g00010
Standardized expressions
Developmental stages
EI
PG
G1
G2
H
T1
T2
C1
C2
S
EP
JP

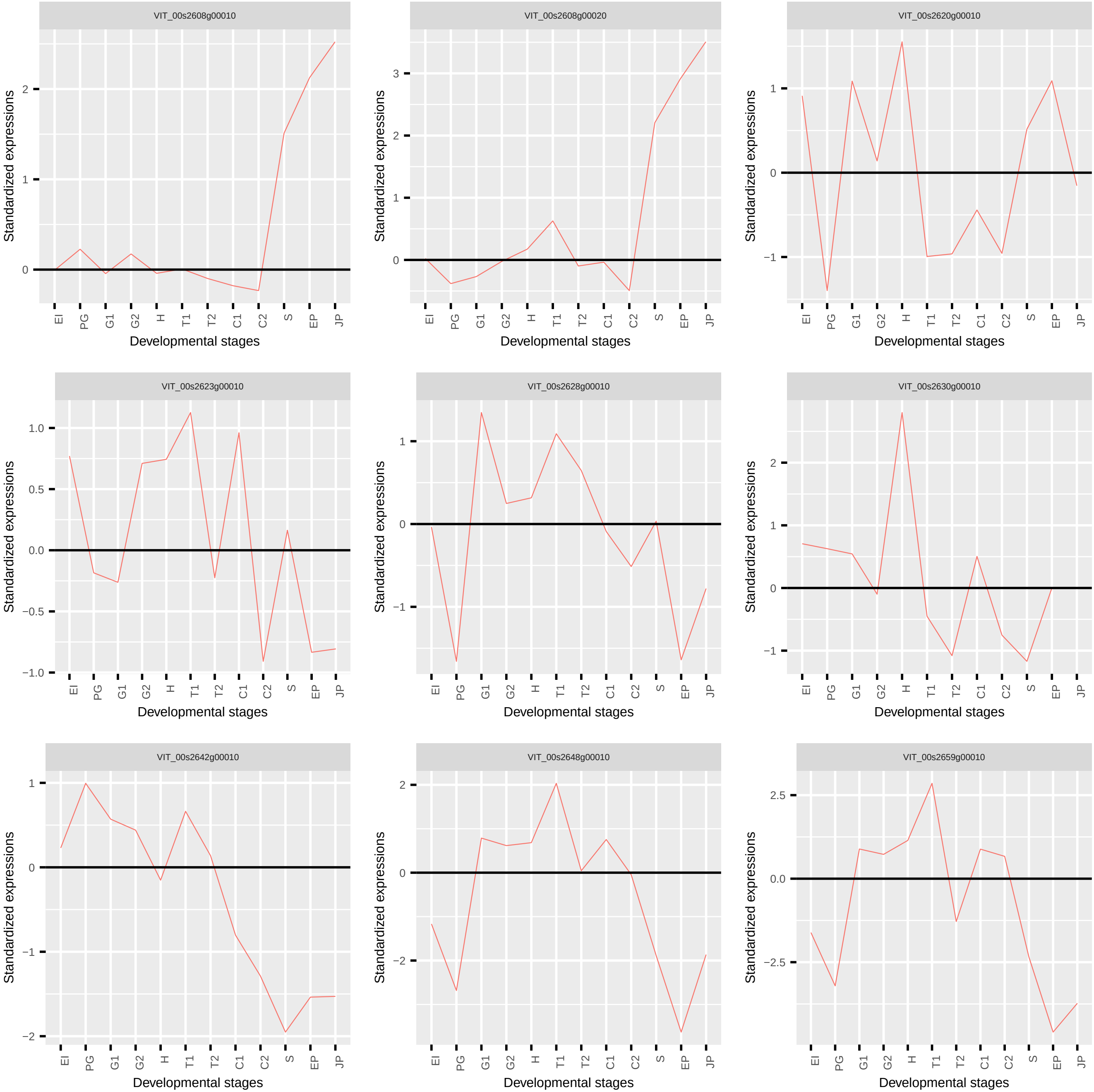
VIT_00s2608g00010
VIT_00s2608g00020
VIT_00s2620g00010
VIT_00s2623g00010
VIT_00s2628g00010
VIT_00s2630g00010
VIT_00s2642g00010
VIT_00s2648g00010
VIT_00s2659g00010
Standardized expressions
Developmental stages
EI PG G1 G2 H T1 T2 C1 C2 S EP JP

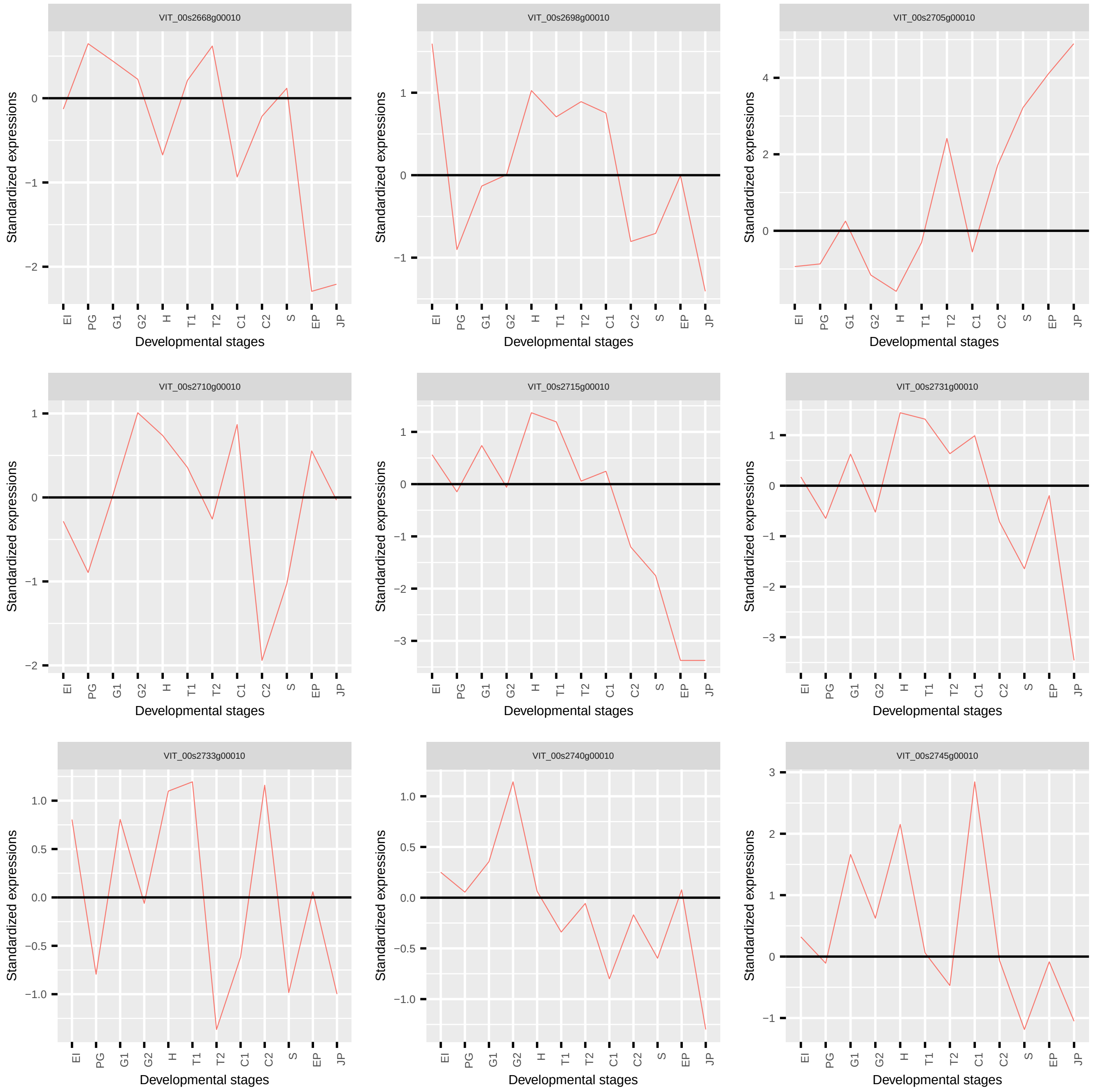
VIT_00s2668g00010
VIT_00s2698g00010
VIT_00s2705g00010
VIT_00s2710g00010
VIT_00s2715g00010
VIT_00s2731g00010
VIT_00s2733g00010
VIT_00s2740g00010
VIT_00s2745g00010
Standardized expressions
Developmental stages
EI PG G1 G2 H T1 T2 C1 C2 S EP JP

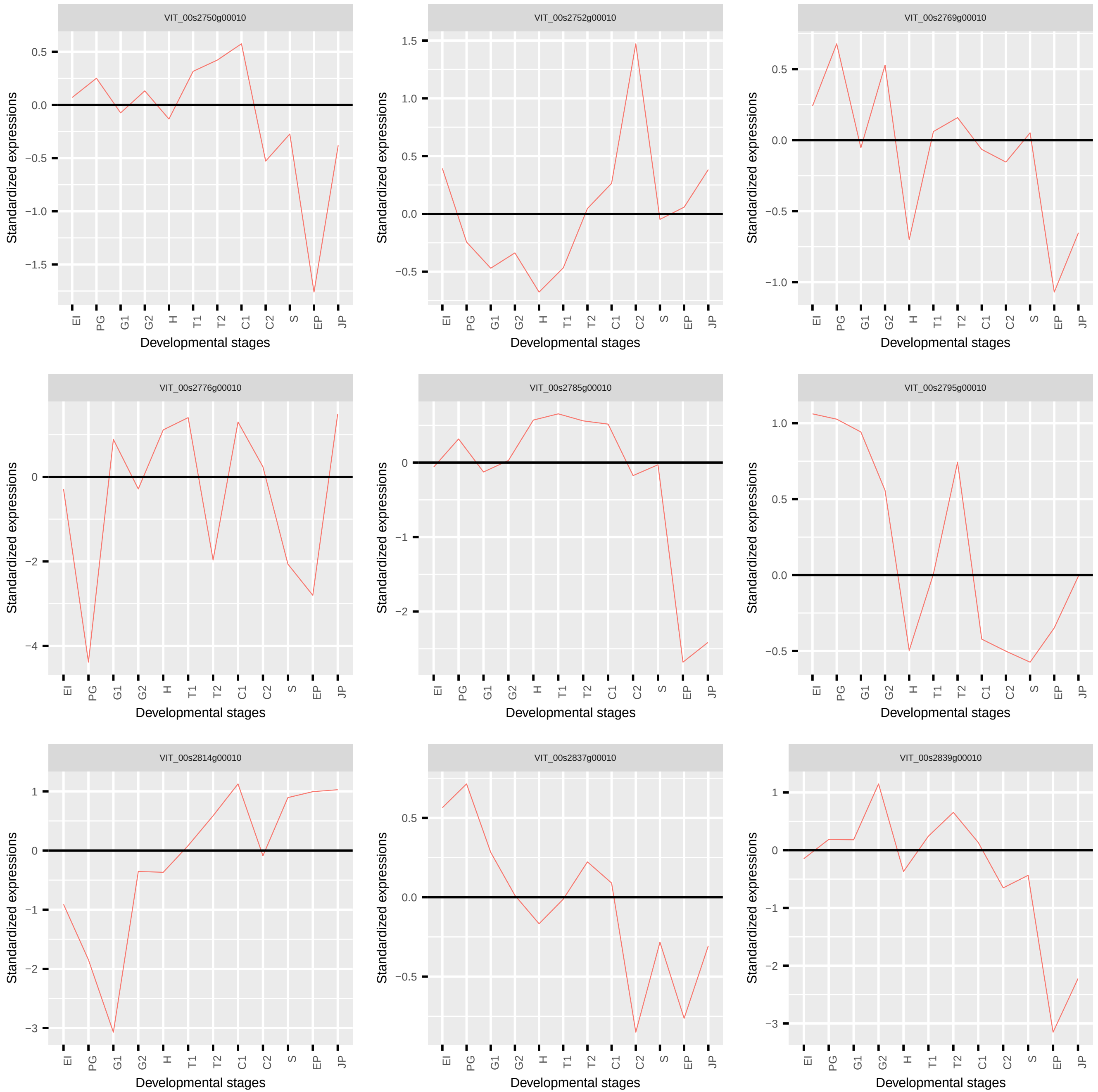
VIT_00s2750g00010
VIT_00s2752g00010
VIT_00s2769g00010
VIT_00s2776g00010
VIT_00s2785g00010
VIT_00s2795g00010
VIT_00s2814g00010
VIT_00s2837g00010
VIT_00s2839g00010
Standardized expressions
Developmental stages
EI PG G1 G2 H T1 T2 C1 C2 S EP JP

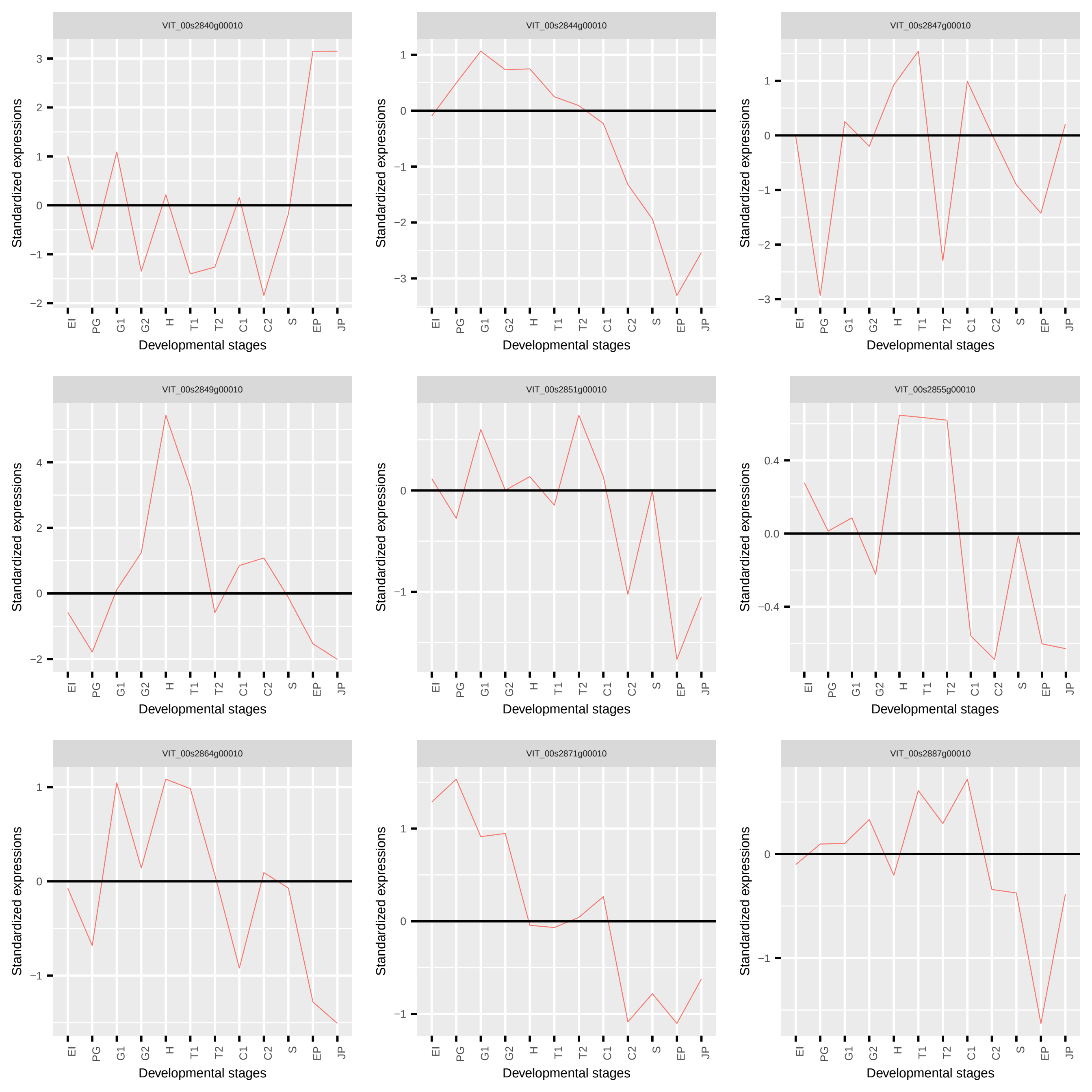
VIT_00s2840g00010
VIT_00s2844g00010
VIT_00s2847g00010
VIT_00s2849g00010
VIT_00s2851g00010
VIT_00s2855g00010
VIT_00s2864g00010
VIT_00s2871g00010
VIT_00s2887g00010
Standardized expressions
Developmental stages
EI
PG
G1
G2
H
T1
T2
C1
C2
S
EP
JP
